# Global Research Trends in ICU Nursing Personnel: A Bibliometric Analysis (2001–2025)

**DOI:** 10.1155/jonm/7601256

**Published:** 2026-08-29

**Authors:** Yadong Song, Ximing Chen, Cen Wu, Yang Shao

**Affiliations:** ^1^ Department of Critical Care Medicine, Shengjing Hospital of China Medical University, Shenyang 110004, China, cmu.edu.cn; ^2^ Innovative Research Center for Integrated Cancer Omics, Shengjing Hospital of China Medical University, Benxi 117004, China, cmu.edu.cn; ^3^ Department of Pulmonary and Critical Care Medicine, Shengjing Hospital of China Medical University, Shenyang 110004, China, cmu.edu.cn; ^4^ Department of Laboratory Medicine, Shengjing Hospital of China Medical University, Shenyang 110004, China, cmu.edu.cn; ^5^ Liaoning Clinical Research Center for Laboratory Medicine, Shenyang 110004, China

**Keywords:** bibliometric analysis, critical care nursing, global landscape, Holt’s linear exponential smoothing, ICU nursing, knowledge structure, research mapping, research trends, the Leiden algorithm, topic clustering

## Abstract

**Background:**

With an aging population, increasing patient complexity, and rising health care burdens in the postpandemic era, ICU nursing personnel research is growing in importance. However, systematic analyses of its knowledge structure, thematic evolution, and international collaboration models remain lacking.

**Objective:**

In this study, we used bibliometric methods to analyze trends, knowledge structures, and collaborative networks in global ICU nursing personnel research from 2001 to 2025.

**Methods:**

We searched PubMed, Web of Science Core Collection, and Scopus for ICU nursing literature from 2001 to 2025. We used descriptive bibliometric analysis to assess the distribution of countries, institutions, authors, journals, and highly cited articles. We constructed an institutional collaboration network and performed topic clustering using the Leiden algorithm based on keyword co‐occurrence networks. We used Holt’s linear exponential smoothing model to predict future publication trends.

**Results:**

We ultimately included 44,011 articles. Global ICU nursing personnel publications showed a steady upward trend, with the United States dominating in terms of publication volume and international collaboration. In a co‐occurrence network analysis using the Leiden algorithm, we identified five core research themes: health care provider attitudes and management (C0), clinical management and patient outcomes (C1), evidence‐based nursing and infection control (C2), pediatric intensive care (C3), and neonatal care (C4). Trend projections indicated that the annual publication output in this field will approach 5000 articles by 2030.

**Conclusion:**

Global ICU nursing personnel research has expanded rapidly, with research attention shifting from clinical‐practice‐oriented topics toward workforce sustainability, organizational support, and nursing management. These bibliometric findings provide a knowledge map of the field and highlight the need for future empirical research and cross‐regional collaboration.

**Implications for Nursing Management:**

Nursing managers may use these mapped trends to inform ICU nursing workforce planning, staffing allocation, organizational support, and burnout prevention. The findings may also guide research prioritization and knowledge translation into locally adaptable nursing management strategies.

## 1. Introduction

Intensive care unit (ICU) nurses are the backbone of critical care services. Their technical proficiency and compassionate care are closely associated with clinical outcomes and patient safety [1]. The core of critical care lies in a “dual competency framework” comprising the precision required for high‐tech interventions such as extracorporeal membrane oxygenation and continuous renal replacement therapy together with the empathy needed to provide psychological support [2, 3]. However, in the 21st century, ICU nursing faces a triple burden characterized by rising rates of critical illness, rapid population aging, and continuous technological innovation [4]. Globally, these pressures manifest as overwhelming workloads, a widening knowledge gap, and a severe occupational health crisis. Data indicate that following the COVID‐19 pandemic, nurse burnout rates have reached 60%–70% [5, 6].

Although there is a global consensus regarding the complexity of ICU care, there are marked differences in research priorities and resource allocation among regions with varying levels of economic development. Research in high‐income countries is often focused on the adoption of smart technologies and refined quality improvement initiatives [7, 8]. By contrast, low‐ and middle‐income countries are grappling with severe shortages of personnel and equipment [9, 10]. Despite a steady increase in academic output over the past 3 decades, existing review studies—primarily narrative reviews and single‐domain meta‐analyses—have often been limited to isolated topics, such as technical specifications of mechanical ventilation or specific burnout interventions [11, 12]. Thus, there is a lack of systematic analysis regarding the overall knowledge structure and evolving trends in global ICU nursing personnel research. Bibliometric analysis can reveal research hotspots, patterns of academic collaboration, and the evolution of research themes through knowledge mapping and network analysis methods, thereby providing evidence‐based support for nursing management and research decision‐making [13].

To systematically identify the development trends and knowledge structure of global ICU nursing personnel research, we integrated the literature data from PubMed, Web of Science Core Collection (WOSCC), and Scopus and applied bibliometric methods to analyze ICU nursing personnel research from 2001 to 2025. Compared with traditional clustering methods based on VOSviewer or the Louvain algorithm, the Leiden algorithm offers superior community connectivity and clustering stability in large‐scale weighted networks, making it more suitable for identifying the knowledge structure of complex medical research topics. The objectives of this study were to (1) characterize the distribution of countries, institutions, and authors in global ICU nursing personnel research; (2) identify core research themes and their temporal evolution; and (3) analyze international research collaboration patterns and future research trends, thereby providing evidence‐based guidance for ICU nursing management and the allocation of research resources.

## 2. Methods

### 2.1. Study Design

In this study, we conducted retrospective bibliometric analysis to systematically evaluate trends and the knowledge structure of global ICU nursing personnel research between 2001 and 2025. We developed a multilayered analytical framework integrating descriptive bibliometric analysis, research collaboration network analysis, topic clustering, and time‐series forecasting.

### 2.2. Data Source and Search Strategy

On March 11, 2026, we conducted a systematic search across three major databases: PubMed, WOSCC, and Scopus. The search strategy targeted broad terms related to intensive care and nursing personnel, so as to capture common management practices across adult, pediatric, and neonatal ICUs. The search strategies for each database were as follows: PubMed: (((“Intensive Care Units”[MeSH Terms] OR “Critical Care”[MeSH Terms]) OR (“ICU”[Title/Abstract] OR “intensive care unit”[Title/Abstract] OR “critical care unit”[Title/Abstract] OR “intensive care”[Title/Abstract] OR “critical care”[Title/Abstract])) AND (((“Nursing Care”[MeSH Terms] OR “Nurses”[MeSH Terms] OR “Nursing”[MeSH Terms]) OR (“nursing”[Title/Abstract] OR “nurse”[Title/Abstract] OR “nursing staff”[Title/Abstract] OR “nursing personnel”[Title/Abstract])))) AND (“2001/01/01”[Date ‐ Publication]: “2025/12/31”[Date ‐ Publication]) AND (“English”[Language]); WOSCC: TS = (“intensive care unit” OR “ICU” OR “critical care unit” OR “intensive care” OR “critical care”) AND TS=(“nursing” OR “nurse” OR “nursing care” OR “nursing staff” OR “nursing personnel”) AND PY=(2001–2025) AND LA = English AND DT = (Article OR Review); Scopus: (TITLE‐ABS‐KEY (“intensive care unit” OR “ICU” OR “critical care unit” OR “intensive care” OR “critical care”) AND TITLE‐ABS‐KEY (“nursing” OR “nurse” OR “nursing care” OR “nursing staff” OR “nursing personnel”)) AND PUBYEAR > 2000 AND PUBYEAR < 2026 AND (LIMIT‐TO (LANGUAGE, “English”)) AND (LIMIT‐TO (DOCTYPE, “ar”) OR LIMIT‐TO (DOCTYPE, “re”)). The search covered the period from January 1, 2001 to December 31, 2025 and included only English‐language literature to ensure consistency and comparability of the data across different databases.

### 2.3. Inclusion and Exclusion Criteria

The inclusion criteria were as follows: (1) studies related to ICU nursing; (2) articles published between 2001 and 2025; (3) original research or review articles; and (4) articles in English.

The exclusion criteria were as follows: (1) nonresearch literature such as conference abstracts, editorials, and commentaries; (2) records with incomplete bibliographic information; and (3) duplicate entries.

### 2.4. Data Extraction and Screening

**FIGURE 1:**
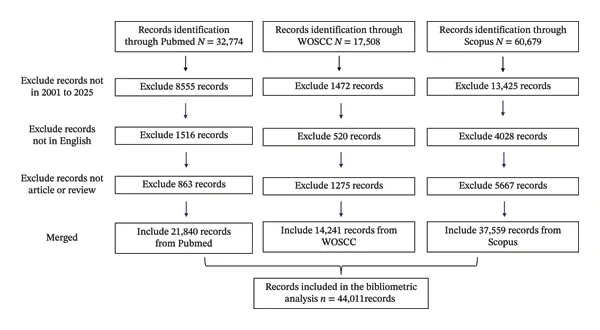
Flowchart of the literature search, screening, and inclusion process.

The literature search and screening process for this study are shown in Figure 1. The initial search yielded a total of 110,961 records (PubMed: 32,774; WOSCC: 17,508; Scopus: 60,679). The screening steps were as follows: (1) exclude records published outside the 2001–2025 timeframe (PubMed: 8555; WOSCC: 1472; Scopus: 13,425); (2) exclude non‐English language literature (PubMed: 1516; WOSCC: 520; Scopus: 4028); and (3) exclude nonoriginal articles or review‐type literature (WOSCC: 1275; Scopus: 5667). For PubMed, we did not include a document‐type restriction in the database search query; instead, manual screening was performed after the search was completed in the database interface, retaining only journal articles and reviews and excluding an additional 863 records.

A multistep combined strategy was used for duplicate detection. First, the raw records from the three databases were merged; case sensitivity, singular/plural forms, and synonym mappings were standardized. Next, precise automatic deduplication was performed based on PubMed Identifiers, supplemented by standardized keyword combinations to identify suspected duplicate records. Finally, author information for suspected duplicate records was verified manually. A total of 44,011 unique records were ultimately retained for all subsequent bibliometric analyses. PubMed records were converted to a standardized XML format using PubMed2XL; field mapping and format standardization were performed on the raw data from WOSCC and Scopus. All data—including title, abstract, author, institution, keywords, publication year, journal, and citation count—were imported into Microsoft Excel 2019.

### 2.5. Analytical Methods

In this study, we applied a multilevel bibliometric analysis framework to conduct a systematic analysis of the included literature. First, Microsoft Excel 2019 was used to calculate the annual publication volume; descriptive statistics and rankings were generated for countries/regions, research institutions, core authors, publishing journals, and the 20 most‐cited articles. The H‐index for high‐output authors was obtained from the Web of Science database; journal impact factors (IFs) and Journal Citation Report (JCR) quartiles were sourced from the LetPub database. Total citation counts were extracted from the Web of Science and Scopus databases based on the cumulative citation counts at the time of data retrieval. Documents were ranked by the total citation count, and the 20 most‐cited documents were selected for thematic analysis and assessment of academic influence. For the collaboration network analysis, on the basis of institutional coauthorship relationships, we extracted copublication records among the 15 leading institutions by publication volume. The intensity of collaboration between institutions was defined according to the frequency of copublications, and an institutional collaboration network was constructed.

#### 2.5.1. Keyword Co‐Occurrence Networks and Cluster Analysis

First, the keyword fields from PubMed, WOSCC, and Scopus were merged to form a raw keyword set (keywords_all_raw). This was followed by systematic standardization and preprocessing, including the following: splitting keywords using the “||” symbol and semicolons (); removing empty values, NaN, and “none”; removing the special character “∗”; retaining only the main subject terms for Medical Subject Heading terms containing “/”; removing parentheses and supplementary information within parentheses; standardizing space formatting; filtering out generic terms and nonsubject terms such as “human,” “humans,” “male,” “female,” “adult,” “patient,” “study,” “method,” “review,” “nurse,” and “nursing”; filtering out expressions with a length ≤ 2, pure numbers, and age range expressions. The subsequent steps were as follows: establish standardized mapping rules (convert to lowercase, replace “&” with “and,” convert hyphens to spaces, and remove nonalphanumeric characters); merge synonyms (e.g., “coronavirus disease 2019,” “covid19,” and “covid 2019” were consolidated into “COVID‐19”; “respiration artificial” was consolidated into “artificial ventilation”); merge singular and plural forms (e.g., risk factor/risk factors, cross‐sectional study/cross‐sectional studies); standardize display labels (e.g., COVID‐19, risk factors, length of stay); and deduplicate keywords within individual articles. This process ultimately yielded a standardized keyword field (keywords_all_clean) (Supporting File: keyword_merge_mapping_table.pdf). When constructing the keyword co‐occurrence network, noninformative generic terms such as “article,” “controlled study,” “priority journal,” “questionnaire,” “case report,” and “United States” were further excluded at the network level. To reduce interference from low‐frequency noise and enhance the stability of the network structure, only keywords with an occurrence frequency ≥ 20 and edges with a co‐occurrence weight ≥ 8 were retained. Subsequently, node importance was comprehensively evaluated based on metrics such as keyword frequency, weight, and network centrality (Supporting File: nodes.pdf and edges.pdf). The 200 leading high‐importance keyword nodes were selected to construct the core keyword co‐occurrence network. We performed Leiden community clustering analysis exclusively on the subnetwork composed of these 200 keywords. Network analysis was conducted using Python‐igraph (version 0.11.2), invoking the Graph.community_leiden() function to implement the Leiden algorithm with a resolution of *γ* = 1, *n* = 2 iterations, and a modularity (*Q*) value of 0.201596 (Supporting File: leiden_network_summary.pdf). Intracommunity co‐occurrence weights accounted for 53.73% of the total weights, and the number of intracommunity edges accounted for 39.65%; both were significantly higher than the theoretical proportion for a random distribution (29.31%), which indicated that the network possessed a well‐interpretable community structure. To avoid interference from high‐frequency background terms in network analysis, although “ICU” and “critical care” were included in the construction of the original network, these terms were not incorporated into the final clustering theme interpretation. To enhance the clarity and readability of the graph, based on the network of the 200 leading core keywords, 110 high‐frequency keywords with co‐occurrence frequencies ≥ 32 were further selected to construct a visualized subnetwork, with edge weights defined according to co‐occurrence frequency (Supporting File: leiden_network_publication_style_params.pdf).

#### 2.5.2. Analysis of the Temporal Evolution of Research Themes

Publications were grouped into 5‐year intervals, and the cumulative co‐occurrence frequency of keywords within each Leiden cluster was calculated for the corresponding time periods (Supporting File: community_period_summary.pdf). Heatmaps were used to visualize the temporal evolution trends of each research theme.

#### 2.5.3. Time‐Series Analysis

Given the nonstationary fluctuations in publication data caused by the COVID‐19 pandemic from 2020 to 2024, Holt’s linear exponential smoothing model was selected to replace the autoregressive integrated moving average model, as the former is better suited for time series with linear trends and no significant seasonality (Supporting File: publication_forecast_notes). Model parameters were set as the smoothing coefficient *α* = 0.25 and trend coefficient *β* = 0.50. The model fit was evaluated using the standard deviation of the residuals (Supporting File: publication_forecast_model_parameters.pdf).

All data processing, network construction, and visualization were performed using Python 3.11.5, primarily with libraries such as pandas, numpy, networkx, matplotlib, and scikit‐learn.

### 2.6. Ethical Considerations

In this study, we exclusively used publicly available secondary metadata retrieved from the PubMed, WOSCC, and Scopus databases. No personally identifiable information was collected, and no direct interaction with human subjects occurred. In accordance with institutional review board standards for secondary data analyses involving nonidentifiable public records, this study was formally exempted from full ethical committee review.

### 2.7. Data Availability

The data that support the findings of this study are available from the corresponding author upon reasonable request.

## 3. Results

### 3.1. Countries, Institutions, Journals, and Distribution of Core Authors

#### 3.1.1. Country/Region Distribution

**FIGURE 2:**
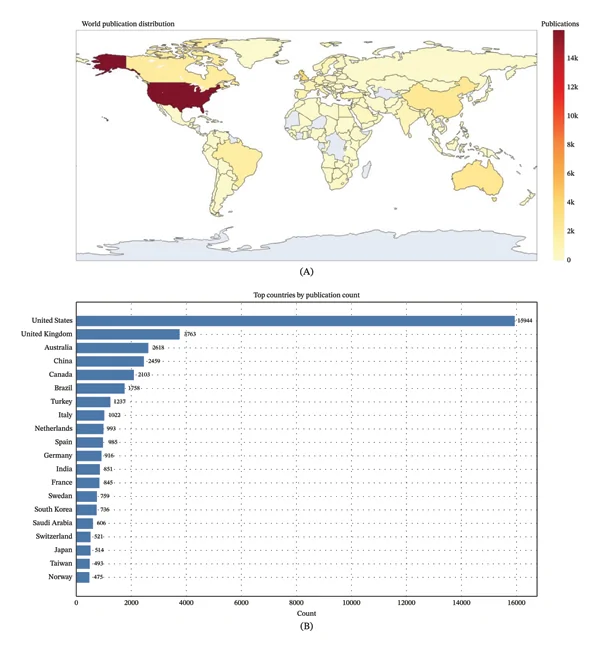
Global distribution of ICU nursing personnel research publications (2001–2025). (A) Geographic heat map showing publication volumes by country. Darker colors indicate higher publication volumes. (B) Twenty countries/regions with the highest number of publications in ICU nursing personnel research.

This study included a total of 44,011 relevant publications in the research field of ICU nursing from 2001 to 2025, covering 165 countries and regions. The geographic distribution is shown in Figure 2A. The United States ranked first in number with 15,944 publications, accounting for 36.22% of the total output; this was followed by the United Kingdom (3763 articles), Australia (2618 articles), China (2459 articles), and Canada (2103 articles). These five countries collectively published 26,887 articles, accounting for 61.1% of the total output (Figure 2B), indicating a marked concentration of research in this field on a global scale.

#### 3.1.2. Distribution of Research Output by Institution

**FIGURE 3:**
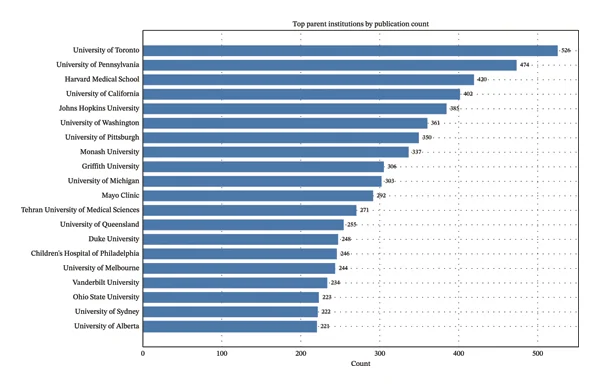
The 20 institutions with the highest production output in ICU nursing personnel research (2001–2025).

Research output at the institutional level is shown in Figure 3. The University of Toronto had the most publications (*n* = 526), followed by the University of Pennsylvania (*n* = 474) and Harvard Medical School (*n* = 420). These three institutions collectively published 1420 articles, accounting for 22.46% of the total output (6320 articles) from the 20 leading high‐output institutions. In terms of publication volume distribution, the three leading institutions published substantially more articles than the remaining institutions; those ranked 4th–20th published between 221 and 402 articles, with the University of California (402 articles), Johns Hopkins University (385 articles), the University of Washington (361 articles), the University of Pittsburgh (350 articles), and Monash University (337 articles) all publishing over 300 articles. The output of the remaining institutions was similar. In terms of geographic distribution, among the 20 leading institutions, 14 were from North America (12 from the United States and two from Canada) and five were from Australia. Tehran University of Medical Sciences (271 articles) was the only institution from the Middle East. According to institution type, the Mayo Clinic (292 articles) and Children’s Hospital of Philadelphia (246 articles) were the only two clinical medical institutions identified; the remainder involved comprehensive universities or medical research institutions.

#### 3.1.3. Journal Distribution and Academic Characteristics

**FIGURE 4:**
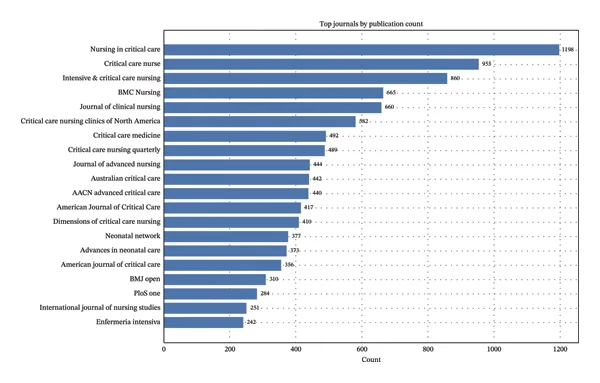
The 20 leading journals by publication output in ICU nursing personnel research (2001–2025).

Globally, a total of 4770 journals published research related to ICU nursing staff between 2001 and 2025 (Figure 4). The three leading journals according to publication volume were *Nursing in Critical Care* (1198 articles), *Critical Care Nurse* (955 articles), and *Intensive & Critical Care Nursing* (860 articles). Together, these journals accounted for 3013 articles, representing 29.4% of the total output (10,247 articles) among the 20 leading journals and constituting the core publications in this field. The top five journals each published over 600 articles, and the journals ranked 6th–20th published between 242 and 582 articles. According to Bradford’s Law, the literature in this field exhibited a typical core journal distribution pattern. Among the 10 leading journals, eight are in the JCR Q1 category, with only *Critical Care Nursing Clinics of North America* and *Critical Care Nursing Quarterly* in the JCR Q2 category (Table 1). In terms of IF, *Critical Care Medicine* exhibited the highest academic influence, with a 5‐year IF of 6.8 and a 2025 IF of 6.9; this was followed by *BMC Nursing* (2025 IF = 4.9) and *Intensive & Critical Care Nursing* (2025 IF = 4.7).

**TABLE 1:** Academic indicators of core journals in global ICU nursing personnel research (2025).

Journal	Publications	IF (5 years)	IF (2025)	JCR
Nursing in critical care	1198	3.4	2.6	1
Critical care nurse	955	2.3	2	1
Intensive and critical care nursing	860	4.4	4.7	1
BMC Nursing	665	4.5	4.9	1
Journal of clinical nursing	660	4.3	3.5	1
Critical care nursing clinics of North America	582	2	1.5	2
Critical care medicine	492	6.8	6.9	1
Critical care nursing quarterly	489	1.2	—	2
Journal of advanced nursing	444	4.2	3.5	1
Australian critical care	442	3.2	2.7	1

#### 3.1.4. Distribution Characteristics of High‐Output Authors

Research on ICU nursing staff involved a total of 98,797 authors. Table 2 lists the 20 most prolific authors. In terms of publication output, Ingrid Egerod ranked first with 63 articles, followed by Louise Rose (53 articles) and Wendy Chaboyer (52 articles). Authors ranked 4th–20th published between 26 and 43 articles. In terms of academic impact, E. Wesley Ely had the highest H‐index (H‐index = 120); although that author only published 36 papers, his work received a high level of citations. J. Randall Curtis (H‐index = 83) and Wendy Chaboyer (H‐index = 55) also demonstrated high levels of academic influence. In terms of institutional distribution, these highly productive authors were primarily affiliated with universities and research institutions in Australia, the United States, Canada, the United Kingdom, and Denmark.

**TABLE 2:** The 20 leading authors in ICU nursing personnel research (publications, H‐index, and affiliated institution).

Author full name	Publications	H‐index	Institution of author
Egerod, Ingrid	63	38	University of Copenhagen
Rose, Louise	53	44	University of St. Michael’s College
Chaboyer, Wendy	52	55	Griffith University
Curtis, J Randall	43	83	University of Washington
Endacott, Ruth	43	33	Plymouth University
Aitken, Leanne M	39	35	Griffith University
Curley, Martha A Q	36	43	University of Pennsylvania
Ely, E Wesley	36	120	Vanderbilt University and Veteran’s Administration
Latour, Jos M	33	34	University of Plymouth
Blackwood, Bronagh	29	45	Queens University Belfast
Olson, DaiWai M	29	42	University of Texas Southwestern Medical Center Dallas
Coyer, Fiona	28	30	The University of Queensland
Happ, Mary Beth	28	29	Ohio State University
Ayed, Ahmad	27	19	Arab American University
Engström, Åsa	27	21	Lulea University of Technology
McGrath, Jacqueline M	27	35	University of Texas
Bloomer, Melissa J	26	22	Deakin University
Currey, Judy	26	21	Deakin University
Gélinas, Céline	26	37	McGill University
Kleinpell, Ruth	26	38	Vanderbilt University

#### 3.1.5. Distribution Characteristics of Highly Cited Publications

Table 3 presents the 20 most‐cited publications identified through the ICU nursing personnel search from 2001 to 2025. These highly cited publications primarily focused on infection prevention guidelines, ICU delirium assessment and management, sedation monitoring, antibiotic stewardship, nursing staffing, end‐of‐life care, and family‐centered care. The most‐cited article was “Guidelines for the Prevention of Intravascular Catheter‐Related Infections,” published in *Clinical Infectious Diseases* in 2002, with 15,379 citations. Several highly cited publications by Ely et al. addressed ICU delirium assessment and sedation‐related management. The interpretation of these highly cited publications requires caution. Given the broad scope of the search strategy, some influential clinical guidelines and clinical‐topic publications were identified because they may provide important evidence frameworks for ICU nursing practice, management, and quality improvement, although they were not designed to directly investigate ICU nursing personnel or nursing workforce outcomes. Therefore, these publications should be considered as reflecting the broader clinical and organizational context that may inform ICU nursing management, rather than as direct empirical evidence on ICU nursing personnel.

**TABLE 3:** The 20 most‐cited publications identified through the ICU nursing personnel search, 2001–2025.

Rank	Year	Title	Journal	Authors	Total citations
1	2002	Guidelines for the prevention of intravascular catheter–related infections	Clinical Infectious Diseases	O’Grady, NP; Alexander, M; Dellinger, EP; Gerberding, JL; Heard, SO; Maki, DG; Masur, H; McCormick, RD; Mermel, LA; Pearson, ML; Raad, II; Randolph, A; Weinstein, RA	15,379
2	2007	Infectious Diseases Society of America/American Thoracic Society Consensus Guidelines on the management of community‐acquired pneumonia in adults	Clinical Infectious Diseases	Mandell L.A.; Wunderink R.G.; Anzueto A.; Bartlett J.G.; Campbell G.D.; Dean N.C.; Dowell S.F.; File Jr. T.M.; Musher D.M.; Niederman M.S.; Torres A.; Whitney C.G.	5411
3	2001	Delirium in mechanically ventilated patients: Validity and reliability of the Confusion Assessment Method for the intensive care unit (CAM‐ICU)	Journal of the American Medical Association	Ely E.W.; Bernard G.R.; Speroff T.; Gautam S.; Dittus R.; May L.; Truman B.; Gordon S.; Margolin R.; Inouye S.K.; Francis J.; Hart R.P.	2698
4	2016	Implementing an Antibiotic Stewardship Program: Guidelines by the Infectious Diseases Society of America and the Society for Healthcare Epidemiology of America	Clinical Infectious Diseases	Barlam, TF; Cosgrove, SE; Abbo, LM; MacDougall, C; Schuetz, AN; Septimus, EJ; Srinivasan, A; Dellit, TH; Falck‐Ytter, YT; Fishman, NO; Hamilton, CW; Jenkins, TC; Lipsett, PA; Malani, PN; May, LS; Moran, GJ; Neuhauser, MM; Newland, JG; Ohl, CA; Samore, MH; Seo, SK; Trivedi, KK	2616
5	2023	2023 Alzheimer’s disease facts and figures	Alzheimer’s and Dementia		2546
6	2001	Evaluation of delirium in critically ill patients: Validation of the Confusion Assessment Method for the intensive care unit (CAM‐ICU)	Critical Care Medicine	Ely E.W.; Margolin R.; Francis J.; May L.; Truman B.; Dittus R.; Speroff T.; Gautam S.; Bernard G.R.; Inouye S.K.	1996
7	2002	Nurse‐staffing levels and the quality of care in hospitals	New England Journal of Medicine	Needleman, J; Buerhaus, P; Mattke, S; Stewart, M; Zelevinsky, K	1951
8	2007	2007 Guideline for Isolation Precautions: Preventing Transmission of Infectious Agents in Health Care Settings	American Journal of Infection Control	Siegel J.D.; Rhinehart E.; Jackson M.; Chiarello L.	1702
9	2012	Breastfeeding and the Use of Human Milk	Pediatrics	Eidelman, AI; Schanler, RJ; Johnston, M; Landers, S; Noble, L; Szucs, K; Viehmann, L; Feldman‐Winter, L; Lawrence, R; Kim, S; Onyema, N	1682
10	2003	Monitoring Sedation Status over Time in ICU Patients: Reliability and Validity of the Richmond Agitation‐Sedation Scale (RASS)	JAMA	Ely E.W.; Truman B.; Shintani A.; Thomason J.W.W.; Wheeler A.P.; Gordon S.; Francis J.; Speroff T.; Gautam S.; Margolin R.; Sessler C.N.; Dittus R.S.; Bernard G.R.	1451
11	2011	The management of community‐acquired pneumonia in infants and children older than 3 months of age: Clinical practice guidelines by the pediatric infectious diseases society and the infectious diseases society of America	Clinical Infectious Diseases	Bradley J.S.; Byington C.L.; Shah S.S.; Alverson B.; Carter E.R.; Harrison C.; Kaplan S.L.; MacE S.E.; McCracken G.H.; Moore M.R.; St Peter S.D.; Stockwell J.A.; Swanson J.T.	1384
12	2014	The effects of advance care planning on end‐of‐life care: A systematic review	Palliative Medicine	Brinkman‐Stoppelenburg, A; Rietjens, JAC; van der Heide, A	1270
13	2017	Guidelines for Family‐Centered Care in the Neonatal, Pediatric, and Adult ICU	Critical Care Medicine	Davidson J.E.; Aslakson R.A.; Long A.C.; Puntillo K.A.; Kross E.K.; Hart J.; Cox C.E.; Wunsch H.; Wickline M.A.; Nunnally M.E.; Netzer G.; Kentish‐Barnes N.; Sprung C.L.; Hartog C.S.; Coombs M.; Gerritsen R.T.; Hopkins R.O.; Franck L.S.; Skrobik Y.; Kon A.A.; Scruth E.A.; Harvey M.A.; Lewis‐Newby M.; White D.B.; Swoboda S.M.; Cooke C.R.; Levy M.M.; Azoulay E.; Curtis J.R.	1180
14	2020	The experiences of health care providers during the COVID‐19 crisis in China: a qualitative study	The Lancet Global Health	Liu Q.; Luo D.; Haase J.E.; Guo Q.; Wang X.Q.; Liu S.; Xia L.; Liu Z.; Yang J.; Yang B.X.	1178
15	2012	Carbapenemases in Klebsiella pneumoniae and Other Enterobacteriaceae: an Evolving Crisis of Global Dimensions	Clinical Microbiology Reviews	Tzouvelekis, LS; Markogiannakis, A; Psichogiou, M; Tassios, PT; Daikos, GL	1136
16	2022	2022 Guideline for the Management of Patients With Spontaneous Intracerebral Hemorrhage: A Guideline From the American Heart Association/American Stroke Association	Stroke	Greenberg S.M.; Ziai W.C.; Cordonnier C.; Dowlatshahi D.; Francis B.; Goldstein J.N.; Hemphill J.C.; Johnson R.; Keigher K.M.; Mack W.J.; Mocco J.; Newton E.J.; Ruff I.M.; Sansing L.H.; Schulman S.; Selim M.H.; Sheth K.N.; Sprigg N.; Sunnerhagen K.S.	1133
17	2007	Clinical practice guidelines for support of the family in the patient‐centered intensive care unit: American College of Critical Care Medicine Task Force 2004–2005	Critical Care Medicine	Davidson J.E.; Powers K.; Hedayat K.M.; Tieszen M.; Kon A.A.; Shepard E.; Spuhler V.; Todres I.D.; Levy M.; Barr J.; Ghandi R.; Hirsch G.; Armstrong D.	986
18	2013	Change in end‐of‐life care for medicare beneficiaries: Site of death, place of care, and health care transitions in 2000, 2005, and 2009	JAMA	Teno J.M.; Gozalo P.L.; Bynum J.P.W.; Leland N.E.; Miller S.C.; Morden N.E.; Scupp T.; Goodman D.C.; Mor V.	873
19	2010	Systematic review of studies on compliance with hand hygiene guidelines in hospital care	Infection Control and Hospital Epidemiology	Erasmus V.; Daha T.J.; Brug H.; Richardus J.H.; Behrendt M.D.; Vos M.C.; Van Beeck E.F.	836
20	2006	The effect of age on the development and outcome of adult sepsis	Critical Care Medicine	Martin G.S.; Mannino D.M.; Moss M.	822

*Note:* Highly cited publications may include clinical guidelines and clinical‐topic papers that may inform ICU nursing management contexts but do not necessarily directly examine ICU nursing personnel.

#### 3.1.6. Institutional Collaboration Networks

**FIGURE 5:**
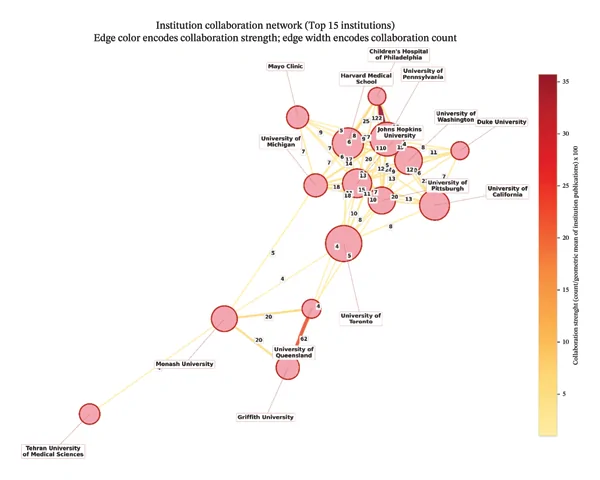
Institutional collaboration networks among the 15 leading institutions with respect to ICU nursing personnel research publications, 2001–2025. Node size represents institutional publication volume; edge color and width denote collaboration intensity and frequency, respectively.

The institutional collaboration network is shown in Figure 5. Overall, the collaboration network in this field exhibited a distinct cluster structure. Institutions in North America collaborate closely with one another. Johns Hopkins University maintains frequent collaborative relationships with Harvard Medical School, the University of Pennsylvania, and other institutions, with the highest frequency of collaboration occurring between Johns Hopkins University and Harvard Medical School (*n* = 122). Australian institutions form a relatively independent collaborative group, with particularly close ties between the University of Queensland and Griffith University (*n* = 62); Monash University also maintains some collaborative links with both of these institutions. The University of Toronto has established collaborative ties across different regions. By contrast, collaboration between the Tehran University of Medical Sciences and other institutions is relatively limited. Overall, our findings showed that institutional collaboration in ICU nursing personnel research is primarily concentrated among a small number of high‐output institutions and exhibits certain regional clustering characteristics.

### 3.2. Trends in Publication Timing and Future Projections

#### 3.2.1. Two‐Stage Growth Characteristics From 2001 to 2025

**FIGURE 6:**
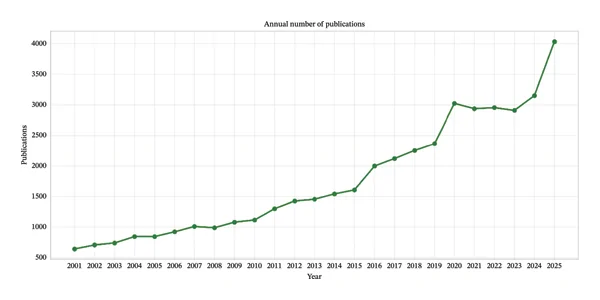
Annual publication trends in ICU nursing personnel research, 2001–2025.

The annual output of ICU nursing personnel research showed a steady upward trend from 2001 to 2025 (Figure 6). The period between 2001 and 2019 marked a phase of steady growth, with the number of publications rising from approximately 650 to 2380. Since 2020, the annual number of publications has increased substantially, exceeding 3000. Although the growth trend fluctuated slightly between 2021 and 2023, it remained at a relatively high level overall. Subsequently, the number of publications continued to rise, reaching a peak in 2025 with more than 4000 publications. Overall, the research output in this field has grown steadily over the past 2 decades and has shown an accelerating upward trend in recent years.

#### 3.2.2. Forecast of Future Publication Trends Based on Holt’s Linear Exponential Smoothing Model (2026–2030)

**FIGURE 7:**
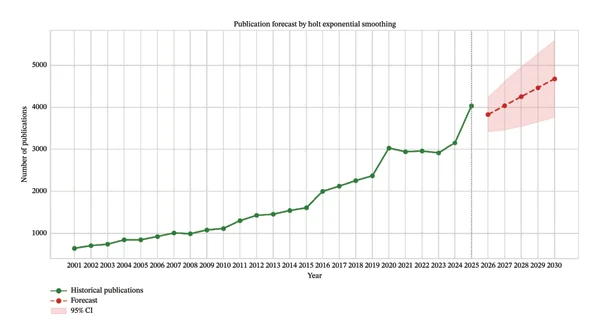
Publications in ICU nursing personnel research: historical data and forecast trends.

Using Holt’s linear exponential smoothing model, we projected future publication trends in the field of ICU nursing (Figure 7). The results indicated that the number of publications in this research field is expected to continue growing over the next 5 years, with an annual publication volume showing a steady upward trend. By 2030, the annual publication volume is projected to approach 5000 articles (Supporting File: publication_forecast_data.pdf). Overall, the projections suggest that research in ICU nursing will remain highly active and continue to grow.

### 3.3. Keyword Co‐Occurrence Networks and Cluster Analysis of Research Topics

#### 3.3.1. Distribution of High‐Frequency Keywords

**FIGURE 8:**
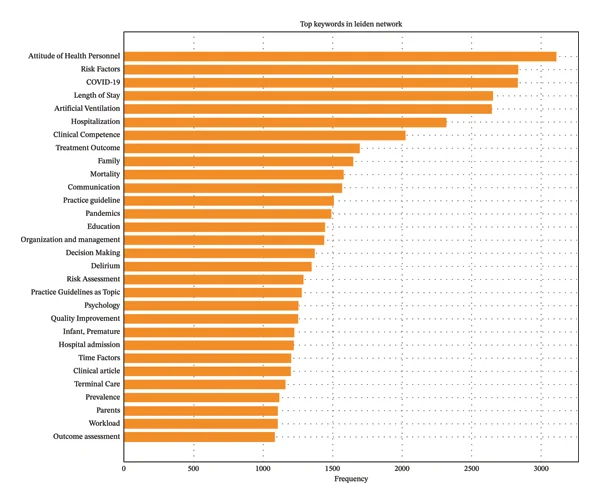
Top keywords in the Leiden network (2001–2025).

To elucidate the knowledge architecture of ICU nursing personnel research, we conducted a Leiden clustering analysis based on a co‐occurrence network of core keywords and visualized the co‐occurrence relationships of high‐frequency keywords (Figure 8). The keywords with the highest frequency of occurrence included “attitude of health personnel,” “risk factors,” and “COVID‐19” (Supporting File: nodes.pdf). Additionally, keywords such as “length of stay,” “artificial ventilation,” “hospitalization,” “clinical competence,” “treatment outcome,” “family,” and “mortality” also appeared with high frequency. Keywords with relatively lower frequency but still strong representativeness included “communication,” “practice guideline,” “pandemics,” “decision making,” “delirium,” “quality improvement,” and “terminal care.”

#### 3.3.2. Keyword Co‐Occurrence Networks and Leiden Clustering Structure

**FIGURE 9:**
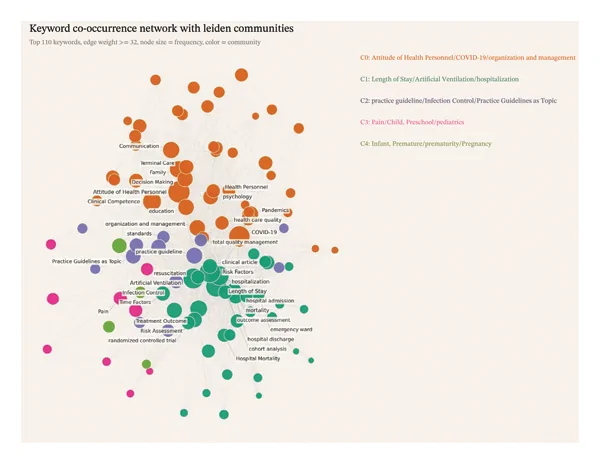
Keyword co‐occurrence network with Leiden clusters, 2001–2025.

We analyzed the keyword co‐occurrence network using the Leiden algorithm, which identified five major clusters (C0–C4) (Figure 9) (Supporting Fil: community_summary.pdf). Cluster C0 primarily included keywords such as attitude of health personnel, COVID‐19, organization and management, and clinical competence, representing research related to the attitudes and management of health care personnel. Cluster C1 included keywords such as length of stay, artificial ventilation, hospitalization, risk factors, and mortality, reflecting research on the clinical management and outcomes of patients in the ICU. Cluster C2 included keywords such as practice guidelines, infection control, standards, and treatment outcomes, representing studies on evidence‐based nursing and infection control. Cluster C3 was primarily composed of keywords such as pain, pediatrics, and preschool children, representing research on pediatric critical care. Cluster C4 included keywords such as premature infants, prematurity, and pregnancy, representing studies on neonatal and perinatal critical care.

#### 3.3.3. Temporal Evolution of Co‐Occurrence Frequencies for Keywords in Each Thematic Cluster

**FIGURE 10:**
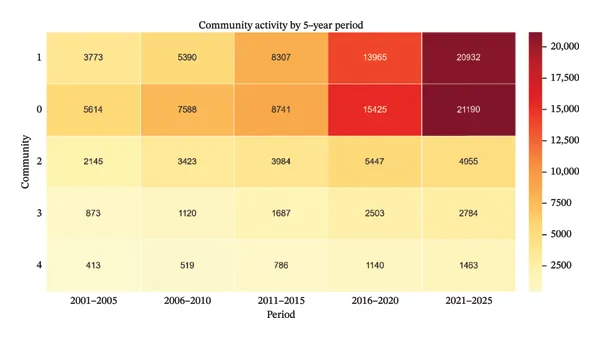
Keyword cluster activity by a 5‐year period during 2001–2025.

Using a 5‐year time window, we analyzed the co‐occurrence frequency of keywords in each cluster (Figure 10). The results showed that the co‐occurrence frequency of keywords in each cluster increased over time. Among them, Clusters C0 and C1 accounted for a high proportion at all time points (Supporting File: community_period_summary.pdf). Cluster C2 showed a significant increase in frequency during the mid‐period. Clusters C3 and C4 were relatively small in scale overall, but they also exhibited an upward trend in the later period. Between 2021 and 2025, the co‐occurrence frequency of keywords in all clusters reached its highest level.

## 4. Discussion

### 4.1. Global Geographic Distribution and Structural Characteristics of Institutional Collaboration Networks

The findings of this study revealed a strong trend toward global concentration in ICU nursing personnel research over the past 2 decades, particularly evident in the fact that institutions in North America and a few institutions in Oceania account for the vast majority of the published literature.

#### 4.1.1. Regional Disparities and Dominance by High‐Output Institutions

High‐income countries such as the United States and Canada dominated the research output in this field. This is closely linked to the substantial investment in research funding, well‐developed research infrastructure, and mature ICU nursing personnel research systems in these countries. By contrast, the proportion of research output from low‐ and middle‐income countries is relatively low [14, 15]. This may reflect the fact that, faced with shortages of basic personnel and equipment, these regions tend to allocate more research resources toward frontline clinical care, lacking dedicated mechanisms for translating research into practice.

#### 4.1.2. Clustered Collaboration Model

Our analysis of international institutional collaboration networks indicated that academic collaboration in this field is primarily concentrated among a small number of high‐output institutions, exhibiting distinct patterns of geographic and institutional clustering (e.g., the close collaboration between Johns Hopkins University and Harvard Medical School) [16, 17]. Compared with previous narrative reviews that have been limited to a single region or a few hospitals, through big‐data analysis spanning 165 countries and regions, our study reveals structural disparities in the global research landscape and highlights the need to strengthen transnational research alliances and multicenter research databases.

### 4.2. Long‐Term Evolution of Research Priorities and the Transition to Sustainability

Our cluster analysis revealed two conceptually distinct but interrelated layers of research focus: clinical‐practice‐oriented themes, including clinical management and patient outcomes (C1) and evidence‐based nursing and infection control (C2), and workforce‐oriented themes, including health care provider attitudes and management (C0). Overall, the thematic evolution of ICU nursing personnel research has undergone a profound shift over the past 25 years, moving from a focus on “technical standards and clinical outcomes” to “staff sustainability and management strategies.” Between 2001 and 2015, research topics were primarily centered on basic life support technical standards, infection control, and clinical outcome indicators (such as mortality rates and length of hospital stay). This pattern reflects the strong emphasis in the early stage of intensive care research on the precision and standardization of high‐tech interventions. Since 2020, driven partly by the COVID‐19 pandemic, burnout and mental health issues among health care workers have become a major focus of academic attention [18]. This shift suggests that the academic community has increasingly recognized the importance of maintaining resilience and sustainability of the ICU workforce alongside patient safety [19]. Compared with earlier meta‐analyses focusing on isolated topics (such as technical specifications of mechanical ventilation or specific burnout interventions), our Leiden‐based clustering analysis over a 25‐year data window provides a broader overview of thematic evolution in this field. The findings indicate an emerging dual focus in modern ICU nursing personnel research, integrating clinical‐practice competencies with humanistic care, workforce sustainability, and nursing management strategies.

### 4.3. Implications for Nursing Management

The bibliometric findings provide evidence‐informed insights for ICU nursing management. However, these implications should be interpreted as being derived from mapped literature trends rather than as direct causal evidence. Overall, the shift from clinically oriented topics toward workforce sustainability suggests that ICU nursing management should integrate workforce planning, staffing, organizational support, burnout prevention, research prioritization, and knowledge translation.

Workforce planning and staffing: Publication growth and workforce‐oriented themes suggest that ICU staffing plans should be aligned with patient complexity, technology intensity, and regional resource constraints [20]. Nursing managers should consider flexible staffing models, skill‐mix planning, succession planning, and competency‐based development strategies across adult, pediatric, and neonatal ICU settings [21].

Organizational support and burnout prevention: The increasing visibility of staff attitudes, mental health, and sustainability themes underscores the need for workload monitoring, psychological support, peer‐support mechanisms, leadership responsiveness, adequate recovery time, and nurse participation in workflow redesign and quality improvement [5, 6, 8].

Research prioritization: Future ICU nursing research should connect clinical‐practice priorities with workforce outcomes, including staffing adequacy, nurse‐sensitive outcomes, burnout and retention, evidence‐based protocol implementation, technology adoption, interprofessional collaboration, and cross‐regional comparisons [14, 15, 17].

Knowledge translation: ICU nursing managers need practical systems to translate research evidence into operational decisions, such as local evidence dashboards, guideline review processes, nurse‐inclusive implementation teams, and indicators linking protocol implementation with staffing effectiveness, nurse well‐being, and patient outcomes [22].

### 4.4. Study Limitations

Although we adopted a multimethodological framework in this study, the following limitations regarding the literature database and language remain. We only included English‐language articles published in PubMed, WoSCC, and Scopus, which may have missed high‐quality regional studies published in other languages. This could affect the comprehensive assessment of research hotspots in non‐English‐speaking countries and regions, to some extent. Regarding an assumption of time‐series stationarity, although we applied Holt’s linear exponential smoothing model to account for nonstationary fluctuations caused by COVID‐19, macrolevel developments in the nursing field over the next 5 years may still be subject to unpredictable influences from public health emergencies or technological advancements. Finally, as a bibliometric study, our findings characterize patterns and trends within the published literature itself; they do not constitute direct empirical evidence of causal relationships with clinical outcomes. The management implications proposed in Section 4.3 should therefore be regarded as evidence‐informed hypotheses warranting further empirical validation, rather than established causal effects.

## 5. Conclusion

Between 2001 and 2025, ICU nursing personnel research demonstrated substantial growth in output and a shift from clinical‐practice‐oriented themes toward workforce sustainability and nursing management strategies. Although this study, which followed a multimethod framework, mapped the knowledge structure and global collaborative networks in this field and offers a valuable complement to previous research, the evidence it provides remains limited to the bibliometric level and should not be interpreted as direct causal evidence of clinical outcomes. Future empirical research is needed to examine whether and how these thematic trends can be translated into effective nursing management strategies, workforce resilience, and health system improvement. These findings provide evidence‐informed directions for nursing managers and policymakers seeking to support the sustainable and equitable allocation of global critical care nursing resources.

## Author Contributions

Conceptualization: Cen Wu and Yadong Song.

Methodology: Cen Wu and Yang Shao.

Investigation: Ximing Chen and Yang Shao.

Visualization: Yang Shao.

Writing–original draft: Ximing Chen and Yadong Song.

Writing–review and editing: Yadong Song, Ximing Chen, Yang Shao, and Cen Wu.

## Funding

No funding was received for this research.

## Conflicts of Interest

The authors declare no conflicts of interest.

## Supporting Information

Additional supporting information can be found online in the Supporting Information section.

## Supporting information


**Supporting Information** The Supporting Information consists of 10 independent PDF files divided into two modules: Leiden keyword co‐occurrence network analysis results and Holt linear exponential smoothing publication forecasting results. All Supporting data were derived from 44,011 valid ICU nursing bibliometric records retrieved from PubMed, Web of Science Core Collection, and Scopus (2001–2025).

## Data Availability

The data that support the findings of this study are available from the corresponding author upon reasonable request.
