# Supplementary material for: Global Research Trends in ICU Nursing Personnel: A Bibliometric Analysis (2001–2025)
Source: J Nurs Manag. 2026 Aug 29;2026:7601256. doi: 10.1155/jonm/7601256 (PMC13525486; doi:10.1155/jonm/7601256)
Supplement: Supplementary file 1 [file JONM-2026-7601256-s001.zip › 1. Supplementary_Material_Guide.docx]

S U P P L E M E N T A R Y	M A T E R I A L	G U I D E

**Supplementary**

**Material**

**Guide**

Contents and Field Definitions of the 10 PDF Files in the supplement material 2026.8.4 Folder

Project: Bibliometric Analysis of Intensive Care Unit (ICU) Literature

Scope: Leiden Network (7 files) and Publication Forecast (3 files)

Date: August 4, 2026	Version: V1.0

Prepared by: Data Analytics Service	2026-08-04

**Contents**

[1. Purpose of This Document	1](#_Toc100000)

[2. Overview of Supplementary Materials	1](#_Toc100001)

[3. Leiden Network Supplementary Materials	1](#_Toc100002)

[3.1 Overall Network Metrics (leiden_network_summary.pdf)	1](#_Toc100003)

[3.2 Keyword Node Attribute Table (nodes.pdf)	1](#_Toc100004)

[3.3 Keyword Co-occurrence Edge Table (edges.pdf)	1](#_Toc100005)

[3.4 Community Summary Table (community_summary.pdf)	1](#_Toc100006)

[3.5 Community-Period Summary Table (community_period_summary.pdf)	1](#_Toc100007)

[3.6 Keyword Merging Mapping Table (keyword_merge_mapping_table.pdf)	1](#_Toc100008)

[3.7 Drawing Parameters of the Publication-Style Network Figure (leiden_network_publication_style_params.pdf)	1](#_Toc100009)

[4. Publication Forecast Supplementary Materials	1](#_Toc100010)

[4.1 Forecast Data Table (publication_forecast_data.pdf)	1](#_Toc100011)

[4.2 Model Parameter Table (publication_forecast_model_parameters.pdf)	1](#_Toc100012)

[4.3 Forecast Notes (publication_forecast_notes.pdf)	1](#_Toc100013)

[5. Usage Notes and Considerations	1](#_Toc100014)

*Note: The Table of Contents is an automatic field. After opening the document, right-click the table of contents and select "Update Field" to refresh the page numbers.*

# **1. Purpose of This Document**

This document provides a file-by-file description of the 10 PDF supplementary materials contained in the supplement material 2026.8.4 folder of the project deliverables. For each file, the document specifies its purpose, the meaning of each field in the corresponding tables, and key data interpretations, so as to facilitate reference during manuscript preparation, reviewer responses, and archiving of the project outputs.

The folder comprises two groups of materials: the Leiden network folder (7 files, pertaining to keyword co-occurrence network and community analysis) and the publication forecast folder (3 files, pertaining to publication output forecasting). All PDF files are printed versions of the corresponding analysis result tables, derived from the analysis master table (PubMed, Scopus, and Web of Science Core Collection, 2001–2025; 44,011 records after deduplication).

Reading guidance: to locate a specific file quickly, use the table of contents; for field definitions, refer to the field description tables in the corresponding chapters; to verify data conventions, refer to Chapter 5 (Usage Notes and Considerations).

# **2. Overview of Supplementary Materials**

The table below summarizes the file names, sizes, and contents of the 10 PDF files, providing an overview of the material structure.

**Table 1. List of Supplementary Material Files**

| **No.** | **File** | **Size** | **Content Summary** |
| --- | --- | --- | --- |
| 1 | Leiden network / leiden_network_summary.pdf | 1 page | Overall scale and quality metrics of the keyword co-occurrence network |
| 2 | Leiden network / nodes.pdf | 6 pages (198 rows) | Keyword node attribute table (frequency, centrality metrics, community assignment) |
| 3 | Leiden network / edges.pdf | 215 pages (11,589 rows) | Co-occurrence relationships between keyword pairs |
| 4 | Leiden network / community_summary.pdf | 2 pages | Profiles and representative keywords of the five thematic communities |
| 5 | Leiden network / community_period_summary.pdf | 1 page | Publication counts of each community across five time periods |
| 6 | Leiden network / keyword_merge_mapping_table.pdf | 3,898 pages (62,365 rows) | Complete record of keyword standardization and merging |
| 7 | Leiden network / leiden_network_publication_style_params.pdf | 1 page | Drawing parameters of the publication-style network figure |
| 8 | publication forecast / publication_forecast_data.pdf | 1 page (30 rows) | Historical publication counts (2001–2025) and forecast values (2026–2030) |
| 9 | publication forecast / publication_forecast_model_parameters.pdf | 1 page | Parameters of the forecasting model (Holt exponential smoothing) |
| 10 | publication forecast / publication_forecast_notes.pdf | 1 page | Methodological notes and limitations of the forecast |

Of the above, keyword_merge_mapping_table.pdf (3,898 pages) and edges.pdf (215 pages) are extensive; they constitute complete, traceable detailed data and may be consulted on demand rather than printed in full.

# **3. Leiden Network Supplementary Materials**

This group of seven files supports the keyword co-occurrence network and Leiden community analysis sections of the manuscript. The network was constructed on the basis of keyword co-occurrence across all included records: keywords with a frequency of at least 20 were retained as nodes, and keyword pairs co-occurring at least 8 times were retained as edges, yielding a network of 200 nodes and 11,591 edges partitioned into five thematic communities.

## **3.1 Overall Network Metrics (leiden_network_summary.pdf)**

This table presents the overall scale and quality metrics of the keyword co-occurrence network and is used to describe the network construction outcomes.

**Field Description**

| **Field** | **Definition** | **Example / Value** |
| --- | --- | --- |
| nodes | Number of network nodes (retained keywords) | 200 |
| edges | Number of network edges (co-occurrence relationships between keyword pairs) | 11,591 |
| communities | Number of communities identified by the Leiden algorithm | 5 |
| modularity | Modularity: a measure of the quality of community partitioning, ranging approximately from 0 to 1; higher values indicate a more distinct community structure | 0.2016 |
| min_node_freq | Minimum node frequency threshold applied during network construction (keywords below this frequency were filtered out) | 20 |
| min_edge_weight | Minimum edge weight threshold applied during network construction (keyword pairs co-occurring below this count were filtered out) | 8 |
| top_nodes_retained | Number of nodes ultimately retained | 200 |

Interpretation: the network comprises 200 keyword nodes and 11,591 co-occurrence edges partitioned into five communities; a modularity of 0.2016 indicates a moderate degree of thematic differentiation among communities.

## **3.2 Keyword Node Attribute Table (nodes.pdf)**

This table reports the statistical metrics of each keyword node (i.e., a vertex of the network), serving as the basis for assessing keyword importance and identifying research hotspots.

**Field Description**

| **Field** | **Definition** | **Example / Value** |
| --- | --- | --- |
| keyword | Keyword (node label) | Length of Stay |
| frequency | Occurrence frequency: number of records containing the keyword | 2,658 |
| degree | Degree centrality: number of other keywords co-occurring with the keyword | 195 |
| weighted_degree | Weighted degree: sum of the weights of all co-occurrence edges incident to the keyword (total co-occurrence strength) | 15,990 |
| pagerank | PageRank centrality: higher scores for keywords connected to important nodes | 0.0193 |
| betweenness | Betweenness centrality: number of shortest paths between keyword pairs that pass through the keyword, reflecting its bridging role | 4,473 |
| closeness | Closeness centrality: reciprocal of the average distance to other nodes; higher values indicate greater centrality | 76.17 |
| eigenvector | Eigenvector centrality: higher scores for keywords connected to high-scoring nodes | 1.0 |
| community | Community ID to which the keyword belongs (0–4, corresponding to the community summary table) | 1 |
| importance_score | Composite importance score (0–1) used to determine whether the keyword label is displayed in the network figure | 0.996 |

Interpretation: the most frequent keyword is Length of Stay (2,658 records), followed by Artificial Ventilation and Attitude of Health Personnel, reflecting the core topics of this research field.

## **3.3 Keyword Co-occurrence Edge Table (edges.pdf)**

This table provides the detailed co-occurrence relationships between keyword pairs (i.e., edges of the network): the co-occurrence of two keywords within the same record constitutes one co-occurrence.

**Field Description**

| **Field** | **Definition** | **Example / Value** |
| --- | --- | --- |
| Source | Source keyword | Pandemics |
| Target | Target keyword | COVID-19 |
| Weight | Co-occurrence count: number of records containing both keywords | 1,413 |
| Type | Edge type: Undirected denotes an undirected co-occurrence relationship | Undirected |

Interpretation: the table is sorted in descending order of co-occurrence count, enabling rapid identification of the most strongly associated keyword pairs, such as Pandemics–COVID-19 (1,413 occurrences) and Length of Stay–hospitalization (619 occurrences).

## **3.4 Community Summary Table (community_summary.pdf)**

This table presents the overall profiles and representative keywords of the five thematic communities, providing the basis for naming the research themes in the manuscript.

**Field Description**

| **Field** | **Definition** | **Example / Value** |
| --- | --- | --- |
| community | Community ID (0–4) | 0 |
| node_count | Number of keywords included in the community | 71 |
| total_frequency | Sum of the frequencies of all keywords in the community (community size) | 58,558 |
| avg_weighted_degree | Average weighted degree of the community (average co-occurrence strength of its keywords) | 3,775 |
| avg_pagerank | Average PageRank score of the community | 0.0051 |
| top_keywords | Representative keywords of the community (separated by double vertical bars) | Attitude of Health Personnel \|\| COVID-19 ... |

The thematic interpretation of each community is provided in Table 2 for reference during manuscript preparation.

**Table 2. Correspondence between Community IDs and Themes**

| **Community** | **Theme** |
| --- | --- |
| 0 | Health care personnel attitudes, COVID-19, organizational management, psychology, education, and communication (clinical humanities and pandemic response) |
| 1 | Length of stay, mechanical ventilation, hospitalization, risk factors, and mortality (critically ill patient outcomes) |
| 2 | Practice guidelines, infection control, quality management, and clinical protocols |
| 3 | Pain, pediatrics, and sedation/analgesia (pediatric analgesia and sedation) |
| 4 | Preterm infants, pregnancy, gestational age, and breastfeeding (perinatal and neonatal care) |

## **3.5 Community-Period Summary Table (community_period_summary.pdf)**

This table reports the number of publications of each community in five time periods (5-year intervals), illustrating the temporal evolution of research themes.

**Field Description**

| **Field** | **Definition** | **Example / Value** |
| --- | --- | --- |
| period | Time period (2001-2005 / 2006-2010 / 2011-2015 / 2016-2020 / 2021-2025) | 2001-2005 |
| community | Community ID (0–4) | 0 |
| count | Number of publications of the community in the given period | 5,614 |

Interpretation: communities 0 and 1 experienced rapid growth in publication output during 2021–2025 (21,190 and 20,932 publications, respectively), indicating that research on health care personnel attitudes and critically ill patient outcomes has intensified in recent years.

## **3.6 Keyword Merging Mapping Table (keyword_merge_mapping_table.pdf)**

This table constitutes the complete record of keyword standardization prior to network analysis: synonyms and variants (e.g., differences in capitalization, plural forms, abbreviations, and aliases) were merged into unified keywords to ensure the traceability of the results. The consistent handling of keyword conventions may be described in the Methods section of the manuscript.

**Field Description**

| **Field** | **Definition** | **Example / Value** |
| --- | --- | --- |
| keyword_raw_cleaned | Original keyword after cleaning | Article |
| keyword_merge_key_raw | Merge key (original form, lowercased) | article |
| keyword_merge_key_alias | Merge key (form after alias normalization) | article |
| keyword_merge_key_final | Merge key (final form used for matching) | article |
| keyword_standard | Standard keyword (canonical term ultimately retained; node label in the network) | Article |
| rows | Occurrence count of the keyword (number of records) | 5,637 |
| merge_rule | Merging rule: kept (retained as is) / case_or_format (differences in capitalization or format only) / singular (plural to singular) / alias (alias merging) | kept |

Interpretation: the table contains 62,365 mapping records in total: 45,329 retained as is, 12,996 unified for capitalization/format, 4,026 converted from plural to singular, and 16 merged by alias. For example, Respiration, Artificial was merged into the standard keyword Artificial Ventilation under the alias rule.

## **3.7 Drawing Parameters of the Publication-Style Network Figure (leiden_network_publication_style_params.pdf)**

This table records the drawing parameters of the network figure (publication style) used in the manuscript, ensuring the reproducibility of the figure.

**Field Description**

| **Field** | **Definition** | **Example / Value** |
| --- | --- | --- |
| top_nodes | Number of nodes included in the figure | 110 |
| label_top_nodes | Number of nodes with displayed labels (only the 36 most important keywords are labeled) | 36 |
| min_edge_weight | Minimum edge weight threshold for drawing | 32 |
| nodes_after_filter | Number of nodes actually drawn after filtering | 110 |
| edges_after_filter | Number of edges actually drawn after filtering | 2,322 |
| layout | Node layout algorithm (spring layout from the networkx library) | networkx spring_layout |
| seed | Random seed (fixed value ensuring a reproducible layout) | 42 |

# **4. Publication Forecast Supplementary Materials**

This group of three files supports the publication output forecasting section of the manuscript. The forecast was generated from the annual publication counts for 2001–2025 using the Holt linear exponential smoothing model (double exponential smoothing with a trend component), projecting publication counts for 2026–2030.

## **4.1 Forecast Data Table (publication_forecast_data.pdf)**

This table provides the complete sequence of observed historical publication counts (2001–2025) and forecast values (2026–2030), serving as the data source for the forecast figures and tables in the manuscript.

**Field Description**

| **Field** | **Definition** | **Example / Value** |
| --- | --- | --- |
| year | Year | 2001 / 2026 |
| publications | Publication count (observed values for historical years; forecast values for forecast years) | 645 / 3,830.8 |
| data_type | Data type: historical (observed) / forecast (projected) | forecast |
| lower_ci | Lower bound of the 95% confidence interval (forecast rows only) | 3,424.0 |
| upper_ci | Upper bound of the 95% confidence interval (forecast rows only) | 4,237.7 |
| horizon | Forecast horizon (1–5, years ahead of 2025; forecast rows only) | 1 |

Interpretation: the forecast projects approximately 3,831 publications in 2026, increasing annually thereafter to approximately 4,679 by 2030; the confidence intervals widen with increasing forecast horizon, reflecting greater uncertainty for more distant projections.

## **4.2 Model Parameter Table (publication_forecast_model_parameters.pdf)**

This table reports all parameters of the forecasting model, supporting the methodological description.

**Field Description**

| **Field** | **Definition** | **Example / Value** |
| --- | --- | --- |
| model | Model name: Holt linear exponential smoothing (double exponential smoothing with a trend component) | Holt linear exponential smoothing |
| alpha | Level smoothing coefficient (range 0–1; higher values indicate greater sensitivity to recent observations) | 0.25 |
| beta | Trend smoothing coefficient | 0.5 |
| level_final | Final level component of the model (current baseline of publication output) | 3,618.7 |
| trend_final | Final trend component of the model (average annual increase of approximately 212 publications) | 212.1 |
| sse | Sum of squared errors (model fitting error) | 1,049,556.3 |
| residual_std | Residual standard deviation (used to construct the forecast confidence intervals) | 207.6 |

## **4.3 Forecast Notes (publication_forecast_notes.pdf)**

This table records the methodological notes and limitations of the forecasting module, for citation in the Methods section of the manuscript.

**Field Description**

| **Field** | **Definition** | **Example / Value** |
| --- | --- | --- |
| historical_year_range | Historical data range used for model fitting | 2001-2025 |
| forecast_year_range | Forecast range | 2026-2030 |
| main_model | Primary model | Holt linear exponential smoothing |
| confidence_interval | Method for constructing confidence intervals | Normal approximation based on residual standard deviation |
| note | Notes: the forecast used the annual publication counts for 2001–2025 only; no ARIMA control model was fitted | See text |

# **5. Usage Notes and Considerations**

The following points should be noted for the correct use of these supplementary materials:

1. Data sources and conventions. All materials were generated from the analysis master table. The data sources comprise PubMed, Scopus, and the Web of Science Core Collection, covering 2001–2025. After article-type filtering (PubMed: journal articles and reviews only) and two rounds of deduplication (by PMID; then by title + authors + journal), a total of 44,008 records were included.

2. Coverage of citation data. Citation-related statistics do not involve citation counts except for the frequency column in nodes.pdf; where citation data are cited in the manuscript, it should be noted that PubMed records do not provide citation counts, and the citation metrics were computed on the basis of Scopus and WoSCC records.

3. Network analysis parameters. Only keywords with a frequency of at least 20 were retained in the network (200 keywords); the community detection results are contingent on these thresholds. The drawing parameters in Section 3.7 apply to the publication-style figure and differ from the network construction parameters (Section 3.1); the two sets of parameters should be distinguished when cited.

4. Large files. keyword_merge_mapping_table.pdf (3,898 pages) and edges.pdf (215 pages) contain complete detailed data and should preferably be consulted on a computer as needed, rather than printed in full.

5. Field layout in PDF tables. Some long field names may wrap across lines in the PDF tables owing to column width constraints (e.g., weighted_degree is displayed over two lines); the authoritative field names are those given in the field description tables of this document.

6. Correspondence with result files. The PDF files are identical in content to the CSV/XLSX files of the same names in the 05_Leiden network and 06_publication forecast directories of the results folder; the PDFs are the printable versions delivered.
