# Supplementary material for: Global Research Trends in ICU Nursing Personnel: A Bibliometric Analysis (2001–2025)
Source: J Nurs Manag. 2026 Aug 29;2026:7601256. doi: 10.1155/jonm/7601256 (PMC13525486; doi:10.1155/jonm/7601256)
Supplement: Supplementary file 1 [file JONM-2026-7601256-s001.zip › 3.1 leiden_network_summary.pdf]

metric	value
nodes	200
edges	11591
communities	5
modularity	0.201596
min_node_freq	20
min_edge_weight	8
top_nodes_retained	200
