# Supplementary material for: Global Research Trends in ICU Nursing Personnel: A Bibliometric Analysis (2001–2025)
Source: J Nurs Manag. 2026 Aug 29;2026:7601256. doi: 10.1155/jonm/7601256 (PMC13525486; doi:10.1155/jonm/7601256)
Supplement: Supplementary file 1 [file JONM-2026-7601256-s001.zip › 3.2 nodes.pdf]

keyword	frequency	degree	weighted_degree	pagerank	betweenness	closeness	eigenvector	community	importance_score
Length of Stay	2658	195	15990	0.0193442	4473	76.1659484	1	1	0.99575
Artificial Ventilation	2648	195	14261	0.0175742	7047	76.7126059	0.814815637	1	0.9905
hospitalization	2321	194	14916	0.0180255	2730	73.9631545	0.936678654	1	0.988
Risk Factors	2839	197	13836	0.016996	2967	71.6290824	0.814400487	1	0.9855
Attitude of Health Personnel	3114	169	12288	0.015158	5086	64.6716192	0.529700962	0	0.9845
COVID-19	2836	181	12555	0.015151	3336	71.7858312	0.774539777	0	0.98075
practice guideline	1512	196	9966	0.0124558	884	63.6171117	0.517889813	2	0.9615
mortality	1582	180	10756	0.0131612	322	68.6798095	0.717352512	1	0.95875
organization and management	1443	183	9034	0.0112135	558	59.4150313	0.478357183	0	0.95125
hospital admission	1225	174	10106	0.0123847	210	66.1528885	0.66431892	1	0.94
psychology	1257	155	8053	0.0099561	496	61.076931	0.429169117	0	0.9335
clinical article	1202	180	7619	0.0097657	588	59.4913769	0.407855567	1	0.92375
Treatment Outcome	1696	192	8925	0.0111906	5	64.8705878	0.5489967	1	0.90375
Clinical Competence	2028	148	6518	0.0084318	186	57.2530434	0.299138508	0	0.89725
Family	1650	147	6863	0.0085595	71	55.3426515	0.352024769	0	0.8905
health care quality	1019	177	6895	0.008729	198	53.838914	0.377655479	0	0.88975
hospital discharge	969	174	7967	0.0099246	123	63.8087891	0.526055255	1	0.88725
education	1448	172	6650	0.0084808	120	51.5020585	0.333521493	0	0.88475
Communication	1572	139	6203	0.0078839	148	54.2974025	0.306800924	0	0.88375
Decision Making	1372	157	6163	0.0078109	121	54.0188295	0.324254303	0	0.87125
resuscitation	1030	167	5862	0.0075427	265	54.040074	0.325340224	1	0.857125
emergency ward	898	170	6776	0.0086	37	55.7171201	0.404657692	1	0.84925
Infection Control	1076	142	5460	0.0072214	469	55.386475	0.299655381	2	0.8485
Risk Assessment	1295	192	6667	0.0085536	1	60.6979303	0.410137551	1	0.842
randomized controlled trial	991	179	5852	0.007644	133	54.2620659	0.332087157	1	0.8405
Health Personnel	1044	145	5270	0.0067629	462	62.8268473	0.30019629	0	0.82575
Terminal Care	1164	134	5383	0.0068282	186	53.6856409	0.299739627	0	0.8245
Practice Guidelines as Topic	1282	148	5076	0.0066897	185	59.8253114	0.278111455	2	0.82025
total quality management	904	174	6013	0.0077489	11	52.5761491	0.326760643	2	0.8195
Time Factors	1206	174	5622	0.0073218	3	60.7176509	0.342578439	1	0.816
Hospital Mortality	1030	143	5465	0.0070075	79	64.5739397	0.408226897	1	0.805125
Pandemics	1496	152	7899	0.0096929	0	68.33176	0.549873005	0	0.8

outcome assessment	1088	189	8365	0.0104371	0	62.611547	0.529813087	1	0.79875
Pain	1037	149	4608	0.0062517	399	51.9737314	0.221903998	3	0.7965
standards	920	161	5500	0.0071241	11	54.7959855	0.298494602	2	0.79475
cohort analysis	1029	178	8478	0.0105056	0	64.9363896	0.58039703	1	0.79375
Delirium	1353	147	4879	0.0064394	47	59.067573	0.314299044	1	0.79125
Child, Preschool	1032	160	4578	0.0061254	282	49.609828	0.253177909	3	0.7895
methodology	1066	154	4843	0.0064414	112	55.9684466	0.263899391	2	0.78925
Sepsis	1014	166	5543	0.0072692	1	53.8076977	0.336435116	1	0.78425
Cross Infection	946	108	4152	0.0057179	640	55.521831	0.232925026	2	0.77575
Infant, Premature	1227	103	4023	0.0054919	466	44.7329635	0.190777796	4	0.77125
Quality Improvement	1255	158	5053	0.00665	2	50.2848668	0.274283124	2	0.76875
Health Knowledge, Attitudes,	1042	117	4229	0.0056581	169	56.6354037	0.205139692	0	0.76725
follow up	899	177	7183	0.0090552	0	56.9339585	0.447992574	1	0.7645
prevalence	1119	176	6166	0.007905	0	60.9521102	0.391173552	1	0.7605
Professional-Family Relations	1052	89	4342	0.0056481	64	54.3660349	0.216285547	0	0.75575
Parents	1109	104	3916	0.0052351	292	46.8228788	0.191602769	0	0.752125
Anxiety	970	149	4835	0.0063105	8	55.456651	0.277532291	0	0.752
prematurity	797	129	4245	0.0057004	131	45.330998	0.222444625	4	0.744
Clinical Protocols	789	160	4158	0.0056566	168	51.4752856	0.23488168	2	0.7375
epidemiology	816	171	6101	0.0077099	0	59.663794	0.406677163	1	0.72425
interpersonal communication	708	139	4847	0.0062593	3	48.625546	0.26078389	0	0.7215
Workload	1109	134	3732	0.0051311	55	47.6120566	0.203234923	0	0.712375
clinical practice	885	179	5534	0.0072013	0	48.9727076	0.295979647	0	0.706
Palliative Care	1007	127	4110	0.0053832	3	48.2626218	0.23342473	0	0.70125
very elderly	722	150	5708	0.0072899	0	57.438011	0.387692507	1	0.70075
interview	738	130	4089	0.0054227	51	47.1582621	0.216156414	0	0.700125
quality of life	1039	160	5159	0.0066618	0	53.1743896	0.313434649	0	0.69175
multicenter study	771	186	5487	0.0071098	0	50.0010972	0.324810701	1	0.689
observational study	772	173	5467	0.0070996	0	53.4683117	0.342347771	1	0.6865
pediatrics	906	161	3813	0.0052	4	41.4344607	0.203714996	3	0.686
palliative therapy	540	134	4325	0.005637	11	47.7482145	0.257028866	0	0.68425
disease severity	661	160	5451	0.0070223	0	56.7588861	0.371776969	1	0.6675
medical education	698	150	4027	0.0053907	1	43.1638185	0.218081623	0	0.659
Job Satisfaction	996	95	2990	0.0042701	86	51.8070941	0.137896075	0	0.6435
attitude to health	753	127	4357	0.0057449	0	51.4321205	0.215463314	0	0.63725

knowledge	876	124	3073	0.0043637	3	41.9463517	0.151699338	0	0.61925
tertiary care center	718	164	4173	0.0055795	0	46.492585	0.248654767	2	0.61375
analgesia	539	123	3146	0.004503	6	46.9893954	0.1621766	3	0.599
Pregnancy	819	123	2826	0.0040035	18	43.6315852	0.16355565	4	0.59225
Quality of Health Care	802	116	2959	0.0041564	3	46.3979177	0.161798919	0	0.5915
Social Support	759	112	3832	0.0051558	0	47.1155419	0.190811232	0	0.59075
Guideline Adherence	657	108	3012	0.0042627	5	50.869057	0.160353217	2	0.59075
Stress, Psychological	849	90	2995	0.0041921	1	46.5748396	0.147390436	0	0.58925
heart rate	490	106	2858	0.0041743	196	41.9777796	0.151732445	1	0.58925
health care delivery	584	145	4107	0.0054311	0	46.4193337	0.244810132	0	0.58925
Reproducibility of Results	863	108	2481	0.0037021	204	39.3218979	0.127821777	3	0.58
caregiver	669	143	3738	0.0050181	0	46.2388166	0.213253635	0	0.570875
perception	902	132	3387	0.004653	0	48.4078324	0.173769709	0	0.57025
university hospital	614	160	3779	0.0050883	0	42.3231407	0.229208028	2	0.57
hypertension	400	108	3297	0.0046061	1	47.9230292	0.214830457	1	0.564125
sedation	482	116	2988	0.0043255	2	56.5971198	0.167411014	3	0.557
medical staff	585	144	3586	0.0048526	0	41.9318212	0.204398115	0	0.551
emergency health service	569	129	3625	0.0048589	0	45.5559075	0.229074692	1	0.5505
emergency care	549	142	3702	0.0049751	0	41.6910249	0.225285125	1	0.55
pneumonia	554	121	3481	0.004805	0	53.4578786	0.240676312	1	0.541
United Kingdom	798	132	3125	0.0043178	0	42.3622204	0.177822677	0	0.53725
depression	612	109	3347	0.0045391	0	49.7526931	0.207290211	0	0.53675
health care cost	529	139	3458	0.0047071	0	53.5818296	0.232648675	1	0.531
Emergency Service, Hospital	690	132	3158	0.0043387	0	45.593116	0.19885659	1	0.53025
Burnout, Professional	675	67	2469	0.0036111	17	50.6781379	0.122732652	0	0.52575
Pilot Projects	669	116	2383	0.0035496	60	39.1890065	0.119714391	3	0.525125
semi structured interview	432	108	2721	0.0038454	47	44.4649178	0.149431342	0	0.5245
health care system	457	128	3459	0.0046806	0	48.0648185	0.220009576	0	0.5225
diabetes mellitus	376	106	2708	0.0039026	22	46.3364414	0.187434332	1	0.50975
pharmacist	430	128	2759	0.0039589	2	35.1647849	0.158584151	0	0.50775
Australia	727	139	2865	0.004038	0	42.2831312	0.164264653	0	0.5
Leadership	738	111	2832	0.0040245	0	41.9313416	0.145685518	0	0.496375
preschool child	448	130	3081	0.004291	0	43.6146586	0.185104786	3	0.48525
hospital readmission	371	115	3275	0.0044428	0	56.1535727	0.25039513	1	0.48525
antibiotic agent	390	130	3088	0.00438	0	42.4733021	0.188959228	1	0.48125

psychological aspect	533	99	2891	0.0040222	0	46.5030766	0.153724003	0	0.471
gestational age	564	105	2806	0.0039921	0	43.0010503	0.166760023	4	0.46525
Pain Measurement	570	74	2130	0.0033041	17	45.094246	0.098102573	3	0.46025
Adaptation, Psychological	695	69	2652	0.0037892	0	46.6445786	0.123787891	0	0.45825
evidence based practice	654	132	2658	0.0038327	0	39.7240617	0.149034383	2	0.45625
burnout	703	84	2576	0.0037192	0	50.8629716	0.135938239	0	0.4515
Postoperative Complications	594	103	2554	0.0037251	0	55.0048579	0.179026953	1	0.442
Heart Arrest	507	119	2714	0.0038688	0	42.2515828	0.166873582	1	0.44175
fever	363	112	2816	0.0040465	0	45.2111758	0.178520833	1	0.4365
Focus Groups	536	83	1940	0.0029758	170	48.6862873	0.094032098	0	0.4355
statistics and numerical data	399	129	2802	0.003942	0	44.0684538	0.176894127	1	0.430625
pilot study	404	120	2180	0.0032749	4	35.7377883	0.120869612	3	0.429125
information processing	426	127	2605	0.0037428	0	36.5251199	0.139812449	0	0.41525
Monitoring, Physiologic	853	98	2164	0.0033134	0	40.4712202	0.1181529	2	0.40875
mental health	497	99	2415	0.0034494	0	50.139945	0.16377571	0	0.397875
health care facility	393	113	2513	0.0035819	0	37.7616264	0.165096674	0	0.3905
Canada	512	124	2312	0.0033767	0	35.4945204	0.133272484	0	0.387
China	617	114	2187	0.0032543	0	42.3653352	0.13531957	0	0.3815
endotracheal intubation	398	104	2350	0.0035364	0	50.1138634	0.1416062	1	0.378
long term care	404	110	2378	0.0034206	0	39.8181334	0.162746034	1	0.376625
therapy	373	123	2399	0.0034855	0	33.9232159	0.153067861	1	0.3755
safety	556	111	2179	0.0033038	0	33.5970647	0.122118228	2	0.3745
dyspnea	319	96	2545	0.0036931	0	45.1465059	0.164849757	1	0.37375
Anti-Bacterial Agents	394	69	1839	0.0029191	84	40.0575624	0.116301643	2	0.37325
Interprofessional Relations	593	77	2156	0.0032642	0	50.0233354	0.097950089	0	0.371875
Workforce	792	87	1983	0.0030826	0	40.8438387	0.103862173	0	0.36925
adverse event	443	114	2186	0.0033186	0	37.5082703	0.136910745	1	0.364375
oxygen saturation	334	90	2297	0.0034519	0	43.2969468	0.141062665	1	0.35425
Program Evaluation	587	87	2008	0.003064	0	43.7106509	0.107805432	0	0.3455
health care planning	337	118	2284	0.0033373	0	34.8171041	0.143643018	0	0.34475
computer assisted tomography	316	97	2265	0.0034062	0	39.0479322	0.141671897	1	0.33925
Needs Assessment	525	90	2027	0.003061	0	42.0025502	0.105720403	0	0.33475
age	350	101	2240	0.0032552	0	42.9551198	0.168967484	1	0.33225
tracheostomy	399	104	2144	0.0032158	0	51.2911443	0.150022531	1	0.328375
cerebrovascular accident	252	89	1932	0.0029824	1	37.5810149	0.13124176	1	0.32325

hospital infection	331	85	2156	0.0032657	0	40.2468357	0.140510629	2	0.31775
Workplace	562	78	1897	0.0029566	0	41.4556147	0.091875432	0	0.31175
Mothers	593	66	1872	0.0029323	0	36.820147	0.08717103	4	0.310875
Personnel Staffing and	579	61	1594	0.0026244	1	38.560851	0.083030949	0	0.3095
home care	305	98	2173	0.0031806	0	39.3946197	0.154373722	1	0.30525
ventilator associated pneumonia	476	84	1905	0.0029646	0	53.4562782	0.128187722	2	0.3015
Postoperative Care	497	94	1883	0.0029683	0	43.1931323	0.117867959	1	0.301375
antiinfective agent	270	76	1833	0.0029173	1	34.3611175	0.111482076	2	0.2985
health care policy	308	110	2109	0.0031354	0	32.5003636	0.13135184	0	0.29525
hospital personnel	345	103	2023	0.0030487	0	32.374345	0.121643008	0	0.29475
respiratory therapist	326	107	2019	0.0030666	0	37.7485286	0.118168056	0	0.292125
hand washing	347	83	1982	0.0030396	0	42.9927816	0.112662392	2	0.28775
Pressure Ulcer	780	68	1721	0.0027044	0	53.3133641	0.126640538	1	0.28725
Medication Errors	547	84	1450	0.0024573	2	29.0416446	0.076950173	2	0.2835
Glasgow coma scale	357	94	1927	0.0029751	0	41.7638181	0.130979706	1	0.28025
heart failure	366	94	1935	0.0029485	0	39.6419033	0.134377638	1	0.278875
Health Services Needs and Demand	449	73	1814	0.0028216	0	44.013608	0.0951734	0	0.26325
Safety Management	400	58	1264	0.0022448	82	37.3883717	0.059834591	2	0.255625
enteric feeding	302	73	1438	0.0024441	9	34.7567335	0.093481651	2	0.24125
Enteral Nutrition	524	57	1216	0.0021968	3	32.309909	0.071549506	2	0.24
Sensitivity and Specificity	418	89	1713	0.0027304	0	34.8617084	0.110716491	1	0.239
Cooperative Behavior	454	68	1654	0.0026625	0	45.5729978	0.078104098	0	0.23675
pain assessment	339	80	1796	0.0028933	0	40.7163571	0.088039253	3	0.23525
hypotension	285	86	1822	0.0029138	0	33.8752776	0.107648212	1	0.2315
Algorithms	443	96	1618	0.002639	0	31.772724	0.098339519	2	0.229375
chronic obstructive lung disease	226	83	1889	0.0028951	0	41.7254186	0.137867587	1	0.22575
ethics	440	75	1651	0.0025892	0	38.2324849	0.096557844	0	0.2245
thematic analysis	296	86	1822	0.0028133	0	34.8270529	0.099500777	0	0.22325
propofol	263	78	1763	0.0029145	0	43.9710689	0.093915767	3	0.222
intensivist	322	100	1785	0.0027767	0	31.7106623	0.11030596	0	0.22075
attitude	576	70	1478	0.0024427	0	41.9941877	0.075199564	0	0.2185
midazolam	253	80	1753	0.0029039	0	41.3723808	0.091147107	3	0.2155
breathing rate	244	76	1812	0.002886	0	36.7751661	0.110045832	1	0.214375
Cardiac Surgical Procedures	377	56	1247	0.0022355	2	36.0162812	0.075388447	1	0.21225
dementia	312	71	1678	0.0026205	0	38.386782	0.124310107	1	0.19475

thorax radiography	227	75	1710	0.0027566	0	38.5226841	0.111070981	1	0.193875
Fatigue	355	89	1595	0.0025606	0	36.0612356	0.099529756	0	0.19375
morphine	244	78	1608	0.0027089	0	33.1682546	0.08292929	3	0.185875
simulation	468	73	1378	0.0023164	0	35.5148136	0.076368236	0	0.18525
teaching hospital	326	97	1590	0.0025611	0	28.9232591	0.101460334	2	0.183125
Intubation, Intratracheal	433	70	1376	0.0023705	0	49.8672254	0.081774967	1	0.18225
Electronic Health Records	415	77	1389	0.0023093	0	36.0566017	0.102834533	1	0.17275
Catheter-Related Infections	333	44	1151	0.0021478	1	38.0440552	0.056237903	2	0.1715
urinary tract infection	272	70	1511	0.0025263	0	35.4493639	0.098637686	2	0.166125
fentanyl	215	66	1490	0.0025824	0	34.6583661	0.07506328	3	0.15975
heart surgery	272	79	1466	0.0024868	0	33.5257678	0.090930089	1	0.158125
acute kidney failure	227	69	1519	0.0024913	0	34.1737819	0.110468519	1	0.157625
OUTCOMES	455	73	1266	0.0021654	0	38.6595159	0.098857401	1	0.15575
Hypnotics and Sedatives	361	52	1322	0.0023135	0	52.2617823	0.081946324	3	0.15275
Logistic Models	356	68	1341	0.0022572	0	45.2154347	0.106236693	1	0.15075
extracorporeal oxygenation	262	78	1419	0.00236	0	38.0941881	0.100260177	1	0.14225
meta analysis	330	73	1330	0.0022444	0	34.0674795	0.100010317	1	0.13675
blood pressure	294	69	1318	0.0023229	0	32.4191782	0.073051998	1	0.13525
Catheterization, Central Venous	382	49	1175	0.0021642	0	36.1894816	0.060595823	2	0.12775
Insulin	366	63	1147	0.0020884	0	31.0694982	0.073717618	2	0.113875
Telemedicine	348	74	1157	0.0020441	0	38.3668528	0.082213325	0	0.1095
physiotherapy	241	80	1277	0.0021903	0	36.9693997	0.093628138	1	0.109
vancomycin	197	58	1194	0.0021792	0	28.4872117	0.073939953	1	0.0945
reliability	342	65	923	0.0018128	0	28.0201558	0.050782307	3	0.09125
Breast Feeding	321	45	1001	0.0019104	0	32.1952783	0.052705107	4	0.09025
microbiology	211	56	1180	0.0021388	0	33.5970166	0.073234003	2	0.08675
Stroke	288	57	967	0.0018432	0	30.3236862	0.072196615	1	0.08025
methicillin resistant Staphylococcus aureus	228	45	1080	0.0020284	0	35.2559998	0.068958718	2	0.07775
