# Supplementary material for: Global Research Trends in ICU Nursing Personnel: A Bibliometric Analysis (2001–2025)
Source: J Nurs Manag. 2026 Aug 29;2026:7601256. doi: 10.1155/jonm/7601256 (PMC13525486; doi:10.1155/jonm/7601256)
Supplement: Supplementary file 1 [file JONM-2026-7601256-s001.zip › 3.3 edges.pdf]

Source	Target	Weight	Type
Pandemics	COVID-19	1413	Undirected
Length of Stay	hospitalization	619	Undirected
Artificial Ventilation	Length of Stay	543	Undirected
Length of Stay	mortality	460	Undirected
Attitude of Health Personnel	Health Knowledge, Attitudes, Practice	440	Undirected
Clinical Competence	Attitude of Health Personnel	438	Undirected
Professional-Family Relations	Family	436	Undirected
Length of Stay	Risk Factors	427	Undirected
Length of Stay	Hospital Mortality	411	Undirected
Infant, Premature	prematurity	410	Undirected
total quality management	Quality Improvement	410	Undirected
Cross Infection	Infection Control	401	Undirected
Practice Guidelines as Topic	practice guideline	396	Undirected
Risk Factors	hospitalization	393	Undirected
Length of Stay	Treatment Outcome	390	Undirected
Risk Factors	prevalence	390	Undirected
Risk Factors	Risk Assessment	385	Undirected
Communication	interpersonal communication	382	Undirected
hospitalization	hospital admission	381	Undirected
hospitalization	mortality	372	Undirected
Length of Stay	hospital admission	370	Undirected
hospitalization	COVID-19	370	Undirected
Anxiety	depression	363	Undirected
Child, Preschool	preschool child	359	Undirected
Length of Stay	cohort analysis	356	Undirected
Length of Stay	hospital discharge	335	Undirected
Palliative Care	Terminal Care	330	Undirected
Length of Stay	outcome assessment	325	Undirected
Pain Measurement	Pain	324	Undirected
Clinical Competence	Health Knowledge, Attitudes, Practice	323	Undirected
Palliative Care	palliative therapy	321	Undirected
hospitalization	cohort analysis	320	Undirected
Family	Attitude of Health Personnel	312	Undirected
Communication	Attitude of Health Personnel	307	Undirected
hospital admission	hospital discharge	304	Undirected
epidemiology	COVID-19	302	Undirected
Artificial Ventilation	hospitalization	298	Undirected
Professional-Family Relations	Attitude of Health Personnel	295	Undirected
Professional-Family Relations	Communication	289	Undirected
Risk Factors	mortality	289	Undirected
Communication	Family	288	Undirected
mortality	hospital admission	287	Undirected
Burnout, Professional	burnout	286	Undirected
hospitalization	hospital discharge	286	Undirected
Attitude of Health Personnel	Terminal Care	285	Undirected
hospital admission	cohort analysis	285	Undirected
Hospital Mortality	mortality	282	Undirected
Artificial Ventilation	Risk Factors	281	Undirected
attitude to health	Health Knowledge, Attitudes, Practice	276	Undirected
Artificial Ventilation	mortality	274	Undirected
Risk Factors	Delirium	274	Undirected
Health Personnel	COVID-19	268	Undirected
Decision Making	Terminal Care	263	Undirected

Clinical Competence	education	262	Undirected
mortality	cohort analysis	260	Undirected
Attitude of Health Personnel	Decision Making	255	Undirected
practice guideline	standards	251	Undirected
Attitude of Health Personnel	Job Satisfaction	248	Undirected
Attitude of Health Personnel	psychology	248	Undirected
Length of Stay	Time Factors	248	Undirected
standards	organization and management	246	Undirected
attitude	knowledge	245	Undirected
Risk Factors	COVID-19	243	Undirected
attitude to health	Attitude of Health Personnel	242	Undirected
hospitalization	outcome assessment	240	Undirected
outcome assessment	mortality	240	Undirected
Parents	Professional-Family Relations	234	Undirected
Artificial Ventilation	Treatment Outcome	230	Undirected
Risk Factors	cohort analysis	230	Undirected
Treatment Outcome	Risk Factors	229	Undirected
prematurity	gestational age	229	Undirected
Pain	analgesia	227	Undirected
Artificial Ventilation	COVID-19	226	Undirected
Family	Terminal Care	225	Undirected
Parents	Infant, Premature	224	Undirected
Treatment Outcome	mortality	223	Undirected
Guideline Adherence	Practice Guidelines as Topic	220	Undirected
Interprofessional Relations	Attitude of Health Personnel	218	Undirected
psychology	organization and management	214	Undirected
hospitalization	follow up	213	Undirected
Job Satisfaction	Burnout, Professional	212	Undirected
Length of Stay	hospital readmission	212	Undirected
Pilot Projects	pilot study	212	Undirected
Anxiety	COVID-19	211	Undirected
Length of Stay	very elderly	211	Undirected
Artificial Ventilation	practice guideline	209	Undirected
Quality of Health Care	health care quality	209	Undirected
Artificial Ventilation	hospital admission	208	Undirected
Treatment Outcome	Time Factors	208	Undirected
Treatment Outcome	hospitalization	208	Undirected
Risk Factors	Pressure Ulcer	207	Undirected
health care quality	organization and management	207	Undirected
Artificial Ventilation	sedation	206	Undirected
Terminal Care	palliative therapy	204	Undirected
Attitude of Health Personnel	Health Personnel	202	Undirected
hospitalization	Hospital Mortality	201	Undirected
hospitalization	very elderly	200	Undirected
Cross Infection	Risk Factors	199	Undirected
Family	psychology	199	Undirected
education	organization and management	198	Undirected
epidemiology	Pandemics	198	Undirected
follow up	hospital discharge	198	Undirected
Infant, Premature	gestational age	197	Undirected
Artificial Ventilation	clinical article	195	Undirected
COVID-19	hospital admission	195	Undirected
hospital discharge	cohort analysis	195	Undirected
Attitude of Health Personnel	Focus Groups	194	Undirected

Health Personnel	psychology	194	Undirected
Length of Stay	Postoperative Complications	194	Undirected
Risk Factors	epidemiology	193	Undirected
mortality	hospital discharge	193	Undirected
Heart Arrest	resuscitation	191	Undirected
hospitalization	emergency ward	191	Undirected
Attitude of Health Personnel	perception	190	Undirected
Length of Stay	Delirium	190	Undirected
Infection Control	hand washing	189	Undirected
Risk Factors	hospital admission	189	Undirected
Health Personnel	Pandemics	187	Undirected
hospitalization	disease severity	187	Undirected
methodology	standards	187	Undirected
Artificial Ventilation	cohort analysis	186	Undirected
hospital admission	disease severity	186	Undirected
mortality	COVID-19	186	Undirected
prevalence	hospitalization	186	Undirected
Family	Decision Making	185	Undirected
Pain	pain assessment	185	Undirected
Risk Factors	Hospital Mortality	185	Undirected
COVID-19	cohort analysis	183	Undirected
Infant, Premature	Mothers	183	Undirected
Communication	Decision Making	182	Undirected
Length of Stay	follow up	182	Undirected
Risk Assessment	Pressure Ulcer	181	Undirected
Artificial Ventilation	outcome assessment	180	Undirected
Length of Stay	COVID-19	180	Undirected
Length of Stay	disease severity	180	Undirected
Professional-Family Relations	Social Support	180	Undirected
Length of Stay	Risk Assessment	179	Undirected
Sepsis	mortality	179	Undirected
very elderly	cohort analysis	179	Undirected
emergency ward	hospital admission	178	Undirected
Attitude of Health Personnel	organization and management	176	Undirected
Length of Stay	health care cost	176	Undirected
Artificial Ventilation	ventilator associated pneumonia	175	Undirected
Risk Factors	Time Factors	175	Undirected
Enteral Nutrition	enteric feeding	174	Undirected
Workforce	Personnel Staffing and Scheduling	174	Undirected
Workload	Personnel Staffing and Scheduling	174	Undirected
practice guideline	clinical practice	174	Undirected
Adaptation, Psychological	Stress, Psychological	173	Undirected
Anti-Bacterial Agents	antiinfective agent	173	Undirected
outcome assessment	hospital admission	173	Undirected
Social Support	Family	172	Undirected
mortality	very elderly	172	Undirected
outcome assessment	cohort analysis	172	Undirected
hospitalization	clinical article	171	Undirected
methodology	organization and management	170	Undirected
mortality	epidemiology	170	Undirected
Treatment Outcome	Risk Assessment	169	Undirected
hospitalization	observational study	169	Undirected
observational study	cohort analysis	169	Undirected
Social Support	Adaptation, Psychological	167	Undirected

outcome assessment	hospital discharge	167	Undirected
Catheterization, Central Venous	Catheter-Related Infections	166	Undirected
prevalence	COVID-19	166	Undirected
psychology	COVID-19	166	Undirected
Artificial Ventilation	Delirium	165	Undirected
health care quality	standards	165	Undirected
mental health	COVID-19	165	Undirected
Length of Stay	observational study	164	Undirected
hospitalization	Pandemics	164	Undirected
Artificial Ventilation	Hypnotics and Sedatives	163	Undirected
follow up	clinical article	163	Undirected
Artificial Ventilation	Time Factors	162	Undirected
Professional-Family Relations	attitude to health	162	Undirected
Risk Factors	Infection Control	162	Undirected
education	medical education	162	Undirected
follow up	hospital admission	162	Undirected
interview	psychology	162	Undirected
Guideline Adherence	Infection Control	161	Undirected
Social Support	Attitude of Health Personnel	161	Undirected
mortality	disease severity	161	Undirected
Communication	Terminal Care	160	Undirected
follow up	cohort analysis	160	Undirected
Artificial Ventilation	disease severity	159	Undirected
Infection Control	COVID-19	159	Undirected
Professional-Family Relations	Terminal Care	159	Undirected
hospital discharge	hospital readmission	159	Undirected
Adaptation, Psychological	Attitude of Health Personnel	158	Undirected
Child, Preschool	pediatrics	158	Undirected
Intubation, Intratracheal	endotracheal intubation	158	Undirected
Length of Stay	emergency ward	156	Undirected
hospitalization	hospital readmission	156	Undirected
Artificial Ventilation	pneumonia	155	Undirected
Communication	Interprofessional Relations	155	Undirected
alth Knowledge, Attitudes, Practi	knowledge	155	Undirected
hospitalization	Risk Assessment	155	Undirected
Artificial Ventilation	randomized controlled trial	154	Undirected
Artificial Ventilation	tracheostomy	154	Undirected
Guideline Adherence	practice guideline	154	Undirected
hospital admission	very elderly	154	Undirected
hospitalization	epidemiology	154	Undirected
Cooperative Behavior	Attitude of Health Personnel	153	Undirected
practice guideline	health care quality	153	Undirected
Artificial Ventilation	Hospital Mortality	152	Undirected
Artificial Ventilation	methodology	152	Undirected
Professional-Family Relations	Decision Making	152	Undirected
Attitude of Health Personnel	Practice Guidelines as Topic	151	Undirected
Length of Stay	pneumonia	151	Undirected
outcome assessment	follow up	151	Undirected
outcome assessment	randomized controlled trial	151	Undirected
Attitude of Health Personnel	Stress, Psychological	150	Undirected
Length of Stay	Sepsis	150	Undirected
Artificial Ventilation	Clinical Protocols	149	Undirected
Artificial Ventilation	hospital discharge	149	Undirected
Cooperative Behavior	Interprofessional Relations	149	Undirected

Pain Measurement	pain assessment	149	Undirected
total quality management	organization and management	149	Undirected
attitude to health	Adaptation, Psychological	148	Undirected
attitude to health	Family	148	Undirected
Risk Factors	outcome assessment	147	Undirected
epidemiology	cohort analysis	147	Undirected
organization and management	psychological aspect	147	Undirected
quality of life	hospitalization	147	Undirected
burnout	COVID-19	146	Undirected
emergency ward	COVID-19	146	Undirected
hospital admission	clinical article	146	Undirected
practice guideline	organization and management	146	Undirected
Cardiac Surgical Procedures	heart surgery	145	Undirected
Risk Factors	Sepsis	145	Undirected
Workplace	Job Satisfaction	145	Undirected
Artificial Ventilation	Pain	144	Undirected
Cross Infection	Guideline Adherence	144	Undirected
Length of Stay	randomized controlled trial	144	Undirected
Risk Factors	very elderly	144	Undirected
Treatment Outcome	Hospital Mortality	144	Undirected
Artificial Ventilation	endotracheal intubation	143	Undirected
Emergency Service, Hospital	emergency ward	143	Undirected
Family	interpersonal communication	143	Undirected
Job Satisfaction	burnout	143	Undirected
emergency ward	emergency health service	143	Undirected
health care quality	health care delivery	143	Undirected
hospitalization	health care cost	143	Undirected
Attitude of Health Personnel	psychological aspect	142	Undirected
Family	caregiver	142	Undirected
Risk Factors	practice guideline	142	Undirected
practice guideline	methodology	142	Undirected
Artificial Ventilation	Intubation, Intratracheal	141	Undirected
COVID-19	health care system	141	Undirected
Emergency Service, Hospital	emergency health service	141	Undirected
Family	Adaptation, Psychological	141	Undirected
depression	COVID-19	141	Undirected
heart rate	blood pressure	141	Undirected
Focus Groups	information processing	139	Undirected
randomized controlled trial	multicenter study	139	Undirected
Risk Factors	Practice Guidelines as Topic	138	Undirected
Treatment Outcome	outcome assessment	138	Undirected
diabetes mellitus	hypertension	138	Undirected
Artificial Ventilation	Sepsis	137	Undirected
Health Services Needs and Demand	Attitude of Health Personnel	137	Undirected
Length of Stay	epidemiology	137	Undirected
Risk Factors	Postoperative Complications	137	Undirected
Risk Factors	disease severity	137	Undirected
quality of life	Length of Stay	137	Undirected
semi structured interview	clinical article	137	Undirected
Treatment Outcome	hospital admission	136	Undirected
interpersonal communication	organization and management	136	Undirected
COVID-19	disease severity	135	Undirected
Clinical Competence	knowledge	135	Undirected
hospitalization	pneumonia	135	Undirected

mortality	follow up	135	Undirected
Artificial Ventilation	resuscitation	134	Undirected
Decision Making	Palliative Care	134	Undirected
Professional-Family Relations	Adaptation, Psychological	134	Undirected
disease severity	cohort analysis	134	Undirected
Anxiety	Pandemics	133	Undirected
Artificial Ventilation	Practice Guidelines as Topic	133	Undirected
Attitude of Health Personnel	Program Evaluation	133	Undirected
Clinical Competence	Practice Guidelines as Topic	132	Undirected
Practice Guidelines as Topic	standards	132	Undirected
Risk Factors	follow up	132	Undirected
Attitude of Health Personnel	Palliative Care	131	Undirected
Parents	Attitude of Health Personnel	131	Undirected
hospital discharge	clinical article	131	Undirected
mortality	observational study	131	Undirected
COVID-19	clinical article	130	Undirected
Length of Stay	prevalence	130	Undirected
Artificial Ventilation	Attitude of Health Personnel	129	Undirected
Burnout, Professional	COVID-19	129	Undirected
Decision Making	ethics	129	Undirected
Risk Factors	hospital discharge	129	Undirected
Risk Factors	methodology	129	Undirected
heart rate	breathing rate	129	Undirected
observational study	hospital admission	129	Undirected
Attitude of Health Personnel	interview	128	Undirected
Hospital Mortality	hospital admission	128	Undirected
Pain Measurement	analgesia	128	Undirected
Parents	psychology	128	Undirected
hospitalization	Delirium	128	Undirected
Anxiety	psychology	127	Undirected
Artificial Ventilation	follow up	127	Undirected
Length of Stay	total quality management	127	Undirected
analgesia	pain assessment	127	Undirected
hospitalization	Sepsis	127	Undirected
total quality management	health care quality	127	Undirected
Infection Control	hospital infection	126	Undirected
Monitoring, Physiologic	methodology	126	Undirected
methodology	psychological aspect	126	Undirected
Risk Assessment	mortality	125	Undirected
Treatment Outcome	methodology	125	Undirected
attitude to health	Social Support	125	Undirected
hospitalization	randomized controlled trial	125	Undirected
Treatment Outcome	cohort analysis	124	Undirected
Treatment Outcome	hospital discharge	124	Undirected
education	practice guideline	124	Undirected
psychology	Pandemics	124	Undirected
psychology	interpersonal communication	124	Undirected
Hospital Mortality	cohort analysis	123	Undirected
Infection Control	practice guideline	123	Undirected
Quality of Health Care	Attitude of Health Personnel	123	Undirected
Safety Management	safety	123	Undirected
Stress, Psychological	psychology	123	Undirected
heart rate	oxygen saturation	123	Undirected
hospitalization	hypertension	123	Undirected

midazolam	fentanyl	123	Undirected
propofol	midazolam	123	Undirected
randomized controlled trial	follow up	123	Undirected
Risk Factors	Logistic Models	122	Undirected
education	standards	122	Undirected
hospitalization	practice guideline	122	Undirected
Attitude of Health Personnel	education	121	Undirected
Risk Factors	Child, Preschool	121	Undirected
Time Factors	Hospital Mortality	121	Undirected
analgesia	sedation	121	Undirected
hospitalization	diabetes mellitus	121	Undirected
prevalence	epidemiology	121	Undirected
Artificial Ventilation	observational study	120	Undirected
Parents	Social Support	120	Undirected
hospital discharge	very elderly	120	Undirected
Artificial Ventilation	Pandemics	119	Undirected
Attitude of Health Personnel	Needs Assessment	119	Undirected
health care delivery	COVID-19	119	Undirected
Attitude of Health Personnel	attitude	118	Undirected
Clinical Competence	Program Evaluation	118	Undirected
Family	Palliative Care	118	Undirected
Length of Stay	Child, Preschool	118	Undirected
epidemiology	hospital admission	118	Undirected
thematic analysis	semi structured interview	118	Undirected
Cross Infection	hand washing	117	Undirected
Pregnancy	gestational age	117	Undirected
Risk Factors	Pandemics	117	Undirected
Treatment Outcome	randomized controlled trial	117	Undirected
psychology	epidemiology	117	Undirected
Guideline Adherence	Attitude of Health Personnel	116	Undirected
Infection Control	Health Personnel	116	Undirected
fever	COVID-19	116	Undirected
follow up	COVID-19	116	Undirected
resuscitation	emergency ward	116	Undirected
Cross Infection	Catheter-Related Infections	115	Undirected
Length of Stay	Quality Improvement	115	Undirected
Length of Stay	clinical article	115	Undirected
Quality Improvement	organization and management	115	Undirected
Workforce	Workload	115	Undirected
resuscitation	practice guideline	115	Undirected
Attitude of Health Personnel	Australia	114	Undirected
Attitude of Health Personnel	Burnout, Professional	114	Undirected
Attitude of Health Personnel	United Kingdom	114	Undirected
Delirium	sedation	114	Undirected
Infant, Premature	Breast Feeding	114	Undirected
Length of Stay	Clinical Protocols	114	Undirected
Risk Factors	multicenter study	114	Undirected
education	psychology	114	Undirected
Clinical Competence	resuscitation	113	Undirected
Cross Infection	hospital infection	113	Undirected
Social Support	Communication	113	Undirected
emergency ward	cohort analysis	113	Undirected
Burnout, Professional	psychology	112	Undirected
Length of Stay	multicenter study	112	Undirected

Risk Assessment	Hospital Mortality	112	Undirected
clinical article	computer assisted tomography	112	Undirected
depression	psychology	112	Undirected
practice guideline	COVID-19	112	Undirected
prematurity	randomized controlled trial	112	Undirected
Artificial Ventilation	analgesia	111	Undirected
Attitude of Health Personnel	Workplace	111	Undirected
Clinical Protocols	practice guideline	111	Undirected
Length of Stay	practice guideline	111	Undirected
Professional-Family Relations	interpersonal communication	111	Undirected
hospitalization	multicenter study	111	Undirected
knowledge	education	111	Undirected
COVID-19	organization and management	110	Undirected
Length of Stay	health care quality	110	Undirected
burnout	psychology	110	Undirected
dyspnea	COVID-19	110	Undirected
emergency ward	hospital discharge	110	Undirected
hospital admission	hospital readmission	110	Undirected
hospital readmission	cohort analysis	110	Undirected
mental health	Pandemics	110	Undirected
oxygen saturation	breathing rate	110	Undirected
Artificial Ventilation	Child, Preschool	109	Undirected
Artificial Ventilation	Risk Assessment	109	Undirected
Cross Infection	Health Personnel	109	Undirected
Risk Assessment	cohort analysis	109	Undirected
pediatrics	preschool child	109	Undirected
practice guideline	hospital admission	109	Undirected
quality of life	Artificial Ventilation	109	Undirected
quality of life	psychology	109	Undirected
Attitude of Health Personnel	resuscitation	108	Undirected
Clinical Competence	Communication	108	Undirected
Length of Stay	organization and management	108	Undirected
Pandemics	health care system	108	Undirected
hypertension	COVID-19	108	Undirected
mortality	hospital readmission	108	Undirected
propofol	fentanyl	108	Undirected
quality of life	follow up	108	Undirected
Anxiety	mental health	107	Undirected
Attitude of Health Personnel	interpersonal communication	107	Undirected
COVID-19	hospital discharge	107	Undirected
Health Personnel	epidemiology	107	Undirected
Sepsis	hospital admission	107	Undirected
hospitalization	organization and management	107	Undirected
prematurity	Mothers	107	Undirected
Attitude of Health Personnel	Workload	106	Undirected
Attitude of Health Personnel	practice guideline	106	Undirected
Communication	organization and management	106	Undirected
Family	Stress, Psychological	106	Undirected
Family	psychological aspect	106	Undirected
Hospital Mortality	Sepsis	106	Undirected
Infection Control	Pandemics	106	Undirected
Length of Stay	tertiary care center	106	Undirected
Parents	prematurity	106	Undirected
emergency health service	organization and management	106	Undirected

emergency ward	clinical article	106	Undirected
hospitalization	health care quality	106	Undirected
mortality	health care quality	106	Undirected
mortality	randomized controlled trial	106	Undirected
Treatment Outcome	Postoperative Complications	105	Undirected
evidence based practice	practice guideline	105	Undirected
health care quality	medical education	105	Undirected
prevalence	mortality	105	Undirected
total quality management	practice guideline	105	Undirected
total quality management	standards	105	Undirected
Attitude of Health Personnel	Leadership	104	Undirected
Decision Making	interpersonal communication	104	Undirected
Decision Making	psychology	104	Undirected
Hospital Mortality	outcome assessment	104	Undirected
Risk Factors	Pregnancy	104	Undirected
Risk Factors	age	104	Undirected
hospitalization	Anxiety	104	Undirected
mortality	practice guideline	104	Undirected
Artificial Ventilation	multicenter study	103	Undirected
Communication	Cooperative Behavior	103	Undirected
Delirium	Postoperative Complications	103	Undirected
Parents	Adaptation, Psychological	103	Undirected
Parents	Communication	103	Undirected
Risk Factors	observational study	103	Undirected
Time Factors	Risk Assessment	103	Undirected
multicenter study	cohort analysis	103	Undirected
quality of life	Palliative Care	103	Undirected
Breast Feeding	Mothers	102	Undirected
Communication	psychology	102	Undirected
Decision Making	interview	102	Undirected
alth Knowledge, Attitudes, Practi	Practice Guidelines as Topic	102	Undirected
Length of Stay	Emergency Service, Hospital	102	Undirected
Length of Stay	diabetes mellitus	102	Undirected
Pandemics	hospital admission	102	Undirected
Risk Factors	pneumonia	102	Undirected
Treatment Outcome	organization and management	102	Undirected
caregiver	psychology	102	Undirected
outcome assessment	practice guideline	102	Undirected
Clinical Competence	Needs Assessment	101	Undirected
Professional-Family Relations	psychology	101	Undirected
Risk Factors	emergency ward	101	Undirected
Workload	COVID-19	101	Undirected
epidemiology	very elderly	101	Undirected
mortality	multicenter study	101	Undirected
quality of life	depression	101	Undirected
Artificial Ventilation	propofol	100	Undirected
Artificial Ventilation	standards	100	Undirected
Attitude of Health Personnel	knowledge	100	Undirected
Clinical Competence	medical education	100	Undirected
Communication	Palliative Care	100	Undirected
Infection Control	Catheter-Related Infections	100	Undirected
Parents	Stress, Psychological	100	Undirected
dyspnea	fever	100	Undirected
education	simulation	100	Undirected

home care	hospital discharge	100	Undirected
interview	clinical article	100	Undirected
interview	semi structured interview	100	Undirected
mortality	organization and management	100	Undirected
Family	organization and management	99	Undirected
Terminal Care	psychology	99	Undirected
United Kingdom	organization and management	99	Undirected
health care cost	mortality	99	Undirected
health care quality	interpersonal communication	99	Undirected
hospital discharge	disease severity	99	Undirected
hospitalization	health care system	99	Undirected
mortality	emergency ward	99	Undirected
mortality	pneumonia	99	Undirected
oxygen saturation	clinical article	99	Undirected
Artificial Ventilation	Decision Making	98	Undirected
Artificial Ventilation	organization and management	98	Undirected
Family	interview	98	Undirected
Guideline Adherence	hand washing	98	Undirected
Infant, Premature	Pregnancy	98	Undirected
Length of Stay	Postoperative Care	98	Undirected
Pandemics	emergency ward	98	Undirected
Parents	Child, Preschool	98	Undirected
Risk Factors	hypertension	98	Undirected
Stress, Psychological	Job Satisfaction	98	Undirected
emergency care	emergency ward	98	Undirected
health care quality	clinical practice	98	Undirected
hospitalization	dyspnea	98	Undirected
mortality	Pandemics	98	Undirected
propofol	sedation	98	Undirected
Artificial Ventilation	clinical practice	97	Undirected
Clinical Competence	organization and management	97	Undirected
Cross Infection	Catheterization, Central Venous	97	Undirected
Emergency Service, Hospital	hospitalization	97	Undirected
Family	Anxiety	97	Undirected
Hypnotics and Sedatives	sedation	97	Undirected
Length of Stay	statistics and numerical data	97	Undirected
Pandemics	health care delivery	97	Undirected
Parents	attitude to health	97	Undirected
Reproducibility of Results	reliability	97	Undirected
Risk Factors	Health Personnel	97	Undirected
Treatment Outcome	follow up	97	Undirected
education	methodology	97	Undirected
health care delivery	health care system	97	Undirected
practice guideline	medical education	97	Undirected
prevalence	cohort analysis	97	Undirected
quality of life	Anxiety	97	Undirected
Artificial Ventilation	Pain Measurement	96	Undirected
Clinical Competence	United Kingdom	96	Undirected
Pain	sedation	96	Undirected
Postoperative Care	Postoperative Complications	96	Undirected
follow up	emergency ward	96	Undirected
midazolam	sedation	96	Undirected
outcome assessment	multicenter study	96	Undirected
psychology	multicenter study	96	Undirected

Clinical Competence	Health Services Needs and Demand	95	Undirected
Cross Infection	Anti-Bacterial Agents	95	Undirected
Cross Infection	ethicillin resistant Staphylococcus aure	95	Undirected
Pandemics	organization and management	95	Undirected
Quality Improvement	health care quality	95	Undirected
Stress, Psychological	Burnout, Professional	95	Undirected
Treatment Outcome	Child, Preschool	95	Undirected
Treatment Outcome	resuscitation	95	Undirected
hospitalization	psychology	95	Undirected
hospitalization	resuscitation	95	Undirected
prevalence	hospital admission	95	Undirected
Anxiety	Pain	94	Undirected
Artificial Ventilation	prevalence	94	Undirected
Attitude of Health Personnel	COVID-19	94	Undirected
Clinical Competence	Decision Making	94	Undirected
Cross Infection	Length of Stay	94	Undirected
Infection Control	Catheterization, Central Venous	94	Undirected
Sepsis	antibiotic agent	94	Undirected
Time Factors	mortality	94	Undirected
depression	Pandemics	94	Undirected
hospitalization	total quality management	94	Undirected
outcome assessment	disease severity	94	Undirected
prevalence	Pandemics	94	Undirected
prevalence	Pressure Ulcer	94	Undirected
psychology	semi structured interview	94	Undirected
Decision Making	organization and management	93	Undirected
Risk Factors	organization and management	93	Undirected
Risk Factors	tertiary care center	93	Undirected
Time Factors	Attitude of Health Personnel	93	Undirected
Workplace	Burnout, Professional	93	Undirected
outcome assessment	health care quality	93	Undirected
outcome assessment	total quality management	93	Undirected
Attitude of Health Personnel	medical staff	92	Undirected
Clinical Competence	Interprofessional Relations	92	Undirected
Clinical Competence	standards	92	Undirected
Family	resuscitation	92	Undirected
Infant, Premature	Pain	92	Undirected
Length of Stay	Glasgow coma scale	92	Undirected
Length of Stay	hypertension	92	Undirected
Risk Factors	psychology	92	Undirected
Social Support	Stress, Psychological	92	Undirected
Treatment Outcome	practice guideline	92	Undirected
attitude to health	Communication	92	Undirected
follow up	hospital readmission	92	Undirected
health care quality	hospital admission	92	Undirected
hospital discharge	organization and management	92	Undirected
hospitalization	fever	92	Undirected
practice guideline	very elderly	92	Undirected
tertiary care center	COVID-19	92	Undirected
Artificial Ventilation	health care quality	91	Undirected
Artificial Ventilation	oxygen saturation	91	Undirected
Child, Preschool	hospitalization	91	Undirected
Practice Guidelines as Topic	Clinical Protocols	91	Undirected
Reproducibility of Results	Sensitivity and Specificity	91	Undirected

Risk Factors	diabetes mellitus	91	Undirected
Sepsis	cohort analysis	91	Undirected
Terminal Care	interpersonal communication	91	Undirected
Time Factors	hospitalization	91	Undirected
Treatment Outcome	Clinical Protocols	91	Undirected
burnout	Pandemics	91	Undirected
education	resuscitation	91	Undirected
emergency care	hospital admission	91	Undirected
fentanyl	morphine	91	Undirected
hospital admission	organization and management	91	Undirected
interview	interpersonal communication	91	Undirected
outcome assessment	emergency ward	91	Undirected
outcome assessment	organization and management	91	Undirected
practice guideline	emergency ward	91	Undirected
psychology	clinical article	91	Undirected
quality of life	Treatment Outcome	91	Undirected
Artificial Ventilation	Communication	90	Undirected
Length of Stay	age	90	Undirected
Length of Stay	emergency health service	90	Undirected
Parents	Decision Making	90	Undirected
Risk Assessment	outcome assessment	90	Undirected
Treatment Outcome	health care quality	90	Undirected
clinical article	disease severity	90	Undirected
diabetes mellitus	mortality	90	Undirected
epidemiology	organization and management	90	Undirected
interview	organization and management	90	Undirected
medical education	clinical practice	90	Undirected
outcome assessment	observational study	90	Undirected
outcome assessment	very elderly	90	Undirected
Artificial Ventilation	chronic obstructive lung disease	89	Undirected
Burnout, Professional	Pandemics	89	Undirected
COVID-19	palliative therapy	89	Undirected
Length of Stay	Logistic Models	89	Undirected
Pain Measurement	Reproducibility of Results	89	Undirected
Quality Improvement	standards	89	Undirected
Sepsis	practice guideline	89	Undirected
hospitalization	caregiver	89	Undirected
observational study	multicenter study	89	Undirected
prematurity	psychology	89	Undirected
prevalence	burnout	89	Undirected
standards	statistics and numerical data	89	Undirected
Artificial Ventilation	Infection Control	88	Undirected
Hospital Mortality	very elderly	88	Undirected
OUTCOMES	mortality	88	Undirected
Pregnancy	Mothers	88	Undirected
Pregnancy	prematurity	88	Undirected
Risk Factors	Anxiety	88	Undirected
Risk Factors	randomized controlled trial	88	Undirected
Treatment Outcome	clinical article	88	Undirected
depression	mental health	88	Undirected
education	Health Personnel	88	Undirected
hospitalization	palliative therapy	88	Undirected
prematurity	clinical article	88	Undirected
resuscitation	hospital admission	88	Undirected

Artificial Ventilation	antibiotic agent	87	Undirected
Artificial Ventilation	heart rate	87	Undirected
Artificial Ventilation	midazolam	87	Undirected
Artificial Ventilation	total quality management	87	Undirected
Attitude of Health Personnel	clinical practice	87	Undirected
Attitude of Health Personnel	ethics	87	Undirected
Delirium	Pain	87	Undirected
Length of Stay	emergency care	87	Undirected
Professional-Family Relations	Stress, Psychological	87	Undirected
Sepsis	Anti-Bacterial Agents	87	Undirected
Stress, Psychological	Anxiety	87	Undirected
Workforce	Attitude of Health Personnel	87	Undirected
attitude to health	psychology	87	Undirected
diabetes mellitus	COVID-19	87	Undirected
heart rate	clinical article	87	Undirected
hospital admission	emergency health service	87	Undirected
hospitalization	tertiary care center	87	Undirected
interview	thematic analysis	87	Undirected
outcome assessment	Sepsis	87	Undirected
prevalence	Risk Assessment	87	Undirected
Artificial Ventilation	very elderly	86	Undirected
Attitude of Health Personnel	Safety Management	86	Undirected
Decision Making	palliative therapy	86	Undirected
Length of Stay	tracheostomy	86	Undirected
Professional-Family Relations	psychological aspect	86	Undirected
Risk Factors	Anti-Bacterial Agents	86	Undirected
Risk Factors	China	86	Undirected
Risk Factors	gestational age	86	Undirected
hypertension	hospital admission	86	Undirected
mortality	antibiotic agent	86	Undirected
quality of life	Job Satisfaction	86	Undirected
randomized controlled trial	meta analysis	86	Undirected
total quality management	education	86	Undirected
Family	COVID-19	85	Undirected
fever	clinical article	85	Undirected
hospitalization	heart failure	85	Undirected
midazolam	morphine	85	Undirected
perception	psychology	85	Undirected
practice guideline	Pandemics	85	Undirected
prevalence	Anxiety	85	Undirected
prevalence	depression	85	Undirected
psychology	very elderly	85	Undirected
Attitude of Health Personnel	total quality management	84	Undirected
COVID-19	very elderly	84	Undirected
Communication	Leadership	84	Undirected
Communication	Quality Improvement	84	Undirected
Cross Infection	microbiology	84	Undirected
Decision Making	resuscitation	84	Undirected
Health Services Needs and Demand	attitude to health	84	Undirected
Stress, Psychological	Infant, Premature	84	Undirected
attitude to health	knowledge	84	Undirected
education	COVID-19	84	Undirected
fever	hospital admission	84	Undirected
follow up	disease severity	84	Undirected

hospitalization	oxygen saturation	84	Undirected
mortality	age	84	Undirected
outcome assessment	hospital readmission	84	Undirected
psychology	palliative therapy	84	Undirected
quality of life	outcome assessment	84	Undirected
Attitude of Health Personnel	Quality Improvement	83	Undirected
Attitude of Health Personnel	standards	83	Undirected
Clinical Competence	simulation	83	Undirected
Delirium	mortality	83	Undirected
alth Knowledge, Attitudes, Practi	Health Personnel	83	Undirected
Job Satisfaction	Workload	83	Undirected
Risk Assessment	hospital admission	83	Undirected
epidemiology	multicenter study	83	Undirected
health care delivery	organization and management	83	Undirected
hospitalization	Palliative Care	83	Undirected
hospitalization	Terminal Care	83	Undirected
hospitalization	prematurity	83	Undirected
outcome assessment	clinical article	83	Undirected
quality of life	palliative therapy	83	Undirected
resuscitation	clinical article	83	Undirected
Clinical Competence	Pilot Projects	82	Undirected
alth Knowledge, Attitudes, Practi	attitude	82	Undirected
Length of Stay	Workload	82	Undirected
Length of Stay	university hospital	82	Undirected
Risk Factors	Workload	82	Undirected
Stroke	cerebrovascular accident	82	Undirected
Telemedicine	COVID-19	82	Undirected
attitude to health	education	82	Undirected
follow up	multicenter study	82	Undirected
hospital admission	antibiotic agent	82	Undirected
hospital admission	university hospital	82	Undirected
hospitalization	emergency care	82	Undirected
pneumonia	hospital admission	82	Undirected
prevalence	Delirium	82	Undirected
quality of life	randomized controlled trial	82	Undirected
Anxiety	burnout	81	Undirected
Clinical Competence	Cooperative Behavior	81	Undirected
Clinical Competence	Workforce	81	Undirected
Decision Making	COVID-19	81	Undirected
Hospital Mortality	hospital discharge	81	Undirected
hospitalization	Infection Control	81	Undirected
hospitalization	clinical practice	81	Undirected
mental health	psychology	81	Undirected
practice guideline	antibiotic agent	81	Undirected
practice guideline	health care delivery	81	Undirected
sedation	practice guideline	81	Undirected
Clinical Competence	practice guideline	80	Undirected
Decision Making	practice guideline	80	Undirected
Family	Needs Assessment	80	Undirected
Health Services Needs and Demand	Social Support	80	Undirected
Hospital Mortality	COVID-19	80	Undirected
Parents	Family	80	Undirected
Treatment Outcome	Sepsis	80	Undirected
education	psychological aspect	80	Undirected

follow up	very elderly	80	Undirected
hospitalization	chronic obstructive lung disease	80	Undirected
observational study	COVID-19	80	Undirected
practice guideline	follow up	80	Undirected
quality of life	COVID-19	80	Undirected
quality of life	caregiver	80	Undirected
randomized controlled trial	clinical article	80	Undirected
resuscitation	emergency health service	80	Undirected
resuscitation	medical education	80	Undirected
tertiary care center	hospital admission	80	Undirected
Artificial Ventilation	dyspnea	79	Undirected
Artificial Ventilation	long term care	79	Undirected
Attitude of Health Personnel	Pilot Projects	79	Undirected
Clinical Competence	Artificial Ventilation	79	Undirected
Communication	education	79	Undirected
Infant, Premature	randomized controlled trial	79	Undirected
Leadership	organization and management	79	Undirected
Risk Assessment	practice guideline	79	Undirected
Risk Factors	depression	79	Undirected
Sepsis	pneumonia	79	Undirected
Treatment Outcome	disease severity	79	Undirected
analgesia	practice guideline	79	Undirected
dyspnea	clinical article	79	Undirected
education	health care quality	79	Undirected
health care quality	methodology	79	Undirected
hospital admission	palliative therapy	79	Undirected
hospitalization	dementia	79	Undirected
hospitalization	home care	79	Undirected
organization and management	university hospital	79	Undirected
Attitude of Health Personnel	Canada	78	Undirected
Communication	perception	78	Undirected
Delirium	Reproducibility of Results	78	Undirected
Infant, Premature	Pain Measurement	78	Undirected
Infection Control	ethicillin resistant Staphylococcus aure	78	Undirected
Length of Stay	Pandemics	78	Undirected
Postoperative Care	Cardiac Surgical Procedures	78	Undirected
attitude to health	psychological aspect	78	Undirected
emergency care	emergency health service	78	Undirected
epidemiology	follow up	78	Undirected
mortality	emergency health service	78	Undirected
observational study	very elderly	78	Undirected
practice guideline	hand washing	78	Undirected
prematurity	hospital discharge	78	Undirected
quality of life	Risk Factors	78	Undirected
quality of life	hospital discharge	78	Undirected
Artificial Ventilation	Monitoring, Physiologic	77	Undirected
Artificial Ventilation	emergency ward	77	Undirected
COVID-19	health care facility	77	Undirected
Infant, Premature	psychology	77	Undirected
Risk Factors	hospital readmission	77	Undirected
Sepsis	resuscitation	77	Undirected
analgesia	morphine	77	Undirected
attitude to health	Health Personnel	77	Undirected
clinical article	thorax radiography	77	Undirected

emergency ward	health care system	77	Undirected
hospitalization	antibiotic agent	77	Undirected
hospitalization	depression	77	Undirected
medical staff	COVID-19	77	Undirected
mortality	resuscitation	77	Undirected
pneumonia	cohort analysis	77	Undirected
prevalence	Health Personnel	77	Undirected
prevalence	outcome assessment	77	Undirected
quality of life	mortality	77	Undirected
Adaptation, Psychological	psychology	76	Undirected
Artificial Ventilation	Anxiety	76	Undirected
Artificial Ventilation	Guideline Adherence	76	Undirected
Attitude of Health Personnel	health care quality	76	Undirected
Child, Preschool	Risk Assessment	76	Undirected
Clinical Competence	Quality of Health Care	76	Undirected
Insulin	Clinical Protocols	76	Undirected
Practice Guidelines as Topic	total quality management	76	Undirected
Sepsis	Infection Control	76	Undirected
epidemiology	emergency ward	76	Undirected
hospitalization	emergency health service	76	Undirected
hospitalization	hospital infection	76	Undirected
hypertension	cohort analysis	76	Undirected
multicenter study	very elderly	76	Undirected
outcome assessment	COVID-19	76	Undirected
Attitude of Health Personnel	Infection Control	75	Undirected
Communication	Adaptation, Psychological	75	Undirected
Cross Infection	Sepsis	75	Undirected
Delirium	cohort analysis	75	Undirected
Guideline Adherence	Health Knowledge, Attitudes, Practice	75	Undirected
Insulin	diabetes mellitus	75	Undirected
Length of Stay	Infection Control	75	Undirected
Practice Guidelines as Topic	Infection Control	75	Undirected
Practice Guidelines as Topic	evidence based practice	75	Undirected
Sepsis	disease severity	75	Undirected
Social Support	psychology	75	Undirected
fentanyl	sedation	75	Undirected
hypertension	clinical article	75	Undirected
multicenter study	organization and management	75	Undirected
perception	education	75	Undirected
practice guideline	randomized controlled trial	75	Undirected
prematurity	Breast Feeding	75	Undirected
prevalence	Burnout, Professional	75	Undirected
prevalence	disease severity	75	Undirected
psychology	randomized controlled trial	75	Undirected
quality of life	Terminal Care	75	Undirected
statistics and numerical data	organization and management	75	Undirected
Artificial Ventilation	epidemiology	74	Undirected
Artificial Ventilation	extracorporeal oxygenation	74	Undirected
Burnout, Professional	Health Personnel	74	Undirected
Delirium	Sensitivity and Specificity	74	Undirected
Family	hospitalization	74	Undirected
Family	perception	74	Undirected
Health Services Needs and Demand	Family	74	Undirected
Infection Control	organization and management	74	Undirected

Length of Stay	antibiotic agent	74	Undirected
Length of Stay	gestational age	74	Undirected
Length of Stay	health care delivery	74	Undirected
Length of Stay	home care	74	Undirected
Length of Stay	long term care	74	Undirected
Monitoring, Physiologic	Time Factors	74	Undirected
Parents	Mothers	74	Undirected
Risk Factors	clinical practice	74	Undirected
Sepsis	follow up	74	Undirected
Time Factors	Infant, Premature	74	Undirected
Time Factors	methodology	74	Undirected
Treatment Outcome	very elderly	74	Undirected
Workforce	COVID-19	74	Undirected
burnout	depression	74	Undirected
clinical article	breathing rate	74	Undirected
health care cost	health care quality	74	Undirected
health care cost	hospital admission	74	Undirected
hospitalization	health care delivery	74	Undirected
interpersonal communication	palliative therapy	74	Undirected
observational study	disease severity	74	Undirected
pharmacist	Medication Errors	74	Undirected
pilot study	clinical article	74	Undirected
practice guideline	emergency health service	74	Undirected
resuscitation	simulation	74	Undirected
Artificial Ventilation	respiratory therapist	73	Undirected
Artificial Ventilation	thorax radiography	73	Undirected
Length of Stay	cerebrovascular accident	73	Undirected
Length of Stay	preschool child	73	Undirected
Risk Assessment	follow up	73	Undirected
Risk Factors	hospital infection	73	Undirected
Terminal Care	health care quality	73	Undirected
antibiotic agent	antiinfective agent	73	Undirected
diabetes mellitus	hospital admission	73	Undirected
health care delivery	hospital admission	73	Undirected
hospitalization	Pain	73	Undirected
hospitalization	long term care	73	Undirected
hypertension	follow up	73	Undirected
medical staff	health care quality	73	Undirected
medical staff	psychology	73	Undirected
multicenter study	hospital admission	73	Undirected
observational study	emergency ward	73	Undirected
outcome assessment	epidemiology	73	Undirected
perception	semi structured interview	73	Undirected
Adaptation, Psychological	Burnout, Professional	72	Undirected
Adaptation, Psychological	psychological aspect	72	Undirected
Artificial Ventilation	hypertension	72	Undirected
Attitude of Health Personnel	China	72	Undirected
Attitude of Health Personnel	semi structured interview	72	Undirected
Clinical Competence	Leadership	72	Undirected
Clinical Competence	attitude to health	72	Undirected
Communication	COVID-19	72	Undirected
Fatigue	COVID-19	72	Undirected
Guideline Adherence	Health Personnel	72	Undirected
alth Knowledge, Attitudes, Practi	psychology	72	Undirected

Job Satisfaction	Leadership	72	Undirected
Leadership	COVID-19	72	Undirected
Practice Guidelines as Topic	very elderly	72	Undirected
Risk Assessment	COVID-19	72	Undirected
Risk Factors	adverse event	72	Undirected
Sepsis	hospital discharge	72	Undirected
Terminal Care	resuscitation	72	Undirected
computer assisted tomography	thorax radiography	72	Undirected
dyspnea	hospital admission	72	Undirected
epidemiology	hospital discharge	72	Undirected
hospital admission	preschool child	72	Undirected
hospitalization	university hospital	72	Undirected
mortality	health care system	72	Undirected
mortality	hypertension	72	Undirected
prematurity	Pain	72	Undirected
Artificial Ventilation	fever	71	Undirected
Artificial Ventilation	physiotherapy	71	Undirected
Decision Making	education	71	Undirected
Pandemics	disease severity	71	Undirected
age	cohort analysis	71	Undirected
age	hospital admission	71	Undirected
diabetes mellitus	cohort analysis	71	Undirected
hospitalization	pediatrics	71	Undirected
medical staff	organization and management	71	Undirected
organization and management	information processing	71	Undirected
practice guideline	interpersonal communication	71	Undirected
quality of life	hospital admission	71	Undirected
very elderly	disease severity	71	Undirected
Artificial Ventilation	Quality Improvement	70	Undirected
Emergency Service, Hospital	organization and management	70	Undirected
Family	Health Personnel	70	Undirected
Guideline Adherence	standards	70	Undirected
Hospital Mortality	Personnel Staffing and Scheduling	70	Undirected
Length of Stay	Anxiety	70	Undirected
Length of Stay	Infant, Premature	70	Undirected
Length of Stay	chronic obstructive lung disease	70	Undirected
Logistic Models	Hospital Mortality	70	Undirected
Monitoring, Physiologic	Practice Guidelines as Topic	70	Undirected
Mothers	psychology	70	Undirected
Pandemics	cohort analysis	70	Undirected
Professional-Family Relations	Infant, Premature	70	Undirected
Professional-Family Relations	Palliative Care	70	Undirected
Quality Improvement	practice guideline	70	Undirected
Quality of Health Care	Terminal Care	70	Undirected
Risk Assessment	Sensitivity and Specificity	70	Undirected
attitude to health	organization and management	70	Undirected
health care quality	palliative therapy	70	Undirected
hospitalization	Electronic Health Records	70	Undirected
mortality	palliative therapy	70	Undirected
organization and management	very elderly	70	Undirected
psychology	follow up	70	Undirected
psychology	statistics and numerical data	70	Undirected
university hospital	cohort analysis	70	Undirected
Attitude of Health Personnel	Pandemics	69	Undirected

Attitude of Health Personnel	Personnel Staffing and Scheduling	69	Undirected
China	COVID-19	69	Undirected
Communication	total quality management	69	Undirected
Cross Infection	urinary tract infection	69	Undirected
Length of Stay	resuscitation	69	Undirected
Quality Improvement	education	69	Undirected
Quality of Health Care	Job Satisfaction	69	Undirected
Treatment Outcome	Heart Arrest	69	Undirected
education	clinical practice	69	Undirected
follow up	computer assisted tomography	69	Undirected
gestational age	clinical article	69	Undirected
health care cost	health care system	69	Undirected
health care quality	COVID-19	69	Undirected
hospital admission	health care system	69	Undirected
medical education	organization and management	69	Undirected
observational study	hospital discharge	69	Undirected
practice guideline	clinical article	69	Undirected
practice guideline	hospital discharge	69	Undirected
prevalence	follow up	69	Undirected
psychology	standards	69	Undirected
tertiary care center	cohort analysis	69	Undirected
Artificial Ventilation	Reproducibility of Results	68	Undirected
Attitude of Health Personnel	pediatrics	68	Undirected
COVID-19	interpersonal communication	68	Undirected
Health Services Needs and Demand	Adaptation, Psychological	68	Undirected
Hospital Mortality	Heart Arrest	68	Undirected
Infection Control	Anti-Bacterial Agents	68	Undirected
Length of Stay	clinical practice	68	Undirected
Length of Stay	sedation	68	Undirected
Pandemics	palliative therapy	68	Undirected
Postoperative Complications	Cardiac Surgical Procedures	68	Undirected
Professional-Family Relations	Needs Assessment	68	Undirected
Professional-Family Relations	organization and management	68	Undirected
Risk Factors	Postoperative Care	68	Undirected
Treatment Outcome	emergency ward	68	Undirected
Workload	Pandemics	68	Undirected
health care quality	health care system	68	Undirected
hospital infection	hand washing	68	Undirected
hospitalization	Decision Making	68	Undirected
hospitalization	Pregnancy	68	Undirected
hospitalization	Quality Improvement	68	Undirected
outcome assessment	resuscitation	68	Undirected
pediatrics	education	68	Undirected
practice guideline	multicenter study	68	Undirected
prevalence	emergency ward	68	Undirected
quality of life	Family	68	Undirected
randomized controlled trial	hospital discharge	68	Undirected
randomized controlled trial	methodology	68	Undirected
very elderly	clinical article	68	Undirected
very elderly	palliative therapy	68	Undirected
Artificial Ventilation	adverse event	67	Undirected
Artificial Ventilation	breathing rate	67	Undirected
Artificial Ventilation	evidence based practice	67	Undirected
Artificial Ventilation	medical education	67	Undirected

Attitude of Health Personnel	information processing	67	Undirected
Communication	interview	67	Undirected
Cross Infection	hospitalization	67	Undirected
Cross Infection	practice guideline	67	Undirected
Delirium	dementia	67	Undirected
Emergency Service, Hospital	Hospital Mortality	67	Undirected
Family	clinical article	67	Undirected
Family	palliative therapy	67	Undirected
alth Knowledge, Attitudes, Practi	education	67	Undirected
Hospital Mortality	observational study	67	Undirected
Length of Stay	Cardiac Surgical Procedures	67	Undirected
Length of Stay	Quality of Health Care	67	Undirected
Length of Stay	psychology	67	Undirected
Length of Stay	ventilator associated pneumonia	67	Undirected
Pain	practice guideline	67	Undirected
Parents	caregiver	67	Undirected
Parents	education	67	Undirected
Practice Guidelines as Topic	Risk Assessment	67	Undirected
Quality Improvement	Clinical Protocols	67	Undirected
Risk Factors	Burnout, Professional	67	Undirected
Sepsis	Catheterization, Central Venous	67	Undirected
Social Support	Mothers	67	Undirected
Time Factors	Child, Preschool	67	Undirected
Time Factors	Emergency Service, Hospital	67	Undirected
clinical article	cohort analysis	67	Undirected
emergency ward	very elderly	67	Undirected
ethics	Terminal Care	67	Undirected
hospital discharge	emergency health service	67	Undirected
hospitalization	age	67	Undirected
hospitalization	computer assisted tomography	67	Undirected
hospitalization	preschool child	67	Undirected
observational study	clinical article	67	Undirected
pneumonia	COVID-19	67	Undirected
quality of life	Burnout, Professional	67	Undirected
safety	organization and management	67	Undirected
total quality management	Clinical Protocols	67	Undirected
Artificial Ventilation	education	66	Undirected
Attitude of Health Personnel	Clinical Protocols	66	Undirected
COVID-19	university hospital	66	Undirected
Emergency Service, Hospital	hospital admission	66	Undirected
Interprofessional Relations	organization and management	66	Undirected
Length of Stay	OUTCOMES	66	Undirected
Length of Stay	adverse event	66	Undirected
Quality of Health Care	Workload	66	Undirected
Risk Assessment	very elderly	66	Undirected
Risk Factors	Sensitivity and Specificity	66	Undirected
Risk Factors	university hospital	66	Undirected
Safety Management	Medication Errors	66	Undirected
Terminal Care	COVID-19	66	Undirected
Time Factors	Clinical Protocols	66	Undirected
Time Factors	Practice Guidelines as Topic	66	Undirected
Treatment Outcome	Infant, Premature	66	Undirected
Treatment Outcome	Postoperative Care	66	Undirected
Workload	Burnout, Professional	66	Undirected

analgesia	fentanyl	66	Undirected
burnout	mental health	66	Undirected
emergency health service	cohort analysis	66	Undirected
health care cost	health care delivery	66	Undirected
hospital readmission	very elderly	66	Undirected
hospitalization	Heart Arrest	66	Undirected
hospitalization	education	66	Undirected
hospitalization	gestational age	66	Undirected
interpersonal communication	psychological aspect	66	Undirected
medical staff	medical education	66	Undirected
propofol	morphine	66	Undirected
psychology	health care quality	66	Undirected
tertiary care center	emergency ward	66	Undirected
Anti-Bacterial Agents	antibiotic agent	65	Undirected
Artificial Ventilation	Cross Infection	65	Undirected
Clinical Competence	Quality Improvement	65	Undirected
Communication	Stress, Psychological	65	Undirected
Cross Infection	Practice Guidelines as Topic	65	Undirected
Hospital Mortality	disease severity	65	Undirected
Infection Control	ventilator associated pneumonia	65	Undirected
Length of Stay	caregiver	65	Undirected
Length of Stay	palliative therapy	65	Undirected
Palliative Care	COVID-19	65	Undirected
Quality of Health Care	Personnel Staffing and Scheduling	65	Undirected
Risk Factors	health care quality	65	Undirected
Social Support	Needs Assessment	65	Undirected
Stress, Psychological	depression	65	Undirected
Time Factors	Workload	65	Undirected
United Kingdom	COVID-19	65	Undirected
Workplace	Leadership	65	Undirected
clinical practice	clinical article	65	Undirected
education	interpersonal communication	65	Undirected
emergency ward	disease severity	65	Undirected
epidemiology	therapy	65	Undirected
health care cost	hospital discharge	65	Undirected
health care planning	organization and management	65	Undirected
health care quality	health care policy	65	Undirected
health care quality	hospital discharge	65	Undirected
Anxiety	clinical article	64	Undirected
Artificial Ventilation	health care cost	64	Undirected
Artificial Ventilation	preschool child	64	Undirected
Attitude of Health Personnel	multicenter study	64	Undirected
COVID-19	multicenter study	64	Undirected
COVID-19	oxygen saturation	64	Undirected
Communication	Health Personnel	64	Undirected
Length of Stay	urinary tract infection	64	Undirected
Monitoring, Physiologic	Risk Factors	64	Undirected
Needs Assessment	organization and management	64	Undirected
Palliative Care	psychology	64	Undirected
Parents	interview	64	Undirected
Risk Assessment	methodology	64	Undirected
Risk Factors	mental health	64	Undirected
Treatment Outcome	Delirium	64	Undirected
Treatment Outcome	Practice Guidelines as Topic	64	Undirected

Treatment Outcome	hospital readmission	64	Undirected
age	very elderly	64	Undirected
emergency care	cohort analysis	64	Undirected
emergency ward	organization and management	64	Undirected
hypertension	hospital discharge	64	Undirected
microbiology	antiinfective agent	64	Undirected
mortality	hospital infection	64	Undirected
mortality	statistics and numerical data	64	Undirected
pediatrics	medical education	64	Undirected
pediatrics	practice guideline	64	Undirected
pneumonia	disease severity	64	Undirected
practice guideline	antiinfective agent	64	Undirected
prevalence	observational study	64	Undirected
psychology	clinical practice	64	Undirected
quality of life	burnout	64	Undirected
resuscitation	COVID-19	64	Undirected
resuscitation	emergency care	64	Undirected
tertiary care center	Infection Control	64	Undirected
tertiary care center	observational study	64	Undirected
tracheostomy	clinical article	64	Undirected
Artificial Ventilation	Pilot Projects	63	Undirected
Artificial Ventilation	Terminal Care	63	Undirected
Attitude of Health Personnel	tertiary care center	63	Undirected
COVID-19	thorax radiography	63	Undirected
Clinical Competence	health care quality	63	Undirected
Clinical Protocols	methodology	63	Undirected
Communication	Focus Groups	63	Undirected
Decision Making	Health Knowledge, Attitudes, Practice	63	Undirected
Decision Making	clinical practice	63	Undirected
Decision Making	perception	63	Undirected
Leadership	Pandemics	63	Undirected
Length of Stay	Australia	63	Undirected
Pain	randomized controlled trial	63	Undirected
Practice Guidelines as Topic	Sepsis	63	Undirected
Risk Factors	Attitude of Health Personnel	63	Undirected
Risk Factors	dementia	63	Undirected
Risk Factors	pediatrics	63	Undirected
Risk Factors	total quality management	63	Undirected
Social Support	Infant, Premature	63	Undirected
caregiver	COVID-19	63	Undirected
emergency ward	clinical practice	63	Undirected
fever	emergency ward	63	Undirected
health care delivery	clinical practice	63	Undirected
health care delivery	emergency ward	63	Undirected
hospital admission	computer assisted tomography	63	Undirected
hospitalization	Postoperative Complications	63	Undirected
hospitalization	health care facility	63	Undirected
hospitalization	heart rate	63	Undirected
interview	education	63	Undirected
medical staff	practice guideline	63	Undirected
mortality	health care delivery	63	Undirected
observational study	follow up	63	Undirected
practice guideline	health care system	63	Undirected
practice guideline	psychology	63	Undirected

prematurity	follow up	63	Undirected
prevalence	multicenter study	63	Undirected
quality of life	prevalence	63	Undirected
sedation	morphine	63	Undirected
total quality management	Infection Control	63	Undirected
Artificial Ventilation	Family	62	Undirected
Artificial Ventilation	psychology	62	Undirected
Attitude of Health Personnel	medical education	62	Undirected
Attitude of Health Personnel	university hospital	62	Undirected
COVID-19	extracorporeal oxygenation	62	Undirected
Clinical Competence	Australia	62	Undirected
Clinical Competence	Focus Groups	62	Undirected
Clinical Competence	Personnel Staffing and Scheduling	62	Undirected
Clinical Competence	Workload	62	Undirected
Clinical Competence	pediatrics	62	Undirected
alth Knowledge, Attitudes, Practi	Infection Control	62	Undirected
Health Personnel	hand washing	62	Undirected
Health Services Needs and Demand	Professional-Family Relations	62	Undirected
Hypnotics and Sedatives	Delirium	62	Undirected
Interprofessional Relations	Leadership	62	Undirected
Length of Stay	Attitude of Health Personnel	62	Undirected
Length of Stay	health care system	62	Undirected
Program Evaluation	health care quality	62	Undirected
Sepsis	clinical article	62	Undirected
Sepsis	emergency ward	62	Undirected
Telemedicine	Pandemics	62	Undirected
Time Factors	organization and management	62	Undirected
Time Factors	randomized controlled trial	62	Undirected
Treatment Outcome	multicenter study	62	Undirected
Workload	burnout	62	Undirected
Workplace	COVID-19	62	Undirected
age	hospital discharge	62	Undirected
caregiver	palliative therapy	62	Undirected
health care delivery	health care policy	62	Undirected
hospitalization	methodology	62	Undirected
interview	clinical practice	62	Undirected
interview	psychological aspect	62	Undirected
mortality	standards	62	Undirected
mortality	therapy	62	Undirected
outcome assessment	health care cost	62	Undirected
total quality management	mortality	62	Undirected
tracheostomy	COVID-19	62	Undirected
Anxiety	Delirium	61	Undirected
Artificial Ventilation	computer assisted tomography	61	Undirected
Artificial Ventilation	fentanyl	61	Undirected
Artificial Ventilation	hospital infection	61	Undirected
Artificial Ventilation	tertiary care center	61	Undirected
Breast Feeding	gestational age	61	Undirected
Clinical Competence	Reproducibility of Results	61	Undirected
Clinical Competence	Time Factors	61	Undirected
Cross Infection	Attitude of Health Personnel	61	Undirected
Decision Making	Pandemics	61	Undirected
Delirium	very elderly	61	Undirected
Guideline Adherence	Quality Improvement	61	Undirected

Job Satisfaction	COVID-19	61	Undirected
Length of Stay	Hypnotics and Sedatives	61	Undirected
Pain	morphine	61	Undirected
Parents	hospitalization	61	Undirected
Program Evaluation	organization and management	61	Undirected
Quality of Health Care	organization and management	61	Undirected
Risk Assessment	Reproducibility of Results	61	Undirected
Risk Assessment	hospital discharge	61	Undirected
Risk Factors	Clinical Protocols	61	Undirected
Risk Factors	burnout	61	Undirected
Risk Factors	cerebrovascular accident	61	Undirected
Sepsis	Catheter-Related Infections	61	Undirected
Stress, Psychological	psychological aspect	61	Undirected
Time Factors	hospital admission	61	Undirected
Treatment Outcome	Attitude of Health Personnel	61	Undirected
Treatment Outcome	Quality Improvement	61	Undirected
Workforce	Pandemics	61	Undirected
Workload	mortality	61	Undirected
education	Pandemics	61	Undirected
education	clinical article	61	Undirected
emergency ward	computer assisted tomography	61	Undirected
endotracheal intubation	clinical article	61	Undirected
fever	hypertension	61	Undirected
heart rate	randomized controlled trial	61	Undirected
hospitalization	acute kidney failure	61	Undirected
hospitalization	cerebrovascular accident	61	Undirected
medical education	COVID-19	61	Undirected
multicenter study	hospital discharge	61	Undirected
outcome assessment	Quality Improvement	61	Undirected
outcome assessment	education	61	Undirected
outcome assessment	emergency health service	61	Undirected
outcome assessment	pneumonia	61	Undirected
practice guideline	palliative therapy	61	Undirected
prevalence	psychology	61	Undirected
safety	practice guideline	61	Undirected
statistics and numerical data	very elderly	61	Undirected
Artificial Ventilation	Glasgow coma scale	60	Undirected
Artificial Ventilation	enteric feeding	60	Undirected
Attitude of Health Personnel	Emergency Service, Hospital	60	Undirected
Attitude of Health Personnel	palliative therapy	60	Undirected
Catheter-Related Infections	urinary tract infection	60	Undirected
Clinical Competence	total quality management	60	Undirected
Communication	psychological aspect	60	Undirected
Delirium	analgesia	60	Undirected
Emergency Service, Hospital	resuscitation	60	Undirected
Health Personnel	organization and management	60	Undirected
Hospital Mortality	epidemiology	60	Undirected
Hypnotics and Sedatives	propofol	60	Undirected
Length of Stay	analgesia	60	Undirected
Length of Stay	prematurity	60	Undirected
Length of Stay	standards	60	Undirected
Pain	Reproducibility of Results	60	Undirected
Risk Factors	Infant, Premature	60	Undirected
Risk Factors	clinical article	60	Undirected

Social Support	Interprofessional Relations	60	Undirected
Social Support	psychological aspect	60	Undirected
Time Factors	Quality Improvement	60	Undirected
Workforce	Quality of Health Care	60	Undirected
caregiver	follow up	60	Undirected
dementia	very elderly	60	Undirected
emergency care	practice guideline	60	Undirected
health care quality	emergency ward	60	Undirected
health care quality	health care planning	60	Undirected
home care	hospital admission	60	Undirected
hospitalization	Glasgow coma scale	60	Undirected
hospitalization	pharmacist	60	Undirected
hospitalization	thorax radiography	60	Undirected
interview	COVID-19	60	Undirected
interview	information processing	60	Undirected
practice guideline	university hospital	60	Undirected
sedation	clinical article	60	Undirected
semi structured interview	interpersonal communication	60	Undirected
Anxiety	caregiver	59	Undirected
Artificial Ventilation	hypotension	59	Undirected
Artificial Ventilation	interpersonal communication	59	Undirected
Artificial Ventilation	prematurity	59	Undirected
Attitude of Health Personnel	Infant, Premature	59	Undirected
Attitude of Health Personnel	Reproducibility of Results	59	Undirected
Burnout, Professional	epidemiology	59	Undirected
Clinical Competence	methodology	59	Undirected
Decision Making	psychological aspect	59	Undirected
Infection Control	antibiotic agent	59	Undirected
Infection Control	epidemiology	59	Undirected
Length of Stay	Anti-Bacterial Agents	59	Undirected
Length of Stay	Pain	59	Undirected
Length of Stay	heart surgery	59	Undirected
Length of Stay	pediatrics	59	Undirected
Length of Stay	pharmacist	59	Undirected
Risk Assessment	Delirium	59	Undirected
Risk Factors	antibiotic agent	59	Undirected
Risk Factors	standards	59	Undirected
Risk Factors	statistics and numerical data	59	Undirected
Sepsis	antiinfective agent	59	Undirected
Sepsis	randomized controlled trial	59	Undirected
Social Support	Risk Factors	59	Undirected
Terminal Care	caregiver	59	Undirected
Terminal Care	hospital admission	59	Undirected
burnout	epidemiology	59	Undirected
evidence based practice	clinical practice	59	Undirected
health care facility	organization and management	59	Undirected
health care quality	Pandemics	59	Undirected
health care system	health care policy	59	Undirected
hospital admission	chronic obstructive lung disease	59	Undirected
hypertension	cerebrovascular accident	59	Undirected
hypertension	emergency ward	59	Undirected
observational study	university hospital	59	Undirected
outcome assessment	standards	59	Undirected
pharmacist	COVID-19	59	Undirected

quality of life	mental health	59	Undirected
resuscitation	health care quality	59	Undirected
standards	psychological aspect	59	Undirected
tertiary care center	Health Personnel	59	Undirected
thematic analysis	COVID-19	59	Undirected
Artificial Ventilation	pain assessment	58	Undirected
Attitude of Health Personnel	Child, Preschool	58	Undirected
COVID-19	computer assisted tomography	58	Undirected
COVID-19	therapy	58	Undirected
Child, Preschool	Practice Guidelines as Topic	58	Undirected
Clinical Protocols	randomized controlled trial	58	Undirected
Decision Making	clinical article	58	Undirected
Decision Making	health care quality	58	Undirected
Delirium	COVID-19	58	Undirected
Family	depression	58	Undirected
Guideline Adherence	Clinical Protocols	58	Undirected
Hypnotics and Sedatives	midazolam	58	Undirected
Infection Control	methodology	58	Undirected
Infection Control	mortality	58	Undirected
Job Satisfaction	psychology	58	Undirected
Length of Stay	Electronic Health Records	58	Undirected
Length of Stay	heart failure	58	Undirected
Monitoring, Physiologic	Treatment Outcome	58	Undirected
Practice Guidelines as Topic	Quality Improvement	58	Undirected
Program Evaluation	total quality management	58	Undirected
Quality Improvement	evidence based practice	58	Undirected
Quality of Health Care	Communication	58	Undirected
Risk Assessment	Postoperative Complications	58	Undirected
Risk Factors	fever	58	Undirected
Risk Factors	meta analysis	58	Undirected
Risk Factors	prematurity	58	Undirected
Stress, Psychological	Mothers	58	Undirected
Terminal Care	organization and management	58	Undirected
Treatment Outcome	standards	58	Undirected
burnout	Health Personnel	58	Undirected
hospital discharge	palliative therapy	58	Undirected
hospitalization	breathing rate	58	Undirected
hypotension	clinical article	58	Undirected
medical staff	Pandemics	58	Undirected
mortality	chronic obstructive lung disease	58	Undirected
mortality	dementia	58	Undirected
organization and management	cohort analysis	58	Undirected
outcome assessment	psychology	58	Undirected
practice guideline	enteric feeding	58	Undirected
psychology	hospital discharge	58	Undirected
quality of life	Decision Making	58	Undirected
resuscitation	hospital discharge	58	Undirected
total quality management	interpersonal communication	58	Undirected
Adaptation, Psychological	Mothers	57	Undirected
Anxiety	Burnout, Professional	57	Undirected
Anxiety	randomized controlled trial	57	Undirected
Artificial Ventilation	university hospital	57	Undirected
COVID-19	emergency health service	57	Undirected
Canada	organization and management	57	Undirected

Clinical Competence	Social Support	57	Undirected
Cross Infection	pneumonia	57	Undirected
Delirium	psychology	57	Undirected
Family	Delirium	57	Undirected
Family	practice guideline	57	Undirected
Health Personnel	emergency ward	57	Undirected
Hospital Mortality	Workload	57	Undirected
Infection Control	urinary tract infection	57	Undirected
Length of Stay	enteric feeding	57	Undirected
Length of Stay	health care facility	57	Undirected
Length of Stay	hospital infection	57	Undirected
Length of Stay	physiotherapy	57	Undirected
Palliative Care	caregiver	57	Undirected
Pandemics	clinical article	57	Undirected
Professional-Family Relations	Health Knowledge, Attitudes, Practice	57	Undirected
Risk Assessment	Quality Improvement	57	Undirected
Risk Factors	Intubation, Intratracheal	57	Undirected
Risk Factors	heart failure	57	Undirected
Sepsis	Quality Improvement	57	Undirected
Terminal Care	very elderly	57	Undirected
Treatment Outcome	Cardiac Surgical Procedures	57	Undirected
analgesia	midazolam	57	Undirected
caregiver	clinical article	57	Undirected
clinical article	palliative therapy	57	Undirected
clinical article	preschool child	57	Undirected
clinical article	university hospital	57	Undirected
emergency care	medical education	57	Undirected
follow up	organization and management	57	Undirected
gestational age	hospital discharge	57	Undirected
hospitalization	mental health	57	Undirected
hospitalization	tracheostomy	57	Undirected
hypotension	hypertension	57	Undirected
interview	practice guideline	57	Undirected
medical staff	interpersonal communication	57	Undirected
mortality	acute kidney failure	57	Undirected
observational study	epidemiology	57	Undirected
outcome assessment	Clinical Protocols	57	Undirected
outcome assessment	clinical practice	57	Undirected
pain assessment	sedation	57	Undirected
perception	interview	57	Undirected
practice guideline	health care facility	57	Undirected
prevalence	Infection Control	57	Undirected
prevalence	mental health	57	Undirected
quality of life	Social Support	57	Undirected
simulation	medical education	57	Undirected
standards	very elderly	57	Undirected
total quality management	Leadership	57	Undirected
Adaptation, Psychological	Infant, Premature	56	Undirected
Anxiety	Health Personnel	56	Undirected
Artificial Ventilation	morphine	56	Undirected
COVID-19	health care policy	56	Undirected
COVID-19	intensivist	56	Undirected
Clinical Competence	Risk Factors	56	Undirected
Family	Focus Groups	56	Undirected

Heart Arrest	hospital admission	56	Undirected
Hospital Mortality	multicenter study	56	Undirected
Pain	heart rate	56	Undirected
Palliative Care	perception	56	Undirected
Parents	Anxiety	56	Undirected
Practice Guidelines as Topic	methodology	56	Undirected
Risk Factors	thicillin resistant Staphylococcus aure	56	Undirected
Social Support	Decision Making	56	Undirected
Social Support	Terminal Care	56	Undirected
Treatment Outcome	COVID-19	56	Undirected
attitude to health	practice guideline	56	Undirected
fever	disease severity	56	Undirected
hospitalization	health care planning	56	Undirected
hospitalization	medical staff	56	Undirected
hospitalization	urinary tract infection	56	Undirected
medical education	health care system	56	Undirected
mortality	Glasgow coma scale	56	Undirected
mortality	antiinfective agent	56	Undirected
mortality	cerebrovascular accident	56	Undirected
mortality	clinical practice	56	Undirected
mortality	health care facility	56	Undirected
mortality	health care planning	56	Undirected
mortality	long term care	56	Undirected
outcome assessment	prematurity	56	Undirected
prematurity	practice guideline	56	Undirected
randomized controlled trial	hospital admission	56	Undirected
resuscitation	palliative therapy	56	Undirected
thematic analysis	clinical article	56	Undirected
total quality management	clinical practice	56	Undirected
Artificial Ventilation	acute kidney failure	55	Undirected
Artificial Ventilation	palliative therapy	55	Undirected
Burnout, Professional	depression	55	Undirected
COVID-19	semi structured interview	55	Undirected
Emergency Service, Hospital	Sepsis	55	Undirected
Health Personnel	multicenter study	55	Undirected
Health Services Needs and Demand	Communication	55	Undirected
Heart Arrest	mortality	55	Undirected
Infant, Premature	Risk Assessment	55	Undirected
Length of Stay	acute kidney failure	55	Undirected
Length of Stay	oxygen saturation	55	Undirected
Pandemics	interpersonal communication	55	Undirected
Pregnancy	resuscitation	55	Undirected
Professional-Family Relations	resuscitation	55	Undirected
Quality Improvement	Catheter-Related Infections	55	Undirected
Risk Factors	Catheterization, Central Venous	55	Undirected
Social Support	Anxiety	55	Undirected
Social Support	hospitalization	55	Undirected
Time Factors	hospital discharge	55	Undirected
Time Factors	very elderly	55	Undirected
Workforce	Job Satisfaction	55	Undirected
attitude to health	Mothers	55	Undirected
attitude to health	Needs Assessment	55	Undirected
emergency care	COVID-19	55	Undirected
emergency care	health care quality	55	Undirected

emergency care	hospital discharge	55	Undirected
emergency ward	hospital readmission	55	Undirected
gestational age	cohort analysis	55	Undirected
health care delivery	medical education	55	Undirected
health care quality	follow up	55	Undirected
hospital discharge	computer assisted tomography	55	Undirected
hypertension	chronic obstructive lung disease	55	Undirected
hypertension	very elderly	55	Undirected
perception	attitude	55	Undirected
practice guideline	disease severity	55	Undirected
prematurity	methodology	55	Undirected
prevalence	hospital discharge	55	Undirected
psychology	hospital admission	55	Undirected
quality of life	clinical article	55	Undirected
randomized controlled trial	pilot study	55	Undirected
resuscitation	follow up	55	Undirected
resuscitation	randomized controlled trial	55	Undirected
safety	standards	55	Undirected
standards	interpersonal communication	55	Undirected
total quality management	health care delivery	55	Undirected
COVID-19	clinical practice	54	Undirected
COVID-19	health care planning	54	Undirected
Clinical Competence	Professional-Family Relations	54	Undirected
Clinical Protocols	Algorithms	54	Undirected
Clinical Protocols	mortality	54	Undirected
Communication	Needs Assessment	54	Undirected
Delirium	practice guideline	54	Undirected
Health Personnel	practice guideline	54	Undirected
Length of Stay	Enteral Nutrition	54	Undirected
Length of Stay	Family	54	Undirected
Length of Stay	Palliative Care	54	Undirected
Length of Stay	dementia	54	Undirected
Logistic Models	Risk Assessment	54	Undirected
Pain	clinical article	54	Undirected
Parents	Pregnancy	54	Undirected
Quality of Health Care	Family	54	Undirected
Reproducibility of Results	methodology	54	Undirected
Risk Assessment	total quality management	54	Undirected
Risk Factors	microbiology	54	Undirected
Stress, Psychological	COVID-19	54	Undirected
Time Factors	cohort analysis	54	Undirected
Treatment Outcome	health care cost	54	Undirected
attitude to health	Decision Making	54	Undirected
attitude to health	Risk Factors	54	Undirected
clinical practice	multicenter study	54	Undirected
dyspnea	emergency ward	54	Undirected
emergency ward	university hospital	54	Undirected
fever	computer assisted tomography	54	Undirected
health care cost	hospital readmission	54	Undirected
health care delivery	health care planning	54	Undirected
hospitalization	interpersonal communication	54	Undirected
hospitalization	statistics and numerical data	54	Undirected
medical staff	clinical practice	54	Undirected
mental health	Health Personnel	54	Undirected

pneumonia	antibiotic agent	54	Undirected
quality of life	multicenter study	54	Undirected
resuscitation	organization and management	54	Undirected
Anti-Bacterial Agents	microbiology	53	Undirected
Artificial Ventilation	Algorithms	53	Undirected
Artificial Ventilation	Heart Arrest	53	Undirected
Artificial Ventilation	home care	53	Undirected
Attitude of Health Personnel	Pain	53	Undirected
Attitude of Health Personnel	Risk Assessment	53	Undirected
Attitude of Health Personnel	methodology	53	Undirected
COVID-19	hospital personnel	53	Undirected
Child, Preschool	hospital admission	53	Undirected
Clinical Competence	Heart Arrest	53	Undirected
Delirium	hospital admission	53	Undirected
Family	semi structured interview	53	Undirected
Hospital Mortality	hospital readmission	53	Undirected
Infection Control	health care facility	53	Undirected
Infection Control	standards	53	Undirected
Leadership	health care quality	53	Undirected
Length of Stay	heart rate	53	Undirected
Length of Stay	meta analysis	53	Undirected
Palliative Care	interpersonal communication	53	Undirected
Pandemics	thematic analysis	53	Undirected
Risk Assessment	Infection Control	53	Undirected
Risk Assessment	disease severity	53	Undirected
Risk Factors	antiinfective agent	53	Undirected
Safety Management	organization and management	53	Undirected
Sensitivity and Specificity	cohort analysis	53	Undirected
Terminal Care	perception	53	Undirected
Time Factors	gestational age	53	Undirected
Workplace	burnout	53	Undirected
caregiver	depression	53	Undirected
caregiver	hospital discharge	53	Undirected
clinical practice	organization and management	53	Undirected
cohort analysis	chronic obstructive lung disease	53	Undirected
diabetes mellitus	follow up	53	Undirected
dyspnea	hypertension	53	Undirected
education	randomized controlled trial	53	Undirected
emergency ward	multicenter study	53	Undirected
fever	thorax radiography	53	Undirected
follow up	Pandemics	53	Undirected
gestational age	randomized controlled trial	53	Undirected
health care quality	information processing	53	Undirected
hospital admission	oxygen saturation	53	Undirected
hospitalization	analgesia	53	Undirected
hospitalization	meta analysis	53	Undirected
hospitalization	therapy	53	Undirected
interview	Health Personnel	53	Undirected
medical education	interpersonal communication	53	Undirected
mortality	fever	53	Undirected
mortality	meta analysis	53	Undirected
mortality	university hospital	53	Undirected
mortality	ventilator associated pneumonia	53	Undirected
observational study	clinical practice	53	Undirected

observational study	practice guideline	53	Undirected
outcome assessment	emergency care	53	Undirected
outcome assessment	palliative therapy	53	Undirected
pediatrics	resuscitation	53	Undirected
perception	knowledge	53	Undirected
prevalence	tertiary care center	53	Undirected
resuscitation	cohort analysis	53	Undirected
Artificial Ventilation	hospital readmission	52	Undirected
Artificial Ventilation	medical staff	52	Undirected
COVID-19	respiratory therapist	52	Undirected
Child, Preschool	Quality Improvement	52	Undirected
Clinical Competence	Guideline Adherence	52	Undirected
Clinical Protocols	organization and management	52	Undirected
Cross Infection	prevalence	52	Undirected
Family	Australia	52	Undirected
Family	Pandemics	52	Undirected
Glasgow coma scale	hospital discharge	52	Undirected
Hospital Mortality	emergency ward	52	Undirected
Hospital Mortality	pneumonia	52	Undirected
Length of Stay	Pressure Ulcer	52	Undirected
Length of Stay	methodology	52	Undirected
Quality Improvement	Delirium	52	Undirected
Quality of Health Care	Palliative Care	52	Undirected
Reproducibility of Results	standards	52	Undirected
Risk Assessment	health care quality	52	Undirected
Risk Assessment	organization and management	52	Undirected
Risk Factors	Cardiac Surgical Procedures	52	Undirected
Risk Factors	Stroke	52	Undirected
Risk Factors	health care cost	52	Undirected
Social Support	organization and management	52	Undirected
Terminal Care	interview	52	Undirected
Time Factors	outcome assessment	52	Undirected
Treatment Outcome	Emergency Service, Hospital	52	Undirected
Treatment Outcome	clinical practice	52	Undirected
Treatment Outcome	pneumonia	52	Undirected
Treatment Outcome	total quality management	52	Undirected
Workplace	psychology	52	Undirected
clinical practice	hospital admission	52	Undirected
education	emergency health service	52	Undirected
epidemiology	disease severity	52	Undirected
fever	Pandemics	52	Undirected
heart rate	resuscitation	52	Undirected
hospital admission	thorax radiography	52	Undirected
hospital discharge	cerebrovascular accident	52	Undirected
hospitalization	Pressure Ulcer	52	Undirected
mortality	clinical article	52	Undirected
pediatrics	emergency ward	52	Undirected
perception	COVID-19	52	Undirected
pharmacist	respiratory therapist	52	Undirected
pneumonia	clinical article	52	Undirected
practice guideline	endotracheal intubation	52	Undirected
prematurity	education	52	Undirected
resuscitation	endotracheal intubation	52	Undirected
tertiary care center	China	52	Undirected

total quality management	emergency ward	52	Undirected
Artificial Ventilation	China	51	Undirected
Artificial Ventilation	therapy	51	Undirected
Child, Preschool	Emergency Service, Hospital	51	Undirected
Clinical Competence	Adaptation, Psychological	51	Undirected
Clinical Protocols	clinical practice	51	Undirected
Communication	Health Knowledge, Attitudes, Practice	51	Undirected
Cooperative Behavior	organization and management	51	Undirected
Family	Health Knowledge, Attitudes, Practice	51	Undirected
Health Services Needs and Demand	Health Knowledge, Attitudes, Practice	51	Undirected
Health Services Needs and Demand	Practice Guidelines as Topic	51	Undirected
Hypnotics and Sedatives	analgesia	51	Undirected
Interprofessional Relations	Health Knowledge, Attitudes, Practice	51	Undirected
Leadership	education	51	Undirected
Length of Stay	fever	51	Undirected
Monitoring, Physiologic	Attitude of Health Personnel	51	Undirected
Monitoring, Physiologic	Reproducibility of Results	51	Undirected
Palliative Care	Pandemics	51	Undirected
Postoperative Complications	hospital discharge	51	Undirected
Postoperative Complications	mortality	51	Undirected
Practice Guidelines as Topic	Algorithms	51	Undirected
Risk Assessment	emergency ward	51	Undirected
Risk Factors	Quality Improvement	51	Undirected
Risk Factors	emergency care	51	Undirected
Risk Factors	health care system	51	Undirected
Risk Factors	ventilator associated pneumonia	51	Undirected
Terminal Care	Pandemics	51	Undirected
Terminal Care	practice guideline	51	Undirected
United Kingdom	Pandemics	51	Undirected
Workplace	Workload	51	Undirected
attitude to health	methodology	51	Undirected
blood pressure	clinical article	51	Undirected
dyspnea	disease severity	51	Undirected
dyspnea	thorax radiography	51	Undirected
ethics	psychology	51	Undirected
follow up	clinical practice	51	Undirected
health care cost	cohort analysis	51	Undirected
health care delivery	health care facility	51	Undirected
health care quality	emergency health service	51	Undirected
hypertension	acute kidney failure	51	Undirected
interpersonal communication	clinical article	51	Undirected
organization and management	palliative therapy	51	Undirected
outcome assessment	health care delivery	51	Undirected
practice guideline	hospital infection	51	Undirected
psychology	information processing	51	Undirected
quality of life	Attitude of Health Personnel	51	Undirected
randomized controlled trial	enteric feeding	51	Undirected
resuscitation	clinical practice	51	Undirected
statistics and numerical data	hospital admission	51	Undirected
teaching hospital	hospital admission	51	Undirected
tertiary care center	Pandemics	51	Undirected
tertiary care center	practice guideline	51	Undirected
total quality management	cohort analysis	51	Undirected
Adaptation, Psychological	Anxiety	50	Undirected

Artificial Ventilation	Health Knowledge, Attitudes, Practice	50	Undirected
Artificial Ventilation	Postoperative Care	50	Undirected
Attitude of Health Personnel	evidence based practice	50	Undirected
Australia	organization and management	50	Undirected
China	Pandemics	50	Undirected
Clinical Competence	Family	50	Undirected
Clinical Protocols	standards	50	Undirected
Communication	Australia	50	Undirected
Decision Making	caregiver	50	Undirected
Decision Making	semi structured interview	50	Undirected
Family	health care quality	50	Undirected
Glasgow coma scale	cohort analysis	50	Undirected
Glasgow coma scale	hospital admission	50	Undirected
Health Services Needs and Demand	organization and management	50	Undirected
Leadership	interpersonal communication	50	Undirected
Length of Stay	depression	50	Undirected
Needs Assessment	education	50	Undirected
Pregnancy	Risk Assessment	50	Undirected
Professional-Family Relations	Child, Preschool	50	Undirected
Quality Improvement	Infection Control	50	Undirected
Quality of Health Care	standards	50	Undirected
Risk Factors	OUTCOMES	50	Undirected
Risk Factors	Stress, Psychological	50	Undirected
Risk Factors	preschool child	50	Undirected
Sepsis	acute kidney failure	50	Undirected
Time Factors	standards	50	Undirected
Treatment Outcome	Insulin	50	Undirected
attitude	education	50	Undirected
blood pressure	breathing rate	50	Undirected
clinical practice	health care system	50	Undirected
diabetes mellitus	chronic obstructive lung disease	50	Undirected
dyspnea	oxygen saturation	50	Undirected
fever	hospital discharge	50	Undirected
health care quality	cohort analysis	50	Undirected
home care	cohort analysis	50	Undirected
hospital admission	cerebrovascular accident	50	Undirected
hospital infection	hospital admission	50	Undirected
hospitalization	Sensitivity and Specificity	50	Undirected
interview	Pandemics	50	Undirected
knowledge	clinical practice	50	Undirected
observational study	hand washing	50	Undirected
outcome assessment	gestational age	50	Undirected
outcome assessment	statistics and numerical data	50	Undirected
pediatrics	clinical practice	50	Undirected
pharmacist	practice guideline	50	Undirected
pneumonia	fever	50	Undirected
practice guideline	epidemiology	50	Undirected
practice guideline	hospital personnel	50	Undirected
prematurity	organization and management	50	Undirected
preschool child	cohort analysis	50	Undirected
prevalence	very elderly	50	Undirected
quality of life	Pandemics	50	Undirected
quality of life	disease severity	50	Undirected
ventilator associated pneumonia	practice guideline	50	Undirected

Artificial Ventilation	blood pressure	49	Undirected
Artificial Ventilation	health care delivery	49	Undirected
Artificial Ventilation	health care facility	49	Undirected
Artificial Ventilation	pediatrics	49	Undirected
COVID-19	age	49	Undirected
COVID-19	breathing rate	49	Undirected
COVID-19	hand washing	49	Undirected
Clinical Competence	Monitoring, Physiologic	49	Undirected
Cross Infection	Quality Improvement	49	Undirected
Decision Making	long term care	49	Undirected
Decision Making	pediatrics	49	Undirected
Hypnotics and Sedatives	Pain	49	Undirected
Length of Stay	Sensitivity and Specificity	49	Undirected
Length of Stay	Terminal Care	49	Undirected
Length of Stay	antiinfective agent	49	Undirected
Length of Stay	therapy	49	Undirected
Parents	psychological aspect	49	Undirected
Risk Factors	health care facility	49	Undirected
Terminal Care	mortality	49	Undirected
Treatment Outcome	heart surgery	49	Undirected
Workforce	Length of Stay	49	Undirected
Workload	organization and management	49	Undirected
diabetes mellitus	hospital discharge	49	Undirected
dyspnea	Fatigue	49	Undirected
dyspnea	hospital discharge	49	Undirected
ethics	COVID-19	49	Undirected
health care delivery	hospital discharge	49	Undirected
health care delivery	palliative therapy	49	Undirected
health care quality	health care facility	49	Undirected
home care	palliative therapy	49	Undirected
hospital admission	health care planning	49	Undirected
hospitalization	Anti-Bacterial Agents	49	Undirected
hospitalization	Clinical Protocols	49	Undirected
hospitalization	Workload	49	Undirected
hospitalization	hypotension	49	Undirected
hospitalization	semi structured interview	49	Undirected
medical education	emergency ward	49	Undirected
medical staff	health care delivery	49	Undirected
cillin resistant Staphylococcus a	vancomycin	49	Undirected
mortality	methodology	49	Undirected
outcome assessment	medical education	49	Undirected
outcome assessment	university hospital	49	Undirected
pneumonia	hospital discharge	49	Undirected
prematurity	cohort analysis	49	Undirected
prematurity	hospital admission	49	Undirected
prematurity	oxygen saturation	49	Undirected
psychology	thematic analysis	49	Undirected
quality of life	Pain	49	Undirected
resuscitation	hypotension	49	Undirected
resuscitation	observational study	49	Undirected
resuscitation	psychology	49	Undirected
safety	health care quality	49	Undirected
standards	emergency health service	49	Undirected
total quality management	medical education	49	Undirected

Anti-Bacterial Agents	mortality	48	Undirected
Artificial Ventilation	diabetes mellitus	48	Undirected
Artificial Ventilation	gestational age	48	Undirected
Artificial Ventilation	health care system	48	Undirected
Artificial Ventilation	pilot study	48	Undirected
Attitude of Health Personnel	thematic analysis	48	Undirected
COVID-19	hospital readmission	48	Undirected
Clinical Competence	Health Personnel	48	Undirected
Clinical Competence	Intubation, Intratracheal	48	Undirected
Decision Making	Leadership	48	Undirected
Emergency Service, Hospital	mortality	48	Undirected
Family	education	48	Undirected
Family	hospital discharge	48	Undirected
alth Knowledge, Attitudes, Practi	China	48	Undirected
alth Knowledge, Attitudes, Practi	Delirium	48	Undirected
Infant, Premature	heart rate	48	Undirected
Infection Control	microbiology	48	Undirected
Infection Control	observational study	48	Undirected
Interprofessional Relations	Job Satisfaction	48	Undirected
Length of Stay	Algorithms	48	Undirected
Length of Stay	Decision Making	48	Undirected
Length of Stay	Heart Arrest	48	Undirected
Length of Stay	Practice Guidelines as Topic	48	Undirected
Length of Stay	education	48	Undirected
Length of Stay	mental health	48	Undirected
Monitoring, Physiologic	heart rate	48	Undirected
Palliative Care	very elderly	48	Undirected
Pandemics	health care policy	48	Undirected
Pandemics	very elderly	48	Undirected
Parents	perception	48	Undirected
Postoperative Care	heart surgery	48	Undirected
Quality Improvement	mortality	48	Undirected
Quality of Health Care	Quality Improvement	48	Undirected
Risk Assessment	epidemiology	48	Undirected
Risk Factors	evidence based practice	48	Undirected
Risk Factors	urinary tract infection	48	Undirected
Terminal Care	Health Personnel	48	Undirected
Time Factors	practice guideline	48	Undirected
Treatment Outcome	Anti-Bacterial Agents	48	Undirected
Treatment Outcome	Pain	48	Undirected
Treatment Outcome	diabetes mellitus	48	Undirected
Treatment Outcome	epidemiology	48	Undirected
Treatment Outcome	observational study	48	Undirected
Treatment Outcome	prevalence	48	Undirected
United Kingdom	education	48	Undirected
Workplace	Stress, Psychological	48	Undirected
age	disease severity	48	Undirected
antibiotic agent	disease severity	48	Undirected
cerebrovascular accident	cohort analysis	48	Undirected
clinical practice	hospital discharge	48	Undirected
disease severity	computer assisted tomography	48	Undirected
education	multicenter study	48	Undirected
emergency care	clinical practice	48	Undirected
emergency care	organization and management	48	Undirected

endotracheal intubation	COVID-19	48	Undirected
health care cost	follow up	48	Undirected
hospital admission	breathing rate	48	Undirected
hospitalization	health care policy	48	Undirected
cillin resistant Staphylococcus a	hospital infection	48	Undirected
mortality	home care	48	Undirected
outcome assessment	Delirium	48	Undirected
outcome assessment	Heart Arrest	48	Undirected
outcome assessment	Postoperative Complications	48	Undirected
pain assessment	morphine	48	Undirected
perception	Health Personnel	48	Undirected
perception	clinical practice	48	Undirected
pneumonia	urinary tract infection	48	Undirected
practice guideline	cohort analysis	48	Undirected
practice guideline	health care planning	48	Undirected
semi structured interview	clinical practice	48	Undirected
tertiary care center	clinical article	48	Undirected
total quality management	pharmacist	48	Undirected
Anti-Bacterial Agents	practice guideline	47	Undirected
Artificial Ventilation	Australia	47	Undirected
Artificial Ventilation	interview	47	Undirected
Artificial Ventilation	meta analysis	47	Undirected
Attitude of Health Personnel	hospital personnel	47	Undirected
Child, Preschool	Reproducibility of Results	47	Undirected
Child, Preschool	cohort analysis	47	Undirected
Communication	health care quality	47	Undirected
Decision Making	Focus Groups	47	Undirected
Delirium	randomized controlled trial	47	Undirected
Emergency Service, Hospital	emergency care	47	Undirected
Family	clinical practice	47	Undirected
Heart Arrest	emergency ward	47	Undirected
Heart Arrest	hospital discharge	47	Undirected
Infection Control	education	47	Undirected
Infection Control	hospital personnel	47	Undirected
Length of Stay	Communication	47	Undirected
Length of Stay	Personnel Staffing and Scheduling	47	Undirected
Length of Stay	computer assisted tomography	47	Undirected
Length of Stay	dyspnea	47	Undirected
Pandemics	emergency health service	47	Undirected
Pandemics	hospital discharge	47	Undirected
Pandemics	semi structured interview	47	Undirected
Parents	Health Personnel	47	Undirected
Parents	interpersonal communication	47	Undirected
Parents	preschool child	47	Undirected
Practice Guidelines as Topic	Enteral Nutrition	47	Undirected
Pregnancy	Breast Feeding	47	Undirected
Professional-Family Relations	Focus Groups	47	Undirected
Quality of Health Care	Leadership	47	Undirected
Risk Assessment	Sepsis	47	Undirected
Risk Factors	education	47	Undirected
Risk Factors	resuscitation	47	Undirected
Sepsis	very elderly	47	Undirected
Social Support	caregiver	47	Undirected
Terminal Care	education	47	Undirected

Time Factors	Pilot Projects	47	Undirected
Treatment Outcome	OUTCOMES	47	Undirected
Treatment Outcome	Pregnancy	47	Undirected
Treatment Outcome	gestational age	47	Undirected
Treatment Outcome	heart failure	47	Undirected
United Kingdom	clinical practice	47	Undirected
United Kingdom	practice guideline	47	Undirected
attitude to health	Stress, Psychological	47	Undirected
dementia	hospital admission	47	Undirected
education	follow up	47	Undirected
education	information processing	47	Undirected
emergency ward	antibiotic agent	47	Undirected
epidemiology	health care delivery	47	Undirected
follow up	thorax radiography	47	Undirected
health care cost	practice guideline	47	Undirected
health care facility	health care system	47	Undirected
heart failure	hospital admission	47	Undirected
heart rate	follow up	47	Undirected
hospitalization	interview	47	Undirected
medical staff	emergency ward	47	Undirected
outcome assessment	health care system	47	Undirected
outcome assessment	methodology	47	Undirected
perception	interpersonal communication	47	Undirected
pharmacist	emergency ward	47	Undirected
practice guideline	therapy	47	Undirected
prevalence	practice guideline	47	Undirected
psychology	emergency ward	47	Undirected
psychology	university hospital	47	Undirected
quality of life	very elderly	47	Undirected
respiratory therapist	intensivist	47	Undirected
sedation	randomized controlled trial	47	Undirected
tertiary care center	mortality	47	Undirected
total quality management	follow up	47	Undirected
total quality management	hospital discharge	47	Undirected
ventilator associated pneumonia	hospital infection	47	Undirected
ventilator associated pneumonia	urinary tract infection	47	Undirected
Artificial Ventilation	caregiver	46	Undirected
Artificial Ventilation	depression	46	Undirected
Artificial Ventilation	statistics and numerical data	46	Undirected
Attitude of Health Personnel	caregiver	46	Undirected
Attitude of Health Personnel	clinical article	46	Undirected
Burnout, Professional	mental health	46	Undirected
Clinical Protocols	health care quality	46	Undirected
Communication	Job Satisfaction	46	Undirected
Communication	hospitalization	46	Undirected
Cross Infection	Risk Assessment	46	Undirected
Cross Infection	mortality	46	Undirected
Decision Making	outcome assessment	46	Undirected
Delirium	propofol	46	Undirected
Focus Groups	interview	46	Undirected
Glasgow coma scale	emergency ward	46	Undirected
Infection Control	antiinfective agent	46	Undirected
Interprofessional Relations	interpersonal communication	46	Undirected
Job Satisfaction	Personnel Staffing and Scheduling	46	Undirected

Length of Stay	breathing rate	46	Undirected
Length of Stay	morphine	46	Undirected
Pain	fentanyl	46	Undirected
Pain	psychology	46	Undirected
Pandemics	health care facility	46	Undirected
Postoperative Complications	cohort analysis	46	Undirected
Practice Guidelines as Topic	organization and management	46	Undirected
Pregnancy	practice guideline	46	Undirected
Professional-Family Relations	Cooperative Behavior	46	Undirected
Risk Assessment	clinical practice	46	Undirected
Risk Factors	Enteral Nutrition	46	Undirected
Risk Factors	Glasgow coma scale	46	Undirected
Risk Factors	long term care	46	Undirected
Safety Management	total quality management	46	Undirected
Sepsis	COVID-19	46	Undirected
Sepsis	total quality management	46	Undirected
Social Support	Health Knowledge, Attitudes, Practice	46	Undirected
Time Factors	Postoperative Complications	46	Undirected
Time Factors	follow up	46	Undirected
Treatment Outcome	prematurity	46	Undirected
United Kingdom	health care quality	46	Undirected
Workforce	Hospital Mortality	46	Undirected
analgesia	propofol	46	Undirected
analgesia	randomized controlled trial	46	Undirected
antibiotic agent	cohort analysis	46	Undirected
caregiver	health care quality	46	Undirected
clinical practice	interpersonal communication	46	Undirected
depression	Health Personnel	46	Undirected
dyspnea	follow up	46	Undirected
fever	antibiotic agent	46	Undirected
gestational age	follow up	46	Undirected
health care quality	hospital personnel	46	Undirected
heart failure	follow up	46	Undirected
heart failure	mortality	46	Undirected
hospitalization	physiotherapy	46	Undirected
knowledge	practice guideline	46	Undirected
medical staff	hospital admission	46	Undirected
mental health	emergency ward	46	Undirected
midazolam	clinical article	46	Undirected
pediatrics	organization and management	46	Undirected
perception	clinical article	46	Undirected
perception	thematic analysis	46	Undirected
pharmacist	organization and management	46	Undirected
pneumonia	emergency ward	46	Undirected
psychology	cohort analysis	46	Undirected
psychology	therapy	46	Undirected
quality of life	Delirium	46	Undirected
quality of life	health care cost	46	Undirected
quality of life	hospital readmission	46	Undirected
randomized controlled trial	COVID-19	46	Undirected
randomized controlled trial	disease severity	46	Undirected
randomized controlled trial	organization and management	46	Undirected
standards	information processing	46	Undirected
statistics and numerical data	hospital discharge	46	Undirected

tertiary care center	Quality Improvement	46	Undirected
total quality management	hospital admission	46	Undirected
total quality management	resuscitation	46	Undirected
vancomycin	clinical article	46	Undirected
Anxiety	Fatigue	45	Undirected
Artificial Ventilation	age	45	Undirected
Artificial Ventilation	heart failure	45	Undirected
Clinical Competence	Job Satisfaction	45	Undirected
Clinical Protocols	analgesia	45	Undirected
Communication	palliative therapy	45	Undirected
Cooperative Behavior	Leadership	45	Undirected
Cross Infection	cohort analysis	45	Undirected
Decision Making	Health Personnel	45	Undirected
Decision Making	mortality	45	Undirected
Decision Making	thematic analysis	45	Undirected
Family	follow up	45	Undirected
alth Knowledge, Attitudes, Practi	Terminal Care	45	Undirected
alth Knowledge, Attitudes, Practi	practice guideline	45	Undirected
Health Services Needs and Demand	Risk Factors	45	Undirected
Hospital Mortality	organization and management	45	Undirected
Infant, Premature	Enteral Nutrition	45	Undirected
Infant, Premature	methodology	45	Undirected
Interprofessional Relations	Decision Making	45	Undirected
Interprofessional Relations	Workplace	45	Undirected
Job Satisfaction	perception	45	Undirected
Length of Stay	endotracheal intubation	45	Undirected
Length of Stay	propofol	45	Undirected
Palliative Care	health care quality	45	Undirected
Palliative Care	hospital admission	45	Undirected
Pandemics	intensivist	45	Undirected
Practice Guidelines as Topic	Pain	45	Undirected
Program Evaluation	Health Knowledge, Attitudes, Practice	45	Undirected
Program Evaluation	Quality Improvement	45	Undirected
Program Evaluation	education	45	Undirected
Risk Factors	Medication Errors	45	Undirected
Risk Factors	chronic obstructive lung disease	45	Undirected
Risk Factors	health care delivery	45	Undirected
Sepsis	observational study	45	Undirected
Terminal Care	psychological aspect	45	Undirected
Treatment Outcome	tertiary care center	45	Undirected
analgesia	clinical article	45	Undirected
caregiver	interpersonal communication	45	Undirected
clinical practice	standards	45	Undirected
depression	follow up	45	Undirected
fever	follow up	45	Undirected
follow up	antibiotic agent	45	Undirected
follow up	cerebrovascular accident	45	Undirected
follow up	therapy	45	Undirected
health care cost	COVID-19	45	Undirected
health care quality	university hospital	45	Undirected
heart rate	hypertension	45	Undirected
hospitalization	Logistic Models	45	Undirected
hospitalization	perception	45	Undirected
hospitalization	standards	45	Undirected

interview	health care quality	45	Undirected
knowledge	medical education	45	Undirected
mortality	education	45	Undirected
mortality	medical staff	45	Undirected
mortality	preschool child	45	Undirected
mortality	psychology	45	Undirected
organization and management	preschool child	45	Undirected
outcome assessment	hypertension	45	Undirected
pediatrics	Health Personnel	45	Undirected
prematurity	heart rate	45	Undirected
quality of life	Fatigue	45	Undirected
quality of life	cohort analysis	45	Undirected
safety	Medication Errors	45	Undirected
sedation	clinical practice	45	Undirected
tertiary care center	epidemiology	45	Undirected
tertiary care center	university hospital	45	Undirected
total quality management	COVID-19	45	Undirected
very elderly	chronic obstructive lung disease	45	Undirected
Artificial Ventilation	emergency care	44	Undirected
Artificial Ventilation	emergency health service	44	Undirected
Attitude of Health Personnel	Pregnancy	44	Undirected
Clinical Competence	Pressure Ulcer	44	Undirected
Clinical Competence	Risk Assessment	44	Undirected
Clinical Protocols	evidence based practice	44	Undirected
Communication	Pandemics	44	Undirected
Cross Infection	epidemiology	44	Undirected
Decision Making	United Kingdom	44	Undirected
Decision Making	medical staff	44	Undirected
Delirium	hospital discharge	44	Undirected
Electronic Health Records	cohort analysis	44	Undirected
Family	Time Factors	44	Undirected
Family	medical staff	44	Undirected
Guideline Adherence	Time Factors	44	Undirected
Hospital Mortality	Postoperative Complications	44	Undirected
Insulin	Algorithms	44	Undirected
Job Satisfaction	interview	44	Undirected
Length of Stay	Pilot Projects	44	Undirected
Palliative Care	education	44	Undirected
Palliative Care	mortality	44	Undirected
Palliative Care	resuscitation	44	Undirected
Pandemics	medical education	44	Undirected
Parents	pediatrics	44	Undirected
Practice Guidelines as Topic	Infant, Premature	44	Undirected
Practice Guidelines as Topic	education	44	Undirected
Professional-Family Relations	interview	44	Undirected
Program Evaluation	Practice Guidelines as Topic	44	Undirected
Quality Improvement	Leadership	44	Undirected
Quality of Health Care	Interprofessional Relations	44	Undirected
Reproducibility of Results	Pilot Projects	44	Undirected
Risk Assessment	Electronic Health Records	44	Undirected
Risk Assessment	multicenter study	44	Undirected
Risk Factors	Pain	44	Undirected
Risk Factors	computer assisted tomography	44	Undirected
Risk Factors	heart surgery	44	Undirected

Risk Factors	home care	44	Undirected
Risk Factors	vancomycin	44	Undirected
Stress, Psychological	burnout	44	Undirected
Stress, Psychological	caregiver	44	Undirected
Time Factors	Sepsis	44	Undirected
Treatment Outcome	education	44	Undirected
attitude to health	Terminal Care	44	Undirected
caregiver	Pandemics	44	Undirected
clinical article	acute kidney failure	44	Undirected
diabetes mellitus	disease severity	44	Undirected
emergency care	psychology	44	Undirected
epidemiology	statistics and numerical data	44	Undirected
follow up	oxygen saturation	44	Undirected
gestational age	resuscitation	44	Undirected
health care cost	health care policy	44	Undirected
health care delivery	interpersonal communication	44	Undirected
health care facility	hospital admission	44	Undirected
health care system	palliative therapy	44	Undirected
hospital discharge	health care system	44	Undirected
hospital discharge	university hospital	44	Undirected
hospitalization	endotracheal intubation	44	Undirected
hypertension	disease severity	44	Undirected
interview	Mothers	44	Undirected
interview	caregiver	44	Undirected
long term care	very elderly	44	Undirected
medical staff	education	44	Undirected
mortality	emergency care	44	Undirected
mortality	health care policy	44	Undirected
multicenter study	statistics and numerical data	44	Undirected
organization and management	hospital personnel	44	Undirected
outcome assessment	pharmacist	44	Undirected
outcome assessment	tertiary care center	44	Undirected
pneumonia	very elderly	44	Undirected
prematurity	observational study	44	Undirected
prevalence	clinical practice	44	Undirected
resuscitation	antibiotic agent	44	Undirected
semi structured interview	palliative therapy	44	Undirected
standards	hospital discharge	44	Undirected
tertiary care center	total quality management	44	Undirected
total quality management	Needs Assessment	44	Undirected
total quality management	evidence based practice	44	Undirected
tracheostomy	hospital discharge	44	Undirected
Adaptation, Psychological	Job Satisfaction	43	Undirected
Anxiety	Mothers	43	Undirected
Artificial Ventilation	Logistic Models	43	Undirected
Artificial Ventilation	United Kingdom	43	Undirected
Artificial Ventilation	Workload	43	Undirected
Attitude of Health Personnel	Anxiety	43	Undirected
Attitude of Health Personnel	Delirium	43	Undirected
Attitude of Health Personnel	respiratory therapist	43	Undirected
COVID-19	antibiotic agent	43	Undirected
COVID-19	chronic obstructive lung disease	43	Undirected
Child, Preschool	Pain Measurement	43	Undirected
Child, Preschool	organization and management	43	Undirected

Clinical Competence	China	43	Undirected
Clinical Competence	emergency care	43	Undirected
Communication	safety	43	Undirected
Cooperative Behavior	Decision Making	43	Undirected
Cross Infection	Treatment Outcome	43	Undirected
Cross Infection	antiinfective agent	43	Undirected
Decision Making	Job Satisfaction	43	Undirected
Decision Making	multicenter study	43	Undirected
Delirium	follow up	43	Undirected
Family	Quality Improvement	43	Undirected
Family	total quality management	43	Undirected
Infant, Premature	hospital discharge	43	Undirected
Job Satisfaction	Pandemics	43	Undirected
Length of Stay	Stroke	43	Undirected
Length of Stay	midazolam	43	Undirected
Monitoring, Physiologic	standards	43	Undirected
OUTCOMES	Hospital Mortality	43	Undirected
Parents	gestational age	43	Undirected
Personnel Staffing and Scheduling	organization and management	43	Undirected
Postoperative Complications	follow up	43	Undirected
Practice Guidelines as Topic	United Kingdom	43	Undirected
Pregnancy	hospital admission	43	Undirected
Pressure Ulcer	Quality Improvement	43	Undirected
Quality Improvement	Catheterization, Central Venous	43	Undirected
Risk Assessment	randomized controlled trial	43	Undirected
Risk Assessment	standards	43	Undirected
Risk Factors	dyspnea	43	Undirected
Risk Factors	endotracheal intubation	43	Undirected
Sepsis	hypotension	43	Undirected
Sepsis	prematurity	43	Undirected
Treatment Outcome	Pain Measurement	43	Undirected
Treatment Outcome	preschool child	43	Undirected
adverse event	cohort analysis	43	Undirected
antibiotic agent	clinical article	43	Undirected
blood pressure	oxygen saturation	43	Undirected
clinical practice	disease severity	43	Undirected
depression	disease severity	43	Undirected
diabetes mellitus	cerebrovascular accident	43	Undirected
emergency care	health care delivery	43	Undirected
emergency ward	health care facility	43	Undirected
emergency ward	oxygen saturation	43	Undirected
epidemiology	health care facility	43	Undirected
heart rate	hospital admission	43	Undirected
hospital discharge	chronic obstructive lung disease	43	Undirected
hospital infection	antibiotic agent	43	Undirected
hospitalization	Health Personnel	43	Undirected
hospitalization	antiinfective agent	43	Undirected
hospitalization	teaching hospital	43	Undirected
knowledge	COVID-19	43	Undirected
knowledge	Health Personnel	43	Undirected
long term care	COVID-19	43	Undirected
medical education	emergency health service	43	Undirected
medical education	hospital admission	43	Undirected
medical staff	standards	43	Undirected

mortality	adverse event	43	Undirected
mortality	computer assisted tomography	43	Undirected
multicenter study	disease severity	43	Undirected
multicenter study	standards	43	Undirected
organization and management	clinical article	43	Undirected
outcome assessment	acute kidney failure	43	Undirected
outcome assessment	preschool child	43	Undirected
oxygen saturation	computer assisted tomography	43	Undirected
pneumonia	thorax radiography	43	Undirected
pneumonia	ventilator associated pneumonia	43	Undirected
practice guideline	health care policy	43	Undirected
practice guideline	preschool child	43	Undirected
tertiary care center	clinical practice	43	Undirected
therapy	cohort analysis	43	Undirected
Adaptation, Psychological	caregiver	42	Undirected
Anti-Bacterial Agents	vancomycin	42	Undirected
Attitude of Health Personnel	emergency care	42	Undirected
Attitude of Health Personnel	epidemiology	42	Undirected
Attitude of Health Personnel	health care delivery	42	Undirected
Australia	practice guideline	42	Undirected
Clinical Competence	Clinical Protocols	42	Undirected
Clinical Competence	clinical practice	42	Undirected
Communication	Practice Guidelines as Topic	42	Undirected
Cross Infection	standards	42	Undirected
Cross Infection	tertiary care center	42	Undirected
Cross Infection	ventilator associated pneumonia	42	Undirected
Decision Making	Canada	42	Undirected
Delirium	disease severity	42	Undirected
Emergency Service, Hospital	Risk Assessment	42	Undirected
Fatigue	Pandemics	42	Undirected
Guideline Adherence	total quality management	42	Undirected
alth Knowledge, Attitudes, Practi	organization and management	42	Undirected
Health Personnel	health care delivery	42	Undirected
Hospital Mortality	Quality Improvement	42	Undirected
Hypnotics and Sedatives	Pain Measurement	42	Undirected
Infection Control	hospital admission	42	Undirected
Leadership	medical education	42	Undirected
Length of Stay	Program Evaluation	42	Undirected
Length of Stay	health care planning	42	Undirected
Length of Stay	teaching hospital	42	Undirected
Monitoring, Physiologic	Postoperative Care	42	Undirected
Monitoring, Physiologic	Risk Assessment	42	Undirected
Pain	methodology	42	Undirected
Palliative Care	Health Personnel	42	Undirected
Pandemics	clinical practice	42	Undirected
Pandemics	respiratory therapist	42	Undirected
Pandemics	university hospital	42	Undirected
Postoperative Complications	very elderly	42	Undirected
Professional-Family Relations	caregiver	42	Undirected
Professional-Family Relations	education	42	Undirected
Program Evaluation	Needs Assessment	42	Undirected
Program Evaluation	Pilot Projects	42	Undirected
Risk Factors	Heart Arrest	42	Undirected
Risk Factors	caregiver	42	Undirected

Risk Factors	sedation	42	Undirected
Sepsis	fever	42	Undirected
Social Support	COVID-19	42	Undirected
Stress, Psychological	Health Personnel	42	Undirected
Terminal Care	clinical article	42	Undirected
Time Factors	Cardiac Surgical Procedures	42	Undirected
Time Factors	Personnel Staffing and Scheduling	42	Undirected
Treatment Outcome	evidence based practice	42	Undirected
Treatment Outcome	hypertension	42	Undirected
Treatment Outcome	psychology	42	Undirected
United Kingdom	methodology	42	Undirected
Workload	perception	42	Undirected
Workplace	Health Personnel	42	Undirected
attitude to health	Infant, Premature	42	Undirected
clinical article	extracorporeal oxygenation	42	Undirected
diabetes mellitus	emergency ward	42	Undirected
diabetes mellitus	very elderly	42	Undirected
dyspnea	computer assisted tomography	42	Undirected
education	emergency ward	42	Undirected
education	university hospital	42	Undirected
emergency ward	breathing rate	42	Undirected
endotracheal intubation	hospital admission	42	Undirected
follow up	acute kidney failure	42	Undirected
follow up	home care	42	Undirected
follow up	medical education	42	Undirected
health care planning	health care system	42	Undirected
health care quality	epidemiology	42	Undirected
health care system	disease severity	42	Undirected
home care	COVID-19	42	Undirected
hospital discharge	preschool child	42	Undirected
hospitalization	Practice Guidelines as Topic	42	Undirected
hospitalization	adverse event	42	Undirected
hospitalization	enteric feeding	42	Undirected
interview	university hospital	42	Undirected
medical education	university hospital	42	Undirected
mortality	tracheostomy	42	Undirected
observational study	pilot study	42	Undirected
pediatrics	hospital admission	42	Undirected
practice guideline	information processing	42	Undirected
practice guideline	psychological aspect	42	Undirected
prematurity	psychological aspect	42	Undirected
prevalence	Child, Preschool	42	Undirected
prevalence	Sepsis	42	Undirected
prevalence	health care system	42	Undirected
prevalence	hospital infection	42	Undirected
psychology	age	42	Undirected
resuscitation	extracorporeal oxygenation	42	Undirected
safety	methodology	42	Undirected
standards	hospital admission	42	Undirected
Artificial Ventilation	Infant, Premature	41	Undirected
Artificial Ventilation	pharmacist	41	Undirected
Artificial Ventilation	vancomycin	41	Undirected
COVID-19	acute kidney failure	41	Undirected
Child, Preschool	Hospital Mortality	41	Undirected

Child, Preschool	outcome assessment	41	Undirected
Communication	caregiver	41	Undirected
Decision Making	hospital admission	41	Undirected
Delirium	midazolam	41	Undirected
Delirium	organization and management	41	Undirected
Electronic Health Records	COVID-19	41	Undirected
Emergency Service, Hospital	cohort analysis	41	Undirected
Family	Child, Preschool	41	Undirected
Family	ethics	41	Undirected
Focus Groups	Terminal Care	41	Undirected
Guideline Adherence	Catheterization, Central Venous	41	Undirected
Heart Arrest	clinical article	41	Undirected
Hospital Mortality	resuscitation	41	Undirected
Hospital Mortality	total quality management	41	Undirected
Leadership	health care delivery	41	Undirected
Length of Stay	hypotension	41	Undirected
Length of Stay	medical staff	41	Undirected
Monitoring, Physiologic	practice guideline	41	Undirected
Pandemics	health care planning	41	Undirected
Postoperative Complications	heart surgery	41	Undirected
Practice Guidelines as Topic	Health Personnel	41	Undirected
Practice Guidelines as Topic	resuscitation	41	Undirected
Professional-Family Relations	Mothers	41	Undirected
Quality Improvement	cohort analysis	41	Undirected
Risk Factors	Workplace	41	Undirected
Social Support	interpersonal communication	41	Undirected
Social Support	interview	41	Undirected
Time Factors	Postoperative Care	41	Undirected
Treatment Outcome	Pilot Projects	41	Undirected
Treatment Outcome	adverse event	41	Undirected
Treatment Outcome	emergency health service	41	Undirected
Treatment Outcome	heart rate	41	Undirected
Treatment Outcome	pediatrics	41	Undirected
Treatment Outcome	university hospital	41	Undirected
Workload	health care quality	41	Undirected
Workplace	Pandemics	41	Undirected
adverse event	randomized controlled trial	41	Undirected
attitude to health	clinical practice	41	Undirected
clinical practice	respiratory therapist	41	Undirected
diabetes mellitus	fever	41	Undirected
diabetes mellitus	pneumonia	41	Undirected
education	pilot study	41	Undirected
emergency care	Pandemics	41	Undirected
emergency care	follow up	41	Undirected
emergency care	health care system	41	Undirected
epidemiology	health care system	41	Undirected
epidemiology	university hospital	41	Undirected
gestational age	hospital admission	41	Undirected
health care cost	randomized controlled trial	41	Undirected
heart failure	hypertension	41	Undirected
home care	organization and management	41	Undirected
hospitalization	Infant, Premature	41	Undirected
hospitalization	Telemedicine	41	Undirected
hypertension	computer assisted tomography	41	Undirected

interview	follow up	41	Undirected
interview	medical education	41	Undirected
long term care	hospital admission	41	Undirected
mental health	follow up	41	Undirected
mortality	dyspnea	41	Undirected
mortality	medical education	41	Undirected
mortality	urinary tract infection	41	Undirected
observational study	Pandemics	41	Undirected
outcome assessment	Terminal Care	41	Undirected
outcome assessment	diabetes mellitus	41	Undirected
outcome assessment	pediatrics	41	Undirected
pain assessment	randomized controlled trial	41	Undirected
pharmacist	Pandemics	41	Undirected
pharmacist	clinical practice	41	Undirected
prematurity	pain assessment	41	Undirected
prevalence	hypertension	41	Undirected
standards	cohort analysis	41	Undirected
total quality management	Terminal Care	41	Undirected
total quality management	respiratory therapist	41	Undirected
tracheostomy	endotracheal intubation	41	Undirected
vancomycin	hospital infection	41	Undirected
Adaptation, Psychological	interview	40	Undirected
Anti-Bacterial Agents	thicillin resistant Staphylococcus aure	40	Undirected
Anxiety	cohort analysis	40	Undirected
Artificial Ventilation	Pressure Ulcer	40	Undirected
Artificial Ventilation	Sensitivity and Specificity	40	Undirected
Attitude of Health Personnel	hospitalization	40	Undirected
Australia	Focus Groups	40	Undirected
Australia	clinical practice	40	Undirected
Child, Preschool	Intubation, Intratracheal	40	Undirected
Child, Preschool	mortality	40	Undirected
Child, Preschool	practice guideline	40	Undirected
Clinical Competence	Delirium	40	Undirected
Clinical Competence	Pain	40	Undirected
Clinical Competence	Terminal Care	40	Undirected
Communication	Anxiety	40	Undirected
Communication	semi structured interview	40	Undirected
Communication	standards	40	Undirected
Cross Infection	methodology	40	Undirected
Cross Infection	total quality management	40	Undirected
Decision Making	emergency ward	40	Undirected
Decision Making	standards	40	Undirected
Emergency Service, Hospital	COVID-19	40	Undirected
Family	Practice Guidelines as Topic	40	Undirected
Family	hospital admission	40	Undirected
Guideline Adherence	Sepsis	40	Undirected
Heart Arrest	cohort analysis	40	Undirected
Heart Arrest	heart rate	40	Undirected
Hospital Mortality	Glasgow coma scale	40	Undirected
Hospital Mortality	follow up	40	Undirected
Hospital Mortality	statistics and numerical data	40	Undirected
Infant, Premature	Quality Improvement	40	Undirected
Infection Control	clinical practice	40	Undirected
Length of Stay	medical education	40	Undirected

Pain	gestational age	40	Undirected
Palliative Care	Health Knowledge, Attitudes, Practice	40	Undirected
Pandemics	hand washing	40	Undirected
Parents	clinical article	40	Undirected
Pilot Projects	randomized controlled trial	40	Undirected
Quality Improvement	Electronic Health Records	40	Undirected
Quality Improvement	Health Personnel	40	Undirected
Risk Assessment	Pandemics	40	Undirected
Risk Assessment	pediatrics	40	Undirected
Risk Assessment	pneumonia	40	Undirected
Risk Assessment	university hospital	40	Undirected
Risk Factors	Emergency Service, Hospital	40	Undirected
Risk Factors	hypotension	40	Undirected
Risk Factors	knowledge	40	Undirected
Sepsis	epidemiology	40	Undirected
Sepsis	urinary tract infection	40	Undirected
Social Support	Job Satisfaction	40	Undirected
Stress, Psychological	ethics	40	Undirected
Time Factors	Logistic Models	40	Undirected
Time Factors	Program Evaluation	40	Undirected
Treatment Outcome	analgesia	40	Undirected
Treatment Outcome	emergency care	40	Undirected
Treatment Outcome	hypotension	40	Undirected
United Kingdom	Needs Assessment	40	Undirected
adverse event	epidemiology	40	Undirected
attitude to health	interview	40	Undirected
attitude to health	standards	40	Undirected
blood pressure	hypertension	40	Undirected
cerebrovascular accident	very elderly	40	Undirected
clinical article	enteric feeding	40	Undirected
clinical practice	palliative therapy	40	Undirected
education	emergency care	40	Undirected
epidemiology	age	40	Undirected
ethics	psychological aspect	40	Undirected
fever	Fatigue	40	Undirected
follow up	health care system	40	Undirected
hand washing	hospital personnel	40	Undirected
health care quality	disease severity	40	Undirected
heart failure	hospital discharge	40	Undirected
heart rate	cohort analysis	40	Undirected
heart rate	emergency ward	40	Undirected
hospital admission	interpersonal communication	40	Undirected
hospitalization	OUTCOMES	40	Undirected
hospitalization	Reproducibility of Results	40	Undirected
hospitalization	ethicillin resistant Staphylococcus aure	40	Undirected
hospitalization	sedation	40	Undirected
hospitalization	vancomycin	40	Undirected
hospitalization	ventilator associated pneumonia	40	Undirected
long term care	hospital discharge	40	Undirected
medical staff	resuscitation	40	Undirected
mental health	epidemiology	40	Undirected
outcome assessment	Glasgow coma scale	40	Undirected
outcome assessment	pilot study	40	Undirected
pediatrics	clinical article	40	Undirected

pediatrics	emergency care	40	Undirected
perception	health care quality	40	Undirected
physiotherapy	hospital discharge	40	Undirected
practice guideline	respiratory therapist	40	Undirected
practice guideline	semi structured interview	40	Undirected
practice guideline	statistics and numerical data	40	Undirected
prevalence	health care facility	40	Undirected
prevalence	pneumonia	40	Undirected
psychology	health care system	40	Undirected
resuscitation	oxygen saturation	40	Undirected
total quality management	psychology	40	Undirected
Algorithms	methodology	39	Undirected
Anxiety	disease severity	39	Undirected
Anxiety	follow up	39	Undirected
Anxiety	hospital discharge	39	Undirected
Attitude of Health Personnel	hand washing	39	Undirected
Child, Preschool	Hypnotics and Sedatives	39	Undirected
China	knowledge	39	Undirected
China	psychology	39	Undirected
Clinical Competence	perception	39	Undirected
Clinical Competence	psychology	39	Undirected
Clinical Protocols	sedation	39	Undirected
Delirium	Clinical Protocols	39	Undirected
Delirium	epidemiology	39	Undirected
Guideline Adherence	evidence based practice	39	Undirected
alth Knowledge, Attitudes, Practi	Infant, Premature	39	Undirected
alth Knowledge, Attitudes, Practi	Pressure Ulcer	39	Undirected
Health Personnel	clinical practice	39	Undirected
Health Personnel	interpersonal communication	39	Undirected
Heart Arrest	practice guideline	39	Undirected
Hospital Mortality	tertiary care center	39	Undirected
Infection Control	Clinical Protocols	39	Undirected
Infection Control	clinical article	39	Undirected
Infection Control	university hospital	39	Undirected
Job Satisfaction	China	39	Undirected
Length of Stay	health care policy	39	Undirected
Length of Stay	safety	39	Undirected
Monitoring, Physiologic	Pilot Projects	39	Undirected
OUTCOMES	outcome assessment	39	Undirected
Pain	dyspnea	39	Undirected
Palliative Care	pediatrics	39	Undirected
Parents	Pain	39	Undirected
Quality Improvement	COVID-19	39	Undirected
Quality Improvement	interpersonal communication	39	Undirected
Reproducibility of Results	pain assessment	39	Undirected
Risk Assessment	emergency care	39	Undirected
Risk Assessment	meta analysis	39	Undirected
Risk Factors	Algorithms	39	Undirected
Risk Factors	Catheter-Related Infections	39	Undirected
Risk Factors	United Kingdom	39	Undirected
Risk Factors	emergency health service	39	Undirected
Sepsis	Clinical Protocols	39	Undirected
Sepsis	multicenter study	39	Undirected
Sepsis	standards	39	Undirected

Social Support	Cooperative Behavior	39	Undirected
Stress, Psychological	Pandemics	39	Undirected
Time Factors	Heart Arrest	39	Undirected
Time Factors	resuscitation	39	Undirected
Treatment Outcome	antibiotic agent	39	Undirected
Treatment Outcome	statistics and numerical data	39	Undirected
Treatment Outcome	tracheostomy	39	Undirected
Workplace	prevalence	39	Undirected
attitude to health	Practice Guidelines as Topic	39	Undirected
attitude to health	total quality management	39	Undirected
caregiver	education	39	Undirected
cohort analysis	breathing rate	39	Undirected
cohort analysis	palliative therapy	39	Undirected
dementia	hypertension	39	Undirected
dyspnea	Pandemics	39	Undirected
education	semi structured interview	39	Undirected
emergency care	epidemiology	39	Undirected
emergency ward	palliative therapy	39	Undirected
emergency ward	thorax radiography	39	Undirected
endotracheal intubation	thorax radiography	39	Undirected
ethics	Pandemics	39	Undirected
ethics	organization and management	39	Undirected
gestational age	observational study	39	Undirected
health care system	emergency health service	39	Undirected
home care	hospital readmission	39	Undirected
hospital admission	acute kidney failure	39	Undirected
hospital admission	antiinfective agent	39	Undirected
hospital discharge	acute kidney failure	39	Undirected
hospitalization	evidence based practice	39	Undirected
hospitalization	medical education	39	Undirected
long term care	home care	39	Undirected
outcome assessment	Electronic Health Records	39	Undirected
outcome assessment	Pandemics	39	Undirected
outcome assessment	adverse event	39	Undirected
outcome assessment	cerebrovascular accident	39	Undirected
outcome assessment	medical staff	39	Undirected
oxygen saturation	thorax radiography	39	Undirected
perception	Pandemics	39	Undirected
pneumonia	chronic obstructive lung disease	39	Undirected
pneumonia	practice guideline	39	Undirected
practice guideline	morphine	39	Undirected
prematurity	resuscitation	39	Undirected
prevalence	China	39	Undirected
prevalence	dyspnea	39	Undirected
prevalence	long term care	39	Undirected
propofol	clinical article	39	Undirected
psychology	preschool child	39	Undirected
quality of life	Risk Assessment	39	Undirected
standards	university hospital	39	Undirected
statistics and numerical data	cohort analysis	39	Undirected
tertiary care center	follow up	39	Undirected
vancomycin	hospital admission	39	Undirected
Adaptation, Psychological	Health Knowledge, Attitudes, Practice	38	Undirected
Adaptation, Psychological	Needs Assessment	38	Undirected

Algorithms	practice guideline	38	Undirected
Anti-Bacterial Agents	pneumonia	38	Undirected
Anxiety	Terminal Care	38	Undirected
Anxiety	dyspnea	38	Undirected
Anxiety	education	38	Undirected
Anxiety	hospital admission	38	Undirected
Artificial Ventilation	Enteral Nutrition	38	Undirected
Artificial Ventilation	Palliative Care	38	Undirected
Australia	Terminal Care	38	Undirected
Australia	hospital admission	38	Undirected
Child, Preschool	clinical article	38	Undirected
Clinical Competence	COVID-19	38	Undirected
Clinical Competence	Infection Control	38	Undirected
Clinical Competence	attitude	38	Undirected
Clinical Competence	psychological aspect	38	Undirected
Cross Infection	hospital admission	38	Undirected
Decision Making	Australia	38	Undirected
Decision Making	knowledge	38	Undirected
Decision Making	total quality management	38	Undirected
Enteral Nutrition	Clinical Protocols	38	Undirected
Family	multicenter study	38	Undirected
Family	standards	38	Undirected
Guideline Adherence	Catheter-Related Infections	38	Undirected
Guideline Adherence	organization and management	38	Undirected
Health Services Needs and Demand	total quality management	38	Undirected
Hospital Mortality	age	38	Undirected
Infant, Premature	caregiver	38	Undirected
Infant, Premature	education	38	Undirected
Infection Control	knowledge	38	Undirected
Leadership	psychology	38	Undirected
Pain	palliative therapy	38	Undirected
Pain Measurement	methodology	38	Undirected
Palliative Care	Pain	38	Undirected
Palliative Care	knowledge	38	Undirected
Parents	Length of Stay	38	Undirected
Parents	Needs Assessment	38	Undirected
Pregnancy	education	38	Undirected
Pressure Ulcer	Reproducibility of Results	38	Undirected
Professional-Family Relations	Australia	38	Undirected
Quality Improvement	adverse event	38	Undirected
Quality of Health Care	Workplace	38	Undirected
Quality of Health Care	total quality management	38	Undirected
Risk Factors	Decision Making	38	Undirected
Sepsis	health care cost	38	Undirected
Treatment Outcome	Decision Making	38	Undirected
Treatment Outcome	Stroke	38	Undirected
Treatment Outcome	therapy	38	Undirected
United Kingdom	standards	38	Undirected
Workforce	Leadership	38	Undirected
analgesia	clinical practice	38	Undirected
analgesia	palliative therapy	38	Undirected
attitude to health	Anxiety	38	Undirected
clinical article	information processing	38	Undirected
depression	dementia	38	Undirected

education	hospital discharge	38	Undirected
endotracheal intubation	methodology	38	Undirected
epidemiology	hospital readmission	38	Undirected
evidence based practice	health care quality	38	Undirected
gestational age	heart rate	38	Undirected
health care cost	very elderly	38	Undirected
health care system	organization and management	38	Undirected
hospital admission	hospital personnel	38	Undirected
hospital infection	antiinfective agent	38	Undirected
hospitalization	Stroke	38	Undirected
hypotension	practice guideline	38	Undirected
intensivist	organization and management	38	Undirected
interpersonal communication	information processing	38	Undirected
interview	standards	38	Undirected
long term care	practice guideline	38	Undirected
methodology	hospital discharge	38	Undirected
mortality	pharmacist	38	Undirected
multicenter study	university hospital	38	Undirected
outcome assessment	interview	38	Undirected
pain assessment	methodology	38	Undirected
pain assessment	practice guideline	38	Undirected
pharmacist	follow up	38	Undirected
pharmacist	intensivist	38	Undirected
practice guideline	intensivist	38	Undirected
prevalence	health care cost	38	Undirected
prevalence	university hospital	38	Undirected
psychology	health care delivery	38	Undirected
quality of life	health care delivery	38	Undirected
quality of life	observational study	38	Undirected
randomized controlled trial	therapy	38	Undirected
resuscitation	respiratory therapist	38	Undirected
safety	COVID-19	38	Undirected
tertiary care center	hand washing	38	Undirected
tertiary care center	medical staff	38	Undirected
tertiary care center	organization and management	38	Undirected
thematic analysis	interpersonal communication	38	Undirected
total quality management	emergency care	38	Undirected
total quality management	palliative therapy	38	Undirected
total quality management	university hospital	38	Undirected
tracheostomy	follow up	38	Undirected
urinary tract infection	hospital infection	38	Undirected
vancomycin	antibiotic agent	38	Undirected
ventilator associated pneumonia	randomized controlled trial	38	Undirected
Adaptation, Psychological	Terminal Care	37	Undirected
Anxiety	palliative therapy	37	Undirected
Anxiety	psychological aspect	37	Undirected
Artificial Ventilation	antiinfective agent	37	Undirected
Artificial Ventilation	knowledge	37	Undirected
Artificial Ventilation	safety	37	Undirected
Attitude of Health Personnel	emergency ward	37	Undirected
COVID-19	hospital infection	37	Undirected
Clinical Competence	Emergency Service, Hospital	37	Undirected
Clinical Competence	interpersonal communication	37	Undirected
Communication	simulation	37	Undirected

Delirium	Pilot Projects	37	Undirected
Delirium	depression	37	Undirected
Emergency Service, Hospital	Quality Improvement	37	Undirected
Family	Risk Factors	37	Undirected
Family	thematic analysis	37	Undirected
Fatigue	depression	37	Undirected
alth Knowledge, Attitudes, Practi	Needs Assessment	37	Undirected
alth Knowledge, Attitudes, Practi	perception	37	Undirected
Health Personnel	medical education	37	Undirected
Health Personnel	semi structured interview	37	Undirected
Infection Control	fever	37	Undirected
Infection Control	pneumonia	37	Undirected
Interprofessional Relations	Adaptation, Psychological	37	Undirected
Length of Stay	Monitoring, Physiologic	37	Undirected
Length of Stay	Pregnancy	37	Undirected
Length of Stay	United Kingdom	37	Undirected
Length of Stay	fentanyl	37	Undirected
Monitoring, Physiologic	Sensitivity and Specificity	37	Undirected
Mothers	gestational age	37	Undirected
Palliative Care	clinical article	37	Undirected
Palliative Care	organization and management	37	Undirected
Parents	hospital discharge	37	Undirected
Postoperative Complications	adverse event	37	Undirected
Practice Guidelines as Topic	Pregnancy	37	Undirected
Practice Guidelines as Topic	Safety Management	37	Undirected
Pregnancy	outcome assessment	37	Undirected
Quality of Health Care	hospitalization	37	Undirected
Risk Assessment	observational study	37	Undirected
Risk Assessment	psychology	37	Undirected
Risk Factors	Adaptation, Psychological	37	Undirected
Risk Factors	Australia	37	Undirected
Risk Factors	Health Knowledge, Attitudes, Practice	37	Undirected
Risk Factors	tracheostomy	37	Undirected
Stress, Psychological	Decision Making	37	Undirected
Time Factors	Enteral Nutrition	37	Undirected
Time Factors	Health Knowledge, Attitudes, Practice	37	Undirected
Time Factors	total quality management	37	Undirected
Treatment Outcome	Workload	37	Undirected
Treatment Outcome	fever	37	Undirected
Treatment Outcome	health care delivery	37	Undirected
Workload	medical staff	37	Undirected
Workload	psychology	37	Undirected
adverse event	very elderly	37	Undirected
attitude to health	Artificial Ventilation	37	Undirected
attitude to health	attitude	37	Undirected
caregiver	hospital admission	37	Undirected
cohort analysis	antiinfective agent	37	Undirected
dementia	palliative therapy	37	Undirected
depression	hospital admission	37	Undirected
depression	randomized controlled trial	37	Undirected
dyspnea	palliative therapy	37	Undirected
education	health care delivery	37	Undirected
emergency care	very elderly	37	Undirected
emergency health service	very elderly	37	Undirected

emergency ward	teaching hospital	37	Undirected
ethics	palliative therapy	37	Undirected
ethics	resuscitation	37	Undirected
evidence based practice	organization and management	37	Undirected
fever	cohort analysis	37	Undirected
health care delivery	emergency health service	37	Undirected
health care delivery	semi structured interview	37	Undirected
health care facility	hospital discharge	37	Undirected
health care quality	teaching hospital	37	Undirected
heart rate	observational study	37	Undirected
home care	very elderly	37	Undirected
hospital discharge	therapy	37	Undirected
hospital discharge	thorax radiography	37	Undirected
hospitalization	Australia	37	Undirected
hospitalization	United Kingdom	37	Undirected
hypotension	hospital admission	37	Undirected
interpersonal communication	emergency health service	37	Undirected
medical education	health care policy	37	Undirected
observational study	health care quality	37	Undirected
oxygen saturation	cohort analysis	37	Undirected
perception	multicenter study	37	Undirected
pharmacist	health care quality	37	Undirected
pneumonia	dyspnea	37	Undirected
prematurity	Pain Measurement	37	Undirected
prematurity	standards	37	Undirected
prevalence	Job Satisfaction	37	Undirected
prevalence	Workload	37	Undirected
quality of life	heart failure	37	Undirected
quality of life	organization and management	37	Undirected
randomized controlled trial	clinical practice	37	Undirected
randomized controlled trial	emergency ward	37	Undirected
randomized controlled trial	oxygen saturation	37	Undirected
resuscitation	methodology	37	Undirected
tertiary care center	psychology	37	Undirected
tertiary care center	statistics and numerical data	37	Undirected
total quality management	health care system	37	Undirected
total quality management	information processing	37	Undirected
total quality management	prematurity	37	Undirected
Anxiety	analgesia	36	Undirected
Artificial Ventilation	Postoperative Complications	36	Undirected
Artificial Ventilation	health care planning	36	Undirected
Attitude of Health Personnel	burnout	36	Undirected
Attitude of Health Personnel	outcome assessment	36	Undirected
Australia	education	36	Undirected
Australia	mortality	36	Undirected
Breast Feeding	hospital discharge	36	Undirected
Clinical Competence	Palliative Care	36	Undirected
Clinical Competence	Treatment Outcome	36	Undirected
Clinical Protocols	hospital admission	36	Undirected
Cross Infection	observational study	36	Undirected
Cross Infection	organization and management	36	Undirected
Decision Making	information processing	36	Undirected
Delirium	knowledge	36	Undirected
Delirium	observational study	36	Undirected

Enteral Nutrition	practice guideline	36	Undirected
Family	Canada	36	Undirected
Focus Groups	psychology	36	Undirected
Glasgow coma scale	clinical article	36	Undirected
Glasgow coma scale	emergency health service	36	Undirected
Glasgow coma scale	follow up	36	Undirected
alth Knowledge, Attitudes, Practi	Pain	36	Undirected
Health Personnel	clinical article	36	Undirected
Health Personnel	follow up	36	Undirected
Health Personnel	health care quality	36	Undirected
Health Services Needs and Demand	Cooperative Behavior	36	Undirected
Heart Arrest	medical education	36	Undirected
Infant, Premature	Pilot Projects	36	Undirected
Infant, Premature	organization and management	36	Undirected
Infection Control	cohort analysis	36	Undirected
Infection Control	emergency ward	36	Undirected
Leadership	interview	36	Undirected
Length of Stay	China	36	Undirected
Length of Stay	Needs Assessment	36	Undirected
Logistic Models	mortality	36	Undirected
Mothers	hospital discharge	36	Undirected
OUTCOMES	hospital admission	36	Undirected
Pain	clinical practice	36	Undirected
Pain	midazolam	36	Undirected
Pain	observational study	36	Undirected
Pain Measurement	sedation	36	Undirected
Pandemics	hospital personnel	36	Undirected
Pilot Projects	clinical article	36	Undirected
Postoperative Care	clinical article	36	Undirected
Postoperative Complications	hospital admission	36	Undirected
Practice Guidelines as Topic	Anti-Bacterial Agents	36	Undirected
Practice Guidelines as Topic	Terminal Care	36	Undirected
Pregnancy	COVID-19	36	Undirected
Pressure Ulcer	knowledge	36	Undirected
Program Evaluation	United Kingdom	36	Undirected
Quality of Health Care	Time Factors	36	Undirected
Risk Assessment	resuscitation	36	Undirected
Risk Factors	Reproducibility of Results	36	Undirected
Risk Factors	acute kidney failure	36	Undirected
Risk Factors	health care policy	36	Undirected
Risk Factors	thorax radiography	36	Undirected
Sensitivity and Specificity	hospital admission	36	Undirected
Sepsis	organization and management	36	Undirected
Sepsis	vancomycin	36	Undirected
Social Support	depression	36	Undirected
Social Support	education	36	Undirected
Treatment Outcome	Glasgow coma scale	36	Undirected
Treatment Outcome	Logistic Models	36	Undirected
Treatment Outcome	acute kidney failure	36	Undirected
United Kingdom	psychological aspect	36	Undirected
United Kingdom	psychology	36	Undirected
Workforce	Australia	36	Undirected
Workforce	United Kingdom	36	Undirected
Workforce	Workplace	36	Undirected

attitude to health	perception	36	Undirected
caregiver	health care delivery	36	Undirected
clinical practice	health care planning	36	Undirected
diabetes mellitus	epidemiology	36	Undirected
dyspnea	breathing rate	36	Undirected
emergency ward	hospital personnel	36	Undirected
epidemiology	emergency health service	36	Undirected
ethics	long term care	36	Undirected
fever	epidemiology	36	Undirected
follow up	palliative therapy	36	Undirected
health care cost	emergency ward	36	Undirected
health care cost	health care planning	36	Undirected
health care cost	medical education	36	Undirected
health care quality	very elderly	36	Undirected
hospital admission	health care policy	36	Undirected
hospital admission	therapy	36	Undirected
hospital readmission	disease severity	36	Undirected
hospitalization	Insulin	36	Undirected
hospitalization	Medication Errors	36	Undirected
hospitalization	pilot study	36	Undirected
interview	knowledge	36	Undirected
long term care	follow up	36	Undirected
medical education	health care planning	36	Undirected
medical education	standards	36	Undirected
medical staff	clinical article	36	Undirected
medical staff	emergency health service	36	Undirected
midazolam	practice guideline	36	Undirected
mortality	intensivist	36	Undirected
observational study	Health Personnel	36	Undirected
observational study	age	36	Undirected
observational study	organization and management	36	Undirected
organization and management	health care policy	36	Undirected
outcome assessment	Pain	36	Undirected
outcome assessment	home care	36	Undirected
outcome assessment	meta analysis	36	Undirected
outcome assessment	sedation	36	Undirected
pediatrics	health care quality	36	Undirected
perception	organization and management	36	Undirected
perception	university hospital	36	Undirected
pilot study	organization and management	36	Undirected
prematurity	analgesia	36	Undirected
prevalence	Hospital Mortality	36	Undirected
prevalence	meta analysis	36	Undirected
prevalence	ethicillin resistant Staphylococcus aure	36	Undirected
propofol	hypotension	36	Undirected
quality of life	Hospital Mortality	36	Undirected
quality of life	epidemiology	36	Undirected
quality of life	long term care	36	Undirected
quality of life	resuscitation	36	Undirected
randomized controlled trial	cohort analysis	36	Undirected
resuscitation	standards	36	Undirected
tertiary care center	hospital discharge	36	Undirected
therapy	organization and management	36	Undirected
total quality management	clinical article	36	Undirected

total quality management	safety	36	Undirected
tracheostomy	cohort analysis	36	Undirected
Artificial Ventilation	Cardiac Surgical Procedures	35	Undirected
Attitude of Health Personnel	Pain Measurement	35	Undirected
Canada	COVID-19	35	Undirected
Child, Preschool	Delirium	35	Undirected
Clinical Competence	Safety Management	35	Undirected
Communication	Pilot Projects	35	Undirected
Communication	Safety Management	35	Undirected
Cooperative Behavior	Job Satisfaction	35	Undirected
Cross Infection	COVID-19	35	Undirected
Decision Making	simulation	35	Undirected
Decision Making	university hospital	35	Undirected
Emergency Service, Hospital	practice guideline	35	Undirected
Glasgow coma scale	computer assisted tomography	35	Undirected
Guideline Adherence	observational study	35	Undirected
Health Personnel	respiratory therapist	35	Undirected
Health Personnel	statistics and numerical data	35	Undirected
Health Personnel	university hospital	35	Undirected
Hospital Mortality	Clinical Protocols	35	Undirected
Hospital Mortality	Electronic Health Records	35	Undirected
Infant, Premature	clinical article	35	Undirected
Infection Control	evidence based practice	35	Undirected
Leadership	evidence based practice	35	Undirected
Leadership	practice guideline	35	Undirected
Length of Stay	Canada	35	Undirected
Length of Stay	Insulin	35	Undirected
Mothers	education	35	Undirected
Pain Measurement	morphine	35	Undirected
Palliative Care	ethics	35	Undirected
Pandemics	extracorporeal oxygenation	35	Undirected
Parents	organization and management	35	Undirected
Postoperative Complications	randomized controlled trial	35	Undirected
Practice Guidelines as Topic	COVID-19	35	Undirected
Pregnancy	follow up	35	Undirected
Program Evaluation	Clinical Protocols	35	Undirected
Quality Improvement	clinical practice	35	Undirected
Risk Assessment	emergency health service	35	Undirected
Risk Assessment	hospital readmission	35	Undirected
Risk Factors	Fatigue	35	Undirected
Risk Factors	medical staff	35	Undirected
Sepsis	Delirium	35	Undirected
Sepsis	gestational age	35	Undirected
Sepsis	hospital readmission	35	Undirected
Social Support	Burnout, Professional	35	Undirected
Terminal Care	semi structured interview	35	Undirected
Time Factors	Decision Making	35	Undirected
Treatment Outcome	Algorithms	35	Undirected
Treatment Outcome	Infection Control	35	Undirected
Treatment Outcome	Program Evaluation	35	Undirected
Treatment Outcome	age	35	Undirected
Treatment Outcome	chronic obstructive lung disease	35	Undirected
Treatment Outcome	long term care	35	Undirected
United Kingdom	mortality	35	Undirected

antibiotic agent	thorax radiography	35	Undirected
antibiotic agent	very elderly	35	Undirected
burnout	Fatigue	35	Undirected
caregiver	semi structured interview	35	Undirected
clinical practice	cohort analysis	35	Undirected
dementia	COVID-19	35	Undirected
dementia	cohort analysis	35	Undirected
depression	cohort analysis	35	Undirected
depression	hospital discharge	35	Undirected
disease severity	palliative therapy	35	Undirected
education	observational study	35	Undirected
emergency care	clinical article	35	Undirected
emergency care	interpersonal communication	35	Undirected
endotracheal intubation	emergency ward	35	Undirected
epidemiology	methodology	35	Undirected
ethics	practice guideline	35	Undirected
evidence based practice	ventilator associated pneumonia	35	Undirected
fentanyl	clinical article	35	Undirected
fentanyl	pain assessment	35	Undirected
fentanyl	practice guideline	35	Undirected
follow up	age	35	Undirected
health care quality	home care	35	Undirected
health care quality	multicenter study	35	Undirected
heart rate	hypotension	35	Undirected
hospital discharge	health care planning	35	Undirected
hospitalization	Mothers	35	Undirected
hospitalization	Postoperative Care	35	Undirected
hospitalization	extracorporeal oxygenation	35	Undirected
hospitalization	hospital personnel	35	Undirected
hospitalization	knowledge	35	Undirected
hypertension	Pandemics	35	Undirected
hypertension	epidemiology	35	Undirected
interview	emergency care	35	Undirected
interview	epidemiology	35	Undirected
long term care	cohort analysis	35	Undirected
long term care	palliative therapy	35	Undirected
medical staff	psychological aspect	35	Undirected
cillin resistant Staphylococcus a	microbiology	35	Undirected
mortality	heart rate	35	Undirected
mortality	thorax radiography	35	Undirected
outcome assessment	Stroke	35	Undirected
outcome assessment	heart rate	35	Undirected
pediatrics	simulation	35	Undirected
pediatrics	standards	35	Undirected
pneumonia	antiinfective agent	35	Undirected
practice guideline	extracorporeal oxygenation	35	Undirected
prevalence	Pain	35	Undirected
prevalence	age	35	Undirected
quality of life	Time Factors	35	Undirected
quality of life	health care quality	35	Undirected
quality of life	practice guideline	35	Undirected
randomized controlled trial	endotracheal intubation	35	Undirected
randomized controlled trial	standards	35	Undirected
respiratory therapist	clinical article	35	Undirected

safety	mortality	35	Undirected
total quality management	Electronic Health Records	35	Undirected
total quality management	hospital readmission	35	Undirected
urinary tract infection	hospital admission	35	Undirected
urinary tract infection	practice guideline	35	Undirected
Adaptation, Psychological	Decision Making	34	Undirected
Anti-Bacterial Agents	hospital admission	34	Undirected
Artificial Ventilation	Quality of Health Care	34	Undirected
Artificial Ventilation	intensivist	34	Undirected
Attitude of Health Personnel	emergency health service	34	Undirected
Attitude of Health Personnel	intensivist	34	Undirected
Australia	COVID-19	34	Undirected
Canada	practice guideline	34	Undirected
Child, Preschool	Sensitivity and Specificity	34	Undirected
Child, Preschool	Sepsis	34	Undirected
Child, Preschool	hospital discharge	34	Undirected
Child, Preschool	psychology	34	Undirected
China	epidemiology	34	Undirected
Clinical Competence	evidence based practice	34	Undirected
Communication	clinical article	34	Undirected
Cross Infection	Health Knowledge, Attitudes, Practice	34	Undirected
Cross Infection	multicenter study	34	Undirected
Decision Making	methodology	34	Undirected
Delirium	clinical article	34	Undirected
Emergency Service, Hospital	very elderly	34	Undirected
Enteral Nutrition	randomized controlled trial	34	Undirected
Family	methodology	34	Undirected
Fatigue	hospital admission	34	Undirected
Fatigue	psychology	34	Undirected
Focus Groups	organization and management	34	Undirected
Guideline Adherence	Program Evaluation	34	Undirected
Health Personnel	health care facility	34	Undirected
Health Personnel	hospital personnel	34	Undirected
Hospital Mortality	Delirium	34	Undirected
Hospital Mortality	emergency health service	34	Undirected
Hospital Mortality	health care quality	34	Undirected
Insulin	mortality	34	Undirected
Leadership	burnout	34	Undirected
Length of Stay	interpersonal communication	34	Undirected
Length of Stay	microbiology	34	Undirected
Medication Errors	practice guideline	34	Undirected
Monitoring, Physiologic	Algorithms	34	Undirected
OUTCOMES	cohort analysis	34	Undirected
Palliative Care	dementia	34	Undirected
Parents	Focus Groups	34	Undirected
Postoperative Care	practice guideline	34	Undirected
Postoperative Complications	hospital readmission	34	Undirected
Practice Guidelines as Topic	Catheterization, Central Venous	34	Undirected
Practice Guidelines as Topic	Delirium	34	Undirected
Pregnancy	clinical article	34	Undirected
Pregnancy	cohort analysis	34	Undirected
Pregnancy	psychology	34	Undirected
Professional-Family Relations	Interprofessional Relations	34	Undirected
Professional-Family Relations	perception	34	Undirected

Risk Assessment	clinical article	34	Undirected
Risk Factors	medical education	34	Undirected
Risk Factors	pharmacist	34	Undirected
Risk Factors	psychological aspect	34	Undirected
Risk Factors	therapy	34	Undirected
Social Support	Pandemics	34	Undirected
Social Support	burnout	34	Undirected
Stress, Psychological	Terminal Care	34	Undirected
Terminal Care	Canada	34	Undirected
Terminal Care	Pain	34	Undirected
Terminal Care	dementia	34	Undirected
Terminal Care	medical staff	34	Undirected
Time Factors	Reproducibility of Results	34	Undirected
Time Factors	clinical article	34	Undirected
Treatment Outcome	Enteral Nutrition	34	Undirected
Treatment Outcome	computer assisted tomography	34	Undirected
Workload	Fatigue	34	Undirected
Workplace	epidemiology	34	Undirected
adverse event	multicenter study	34	Undirected
attitude to health	Pain	34	Undirected
attitude to health	Program Evaluation	34	Undirected
caregiver	Health Personnel	34	Undirected
caregiver	home care	34	Undirected
caregiver	randomized controlled trial	34	Undirected
cohort analysis	acute kidney failure	34	Undirected
dementia	psychology	34	Undirected
depression	clinical article	34	Undirected
disease severity	thorax radiography	34	Undirected
dyspnea	very elderly	34	Undirected
education	palliative therapy	34	Undirected
emergency care	health care facility	34	Undirected
emergency health service	disease severity	34	Undirected
emergency ward	health care planning	34	Undirected
emergency ward	respiratory therapist	34	Undirected
evidence based practice	randomized controlled trial	34	Undirected
fever	vancomycin	34	Undirected
fever	very elderly	34	Undirected
follow up	endotracheal intubation	34	Undirected
follow up	interpersonal communication	34	Undirected
follow up	university hospital	34	Undirected
health care cost	clinical practice	34	Undirected
health care cost	disease severity	34	Undirected
heart failure	cohort analysis	34	Undirected
heart failure	diabetes mellitus	34	Undirected
hospital discharge	extracorporeal oxygenation	34	Undirected
hospital infection	cohort analysis	34	Undirected
hospitalization	Algorithms	34	Undirected
hospitalization	China	34	Undirected
hypertension	oxygen saturation	34	Undirected
hypotension	computer assisted tomography	34	Undirected
hypotension	emergency ward	34	Undirected
interview	emergency ward	34	Undirected
long term care	emergency care	34	Undirected
medical staff	health care system	34	Undirected

morphine	clinical article	34	Undirected
mortality	extracorporeal oxygenation	34	Undirected
mortality	heart surgery	34	Undirected
mortality	hypotension	34	Undirected
multicenter study	clinical article	34	Undirected
multicenter study	information processing	34	Undirected
outcome assessment	Australia	34	Undirected
outcome assessment	age	34	Undirected
outcome assessment	health care facility	34	Undirected
outcome assessment	information processing	34	Undirected
outcome assessment	mental health	34	Undirected
pediatrics	Quality Improvement	34	Undirected
pediatrics	observational study	34	Undirected
pneumonia	follow up	34	Undirected
practice guideline	meta analysis	34	Undirected
prematurity	health care quality	34	Undirected
prevalence	dementia	34	Undirected
prevalence	diabetes mellitus	34	Undirected
prevalence	health care quality	34	Undirected
prevalence	randomized controlled trial	34	Undirected
quality of life	Adaptation, Psychological	34	Undirected
randomized controlled trial	very elderly	34	Undirected
resuscitation	multicenter study	34	Undirected
semi structured interview	information processing	34	Undirected
semi structured interview	multicenter study	34	Undirected
tertiary care center	knowledge	34	Undirected
thematic analysis	clinical practice	34	Undirected
therapy	very elderly	34	Undirected
total quality management	Algorithms	34	Undirected
total quality management	emergency health service	34	Undirected
total quality management	health care cost	34	Undirected
total quality management	medical staff	34	Undirected
tracheostomy	hospital admission	34	Undirected
Anxiety	epidemiology	33	Undirected
Anxiety	interview	33	Undirected
Artificial Ventilation	Health Personnel	33	Undirected
Attitude of Health Personnel	long term care	33	Undirected
Attitude of Health Personnel	pharmacist	33	Undirected
Attitude of Health Personnel	safety	33	Undirected
Burnout, Professional	Fatigue	33	Undirected
Burnout, Professional	Leadership	33	Undirected
Child, Preschool	total quality management	33	Undirected
China	Health Personnel	33	Undirected
Clinical Competence	Canada	33	Undirected
Clinical Protocols	emergency ward	33	Undirected
Clinical Protocols	hospital discharge	33	Undirected
Clinical Protocols	medical education	33	Undirected
Communication	Child, Preschool	33	Undirected
Communication	Time Factors	33	Undirected
Communication	medical staff	33	Undirected
Communication	practice guideline	33	Undirected
Decision Making	Practice Guidelines as Topic	33	Undirected
Decision Making	epidemiology	33	Undirected
Decision Making	prematurity	33	Undirected

Emergency Service, Hospital	epidemiology	33	Undirected
Family	Pilot Projects	33	Undirected
Family	long term care	33	Undirected
Family	pediatrics	33	Undirected
Focus Groups	perception	33	Undirected
alth Knowledge, Attitudes, Practi	Pilot Projects	33	Undirected
alth Knowledge, Attitudes, Practi	Reproducibility of Results	33	Undirected
alth Knowledge, Attitudes, Practi	evidence based practice	33	Undirected
Health Services Needs and Demand	Workload	33	Undirected
Heart Arrest	education	33	Undirected
Heart Arrest	emergency health service	33	Undirected
Hospital Mortality	Australia	33	Undirected
Intubation, Intratracheal	methodology	33	Undirected
Job Satisfaction	Health Personnel	33	Undirected
Job Satisfaction	education	33	Undirected
Length of Stay	extracorporeal oxygenation	33	Undirected
Monitoring, Physiologic	hospitalization	33	Undirected
Monitoring, Physiologic	organization and management	33	Undirected
Palliative Care	practice guideline	33	Undirected
Parents	semi structured interview	33	Undirected
Practice Guidelines as Topic	clinical practice	33	Undirected
Practice Guidelines as Topic	pediatrics	33	Undirected
Pregnancy	organization and management	33	Undirected
Professional-Family Relations	ethics	33	Undirected
Professional-Family Relations	methodology	33	Undirected
Quality Improvement	hospital discharge	33	Undirected
Quality of Health Care	Decision Making	33	Undirected
Quality of Health Care	Hospital Mortality	33	Undirected
Risk Assessment	prematurity	33	Undirected
Risk Factors	Electronic Health Records	33	Undirected
Risk Factors	enteric feeding	33	Undirected
Sepsis	heart rate	33	Undirected
Sepsis	hospital infection	33	Undirected
Sepsis	hypertension	33	Undirected
Telemedicine	organization and management	33	Undirected
Terminal Care	standards	33	Undirected
Time Factors	age	33	Undirected
Time Factors	multicenter study	33	Undirected
Treatment Outcome	medical education	33	Undirected
Treatment Outcome	safety	33	Undirected
Workload	Leadership	33	Undirected
Workload	university hospital	33	Undirected
adverse event	endotracheal intubation	33	Undirected
attitude to health	Guideline Adherence	33	Undirected
attitude to health	Length of Stay	33	Undirected
attitude to health	multicenter study	33	Undirected
burnout	multicenter study	33	Undirected
caregiver	multicenter study	33	Undirected
caregiver	practice guideline	33	Undirected
depression	hypertension	33	Undirected
emergency care	multicenter study	33	Undirected
endotracheal intubation	oxygen saturation	33	Undirected
fever	practice guideline	33	Undirected
health care cost	home care	33	Undirected

health care delivery	university hospital	33	Undirected
health care quality	clinical article	33	Undirected
heart failure	hospital readmission	33	Undirected
home care	clinical article	33	Undirected
hospital admission	intensivist	33	Undirected
hospital discharge	interpersonal communication	33	Undirected
hospitalization	Fatigue	33	Undirected
hospitalization	midazolam	33	Undirected
information processing	psychological aspect	33	Undirected
interview	methodology	33	Undirected
knowledge	emergency ward	33	Undirected
knowledge	simulation	33	Undirected
knowledge	ventilator associated pneumonia	33	Undirected
medical education	multicenter study	33	Undirected
methodology	emergency health service	33	Undirected
methodology	information processing	33	Undirected
microbiology	hospital infection	33	Undirected
mortality	depression	33	Undirected
mortality	oxygen saturation	33	Undirected
observational study	emergency health service	33	Undirected
observational study	hospital readmission	33	Undirected
observational study	preschool child	33	Undirected
outcome assessment	Workload	33	Undirected
outcome assessment	analgesia	33	Undirected
outcome assessment	caregiver	33	Undirected
outcome assessment	depression	33	Undirected
outcome assessment	oxygen saturation	33	Undirected
pain assessment	clinical article	33	Undirected
pediatrics	cohort analysis	33	Undirected
perception	Leadership	33	Undirected
perception	practice guideline	33	Undirected
pharmacist	clinical article	33	Undirected
pneumonia	observational study	33	Undirected
prematurity	caregiver	33	Undirected
prematurity	clinical practice	33	Undirected
prematurity	interview	33	Undirected
prematurity	mortality	33	Undirected
prevalence	Anti-Bacterial Agents	33	Undirected
prevalence	hospital readmission	33	Undirected
propofol	practice guideline	33	Undirected
quality of life	clinical practice	33	Undirected
quality of life	education	33	Undirected
quality of life	health care system	33	Undirected
quality of life	home care	33	Undirected
randomized controlled trial	heart surgery	33	Undirected
randomized controlled trial	hospital readmission	33	Undirected
resuscitation	Pandemics	33	Undirected
resuscitation	computer assisted tomography	33	Undirected
resuscitation	psychological aspect	33	Undirected
semi structured interview	organization and management	33	Undirected
simulation	COVID-19	33	Undirected
tertiary care center	gestational age	33	Undirected
therapy	clinical article	33	Undirected
total quality management	adverse event	33	Undirected

total quality management	statistics and numerical data	33	Undirected
ventilator associated pneumonia	endotracheal intubation	33	Undirected
Artificial Ventilation	hand washing	32	Undirected
Artificial Ventilation	perception	32	Undirected
Attitude of Health Personnel	Telemedicine	32	Undirected
Australia	United Kingdom	32	Undirected
Catheterization, Central Venous	adverse event	32	Undirected
Child, Preschool	Pain	32	Undirected
Clinical Competence	Infant, Premature	32	Undirected
Clinical Protocols	clinical article	32	Undirected
Communication	Risk Factors	32	Undirected
Communication	tertiary care center	32	Undirected
Cooperative Behavior	Health Knowledge, Attitudes, Practice	32	Undirected
Cooperative Behavior	interpersonal communication	32	Undirected
Cross Infection	Child, Preschool	32	Undirected
Cross Infection	Hospital Mortality	32	Undirected
Cross Infection	hospital personnel	32	Undirected
Cross Infection	very elderly	32	Undirected
Decision Making	health care delivery	32	Undirected
Decision Making	hospital discharge	32	Undirected
Delirium	education	32	Undirected
Delirium	fentanyl	32	Undirected
Electronic Health Records	emergency ward	32	Undirected
Emergency Service, Hospital	Heart Arrest	32	Undirected
Enteral Nutrition	methodology	32	Undirected
Family	attitude	32	Undirected
Family	health care delivery	32	Undirected
Fatigue	clinical article	32	Undirected
Glasgow coma scale	disease severity	32	Undirected
Glasgow coma scale	very elderly	32	Undirected
Guideline Adherence	Risk Factors	32	Undirected
alth Knowledge, Attitudes, Practi	Focus Groups	32	Undirected
Health Services Needs and Demand	Interprofessional Relations	32	Undirected
Heart Arrest	hypotension	32	Undirected
Hospital Mortality	randomized controlled trial	32	Undirected
Infant, Premature	follow up	32	Undirected
Infection Control	follow up	32	Undirected
Infection Control	health care delivery	32	Undirected
Interprofessional Relations	Burnout, Professional	32	Undirected
Interprofessional Relations	Focus Groups	32	Undirected
Job Satisfaction	Fatigue	32	Undirected
Length of Stay	intensivist	32	Undirected
Length of Stay	pilot study	32	Undirected
Length of Stay	thorax radiography	32	Undirected
Monitoring, Physiologic	blood pressure	32	Undirected
Palliative Care	dyspnea	32	Undirected
Palliative Care	home care	32	Undirected
Parents	Quality Improvement	32	Undirected
Parents	United Kingdom	32	Undirected
Practice Guidelines as Topic	Intubation, Intratracheal	32	Undirected
Practice Guidelines as Topic	analgesia	32	Undirected
Quality Improvement	hospital admission	32	Undirected
Quality Improvement	psychology	32	Undirected
Quality of Health Care	perception	32	Undirected

Risk Assessment	adverse event	32	Undirected
Risk Assessment	health care delivery	32	Undirected
Risk Factors	Personnel Staffing and Scheduling	32	Undirected
Risk Factors	hospital personnel	32	Undirected
Risk Factors	safety	32	Undirected
Safety Management	standards	32	Undirected
Sepsis	health care quality	32	Undirected
Social Support	Length of Stay	32	Undirected
Social Support	Workload	32	Undirected
Social Support	Workplace	32	Undirected
Stress, Psychological	Workload	32	Undirected
Terminal Care	dyspnea	32	Undirected
Terminal Care	health care delivery	32	Undirected
Terminal Care	long term care	32	Undirected
Time Factors	Algorithms	32	Undirected
Treatment Outcome	Terminal Care	32	Undirected
Treatment Outcome	cerebrovascular accident	32	Undirected
United Kingdom	hospital admission	32	Undirected
United Kingdom	interview	32	Undirected
Workload	clinical article	32	Undirected
antibiotic agent	computer assisted tomography	32	Undirected
attitude to health	interpersonal communication	32	Undirected
cerebrovascular accident	computer assisted tomography	32	Undirected
clinical practice	antibiotic agent	32	Undirected
clinical practice	health care policy	32	Undirected
clinical practice	intensivist	32	Undirected
clinical practice	university hospital	32	Undirected
depression	epidemiology	32	Undirected
diabetes mellitus	dementia	32	Undirected
diabetes mellitus	dyspnea	32	Undirected
education	epidemiology	32	Undirected
education	hospital personnel	32	Undirected
emergency care	disease severity	32	Undirected
emergency ward	health care policy	32	Undirected
epidemiology	clinical article	32	Undirected
epidemiology	hospital infection	32	Undirected
evidence based practice	standards	32	Undirected
follow up	breathing rate	32	Undirected
follow up	emergency health service	32	Undirected
health care cost	palliative therapy	32	Undirected
health care planning	health care policy	32	Undirected
health care quality	semi structured interview	32	Undirected
health care quality	statistics and numerical data	32	Undirected
heart failure	Practice Guidelines as Topic	32	Undirected
heart failure	Sepsis	32	Undirected
heart failure	chronic obstructive lung disease	32	Undirected
heart rate	computer assisted tomography	32	Undirected
hospital admission	extracorporeal oxygenation	32	Undirected
hospital discharge	antibiotic agent	32	Undirected
hospitalization	Canada	32	Undirected
hospitalization	morphine	32	Undirected
hospitalization	pain assessment	32	Undirected
hospitalization	safety	32	Undirected
interpersonal communication	university hospital	32	Undirected

interview	health care system	32	Undirected
knowledge	psychology	32	Undirected
medical education	clinical article	32	Undirected
medical staff	emergency care	32	Undirected
mental health	hospital admission	32	Undirected
mental health	hospital discharge	32	Undirected
methodology	interpersonal communication	32	Undirected
microbiology	antibiotic agent	32	Undirected
mortality	Stroke	32	Undirected
mortality	endotracheal intubation	32	Undirected
mortality	interpersonal communication	32	Undirected
multicenter study	age	32	Undirected
multicenter study	hospital readmission	32	Undirected
observational study	emergency care	32	Undirected
observational study	psychology	32	Undirected
observational study	randomized controlled trial	32	Undirected
observational study	statistics and numerical data	32	Undirected
outcome assessment	health care policy	32	Undirected
pediatrics	mortality	32	Undirected
pediatrics	multicenter study	32	Undirected
pediatrics	palliative therapy	32	Undirected
perception	caregiver	32	Undirected
perception	palliative therapy	32	Undirected
pharmacist	health care system	32	Undirected
pneumonia	epidemiology	32	Undirected
propofol	randomized controlled trial	32	Undirected
psychology	medical education	32	Undirected
psychology	pilot study	32	Undirected
randomized controlled trial	preschool child	32	Undirected
resuscitation	breathing rate	32	Undirected
resuscitation	disease severity	32	Undirected
sedation	endotracheal intubation	32	Undirected
tertiary care center	multicenter study	32	Undirected
tertiary care center	resuscitation	32	Undirected
total quality management	Health Personnel	32	Undirected
total quality management	gestational age	32	Undirected
total quality management	health care planning	32	Undirected
university hospital	hospital personnel	32	Undirected
urinary tract infection	antibiotic agent	32	Undirected
vancomycin	antiinfective agent	32	Undirected
ventilator associated pneumonia	antibiotic agent	32	Undirected
ventilator associated pneumonia	health care quality	32	Undirected
very elderly	antiinfective agent	32	Undirected
Anti-Bacterial Agents	cohort analysis	31	Undirected
Anxiety	practice guideline	31	Undirected
Artificial Ventilation	Anti-Bacterial Agents	31	Undirected
Attitude of Health Personnel	Mothers	31	Undirected
Australia	Pandemics	31	Undirected
Australia	multicenter study	31	Undirected
Canada	standards	31	Undirected
Child, Preschool	Cardiac Surgical Procedures	31	Undirected
Child, Preschool	Decision Making	31	Undirected
Child, Preschool	Terminal Care	31	Undirected
Child, Preschool	methodology	31	Undirected

Child, Preschool	resuscitation	31	Undirected
Child, Preschool	statistics and numerical data	31	Undirected
China	attitude	31	Undirected
Clinical Protocols	multicenter study	31	Undirected
Cooperative Behavior	total quality management	31	Undirected
Cross Infection	health care facility	31	Undirected
Decision Making	Pregnancy	31	Undirected
Decision Making	emergency care	31	Undirected
Decision Making	emergency health service	31	Undirected
Decision Making	health care system	31	Undirected
Delirium	caregiver	31	Undirected
Delirium	hypertension	31	Undirected
Emergency Service, Hospital	total quality management	31	Undirected
Focus Groups	education	31	Undirected
Glasgow coma scale	breathing rate	31	Undirected
Glasgow coma scale	heart rate	31	Undirected
Guideline Adherence	education	31	Undirected
Guideline Adherence	hospital personnel	31	Undirected
Health Personnel	cohort analysis	31	Undirected
Health Personnel	information processing	31	Undirected
Health Personnel	standards	31	Undirected
Heart Arrest	breathing rate	31	Undirected
Hospital Mortality	health care cost	31	Undirected
Infection Control	medical education	31	Undirected
Interprofessional Relations	Program Evaluation	31	Undirected
Length of Stay	Telemedicine	31	Undirected
Mothers	depression	31	Undirected
Pain	perception	31	Undirected
Pain Measurement	Delirium	31	Undirected
Pain Measurement	heart rate	31	Undirected
Palliative Care	attitude	31	Undirected
Pandemics	therapy	31	Undirected
Pilot Projects	education	31	Undirected
Postoperative Care	outcome assessment	31	Undirected
Practice Guidelines as Topic	Catheter-Related Infections	31	Undirected
Pregnancy	epidemiology	31	Undirected
Pregnancy	hospital discharge	31	Undirected
Pregnancy	tertiary care center	31	Undirected
Professional-Family Relations	United Kingdom	31	Undirected
Quality Improvement	health care delivery	31	Undirected
Quality Improvement	medical education	31	Undirected
Quality Improvement	pharmacist	31	Undirected
Quality of Health Care	health care delivery	31	Undirected
Reproducibility of Results	psychology	31	Undirected
Risk Assessment	China	31	Undirected
Risk Assessment	United Kingdom	31	Undirected
Risk Assessment	Workload	31	Undirected
Risk Assessment	gestational age	31	Undirected
Risk Assessment	tertiary care center	31	Undirected
Risk Factors	Job Satisfaction	31	Undirected
Risk Factors	Safety Management	31	Undirected
Social Support	Leadership	31	Undirected
Social Support	prematurity	31	Undirected
Stress, Psychological	Risk Assessment	31	Undirected

Time Factors	Catheterization, Central Venous	31	Undirected
Time Factors	Infection Control	31	Undirected
Treatment Outcome	Intubation, Intratracheal	31	Undirected
Treatment Outcome	Leadership	31	Undirected
Treatment Outcome	Quality of Health Care	31	Undirected
Treatment Outcome	United Kingdom	31	Undirected
Treatment Outcome	endotracheal intubation	31	Undirected
Treatment Outcome	health care system	31	Undirected
United Kingdom	health care delivery	31	Undirected
Workforce	Burnout, Professional	31	Undirected
Workforce	Time Factors	31	Undirected
Workload	epidemiology	31	Undirected
adverse event	practice guideline	31	Undirected
analgesia	psychology	31	Undirected
attitude to health	Infection Control	31	Undirected
attitude to health	United Kingdom	31	Undirected
caregiver	organization and management	31	Undirected
caregiver	very elderly	31	Undirected
clinical practice	hand washing	31	Undirected
depression	psychological aspect	31	Undirected
diabetes mellitus	clinical article	31	Undirected
emergency ward	interpersonal communication	31	Undirected
emergency ward	preschool child	31	Undirected
fever	hypotension	31	Undirected
fever	oxygen saturation	31	Undirected
follow up	health care delivery	31	Undirected
follow up	preschool child	31	Undirected
follow up	semi structured interview	31	Undirected
gestational age	mortality	31	Undirected
health care cost	Clinical Protocols	31	Undirected
health care cost	emergency care	31	Undirected
health care cost	organization and management	31	Undirected
health care facility	emergency health service	31	Undirected
health care facility	interpersonal communication	31	Undirected
health care planning	disease severity	31	Undirected
health care planning	emergency health service	31	Undirected
health care quality	hospital readmission	31	Undirected
heart failure	outcome assessment	31	Undirected
heart rate	hospital discharge	31	Undirected
heart rate	methodology	31	Undirected
heart rate	pain assessment	31	Undirected
heart surgery	clinical article	31	Undirected
hospital readmission	health care system	31	Undirected
hospitalization	blood pressure	31	Undirected
hospitalization	fentanyl	31	Undirected
hospitalization	information processing	31	Undirected
hospitalization	propofol	31	Undirected
interview	medical staff	31	Undirected
long term care	organization and management	31	Undirected
long term care	resuscitation	31	Undirected
medical staff	health care facility	31	Undirected
medical staff	university hospital	31	Undirected
mental health	cohort analysis	31	Undirected
mortality	breathing rate	31	Undirected

mortality	caregiver	31	Undirected
mortality	ethicillin resistant Staphylococcus aure	31	Undirected
multicenter study	preschool child	31	Undirected
observational study	hypertension	31	Undirected
observational study	medical education	31	Undirected
observational study	oxygen saturation	31	Undirected
outcome assessment	respiratory therapist	31	Undirected
outcome assessment	therapy	31	Undirected
oxygen saturation	disease severity	31	Undirected
pediatrics	psychology	31	Undirected
pharmacist	hospital admission	31	Undirected
pharmacist	medical education	31	Undirected
pneumonia	hypertension	31	Undirected
pneumonia	randomized controlled trial	31	Undirected
practice guideline	heart surgery	31	Undirected
prevalence	antibiotic agent	31	Undirected
prevalence	clinical article	31	Undirected
prevalence	health care delivery	31	Undirected
prevalence	urinary tract infection	31	Undirected
psychology	hospital personnel	31	Undirected
psychology	intensivist	31	Undirected
quality of life	dementia	31	Undirected
randomized controlled trial	medical education	31	Undirected
resuscitation	epidemiology	31	Undirected
safety	interpersonal communication	31	Undirected
statistics and numerical data	preschool child	31	Undirected
teaching hospital	organization and management	31	Undirected
tertiary care center	disease severity	31	Undirected
tertiary care center	education	31	Undirected
tertiary care center	emergency care	31	Undirected
total quality management	epidemiology	31	Undirected
total quality management	methodology	31	Undirected
total quality management	multicenter study	31	Undirected
total quality management	pediatrics	31	Undirected
tracheostomy	practice guideline	31	Undirected
ventilator associated pneumonia	methodology	31	Undirected
Adaptation, Psychological	organization and management	30	Undirected
Anxiety	sedation	30	Undirected
Artificial Ventilation	Canada	30	Undirected
Artificial Ventilation	Fatigue	30	Undirected
Artificial Ventilation	OUTCOMES	30	Undirected
Artificial Ventilation	heart surgery	30	Undirected
Attitude of Health Personnel	Sepsis	30	Undirected
Attitude of Health Personnel	prevalence	30	Undirected
COVID-19	cerebrovascular accident	30	Undirected
COVID-19	physiotherapy	30	Undirected
Canada	psychology	30	Undirected
Child, Preschool	Canada	30	Undirected
Child, Preschool	Catheterization, Central Venous	30	Undirected
Child, Preschool	Pressure Ulcer	30	Undirected
Child, Preschool	tertiary care center	30	Undirected
Clinical Competence	Sepsis	30	Undirected
Clinical Protocols	Pilot Projects	30	Undirected
Clinical Protocols	resuscitation	30	Undirected

Communication	Mothers	30	Undirected
Communication	pediatrics	30	Undirected
Communication	resuscitation	30	Undirected
Delirium	clinical practice	30	Undirected
Delirium	morphine	30	Undirected
Emergency Service, Hospital	education	30	Undirected
Emergency Service, Hospital	health care quality	30	Undirected
Emergency Service, Hospital	multicenter study	30	Undirected
Fatigue	follow up	30	Undirected
alth Knowledge, Attitudes, Practi	clinical practice	30	Undirected
alth Knowledge, Attitudes, Practi	standards	30	Undirected
Health Personnel	palliative therapy	30	Undirected
Health Services Needs and Demand	United Kingdom	30	Undirected
Heart Arrest	disease severity	30	Undirected
Heart Arrest	extracorporeal oxygenation	30	Undirected
Hospital Mortality	Terminal Care	30	Undirected
Infant, Premature	oxygen saturation	30	Undirected
Infant, Premature	psychological aspect	30	Undirected
Infection Control	health care quality	30	Undirected
Interprofessional Relations	psychology	30	Undirected
Job Satisfaction	health care quality	30	Undirected
Leadership	emergency ward	30	Undirected
Leadership	semi structured interview	30	Undirected
Length of Stay	Leadership	30	Undirected
Length of Stay	evidence based practice	30	Undirected
Length of Stay	interview	30	Undirected
Length of Stay	pain assessment	30	Undirected
Palliative Care	Anxiety	30	Undirected
Palliative Care	Practice Guidelines as Topic	30	Undirected
Palliative Care	health care delivery	30	Undirected
Pandemics	home care	30	Undirected
Parents	Palliative Care	30	Undirected
Pilot Projects	psychology	30	Undirected
Postoperative Complications	clinical article	30	Undirected
Practice Guidelines as Topic	health care quality	30	Undirected
Program Evaluation	methodology	30	Undirected
Quality Improvement	Medication Errors	30	Undirected
Quality Improvement	statistics and numerical data	30	Undirected
Quality of Health Care	mortality	30	Undirected
Reproducibility of Results	China	30	Undirected
Risk Assessment	age	30	Undirected
Risk Assessment	health care cost	30	Undirected
Risk Factors	heart rate	30	Undirected
Sensitivity and Specificity	very elderly	30	Undirected
Stress, Psychological	Pain	30	Undirected
Telemedicine	health care delivery	30	Undirected
Terminal Care	cohort analysis	30	Undirected
Terminal Care	home care	30	Undirected
Time Factors	epidemiology	30	Undirected
Treatment Outcome	Pressure Ulcer	30	Undirected
Workload	Needs Assessment	30	Undirected
Workload	clinical practice	30	Undirected
Workload	interview	30	Undirected
Workload	observational study	30	Undirected

Workload	practice guideline	30	Undirected
analgesia	hospital admission	30	Undirected
attitude	psychology	30	Undirected
blood pressure	follow up	30	Undirected
blood pressure	hypotension	30	Undirected
caregiver	dementia	30	Undirected
caregiver	health care system	30	Undirected
clinical practice	information processing	30	Undirected
dementia	hospital discharge	30	Undirected
dyspnea	cohort analysis	30	Undirected
education	extracorporeal oxygenation	30	Undirected
education	thematic analysis	30	Undirected
emergency care	hospital readmission	30	Undirected
emergency ward	cerebrovascular accident	30	Undirected
emergency ward	semi structured interview	30	Undirected
epidemiology	standards	30	Undirected
follow up	physiotherapy	30	Undirected
health care cost	Terminal Care	30	Undirected
health care facility	cohort analysis	30	Undirected
health care quality	randomized controlled trial	30	Undirected
heart failure	very elderly	30	Undirected
hospital readmission	palliative therapy	30	Undirected
hypertension	breathing rate	30	Undirected
hypertension	hospital readmission	30	Undirected
hypotension	fentanyl	30	Undirected
interpersonal communication	hospital personnel	30	Undirected
interview	palliative therapy	30	Undirected
long term care	clinical article	30	Undirected
long term care	health care facility	30	Undirected
medical education	palliative therapy	30	Undirected
medical staff	follow up	30	Undirected
medical staff	hospital personnel	30	Undirected
medical staff	multicenter study	30	Undirected
methodology	enteric feeding	30	Undirected
methodology	preschool child	30	Undirected
multicenter study	emergency health service	30	Undirected
multicenter study	interpersonal communication	30	Undirected
multicenter study	therapy	30	Undirected
observational study	antibiotic agent	30	Undirected
organization and management	disease severity	30	Undirected
outcome assessment	antibiotic agent	30	Undirected
outcome assessment	chronic obstructive lung disease	30	Undirected
outcome assessment	endotracheal intubation	30	Undirected
outcome assessment	interpersonal communication	30	Undirected
outcome assessment	long term care	30	Undirected
perception	burnout	30	Undirected
perception	medical education	30	Undirected
pharmacist	cohort analysis	30	Undirected
pharmacist	health care delivery	30	Undirected
physiotherapy	clinical article	30	Undirected
physiotherapy	hospital admission	30	Undirected
pilot study	multicenter study	30	Undirected
practice guideline	physiotherapy	30	Undirected
prematurity	Quality Improvement	30	Undirected

prematurity	breathing rate	30	Undirected
prevalence	emergency care	30	Undirected
prevalence	knowledge	30	Undirected
quality of life	meta analysis	30	Undirected
randomized controlled trial	interpersonal communication	30	Undirected
resuscitation	health care delivery	30	Undirected
resuscitation	health care planning	30	Undirected
resuscitation	hypertension	30	Undirected
resuscitation	interpersonal communication	30	Undirected
safety	education	30	Undirected
standards	hand washing	30	Undirected
standards	hospital personnel	30	Undirected
tertiary care center	Sepsis	30	Undirected
university hospital	disease severity	30	Undirected
university hospital	information processing	30	Undirected
urinary tract infection	cohort analysis	30	Undirected
Adaptation, Psychological	COVID-19	29	Undirected
Adaptation, Psychological	depression	29	Undirected
Anti-Bacterial Agents	hospital infection	29	Undirected
Anxiety	emergency ward	29	Undirected
Anxiety	perception	29	Undirected
Artificial Ventilation	information processing	29	Undirected
Attitude of Health Personnel	Heart Arrest	29	Undirected
Attitude of Health Personnel	Logistic Models	29	Undirected
Attitude of Health Personnel	Postoperative Care	29	Undirected
Attitude of Health Personnel	analgesia	29	Undirected
Attitude of Health Personnel	health care facility	29	Undirected
Attitude of Health Personnel	observational study	29	Undirected
Australia	psychology	29	Undirected
Australia	standards	29	Undirected
Burnout, Professional	Terminal Care	29	Undirected
Canada	Pandemics	29	Undirected
Canada	cohort analysis	29	Undirected
Canada	mortality	29	Undirected
Child, Preschool	Infection Control	29	Undirected
Clinical Competence	medical staff	29	Undirected
Clinical Competence	tertiary care center	29	Undirected
Clinical Protocols	follow up	29	Undirected
Communication	Program Evaluation	29	Undirected
Communication	Workplace	29	Undirected
Cross Infection	Time Factors	29	Undirected
Cross Infection	antibiotic agent	29	Undirected
Decision Making	follow up	29	Undirected
Delirium	Cardiac Surgical Procedures	29	Undirected
Delirium	mental health	29	Undirected
Emergency Service, Hospital	standards	29	Undirected
Family	information processing	29	Undirected
Guideline Adherence	tertiary care center	29	Undirected
alth Knowledge, Attitudes, Practi	Quality Improvement	29	Undirected
Health Personnel	thematic analysis	29	Undirected
Health Services Needs and Demand	Safety Management	29	Undirected
Infant, Premature	Reproducibility of Results	29	Undirected
Infant, Premature	outcome assessment	29	Undirected
Infection Control	health care system	29	Undirected

Infection Control	multicenter study	29	Undirected
Infection Control	vancomycin	29	Undirected
Interprofessional Relations	Practice Guidelines as Topic	29	Undirected
Interprofessional Relations	Terminal Care	29	Undirected
Interprofessional Relations	total quality management	29	Undirected
Intubation, Intratracheal	tracheostomy	29	Undirected
Job Satisfaction	organization and management	29	Undirected
Leadership	Health Personnel	29	Undirected
Leadership	emergency care	29	Undirected
Length of Stay	Reproducibility of Results	29	Undirected
Length of Stay	hospital personnel	29	Undirected
Length of Stay	respiratory therapist	29	Undirected
Medication Errors	health care quality	29	Undirected
Monitoring, Physiologic	Child, Preschool	29	Undirected
Monitoring, Physiologic	Clinical Protocols	29	Undirected
Monitoring, Physiologic	Infant, Premature	29	Undirected
Mothers	clinical article	29	Undirected
Pain	Clinical Protocols	29	Undirected
Pain	multicenter study	29	Undirected
Palliative Care	hospital discharge	29	Undirected
Parents	Breast Feeding	29	Undirected
Parents	mental health	29	Undirected
Personnel Staffing and Scheduling	Needs Assessment	29	Undirected
Practice Guidelines as Topic	Insulin	29	Undirected
Practice Guidelines as Topic	Pandemics	29	Undirected
Professional-Family Relations	Practice Guidelines as Topic	29	Undirected
Quality Improvement	Terminal Care	29	Undirected
Quality Improvement	university hospital	29	Undirected
Quality of Health Care	United Kingdom	29	Undirected
Quality of Health Care	practice guideline	29	Undirected
Quality of Health Care	psychology	29	Undirected
Reproducibility of Results	Glasgow coma scale	29	Undirected
Reproducibility of Results	cohort analysis	29	Undirected
Risk Factors	Insulin	29	Undirected
Risk Factors	hand washing	29	Undirected
Sensitivity and Specificity	observational study	29	Undirected
Sepsis	chronic obstructive lung disease	29	Undirected
Sepsis	emergency care	29	Undirected
Social Support	mental health	29	Undirected
Stress, Psychological	Fatigue	29	Undirected
Terminal Care	attitude	29	Undirected
Time Factors	Delirium	29	Undirected
Time Factors	United Kingdom	29	Undirected
Time Factors	health care quality	29	Undirected
Time Factors	psychology	29	Undirected
Treatment Outcome	Anxiety	29	Undirected
Treatment Outcome	Australia	29	Undirected
Treatment Outcome	medical staff	29	Undirected
United Kingdom	Health Personnel	29	Undirected
Workload	Reproducibility of Results	29	Undirected
age	preschool child	29	Undirected
attitude to health	COVID-19	29	Undirected
blood pressure	emergency ward	29	Undirected
breathing rate	thorax radiography	29	Undirected

cerebrovascular accident	chronic obstructive lung disease	29	Undirected
dyspnea	heart rate	29	Undirected
emergency care	home care	29	Undirected
emergency care	semi structured interview	29	Undirected
epidemiology	preschool child	29	Undirected
ethics	interview	29	Undirected
ethics	standards	29	Undirected
follow up	chronic obstructive lung disease	29	Undirected
gestational age	oxygen saturation	29	Undirected
health care cost	pharmacist	29	Undirected
heart rate	COVID-19	29	Undirected
home care	disease severity	29	Undirected
home care	emergency ward	29	Undirected
hospitalization	heart surgery	29	Undirected
hypotension	follow up	29	Undirected
hypotension	hospital discharge	29	Undirected
long term care	Pandemics	29	Undirected
long term care	dementia	29	Undirected
long term care	health care quality	29	Undirected
medical education	hospital personnel	29	Undirected
medical education	information processing	29	Undirected
medical staff	epidemiology	29	Undirected
cillin resistant Staphylococcus a	hospital admission	29	Undirected
methodology	university hospital	29	Undirected
morphine	randomized controlled trial	29	Undirected
mortality	Electronic Health Records	29	Undirected
mortality	teaching hospital	29	Undirected
outcome assessment	United Kingdom	29	Undirected
outcome assessment	health care planning	29	Undirected
outcome assessment	hypotension	29	Undirected
outcome assessment	intensivist	29	Undirected
outcome assessment	teaching hospital	29	Undirected
pediatrics	Terminal Care	29	Undirected
pharmacist	interpersonal communication	29	Undirected
pilot study	methodology	29	Undirected
pneumonia	hospital readmission	29	Undirected
prematurity	enteric feeding	29	Undirected
prevalence	Sensitivity and Specificity	29	Undirected
psychology	disease severity	29	Undirected
quality of life	Communication	29	Undirected
quality of life	Sepsis	29	Undirected
quality of life	emergency ward	29	Undirected
quality of life	interpersonal communication	29	Undirected
quality of life	perception	29	Undirected
randomized controlled trial	breathing rate	29	Undirected
respiratory therapist	organization and management	29	Undirected
resuscitation	Health Personnel	29	Undirected
resuscitation	very elderly	29	Undirected
sedation	observational study	29	Undirected
tertiary care center	very elderly	29	Undirected
thematic analysis	information processing	29	Undirected
total quality management	Pandemics	29	Undirected
urinary tract infection	very elderly	29	Undirected
vancomycin	computer assisted tomography	29	Undirected

vancomycin	thorax radiography	29	Undirected
Anxiety	hypertension	28	Undirected
Anxiety	mortality	28	Undirected
Anxiety	observational study	28	Undirected
Artificial Ventilation	Stress, Psychological	28	Undirected
Artificial Ventilation	dementia	28	Undirected
Artificial Ventilation	psychological aspect	28	Undirected
Artificial Ventilation	semi structured interview	28	Undirected
Child, Preschool	epidemiology	28	Undirected
Child, Preschool	multicenter study	28	Undirected
Clinical Competence	Child, Preschool	28	Undirected
Clinical Competence	Pain Measurement	28	Undirected
Clinical Competence	multicenter study	28	Undirected
Communication	United Kingdom	28	Undirected
Communication	ethics	28	Undirected
Communication	information processing	28	Undirected
Communication	multicenter study	28	Undirected
Cooperative Behavior	Family	28	Undirected
Cross Infection	Clinical Protocols	28	Undirected
Cross Infection	Personnel Staffing and Scheduling	28	Undirected
Cross Infection	education	28	Undirected
Decision Making	Anxiety	28	Undirected
Decision Making	Workload	28	Undirected
Decision Making	observational study	28	Undirected
Decision Making	very elderly	28	Undirected
Delirium	meta analysis	28	Undirected
Delirium	perception	28	Undirected
Emergency Service, Hospital	Australia	28	Undirected
Family	Pain	28	Undirected
Family	United Kingdom	28	Undirected
Family	very elderly	28	Undirected
Focus Groups	Health Personnel	28	Undirected
Guideline Adherence	ventilator associated pneumonia	28	Undirected
alth Knowledge, Attitudes, Practi	Pain Measurement	28	Undirected
Heart Arrest	COVID-19	28	Undirected
Hypnotics and Sedatives	Clinical Protocols	28	Undirected
Hypnotics and Sedatives	fentanyl	28	Undirected
Insulin	practice guideline	28	Undirected
Leadership	clinical practice	28	Undirected
Length of Stay	Pain Measurement	28	Undirected
Length of Stay	information processing	28	Undirected
Logistic Models	cohort analysis	28	Undirected
Monitoring, Physiologic	Heart Arrest	28	Undirected
Pain	hospital admission	28	Undirected
Pain Measurement	randomized controlled trial	28	Undirected
Pandemics	thorax radiography	28	Undirected
Parents	Terminal Care	28	Undirected
Personnel Staffing and Scheduling	COVID-19	28	Undirected
Pilot Projects	organization and management	28	Undirected
Postoperative Care	Clinical Protocols	28	Undirected
Postoperative Care	methodology	28	Undirected
Practice Guidelines as Topic	Hypnotics and Sedatives	28	Undirected
Practice Guidelines as Topic	Pressure Ulcer	28	Undirected
Pressure Ulcer	cohort analysis	28	Undirected

Professional-Family Relations	Quality Improvement	28	Undirected
Professional-Family Relations	Time Factors	28	Undirected
Reproducibility of Results	very elderly	28	Undirected
Risk Assessment	Anxiety	28	Undirected
Risk Assessment	Clinical Protocols	28	Undirected
Risk Assessment	Health Personnel	28	Undirected
Risk Assessment	Safety Management	28	Undirected
Risk Assessment	safety	28	Undirected
Risk Factors	Canada	28	Undirected
Safety Management	methodology	28	Undirected
Sensitivity and Specificity	mortality	28	Undirected
Sepsis	computer assisted tomography	28	Undirected
Sepsis	evidence based practice	28	Undirected
Sepsis	methodology	28	Undirected
Sepsis	preschool child	28	Undirected
Social Support	Breast Feeding	28	Undirected
Social Support	Program Evaluation	28	Undirected
Social Support	follow up	28	Undirected
Social Support	hospital discharge	28	Undirected
Stress, Psychological	Pilot Projects	28	Undirected
Stroke	hospital discharge	28	Undirected
Terminal Care	clinical practice	28	Undirected
Terminal Care	emergency ward	28	Undirected
Time Factors	Intubation, Intratracheal	28	Undirected
Time Factors	heart rate	28	Undirected
Time Factors	prematurity	28	Undirected
Time Factors	preschool child	28	Undirected
Time Factors	tertiary care center	28	Undirected
Treatment Outcome	Guideline Adherence	28	Undirected
Treatment Outcome	Hypnotics and Sedatives	28	Undirected
United Kingdom	Infection Control	28	Undirected
Workload	emergency ward	28	Undirected
Workload	multicenter study	28	Undirected
adverse event	COVID-19	28	Undirected
attitude to health	hand washing	28	Undirected
blood pressure	computer assisted tomography	28	Undirected
burnout	intensivist	28	Undirected
caregiver	long term care	28	Undirected
caregiver	observational study	28	Undirected
clinical practice	emergency health service	28	Undirected
dementia	disease severity	28	Undirected
diabetes mellitus	Pandemics	28	Undirected
diabetes mellitus	observational study	28	Undirected
education	teaching hospital	28	Undirected
emergency ward	age	28	Undirected
emergency ward	standards	28	Undirected
ethics	interpersonal communication	28	Undirected
fentanyl	endotracheal intubation	28	Undirected
follow up	standards	28	Undirected
gestational age	practice guideline	28	Undirected
health care cost	health care facility	28	Undirected
health care cost	pneumonia	28	Undirected
health care delivery	disease severity	28	Undirected
health care delivery	hospital personnel	28	Undirected

health care delivery	hospital readmission	28	Undirected
health care facility	hand washing	28	Undirected
health care quality	psychological aspect	28	Undirected
health care system	cohort analysis	28	Undirected
health care system	interpersonal communication	28	Undirected
heart failure	clinical article	28	Undirected
heart failure	practice guideline	28	Undirected
hospital discharge	psychological aspect	28	Undirected
hospital infection	hospital personnel	28	Undirected
hospitalization	hand washing	28	Undirected
hospitalization	psychological aspect	28	Undirected
intensivist	university hospital	28	Undirected
interview	multicenter study	28	Undirected
interview	pharmacist	28	Undirected
knowledge	multicenter study	28	Undirected
medical education	intensivist	28	Undirected
medical staff	pharmacist	28	Undirected
cillin resistant Staphylococcus a	antibiotic agent	28	Undirected
cillin resistant Staphylococcus a	antiinfective agent	28	Undirected
methodology	multicenter study	28	Undirected
midazolam	pain assessment	28	Undirected
observational study	chronic obstructive lung disease	28	Undirected
outcome assessment	Anxiety	28	Undirected
outcome assessment	evidence based practice	28	Undirected
outcome assessment	hospital infection	28	Undirected
oxygen saturation	hospital discharge	28	Undirected
pediatrics	Canada	28	Undirected
pediatrics	Heart Arrest	28	Undirected
pediatrics	Pain	28	Undirected
pharmacist	antibiotic agent	28	Undirected
pilot study	cohort analysis	28	Undirected
pneumonia	hospital infection	28	Undirected
pneumonia	thicillin resistant Staphylococcus aure	28	Undirected
practice guideline	computer assisted tomography	28	Undirected
practice guideline	home care	28	Undirected
practice guideline	teaching hospital	28	Undirected
prevalence	total quality management	28	Undirected
quality of life	attitude to health	28	Undirected
respiratory therapist	interpersonal communication	28	Undirected
tertiary care center	interpersonal communication	28	Undirected
total quality management	Catheter-Related Infections	28	Undirected
total quality management	preschool child	28	Undirected
tracheostomy	Pandemics	28	Undirected
Adaptation, Psychological	Workplace	27	Undirected
Algorithms	organization and management	27	Undirected
Artificial Ventilation	cerebrovascular accident	27	Undirected
Artificial Ventilation	thicillin resistant Staphylococcus aure	27	Undirected
Attitude of Health Personnel	mortality	27	Undirected
Canada	clinical practice	27	Undirected
Canada	methodology	27	Undirected
Child, Preschool	Clinical Protocols	27	Undirected
Child, Preschool	Enteral Nutrition	27	Undirected
Child, Preschool	Pilot Projects	27	Undirected
Child, Preschool	standards	27	Undirected

China	disease severity	27	Undirected
China	hospital admission	27	Undirected
Clinical Competence	Catheterization, Central Venous	27	Undirected
Clinical Competence	Length of Stay	27	Undirected
Clinical Competence	hospitalization	27	Undirected
Clinical Protocols	education	27	Undirected
Cross Infection	Logistic Models	27	Undirected
Decision Making	Quality Improvement	27	Undirected
Decision Making	intensivist	27	Undirected
Decision Making	pharmacist	27	Undirected
Delirium	Pandemics	27	Undirected
Delirium	heart surgery	27	Undirected
Electronic Health Records	hospital admission	27	Undirected
Emergency Service, Hospital	hospital discharge	27	Undirected
Emergency Service, Hospital	outcome assessment	27	Undirected
Emergency Service, Hospital	pediatrics	27	Undirected
Family	Interprofessional Relations	27	Undirected
Family	Reproducibility of Results	27	Undirected
Family	outcome assessment	27	Undirected
Glasgow coma scale	observational study	27	Undirected
Guideline Adherence	multicenter study	27	Undirected
Guideline Adherence	statistics and numerical data	27	Undirected
alth Knowledge, Attitudes, Practi	Australia	27	Undirected
alth Knowledge, Attitudes, Practi	Clinical Protocols	27	Undirected
alth Knowledge, Attitudes, Practi	United Kingdom	27	Undirected
alth Knowledge, Attitudes, Practi	methodology	27	Undirected
Heart Arrest	emergency care	27	Undirected
Heart Arrest	observational study	27	Undirected
Hospital Mortality	Pandemics	27	Undirected
Hospital Mortality	diabetes mellitus	27	Undirected
Hospital Mortality	standards	27	Undirected
Hospital Mortality	university hospital	27	Undirected
Hypnotics and Sedatives	methodology	27	Undirected
Infant, Premature	pain assessment	27	Undirected
Infant, Premature	standards	27	Undirected
Infection Control	randomized controlled trial	27	Undirected
Length of Stay	Intubation, Intratracheal	27	Undirected
Length of Stay	thicillin resistant Staphylococcus aure	27	Undirected
Medication Errors	observational study	27	Undirected
Monitoring, Physiologic	Quality Improvement	27	Undirected
Monitoring, Physiologic	Workload	27	Undirected
OUTCOMES	hospital discharge	27	Undirected
Pain	organization and management	27	Undirected
Palliative Care	semi structured interview	27	Undirected
Palliative Care	total quality management	27	Undirected
Parents	Risk Assessment	27	Undirected
Parents	Time Factors	27	Undirected
Postoperative Care	mortality	27	Undirected
Postoperative Complications	pneumonia	27	Undirected
Practice Guidelines as Topic	Pain Measurement	27	Undirected
Pressure Ulcer	COVID-19	27	Undirected
Professional-Family Relations	Quality of Health Care	27	Undirected
Professional-Family Relations	palliative therapy	27	Undirected
Program Evaluation	Hospital Mortality	27	Undirected

Program Evaluation	Infant, Premature	27	Undirected
Program Evaluation	Infection Control	27	Undirected
Program Evaluation	Risk Assessment	27	Undirected
Quality Improvement	information processing	27	Undirected
Quality of Health Care	Risk Assessment	27	Undirected
Quality of Health Care	Safety Management	27	Undirected
Risk Assessment	Needs Assessment	27	Undirected
Risk Assessment	hospital infection	27	Undirected
Risk Factors	health care planning	27	Undirected
Risk Factors	information processing	27	Undirected
Risk Factors	oxygen saturation	27	Undirected
Risk Factors	palliative therapy	27	Undirected
Risk Factors	propofol	27	Undirected
Safety Management	Workload	27	Undirected
Safety Management	practice guideline	27	Undirected
Sensitivity and Specificity	methodology	27	Undirected
Sepsis	clinical practice	27	Undirected
Sepsis	diabetes mellitus	27	Undirected
Sepsis	pharmacist	27	Undirected
Sepsis	therapy	27	Undirected
Social Support	Artificial Ventilation	27	Undirected
Social Support	Palliative Care	27	Undirected
Social Support	health care quality	27	Undirected
Stress, Psychological	hospitalization	27	Undirected
Telemedicine	health care quality	27	Undirected
Terminal Care	hospital discharge	27	Undirected
Time Factors	Pregnancy	27	Undirected
Time Factors	Terminal Care	27	Undirected
Time Factors	disease severity	27	Undirected
Treatment Outcome	Canada	27	Undirected
Treatment Outcome	Family	27	Undirected
Treatment Outcome	enteric feeding	27	Undirected
Treatment Outcome	extracorporeal oxygenation	27	Undirected
Treatment Outcome	oxygen saturation	27	Undirected
Treatment Outcome	pilot study	27	Undirected
United Kingdom	total quality management	27	Undirected
Workload	Health Personnel	27	Undirected
Workload	education	27	Undirected
Workload	health care facility	27	Undirected
Workload	hospital admission	27	Undirected
Workload	semi structured interview	27	Undirected
Workplace	Personnel Staffing and Scheduling	27	Undirected
Workplace	organization and management	27	Undirected
adverse event	Catheter-Related Infections	27	Undirected
attitude	Health Personnel	27	Undirected
attitude to health	health care quality	27	Undirected
attitude to health	medical staff	27	Undirected
caregiver	disease severity	27	Undirected
caregiver	emergency ward	27	Undirected
cerebrovascular accident	acute kidney failure	27	Undirected
cerebrovascular accident	clinical article	27	Undirected
clinical article	chronic obstructive lung disease	27	Undirected
clinical practice	very elderly	27	Undirected
cohort analysis	computer assisted tomography	27	Undirected

cohort analysis	thorax radiography	27	Undirected
disease severity	acute kidney failure	27	Undirected
dyspnea	acute kidney failure	27	Undirected
education	respiratory therapist	27	Undirected
education	therapy	27	Undirected
emergency care	randomized controlled trial	27	Undirected
emergency health service	computer assisted tomography	27	Undirected
endotracheal intubation	computer assisted tomography	27	Undirected
epidemiology	health care planning	27	Undirected
epidemiology	health care policy	27	Undirected
epidemiology	interpersonal communication	27	Undirected
evidence based practice	education	27	Undirected
fever	antiinfective agent	27	Undirected
fever	breathing rate	27	Undirected
gestational age	disease severity	27	Undirected
health care delivery	home care	27	Undirected
health care facility	disease severity	27	Undirected
health care facility	health care policy	27	Undirected
health care facility	very elderly	27	Undirected
health care planning	interpersonal communication	27	Undirected
health care quality	intensivist	27	Undirected
heart failure	dyspnea	27	Undirected
heart rate	disease severity	27	Undirected
heart rate	emergency health service	27	Undirected
hospital discharge	health care policy	27	Undirected
hypertension	randomized controlled trial	27	Undirected
interview	hospital discharge	27	Undirected
knowledge	pharmacist	27	Undirected
long term care	clinical practice	27	Undirected
medical staff	observational study	27	Undirected
mortality	sedation	27	Undirected
mortality	vancomycin	27	Undirected
observational study	adverse event	27	Undirected
observational study	antiinfective agent	27	Undirected
observational study	health care delivery	27	Undirected
observational study	interpersonal communication	27	Undirected
outcome assessment	Canada	27	Undirected
outcome assessment	Leadership	27	Undirected
outcome assessment	tracheostomy	27	Undirected
perception	emergency ward	27	Undirected
perception	health care delivery	27	Undirected
perception	pharmacist	27	Undirected
pilot study	clinical practice	27	Undirected
pneumonia	acute kidney failure	27	Undirected
pneumonia	cerebrovascular accident	27	Undirected
pneumonia	oxygen saturation	27	Undirected
pneumonia	tracheostomy	27	Undirected
pneumonia	vancomycin	27	Undirected
prevalence	Fatigue	27	Undirected
prevalence	antiinfective agent	27	Undirected
prevalence	chronic obstructive lung disease	27	Undirected
prevalence	education	27	Undirected
prevalence	fever	27	Undirected
prevalence	microbiology	27	Undirected

prevalence	preschool child	27	Undirected
propofol	mortality	27	Undirected
quality of life	Health Personnel	27	Undirected
quality of life	Postoperative Complications	27	Undirected
quality of life	physiotherapy	27	Undirected
randomized controlled trial	physiotherapy	27	Undirected
resuscitation	health care system	27	Undirected
safety	clinical practice	27	Undirected
sedation	cohort analysis	27	Undirected
semi structured interview	health care system	27	Undirected
simulation	clinical practice	27	Undirected
simulation	respiratory therapist	27	Undirected
standards	health care policy	27	Undirected
standards	preschool child	27	Undirected
statistics and numerical data	university hospital	27	Undirected
tertiary care center	Australia	27	Undirected
tertiary care center	perception	27	Undirected
total quality management	Medication Errors	27	Undirected
total quality management	caregiver	27	Undirected
total quality management	interview	27	Undirected
ventilator associated pneumonia	standards	27	Undirected
very elderly	university hospital	27	Undirected
Adaptation, Psychological	Focus Groups	26	Undirected
Anti-Bacterial Agents	very elderly	26	Undirected
Anxiety	health care delivery	26	Undirected
Anxiety	semi structured interview	26	Undirected
Artificial Ventilation	mental health	26	Undirected
Australia	emergency care	26	Undirected
Australia	emergency ward	26	Undirected
Child, Preschool	observational study	26	Undirected
Child, Preschool	randomized controlled trial	26	Undirected
China	multicenter study	26	Undirected
Clinical Competence	safety	26	Undirected
Clinical Protocols	COVID-19	26	Undirected
Clinical Protocols	health care delivery	26	Undirected
Clinical Protocols	health care planning	26	Undirected
Communication	methodology	26	Undirected
Communication	randomized controlled trial	26	Undirected
Communication	thematic analysis	26	Undirected
Cooperative Behavior	Adaptation, Psychological	26	Undirected
Cooperative Behavior	Needs Assessment	26	Undirected
Cross Infection	Workload	26	Undirected
Cross Infection	university hospital	26	Undirected
Cross Infection	vancomycin	26	Undirected
Decision Making	medical education	26	Undirected
Decision Making	randomized controlled trial	26	Undirected
Delirium	China	26	Undirected
Delirium	palliative therapy	26	Undirected
Delirium	pharmacist	26	Undirected
Family	Emergency Service, Hospital	26	Undirected
alth Knowledge, Attitudes, Practi	psychological aspect	26	Undirected
Health Personnel	health care system	26	Undirected
Health Personnel	hospital admission	26	Undirected
Health Personnel	hospital infection	26	Undirected

Health Services Needs and Demand	Parents	26	Undirected
Health Services Needs and Demand	Program Evaluation	26	Undirected
Health Services Needs and Demand	Time Factors	26	Undirected
Heart Arrest	organization and management	26	Undirected
Hospital Mortality	emergency care	26	Undirected
Hospital Mortality	palliative therapy	26	Undirected
Infant, Premature	Needs Assessment	26	Undirected
Infant, Premature	resuscitation	26	Undirected
Job Satisfaction	Focus Groups	26	Undirected
Logistic Models	hospital admission	26	Undirected
Mothers	psychological aspect	26	Undirected
Needs Assessment	psychological aspect	26	Undirected
Pain	cohort analysis	26	Undirected
Pain	depression	26	Undirected
Pain	follow up	26	Undirected
Pain	knowledge	26	Undirected
Pain	oxygen saturation	26	Undirected
Pain	propofol	26	Undirected
Palliative Care	Quality Improvement	26	Undirected
Palliative Care	outcome assessment	26	Undirected
Pandemics	multicenter study	26	Undirected
Parents	Health Knowledge, Attitudes, Practice	26	Undirected
Postoperative Care	Risk Assessment	26	Undirected
Postoperative Care	follow up	26	Undirected
Pregnancy	health care quality	26	Undirected
Professional-Family Relations	Anxiety	26	Undirected
Professional-Family Relations	medical staff	26	Undirected
Quality of Health Care	Health Knowledge, Attitudes, Practice	26	Undirected
Quality of Health Care	Risk Factors	26	Undirected
Risk Assessment	education	26	Undirected
Risk Assessment	heart surgery	26	Undirected
Risk Assessment	medical staff	26	Undirected
Risk Assessment	preschool child	26	Undirected
Risk Assessment	statistics and numerical data	26	Undirected
Safety Management	health care quality	26	Undirected
Sensitivity and Specificity	emergency ward	26	Undirected
Social Support	Focus Groups	26	Undirected
Stress, Psychological	Canada	26	Undirected
Terminal Care	follow up	26	Undirected
Terminal Care	multicenter study	26	Undirected
Time Factors	education	26	Undirected
Time Factors	statistics and numerical data	26	Undirected
Treatment Outcome	antiinfective agent	26	Undirected
Treatment Outcome	blood pressure	26	Undirected
Treatment Outcome	interpersonal communication	26	Undirected
Treatment Outcome	meta analysis	26	Undirected
Treatment Outcome	palliative therapy	26	Undirected
United Kingdom	Personnel Staffing and Scheduling	26	Undirected
United Kingdom	Terminal Care	26	Undirected
United Kingdom	medical staff	26	Undirected
Workforce	Risk Factors	26	Undirected
adverse event	follow up	26	Undirected
adverse event	hospital admission	26	Undirected
adverse event	hospital discharge	26	Undirected

analgesia	methodology	26	Undirected
analgesia	multicenter study	26	Undirected
analgesia	organization and management	26	Undirected
attitude	clinical practice	26	Undirected
attitude to health	Australia	26	Undirected
attitude to health	Cooperative Behavior	26	Undirected
attitude to health	Pilot Projects	26	Undirected
attitude to health	resuscitation	26	Undirected
burnout	health care system	26	Undirected
clinical practice	statistics and numerical data	26	Undirected
dementia	follow up	26	Undirected
diabetes mellitus	hospital readmission	26	Undirected
disease severity	preschool child	26	Undirected
dyspnea	chronic obstructive lung disease	26	Undirected
dyspnea	epidemiology	26	Undirected
education	reliability	26	Undirected
emergency care	health care policy	26	Undirected
emergency care	palliative therapy	26	Undirected
emergency ward	chronic obstructive lung disease	26	Undirected
emergency ward	information processing	26	Undirected
emergency ward	therapy	26	Undirected
endotracheal intubation	cohort analysis	26	Undirected
epidemiology	hospital personnel	26	Undirected
evidence based practice	knowledge	26	Undirected
fever	preschool child	26	Undirected
health care cost	multicenter study	26	Undirected
heart failure	COVID-19	26	Undirected
heart failure	Terminal Care	26	Undirected
heart surgery	cohort analysis	26	Undirected
home care	age	26	Undirected
hospital admission	information processing	26	Undirected
hospitalization	Breast Feeding	26	Undirected
hospitalization	microbiology	26	Undirected
hypotension	endotracheal intubation	26	Undirected
intensivist	clinical article	26	Undirected
interview	resuscitation	26	Undirected
knowledge	interpersonal communication	26	Undirected
long term care	interpersonal communication	26	Undirected
medical education	disease severity	26	Undirected
medical education	health care facility	26	Undirected
medical education	respiratory therapist	26	Undirected
medical education	semi structured interview	26	Undirected
methodology	clinical practice	26	Undirected
microbiology	very elderly	26	Undirected
mortality	hospital personnel	26	Undirected
multicenter study	respiratory therapist	26	Undirected
observational study	endotracheal intubation	26	Undirected
observational study	information processing	26	Undirected
observational study	standards	26	Undirected
outcome assessment	Infection Control	26	Undirected
outcome assessment	dyspnea	26	Undirected
palliative therapy	chronic obstructive lung disease	26	Undirected
pediatrics	COVID-19	26	Undirected
pediatrics	emergency health service	26	Undirected

pediatrics	hospital discharge	26	Undirected
pediatrics	pharmacist	26	Undirected
pilot study	hospital admission	26	Undirected
prematurity	disease severity	26	Undirected
prematurity	multicenter study	26	Undirected
prematurity	pilot study	26	Undirected
prevalence	United Kingdom	26	Undirected
prevalence	palliative therapy	26	Undirected
prevalence	vancomycin	26	Undirected
psychology	emergency health service	26	Undirected
psychology	health care facility	26	Undirected
quality of life	dyspnea	26	Undirected
quality of life	ethics	26	Undirected
randomized controlled trial	antibiotic agent	26	Undirected
resuscitation	preschool child	26	Undirected
resuscitation	therapy	26	Undirected
sedation	hospital admission	26	Undirected
standards	enteric feeding	26	Undirected
teaching hospital	multicenter study	26	Undirected
tertiary care center	interview	26	Undirected
tertiary care center	pneumonia	26	Undirected
tertiary care center	preschool child	26	Undirected
thematic analysis	organization and management	26	Undirected
therapy	disease severity	26	Undirected
total quality management	Catheterization, Central Venous	26	Undirected
total quality management	Delirium	26	Undirected
total quality management	antibiotic agent	26	Undirected
total quality management	health care policy	26	Undirected
total quality management	randomized controlled trial	26	Undirected
total quality management	therapy	26	Undirected
urinary tract infection	antiinfective agent	26	Undirected
ventilator associated pneumonia	hand washing	26	Undirected
Adaptation, Psychological	education	25	Undirected
Anti-Bacterial Agents	fever	25	Undirected
Anti-Bacterial Agents	urinary tract infection	25	Undirected
Anxiety	clinical practice	25	Undirected
Anxiety	interpersonal communication	25	Undirected
Anxiety	organization and management	25	Undirected
Anxiety	resuscitation	25	Undirected
Anxiety	thematic analysis	25	Undirected
Artificial Ventilation	Pregnancy	25	Undirected
Artificial Ventilation	health care policy	25	Undirected
Attitude of Health Personnel	Medication Errors	25	Undirected
Attitude of Health Personnel	follow up	25	Undirected
Attitude of Health Personnel	prematurity	25	Undirected
Australia	health care quality	25	Undirected
Breast Feeding	randomized controlled trial	25	Undirected
COVID-19	information processing	25	Undirected
Canada	clinical article	25	Undirected
Canada	epidemiology	25	Undirected
Child, Preschool	Heart Arrest	25	Undirected
Child, Preschool	United Kingdom	25	Undirected
Child, Preschool	age	25	Undirected
Child, Preschool	caregiver	25	Undirected

Child, Preschool	emergency ward	25	Undirected
China	clinical practice	25	Undirected
China	organization and management	25	Undirected
Clinical Competence	Pandemics	25	Undirected
Clinical Competence	Stress, Psychological	25	Undirected
Communication	Canada	25	Undirected
Communication	Emergency Service, Hospital	25	Undirected
Communication	Infant, Premature	25	Undirected
Communication	depression	25	Undirected
Cooperative Behavior	Practice Guidelines as Topic	25	Undirected
Cross Infection	outcome assessment	25	Undirected
Decision Making	Risk Assessment	25	Undirected
Decision Making	attitude	25	Undirected
Delirium	multicenter study	25	Undirected
Emergency Service, Hospital	Pandemics	25	Undirected
Family	Program Evaluation	25	Undirected
Family	mental health	25	Undirected
Focus Groups	thematic analysis	25	Undirected
alth Knowledge, Attitudes, Practi	medical staff	25	Undirected
alth Knowledge, Attitudes, Practi	tertiary care center	25	Undirected
alth Knowledge, Attitudes, Practi	total quality management	25	Undirected
Health Personnel	therapy	25	Undirected
Health Services Needs and Demand	Personnel Staffing and Scheduling	25	Undirected
Heart Arrest	Clinical Protocols	25	Undirected
Hospital Mortality	Anti-Bacterial Agents	25	Undirected
Hospital Mortality	chronic obstructive lung disease	25	Undirected
Infant, Premature	analgesia	25	Undirected
Infection Control	safety	25	Undirected
Interprofessional Relations	Quality Improvement	25	Undirected
Interprofessional Relations	United Kingdom	25	Undirected
Intubation, Intratracheal	resuscitation	25	Undirected
Job Satisfaction	psychological aspect	25	Undirected
Leadership	health care system	25	Undirected
Leadership	thematic analysis	25	Undirected
Length of Stay	Health Personnel	25	Undirected
Length of Stay	blood pressure	25	Undirected
Length of Stay	semi structured interview	25	Undirected
Length of Stay	vancomycin	25	Undirected
Medication Errors	organization and management	25	Undirected
Mothers	caregiver	25	Undirected
Pain	COVID-19	25	Undirected
Pain	Intubation, Intratracheal	25	Undirected
Pain	caregiver	25	Undirected
Pain	disease severity	25	Undirected
Pain Measurement	gestational age	25	Undirected
Palliative Care	Hospital Mortality	25	Undirected
Palliative Care	analgesia	25	Undirected
Palliative Care	cohort analysis	25	Undirected
Pandemics	age	25	Undirected
Parents	Pilot Projects	25	Undirected
Parents	Risk Factors	25	Undirected
Pilot Projects	methodology	25	Undirected
Postoperative Care	Delirium	25	Undirected
Postoperative Care	Practice Guidelines as Topic	25	Undirected

Postoperative Complications	hypotension	25	Undirected
Practice Guidelines as Topic	antiinfective agent	25	Undirected
Pregnancy	Quality Improvement	25	Undirected
Pregnancy	health care delivery	25	Undirected
Pressure Ulcer	China	25	Undirected
Pressure Ulcer	organization and management	25	Undirected
Professional-Family Relations	practice guideline	25	Undirected
Professional-Family Relations	standards	25	Undirected
Quality Improvement	observational study	25	Undirected
Quality Improvement	simulation	25	Undirected
Risk Assessment	Algorithms	25	Undirected
Risk Assessment	diabetes mellitus	25	Undirected
Risk Assessment	hospital personnel	25	Undirected
Risk Factors	analgesia	25	Undirected
Sepsis	Heart Arrest	25	Undirected
Sepsis	adverse event	25	Undirected
Sepsis	age	25	Undirected
Sepsis	cerebrovascular accident	25	Undirected
Sepsis	knowledge	25	Undirected
Sepsis	ventilator associated pneumonia	25	Undirected
Social Support	Pregnancy	25	Undirected
Social Support	health care delivery	25	Undirected
Social Support	health care system	25	Undirected
Social Support	prevalence	25	Undirected
Time Factors	COVID-19	25	Undirected
Time Factors	Hypnotics and Sedatives	25	Undirected
Time Factors	Pain Measurement	25	Undirected
Time Factors	adverse event	25	Undirected
Time Factors	university hospital	25	Undirected
Treatment Outcome	dyspnea	25	Undirected
Treatment Outcome	health care facility	25	Undirected
Treatment Outcome	psychological aspect	25	Undirected
Treatment Outcome	urinary tract infection	25	Undirected
United Kingdom	Quality Improvement	25	Undirected
United Kingdom	health care policy	25	Undirected
United Kingdom	resuscitation	25	Undirected
Workforce	Focus Groups	25	Undirected
Workforce	burnout	25	Undirected
Workplace	education	25	Undirected
adverse event	heart surgery	25	Undirected
analgesia	disease severity	25	Undirected
attitude to health	hospitalization	25	Undirected
burnout	health care quality	25	Undirected
caregiver	medical staff	25	Undirected
cerebrovascular accident	organization and management	25	Undirected
clinical practice	therapy	25	Undirected
dementia	health care quality	25	Undirected
diabetes mellitus	acute kidney failure	25	Undirected
diabetes mellitus	antibiotic agent	25	Undirected
disease severity	breathing rate	25	Undirected
disease severity	chronic obstructive lung disease	25	Undirected
dyspnea	hypotension	25	Undirected
education	hospital admission	25	Undirected
education	statistics and numerical data	25	Undirected

emergency care	standards	25	Undirected
emergency ward	statistics and numerical data	25	Undirected
epidemiology	randomized controlled trial	25	Undirected
epidemiology	thematic analysis	25	Undirected
evidence based practice	Catheter-Related Infections	25	Undirected
evidence based practice	clinical article	25	Undirected
evidence based practice	mortality	25	Undirected
gestational age	breathing rate	25	Undirected
health care cost	dementia	25	Undirected
health care delivery	cohort analysis	25	Undirected
health care planning	palliative therapy	25	Undirected
health care quality	pilot study	25	Undirected
heart failure	Hospital Mortality	25	Undirected
heart failure	acute kidney failure	25	Undirected
heart failure	organization and management	25	Undirected
heart failure	pneumonia	25	Undirected
heart rate	endotracheal intubation	25	Undirected
heart rate	thorax radiography	25	Undirected
home care	emergency health service	25	Undirected
hospital readmission	chronic obstructive lung disease	25	Undirected
hypertension	thorax radiography	25	Undirected
hypotension	acute kidney failure	25	Undirected
hypotension	antibiotic agent	25	Undirected
hypotension	midazolam	25	Undirected
hypotension	randomized controlled trial	25	Undirected
interpersonal communication	very elderly	25	Undirected
interview	therapy	25	Undirected
medical staff	statistics and numerical data	25	Undirected
mental health	health care system	25	Undirected
meta analysis	hospital admission	25	Undirected
methodology	cohort analysis	25	Undirected
methodology	microbiology	25	Undirected
midazolam	endotracheal intubation	25	Undirected
mortality	microbiology	25	Undirected
mortality	physiotherapy	25	Undirected
observational study	palliative therapy	25	Undirected
outcome assessment	Health Personnel	25	Undirected
outcome assessment	dementia	25	Undirected
outcome assessment	enteric feeding	25	Undirected
outcome assessment	perception	25	Undirected
perception	epidemiology	25	Undirected
pneumonia	heart rate	25	Undirected
practice guideline	hospital readmission	25	Undirected
prematurity	Anxiety	25	Undirected
prematurity	health care cost	25	Undirected
prematurity	statistics and numerical data	25	Undirected
quality of life	Workload	25	Undirected
resuscitation	health care facility	25	Undirected
sedation	disease severity	25	Undirected
semi structured interview	respiratory therapist	25	Undirected
standards	health care facility	25	Undirected
statistics and numerical data	hospital readmission	25	Undirected
tertiary care center	health care cost	25	Undirected
tertiary care center	medical education	25	Undirected

thematic analysis	emergency ward	25	Undirected
thematic analysis	palliative therapy	25	Undirected
therapy	extracorporeal oxygenation	25	Undirected
total quality management	observational study	25	Undirected
total quality management	perception	25	Undirected
total quality management	very elderly	25	Undirected
tracheostomy	oxygen saturation	25	Undirected
urinary tract infection	hospital discharge	25	Undirected
Adaptation, Psychological	Child, Preschool	24	Undirected
Anxiety	heart rate	24	Undirected
Anxiety	multicenter study	24	Undirected
Anxiety	very elderly	24	Undirected
Attitude of Health Personnel	health care system	24	Undirected
Attitude of Health Personnel	hospital admission	24	Undirected
Attitude of Health Personnel	sedation	24	Undirected
Attitude of Health Personnel	statistics and numerical data	24	Undirected
Australia	Canada	24	Undirected
Australia	hospital discharge	24	Undirected
COVID-19	meta analysis	24	Undirected
Canada	education	24	Undirected
Catheterization, Central Venous	methodology	24	Undirected
Child, Preschool	Postoperative Care	24	Undirected
Child, Preschool	Postoperative Complications	24	Undirected
Child, Preschool	follow up	24	Undirected
Clinical Competence	interview	24	Undirected
Clinical Protocols	fentanyl	24	Undirected
Clinical Protocols	midazolam	24	Undirected
Clinical Protocols	safety	24	Undirected
Communication	Burnout, Professional	24	Undirected
Communication	Pain	24	Undirected
Communication	Workload	24	Undirected
Cooperative Behavior	Program Evaluation	24	Undirected
Cooperative Behavior	Workplace	24	Undirected
Decision Making	Infant, Premature	24	Undirected
Decision Making	preschool child	24	Undirected
Delirium	emergency ward	24	Undirected
Delirium	reliability	24	Undirected
Delirium	therapy	24	Undirected
Electronic Health Records	Pandemics	24	Undirected
Electronic Health Records	health care system	24	Undirected
Emergency Service, Hospital	Decision Making	24	Undirected
Enteral Nutrition	standards	24	Undirected
Family	Infant, Premature	24	Undirected
Glasgow coma scale	oxygen saturation	24	Undirected
Guideline Adherence	Risk Assessment	24	Undirected
Guideline Adherence	hospital infection	24	Undirected
alth Knowledge, Attitudes, Practi	Canada	24	Undirected
alth Knowledge, Attitudes, Practi	multicenter study	24	Undirected
Health Personnel	emergency health service	24	Undirected
Health Services Needs and Demand	psychological aspect	24	Undirected
Heart Arrest	hypertension	24	Undirected
Hospital Mortality	hypertension	24	Undirected
Infant, Premature	Canada	24	Undirected
Infant, Premature	China	24	Undirected

Infant, Premature	Sepsis	24	Undirected
Infant, Premature	practice guideline	24	Undirected
Infection Control	China	24	Undirected
Infection Control	disease severity	24	Undirected
Infection Control	medical staff	24	Undirected
Interprofessional Relations	education	24	Undirected
Interprofessional Relations	health care quality	24	Undirected
Length of Stay	Stress, Psychological	24	Undirected
Monitoring, Physiologic	Postoperative Complications	24	Undirected
Pain	Fatigue	24	Undirected
Pain	Glasgow coma scale	24	Undirected
Pandemics	antibiotic agent	24	Undirected
Pandemics	computer assisted tomography	24	Undirected
Parents	Program Evaluation	24	Undirected
Parents	methodology	24	Undirected
Parents	total quality management	24	Undirected
Postoperative Care	hospital discharge	24	Undirected
Postoperative Complications	cerebrovascular accident	24	Undirected
Practice Guidelines as Topic	Australia	24	Undirected
Practice Guidelines as Topic	Needs Assessment	24	Undirected
Practice Guidelines as Topic	psychology	24	Undirected
Pressure Ulcer	Sensitivity and Specificity	24	Undirected
Pressure Ulcer	attitude	24	Undirected
Professional-Family Relations	Health Personnel	24	Undirected
Professional-Family Relations	Program Evaluation	24	Undirected
Professional-Family Relations	hospitalization	24	Undirected
Quality Improvement	Enteral Nutrition	24	Undirected
Quality Improvement	emergency health service	24	Undirected
Quality Improvement	emergency ward	24	Undirected
Quality Improvement	gestational age	24	Undirected
Quality Improvement	health care cost	24	Undirected
Quality Improvement	health care planning	24	Undirected
Quality Improvement	resuscitation	24	Undirected
Quality of Health Care	outcome assessment	24	Undirected
Reproducibility of Results	multicenter study	24	Undirected
Risk Assessment	Cardiac Surgical Procedures	24	Undirected
Risk Assessment	Medication Errors	24	Undirected
Risk Assessment	fever	24	Undirected
Risk Factors	Program Evaluation	24	Undirected
Risk Factors	interpersonal communication	24	Undirected
Sepsis	Electronic Health Records	24	Undirected
Sepsis	Insulin	24	Undirected
Sepsis	enteric feeding	24	Undirected
Social Support	clinical article	24	Undirected
Stress, Psychological	epidemiology	24	Undirected
Stroke	hypertension	24	Undirected
Time Factors	Adaptation, Psychological	24	Undirected
Time Factors	psychological aspect	24	Undirected
Treatment Outcome	Palliative Care	24	Undirected
Treatment Outcome	Pandemics	24	Undirected
Treatment Outcome	depression	24	Undirected
Treatment Outcome	propofol	24	Undirected
United Kingdom	epidemiology	24	Undirected
United Kingdom	medical education	24	Undirected

United Kingdom	multicenter study	24	Undirected
Workload	China	24	Undirected
Workload	health care cost	24	Undirected
adverse event	clinical article	24	Undirected
adverse event	organization and management	24	Undirected
analgesia	follow up	24	Undirected
attitude to health	medical education	24	Undirected
blood pressure	hospital admission	24	Undirected
blood pressure	methodology	24	Undirected
cohort analysis	extracorporeal oxygenation	24	Undirected
dementia	chronic obstructive lung disease	24	Undirected
depression	very elderly	24	Undirected
diabetes mellitus	computer assisted tomography	24	Undirected
dyspnea	observational study	24	Undirected
education	age	24	Undirected
education	hand washing	24	Undirected
education	pharmacist	24	Undirected
education	preschool child	24	Undirected
education	ventilator associated pneumonia	24	Undirected
emergency health service	hospital personnel	24	Undirected
endotracheal intubation	hospital discharge	24	Undirected
epidemiology	microbiology	24	Undirected
evidence based practice	follow up	24	Undirected
evidence based practice	meta analysis	24	Undirected
follow up	methodology	24	Undirected
follow up	pilot study	24	Undirected
follow up	vancomycin	24	Undirected
hand washing	antiinfective agent	24	Undirected
health care cost	Pandemics	24	Undirected
health care cost	antibiotic agent	24	Undirected
health care cost	diabetes mellitus	24	Undirected
health care cost	epidemiology	24	Undirected
health care cost	hospital infection	24	Undirected
health care cost	long term care	24	Undirected
health care cost	medical staff	24	Undirected
health care delivery	thematic analysis	24	Undirected
health care system	very elderly	24	Undirected
heart failure	disease severity	24	Undirected
heart failure	emergency ward	24	Undirected
heart rate	very elderly	24	Undirected
hospitalization	Pilot Projects	24	Undirected
hypotension	oxygen saturation	24	Undirected
hypotension	sedation	24	Undirected
intensivist	interpersonal communication	24	Undirected
knowledge	resuscitation	24	Undirected
medical staff	Health Personnel	24	Undirected
medical staff	semi structured interview	24	Undirected
medical staff	teaching hospital	24	Undirected
mortality	enteric feeding	24	Undirected
mortality	midazolam	24	Undirected
observational study	hospital infection	24	Undirected
outcome assessment	fentanyl	24	Undirected
outcome assessment	heart surgery	24	Undirected
outcome assessment	morphine	24	Undirected

outcome assessment	physiotherapy	24	Undirected
outcome assessment	simulation	24	Undirected
outcome assessment	ventilator associated pneumonia	24	Undirected
pain assessment	clinical practice	24	Undirected
pediatrics	methodology	24	Undirected
pharmacist	Health Personnel	24	Undirected
pharmacist	health care planning	24	Undirected
pharmacist	hospital discharge	24	Undirected
pharmacist	semi structured interview	24	Undirected
pharmacist	university hospital	24	Undirected
pneumonia	computer assisted tomography	24	Undirected
pneumonia	resuscitation	24	Undirected
practice guideline	pilot study	24	Undirected
practice guideline	vancomycin	24	Undirected
prematurity	medical education	24	Undirected
prevalence	gestational age	24	Undirected
prevalence	pharmacist	24	Undirected
prevalence	prematurity	24	Undirected
prevalence	teaching hospital	24	Undirected
propofol	endotracheal intubation	24	Undirected
propofol	resuscitation	24	Undirected
quality of life	therapy	24	Undirected
safety	Pandemics	24	Undirected
safety	adverse event	24	Undirected
safety	emergency ward	24	Undirected
semi structured interview	university hospital	24	Undirected
simulation	organization and management	24	Undirected
simulation	randomized controlled trial	24	Undirected
teaching hospital	cohort analysis	24	Undirected
tertiary care center	semi structured interview	24	Undirected
thematic analysis	respiratory therapist	24	Undirected
total quality management	Heart Arrest	24	Undirected
total quality management	Pressure Ulcer	24	Undirected
total quality management	hospital personnel	24	Undirected
tracheostomy	computer assisted tomography	24	Undirected
tracheostomy	observational study	24	Undirected
vancomycin	emergency ward	24	Undirected
very elderly	computer assisted tomography	24	Undirected
Algorithms	mortality	23	Undirected
Anxiety	Postoperative Complications	23	Undirected
Anxiety	health care system	23	Undirected
Artificial Ventilation	Emergency Service, Hospital	23	Undirected
Artificial Ventilation	Insulin	23	Undirected
Artificial Ventilation	Workforce	23	Undirected
Attitude of Health Personnel	Fatigue	23	Undirected
Attitude of Health Personnel	Intubation, Intratracheal	23	Undirected
Attitude of Health Personnel	mental health	23	Undirected
Attitude of Health Personnel	very elderly	23	Undirected
Australia	Delirium	23	Undirected
Burnout, Professional	China	23	Undirected
Canada	interpersonal communication	23	Undirected
Canada	resuscitation	23	Undirected
China	mortality	23	Undirected
Clinical Competence	Medication Errors	23	Undirected

Clinical Competence	Workplace	23	Undirected
Clinical Protocols	Cardiac Surgical Procedures	23	Undirected
Clinical Protocols	disease severity	23	Undirected
Clinical Protocols	pharmacist	23	Undirected
Communication	attitude	23	Undirected
Communication	burnout	23	Undirected
Communication	clinical practice	23	Undirected
Communication	follow up	23	Undirected
Communication	medical education	23	Undirected
Communication	outcome assessment	23	Undirected
Cross Infection	clinical article	23	Undirected
Decision Making	Clinical Protocols	23	Undirected
Decision Making	Pain	23	Undirected
Decision Making	evidence based practice	23	Undirected
Decision Making	health care planning	23	Undirected
Decision Making	therapy	23	Undirected
Delirium	pain assessment	23	Undirected
Delirium	physiotherapy	23	Undirected
Emergency Service, Hospital	Needs Assessment	23	Undirected
Emergency Service, Hospital	observational study	23	Undirected
Emergency Service, Hospital	preschool child	23	Undirected
Family	intensivist	23	Undirected
Family	mortality	23	Undirected
Guideline Adherence	clinical practice	23	Undirected
Guideline Adherence	hospitalization	23	Undirected
Guideline Adherence	university hospital	23	Undirected
Health Services Needs and Demand	Decision Making	23	Undirected
Health Services Needs and Demand	Length of Stay	23	Undirected
Heart Arrest	simulation	23	Undirected
Hypnotics and Sedatives	practice guideline	23	Undirected
Infant, Premature	Anxiety	23	Undirected
Infant, Premature	cohort analysis	23	Undirected
Infection Control	emergency care	23	Undirected
Infection Control	health care policy	23	Undirected
Interprofessional Relations	Australia	23	Undirected
Interprofessional Relations	Health Personnel	23	Undirected
Interprofessional Relations	Workload	23	Undirected
Intubation, Intratracheal	adverse event	23	Undirected
Job Satisfaction	depression	23	Undirected
Job Satisfaction	epidemiology	23	Undirected
Job Satisfaction	mental health	23	Undirected
Leadership	university hospital	23	Undirected
Length of Stay	Fatigue	23	Undirected
Length of Stay	Guideline Adherence	23	Undirected
Logistic Models	Pressure Ulcer	23	Undirected
Medication Errors	adverse event	23	Undirected
Medication Errors	clinical practice	23	Undirected
Medication Errors	standards	23	Undirected
Mothers	semi structured interview	23	Undirected
Needs Assessment	Focus Groups	23	Undirected
OUTCOMES	Sepsis	23	Undirected
Pain	Canada	23	Undirected
Pain	Pilot Projects	23	Undirected
Pain	breathing rate	23	Undirected

Pain	education	23	Undirected
Pain	hospital discharge	23	Undirected
Pain	interview	23	Undirected
Pain	very elderly	23	Undirected
Pain Measurement	psychology	23	Undirected
Palliative Care	Delirium	23	Undirected
Pandemics	endotracheal intubation	23	Undirected
Pandemics	physiotherapy	23	Undirected
Parents	COVID-19	23	Undirected
Parents	Cooperative Behavior	23	Undirected
Parents	Practice Guidelines as Topic	23	Undirected
Parents	depression	23	Undirected
Parents	practice guideline	23	Undirected
Parents	randomized controlled trial	23	Undirected
Personnel Staffing and Scheduling	Leadership	23	Undirected
Personnel Staffing and Scheduling	health care quality	23	Undirected
Personnel Staffing and Scheduling	standards	23	Undirected
Pilot Projects	heart rate	23	Undirected
Postoperative Complications	hypertension	23	Undirected
Practice Guidelines as Topic	Hospital Mortality	23	Undirected
Pregnancy	mortality	23	Undirected
Pregnancy	randomized controlled trial	23	Undirected
Pregnancy	total quality management	23	Undirected
Pressure Ulcer	adverse event	23	Undirected
Pressure Ulcer	methodology	23	Undirected
Pressure Ulcer	very elderly	23	Undirected
Professional-Family Relations	long term care	23	Undirected
Quality Improvement	Focus Groups	23	Undirected
Quality Improvement	follow up	23	Undirected
Quality Improvement	medical staff	23	Undirected
Quality Improvement	multicenter study	23	Undirected
Quality Improvement	respiratory therapist	23	Undirected
Quality of Health Care	Child, Preschool	23	Undirected
Quality of Health Care	Needs Assessment	23	Undirected
Reproducibility of Results	education	23	Undirected
Risk Assessment	Pain	23	Undirected
Risk Assessment	depression	23	Undirected
Risk Assessment	health care policy	23	Undirected
Risk Assessment	hypertension	23	Undirected
Risk Factors	Hypnotics and Sedatives	23	Undirected
Risk Factors	Terminal Care	23	Undirected
Risk Factors	teaching hospital	23	Undirected
Safety Management	Personnel Staffing and Scheduling	23	Undirected
Sensitivity and Specificity	multicenter study	23	Undirected
Sepsis	Postoperative Complications	23	Undirected
Sepsis	education	23	Undirected
Sepsis	emergency health service	23	Undirected
Sepsis	pediatrics	23	Undirected
Sepsis	thorax radiography	23	Undirected
Sepsis	university hospital	23	Undirected
Social Support	Australia	23	Undirected
Social Support	medical staff	23	Undirected
Social Support	semi structured interview	23	Undirected
Telemedicine	mortality	23	Undirected

Terminal Care	depression	23	Undirected
Terminal Care	knowledge	23	Undirected
Terminal Care	medical education	23	Undirected
Time Factors	Canada	23	Undirected
Time Factors	Needs Assessment	23	Undirected
Time Factors	Pain	23	Undirected
Time Factors	clinical practice	23	Undirected
Time Factors	emergency health service	23	Undirected
Time Factors	observational study	23	Undirected
Time Factors	pediatrics	23	Undirected
Time Factors	safety	23	Undirected
Treatment Outcome	Reproducibility of Results	23	Undirected
Treatment Outcome	caregiver	23	Undirected
Treatment Outcome	ethics	23	Undirected
Treatment Outcome	health care planning	23	Undirected
United Kingdom	Clinical Protocols	23	Undirected
United Kingdom	Leadership	23	Undirected
United Kingdom	hospital discharge	23	Undirected
Workforce	Stress, Psychological	23	Undirected
Workforce	education	23	Undirected
Workload	Clinical Protocols	23	Undirected
Workload	Infection Control	23	Undirected
Workload	United Kingdom	23	Undirected
Workload	mental health	23	Undirected
Workload	safety	23	Undirected
acute kidney failure	computer assisted tomography	23	Undirected
age	statistics and numerical data	23	Undirected
analgesia	heart rate	23	Undirected
attitude to health	Cross Infection	23	Undirected
attitude to health	Time Factors	23	Undirected
breathing rate	computer assisted tomography	23	Undirected
caregiver	resuscitation	23	Undirected
depression	observational study	23	Undirected
education	health care facility	23	Undirected
education	health care system	23	Undirected
emergency care	Health Personnel	23	Undirected
emergency care	health care planning	23	Undirected
emergency care	statistics and numerical data	23	Undirected
emergency ward	acute kidney failure	23	Undirected
endotracheal intubation	breathing rate	23	Undirected
endotracheal intubation	clinical practice	23	Undirected
endotracheal intubation	standards	23	Undirected
ethics	clinical practice	23	Undirected
ethics	health care quality	23	Undirected
evidence based practice	COVID-19	23	Undirected
evidence based practice	hospital admission	23	Undirected
follow up	extracorporeal oxygenation	23	Undirected
gestational age	psychology	23	Undirected
health care delivery	standards	23	Undirected
health care quality	hand washing	23	Undirected
health care quality	respiratory therapist	23	Undirected
heart failure	extracorporeal oxygenation	23	Undirected
heart failure	palliative therapy	23	Undirected
heart rate	sedation	23	Undirected

home care	health care system	23	Undirected
hospital admission	enteric feeding	23	Undirected
hypotension	thorax radiography	23	Undirected
intensivist	extracorporeal oxygenation	23	Undirected
interpersonal communication	health care policy	23	Undirected
interview	health care delivery	23	Undirected
knowledge	Pandemics	23	Undirected
knowledge	observational study	23	Undirected
long term care	disease severity	23	Undirected
long term care	epidemiology	23	Undirected
long term care	health care delivery	23	Undirected
medical staff	health care planning	23	Undirected
medical staff	information processing	23	Undirected
medical staff	respiratory therapist	23	Undirected
mental health	clinical article	23	Undirected
cillin resistant Staphylococcus a	hand washing	23	Undirected
microbiology	hospital admission	23	Undirected
midazolam	hospital admission	23	Undirected
midazolam	randomized controlled trial	23	Undirected
multicenter study	hospital infection	23	Undirected
multicenter study	palliative therapy	23	Undirected
observational study	teaching hospital	23	Undirected
outcome assessment	computer assisted tomography	23	Undirected
pain assessment	observational study	23	Undirected
pediatrics	Leadership	23	Undirected
pediatrics	caregiver	23	Undirected
pediatrics	evidence based practice	23	Undirected
pediatrics	medical staff	23	Undirected
pediatrics	sedation	23	Undirected
perception	medical staff	23	Undirected
pharmacist	epidemiology	23	Undirected
pharmacist	hospital personnel	23	Undirected
pharmacist	information processing	23	Undirected
pharmacist	palliative therapy	23	Undirected
pharmacist	thematic analysis	23	Undirected
pneumonia	breathing rate	23	Undirected
pneumonia	dementia	23	Undirected
practice guideline	thematic analysis	23	Undirected
prematurity	epidemiology	23	Undirected
prematurity	interpersonal communication	23	Undirected
prematurity	semi structured interview	23	Undirected
prevalence	organization and management	23	Undirected
prevalence	pediatrics	23	Undirected
prevalence	resuscitation	23	Undirected
quality of life	Stress, Psychological	23	Undirected
quality of life	chronic obstructive lung disease	23	Undirected
randomized controlled trial	statistics and numerical data	23	Undirected
resuscitation	health care policy	23	Undirected
safety	medical education	23	Undirected
sedation	multicenter study	23	Undirected
simulation	Pandemics	23	Undirected
simulation	health care quality	23	Undirected
statistics and numerical data	emergency health service	23	Undirected
teaching hospital	clinical practice	23	Undirected

tertiary care center	Delirium	23	Undirected
tertiary care center	antibiotic agent	23	Undirected
tertiary care center	health care quality	23	Undirected
tertiary care center	prematurity	23	Undirected
tertiary care center	randomized controlled trial	23	Undirected
tracheostomy	hypertension	23	Undirected
Adaptation, Psychological	methodology	22	Undirected
Anxiety	therapy	22	Undirected
Artificial Ventilation	Program Evaluation	22	Undirected
Artificial Ventilation	reliability	22	Undirected
Attitude of Health Personnel	Insulin	22	Undirected
Attitude of Health Personnel	Pressure Ulcer	22	Undirected
Australia	total quality management	22	Undirected
Breast Feeding	psychology	22	Undirected
Canada	hospital admission	22	Undirected
Child, Preschool	Anti-Bacterial Agents	22	Undirected
Child, Preschool	Health Personnel	22	Undirected
Child, Preschool	Workload	22	Undirected
Child, Preschool	education	22	Undirected
Child, Preschool	pneumonia	22	Undirected
China	clinical article	22	Undirected
China	cohort analysis	22	Undirected
China	practice guideline	22	Undirected
Clinical Competence	Cardiac Surgical Procedures	22	Undirected
Clinical Competence	Cross Infection	22	Undirected
Clinical Competence	emergency ward	22	Undirected
Clinical Competence	information processing	22	Undirected
Clinical Protocols	propofol	22	Undirected
Clinical Protocols	university hospital	22	Undirected
Clinical Protocols	ventilator associated pneumonia	22	Undirected
Cooperative Behavior	Quality Improvement	22	Undirected
Decision Making	health care policy	22	Undirected
Delirium	cerebrovascular accident	22	Undirected
Electronic Health Records	hospital readmission	22	Undirected
Emergency Service, Hospital	tertiary care center	22	Undirected
Family	Pregnancy	22	Undirected
Family	cohort analysis	22	Undirected
Family	epidemiology	22	Undirected
Family	medical education	22	Undirected
Family	randomized controlled trial	22	Undirected
Fatigue	health care system	22	Undirected
Guideline Adherence	resuscitation	22	Undirected
Health Personnel	disease severity	22	Undirected
Health Services Needs and Demand	Quality of Health Care	22	Undirected
Heart Arrest	oxygen saturation	22	Undirected
Hospital Mortality	Cardiac Surgical Procedures	22	Undirected
Hospital Mortality	antibiotic agent	22	Undirected
Hospital Mortality	medical staff	22	Undirected
Hospital Mortality	practice guideline	22	Undirected
Infant, Premature	interview	22	Undirected
Infection Control	teaching hospital	22	Undirected
Insulin	Workload	22	Undirected
Insulin	randomized controlled trial	22	Undirected
Interprofessional Relations	Safety Management	22	Undirected

Interprofessional Relations	psychological aspect	22	Undirected
Intubation, Intratracheal	ventilator associated pneumonia	22	Undirected
Job Satisfaction	Safety Management	22	Undirected
Leadership	health care policy	22	Undirected
Leadership	medical staff	22	Undirected
Length of Stay	Breast Feeding	22	Undirected
Logistic Models	very elderly	22	Undirected
Monitoring, Physiologic	Health Knowledge, Attitudes, Practice	22	Undirected
Monitoring, Physiologic	safety	22	Undirected
Mothers	randomized controlled trial	22	Undirected
Needs Assessment	Pilot Projects	22	Undirected
OUTCOMES	Delirium	22	Undirected
OUTCOMES	safety	22	Undirected
Pain	blood pressure	22	Undirected
Pain	standards	22	Undirected
Palliative Care	Needs Assessment	22	Undirected
Palliative Care	clinical practice	22	Undirected
Palliative Care	observational study	22	Undirected
Pandemics	hospital readmission	22	Undirected
Parents	Canada	22	Undirected
Parents	ethics	22	Undirected
Parents	follow up	22	Undirected
Parents	multicenter study	22	Undirected
Postoperative Care	analgesia	22	Undirected
Postoperative Care	morphine	22	Undirected
Postoperative Complications	practice guideline	22	Undirected
Practice Guidelines as Topic	preschool child	22	Undirected
Practice Guidelines as Topic	statistics and numerical data	22	Undirected
Pregnancy	Anxiety	22	Undirected
Pregnancy	methodology	22	Undirected
Pressure Ulcer	standards	22	Undirected
Professional-Family Relations	Canada	22	Undirected
Quality Improvement	epidemiology	22	Undirected
Quality Improvement	safety	22	Undirected
Quality of Health Care	Burnout, Professional	22	Undirected
Quality of Health Care	COVID-19	22	Undirected
Quality of Health Care	Cooperative Behavior	22	Undirected
Quality of Health Care	Emergency Service, Hospital	22	Undirected
Quality of Health Care	hospital admission	22	Undirected
Quality of Health Care	palliative therapy	22	Undirected
Reproducibility of Results	organization and management	22	Undirected
Risk Assessment	Glasgow coma scale	22	Undirected
Risk Assessment	antibiotic agent	22	Undirected
Risk Assessment	heart rate	22	Undirected
Risk Factors	extracorporeal oxygenation	22	Undirected
Risk Factors	fentanyl	22	Undirected
Risk Factors	midazolam	22	Undirected
Risk Factors	respiratory therapist	22	Undirected
Sensitivity and Specificity	preschool child	22	Undirected
Sensitivity and Specificity	standards	22	Undirected
Sepsis	Algorithms	22	Undirected
Sepsis	United Kingdom	22	Undirected
Sepsis	microbiology	22	Undirected
Social Support	palliative therapy	22	Undirected

Social Support	practice guideline	22	Undirected
Stress, Psychological	Palliative Care	22	Undirected
Stress, Psychological	interpersonal communication	22	Undirected
Terminal Care	university hospital	22	Undirected
Time Factors	Anti-Bacterial Agents	22	Undirected
Time Factors	Australia	22	Undirected
Time Factors	emergency ward	22	Undirected
Time Factors	prevalence	22	Undirected
Treatment Outcome	breathing rate	22	Undirected
Treatment Outcome	thorax radiography	22	Undirected
Treatment Outcome	ventilator associated pneumonia	22	Undirected
United Kingdom	information processing	22	Undirected
United Kingdom	interpersonal communication	22	Undirected
Workforce	Decision Making	22	Undirected
Workload	health care system	22	Undirected
Workplace	Anxiety	22	Undirected
Workplace	emergency ward	22	Undirected
Workplace	mental health	22	Undirected
adverse event	therapy	22	Undirected
analgesia	standards	22	Undirected
attitude to health	Focus Groups	22	Undirected
attitude to health	emergency ward	22	Undirected
attitude to health	tertiary care center	22	Undirected
blood pressure	randomized controlled trial	22	Undirected
blood pressure	thorax radiography	22	Undirected
burnout	caregiver	22	Undirected
cerebrovascular accident	disease severity	22	Undirected
clinical practice	hospital personnel	22	Undirected
cohort analysis	health care policy	22	Undirected
depression	health care quality	22	Undirected
depression	health care system	22	Undirected
depression	palliative therapy	22	Undirected
diabetes mellitus	randomized controlled trial	22	Undirected
disease severity	antiinfective agent	22	Undirected
dyspnea	resuscitation	22	Undirected
education	intensivist	22	Undirected
emergency care	antibiotic agent	22	Undirected
emergency health service	breathing rate	22	Undirected
emergency ward	hand washing	22	Undirected
endotracheal intubation	disease severity	22	Undirected
epidemiology	chronic obstructive lung disease	22	Undirected
epidemiology	clinical practice	22	Undirected
epidemiology	information processing	22	Undirected
ethics	education	22	Undirected
ethics	pediatrics	22	Undirected
evidence based practice	emergency ward	22	Undirected
evidence based practice	psychology	22	Undirected
follow up	enteric feeding	22	Undirected
follow up	statistics and numerical data	22	Undirected
heart failure	Palliative Care	22	Undirected
heart rate	practice guideline	22	Undirected
heart surgery	organization and management	22	Undirected
hospital discharge	antiinfective agent	22	Undirected
hospital discharge	enteric feeding	22	Undirected

hospital infection	clinical article	22	Undirected
hospitalization	Leadership	22	Undirected
hospitalization	thematic analysis	22	Undirected
hypotension	disease severity	22	Undirected
interview	health care facility	22	Undirected
knowledge	reliability	22	Undirected
long term care	psychology	22	Undirected
long term care	randomized controlled trial	22	Undirected
medical education	teaching hospital	22	Undirected
medical staff	disease severity	22	Undirected
medical staff	intensivist	22	Undirected
microbiology	cohort analysis	22	Undirected
midazolam	observational study	22	Undirected
mortality	mental health	22	Undirected
multicenter study	antibiotic agent	22	Undirected
multicenter study	hand washing	22	Undirected
observational study	breathing rate	22	Undirected
outcome assessment	breathing rate	22	Undirected
outcome assessment	fever	22	Undirected
oxygen saturation	very elderly	22	Undirected
pediatrics	Medication Errors	22	Undirected
pediatrics	analgesia	22	Undirected
pediatrics	health care delivery	22	Undirected
pediatrics	knowledge	22	Undirected
pediatrics	respiratory therapist	22	Undirected
pediatrics	semi structured interview	22	Undirected
perception	information processing	22	Undirected
perception	respiratory therapist	22	Undirected
pharmacist	Electronic Health Records	22	Undirected
pharmacist	antiinfective agent	22	Undirected
pharmacist	psychology	22	Undirected
physiotherapy	cohort analysis	22	Undirected
pilot study	emergency ward	22	Undirected
pneumonia	multicenter study	22	Undirected
practice guideline	oxygen saturation	22	Undirected
preschool child	enteric feeding	22	Undirected
prevalence	Logistic Models	22	Undirected
prevalence	caregiver	22	Undirected
prevalence	sedation	22	Undirected
prevalence	statistics and numerical data	22	Undirected
psychology	health care policy	22	Undirected
psychology	home care	22	Undirected
psychology	respiratory therapist	22	Undirected
quality of life	China	22	Undirected
quality of life	adverse event	22	Undirected
quality of life	analgesia	22	Undirected
quality of life	emergency care	22	Undirected
randomized controlled trial	Pandemics	22	Undirected
randomized controlled trial	cerebrovascular accident	22	Undirected
randomized controlled trial	health care system	22	Undirected
resuscitation	midazolam	22	Undirected
safety	emergency health service	22	Undirected
safety	medical staff	22	Undirected
safety	randomized controlled trial	22	Undirected

sedation	health care quality	22	Undirected
sedation	standards	22	Undirected
teaching hospital	COVID-19	22	Undirected
tertiary care center	health care delivery	22	Undirected
tertiary care center	hypertension	22	Undirected
tertiary care center	pediatrics	22	Undirected
tertiary care center	pharmacist	22	Undirected
tertiary care center	teaching hospital	22	Undirected
thematic analysis	medical education	22	Undirected
therapy	emergency health service	22	Undirected
total quality management	intensivist	22	Undirected
total quality management	knowledge	22	Undirected
total quality management	thematic analysis	22	Undirected
tracheostomy	adverse event	22	Undirected
tracheostomy	education	22	Undirected
tracheostomy	thorax radiography	22	Undirected
university hospital	palliative therapy	22	Undirected
Anti-Bacterial Agents	hospital discharge	21	Undirected
Anxiety	Pilot Projects	21	Undirected
Anxiety	knowledge	21	Undirected
Artificial Ventilation	ethics	21	Undirected
Attitude of Health Personnel	pilot study	21	Undirected
Australia	epidemiology	21	Undirected
Australia	information processing	21	Undirected
Australia	interview	21	Undirected
Australia	medical staff	21	Undirected
Australia	methodology	21	Undirected
Australia	perception	21	Undirected
Canada	Health Personnel	21	Undirected
Canada	emergency ward	21	Undirected
Canada	multicenter study	21	Undirected
Canada	psychological aspect	21	Undirected
Canada	respiratory therapist	21	Undirected
Child, Preschool	Program Evaluation	21	Undirected
Child, Preschool	fever	21	Undirected
Child, Preschool	sedation	21	Undirected
Child, Preschool	tracheostomy	21	Undirected
China	medical staff	21	Undirected
Clinical Competence	Pregnancy	21	Undirected
Clinical Protocols	enteric feeding	21	Undirected
Clinical Protocols	respiratory therapist	21	Undirected
Clinical Protocols	tracheostomy	21	Undirected
Communication	Reproducibility of Results	21	Undirected
Communication	emergency care	21	Undirected
Communication	hospital admission	21	Undirected
Communication	observational study	21	Undirected
Communication	pharmacist	21	Undirected
Communication	very elderly	21	Undirected
Cooperative Behavior	Focus Groups	21	Undirected
Cooperative Behavior	education	21	Undirected
Cross Infection	evidence based practice	21	Undirected
Cross Infection	health care quality	21	Undirected
Decision Making	Heart Arrest	21	Undirected
Decision Making	depression	21	Undirected

Delirium	university hospital	21	Undirected
Electronic Health Records	very elderly	21	Undirected
Emergency Service, Hospital	Practice Guidelines as Topic	21	Undirected
Emergency Service, Hospital	Reproducibility of Results	21	Undirected
Emergency Service, Hospital	Workload	21	Undirected
Emergency Service, Hospital	psychology	21	Undirected
Emergency Service, Hospital	statistics and numerical data	21	Undirected
Enteral Nutrition	Algorithms	21	Undirected
Family	analgesia	21	Undirected
Family	preschool child	21	Undirected
Family	university hospital	21	Undirected
Fatigue	hospital discharge	21	Undirected
Focus Groups	Delirium	21	Undirected
Focus Groups	practice guideline	21	Undirected
Glasgow coma scale	endotracheal intubation	21	Undirected
Glasgow coma scale	randomized controlled trial	21	Undirected
Health Services Needs and Demand	Terminal Care	21	Undirected
Heart Arrest	health care quality	21	Undirected
Hospital Mortality	Algorithms	21	Undirected
Hospital Mortality	dementia	21	Undirected
Hypnotics and Sedatives	Reproducibility of Results	21	Undirected
Hypnotics and Sedatives	randomized controlled trial	21	Undirected
Infant, Premature	United Kingdom	21	Undirected
Infant, Premature	clinical practice	21	Undirected
Infection Control	Leadership	21	Undirected
Infection Control	psychology	21	Undirected
Insulin	Medication Errors	21	Undirected
Intubation, Intratracheal	randomized controlled trial	21	Undirected
Job Satisfaction	clinical practice	21	Undirected
Job Satisfaction	multicenter study	21	Undirected
Leadership	health care planning	21	Undirected
Leadership	pharmacist	21	Undirected
Length of Stay	knowledge	21	Undirected
Logistic Models	Delirium	21	Undirected
Medication Errors	university hospital	21	Undirected
Monitoring, Physiologic	Cardiac Surgical Procedures	21	Undirected
Monitoring, Physiologic	Hypnotics and Sedatives	21	Undirected
Monitoring, Physiologic	Insulin	21	Undirected
Monitoring, Physiologic	resuscitation	21	Undirected
OUTCOMES	Risk Assessment	21	Undirected
Pain	dementia	21	Undirected
Pain	emergency ward	21	Undirected
Pain	mortality	21	Undirected
Pain Measurement	Canada	21	Undirected
Pain Measurement	Cardiac Surgical Procedures	21	Undirected
Pain Measurement	Clinical Protocols	21	Undirected
Pain Measurement	fentanyl	21	Undirected
Pain Measurement	organization and management	21	Undirected
Parents	health care quality	21	Undirected
Personnel Staffing and Scheduling	Quality Improvement	21	Undirected
Personnel Staffing and Scheduling	mortality	21	Undirected
Postoperative Care	hospital admission	21	Undirected
Practice Guidelines as Topic	Pilot Projects	21	Undirected
Practice Guidelines as Topic	mortality	21	Undirected

Pregnancy	Australia	21	Undirected
Pregnancy	clinical practice	21	Undirected
Pregnancy	disease severity	21	Undirected
Pregnancy	heart rate	21	Undirected
Pregnancy	hypertension	21	Undirected
Professional-Family Relations	pediatrics	21	Undirected
Professional-Family Relations	total quality management	21	Undirected
Program Evaluation	Leadership	21	Undirected
Program Evaluation	resuscitation	21	Undirected
Quality Improvement	Cardiac Surgical Procedures	21	Undirected
Quality Improvement	Pandemics	21	Undirected
Quality of Health Care	Health Personnel	21	Undirected
Quality of Health Care	Practice Guidelines as Topic	21	Undirected
Quality of Health Care	Reproducibility of Results	21	Undirected
Quality of Health Care	medical staff	21	Undirected
Risk Assessment	cerebrovascular accident	21	Undirected
Risk Assessment	health care facility	21	Undirected
Risk Factors	Breast Feeding	21	Undirected
Risk Factors	Mothers	21	Undirected
Sensitivity and Specificity	disease severity	21	Undirected
Social Support	methodology	21	Undirected
Stress, Psychological	mental health	21	Undirected
Stress, Psychological	perception	21	Undirected
Stress, Psychological	prematurity	21	Undirected
Stroke	hospital admission	21	Undirected
Telemedicine	health care system	21	Undirected
Terminal Care	health care system	21	Undirected
Terminal Care	intensivist	21	Undirected
Terminal Care	morphine	21	Undirected
Time Factors	Catheter-Related Infections	21	Undirected
Time Factors	Palliative Care	21	Undirected
Time Factors	Stroke	21	Undirected
Time Factors	tracheostomy	21	Undirected
Treatment Outcome	home care	21	Undirected
Treatment Outcome	microbiology	21	Undirected
Treatment Outcome	pharmacist	21	Undirected
Treatment Outcome	physiotherapy	21	Undirected
United Kingdom	cohort analysis	21	Undirected
Workforce	Family	21	Undirected
Workforce	pediatrics	21	Undirected
Workload	Canada	21	Undirected
Workload	health care delivery	21	Undirected
Workload	total quality management	21	Undirected
Workplace	China	21	Undirected
acute kidney failure	chronic obstructive lung disease	21	Undirected
adverse event	emergency ward	21	Undirected
analgesia	health care quality	21	Undirected
analgesia	physiotherapy	21	Undirected
attitude to health	Treatment Outcome	21	Undirected
attitude to health	hospital personnel	21	Undirected
attitude to health	prematurity	21	Undirected
attitude to health	university hospital	21	Undirected
blood pressure	COVID-19	21	Undirected
blood pressure	hospital discharge	21	Undirected

blood pressure	practice guideline	21	Undirected
blood pressure	resuscitation	21	Undirected
burnout	education	21	Undirected
burnout	health care delivery	21	Undirected
caregiver	clinical practice	21	Undirected
caregiver	cohort analysis	21	Undirected
caregiver	emergency care	21	Undirected
caregiver	thematic analysis	21	Undirected
dementia	Pandemics	21	Undirected
dementia	cerebrovascular accident	21	Undirected
dementia	organization and management	21	Undirected
depression	age	21	Undirected
depression	multicenter study	21	Undirected
diabetes mellitus	depression	21	Undirected
dyspnea	depression	21	Undirected
education	Medication Errors	21	Undirected
education	health care planning	21	Undirected
education	very elderly	21	Undirected
emergency care	cerebrovascular accident	21	Undirected
emergency care	computer assisted tomography	21	Undirected
emergency care	hospital personnel	21	Undirected
emergency health service	clinical article	21	Undirected
emergency health service	preschool child	21	Undirected
emergency ward	antiinfective agent	21	Undirected
endotracheal intubation	antibiotic agent	21	Undirected
endotracheal intubation	multicenter study	21	Undirected
epidemiology	computer assisted tomography	21	Undirected
epidemiology	medical education	21	Undirected
epidemiology	respiratory therapist	21	Undirected
ethics	methodology	21	Undirected
evidence based practice	analgesia	21	Undirected
evidence based practice	emergency care	21	Undirected
evidence based practice	therapy	21	Undirected
fentanyl	randomized controlled trial	21	Undirected
fever	acute kidney failure	21	Undirected
fever	observational study	21	Undirected
follow up	health care facility	21	Undirected
follow up	psychological aspect	21	Undirected
gestational age	clinical practice	21	Undirected
health care cost	Postoperative Complications	21	Undirected
health care cost	statistics and numerical data	21	Undirected
health care delivery	respiratory therapist	21	Undirected
health care planning	cohort analysis	21	Undirected
health care quality	antibiotic agent	21	Undirected
health care quality	cerebrovascular accident	21	Undirected
health care quality	hospital infection	21	Undirected
health care quality	thematic analysis	21	Undirected
heart failure	resuscitation	21	Undirected
heart rate	multicenter study	21	Undirected
heart surgery	hospital discharge	21	Undirected
home care	health care policy	21	Undirected
hospital admission	hand washing	21	Undirected
hospital infection	hospital discharge	21	Undirected
hospital readmission	cerebrovascular accident	21	Undirected

hospital readmission	organization and management	21	Undirected
hospitalization	Enteral Nutrition	21	Undirected
hypertension	endotracheal intubation	21	Undirected
interview	Canada	21	Undirected
interview	observational study	21	Undirected
interview	preschool child	21	Undirected
knowledge	epidemiology	21	Undirected
long term care	multicenter study	21	Undirected
medical staff	very elderly	21	Undirected
mental health	health care delivery	21	Undirected
mental health	randomized controlled trial	21	Undirected
microbiology	multicenter study	21	Undirected
midazolam	clinical practice	21	Undirected
multicenter study	health care system	21	Undirected
multicenter study	intensivist	21	Undirected
outcome assessment	Fatigue	21	Undirected
outcome assessment	extracorporeal oxygenation	21	Undirected
outcome assessment	hospital personnel	21	Undirected
outcome assessment	semi structured interview	21	Undirected
pediatrics	follow up	21	Undirected
pilot study	COVID-19	21	Undirected
pilot study	very elderly	21	Undirected
pneumonia	clinical practice	21	Undirected
pneumonia	physiotherapy	21	Undirected
practice guideline	age	21	Undirected
prematurity	evidence based practice	21	Undirected
prematurity	information processing	21	Undirected
prevalence	Australia	21	Undirected
prevalence	evidence based practice	21	Undirected
propofol	pain assessment	21	Undirected
psychology	health care planning	21	Undirected
quality of life	evidence based practice	21	Undirected
quality of life	hypertension	21	Undirected
randomized controlled trial	chronic obstructive lung disease	21	Undirected
randomized controlled trial	hand washing	21	Undirected
randomized controlled trial	palliative therapy	21	Undirected
respiratory therapist	cohort analysis	21	Undirected
resuscitation	hospital personnel	21	Undirected
resuscitation	university hospital	21	Undirected
safety	pharmacist	21	Undirected
sedation	COVID-19	21	Undirected
sedation	methodology	21	Undirected
sedation	physiotherapy	21	Undirected
simulation	interpersonal communication	21	Undirected
standards	palliative therapy	21	Undirected
teaching hospital	semi structured interview	21	Undirected
tertiary care center	Workload	21	Undirected
tertiary care center	emergency health service	21	Undirected
tertiary care center	fever	21	Undirected
tertiary care center	respiratory therapist	21	Undirected
therapy	preschool child	21	Undirected
total quality management	Canada	21	Undirected
total quality management	semi structured interview	21	Undirected
tracheostomy	Glasgow coma scale	21	Undirected

university hospital	preschool child	21	Undirected
urinary tract infection	follow up	21	Undirected
urinary tract infection	microbiology	21	Undirected
ventilator associated pneumonia	COVID-19	21	Undirected
ventilator associated pneumonia	:thicillin resistant Staphylococcus aure	21	Undirected
ventilator associated pneumonia	multicenter study	21	Undirected
ventilator associated pneumonia	observational study	21	Undirected
very elderly	acute kidney failure	21	Undirected
Algorithms	Electronic Health Records	20	Undirected
Anti-Bacterial Agents	emergency ward	20	Undirected
Anxiety	Pain Measurement	20	Undirected
Anxiety	medical staff	20	Undirected
Artificial Ventilation	Stroke	20	Undirected
Artificial Ventilation	teaching hospital	20	Undirected
Artificial Ventilation	urinary tract infection	20	Undirected
Attitude of Health Personnel	Hypnotics and Sedatives	20	Undirected
Attitude of Health Personnel	health care planning	20	Undirected
Australia	follow up	20	Undirected
Australia	health care delivery	20	Undirected
Australia	interpersonal communication	20	Undirected
Australia	statistics and numerical data	20	Undirected
Cardiac Surgical Procedures	adverse event	20	Undirected
Cardiac Surgical Procedures	methodology	20	Undirected
Catheter-Related Infections	practice guideline	20	Undirected
Child, Preschool	analgesia	20	Undirected
Child, Preschool	disease severity	20	Undirected
Child, Preschool	interview	20	Undirected
Clinical Competence	emergency health service	20	Undirected
Clinical Competence	health care delivery	20	Undirected
Clinical Competence	observational study	20	Undirected
Clinical Competence	tracheostomy	20	Undirected
Communication	Telemedicine	20	Undirected
Cooperative Behavior	psychological aspect	20	Undirected
Cross Infection	China	20	Undirected
Cross Infection	Pandemics	20	Undirected
Decision Making	Delirium	20	Undirected
Decision Making	burnout	20	Undirected
Decision Making	cohort analysis	20	Undirected
Delirium	age	20	Undirected
Emergency Service, Hospital	medical staff	20	Undirected
Enteral Nutrition	clinical article	20	Undirected
Family	Workload	20	Undirected
Family	emergency ward	20	Undirected
Family	health care policy	20	Undirected
Family	home care	20	Undirected
Family	observational study	20	Undirected
Family	prematurity	20	Undirected
Fatigue	palliative therapy	20	Undirected
Focus Groups	psychological aspect	20	Undirected
Focus Groups	standards	20	Undirected
Glasgow coma scale	hypertension	20	Undirected
Guideline Adherence	Algorithms	20	Undirected
Guideline Adherence	Hospital Mortality	20	Undirected
Guideline Adherence	health care quality	20	Undirected

Guideline Adherence	information processing	20	Undirected
alth Knowledge, Attitudes, Practi	interview	20	Undirected
alth Knowledge, Attitudes, Practi	resuscitation	20	Undirected
Health Services Needs and Demand	methodology	20	Undirected
Heart Arrest	clinical practice	20	Undirected
Heart Arrest	follow up	20	Undirected
Heart Arrest	randomized controlled trial	20	Undirected
Hospital Mortality	Sensitivity and Specificity	20	Undirected
Hospital Mortality	United Kingdom	20	Undirected
Hospital Mortality	home care	20	Undirected
Hospital Mortality	intensivist	20	Undirected
Hospital Mortality	meta analysis	20	Undirected
Hospital Mortality	therapy	20	Undirected
Hospital Mortality	ventilator associated pneumonia	20	Undirected
Infant, Premature	enteric feeding	20	Undirected
Infant, Premature	hospital admission	20	Undirected
Infant, Premature	multicenter study	20	Undirected
Infection Control	endotracheal intubation	20	Undirected
Job Satisfaction	tertiary care center	20	Undirected
Leadership	emergency health service	20	Undirected
Leadership	resuscitation	20	Undirected
Logistic Models	Personnel Staffing and Scheduling	20	Undirected
Monitoring, Physiologic	Intubation, Intratracheal	20	Undirected
Mothers	hospital admission	20	Undirected
Needs Assessment	psychology	20	Undirected
Pain	interpersonal communication	20	Undirected
Pain	psychological aspect	20	Undirected
Pain	tracheostomy	20	Undirected
Pain	university hospital	20	Undirected
Pain Measurement	Intubation, Intratracheal	20	Undirected
Pain Measurement	practice guideline	20	Undirected
Palliative Care	morphine	20	Undirected
Pandemics	hospital infection	20	Undirected
Pandemics	oxygen saturation	20	Undirected
Parents	Australia	20	Undirected
Parents	medical staff	20	Undirected
Personnel Staffing and Scheduling	Burnout, Professional	20	Undirected
Personnel Staffing and Scheduling	Pandemics	20	Undirected
Postoperative Care	heart rate	20	Undirected
Postoperative Care	organization and management	20	Undirected
Postoperative Complications	epidemiology	20	Undirected
Postoperative Complications	propofol	20	Undirected
Postoperative Complications	tracheostomy	20	Undirected
Practice Guidelines as Topic	Heart Arrest	20	Undirected
Practice Guidelines as Topic	Leadership	20	Undirected
Practice Guidelines as Topic	hospital discharge	20	Undirected
Practice Guidelines as Topic	safety	20	Undirected
Pregnancy	Health Personnel	20	Undirected
Pregnancy	health care cost	20	Undirected
Pressure Ulcer	epidemiology	20	Undirected
Professional-Family Relations	Length of Stay	20	Undirected
Quality Improvement	Intubation, Intratracheal	20	Undirected
Quality Improvement	Pilot Projects	20	Undirected
Quality Improvement	Postoperative Complications	20	Undirected

Quality Improvement	interview	20	Undirected
Quality Improvement	perception	20	Undirected
Quality Improvement	very elderly	20	Undirected
Quality of Health Care	Focus Groups	20	Undirected
Quality of Health Care	interpersonal communication	20	Undirected
Reproducibility of Results	preschool child	20	Undirected
Risk Assessment	Australia	20	Undirected
Risk Assessment	computer assisted tomography	20	Undirected
Risk Assessment	hand washing	20	Undirected
Risk Assessment	hypotension	20	Undirected
Risk Assessment	pharmacist	20	Undirected
Risk Factors	Needs Assessment	20	Undirected
Risk Factors	blood pressure	20	Undirected
Risk Factors	breathing rate	20	Undirected
Risk Factors	perception	20	Undirected
Safety Management	Leadership	20	Undirected
Sensitivity and Specificity	reliability	20	Undirected
Sepsis	Glasgow coma scale	20	Undirected
Sepsis	medical education	20	Undirected
Sepsis	medical staff	20	Undirected
Sepsis	meta analysis	20	Undirected
Social Support	Health Personnel	20	Undirected
Social Support	United Kingdom	20	Undirected
Stress, Psychological	Health Knowledge, Attitudes, Practice	20	Undirected
Stress, Psychological	interview	20	Undirected
Stress, Psychological	organization and management	20	Undirected
Telemedicine	follow up	20	Undirected
Terminal Care	analgesia	20	Undirected
Terminal Care	disease severity	20	Undirected
Terminal Care	epidemiology	20	Undirected
Terminal Care	thematic analysis	20	Undirected
Time Factors	Glasgow coma scale	20	Undirected
Time Factors	Pressure Ulcer	20	Undirected
Time Factors	Sensitivity and Specificity	20	Undirected
Time Factors	health care cost	20	Undirected
Time Factors	long term care	20	Undirected
Treatment Outcome	Catheterization, Central Venous	20	Undirected
Treatment Outcome	Health Personnel	20	Undirected
United Kingdom	Canada	20	Undirected
Workforce	Needs Assessment	20	Undirected
Workload	Focus Groups	20	Undirected
Workload	cohort analysis	20	Undirected
Workload	information processing	20	Undirected
Workplace	health care facility	20	Undirected
analgesia	cohort analysis	20	Undirected
analgesia	observational study	20	Undirected
attitude	multicenter study	20	Undirected
attitude to health	Child, Preschool	20	Undirected
attitude to health	Delirium	20	Undirected
attitude to health	Palliative Care	20	Undirected
attitude to health	Risk Assessment	20	Undirected
attitude to health	emergency care	20	Undirected
attitude to health	epidemiology	20	Undirected
attitude to health	follow up	20	Undirected

attitude to health	information processing	20	Undirected
attitude to health	pilot study	20	Undirected
burnout	emergency ward	20	Undirected
burnout	medical staff	20	Undirected
caregiver	hospital readmission	20	Undirected
caregiver	mental health	20	Undirected
caregiver	psychological aspect	20	Undirected
dementia	age	20	Undirected
dementia	hospital readmission	20	Undirected
dementia	randomized controlled trial	20	Undirected
depression	emergency ward	20	Undirected
depression	health care delivery	20	Undirected
diabetes mellitus	age	20	Undirected
diabetes mellitus	oxygen saturation	20	Undirected
diabetes mellitus	practice guideline	20	Undirected
emergency care	simulation	20	Undirected
emergency care	university hospital	20	Undirected
emergency health service	university hospital	20	Undirected
endotracheal intubation	medical education	20	Undirected
epidemiology	palliative therapy	20	Undirected
evidence based practice	health care delivery	20	Undirected
fever	microbiology	20	Undirected
fever	resuscitation	20	Undirected
follow up	hand washing	20	Undirected
follow up	meta analysis	20	Undirected
hand washing	antibiotic agent	20	Undirected
health care cost	Infection Control	20	Undirected
health care cost	caregiver	20	Undirected
health care cost	depression	20	Undirected
health care delivery	clinical article	20	Undirected
health care facility	health care planning	20	Undirected
health care policy	palliative therapy	20	Undirected
health care quality	reliability	20	Undirected
heart failure	Risk Assessment	20	Undirected
heart failure	depression	20	Undirected
heart failure	health care cost	20	Undirected
heart failure	heart rate	20	Undirected
home care	chronic obstructive lung disease	20	Undirected
hospital discharge	breathing rate	20	Undirected
hospital infection	health care facility	20	Undirected
hospital infection	university hospital	20	Undirected
hospital infection	very elderly	20	Undirected
hospital readmission	clinical article	20	Undirected
hospital readmission	university hospital	20	Undirected
hospitalization	respiratory therapist	20	Undirected
hypertension	age	20	Undirected
hypotension	cohort analysis	20	Undirected
hypotension	morphine	20	Undirected
intensivist	palliative therapy	20	Undirected
interpersonal communication	disease severity	20	Undirected
knowledge	clinical article	20	Undirected
knowledge	follow up	20	Undirected
long term care	hospital readmission	20	Undirected
long term care	observational study	20	Undirected

medical education	hospital discharge	20	Undirected
medical staff	health care policy	20	Undirected
medical staff	methodology	20	Undirected
cillin resistant Staphylococcus a	cohort analysis	20	Undirected
methodology	health care facility	20	Undirected
methodology	heart surgery	20	Undirected
methodology	hospital admission	20	Undirected
microbiology	clinical article	20	Undirected
morphine	hospital admission	20	Undirected
mortality	information processing	20	Undirected
multicenter study	cerebrovascular accident	20	Undirected
multicenter study	enteric feeding	20	Undirected
observational study	cerebrovascular accident	20	Undirected
observational study	extracorporeal oxygenation	20	Undirected
observational study	health care facility	20	Undirected
observational study	heart surgery	20	Undirected
observational study	home care	20	Undirected
observational study	hospital personnel	20	Undirected
observational study	simulation	20	Undirected
outcome assessment	Reproducibility of Results	20	Undirected
outcome assessment	propofol	20	Undirected
outcome assessment	safety	20	Undirected
pediatrics	Pressure Ulcer	20	Undirected
pediatrics	information processing	20	Undirected
perception	safety	20	Undirected
perception	teaching hospital	20	Undirected
pharmacist	health care policy	20	Undirected
pharmacist	teaching hospital	20	Undirected
pilot study	standards	20	Undirected
pneumonia	Glasgow coma scale	20	Undirected
pneumonia	emergency health service	20	Undirected
pneumonia	preschool child	20	Undirected
practice guideline	acute kidney failure	20	Undirected
practice guideline	cerebrovascular accident	20	Undirected
practice guideline	hypertension	20	Undirected
practice guideline	thicillin resistant Staphylococcus aure	20	Undirected
prematurity	Clinical Protocols	20	Undirected
prematurity	Enteral Nutrition	20	Undirected
prematurity	medical staff	20	Undirected
prevalence	Clinical Protocols	20	Undirected
prevalence	Medication Errors	20	Undirected
prevalence	adverse event	20	Undirected
prevalence	heart failure	20	Undirected
prevalence	hospital personnel	20	Undirected
propofol	clinical practice	20	Undirected
propofol	hospital admission	20	Undirected
propofol	oxygen saturation	20	Undirected
psychology	psychological aspect	20	Undirected
quality of life	OUTCOMES	20	Undirected
quality of life	Parents	20	Undirected
quality of life	interview	20	Undirected
quality of life	pediatrics	20	Undirected
quality of life	psychological aspect	20	Undirected
quality of life	total quality management	20	Undirected

quality of life	tracheostomy	20	Undirected
respiratory therapist	extracorporeal oxygenation	20	Undirected
sedation	preschool child	20	Undirected
simulation	emergency ward	20	Undirected
statistics and numerical data	disease severity	20	Undirected
statistics and numerical data	interpersonal communication	20	Undirected
teaching hospital	clinical article	20	Undirected
teaching hospital	university hospital	20	Undirected
tertiary care center	Anxiety	20	Undirected
tertiary care center	Terminal Care	20	Undirected
tertiary care center	age	20	Undirected
tertiary care center	hospital personnel	20	Undirected
tertiary care center	urinary tract infection	20	Undirected
tertiary care center	ventilator associated pneumonia	20	Undirected
therapy	palliative therapy	20	Undirected
total quality management	disease severity	20	Undirected
total quality management	hand washing	20	Undirected
total quality management	pilot study	20	Undirected
total quality management	teaching hospital	20	Undirected
tracheostomy	disease severity	20	Undirected
tracheostomy	emergency ward	20	Undirected
tracheostomy	fever	20	Undirected
urinary tract infection	ethicillin resistant Staphylococcus aure	20	Undirected
vancomycin	cohort analysis	20	Undirected
vancomycin	hospital discharge	20	Undirected
ventilator associated pneumonia	hospital admission	20	Undirected
ventilator associated pneumonia	microbiology	20	Undirected
Adaptation, Psychological	hospitalization	19	Undirected
Algorithms	resuscitation	19	Undirected
Anxiety	China	19	Undirected
Anxiety	dementia	19	Undirected
Anxiety	health care quality	19	Undirected
Artificial Ventilation	Electronic Health Records	19	Undirected
Artificial Ventilation	hospital personnel	19	Undirected
Artificial Ventilation	thematic analysis	19	Undirected
Attitude of Health Personnel	health care policy	19	Undirected
Attitude of Health Personnel	preschool child	19	Undirected
Attitude of Health Personnel	randomized controlled trial	19	Undirected
Attitude of Health Personnel	teaching hospital	19	Undirected
Australia	Pilot Projects	19	Undirected
Australia	Workload	19	Undirected
Australia	cohort analysis	19	Undirected
Australia	emergency health service	19	Undirected
Canada	follow up	19	Undirected
Canada	health care quality	19	Undirected
Catheter-Related Infections	organization and management	19	Undirected
Child, Preschool	Catheter-Related Infections	19	Undirected
Child, Preschool	Logistic Models	19	Undirected
Child, Preschool	Needs Assessment	19	Undirected
Child, Preschool	Palliative Care	19	Undirected
Child, Preschool	emergency health service	19	Undirected
Child, Preschool	psychological aspect	19	Undirected
Clinical Competence	Enteral Nutrition	19	Undirected
Clinical Competence	clinical article	19	Undirected

Clinical Competence	pilot study	19	Undirected
Clinical Competence	ventilator associated pneumonia	19	Undirected
Clinical Protocols	cohort analysis	19	Undirected
Clinical Protocols	emergency health service	19	Undirected
Communication	emergency health service	19	Undirected
Communication	health care delivery	19	Undirected
Communication	intensivist	19	Undirected
Communication	knowledge	19	Undirected
Communication	long term care	19	Undirected
Communication	respiratory therapist	19	Undirected
Communication	statistics and numerical data	19	Undirected
Cross Infection	Program Evaluation	19	Undirected
Cross Infection	Workforce	19	Undirected
Cross Infection	follow up	19	Undirected
Cross Infection	health care delivery	19	Undirected
Cross Infection	randomized controlled trial	19	Undirected
Cross Infection	statistics and numerical data	19	Undirected
Cross Infection	teaching hospital	19	Undirected
Decision Making	disease severity	19	Undirected
Decision Making	gestational age	19	Undirected
Delirium	Stroke	19	Undirected
Delirium	adverse event	19	Undirected
Delirium	methodology	19	Undirected
Electronic Health Records	follow up	19	Undirected
Emergency Service, Hospital	Sensitivity and Specificity	19	Undirected
Emergency Service, Hospital	follow up	19	Undirected
Enteral Nutrition	Breast Feeding	19	Undirected
Family	emergency health service	19	Undirected
Family	evidence based practice	19	Undirected
Family	prevalence	19	Undirected
Family	sedation	19	Undirected
Family	tertiary care center	19	Undirected
Focus Groups	semi structured interview	19	Undirected
Glasgow coma scale	clinical practice	19	Undirected
Glasgow coma scale	practice guideline	19	Undirected
Glasgow coma scale	resuscitation	19	Undirected
Guideline Adherence	medical education	19	Undirected
Guideline Adherence	medical staff	19	Undirected
Guideline Adherence	psychology	19	Undirected
alth Knowledge, Attitudes, Practi	COVID-19	19	Undirected
alth Knowledge, Attitudes, Practi	Pregnancy	19	Undirected
alth Knowledge, Attitudes, Practi	Workload	19	Undirected
Hospital Mortality	adverse event	19	Undirected
Hospital Mortality	heart rate	19	Undirected
Hospital Mortality	tracheostomy	19	Undirected
Hypnotics and Sedatives	Pilot Projects	19	Undirected
Infant, Premature	Clinical Protocols	19	Undirected
Infection Control	long term care	19	Undirected
Insulin	clinical article	19	Undirected
Insulin	hospital admission	19	Undirected
Interprofessional Relations	Pilot Projects	19	Undirected
Job Satisfaction	emergency ward	19	Undirected
Job Satisfaction	health care delivery	19	Undirected
Job Satisfaction	medical staff	19	Undirected

Leadership	Heart Arrest	19	Undirected
Leadership	hospital admission	19	Undirected
Leadership	multicenter study	19	Undirected
Leadership	simulation	19	Undirected
Length of Stay	Cooperative Behavior	19	Undirected
Logistic Models	Workload	19	Undirected
Monitoring, Physiologic	Decision Making	19	Undirected
Monitoring, Physiologic	Safety Management	19	Undirected
Monitoring, Physiologic	Sepsis	19	Undirected
Monitoring, Physiologic	United Kingdom	19	Undirected
Mothers	follow up	19	Undirected
Mothers	interpersonal communication	19	Undirected
Needs Assessment	Leadership	19	Undirected
Needs Assessment	standards	19	Undirected
OUTCOMES	Quality Improvement	19	Undirected
OUTCOMES	epidemiology	19	Undirected
Pain	hypertension	19	Undirected
Pain	physiotherapy	19	Undirected
Pain Measurement	midazolam	19	Undirected
Palliative Care	follow up	19	Undirected
Palliative Care	medical education	19	Undirected
Palliative Care	standards	19	Undirected
Parents	Artificial Ventilation	19	Undirected
Parents	resuscitation	19	Undirected
Postoperative Care	Pilot Projects	19	Undirected
Postoperative Care	propofol	19	Undirected
Practice Guidelines as Topic	Canada	19	Undirected
Practice Guidelines as Topic	Postoperative Complications	19	Undirected
Practice Guidelines as Topic	emergency health service	19	Undirected
Practice Guidelines as Topic	hospital admission	19	Undirected
Practice Guidelines as Topic	sedation	19	Undirected
Pregnancy	interview	19	Undirected
Pressure Ulcer	Intubation, Intratracheal	19	Undirected
Professional-Family Relations	Emergency Service, Hospital	19	Undirected
Professional-Family Relations	prematurity	19	Undirected
Program Evaluation	outcome assessment	19	Undirected
Program Evaluation	standards	19	Undirected
Quality Improvement	Algorithms	19	Undirected
Quality Improvement	Canada	19	Undirected
Quality Improvement	Heart Arrest	19	Undirected
Quality Improvement	sedation	19	Undirected
Quality Improvement	therapy	19	Undirected
Quality of Health Care	safety	19	Undirected
Risk Assessment	Pilot Projects	19	Undirected
Risk Assessment	chronic obstructive lung disease	19	Undirected
Risk Assessment	health care system	19	Undirected
Risk Assessment	long term care	19	Undirected
Risk Assessment	reliability	19	Undirected
Risk Factors	interview	19	Undirected
Safety Management	Infection Control	19	Undirected
Sensitivity and Specificity	epidemiology	19	Undirected
Sepsis	breathing rate	19	Undirected
Sepsis	health care facility	19	Undirected
Sepsis	health care system	19	Undirected

Sepsis	long term care	19	Undirected
Social Support	Child, Preschool	19	Undirected
Social Support	Practice Guidelines as Topic	19	Undirected
Social Support	Quality of Health Care	19	Undirected
Stress, Psychological	Child, Preschool	19	Undirected
Stress, Psychological	Focus Groups	19	Undirected
Stroke	cohort analysis	19	Undirected
Stroke	computer assisted tomography	19	Undirected
Stroke	very elderly	19	Undirected
Terminal Care	emergency care	19	Undirected
Terminal Care	statistics and numerical data	19	Undirected
Time Factors	Electronic Health Records	19	Undirected
Time Factors	Stress, Psychological	19	Undirected
Time Factors	medical staff	19	Undirected
Time Factors	pneumonia	19	Undirected
Treatment Outcome	Communication	19	Undirected
Treatment Outcome	Sensitivity and Specificity	19	Undirected
Treatment Outcome	sedation	19	Undirected
Treatment Outcome	teaching hospital	19	Undirected
United Kingdom	Pilot Projects	19	Undirected
United Kingdom	emergency ward	19	Undirected
United Kingdom	health care cost	19	Undirected
Workforce	Health Knowledge, Attitudes, Practice	19	Undirected
Workforce	Logistic Models	19	Undirected
Workforce	perception	19	Undirected
Workload	interpersonal communication	19	Undirected
Workplace	Safety Management	19	Undirected
Workplace	perception	19	Undirected
adverse event	disease severity	19	Undirected
adverse event	hospital readmission	19	Undirected
age	emergency health service	19	Undirected
attitude to health	Canada	19	Undirected
attitude to health	caregiver	19	Undirected
attitude to health	hospital discharge	19	Undirected
attitude to health	semi structured interview	19	Undirected
blood pressure	disease severity	19	Undirected
burnout	randomized controlled trial	19	Undirected
caregiver	cerebrovascular accident	19	Undirected
caregiver	therapy	19	Undirected
clinical article	antiinfective agent	19	Undirected
clinical practice	antiinfective agent	19	Undirected
cohort analysis	enteric feeding	19	Undirected
dementia	emergency ward	19	Undirected
dementia	observational study	19	Undirected
dyspnea	tracheostomy	19	Undirected
education	depression	19	Undirected
education	mental health	19	Undirected
emergency care	information processing	19	Undirected
emergency health service	psychological aspect	19	Undirected
emergency ward	hospital infection	19	Undirected
emergency ward	meta analysis	19	Undirected
endotracheal intubation	respiratory therapist	19	Undirected
ethics	perception	19	Undirected
evidence based practice	Catheterization, Central Venous	19	Undirected

evidence based practice	respiratory therapist	19	Undirected
fentanyl	observational study	19	Undirected
follow up	information processing	19	Undirected
follow up	thematic analysis	19	Undirected
gestational age	education	19	Undirected
gestational age	enteric feeding	19	Undirected
gestational age	pain assessment	19	Undirected
hand washing	organization and management	19	Undirected
hand washing	university hospital	19	Undirected
health care cost	observational study	19	Undirected
health care delivery	information processing	19	Undirected
health care delivery	intensivist	19	Undirected
health care delivery	very elderly	19	Undirected
health care facility	hospital personnel	19	Undirected
heart failure	dementia	19	Undirected
heart failure	multicenter study	19	Undirected
heart rate	fever	19	Undirected
hospital admission	respiratory therapist	19	Undirected
hospital readmission	acute kidney failure	19	Undirected
hospital readmission	therapy	19	Undirected
hypertension	health care delivery	19	Undirected
hypertension	therapy	19	Undirected
hypotension	breathing rate	19	Undirected
intensivist	cohort analysis	19	Undirected
interview	hospital personnel	19	Undirected
interview	respiratory therapist	19	Undirected
knowledge	health care delivery	19	Undirected
knowledge	organization and management	19	Undirected
knowledge	university hospital	19	Undirected
long term care	health care system	19	Undirected
long term care	psychological aspect	19	Undirected
medical education	hand washing	19	Undirected
medical education	methodology	19	Undirected
mental health	disease severity	19	Undirected
mental health	organization and management	19	Undirected
observational study	health care system	19	Undirected
observational study	microbiology	19	Undirected
observational study	reliability	19	Undirected
observational study	therapy	19	Undirected
organization and management	antiinfective agent	19	Undirected
outcome assessment	Pilot Projects	19	Undirected
outcome assessment	Sensitivity and Specificity	19	Undirected
pediatrics	perception	19	Undirected
pediatrics	university hospital	19	Undirected
pharmacist	disease severity	19	Undirected
pharmacist	hospital readmission	19	Undirected
pharmacist	standards	19	Undirected
pilot study	interpersonal communication	19	Undirected
pneumonia	Pandemics	19	Undirected
pneumonia	age	19	Undirected
pneumonia	emergency care	19	Undirected
pneumonia	health care quality	19	Undirected
practice guideline	thorax radiography	19	Undirected
prevalence	Pregnancy	19	Undirected

prevalence	Quality Improvement	19	Undirected
prevalence	acute kidney failure	19	Undirected
prevalence	computer assisted tomography	19	Undirected
prevalence	emergency health service	19	Undirected
prevalence	health care policy	19	Undirected
prevalence	medical education	19	Undirected
propofol	heart rate	19	Undirected
propofol	observational study	19	Undirected
quality of life	Heart Arrest	19	Undirected
quality of life	Quality of Health Care	19	Undirected
quality of life	health care planning	19	Undirected
quality of life	semi structured interview	19	Undirected
randomized controlled trial	hospital infection	19	Undirected
randomized controlled trial	psychological aspect	19	Undirected
randomized controlled trial	university hospital	19	Undirected
respiratory therapist	university hospital	19	Undirected
resuscitation	acute kidney failure	19	Undirected
resuscitation	thorax radiography	19	Undirected
safety	hospital personnel	19	Undirected
sedation	follow up	19	Undirected
sedation	palliative therapy	19	Undirected
semi structured interview	hospital admission	19	Undirected
simulation	therapy	19	Undirected
standards	clinical article	19	Undirected
therapy	health care system	19	Undirected
total quality management	Pain	19	Undirected
total quality management	sedation	19	Undirected
tracheostomy	preschool child	19	Undirected
tracheostomy	resuscitation	19	Undirected
university hospital	psychological aspect	19	Undirected
urinary tract infection	emergency ward	19	Undirected
vancomycin	disease severity	19	Undirected
ventilator associated pneumonia	cohort analysis	19	Undirected
ventilator associated pneumonia	sedation	19	Undirected
Adaptation, Psychological	Workload	18	Undirected
Adaptation, Psychological	prematurity	18	Undirected
Algorithms	COVID-19	18	Undirected
Algorithms	education	18	Undirected
Anxiety	medical education	18	Undirected
Anxiety	meta analysis	18	Undirected
Anxiety	university hospital	18	Undirected
Artificial Ventilation	Focus Groups	18	Undirected
Artificial Ventilation	simulation	18	Undirected
Attitude of Health Personnel	Breast Feeding	18	Undirected
Attitude of Health Personnel	Electronic Health Records	18	Undirected
Attitude of Health Personnel	gestational age	18	Undirected
Attitude of Health Personnel	hospital discharge	18	Undirected
Australia	Clinical Protocols	18	Undirected
Australia	observational study	18	Undirected
Australia	psychological aspect	18	Undirected
Australia	very elderly	18	Undirected
Canada	information processing	18	Undirected
Cardiac Surgical Procedures	organization and management	18	Undirected
Catheterization, Central Venous	practice guideline	18	Undirected

Catheterization, Central Venous	randomized controlled trial	18	Undirected
Catheterization, Central Venous	standards	18	Undirected
Child, Preschool	Australia	18	Undirected
Child, Preschool	midazolam	18	Undirected
Clinical Competence	endotracheal intubation	18	Undirected
Clinical Competence	hospital personnel	18	Undirected
Clinical Protocols	antibiotic agent	18	Undirected
Clinical Protocols	diabetes mellitus	18	Undirected
Clinical Protocols	epidemiology	18	Undirected
Clinical Protocols	pilot study	18	Undirected
Communication	Delirium	18	Undirected
Communication	hospital discharge	18	Undirected
Communication	mortality	18	Undirected
Communication	pilot study	18	Undirected
Communication	university hospital	18	Undirected
Cooperative Behavior	United Kingdom	18	Undirected
Decision Making	Electronic Health Records	18	Undirected
Delirium	hospital readmission	18	Undirected
Delirium	standards	18	Undirected
Emergency Service, Hospital	Health Knowledge, Attitudes, Practice	18	Undirected
Emergency Service, Hospital	Palliative Care	18	Undirected
Emergency Service, Hospital	interpersonal communication	18	Undirected
Emergency Service, Hospital	medical education	18	Undirected
Enteral Nutrition	gestational age	18	Undirected
Family	Leadership	18	Undirected
Family	Mothers	18	Undirected
Family	disease severity	18	Undirected
Fatigue	epidemiology	18	Undirected
Fatigue	hypertension	18	Undirected
Focus Groups	interpersonal communication	18	Undirected
Guideline Adherence	Australia	18	Undirected
Guideline Adherence	Enteral Nutrition	18	Undirected
Guideline Adherence	knowledge	18	Undirected
Guideline Adherence	methodology	18	Undirected
alth Knowledge, Attitudes, Practi	Catheterization, Central Venous	18	Undirected
alth Knowledge, Attitudes, Practi	Mothers	18	Undirected
alth Knowledge, Attitudes, Practi	hand washing	18	Undirected
Health Personnel	thicillin resistant Staphylococcus aure	18	Undirected
Health Personnel	simulation	18	Undirected
Health Services Needs and Demand	Palliative Care	18	Undirected
Health Services Needs and Demand	education	18	Undirected
Heart Arrest	cerebrovascular accident	18	Undirected
Heart Arrest	endotracheal intubation	18	Undirected
Heart Arrest	medical staff	18	Undirected
Heart Arrest	methodology	18	Undirected
Hospital Mortality	dyspnea	18	Undirected
Hospital Mortality	health care delivery	18	Undirected
Hypnotics and Sedatives	morphine	18	Undirected
Infant, Premature	total quality management	18	Undirected
Infection Control	hospital discharge	18	Undirected
Infection Control	respiratory therapist	18	Undirected
Infection Control	resuscitation	18	Undirected
Infection Control	simulation	18	Undirected
Interprofessional Relations	Needs Assessment	18	Undirected

Interprofessional Relations	Stress, Psychological	18	Undirected
Job Satisfaction	Anxiety	18	Undirected
Job Satisfaction	Australia	18	Undirected
Job Satisfaction	interpersonal communication	18	Undirected
Job Satisfaction	knowledge	18	Undirected
Job Satisfaction	outcome assessment	18	Undirected
Job Satisfaction	total quality management	18	Undirected
Length of Stay	Job Satisfaction	18	Undirected
Length of Stay	reliability	18	Undirected
Medication Errors	information processing	18	Undirected
Medication Errors	medical education	18	Undirected
Medication Errors	morphine	18	Undirected
Monitoring, Physiologic	evidence based practice	18	Undirected
Mothers	organization and management	18	Undirected
Needs Assessment	Terminal Care	18	Undirected
OUTCOMES	education	18	Undirected
OUTCOMES	pneumonia	18	Undirected
Pain	endotracheal intubation	18	Undirected
Pain	fever	18	Undirected
Pain	hypotension	18	Undirected
Palliative Care	depression	18	Undirected
Palliative Care	disease severity	18	Undirected
Palliative Care	emergency ward	18	Undirected
Pandemics	information processing	18	Undirected
Parents	Pain Measurement	18	Undirected
Parents	thematic analysis	18	Undirected
Pilot Projects	caregiver	18	Undirected
Pilot Projects	resuscitation	18	Undirected
Postoperative Care	Pain Measurement	18	Undirected
Postoperative Care	Quality Improvement	18	Undirected
Postoperative Complications	observational study	18	Undirected
Practice Guidelines as Topic	Cardiac Surgical Procedures	18	Undirected
Practice Guidelines as Topic	Focus Groups	18	Undirected
Practice Guidelines as Topic	Medication Errors	18	Undirected
Practice Guidelines as Topic	tertiary care center	18	Undirected
Pregnancy	Sepsis	18	Undirected
Pressure Ulcer	multicenter study	18	Undirected
Professional-Family Relations	Pilot Projects	18	Undirected
Professional-Family Relations	health care quality	18	Undirected
Quality Improvement	emergency care	18	Undirected
Quality Improvement	hospital readmission	18	Undirected
Quality Improvement	preschool child	18	Undirected
Quality of Health Care	Logistic Models	18	Undirected
Quality of Health Care	Pilot Projects	18	Undirected
Quality of Health Care	clinical practice	18	Undirected
Quality of Health Care	education	18	Undirected
Quality of Health Care	health care cost	18	Undirected
Reproducibility of Results	clinical practice	18	Undirected
Reproducibility of Results	hospital admission	18	Undirected
Reproducibility of Results	observational study	18	Undirected
Reproducibility of Results	practice guideline	18	Undirected
Risk Assessment	Anti-Bacterial Agents	18	Undirected
Risk Assessment	Stroke	18	Undirected
Risk Assessment	caregiver	18	Undirected

Risk Assessment	pilot study	18	Undirected
Risk Factors	morphine	18	Undirected
Risk Factors	pilot study	18	Undirected
Safety Management	interpersonal communication	18	Undirected
Sensitivity and Specificity	Glasgow coma scale	18	Undirected
Sepsis	Sensitivity and Specificity	18	Undirected
Sepsis	statistics and numerical data	18	Undirected
Sepsis	tracheostomy	18	Undirected
Social Support	Time Factors	18	Undirected
Stress, Psychological	Needs Assessment	18	Undirected
Stress, Psychological	randomized controlled trial	18	Undirected
Telemedicine	intensivist	18	Undirected
Telemedicine	practice guideline	18	Undirected
Terminal Care	health care planning	18	Undirected
Terminal Care	observational study	18	Undirected
Time Factors	Breast Feeding	18	Undirected
Time Factors	heart surgery	18	Undirected
Time Factors	sedation	18	Undirected
Treatment Outcome	Electronic Health Records	18	Undirected
Treatment Outcome	Health Knowledge, Attitudes, Practice	18	Undirected
Treatment Outcome	Personnel Staffing and Scheduling	18	Undirected
Treatment Outcome	fentanyl	18	Undirected
Treatment Outcome	hospital infection	18	Undirected
Treatment Outcome	morphine	18	Undirected
United Kingdom	follow up	18	Undirected
Workforce	Interprofessional Relations	18	Undirected
Workforce	mortality	18	Undirected
Workload	Electronic Health Records	18	Undirected
Workload	Pilot Projects	18	Undirected
Workload	medical education	18	Undirected
adverse event	meta analysis	18	Undirected
analgesia	COVID-19	18	Undirected
analgesia	caregiver	18	Undirected
analgesia	resuscitation	18	Undirected
antibiotic agent	enteric feeding	18	Undirected
antibiotic agent	organization and management	18	Undirected
attitude to health	Pain Measurement	18	Undirected
attitude to health	randomized controlled trial	18	Undirected
attitude to health	statistics and numerical data	18	Undirected
caregiver	medical education	18	Undirected
caregiver	preschool child	18	Undirected
clinical practice	enteric feeding	18	Undirected
clinical practice	health care facility	18	Undirected
clinical practice	preschool child	18	Undirected
diabetes mellitus	Glasgow coma scale	18	Undirected
diabetes mellitus	breathing rate	18	Undirected
diabetes mellitus	health care quality	18	Undirected
diabetes mellitus	thorax radiography	18	Undirected
disease severity	enteric feeding	18	Undirected
dyspnea	antibiotic agent	18	Undirected
dyspnea	emergency care	18	Undirected
dyspnea	endotracheal intubation	18	Undirected
dyspnea	health care system	18	Undirected
dyspnea	multicenter study	18	Undirected

dyspnea	practice guideline	18	Undirected
education	heart surgery	18	Undirected
emergency health service	palliative therapy	18	Undirected
endotracheal intubation	emergency health service	18	Undirected
epidemiology	cerebrovascular accident	18	Undirected
epidemiology	extracorporeal oxygenation	18	Undirected
epidemiology	hand washing	18	Undirected
ethics	Health Personnel	18	Undirected
ethics	mortality	18	Undirected
evidence based practice	health care planning	18	Undirected
evidence based practice	health care system	18	Undirected
evidence based practice	hospital discharge	18	Undirected
evidence based practice	medical education	18	Undirected
evidence based practice	methodology	18	Undirected
fentanyl	cohort analysis	18	Undirected
fever	Health Personnel	18	Undirected
fever	hospital infection	18	Undirected
fever	university hospital	18	Undirected
follow up	health care planning	18	Undirected
follow up	hospital personnel	18	Undirected
gestational age	epidemiology	18	Undirected
health care cost	acute kidney failure	18	Undirected
health care cost	clinical article	18	Undirected
health care cost	education	18	Undirected
health care cost	hypertension	18	Undirected
health care cost	resuscitation	18	Undirected
health care delivery	multicenter study	18	Undirected
health care facility	clinical article	18	Undirected
health care system	information processing	18	Undirected
heart failure	Delirium	18	Undirected
heart failure	Heart Arrest	18	Undirected
heart failure	cerebrovascular accident	18	Undirected
heart failure	randomized controlled trial	18	Undirected
home care	interpersonal communication	18	Undirected
hospital discharge	information processing	18	Undirected
hospitalization	Needs Assessment	18	Undirected
hospitalization	intensivist	18	Undirected
hypertension	health care system	18	Undirected
hypotension	clinical practice	18	Undirected
information processing	cohort analysis	18	Undirected
intensivist	disease severity	18	Undirected
intensivist	health care system	18	Undirected
interview	hospital admission	18	Undirected
knowledge	analgesia	18	Undirected
knowledge	hand washing	18	Undirected
knowledge	health care facility	18	Undirected
knowledge	thematic analysis	18	Undirected
long term care	emergency ward	18	Undirected
long term care	methodology	18	Undirected
medical education	extracorporeal oxygenation	18	Undirected
medical staff	cohort analysis	18	Undirected
medical staff	hand washing	18	Undirected
medical staff	hospital discharge	18	Undirected
medical staff	hospital infection	18	Undirected

medical staff	palliative therapy	18	Undirected
mental health	hypertension	18	Undirected
mortality	Health Personnel	18	Undirected
mortality	fentanyl	18	Undirected
multicenter study	hospital personnel	18	Undirected
observational study	acute kidney failure	18	Undirected
observational study	enteric feeding	18	Undirected
observational study	respiratory therapist	18	Undirected
outcome assessment	Insulin	18	Undirected
outcome assessment	Medication Errors	18	Undirected
outcome assessment	Telemedicine	18	Undirected
pain assessment	breathing rate	18	Undirected
pediatrics	antibiotic agent	18	Undirected
pediatrics	interpersonal communication	18	Undirected
pediatrics	morphine	18	Undirected
pediatrics	pilot study	18	Undirected
pediatrics	safety	18	Undirected
pediatrics	statistics and numerical data	18	Undirected
perception	Medication Errors	18	Undirected
perception	health care system	18	Undirected
perception	resuscitation	18	Undirected
physiotherapy	organization and management	18	Undirected
pilot study	disease severity	18	Undirected
pilot study	hospital discharge	18	Undirected
pilot study	statistics and numerical data	18	Undirected
pneumonia	methodology	18	Undirected
prematurity	health care delivery	18	Undirected
preschool child	psychological aspect	18	Undirected
prevalence	Decision Making	18	Undirected
prevalence	Practice Guidelines as Topic	18	Undirected
prevalence	hand washing	18	Undirected
prevalence	home care	18	Undirected
prevalence	reliability	18	Undirected
prevalence	therapy	18	Undirected
propofol	cohort analysis	18	Undirected
propofol	disease severity	18	Undirected
quality of life	cerebrovascular accident	18	Undirected
quality of life	health care policy	18	Undirected
quality of life	medical education	18	Undirected
quality of life	pain assessment	18	Undirected
randomized controlled trial	information processing	18	Undirected
respiratory therapist	information processing	18	Undirected
safety	information processing	18	Undirected
safety	simulation	18	Undirected
sedation	interpersonal communication	18	Undirected
semi structured interview	intensivist	18	Undirected
simulation	practice guideline	18	Undirected
standards	antiinfective agent	18	Undirected
teaching hospital	antibiotic agent	18	Undirected
teaching hospital	hospital discharge	18	Undirected
teaching hospital	hospital personnel	18	Undirected
teaching hospital	standards	18	Undirected
tertiary care center	Catheter-Related Infections	18	Undirected
tertiary care center	Medication Errors	18	Undirected

tertiary care center	health care system	18	Undirected
tertiary care center	standards	18	Undirected
thematic analysis	health care system	18	Undirected
total quality management	endotracheal intubation	18	Undirected
total quality management	home care	18	Undirected
total quality management	simulation	18	Undirected
total quality management	urinary tract infection	18	Undirected
total quality management	ventilator associated pneumonia	18	Undirected
tracheostomy	clinical practice	18	Undirected
tracheostomy	home care	18	Undirected
tracheostomy	sedation	18	Undirected
tracheostomy	ventilator associated pneumonia	18	Undirected
urinary tract infection	epidemiology	18	Undirected
vancomycin	COVID-19	18	Undirected
ventilator associated pneumonia	thorax radiography	18	Undirected
very elderly	breathing rate	18	Undirected
Adaptation, Psychological	Pandemics	17	Undirected
Algorithms	clinical article	17	Undirected
Algorithms	epidemiology	17	Undirected
Anti-Bacterial Agents	disease severity	17	Undirected
Anti-Bacterial Agents	standards	17	Undirected
Anxiety	fever	17	Undirected
Anxiety	morphine	17	Undirected
Anxiety	pain assessment	17	Undirected
Anxiety	pharmacist	17	Undirected
Anxiety	propofol	17	Undirected
Artificial Ventilation	Cooperative Behavior	17	Undirected
Artificial Ventilation	microbiology	17	Undirected
Attitude of Health Personnel	Cardiac Surgical Procedures	17	Undirected
Attitude of Health Personnel	age	17	Undirected
Australia	Health Personnel	17	Undirected
Australia	resuscitation	17	Undirected
Australia	thematic analysis	17	Undirected
Breast Feeding	clinical article	17	Undirected
Breast Feeding	follow up	17	Undirected
COVID-19	preschool child	17	Undirected
COVID-19	psychological aspect	17	Undirected
Canada	health care delivery	17	Undirected
Canada	long term care	17	Undirected
Canada	preschool child	17	Undirected
Cardiac Surgical Procedures	morphine	17	Undirected
Cardiac Surgical Procedures	practice guideline	17	Undirected
Child, Preschool	adverse event	17	Undirected
Child, Preschool	health care quality	17	Undirected
Child, Preschool	therapy	17	Undirected
Child, Preschool	university hospital	17	Undirected
China	burnout	17	Undirected
China	depression	17	Undirected
China	emergency ward	17	Undirected
China	follow up	17	Undirected
Clinical Competence	Algorithms	17	Undirected
Clinical Competence	Burnout, Professional	17	Undirected
Clinical Competence	Catheter-Related Infections	17	Undirected
Clinical Competence	Hospital Mortality	17	Undirected

Clinical Competence	Sensitivity and Specificity	17	Undirected
Clinical Competence	caregiver	17	Undirected
Clinical Competence	randomized controlled trial	17	Undirected
Clinical Protocols	emergency care	17	Undirected
Clinical Protocols	health care facility	17	Undirected
Clinical Protocols	medical staff	17	Undirected
Clinical Protocols	psychology	17	Undirected
Communication	Electronic Health Records	17	Undirected
Communication	Medication Errors	17	Undirected
Communication	health care facility	17	Undirected
Cooperative Behavior	Safety Management	17	Undirected
Cooperative Behavior	Terminal Care	17	Undirected
Cooperative Behavior	health care quality	17	Undirected
Cross Infection	clinical practice	17	Undirected
Cross Infection	hospital discharge	17	Undirected
Decision Making	Algorithms	17	Undirected
Decision Making	Mothers	17	Undirected
Decision Making	Pilot Projects	17	Undirected
Decision Making	Reproducibility of Results	17	Undirected
Delirium	evidence based practice	17	Undirected
Delirium	pilot study	17	Undirected
Delirium	pneumonia	17	Undirected
Electronic Health Records	hospital discharge	17	Undirected
Electronic Health Records	practice guideline	17	Undirected
Emergency Service, Hospital	Canada	17	Undirected
Emergency Service, Hospital	Electronic Health Records	17	Undirected
Emergency Service, Hospital	Health Personnel	17	Undirected
Emergency Service, Hospital	United Kingdom	17	Undirected
Emergency Service, Hospital	disease severity	17	Undirected
Emergency Service, Hospital	health care system	17	Undirected
Enteral Nutrition	Pilot Projects	17	Undirected
Family	Clinical Protocols	17	Undirected
Family	emergency care	17	Undirected
Family	health care facility	17	Undirected
Family	knowledge	17	Undirected
Family	respiratory therapist	17	Undirected
Fatigue	disease severity	17	Undirected
Fatigue	emergency ward	17	Undirected
Focus Groups	Canada	17	Undirected
Focus Groups	clinical article	17	Undirected
Focus Groups	health care quality	17	Undirected
Glasgow coma scale	cerebrovascular accident	17	Undirected
Glasgow coma scale	emergency care	17	Undirected
Guideline Adherence	Delirium	17	Undirected
Guideline Adherence	United Kingdom	17	Undirected
alth Knowledge, Attitudes, Practi	Catheter-Related Infections	17	Undirected
alth Knowledge, Attitudes, Practi	epidemiology	17	Undirected
alth Knowledge, Attitudes, Practi	health care quality	17	Undirected
Health Personnel	health care planning	17	Undirected
Health Services Needs and Demand	Leadership	17	Undirected
Health Services Needs and Demand	health care quality	17	Undirected
Heart Arrest	dyspnea	17	Undirected
Heart Arrest	multicenter study	17	Undirected
Heart Arrest	palliative therapy	17	Undirected

Heart Arrest	tracheostomy	17	Undirected
Hospital Mortality	acute kidney failure	17	Undirected
Hospital Mortality	cerebrovascular accident	17	Undirected
Hospital Mortality	long term care	17	Undirected
Hospital Mortality	methodology	17	Undirected
Infant, Premature	Health Personnel	17	Undirected
Infant, Premature	Intubation, Intratracheal	17	Undirected
Infant, Premature	Sensitivity and Specificity	17	Undirected
Infant, Premature	tertiary care center	17	Undirected
Infection Control	dyspnea	17	Undirected
Interprofessional Relations	Palliative Care	17	Undirected
Intubation, Intratracheal	heart rate	17	Undirected
Job Satisfaction	Terminal Care	17	Undirected
Job Satisfaction	ethics	17	Undirected
Job Satisfaction	reliability	17	Undirected
Job Satisfaction	semi structured interview	17	Undirected
Job Satisfaction	university hospital	17	Undirected
Leadership	epidemiology	17	Undirected
Leadership	health care facility	17	Undirected
Leadership	standards	17	Undirected
Length of Stay	Adaptation, Psychological	17	Undirected
Length of Stay	Medication Errors	17	Undirected
Length of Stay	hand washing	17	Undirected
Length of Stay	perception	17	Undirected
Logistic Models	Infection Control	17	Undirected
Logistic Models	Sepsis	17	Undirected
Medication Errors	teaching hospital	17	Undirected
Monitoring, Physiologic	clinical article	17	Undirected
Mothers	Pilot Projects	17	Undirected
Mothers	mental health	17	Undirected
OUTCOMES	COVID-19	17	Undirected
OUTCOMES	Postoperative Complications	17	Undirected
OUTCOMES	disease severity	17	Undirected
Pain	Pandemics	17	Undirected
Pain	evidence based practice	17	Undirected
Pain	health care quality	17	Undirected
Pain	medical education	17	Undirected
Pain Measurement	Pilot Projects	17	Undirected
Pain Measurement	clinical article	17	Undirected
Palliative Care	Reproducibility of Results	17	Undirected
Palliative Care	health care cost	17	Undirected
Palliative Care	health care system	17	Undirected
Palliative Care	interview	17	Undirected
Palliative Care	long term care	17	Undirected
Parents	information processing	17	Undirected
Parents	outcome assessment	17	Undirected
Postoperative Care	randomized controlled trial	17	Undirected
Postoperative Complications	depression	17	Undirected
Practice Guidelines as Topic	Breast Feeding	17	Undirected
Practice Guidelines as Topic	outcome assessment	17	Undirected
Practice Guidelines as Topic	prematurity	17	Undirected
Practice Guidelines as Topic	randomized controlled trial	17	Undirected
Pregnancy	mental health	17	Undirected
Pregnancy	multicenter study	17	Undirected

Pressure Ulcer	randomized controlled trial	17	Undirected
Quality Improvement	caregiver	17	Undirected
Quality Improvement	clinical article	17	Undirected
Quality Improvement	palliative therapy	17	Undirected
Quality of Health Care	Australia	17	Undirected
Quality of Health Care	Canada	17	Undirected
Quality of Health Care	Pregnancy	17	Undirected
Reproducibility of Results	COVID-19	17	Undirected
Reproducibility of Results	Health Personnel	17	Undirected
Reproducibility of Results	resuscitation	17	Undirected
Risk Assessment	Heart Arrest	17	Undirected
Risk Assessment	Intubation, Intratracheal	17	Undirected
Risk Assessment	Personnel Staffing and Scheduling	17	Undirected
Risk Assessment	acute kidney failure	17	Undirected
Risk Assessment	health care planning	17	Undirected
Risk Factors	Pilot Projects	17	Undirected
Risk Factors	physiotherapy	17	Undirected
Safety Management	Clinical Protocols	17	Undirected
Safety Management	Quality Improvement	17	Undirected
Sensitivity and Specificity	clinical article	17	Undirected
Sensitivity and Specificity	follow up	17	Undirected
Sepsis	caregiver	17	Undirected
Sepsis	dyspnea	17	Undirected
Social Support	Canada	17	Undirected
Social Support	China	17	Undirected
Stress, Psychological	Pregnancy	17	Undirected
Stress, Psychological	education	17	Undirected
Stress, Psychological	medical staff	17	Undirected
Stroke	practice guideline	17	Undirected
Telemedicine	emergency ward	17	Undirected
Telemedicine	health care planning	17	Undirected
Terminal Care	burnout	17	Undirected
Terminal Care	emergency health service	17	Undirected
Terminal Care	methodology	17	Undirected
Terminal Care	preschool child	17	Undirected
Time Factors	Anxiety	17	Undirected
Time Factors	Health Personnel	17	Undirected
Time Factors	Medication Errors	17	Undirected
Treatment Outcome	Catheter-Related Infections	17	Undirected
Treatment Outcome	Stress, Psychological	17	Undirected
Treatment Outcome	information processing	17	Undirected
Treatment Outcome	intensivist	17	Undirected
Treatment Outcome	pain assessment	17	Undirected
United Kingdom	pediatrics	17	Undirected
United Kingdom	safety	17	Undirected
Workforce	Canada	17	Undirected
Workforce	Emergency Service, Hospital	17	Undirected
Workforce	Health Personnel	17	Undirected
Workforce	Pregnancy	17	Undirected
Workforce	hospitalization	17	Undirected
Workload	Anxiety	17	Undirected
Workload	Medication Errors	17	Undirected
Workload	pharmacist	17	Undirected
Workload	thematic analysis	17	Undirected

Workplace	tertiary care center	17	Undirected
adverse event	health care quality	17	Undirected
age	organization and management	17	Undirected
analgesia	education	17	Undirected
analgesia	emergency ward	17	Undirected
analgesia	heart surgery	17	Undirected
antibiotic agent	preschool child	17	Undirected
attitude	COVID-19	17	Undirected
attitude	palliative therapy	17	Undirected
attitude to health	palliative therapy	17	Undirected
attitude to health	pharmacist	17	Undirected
burnout	interpersonal communication	17	Undirected
burnout	psychological aspect	17	Undirected
caregiver	health care facility	17	Undirected
cerebrovascular accident	emergency health service	17	Undirected
clinical practice	age	17	Undirected
clinical practice	hospital infection	17	Undirected
dementia	home care	17	Undirected
depression	organization and management	17	Undirected
depression	practice guideline	17	Undirected
diabetes mellitus	health care delivery	17	Undirected
diabetes mellitus	health care system	17	Undirected
diabetes mellitus	heart rate	17	Undirected
disease severity	health care policy	17	Undirected
dyspnea	hospital readmission	17	Undirected
dyspnea	vancomycin	17	Undirected
education	health care policy	17	Undirected
emergency care	pilot study	17	Undirected
epidemiology	acute kidney failure	17	Undirected
epidemiology	home care	17	Undirected
epidemiology	intensivist	17	Undirected
epidemiology	semi structured interview	17	Undirected
ethics	medical staff	17	Undirected
ethics	multicenter study	17	Undirected
evidence based practice	Algorithms	17	Undirected
evidence based practice	Health Personnel	17	Undirected
evidence based practice	interpersonal communication	17	Undirected
fentanyl	clinical practice	17	Undirected
fever	endotracheal intubation	17	Undirected
fever	urinary tract infection	17	Undirected
follow up	teaching hospital	17	Undirected
gestational age	organization and management	17	Undirected
hand washing	clinical article	17	Undirected
health care cost	chronic obstructive lung disease	17	Undirected
health care cost	hospital personnel	17	Undirected
health care facility	psychological aspect	17	Undirected
health care planning	very elderly	17	Undirected
health care quality	antiinfective agent	17	Undirected
health care system	hospital personnel	17	Undirected
health care system	university hospital	17	Undirected
heart failure	hypotension	17	Undirected
heart rate	preschool child	17	Undirected
home care	cerebrovascular accident	17	Undirected
home care	health care facility	17	Undirected

hospital personnel	cohort analysis	17	Undirected
hospital readmission	antibiotic agent	17	Undirected
hospitalization	Pain Measurement	17	Undirected
hypertension	university hospital	17	Undirected
hypertension	vancomycin	17	Undirected
hypotension	adverse event	17	Undirected
hypotension	vancomycin	17	Undirected
hypotension	very elderly	17	Undirected
interpersonal communication	cohort analysis	17	Undirected
interview	burnout	17	Undirected
interview	evidence based practice	17	Undirected
interview	reliability	17	Undirected
knowledge	emergency care	17	Undirected
knowledge	mortality	17	Undirected
knowledge	palliative therapy	17	Undirected
knowledge	pilot study	17	Undirected
knowledge	teaching hospital	17	Undirected
long term care	education	17	Undirected
long term care	emergency health service	17	Undirected
long term care	health care policy	17	Undirected
medical education	cohort analysis	17	Undirected
meta analysis	therapy	17	Undirected
cillin resistant Staphylococcus a	clinical article	17	Undirected
microbiology	hand washing	17	Undirected
morphine	clinical practice	17	Undirected
morphine	cohort analysis	17	Undirected
morphine	palliative therapy	17	Undirected
mortality	Cardiac Surgical Procedures	17	Undirected
observational study	intensivist	17	Undirected
observational study	thicillin resistant Staphylococcus aure	17	Undirected
outcome assessment	antiinfective agent	17	Undirected
outcome assessment	midazolam	17	Undirected
outcome assessment	pain assessment	17	Undirected
pediatrics	Delirium	17	Undirected
pediatrics	Intubation, Intratracheal	17	Undirected
pediatrics	extracorporeal oxygenation	17	Undirected
pediatrics	health care planning	17	Undirected
pediatrics	intensivist	17	Undirected
pediatrics	interview	17	Undirected
pediatrics	therapy	17	Undirected
perception	Canada	17	Undirected
perception	Mothers	17	Undirected
perception	follow up	17	Undirected
perception	health care facility	17	Undirected
perception	mental health	17	Undirected
pharmacist	observational study	17	Undirected
pneumonia	endotracheal intubation	17	Undirected
pneumonia	hypotension	17	Undirected
practice guideline	microbiology	17	Undirected
prematurity	Reproducibility of Results	17	Undirected
prematurity	antibiotic agent	17	Undirected
prematurity	endotracheal intubation	17	Undirected
prematurity	meta analysis	17	Undirected
prematurity	therapy	17	Undirected

prevalence	Glasgow coma scale	17	Undirected
prevalence	Postoperative Complications	17	Undirected
prevalence	Terminal Care	17	Undirected
prevalence	analgesia	17	Undirected
prevalence	cerebrovascular accident	17	Undirected
prevalence	oxygen saturation	17	Undirected
prevalence	pilot study	17	Undirected
prevalence	tracheostomy	17	Undirected
prevalence	ventilator associated pneumonia	17	Undirected
propofol	hypertension	17	Undirected
psychology	physiotherapy	17	Undirected
psychology	reliability	17	Undirected
psychology	teaching hospital	17	Undirected
quality of life	Workplace	17	Undirected
quality of life	age	17	Undirected
quality of life	methodology	17	Undirected
quality of life	prematurity	17	Undirected
randomized controlled trial	microbiology	17	Undirected
reliability	clinical article	17	Undirected
resuscitation	hospital readmission	17	Undirected
resuscitation	pilot study	17	Undirected
resuscitation	sedation	17	Undirected
resuscitation	statistics and numerical data	17	Undirected
safety	health care facility	17	Undirected
safety	multicenter study	17	Undirected
sedation	breathing rate	17	Undirected
sedation	computer assisted tomography	17	Undirected
sedation	university hospital	17	Undirected
simulation	clinical article	17	Undirected
teaching hospital	statistics and numerical data	17	Undirected
tertiary care center	Clinical Protocols	17	Undirected
tertiary care center	Pain	17	Undirected
tertiary care center	adverse event	17	Undirected
tertiary care center	burnout	17	Undirected
tertiary care center	depression	17	Undirected
tertiary care center	dyspnea	17	Undirected
tertiary care center	health care planning	17	Undirected
tertiary care center	microbiology	17	Undirected
tertiary care center	palliative therapy	17	Undirected
thematic analysis	university hospital	17	Undirected
total quality management	Anxiety	17	Undirected
total quality management	health care facility	17	Undirected
total quality management	tracheostomy	17	Undirected
tracheostomy	extracorporeal oxygenation	17	Undirected
tracheostomy	hospital readmission	17	Undirected
tracheostomy	randomized controlled trial	17	Undirected
urinary tract infection	vancomycin	17	Undirected
ventilator associated pneumonia	chronic obstructive lung disease	17	Undirected
Adaptation, Psychological	Health Personnel	16	Undirected
Adaptation, Psychological	Pregnancy	16	Undirected
Adaptation, Psychological	burnout	16	Undirected
Algorithms	clinical practice	16	Undirected
Algorithms	disease severity	16	Undirected
Anti-Bacterial Agents	clinical article	16	Undirected

Anti-Bacterial Agents	organization and management	16	Undirected
Anxiety	Infection Control	16	Undirected
Anxiety	blood pressure	16	Undirected
Anxiety	gestational age	16	Undirected
Anxiety	methodology	16	Undirected
Anxiety	pilot study	16	Undirected
Artificial Ventilation	Interprofessional Relations	16	Undirected
Artificial Ventilation	Needs Assessment	16	Undirected
Attitude of Health Personnel	Algorithms	16	Undirected
Attitude of Health Personnel	Hospital Mortality	16	Undirected
Attitude of Health Personnel	cohort analysis	16	Undirected
Attitude of Health Personnel	simulation	16	Undirected
Australia	Needs Assessment	16	Undirected
Burnout, Professional	multicenter study	16	Undirected
Canada	emergency health service	16	Undirected
Canada	medical staff	16	Undirected
Catheter-Related Infections	randomized controlled trial	16	Undirected
Child, Preschool	COVID-19	16	Undirected
Child, Preschool	Glasgow coma scale	16	Undirected
Child, Preschool	fentanyl	16	Undirected
Child, Preschool	interpersonal communication	16	Undirected
Clinical Competence	Parents	16	Undirected
Clinical Competence	follow up	16	Undirected
Clinical Competence	therapy	16	Undirected
Clinical Protocols	Postoperative Complications	16	Undirected
Clinical Protocols	adverse event	16	Undirected
Clinical Protocols	gestational age	16	Undirected
Clinical Protocols	health care system	16	Undirected
Clinical Protocols	heart surgery	16	Undirected
Clinical Protocols	morphine	16	Undirected
Clinical Protocols	observational study	16	Undirected
Clinical Protocols	pneumonia	16	Undirected
Communication	Clinical Protocols	16	Undirected
Communication	OUTCOMES	16	Undirected
Communication	Pregnancy	16	Undirected
Communication	evidence based practice	16	Undirected
Cross Infection	Safety Management	16	Undirected
Cross Infection	United Kingdom	16	Undirected
Cross Infection	health care cost	16	Undirected
Cross Infection	information processing	16	Undirected
Cross Infection	medical staff	16	Undirected
Decision Making	Hospital Mortality	16	Undirected
Decision Making	Needs Assessment	16	Undirected
Decision Making	Sepsis	16	Undirected
Decision Making	dementia	16	Undirected
Decision Making	health care facility	16	Undirected
Delirium	Health Personnel	16	Undirected
Delirium	health care quality	16	Undirected
Delirium	long term care	16	Undirected
Delirium	medical staff	16	Undirected
Delirium	preschool child	16	Undirected
Electronic Health Records	university hospital	16	Undirected
Emergency Service, Hospital	Clinical Protocols	16	Undirected
Emergency Service, Hospital	clinical article	16	Undirected

Emergency Service, Hospital	fever	16	Undirected
Emergency Service, Hospital	methodology	16	Undirected
Enteral Nutrition	evidence based practice	16	Undirected
Enteral Nutrition	mortality	16	Undirected
Family	Job Satisfaction	16	Undirected
Family	Risk Assessment	16	Undirected
Family	hospital personnel	16	Undirected
Fatigue	cohort analysis	16	Undirected
Focus Groups	Leadership	16	Undirected
Guideline Adherence	COVID-19	16	Undirected
Guideline Adherence	Canada	16	Undirected
Guideline Adherence	Focus Groups	16	Undirected
Guideline Adherence	randomized controlled trial	16	Undirected
alth Knowledge, Attitudes, Practi	Breast Feeding	16	Undirected
alth Knowledge, Attitudes, Practi	Intubation, Intratracheal	16	Undirected
alth Knowledge, Attitudes, Practi	Risk Assessment	16	Undirected
alth Knowledge, Attitudes, Practi	Safety Management	16	Undirected
alth Knowledge, Attitudes, Practi	Sepsis	16	Undirected
alth Knowledge, Attitudes, Practi	pediatrics	16	Undirected
alth Knowledge, Attitudes, Practi	university hospital	16	Undirected
Health Personnel	health care policy	16	Undirected
Health Personnel	intensivist	16	Undirected
Health Personnel	teaching hospital	16	Undirected
Health Services Needs and Demand	Risk Assessment	16	Undirected
Health Services Needs and Demand	Stress, Psychological	16	Undirected
Heart Arrest	adverse event	16	Undirected
Heart Arrest	respiratory therapist	16	Undirected
Heart Arrest	very elderly	16	Undirected
Hospital Mortality	Canada	16	Undirected
Hospital Mortality	Needs Assessment	16	Undirected
Hospital Mortality	antiinfective agent	16	Undirected
Hospital Mortality	hospital infection	16	Undirected
Hypnotics and Sedatives	Anxiety	16	Undirected
Infant, Premature	mortality	16	Undirected
Infant, Premature	statistics and numerical data	16	Undirected
Infection Control	Canada	16	Undirected
Infection Control	extracorporeal oxygenation	16	Undirected
Infection Control	very elderly	16	Undirected
Insulin	Postoperative Complications	16	Undirected
Interprofessional Relations	Personnel Staffing and Scheduling	16	Undirected
Interprofessional Relations	interview	16	Undirected
Interprofessional Relations	pharmacist	16	Undirected
Intubation, Intratracheal	practice guideline	16	Undirected
Job Satisfaction	Pilot Projects	16	Undirected
Job Satisfaction	pharmacist	16	Undirected
Leadership	safety	16	Undirected
Logistic Models	Pregnancy	16	Undirected
Logistic Models	Sensitivity and Specificity	16	Undirected
Logistic Models	hospital discharge	16	Undirected
Logistic Models	multicenter study	16	Undirected
Logistic Models	organization and management	16	Undirected
Medication Errors	statistics and numerical data	16	Undirected
Monitoring, Physiologic	Hospital Mortality	16	Undirected
Monitoring, Physiologic	fever	16	Undirected

Needs Assessment	Reproducibility of Results	16	Undirected
Needs Assessment	caregiver	16	Undirected
OUTCOMES	follow up	16	Undirected
Pain	Sensitivity and Specificity	16	Undirected
Pain	adverse event	16	Undirected
Pain	attitude	16	Undirected
Pain	preschool child	16	Undirected
Pain Measurement	very elderly	16	Undirected
Palliative Care	Focus Groups	16	Undirected
Palliative Care	Pregnancy	16	Undirected
Pandemics	pilot study	16	Undirected
Parents	Treatment Outcome	16	Undirected
Parents	analgesia	16	Undirected
Parents	clinical practice	16	Undirected
Parents	home care	16	Undirected
Parents	hospital admission	16	Undirected
Parents	tertiary care center	16	Undirected
Postoperative Care	Hospital Mortality	16	Undirected
Postoperative Care	health care quality	16	Undirected
Postoperative Care	hypotension	16	Undirected
Postoperative Care	pediatrics	16	Undirected
Postoperative Care	standards	16	Undirected
Postoperative Complications	acute kidney failure	16	Undirected
Postoperative Complications	diabetes mellitus	16	Undirected
Postoperative Complications	heart rate	16	Undirected
Postoperative Complications	meta analysis	16	Undirected
Postoperative Complications	multicenter study	16	Undirected
Practice Guidelines as Topic	antibiotic agent	16	Undirected
Practice Guidelines as Topic	gestational age	16	Undirected
Pregnancy	evidence based practice	16	Undirected
Pregnancy	fever	16	Undirected
Pregnancy	health care planning	16	Undirected
Pregnancy	observational study	16	Undirected
Pregnancy	standards	16	Undirected
Pressure Ulcer	education	16	Undirected
Pressure Ulcer	meta analysis	16	Undirected
Professional-Family Relations	Artificial Ventilation	16	Undirected
Professional-Family Relations	COVID-19	16	Undirected
Professional-Family Relations	Pregnancy	16	Undirected
Professional-Family Relations	depression	16	Undirected
Professional-Family Relations	multicenter study	16	Undirected
Program Evaluation	Algorithms	16	Undirected
Program Evaluation	practice guideline	16	Undirected
Program Evaluation	psychology	16	Undirected
Quality Improvement	Reproducibility of Results	16	Undirected
Quality Improvement	health care system	16	Undirected
Quality Improvement	hospital personnel	16	Undirected
Quality Improvement	knowledge	16	Undirected
Quality Improvement	methodology	16	Undirected
Quality Improvement	pilot study	16	Undirected
Quality Improvement	urinary tract infection	16	Undirected
Quality of Health Care	Pandemics	16	Undirected
Quality of Health Care	emergency health service	16	Undirected
Quality of Health Care	interview	16	Undirected

Quality of Health Care	medical education	16	Undirected
Reproducibility of Results	Algorithms	16	Undirected
Reproducibility of Results	mortality	16	Undirected
Risk Assessment	Canada	16	Undirected
Risk Assessment	analgesia	16	Undirected
Risk Assessment	enteric feeding	16	Undirected
Risk Assessment	evidence based practice	16	Undirected
Risk Assessment	information processing	16	Undirected
Risk Assessment	perception	16	Undirected
Risk Factors	Palliative Care	16	Undirected
Sensitivity and Specificity	COVID-19	16	Undirected
Sensitivity and Specificity	gestational age	16	Undirected
Sensitivity and Specificity	hypertension	16	Undirected
Sepsis	health care delivery	16	Undirected
Sepsis	health care planning	16	Undirected
Sepsis	interview	16	Undirected
Sepsis	ethicillin resistant Staphylococcus aure	16	Undirected
Sepsis	physiotherapy	16	Undirected
Social Support	hospital admission	16	Undirected
Social Support	perception	16	Undirected
Stress, Psychological	Australia	16	Undirected
Stress, Psychological	gestational age	16	Undirected
Stroke	disease severity	16	Undirected
Stroke	follow up	16	Undirected
Telemedicine	caregiver	16	Undirected
Telemedicine	hospital admission	16	Undirected
Telemedicine	medical education	16	Undirected
Telemedicine	medical staff	16	Undirected
Terminal Care	Leadership	16	Undirected
Terminal Care	chronic obstructive lung disease	16	Undirected
Terminal Care	information processing	16	Undirected
Terminal Care	sedation	16	Undirected
Time Factors	Safety Management	16	Undirected
Time Factors	cerebrovascular accident	16	Undirected
Time Factors	enteric feeding	16	Undirected
Time Factors	hospital readmission	16	Undirected
Time Factors	interview	16	Undirected
Time Factors	pilot study	16	Undirected
Treatment Outcome	Telemedicine	16	Undirected
Treatment Outcome	health care policy	16	Undirected
Treatment Outcome	midazolam	16	Undirected
Treatment Outcome	respiratory therapist	16	Undirected
United Kingdom	home care	16	Undirected
Workforce	Child, Preschool	16	Undirected
Workforce	Pilot Projects	16	Undirected
Workforce	Reproducibility of Results	16	Undirected
Workload	Delirium	16	Undirected
Workload	adverse event	16	Undirected
Workload	health care policy	16	Undirected
Workload	intensivist	16	Undirected
adverse event	hypertension	16	Undirected
age	hospital readmission	16	Undirected
analgesia	Cardiac Surgical Procedures	16	Undirected
analgesia	hospital discharge	16	Undirected

analgesia	mortality	16	Undirected
analgesia	preschool child	16	Undirected
analgesia	university hospital	16	Undirected
antibiotic agent	health care policy	16	Undirected
attitude	resuscitation	16	Undirected
attitude to health	Job Satisfaction	16	Undirected
attitude to health	Pregnancy	16	Undirected
attitude to health	Quality of Health Care	16	Undirected
attitude to health	analgesia	16	Undirected
blood pressure	fever	16	Undirected
burnout	pharmacist	16	Undirected
burnout	respiratory therapist	16	Undirected
burnout	university hospital	16	Undirected
caregiver	pilot study	16	Undirected
caregiver	university hospital	16	Undirected
cerebrovascular accident	university hospital	16	Undirected
computer assisted tomography	extracorporeal oxygenation	16	Undirected
depression	cerebrovascular accident	16	Undirected
depression	interpersonal communication	16	Undirected
diabetes mellitus	Stroke	16	Undirected
diabetes mellitus	multicenter study	16	Undirected
diabetes mellitus	urinary tract infection	16	Undirected
dyspnea	caregiver	16	Undirected
dyspnea	dementia	16	Undirected
dyspnea	emergency health service	16	Undirected
education	cohort analysis	16	Undirected
emergency care	age	16	Undirected
emergency care	mental health	16	Undirected
emergency care	respiratory therapist	16	Undirected
epidemiology	antibiotic agent	16	Undirected
epidemiology	teaching hospital	16	Undirected
ethics	emergency health service	16	Undirected
ethics	semi structured interview	16	Undirected
evidence based practice	caregiver	16	Undirected
evidence based practice	resuscitation	16	Undirected
evidence based practice	sedation	16	Undirected
fentanyl	oxygen saturation	16	Undirected
fever	emergency care	16	Undirected
follow up	hospital infection	16	Undirected
gestational age	caregiver	16	Undirected
gestational age	endotracheal intubation	16	Undirected
gestational age	health care quality	16	Undirected
health care cost	Delirium	16	Undirected
health care cost	age	16	Undirected
health care delivery	hospital infection	16	Undirected
health care quality	endotracheal intubation	16	Undirected
health care quality	preschool child	16	Undirected
health care system	antibiotic agent	16	Undirected
health care system	chronic obstructive lung disease	16	Undirected
health care system	clinical article	16	Undirected
heart failure	caregiver	16	Undirected
heart failure	tertiary care center	16	Undirected
heart rate	midazolam	16	Undirected
heart surgery	standards	16	Undirected

home care	clinical practice	16	Undirected
hospitalization	ethics	16	Undirected
hospitalization	reliability	16	Undirected
hypertension	antibiotic agent	16	Undirected
hypertension	clinical practice	16	Undirected
interview	mortality	16	Undirected
interview	very elderly	16	Undirected
knowledge	Medication Errors	16	Undirected
knowledge	semi structured interview	16	Undirected
long term care	fever	16	Undirected
medical education	statistics and numerical data	16	Undirected
medical staff	randomized controlled trial	16	Undirected
mental health	hospital personnel	16	Undirected
mental health	multicenter study	16	Undirected
meta analysis	hospital discharge	16	Undirected
cillin resistant Staphylococcus a	emergency ward	16	Undirected
microbiology	organization and management	16	Undirected
midazolam	hypertension	16	Undirected
morphine	endotracheal intubation	16	Undirected
mortality	Fatigue	16	Undirected
mortality	Medication Errors	16	Undirected
multicenter study	oxygen saturation	16	Undirected
observational study	physiotherapy	16	Undirected
observational study	thorax radiography	16	Undirected
observational study	urinary tract infection	16	Undirected
organization and management	extracorporeal oxygenation	16	Undirected
outcome assessment	Algorithms	16	Undirected
outcome assessment	Enteral Nutrition	16	Undirected
outcome assessment	burnout	16	Undirected
outcome assessment	knowledge	16	Undirected
outcome assessment	urinary tract infection	16	Undirected
oxygen saturation	emergency health service	16	Undirected
pain assessment	disease severity	16	Undirected
pain assessment	multicenter study	16	Undirected
pain assessment	oxygen saturation	16	Undirected
pediatrics	Clinical Protocols	16	Undirected
pediatrics	Reproducibility of Results	16	Undirected
pediatrics	fever	16	Undirected
perception	China	16	Undirected
perception	observational study	16	Undirected
pilot study	health care delivery	16	Undirected
pneumonia	Stroke	16	Undirected
pneumonia	blood pressure	16	Undirected
pneumonia	home care	16	Undirected
prematurity	Pilot Projects	16	Undirected
prematurity	thematic analysis	16	Undirected
preschool child	extracorporeal oxygenation	16	Undirected
prevalence	Emergency Service, Hospital	16	Undirected
prevalence	intensivist	16	Undirected
prevalence	psychological aspect	16	Undirected
prevalence	standards	16	Undirected
propofol	hospital discharge	16	Undirected
psychology	hand washing	16	Undirected
psychology	methodology	16	Undirected

quality of life	Clinical Protocols	16	Undirected
quality of life	heart rate	16	Undirected
quality of life	intensivist	16	Undirected
quality of life	pneumonia	16	Undirected
quality of life	statistics and numerical data	16	Undirected
quality of life	university hospital	16	Undirected
randomized controlled trial	antiinfective agent	16	Undirected
randomized controlled trial	health care delivery	16	Undirected
reliability	emergency ward	16	Undirected
reliability	multicenter study	16	Undirected
resuscitation	adverse event	16	Undirected
resuscitation	age	16	Undirected
resuscitation	cerebrovascular accident	16	Undirected
resuscitation	depression	16	Undirected
sedation	hypertension	16	Undirected
sedation	respiratory therapist	16	Undirected
semi structured interview	health care policy	16	Undirected
standards	intensivist	16	Undirected
statistics and numerical data	clinical article	16	Undirected
statistics and numerical data	hospital personnel	16	Undirected
teaching hospital	hand washing	16	Undirected
teaching hospital	interpersonal communication	16	Undirected
tertiary care center	Glasgow coma scale	16	Undirected
tertiary care center	Postoperative Complications	16	Undirected
tertiary care center	attitude	16	Undirected
tertiary care center	mental health	16	Undirected
thematic analysis	therapy	16	Undirected
total quality management	Pilot Projects	16	Undirected
total quality management	antiinfective agent	16	Undirected
total quality management	heart surgery	16	Undirected
tracheostomy	breathing rate	16	Undirected
tracheostomy	emergency care	16	Undirected
tracheostomy	very elderly	16	Undirected
urinary tract infection	acute kidney failure	16	Undirected
vancomycin	acute kidney failure	16	Undirected
ventilator associated pneumonia	clinical article	16	Undirected
ventilator associated pneumonia	clinical practice	16	Undirected
ventilator associated pneumonia	medical education	16	Undirected
ventilator associated pneumonia	vancomycin	16	Undirected
Adaptation, Psychological	interpersonal communication	15	Undirected
Algorithms	analgesia	15	Undirected
Algorithms	health care quality	15	Undirected
Algorithms	heart rate	15	Undirected
Anxiety	chronic obstructive lung disease	15	Undirected
Anxiety	hospital readmission	15	Undirected
Artificial Ventilation	Medication Errors	15	Undirected
Artificial Ventilation	Telemedicine	15	Undirected
Australia	Leadership	15	Undirected
Australia	Personnel Staffing and Scheduling	15	Undirected
Australia	Quality Improvement	15	Undirected
Australia	clinical article	15	Undirected
Australia	safety	15	Undirected
Australia	semi structured interview	15	Undirected
Breast Feeding	statistics and numerical data	15	Undirected

Burnout, Professional	Focus Groups	15	Undirected
Burnout, Professional	perception	15	Undirected
Canada	health care planning	15	Undirected
Canada	randomized controlled trial	15	Undirected
Cardiac Surgical Procedures	randomized controlled trial	15	Undirected
Cardiac Surgical Procedures	standards	15	Undirected
Catheterization, Central Venous	organization and management	15	Undirected
Child, Preschool	Anxiety	15	Undirected
Child, Preschool	clinical practice	15	Undirected
Child, Preschool	morphine	15	Undirected
Child, Preschool	very elderly	15	Undirected
China	evidence based practice	15	Undirected
China	fever	15	Undirected
China	pneumonia	15	Undirected
China	randomized controlled trial	15	Undirected
Clinical Competence	Postoperative Care	15	Undirected
Clinical Competence	Telemedicine	15	Undirected
Clinical Competence	extracorporeal oxygenation	15	Undirected
Clinical Competence	respiratory therapist	15	Undirected
Clinical Competence	statistics and numerical data	15	Undirected
Clinical Protocols	Catheter-Related Infections	15	Undirected
Clinical Protocols	Pandemics	15	Undirected
Clinical Protocols	extracorporeal oxygenation	15	Undirected
Clinical Protocols	interpersonal communication	15	Undirected
Clinical Protocols	teaching hospital	15	Undirected
Communication	Personnel Staffing and Scheduling	15	Undirected
Communication	Postoperative Care	15	Undirected
Communication	preschool child	15	Undirected
Cross Infection	health care policy	15	Undirected
Cross Infection	long term care	15	Undirected
Decision Making	mental health	15	Undirected
Decision Making	safety	15	Undirected
Decision Making	tertiary care center	15	Undirected
Delirium	Glasgow coma scale	15	Undirected
Delirium	hypotension	15	Undirected
Delirium	statistics and numerical data	15	Undirected
Electronic Health Records	health care quality	15	Undirected
Electronic Health Records	multicenter study	15	Undirected
Electronic Health Records	organization and management	15	Undirected
Emergency Service, Hospital	Logistic Models	15	Undirected
Emergency Service, Hospital	Program Evaluation	15	Undirected
Emergency Service, Hospital	clinical practice	15	Undirected
Emergency Service, Hospital	perception	15	Undirected
Emergency Service, Hospital	pharmacist	15	Undirected
Emergency Service, Hospital	safety	15	Undirected
Family	Workplace	15	Undirected
Family	age	15	Undirected
Family	health care system	15	Undirected
Fatigue	randomized controlled trial	15	Undirected
Fatigue	very elderly	15	Undirected
Glasgow coma scale	blood pressure	15	Undirected
Glasgow coma scale	epidemiology	15	Undirected
Glasgow coma scale	hypotension	15	Undirected
Glasgow coma scale	sedation	15	Undirected

Glasgow coma scale	university hospital	15	Undirected
Guideline Adherence	Leadership	15	Undirected
Guideline Adherence	analgesia	15	Undirected
Guideline Adherence	follow up	15	Undirected
Guideline Adherence	pediatrics	15	Undirected
alth Knowledge, Attitudes, Practi	Leadership	15	Undirected
alth Knowledge, Attitudes, Practi	Medication Errors	15	Undirected
alth Knowledge, Attitudes, Practi	hospital personnel	15	Undirected
alth Knowledge, Attitudes, Practi	statistics and numerical data	15	Undirected
Health Personnel	age	15	Undirected
Health Services Needs and Demand	Artificial Ventilation	15	Undirected
Health Services Needs and Demand	Clinical Protocols	15	Undirected
Health Services Needs and Demand	Monitoring, Physiologic	15	Undirected
Health Services Needs and Demand	interpersonal communication	15	Undirected
Heart Arrest	fever	15	Undirected
Heart Arrest	propofol	15	Undirected
Hospital Mortality	Infection Control	15	Undirected
Hospital Mortality	breathing rate	15	Undirected
Hospital Mortality	oxygen saturation	15	Undirected
Hospital Mortality	pharmacist	15	Undirected
Hospital Mortality	safety	15	Undirected
Hypnotics and Sedatives	Intubation, Intratracheal	15	Undirected
Hypnotics and Sedatives	Quality Improvement	15	Undirected
Hypnotics and Sedatives	multicenter study	15	Undirected
Infant, Premature	epidemiology	15	Undirected
Infant, Premature	pilot study	15	Undirected
Infection Control	health care planning	15	Undirected
Infection Control	information processing	15	Undirected
Infection Control	interview	15	Undirected
Infection Control	pharmacist	15	Undirected
Infection Control	tracheostomy	15	Undirected
Insulin	disease severity	15	Undirected
Insulin	hospital discharge	15	Undirected
Insulin	methodology	15	Undirected
Interprofessional Relations	Canada	15	Undirected
Interprofessional Relations	perception	15	Undirected
Interprofessional Relations	standards	15	Undirected
Leadership	Clinical Protocols	15	Undirected
Leadership	intensivist	15	Undirected
Leadership	palliative therapy	15	Undirected
Leadership	respiratory therapist	15	Undirected
Logistic Models	China	15	Undirected
Logistic Models	outcome assessment	15	Undirected
Medication Errors	clinical article	15	Undirected
Medication Errors	emergency ward	15	Undirected
Medication Errors	hospital admission	15	Undirected
Medication Errors	interpersonal communication	15	Undirected
Monitoring, Physiologic	Glasgow coma scale	15	Undirected
Monitoring, Physiologic	Pain	15	Undirected
Monitoring, Physiologic	Telemedicine	15	Undirected
Monitoring, Physiologic	gestational age	15	Undirected
Monitoring, Physiologic	randomized controlled trial	15	Undirected
Mothers	knowledge	15	Undirected
Mothers	statistics and numerical data	15	Undirected

Needs Assessment	Pain	15	Undirected
Needs Assessment	health care quality	15	Undirected
Needs Assessment	resuscitation	15	Undirected
OUTCOMES	adverse event	15	Undirected
OUTCOMES	very elderly	15	Undirected
Pain	Health Personnel	15	Undirected
Pain	antibiotic agent	15	Undirected
Pain	pilot study	15	Undirected
Pain	statistics and numerical data	15	Undirected
Pain Measurement	Glasgow coma scale	15	Undirected
Palliative Care	tertiary care center	15	Undirected
Palliative Care	thematic analysis	15	Undirected
Palliative Care	university hospital	15	Undirected
Parents	Pandemics	15	Undirected
Parents	Quality of Health Care	15	Undirected
Parents	Telemedicine	15	Undirected
Parents	cohort analysis	15	Undirected
Pilot Projects	health care quality	15	Undirected
Pilot Projects	multicenter study	15	Undirected
Pilot Projects	observational study	15	Undirected
Pilot Projects	standards	15	Undirected
Postoperative Care	Infection Control	15	Undirected
Postoperative Care	resuscitation	15	Undirected
Practice Guidelines as Topic	Stroke	15	Undirected
Practice Guidelines as Topic	enteric feeding	15	Undirected
Practice Guidelines as Topic	palliative therapy	15	Undirected
Pregnancy	Canada	15	Undirected
Pregnancy	Pain	15	Undirected
Pregnancy	Pandemics	15	Undirected
Pregnancy	United Kingdom	15	Undirected
Pregnancy	psychological aspect	15	Undirected
Pregnancy	semi structured interview	15	Undirected
Pressure Ulcer	hospital admission	15	Undirected
Professional-Family Relations	clinical article	15	Undirected
Professional-Family Relations	semi structured interview	15	Undirected
Program Evaluation	Australia	15	Undirected
Program Evaluation	Heart Arrest	15	Undirected
Program Evaluation	Pregnancy	15	Undirected
Program Evaluation	evidence based practice	15	Undirected
Quality Improvement	Breast Feeding	15	Undirected
Quality Improvement	Pain	15	Undirected
Quality Improvement	heart surgery	15	Undirected
Quality Improvement	tracheostomy	15	Undirected
Quality of Health Care	Pressure Ulcer	15	Undirected
Quality of Health Care	Stress, Psychological	15	Undirected
Quality of Health Care	prevalence	15	Undirected
Reproducibility of Results	clinical article	15	Undirected
Reproducibility of Results	heart rate	15	Undirected
Reproducibility of Results	perception	15	Undirected
Reproducibility of Results	statistics and numerical data	15	Undirected
Risk Assessment	Insulin	15	Undirected
Risk Assessment	Pain Measurement	15	Undirected
Risk Assessment	medical education	15	Undirected
Risk Assessment	mental health	15	Undirected

Risk Assessment	ethicillin resistant Staphylococcus aure	15	Undirected
Risk Assessment	psychological aspect	15	Undirected
Risk Assessment	teaching hospital	15	Undirected
Risk Assessment	therapy	15	Undirected
Risk Assessment	ventilator associated pneumonia	15	Undirected
Risk Factors	semi structured interview	15	Undirected
Safety Management	education	15	Undirected
Sensitivity and Specificity	Algorithms	15	Undirected
Sensitivity and Specificity	Electronic Health Records	15	Undirected
Sensitivity and Specificity	practice guideline	15	Undirected
Sensitivity and Specificity	university hospital	15	Undirected
Sepsis	Health Personnel	15	Undirected
Sepsis	dementia	15	Undirected
Sepsis	oxygen saturation	15	Undirected
Sepsis	psychology	15	Undirected
Social Support	Fatigue	15	Undirected
Social Support	Treatment Outcome	15	Undirected
Social Support	clinical practice	15	Undirected
Social Support	health care planning	15	Undirected
Social Support	heart failure	15	Undirected
Social Support	total quality management	15	Undirected
Stress, Psychological	hospital discharge	15	Undirected
Stress, Psychological	therapy	15	Undirected
Terminal Care	Pilot Projects	15	Undirected
Time Factors	Insulin	15	Undirected
Time Factors	OUTCOMES	15	Undirected
Time Factors	emergency care	15	Undirected
Time Factors	heart failure	15	Undirected
Treatment Outcome	China	15	Undirected
Treatment Outcome	hospital personnel	15	Undirected
Treatment Outcome	interview	15	Undirected
United Kingdom	emergency care	15	Undirected
United Kingdom	emergency health service	15	Undirected
United Kingdom	health care system	15	Undirected
United Kingdom	observational study	15	Undirected
United Kingdom	palliative therapy	15	Undirected
United Kingdom	semi structured interview	15	Undirected
Workforce	Adaptation, Psychological	15	Undirected
Workforce	Communication	15	Undirected
Workforce	Telemedicine	15	Undirected
Workforce	epidemiology	15	Undirected
Workload	Algorithms	15	Undirected
Workload	Quality Improvement	15	Undirected
Workload	emergency care	15	Undirected
Workload	emergency health service	15	Undirected
Workload	health care planning	15	Undirected
Workload	resuscitation	15	Undirected
Workplace	Australia	15	Undirected
Workplace	Focus Groups	15	Undirected
Workplace	Infection Control	15	Undirected
Workplace	Risk Assessment	15	Undirected
Workplace	depression	15	Undirected
Workplace	health care quality	15	Undirected
acute kidney failure	thorax radiography	15	Undirected

adverse event	clinical practice	15	Undirected
adverse event	preschool child	15	Undirected
adverse event	psychology	15	Undirected
age	antibiotic agent	15	Undirected
age	chronic obstructive lung disease	15	Undirected
analgesia	breathing rate	15	Undirected
analgesia	endotracheal intubation	15	Undirected
analgesia	hypotension	15	Undirected
analgesia	medical education	15	Undirected
analgesia	medical staff	15	Undirected
antibiotic agent	chronic obstructive lung disease	15	Undirected
antiinfective agent	thorax radiography	15	Undirected
attitude	emergency ward	15	Undirected
attitude to health	Workload	15	Undirected
attitude to health	depression	15	Undirected
attitude to health	health care facility	15	Undirected
burnout	hospital personnel	15	Undirected
burnout	organization and management	15	Undirected
burnout	reliability	15	Undirected
caregiver	epidemiology	15	Undirected
caregiver	health care policy	15	Undirected
clinical practice	heart surgery	15	Undirected
clinical practice	hospital readmission	15	Undirected
clinical practice	physiotherapy	15	Undirected
dementia	clinical article	15	Undirected
dementia	mental health	15	Undirected
depression	chronic obstructive lung disease	15	Undirected
depression	hospital personnel	15	Undirected
diabetes mellitus	tracheostomy	15	Undirected
dyspnea	Health Personnel	15	Undirected
dyspnea	sedation	15	Undirected
education	adverse event	15	Undirected
education	disease severity	15	Undirected
emergency care	preschool child	15	Undirected
endotracheal intubation	extracorporeal oxygenation	15	Undirected
endotracheal intubation	very elderly	15	Undirected
epidemiology	endotracheal intubation	15	Undirected
epidemiology	psychological aspect	15	Undirected
ethics	burnout	15	Undirected
evidence based practice	Pandemics	15	Undirected
evidence based practice	antibiotic agent	15	Undirected
evidence based practice	cohort analysis	15	Undirected
evidence based practice	multicenter study	15	Undirected
evidence based practice	palliative therapy	15	Undirected
evidence based practice	university hospital	15	Undirected
fever	cerebrovascular accident	15	Undirected
fever	clinical practice	15	Undirected
fever	randomized controlled trial	15	Undirected
follow up	intensivist	15	Undirected
gestational age	analgesia	15	Undirected
gestational age	multicenter study	15	Undirected
hand washing	cohort analysis	15	Undirected
health care cost	computer assisted tomography	15	Undirected
health care cost	emergency health service	15	Undirected

health care cost	evidence based practice	15	Undirected
health care cost	physiotherapy	15	Undirected
health care cost	psychology	15	Undirected
health care cost	university hospital	15	Undirected
health care delivery	antibiotic agent	15	Undirected
health care delivery	psychological aspect	15	Undirected
health care delivery	statistics and numerical data	15	Undirected
health care delivery	therapy	15	Undirected
health care system	computer assisted tomography	15	Undirected
heart failure	observational study	15	Undirected
heart rate	adverse event	15	Undirected
heart rate	chronic obstructive lung disease	15	Undirected
heart rate	clinical practice	15	Undirected
heart rate	fentanyl	15	Undirected
heart rate	pilot study	15	Undirected
heart surgery	extracorporeal oxygenation	15	Undirected
home care	antibiotic agent	15	Undirected
home care	multicenter study	15	Undirected
hospital infection	disease severity	15	Undirected
hospital readmission	emergency health service	15	Undirected
hospitalization	Health Knowledge, Attitudes, Practice	15	Undirected
hypertension	emergency health service	15	Undirected
hypotension	emergency health service	15	Undirected
information processing	palliative therapy	15	Undirected
interpersonal communication	preschool child	15	Undirected
interview	analgesia	15	Undirected
interview	depression	15	Undirected
interview	gestational age	15	Undirected
interview	health care policy	15	Undirected
interview	mental health	15	Undirected
interview	simulation	15	Undirected
knowledge	caregiver	15	Undirected
knowledge	health care quality	15	Undirected
long term care	age	15	Undirected
long term care	antibiotic agent	15	Undirected
medical education	heart surgery	15	Undirected
medical staff	thematic analysis	15	Undirected
mental health	palliative therapy	15	Undirected
mental health	practice guideline	15	Undirected
mental health	very elderly	15	Undirected
cillin resistant Staphylococcus a	thorax radiography	15	Undirected
methodology	age	15	Undirected
methodology	clinical article	15	Undirected
microbiology	hospital discharge	15	Undirected
microbiology	statistics and numerical data	15	Undirected
midazolam	hospital discharge	15	Undirected
midazolam	palliative therapy	15	Undirected
morphine	heart surgery	15	Undirected
mortality	blood pressure	15	Undirected
mortality	morphine	15	Undirected
multicenter study	health care facility	15	Undirected
observational study	mental health	15	Undirected
outcome assessment	blood pressure	15	Undirected
outcome assessment	microbiology	15	Undirected

outcome assessment	reliability	15	Undirected
outcome assessment	thorax radiography	15	Undirected
oxygen saturation	acute kidney failure	15	Undirected
oxygen saturation	chronic obstructive lung disease	15	Undirected
oxygen saturation	extracorporeal oxygenation	15	Undirected
pediatrics	Enteral Nutrition	15	Undirected
pediatrics	fentanyl	15	Undirected
pediatrics	long term care	15	Undirected
pediatrics	randomized controlled trial	15	Undirected
pediatrics	tracheostomy	15	Undirected
perception	reliability	15	Undirected
perception	therapy	15	Undirected
pharmacist	multicenter study	15	Undirected
pharmacist	pilot study	15	Undirected
pharmacist	randomized controlled trial	15	Undirected
pharmacist	resuscitation	15	Undirected
pharmacist	sedation	15	Undirected
pharmacist	very elderly	15	Undirected
pneumonia	enteric feeding	15	Undirected
prematurity	health care policy	15	Undirected
prevalence	Health Knowledge, Attitudes, Practice	15	Undirected
prevalence	Heart Arrest	15	Undirected
prevalence	OUTCOMES	15	Undirected
prevalence	enteric feeding	15	Undirected
prevalence	heart rate	15	Undirected
prevalence	information processing	15	Undirected
prevalence	methodology	15	Undirected
prevalence	pain assessment	15	Undirected
quality of life	Health Services Needs and Demand	15	Undirected
quality of life	Postoperative Care	15	Undirected
quality of life	knowledge	15	Undirected
quality of life	pilot study	15	Undirected
randomized controlled trial	acute kidney failure	15	Undirected
randomized controlled trial	teaching hospital	15	Undirected
reliability	clinical practice	15	Undirected
reliability	standards	15	Undirected
resuscitation	chronic obstructive lung disease	15	Undirected
resuscitation	intensivist	15	Undirected
sedation	emergency ward	15	Undirected
sedation	epidemiology	15	Undirected
sedation	organization and management	15	Undirected
sedation	oxygen saturation	15	Undirected
sedation	therapy	15	Undirected
semi structured interview	health care planning	15	Undirected
semi structured interview	therapy	15	Undirected
teaching hospital	emergency health service	15	Undirected
teaching hospital	hospital infection	15	Undirected
tertiary care center	Anti-Bacterial Agents	15	Undirected
tertiary care center	Heart Arrest	15	Undirected
tertiary care center	antiinfective agent	15	Undirected
tertiary care center	computer assisted tomography	15	Undirected
tertiary care center	evidence based practice	15	Undirected
tertiary care center	hospital readmission	15	Undirected
tertiary care center	oxygen saturation	15	Undirected

therapy	information processing	15	Undirected
thorax radiography	chronic obstructive lung disease	15	Undirected
total quality management	Personnel Staffing and Scheduling	15	Undirected
total quality management	long term care	15	Undirected
tracheostomy	acute kidney failure	15	Undirected
tracheostomy	chronic obstructive lung disease	15	Undirected
tracheostomy	palliative therapy	15	Undirected
tracheostomy	physiotherapy	15	Undirected
tracheostomy	therapy	15	Undirected
urinary tract infection	hospital readmission	15	Undirected
ventilator associated pneumonia	hospital discharge	15	Undirected
very elderly	enteric feeding	15	Undirected
very elderly	extracorporeal oxygenation	15	Undirected
very elderly	information processing	15	Undirected
very elderly	preschool child	15	Undirected
Adaptation, Psychological	Palliative Care	14	Undirected
Adaptation, Psychological	mental health	14	Undirected
Algorithms	hospital admission	14	Undirected
Algorithms	observational study	14	Undirected
Algorithms	standards	14	Undirected
Anti-Bacterial Agents	epidemiology	14	Undirected
Anxiety	health care cost	14	Undirected
Anxiety	heart surgery	14	Undirected
Anxiety	intensivist	14	Undirected
Artificial Ventilation	attitude	14	Undirected
Attitude of Health Personnel	Catheter-Related Infections	14	Undirected
Attitude of Health Personnel	Catheterization, Central Venous	14	Undirected
Attitude of Health Personnel	disease severity	14	Undirected
Attitude of Health Personnel	hospital infection	14	Undirected
Australia	Burnout, Professional	14	Undirected
Australia	Heart Arrest	14	Undirected
Australia	Infection Control	14	Undirected
Australia	Pain	14	Undirected
Australia	Reproducibility of Results	14	Undirected
Australia	disease severity	14	Undirected
Australia	teaching hospital	14	Undirected
Breast Feeding	caregiver	14	Undirected
Breast Feeding	education	14	Undirected
Breast Feeding	practice guideline	14	Undirected
Burnout, Professional	Pilot Projects	14	Undirected
Burnout, Professional	intensivist	14	Undirected
COVID-19	heart surgery	14	Undirected
Canada	semi structured interview	14	Undirected
Cardiac Surgical Procedures	cohort analysis	14	Undirected
Cardiac Surgical Procedures	hospital discharge	14	Undirected
Catheter-Related Infections	health care quality	14	Undirected
Catheter-Related Infections	microbiology	14	Undirected
Child, Preschool	Focus Groups	14	Undirected
Child, Preschool	Personnel Staffing and Scheduling	14	Undirected
Child, Preschool	emergency care	14	Undirected
Child, Preschool	pain assessment	14	Undirected
China	education	14	Undirected
China	health care system	14	Undirected
China	hospital discharge	14	Undirected

China	medical education	14	Undirected
Clinical Competence	Anxiety	14	Undirected
Clinical Competence	prematurity	14	Undirected
Clinical Competence	university hospital	14	Undirected
Clinical Protocols	Canada	14	Undirected
Clinical Protocols	antiinfective agent	14	Undirected
Clinical Protocols	information processing	14	Undirected
Communication	hospital personnel	14	Undirected
Cooperative Behavior	Personnel Staffing and Scheduling	14	Undirected
Cross Infection	Infant, Premature	14	Undirected
Cross Infection	emergency care	14	Undirected
Cross Infection	psychology	14	Undirected
Decision Making	Burnout, Professional	14	Undirected
Decision Making	analgesia	14	Undirected
Decision Making	home care	14	Undirected
Decision Making	pilot study	14	Undirected
Decision Making	respiratory therapist	14	Undirected
Decision Making	sedation	14	Undirected
Delirium	Algorithms	14	Undirected
Delirium	Electronic Health Records	14	Undirected
Delirium	interview	14	Undirected
Electronic Health Records	observational study	14	Undirected
Emergency Service, Hospital	Glasgow coma scale	14	Undirected
Emergency Service, Hospital	therapy	14	Undirected
Enteral Nutrition	multicenter study	14	Undirected
Family	Heart Arrest	14	Undirected
Family	Infection Control	14	Undirected
Family	dyspnea	14	Undirected
Family	health care cost	14	Undirected
Family	therapy	14	Undirected
Fatigue	Health Personnel	14	Undirected
Fatigue	multicenter study	14	Undirected
Focus Groups	COVID-19	14	Undirected
Glasgow coma scale	age	14	Undirected
Glasgow coma scale	fever	14	Undirected
Glasgow coma scale	multicenter study	14	Undirected
Glasgow coma scale	thorax radiography	14	Undirected
Guideline Adherence	cohort analysis	14	Undirected
Guideline Adherence	urinary tract infection	14	Undirected
alth Knowledge, Attitudes, Practi	Enteral Nutrition	14	Undirected
alth Knowledge, Attitudes, Practi	information processing	14	Undirected
Health Personnel	randomized controlled trial	14	Undirected
Health Services Needs and Demand	Focus Groups	14	Undirected
Health Services Needs and Demand	Job Satisfaction	14	Undirected
Health Services Needs and Demand	Needs Assessment	14	Undirected
Health Services Needs and Demand	practice guideline	14	Undirected
Heart Arrest	Glasgow coma scale	14	Undirected
Heart Arrest	acute kidney failure	14	Undirected
Heart Arrest	epidemiology	14	Undirected
Heart Arrest	interpersonal communication	14	Undirected
Heart Arrest	preschool child	14	Undirected
Heart Arrest	university hospital	14	Undirected
Hospital Mortality	Stroke	14	Undirected
Hospital Mortality	clinical article	14	Undirected

Hospital Mortality	health care system	14	Undirected
Hospital Mortality	preschool child	14	Undirected
Hypnotics and Sedatives	Algorithms	14	Undirected
Hypnotics and Sedatives	pilot study	14	Undirected
Hypnotics and Sedatives	standards	14	Undirected
Infant, Premature	Australia	14	Undirected
Infant, Premature	evidence based practice	14	Undirected
Infant, Premature	perception	14	Undirected
Infection Control	adverse event	14	Undirected
Infection Control	caregiver	14	Undirected
Infection Control	interpersonal communication	14	Undirected
Infection Control	statistics and numerical data	14	Undirected
Insulin	Cardiac Surgical Procedures	14	Undirected
Insulin	antibiotic agent	14	Undirected
Insulin	health care quality	14	Undirected
Insulin	morphine	14	Undirected
Insulin	propofol	14	Undirected
Intubation, Intratracheal	cohort analysis	14	Undirected
Intubation, Intratracheal	standards	14	Undirected
Job Satisfaction	Quality Improvement	14	Undirected
Job Satisfaction	Risk Assessment	14	Undirected
Job Satisfaction	health care system	14	Undirected
Leadership	Canada	14	Undirected
Leadership	clinical article	14	Undirected
Length of Stay	Health Knowledge, Attitudes, Practice	14	Undirected
Length of Stay	Interprofessional Relations	14	Undirected
Length of Stay	Mothers	14	Undirected
Length of Stay	psychological aspect	14	Undirected
Logistic Models	Cardiac Surgical Procedures	14	Undirected
Medication Errors	multicenter study	14	Undirected
Monitoring, Physiologic	Guideline Adherence	14	Undirected
Monitoring, Physiologic	Stroke	14	Undirected
Monitoring, Physiologic	health care quality	14	Undirected
Monitoring, Physiologic	propofol	14	Undirected
Monitoring, Physiologic	total quality management	14	Undirected
Mothers	COVID-19	14	Undirected
Needs Assessment	emergency health service	14	Undirected
Needs Assessment	methodology	14	Undirected
OUTCOMES	health care quality	14	Undirected
OUTCOMES	observational study	14	Undirected
Pain	medical staff	14	Undirected
Pain	reliability	14	Undirected
Pain	resuscitation	14	Undirected
Pain Measurement	Algorithms	14	Undirected
Palliative Care	Australia	14	Undirected
Palliative Care	Canada	14	Undirected
Palliative Care	Clinical Protocols	14	Undirected
Palliative Care	United Kingdom	14	Undirected
Palliative Care	chronic obstructive lung disease	14	Undirected
Palliative Care	health care planning	14	Undirected
Palliative Care	psychological aspect	14	Undirected
Pandemics	breathing rate	14	Undirected
Pandemics	psychological aspect	14	Undirected
Parents	evidence based practice	14	Undirected

Parents	palliative therapy	14	Undirected
Parents	standards	14	Undirected
Personnel Staffing and Scheduling	safety	14	Undirected
Pilot Projects	cohort analysis	14	Undirected
Pilot Projects	statistics and numerical data	14	Undirected
Postoperative Care	Insulin	14	Undirected
Postoperative Care	blood pressure	14	Undirected
Postoperative Care	clinical practice	14	Undirected
Postoperative Care	hypertension	14	Undirected
Postoperative Care	total quality management	14	Undirected
Postoperative Care	tracheostomy	14	Undirected
Postoperative Care	university hospital	14	Undirected
Postoperative Complications	analgesia	14	Undirected
Postoperative Complications	disease severity	14	Undirected
Postoperative Complications	emergency ward	14	Undirected
Postoperative Complications	fentanyl	14	Undirected
Postoperative Complications	methodology	14	Undirected
Postoperative Complications	standards	14	Undirected
Practice Guidelines as Topic	Reproducibility of Results	14	Undirected
Practice Guidelines as Topic	follow up	14	Undirected
Practice Guidelines as Topic	knowledge	14	Undirected
Practice Guidelines as Topic	multicenter study	14	Undirected
Practice Guidelines as Topic	ventilator associated pneumonia	14	Undirected
Pregnancy	Pilot Projects	14	Undirected
Pregnancy	diabetes mellitus	14	Undirected
Pregnancy	knowledge	14	Undirected
Pregnancy	therapy	14	Undirected
Pressure Ulcer	Electronic Health Records	14	Undirected
Pressure Ulcer	clinical article	14	Undirected
Pressure Ulcer	evidence based practice	14	Undirected
Professional-Family Relations	clinical practice	14	Undirected
Program Evaluation	Catheterization, Central Venous	14	Undirected
Program Evaluation	Health Personnel	14	Undirected
Program Evaluation	Job Satisfaction	14	Undirected
Program Evaluation	follow up	14	Undirected
Program Evaluation	pediatrics	14	Undirected
Quality Improvement	Anti-Bacterial Agents	14	Undirected
Quality Improvement	China	14	Undirected
Quality Improvement	Telemedicine	14	Undirected
Quality Improvement	endotracheal intubation	14	Undirected
Quality Improvement	hand washing	14	Undirected
Quality Improvement	intensivist	14	Undirected
Quality of Health Care	Pain	14	Undirected
Quality of Health Care	Program Evaluation	14	Undirected
Quality of Health Care	statistics and numerical data	14	Undirected
Reproducibility of Results	Intubation, Intratracheal	14	Undirected
Reproducibility of Results	emergency care	14	Undirected
Risk Assessment	Leadership	14	Undirected
Risk Assessment	antiinfective agent	14	Undirected
Risk Assessment	breathing rate	14	Undirected
Risk Assessment	home care	14	Undirected
Risk Assessment	oxygen saturation	14	Undirected
Risk Assessment	palliative therapy	14	Undirected
Risk Assessment	sedation	14	Undirected

Risk Assessment	simulation	14	Undirected
Risk Factors	reliability	14	Undirected
Safety Management	Needs Assessment	14	Undirected
Sensitivity and Specificity	heart rate	14	Undirected
Sensitivity and Specificity	randomized controlled trial	14	Undirected
Sepsis	blood pressure	14	Undirected
Sepsis	depression	14	Undirected
Sepsis	endotracheal intubation	14	Undirected
Sepsis	extracorporeal oxygenation	14	Undirected
Sepsis	health care policy	14	Undirected
Sepsis	sedation	14	Undirected
Social Support	emergency ward	14	Undirected
Social Support	gestational age	14	Undirected
Social Support	home care	14	Undirected
Social Support	information processing	14	Undirected
Social Support	long term care	14	Undirected
Social Support	mortality	14	Undirected
Stress, Psychological	China	14	Undirected
Stress, Psychological	clinical article	14	Undirected
Stress, Psychological	pediatrics	14	Undirected
Telemedicine	clinical practice	14	Undirected
Telemedicine	hospital discharge	14	Undirected
Terminal Care	Heart Arrest	14	Undirected
Terminal Care	health care policy	14	Undirected
Terminal Care	pharmacist	14	Undirected
Terminal Care	randomized controlled trial	14	Undirected
Treatment Outcome	Needs Assessment	14	Undirected
Treatment Outcome	mental health	14	Undirected
Treatment Outcome	ethicillin resistant Staphylococcus aure	14	Undirected
United Kingdom	Focus Groups	14	Undirected
United Kingdom	health care planning	14	Undirected
United Kingdom	hospital personnel	14	Undirected
United Kingdom	pharmacist	14	Undirected
United Kingdom	thematic analysis	14	Undirected
Workforce	Terminal Care	14	Undirected
Workforce	organization and management	14	Undirected
Workforce	prevalence	14	Undirected
Workload	follow up	14	Undirected
Workload	pediatrics	14	Undirected
Workload	randomized controlled trial	14	Undirected
Workload	reliability	14	Undirected
Workload	standards	14	Undirected
Workplace	Emergency Service, Hospital	14	Undirected
Workplace	interview	14	Undirected
Workplace	knowledge	14	Undirected
Workplace	university hospital	14	Undirected
acute kidney failure	extracorporeal oxygenation	14	Undirected
adverse event	Pandemics	14	Undirected
age	palliative therapy	14	Undirected
analgesia	therapy	14	Undirected
attitude to health	Pandemics	14	Undirected
attitude to health	Quality Improvement	14	Undirected
attitude to health	health care delivery	14	Undirected
attitude to health	hospital admission	14	Undirected

attitude to health	safety	14	Undirected
attitude to health	thematic analysis	14	Undirected
blood pressure	endotracheal intubation	14	Undirected
breathing rate	extracorporeal oxygenation	14	Undirected
burnout	medical education	14	Undirected
caregiver	Fatigue	14	Undirected
caregiver	health care planning	14	Undirected
caregiver	hospital personnel	14	Undirected
caregiver	hypertension	14	Undirected
clinical practice	extracorporeal oxygenation	14	Undirected
dementia	multicenter study	14	Undirected
depression	clinical practice	14	Undirected
depression	hospital readmission	14	Undirected
disease severity	extracorporeal oxygenation	14	Undirected
dyspnea	blood pressure	14	Undirected
dyspnea	university hospital	14	Undirected
education	Electronic Health Records	14	Undirected
education	endotracheal intubation	14	Undirected
emergency care	teaching hospital	14	Undirected
emergency care	thematic analysis	14	Undirected
emergency health service	health care policy	14	Undirected
emergency ward	intensivist	14	Undirected
endotracheal intubation	organization and management	14	Undirected
epidemiology	pilot study	14	Undirected
ethics	United Kingdom	14	Undirected
evidence based practice	health care policy	14	Undirected
fever	hospital readmission	14	Undirected
follow up	antiinfective agent	14	Undirected
follow up	health care policy	14	Undirected
follow up	respiratory therapist	14	Undirected
gestational age	hospital readmission	14	Undirected
gestational age	meta analysis	14	Undirected
health care cost	Leadership	14	Undirected
health care cost	Medication Errors	14	Undirected
health care cost	cerebrovascular accident	14	Undirected
health care cost	gestational age	14	Undirected
health care cost	standards	14	Undirected
health care delivery	cerebrovascular accident	14	Undirected
health care delivery	computer assisted tomography	14	Undirected
health care facility	antibiotic agent	14	Undirected
health care facility	statistics and numerical data	14	Undirected
health care quality	hypertension	14	Undirected
heart failure	Postoperative Complications	14	Undirected
heart failure	emergency care	14	Undirected
heart rate	Fatigue	14	Undirected
heart rate	emergency care	14	Undirected
heart rate	therapy	14	Undirected
heart surgery	hospital admission	14	Undirected
heart surgery	university hospital	14	Undirected
heart surgery	very elderly	14	Undirected
home care	health care planning	14	Undirected
home care	standards	14	Undirected
home care	therapy	14	Undirected
hospital discharge	hospital personnel	14	Undirected

hospital discharge	intensivist	14	Undirected
hospital personnel	health care policy	14	Undirected
hospitalization	Program Evaluation	14	Undirected
hypertension	heart surgery	14	Undirected
hypertension	multicenter study	14	Undirected
hypertension	palliative therapy	14	Undirected
hypotension	COVID-19	14	Undirected
hypotension	cerebrovascular accident	14	Undirected
hypotension	methodology	14	Undirected
information processing	disease severity	14	Undirected
interview	attitude	14	Undirected
interview	emergency health service	14	Undirected
interview	intensivist	14	Undirected
interview	long term care	14	Undirected
interview	pilot study	14	Undirected
interview	randomized controlled trial	14	Undirected
interview	teaching hospital	14	Undirected
knowledge	sedation	14	Undirected
long term care	antiinfective agent	14	Undirected
long term care	statistics and numerical data	14	Undirected
medical education	therapy	14	Undirected
medical staff	Medication Errors	14	Undirected
mental health	clinical practice	14	Undirected
mental health	hospital readmission	14	Undirected
mental health	thematic analysis	14	Undirected
meta analysis	clinical practice	14	Undirected
meta analysis	hospital readmission	14	Undirected
cillin resistant Staphylococcus a	very elderly	14	Undirected
methodology	acute kidney failure	14	Undirected
methodology	meta analysis	14	Undirected
midazolam	COVID-19	14	Undirected
midazolam	follow up	14	Undirected
midazolam	preschool child	14	Undirected
morphine	disease severity	14	Undirected
morphine	hypertension	14	Undirected
morphine	preschool child	14	Undirected
observational study	computer assisted tomography	14	Undirected
observational study	meta analysis	14	Undirected
observational study	morphine	14	Undirected
outcome assessment	Anti-Bacterial Agents	14	Undirected
outcome assessment	Cardiac Surgical Procedures	14	Undirected
outcome assessment	Personnel Staffing and Scheduling	14	Undirected
outcome assessment	hand washing	14	Undirected
outcome assessment	thicillin resistant Staphylococcus aure	14	Undirected
outcome assessment	psychological aspect	14	Undirected
pain assessment	hospital admission	14	Undirected
pain assessment	preschool child	14	Undirected
pediatrics	Anti-Bacterial Agents	14	Undirected
pediatrics	China	14	Undirected
pediatrics	Infection Control	14	Undirected
pediatrics	Sensitivity and Specificity	14	Undirected
pediatrics	Telemedicine	14	Undirected
pediatrics	age	14	Undirected
pediatrics	antiinfective agent	14	Undirected

pediatrics	epidemiology	14	Undirected
pediatrics	gestational age	14	Undirected
pediatrics	heart rate	14	Undirected
perception	analgesia	14	Undirected
perception	emergency care	14	Undirected
perception	evidence based practice	14	Undirected
pharmacist	emergency care	14	Undirected
pharmacist	emergency health service	14	Undirected
pharmacist	health care facility	14	Undirected
pilot study	university hospital	14	Undirected
practice guideline	chronic obstructive lung disease	14	Undirected
prematurity	Canada	14	Undirected
prematurity	Health Personnel	14	Undirected
prematurity	home care	14	Undirected
prematurity	university hospital	14	Undirected
prevalence	Leadership	14	Undirected
prevalence	Palliative Care	14	Undirected
prevalence	Stress, Psychological	14	Undirected
prevalence	hypotension	14	Undirected
prevalence	medical staff	14	Undirected
prevalence	perception	14	Undirected
prevalence	thorax radiography	14	Undirected
propofol	COVID-19	14	Undirected
propofol	blood pressure	14	Undirected
propofol	follow up	14	Undirected
propofol	pharmacist	14	Undirected
psychological aspect	palliative therapy	14	Undirected
psychology	cerebrovascular accident	14	Undirected
quality of life	Pilot Projects	14	Undirected
quality of life	United Kingdom	14	Undirected
quality of life	pharmacist	14	Undirected
randomized controlled trial	intensivist	14	Undirected
respiratory therapist	hospital personnel	14	Undirected
respiratory therapist	preschool child	14	Undirected
respiratory therapist	therapy	14	Undirected
resuscitation	fentanyl	14	Undirected
resuscitation	semi structured interview	14	Undirected
safety	epidemiology	14	Undirected
sedation	adverse event	14	Undirected
simulation	extracorporeal oxygenation	14	Undirected
simulation	pilot study	14	Undirected
standards	health care planning	14	Undirected
statistics and numerical data	health care planning	14	Undirected
tertiary care center	Canada	14	Undirected
tertiary care center	Mothers	14	Undirected
tertiary care center	Reproducibility of Results	14	Undirected
tertiary care center	health care facility	14	Undirected
tertiary care center	heart surgery	14	Undirected
tertiary care center	hospital infection	14	Undirected
tertiary care center	pilot study	14	Undirected
thematic analysis	health care facility	14	Undirected
thematic analysis	multicenter study	14	Undirected
therapy	interpersonal communication	14	Undirected
total quality management	Fatigue	14	Undirected

total quality management	Intubation, Intratracheal	14	Undirected
total quality management	burnout	14	Undirected
tracheostomy	antibiotic agent	14	Undirected
tracheostomy	heart rate	14	Undirected
tracheostomy	multicenter study	14	Undirected
urinary tract infection	COVID-19	14	Undirected
urinary tract infection	health care quality	14	Undirected
vancomycin	microbiology	14	Undirected
ventilator associated pneumonia	epidemiology	14	Undirected
ventilator associated pneumonia	health care delivery	14	Undirected
very elderly	thorax radiography	14	Undirected
Adaptation, Psychological	Fatigue	13	Undirected
Adaptation, Psychological	Pilot Projects	13	Undirected
Adaptation, Psychological	pediatrics	13	Undirected
Algorithms	Pilot Projects	13	Undirected
Algorithms	diabetes mellitus	13	Undirected
Algorithms	sedation	13	Undirected
Anti-Bacterial Agents	clinical practice	13	Undirected
Anti-Bacterial Agents	pharmacist	13	Undirected
Anxiety	Canada	13	Undirected
Anxiety	Needs Assessment	13	Undirected
Anxiety	health care facility	13	Undirected
Anxiety	midazolam	13	Undirected
Anxiety	physiotherapy	13	Undirected
Artificial Ventilation	Personnel Staffing and Scheduling	13	Undirected
Attitude of Health Personnel	Sensitivity and Specificity	13	Undirected
Attitude of Health Personnel	adverse event	13	Undirected
Attitude of Health Personnel	depression	13	Undirected
Attitude of Health Personnel	ventilator associated pneumonia	13	Undirected
Australia	Anxiety	13	Undirected
Australia	Postoperative Complications	13	Undirected
Australia	attitude	13	Undirected
Australia	evidence based practice	13	Undirected
Australia	medical education	13	Undirected
Australia	randomized controlled trial	13	Undirected
Burnout, Professional	psychological aspect	13	Undirected
Canada	disease severity	13	Undirected
Canada	hospital discharge	13	Undirected
Canada	observational study	13	Undirected
Canada	very elderly	13	Undirected
Catheter-Related Infections	methodology	13	Undirected
Catheter-Related Infections	standards	13	Undirected
Child, Preschool	Algorithms	13	Undirected
Child, Preschool	China	13	Undirected
Child, Preschool	Health Knowledge, Attitudes, Practice	13	Undirected
Child, Preschool	Medication Errors	13	Undirected
Child, Preschool	antiinfective agent	13	Undirected
Child, Preschool	extracorporeal oxygenation	13	Undirected
Child, Preschool	health care cost	13	Undirected
Child, Preschool	heart rate	13	Undirected
Child, Preschool	home care	13	Undirected
Child, Preschool	medical education	13	Undirected
Child, Preschool	respiratory therapist	13	Undirected
China	analgesia	13	Undirected

China	sedation	13	Undirected
Clinical Competence	ethics	13	Undirected
Clinical Competence	mortality	13	Undirected
Clinical Competence	outcome assessment	13	Undirected
Clinical Competence	palliative therapy	13	Undirected
Clinical Competence	teaching hospital	13	Undirected
Clinical Protocols	Catheterization, Central Venous	13	Undirected
Clinical Protocols	hospital readmission	13	Undirected
Clinical Protocols	hypotension	13	Undirected
Clinical Protocols	intensivist	13	Undirected
Clinical Protocols	pain assessment	13	Undirected
Communication	Algorithms	13	Undirected
Communication	Infection Control	13	Undirected
Communication	Pain Measurement	13	Undirected
Communication	cohort analysis	13	Undirected
Communication	epidemiology	13	Undirected
Communication	health care planning	13	Undirected
Communication	reliability	13	Undirected
Communication	tracheostomy	13	Undirected
Cooperative Behavior	Clinical Protocols	13	Undirected
Cooperative Behavior	Palliative Care	13	Undirected
Cooperative Behavior	Pilot Projects	13	Undirected
Cooperative Behavior	interview	13	Undirected
Cooperative Behavior	medical staff	13	Undirected
Cross Infection	fever	13	Undirected
Cross Infection	medical education	13	Undirected
Decision Making	Infection Control	13	Undirected
Decision Making	extracorporeal oxygenation	13	Undirected
Decision Making	hospital personnel	13	Undirected
Decision Making	teaching hospital	13	Undirected
Delirium	Canada	13	Undirected
Delirium	dyspnea	13	Undirected
Delirium	interpersonal communication	13	Undirected
Delirium	respiratory therapist	13	Undirected
Delirium	tracheostomy	13	Undirected
Electronic Health Records	clinical practice	13	Undirected
Electronic Health Records	disease severity	13	Undirected
Electronic Health Records	emergency care	13	Undirected
Electronic Health Records	information processing	13	Undirected
Electronic Health Records	interpersonal communication	13	Undirected
Emergency Service, Hospital	Anti-Bacterial Agents	13	Undirected
Emergency Service, Hospital	Delirium	13	Undirected
Emergency Service, Hospital	Telemedicine	13	Undirected
Emergency Service, Hospital	dyspnea	13	Undirected
Emergency Service, Hospital	heart rate	13	Undirected
Emergency Service, Hospital	hospital personnel	13	Undirected
Emergency Service, Hospital	university hospital	13	Undirected
Enteral Nutrition	education	13	Undirected
Enteral Nutrition	knowledge	13	Undirected
Enteral Nutrition	preschool child	13	Undirected
Family	Burnout, Professional	13	Undirected
Family	China	13	Undirected
Family	Sepsis	13	Undirected
Family	heart failure	13	Undirected

Family	pilot study	13	Undirected
Fatigue	age	13	Undirected
Fatigue	computer assisted tomography	13	Undirected
Fatigue	mental health	13	Undirected
Focus Groups	evidence based practice	13	Undirected
Glasgow coma scale	chronic obstructive lung disease	13	Undirected
Glasgow coma scale	home care	13	Undirected
Guideline Adherence	Child, Preschool	13	Undirected
Guideline Adherence	Emergency Service, Hospital	13	Undirected
Guideline Adherence	Heart Arrest	13	Undirected
Guideline Adherence	Intubation, Intratracheal	13	Undirected
Guideline Adherence	outcome assessment	13	Undirected
alth Knowledge, Attitudes, Practi	Burnout, Professional	13	Undirected
Health Personnel	very elderly	13	Undirected
Health Services Needs and Demand	COVID-19	13	Undirected
Heart Arrest	blood pressure	13	Undirected
Heart Arrest	computer assisted tomography	13	Undirected
Heart Arrest	diabetes mellitus	13	Undirected
Heart Arrest	psychology	13	Undirected
Heart Arrest	standards	13	Undirected
Heart Arrest	statistics and numerical data	13	Undirected
Heart Arrest	therapy	13	Undirected
Hospital Mortality	Hypnotics and Sedatives	13	Undirected
Hospital Mortality	education	13	Undirected
Hospital Mortality	fever	13	Undirected
Hospital Mortality	health care planning	13	Undirected
Hospital Mortality	hypotension	13	Undirected
Hospital Mortality	propofol	13	Undirected
Hospital Mortality	urinary tract infection	13	Undirected
Infant, Premature	depression	13	Undirected
Infant, Premature	health care cost	13	Undirected
Infant, Premature	health care quality	13	Undirected
Infant, Premature	observational study	13	Undirected
Infant, Premature	safety	13	Undirected
Infection Control	computer assisted tomography	13	Undirected
Infection Control	preschool child	13	Undirected
Infection Control	thematic analysis	13	Undirected
Insulin	COVID-19	13	Undirected
Insulin	clinical practice	13	Undirected
Insulin	cohort analysis	13	Undirected
Insulin	hypertension	13	Undirected
Insulin	midazolam	13	Undirected
Insulin	pharmacist	13	Undirected
Interprofessional Relations	Clinical Protocols	13	Undirected
Interprofessional Relations	Risk Assessment	13	Undirected
Intubation, Intratracheal	Cardiac Surgical Procedures	13	Undirected
Intubation, Intratracheal	propofol	13	Undirected
Job Satisfaction	Reproducibility of Results	13	Undirected
Job Satisfaction	caregiver	13	Undirected
Job Satisfaction	clinical article	13	Undirected
Leadership	mortality	13	Undirected
Leadership	psychological aspect	13	Undirected
Length of Stay	Catheterization, Central Venous	13	Undirected
Logistic Models	observational study	13	Undirected

Logistic Models	statistics and numerical data	13	Undirected
Logistic Models	tertiary care center	13	Undirected
Medication Errors	antiinfective agent	13	Undirected
Monitoring, Physiologic	Delirium	13	Undirected
Monitoring, Physiologic	Emergency Service, Hospital	13	Undirected
Monitoring, Physiologic	pilot study	13	Undirected
Monitoring, Physiologic	prematurity	13	Undirected
Mothers	health care quality	13	Undirected
Needs Assessment	Quality Improvement	13	Undirected
Needs Assessment	information processing	13	Undirected
Needs Assessment	interpersonal communication	13	Undirected
Needs Assessment	practice guideline	13	Undirected
OUTCOMES	Glasgow coma scale	13	Undirected
OUTCOMES	age	13	Undirected
OUTCOMES	hospital readmission	13	Undirected
OUTCOMES	pediatrics	13	Undirected
Pain	epidemiology	13	Undirected
Pain Measurement	Sensitivity and Specificity	13	Undirected
Pain Measurement	multicenter study	13	Undirected
Pain Measurement	preschool child	13	Undirected
Pain Measurement	propofol	13	Undirected
Pain Measurement	standards	13	Undirected
Palliative Care	Pilot Projects	13	Undirected
Palliative Care	age	13	Undirected
Palliative Care	emergency care	13	Undirected
Palliative Care	epidemiology	13	Undirected
Palliative Care	evidence based practice	13	Undirected
Palliative Care	hospital readmission	13	Undirected
Palliative Care	midazolam	13	Undirected
Palliative Care	multicenter study	13	Undirected
Palliative Care	randomized controlled trial	13	Undirected
Palliative Care	therapy	13	Undirected
Pandemics	acute kidney failure	13	Undirected
Pandemics	cerebrovascular accident	13	Undirected
Pandemics	chronic obstructive lung disease	13	Undirected
Personnel Staffing and Scheduling	medical staff	13	Undirected
Pilot Projects	hospital discharge	13	Undirected
Pilot Projects	very elderly	13	Undirected
Postoperative Care	United Kingdom	13	Undirected
Postoperative Care	cohort analysis	13	Undirected
Postoperative Care	evidence based practice	13	Undirected
Postoperative Care	fentanyl	13	Undirected
Postoperative Care	observational study	13	Undirected
Postoperative Care	safety	13	Undirected
Postoperative Complications	age	13	Undirected
Postoperative Complications	antibiotic agent	13	Undirected
Postoperative Complications	health care quality	13	Undirected
Postoperative Complications	midazolam	13	Undirected
Postoperative Complications	statistics and numerical data	13	Undirected
Practice Guidelines as Topic	emergency ward	13	Undirected
Practice Guidelines as Topic	health care policy	13	Undirected
Practice Guidelines as Topic	information processing	13	Undirected
Practice Guidelines as Topic	interpersonal communication	13	Undirected
Practice Guidelines as Topic	observational study	13	Undirected

Practice Guidelines as Topic	urinary tract infection	13	Undirected
Pregnancy	Focus Groups	13	Undirected
Pregnancy	caregiver	13	Undirected
Pregnancy	dyspnea	13	Undirected
Pregnancy	meta analysis	13	Undirected
Pregnancy	perception	13	Undirected
Pregnancy	statistics and numerical data	13	Undirected
Pressure Ulcer	Algorithms	13	Undirected
Pressure Ulcer	health care quality	13	Undirected
Professional-Family Relations	Pain	13	Undirected
Professional-Family Relations	Treatment Outcome	13	Undirected
Professional-Family Relations	preschool child	13	Undirected
Professional-Family Relations	randomized controlled trial	13	Undirected
Program Evaluation	Focus Groups	13	Undirected
Program Evaluation	Safety Management	13	Undirected
Quality Improvement	Pain Measurement	13	Undirected
Quality Improvement	disease severity	13	Undirected
Quality Improvement	randomized controlled trial	13	Undirected
Quality of Health Care	Heart Arrest	13	Undirected
Quality of Health Care	emergency care	13	Undirected
Quality of Health Care	evidence based practice	13	Undirected
Quality of Health Care	information processing	13	Undirected
Quality of Health Care	pediatrics	13	Undirected
Reproducibility of Results	emergency ward	13	Undirected
Reproducibility of Results	pilot study	13	Undirected
Reproducibility of Results	sedation	13	Undirected
Reproducibility of Results	university hospital	13	Undirected
Risk Assessment	Hypnotics and Sedatives	13	Undirected
Risk Assessment	fentanyl	13	Undirected
Risk Assessment	interview	13	Undirected
Risk Assessment	knowledge	13	Undirected
Risk Assessment	microbiology	13	Undirected
Risk Assessment	urinary tract infection	13	Undirected
Risk Factors	Pain Measurement	13	Undirected
Risk Factors	intensivist	13	Undirected
Risk Factors	pain assessment	13	Undirected
Sepsis	Australia	13	Undirected
Sepsis	heart surgery	13	Undirected
Sepsis	palliative therapy	13	Undirected
Sepsis	pilot study	13	Undirected
Social Support	epidemiology	13	Undirected
Social Support	health care cost	13	Undirected
Social Support	health care facility	13	Undirected
Stress, Psychological	Reproducibility of Results	13	Undirected
Stress, Psychological	United Kingdom	13	Undirected
Stress, Psychological	follow up	13	Undirected
Stroke	clinical article	13	Undirected
Stroke	randomized controlled trial	13	Undirected
Telemedicine	cohort analysis	13	Undirected
Telemedicine	disease severity	13	Undirected
Terminal Care	Clinical Protocols	13	Undirected
Terminal Care	midazolam	13	Undirected
Time Factors	Interprofessional Relations	13	Undirected
Time Factors	antiinfective agent	13	Undirected

Time Factors	caregiver	13	Undirected
Time Factors	diabetes mellitus	13	Undirected
Time Factors	interpersonal communication	13	Undirected
Time Factors	propofol	13	Undirected
Treatment Outcome	Fatigue	13	Undirected
Treatment Outcome	Interprofessional Relations	13	Undirected
Treatment Outcome	Mothers	13	Undirected
Treatment Outcome	burnout	13	Undirected
Treatment Outcome	knowledge	13	Undirected
United Kingdom	Algorithms	13	Undirected
United Kingdom	Heart Arrest	13	Undirected
United Kingdom	mental health	13	Undirected
United Kingdom	preschool child	13	Undirected
United Kingdom	randomized controlled trial	13	Undirected
Workforce	Program Evaluation	13	Undirected
Workforce	Quality Improvement	13	Undirected
Workforce	Risk Assessment	13	Undirected
Workload	caregiver	13	Undirected
Workload	statistics and numerical data	13	Undirected
Workplace	Decision Making	13	Undirected
Workplace	Fatigue	13	Undirected
Workplace	health care system	13	Undirected
Workplace	medical staff	13	Undirected
Workplace	practice guideline	13	Undirected
Workplace	psychological aspect	13	Undirected
Workplace	safety	13	Undirected
acute kidney failure	breathing rate	13	Undirected
adverse event	extracorporeal oxygenation	13	Undirected
adverse event	oxygen saturation	13	Undirected
age	interpersonal communication	13	Undirected
analgesia	Glasgow coma scale	13	Undirected
analgesia	adverse event	13	Undirected
analgesia	very elderly	13	Undirected
antiinfective agent	computer assisted tomography	13	Undirected
attitude	analgesia	13	Undirected
attitude	clinical article	13	Undirected
attitude	practice guideline	13	Undirected
attitude to health	Emergency Service, Hospital	13	Undirected
attitude to health	Reproducibility of Results	13	Undirected
attitude to health	outcome assessment	13	Undirected
blood pressure	acute kidney failure	13	Undirected
blood pressure	midazolam	13	Undirected
breathing rate	chronic obstructive lung disease	13	Undirected
burnout	health care policy	13	Undirected
burnout	mortality	13	Undirected
burnout	semi structured interview	13	Undirected
caregiver	pharmacist	13	Undirected
caregiver	physiotherapy	13	Undirected
clinical article	hospital personnel	13	Undirected
dementia	clinical practice	13	Undirected
depression	meta analysis	13	Undirected
diabetes mellitus	Health Personnel	13	Undirected
diabetes mellitus	organization and management	13	Undirected
diabetes mellitus	palliative therapy	13	Undirected

dyspnea	cerebrovascular accident	13	Undirected
education	hospital infection	13	Undirected
education	sedation	13	Undirected
emergency care	chronic obstructive lung disease	13	Undirected
emergency care	methodology	13	Undirected
emergency care	oxygen saturation	13	Undirected
emergency ward	extracorporeal oxygenation	13	Undirected
endotracheal intubation	physiotherapy	13	Undirected
endotracheal intubation	therapy	13	Undirected
epidemiology	antiinfective agent	13	Undirected
epidemiology	meta analysis	13	Undirected
epidemiology	oxygen saturation	13	Undirected
ethics	Leadership	13	Undirected
ethics	Pain	13	Undirected
ethics	Pregnancy	13	Undirected
ethics	health care policy	13	Undirected
evidence based practice	disease severity	13	Undirected
evidence based practice	semi structured interview	13	Undirected
fentanyl	preschool child	13	Undirected
fever	chronic obstructive lung disease	13	Undirected
fever	depression	13	Undirected
fever	hand washing	13	Undirected
fever	health care delivery	13	Undirected
fever	multicenter study	13	Undirected
gestational age	adverse event	13	Undirected
gestational age	methodology	13	Undirected
health care cost	Telemedicine	13	Undirected
health care cost	antiinfective agent	13	Undirected
health care cost	heart surgery	13	Undirected
health care cost	methodology	13	Undirected
health care cost	tracheostomy	13	Undirected
health care cost	ventilator associated pneumonia	13	Undirected
health care delivery	age	13	Undirected
health care delivery	teaching hospital	13	Undirected
health care planning	antibiotic agent	13	Undirected
health care planning	intensivist	13	Undirected
health care quality	chronic obstructive lung disease	13	Undirected
health care system	cerebrovascular accident	13	Undirected
heart failure	Decision Making	13	Undirected
heart failure	Postoperative Care	13	Undirected
heart failure	epidemiology	13	Undirected
heart failure	fever	13	Undirected
heart failure	health care quality	13	Undirected
heart failure	standards	13	Undirected
heart failure	total quality management	13	Undirected
heart rate	hospital readmission	13	Undirected
heart rate	morphine	13	Undirected
heart rate	psychology	13	Undirected
heart surgery	preschool child	13	Undirected
home care	preschool child	13	Undirected
hospital discharge	respiratory therapist	13	Undirected
hospital infection	health care system	13	Undirected
hospital infection	hospital readmission	13	Undirected
hospital personnel	information processing	13	Undirected

hospital readmission	health care policy	13	Undirected
hospitalization	Hypnotics and Sedatives	13	Undirected
hospitalization	attitude	13	Undirected
hypotension	observational study	13	Undirected
interview	China	13	Undirected
knowledge	Catheter-Related Infections	13	Undirected
knowledge	cohort analysis	13	Undirected
knowledge	randomized controlled trial	13	Undirected
long term care	Health Personnel	13	Undirected
long term care	chronic obstructive lung disease	13	Undirected
medical education	hospital infection	13	Undirected
medical staff	cerebrovascular accident	13	Undirected
medical staff	oxygen saturation	13	Undirected
medical staff	sedation	13	Undirected
mental health	health care quality	13	Undirected
mental health	therapy	13	Undirected
meta analysis	cohort analysis	13	Undirected
meta analysis	disease severity	13	Undirected
cillin resistant Staphylococcus a	disease severity	13	Undirected
cillin resistant Staphylococcus a	teaching hospital	13	Undirected
methodology	antiinfective agent	13	Undirected
methodology	disease severity	13	Undirected
methodology	palliative therapy	13	Undirected
methodology	very elderly	13	Undirected
microbiology	preschool child	13	Undirected
midazolam	computer assisted tomography	13	Undirected
midazolam	disease severity	13	Undirected
midazolam	emergency ward	13	Undirected
morphine	follow up	13	Undirected
morphine	hospital discharge	13	Undirected
multicenter study	acute kidney failure	13	Undirected
multicenter study	chronic obstructive lung disease	13	Undirected
outcome assessment	China	13	Undirected
pain assessment	cohort analysis	13	Undirected
pain assessment	endotracheal intubation	13	Undirected
pain assessment	organization and management	13	Undirected
pain assessment	psychology	13	Undirected
pediatrics	Pain Measurement	13	Undirected
pediatrics	physiotherapy	13	Undirected
perception	depression	13	Undirected
perception	mortality	13	Undirected
pharmacist	depression	13	Undirected
pharmacist	enteric feeding	13	Undirected
pharmacist	morphine	13	Undirected
physiotherapy	disease severity	13	Undirected
physiotherapy	hospital readmission	13	Undirected
pilot study	medical education	13	Undirected
pilot study	oxygen saturation	13	Undirected
pilot study	respiratory therapist	13	Undirected
pneumonia	health care system	13	Undirected
pneumonia	long term care	13	Undirected
pneumonia	microbiology	13	Undirected
pneumonia	standards	13	Undirected
practice guideline	breathing rate	13	Undirected

prematurity	COVID-19	13	Undirected
prematurity	depression	13	Undirected
prematurity	fentanyl	13	Undirected
prematurity	palliative therapy	13	Undirected
preschool child	computer assisted tomography	13	Undirected
prevalence	attitude	13	Undirected
prevalence	health care planning	13	Undirected
prevalence	interview	13	Undirected
prevalence	safety	13	Undirected
propofol	adverse event	13	Undirected
propofol	multicenter study	13	Undirected
quality of life	Canada	13	Undirected
quality of life	Clinical Competence	13	Undirected
quality of life	Emergency Service, Hospital	13	Undirected
quality of life	acute kidney failure	13	Undirected
quality of life	hospital personnel	13	Undirected
quality of life	sedation	13	Undirected
quality of life	standards	13	Undirected
randomized controlled trial	age	13	Undirected
randomized controlled trial	health care planning	13	Undirected
randomized controlled trial	hospital personnel	13	Undirected
randomized controlled trial	respiratory therapist	13	Undirected
randomized controlled trial	semi structured interview	13	Undirected
respiratory therapist	emergency health service	13	Undirected
respiratory therapist	health care planning	13	Undirected
resuscitation	dementia	13	Undirected
resuscitation	home care	13	Undirected
resuscitation	teaching hospital	13	Undirected
safety	health care delivery	13	Undirected
safety	hospital admission	13	Undirected
sedation	heart surgery	13	Undirected
semi structured interview	health care facility	13	Undirected
semi structured interview	hospital discharge	13	Undirected
standards	antibiotic agent	13	Undirected
standards	respiratory therapist	13	Undirected
tertiary care center	breathing rate	13	Undirected
tertiary care center	cerebrovascular accident	13	Undirected
tertiary care center	chronic obstructive lung disease	13	Undirected
tertiary care center	diabetes mellitus	13	Undirected
tertiary care center	information processing	13	Undirected
tertiary care center	thematic analysis	13	Undirected
total quality management	Anti-Bacterial Agents	13	Undirected
total quality management	Breast Feeding	13	Undirected
total quality management	Postoperative Complications	13	Undirected
total quality management	enteric feeding	13	Undirected
tracheostomy	caregiver	13	Undirected
tracheostomy	hypotension	13	Undirected
tracheostomy	respiratory therapist	13	Undirected
university hospital	chronic obstructive lung disease	13	Undirected
urinary tract infection	clinical article	13	Undirected
urinary tract infection	hand washing	13	Undirected
urinary tract infection	hypertension	13	Undirected
urinary tract infection	randomized controlled trial	13	Undirected
vancomycin	very elderly	13	Undirected

ventilator associated pneumonia	antiinfective agent	13	Undirected
ventilator associated pneumonia	organization and management	13	Undirected
Adaptation, Psychological	Australia	12	Undirected
Adaptation, Psychological	China	12	Undirected
Adaptation, Psychological	Risk Assessment	12	Undirected
Adaptation, Psychological	hospital discharge	12	Undirected
Adaptation, Psychological	preschool child	12	Undirected
Algorithms	Pandemics	12	Undirected
Algorithms	emergency health service	12	Undirected
Algorithms	emergency ward	12	Undirected
Algorithms	hospital discharge	12	Undirected
Algorithms	medical education	12	Undirected
Algorithms	simulation	12	Undirected
Anti-Bacterial Agents	observational study	12	Undirected
Anti-Bacterial Agents	thorax radiography	12	Undirected
Anxiety	Cardiac Surgical Procedures	12	Undirected
Anxiety	Clinical Protocols	12	Undirected
Anxiety	Sensitivity and Specificity	12	Undirected
Anxiety	diabetes mellitus	12	Undirected
Anxiety	fentanyl	12	Undirected
Anxiety	hospital personnel	12	Undirected
Anxiety	oxygen saturation	12	Undirected
Attitude of Health Personnel	Enteral Nutrition	12	Undirected
Attitude of Health Personnel	therapy	12	Undirected
Australia	Pressure Ulcer	12	Undirected
Australia	health care policy	12	Undirected
Australia	pilot study	12	Undirected
Burnout, Professional	caregiver	12	Undirected
Burnout, Professional	health care system	12	Undirected
COVID-19	enteric feeding	12	Undirected
Canada	Medication Errors	12	Undirected
Canada	Pilot Projects	12	Undirected
Catheterization, Central Venous	antibiotic agent	12	Undirected
Child, Preschool	Infant, Premature	12	Undirected
Child, Preschool	OUTCOMES	12	Undirected
Child, Preschool	endotracheal intubation	12	Undirected
Child, Preschool	enteric feeding	12	Undirected
Child, Preschool	microbiology	12	Undirected
Child, Preschool	pilot study	12	Undirected
Child, Preschool	propofol	12	Undirected
China	Catheterization, Central Venous	12	Undirected
China	Fatigue	12	Undirected
China	Intubation, Intratracheal	12	Undirected
China	Sensitivity and Specificity	12	Undirected
China	caregiver	12	Undirected
China	observational study	12	Undirected
Clinical Competence	prevalence	12	Undirected
Clinical Protocols	Glasgow coma scale	12	Undirected
Clinical Protocols	Intubation, Intratracheal	12	Undirected
Clinical Protocols	Medication Errors	12	Undirected
Clinical Protocols	cerebrovascular accident	12	Undirected
Clinical Protocols	endotracheal intubation	12	Undirected
Clinical Protocols	hospital infection	12	Undirected
Clinical Protocols	preschool child	12	Undirected

Clinical Protocols	urinary tract infection	12	Undirected
Communication	emergency ward	12	Undirected
Communication	health care system	12	Undirected
Communication	prematurity	12	Undirected
Cooperative Behavior	Emergency Service, Hospital	12	Undirected
Cooperative Behavior	Time Factors	12	Undirected
Cooperative Behavior	evidence based practice	12	Undirected
Cross Infection	emergency ward	12	Undirected
Cross Infection	gestational age	12	Undirected
Cross Infection	safety	12	Undirected
Decision Making	China	12	Undirected
Decision Making	Postoperative Care	12	Undirected
Delirium	diabetes mellitus	12	Undirected
Delirium	emergency care	12	Undirected
Delirium	health care system	12	Undirected
Delirium	heart rate	12	Undirected
Delirium	information processing	12	Undirected
Delirium	oxygen saturation	12	Undirected
Electronic Health Records	palliative therapy	12	Undirected
Emergency Service, Hospital	Burnout, Professional	12	Undirected
Emergency Service, Hospital	antibiotic agent	12	Undirected
Emergency Service, Hospital	computer assisted tomography	12	Undirected
Emergency Service, Hospital	health care facility	12	Undirected
Emergency Service, Hospital	heart failure	12	Undirected
Emergency Service, Hospital	interview	12	Undirected
Enteral Nutrition	very elderly	12	Undirected
Family	antibiotic agent	12	Undirected
Family	health care planning	12	Undirected
Family	reliability	12	Undirected
Family	teaching hospital	12	Undirected
Fatigue	thorax radiography	12	Undirected
Focus Groups	Pandemics	12	Undirected
Focus Groups	university hospital	12	Undirected
Glasgow coma scale	COVID-19	12	Undirected
Glasgow coma scale	Stroke	12	Undirected
Glasgow coma scale	acute kidney failure	12	Undirected
Glasgow coma scale	preschool child	12	Undirected
Guideline Adherence	Insulin	12	Undirected
Guideline Adherence	Logistic Models	12	Undirected
Guideline Adherence	Reproducibility of Results	12	Undirected
Guideline Adherence	hospital admission	12	Undirected
Guideline Adherence	sedation	12	Undirected
alth Knowledge, Attitudes, Practi	Anxiety	12	Undirected
alth Knowledge, Attitudes, Practi	emergency ward	12	Undirected
alth Knowledge, Attitudes, Practi	pilot study	12	Undirected
alth Knowledge, Attitudes, Practi	randomized controlled trial	12	Undirected
Health Personnel	microbiology	12	Undirected
Health Personnel	pilot study	12	Undirected
Health Services Needs and Demand	Infant, Premature	12	Undirected
Health Services Needs and Demand	Treatment Outcome	12	Undirected
Health Services Needs and Demand	Workforce	12	Undirected
Heart Arrest	Postoperative Complications	12	Undirected
Heart Arrest	fentanyl	12	Undirected
Heart Arrest	heart surgery	12	Undirected

Heart Arrest	intensivist	12	Undirected
Heart Arrest	midazolam	12	Undirected
Hospital Mortality	computer assisted tomography	12	Undirected
Hospital Mortality	enteric feeding	12	Undirected
Hospital Mortality	extracorporeal oxygenation	12	Undirected
Hospital Mortality	health care facility	12	Undirected
Hospital Mortality	psychology	12	Undirected
Hypnotics and Sedatives	clinical article	12	Undirected
Infant, Premature	disease severity	12	Undirected
Infection Control	attitude	12	Undirected
Infection Control	emergency health service	12	Undirected
Infection Control	semi structured interview	12	Undirected
Infection Control	thorax radiography	12	Undirected
Insulin	follow up	12	Undirected
Insulin	observational study	12	Undirected
Insulin	safety	12	Undirected
Insulin	total quality management	12	Undirected
Interprofessional Relations	Child, Preschool	12	Undirected
Interprofessional Relations	Reproducibility of Results	12	Undirected
Interprofessional Relations	practice guideline	12	Undirected
Job Satisfaction	Health Knowledge, Attitudes, Practice	12	Undirected
Job Satisfaction	standards	12	Undirected
Job Satisfaction	thematic analysis	12	Undirected
Leadership	depression	12	Undirected
Leadership	hospital personnel	12	Undirected
Leadership	knowledge	12	Undirected
Leadership	therapy	12	Undirected
Length of Stay	burnout	12	Undirected
Logistic Models	Postoperative Complications	12	Undirected
Logistic Models	disease severity	12	Undirected
Logistic Models	follow up	12	Undirected
Medication Errors	antibiotic agent	12	Undirected
Medication Errors	health care planning	12	Undirected
Monitoring, Physiologic	COVID-19	12	Undirected
Monitoring, Physiologic	Pain Measurement	12	Undirected
Monitoring, Physiologic	Quality of Health Care	12	Undirected
Monitoring, Physiologic	hypotension	12	Undirected
Monitoring, Physiologic	mortality	12	Undirected
Monitoring, Physiologic	pediatrics	12	Undirected
Monitoring, Physiologic	sedation	12	Undirected
Monitoring, Physiologic	very elderly	12	Undirected
Needs Assessment	Clinical Protocols	12	Undirected
Needs Assessment	Mothers	12	Undirected
Needs Assessment	long term care	12	Undirected
OUTCOMES	Stroke	12	Undirected
OUTCOMES	attitude	12	Undirected
OUTCOMES	emergency ward	12	Undirected
OUTCOMES	standards	12	Undirected
Pain	China	12	Undirected
Pain	Mothers	12	Undirected
Pain	heart surgery	12	Undirected
Pain	mental health	12	Undirected
Pain	safety	12	Undirected
Pain Measurement	blood pressure	12	Undirected

Pain Measurement	perception	12	Undirected
Palliative Care	Hypnotics and Sedatives	12	Undirected
Palliative Care	burnout	12	Undirected
Palliative Care	intensivist	12	Undirected
Palliative Care	methodology	12	Undirected
Palliative Care	statistics and numerical data	12	Undirected
Pandemics	preschool child	12	Undirected
Pandemics	teaching hospital	12	Undirected
Parents	China	12	Undirected
Parents	Reproducibility of Results	12	Undirected
Parents	health care delivery	12	Undirected
Parents	statistics and numerical data	12	Undirected
Parents	university hospital	12	Undirected
Personnel Staffing and Scheduling	Canada	12	Undirected
Personnel Staffing and Scheduling	Fatigue	12	Undirected
Personnel Staffing and Scheduling	Pilot Projects	12	Undirected
Personnel Staffing and Scheduling	pediatrics	12	Undirected
Personnel Staffing and Scheduling	practice guideline	12	Undirected
Personnel Staffing and Scheduling	statistics and numerical data	12	Undirected
Pilot Projects	follow up	12	Undirected
Pilot Projects	psychological aspect	12	Undirected
Postoperative Care	Sepsis	12	Undirected
Postoperative Care	antibiotic agent	12	Undirected
Postoperative Care	sedation	12	Undirected
Postoperative Complications	resuscitation	12	Undirected
Practice Guidelines as Topic	emergency care	12	Undirected
Practice Guidelines as Topic	endotracheal intubation	12	Undirected
Practice Guidelines as Topic	epidemiology	12	Undirected
Practice Guidelines as Topic	health care delivery	12	Undirected
Practice Guidelines as Topic	pharmacist	12	Undirected
Pregnancy	Clinical Protocols	12	Undirected
Pregnancy	antibiotic agent	12	Undirected
Pregnancy	computer assisted tomography	12	Undirected
Pressure Ulcer	Clinical Protocols	12	Undirected
Pressure Ulcer	practice guideline	12	Undirected
Pressure Ulcer	statistics and numerical data	12	Undirected
Pressure Ulcer	university hospital	12	Undirected
Professional-Family Relations	Risk Assessment	12	Undirected
Professional-Family Relations	Risk Factors	12	Undirected
Professional-Family Relations	Workforce	12	Undirected
Professional-Family Relations	emergency health service	12	Undirected
Professional-Family Relations	hospital admission	12	Undirected
Professional-Family Relations	information processing	12	Undirected
Program Evaluation	Mothers	12	Undirected
Program Evaluation	Personnel Staffing and Scheduling	12	Undirected
Program Evaluation	Terminal Care	12	Undirected
Program Evaluation	medical education	12	Undirected
Quality Improvement	antibiotic agent	12	Undirected
Quality Improvement	health care policy	12	Undirected
Quality Improvement	pneumonia	12	Undirected
Quality Improvement	semi structured interview	12	Undirected
Quality Improvement	thematic analysis	12	Undirected
Quality of Health Care	Clinical Protocols	12	Undirected
Quality of Health Care	cohort analysis	12	Undirected

Quality of Health Care	tertiary care center	12	Undirected
Quality of Health Care	university hospital	12	Undirected
Reproducibility of Results	Canada	12	Undirected
Reproducibility of Results	Electronic Health Records	12	Undirected
Reproducibility of Results	gestational age	12	Undirected
Reproducibility of Results	health care quality	12	Undirected
Reproducibility of Results	interpersonal communication	12	Undirected
Reproducibility of Results	safety	12	Undirected
Risk Assessment	Catheter-Related Infections	12	Undirected
Risk Assessment	Catheterization, Central Venous	12	Undirected
Risk Assessment	Enteral Nutrition	12	Undirected
Risk Assessment	Focus Groups	12	Undirected
Risk Assessment	dementia	12	Undirected
Risk Assessment	dyspnea	12	Undirected
Risk Assessment	morphine	12	Undirected
Risk Factors	Focus Groups	12	Undirected
Risk Factors	simulation	12	Undirected
Safety Management	United Kingdom	12	Undirected
Sensitivity and Specificity	Health Personnel	12	Undirected
Sensitivity and Specificity	blood pressure	12	Undirected
Sensitivity and Specificity	clinical practice	12	Undirected
Sensitivity and Specificity	fever	12	Undirected
Sensitivity and Specificity	pain assessment	12	Undirected
Sepsis	Pandemics	12	Undirected
Sepsis	Telemedicine	12	Undirected
Sepsis	Terminal Care	12	Undirected
Sepsis	home care	12	Undirected
Sepsis	interpersonal communication	12	Undirected
Sepsis	mental health	12	Undirected
Sepsis	propofol	12	Undirected
Social Support	Pain	12	Undirected
Social Support	Risk Assessment	12	Undirected
Social Support	randomized controlled trial	12	Undirected
Stress, Psychological	Pain Measurement	12	Undirected
Stress, Psychological	Program Evaluation	12	Undirected
Stress, Psychological	semi structured interview	12	Undirected
Stroke	health care quality	12	Undirected
Stroke	organization and management	12	Undirected
Telemedicine	Electronic Health Records	12	Undirected
Telemedicine	health care policy	12	Undirected
Telemedicine	interpersonal communication	12	Undirected
Terminal Care	Delirium	12	Undirected
Terminal Care	hospital readmission	12	Undirected
Time Factors	Focus Groups	12	Undirected
Time Factors	Job Satisfaction	12	Undirected
Time Factors	analgesia	12	Undirected
Time Factors	antibiotic agent	12	Undirected
Time Factors	evidence based practice	12	Undirected
Time Factors	health care delivery	12	Undirected
Time Factors	health care facility	12	Undirected
Time Factors	hypotension	12	Undirected
Time Factors	therapy	12	Undirected
Treatment Outcome	Breast Feeding	12	Undirected
Treatment Outcome	Job Satisfaction	12	Undirected

Treatment Outcome	dementia	12	Undirected
Treatment Outcome	perception	12	Undirected
Treatment Outcome	simulation	12	Undirected
Treatment Outcome	vancomycin	12	Undirected
United Kingdom	Anti-Bacterial Agents	12	Undirected
United Kingdom	Anxiety	12	Undirected
United Kingdom	antibiotic agent	12	Undirected
United Kingdom	perception	12	Undirected
United Kingdom	prematurity	12	Undirected
Workforce	Infection Control	12	Undirected
Workforce	Safety Management	12	Undirected
Workforce	Treatment Outcome	12	Undirected
Workforce	emergency ward	12	Undirected
Workforce	health care cost	12	Undirected
Workforce	health care system	12	Undirected
Workforce	psychology	12	Undirected
Workload	evidence based practice	12	Undirected
Workload	hospital discharge	12	Undirected
Workload	hospital personnel	12	Undirected
Workload	knowledge	12	Undirected
Workload	methodology	12	Undirected
Workload	psychological aspect	12	Undirected
Workplace	Health Knowledge, Attitudes, Practice	12	Undirected
Workplace	clinical practice	12	Undirected
Workplace	interpersonal communication	12	Undirected
adverse event	Health Personnel	12	Undirected
adverse event	antibiotic agent	12	Undirected
adverse event	breathing rate	12	Undirected
adverse event	urinary tract infection	12	Undirected
analgesia	emergency care	12	Undirected
analgesia	hypertension	12	Undirected
analgesia	information processing	12	Undirected
antibiotic agent	emergency health service	12	Undirected
antibiotic agent	palliative therapy	12	Undirected
attitude	reliability	12	Undirected
attitude to health	Workplace	12	Undirected
attitude to health	emergency health service	12	Undirected
attitude to health	ethics	12	Undirected
attitude to health	evidence based practice	12	Undirected
attitude to health	mortality	12	Undirected
attitude to health	observational study	12	Undirected
attitude to health	reliability	12	Undirected
blood pressure	sedation	12	Undirected
burnout	observational study	12	Undirected
burnout	standards	12	Undirected
caregiver	emergency health service	12	Undirected
caregiver	information processing	12	Undirected
caregiver	pain assessment	12	Undirected
caregiver	respiratory therapist	12	Undirected
caregiver	standards	12	Undirected
cerebrovascular accident	antibiotic agent	12	Undirected
cerebrovascular accident	palliative therapy	12	Undirected
clinical practice	thorax radiography	12	Undirected
computer assisted tomography	chronic obstructive lung disease	12	Undirected

computer assisted tomography	palliative therapy	12	Undirected
dementia	practice guideline	12	Undirected
depression	medical education	12	Undirected
depression	university hospital	12	Undirected
diabetes mellitus	Electronic Health Records	12	Undirected
diabetes mellitus	emergency health service	12	Undirected
diabetes mellitus	hypotension	12	Undirected
diabetes mellitus	mental health	12	Undirected
diabetes mellitus	psychology	12	Undirected
diabetes mellitus	resuscitation	12	Undirected
diabetes mellitus	therapy	12	Undirected
dyspnea	clinical practice	12	Undirected
dyspnea	morphine	12	Undirected
dyspnea	pharmacist	12	Undirected
dyspnea	psychology	12	Undirected
dyspnea	therapy	12	Undirected
emergency care	endotracheal intubation	12	Undirected
emergency care	physiotherapy	12	Undirected
emergency care	therapy	12	Undirected
emergency health service	information processing	12	Undirected
emergency ward	physiotherapy	12	Undirected
endotracheal intubation	chronic obstructive lung disease	12	Undirected
endotracheal intubation	hospital infection	12	Undirected
endotracheal intubation	interpersonal communication	12	Undirected
ethics	Anxiety	12	Undirected
ethics	information processing	12	Undirected
ethics	prematurity	12	Undirected
evidence based practice	Pilot Projects	12	Undirected
evidence based practice	enteric feeding	12	Undirected
evidence based practice	hand washing	12	Undirected
fentanyl	antibiotic agent	12	Undirected
fentanyl	follow up	12	Undirected
fentanyl	hospital admission	12	Undirected
fever	emergency health service	12	Undirected
fever	palliative therapy	12	Undirected
gestational age	antibiotic agent	12	Undirected
gestational age	health care delivery	12	Undirected
gestational age	hypertension	12	Undirected
gestational age	pilot study	12	Undirected
gestational age	statistics and numerical data	12	Undirected
gestational age	therapy	12	Undirected
hand washing	health care system	12	Undirected
health care cost	preschool child	12	Undirected
health care cost	safety	12	Undirected
health care cost	urinary tract infection	12	Undirected
health care delivery	chronic obstructive lung disease	12	Undirected
health care facility	hospital readmission	12	Undirected
health care facility	information processing	12	Undirected
health care planning	hospital personnel	12	Undirected
health care planning	university hospital	12	Undirected
health care quality	age	12	Undirected
health care quality	microbiology	12	Undirected
heart failure	Fatigue	12	Undirected
heart failure	Pandemics	12	Undirected

heart failure	clinical practice	12	Undirected
heart rate	acute kidney failure	12	Undirected
heart rate	epidemiology	12	Undirected
heart rate	extracorporeal oxygenation	12	Undirected
heart surgery	disease severity	12	Undirected
heart surgery	multicenter study	12	Undirected
hospital infection	thorax radiography	12	Undirected
hospital personnel	disease severity	12	Undirected
hospital readmission	health care planning	12	Undirected
hospital readmission	intensivist	12	Undirected
hospitalization	Cardiac Surgical Procedures	12	Undirected
hospitalization	Personnel Staffing and Scheduling	12	Undirected
hypertension	home care	12	Undirected
hypertension	organization and management	12	Undirected
hypotension	multicenter study	12	Undirected
intensivist	emergency health service	12	Undirected
interview	health care planning	12	Undirected
knowledge	information processing	12	Undirected
knowledge	medical staff	12	Undirected
knowledge	therapy	12	Undirected
long term care	enteric feeding	12	Undirected
long term care	health care planning	12	Undirected
long term care	hospital infection	12	Undirected
long term care	medical education	12	Undirected
medical education	antibiotic agent	12	Undirected
medical education	preschool child	12	Undirected
medical staff	pilot study	12	Undirected
medical staff	simulation	12	Undirected
mental health	emergency health service	12	Undirected
mental health	medical education	12	Undirected
mental health	psychological aspect	12	Undirected
meta analysis	organization and management	12	Undirected
cillin resistant Staphylococcus a	clinical practice	12	Undirected
methodology	extracorporeal oxygenation	12	Undirected
microbiology	standards	12	Undirected
midazolam	cohort analysis	12	Undirected
morphine	physiotherapy	12	Undirected
mortality	hand washing	12	Undirected
multicenter study	antiinfective agent	12	Undirected
multicenter study	psychological aspect	12	Undirected
observational study	vancomycin	12	Undirected
oxygen saturation	hospital readmission	12	Undirected
oxygen saturation	preschool child	12	Undirected
oxygen saturation	respiratory therapist	12	Undirected
oxygen saturation	therapy	12	Undirected
pain assessment	health care quality	12	Undirected
pain assessment	reliability	12	Undirected
pediatrics	Electronic Health Records	12	Undirected
pediatrics	Needs Assessment	12	Undirected
pediatrics	attitude	12	Undirected
pediatrics	burnout	12	Undirected
pediatrics	disease severity	12	Undirected
pediatrics	health care policy	12	Undirected
pediatrics	health care system	12	Undirected

perception	hospital admission	12	Undirected
perception	hospital discharge	12	Undirected
perception	hospital personnel	12	Undirected
perception	simulation	12	Undirected
perception	standards	12	Undirected
pharmacist	adverse event	12	Undirected
pharmacist	therapy	12	Undirected
pharmacist	vancomycin	12	Undirected
pilot study	information processing	12	Undirected
pilot study	psychological aspect	12	Undirected
pneumonia	education	12	Undirected
pneumonia	extracorporeal oxygenation	12	Undirected
pneumonia	health care delivery	12	Undirected
pneumonia	university hospital	12	Undirected
prematurity	health care facility	12	Undirected
prematurity	health care planning	12	Undirected
prematurity	knowledge	12	Undirected
prematurity	pediatrics	12	Undirected
prematurity	perception	12	Undirected
preschool child	antiinfective agent	12	Undirected
prevalence	Canada	12	Undirected
prevalence	Electronic Health Records	12	Undirected
prevalence	Postoperative Care	12	Undirected
prevalence	endotracheal intubation	12	Undirected
propofol	methodology	12	Undirected
psychology	meta analysis	12	Undirected
quality of life	Cardiac Surgical Procedures	12	Undirected
quality of life	Child, Preschool	12	Undirected
quality of life	Mothers	12	Undirected
quality of life	Professional-Family Relations	12	Undirected
quality of life	attitude	12	Undirected
quality of life	diabetes mellitus	12	Undirected
quality of life	enteric feeding	12	Undirected
quality of life	extracorporeal oxygenation	12	Undirected
quality of life	oxygen saturation	12	Undirected
randomized controlled trial	home care	12	Undirected
respiratory therapist	health care policy	12	Undirected
safety	clinical article	12	Undirected
safety	emergency care	12	Undirected
safety	health care policy	12	Undirected
safety	hospital discharge	12	Undirected
safety	pilot study	12	Undirected
safety	university hospital	12	Undirected
sedation	hospital discharge	12	Undirected
sedation	meta analysis	12	Undirected
sedation	pilot study	12	Undirected
sedation	psychology	12	Undirected
sedation	reliability	12	Undirected
semi structured interview	cohort analysis	12	Undirected
semi structured interview	preschool child	12	Undirected
simulation	emergency health service	12	Undirected
standards	disease severity	12	Undirected
statistics and numerical data	hand washing	12	Undirected
statistics and numerical data	information processing	12	Undirected

statistics and numerical data	intensivist	12	Undirected
statistics and numerical data	palliative therapy	12	Undirected
statistics and numerical data	respiratory therapist	12	Undirected
teaching hospital	very elderly	12	Undirected
tertiary care center	Sensitivity and Specificity	12	Undirected
tertiary care center	endotracheal intubation	12	Undirected
tertiary care center	extracorporeal oxygenation	12	Undirected
tertiary care center	health care policy	12	Undirected
tertiary care center	intensivist	12	Undirected
tertiary care center	therapy	12	Undirected
therapy	cerebrovascular accident	12	Undirected
therapy	university hospital	12	Undirected
thorax radiography	extracorporeal oxygenation	12	Undirected
total quality management	Cardiac Surgical Procedures	12	Undirected
total quality management	analgesia	12	Undirected
total quality management	cerebrovascular accident	12	Undirected
total quality management	chronic obstructive lung disease	12	Undirected
total quality management	hospital infection	12	Undirected
total quality management	hypertension	12	Undirected
total quality management	pneumonia	12	Undirected
tracheostomy	epidemiology	12	Undirected
tracheostomy	long term care	12	Undirected
urinary tract infection	disease severity	12	Undirected
urinary tract infection	multicenter study	12	Undirected
urinary tract infection	organization and management	12	Undirected
vancomycin	hand washing	12	Undirected
ventilator associated pneumonia	enteric feeding	12	Undirected
ventilator associated pneumonia	respiratory therapist	12	Undirected
very elderly	health care policy	12	Undirected
Adaptation, Psychological	Pain	11	Undirected
Algorithms	enteric feeding	11	Undirected
Algorithms	randomized controlled trial	11	Undirected
Anti-Bacterial Agents	Health Personnel	11	Undirected
Anti-Bacterial Agents	long term care	11	Undirected
Anti-Bacterial Agents	preschool child	11	Undirected
Anti-Bacterial Agents	ventilator associated pneumonia	11	Undirected
Anxiety	Leadership	11	Undirected
Anxiety	age	11	Undirected
Anxiety	health care policy	11	Undirected
Anxiety	home care	11	Undirected
Anxiety	reliability	11	Undirected
Anxiety	statistics and numerical data	11	Undirected
Artificial Ventilation	Safety Management	11	Undirected
Attitude of Health Personnel	dementia	11	Undirected
Attitude of Health Personnel	health care cost	11	Undirected
Attitude of Health Personnel	pain assessment	11	Undirected
Australia	Catheterization, Central Venous	11	Undirected
Australia	Medication Errors	11	Undirected
Australia	Telemedicine	11	Undirected
Australia	burnout	11	Undirected
Australia	knowledge	11	Undirected
Australia	prematurity	11	Undirected
Breast Feeding	enteric feeding	11	Undirected
Breast Feeding	hospital readmission	11	Undirected

Breast Feeding	interview	11	Undirected
Burnout, Professional	health care quality	11	Undirected
Canada	caregiver	11	Undirected
Canada	emergency care	11	Undirected
Canada	health care policy	11	Undirected
Canada	health care system	11	Undirected
Cardiac Surgical Procedures	clinical article	11	Undirected
Cardiac Surgical Procedures	education	11	Undirected
Cardiac Surgical Procedures	very elderly	11	Undirected
Catheter-Related Infections	COVID-19	11	Undirected
Child, Preschool	Mothers	11	Undirected
Child, Preschool	health care facility	11	Undirected
Child, Preschool	heart surgery	11	Undirected
Child, Preschool	palliative therapy	11	Undirected
Child, Preschool	semi structured interview	11	Undirected
China	dyspnea	11	Undirected
China	hospital infection	11	Undirected
China	ventilator associated pneumonia	11	Undirected
Clinical Competence	Logistic Models	11	Undirected
Clinical Competence	reliability	11	Undirected
Clinical Competence	semi structured interview	11	Undirected
Clinical Protocols	Anti-Bacterial Agents	11	Undirected
Clinical Protocols	hand washing	11	Undirected
Clinical Protocols	health care policy	11	Undirected
Clinical Protocols	long term care	11	Undirected
Clinical Protocols	palliative therapy	11	Undirected
Communication	disease severity	11	Undirected
Communication	prevalence	11	Undirected
Cooperative Behavior	Burnout, Professional	11	Undirected
Cooperative Behavior	Workload	11	Undirected
Cooperative Behavior	hospitalization	11	Undirected
Cooperative Behavior	medical education	11	Undirected
Cooperative Behavior	perception	11	Undirected
Cooperative Behavior	practice guideline	11	Undirected
Cross Infection	Australia	11	Undirected
Cross Infection	Canada	11	Undirected
Cross Infection	adverse event	11	Undirected
Cross Infection	diabetes mellitus	11	Undirected
Cross Infection	disease severity	11	Undirected
Cross Infection	health care system	11	Undirected
Cross Infection	knowledge	11	Undirected
Cross Infection	preschool child	11	Undirected
Decision Making	Cardiac Surgical Procedures	11	Undirected
Decision Making	Pain Measurement	11	Undirected
Decision Making	Sensitivity and Specificity	11	Undirected
Decision Making	antibiotic agent	11	Undirected
Decision Making	health care cost	11	Undirected
Decision Making	reliability	11	Undirected
Decision Making	tracheostomy	11	Undirected
Delirium	attitude	11	Undirected
Delirium	fever	11	Undirected
Delirium	intensivist	11	Undirected
Delirium	urinary tract infection	11	Undirected
Electronic Health Records	health care delivery	11	Undirected

Emergency Service, Hospital	Algorithms	11	Undirected
Emergency Service, Hospital	Focus Groups	11	Undirected
Emergency Service, Hospital	Leadership	11	Undirected
Emergency Service, Hospital	Personnel Staffing and Scheduling	11	Undirected
Emergency Service, Hospital	Pilot Projects	11	Undirected
Emergency Service, Hospital	Terminal Care	11	Undirected
Emergency Service, Hospital	breathing rate	11	Undirected
Emergency Service, Hospital	health care cost	11	Undirected
Emergency Service, Hospital	health care delivery	11	Undirected
Emergency Service, Hospital	health care planning	11	Undirected
Emergency Service, Hospital	teaching hospital	11	Undirected
Family	dementia	11	Undirected
Family	pharmacist	11	Undirected
Family	statistics and numerical data	11	Undirected
Family	tracheostomy	11	Undirected
Fatigue	hospital readmission	11	Undirected
Fatigue	oxygen saturation	11	Undirected
Focus Groups	clinical practice	11	Undirected
Focus Groups	multicenter study	11	Undirected
Glasgow coma scale	antibiotic agent	11	Undirected
Glasgow coma scale	dementia	11	Undirected
Glasgow coma scale	hospital readmission	11	Undirected
Glasgow coma scale	organization and management	11	Undirected
Guideline Adherence	Cooperative Behavior	11	Undirected
Guideline Adherence	Medication Errors	11	Undirected
Guideline Adherence	Quality of Health Care	11	Undirected
Guideline Adherence	Safety Management	11	Undirected
Guideline Adherence	Workload	11	Undirected
Guideline Adherence	health care facility	11	Undirected
Guideline Adherence	interview	11	Undirected
alth Knowledge, Attitudes, Practi	emergency care	11	Undirected
alth Knowledge, Attitudes, Practi	interpersonal communication	11	Undirected
alth Knowledge, Attitudes, Practi	ventilator associated pneumonia	11	Undirected
Health Personnel	antibiotic agent	11	Undirected
Health Services Needs and Demand	Australia	11	Undirected
Health Services Needs and Demand	Guideline Adherence	11	Undirected
Health Services Needs and Demand	Mothers	11	Undirected
Health Services Needs and Demand	hospital discharge	11	Undirected
Health Services Needs and Demand	standards	11	Undirected
Heart Arrest	chronic obstructive lung disease	11	Undirected
Heart Arrest	knowledge	11	Undirected
Heart Arrest	long term care	11	Undirected
Hospital Mortality	Insulin	11	Undirected
Hospital Mortality	Pilot Projects	11	Undirected
Hospital Mortality	Reproducibility of Results	11	Undirected
Hospital Mortality	pediatrics	11	Undirected
Hospital Mortality	sedation	11	Undirected
Hypnotics and Sedatives	heart rate	11	Undirected
Hypnotics and Sedatives	pain assessment	11	Undirected
Infant, Premature	Catheterization, Central Venous	11	Undirected
Infant, Premature	Telemedicine	11	Undirected
Infant, Premature	meta analysis	11	Undirected
Infection Control	Fatigue	11	Undirected
Infection Control	diabetes mellitus	11	Undirected

Infection Control	hypertension	11	Undirected
Infection Control	intensivist	11	Undirected
Insulin	Pilot Projects	11	Undirected
Insulin	emergency ward	11	Undirected
Insulin	fentanyl	11	Undirected
Insulin	hypotension	11	Undirected
Insulin	multicenter study	11	Undirected
Insulin	organization and management	11	Undirected
Interprofessional Relations	Emergency Service, Hospital	11	Undirected
Interprofessional Relations	emergency care	11	Undirected
Interprofessional Relations	evidence based practice	11	Undirected
Interprofessional Relations	medical education	11	Undirected
Interprofessional Relations	medical staff	11	Undirected
Interprofessional Relations	simulation	11	Undirected
Intubation, Intratracheal	analgesia	11	Undirected
Intubation, Intratracheal	multicenter study	11	Undirected
Intubation, Intratracheal	organization and management	11	Undirected
Intubation, Intratracheal	respiratory therapist	11	Undirected
Job Satisfaction	age	11	Undirected
Job Satisfaction	hospital personnel	11	Undirected
Job Satisfaction	intensivist	11	Undirected
Job Satisfaction	medical education	11	Undirected
Leadership	hand washing	11	Undirected
Leadership	information processing	11	Undirected
Length of Stay	Catheter-Related Infections	11	Undirected
Length of Stay	simulation	11	Undirected
Logistic Models	Quality Improvement	11	Undirected
Logistic Models	age	11	Undirected
Medication Errors	disease severity	11	Undirected
Medication Errors	emergency care	11	Undirected
Medication Errors	health care delivery	11	Undirected
Medication Errors	health care policy	11	Undirected
Medication Errors	health care system	11	Undirected
Medication Errors	methodology	11	Undirected
Medication Errors	simulation	11	Undirected
Medication Errors	vancomycin	11	Undirected
Monitoring, Physiologic	Infection Control	11	Undirected
Monitoring, Physiologic	Program Evaluation	11	Undirected
Monitoring, Physiologic	epidemiology	11	Undirected
Monitoring, Physiologic	heart failure	11	Undirected
Monitoring, Physiologic	outcome assessment	11	Undirected
Mothers	clinical practice	11	Undirected
Mothers	information processing	11	Undirected
Mothers	practice guideline	11	Undirected
Needs Assessment	Health Personnel	11	Undirected
Needs Assessment	Infection Control	11	Undirected
OUTCOMES	burnout	11	Undirected
OUTCOMES	multicenter study	11	Undirected
OUTCOMES	perception	11	Undirected
OUTCOMES	practice guideline	11	Undirected
OUTCOMES	statistics and numerical data	11	Undirected
Pain	Postoperative Complications	11	Undirected
Pain	health care planning	11	Undirected
Pain	information processing	11	Undirected

Pain	therapy	11	Undirected
Pain Measurement	China	11	Undirected
Pain Measurement	clinical practice	11	Undirected
Pain Measurement	reliability	11	Undirected
Palliative Care	Heart Arrest	11	Undirected
Palliative Care	Job Satisfaction	11	Undirected
Palliative Care	Leadership	11	Undirected
Palliative Care	information processing	11	Undirected
Palliative Care	sedation	11	Undirected
Parents	heart rate	11	Undirected
Parents	knowledge	11	Undirected
Parents	long term care	11	Undirected
Parents	mortality	11	Undirected
Parents	prevalence	11	Undirected
Personnel Staffing and Scheduling	Focus Groups	11	Undirected
Personnel Staffing and Scheduling	Telemedicine	11	Undirected
Personnel Staffing and Scheduling	education	11	Undirected
Pilot Projects	Cardiac Surgical Procedures	11	Undirected
Pilot Projects	depression	11	Undirected
Pilot Projects	interpersonal communication	11	Undirected
Pilot Projects	university hospital	11	Undirected
Postoperative Care	Australia	11	Undirected
Postoperative Care	computer assisted tomography	11	Undirected
Postoperative Care	education	11	Undirected
Postoperative Care	very elderly	11	Undirected
Postoperative Complications	Stroke	11	Undirected
Postoperative Complications	evidence based practice	11	Undirected
Postoperative Complications	organization and management	11	Undirected
Postoperative Complications	psychology	11	Undirected
Postoperative Complications	safety	11	Undirected
Postoperative Complications	university hospital	11	Undirected
Postoperative Complications	urinary tract infection	11	Undirected
Practice Guidelines as Topic	Personnel Staffing and Scheduling	11	Undirected
Practice Guidelines as Topic	fever	11	Undirected
Practice Guidelines as Topic	medical education	11	Undirected
Practice Guidelines as Topic	medical staff	11	Undirected
Practice Guidelines as Topic	tracheostomy	11	Undirected
Practice Guidelines as Topic	university hospital	11	Undirected
Pregnancy	Infection Control	11	Undirected
Pregnancy	depression	11	Undirected
Pregnancy	hypotension	11	Undirected
Pregnancy	medical education	11	Undirected
Pregnancy	palliative therapy	11	Undirected
Pregnancy	thematic analysis	11	Undirected
Pressure Ulcer	clinical practice	11	Undirected
Pressure Ulcer	preschool child	11	Undirected
Professional-Family Relations	Clinical Protocols	11	Undirected
Professional-Family Relations	Pandemics	11	Undirected
Professional-Family Relations	attitude	11	Undirected
Professional-Family Relations	hospital discharge	11	Undirected
Program Evaluation	Breast Feeding	11	Undirected
Quality Improvement	analgesia	11	Undirected
Quality Improvement	enteric feeding	11	Undirected
Quality of Health Care	Infant, Premature	11	Undirected

Quality of Health Care	Sepsis	11	Undirected
Quality of Health Care	caregiver	11	Undirected
Quality of Health Care	health care planning	11	Undirected
Quality of Health Care	multicenter study	11	Undirected
Quality of Health Care	very elderly	11	Undirected
Reproducibility of Results	Leadership	11	Undirected
Reproducibility of Results	emergency health service	11	Undirected
Reproducibility of Results	hospital discharge	11	Undirected
Reproducibility of Results	medical staff	11	Undirected
Reproducibility of Results	randomized controlled trial	11	Undirected
Risk Assessment	Burnout, Professional	11	Undirected
Risk Assessment	burnout	11	Undirected
Risk Factors	Leadership	11	Undirected
Risk Factors	attitude	11	Undirected
Risk Factors	thematic analysis	11	Undirected
Sensitivity and Specificity	organization and management	11	Undirected
Sensitivity and Specificity	sedation	11	Undirected
Sepsis	Enteral Nutrition	11	Undirected
Sepsis	Pilot Projects	11	Undirected
Sepsis	Reproducibility of Results	11	Undirected
Sepsis	intensivist	11	Undirected
Sepsis	teaching hospital	11	Undirected
Social Support	Delirium	11	Undirected
Social Support	disease severity	11	Undirected
Social Support	hospital readmission	11	Undirected
Social Support	resuscitation	11	Undirected
Social Support	thematic analysis	11	Undirected
Stress, Psychological	Emergency Service, Hospital	11	Undirected
Stress, Psychological	outcome assessment	11	Undirected
Stress, Psychological	standards	11	Undirected
Stroke	methodology	11	Undirected
Stroke	multicenter study	11	Undirected
Telemedicine	epidemiology	11	Undirected
Telemedicine	health care facility	11	Undirected
Telemedicine	home care	11	Undirected
Telemedicine	randomized controlled trial	11	Undirected
Terminal Care	Fatigue	11	Undirected
Terminal Care	hospital personnel	11	Undirected
Time Factors	fever	11	Undirected
Time Factors	health care planning	11	Undirected
Time Factors	hospital personnel	11	Undirected
Time Factors	hypertension	11	Undirected
Time Factors	microbiology	11	Undirected
Time Factors	midazolam	11	Undirected
Time Factors	respiratory therapist	11	Undirected
United Kingdom	Pain	11	Undirected
United Kingdom	disease severity	11	Undirected
United Kingdom	evidence based practice	11	Undirected
United Kingdom	knowledge	11	Undirected
United Kingdom	statistics and numerical data	11	Undirected
United Kingdom	tracheostomy	11	Undirected
United Kingdom	very elderly	11	Undirected
Workforce	health care delivery	11	Undirected
Workforce	pharmacist	11	Undirected

Workforce	practice guideline	11	Undirected
Workload	Heart Arrest	11	Undirected
Workplace	Quality Improvement	11	Undirected
Workplace	Reproducibility of Results	11	Undirected
Workplace	United Kingdom	11	Undirected
Workplace	hand washing	11	Undirected
Workplace	health care delivery	11	Undirected
Workplace	total quality management	11	Undirected
adverse event	acute kidney failure	11	Undirected
adverse event	chronic obstructive lung disease	11	Undirected
age	cerebrovascular accident	11	Undirected
age	health care planning	11	Undirected
age	psychological aspect	11	Undirected
age	university hospital	11	Undirected
analgesia	Fatigue	11	Undirected
analgesia	blood pressure	11	Undirected
analgesia	interpersonal communication	11	Undirected
analgesia	long term care	11	Undirected
analgesia	oxygen saturation	11	Undirected
analgesia	pharmacist	11	Undirected
analgesia	statistics and numerical data	11	Undirected
attitude	semi structured interview	11	Undirected
attitude to health	clinical article	11	Undirected
attitude to health	health care system	11	Undirected
attitude to health	prevalence	11	Undirected
burnout	clinical article	11	Undirected
burnout	cohort analysis	11	Undirected
burnout	practice guideline	11	Undirected
burnout	thematic analysis	11	Undirected
caregiver	age	11	Undirected
caregiver	fever	11	Undirected
caregiver	hand washing	11	Undirected
caregiver	intensivist	11	Undirected
clinical article	health care policy	11	Undirected
clinical practice	chronic obstructive lung disease	11	Undirected
clinical practice	psychological aspect	11	Undirected
depression	computer assisted tomography	11	Undirected
depression	health care policy	11	Undirected
depression	pain assessment	11	Undirected
diabetes mellitus	Fatigue	11	Undirected
diabetes mellitus	emergency care	11	Undirected
diabetes mellitus	thicillin resistant Staphylococcus aure	11	Undirected
diabetes mellitus	microbiology	11	Undirected
diabetes mellitus	university hospital	11	Undirected
dyspnea	extracorporeal oxygenation	11	Undirected
dyspnea	health care delivery	11	Undirected
dyspnea	home care	11	Undirected
dyspnea	long term care	11	Undirected
education	dementia	11	Undirected
education	pain assessment	11	Undirected
emergency care	breathing rate	11	Undirected
emergency care	extracorporeal oxygenation	11	Undirected
emergency care	hypertension	11	Undirected
emergency care	intensivist	11	Undirected

emergency care	thorax radiography	11	Undirected
emergency health service	extracorporeal oxygenation	11	Undirected
emergency health service	thorax radiography	11	Undirected
emergency ward	microbiology	11	Undirected
endotracheal intubation	meta analysis	11	Undirected
ethics	Canada	11	Undirected
ethics	Focus Groups	11	Undirected
ethics	caregiver	11	Undirected
ethics	depression	11	Undirected
ethics	hospital personnel	11	Undirected
evidence based practice	emergency health service	11	Undirected
evidence based practice	hospital infection	11	Undirected
evidence based practice	observational study	11	Undirected
evidence based practice	pharmacist	11	Undirected
fentanyl	computer assisted tomography	11	Undirected
fever	health care facility	11	Undirected
fever	home care	11	Undirected
fever	ethicillin resistant Staphylococcus aure	11	Undirected
fever	morphine	11	Undirected
fever	ventilator associated pneumonia	11	Undirected
follow up	heart surgery	11	Undirected
gestational age	COVID-19	11	Undirected
gestational age	thorax radiography	11	Undirected
health care cost	Anti-Bacterial Agents	11	Undirected
health care cost	Electronic Health Records	11	Undirected
health care cost	enteric feeding	11	Undirected
health care delivery	hand washing	11	Undirected
health care delivery	heart surgery	11	Undirected
health care delivery	methodology	11	Undirected
health care delivery	preschool child	11	Undirected
health care facility	computer assisted tomography	11	Undirected
health care facility	palliative therapy	11	Undirected
health care facility	preschool child	11	Undirected
heart failure	heart surgery	11	Undirected
heart failure	home care	11	Undirected
heart failure	psychology	11	Undirected
heart failure	thorax radiography	11	Undirected
heart failure	urinary tract infection	11	Undirected
heart rate	education	11	Undirected
heart surgery	hospital readmission	11	Undirected
home care	methodology	11	Undirected
home care	physiotherapy	11	Undirected
hospital admission	psychological aspect	11	Undirected
hospital personnel	palliative therapy	11	Undirected
hypertension	health care facility	11	Undirected
hypertension	physiotherapy	11	Undirected
hypotension	health care quality	11	Undirected
hypotension	therapy	11	Undirected
intensivist	health care policy	11	Undirected
intensivist	therapy	11	Undirected
intensivist	very elderly	11	Undirected
interview	hand washing	11	Undirected
interview	hospital readmission	11	Undirected
knowledge	antibiotic agent	11	Undirected

knowledge	health care system	11	Undirected
knowledge	hospital infection	11	Undirected
knowledge	pneumonia	11	Undirected
knowledge	respiratory therapist	11	Undirected
long term care	depression	11	Undirected
medical education	chronic obstructive lung disease	11	Undirected
medical education	physiotherapy	11	Undirected
medical staff	antibiotic agent	11	Undirected
medical staff	endotracheal intubation	11	Undirected
medical staff	extracorporeal oxygenation	11	Undirected
medical staff	physiotherapy	11	Undirected
medical staff	ventilator associated pneumonia	11	Undirected
mental health	health care facility	11	Undirected
mental health	health care policy	11	Undirected
meta analysis	antibiotic agent	11	Undirected
meta analysis	hospital infection	11	Undirected
cillin resistant Staphylococcus a	follow up	11	Undirected
cillin resistant Staphylococcus a	university hospital	11	Undirected
methodology	cerebrovascular accident	11	Undirected
methodology	computer assisted tomography	11	Undirected
methodology	health care planning	11	Undirected
methodology	teaching hospital	11	Undirected
midazolam	breathing rate	11	Undirected
midazolam	epidemiology	11	Undirected
midazolam	health care quality	11	Undirected
midazolam	oxygen saturation	11	Undirected
morphine	COVID-19	11	Undirected
morphine	health care quality	11	Undirected
morphine	methodology	11	Undirected
mortality	pain assessment	11	Undirected
mortality	psychological aspect	11	Undirected
mortality	respiratory therapist	11	Undirected
multicenter study	extracorporeal oxygenation	11	Undirected
multicenter study	health care planning	11	Undirected
observational study	health care policy	11	Undirected
organization and management	chronic obstructive lung disease	11	Undirected
outcome assessment	Breast Feeding	11	Undirected
outcome assessment	Pain Measurement	11	Undirected
outcome assessment	thematic analysis	11	Undirected
outcome assessment	vancomycin	11	Undirected
oxygen saturation	university hospital	11	Undirected
pain assessment	palliative therapy	11	Undirected
pain assessment	physiotherapy	11	Undirected
pain assessment	standards	11	Undirected
palliative therapy	extracorporeal oxygenation	11	Undirected
pediatrics	Pandemics	11	Undirected
pediatrics	endotracheal intubation	11	Undirected
pediatrics	enteric feeding	11	Undirected
pediatrics	health care cost	11	Undirected
perception	intensivist	11	Undirected
pharmacist	midazolam	11	Undirected
pharmacist	statistics and numerical data	11	Undirected
physiotherapy	chronic obstructive lung disease	11	Undirected
physiotherapy	health care system	11	Undirected

physiotherapy	preschool child	11	Undirected
physiotherapy	very elderly	11	Undirected
pilot study	emergency health service	11	Undirected
pilot study	preschool child	11	Undirected
pilot study	semi structured interview	11	Undirected
pneumonia	organization and management	11	Undirected
pneumonia	statistics and numerical data	11	Undirected
prematurity	China	11	Undirected
prematurity	Infection Control	11	Undirected
prematurity	adverse event	11	Undirected
prematurity	hospital infection	11	Undirected
prematurity	hospital readmission	11	Undirected
prematurity	mental health	11	Undirected
prematurity	midazolam	11	Undirected
prematurity	morphine	11	Undirected
prematurity	pneumonia	11	Undirected
prematurity	sedation	11	Undirected
preschool child	palliative therapy	11	Undirected
prevalence	Infant, Premature	11	Undirected
prevalence	Pain Measurement	11	Undirected
prevalence	Stroke	11	Undirected
prevalence	Telemedicine	11	Undirected
prevalence	extracorporeal oxygenation	11	Undirected
prevalence	midazolam	11	Undirected
propofol	breathing rate	11	Undirected
propofol	emergency ward	11	Undirected
propofol	health care quality	11	Undirected
propofol	heart surgery	11	Undirected
propofol	tracheostomy	11	Undirected
psychology	hospital readmission	11	Undirected
psychology	hypertension	11	Undirected
quality of life	Glasgow coma scale	11	Undirected
quality of life	Needs Assessment	11	Undirected
quality of life	Reproducibility of Results	11	Undirected
quality of life	Telemedicine	11	Undirected
quality of life	emergency health service	11	Undirected
quality of life	endotracheal intubation	11	Undirected
quality of life	thematic analysis	11	Undirected
reliability	information processing	11	Undirected
reliability	university hospital	11	Undirected
respiratory therapist	disease severity	11	Undirected
respiratory therapist	palliative therapy	11	Undirected
resuscitation	Fatigue	11	Undirected
resuscitation	heart surgery	11	Undirected
resuscitation	information processing	11	Undirected
resuscitation	meta analysis	11	Undirected
resuscitation	physiotherapy	11	Undirected
resuscitation	reliability	11	Undirected
resuscitation	thematic analysis	11	Undirected
safety	Canada	11	Undirected
safety	Health Personnel	11	Undirected
safety	caregiver	11	Undirected
safety	cohort analysis	11	Undirected
safety	follow up	11	Undirected

safety	observational study	11	Undirected
safety	resuscitation	11	Undirected
safety	statistics and numerical data	11	Undirected
sedation	medical education	11	Undirected
sedation	semi structured interview	11	Undirected
sedation	statistics and numerical data	11	Undirected
semi structured interview	hospital personnel	11	Undirected
tertiary care center	Leadership	11	Undirected
tertiary care center	fentanyl	11	Undirected
tertiary care center	hypotension	11	Undirected
tertiary care center	safety	11	Undirected
tertiary care center	sedation	11	Undirected
tertiary care center	simulation	11	Undirected
thematic analysis	health care planning	11	Undirected
thematic analysis	intensivist	11	Undirected
therapy	acute kidney failure	11	Undirected
therapy	antibiotic agent	11	Undirected
therapy	computer assisted tomography	11	Undirected
total quality management	Focus Groups	11	Undirected
total quality management	extracorporeal oxygenation	11	Undirected
total quality management	mental health	11	Undirected
vancomycin	clinical practice	11	Undirected
ventilator associated pneumonia	hypertension	11	Undirected
ventilator associated pneumonia	meta analysis	11	Undirected
Adaptation, Psychological	Practice Guidelines as Topic	10	Undirected
Adaptation, Psychological	Program Evaluation	10	Undirected
Adaptation, Psychological	United Kingdom	10	Undirected
Algorithms	cohort analysis	10	Undirected
Algorithms	follow up	10	Undirected
Algorithms	interpersonal communication	10	Undirected
Algorithms	medical staff	10	Undirected
Anti-Bacterial Agents	computer assisted tomography	10	Undirected
Anti-Bacterial Agents	follow up	10	Undirected
Anxiety	Telemedicine	10	Undirected
Anxiety	evidence based practice	10	Undirected
Anxiety	hypotension	10	Undirected
Anxiety	information processing	10	Undirected
Anxiety	preschool child	10	Undirected
Artificial Ventilation	Breast Feeding	10	Undirected
Artificial Ventilation	Catheterization, Central Venous	10	Undirected
Artificial Ventilation	Leadership	10	Undirected
Attitude of Health Personnel	fever	10	Undirected
Attitude of Health Personnel	home care	10	Undirected
Attitude of Health Personnel	physiotherapy	10	Undirected
Attitude of Health Personnel	pneumonia	10	Undirected
Attitude of Health Personnel	reliability	10	Undirected
Attitude of Health Personnel	tracheostomy	10	Undirected
Australia	Sensitivity and Specificity	10	Undirected
Australia	adverse event	10	Undirected
Australia	health care facility	10	Undirected
Australia	health care system	10	Undirected
Australia	heart surgery	10	Undirected
Breast Feeding	Clinical Protocols	10	Undirected
Breast Feeding	analgesia	10	Undirected

Breast Feeding	cohort analysis	10	Undirected
Breast Feeding	evidence based practice	10	Undirected
Burnout, Professional	interview	10	Undirected
Burnout, Professional	medical staff	10	Undirected
Burnout, Professional	organization and management	10	Undirected
Canada	medical education	10	Undirected
Cardiac Surgical Procedures	blood pressure	10	Undirected
Catheter-Related Infections	hand washing	10	Undirected
Catheterization, Central Venous	health care quality	10	Undirected
Catheterization, Central Venous	microbiology	10	Undirected
Child, Preschool	antibiotic agent	10	Undirected
Child, Preschool	computer assisted tomography	10	Undirected
Child, Preschool	heart failure	10	Undirected
Child, Preschool	perception	10	Undirected
Child, Preschool	safety	10	Undirected
Child, Preschool	urinary tract infection	10	Undirected
China	health care quality	10	Undirected
China	mental health	10	Undirected
China	pain assessment	10	Undirected
China	respiratory therapist	10	Undirected
China	semi structured interview	10	Undirected
China	statistics and numerical data	10	Undirected
China	therapy	10	Undirected
China	university hospital	10	Undirected
Clinical Competence	adverse event	10	Undirected
Clinical Competence	antibiotic agent	10	Undirected
Clinical Competence	health care facility	10	Undirected
Clinical Competence	health care system	10	Undirected
Clinical Competence	hospital admission	10	Undirected
Clinical Competence	intensivist	10	Undirected
Clinical Competence	pain assessment	10	Undirected
Clinical Competence	thematic analysis	10	Undirected
Clinical Protocols	Sensitivity and Specificity	10	Undirected
Clinical Protocols	hospital personnel	10	Undirected
Clinical Protocols	meta analysis	10	Undirected
Clinical Protocols	very elderly	10	Undirected
Communication	Hypnotics and Sedatives	10	Undirected
Communication	Intubation, Intratracheal	10	Undirected
Communication	Risk Assessment	10	Undirected
Communication	teaching hospital	10	Undirected
Cooperative Behavior	Child, Preschool	10	Undirected
Cooperative Behavior	Telemedicine	10	Undirected
Cooperative Behavior	emergency care	10	Undirected
Cooperative Behavior	methodology	10	Undirected
Cooperative Behavior	psychology	10	Undirected
Cooperative Behavior	standards	10	Undirected
Cooperative Behavior	university hospital	10	Undirected
Cross Infection	respiratory therapist	10	Undirected
Decision Making	Intubation, Intratracheal	10	Undirected
Decision Making	antiinfective agent	10	Undirected
Decision Making	heart rate	10	Undirected
Decision Making	statistics and numerical data	10	Undirected
Delirium	Fatigue	10	Undirected
Delirium	acute kidney failure	10	Undirected

Delirium	computer assisted tomography	10	Undirected
Delirium	home care	10	Undirected
Delirium	semi structured interview	10	Undirected
Electronic Health Records	clinical article	10	Undirected
Electronic Health Records	health care planning	10	Undirected
Electronic Health Records	hypertension	10	Undirected
Electronic Health Records	intensivist	10	Undirected
Electronic Health Records	randomized controlled trial	10	Undirected
Emergency Service, Hospital	China	10	Undirected
Emergency Service, Hospital	health care policy	10	Undirected
Emergency Service, Hospital	home care	10	Undirected
Emergency Service, Hospital	hospital readmission	10	Undirected
Emergency Service, Hospital	information processing	10	Undirected
Emergency Service, Hospital	knowledge	10	Undirected
Emergency Service, Hospital	mental health	10	Undirected
Emergency Service, Hospital	pilot study	10	Undirected
Emergency Service, Hospital	psychological aspect	10	Undirected
Enteral Nutrition	Reproducibility of Results	10	Undirected
Enteral Nutrition	cohort analysis	10	Undirected
Family	burnout	10	Undirected
Family	extracorporeal oxygenation	10	Undirected
Family	physiotherapy	10	Undirected
Fatigue	chronic obstructive lung disease	10	Undirected
Fatigue	interpersonal communication	10	Undirected
Fatigue	observational study	10	Undirected
Fatigue	university hospital	10	Undirected
Focus Groups	caregiver	10	Undirected
Focus Groups	respiratory therapist	10	Undirected
Focus Groups	therapy	10	Undirected
Glasgow coma scale	Electronic Health Records	10	Undirected
Glasgow coma scale	health care system	10	Undirected
Glasgow coma scale	pain assessment	10	Undirected
Guideline Adherence	Anti-Bacterial Agents	10	Undirected
Guideline Adherence	China	10	Undirected
Guideline Adherence	Communication	10	Undirected
Guideline Adherence	Hypnotics and Sedatives	10	Undirected
Guideline Adherence	Interprofessional Relations	10	Undirected
Guideline Adherence	Pandemics	10	Undirected
Guideline Adherence	epidemiology	10	Undirected
Guideline Adherence	mortality	10	Undirected
Guideline Adherence	teaching hospital	10	Undirected
alth Knowledge, Attitudes, Practi	caregiver	10	Undirected
alth Knowledge, Attitudes, Practi	health care facility	10	Undirected
alth Knowledge, Attitudes, Practi	palliative therapy	10	Undirected
alth Knowledge, Attitudes, Practi	prematurity	10	Undirected
Health Personnel	hypertension	10	Undirected
Health Personnel	psychological aspect	10	Undirected
Health Personnel	reliability	10	Undirected
Health Services Needs and Demand	Canada	10	Undirected
Health Services Needs and Demand	Health Personnel	10	Undirected
Health Services Needs and Demand	Infection Control	10	Undirected
Health Services Needs and Demand	Pandemics	10	Undirected
Health Services Needs and Demand	hospitalization	10	Undirected
Health Services Needs and Demand	medical education	10	Undirected

Health Services Needs and Demand	psychology	10	Undirected
Heart Arrest	Algorithms	10	Undirected
Heart Arrest	Pandemics	10	Undirected
Heart Arrest	health care planning	10	Undirected
Heart Arrest	information processing	10	Undirected
Heart Arrest	pneumonia	10	Undirected
Heart Arrest	safety	10	Undirected
Hospital Mortality	Pressure Ulcer	10	Undirected
Hospital Mortality	fentanyl	10	Undirected
Hospital Mortality	gestational age	10	Undirected
Hospital Mortality	midazolam	10	Undirected
Hospital Mortality	teaching hospital	10	Undirected
Hypnotics and Sedatives	observational study	10	Undirected
Infant, Premature	Infection Control	10	Undirected
Infant, Premature	semi structured interview	10	Undirected
Infant, Premature	therapy	10	Undirected
Infection Control	Algorithms	10	Undirected
Infection Control	gestational age	10	Undirected
Infection Control	meta analysis	10	Undirected
Infection Control	palliative therapy	10	Undirected
Insulin	Electronic Health Records	10	Undirected
Insulin	Enteral Nutrition	10	Undirected
Interprofessional Relations	Pregnancy	10	Undirected
Interprofessional Relations	ethics	10	Undirected
Interprofessional Relations	methodology	10	Undirected
Intubation, Intratracheal	clinical article	10	Undirected
Intubation, Intratracheal	epidemiology	10	Undirected
Intubation, Intratracheal	knowledge	10	Undirected
Intubation, Intratracheal	morphine	10	Undirected
Intubation, Intratracheal	observational study	10	Undirected
Job Satisfaction	Telemedicine	10	Undirected
Job Satisfaction	United Kingdom	10	Undirected
Job Satisfaction	mortality	10	Undirected
Leadership	hospital discharge	10	Undirected
Leadership	mental health	10	Undirected
Length of Stay	thematic analysis	10	Undirected
Logistic Models	Decision Making	10	Undirected
Logistic Models	Intubation, Intratracheal	10	Undirected
Logistic Models	Practice Guidelines as Topic	10	Undirected
Logistic Models	epidemiology	10	Undirected
Logistic Models	pneumonia	10	Undirected
Medication Errors	follow up	10	Undirected
Medication Errors	resuscitation	10	Undirected
Monitoring, Physiologic	Catheterization, Central Venous	10	Undirected
Monitoring, Physiologic	Communication	10	Undirected
Monitoring, Physiologic	Enteral Nutrition	10	Undirected
Monitoring, Physiologic	analgesia	10	Undirected
Monitoring, Physiologic	information processing	10	Undirected
Monitoring, Physiologic	oxygen saturation	10	Undirected
Mothers	methodology	10	Undirected
Needs Assessment	Burnout, Professional	10	Undirected
Needs Assessment	health care delivery	10	Undirected
Needs Assessment	health care policy	10	Undirected
Needs Assessment	hospital admission	10	Undirected

Needs Assessment	hospital discharge	10	Undirected
Needs Assessment	interview	10	Undirected
Needs Assessment	medical staff	10	Undirected
Needs Assessment	mortality	10	Undirected
OUTCOMES	Heart Arrest	10	Undirected
OUTCOMES	Job Satisfaction	10	Undirected
OUTCOMES	health care cost	10	Undirected
OUTCOMES	prematurity	10	Undirected
OUTCOMES	resuscitation	10	Undirected
OUTCOMES	therapy	10	Undirected
OUTCOMES	total quality management	10	Undirected
OUTCOMES	tracheostomy	10	Undirected
Pain	Algorithms	10	Undirected
Pain	age	10	Undirected
Pain	health care policy	10	Undirected
Pain	long term care	10	Undirected
Pain	meta analysis	10	Undirected
Palliative Care	Fatigue	10	Undirected
Palliative Care	Risk Assessment	10	Undirected
Palliative Care	health care policy	10	Undirected
Palliative Care	pharmacist	10	Undirected
Palliative Care	prematurity	10	Undirected
Palliative Care	preschool child	10	Undirected
Pandemics	meta analysis	10	Undirected
Parents	emergency ward	10	Undirected
Parents	pain assessment	10	Undirected
Personnel Staffing and Scheduling	Infection Control	10	Undirected
Pilot Projects	disease severity	10	Undirected
Pilot Projects	emergency ward	10	Undirected
Pilot Projects	hospital admission	10	Undirected
Postoperative Care	Anxiety	10	Undirected
Postoperative Care	China	10	Undirected
Postoperative Care	health care cost	10	Undirected
Postoperative Care	hospital readmission	10	Undirected
Postoperative Care	intensivist	10	Undirected
Postoperative Care	interpersonal communication	10	Undirected
Postoperative Care	pain assessment	10	Undirected
Postoperative Care	pneumonia	10	Undirected
Postoperative Care	statistics and numerical data	10	Undirected
Postoperative Complications	China	10	Undirected
Postoperative Complications	clinical practice	10	Undirected
Postoperative Complications	home care	10	Undirected
Postoperative Complications	intensivist	10	Undirected
Postoperative Complications	physiotherapy	10	Undirected
Postoperative Complications	sedation	10	Undirected
Practice Guidelines as Topic	China	10	Undirected
Practice Guidelines as Topic	Workload	10	Undirected
Practice Guidelines as Topic	cohort analysis	10	Undirected
Practice Guidelines as Topic	diabetes mellitus	10	Undirected
Practice Guidelines as Topic	disease severity	10	Undirected
Practice Guidelines as Topic	hand washing	10	Undirected
Practice Guidelines as Topic	health care planning	10	Undirected
Practice Guidelines as Topic	hospital readmission	10	Undirected
Practice Guidelines as Topic	interview	10	Undirected

Practice Guidelines as Topic	pneumonia	10	Undirected
Pregnancy	Needs Assessment	10	Undirected
Pregnancy	Telemedicine	10	Undirected
Pregnancy	adverse event	10	Undirected
Pregnancy	emergency ward	10	Undirected
Pregnancy	health care system	10	Undirected
Pregnancy	hospital readmission	10	Undirected
Pregnancy	information processing	10	Undirected
Pregnancy	medical staff	10	Undirected
Pregnancy	pediatrics	10	Undirected
Pregnancy	simulation	10	Undirected
Pressure Ulcer	Cardiac Surgical Procedures	10	Undirected
Pressure Ulcer	Pilot Projects	10	Undirected
Pressure Ulcer	age	10	Undirected
Pressure Ulcer	endotracheal intubation	10	Undirected
Pressure Ulcer	reliability	10	Undirected
Professional-Family Relations	follow up	10	Undirected
Professional-Family Relations	hospital personnel	10	Undirected
Professional-Family Relations	outcome assessment	10	Undirected
Professional-Family Relations	thematic analysis	10	Undirected
Professional-Family Relations	university hospital	10	Undirected
Program Evaluation	Reproducibility of Results	10	Undirected
Program Evaluation	hospital discharge	10	Undirected
Program Evaluation	psychological aspect	10	Undirected
Quality Improvement	Burnout, Professional	10	Undirected
Quality Improvement	burnout	10	Undirected
Quality Improvement	teaching hospital	10	Undirected
Quality Improvement	ventilator associated pneumonia	10	Undirected
Quality of Health Care	China	10	Undirected
Quality of Health Care	Stroke	10	Undirected
Quality of Health Care	Telemedicine	10	Undirected
Quality of Health Care	adverse event	10	Undirected
Quality of Health Care	burnout	10	Undirected
Quality of Health Care	epidemiology	10	Undirected
Quality of Health Care	hospital discharge	10	Undirected
Quality of Health Care	hospital readmission	10	Undirected
Quality of Health Care	long term care	10	Undirected
Quality of Health Care	methodology	10	Undirected
Quality of Health Care	resuscitation	10	Undirected
Reproducibility of Results	analgesia	10	Undirected
Reproducibility of Results	attitude	10	Undirected
Reproducibility of Results	blood pressure	10	Undirected
Reproducibility of Results	caregiver	10	Undirected
Reproducibility of Results	epidemiology	10	Undirected
Reproducibility of Results	knowledge	10	Undirected
Risk Assessment	Terminal Care	10	Undirected
Risk Assessment	intensivist	10	Undirected
Safety Management	Canada	10	Undirected
Safety Management	Pilot Projects	10	Undirected
Safety Management	hospital personnel	10	Undirected
Safety Management	perception	10	Undirected
Safety Management	pharmacist	10	Undirected
Sensitivity and Specificity	Pilot Projects	10	Undirected
Sensitivity and Specificity	Stroke	10	Undirected

Sensitivity and Specificity	diabetes mellitus	10	Undirected
Sensitivity and Specificity	oxygen saturation	10	Undirected
Sensitivity and Specificity	pneumonia	10	Undirected
Sensitivity and Specificity	resuscitation	10	Undirected
Sepsis	Anxiety	10	Undirected
Sepsis	China	10	Undirected
Sepsis	Fatigue	10	Undirected
Sepsis	perception	10	Undirected
Sepsis	respiratory therapist	10	Undirected
Sepsis	simulation	10	Undirected
Social Support	Pilot Projects	10	Undirected
Social Support	Safety Management	10	Undirected
Social Support	health care policy	10	Undirected
Stress, Psychological	heart rate	10	Undirected
Stress, Psychological	pilot study	10	Undirected
Stroke	emergency ward	10	Undirected
Stroke	health care delivery	10	Undirected
Telemedicine	emergency health service	10	Undirected
Telemedicine	hospital readmission	10	Undirected
Telemedicine	pharmacist	10	Undirected
Telemedicine	psychology	10	Undirected
Telemedicine	resuscitation	10	Undirected
Terminal Care	Infection Control	10	Undirected
Terminal Care	pilot study	10	Undirected
Terminal Care	therapy	10	Undirected
Time Factors	China	10	Undirected
Time Factors	Mothers	10	Undirected
Time Factors	endotracheal intubation	10	Undirected
Time Factors	home care	10	Undirected
Time Factors	information processing	10	Undirected
Time Factors	medical education	10	Undirected
Time Factors	oxygen saturation	10	Undirected
Time Factors	simulation	10	Undirected
Treatment Outcome	Cooperative Behavior	10	Undirected
Treatment Outcome	Medication Errors	10	Undirected
United Kingdom	Delirium	10	Undirected
United Kingdom	hospital readmission	10	Undirected
United Kingdom	university hospital	10	Undirected
Workforce	Cooperative Behavior	10	Undirected
Workforce	Practice Guidelines as Topic	10	Undirected
Workforce	Sepsis	10	Undirected
Workforce	tertiary care center	10	Undirected
Workload	depression	10	Undirected
Workload	hand washing	10	Undirected
Workload	palliative therapy	10	Undirected
Workload	respiratory therapist	10	Undirected
Workload	ventilator associated pneumonia	10	Undirected
Workplace	cohort analysis	10	Undirected
Workplace	hospital personnel	10	Undirected
Workplace	standards	10	Undirected
adverse event	computer assisted tomography	10	Undirected
adverse event	enteric feeding	10	Undirected
adverse event	medical education	10	Undirected
analgesia	Pandemics	10	Undirected

analgesia	dyspnea	10	Undirected
analgesia	enteric feeding	10	Undirected
analgesia	health care planning	10	Undirected
analgesia	pilot study	10	Undirected
analgesia	semi structured interview	10	Undirected
analgesia	teaching hospital	10	Undirected
antibiotic agent	breathing rate	10	Undirected
attitude	cohort analysis	10	Undirected
attitude	medical education	10	Undirected
attitude	teaching hospital	10	Undirected
attitude to health	Fatigue	10	Undirected
attitude to health	Leadership	10	Undirected
attitude to health	Postoperative Care	10	Undirected
attitude to health	respiratory therapist	10	Undirected
blood pressure	cerebrovascular accident	10	Undirected
blood pressure	cohort analysis	10	Undirected
blood pressure	very elderly	10	Undirected
burnout	Canada	10	Undirected
burnout	age	10	Undirected
burnout	clinical practice	10	Undirected
burnout	follow up	10	Undirected
burnout	information processing	10	Undirected
burnout	meta analysis	10	Undirected
caregiver	Stroke	10	Undirected
caregiver	chronic obstructive lung disease	10	Undirected
caregiver	enteric feeding	10	Undirected
caregiver	meta analysis	10	Undirected
caregiver	methodology	10	Undirected
clinical practice	breathing rate	10	Undirected
clinical practice	cerebrovascular accident	10	Undirected
clinical practice	computer assisted tomography	10	Undirected
clinical practice	oxygen saturation	10	Undirected
dementia	emergency care	10	Undirected
dementia	epidemiology	10	Undirected
depression	Stroke	10	Undirected
depression	home care	10	Undirected
depression	intensivist	10	Undirected
diabetes mellitus	Anti-Bacterial Agents	10	Undirected
diabetes mellitus	antiinfective agent	10	Undirected
diabetes mellitus	clinical practice	10	Undirected
diabetes mellitus	heart surgery	10	Undirected
diabetes mellitus	home care	10	Undirected
diabetes mellitus	pharmacist	10	Undirected
dyspnea	Glasgow coma scale	10	Undirected
dyspnea	age	10	Undirected
dyspnea	medical education	10	Undirected
dyspnea	medical staff	10	Undirected
dyspnea	mental health	10	Undirected
education	Catheterization, Central Venous	10	Undirected
education	cerebrovascular accident	10	Undirected
education	enteric feeding	10	Undirected
education	home care	10	Undirected
education	meta analysis	10	Undirected
emergency care	hand washing	10	Undirected

emergency care	reliability	10	Undirected
emergency health service	chronic obstructive lung disease	10	Undirected
emergency ward	methodology	10	Undirected
emergency ward	psychological aspect	10	Undirected
endotracheal intubation	preschool child	10	Undirected
ethics	Health Knowledge, Attitudes, Practice	10	Undirected
ethics	health care delivery	10	Undirected
ethics	hospital admission	10	Undirected
ethics	medical education	10	Undirected
ethics	thematic analysis	10	Undirected
evidence based practice	Canada	10	Undirected
evidence based practice	Electronic Health Records	10	Undirected
evidence based practice	epidemiology	10	Undirected
evidence based practice	pain assessment	10	Undirected
evidence based practice	pilot study	10	Undirected
evidence based practice	safety	10	Undirected
evidence based practice	thematic analysis	10	Undirected
evidence based practice	very elderly	10	Undirected
fentanyl	breathing rate	10	Undirected
fentanyl	epidemiology	10	Undirected
fentanyl	heart surgery	10	Undirected
fever	extracorporeal oxygenation	10	Undirected
fever	health care system	10	Undirected
fever	methodology	10	Undirected
fever	midazolam	10	Undirected
follow up	microbiology	10	Undirected
gestational age	Algorithms	10	Undirected
gestational age	Canada	10	Undirected
gestational age	interpersonal communication	10	Undirected
gestational age	medical staff	10	Undirected
gestational age	respiratory therapist	10	Undirected
gestational age	university hospital	10	Undirected
hand washing	information processing	10	Undirected
health care cost	Stroke	10	Undirected
health care cost	adverse event	10	Undirected
health care cost	burnout	10	Undirected
health care cost	hand washing	10	Undirected
health care cost	information processing	10	Undirected
health care cost	intensivist	10	Undirected
health care cost	thorax radiography	10	Undirected
health care planning	therapy	10	Undirected
health care quality	enteric feeding	10	Undirected
health care quality	extracorporeal oxygenation	10	Undirected
health care quality	heart surgery	10	Undirected
health care quality	therapy	10	Undirected
heart failure	Anxiety	10	Undirected
heart failure	Pregnancy	10	Undirected
heart failure	age	10	Undirected
heart failure	antibiotic agent	10	Undirected
heart failure	education	10	Undirected
heart failure	health care system	10	Undirected
heart failure	therapy	10	Undirected
heart rate	antibiotic agent	10	Undirected
heart rate	cerebrovascular accident	10	Undirected

heart rate	health care quality	10	Undirected
heart rate	heart surgery	10	Undirected
heart rate	medical staff	10	Undirected
heart surgery	age	10	Undirected
heart surgery	intensivist	10	Undirected
heart surgery	therapy	10	Undirected
home care	university hospital	10	Undirected
hospital infection	chronic obstructive lung disease	10	Undirected
hospital infection	health care planning	10	Undirected
hospital infection	health care policy	10	Undirected
hospital infection	statistics and numerical data	10	Undirected
hospital readmission	antiinfective agent	10	Undirected
hospital readmission	breathing rate	10	Undirected
hospital readmission	computer assisted tomography	10	Undirected
hospitalization	Catheterization, Central Venous	10	Undirected
hospitalization	Job Satisfaction	10	Undirected
hypertension	extracorporeal oxygenation	10	Undirected
hypotension	chronic obstructive lung disease	10	Undirected
hypotension	depression	10	Undirected
hypotension	medical education	10	Undirected
hypotension	meta analysis	10	Undirected
hypotension	physiotherapy	10	Undirected
hypotension	urinary tract infection	10	Undirected
information processing	antiinfective agent	10	Undirected
interpersonal communication	antibiotic agent	10	Undirected
interview	Fatigue	10	Undirected
interview	cohort analysis	10	Undirected
interview	home care	10	Undirected
interview	statistics and numerical data	10	Undirected
knowledge	emergency health service	10	Undirected
knowledge	standards	10	Undirected
knowledge	tracheostomy	10	Undirected
knowledge	urinary tract infection	10	Undirected
long term care	cerebrovascular accident	10	Undirected
long term care	information processing	10	Undirected
long term care	semi structured interview	10	Undirected
long term care	standards	10	Undirected
medical education	cerebrovascular accident	10	Undirected
medical education	meta analysis	10	Undirected
medical staff	fever	10	Undirected
medical staff	heart surgery	10	Undirected
medical staff	hypertension	10	Undirected
mental health	age	10	Undirected
mental health	interpersonal communication	10	Undirected
mental health	meta analysis	10	Undirected
mental health	semi structured interview	10	Undirected
meta analysis	standards	10	Undirected
cillin resistant Staphylococcus a	hospital personnel	10	Undirected
cillin resistant Staphylococcus a	multicenter study	10	Undirected
methodology	health care system	10	Undirected
methodology	physiotherapy	10	Undirected
midazolam	heart surgery	10	Undirected
midazolam	multicenter study	10	Undirected
morphine	antibiotic agent	10	Undirected

morphine	health care planning	10	Undirected
morphine	multicenter study	10	Undirected
morphine	university hospital	10	Undirected
multicenter study	breathing rate	10	Undirected
multicenter study	computer assisted tomography	10	Undirected
multicenter study	physiotherapy	10	Undirected
observational study	health care planning	10	Undirected
outcome assessment	Catheterization, Central Venous	10	Undirected
outcome assessment	Hypnotics and Sedatives	10	Undirected
outcome assessment	Needs Assessment	10	Undirected
pain assessment	follow up	10	Undirected
pain assessment	heart surgery	10	Undirected
pain assessment	very elderly	10	Undirected
pediatrics	Cardiac Surgical Procedures	10	Undirected
pediatrics	Postoperative Complications	10	Undirected
pediatrics	health care facility	10	Undirected
pediatrics	heart surgery	10	Undirected
pediatrics	home care	10	Undirected
pediatrics	pain assessment	10	Undirected
pediatrics	thematic analysis	10	Undirected
pediatrics	thorax radiography	10	Undirected
pediatrics	ventilator associated pneumonia	10	Undirected
perception	Clinical Protocols	10	Undirected
perception	Pilot Projects	10	Undirected
perception	age	10	Undirected
perception	health care policy	10	Undirected
perception	home care	10	Undirected
perception	pain assessment	10	Undirected
perception	pilot study	10	Undirected
perception	randomized controlled trial	10	Undirected
perception	statistics and numerical data	10	Undirected
pharmacist	hand washing	10	Undirected
pharmacist	urinary tract infection	10	Undirected
physiotherapy	oxygen saturation	10	Undirected
physiotherapy	palliative therapy	10	Undirected
physiotherapy	university hospital	10	Undirected
pilot study	thematic analysis	10	Undirected
pneumonia	palliative therapy	10	Undirected
pneumonia	teaching hospital	10	Undirected
prematurity	Algorithms	10	Undirected
prematurity	preschool child	10	Undirected
prematurity	reliability	10	Undirected
prematurity	thorax radiography	10	Undirected
prevalence	interpersonal communication	10	Undirected
propofol	Glasgow coma scale	10	Undirected
propofol	Medication Errors	10	Undirected
propofol	antibiotic agent	10	Undirected
propofol	computer assisted tomography	10	Undirected
propofol	dyspnea	10	Undirected
propofol	epidemiology	10	Undirected
quality of life	Australia	10	Undirected
quality of life	Health Knowledge, Attitudes, Practice	10	Undirected
quality of life	Infection Control	10	Undirected
quality of life	Pregnancy	10	Undirected

quality of life	Quality Improvement	10	Undirected
quality of life	Workforce	10	Undirected
quality of life	computer assisted tomography	10	Undirected
quality of life	fever	10	Undirected
quality of life	gestational age	10	Undirected
quality of life	medical staff	10	Undirected
randomized controlled trial	emergency health service	10	Undirected
randomized controlled trial	extracorporeal oxygenation	10	Undirected
randomized controlled trial	health care policy	10	Undirected
reliability	COVID-19	10	Undirected
reliability	preschool child	10	Undirected
resuscitation	morphine	10	Undirected
safety	Algorithms	10	Undirected
safety	Pilot Projects	10	Undirected
safety	antibiotic agent	10	Undirected
safety	disease severity	10	Undirected
safety	heart rate	10	Undirected
safety	knowledge	10	Undirected
safety	midazolam	10	Undirected
safety	teaching hospital	10	Undirected
sedation	intensivist	10	Undirected
simulation	intensivist	10	Undirected
simulation	methodology	10	Undirected
simulation	standards	10	Undirected
simulation	thematic analysis	10	Undirected
standards	extracorporeal oxygenation	10	Undirected
standards	hospital readmission	10	Undirected
standards	physiotherapy	10	Undirected
statistics and numerical data	antiinfective agent	10	Undirected
teaching hospital	disease severity	10	Undirected
teaching hospital	information processing	10	Undirected
teaching hospital	intensivist	10	Undirected
tertiary care center	Breast Feeding	10	Undirected
tertiary care center	Catheterization, Central Venous	10	Undirected
tertiary care center	heart rate	10	Undirected
tertiary care center	morphine	10	Undirected
tertiary care center	thorax radiography	10	Undirected
tertiary care center	tracheostomy	10	Undirected
thematic analysis	health care policy	10	Undirected
thematic analysis	teaching hospital	10	Undirected
therapy	enteric feeding	10	Undirected
total quality management	Enteral Nutrition	10	Undirected
total quality management	Pain Measurement	10	Undirected
total quality management	fever	10	Undirected
total quality management	oxygen saturation	10	Undirected
total quality management	thorax radiography	10	Undirected
tracheostomy	cerebrovascular accident	10	Undirected
tracheostomy	health care quality	10	Undirected
tracheostomy	interpersonal communication	10	Undirected
tracheostomy	methodology	10	Undirected
tracheostomy	standards	10	Undirected
university hospital	antiinfective agent	10	Undirected
urinary tract infection	cerebrovascular accident	10	Undirected
vancomycin	teaching hospital	10	Undirected

vancomycin	university hospital	10	Undirected
ventilator associated pneumonia	adverse event	10	Undirected
ventilator associated pneumonia	disease severity	10	Undirected
ventilator associated pneumonia	follow up	10	Undirected
ventilator associated pneumonia	preschool child	10	Undirected
ventilator associated pneumonia	university hospital	10	Undirected
Adaptation, Psychological	Canada	9	Undirected
Adaptation, Psychological	clinical article	9	Undirected
Algorithms	Medication Errors	9	Undirected
Algorithms	health care system	9	Undirected
Algorithms	midazolam	9	Undirected
Algorithms	morphine	9	Undirected
Algorithms	multicenter study	9	Undirected
Algorithms	oxygen saturation	9	Undirected
Algorithms	tracheostomy	9	Undirected
Algorithms	university hospital	9	Undirected
Anti-Bacterial Agents	COVID-19	9	Undirected
Anti-Bacterial Agents	hand washing	9	Undirected
Anti-Bacterial Agents	hospital readmission	9	Undirected
Anti-Bacterial Agents	hypotension	9	Undirected
Anti-Bacterial Agents	methodology	9	Undirected
Anti-Bacterial Agents	multicenter study	9	Undirected
Anxiety	Reproducibility of Results	9	Undirected
Anxiety	breathing rate	9	Undirected
Anxiety	hand washing	9	Undirected
Artificial Ventilation	Job Satisfaction	9	Undirected
Artificial Ventilation	burnout	9	Undirected
Attitude of Health Personnel	Stroke	9	Undirected
Attitude of Health Personnel	antibiotic agent	9	Undirected
Australia	Enteral Nutrition	9	Undirected
Australia	analgesia	9	Undirected
Australia	depression	9	Undirected
Australia	endotracheal intubation	9	Undirected
Australia	heart rate	9	Undirected
Australia	pharmacist	9	Undirected
Australia	tracheostomy	9	Undirected
Breast Feeding	Pain	9	Undirected
Burnout, Professional	education	9	Undirected
Burnout, Professional	emergency ward	9	Undirected
Burnout, Professional	hospital personnel	9	Undirected
Burnout, Professional	randomized controlled trial	9	Undirected
Canada	analgesia	9	Undirected
Canada	pharmacist	9	Undirected
Canada	statistics and numerical data	9	Undirected
Canada	thematic analysis	9	Undirected
Cardiac Surgical Procedures	medical education	9	Undirected
Cardiac Surgical Procedures	multicenter study	9	Undirected
Catheter-Related Infections	epidemiology	9	Undirected
Catheter-Related Infections	multicenter study	9	Undirected
Catheterization, Central Venous	thorax radiography	9	Undirected
Child, Preschool	Electronic Health Records	9	Undirected
Child, Preschool	Pandemics	9	Undirected
Child, Preschool	Safety Management	9	Undirected
Child, Preschool	ethics	9	Undirected

Child, Preschool	health care delivery	9	Undirected
Child, Preschool	health care planning	9	Undirected
Child, Preschool	physiotherapy	9	Undirected
Child, Preschool	prematurity	9	Undirected
Child, Preschool	ventilator associated pneumonia	9	Undirected
China	endotracheal intubation	9	Undirected
China	health care delivery	9	Undirected
China	health care policy	9	Undirected
China	resuscitation	9	Undirected
China	very elderly	9	Undirected
Clinical Competence	Stroke	9	Undirected
Clinical Competence	heart failure	9	Undirected
Clinical Competence	very elderly	9	Undirected
Clinical Protocols	Mothers	9	Undirected
Clinical Protocols	Stroke	9	Undirected
Clinical Protocols	computer assisted tomography	9	Undirected
Clinical Protocols	statistics and numerical data	9	Undirected
Clinical Protocols	therapy	9	Undirected
Communication	Heart Arrest	9	Undirected
Communication	analgesia	9	Undirected
Communication	health care cost	9	Undirected
Communication	mental health	9	Undirected
Communication	sedation	9	Undirected
Cooperative Behavior	Australia	9	Undirected
Cooperative Behavior	health care cost	9	Undirected
Cooperative Behavior	resuscitation	9	Undirected
Cross Infection	health care planning	9	Undirected
Cross Infection	heart failure	9	Undirected
Cross Infection	pediatrics	9	Undirected
Cross Infection	simulation	9	Undirected
Decision Making	Glasgow coma scale	9	Undirected
Decision Making	Program Evaluation	9	Undirected
Decision Making	Telemedicine	9	Undirected
Decision Making	breathing rate	9	Undirected
Decision Making	dyspnea	9	Undirected
Decision Making	midazolam	9	Undirected
Delirium	breathing rate	9	Undirected
Delirium	chronic obstructive lung disease	9	Undirected
Delirium	health care delivery	9	Undirected
Delirium	health care policy	9	Undirected
Delirium	medical education	9	Undirected
Delirium	safety	9	Undirected
Electronic Health Records	antibiotic agent	9	Undirected
Electronic Health Records	emergency health service	9	Undirected
Electronic Health Records	pilot study	9	Undirected
Electronic Health Records	respiratory therapist	9	Undirected
Electronic Health Records	urinary tract infection	9	Undirected
Emergency Service, Hospital	Safety Management	9	Undirected
Emergency Service, Hospital	adverse event	9	Undirected
Emergency Service, Hospital	endotracheal intubation	9	Undirected
Emergency Service, Hospital	hypotension	9	Undirected
Emergency Service, Hospital	oxygen saturation	9	Undirected
Emergency Service, Hospital	palliative therapy	9	Undirected
Emergency Service, Hospital	pneumonia	9	Undirected

Emergency Service, Hospital	randomized controlled trial	9	Undirected
Enteral Nutrition	clinical practice	9	Undirected
Enteral Nutrition	follow up	9	Undirected
Enteral Nutrition	meta analysis	9	Undirected
Enteral Nutrition	pneumonia	9	Undirected
Enteral Nutrition	therapy	9	Undirected
Family	Fatigue	9	Undirected
Family	Glasgow coma scale	9	Undirected
Family	Postoperative Care	9	Undirected
Family	Telemedicine	9	Undirected
Family	gestational age	9	Undirected
Family	morphine	9	Undirected
Family	pain assessment	9	Undirected
Fatigue	emergency care	9	Undirected
Fatigue	health care quality	9	Undirected
Focus Groups	Medication Errors	9	Undirected
Focus Groups	Mothers	9	Undirected
Focus Groups	Pain	9	Undirected
Focus Groups	palliative therapy	9	Undirected
Glasgow coma scale	adverse event	9	Undirected
Glasgow coma scale	methodology	9	Undirected
Glasgow coma scale	midazolam	9	Undirected
Glasgow coma scale	morphine	9	Undirected
Glasgow coma scale	palliative therapy	9	Undirected
Glasgow coma scale	physiotherapy	9	Undirected
Glasgow coma scale	statistics and numerical data	9	Undirected
Guideline Adherence	Infant, Premature	9	Undirected
Guideline Adherence	Pain	9	Undirected
Guideline Adherence	Pain Measurement	9	Undirected
Guideline Adherence	Pilot Projects	9	Undirected
Guideline Adherence	clinical article	9	Undirected
Guideline Adherence	emergency ward	9	Undirected
Guideline Adherence	health care planning	9	Undirected
Guideline Adherence	interpersonal communication	9	Undirected
Guideline Adherence	pneumonia	9	Undirected
Guideline Adherence	psychological aspect	9	Undirected
Guideline Adherence	simulation	9	Undirected
alth Knowledge, Attitudes, Practi	Cardiac Surgical Procedures	9	Undirected
alth Knowledge, Attitudes, Practi	analgesia	9	Undirected
alth Knowledge, Attitudes, Practi	medical education	9	Undirected
alth Knowledge, Attitudes, Practi	pharmacist	9	Undirected
Health Personnel	hospital discharge	9	Undirected
Health Personnel	methodology	9	Undirected
Health Personnel	preschool child	9	Undirected
Health Services Needs and Demand	Anxiety	9	Undirected
Health Services Needs and Demand	Child, Preschool	9	Undirected
Health Services Needs and Demand	Emergency Service, Hospital	9	Undirected
Health Services Needs and Demand	Pain	9	Undirected
Health Services Needs and Demand	Workplace	9	Undirected
Health Services Needs and Demand	emergency health service	9	Undirected
Health Services Needs and Demand	health care policy	9	Undirected
Health Services Needs and Demand	mortality	9	Undirected
Health Services Needs and Demand	prevalence	9	Undirected
Heart Arrest	Sensitivity and Specificity	9	Undirected

Heart Arrest	evidence based practice	9	Undirected
Heart Arrest	hospital personnel	9	Undirected
Heart Arrest	hospital readmission	9	Undirected
Heart Arrest	sedation	9	Undirected
Hospital Mortality	China	9	Undirected
Hospital Mortality	Safety Management	9	Undirected
Hospital Mortality	Telemedicine	9	Undirected
Hospital Mortality	endotracheal intubation	9	Undirected
Hospital Mortality	health care policy	9	Undirected
Hospital Mortality	mental health	9	Undirected
Hypnotics and Sedatives	Terminal Care	9	Undirected
Hypnotics and Sedatives	total quality management	9	Undirected
Infant, Premature	Anti-Bacterial Agents	9	Undirected
Infant, Premature	Focus Groups	9	Undirected
Infant, Premature	breathing rate	9	Undirected
Infection Control	Postoperative Complications	9	Undirected
Infection Control	chronic obstructive lung disease	9	Undirected
Infection Control	hospital readmission	9	Undirected
Insulin	standards	9	Undirected
Interprofessional Relations	Infant, Premature	9	Undirected
Interprofessional Relations	resuscitation	9	Undirected
Interprofessional Relations	university hospital	9	Undirected
Intubation, Intratracheal	Pilot Projects	9	Undirected
Intubation, Intratracheal	blood pressure	9	Undirected
Intubation, Intratracheal	emergency ward	9	Undirected
Intubation, Intratracheal	evidence based practice	9	Undirected
Intubation, Intratracheal	pneumonia	9	Undirected
Intubation, Intratracheal	sedation	9	Undirected
Intubation, Intratracheal	simulation	9	Undirected
Job Satisfaction	Canada	9	Undirected
Job Satisfaction	attitude	9	Undirected
Job Satisfaction	randomized controlled trial	9	Undirected
Leadership	cerebrovascular accident	9	Undirected
Leadership	follow up	9	Undirected
Leadership	observational study	9	Undirected
Logistic Models	Anti-Bacterial Agents	9	Undirected
Logistic Models	Australia	9	Undirected
Logistic Models	Clinical Protocols	9	Undirected
Logistic Models	Health Knowledge, Attitudes, Practice	9	Undirected
Logistic Models	Health Personnel	9	Undirected
Logistic Models	Infant, Premature	9	Undirected
Logistic Models	Postoperative Care	9	Undirected
Logistic Models	gestational age	9	Undirected
Logistic Models	resuscitation	9	Undirected
Medication Errors	Health Personnel	9	Undirected
Medication Errors	hospital personnel	9	Undirected
Medication Errors	midazolam	9	Undirected
Monitoring, Physiologic	Fatigue	9	Undirected
Monitoring, Physiologic	Pregnancy	9	Undirected
Monitoring, Physiologic	clinical practice	9	Undirected
Monitoring, Physiologic	computer assisted tomography	9	Undirected
Monitoring, Physiologic	diabetes mellitus	9	Undirected
Monitoring, Physiologic	hospital discharge	9	Undirected
Monitoring, Physiologic	preschool child	9	Undirected

Monitoring, Physiologic	psychological aspect	9	Undirected
Needs Assessment	clinical article	9	Undirected
Needs Assessment	emergency care	9	Undirected
Needs Assessment	medical education	9	Undirected
Needs Assessment	palliative therapy	9	Undirected
Needs Assessment	teaching hospital	9	Undirected
OUTCOMES	Leadership	9	Undirected
OUTCOMES	Pain	9	Undirected
OUTCOMES	Workload	9	Undirected
OUTCOMES	health care delivery	9	Undirected
OUTCOMES	tertiary care center	9	Undirected
Pain	chronic obstructive lung disease	9	Undirected
Pain	diabetes mellitus	9	Undirected
Pain	pneumonia	9	Undirected
Pain	semi structured interview	9	Undirected
Pain Measurement	breathing rate	9	Undirected
Pain Measurement	heart surgery	9	Undirected
Pain Measurement	observational study	9	Undirected
Pain Measurement	oxygen saturation	9	Undirected
Palliative Care	China	9	Undirected
Palliative Care	Mothers	9	Undirected
Palliative Care	emergency health service	9	Undirected
Palliative Care	mental health	9	Undirected
Parents	OUTCOMES	9	Undirected
Parents	epidemiology	9	Undirected
Parents	medical education	9	Undirected
Parents	pilot study	9	Undirected
Pilot Projects	Health Personnel	9	Undirected
Pilot Projects	emergency care	9	Undirected
Pilot Projects	oxygen saturation	9	Undirected
Pilot Projects	preschool child	9	Undirected
Pilot Projects	sedation	9	Undirected
Pilot Projects	simulation	9	Undirected
Postoperative Care	Glasgow coma scale	9	Undirected
Postoperative Care	Needs Assessment	9	Undirected
Postoperative Care	Pain	9	Undirected
Postoperative Care	adverse event	9	Undirected
Postoperative Care	diabetes mellitus	9	Undirected
Postoperative Care	endotracheal intubation	9	Undirected
Postoperative Care	health care delivery	9	Undirected
Postoperative Care	midazolam	9	Undirected
Postoperative Complications	COVID-19	9	Undirected
Postoperative Complications	Sensitivity and Specificity	9	Undirected
Postoperative Complications	computer assisted tomography	9	Undirected
Postoperative Complications	fever	9	Undirected
Postoperative Complications	morphine	9	Undirected
Practice Guidelines as Topic	adverse event	9	Undirected
Practice Guidelines as Topic	caregiver	9	Undirected
Practice Guidelines as Topic	home care	9	Undirected
Practice Guidelines as Topic	ethicillin resistant Staphylococcus aure	9	Undirected
Practice Guidelines as Topic	respiratory therapist	9	Undirected
Practice Guidelines as Topic	therapy	9	Undirected
Pregnancy	Postoperative Complications	9	Undirected
Pregnancy	oxygen saturation	9	Undirected

Pregnancy	pilot study	9	Undirected
Pressure Ulcer	Pandemics	9	Undirected
Professional-Family Relations	emergency care	9	Undirected
Program Evaluation	Delirium	9	Undirected
Program Evaluation	Medication Errors	9	Undirected
Program Evaluation	Telemedicine	9	Undirected
Program Evaluation	health care planning	9	Undirected
Program Evaluation	mortality	9	Undirected
Program Evaluation	randomized controlled trial	9	Undirected
Program Evaluation	statistics and numerical data	9	Undirected
Quality Improvement	antiinfective agent	9	Undirected
Quality of Health Care	Infection Control	9	Undirected
Quality of Health Care	emergency ward	9	Undirected
Quality of Health Care	psychological aspect	9	Undirected
Reproducibility of Results	Mothers	9	Undirected
Reproducibility of Results	Postoperative Complications	9	Undirected
Risk Assessment	Breast Feeding	9	Undirected
Risk Assessment	Fatigue	9	Undirected
Risk Assessment	blood pressure	9	Undirected
Risk Assessment	endotracheal intubation	9	Undirected
Risk Assessment	interpersonal communication	9	Undirected
Risk Assessment	propofol	9	Undirected
Risk Assessment	respiratory therapist	9	Undirected
Risk Assessment	semi structured interview	9	Undirected
Risk Assessment	thorax radiography	9	Undirected
Risk Factors	Telemedicine	9	Undirected
Risk Factors	ethics	9	Undirected
Safety Management	COVID-19	9	Undirected
Safety Management	Pandemics	9	Undirected
Safety Management	health care policy	9	Undirected
Sensitivity and Specificity	emergency health service	9	Undirected
Sensitivity and Specificity	hospital discharge	9	Undirected
Sensitivity and Specificity	medical education	9	Undirected
Sensitivity and Specificity	pilot study	9	Undirected
Sepsis	Pain	9	Undirected
Sepsis	analgesia	9	Undirected
Sepsis	attitude	9	Undirected
Sepsis	hand washing	9	Undirected
Sepsis	safety	9	Undirected
Sepsis	semi structured interview	9	Undirected
Social Support	Personnel Staffing and Scheduling	9	Undirected
Social Support	Workforce	9	Undirected
Social Support	age	9	Undirected
Social Support	cohort analysis	9	Undirected
Social Support	emergency care	9	Undirected
Social Support	ethics	9	Undirected
Social Support	multicenter study	9	Undirected
Stress, Psychological	Leadership	9	Undirected
Stress, Psychological	Practice Guidelines as Topic	9	Undirected
Stress, Psychological	methodology	9	Undirected
Stroke	epidemiology	9	Undirected
Stroke	therapy	9	Undirected
Telemedicine	Pilot Projects	9	Undirected
Telemedicine	education	9	Undirected

Telemedicine	pilot study	9	Undirected
Terminal Care	Reproducibility of Results	9	Undirected
Terminal Care	age	9	Undirected
Terminal Care	antibiotic agent	9	Undirected
Terminal Care	cerebrovascular accident	9	Undirected
Terminal Care	mental health	9	Undirected
Terminal Care	respiratory therapist	9	Undirected
Time Factors	Pandemics	9	Undirected
Time Factors	blood pressure	9	Undirected
Time Factors	chronic obstructive lung disease	9	Undirected
Time Factors	computer assisted tomography	9	Undirected
Time Factors	depression	9	Undirected
Time Factors	mental health	9	Undirected
Time Factors	meta analysis	9	Undirected
Time Factors	pain assessment	9	Undirected
Time Factors	palliative therapy	9	Undirected
Time Factors	perception	9	Undirected
Time Factors	urinary tract infection	9	Undirected
Treatment Outcome	thematic analysis	9	Undirected
United Kingdom	Burnout, Professional	9	Undirected
United Kingdom	Intubation, Intratracheal	9	Undirected
United Kingdom	Reproducibility of Results	9	Undirected
United Kingdom	clinical article	9	Undirected
United Kingdom	depression	9	Undirected
United Kingdom	intensivist	9	Undirected
United Kingdom	pilot study	9	Undirected
United Kingdom	sedation	9	Undirected
Workforce	China	9	Undirected
Workforce	Fatigue	9	Undirected
Workforce	Guideline Adherence	9	Undirected
Workforce	Heart Arrest	9	Undirected
Workforce	knowledge	9	Undirected
Workforce	medical education	9	Undirected
Workload	Sensitivity and Specificity	9	Undirected
Workload	Terminal Care	9	Undirected
Workload	disease severity	9	Undirected
Workload	teaching hospital	9	Undirected
Workplace	emergency care	9	Undirected
Workplace	ethics	9	Undirected
Workplace	follow up	9	Undirected
Workplace	hospitalization	9	Undirected
Workplace	multicenter study	9	Undirected
Workplace	statistics and numerical data	9	Undirected
Workplace	teaching hospital	9	Undirected
adverse event	information processing	9	Undirected
adverse event	pilot study	9	Undirected
adverse event	respiratory therapist	9	Undirected
adverse event	thorax radiography	9	Undirected
adverse event	university hospital	9	Undirected
age	clinical article	9	Undirected
age	enteric feeding	9	Undirected
analgesia	health care system	9	Undirected
analgesia	mental health	9	Undirected
analgesia	reliability	9	Undirected

antibiotic agent	acute kidney failure	9	Undirected
antibiotic agent	hospital personnel	9	Undirected
antibiotic agent	information processing	9	Undirected
attitude	caregiver	9	Undirected
attitude	health care quality	9	Undirected
attitude	hospital personnel	9	Undirected
attitude	observational study	9	Undirected
attitude	pharmacist	9	Undirected
attitude	psychological aspect	9	Undirected
attitude to health	Breast Feeding	9	Undirected
attitude to health	China	9	Undirected
attitude to health	Clinical Protocols	9	Undirected
attitude to health	cohort analysis	9	Undirected
attitude to health	health care planning	9	Undirected
attitude to health	hospital infection	9	Undirected
attitude to health	mental health	9	Undirected
attitude to health	pediatrics	9	Undirected
attitude to health	tracheostomy	9	Undirected
attitude to health	ventilator associated pneumonia	9	Undirected
blood pressure	adverse event	9	Undirected
blood pressure	fentanyl	9	Undirected
blood pressure	pain assessment	9	Undirected
burnout	palliative therapy	9	Undirected
caregiver	Electronic Health Records	9	Undirected
caregiver	morphine	9	Undirected
caregiver	reliability	9	Undirected
cerebrovascular accident	information processing	9	Undirected
dementia	health care facility	9	Undirected
dementia	information processing	9	Undirected
depression	pilot study	9	Undirected
depression	sedation	9	Undirected
diabetes mellitus	Medication Errors	9	Undirected
diabetes mellitus	blood pressure	9	Undirected
diabetes mellitus	endotracheal intubation	9	Undirected
diabetes mellitus	health care facility	9	Undirected
diabetes mellitus	health care policy	9	Undirected
diabetes mellitus	long term care	9	Undirected
diabetes mellitus	teaching hospital	9	Undirected
diabetes mellitus	vancomycin	9	Undirected
disease severity	psychological aspect	9	Undirected
dyspnea	fentanyl	9	Undirected
dyspnea	midazolam	9	Undirected
dyspnea	randomized controlled trial	9	Undirected
education	Catheter-Related Infections	9	Undirected
education	acute kidney failure	9	Undirected
emergency care	hospital infection	9	Undirected
emergency care	urinary tract infection	9	Undirected
endotracheal intubation	age	9	Undirected
endotracheal intubation	statistics and numerical data	9	Undirected
epidemiology	enteric feeding	9	Undirected
epidemiology	thorax radiography	9	Undirected
ethics	disease severity	9	Undirected
ethics	health care system	9	Undirected
ethics	outcome assessment	9	Undirected

ethics	university hospital	9	Undirected
evidence based practice	Mothers	9	Undirected
evidence based practice	Stroke	9	Undirected
evidence based practice	cerebrovascular accident	9	Undirected
evidence based practice	gestational age	9	Undirected
evidence based practice	information processing	9	Undirected
fentanyl	adverse event	9	Undirected
fentanyl	disease severity	9	Undirected
fentanyl	hospital discharge	9	Undirected
fentanyl	multicenter study	9	Undirected
fentanyl	physiotherapy	9	Undirected
fever	Stroke	9	Undirected
fever	age	9	Undirected
fever	enteric feeding	9	Undirected
fever	physiotherapy	9	Undirected
fever	psychology	9	Undirected
fever	therapy	9	Undirected
gestational age	emergency ward	9	Undirected
gestational age	fentanyl	9	Undirected
gestational age	health care policy	9	Undirected
gestational age	home care	9	Undirected
gestational age	knowledge	9	Undirected
gestational age	mental health	9	Undirected
gestational age	semi structured interview	9	Undirected
gestational age	standards	9	Undirected
gestational age	thematic analysis	9	Undirected
health care cost	Glasgow coma scale	9	Undirected
health care cost	Heart Arrest	9	Undirected
health care cost	interpersonal communication	9	Undirected
health care cost	knowledge	9	Undirected
health care delivery	physiotherapy	9	Undirected
health care facility	antiinfective agent	9	Undirected
health care facility	respiratory therapist	9	Undirected
health care facility	therapy	9	Undirected
health care facility	university hospital	9	Undirected
health care planning	antiinfective agent	9	Undirected
health care planning	computer assisted tomography	9	Undirected
health care quality	computer assisted tomography	9	Undirected
health care quality	oxygen saturation	9	Undirected
health care quality	vancomycin	9	Undirected
heart failure	Algorithms	9	Undirected
heart failure	Health Knowledge, Attitudes, Practice	9	Undirected
heart failure	Quality Improvement	9	Undirected
heart failure	oxygen saturation	9	Undirected
heart rate	caregiver	9	Undirected
heart rate	depression	9	Undirected
heart rate	statistics and numerical data	9	Undirected
heart surgery	acute kidney failure	9	Undirected
heart surgery	cerebrovascular accident	9	Undirected
home care	information processing	9	Undirected
home care	medical education	9	Undirected
home care	semi structured interview	9	Undirected
hospital infection	respiratory therapist	9	Undirected
hospital personnel	psychological aspect	9	Undirected

hospital readmission	interpersonal communication	9	Undirected
hospital readmission	preschool child	9	Undirected
hospitalization	Catheter-Related Infections	9	Undirected
hospitalization	Focus Groups	9	Undirected
hospitalization	simulation	9	Undirected
hypertension	medical education	9	Undirected
hypotension	antiinfective agent	9	Undirected
hypotension	epidemiology	9	Undirected
hypotension	heart surgery	9	Undirected
information processing	health care policy	9	Undirected
intensivist	antibiotic agent	9	Undirected
intensivist	information processing	9	Undirected
interview	sedation	9	Undirected
knowledge	Catheterization, Central Venous	9	Undirected
knowledge	dyspnea	9	Undirected
knowledge	health care policy	9	Undirected
knowledge	hospital personnel	9	Undirected
knowledge	hospital readmission	9	Undirected
knowledge	meta analysis	9	Undirected
long term care	computer assisted tomography	9	Undirected
long term care	microbiology	9	Undirected
long term care	therapy	9	Undirected
medical education	computer assisted tomography	9	Undirected
medical education	very elderly	9	Undirected
medical staff	antiinfective agent	9	Undirected
medical staff	enteric feeding	9	Undirected
medical staff	mental health	9	Undirected
mental health	home care	9	Undirected
meta analysis	multicenter study	9	Undirected
meta analysis	palliative therapy	9	Undirected
cillin resistant Staphylococcus a	hospital discharge	9	Undirected
methodology	COVID-19	9	Undirected
methodology	hand washing	9	Undirected
microbiology	disease severity	9	Undirected
microbiology	university hospital	9	Undirected
midazolam	antibiotic agent	9	Undirected
midazolam	reliability	9	Undirected
morphine	breathing rate	9	Undirected
morphine	hospital readmission	9	Undirected
morphine	oxygen saturation	9	Undirected
morphine	standards	9	Undirected
mortality	thematic analysis	9	Undirected
multicenter study	thorax radiography	9	Undirected
observational study	Catheter-Related Infections	9	Undirected
organization and management	enteric feeding	9	Undirected
outcome assessment	Pressure Ulcer	9	Undirected
outcome assessment	Safety Management	9	Undirected
oxygen saturation	antibiotic agent	9	Undirected
pain assessment	emergency ward	9	Undirected
pain assessment	health care planning	9	Undirected
pediatrics	Anxiety	9	Undirected
pediatrics	Glasgow coma scale	9	Undirected
pediatrics	Pilot Projects	9	Undirected
pediatrics	blood pressure	9	Undirected

pediatrics	dyspnea	9	Undirected
pediatrics	midazolam	9	Undirected
pediatrics	reliability	9	Undirected
pediatrics	teaching hospital	9	Undirected
perception	Fatigue	9	Undirected
perception	Infection Control	9	Undirected
perception	hand washing	9	Undirected
perception	methodology	9	Undirected
perception	psychological aspect	9	Undirected
pharmacist	fever	9	Undirected
pharmacist	hospital infection	9	Undirected
pharmacist	mental health	9	Undirected
physiotherapy	computer assisted tomography	9	Undirected
physiotherapy	respiratory therapist	9	Undirected
physiotherapy	thorax radiography	9	Undirected
pilot study	enteric feeding	9	Undirected
pilot study	health care facility	9	Undirected
pilot study	health care planning	9	Undirected
pilot study	intensivist	9	Undirected
pilot study	teaching hospital	9	Undirected
pilot study	therapy	9	Undirected
pneumonia	Electronic Health Records	9	Undirected
pneumonia	Health Personnel	9	Undirected
pneumonia	adverse event	9	Undirected
pneumonia	hand washing	9	Undirected
pneumonia	health care facility	9	Undirected
pneumonia	medical staff	9	Undirected
pneumonia	meta analysis	9	Undirected
pneumonia	respiratory therapist	9	Undirected
practice guideline	reliability	9	Undirected
prematurity	emergency care	9	Undirected
prematurity	teaching hospital	9	Undirected
prematurity	vancomycin	9	Undirected
preschool child	thorax radiography	9	Undirected
prevalence	Algorithms	9	Undirected
prevalence	Insulin	9	Undirected
prevalence	Pilot Projects	9	Undirected
prevalence	Reproducibility of Results	9	Undirected
prevalence	heart surgery	9	Undirected
prevalence	physiotherapy	9	Undirected
propofol	acute kidney failure	9	Undirected
propofol	extracorporeal oxygenation	9	Undirected
propofol	fever	9	Undirected
propofol	university hospital	9	Undirected
psychology	heart surgery	9	Undirected
quality of life	Leadership	9	Undirected
quality of life	Logistic Models	9	Undirected
quality of life	Practice Guidelines as Topic	9	Undirected
quality of life	Pressure Ulcer	9	Undirected
quality of life	antibiotic agent	9	Undirected
quality of life	health care facility	9	Undirected
quality of life	preschool child	9	Undirected
quality of life	reliability	9	Undirected
quality of life	respiratory therapist	9	Undirected

randomized controlled trial	health care facility	9	Undirected
randomized controlled trial	thematic analysis	9	Undirected
randomized controlled trial	thorax radiography	9	Undirected
reliability	cohort analysis	9	Undirected
reliability	hospital admission	9	Undirected
reliability	medical education	9	Undirected
reliability	methodology	9	Undirected
reliability	pilot study	9	Undirected
respiratory therapist	breathing rate	9	Undirected
respiratory therapist	hand washing	9	Undirected
resuscitation	antiinfective agent	9	Undirected
safety	gestational age	9	Undirected
safety	propofol	9	Undirected
safety	psychology	9	Undirected
safety	reliability	9	Undirected
safety	respiratory therapist	9	Undirected
sedation	Pandemics	9	Undirected
sedation	thorax radiography	9	Undirected
semi structured interview	emergency health service	9	Undirected
semi structured interview	very elderly	9	Undirected
simulation	endotracheal intubation	9	Undirected
simulation	epidemiology	9	Undirected
simulation	hospital admission	9	Undirected
standards	cerebrovascular accident	9	Undirected
statistics and numerical data	cerebrovascular accident	9	Undirected
teaching hospital	age	9	Undirected
teaching hospital	enteric feeding	9	Undirected
teaching hospital	health care facility	9	Undirected
teaching hospital	health care planning	9	Undirected
teaching hospital	therapy	9	Undirected
tertiary care center	Burnout, Professional	9	Undirected
tertiary care center	Electronic Health Records	9	Undirected
tertiary care center	Personnel Staffing and Scheduling	9	Undirected
tertiary care center	Pressure Ulcer	9	Undirected
tertiary care center	acute kidney failure	9	Undirected
tertiary care center	caregiver	9	Undirected
tertiary care center	enteric feeding	9	Undirected
tertiary care center	home care	9	Undirected
tertiary care center	ethicillin resistant Staphylococcus aure	9	Undirected
thematic analysis	cohort analysis	9	Undirected
therapy	breathing rate	9	Undirected
total quality management	Telemedicine	9	Undirected
total quality management	dementia	9	Undirected
total quality management	heart rate	9	Undirected
total quality management	hypotension	9	Undirected
total quality management	morphine	9	Undirected
total quality management	physiotherapy	9	Undirected
total quality management	reliability	9	Undirected
tracheostomy	blood pressure	9	Undirected
tracheostomy	enteric feeding	9	Undirected
tracheostomy	medical education	9	Undirected
tracheostomy	organization and management	9	Undirected
tracheostomy	vancomycin	9	Undirected
university hospital	computer assisted tomography	9	Undirected

urinary tract infection	health care facility	9	Undirected
vancomycin	breathing rate	9	Undirected
ventilator associated pneumonia	Catheter-Related Infections	9	Undirected
ventilator associated pneumonia	hospital personnel	9	Undirected
ventilator associated pneumonia	information processing	9	Undirected
Adaptation, Psychological	Emergency Service, Hospital	8	Undirected
Adaptation, Psychological	Personnel Staffing and Scheduling	8	Undirected
Adaptation, Psychological	perception	8	Undirected
Adaptation, Psychological	prevalence	8	Undirected
Adaptation, Psychological	semi structured interview	8	Undirected
Algorithms	antibiotic agent	8	Undirected
Algorithms	blood pressure	8	Undirected
Algorithms	fentanyl	8	Undirected
Algorithms	health care planning	8	Undirected
Algorithms	pain assessment	8	Undirected
Algorithms	pneumonia	8	Undirected
Algorithms	preschool child	8	Undirected
Anti-Bacterial Agents	Catheterization, Central Venous	8	Undirected
Anti-Bacterial Agents	education	8	Undirected
Anti-Bacterial Agents	randomized controlled trial	8	Undirected
Anti-Bacterial Agents	university hospital	8	Undirected
Anxiety	Focus Groups	8	Undirected
Anxiety	Glasgow coma scale	8	Undirected
Anxiety	adverse event	8	Undirected
Anxiety	computer assisted tomography	8	Undirected
Anxiety	long term care	8	Undirected
Anxiety	teaching hospital	8	Undirected
Anxiety	thorax radiography	8	Undirected
Artificial Ventilation	Adaptation, Psychological	8	Undirected
Attitude of Health Personnel	Postoperative Complications	8	Undirected
Attitude of Health Personnel	endotracheal intubation	8	Undirected
Attitude of Health Personnel	morphine	8	Undirected
Australia	Cardiac Surgical Procedures	8	Undirected
Australia	Intubation, Intratracheal	8	Undirected
Australia	Mothers	8	Undirected
Australia	Pain Measurement	8	Undirected
Australia	health care planning	8	Undirected
Australia	hospital readmission	8	Undirected
Australia	intensivist	8	Undirected
Australia	palliative therapy	8	Undirected
Australia	physiotherapy	8	Undirected
Australia	pneumonia	8	Undirected
Australia	preschool child	8	Undirected
Australia	therapy	8	Undirected
Breast Feeding	knowledge	8	Undirected
Burnout, Professional	Canada	8	Undirected
Burnout, Professional	age	8	Undirected
Burnout, Professional	health care delivery	8	Undirected
Burnout, Professional	interpersonal communication	8	Undirected
Burnout, Professional	standards	8	Undirected
Burnout, Professional	thematic analysis	8	Undirected
COVID-19	antiinfective agent	8	Undirected
COVID-19	microbiology	8	Undirected
Canada	antibiotic agent	8	Undirected

Canada	health care facility	8	Undirected
Canada	hospital personnel	8	Undirected
Canada	intensivist	8	Undirected
Canada	pain assessment	8	Undirected
Canada	palliative therapy	8	Undirected
Canada	pilot study	8	Undirected
Canada	pneumonia	8	Undirected
Cardiac Surgical Procedures	sedation	8	Undirected
Catheter-Related Infections	cohort analysis	8	Undirected
Catheter-Related Infections	follow up	8	Undirected
Catheter-Related Infections	statistics and numerical data	8	Undirected
Catheter-Related Infections	university hospital	8	Undirected
Catheter-Related Infections	very elderly	8	Undirected
Catheterization, Central Venous	COVID-19	8	Undirected
Catheterization, Central Venous	cohort analysis	8	Undirected
Catheterization, Central Venous	follow up	8	Undirected
Catheterization, Central Venous	hand washing	8	Undirected
Catheterization, Central Venous	multicenter study	8	Undirected
Catheterization, Central Venous	very elderly	8	Undirected
Child, Preschool	Insulin	8	Undirected
Child, Preschool	Telemedicine	8	Undirected
Child, Preschool	evidence based practice	8	Undirected
Child, Preschool	hospital infection	8	Undirected
Child, Preschool	information processing	8	Undirected
Child, Preschool	long term care	8	Undirected
Child, Preschool	oxygen saturation	8	Undirected
Child, Preschool	simulation	8	Undirected
China	Anti-Bacterial Agents	8	Undirected
China	antibiotic agent	8	Undirected
China	hypertension	8	Undirected
China	meta analysis	8	Undirected
China	reliability	8	Undirected
China	teaching hospital	8	Undirected
China	thematic analysis	8	Undirected
Clinical Competence	Breast Feeding	8	Undirected
Clinical Competence	Fatigue	8	Undirected
Clinical Competence	analgesia	8	Undirected
Clinical Competence	epidemiology	8	Undirected
Clinical Competence	health care cost	8	Undirected
Clinical Competence	health care policy	8	Undirected
Clinical Competence	heart surgery	8	Undirected
Clinical Competence	long term care	8	Undirected
Clinical Competence	preschool child	8	Undirected
Clinical Protocols	Health Personnel	8	Undirected
Clinical Protocols	acute kidney failure	8	Undirected
Clinical Protocols	caregiver	8	Undirected
Clinical Protocols	heart rate	8	Undirected
Clinical Protocols	interview	8	Undirected
Communication	Fatigue	8	Undirected
Communication	Hospital Mortality	8	Undirected
Cooperative Behavior	Canada	8	Undirected
Cooperative Behavior	Health Personnel	8	Undirected
Cooperative Behavior	Pregnancy	8	Undirected
Cooperative Behavior	Stress, Psychological	8	Undirected

Cooperative Behavior	health care delivery	8	Undirected
Cooperative Behavior	information processing	8	Undirected
Cross Infection	Cooperative Behavior	8	Undirected
Cross Infection	Family	8	Undirected
Cross Infection	Intubation, Intratracheal	8	Undirected
Cross Infection	Leadership	8	Undirected
Cross Infection	Pressure Ulcer	8	Undirected
Cross Infection	Quality of Health Care	8	Undirected
Cross Infection	Sensitivity and Specificity	8	Undirected
Cross Infection	interview	8	Undirected
Cross Infection	prematurity	8	Undirected
Cross Infection	thorax radiography	8	Undirected
Decision Making	Medication Errors	8	Undirected
Decision Making	OUTCOMES	8	Undirected
Decision Making	acute kidney failure	8	Undirected
Decision Making	adverse event	8	Undirected
Decision Making	age	8	Undirected
Decision Making	endotracheal intubation	8	Undirected
Delirium	emergency health service	8	Undirected
Delirium	endotracheal intubation	8	Undirected
Delirium	health care planning	8	Undirected
Delirium	psychological aspect	8	Undirected
Delirium	resuscitation	8	Undirected
Electronic Health Records	Catheter-Related Infections	8	Undirected
Electronic Health Records	epidemiology	8	Undirected
Emergency Service, Hospital	Fatigue	8	Undirected
Emergency Service, Hospital	Pain	8	Undirected
Emergency Service, Hospital	burnout	8	Undirected
Emergency Service, Hospital	simulation	8	Undirected
Enteral Nutrition	Canada	8	Undirected
Enteral Nutrition	Intubation, Intratracheal	8	Undirected
Enteral Nutrition	Mothers	8	Undirected
Enteral Nutrition	Sensitivity and Specificity	8	Undirected
Enteral Nutrition	adverse event	8	Undirected
Enteral Nutrition	attitude	8	Undirected
Enteral Nutrition	hospital discharge	8	Undirected
Enteral Nutrition	observational study	8	Undirected
Enteral Nutrition	pilot study	8	Undirected
Family	Breast Feeding	8	Undirected
Family	OUTCOMES	8	Undirected
Family	cerebrovascular accident	8	Undirected
Family	hospital readmission	8	Undirected
Fatigue	breathing rate	8	Undirected
Fatigue	health care delivery	8	Undirected
Fatigue	home care	8	Undirected
Fatigue	meta analysis	8	Undirected
Fatigue	organization and management	8	Undirected
Fatigue	semi structured interview	8	Undirected
Fatigue	therapy	8	Undirected
Focus Groups	Clinical Protocols	8	Undirected
Focus Groups	Infection Control	8	Undirected
Focus Groups	emergency ward	8	Undirected
Focus Groups	medical education	8	Undirected
Focus Groups	pharmacist	8	Undirected

Glasgow coma scale	long term care	8	Undirected
Glasgow coma scale	pilot study	8	Undirected
Glasgow coma scale	semi structured interview	8	Undirected
Glasgow coma scale	standards	8	Undirected
Glasgow coma scale	therapy	8	Undirected
Guideline Adherence	Cardiac Surgical Procedures	8	Undirected
Guideline Adherence	Personnel Staffing and Scheduling	8	Undirected
Guideline Adherence	Workplace	8	Undirected
Guideline Adherence	antiinfective agent	8	Undirected
Guideline Adherence	emergency health service	8	Undirected
Guideline Adherence	hospital discharge	8	Undirected
Guideline Adherence	safety	8	Undirected
alth Knowledge, Attitudes, Practi	Personnel Staffing and Scheduling	8	Undirected
alth Knowledge, Attitudes, Practi	emergency health service	8	Undirected
alth Knowledge, Attitudes, Practi	health care delivery	8	Undirected
alth Knowledge, Attitudes, Practi	pain assessment	8	Undirected
alth Knowledge, Attitudes, Practi	reliability	8	Undirected
alth Knowledge, Attitudes, Practi	semi structured interview	8	Undirected
Health Services Needs and Demand	Burnout, Professional	8	Undirected
Health Services Needs and Demand	Pilot Projects	8	Undirected
Health Services Needs and Demand	evidence based practice	8	Undirected
Health Services Needs and Demand	health care delivery	8	Undirected
Health Services Needs and Demand	home care	8	Undirected
Heart Arrest	Canada	8	Undirected
Heart Arrest	Medication Errors	8	Undirected
Heart Arrest	Pilot Projects	8	Undirected
Heart Arrest	antibiotic agent	8	Undirected
Heart Arrest	health care delivery	8	Undirected
Heart Arrest	pharmacist	8	Undirected
Heart Arrest	thorax radiography	8	Undirected
Hospital Mortality	Anxiety	8	Undirected
Hospital Mortality	Fatigue	8	Undirected
Hospital Mortality	blood pressure	8	Undirected
Hospital Mortality	caregiver	8	Undirected
Hospital Mortality	depression	8	Undirected
Hospital Mortality	heart surgery	8	Undirected
Hospital Mortality	prematurity	8	Undirected
Hypnotics and Sedatives	adverse event	8	Undirected
Hypnotics and Sedatives	blood pressure	8	Undirected
Hypnotics and Sedatives	cohort analysis	8	Undirected
Hypnotics and Sedatives	hypotension	8	Undirected
Hypnotics and Sedatives	organization and management	8	Undirected
Hypnotics and Sedatives	safety	8	Undirected
Hypnotics and Sedatives	statistics and numerical data	8	Undirected
Infant, Premature	adverse event	8	Undirected
Infant, Premature	interpersonal communication	8	Undirected
Infant, Premature	university hospital	8	Undirected
Infection Control	Cardiac Surgical Procedures	8	Undirected
Infection Control	Sensitivity and Specificity	8	Undirected
Infection Control	psychological aspect	8	Undirected
Insulin	Quality Improvement	8	Undirected
Insulin	education	8	Undirected
Insulin	fever	8	Undirected
Insulin	health care cost	8	Undirected

Insulin	hospital personnel	8	Undirected
Insulin	pediatrics	8	Undirected
Insulin	pilot study	8	Undirected
Insulin	teaching hospital	8	Undirected
Interprofessional Relations	Algorithms	8	Undirected
Interprofessional Relations	Medication Errors	8	Undirected
Interprofessional Relations	health care delivery	8	Undirected
Interprofessional Relations	hospital personnel	8	Undirected
Interprofessional Relations	information processing	8	Undirected
Interprofessional Relations	intensivist	8	Undirected
Interprofessional Relations	knowledge	8	Undirected
Interprofessional Relations	pediatrics	8	Undirected
Interprofessional Relations	respiratory therapist	8	Undirected
Intubation, Intratracheal	COVID-19	8	Undirected
Intubation, Intratracheal	Glasgow coma scale	8	Undirected
Intubation, Intratracheal	education	8	Undirected
Intubation, Intratracheal	fentanyl	8	Undirected
Intubation, Intratracheal	gestational age	8	Undirected
Intubation, Intratracheal	midazolam	8	Undirected
Intubation, Intratracheal	mortality	8	Undirected
Job Satisfaction	evidence based practice	8	Undirected
Job Satisfaction	follow up	8	Undirected
Job Satisfaction	health care policy	8	Undirected
Job Satisfaction	information processing	8	Undirected
Job Satisfaction	pediatrics	8	Undirected
Job Satisfaction	practice guideline	8	Undirected
Job Satisfaction	respiratory therapist	8	Undirected
Job Satisfaction	safety	8	Undirected
Job Satisfaction	teaching hospital	8	Undirected
Leadership	China	8	Undirected
Leadership	Medication Errors	8	Undirected
Leadership	Telemedicine	8	Undirected
Leadership	cohort analysis	8	Undirected
Length of Stay	attitude	8	Undirected
Length of Stay	ethics	8	Undirected
Logistic Models	Burnout, Professional	8	Undirected
Logistic Models	Program Evaluation	8	Undirected
Logistic Models	Reproducibility of Results	8	Undirected
Logistic Models	Terminal Care	8	Undirected
Logistic Models	emergency health service	8	Undirected
Logistic Models	hand washing	8	Undirected
Logistic Models	methodology	8	Undirected
Logistic Models	pediatrics	8	Undirected
Logistic Models	practice guideline	8	Undirected
Logistic Models	preschool child	8	Undirected
Medication Errors	epidemiology	8	Undirected
Medication Errors	hospital readmission	8	Undirected
Medication Errors	pilot study	8	Undirected
Monitoring, Physiologic	Anxiety	8	Undirected
Monitoring, Physiologic	Australia	8	Undirected
Monitoring, Physiologic	Family	8	Undirected
Monitoring, Physiologic	Focus Groups	8	Undirected
Monitoring, Physiologic	Needs Assessment	8	Undirected
Monitoring, Physiologic	cohort analysis	8	Undirected

Monitoring, Physiologic	education	8	Undirected
Monitoring, Physiologic	medical education	8	Undirected
Monitoring, Physiologic	observational study	8	Undirected
Monitoring, Physiologic	prevalence	8	Undirected
Monitoring, Physiologic	statistics and numerical data	8	Undirected
Mothers	thematic analysis	8	Undirected
Needs Assessment	clinical practice	8	Undirected
Needs Assessment	health care facility	8	Undirected
Needs Assessment	knowledge	8	Undirected
Needs Assessment	multicenter study	8	Undirected
Needs Assessment	very elderly	8	Undirected
OUTCOMES	Clinical Protocols	8	Undirected
OUTCOMES	cerebrovascular accident	8	Undirected
OUTCOMES	clinical practice	8	Undirected
OUTCOMES	dementia	8	Undirected
OUTCOMES	diabetes mellitus	8	Undirected
OUTCOMES	organization and management	8	Undirected
OUTCOMES	preschool child	8	Undirected
OUTCOMES	sedation	8	Undirected
OUTCOMES	simulation	8	Undirected
OUTCOMES	university hospital	8	Undirected
Pain	Cardiac Surgical Procedures	8	Undirected
Pain	Heart Arrest	8	Undirected
Pain	Infection Control	8	Undirected
Pain	cerebrovascular accident	8	Undirected
Pain	computer assisted tomography	8	Undirected
Pain	emergency care	8	Undirected
Pain	pharmacist	8	Undirected
Pain Measurement	adverse event	8	Undirected
Pain Measurement	endotracheal intubation	8	Undirected
Pain Measurement	medical staff	8	Undirected
Palliative Care	Burnout, Professional	8	Undirected
Palliative Care	Electronic Health Records	8	Undirected
Palliative Care	Program Evaluation	8	Undirected
Palliative Care	gestational age	8	Undirected
Palliative Care	pneumonia	8	Undirected
Pandemics	heart surgery	8	Undirected
Parents	Interprofessional Relations	8	Undirected
Parents	attitude	8	Undirected
Parents	health care planning	8	Undirected
Parents	sedation	8	Undirected
Personnel Staffing and Scheduling	Postoperative Complications	8	Undirected
Personnel Staffing and Scheduling	perception	8	Undirected
Personnel Staffing and Scheduling	pharmacist	8	Undirected
Pilot Projects	COVID-19	8	Undirected
Pilot Projects	Electronic Health Records	8	Undirected
Pilot Projects	Medication Errors	8	Undirected
Pilot Projects	blood pressure	8	Undirected
Pilot Projects	diabetes mellitus	8	Undirected
Pilot Projects	knowledge	8	Undirected
Pilot Projects	midazolam	8	Undirected
Pilot Projects	practice guideline	8	Undirected
Postoperative Care	Hypnotics and Sedatives	8	Undirected
Postoperative Care	Pressure Ulcer	8	Undirected

Postoperative Care	disease severity	8	Undirected
Postoperative Care	enteric feeding	8	Undirected
Postoperative Care	hospital infection	8	Undirected
Postoperative Care	multicenter study	8	Undirected
Postoperative Care	preschool child	8	Undirected
Postoperative Complications	Intubation, Intratracheal	8	Undirected
Postoperative Complications	Pilot Projects	8	Undirected
Postoperative Complications	chronic obstructive lung disease	8	Undirected
Postoperative Complications	health care delivery	8	Undirected
Postoperative Complications	long term care	8	Undirected
Postoperative Complications	mental health	8	Undirected
Postoperative Complications	pilot study	8	Undirected
Practice Guidelines as Topic	Electronic Health Records	8	Undirected
Practice Guidelines as Topic	Mothers	8	Undirected
Practice Guidelines as Topic	blood pressure	8	Undirected
Practice Guidelines as Topic	extracorporeal oxygenation	8	Undirected
Practice Guidelines as Topic	heart rate	8	Undirected
Practice Guidelines as Topic	long term care	8	Undirected
Pregnancy	Algorithms	8	Undirected
Pregnancy	China	8	Undirected
Pregnancy	Terminal Care	8	Undirected
Pregnancy	Workload	8	Undirected
Pregnancy	analgesia	8	Undirected
Pregnancy	attitude	8	Undirected
Pregnancy	health care facility	8	Undirected
Pregnancy	health care policy	8	Undirected
Pregnancy	interpersonal communication	8	Undirected
Pregnancy	safety	8	Undirected
Pressure Ulcer	Postoperative Complications	8	Undirected
Pressure Ulcer	follow up	8	Undirected
Pressure Ulcer	perception	8	Undirected
Pressure Ulcer	pilot study	8	Undirected
Pressure Ulcer	psychology	8	Undirected
Professional-Family Relations	Burnout, Professional	8	Undirected
Professional-Family Relations	cohort analysis	8	Undirected
Professional-Family Relations	gestational age	8	Undirected
Professional-Family Relations	health care delivery	8	Undirected
Professional-Family Relations	medical education	8	Undirected
Program Evaluation	Canada	8	Undirected
Program Evaluation	Pressure Ulcer	8	Undirected
Program Evaluation	Stroke	8	Undirected
Program Evaluation	home care	8	Undirected
Program Evaluation	hospital personnel	8	Undirected
Program Evaluation	multicenter study	8	Undirected
Program Evaluation	perception	8	Undirected
Program Evaluation	pharmacist	8	Undirected
Program Evaluation	tertiary care center	8	Undirected
Quality Improvement	Fatigue	8	Undirected
Quality Improvement	Mothers	8	Undirected
Quality Improvement	Stroke	8	Undirected
Quality Improvement	extracorporeal oxygenation	8	Undirected
Quality Improvement	hypertension	8	Undirected
Quality of Health Care	Cardiac Surgical Procedures	8	Undirected
Quality of Health Care	Electronic Health Records	8	Undirected

Quality of Health Care	Fatigue	8	Undirected
Quality of Health Care	Medication Errors	8	Undirected
Quality of Health Care	Mothers	8	Undirected
Quality of Health Care	Postoperative Complications	8	Undirected
Quality of Health Care	health care facility	8	Undirected
Quality of Health Care	health care system	8	Undirected
Quality of Health Care	observational study	8	Undirected
Reproducibility of Results	Catheterization, Central Venous	8	Undirected
Reproducibility of Results	Pandemics	8	Undirected
Reproducibility of Results	oxygen saturation	8	Undirected
Risk Assessment	Mothers	8	Undirected
Risk Assessment	attitude	8	Undirected
Risk Assessment	midazolam	8	Undirected
Risk Assessment	pain assessment	8	Undirected
Risk Assessment	vancomycin	8	Undirected
Safety Management	Algorithms	8	Undirected
Safety Management	Focus Groups	8	Undirected
Safety Management	adverse event	8	Undirected
Safety Management	clinical practice	8	Undirected
Safety Management	evidence based practice	8	Undirected
Safety Management	teaching hospital	8	Undirected
Sensitivity and Specificity	Intubation, Intratracheal	8	Undirected
Sensitivity and Specificity	Pandemics	8	Undirected
Sensitivity and Specificity	age	8	Undirected
Sensitivity and Specificity	cerebrovascular accident	8	Undirected
Sensitivity and Specificity	computer assisted tomography	8	Undirected
Sensitivity and Specificity	dyspnea	8	Undirected
Sensitivity and Specificity	emergency care	8	Undirected
Sensitivity and Specificity	endotracheal intubation	8	Undirected
Sensitivity and Specificity	thorax radiography	8	Undirected
Sepsis	Breast Feeding	8	Undirected
Sepsis	Leadership	8	Undirected
Sepsis	Needs Assessment	8	Undirected
Sepsis	Personnel Staffing and Scheduling	8	Undirected
Sepsis	Pressure Ulcer	8	Undirected
Sepsis	Safety Management	8	Undirected
Sepsis	Workload	8	Undirected
Sepsis	midazolam	8	Undirected
Social Support	Algorithms	8	Undirected
Social Support	Quality Improvement	8	Undirected
Social Support	analgesia	8	Undirected
Social Support	cerebrovascular accident	8	Undirected
Social Support	evidence based practice	8	Undirected
Social Support	observational study	8	Undirected
Social Support	outcome assessment	8	Undirected
Social Support	standards	8	Undirected
Social Support	university hospital	8	Undirected
Stress, Psychological	clinical practice	8	Undirected
Stress, Psychological	health care delivery	8	Undirected
Stress, Psychological	hospital personnel	8	Undirected
Stress, Psychological	information processing	8	Undirected
Stress, Psychological	practice guideline	8	Undirected
Stress, Psychological	preschool child	8	Undirected
Stress, Psychological	resuscitation	8	Undirected

Stress, Psychological	very elderly	8	Undirected
Stroke	emergency care	8	Undirected
Stroke	observational study	8	Undirected
Stroke	standards	8	Undirected
Telemedicine	Health Personnel	8	Undirected
Telemedicine	multicenter study	8	Undirected
Terminal Care	computer assisted tomography	8	Undirected
Terminal Care	extracorporeal oxygenation	8	Undirected
Terminal Care	propofol	8	Undirected
Time Factors	Leadership	8	Undirected
Time Factors	Telemedicine	8	Undirected
Time Factors	dementia	8	Undirected
Time Factors	hand washing	8	Undirected
Time Factors	pharmacist	8	Undirected
Time Factors	teaching hospital	8	Undirected
Treatment Outcome	Adaptation, Psychological	8	Undirected
Treatment Outcome	Burnout, Professional	8	Undirected
United Kingdom	Pain Measurement	8	Undirected
United Kingdom	Postoperative Complications	8	Undirected
United Kingdom	long term care	8	Undirected
United Kingdom	microbiology	8	Undirected
United Kingdom	therapy	8	Undirected
Workforce	Clinical Protocols	8	Undirected
Workforce	Monitoring, Physiologic	8	Undirected
Workforce	OUTCOMES	8	Undirected
Workforce	Postoperative Care	8	Undirected
Workforce	caregiver	8	Undirected
Workforce	health care policy	8	Undirected
Workforce	health care quality	8	Undirected
Workforce	hospital admission	8	Undirected
Workforce	intensivist	8	Undirected
Workforce	outcome assessment	8	Undirected
Workforce	respiratory therapist	8	Undirected
Workforce	resuscitation	8	Undirected
Workforce	thematic analysis	8	Undirected
Workload	attitude	8	Undirected
Workload	hospital infection	8	Undirected
Workload	long term care	8	Undirected
Workload	pilot study	8	Undirected
Workload	pneumonia	8	Undirected
Workload	simulation	8	Undirected
Workload	therapy	8	Undirected
Workload	very elderly	8	Undirected
Workplace	Canada	8	Undirected
Workplace	evidence based practice	8	Undirected
Workplace	outcome assessment	8	Undirected
Workplace	semi structured interview	8	Undirected
adverse event	age	8	Undirected
adverse event	statistics and numerical data	8	Undirected
age	antiinfective agent	8	Undirected
age	health care system	8	Undirected
age	hospital infection	8	Undirected
age	hospital personnel	8	Undirected
age	standards	8	Undirected

analgesia	Health Personnel	8	Undirected
analgesia	depression	8	Undirected
analgesia	emergency health service	8	Undirected
analgesia	epidemiology	8	Undirected
analgesia	health care delivery	8	Undirected
antibiotic agent	university hospital	8	Undirected
antiinfective agent	health care policy	8	Undirected
attitude	Pandemics	8	Undirected
attitude	epidemiology	8	Undirected
attitude	health care facility	8	Undirected
attitude	hospital discharge	8	Undirected
attitude	information processing	8	Undirected
attitude	medical staff	8	Undirected
attitude	mortality	8	Undirected
attitude	respiratory therapist	8	Undirected
attitude	sedation	8	Undirected
attitude to health	Algorithms	8	Undirected
attitude to health	Monitoring, Physiologic	8	Undirected
attitude to health	Sepsis	8	Undirected
attitude to health	antibiotic agent	8	Undirected
attitude to health	health care policy	8	Undirected
attitude to health	long term care	8	Undirected
attitude to health	therapy	8	Undirected
blood pressure	clinical practice	8	Undirected
blood pressure	emergency care	8	Undirected
blood pressure	emergency health service	8	Undirected
blood pressure	heart surgery	8	Undirected
blood pressure	mental health	8	Undirected
blood pressure	multicenter study	8	Undirected
burnout	disease severity	8	Undirected
burnout	evidence based practice	8	Undirected
burnout	health care facility	8	Undirected
burnout	knowledge	8	Undirected
caregiver	Medication Errors	8	Undirected
caregiver	adverse event	8	Undirected
caregiver	computer assisted tomography	8	Undirected
caregiver	hospital infection	8	Undirected
caregiver	statistics and numerical data	8	Undirected
clinical practice	acute kidney failure	8	Undirected
cohort analysis	psychological aspect	8	Undirected
dementia	antibiotic agent	8	Undirected
dementia	health care planning	8	Undirected
dementia	health care system	8	Undirected
dementia	physiotherapy	8	Undirected
dementia	statistics and numerical data	8	Undirected
dementia	urinary tract infection	8	Undirected
depression	physiotherapy	8	Undirected
diabetes mellitus	adverse event	8	Undirected
diabetes mellitus	health care planning	8	Undirected
diabetes mellitus	hospital infection	8	Undirected
diabetes mellitus	medical education	8	Undirected
diabetes mellitus	statistics and numerical data	8	Undirected
dyspnea	antiinfective agent	8	Undirected
dyspnea	pain assessment	8	Undirected

dyspnea	physiotherapy	8	Undirected
education	antibiotic agent	8	Undirected
education	fever	8	Undirected
education	hospital readmission	8	Undirected
education	hypertension	8	Undirected
endotracheal intubation	enteric feeding	8	Undirected
endotracheal intubation	university hospital	8	Undirected
enteric feeding	computer assisted tomography	8	Undirected
enteric feeding	extracorporeal oxygenation	8	Undirected
ethics	Delirium	8	Undirected
ethics	attitude	8	Undirected
ethics	clinical article	8	Undirected
ethics	gestational age	8	Undirected
ethics	health care planning	8	Undirected
ethics	randomized controlled trial	8	Undirected
evidence based practice	adverse event	8	Undirected
evidence based practice	attitude	8	Undirected
evidence based practice	health care facility	8	Undirected
evidence based practice	intensivist	8	Undirected
evidence based practice	long term care	8	Undirected
fentanyl	COVID-19	8	Undirected
fentanyl	hypertension	8	Undirected
fentanyl	palliative therapy	8	Undirected
fentanyl	pilot study	8	Undirected
fever	health care policy	8	Undirected
fever	hospital personnel	8	Undirected
fever	mental health	8	Undirected
fever	meta analysis	8	Undirected
fever	respiratory therapist	8	Undirected
gestational age	Pilot Projects	8	Undirected
gestational age	age	8	Undirected
gestational age	depression	8	Undirected
gestational age	fever	8	Undirected
hand washing	disease severity	8	Undirected
hand washing	health care policy	8	Undirected
health care cost	Algorithms	8	Undirected
health care cost	Cardiac Surgical Procedures	8	Undirected
health care cost	China	8	Undirected
health care cost	Fatigue	8	Undirected
health care cost	Health Personnel	8	Undirected
health care cost	analgesia	8	Undirected
health care cost	endotracheal intubation	8	Undirected
health care cost	mental health	8	Undirected
health care cost	meta analysis	8	Undirected
health care cost	microbiology	8	Undirected
health care cost	morphine	8	Undirected
health care cost	propofol	8	Undirected
health care cost	sedation	8	Undirected
health care cost	teaching hospital	8	Undirected
health care delivery	antiinfective agent	8	Undirected
health care planning	clinical article	8	Undirected
health care planning	extracorporeal oxygenation	8	Undirected
health care planning	information processing	8	Undirected
health care planning	preschool child	8	Undirected

health care system	breathing rate	8	Undirected
heart failure	Clinical Protocols	8	Undirected
heart failure	OUTCOMES	8	Undirected
heart failure	Pain	8	Undirected
heart failure	Telemedicine	8	Undirected
heart failure	computer assisted tomography	8	Undirected
heart failure	health care delivery	8	Undirected
heart failure	medical education	8	Undirected
heart failure	meta analysis	8	Undirected
heart failure	psychological aspect	8	Undirected
heart rate	Stroke	8	Undirected
heart rate	information processing	8	Undirected
heart rate	university hospital	8	Undirected
heart surgery	health care system	8	Undirected
heart surgery	statistics and numerical data	8	Undirected
home care	enteric feeding	8	Undirected
home care	statistics and numerical data	8	Undirected
hospital infection	organization and management	8	Undirected
hypertension	enteric feeding	8	Undirected
hypertension	health care policy	8	Undirected
hypertension	hospital infection	8	Undirected
hypertension	methodology	8	Undirected
hypotension	age	8	Undirected
hypotension	emergency care	8	Undirected
hypotension	extracorporeal oxygenation	8	Undirected
hypotension	health care system	8	Undirected
information processing	preschool child	8	Undirected
intensivist	acute kidney failure	8	Undirected
intensivist	preschool child	8	Undirected
intensivist	thorax radiography	8	Undirected
interview	Medication Errors	8	Undirected
interview	disease severity	8	Undirected
interview	microbiology	8	Undirected
interview	safety	8	Undirected
knowledge	Fatigue	8	Undirected
knowledge	enteric feeding	8	Undirected
knowledge	hospital admission	8	Undirected
knowledge	hospital discharge	8	Undirected
knowledge	mental health	8	Undirected
knowledge	pain assessment	8	Undirected
long term care	midazolam	8	Undirected
long term care	pharmacist	8	Undirected
long term care	preschool child	8	Undirected
long term care	university hospital	8	Undirected
medical education	age	8	Undirected
medical education	antiinfective agent	8	Undirected
medical education	hospital readmission	8	Undirected
medical education	thorax radiography	8	Undirected
medical staff	computer assisted tomography	8	Undirected
medical staff	depression	8	Undirected
medical staff	hospital readmission	8	Undirected
medical staff	pain assessment	8	Undirected
medical staff	reliability	8	Undirected
medical staff	therapy	8	Undirected

medical staff	thorax radiography	8	Undirected
mental health	university hospital	8	Undirected
meta analysis	acute kidney failure	8	Undirected
meta analysis	antiinfective agent	8	Undirected
meta analysis	enteric feeding	8	Undirected
meta analysis	health care system	8	Undirected
cillin resistant Staphylococcus a	breathing rate	8	Undirected
cillin resistant Staphylococcus a	epidemiology	8	Undirected
methodology	health care policy	8	Undirected
microbiology	health care facility	8	Undirected
midazolam	adverse event	8	Undirected
midazolam	methodology	8	Undirected
midazolam	organization and management	8	Undirected
midazolam	pilot study	8	Undirected
midazolam	university hospital	8	Undirected
midazolam	vancomycin	8	Undirected
morphine	computer assisted tomography	8	Undirected
morphine	emergency ward	8	Undirected
morphine	health care policy	8	Undirected
morphine	organization and management	8	Undirected
morphine	pilot study	8	Undirected
morphine	vancomycin	8	Undirected
mortality	pilot study	8	Undirected
mortality	semi structured interview	8	Undirected
mortality	simulation	8	Undirected
observational study	psychological aspect	8	Undirected
observational study	semi structured interview	8	Undirected
observational study	thematic analysis	8	Undirected
organization and management	breathing rate	8	Undirected
outcome assessment	Intubation, Intratracheal	8	Undirected
pain assessment	dementia	8	Undirected
pain assessment	epidemiology	8	Undirected
pain assessment	psychological aspect	8	Undirected
pediatrics	Catheterization, Central Venous	8	Undirected
pediatrics	hypotension	8	Undirected
pediatrics	pneumonia	8	Undirected
pediatrics	propofol	8	Undirected
pediatrics	urinary tract infection	8	Undirected
pediatrics	vancomycin	8	Undirected
perception	Telemedicine	8	Undirected
perception	adverse event	8	Undirected
perception	emergency health service	8	Undirected
perception	gestational age	8	Undirected
perception	physiotherapy	8	Undirected
perception	sedation	8	Undirected
perception	very elderly	8	Undirected
pharmacist	extracorporeal oxygenation	8	Undirected
pharmacist	fentanyl	8	Undirected
pharmacist	physiotherapy	8	Undirected
pharmacist	simulation	8	Undirected
physiotherapy	breathing rate	8	Undirected
physiotherapy	cerebrovascular accident	8	Undirected
physiotherapy	health care planning	8	Undirected
physiotherapy	interpersonal communication	8	Undirected

pilot study	breathing rate	8	Undirected
pilot study	endotracheal intubation	8	Undirected
pilot study	palliative therapy	8	Undirected
pneumonia	information processing	8	Undirected
pneumonia	therapy	8	Undirected
prematurity	Needs Assessment	8	Undirected
prematurity	Sensitivity and Specificity	8	Undirected
prematurity	age	8	Undirected
prematurity	hand washing	8	Undirected
prematurity	hypertension	8	Undirected
prematurity	simulation	8	Undirected
preschool child	acute kidney failure	8	Undirected
preschool child	breathing rate	8	Undirected
prevalence	fentanyl	8	Undirected
prevalence	propofol	8	Undirected
prevalence	respiratory therapist	8	Undirected
prevalence	semi structured interview	8	Undirected
propofol	health care system	8	Undirected
propofol	medical education	8	Undirected
propofol	physiotherapy	8	Undirected
propofol	pilot study	8	Undirected
propofol	thorax radiography	8	Undirected
propofol	vancomycin	8	Undirected
psychological aspect	chronic obstructive lung disease	8	Undirected
psychology	antibiotic agent	8	Undirected
psychology	hospital infection	8	Undirected
quality of life	Focus Groups	8	Undirected
quality of life	Infant, Premature	8	Undirected
quality of life	Interprofessional Relations	8	Undirected
quality of life	Stroke	8	Undirected
quality of life	blood pressure	8	Undirected
quality of life	fentanyl	8	Undirected
quality of life	hand washing	8	Undirected
reliability	disease severity	8	Undirected
reliability	interpersonal communication	8	Undirected
reliability	organization and management	8	Undirected
respiratory therapist	health care system	8	Undirected
resuscitation	Electronic Health Records	8	Undirected
safety	hand washing	8	Undirected
safety	health care planning	8	Undirected
safety	health care system	8	Undirected
safety	oxygen saturation	8	Undirected
safety	ventilator associated pneumonia	8	Undirected
sedation	antibiotic agent	8	Undirected
sedation	health care planning	8	Undirected
sedation	very elderly	8	Undirected
semi structured interview	disease severity	8	Undirected
semi structured interview	physiotherapy	8	Undirected
simulation	hospital personnel	8	Undirected
simulation	information processing	8	Undirected
simulation	multicenter study	8	Undirected
simulation	preschool child	8	Undirected
simulation	semi structured interview	8	Undirected
standards	acute kidney failure	8	Undirected

standards	health care system	8	Undirected
standards	hospital infection	8	Undirected
teaching hospital	health care system	8	Undirected
teaching hospital	heart surgery	8	Undirected
tertiary care center	Focus Groups	8	Undirected
tertiary care center	Needs Assessment	8	Undirected
tertiary care center	Pain Measurement	8	Undirected
tertiary care center	Pilot Projects	8	Undirected
thematic analysis	very elderly	8	Undirected
total quality management	Reproducibility of Results	8	Undirected
total quality management	Sensitivity and Specificity	8	Undirected
total quality management	Stroke	8	Undirected
total quality management	computer assisted tomography	8	Undirected
total quality management	pain assessment	8	Undirected
tracheostomy	Health Personnel	8	Undirected
tracheostomy	depression	8	Undirected
tracheostomy	fentanyl	8	Undirected
tracheostomy	hospital infection	8	Undirected
tracheostomy	meta analysis	8	Undirected
tracheostomy	midazolam	8	Undirected
tracheostomy	statistics and numerical data	8	Undirected
university hospital	acute kidney failure	8	Undirected
university hospital	enteric feeding	8	Undirected
university hospital	extracorporeal oxygenation	8	Undirected
urinary tract infection	chronic obstructive lung disease	8	Undirected
urinary tract infection	clinical practice	8	Undirected
urinary tract infection	pilot study	8	Undirected
vancomycin	chronic obstructive lung disease	8	Undirected
vancomycin	endotracheal intubation	8	Undirected
vancomycin	enteric feeding	8	Undirected
vancomycin	oxygen saturation	8	Undirected
ventilator associated pneumonia	age	8	Undirected
ventilator associated pneumonia	cerebrovascular accident	8	Undirected
ventilator associated pneumonia	extracorporeal oxygenation	8	Undirected
ventilator associated pneumonia	oxygen saturation	8	Undirected
ventilator associated pneumonia	statistics and numerical data	8	Undirected
