# Supplementary material for: Global Research Trends in ICU Nursing Personnel: A Bibliometric Analysis (2001–2025)
Source: J Nurs Manag. 2026 Aug 29;2026:7601256. doi: 10.1155/jonm/7601256 (PMC13525486; doi:10.1155/jonm/7601256)
Supplement: Supplementary file 1 [file JONM-2026-7601256-s001.zip › 3.4 community_summary.pdf]

community	node_count	total_frequency	avg_weighted_degree	avg_pagerank
1	74	52367	4230.973	0.005655
0	71	58558	3775.2817	0.005085
2	32	19954	2928.7812	0.004182
3	17	8967	2473.1176	0.003686
4	6	4321	2795.5	0.004005

top_keywords
Length of Stay || Artificial Ventilation || hospitalization || Risk Factors || mortality || hospital admission || clinical article || Treatment Outcome || hospital discharge || Attitude of Health Personnel || COVID-19 || organization and management || psychology || Clinical Competence || Family || health care quality || education || Communication || practice guideline || Infection Control || Practice Guidelines as Topic || total quality management || standards || methodology || Cross Infection || Quality Improvement || Clinical Protocols || tertiary care center
Pain || Child, Preschool || pediatrics || analgesia || Reproducibility of Results || sedation || Pilot Projects || preschool child || Pain Measurement || pilot study
Infant, Premature || prematurity || Pregnancy || gestational age || Mothers || Breast
