# Supplementary material for: Global Research Trends in ICU Nursing Personnel: A Bibliometric Analysis (2001–2025)
Source: J Nurs Manag. 2026 Aug 29;2026:7601256. doi: 10.1155/jonm/7601256 (PMC13525486; doi:10.1155/jonm/7601256)
Supplement: Supplementary file 1 [file JONM-2026-7601256-s001.zip › 3.5 community_period_summary.pdf]

period	community	count
2001-2005	0	5614
2001-2005	1	3773
2001-2005	2	2145
2001-2005	3	873
2001-2005	4	413
2006-2010	0	7588
2006-2010	1	5390
2006-2010	2	3423
2006-2010	3	1120
2006-2010	4	519
2011-2015	0	8741
2011-2015	1	8307
2011-2015	2	3984
2011-2015	3	1687
2011-2015	4	786
2016-2020	0	15425
2016-2020	1	13965
2016-2020	2	5447
2016-2020	3	2503
2016-2020	4	1140
2021-2025	0	21190
2021-2025	1	20932
2021-2025	2	4955
2021-2025	3	2784
2021-2025	4	1463
