# Supplementary material for: Global Research Trends in ICU Nursing Personnel: A Bibliometric Analysis (2001–2025)
Source: J Nurs Manag. 2026 Aug 29;2026:7601256. doi: 10.1155/jonm/7601256 (PMC13525486; doi:10.1155/jonm/7601256)
Supplement: Supplementary file 1 [file JONM-2026-7601256-s001.zip › 3.6 keyword_merge_mapping_table.pdf]

keyword_raw_cleaned	keyword_merge_key_raw	keyword_merge_key_alias	keyword_merge_key_final	keyword_standard	rows	merge_rule
Article	article	article	article	Article	5637	kept
article	article	article	article	Article	4843	case_or_format
Infant, Newborn	infant newborn	infant newborn	infant newborn	Infant, Newborn	4593	kept
Surveys and Questionnaires	surveys and questionnaires	surveys and questionnaires	surveys and questionnaires	Surveys and Questionnaires	3928	kept
controlled study	controlled study	controlled study	controlled study	controlled study	3502	kept
United States	united states	united states	united states	United States	3400	kept
COVID-19	covid 19	covid 19	covid 19	COVID-19	3362	kept
major clinical study	major clinical study	major clinical study	major clinical study	major clinical study	3257	kept
Attitude of Health Personnel	attitude of health personnel	attitude of health personnel	attitude of health personnel	Attitude of Health Personnel	2975	kept
Cross-Sectional Studies	cross sectional studies	cross sectional studies	cross sectional study	Cross-Sectional Studies	2672	singular
Retrospective Studies	retrospective studies	retrospective studies	retrospective study	Retrospective Studies	2642	singular
priority journal	priority journal	priority journal	priority journal	priority journal	2413	kept
questionnaire	questionnaire	questionnaire	questionnaire	questionnaire	2352	kept
Prospective Studies	prospective studies	prospective studies	prospective study	Prospective Studies	2331	singular
Aged, 80 and over	aged 80 and over	aged 80 and over	aged 80 and over	Aged, 80 and over	2077	kept

health care personnel	health care personnel	health care personnel	health care personnel	health care personnel	2043	kept
Clinical Competence	clinical competence	clinical competence	clinical competence	Clinical Competence	1984	kept
newborn	newborn	newborn	newborn	newborn	1968	kept
Risk Factors	risk factors	risk factors	risk factor	Risk Factors	1869	singular
Respiration, Artificial	respiration artificial	artificial ventilation	artificial ventilation	Artificial Ventilation	1861	alias
procedures	procedures	procedures	procedure	procedures	1824	singular
hospitalization	hospitalization	hospitalization	hospitalization	hospitalization	1713	kept
Delirium	delirium	delirium	delirium	Delirium	1691	kept
artificial ventilation	artificial ventilation	artificial ventilation	artificial ventilation	Artificial Ventilation	1689	case_or_format
physician	physician	physician	physician	physician	1683	kept
length of stay	length of stay	length of stay	length of stay	Length of Stay	1637	case_or_format
practice guideline	practice guideline	practice guideline	practice guideline	practice guideline	1529	kept
cross-sectional study	cross sectional study	cross sectional study	cross sectional study	Cross-Sectional Studies	1527	case_or_format
retrospective study	retrospective study	retrospective study	retrospective study	Retrospective Studies	1509	case_or_format
Length of Stay	length of stay	length of stay	length of stay	Length of Stay	1498	kept
Communication	communication	communication	communication	Communication	1469	kept

risk factor	risk factor	risk factor	risk factor	Risk Factors	1464	case_or_format
organization and management	organization and management	organization and management	organization and management	organization and management	1447	kept
Family	family	family	family	Family	1349	kept
mortality	mortality	mortality	mortality	mortality	1349	kept
coronavirus disease 2019	coronavirus disease 2019	covid 19	covid 19	COVID-19	1345	alias
prospective study	prospective study	prospective study	prospective study	Prospective Studies	1336	case_or_format
Cross Infection	cross infection	cross infection	cross infection	Cross Infection	1307	kept
Practice Guidelines as Topic	practice guidelines as topic	practice guidelines as topic	practice guidelines as topic	Practice Guidelines as Topic	1280	kept
Infant, Premature	infant premature	infant premature	infant premature	Infant, Premature	1259	kept
psychology	psychology	psychology	psychology	psychology	1252	kept
hospital admission	hospital admission	hospital admission	hospital admission	hospital admission	1235	kept
clinical article	clinical article	clinical article	clinical article	clinical article	1202	kept
Treatment Outcome	treatment outcome	treatment outcome	treatment outcome	Treatment Outcome	1201	kept
Time Factors	time factors	time factors	time factor	Time Factors	1191	singular
education	education	education	education	education	1156	kept
Pandemics	pandemics	pandemics	pandemic	Pandemics	1125	singular

pandemic	pandemic	pandemic	pandemic	Pandemics	1122	case_or_format
Parents	parents	parents	parent	Parents	1119	singular
Monitoring, Physiologic	monitoring physiologic	monitoring physiologic	monitoring physiologic	Monitoring, Physiologic	1086	kept
Hospitalization	hospitalization	hospitalization	hospitalization	hospitalization	1065	case_or_format
methodology	methodology	methodology	methodology	methodology	1061	kept
outcome assessment	outcome assessment	outcome assessment	outcome assessment	outcome assessment	1059	kept
Terminal Care	terminal care	terminal care	terminal care	Terminal Care	1055	kept
Health Knowledge, Attitudes, Practice	health knowledge attitudes practice	health knowledge attitudes practice	health knowledge attitudes practice	Health Knowledge, Attitudes, Practice	1039	kept
health care quality	health care quality	health care quality	health care quality	health care quality	1037	kept
Stress, Psychological	stress psychological	stress psychological	stress psychological	Stress, Psychological	1037	kept
cohort analysis	cohort analysis	cohort analysis	cohort analysis	cohort analysis	1034	kept
Child, Preschool	child preschool	child preschool	child preschool	Child, Preschool	1032	kept
Professional-Family Relations	professional family relations	professional family relations	professional family relations	Professional-Family Relations	1023	kept
Health Personnel	health personnel	health personnel	health personnel	Health Personnel	1011	kept
SARS-CoV-2	sars cov 2	sars cov 2	sars cov 2	SARS-CoV-2	1007	kept
Pressure Ulcer	pressure ulcer	pressure ulcer	pressure ulcer	Pressure Ulcer	997	kept

hospital discharge	hospital discharge	hospital discharge	hospital discharge	hospital discharge	995	kept
randomized controlled trial	randomized controlled trial	randomized controlled trial	randomized controlled trial	randomized controlled trial	963	kept
Pain	pain	pain	pain	Pain	955	kept
Sepsis	sepsis	sepsis	sepsis	Sepsis	954	kept
Quality Improvement	quality improvement	quality improvement	quality improvement	Quality Improvement	938	kept
Australia	australia	australia	australia	Australia	922	kept
Burnout, Professional	burnout professional	burnout professional	burnout professional	Burnout, Professional	915	kept
Workload	workload	workload	workload	Workload	908	kept
emergency ward	emergency ward	emergency ward	emergency ward	emergency ward	898	kept
resuscitation	resuscitation	resuscitation	resuscitation	resuscitation	892	kept
United Kingdom	united kingdom	united kingdom	united kingdom	United Kingdom	887	kept
treatment outcome	treatment outcome	treatment outcome	treatment outcome	Treatment Outcome	882	case_or_format
case report	case report	case report	case report	case report	880	kept
follow up	follow up	follow up	follow up	follow up	870	kept
prematurity	prematurity	prematurity	prematurity	prematurity	860	kept
clinical practice	clinical practice	clinical practice	clinical practice	clinical practice	858	kept

delirium	delirium	delirium	delirium	Delirium	858	case_or_format
Risk Assessment	risk assessment	risk assessment	risk assessment	Risk Assessment	854	kept
Reproducibility of Results	reproducibility of results	reproducibility of results	reproducibility of results	Reproducibility of Results	850	kept
Hospital Mortality	hospital mortality	hospital mortality	hospital mortality	Hospital Mortality	835	kept
clinical trial	clinical trial	clinical trial	clinical trial	clinical trial	833	kept
health personnel attitude	health personnel attitude	attitude of health personnel	attitude of health personnel	Attitude of Health Personnel	817	alias
China	china	china	china	China	814	kept
Palliative Care	palliative care	palliative care	palliative care	Palliative Care	803	kept
multicenter study	multicenter study	multicenter study	multicenter study	multicenter study	788	kept
sepsis	sepsis	sepsis	sepsis	Sepsis	787	case_or_format
Decision Making	decision making	decision making	decision making	Decision Making	786	kept
prevalence	prevalence	prevalence	prevalence	prevalence	780	kept
Physicians	physicians	physicians	physician	physician	776	singular
Pregnancy	pregnancy	pregnancy	pregnancy	Pregnancy	762	kept
Quality of Health Care	quality of health care	quality of health care	quality of health care	Quality of Health Care	760	kept
total quality management	total quality management	total quality management	total quality management	total quality management	755	kept

Cohort Studies	cohort studies	cohort studies	cohort study	Cohort Studies	752	singular
observational study	observational study	observational study	observational study	observational study	750	kept
Anxiety	anxiety	anxiety	anxiety	Anxiety	746	kept
family	family	family	family	Family	744	case_or_format
Incidence	incidence	incidence	incidence	Incidence	739	kept
risk assessment	risk assessment	risk assessment	risk assessment	Risk Assessment	736	case_or_format
Emergency Service, Hospital	emergency service hospital	emergency service hospital	emergency service hospital	Emergency Service, Hospital	735	kept
Infection Control	infection control	infection control	infection control	Infection Control	729	kept
interpersonal communication	interpersonal communication	interpersonal communication	interpersonal communication	interpersonal communication	725	kept
epidemiology	epidemiology	epidemiology	epidemiology	epidemiology	724	kept
very elderly	very elderly	very elderly	very elderly	very elderly	722	kept
Job Satisfaction	job satisfaction	job satisfaction	job satisfaction	Job Satisfaction	708	kept
comparative study	comparative study	comparative study	comparative study	comparative study	706	kept
Workforce	workforce	workforce	workforce	Workforce	704	kept
medical education	medical education	medical education	medical education	medical education	702	kept
Adaptation, Psychological	adaptation psychological	adaptation psychological	adaptation psychological	Adaptation, Psychological	695	kept

interview	interview	interview	interview	interview	695	kept
incidence	incidence	incidence	incidence	Incidence	692	case_or_format
Iran	iran	iran	iran	Iran	683	kept
decision making	decision making	decision making	decision making	Decision Making	681	case_or_format
infection control	infection control	infection control	infection control	Infection Control	676	case_or_format
Pilot Projects	pilot projects	pilot projects	pilot project	Pilot Projects	668	singular
Guideline Adherence	guideline adherence	guideline adherence	guideline adherence	Guideline Adherence	666	kept
Canada	canada	canada	canada	Canada	661	kept
disease severity	disease severity	disease severity	disease severity	disease severity	661	kept
quality of life	quality of life	quality of life	quality of life	quality of life	655	kept
Pain Measurement	pain measurement	pain measurement	pain measurement	Pain Measurement	650	kept
Postoperative Complications	postoperative complications	postoperative complications	postoperative complication	Postoperative Complications	649	singular
university hospital	university hospital	university hospital	university hospital	university hospital	625	kept
Personnel Staffing and Scheduling	personnel staffing and scheduling	personnel staffing and scheduling	personnel staffing and scheduling	Personnel Staffing and Scheduling	620	kept
anxiety	anxiety	anxiety	anxiety	Anxiety	619	case_or_format
Severity of Illness Index	severity of illness index	severity of illness index	severity of illness index	Severity of Illness Index	618	kept

terminal care	terminal care	terminal care	terminal care	Terminal Care	610	case_or_format
emergency health service	emergency health service	emergency health service	emergency health service	emergency health service	609	kept
pain	pain	pain	pain	Pain	609	case_or_format
palliative therapy	palliative therapy	palliative therapy	palliative therapy	palliative therapy	603	kept
health care delivery	health care delivery	health care delivery	health care delivery	health care delivery	591	kept
Interprofessional Relations	interprofessional relations	interprofessional relations	interprofessional relations	Interprofessional Relations	576	kept
Program Evaluation	program evaluation	program evaluation	program evaluation	Program Evaluation	575	kept
Social Support	social support	social support	social support	Social Support	573	kept
medical staff	medical staff	medical staff	medical staff	medical staff	567	kept
tertiary care center	tertiary care center	tertiary care center	tertiary care center	tertiary care center	558	kept
Quality of Life	quality of life	quality of life	quality of life	quality of life	557	case_or_format
burnout	burnout	burnout	burnout	burnout	553	kept
Enteral Nutrition	enteral nutrition	enteral nutrition	enteral nutrition	Enteral Nutrition	551	kept
Prevalence	prevalence	prevalence	prevalence	prevalence	548	case_or_format
Medical Staff, Hospital	medical staff hospital	medical staff hospital	medical staff hospital	Medical Staff, Hospital	543	kept
perception	perception	perception	perception	perception	542	kept

Workplace	workplace	workplace	workplace	Workplace	541	kept
psychological aspect	psychological aspect	psychological aspect	psychological aspect	psychological aspect	533	kept
Intubation, Intratracheal	intubation intratracheal	intubation intratracheal	intubation intratracheal	Intubation, Intratracheal	532	kept
emergency care	emergency care	emergency care	emergency care	emergency care	531	kept
knowledge	knowledge	knowledge	knowledge	knowledge	522	kept
Mothers	mothers	mothers	mother	Mothers	522	singular
Clinical Protocols	clinical protocols	clinical protocols	clinical protocol	Clinical Protocols	513	singular
Evidence-Based Medicine	evidence based medicine	evidence based medicine	evidence based medicine	Evidence-Based Medicine	501	kept
Focus Groups	focus groups	focus groups	focus group	Focus Groups	501	singular
clinical competence	clinical competence	clinical competence	clinical competence	Clinical Competence	499	case_or_format
clinical outcome	clinical outcome	clinical outcome	clinical outcome	clinical outcome	495	kept
Brazil	brazil	brazil	brazil	Brazil	494	kept
Catheterization, Central Venous	catheterization central venous	catheterization central venous	catheterization central venous	Catheterization, Central Venous	493	kept
Outcome Assessment, Health Care	outcome assessment health care	outcome assessment health care	outcome assessment health care	Outcome Assessment, Health Care	493	kept
Psychometrics	psychometrics	psychometrics	psychometric	Psychometrics	490	singular
training	training	training	training	training	490	kept

workload	workload	workload	workload	Workload	485	case_or_format
pathophysiology	pathophysiology	pathophysiology	pathophysiology	pathophysiology	484	kept
Italy	italy	italy	italy	Italy	475	kept
Needs Assessment	needs assessment	needs assessment	needs assessment	Needs Assessment	474	kept
Pain Management	pain management	pain management	pain management	Pain Management	474	kept
Prognosis	prognosis	prognosis	prognosis	Prognosis	474	kept
standards	standards	standards	standard	standards	473	singular
Hospitals, University	hospitals university	hospitals university	hospitals university	Hospitals, University	472	kept
comorbidity	comorbidity	comorbidity	comorbidity	comorbidity	470	kept
Pneumonia, Ventilator-Associated	pneumonia ventilator associated	pneumonia ventilator associated	pneumonia ventilator associated	Pneumonia, Ventilator-Associated	466	kept
Leadership	leadership	leadership	leadership	Leadership	465	kept
depression	depression	depression	depression	depression	464	kept
APACHE	apache	apache	apache	APACHE	462	kept
Resuscitation	resuscitation	resuscitation	resuscitation	resuscitation	460	case_or_format
caregiver	caregiver	caregiver	caregiver	caregiver	452	kept
analgesia	analgesia	analgesia	analgesia	analgesia	451	kept

Cooperative Behavior	cooperative behavior	cooperative behavior	cooperative behavior	Cooperative Behavior	451	kept
health care cost	health care cost	health care cost	health care cost	health care cost	451	kept
health care system	health care system	health care system	health care system	health care system	451	kept
Health Services Needs and Demand	health services needs and demand	health services needs and demand	health services needs and demand	Health Services Needs and Demand	450	kept
preschool child	preschool child	preschool child	preschool child	preschool child	448	kept
Catheter-Related Infections	catheter related infections	catheter related infections	catheter related infection	Catheter-Related Infections	447	singular
Wounds and Injuries	wounds and injuries	wounds and injuries	wounds and injuries	Wounds and Injuries	447	kept
standard	standard	standard	standard	standards	442	case_or_format
Turkey	turkey	turkey	turkey	Turkey	442	kept
pediatrics	pediatrics	pediatrics	pediatric	pediatrics	440	singular
Pediatrics	pediatrics	pediatrics	pediatric	pediatrics	439	singular
Inservice Training	inservice training	inservice training	inservice training	Inservice Training	434	kept
Postoperative Care	postoperative care	postoperative care	postoperative care	Postoperative Care	432	kept
information processing	information processing	information processing	information processing	information processing	430	kept
Cardiac Surgical Procedures	cardiac surgical procedures	cardiac surgical procedures	cardiac surgical procedure	Cardiac Surgical Procedures	427	singular
attitude to health	attitude to health	attitude to health	attitude to health	attitude to health	426	kept

clinical protocol	clinical protocol	clinical protocol	clinical protocol	Clinical Protocols	425	case_or_format
Medication Errors	medication errors	medication errors	medication error	Medication Errors	425	singular
prognosis	prognosis	prognosis	prognosis	Prognosis	425	case_or_format
semi structured interview	semi structured interview	semi structured interview	semi structured interview	semi structured interview	425	kept
Anti-Bacterial Agents	anti bacterial agents	anti bacterial agents	anti bacterial agents	Anti-Bacterial Agents	424	kept
Hypnotics and Sedatives	hypnotics and sedatives	hypnotics and sedatives	hypnotics and sedatives	Hypnotics and Sedatives	424	kept
Germany	germany	germany	germany	Germany	422	kept
pneumonia	pneumonia	pneumonia	pneumonia	pneumonia	418	kept
Heart Arrest	heart arrest	heart arrest	heart arrest	Heart Arrest	415	kept
Health Care Surveys	health care surveys	health care surveys	health care survey	Health Care Surveys	414	singular
leadership	leadership	leadership	leadership	Leadership	413	case_or_format
Infant, Premature, Diseases	infant premature diseases	infant premature diseases	infant premature diseases	Infant, Premature, Diseases	411	kept
mental health	mental health	mental health	mental health	mental health	411	kept
Attitude to Health	attitude to health	attitude to health	attitude to health	attitude to health	408	case_or_format
Empathy	empathy	empathy	empathy	Empathy	408	kept
complication	complication	complication	complication	complication	404	kept

statistics and numerical data	statistics and numerical data	statistics and numerical data	statistics and numerical data	statistics and numerical data	404	kept
pilot study	pilot study	pilot study	pilot study	pilot study	403	kept
Respiratory Insufficiency	respiratory insufficiency	respiratory insufficiency	respiratory insufficiency	Respiratory Insufficiency	402	kept
Depression	depression	depression	depression	depression	400	case_or_format
endotracheal intubation	endotracheal intubation	endotracheal intubation	endotracheal intubation	endotracheal intubation	400	kept
sedation	sedation	sedation	sedation	sedation	400	kept
Safety Management	safety management	safety management	safety management	Safety Management	399	kept
pharmacist	pharmacist	pharmacist	pharmacist	pharmacist	398	kept
Knowledge	knowledge	knowledge	knowledge	knowledge	395	case_or_format
prevention and control	prevention and control	prevention and control	prevention and control	prevention and control	393	kept
Medical Errors	medical errors	medical errors	medical error	Medical Errors	391	singular
antibiotic agent	antibiotic agent	antibiotic agent	antibiotic agent	antibiotic agent	390	kept
Burns	burns	burns	burn	Burns	390	singular
health care facility	health care facility	health care facility	health care facility	health care facility	388	kept
gestational age	gestational age	gestational age	gestational age	gestational age	387	kept
scoring system	scoring system	scoring system	scoring system	scoring system	387	kept

hospital readmission	hospital readmission	hospital readmission	hospital readmission	hospital readmission	386	kept
Delivery of Health Care	delivery of health care	delivery of health care	delivery of health care	Delivery of Health Care	385	kept
hypertension	hypertension	hypertension	hypertension	hypertension	385	kept
Cardiopulmonary Resuscitation	cardiopulmonary resuscitation	cardiopulmonary resuscitation	cardiopulmonary resuscitation	Cardiopulmonary Resuscitation	382	kept
health survey	health survey	health survey	health survey	health survey	382	kept
simulation	simulation	simulation	simulation	simulation	379	kept
Ventilator Weaning	ventilator weaning	ventilator weaning	ventilator weaning	Ventilator Weaning	379	kept
heart rate	heart rate	heart rate	heart rate	heart rate	375	kept
hand washing	hand washing	hand washing	hand washing	hand washing	373	kept
heart arrest	heart arrest	heart arrest	heart arrest	Heart Arrest	373	case_or_format
job satisfaction	job satisfaction	job satisfaction	job satisfaction	Job Satisfaction	367	case_or_format
Stroke	stroke	stroke	stroke	Stroke	367	kept
Follow-Up Studies	follow up studies	follow up studies	follow up studies	Follow-Up Studies	365	kept
Perception	perception	perception	perception	perception	364	case_or_format
Acute Disease	acute disease	acute disease	acute disease	Acute Disease	361	kept
awareness	awareness	awareness	awareness	awareness	361	kept

hospital mortality	hospital mortality	hospital mortality	hospital mortality	Hospital Mortality	358	case_or_format
pregnancy	pregnancy	pregnancy	pregnancy	Pregnancy	358	case_or_format
health service	health service	health service	health service	health service	357	kept
Organizational Culture	organizational culture	organizational culture	organizational culture	Organizational Culture	357	kept
decubitus	decubitus	decubitus	decubitus	decubitus	356	kept
Betacoronavirus	betacoronavirus	betacoronavirus	betacoronavirus	Betacoronavirus	355	kept
Logistic Models	logistic models	logistic models	logistic models	Logistic Models	355	kept
Brain Injuries	brain injuries	brain injuries	brain injury	Brain Injuries	352	singular
Pneumonia, Viral	pneumonia viral	pneumonia viral	pneumonia viral	Pneumonia, Viral	352	kept
Sweden	sweden	sweden	sweden	Sweden	352	kept
Caregivers	caregivers	caregivers	caregiver	caregiver	351	singular
physiology	physiology	physiology	physiology	physiology	350	kept
Procedural Sedation	procedural sedation	procedural sedation	procedural sedation	Procedural Sedation	349	kept
hospital personnel	hospital personnel	hospital personnel	hospital personnel	hospital personnel	348	kept
Pneumonia	pneumonia	pneumonia	pneumonia	pneumonia	348	case_or_format
safety	safety	safety	safety	safety	348	kept

cross infection	cross infection	cross infection	cross infection	Cross Infection	347	case_or_format
mortality rate	mortality rate	mortality rate	mortality rate	mortality rate	346	kept
Netherlands	netherlands	netherlands	netherlands	Netherlands	346	kept
respiratory failure	respiratory failure	respiratory failure	respiratory failure	respiratory failure	346	kept
ethics	ethics	ethics	ethic	ethics	343	singular
Coronavirus Infections	coronavirus infections	coronavirus infections	coronavirus infection	Coronavirus Infections	342	singular
Tertiary Care Centers	tertiary care centers	tertiary care centers	tertiary care center	tertiary care center	341	singular
age	age	age	age	age	337	kept
death	death	death	death	death	337	kept
health care planning	health care planning	health care planning	health care planning	health care planning	337	kept
therapy	therapy	therapy	therapy	therapy	336	kept
morbidity	morbidity	morbidity	morbidity	morbidity	335	kept
tracheostomy	tracheostomy	tracheostomy	tracheostomy	tracheostomy	335	kept
health care	health care	health care	health care	health care	334	kept
France	france	france	france	France	333	kept
Education	education	education	education	education	332	case_or_format

hospital infection	hospital infection	hospital infection	hospital infection	hospital infection	330	kept
teaching hospital	teaching hospital	teaching hospital	teaching hospital	teaching hospital	330	kept
teamwork	teamwork	teamwork	teamwork	teamwork	329	kept
fever	fever	fever	fever	fever	328	kept
Hospitals, Teaching	hospitals teaching	hospitals teaching	hospitals teaching	Hospitals, Teaching	327	kept
Program Development	program development	program development	program development	Program Development	327	kept
Questionnaires	questionnaires	questionnaires	questionnaire	questionnaire	326	singular
Algorithms	algorithms	algorithms	algorithm	Algorithms	325	singular
diabetes mellitus	diabetes mellitus	diabetes mellitus	diabetes mellitus	diabetes mellitus	323	kept
Hyperglycemia	hyperglycemia	hyperglycemia	hyperglycemia	Hyperglycemia	323	kept
Randomized Controlled Trials as Topic	randomized controlled trials as topic	randomized controlled trials as topic	randomized controlled trials as topic	Randomized Controlled Trials as Topic	321	kept
respiratory therapist	respiratory therapist	respiratory therapist	respiratory therapist	respiratory therapist	320	kept
Age Factors	age factors	age factors	age factors	Age Factors	319	kept
Saudi Arabia	saudi arabia	saudi arabia	saudi arabia	Saudi Arabia	319	kept
Sleep	sleep	sleep	sleep	Sleep	317	kept
computer assisted tomography	computer assisted tomography	computer assisted tomography	computer assisted tomography	computer assisted tomography	316	kept

protocol compliance	protocol compliance	protocol compliance	protocol compliance	protocol compliance	316	kept
enteric feeding	enteric feeding	enteric feeding	enteric feeding	enteric feeding	315	kept
long term care	long term care	long term care	long term care	long term care	315	kept
home care	home care	home care	home care	home care	314	kept
oxygen saturation	oxygen saturation	oxygen saturation	oxygen saturation	oxygen saturation	314	kept
health care policy	health care policy	health care policy	health care policy	health care policy	313	kept
work environment	work environment	work environment	work environment	work environment	313	kept
Mechanical ventilation	mechanical ventilation	mechanical ventilation	mechanical ventilation	Mechanical ventilation	312	kept
New Zealand	new zealand	new zealand	new zealand	New Zealand	312	kept
Infusions, Intravenous	infusions intravenous	infusions intravenous	infusions intravenous	Infusions, Intravenous	311	kept
intensivist	intensivist	intensivist	intensivist	intensivist	311	kept
adverse event	adverse event	adverse event	adverse event	adverse event	308	kept
Telemedicine	telemedicine	telemedicine	telemedicine	Telemedicine	308	kept
Insulin	insulin	insulin	insulin	Insulin	307	kept
Quality Assurance, Health Care	quality assurance health care	quality assurance health care	quality assurance health care	Quality Assurance, Health Care	307	kept
heart failure	heart failure	heart failure	heart failure	heart failure	306	kept

pain assessment	pain assessment	pain assessment	pain assessment	pain assessment	305	kept
social support	social support	social support	social support	Social Support	305	case_or_format
Spain	spain	spain	spain	Spain	305	kept
Stress Disorders, Post-Traumatic	stress disorders post traumatic	stress disorders post traumatic	stress disorders post traumatic	Stress Disorders, Post-Traumatic	305	kept
evidence based practice	evidence based practice	evidence based practice	evidence based practice	evidence based practice	304	kept
reproducibility	reproducibility	reproducibility	reproducibility	reproducibility	304	kept
Infant Care	infant care	infant care	infant care	Infant Care	303	kept
ventilator associated pneumonia	ventilator associated pneumonia	ventilator associated pneumonia	ventilator associated pneumonia	ventilator associated pneumonia	302	kept
Catheterization, Peripheral	catheterization peripheral	catheterization peripheral	catheterization peripheral	Catheterization, Peripheral	301	kept
Academic Medical Centers	academic medical centers	academic medical centers	academic medical center	Academic Medical Centers	300	singular
India	india	india	india	India	298	kept
England	england	england	england	England	297	kept
Hospitals, Pediatric	hospitals pediatric	hospitals pediatric	hospitals pediatric	Hospitals, Pediatric	297	kept
medication error	medication error	medication error	medication error	Medication Errors	294	case_or_format
mental stress	mental stress	mental stress	mental stress	mental stress	294	kept
injury	injury	injury	injury	injury	293	kept

dementia	dementia	dementia	dementia	dementia	292	kept
Emergency Medical Services	emergency medical services	emergency medical services	emergency medical service	Emergency Medical Services	292	singular
Blood Glucose	blood glucose	blood glucose	blood glucose	Blood Glucose	290	kept
heart surgery	heart surgery	heart surgery	heart surgery	heart surgery	290	kept
demography	demography	demography	demography	demography	289	kept
Neoplasms	neoplasms	neoplasms	neoplasm	Neoplasms	289	singular
statistics	statistics	statistics	statistics	statistics	288	kept
Triage	triage	triage	triage	Triage	287	kept
economics	economics	economics	economic	economics	285	singular
quality improvement	quality improvement	quality improvement	quality improvement	Quality Improvement	285	case_or_format
Feasibility Studies	feasibility studies	feasibility studies	feasibility study	Feasibility Studies	284	singular
resident	resident	resident	resident	resident	283	kept
antiinfective agent	antiinfective agent	antiinfective agent	antiinfective agent	antiinfective agent	281	kept
extracorporeal oxygenation	extracorporeal oxygenation	extracorporeal oxygenation	extracorporeal oxygenation	extracorporeal oxygenation	281	kept
thematic analysis	thematic analysis	thematic analysis	thematic analysis	thematic analysis	281	kept
Tracheostomy	tracheostomy	tracheostomy	tracheostomy	tracheostomy	281	case_or_format

Attitude to Death	attitude to death	attitude to death	attitude to death	Attitude to Death	280	kept
consultation	consultation	consultation	consultation	consultation	279	kept
dyspnea	dyspnea	dyspnea	dyspnea	dyspnea	279	kept
Severe acute respiratory syndrome coronavirus 2	severe acute respiratory syndrome coronavirus 2	severe acute respiratory syndrome coronavirus 2	severe acute respiratory syndrome coronavirus 2	Severe acute respiratory syndrome coronavirus 2	279	kept
demographics	demographics	demographics	demographics	demographics	278	kept
infection prevention	infection prevention	infection prevention	infection prevention	infection prevention	277	kept
Likert scale	likert scale	likert scale	likert scale	Likert scale	277	kept
postoperative complication	postoperative complication	postoperative complication	postoperative complication	Postoperative Complications	276	case_or_format
Gestational Age	gestational age	gestational age	gestational age	gestational age	275	case_or_format
Data Collection	data collection	data collection	data collection	Data Collection	274	kept
Morals	morals	morals	moral	Morals	274	singular
cerebrovascular accident	cerebrovascular accident	cerebrovascular accident	cerebrovascular accident	cerebrovascular accident	273	kept
Extracorporeal Membrane Oxygenation	extracorporeal membrane oxygenation	extracorporeal membrane oxygenation	extracorporeal membrane oxygenation	Extracorporeal Membrane Oxygenation	273	kept
multidisciplinary team	multidisciplinary team	multidisciplinary team	multidisciplinary team	multidisciplinary team	273	kept
palliative care	palliative care	palliative care	palliative care	Palliative Care	270	case_or_format
Heart Failure	heart failure	heart failure	heart failure	heart failure	268	case_or_format

Glasgow coma scale	glasgow coma scale	glasgow coma scale	glasgow coma scale	Glasgow coma scale	267	kept
Europe	europe	europe	europe	Europe	265	kept
Taiwan	taiwan	taiwan	taiwan	Taiwan	265	kept
reliability	reliability	reliability	reliability	reliability	264	kept
stress	stress	stress	stress	stress	264	kept
checklist	checklist	checklist	checklist	checklist	263	kept
postoperative care	postoperative care	postoperative care	postoperative care	Postoperative Care	262	case_or_format
body mass	body mass	body mass	body mass	body mass	259	kept
clinical decision making	clinical decision making	clinical decision making	clinical decision making	clinical decision making	259	kept
oxygen therapy	oxygen therapy	oxygen therapy	oxygen therapy	oxygen therapy	259	kept
mother	mother	mother	mother	Mothers	258	case_or_format
physiotherapist	physiotherapist	physiotherapist	physiotherapist	physiotherapist	258	kept
work experience	work experience	work experience	work experience	work experience	258	kept
Palliative care	palliative care	palliative care	palliative care	Palliative Care	256	case_or_format
Breast Feeding	breast feeding	breast feeding	breast feeding	Breast Feeding	254	kept
hypotension	hypotension	hypotension	hypotension	hypotension	254	kept

quality control	quality control	quality control	quality control	quality control	253	kept
Sensitivity and Specificity	sensitivity and specificity	sensitivity and specificity	sensitivity and specificity	Sensitivity and Specificity	253	kept
sensitivity and specificity	sensitivity and specificity	sensitivity and specificity	sensitivity and specificity	Sensitivity and Specificity	253	case_or_format
Documentation	documentation	documentation	documentation	Documentation	252	kept
Longitudinal Studies	longitudinal studies	longitudinal studies	longitudinal study	Longitudinal Studies	252	singular
monitoring	monitoring	monitoring	monitoring	monitoring	252	kept
Trauma Centers	trauma centers	trauma centers	trauma center	Trauma Centers	252	singular
satisfaction	satisfaction	satisfaction	satisfaction	satisfaction	251	kept
skill	skill	skill	skill	skill	250	kept
extubation	extubation	extubation	extubation	extubation	249	kept
acute kidney failure	acute kidney failure	acute kidney failure	acute kidney failure	acute kidney failure	248	kept
Interdisciplinary Communication	interdisciplinary communication	interdisciplinary communication	interdisciplinary communication	Interdisciplinary Communication	247	kept
septic shock	septic shock	septic shock	septic shock	septic shock	247	kept
Early Ambulation	early ambulation	early ambulation	early ambulation	Early Ambulation	246	kept
Total Quality Management	total quality management	total quality management	total quality management	total quality management	246	case_or_format
breathing rate	breathing rate	breathing rate	breathing rate	breathing rate	245	kept

intubation	intubation	intubation	intubation	intubation	245	kept
clinical feature	clinical feature	clinical feature	clinical feature	clinical feature	244	kept
Mortality	mortality	mortality	mortality	mortality	244	case_or_format
parent	parent	parent	parent	Parents	244	case_or_format
Health Facility Environment	health facility environment	health facility environment	health facility environment	Health Facility Environment	242	kept
Referral and Consultation	referral and consultation	referral and consultation	referral and consultation	Referral and Consultation	242	kept
chronic obstructive lung disease	chronic obstructive lung disease	chronic obstructive lung disease	chronic obstructive lung disease	chronic obstructive lung disease	241	kept
electronic medical record	electronic medical record	electronic medical record	electronic medical record	electronic medical record	241	kept
catheter infection	catheter infection	catheter infection	catheter infection	catheter infection	239	kept
medical decision making	medical decision making	medical decision making	medical decision making	medical decision making	239	kept
controlled clinical trial	controlled clinical trial	controlled clinical trial	controlled clinical trial	controlled clinical trial	238	kept
physiotherapy	physiotherapy	physiotherapy	physiotherapy	physiotherapy	238	kept
evidence based medicine	evidence based medicine	evidence based medicine	evidence based medicine	Evidence-Based Medicine	237	case_or_format
Evidence-Based Practice	evidence based practice	evidence based practice	evidence based practice	evidence based practice	237	case_or_format
health care utilization	health care utilization	health care utilization	health care utilization	health care utilization	237	kept
Jordan	jordan	jordan	jordan	Jordan	237	kept

Internet	internet	internet	internet	Internet	236	kept
Electrocardiography	electrocardiography	electrocardiography	electrocardiography	Electrocardiography	235	kept
propofol	propofol	propofol	propofol	propofol	235	kept
content analysis	content analysis	content analysis	content analysis	content analysis	234	kept
attitude	attitude	attitude	attitude	attitude	233	kept
surgery	surgery	surgery	surgery	surgery	232	kept
Analgesics, Opioid	analgesics opioid	analgesics opioid	analgesics opioid	Analgesics, Opioid	231	kept
Heart Defects, Congenital	heart defects congenital	heart defects congenital	heart defects congenital	Heart Defects, Congenital	231	kept
medical record	medical record	medical record	medical record	medical record	231	kept
organization	organization	organization	organization	organization	231	kept
telemedicine	telemedicine	telemedicine	telemedicine	Telemedicine	231	case_or_format
mechanical ventilation	mechanical ventilation	mechanical ventilation	mechanical ventilation	Mechanical ventilation	230	case_or_format
medical ethics	medical ethics	medical ethics	medical ethics	medical ethics	229	kept
algorithm	algorithm	algorithm	algorithm	Algorithms	228	case_or_format
diagnosis	diagnosis	diagnosis	diagnosis	diagnosis	228	kept
prescription	prescription	prescription	prescription	prescription	228	kept

workplace	workplace	workplace	workplace	Workplace	228	case_or_format
devices	devices	devices	device	devices	227	singular
Noise	noise	noise	noise	Noise	227	kept
Organizational Innovation	organizational innovation	organizational innovation	organizational innovation	Organizational Innovation	227	kept
thorax radiography	thorax radiography	thorax radiography	thorax radiography	thorax radiography	227	kept
Analgesia	analgesia	analgesia	analgesia	analgesia	226	case_or_format
meta analysis	meta analysis	meta analysis	meta analysis	meta analysis	226	kept
Electronic Health Records	electronic health records	electronic health records	electronic health record	Electronic Health Records	225	singular
Equipment Design	equipment design	equipment design	equipment design	Equipment Design	225	kept
insulin	insulin	insulin	insulin	Insulin	225	case_or_format
Staphylococcal Infections	staphylococcal infections	staphylococcal infections	staphylococcal infections	Staphylococcal Infections	225	kept
descriptive research	descriptive research	descriptive research	descriptive research	descriptive research	224	kept
health care organization	health care organization	health care organization	health care organization	health care organization	224	kept
Burnout	burnout	burnout	burnout	burnout	223	case_or_format
Death	death	death	death	death	223	case_or_format
feasibility study	feasibility study	feasibility study	feasibility study	Feasibility Studies	223	case_or_format

Japan	japan	japan	japan	Japan	223	kept
medical society	medical society	medical society	medical society	medical society	223	kept
Predictive Value of Tests	predictive value of tests	predictive value of tests	predictive value of tests	Predictive Value of Tests	223	kept
Comorbidity	comorbidity	comorbidity	comorbidity	comorbidity	222	case_or_format
electronic health record	electronic health record	electronic health record	electronic health record	Electronic Health Records	221	case_or_format
Norway	norway	norway	norway	Norway	221	kept
Withholding Treatment	withholding treatment	withholding treatment	withholding treatment	Withholding Treatment	221	kept
Cost-Benefit Analysis	cost benefit analysis	cost benefit analysis	cost benefit analysis	Cost-Benefit Analysis	220	kept
Fatigue	fatigue	fatigue	fatigue	Fatigue	220	kept
virus pneumonia	virus pneumonia	virus pneumonia	virus pneumonia	virus pneumonia	220	kept
Case-Control Studies	case control studies	case control studies	case control study	Case-Control Studies	219	singular
child parent relation	child parent relation	child parent relation	child parent relation	child parent relation	219	kept
fatigue	fatigue	fatigue	fatigue	Fatigue	218	case_or_format
glucose blood level	glucose blood level	glucose blood level	glucose blood level	glucose blood level	218	kept
midazolam	midazolam	midazolam	midazolam	midazolam	218	kept
Quality improvement	quality improvement	quality improvement	quality improvement	Quality Improvement	218	case_or_format

Suction	suction	suction	suction	Suction	218	kept
time	time	time	time	time	218	kept
epidemic	epidemic	epidemic	epidemic	epidemic	217	kept
Emotions	emotions	emotions	emotion	Emotions	216	singular
personnel management	personnel management	personnel management	personnel management	personnel management	216	kept
Hospital Units	hospital units	hospital units	hospital units	Hospital Units	215	kept
communication	communication	communication	communication	Communication	213	case_or_format
medical specialist	medical specialist	medical specialist	medical specialist	medical specialist	213	kept
newborn care	newborn care	newborn care	newborn care	newborn care	213	kept
Personnel, Hospital	personnel hospital	personnel hospital	personnel hospital	Personnel, Hospital	213	kept
prediction	prediction	prediction	prediction	prediction	213	kept
data analysis	data analysis	data analysis	data analysis	data analysis	212	kept
hospital emergency service	hospital emergency service	hospital emergency service	hospital emergency service	hospital emergency service	211	kept
Hospital Rapid Response Team	hospital rapid response team	hospital rapid response team	hospital rapid response team	Hospital Rapid Response Team	211	kept
Hypoglycemia	hypoglycemia	hypoglycemia	hypoglycemia	Hypoglycemia	211	kept
Oxygen Inhalation Therapy	oxygen inhalation therapy	oxygen inhalation therapy	oxygen inhalation therapy	Oxygen Inhalation Therapy	211	kept

Professional Autonomy	professional autonomy	professional autonomy	professional autonomy	Professional Autonomy	211	kept
Restraint, Physical	restraint physical	restraint physical	restraint physical	Restraint, Physical	211	kept
antibiotic therapy	antibiotic therapy	antibiotic therapy	antibiotic therapy	antibiotic therapy	210	kept
consensus	consensus	consensus	consensus	consensus	210	kept
microbiology	microbiology	microbiology	microbiology	microbiology	210	kept
morphine	morphine	morphine	morphine	morphine	210	kept
Skin Care	skin care	skin care	skin care	Skin Care	210	kept
adult respiratory distress syndrome	adult respiratory distress syndrome	adult respiratory distress syndrome	adult respiratory distress syndrome	adult respiratory distress syndrome	209	kept
coronary care unit	coronary care unit	coronary care unit	coronary care unit	coronary care unit	209	kept
Letter	letter	letter	letter	Letter	209	kept
posttraumatic stress disorder	posttraumatic stress disorder	posttraumatic stress disorder	posttraumatic stress disorder	posttraumatic stress disorder	209	kept
qualitative analysis	qualitative analysis	qualitative analysis	qualitative analysis	qualitative analysis	209	kept
Staphylococcus aureus	staphylococcus aureus	staphylococcus aureus	staphylococcus aureus	Staphylococcus aureus	209	kept
time factor	time factor	time factor	time factor	Time Factors	209	case_or_format
Analysis of Variance	analysis of variance	analysis of variance	analysis of variance	Analysis of Variance	208	kept
bleeding	bleeding	bleeding	bleeding	bleeding	208	kept

clinical assessment	clinical assessment	clinical assessment	clinical assessment	clinical assessment	208	kept
Neonatology	neonatology	neonatology	neonatology	Neonatology	208	kept
drug safety	drug safety	drug safety	drug safety	drug safety	207	kept
emergency medicine	emergency medicine	emergency medicine	emergency medicine	emergency medicine	207	kept
Respiratory Distress Syndrome	respiratory distress syndrome	respiratory distress syndrome	respiratory distress syndrome	Respiratory Distress Syndrome	207	kept
Checklist	checklist	checklist	checklist	checklist	206	case_or_format
Multivariate Analysis	multivariate analysis	multivariate analysis	multivariate analysis	Multivariate Analysis	206	kept
blood transfusion	blood transfusion	blood transfusion	blood transfusion	blood transfusion	205	kept
Coronary Care Units	coronary care units	coronary care units	coronary care unit	coronary care unit	205	singular
Coronavirus infection	coronavirus infection	coronavirus infection	coronavirus infection	Coronavirus Infections	205	case_or_format
Educational Measurement	educational measurement	educational measurement	educational measurement	Educational Measurement	204	kept
Fluid Therapy	fluid therapy	fluid therapy	fluid therapy	Fluid Therapy	204	kept
Tissue and Organ Procurement	tissue and organ procurement	tissue and organ procurement	tissue and organ procurement	Tissue and Organ Procurement	203	kept
vital sign	vital sign	vital sign	vital sign	vital sign	203	kept
treatment duration	treatment duration	treatment duration	treatment duration	treatment duration	202	kept
Simulation Training	simulation training	simulation training	simulation training	Simulation Training	201	kept

traumatic brain injury	traumatic brain injury	traumatic brain injury	traumatic brain injury	traumatic brain injury	201	kept
analgesic agent	analgesic agent	analgesic agent	analgesic agent	analgesic agent	200	kept
Acute Kidney Injury	acute kidney injury	acute kidney injury	acute kidney injury	Acute Kidney Injury	199	kept
Bacteremia	bacteremia	bacteremia	bacteremia	Bacteremia	199	kept
neonatology	neonatology	neonatology	neonatology	Neonatology	199	case_or_format
learning	learning	learning	learning	learning	198	kept
personal experience	personal experience	personal experience	personal experience	personal experience	198	kept
glucose	glucose	glucose	glucose	glucose	197	kept
health care survey	health care survey	health care survey	health care survey	Health Care Surveys	197	case_or_format
Emergencies	emergencies	emergencies	emergency	Emergencies	196	singular
medical error	medical error	medical error	medical error	Medical Errors	196	case_or_format
public health	public health	public health	public health	public health	196	kept
public hospital	public hospital	public hospital	public hospital	public hospital	196	kept
urinary tract infection	urinary tract infection	urinary tract infection	urinary tract infection	urinary tract infection	196	kept
noninvasive ventilation	noninvasive ventilation	noninvasive ventilation	noninvasive ventilation	noninvasive ventilation	195	kept
Safety	safety	safety	safety	safety	195	case_or_format

anesthesia	anesthesia	anesthesia	anesthesia	anesthesia	194	kept
fentanyl	fentanyl	fentanyl	fentanyl	fentanyl	194	kept
cognition	cognition	cognition	cognition	cognition	193	kept
Shock, Septic	shock septic	shock septic	shock septic	Shock, Septic	192	kept
clinical effectiveness	clinical effectiveness	clinical effectiveness	clinical effectiveness	clinical effectiveness	191	kept
infection risk	infection risk	infection risk	infection risk	infection risk	191	kept
job stress	job stress	job stress	job stress	job stress	191	kept
Midwifery	midwifery	midwifery	midwifery	Midwifery	191	kept
Neonatal	neonatal	neonatal	neonatal	Neonatal	191	kept
sleep	sleep	sleep	sleep	Sleep	191	case_or_format
Dementia	dementia	dementia	dementia	dementia	190	case_or_format
OUTCOMES	outcomes	outcomes	outcome	OUTCOMES	190	singular
Editorial	editorial	editorial	editorial	Editorial	189	kept
health program	health program	health program	health program	health program	189	kept
Intubation, Gastrointestinal	intubation gastrointestinal	intubation gastrointestinal	intubation gastrointestinal	Intubation, Gastrointestinal	189	kept
Postoperative Pain	postoperative pain	postoperative pain	postoperative pain	Postoperative Pain	189	kept

Recovery Room	recovery room	recovery room	recovery room	Recovery Room	189	kept
emotion	emotion	emotion	emotion	Emotions	188	case_or_format
Survival Rate	survival rate	survival rate	survival rate	Survival Rate	188	kept
vancomycin	vancomycin	vancomycin	vancomycin	vancomycin	188	kept
cost effectiveness analysis	cost effectiveness analysis	cost effectiveness analysis	cost effectiveness analysis	cost effectiveness analysis	187	kept
Drug Monitoring	drug monitoring	drug monitoring	drug monitoring	Drug Monitoring	187	kept
Stress	stress	stress	stress	stress	187	case_or_format
Benchmarking	benchmarking	benchmarking	benchmarking	Benchmarking	186	kept
central venous catheterization	central venous catheterization	central venous catheterization	central venous catheterization	central venous catheterization	186	kept
Self Efficacy	self efficacy	self efficacy	self efficacy	Self Efficacy	186	kept
blood pressure	blood pressure	blood pressure	blood pressure	blood pressure	185	kept
Internship and Residency	internship and residency	internship and residency	internship and residency	Internship and Residency	185	kept
Medicare	medicare	medicare	medicare	Medicare	185	kept
Myocardial Infarction	myocardial infarction	myocardial infarction	myocardial infarction	Myocardial Infarction	185	kept
Oxygen	oxygen	oxygen	oxygen	Oxygen	185	kept
Point-of-Care Systems	point of care systems	point of care systems	point of care system	Point-of-Care Systems	185	singular

Switzerland	switzerland	switzerland	switzerland	Switzerland	185	kept
brain hemorrhage	brain hemorrhage	brain hemorrhage	brain hemorrhage	brain hemorrhage	184	kept
disease transmission	disease transmission	disease transmission	disease transmission	disease transmission	184	kept
Glasgow Coma Scale	glasgow coma scale	glasgow coma scale	glasgow coma scale	Glasgow coma scale	184	case_or_format
predictive value	predictive value	predictive value	predictive value	predictive value	184	kept
California	california	california	california	California	183	kept
Disease Outbreaks	disease outbreaks	disease outbreaks	disease outbreak	Disease Outbreaks	183	singular
Ontario	ontario	ontario	ontario	Ontario	183	kept
physician attitude	physician attitude	physician attitude	physician attitude	physician attitude	183	kept
South Africa	south africa	south africa	south africa	South Africa	183	kept
mobilization	mobilization	mobilization	mobilization	mobilization	182	kept
staff training	staff training	staff training	staff training	staff training	182	kept
Survivors	survivors	survivors	survivor	Survivors	182	singular
Catheters, Indwelling	catheters indwelling	catheters indwelling	catheters indwelling	Catheters, Indwelling	181	kept
methicillin resistant Staphylococcus aureus	methicillin resistant staphylococcus aureus	methicillin resistant staphylococcus aureus	methicillin resistant staphylococcus aureus	methicillin resistant Staphylococcus aureus	181	kept
Practice Patterns, Physicians’	practice patterns physicians	practice patterns physicians	practice patterns physicians	Practice Patterns, Physicians’	181	kept

Infant, Very Low Birth Weight	infant very low birth weight	infant very low birth weight	infant very low birth weight	Infant, Very Low Birth Weight	180	kept
positive end expiratory pressure	positive end expiratory pressure	positive end expiratory pressure	positive end expiratory pressure	positive end expiratory pressure	180	kept
Aftercare	aftercare	aftercare	aftercare	Aftercare	179	kept
infection	infection	infection	infection	infection	179	kept
Mass Screening	mass screening	mass screening	mass screening	Mass Screening	179	kept
validity	validity	validity	validity	validity	179	kept
Chronic Disease	chronic disease	chronic disease	chronic disease	Chronic Disease	178	kept
Heart Rate	heart rate	heart rate	heart rate	heart rate	178	case_or_format
Outcome and Process Assessment, Health Care	outcome and process assessment health care	outcome and process assessment health care	outcome and process assessment health care	Outcome and Process Assessment, Health Care	178	kept
hypoglycemia	hypoglycemia	hypoglycemia	hypoglycemia	Hypoglycemia	177	case_or_format
IMPACT	impact	impact	impact	IMPACT	177	kept
Models, Organizational	models organizational	models organizational	models organizational	Models, Organizational	177	kept
Quality Indicators, Health Care	quality indicators health care	quality indicators health care	quality indicators health care	Quality Indicators, Health Care	177	kept
survival rate	survival rate	survival rate	survival rate	Survival Rate	177	case_or_format
Airway Extubation	airway extubation	airway extubation	airway extubation	Airway Extubation	176	kept
birth weight	birth weight	birth weight	birth weight	birth weight	176	kept

cardiovascular disease	cardiovascular disease	cardiovascular disease	cardiovascular disease	cardiovascular disease	176	kept
Hypoglycemic Agents	hypoglycemic agents	hypoglycemic agents	hypoglycemic agents	Hypoglycemic Agents	176	kept
medical record review	medical record review	medical record review	medical record review	medical record review	176	kept
ward	ward	ward	ward	ward	176	kept
Attitude	attitude	attitude	attitude	attitude	175	case_or_format
Hand Hygiene	hand hygiene	hand hygiene	hand hygiene	Hand Hygiene	175	kept
hyperglycemia	hyperglycemia	hyperglycemia	hyperglycemia	Hyperglycemia	175	case_or_format
intervention study	intervention study	intervention study	intervention study	intervention study	175	kept
education program	education program	education program	education program	education program	174	kept
parenteral nutrition	parenteral nutrition	parenteral nutrition	parenteral nutrition	parenteral nutrition	174	kept
Pseudomonas aeruginosa	pseudomonas aeruginosa	pseudomonas aeruginosa	pseudomonas aeruginosa	Pseudomonas aeruginosa	174	kept
simulation training	simulation training	simulation training	simulation training	Simulation Training	174	case_or_format
self report	self report	self report	self report	self report	173	kept
survival	survival	survival	survival	survival	173	kept
validation study	validation study	validation study	validation study	validation study	173	kept
Burn Units	burn units	burn units	burn unit	Burn Units	172	singular

breast feeding	breast feeding	breast feeding	breast feeding	Breast Feeding	171	case_or_format
clinical evaluation	clinical evaluation	clinical evaluation	clinical evaluation	clinical evaluation	171	kept
cesarean section	cesarean section	cesarean section	cesarean section	cesarean section	170	kept
Organizational Policy	organizational policy	organizational policy	organizational policy	Organizational Policy	170	kept
Republic of Korea	republic of korea	republic of korea	republic of korea	Republic of Korea	170	kept
Anesthesia	anesthesia	anesthesia	anesthesia	anesthesia	169	case_or_format
informed consent	informed consent	informed consent	informed consent	informed consent	169	kept
machine learning	machine learning	machine learning	machine learning	machine learning	169	kept
medical history	medical history	medical history	medical history	medical history	169	kept
moral distress	moral distress	moral distress	moral distress	moral distress	169	kept
neurosurgery	neurosurgery	neurosurgery	neurosurgery	neurosurgery	169	kept
Newborn	newborn	newborn	newborn	newborn	169	case_or_format
Statistics, Nonparametric	statistics nonparametric	statistics nonparametric	statistics nonparametric	Statistics, Nonparametric	169	kept
blood	blood	blood	blood	blood	168	kept
distress syndrome	distress syndrome	distress syndrome	distress syndrome	distress syndrome	168	kept
educational status	educational status	educational status	educational status	educational status	168	kept

hemodynamics	hemodynamics	hemodynamics	hemodynamic	hemodynamics	168	singular
professional knowledge	professional knowledge	professional knowledge	professional knowledge	professional knowledge	168	kept
Belgium	belgium	belgium	belgium	Belgium	167	kept
chronic disease	chronic disease	chronic disease	chronic disease	Chronic Disease	167	case_or_format
clinician	clinician	clinician	clinician	clinician	167	kept
Denmark	denmark	denmark	denmark	Denmark	167	kept
medical practice	medical practice	medical practice	medical practice	medical practice	167	kept
Mother-Child Relations	mother child relations	mother child relations	mother child relation	Mother-Child Relations	167	singular
obesity	obesity	obesity	obesity	obesity	167	kept
opiate	opiate	opiate	opiate	opiate	167	kept
oxygen	oxygen	oxygen	oxygen	Oxygen	167	case_or_format
renal replacement therapy	renal replacement therapy	renal replacement therapy	renal replacement therapy	renal replacement therapy	167	kept
nuclear magnetic resonance imaging	nuclear magnetic resonance imaging	nuclear magnetic resonance imaging	nuclear magnetic resonance imaging	nuclear magnetic resonance imaging	166	kept
Oral Hygiene	oral hygiene	oral hygiene	oral hygiene	Oral Hygiene	166	kept
rapid response team	rapid response team	rapid response team	rapid response team	rapid response team	166	kept
teaching	teaching	teaching	teaching	teaching	166	kept

antibiotic resistance	antibiotic resistance	antibiotic resistance	antibiotic resistance	antibiotic resistance	165	kept
Chi-Square Distribution	chi square distribution	chi square distribution	chi square distribution	Chi-Square Distribution	165	kept
Clinical Alarms	clinical alarms	clinical alarms	clinical alarm	Clinical Alarms	165	singular
deterioration	deterioration	deterioration	deterioration	deterioration	165	kept
disease course	disease course	disease course	disease course	disease course	165	kept
Home Care Services	home care services	home care services	home care services	Home Care Services	165	kept
hemodialysis	hemodialysis	hemodialysis	hemodialysis	hemodialysis	164	kept
medicare	medicare	medicare	medicare	Medicare	164	case_or_format
State Medicine	state medicine	state medicine	state medicine	State Medicine	164	kept
vaccination	vaccination	vaccination	vaccination	vaccination	164	kept
acute disease	acute disease	acute disease	acute disease	Acute Disease	163	case_or_format
cognitive defect	cognitive defect	cognitive defect	cognitive defect	cognitive defect	163	kept
hospital management	hospital management	hospital management	hospital management	hospital management	163	kept
Analgesics	analgesics	analgesics	analgesic	Analgesics	162	singular
emotional stress	emotional stress	emotional stress	emotional stress	emotional stress	162	kept
psychometry	psychometry	psychometry	psychometry	psychometry	162	kept

quantitative analysis	quantitative analysis	quantitative analysis	quantitative analysis	quantitative analysis	162	kept
Perioperative Care	perioperative care	perioperative care	perioperative care	Perioperative Care	161	kept
seizure	seizure	seizure	seizure	seizure	161	kept
blood culture	blood culture	blood culture	blood culture	blood culture	160	kept
daily life activity	daily life activity	daily life activity	daily life activity	daily life activity	160	kept
electrocardiography	electrocardiography	electrocardiography	electrocardiography	Electrocardiography	160	case_or_format
Spirituality	spirituality	spirituality	spirituality	Spirituality	160	kept
Community-Acquired Infections	community acquired infections	community acquired infections	community acquired infection	Community-Acquired Infections	159	singular
Personnel Turnover	personnel turnover	personnel turnover	personnel turnover	Personnel Turnover	159	kept
Prevention	prevention	prevention	prevention	Prevention	159	kept
public relations	public relations	public relations	public relations	public relations	159	kept
vomiting	vomiting	vomiting	vomiting	vomiting	159	kept
Bereavement	bereavement	bereavement	bereavement	Bereavement	158	kept
Health Services Research	health services research	health services research	health services research	Health Services Research	158	kept
catheterization	catheterization	catheterization	catheterization	catheterization	157	kept
coughing	coughing	coughing	coughing	coughing	157	kept

postoperative period	postoperative period	postoperative period	postoperative period	postoperative period	157	kept
anticoagulant agent	anticoagulant agent	anticoagulant agent	anticoagulant agent	anticoagulant agent	156	kept
needs assessment	needs assessment	needs assessment	needs assessment	Needs Assessment	156	case_or_format
diarrhea	diarrhea	diarrhea	diarrhea	diarrhea	155	kept
Occupational Diseases	occupational diseases	occupational diseases	occupational disease	Occupational Diseases	155	singular
physical examination	physical examination	physical examination	physical examination	physical examination	155	kept
Certification	certification	certification	certification	Certification	154	kept
Diagnosis, Differential	diagnosis differential	diagnosis differential	diagnosis differential	Diagnosis, Differential	154	kept
health education	health education	health education	health education	health education	154	kept
heart arrhythmia	heart arrhythmia	heart arrhythmia	heart arrhythmia	heart arrhythmia	154	kept
rehabilitation	rehabilitation	rehabilitation	rehabilitation	rehabilitation	154	kept
statistical model	statistical model	statistical model	statistical model	statistical model	154	kept
end-of-life care	end of life care	end of life care	end of life care	end-of-life care	153	kept
exercise	exercise	exercise	exercise	exercise	153	kept
longitudinal study	longitudinal study	longitudinal study	longitudinal study	Longitudinal Studies	153	case_or_format
mental disease	mental disease	mental disease	mental disease	mental disease	153	kept

nutritional support	nutritional support	nutritional support	nutritional support	nutritional support	153	kept
Psychomotor Agitation	psychomotor agitation	psychomotor agitation	psychomotor agitation	Psychomotor Agitation	153	kept
tertiary health care	tertiary health care	tertiary health care	tertiary health care	tertiary health care	153	kept
Delphi Technique	delphi technique	delphi technique	delphi technique	Delphi Technique	152	kept
Forecasting	forecasting	forecasting	forecasting	Forecasting	152	kept
Klebsiella pneumoniae	klebsiella pneumoniae	klebsiella pneumoniae	klebsiella pneumoniae	Klebsiella pneumoniae	152	kept
medical documentation	medical documentation	medical documentation	medical documentation	medical documentation	152	kept
paracetamol	paracetamol	paracetamol	paracetamol	paracetamol	152	kept
Risk Management	risk management	risk management	risk management	Risk Management	152	kept
Child Development	child development	child development	child development	Child Development	151	kept
Hospital Costs	hospital costs	hospital costs	hospital cost	Hospital Costs	151	singular
Ireland	ireland	ireland	ireland	Ireland	151	kept
Urinary Tract Infections	urinary tract infections	urinary tract infections	urinary tract infection	urinary tract infection	151	singular
coping behavior	coping behavior	coping behavior	coping behavior	coping behavior	150	kept
Decision Support Systems, Clinical	decision support systems clinical	decision support systems clinical	decision support systems clinical	Decision Support Systems, Clinical	150	kept
Outcome Assessment	outcome assessment	outcome assessment	outcome assessment	outcome assessment	150	case_or_format

survivor	survivor	survivor	survivor	Survivors	150	case_or_format
dexmedetomidine	dexmedetomidine	dexmedetomidine	dexmedetomidine	dexmedetomidine	149	kept
drug therapy	drug therapy	drug therapy	drug therapy	drug therapy	149	kept
motivation	motivation	motivation	motivation	motivation	149	kept
prevention	prevention	prevention	prevention	Prevention	149	case_or_format
Risk factors	risk factors	risk factors	risk factor	Risk Factors	149	singular
body temperature	body temperature	body temperature	body temperature	body temperature	148	kept
brain injury	brain injury	brain injury	brain injury	Brain Injuries	148	case_or_format
echography	echography	echography	echography	echography	148	kept
Electroencephalography	electroencephalography	electroencephalography	electroencephalography	Electroencephalography	148	kept
interdisciplinary communication	interdisciplinary communication	interdisciplinary communication	interdisciplinary communication	Interdisciplinary Communication	148	case_or_format
laboratory test	laboratory test	laboratory test	laboratory test	laboratory test	148	kept
respiratory distress	respiratory distress	respiratory distress	respiratory distress	respiratory distress	148	kept
Chlorhexidine	chlorhexidine	chlorhexidine	chlorhexidine	Chlorhexidine	147	kept
health care access	health care access	health care access	health care access	health care access	147	kept
hospital care	hospital care	hospital care	hospital care	hospital care	147	kept

Hypothermia, Induced	hypothermia induced	hypothermia induced	hypothermia induced	Hypothermia, Induced	147	kept
Infection control	infection control	infection control	infection control	Infection Control	147	case_or_format
morality	morality	morality	morality	morality	147	kept
noradrenalin	noradrenalin	noradrenalin	noradrenalin	noradrenalin	147	kept
nutrition	nutrition	nutrition	nutrition	nutrition	147	kept
frailty	frailty	frailty	frailty	frailty	146	kept
health insurance	health insurance	health insurance	health insurance	health insurance	146	kept
self concept	self concept	self concept	self concept	self concept	146	kept
C reactive protein	c reactive protein	c reactive protein	c reactive protein	C reactive protein	145	kept
classification	classification	classification	classification	classification	145	kept
documentation	documentation	documentation	documentation	Documentation	145	case_or_format
intravenous drug administration	intravenous drug administration	intravenous drug administration	intravenous drug administration	intravenous drug administration	145	kept
medical information	medical information	medical information	medical information	medical information	145	kept
medical student	medical student	medical student	medical student	medical student	145	kept
responsibility	responsibility	responsibility	responsibility	responsibility	145	kept
agitation	agitation	agitation	agitation	agitation	144	kept

Child, Hospitalized	child hospitalized	child hospitalized	child hospitalized	Child, Hospitalized	144	kept
Frailty	frailty	frailty	frailty	frailty	144	case_or_format
corticosteroid	corticosteroid	corticosteroid	corticosteroid	corticosteroid	143	kept
cost benefit analysis	cost benefit analysis	cost benefit analysis	cost benefit analysis	Cost-Benefit Analysis	143	case_or_format
Early Diagnosis	early diagnosis	early diagnosis	early diagnosis	Early Diagnosis	143	kept
empathy	empathy	empathy	empathy	Empathy	143	case_or_format
evaluation	evaluation	evaluation	evaluation	evaluation	143	kept
systolic blood pressure	systolic blood pressure	systolic blood pressure	systolic blood pressure	systolic blood pressure	143	kept
etiology	etiology	etiology	etiology	etiology	142	kept
Infant, Newborn, Diseases	infant newborn diseases	infant newborn diseases	infant newborn diseases	Infant, Newborn, Diseases	142	kept
Occupational Health	occupational health	occupational health	occupational health	Occupational Health	142	kept
Occupational Stress	occupational stress	occupational stress	occupational stress	Occupational Stress	142	kept
Professional Competence	professional competence	professional competence	professional competence	Professional Competence	142	kept
sleep quality	sleep quality	sleep quality	sleep quality	sleep quality	142	kept
Cardiovascular Diseases	cardiovascular diseases	cardiovascular diseases	cardiovascular disease	cardiovascular disease	141	singular
Charlson Comorbidity Index	charlson comorbidity index	charlson comorbidity index	charlson comorbidity index	Charlson Comorbidity Index	141	kept

Databases, Factual	databases factual	databases factual	databases factual	Databases, Factual	141	kept
heart infarction	heart infarction	heart infarction	heart infarction	heart infarction	141	kept
Life Support Care	life support care	life support care	life support care	Life Support Care	141	kept
neonatal	neonatal	neonatal	neonatal	Neonatal	141	case_or_format
Pressure ulcer	pressure ulcer	pressure ulcer	pressure ulcer	Pressure Ulcer	141	case_or_format
Anticoagulants	anticoagulants	anticoagulants	anticoagulant	Anticoagulants	140	singular
manpower	manpower	manpower	manpower	manpower	140	kept
MORTALITY	mortality	mortality	mortality	mortality	140	case_or_format
pediatric hospital	pediatric hospital	pediatric hospital	pediatric hospital	pediatric hospital	140	kept
wellbeing	wellbeing	wellbeing	wellbeing	wellbeing	140	kept
anesthesiologist	anesthesiologist	anesthesiologist	anesthesiologist	anesthesiologist	139	kept
Fever	fever	fever	fever	fever	139	case_or_format
malnutrition	malnutrition	malnutrition	malnutrition	malnutrition	139	kept
Mental Disorders	mental disorders	mental disorders	mental disorder	Mental Disorders	139	singular
Parent-Child Relations	parent child relations	parent child relations	parent child relation	Parent-Child Relations	139	singular
surgeon	surgeon	surgeon	surgeon	surgeon	139	kept

central venous catheter	central venous catheter	central venous catheter	central venous catheter	central venous catheter	138	kept
chlorhexidine	chlorhexidine	chlorhexidine	chlorhexidine	Chlorhexidine	138	case_or_format
cooperation	cooperation	cooperation	cooperation	cooperation	138	kept
Escherichia coli	escherichia coli	escherichia coli	escherichia coli	Escherichia coli	138	kept
Continuous Positive Airway Pressure	continuous positive airway pressure	continuous positive airway pressure	continuous positive airway pressure	Continuous Positive Airway Pressure	137	kept
differential diagnosis	differential diagnosis	differential diagnosis	differential diagnosis	differential diagnosis	137	kept
End-of-life care	end of life care	end of life care	end of life care	end-of-life care	137	case_or_format
instrumentation	instrumentation	instrumentation	instrumentation	instrumentation	137	kept
medical care	medical care	medical care	medical care	medical care	137	kept
neurologic disease	neurologic disease	neurologic disease	neurologic disease	neurologic disease	137	kept
Rehabilitation	rehabilitation	rehabilitation	rehabilitation	rehabilitation	137	case_or_format
risk reduction	risk reduction	risk reduction	risk reduction	risk reduction	137	kept
Self Report	self report	self report	self report	self report	137	case_or_format
Vital Signs	vital signs	vital signs	vital sign	vital sign	137	singular
child care	child care	child care	child care	child care	136	kept
Delphi study	delphi study	delphi study	delphi study	Delphi study	136	kept

disease association	disease association	disease association	disease association	disease association	136	kept
Ethics	ethics	ethics	ethic	ethics	136	singular
multiple organ failure	multiple organ failure	multiple organ failure	multiple organ failure	multiple organ failure	136	kept
pain measurement	pain measurement	pain measurement	pain measurement	Pain Measurement	136	case_or_format
paramedical personnel	paramedical personnel	paramedical personnel	paramedical personnel	paramedical personnel	136	kept
quasi experimental study	quasi experimental study	quasi experimental study	quasi experimental study	quasi experimental study	136	kept
Blood Pressure	blood pressure	blood pressure	blood pressure	blood pressure	135	case_or_format
Health Resources	health resources	health resources	health resources	Health Resources	135	kept
Heart Diseases	heart diseases	heart diseases	heart disease	Heart Diseases	135	singular
hospital cost	hospital cost	hospital cost	hospital cost	Hospital Costs	135	case_or_format
Hypothermia	hypothermia	hypothermia	hypothermia	Hypothermia	135	kept
occupational exposure	occupational exposure	occupational exposure	occupational exposure	occupational exposure	135	kept
Odds Ratio	odds ratio	odds ratio	odds ratio	Odds Ratio	135	kept
receiver operating characteristic	receiver operating characteristic	receiver operating characteristic	receiver operating characteristic	receiver operating characteristic	135	kept
sex difference	sex difference	sex difference	sex difference	sex difference	135	kept
burn	burn	burn	burn	Burns	134	case_or_format

Efficiency, Organizational	efficiency organizational	efficiency organizational	efficiency organizational	Efficiency, Organizational	134	kept
heparin	heparin	heparin	heparin	heparin	134	kept
Long-Term Care	long term care	long term care	long term care	long term care	134	case_or_format
MANAGEMENT	management	management	management	MANAGEMENT	134	kept
Pediatric	pediatric	pediatric	pediatric	pediatrics	134	case_or_format
perioperative period	perioperative period	perioperative period	perioperative period	perioperative period	134	kept
Sedation	sedation	sedation	sedation	sedation	134	case_or_format
experience	experience	experience	experience	experience	133	kept
Simulation	simulation	simulation	simulation	simulation	133	case_or_format
treatment withdrawal	treatment withdrawal	treatment withdrawal	treatment withdrawal	treatment withdrawal	133	kept
Ventilator-associated pneumonia	ventilator associated pneumonia	ventilator associated pneumonia	ventilator associated pneumonia	ventilator associated pneumonia	133	case_or_format
Activities of Daily Living	activities of daily living	activities of daily living	activities of daily living	Activities of Daily Living	132	kept
bacteremia	bacteremia	bacteremia	bacteremia	Bacteremia	132	case_or_format
Brain Injuries, Traumatic	brain injuries traumatic	brain injuries traumatic	brain injuries traumatic	Brain Injuries, Traumatic	132	kept
Grief	grief	grief	grief	Grief	132	kept
Learning	learning	learning	learning	learning	132	case_or_format

physiologic monitoring	physiologic monitoring	physiologic monitoring	physiologic monitoring	physiologic monitoring	132	kept
Pregnancy Complications	pregnancy complications	pregnancy complications	pregnancy complication	Pregnancy Complications	132	singular
religion	religion	religion	religion	religion	132	kept
Sex Factors	sex factors	sex factors	sex factor	Sex Factors	132	singular
Anesthesiology	anesthesiology	anesthesiology	anesthesiology	Anesthesiology	131	kept
Clinical Decision-Making	clinical decision making	clinical decision making	clinical decision making	clinical decision making	131	case_or_format
Consensus	consensus	consensus	consensus	consensus	131	case_or_format
Hospitals, Community	hospitals community	hospitals community	hospitals community	Hospitals, Community	131	kept
operating room	operating room	operating room	operating room	operating room	131	kept
Oximetry	oximetry	oximetry	oximetry	Oximetry	131	kept
severity of illness index	severity of illness index	severity of illness index	severity of illness index	Severity of Illness Index	131	case_or_format
Survival Analysis	survival analysis	survival analysis	survival analysis	Survival Analysis	131	kept
Bacterial Infections	bacterial infections	bacterial infections	bacterial infection	Bacterial Infections	130	singular
Hemodynamics	hemodynamics	hemodynamics	hemodynamic	hemodynamics	130	singular
social worker	social worker	social worker	social worker	social worker	130	kept
Subacute Care	subacute care	subacute care	subacute care	Subacute Care	130	kept

ventilator weaning	ventilator weaning	ventilator weaning	ventilator weaning	Ventilator Weaning	130	case_or_format
care behavior	care behavior	care behavior	care behavior	care behavior	129	kept
case control study	case control study	case control study	case control study	Case-Control Studies	129	case_or_format
creatinine	creatinine	creatinine	creatinine	creatinine	129	kept
general practitioner	general practitioner	general practitioner	general practitioner	general practitioner	129	kept
hemoglobin	hemoglobin	hemoglobin	hemoglobin	hemoglobin	129	kept
Motivation	motivation	motivation	motivation	motivation	129	case_or_format
cause of death	cause of death	cause of death	cause of death	cause of death	128	kept
Critical Pathways	critical pathways	critical pathways	critical pathway	Critical Pathways	128	singular
Guidelines as Topic	guidelines as topic	guidelines as topic	guidelines as topic	Guidelines as Topic	128	kept
in service training	in service training	in service training	in service training	in service training	128	kept
professional practice	professional practice	professional practice	professional practice	professional practice	128	kept
tachycardia	tachycardia	tachycardia	tachycardia	tachycardia	128	kept
bloodstream infection	bloodstream infection	bloodstream infection	bloodstream infection	bloodstream infection	127	kept
evidence-based practice	evidence based practice	evidence based practice	evidence based practice	evidence based practice	127	case_or_format
hospital bed capacity	hospital bed capacity	hospital bed capacity	hospital bed capacity	hospital bed capacity	127	kept

medical research	medical research	medical research	medical research	medical research	127	kept
Pulmonary Disease, Chronic Obstructive	pulmonary disease chronic obstructive	pulmonary disease chronic obstructive	pulmonary disease chronic obstructive	Pulmonary Disease, Chronic Obstructive	127	kept
Acinetobacter baumannii	acinetobacter baumannii	acinetobacter baumannii	acinetobacter baumannii	Acinetobacter baumannii	126	kept
cost control	cost control	cost control	cost control	cost control	126	kept
emergency	emergency	emergency	emergency	Emergencies	126	case_or_format
Hand Disinfection	hand disinfection	hand disinfection	hand disinfection	Hand Disinfection	126	kept
health care need	health care need	health care need	health care need	health care need	126	kept
Hospitals, Public	hospitals public	hospitals public	hospitals public	Hospitals, Public	126	kept
neonatologist	neonatologist	neonatologist	neonatologist	neonatologist	126	kept
polymerase chain reaction	polymerase chain reaction	polymerase chain reaction	polymerase chain reaction	polymerase chain reaction	126	kept
standardization	standardization	standardization	standardization	standardization	126	kept
Developing Countries	developing countries	developing countries	developing country	Developing Countries	125	singular
fear	fear	fear	fear	fear	125	kept
Health Care Costs	health care costs	health care costs	health care cost	health care cost	125	singular
mouth hygiene	mouth hygiene	mouth hygiene	mouth hygiene	mouth hygiene	125	kept
noise	noise	noise	noise	Noise	125	case_or_format

occupational health	occupational health	occupational health	occupational health	Occupational Health	125	case_or_format
anesthesist	anesthesist	anesthesist	anesthesist	anesthesist	124	kept
chronic kidney failure	chronic kidney failure	chronic kidney failure	chronic kidney failure	chronic kidney failure	124	kept
Great Britain	great britain	great britain	great britain	Great Britain	124	kept
Nutritional Support	nutritional support	nutritional support	nutritional support	nutritional support	124	case_or_format
QUALITY	quality	quality	quality	QUALITY	124	kept
Central Venous Catheters	central venous catheters	central venous catheters	central venous catheter	central venous catheter	123	singular
convenience sample	convenience sample	convenience sample	convenience sample	convenience sample	123	kept
hypothermia	hypothermia	hypothermia	hypothermia	Hypothermia	123	case_or_format
physiological stress	physiological stress	physiological stress	physiological stress	physiological stress	123	kept
rating scale	rating scale	rating scale	rating scale	rating scale	123	kept
sedative agent	sedative agent	sedative agent	sedative agent	sedative agent	123	kept
Sleep Wake Disorders	sleep wake disorders	sleep wake disorders	sleep wake disorders	Sleep Wake Disorders	123	kept
echocardiography	echocardiography	echocardiography	echocardiography	echocardiography	122	kept
Hand hygiene	hand hygiene	hand hygiene	hand hygiene	Hand Hygiene	122	case_or_format
headache	headache	headache	headache	headache	122	kept

injury scale	injury scale	injury scale	injury scale	injury scale	122	kept
Injury Severity Score	injury severity score	injury severity score	injury severity score	Injury Severity Score	122	kept
Methicillin-Resistant Staphylococcus aureus	methicillin resistant staphylococcus aureus	methicillin resistant staphylococcus aureus	methicillin resistant staphylococcus aureus	methicillin resistant Staphylococcus aureus	122	case_or_format
shock	shock	shock	shock	shock	122	kept
assisted ventilation	assisted ventilation	assisted ventilation	assisted ventilation	assisted ventilation	121	kept
bradycardia	bradycardia	bradycardia	bradycardia	bradycardia	121	kept
continuing education	continuing education	continuing education	continuing education	continuing education	121	kept
Cross-Over Studies	cross over studies	cross over studies	cross over studies	Cross-Over Studies	121	kept
drug efficacy	drug efficacy	drug efficacy	drug efficacy	drug efficacy	121	kept
government	government	government	government	government	121	kept
Hypoxia	hypoxia	hypoxia	hypoxia	Hypoxia	121	kept
kangaroo care	kangaroo care	kangaroo care	kangaroo care	kangaroo care	121	kept
Mental Health	mental health	mental health	mental health	mental health	121	case_or_format
Moral distress	moral distress	moral distress	moral distress	moral distress	121	case_or_format
Neonate	neonate	neonate	neonate	Neonate	121	kept
pediatrician	pediatrician	pediatrician	pediatrician	pediatrician	121	kept

publication	publication	publication	publication	publication	121	kept
Regression Analysis	regression analysis	regression analysis	regression analysis	Regression Analysis	121	kept
Staff Development	staff development	staff development	staff development	Staff Development	121	kept
very low birth weight	very low birth weight	very low birth weight	very low birth weight	very low birth weight	121	kept
Victoria	victoria	victoria	victoria	Victoria	121	kept
World Health Organization	world health organization	world health organization	world health organization	World Health Organization	121	kept
Compassion Fatigue	compassion fatigue	compassion fatigue	compassion fatigue	Compassion Fatigue	120	kept
content validity	content validity	content validity	content validity	content validity	120	kept
Ethiopia	ethiopia	ethiopia	ethiopia	Ethiopia	120	kept
hospital department	hospital department	hospital department	hospital department	hospital department	120	kept
Operating Rooms	operating rooms	operating rooms	operating room	operating room	120	singular
Personal Satisfaction	personal satisfaction	personal satisfaction	personal satisfaction	Personal Satisfaction	120	kept
screening	screening	screening	screening	screening	120	kept
Ultrasonography	ultrasonography	ultrasonography	ultrasonography	Ultrasonography	120	kept
lung embolism	lung embolism	lung embolism	lung embolism	lung embolism	119	kept
Malnutrition	malnutrition	malnutrition	malnutrition	malnutrition	119	case_or_format

metabolism	metabolism	metabolism	metabolism	metabolism	119	kept
physician assistant	physician assistant	physician assistant	physician assistant	physician assistant	119	kept
Renal Dialysis	renal dialysis	renal dialysis	renal dialysis	Renal Dialysis	119	kept
videorecording	videorecording	videorecording	videorecording	videorecording	119	kept
artificial intelligence	artificial intelligence	artificial intelligence	artificial intelligence	artificial intelligence	118	kept
bacterial colonization	bacterial colonization	bacterial colonization	bacterial colonization	bacterial colonization	118	kept
grounded theory	grounded theory	grounded theory	grounded theory	grounded theory	118	kept
Hospice Care	hospice care	hospice care	hospice care	Hospice Care	118	kept
Survey	survey	survey	survey	Survey	118	kept
continuous renal replacement therapy	continuous renal replacement therapy	continuous renal replacement therapy	continuous renal replacement therapy	continuous renal replacement therapy	117	kept
early diagnosis	early diagnosis	early diagnosis	early diagnosis	Early Diagnosis	117	case_or_format
Health Services Accessibility	health services accessibility	health services accessibility	health services accessibility	Health Services Accessibility	117	kept
Nervous System Diseases	nervous system diseases	nervous system diseases	nervous system diseases	Nervous System Diseases	117	kept
PERCEPTIONS	perceptions	perceptions	perception	perception	117	singular
professional competence	professional competence	professional competence	professional competence	Professional Competence	117	case_or_format
professional standard	professional standard	professional standard	professional standard	professional standard	117	kept

Sequential Organ Failure Assessment Score	sequential organ failure assessment score	sequential organ failure assessment score	sequential organ failure assessment score	Sequential Organ Failure Assessment Score	117	kept
communication skill	communication skill	communication skill	communication skill	communication skill	116	kept
data base	data base	data base	data base	data base	116	kept
fluid therapy	fluid therapy	fluid therapy	fluid therapy	Fluid Therapy	116	case_or_format
Geriatric Assessment	geriatric assessment	geriatric assessment	geriatric assessment	Geriatric Assessment	116	kept
Medline	medline	medline	medline	Medline	116	kept
Air Ambulances	air ambulances	air ambulances	air ambulance	Air Ambulances	115	singular
anemia	anemia	anemia	anemia	anemia	115	kept
Diabetes Mellitus	diabetes mellitus	diabetes mellitus	diabetes mellitus	diabetes mellitus	115	case_or_format
Egypt	egypt	egypt	egypt	Egypt	115	kept
Finland	finland	finland	finland	Finland	115	kept
hypnotic sedative agent	hypnotic sedative agent	hypnotic sedative agent	hypnotic sedative agent	hypnotic sedative agent	115	kept
Renal Replacement Therapy	renal replacement therapy	renal replacement therapy	renal replacement therapy	renal replacement therapy	115	case_or_format
risk factors	risk factors	risk factors	risk factor	Risk Factors	115	singular
Socioeconomic Factors	socioeconomic factors	socioeconomic factors	socioeconomic factors	Socioeconomic Factors	115	kept
Urinary Catheterization	urinary catheterization	urinary catheterization	urinary catheterization	Urinary Catheterization	115	kept

workforce	workforce	workforce	workforce	Workforce	115	case_or_format
Hemorrhage	hemorrhage	hemorrhage	hemorrhage	Hemorrhage	114	kept
in-hospital mortality	in hospital mortality	in hospital mortality	in hospital mortality	in-hospital mortality	114	kept
Note	note	note	note	Note	114	kept
Teaching	teaching	teaching	teaching	teaching	114	case_or_format
Ventilators, Mechanical	ventilators mechanical	ventilators mechanical	ventilators mechanical	Ventilators, Mechanical	114	kept
asthma	asthma	asthma	asthma	asthma	113	kept
behavior	behavior	behavior	behavior	behavior	113	kept
Communication Barriers	communication barriers	communication barriers	communication barrier	Communication Barriers	113	singular
diagnostic test accuracy study	diagnostic test accuracy study	diagnostic test accuracy study	diagnostic test accuracy study	diagnostic test accuracy study	113	kept
heart disease	heart disease	heart disease	heart disease	Heart Diseases	113	case_or_format
hygiene	hygiene	hygiene	hygiene	hygiene	113	kept
interrater reliability	interrater reliability	interrater reliability	interrater reliability	interrater reliability	113	kept
phenomenology	phenomenology	phenomenology	phenomenology	phenomenology	113	kept
risk management	risk management	risk management	risk management	Risk Management	113	case_or_format
sensitivity analysis	sensitivity analysis	sensitivity analysis	sensitivity analysis	sensitivity analysis	113	kept

gender	gender	gender	gender	gender	112	kept
Texas	texas	texas	texas	Texas	112	kept
brain death	brain death	brain death	brain death	brain death	111	kept
Israel	israel	israel	israel	Israel	111	kept
Kangaroo-Mother Care Method	kangaroo mother care method	kangaroo mother care method	kangaroo mother care method	Kangaroo-Mother Care Method	111	kept
neonate	neonate	neonate	neonate	Neonate	111	case_or_format
Organizational Objectives	organizational objectives	organizational objectives	organizational objectives	Organizational Objectives	111	kept
Preceptorship	preceptorship	preceptorship	preceptorship	Preceptorship	111	kept
Sleep Deprivation	sleep deprivation	sleep deprivation	sleep deprivation	Sleep Deprivation	111	kept
Beds	beds	beds	bed	Beds	110	singular
hospice care	hospice care	hospice care	hospice care	Hospice Care	110	case_or_format
Hospital Design and Construction	hospital design and construction	hospital design and construction	hospital design and construction	Hospital Design and Construction	110	kept
ketamine	ketamine	ketamine	ketamine	ketamine	110	kept
Medical Records	medical records	medical records	medical record	medical record	110	singular
primary medical care	primary medical care	primary medical care	primary medical care	primary medical care	110	kept
professional burnout	professional burnout	professional burnout	professional burnout	professional burnout	110	kept

respiratory care	respiratory care	respiratory care	respiratory care	respiratory care	110	kept
Resuscitation Orders	resuscitation orders	resuscitation orders	resuscitation orders	Resuscitation Orders	110	kept
Task Performance and Analysis	task performance and analysis	task performance and analysis	task performance and analysis	Task Performance and Analysis	110	kept
blood sampling	blood sampling	blood sampling	blood sampling	blood sampling	109	kept
Disaster Planning	disaster planning	disaster planning	disaster planning	Disaster Planning	109	kept
drug effect	drug effect	drug effect	drug effect	drug effect	109	kept
functional status	functional status	functional status	functional status	functional status	109	kept
general anesthesia	general anesthesia	general anesthesia	general anesthesia	general anesthesia	109	kept
Informed Consent	informed consent	informed consent	informed consent	informed consent	109	case_or_format
Observer Variation	observer variation	observer variation	observer variation	Observer Variation	109	kept
Pennsylvania	pennsylvania	pennsylvania	pennsylvania	Pennsylvania	109	kept
Power, Psychological	power psychological	power psychological	power psychological	Power, Psychological	109	kept
Registries	registries	registries	registry	Registries	109	singular
Resilience, Psychological	resilience psychological	resilience psychological	resilience psychological	Resilience, Psychological	109	kept
Skin	skin	skin	skin	Skin	109	kept
steroid	steroid	steroid	steroid	steroid	109	kept

transplantation	transplantation	transplantation	transplantation	transplantation	109	kept
Anthropology, Cultural	anthropology cultural	anthropology cultural	anthropology cultural	Anthropology, Cultural	108	kept
health status	health status	health status	health status	health status	108	kept
marriage	marriage	marriage	marriage	marriage	108	kept
model	model	model	model	model	108	kept
national health service	national health service	national health service	national health service	national health service	108	kept
occupation	occupation	occupation	occupation	occupation	108	kept
organ donor	organ donor	organ donor	organ donor	organ donor	108	kept
pediatric	pediatric	pediatric	pediatric	pediatrics	108	case_or_format
school child	school child	school child	school child	school child	108	kept
treatment planning	treatment planning	treatment planning	treatment planning	treatment planning	108	kept
vascular access	vascular access	vascular access	vascular access	vascular access	108	kept
dietitian	dietitian	dietitian	dietitian	dietitian	107	kept
electrocardiogram	electrocardiogram	electrocardiogram	electrocardiogram	electrocardiogram	107	kept
Equipment Failure	equipment failure	equipment failure	equipment failure	Equipment Failure	107	kept
Greece	greece	greece	greece	Greece	107	kept

hypoxia	hypoxia	hypoxia	hypoxia	Hypoxia	107	case_or_format
midwife	midwife	midwife	midwife	midwife	107	kept
Narration	narration	narration	narration	Narration	107	kept
postoperative pain	postoperative pain	postoperative pain	postoperative pain	Postoperative Pain	107	case_or_format
Respiratory Distress Syndrome, Newborn	respiratory distress syndrome newborn	respiratory distress syndrome newborn	respiratory distress syndrome newborn	Respiratory Distress Syndrome, Newborn	107	kept
ROC Curve	roc curve	roc curve	roc curve	ROC Curve	107	kept
self care	self care	self care	self care	self care	107	kept
sodium chloride	sodium chloride	sodium chloride	sodium chloride	sodium chloride	107	kept
Arrhythmias, Cardiac	arrhythmias cardiac	arrhythmias cardiac	arrhythmias cardiac	Arrhythmias, Cardiac	106	kept
Brain Death	brain death	brain death	brain death	brain death	106	case_or_format
Cognition	cognition	cognition	cognition	cognition	106	case_or_format
emergency physician	emergency physician	emergency physician	emergency physician	emergency physician	106	kept
isolation and purification	isolation and purification	isolation and purification	isolation and purification	isolation and purification	106	kept
Manikins	manikins	manikins	manikin	Manikins	106	singular
New South Wales	new south wales	new south wales	new south wales	New South Wales	106	kept
Self Care	self care	self care	self care	self care	106	case_or_format

disinfection	disinfection	disinfection	disinfection	disinfection	105	kept
electroencephalography	electroencephalography	electroencephalography	electroencephalography	Electroencephalography	105	case_or_format
Obesity	obesity	obesity	obesity	obesity	105	case_or_format
Positive-Pressure Respiration	positive pressure respiration	positive pressure respiration	positive pressure respiration	Positive-Pressure Respiration	105	kept
Professional Role	professional role	professional role	professional role	Professional Role	105	kept
staff	staff	staff	staff	staff	105	kept
Training	training	training	training	training	105	case_or_format
Blood Transfusion	blood transfusion	blood transfusion	blood transfusion	blood transfusion	104	case_or_format
Clinical Deterioration	clinical deterioration	clinical deterioration	clinical deterioration	Clinical Deterioration	104	kept
compassion fatigue	compassion fatigue	compassion fatigue	compassion fatigue	Compassion Fatigue	104	case_or_format
Disease Progression	disease progression	disease progression	disease progression	Disease Progression	104	kept
dying	dying	dying	dying	dying	104	kept
Educational Status	educational status	educational status	educational status	educational status	104	case_or_format
History, 20th Century	history 20th century	history 20th century	history 20th century	History, 20th Century	104	kept
hypertensive factor	hypertensive factor	hypertensive factor	hypertensive factor	hypertensive factor	104	kept
Posture	posture	posture	posture	Posture	104	kept

surgical ward	surgical ward	surgical ward	surgical ward	surgical ward	104	kept
Coronary Artery Bypass	coronary artery bypass	coronary artery bypass	coronary artery bypass	Coronary Artery Bypass	103	kept
COVID-19 pandemic	covid 19 pandemic	covid 19 pandemic	covid 19 pandemic	COVID-19 pandemic	103	kept
health practitioner	health practitioner	health practitioner	health practitioner	health practitioner	103	kept
Midwestern United States	midwestern united states	midwestern united states	midwestern united states	Midwestern United States	103	kept
Music Therapy	music therapy	music therapy	music therapy	Music Therapy	103	kept
New York	new york	new york	new york	New York	103	kept
Quality of life	quality of life	quality of life	quality of life	quality of life	103	case_or_format
Seizures	seizures	seizures	seizure	seizure	103	singular
adverse effects	adverse effects	adverse effects	adverse effect	adverse effects	102	singular
age distribution	age distribution	age distribution	age distribution	age distribution	102	kept
Anti-Infective Agents, Local	anti infective agents local	anti infective agents local	anti infective agents local	Anti-Infective Agents, Local	102	kept
Decision Making, Organizational	decision making organizational	decision making organizational	decision making organizational	Decision Making, Organizational	102	kept
Fathers	fathers	fathers	father	Fathers	102	singular
hospice	hospice	hospice	hospice	hospice	102	kept
leukocyte count	leukocyte count	leukocyte count	leukocyte count	leukocyte count	102	kept

mean arterial pressure	mean arterial pressure	mean arterial pressure	mean arterial pressure	mean arterial pressure	102	kept
Neonatal Abstinence Syndrome	neonatal abstinence syndrome	neonatal abstinence syndrome	neonatal abstinence syndrome	Neonatal Abstinence Syndrome	102	kept
nutritional status	nutritional status	nutritional status	nutritional status	nutritional status	102	kept
pathology	pathology	pathology	pathology	pathology	102	kept
pressure injury	pressure injury	pressure injury	pressure injury	pressure injury	102	kept
surgical infection	surgical infection	surgical infection	surgical infection	surgical infection	102	kept
advance care planning	advance care planning	advance care planning	advance care planning	advance care planning	101	kept
clinical audit	clinical audit	clinical audit	clinical audit	clinical audit	101	kept
community hospital	community hospital	community hospital	community hospital	community hospital	101	kept
confusion	confusion	confusion	confusion	confusion	101	kept
Emergency Treatment	emergency treatment	emergency treatment	emergency treatment	Emergency Treatment	101	kept
Hospital Bed Capacity	hospital bed capacity	hospital bed capacity	hospital bed capacity	hospital bed capacity	101	case_or_format
kidney failure	kidney failure	kidney failure	kidney failure	kidney failure	101	kept
pressure ulcer	pressure ulcer	pressure ulcer	pressure ulcer	Pressure Ulcer	101	case_or_format
respiratory tract disease	respiratory tract disease	respiratory tract disease	respiratory tract disease	respiratory tract disease	101	kept
structured questionnaire	structured questionnaire	structured questionnaire	structured questionnaire	structured questionnaire	101	kept

Apgar score	apgar score	apgar score	apgar score	Apgar score	100	kept
ceftriaxone	ceftriaxone	ceftriaxone	ceftriaxone	ceftriaxone	100	kept
Coma	coma	coma	coma	Coma	100	kept
deep vein thrombosis	deep vein thrombosis	deep vein thrombosis	deep vein thrombosis	deep vein thrombosis	100	kept
Equipment Contamination	equipment contamination	equipment contamination	equipment contamination	Equipment Contamination	100	kept
multivariate analysis	multivariate analysis	multivariate analysis	multivariate analysis	Multivariate Analysis	100	case_or_format
Pain management	pain management	pain management	pain management	Pain Management	100	case_or_format
Physical Therapy Modalities	physical therapy modalities	physical therapy modalities	physical therapy modalities	Physical Therapy Modalities	100	kept
prone position	prone position	prone position	prone position	prone position	100	kept
pulse oximetry	pulse oximetry	pulse oximetry	pulse oximetry	pulse oximetry	100	kept
Societies, Medical	societies medical	societies medical	societies medical	Societies, Medical	100	kept
aging	aging	aging	aging	aging	99	kept
Cesarean Section	cesarean section	cesarean section	cesarean section	cesarean section	99	case_or_format
congestive heart failure	congestive heart failure	congestive heart failure	congestive heart failure	congestive heart failure	99	kept
Covid-19	covid 19	covid 19	covid 19	COVID-19	99	case_or_format
dysphagia	dysphagia	dysphagia	dysphagia	dysphagia	99	kept

evaluation study	evaluation study	evaluation study	evaluation study	evaluation study	99	kept
healthcare associated infection	healthcare associated infection	healthcare associated infection	healthcare associated infection	healthcare associated infection	99	kept
intermethod comparison	intermethod comparison	intermethod comparison	intermethod comparison	intermethod comparison	99	kept
neurology	neurology	neurology	neurology	neurology	99	kept
respiratory distress syndrome	respiratory distress syndrome	respiratory distress syndrome	respiratory distress syndrome	Respiratory Distress Syndrome	99	case_or_format
Trauma	trauma	trauma	trauma	Trauma	99	kept
validation process	validation process	validation process	validation process	validation process	99	kept
antibiotic sensitivity	antibiotic sensitivity	antibiotic sensitivity	antibiotic sensitivity	antibiotic sensitivity	98	kept
correlation analysis	correlation analysis	correlation analysis	correlation analysis	correlation analysis	98	kept
diagnostic imaging	diagnostic imaging	diagnostic imaging	diagnostic imaging	diagnostic imaging	98	kept
Emergency department	emergency department	emergency department	emergency department	Emergency department	98	kept
Emergency Medicine	emergency medicine	emergency medicine	emergency medicine	emergency medicine	98	case_or_format
feedback system	feedback system	feedback system	feedback system	feedback system	98	kept
health services research	health services research	health services research	health services research	Health Services Research	98	case_or_format
hospital subdivisions and components	hospital subdivisions and components	hospital subdivisions and components	hospital subdivisions and components	hospital subdivisions and components	98	kept
Infant Mortality	infant mortality	infant mortality	infant mortality	Infant Mortality	98	kept

infection rate	infection rate	infection rate	infection rate	infection rate	98	kept
physical activity	physical activity	physical activity	physical activity	physical activity	98	kept
Practice Guidelines	practice guidelines	practice guidelines	practice guideline	practice guideline	98	singular
Prone Position	prone position	prone position	prone position	prone position	98	case_or_format
sleep disorder	sleep disorder	sleep disorder	sleep disorder	sleep disorder	98	kept
workflow	workflow	workflow	workflow	workflow	98	kept
acute respiratory failure	acute respiratory failure	acute respiratory failure	acute respiratory failure	acute respiratory failure	97	kept
advanced practice provider	advanced practice provider	advanced practice provider	advanced practice provider	advanced practice provider	97	kept
anesthesiology	anesthesiology	anesthesiology	anesthesiology	Anesthesiology	97	case_or_format
Barriers	barriers	barriers	barrier	Barriers	97	singular
Cardiac surgery	cardiac surgery	cardiac surgery	cardiac surgery	Cardiac surgery	97	kept
consciousness	consciousness	consciousness	consciousness	consciousness	97	kept
continuous infusion	continuous infusion	continuous infusion	continuous infusion	continuous infusion	97	kept
Delivery, Obstetric	delivery obstetric	delivery obstetric	delivery obstetric	Delivery, Obstetric	97	kept
Enteral nutrition	enteral nutrition	enteral nutrition	enteral nutrition	Enteral Nutrition	97	case_or_format
general hospital	general hospital	general hospital	general hospital	general hospital	97	kept

Observation	observation	observation	observation	Observation	97	kept
piperacillin plus tazobactam	piperacillin plus tazobactam	piperacillin plus tazobactam	piperacillin plus tazobactam	piperacillin plus tazobactam	97	kept
Premature Birth	premature birth	premature birth	premature birth	Premature Birth	97	kept
socioeconomics	socioeconomics	socioeconomics	socioeconomic	socioeconomics	97	singular
surgical technique	surgical technique	surgical technique	surgical technique	surgical technique	97	kept
trends	trends	trends	trends	trends	97	kept
Blood Gas Analysis	blood gas analysis	blood gas analysis	blood gas analysis	Blood Gas Analysis	96	kept
Decision Trees	decision trees	decision trees	decision tree	Decision Trees	96	singular
Developmental Disabilities	developmental disabilities	developmental disabilities	developmental disabilities	Developmental Disabilities	96	kept
disease exacerbation	disease exacerbation	disease exacerbation	disease exacerbation	disease exacerbation	96	kept
dopamine	dopamine	dopamine	dopamine	dopamine	96	kept
Evidence-based practice	evidence based practice	evidence based practice	evidence based practice	evidence based practice	96	case_or_format
Fecal Incontinence	fecal incontinence	fecal incontinence	fecal incontinence	Fecal Incontinence	96	kept
life sustaining treatment	life sustaining treatment	life sustaining treatment	life sustaining treatment	life sustaining treatment	96	kept
Neonatal Screening	neonatal screening	neonatal screening	neonatal screening	Neonatal Screening	96	kept
qualitative	qualitative	qualitative	qualitative	qualitative	96	kept

treatment indication	treatment indication	treatment indication	treatment indication	treatment indication	96	kept
adaptive behavior	adaptive behavior	adaptive behavior	adaptive behavior	adaptive behavior	95	kept
blood gas analysis	blood gas analysis	blood gas analysis	blood gas analysis	Blood Gas Analysis	95	case_or_format
breast milk	breast milk	breast milk	breast milk	breast milk	95	kept
Catheterization	catheterization	catheterization	catheterization	catheterization	95	case_or_format
Conflict, Psychological	conflict psychological	conflict psychological	conflict psychological	Conflict, Psychological	95	kept
congenital heart disease	congenital heart disease	congenital heart disease	congenital heart disease	congenital heart disease	95	kept
coronary artery bypass graft	coronary artery bypass graft	coronary artery bypass graft	coronary artery bypass graft	coronary artery bypass graft	95	kept
drug use	drug use	drug use	drug use	drug use	95	kept
gentamicin	gentamicin	gentamicin	gentamicin	gentamicin	95	kept
Health Promotion	health promotion	health promotion	health promotion	Health Promotion	95	kept
hypoxemia	hypoxemia	hypoxemia	hypoxemia	hypoxemia	95	kept
implementation science	implementation science	implementation science	implementation science	implementation science	95	kept
Infant, Extremely Premature	infant extremely premature	infant extremely premature	infant extremely premature	Infant, Extremely Premature	95	kept
nausea	nausea	nausea	nausea	nausea	95	kept
program development	program development	program development	program development	Program Development	95	case_or_format

Respiratory Therapy	respiratory therapy	respiratory therapy	respiratory therapy	Respiratory Therapy	95	kept
analysis of variance	analysis of variance	analysis of variance	analysis of variance	Analysis of Variance	94	case_or_format
decision support system	decision support system	decision support system	decision support system	decision support system	94	kept
induced hypothermia	induced hypothermia	induced hypothermia	induced hypothermia	induced hypothermia	94	kept
low birth weight	low birth weight	low birth weight	low birth weight	low birth weight	94	kept
meropenem	meropenem	meropenem	meropenem	meropenem	94	kept
Outcomes	outcomes	outcomes	outcome	OUTCOMES	94	singular
Pregnancy Outcome	pregnancy outcome	pregnancy outcome	pregnancy outcome	Pregnancy Outcome	94	kept
Quality of care	quality of care	quality of care	quality of care	Quality of care	94	kept
sociodemographics	sociodemographics	sociodemographics	sociodemographics	sociodemographics	94	kept
survey	survey	survey	survey	Survey	94	case_or_format
thorax surgery	thorax surgery	thorax surgery	thorax surgery	thorax surgery	94	kept
ventilator	ventilator	ventilator	ventilator	ventilator	94	kept
certification	certification	certification	certification	Certification	93	case_or_format
Decision making	decision making	decision making	decision making	Decision Making	93	case_or_format
Dyspnea	dyspnea	dyspnea	dyspnea	dyspnea	93	case_or_format

Linear Models	linear models	linear models	linear models	Linear Models	93	kept
Models, Educational	models educational	models educational	models educational	Models, Educational	93	kept
Multiple Organ Failure	multiple organ failure	multiple organ failure	multiple organ failure	multiple organ failure	93	case_or_format
Occupational Exposure	occupational exposure	occupational exposure	occupational exposure	occupational exposure	93	case_or_format
Pandemic	pandemic	pandemic	pandemic	Pandemics	93	case_or_format
pediatric ward	pediatric ward	pediatric ward	pediatric ward	pediatric ward	93	kept
Poland	poland	poland	poland	Poland	93	kept
rehabilitation care	rehabilitation care	rehabilitation care	rehabilitation care	rehabilitation care	93	kept
rehabilitation center	rehabilitation center	rehabilitation center	rehabilitation center	rehabilitation center	93	kept
skin care	skin care	skin care	skin care	Skin Care	93	case_or_format
Body Temperature	body temperature	body temperature	body temperature	body temperature	92	case_or_format
breathing	breathing	breathing	breathing	breathing	92	kept
CARE	care	care	care	CARE	92	kept
Complications	complications	complications	complication	complication	92	singular
Coronavirus	coronavirus	coronavirus	coronavirus	Coronavirus	92	kept
data analysis software	data analysis software	data analysis software	data analysis software	data analysis software	92	kept

Hospitals, Urban	hospitals urban	hospitals urban	hospitals urban	Hospitals, Urban	92	kept
invasive ventilation	invasive ventilation	invasive ventilation	invasive ventilation	invasive ventilation	92	kept
neoplasm	neoplasm	neoplasm	neoplasm	Neoplasms	92	case_or_format
Neurology	neurology	neurology	neurology	neurology	92	case_or_format
Nutritional Status	nutritional status	nutritional status	nutritional status	nutritional status	92	case_or_format
stroke	stroke	stroke	stroke	Stroke	92	case_or_format
suction	suction	suction	suction	Suction	92	case_or_format
Advance Care Planning	advance care planning	advance care planning	advance care planning	advance care planning	91	case_or_format
conscious sedation	conscious sedation	conscious sedation	conscious sedation	conscious sedation	91	kept
equipment design	equipment design	equipment design	equipment design	Equipment Design	91	case_or_format
Factor Analysis, Statistical	factor analysis statistical	factor analysis statistical	factor analysis statistical	Factor Analysis, Statistical	91	kept
Medical Futility	medical futility	medical futility	medical futility	Medical Futility	91	kept
Primary Health Care	primary health care	primary health care	primary health care	Primary Health Care	91	kept
Recovery of Function	recovery of function	recovery of function	recovery of function	Recovery of Function	91	kept
relative	relative	relative	relative	relative	91	kept
Richmond Agitation Sedation Scale	richmond agitation sedation scale	richmond agitation sedation scale	richmond agitation sedation scale	Richmond Agitation Sedation Scale	91	kept

RISK-FACTORS	risk factors	risk factors	risk factor	Risk Factors	91	singular
smoking	smoking	smoking	smoking	smoking	91	kept
transitional care	transitional care	transitional care	transitional care	transitional care	91	kept
visual analog scale	visual analog scale	visual analog scale	visual analog scale	visual analog scale	91	kept
albumin	albumin	albumin	albumin	albumin	90	kept
body weight	body weight	body weight	body weight	body weight	90	kept
Cardiology	cardiology	cardiology	cardiology	Cardiology	90	kept
care bundle	care bundle	care bundle	care bundle	care bundle	90	kept
clinical pathway	clinical pathway	clinical pathway	clinical pathway	clinical pathway	90	kept
falling	falling	falling	falling	falling	90	kept
high risk population	high risk population	high risk population	high risk population	high risk population	90	kept
private hospital	private hospital	private hospital	private hospital	private hospital	90	kept
Retinopathy of Prematurity	retinopathy of prematurity	retinopathy of prematurity	retinopathy of prematurity	Retinopathy of Prematurity	90	kept
Assessment	assessment	assessment	assessment	Assessment	89	kept
Attitudes	attitudes	attitudes	attitude	attitude	89	singular
elective surgery	elective surgery	elective surgery	elective surgery	elective surgery	89	kept

enteral nutrition	enteral nutrition	enteral nutrition	enteral nutrition	Enteral Nutrition	89	case_or_format
family centered care	family centered care	family centered care	family centered care	family centered care	89	kept
health promotion	health promotion	health promotion	health promotion	Health Promotion	89	case_or_format
Hypotension	hypotension	hypotension	hypotension	hypotension	89	case_or_format
internal medicine	internal medicine	internal medicine	internal medicine	internal medicine	89	kept
Lung Diseases	lung diseases	lung diseases	lung disease	Lung Diseases	89	singular
Medical Audit	medical audit	medical audit	medical audit	Medical Audit	89	kept
Mentors	mentors	mentors	mentor	Mentors	89	singular
Personal Protective Equipment	personal protective equipment	personal protective equipment	personal protective equipment	Personal Protective Equipment	89	kept
pneumothorax	pneumothorax	pneumothorax	pneumothorax	pneumothorax	89	kept
Queensland	queensland	queensland	queensland	Queensland	89	kept
Thailand	thailand	thailand	thailand	Thailand	89	kept
Work Schedule Tolerance	work schedule tolerance	work schedule tolerance	work schedule tolerance	Work Schedule Tolerance	89	kept
antimicrobial stewardship	antimicrobial stewardship	antimicrobial stewardship	antimicrobial stewardship	antimicrobial stewardship	88	kept
atrial fibrillation	atrial fibrillation	atrial fibrillation	atrial fibrillation	atrial fibrillation	88	kept
clinical research	clinical research	clinical research	clinical research	clinical research	88	kept

dialysis	dialysis	dialysis	dialysis	dialysis	88	kept
drug withdrawal	drug withdrawal	drug withdrawal	drug withdrawal	drug withdrawal	88	kept
Hospitals, General	hospitals general	hospitals general	hospitals general	Hospitals, General	88	kept
Intracranial Hypertension	intracranial hypertension	intracranial hypertension	intracranial hypertension	Intracranial Hypertension	88	kept
Massage	massage	massage	massage	Massage	88	kept
occupational therapy	occupational therapy	occupational therapy	occupational therapy	occupational therapy	88	kept
Propofol	propofol	propofol	propofol	propofol	88	case_or_format
statistical analysis	statistical analysis	statistical analysis	statistical analysis	statistical analysis	88	kept
subarachnoid hemorrhage	subarachnoid hemorrhage	subarachnoid hemorrhage	subarachnoid hemorrhage	subarachnoid hemorrhage	88	kept
task performance	task performance	task performance	task performance	task performance	88	kept
correlation coefficient	correlation coefficient	correlation coefficient	correlation coefficient	correlation coefficient	87	kept
Costs and Cost Analysis	costs and cost analysis	costs and cost analysis	costs and cost analysis	Costs and Cost Analysis	87	kept
Dexmedetomidine	dexmedetomidine	dexmedetomidine	dexmedetomidine	dexmedetomidine	87	case_or_format
Diarrhea	diarrhea	diarrhea	diarrhea	diarrhea	87	case_or_format
Medication errors	medication errors	medication errors	medication error	Medication Errors	87	singular
narcotic analgesic agent	narcotic analgesic agent	narcotic analgesic agent	narcotic analgesic agent	narcotic analgesic agent	87	kept

neurologic examination	neurologic examination	neurologic examination	neurologic examination	neurologic examination	87	kept
Organizational Case Studies	organizational case studies	organizational case studies	organizational case studies	Organizational Case Studies	87	kept
Physician Assistants	physician assistants	physician assistants	physician assistant	physician assistant	87	singular
Teaching Rounds	teaching rounds	teaching rounds	teaching round	Teaching Rounds	87	singular
Tissue Donors	tissue donors	tissue donors	tissue donor	Tissue Donors	87	singular
Career Choice	career choice	career choice	career choice	Career Choice	86	kept
ciprofloxacin	ciprofloxacin	ciprofloxacin	ciprofloxacin	ciprofloxacin	86	kept
coronary artery disease	coronary artery disease	coronary artery disease	coronary artery disease	coronary artery disease	86	kept
developing country	developing country	developing country	developing country	Developing Countries	86	case_or_format
diagnostic accuracy	diagnostic accuracy	diagnostic accuracy	diagnostic accuracy	diagnostic accuracy	86	kept
Disease Management	disease management	disease management	disease management	Disease Management	86	kept
Drug Resistance, Multiple, Bacterial	drug resistance multiple bacterial	drug resistance multiple bacterial	drug resistance multiple bacterial	Drug Resistance, Multiple, Bacterial	86	kept
fluid resuscitation	fluid resuscitation	fluid resuscitation	fluid resuscitation	fluid resuscitation	86	kept
internal consistency	internal consistency	internal consistency	internal consistency	internal consistency	86	kept
Noninvasive Ventilation	noninvasive ventilation	noninvasive ventilation	noninvasive ventilation	noninvasive ventilation	86	case_or_format
occupational therapist	occupational therapist	occupational therapist	occupational therapist	occupational therapist	86	kept

oncology	oncology	oncology	oncology	oncology	86	kept
outcomes	outcomes	outcomes	outcome	OUTCOMES	86	singular
Physical Examination	physical examination	physical examination	physical examination	physical examination	86	case_or_format
Practice	practice	practice	practice	Practice	86	kept
prenatal care	prenatal care	prenatal care	prenatal care	prenatal care	86	kept
respiration control	respiration control	respiration control	respiration control	respiration control	86	kept
Cardiopulmonary resuscitation	cardiopulmonary resuscitation	cardiopulmonary resuscitation	cardiopulmonary resuscitation	Cardiopulmonary Resuscitation	85	case_or_format
health care management	health care management	health care management	health care management	health care management	85	kept
Implementation	implementation	implementation	implementation	Implementation	85	kept
intracranial pressure	intracranial pressure	intracranial pressure	intracranial pressure	intracranial pressure	85	kept
legal aspect	legal aspect	legal aspect	legal aspect	legal aspect	85	kept
logistic regression analysis	logistic regression analysis	logistic regression analysis	logistic regression analysis	logistic regression analysis	85	kept
Airway Management	airway management	airway management	airway management	Airway Management	84	kept
coma	coma	coma	coma	Coma	84	case_or_format
drug monitoring	drug monitoring	drug monitoring	drug monitoring	Drug Monitoring	84	case_or_format
epinephrine	epinephrine	epinephrine	epinephrine	epinephrine	84	kept

Fatal Outcome	fatal outcome	fatal outcome	fatal outcome	Fatal Outcome	84	kept
Fear	fear	fear	fear	fear	84	case_or_format
glycemic control	glycemic control	glycemic control	glycemic control	glycemic control	84	kept
head injury	head injury	head injury	head injury	head injury	84	kept
Health Policy	health policy	health policy	health policy	Health Policy	84	kept
Health Status	health status	health status	health status	health status	84	case_or_format
Infant, Low Birth Weight	infant low birth weight	infant low birth weight	infant low birth weight	Infant, Low Birth Weight	84	kept
Ohio	ohio	ohio	ohio	Ohio	84	kept
social media	social media	social media	social media	social media	84	kept
Surgical Procedures, Operative	surgical procedures operative	surgical procedures operative	surgical procedures operative	Surgical Procedures, Operative	84	kept
Workflow	workflow	workflow	workflow	workflow	84	case_or_format
Accidental Falls	accidental falls	accidental falls	accidental falls	Accidental Falls	83	kept
accreditation	accreditation	accreditation	accreditation	accreditation	83	kept
acute care	acute care	acute care	acute care	acute care	83	kept
bronchoscopy	bronchoscopy	bronchoscopy	bronchoscopy	bronchoscopy	83	kept
confidence interval	confidence interval	confidence interval	confidence interval	confidence interval	83	kept

Diffusion of Innovation	diffusion of innovation	diffusion of innovation	diffusion of innovation	Diffusion of Innovation	83	kept
disease surveillance	disease surveillance	disease surveillance	disease surveillance	disease surveillance	83	kept
Guidelines	guidelines	guidelines	guideline	Guidelines	83	singular
lorazepam	lorazepam	lorazepam	lorazepam	lorazepam	83	kept
Neurosurgery	neurosurgery	neurosurgery	neurosurgery	neurosurgery	83	case_or_format
operation duration	operation duration	operation duration	operation duration	operation duration	83	kept
parents	parents	parents	parent	Parents	83	singular
professional development	professional development	professional development	professional development	professional development	83	kept
Respiration	respiration	respiration	respiration	Respiration	83	kept
Singapore	singapore	singapore	singapore	Singapore	83	kept
statistical significance	statistical significance	statistical significance	statistical significance	statistical significance	83	kept
tidal volume	tidal volume	tidal volume	tidal volume	tidal volume	83	kept
Vaccination	vaccination	vaccination	vaccination	vaccination	83	case_or_format
Advance Directives	advance directives	advance directives	advance directive	Advance Directives	82	singular
Biomarkers	biomarkers	biomarkers	biomarker	Biomarkers	82	singular
Cost of Illness	cost of illness	cost of illness	cost of illness	Cost of Illness	82	kept

dexamethasone	dexamethasone	dexamethasone	dexamethasone	dexamethasone	82	kept
manager	manager	manager	manager	manager	82	kept
Morphine	morphine	morphine	morphine	morphine	82	case_or_format
mother child relation	mother child relation	mother child relation	mother child relation	Mother-Child Relations	82	case_or_format
newborn mortality	newborn mortality	newborn mortality	newborn mortality	newborn mortality	82	kept
outcomes research	outcomes research	outcomes research	outcomes research	outcomes research	82	kept
Parenteral Nutrition	parenteral nutrition	parenteral nutrition	parenteral nutrition	parenteral nutrition	82	case_or_format
Perinatal Care	perinatal care	perinatal care	perinatal care	Perinatal Care	82	kept
Pneumonia, Bacterial	pneumonia bacterial	pneumonia bacterial	pneumonia bacterial	Pneumonia, Bacterial	82	kept
Postoperative Period	postoperative period	postoperative period	postoperative period	postoperative period	82	case_or_format
sample size	sample size	sample size	sample size	sample size	82	kept
Single-Blind Method	single blind method	single blind method	single blind method	Single-Blind Method	82	kept
Telemetry	telemetry	telemetry	telemetry	Telemetry	82	kept
Video Recording	video recording	video recording	video recording	Video Recording	82	kept
Violence	violence	violence	violence	Violence	82	kept
virus transmission	virus transmission	virus transmission	virus transmission	virus transmission	82	kept

alcohol	alcohol	alcohol	alcohol	alcohol	81	kept
bacterium culture	bacterium culture	bacterium culture	bacterium culture	bacterium culture	81	kept
Bandages	bandages	bandages	bandage	Bandages	81	singular
Cause of Death	cause of death	cause of death	cause of death	cause of death	81	case_or_format
convalescence	convalescence	convalescence	convalescence	convalescence	81	kept
Counseling	counseling	counseling	counseling	Counseling	81	kept
Ergonomics	ergonomics	ergonomics	ergonomic	Ergonomics	81	singular
influenza	influenza	influenza	influenza	influenza	81	kept
Neurologic Examination	neurologic examination	neurologic examination	neurologic examination	neurologic examination	81	case_or_format
non insulin dependent diabetes mellitus	non insulin dependent diabetes mellitus	non insulin dependent diabetes mellitus	non insulin dependent diabetes mellitus	non insulin dependent diabetes mellitus	81	kept
practice	practice	practice	practice	Practice	81	case_or_format
resource allocation	resource allocation	resource allocation	resource allocation	resource allocation	81	kept
Touch	touch	touch	touch	Touch	81	kept
Trust	trust	trust	trust	Trust	81	kept
utilization review	utilization review	utilization review	utilization review	utilization review	81	kept
wound care	wound care	wound care	wound care	wound care	81	kept

Awareness	awareness	awareness	awareness	awareness	80	case_or_format
biological marker	biological marker	biological marker	biological marker	biological marker	80	kept
Brain	brain	brain	brain	Brain	80	kept
COMMUNICATION	communication	communication	communication	Communication	80	case_or_format
Computer-Assisted Instruction	computer assisted instruction	computer assisted instruction	computer assisted instruction	Computer-Assisted Instruction	80	kept
Deglutition Disorders	deglutition disorders	deglutition disorders	deglutition disorder	Deglutition Disorders	80	singular
emergency department	emergency department	emergency department	emergency department	Emergency department	80	case_or_format
genetics	genetics	genetics	genetics	genetics	80	kept
Infant Behavior	infant behavior	infant behavior	infant behavior	Infant Behavior	80	kept
Job satisfaction	job satisfaction	job satisfaction	job satisfaction	Job Satisfaction	80	case_or_format
Length of stay	length of stay	length of stay	length of stay	Length of Stay	80	case_or_format
mortality risk	mortality risk	mortality risk	mortality risk	mortality risk	80	kept
multidrug resistance	multidrug resistance	multidrug resistance	multidrug resistance	multidrug resistance	80	kept
organ transplantation	organ transplantation	organ transplantation	organ transplantation	organ transplantation	80	kept
Parenting	parenting	parenting	parenting	Parenting	80	kept
South Korea	south korea	south korea	south korea	South Korea	80	kept

thorax pain	thorax pain	thorax pain	thorax pain	thorax pain	80	kept
abdominal pain	abdominal pain	abdominal pain	abdominal pain	abdominal pain	79	kept
acute coronary syndrome	acute coronary syndrome	acute coronary syndrome	acute coronary syndrome	acute coronary syndrome	79	kept
adverse outcome	adverse outcome	adverse outcome	adverse outcome	adverse outcome	79	kept
cardiac surgery	cardiac surgery	cardiac surgery	cardiac surgery	Cardiac surgery	79	case_or_format
Cerebral Hemorrhage	cerebral hemorrhage	cerebral hemorrhage	cerebral hemorrhage	Cerebral Hemorrhage	79	kept
Compliance	compliance	compliance	compliance	Compliance	79	kept
conceptual framework	conceptual framework	conceptual framework	conceptual framework	conceptual framework	79	kept
depersonalization	depersonalization	depersonalization	depersonalization	depersonalization	79	kept
Double-Blind Method	double blind method	double blind method	double blind method	Double-Blind Method	79	kept
ethical dilemma	ethical dilemma	ethical dilemma	ethical dilemma	ethical dilemma	79	kept
Healthcare workers	healthcare workers	healthcare workers	healthcare worker	Healthcare workers	79	singular
History, 21st Century	history 21st century	history 21st century	history 21st century	History, 21st Century	79	kept
lactic acid	lactic acid	lactic acid	lactic acid	lactic acid	79	kept
medical personnel	medical personnel	medical personnel	medical personnel	medical personnel	79	kept
oxygenation	oxygenation	oxygenation	oxygenation	oxygenation	79	kept

psychologist	psychologist	psychologist	psychologist	psychologist	79	kept
reimbursement	reimbursement	reimbursement	reimbursement	reimbursement	79	kept
research	research	research	research	research	79	kept
spiritual care	spiritual care	spiritual care	spiritual care	spiritual care	79	kept
trust	trust	trust	trust	Trust	79	case_or_format
utilization	utilization	utilization	utilization	utilization	79	kept
attitude to death	attitude to death	attitude to death	attitude to death	Attitude to Death	78	case_or_format
bereavement	bereavement	bereavement	bereavement	Bereavement	78	case_or_format
blood glucose monitoring	blood glucose monitoring	blood glucose monitoring	blood glucose monitoring	blood glucose monitoring	78	kept
brain ischemia	brain ischemia	brain ischemia	brain ischemia	brain ischemia	78	kept
cardiogenic shock	cardiogenic shock	cardiogenic shock	cardiogenic shock	cardiogenic shock	78	kept
Computer Simulation	computer simulation	computer simulation	computer simulation	Computer Simulation	78	kept
Conscious Sedation	conscious sedation	conscious sedation	conscious sedation	conscious sedation	78	case_or_format
Cost Savings	cost savings	cost savings	cost saving	Cost Savings	78	singular
Device Removal	device removal	device removal	device removal	Device Removal	78	kept
Health Plan Implementation	health plan implementation	health plan implementation	health plan implementation	Health Plan Implementation	78	kept

heart output	heart output	heart output	heart output	heart output	78	kept
insomnia	insomnia	insomnia	insomnia	insomnia	78	kept
Intention	intention	intention	intention	Intention	78	kept
Models, Psychological	models psychological	models psychological	models psychological	Models, Psychological	78	kept
Object Attachment	object attachment	object attachment	object attachment	Object Attachment	78	kept
Pharmacists	pharmacists	pharmacists	pharmacist	pharmacist	78	singular
Premature	premature	premature	premature	Premature	78	kept
symptom	symptom	symptom	symptom	symptom	78	kept
working time	working time	working time	working time	working time	78	kept
Age Distribution	age distribution	age distribution	age distribution	age distribution	77	case_or_format
anxiety disorder	anxiety disorder	anxiety disorder	anxiety disorder	anxiety disorder	77	kept
Bacteria	bacteria	bacteria	bacteria	Bacteria	77	kept
benzodiazepine	benzodiazepine	benzodiazepine	benzodiazepine	benzodiazepine	77	kept
Birth Weight	birth weight	birth weight	birth weight	birth weight	77	case_or_format
Causality	causality	causality	causality	Causality	77	kept
clinical handover	clinical handover	clinical handover	clinical handover	clinical handover	77	kept

comfort	comfort	comfort	comfort	comfort	77	kept
cost	cost	cost	cost	cost	77	kept
Decision Support Techniques	decision support techniques	decision support techniques	decision support technique	Decision Support Techniques	77	singular
dobutamine	dobutamine	dobutamine	dobutamine	dobutamine	77	kept
empowerment	empowerment	empowerment	empowerment	empowerment	77	kept
Family-centered care	family centered care	family centered care	family centered care	family centered care	77	case_or_format
HEALTH-CARE	health care	health care	health care	health care	77	case_or_format
Infection	infection	infection	infection	infection	77	case_or_format
management	management	management	management	MANAGEMENT	77	case_or_format
Morbidity	morbidity	morbidity	morbidity	morbidity	77	case_or_format
Multiple Trauma	multiple trauma	multiple trauma	multiple trauma	Multiple Trauma	77	kept
postoperative delirium	postoperative delirium	postoperative delirium	postoperative delirium	postoperative delirium	77	kept
prophylaxis	prophylaxis	prophylaxis	prophylaxis	prophylaxis	77	kept
quality of care	quality of care	quality of care	quality of care	Quality of care	77	case_or_format
resilience	resilience	resilience	resilience	resilience	77	kept
ventilator-associated pneumonia	ventilator associated pneumonia	ventilator associated pneumonia	ventilator associated pneumonia	ventilator associated pneumonia	77	case_or_format

weaning	weaning	weaning	weaning	weaning	77	kept
cefepime	cefepime	cefepime	cefepime	cefepime	76	kept
early warning score	early warning score	early warning score	early warning score	early warning score	76	kept
employment	employment	employment	employment	employment	76	kept
End of life	end of life	end of life	end of life	End of life	76	kept
end of life	end of life	end of life	end of life	End of life	76	case_or_format
Enterocolitis, Necrotizing	enterocolitis necrotizing	enterocolitis necrotizing	enterocolitis necrotizing	Enterocolitis, Necrotizing	76	kept
family counseling	family counseling	family counseling	family counseling	family counseling	76	kept
father	father	father	father	Fathers	76	case_or_format
Grounded Theory	grounded theory	grounded theory	grounded theory	grounded theory	76	case_or_format
Models, Theoretical	models theoretical	models theoretical	models theoretical	Models, Theoretical	76	kept
point of care testing	point of care testing	point of care testing	point of care testing	point of care testing	76	kept
Resilience	resilience	resilience	resilience	resilience	76	case_or_format
respiratory tract infection	respiratory tract infection	respiratory tract infection	respiratory tract infection	respiratory tract infection	76	kept
Software	software	software	software	Software	76	kept
Teamwork	teamwork	teamwork	teamwork	teamwork	76	case_or_format

Venous Thrombosis	venous thrombosis	venous thrombosis	venous thrombosis	Venous Thrombosis	76	kept
counseling	counseling	counseling	counseling	Counseling	75	case_or_format
Drug Administration Schedule	drug administration schedule	drug administration schedule	drug administration schedule	Drug Administration Schedule	75	kept
drug megadose	drug megadose	drug megadose	drug megadose	drug megadose	75	kept
Environment	environment	environment	environment	Environment	75	kept
Goals	goals	goals	goals	Goals	75	kept
haloperidol	haloperidol	haloperidol	haloperidol	haloperidol	75	kept
immunosuppressive treatment	immunosuppressive treatment	immunosuppressive treatment	immunosuppressive treatment	immunosuppressive treatment	75	kept
inflammation	inflammation	inflammation	inflammation	inflammation	75	kept
Military Personnel	military personnel	military personnel	military personnel	Military Personnel	75	kept
music therapy	music therapy	music therapy	music therapy	Music Therapy	75	case_or_format
Pharmaceutical Preparations	pharmaceutical preparations	pharmaceutical preparations	pharmaceutical preparations	Pharmaceutical Preparations	75	kept
pharmacy	pharmacy	pharmacy	pharmacy	pharmacy	75	kept
Preoperative Care	preoperative care	preoperative care	preoperative care	Preoperative Care	75	kept
Pressure injury	pressure injury	pressure injury	pressure injury	pressure injury	75	case_or_format
Qualitative	qualitative	qualitative	qualitative	qualitative	75	case_or_format

risk	risk	risk	risk	risk	75	kept
Vasoconstrictor Agents	vasoconstrictor agents	vasoconstrictor agents	vasoconstrictor agent	Vasoconstrictor Agents	75	singular
air medical transport	air medical transport	air medical transport	air medical transport	air medical transport	74	kept
Artificial Intelligence	artificial intelligence	artificial intelligence	artificial intelligence	artificial intelligence	74	case_or_format
Cardiac arrest	cardiac arrest	cardiac arrest	cardiac arrest	Cardiac arrest	74	kept
cardiology	cardiology	cardiology	cardiology	Cardiology	74	case_or_format
diastolic blood pressure	diastolic blood pressure	diastolic blood pressure	diastolic blood pressure	diastolic blood pressure	74	kept
Drainage	drainage	drainage	drainage	Drainage	74	kept
emergency treatment	emergency treatment	emergency treatment	emergency treatment	Emergency Treatment	74	case_or_format
equipment	equipment	equipment	equipment	equipment	74	kept
ethnicity	ethnicity	ethnicity	ethnicity	ethnicity	74	kept
grief	grief	grief	grief	Grief	74	case_or_format
Health care	health care	health care	health care	health care	74	case_or_format
Mental health	mental health	mental health	mental health	mental health	74	case_or_format
Microbial Sensitivity Tests	microbial sensitivity tests	microbial sensitivity tests	microbial sensitivity test	Microbial Sensitivity Tests	74	singular
pain management	pain management	pain management	pain management	Pain Management	74	case_or_format

Proportional Hazards Models	proportional hazards models	proportional hazards models	proportional hazards model	Proportional Hazards Models	74	singular
protective equipment	protective equipment	protective equipment	protective equipment	protective equipment	74	kept
pulmonary hypertension	pulmonary hypertension	pulmonary hypertension	pulmonary hypertension	pulmonary hypertension	74	kept
software	software	software	software	Software	74	case_or_format
Substance Withdrawal Syndrome	substance withdrawal syndrome	substance withdrawal syndrome	substance withdrawal syndrome	Substance Withdrawal Syndrome	74	kept
Time and Motion Studies	time and motion studies	time and motion studies	time and motion study	Time and Motion Studies	74	singular
acetylsalicylic acid	acetylsalicylic acid	acetylsalicylic acid	acetylsalicylic acid	acetylsalicylic acid	73	kept
Alberta	alberta	alberta	alberta	Alberta	73	kept
continuous positive airway pressure	continuous positive airway pressure	continuous positive airway pressure	continuous positive airway pressure	Continuous Positive Airway Pressure	73	case_or_format
Equipment and Supplies	equipment and supplies	equipment and supplies	equipment and supplies	Equipment and Supplies	73	kept
Frail Elderly	frail elderly	frail elderly	frail elderly	Frail Elderly	73	kept
Infusion Pumps	infusion pumps	infusion pumps	infusion pump	Infusion Pumps	73	singular
inotropic agent	inotropic agent	inotropic agent	inotropic agent	inotropic agent	73	kept
Intubation	intubation	intubation	intubation	intubation	73	case_or_format
job performance	job performance	job performance	job performance	job performance	73	kept
lung disease	lung disease	lung disease	lung disease	Lung Diseases	73	case_or_format

Michigan	michigan	michigan	michigan	Michigan	73	kept
necrotizing enterocolitis	necrotizing enterocolitis	necrotizing enterocolitis	necrotizing enterocolitis	necrotizing enterocolitis	73	kept
Postnatal Care	postnatal care	postnatal care	postnatal care	Postnatal Care	73	kept
Prenatal Care	prenatal care	prenatal care	prenatal care	prenatal care	73	case_or_format
Subarachnoid Hemorrhage	subarachnoid hemorrhage	subarachnoid hemorrhage	subarachnoid hemorrhage	subarachnoid hemorrhage	73	case_or_format
Tomography, X-Ray Computed	tomography x ray computed	tomography x ray computed	tomography x ray computed	Tomography, X-Ray Computed	73	kept
accuracy	accuracy	accuracy	accuracy	accuracy	72	kept
administrative personnel	administrative personnel	administrative personnel	administrative personnel	administrative personnel	72	kept
apnea	apnea	apnea	apnea	apnea	72	kept
ATTITUDES	attitudes	attitudes	attitude	attitude	72	singular
barriers	barriers	barriers	barrier	Barriers	72	singular
Cinahl	cinahl	cinahl	cinahl	Cinahl	72	kept
clinical practice guideline	clinical practice guideline	clinical practice guideline	clinical practice guideline	clinical practice guideline	72	kept
community care	community care	community care	community care	community care	72	kept
Dermatitis	dermatitis	dermatitis	dermatitis	Dermatitis	72	kept
EDUCATION	education	education	education	education	72	case_or_format

Iatrogenic Disease	iatrogenic disease	iatrogenic disease	iatrogenic disease	Iatrogenic Disease	72	kept
Intracranial Pressure	intracranial pressure	intracranial pressure	intracranial pressure	intracranial pressure	72	case_or_format
Medical Records Systems, Computerized	medical records systems computerized	medical records systems computerized	medical records systems computerized	Medical Records Systems, Computerized	72	kept
Midazolam	midazolam	midazolam	midazolam	midazolam	72	case_or_format
nutritional assessment	nutritional assessment	nutritional assessment	nutritional assessment	nutritional assessment	72	kept
Pakistan	pakistan	pakistan	pakistan	Pakistan	72	kept
Premature infant	premature infant	premature infant	premature infant	Premature infant	72	kept
RISK	risk	risk	risk	risk	72	case_or_format
Surgery	surgery	surgery	surgery	surgery	72	case_or_format
Technology	technology	technology	technology	Technology	72	kept
technology	technology	technology	technology	Technology	72	case_or_format
traffic accident	traffic accident	traffic accident	traffic accident	traffic accident	72	kept
User-Computer Interface	user computer interface	user computer interface	user computer interface	User-Computer Interface	72	kept
venous thromboembolism	venous thromboembolism	venous thromboembolism	venous thromboembolism	venous thromboembolism	72	kept
Anti-Infective Agents	anti infective agents	anti infective agents	anti infective agents	Anti-Infective Agents	71	kept
bacterial infection	bacterial infection	bacterial infection	bacterial infection	Bacterial Infections	71	case_or_format

child hospitalization	child hospitalization	child hospitalization	child hospitalization	child hospitalization	71	kept
conflict	conflict	conflict	conflict	conflict	71	kept
Confusion	confusion	confusion	confusion	confusion	71	case_or_format
Data Interpretation, Statistical	data interpretation statistical	data interpretation statistical	data interpretation statistical	Data Interpretation, Statistical	71	kept
Decision-making	decision making	decision making	decision making	Decision Making	71	case_or_format
disability	disability	disability	disability	disability	71	kept
Drug-Related Side Effects and Adverse Reactions	drug related side effects and adverse reactions	drug related side effects and adverse reactions	drug related side effects and adverse reactions	Drug-Related Side Effects and Adverse Reactions	71	kept
Epidemiology	epidemiology	epidemiology	epidemiology	epidemiology	71	case_or_format
exploratory research	exploratory research	exploratory research	exploratory research	exploratory research	71	kept
Extracorporeal membrane oxygenation	extracorporeal membrane oxygenation	extracorporeal membrane oxygenation	extracorporeal membrane oxygenation	Extracorporeal Membrane Oxygenation	71	case_or_format
Management	management	management	management	MANAGEMENT	71	case_or_format
medicaid	medicaid	medicaid	medicaid	medicaid	71	kept
Monitoring	monitoring	monitoring	monitoring	monitoring	71	case_or_format
policy	policy	policy	policy	policy	71	kept
Shock	shock	shock	shock	shock	71	case_or_format
social status	social status	social status	social status	social status	71	kept

aftercare	aftercare	aftercare	aftercare	Aftercare	70	case_or_format
Education, Medical, Continuing	education medical continuing	education medical continuing	education medical continuing	Education, Medical, Continuing	70	kept
high flow nasal cannula therapy	high flow nasal cannula therapy	high flow nasal cannula therapy	high flow nasal cannula therapy	high flow nasal cannula therapy	70	kept
history	history	history	history	history	70	kept
hospital information system	hospital information system	hospital information system	hospital information system	hospital information system	70	kept
medical technology	medical technology	medical technology	medical technology	medical technology	70	kept
Pressure	pressure	pressure	pressure	Pressure	70	kept
Risk	risk	risk	risk	risk	70	case_or_format
unclassified drug	unclassified drug	unclassified drug	unclassified drug	unclassified drug	70	kept
Validity	validity	validity	validity	validity	70	case_or_format
Wound Healing	wound healing	wound healing	wound healing	Wound Healing	70	kept
aggression	aggression	aggression	aggression	aggression	69	kept
area under the curve	area under the curve	area under the curve	area under the curve	area under the curve	69	kept
attitudes	attitudes	attitudes	attitude	attitude	69	singular
Baths	baths	baths	bath	Baths	69	singular
Benzodiazepines	benzodiazepines	benzodiazepines	benzodiazepine	benzodiazepine	69	singular

Cronbach alpha coefficient	cronbach alpha coefficient	cronbach alpha coefficient	cronbach alpha coefficient	Cronbach alpha coefficient	69	kept
cultural factor	cultural factor	cultural factor	cultural factor	cultural factor	69	kept
Disinfection	disinfection	disinfection	disinfection	disinfection	69	case_or_format
healthcare workers	healthcare workers	healthcare workers	healthcare worker	Healthcare workers	69	singular
neuroleptic agent	neuroleptic agent	neuroleptic agent	neuroleptic agent	neuroleptic agent	69	kept
Phlebotomy	phlebotomy	phlebotomy	phlebotomy	Phlebotomy	69	kept
Portugal	portugal	portugal	portugal	Portugal	69	kept
program evaluation	program evaluation	program evaluation	program evaluation	Program Evaluation	69	case_or_format
Risk assessment	risk assessment	risk assessment	risk assessment	Risk Assessment	69	case_or_format
ultrasound	ultrasound	ultrasound	ultrasound	ultrasound	69	kept
wound healing	wound healing	wound healing	wound healing	Wound Healing	69	case_or_format
Ambulatory Care	ambulatory care	ambulatory care	ambulatory care	Ambulatory Care	68	kept
Atrial Fibrillation	atrial fibrillation	atrial fibrillation	atrial fibrillation	atrial fibrillation	68	case_or_format
Body Mass Index	body mass index	body mass index	body mass index	Body Mass Index	68	kept
Consciousness	consciousness	consciousness	consciousness	consciousness	68	case_or_format
e-mail	e mail	e mail	e mail	e-mail	68	kept

Hong Kong	hong kong	hong kong	hong kong	Hong Kong	68	kept
hospitalized child	hospitalized child	hospitalized child	hospitalized child	hospitalized child	68	kept
hydrocortisone	hydrocortisone	hydrocortisone	hydrocortisone	hydrocortisone	68	kept
job experience	job experience	job experience	job experience	job experience	68	kept
Life Change Events	life change events	life change events	life change events	Life Change Events	68	kept
organizational culture	organizational culture	organizational culture	organizational culture	Organizational Culture	68	case_or_format
tachypnea	tachypnea	tachypnea	tachypnea	tachypnea	68	kept
trauma	trauma	trauma	trauma	Trauma	68	case_or_format
Acute Coronary Syndrome	acute coronary syndrome	acute coronary syndrome	acute coronary syndrome	acute coronary syndrome	67	case_or_format
amikacin	amikacin	amikacin	amikacin	amikacin	67	kept
Anesthesia Recovery Period	anesthesia recovery period	anesthesia recovery period	anesthesia recovery period	Anesthesia Recovery Period	67	kept
Bed Occupancy	bed occupancy	bed occupancy	bed occupancy	Bed Occupancy	67	kept
benzodiazepine derivative	benzodiazepine derivative	benzodiazepine derivative	benzodiazepine derivative	benzodiazepine derivative	67	kept
chlorhexidine gluconate	chlorhexidine gluconate	chlorhexidine gluconate	chlorhexidine gluconate	chlorhexidine gluconate	67	kept
comparative effectiveness	comparative effectiveness	comparative effectiveness	comparative effectiveness	comparative effectiveness	67	kept
creatinine blood level	creatinine blood level	creatinine blood level	creatinine blood level	creatinine blood level	67	kept

Culture	culture	culture	culture	Culture	67	kept
Elderly	elderly	elderly	elderly	Elderly	67	kept
emergency surgery	emergency surgery	emergency surgery	emergency surgery	emergency surgery	67	kept
environmental factor	environmental factor	environmental factor	environmental factor	environmental factor	67	kept
EXPERIENCES	experiences	experiences	experience	experience	67	singular
feeding	feeding	feeding	feeding	feeding	67	kept
gastrointestinal hemorrhage	gastrointestinal hemorrhage	gastrointestinal hemorrhage	gastrointestinal hemorrhage	gastrointestinal hemorrhage	67	kept
Heart-Assist Devices	heart assist devices	heart assist devices	heart assist device	Heart-Assist Devices	67	singular
Hemofiltration	hemofiltration	hemofiltration	hemofiltration	Hemofiltration	67	kept
hip fracture	hip fracture	hip fracture	hip fracture	hip fracture	67	kept
Korea	korea	korea	korea	Korea	67	kept
London	london	london	london	London	67	kept
middle income country	middle income country	middle income country	middle income country	middle income country	67	kept
Obstetrics	obstetrics	obstetrics	obstetric	Obstetrics	67	singular
Outcome and Process Assessment	outcome and process assessment	outcome and process assessment	outcome and process assessment	Outcome and Process Assessment	67	kept
Pain assessment	pain assessment	pain assessment	pain assessment	pain assessment	67	case_or_format

Physician's Role	physician s role	physician s role	physician s role	Physician's Role	67	kept
primary health care	primary health care	primary health care	primary health care	Primary Health Care	67	case_or_format
secondary analysis	secondary analysis	secondary analysis	secondary analysis	secondary analysis	67	kept
side effect	side effect	side effect	side effect	side effect	67	kept
Stress, Physiological	stress physiological	stress physiological	stress physiological	Stress, Physiological	67	kept
Systemic Inflammatory Response Syndrome	systemic inflammatory response syndrome	systemic inflammatory response syndrome	systemic inflammatory response syndrome	Systemic Inflammatory Response Syndrome	67	kept
Uncertainty	uncertainty	uncertainty	uncertainty	Uncertainty	67	kept
Acute care	acute care	acute care	acute care	acute care	66	case_or_format
anticoagulant therapy	anticoagulant therapy	anticoagulant therapy	anticoagulant therapy	anticoagulant therapy	66	kept
crossover procedure	crossover procedure	crossover procedure	crossover procedure	crossover procedure	66	kept
decision-making	decision making	decision making	decision making	Decision Making	66	case_or_format
Hygiene	hygiene	hygiene	hygiene	hygiene	66	case_or_format
indwelling catheter	indwelling catheter	indwelling catheter	indwelling catheter	indwelling catheter	66	kept
Liver Transplantation	liver transplantation	liver transplantation	liver transplantation	Liver Transplantation	66	kept
Maslach Burnout Inventory	maslach burnout inventory	maslach burnout inventory	maslach burnout inventory	Maslach Burnout Inventory	66	kept
medical audit	medical audit	medical audit	medical audit	Medical Audit	66	case_or_format

MLCS	mlcs	mlcs	mlcs	MLCS	66	kept
MLOWN	mlown	mlown	mlown	MLOWN	66	kept
New York City	new york city	new york city	new york city	New York City	66	kept
occupational hazard	occupational hazard	occupational hazard	occupational hazard	occupational hazard	66	kept
population research	population research	population research	population research	population research	66	kept
preterm infant	preterm infant	preterm infant	preterm infant	preterm infant	66	kept
register	register	register	register	register	66	kept
Research	research	research	research	research	66	case_or_format
Shock, Cardiogenic	shock cardiogenic	shock cardiogenic	shock cardiogenic	Shock, Cardiogenic	66	kept
treatment failure	treatment failure	treatment failure	treatment failure	treatment failure	66	kept
Acute kidney injury	acute kidney injury	acute kidney injury	acute kidney injury	Acute Kidney Injury	65	case_or_format
Aging	aging	aging	aging	aging	65	case_or_format
all cause mortality	all cause mortality	all cause mortality	all cause mortality	all cause mortality	65	kept
ambulance	ambulance	ambulance	ambulance	ambulance	65	kept
ampicillin	ampicillin	ampicillin	ampicillin	ampicillin	65	kept
Asthma	asthma	asthma	asthma	asthma	65	case_or_format

Austria	austria	austria	austria	Austria	65	kept
beta adrenergic receptor blocking agent	beta adrenergic receptor blocking agent	beta adrenergic receptor blocking agent	beta adrenergic receptor blocking agent	beta adrenergic receptor blocking agent	65	kept
breastfeeding	breastfeeding	breastfeeding	breastfeeding	breastfeeding	65	kept
cardiopulmonary bypass	cardiopulmonary bypass	cardiopulmonary bypass	cardiopulmonary bypass	cardiopulmonary bypass	65	kept
community acquired pneumonia	community acquired pneumonia	community acquired pneumonia	community acquired pneumonia	community acquired pneumonia	65	kept
Deep Sedation	deep sedation	deep sedation	deep sedation	Deep Sedation	65	kept
diuretic agent	diuretic agent	diuretic agent	diuretic agent	diuretic agent	65	kept
Energy Intake	energy intake	energy intake	energy intake	Energy Intake	65	kept
furosemide	furosemide	furosemide	furosemide	furosemide	65	kept
geriatric assessment	geriatric assessment	geriatric assessment	geriatric assessment	Geriatric Assessment	65	case_or_format
Interpersonal Relations	interpersonal relations	interpersonal relations	interpersonal relations	Interpersonal Relations	65	kept
Neonates	neonates	neonates	neonate	Neonate	65	singular
personnel shortage	personnel shortage	personnel shortage	personnel shortage	personnel shortage	65	kept
Preterm	preterm	preterm	preterm	Preterm	65	kept
Respiratory Distress Syndrome, Adult	respiratory distress syndrome adult	respiratory distress syndrome adult	respiratory distress syndrome adult	Respiratory Distress Syndrome, Adult	65	kept
sampling	sampling	sampling	sampling	sampling	65	kept

telemetry	telemetry	telemetry	telemetry	Telemetry	65	case_or_format
anesthetic agent	anesthetic agent	anesthetic agent	anesthetic agent	anesthetic agent	64	kept
aspiration pneumonia	aspiration pneumonia	aspiration pneumonia	aspiration pneumonia	aspiration pneumonia	64	kept
competence	competence	competence	competence	competence	64	kept
cotrimoxazole	cotrimoxazole	cotrimoxazole	cotrimoxazole	cotrimoxazole	64	kept
Geriatrics	geriatrics	geriatrics	geriatric	Geriatrics	64	singular
geriatrics	geriatrics	geriatrics	geriatric	Geriatrics	64	singular
infusion fluid	infusion fluid	infusion fluid	infusion fluid	infusion fluid	64	kept
lidocaine	lidocaine	lidocaine	lidocaine	lidocaine	64	kept
living will	living will	living will	living will	living will	64	kept
Malaysia	malaysia	malaysia	malaysia	Malaysia	64	kept
Meta-analysis	meta analysis	meta analysis	meta analysis	meta analysis	64	case_or_format
Nutrition Assessment	nutrition assessment	nutrition assessment	nutrition assessment	Nutrition Assessment	64	kept
oxygen consumption	oxygen consumption	oxygen consumption	oxygen consumption	oxygen consumption	64	kept
PERFORMANCE	performance	performance	performance	PERFORMANCE	64	kept
Pneumonia, Aspiration	pneumonia aspiration	pneumonia aspiration	pneumonia aspiration	Pneumonia, Aspiration	64	kept

Preterm infant	preterm infant	preterm infant	preterm infant	preterm infant	64	case_or_format
PREVALENCE	prevalence	prevalence	prevalence	prevalence	64	case_or_format
uncertainty	uncertainty	uncertainty	uncertainty	Uncertainty	64	case_or_format
virology	virology	virology	virology	virology	64	kept
Alzheimer disease	alzheimer disease	alzheimer disease	alzheimer disease	Alzheimer disease	63	kept
cannulation	cannulation	cannulation	cannulation	cannulation	63	kept
Critically ill	critically ill	critically ill	critically ill	Critically ill	63	kept
erythrocyte transfusion	erythrocyte transfusion	erythrocyte transfusion	erythrocyte transfusion	erythrocyte transfusion	63	kept
groups by age	groups by age	groups by age	groups by age	groups by age	63	kept
heart transplantation	heart transplantation	heart transplantation	heart transplantation	heart transplantation	63	kept
Hypertension	hypertension	hypertension	hypertension	hypertension	63	case_or_format
infant care	infant care	infant care	infant care	Infant Care	63	case_or_format
language	language	language	language	language	63	kept
Machine Learning	machine learning	machine learning	machine learning	machine learning	63	case_or_format
malignant neoplasm	malignant neoplasm	malignant neoplasm	malignant neoplasm	malignant neoplasm	63	kept
Mindfulness	mindfulness	mindfulness	mindfulness	Mindfulness	63	kept

Neurosurgical Procedures	neurosurgical procedures	neurosurgical procedures	neurosurgical procedure	Neurosurgical Procedures	63	singular
pretest posttest design	pretest posttest design	pretest posttest design	pretest posttest design	pretest posttest design	63	kept
propensity score	propensity score	propensity score	propensity score	propensity score	63	kept
skin	skin	skin	skin	Skin	63	case_or_format
Surgical Wound Infection	surgical wound infection	surgical wound infection	surgical wound infection	Surgical Wound Infection	63	kept
telehealth	telehealth	telehealth	telehealth	telehealth	63	kept
Thinking	thinking	thinking	thinking	Thinking	63	kept
thrombosis	thrombosis	thrombosis	thrombosis	thrombosis	63	kept
ambulatory care	ambulatory care	ambulatory care	ambulatory care	Ambulatory Care	62	case_or_format
Artificial intelligence	artificial intelligence	artificial intelligence	artificial intelligence	artificial intelligence	62	case_or_format
BURNOUT	burnout	burnout	burnout	burnout	62	case_or_format
cardiologist	cardiologist	cardiologist	cardiologist	cardiologist	62	kept
child development	child development	child development	child development	Child Development	62	case_or_format
clinical decision support system	clinical decision support system	clinical decision support system	clinical decision support system	clinical decision support system	62	kept
expectation	expectation	expectation	expectation	expectation	62	kept
Iraq	iraq	iraq	iraq	Iraq	62	kept

kidney disease	kidney disease	kidney disease	kidney disease	kidney disease	62	kept
Lung	lung	lung	lung	Lung	62	kept
massage	massage	massage	massage	Massage	62	case_or_format
Medication Systems, Hospital	medication systems hospital	medication systems hospital	medication systems hospital	Medication Systems, Hospital	62	kept
meta-analysis	meta analysis	meta analysis	meta analysis	meta analysis	62	case_or_format
Multicenter Studies as Topic	multicenter studies as topic	multicenter studies as topic	multicenter studies as topic	Multicenter Studies as Topic	62	kept
Nutrition	nutrition	nutrition	nutrition	nutrition	62	case_or_format
Preterm infants	preterm infants	preterm infants	preterm infant	preterm infant	62	singular
recovery room	recovery room	recovery room	recovery room	Recovery Room	62	case_or_format
SATISFACTION	satisfaction	satisfaction	satisfaction	satisfaction	62	case_or_format
Terminal care	terminal care	terminal care	terminal care	Terminal Care	62	case_or_format
vaginal delivery	vaginal delivery	vaginal delivery	vaginal delivery	vaginal delivery	62	kept
attention	attention	attention	attention	attention	61	kept
Blood Pressure Determination	blood pressure determination	blood pressure determination	blood pressure determination	Blood Pressure Determination	61	kept
caloric intake	caloric intake	caloric intake	caloric intake	caloric intake	61	kept
case study	case study	case study	case study	case study	61	kept

child health	child health	child health	child health	child health	61	kept
ECMO	ecmo	ecmo	ecmo	ECMO	61	kept
elderly care	elderly care	elderly care	elderly care	elderly care	61	kept
family-centered care	family centered care	family centered care	family centered care	family centered care	61	case_or_format
fatality	fatality	fatality	fatality	fatality	61	kept
kidney function	kidney function	kidney function	kidney function	kidney function	61	kept
levofloxacin	levofloxacin	levofloxacin	levofloxacin	levofloxacin	61	kept
Magnetic Resonance Imaging	magnetic resonance imaging	magnetic resonance imaging	magnetic resonance imaging	Magnetic Resonance Imaging	61	kept
medical literature	medical literature	medical literature	medical literature	medical literature	61	kept
pregnant woman	pregnant woman	pregnant woman	pregnant woman	pregnant woman	61	kept
psychological resilience	psychological resilience	psychological resilience	psychological resilience	psychological resilience	61	kept
psychological well-being	psychological well being	psychological well being	psychological well being	psychological well-being	61	kept
Reliability	reliability	reliability	reliability	reliability	61	case_or_format
restlessness	restlessness	restlessness	restlessness	restlessness	61	kept
self evaluation	self evaluation	self evaluation	self evaluation	self evaluation	61	kept
shared decision making	shared decision making	shared decision making	shared decision making	shared decision making	61	kept

UNIT	unit	unit	unit	UNIT	61	kept
weakness	weakness	weakness	weakness	weakness	61	kept
Bed Rest	bed rest	bed rest	bed rest	Bed Rest	60	kept
clinical examination	clinical examination	clinical examination	clinical examination	clinical examination	60	kept
drug cost	drug cost	drug cost	drug cost	drug cost	60	kept
Education, Continuing	education continuing	education continuing	education continuing	Education, Continuing	60	kept
Feedback	feedback	feedback	feedback	Feedback	60	kept
Florida	florida	florida	florida	Florida	60	kept
global health	global health	global health	global health	global health	60	kept
Gram negative bacterium	gram negative bacterium	gram negative bacterium	gram negative bacterium	Gram negative bacterium	60	kept
Health Education	health education	health education	health education	health education	60	case_or_format
Hospital Administration	hospital administration	hospital administration	hospital administration	Hospital Administration	60	kept
Hospital Information Systems	hospital information systems	hospital information systems	hospital information system	hospital information system	60	singular
hospital policy	hospital policy	hospital policy	hospital policy	hospital policy	60	kept
medication therapy management	medication therapy management	medication therapy management	medication therapy management	medication therapy management	60	kept
obstetric delivery	obstetric delivery	obstetric delivery	obstetric delivery	obstetric delivery	60	kept

Pharmacy Service, Hospital	pharmacy service hospital	pharmacy service hospital	pharmacy service hospital	Pharmacy Service, Hospital	60	kept
Spinal Cord Injuries	spinal cord injuries	spinal cord injuries	spinal cord injury	Spinal Cord Injuries	60	singular
total parenteral nutrition	total parenteral nutrition	total parenteral nutrition	total parenteral nutrition	total parenteral nutrition	60	kept
anticonvulsive agent	anticonvulsive agent	anticonvulsive agent	anticonvulsive agent	anticonvulsive agent	59	kept
antihypertensive agent	antihypertensive agent	antihypertensive agent	antihypertensive agent	antihypertensive agent	59	kept
arterial gas	arterial gas	arterial gas	arterial gas	arterial gas	59	kept
clinical assessment tool	clinical assessment tool	clinical assessment tool	clinical assessment tool	clinical assessment tool	59	kept
Cognitive Dysfunction	cognitive dysfunction	cognitive dysfunction	cognitive dysfunction	Cognitive Dysfunction	59	kept
digestive tract intubation	digestive tract intubation	digestive tract intubation	digestive tract intubation	digestive tract intubation	59	kept
disease management	disease management	disease management	disease management	Disease Management	59	case_or_format
dizziness	dizziness	dizziness	dizziness	dizziness	59	kept
Glucose	glucose	glucose	glucose	glucose	59	case_or_format
health care practice	health care practice	health care practice	health care practice	health care practice	59	kept
Health Status Indicators	health status indicators	health status indicators	health status indicator	Health Status Indicators	59	singular
imipenem	imipenem	imipenem	imipenem	imipenem	59	kept
injury severity	injury severity	injury severity	injury severity	injury severity	59	kept

Intermediate Care Facilities	intermediate care facilities	intermediate care facilities	intermediate care facility	Intermediate Care Facilities	59	singular
Muscle Weakness	muscle weakness	muscle weakness	muscle weakness	Muscle Weakness	59	kept
PREVENTION	prevention	prevention	prevention	Prevention	59	case_or_format
psychosocial care	psychosocial care	psychosocial care	psychosocial care	psychosocial care	59	kept
Reference Values	reference values	reference values	reference value	Reference Values	59	singular
regression analysis	regression analysis	regression analysis	regression analysis	Regression Analysis	59	case_or_format
retrolental fibroplasia	retrolental fibroplasia	retrolental fibroplasia	retrolental fibroplasia	retrolental fibroplasia	59	kept
SAFETY	safety	safety	safety	safety	59	case_or_format
Alarm fatigue	alarm fatigue	alarm fatigue	alarm fatigue	Alarm fatigue	58	kept
Breastfeeding	breastfeeding	breastfeeding	breastfeeding	breastfeeding	58	case_or_format
Bronchopulmonary Dysplasia	bronchopulmonary dysplasia	bronchopulmonary dysplasia	bronchopulmonary dysplasia	Bronchopulmonary Dysplasia	58	kept
child health care	child health care	child health care	child health care	child health care	58	kept
cultural anthropology	cultural anthropology	cultural anthropology	cultural anthropology	cultural anthropology	58	kept
data extraction	data extraction	data extraction	data extraction	data extraction	58	kept
general surgery	general surgery	general surgery	general surgery	general surgery	58	kept
Health personnel	health personnel	health personnel	health personnel	Health Personnel	58	case_or_format

health workforce	health workforce	health workforce	health workforce	health workforce	58	kept
hospital organization	hospital organization	hospital organization	hospital organization	hospital organization	58	kept
implementation	implementation	implementation	implementation	Implementation	58	case_or_format
muscle weakness	muscle weakness	muscle weakness	muscle weakness	Muscle Weakness	58	case_or_format
neonates	neonates	neonates	neonate	Neonate	58	singular
Pulmonary Embolism	pulmonary embolism	pulmonary embolism	pulmonary embolism	Pulmonary Embolism	58	kept
Substance-Related Disorders	substance related disorders	substance related disorders	substance related disorders	Substance-Related Disorders	58	kept
temperature	temperature	temperature	temperature	temperature	58	kept
Allied Health Personnel	allied health personnel	allied health personnel	allied health personnel	Allied Health Personnel	57	kept
Anesthesia, General	anesthesia general	anesthesia general	anesthesia general	Anesthesia, General	57	kept
cefotaxime	cefotaxime	cefotaxime	cefotaxime	cefotaxime	57	kept
Competency-Based Education	competency based education	competency based education	competency based education	Competency-Based Education	57	kept
Disasters	disasters	disasters	disaster	Disasters	57	singular
disease burden	disease burden	disease burden	disease burden	disease burden	57	kept
electroencephalogram	electroencephalogram	electroencephalogram	electroencephalogram	electroencephalogram	57	kept
End-of-life	end of life	end of life	end of life	End of life	57	case_or_format

endotracheal tube	endotracheal tube	endotracheal tube	endotracheal tube	endotracheal tube	57	kept
ethnography	ethnography	ethnography	ethnography	ethnography	57	kept
factual database	factual database	factual database	factual database	factual database	57	kept
financial management	financial management	financial management	financial management	financial management	57	kept
health behavior	health behavior	health behavior	health behavior	health behavior	57	kept
Language	language	language	language	language	57	case_or_format
mass screening	mass screening	mass screening	mass screening	Mass Screening	57	case_or_format
medical information system	medical information system	medical information system	medical information system	medical information system	57	kept
neuroimaging	neuroimaging	neuroimaging	neuroimaging	neuroimaging	57	kept
physical restraint	physical restraint	physical restraint	physical restraint	physical restraint	57	kept
positive end expiratory pressure ventilation	positive end expiratory pressure ventilation	positive end expiratory pressure ventilation	positive end expiratory pressure ventilation	positive end expiratory pressure ventilation	57	kept
premature labor	premature labor	premature labor	premature labor	premature labor	57	kept
preterm infants	preterm infants	preterm infants	preterm infant	preterm infant	57	singular
Problem-Based Learning	problem based learning	problem based learning	problem based learning	Problem-Based Learning	57	kept
Quality of health care	quality of health care	quality of health care	quality of health care	Quality of Health Care	57	case_or_format
radiography	radiography	radiography	radiography	radiography	57	kept

social work	social work	social work	social work	social work	57	kept
verbal communication	verbal communication	verbal communication	verbal communication	verbal communication	57	kept
Acinetobacter Infections	acinetobacter infections	acinetobacter infections	acinetobacter infection	Acinetobacter Infections	56	singular
adrenalin	adrenalin	adrenalin	adrenalin	adrenalin	56	kept
Anemia	anemia	anemia	anemia	anemia	56	case_or_format
automation	automation	automation	automation	automation	56	kept
azithromycin	azithromycin	azithromycin	azithromycin	azithromycin	56	kept
bacterium isolate	bacterium isolate	bacterium isolate	bacterium isolate	bacterium isolate	56	kept
Candida	candida	candida	candida	Candida	56	kept
Catheters	catheters	catheters	catheter	Catheters	56	singular
Cochrane Library	cochrane library	cochrane library	cochrane library	Cochrane Library	56	kept
D dimer	d dimer	d dimer	d dimer	D dimer	56	kept
delivery room	delivery room	delivery room	delivery room	delivery room	56	kept
Early Warning Score	early warning score	early warning score	early warning score	early warning score	56	case_or_format
Embase	embase	embase	embase	Embase	56	kept
Empirical Approach	empirical approach	empirical approach	empirical approach	Empirical Approach	56	kept

END	end	end	end	END	56	kept
Environmental Exposure	environmental exposure	environmental exposure	environmental exposure	Environmental Exposure	56	kept
Health Services for the Aged	health services for the aged	health services for the aged	health services for the aged	Health Services for the Aged	56	kept
health worker	health worker	health worker	health worker	health worker	56	kept
hemodynamic monitoring	hemodynamic monitoring	hemodynamic monitoring	hemodynamic monitoring	hemodynamic monitoring	56	kept
Holistic Health	holistic health	holistic health	holistic health	Holistic Health	56	kept
Maternal Behavior	maternal behavior	maternal behavior	maternal behavior	Maternal Behavior	56	kept
Minnesota	minnesota	minnesota	minnesota	Minnesota	56	kept
North America	north america	north america	north america	North America	56	kept
preoperative care	preoperative care	preoperative care	preoperative care	Preoperative Care	56	case_or_format
privacy	privacy	privacy	privacy	privacy	56	kept
Quality	quality	quality	quality	QUALITY	56	case_or_format
rural area	rural area	rural area	rural area	rural area	56	kept
Self Concept	self concept	self concept	self concept	self concept	56	case_or_format
Simplified Acute Physiology Score	simplified acute physiology score	simplified acute physiology score	simplified acute physiology score	Simplified Acute Physiology Score	56	kept
Urinary Incontinence	urinary incontinence	urinary incontinence	urinary incontinence	Urinary Incontinence	56	kept

Ventilation	ventilation	ventilation	ventilation	Ventilation	56	kept
wound infection	wound infection	wound infection	wound infection	wound infection	56	kept
acute heart infarction	acute heart infarction	acute heart infarction	acute heart infarction	acute heart infarction	55	kept
Africa	africa	africa	africa	Africa	55	kept
bed	bed	bed	bed	Beds	55	case_or_format
bicarbonate	bicarbonate	bicarbonate	bicarbonate	bicarbonate	55	kept
blood pressure measurement	blood pressure measurement	blood pressure measurement	blood pressure measurement	blood pressure measurement	55	kept
Blood Specimen Collection	blood specimen collection	blood specimen collection	blood specimen collection	Blood Specimen Collection	55	kept
bronchiolitis	bronchiolitis	bronchiolitis	bronchiolitis	bronchiolitis	55	kept
Case Management	case management	case management	case management	Case Management	55	kept
consensus development	consensus development	consensus development	consensus development	consensus development	55	kept
Delivery of Health Care, Integrated	delivery of health care integrated	delivery of health care integrated	delivery of health care integrated	Delivery of Health Care, Integrated	55	kept
diagnostic test	diagnostic test	diagnostic test	diagnostic test	diagnostic test	55	kept
disease duration	disease duration	disease duration	disease duration	disease duration	55	kept
Drug Resistance, Bacterial	drug resistance bacterial	drug resistance bacterial	drug resistance bacterial	Drug Resistance, Bacterial	55	kept
Evaluation	evaluation	evaluation	evaluation	evaluation	55	case_or_format

extracorporeal membrane oxygenation	extracorporeal membrane oxygenation	extracorporeal membrane oxygenation	extracorporeal membrane oxygenation	Extracorporeal Membrane Oxygenation	55	case_or_format
Global Health	global health	global health	global health	global health	55	case_or_format
Hand	hand	hand	hand	Hand	55	kept
Healthcare Disparities	healthcare disparities	healthcare disparities	healthcare disparity	Healthcare Disparities	55	singular
hospital design	hospital design	hospital design	hospital design	hospital design	55	kept
hospital service	hospital service	hospital service	hospital service	hospital service	55	kept
hospitalization cost	hospitalization cost	hospitalization cost	hospitalization cost	hospitalization cost	55	kept
Hypoxia-Ischemia, Brain	hypoxia ischemia brain	hypoxia ischemia brain	hypoxia ischemia brain	Hypoxia-Ischemia, Brain	55	kept
infusion pump	infusion pump	infusion pump	infusion pump	Infusion Pumps	55	case_or_format
Interior Design and Furnishings	interior design and furnishings	interior design and furnishings	interior design and furnishings	Interior Design and Furnishings	55	kept
Job Description	job description	job description	job description	Job Description	55	kept
JOB-SATISFACTION	job satisfaction	job satisfaction	job satisfaction	Job Satisfaction	55	case_or_format
medication errors	medication errors	medication errors	medication error	Medication Errors	55	singular
methylprednisolone	methylprednisolone	methylprednisolone	methylprednisolone	methylprednisolone	55	kept
multiple trauma	multiple trauma	multiple trauma	multiple trauma	Multiple Trauma	55	case_or_format
nonsteroid antiinflammatory agent	nonsteroid antiinflammatory agent	nonsteroid antiinflammatory agent	nonsteroid antiinflammatory agent	nonsteroid antiinflammatory agent	55	kept

North Carolina	north carolina	north carolina	north carolina	North Carolina	55	kept
observation	observation	observation	observation	Observation	55	case_or_format
PALLIATIVE CARE	palliative care	palliative care	palliative care	Palliative Care	55	case_or_format
perinatal care	perinatal care	perinatal care	perinatal care	Perinatal Care	55	case_or_format
procalcitonin	procalcitonin	procalcitonin	procalcitonin	procalcitonin	55	kept
Psychiatric Status Rating Scales	psychiatric status rating scales	psychiatric status rating scales	psychiatric status rating scales	Psychiatric Status Rating Scales	55	kept
quantitative study	quantitative study	quantitative study	quantitative study	quantitative study	55	kept
rescue personnel	rescue personnel	rescue personnel	rescue personnel	rescue personnel	55	kept
residency education	residency education	residency education	residency education	residency education	55	kept
Scotland	scotland	scotland	scotland	Scotland	55	kept
Self-Assessment	self assessment	self assessment	self assessment	Self-Assessment	55	kept
Students, Medical	students medical	students medical	students medical	Students, Medical	55	kept
thermoregulation	thermoregulation	thermoregulation	thermoregulation	thermoregulation	55	kept
vasoactive agent	vasoactive agent	vasoactive agent	vasoactive agent	vasoactive agent	55	kept
Vital signs	vital signs	vital signs	vital sign	vital sign	55	singular
Automation	automation	automation	automation	automation	54	case_or_format

cancer chemotherapy	cancer chemotherapy	cancer chemotherapy	cancer chemotherapy	cancer chemotherapy	54	kept
cancer surgery	cancer surgery	cancer surgery	cancer surgery	cancer surgery	54	kept
Cardiac Output	cardiac output	cardiac output	cardiac output	Cardiac Output	54	kept
Cognition Disorders	cognition disorders	cognition disorders	cognition disorder	Cognition Disorders	54	singular
constipation	constipation	constipation	constipation	constipation	54	kept
critically ill	critically ill	critically ill	critically ill	Critically ill	54	case_or_format
elderly	elderly	elderly	elderly	Elderly	54	case_or_format
Elective Surgical Procedures	elective surgical procedures	elective surgical procedures	elective surgical procedures	Elective Surgical Procedures	54	kept
emotional intelligence	emotional intelligence	emotional intelligence	emotional intelligence	emotional intelligence	54	kept
environment	environment	environment	environment	Environment	54	case_or_format
hydroxychloroquine	hydroxychloroquine	hydroxychloroquine	hydroxychloroquine	hydroxychloroquine	54	kept
hyponatremia	hyponatremia	hyponatremia	hyponatremia	hyponatremia	54	kept
immunization	immunization	immunization	immunization	immunization	54	kept
influenza vaccine	influenza vaccine	influenza vaccine	influenza vaccine	influenza vaccine	54	kept
interleukin 6	interleukin 6	interleukin 6	interleukin 6	interleukin 6	54	kept
oximetry	oximetry	oximetry	oximetry	Oximetry	54	case_or_format

Personnel Selection	personnel selection	personnel selection	personnel selection	Personnel Selection	54	kept
preterm	preterm	preterm	preterm	Preterm	54	case_or_format
Sleep quality	sleep quality	sleep quality	sleep quality	sleep quality	54	case_or_format
Systems Analysis	systems analysis	systems analysis	systems analysis	Systems Analysis	54	kept
urine volume	urine volume	urine volume	urine volume	urine volume	54	kept
work	work	work	work	work	54	kept
Adverse events	adverse events	adverse events	adverse event	adverse event	53	singular
bacterium contamination	bacterium contamination	bacterium contamination	bacterium contamination	bacterium contamination	53	kept
brain disease	brain disease	brain disease	brain disease	brain disease	53	kept
ceftazidime	ceftazidime	ceftazidime	ceftazidime	ceftazidime	53	kept
Combined Modality Therapy	combined modality therapy	combined modality therapy	combined modality therapy	Combined Modality Therapy	53	kept
conversation	conversation	conversation	conversation	conversation	53	kept
correlational study	correlational study	correlational study	correlational study	correlational study	53	kept
Drug Therapy	drug therapy	drug therapy	drug therapy	drug therapy	53	case_or_format
early intervention	early intervention	early intervention	early intervention	early intervention	53	kept
Enterobacteriaceae	enterobacteriaceae	enterobacteriaceae	enterobacteriaceae	Enterobacteriaceae	53	kept

Equipment and Supplies, Hospital	equipment and supplies hospital	equipment and supplies hospital	equipment and supplies hospital	Equipment and Supplies, Hospital	53	kept
Ethics, Medical	ethics medical	ethics medical	ethics medical	Ethics, Medical	53	kept
Health Workforce	health workforce	health workforce	health workforce	health workforce	53	case_or_format
hospital bed	hospital bed	hospital bed	hospital bed	hospital bed	53	kept
hospital bed utilization	hospital bed utilization	hospital bed utilization	hospital bed utilization	hospital bed utilization	53	kept
infant mortality	infant mortality	infant mortality	infant mortality	Infant Mortality	53	case_or_format
Intervention	intervention	intervention	intervention	Intervention	53	kept
Lighting	lighting	lighting	lighting	Lighting	53	kept
metronidazole	metronidazole	metronidazole	metronidazole	metronidazole	53	kept
Nigeria	nigeria	nigeria	nigeria	Nigeria	53	kept
night shift	night shift	night shift	night shift	night shift	53	kept
Organ donation	organ donation	organ donation	organ donation	Organ donation	53	kept
Organ Transplantation	organ transplantation	organ transplantation	organ transplantation	organ transplantation	53	case_or_format
Outcome	outcome	outcome	outcome	OUTCOMES	53	case_or_format
potassium	potassium	potassium	potassium	potassium	53	kept
protocol	protocol	protocol	protocol	protocol	53	kept

puerperium	puerperium	puerperium	puerperium	puerperium	53	kept
Quebec	quebec	quebec	quebec	Quebec	53	kept
race	race	race	race	race	53	kept
reverse transcription polymerase chain reaction	reverse transcription polymerase chain reaction	reverse transcription polymerase chain reaction	reverse transcription polymerase chain reaction	reverse transcription polymerase chain reaction	53	kept
safety culture	safety culture	safety culture	safety culture	safety culture	53	kept
Self-efficacy	self efficacy	self efficacy	self efficacy	Self Efficacy	53	case_or_format
social interaction	social interaction	social interaction	social interaction	social interaction	53	kept
thrombocytopenia	thrombocytopenia	thrombocytopenia	thrombocytopenia	thrombocytopenia	53	kept
Thrombosis	thrombosis	thrombosis	thrombosis	thrombosis	53	case_or_format
tracheotomy	tracheotomy	tracheotomy	tracheotomy	tracheotomy	53	kept
Transitional Care	transitional care	transitional care	transitional care	transitional care	53	case_or_format
violence	violence	violence	violence	Violence	53	case_or_format
Weaning	weaning	weaning	weaning	weaning	53	case_or_format
Writing	writing	writing	writing	Writing	53	kept
acute respiratory distress syndrome	acute respiratory distress syndrome	acute respiratory distress syndrome	acute respiratory distress syndrome	acute respiratory distress syndrome	52	kept
Administration, Oral	administration oral	administration oral	administration oral	Administration, Oral	52	kept

alarm monitor	alarm monitor	alarm monitor	alarm monitor	alarm monitor	52	kept
animal	animal	animal	animal	animal	52	kept
antivirus agent	antivirus agent	antivirus agent	antivirus agent	antivirus agent	52	kept
aspiration	aspiration	aspiration	aspiration	aspiration	52	kept
Brain Ischemia	brain ischemia	brain ischemia	brain ischemia	brain ischemia	52	case_or_format
Caucasian	caucasian	caucasian	caucasian	Caucasian	52	kept
Clinical Trials as Topic	clinical trials as topic	clinical trials as topic	clinical trials as topic	Clinical Trials as Topic	52	kept
Colombia	colombia	colombia	colombia	Colombia	52	kept
Confidence Intervals	confidence intervals	confidence intervals	confidence interval	confidence interval	52	singular
construct validity	construct validity	construct validity	construct validity	construct validity	52	kept
Continuous Renal Replacement Therapy	continuous renal replacement therapy	continuous renal replacement therapy	continuous renal replacement therapy	continuous renal replacement therapy	52	case_or_format
Coronary Disease	coronary disease	coronary disease	coronary disease	Coronary Disease	52	kept
dehydration	dehydration	dehydration	dehydration	dehydration	52	kept
Diabetes Mellitus, Type 2	diabetes mellitus type 2	diabetes mellitus type 2	diabetes mellitus type 2	Diabetes Mellitus, Type 2	52	kept
Diagnosis-Related Groups	diagnosis related groups	diagnosis related groups	diagnosis related group	Diagnosis-Related Groups	52	singular
dipeptidyl carboxypeptidase inhibitor	dipeptidyl carboxypeptidase inhibitor	dipeptidyl carboxypeptidase inhibitor	dipeptidyl carboxypeptidase inhibitor	dipeptidyl carboxypeptidase inhibitor	52	kept

disaster planning	disaster planning	disaster planning	disaster planning	Disaster Planning	52	case_or_format
double blind procedure	double blind procedure	double blind procedure	double blind procedure	double blind procedure	52	kept
health care availability	health care availability	health care availability	health care availability	health care availability	52	kept
immunology	immunology	immunology	immunology	immunology	52	kept
Infections	infections	infections	infection	infection	52	singular
liver cirrhosis	liver cirrhosis	liver cirrhosis	liver cirrhosis	liver cirrhosis	52	kept
low molecular weight heparin	low molecular weight heparin	low molecular weight heparin	low molecular weight heparin	low molecular weight heparin	52	kept
lung edema	lung edema	lung edema	lung edema	lung edema	52	kept
lung transplantation	lung transplantation	lung transplantation	lung transplantation	lung transplantation	52	kept
newborn disease	newborn disease	newborn disease	newborn disease	newborn disease	52	kept
Oral care	oral care	oral care	oral care	Oral care	52	kept
Oxygen Consumption	oxygen consumption	oxygen consumption	oxygen consumption	oxygen consumption	52	case_or_format
pregnancy complication	pregnancy complication	pregnancy complication	pregnancy complication	Pregnancy Complications	52	case_or_format
Primary Prevention	primary prevention	primary prevention	primary prevention	Primary Prevention	52	kept
rank sum test	rank sum test	rank sum test	rank sum test	rank sum test	52	kept
unconsciousness	unconsciousness	unconsciousness	unconsciousness	unconsciousness	52	kept

vulnerable population	vulnerable population	vulnerable population	vulnerable population	vulnerable population	52	kept
adverse device effect	adverse device effect	adverse device effect	adverse device effect	adverse device effect	51	kept
anticoagulation	anticoagulation	anticoagulation	anticoagulation	anticoagulation	51	kept
bladder catheterization	bladder catheterization	bladder catheterization	bladder catheterization	bladder catheterization	51	kept
Career Mobility	career mobility	career mobility	career mobility	Career Mobility	51	kept
chronic lung disease	chronic lung disease	chronic lung disease	chronic lung disease	chronic lung disease	51	kept
Death and Euthanasia	death and euthanasia	death and euthanasia	death and euthanasia	Death and Euthanasia	51	kept
drug administration	drug administration	drug administration	drug administration	drug administration	51	kept
Health Care Rationing	health care rationing	health care rationing	health care rationing	Health Care Rationing	51	kept
heart catheterization	heart catheterization	heart catheterization	heart catheterization	heart catheterization	51	kept
Judgment	judgment	judgment	judgment	Judgment	51	kept
linezolid	linezolid	linezolid	linezolid	linezolid	51	kept
low income country	low income country	low income country	low income country	low income country	51	kept
mechanical ventilator	mechanical ventilator	mechanical ventilator	mechanical ventilator	mechanical ventilator	51	kept
neonatal respiratory distress syndrome	neonatal respiratory distress syndrome	neonatal respiratory distress syndrome	neonatal respiratory distress syndrome	neonatal respiratory distress syndrome	51	kept
Obesity, Morbid	obesity morbid	obesity morbid	obesity morbid	Obesity, Morbid	51	kept

Occupational health	occupational health	occupational health	occupational health	Occupational Health	51	case_or_format
preeclampsia	preeclampsia	preeclampsia	preeclampsia	preeclampsia	51	kept
Premature infants	premature infants	premature infants	premature infant	Premature infant	51	singular
remifentanil	remifentanil	remifentanil	remifentanil	remifentanil	51	kept
sleep deprivation	sleep deprivation	sleep deprivation	sleep deprivation	Sleep Deprivation	51	case_or_format
Sucking Behavior	sucking behavior	sucking behavior	sucking behavior	Sucking Behavior	51	kept
telephone interview	telephone interview	telephone interview	telephone interview	telephone interview	51	kept
touch	touch	touch	touch	Touch	51	case_or_format
Traumatic brain injury	traumatic brain injury	traumatic brain injury	traumatic brain injury	traumatic brain injury	51	case_or_format
virtual reality	virtual reality	virtual reality	virtual reality	virtual reality	51	kept
Biomedical Research	biomedical research	biomedical research	biomedical research	Biomedical Research	50	kept
Body Temperature Regulation	body temperature regulation	body temperature regulation	body temperature regulation	Body Temperature Regulation	50	kept
Boston	boston	boston	boston	Boston	50	kept
bronchodilating agent	bronchodilating agent	bronchodilating agent	bronchodilating agent	bronchodilating agent	50	kept
carbon dioxide	carbon dioxide	carbon dioxide	carbon dioxide	carbon dioxide	50	kept
cerebrovascular disease	cerebrovascular disease	cerebrovascular disease	cerebrovascular disease	cerebrovascular disease	50	kept

child behavior	child behavior	child behavior	child behavior	child behavior	50	kept
Clinical deterioration	clinical deterioration	clinical deterioration	clinical deterioration	Clinical Deterioration	50	case_or_format
coronavirus	coronavirus	coronavirus	coronavirus	Coronavirus	50	case_or_format
Developmental care	developmental care	developmental care	developmental care	Developmental care	50	kept
Diabetes Mellitus, Type 1	diabetes mellitus type 1	diabetes mellitus type 1	diabetes mellitus type 1	Diabetes Mellitus, Type 1	50	kept
Drug Prescriptions	drug prescriptions	drug prescriptions	drug prescriptions	Drug Prescriptions	50	kept
Education, Medical	education medical	education medical	education medical	Education, Medical	50	kept
healthcare professionals	healthcare professionals	healthcare professionals	healthcare professional	healthcare professionals	50	singular
hematocrit	hematocrit	hematocrit	hematocrit	hematocrit	50	kept
Hip Fractures	hip fractures	hip fractures	hip fracture	hip fracture	50	singular
hypertensive agent	hypertensive agent	hypertensive agent	hypertensive agent	hypertensive agent	50	kept
lung dysplasia	lung dysplasia	lung dysplasia	lung dysplasia	lung dysplasia	50	kept
Massachusetts	massachusetts	massachusetts	massachusetts	Massachusetts	50	kept
medical informatics	medical informatics	medical informatics	medical informatics	medical informatics	50	kept
myalgia	myalgia	myalgia	myalgia	myalgia	50	kept
Physician's Practice Patterns	physician s practice patterns	physician s practice patterns	physician s practice patterns	Physician's Practice Patterns	50	kept

platelet count	platelet count	platelet count	platelet count	platelet count	50	kept
premature infant	premature infant	premature infant	premature infant	Premature infant	50	case_or_format
Probability	probability	probability	probability	Probability	50	kept
quality	quality	quality	quality	QUALITY	50	case_or_format
Respiratory Tract Infections	respiratory tract infections	respiratory tract infections	respiratory tract infection	respiratory tract infection	50	singular
spinal cord injury	spinal cord injury	spinal cord injury	spinal cord injury	Spinal Cord Injuries	50	case_or_format
theoretical model	theoretical model	theoretical model	theoretical model	theoretical model	50	kept
workplace violence	workplace violence	workplace violence	workplace violence	workplace violence	50	kept
Aggression	aggression	aggression	aggression	aggression	49	case_or_format
Bottle Feeding	bottle feeding	bottle feeding	bottle feeding	Bottle Feeding	49	kept
breathing exercise	breathing exercise	breathing exercise	breathing exercise	breathing exercise	49	kept
cardiopulmonary resuscitation	cardiopulmonary resuscitation	cardiopulmonary resuscitation	cardiopulmonary resuscitation	Cardiopulmonary Resuscitation	49	case_or_format
central venous pressure	central venous pressure	central venous pressure	central venous pressure	central venous pressure	49	kept
clinical study	clinical study	clinical study	clinical study	clinical study	49	kept
diabetic ketoacidosis	diabetic ketoacidosis	diabetic ketoacidosis	diabetic ketoacidosis	diabetic ketoacidosis	49	kept
disaster	disaster	disaster	disaster	Disasters	49	case_or_format

Family Health	family health	family health	family health	Family Health	49	kept
Fibrinolytic Agents	fibrinolytic agents	fibrinolytic agents	fibrinolytic agent	Fibrinolytic Agents	49	singular
Handwashing	handwashing	handwashing	handwashing	Handwashing	49	kept
Healthcare	healthcare	healthcare	healthcare	Healthcare	49	kept
hemoglobin blood level	hemoglobin blood level	hemoglobin blood level	hemoglobin blood level	hemoglobin blood level	49	kept
Hermeneutics	hermeneutics	hermeneutics	hermeneutic	Hermeneutics	49	singular
Hospitals, Veterans	hospitals veterans	hospitals veterans	hospitals veterans	Hospitals, Veterans	49	kept
Inflammation	inflammation	inflammation	inflammation	inflammation	49	case_or_format
Information Dissemination	information dissemination	information dissemination	information dissemination	Information Dissemination	49	kept
interprofessional education	interprofessional education	interprofessional education	interprofessional education	interprofessional education	49	kept
liver transplantation	liver transplantation	liver transplantation	liver transplantation	Liver Transplantation	49	case_or_format
lung ventilation	lung ventilation	lung ventilation	lung ventilation	lung ventilation	49	kept
nomenclature	nomenclature	nomenclature	nomenclature	nomenclature	49	kept
percutaneous coronary intervention	percutaneous coronary intervention	percutaneous coronary intervention	percutaneous coronary intervention	percutaneous coronary intervention	49	kept
PHYSICIANS	physicians	physicians	physician	physician	49	singular
placebo	placebo	placebo	placebo	placebo	49	kept

pregnancy outcome	pregnancy outcome	pregnancy outcome	pregnancy outcome	Pregnancy Outcome	49	case_or_format
pressure	pressure	pressure	pressure	Pressure	49	case_or_format
Problem Solving	problem solving	problem solving	problem solving	Problem Solving	49	kept
Pseudomonas Infections	pseudomonas infections	pseudomonas infections	pseudomonas infection	Pseudomonas Infections	49	singular
public health service	public health service	public health service	public health service	public health service	49	kept
Role	role	role	role	Role	49	kept
speech language pathologist	speech language pathologist	speech language pathologist	speech language pathologist	speech language pathologist	49	kept
Tanzania	tanzania	tanzania	tanzania	Tanzania	49	kept
antidiabetic agent	antidiabetic agent	antidiabetic agent	antidiabetic agent	antidiabetic agent	48	kept
antineoplastic agent	antineoplastic agent	antineoplastic agent	antineoplastic agent	antineoplastic agent	48	kept
bacterial transmission	bacterial transmission	bacterial transmission	bacterial transmission	bacterial transmission	48	kept
bacterium identification	bacterium identification	bacterium identification	bacterium identification	bacterium identification	48	kept
carbapenem	carbapenem	carbapenem	carbapenem	carbapenem	48	kept
Compassion fatigue	compassion fatigue	compassion fatigue	compassion fatigue	Compassion Fatigue	48	case_or_format
Complementary Therapies	complementary therapies	complementary therapies	complementary therapy	Complementary Therapies	48	singular
consciousness disorder	consciousness disorder	consciousness disorder	consciousness disorder	consciousness disorder	48	kept

deep sedation	deep sedation	deep sedation	deep sedation	Deep Sedation	48	case_or_format
Depression, Postpartum	depression postpartum	depression postpartum	depression postpartum	Depression, Postpartum	48	kept
Diagnosis	diagnosis	diagnosis	diagnosis	diagnosis	48	case_or_format
Education, Medical, Graduate	education medical graduate	education medical graduate	education medical graduate	Education, Medical, Graduate	48	kept
Enterococcus	enterococcus	enterococcus	enterococcus	Enterococcus	48	kept
frail elderly	frail elderly	frail elderly	frail elderly	Frail Elderly	48	case_or_format
funding	funding	funding	funding	funding	48	kept
gastrointestinal disease	gastrointestinal disease	gastrointestinal disease	gastrointestinal disease	gastrointestinal disease	48	kept
hospital pharmacy	hospital pharmacy	hospital pharmacy	hospital pharmacy	hospital pharmacy	48	kept
Incubators, Infant	incubators infant	incubators infant	incubators infant	Incubators, Infant	48	kept
Kaplan-Meier Estimate	kaplan meier estimate	kaplan meier estimate	kaplan meier estimate	Kaplan-Meier Estimate	48	kept
low drug dose	low drug dose	low drug dose	low drug dose	low drug dose	48	kept
Masks	masks	masks	mask	Masks	48	singular
Musculoskeletal Diseases	musculoskeletal diseases	musculoskeletal diseases	musculoskeletal disease	Musculoskeletal Diseases	48	singular
nasopharyngeal swab	nasopharyngeal swab	nasopharyngeal swab	nasopharyngeal swab	nasopharyngeal swab	48	kept
neuromuscular blocking agent	neuromuscular blocking agent	neuromuscular blocking agent	neuromuscular blocking agent	neuromuscular blocking agent	48	kept

newborn sepsis	newborn sepsis	newborn sepsis	newborn sepsis	newborn sepsis	48	kept
obstetrics	obstetrics	obstetrics	obstetric	Obstetrics	48	singular
occupational disease	occupational disease	occupational disease	occupational disease	Occupational Diseases	48	case_or_format
organ donation	organ donation	organ donation	organ donation	Organ donation	48	case_or_format
overall survival	overall survival	overall survival	overall survival	overall survival	48	kept
Paediatric	paediatric	paediatric	paediatric	Paediatric	48	kept
resource management	resource management	resource management	resource management	resource management	48	kept
Satisfaction	satisfaction	satisfaction	satisfaction	satisfaction	48	case_or_format
shift work	shift work	shift work	shift work	shift work	48	kept
sodium	sodium	sodium	sodium	sodium	48	kept
stakeholder engagement	stakeholder engagement	stakeholder engagement	stakeholder engagement	stakeholder engagement	48	kept
Streptococcus pneumoniae	streptococcus pneumoniae	streptococcus pneumoniae	streptococcus pneumoniae	Streptococcus pneumoniae	48	kept
STRESS	stress	stress	stress	stress	48	case_or_format
Weight Gain	weight gain	weight gain	weight gain	Weight Gain	48	kept
airway	airway	airway	airway	airway	47	kept
Aortic Aneurysm, Abdominal	aortic aneurysm abdominal	aortic aneurysm abdominal	aortic aneurysm abdominal	Aortic Aneurysm, Abdominal	47	kept

Biomedical Technology	biomedical technology	biomedical technology	biomedical technology	Biomedical Technology	47	kept
Carbapenems	carbapenems	carbapenems	carbapenem	carbapenem	47	singular
clinical pharmacist	clinical pharmacist	clinical pharmacist	clinical pharmacist	clinical pharmacist	47	kept
Dose-Response Relationship, Drug	dose response relationship drug	dose response relationship drug	dose response relationship drug	Dose-Response Relationship, Drug	47	kept
drug	drug	drug	drug	drug	47	kept
Employment	employment	employment	employment	employment	47	case_or_format
epileptic state	epileptic state	epileptic state	epileptic state	epileptic state	47	kept
Family Relations	family relations	family relations	family relation	Family Relations	47	singular
Heparin	heparin	heparin	heparin	heparin	47	case_or_format
Homes for the Aged	homes for the aged	homes for the aged	homes for the aged	Homes for the Aged	47	kept
Hydrocortisone	hydrocortisone	hydrocortisone	hydrocortisone	hydrocortisone	47	case_or_format
Indonesia	indonesia	indonesia	indonesia	Indonesia	47	kept
Internationality	internationality	internationality	internationality	Internationality	47	kept
KNOWLEDGE	knowledge	knowledge	knowledge	knowledge	47	case_or_format
Lung Neoplasms	lung neoplasms	lung neoplasms	lung neoplasms	Lung Neoplasms	47	kept
medical school	medical school	medical school	medical school	medical school	47	kept

muscle strength	muscle strength	muscle strength	muscle strength	muscle strength	47	kept
narrative	narrative	narrative	narrative	narrative	47	kept
neurologist	neurologist	neurologist	neurologist	neurologist	47	kept
Nosocomial infection	nosocomial infection	nosocomial infection	nosocomial infection	Nosocomial infection	47	kept
Nutritional Requirements	nutritional requirements	nutritional requirements	nutritional requirement	Nutritional Requirements	47	singular
occupational safety	occupational safety	occupational safety	occupational safety	occupational safety	47	kept
occupational stress	occupational stress	occupational stress	occupational stress	Occupational Stress	47	case_or_format
Older adults	older adults	older adults	older adult	Older adults	47	singular
Paediatrics	paediatrics	paediatrics	paediatric	Paediatric	47	singular
real time polymerase chain reaction	real time polymerase chain reaction	real time polymerase chain reaction	real time polymerase chain reaction	real time polymerase chain reaction	47	kept
screening test	screening test	screening test	screening test	screening test	47	kept
Sex Distribution	sex distribution	sex distribution	sex distribution	Sex Distribution	47	kept
Sleep Quality	sleep quality	sleep quality	sleep quality	sleep quality	47	case_or_format
social distancing	social distancing	social distancing	social distancing	social distancing	47	kept
sufentanil	sufentanil	sufentanil	sufentanil	sufentanil	47	kept
Thoracic Surgical Procedures	thoracic surgical procedures	thoracic surgical procedures	thoracic surgical procedures	Thoracic Surgical Procedures	47	kept

Time-to-Treatment	time to treatment	time to treatment	time to treatment	Time-to-Treatment	47	kept
Translations	translations	translations	translation	Translations	47	singular
withdrawal syndrome	withdrawal syndrome	withdrawal syndrome	withdrawal syndrome	withdrawal syndrome	47	kept
alarm fatigue	alarm fatigue	alarm fatigue	alarm fatigue	Alarm fatigue	46	case_or_format
Behavior	behavior	behavior	behavior	behavior	46	case_or_format
blood pressure monitoring	blood pressure monitoring	blood pressure monitoring	blood pressure monitoring	blood pressure monitoring	46	kept
Consumer Behavior	consumer behavior	consumer behavior	consumer behavior	Consumer Behavior	46	kept
coping	coping	coping	coping	coping	46	kept
dose response	dose response	dose response	dose response	dose response	46	kept
Emergency medicine	emergency medicine	emergency medicine	emergency medicine	emergency medicine	46	case_or_format
Exercise	exercise	exercise	exercise	exercise	46	case_or_format
Fentanyl	fentanyl	fentanyl	fentanyl	fentanyl	46	case_or_format
immunoglobulin	immunoglobulin	immunoglobulin	immunoglobulin	immunoglobulin	46	kept
income	income	income	income	income	46	kept
mindfulness	mindfulness	mindfulness	mindfulness	Mindfulness	46	case_or_format
newborn morbidity	newborn morbidity	newborn morbidity	newborn morbidity	newborn morbidity	46	kept

Norepinephrine	norepinephrine	norepinephrine	norepinephrine	Norepinephrine	46	kept
numeric rating scale	numeric rating scale	numeric rating scale	numeric rating scale	numeric rating scale	46	kept
paediatrics	paediatrics	paediatrics	paediatric	Paediatric	46	singular
performance	performance	performance	performance	PERFORMANCE	46	case_or_format
Periodicals as Topic	periodicals as topic	periodicals as topic	periodicals as topic	Periodicals as Topic	46	kept
Pressure ulcers	pressure ulcers	pressure ulcers	pressure ulcer	Pressure Ulcer	46	singular
quarantine	quarantine	quarantine	quarantine	quarantine	46	kept
recurrent disease	recurrent disease	recurrent disease	recurrent disease	recurrent disease	46	kept
Rehabilitation Centers	rehabilitation centers	rehabilitation centers	rehabilitation center	rehabilitation center	46	singular
scientist	scientist	scientist	scientist	scientist	46	kept
single blind procedure	single blind procedure	single blind procedure	single blind procedure	single blind procedure	46	kept
Southeastern United States	southeastern united states	southeastern united states	southeastern united states	Southeastern United States	46	kept
therapy effect	therapy effect	therapy effect	therapy effect	therapy effect	46	kept
Trauma Severity Indices	trauma severity indices	trauma severity indices	trauma severity indices	Trauma Severity Indices	46	kept
tuberculosis	tuberculosis	tuberculosis	tuberculosis	tuberculosis	46	kept
Washington	washington	washington	washington	Washington	46	kept

Workplace Violence	workplace violence	workplace violence	workplace violence	workplace violence	46	case_or_format
Antineoplastic Agents	antineoplastic agents	antineoplastic agents	antineoplastic agent	antineoplastic agent	45	singular
Apnea	apnea	apnea	apnea	apnea	45	case_or_format
Carbon Dioxide	carbon dioxide	carbon dioxide	carbon dioxide	carbon dioxide	45	case_or_format
Carrier State	carrier state	carrier state	carrier state	Carrier State	45	kept
Clostridium difficile infection	clostridium difficile infection	clostridium difficile infection	clostridium difficile infection	Clostridium difficile infection	45	kept
congenital heart malformation	congenital heart malformation	congenital heart malformation	congenital heart malformation	congenital heart malformation	45	kept
decision tree	decision tree	decision tree	decision tree	Decision Trees	45	case_or_format
DECISION-MAKING	decision making	decision making	decision making	Decision Making	45	case_or_format
diet therapy	diet therapy	diet therapy	diet therapy	diet therapy	45	kept
end-of-life	end of life	end of life	end of life	End of life	45	case_or_format
epilepsy	epilepsy	epilepsy	epilepsy	epilepsy	45	kept
Family-centred care	family centred care	family centred care	family centred care	Family-centred care	45	kept
family-centred care	family centred care	family centred care	family centred care	Family-centred care	45	case_or_format
Fellowships and Scholarships	fellowships and scholarships	fellowships and scholarships	fellowships and scholarships	Fellowships and Scholarships	45	kept
forecasting	forecasting	forecasting	forecasting	Forecasting	45	case_or_format

genetic transcription	genetic transcription	genetic transcription	genetic transcription	genetic transcription	45	kept
heart ventricle tachycardia	heart ventricle tachycardia	heart ventricle tachycardia	heart ventricle tachycardia	heart ventricle tachycardia	45	kept
hydromorphone	hydromorphone	hydromorphone	hydromorphone	hydromorphone	45	kept
iatrogenic disease	iatrogenic disease	iatrogenic disease	iatrogenic disease	Iatrogenic Disease	45	case_or_format
ICD-10	icd 10	icd 10	icd 10	ICD-10	45	kept
insulin treatment	insulin treatment	insulin treatment	insulin treatment	insulin treatment	45	kept
Klebsiella Infections	klebsiella infections	klebsiella infections	klebsiella infection	Klebsiella Infections	45	singular
medical procedures	medical procedures	medical procedures	medical procedures	medical procedures	45	kept
medication compliance	medication compliance	medication compliance	medication compliance	medication compliance	45	kept
minimum inhibitory concentration	minimum inhibitory concentration	minimum inhibitory concentration	minimum inhibitory concentration	minimum inhibitory concentration	45	kept
multivariate logistic regression analysis	multivariate logistic regression analysis	multivariate logistic regression analysis	multivariate logistic regression analysis	multivariate logistic regression analysis	45	kept
neonatal abstinence syndrome	neonatal abstinence syndrome	neonatal abstinence syndrome	neonatal abstinence syndrome	Neonatal Abstinence Syndrome	45	case_or_format
penicillin derivative	penicillin derivative	penicillin derivative	penicillin derivative	penicillin derivative	45	kept
pleura effusion	pleura effusion	pleura effusion	pleura effusion	pleura effusion	45	kept
postpartum hemorrhage	postpartum hemorrhage	postpartum hemorrhage	postpartum hemorrhage	postpartum hemorrhage	45	kept
Propensity Score	propensity score	propensity score	propensity score	propensity score	45	case_or_format

Quality Control	quality control	quality control	quality control	quality control	45	case_or_format
Questionnaire	questionnaire	questionnaire	questionnaire	questionnaire	45	case_or_format
quinoline derived antiinfective agent	quinoline derived antiinfective agent	quinoline derived antiinfective agent	quinoline derived antiinfective agent	quinoline derived antiinfective agent	45	kept
sex ratio	sex ratio	sex ratio	sex ratio	sex ratio	45	kept
sleep time	sleep time	sleep time	sleep time	sleep time	45	kept
staffing	staffing	staffing	staffing	staffing	45	kept
theoretical study	theoretical study	theoretical study	theoretical study	theoretical study	45	kept
time series analysis	time series analysis	time series analysis	time series analysis	time series analysis	45	kept
Translating	translating	translating	translating	Translating	45	kept
Truth Disclosure	truth disclosure	truth disclosure	truth disclosure	Truth Disclosure	45	kept
urban area	urban area	urban area	urban area	urban area	45	kept
work schedule	work schedule	work schedule	work schedule	work schedule	45	kept
adverse events	adverse events	adverse events	adverse event	adverse event	44	singular
African American	african american	african american	african american	African American	44	kept
Argentina	argentina	argentina	argentina	Argentina	44	kept
assessment	assessment	assessment	assessment	Assessment	44	case_or_format

ASSOCIATION	association	association	association	ASSOCIATION	44	kept
cardiopulmonary arrest	cardiopulmonary arrest	cardiopulmonary arrest	cardiopulmonary arrest	cardiopulmonary arrest	44	kept
Care	care	care	care	CARE	44	case_or_format
clonidine	clonidine	clonidine	clonidine	clonidine	44	kept
compliance	compliance	compliance	compliance	Compliance	44	case_or_format
diazepam	diazepam	diazepam	diazepam	diazepam	44	kept
geriatric care	geriatric care	geriatric care	geriatric care	geriatric care	44	kept
health care disparity	health care disparity	health care disparity	health care disparity	health care disparity	44	kept
Heart failure	heart failure	heart failure	heart failure	heart failure	44	case_or_format
Hispanic	hispanic	hispanic	hispanic	Hispanic	44	kept
hospital equipment	hospital equipment	hospital equipment	hospital equipment	hospital equipment	44	kept
hospital planning	hospital planning	hospital planning	hospital planning	hospital planning	44	kept
infant feeding	infant feeding	infant feeding	infant feeding	infant feeding	44	kept
Kenya	kenya	kenya	kenya	Kenya	44	kept
Lactation	lactation	lactation	lactation	Lactation	44	kept
laparotomy	laparotomy	laparotomy	laparotomy	laparotomy	44	kept

Mexico	mexico	mexico	mexico	Mexico	44	kept
Military Medicine	military medicine	military medicine	military medicine	Military Medicine	44	kept
Mini Mental State Examination	mini mental state examination	mini mental state examination	mini mental state examination	Mini Mental State Examination	44	kept
MODEL	model	model	model	model	44	case_or_format
Music	music	music	music	Music	44	kept
odds ratio	odds ratio	odds ratio	odds ratio	Odds Ratio	44	case_or_format
pain intensity	pain intensity	pain intensity	pain intensity	pain intensity	44	kept
philosophy	philosophy	philosophy	philosophy	philosophy	44	kept
Policy	policy	policy	policy	policy	44	case_or_format
Polysomnography	polysomnography	polysomnography	polysomnography	Polysomnography	44	kept
Population Surveillance	population surveillance	population surveillance	population surveillance	Population Surveillance	44	kept
premature	premature	premature	premature	Premature	44	case_or_format
problem solving	problem solving	problem solving	problem solving	Problem Solving	44	case_or_format
psychosis	psychosis	psychosis	psychosis	psychosis	44	kept
SARS-CoV-2 vaccine	sars cov 2 vaccine	sars cov 2 vaccine	sars cov 2 vaccine	SARS-CoV-2 vaccine	44	kept
severe acute respiratory syndrome	severe acute respiratory syndrome	severe acute respiratory syndrome	severe acute respiratory syndrome	severe acute respiratory syndrome	44	kept

Terminally Ill	terminally ill	terminally ill	terminally ill	Terminally Ill	44	kept
Terminology as Topic	terminology as topic	terminology as topic	terminology as topic	Terminology as Topic	44	kept
Traumatology	traumatology	traumatology	traumatology	Traumatology	44	kept
treatment response	treatment response	treatment response	treatment response	treatment response	44	kept
turkey	turkey	turkey	turkey	Turkey	44	case_or_format
Ultrasonography, Interventional	ultrasonography interventional	ultrasonography interventional	ultrasonography interventional	Ultrasonography, Interventional	44	kept
urea nitrogen blood level	urea nitrogen blood level	urea nitrogen blood level	urea nitrogen blood level	urea nitrogen blood level	44	kept
Venous Thromboembolism	venous thromboembolism	venous thromboembolism	venous thromboembolism	venous thromboembolism	44	case_or_format
Web of Science	web of science	web of science	web of science	Web of Science	44	kept
x-ray computed tomography	x ray computed tomography	x ray computed tomography	x ray computed tomography	x-ray computed tomography	44	kept
Abdomen	abdomen	abdomen	abdomen	Abdomen	43	kept
Administration, Intravenous	administration intravenous	administration intravenous	administration intravenous	Administration, Intravenous	43	kept
alcohol consumption	alcohol consumption	alcohol consumption	alcohol consumption	alcohol consumption	43	kept
antimicrobial therapy	antimicrobial therapy	antimicrobial therapy	antimicrobial therapy	antimicrobial therapy	43	kept
Asphyxia Neonatorum	asphyxia neonatorum	asphyxia neonatorum	asphyxia neonatorum	Asphyxia Neonatorum	43	kept
Attitude to Computers	attitude to computers	attitude to computers	attitude to computers	Attitude to Computers	43	kept

Blood pressure	blood pressure	blood pressure	blood pressure	blood pressure	43	case_or_format
chronic pain	chronic pain	chronic pain	chronic pain	chronic pain	43	kept
clindamycin	clindamycin	clindamycin	clindamycin	clindamycin	43	kept
clinical education	clinical education	clinical education	clinical education	clinical education	43	kept
computer program	computer program	computer program	computer program	computer program	43	kept
debridement	debridement	debridement	debridement	debridement	43	kept
Diabetic Ketoacidosis	diabetic ketoacidosis	diabetic ketoacidosis	diabetic ketoacidosis	diabetic ketoacidosis	43	case_or_format
Dissent and Disputes	dissent and disputes	dissent and disputes	dissent and disputes	Dissent and Disputes	43	kept
Echocardiography	echocardiography	echocardiography	echocardiography	echocardiography	43	case_or_format
electrolyte disturbance	electrolyte disturbance	electrolyte disturbance	electrolyte disturbance	electrolyte disturbance	43	kept
Emergency	emergency	emergency	emergency	Emergencies	43	case_or_format
ergonomics	ergonomics	ergonomics	ergonomic	Ergonomics	43	singular
EXPERIENCE	experience	experience	experience	experience	43	case_or_format
factor analysis	factor analysis	factor analysis	factor analysis	factor analysis	43	kept
feces incontinence	feces incontinence	feces incontinence	feces incontinence	feces incontinence	43	kept
Feeding Behavior	feeding behavior	feeding behavior	feeding behavior	Feeding Behavior	43	kept

Grounded theory	grounded theory	grounded theory	grounded theory	grounded theory	43	case_or_format
GUIDELINES	guidelines	guidelines	guideline	Guidelines	43	singular
hematology	hematology	hematology	hematology	hematology	43	kept
holistic care	holistic care	holistic care	holistic care	holistic care	43	kept
Infant, Extremely Low Birth Weight	infant extremely low birth weight	infant extremely low birth weight	infant extremely low birth weight	Infant, Extremely Low Birth Weight	43	kept
Interviews	interviews	interviews	interview	interview	43	singular
Kidney Failure, Chronic	kidney failure chronic	kidney failure chronic	kidney failure chronic	Kidney Failure, Chronic	43	kept
Medicaid	medicaid	medicaid	medicaid	medicaid	43	case_or_format
methadone	methadone	methadone	methadone	methadone	43	kept
Myocardial Ischemia	myocardial ischemia	myocardial ischemia	myocardial ischemia	Myocardial Ischemia	43	kept
Neonatologists	neonatologists	neonatologists	neonatologist	neonatologist	43	singular
newborn death	newborn death	newborn death	newborn death	newborn death	43	kept
nociception	nociception	nociception	nociception	nociception	43	kept
participation	participation	participation	participation	participation	43	kept
personalized medicine	personalized medicine	personalized medicine	personalized medicine	personalized medicine	43	kept
postgraduate education	postgraduate education	postgraduate education	postgraduate education	postgraduate education	43	kept

Professional Practice	professional practice	professional practice	professional practice	professional practice	43	case_or_format
proton pump inhibitor	proton pump inhibitor	proton pump inhibitor	proton pump inhibitor	proton pump inhibitor	43	kept
Psychological Distress	psychological distress	psychological distress	psychological distress	Psychological Distress	43	kept
Relatives	relatives	relatives	relative	relative	43	singular
Serratia marcescens	serratia marcescens	serratia marcescens	serratia marcescens	Serratia marcescens	43	kept
sore throat	sore throat	sore throat	sore throat	sore throat	43	kept
Statistics as Topic	statistics as topic	statistics as topic	statistics as topic	Statistics as Topic	43	kept
Surveillance	surveillance	surveillance	surveillance	Surveillance	43	kept
Syndrome	syndrome	syndrome	syndrome	Syndrome	43	kept
time to treatment	time to treatment	time to treatment	time to treatment	Time-to-Treatment	43	case_or_format
Tracheotomy	tracheotomy	tracheotomy	tracheotomy	tracheotomy	43	case_or_format
urinalysis	urinalysis	urinalysis	urinalysis	urinalysis	43	kept
warfarin	warfarin	warfarin	warfarin	warfarin	43	kept
anesthesia induction	anesthesia induction	anesthesia induction	anesthesia induction	anesthesia induction	42	kept
antifungal agent	antifungal agent	antifungal agent	antifungal agent	antifungal agent	42	kept
behavior change	behavior change	behavior change	behavior change	behavior change	42	kept

beta-Lactamases	beta lactamases	beta lactamases	beta lactamase	beta-Lactamases	42	singular
bilirubin	bilirubin	bilirubin	bilirubin	bilirubin	42	kept
blood clotting disorder	blood clotting disorder	blood clotting disorder	blood clotting disorder	blood clotting disorder	42	kept
body position	body position	body position	body position	body position	42	kept
Body Weight	body weight	body weight	body weight	body weight	42	case_or_format
Bronchiolitis	bronchiolitis	bronchiolitis	bronchiolitis	bronchiolitis	42	case_or_format
Burnout, Psychological	burnout psychological	burnout psychological	burnout psychological	Burnout, Psychological	42	kept
Cardiopulmonary Bypass	cardiopulmonary bypass	cardiopulmonary bypass	cardiopulmonary bypass	cardiopulmonary bypass	42	case_or_format
Child Health Services	child health services	child health services	child health services	Child Health Services	42	kept
circadian rhythm	circadian rhythm	circadian rhythm	circadian rhythm	circadian rhythm	42	kept
collaborative care team	collaborative care team	collaborative care team	collaborative care team	collaborative care team	42	kept
communication barrier	communication barrier	communication barrier	communication barrier	Communication Barriers	42	case_or_format
Congresses as Topic	congresses as topic	congresses as topic	congresses as topic	Congresses as Topic	42	kept
Consciousness Disorders	consciousness disorders	consciousness disorders	consciousness disorder	consciousness disorder	42	singular
consciousness level	consciousness level	consciousness level	consciousness level	consciousness level	42	kept
craniotomy	craniotomy	craniotomy	craniotomy	craniotomy	42	kept

disease classification	disease classification	disease classification	disease classification	disease classification	42	kept
disease control	disease control	disease control	disease control	disease control	42	kept
fluconazole	fluconazole	fluconazole	fluconazole	fluconazole	42	kept
health disparity	health disparity	health disparity	health disparity	health disparity	42	kept
Heart Transplantation	heart transplantation	heart transplantation	heart transplantation	heart transplantation	42	case_or_format
heart ventricle fibrillation	heart ventricle fibrillation	heart ventricle fibrillation	heart ventricle fibrillation	heart ventricle fibrillation	42	kept
Hospital Departments	hospital departments	hospital departments	hospital department	hospital department	42	singular
hospital sector	hospital sector	hospital sector	hospital sector	hospital sector	42	kept
Implementation science	implementation science	implementation science	implementation science	implementation science	42	case_or_format
intermediate care unit	intermediate care unit	intermediate care unit	intermediate care unit	intermediate care unit	42	kept
maternal mortality	maternal mortality	maternal mortality	maternal mortality	maternal mortality	42	kept
Medical Order Entry Systems	medical order entry systems	medical order entry systems	medical order entry system	Medical Order Entry Systems	42	singular
medical profession	medical profession	medical profession	medical profession	medical profession	42	kept
memory	memory	memory	memory	memory	42	kept
metabolic acidosis	metabolic acidosis	metabolic acidosis	metabolic acidosis	metabolic acidosis	42	kept
Obstetric Labor Complications	obstetric labor complications	obstetric labor complications	obstetric labor complications	Obstetric Labor Complications	42	kept

oral care	oral care	oral care	oral care	Oral care	42	case_or_format
Organ Dysfunction Scores	organ dysfunction scores	organ dysfunction scores	organ dysfunction score	Organ Dysfunction Scores	42	singular
Pain, Postoperative	pain postoperative	pain postoperative	pain postoperative	Pain, Postoperative	42	kept
pediatric surgery	pediatric surgery	pediatric surgery	pediatric surgery	pediatric surgery	42	kept
perioperative care	perioperative care	perioperative care	perioperative care	Perioperative Care	42	case_or_format
personality	personality	personality	personality	personality	42	kept
preoperative evaluation	preoperative evaluation	preoperative evaluation	preoperative evaluation	preoperative evaluation	42	kept
pulse rate	pulse rate	pulse rate	pulse rate	pulse rate	42	kept
Randomized controlled trial	randomized controlled trial	randomized controlled trial	randomized controlled trial	randomized controlled trial	42	case_or_format
Recurrence	recurrence	recurrence	recurrence	Recurrence	42	kept
reoperation	reoperation	reoperation	reoperation	reoperation	42	kept
Staphylococcus infection	staphylococcus infection	staphylococcus infection	staphylococcus infection	Staphylococcus infection	42	kept
Surgeons	surgeons	surgeons	surgeon	surgeon	42	singular
surgical mortality	surgical mortality	surgical mortality	surgical mortality	surgical mortality	42	kept
systemic inflammatory response syndrome	systemic inflammatory response syndrome	systemic inflammatory response syndrome	systemic inflammatory response syndrome	Systemic Inflammatory Response Syndrome	42	case_or_format
Temperature	temperature	temperature	temperature	temperature	42	case_or_format

United States Department of Veterans Affairs	united states department of veterans affairs	united states department of veterans affairs	united states department of veterans affairs	United States Department of Veterans Affairs	42	kept
videoconferencing	videoconferencing	videoconferencing	videoconferencing	videoconferencing	42	kept
Wales	wales	wales	wales	Wales	42	kept
amiodarone	amiodarone	amiodarone	amiodarone	amiodarone	41	kept
burn unit	burn unit	burn unit	burn unit	Burn Units	41	case_or_format
carbapenem derivative	carbapenem derivative	carbapenem derivative	carbapenem derivative	carbapenem derivative	41	kept
career	career	career	career	career	41	kept
chi square test	chi square test	chi square test	chi square test	chi square test	41	kept
clinical deterioration	clinical deterioration	clinical deterioration	clinical deterioration	Clinical Deterioration	41	case_or_format
Collaboration	collaboration	collaboration	collaboration	Collaboration	41	kept
computed tomographic angiography	computed tomographic angiography	computed tomographic angiography	computed tomographic angiography	computed tomographic angiography	41	kept
drug dose titration	drug dose titration	drug dose titration	drug dose titration	drug dose titration	41	kept
Efficiency	efficiency	efficiency	efficiency	Efficiency	41	kept
ethnology	ethnology	ethnology	ethnology	ethnology	41	kept
facial expression	facial expression	facial expression	facial expression	facial expression	41	kept
family study	family study	family study	family study	family study	41	kept

fresh frozen plasma	fresh frozen plasma	fresh frozen plasma	fresh frozen plasma	fresh frozen plasma	41	kept
Gastrointestinal Hemorrhage	gastrointestinal hemorrhage	gastrointestinal hemorrhage	gastrointestinal hemorrhage	gastrointestinal hemorrhage	41	case_or_format
general practice	general practice	general practice	general practice	general practice	41	kept
geographic distribution	geographic distribution	geographic distribution	geographic distribution	geographic distribution	41	kept
health center	health center	health center	health center	health center	41	kept
hyperkalemia	hyperkalemia	hyperkalemia	hyperkalemia	hyperkalemia	41	kept
hypokalemia	hypokalemia	hypokalemia	hypokalemia	hypokalemia	41	kept
IMPLEMENTATION	implementation	implementation	implementation	Implementation	41	case_or_format
Insulin Infusion Systems	insulin infusion systems	insulin infusion systems	insulin infusion system	Insulin Infusion Systems	41	singular
international cooperation	international cooperation	international cooperation	international cooperation	international cooperation	41	kept
intersectoral collaboration	intersectoral collaboration	intersectoral collaboration	intersectoral collaboration	intersectoral collaboration	41	kept
law	law	law	law	law	41	kept
liver disease	liver disease	liver disease	liver disease	liver disease	41	kept
Machine learning	machine learning	machine learning	machine learning	machine learning	41	case_or_format
macrolide	macrolide	macrolide	macrolide	macrolide	41	kept
methicillin resistant Staphylococcus aureus infection	methicillin resistant staphylococcus aureus infection	methicillin resistant staphylococcus aureus infection	methicillin resistant staphylococcus aureus infection	methicillin resistant Staphylococcus aureus infection	41	kept

Middle East	middle east	middle east	middle east	Middle East	41	kept
Neuromuscular Blocking Agents	neuromuscular blocking agents	neuromuscular blocking agents	neuromuscular blocking agent	neuromuscular blocking agent	41	singular
newborn screening	newborn screening	newborn screening	newborn screening	newborn screening	41	kept
Observational Studies as Topic	observational studies as topic	observational studies as topic	observational studies as topic	Observational Studies as Topic	41	kept
obstetrician	obstetrician	obstetrician	obstetrician	obstetrician	41	kept
out of hospital cardiac arrest	out of hospital cardiac arrest	out of hospital cardiac arrest	out of hospital cardiac arrest	out of hospital cardiac arrest	41	kept
phenobarbital	phenobarbital	phenobarbital	phenobarbital	phenobarbital	41	kept
Physical restraint	physical restraint	physical restraint	physical restraint	physical restraint	41	case_or_format
Psychiatric Department, Hospital	psychiatric department hospital	psychiatric department hospital	psychiatric department hospital	Psychiatric Department, Hospital	41	kept
psychiatrist	psychiatrist	psychiatrist	psychiatrist	psychiatrist	41	kept
Respiratory failure	respiratory failure	respiratory failure	respiratory failure	respiratory failure	41	case_or_format
rifampicin	rifampicin	rifampicin	rifampicin	rifampicin	41	kept
Septic shock	septic shock	septic shock	septic shock	septic shock	41	case_or_format
SURGERY	surgery	surgery	surgery	surgery	41	case_or_format
Surgery Department, Hospital	surgery department hospital	surgery department hospital	surgery department hospital	Surgery Department, Hospital	41	kept
adverse drug reaction	adverse drug reaction	adverse drug reaction	adverse drug reaction	adverse drug reaction	40	kept

airway obstruction	airway obstruction	airway obstruction	airway obstruction	airway obstruction	40	kept
amoxicillin	amoxicillin	amoxicillin	amoxicillin	amoxicillin	40	kept
arousal	arousal	arousal	arousal	arousal	40	kept
bacterium isolation	bacterium isolation	bacterium isolation	bacterium isolation	bacterium isolation	40	kept
benchmarking	benchmarking	benchmarking	benchmarking	Benchmarking	40	case_or_format
Bullying	bullying	bullying	bullying	Bullying	40	kept
Chile	chile	chile	chile	Chile	40	kept
Cluster Analysis	cluster analysis	cluster analysis	cluster analysis	Cluster Analysis	40	kept
cost of illness	cost of illness	cost of illness	cost of illness	Cost of Illness	40	case_or_format
Cross-Cultural Comparison	cross cultural comparison	cross cultural comparison	cross cultural comparison	Cross-Cultural Comparison	40	kept
Cuidados críticos	cuidados cr ticos	cuidados cr ticos	cuidados cr ticos	Cuidados críticos	40	kept
Early mobilization	early mobilization	early mobilization	early mobilization	Early mobilization	40	kept
Emergence Delirium	emergence delirium	emergence delirium	emergence delirium	Emergence Delirium	40	kept
employment status	employment status	employment status	employment status	employment status	40	kept
Enfermería	enfermer a	enfermer a	enfermer a	Enfermería	40	kept
error	error	error	error	error	40	kept

Experiences	experiences	experiences	experience	experience	40	singular
Gram-Positive Bacterial Infections	gram positive bacterial infections	gram positive bacterial infections	gram positive bacterial infections	Gram-Positive Bacterial Infections	40	kept
healthcare	healthcare	healthcare	healthcare	Healthcare	40	case_or_format
Heart	heart	heart	heart	Heart	40	kept
Helping Behavior	helping behavior	helping behavior	helping behavior	Helping Behavior	40	kept
Hospital Anxiety and Depression Scale	hospital anxiety and depression scale	hospital anxiety and depression scale	hospital anxiety and depression scale	Hospital Anxiety and Depression Scale	40	kept
Hospitals, Private	hospitals private	hospitals private	hospitals private	Hospitals, Private	40	kept
immobility	immobility	immobility	immobility	immobility	40	kept
immobilization	immobilization	immobilization	immobilization	immobilization	40	kept
INTERVENTIONS	interventions	interventions	intervention	Intervention	40	singular
life expectancy	life expectancy	life expectancy	life expectancy	life expectancy	40	kept
lung lavage	lung lavage	lung lavage	lung lavage	lung lavage	40	kept
maternal care	maternal care	maternal care	maternal care	maternal care	40	kept
Medical Staff	medical staff	medical staff	medical staff	medical staff	40	case_or_format
mental health service	mental health service	mental health service	mental health service	mental health service	40	kept
microbiological examination	microbiological examination	microbiological examination	microbiological examination	microbiological examination	40	kept

needlestick injury	needlestick injury	needlestick injury	needlestick injury	needlestick injury	40	kept
nonbiological model	nonbiological model	nonbiological model	nonbiological model	nonbiological model	40	kept
pathogenesis	pathogenesis	pathogenesis	pathogenesis	pathogenesis	40	kept
predictive model	predictive model	predictive model	predictive model	predictive model	40	kept
Pregnancy Complications, Infectious	pregnancy complications infectious	pregnancy complications infectious	pregnancy complications infectious	Pregnancy Complications, Infectious	40	kept
Proxy	proxy	proxy	proxy	Proxy	40	kept
remdesivir	remdesivir	remdesivir	remdesivir	remdesivir	40	kept
Reoperation	reoperation	reoperation	reoperation	reoperation	40	case_or_format
Respiratory Rate	respiratory rate	respiratory rate	respiratory rate	Respiratory Rate	40	kept
Screening	screening	screening	screening	screening	40	case_or_format
secondary traumatic stress	secondary traumatic stress	secondary traumatic stress	secondary traumatic stress	secondary traumatic stress	40	kept
shift worker	shift worker	shift worker	shift worker	shift worker	40	kept
social psychology	social psychology	social psychology	social psychology	social psychology	40	kept
supine position	supine position	supine position	supine position	supine position	40	kept
Thirst	thirst	thirst	thirst	Thirst	40	kept
Uganda	uganda	uganda	uganda	Uganda	40	kept

university	university	university	university	university	40	kept
vasoconstrictor agent	vasoconstrictor agent	vasoconstrictor agent	vasoconstrictor agent	Vasoconstrictor Agents	40	case_or_format
Wisconsin	wisconsin	wisconsin	wisconsin	Wisconsin	40	kept
Wounds, Nonpenetrating	wounds nonpenetrating	wounds nonpenetrating	wounds nonpenetrating	Wounds, Nonpenetrating	40	kept
ARDS	ards	ards	ards	ARDS	39	kept
body posture	body posture	body posture	body posture	body posture	39	kept
care	care	care	care	CARE	39	case_or_format
caregiver burden	caregiver burden	caregiver burden	caregiver burden	caregiver burden	39	kept
cefazolin	cefazolin	cefazolin	cefazolin	cefazolin	39	kept
Chinese	chinese	chinese	chinese	Chinese	39	kept
complications	complications	complications	complication	complication	39	singular
Congenital Abnormalities	congenital abnormalities	congenital abnormalities	congenital abnormality	Congenital Abnormalities	39	singular
Content analysis	content analysis	content analysis	content analysis	content analysis	39	case_or_format
Craniocerebral Trauma	craniocerebral trauma	craniocerebral trauma	craniocerebral trauma	Craniocerebral Trauma	39	kept
Discharge	discharge	discharge	discharge	Discharge	39	kept
early mobilization	early mobilization	early mobilization	early mobilization	Early mobilization	39	case_or_format

economic aspect	economic aspect	economic aspect	economic aspect	economic aspect	39	kept
electrolyte	electrolyte	electrolyte	electrolyte	electrolyte	39	kept
emotionality	emotionality	emotionality	emotionality	emotionality	39	kept
Enterobacteriaceae Infections	enterobacteriaceae infections	enterobacteriaceae infections	enterobacteriaceae infection	Enterobacteriaceae Infections	39	singular
erythema	erythema	erythema	erythema	erythema	39	kept
erythromycin	erythromycin	erythromycin	erythromycin	erythromycin	39	kept
Gram-Negative Bacteria	gram negative bacteria	gram negative bacteria	gram negative bacteria	Gram-Negative Bacteria	39	kept
Gram-Negative Bacterial Infections	gram negative bacterial infections	gram negative bacterial infections	gram negative bacterial infections	Gram-Negative Bacterial Infections	39	kept
growth, development and aging	growth development and aging	growth development and aging	growth development and aging	growth, development and aging	39	kept
Health Surveys	health surveys	health surveys	health survey	health survey	39	singular
heart assist device	heart assist device	heart assist device	heart assist device	Heart-Assist Devices	39	case_or_format
hospital administrator	hospital administrator	hospital administrator	hospital administrator	hospital administrator	39	kept
Hospitals, Rural	hospitals rural	hospitals rural	hospitals rural	Hospitals, Rural	39	kept
hypercapnia	hypercapnia	hypercapnia	hypercapnia	hypercapnia	39	kept
Hypertension, Pulmonary	hypertension pulmonary	hypertension pulmonary	hypertension pulmonary	Hypertension, Pulmonary	39	kept
hypoxic ischemic encephalopathy	hypoxic ischemic encephalopathy	hypoxic ischemic encephalopathy	hypoxic ischemic encephalopathy	hypoxic ischemic encephalopathy	39	kept

immunoglobulin G	immunoglobulin g	immunoglobulin g	immunoglobulin g	immunoglobulin G	39	kept
invasive procedure	invasive procedure	invasive procedure	invasive procedure	invasive procedure	39	kept
medical assessment	medical assessment	medical assessment	medical assessment	medical assessment	39	kept
Memory	memory	memory	memory	memory	39	case_or_format
Models, Statistical	models statistical	models statistical	models statistical	Models, Statistical	39	kept
neonatal care	neonatal care	neonatal care	neonatal care	neonatal care	39	kept
neuromuscular blocking	neuromuscular blocking	neuromuscular blocking	neuromuscular blocking	neuromuscular blocking	39	kept
phenytoin	phenytoin	phenytoin	phenytoin	phenytoin	39	kept
Postpartum Period	postpartum period	postpartum period	postpartum period	Postpartum Period	39	kept
Religion	religion	religion	religion	religion	39	case_or_format
rocuronium	rocuronium	rocuronium	rocuronium	rocuronium	39	kept
Rural Population	rural population	rural population	rural population	Rural Population	39	kept
specialization	specialization	specialization	specialization	specialization	39	kept
survival analysis	survival analysis	survival analysis	survival analysis	Survival Analysis	39	case_or_format
univariate analysis	univariate analysis	univariate analysis	univariate analysis	univariate analysis	39	kept
VALIDATION	validation	validation	validation	VALIDATION	39	kept

vasopressin	vasopressin	vasopressin	vasopressin	vasopressin	39	kept
Vomiting	vomiting	vomiting	vomiting	vomiting	39	case_or_format
abdominal surgery	abdominal surgery	abdominal surgery	abdominal surgery	abdominal surgery	38	kept
amoxicillin plus clavulanic acid	amoxicillin plus clavulanic acid	amoxicillin plus clavulanic acid	amoxicillin plus clavulanic acid	amoxicillin plus clavulanic acid	38	kept
anamnesis	anamnesis	anamnesis	anamnesis	anamnesis	38	kept
antidepressant agent	antidepressant agent	antidepressant agent	antidepressant agent	antidepressant agent	38	kept
Antipsychotic Agents	antipsychotic agents	antipsychotic agents	antipsychotic agents	Antipsychotic Agents	38	kept
Aromatherapy	aromatherapy	aromatherapy	aromatherapy	Aromatherapy	38	kept
aspartate aminotransferase	aspartate aminotransferase	aspartate aminotransferase	aspartate aminotransferase	aspartate aminotransferase	38	kept
Brain Diseases	brain diseases	brain diseases	brain disease	brain disease	38	singular
British Columbia	british columbia	british columbia	british columbia	British Columbia	38	kept
cancer	cancer	cancer	cancer	cancer	38	kept
cephalosporin derivative	cephalosporin derivative	cephalosporin derivative	cephalosporin derivative	cephalosporin derivative	38	kept
community acquired infection	community acquired infection	community acquired infection	community acquired infection	Community-Acquired Infections	38	case_or_format
Continuous renal replacement therapy	continuous renal replacement therapy	continuous renal replacement therapy	continuous renal replacement therapy	continuous renal replacement therapy	38	case_or_format
Cultural Competency	cultural competency	cultural competency	cultural competency	Cultural Competency	38	kept

device removal	device removal	device removal	device removal	Device Removal	38	case_or_format
Euthanasia	euthanasia	euthanasia	euthanasia	Euthanasia	38	kept
extremely low birth weight	extremely low birth weight	extremely low birth weight	extremely low birth weight	extremely low birth weight	38	kept
food intake	food intake	food intake	food intake	food intake	38	kept
Group Processes	group processes	group processes	group processes	Group Processes	38	kept
hand hygiene	hand hygiene	hand hygiene	hand hygiene	Hand Hygiene	38	case_or_format
Health Care Reform	health care reform	health care reform	health care reform	Health Care Reform	38	kept
Health Facilities	health facilities	health facilities	health facilities	Health Facilities	38	kept
health personnel	health personnel	health personnel	health personnel	Health Personnel	38	case_or_format
Healthcare professionals	healthcare professionals	healthcare professionals	healthcare professional	healthcare professionals	38	singular
Hyponatremia	hyponatremia	hyponatremia	hyponatremia	hyponatremia	38	case_or_format
Infant Nutritional Physiological Phenomena	infant nutritional physiological phenomena	infant nutritional physiological phenomena	infant nutritional physiological phenomena	Infant Nutritional Physiological Phenomena	38	kept
insulin infusion	insulin infusion	insulin infusion	insulin infusion	insulin infusion	38	kept
International Cooperation	international cooperation	international cooperation	international cooperation	international cooperation	38	case_or_format
Mass Casualty Incidents	mass casualty incidents	mass casualty incidents	mass casualty incident	Mass Casualty Incidents	38	singular
Maternal Health Services	maternal health services	maternal health services	maternal health service	Maternal Health Services	38	singular

MRSA	mrsa	mrsa	mrsa	MRSA	38	kept
Nepal	nepal	nepal	nepal	Nepal	38	kept
parental stress	parental stress	parental stress	parental stress	parental stress	38	kept
Parenteral Nutrition, Total	parenteral nutrition total	parenteral nutrition total	parenteral nutrition total	Parenteral Nutrition, Total	38	kept
person centered care	person centered care	person centered care	person centered care	person centered care	38	kept
phlebotomy	phlebotomy	phlebotomy	phlebotomy	Phlebotomy	38	case_or_format
physicians	physicians	physicians	physician	physician	38	singular
polysomnography	polysomnography	polysomnography	polysomnography	Polysomnography	38	case_or_format
postoperative hemorrhage	postoperative hemorrhage	postoperative hemorrhage	postoperative hemorrhage	postoperative hemorrhage	38	kept
Postpartum Hemorrhage	postpartum hemorrhage	postpartum hemorrhage	postpartum hemorrhage	postpartum hemorrhage	38	case_or_format
Privacy	privacy	privacy	privacy	privacy	38	case_or_format
probability	probability	probability	probability	Probability	38	case_or_format
psychiatry	psychiatry	psychiatry	psychiatry	psychiatry	38	kept
Pulmonary Edema	pulmonary edema	pulmonary edema	pulmonary edema	Pulmonary Edema	38	kept
residential care	residential care	residential care	residential care	residential care	38	kept
respiratory tract intubation	respiratory tract intubation	respiratory tract intubation	respiratory tract intubation	respiratory tract intubation	38	kept

sevoflurane	sevoflurane	sevoflurane	sevoflurane	sevoflurane	38	kept
Sucrose	sucrose	sucrose	sucrose	Sucrose	38	kept
SUPPORT	support	support	support	SUPPORT	38	kept
support group	support group	support group	support group	support group	38	kept
SYSTEM	system	system	system	SYSTEM	38	kept
Thrombolytic Therapy	thrombolytic therapy	thrombolytic therapy	thrombolytic therapy	Thrombolytic Therapy	38	kept
Tidal Volume	tidal volume	tidal volume	tidal volume	tidal volume	38	case_or_format
topical antiinfective agent	topical antiinfective agent	topical antiinfective agent	topical antiinfective agent	topical antiinfective agent	38	kept
treatment contraindication	treatment contraindication	treatment contraindication	treatment contraindication	treatment contraindication	38	kept
urine incontinence	urine incontinence	urine incontinence	urine incontinence	urine incontinence	38	kept
WORK	work	work	work	work	38	case_or_format
Acute respiratory distress syndrome	acute respiratory distress syndrome	acute respiratory distress syndrome	acute respiratory distress syndrome	acute respiratory distress syndrome	37	case_or_format
antibiotic prophylaxis	antibiotic prophylaxis	antibiotic prophylaxis	antibiotic prophylaxis	antibiotic prophylaxis	37	kept
Apgar Score	apgar score	apgar score	apgar score	Apgar score	37	case_or_format
Asia	asia	asia	asia	Asia	37	kept
bacterial strain	bacterial strain	bacterial strain	bacterial strain	bacterial strain	37	kept

blood cell count	blood cell count	blood cell count	blood cell count	blood cell count	37	kept
brain edema	brain edema	brain edema	brain edema	brain edema	37	kept
Brain Neoplasms	brain neoplasms	brain neoplasms	brain neoplasms	Brain Neoplasms	37	kept
Catheterization, Swan-Ganz	catheterization swan ganz	catheterization swan ganz	catheterization swan ganz	Catheterization, Swan-Ganz	37	kept
cleaning	cleaning	cleaning	cleaning	cleaning	37	kept
clergy	clergy	clergy	clergy	clergy	37	kept
Constipation	constipation	constipation	constipation	constipation	37	case_or_format
diagnostic procedure	diagnostic procedure	diagnostic procedure	diagnostic procedure	diagnostic procedure	37	kept
Environmental Monitoring	environmental monitoring	environmental monitoring	environmental monitoring	Environmental Monitoring	37	kept
exploratory factor analysis	exploratory factor analysis	exploratory factor analysis	exploratory factor analysis	exploratory factor analysis	37	kept
family relation	family relation	family relation	family relation	Family Relations	37	case_or_format
gabapentin	gabapentin	gabapentin	gabapentin	gabapentin	37	kept
Illinois	illinois	illinois	illinois	Illinois	37	kept
INTERVENTION	intervention	intervention	intervention	Intervention	37	case_or_format
intoxication	intoxication	intoxication	intoxication	intoxication	37	kept
ischemic heart disease	ischemic heart disease	ischemic heart disease	ischemic heart disease	ischemic heart disease	37	kept

lactation	lactation	lactation	lactation	Lactation	37	case_or_format
lung infection	lung infection	lung infection	lung infection	lung infection	37	kept
Meta-Analysis as Topic	meta analysis as topic	meta analysis as topic	meta analysis as topic	Meta-Analysis as Topic	37	kept
Methicillin Resistance	methicillin resistance	methicillin resistance	methicillin resistance	Methicillin Resistance	37	kept
music	music	music	music	Music	37	case_or_format
neuromonitoring	neuromonitoring	neuromonitoring	neuromonitoring	neuromonitoring	37	kept
Occupational stress	occupational stress	occupational stress	occupational stress	Occupational Stress	37	case_or_format
Personality	personality	personality	personality	personality	37	case_or_format
phenylephrine	phenylephrine	phenylephrine	phenylephrine	phenylephrine	37	kept
Pneumothorax	pneumothorax	pneumothorax	pneumothorax	pneumothorax	37	case_or_format
primary prevention	primary prevention	primary prevention	primary prevention	Primary Prevention	37	case_or_format
procedural pain	procedural pain	procedural pain	procedural pain	procedural pain	37	kept
productivity	productivity	productivity	productivity	productivity	37	kept
Protocol	protocol	protocol	protocol	protocol	37	case_or_format
QUALITY-OF-LIFE	quality of life	quality of life	quality of life	quality of life	37	case_or_format
Radiography	radiography	radiography	radiography	radiography	37	case_or_format

return of spontaneous circulation	return of spontaneous circulation	return of spontaneous circulation	return of spontaneous circulation	return of spontaneous circulation	37	kept
Self-Help Groups	self help groups	self help groups	self help groups	Self-Help Groups	37	kept
Specialization	specialization	specialization	specialization	specialization	37	case_or_format
Students	students	students	student	Students	37	singular
surge capacity	surge capacity	surge capacity	surge capacity	surge capacity	37	kept
swallowing	swallowing	swallowing	swallowing	swallowing	37	kept
Technology Assessment, Biomedical	technology assessment biomedical	technology assessment biomedical	technology assessment biomedical	Technology Assessment, Biomedical	37	kept
Tennessee	tennessee	tennessee	tennessee	Tennessee	37	kept
Thoracic Surgery	thoracic surgery	thoracic surgery	thoracic surgery	Thoracic Surgery	37	kept
Validation	validation	validation	validation	VALIDATION	37	case_or_format
vital signs	vital signs	vital signs	vital sign	vital sign	37	singular
access to information	access to information	access to information	access to information	access to information	36	kept
Acute Pain	acute pain	acute pain	acute pain	Acute Pain	36	kept
adaptation	adaptation	adaptation	adaptation	adaptation	36	kept
alternative medicine	alternative medicine	alternative medicine	alternative medicine	alternative medicine	36	kept
Ambulances	ambulances	ambulances	ambulance	ambulance	36	singular

Anesthesiologists	anesthesiologists	anesthesiologists	anesthesiologist	anesthesiologist	36	singular
asepsis	asepsis	asepsis	asepsis	asepsis	36	kept
Awards and Prizes	awards and prizes	awards and prizes	awards and prizes	Awards and Prizes	36	kept
best practice	best practice	best practice	best practice	best practice	36	kept
body weight loss	body weight loss	body weight loss	body weight loss	body weight loss	36	kept
Brain death	brain death	brain death	brain death	brain death	36	case_or_format
cephalosporin	cephalosporin	cephalosporin	cephalosporin	cephalosporin	36	kept
chi square distribution	chi square distribution	chi square distribution	chi square distribution	Chi-Square Distribution	36	case_or_format
Choice Behavior	choice behavior	choice behavior	choice behavior	Choice Behavior	36	kept
Clostridioides difficile	clostridioides difficile	clostridioides difficile	clostridioides difficile	Clostridioides difficile	36	kept
communication disorder	communication disorder	communication disorder	communication disorder	communication disorder	36	kept
community	community	community	community	community	36	kept
Competence	competence	competence	competence	competence	36	case_or_format
comprehension	comprehension	comprehension	comprehension	comprehension	36	kept
confidentiality	confidentiality	confidentiality	confidentiality	confidentiality	36	kept
Congenital heart disease	congenital heart disease	congenital heart disease	congenital heart disease	congenital heart disease	36	case_or_format

conservative treatment	conservative treatment	conservative treatment	conservative treatment	conservative treatment	36	kept
do not resuscitate order	do not resuscitate order	do not resuscitate order	do not resuscitate order	do not resuscitate order	36	kept
donor	donor	donor	donor	donor	36	kept
Drug Utilization	drug utilization	drug utilization	drug utilization	Drug Utilization	36	kept
Education, Distance	education distance	education distance	education distance	Education, Distance	36	kept
Employee Performance Appraisal	employee performance appraisal	employee performance appraisal	employee performance appraisal	Employee Performance Appraisal	36	kept
end stage renal disease	end stage renal disease	end stage renal disease	end stage renal disease	end stage renal disease	36	kept
Enterobacter cloacae	enterobacter cloacae	enterobacter cloacae	enterobacter cloacae	Enterobacter cloacae	36	kept
Equipment Safety	equipment safety	equipment safety	equipment safety	Equipment Safety	36	kept
Families	families	families	family	Family	36	singular
family attitude	family attitude	family attitude	family attitude	family attitude	36	kept
fluid balance	fluid balance	fluid balance	fluid balance	fluid balance	36	kept
Ghana	ghana	ghana	ghana	Ghana	36	kept
health economics	health economics	health economics	health economics	health economics	36	kept
Hospitalists	hospitalists	hospitalists	hospitalist	Hospitalists	36	singular
information retrieval	information retrieval	information retrieval	information retrieval	information retrieval	36	kept

integrated health care system	integrated health care system	integrated health care system	integrated health care system	integrated health care system	36	kept
intracranial hypertension	intracranial hypertension	intracranial hypertension	intracranial hypertension	Intracranial Hypertension	36	case_or_format
Maryland	maryland	maryland	maryland	Maryland	36	kept
medical expert	medical expert	medical expert	medical expert	medical expert	36	kept
Medical History Taking	medical history taking	medical history taking	medical history taking	Medical History Taking	36	kept
metabolic disorder	metabolic disorder	metabolic disorder	metabolic disorder	metabolic disorder	36	kept
Mobile Applications	mobile applications	mobile applications	mobile application	Mobile Applications	36	singular
Neonatal care	neonatal care	neonatal care	neonatal care	neonatal care	36	case_or_format
nephrology	nephrology	nephrology	nephrology	nephrology	36	kept
nonparametric test	nonparametric test	nonparametric test	nonparametric test	nonparametric test	36	kept
OF-LIFE CARE	of life care	of life care	of life care	OF-LIFE CARE	36	kept
olanzapine	olanzapine	olanzapine	olanzapine	olanzapine	36	kept
older adults	older adults	older adults	older adult	Older adults	36	singular
outcome	outcome	outcome	outcome	OUTCOMES	36	case_or_format
Poisoning	poisoning	poisoning	poisoning	Poisoning	36	kept
pressure ulcers	pressure ulcers	pressure ulcers	pressure ulcer	Pressure Ulcer	36	singular

pruritus	pruritus	pruritus	pruritus	pruritus	36	kept
psychological care	psychological care	psychological care	psychological care	psychological care	36	kept
Respiration Disorders	respiration disorders	respiration disorders	respiration disorders	Respiration Disorders	36	kept
Robotics	robotics	robotics	robotics	Robotics	36	kept
salary	salary	salary	salary	salary	36	kept
self-efficacy	self efficacy	self efficacy	self efficacy	Self Efficacy	36	case_or_format
Severe Acute Respiratory Syndrome	severe acute respiratory syndrome	severe acute respiratory syndrome	severe acute respiratory syndrome	severe acute respiratory syndrome	36	case_or_format
Surge Capacity	surge capacity	surge capacity	surge capacity	surge capacity	36	case_or_format
transesophageal echocardiography	transesophageal echocardiography	transesophageal echocardiography	transesophageal echocardiography	transesophageal echocardiography	36	kept
vein puncture	vein puncture	vein puncture	vein puncture	vein puncture	36	kept
Veterans	veterans	veterans	veteran	Veterans	36	singular
Virginia	virginia	virginia	virginia	Virginia	36	kept
Working Conditions	working conditions	working conditions	working conditions	Working Conditions	36	kept
Anxiety Disorders	anxiety disorders	anxiety disorders	anxiety disorder	anxiety disorder	35	singular
atelectasis	atelectasis	atelectasis	atelectasis	atelectasis	35	kept
Bias	bias	bias	bias	Bias	35	kept

Blood Glucose Self-Monitoring	blood glucose self monitoring	blood glucose self monitoring	blood glucose self monitoring	Blood Glucose Self-Monitoring	35	kept
breast cancer	breast cancer	breast cancer	breast cancer	breast cancer	35	kept
Candida albicans	candida albicans	candida albicans	candida albicans	Candida albicans	35	kept
cardiac arrest	cardiac arrest	cardiac arrest	cardiac arrest	Cardiac arrest	35	case_or_format
catheter removal	catheter removal	catheter removal	catheter removal	catheter removal	35	kept
childbirth	childbirth	childbirth	childbirth	childbirth	35	kept
Circadian Rhythm	circadian rhythm	circadian rhythm	circadian rhythm	circadian rhythm	35	case_or_format
Clinical competence	clinical competence	clinical competence	clinical competence	Clinical Competence	35	case_or_format
colistin	colistin	colistin	colistin	colistin	35	kept
communicable disease	communicable disease	communicable disease	communicable disease	communicable disease	35	kept
congenital malformation	congenital malformation	congenital malformation	congenital malformation	congenital malformation	35	kept
Craniotomy	craniotomy	craniotomy	craniotomy	craniotomy	35	case_or_format
Cues	cues	cues	cues	Cues	35	kept
Cultural Diversity	cultural diversity	cultural diversity	cultural diversity	Cultural Diversity	35	kept
Delivery Rooms	delivery rooms	delivery rooms	delivery room	delivery room	35	singular
device	device	device	device	devices	35	case_or_format

digoxin	digoxin	digoxin	digoxin	digoxin	35	kept
disseminated intravascular clotting	disseminated intravascular clotting	disseminated intravascular clotting	disseminated intravascular clotting	disseminated intravascular clotting	35	kept
Drug Therapy, Combination	drug therapy combination	drug therapy combination	drug therapy combination	Drug Therapy, Combination	35	kept
Emotional Intelligence	emotional intelligence	emotional intelligence	emotional intelligence	emotional intelligence	35	case_or_format
emotional support	emotional support	emotional support	emotional support	emotional support	35	kept
employee	employee	employee	employee	employee	35	kept
erratum	erratum	erratum	erratum	erratum	35	kept
Evaluation Studies as Topic	evaluation studies as topic	evaluation studies as topic	evaluation studies as topic	Evaluation Studies as Topic	35	kept
Extravasation of Diagnostic and Therapeutic Materials	extravasation of diagnostic and therapeutic materials	extravasation of diagnostic and therapeutic materials	extravasation of diagnostic and therapeutic materials	Extravasation of Diagnostic and Therapeutic Materials	35	kept
gastrointestinal symptom	gastrointestinal symptom	gastrointestinal symptom	gastrointestinal symptom	gastrointestinal symptom	35	kept
Hospitals, Military	hospitals military	hospitals military	hospitals military	Hospitals, Military	35	kept
hydrocephalus	hydrocephalus	hydrocephalus	hydrocephalus	hydrocephalus	35	kept
hyperlipidemia	hyperlipidemia	hyperlipidemia	hyperlipidemia	hyperlipidemia	35	kept
information dissemination	information dissemination	information dissemination	information dissemination	Information Dissemination	35	case_or_format
information technology	information technology	information technology	information technology	information technology	35	kept
laryngoscopy	laryngoscopy	laryngoscopy	laryngoscopy	laryngoscopy	35	kept

Lebanon	lebanon	lebanon	lebanon	Lebanon	35	kept
literature	literature	literature	literature	literature	35	kept
measurement	measurement	measurement	measurement	measurement	35	kept
Medical computing	medical computing	medical computing	medical computing	Medical computing	35	kept
medical service	medical service	medical service	medical service	medical service	35	kept
melatonin	melatonin	melatonin	melatonin	melatonin	35	kept
Mental Health Services	mental health services	mental health services	mental health service	mental health service	35	singular
nausea and vomiting	nausea and vomiting	nausea and vomiting	nausea and vomiting	nausea and vomiting	35	kept
New England	new england	new england	new england	New England	35	kept
Night Care	night care	night care	night care	Night Care	35	kept
Nonverbal Communication	nonverbal communication	nonverbal communication	nonverbal communication	Nonverbal Communication	35	kept
pediatric anesthesia	pediatric anesthesia	pediatric anesthesia	pediatric anesthesia	pediatric anesthesia	35	kept
Peer Group	peer group	peer group	peer group	Peer Group	35	kept
pethidine	pethidine	pethidine	pethidine	pethidine	35	kept
pharmaceutical care	pharmaceutical care	pharmaceutical care	pharmaceutical care	pharmaceutical care	35	kept
Phenomenology	phenomenology	phenomenology	phenomenology	phenomenology	35	case_or_format

Photography	photography	photography	photography	Photography	35	kept
Planning Techniques	planning techniques	planning techniques	planning techniques	Planning Techniques	35	kept
polypharmacy	polypharmacy	polypharmacy	polypharmacy	polypharmacy	35	kept
Power	power	power	power	Power	35	kept
prednisone	prednisone	prednisone	prednisone	prednisone	35	kept
risk benefit analysis	risk benefit analysis	risk benefit analysis	risk benefit analysis	risk benefit analysis	35	kept
Salaries and Fringe Benefits	salaries and fringe benefits	salaries and fringe benefits	salaries and fringe benefits	Salaries and Fringe Benefits	35	kept
Shift Work Schedule	shift work schedule	shift work schedule	shift work schedule	Shift Work Schedule	35	kept
smoking cessation	smoking cessation	smoking cessation	smoking cessation	smoking cessation	35	kept
social behavior	social behavior	social behavior	social behavior	social behavior	35	kept
social determinants of health	social determinants of health	social determinants of health	social determinants of health	social determinants of health	35	kept
special situation for pharmacovigilance	special situation for pharmacovigilance	special situation for pharmacovigilance	special situation for pharmacovigilance	special situation for pharmacovigilance	35	kept
speech	speech	speech	speech	speech	35	kept
STAFF	staff	staff	staff	staff	35	case_or_format
sultamicillin	sultamicillin	sultamicillin	sultamicillin	sultamicillin	35	kept
transthoracic echocardiography	transthoracic echocardiography	transthoracic echocardiography	transthoracic echocardiography	transthoracic echocardiography	35	kept

traumatology	traumatology	traumatology	traumatology	Traumatology	35	case_or_format
Treatment	treatment	treatment	treatment	Treatment	35	kept
wakefulness	wakefulness	wakefulness	wakefulness	wakefulness	35	kept
walking	walking	walking	walking	walking	35	kept
writing	writing	writing	writing	Writing	35	case_or_format
Accidents, Traffic	accidents traffic	accidents traffic	accidents traffic	Accidents, Traffic	34	kept
aerosol	aerosol	aerosol	aerosol	aerosol	34	kept
Agitation	agitation	agitation	agitation	agitation	34	case_or_format
airway pressure	airway pressure	airway pressure	airway pressure	airway pressure	34	kept
Arizona	arizona	arizona	arizona	Arizona	34	kept
atropine	atropine	atropine	atropine	atropine	34	kept
BARRIERS	barriers	barriers	barrier	Barriers	34	singular
Blood	blood	blood	blood	blood	34	case_or_format
brain tumor	brain tumor	brain tumor	brain tumor	brain tumor	34	kept
calculation	calculation	calculation	calculation	calculation	34	kept
Cancer	cancer	cancer	cancer	cancer	34	case_or_format

case management	case management	case management	case management	Case Management	34	case_or_format
Cerebrovascular Circulation	cerebrovascular circulation	cerebrovascular circulation	cerebrovascular circulation	Cerebrovascular Circulation	34	kept
chemically induced disorder	chemically induced disorder	chemically induced disorder	chemically induced disorder	chemically induced disorder	34	kept
childhood disease	childhood disease	childhood disease	childhood disease	childhood disease	34	kept
clopidogrel	clopidogrel	clopidogrel	clopidogrel	clopidogrel	34	kept
COLLABORATION	collaboration	collaboration	collaboration	Collaboration	34	case_or_format
COVID-19 testing	covid 19 testing	covid 19 testing	covid 19 testing	COVID-19 testing	34	kept
DEATH	death	death	death	death	34	case_or_format
Decision support systems	decision support systems	decision support systems	decision support system	decision support system	34	singular
defibrillation	defibrillation	defibrillation	defibrillation	defibrillation	34	kept
ethical decision making	ethical decision making	ethical decision making	ethical decision making	ethical decision making	34	kept
Father-Child Relations	father child relations	father child relations	father child relation	Father-Child Relations	34	singular
fraction of inspired oxygen	fraction of inspired oxygen	fraction of inspired oxygen	fraction of inspired oxygen	fraction of inspired oxygen	34	kept
hallucination	hallucination	hallucination	hallucination	hallucination	34	kept
hand disinfection	hand disinfection	hand disinfection	hand disinfection	Hand Disinfection	34	case_or_format
Heart Valve Prosthesis Implantation	heart valve prosthesis implantation	heart valve prosthesis implantation	heart valve prosthesis implantation	Heart Valve Prosthesis Implantation	34	kept

hematoma	hematoma	hematoma	hematoma	hematoma	34	kept
high dependency unit	high dependency unit	high dependency unit	high dependency unit	high dependency unit	34	kept
hospital hygiene	hospital hygiene	hospital hygiene	hospital hygiene	hospital hygiene	34	kept
Internal Medicine	internal medicine	internal medicine	internal medicine	internal medicine	34	case_or_format
Intracranial Hemorrhages	intracranial hemorrhages	intracranial hemorrhages	intracranial hemorrhage	Intracranial Hemorrhages	34	singular
Labor, Obstetric	labor obstetric	labor obstetric	labor obstetric	Labor, Obstetric	34	kept
lactate blood level	lactate blood level	lactate blood level	lactate blood level	lactate blood level	34	kept
lethargy	lethargy	lethargy	lethargy	lethargy	34	kept
linear regression analysis	linear regression analysis	linear regression analysis	linear regression analysis	linear regression analysis	34	kept
lumbar puncture	lumbar puncture	lumbar puncture	lumbar puncture	lumbar puncture	34	kept
Malawi	malawi	malawi	malawi	Malawi	34	kept
Malpractice	malpractice	malpractice	malpractice	Malpractice	34	kept
malpractice	malpractice	malpractice	malpractice	Malpractice	34	case_or_format
managed care	managed care	managed care	managed care	managed care	34	kept
maternal hypertension	maternal hypertension	maternal hypertension	maternal hypertension	maternal hypertension	34	kept
medical device	medical device	medical device	medical device	medical device	34	kept

milrinone	milrinone	milrinone	milrinone	milrinone	34	kept
mothers	mothers	mothers	mother	Mothers	34	singular
National Health Programs	national health programs	national health programs	national health programs	National Health Programs	34	kept
operative blood loss	operative blood loss	operative blood loss	operative blood loss	operative blood loss	34	kept
oxytocin	oxytocin	oxytocin	oxytocin	oxytocin	34	kept
Phlebitis	phlebitis	phlebitis	phlebitis	Phlebitis	34	kept
phlebitis	phlebitis	phlebitis	phlebitis	Phlebitis	34	case_or_format
phototherapy	phototherapy	phototherapy	phototherapy	phototherapy	34	kept
predictor variable	predictor variable	predictor variable	predictor variable	predictor variable	34	kept
PROGRAM	program	program	program	PROGRAM	34	kept
Public Health	public health	public health	public health	public health	34	case_or_format
purposive sample	purposive sample	purposive sample	purposive sample	purposive sample	34	kept
recognition	recognition	recognition	recognition	recognition	34	kept
respiratory function	respiratory function	respiratory function	respiratory function	respiratory function	34	kept
Respiratory Tract Diseases	respiratory tract diseases	respiratory tract diseases	respiratory tract disease	respiratory tract disease	34	singular
schizophrenia	schizophrenia	schizophrenia	schizophrenia	schizophrenia	34	kept

social aspect	social aspect	social aspect	social aspect	social aspect	34	kept
Sound	sound	sound	sound	Sound	34	kept
Staphylococcus epidermidis	staphylococcus epidermidis	staphylococcus epidermidis	staphylococcus epidermidis	Staphylococcus epidermidis	34	kept
subdural hematoma	subdural hematoma	subdural hematoma	subdural hematoma	subdural hematoma	34	kept
supply and distribution	supply and distribution	supply and distribution	supply and distribution	supply and distribution	34	kept
teleconsultation	teleconsultation	teleconsultation	teleconsultation	teleconsultation	34	kept
thrombosis prevention	thrombosis prevention	thrombosis prevention	thrombosis prevention	thrombosis prevention	34	kept
trend study	trend study	trend study	trend study	trend study	34	kept
Vasodilator Agents	vasodilator agents	vasodilator agents	vasodilator agent	Vasodilator Agents	34	singular
ventilation	ventilation	ventilation	ventilation	Ventilation	34	case_or_format
work engagement	work engagement	work engagement	work engagement	work engagement	34	kept
Accreditation	accreditation	accreditation	accreditation	accreditation	33	case_or_format
alanine aminotransferase	alanine aminotransferase	alanine aminotransferase	alanine aminotransferase	alanine aminotransferase	33	kept
Antibiotics	antibiotics	antibiotics	antibiotic	Antibiotics	33	singular
Anticonvulsants	anticonvulsants	anticonvulsants	anticonvulsants	Anticonvulsants	33	kept
Asian	asian	asian	asian	Asian	33	kept

Braden Scale	braden scale	braden scale	braden scale	Braden Scale	33	kept
Cannula	cannula	cannula	cannula	Cannula	33	kept
caregivers	caregivers	caregivers	caregiver	caregiver	33	singular
clinical decision-making	clinical decision making	clinical decision making	clinical decision making	clinical decision making	33	case_or_format
Drug Therapy, Computer-Assisted	drug therapy computer assisted	drug therapy computer assisted	drug therapy computer assisted	Drug Therapy, Computer-Assisted	33	kept
DSM-5	dsm 5	dsm 5	dsm 5	DSM-5	33	kept
edema	edema	edema	edema	edema	33	kept
Ethnicity	ethnicity	ethnicity	ethnicity	ethnicity	33	case_or_format
Family members	family members	family members	family member	Family members	33	singular
fibrinolytic agent	fibrinolytic agent	fibrinolytic agent	fibrinolytic agent	Fibrinolytic Agents	33	case_or_format
fracture	fracture	fracture	fracture	fracture	33	kept
glyceryl trinitrate	glyceryl trinitrate	glyceryl trinitrate	glyceryl trinitrate	glyceryl trinitrate	33	kept
heart	heart	heart	heart	Heart	33	case_or_format
hospital personnel management	hospital personnel management	hospital personnel management	hospital personnel management	hospital personnel management	33	kept
international normalized ratio	international normalized ratio	international normalized ratio	international normalized ratio	international normalized ratio	33	kept
interventional radiology	interventional radiology	interventional radiology	interventional radiology	interventional radiology	33	kept

Kaplan Meier method	kaplan meier method	kaplan meier method	kaplan meier method	Kaplan Meier method	33	kept
leukocytosis	leukocytosis	leukocytosis	leukocytosis	leukocytosis	33	kept
lifestyle	lifestyle	lifestyle	lifestyle	lifestyle	33	kept
low back pain	low back pain	low back pain	low back pain	low back pain	33	kept
lung function	lung function	lung function	lung function	lung function	33	kept
neurosurgeon	neurosurgeon	neurosurgeon	neurosurgeon	neurosurgeon	33	kept
Out-of-Hospital Cardiac Arrest	out of hospital cardiac arrest	out of hospital cardiac arrest	out of hospital cardiac arrest	out of hospital cardiac arrest	33	case_or_format
paediatric	paediatric	paediatric	paediatric	Paediatric	33	case_or_format
penicillin G	penicillin g	penicillin g	penicillin g	penicillin G	33	kept
personal protective equipment	personal protective equipment	personal protective equipment	personal protective equipment	Personal Protective Equipment	33	case_or_format
professional	professional	professional	professional	professional	33	kept
professionalism	professionalism	professionalism	professionalism	professionalism	33	kept
proportional hazards model	proportional hazards model	proportional hazards model	proportional hazards model	Proportional Hazards Models	33	case_or_format
psychotherapy	psychotherapy	psychotherapy	psychotherapy	psychotherapy	33	kept
quality of health care	quality of health care	quality of health care	quality of health care	Quality of Health Care	33	case_or_format
RANDOMIZED CONTROLLED-TRIAL	randomized controlled trial	randomized controlled trial	randomized controlled trial	randomized controlled trial	33	case_or_format

Resource Allocation	resource allocation	resource allocation	resource allocation	resource allocation	33	case_or_format
Respiratory Care Units	respiratory care units	respiratory care units	respiratory care units	Respiratory Care Units	33	kept
Risk management	risk management	risk management	risk management	Risk Management	33	case_or_format
SCALE	scale	scale	scale	SCALE	33	kept
Seasons	seasons	seasons	season	Seasons	33	singular
Skin Diseases	skin diseases	skin diseases	skin disease	Skin Diseases	33	singular
ST segment elevation myocardial infarction	st segment elevation myocardial infarction	st segment elevation myocardial infarction	st segment elevation myocardial infarction	ST segment elevation myocardial infarction	33	kept
Student t test	student t test	student t test	student t test	Student t test	33	kept
sucrose	sucrose	sucrose	sucrose	Sucrose	33	case_or_format
Supine Position	supine position	supine position	supine position	supine position	33	case_or_format
SYMPTOMS	symptoms	symptoms	symptom	symptom	33	singular
Therapeutic Touch	therapeutic touch	therapeutic touch	therapeutic touch	Therapeutic Touch	33	kept
thirst	thirst	thirst	thirst	Thirst	33	case_or_format
thromboembolism	thromboembolism	thromboembolism	thromboembolism	thromboembolism	33	kept
tracheobronchial toilet	tracheobronchial toilet	tracheobronchial toilet	tracheobronchial toilet	tracheobronchial toilet	33	kept
urea	urea	urea	urea	urea	33	kept

Vancomycin	vancomycin	vancomycin	vancomycin	vancomycin	33	case_or_format
vancomycin resistant Enterococcus	vancomycin resistant enterococcus	vancomycin resistant enterococcus	vancomycin resistant enterococcus	vancomycin resistant Enterococcus	33	kept
Videotape Recording	videotape recording	videotape recording	videotape recording	Videotape Recording	33	kept
abdominal radiography	abdominal radiography	abdominal radiography	abdominal radiography	abdominal radiography	32	kept
Acidosis	acidosis	acidosis	acidosis	Acidosis	32	kept
ADVERSE DRUG EVENTS	adverse drug events	adverse drug events	adverse drug event	ADVERSE DRUG EVENTS	32	singular
alcoholism	alcoholism	alcoholism	alcoholism	alcoholism	32	kept
aminoglycoside	aminoglycoside	aminoglycoside	aminoglycoside	aminoglycoside	32	kept
autopsy	autopsy	autopsy	autopsy	autopsy	32	kept
bacterial pneumonia	bacterial pneumonia	bacterial pneumonia	bacterial pneumonia	bacterial pneumonia	32	kept
Barthel index	barthel index	barthel index	barthel index	Barthel index	32	kept
blunt trauma	blunt trauma	blunt trauma	blunt trauma	blunt trauma	32	kept
bone marrow transplantation	bone marrow transplantation	bone marrow transplantation	bone marrow transplantation	bone marrow transplantation	32	kept
Bradycardia	bradycardia	bradycardia	bradycardia	bradycardia	32	case_or_format
calibration	calibration	calibration	calibration	calibration	32	kept
cardiovascular risk	cardiovascular risk	cardiovascular risk	cardiovascular risk	cardiovascular risk	32	kept

cardiovascular surgery	cardiovascular surgery	cardiovascular surgery	cardiovascular surgery	cardiovascular surgery	32	kept
Conflict	conflict	conflict	conflict	conflict	32	case_or_format
Confusion Assessment Method	confusion assessment method	confusion assessment method	confusion assessment method	Confusion Assessment Method	32	kept
Coronary Artery Disease	coronary artery disease	coronary artery disease	coronary artery disease	coronary artery disease	32	case_or_format
Diagnostic Tests, Routine	diagnostic tests routine	diagnostic tests routine	diagnostic tests routine	Diagnostic Tests, Routine	32	kept
doxycycline	doxycycline	doxycycline	doxycycline	doxycycline	32	kept
drug substitution	drug substitution	drug substitution	drug substitution	drug substitution	32	kept
Electronic health records	electronic health records	electronic health records	electronic health record	Electronic Health Records	32	singular
enoxaparin	enoxaparin	enoxaparin	enoxaparin	enoxaparin	32	kept
Enterococcus faecalis	enterococcus faecalis	enterococcus faecalis	enterococcus faecalis	Enterococcus faecalis	32	kept
enzyme linked immunosorbent assay	enzyme linked immunosorbent assay	enzyme linked immunosorbent assay	enzyme linked immunosorbent assay	enzyme linked immunosorbent assay	32	kept
Exercise Therapy	exercise therapy	exercise therapy	exercise therapy	Exercise Therapy	32	kept
experiences	experiences	experiences	experience	experience	32	singular
Focus groups	focus groups	focus groups	focus group	Focus Groups	32	singular
gastroesophageal reflux	gastroesophageal reflux	gastroesophageal reflux	gastroesophageal reflux	gastroesophageal reflux	32	kept
Health care workers	health care workers	health care workers	health care worker	Health care workers	32	singular

hematologic disease	hematologic disease	hematologic disease	hematologic disease	hematologic disease	32	kept
hematologic malignancy	hematologic malignancy	hematologic malignancy	hematologic malignancy	hematologic malignancy	32	kept
hospital physician	hospital physician	hospital physician	hospital physician	hospital physician	32	kept
Indiana	indiana	indiana	indiana	Indiana	32	kept
Injections, Intravenous	injections intravenous	injections intravenous	injections intravenous	Injections, Intravenous	32	kept
intervention	intervention	intervention	intervention	Intervention	32	case_or_format
ischemic stroke	ischemic stroke	ischemic stroke	ischemic stroke	ischemic stroke	32	kept
lactate dehydrogenase	lactate dehydrogenase	lactate dehydrogenase	lactate dehydrogenase	lactate dehydrogenase	32	kept
lockdown	lockdown	lockdown	lockdown	lockdown	32	kept
Lung Transplantation	lung transplantation	lung transplantation	lung transplantation	lung transplantation	32	case_or_format
married person	married person	married person	married person	married person	32	kept
Medical emergency team	medical emergency team	medical emergency team	medical emergency team	Medical emergency team	32	kept
mental health care	mental health care	mental health care	mental health care	mental health care	32	kept
mentoring	mentoring	mentoring	mentoring	mentoring	32	kept
multidisciplinary	multidisciplinary	multidisciplinary	multidisciplinary	multidisciplinary	32	kept
NEEDS	needs	needs	need	NEEDS	32	singular

neuromuscular disease	neuromuscular disease	neuromuscular disease	neuromuscular disease	neuromuscular disease	32	kept
neuroprotection	neuroprotection	neuroprotection	neuroprotection	neuroprotection	32	kept
neurorehabilitation	neurorehabilitation	neurorehabilitation	neurorehabilitation	neurorehabilitation	32	kept
Nosocomial infections	nosocomial infections	nosocomial infections	nosocomial infection	Nosocomial infection	32	singular
occupational accident	occupational accident	occupational accident	occupational accident	occupational accident	32	kept
Occupational Injuries	occupational injuries	occupational injuries	occupational injury	Occupational Injuries	32	singular
online system	online system	online system	online system	online system	32	kept
Opioid-Related Disorders	opioid related disorders	opioid related disorders	opioid related disorders	Opioid-Related Disorders	32	kept
Oral Health	oral health	oral health	oral health	Oral Health	32	kept
organizational structure	organizational structure	organizational structure	organizational structure	organizational structure	32	kept
patent ductus arteriosus	patent ductus arteriosus	patent ductus arteriosus	patent ductus arteriosus	patent ductus arteriosus	32	kept
peritoneal dialysis	peritoneal dialysis	peritoneal dialysis	peritoneal dialysis	peritoneal dialysis	32	kept
Personal Autonomy	personal autonomy	personal autonomy	personal autonomy	Personal Autonomy	32	kept
Physical Therapists	physical therapists	physical therapists	physical therapist	Physical Therapists	32	singular
preoperative period	preoperative period	preoperative period	preoperative period	preoperative period	32	kept
Professional Staff Committees	professional staff committees	professional staff committees	professional staff committees	Professional Staff Committees	32	kept

protein blood level	protein blood level	protein blood level	protein blood level	protein blood level	32	kept
PTSD	ptsd	ptsd	ptsd	PTSD	32	kept
quetiapine	quetiapine	quetiapine	quetiapine	quetiapine	32	kept
radiologist	radiologist	radiologist	radiologist	radiologist	32	kept
radiology	radiology	radiology	radiology	radiology	32	kept
rash	rash	rash	rash	rash	32	kept
reference value	reference value	reference value	reference value	Reference Values	32	case_or_format
relatives	relatives	relatives	relative	relative	32	singular
Respiratory Mechanics	respiratory mechanics	respiratory mechanics	respiratory mechanics	Respiratory Mechanics	32	kept
Smoking	smoking	smoking	smoking	smoking	32	case_or_format
social isolation	social isolation	social isolation	social isolation	social isolation	32	kept
Staphylococcus	staphylococcus	staphylococcus	staphylococcus	Staphylococcus	32	kept
sucking	sucking	sucking	sucking	sucking	32	kept
surgical training	surgical training	surgical training	surgical training	surgical training	32	kept
thinking	thinking	thinking	thinking	Thinking	32	case_or_format
tocilizumab	tocilizumab	tocilizumab	tocilizumab	tocilizumab	32	kept

treatment refusal	treatment refusal	treatment refusal	treatment refusal	treatment refusal	32	kept
Unconsciousness	unconsciousness	unconsciousness	unconsciousness	unconsciousness	32	case_or_format
Unidades de cuidados intensivos	unidades de cuidados intensivos	unidades de cuidados intensivos	unidades de cuidados intensivos	Unidades de cuidados intensivos	32	kept
United Arab Emirates	united arab emirates	united arab emirates	united arab emirates	United Arab Emirates	32	kept
Validation studies	validation studies	validation studies	validation study	validation study	32	singular
Vascular Access Devices	vascular access devices	vascular access devices	vascular access device	Vascular Access Devices	32	singular
acute kidney injury	acute kidney injury	acute kidney injury	acute kidney injury	Acute Kidney Injury	31	case_or_format
anesthesia level	anesthesia level	anesthesia level	anesthesia level	anesthesia level	31	kept
anosmia	anosmia	anosmia	anosmia	anosmia	31	kept
Aortic Valve Stenosis	aortic valve stenosis	aortic valve stenosis	aortic valve stenosis	Aortic Valve Stenosis	31	kept
blood oxygen tension	blood oxygen tension	blood oxygen tension	blood oxygen tension	blood oxygen tension	31	kept
bottle feeding	bottle feeding	bottle feeding	bottle feeding	Bottle Feeding	31	case_or_format
C-Reactive Protein	c reactive protein	c reactive protein	c reactive protein	C reactive protein	31	case_or_format
Case report	case report	case report	case report	case report	31	case_or_format
Child Abuse	child abuse	child abuse	child abuse	Child Abuse	31	kept
child death	child death	child death	child death	child death	31	kept

childhood mortality	childhood mortality	childhood mortality	childhood mortality	childhood mortality	31	kept
compassion satisfaction	compassion satisfaction	compassion satisfaction	compassion satisfaction	compassion satisfaction	31	kept
Comprehension	comprehension	comprehension	comprehension	comprehension	31	case_or_format
concept analysis	concept analysis	concept analysis	concept analysis	concept analysis	31	kept
dermatitis	dermatitis	dermatitis	dermatitis	Dermatitis	31	case_or_format
diagnostic value	diagnostic value	diagnostic value	diagnostic value	diagnostic value	31	kept
disease predisposition	disease predisposition	disease predisposition	disease predisposition	disease predisposition	31	kept
e-learning	e learning	e learning	e learning	e-learning	31	kept
electronic health records	electronic health records	electronic health records	electronic health record	Electronic Health Records	31	singular
Emergency medical services	emergency medical services	emergency medical services	emergency medical service	Emergency Medical Services	31	singular
Empowerment	empowerment	empowerment	empowerment	empowerment	31	case_or_format
ERRORS	errors	errors	error	error	31	singular
Eye Diseases	eye diseases	eye diseases	eye disease	Eye Diseases	31	singular
functional assessment	functional assessment	functional assessment	functional assessment	functional assessment	31	kept
Gastrointestinal Diseases	gastrointestinal diseases	gastrointestinal diseases	gastrointestinal disease	gastrointestinal disease	31	singular
gastrostomy	gastrostomy	gastrostomy	gastrostomy	gastrostomy	31	kept

General Surgery	general surgery	general surgery	general surgery	general surgery	31	case_or_format
health care personnel management	health care personnel management	health care personnel management	health care personnel management	health care personnel management	31	kept
heart muscle ischemia	heart muscle ischemia	heart muscle ischemia	heart muscle ischemia	heart muscle ischemia	31	kept
hemoglobin A1c	hemoglobin a1c	hemoglobin a1c	hemoglobin a1c	hemoglobin A1c	31	kept
HIV Infections	hiv infections	hiv infections	hiv infections	HIV Infections	31	kept
Homeostasis	homeostasis	homeostasis	homeostasis	Homeostasis	31	kept
Hyperoxia	hyperoxia	hyperoxia	hyperoxia	Hyperoxia	31	kept
hyperthermia	hyperthermia	hyperthermia	hyperthermia	hyperthermia	31	kept
immune response	immune response	immune response	immune response	immune response	31	kept
Infection prevention	infection prevention	infection prevention	infection prevention	infection prevention	31	case_or_format
information system	information system	information system	information system	information system	31	kept
irritability	irritability	irritability	irritability	irritability	31	kept
loading drug dose	loading drug dose	loading drug dose	loading drug dose	loading drug dose	31	kept
lung cancer	lung cancer	lung cancer	lung cancer	lung cancer	31	kept
medical device complication	medical device complication	medical device complication	medical device complication	medical device complication	31	kept
Medical education	medical education	medical education	medical education	medical education	31	case_or_format

medical instrumentation	medical instrumentation	medical instrumentation	medical instrumentation	medical instrumentation	31	kept
Melatonin	melatonin	melatonin	melatonin	melatonin	31	case_or_format
Morale	morale	morale	morale	Morale	31	kept
motor performance	motor performance	motor performance	motor performance	motor performance	31	kept
nasogastric tube	nasogastric tube	nasogastric tube	nasogastric tube	nasogastric tube	31	kept
neutropenia	neutropenia	neutropenia	neutropenia	neutropenia	31	kept
nose feeding	nose feeding	nose feeding	nose feeding	nose feeding	31	kept
nose smear	nose smear	nose smear	nose smear	nose smear	31	kept
Outbreak	outbreak	outbreak	outbreak	Outbreak	31	kept
Pancreatitis	pancreatitis	pancreatitis	pancreatitis	Pancreatitis	31	kept
Parturition	parturition	parturition	parturition	Parturition	31	kept
Perceived Stress Scale	perceived stress scale	perceived stress scale	perceived stress scale	Perceived Stress Scale	31	kept
Performance	performance	performance	performance	PERFORMANCE	31	case_or_format
Personal protective equipment	personal protective equipment	personal protective equipment	personal protective equipment	Personal Protective Equipment	31	case_or_format
postoperative infection	postoperative infection	postoperative infection	postoperative infection	postoperative infection	31	kept
Prematurity	prematurity	prematurity	prematurity	prematurity	31	case_or_format

PRETERM INFANTS	preterm infants	preterm infants	preterm infant	preterm infant	31	singular
Procedural Pain	procedural pain	procedural pain	procedural pain	procedural pain	31	case_or_format
Protective Clothing	protective clothing	protective clothing	protective clothing	Protective Clothing	31	kept
Psychological distress	psychological distress	psychological distress	psychological distress	Psychological Distress	31	case_or_format
psychotropic agent	psychotropic agent	psychotropic agent	psychotropic agent	psychotropic agent	31	kept
Public health	public health	public health	public health	public health	31	case_or_format
randomization	randomization	randomization	randomization	randomization	31	kept
Rapid response team	rapid response team	rapid response team	rapid response team	rapid response team	31	case_or_format
Recombinant Proteins	recombinant proteins	recombinant proteins	recombinant protein	Recombinant Proteins	31	singular
social network	social network	social network	social network	social network	31	kept
Social Work	social work	social work	social work	social work	31	case_or_format
Standard of Care	standard of care	standard of care	standard of care	Standard of Care	31	kept
STRATEGIES	strategies	strategies	strategy	STRATEGIES	31	singular
Surveys	surveys	surveys	survey	Survey	31	singular
Syringes	syringes	syringes	syringe	Syringes	31	singular
teaching round	teaching round	teaching round	teaching round	Teaching Rounds	31	case_or_format

Time Management	time management	time management	time management	Time Management	31	kept
Treatment Failure	treatment failure	treatment failure	treatment failure	treatment failure	31	case_or_format
Vascular Surgical Procedures	vascular surgical procedures	vascular surgical procedures	vascular surgical procedures	Vascular Surgical Procedures	31	kept
vascularization	vascularization	vascularization	vascularization	vascularization	31	kept
voice	voice	voice	voice	voice	31	kept
Water-Electrolyte Imbalance	water electrolyte imbalance	water electrolyte imbalance	water electrolyte imbalance	Water-Electrolyte Imbalance	31	kept
Wound Infection	wound infection	wound infection	wound infection	wound infection	31	case_or_format
abdominal distension	abdominal distension	abdominal distension	abdominal distension	abdominal distension	30	kept
ADVERSE EVENTS	adverse events	adverse events	adverse event	adverse event	30	singular
Alarm systems	alarm systems	alarm systems	alarm system	Alarm systems	30	singular
Anti-Inflammatory Agents	anti inflammatory agents	anti inflammatory agents	anti inflammatory agents	Anti-Inflammatory Agents	30	kept
bioethics	bioethics	bioethics	bioethics	bioethics	30	kept
Capnography	capnography	capnography	capnography	Capnography	30	kept
cardioversion	cardioversion	cardioversion	cardioversion	cardioversion	30	kept
catheter related bloodstream infection	catheter related bloodstream infection	catheter related bloodstream infection	catheter related bloodstream infection	catheter related bloodstream infection	30	kept
chemotherapy	chemotherapy	chemotherapy	chemotherapy	chemotherapy	30	kept

childhood cancer	childhood cancer	childhood cancer	childhood cancer	childhood cancer	30	kept
Communicable Diseases	communicable diseases	communicable diseases	communicable disease	communicable disease	30	singular
Coping	coping	coping	coping	coping	30	case_or_format
Depressive Disorder	depressive disorder	depressive disorder	depressive disorder	Depressive Disorder	30	kept
device failure	device failure	device failure	device failure	device failure	30	kept
Diabetes Complications	diabetes complications	diabetes complications	diabetes complications	Diabetes Complications	30	kept
Diagnostic Errors	diagnostic errors	diagnostic errors	diagnostic error	Diagnostic Errors	30	singular
Doctors	doctors	doctors	doctor	Doctors	30	singular
drug infusion	drug infusion	drug infusion	drug infusion	drug infusion	30	kept
Drug Interactions	drug interactions	drug interactions	drug interaction	Drug Interactions	30	singular
dyslipidemia	dyslipidemia	dyslipidemia	dyslipidemia	dyslipidemia	30	kept
Effectiveness	effectiveness	effectiveness	effectiveness	Effectiveness	30	kept
electrolyte balance	electrolyte balance	electrolyte balance	electrolyte balance	electrolyte balance	30	kept
Emotional intelligence	emotional intelligence	emotional intelligence	emotional intelligence	emotional intelligence	30	case_or_format
EPIDEMIOLOGY	epidemiology	epidemiology	epidemiology	epidemiology	30	case_or_format
Facial Expression	facial expression	facial expression	facial expression	facial expression	30	case_or_format

guidelines	guidelines	guidelines	guideline	Guidelines	30	singular
Guillain-Barre Syndrome	guillain barre syndrome	guillain barre syndrome	guillain barre syndrome	Guillain-Barre Syndrome	30	kept
Health care professionals	health care professionals	health care professionals	health care professional	Health care professionals	30	singular
heart function	heart function	heart function	heart function	heart function	30	kept
heart left ventricle ejection fraction	heart left ventricle ejection fraction	heart left ventricle ejection fraction	heart left ventricle ejection fraction	heart left ventricle ejection fraction	30	kept
home	home	home	home	home	30	kept
hydration	hydration	hydration	hydration	hydration	30	kept
hysterectomy	hysterectomy	hysterectomy	hysterectomy	hysterectomy	30	kept
influenza vaccination	influenza vaccination	influenza vaccination	influenza vaccination	influenza vaccination	30	kept
insulin dependent diabetes mellitus	insulin dependent diabetes mellitus	insulin dependent diabetes mellitus	insulin dependent diabetes mellitus	insulin dependent diabetes mellitus	30	kept
Internship, Nonmedical	internship nonmedical	internship nonmedical	internship nonmedical	Internship, Nonmedical	30	kept
labor	labor	labor	labor	labor	30	kept
Laryngoscopy	laryngoscopy	laryngoscopy	laryngoscopy	laryngoscopy	30	case_or_format
lung	lung	lung	lung	Lung	30	case_or_format
lung function test	lung function test	lung function test	lung function test	lung function test	30	kept
magnesium sulfate	magnesium sulfate	magnesium sulfate	magnesium sulfate	magnesium sulfate	30	kept

meconium aspiration	meconium aspiration	meconium aspiration	meconium aspiration	meconium aspiration	30	kept
Medical Informatics	medical informatics	medical informatics	medical informatics	medical informatics	30	case_or_format
nebulization	nebulization	nebulization	nebulization	nebulization	30	kept
Needlestick Injuries	needlestick injuries	needlestick injuries	needlestick injury	needlestick injury	30	singular
Noise, Occupational	noise occupational	noise occupational	noise occupational	Noise, Occupational	30	kept
Occupational Therapy	occupational therapy	occupational therapy	occupational therapy	occupational therapy	30	case_or_format
Oman	oman	oman	oman	Oman	30	kept
orthopedic surgery	orthopedic surgery	orthopedic surgery	orthopedic surgery	orthopedic surgery	30	kept
osteoporosis	osteoporosis	osteoporosis	osteoporosis	osteoporosis	30	kept
pancreatitis	pancreatitis	pancreatitis	pancreatitis	Pancreatitis	30	case_or_format
parental attitude	parental attitude	parental attitude	parental attitude	parental attitude	30	kept
peer group	peer group	peer group	peer group	Peer Group	30	case_or_format
perinatal asphyxia	perinatal asphyxia	perinatal asphyxia	perinatal asphyxia	perinatal asphyxia	30	kept
psychological adjustment	psychological adjustment	psychological adjustment	psychological adjustment	psychological adjustment	30	kept
Pulmonary Artery	pulmonary artery	pulmonary artery	pulmonary artery	Pulmonary Artery	30	kept
RELIABILITY	reliability	reliability	reliability	reliability	30	case_or_format

Respiratory Syncytial Virus Infections	respiratory syncytial virus infections	respiratory syncytial virus infections	respiratory syncytial virus infection	Respiratory Syncytial Virus Infections	30	singular
Scopus	scopus	scopus	scopus	Scopus	30	kept
serology	serology	serology	serology	serology	30	kept
sex	sex	sex	sex	sex	30	kept
Sleep Initiation and Maintenance Disorders	sleep initiation and maintenance disorders	sleep initiation and maintenance disorders	sleep initiation and maintenance disorders	Sleep Initiation and Maintenance Disorders	30	kept
Social Perception	social perception	social perception	social perception	Social Perception	30	kept
Social support	social support	social support	social support	Social Support	30	case_or_format
Specimen Handling	specimen handling	specimen handling	specimen handling	Specimen Handling	30	kept
sternotomy	sternotomy	sternotomy	sternotomy	sternotomy	30	kept
structured interview	structured interview	structured interview	structured interview	structured interview	30	kept
suicide	suicide	suicide	suicide	suicide	30	kept
survival time	survival time	survival time	survival time	survival time	30	kept
syndrome	syndrome	syndrome	syndrome	Syndrome	30	case_or_format
Telephone	telephone	telephone	telephone	Telephone	30	kept
test retest reliability	test retest reliability	test retest reliability	test retest reliability	test retest reliability	30	kept
Unnecessary Procedures	unnecessary procedures	unnecessary procedures	unnecessary procedure	Unnecessary Procedures	30	singular

urinary catheter	urinary catheter	urinary catheter	urinary catheter	urinary catheter	30	kept
VALIDITY	validity	validity	validity	validity	30	case_or_format
Wakefulness	wakefulness	wakefulness	wakefulness	wakefulness	30	case_or_format
Work environment	work environment	work environment	work environment	work environment	30	case_or_format
Abnormalities, Multiple	abnormalities multiple	abnormalities multiple	abnormalities multiple	Abnormalities, Multiple	29	kept
Acetaminophen	acetaminophen	acetaminophen	acetaminophen	Acetaminophen	29	kept
aciclovir	aciclovir	aciclovir	aciclovir	aciclovir	29	kept
Advanced Cardiac Life Support	advanced cardiac life support	advanced cardiac life support	advanced cardiac life support	Advanced Cardiac Life Support	29	kept
alertness	alertness	alertness	alertness	alertness	29	kept
antithrombocytic agent	antithrombocytic agent	antithrombocytic agent	antithrombocytic agent	antithrombocytic agent	29	kept
assisted circulation	assisted circulation	assisted circulation	assisted circulation	assisted circulation	29	kept
audiovisual equipment	audiovisual equipment	audiovisual equipment	audiovisual equipment	audiovisual equipment	29	kept
bath	bath	bath	bath	Baths	29	case_or_format
beta lactam antibiotic	beta lactam antibiotic	beta lactam antibiotic	beta lactam antibiotic	beta lactam antibiotic	29	kept
Breast Milk Expression	breast milk expression	breast milk expression	breast milk expression	Breast Milk Expression	29	kept
Bronchoscopy	bronchoscopy	bronchoscopy	bronchoscopy	bronchoscopy	29	case_or_format

cardiomyopathy	cardiomyopathy	cardiomyopathy	cardiomyopathy	cardiomyopathy	29	kept
Chest Tubes	chest tubes	chest tubes	chest tube	Chest Tubes	29	singular
chill	chill	chill	chill	chill	29	kept
cluster analysis	cluster analysis	cluster analysis	cluster analysis	Cluster Analysis	29	case_or_format
coagulase negative Staphylococcus	coagulase negative staphylococcus	coagulase negative staphylococcus	coagulase negative staphylococcus	coagulase negative Staphylococcus	29	kept
Communicable Disease Control	communicable disease control	communicable disease control	communicable disease control	Communicable Disease Control	29	kept
Cost	cost	cost	cost	cost	29	case_or_format
Culturally Competent Care	culturally competent care	culturally competent care	culturally competent care	Culturally Competent Care	29	kept
cyanosis	cyanosis	cyanosis	cyanosis	cyanosis	29	kept
Developing countries	developing countries	developing countries	developing country	Developing Countries	29	singular
device safety	device safety	device safety	device safety	device safety	29	kept
diagnosis related group	diagnosis related group	diagnosis related group	diagnosis related group	Diagnosis-Related Groups	29	case_or_format
diagnostic error	diagnostic error	diagnostic error	diagnostic error	Diagnostic Errors	29	case_or_format
disorientation	disorientation	disorientation	disorientation	disorientation	29	kept
drug hypersensitivity	drug hypersensitivity	drug hypersensitivity	drug hypersensitivity	drug hypersensitivity	29	kept
Electronic health record	electronic health record	electronic health record	electronic health record	Electronic Health Records	29	case_or_format

Emergency Medical Technicians	emergency medical technicians	emergency medical technicians	emergency medical technician	Emergency Medical Technicians	29	singular
ertapenem	ertapenem	ertapenem	ertapenem	ertapenem	29	kept
Erythrocyte Transfusion	erythrocyte transfusion	erythrocyte transfusion	erythrocyte transfusion	erythrocyte transfusion	29	case_or_format
Euthanasia, Passive	euthanasia passive	euthanasia passive	euthanasia passive	Euthanasia, Passive	29	kept
Experience	experience	experience	experience	experience	29	case_or_format
face validity	face validity	face validity	face validity	face validity	29	kept
feeding behavior	feeding behavior	feeding behavior	feeding behavior	Feeding Behavior	29	case_or_format
Gastroschisis	gastroschisis	gastroschisis	gastroschisis	Gastroschisis	29	kept
Head and Neck Neoplasms	head and neck neoplasms	head and neck neoplasms	head and neck neoplasms	Head and Neck Neoplasms	29	kept
health literacy	health literacy	health literacy	health literacy	health literacy	29	kept
home for the aged	home for the aged	home for the aged	home for the aged	home for the aged	29	kept
immunosuppressive agent	immunosuppressive agent	immunosuppressive agent	immunosuppressive agent	immunosuppressive agent	29	kept
Jaundice, Neonatal	jaundice neonatal	jaundice neonatal	jaundice neonatal	Jaundice, Neonatal	29	kept
legislation and jurisprudence	legislation and jurisprudence	legislation and jurisprudence	legislation and jurisprudence	legislation and jurisprudence	29	kept
life event	life event	life event	life event	life event	29	kept
literature review	literature review	literature review	literature review	literature review	29	kept

liver failure	liver failure	liver failure	liver failure	liver failure	29	kept
MECHANICAL VENTILATION	mechanical ventilation	mechanical ventilation	mechanical ventilation	Mechanical ventilation	29	case_or_format
Medical Oncology	medical oncology	medical oncology	medical oncology	Medical Oncology	29	kept
Mother	mother	mother	mother	Mothers	29	case_or_format
multiple choice test	multiple choice test	multiple choice test	multiple choice test	multiple choice test	29	kept
multiple regression	multiple regression	multiple regression	multiple regression	multiple regression	29	kept
natural language processing	natural language processing	natural language processing	natural language processing	natural language processing	29	kept
nonverbal communication	nonverbal communication	nonverbal communication	nonverbal communication	Nonverbal Communication	29	case_or_format
note	note	note	note	Note	29	case_or_format
Nutrition Therapy	nutrition therapy	nutrition therapy	nutrition therapy	Nutrition Therapy	29	kept
operating room personnel	operating room personnel	operating room personnel	operating room personnel	operating room personnel	29	kept
oral hygiene	oral hygiene	oral hygiene	oral hygiene	Oral Hygiene	29	case_or_format
oxycodone	oxycodone	oxycodone	oxycodone	oxycodone	29	kept
pediatric emergency medicine	pediatric emergency medicine	pediatric emergency medicine	pediatric emergency medicine	pediatric emergency medicine	29	kept
percutaneous endoscopic gastrostomy	percutaneous endoscopic gastrostomy	percutaneous endoscopic gastrostomy	percutaneous endoscopic gastrostomy	percutaneous endoscopic gastrostomy	29	kept
Personnel Administration, Hospital	personnel administration hospital	personnel administration hospital	personnel administration hospital	Personnel Administration, Hospital	29	kept

point of care system	point of care system	point of care system	point of care system	Point-of-Care Systems	29	case_or_format
postnatal care	postnatal care	postnatal care	postnatal care	Postnatal Care	29	case_or_format
postoperative	postoperative	postoperative	postoperative	postoperative	29	kept
Postoperative care	postoperative care	postoperative care	postoperative care	Postoperative Care	29	case_or_format
Professional-family relations	professional family relations	professional family relations	professional family relations	Professional-Family Relations	29	case_or_format
prothrombin time	prothrombin time	prothrombin time	prothrombin time	prothrombin time	29	kept
psychologic assessment	psychologic assessment	psychologic assessment	psychologic assessment	psychologic assessment	29	kept
Psychological Theory	psychological theory	psychological theory	psychological theory	Psychological Theory	29	kept
PsycINFO	psycinfo	psycinfo	psycinfo	PsycINFO	29	kept
quality improvement study	quality improvement study	quality improvement study	quality improvement study	quality improvement study	29	kept
Respiratory Function Tests	respiratory function tests	respiratory function tests	respiratory function tests	Respiratory Function Tests	29	kept
rib fracture	rib fracture	rib fracture	rib fracture	rib fracture	29	kept
Risk Reduction Behavior	risk reduction behavior	risk reduction behavior	risk reduction behavior	Risk Reduction Behavior	29	kept
salbutamol	salbutamol	salbutamol	salbutamol	salbutamol	29	kept
sodium blood level	sodium blood level	sodium blood level	sodium blood level	sodium blood level	29	kept
soft tissue infection	soft tissue infection	soft tissue infection	soft tissue infection	soft tissue infection	29	kept

student	student	student	student	Students	29	case_or_format
subacute care	subacute care	subacute care	subacute care	Subacute Care	29	case_or_format
Survival	survival	survival	survival	survival	29	case_or_format
symptomatology	symptomatology	symptomatology	symptomatology	symptomatology	29	kept
TEAMWORK	teamwork	teamwork	teamwork	teamwork	29	case_or_format
Tetanus	tetanus	tetanus	tetanus	Tetanus	29	kept
tetracycline	tetracycline	tetracycline	tetracycline	tetracycline	29	kept
time management	time management	time management	time management	Time Management	29	case_or_format
travel	travel	travel	travel	travel	29	kept
treatment	treatment	treatment	treatment	Treatment	29	case_or_format
unindexed drug	unindexed drug	unindexed drug	unindexed drug	unindexed drug	29	kept
Urinary Catheters	urinary catheters	urinary catheters	urinary catheter	urinary catheter	29	singular
vecuronium	vecuronium	vecuronium	vecuronium	vecuronium	29	kept
workshop	workshop	workshop	workshop	workshop	29	kept
Adrenal Cortex Hormones	adrenal cortex hormones	adrenal cortex hormones	adrenal cortex hormones	Adrenal Cortex Hormones	28	kept
Airway Obstruction	airway obstruction	airway obstruction	airway obstruction	airway obstruction	28	case_or_format

Alzheimer Disease	alzheimer disease	alzheimer disease	alzheimer disease	Alzheimer disease	28	case_or_format
Ambulatory Surgical Procedures	ambulatory surgical procedures	ambulatory surgical procedures	ambulatory surgical procedures	Ambulatory Surgical Procedures	28	kept
Anesthetics	anesthetics	anesthetics	anesthetics	Anesthetics	28	kept
antiarrhythmic agent	antiarrhythmic agent	antiarrhythmic agent	antiarrhythmic agent	antiarrhythmic agent	28	kept
Antiviral Agents	antiviral agents	antiviral agents	antiviral agents	Antiviral Agents	28	kept
aztreonam	aztreonam	aztreonam	aztreonam	aztreonam	28	kept
Bibliometrics	bibliometrics	bibliometrics	bibliometric	Bibliometrics	28	singular
blood clotting	blood clotting	blood clotting	blood clotting	blood clotting	28	kept
Blood Coagulation Disorders	blood coagulation disorders	blood coagulation disorders	blood coagulation disorders	Blood Coagulation Disorders	28	kept
Cardiotonic Agents	cardiotonic agents	cardiotonic agents	cardiotonic agent	Cardiotonic Agents	28	singular
cardiovascular agent	cardiovascular agent	cardiovascular agent	cardiovascular agent	cardiovascular agent	28	kept
caregiver support	caregiver support	caregiver support	caregiver support	caregiver support	28	kept
catheter	catheter	catheter	catheter	Catheters	28	case_or_format
catheter occlusion	catheter occlusion	catheter occlusion	catheter occlusion	catheter occlusion	28	kept
chemistry	chemistry	chemistry	chemistry	chemistry	28	kept
CLABSI	clabsi	clabsi	clabsi	CLABSI	28	kept

Clinical trial	clinical trial	clinical trial	clinical trial	clinical trial	28	case_or_format
Confidentiality	confidentiality	confidentiality	confidentiality	confidentiality	28	case_or_format
COVID-19 Vaccines	covid 19 vaccines	covid 19 vaccines	covid 19 vaccine	COVID-19 Vaccines	28	singular
crowding	crowding	crowding	crowding	crowding	28	kept
Cytokines	cytokines	cytokines	cytokine	Cytokines	28	singular
dietary intake	dietary intake	dietary intake	dietary intake	dietary intake	28	kept
Disease Transmission, Infectious	disease transmission infectious	disease transmission infectious	disease transmission infectious	Disease Transmission, Infectious	28	kept
Diuretics	diuretics	diuretics	diuretics	Diuretics	28	kept
drug dose increase	drug dose increase	drug dose increase	drug dose increase	drug dose increase	28	kept
drug effects	drug effects	drug effects	drug effect	drug effect	28	singular
Electric Countershock	electric countershock	electric countershock	electric countershock	Electric Countershock	28	kept
Emergency care	emergency care	emergency care	emergency care	emergency care	28	case_or_format
Epilepsy	epilepsy	epilepsy	epilepsy	epilepsy	28	case_or_format
Epinephrine	epinephrine	epinephrine	epinephrine	epinephrine	28	case_or_format
Ethanol	ethanol	ethanol	ethanol	Ethanol	28	kept
euthanasia	euthanasia	euthanasia	euthanasia	Euthanasia	28	case_or_format

family health	family health	family health	family health	Family Health	28	case_or_format
family support	family support	family support	family support	family support	28	kept
frustration	frustration	frustration	frustration	frustration	28	kept
hand	hand	hand	hand	Hand	28	case_or_format
health policy	health policy	health policy	health policy	Health Policy	28	case_or_format
helicopter	helicopter	helicopter	helicopter	helicopter	28	kept
hemorrhagic shock	hemorrhagic shock	hemorrhagic shock	hemorrhagic shock	hemorrhagic shock	28	kept
hepatitis B	hepatitis b	hepatitis b	hepatitis b	hepatitis B	28	kept
histopathology	histopathology	histopathology	histopathology	histopathology	28	kept
Hope	hope	hope	hope	Hope	28	kept
Hospices	hospices	hospices	hospice	hospice	28	singular
Hospitals, District	hospitals district	hospitals district	hospitals district	Hospitals, District	28	kept
Humidity	humidity	humidity	humidity	Humidity	28	kept
hydroxymethylglutaryl coenzyme A reductase inhibitor	hydroxymethylglutaryl coenzyme a reductase inhibitor	hydroxymethylglutaryl coenzyme a reductase inhibitor	hydroxymethylglutaryl coenzyme a reductase inhibitor	hydroxymethylglutaryl coenzyme A reductase inhibitor	28	kept
Hypoplastic Left Heart Syndrome	hypoplastic left heart syndrome	hypoplastic left heart syndrome	hypoplastic left heart syndrome	Hypoplastic Left Heart Syndrome	28	kept
inferential statistics	inferential statistics	inferential statistics	inferential statistics	inferential statistics	28	kept

instrument development	instrument development	instrument development	instrument development	instrument development	28	kept
Internal-External Control	internal external control	internal external control	internal external control	Internal-External Control	28	kept
Ischemia	ischemia	ischemia	ischemia	Ischemia	28	kept
isolation	isolation	isolation	isolation	isolation	28	kept
Ketamine	ketamine	ketamine	ketamine	ketamine	28	case_or_format
knowledge gap	knowledge gap	knowledge gap	knowledge gap	knowledge gap	28	kept
Laparoscopy	laparoscopy	laparoscopy	laparoscopy	Laparoscopy	28	kept
Latin America	latin america	latin america	latin america	Latin America	28	kept
lifestyle modification	lifestyle modification	lifestyle modification	lifestyle modification	lifestyle modification	28	kept
Low Back Pain	low back pain	low back pain	low back pain	low back pain	28	case_or_format
maternal morbidity	maternal morbidity	maternal morbidity	maternal morbidity	maternal morbidity	28	kept
measurement accuracy	measurement accuracy	measurement accuracy	measurement accuracy	measurement accuracy	28	kept
medical device contamination	medical device contamination	medical device contamination	medical device contamination	medical device contamination	28	kept
Medication error	medication error	medication error	medication error	Medication Errors	28	case_or_format
Natural Language Processing	natural language processing	natural language processing	natural language processing	natural language processing	28	case_or_format
near infrared spectroscopy	near infrared spectroscopy	near infrared spectroscopy	near infrared spectroscopy	near infrared spectroscopy	28	kept

Needs assessment	needs assessment	needs assessment	needs assessment	Needs Assessment	28	case_or_format
nitric oxide	nitric oxide	nitric oxide	nitric oxide	nitric oxide	28	kept
Pain measurement	pain measurement	pain measurement	pain measurement	Pain Measurement	28	case_or_format
Parkinson disease	parkinson disease	parkinson disease	parkinson disease	Parkinson disease	28	kept
peripherally inserted central venous catheter	peripherally inserted central venous catheter	peripherally inserted central venous catheter	peripherally inserted central venous catheter	peripherally inserted central venous catheter	28	kept
potassium blood level	potassium blood level	potassium blood level	potassium blood level	potassium blood level	28	kept
Practices	practices	practices	practice	Practice	28	singular
psychiatric department	psychiatric department	psychiatric department	psychiatric department	psychiatric department	28	kept
Reminder Systems	reminder systems	reminder systems	reminder system	Reminder Systems	28	singular
root cause analysis	root cause analysis	root cause analysis	root cause analysis	root cause analysis	28	kept
Sarcopenia	sarcopenia	sarcopenia	sarcopenia	Sarcopenia	28	kept
scope of practice	scope of practice	scope of practice	scope of practice	scope of practice	28	kept
Sleep Apnea, Obstructive	sleep apnea obstructive	sleep apnea obstructive	sleep apnea obstructive	Sleep Apnea, Obstructive	28	kept
social environment	social environment	social environment	social environment	social environment	28	kept
Sodium Chloride	sodium chloride	sodium chloride	sodium chloride	sodium chloride	28	case_or_format
sound	sound	sound	sound	Sound	28	case_or_format

Spiritual care	spiritual care	spiritual care	spiritual care	spiritual care	28	case_or_format
Spouses	spouses	spouses	spouse	Spouses	28	singular
substance abuse	substance abuse	substance abuse	substance abuse	substance abuse	28	kept
telecommunication	telecommunication	telecommunication	telecommunication	telecommunication	28	kept
tissue plasminogen activator	tissue plasminogen activator	tissue plasminogen activator	tissue plasminogen activator	tissue plasminogen activator	28	kept
Treatment Refusal	treatment refusal	treatment refusal	treatment refusal	treatment refusal	28	case_or_format
Virtual Reality	virtual reality	virtual reality	virtual reality	virtual reality	28	case_or_format
Virtual reality	virtual reality	virtual reality	virtual reality	virtual reality	28	case_or_format
waste management	waste management	waste management	waste management	waste management	28	kept
air conditioning	air conditioning	air conditioning	air conditioning	air conditioning	27	kept
Airway management	airway management	airway management	airway management	Airway Management	27	case_or_format
alert fatigue	alert fatigue	alert fatigue	alert fatigue	alert fatigue	27	kept
Attention	attention	attention	attention	attention	27	case_or_format
body height	body height	body height	body height	body height	27	kept
body weight gain	body weight gain	body weight gain	body weight gain	body weight gain	27	kept
brain infarction	brain infarction	brain infarction	brain infarction	brain infarction	27	kept

cancer radiotherapy	cancer radiotherapy	cancer radiotherapy	cancer radiotherapy	cancer radiotherapy	27	kept
Cardiology Service, Hospital	cardiology service hospital	cardiology service hospital	cardiology service hospital	Cardiology Service, Hospital	27	kept
Clostridium Infections	clostridium infections	clostridium infections	clostridium infection	Clostridium Infections	27	singular
cognitive behavioral therapy	cognitive behavioral therapy	cognitive behavioral therapy	cognitive behavioral therapy	cognitive behavioral therapy	27	kept
Computers	computers	computers	computer	Computers	27	singular
Consultants	consultants	consultants	consultants	Consultants	27	kept
contamination	contamination	contamination	contamination	contamination	27	kept
Cost Control	cost control	cost control	cost control	cost control	27	case_or_format
COVID‑19	covid 19	covid 19	covid 19	COVID-19	27	case_or_format
critical thinking	critical thinking	critical thinking	critical thinking	critical thinking	27	kept
crying	crying	crying	crying	crying	27	kept
crystalloid	crystalloid	crystalloid	crystalloid	crystalloid	27	kept
Delphi technique	delphi technique	delphi technique	delphi technique	Delphi Technique	27	case_or_format
disk diffusion	disk diffusion	disk diffusion	disk diffusion	disk diffusion	27	kept
drug dependence	drug dependence	drug dependence	drug dependence	drug dependence	27	kept
Electronics	electronics	electronics	electronic	Electronics	27	singular

emotional exhaustion	emotional exhaustion	emotional exhaustion	emotional exhaustion	emotional exhaustion	27	kept
enhanced recovery after surgery	enhanced recovery after surgery	enhanced recovery after surgery	enhanced recovery after surgery	enhanced recovery after surgery	27	kept
exhaustion	exhaustion	exhaustion	exhaustion	exhaustion	27	kept
Feeding Methods	feeding methods	feeding methods	feeding method	Feeding Methods	27	singular
Fractures, Bone	fractures bone	fractures bone	fractures bone	Fractures, Bone	27	kept
Gastrostomy	gastrostomy	gastrostomy	gastrostomy	gastrostomy	27	case_or_format
glucocorticoid	glucocorticoid	glucocorticoid	glucocorticoid	glucocorticoid	27	kept
health equity	health equity	health equity	health equity	health equity	27	kept
Healthcare-associated infections	healthcare associated infections	healthcare associated infections	healthcare associated infection	healthcare associated infection	27	singular
heart rhythm	heart rhythm	heart rhythm	heart rhythm	heart rhythm	27	kept
Hematologic Neoplasms	hematologic neoplasms	hematologic neoplasms	hematologic neoplasms	Hematologic Neoplasms	27	kept
hemofiltration	hemofiltration	hemofiltration	hemofiltration	Hemofiltration	27	case_or_format
hospital acquired pneumonia	hospital acquired pneumonia	hospital acquired pneumonia	hospital acquired pneumonia	hospital acquired pneumonia	27	kept
Influenza A Virus, H1N1 Subtype	influenza a virus h1n1 subtype	influenza a virus h1n1 subtype	influenza a virus h1n1 subtype	Influenza A Virus, H1N1 Subtype	27	kept
Interinstitutional Relations	interinstitutional relations	interinstitutional relations	interinstitutional relations	Interinstitutional Relations	27	kept
Intra-Abdominal Hypertension	intra abdominal hypertension	intra abdominal hypertension	intra abdominal hypertension	Intra-Abdominal Hypertension	27	kept

Islam	islam	islam	islam	Islam	27	kept
Joint Commission on Accreditation of Healthcare	joint commission on accreditation of healthcare	joint commission on accreditation of healthcare	joint commission on accreditation of healthcare	Joint Commission on Accreditation of Healthcare	27	kept
Kangaroo mother care	kangaroo mother care	kangaroo mother care	kangaroo mother care	Kangaroo mother care	27	kept
kidney transplantation	kidney transplantation	kidney transplantation	kidney transplantation	kidney transplantation	27	kept
leisure	leisure	leisure	leisure	leisure	27	kept
Light	light	light	light	Light	27	kept
local anesthetic agent	local anesthetic agent	local anesthetic agent	local anesthetic agent	local anesthetic agent	27	kept
lower respiratory tract infection	lower respiratory tract infection	lower respiratory tract infection	lower respiratory tract infection	lower respiratory tract infection	27	kept
lymphocyte count	lymphocyte count	lymphocyte count	lymphocyte count	lymphocyte count	27	kept
maternal behavior	maternal behavior	maternal behavior	maternal behavior	Maternal Behavior	27	case_or_format
medication safety	medication safety	medication safety	medication safety	medication safety	27	kept
Mentoring	mentoring	mentoring	mentoring	mentoring	27	case_or_format
metoclopramide	metoclopramide	metoclopramide	metoclopramide	metoclopramide	27	kept
Motor Activity	motor activity	motor activity	motor activity	Motor Activity	27	kept
Neuropsychological Tests	neuropsychological tests	neuropsychological tests	neuropsychological test	Neuropsychological Tests	27	singular
newborn infection	newborn infection	newborn infection	newborn infection	newborn infection	27	kept

nomogram	nomogram	nomogram	nomogram	nomogram	27	kept
observer variation	observer variation	observer variation	observer variation	Observer Variation	27	case_or_format
oral drug administration	oral drug administration	oral drug administration	oral drug administration	oral drug administration	27	kept
oxygen supply	oxygen supply	oxygen supply	oxygen supply	oxygen supply	27	kept
Palestine	palestine	palestine	palestine	Palestine	27	kept
Parent	parent	parent	parent	Parents	27	case_or_format
parental behavior	parental behavior	parental behavior	parental behavior	parental behavior	27	kept
Parenteral nutrition	parenteral nutrition	parenteral nutrition	parenteral nutrition	parenteral nutrition	27	case_or_format
perinatal period	perinatal period	perinatal period	perinatal period	perinatal period	27	kept
peripheral vascular disease	peripheral vascular disease	peripheral vascular disease	peripheral vascular disease	peripheral vascular disease	27	kept
personal hygiene	personal hygiene	personal hygiene	personal hygiene	personal hygiene	27	kept
Personnel Management	personnel management	personnel management	personnel management	personnel management	27	case_or_format
Phototherapy	phototherapy	phototherapy	phototherapy	phototherapy	27	case_or_format
Physician	physician	physician	physician	physician	27	case_or_format
Pittsburgh Sleep Quality Index	pittsburgh sleep quality index	pittsburgh sleep quality index	pittsburgh sleep quality index	Pittsburgh Sleep Quality Index	27	kept
population	population	population	population	population	27	kept

postgraduate student	postgraduate student	postgraduate student	postgraduate student	postgraduate student	27	kept
povidone iodine	povidone iodine	povidone iodine	povidone iodine	povidone iodine	27	kept
premature infants	premature infants	premature infants	premature infant	Premature infant	27	singular
Process Assessment, Health Care	process assessment health care	process assessment health care	process assessment health care	Process Assessment, Health Care	27	kept
Pseudomonas	pseudomonas	pseudomonas	pseudomonas	Pseudomonas	27	kept
psychological rating scale	psychological rating scale	psychological rating scale	psychological rating scale	psychological rating scale	27	kept
pulmonary artery	pulmonary artery	pulmonary artery	pulmonary artery	Pulmonary Artery	27	case_or_format
Punctures	punctures	punctures	puncture	Punctures	27	singular
resource limited setting	resource limited setting	resource limited setting	resource limited setting	resource limited setting	27	kept
respiration depression	respiration depression	respiration depression	respiration depression	respiration depression	27	kept
respiratory syncytial virus infection	respiratory syncytial virus infection	respiratory syncytial virus infection	respiratory syncytial virus infection	Respiratory Syncytial Virus Infections	27	case_or_format
Respiratory therapy	respiratory therapy	respiratory therapy	respiratory therapy	Respiratory Therapy	27	case_or_format
return to work	return to work	return to work	return to work	return to work	27	kept
Risk factor	risk factor	risk factor	risk factor	Risk Factors	27	case_or_format
risperidone	risperidone	risperidone	risperidone	risperidone	27	kept
Sleep Disorders, Circadian Rhythm	sleep disorders circadian rhythm	sleep disorders circadian rhythm	sleep disorders circadian rhythm	Sleep Disorders, Circadian Rhythm	27	kept

suxamethonium	suxamethonium	suxamethonium	suxamethonium	suxamethonium	27	kept
Tissue Plasminogen Activator	tissue plasminogen activator	tissue plasminogen activator	tissue plasminogen activator	tissue plasminogen activator	27	case_or_format
Transfer	transfer	transfer	transfer	Transfer	27	kept
Transition	transition	transition	transition	Transition	27	kept
triage	triage	triage	triage	Triage	27	case_or_format
turnover rate	turnover rate	turnover rate	turnover rate	turnover rate	27	kept
Utilization Review	utilization review	utilization review	utilization review	utilization review	27	case_or_format
well-being	well being	well being	well being	well-being	27	kept
acidosis	acidosis	acidosis	acidosis	Acidosis	26	case_or_format
acute heart failure	acute heart failure	acute heart failure	acute heart failure	acute heart failure	26	kept
Ambulatory Care Facilities	ambulatory care facilities	ambulatory care facilities	ambulatory care facilities	Ambulatory Care Facilities	26	kept
Anesthetics, Local	anesthetics local	anesthetics local	anesthetics local	Anesthetics, Local	26	kept
Antihypertensive Agents	antihypertensive agents	antihypertensive agents	antihypertensive agent	antihypertensive agent	26	singular
aromatherapy	aromatherapy	aromatherapy	aromatherapy	Aromatherapy	26	case_or_format
backache	backache	backache	backache	backache	26	kept
Baltimore	baltimore	baltimore	baltimore	Baltimore	26	kept

bandage	bandage	bandage	bandage	Bandages	26	case_or_format
behavior disorder	behavior disorder	behavior disorder	behavior disorder	behavior disorder	26	kept
Bioethics	bioethics	bioethics	bioethics	bioethics	26	case_or_format
birth	birth	birth	birth	birth	26	kept
Birthing Centers	birthing centers	birthing centers	birthing center	Birthing Centers	26	singular
bispectral index	bispectral index	bispectral index	bispectral index	bispectral index	26	kept
blood analysis	blood analysis	blood analysis	blood analysis	blood analysis	26	kept
blood clot lysis	blood clot lysis	blood clot lysis	blood clot lysis	blood clot lysis	26	kept
brain natriuretic peptide	brain natriuretic peptide	brain natriuretic peptide	brain natriuretic peptide	brain natriuretic peptide	26	kept
cancer center	cancer center	cancer center	cancer center	cancer center	26	kept
cancer staging	cancer staging	cancer staging	cancer staging	cancer staging	26	kept
Candidiasis	candidiasis	candidiasis	candidiasis	Candidiasis	26	kept
Child Advocacy	child advocacy	child advocacy	child advocacy	Child Advocacy	26	kept
Clinical decision-making	clinical decision making	clinical decision making	clinical decision making	clinical decision making	26	case_or_format
clinical pharmacy	clinical pharmacy	clinical pharmacy	clinical pharmacy	clinical pharmacy	26	kept
Clostridium difficile	clostridium difficile	clostridium difficile	clostridium difficile	Clostridium difficile	26	kept

Colectomy	colectomy	colectomy	colectomy	Colectomy	26	kept
COMPLICATIONS	complications	complications	complication	complication	26	singular
computer simulation	computer simulation	computer simulation	computer simulation	Computer Simulation	26	case_or_format
Cross infection	cross infection	cross infection	cross infection	Cross Infection	26	case_or_format
Demography	demography	demography	demography	demography	26	case_or_format
DEPRESSION	depression	depression	depression	depression	26	case_or_format
Diaries as Topic	diaries as topic	diaries as topic	diaries as topic	Diaries as Topic	26	kept
Disability Evaluation	disability evaluation	disability evaluation	disability evaluation	Disability Evaluation	26	kept
drug dose reduction	drug dose reduction	drug dose reduction	drug dose reduction	drug dose reduction	26	kept
drug overdose	drug overdose	drug overdose	drug overdose	drug overdose	26	kept
Electrophoresis, Gel, Pulsed-Field	electrophoresis gel pulsed field	electrophoresis gel pulsed field	electrophoresis gel pulsed field	Electrophoresis, Gel, Pulsed-Field	26	kept
End of life care	end of life care	end of life care	end of life care	end-of-life care	26	case_or_format
Energy Metabolism	energy metabolism	energy metabolism	energy metabolism	Energy Metabolism	26	kept
Ethics, Clinical	ethics clinical	ethics clinical	ethics clinical	Ethics, Clinical	26	kept
evidence based practice center	evidence based practice center	evidence based practice center	evidence based practice center	evidence based practice center	26	kept
exposure	exposure	exposure	exposure	exposure	26	kept

families	families	families	family	Family	26	singular
family members	family members	family members	family member	Family members	26	singular
fluid intake	fluid intake	fluid intake	fluid intake	fluid intake	26	kept
functional disease	functional disease	functional disease	functional disease	functional disease	26	kept
Gram negative infection	gram negative infection	gram negative infection	gram negative infection	Gram negative infection	26	kept
Health Priorities	health priorities	health priorities	health priorities	Health Priorities	26	kept
hemostasis	hemostasis	hemostasis	hemostasis	hemostasis	26	kept
high income country	high income country	high income country	high income country	high income country	26	kept
ibuprofen	ibuprofen	ibuprofen	ibuprofen	ibuprofen	26	kept
illumination	illumination	illumination	illumination	illumination	26	kept
Immobilization	immobilization	immobilization	immobilization	immobilization	26	case_or_format
Infant Welfare	infant welfare	infant welfare	infant welfare	Infant Welfare	26	kept
influencing factors	influencing factors	influencing factors	influencing factor	influencing factors	26	singular
insurance	insurance	insurance	insurance	insurance	26	kept
laboratory personnel	laboratory personnel	laboratory personnel	laboratory personnel	laboratory personnel	26	kept
lung injury	lung injury	lung injury	lung injury	lung injury	26	kept

Maternal Mortality	maternal mortality	maternal mortality	maternal mortality	maternal mortality	26	case_or_format
maternity ward	maternity ward	maternity ward	maternity ward	maternity ward	26	kept
microbial sensitivity test	microbial sensitivity test	microbial sensitivity test	microbial sensitivity test	Microbial Sensitivity Tests	26	case_or_format
Missouri	missouri	missouri	missouri	Missouri	26	kept
mobile application	mobile application	mobile application	mobile application	Mobile Applications	26	case_or_format
monotherapy	monotherapy	monotherapy	monotherapy	monotherapy	26	kept
moxifloxacin	moxifloxacin	moxifloxacin	moxifloxacin	moxifloxacin	26	kept
Multidisciplinary	multidisciplinary	multidisciplinary	multidisciplinary	multidisciplinary	26	case_or_format
Music therapy	music therapy	music therapy	music therapy	Music Therapy	26	case_or_format
naloxone	naloxone	naloxone	naloxone	naloxone	26	kept
Nose	nose	nose	nose	Nose	26	kept
nutritional requirement	nutritional requirement	nutritional requirement	nutritional requirement	Nutritional Requirements	26	case_or_format
Oral hygiene	oral hygiene	oral hygiene	oral hygiene	Oral Hygiene	26	case_or_format
organizational policy	organizational policy	organizational policy	organizational policy	Organizational Policy	26	case_or_format
perfusion	perfusion	perfusion	perfusion	perfusion	26	kept
point of care ultrasound	point of care ultrasound	point of care ultrasound	point of care ultrasound	point of care ultrasound	26	kept

Point-of-Care Testing	point of care testing	point of care testing	point of care testing	point of care testing	26	case_or_format
PREDICTORS	predictors	predictors	predictor	PREDICTORS	26	singular
Pregnancy, High-Risk	pregnancy high risk	pregnancy high risk	pregnancy high risk	Pregnancy, High-Risk	26	kept
protein intake	protein intake	protein intake	protein intake	protein intake	26	kept
Protocols	protocols	protocols	protocol	protocol	26	singular
Pulmonary Gas Exchange	pulmonary gas exchange	pulmonary gas exchange	pulmonary gas exchange	Pulmonary Gas Exchange	26	kept
pulmonology	pulmonology	pulmonology	pulmonology	pulmonology	26	kept
quinolone derivative	quinolone derivative	quinolone derivative	quinolone derivative	quinolone derivative	26	kept
Rapid response system	rapid response system	rapid response system	rapid response system	Rapid response system	26	kept
Reflex, Pupillary	reflex pupillary	reflex pupillary	reflex pupillary	Reflex, Pupillary	26	kept
Reimbursement Mechanisms	reimbursement mechanisms	reimbursement mechanisms	reimbursement mechanisms	Reimbursement Mechanisms	26	kept
Residence Characteristics	residence characteristics	residence characteristics	residence characteristics	Residence Characteristics	26	kept
respect	respect	respect	respect	respect	26	kept
Respiratory Aspiration	respiratory aspiration	respiratory aspiration	respiratory aspiration	Respiratory Aspiration	26	kept
Rural Health Services	rural health services	rural health services	rural health services	Rural Health Services	26	kept
rural population	rural population	rural population	rural population	Rural Population	26	case_or_format

scientific literature	scientific literature	scientific literature	scientific literature	scientific literature	26	kept
serum albumin	serum albumin	serum albumin	serum albumin	serum albumin	26	kept
Signal Processing, Computer-Assisted	signal processing computer assisted	signal processing computer assisted	signal processing computer assisted	Signal Processing, Computer-Assisted	26	kept
SIMULATION	simulation	simulation	simulation	simulation	26	case_or_format
South and Central America	south and central america	south and central america	south and central america	South and Central America	26	kept
speech therapy	speech therapy	speech therapy	speech therapy	speech therapy	26	kept
spirituality	spirituality	spirituality	spirituality	Spirituality	26	case_or_format
teacher	teacher	teacher	teacher	teacher	26	kept
thorax injury	thorax injury	thorax injury	thorax injury	thorax injury	26	kept
tigecycline	tigecycline	tigecycline	tigecycline	tigecycline	26	kept
tooth brushing	tooth brushing	tooth brushing	tooth brushing	tooth brushing	26	kept
Toothbrushing	toothbrushing	toothbrushing	toothbrushing	Toothbrushing	26	kept
veno-venous ECMO	veno venous ecmo	veno venous ecmo	veno venous ecmo	veno-venous ECMO	26	kept
Vulnerable Populations	vulnerable populations	vulnerable populations	vulnerable population	vulnerable population	26	singular
Walking	walking	walking	walking	walking	26	case_or_format
world health organization	world health organization	world health organization	world health organization	World Health Organization	26	case_or_format

wound	wound	wound	wound	wound	26	kept
abdominal infection	abdominal infection	abdominal infection	abdominal infection	abdominal infection	25	kept
Absenteeism	absenteeism	absenteeism	absenteeism	Absenteeism	25	kept
accident prevention	accident prevention	accident prevention	accident prevention	accident prevention	25	kept
Acinetobacter	acinetobacter	acinetobacter	acinetobacter	Acinetobacter	25	kept
aminoglycoside antibiotic agent	aminoglycoside antibiotic agent	aminoglycoside antibiotic agent	aminoglycoside antibiotic agent	aminoglycoside antibiotic agent	25	kept
angiography	angiography	angiography	angiography	angiography	25	kept
Antimicrobial Stewardship	antimicrobial stewardship	antimicrobial stewardship	antimicrobial stewardship	antimicrobial stewardship	25	case_or_format
ANXIETY	anxiety	anxiety	anxiety	Anxiety	25	case_or_format
arterial oxygen tension	arterial oxygen tension	arterial oxygen tension	arterial oxygen tension	arterial oxygen tension	25	kept
arterial pressure	arterial pressure	arterial pressure	arterial pressure	arterial pressure	25	kept
bacterial growth	bacterial growth	bacterial growth	bacterial growth	bacterial growth	25	kept
bed rest	bed rest	bed rest	bed rest	Bed Rest	25	case_or_format
Biological Markers	biological markers	biological markers	biological marker	biological marker	25	singular
Black or African American	black or african american	black or african american	black or african american	Black or African American	25	kept
Black person	black person	black person	black person	Black person	25	kept

brain	brain	brain	brain	Brain	25	case_or_format
cancer palliative therapy	cancer palliative therapy	cancer palliative therapy	cancer palliative therapy	cancer palliative therapy	25	kept
Candida auris	candida auris	candida auris	candida auris	Candida auris	25	kept
Cardiac Output, Low	cardiac output low	cardiac output low	cardiac output low	Cardiac Output, Low	25	kept
cardiopulmonary insufficiency	cardiopulmonary insufficiency	cardiopulmonary insufficiency	cardiopulmonary insufficiency	cardiopulmonary insufficiency	25	kept
Cardiovascular Surgical Procedures	cardiovascular surgical procedures	cardiovascular surgical procedures	cardiovascular surgical procedures	Cardiovascular Surgical Procedures	25	kept
cefuroxime	cefuroxime	cefuroxime	cefuroxime	cefuroxime	25	kept
Central venous catheter	central venous catheter	central venous catheter	central venous catheter	central venous catheter	25	case_or_format
circulation	circulation	circulation	circulation	circulation	25	kept
clinical observation	clinical observation	clinical observation	clinical observation	clinical observation	25	kept
collaboration	collaboration	collaboration	collaboration	Collaboration	25	case_or_format
Colorado	colorado	colorado	colorado	Colorado	25	kept
computer system	computer system	computer system	computer system	computer system	25	kept
Computers, Handheld	computers handheld	computers handheld	computers handheld	Computers, Handheld	25	kept
confirmatory factor analysis	confirmatory factor analysis	confirmatory factor analysis	confirmatory factor analysis	confirmatory factor analysis	25	kept
CONFUSION ASSESSMENT METHOD	confusion assessment method	confusion assessment method	confusion assessment method	Confusion Assessment Method	25	case_or_format

Controlled Before-After Studies	controlled before after studies	controlled before after studies	controlled before after studies	Controlled Before-After Studies	25	kept
coordination	coordination	coordination	coordination	coordination	25	kept
Crying	crying	crying	crying	crying	25	case_or_format
Cultural Characteristics	cultural characteristics	cultural characteristics	cultural characteristics	Cultural Characteristics	25	kept
DELIRIUM	delirium	delirium	delirium	Delirium	25	case_or_format
Delphi method	delphi method	delphi method	delphi method	Delphi method	25	kept
diet	diet	diet	diet	diet	25	kept
Dogs	dogs	dogs	dog	Dogs	25	singular
dose calculation	dose calculation	dose calculation	dose calculation	dose calculation	25	kept
drug delivery system	drug delivery system	drug delivery system	drug delivery system	drug delivery system	25	kept
economic evaluation	economic evaluation	economic evaluation	economic evaluation	economic evaluation	25	kept
endocarditis	endocarditis	endocarditis	endocarditis	endocarditis	25	kept
ethnic group	ethnic group	ethnic group	ethnic group	ethnic group	25	kept
Ethnography	ethnography	ethnography	ethnography	ethnography	25	case_or_format
eye care	eye care	eye care	eye care	eye care	25	kept
Facilitators	facilitators	facilitators	facilitator	Facilitators	25	singular

fetus	fetus	fetus	fetus	fetus	25	kept
first aid	first aid	first aid	first aid	first aid	25	kept
flow rate	flow rate	flow rate	flow rate	flow rate	25	kept
gold standard	gold standard	gold standard	gold standard	gold standard	25	kept
gunshot injury	gunshot injury	gunshot injury	gunshot injury	gunshot injury	25	kept
hand sanitizer	hand sanitizer	hand sanitizer	hand sanitizer	hand sanitizer	25	kept
Handover	handover	handover	handover	Handover	25	kept
Health Expenditures	health expenditures	health expenditures	health expenditure	Health Expenditures	25	singular
Heel	heel	heel	heel	Heel	25	kept
Hematopoietic Stem Cell Transplantation	hematopoietic stem cell transplantation	hematopoietic stem cell transplantation	hematopoietic stem cell transplantation	Hematopoietic Stem Cell Transplantation	25	kept
hemolysis	hemolysis	hemolysis	hemolysis	hemolysis	25	kept
ICD-9	icd 9	icd 9	icd 9	ICD-9	25	kept
imaging	imaging	imaging	imaging	imaging	25	kept
Influenza Vaccines	influenza vaccines	influenza vaccines	influenza vaccine	influenza vaccine	25	singular
infusion	infusion	infusion	infusion	infusion	25	kept
instrument sterilization	instrument sterilization	instrument sterilization	instrument sterilization	instrument sterilization	25	kept

Interdepartmental Relations	interdepartmental relations	interdepartmental relations	interdepartmental relations	Interdepartmental Relations	25	kept
interprofessional collaboration	interprofessional collaboration	interprofessional collaboration	interprofessional collaboration	interprofessional collaboration	25	kept
intraoperative period	intraoperative period	intraoperative period	intraoperative period	intraoperative period	25	kept
Kidney Diseases	kidney diseases	kidney diseases	kidney disease	kidney disease	25	singular
Long-term care	long term care	long term care	long term care	long term care	25	case_or_format
magnesium	magnesium	magnesium	magnesium	magnesium	25	kept
Mental Competency	mental competency	mental competency	mental competency	Mental Competency	25	kept
mental disease assessment	mental disease assessment	mental disease assessment	mental disease assessment	mental disease assessment	25	kept
mobile phone	mobile phone	mobile phone	mobile phone	mobile phone	25	kept
Mouth	mouth	mouth	mouth	Mouth	25	kept
multiple linear regression analysis	multiple linear regression analysis	multiple linear regression analysis	multiple linear regression analysis	multiple linear regression analysis	25	kept
musculoskeletal disease	musculoskeletal disease	musculoskeletal disease	musculoskeletal disease	Musculoskeletal Diseases	25	case_or_format
Negotiating	negotiating	negotiating	negotiating	Negotiating	25	kept
neuroscience	neuroscience	neuroscience	neuroscience	neuroscience	25	kept
PACU	pacu	pacu	pacu	PACU	25	kept
pediatric cardiology	pediatric cardiology	pediatric cardiology	pediatric cardiology	pediatric cardiology	25	kept

Physical Stimulation	physical stimulation	physical stimulation	physical stimulation	Physical Stimulation	25	kept
piperacillin	piperacillin	piperacillin	piperacillin	piperacillin	25	kept
postoperative analgesia	postoperative analgesia	postoperative analgesia	postoperative analgesia	postoperative analgesia	25	kept
Postoperative delirium	postoperative delirium	postoperative delirium	postoperative delirium	postoperative delirium	25	case_or_format
protein	protein	protein	protein	protein	25	kept
Psychotic Disorders	psychotic disorders	psychotic disorders	psychotic disorder	Psychotic Disorders	25	singular
Remote Consultation	remote consultation	remote consultation	remote consultation	Remote Consultation	25	kept
Renal Insufficiency	renal insufficiency	renal insufficiency	renal insufficiency	Renal Insufficiency	25	kept
Research Personnel	research personnel	research personnel	research personnel	Research Personnel	25	kept
respiratory arrest	respiratory arrest	respiratory arrest	respiratory arrest	respiratory arrest	25	kept
Right to Die	right to die	right to die	right to die	Right to Die	25	kept
Sampling Studies	sampling studies	sampling studies	sampling studies	Sampling Studies	25	kept
Scale	scale	scale	scale	SCALE	25	case_or_format
Seroepidemiologic Studies	seroepidemiologic studies	seroepidemiologic studies	seroepidemiologic studies	Seroepidemiologic Studies	25	kept
Short Form 36	short form 36	short form 36	short form 36	Short Form 36	25	kept
skin infection	skin infection	skin infection	skin infection	skin infection	25	kept

stress management	stress management	stress management	stress management	stress management	25	kept
study design	study design	study design	study design	study design	25	kept
symptom assessment	symptom assessment	symptom assessment	symptom assessment	symptom assessment	25	kept
Team	team	team	team	Team	25	kept
thoracotomy	thoracotomy	thoracotomy	thoracotomy	thoracotomy	25	kept
tobramycin	tobramycin	tobramycin	tobramycin	tobramycin	25	kept
Trachea	trachea	trachea	trachea	Trachea	25	kept
tracheal aspiration procedure	tracheal aspiration procedure	tracheal aspiration procedure	tracheal aspiration procedure	tracheal aspiration procedure	25	kept
transient ischemic attack	transient ischemic attack	transient ischemic attack	transient ischemic attack	transient ischemic attack	25	kept
tumor necrosis factor	tumor necrosis factor	tumor necrosis factor	tumor necrosis factor	tumor necrosis factor	25	kept
UNITED-STATES	united states	united states	united states	United States	25	case_or_format
unplanned extubation	unplanned extubation	unplanned extubation	unplanned extubation	unplanned extubation	25	kept
urine culture	urine culture	urine culture	urine culture	urine culture	25	kept
urine retention	urine retention	urine retention	urine retention	urine retention	25	kept
virus load	virus load	virus load	virus load	virus load	25	kept
water	water	water	water	water	25	kept

Water-Electrolyte Balance	water electrolyte balance	water electrolyte balance	water electrolyte balance	Water-Electrolyte Balance	25	kept
weight gain	weight gain	weight gain	weight gain	Weight Gain	25	case_or_format
Well-being	well being	well being	well being	well-being	25	case_or_format
Western Australia	western australia	western australia	western australia	Western Australia	25	kept
Abdominal Injuries	abdominal injuries	abdominal injuries	abdominal injury	Abdominal Injuries	24	singular
absenteeism	absenteeism	absenteeism	absenteeism	Absenteeism	24	case_or_format
Actigraphy	actigraphy	actigraphy	actigraphy	Actigraphy	24	kept
activated partial thromboplastin time	activated partial thromboplastin time	activated partial thromboplastin time	activated partial thromboplastin time	activated partial thromboplastin time	24	kept
acute lung injury	acute lung injury	acute lung injury	acute lung injury	acute lung injury	24	kept
Adherence	adherence	adherence	adherence	Adherence	24	kept
Advisory Committees	advisory committees	advisory committees	advisory committee	Advisory Committees	24	singular
anaphylaxis	anaphylaxis	anaphylaxis	anaphylaxis	anaphylaxis	24	kept
Anti-Arrhythmia Agents	anti arrhythmia agents	anti arrhythmia agents	anti arrhythmia agents	Anti-Arrhythmia Agents	24	kept
Antimicrobial resistance	antimicrobial resistance	antimicrobial resistance	antimicrobial resistance	Antimicrobial resistance	24	kept
antisepsis	antisepsis	antisepsis	antisepsis	antisepsis	24	kept
anxiolytic agent	anxiolytic agent	anxiolytic agent	anxiolytic agent	anxiolytic agent	24	kept

Arabs	arabs	arabs	arab	Arabs	24	singular
bilevel positive airway pressure	bilevel positive airway pressure	bilevel positive airway pressure	bilevel positive airway pressure	bilevel positive airway pressure	24	kept
blood volume	blood volume	blood volume	blood volume	blood volume	24	kept
body surface	body surface	body surface	body surface	body surface	24	kept
Breast Neoplasms	breast neoplasms	breast neoplasms	breast neoplasms	Breast Neoplasms	24	kept
calcium	calcium	calcium	calcium	calcium	24	kept
cancer survival	cancer survival	cancer survival	cancer survival	cancer survival	24	kept
caring	caring	caring	caring	caring	24	kept
cerebral palsy	cerebral palsy	cerebral palsy	cerebral palsy	cerebral palsy	24	kept
Clinical alarms	clinical alarms	clinical alarms	clinical alarm	Clinical Alarms	24	singular
Clinical decision support	clinical decision support	clinical decision support	clinical decision support	Clinical decision support	24	kept
Colorectal Neoplasms	colorectal neoplasms	colorectal neoplasms	colorectal neoplasms	Colorectal Neoplasms	24	kept
Comfort	comfort	comfort	comfort	comfort	24	case_or_format
Concept analysis	concept analysis	concept analysis	concept analysis	concept analysis	24	case_or_format
Coping strategies	coping strategies	coping strategies	coping strategy	Coping strategies	24	singular
Coronavirus infections	coronavirus infections	coronavirus infections	coronavirus infection	Coronavirus Infections	24	singular

Deglutition	deglutition	deglutition	deglutition	Deglutition	24	kept
delirium assessment	delirium assessment	delirium assessment	delirium assessment	delirium assessment	24	kept
Diagnosis, Computer-Assisted	diagnosis computer assisted	diagnosis computer assisted	diagnosis computer assisted	Diagnosis, Computer-Assisted	24	kept
digital health	digital health	digital health	digital health	digital health	24	kept
discharge	discharge	discharge	discharge	Discharge	24	case_or_format
disease assessment	disease assessment	disease assessment	disease assessment	disease assessment	24	kept
drug formulation	drug formulation	drug formulation	drug formulation	drug formulation	24	kept
Drug Hypersensitivity	drug hypersensitivity	drug hypersensitivity	drug hypersensitivity	drug hypersensitivity	24	case_or_format
drug indication	drug indication	drug indication	drug indication	drug indication	24	kept
Drug Labeling	drug labeling	drug labeling	drug labeling	Drug Labeling	24	kept
E-learning	e learning	e learning	e learning	e-learning	24	case_or_format
Enterobacteriaceae infection	enterobacteriaceae infection	enterobacteriaceae infection	enterobacteriaceae infection	Enterobacteriaceae Infections	24	case_or_format
Enterococcus faecium	enterococcus faecium	enterococcus faecium	enterococcus faecium	Enterococcus faecium	24	kept
experimental study	experimental study	experimental study	experimental study	experimental study	24	kept
FAILURE	failure	failure	failure	FAILURE	24	kept
ferritin	ferritin	ferritin	ferritin	ferritin	24	kept

focus groups	focus groups	focus groups	focus group	Focus Groups	24	singular
guilt	guilt	guilt	guilt	guilt	24	kept
health services	health services	health services	health service	health service	24	singular
Health services research	health services research	health services research	health services research	Health Services Research	24	case_or_format
heart stroke volume	heart stroke volume	heart stroke volume	heart stroke volume	heart stroke volume	24	kept
Hospitals, Maternity	hospitals maternity	hospitals maternity	hospitals maternity	Hospitals, Maternity	24	kept
immune deficiency	immune deficiency	immune deficiency	immune deficiency	immune deficiency	24	kept
intention to treat analysis	intention to treat analysis	intention to treat analysis	intention to treat analysis	intention to treat analysis	24	kept
International Classification of Diseases	international classification of diseases	international classification of diseases	international classification of disease	International Classification of Diseases	24	singular
Interprofessional collaboration	interprofessional collaboration	interprofessional collaboration	interprofessional collaboration	interprofessional collaboration	24	case_or_format
Intra-Aortic Balloon Pumping	intra aortic balloon pumping	intra aortic balloon pumping	intra aortic balloon pumping	Intra-Aortic Balloon Pumping	24	kept
Intuition	intuition	intuition	intuition	Intuition	24	kept
isoflurane	isoflurane	isoflurane	isoflurane	isoflurane	24	kept
levetiracetam	levetiracetam	levetiracetam	levetiracetam	levetiracetam	24	kept
lung infiltrate	lung infiltrate	lung infiltrate	lung infiltrate	lung infiltrate	24	kept
maternal welfare	maternal welfare	maternal welfare	maternal welfare	maternal welfare	24	kept

Medical staff	medical staff	medical staff	medical staff	medical staff	24	case_or_format
minimally invasive surgery	minimally invasive surgery	minimally invasive surgery	minimally invasive surgery	minimally invasive surgery	24	kept
national health organization	national health organization	national health organization	national health organization	national health organization	24	kept
National Institutes of Health Stroke Scale	national institutes of health stroke scale	national institutes of health stroke scale	national institutes of health stroke scale	National Institutes of Health Stroke Scale	24	kept
Non-invasive ventilation	non invasive ventilation	non invasive ventilation	non invasive ventilation	Non-invasive ventilation	24	kept
Observational study	observational study	observational study	observational study	observational study	24	case_or_format
opiate addiction	opiate addiction	opiate addiction	opiate addiction	opiate addiction	24	kept
oral health	oral health	oral health	oral health	Oral Health	24	case_or_format
organ dysfunction score	organ dysfunction score	organ dysfunction score	organ dysfunction score	Organ Dysfunction Scores	24	case_or_format
orientation	orientation	orientation	orientation	orientation	24	kept
orthopedics	orthopedics	orthopedics	orthopedics	orthopedics	24	kept
oxygen desaturation	oxygen desaturation	oxygen desaturation	oxygen desaturation	oxygen desaturation	24	kept
PEOPLE	people	people	people	PEOPLE	24	kept
Personhood	personhood	personhood	personhood	Personhood	24	kept
PERSPECTIVES	perspectives	perspectives	perspective	PERSPECTIVES	24	singular
photography	photography	photography	photography	Photography	24	case_or_format

positive pressure ventilation	positive pressure ventilation	positive pressure ventilation	positive pressure ventilation	positive pressure ventilation	24	kept
Prenatal Diagnosis	prenatal diagnosis	prenatal diagnosis	prenatal diagnosis	Prenatal Diagnosis	24	kept
Pressure injuries	pressure injuries	pressure injuries	pressure injury	pressure injury	24	singular
Prone position	prone position	prone position	prone position	prone position	24	case_or_format
psychological distress	psychological distress	psychological distress	psychological distress	Psychological Distress	24	case_or_format
Psychological stress	psychological stress	psychological stress	psychological stress	Psychological stress	24	kept
Quality indicators	quality indicators	quality indicators	quality indicator	Quality indicators	24	singular
Radiography, Thoracic	radiography thoracic	radiography thoracic	radiography thoracic	Radiography, Thoracic	24	kept
random forest	random forest	random forest	random forest	random forest	24	kept
Rare Diseases	rare diseases	rare diseases	rare disease	Rare Diseases	24	singular
Review Literature as Topic	review literature as topic	review literature as topic	review literature as topic	Review Literature as Topic	24	kept
Risk Adjustment	risk adjustment	risk adjustment	risk adjustment	Risk Adjustment	24	kept
role playing	role playing	role playing	role playing	role playing	24	kept
seroprevalence	seroprevalence	seroprevalence	seroprevalence	seroprevalence	24	kept
skin disease	skin disease	skin disease	skin disease	Skin Diseases	24	case_or_format
Smoking Cessation	smoking cessation	smoking cessation	smoking cessation	smoking cessation	24	case_or_format

stroke unit	stroke unit	stroke unit	stroke unit	stroke unit	24	kept
Support	support	support	support	SUPPORT	24	case_or_format
support	support	support	support	SUPPORT	24	case_or_format
syringe	syringe	syringe	syringe	Syringes	24	case_or_format
system analysis	system analysis	system analysis	system analysis	system analysis	24	kept
theory	theory	theory	theory	theory	24	kept
TIME	time	time	time	time	24	case_or_format
tramadol	tramadol	tramadol	tramadol	tramadol	24	kept
vascular surgery	vascular surgery	vascular surgery	vascular surgery	vascular surgery	24	kept
vasodilator agent	vasodilator agent	vasodilator agent	vasodilator agent	Vasodilator Agents	24	case_or_format
virus infection	virus infection	virus infection	virus infection	virus infection	24	kept
whole genome sequencing	whole genome sequencing	whole genome sequencing	whole genome sequencing	whole genome sequencing	24	kept
accident	accident	accident	accident	accident	23	kept
achievement	achievement	achievement	achievement	achievement	23	kept
Administration, Inhalation	administration inhalation	administration inhalation	administration inhalation	Administration, Inhalation	23	kept
advanced cancer	advanced cancer	advanced cancer	advanced cancer	advanced cancer	23	kept

Affect	affect	affect	affect	Affect	23	kept
After-Hours Care	after hours care	after hours care	after hours care	After-Hours Care	23	kept
airway management	airway management	airway management	airway management	Airway Management	23	case_or_format
American Society of Anaesthesiologists score	american society of anaesthesiologists score	american society of anaesthesiologists score	american society of anaesthesiologists score	American Society of Anaesthesiologists score	23	kept
analytic method	analytic method	analytic method	analytic method	analytic method	23	kept
anger	anger	anger	anger	anger	23	kept
angiotensin receptor antagonist	angiotensin receptor antagonist	angiotensin receptor antagonist	angiotensin receptor antagonist	angiotensin receptor antagonist	23	kept
anthropometry	anthropometry	anthropometry	anthropometry	anthropometry	23	kept
Antifungal Agents	antifungal agents	antifungal agents	antifungal agent	antifungal agent	23	singular
Antimicrobial stewardship	antimicrobial stewardship	antimicrobial stewardship	antimicrobial stewardship	antimicrobial stewardship	23	case_or_format
Arteries	arteries	arteries	artery	Arteries	23	singular
Arthroplasty, Replacement, Hip	arthroplasty replacement hip	arthroplasty replacement hip	arthroplasty replacement hip	Arthroplasty, Replacement, Hip	23	kept
assisted living facility	assisted living facility	assisted living facility	assisted living facility	assisted living facility	23	kept
Auscultation	auscultation	auscultation	auscultation	Auscultation	23	kept
Behavior Therapy	behavior therapy	behavior therapy	behavior therapy	Behavior Therapy	23	kept
BLOOD-STREAM INFECTIONS	blood stream infections	blood stream infections	blood stream infection	BLOOD-STREAM INFECTIONS	23	singular

cancer therapy	cancer therapy	cancer therapy	cancer therapy	cancer therapy	23	kept
candidiasis	candidiasis	candidiasis	candidiasis	Candidiasis	23	case_or_format
capacity building	capacity building	capacity building	capacity building	capacity building	23	kept
cardiac surgeon	cardiac surgeon	cardiac surgeon	cardiac surgeon	cardiac surgeon	23	kept
Central Nervous System Diseases	central nervous system diseases	central nervous system diseases	central nervous system disease	Central Nervous System Diseases	23	singular
Chest Pain	chest pain	chest pain	chest pain	Chest Pain	23	kept
Chicago	chicago	chicago	chicago	Chicago	23	kept
child abuse	child abuse	child abuse	child abuse	Child Abuse	23	case_or_format
Cognitive impairment	cognitive impairment	cognitive impairment	cognitive impairment	Cognitive impairment	23	kept
Community-Institutional Relations	community institutional relations	community institutional relations	community institutional relations	Community-Institutional Relations	23	kept
consumer	consumer	consumer	consumer	consumer	23	kept
corticosteroid therapy	corticosteroid therapy	corticosteroid therapy	corticosteroid therapy	corticosteroid therapy	23	kept
Cuidados intensivos	cuidados intensivos	cuidados intensivos	cuidados intensivos	Cuidados intensivos	23	kept
daptomycin	daptomycin	daptomycin	daptomycin	daptomycin	23	kept
development	development	development	development	development	23	kept
Diabetes, Gestational	diabetes gestational	diabetes gestational	diabetes gestational	Diabetes, Gestational	23	kept

Diseases	diseases	diseases	disease	Diseases	23	singular
drug interaction	drug interaction	drug interaction	drug interaction	Drug Interactions	23	case_or_format
Dysphagia	dysphagia	dysphagia	dysphagia	dysphagia	23	case_or_format
Early warning score	early warning score	early warning score	early warning score	early warning score	23	case_or_format
EMERGENCY-DEPARTMENT	emergency department	emergency department	emergency department	Emergency department	23	case_or_format
end of life care	end of life care	end of life care	end of life care	end-of-life care	23	case_or_format
endoscopy	endoscopy	endoscopy	endoscopy	endoscopy	23	kept
Enterocolitis, Pseudomembranous	enterocolitis pseudomembranous	enterocolitis pseudomembranous	enterocolitis pseudomembranous	Enterocolitis, Pseudomembranous	23	kept
escalation of care	escalation of care	escalation of care	escalation of care	escalation of care	23	kept
extravasation	extravasation	extravasation	extravasation	extravasation	23	kept
Family needs	family needs	family needs	family need	Family needs	23	singular
family stress	family stress	family stress	family stress	family stress	23	kept
febrile neutropenia	febrile neutropenia	febrile neutropenia	febrile neutropenia	febrile neutropenia	23	kept
fetus monitoring	fetus monitoring	fetus monitoring	fetus monitoring	fetus monitoring	23	kept
fibrinolytic therapy	fibrinolytic therapy	fibrinolytic therapy	fibrinolytic therapy	fibrinolytic therapy	23	kept
Guideline	guideline	guideline	guideline	Guidelines	23	case_or_format

Guilt	guilt	guilt	guilt	guilt	23	case_or_format
Health policy	health policy	health policy	health policy	Health Policy	23	case_or_format
Health Services	health services	health services	health service	health service	23	singular
Health services	health services	health services	health service	health service	23	singular
hemodynamic parameters	hemodynamic parameters	hemodynamic parameters	hemodynamic parameters	hemodynamic parameters	23	kept
HOSPITAL MORTALITY	hospital mortality	hospital mortality	hospital mortality	Hospital Mortality	23	case_or_format
hypervolemia	hypervolemia	hypervolemia	hypervolemia	hypervolemia	23	kept
hypnotic agent	hypnotic agent	hypnotic agent	hypnotic agent	hypnotic agent	23	kept
hypovolemia	hypovolemia	hypovolemia	hypovolemia	hypovolemia	23	kept
incident report	incident report	incident report	incident report	incident report	23	kept
Infection prevention and control	infection prevention and control	infection prevention and control	infection prevention and control	Infection prevention and control	23	kept
INFECTIONS	infections	infections	infection	infection	23	singular
influenza A	influenza a	influenza a	influenza a	influenza A	23	kept
integrative review	integrative review	integrative review	integrative review	integrative review	23	kept
Interprofessional	interprofessional	interprofessional	interprofessional	Interprofessional	23	kept
Intersectoral Collaboration	intersectoral collaboration	intersectoral collaboration	intersectoral collaboration	intersectoral collaboration	23	case_or_format

kinesiotherapy	kinesiotherapy	kinesiotherapy	kinesiotherapy	kinesiotherapy	23	kept
Learning systems	learning systems	learning systems	learning systems	Learning systems	23	kept
lower limb	lower limb	lower limb	lower limb	lower limb	23	kept
manikin	manikin	manikin	manikin	Manikins	23	case_or_format
mass disaster	mass disaster	mass disaster	mass disaster	mass disaster	23	kept
medical emergency team	medical emergency team	medical emergency team	medical emergency team	Medical emergency team	23	case_or_format
medical examination	medical examination	medical examination	medical examination	medical examination	23	kept
meningitis	meningitis	meningitis	meningitis	meningitis	23	kept
metastasis	metastasis	metastasis	metastasis	metastasis	23	kept
Mobility	mobility	mobility	mobility	Mobility	23	kept
Mobility Limitation	mobility limitation	mobility limitation	mobility limitation	Mobility Limitation	23	kept
Monitoring, Intraoperative	monitoring intraoperative	monitoring intraoperative	monitoring intraoperative	Monitoring, Intraoperative	23	kept
multidisciplinary care	multidisciplinary care	multidisciplinary care	multidisciplinary care	multidisciplinary care	23	kept
multiple chronic conditions	multiple chronic conditions	multiple chronic conditions	multiple chronic conditions	multiple chronic conditions	23	kept
Neuromuscular Blockade	neuromuscular blockade	neuromuscular blockade	neuromuscular blockade	Neuromuscular Blockade	23	kept
nitroprusside sodium	nitroprusside sodium	nitroprusside sodium	nitroprusside sodium	nitroprusside sodium	23	kept

open ended questionnaire	open ended questionnaire	open ended questionnaire	open ended questionnaire	open ended questionnaire	23	kept
Organizational culture	organizational culture	organizational culture	organizational culture	Organizational Culture	23	case_or_format
oxacillin	oxacillin	oxacillin	oxacillin	oxacillin	23	kept
pathogenicity	pathogenicity	pathogenicity	pathogenicity	pathogenicity	23	kept
Pediatricians	pediatricians	pediatricians	pediatrician	pediatrician	23	singular
peer review	peer review	peer review	peer review	peer review	23	kept
plasmapheresis	plasmapheresis	plasmapheresis	plasmapheresis	plasmapheresis	23	kept
Plastic Surgery Procedures	plastic surgery procedures	plastic surgery procedures	plastic surgery procedures	Plastic Surgery Procedures	23	kept
Post-traumatic stress disorder	post traumatic stress disorder	post traumatic stress disorder	post traumatic stress disorder	Post-traumatic stress disorder	23	kept
poverty	poverty	poverty	poverty	poverty	23	kept
prediction model	prediction model	prediction model	prediction model	prediction model	23	kept
prenatal diagnosis	prenatal diagnosis	prenatal diagnosis	prenatal diagnosis	Prenatal Diagnosis	23	case_or_format
protective glasses	protective glasses	protective glasses	protective glasses	protective glasses	23	kept
proxy	proxy	proxy	proxy	Proxy	23	case_or_format
Psychological	psychological	psychological	psychological	Psychological	23	kept
pulmonary rehabilitation	pulmonary rehabilitation	pulmonary rehabilitation	pulmonary rehabilitation	pulmonary rehabilitation	23	kept

Quality control	quality control	quality control	quality control	quality control	23	case_or_format
Renal replacement therapy	renal replacement therapy	renal replacement therapy	renal replacement therapy	renal replacement therapy	23	case_or_format
respiratory	respiratory	respiratory	respiratory	respiratory	23	kept
Safety culture	safety culture	safety culture	safety culture	safety culture	23	case_or_format
Short Survey	short survey	short survey	short survey	Short Survey	23	kept
Simulation training	simulation training	simulation training	simulation training	Simulation Training	23	case_or_format
sleep disordered breathing	sleep disordered breathing	sleep disordered breathing	sleep disordered breathing	sleep disordered breathing	23	kept
somnolence	somnolence	somnolence	somnolence	somnolence	23	kept
Stevens-Johnson Syndrome	stevens johnson syndrome	stevens johnson syndrome	stevens johnson syndrome	Stevens-Johnson Syndrome	23	kept
Stomach	stomach	stomach	stomach	Stomach	23	kept
Suicide	suicide	suicide	suicide	suicide	23	case_or_format
Surveys and questionnaires	surveys and questionnaires	surveys and questionnaires	surveys and questionnaires	Surveys and Questionnaires	23	case_or_format
Teaching Materials	teaching materials	teaching materials	teaching materials	Teaching Materials	23	kept
telephone	telephone	telephone	telephone	Telephone	23	case_or_format
Transitional care	transitional care	transitional care	transitional care	transitional care	23	case_or_format
Tuberculosis	tuberculosis	tuberculosis	tuberculosis	tuberculosis	23	case_or_format

Ultrasound	ultrasound	ultrasound	ultrasound	ultrasound	23	case_or_format
usability	usability	usability	usability	usability	23	kept
vaccine	vaccine	vaccine	vaccine	vaccine	23	kept
valproic acid	valproic acid	valproic acid	valproic acid	valproic acid	23	kept
VENTILATOR-ASSOCIATED PNEUMONIA	ventilator associated pneumonia	ventilator associated pneumonia	ventilator associated pneumonia	ventilator associated pneumonia	23	case_or_format
veteran	veteran	veteran	veteran	Veterans	23	case_or_format
Vietnam	vietnam	vietnam	vietnam	Vietnam	23	kept
Waiting Lists	waiting lists	waiting lists	waiting list	Waiting Lists	23	singular
Work	work	work	work	work	23	case_or_format
abdomen	abdomen	abdomen	abdomen	Abdomen	22	case_or_format
Acoustic Stimulation	acoustic stimulation	acoustic stimulation	acoustic stimulation	Acoustic Stimulation	22	kept
ADMISSION	admission	admission	admission	ADMISSION	22	kept
amphotericin B	amphotericin b	amphotericin b	amphotericin b	amphotericin B	22	kept
Anaphylaxis	anaphylaxis	anaphylaxis	anaphylaxis	anaphylaxis	22	case_or_format
Anecdotes as Topic	anecdotes as topic	anecdotes as topic	anecdotes as topic	Anecdotes as Topic	22	kept
Arrhythmia	arrhythmia	arrhythmia	arrhythmia	Arrhythmia	22	kept

Audiovisual Aids	audiovisual aids	audiovisual aids	audiovisual aid	Audiovisual Aids	22	singular
Audit	audit	audit	audit	Audit	22	kept
bariatric surgery	bariatric surgery	bariatric surgery	bariatric surgery	bariatric surgery	22	kept
beta lactamase	beta lactamase	beta lactamase	beta lactamase	beta-Lactamases	22	case_or_format
biomedical technology assessment	biomedical technology assessment	biomedical technology assessment	biomedical technology assessment	biomedical technology assessment	22	kept
breast milk expression	breast milk expression	breast milk expression	breast milk expression	Breast Milk Expression	22	case_or_format
burnout syndrome	burnout syndrome	burnout syndrome	burnout syndrome	burnout syndrome	22	kept
cardiovascular mortality	cardiovascular mortality	cardiovascular mortality	cardiovascular mortality	cardiovascular mortality	22	kept
CHALLENGES	challenges	challenges	challenge	CHALLENGES	22	singular
Classification	classification	classification	classification	classification	22	case_or_format
clinical reasoning	clinical reasoning	clinical reasoning	clinical reasoning	clinical reasoning	22	kept
Colony Count, Microbial	colony count microbial	colony count microbial	colony count microbial	Colony Count, Microbial	22	kept
Compartment Syndromes	compartment syndromes	compartment syndromes	compartment syndrome	Compartment Syndromes	22	singular
computer interface	computer interface	computer interface	computer interface	computer interface	22	kept
convulsion	convulsion	convulsion	convulsion	convulsion	22	kept
cystic fibrosis	cystic fibrosis	cystic fibrosis	cystic fibrosis	cystic fibrosis	22	kept

Debridement	debridement	debridement	debridement	debridement	22	case_or_format
Dermatitis, Occupational	dermatitis occupational	dermatitis occupational	dermatitis occupational	Dermatitis, Occupational	22	kept
developed country	developed country	developed country	developed country	developed country	22	kept
Dialysis	dialysis	dialysis	dialysis	dialysis	22	case_or_format
disinfectant agent	disinfectant agent	disinfectant agent	disinfectant agent	disinfectant agent	22	kept
DISTRESS	distress	distress	distress	DISTRESS	22	kept
Dog Diseases	dog diseases	dog diseases	dog disease	Dog Diseases	22	singular
drug utilization	drug utilization	drug utilization	drug utilization	Drug Utilization	22	case_or_format
Dry Eye Syndromes	dry eye syndromes	dry eye syndromes	dry eye syndrome	Dry Eye Syndromes	22	singular
educational model	educational model	educational model	educational model	educational model	22	kept
estimated glomerular filtration rate	estimated glomerular filtration rate	estimated glomerular filtration rate	estimated glomerular filtration rate	estimated glomerular filtration rate	22	kept
Ethical Dilemmas	ethical dilemmas	ethical dilemmas	ethical dilemma	ethical dilemma	22	singular
facilitators	facilitators	facilitators	facilitator	Facilitators	22	singular
Family centered care	family centered care	family centered care	family centered care	family centered care	22	case_or_format
family decision making	family decision making	family decision making	family decision making	family decision making	22	kept
father child relation	father child relation	father child relation	father child relation	Father-Child Relations	22	case_or_format

Forms and Records Control	forms and records control	forms and records control	forms and records control	Forms and Records Control	22	kept
genotype	genotype	genotype	genotype	genotype	22	kept
geriatrician	geriatrician	geriatrician	geriatrician	geriatrician	22	kept
handover	handover	handover	handover	Handover	22	case_or_format
healing	healing	healing	healing	healing	22	kept
heart tamponade	heart tamponade	heart tamponade	heart tamponade	heart tamponade	22	kept
humidity	humidity	humidity	humidity	Humidity	22	case_or_format
hypocalcemia	hypocalcemia	hypocalcemia	hypocalcemia	hypocalcemia	22	kept
hypothyroidism	hypothyroidism	hypothyroidism	hypothyroidism	hypothyroidism	22	kept
Implementation Science	implementation science	implementation science	implementation science	implementation science	22	case_or_format
Information	information	information	information	Information	22	kept
Integrative review	integrative review	integrative review	integrative review	integrative review	22	case_or_format
Internet of things	internet of things	internet of things	internet of thing	Internet of things	22	singular
interventions	interventions	interventions	intervention	Intervention	22	singular
labor induction	labor induction	labor induction	labor induction	labor induction	22	kept
Lifting	lifting	lifting	lifting	Lifting	22	kept

Medical errors	medical errors	medical errors	medical error	Medical Errors	22	singular
Medical informatics	medical informatics	medical informatics	medical informatics	medical informatics	22	case_or_format
medical leave	medical leave	medical leave	medical leave	medical leave	22	kept
medicolegal aspect	medicolegal aspect	medicolegal aspect	medicolegal aspect	medicolegal aspect	22	kept
mentor	mentor	mentor	mentor	Mentors	22	case_or_format
meticillin	meticillin	meticillin	meticillin	meticillin	22	kept
military medicine	military medicine	military medicine	military medicine	Military Medicine	22	case_or_format
Mixed methods	mixed methods	mixed methods	mixed method	Mixed methods	22	singular
Myocardial infarction	myocardial infarction	myocardial infarction	myocardial infarction	Myocardial Infarction	22	case_or_format
Narcotics	narcotics	narcotics	narcotics	Narcotics	22	kept
Nephrology	nephrology	nephrology	nephrology	nephrology	22	case_or_format
Neurosciences	neurosciences	neurosciences	neuroscience	neuroscience	22	singular
night	night	night	night	night	22	kept
noise reduction	noise reduction	noise reduction	noise reduction	noise reduction	22	kept
Noninvasive ventilation	noninvasive ventilation	noninvasive ventilation	noninvasive ventilation	noninvasive ventilation	22	case_or_format
Northern Ireland	northern ireland	northern ireland	northern ireland	Northern Ireland	22	kept

oncologist	oncologist	oncologist	oncologist	oncologist	22	kept
Oregon	oregon	oregon	oregon	Oregon	22	kept
Parental stress	parental stress	parental stress	parental stress	parental stress	22	case_or_format
partial thromboplastin time	partial thromboplastin time	partial thromboplastin time	partial thromboplastin time	partial thromboplastin time	22	kept
perfusionist	perfusionist	perfusionist	perfusionist	perfusionist	22	kept
Perinatal Death	perinatal death	perinatal death	perinatal death	Perinatal Death	22	kept
peroperative complication	peroperative complication	peroperative complication	peroperative complication	peroperative complication	22	kept
Physiotherapy	physiotherapy	physiotherapy	physiotherapy	physiotherapy	22	case_or_format
potassium chloride	potassium chloride	potassium chloride	potassium chloride	potassium chloride	22	kept
Pre-Eclampsia	pre eclampsia	pre eclampsia	pre eclampsia	Pre-Eclampsia	22	kept
Preterm birth	preterm birth	preterm birth	preterm birth	Preterm birth	22	kept
preventive health service	preventive health service	preventive health service	preventive health service	preventive health service	22	kept
psychological safety	psychological safety	psychological safety	psychological safety	psychological safety	22	kept
psychometrics	psychometrics	psychometrics	psychometric	Psychometrics	22	singular
psychotrauma	psychotrauma	psychotrauma	psychotrauma	psychotrauma	22	kept
public sector	public sector	public sector	public sector	public sector	22	kept

quality adjusted life year	quality adjusted life year	quality adjusted life year	quality adjusted life year	quality adjusted life year	22	kept
Rankin scale	rankin scale	rankin scale	rankin scale	Rankin scale	22	kept
RELATIVES	relatives	relatives	relative	relative	22	singular
Retention	retention	retention	retention	Retention	22	kept
safety climate	safety climate	safety climate	safety climate	safety climate	22	kept
sensory stimulation	sensory stimulation	sensory stimulation	sensory stimulation	sensory stimulation	22	kept
Shift work	shift work	shift work	shift work	shift work	22	case_or_format
Skills	skills	skills	skill	skill	22	singular
sleep pattern	sleep pattern	sleep pattern	sleep pattern	sleep pattern	22	kept
soap	soap	soap	soap	soap	22	kept
spouse	spouse	spouse	spouse	Spouses	22	case_or_format
sputum culture	sputum culture	sputum culture	sputum culture	sputum culture	22	kept
Stroke Volume	stroke volume	stroke volume	stroke volume	Stroke Volume	22	kept
surfactant	surfactant	surfactant	surfactant	surfactant	22	kept
surgical approach	surgical approach	surgical approach	surgical approach	surgical approach	22	kept
surveillance	surveillance	surveillance	surveillance	Surveillance	22	case_or_format

SURVIVAL	survival	survival	survival	survival	22	case_or_format
THERAPY	therapy	therapy	therapy	therapy	22	case_or_format
thrombectomy	thrombectomy	thrombectomy	thrombectomy	thrombectomy	22	kept
tranexamic acid	tranexamic acid	tranexamic acid	tranexamic acid	tranexamic acid	22	kept
transcatheter aortic valve implantation	transcatheter aortic valve implantation	transcatheter aortic valve implantation	transcatheter aortic valve implantation	transcatheter aortic valve implantation	22	kept
transmission	transmission	transmission	transmission	transmission	22	kept
tremor	tremor	tremor	tremor	tremor	22	kept
UNITS	units	units	unit	UNIT	22	singular
urban hospital	urban hospital	urban hospital	urban hospital	urban hospital	22	kept
validation	validation	validation	validation	VALIDATION	22	case_or_format
VAP	vap	vap	vap	VAP	22	kept
Ventilator associated pneumonia	ventilator associated pneumonia	ventilator associated pneumonia	ventilator associated pneumonia	ventilator associated pneumonia	22	case_or_format
volunteer	volunteer	volunteer	volunteer	volunteer	22	kept
war	war	war	war	war	22	kept
wheezing	wheezing	wheezing	wheezing	wheezing	22	kept
Wounds, Gunshot	wounds gunshot	wounds gunshot	wounds gunshot	Wounds, Gunshot	22	kept

X ray	x ray	x ray	x ray	X ray	22	kept
Accidents	accidents	accidents	accident	accident	21	singular
Acute respiratory failure	acute respiratory failure	acute respiratory failure	acute respiratory failure	acute respiratory failure	21	case_or_format
alarm monitoring	alarm monitoring	alarm monitoring	alarm monitoring	alarm monitoring	21	kept
allergy	allergy	allergy	allergy	allergy	21	kept
Analgesics, Non-Narcotic	analgesics non narcotic	analgesics non narcotic	analgesics non narcotic	Analgesics, Non-Narcotic	21	kept
Anesthetics, Intravenous	anesthetics intravenous	anesthetics intravenous	anesthetics intravenous	Anesthetics, Intravenous	21	kept
Angioplasty, Balloon, Coronary	angioplasty balloon coronary	angioplasty balloon coronary	angioplasty balloon coronary	Angioplasty, Balloon, Coronary	21	kept
ascorbic acid	ascorbic acid	ascorbic acid	ascorbic acid	ascorbic acid	21	kept
Autonomy	autonomy	autonomy	autonomy	Autonomy	21	kept
Bangladesh	bangladesh	bangladesh	bangladesh	Bangladesh	21	kept
Bedding and Linens	bedding and linens	bedding and linens	bedding and linens	Bedding and Linens	21	kept
bibliometrics	bibliometrics	bibliometrics	bibliometric	Bibliometrics	21	singular
Blood Coagulation	blood coagulation	blood coagulation	blood coagulation	Blood Coagulation	21	kept
Blood Component Transfusion	blood component transfusion	blood component transfusion	blood component transfusion	Blood Component Transfusion	21	kept
Blood Vessel Prosthesis Implantation	blood vessel prosthesis implantation	blood vessel prosthesis implantation	blood vessel prosthesis implantation	Blood Vessel Prosthesis Implantation	21	kept

Bronchodilator Agents	bronchodilator agents	bronchodilator agents	bronchodilator agents	Bronchodilator Agents	21	kept
capnometry	capnometry	capnometry	capnometry	capnometry	21	kept
carbamazepine	carbamazepine	carbamazepine	carbamazepine	carbamazepine	21	kept
Cardiac Catheterization	cardiac catheterization	cardiac catheterization	cardiac catheterization	Cardiac Catheterization	21	kept
case fatality rate	case fatality rate	case fatality rate	case fatality rate	case fatality rate	21	kept
Central venous catheters	central venous catheters	central venous catheters	central venous catheter	central venous catheter	21	singular
cholinergic receptor blocking agent	cholinergic receptor blocking agent	cholinergic receptor blocking agent	cholinergic receptor blocking agent	cholinergic receptor blocking agent	21	kept
chronic liver disease	chronic liver disease	chronic liver disease	chronic liver disease	chronic liver disease	21	kept
Clinical research	clinical research	clinical research	clinical research	clinical research	21	case_or_format
Community Health Services	community health services	community health services	community health services	Community Health Services	21	kept
Confidence	confidence	confidence	confidence	Confidence	21	kept
contact examination	contact examination	contact examination	contact examination	contact examination	21	kept
CPR	cpr	cpr	cpr	CPR	21	kept
Crowding	crowding	crowding	crowding	crowding	21	case_or_format
cytokine	cytokine	cytokine	cytokine	Cytokines	21	case_or_format
deep learning	deep learning	deep learning	deep learning	deep learning	21	kept

delivery	delivery	delivery	delivery	delivery	21	kept
Deterioration	deterioration	deterioration	deterioration	deterioration	21	case_or_format
Disseminated Intravascular Coagulation	disseminated intravascular coagulation	disseminated intravascular coagulation	disseminated intravascular coagulation	Disseminated Intravascular Coagulation	21	kept
dog	dog	dog	dog	Dogs	21	case_or_format
Drug Compounding	drug compounding	drug compounding	drug compounding	Drug Compounding	21	kept
East Asian People	east asian people	east asian people	east asian people	East Asian People	21	kept
Enteral feeding	enteral feeding	enteral feeding	enteral feeding	Enteral feeding	21	kept
Enterobacter	enterobacter	enterobacter	enterobacter	Enterobacter	21	kept
environmental exposure	environmental exposure	environmental exposure	environmental exposure	Environmental Exposure	21	case_or_format
EVENTS	events	events	event	EVENTS	21	singular
Family presence	family presence	family presence	family presence	Family presence	21	kept
fibrinogen	fibrinogen	fibrinogen	fibrinogen	fibrinogen	21	kept
fluoroscopy	fluoroscopy	fluoroscopy	fluoroscopy	fluoroscopy	21	kept
forced expiratory volume	forced expiratory volume	forced expiratory volume	forced expiratory volume	forced expiratory volume	21	kept
genetic screening	genetic screening	genetic screening	genetic screening	genetic screening	21	kept
geography	geography	geography	geography	geography	21	kept

gynecology	gynecology	gynecology	gynecology	gynecology	21	kept
Haloperidol	haloperidol	haloperidol	haloperidol	haloperidol	21	case_or_format
Hand Dermatoses	hand dermatoses	hand dermatoses	hand dermatoses	Hand Dermatoses	21	kept
head circumference	head circumference	head circumference	head circumference	head circumference	21	kept
health belief	health belief	health belief	health belief	health belief	21	kept
heart rehabilitation	heart rehabilitation	heart rehabilitation	heart rehabilitation	heart rehabilitation	21	kept
Hemodynamic Processes	hemodynamic processes	hemodynamic processes	hemodynamic processes	Hemodynamic Processes	21	kept
hepatitis C	hepatitis c	hepatitis c	hepatitis c	hepatitis C	21	kept
histamine H2 receptor antagonist	histamine h2 receptor antagonist	histamine h2 receptor antagonist	histamine h2 receptor antagonist	histamine H2 receptor antagonist	21	kept
histology	histology	histology	histology	histology	21	kept
hope	hope	hope	hope	Hope	21	case_or_format
Hospital Charges	hospital charges	hospital charges	hospital charge	Hospital Charges	21	singular
Hydrocephalus	hydrocephalus	hydrocephalus	hydrocephalus	hydrocephalus	21	case_or_format
hypernatremia	hypernatremia	hypernatremia	hypernatremia	hypernatremia	21	kept
Infant, newborn	infant newborn	infant newborn	infant newborn	Infant, Newborn	21	case_or_format
INFORMATION	information	information	information	Information	21	case_or_format

information	information	information	information	Information	21	case_or_format
Intracranial Aneurysm	intracranial aneurysm	intracranial aneurysm	intracranial aneurysm	Intracranial Aneurysm	21	kept
intracranial pressure monitoring	intracranial pressure monitoring	intracranial pressure monitoring	intracranial pressure monitoring	intracranial pressure monitoring	21	kept
Intraoperative Complications	intraoperative complications	intraoperative complications	intraoperative complications	Intraoperative Complications	21	kept
ischemia	ischemia	ischemia	ischemia	Ischemia	21	case_or_format
jaundice	jaundice	jaundice	jaundice	jaundice	21	kept
laboratory	laboratory	laboratory	laboratory	laboratory	21	kept
light	light	light	light	Light	21	case_or_format
lowest income group	lowest income group	lowest income group	lowest income group	lowest income group	21	kept
mental capacity	mental capacity	mental capacity	mental capacity	mental capacity	21	kept
nephrologist	nephrologist	nephrologist	nephrologist	nephrologist	21	kept
New Jersey	new jersey	new jersey	new jersey	New Jersey	21	kept
older people	older people	older people	older people	older people	21	kept
open study	open study	open study	open study	open study	21	kept
PAIN	pain	pain	pain	Pain	21	case_or_format
Pastoral Care	pastoral care	pastoral care	pastoral care	Pastoral Care	21	kept

Perceptions	perceptions	perceptions	perception	perception	21	singular
Perioperative care	perioperative care	perioperative care	perioperative care	Perioperative Care	21	case_or_format
persistent vegetative state	persistent vegetative state	persistent vegetative state	persistent vegetative state	persistent vegetative state	21	kept
post-traumatic stress disorder	post traumatic stress disorder	post traumatic stress disorder	post traumatic stress disorder	Post-traumatic stress disorder	21	case_or_format
postanesthesia care unit	postanesthesia care unit	postanesthesia care unit	postanesthesia care unit	postanesthesia care unit	21	kept
Postoperative complications	postoperative complications	postoperative complications	postoperative complication	Postoperative Complications	21	singular
prediction and forecasting	prediction and forecasting	prediction and forecasting	prediction and forecasting	prediction and forecasting	21	kept
Pregnancy Complications, Cardiovascular	pregnancy complications cardiovascular	pregnancy complications cardiovascular	pregnancy complications cardiovascular	Pregnancy Complications, Cardiovascular	21	kept
preventive medicine	preventive medicine	preventive medicine	preventive medicine	preventive medicine	21	kept
principal component analysis	principal component analysis	principal component analysis	principal component analysis	principal component analysis	21	kept
psychological	psychological	psychological	psychological	Psychological	21	case_or_format
Publishing	publishing	publishing	publishing	Publishing	21	kept
Rapid response teams	rapid response teams	rapid response teams	rapid response team	rapid response team	21	singular
Readmission	readmission	readmission	readmission	Readmission	21	kept
Recovery	recovery	recovery	recovery	Recovery	21	kept
Reference Standards	reference standards	reference standards	reference standard	Reference Standards	21	singular

rehabilitation medicine	rehabilitation medicine	rehabilitation medicine	rehabilitation medicine	rehabilitation medicine	21	kept
respiratory acidosis	respiratory acidosis	respiratory acidosis	respiratory acidosis	respiratory acidosis	21	kept
respiratory system	respiratory system	respiratory system	respiratory system	respiratory system	21	kept
rhinorrhea	rhinorrhea	rhinorrhea	rhinorrhea	rhinorrhea	21	kept
Rotation	rotation	rotation	rotation	Rotation	21	kept
septicemia	septicemia	septicemia	septicemia	septicemia	21	kept
Shock, Hemorrhagic	shock hemorrhagic	shock hemorrhagic	shock hemorrhagic	Shock, Hemorrhagic	21	kept
skin defect	skin defect	skin defect	skin defect	skin defect	21	kept
sleep waking cycle	sleep waking cycle	sleep waking cycle	sleep waking cycle	sleep waking cycle	21	kept
Southwestern United States	southwestern united states	southwestern united states	southwestern united states	Southwestern United States	21	kept
Sri Lanka	sri lanka	sri lanka	sri lanka	Sri Lanka	21	kept
surveys and questionnaires	surveys and questionnaires	surveys and questionnaires	surveys and questionnaires	Surveys and Questionnaires	21	case_or_format
Tachycardia	tachycardia	tachycardia	tachycardia	tachycardia	21	case_or_format
tactile stimulation	tactile stimulation	tactile stimulation	tactile stimulation	tactile stimulation	21	kept
temperature measurement	temperature measurement	temperature measurement	temperature measurement	temperature measurement	21	kept
therapy delay	therapy delay	therapy delay	therapy delay	therapy delay	21	kept

thorax	thorax	thorax	thorax	thorax	21	kept
trachea	trachea	trachea	trachea	Trachea	21	case_or_format
troponin	troponin	troponin	troponin	troponin	21	kept
Turnover intention	turnover intention	turnover intention	turnover intention	Turnover intention	21	kept
Twins	twins	twins	twin	Twins	21	singular
Urban Population	urban population	urban population	urban population	Urban Population	21	kept
Urinary Bladder	urinary bladder	urinary bladder	urinary bladder	Urinary Bladder	21	kept
Usability	usability	usability	usability	usability	21	case_or_format
Utah	utah	utah	utah	Utah	21	kept
Validation study	validation study	validation study	validation study	validation study	21	case_or_format
vitamin D	vitamin d	vitamin d	vitamin d	vitamin D	21	kept
wound dressing	wound dressing	wound dressing	wound dressing	wound dressing	21	kept
adoption	adoption	adoption	adoption	adoption	20	kept
Advance care planning	advance care planning	advance care planning	advance care planning	advance care planning	20	case_or_format
Afghanistan	afghanistan	afghanistan	afghanistan	Afghanistan	20	kept
alcohol abuse	alcohol abuse	alcohol abuse	alcohol abuse	alcohol abuse	20	kept

American Heart Association	american heart association	american heart association	american heart association	American Heart Association	20	kept
Analgesia, Epidural	analgesia epidural	analgesia epidural	analgesia epidural	Analgesia, Epidural	20	kept
anorexia	anorexia	anorexia	anorexia	anorexia	20	kept
apixaban	apixaban	apixaban	apixaban	apixaban	20	kept
arterial oxygen saturation	arterial oxygen saturation	arterial oxygen saturation	arterial oxygen saturation	arterial oxygen saturation	20	kept
Arterial Pressure	arterial pressure	arterial pressure	arterial pressure	arterial pressure	20	case_or_format
Bacterial Proteins	bacterial proteins	bacterial proteins	bacterial protein	Bacterial Proteins	20	singular
bacterium	bacterium	bacterium	bacterium	bacterium	20	kept
Bayes Theorem	bayes theorem	bayes theorem	bayes theorem	Bayes Theorem	20	kept
breast feeding education	breast feeding education	breast feeding education	breast feeding education	breast feeding education	20	kept
bupivacaine	bupivacaine	bupivacaine	bupivacaine	bupivacaine	20	kept
Cardiac Tamponade	cardiac tamponade	cardiac tamponade	cardiac tamponade	Cardiac Tamponade	20	kept
Cardiovascular Agents	cardiovascular agents	cardiovascular agents	cardiovascular agent	cardiovascular agent	20	singular
catheter complication	catheter complication	catheter complication	catheter complication	catheter complication	20	kept
Catheter-related infections	catheter related infections	catheter related infections	catheter related infection	Catheter-Related Infections	20	singular
Cerebral Infarction	cerebral infarction	cerebral infarction	cerebral infarction	Cerebral Infarction	20	kept

Chaplaincy Service, Hospital	chaplaincy service hospital	chaplaincy service hospital	chaplaincy service hospital	Chaplaincy Service, Hospital	20	kept
Chronic obstructive pulmonary disease	chronic obstructive pulmonary disease	chronic obstructive pulmonary disease	chronic obstructive pulmonary disease	Chronic obstructive pulmonary disease	20	kept
cisatracurium	cisatracurium	cisatracurium	cisatracurium	cisatracurium	20	kept
clarithromycin	clarithromycin	clarithromycin	clarithromycin	clarithromycin	20	kept
climate	climate	climate	climate	climate	20	kept
coinfection	coinfection	coinfection	coinfection	coinfection	20	kept
colorectal cancer	colorectal cancer	colorectal cancer	colorectal cancer	colorectal cancer	20	kept
computerized provider order entry	computerized provider order entry	computerized provider order entry	computerized provider order entry	computerized provider order entry	20	kept
conjunctivitis	conjunctivitis	conjunctivitis	conjunctivitis	conjunctivitis	20	kept
Coronavirus disease 2019	coronavirus disease 2019	covid 19	covid 19	COVID-19	20	alias
Cost-effectiveness	cost effectiveness	cost effectiveness	cost effectiveness	Cost-effectiveness	20	kept
Costs	costs	costs	cost	cost	20	singular
covid-19	covid 19	covid 19	covid 19	COVID-19	20	case_or_format
Croatia	croatia	croatia	croatia	Croatia	20	kept
diabetes	diabetes	diabetes	diabetes	diabetes	20	kept
Diagnostic and Statistical Manual of Mental Disorders	diagnostic and statistical manual of mental disorders	diagnostic and statistical manual of mental disorders	diagnostic and statistical manual of mental disorder	Diagnostic and Statistical Manual of Mental Disorders	20	singular

Diary	diary	diary	diary	Diary	20	kept
Dietary Proteins	dietary proteins	dietary proteins	dietary proteins	Dietary Proteins	20	kept
Disinfectants	disinfectants	disinfectants	disinfectant	Disinfectants	20	singular
Dying	dying	dying	dying	dying	20	case_or_format
Ear Protective Devices	ear protective devices	ear protective devices	ear protective device	Ear Protective Devices	20	singular
Endotracheal tube	endotracheal tube	endotracheal tube	endotracheal tube	endotracheal tube	20	case_or_format
Endovascular Procedures	endovascular procedures	endovascular procedures	endovascular procedures	Endovascular Procedures	20	kept
English	english	english	english	English	20	kept
Escherichia coli infection	escherichia coli infection	escherichia coli infection	escherichia coli infection	Escherichia coli infection	20	kept
etomidate	etomidate	etomidate	etomidate	etomidate	20	kept
face-to-face interview	face to face interview	face to face interview	face to face interview	face-to-face interview	20	kept
family medicine	family medicine	family medicine	family medicine	family medicine	20	kept
Fasciitis, Necrotizing	fasciitis necrotizing	fasciitis necrotizing	fasciitis necrotizing	Fasciitis, Necrotizing	20	kept
friend	friend	friend	friend	friend	20	kept
gastroenterology	gastroenterology	gastroenterology	gastroenterology	gastroenterology	20	kept
Generalized Anxiety Disorder-7	generalized anxiety disorder 7	generalized anxiety disorder 7	generalized anxiety disorder 7	Generalized Anxiety Disorder-7	20	kept

Gloves, Protective	gloves protective	gloves protective	gloves protective	Gloves, Protective	20	kept
good clinical practice	good clinical practice	good clinical practice	good clinical practice	good clinical practice	20	kept
Haemophilus influenzae	haemophilus influenzae	haemophilus influenzae	haemophilus influenzae	Haemophilus influenzae	20	kept
Health Care and Public Health	health care and public health	health care and public health	health care and public health	Health Care and Public Health	20	kept
health hazard	health hazard	health hazard	health hazard	health hazard	20	kept
Healthcare-associated infection	healthcare associated infection	healthcare associated infection	healthcare associated infection	healthcare associated infection	20	case_or_format
Hemoglobins	hemoglobins	hemoglobins	hemoglobin	hemoglobin	20	singular
Hepatitis B	hepatitis b	hepatitis b	hepatitis b	hepatitis B	20	case_or_format
high-fidelity simulation	high fidelity simulation	high fidelity simulation	high fidelity simulation	high-fidelity simulation	20	kept
Home Care Services, Hospital-Based	home care services hospital based	home care services hospital based	home care services hospital based	Home Care Services, Hospital-Based	20	kept
Hot Temperature	hot temperature	hot temperature	hot temperature	Hot Temperature	20	kept
Hydrogen-Ion Concentration	hydrogen ion concentration	hydrogen ion concentration	hydrogen ion concentration	Hydrogen-Ion Concentration	20	kept
hyperbilirubinemia	hyperbilirubinemia	hyperbilirubinemia	hyperbilirubinemia	hyperbilirubinemia	20	kept
immunoglobulin M	immunoglobulin m	immunoglobulin m	immunoglobulin m	immunoglobulin M	20	kept
INFECTION	infection	infection	infection	infection	20	case_or_format
Information Storage and Retrieval	information storage and retrieval	information storage and retrieval	information storage and retrieval	Information Storage and Retrieval	20	kept

intestine obstruction	intestine obstruction	intestine obstruction	intestine obstruction	intestine obstruction	20	kept
Ischemic Stroke	ischemic stroke	ischemic stroke	ischemic stroke	ischemic stroke	20	case_or_format
kidney dysfunction	kidney dysfunction	kidney dysfunction	kidney dysfunction	kidney dysfunction	20	kept
Klebsiella	klebsiella	klebsiella	klebsiella	Klebsiella	20	kept
Kruskal Wallis test	kruskal wallis test	kruskal wallis test	kruskal wallis test	Kruskal Wallis test	20	kept
Labor, Induced	labor induced	labor induced	labor induced	Labor, Induced	20	kept
learning environment	learning environment	learning environment	learning environment	learning environment	20	kept
Life Style	life style	life style	life style	Life Style	20	kept
liver function test	liver function test	liver function test	liver function test	liver function test	20	kept
maternal age	maternal age	maternal age	maternal age	maternal age	20	kept
Medication safety	medication safety	medication safety	medication safety	medication safety	20	case_or_format
MEMBERS	members	members	member	MEMBERS	20	singular
MENTAL-HEALTH	mental health	mental health	mental health	mental health	20	case_or_format
metformin	metformin	metformin	metformin	metformin	20	kept
MORAL DISTRESS	moral distress	moral distress	moral distress	moral distress	20	case_or_format
Movement	movement	movement	movement	Movement	20	kept

Multi-Institutional Systems	multi institutional systems	multi institutional systems	multi institutional systems	Multi-Institutional Systems	20	kept
Neonatal Sepsis	neonatal sepsis	neonatal sepsis	neonatal sepsis	Neonatal Sepsis	20	kept
nephrotoxicity	nephrotoxicity	nephrotoxicity	nephrotoxicity	nephrotoxicity	20	kept
object relation	object relation	object relation	object relation	object relation	20	kept
Obstetrics and Gynecology Department, Hospital	obstetrics and gynecology department hospital	obstetrics and gynecology department hospital	obstetrics and gynecology department hospital	Obstetrics and Gynecology Department, Hospital	20	kept
oliguria	oliguria	oliguria	oliguria	oliguria	20	kept
Oral health	oral health	oral health	oral health	Oral Health	20	case_or_format
osteomyelitis	osteomyelitis	osteomyelitis	osteomyelitis	osteomyelitis	20	kept
Outcome assessment	outcome assessment	outcome assessment	outcome assessment	outcome assessment	20	case_or_format
Pacemaker, Artificial	pacemaker artificial	pacemaker artificial	pacemaker artificial	Pacemaker, Artificial	20	kept
pandemics	pandemics	pandemics	pandemic	Pandemics	20	singular
paralysis	paralysis	paralysis	paralysis	paralysis	20	kept
parenting	parenting	parenting	parenting	Parenting	20	case_or_format
Persistent Vegetative State	persistent vegetative state	persistent vegetative state	persistent vegetative state	persistent vegetative state	20	case_or_format
Personnel Loyalty	personnel loyalty	personnel loyalty	personnel loyalty	Personnel Loyalty	20	kept
phenotype	phenotype	phenotype	phenotype	phenotype	20	kept

Philippines	philippines	philippines	philippines	Philippines	20	kept
physical performance	physical performance	physical performance	physical performance	physical performance	20	kept
politics	politics	politics	politics	politics	20	kept
Poverty	poverty	poverty	poverty	poverty	20	case_or_format
Premature birth	premature birth	premature birth	premature birth	Premature Birth	20	case_or_format
PREMATURE-INFANTS	premature infants	premature infants	premature infant	Premature infant	20	singular
process optimization	process optimization	process optimization	process optimization	process optimization	20	kept
Puerperal Disorders	puerperal disorders	puerperal disorders	puerperal disorder	Puerperal Disorders	20	singular
pulsed field gel electrophoresis	pulsed field gel electrophoresis	pulsed field gel electrophoresis	pulsed field gel electrophoresis	pulsed field gel electrophoresis	20	kept
quality in health care	quality in health care	quality in health care	quality in health care	quality in health care	20	kept
Racial Groups	racial groups	racial groups	racial groups	Racial Groups	20	kept
radiation exposure	radiation exposure	radiation exposure	radiation exposure	radiation exposure	20	kept
recovery	recovery	recovery	recovery	Recovery	20	case_or_format
regional anesthesia	regional anesthesia	regional anesthesia	regional anesthesia	regional anesthesia	20	kept
research ethics	research ethics	research ethics	research ethics	research ethics	20	kept
rituximab	rituximab	rituximab	rituximab	rituximab	20	kept

rural health care	rural health care	rural health care	rural health care	rural health care	20	kept
réanimation	r animation	r animation	r animation	réanimation	20	kept
Safety management	safety management	safety management	safety management	Safety Management	20	case_or_format
sarcopenia	sarcopenia	sarcopenia	sarcopenia	Sarcopenia	20	case_or_format
secondary care center	secondary care center	secondary care center	secondary care center	secondary care center	20	kept
Serratia Infections	serratia infections	serratia infections	serratia infection	Serratia Infections	20	singular
sex factor	sex factor	sex factor	sex factor	Sex Factors	20	case_or_format
Social Responsibility	social responsibility	social responsibility	social responsibility	Social Responsibility	20	kept
Software Design	software design	software design	software design	Software Design	20	kept
Spinal Fractures	spinal fractures	spinal fractures	spinal fracture	Spinal Fractures	20	singular
Spiritual Therapies	spiritual therapies	spiritual therapies	spiritual therapies	Spiritual Therapies	20	kept
sputum	sputum	sputum	sputum	sputum	20	kept
Stenotrophomonas maltophilia	stenotrophomonas maltophilia	stenotrophomonas maltophilia	stenotrophomonas maltophilia	Stenotrophomonas maltophilia	20	kept
structural equation modeling	structural equation modeling	structural equation modeling	structural equation modeling	structural equation modeling	20	kept
sweating	sweating	sweating	sweating	sweating	20	kept
Transplantation	transplantation	transplantation	transplantation	transplantation	20	case_or_format

veno-arterial ECMO	veno arterial ecmo	veno arterial ecmo	veno arterial ecmo	veno-arterial ECMO	20	kept
vitamin	vitamin	vitamin	vitamin	vitamin	20	kept
warming	warming	warming	warming	warming	20	kept
acetylcysteine	acetylcysteine	acetylcysteine	acetylcysteine	acetylcysteine	19	kept
Acute coronary syndrome	acute coronary syndrome	acute coronary syndrome	acute coronary syndrome	acute coronary syndrome	19	case_or_format
Adhesives	adhesives	adhesives	adhesive	Adhesives	19	singular
Aircraft	aircraft	aircraft	aircraft	Aircraft	19	kept
alteplase	alteplase	alteplase	alteplase	alteplase	19	kept
Antibiotic resistance	antibiotic resistance	antibiotic resistance	antibiotic resistance	antibiotic resistance	19	case_or_format
atracurium besilate	atracurium besilate	atracurium besilate	atracurium besilate	atracurium besilate	19	kept
audio recording	audio recording	audio recording	audio recording	audio recording	19	kept
Australia and New Zealand	australia and new zealand	australia and new zealand	australia and new zealand	Australia and New Zealand	19	kept
avoidance behavior	avoidance behavior	avoidance behavior	avoidance behavior	avoidance behavior	19	kept
awards and prizes	awards and prizes	awards and prizes	awards and prizes	Awards and Prizes	19	case_or_format
Bilirubin	bilirubin	bilirubin	bilirubin	bilirubin	19	case_or_format
breathing mechanics	breathing mechanics	breathing mechanics	breathing mechanics	breathing mechanics	19	kept

breathing muscle	breathing muscle	breathing muscle	breathing muscle	breathing muscle	19	kept
budget	budget	budget	budget	budget	19	kept
Burnout syndrome	burnout syndrome	burnout syndrome	burnout syndrome	burnout syndrome	19	case_or_format
calcium channel blocking agent	calcium channel blocking agent	calcium channel blocking agent	calcium channel blocking agent	calcium channel blocking agent	19	kept
Calibration	calibration	calibration	calibration	calibration	19	case_or_format
CANCER	cancer	cancer	cancer	cancer	19	case_or_format
cannula	cannula	cannula	cannula	Cannula	19	case_or_format
Cardiac output	cardiac output	cardiac output	cardiac output	Cardiac Output	19	case_or_format
CARDIAC-ARREST	cardiac arrest	cardiac arrest	cardiac arrest	Cardiac arrest	19	case_or_format
Caring	caring	caring	caring	caring	19	case_or_format
Clinical Audit	clinical audit	clinical audit	clinical audit	clinical audit	19	case_or_format
codeine	codeine	codeine	codeine	codeine	19	kept
colonoscopy	colonoscopy	colonoscopy	colonoscopy	colonoscopy	19	kept
compassion	compassion	compassion	compassion	compassion	19	kept
Comprehensive Health Care	comprehensive health care	comprehensive health care	comprehensive health care	Comprehensive Health Care	19	kept
creatinine clearance	creatinine clearance	creatinine clearance	creatinine clearance	creatinine clearance	19	kept

decontamination	decontamination	decontamination	decontamination	decontamination	19	kept
defibrillator	defibrillator	defibrillator	defibrillator	defibrillator	19	kept
desensitization	desensitization	desensitization	desensitization	desensitization	19	kept
Diabetes	diabetes	diabetes	diabetes	diabetes	19	case_or_format
Disclosure	disclosure	disclosure	disclosure	Disclosure	19	kept
District of Columbia	district of columbia	district of columbia	district of columbia	District of Columbia	19	kept
Down syndrome	down syndrome	down syndrome	down syndrome	Down syndrome	19	kept
drug mechanism	drug mechanism	drug mechanism	drug mechanism	drug mechanism	19	kept
Early mobilisation	early mobilisation	early mobilisation	early mobilisation	Early mobilisation	19	kept
Electromyography	electromyography	electromyography	electromyography	Electromyography	19	kept
Electronic medical record	electronic medical record	electronic medical record	electronic medical record	electronic medical record	19	case_or_format
end tidal carbon dioxide tension	end tidal carbon dioxide tension	end tidal carbon dioxide tension	end tidal carbon dioxide tension	end tidal carbon dioxide tension	19	kept
Endarterectomy, Carotid	endarterectomy carotid	endarterectomy carotid	endarterectomy carotid	Endarterectomy, Carotid	19	kept
Ethical climate	ethical climate	ethical climate	ethical climate	Ethical climate	19	kept
Facility Design and Construction	facility design and construction	facility design and construction	facility design and construction	Facility Design and Construction	19	kept
fathers	fathers	fathers	father	Fathers	19	singular

foreign body	foreign body	foreign body	foreign body	foreign body	19	kept
functional status assessment	functional status assessment	functional status assessment	functional status assessment	functional status assessment	19	kept
Gastroesophageal Reflux	gastroesophageal reflux	gastroesophageal reflux	gastroesophageal reflux	gastroesophageal reflux	19	case_or_format
general condition deterioration	general condition deterioration	general condition deterioration	general condition deterioration	general condition deterioration	19	kept
Genetic Testing	genetic testing	genetic testing	genetic testing	Genetic Testing	19	kept
glomerulus filtration rate	glomerulus filtration rate	glomerulus filtration rate	glomerulus filtration rate	glomerulus filtration rate	19	kept
Graft Rejection	graft rejection	graft rejection	graft rejection	Graft Rejection	19	kept
Health Behavior	health behavior	health behavior	health behavior	health behavior	19	case_or_format
Health professionals	health professionals	health professionals	health professional	Health professionals	19	singular
Health resources	health resources	health resources	health resources	Health Resources	19	case_or_format
health risk	health risk	health risk	health risk	health risk	19	kept
Healthcare-Associated Pneumonia	healthcare associated pneumonia	healthcare associated pneumonia	healthcare associated pneumonia	Healthcare-Associated Pneumonia	19	kept
heart atrium fibrillation	heart atrium fibrillation	heart atrium fibrillation	heart atrium fibrillation	heart atrium fibrillation	19	kept
heel	heel	heel	heel	Heel	19	case_or_format
Hemorrhagic Fever, Ebola	hemorrhagic fever ebola	hemorrhagic fever ebola	hemorrhagic fever ebola	Hemorrhagic Fever, Ebola	19	kept
hermeneutics	hermeneutics	hermeneutics	hermeneutic	Hermeneutics	19	singular

Hernia, Diaphragmatic	hernia diaphragmatic	hernia diaphragmatic	hernia diaphragmatic	Hernia, Diaphragmatic	19	kept
History, 19th Century	history 19th century	history 19th century	history 19th century	History, 19th Century	19	kept
home visit	home visit	home visit	home visit	home visit	19	kept
hospital charge	hospital charge	hospital charge	hospital charge	Hospital Charges	19	case_or_format
Hospital-acquired infections	hospital acquired infections	hospital acquired infections	hospital acquired infection	Hospital-acquired infections	19	singular
household income	household income	household income	household income	household income	19	kept
Hypercapnia	hypercapnia	hypercapnia	hypercapnia	hypercapnia	19	case_or_format
hyperoxia	hyperoxia	hyperoxia	hyperoxia	Hyperoxia	19	case_or_format
Iceland	iceland	iceland	iceland	Iceland	19	kept
Income	income	income	income	income	19	case_or_format
infection control practitioner	infection control practitioner	infection control practitioner	infection control practitioner	infection control practitioner	19	kept
Information systems	information systems	information systems	information system	information system	19	singular
institutionalization	institutionalization	institutionalization	institutionalization	institutionalization	19	kept
Intermediate care	intermediate care	intermediate care	intermediate care	Intermediate care	19	kept
Intervention Studies	intervention studies	intervention studies	intervention study	intervention study	19	singular
interventional ultrasonography	interventional ultrasonography	interventional ultrasonography	interventional ultrasonography	interventional ultrasonography	19	kept

interviews	interviews	interviews	interview	interview	19	singular
Kentucky	kentucky	kentucky	kentucky	Kentucky	19	kept
kidney injury	kidney injury	kidney injury	kidney injury	kidney injury	19	kept
LIFE	life	life	life	LIFE	19	kept
Literature review	literature review	literature review	literature review	literature review	19	case_or_format
Liver Failure, Acute	liver failure acute	liver failure acute	liver failure acute	Liver Failure, Acute	19	kept
Loneliness	loneliness	loneliness	loneliness	Loneliness	19	kept
low risk population	low risk population	low risk population	low risk population	low risk population	19	kept
lymphocytopenia	lymphocytopenia	lymphocytopenia	lymphocytopenia	lymphocytopenia	19	kept
mass communication	mass communication	mass communication	mass communication	mass communication	19	kept
MEDLINE	medline	medline	medline	Medline	19	case_or_format
Mental Recall	mental recall	mental recall	mental recall	Mental Recall	19	kept
minocycline	minocycline	minocycline	minocycline	minocycline	19	kept
Mobile Health Units	mobile health units	mobile health units	mobile health units	Mobile Health Units	19	kept
mood disorder	mood disorder	mood disorder	mood disorder	mood disorder	19	kept
morbid obesity	morbid obesity	morbid obesity	morbid obesity	morbid obesity	19	kept

morphine sulfate	morphine sulfate	morphine sulfate	morphine sulfate	morphine sulfate	19	kept
mouthwash	mouthwash	mouthwash	mouthwash	mouthwash	19	kept
Mouthwashes	mouthwashes	mouthwashes	mouthwash	mouthwash	19	singular
muscle atrophy	muscle atrophy	muscle atrophy	muscle atrophy	muscle atrophy	19	kept
Mycobacterium tuberculosis	mycobacterium tuberculosis	mycobacterium tuberculosis	mycobacterium tuberculosis	Mycobacterium tuberculosis	19	kept
mycosis	mycosis	mycosis	mycosis	mycosis	19	kept
myocarditis	myocarditis	myocarditis	myocarditis	myocarditis	19	kept
Neurophysiological Monitoring	neurophysiological monitoring	neurophysiological monitoring	neurophysiological monitoring	Neurophysiological Monitoring	19	kept
neurophysiology	neurophysiology	neurophysiology	neurophysiology	neurophysiology	19	kept
Neutropenia	neutropenia	neutropenia	neutropenia	neutropenia	19	case_or_format
newborns	newborns	newborns	newborn	newborn	19	singular
Nomograms	nomograms	nomograms	nomogram	nomogram	19	singular
non ST segment elevation myocardial infarction	non st segment elevation myocardial infarction	non st segment elevation myocardial infarction	non st segment elevation myocardial infarction	non ST segment elevation myocardial infarction	19	kept
Nutrition Disorders	nutrition disorders	nutrition disorders	nutrition disorders	Nutrition Disorders	19	kept
OLDER-ADULTS	older adults	older adults	older adult	Older adults	19	singular
open heart surgery	open heart surgery	open heart surgery	open heart surgery	open heart surgery	19	kept

oseltamivir	oseltamivir	oseltamivir	oseltamivir	oseltamivir	19	kept
outbreak	outbreak	outbreak	outbreak	Outbreak	19	case_or_format
outcome variable	outcome variable	outcome variable	outcome variable	outcome variable	19	kept
pediatric surgeon	pediatric surgeon	pediatric surgeon	pediatric surgeon	pediatric surgeon	19	kept
penetrating trauma	penetrating trauma	penetrating trauma	penetrating trauma	penetrating trauma	19	kept
peptic ulcer	peptic ulcer	peptic ulcer	peptic ulcer	peptic ulcer	19	kept
Percutaneous Coronary Intervention	percutaneous coronary intervention	percutaneous coronary intervention	percutaneous coronary intervention	percutaneous coronary intervention	19	case_or_format
Polymerase Chain Reaction	polymerase chain reaction	polymerase chain reaction	polymerase chain reaction	polymerase chain reaction	19	case_or_format
preterm birth	preterm birth	preterm birth	preterm birth	Preterm birth	19	case_or_format
program effectiveness	program effectiveness	program effectiveness	program effectiveness	program effectiveness	19	kept
prostacyclin	prostacyclin	prostacyclin	prostacyclin	prostacyclin	19	kept
protective clothing	protective clothing	protective clothing	protective clothing	Protective Clothing	19	case_or_format
Psychology, Child	psychology child	psychology child	psychology child	Psychology, Child	19	kept
Quality assurance	quality assurance	quality assurance	quality assurance	Quality assurance	19	kept
RATES	rates	rates	rate	RATES	19	singular
relaxation training	relaxation training	relaxation training	relaxation training	relaxation training	19	kept

Remifentanil	remifentanil	remifentanil	remifentanil	remifentanil	19	case_or_format
Retinopathy of prematurity	retinopathy of prematurity	retinopathy of prematurity	retinopathy of prematurity	Retinopathy of Prematurity	19	case_or_format
Ringer lactate solution	ringer lactate solution	ringer lactate solution	ringer lactate solution	Ringer lactate solution	19	kept
robot assisted surgery	robot assisted surgery	robot assisted surgery	robot assisted surgery	robot assisted surgery	19	kept
Rwanda	rwanda	rwanda	rwanda	Rwanda	19	kept
Saliva	saliva	saliva	saliva	Saliva	19	kept
Sample Size	sample size	sample size	sample size	sample size	19	case_or_format
secondary prevention	secondary prevention	secondary prevention	secondary prevention	secondary prevention	19	kept
Semantics	semantics	semantics	semantics	Semantics	19	kept
shivering	shivering	shivering	shivering	shivering	19	kept
single drug dose	single drug dose	single drug dose	single drug dose	single drug dose	19	kept
social care	social care	social care	social care	social care	19	kept
Social Media	social media	social media	social media	social media	19	case_or_format
soldier	soldier	soldier	soldier	soldier	19	kept
spirometry	spirometry	spirometry	spirometry	spirometry	19	kept
standing	standing	standing	standing	standing	19	kept

State Trait Anxiety Inventory	state trait anxiety inventory	state trait anxiety inventory	state trait anxiety inventory	State Trait Anxiety Inventory	19	kept
stigma	stigma	stigma	stigma	stigma	19	kept
Sudden Infant Death	sudden infant death	sudden infant death	sudden infant death	Sudden Infant Death	19	kept
Symptom Assessment	symptom assessment	symptom assessment	symptom assessment	symptom assessment	19	case_or_format
Therapeutic Irrigation	therapeutic irrigation	therapeutic irrigation	therapeutic irrigation	Therapeutic Irrigation	19	kept
Thermodilution	thermodilution	thermodilution	thermodilution	Thermodilution	19	kept
Third-Party Consent	third party consent	third party consent	third party consent	Third-Party Consent	19	kept
thrombocyte transfusion	thrombocyte transfusion	thrombocyte transfusion	thrombocyte transfusion	thrombocyte transfusion	19	kept
Transfusion Reaction	transfusion reaction	transfusion reaction	transfusion reaction	Transfusion Reaction	19	kept
transition	transition	transition	transition	Transition	19	case_or_format
Travel	travel	travel	travel	travel	19	case_or_format
vacuum assisted closure	vacuum assisted closure	vacuum assisted closure	vacuum assisted closure	vacuum assisted closure	19	kept
vein catheterization	vein catheterization	vein catheterization	vein catheterization	vein catheterization	19	kept
Very low birth weight	very low birth weight	very low birth weight	very low birth weight	very low birth weight	19	case_or_format
victim	victim	victim	victim	victim	19	kept
vignette	vignette	vignette	vignette	vignette	19	kept

virus RNA	virus rna	virus rna	virus rna	virus RNA	19	kept
visual impairment	visual impairment	visual impairment	visual impairment	visual impairment	19	kept
Voice	voice	voice	voice	voice	19	case_or_format
voriconazole	voriconazole	voriconazole	voriconazole	voriconazole	19	kept
vulnerability	vulnerability	vulnerability	vulnerability	vulnerability	19	kept
xerostomia	xerostomia	xerostomia	xerostomia	xerostomia	19	kept
ABCDEF bundle	abcdef bundle	abcdef bundle	abcdef bundle	ABCDEF bundle	18	kept
Acute Lung Injury	acute lung injury	acute lung injury	acute lung injury	acute lung injury	18	case_or_format
advanced cardiac life support	advanced cardiac life support	advanced cardiac life support	advanced cardiac life support	Advanced Cardiac Life Support	18	case_or_format
Adverse Drug Reaction Reporting Systems	adverse drug reaction reporting systems	adverse drug reaction reporting systems	adverse drug reaction reporting systems	Adverse Drug Reaction Reporting Systems	18	kept
albumin blood level	albumin blood level	albumin blood level	albumin blood level	albumin blood level	18	kept
Anaesthesia	anaesthesia	anaesthesia	anaesthesia	Anaesthesia	18	kept
anaesthesia	anaesthesia	anaesthesia	anaesthesia	Anaesthesia	18	case_or_format
Anesthetics, Inhalation	anesthetics inhalation	anesthetics inhalation	anesthetics inhalation	Anesthetics, Inhalation	18	kept
animal experiment	animal experiment	animal experiment	animal experiment	animal experiment	18	kept
Anoxia	anoxia	anoxia	anoxia	Anoxia	18	kept

anoxia	anoxia	anoxia	anoxia	Anoxia	18	case_or_format
antibiotics	antibiotics	antibiotics	antibiotic	Antibiotics	18	singular
Area Under Curve	area under curve	area under curve	area under curve	Area Under Curve	18	kept
artery catheterization	artery catheterization	artery catheterization	artery catheterization	artery catheterization	18	kept
artificial heart pacemaker	artificial heart pacemaker	artificial heart pacemaker	artificial heart pacemaker	artificial heart pacemaker	18	kept
artificial milk	artificial milk	artificial milk	artificial milk	artificial milk	18	kept
ascites	ascites	ascites	ascites	ascites	18	kept
asphyxia	asphyxia	asphyxia	asphyxia	asphyxia	18	kept
Beck Depression Inventory	beck depression inventory	beck depression inventory	beck depression inventory	Beck Depression Inventory	18	kept
Bioethics and Professional Ethics	bioethics and professional ethics	bioethics and professional ethics	bioethics and professional ethics	Bioethics and Professional Ethics	18	kept
blood flow velocity	blood flow velocity	blood flow velocity	blood flow velocity	blood flow velocity	18	kept
blood pressure regulation	blood pressure regulation	blood pressure regulation	blood pressure regulation	blood pressure regulation	18	kept
body fluid	body fluid	body fluid	body fluid	body fluid	18	kept
body movement	body movement	body movement	body movement	body movement	18	kept
Braden scale	braden scale	braden scale	braden scale	Braden Scale	18	case_or_format
brain circulation	brain circulation	brain circulation	brain circulation	brain circulation	18	kept

brain function	brain function	brain function	brain function	brain function	18	kept
bullying	bullying	bullying	bullying	Bullying	18	case_or_format
cancer prognosis	cancer prognosis	cancer prognosis	cancer prognosis	cancer prognosis	18	kept
carbapenem-resistant Enterobacteriaceae	carbapenem resistant enterobacteriaceae	carbapenem resistant enterobacteriaceae	carbapenem resistant enterobacteriaceae	carbapenem-resistant Enterobacteriaceae	18	kept
cefoxitin	cefoxitin	cefoxitin	cefoxitin	cefoxitin	18	kept
cellulitis	cellulitis	cellulitis	cellulitis	cellulitis	18	kept
cerebrospinal fluid	cerebrospinal fluid	cerebrospinal fluid	cerebrospinal fluid	cerebrospinal fluid	18	kept
Cerebrovascular Accident	cerebrovascular accident	cerebrovascular accident	cerebrovascular accident	cerebrovascular accident	18	case_or_format
challenges	challenges	challenges	challenge	CHALLENGES	18	singular
city	city	city	city	city	18	kept
clinical alarms	clinical alarms	clinical alarms	clinical alarm	Clinical Alarms	18	singular
Clinical decision support systems	clinical decision support systems	clinical decision support systems	clinical decision support system	clinical decision support system	18	singular
Clinical Frailty Scale	clinical frailty scale	clinical frailty scale	clinical frailty scale	Clinical Frailty Scale	18	kept
Clinical practice	clinical practice	clinical practice	clinical practice	clinical practice	18	case_or_format
Competency	competency	competency	competency	Competency	18	kept
computer	computer	computer	computer	Computers	18	case_or_format

Concept Formation	concept formation	concept formation	concept formation	Concept Formation	18	kept
constructive feedback	constructive feedback	constructive feedback	constructive feedback	constructive feedback	18	kept
coronary angiography	coronary angiography	coronary angiography	coronary angiography	coronary angiography	18	kept
Cough	cough	cough	cough	Cough	18	kept
COVID-19 Testing	covid 19 testing	covid 19 testing	covid 19 testing	COVID-19 testing	18	case_or_format
CPAP	cpap	cpap	cpap	CPAP	18	kept
Creatinine	creatinine	creatinine	creatinine	creatinine	18	case_or_format
Cross-sectional studies	cross sectional studies	cross sectional studies	cross sectional study	Cross-Sectional Studies	18	singular
Cryotherapy	cryotherapy	cryotherapy	cryotherapy	Cryotherapy	18	kept
cultural competence	cultural competence	cultural competence	cultural competence	cultural competence	18	kept
Cyprus	cyprus	cyprus	cyprus	Cyprus	18	kept
Dehydration	dehydration	dehydration	dehydration	dehydration	18	case_or_format
Delphi	delphi	delphi	delphi	Delphi	18	kept
delusion	delusion	delusion	delusion	delusion	18	kept
Dental Plaque	dental plaque	dental plaque	dental plaque	Dental Plaque	18	kept
Diaper Rash	diaper rash	diaper rash	diaper rash	Diaper Rash	18	kept

diphenhydramine	diphenhydramine	diphenhydramine	diphenhydramine	diphenhydramine	18	kept
diuresis	diuresis	diuresis	diuresis	diuresis	18	kept
drug blood level	drug blood level	drug blood level	drug blood level	drug blood level	18	kept
drug contraindication	drug contraindication	drug contraindication	drug contraindication	drug contraindication	18	kept
drug labeling	drug labeling	drug labeling	drug labeling	Drug Labeling	18	case_or_format
drug metabolism	drug metabolism	drug metabolism	drug metabolism	drug metabolism	18	kept
early ambulation	early ambulation	early ambulation	early ambulation	Early Ambulation	18	case_or_format
electronic prescribing	electronic prescribing	electronic prescribing	electronic prescribing	electronic prescribing	18	kept
EMERGENCY	emergency	emergency	emergency	Emergencies	18	case_or_format
Errors	errors	errors	error	error	18	singular
Evidence-based medicine	evidence based medicine	evidence based medicine	evidence based medicine	Evidence-Based Medicine	18	case_or_format
Feasibility	feasibility	feasibility	feasibility	Feasibility	18	kept
Fetal Monitoring	fetal monitoring	fetal monitoring	fetal monitoring	Fetal Monitoring	18	kept
fetus death	fetus death	fetus death	fetus death	fetus death	18	kept
Frustration	frustration	frustration	frustration	frustration	18	case_or_format
Gastric Emptying	gastric emptying	gastric emptying	gastric emptying	Gastric Emptying	18	kept

Georgia	georgia	georgia	georgia	Georgia	18	kept
Global health	global health	global health	global health	global health	18	case_or_format
Glycemic Control	glycemic control	glycemic control	glycemic control	glycemic control	18	case_or_format
Glycemic control	glycemic control	glycemic control	glycemic control	glycemic control	18	case_or_format
grip strength	grip strength	grip strength	grip strength	grip strength	18	kept
Haiti	haiti	haiti	haiti	Haiti	18	kept
Hallucinations	hallucinations	hallucinations	hallucination	hallucination	18	singular
Health care costs	health care costs	health care costs	health care cost	health care cost	18	singular
health care workers	health care workers	health care workers	health care worker	Health care workers	18	singular
Hemodiafiltration	hemodiafiltration	hemodiafiltration	hemodiafiltration	Hemodiafiltration	18	kept
hemoptysis	hemoptysis	hemoptysis	hemoptysis	hemoptysis	18	kept
hepatitis B vaccine	hepatitis b vaccine	hepatitis b vaccine	hepatitis b vaccine	hepatitis B vaccine	18	kept
Hospital data processing	hospital data processing	hospital data processing	hospital data processing	Hospital data processing	18	kept
hospital medicine	hospital medicine	hospital medicine	hospital medicine	hospital medicine	18	kept
hypertriglyceridemia	hypertriglyceridemia	hypertriglyceridemia	hypertriglyceridemia	hypertriglyceridemia	18	kept
Hypokalemia	hypokalemia	hypokalemia	hypokalemia	hypokalemia	18	case_or_format

immunotherapy	immunotherapy	immunotherapy	immunotherapy	immunotherapy	18	kept
independent variable	independent variable	independent variable	independent variable	independent variable	18	kept
infant nutrition	infant nutrition	infant nutrition	infant nutrition	infant nutrition	18	kept
Information management	information management	information management	information management	Information management	18	kept
infusion rate	infusion rate	infusion rate	infusion rate	infusion rate	18	kept
inhalational drug administration	inhalational drug administration	inhalational drug administration	inhalational drug administration	inhalational drug administration	18	kept
institutional care	institutional care	institutional care	institutional care	institutional care	18	kept
interprofessional	interprofessional	interprofessional	interprofessional	Interprofessional	18	case_or_format
Interprofessional relations	interprofessional relations	interprofessional relations	interprofessional relations	Interprofessional Relations	18	case_or_format
Intraoperative Care	intraoperative care	intraoperative care	intraoperative care	Intraoperative Care	18	kept
intravenous catheter	intravenous catheter	intravenous catheter	intravenous catheter	intravenous catheter	18	kept
Iraq War, 2003-2011	iraq war 2003 2011	iraq war 2003 2011	iraq war 2003 2011	Iraq War, 2003-2011	18	kept
Kidney	kidney	kidney	kidney	Kidney	18	kept
labetalol	labetalol	labetalol	labetalol	labetalol	18	kept
laboratory diagnosis	laboratory diagnosis	laboratory diagnosis	laboratory diagnosis	laboratory diagnosis	18	kept
laceration	laceration	laceration	laceration	laceration	18	kept

laparoscopic surgery	laparoscopic surgery	laparoscopic surgery	laparoscopic surgery	laparoscopic surgery	18	kept
leukopenia	leukopenia	leukopenia	leukopenia	leukopenia	18	kept
liver dysfunction	liver dysfunction	liver dysfunction	liver dysfunction	liver dysfunction	18	kept
Lorazepam	lorazepam	lorazepam	lorazepam	lorazepam	18	case_or_format
lung compliance	lung compliance	lung compliance	lung compliance	lung compliance	18	kept
maintenance therapy	maintenance therapy	maintenance therapy	maintenance therapy	maintenance therapy	18	kept
malaise	malaise	malaise	malaise	malaise	18	kept
Manitoba	manitoba	manitoba	manitoba	Manitoba	18	kept
maternal stress	maternal stress	maternal stress	maternal stress	maternal stress	18	kept
medical errors	medical errors	medical errors	medical error	Medical Errors	18	singular
medical order	medical order	medical order	medical order	medical order	18	kept
meta-synthesis	meta synthesis	meta synthesis	meta synthesis	meta-synthesis	18	kept
motor dysfunction	motor dysfunction	motor dysfunction	motor dysfunction	motor dysfunction	18	kept
muscle relaxation	muscle relaxation	muscle relaxation	muscle relaxation	muscle relaxation	18	kept
Muscle, Skeletal	muscle skeletal	muscle skeletal	muscle skeletal	Muscle, Skeletal	18	kept
narcotic agent	narcotic agent	narcotic agent	narcotic agent	narcotic agent	18	kept

neonatal resuscitation	neonatal resuscitation	neonatal resuscitation	neonatal resuscitation	neonatal resuscitation	18	kept
neurological complication	neurological complication	neurological complication	neurological complication	neurological complication	18	kept
newborn hypoxia	newborn hypoxia	newborn hypoxia	newborn hypoxia	newborn hypoxia	18	kept
newborn period	newborn period	newborn period	newborn period	newborn period	18	kept
Nitric Oxide	nitric oxide	nitric oxide	nitric oxide	nitric oxide	18	case_or_format
nosocomial infection	nosocomial infection	nosocomial infection	nosocomial infection	Nosocomial infection	18	case_or_format
Older people	older people	older people	older people	older people	18	case_or_format
ondansetron	ondansetron	ondansetron	ondansetron	ondansetron	18	kept
ophthalmology	ophthalmology	ophthalmology	ophthalmology	ophthalmology	18	kept
Oropharynx	oropharynx	oropharynx	oropharynx	Oropharynx	18	kept
otorhinolaryngology	otorhinolaryngology	otorhinolaryngology	otorhinolaryngology	otorhinolaryngology	18	kept
oxygenation index	oxygenation index	oxygenation index	oxygenation index	oxygenation index	18	kept
Parental Consent	parental consent	parental consent	parental consent	Parental Consent	18	kept
PARENTS	parents	parents	parent	Parents	18	singular
parity	parity	parity	parity	parity	18	kept
participant observation	participant observation	participant observation	participant observation	participant observation	18	kept

pharmacology	pharmacology	pharmacology	pharmacology	pharmacology	18	kept
Physiology	physiology	physiology	physiology	physiology	18	case_or_format
plasma exchange	plasma exchange	plasma exchange	plasma exchange	plasma exchange	18	kept
Pneumococcus vaccine	pneumococcus vaccine	pneumococcus vaccine	pneumococcus vaccine	Pneumococcus vaccine	18	kept
postoperative complications	postoperative complications	postoperative complications	postoperative complication	Postoperative Complications	18	singular
Posttraumatic Growth, Psychological	posttraumatic growth psychological	posttraumatic growth psychological	posttraumatic growth psychological	Posttraumatic Growth, Psychological	18	kept
practices	practices	practices	practice	Practice	18	singular
Precision Medicine	precision medicine	precision medicine	precision medicine	Precision Medicine	18	kept
prednisolone	prednisolone	prednisolone	prednisolone	prednisolone	18	kept
pressure injuries	pressure injuries	pressure injuries	pressure injury	pressure injury	18	singular
probiotic agent	probiotic agent	probiotic agent	probiotic agent	probiotic agent	18	kept
prostate cancer	prostate cancer	prostate cancer	prostate cancer	prostate cancer	18	kept
Protein C	protein c	protein c	protein c	Protein C	18	kept
Pseudomonas infection	pseudomonas infection	pseudomonas infection	pseudomonas infection	Pseudomonas Infections	18	case_or_format
Psychiatry	psychiatry	psychiatry	psychiatry	psychiatry	18	case_or_format
puncture	puncture	puncture	puncture	Punctures	18	case_or_format

range of motion	range of motion	range of motion	range of motion	range of motion	18	kept
rare disease	rare disease	rare disease	rare disease	Rare Diseases	18	case_or_format
registration	registration	registration	registration	registration	18	kept
remote sensing	remote sensing	remote sensing	remote sensing	remote sensing	18	kept
Respect	respect	respect	respect	respect	18	case_or_format
Respiratory insufficiency	respiratory insufficiency	respiratory insufficiency	respiratory insufficiency	Respiratory Insufficiency	18	case_or_format
Respiratory Sounds	respiratory sounds	respiratory sounds	respiratory sounds	Respiratory Sounds	18	kept
Rewarming	rewarming	rewarming	rewarming	Rewarming	18	kept
Sedation Agitation Scale	sedation agitation scale	sedation agitation scale	sedation agitation scale	Sedation Agitation Scale	18	kept
Self-care	self care	self care	self care	self care	18	case_or_format
short survey	short survey	short survey	short survey	Short Survey	18	case_or_format
sibling	sibling	sibling	sibling	sibling	18	kept
Silicones	silicones	silicones	silicone	Silicones	18	singular
Sleep deprivation	sleep deprivation	sleep deprivation	sleep deprivation	Sleep Deprivation	18	case_or_format
Staffing	staffing	staffing	staffing	staffing	18	case_or_format
Stevens Johnson syndrome	stevens johnson syndrome	stevens johnson syndrome	stevens johnson syndrome	Stevens-Johnson Syndrome	18	case_or_format

Stroke Rehabilitation	stroke rehabilitation	stroke rehabilitation	stroke rehabilitation	Stroke Rehabilitation	18	kept
substance use	substance use	substance use	substance use	substance use	18	kept
sudden death	sudden death	sudden death	sudden death	sudden death	18	kept
surgical risk	surgical risk	surgical risk	surgical risk	surgical risk	18	kept
synthesis	synthesis	synthesis	synthesis	synthesis	18	kept
telemonitoring	telemonitoring	telemonitoring	telemonitoring	telemonitoring	18	kept
Thematic analysis	thematic analysis	thematic analysis	thematic analysis	thematic analysis	18	case_or_format
thorax drainage	thorax drainage	thorax drainage	thorax drainage	thorax drainage	18	kept
throat culture	throat culture	throat culture	throat culture	throat culture	18	kept
Thrombocytopenia	thrombocytopenia	thrombocytopenia	thrombocytopenia	thrombocytopenia	18	case_or_format
toxic epidermal necrolysis	toxic epidermal necrolysis	toxic epidermal necrolysis	toxic epidermal necrolysis	toxic epidermal necrolysis	18	kept
traffic and transport	traffic and transport	traffic and transport	traffic and transport	traffic and transport	18	kept
translational research	translational research	translational research	translational research	translational research	18	kept
ulcer	ulcer	ulcer	ulcer	ulcer	18	kept
Unidad de Cuidados Intensivos	unidad de cuidados intensivos	unidad de cuidados intensivos	unidad de cuidados intensivos	Unidad de Cuidados Intensivos	18	kept
unnecessary procedure	unnecessary procedure	unnecessary procedure	unnecessary procedure	Unnecessary Procedures	18	case_or_format

Unplanned extubation	unplanned extubation	unplanned extubation	unplanned extubation	unplanned extubation	18	case_or_format
vein thrombosis	vein thrombosis	vein thrombosis	vein thrombosis	vein thrombosis	18	kept
Wellbeing	wellbeing	wellbeing	wellbeing	wellbeing	18	case_or_format
working conditions	working conditions	working conditions	working conditions	Working Conditions	18	case_or_format
abdominal injury	abdominal injury	abdominal injury	abdominal injury	Abdominal Injuries	17	case_or_format
acute myocardial infarction	acute myocardial infarction	acute myocardial infarction	acute myocardial infarction	acute myocardial infarction	17	kept
Acute pain	acute pain	acute pain	acute pain	Acute Pain	17	case_or_format
Adaptation	adaptation	adaptation	adaptation	adaptation	17	case_or_format
Adaptation, Physiological	adaptation physiological	adaptation physiological	adaptation physiological	Adaptation, Physiological	17	kept
Adverse event	adverse event	adverse event	adverse event	adverse event	17	case_or_format
Alcohol Withdrawal Delirium	alcohol withdrawal delirium	alcohol withdrawal delirium	alcohol withdrawal delirium	Alcohol Withdrawal Delirium	17	kept
Altruism	altruism	altruism	altruism	Altruism	17	kept
army	army	army	army	army	17	kept
art	art	art	art	art	17	kept
atorvastatin	atorvastatin	atorvastatin	atorvastatin	atorvastatin	17	kept
autoimmune disease	autoimmune disease	autoimmune disease	autoimmune disease	autoimmune disease	17	kept

bacterial endocarditis	bacterial endocarditis	bacterial endocarditis	bacterial endocarditis	bacterial endocarditis	17	kept
bacterial load	bacterial load	bacterial load	bacterial load	bacterial load	17	kept
battle injury	battle injury	battle injury	battle injury	battle injury	17	kept
behavior therapy	behavior therapy	behavior therapy	behavior therapy	Behavior Therapy	17	case_or_format
Behavioral Pain Scale	behavioral pain scale	behavioral pain scale	behavioral pain scale	Behavioral Pain Scale	17	kept
BIRTH-WEIGHT INFANTS	birth weight infants	birth weight infants	birth weight infant	BIRTH-WEIGHT INFANTS	17	singular
Blood-Borne Pathogens	blood borne pathogens	blood borne pathogens	blood borne pathogens	Blood-Borne Pathogens	17	kept
Bloodstream infection	bloodstream infection	bloodstream infection	bloodstream infection	bloodstream infection	17	case_or_format
cancer diagnosis	cancer diagnosis	cancer diagnosis	cancer diagnosis	cancer diagnosis	17	kept
CARDIOPULMONARY-RESUSCITATION	cardiopulmonary resuscitation	cardiopulmonary resuscitation	cardiopulmonary resuscitation	Cardiopulmonary Resuscitation	17	case_or_format
cardiotonic agent	cardiotonic agent	cardiotonic agent	cardiotonic agent	Cardiotonic Agents	17	case_or_format
Care bundle	care bundle	care bundle	care bundle	care bundle	17	case_or_format
catecholamine	catecholamine	catecholamine	catecholamine	catecholamine	17	kept
central nervous system	central nervous system	central nervous system	central nervous system	central nervous system	17	kept
Central Venous Pressure	central venous pressure	central venous pressure	central venous pressure	central venous pressure	17	case_or_format
Child Behavior	child behavior	child behavior	child behavior	child behavior	17	case_or_format

chorioamnionitis	chorioamnionitis	chorioamnionitis	chorioamnionitis	chorioamnionitis	17	kept
Chronic Pain	chronic pain	chronic pain	chronic pain	chronic pain	17	case_or_format
Citric Acid	citric acid	citric acid	citric acid	Citric Acid	17	kept
Clinical reasoning	clinical reasoning	clinical reasoning	clinical reasoning	clinical reasoning	17	case_or_format
clinical supervision	clinical supervision	clinical supervision	clinical supervision	clinical supervision	17	kept
colony forming unit	colony forming unit	colony forming unit	colony forming unit	colony forming unit	17	kept
colostomy	colostomy	colostomy	colostomy	colostomy	17	kept
competency	competency	competency	competency	Competency	17	case_or_format
Computer Systems	computer systems	computer systems	computer system	computer system	17	singular
confidence	confidence	confidence	confidence	Confidence	17	case_or_format
contrast medium	contrast medium	contrast medium	contrast medium	contrast medium	17	kept
COPD	copd	copd	copd	COPD	17	kept
COVID	covid	covid	covid	COVID	17	kept
creatine kinase	creatine kinase	creatine kinase	creatine kinase	creatine kinase	17	kept
Crisis Intervention	crisis intervention	crisis intervention	crisis intervention	Crisis Intervention	17	kept
CRITICALLY-ILL	critically ill	critically ill	critically ill	Critically ill	17	case_or_format

Cross-sectional study	cross sectional study	cross sectional study	cross sectional study	Cross-Sectional Studies	17	case_or_format
data collection method	data collection method	data collection method	data collection method	data collection method	17	kept
Defibrillators	defibrillators	defibrillators	defibrillator	defibrillator	17	singular
Defibrillators, Implantable	defibrillators implantable	defibrillators implantable	defibrillators implantable	Defibrillators, Implantable	17	kept
developmental disorder	developmental disorder	developmental disorder	developmental disorder	developmental disorder	17	kept
diet supplementation	diet supplementation	diet supplementation	diet supplementation	diet supplementation	17	kept
Dietary Supplements	dietary supplements	dietary supplements	dietary supplement	Dietary Supplements	17	singular
disaster medicine	disaster medicine	disaster medicine	disaster medicine	disaster medicine	17	kept
disease severity assessment	disease severity assessment	disease severity assessment	disease severity assessment	disease severity assessment	17	kept
Drug Overdose	drug overdose	drug overdose	drug overdose	drug overdose	17	case_or_format
Earthquakes	earthquakes	earthquakes	earthquake	Earthquakes	17	singular
educational technology	educational technology	educational technology	educational technology	educational technology	17	kept
Electronic Data Processing	electronic data processing	electronic data processing	electronic data processing	Electronic Data Processing	17	kept
engagement	engagement	engagement	engagement	engagement	17	kept
erythrocyte	erythrocyte	erythrocyte	erythrocyte	erythrocyte	17	kept
ethnic difference	ethnic difference	ethnic difference	ethnic difference	ethnic difference	17	kept

European Quality of Life 5 Dimensions questionnaire	european quality of life 5 dimensions questionnaire	european quality of life 5 dimensions questionnaire	european quality of life 5 dimensions questionnaire	European Quality of Life 5 Dimensions questionnaire	17	kept
Faculty, Medical	faculty medical	faculty medical	faculty medical	Faculty, Medical	17	kept
FAMILIES	families	families	family	Family	17	singular
femoral artery	femoral artery	femoral artery	femoral artery	femoral artery	17	kept
Fisher exact test	fisher exact test	fisher exact test	fisher exact test	Fisher exact test	17	kept
frontline staff	frontline staff	frontline staff	frontline staff	frontline staff	17	kept
gene mutation	gene mutation	gene mutation	gene mutation	gene mutation	17	kept
Genotype	genotype	genotype	genotype	genotype	17	case_or_format
Gram positive bacterium	gram positive bacterium	gram positive bacterium	gram positive bacterium	Gram positive bacterium	17	kept
Hand hygiene compliance	hand hygiene compliance	hand hygiene compliance	hand hygiene compliance	Hand hygiene compliance	17	kept
handoff	handoff	handoff	handoff	handoff	17	kept
Health Communication	health communication	health communication	health communication	Health Communication	17	kept
Health education	health education	health education	health education	health education	17	case_or_format
Health Literacy	health literacy	health literacy	health literacy	health literacy	17	case_or_format
Health risks	health risks	health risks	health risk	health risk	17	singular
healthy work environment	healthy work environment	healthy work environment	healthy work environment	healthy work environment	17	kept

hearing impairment	hearing impairment	hearing impairment	hearing impairment	hearing impairment	17	kept
Hearing Loss	hearing loss	hearing loss	hearing loss	Hearing Loss	17	kept
heart ejection fraction	heart ejection fraction	heart ejection fraction	heart ejection fraction	heart ejection fraction	17	kept
home quarantine	home quarantine	home quarantine	home quarantine	home quarantine	17	kept
Hospital Restructuring	hospital restructuring	hospital restructuring	hospital restructuring	Hospital Restructuring	17	kept
Hospitals, Psychiatric	hospitals psychiatric	hospitals psychiatric	hospitals psychiatric	Hospitals, Psychiatric	17	kept
Hungary	hungary	hungary	hungary	Hungary	17	kept
hydrogen peroxide	hydrogen peroxide	hydrogen peroxide	hydrogen peroxide	hydrogen peroxide	17	kept
Hypoxia, Brain	hypoxia brain	hypoxia brain	hypoxia brain	Hypoxia, Brain	17	kept
incontinence	incontinence	incontinence	incontinence	incontinence	17	kept
incubation time	incubation time	incubation time	incubation time	incubation time	17	kept
Indian	indian	indian	indian	Indian	17	kept
Infant Formula	infant formula	infant formula	infant formula	Infant Formula	17	kept
Information Services	information services	information services	information service	Information Services	17	singular
Information technology	information technology	information technology	information technology	information technology	17	case_or_format
injuries	injuries	injuries	injury	injury	17	singular

internal jugular vein	internal jugular vein	internal jugular vein	internal jugular vein	internal jugular vein	17	kept
Interventions	interventions	interventions	intervention	Intervention	17	singular
Interview	interview	interview	interview	interview	17	case_or_format
intrauterine growth retardation	intrauterine growth retardation	intrauterine growth retardation	intrauterine growth retardation	intrauterine growth retardation	17	kept
Inventions	inventions	inventions	invention	Inventions	17	singular
investment	investment	investment	investment	investment	17	kept
Kangaroo care	kangaroo care	kangaroo care	kangaroo care	kangaroo care	17	case_or_format
Laparotomy	laparotomy	laparotomy	laparotomy	laparotomy	17	case_or_format
Latent Class Analysis	latent class analysis	latent class analysis	latent class analysis	Latent Class Analysis	17	kept
leukemia	leukemia	leukemia	leukemia	leukemia	17	kept
Liver Failure	liver failure	liver failure	liver failure	liver failure	17	case_or_format
long-term care	long term care	long term care	long term care	long term care	17	case_or_format
major depression	major depression	major depression	major depression	major depression	17	kept
mannitol	mannitol	mannitol	mannitol	mannitol	17	kept
medical devices	medical devices	medical devices	medical device	medical device	17	singular
MEDICAL EMERGENCY TEAM	medical emergency team	medical emergency team	medical emergency team	Medical emergency team	17	case_or_format

Medical Laboratory Science	medical laboratory science	medical laboratory science	medical laboratory science	Medical Laboratory Science	17	kept
Minority Groups	minority groups	minority groups	minority group	Minority Groups	17	singular
mitral valve regurgitation	mitral valve regurgitation	mitral valve regurgitation	mitral valve regurgitation	mitral valve regurgitation	17	kept
Models, Biological	models biological	models biological	models biological	Models, Biological	17	kept
Modified Early Warning Score	modified early warning score	modified early warning score	modified early warning score	Modified Early Warning Score	17	kept
MOTHERS	mothers	mothers	mother	Mothers	17	singular
multimodality cancer therapy	multimodality cancer therapy	multimodality cancer therapy	multimodality cancer therapy	multimodality cancer therapy	17	kept
muscle relaxant agent	muscle relaxant agent	muscle relaxant agent	muscle relaxant agent	muscle relaxant agent	17	kept
muscle tone	muscle tone	muscle tone	muscle tone	muscle tone	17	kept
National Early Warning Score	national early warning score	national early warning score	national early warning score	National Early Warning Score	17	kept
neck pain	neck pain	neck pain	neck pain	neck pain	17	kept
Needs	needs	needs	need	NEEDS	17	singular
non invasive procedure	non invasive procedure	non invasive procedure	non invasive procedure	non invasive procedure	17	kept
Obstetric Labor, Premature	obstetric labor premature	obstetric labor premature	obstetric labor premature	Obstetric Labor, Premature	17	kept
pain severity	pain severity	pain severity	pain severity	pain severity	17	kept
PDSA	pdsa	pdsa	pdsa	PDSA	17	kept

pelvis fracture	pelvis fracture	pelvis fracture	pelvis fracture	pelvis fracture	17	kept
Perfusion	perfusion	perfusion	perfusion	perfusion	17	case_or_format
Philadelphia	philadelphia	philadelphia	philadelphia	Philadelphia	17	kept
Physical therapy	physical therapy	physical therapy	physical therapy	Physical therapy	17	kept
Policy Making	policy making	policy making	policy making	Policy Making	17	kept
Postoperative	postoperative	postoperative	postoperative	postoperative	17	case_or_format
Postoperative Nausea and Vomiting	postoperative nausea and vomiting	postoperative nausea and vomiting	postoperative nausea and vomiting	Postoperative Nausea and Vomiting	17	kept
postoperative nausea and vomiting	postoperative nausea and vomiting	postoperative nausea and vomiting	postoperative nausea and vomiting	Postoperative Nausea and Vomiting	17	case_or_format
Premedication	premedication	premedication	premedication	Premedication	17	kept
premedication	premedication	premedication	premedication	Premedication	17	case_or_format
Principle-Based Ethics	principle based ethics	principle based ethics	principle based ethics	Principle-Based Ethics	17	kept
Process Assessment	process assessment	process assessment	process assessment	Process Assessment	17	kept
professional autonomy	professional autonomy	professional autonomy	professional autonomy	Professional Autonomy	17	case_or_format
propranolol	propranolol	propranolol	propranolol	propranolol	17	kept
Proteus mirabilis	proteus mirabilis	proteus mirabilis	proteus mirabilis	Proteus mirabilis	17	kept
Psychomotor Performance	psychomotor performance	psychomotor performance	psychomotor performance	Psychomotor Performance	17	kept

pulmonary aspiration	pulmonary aspiration	pulmonary aspiration	pulmonary aspiration	pulmonary aspiration	17	kept
pupil reflex	pupil reflex	pupil reflex	pupil reflex	pupil reflex	17	kept
Qualitative content analysis	qualitative content analysis	qualitative content analysis	qualitative content analysis	Qualitative content analysis	17	kept
ranitidine	ranitidine	ranitidine	ranitidine	ranitidine	17	kept
Relaxation Therapy	relaxation therapy	relaxation therapy	relaxation therapy	Relaxation Therapy	17	kept
Residential Facilities	residential facilities	residential facilities	residential facilities	Residential Facilities	17	kept
RESIDENTS	residents	residents	resident	resident	17	singular
Respiratory distress syndrome	respiratory distress syndrome	respiratory distress syndrome	respiratory distress syndrome	Respiratory Distress Syndrome	17	case_or_format
robotics	robotics	robotics	robotics	Robotics	17	case_or_format
rotation	rotation	rotation	rotation	Rotation	17	case_or_format
SARS coronavirus	sars coronavirus	sars coronavirus	sars coronavirus	SARS coronavirus	17	kept
season	season	season	season	Seasons	17	case_or_format
Sentinel Surveillance	sentinel surveillance	sentinel surveillance	sentinel surveillance	Sentinel Surveillance	17	kept
seroepidemiology	seroepidemiology	seroepidemiology	seroepidemiology	seroepidemiology	17	kept
Serum Albumin	serum albumin	serum albumin	serum albumin	serum albumin	17	case_or_format
shift schedule	shift schedule	shift schedule	shift schedule	shift schedule	17	kept

six minute walk test	six minute walk test	six minute walk test	six minute walk test	six minute walk test	17	kept
Skin Temperature	skin temperature	skin temperature	skin temperature	Skin Temperature	17	kept
sociology	sociology	sociology	sociology	sociology	17	kept
South Carolina	south carolina	south carolina	south carolina	South Carolina	17	kept
Specialties, Surgical	specialties surgical	specialties surgical	specialties surgical	Specialties, Surgical	17	kept
Spectroscopy, Near-Infrared	spectroscopy near infrared	spectroscopy near infrared	spectroscopy near infrared	Spectroscopy, Near-Infrared	17	kept
spinal anesthesia	spinal anesthesia	spinal anesthesia	spinal anesthesia	spinal anesthesia	17	kept
Sternotomy	sternotomy	sternotomy	sternotomy	sternotomy	17	case_or_format
STUDENTS	students	students	student	Students	17	singular
terrorism	terrorism	terrorism	terrorism	terrorism	17	kept
thiopental	thiopental	thiopental	thiopental	thiopental	17	kept
thrombocyte count	thrombocyte count	thrombocyte count	thrombocyte count	thrombocyte count	17	kept
tooth plaque	tooth plaque	tooth plaque	tooth plaque	tooth plaque	17	kept
translating	translating	translating	translating	Translating	17	case_or_format
Transport	transport	transport	transport	Transport	17	kept
triacylglycerol	triacylglycerol	triacylglycerol	triacylglycerol	triacylglycerol	17	kept

Universities	universities	universities	university	university	17	singular
urine	urine	urine	urine	urine	17	kept
Vascular Diseases	vascular diseases	vascular diseases	vascular disease	Vascular Diseases	17	singular
virus antibody	virus antibody	virus antibody	virus antibody	virus antibody	17	kept
vision	vision	vision	vision	vision	17	kept
Wearable Electronic Devices	wearable electronic devices	wearable electronic devices	wearable electronic device	Wearable Electronic Devices	17	singular
Work Engagement	work engagement	work engagement	work engagement	work engagement	17	case_or_format
work-life balance	work life balance	work life balance	work life balance	work-life balance	17	kept
Working conditions	working conditions	working conditions	working conditions	Working Conditions	17	case_or_format
Accidents, Occupational	accidents occupational	accidents occupational	accidents occupational	Accidents, Occupational	16	kept
Acinetobacter infection	acinetobacter infection	acinetobacter infection	acinetobacter infection	Acinetobacter Infections	16	case_or_format
acquired immune deficiency syndrome	acquired immune deficiency syndrome	acquired immune deficiency syndrome	acquired immune deficiency syndrome	acquired immune deficiency syndrome	16	kept
adherence	adherence	adherence	adherence	Adherence	16	case_or_format
Adrenergic beta-Antagonists	adrenergic beta antagonists	adrenergic beta antagonists	adrenergic beta antagonists	Adrenergic beta-Antagonists	16	kept
advance directives	advance directives	advance directives	advance directive	Advance Directives	16	singular
advanced life support	advanced life support	advanced life support	advanced life support	advanced life support	16	kept

Afghan Campaign 2001-	afghan campaign 2001	afghan campaign 2001	afghan campaign 2001	Afghan Campaign 2001-	16	kept
ageusia	ageusia	ageusia	ageusia	ageusia	16	kept
air embolism	air embolism	air embolism	air embolism	air embolism	16	kept
aircraft	aircraft	aircraft	aircraft	Aircraft	16	case_or_format
Anesthesia, Obstetrical	anesthesia obstetrical	anesthesia obstetrical	anesthesia obstetrical	Anesthesia, Obstetrical	16	kept
angina pectoris	angina pectoris	angina pectoris	angina pectoris	angina pectoris	16	kept
Anthropometry	anthropometry	anthropometry	anthropometry	anthropometry	16	case_or_format
Antibiotic Prophylaxis	antibiotic prophylaxis	antibiotic prophylaxis	antibiotic prophylaxis	antibiotic prophylaxis	16	case_or_format
Antibodies, Viral	antibodies viral	antibodies viral	antibodies viral	Antibodies, Viral	16	kept
antiemetic agent	antiemetic agent	antiemetic agent	antiemetic agent	antiemetic agent	16	kept
Aortic Dissection	aortic dissection	aortic dissection	aortic dissection	Aortic Dissection	16	kept
apathy	apathy	apathy	apathy	apathy	16	kept
Arousal	arousal	arousal	arousal	arousal	16	case_or_format
Augmentative and alternative communication	augmentative and alternative communication	augmentative and alternative communication	augmentative and alternative communication	Augmentative and alternative communication	16	kept
automutilation	automutilation	automutilation	automutilation	automutilation	16	kept
Bacillus	bacillus	bacillus	bacillus	Bacillus	16	kept

beta lactam	beta lactam	beta lactam	beta lactam	beta lactam	16	kept
bipolar disorder	bipolar disorder	bipolar disorder	bipolar disorder	bipolar disorder	16	kept
Blood Culture	blood culture	blood culture	blood culture	blood culture	16	case_or_format
brain damage	brain damage	brain damage	brain damage	brain damage	16	kept
Brain injury	brain injury	brain injury	brain injury	Brain Injuries	16	case_or_format
bronchospasm	bronchospasm	bronchospasm	bronchospasm	bronchospasm	16	kept
Cardiac Care Facilities	cardiac care facilities	cardiac care facilities	cardiac care facilities	Cardiac Care Facilities	16	kept
Cardiogenic shock	cardiogenic shock	cardiogenic shock	cardiogenic shock	cardiogenic shock	16	case_or_format
cardiovascular system	cardiovascular system	cardiovascular system	cardiovascular system	cardiovascular system	16	kept
caspofungin	caspofungin	caspofungin	caspofungin	caspofungin	16	kept
catheter associated urinary tract infection	catheter associated urinary tract infection	catheter associated urinary tract infection	catheter associated urinary tract infection	catheter associated urinary tract infection	16	kept
catheter care	catheter care	catheter care	catheter care	catheter care	16	kept
central nervous system infection	central nervous system infection	central nervous system infection	central nervous system infection	central nervous system infection	16	kept
change management	change management	change management	change management	change management	16	kept
chest tube	chest tube	chest tube	chest tube	Chest Tubes	16	case_or_format
child growth	child growth	child growth	child growth	child growth	16	kept

childhood injury	childhood injury	childhood injury	childhood injury	childhood injury	16	kept
Christianity	christianity	christianity	christianity	Christianity	16	kept
chronic respiratory failure	chronic respiratory failure	chronic respiratory failure	chronic respiratory failure	chronic respiratory failure	16	kept
Civil Defense	civil defense	civil defense	civil defense	Civil Defense	16	kept
Clergy	clergy	clergy	clergy	clergy	16	case_or_format
clinical ethics	clinical ethics	clinical ethics	clinical ethics	clinical ethics	16	kept
communicable disease control	communicable disease control	communicable disease control	communicable disease control	Communicable Disease Control	16	case_or_format
Conference Paper	conference paper	conference paper	conference paper	Conference Paper	16	kept
Consumer Satisfaction	consumer satisfaction	consumer satisfaction	consumer satisfaction	Consumer Satisfaction	16	kept
Continuing education	continuing education	continuing education	continuing education	continuing education	16	case_or_format
continuous glucose monitoring	continuous glucose monitoring	continuous glucose monitoring	continuous glucose monitoring	continuous glucose monitoring	16	kept
control group	control group	control group	control group	control group	16	kept
Coping Skills	coping skills	coping skills	coping skills	Coping Skills	16	kept
coping strategies	coping strategies	coping strategies	coping strategy	Coping strategies	16	singular
core temperature	core temperature	core temperature	core temperature	core temperature	16	kept
crackle	crackle	crackle	crackle	crackle	16	kept

creativity	creativity	creativity	creativity	creativity	16	kept
Credentialing	credentialing	credentialing	credentialing	Credentialing	16	kept
death and dying	death and dying	death and dying	death and dying	death and dying	16	kept
Debriefing	debriefing	debriefing	debriefing	Debriefing	16	kept
Decontamination	decontamination	decontamination	decontamination	decontamination	16	case_or_format
dentist	dentist	dentist	dentist	dentist	16	kept
developmental care	developmental care	developmental care	developmental care	Developmental care	16	case_or_format
Drug Dosage Calculations	drug dosage calculations	drug dosage calculations	drug dosage calculations	Drug Dosage Calculations	16	kept
drug exposure	drug exposure	drug exposure	drug exposure	drug exposure	16	kept
drug information	drug information	drug information	drug information	drug information	16	kept
Edema	edema	edema	edema	edema	16	case_or_format
electric activity	electric activity	electric activity	electric activity	electric activity	16	kept
electrocardiography monitoring	electrocardiography monitoring	electrocardiography monitoring	electrocardiography monitoring	electrocardiography monitoring	16	kept
Elixhauser comorbidity index	elixhauser comorbidity index	elixhauser comorbidity index	elixhauser comorbidity index	Elixhauser comorbidity index	16	kept
emergency medical services	emergency medical services	emergency medical services	emergency medical service	Emergency Medical Services	16	singular
energy expenditure	energy expenditure	energy expenditure	energy expenditure	energy expenditure	16	kept

Enhanced Recovery After Surgery	enhanced recovery after surgery	enhanced recovery after surgery	enhanced recovery after surgery	enhanced recovery after surgery	16	case_or_format
Environmental Microbiology	environmental microbiology	environmental microbiology	environmental microbiology	Environmental Microbiology	16	kept
epidural anesthesia	epidural anesthesia	epidural anesthesia	epidural anesthesia	epidural anesthesia	16	kept
Equipment Failure Analysis	equipment failure analysis	equipment failure analysis	equipment failure analysis	Equipment Failure Analysis	16	kept
Equivalence Trials as Topic	equivalence trials as topic	equivalence trials as topic	equivalence trials as topic	Equivalence Trials as Topic	16	kept
erythrocyte concentrate	erythrocyte concentrate	erythrocyte concentrate	erythrocyte concentrate	erythrocyte concentrate	16	kept
Ethics Consultation	ethics consultation	ethics consultation	ethics consultation	Ethics Consultation	16	kept
experiential learning	experiential learning	experiential learning	experiential learning	experiential learning	16	kept
extracorporeal circulation	extracorporeal circulation	extracorporeal circulation	extracorporeal circulation	extracorporeal circulation	16	kept
Extubation	extubation	extubation	extubation	extubation	16	case_or_format
face injury	face injury	face injury	face injury	face injury	16	kept
Factor analysis	factor analysis	factor analysis	factor analysis	factor analysis	16	case_or_format
failure to rescue	failure to rescue	failure to rescue	failure to rescue	failure to rescue	16	kept
Fallot tetralogy	fallot tetralogy	fallot tetralogy	fallot tetralogy	Fallot tetralogy	16	kept
False Positive Reactions	false positive reactions	false positive reactions	false positive reactions	False Positive Reactions	16	kept
family satisfaction	family satisfaction	family satisfaction	family satisfaction	family satisfaction	16	kept

fetus echography	fetus echography	fetus echography	fetus echography	fetus echography	16	kept
flumazenil	flumazenil	flumazenil	flumazenil	flumazenil	16	kept
Follow-up	follow up	follow up	follow up	follow up	16	case_or_format
Fomites	fomites	fomites	fomite	Fomites	16	singular
gesture	gesture	gesture	gesture	gesture	16	kept
gluconate calcium	gluconate calcium	gluconate calcium	gluconate calcium	gluconate calcium	16	kept
ground glass opacity	ground glass opacity	ground glass opacity	ground glass opacity	ground glass opacity	16	kept
happiness	happiness	happiness	happiness	happiness	16	kept
Health Facility Size	health facility size	health facility size	health facility size	Health Facility Size	16	kept
Health information technology	health information technology	health information technology	health information technology	Health information technology	16	kept
Health Planning	health planning	health planning	health planning	Health Planning	16	kept
Health Planning Guidelines	health planning guidelines	health planning guidelines	health planning guidelines	Health Planning Guidelines	16	kept
healthcare quality improvement	healthcare quality improvement	healthcare quality improvement	healthcare quality improvement	healthcare quality improvement	16	kept
heart valve replacement	heart valve replacement	heart valve replacement	heart valve replacement	heart valve replacement	16	kept
Hematologic Tests	hematologic tests	hematologic tests	hematologic tests	Hematologic Tests	16	kept
High Fidelity Simulation Training	high fidelity simulation training	high fidelity simulation training	high fidelity simulation training	High Fidelity Simulation Training	16	kept

Hispanic or Latino	hispanic or latino	hispanic or latino	hispanic or latino	Hispanic or Latino	16	kept
Home Childbirth	home childbirth	home childbirth	home childbirth	Home Childbirth	16	kept
Horowitz index	horowitz index	horowitz index	horowitz index	Horowitz index	16	kept
Hospital Communication Systems	hospital communication systems	hospital communication systems	hospital communication systems	Hospital Communication Systems	16	kept
Hospital-acquired infection	hospital acquired infection	hospital acquired infection	hospital acquired infection	Hospital-acquired infections	16	case_or_format
Hospitals, Special	hospitals special	hospitals special	hospitals special	Hospitals, Special	16	kept
House Calls	house calls	house calls	house calls	House Calls	16	kept
hydralazine	hydralazine	hydralazine	hydralazine	hydralazine	16	kept
Hyperbilirubinemia, Neonatal	hyperbilirubinemia neonatal	hyperbilirubinemia neonatal	hyperbilirubinemia neonatal	Hyperbilirubinemia, Neonatal	16	kept
IMPROVEMENT	improvement	improvement	improvement	IMPROVEMENT	16	kept
infectious disease	infectious disease	infectious disease	infectious disease	infectious disease	16	kept
infectious diseases	infectious diseases	infectious diseases	infectious disease	infectious disease	16	singular
Influenza	influenza	influenza	influenza	influenza	16	case_or_format
Instrument development	instrument development	instrument development	instrument development	instrument development	16	case_or_format
instrument validation	instrument validation	instrument validation	instrument validation	instrument validation	16	kept
internist	internist	internist	internist	internist	16	kept

intimacy	intimacy	intimacy	intimacy	intimacy	16	kept
Intracerebral hemorrhage	intracerebral hemorrhage	intracerebral hemorrhage	intracerebral hemorrhage	Intracerebral hemorrhage	16	kept
intravascular catheter	intravascular catheter	intravascular catheter	intravascular catheter	intravascular catheter	16	kept
Job stress	job stress	job stress	job stress	job stress	16	case_or_format
Kuwait	kuwait	kuwait	kuwait	Kuwait	16	kept
Lacerations	lacerations	lacerations	laceration	laceration	16	singular
laparoscopy	laparoscopy	laparoscopy	laparoscopy	Laparoscopy	16	case_or_format
law suit	law suit	law suit	law suit	law suit	16	kept
LENGTH-OF-STAY	length of stay	length of stay	length of stay	Length of Stay	16	case_or_format
lipid emulsion	lipid emulsion	lipid emulsion	lipid emulsion	lipid emulsion	16	kept
live birth	live birth	live birth	live birth	live birth	16	kept
Liver Diseases	liver diseases	liver diseases	liver disease	liver disease	16	singular
Long QT Syndrome	long qt syndrome	long qt syndrome	long qt syndrome	Long QT Syndrome	16	kept
Los Angeles	los angeles	los angeles	los angeles	Los Angeles	16	kept
Low birth weight	low birth weight	low birth weight	low birth weight	low birth weight	16	case_or_format
lung surfactant	lung surfactant	lung surfactant	lung surfactant	lung surfactant	16	kept

lymphoma	lymphoma	lymphoma	lymphoma	lymphoma	16	kept
Mandatory Reporting	mandatory reporting	mandatory reporting	mandatory reporting	Mandatory Reporting	16	kept
Manometry	manometry	manometry	manometry	Manometry	16	kept
mask	mask	mask	mask	Masks	16	case_or_format
Medically Underserved Area	medically underserved area	medically underserved area	medically underserved area	Medically Underserved Area	16	kept
Medication	medication	medication	medication	Medication	16	kept
mHealth	mhealth	mhealth	mhealth	mHealth	16	kept
Mid-Atlantic Region	mid atlantic region	mid atlantic region	mid atlantic region	Mid-Atlantic Region	16	kept
midwifery	midwifery	midwifery	midwifery	Midwifery	16	case_or_format
migration	migration	migration	migration	migration	16	kept
Minimally Invasive Surgical Procedures	minimally invasive surgical procedures	minimally invasive surgical procedures	minimally invasive surgical procedures	Minimally Invasive Surgical Procedures	16	kept
mobility	mobility	mobility	mobility	Mobility	16	case_or_format
Mouth Mucosa	mouth mucosa	mouth mucosa	mouth mucosa	Mouth Mucosa	16	kept
Multidisciplinary team	multidisciplinary team	multidisciplinary team	multidisciplinary team	multidisciplinary team	16	case_or_format
multiple pregnancy	multiple pregnancy	multiple pregnancy	multiple pregnancy	multiple pregnancy	16	kept
NAS	nas	nas	nas	NAS	16	kept

necrosis	necrosis	necrosis	necrosis	necrosis	16	kept
Necrotizing enterocolitis	necrotizing enterocolitis	necrotizing enterocolitis	necrotizing enterocolitis	necrotizing enterocolitis	16	case_or_format
neurotoxicity	neurotoxicity	neurotoxicity	neurotoxicity	neurotoxicity	16	kept
noninvasive positive pressure ventilation	noninvasive positive pressure ventilation	noninvasive positive pressure ventilation	noninvasive positive pressure ventilation	noninvasive positive pressure ventilation	16	kept
nose	nose	nose	nose	Nose	16	case_or_format
nose obstruction	nose obstruction	nose obstruction	nose obstruction	nose obstruction	16	kept
nosocomial infections	nosocomial infections	nosocomial infections	nosocomial infection	Nosocomial infection	16	singular
obstetric anesthesia	obstetric anesthesia	obstetric anesthesia	obstetric anesthesia	obstetric anesthesia	16	kept
Organ transplantation	organ transplantation	organ transplantation	organ transplantation	organ transplantation	16	case_or_format
Orthopedic Procedures	orthopedic procedures	orthopedic procedures	orthopedic procedures	Orthopedic Procedures	16	kept
overall response rate	overall response rate	overall response rate	overall response rate	overall response rate	16	kept
oxidative stress	oxidative stress	oxidative stress	oxidative stress	oxidative stress	16	kept
Pamphlets	pamphlets	pamphlets	pamphlet	Pamphlets	16	singular
Parity	parity	parity	parity	parity	16	case_or_format
perceptions	perceptions	perceptions	perception	perception	16	singular
perinatal death	perinatal death	perinatal death	perinatal death	Perinatal Death	16	case_or_format

peritonitis	peritonitis	peritonitis	peritonitis	peritonitis	16	kept
physical disability	physical disability	physical disability	physical disability	physical disability	16	kept
physical therapy	physical therapy	physical therapy	physical therapy	Physical therapy	16	case_or_format
Plasmapheresis	plasmapheresis	plasmapheresis	plasmapheresis	plasmapheresis	16	case_or_format
pregnancy diabetes mellitus	pregnancy diabetes mellitus	pregnancy diabetes mellitus	pregnancy diabetes mellitus	pregnancy diabetes mellitus	16	kept
primary care	primary care	primary care	primary care	primary care	16	kept
Probiotics	probiotics	probiotics	probiotics	Probiotics	16	kept
Professional	professional	professional	professional	professional	16	case_or_format
professional quality of life	professional quality of life	professional quality of life	professional quality of life	professional quality of life	16	kept
psychological model	psychological model	psychological model	psychological model	psychological model	16	kept
publishing	publishing	publishing	publishing	Publishing	16	case_or_format
Pulmonary Medicine	pulmonary medicine	pulmonary medicine	pulmonary medicine	Pulmonary Medicine	16	kept
QT prolongation	qt prolongation	qt prolongation	qt prolongation	QT prolongation	16	kept
qualitative approaches	qualitative approaches	qualitative approaches	qualitative approach	qualitative approaches	16	singular
recommended drug dose	recommended drug dose	recommended drug dose	recommended drug dose	recommended drug dose	16	kept
Reflection	reflection	reflection	reflection	Reflection	16	kept

remission	remission	remission	remission	remission	16	kept
residential home	residential home	residential home	residential home	residential home	16	kept
residual volume	residual volume	residual volume	residual volume	residual volume	16	kept
room temperature	room temperature	room temperature	room temperature	room temperature	16	kept
ropivacaine	ropivacaine	ropivacaine	ropivacaine	ropivacaine	16	kept
Safety engineering	safety engineering	safety engineering	safety engineering	Safety engineering	16	kept
seroconversion	seroconversion	seroconversion	seroconversion	seroconversion	16	kept
skin temperature	skin temperature	skin temperature	skin temperature	Skin Temperature	16	case_or_format
sleep hygiene	sleep hygiene	sleep hygiene	sleep hygiene	sleep hygiene	16	kept
Sleep Stages	sleep stages	sleep stages	sleep stage	Sleep Stages	16	singular
Smartphone	smartphone	smartphone	smartphone	Smartphone	16	kept
Social Values	social values	social values	social value	Social Values	16	singular
sound intensity	sound intensity	sound intensity	sound intensity	sound intensity	16	kept
speech disorder	speech disorder	speech disorder	speech disorder	speech disorder	16	kept
spine surgery	spine surgery	spine surgery	spine surgery	spine surgery	16	kept
Staff	staff	staff	staff	staff	16	case_or_format

stillbirth	stillbirth	stillbirth	stillbirth	stillbirth	16	kept
strategic planning	strategic planning	strategic planning	strategic planning	strategic planning	16	kept
Subarachnoid hemorrhage	subarachnoid hemorrhage	subarachnoid hemorrhage	subarachnoid hemorrhage	subarachnoid hemorrhage	16	case_or_format
Suicide, Attempted	suicide attempted	suicide attempted	suicide attempted	Suicide, Attempted	16	kept
surgical drainage	surgical drainage	surgical drainage	surgical drainage	surgical drainage	16	kept
swelling	swelling	swelling	swelling	swelling	16	kept
teicoplanin	teicoplanin	teicoplanin	teicoplanin	teicoplanin	16	kept
terminal disease	terminal disease	terminal disease	terminal disease	terminal disease	16	kept
third trimester pregnancy	third trimester pregnancy	third trimester pregnancy	third trimester pregnancy	third trimester pregnancy	16	kept
Thoracic Injuries	thoracic injuries	thoracic injuries	thoracic injuries	Thoracic Injuries	16	kept
thrombocyte	thrombocyte	thrombocyte	thrombocyte	thrombocyte	16	kept
Thromboembolism	thromboembolism	thromboembolism	thromboembolism	thromboembolism	16	case_or_format
transcultural care	transcultural care	transcultural care	transcultural care	transcultural care	16	kept
Tuberculosis, Pulmonary	tuberculosis pulmonary	tuberculosis pulmonary	tuberculosis pulmonary	Tuberculosis, Pulmonary	16	kept
ULCERS	ulcers	ulcers	ulcer	ulcer	16	singular
valvular heart disease	valvular heart disease	valvular heart disease	valvular heart disease	valvular heart disease	16	kept

Vasospasm, Intracranial	vasospasm intracranial	vasospasm intracranial	vasospasm intracranial	Vasospasm, Intracranial	16	kept
Ventilator weaning	ventilator weaning	ventilator weaning	ventilator weaning	Ventilator Weaning	16	case_or_format
veterinary medicine	veterinary medicine	veterinary medicine	veterinary medicine	veterinary medicine	16	kept
Videoconferencing	videoconferencing	videoconferencing	videoconferencing	videoconferencing	16	case_or_format
Viet Nam	viet nam	viet nam	viet nam	Viet Nam	16	kept
Vocabulary, Controlled	vocabulary controlled	vocabulary controlled	vocabulary controlled	Vocabulary, Controlled	16	kept
Water	water	water	water	water	16	case_or_format
Workplace violence	workplace violence	workplace violence	workplace violence	workplace violence	16	case_or_format
zinc	zinc	zinc	zinc	zinc	16	kept
Abdominal compartment syndrome	abdominal compartment syndrome	abdominal compartment syndrome	abdominal compartment syndrome	Abdominal compartment syndrome	15	kept
acute myeloid leukemia	acute myeloid leukemia	acute myeloid leukemia	acute myeloid leukemia	acute myeloid leukemia	15	kept
acute stress disorder	acute stress disorder	acute stress disorder	acute stress disorder	acute stress disorder	15	kept
Administration	administration	administration	administration	Administration	15	kept
Advance Directive Adherence	advance directive adherence	advance directive adherence	advance directive adherence	Advance Directive Adherence	15	kept
aerosol generating procedure	aerosol generating procedure	aerosol generating procedure	aerosol generating procedure	aerosol generating procedure	15	kept
Aerosols	aerosols	aerosols	aerosol	aerosol	15	singular

ambient air	ambient air	ambient air	ambient air	ambient air	15	kept
ambulatory surgery	ambulatory surgery	ambulatory surgery	ambulatory surgery	ambulatory surgery	15	kept
amyotrophic lateral sclerosis	amyotrophic lateral sclerosis	amyotrophic lateral sclerosis	amyotrophic lateral sclerosis	amyotrophic lateral sclerosis	15	kept
Anesthesia, Spinal	anesthesia spinal	anesthesia spinal	anesthesia spinal	Anesthesia, Spinal	15	kept
angioneurotic edema	angioneurotic edema	angioneurotic edema	angioneurotic edema	angioneurotic edema	15	kept
Angioplasty	angioplasty	angioplasty	angioplasty	Angioplasty	15	kept
Anticoagulation	anticoagulation	anticoagulation	anticoagulation	anticoagulation	15	case_or_format
antihistaminic agent	antihistaminic agent	antihistaminic agent	antihistaminic agent	antihistaminic agent	15	kept
antiviral therapy	antiviral therapy	antiviral therapy	antiviral therapy	antiviral therapy	15	kept
Anxiety, Separation	anxiety separation	anxiety separation	anxiety separation	Anxiety, Separation	15	kept
aphasia	aphasia	aphasia	aphasia	aphasia	15	kept
arterial carbon dioxide tension	arterial carbon dioxide tension	arterial carbon dioxide tension	arterial carbon dioxide tension	arterial carbon dioxide tension	15	kept
auditory stimulation	auditory stimulation	auditory stimulation	auditory stimulation	auditory stimulation	15	kept
autism	autism	autism	autism	autism	15	kept
bicarbonate blood level	bicarbonate blood level	bicarbonate blood level	bicarbonate blood level	bicarbonate blood level	15	kept
blast injury	blast injury	blast injury	blast injury	blast injury	15	kept

blister	blister	blister	blister	blister	15	kept
blood carbon dioxide tension	blood carbon dioxide tension	blood carbon dioxide tension	blood carbon dioxide tension	blood carbon dioxide tension	15	kept
Blood Flow Velocity	blood flow velocity	blood flow velocity	blood flow velocity	blood flow velocity	15	case_or_format
blood gas	blood gas	blood gas	blood gas	blood gas	15	kept
Brain Edema	brain edema	brain edema	brain edema	brain edema	15	case_or_format
Budgets	budgets	budgets	budget	budget	15	singular
cadaver	cadaver	cadaver	cadaver	cadaver	15	kept
Canadian	canadian	canadian	canadian	Canadian	15	kept
capnography	capnography	capnography	capnography	Capnography	15	case_or_format
cardiac	cardiac	cardiac	cardiac	cardiac	15	kept
Cardiac Pacing, Artificial	cardiac pacing artificial	cardiac pacing artificial	cardiac pacing artificial	Cardiac Pacing, Artificial	15	kept
career mobility	career mobility	career mobility	career mobility	Career Mobility	15	case_or_format
case manager	case manager	case manager	case manager	case manager	15	kept
case mix	case mix	case mix	case mix	case mix	15	kept
Cat Diseases	cat diseases	cat diseases	cat disease	Cat Diseases	15	singular
Cell Phone	cell phone	cell phone	cell phone	Cell Phone	15	kept

central venous catheters	central venous catheters	central venous catheters	central venous catheter	central venous catheter	15	singular
Cervical Vertebrae	cervical vertebrae	cervical vertebrae	cervical vertebrae	Cervical Vertebrae	15	kept
Challenges	challenges	challenges	challenge	CHALLENGES	15	singular
chemically induced	chemically induced	chemically induced	chemically induced	chemically induced	15	kept
chickenpox	chickenpox	chickenpox	chickenpox	chickenpox	15	kept
cigarette smoking	cigarette smoking	cigarette smoking	cigarette smoking	cigarette smoking	15	kept
CLIMATE	climate	climate	climate	climate	15	case_or_format
cognitive therapy	cognitive therapy	cognitive therapy	cognitive therapy	cognitive therapy	15	kept
Coinfection	coinfection	coinfection	coinfection	coinfection	15	case_or_format
Competencies	competencies	competencies	competency	Competency	15	singular
Consciousness Monitors	consciousness monitors	consciousness monitors	consciousness monitor	Consciousness Monitors	15	singular
Continuous monitoring	continuous monitoring	continuous monitoring	continuous monitoring	Continuous monitoring	15	kept
Continuous positive airway pressure	continuous positive airway pressure	continuous positive airway pressure	continuous positive airway pressure	Continuous Positive Airway Pressure	15	case_or_format
Contraindications	contraindications	contraindications	contraindication	Contraindications	15	singular
Convalescence	convalescence	convalescence	convalescence	convalescence	15	case_or_format
Cost analysis	cost analysis	cost analysis	cost analysis	Cost analysis	15	kept

Critical thinking	critical thinking	critical thinking	critical thinking	critical thinking	15	case_or_format
Crush Injuries	crush injuries	crush injuries	crush injury	Crush Injuries	15	singular
cyclophosphamide	cyclophosphamide	cyclophosphamide	cyclophosphamide	cyclophosphamide	15	kept
cytokine release syndrome	cytokine release syndrome	cytokine release syndrome	cytokine release syndrome	cytokine release syndrome	15	kept
data mining	data mining	data mining	data mining	data mining	15	kept
Decision Making, Computer-Assisted	decision making computer assisted	decision making computer assisted	decision making computer assisted	Decision Making, Computer-Assisted	15	kept
Deep learning	deep learning	deep learning	deep learning	deep learning	15	case_or_format
delayed diagnosis	delayed diagnosis	delayed diagnosis	delayed diagnosis	delayed diagnosis	15	kept
DEMENTIA	dementia	dementia	dementia	dementia	15	case_or_format
Development	development	development	development	development	15	case_or_format
device infection	device infection	device infection	device infection	device infection	15	kept
dignity	dignity	dignity	dignity	dignity	15	kept
disaster preparedness	disaster preparedness	disaster preparedness	disaster preparedness	disaster preparedness	15	kept
DNA, Bacterial	dna bacterial	dna bacterial	dna bacterial	DNA, Bacterial	15	kept
DOUBLE-BLIND	double blind	double blind	double blind	DOUBLE-BLIND	15	kept
drug derivative	drug derivative	drug derivative	drug derivative	drug derivative	15	kept

drug dose escalation	drug dose escalation	drug dose escalation	drug dose escalation	drug dose escalation	15	kept
drug dose regimen	drug dose regimen	drug dose regimen	drug dose regimen	drug dose regimen	15	kept
drug surveillance program	drug surveillance program	drug surveillance program	drug surveillance program	drug surveillance program	15	kept
earthquake	earthquake	earthquake	earthquake	Earthquakes	15	case_or_format
Economics	economics	economics	economic	economics	15	singular
effect size	effect size	effect size	effect size	effect size	15	kept
electricity	electricity	electricity	electricity	electricity	15	kept
electrophysiology	electrophysiology	electrophysiology	electrophysiology	electrophysiology	15	kept
Emigrants and Immigrants	emigrants and immigrants	emigrants and immigrants	emigrants and immigrants	Emigrants and Immigrants	15	kept
emotional well-being	emotional well being	emotional well being	emotional well being	emotional well-being	15	kept
Endotracheal intubation	endotracheal intubation	endotracheal intubation	endotracheal intubation	endotracheal intubation	15	case_or_format
Endotracheal suctioning	endotracheal suctioning	endotracheal suctioning	endotracheal suctioning	Endotracheal suctioning	15	kept
endovascular surgery	endovascular surgery	endovascular surgery	endovascular surgery	endovascular surgery	15	kept
ENVIRONMENT	environment	environment	environment	Environment	15	case_or_format
Environment Design	environment design	environment design	environment design	Environment Design	15	kept
environmental monitoring	environmental monitoring	environmental monitoring	environmental monitoring	Environmental Monitoring	15	case_or_format

Epidemics	epidemics	epidemics	epidemic	epidemic	15	singular
epidemiological data	epidemiological data	epidemiological data	epidemiological data	epidemiological data	15	kept
epistaxis	epistaxis	epistaxis	epistaxis	epistaxis	15	kept
Equipment	equipment	equipment	equipment	equipment	15	case_or_format
Ethical conflict	ethical conflict	ethical conflict	ethical conflict	Ethical conflict	15	kept
Expert Testimony	expert testimony	expert testimony	expert testimony	Expert Testimony	15	kept
extended spectrum beta lactamase	extended spectrum beta lactamase	extended spectrum beta lactamase	extended spectrum beta lactamase	extended spectrum beta lactamase	15	kept
eye tracking	eye tracking	eye tracking	eye tracking	eye tracking	15	kept
factorial analysis	factorial analysis	factorial analysis	factorial analysis	factorial analysis	15	kept
faintness	faintness	faintness	faintness	faintness	15	kept
false positive result	false positive result	false positive result	false positive result	false positive result	15	kept
family interaction	family interaction	family interaction	family interaction	family interaction	15	kept
family presence	family presence	family presence	family presence	Family presence	15	case_or_format
Fat Emulsions, Intravenous	fat emulsions intravenous	fat emulsions intravenous	fat emulsions intravenous	Fat Emulsions, Intravenous	15	kept
feces analysis	feces analysis	feces analysis	feces analysis	feces analysis	15	kept
femoral vein	femoral vein	femoral vein	femoral vein	femoral vein	15	kept

Fetal Viability	fetal viability	fetal viability	fetal viability	Fetal Viability	15	kept
fetus heart rate	fetus heart rate	fetus heart rate	fetus heart rate	fetus heart rate	15	kept
food and drug administration	food and drug administration	food and drug administration	food and drug administration	food and drug administration	15	kept
gastroenteritis	gastroenteritis	gastroenteritis	gastroenteritis	gastroenteritis	15	kept
gastrointestinal infection	gastrointestinal infection	gastrointestinal infection	gastrointestinal infection	gastrointestinal infection	15	kept
gestational diabetes	gestational diabetes	gestational diabetes	gestational diabetes	gestational diabetes	15	kept
guideline	guideline	guideline	guideline	Guidelines	15	case_or_format
Handoff	handoff	handoff	handoff	handoff	15	case_or_format
Happiness	happiness	happiness	happiness	happiness	15	case_or_format
health care manpower	health care manpower	health care manpower	health care manpower	health care manpower	15	kept
healthcare-associated infections	healthcare associated infections	healthcare associated infections	healthcare associated infection	healthcare associated infection	15	singular
heart atrium septum defect	heart atrium septum defect	heart atrium septum defect	heart atrium septum defect	heart atrium septum defect	15	kept
Heart Failure, Congestive	heart failure congestive	heart failure congestive	heart failure congestive	Heart Failure, Congestive	15	kept
heart rate variability	heart rate variability	heart rate variability	heart rate variability	heart rate variability	15	kept
heart ventricle septum defect	heart ventricle septum defect	heart ventricle septum defect	heart ventricle septum defect	heart ventricle septum defect	15	kept
Heart Ventricles	heart ventricles	heart ventricles	heart ventricle	Heart Ventricles	15	singular

hematopoietic stem cell transplantation	hematopoietic stem cell transplantation	hematopoietic stem cell transplantation	hematopoietic stem cell transplantation	Hematopoietic Stem Cell Transplantation	15	case_or_format
hematothorax	hematothorax	hematothorax	hematothorax	hematothorax	15	kept
hemiplegia	hemiplegia	hemiplegia	hemiplegia	hemiplegia	15	kept
hepatic encephalopathy	hepatic encephalopathy	hepatic encephalopathy	hepatic encephalopathy	hepatic encephalopathy	15	kept
Hospital Planning	hospital planning	hospital planning	hospital planning	hospital planning	15	case_or_format
hospital-acquired infections	hospital acquired infections	hospital acquired infections	hospital acquired infection	Hospital-acquired infections	15	singular
hospitalized infant	hospitalized infant	hospitalized infant	hospitalized infant	hospitalized infant	15	kept
Hyperkalemia	hyperkalemia	hyperkalemia	hyperkalemia	hyperkalemia	15	case_or_format
Hypernatremia	hypernatremia	hypernatremia	hypernatremia	hypernatremia	15	case_or_format
ileostomy	ileostomy	ileostomy	ileostomy	ileostomy	15	kept
immunity	immunity	immunity	immunity	immunity	15	kept
Incontinence-associated dermatitis	incontinence associated dermatitis	incontinence associated dermatitis	incontinence associated dermatitis	Incontinence-associated dermatitis	15	kept
incontinence-associated dermatitis	incontinence associated dermatitis	incontinence associated dermatitis	incontinence associated dermatitis	Incontinence-associated dermatitis	15	case_or_format
Influenza A virus	influenza a virus	influenza a virus	influenza a virus	Influenza A virus	15	kept
Injections, Subcutaneous	injections subcutaneous	injections subcutaneous	injections subcutaneous	Injections, Subcutaneous	15	kept
Injury	injury	injury	injury	injury	15	case_or_format

insulin glargine	insulin glargine	insulin glargine	insulin glargine	insulin glargine	15	kept
insulin resistance	insulin resistance	insulin resistance	insulin resistance	insulin resistance	15	kept
interdisciplinary research	interdisciplinary research	interdisciplinary research	interdisciplinary research	interdisciplinary research	15	kept
Intergenerational Relations	intergenerational relations	intergenerational relations	intergenerational relations	Intergenerational Relations	15	kept
Interprofessional education	interprofessional education	interprofessional education	interprofessional education	interprofessional education	15	case_or_format
Interruptions	interruptions	interruptions	interruption	Interruptions	15	singular
interstitial lung disease	interstitial lung disease	interstitial lung disease	interstitial lung disease	interstitial lung disease	15	kept
Intra-abdominal hypertension	intra abdominal hypertension	intra abdominal hypertension	intra abdominal hypertension	Intra-Abdominal Hypertension	15	case_or_format
Intracranial pressure	intracranial pressure	intracranial pressure	intracranial pressure	intracranial pressure	15	case_or_format
iron	iron	iron	iron	iron	15	kept
Ischemic stroke	ischemic stroke	ischemic stroke	ischemic stroke	ischemic stroke	15	case_or_format
Isoflurane	isoflurane	isoflurane	isoflurane	isoflurane	15	case_or_format
isolation facility	isolation facility	isolation facility	isolation facility	isolation facility	15	kept
Japanese	japanese	japanese	japanese	Japanese	15	kept
justice	justice	justice	justice	justice	15	kept
Kidney Failure, Acute	kidney failure acute	kidney failure acute	kidney failure acute	Kidney Failure, Acute	15	kept

lived experience	lived experience	lived experience	lived experience	lived experience	15	kept
lopinavir plus ritonavir	lopinavir plus ritonavir	lopinavir plus ritonavir	lopinavir plus ritonavir	lopinavir plus ritonavir	15	kept
Lung Injury	lung injury	lung injury	lung injury	lung injury	15	case_or_format
maternal health service	maternal health service	maternal health service	maternal health service	Maternal Health Services	15	case_or_format
medical assistant	medical assistant	medical assistant	medical assistant	medical assistant	15	kept
medical director	medical director	medical director	medical director	medical director	15	kept
memory disorder	memory disorder	memory disorder	memory disorder	memory disorder	15	kept
Metabolic Clearance Rate	metabolic clearance rate	metabolic clearance rate	metabolic clearance rate	Metabolic Clearance Rate	15	kept
Microcirculation	microcirculation	microcirculation	microcirculation	Microcirculation	15	kept
Midwives	midwives	midwives	midwives	Midwives	15	kept
Milk Banks	milk banks	milk banks	milk bank	Milk Banks	15	singular
moisture	moisture	moisture	moisture	moisture	15	kept
monoclonal antibody	monoclonal antibody	monoclonal antibody	monoclonal antibody	monoclonal antibody	15	kept
Muscle Strength	muscle strength	muscle strength	muscle strength	muscle strength	15	case_or_format
named inventories, questionnaires and rating scales	named inventories questionnaires and rating scales	named inventories questionnaires and rating scales	named inventories questionnaires and rating scales	named inventories, questionnaires and rating scales	15	kept
Natural language processing	natural language processing	natural language processing	natural language processing	natural language processing	15	case_or_format

Nausea	nausea	nausea	nausea	nausea	15	case_or_format
Negative-Pressure Wound Therapy	negative pressure wound therapy	negative pressure wound therapy	negative pressure wound therapy	Negative-Pressure Wound Therapy	15	kept
negligence	negligence	negligence	negligence	negligence	15	kept
nerve cell differentiation	nerve cell differentiation	nerve cell differentiation	nerve cell differentiation	nerve cell differentiation	15	kept
neuropathic pain	neuropathic pain	neuropathic pain	neuropathic pain	neuropathic pain	15	kept
neuropsychological test	neuropsychological test	neuropsychological test	neuropsychological test	Neuropsychological Tests	15	case_or_format
nutrition support	nutrition support	nutrition support	nutrition support	nutrition support	15	kept
obstetric hemorrhage	obstetric hemorrhage	obstetric hemorrhage	obstetric hemorrhage	obstetric hemorrhage	15	kept
Occlusive Dressings	occlusive dressings	occlusive dressings	occlusive dressing	Occlusive Dressings	15	singular
Oncology	oncology	oncology	oncology	oncology	15	case_or_format
oncology ward	oncology ward	oncology ward	oncology ward	oncology ward	15	kept
open surgery	open surgery	open surgery	open surgery	open surgery	15	kept
opioids	opioids	opioids	opioid	opioids	15	singular
Oxygen Saturation	oxygen saturation	oxygen saturation	oxygen saturation	oxygen saturation	15	case_or_format
pantoprazole	pantoprazole	pantoprazole	pantoprazole	pantoprazole	15	kept
Parkinson Disease	parkinson disease	parkinson disease	parkinson disease	Parkinson disease	15	case_or_format

pericardial effusion	pericardial effusion	pericardial effusion	pericardial effusion	pericardial effusion	15	kept
perinatology	perinatology	perinatology	perinatology	perinatology	15	kept
peripherally inserted central catheter	peripherally inserted central catheter	peripherally inserted central catheter	peripherally inserted central catheter	peripherally inserted central catheter	15	kept
pharmacokinetics	pharmacokinetics	pharmacokinetics	pharmacokinetic	pharmacokinetics	15	singular
phase 2 clinical trial	phase 2 clinical trial	phase 2 clinical trial	phase 2 clinical trial	phase 2 clinical trial	15	kept
physician assistants	physician assistants	physician assistants	physician assistant	physician assistant	15	singular
PICS	pics	pics	pics	PICS	15	kept
Plant Oils	plant oils	plant oils	plant oils	Plant Oils	15	kept
plastic surgery	plastic surgery	plastic surgery	plastic surgery	plastic surgery	15	kept
post hoc analysis	post hoc analysis	post hoc analysis	post hoc analysis	post hoc analysis	15	kept
Preparedness	preparedness	preparedness	preparedness	Preparedness	15	kept
Professional competence	professional competence	professional competence	professional competence	Professional Competence	15	case_or_format
PROFESSIONALS	professionals	professionals	professional	professional	15	singular
pseudomonic acid	pseudomonic acid	pseudomonic acid	pseudomonic acid	pseudomonic acid	15	kept
psychologic test	psychologic test	psychologic test	psychologic test	psychologic test	15	kept
psychological stress	psychological stress	psychological stress	psychological stress	Psychological stress	15	case_or_format

Psychotherapy	psychotherapy	psychotherapy	psychotherapy	psychotherapy	15	case_or_format
Pulmonary Atelectasis	pulmonary atelectasis	pulmonary atelectasis	pulmonary atelectasis	Pulmonary Atelectasis	15	kept
Pulmonary Surfactants	pulmonary surfactants	pulmonary surfactants	pulmonary surfactants	Pulmonary Surfactants	15	kept
Qatar	qatar	qatar	qatar	Qatar	15	kept
quality of life assessment	quality of life assessment	quality of life assessment	quality of life assessment	quality of life assessment	15	kept
Quarantine	quarantine	quarantine	quarantine	quarantine	15	case_or_format
race difference	race difference	race difference	race difference	race difference	15	kept
racism	racism	racism	racism	racism	15	kept
Remote monitoring	remote monitoring	remote monitoring	remote monitoring	Remote monitoring	15	kept
rest	rest	rest	rest	rest	15	kept
Risk stratification	risk stratification	risk stratification	risk stratification	Risk stratification	15	kept
rivaroxaban	rivaroxaban	rivaroxaban	rivaroxaban	rivaroxaban	15	kept
Sacrococcygeal Region	sacrococcygeal region	sacrococcygeal region	sacrococcygeal region	Sacrococcygeal Region	15	kept
sadness	sadness	sadness	sadness	sadness	15	kept
salvage therapy	salvage therapy	salvage therapy	salvage therapy	salvage therapy	15	kept
SARS-CoV-2 antibody	sars cov 2 antibody	sars cov 2 antibody	sars cov 2 antibody	SARS-CoV-2 antibody	15	kept

SARS-CoV-2 Omicron	sars cov 2 omicron	sars cov 2 omicron	sars cov 2 omicron	SARS-CoV-2 Omicron	15	kept
Sedatives	sedatives	sedatives	sedative	Sedatives	15	singular
self esteem	self esteem	self esteem	self esteem	self esteem	15	kept
self-care	self care	self care	self care	self care	15	case_or_format
Self-Injurious Behavior	self injurious behavior	self injurious behavior	self injurious behavior	Self-Injurious Behavior	15	kept
Sickness Impact Profile	sickness impact profile	sickness impact profile	sickness impact profile	Sickness Impact Profile	15	kept
signal processing	signal processing	signal processing	signal processing	signal processing	15	kept
sitting	sitting	sitting	sitting	sitting	15	kept
SKILLS	skills	skills	skill	skill	15	singular
skin injury	skin injury	skin injury	skin injury	skin injury	15	kept
Skin-to-skin contact	skin to skin contact	skin to skin contact	skin to skin contact	Skin-to-skin contact	15	kept
social stigma	social stigma	social stigma	social stigma	social stigma	15	kept
spontaneous abortion	spontaneous abortion	spontaneous abortion	spontaneous abortion	spontaneous abortion	15	kept
stomach	stomach	stomach	stomach	Stomach	15	case_or_format
Streptococcal Infections	streptococcal infections	streptococcal infections	streptococcal infections	Streptococcal Infections	15	kept
Streptococcus agalactiae	streptococcus agalactiae	streptococcus agalactiae	streptococcus agalactiae	Streptococcus agalactiae	15	kept

Streptococcus pyogenes	streptococcus pyogenes	streptococcus pyogenes	streptococcus pyogenes	Streptococcus pyogenes	15	kept
stridor	stridor	stridor	stridor	stridor	15	kept
Sufentanil	sufentanil	sufentanil	sufentanil	sufentanil	15	case_or_format
SURVEILLANCE	surveillance	surveillance	surveillance	Surveillance	15	case_or_format
tacrolimus	tacrolimus	tacrolimus	tacrolimus	tacrolimus	15	kept
teleconference	teleconference	teleconference	teleconference	teleconference	15	kept
Tertiary Healthcare	tertiary healthcare	tertiary healthcare	tertiary healthcare	Tertiary Healthcare	15	kept
Text Messaging	text messaging	text messaging	text messaging	Text Messaging	15	kept
Thoracostomy	thoracostomy	thoracostomy	thoracostomy	Thoracostomy	15	kept
thoracostomy	thoracostomy	thoracostomy	thoracostomy	Thoracostomy	15	case_or_format
Thorax	thorax	thorax	thorax	thorax	15	case_or_format
time of death	time of death	time of death	time of death	time of death	15	kept
trace element	trace element	trace element	trace element	trace element	15	kept
transfusion	transfusion	transfusion	transfusion	transfusion	15	kept
trauma surgeon	trauma surgeon	trauma surgeon	trauma surgeon	trauma surgeon	15	kept
TRIAL	trial	trial	trial	TRIAL	15	kept

Unidad de cuidados intensivos	unidad de cuidados intensivos	unidad de cuidados intensivos	unidad de cuidados intensivos	Unidad de Cuidados Intensivos	15	case_or_format
upper respiratory tract infection	upper respiratory tract infection	upper respiratory tract infection	upper respiratory tract infection	upper respiratory tract infection	15	kept
Ventilator	ventilator	ventilator	ventilator	ventilator	15	case_or_format
verbal hostility	verbal hostility	verbal hostility	verbal hostility	verbal hostility	15	kept
Volunteers	volunteers	volunteers	volunteer	volunteer	15	singular
Warfare	warfare	warfare	warfare	Warfare	15	kept
Warfarin	warfarin	warfarin	warfarin	warfarin	15	case_or_format
Weights and Measures	weights and measures	weights and measures	weights and measures	Weights and Measures	15	kept
Work Performance	work performance	work performance	work performance	Work Performance	15	kept
WORK-ENVIRONMENT	work environment	work environment	work environment	work environment	15	case_or_format
wound dehiscence	wound dehiscence	wound dehiscence	wound dehiscence	wound dehiscence	15	kept
Wounds and injuries	wounds and injuries	wounds and injuries	wounds and injuries	Wounds and Injuries	15	case_or_format
Zambia	zambia	zambia	zambia	Zambia	15	kept
Acoustic noise	acoustic noise	acoustic noise	acoustic noise	Acoustic noise	14	kept
Acoustics	acoustics	acoustics	acoustics	Acoustics	14	kept
Acute renal failure	acute renal failure	acute renal failure	acute renal failure	Acute renal failure	14	kept

Advanced practice	advanced practice	advanced practice	advanced practice	Advanced practice	14	kept
Alcohols	alcohols	alcohols	alcohol	alcohol	14	singular
alfentanil	alfentanil	alfentanil	alfentanil	alfentanil	14	kept
Algorithm	algorithm	algorithm	algorithm	Algorithms	14	case_or_format
analgesic activity	analgesic activity	analgesic activity	analgesic activity	analgesic activity	14	kept
Anger	anger	anger	anger	anger	14	case_or_format
antipyretic agent	antipyretic agent	antipyretic agent	antipyretic agent	antipyretic agent	14	kept
Aortic Rupture	aortic rupture	aortic rupture	aortic rupture	Aortic Rupture	14	kept
Appointments and Schedules	appointments and schedules	appointments and schedules	appointments and schedules	Appointments and Schedules	14	kept
artery puncture	artery puncture	artery puncture	artery puncture	artery puncture	14	kept
arthralgia	arthralgia	arthralgia	arthralgia	arthralgia	14	kept
association	association	association	association	ASSOCIATION	14	case_or_format
Atrial fibrillation	atrial fibrillation	atrial fibrillation	atrial fibrillation	atrial fibrillation	14	case_or_format
Australian	australian	australian	australian	Australian	14	kept
autonomy	autonomy	autonomy	autonomy	Autonomy	14	case_or_format
Autopsy	autopsy	autopsy	autopsy	autopsy	14	case_or_format

Back Pain	back pain	back pain	back pain	Back Pain	14	kept
bacterium detection	bacterium detection	bacterium detection	bacterium detection	bacterium detection	14	kept
bag mask ventilation	bag mask ventilation	bag mask ventilation	bag mask ventilation	bag mask ventilation	14	kept
BEHAVIOR	behavior	behavior	behavior	behavior	14	case_or_format
Behaviour	behaviour	behaviour	behaviour	Behaviour	14	kept
bereavement support	bereavement support	bereavement support	bereavement support	bereavement support	14	kept
bilirubin blood level	bilirubin blood level	bilirubin blood level	bilirubin blood level	bilirubin blood level	14	kept
Biomedical engineering	biomedical engineering	biomedical engineering	biomedical engineering	Biomedical engineering	14	kept
biomedical engineering	biomedical engineering	biomedical engineering	biomedical engineering	Biomedical engineering	14	case_or_format
Blast Injuries	blast injuries	blast injuries	blast injury	blast injury	14	singular
Blood transfusion	blood transfusion	blood transfusion	blood transfusion	blood transfusion	14	case_or_format
Blood Volume	blood volume	blood volume	blood volume	blood volume	14	case_or_format
Body Surface Area	body surface area	body surface area	body surface area	Body Surface Area	14	kept
brain blood flow	brain blood flow	brain blood flow	brain blood flow	brain blood flow	14	kept
brain development	brain development	brain development	brain development	brain development	14	kept
brain hematoma	brain hematoma	brain hematoma	brain hematoma	brain hematoma	14	kept

brain vasospasm	brain vasospasm	brain vasospasm	brain vasospasm	brain vasospasm	14	kept
bronchiectasis	bronchiectasis	bronchiectasis	bronchiectasis	bronchiectasis	14	kept
Cancer Care Facilities	cancer care facilities	cancer care facilities	cancer care facilities	Cancer Care Facilities	14	kept
cancer mortality	cancer mortality	cancer mortality	cancer mortality	cancer mortality	14	kept
cancer pain	cancer pain	cancer pain	cancer pain	cancer pain	14	kept
candidemia	candidemia	candidemia	candidemia	candidemia	14	kept
captopril	captopril	captopril	captopril	captopril	14	kept
carbapenemase	carbapenemase	carbapenemase	carbapenemase	carbapenemase	14	kept
Carcinoma, Non-Small-Cell Lung	carcinoma non small cell lung	carcinoma non small cell lung	carcinoma non small cell lung	Carcinoma, Non-Small-Cell Lung	14	kept
cardiovascular function	cardiovascular function	cardiovascular function	cardiovascular function	cardiovascular function	14	kept
Caregiver	caregiver	caregiver	caregiver	caregiver	14	case_or_format
Cerebrovascular Disorders	cerebrovascular disorders	cerebrovascular disorders	cerebrovascular disorders	Cerebrovascular Disorders	14	kept
chloride	chloride	chloride	chloride	chloride	14	kept
cholecystectomy	cholecystectomy	cholecystectomy	cholecystectomy	cholecystectomy	14	kept
cleaning staff	cleaning staff	cleaning staff	cleaning staff	cleaning staff	14	kept
climate change	climate change	climate change	climate change	climate change	14	kept

Cognitive Behavioral Therapy	cognitive behavioral therapy	cognitive behavioral therapy	cognitive behavioral therapy	cognitive behavioral therapy	14	case_or_format
COLONIZATION	colonization	colonization	colonization	COLONIZATION	14	kept
Colonization	colonization	colonization	colonization	COLONIZATION	14	case_or_format
colorectal surgery	colorectal surgery	colorectal surgery	colorectal surgery	colorectal surgery	14	kept
Compassion satisfaction	compassion satisfaction	compassion satisfaction	compassion satisfaction	compassion satisfaction	14	case_or_format
COMPETENCE	competence	competence	competence	competence	14	case_or_format
Computer Literacy	computer literacy	computer literacy	computer literacy	Computer Literacy	14	kept
concept formation	concept formation	concept formation	concept formation	Concept Formation	14	case_or_format
conference paper	conference paper	conference paper	conference paper	Conference Paper	14	case_or_format
contusion	contusion	contusion	contusion	contusion	14	kept
coronary artery bypass surgery	coronary artery bypass surgery	coronary artery bypass surgery	coronary artery bypass surgery	coronary artery bypass surgery	14	kept
Coronavirinae	coronavirinae	coronavirinae	coronavirinae	Coronavirinae	14	kept
COVID-19 Drug Treatment	covid 19 drug treatment	covid 19 drug treatment	covid 19 drug treatment	COVID-19 Drug Treatment	14	kept
CULTURE	culture	culture	culture	Culture	14	case_or_format
Data collection	data collection	data collection	data collection	Data Collection	14	case_or_format
Data Display	data display	data display	data display	Data Display	14	kept

Data Mining	data mining	data mining	data mining	data mining	14	case_or_format
data synthesis	data synthesis	data synthesis	data synthesis	data synthesis	14	kept
Death, Sudden, Cardiac	death sudden cardiac	death sudden cardiac	death sudden cardiac	Death, Sudden, Cardiac	14	kept
Decompression, Surgical	decompression surgical	decompression surgical	decompression surgical	Decompression, Surgical	14	kept
decompressive craniectomy	decompressive craniectomy	decompressive craniectomy	decompressive craniectomy	decompressive craniectomy	14	kept
Delusions	delusions	delusions	delusion	delusion	14	singular
Dental Care	dental care	dental care	dental care	Dental Care	14	kept
dental procedure	dental procedure	dental procedure	dental procedure	dental procedure	14	kept
Dexamethasone	dexamethasone	dexamethasone	dexamethasone	dexamethasone	14	case_or_format
Diabetes mellitus	diabetes mellitus	diabetes mellitus	diabetes mellitus	diabetes mellitus	14	case_or_format
Diaries	diaries	diaries	diary	Diary	14	singular
Digestive System Surgical Procedures	digestive system surgical procedures	digestive system surgical procedures	digestive system surgical procedures	Digestive System Surgical Procedures	14	kept
Discharge planning	discharge planning	discharge planning	discharge planning	Discharge planning	14	kept
Dopamine	dopamine	dopamine	dopamine	dopamine	14	case_or_format
drug absorption	drug absorption	drug absorption	drug absorption	drug absorption	14	kept
drug half life	drug half life	drug half life	drug half life	drug half life	14	kept

Drug Resistance, Microbial	drug resistance microbial	drug resistance microbial	drug resistance microbial	Drug Resistance, Microbial	14	kept
Drug Utilization Review	drug utilization review	drug utilization review	drug utilization review	Drug Utilization Review	14	kept
Early Medical Intervention	early medical intervention	early medical intervention	early medical intervention	Early Medical Intervention	14	kept
electromyography	electromyography	electromyography	electromyography	Electromyography	14	case_or_format
emergence agitation	emergence agitation	emergence agitation	emergence agitation	emergence agitation	14	kept
Emergency service	emergency service	emergency service	emergency service	Emergency service	14	kept
emotional attachment	emotional attachment	emotional attachment	emotional attachment	emotional attachment	14	kept
Emotional exhaustion	emotional exhaustion	emotional exhaustion	emotional exhaustion	emotional exhaustion	14	case_or_format
encephalitis	encephalitis	encephalitis	encephalitis	encephalitis	14	kept
End-of-life decisions	end of life decisions	end of life decisions	end of life decision	End-of-life decisions	14	singular
epidural analgesia	epidural analgesia	epidural analgesia	epidural analgesia	epidural analgesia	14	kept
European Union	european union	european union	european union	European Union	14	kept
explosion	explosion	explosion	explosion	explosion	14	kept
Eye Protective Devices	eye protective devices	eye protective devices	eye protective device	Eye Protective Devices	14	singular
facilitation	facilitation	facilitation	facilitation	facilitation	14	kept
falls	falls	falls	fall	falls	14	singular

Family care	family care	family care	family care	Family care	14	kept
family engagement	family engagement	family engagement	family engagement	family engagement	14	kept
family history	family history	family history	family history	family history	14	kept
Family support	family support	family support	family support	family support	14	case_or_format
fetus distress	fetus distress	fetus distress	fetus distress	fetus distress	14	kept
forward heart failure	forward heart failure	forward heart failure	forward heart failure	forward heart failure	14	kept
Free Tissue Flaps	free tissue flaps	free tissue flaps	free tissue flaps	Free Tissue Flaps	14	kept
Friends	friends	friends	friend	friend	14	singular
Functional Independence Measure	functional independence measure	functional independence measure	functional independence measure	Functional Independence Measure	14	kept
gastrointestinal tract	gastrointestinal tract	gastrointestinal tract	gastrointestinal tract	gastrointestinal tract	14	kept
Genomics	genomics	genomics	genomics	Genomics	14	kept
glioblastoma	glioblastoma	glioblastoma	glioblastoma	glioblastoma	14	kept
glycerol	glycerol	glycerol	glycerol	glycerol	14	kept
hazard ratio	hazard ratio	hazard ratio	hazard ratio	hazard ratio	14	kept
health care industry	health care industry	health care industry	health care industry	health care industry	14	kept
Health Services Misuse	health services misuse	health services misuse	health services misuse	Health Services Misuse	14	kept

Healthcare worker	healthcare worker	healthcare worker	healthcare worker	Healthcare workers	14	case_or_format
Heart rate	heart rate	heart rate	heart rate	heart rate	14	case_or_format
helplessness	helplessness	helplessness	helplessness	helplessness	14	kept
Hematoma	hematoma	hematoma	hematoma	hematoma	14	case_or_format
hematuria	hematuria	hematuria	hematuria	hematuria	14	kept
hospital waste	hospital waste	hospital waste	hospital waste	hospital waste	14	kept
hypoplastic left heart syndrome	hypoplastic left heart syndrome	hypoplastic left heart syndrome	hypoplastic left heart syndrome	Hypoplastic Left Heart Syndrome	14	case_or_format
Hypovolemia	hypovolemia	hypovolemia	hypovolemia	hypovolemia	14	case_or_format
Hysterectomy	hysterectomy	hysterectomy	hysterectomy	hysterectomy	14	case_or_format
Immunization	immunization	immunization	immunization	immunization	14	case_or_format
Immunoglobulin G	immunoglobulin g	immunoglobulin g	immunoglobulin g	immunoglobulin G	14	case_or_format
immunoglobulin G antibody	immunoglobulin g antibody	immunoglobulin g antibody	immunoglobulin g antibody	immunoglobulin G antibody	14	kept
Immunosuppression	immunosuppression	immunosuppression	immunosuppression	Immunosuppression	14	kept
Immunosuppression Therapy	immunosuppression therapy	immunosuppression therapy	immunosuppression therapy	Immunosuppression Therapy	14	kept
Impact of Events Scale	impact of events scale	impact of events scale	impact of events scale	Impact of Events Scale	14	kept
in situ simulation	in situ simulation	in situ simulation	in situ simulation	in situ simulation	14	kept

inappropriate prescribing	inappropriate prescribing	inappropriate prescribing	inappropriate prescribing	inappropriate prescribing	14	kept
indirect calorimetry	indirect calorimetry	indirect calorimetry	indirect calorimetry	indirect calorimetry	14	kept
institutional review	institutional review	institutional review	institutional review	institutional review	14	kept
Intellectual Disability	intellectual disability	intellectual disability	intellectual disability	Intellectual Disability	14	kept
Intestinal Perforation	intestinal perforation	intestinal perforation	intestinal perforation	Intestinal Perforation	14	kept
intrapartum care	intrapartum care	intrapartum care	intrapartum care	intrapartum care	14	kept
intuition	intuition	intuition	intuition	Intuition	14	case_or_format
invention	invention	invention	invention	Inventions	14	case_or_format
Kidney Transplantation	kidney transplantation	kidney transplantation	kidney transplantation	kidney transplantation	14	case_or_format
lactation consultant	lactation consultant	lactation consultant	lactation consultant	lactation consultant	14	kept
lactic acidosis	lactic acidosis	lactic acidosis	lactic acidosis	lactic acidosis	14	kept
Laryngeal Masks	laryngeal masks	laryngeal masks	laryngeal mask	Laryngeal Masks	14	singular
LEADERSHIP	leadership	leadership	leadership	Leadership	14	case_or_format
life satisfaction	life satisfaction	life satisfaction	life satisfaction	life satisfaction	14	kept
Lived experience	lived experience	lived experience	lived experience	lived experience	14	case_or_format
Liver Cirrhosis	liver cirrhosis	liver cirrhosis	liver cirrhosis	liver cirrhosis	14	case_or_format

local anesthesia	local anesthesia	local anesthesia	local anesthesia	local anesthesia	14	kept
long QT syndrome	long qt syndrome	long qt syndrome	long qt syndrome	Long QT Syndrome	14	case_or_format
loss of appetite	loss of appetite	loss of appetite	loss of appetite	loss of appetite	14	kept
Louisiana	louisiana	louisiana	louisiana	Louisiana	14	kept
Lower Extremity	lower extremity	lower extremity	lower extremity	Lower Extremity	14	kept
magnetic resonance angiography	magnetic resonance angiography	magnetic resonance angiography	magnetic resonance angiography	magnetic resonance angiography	14	kept
matrix assisted laser desorption ionization time of flight mass	matrix assisted laser desorption ionization time of flight mass	matrix assisted laser desorption ionization time of flight mass	matrix assisted laser desorption ionization time of flight mass	matrix assisted laser desorption ionization time of flight mass	14	kept
mediation analysis	mediation analysis	mediation analysis	mediation analysis	mediation analysis	14	kept
medical device related pressure ulcer	medical device related pressure ulcer	medical device related pressure ulcer	medical device related pressure ulcer	medical device related pressure ulcer	14	kept
methicillin susceptible Staphylococcus aureus	methicillin susceptible staphylococcus aureus	methicillin susceptible staphylococcus aureus	methicillin susceptible staphylococcus aureus	methicillin susceptible Staphylococcus aureus	14	kept
methotrexate	methotrexate	methotrexate	methotrexate	methotrexate	14	kept
mild cognitive impairment	mild cognitive impairment	mild cognitive impairment	mild cognitive impairment	mild cognitive impairment	14	kept
mixed infection	mixed infection	mixed infection	mixed infection	mixed infection	14	kept
Moral Obligations	moral obligations	moral obligations	moral obligation	Moral Obligations	14	singular
morning shift	morning shift	morning shift	morning shift	morning shift	14	kept
mouth cavity	mouth cavity	mouth cavity	mouth cavity	mouth cavity	14	kept

multilocus sequence typing	multilocus sequence typing	multilocus sequence typing	multilocus sequence typing	multilocus sequence typing	14	kept
national health insurance	national health insurance	national health insurance	national health insurance	national health insurance	14	kept
Naval Medicine	naval medicine	naval medicine	naval medicine	Naval Medicine	14	kept
Neonatal monitoring	neonatal monitoring	neonatal monitoring	neonatal monitoring	Neonatal monitoring	14	kept
Neurodevelopment	neurodevelopment	neurodevelopment	neurodevelopment	Neurodevelopment	14	kept
neutrophil count	neutrophil count	neutrophil count	neutrophil count	neutrophil count	14	kept
New York Heart Association class	new york heart association class	new york heart association class	new york heart association class	New York Heart Association class	14	kept
newborn jaundice	newborn jaundice	newborn jaundice	newborn jaundice	newborn jaundice	14	kept
newborn surgery	newborn surgery	newborn surgery	newborn surgery	newborn surgery	14	kept
Newcastle-Ottawa scale	newcastle ottawa scale	newcastle ottawa scale	newcastle ottawa scale	Newcastle-Ottawa scale	14	kept
nimodipine	nimodipine	nimodipine	nimodipine	nimodipine	14	kept
Nova Scotia	nova scotia	nova scotia	nova scotia	Nova Scotia	14	kept
Occupational risks	occupational risks	occupational risks	occupational risk	Occupational risks	14	singular
Oklahoma	oklahoma	oklahoma	oklahoma	Oklahoma	14	kept
omeprazole	omeprazole	omeprazole	omeprazole	omeprazole	14	kept
online questionnaire	online questionnaire	online questionnaire	online questionnaire	online questionnaire	14	kept

optimism	optimism	optimism	optimism	optimism	14	kept
Oxygenation	oxygenation	oxygenation	oxygenation	oxygenation	14	case_or_format
palliative	palliative	palliative	palliative	palliative	14	kept
palpation	palpation	palpation	palpation	palpation	14	kept
parallel design	parallel design	parallel design	parallel design	parallel design	14	kept
parenthood	parenthood	parenthood	parenthood	parenthood	14	kept
paresthesia	paresthesia	paresthesia	paresthesia	paresthesia	14	kept
pediatric oncology	pediatric oncology	pediatric oncology	pediatric oncology	pediatric oncology	14	kept
Pelvic Bones	pelvic bones	pelvic bones	pelvic bones	Pelvic Bones	14	kept
Peptic Ulcer	peptic ulcer	peptic ulcer	peptic ulcer	peptic ulcer	14	case_or_format
Perinatal Mortality	perinatal mortality	perinatal mortality	perinatal mortality	Perinatal Mortality	14	kept
personal autonomy	personal autonomy	personal autonomy	personal autonomy	Personal Autonomy	14	case_or_format
Peru	peru	peru	peru	Peru	14	kept
physical well-being	physical well being	physical well being	physical well being	physical well-being	14	kept
PHYSICIAN ORDER ENTRY	physician order entry	physician order entry	physician order entry	PHYSICIAN ORDER ENTRY	14	kept
PNEUMONIA	pneumonia	pneumonia	pneumonia	pneumonia	14	case_or_format

post exposure prophylaxis	post exposure prophylaxis	post exposure prophylaxis	post exposure prophylaxis	post exposure prophylaxis	14	kept
Prediction	prediction	prediction	prediction	prediction	14	case_or_format
predictors	predictors	predictors	predictor	PREDICTORS	14	singular
private sector	private sector	private sector	private sector	private sector	14	kept
problem based learning	problem based learning	problem based learning	problem based learning	Problem-Based Learning	14	case_or_format
program acceptability	program acceptability	program acceptability	program acceptability	program acceptability	14	kept
prospective payment	prospective payment	prospective payment	prospective payment	prospective payment	14	kept
PROTOCOL	protocol	protocol	protocol	protocol	14	case_or_format
psoriasis	psoriasis	psoriasis	psoriasis	psoriasis	14	kept
psychiatric treatment	psychiatric treatment	psychiatric treatment	psychiatric treatment	psychiatric treatment	14	kept
Psychological resilience	psychological resilience	psychological resilience	psychological resilience	psychological resilience	14	case_or_format
psychosocial intervention	psychosocial intervention	psychosocial intervention	psychosocial intervention	psychosocial intervention	14	kept
public policy	public policy	public policy	public policy	public policy	14	kept
pulmonologist	pulmonologist	pulmonologist	pulmonologist	pulmonologist	14	kept
Pupil	pupil	pupil	pupil	Pupil	14	kept
quality indicators	quality indicators	quality indicators	quality indicator	Quality indicators	14	singular

radiology department	radiology department	radiology department	radiology department	radiology department	14	kept
Randomised controlled trial	randomised controlled trial	randomised controlled trial	randomised controlled trial	Randomised controlled trial	14	kept
reading	reading	reading	reading	reading	14	kept
real time reverse transcription polymerase chain	real time reverse transcription polymerase chain	real time reverse transcription polymerase chain	real time reverse transcription polymerase chain	real time reverse transcription polymerase chain	14	kept
recall	recall	recall	recall	recall	14	kept
RECOGNITION	recognition	recognition	recognition	recognition	14	case_or_format
Rectum	rectum	rectum	rectum	Rectum	14	kept
REDUCTION	reduction	reduction	reduction	REDUCTION	14	kept
Regional Medical Programs	regional medical programs	regional medical programs	regional medical programs	Regional Medical Programs	14	kept
Religion and Medicine	religion and medicine	religion and medicine	religion and medicine	Religion and Medicine	14	kept
RESISTANT STAPHYLOCOCCUS-AUREUS	resistant staphylococcus aureus	resistant staphylococcus aureus	resistant staphylococcus aureus	RESISTANT STAPHYLOCOCCUS-AUREUS	14	kept
Resource allocation	resource allocation	resource allocation	resource allocation	resource allocation	14	case_or_format
respiration	respiration	respiration	respiration	Respiration	14	case_or_format
respiratory therapy	respiratory therapy	respiratory therapy	respiratory therapy	Respiratory Therapy	14	case_or_format
rhabdomyolysis	rhabdomyolysis	rhabdomyolysis	rhabdomyolysis	rhabdomyolysis	14	kept
Rib Fractures	rib fractures	rib fractures	rib fracture	rib fracture	14	singular

Role Playing	role playing	role playing	role playing	role playing	14	case_or_format
Saline Solution	saline solution	saline solution	saline solution	Saline Solution	14	kept
SARS-COV-2	sars cov 2	sars cov 2	sars cov 2	SARS-CoV-2	14	case_or_format
Schizophrenia	schizophrenia	schizophrenia	schizophrenia	schizophrenia	14	case_or_format
search engine	search engine	search engine	search engine	search engine	14	kept
SEDATION	sedation	sedation	sedation	sedation	14	case_or_format
Self-rating Anxiety Scale	self rating anxiety scale	self rating anxiety scale	self rating anxiety scale	Self-rating Anxiety Scale	14	kept
SEVERITY	severity	severity	severity	SEVERITY	14	kept
simvastatin	simvastatin	simvastatin	simvastatin	simvastatin	14	kept
Skin care	skin care	skin care	skin care	Skin Care	14	case_or_format
skin examination	skin examination	skin examination	skin examination	skin examination	14	kept
social distance	social distance	social distance	social distance	social distance	14	kept
Social Environment	social environment	social environment	social environment	social environment	14	case_or_format
Social Stigma	social stigma	social stigma	social stigma	social stigma	14	case_or_format
social welfare	social welfare	social welfare	social welfare	social welfare	14	kept
solid malignant neoplasm	solid malignant neoplasm	solid malignant neoplasm	solid malignant neoplasm	solid malignant neoplasm	14	kept

Speech	speech	speech	speech	speech	14	case_or_format
Spinal Injuries	spinal injuries	spinal injuries	spinal injuries	Spinal Injuries	14	kept
Sputum	sputum	sputum	sputum	sputum	14	case_or_format
steroid therapy	steroid therapy	steroid therapy	steroid therapy	steroid therapy	14	kept
Stillbirth	stillbirth	stillbirth	stillbirth	stillbirth	14	case_or_format
suicide attempt	suicide attempt	suicide attempt	suicide attempt	suicide attempt	14	kept
sustainability	sustainability	sustainability	sustainability	sustainability	14	kept
SYSTEMS	systems	systems	system	SYSTEM	14	singular
Systems Integration	systems integration	systems integration	systems integration	Systems Integration	14	kept
Team training	team training	team training	team training	Team training	14	kept
Terrorism	terrorism	terrorism	terrorism	terrorism	14	case_or_format
Tertiary Health Care	tertiary health care	tertiary health care	tertiary health care	tertiary health care	14	case_or_format
therapeutic hypothermia	therapeutic hypothermia	therapeutic hypothermia	therapeutic hypothermia	therapeutic hypothermia	14	kept
thoracic surgery	thoracic surgery	thoracic surgery	thoracic surgery	Thoracic Surgery	14	case_or_format
Thrombectomy	thrombectomy	thrombectomy	thrombectomy	thrombectomy	14	case_or_format
Thrombolysis	thrombolysis	thrombolysis	thrombolysis	Thrombolysis	14	kept

tozinameran	tozinameran	tozinameran	tozinameran	tozinameran	14	kept
Transfusion	transfusion	transfusion	transfusion	transfusion	14	case_or_format
treatment interruption	treatment interruption	treatment interruption	treatment interruption	treatment interruption	14	kept
troponin I	troponin i	troponin i	troponin i	troponin I	14	kept
turnover	turnover	turnover	turnover	turnover	14	kept
twins	twins	twins	twin	Twins	14	singular
underweight	underweight	underweight	underweight	underweight	14	kept
United States Food and Drug Administration	united states food and drug administration	united states food and drug administration	united states food and drug administration	United States Food and Drug Administration	14	kept
uremia	uremia	uremia	uremia	uremia	14	kept
urticaria	urticaria	urticaria	urticaria	urticaria	14	kept
vaccination coverage	vaccination coverage	vaccination coverage	vaccination coverage	vaccination coverage	14	kept
vaccine hesitancy	vaccine hesitancy	vaccine hesitancy	vaccine hesitancy	vaccine hesitancy	14	kept
WITHDRAWAL	withdrawal	withdrawal	withdrawal	WITHDRAWAL	14	kept
women's health	women s health	women s health	women s health	women's health	14	kept
work capacity	work capacity	work capacity	work capacity	work capacity	14	kept
Wounds, Penetrating	wounds penetrating	wounds penetrating	wounds penetrating	Wounds, Penetrating	14	kept

2 propanol	2 propanol	2 propanol	2 propanol	2 propanol	13	kept
abnormal respiratory sound	abnormal respiratory sound	abnormal respiratory sound	abnormal respiratory sound	abnormal respiratory sound	13	kept
acid base balance	acid base balance	acid base balance	acid base balance	acid base balance	13	kept
Acid-Base Equilibrium	acid base equilibrium	acid base equilibrium	acid base equilibrium	Acid-Base Equilibrium	13	kept
actimetry	actimetry	actimetry	actimetry	actimetry	13	kept
acute pancreatitis	acute pancreatitis	acute pancreatitis	acute pancreatitis	acute pancreatitis	13	kept
Administration, Cutaneous	administration cutaneous	administration cutaneous	administration cutaneous	Administration, Cutaneous	13	kept
Adrenergic alpha-Agonists	adrenergic alpha agonists	adrenergic alpha agonists	adrenergic alpha agonists	Adrenergic alpha-Agonists	13	kept
ADVANCED DEMENTIA	advanced dementia	advanced dementia	advanced dementia	ADVANCED DEMENTIA	13	kept
advanced practice	advanced practice	advanced practice	advanced practice	Advanced practice	13	case_or_format
advisory committee	advisory committee	advisory committee	advisory committee	Advisory Committees	13	case_or_format
Advocacy	advocacy	advocacy	advocacy	Advocacy	13	kept
Aerospace Medicine	aerospace medicine	aerospace medicine	aerospace medicine	Aerospace Medicine	13	kept
Alabama	alabama	alabama	alabama	Alabama	13	kept
Alarm management	alarm management	alarm management	alarm management	Alarm management	13	kept
allergic reaction	allergic reaction	allergic reaction	allergic reaction	allergic reaction	13	kept

amnesia	amnesia	amnesia	amnesia	amnesia	13	kept
Angina, Unstable	angina unstable	angina unstable	angina unstable	Angina, Unstable	13	kept
animal model	animal model	animal model	animal model	animal model	13	kept
Anti-Inflammatory Agents, Non-Steroidal	anti inflammatory agents non steroidal	anti inflammatory agents non steroidal	anti inflammatory agents non steroidal	Anti-Inflammatory Agents, Non-Steroidal	13	kept
Antibodies, Monoclonal	antibodies monoclonal	antibodies monoclonal	antibodies monoclonal	Antibodies, Monoclonal	13	kept
Aortic Valve	aortic valve	aortic valve	aortic valve	Aortic Valve	13	kept
Aspiration	aspiration	aspiration	aspiration	aspiration	13	case_or_format
asymptomatic infection	asymptomatic infection	asymptomatic infection	asymptomatic infection	asymptomatic infection	13	kept
atmosphere	atmosphere	atmosphere	atmosphere	atmosphere	13	kept
augmented reality	augmented reality	augmented reality	augmented reality	augmented reality	13	kept
Autonomic Nervous System Diseases	autonomic nervous system diseases	autonomic nervous system diseases	autonomic nervous system diseases	Autonomic Nervous System Diseases	13	kept
behavior assessment	behavior assessment	behavior assessment	behavior assessment	behavior assessment	13	kept
Biochemical engineering	biochemical engineering	biochemical engineering	biochemical engineering	Biochemical engineering	13	kept
bivariate analysis	bivariate analysis	bivariate analysis	bivariate analysis	bivariate analysis	13	kept
Blood Chemical Analysis	blood chemical analysis	blood chemical analysis	blood chemical analysis	Blood Chemical Analysis	13	kept
blood flow	blood flow	blood flow	blood flow	blood flow	13	kept

bolus injection	bolus injection	bolus injection	bolus injection	bolus injection	13	kept
Cadaver	cadaver	cadaver	cadaver	cadaver	13	case_or_format
Capacity Building	capacity building	capacity building	capacity building	capacity building	13	case_or_format
carbapenem resistance	carbapenem resistance	carbapenem resistance	carbapenem resistance	carbapenem resistance	13	kept
carbon dioxide tension	carbon dioxide tension	carbon dioxide tension	carbon dioxide tension	carbon dioxide tension	13	kept
Cardiac surgical procedures	cardiac surgical procedures	cardiac surgical procedures	cardiac surgical procedure	Cardiac Surgical Procedures	13	singular
Cardiomyopathies	cardiomyopathies	cardiomyopathies	cardiomyopathy	cardiomyopathy	13	singular
care management	care management	care management	care management	care management	13	kept
catatonia	catatonia	catatonia	catatonia	catatonia	13	kept
Catheter-associated urinary tract infection	catheter associated urinary tract infection	catheter associated urinary tract infection	catheter associated urinary tract infection	catheter associated urinary tract infection	13	case_or_format
Cats	cats	cats	cat	Cats	13	singular
Ceremonial Behavior	ceremonial behavior	ceremonial behavior	ceremonial behavior	Ceremonial Behavior	13	kept
Children with Disabilities	children with disabilities	children with disabilities	children with disabilities	Children with Disabilities	13	kept
chloramphenicol	chloramphenicol	chloramphenicol	chloramphenicol	chloramphenicol	13	kept
chlorpromazine	chlorpromazine	chlorpromazine	chlorpromazine	chlorpromazine	13	kept
cholestasis	cholestasis	cholestasis	cholestasis	cholestasis	13	kept

chronic kidney disease	chronic kidney disease	chronic kidney disease	chronic kidney disease	chronic kidney disease	13	kept
citric acid	citric acid	citric acid	citric acid	Citric Acid	13	case_or_format
Clinical Laboratory Techniques	clinical laboratory techniques	clinical laboratory techniques	clinical laboratory techniques	Clinical Laboratory Techniques	13	kept
Clinical protocols	clinical protocols	clinical protocols	clinical protocol	Clinical Protocols	13	singular
clinical skills	clinical skills	clinical skills	clinical skill	clinical skills	13	singular
clinical trials	clinical trials	clinical trials	clinical trial	clinical trial	13	singular
clonazepam	clonazepam	clonazepam	clonazepam	clonazepam	13	kept
cocaine	cocaine	cocaine	cocaine	cocaine	13	kept
Codes of Ethics	codes of ethics	codes of ethics	codes of ethics	Codes of Ethics	13	kept
Colostrum	colostrum	colostrum	colostrum	Colostrum	13	kept
combination drug therapy	combination drug therapy	combination drug therapy	combination drug therapy	combination drug therapy	13	kept
Communication Devices for People with Disabilities	communication devices for people with disabilities	communication devices for people with disabilities	communication devices for people with disabilities	Communication Devices for People with Disabilities	13	kept
Compassion	compassion	compassion	compassion	compassion	13	case_or_format
COMPASSION FATIGUE	compassion fatigue	compassion fatigue	compassion fatigue	Compassion Fatigue	13	case_or_format
conceptual model	conceptual model	conceptual model	conceptual model	conceptual model	13	kept
continuous hemofiltration	continuous hemofiltration	continuous hemofiltration	continuous hemofiltration	continuous hemofiltration	13	kept

CONTROLLED-TRIAL	controlled trial	controlled trial	controlled trial	CONTROLLED-TRIAL	13	kept
Coronary Angiography	coronary angiography	coronary angiography	coronary angiography	coronary angiography	13	case_or_format
Correlation of Data	correlation of data	correlation of data	correlation of data	Correlation of Data	13	kept
COST	cost	cost	cost	cost	13	case_or_format
costs	costs	costs	cost	cost	13	singular
CPOT	cpot	cpot	cpot	CPOT	13	kept
culture	culture	culture	culture	Culture	13	case_or_format
Czech Republic	czech republic	czech republic	czech republic	Czech Republic	13	kept
Data mining	data mining	data mining	data mining	data mining	13	case_or_format
data quality	data quality	data quality	data quality	data quality	13	kept
debriefing	debriefing	debriefing	debriefing	Debriefing	13	case_or_format
defense mechanism	defense mechanism	defense mechanism	defense mechanism	defense mechanism	13	kept
diaphoresis	diaphoresis	diaphoresis	diaphoresis	diaphoresis	13	kept
diaphragm	diaphragm	diaphragm	diaphragm	diaphragm	13	kept
digital technology	digital technology	digital technology	digital technology	digital technology	13	kept
discharge planning	discharge planning	discharge planning	discharge planning	Discharge planning	13	case_or_format

DOCUMENTATION	documentation	documentation	documentation	Documentation	13	case_or_format
doxorubicin	doxorubicin	doxorubicin	doxorubicin	doxorubicin	13	kept
Dreams	dreams	dreams	dream	Dreams	13	singular
drotrecogin	drotrecogin	drotrecogin	drotrecogin	drotrecogin	13	kept
drowsiness	drowsiness	drowsiness	drowsiness	drowsiness	13	kept
Drug administration	drug administration	drug administration	drug administration	drug administration	13	case_or_format
drug combination	drug combination	drug combination	drug combination	drug combination	13	kept
drug elimination	drug elimination	drug elimination	drug elimination	drug elimination	13	kept
drug intermittent therapy	drug intermittent therapy	drug intermittent therapy	drug intermittent therapy	drug intermittent therapy	13	kept
Drug Resistance, Multiple	drug resistance multiple	drug resistance multiple	drug resistance multiple	Drug Resistance, Multiple	13	kept
DSM-IV	dsm iv	dsm iv	dsm iv	DSM-IV	13	kept
Ebola hemorrhagic fever	ebola hemorrhagic fever	ebola hemorrhagic fever	ebola hemorrhagic fever	Ebola hemorrhagic fever	13	kept
Echocardiography, Transesophageal	echocardiography transesophageal	echocardiography transesophageal	echocardiography transesophageal	Echocardiography, Transesophageal	13	kept
eclampsia	eclampsia	eclampsia	eclampsia	eclampsia	13	kept
EFFICACY	efficacy	efficacy	efficacy	EFFICACY	13	kept
EHR	ehr	ehr	ehr	EHR	13	kept

Electrodes	electrodes	electrodes	electrode	Electrodes	13	singular
Electrolytes	electrolytes	electrolytes	electrolyte	electrolyte	13	singular
emergency department visit	emergency department visit	emergency department visit	emergency department visit	emergency department visit	13	kept
emergency service	emergency service	emergency service	emergency service	Emergency service	13	case_or_format
Enterobacterales	enterobacterales	enterobacterales	enterobacterales	Enterobacterales	13	kept
ethical issues	ethical issues	ethical issues	ethical issues	ethical issues	13	kept
Existentialism	existentialism	existentialism	existentialism	Existentialism	13	kept
experimental design	experimental design	experimental design	experimental design	experimental design	13	kept
eye protection	eye protection	eye protection	eye protection	eye protection	13	kept
Factors	factors	factors	factor	Factors	13	singular
false aneurysm	false aneurysm	false aneurysm	false aneurysm	false aneurysm	13	kept
FAMILY	family	family	family	Family	13	case_or_format
Family integrated care	family integrated care	family integrated care	family integrated care	Family integrated care	13	kept
family integrated care	family integrated care	family integrated care	family integrated care	Family integrated care	13	case_or_format
fatty acid desaturation	fatty acid desaturation	fatty acid desaturation	fatty acid desaturation	fatty acid desaturation	13	kept
femur fracture	femur fracture	femur fracture	femur fracture	femur fracture	13	kept

Fetal Death	fetal death	fetal death	fetal death	Fetal Death	13	kept
flushing	flushing	flushing	flushing	flushing	13	kept
Focus group	focus group	focus group	focus group	Focus Groups	13	case_or_format
follow-up	follow up	follow up	follow up	follow up	13	case_or_format
gastrointestinal endoscopy	gastrointestinal endoscopy	gastrointestinal endoscopy	gastrointestinal endoscopy	gastrointestinal endoscopy	13	kept
genetic disorder	genetic disorder	genetic disorder	genetic disorder	genetic disorder	13	kept
geriatric medicine	geriatric medicine	geriatric medicine	geriatric medicine	geriatric medicine	13	kept
Glasgow outcome scale	glasgow outcome scale	glasgow outcome scale	glasgow outcome scale	Glasgow outcome scale	13	kept
Glottis	glottis	glottis	glottis	Glottis	13	kept
glove	glove	glove	glove	glove	13	kept
Glycemic Index	glycemic index	glycemic index	glycemic index	Glycemic Index	13	kept
government regulation	government regulation	government regulation	government regulation	government regulation	13	kept
graft rejection	graft rejection	graft rejection	graft rejection	Graft Rejection	13	case_or_format
Guideline adherence	guideline adherence	guideline adherence	guideline adherence	Guideline Adherence	13	case_or_format
gynecologist	gynecologist	gynecologist	gynecologist	gynecologist	13	kept
Hand washing	hand washing	hand washing	hand washing	hand washing	13	case_or_format

health care associated pneumonia	health care associated pneumonia	health care associated pneumonia	health care associated pneumonia	health care associated pneumonia	13	kept
health outcome	health outcome	health outcome	health outcome	health outcome	13	kept
health professionals	health professionals	health professionals	health professional	Health professionals	13	singular
heart center	heart center	heart center	heart center	heart center	13	kept
heart death	heart death	heart death	heart death	heart death	13	kept
heart palpitation	heart palpitation	heart palpitation	heart palpitation	heart palpitation	13	kept
heart right ventricle failure	heart right ventricle failure	heart right ventricle failure	heart right ventricle failure	heart right ventricle failure	13	kept
Hematocrit	hematocrit	hematocrit	hematocrit	hematocrit	13	case_or_format
Hepatectomy	hepatectomy	hepatectomy	hepatectomy	Hepatectomy	13	kept
High-fidelity simulation	high fidelity simulation	high fidelity simulation	high fidelity simulation	high-fidelity simulation	13	case_or_format
history of medicine	history of medicine	history of medicine	history of medicine	history of medicine	13	kept
HIV	hiv	hiv	hiv	HIV	13	kept
home oxygen therapy	home oxygen therapy	home oxygen therapy	home oxygen therapy	home oxygen therapy	13	kept
homeostasis	homeostasis	homeostasis	homeostasis	Homeostasis	13	case_or_format
household	household	household	household	household	13	kept
hypoalbuminemia	hypoalbuminemia	hypoalbuminemia	hypoalbuminemia	hypoalbuminemia	13	kept

incubator	incubator	incubator	incubator	incubator	13	kept
infection prevention and control	infection prevention and control	infection prevention and control	infection prevention and control	Infection prevention and control	13	case_or_format
Influencing factors	influencing factors	influencing factors	influencing factor	influencing factors	13	singular
Information Systems	information systems	information systems	information system	information system	13	singular
infusion pumps	infusion pumps	infusion pumps	infusion pump	Infusion Pumps	13	singular
injection	injection	injection	injection	injection	13	kept
Insulin Resistance	insulin resistance	insulin resistance	insulin resistance	insulin resistance	13	case_or_format
Insurance, Health	insurance health	insurance health	insurance health	Insurance, Health	13	kept
Insurance, Health, Reimbursement	insurance health reimbursement	insurance health reimbursement	insurance health reimbursement	Insurance, Health, Reimbursement	13	kept
Intention to leave	intention to leave	intention to leave	intention to leave	Intention to leave	13	kept
interdisciplinary	interdisciplinary	interdisciplinary	interdisciplinary	interdisciplinary	13	kept
Intermittent Positive-Pressure Ventilation	intermittent positive pressure ventilation	intermittent positive pressure ventilation	intermittent positive pressure ventilation	Intermittent Positive-Pressure Ventilation	13	kept
Interrupted Time Series Analysis	interrupted time series analysis	interrupted time series analysis	interrupted time series analysis	Interrupted Time Series Analysis	13	kept
Intestinal Obstruction	intestinal obstruction	intestinal obstruction	intestinal obstruction	Intestinal Obstruction	13	kept
Ischemic Attack, Transient	ischemic attack transient	ischemic attack transient	ischemic attack transient	Ischemic Attack, Transient	13	kept
Job performance	job performance	job performance	job performance	job performance	13	case_or_format

ketorolac	ketorolac	ketorolac	ketorolac	ketorolac	13	kept
Klebsiella pneumoniae infection	klebsiella pneumoniae infection	klebsiella pneumoniae infection	klebsiella pneumoniae infection	Klebsiella pneumoniae infection	13	kept
Lactic Acid	lactic acid	lactic acid	lactic acid	lactic acid	13	case_or_format
least absolute shrinkage and selection operator	least absolute shrinkage and selection operator	least absolute shrinkage and selection operator	least absolute shrinkage and selection operator	least absolute shrinkage and selection operator	13	kept
Leukocyte Count	leukocyte count	leukocyte count	leukocyte count	leukocyte count	13	case_or_format
Liability, Legal	liability legal	liability legal	liability legal	Liability, Legal	13	kept
licensing	licensing	licensing	licensing	licensing	13	kept
lipid	lipid	lipid	lipid	lipid	13	kept
locomotion	locomotion	locomotion	locomotion	locomotion	13	kept
long term survival	long term survival	long term survival	long term survival	long term survival	13	kept
LONG-TERM-CARE	long term care	long term care	long term care	long term care	13	case_or_format
lung burn	lung burn	lung burn	lung burn	lung burn	13	kept
lung complication	lung complication	lung complication	lung complication	lung complication	13	kept
lung hemorrhage	lung hemorrhage	lung hemorrhage	lung hemorrhage	lung hemorrhage	13	kept
lung tumor	lung tumor	lung tumor	lung tumor	lung tumor	13	kept
Mannitol	mannitol	mannitol	mannitol	mannitol	13	case_or_format

mathematical model	mathematical model	mathematical model	mathematical model	mathematical model	13	kept
medical fee	medical fee	medical fee	medical fee	medical fee	13	kept
Medication Systems	medication systems	medication systems	medication systems	Medication Systems	13	kept
meditation	meditation	meditation	meditation	meditation	13	kept
Memory Disorders	memory disorders	memory disorders	memory disorder	memory disorder	13	singular
METAANALYSIS	metaanalysis	metaanalysis	metaanalysis	METAANALYSIS	13	kept
metoprolol	metoprolol	metoprolol	metoprolol	metoprolol	13	kept
microbial contamination	microbial contamination	microbial contamination	microbial contamination	microbial contamination	13	kept
Missed care	missed care	missed care	missed care	Missed care	13	kept
motor activity	motor activity	motor activity	motor activity	Motor Activity	13	case_or_format
mouth disease	mouth disease	mouth disease	mouth disease	mouth disease	13	kept
Mouth Neoplasms	mouth neoplasms	mouth neoplasms	mouth neoplasms	Mouth Neoplasms	13	kept
Multilocus Sequence Typing	multilocus sequence typing	multilocus sequence typing	multilocus sequence typing	multilocus sequence typing	13	case_or_format
muscle mass	muscle mass	muscle mass	muscle mass	muscle mass	13	kept
necrotizing fasciitis	necrotizing fasciitis	necrotizing fasciitis	necrotizing fasciitis	necrotizing fasciitis	13	kept
negative pressure ventilation	negative pressure ventilation	negative pressure ventilation	negative pressure ventilation	negative pressure ventilation	13	kept

nervous system	nervous system	nervous system	nervous system	nervous system	13	kept
network analysis	network analysis	network analysis	network analysis	network analysis	13	kept
neurodevelopment	neurodevelopment	neurodevelopment	neurodevelopment	Neurodevelopment	13	case_or_format
Nutritional support	nutritional support	nutritional support	nutritional support	nutritional support	13	case_or_format
OF-LIFE	of life	of life	of life	OF-LIFE	13	kept
Operating room	operating room	operating room	operating room	operating room	13	case_or_format
Operative Time	operative time	operative time	operative time	Operative Time	13	kept
Opioids	opioids	opioids	opioid	opioids	13	singular
Organization	organization	organization	organization	organization	13	case_or_format
oropharynx	oropharynx	oropharynx	oropharynx	Oropharynx	13	case_or_format
otolaryngologist	otolaryngologist	otolaryngologist	otolaryngologist	otolaryngologist	13	kept
OUTBREAK	outbreak	outbreak	outbreak	Outbreak	13	case_or_format
Overweight	overweight	overweight	overweight	Overweight	13	kept
oxygen concentration	oxygen concentration	oxygen concentration	oxygen concentration	oxygen concentration	13	kept
oxygen tension	oxygen tension	oxygen tension	oxygen tension	oxygen tension	13	kept
Oxygen therapy	oxygen therapy	oxygen therapy	oxygen therapy	oxygen therapy	13	case_or_format

Oxytocin	oxytocin	oxytocin	oxytocin	oxytocin	13	case_or_format
Pain Perception	pain perception	pain perception	pain perception	Pain Perception	13	kept
palliative medicine	palliative medicine	palliative medicine	palliative medicine	palliative medicine	13	kept
paramedical education	paramedical education	paramedical education	paramedical education	paramedical education	13	kept
paraplegia	paraplegia	paraplegia	paraplegia	paraplegia	13	kept
PATTERNS	patterns	patterns	pattern	PATTERNS	13	singular
perceived stress	perceived stress	perceived stress	perceived stress	perceived stress	13	kept
Perinatology	perinatology	perinatology	perinatology	perinatology	13	case_or_format
Persistent Fetal Circulation Syndrome	persistent fetal circulation syndrome	persistent fetal circulation syndrome	persistent fetal circulation syndrome	Persistent Fetal Circulation Syndrome	13	kept
person-centred care	person centred care	person centred care	person centred care	person-centred care	13	kept
personhood	personhood	personhood	personhood	Personhood	13	case_or_format
pharmacoeconomics	pharmacoeconomics	pharmacoeconomics	pharmacoeconomics	pharmacoeconomics	13	kept
physical restraints	physical restraints	physical restraints	physical restraint	physical restraint	13	singular
Physician assistant	physician assistant	physician assistant	physician assistant	physician assistant	13	case_or_format
Physician Executives	physician executives	physician executives	physician executives	Physician Executives	13	kept
Piperidines	piperidines	piperidines	piperidines	Piperidines	13	kept

Pneumonectomy	pneumonectomy	pneumonectomy	pneumonectomy	Pneumonectomy	13	kept
poliomyelitis	poliomyelitis	poliomyelitis	poliomyelitis	poliomyelitis	13	kept
Polypharmacy	polypharmacy	polypharmacy	polypharmacy	polypharmacy	13	case_or_format
Postoperative Cognitive Complications	postoperative cognitive complications	postoperative cognitive complications	postoperative cognitive complications	Postoperative Cognitive Complications	13	kept
Postoperative Hemorrhage	postoperative hemorrhage	postoperative hemorrhage	postoperative hemorrhage	postoperative hemorrhage	13	case_or_format
Posttraumatic stress disorder	posttraumatic stress disorder	posttraumatic stress disorder	posttraumatic stress disorder	posttraumatic stress disorder	13	case_or_format
Potassium	potassium	potassium	potassium	potassium	13	case_or_format
premature birth	premature birth	premature birth	premature birth	Premature Birth	13	case_or_format
Presenteeism	presenteeism	presenteeism	presenteeism	Presenteeism	13	kept
primary graft dysfunction	primary graft dysfunction	primary graft dysfunction	primary graft dysfunction	primary graft dysfunction	13	kept
Principal Component Analysis	principal component analysis	principal component analysis	principal component analysis	principal component analysis	13	case_or_format
Professional autonomy	professional autonomy	professional autonomy	professional autonomy	Professional Autonomy	13	case_or_format
Professional burnout	professional burnout	professional burnout	professional burnout	professional burnout	13	case_or_format
Professional practice	professional practice	professional practice	professional practice	professional practice	13	case_or_format
Professionalism	professionalism	professionalism	professionalism	professionalism	13	case_or_format
prognostic assessment	prognostic assessment	prognostic assessment	prognostic assessment	prognostic assessment	13	kept

prolonged mechanical ventilation	prolonged mechanical ventilation	prolonged mechanical ventilation	prolonged mechanical ventilation	prolonged mechanical ventilation	13	kept
prone ventilation	prone ventilation	prone ventilation	prone ventilation	prone ventilation	13	kept
prothrombin complex	prothrombin complex	prothrombin complex	prothrombin complex	prothrombin complex	13	kept
Psychological Tests	psychological tests	psychological tests	psychological tests	Psychological Tests	13	kept
Psychometric properties	psychometric properties	psychometric properties	psychometric properties	Psychometric properties	13	kept
Pulse	pulse	pulse	pulse	Pulse	13	kept
quadriplegia	quadriplegia	quadriplegia	quadriplegia	quadriplegia	13	kept
radial artery	radial artery	radial artery	radial artery	radial artery	13	kept
radiodiagnosis	radiodiagnosis	radiodiagnosis	radiodiagnosis	radiodiagnosis	13	kept
Radiology	radiology	radiology	radiology	radiology	13	case_or_format
RANDOMIZED-TRIAL	randomized trial	randomized trial	randomized trial	RANDOMIZED-TRIAL	13	kept
readmission	readmission	readmission	readmission	Readmission	13	case_or_format
reinforcement	reinforcement	reinforcement	reinforcement	reinforcement	13	kept
Respiration, artificial	respiration artificial	artificial ventilation	artificial ventilation	Artificial Ventilation	13	alias
Respiratory care	respiratory care	respiratory care	respiratory care	respiratory care	13	case_or_format
RESUSCITATION	resuscitation	resuscitation	resuscitation	resuscitation	13	case_or_format

rheumatoid arthritis	rheumatoid arthritis	rheumatoid arthritis	rheumatoid arthritis	rheumatoid arthritis	13	kept
Rural	rural	rural	rural	Rural	13	kept
Saline Solution, Hypertonic	saline solution hypertonic	saline solution hypertonic	saline solution hypertonic	Saline Solution, Hypertonic	13	kept
scoliosis	scoliosis	scoliosis	scoliosis	scoliosis	13	kept
secondary health care	secondary health care	secondary health care	secondary health care	secondary health care	13	kept
Secondary traumatic stress	secondary traumatic stress	secondary traumatic stress	secondary traumatic stress	secondary traumatic stress	13	case_or_format
SERVICES	services	services	service	SERVICES	13	singular
severe pneumonia	severe pneumonia	severe pneumonia	severe pneumonia	severe pneumonia	13	kept
shame	shame	shame	shame	shame	13	kept
simulator	simulator	simulator	simulator	simulator	13	kept
Skin Transplantation	skin transplantation	skin transplantation	skin transplantation	Skin Transplantation	13	kept
skin-to-skin care	skin to skin care	skin to skin care	skin to skin care	skin-to-skin care	13	kept
SLEEP	sleep	sleep	sleep	Sleep	13	case_or_format
small for date infant	small for date infant	small for date infant	small for date infant	small for date infant	13	kept
Social Behavior	social behavior	social behavior	social behavior	social behavior	13	case_or_format
Sodium	sodium	sodium	sodium	sodium	13	case_or_format

spironolactone	spironolactone	spironolactone	spironolactone	spironolactone	13	kept
Stents	stents	stents	stent	Stents	13	singular
stoma	stoma	stoma	stoma	stoma	13	kept
stomach emptying	stomach emptying	stomach emptying	stomach emptying	stomach emptying	13	kept
stroke rehabilitation	stroke rehabilitation	stroke rehabilitation	stroke rehabilitation	Stroke Rehabilitation	13	case_or_format
sucralfate	sucralfate	sucralfate	sucralfate	sucralfate	13	kept
Sudan	sudan	sudan	sudan	Sudan	13	kept
survivors	survivors	survivors	survivor	Survivors	13	singular
Tape Recording	tape recording	tape recording	tape recording	Tape Recording	13	kept
tazobactam	tazobactam	tazobactam	tazobactam	tazobactam	13	kept
TEAM	team	team	team	Team	13	case_or_format
text messaging	text messaging	text messaging	text messaging	Text Messaging	13	case_or_format
Therapy	therapy	therapy	therapy	therapy	13	case_or_format
Therapy, Computer-Assisted	therapy computer assisted	therapy computer assisted	therapy computer assisted	Therapy, Computer-Assisted	13	kept
Thyroid Crisis	thyroid crisis	thyroid crisis	thyroid crisis	Thyroid Crisis	13	kept
tissue perfusion	tissue perfusion	tissue perfusion	tissue perfusion	tissue perfusion	13	kept

TOOL	tool	tool	tool	TOOL	13	kept
transluminal coronary angioplasty	transluminal coronary angioplasty	transluminal coronary angioplasty	transluminal coronary angioplasty	transluminal coronary angioplasty	13	kept
transplant coordinator	transplant coordinator	transplant coordinator	transplant coordinator	transplant coordinator	13	kept
Transportation	transportation	transportation	transportation	Transportation	13	kept
tube	tube	tube	tube	tube	13	kept
Tube feeding	tube feeding	tube feeding	tube feeding	Tube feeding	13	kept
Unidades de Cuidados Intensivos	unidades de cuidados intensivos	unidades de cuidados intensivos	unidades de cuidados intensivos	Unidades de cuidados intensivos	13	case_or_format
upper limb	upper limb	upper limb	upper limb	upper limb	13	kept
urea blood level	urea blood level	urea blood level	urea blood level	urea blood level	13	kept
vascular access device	vascular access device	vascular access device	vascular access device	Vascular Access Devices	13	case_or_format
vascular disease	vascular disease	vascular disease	vascular disease	Vascular Diseases	13	case_or_format
vasospasm	vasospasm	vasospasm	vasospasm	vasospasm	13	kept
Vital sign	vital sign	vital sign	vital sign	vital sign	13	case_or_format
waste disposal	waste disposal	waste disposal	waste disposal	waste disposal	13	kept
Xerostomia	xerostomia	xerostomia	xerostomia	xerostomia	13	case_or_format
2-Propanol	2 propanol	2 propanol	2 propanol	2 propanol	12	case_or_format

Abdominal Pain	abdominal pain	abdominal pain	abdominal pain	abdominal pain	12	case_or_format
Academies and Institutes	academies and institutes	academies and institutes	academies and institutes	Academies and Institutes	12	kept
Accuracy	accuracy	accuracy	accuracy	accuracy	12	case_or_format
activities of daily living	activities of daily living	activities of daily living	activities of daily living	Activities of Daily Living	12	case_or_format
acute liver failure	acute liver failure	acute liver failure	acute liver failure	acute liver failure	12	kept
acute lymphoblastic leukemia	acute lymphoblastic leukemia	acute lymphoblastic leukemia	acute lymphoblastic leukemia	acute lymphoblastic leukemia	12	kept
Acute myocardial infarction	acute myocardial infarction	acute myocardial infarction	acute myocardial infarction	acute myocardial infarction	12	case_or_format
African Americans	african americans	african americans	african american	African American	12	singular
ageism	ageism	ageism	ageism	ageism	12	kept
AKI	aki	aki	aki	AKI	12	kept
alcohol withdrawal syndrome	alcohol withdrawal syndrome	alcohol withdrawal syndrome	alcohol withdrawal syndrome	alcohol withdrawal syndrome	12	kept
Alert Fatigue, Health Personnel	alert fatigue health personnel	alert fatigue health personnel	alert fatigue health personnel	Alert Fatigue, Health Personnel	12	kept
amino terminal pro brain natriuretic peptide	amino terminal pro brain natriuretic peptide	amino terminal pro brain natriuretic peptide	amino terminal pro brain natriuretic peptide	amino terminal pro brain natriuretic peptide	12	kept
amlodipine	amlodipine	amlodipine	amlodipine	amlodipine	12	kept
analytical research	analytical research	analytical research	analytical research	analytical research	12	kept
Anesthesia, Conduction	anesthesia conduction	anesthesia conduction	anesthesia conduction	Anesthesia, Conduction	12	kept

Anesthetists	anesthetists	anesthetists	anesthetist	Anesthetists	12	singular
Angiotensin-Converting Enzyme Inhibitors	angiotensin converting enzyme inhibitors	angiotensin converting enzyme inhibitors	angiotensin converting enzyme inhibitor	Angiotensin-Converting Enzyme Inhibitors	12	singular
antidote	antidote	antidote	antidote	antidote	12	kept
Antisepsis	antisepsis	antisepsis	antisepsis	antisepsis	12	case_or_format
anuria	anuria	anuria	anuria	anuria	12	kept
aorta balloon	aorta balloon	aorta balloon	aorta balloon	aorta balloon	12	kept
aortic dissection	aortic dissection	aortic dissection	aortic dissection	Aortic Dissection	12	case_or_format
aortic valve	aortic valve	aortic valve	aortic valve	Aortic Valve	12	case_or_format
arteriovenous fistula	arteriovenous fistula	arteriovenous fistula	arteriovenous fistula	arteriovenous fistula	12	kept
Artifacts	artifacts	artifacts	artifact	Artifacts	12	singular
artificial embolization	artificial embolization	artificial embolization	artificial embolization	artificial embolization	12	kept
artificial feeding	artificial feeding	artificial feeding	artificial feeding	artificial feeding	12	kept
attitude to illness	attitude to illness	attitude to illness	attitude to illness	attitude to illness	12	kept
audit	audit	audit	audit	Audit	12	case_or_format
Augmented reality	augmented reality	augmented reality	augmented reality	augmented reality	12	case_or_format
Autonomic Nervous System	autonomic nervous system	autonomic nervous system	autonomic nervous system	Autonomic Nervous System	12	kept

bacterial gene	bacterial gene	bacterial gene	bacterial gene	bacterial gene	12	kept
Bacterial Load	bacterial load	bacterial load	bacterial load	bacterial load	12	case_or_format
bacterial meningitis	bacterial meningitis	bacterial meningitis	bacterial meningitis	bacterial meningitis	12	kept
bacterial virulence	bacterial virulence	bacterial virulence	bacterial virulence	bacterial virulence	12	kept
barbituric acid derivative	barbituric acid derivative	barbituric acid derivative	barbituric acid derivative	barbituric acid derivative	12	kept
barrier	barrier	barrier	barrier	Barriers	12	case_or_format
Bayes theorem	bayes theorem	bayes theorem	bayes theorem	Bayes Theorem	12	case_or_format
betamethasone	betamethasone	betamethasone	betamethasone	betamethasone	12	kept
biochemistry	biochemistry	biochemistry	biochemistry	biochemistry	12	kept
bioengineering	bioengineering	bioengineering	bioengineering	bioengineering	12	kept
Bioethical Issues	bioethical issues	bioethical issues	bioethical issues	Bioethical Issues	12	kept
biomechanics	biomechanics	biomechanics	biomechanics	biomechanics	12	kept
brain stem	brain stem	brain stem	brain stem	brain stem	12	kept
buprenorphine	buprenorphine	buprenorphine	buprenorphine	buprenorphine	12	kept
care transitions	care transitions	care transitions	care transition	care transitions	12	singular
Carotid Stenosis	carotid stenosis	carotid stenosis	carotid stenosis	Carotid Stenosis	12	kept

Central venous catheterization	central venous catheterization	central venous catheterization	central venous catheterization	central venous catheterization	12	case_or_format
Cerebral Palsy	cerebral palsy	cerebral palsy	cerebral palsy	cerebral palsy	12	case_or_format
Cerebral Ventricles	cerebral ventricles	cerebral ventricles	cerebral ventricles	Cerebral Ventricles	12	kept
cervical spine	cervical spine	cervical spine	cervical spine	cervical spine	12	kept
checklists	checklists	checklists	checklist	checklist	12	singular
chronic critical illness	chronic critical illness	chronic critical illness	chronic critical illness	chronic critical illness	12	kept
cilastatin plus imipenem	cilastatin plus imipenem	cilastatin plus imipenem	cilastatin plus imipenem	cilastatin plus imipenem	12	kept
clinical guidelines	clinical guidelines	clinical guidelines	clinical guideline	clinical guidelines	12	singular
clinical laboratory	clinical laboratory	clinical laboratory	clinical laboratory	clinical laboratory	12	kept
Clinical Pharmacy Information Systems	clinical pharmacy information systems	clinical pharmacy information systems	clinical pharmacy information systems	Clinical Pharmacy Information Systems	12	kept
Clinical practice guidelines	clinical practice guidelines	clinical practice guidelines	clinical practice guideline	clinical practice guideline	12	singular
clinical practice guidelines	clinical practice guidelines	clinical practice guidelines	clinical practice guideline	clinical practice guideline	12	singular
Clinical Reasoning	clinical reasoning	clinical reasoning	clinical reasoning	clinical reasoning	12	case_or_format
Clonidine	clonidine	clonidine	clonidine	clonidine	12	case_or_format
colostrum	colostrum	colostrum	colostrum	Colostrum	12	case_or_format
commercial phenomena	commercial phenomena	commercial phenomena	commercial phenomena	commercial phenomena	12	kept

Community-acquired pneumonia	community acquired pneumonia	community acquired pneumonia	community acquired pneumonia	community acquired pneumonia	12	case_or_format
Computer Communication Networks	computer communication networks	computer communication networks	computer communication networks	Computer Communication Networks	12	kept
congenital disorder	congenital disorder	congenital disorder	congenital disorder	congenital disorder	12	kept
Conscious sedation	conscious sedation	conscious sedation	conscious sedation	conscious sedation	12	case_or_format
cooling	cooling	cooling	cooling	cooling	12	kept
Corneal Diseases	corneal diseases	corneal diseases	corneal diseases	Corneal Diseases	12	kept
coworker	coworker	coworker	coworker	coworker	12	kept
craniectomy	craniectomy	craniectomy	craniectomy	craniectomy	12	kept
crisis intervention	crisis intervention	crisis intervention	crisis intervention	Crisis Intervention	12	case_or_format
cross validation	cross validation	cross validation	cross validation	cross validation	12	kept
cross-sectional survey	cross sectional survey	cross sectional survey	cross sectional survey	cross-sectional survey	12	kept
current smoker	current smoker	current smoker	current smoker	current smoker	12	kept
cytokine storm	cytokine storm	cytokine storm	cytokine storm	cytokine storm	12	kept
Death and dying	death and dying	death and dying	death and dying	death and dying	12	case_or_format
death certificate	death certificate	death certificate	death certificate	death certificate	12	kept
Decision Making, Shared	decision making shared	decision making shared	decision making shared	Decision Making, Shared	12	kept

Decision support	decision support	decision support	decision support	Decision support	12	kept
Diaphragm	diaphragm	diaphragm	diaphragm	diaphragm	12	case_or_format
Disaster	disaster	disaster	disaster	Disasters	12	case_or_format
DISEASE	disease	disease	disease	Diseases	12	case_or_format
Disposable Equipment	disposable equipment	disposable equipment	disposable equipment	Disposable Equipment	12	kept
distress	distress	distress	distress	DISTRESS	12	case_or_format
Doppler flowmetry	doppler flowmetry	doppler flowmetry	doppler flowmetry	Doppler flowmetry	12	kept
drug induced disease	drug induced disease	drug induced disease	drug induced disease	drug induced disease	12	kept
drug intoxication	drug intoxication	drug intoxication	drug intoxication	drug intoxication	12	kept
Early Intervention, Educational	early intervention educational	early intervention educational	early intervention educational	Early Intervention, Educational	12	kept
early mobilisation	early mobilisation	early mobilisation	early mobilisation	Early mobilisation	12	case_or_format
early rehabilitation	early rehabilitation	early rehabilitation	early rehabilitation	early rehabilitation	12	kept
Educational Technology	educational technology	educational technology	educational technology	educational technology	12	case_or_format
EEG	eeg	eeg	eeg	EEG	12	kept
Electric Stimulation Therapy	electric stimulation therapy	electric stimulation therapy	electric stimulation therapy	Electric Stimulation Therapy	12	kept
electrostimulation	electrostimulation	electrostimulation	electrostimulation	electrostimulation	12	kept

Embolism, Air	embolism air	embolism air	embolism air	Embolism, Air	12	kept
emotions	emotions	emotions	emotion	Emotions	12	singular
Empirical Research	empirical research	empirical research	empirical research	Empirical Research	12	kept
empyema	empyema	empyema	empyema	empyema	12	kept
Endoscopy	endoscopy	endoscopy	endoscopy	endoscopy	12	case_or_format
enema	enema	enema	enema	enema	12	kept
enteral feeding	enteral feeding	enteral feeding	enteral feeding	Enteral feeding	12	case_or_format
Epidemiological Monitoring	epidemiological monitoring	epidemiological monitoring	epidemiological monitoring	Epidemiological Monitoring	12	kept
epidural hematoma	epidural hematoma	epidural hematoma	epidural hematoma	epidural hematoma	12	kept
erythropoietin	erythropoietin	erythropoietin	erythropoietin	erythropoietin	12	kept
Escherichia coli Infections	escherichia coli infections	escherichia coli infections	escherichia coli infection	Escherichia coli infection	12	singular
essential oil	essential oil	essential oil	essential oil	essential oil	12	kept
Ethics Committees, Research	ethics committees research	ethics committees research	ethics committees research	Ethics Committees, Research	12	kept
Explosions	explosions	explosions	explosion	explosion	12	singular
face mask	face mask	face mask	face mask	face mask	12	kept
Facial Injuries	facial injuries	facial injuries	facial injuries	Facial Injuries	12	kept

facilitated tucking	facilitated tucking	facilitated tucking	facilitated tucking	facilitated tucking	12	kept
false negative result	false negative result	false negative result	false negative result	false negative result	12	kept
Family engagement	family engagement	family engagement	family engagement	family engagement	12	case_or_format
family functioning	family functioning	family functioning	family functioning	family functioning	12	kept
Family satisfaction	family satisfaction	family satisfaction	family satisfaction	family satisfaction	12	case_or_format
fasting	fasting	fasting	fasting	fasting	12	kept
Feces	feces	feces	feces	Feces	12	kept
feces	feces	feces	feces	Feces	12	case_or_format
Feeding and Eating Disorders	feeding and eating disorders	feeding and eating disorders	feeding and eating disorders	Feeding and Eating Disorders	12	kept
feeding apparatus	feeding apparatus	feeding apparatus	feeding apparatus	feeding apparatus	12	kept
femoral neck fracture	femoral neck fracture	femoral neck fracture	femoral neck fracture	femoral neck fracture	12	kept
Fetus	fetus	fetus	fetus	fetus	12	case_or_format
fetus outcome	fetus outcome	fetus outcome	fetus outcome	fetus outcome	12	kept
FOLLOW-UP	follow up	follow up	follow up	follow up	12	case_or_format
Foot	foot	foot	foot	Foot	12	kept
fosfomycin	fosfomycin	fosfomycin	fosfomycin	fosfomycin	12	kept

frequency analysis	frequency analysis	frequency analysis	frequency analysis	frequency analysis	12	kept
Futile care	futile care	futile care	futile care	Futile care	12	kept
gastric residual volume	gastric residual volume	gastric residual volume	gastric residual volume	gastric residual volume	12	kept
Gastrointestinal Tract	gastrointestinal tract	gastrointestinal tract	gastrointestinal tract	gastrointestinal tract	12	case_or_format
Gels	gels	gels	gel	Gels	12	singular
Gender Identity	gender identity	gender identity	gender identity	Gender Identity	12	kept
gene expression	gene expression	gene expression	gene expression	gene expression	12	kept
gene sequence	gene sequence	gene sequence	gene sequence	gene sequence	12	kept
Genetic Diseases, Inborn	genetic diseases inborn	genetic diseases inborn	genetic diseases inborn	Genetic Diseases, Inborn	12	kept
geriatric	geriatric	geriatric	geriatric	Geriatrics	12	case_or_format
Government Regulation	government regulation	government regulation	government regulation	government regulation	12	case_or_format
group process	group process	group process	group process	group process	12	kept
Guillain Barre syndrome	guillain barre syndrome	guillain barre syndrome	guillain barre syndrome	Guillain-Barre Syndrome	12	case_or_format
Head	head	head	head	Head	12	kept
health care professionals	health care professionals	health care professionals	health care professional	Health care professionals	12	singular
health care providers	health care providers	health care providers	health care provider	health care providers	12	singular

health-related quality of life	health related quality of life	health related quality of life	health related quality of life	health-related quality of life	12	kept
Healthy work environment	healthy work environment	healthy work environment	healthy work environment	healthy work environment	12	case_or_format
hemiparesis	hemiparesis	hemiparesis	hemiparesis	hemiparesis	12	kept
heparin induced thrombocytopenia	heparin induced thrombocytopenia	heparin induced thrombocytopenia	heparin induced thrombocytopenia	heparin induced thrombocytopenia	12	kept
Hernias, Diaphragmatic, Congenital	hernias diaphragmatic congenital	hernias diaphragmatic congenital	hernias diaphragmatic congenital	Hernias, Diaphragmatic, Congenital	12	kept
high risk pregnancy	high risk pregnancy	high risk pregnancy	high risk pregnancy	high risk pregnancy	12	kept
Home care	home care	home care	home care	home care	12	case_or_format
Hospice	hospice	hospice	hospice	hospice	12	case_or_format
Hospital mortality	hospital mortality	hospital mortality	hospital mortality	Hospital Mortality	12	case_or_format
Hospitals, Religious	hospitals religious	hospitals religious	hospitals religious	Hospitals, Religious	12	kept
hyperbaric oxygen therapy	hyperbaric oxygen therapy	hyperbaric oxygen therapy	hyperbaric oxygen therapy	hyperbaric oxygen therapy	12	kept
hypophosphatemia	hypophosphatemia	hypophosphatemia	hypophosphatemia	hypophosphatemia	12	kept
hypoxemic respiratory failure	hypoxemic respiratory failure	hypoxemic respiratory failure	hypoxemic respiratory failure	hypoxemic respiratory failure	12	kept
identity	identity	identity	identity	identity	12	kept
image analysis	image analysis	image analysis	image analysis	image analysis	12	kept
Immunosuppressive Agents	immunosuppressive agents	immunosuppressive agents	immunosuppressive agent	immunosuppressive agent	12	singular

infectious agent	infectious agent	infectious agent	infectious agent	infectious agent	12	kept
Infusion pumps	infusion pumps	infusion pumps	infusion pump	Infusion Pumps	12	singular
Infusions, Intraosseous	infusions intraosseous	infusions intraosseous	infusions intraosseous	Infusions, Intraosseous	12	kept
inhalation	inhalation	inhalation	inhalation	inhalation	12	kept
INJURY	injury	injury	injury	injury	12	case_or_format
innovation	innovation	innovation	innovation	innovation	12	kept
Insurance Coverage	insurance coverage	insurance coverage	insurance coverage	Insurance Coverage	12	kept
intellectual impairment	intellectual impairment	intellectual impairment	intellectual impairment	intellectual impairment	12	kept
Intensive insulin therapy	intensive insulin therapy	intensive insulin therapy	intensive insulin therapy	Intensive insulin therapy	12	kept
Interprofessional Education	interprofessional education	interprofessional education	interprofessional education	interprofessional education	12	case_or_format
Interview, Psychological	interview psychological	interview psychological	interview psychological	Interview, Psychological	12	kept
intestine perforation	intestine perforation	intestine perforation	intestine perforation	intestine perforation	12	kept
Intravenous	intravenous	intravenous	intravenous	Intravenous	12	kept
Iowa	iowa	iowa	iowa	Iowa	12	kept
isoniazid	isoniazid	isoniazid	isoniazid	isoniazid	12	kept
Isotonic Solutions	isotonic solutions	isotonic solutions	isotonic solution	Isotonic Solutions	12	singular

Jordanian	jordanian	jordanian	jordanian	Jordanian	12	kept
Jugular Veins	jugular veins	jugular veins	jugular vein	Jugular Veins	12	singular
knowledge base	knowledge base	knowledge base	knowledge base	knowledge base	12	kept
Knowledge translation	knowledge translation	knowledge translation	knowledge translation	Knowledge translation	12	kept
laryngoscope	laryngoscope	laryngoscope	laryngoscope	laryngoscope	12	kept
law enforcement	law enforcement	law enforcement	law enforcement	law enforcement	12	kept
laxative	laxative	laxative	laxative	laxative	12	kept
Likelihood Functions	likelihood functions	likelihood functions	likelihood functions	Likelihood Functions	12	kept
Liver Neoplasms	liver neoplasms	liver neoplasms	liver neoplasms	Liver Neoplasms	12	kept
long COVID	long covid	long covid	long covid	long COVID	12	kept
lung artery pressure	lung artery pressure	lung artery pressure	lung artery pressure	lung artery pressure	12	kept
Lung transplantation	lung transplantation	lung transplantation	lung transplantation	lung transplantation	12	case_or_format
malignant neoplastic disease	malignant neoplastic disease	malignant neoplastic disease	malignant neoplastic disease	malignant neoplastic disease	12	kept
Maternal Age	maternal age	maternal age	maternal age	maternal age	12	case_or_format
mechanical thrombectomy	mechanical thrombectomy	mechanical thrombectomy	mechanical thrombectomy	mechanical thrombectomy	12	kept
Meconium Aspiration Syndrome	meconium aspiration syndrome	meconium aspiration syndrome	meconium aspiration syndrome	Meconium Aspiration Syndrome	12	kept

Medical error	medical error	medical error	medical error	Medical Errors	12	case_or_format
medication	medication	medication	medication	Medication	12	case_or_format
medication administration errors	medication administration errors	medication administration errors	medication administration error	medication administration errors	12	singular
MEDICATION ERRORS	medication errors	medication errors	medication error	Medication Errors	12	singular
Mental Fatigue	mental fatigue	mental fatigue	mental fatigue	Mental Fatigue	12	kept
micafungin	micafungin	micafungin	micafungin	micafungin	12	kept
military personnel	military personnel	military personnel	military personnel	Military Personnel	12	case_or_format
milk	milk	milk	milk	milk	12	kept
minimally invasive cardiac surgery	minimally invasive cardiac surgery	minimally invasive cardiac surgery	minimally invasive cardiac surgery	minimally invasive cardiac surgery	12	kept
mixed methods	mixed methods	mixed methods	mixed method	Mixed methods	12	singular
molecular epidemiology	molecular epidemiology	molecular epidemiology	molecular epidemiology	molecular epidemiology	12	kept
monitor	monitor	monitor	monitor	monitor	12	kept
Monitoring, Ambulatory	monitoring ambulatory	monitoring ambulatory	monitoring ambulatory	Monitoring, Ambulatory	12	kept
Moral resilience	moral resilience	moral resilience	moral resilience	Moral resilience	12	kept
moral resilience	moral resilience	moral resilience	moral resilience	Moral resilience	12	case_or_format
moral sensitivity	moral sensitivity	moral sensitivity	moral sensitivity	moral sensitivity	12	kept

mouth	mouth	mouth	mouth	Mouth	12	case_or_format
MULTICENTER	multicenter	multicenter	multicenter	MULTICENTER	12	kept
multihospital system	multihospital system	multihospital system	multihospital system	multihospital system	12	kept
muscle hypotonia	muscle hypotonia	muscle hypotonia	muscle hypotonia	muscle hypotonia	12	kept
Muscular Diseases	muscular diseases	muscular diseases	muscular diseases	Muscular Diseases	12	kept
musculoskeletal pain	musculoskeletal pain	musculoskeletal pain	musculoskeletal pain	musculoskeletal pain	12	kept
Mycoses	mycoses	mycoses	mycoses	Mycoses	12	kept
near miss	near miss	near miss	near miss	near miss	12	kept
Neonatal mortality	neonatal mortality	neonatal mortality	neonatal mortality	Neonatal mortality	12	kept
neonatal mortality	neonatal mortality	neonatal mortality	neonatal mortality	Neonatal mortality	12	case_or_format
neonatal palliative care	neonatal palliative care	neonatal palliative care	neonatal palliative care	neonatal palliative care	12	kept
Neonatal resuscitation	neonatal resuscitation	neonatal resuscitation	neonatal resuscitation	neonatal resuscitation	12	case_or_format
Nerve Block	nerve block	nerve block	nerve block	Nerve Block	12	kept
nerve block	nerve block	nerve block	nerve block	Nerve Block	12	case_or_format
Neurodevelopmental Disorders	neurodevelopmental disorders	neurodevelopmental disorders	neurodevelopmental disorder	Neurodevelopmental Disorders	12	singular
Neuroprotection	neuroprotection	neuroprotection	neuroprotection	neuroprotection	12	case_or_format

neutrophil	neutrophil	neutrophil	neutrophil	neutrophil	12	kept
Newborn infant	newborn infant	newborn infant	newborn infant	Newborn infant	12	kept
NIDCAP	nidcap	nidcap	nidcap	NIDCAP	12	kept
nifedipine	nifedipine	nifedipine	nifedipine	nifedipine	12	kept
nightmare	nightmare	nightmare	nightmare	nightmare	12	kept
Nosocomial	nosocomial	nosocomial	nosocomial	Nosocomial	12	kept
NOSOCOMIAL INFECTIONS	nosocomial infections	nosocomial infections	nosocomial infection	Nosocomial infection	12	singular
obsessive compulsive disorder	obsessive compulsive disorder	obsessive compulsive disorder	obsessive compulsive disorder	obsessive compulsive disorder	12	kept
Occupational exposure	occupational exposure	occupational exposure	occupational exposure	occupational exposure	12	case_or_format
Oils, Volatile	oils volatile	oils volatile	oils volatile	Oils, Volatile	12	kept
Oliguria	oliguria	oliguria	oliguria	oliguria	12	case_or_format
online analysis	online analysis	online analysis	online analysis	online analysis	12	kept
ophthalmoscopy	ophthalmoscopy	ophthalmoscopy	ophthalmoscopy	ophthalmoscopy	12	kept
Orientation	orientation	orientation	orientation	orientation	12	case_or_format
Otolaryngology	otolaryngology	otolaryngology	otolaryngology	Otolaryngology	12	kept
performance measurement system	performance measurement system	performance measurement system	performance measurement system	performance measurement system	12	kept

Peripheral	peripheral	peripheral	peripheral	Peripheral	12	kept
peroperative care	peroperative care	peroperative care	peroperative care	peroperative care	12	kept
Personality Inventory	personality inventory	personality inventory	personality inventory	Personality Inventory	12	kept
pertussis	pertussis	pertussis	pertussis	pertussis	12	kept
Physical activity	physical activity	physical activity	physical activity	physical activity	12	case_or_format
physical disease	physical disease	physical disease	physical disease	physical disease	12	kept
PICC	picc	picc	picc	PICC	12	kept
placenta	placenta	placenta	placenta	placenta	12	kept
Platelet Aggregation Inhibitors	platelet aggregation inhibitors	platelet aggregation inhibitors	platelet aggregation inhibitors	Platelet Aggregation Inhibitors	12	kept
Politics	politics	politics	politics	politics	12	case_or_format
Polyneuropathies	polyneuropathies	polyneuropathies	polyneuropathy	Polyneuropathies	12	singular
population density	population density	population density	population density	population density	12	kept
postanesthesia care	postanesthesia care	postanesthesia care	postanesthesia care	postanesthesia care	12	kept
POSTOPERATIVE DELIRIUM	postoperative delirium	postoperative delirium	postoperative delirium	postoperative delirium	12	case_or_format
posttraumatic growth	posttraumatic growth	posttraumatic growth	posttraumatic growth	posttraumatic growth	12	kept
Pragmatic Clinical Trials as Topic	pragmatic clinical trials as topic	pragmatic clinical trials as topic	pragmatic clinical trials as topic	Pragmatic Clinical Trials as Topic	12	kept

pregabalin	pregabalin	pregabalin	pregabalin	pregabalin	12	kept
Preoperative Period	preoperative period	preoperative period	preoperative period	preoperative period	12	case_or_format
prescription drug	prescription drug	prescription drug	prescription drug	prescription drug	12	kept
Prevention and control	prevention and control	prevention and control	prevention and control	prevention and control	12	case_or_format
professional issues	professional issues	professional issues	professional issues	professional issues	12	kept
prostaglandin E2	prostaglandin e2	prostaglandin e2	prostaglandin e2	prostaglandin E2	12	kept
Psychological Trauma	psychological trauma	psychological trauma	psychological trauma	Psychological Trauma	12	kept
Psychology	psychology	psychology	psychology	psychology	12	case_or_format
Psychotropic Drugs	psychotropic drugs	psychotropic drugs	psychotropic drugs	Psychotropic Drugs	12	kept
Pulmonary Wedge Pressure	pulmonary wedge pressure	pulmonary wedge pressure	pulmonary wedge pressure	Pulmonary Wedge Pressure	12	kept
pulse oximeter	pulse oximeter	pulse oximeter	pulse oximeter	pulse oximeter	12	kept
rapid response system	rapid response system	rapid response system	rapid response system	Rapid response system	12	case_or_format
Rapid response systems	rapid response systems	rapid response systems	rapid response system	Rapid response system	12	singular
recombinant protein	recombinant protein	recombinant protein	recombinant protein	Recombinant Proteins	12	case_or_format
redeployment	redeployment	redeployment	redeployment	redeployment	12	kept
Refeeding Syndrome	refeeding syndrome	refeeding syndrome	refeeding syndrome	Refeeding Syndrome	12	kept

refeeding syndrome	refeeding syndrome	refeeding syndrome	refeeding syndrome	Refeeding Syndrome	12	case_or_format
reminder system	reminder system	reminder system	reminder system	Reminder Systems	12	case_or_format
Renal Insufficiency, Chronic	renal insufficiency chronic	renal insufficiency chronic	renal insufficiency chronic	Renal Insufficiency, Chronic	12	kept
resistance training	resistance training	resistance training	resistance training	resistance training	12	kept
Restraint	restraint	restraint	restraint	Restraint	12	kept
retirement	retirement	retirement	retirement	retirement	12	kept
Reward	reward	reward	reward	Reward	12	kept
reward	reward	reward	reward	Reward	12	case_or_format
Safety-net Providers	safety net providers	safety net providers	safety net providers	Safety-net Providers	12	kept
Scandinavia	scandinavia	scandinavia	scandinavia	Scandinavia	12	kept
Scandinavian and Nordic Countries	scandinavian and nordic countries	scandinavian and nordic countries	scandinavian and nordic countries	Scandinavian and Nordic Countries	12	kept
Self-Management	self management	self management	self management	Self-Management	12	kept
Sensation	sensation	sensation	sensation	Sensation	12	kept
sinus rhythm	sinus rhythm	sinus rhythm	sinus rhythm	sinus rhythm	12	kept
skin decontamination	skin decontamination	skin decontamination	skin decontamination	skin decontamination	12	kept
skin irritation	skin irritation	skin irritation	skin irritation	skin irritation	12	kept

Skin Physiological Phenomena	skin physiological phenomena	skin physiological phenomena	skin physiological phenomena	Skin Physiological Phenomena	12	kept
Sleep disturbance	sleep disturbance	sleep disturbance	sleep disturbance	Sleep disturbance	12	kept
smartphone	smartphone	smartphone	smartphone	Smartphone	12	case_or_format
Social Isolation	social isolation	social isolation	social isolation	social isolation	12	case_or_format
social security	social security	social security	social security	social security	12	kept
Socialization	socialization	socialization	socialization	Socialization	12	kept
spasticity	spasticity	spasticity	spasticity	spasticity	12	kept
Spirometry	spirometry	spirometry	spirometry	spirometry	12	case_or_format
stakeholder participation	stakeholder participation	stakeholder participation	stakeholder participation	stakeholder participation	12	kept
Standards	standards	standards	standard	standards	12	singular
stomach paresis	stomach paresis	stomach paresis	stomach paresis	stomach paresis	12	kept
stress assessment	stress assessment	stress assessment	stress assessment	stress assessment	12	kept
students	students	students	student	Students	12	singular
summer	summer	summer	summer	summer	12	kept
support vector machine	support vector machine	support vector machine	support vector machine	support vector machine	12	kept
Surgical Flaps	surgical flaps	surgical flaps	surgical flaps	Surgical Flaps	12	kept

symptom management	symptom management	symptom management	symptom management	symptom management	12	kept
systemic lupus erythematosus	systemic lupus erythematosus	systemic lupus erythematosus	systemic lupus erythematosus	systemic lupus erythematosus	12	kept
team training	team training	team training	team training	Team training	12	case_or_format
technique	technique	technique	technique	technique	12	kept
Teratoma	teratoma	teratoma	teratoma	Teratoma	12	kept
Therapeutic hypothermia	therapeutic hypothermia	therapeutic hypothermia	therapeutic hypothermia	therapeutic hypothermia	12	case_or_format
thermodilution	thermodilution	thermodilution	thermodilution	Thermodilution	12	case_or_format
Thermography	thermography	thermography	thermography	Thermography	12	kept
Thermometers	thermometers	thermometers	thermometer	Thermometers	12	singular
thoracic surgeon	thoracic surgeon	thoracic surgeon	thoracic surgeon	thoracic surgeon	12	kept
thoracocentesis	thoracocentesis	thoracocentesis	thoracocentesis	thoracocentesis	12	kept
thromboelastography	thromboelastography	thromboelastography	thromboelastography	thromboelastography	12	kept
thrombolysis	thrombolysis	thrombolysis	thrombolysis	Thrombolysis	12	case_or_format
Training Support	training support	training support	training support	Training Support	12	kept
Transcatheter Aortic Valve Replacement	transcatheter aortic valve replacement	transcatheter aortic valve replacement	transcatheter aortic valve replacement	Transcatheter Aortic Valve Replacement	12	kept
tube feeding	tube feeding	tube feeding	tube feeding	Tube feeding	12	case_or_format

Tunisia	tunisia	tunisia	tunisia	Tunisia	12	kept
Urinary tract infection	urinary tract infection	urinary tract infection	urinary tract infection	urinary tract infection	12	case_or_format
urosepsis	urosepsis	urosepsis	urosepsis	urosepsis	12	kept
Vancomycin Resistance	vancomycin resistance	vancomycin resistance	vancomycin resistance	Vancomycin Resistance	12	kept
Vascular access	vascular access	vascular access	vascular access	vascular access	12	case_or_format
Vascular access devices	vascular access devices	vascular access devices	vascular access device	Vascular Access Devices	12	singular
vibration	vibration	vibration	vibration	vibration	12	kept
Virus Diseases	virus diseases	virus diseases	virus diseases	Virus Diseases	12	kept
Visitation	visitation	visitation	visitation	Visitation	12	kept
Vitamins	vitamins	vitamins	vitamin	vitamin	12	singular
vocational education	vocational education	vocational education	vocational education	vocational education	12	kept
weight reduction	weight reduction	weight reduction	weight reduction	weight reduction	12	kept
Whole Genome Sequencing	whole genome sequencing	whole genome sequencing	whole genome sequencing	whole genome sequencing	12	case_or_format
withdrawal	withdrawal	withdrawal	withdrawal	WITHDRAWAL	12	case_or_format
worker	worker	worker	worker	worker	12	kept
WORKLOAD	workload	workload	workload	Workload	12	case_or_format

yoga	yoga	yoga	yoga	yoga	12	kept
academic achievement	academic achievement	academic achievement	academic achievement	academic achievement	11	kept
ACCURACY	accuracy	accuracy	accuracy	accuracy	11	case_or_format
acid aspiration	acid aspiration	acid aspiration	acid aspiration	acid aspiration	11	kept
action research	action research	action research	action research	action research	11	kept
acute care surgery	acute care surgery	acute care surgery	acute care surgery	acute care surgery	11	kept
ADVANCE DIRECTIVES	advance directives	advance directives	advance directive	Advance Directives	11	singular
Ageism	ageism	ageism	ageism	ageism	11	case_or_format
AHA Scientific Statements	aha scientific statements	aha scientific statements	aha scientific statements	AHA Scientific Statements	11	kept
alanine aminotransferase blood level	alanine aminotransferase blood level	alanine aminotransferase blood level	alanine aminotransferase blood level	alanine aminotransferase blood level	11	kept
Alarms	alarms	alarms	alarm	Alarms	11	singular
Alcoholism	alcoholism	alcoholism	alcoholism	alcoholism	11	case_or_format
Alzheimer's disease	alzheimer s disease	alzheimer s disease	alzheimer s disease	Alzheimer's disease	11	kept
amantadine	amantadine	amantadine	amantadine	amantadine	11	kept
Amiodarone	amiodarone	amiodarone	amiodarone	amiodarone	11	case_or_format
analogs and derivatives	analogs and derivatives	analogs and derivatives	analogs and derivatives	analogs and derivatives	11	kept

Anemia, Sickle Cell	anemia sickle cell	anemia sickle cell	anemia sickle cell	Anemia, Sickle Cell	11	kept
Anesthesia, Inhalation	anesthesia inhalation	anesthesia inhalation	anesthesia inhalation	Anesthesia, Inhalation	11	kept
Angina Pectoris	angina pectoris	angina pectoris	angina pectoris	angina pectoris	11	case_or_format
Angiography	angiography	angiography	angiography	angiography	11	case_or_format
Anti-Anxiety Agents	anti anxiety agents	anti anxiety agents	anti anxiety agents	Anti-Anxiety Agents	11	kept
antihypertensive therapy	antihypertensive therapy	antihypertensive therapy	antihypertensive therapy	antihypertensive therapy	11	kept
Aortic Aneurysm, Thoracic	aortic aneurysm thoracic	aortic aneurysm thoracic	aortic aneurysm thoracic	Aortic Aneurysm, Thoracic	11	kept
arterial pH	arterial ph	arterial ph	arterial ph	arterial pH	11	kept
artery	artery	artery	artery	Arteries	11	case_or_format
artificial	artificial	artificial	artificial	artificial	11	kept
artificial neural network	artificial neural network	artificial neural network	artificial neural network	artificial neural network	11	kept
Atención de enfermería	atenci n de enfermer a	atenci n de enfermer a	atenci n de enfermer a	Atención de enfermería	11	kept
attitude scale	attitude scale	attitude scale	attitude scale	attitude scale	11	kept
bacterial count	bacterial count	bacterial count	bacterial count	bacterial count	11	kept
Barrier	barrier	barrier	barrier	Barriers	11	case_or_format
benzodiazepines	benzodiazepines	benzodiazepines	benzodiazepine	benzodiazepine	11	singular

best practices	best practices	best practices	best practice	best practice	11	singular
beta-Lactam Resistance	beta lactam resistance	beta lactam resistance	beta lactam resistance	beta-Lactam Resistance	11	kept
biofilm	biofilm	biofilm	biofilm	biofilm	11	kept
Biomechanical Phenomena	biomechanical phenomena	biomechanical phenomena	biomechanical phenomena	Biomechanical Phenomena	11	kept
biopsy	biopsy	biopsy	biopsy	biopsy	11	kept
bladder	bladder	bladder	bladder	bladder	11	kept
blood clotting time	blood clotting time	blood clotting time	blood clotting time	blood clotting time	11	kept
Bone Marrow Transplantation	bone marrow transplantation	bone marrow transplantation	bone marrow transplantation	bone marrow transplantation	11	case_or_format
Botulism	botulism	botulism	botulism	Botulism	11	kept
brain dysfunction	brain dysfunction	brain dysfunction	brain dysfunction	brain dysfunction	11	kept
brain hypoxia	brain hypoxia	brain hypoxia	brain hypoxia	brain hypoxia	11	kept
Breast milk	breast milk	breast milk	breast milk	breast milk	11	case_or_format
bronchitis	bronchitis	bronchitis	bronchitis	bronchitis	11	kept
Bronchopulmonary dysplasia	bronchopulmonary dysplasia	bronchopulmonary dysplasia	bronchopulmonary dysplasia	Bronchopulmonary Dysplasia	11	case_or_format
bronchopulmonary dysplasia	bronchopulmonary dysplasia	bronchopulmonary dysplasia	bronchopulmonary dysplasia	Bronchopulmonary Dysplasia	11	case_or_format
BUNDLE	bundle	bundle	bundle	BUNDLE	11	kept

Bundle	bundle	bundle	bundle	BUNDLE	11	case_or_format
burns	burns	burns	burn	Burns	11	singular
Cambodia	cambodia	cambodia	cambodia	Cambodia	11	kept
carbohydrate	carbohydrate	carbohydrate	carbohydrate	carbohydrate	11	kept
Cardiac	cardiac	cardiac	cardiac	cardiac	11	case_or_format
cardiac surgical procedures	cardiac surgical procedures	cardiac surgical procedures	cardiac surgical procedure	Cardiac Surgical Procedures	11	singular
Cardiopulmonary arrest	cardiopulmonary arrest	cardiopulmonary arrest	cardiopulmonary arrest	cardiopulmonary arrest	11	case_or_format
Cardiovascular diseases	cardiovascular diseases	cardiovascular diseases	cardiovascular disease	cardiovascular disease	11	singular
care coordinator	care coordinator	care coordinator	care coordinator	care coordinator	11	kept
CAREGIVERS	caregivers	caregivers	caregiver	caregiver	11	singular
Catheter Obstruction	catheter obstruction	catheter obstruction	catheter obstruction	Catheter Obstruction	11	kept
Central Nervous System Depressants	central nervous system depressants	central nervous system depressants	central nervous system depressants	Central Nervous System Depressants	11	kept
Cephalosporins	cephalosporins	cephalosporins	cephalosporin	cephalosporin	11	singular
Cerebrospinal Fluid	cerebrospinal fluid	cerebrospinal fluid	cerebrospinal fluid	cerebrospinal fluid	11	case_or_format
cerebrospinal fluid analysis	cerebrospinal fluid analysis	cerebrospinal fluid analysis	cerebrospinal fluid analysis	cerebrospinal fluid analysis	11	kept
chest tightness	chest tightness	chest tightness	chest tightness	chest tightness	11	kept

child advocacy	child advocacy	child advocacy	child advocacy	Child Advocacy	11	case_or_format
Chronic disease	chronic disease	chronic disease	chronic disease	Chronic Disease	11	case_or_format
chronic obstructive pulmonary disease	chronic obstructive pulmonary disease	chronic obstructive pulmonary disease	chronic obstructive pulmonary disease	Chronic obstructive pulmonary disease	11	case_or_format
chronic respiratory tract disease	chronic respiratory tract disease	chronic respiratory tract disease	chronic respiratory tract disease	chronic respiratory tract disease	11	kept
Circadian rhythm	circadian rhythm	circadian rhythm	circadian rhythm	circadian rhythm	11	case_or_format
Cities	cities	cities	city	city	11	singular
civil defense	civil defense	civil defense	civil defense	Civil Defense	11	case_or_format
clinical decision support	clinical decision support	clinical decision support	clinical decision support	Clinical decision support	11	case_or_format
clinical outcomes	clinical outcomes	clinical outcomes	clinical outcome	clinical outcome	11	singular
clinical significance	clinical significance	clinical significance	clinical significance	clinical significance	11	kept
cognitive impairment	cognitive impairment	cognitive impairment	cognitive impairment	Cognitive impairment	11	case_or_format
colon resection	colon resection	colon resection	colon resection	colon resection	11	kept
Color	color	color	color	Color	11	kept
Communicable Diseases, Emerging	communicable diseases emerging	communicable diseases emerging	communicable diseases emerging	Communicable Diseases, Emerging	11	kept
Communication barriers	communication barriers	communication barriers	communication barrier	Communication Barriers	11	singular
competency-based education	competency based education	competency based education	competency based education	Competency-Based Education	11	case_or_format

conflict management	conflict management	conflict management	conflict management	conflict management	11	kept
confounding variable	confounding variable	confounding variable	confounding variable	confounding variable	11	kept
Conscience	conscience	conscience	conscience	Conscience	11	kept
Contact Tracing	contact tracing	contact tracing	contact tracing	Contact Tracing	11	kept
control	control	control	control	control	11	kept
Coordination	coordination	coordination	coordination	coordination	11	case_or_format
copper	copper	copper	copper	copper	11	kept
corticosteroid derivative	corticosteroid derivative	corticosteroid derivative	corticosteroid derivative	corticosteroid derivative	11	kept
Crohn disease	crohn disease	crohn disease	crohn disease	Crohn disease	11	kept
cross-sectional studies	cross sectional studies	cross sectional studies	cross sectional study	Cross-Sectional Studies	11	singular
CRRT	crrt	crrt	crrt	CRRT	11	kept
Current Procedural Terminology	current procedural terminology	current procedural terminology	current procedural terminology	Current Procedural Terminology	11	kept
Cystic Fibrosis	cystic fibrosis	cystic fibrosis	cystic fibrosis	cystic fibrosis	11	case_or_format
Databases, Bibliographic	databases bibliographic	databases bibliographic	databases bibliographic	Databases, Bibliographic	11	kept
day care	day care	day care	day care	day care	11	kept
defecation	defecation	defecation	defecation	defecation	11	kept

degenerative disease	degenerative disease	degenerative disease	degenerative disease	degenerative disease	11	kept
Delivery of health care	delivery of health care	delivery of health care	delivery of health care	Delivery of Health Care	11	case_or_format
dental caries	dental caries	dental caries	dental caries	dental caries	11	kept
Depersonalization	depersonalization	depersonalization	depersonalization	depersonalization	11	case_or_format
Depression, Anxiety and Stress Scale-21	depression anxiety and stress scale 21	depression anxiety and stress scale 21	depression anxiety and stress scale 21	Depression, Anxiety and Stress Scale-21	11	kept
Dermatitis, Irritant	dermatitis irritant	dermatitis irritant	dermatitis irritant	Dermatitis, Irritant	11	kept
DETERMINANTS	determinants	determinants	determinant	DETERMINANTS	11	singular
Developed Countries	developed countries	developed countries	developed country	developed country	11	singular
developmental delay	developmental delay	developmental delay	developmental delay	developmental delay	11	kept
Diagnostic Techniques and Procedures	diagnostic techniques and procedures	diagnostic techniques and procedures	diagnostic techniques and procedures	Diagnostic Techniques and Procedures	11	kept
diclofenac	diclofenac	diclofenac	diclofenac	diclofenac	11	kept
Dietetics	dietetics	dietetics	dietetics	Dietetics	11	kept
dipyrone	dipyrone	dipyrone	dipyrone	dipyrone	11	kept
disaster management	disaster management	disaster management	disaster management	disaster management	11	kept
Disease Reservoirs	disease reservoirs	disease reservoirs	disease reservoirs	Disease Reservoirs	11	kept
Drug Administration Routes	drug administration routes	drug administration routes	drug administration route	Drug Administration Routes	11	singular

drug distribution	drug distribution	drug distribution	drug distribution	drug distribution	11	kept
drug industry	drug industry	drug industry	drug industry	drug industry	11	kept
drug potentiation	drug potentiation	drug potentiation	drug potentiation	drug potentiation	11	kept
drug storage	drug storage	drug storage	drug storage	drug storage	11	kept
drug synthesis	drug synthesis	drug synthesis	drug synthesis	drug synthesis	11	kept
drug tolerability	drug tolerability	drug tolerability	drug tolerability	drug tolerability	11	kept
Drug Tolerance	drug tolerance	drug tolerance	drug tolerance	Drug Tolerance	11	kept
dwell time	dwell time	dwell time	dwell time	dwell time	11	kept
early mobility	early mobility	early mobility	early mobility	early mobility	11	kept
early warning system	early warning system	early warning system	early warning system	early warning system	11	kept
eating	eating	eating	eating	eating	11	kept
echinocandin	echinocandin	echinocandin	echinocandin	echinocandin	11	kept
efficiency	efficiency	efficiency	efficiency	Efficiency	11	case_or_format
Electromagnetic Phenomena	electromagnetic phenomena	electromagnetic phenomena	electromagnetic phenomena	Electromagnetic Phenomena	11	kept
electronic medical record system	electronic medical record system	electronic medical record system	electronic medical record system	electronic medical record system	11	kept
Emergency Department	emergency department	emergency department	emergency department	Emergency department	11	case_or_format

Emergency departments	emergency departments	emergency departments	emergency department	Emergency department	11	singular
emotional stability	emotional stability	emotional stability	emotional stability	emotional stability	11	kept
energy metabolism	energy metabolism	energy metabolism	energy metabolism	Energy Metabolism	11	case_or_format
environmental sustainability	environmental sustainability	environmental sustainability	environmental sustainability	environmental sustainability	11	kept
epidemiological monitoring	epidemiological monitoring	epidemiological monitoring	epidemiological monitoring	Epidemiological Monitoring	11	case_or_format
Error	error	error	error	error	11	case_or_format
erythrocyte count	erythrocyte count	erythrocyte count	erythrocyte count	erythrocyte count	11	kept
Esophagus	esophagus	esophagus	esophagus	Esophagus	11	kept
ethical climate	ethical climate	ethical climate	ethical climate	Ethical climate	11	case_or_format
ethical conflict	ethical conflict	ethical conflict	ethical conflict	Ethical conflict	11	case_or_format
Ethical issues	ethical issues	ethical issues	ethical issues	ethical issues	11	case_or_format
Evidence-based	evidence based	evidence based	evidence based	Evidence-based	11	kept
evidence-based medicine	evidence based medicine	evidence based medicine	evidence based medicine	Evidence-Based Medicine	11	case_or_format
evidence‐based practice	evidence based practice	evidence based practice	evidence based practice	evidence based practice	11	case_or_format
executive function	executive function	executive function	executive function	executive function	11	kept
Eye care	eye care	eye care	eye care	eye care	11	case_or_format

Falls	falls	falls	fall	falls	11	singular
FAMILY-MEMBERS	family members	family members	family member	Family members	11	singular
famotidine	famotidine	famotidine	famotidine	famotidine	11	kept
Father	father	father	father	Fathers	11	case_or_format
FATIGUE	fatigue	fatigue	fatigue	Fatigue	11	case_or_format
feasibility	feasibility	feasibility	feasibility	Feasibility	11	case_or_format
Feedback, Psychological	feedback psychological	feedback psychological	feedback psychological	Feedback, Psychological	11	kept
Feeding	feeding	feeding	feeding	feeding	11	case_or_format
feeding intolerance	feeding intolerance	feeding intolerance	feeding intolerance	feeding intolerance	11	kept
Fetal Diseases	fetal diseases	fetal diseases	fetal diseases	Fetal Diseases	11	kept
Fires	fires	fires	fire	Fires	11	singular
flu like syndrome	flu like syndrome	flu like syndrome	flu like syndrome	flu like syndrome	11	kept
focused ethnography	focused ethnography	focused ethnography	focused ethnography	focused ethnography	11	kept
folic acid	folic acid	folic acid	folic acid	folic acid	11	kept
Food, Formulated	food formulated	food formulated	food formulated	Food, Formulated	11	kept
Foreign Bodies	foreign bodies	foreign bodies	foreign body	foreign body	11	singular

free tissue graft	free tissue graft	free tissue graft	free tissue graft	free tissue graft	11	kept
friction	friction	friction	friction	friction	11	kept
fungal colonization	fungal colonization	fungal colonization	fungal colonization	fungal colonization	11	kept
futility	futility	futility	futility	futility	11	kept
Gastrointestinal Microbiome	gastrointestinal microbiome	gastrointestinal microbiome	gastrointestinal microbiome	Gastrointestinal Microbiome	11	kept
genetic analysis	genetic analysis	genetic analysis	genetic analysis	genetic analysis	11	kept
Geography	geography	geography	geography	geography	11	case_or_format
Geriatric	geriatric	geriatric	geriatric	Geriatrics	11	case_or_format
Glucocorticoids	glucocorticoids	glucocorticoids	glucocorticoid	glucocorticoid	11	singular
glycopyrronium	glycopyrronium	glycopyrronium	glycopyrronium	glycopyrronium	11	kept
goal attainment	goal attainment	goal attainment	goal attainment	goal attainment	11	kept
Goals of care	goals of care	goals of care	goals of care	Goals of care	11	kept
goals of care	goals of care	goals of care	goals of care	Goals of care	11	case_or_format
graft recipient	graft recipient	graft recipient	graft recipient	graft recipient	11	kept
Gram positive infection	gram positive infection	gram positive infection	gram positive infection	Gram positive infection	11	kept
harm reduction	harm reduction	harm reduction	harm reduction	harm reduction	11	kept

Healthcare providers	healthcare providers	healthcare providers	healthcare provider	Healthcare providers	11	singular
heart graft	heart graft	heart graft	heart graft	heart graft	11	kept
Heart Valve Diseases	heart valve diseases	heart valve diseases	heart valve diseases	Heart Valve Diseases	11	kept
heat	heat	heat	heat	heat	11	kept
Heat Stress Disorders	heat stress disorders	heat stress disorders	heat stress disorders	Heat Stress Disorders	11	kept
Hematologic Diseases	hematologic diseases	hematologic diseases	hematologic disease	hematologic disease	11	singular
Hemodynamic Monitoring	hemodynamic monitoring	hemodynamic monitoring	hemodynamic monitoring	hemodynamic monitoring	11	case_or_format
hemostatic agent	hemostatic agent	hemostatic agent	hemostatic agent	hemostatic agent	11	kept
Hepatitis B virus	hepatitis b virus	hepatitis b virus	hepatitis b virus	Hepatitis B virus	11	kept
hepatomegaly	hepatomegaly	hepatomegaly	hepatomegaly	hepatomegaly	11	kept
Hernia, Umbilical	hernia umbilical	hernia umbilical	hernia umbilical	Hernia, Umbilical	11	kept
high volume hospital	high volume hospital	high volume hospital	high volume hospital	high volume hospital	11	kept
hip replacement	hip replacement	hip replacement	hip replacement	hip replacement	11	kept
home environment	home environment	home environment	home environment	home environment	11	kept
hopelessness	hopelessness	hopelessness	hopelessness	hopelessness	11	kept
Hospital Administrators	hospital administrators	hospital administrators	hospital administrator	hospital administrator	11	singular

Hospital care	hospital care	hospital care	hospital care	hospital care	11	case_or_format
Hospital rapid response team	hospital rapid response team	hospital rapid response team	hospital rapid response team	Hospital Rapid Response Team	11	case_or_format
hospital utilization	hospital utilization	hospital utilization	hospital utilization	hospital utilization	11	kept
hospital-acquired infection	hospital acquired infection	hospital acquired infection	hospital acquired infection	Hospital-acquired infections	11	case_or_format
humidifier	humidifier	humidifier	humidifier	humidifier	11	kept
hunger	hunger	hunger	hunger	hunger	11	kept
Hyperbilirubinemia	hyperbilirubinemia	hyperbilirubinemia	hyperbilirubinemia	hyperbilirubinemia	11	case_or_format
hypercholesterolemia	hypercholesterolemia	hypercholesterolemia	hypercholesterolemia	hypercholesterolemia	11	kept
hypomagnesemia	hypomagnesemia	hypomagnesemia	hypomagnesemia	hypomagnesemia	11	kept
hypothesis	hypothesis	hypothesis	hypothesis	hypothesis	11	kept
image quality	image quality	image quality	image quality	image quality	11	kept
immunoassay	immunoassay	immunoassay	immunoassay	immunoassay	11	kept
immunoglobulin A	immunoglobulin a	immunoglobulin a	immunoglobulin a	immunoglobulin A	11	kept
Immunoglobulins, Intravenous	immunoglobulins intravenous	immunoglobulins intravenous	immunoglobulins intravenous	Immunoglobulins, Intravenous	11	kept
Inappropriate Prescribing	inappropriate prescribing	inappropriate prescribing	inappropriate prescribing	inappropriate prescribing	11	case_or_format
individualization	individualization	individualization	individualization	individualization	11	kept

indometacin	indometacin	indometacin	indometacin	indometacin	11	kept
inflammatory bowel disease	inflammatory bowel disease	inflammatory bowel disease	inflammatory bowel disease	inflammatory bowel disease	11	kept
Influenza virus A H1N1	influenza virus a h1n1	influenza virus a h1n1	influenza virus a h1n1	Influenza virus A H1N1	11	kept
Information Seeking Behavior	information seeking behavior	information seeking behavior	information seeking behavior	Information Seeking Behavior	11	kept
Injections	injections	injections	injection	injection	11	singular
Insomnia Severity Index	insomnia severity index	insomnia severity index	insomnia severity index	Insomnia Severity Index	11	kept
Insurance Claim Review	insurance claim review	insurance claim review	insurance claim review	Insurance Claim Review	11	kept
integration	integration	integration	integration	integration	11	kept
intention to leave	intention to leave	intention to leave	intention to leave	Intention to leave	11	case_or_format
Interaction	interaction	interaction	interaction	Interaction	11	kept
interdisciplinary education	interdisciplinary education	interdisciplinary education	interdisciplinary education	interdisciplinary education	11	kept
Intra-abdominal pressure	intra abdominal pressure	intra abdominal pressure	intra abdominal pressure	Intra-abdominal pressure	11	kept
intrarater reliability	intrarater reliability	intrarater reliability	intrarater reliability	intrarater reliability	11	kept
Italian	italian	italian	italian	Italian	11	kept
job security	job security	job security	job security	job security	11	kept
kangaroo mother care	kangaroo mother care	kangaroo mother care	kangaroo mother care	Kangaroo mother care	11	case_or_format

kidney	kidney	kidney	kidney	Kidney	11	case_or_format
knowledge translation	knowledge translation	knowledge translation	knowledge translation	Knowledge translation	11	case_or_format
labor complication	labor complication	labor complication	labor complication	labor complication	11	kept
Laboratories, Hospital	laboratories hospital	laboratories hospital	laboratories hospital	Laboratories, Hospital	11	kept
laryngeal mask	laryngeal mask	laryngeal mask	laryngeal mask	Laryngeal Masks	11	case_or_format
Laryngoscopes	laryngoscopes	laryngoscopes	laryngoscope	laryngoscope	11	singular
learning curve	learning curve	learning curve	learning curve	learning curve	11	kept
leg	leg	leg	leg	leg	11	kept
Legionella pneumophila	legionella pneumophila	legionella pneumophila	legionella pneumophila	Legionella pneumophila	11	kept
Level of consciousness	level of consciousness	level of consciousness	level of consciousness	Level of consciousness	11	kept
Lidocaine	lidocaine	lidocaine	lidocaine	lidocaine	11	case_or_format
life cycle	life cycle	life cycle	life cycle	life cycle	11	kept
Life Expectancy	life expectancy	life expectancy	life expectancy	life expectancy	11	case_or_format
liver cell carcinoma	liver cell carcinoma	liver cell carcinoma	liver cell carcinoma	liver cell carcinoma	11	kept
Liver transplantation	liver transplantation	liver transplantation	liver transplantation	Liver Transplantation	11	case_or_format
loneliness	loneliness	loneliness	loneliness	Loneliness	11	case_or_format

lung clearance	lung clearance	lung clearance	lung clearance	lung clearance	11	kept
lung gas exchange	lung gas exchange	lung gas exchange	lung gas exchange	lung gas exchange	11	kept
major surgery	major surgery	major surgery	major surgery	major surgery	11	kept
malaria	malaria	malaria	malaria	malaria	11	kept
Malignant Hyperthermia	malignant hyperthermia	malignant hyperthermia	malignant hyperthermia	Malignant Hyperthermia	11	kept
malignant hyperthermia	malignant hyperthermia	malignant hyperthermia	malignant hyperthermia	Malignant Hyperthermia	11	case_or_format
Mechanical Ventilation	mechanical ventilation	mechanical ventilation	mechanical ventilation	Mechanical ventilation	11	case_or_format
meconium	meconium	meconium	meconium	meconium	11	kept
medical futility	medical futility	medical futility	medical futility	Medical Futility	11	case_or_format
Medication Therapy Management	medication therapy management	medication therapy management	medication therapy management	medication therapy management	11	case_or_format
mental workload	mental workload	mental workload	mental workload	mental workload	11	kept
meta‑analysis	meta analysis	meta analysis	meta analysis	meta analysis	11	case_or_format
Methadone	methadone	methadone	methadone	methadone	11	case_or_format
microbial colonization	microbial colonization	microbial colonization	microbial colonization	microbial colonization	11	kept
microbiome	microbiome	microbiome	microbiome	microbiome	11	kept
minimally invasive procedure	minimally invasive procedure	minimally invasive procedure	minimally invasive procedure	minimally invasive procedure	11	kept

Mitral Valve Insufficiency	mitral valve insufficiency	mitral valve insufficiency	mitral valve insufficiency	Mitral Valve Insufficiency	11	kept
Molecular Epidemiology	molecular epidemiology	molecular epidemiology	molecular epidemiology	molecular epidemiology	11	case_or_format
Montreal cognitive assessment	montreal cognitive assessment	montreal cognitive assessment	montreal cognitive assessment	Montreal cognitive assessment	11	kept
Moral sensitivity	moral sensitivity	moral sensitivity	moral sensitivity	moral sensitivity	11	case_or_format
multidrug resistant bacterium	multidrug resistant bacterium	multidrug resistant bacterium	multidrug resistant bacterium	multidrug resistant bacterium	11	kept
multivitamin	multivitamin	multivitamin	multivitamin	multivitamin	11	kept
mycophenolate mofetil	mycophenolate mofetil	mycophenolate mofetil	mycophenolate mofetil	mycophenolate mofetil	11	kept
Myocardial Contraction	myocardial contraction	myocardial contraction	myocardial contraction	Myocardial Contraction	11	kept
myopathy	myopathy	myopathy	myopathy	myopathy	11	kept
Natriuretic Peptide, Brain	natriuretic peptide brain	natriuretic peptide brain	natriuretic peptide brain	Natriuretic Peptide, Brain	11	kept
Nebraska	nebraska	nebraska	nebraska	Nebraska	11	kept
Nebulizers and Vaporizers	nebulizers and vaporizers	nebulizers and vaporizers	nebulizers and vaporizers	Nebulizers and Vaporizers	11	kept
Neonatal abstinence syndrome	neonatal abstinence syndrome	neonatal abstinence syndrome	neonatal abstinence syndrome	Neonatal Abstinence Syndrome	11	case_or_format
Neonatal pain	neonatal pain	neonatal pain	neonatal pain	Neonatal pain	11	kept
neostigmine	neostigmine	neostigmine	neostigmine	neostigmine	11	kept
Neural Networks, Computer	neural networks computer	neural networks computer	neural networks computer	Neural Networks, Computer	11	kept

Neurological Rehabilitation	neurological rehabilitation	neurological rehabilitation	neurological rehabilitation	Neurological Rehabilitation	11	kept
Neuromuscular Diseases	neuromuscular diseases	neuromuscular diseases	neuromuscular disease	neuromuscular disease	11	singular
neuromuscular electrical stimulation	neuromuscular electrical stimulation	neuromuscular electrical stimulation	neuromuscular electrical stimulation	neuromuscular electrical stimulation	11	kept
newborn monitoring	newborn monitoring	newborn monitoring	newborn monitoring	newborn monitoring	11	kept
nitrous oxide	nitrous oxide	nitrous oxide	nitrous oxide	nitrous oxide	11	kept
Nociception	nociception	nociception	nociception	nociception	11	case_or_format
Non-Randomized Controlled Trials as Topic	non randomized controlled trials as topic	non randomized controlled trials as topic	non randomized controlled trials as topic	Non-Randomized Controlled Trials as Topic	11	kept
nystatin	nystatin	nystatin	nystatin	nystatin	11	kept
occupational burnout	occupational burnout	occupational burnout	occupational burnout	occupational burnout	11	kept
Oral feeding	oral feeding	oral feeding	oral feeding	Oral feeding	11	kept
oral feeding	oral feeding	oral feeding	oral feeding	Oral feeding	11	case_or_format
orthostatic hypotension	orthostatic hypotension	orthostatic hypotension	orthostatic hypotension	orthostatic hypotension	11	kept
osteosynthesis	osteosynthesis	osteosynthesis	osteosynthesis	osteosynthesis	11	kept
Palpation	palpation	palpation	palpation	palpation	11	case_or_format
parent counseling	parent counseling	parent counseling	parent counseling	parent counseling	11	kept
Paris	paris	paris	paris	Paris	11	kept

PARTICIPATION	participation	participation	participation	participation	11	case_or_format
Paternal Behavior	paternal behavior	paternal behavior	paternal behavior	Paternal Behavior	11	kept
pediatric palliative care	pediatric palliative care	pediatric palliative care	pediatric palliative care	pediatric palliative care	11	kept
Pharmacist	pharmacist	pharmacist	pharmacist	pharmacist	11	case_or_format
phlebotomist	phlebotomist	phlebotomist	phlebotomist	phlebotomist	11	kept
phylogeny	phylogeny	phylogeny	phylogeny	phylogeny	11	kept
Physical	physical	physical	physical	Physical	11	kept
physical	physical	physical	physical	Physical	11	case_or_format
physical stress	physical stress	physical stress	physical stress	physical stress	11	kept
Physiological parameters	physiological parameters	physiological parameters	physiological parameter	Physiological parameters	11	singular
place of death	place of death	place of death	place of death	place of death	11	kept
Placenta	placenta	placenta	placenta	placenta	11	case_or_format
planning	planning	planning	planning	planning	11	kept
Pneumococcal Vaccines	pneumococcal vaccines	pneumococcal vaccines	pneumococcal vaccine	Pneumococcal Vaccines	11	singular
Pneumonia, Staphylococcal	pneumonia staphylococcal	pneumonia staphylococcal	pneumonia staphylococcal	Pneumonia, Staphylococcal	11	kept
point-of-care ultrasound	point of care ultrasound	point of care ultrasound	point of care ultrasound	point of care ultrasound	11	case_or_format

Polyurethanes	polyurethanes	polyurethanes	polyurethane	Polyurethanes	11	singular
postnatal depression	postnatal depression	postnatal depression	postnatal depression	postnatal depression	11	kept
powerlessness	powerlessness	powerlessness	powerlessness	powerlessness	11	kept
Precipitating Factors	precipitating factors	precipitating factors	precipitating factors	Precipitating Factors	11	kept
Prenatal Exposure Delayed Effects	prenatal exposure delayed effects	prenatal exposure delayed effects	prenatal exposure delayed effects	Prenatal Exposure Delayed Effects	11	kept
presenteeism	presenteeism	presenteeism	presenteeism	Presenteeism	11	case_or_format
pressure measurement	pressure measurement	pressure measurement	pressure measurement	pressure measurement	11	kept
pressure support ventilation	pressure support ventilation	pressure support ventilation	pressure support ventilation	pressure support ventilation	11	kept
process development	process development	process development	process development	process development	11	kept
prognostication	prognostication	prognostication	prognostication	prognostication	11	kept
Prospective Payment System	prospective payment system	prospective payment system	prospective payment system	Prospective Payment System	11	kept
protection	protection	protection	protection	protection	11	kept
psychometric	psychometric	psychometric	psychometric	Psychometrics	11	case_or_format
public-private partnership	public private partnership	public private partnership	public private partnership	public-private partnership	11	kept
Quadriplegia	quadriplegia	quadriplegia	quadriplegia	quadriplegia	11	case_or_format
QUALITATIVE CONTENT-ANALYSIS	qualitative content analysis	qualitative content analysis	qualitative content analysis	Qualitative content analysis	11	case_or_format

qualitative studies	qualitative studies	qualitative studies	qualitative studies	qualitative studies	11	kept
quality improvement project	quality improvement project	quality improvement project	quality improvement project	quality improvement project	11	kept
Quality management	quality management	quality management	quality management	Quality management	11	kept
quinolone	quinolone	quinolone	quinolone	quinolone	11	kept
Radial Artery	radial artery	radial artery	radial artery	radial artery	11	case_or_format
radiological technologist	radiological technologist	radiological technologist	radiological technologist	radiological technologist	11	kept
RASS	rass	rass	rass	RASS	11	kept
recurrent infection	recurrent infection	recurrent infection	recurrent infection	recurrent infection	11	kept
regionalization	regionalization	regionalization	regionalization	regionalization	11	kept
rehydration	rehydration	rehydration	rehydration	rehydration	11	kept
reperfusion injury	reperfusion injury	reperfusion injury	reperfusion injury	reperfusion injury	11	kept
Reporting	reporting	reporting	reporting	Reporting	11	kept
Resources	resources	resources	resource	Resources	11	singular
response time	response time	response time	response time	response time	11	kept
RESPONSES	responses	responses	response	RESPONSES	11	singular
restraint	restraint	restraint	restraint	Restraint	11	case_or_format

retinopathy	retinopathy	retinopathy	retinopathy	retinopathy	11	kept
retrospective studies	retrospective studies	retrospective studies	retrospective study	Retrospective Studies	11	singular
ribavirin	ribavirin	ribavirin	ribavirin	ribavirin	11	kept
risk prediction	risk prediction	risk prediction	risk prediction	risk prediction	11	kept
Rooming-in Care	rooming in care	rooming in care	rooming in care	Rooming-in Care	11	kept
safety procedure	safety procedure	safety procedure	safety procedure	safety procedure	11	kept
San Francisco	san francisco	san francisco	san francisco	San Francisco	11	kept
sanitation	sanitation	sanitation	sanitation	sanitation	11	kept
seasonal variation	seasonal variation	seasonal variation	seasonal variation	seasonal variation	11	kept
self help	self help	self help	self help	self help	11	kept
sensation	sensation	sensation	sensation	Sensation	11	case_or_format
serotonin uptake inhibitor	serotonin uptake inhibitor	serotonin uptake inhibitor	serotonin uptake inhibitor	serotonin uptake inhibitor	11	kept
Sex Characteristics	sex characteristics	sex characteristics	sex characteristics	Sex Characteristics	11	kept
Shivering	shivering	shivering	shivering	shivering	11	case_or_format
Sierra Leone	sierra leone	sierra leone	sierra leone	Sierra Leone	11	kept
skin biopsy	skin biopsy	skin biopsy	skin biopsy	skin biopsy	11	kept

skin-to-skin contact	skin to skin contact	skin to skin contact	skin to skin contact	Skin-to-skin contact	11	case_or_format
sleep apnea syndrome	sleep apnea syndrome	sleep apnea syndrome	sleep apnea syndrome	sleep apnea syndrome	11	kept
Slovenia	slovenia	slovenia	slovenia	Slovenia	11	kept
Soaps	soaps	soaps	soap	soap	11	singular
Social Determinants of Health	social determinants of health	social determinants of health	social determinants of health	social determinants of health	11	case_or_format
solutio placentae	solutio placentae	solutio placentae	solutio placentae	solutio placentae	11	kept
South Australia	south australia	south australia	south australia	South Australia	11	kept
spinal cord compression	spinal cord compression	spinal cord compression	spinal cord compression	spinal cord compression	11	kept
Spinal Puncture	spinal puncture	spinal puncture	spinal puncture	Spinal Puncture	11	kept
spine injury	spine injury	spine injury	spine injury	spine injury	11	kept
ST segment elevation	st segment elevation	st segment elevation	st segment elevation	ST segment elevation	11	kept
statistical bias	statistical bias	statistical bias	statistical bias	statistical bias	11	kept
subcutaneous emphysema	subcutaneous emphysema	subcutaneous emphysema	subcutaneous emphysema	subcutaneous emphysema	11	kept
sudden cardiac death	sudden cardiac death	sudden cardiac death	sudden cardiac death	sudden cardiac death	11	kept
Suicide, Assisted	suicide assisted	suicide assisted	suicide assisted	Suicide, Assisted	11	kept
Surgical Tape	surgical tape	surgical tape	surgical tape	Surgical Tape	11	kept

systemic therapy	systemic therapy	systemic therapy	systemic therapy	systemic therapy	11	kept
team building	team building	team building	team building	team building	11	kept
Telehealth	telehealth	telehealth	telehealth	telehealth	11	case_or_format
Tetralogy of Fallot	tetralogy of fallot	tetralogy of fallot	tetralogy of fallot	Tetralogy of Fallot	11	kept
Textbooks as Topic	textbooks as topic	textbooks as topic	textbooks as topic	Textbooks as Topic	11	kept
Therapeutics	therapeutics	therapeutics	therapeutic	Therapeutics	11	singular
Thoracic surgery	thoracic surgery	thoracic surgery	thoracic surgery	Thoracic Surgery	11	case_or_format
Tissue and organ procurement	tissue and organ procurement	tissue and organ procurement	tissue and organ procurement	Tissue and Organ Procurement	11	case_or_format
tissue and organ procurement	tissue and organ procurement	tissue and organ procurement	tissue and organ procurement	Tissue and Organ Procurement	11	case_or_format
tissue injury	tissue injury	tissue injury	tissue injury	tissue injury	11	kept
Tobacco Use Disorder	tobacco use disorder	tobacco use disorder	tobacco use disorder	Tobacco Use Disorder	11	kept
torsade des pointes	torsade des pointes	torsade des pointes	torsade des pointes	torsade des pointes	11	kept
toxicology	toxicology	toxicology	toxicology	toxicology	11	kept
Tracheal Stenosis	tracheal stenosis	tracheal stenosis	tracheal stenosis	Tracheal Stenosis	11	kept
Translation	translation	translation	translation	Translations	11	case_or_format
Trauma care	trauma care	trauma care	trauma care	Trauma care	11	kept

trazodone	trazodone	trazodone	trazodone	trazodone	11	kept
Tumor Lysis Syndrome	tumor lysis syndrome	tumor lysis syndrome	tumor lysis syndrome	Tumor Lysis Syndrome	11	kept
turnaround time	turnaround time	turnaround time	turnaround time	turnaround time	11	kept
turnover intention	turnover intention	turnover intention	turnover intention	Turnover intention	11	case_or_format
Ultrafiltration	ultrafiltration	ultrafiltration	ultrafiltration	Ultrafiltration	11	kept
ultrafiltration	ultrafiltration	ultrafiltration	ultrafiltration	Ultrafiltration	11	case_or_format
ultrasonography	ultrasonography	ultrasonography	ultrasonography	Ultrasonography	11	case_or_format
umbilical cord	umbilical cord	umbilical cord	umbilical cord	umbilical cord	11	kept
unemployment	unemployment	unemployment	unemployment	unemployment	11	kept
Unidades de Terapia Intensiva	unidades de terapia intensiva	unidades de terapia intensiva	unidades de terapia intensiva	Unidades de Terapia Intensiva	11	kept
Universal Precautions	universal precautions	universal precautions	universal precaution	Universal Precautions	11	singular
unspecified side effect	unspecified side effect	unspecified side effect	unspecified side effect	unspecified side effect	11	kept
Urinary Bladder Neoplasms	urinary bladder neoplasms	urinary bladder neoplasms	urinary bladder neoplasms	Urinary Bladder Neoplasms	11	kept
urine sampling	urine sampling	urine sampling	urine sampling	urine sampling	11	kept
urology	urology	urology	urology	urology	11	kept
usability testing	usability testing	usability testing	usability testing	usability testing	11	kept

virus detection	virus detection	virus detection	virus detection	virus detection	11	kept
vitamin K group	vitamin k group	vitamin k group	vitamin k group	vitamin K group	11	kept
waveform	waveform	waveform	waveform	waveform	11	kept
Wireless Technology	wireless technology	wireless technology	wireless technology	Wireless Technology	11	kept
witness	witness	witness	witness	witness	11	kept
WOMEN	women	women	women	WOMEN	11	kept
wound drainage	wound drainage	wound drainage	wound drainage	wound drainage	11	kept
abdominal compartment syndrome	abdominal compartment syndrome	abdominal compartment syndrome	abdominal compartment syndrome	Abdominal compartment syndrome	10	case_or_format
Action research	action research	action research	action research	action research	10	case_or_format
acupuncture	acupuncture	acupuncture	acupuncture	acupuncture	10	kept
acute hypoxemic respiratory failure	acute hypoxemic respiratory failure	acute hypoxemic respiratory failure	acute hypoxemic respiratory failure	acute hypoxemic respiratory failure	10	kept
acute ischemic stroke	acute ischemic stroke	acute ischemic stroke	acute ischemic stroke	acute ischemic stroke	10	kept
Acute lung injury	acute lung injury	acute lung injury	acute lung injury	acute lung injury	10	case_or_format
addiction	addiction	addiction	addiction	addiction	10	kept
Adenoidectomy	adenoidectomy	adenoidectomy	adenoidectomy	Adenoidectomy	10	kept
adhesive agent	adhesive agent	adhesive agent	adhesive agent	adhesive agent	10	kept

adjuvant therapy	adjuvant therapy	adjuvant therapy	adjuvant therapy	adjuvant therapy	10	kept
ADL disability	adl disability	adl disability	adl disability	ADL disability	10	kept
administration	administration	administration	administration	Administration	10	case_or_format
Advanced practice provider	advanced practice provider	advanced practice provider	advanced practice provider	advanced practice provider	10	case_or_format
airplane crew	airplane crew	airplane crew	airplane crew	airplane crew	10	kept
alarms	alarms	alarms	alarm	Alarms	10	singular
Albumins	albumins	albumins	albumin	albumin	10	singular
alcohol intoxication	alcohol intoxication	alcohol intoxication	alcohol intoxication	alcohol intoxication	10	kept
alkaline phosphatase	alkaline phosphatase	alkaline phosphatase	alkaline phosphatase	alkaline phosphatase	10	kept
alkalosis	alkalosis	alkalosis	alkalosis	alkalosis	10	kept
alprazolam	alprazolam	alprazolam	alprazolam	alprazolam	10	kept
American Indian	american indian	american indian	american indian	American Indian	10	kept
ANESTHESIA	anesthesia	anesthesia	anesthesia	anesthesia	10	case_or_format
aneurysm	aneurysm	aneurysm	aneurysm	aneurysm	10	kept
anion gap	anion gap	anion gap	anion gap	anion gap	10	kept
antibiotic stewardship	antibiotic stewardship	antibiotic stewardship	antibiotic stewardship	antibiotic stewardship	10	kept

anticonvulsant therapy	anticonvulsant therapy	anticonvulsant therapy	anticonvulsant therapy	anticonvulsant therapy	10	kept
Antidotes	antidotes	antidotes	antidote	antidote	10	singular
antifungal resistance	antifungal resistance	antifungal resistance	antifungal resistance	antifungal resistance	10	kept
antifungal susceptibility	antifungal susceptibility	antifungal susceptibility	antifungal susceptibility	antifungal susceptibility	10	kept
antifungal therapy	antifungal therapy	antifungal therapy	antifungal therapy	antifungal therapy	10	kept
antiretroviral therapy	antiretroviral therapy	antiretroviral therapy	antiretroviral therapy	antiretroviral therapy	10	kept
anxiety assessment	anxiety assessment	anxiety assessment	anxiety assessment	anxiety assessment	10	kept
aorta valve replacement	aorta valve replacement	aorta valve replacement	aorta valve replacement	aorta valve replacement	10	kept
Aorta, Thoracic	aorta thoracic	aorta thoracic	aorta thoracic	Aorta, Thoracic	10	kept
apheresis	apheresis	apheresis	apheresis	apheresis	10	kept
appendicitis	appendicitis	appendicitis	appendicitis	appendicitis	10	kept
Arkansas	arkansas	arkansas	arkansas	Arkansas	10	kept
Arteriovenous Shunt, Surgical	arteriovenous shunt surgical	arteriovenous shunt surgical	arteriovenous shunt surgical	Arteriovenous Shunt, Surgical	10	kept
Arthroplasty, Replacement, Knee	arthroplasty replacement knee	arthroplasty replacement knee	arthroplasty replacement knee	Arthroplasty, Replacement, Knee	10	kept
artifact	artifact	artifact	artifact	Artifacts	10	case_or_format
Artificial	artificial	artificial	artificial	artificial	10	case_or_format

Artificial respiration	artificial respiration	artificial respiration	artificial respiration	Artificial respiration	10	kept
aspartate aminotransferase blood level	aspartate aminotransferase blood level	aspartate aminotransferase blood level	aspartate aminotransferase blood level	aspartate aminotransferase blood level	10	kept
asystole	asystole	asystole	asystole	asystole	10	kept
auscultation	auscultation	auscultation	auscultation	Auscultation	10	case_or_format
baclofen	baclofen	baclofen	baclofen	baclofen	10	kept
BACTEREMIA	bacteremia	bacteremia	bacteremia	Bacteremia	10	case_or_format
bacterial protein	bacterial protein	bacterial protein	bacterial protein	Bacterial Proteins	10	case_or_format
basic life support	basic life support	basic life support	basic life support	basic life support	10	kept
beta lactamase inhibitor	beta lactamase inhibitor	beta lactamase inhibitor	beta lactamase inhibitor	beta lactamase inhibitor	10	kept
Biopsy	biopsy	biopsy	biopsy	biopsy	10	case_or_format
blood glucose	blood glucose	blood glucose	blood glucose	Blood Glucose	10	case_or_format
Blood Loss, Surgical	blood loss surgical	blood loss surgical	blood loss surgical	Blood Loss, Surgical	10	kept
blood transfusion reaction	blood transfusion reaction	blood transfusion reaction	blood transfusion reaction	blood transfusion reaction	10	kept
bloodborne bacterium	bloodborne bacterium	bloodborne bacterium	bloodborne bacterium	bloodborne bacterium	10	kept
body composition	body composition	body composition	body composition	body composition	10	kept
Body Height	body height	body height	body height	body height	10	case_or_format

Body mass index	body mass index	body mass index	body mass index	Body Mass Index	10	case_or_format
brain angiography	brain angiography	brain angiography	brain angiography	brain angiography	10	kept
breathing disorder	breathing disorder	breathing disorder	breathing disorder	breathing disorder	10	kept
bubble continuous positive airway pressure	bubble continuous positive airway pressure	bubble continuous positive airway pressure	bubble continuous positive airway pressure	bubble continuous positive airway pressure	10	kept
Burn	burn	burn	burn	Burns	10	case_or_format
Calcium Channel Blockers	calcium channel blockers	calcium channel blockers	calcium channel blocker	Calcium Channel Blockers	10	singular
cancer recurrence	cancer recurrence	cancer recurrence	cancer recurrence	cancer recurrence	10	kept
Candidemia	candidemia	candidemia	candidemia	candidemia	10	case_or_format
carbapenem resistant Klebsiella pneumoniae	carbapenem resistant klebsiella pneumoniae	carbapenem resistant klebsiella pneumoniae	carbapenem resistant klebsiella pneumoniae	carbapenem resistant Klebsiella pneumoniae	10	kept
carbapenemase producing Enterobacteriaceae	carbapenemase producing enterobacteriaceae	carbapenemase producing enterobacteriaceae	carbapenemase producing enterobacteriaceae	carbapenemase producing Enterobacteriaceae	10	kept
CARDIAC-SURGERY	cardiac surgery	cardiac surgery	cardiac surgery	Cardiac surgery	10	case_or_format
cardiology service	cardiology service	cardiology service	cardiology service	cardiology service	10	kept
Cardiomyopathy, Dilated	cardiomyopathy dilated	cardiomyopathy dilated	cardiomyopathy dilated	Cardiomyopathy, Dilated	10	kept
Cardiovascular	cardiovascular	cardiovascular	cardiovascular	Cardiovascular	10	kept
Cardiovascular disease	cardiovascular disease	cardiovascular disease	cardiovascular disease	cardiovascular disease	10	case_or_format
Cardiovascular surgery	cardiovascular surgery	cardiovascular surgery	cardiovascular surgery	cardiovascular surgery	10	case_or_format

Case study	case study	case study	case study	case study	10	case_or_format
Catheter Ablation	catheter ablation	catheter ablation	catheter ablation	Catheter Ablation	10	kept
CAUTI	cauti	cauti	cauti	CAUTI	10	kept
Centers for Disease Control and Prevention, U.S	centers for disease control and prevention u.s	centers for disease control and prevention u.s	centers for disease control and prevention u.s	Centers for Disease Control and Prevention, U.S	10	kept
Central line	central line	central line	central line	Central line	10	kept
Central line-associated bloodstream infections	central line associated bloodstream infections	central line associated bloodstream infections	central line associated bloodstream infection	Central line-associated bloodstream infections	10	singular
Central venous	central venous	central venous	central venous	Central venous	10	kept
cerebrospinal fluid drainage	cerebrospinal fluid drainage	cerebrospinal fluid drainage	cerebrospinal fluid drainage	cerebrospinal fluid drainage	10	kept
ChatGPT	chatgpt	chatgpt	chatgpt	ChatGPT	10	kept
chemoprophylaxis	chemoprophylaxis	chemoprophylaxis	chemoprophylaxis	chemoprophylaxis	10	kept
child safety	child safety	child safety	child safety	child safety	10	kept
Child Welfare	child welfare	child welfare	child welfare	Child Welfare	10	kept
chloral hydrate	chloral hydrate	chloral hydrate	chloral hydrate	chloral hydrate	10	kept
cholesterol	cholesterol	cholesterol	cholesterol	cholesterol	10	kept
Chorioamnionitis	chorioamnionitis	chorioamnionitis	chorioamnionitis	chorioamnionitis	10	case_or_format
Cirrhosis	cirrhosis	cirrhosis	cirrhosis	Cirrhosis	10	kept

cisplatin	cisplatin	cisplatin	cisplatin	cisplatin	10	kept
Citrobacter koseri	citrobacter koseri	citrobacter koseri	citrobacter koseri	Citrobacter koseri	10	kept
Clinical ethics	clinical ethics	clinical ethics	clinical ethics	clinical ethics	10	case_or_format
CLINICAL-PRACTICE	clinical practice	clinical practice	clinical practice	clinical practice	10	case_or_format
Cluster analysis	cluster analysis	cluster analysis	cluster analysis	Cluster Analysis	10	case_or_format
coding	coding	coding	coding	coding	10	kept
colloid	colloid	colloid	colloid	colloid	10	kept
colonization	colonization	colonization	colonization	COLONIZATION	10	case_or_format
Communication Disorders	communication disorders	communication disorders	communication disorder	communication disorder	10	singular
COMMUNITY	community	community	community	community	10	case_or_format
Community Participation	community participation	community participation	community participation	Community Participation	10	kept
compression	compression	compression	compression	compression	10	kept
Computer User Training	computer user training	computer user training	computer user training	Computer User Training	10	kept
CONTAMINATION	contamination	contamination	contamination	contamination	10	case_or_format
Continuous Glucose Monitoring	continuous glucose monitoring	continuous glucose monitoring	continuous glucose monitoring	continuous glucose monitoring	10	case_or_format
contrast medium extravasation	contrast medium extravasation	contrast medium extravasation	contrast medium extravasation	contrast medium extravasation	10	kept

Cost effectiveness	cost effectiveness	cost effectiveness	cost effectiveness	Cost-effectiveness	10	case_or_format
cost utility analysis	cost utility analysis	cost utility analysis	cost utility analysis	cost utility analysis	10	kept
COST-EFFECTIVENESS	cost effectiveness	cost effectiveness	cost effectiveness	Cost-effectiveness	10	case_or_format
COVID-19 pneumonia	covid 19 pneumonia	covid 19 pneumonia	covid 19 pneumonia	COVID-19 pneumonia	10	kept
cultural diversity	cultural diversity	cultural diversity	cultural diversity	Cultural Diversity	10	case_or_format
cumulative incidence	cumulative incidence	cumulative incidence	cumulative incidence	cumulative incidence	10	kept
Cytomegalovirus	cytomegalovirus	cytomegalovirus	cytomegalovirus	Cytomegalovirus	10	kept
Cytoreduction Surgical Procedures	cytoreduction surgical procedures	cytoreduction surgical procedures	cytoreduction surgical procedures	Cytoreduction Surgical Procedures	10	kept
cytoreductive surgery	cytoreductive surgery	cytoreductive surgery	cytoreductive surgery	cytoreductive surgery	10	kept
dabigatran	dabigatran	dabigatran	dabigatran	dabigatran	10	kept
data accuracy	data accuracy	data accuracy	data accuracy	data accuracy	10	kept
decannulation	decannulation	decannulation	decannulation	decannulation	10	kept
decompression	decompression	decompression	decompression	decompression	10	kept
decompression surgery	decompression surgery	decompression surgery	decompression surgery	decompression surgery	10	kept
Decompressive Craniectomy	decompressive craniectomy	decompressive craniectomy	decompressive craniectomy	decompressive craniectomy	10	case_or_format
Deglutition disorders	deglutition disorders	deglutition disorders	deglutition disorder	Deglutition Disorders	10	singular

Delegation, Professional	delegation professional	delegation professional	delegation professional	Delegation, Professional	10	kept
desflurane	desflurane	desflurane	desflurane	desflurane	10	kept
DIAGNOSIS	diagnosis	diagnosis	diagnosis	diagnosis	10	case_or_format
Diagnostic Techniques, Ophthalmological	diagnostic techniques ophthalmological	diagnostic techniques ophthalmological	diagnostic techniques ophthalmological	Diagnostic Techniques, Ophthalmological	10	kept
disease registry	disease registry	disease registry	disease registry	disease registry	10	kept
Diseases in Twins	diseases in twins	diseases in twins	diseases in twins	Diseases in Twins	10	kept
disorders of higher cerebral function	disorders of higher cerebral function	disorders of higher cerebral function	disorders of higher cerebral function	disorders of higher cerebral function	10	kept
DNA extraction	dna extraction	dna extraction	dna extraction	DNA extraction	10	kept
Doppler echography	doppler echography	doppler echography	doppler echography	Doppler echography	10	kept
drug abuse	drug abuse	drug abuse	drug abuse	drug abuse	10	kept
drug eruption	drug eruption	drug eruption	drug eruption	drug eruption	10	kept
drug incompatibility	drug incompatibility	drug incompatibility	drug incompatibility	drug incompatibility	10	kept
dry skin	dry skin	dry skin	dry skin	dry skin	10	kept
Ductus Arteriosus, Patent	ductus arteriosus patent	ductus arteriosus patent	ductus arteriosus patent	Ductus Arteriosus, Patent	10	kept
Early intervention	early intervention	early intervention	early intervention	early intervention	10	case_or_format
Early mobility	early mobility	early mobility	early mobility	early mobility	10	case_or_format

Eating	eating	eating	eating	eating	10	case_or_format
Eclampsia	eclampsia	eclampsia	eclampsia	eclampsia	10	case_or_format
education and training	education and training	education and training	education and training	education and training	10	kept
Education, Medical, Undergraduate	education medical undergraduate	education medical undergraduate	education medical undergraduate	Education, Medical, Undergraduate	10	kept
Education, Nonprofessional	education nonprofessional	education nonprofessional	education nonprofessional	Education, Nonprofessional	10	kept
Educational program	educational program	educational program	educational program	Educational program	10	kept
Electrocardiogram	electrocardiogram	electrocardiogram	electrocardiogram	electrocardiogram	10	case_or_format
electronics	electronics	electronics	electronic	Electronics	10	singular
Embolization, Therapeutic	embolization therapeutic	embolization therapeutic	embolization therapeutic	Embolization, Therapeutic	10	kept
Emigration and Immigration	emigration and immigration	emigration and immigration	emigration and immigration	Emigration and Immigration	10	kept
emotional disorder	emotional disorder	emotional disorder	emotional disorder	emotional disorder	10	kept
End Stage Liver Disease	end stage liver disease	end stage liver disease	end stage liver disease	End Stage Liver Disease	10	kept
end-of-life decisions	end of life decisions	end of life decisions	end of life decision	End-of-life decisions	10	singular
endocrine disease	endocrine disease	endocrine disease	endocrine disease	endocrine disease	10	kept
Enfermera	enfermera	enfermera	enfermera	Enfermera	10	kept
enterocutaneous fistula	enterocutaneous fistula	enterocutaneous fistula	enterocutaneous fistula	enterocutaneous fistula	10	kept

epileptic discharge	epileptic discharge	epileptic discharge	epileptic discharge	epileptic discharge	10	kept
erythrocyte sedimentation rate	erythrocyte sedimentation rate	erythrocyte sedimentation rate	erythrocyte sedimentation rate	erythrocyte sedimentation rate	10	kept
esmolol	esmolol	esmolol	esmolol	esmolol	10	kept
Esophageal Neoplasms	esophageal neoplasms	esophageal neoplasms	esophageal neoplasms	Esophageal Neoplasms	10	kept
Ethics, Research	ethics research	ethics research	ethics research	Ethics, Research	10	kept
Ethnic Groups	ethnic groups	ethnic groups	ethnic group	ethnic group	10	singular
European	european	european	european	European	10	kept
exercise test	exercise test	exercise test	exercise test	exercise test	10	kept
expert system	expert system	expert system	expert system	expert system	10	kept
Expert Systems	expert systems	expert systems	expert system	expert system	10	singular
face	face	face	face	face	10	kept
Failure to rescue	failure to rescue	failure to rescue	failure to rescue	failure to rescue	10	case_or_format
Familia	familia	familia	familia	Familia	10	kept
Family caregivers	family caregivers	family caregivers	family caregiver	Family caregivers	10	singular
Family centred care	family centred care	family centred care	family centred care	Family-centred care	10	case_or_format
family involvement	family involvement	family involvement	family involvement	family involvement	10	kept

Family Practice	family practice	family practice	family practice	Family Practice	10	kept
Family relations	family relations	family relations	family relation	Family Relations	10	singular
fetal well being	fetal well being	fetal well being	fetal well being	fetal well being	10	kept
field hospital	field hospital	field hospital	field hospital	field hospital	10	kept
finance	finance	finance	finance	finance	10	kept
fistula	fistula	fistula	fistula	fistula	10	kept
Fluid overload	fluid overload	fluid overload	fluid overload	Fluid overload	10	kept
forced vital capacity	forced vital capacity	forced vital capacity	forced vital capacity	forced vital capacity	10	kept
forearm	forearm	forearm	forearm	forearm	10	kept
fracture fixation	fracture fixation	fracture fixation	fracture fixation	fracture fixation	10	kept
Funeral Rites	funeral rites	funeral rites	funeral rites	Funeral Rites	10	kept
furniture	furniture	furniture	furniture	furniture	10	kept
Gamification	gamification	gamification	gamification	Gamification	10	kept
gamma interferon	gamma interferon	gamma interferon	gamma interferon	gamma interferon	10	kept
Gastric Bypass	gastric bypass	gastric bypass	gastric bypass	Gastric Bypass	10	kept
Gastric residual volume	gastric residual volume	gastric residual volume	gastric residual volume	gastric residual volume	10	case_or_format

gastrointestinal agent	gastrointestinal agent	gastrointestinal agent	gastrointestinal agent	gastrointestinal agent	10	kept
Gastrointestinal Motility	gastrointestinal motility	gastrointestinal motility	gastrointestinal motility	Gastrointestinal Motility	10	kept
gastroschisis	gastroschisis	gastroschisis	gastroschisis	Gastroschisis	10	case_or_format
Gender	gender	gender	gender	gender	10	case_or_format
Genetic Counseling	genetic counseling	genetic counseling	genetic counseling	Genetic Counseling	10	kept
geriatric rehabilitation	geriatric rehabilitation	geriatric rehabilitation	geriatric rehabilitation	geriatric rehabilitation	10	kept
glioma	glioma	glioma	glioma	glioma	10	kept
glycopeptide	glycopeptide	glycopeptide	glycopeptide	glycopeptide	10	kept
graft failure	graft failure	graft failure	graft failure	graft failure	10	kept
gross national product	gross national product	gross national product	gross national product	gross national product	10	kept
Growth	growth	growth	growth	Growth	10	kept
Guatemala	guatemala	guatemala	guatemala	Guatemala	10	kept
Hand Sanitizers	hand sanitizers	hand sanitizers	hand sanitizer	hand sanitizer	10	singular
health care concepts	health care concepts	health care concepts	health care concepts	health care concepts	10	kept
health care financing	health care financing	health care financing	health care financing	health care financing	10	kept
Health care-associated infections	health care associated infections	health care associated infections	health care associated infection	Health care-associated infections	10	singular

healthcare providers	healthcare providers	healthcare providers	healthcare provider	Healthcare providers	10	singular
Hearing Tests	hearing tests	hearing tests	hearing test	Hearing Tests	10	singular
heart atrium flutter	heart atrium flutter	heart atrium flutter	heart atrium flutter	heart atrium flutter	10	kept
heart valve surgery	heart valve surgery	heart valve surgery	heart valve surgery	heart valve surgery	10	kept
Hemodialysis	hemodialysis	hemodialysis	hemodialysis	hemodialysis	10	case_or_format
Hepatitis C	hepatitis c	hepatitis c	hepatitis c	hepatitis C	10	case_or_format
Hepatitis C virus	hepatitis c virus	hepatitis c virus	hepatitis c virus	Hepatitis C virus	10	kept
high fidelity simulation training	high fidelity simulation training	high fidelity simulation training	high fidelity simulation training	High Fidelity Simulation Training	10	case_or_format
High-Frequency Ventilation	high frequency ventilation	high frequency ventilation	high frequency ventilation	High-Frequency Ventilation	10	kept
homicide	homicide	homicide	homicide	homicide	10	kept
Hospital acquired infections	hospital acquired infections	hospital acquired infections	hospital acquired infection	Hospital-acquired infections	10	singular
hospital building	hospital building	hospital building	hospital building	hospital building	10	kept
hospital laboratory	hospital laboratory	hospital laboratory	hospital laboratory	hospital laboratory	10	kept
hospital pharmacist	hospital pharmacist	hospital pharmacist	hospital pharmacist	hospital pharmacist	10	kept
hospital to home transition	hospital to home transition	hospital to home transition	hospital to home transition	hospital to home transition	10	kept
hospitalisation	hospitalisation	hospitalisation	hospitalisation	hospitalisation	10	kept

Hospitalized	hospitalized	hospitalized	hospitalized	Hospitalized	10	kept
hydrochlorothiazide	hydrochlorothiazide	hydrochlorothiazide	hydrochlorothiazide	hydrochlorothiazide	10	kept
hypercalcemia	hypercalcemia	hypercalcemia	hypercalcemia	hypercalcemia	10	kept
hypersensitivity	hypersensitivity	hypersensitivity	hypersensitivity	hypersensitivity	10	kept
Hyperthermia, Induced	hyperthermia induced	hyperthermia induced	hyperthermia induced	Hyperthermia, Induced	10	kept
hypertransaminasemia	hypertransaminasemia	hypertransaminasemia	hypertransaminasemia	hypertransaminasemia	10	kept
identifiable information	identifiable information	identifiable information	identifiable information	identifiable information	10	kept
ileus	ileus	ileus	ileus	ileus	10	kept
Immunization Programs	immunization programs	immunization programs	immunization programs	Immunization Programs	10	kept
implantation	implantation	implantation	implantation	implantation	10	kept
in vitro study	in vitro study	in vitro study	in vitro study	in vitro study	10	kept
In-hospital cardiac arrest	in hospital cardiac arrest	in hospital cardiac arrest	in hospital cardiac arrest	In-hospital cardiac arrest	10	kept
independent living	independent living	independent living	independent living	independent living	10	kept
Infant Equipment	infant equipment	infant equipment	infant equipment	Infant Equipment	10	kept
Infant Food	infant food	infant food	infant food	Infant Food	10	kept
Infant Nutrition Disorders	infant nutrition disorders	infant nutrition disorders	infant nutrition disorders	Infant Nutrition Disorders	10	kept

Infant, premature	infant premature	infant premature	infant premature	Infant, Premature	10	case_or_format
infectious complication	infectious complication	infectious complication	infectious complication	infectious complication	10	kept
Influenza virus	influenza virus	influenza virus	influenza virus	Influenza virus	10	kept
Information use	information use	information use	information use	Information use	10	kept
Infusions, Parenteral	infusions parenteral	infusions parenteral	infusions parenteral	Infusions, Parenteral	10	kept
Instillation, Drug	instillation drug	instillation drug	instillation drug	Instillation, Drug	10	kept
Institutionalization	institutionalization	institutionalization	institutionalization	institutionalization	10	case_or_format
intensive	intensive	intensive	intensive	intensive	10	kept
Intensivist	intensivist	intensivist	intensivist	intensivist	10	case_or_format
interleukin 1beta	interleukin 1beta	interleukin 1beta	interleukin 1beta	interleukin 1beta	10	kept
Intestines	intestines	intestines	intestine	Intestines	10	singular
intraabdominal hypertension	intraabdominal hypertension	intraabdominal hypertension	intraabdominal hypertension	intraabdominal hypertension	10	kept
invasive mechanical ventilation	invasive mechanical ventilation	invasive mechanical ventilation	invasive mechanical ventilation	invasive mechanical ventilation	10	kept
isotonic solution	isotonic solution	isotonic solution	isotonic solution	Isotonic Solutions	10	case_or_format
ISSUES	issues	issues	issues	ISSUES	10	kept
Jejunostomy	jejunostomy	jejunostomy	jejunostomy	Jejunostomy	10	kept

jugular vein	jugular vein	jugular vein	jugular vein	Jugular Veins	10	case_or_format
language development	language development	language development	language development	language development	10	kept
larynx spasm	larynx spasm	larynx spasm	larynx spasm	larynx spasm	10	kept
latent tuberculosis	latent tuberculosis	latent tuberculosis	latent tuberculosis	latent tuberculosis	10	kept
Latvia	latvia	latvia	latvia	Latvia	10	kept
lavender oil	lavender oil	lavender oil	lavender oil	lavender oil	10	kept
levosimendan	levosimendan	levosimendan	levosimendan	levosimendan	10	kept
Life support care	life support care	life support care	life support care	Life Support Care	10	case_or_format
lifespan	lifespan	lifespan	lifespan	lifespan	10	kept
lisinopril	lisinopril	lisinopril	lisinopril	lisinopril	10	kept
liver graft	liver graft	liver graft	liver graft	liver graft	10	kept
loose feces	loose feces	loose feces	loose feces	loose feces	10	kept
LOW-BIRTH-WEIGHT	low birth weight	low birth weight	low birth weight	low birth weight	10	case_or_format
lung auscultation	lung auscultation	lung auscultation	lung auscultation	lung auscultation	10	kept
Lung cancer	lung cancer	lung cancer	lung cancer	lung cancer	10	case_or_format
lung congestion	lung congestion	lung congestion	lung congestion	lung congestion	10	kept

Lupus Erythematosus, Systemic	lupus erythematosus systemic	lupus erythematosus systemic	lupus erythematosus systemic	Lupus Erythematosus, Systemic	10	kept
Maternal	maternal	maternal	maternal	Maternal	10	kept
Maternal Welfare	maternal welfare	maternal welfare	maternal welfare	maternal welfare	10	case_or_format
measles	measles	measles	measles	measles	10	kept
measurement error	measurement error	measurement error	measurement error	measurement error	10	kept
Medical Emergency Team	medical emergency team	medical emergency team	medical emergency team	Medical emergency team	10	case_or_format
Medical futility	medical futility	medical futility	medical futility	Medical Futility	10	case_or_format
Medical imaging	medical imaging	medical imaging	medical imaging	Medical imaging	10	kept
Medical personnel	medical personnel	medical personnel	medical personnel	medical personnel	10	case_or_format
Medication Adherence	medication adherence	medication adherence	medication adherence	Medication Adherence	10	kept
medication administration	medication administration	medication administration	medication administration	medication administration	10	kept
medication management	medication management	medication management	medication management	medication management	10	kept
Meningitis, Bacterial	meningitis bacterial	meningitis bacterial	meningitis bacterial	Meningitis, Bacterial	10	kept
mental deficiency	mental deficiency	mental deficiency	mental deficiency	mental deficiency	10	kept
Mental workload	mental workload	mental workload	mental workload	mental workload	10	case_or_format
metabolic encephalopathy	metabolic encephalopathy	metabolic encephalopathy	metabolic encephalopathy	metabolic encephalopathy	10	kept

Microbiota	microbiota	microbiota	microbiota	Microbiota	10	kept
misoprostol	misoprostol	misoprostol	misoprostol	misoprostol	10	kept
missed care	missed care	missed care	missed care	Missed care	10	case_or_format
mitral valve repair	mitral valve repair	mitral valve repair	mitral valve repair	mitral valve repair	10	kept
Models, Anatomic	models anatomic	models anatomic	models anatomic	Models, Anatomic	10	kept
Mongolia	mongolia	mongolia	mongolia	Mongolia	10	kept
moral injury	moral injury	moral injury	moral injury	moral injury	10	kept
MORBIDITY	morbidity	morbidity	morbidity	morbidity	10	case_or_format
multiplex polymerase chain reaction	multiplex polymerase chain reaction	multiplex polymerase chain reaction	multiplex polymerase chain reaction	multiplex polymerase chain reaction	10	kept
myocardial infarction	myocardial infarction	myocardial infarction	myocardial infarction	Myocardial Infarction	10	case_or_format
Nasogastric tube	nasogastric tube	nasogastric tube	nasogastric tube	nasogastric tube	10	case_or_format
Near Miss, Healthcare	near miss healthcare	near miss healthcare	near miss healthcare	Near Miss, Healthcare	10	kept
nebulizer	nebulizer	nebulizer	nebulizer	nebulizer	10	kept
Necrosis	necrosis	necrosis	necrosis	necrosis	10	case_or_format
Neoplasm Metastasis	neoplasm metastasis	neoplasm metastasis	neoplasm metastasis	Neoplasm Metastasis	10	kept
nervous system development	nervous system development	nervous system development	nervous system development	nervous system development	10	kept

neutrophil lymphocyte ratio	neutrophil lymphocyte ratio	neutrophil lymphocyte ratio	neutrophil lymphocyte ratio	neutrophil lymphocyte ratio	10	kept
newborn apnea	newborn apnea	newborn apnea	newborn apnea	newborn apnea	10	kept
Newborn care	newborn care	newborn care	newborn care	newborn care	10	case_or_format
Newborns	newborns	newborns	newborn	newborn	10	singular
nicardipine	nicardipine	nicardipine	nicardipine	nicardipine	10	kept
night sleep	night sleep	night sleep	night sleep	night sleep	10	kept
obstetric procedure	obstetric procedure	obstetric procedure	obstetric procedure	obstetric procedure	10	kept
Opioid	opioid	opioid	opioid	opioids	10	case_or_format
organisation of health services	organisation of health services	organisation of health services	organisation of health services	organisation of health services	10	kept
orthopedic surgeon	orthopedic surgeon	orthopedic surgeon	orthopedic surgeon	orthopedic surgeon	10	kept
Orthopedics	orthopedics	orthopedics	orthopedics	orthopedics	10	case_or_format
oxygen mask	oxygen mask	oxygen mask	oxygen mask	oxygen mask	10	kept
Oxygen saturation	oxygen saturation	oxygen saturation	oxygen saturation	oxygen saturation	10	case_or_format
Pacifiers	pacifiers	pacifiers	pacifier	Pacifiers	10	singular
Pain, Procedural	pain procedural	pain procedural	pain procedural	Pain, Procedural	10	kept
palivizumab	palivizumab	palivizumab	palivizumab	palivizumab	10	kept

Pancreatectomy	pancreatectomy	pancreatectomy	pancreatectomy	Pancreatectomy	10	kept
Pathophysiology	pathophysiology	pathophysiology	pathophysiology	pathophysiology	10	case_or_format
pediatric advanced life support	pediatric advanced life support	pediatric advanced life support	pediatric advanced life support	pediatric advanced life support	10	kept
pediatric delirium	pediatric delirium	pediatric delirium	pediatric delirium	pediatric delirium	10	kept
Percutaneous coronary intervention	percutaneous coronary intervention	percutaneous coronary intervention	percutaneous coronary intervention	percutaneous coronary intervention	10	case_or_format
performance indicator	performance indicator	performance indicator	performance indicator	performance indicator	10	kept
pericardiocentesis	pericardiocentesis	pericardiocentesis	pericardiocentesis	pericardiocentesis	10	kept
perinatal mortality	perinatal mortality	perinatal mortality	perinatal mortality	Perinatal Mortality	10	case_or_format
peripheral occlusive artery disease	peripheral occlusive artery disease	peripheral occlusive artery disease	peripheral occlusive artery disease	peripheral occlusive artery disease	10	kept
persistent pulmonary hypertension	persistent pulmonary hypertension	persistent pulmonary hypertension	persistent pulmonary hypertension	persistent pulmonary hypertension	10	kept
Person-centred care	person centred care	person centred care	person centred care	person-centred care	10	case_or_format
personal satisfaction	personal satisfaction	personal satisfaction	personal satisfaction	Personal Satisfaction	10	case_or_format
personality disorder	personality disorder	personality disorder	personality disorder	personality disorder	10	kept
personnel staffing and scheduling	personnel staffing and scheduling	personnel staffing and scheduling	personnel staffing and scheduling	Personnel Staffing and Scheduling	10	case_or_format
Personnel training	personnel training	personnel training	personnel training	Personnel training	10	kept
PERSPECTIVE	perspective	perspective	perspective	PERSPECTIVES	10	case_or_format

pharmacodynamics	pharmacodynamics	pharmacodynamics	pharmacodynamics	pharmacodynamics	10	kept
phase 1 clinical trial	phase 1 clinical trial	phase 1 clinical trial	phase 1 clinical trial	phase 1 clinical trial	10	kept
Phenotype	phenotype	phenotype	phenotype	phenotype	10	case_or_format
Phylogeny	phylogeny	phylogeny	phylogeny	phylogeny	10	case_or_format
physical capacity	physical capacity	physical capacity	physical capacity	physical capacity	10	kept
physical disease by body function	physical disease by body function	physical disease by body function	physical disease by body function	physical disease by body function	10	kept
Physical restraints	physical restraints	physical restraints	physical restraint	physical restraint	10	singular
physical violence	physical violence	physical violence	physical violence	physical violence	10	kept
Physiologic monitoring	physiologic monitoring	physiologic monitoring	physiologic monitoring	physiologic monitoring	10	case_or_format
placenta previa	placenta previa	placenta previa	placenta previa	placenta previa	10	kept
Plagiocephaly, Nonsynostotic	plagiocephaly nonsynostotic	plagiocephaly nonsynostotic	plagiocephaly nonsynostotic	Plagiocephaly, Nonsynostotic	10	kept
pneumoperitoneum	pneumoperitoneum	pneumoperitoneum	pneumoperitoneum	pneumoperitoneum	10	kept
Poisson Distribution	poisson distribution	poisson distribution	poisson distribution	Poisson Distribution	10	kept
police	police	police	police	police	10	kept
Poliomyelitis	poliomyelitis	poliomyelitis	poliomyelitis	poliomyelitis	10	case_or_format
polyneuropathy	polyneuropathy	polyneuropathy	polyneuropathy	Polyneuropathies	10	case_or_format

population health	population health	population health	population health	population health	10	kept
practice environment	practice environment	practice environment	practice environment	practice environment	10	kept
Practice guidelines	practice guidelines	practice guidelines	practice guideline	practice guideline	10	singular
predictive modeling	predictive modeling	predictive modeling	predictive modeling	predictive modeling	10	kept
predictive validity	predictive validity	predictive validity	predictive validity	predictive validity	10	kept
Prejudice	prejudice	prejudice	prejudice	Prejudice	10	kept
preliminary data	preliminary data	preliminary data	preliminary data	preliminary data	10	kept
prenatal period	prenatal period	prenatal period	prenatal period	prenatal period	10	kept
preparedness	preparedness	preparedness	preparedness	Preparedness	10	case_or_format
prescribing error	prescribing error	prescribing error	prescribing error	prescribing error	10	kept
primipara	primipara	primipara	primipara	primipara	10	kept
private health insurance	private health insurance	private health insurance	private health insurance	private health insurance	10	kept
professional ethics	professional ethics	professional ethics	professional ethics	professional ethics	10	kept
prostaglandin	prostaglandin	prostaglandin	prostaglandin	prostaglandin	10	kept
prostate hypertrophy	prostate hypertrophy	prostate hypertrophy	prostate hypertrophy	prostate hypertrophy	10	kept
Protective Devices	protective devices	protective devices	protective devices	Protective Devices	10	kept

protocols & guidelines	protocols and guidelines	protocols and guidelines	protocols and guidelines	protocols & guidelines	10	kept
Psychosocial Support Systems	psychosocial support systems	psychosocial support systems	psychosocial support systems	Psychosocial Support Systems	10	kept
Public Opinion	public opinion	public opinion	public opinion	Public Opinion	10	kept
Pulse oximetry	pulse oximetry	pulse oximetry	pulse oximetry	pulse oximetry	10	case_or_format
qualitative content analysis	qualitative content analysis	qualitative content analysis	qualitative content analysis	Qualitative content analysis	10	case_or_format
Qualitative methods	qualitative methods	qualitative methods	qualitative methods	Qualitative methods	10	kept
quality measurement	quality measurement	quality measurement	quality measurement	quality measurement	10	kept
Quality-Adjusted Life Years	quality adjusted life years	quality adjusted life years	quality adjusted life year	quality adjusted life year	10	singular
QUALITY-OF-CARE	quality of care	quality of care	quality of care	Quality of care	10	case_or_format
QUESTIONNAIRE	questionnaire	questionnaire	questionnaire	questionnaire	10	case_or_format
questionnaires	questionnaires	questionnaires	questionnaire	questionnaire	10	singular
reaction time	reaction time	reaction time	reaction time	reaction time	10	kept
Recommendations	recommendations	recommendations	recommendation	Recommendations	10	singular
reconstructive surgery	reconstructive surgery	reconstructive surgery	reconstructive surgery	reconstructive surgery	10	kept
recording	recording	recording	recording	recording	10	kept
rectum	rectum	rectum	rectum	Rectum	10	case_or_format

recurrence risk	recurrence risk	recurrence risk	recurrence risk	recurrence risk	10	kept
recycling	recycling	recycling	recycling	recycling	10	kept
regression model	regression model	regression model	regression model	regression model	10	kept
Research Support as Topic	research support as topic	research support as topic	research support as topic	Research Support as Topic	10	kept
RESISTANCE	resistance	resistance	resistance	RESISTANCE	10	kept
Resource utilization	resource utilization	resource utilization	resource utilization	Resource utilization	10	kept
Respiratory	respiratory	respiratory	respiratory	respiratory	10	case_or_format
respiratory tract parameters	respiratory tract parameters	respiratory tract parameters	respiratory tract parameters	respiratory tract parameters	10	kept
risk model	risk model	risk model	risk model	risk model	10	kept
RNA, Viral	rna viral	rna viral	rna viral	RNA, Viral	10	kept
Robotic Surgical Procedures	robotic surgical procedures	robotic surgical procedures	robotic surgical procedures	Robotic Surgical Procedures	10	kept
sacrum	sacrum	sacrum	sacrum	sacrum	10	kept
Scale development	scale development	scale development	scale development	Scale development	10	kept
SCORE	score	score	score	SCORE	10	kept
Second victim	second victim	second victim	second victim	Second victim	10	kept
Secondary Prevention	secondary prevention	secondary prevention	secondary prevention	secondary prevention	10	case_or_format

Self-confidence	self confidence	self confidence	self confidence	Self-confidence	10	kept
self-management	self management	self management	self management	Self-Management	10	case_or_format
Self-rating Depression Scale	self rating depression scale	self rating depression scale	self rating depression scale	Self-rating Depression Scale	10	kept
Sensitivity and specificity	sensitivity and specificity	sensitivity and specificity	sensitivity and specificity	Sensitivity and Specificity	10	case_or_format
sensor	sensor	sensor	sensor	sensor	10	kept
severe sepsis	severe sepsis	severe sepsis	severe sepsis	severe sepsis	10	kept
shared decision-making	shared decision making	shared decision making	shared decision making	shared decision making	10	case_or_format
signs and symptoms	signs and symptoms	signs and symptoms	signs and symptoms	signs and symptoms	10	kept
Simulation-based training	simulation based training	simulation based training	simulation based training	Simulation-based training	10	kept
sinus tachycardia	sinus tachycardia	sinus tachycardia	sinus tachycardia	sinus tachycardia	10	kept
skeletal muscle	skeletal muscle	skeletal muscle	skeletal muscle	skeletal muscle	10	kept
skin manifestation	skin manifestation	skin manifestation	skin manifestation	skin manifestation	10	kept
skin protection	skin protection	skin protection	skin protection	skin protection	10	kept
skin-to-skin	skin to skin	skin to skin	skin to skin	skin-to-skin	10	kept
social acceptance	social acceptance	social acceptance	social acceptance	social acceptance	10	kept
Social Class	social class	social class	social class	Social Class	10	kept

Social Identification	social identification	social identification	social identification	Social Identification	10	kept
Social Justice	social justice	social justice	social justice	Social Justice	10	kept
Social Networking	social networking	social networking	social networking	Social Networking	10	kept
social participation	social participation	social participation	social participation	social participation	10	kept
socialization	socialization	socialization	socialization	Socialization	10	case_or_format
Spinal Fusion	spinal fusion	spinal fusion	spinal fusion	Spinal Fusion	10	kept
spine fracture	spine fracture	spine fracture	spine fracture	spine fracture	10	kept
Stakeholder Participation	stakeholder participation	stakeholder participation	stakeholder participation	stakeholder participation	10	case_or_format
stimulation	stimulation	stimulation	stimulation	stimulation	10	kept
Stomatitis	stomatitis	stomatitis	stomatitis	Stomatitis	10	kept
Strategies	strategies	strategies	strategy	STRATEGIES	10	singular
Stressors	stressors	stressors	stressor	Stressors	10	singular
suffering	suffering	suffering	suffering	suffering	10	kept
suicidal ideation	suicidal ideation	suicidal ideation	suicidal ideation	suicidal ideation	10	kept
sulbactam	sulbactam	sulbactam	sulbactam	sulbactam	10	kept
sulfonamide	sulfonamide	sulfonamide	sulfonamide	sulfonamide	10	kept

superinfection	superinfection	superinfection	superinfection	superinfection	10	kept
surface property	surface property	surface property	surface property	surface property	10	kept
tension	tension	tension	tension	tension	10	kept
tertiary education	tertiary education	tertiary education	tertiary education	tertiary education	10	kept
tetanus	tetanus	tetanus	tetanus	Tetanus	10	case_or_format
thiamine	thiamine	thiamine	thiamine	thiamine	10	kept
Thoracotomy	thoracotomy	thoracotomy	thoracotomy	thoracotomy	10	case_or_format
threat	threat	threat	threat	threat	10	kept
thrombophlebitis	thrombophlebitis	thrombophlebitis	thrombophlebitis	thrombophlebitis	10	kept
Time	time	time	time	time	10	case_or_format
Tissue and Organ Harvesting	tissue and organ harvesting	tissue and organ harvesting	tissue and organ harvesting	Tissue and Organ Harvesting	10	kept
tonic clonic seizure	tonic clonic seizure	tonic clonic seizure	tonic clonic seizure	tonic clonic seizure	10	kept
Tonsillectomy	tonsillectomy	tonsillectomy	tonsillectomy	Tonsillectomy	10	kept
total knee arthroplasty	total knee arthroplasty	total knee arthroplasty	total knee arthroplasty	total knee arthroplasty	10	kept
tracheal extubation	tracheal extubation	tracheal extubation	tracheal extubation	tracheal extubation	10	kept
tracheoesophageal fistula	tracheoesophageal fistula	tracheoesophageal fistula	tracheoesophageal fistula	tracheoesophageal fistula	10	kept

Tramadol	tramadol	tramadol	tramadol	tramadol	10	case_or_format
transcranial Doppler ultrasonography	transcranial doppler ultrasonography	transcranial doppler ultrasonography	transcranial doppler ultrasonography	transcranial Doppler ultrasonography	10	kept
Transplantation, Autologous	transplantation autologous	transplantation autologous	transplantation autologous	Transplantation, Autologous	10	kept
transthyretin	transthyretin	transthyretin	transthyretin	transthyretin	10	kept
tube removal	tube removal	tube removal	tube removal	tube removal	10	kept
Ukraine	ukraine	ukraine	ukraine	Ukraine	10	kept
ulcerative colitis	ulcerative colitis	ulcerative colitis	ulcerative colitis	ulcerative colitis	10	kept
Ultrasonography, Doppler, Transcranial	ultrasonography doppler transcranial	ultrasonography doppler transcranial	ultrasonography doppler transcranial	Ultrasonography, Doppler, Transcranial	10	kept
Ultrasonography, Prenatal	ultrasonography prenatal	ultrasonography prenatal	ultrasonography prenatal	Ultrasonography, Prenatal	10	kept
user-centered design	user centered design	user centered design	user centered design	user-centered design	10	kept
Vaccine	vaccine	vaccine	vaccine	vaccine	10	case_or_format
Vaccines	vaccines	vaccines	vaccine	vaccine	10	singular
vascular access devices	vascular access devices	vascular access devices	vascular access device	Vascular Access Devices	10	singular
vasoconstriction	vasoconstriction	vasoconstriction	vasoconstriction	vasoconstriction	10	kept
Vasopressins	vasopressins	vasopressins	vasopressin	vasopressin	10	singular
Veins	veins	veins	vein	Veins	10	singular

Ventilator-Induced Lung Injury	ventilator induced lung injury	ventilator induced lung injury	ventilator induced lung injury	Ventilator-Induced Lung Injury	10	kept
verapamil	verapamil	verapamil	verapamil	verapamil	10	kept
vertical transmission	vertical transmission	vertical transmission	vertical transmission	vertical transmission	10	kept
videolaryngoscopy	videolaryngoscopy	videolaryngoscopy	videolaryngoscopy	videolaryngoscopy	10	kept
visual disorder	visual disorder	visual disorder	visual disorder	visual disorder	10	kept
vitamin supplementation	vitamin supplementation	vitamin supplementation	vitamin supplementation	vitamin supplementation	10	kept
War	war	war	war	war	10	case_or_format
West Virginia	west virginia	west virginia	west virginia	West Virginia	10	kept
White People	white people	white people	white people	White People	10	kept
Whooping Cough	whooping cough	whooping cough	whooping cough	Whooping Cough	10	kept
wireless communication	wireless communication	wireless communication	wireless communication	wireless communication	10	kept
Withdrawal	withdrawal	withdrawal	withdrawal	WITHDRAWAL	10	case_or_format
Work engagement	work engagement	work engagement	work engagement	work engagement	10	case_or_format
Work stress	work stress	work stress	work stress	Work stress	10	kept
WORKING	working	working	working	WORKING	10	kept
workplace bullying	workplace bullying	workplace bullying	workplace bullying	workplace bullying	10	kept

Yemen	yemen	yemen	yemen	Yemen	10	kept
2009 H1N1 influenza	2009 h1n1 influenza	2009 h1n1 influenza	2009 h1n1 influenza	2009 H1N1 influenza	9	kept
Abbreviated Injury Scale	abbreviated injury scale	abbreviated injury scale	abbreviated injury scale	Abbreviated Injury Scale	9	kept
Abortion, Induced	abortion induced	abortion induced	abortion induced	Abortion, Induced	9	kept
abscess	abscess	abscess	abscess	abscess	9	kept
Acceptability	acceptability	acceptability	acceptability	Acceptability	9	kept
acceptability	acceptability	acceptability	acceptability	Acceptability	9	case_or_format
access	access	access	access	access	9	kept
Access to Information	access to information	access to information	access to information	access to information	9	case_or_format
acute care setting	acute care setting	acute care setting	acute care setting	acute care setting	9	kept
Acute ischemic stroke	acute ischemic stroke	acute ischemic stroke	acute ischemic stroke	acute ischemic stroke	9	case_or_format
acute pain	acute pain	acute pain	acute pain	Acute Pain	9	case_or_format
Advance directives	advance directives	advance directives	advance directive	Advance Directives	9	singular
Airway	airway	airway	airway	airway	9	case_or_format
alarm management	alarm management	alarm management	alarm management	Alarm management	9	case_or_format
alcohol withdrawal	alcohol withdrawal	alcohol withdrawal	alcohol withdrawal	alcohol withdrawal	9	kept

ambulance transportation	ambulance transportation	ambulance transportation	ambulance transportation	ambulance transportation	9	kept
aminophylline	aminophylline	aminophylline	aminophylline	aminophylline	9	kept
amputation	amputation	amputation	amputation	amputation	9	kept
Anaesthesiology	anaesthesiology	anaesthesiology	anaesthesiology	Anaesthesiology	9	kept
ANALGESIA	analgesia	analgesia	analgesia	analgesia	9	case_or_format
anastomosis	anastomosis	anastomosis	anastomosis	anastomosis	9	kept
andexanet alfa	andexanet alfa	andexanet alfa	andexanet alfa	andexanet alfa	9	kept
Anesthesia, Epidural	anesthesia epidural	anesthesia epidural	anesthesia epidural	Anesthesia, Epidural	9	kept
anesthetic recovery	anesthetic recovery	anesthetic recovery	anesthetic recovery	anesthetic recovery	9	kept
Aneurysm, Ruptured	aneurysm ruptured	aneurysm ruptured	aneurysm ruptured	Aneurysm, Ruptured	9	kept
Angioedema	angioedema	angioedema	angioedema	Angioedema	9	kept
anidulafungin	anidulafungin	anidulafungin	anidulafungin	anidulafungin	9	kept
Antibiotic	antibiotic	antibiotic	antibiotic	Antibiotics	9	case_or_format
antiinflammatory agent	antiinflammatory agent	antiinflammatory agent	antiinflammatory agent	antiinflammatory agent	9	kept
antilipemic agent	antilipemic agent	antilipemic agent	antilipemic agent	antilipemic agent	9	kept
antimicrobial resistance	antimicrobial resistance	antimicrobial resistance	antimicrobial resistance	Antimicrobial resistance	9	case_or_format

Antioxidants	antioxidants	antioxidants	antioxidant	Antioxidants	9	singular
aripiprazole	aripiprazole	aripiprazole	aripiprazole	aripiprazole	9	kept
Arm	arm	arm	arm	Arm	9	kept
arm	arm	arm	arm	Arm	9	case_or_format
artery catheter	artery catheter	artery catheter	artery catheter	artery catheter	9	kept
Asian People	asian people	asian people	asian people	Asian People	9	kept
Assertiveness	assertiveness	assertiveness	assertiveness	Assertiveness	9	kept
Assessment tool	assessment tool	assessment tool	assessment tool	Assessment tool	9	kept
Attachment	attachment	attachment	attachment	Attachment	9	kept
attitude assessment	attitude assessment	attitude assessment	attitude assessment	attitude assessment	9	kept
Attitude of health personnel	attitude of health personnel	attitude of health personnel	attitude of health personnel	Attitude of Health Personnel	9	case_or_format
Attitude to death	attitude to death	attitude to death	attitude to death	Attitude to Death	9	case_or_format
autoregulation	autoregulation	autoregulation	autoregulation	autoregulation	9	kept
Bacterial Typing Techniques	bacterial typing techniques	bacterial typing techniques	bacterial typing techniques	Bacterial Typing Techniques	9	kept
barotrauma	barotrauma	barotrauma	barotrauma	barotrauma	9	kept
Basic life support	basic life support	basic life support	basic life support	basic life support	9	case_or_format

Behavior Observation Techniques	behavior observation techniques	behavior observation techniques	behavior observation techniques	Behavior Observation Techniques	9	kept
beliefs	beliefs	beliefs	belief	beliefs	9	singular
beta-Lactams	beta lactams	beta lactams	beta lactam	beta lactam	9	singular
bibliographic database	bibliographic database	bibliographic database	bibliographic database	bibliographic database	9	kept
biological model	biological model	biological model	biological model	biological model	9	kept
BIRTH	birth	birth	birth	birth	9	case_or_format
Bladder Exstrophy	bladder exstrophy	bladder exstrophy	bladder exstrophy	Bladder Exstrophy	9	kept
Bleeding	bleeding	bleeding	bleeding	bleeding	9	case_or_format
blood bank	blood bank	blood bank	blood bank	blood bank	9	kept
Blood Coagulation Factors	blood coagulation factors	blood coagulation factors	blood coagulation factors	Blood Coagulation Factors	9	kept
Blood Component Removal	blood component removal	blood component removal	blood component removal	Blood Component Removal	9	kept
blood examination	blood examination	blood examination	blood examination	blood examination	9	kept
blood gas parameters	blood gas parameters	blood gas parameters	blood gas parameters	blood gas parameters	9	kept
Blood glucose	blood glucose	blood glucose	blood glucose	Blood Glucose	9	case_or_format
blood purification	blood purification	blood purification	blood purification	blood purification	9	kept
blood vessel injury	blood vessel injury	blood vessel injury	blood vessel injury	blood vessel injury	9	kept

Bluetooth	bluetooth	bluetooth	bluetooth	Bluetooth	9	kept
blurred vision	blurred vision	blurred vision	blurred vision	blurred vision	9	kept
Body temperature	body temperature	body temperature	body temperature	body temperature	9	case_or_format
Bonding	bonding	bonding	bonding	Bonding	9	kept
Bordetella pertussis	bordetella pertussis	bordetella pertussis	bordetella pertussis	Bordetella pertussis	9	kept
Brain Chemistry	brain chemistry	brain chemistry	brain chemistry	Brain Chemistry	9	kept
Brain Damage, Chronic	brain damage chronic	brain damage chronic	brain damage chronic	Brain Damage, Chronic	9	kept
brain depth stimulation	brain depth stimulation	brain depth stimulation	brain depth stimulation	brain depth stimulation	9	kept
brain hernia	brain hernia	brain hernia	brain hernia	brain hernia	9	kept
Bronchiolitis, Viral	bronchiolitis viral	bronchiolitis viral	bronchiolitis viral	Bronchiolitis, Viral	9	kept
Buddhism	buddhism	buddhism	buddhism	Buddhism	9	kept
calcium chloride	calcium chloride	calcium chloride	calcium chloride	calcium chloride	9	kept
calorimetry	calorimetry	calorimetry	calorimetry	calorimetry	9	kept
Calorimetry, Indirect	calorimetry indirect	calorimetry indirect	calorimetry indirect	Calorimetry, Indirect	9	kept
cancer registry	cancer registry	cancer registry	cancer registry	cancer registry	9	kept
cannabis	cannabis	cannabis	cannabis	cannabis	9	kept

carbon footprint	carbon footprint	carbon footprint	carbon footprint	carbon footprint	9	kept
cardiac anesthesia	cardiac anesthesia	cardiac anesthesia	cardiac anesthesia	cardiac anesthesia	9	kept
Cardiac Rehabilitation	cardiac rehabilitation	cardiac rehabilitation	cardiac rehabilitation	Cardiac Rehabilitation	9	kept
cardiopulmonary function	cardiopulmonary function	cardiopulmonary function	cardiopulmonary function	cardiopulmonary function	9	kept
Cardiovascular System	cardiovascular system	cardiovascular system	cardiovascular system	cardiovascular system	9	case_or_format
Care bundles	care bundles	care bundles	care bundle	care bundle	9	singular
Care coordination	care coordination	care coordination	care coordination	Care coordination	9	kept
care coordination	care coordination	care coordination	care coordination	Care coordination	9	case_or_format
catheter ablation	catheter ablation	catheter ablation	catheter ablation	Catheter Ablation	9	case_or_format
cauterization	cauterization	cauterization	cauterization	cauterization	9	kept
CCU	ccu	ccu	ccu	CCU	9	kept
CD4 lymphocyte count	cd4 lymphocyte count	cd4 lymphocyte count	cd4 lymphocyte count	CD4 lymphocyte count	9	kept
central nervous system disease	central nervous system disease	central nervous system disease	central nervous system disease	Central Nervous System Diseases	9	case_or_format
CHD	chd	chd	chd	CHD	9	kept
CHECKLIST	checklist	checklist	checklist	checklist	9	case_or_format
Chemoprevention	chemoprevention	chemoprevention	chemoprevention	Chemoprevention	9	kept

Chemotherapy	chemotherapy	chemotherapy	chemotherapy	chemotherapy	9	case_or_format
Child Pugh score	child pugh score	child pugh score	child pugh score	Child Pugh score	9	kept
CHLORHEXIDINE	chlorhexidine	chlorhexidine	chlorhexidine	Chlorhexidine	9	case_or_format
chloride blood level	chloride blood level	chloride blood level	chloride blood level	chloride blood level	9	kept
chloroquine	chloroquine	chloroquine	chloroquine	chloroquine	9	kept
Chronic critical illness	chronic critical illness	chronic critical illness	chronic critical illness	chronic critical illness	9	case_or_format
Chronic pain	chronic pain	chronic pain	chronic pain	chronic pain	9	case_or_format
Chylothorax	chylothorax	chylothorax	chylothorax	Chylothorax	9	kept
clavulanic acid	clavulanic acid	clavulanic acid	clavulanic acid	clavulanic acid	9	kept
cleft palate	cleft palate	cleft palate	cleft palate	cleft palate	9	kept
Clinical audit	clinical audit	clinical audit	clinical audit	clinical audit	9	case_or_format
Clinical competency	clinical competency	clinical competency	clinical competency	Clinical competency	9	kept
Clinical decision making	clinical decision making	clinical decision making	clinical decision making	clinical decision making	9	case_or_format
Clinical guidelines	clinical guidelines	clinical guidelines	clinical guideline	clinical guidelines	9	singular
Clinical judgment	clinical judgment	clinical judgment	clinical judgment	Clinical judgment	9	kept
clinical pharmacology	clinical pharmacology	clinical pharmacology	clinical pharmacology	clinical pharmacology	9	kept

Clinical trials	clinical trials	clinical trials	clinical trial	clinical trial	9	singular
Cohort study	cohort study	cohort study	cohort study	Cohort Studies	9	case_or_format
coil embolization	coil embolization	coil embolization	coil embolization	coil embolization	9	kept
colchicine	colchicine	colchicine	colchicine	colchicine	9	kept
collaborative learning	collaborative learning	collaborative learning	collaborative learning	collaborative learning	9	kept
college	college	college	college	college	9	kept
Communication skills	communication skills	communication skills	communication skill	communication skill	9	singular
Community Networks	community networks	community networks	community networks	Community Networks	9	kept
compensation	compensation	compensation	compensation	compensation	9	kept
Complementary therapies	complementary therapies	complementary therapies	complementary therapy	Complementary Therapies	9	singular
Computational Biology	computational biology	computational biology	computational biology	Computational Biology	9	kept
computer assisted diagnosis	computer assisted diagnosis	computer assisted diagnosis	computer assisted diagnosis	computer assisted diagnosis	9	kept
concurrent validity	concurrent validity	concurrent validity	concurrent validity	concurrent validity	9	kept
conflict of interest	conflict of interest	conflict of interest	conflict of interest	conflict of interest	9	kept
congenital diaphragm hernia	congenital diaphragm hernia	congenital diaphragm hernia	congenital diaphragm hernia	congenital diaphragm hernia	9	kept
Contamination	contamination	contamination	contamination	contamination	9	case_or_format

Continuity of care	continuity of care	continuity of care	continuity of care	Continuity of care	9	kept
continuous electroencephalography	continuous electroencephalography	continuous electroencephalography	continuous electroencephalography	continuous electroencephalography	9	kept
Corneal Injuries	corneal injuries	corneal injuries	corneal injury	Corneal Injuries	9	singular
Coronary care unit	coronary care unit	coronary care unit	coronary care unit	coronary care unit	9	case_or_format
COSTS	costs	costs	cost	cost	9	singular
Costs and cost analysis	costs and cost analysis	costs and cost analysis	costs and cost analysis	Costs and Cost Analysis	9	case_or_format
counselor	counselor	counselor	counselor	counselor	9	kept
course content	course content	course content	course content	course content	9	kept
COVID-19 nucleic acid testing	covid 19 nucleic acid testing	covid 19 nucleic acid testing	covid 19 nucleic acid testing	COVID-19 nucleic acid testing	9	kept
Creativity	creativity	creativity	creativity	creativity	9	case_or_format
Crew resource management	crew resource management	crew resource management	crew resource management	Crew resource management	9	kept
criterion related validity	criterion related validity	criterion related validity	criterion related validity	criterion related validity	9	kept
Cross-sectional survey	cross sectional survey	cross sectional survey	cross sectional survey	cross-sectional survey	9	case_or_format
Cultural competence	cultural competence	cultural competence	cultural competence	cultural competence	9	case_or_format
Cultural diversity	cultural diversity	cultural diversity	cultural diversity	Cultural Diversity	9	case_or_format
cultural safety	cultural safety	cultural safety	cultural safety	cultural safety	9	kept

cultural sensitivity	cultural sensitivity	cultural sensitivity	cultural sensitivity	cultural sensitivity	9	kept
custody	custody	custody	custody	custody	9	kept
cyclosporin	cyclosporin	cyclosporin	cyclosporin	cyclosporin	9	kept
cyclosporine	cyclosporine	cyclosporine	cyclosporine	cyclosporine	9	kept
Data Accuracy	data accuracy	data accuracy	data accuracy	data accuracy	9	case_or_format
data interpretation	data interpretation	data interpretation	data interpretation	data interpretation	9	kept
death anxiety	death anxiety	death anxiety	death anxiety	death anxiety	9	kept
decision support	decision support	decision support	decision support	Decision support	9	case_or_format
Decision trees	decision trees	decision trees	decision tree	Decision Trees	9	singular
dengue	dengue	dengue	dengue	dengue	9	kept
device maintenance	device maintenance	device maintenance	device maintenance	device maintenance	9	kept
Diagnostic Techniques, Neurological	diagnostic techniques neurological	diagnostic techniques neurological	diagnostic techniques neurological	Diagnostic Techniques, Neurological	9	kept
diet restriction	diet restriction	diet restriction	diet restriction	diet restriction	9	kept
Dignity	dignity	dignity	dignity	dignity	9	case_or_format
Dilatation	dilatation	dilatation	dilatation	Dilatation	9	kept
Direct Service Costs	direct service costs	direct service costs	direct service costs	Direct Service Costs	9	kept

disabled person	disabled person	disabled person	disabled person	disabled person	9	kept
discomfort	discomfort	discomfort	discomfort	discomfort	9	kept
disposable equipment	disposable equipment	disposable equipment	disposable equipment	Disposable Equipment	9	case_or_format
Diuretics, Osmotic	diuretics osmotic	diuretics osmotic	diuretics osmotic	Diuretics, Osmotic	9	kept
DNA	dna	dna	dna	DNA	9	kept
DNA transcription	dna transcription	dna transcription	dna transcription	DNA transcription	9	kept
do-not-resuscitate	do not resuscitate	do not resuscitate	do not resuscitate	do-not-resuscitate	9	kept
Dobutamine	dobutamine	dobutamine	dobutamine	dobutamine	9	case_or_format
DOCTORS	doctors	doctors	doctor	Doctors	9	singular
Doppler ultrasonography	doppler ultrasonography	doppler ultrasonography	doppler ultrasonography	Doppler ultrasonography	9	kept
drug choice	drug choice	drug choice	drug choice	drug choice	9	kept
drug clearance	drug clearance	drug clearance	drug clearance	drug clearance	9	kept
Drug Costs	drug costs	drug costs	drug cost	drug cost	9	singular
drug resistance	drug resistance	drug resistance	drug resistance	drug resistance	9	kept
drug shortage	drug shortage	drug shortage	drug shortage	drug shortage	9	kept
dry cough	dry cough	dry cough	dry cough	dry cough	9	kept

Duchenne muscular dystrophy	duchenne muscular dystrophy	duchenne muscular dystrophy	duchenne muscular dystrophy	Duchenne muscular dystrophy	9	kept
Early warning scores	early warning scores	early warning scores	early warning score	early warning score	9	singular
ECOG Performance Status	ecog performance status	ecog performance status	ecog performance status	ECOG Performance Status	9	kept
Educational intervention	educational intervention	educational intervention	educational intervention	Educational intervention	9	kept
efficacy	efficacy	efficacy	efficacy	EFFICACY	9	case_or_format
electroencephalography monitoring	electroencephalography monitoring	electroencephalography monitoring	electroencephalography monitoring	electroencephalography monitoring	9	kept
Embolism, Amniotic Fluid	embolism amniotic fluid	embolism amniotic fluid	embolism amniotic fluid	Embolism, Amniotic Fluid	9	kept
Emergency Medical Service Communication [illegible]	emergency medical service communication [illegible]	emergency medical service communication [illegible]	emergency medical service communication [illegible]	Emergency Medical Service Communication [illegible]	9	kept
emotion regulation	emotion regulation	emotion regulation	emotion regulation	emotion regulation	9	kept
Encephalitis	encephalitis	encephalitis	encephalitis	encephalitis	9	case_or_format
Encephalopathy	encephalopathy	encephalopathy	encephalopathy	Encephalopathy	9	kept
endemic disease	endemic disease	endemic disease	endemic disease	endemic disease	9	kept
Endocarditis, Bacterial	endocarditis bacterial	endocarditis bacterial	endocarditis bacterial	Endocarditis, Bacterial	9	kept
endoscopic retrograde cholangiopancreatograp[illegible]	endoscopic retrograde cholangiopancreatograp[illegible]	endoscopic retrograde cholangiopancreatograp[illegible]	endoscopic retrograde cholangiopancreatograp[illegible]	endoscopic retrograde cholangiopancreatograp[illegible]	9	kept
Endoscopy, Gastrointestinal	endoscopy gastrointestinal	endoscopy gastrointestinal	endoscopy gastrointestinal	Endoscopy, Gastrointestinal	9	kept
Endotracheal suction	endotracheal suction	endotracheal suction	endotracheal suction	Endotracheal suction	9	kept

energy	energy	energy	energy	energy	9	kept
Enterobacter aerogenes	enterobacter aerogenes	enterobacter aerogenes	enterobacter aerogenes	Enterobacter aerogenes	9	kept
Environment, Controlled	environment controlled	environment controlled	environment controlled	Environment, Controlled	9	kept
Environmental factors	environmental factors	environmental factors	environmental factor	environmental factor	9	singular
environmental impact	environmental impact	environmental impact	environmental impact	environmental impact	9	kept
Episiotomy	episiotomy	episiotomy	episiotomy	Episiotomy	9	kept
episiotomy	episiotomy	episiotomy	episiotomy	Episiotomy	9	case_or_format
ethical sensitivity	ethical sensitivity	ethical sensitivity	ethical sensitivity	ethical sensitivity	9	kept
Ethics, Institutional	ethics institutional	ethics institutional	ethics institutional	Ethics, Institutional	9	kept
European Quality of Life 5 Dimensions 5 Level questionnaire	european quality of life 5 dimensions 5 level questionnaire	european quality of life 5 dimensions 5 level questionnaire	european quality of life 5 dimensions 5 level questionnaire	European Quality of Life 5 Dimensions 5 Level questionnaire	9	kept
Evidence based practice	evidence based practice	evidence based practice	evidence based practice	evidence based practice	9	case_or_format
expert witness	expert witness	expert witness	expert witness	expert witness	9	kept
extended spectrum beta lactamase producing Enterobacteriaceae	extended spectrum beta lactamase producing enterobacteriaceae	extended spectrum beta lactamase producing enterobacteriaceae	extended spectrum beta lactamase producing enterobacteriaceae	extended spectrum beta lactamase producing Enterobacteriaceae	9	kept
extreme prematurity	extreme prematurity	extreme prematurity	extreme prematurity	extreme prematurity	9	kept
extremely premature birth	extremely premature birth	extremely premature birth	extremely premature birth	extremely premature birth	9	kept
eye disease	eye disease	eye disease	eye disease	Eye Diseases	9	case_or_format

eye examination	eye examination	eye examination	eye examination	eye examination	9	kept
Eye Injuries	eye injuries	eye injuries	eye injury	Eye Injuries	9	singular
Eye Movements	eye movements	eye movements	eye movement	Eye Movements	9	singular
Facilitated Tucking	facilitated tucking	facilitated tucking	facilitated tucking	facilitated tucking	9	case_or_format
factors	factors	factors	factor	Factors	9	singular
Faculty	faculty	faculty	faculty	Faculty	9	kept
Family caregiver	family caregiver	family caregiver	family caregiver	Family caregivers	9	case_or_format
family caregivers	family caregivers	family caregivers	family caregiver	Family caregivers	9	singular
family coping	family coping	family coping	family coping	family coping	9	kept
Family involvement	family involvement	family involvement	family involvement	family involvement	9	case_or_format
family participation	family participation	family participation	family participation	family participation	9	kept
family therapy	family therapy	family therapy	family therapy	family therapy	9	kept
fasciotomy	fasciotomy	fasciotomy	fasciotomy	fasciotomy	9	kept
fear of death	fear of death	fear of death	fear of death	fear of death	9	kept
Femoral Artery	femoral artery	femoral artery	femoral artery	femoral artery	9	case_or_format
fiberoptic bronchoscopy	fiberoptic bronchoscopy	fiberoptic bronchoscopy	fiberoptic bronchoscopy	fiberoptic bronchoscopy	9	kept

Filtration	filtration	filtration	filtration	Filtration	9	kept
financial stress	financial stress	financial stress	financial stress	financial stress	9	kept
First Aid	first aid	first aid	first aid	first aid	9	case_or_format
fludrocortisone	fludrocortisone	fludrocortisone	fludrocortisone	fludrocortisone	9	kept
Fluoroquinolones	fluoroquinolones	fluoroquinolones	fluoroquinolones	Fluoroquinolones	9	kept
fomite	fomite	fomite	fomite	Fomites	9	case_or_format
food	food	food	food	food	9	kept
Foreign-Body Migration	foreign body migration	foreign body migration	foreign body migration	Foreign-Body Migration	9	kept
forensic medicine	forensic medicine	forensic medicine	forensic medicine	forensic medicine	9	kept
Fracture Fixation, Internal	fracture fixation internal	fracture fixation internal	fracture fixation internal	Fracture Fixation, Internal	9	kept
gas exchange	gas exchange	gas exchange	gas exchange	gas exchange	9	kept
gastrointestinal motility	gastrointestinal motility	gastrointestinal motility	gastrointestinal motility	Gastrointestinal Motility	9	case_or_format
gastrointestinal surgery	gastrointestinal surgery	gastrointestinal surgery	gastrointestinal surgery	gastrointestinal surgery	9	kept
general ward	general ward	general ward	general ward	general ward	9	kept
Genetics, Medical	genetics medical	genetics medical	genetics medical	Genetics, Medical	9	kept
genomics	genomics	genomics	genomics	Genomics	9	case_or_format

geriatric disorder	geriatric disorder	geriatric disorder	geriatric disorder	geriatric disorder	9	kept
German	german	german	german	German	9	kept
GRADE	grade	grade	grade	GRADE	9	kept
GRADE approach	grade approach	grade approach	grade approach	GRADE approach	9	kept
Graft Survival	graft survival	graft survival	graft survival	Graft Survival	9	kept
graft survival	graft survival	graft survival	graft survival	Graft Survival	9	case_or_format
Gram staining	gram staining	gram staining	gram staining	Gram staining	9	kept
granulation tissue	granulation tissue	granulation tissue	granulation tissue	granulation tissue	9	kept
growth	growth	growth	growth	Growth	9	case_or_format
HAI	hai	hai	hai	HAI	9	kept
Hawaii	hawaii	hawaii	hawaii	Hawaii	9	kept
head and neck cancer	head and neck cancer	head and neck cancer	head and neck cancer	head and neck cancer	9	kept
Health care providers	health care providers	health care providers	health care provider	health care providers	9	singular
Health care surveys	health care surveys	health care surveys	health care survey	Health Care Surveys	9	singular
Health care worker	health care worker	health care worker	health care worker	Health care workers	9	case_or_format
health care worker	health care worker	health care worker	health care worker	Health care workers	9	case_or_format

health informatics	health informatics	health informatics	health informatics	health informatics	9	kept
health resources	health resources	health resources	health resources	Health Resources	9	case_or_format
Health-related quality of life	health related quality of life	health related quality of life	health related quality of life	health-related quality of life	9	case_or_format
Healthcare personnel	healthcare personnel	healthcare personnel	healthcare personnel	Healthcare personnel	9	kept
Healthcare professional	healthcare professional	healthcare professional	healthcare professional	healthcare professionals	9	case_or_format
Healthcare quality	healthcare quality	healthcare quality	healthcare quality	Healthcare quality	9	kept
Healthcare quality improvement	healthcare quality improvement	healthcare quality improvement	healthcare quality improvement	healthcare quality improvement	9	case_or_format
healthcare system	healthcare system	healthcare system	healthcare system	healthcare system	9	kept
healthcare worker	healthcare worker	healthcare worker	healthcare worker	Healthcare workers	9	case_or_format
hearing	hearing	hearing	hearing	hearing	9	kept
heart muscle contractility	heart muscle contractility	heart muscle contractility	heart muscle contractility	heart muscle contractility	9	kept
Heart Valves	heart valves	heart valves	heart valve	Heart Valves	9	singular
Helicobacter pylori	helicobacter pylori	helicobacter pylori	helicobacter pylori	Helicobacter pylori	9	kept
HELLP syndrome	hellp syndrome	hellp syndrome	hellp syndrome	HELLP syndrome	9	kept
Hematology	hematology	hematology	hematology	hematology	9	case_or_format
hemodiafiltration	hemodiafiltration	hemodiafiltration	hemodiafiltration	Hemodiafiltration	9	case_or_format

hemoglobin determination	hemoglobin determination	hemoglobin determination	hemoglobin determination	hemoglobin determination	9	kept
Hemolysis	hemolysis	hemolysis	hemolysis	hemolysis	9	case_or_format
Hemostasis	hemostasis	hemostasis	hemostasis	hemostasis	9	case_or_format
heparinization	heparinization	heparinization	heparinization	heparinization	9	kept
hepatitis	hepatitis	hepatitis	hepatitis	hepatitis	9	kept
high frequency oscillatory ventilation	high frequency oscillatory ventilation	high frequency oscillatory ventilation	high frequency oscillatory ventilation	high frequency oscillatory ventilation	9	kept
high performance liquid chromatography	high performance liquid chromatography	high performance liquid chromatography	high performance liquid chromatography	high performance liquid chromatography	9	kept
high risk infant	high risk infant	high risk infant	high risk infant	high risk infant	9	kept
hoarseness	hoarseness	hoarseness	hoarseness	hoarseness	9	kept
Homicide	homicide	homicide	homicide	homicide	9	case_or_format
Hospital Bed Capacity, 500 and over	hospital bed capacity 500 and over	hospital bed capacity 500 and over	hospital bed capacity 500 and over	Hospital Bed Capacity, 500 and over	9	kept
HOSPITAL CARDIAC-ARREST	hospital cardiac arrest	hospital cardiac arrest	hospital cardiac arrest	HOSPITAL CARDIAC-ARREST	9	kept
hospital volume	hospital volume	hospital volume	hospital volume	hospital volume	9	kept
Hospitals, High-Volume	hospitals high volume	hospitals high volume	hospitals high volume	Hospitals, High-Volume	9	kept
Hostility	hostility	hostility	hostility	Hostility	9	kept
housekeeping	housekeeping	housekeeping	housekeeping	housekeeping	9	kept

Housekeeping, Hospital	housekeeping hospital	housekeeping hospital	housekeeping hospital	Housekeeping, Hospital	9	kept
hyperbaric oxygen	hyperbaric oxygen	hyperbaric oxygen	hyperbaric oxygen	hyperbaric oxygen	9	kept
Hyperglycaemia	hyperglycaemia	hyperglycaemia	hyperglycaemia	Hyperglycaemia	9	kept
hyperthermic intraperitoneal chemotherapy	hyperthermic intraperitoneal chemotherapy	hyperthermic intraperitoneal chemotherapy	hyperthermic intraperitoneal chemotherapy	hyperthermic intraperitoneal chemotherapy	9	kept
hypertrophic cardiomyopathy	hypertrophic cardiomyopathy	hypertrophic cardiomyopathy	hypertrophic cardiomyopathy	hypertrophic cardiomyopathy	9	kept
hyperventilation	hyperventilation	hyperventilation	hyperventilation	hyperventilation	9	kept
Hypnotics and sedatives	hypnotics and sedatives	hypnotics and sedatives	hypnotics and sedatives	Hypnotics and Sedatives	9	case_or_format
Hypocalcemia	hypocalcemia	hypocalcemia	hypocalcemia	hypocalcemia	9	case_or_format
ICD-10-CM	icd 10 cm	icd 10 cm	icd 10 cm	ICD-10-CM	9	kept
illicit drug	illicit drug	illicit drug	illicit drug	illicit drug	9	kept
ILLNESS	illness	illness	illness	ILLNESS	9	kept
illness trajectory	illness trajectory	illness trajectory	illness trajectory	illness trajectory	9	kept
Imagery, Psychotherapy	imagery psychotherapy	imagery psychotherapy	imagery psychotherapy	Imagery, Psychotherapy	9	kept
immune system	immune system	immune system	immune system	immune system	9	kept
Immunotherapy	immunotherapy	immunotherapy	immunotherapy	immunotherapy	9	case_or_format
In-hospital mortality	in hospital mortality	in hospital mortality	in hospital mortality	in-hospital mortality	9	case_or_format

incontinence associated dermatitis	incontinence associated dermatitis	incontinence associated dermatitis	incontinence associated dermatitis	Incontinence-associated dermatitis	9	case_or_format
Independent Living	independent living	independent living	independent living	independent living	9	case_or_format
indwelling urinary catheter	indwelling urinary catheter	indwelling urinary catheter	indwelling urinary catheter	indwelling urinary catheter	9	kept
Infection Control Practitioners	infection control practitioners	infection control practitioners	infection control practitioner	infection control practitioner	9	singular
Infectious Disease Transmission, Vertical	infectious disease transmission vertical	infectious disease transmission vertical	infectious disease transmission vertical	Infectious Disease Transmission, Vertical	9	kept
information science	information science	information science	information science	information science	9	kept
infrastructure	infrastructure	infrastructure	infrastructure	infrastructure	9	kept
infusion therapy	infusion therapy	infusion therapy	infusion therapy	infusion therapy	9	kept
Infusions	infusions	infusions	infusion	infusion	9	singular
inguinal region	inguinal region	inguinal region	inguinal region	inguinal region	9	kept
inhalation anesthetic agent	inhalation anesthetic agent	inhalation anesthetic agent	inhalation anesthetic agent	inhalation anesthetic agent	9	kept
Insufflation	insufflation	insufflation	insufflation	Insufflation	9	kept
insulin aspart	insulin aspart	insulin aspart	insulin aspart	insulin aspart	9	kept
integrative medicine	integrative medicine	integrative medicine	integrative medicine	integrative medicine	9	kept
interleukin 10	interleukin 10	interleukin 10	interleukin 10	interleukin 10	9	kept
Intermittent Pneumatic Compression Devices	intermittent pneumatic compression devices	intermittent pneumatic compression devices	intermittent pneumatic compression device	Intermittent Pneumatic Compression Devices	9	singular

intermittent positive pressure ventilation	intermittent positive pressure ventilation	intermittent positive pressure ventilation	intermittent positive pressure ventilation	Intermittent Positive-Pressure Ventilation	9	case_or_format
interprofessional communication	interprofessional communication	interprofessional communication	interprofessional communication	interprofessional communication	9	kept
Intestinal Diseases	intestinal diseases	intestinal diseases	intestinal diseases	Intestinal Diseases	9	kept
Intestinal Fistula	intestinal fistula	intestinal fistula	intestinal fistula	Intestinal Fistula	9	kept
intestine ischemia	intestine ischemia	intestine ischemia	intestine ischemia	intestine ischemia	9	kept
intracranial aneurysm	intracranial aneurysm	intracranial aneurysm	intracranial aneurysm	Intracranial Aneurysm	9	case_or_format
intratracheal	intratracheal	intratracheal	intratracheal	intratracheal	9	kept
keratitis	keratitis	keratitis	keratitis	keratitis	9	kept
Klebsiella oxytoca	klebsiella oxytoca	klebsiella oxytoca	klebsiella oxytoca	Klebsiella oxytoca	9	kept
Laryngismus	laryngismus	laryngismus	laryngismus	Laryngismus	9	kept
Larynx	larynx	larynx	larynx	Larynx	9	kept
learning style	learning style	learning style	learning style	learning style	9	kept
Leg	leg	leg	leg	leg	9	case_or_format
Leg Injuries	leg injuries	leg injuries	leg injury	leg injury	9	singular
leg injury	leg injury	leg injury	leg injury	leg injury	9	kept
Legionella	legionella	legionella	legionella	Legionella	9	kept

life threat	life threat	life threat	life threat	life threat	9	kept
light exposure	light exposure	light exposure	light exposure	light exposure	9	kept
limb weakness	limb weakness	limb weakness	limb weakness	limb weakness	9	kept
Limited English Proficiency	limited english proficiency	limited english proficiency	limited english proficiency	Limited English Proficiency	9	kept
Liver	liver	liver	liver	Liver	9	kept
liver enzyme	liver enzyme	liver enzyme	liver enzyme	liver enzyme	9	kept
liver function	liver function	liver function	liver function	liver function	9	kept
liver injury	liver injury	liver injury	liver injury	liver injury	9	kept
lung consolidation	lung consolidation	lung consolidation	lung consolidation	lung consolidation	9	kept
lung fibrosis	lung fibrosis	lung fibrosis	lung fibrosis	lung fibrosis	9	kept
lung insufficiency	lung insufficiency	lung insufficiency	lung insufficiency	lung insufficiency	9	kept
lung parenchyma	lung parenchyma	lung parenchyma	lung parenchyma	lung parenchyma	9	kept
lymphocyte	lymphocyte	lymphocyte	lymphocyte	lymphocyte	9	kept
Major Depressive Disorder	major depressive disorder	major depressive disorder	major depressive disorder	Major Depressive Disorder	9	kept
manual ventilation	manual ventilation	manual ventilation	manual ventilation	manual ventilation	9	kept
Maternal health	maternal health	maternal health	maternal health	Maternal health	9	kept

Maternal mortality	maternal mortality	maternal mortality	maternal mortality	maternal mortality	9	case_or_format
Measurement	measurement	measurement	measurement	measurement	9	case_or_format
Mechanical	mechanical	mechanical	mechanical	Mechanical	9	kept
Mechanical thrombectomy	mechanical thrombectomy	mechanical thrombectomy	mechanical thrombectomy	mechanical thrombectomy	9	case_or_format
medical device-related pressure injury	medical device related pressure injury	medical device related pressure injury	medical device related pressure injury	medical device-related pressure injury	9	kept
medical parameters	medical parameters	medical parameters	medical parameters	medical parameters	9	kept
Medical Subject Headings	medical subject headings	medical subject headings	medical subject headings	Medical Subject Headings	9	kept
medically uninsured	medically uninsured	medically uninsured	medically uninsured	medically uninsured	9	kept
Meditation	meditation	meditation	meditation	meditation	9	case_or_format
melanoma	melanoma	melanoma	melanoma	melanoma	9	kept
Memorial Delirium Assessment Scale	memorial delirium assessment scale	memorial delirium assessment scale	memorial delirium assessment scale	Memorial Delirium Assessment Scale	9	kept
Meningitis	meningitis	meningitis	meningitis	meningitis	9	case_or_format
mental health care personnel	mental health care personnel	mental health care personnel	mental health care personnel	mental health care personnel	9	kept
mental hospital	mental hospital	mental hospital	mental hospital	mental hospital	9	kept
microclimate	microclimate	microclimate	microclimate	microclimate	9	kept
middle cerebral artery	middle cerebral artery	middle cerebral artery	middle cerebral artery	middle cerebral artery	9	kept

Midwife	midwife	midwife	midwife	midwife	9	case_or_format
mitigation	mitigation	mitigation	mitigation	mitigation	9	kept
mixed-methods	mixed methods	mixed methods	mixed method	Mixed methods	9	singular
MODELS	models	models	model	model	9	singular
mood	mood	mood	mood	mood	9	kept
Mortality rate	mortality rate	mortality rate	mortality rate	mortality rate	9	case_or_format
Motor Skills	motor skills	motor skills	motor skills	Motor Skills	9	kept
Mouth Diseases	mouth diseases	mouth diseases	mouth disease	mouth disease	9	singular
multilevel analysis	multilevel analysis	multilevel analysis	multilevel analysis	multilevel analysis	9	kept
multiple cycle treatment	multiple cycle treatment	multiple cycle treatment	multiple cycle treatment	multiple cycle treatment	9	kept
muscle disease	muscle disease	muscle disease	muscle disease	muscle disease	9	kept
muscle rigidity	muscle rigidity	muscle rigidity	muscle rigidity	muscle rigidity	9	kept
nalbuphine	nalbuphine	nalbuphine	nalbuphine	nalbuphine	9	kept
Natural language processing systems	natural language processing systems	natural language processing systems	natural language processing systems	Natural language processing systems	9	kept
needle	needle	needle	needle	needle	9	kept
needs	needs	needs	need	NEEDS	9	singular

Neonatal palliative care	neonatal palliative care	neonatal palliative care	neonatal palliative care	neonatal palliative care	9	case_or_format
NEONATOLOGY	neonatology	neonatology	neonatology	Neonatology	9	case_or_format
Neoplasm Staging	neoplasm staging	neoplasm staging	neoplasm staging	Neoplasm Staging	9	kept
neuropathy	neuropathy	neuropathy	neuropathy	neuropathy	9	kept
neuropsychiatric inventory	neuropsychiatric inventory	neuropsychiatric inventory	neuropsychiatric inventory	neuropsychiatric inventory	9	kept
neuropsychology	neuropsychology	neuropsychology	neuropsychology	neuropsychology	9	kept
nullipara	nullipara	nullipara	nullipara	nullipara	9	kept
nutrition supplement	nutrition supplement	nutrition supplement	nutrition supplement	nutrition supplement	9	kept
nutritional deficiency	nutritional deficiency	nutritional deficiency	nutritional deficiency	nutritional deficiency	9	kept
observation unit	observation unit	observation unit	observation unit	observation unit	9	kept
Obstructive sleep apnea	obstructive sleep apnea	obstructive sleep apnea	obstructive sleep apnea	Obstructive sleep apnea	9	kept
OCCUPATIONAL STRESS	occupational stress	occupational stress	occupational stress	Occupational Stress	9	case_or_format
Occupational therapy	occupational therapy	occupational therapy	occupational therapy	occupational therapy	9	case_or_format
octreotide	octreotide	octreotide	octreotide	octreotide	9	kept
OF-LIFE DECISIONS	of life decisions	of life decisions	of life decisions	OF-LIFE DECISIONS	9	kept
Older adult	older adult	older adult	older adult	Older adults	9	case_or_format

OLDER-PEOPLE	older people	older people	older people	older people	9	case_or_format
omega 3 fatty acid	omega 3 fatty acid	omega 3 fatty acid	omega 3 fatty acid	omega 3 fatty acid	9	kept
OPERATING-ROOM	operating room	operating room	operating room	operating room	9	case_or_format
ophthalmologist	ophthalmologist	ophthalmologist	ophthalmologist	ophthalmologist	9	kept
Ophthalmoscopy	ophthalmoscopy	ophthalmoscopy	ophthalmoscopy	ophthalmoscopy	9	case_or_format
opportunistic infection	opportunistic infection	opportunistic infection	opportunistic infection	opportunistic infection	9	kept
oral antidiabetic agent	oral antidiabetic agent	oral antidiabetic agent	oral antidiabetic agent	oral antidiabetic agent	9	kept
Organizational commitment	organizational commitment	organizational commitment	organizational commitment	Organizational commitment	9	kept
oropharyngeal swab	oropharyngeal swab	oropharyngeal swab	oropharyngeal swab	oropharyngeal swab	9	kept
ostomy	ostomy	ostomy	ostomy	ostomy	9	kept
Oxytocics	oxytocics	oxytocics	oxytocics	Oxytocics	9	kept
paraveterinary worker	paraveterinary worker	paraveterinary worker	paraveterinary worker	paraveterinary worker	9	kept
parental consent	parental consent	parental consent	parental consent	Parental Consent	9	case_or_format
Partial Pressure	partial pressure	partial pressure	partial pressure	Partial Pressure	9	kept
Partial Thromboplastin Time	partial thromboplastin time	partial thromboplastin time	partial thromboplastin time	partial thromboplastin time	9	case_or_format
paternalism	paternalism	paternalism	paternalism	paternalism	9	kept

Pediatric trauma	pediatric trauma	pediatric trauma	pediatric trauma	Pediatric trauma	9	kept
Perceived stress	perceived stress	perceived stress	perceived stress	perceived stress	9	case_or_format
perineum	perineum	perineum	perineum	perineum	9	kept
Peripherally inserted central catheter	peripherally inserted central catheter	peripherally inserted central catheter	peripherally inserted central catheter	peripherally inserted central catheter	9	case_or_format
Peritoneal dialysis	peritoneal dialysis	peritoneal dialysis	peritoneal dialysis	peritoneal dialysis	9	case_or_format
Peritoneal Neoplasms	peritoneal neoplasms	peritoneal neoplasms	peritoneal neoplasms	Peritoneal Neoplasms	9	kept
persuasive communication	persuasive communication	persuasive communication	persuasive communication	persuasive communication	9	kept
pharmacists	pharmacists	pharmacists	pharmacist	pharmacist	9	singular
Pharmacokinetics	pharmacokinetics	pharmacokinetics	pharmacokinetic	pharmacokinetics	9	singular
Pharmacy	pharmacy	pharmacy	pharmacy	pharmacy	9	case_or_format
pharmacy education	pharmacy education	pharmacy education	pharmacy education	pharmacy education	9	kept
phosphorus	phosphorus	phosphorus	phosphorus	phosphorus	9	kept
physical function	physical function	physical function	physical function	physical function	9	kept
Physical Therapy Specialty	physical therapy specialty	physical therapy specialty	physical therapy specialty	Physical Therapy Specialty	9	kept
PHYSICIAN ASSISTANTS	physician assistants	physician assistants	physician assistant	physician assistant	9	singular
Placebos	placebos	placebos	placebo	placebo	9	singular

Plasma	plasma	plasma	plasma	Plasma	9	kept
Plasma Exchange	plasma exchange	plasma exchange	plasma exchange	plasma exchange	9	case_or_format
plasma transfusion	plasma transfusion	plasma transfusion	plasma transfusion	plasma transfusion	9	kept
POCUS	pocus	pocus	pocus	POCUS	9	kept
Point-of-care testing	point of care testing	point of care testing	point of care testing	point of care testing	9	case_or_format
polymyxin B	polymyxin b	polymyxin b	polymyxin b	polymyxin B	9	kept
polyurethan	polyurethan	polyurethan	polyurethan	polyurethan	9	kept
portal hypertension	portal hypertension	portal hypertension	portal hypertension	portal hypertension	9	kept
positron emission tomography	positron emission tomography	positron emission tomography	positron emission tomography	positron emission tomography	9	kept
post-traumatic growth	post traumatic growth	post traumatic growth	post traumatic growth	post-traumatic growth	9	kept
posthumous care	posthumous care	posthumous care	posthumous care	posthumous care	9	kept
POSTTRAUMATIC-STRESS-DISORDER	posttraumatic stress disorder	posttraumatic stress disorder	posttraumatic stress disorder	posttraumatic stress disorder	9	case_or_format
power supply	power supply	power supply	power supply	power supply	9	kept
Predictors	predictors	predictors	predictor	PREDICTORS	9	singular
Prescription Drugs	prescription drugs	prescription drugs	prescription drug	prescription drug	9	singular
pressure ulcer prevention	pressure ulcer prevention	pressure ulcer prevention	pressure ulcer prevention	pressure ulcer prevention	9	kept

Preterm neonates	preterm neonates	preterm neonates	preterm neonate	Preterm neonates	9	singular
Primary care	primary care	primary care	primary care	primary care	9	case_or_format
PROCEDURAL PAIN	procedural pain	procedural pain	procedural pain	procedural pain	9	case_or_format
Procedural pain	procedural pain	procedural pain	procedural pain	procedural pain	9	case_or_format
profit	profit	profit	profit	profit	9	kept
prone positioning	prone positioning	prone positioning	prone positioning	prone positioning	9	kept
Prophylaxis	prophylaxis	prophylaxis	prophylaxis	prophylaxis	9	case_or_format
Prosthesis-Related Infections	prosthesis related infections	prosthesis related infections	prosthesis related infections	Prosthesis-Related Infections	9	kept
protective equipment shortage	protective equipment shortage	protective equipment shortage	protective equipment shortage	protective equipment shortage	9	kept
protective glove	protective glove	protective glove	protective glove	protective glove	9	kept
protein C	protein c	protein c	protein c	Protein C	9	case_or_format
protein expression	protein expression	protein expression	protein expression	protein expression	9	kept
Proton Pump Inhibitors	proton pump inhibitors	proton pump inhibitors	proton pump inhibitor	proton pump inhibitor	9	singular
Psychology, Educational	psychology educational	psychology educational	psychology educational	Psychology, Educational	9	kept
public opinion	public opinion	public opinion	public opinion	Public Opinion	9	case_or_format
pulmonary artery catheter	pulmonary artery catheter	pulmonary artery catheter	pulmonary artery catheter	pulmonary artery catheter	9	kept

pulmonary embolism	pulmonary embolism	pulmonary embolism	pulmonary embolism	Pulmonary Embolism	9	case_or_format
Pylorus	pylorus	pylorus	pylorus	Pylorus	9	kept
Pyrazoles	pyrazoles	pyrazoles	pyrazoles	Pyrazoles	9	kept
Pyridones	pyridones	pyridones	pyridones	Pyridones	9	kept
QT interval	qt interval	qt interval	qt interval	QT interval	9	kept
quality and safety	quality and safety	quality and safety	quality and safety	quality and safety	9	kept
Quality care	quality care	quality care	quality care	Quality care	9	kept
Quality in health care	quality in health care	quality in health care	quality in health care	quality in health care	9	case_or_format
quantitative research	quantitative research	quantitative research	quantitative research	quantitative research	9	kept
Racism	racism	racism	racism	racism	9	case_or_format
Radiology, Interventional	radiology interventional	radiology interventional	radiology interventional	Radiology, Interventional	9	kept
rapid response systems	rapid response systems	rapid response systems	rapid response system	Rapid response system	9	singular
reassurance	reassurance	reassurance	reassurance	reassurance	9	kept
Recognition	recognition	recognition	recognition	recognition	9	case_or_format
Rectal Neoplasms	rectal neoplasms	rectal neoplasms	rectal neoplasm	Rectal Neoplasms	9	singular
Reintubation	reintubation	reintubation	reintubation	Reintubation	9	kept

relapse	relapse	relapse	relapse	relapse	9	kept
relationships	relationships	relationships	relationship	relationships	9	singular
Religion and Psychology	religion and psychology	religion and psychology	religion and psychology	Religion and Psychology	9	kept
remimazolam	remimazolam	remimazolam	remimazolam	remimazolam	9	kept
renin angiotensin aldosterone system	renin angiotensin aldosterone system	renin angiotensin aldosterone system	renin angiotensin aldosterone system	renin angiotensin aldosterone system	9	kept
Reperfusion	reperfusion	reperfusion	reperfusion	Reperfusion	9	kept
reporting	reporting	reporting	reporting	Reporting	9	case_or_format
reproducibility of results	reproducibility of results	reproducibility of results	reproducibility of results	Reproducibility of Results	9	case_or_format
research implementation	research implementation	research implementation	research implementation	research implementation	9	kept
Respiratory distress	respiratory distress	respiratory distress	respiratory distress	respiratory distress	9	case_or_format
Respiratory syncytial virus	respiratory syncytial virus	respiratory syncytial virus	respiratory syncytial virus	Respiratory syncytial virus	9	kept
Rest	rest	rest	rest	rest	9	case_or_format
Return to Work	return to work	return to work	return to work	return to work	9	case_or_format
right to die	right to die	right to die	right to die	Right to Die	9	case_or_format
Risk perception	risk perception	risk perception	risk perception	Risk perception	9	kept
Rivaroxaban	rivaroxaban	rivaroxaban	rivaroxaban	rivaroxaban	9	case_or_format

Romania	romania	romania	romania	Romania	9	kept
Rome	rome	rome	rome	Rome	9	kept
ScienceDirect	sciencedirect	sciencedirect	sciencedirect	ScienceDirect	9	kept
Seguridad del paciente	seguridad del paciente	seguridad del paciente	seguridad del paciente	Seguridad del paciente	9	kept
selenium	selenium	selenium	selenium	selenium	9	kept
severe acute respiratory syndrome coronavirus 2	severe acute respiratory syndrome coronavirus 2	severe acute respiratory syndrome coronavirus 2	severe acute respiratory syndrome coronavirus 2	Severe acute respiratory syndrome coronavirus 2	9	case_or_format
Severity of illness	severity of illness	severity of illness	severity of illness	Severity of illness	9	kept
Sevoflurane	sevoflurane	sevoflurane	sevoflurane	sevoflurane	9	case_or_format
sexual behavior	sexual behavior	sexual behavior	sexual behavior	sexual behavior	9	kept
sexually transmitted disease	sexually transmitted disease	sexually transmitted disease	sexually transmitted disease	sexually transmitted disease	9	kept
Shame	shame	shame	shame	shame	9	case_or_format
Siblings	siblings	siblings	sibling	sibling	9	singular
simulation-based training	simulation based training	simulation based training	simulation based training	Simulation-based training	9	case_or_format
single	single	single	single	single	9	kept
situation awareness	situation awareness	situation awareness	situation awareness	situation awareness	9	kept
skin color	skin color	skin color	skin color	skin color	9	kept

skin necrosis	skin necrosis	skin necrosis	skin necrosis	skin necrosis	9	kept
skin transplantation	skin transplantation	skin transplantation	skin transplantation	Skin Transplantation	9	case_or_format
skin ulcer	skin ulcer	skin ulcer	skin ulcer	skin ulcer	9	kept
Sleepiness	sleepiness	sleepiness	sleepiness	Sleepiness	9	kept
Slovakia	slovakia	slovakia	slovakia	Slovakia	9	kept
Snake Bites	snake bites	snake bites	snake bites	Snake Bites	9	kept
social life	social life	social life	social life	social life	9	kept
Social network analysis	social network analysis	social network analysis	social network analysis	Social network analysis	9	kept
SOCIAL SUPPORT	social support	social support	social support	Social Support	9	case_or_format
Soft Tissue Infections	soft tissue infections	soft tissue infections	soft tissue infection	soft tissue infection	9	singular
Sound Spectrography	sound spectrography	sound spectrography	sound spectrography	Sound Spectrography	9	kept
Spinal Dysraphism	spinal dysraphism	spinal dysraphism	spinal dysraphism	Spinal Dysraphism	9	kept
Standard precautions	standard precautions	standard precautions	standard precautions	Standard precautions	9	kept
STAPHYLOCOCCUS-AUREUS	staphylococcus aureus	staphylococcus aureus	staphylococcus aureus	Staphylococcus aureus	9	case_or_format
stem cell transplantation	stem cell transplantation	stem cell transplantation	stem cell transplantation	stem cell transplantation	9	kept
Stereotyping	stereotyping	stereotyping	stereotyping	Stereotyping	9	kept

Stethoscopes	stethoscopes	stethoscopes	stethoscope	Stethoscopes	9	singular
stomach juice	stomach juice	stomach juice	stomach juice	stomach juice	9	kept
storytelling	storytelling	storytelling	storytelling	storytelling	9	kept
Streptococcus infection	streptococcus infection	streptococcus infection	streptococcus infection	Streptococcus infection	9	kept
Stress disorders	stress disorders	stress disorders	stress disorder	Stress disorders	9	singular
Subclavian Vein	subclavian vein	subclavian vein	subclavian vein	Subclavian Vein	9	kept
subclavian vein	subclavian vein	subclavian vein	subclavian vein	Subclavian Vein	9	case_or_format
suicidal behavior	suicidal behavior	suicidal behavior	suicidal behavior	suicidal behavior	9	kept
sulfadiazine silver	sulfadiazine silver	sulfadiazine silver	sulfadiazine silver	sulfadiazine silver	9	kept
supplementation	supplementation	supplementation	supplementation	supplementation	9	kept
survivorship	survivorship	survivorship	survivorship	survivorship	9	kept
SUSTAINING TREATMENT	sustaining treatment	sustaining treatment	sustaining treatment	SUSTAINING TREATMENT	9	kept
Swan Ganz catheter	swan ganz catheter	swan ganz catheter	swan ganz catheter	Swan Ganz catheter	9	kept
Symptoms	symptoms	symptoms	symptom	symptom	9	singular
symptoms	symptoms	symptoms	symptom	symptom	9	singular
telecare	telecare	telecare	telecare	telecare	9	kept

Telecommunications	telecommunications	telecommunications	telecommunication	telecommunication	9	singular
theophylline	theophylline	theophylline	theophylline	theophylline	9	kept
Thermoregulation	thermoregulation	thermoregulation	thermoregulation	thermoregulation	9	case_or_format
three dimensional printing	three dimensional printing	three dimensional printing	three dimensional printing	three dimensional printing	9	kept
thromboelastometry	thromboelastometry	thromboelastometry	thromboelastometry	thromboelastometry	9	kept
ticagrelor	ticagrelor	ticagrelor	ticagrelor	ticagrelor	9	kept
tissue oxygenation	tissue oxygenation	tissue oxygenation	tissue oxygenation	tissue oxygenation	9	kept
tonsillectomy	tonsillectomy	tonsillectomy	tonsillectomy	Tonsillectomy	9	case_or_format
Tracheal intubation	tracheal intubation	tracheal intubation	tracheal intubation	Tracheal intubation	9	kept
tracheostomy tube	tracheostomy tube	tracheostomy tube	tracheostomy tube	tracheostomy tube	9	kept
Training needs	training needs	training needs	training needs	Training needs	9	kept
transfer	transfer	transfer	transfer	Transfer	9	case_or_format
Translational Research, Biomedical	translational research biomedical	translational research biomedical	translational research biomedical	Translational Research, Biomedical	9	kept
Transmission	transmission	transmission	transmission	transmission	9	case_or_format
transport	transport	transport	transport	Transport	9	case_or_format
Transposition of Great Vessels	transposition of great vessels	transposition of great vessels	transposition of great vessels	Transposition of Great Vessels	9	kept

TRAUMA	trauma	trauma	trauma	Trauma	9	case_or_format
trauma surgery	trauma surgery	trauma surgery	trauma surgery	trauma surgery	9	kept
Turnover	turnover	turnover	turnover	turnover	9	case_or_format
Twins, Conjoined	twins conjoined	twins conjoined	twins conjoined	Twins, Conjoined	9	kept
Ultrasonography, Doppler	ultrasonography doppler	ultrasonography doppler	ultrasonography doppler	Ultrasonography, Doppler	9	kept
umbrella review	umbrella review	umbrella review	umbrella review	umbrella review	9	kept
United States Centers for Medicare and Medicaid Services	united states centers for medicare and medicaid services	united states centers for medicare and medicaid services	united states centers for medicare and medicaid services	United States Centers for Medicare and Medicaid Services	9	kept
universal precaution	universal precaution	universal precaution	universal precaution	Universal Precautions	9	case_or_format
Upper Extremity	upper extremity	upper extremity	upper extremity	Upper Extremity	9	kept
upper gastrointestinal bleeding	upper gastrointestinal bleeding	upper gastrointestinal bleeding	upper gastrointestinal bleeding	upper gastrointestinal bleeding	9	kept
Urban Health Services	urban health services	urban health services	urban health services	Urban Health Services	9	kept
Urology	urology	urology	urology	urology	9	case_or_format
vascular patency	vascular patency	vascular patency	vascular patency	vascular patency	9	kept
vein	vein	vein	vein	Veins	9	case_or_format
vein occlusion	vein occlusion	vein occlusion	vein occlusion	vein occlusion	9	kept
Ventilator-associated	ventilator associated	ventilator associated	ventilator associated	Ventilator-associated	9	kept

Ventilators	ventilators	ventilators	ventilator	ventilator	9	singular
Verbal Behavior	verbal behavior	verbal behavior	verbal behavior	Verbal Behavior	9	kept
veterinarian	veterinarian	veterinarian	veterinarian	veterinarian	9	kept
video assisted thoracoscopic surgery	video assisted thoracoscopic surgery	video assisted thoracoscopic surgery	video assisted thoracoscopic surgery	video assisted thoracoscopic surgery	9	kept
Video Games	video games	video games	video game	Video Games	9	singular
virus vaccine	virus vaccine	virus vaccine	virus vaccine	virus vaccine	9	kept
visibility	visibility	visibility	visibility	visibility	9	kept
VITAL SIGNS	vital signs	vital signs	vital sign	vital sign	9	singular
voluntary reporting	voluntary reporting	voluntary reporting	voluntary reporting	voluntary reporting	9	kept
War-Related Injuries	war related injuries	war related injuries	war related injuries	War-Related Injuries	9	kept
white noise	white noise	white noise	white noise	white noise	9	kept
Women	women	women	women	WOMEN	9	case_or_format
WORK ENVIRONMENTS	work environments	work environments	work environment	work environment	9	singular
Work of Breathing	work of breathing	work of breathing	work of breathing	Work of Breathing	9	kept
Work performance	work performance	work performance	work performance	Work Performance	9	case_or_format
Work-related stress	work related stress	work related stress	work related stress	Work-related stress	9	kept

work-related stress	work related stress	work related stress	work related stress	Work-related stress	9	case_or_format
WORKERS	workers	workers	worker	worker	9	singular
Working environment	working environment	working environment	working environment	Working environment	9	kept
World Health	world health	world health	world health	World Health	9	kept
wound closure	wound closure	wound closure	wound closure	wound closure	9	kept
Abbreviations as Topic	abbreviations as topic	abbreviations as topic	abbreviations as topic	Abbreviations as Topic	8	kept
abdominal cavity	abdominal cavity	abdominal cavity	abdominal cavity	abdominal cavity	8	kept
abdominal drainage	abdominal drainage	abdominal drainage	abdominal drainage	abdominal drainage	8	kept
abdominal tenderness	abdominal tenderness	abdominal tenderness	abdominal tenderness	abdominal tenderness	8	kept
Abstract	abstract	abstract	abstract	Abstract	8	kept
ACTIVATION	activation	activation	activation	ACTIVATION	8	kept
active euthanasia	active euthanasia	active euthanasia	active euthanasia	active euthanasia	8	kept
Acupressure	acupressure	acupressure	acupressure	Acupressure	8	kept
acute	acute	acute	acute	acute	8	kept
ACUTE MYOCARDIAL-INFARCTION	acute myocardial infarction	acute myocardial infarction	acute myocardial infarction	acute myocardial infarction	8	case_or_format
administration and dosage	administration and dosage	administration and dosage	administration and dosage	administration and dosage	8	kept

administrative claims	administrative claims	administrative claims	administrative claims	administrative claims	8	kept
affect	affect	affect	affect	Affect	8	case_or_format
Africa south of the Sahara	africa south of the sahara	africa south of the sahara	africa south of the sahara	Africa south of the Sahara	8	kept
Air Pollution, Indoor	air pollution indoor	air pollution indoor	air pollution indoor	Air Pollution, Indoor	8	kept
Airway extubation	airway extubation	airway extubation	airway extubation	Airway Extubation	8	case_or_format
albumin level	albumin level	albumin level	albumin level	albumin level	8	kept
Alcohol Drinking	alcohol drinking	alcohol drinking	alcohol drinking	Alcohol Drinking	8	kept
Alcohol-Related Disorders	alcohol related disorders	alcohol related disorders	alcohol related disorders	Alcohol-Related Disorders	8	kept
algorithms	algorithms	algorithms	algorithm	Algorithms	8	singular
Alkalosis	alkalosis	alkalosis	alkalosis	alkalosis	8	case_or_format
allopurinol	allopurinol	allopurinol	allopurinol	allopurinol	8	kept
alpha 2 adrenergic receptor stimulating agent	alpha 2 adrenergic receptor stimulating agent	alpha 2 adrenergic receptor stimulating agent	alpha 2 adrenergic receptor stimulating agent	alpha 2 adrenergic receptor stimulating agent	8	kept
altruism	altruism	altruism	altruism	Altruism	8	case_or_format
Amphotericin B	amphotericin b	amphotericin b	amphotericin b	amphotericin B	8	case_or_format
Antacids	antacids	antacids	antacids	Antacids	8	kept
antibody titer	antibody titer	antibody titer	antibody titer	antibody titer	8	kept

antimicrobial activity	antimicrobial activity	antimicrobial activity	antimicrobial activity	antimicrobial activity	8	kept
Antipyretics	antipyretics	antipyretics	antipyretics	Antipyretics	8	kept
antivitamin K	antivitamin k	antivitamin k	antivitamin k	antivitamin K	8	kept
Aorta	aorta	aorta	aorta	Aorta	8	kept
aortic stenosis	aortic stenosis	aortic stenosis	aortic stenosis	aortic stenosis	8	kept
aortic valve replacement	aortic valve replacement	aortic valve replacement	aortic valve replacement	aortic valve replacement	8	kept
appetite disorder	appetite disorder	appetite disorder	appetite disorder	appetite disorder	8	kept
architecture	architecture	architecture	architecture	architecture	8	kept
arterial line	arterial line	arterial line	arterial line	arterial line	8	kept
Asepsis	asepsis	asepsis	asepsis	asepsis	8	case_or_format
Asian Continental Ancestry Group	asian continental ancestry group	asian continental ancestry group	asian continental ancestry group	Asian Continental Ancestry Group	8	kept
Aspergillus	aspergillus	aspergillus	aspergillus	Aspergillus	8	kept
assault	assault	assault	assault	assault	8	kept
Assisted Circulation	assisted circulation	assisted circulation	assisted circulation	assisted circulation	8	case_or_format
Assisted Living Facilities	assisted living facilities	assisted living facilities	assisted living facility	assisted living facility	8	singular
attention disturbance	attention disturbance	attention disturbance	attention disturbance	attention disturbance	8	kept

aviation	aviation	aviation	aviation	aviation	8	kept
avibactam plus ceftazidime	avibactam plus ceftazidime	avibactam plus ceftazidime	avibactam plus ceftazidime	avibactam plus ceftazidime	8	kept
baby	baby	baby	baby	baby	8	kept
Bandages, Hydrocolloid	bandages hydrocolloid	bandages hydrocolloid	bandages hydrocolloid	Bandages, Hydrocolloid	8	kept
bedtime dosage	bedtime dosage	bedtime dosage	bedtime dosage	bedtime dosage	8	kept
behaviour	behaviour	behaviour	behaviour	Behaviour	8	case_or_format
Beliefs	beliefs	beliefs	belief	beliefs	8	singular
beneficence	beneficence	beneficence	beneficence	beneficence	8	kept
Best practices	best practices	best practices	best practice	best practice	8	singular
beta adrenergic receptor stimulating agent	beta adrenergic receptor stimulating agent	beta adrenergic receptor stimulating agent	beta adrenergic receptor stimulating agent	beta adrenergic receptor stimulating agent	8	kept
bibliometric analysis	bibliometric analysis	bibliometric analysis	bibliometric analysis	bibliometric analysis	8	kept
big data	big data	big data	big data	big data	8	kept
biochemical analysis	biochemical analysis	biochemical analysis	biochemical analysis	biochemical analysis	8	kept
Biofilms	biofilms	biofilms	biofilm	biofilm	8	singular
biosafety	biosafety	biosafety	biosafety	biosafety	8	kept
birth setting	birth setting	birth setting	birth setting	birth setting	8	kept

Bispectral index	bispectral index	bispectral index	bispectral index	bispectral index	8	case_or_format
bladder cancer	bladder cancer	bladder cancer	bladder cancer	bladder cancer	8	kept
blindness	blindness	blindness	blindness	blindness	8	kept
blood clotting factor 10a	blood clotting factor 10a	blood clotting factor 10a	blood clotting factor 10a	blood clotting factor 10a	8	kept
blood clotting test	blood clotting test	blood clotting test	blood clotting test	blood clotting test	8	kept
Blood Coagulation Tests	blood coagulation tests	blood coagulation tests	blood coagulation tests	Blood Coagulation Tests	8	kept
blood level	blood level	blood level	blood level	blood level	8	kept
blood pH	blood ph	blood ph	blood ph	blood pH	8	kept
Blood Pressure Monitors	blood pressure monitors	blood pressure monitors	blood pressure monitor	Blood Pressure Monitors	8	singular
BLOOD-STREAM INFECTION	blood stream infection	blood stream infection	blood stream infection	BLOOD-STREAM INFECTIONS	8	case_or_format
Body Fluids	body fluids	body fluids	body fluid	body fluid	8	singular
book	book	book	book	book	8	kept
botulism	botulism	botulism	botulism	Botulism	8	case_or_format
brain contusion	brain contusion	brain contusion	brain contusion	brain contusion	8	kept
Brain injuries	brain injuries	brain injuries	brain injury	Brain Injuries	8	singular
brain perfusion	brain perfusion	brain perfusion	brain perfusion	brain perfusion	8	kept

breast tumor	breast tumor	breast tumor	breast tumor	breast tumor	8	kept
breathing pattern	breathing pattern	breathing pattern	breathing pattern	breathing pattern	8	kept
Brunei	brunei	brunei	brunei	Brunei	8	kept
Bupivacaine	bupivacaine	bupivacaine	bupivacaine	bupivacaine	8	case_or_format
Burn unit	burn unit	burn unit	burn unit	Burn Units	8	case_or_format
Burns, Inhalation	burns inhalation	burns inhalation	burns inhalation	Burns, Inhalation	8	kept
bypass surgery	bypass surgery	bypass surgery	bypass surgery	bypass surgery	8	kept
cadaver donor	cadaver donor	cadaver donor	cadaver donor	cadaver donor	8	kept
caffeine	caffeine	caffeine	caffeine	caffeine	8	kept
Calcium	calcium	calcium	calcium	calcium	8	case_or_format
cancer risk	cancer risk	cancer risk	cancer risk	cancer risk	8	kept
Candida parapsilosis	candida parapsilosis	candida parapsilosis	candida parapsilosis	Candida parapsilosis	8	kept
Capillaries	capillaries	capillaries	capillary	Capillaries	8	singular
capillary leak syndrome	capillary leak syndrome	capillary leak syndrome	capillary leak syndrome	capillary leak syndrome	8	kept
cardiotoxicity	cardiotoxicity	cardiotoxicity	cardiotoxicity	cardiotoxicity	8	kept
cardiovascular	cardiovascular	cardiovascular	cardiovascular	Cardiovascular	8	case_or_format

Care management	care management	care management	care management	care management	8	case_or_format
Caring behavior	caring behavior	caring behavior	caring behavior	Caring behavior	8	kept
cat	cat	cat	cat	Cats	8	case_or_format
Catatonia	catatonia	catatonia	catatonia	catatonia	8	case_or_format
Catheter	catheter	catheter	catheter	Catheters	8	case_or_format
Catheter-related bloodstream infection	catheter related bloodstream infection	catheter related bloodstream infection	catheter related bloodstream infection	catheter related bloodstream infection	8	case_or_format
cefalexin	cefalexin	cefalexin	cefalexin	cefalexin	8	kept
Censuses	censuses	censuses	censuses	Censuses	8	kept
central line-associated bloodstream infection	central line associated bloodstream infection	central line associated bloodstream infection	central line associated bloodstream infection	Central line-associated bloodstream infections	8	case_or_format
centralization	centralization	centralization	centralization	centralization	8	kept
cephalosporin resistance	cephalosporin resistance	cephalosporin resistance	cephalosporin resistance	cephalosporin resistance	8	kept
cerebral perfusion pressure	cerebral perfusion pressure	cerebral perfusion pressure	cerebral perfusion pressure	cerebral perfusion pressure	8	kept
Child development	child development	child development	child development	Child Development	8	case_or_format
Child Health	child health	child health	child health	child health	8	case_or_format
Child health	child health	child health	child health	child health	8	case_or_format
child nutrition	child nutrition	child nutrition	child nutrition	child nutrition	8	kept

chronic fatigue syndrome	chronic fatigue syndrome	chronic fatigue syndrome	chronic fatigue syndrome	chronic fatigue syndrome	8	kept
Chronobiology Disorders	chronobiology disorders	chronobiology disorders	chronobiology disorders	Chronobiology Disorders	8	kept
Circadian rhythms	circadian rhythms	circadian rhythms	circadian rhythm	circadian rhythm	8	singular
CLASSIFICATION	classification	classification	classification	classification	8	case_or_format
Clinical handover	clinical handover	clinical handover	clinical handover	clinical handover	8	case_or_format
clinical judgment	clinical judgment	clinical judgment	clinical judgment	Clinical judgment	8	case_or_format
clinical monitoring	clinical monitoring	clinical monitoring	clinical monitoring	clinical monitoring	8	kept
Clinical outcomes	clinical outcomes	clinical outcomes	clinical outcome	clinical outcome	8	singular
clinical procedures	clinical procedures	clinical procedures	clinical procedure	clinical procedures	8	singular
clinical psychology	clinical psychology	clinical psychology	clinical psychology	clinical psychology	8	kept
Clinical Trial	clinical trial	clinical trial	clinical trial	clinical trial	8	case_or_format
CLINICAL-TRIAL	clinical trial	clinical trial	clinical trial	clinical trial	8	case_or_format
Coagulopathy	coagulopathy	coagulopathy	coagulopathy	Coagulopathy	8	kept
COGNITIVE IMPAIRMENT	cognitive impairment	cognitive impairment	cognitive impairment	Cognitive impairment	8	case_or_format
cognitive model	cognitive model	cognitive model	cognitive model	cognitive model	8	kept
Cognitive Therapy	cognitive therapy	cognitive therapy	cognitive therapy	cognitive therapy	8	case_or_format

Coma, Post-Head Injury	coma post head injury	coma post head injury	coma post head injury	Coma, Post-Head Injury	8	kept
communication aid	communication aid	communication aid	communication aid	communication aid	8	kept
communication barriers	communication barriers	communication barriers	communication barrier	Communication Barriers	8	singular
COMMUNITY-ACQUIRED PNEUMONIA	community acquired pneumonia	community acquired pneumonia	community acquired pneumonia	community acquired pneumonia	8	case_or_format
community-acquired pneumonia	community acquired pneumonia	community acquired pneumonia	community acquired pneumonia	community acquired pneumonia	8	case_or_format
compartment syndrome	compartment syndrome	compartment syndrome	compartment syndrome	Compartment Syndromes	8	case_or_format
Complex interventions	complex interventions	complex interventions	complex intervention	Complex interventions	8	singular
Complexity	complexity	complexity	complexity	Complexity	8	kept
Computer simulation	computer simulation	computer simulation	computer simulation	Computer Simulation	8	case_or_format
confusion assessment method	confusion assessment method	confusion assessment method	confusion assessment method	Confusion Assessment Method	8	case_or_format
Consensus Statements as Topic	consensus statements as topic	consensus statements as topic	consensus statements as topic	Consensus Statements as Topic	8	kept
CONSEQUENCES	consequences	consequences	consequence	CONSEQUENCES	8	singular
constant comparative method	constant comparative method	constant comparative method	constant comparative method	constant comparative method	8	kept
Consumer Health Information	consumer health information	consumer health information	consumer health information	Consumer Health Information	8	kept
Continuous glucose monitoring	continuous glucose monitoring	continuous glucose monitoring	continuous glucose monitoring	continuous glucose monitoring	8	case_or_format
continuous hemodiafiltration	continuous hemodiafiltration	continuous hemodiafiltration	continuous hemodiafiltration	continuous hemodiafiltration	8	kept

Contrast Media	contrast media	contrast media	contrast media	Contrast Media	8	kept
control charts	control charts	control charts	control chart	control charts	8	singular
control system	control system	control system	control system	control system	8	kept
Corticosteroids	corticosteroids	corticosteroids	corticosteroid	corticosteroid	8	singular
Corynebacterium	corynebacterium	corynebacterium	corynebacterium	Corynebacterium	8	kept
cost analysis	cost analysis	cost analysis	cost analysis	Cost analysis	8	case_or_format
COVID 19	covid 19	covid 19	covid 19	COVID-19	8	case_or_format
cream	cream	cream	cream	cream	8	kept
cryoanesthesia	cryoanesthesia	cryoanesthesia	cryoanesthesia	cryoanesthesia	8	kept
cryoprecipitate	cryoprecipitate	cryoprecipitate	cryoprecipitate	cryoprecipitate	8	kept
Cyanosis	cyanosis	cyanosis	cyanosis	cyanosis	8	case_or_format
cystectomy	cystectomy	cystectomy	cystectomy	cystectomy	8	kept
cytomegalovirus infection	cytomegalovirus infection	cytomegalovirus infection	cytomegalovirus infection	cytomegalovirus infection	8	kept
data quality assessment	data quality assessment	data quality assessment	data quality assessment	data quality assessment	8	kept
Database Management Systems	database management systems	database management systems	database management system	Database Management Systems	8	singular
DEATHS	deaths	deaths	death	death	8	singular

deceased donor	deceased donor	deceased donor	deceased donor	deceased donor	8	kept
decreased appetite	decreased appetite	decreased appetite	decreased appetite	decreased appetite	8	kept
Delayed Diagnosis	delayed diagnosis	delayed diagnosis	delayed diagnosis	delayed diagnosis	8	case_or_format
Delirium management	delirium management	delirium management	delirium management	Delirium management	8	kept
delirium tremens	delirium tremens	delirium tremens	delirium tremens	delirium tremens	8	kept
dependent variable	dependent variable	dependent variable	dependent variable	dependent variable	8	kept
Depressive Disorder, Major	depressive disorder major	depressive disorder major	depressive disorder major	Depressive Disorder, Major	8	kept
developing world	developing world	developing world	developing world	developing world	8	kept
Diabetic Angiopathies	diabetic angiopathies	diabetic angiopathies	diabetic angiopathies	Diabetic Angiopathies	8	kept
Diagnostic Imaging	diagnostic imaging	diagnostic imaging	diagnostic imaging	diagnostic imaging	8	case_or_format
diffusion weighted imaging	diffusion weighted imaging	diffusion weighted imaging	diffusion weighted imaging	diffusion weighted imaging	8	kept
digestive system cancer	digestive system cancer	digestive system cancer	digestive system cancer	digestive system cancer	8	kept
dilatation	dilatation	dilatation	dilatation	Dilatation	8	case_or_format
diltiazem	diltiazem	diltiazem	diltiazem	diltiazem	8	kept
dilution	dilution	dilution	dilution	dilution	8	kept
direct laryngoscopy	direct laryngoscopy	direct laryngoscopy	direct laryngoscopy	direct laryngoscopy	8	kept

Disaster medicine	disaster medicine	disaster medicine	disaster medicine	disaster medicine	8	case_or_format
disease activity	disease activity	disease activity	disease activity	disease activity	8	kept
disease carrier	disease carrier	disease carrier	disease carrier	disease carrier	8	kept
disease marker	disease marker	disease marker	disease marker	disease marker	8	kept
Disease Models, Animal	disease models animal	disease models animal	disease models animal	Disease Models, Animal	8	kept
disease risk assessment	disease risk assessment	disease risk assessment	disease risk assessment	disease risk assessment	8	kept
disease spread	disease spread	disease spread	disease spread	disease spread	8	kept
disparities	disparities	disparities	disparity	disparities	8	singular
Down Syndrome	down syndrome	down syndrome	down syndrome	Down syndrome	8	case_or_format
drawing	drawing	drawing	drawing	drawing	8	kept
drug administration route	drug administration route	drug administration route	drug administration route	Drug Administration Routes	8	case_or_format
drug dose	drug dose	drug dose	drug dose	drug dose	8	kept
Drug Incompatibility	drug incompatibility	drug incompatibility	drug incompatibility	drug incompatibility	8	case_or_format
Dry eye syndromes	dry eye syndromes	dry eye syndromes	dry eye syndrome	Dry Eye Syndromes	8	singular
Dyssomnias	dyssomnias	dyssomnias	dyssomnia	Dyssomnias	8	singular
ear nose throat surgery	ear nose throat surgery	ear nose throat surgery	ear nose throat surgery	ear nose throat surgery	8	kept

eating disorder	eating disorder	eating disorder	eating disorder	eating disorder	8	kept
ecchymosis	ecchymosis	ecchymosis	ecchymosis	ecchymosis	8	kept
Economics, Hospital	economics hospital	economics hospital	economics hospital	Economics, Hospital	8	kept
ectopic pregnancy	ectopic pregnancy	ectopic pregnancy	ectopic pregnancy	ectopic pregnancy	8	kept
eczema	eczema	eczema	eczema	eczema	8	kept
Edinburgh Postnatal Depression Scale	edinburgh postnatal depression scale	edinburgh postnatal depression scale	edinburgh postnatal depression scale	Edinburgh Postnatal Depression Scale	8	kept
editorial	editorial	editorial	editorial	Editorial	8	case_or_format
edoxaban	edoxaban	edoxaban	edoxaban	edoxaban	8	kept
electrode	electrode	electrode	electrode	Electrodes	8	case_or_format
eligibility criteria	eligibility criteria	eligibility criteria	eligibility criteria	eligibility criteria	8	kept
emergency room	emergency room	emergency room	emergency room	emergency room	8	kept
EMLA	emla	emla	emla	EMLA	8	kept
empiricism	empiricism	empiricism	empiricism	empiricism	8	kept
employer	employer	employer	employer	employer	8	kept
end stage liver disease	end stage liver disease	end stage liver disease	end stage liver disease	End Stage Liver Disease	8	case_or_format
endocrinology	endocrinology	endocrinology	endocrinology	endocrinology	8	kept

endovascular aneurysm repair	endovascular aneurysm repair	endovascular aneurysm repair	endovascular aneurysm repair	endovascular aneurysm repair	8	kept
Engagement	engagement	engagement	engagement	engagement	8	case_or_format
ENTERAL NUTRITION	enteral nutrition	enteral nutrition	enteral nutrition	Enteral Nutrition	8	case_or_format
environmental temperature	environmental temperature	environmental temperature	environmental temperature	environmental temperature	8	kept
enzymology	enzymology	enzymology	enzymology	enzymology	8	kept
ephedrine	ephedrine	ephedrine	ephedrine	ephedrine	8	kept
Epidemiologic Methods	epidemiologic methods	epidemiologic methods	epidemiologic methods	Epidemiologic Methods	8	kept
ERAS	eras	eras	era	ERAS	8	singular
ESBL	esbl	esbl	esbl	ESBL	8	kept
esketamine	esketamine	esketamine	esketamine	esketamine	8	kept
Esophagectomy	esophagectomy	esophagectomy	esophagectomy	Esophagectomy	8	kept
esophagus cancer	esophagus cancer	esophagus cancer	esophagus cancer	esophagus cancer	8	kept
esophagus intubation	esophagus intubation	esophagus intubation	esophagus intubation	esophagus intubation	8	kept
esophagus resection	esophagus resection	esophagus resection	esophagus resection	esophagus resection	8	kept
ETHICS	ethics	ethics	ethic	ethics	8	singular
etiracetam	etiracetam	etiracetam	etiracetam	etiracetam	8	kept

evening shift	evening shift	evening shift	evening shift	evening shift	8	kept
ex-smoker	ex smoker	ex smoker	ex smoker	ex-smoker	8	kept
explanatory variable	explanatory variable	explanatory variable	explanatory variable	explanatory variable	8	kept
eye drops	eye drops	eye drops	eye drops	eye drops	8	kept
Eye-Tracking Technology	eye tracking technology	eye tracking technology	eye tracking technology	Eye-Tracking Technology	8	kept
FACILITIES	facilities	facilities	facility	FACILITIES	8	singular
failure mode and effects analysis	failure mode and effects analysis	failure mode and effects analysis	failure mode and effects analysis	failure mode and effects analysis	8	kept
fall risk	fall risk	fall risk	fall risk	fall risk	8	kept
fall risk assessment	fall risk assessment	fall risk assessment	fall risk assessment	fall risk assessment	8	kept
False alarms	false alarms	false alarms	false alarm	False alarms	8	singular
family life	family life	family life	family life	family life	8	kept
family needs	family needs	family needs	family need	Family needs	8	singular
family planning	family planning	family planning	family planning	family planning	8	kept
Family Support	family support	family support	family support	family support	8	case_or_format
family visitation	family visitation	family visitation	family visitation	family visitation	8	kept
FAMILY-CENTERED CARE	family centered care	family centered care	family centered care	family centered care	8	case_or_format

Family-witnessed resuscitation	family witnessed resuscitation	family witnessed resuscitation	family witnessed resuscitation	Family-witnessed resuscitation	8	kept
Fasting	fasting	fasting	fasting	fasting	8	case_or_format
Fecal incontinence	fecal incontinence	fecal incontinence	fecal incontinence	Fecal Incontinence	8	case_or_format
Fee-for-Service Plans	fee for service plans	fee for service plans	fee for service plans	Fee-for-Service Plans	8	kept
fetus disease	fetus disease	fetus disease	fetus disease	fetus disease	8	kept
fetus weight	fetus weight	fetus weight	fetus weight	fetus weight	8	kept
fibrinogen blood level	fibrinogen blood level	fibrinogen blood level	fibrinogen blood level	fibrinogen blood level	8	kept
fibrinolysis	fibrinolysis	fibrinolysis	fibrinolysis	fibrinolysis	8	kept
field work	field work	field work	field work	field work	8	kept
finger	finger	finger	finger	finger	8	kept
fire	fire	fire	fire	Fires	8	case_or_format
fish oil	fish oil	fish oil	fish oil	fish oil	8	kept
focus group	focus group	focus group	focus group	Focus Groups	8	case_or_format
formation	formation	formation	formation	formation	8	kept
fosphenytoin sodium	fosphenytoin sodium	fosphenytoin sodium	fosphenytoin sodium	fosphenytoin sodium	8	kept
Fracture Fixation	fracture fixation	fracture fixation	fracture fixation	fracture fixation	8	case_or_format

FRAILTY	frailty	frailty	frailty	frailty	8	case_or_format
frequency	frequency	frequency	frequency	frequency	8	kept
functional magnetic resonance imaging	functional magnetic resonance imaging	functional magnetic resonance imaging	functional magnetic resonance imaging	functional magnetic resonance imaging	8	kept
Functional status	functional status	functional status	functional status	functional status	8	case_or_format
Fungemia	fungemia	fungemia	fungemia	Fungemia	8	kept
gastrectomy	gastrectomy	gastrectomy	gastrectomy	gastrectomy	8	kept
Gastrointestinal Contents	gastrointestinal contents	gastrointestinal contents	gastrointestinal contents	Gastrointestinal Contents	8	kept
gel	gel	gel	gel	Gels	8	case_or_format
gene	gene	gene	gene	gene	8	kept
generalized anxiety disorder	generalized anxiety disorder	generalized anxiety disorder	generalized anxiety disorder	generalized anxiety disorder	8	kept
genetic counseling	genetic counseling	genetic counseling	genetic counseling	Genetic Counseling	8	case_or_format
Genetic Predisposition to Disease	genetic predisposition to disease	genetic predisposition to disease	genetic predisposition to disease	Genetic Predisposition to Disease	8	kept
geriatric trauma	geriatric trauma	geriatric trauma	geriatric trauma	geriatric trauma	8	kept
gerontology	gerontology	gerontology	gerontology	gerontology	8	kept
Gestational age	gestational age	gestational age	gestational age	gestational age	8	case_or_format
glucagon	glucagon	glucagon	glucagon	glucagon	8	kept

glutamine	glutamine	glutamine	glutamine	glutamine	8	kept
Goal programming	goal programming	goal programming	goal programming	Goal programming	8	kept
graduate medical education	graduate medical education	graduate medical education	graduate medical education	graduate medical education	8	kept
Gram negative sepsis	gram negative sepsis	gram negative sepsis	gram negative sepsis	Gram negative sepsis	8	kept
grey literature	grey literature	grey literature	grey literature	grey literature	8	kept
Growth Disorders	growth disorders	growth disorders	growth disorder	Growth Disorders	8	singular
Half-Life	half life	half life	half life	Half-Life	8	kept
Hand disinfection	hand disinfection	hand disinfection	hand disinfection	Hand Disinfection	8	case_or_format
hand eczema	hand eczema	hand eczema	hand eczema	hand eczema	8	kept
head	head	head	head	Head	8	case_or_format
head position	head position	head position	head position	head position	8	kept
Headache	headache	headache	headache	headache	8	case_or_format
health & safety	health and safety	health and safety	health and safety	health & safety	8	kept
health care costs	health care costs	health care costs	health care cost	health care cost	8	singular
Health communication	health communication	health communication	health communication	Health Communication	8	case_or_format
health disparities	health disparities	health disparities	health disparity	health disparity	8	singular

health impact assessment	health impact assessment	health impact assessment	health impact assessment	health impact assessment	8	kept
Health knowledge	health knowledge	health knowledge	health knowledge	Health knowledge	8	kept
Health Occupations	health occupations	health occupations	health occupations	Health Occupations	8	kept
Health workers	health workers	health workers	health worker	health worker	8	singular
Healthcare costs	healthcare costs	healthcare costs	healthcare cost	Healthcare costs	8	singular
Healthcare utilization	healthcare utilization	healthcare utilization	healthcare utilization	Healthcare utilization	8	kept
Heart arrest	heart arrest	heart arrest	heart arrest	Heart Arrest	8	case_or_format
heart contraction	heart contraction	heart contraction	heart contraction	heart contraction	8	kept
Heart Failure, Systolic	heart failure systolic	heart failure systolic	heart failure systolic	Heart Failure, Systolic	8	kept
heart hemodynamics	heart hemodynamics	heart hemodynamics	heart hemodynamics	heart hemodynamics	8	kept
heart pacing	heart pacing	heart pacing	heart pacing	heart pacing	8	kept
Heart rate variability	heart rate variability	heart rate variability	heart rate variability	heart rate variability	8	case_or_format
heart ventricle arrhythmia	heart ventricle arrhythmia	heart ventricle arrhythmia	heart ventricle arrhythmia	heart ventricle arrhythmia	8	kept
heat stroke	heat stroke	heat stroke	heat stroke	heat stroke	8	kept
help seeking behavior	help seeking behavior	help seeking behavior	help seeking behavior	help seeking behavior	8	kept
Heparin, Low-Molecular-Weight	heparin low molecular weight	heparin low molecular weight	heparin low molecular weight	Heparin, Low-Molecular-Weight	8	kept

high frequency ventilation	high frequency ventilation	high frequency ventilation	high frequency ventilation	High-Frequency Ventilation	8	case_or_format
HIGH-FIDELITY SIMULATION	high fidelity simulation	high fidelity simulation	high fidelity simulation	high-fidelity simulation	8	case_or_format
Hirschsprung Disease	hirschsprung disease	hirschsprung disease	hirschsprung disease	Hirschsprung Disease	8	kept
History	history	history	history	history	8	case_or_format
HOME	home	home	home	home	8	case_or_format
Home care services	home care services	home care services	home care services	Home Care Services	8	case_or_format
horse	horse	horse	horse	horse	8	kept
HOSPICE	hospice	hospice	hospice	hospice	8	case_or_format
Hospital acquired infection	hospital acquired infection	hospital acquired infection	hospital acquired infection	Hospital-acquired infections	8	case_or_format
hospital billing	hospital billing	hospital billing	hospital billing	hospital billing	8	kept
Hospital costs	hospital costs	hospital costs	hospital cost	Hospital Costs	8	singular
Hospital discharge	hospital discharge	hospital discharge	hospital discharge	hospital discharge	8	case_or_format
Hospital environment	hospital environment	hospital environment	hospital environment	Hospital environment	8	kept
Hospital medicine	hospital medicine	hospital medicine	hospital medicine	hospital medicine	8	case_or_format
Hospital Records	hospital records	hospital records	hospital record	Hospital Records	8	singular
Hospital settings	hospital settings	hospital settings	hospital setting	Hospital settings	8	singular

Hospital-acquired pressure ulcers	hospital acquired pressure ulcers	hospital acquired pressure ulcers	hospital acquired pressure ulcer	Hospital-acquired pressure ulcers	8	singular
Hospitalist	hospitalist	hospitalist	hospitalist	Hospitalists	8	case_or_format
Hospitals, County	hospitals county	hospitals county	hospitals county	Hospitals, County	8	kept
Hospitals, State	hospitals state	hospitals state	hospitals state	Hospitals, State	8	kept
Hospitals, Voluntary	hospitals voluntary	hospitals voluntary	hospitals voluntary	Hospitals, Voluntary	8	kept
housing	housing	housing	housing	housing	8	kept
Hydroxychloroquine	hydroxychloroquine	hydroxychloroquine	hydroxychloroquine	hydroxychloroquine	8	case_or_format
Hyperinsulinism	hyperinsulinism	hyperinsulinism	hyperinsulinism	Hyperinsulinism	8	kept
hypermetabolism	hypermetabolism	hypermetabolism	hypermetabolism	hypermetabolism	8	kept
hypnosis	hypnosis	hypnosis	hypnosis	hypnosis	8	kept
hypobaric chamber	hypobaric chamber	hypobaric chamber	hypobaric chamber	hypobaric chamber	8	kept
hypocapnia	hypocapnia	hypocapnia	hypocapnia	hypocapnia	8	kept
Hypothyroidism	hypothyroidism	hypothyroidism	hypothyroidism	hypothyroidism	8	case_or_format
ICDSC	icdsc	icdsc	icdsc	ICDSC	8	kept
Idaho	idaho	idaho	idaho	Idaho	8	kept
Ileostomy	ileostomy	ileostomy	ileostomy	ileostomy	8	case_or_format

Immune System	immune system	immune system	immune system	immune system	8	case_or_format
immunoglobulin E	immunoglobulin e	immunoglobulin e	immunoglobulin e	immunoglobulin E	8	kept
Incivility	incivility	incivility	incivility	Incivility	8	kept
infant welfare	infant welfare	infant welfare	infant welfare	Infant Welfare	8	case_or_format
infectious disease specialist	infectious disease specialist	infectious disease specialist	infectious disease specialist	infectious disease specialist	8	kept
infirmier	infirmier	infirmier	infirmier	infirmier	8	kept
Inflammation Mediators	inflammation mediators	inflammation mediators	inflammation mediators	Inflammation Mediators	8	kept
influencing factor	influencing factor	influencing factor	influencing factor	influencing factors	8	case_or_format
informatics	informatics	informatics	informatics	informatics	8	kept
Information Management	information management	information management	information management	Information management	8	case_or_format
Information Technology	information technology	information technology	information technology	information technology	8	case_or_format
Informed consent	informed consent	informed consent	informed consent	informed consent	8	case_or_format
Injections, Spinal	injections spinal	injections spinal	injections spinal	Injections, Spinal	8	kept
innate immunity	innate immunity	innate immunity	innate immunity	innate immunity	8	kept
Innovation	innovation	innovation	innovation	innovation	8	case_or_format
instrument	instrument	instrument	instrument	instrument	8	kept

insulin sensitivity	insulin sensitivity	insulin sensitivity	insulin sensitivity	insulin sensitivity	8	kept
Interdisciplinary	interdisciplinary	interdisciplinary	interdisciplinary	interdisciplinary	8	case_or_format
Interdisciplinary communication	interdisciplinary communication	interdisciplinary communication	interdisciplinary communication	Interdisciplinary Communication	8	case_or_format
Interdisciplinary Studies	interdisciplinary studies	interdisciplinary studies	interdisciplinary studies	Interdisciplinary Studies	8	kept
interdisciplinary team	interdisciplinary team	interdisciplinary team	interdisciplinary team	interdisciplinary team	8	kept
Interleukin-6	interleukin 6	interleukin 6	interleukin 6	interleukin 6	8	case_or_format
Internal medicine	internal medicine	internal medicine	internal medicine	internal medicine	8	case_or_format
internal validity	internal validity	internal validity	internal validity	internal validity	8	kept
Internet of Things	internet of things	internet of things	internet of thing	Internet of things	8	singular
interprofessional care	interprofessional care	interprofessional care	interprofessional care	interprofessional care	8	kept
Interprofessional communication	interprofessional communication	interprofessional communication	interprofessional communication	interprofessional communication	8	case_or_format
interprofessional relations	interprofessional relations	interprofessional relations	interprofessional relations	Interprofessional Relations	8	case_or_format
interstitial pneumonia	interstitial pneumonia	interstitial pneumonia	interstitial pneumonia	interstitial pneumonia	8	kept
intestine sound	intestine sound	intestine sound	intestine sound	intestine sound	8	kept
intravenous	intravenous	intravenous	intravenous	Intravenous	8	case_or_format
intravenous administration	intravenous administration	intravenous administration	intravenous administration	intravenous administration	8	kept

ipratropium bromide	ipratropium bromide	ipratropium bromide	ipratropium bromide	ipratropium bromide	8	kept
isoprenaline	isoprenaline	isoprenaline	isoprenaline	isoprenaline	8	kept
Kangaroo Mother Care	kangaroo mother care	kangaroo mother care	kangaroo mother care	Kangaroo mother care	8	case_or_format
Kansas	kansas	kansas	kansas	Kansas	8	kept
kappa statistics	kappa statistics	kappa statistics	kappa statistics	kappa statistics	8	kept
Keratitis	keratitis	keratitis	keratitis	keratitis	8	case_or_format
kidney graft	kidney graft	kidney graft	kidney graft	kidney graft	8	kept
laboratory technique	laboratory technique	laboratory technique	laboratory technique	laboratory technique	8	kept
Lactation Disorders	lactation disorders	lactation disorders	lactation disorder	Lactation Disorders	8	singular
lactulose	lactulose	lactulose	lactulose	lactulose	8	kept
legal liability	legal liability	legal liability	legal liability	legal liability	8	kept
leukocyte	leukocyte	leukocyte	leukocyte	leukocyte	8	kept
LEVEL	level	level	level	LEVEL	8	kept
life	life	life	life	LIFE	8	case_or_format
limb injury	limb injury	limb injury	limb injury	limb injury	8	kept
limb ischemia	limb ischemia	limb ischemia	limb ischemia	limb ischemia	8	kept

limited English proficiency	limited english proficiency	limited english proficiency	limited english proficiency	Limited English Proficiency	8	case_or_format
Lipids	lipids	lipids	lipid	lipid	8	singular
liquorrhea	liquorrhea	liquorrhea	liquorrhea	liquorrhea	8	kept
Lived experiences	lived experiences	lived experiences	lived experience	lived experience	8	singular
Liver failure	liver failure	liver failure	liver failure	liver failure	8	case_or_format
Living Donors	living donors	living donors	living donor	Living Donors	8	singular
LMIC	lmic	lmic	lmic	LMIC	8	kept
Logic	logic	logic	logic	Logic	8	kept
Low back pain	low back pain	low back pain	low back pain	low back pain	8	case_or_format
low- and middle-income countries	low and middle income countries	low and middle income countries	low and middle income country	low- and middle-income countries	8	singular
lung abscess	lung abscess	lung abscess	lung abscess	lung abscess	8	kept
Lung Diseases, Obstructive	lung diseases obstructive	lung diseases obstructive	lung diseases obstructive	Lung Diseases, Obstructive	8	kept
lung metastasis	lung metastasis	lung metastasis	lung metastasis	lung metastasis	8	kept
lung resection	lung resection	lung resection	lung resection	lung resection	8	kept
lymphadenopathy	lymphadenopathy	lymphadenopathy	lymphadenopathy	lymphadenopathy	8	kept
macrosomia	macrosomia	macrosomia	macrosomia	macrosomia	8	kept

Magnesium Sulfate	magnesium sulfate	magnesium sulfate	magnesium sulfate	magnesium sulfate	8	case_or_format
mandatory reporting	mandatory reporting	mandatory reporting	mandatory reporting	Mandatory Reporting	8	case_or_format
Marital Status	marital status	marital status	marital status	Marital Status	8	kept
Materials Testing	materials testing	materials testing	materials testing	Materials Testing	8	kept
maternal attitude	maternal attitude	maternal attitude	maternal attitude	maternal attitude	8	kept
maternal death	maternal death	maternal death	maternal death	maternal death	8	kept
Mechanical circulatory support	mechanical circulatory support	mechanical circulatory support	mechanical circulatory support	Mechanical circulatory support	8	kept
Medical device	medical device	medical device	medical device	medical device	8	case_or_format
Medical device-related pressure injuries	medical device related pressure injuries	medical device related pressure injuries	medical device related pressure injury	medical device-related pressure injury	8	singular
Medical Informatics Applications	medical informatics applications	medical informatics applications	medical informatics applications	Medical Informatics Applications	8	kept
Medical students	medical students	medical students	medical student	medical student	8	singular
Medically Uninsured	medically uninsured	medically uninsured	medically uninsured	medically uninsured	8	case_or_format
Medication management	medication management	medication management	medication management	medication management	8	case_or_format
mental deterioration	mental deterioration	mental deterioration	mental deterioration	mental deterioration	8	kept
Meta-synthesis	meta synthesis	meta synthesis	meta synthesis	meta-synthesis	8	case_or_format
Mexican Americans	mexican americans	mexican americans	mexican americans	Mexican Americans	8	kept

Micrococcus	micrococcus	micrococcus	micrococcus	Micrococcus	8	kept
Microdialysis	microdialysis	microdialysis	microdialysis	Microdialysis	8	kept
microdialysis	microdialysis	microdialysis	microdialysis	Microdialysis	8	case_or_format
microorganism	microorganism	microorganism	microorganism	microorganism	8	kept
microscopy	microscopy	microscopy	microscopy	microscopy	8	kept
Microsurgery	microsurgery	microsurgery	microsurgery	Microsurgery	8	kept
Middle East respiratory syndrome coronavirus	middle east respiratory syndrome coronavirus	middle east respiratory syndrome coronavirus	middle east respiratory syndrome coronavirus	Middle East respiratory syndrome coronavirus	8	kept
military deployment	military deployment	military deployment	military deployment	military deployment	8	kept
Milk	milk	milk	milk	milk	8	case_or_format
Mitral Valve	mitral valve	mitral valve	mitral valve	Mitral Valve	8	kept
Mobilisation	mobilisation	mobilisation	mobilisation	Mobilisation	8	kept
Moral Distress	moral distress	moral distress	moral distress	moral distress	8	case_or_format
Morocco	morocco	morocco	morocco	Morocco	8	kept
Mortality prediction	mortality prediction	mortality prediction	mortality prediction	Mortality prediction	8	kept
motor vehicle	motor vehicle	motor vehicle	motor vehicle	motor vehicle	8	kept
multidrug resistant Gram negative bacterium	multidrug resistant gram negative bacterium	multidrug resistant gram negative bacterium	multidrug resistant gram negative bacterium	multidrug resistant Gram negative bacterium	8	kept

Multimedia	multimedia	multimedia	multimedia	Multimedia	8	kept
Multimorbidity	multimorbidity	multimorbidity	multimorbidity	Multimorbidity	8	kept
multiple myeloma	multiple myeloma	multiple myeloma	multiple myeloma	multiple myeloma	8	kept
Muscle weakness	muscle weakness	muscle weakness	muscle weakness	Muscle Weakness	8	case_or_format
named groups by occupation	named groups by occupation	named groups by occupation	named groups by occupation	named groups by occupation	8	kept
national early warning score	national early warning score	national early warning score	national early warning score	National Early Warning Score	8	case_or_format
national lockdown	national lockdown	national lockdown	national lockdown	national lockdown	8	kept
neonatal pain	neonatal pain	neonatal pain	neonatal pain	Neonatal pain	8	case_or_format
Neonatal transport	neonatal transport	neonatal transport	neonatal transport	Neonatal transport	8	kept
Neonatal unit	neonatal unit	neonatal unit	neonatal unit	Neonatal unit	8	kept
Neonatal units	neonatal units	neonatal units	neonatal unit	Neonatal unit	8	singular
nervousness	nervousness	nervousness	nervousness	nervousness	8	kept
Network analysis	network analysis	network analysis	network analysis	network analysis	8	case_or_format
Neurorehabilitation	neurorehabilitation	neurorehabilitation	neurorehabilitation	neurorehabilitation	8	case_or_format
Neutrophils	neutrophils	neutrophils	neutrophil	neutrophil	8	singular
noise measurement	noise measurement	noise measurement	noise measurement	noise measurement	8	kept

non invasive measurement	non invasive measurement	non invasive measurement	non invasive measurement	non invasive measurement	8	kept
non small cell lung cancer	non small cell lung cancer	non small cell lung cancer	non small cell lung cancer	non small cell lung cancer	8	kept
non-invasive ventilation	non invasive ventilation	non invasive ventilation	non invasive ventilation	Non-invasive ventilation	8	case_or_format
nonalcoholic fatty liver	nonalcoholic fatty liver	nonalcoholic fatty liver	nonalcoholic fatty liver	nonalcoholic fatty liver	8	kept
nosocomial transmission	nosocomial transmission	nosocomial transmission	nosocomial transmission	nosocomial transmission	8	kept
nucleotide sequence	nucleotide sequence	nucleotide sequence	nucleotide sequence	nucleotide sequence	8	kept
nutrition assessment	nutrition assessment	nutrition assessment	nutrition assessment	Nutrition Assessment	8	case_or_format
obstructive sleep apnea	obstructive sleep apnea	obstructive sleep apnea	obstructive sleep apnea	Obstructive sleep apnea	8	case_or_format
occlusion	occlusion	occlusion	occlusion	occlusion	8	kept
occupational eczema	occupational eczema	occupational eczema	occupational eczema	occupational eczema	8	kept
occupational health service	occupational health service	occupational health service	occupational health service	occupational health service	8	kept
offender	offender	offender	offender	offender	8	kept
olive oil	olive oil	olive oil	olive oil	olive oil	8	kept
opioid	opioid	opioid	opioid	opioids	8	case_or_format
Optimization	optimization	optimization	optimization	Optimization	8	kept
Oral	oral	oral	oral	Oral	8	kept

Organisation	organisation	organisation	organisation	Organisation	8	kept
Organizational climate	organizational climate	organizational climate	organizational climate	Organizational climate	8	kept
organizational climate	organizational climate	organizational climate	organizational climate	Organizational climate	8	case_or_format
organizational commitment	organizational commitment	organizational commitment	organizational commitment	Organizational commitment	8	case_or_format
organizational efficiency	organizational efficiency	organizational efficiency	organizational efficiency	organizational efficiency	8	kept
Orthotic Devices	orthotic devices	orthotic devices	orthotic devices	Orthotic Devices	8	kept
Otorhinolaryngologic Surgical Procedures	otorhinolaryngologic surgical procedures	otorhinolaryngologic surgical procedures	otorhinolaryngologic surgical procedures	Otorhinolaryngologic Surgical Procedures	8	kept
out-of-hours care	out of hours care	out of hours care	out of hours care	out-of-hours care	8	kept
Ownership	ownership	ownership	ownership	Ownership	8	kept
Oxidative Stress	oxidative stress	oxidative stress	oxidative stress	oxidative stress	8	case_or_format
Palliative medicine	palliative medicine	palliative medicine	palliative medicine	palliative medicine	8	case_or_format
pallor	pallor	pallor	pallor	pallor	8	kept
panic	panic	panic	panic	panic	8	kept
paracentesis	paracentesis	paracentesis	paracentesis	paracentesis	8	kept
parameters	parameters	parameters	parameter	parameters	8	singular
Participatory design	participatory design	participatory design	participatory design	Participatory design	8	kept

Paternalism	paternalism	paternalism	paternalism	paternalism	8	case_or_format
path analysis	path analysis	path analysis	path analysis	path analysis	8	kept
pathologist	pathologist	pathologist	pathologist	pathologist	8	kept
peak inspiratory pressure	peak inspiratory pressure	peak inspiratory pressure	peak inspiratory pressure	peak inspiratory pressure	8	kept
Pediatría	pediatr a	pediatr a	pediatr a	Pediatría	8	kept
Peer Review	peer review	peer review	peer review	peer review	8	case_or_format
pelvis	pelvis	pelvis	pelvis	pelvis	8	kept
penicillin resistance	penicillin resistance	penicillin resistance	penicillin resistance	penicillin resistance	8	kept
pentobarbital	pentobarbital	pentobarbital	pentobarbital	pentobarbital	8	kept
Perceived barriers	perceived barriers	perceived barriers	perceived barrier	Perceived barriers	8	singular
perfusion pressure	perfusion pressure	perfusion pressure	perfusion pressure	perfusion pressure	8	kept
Perineum	perineum	perineum	perineum	perineum	8	case_or_format
peripheral edema	peripheral edema	peripheral edema	peripheral edema	peripheral edema	8	kept
Peritoneal Dialysis	peritoneal dialysis	peritoneal dialysis	peritoneal dialysis	peritoneal dialysis	8	case_or_format
Persian	persian	persian	persian	Persian	8	kept
Personnel	personnel	personnel	personnel	Personnel	8	kept

Personnel management	personnel management	personnel management	personnel management	personnel management	8	case_or_format
Persons with Disabilities	persons with disabilities	persons with disabilities	persons with disabilities	Persons with Disabilities	8	kept
perspectives	perspectives	perspectives	perspective	PERSPECTIVES	8	singular
PEWS	pews	pews	pew	PEWS	8	singular
Pharmacology	pharmacology	pharmacology	pharmacology	pharmacology	8	case_or_format
pharmacovigilance	pharmacovigilance	pharmacovigilance	pharmacovigilance	pharmacovigilance	8	kept
pharmacy technician	pharmacy technician	pharmacy technician	pharmacy technician	pharmacy technician	8	kept
Pharynx	pharynx	pharynx	pharynx	Pharynx	8	kept
phase 3 clinical trial	phase 3 clinical trial	phase 3 clinical trial	phase 3 clinical trial	phase 3 clinical trial	8	kept
Phenytoin	phenytoin	phenytoin	phenytoin	phenytoin	8	case_or_format
Physical Distancing	physical distancing	physical distancing	physical distancing	Physical Distancing	8	kept
Physicians, Family	physicians family	physicians family	physicians family	Physicians, Family	8	kept
Physiologic	physiologic	physiologic	physiologic	Physiologic	8	kept
Physiological models	physiological models	physiological models	physiological models	Physiological models	8	kept
Pierre Robin syndrome	pierre robin syndrome	pierre robin syndrome	pierre robin syndrome	Pierre Robin syndrome	8	kept
Pilot study	pilot study	pilot study	pilot study	pilot study	8	case_or_format

Piperacillin	piperacillin	piperacillin	piperacillin	piperacillin	8	case_or_format
pneumococcal infection	pneumococcal infection	pneumococcal infection	pneumococcal infection	pneumococcal infection	8	kept
Pneumococcal Infections	pneumococcal infections	pneumococcal infections	pneumococcal infection	pneumococcal infection	8	singular
point prevalence	point prevalence	point prevalence	point prevalence	point prevalence	8	kept
Polyethylene	polyethylene	polyethylene	polyethylene	Polyethylene	8	kept
polymyxin	polymyxin	polymyxin	polymyxin	polymyxin	8	kept
polypeptide antibiotic agent	polypeptide antibiotic agent	polypeptide antibiotic agent	polypeptide antibiotic agent	polypeptide antibiotic agent	8	kept
POPULATION	population	population	population	population	8	case_or_format
postoperative cognitive dysfunction	postoperative cognitive dysfunction	postoperative cognitive dysfunction	postoperative cognitive dysfunction	postoperative cognitive dysfunction	8	kept
PPE	ppe	ppe	ppe	PPE	8	kept
pragmatic trial	pragmatic trial	pragmatic trial	pragmatic trial	pragmatic trial	8	kept
PREFERENCES	preferences	preferences	preference	PREFERENCES	8	singular
pregnancy termination	pregnancy termination	pregnancy termination	pregnancy termination	pregnancy termination	8	kept
Premature baby	premature baby	premature baby	premature baby	Premature baby	8	kept
Premature newborn	premature newborn	premature newborn	premature newborn	Premature newborn	8	kept
prescribing practice	prescribing practice	prescribing practice	prescribing practice	prescribing practice	8	kept

pressure injury prevention	pressure injury prevention	pressure injury prevention	pressure injury prevention	pressure injury prevention	8	kept
Pressure ulcer prevention	pressure ulcer prevention	pressure ulcer prevention	pressure ulcer prevention	pressure ulcer prevention	8	case_or_format
private practice	private practice	private practice	private practice	private practice	8	kept
Private Sector	private sector	private sector	private sector	private sector	8	case_or_format
process control	process control	process control	process control	process control	8	kept
professional identity	professional identity	professional identity	professional identity	professional identity	8	kept
Professional quality of life	professional quality of life	professional quality of life	professional quality of life	professional quality of life	8	case_or_format
program feasibility	program feasibility	program feasibility	program feasibility	program feasibility	8	kept
Prolonged mechanical ventilation	prolonged mechanical ventilation	prolonged mechanical ventilation	prolonged mechanical ventilation	prolonged mechanical ventilation	8	case_or_format
promethazine	promethazine	promethazine	promethazine	promethazine	8	kept
psychological counseling	psychological counseling	psychological counseling	psychological counseling	psychological counseling	8	kept
Psychological empowerment	psychological empowerment	psychological empowerment	psychological empowerment	Psychological empowerment	8	kept
psychometric properties	psychometric properties	psychometric properties	psychometric properties	Psychometric properties	8	case_or_format
public health insurance	public health insurance	public health insurance	public health insurance	public health insurance	8	kept
Public Policy	public policy	public policy	public policy	public policy	8	case_or_format
puerperal disorder	puerperal disorder	puerperal disorder	puerperal disorder	Puerperal Disorders	8	case_or_format

pyelonephritis	pyelonephritis	pyelonephritis	pyelonephritis	pyelonephritis	8	kept
QRS complex	qrs complex	qrs complex	qrs complex	QRS complex	8	kept
qualitative design	qualitative design	qualitative design	qualitative design	qualitative design	8	kept
qualitative methods	qualitative methods	qualitative methods	qualitative methods	Qualitative methods	8	case_or_format
Qualitative studies	qualitative studies	qualitative studies	qualitative studies	qualitative studies	8	case_or_format
Quality assessment	quality assessment	quality assessment	quality assessment	Quality assessment	8	kept
quality assurance	quality assurance	quality assurance	quality assurance	Quality assurance	8	case_or_format
Quality of healthcare	quality of healthcare	quality of healthcare	quality of healthcare	Quality of healthcare	8	kept
questionnaire survey	questionnaire survey	questionnaire survey	questionnaire survey	questionnaire survey	8	kept
racial disparity	racial disparity	racial disparity	racial disparity	racial disparity	8	kept
radiation dose	radiation dose	radiation dose	radiation dose	radiation dose	8	kept
Radiography, Abdominal	radiography abdominal	radiography abdominal	radiography abdominal	Radiography, Abdominal	8	kept
ramelteon	ramelteon	ramelteon	ramelteon	ramelteon	8	kept
Random Allocation	random allocation	random allocation	random allocation	Random Allocation	8	kept
random sample	random sample	random sample	random sample	random sample	8	kept
Randomized Controlled Trial	randomized controlled trial	randomized controlled trial	randomized controlled trial	randomized controlled trial	8	case_or_format

rapid response teams	rapid response teams	rapid response teams	rapid response team	rapid response team	8	singular
Reaction Time	reaction time	reaction time	reaction time	reaction time	8	case_or_format
Reading	reading	reading	reading	reading	8	case_or_format
recommendation	recommendation	recommendation	recommendation	Recommendations	8	case_or_format
Reconstructive Surgical Procedures	reconstructive surgical procedures	reconstructive surgical procedures	reconstructive surgical procedures	Reconstructive Surgical Procedures	8	kept
RECOVERY	recovery	recovery	recovery	Recovery	8	case_or_format
rectal swab	rectal swab	rectal swab	rectal swab	rectal swab	8	kept
REM sleep	rem sleep	rem sleep	rem sleep	REM sleep	8	kept
Rescue Work	rescue work	rescue work	rescue work	Rescue Work	8	kept
residence characteristics	residence characteristics	residence characteristics	residence characteristics	Residence Characteristics	8	case_or_format
Resident education	resident education	resident education	resident education	Resident education	8	kept
RESILIENCE	resilience	resilience	resilience	resilience	8	case_or_format
resource shortage	resource shortage	resource shortage	resource shortage	resource shortage	8	kept
resource use	resource use	resource use	resource use	resource use	8	kept
Resource-Limited Settings	resource limited settings	resource limited settings	resource limited setting	resource limited setting	8	singular
Respiratory Physiological Phenomena	respiratory physiological phenomena	respiratory physiological phenomena	respiratory physiological phenomena	Respiratory Physiological Phenomena	8	kept

Respiratory Protective Devices	respiratory protective devices	respiratory protective devices	respiratory protective devices	Respiratory Protective Devices	8	kept
Respiratory syncytial pneumovirus	respiratory syncytial pneumovirus	respiratory syncytial pneumovirus	respiratory syncytial pneumovirus	Respiratory syncytial pneumovirus	8	kept
respiratory syncytial virus	respiratory syncytial virus	respiratory syncytial virus	respiratory syncytial virus	Respiratory syncytial virus	8	case_or_format
Respiratory System	respiratory system	respiratory system	respiratory system	respiratory system	8	case_or_format
RESPIRATORY-DISTRESS-SYNDROME	respiratory distress syndrome	respiratory distress syndrome	respiratory distress syndrome	Respiratory Distress Syndrome	8	case_or_format
retention	retention	retention	retention	Retention	8	case_or_format
retinol	retinol	retinol	retinol	retinol	8	kept
retinopathy of prematurity	retinopathy of prematurity	retinopathy of prematurity	retinopathy of prematurity	Retinopathy of Prematurity	8	case_or_format
RNA, Ribosomal, 16S	rna ribosomal 16s	rna ribosomal 16s	rna ribosomal 16s	RNA, Ribosomal, 16S	8	kept
Root Cause Analysis	root cause analysis	root cause analysis	root cause analysis	root cause analysis	8	case_or_format
rural health	rural health	rural health	rural health	rural health	8	kept
Safety climate	safety climate	safety climate	safety climate	safety climate	8	case_or_format
safety net hospital	safety net hospital	safety net hospital	safety net hospital	safety net hospital	8	kept
Salvage Therapy	salvage therapy	salvage therapy	salvage therapy	salvage therapy	8	case_or_format
sample	sample	sample	sample	sample	8	kept
scale	scale	scale	scale	SCALE	8	case_or_format

Science	science	science	science	Science	8	kept
Scope of practice	scope of practice	scope of practice	scope of practice	scope of practice	8	case_or_format
seasonal influenza	seasonal influenza	seasonal influenza	seasonal influenza	seasonal influenza	8	kept
Security Measures	security measures	security measures	security measures	Security Measures	8	kept
seizures	seizures	seizures	seizure	seizure	8	singular
SENSITIVITY	sensitivity	sensitivity	sensitivity	SENSITIVITY	8	kept
sensitivity	sensitivity	sensitivity	sensitivity	SENSITIVITY	8	case_or_format
sensory dysfunction	sensory dysfunction	sensory dysfunction	sensory dysfunction	sensory dysfunction	8	kept
Shared decision-making	shared decision making	shared decision making	shared decision making	shared decision making	8	case_or_format
short bowel syndrome	short bowel syndrome	short bowel syndrome	short bowel syndrome	short bowel syndrome	8	kept
Sick Leave	sick leave	sick leave	sick leave	Sick Leave	8	kept
signal transduction	signal transduction	signal transduction	signal transduction	signal transduction	8	kept
sildenafil	sildenafil	sildenafil	sildenafil	sildenafil	8	kept
Situation awareness	situation awareness	situation awareness	situation awareness	situation awareness	8	case_or_format
skill mix	skill mix	skill mix	skill mix	skill mix	8	kept
Skin Tests	skin tests	skin tests	skin test	Skin Tests	8	singular

Skin-to-skin care	skin to skin care	skin to skin care	skin to skin care	skin-to-skin care	8	case_or_format
Sleep Apnea Syndromes	sleep apnea syndromes	sleep apnea syndromes	sleep apnea syndrome	sleep apnea syndrome	8	singular
sleep stage	sleep stage	sleep stage	sleep stage	Sleep Stages	8	case_or_format
slurred speech	slurred speech	slurred speech	slurred speech	slurred speech	8	kept
Smoke Inhalation Injury	smoke inhalation injury	smoke inhalation injury	smoke inhalation injury	Smoke Inhalation Injury	8	kept
snakebite	snakebite	snakebite	snakebite	snakebite	8	kept
social class	social class	social class	social class	Social Class	8	case_or_format
Social media	social media	social media	social media	social media	8	case_or_format
Social Workers	social workers	social workers	social worker	social worker	8	singular
SOCIETY	society	society	society	SOCIETY	8	kept
soft tissue injury	soft tissue injury	soft tissue injury	soft tissue injury	soft tissue injury	8	kept
Solutions	solutions	solutions	solutions	Solutions	8	kept
sorbitol	sorbitol	sorbitol	sorbitol	sorbitol	8	kept
space	space	space	space	space	8	kept
Spaniard	spaniard	spaniard	spaniard	Spaniard	8	kept
Spanish	spanish	spanish	spanish	Spanish	8	kept

specificity	specificity	specificity	specificity	specificity	8	kept
Speech Therapy	speech therapy	speech therapy	speech therapy	speech therapy	8	case_or_format
Spouse Abuse	spouse abuse	spouse abuse	spouse abuse	Spouse Abuse	8	kept
ST Elevation Myocardial Infarction	st elevation myocardial infarction	st elevation myocardial infarction	st elevation myocardial infarction	ST Elevation Myocardial Infarction	8	kept
Staphylococcus capitis	staphylococcus capitis	staphylococcus capitis	staphylococcus capitis	Staphylococcus capitis	8	kept
STAY	stay	stay	stay	STAY	8	kept
Sterilization	sterilization	sterilization	sterilization	Sterilization	8	kept
stethoscope	stethoscope	stethoscope	stethoscope	Stethoscopes	8	case_or_format
stomach distension	stomach distension	stomach distension	stomach distension	stomach distension	8	kept
stomach tube	stomach tube	stomach tube	stomach tube	stomach tube	8	kept
Streptococcus	streptococcus	streptococcus	streptococcus	Streptococcus	8	kept
stress disorders	stress disorders	stress disorders	stress disorder	Stress disorders	8	singular
Stress Disorders, Traumatic, Acute	stress disorders traumatic acute	stress disorders traumatic acute	stress disorders traumatic acute	Stress Disorders, Traumatic, Acute	8	kept
Structural equation modeling	structural equation modeling	structural equation modeling	structural equation modeling	structural equation modeling	8	case_or_format
Sub-Saharan Africa	sub saharan africa	sub saharan africa	sub saharan africa	Sub-Saharan Africa	8	kept
sugammadex	sugammadex	sugammadex	sugammadex	sugammadex	8	kept

surgical debridement	surgical debridement	surgical debridement	surgical debridement	surgical debridement	8	kept
survival prediction	survival prediction	survival prediction	survival prediction	survival prediction	8	kept
Survivorship	survivorship	survivorship	survivorship	survivorship	8	case_or_format
Symbolism	symbolism	symbolism	symbolism	Symbolism	8	kept
synchronized intermittent mandatory ventilation	synchronized intermittent mandatory ventilation	synchronized intermittent mandatory ventilation	synchronized intermittent mandatory ventilation	synchronized intermittent mandatory ventilation	8	kept
systemic vascular resistance	systemic vascular resistance	systemic vascular resistance	systemic vascular resistance	systemic vascular resistance	8	kept
takotsubo cardiomyopathy	takotsubo cardiomyopathy	takotsubo cardiomyopathy	takotsubo cardiomyopathy	takotsubo cardiomyopathy	8	kept
techniques and procedures	techniques and procedures	techniques and procedures	techniques and procedure	techniques and procedures	8	singular
TECHNOLOGY	technology	technology	technology	Technology	8	case_or_format
Terminally ill	terminally ill	terminally ill	terminally ill	Terminally Ill	8	case_or_format
thorax wall	thorax wall	thorax wall	thorax wall	thorax wall	8	kept
thrombotic thrombocytopenic purpura	thrombotic thrombocytopenic purpura	thrombotic thrombocytopenic purpura	thrombotic thrombocytopenic purpura	thrombotic thrombocytopenic purpura	8	kept
thyroid hormone	thyroid hormone	thyroid hormone	thyroid hormone	thyroid hormone	8	kept
ticarcillin	ticarcillin	ticarcillin	ticarcillin	ticarcillin	8	kept
time perception	time perception	time perception	time perception	time perception	8	kept
topic areas	topic areas	topic areas	topic areas	topic areas	8	kept

total body surface area	total body surface area	total body surface area	total body surface area	total body surface area	8	kept
trachea stenosis	trachea stenosis	trachea stenosis	trachea stenosis	trachea stenosis	8	kept
TRANSFERS	transfers	transfers	transfer	Transfer	8	singular
transition to adult care	transition to adult care	transition to adult care	transition to adult care	transition to adult care	8	kept
transitions of care	transitions of care	transitions of care	transitions of care	transitions of care	8	kept
trauma-informed care	trauma informed care	trauma informed care	trauma informed care	trauma-informed care	8	kept
Treatment outcome	treatment outcome	treatment outcome	treatment outcome	Treatment Outcome	8	case_or_format
treatment protocol	treatment protocol	treatment protocol	treatment protocol	treatment protocol	8	kept
triangulation	triangulation	triangulation	triangulation	triangulation	8	kept
tuberculin test	tuberculin test	tuberculin test	tuberculin test	tuberculin test	8	kept
tuberculostatic agent	tuberculostatic agent	tuberculostatic agent	tuberculostatic agent	tuberculostatic agent	8	kept
tumor necrosis factor alpha	tumor necrosis factor alpha	tumor necrosis factor alpha	tumor necrosis factor alpha	tumor necrosis factor alpha	8	kept
Tumor Necrosis Factor-alpha	tumor necrosis factor alpha	tumor necrosis factor alpha	tumor necrosis factor alpha	tumor necrosis factor alpha	8	case_or_format
tumor recurrence	tumor recurrence	tumor recurrence	tumor recurrence	tumor recurrence	8	kept
twin pregnancy	twin pregnancy	twin pregnancy	twin pregnancy	twin pregnancy	8	kept
Umbilical Cord	umbilical cord	umbilical cord	umbilical cord	umbilical cord	8	case_or_format

Umbilical Veins	umbilical veins	umbilical veins	umbilical vein	Umbilical Veins	8	singular
undergraduate student	undergraduate student	undergraduate student	undergraduate student	undergraduate student	8	kept
unstable angina pectoris	unstable angina pectoris	unstable angina pectoris	unstable angina pectoris	unstable angina pectoris	8	kept
upper respiratory tract obstruction	upper respiratory tract obstruction	upper respiratory tract obstruction	upper respiratory tract obstruction	upper respiratory tract obstruction	8	kept
Urine	urine	urine	urine	urine	8	case_or_format
Urodynamics	urodynamics	urodynamics	urodynamics	Urodynamics	8	kept
User experience	user experience	user experience	user experience	User experience	8	kept
Vaccination Coverage	vaccination coverage	vaccination coverage	vaccination coverage	vaccination coverage	8	case_or_format
Vancomycin-Resistant Enterococci	vancomycin resistant enterococci	vancomycin resistant enterococci	vancomycin resistant enterococci	Vancomycin-Resistant Enterococci	8	kept
vascular resistance	vascular resistance	vascular resistance	vascular resistance	vascular resistance	8	kept
vasodilatation	vasodilatation	vasodilatation	vasodilatation	vasodilatation	8	kept
Venous thromboembolism	venous thromboembolism	venous thromboembolism	venous thromboembolism	venous thromboembolism	8	case_or_format
VENTILATION	ventilation	ventilation	ventilation	Ventilation	8	case_or_format
ventilator induced lung injury	ventilator induced lung injury	ventilator induced lung injury	ventilator induced lung injury	Ventilator-Induced Lung Injury	8	case_or_format
veterinary clinic	veterinary clinic	veterinary clinic	veterinary clinic	veterinary clinic	8	kept
Vibration	vibration	vibration	vibration	vibration	8	case_or_format

Visiting	visiting	visiting	visiting	Visiting	8	kept
Visual Analog Scale	visual analog scale	visual analog scale	visual analog scale	visual analog scale	8	case_or_format
visual attention	visual attention	visual attention	visual attention	visual attention	8	kept
Vitamin D	vitamin d	vitamin d	vitamin d	vitamin D	8	case_or_format
vitamin D deficiency	vitamin d deficiency	vitamin d deficiency	vitamin d deficiency	vitamin D deficiency	8	kept
vocalization	vocalization	vocalization	vocalization	vocalization	8	kept
WanFang Database	wanfang database	wanfang database	wanfang database	WanFang Database	8	kept
Wearable	wearable	wearable	wearable	Wearable	8	kept
weight bearing	weight bearing	weight bearing	weight bearing	weight bearing	8	kept
Weight Loss	weight loss	weight loss	weight loss	Weight Loss	8	kept
Wit and Humor as Topic	wit and humor as topic	wit and humor as topic	wit and humor as topic	Wit and Humor as Topic	8	kept
Withholding treatment	withholding treatment	withholding treatment	withholding treatment	Withholding Treatment	8	case_or_format
Women's Health	women s health	women s health	women s health	women's health	8	case_or_format
Work-Life Balance	work life balance	work life balance	work life balance	work-life balance	8	case_or_format
Wound healing	wound healing	wound healing	wound healing	Wound Healing	8	case_or_format
wounds and injuries	wounds and injuries	wounds and injuries	wounds and injuries	Wounds and Injuries	8	case_or_format

Youden index	youden index	youden index	youden index	Youden index	8	kept
Zimbabwe	zimbabwe	zimbabwe	zimbabwe	Zimbabwe	8	kept
ABCDE bundle	abcde bundle	abcde bundle	abcde bundle	ABCDE bundle	7	kept
abdominal aorta aneurysm	abdominal aorta aneurysm	abdominal aorta aneurysm	abdominal aorta aneurysm	abdominal aorta aneurysm	7	kept
abdominal disease	abdominal disease	abdominal disease	abdominal disease	abdominal disease	7	kept
accident & emergency medicine	accident and emergency medicine	accident and emergency medicine	accident and emergency medicine	accident & emergency medicine	7	kept
Acquired Immunodeficiency Syndrome	acquired immunodeficiency syndrome	acquired immunodeficiency syndrome	acquired immunodeficiency syndrome	Acquired Immunodeficiency Syndrome	7	kept
ACQUISITION	acquisition	acquisition	acquisition	ACQUISITION	7	kept
active surveillance	active surveillance	active surveillance	active surveillance	active surveillance	7	kept
Activities of daily living	activities of daily living	activities of daily living	activities of daily living	Activities of Daily Living	7	case_or_format
acute abdomen	acute abdomen	acute abdomen	acute abdomen	acute abdomen	7	kept
acute leukemia	acute leukemia	acute leukemia	acute leukemia	acute leukemia	7	kept
Acute liver failure	acute liver failure	acute liver failure	acute liver failure	acute liver failure	7	case_or_format
acute renal failure	acute renal failure	acute renal failure	acute renal failure	Acute renal failure	7	case_or_format
Acute Respiratory Distress Syndrome	acute respiratory distress syndrome	acute respiratory distress syndrome	acute respiratory distress syndrome	acute respiratory distress syndrome	7	case_or_format
adenosine	adenosine	adenosine	adenosine	adenosine	7	kept

adenosine triphosphate	adenosine triphosphate	adenosine triphosphate	adenosine triphosphate	adenosine triphosphate	7	kept
ADHERENCE	adherence	adherence	adherence	Adherence	7	case_or_format
Adult respiratory distress syndrome	adult respiratory distress syndrome	adult respiratory distress syndrome	adult respiratory distress syndrome	adult respiratory distress syndrome	7	case_or_format
Advanced life support	advanced life support	advanced life support	advanced life support	advanced life support	7	case_or_format
advanced practice providers	advanced practice providers	advanced practice providers	advanced practice provider	advanced practice provider	7	singular
Adverse drug events	adverse drug events	adverse drug events	adverse drug event	ADVERSE DRUG EVENTS	7	singular
Adverse effects	adverse effects	adverse effects	adverse effect	adverse effects	7	singular
aeration	aeration	aeration	aeration	aeration	7	kept
Affective Symptoms	affective symptoms	affective symptoms	affective symptoms	Affective Symptoms	7	kept
Africa South of the Sahara	africa south of the sahara	africa south of the sahara	africa south of the sahara	Africa south of the Sahara	7	case_or_format
agar	agar	agar	agar	agar	7	kept
AGE	age	age	age	age	7	case_or_format
Age	age	age	age	age	7	case_or_format
AIDS-Related Opportunistic Infections	aids related opportunistic infections	aids related opportunistic infections	aids related opportunistic infections	AIDS-Related Opportunistic Infections	7	kept
Air	air	air	air	Air	7	kept
Air Pollutants, Occupational	air pollutants occupational	air pollutants occupational	air pollutants occupational	Air Pollutants, Occupational	7	kept

air sampling	air sampling	air sampling	air sampling	air sampling	7	kept
airborne transmission	airborne transmission	airborne transmission	airborne transmission	airborne transmission	7	kept
altered state of consciousness	altered state of consciousness	altered state of consciousness	altered state of consciousness	altered state of consciousness	7	kept
ambivalence	ambivalence	ambivalence	ambivalence	ambivalence	7	kept
Ambulance	ambulance	ambulance	ambulance	ambulance	7	case_or_format
amitriptyline	amitriptyline	amitriptyline	amitriptyline	amitriptyline	7	kept
amphotericin	amphotericin	amphotericin	amphotericin	amphotericin	7	kept
Anaemia	anaemia	anaemia	anaemia	Anaemia	7	kept
analytical error	analytical error	analytical error	analytical error	analytical error	7	kept
analytical parameters	analytical parameters	analytical parameters	analytical parameters	analytical parameters	7	kept
anastomosis leakage	anastomosis leakage	anastomosis leakage	anastomosis leakage	anastomosis leakage	7	kept
angiocardiography	angiocardiography	angiocardiography	angiocardiography	angiocardiography	7	kept
angioplasty	angioplasty	angioplasty	angioplasty	Angioplasty	7	case_or_format
Angioplasty, Balloon	angioplasty balloon	angioplasty balloon	angioplasty balloon	Angioplasty, Balloon	7	kept
angiotensin converting enzyme 2	angiotensin converting enzyme 2	angiotensin converting enzyme 2	angiotensin converting enzyme 2	angiotensin converting enzyme 2	7	kept
animal tissue	animal tissue	animal tissue	animal tissue	animal tissue	7	kept

Animalia	animalia	animalia	animalia	Animalia	7	kept
antacid agent	antacid agent	antacid agent	antacid agent	antacid agent	7	kept
ANTECEDENTS	antecedents	antecedents	antecedents	ANTECEDENTS	7	kept
antepartum hemorrhage	antepartum hemorrhage	antepartum hemorrhage	antepartum hemorrhage	antepartum hemorrhage	7	kept
Anti-Asthmatic Agents	anti asthmatic agents	anti asthmatic agents	anti asthmatic agents	Anti-Asthmatic Agents	7	kept
Anti-Ulcer Agents	anti ulcer agents	anti ulcer agents	anti ulcer agent	Anti-Ulcer Agents	7	singular
antibacterial activity	antibacterial activity	antibacterial activity	antibacterial activity	antibacterial activity	7	kept
Antidepressive Agents	antidepressive agents	antidepressive agents	antidepressive agents	Antidepressive Agents	7	kept
Antiemetics	antiemetics	antiemetics	antiemetics	Antiemetics	7	kept
antioxidant	antioxidant	antioxidant	antioxidant	Antioxidants	7	case_or_format
antiparkinson agent	antiparkinson agent	antiparkinson agent	antiparkinson agent	antiparkinson agent	7	kept
Antipsychotics	antipsychotics	antipsychotics	antipsychotic	Antipsychotics	7	singular
aorta valve stenosis	aorta valve stenosis	aorta valve stenosis	aorta valve stenosis	aorta valve stenosis	7	kept
Aorta, Abdominal	aorta abdominal	aorta abdominal	aorta abdominal	Aorta, Abdominal	7	kept
Aortic Diseases	aortic diseases	aortic diseases	aortic disease	Aortic Diseases	7	singular
Aortic stenosis	aortic stenosis	aortic stenosis	aortic stenosis	aortic stenosis	7	case_or_format

apoptosis	apoptosis	apoptosis	apoptosis	apoptosis	7	kept
apparent life threatening event	apparent life threatening event	apparent life threatening event	apparent life threatening event	apparent life threatening event	7	kept
aprotinin	aprotinin	aprotinin	aprotinin	aprotinin	7	kept
Arab	arab	arab	arab	Arabs	7	case_or_format
argipressin	argipressin	argipressin	argipressin	argipressin	7	kept
arrhythmia	arrhythmia	arrhythmia	arrhythmia	Arrhythmia	7	case_or_format
Arrhythmias	arrhythmias	arrhythmias	arrhythmia	Arrhythmia	7	singular
arterial blood	arterial blood	arterial blood	arterial blood	arterial blood	7	kept
arthritis	arthritis	arthritis	arthritis	arthritis	7	kept
artificial respiration	artificial respiration	artificial respiration	artificial respiration	Artificial respiration	7	case_or_format
Aspergillosis	aspergillosis	aspergillosis	aspergillosis	Aspergillosis	7	kept
Aspiration pneumonia	aspiration pneumonia	aspiration pneumonia	aspiration pneumonia	aspiration pneumonia	7	case_or_format
ataxia	ataxia	ataxia	ataxia	ataxia	7	kept
attachment	attachment	attachment	attachment	Attachment	7	case_or_format
attention deficit disorder	attention deficit disorder	attention deficit disorder	attention deficit disorder	attention deficit disorder	7	kept
attitude of health personnel	attitude of health personnel	attitude of health personnel	attitude of health personnel	Attitude of Health Personnel	7	case_or_format

attitude to computers	attitude to computers	attitude to computers	attitude to computers	Attitude to Computers	7	case_or_format
Auditory Perception	auditory perception	auditory perception	auditory perception	Auditory Perception	7	kept
Automatic Data Processing	automatic data processing	automatic data processing	automatic data processing	Automatic Data Processing	7	kept
autonomic nervous system	autonomic nervous system	autonomic nervous system	autonomic nervous system	Autonomic Nervous System	7	case_or_format
AUTONOMY	autonomy	autonomy	autonomy	Autonomy	7	case_or_format
Axilla	axilla	axilla	axilla	Axilla	7	kept
Bacillus cereus	bacillus cereus	bacillus cereus	bacillus cereus	Bacillus cereus	7	kept
bacterial genome	bacterial genome	bacterial genome	bacterial genome	bacterial genome	7	kept
bacterium carrier	bacterium carrier	bacterium carrier	bacterium carrier	bacterium carrier	7	kept
bacterium colony	bacterium colony	bacterium colony	bacterium colony	bacterium colony	7	kept
balloon dilatation	balloon dilatation	balloon dilatation	balloon dilatation	balloon dilatation	7	kept
Bariatric Surgery	bariatric surgery	bariatric surgery	bariatric surgery	bariatric surgery	7	case_or_format
baricitinib	baricitinib	baricitinib	baricitinib	baricitinib	7	kept
Barriers and facilitators	barriers and facilitators	barriers and facilitators	barriers and facilitators	Barriers and facilitators	7	kept
BCG vaccine	bcg vaccine	bcg vaccine	bcg vaccine	BCG vaccine	7	kept
BELIEFS	beliefs	beliefs	belief	beliefs	7	singular

Benzodiazepine	benzodiazepine	benzodiazepine	benzodiazepine	benzodiazepine	7	case_or_format
Big data	big data	big data	big data	big data	7	case_or_format
Biomedical and Behavioral Research	biomedical and behavioral research	biomedical and behavioral research	biomedical and behavioral research	Biomedical and Behavioral Research	7	kept
Biomedical Engineering	biomedical engineering	biomedical engineering	biomedical engineering	Biomedical engineering	7	case_or_format
Biomedical equipment	biomedical equipment	biomedical equipment	biomedical equipment	Biomedical equipment	7	kept
Biomedical technology	biomedical technology	biomedical technology	biomedical technology	Biomedical Technology	7	case_or_format
Birth Injuries	birth injuries	birth injuries	birth injury	Birth Injuries	7	singular
Blood Circulation	blood circulation	blood circulation	blood circulation	Blood Circulation	7	kept
blood clotting factor	blood clotting factor	blood clotting factor	blood clotting factor	blood clotting factor	7	kept
blood clotting factor 10a inhibitor	blood clotting factor 10a inhibitor	blood clotting factor 10a inhibitor	blood clotting factor 10a inhibitor	blood clotting factor 10a inhibitor	7	kept
blood component therapy	blood component therapy	blood component therapy	blood component therapy	blood component therapy	7	kept
Blood Gas Monitoring, Transcutaneous	blood gas monitoring transcutaneous	blood gas monitoring transcutaneous	blood gas monitoring transcutaneous	Blood Gas Monitoring, Transcutaneous	7	kept
Body Composition	body composition	body composition	body composition	body composition	7	case_or_format
bone density	bone density	bone density	bone density	bone density	7	kept
bone infection	bone infection	bone infection	bone infection	bone infection	7	kept
bone marrow biopsy	bone marrow biopsy	bone marrow biopsy	bone marrow biopsy	bone marrow biopsy	7	kept

Bone Neoplasms	bone neoplasms	bone neoplasms	bone neoplasms	Bone Neoplasms	7	kept
bootstrapping	bootstrapping	bootstrapping	bootstrapping	bootstrapping	7	kept
BRADEN SCALE	braden scale	braden scale	braden scale	Braden Scale	7	case_or_format
brain atrophy	brain atrophy	brain atrophy	brain atrophy	brain atrophy	7	kept
brain embolism	brain embolism	brain embolism	brain embolism	brain embolism	7	kept
brain ventricle peritoneum shunt	brain ventricle peritoneum shunt	brain ventricle peritoneum shunt	brain ventricle peritoneum shunt	brain ventricle peritoneum shunt	7	kept
Brazilian	brazilian	brazilian	brazilian	Brazilian	7	kept
Breast feeding	breast feeding	breast feeding	breast feeding	Breast Feeding	7	case_or_format
Breathing Exercises	breathing exercises	breathing exercises	breathing exercise	breathing exercise	7	singular
Brief Resilience Scale	brief resilience scale	brief resilience scale	brief resilience scale	Brief Resilience Scale	7	kept
bundle	bundle	bundle	bundle	BUNDLE	7	case_or_format
Burn care	burn care	burn care	burn care	Burn care	7	kept
Calcitonin	calcitonin	calcitonin	calcitonin	Calcitonin	7	kept
calcium blood level	calcium blood level	calcium blood level	calcium blood level	calcium blood level	7	kept
Candida tropicalis	candida tropicalis	candida tropicalis	candida tropicalis	Candida tropicalis	7	kept
Candidiasis, Invasive	candidiasis invasive	candidiasis invasive	candidiasis invasive	Candidiasis, Invasive	7	kept

Capacity building	capacity building	capacity building	capacity building	capacity building	7	case_or_format
capillary blood	capillary blood	capillary blood	capillary blood	capillary blood	7	kept
cardiomegaly	cardiomegaly	cardiomegaly	cardiomegaly	cardiomegaly	7	kept
cardiorenal syndrome	cardiorenal syndrome	cardiorenal syndrome	cardiorenal syndrome	cardiorenal syndrome	7	kept
Cardiothoracic surgery	cardiothoracic surgery	cardiothoracic surgery	cardiothoracic surgery	Cardiothoracic surgery	7	kept
cardiovascular system examination	cardiovascular system examination	cardiovascular system examination	cardiovascular system examination	cardiovascular system examination	7	kept
care delivery	care delivery	care delivery	care delivery	care delivery	7	kept
caregiving	caregiving	caregiving	caregiving	caregiving	7	kept
carvedilol	carvedilol	carvedilol	carvedilol	carvedilol	7	kept
catheter-associated urinary tract infection	catheter associated urinary tract infection	catheter associated urinary tract infection	catheter associated urinary tract infection	catheter associated urinary tract infection	7	case_or_format
catheter-related infections	catheter related infections	catheter related infections	catheter related infection	Catheter-Related Infections	7	singular
CD4+ T lymphocyte	cd4 t lymphocyte	cd4 t lymphocyte	cd4 t lymphocyte	CD4+ T lymphocyte	7	kept
Cefepime	cefepime	cefepime	cefepime	cefepime	7	case_or_format
cell phone use	cell phone use	cell phone use	cell phone use	cell phone use	7	kept
cell therapy	cell therapy	cell therapy	cell therapy	cell therapy	7	kept
CENTERED CARE	centered care	centered care	centered care	CENTERED CARE	7	kept

Centers for Disease Control and Prevention	centers for disease control and prevention	centers for disease control and prevention	centers for disease control and prevention	Centers for Disease Control and Prevention	7	kept
Central Venous	central venous	central venous	central venous	Central venous	7	case_or_format
Central venous pressure	central venous pressure	central venous pressure	central venous pressure	central venous pressure	7	case_or_format
Cerebral Angiography	cerebral angiography	cerebral angiography	cerebral angiography	Cerebral Angiography	7	kept
Cerebral Hemorrhage, Traumatic	cerebral hemorrhage traumatic	cerebral hemorrhage traumatic	cerebral hemorrhage traumatic	Cerebral Hemorrhage, Traumatic	7	kept
cervical spine fracture	cervical spine fracture	cervical spine fracture	cervical spine fracture	cervical spine fracture	7	kept
Change management	change management	change management	change management	change management	7	case_or_format
Child Nutrition Disorders	child nutrition disorders	child nutrition disorders	child nutrition disorders	Child Nutrition Disorders	7	kept
childhood trauma	childhood trauma	childhood trauma	childhood trauma	childhood trauma	7	kept
Chlorhexidine gluconate	chlorhexidine gluconate	chlorhexidine gluconate	chlorhexidine gluconate	chlorhexidine gluconate	7	case_or_format
cholecystitis	cholecystitis	cholecystitis	cholecystitis	cholecystitis	7	kept
chylothorax	chylothorax	chylothorax	chylothorax	Chylothorax	7	case_or_format
cleft lip	cleft lip	cleft lip	cleft lip	cleft lip	7	kept
Cleft Palate	cleft palate	cleft palate	cleft palate	cleft palate	7	case_or_format
Clinical Clerkship	clinical clerkship	clinical clerkship	clinical clerkship	Clinical Clerkship	7	kept
Clinical Coding	clinical coding	clinical coding	clinical coding	Clinical Coding	7	kept

Clinical decision support system	clinical decision support system	clinical decision support system	clinical decision support system	clinical decision support system	7	case_or_format
clinical decision support systems	clinical decision support systems	clinical decision support systems	clinical decision support system	clinical decision support system	7	singular
CLINICAL DETERIORATION	clinical deterioration	clinical deterioration	clinical deterioration	Clinical Deterioration	7	case_or_format
Clinical management	clinical management	clinical management	clinical management	Clinical management	7	kept
clinical medicine	clinical medicine	clinical medicine	clinical medicine	clinical medicine	7	kept
Clinical outcome	clinical outcome	clinical outcome	clinical outcome	clinical outcome	7	case_or_format
Clinical practice guideline	clinical practice guideline	clinical practice guideline	clinical practice guideline	clinical practice guideline	7	case_or_format
clinical protocols	clinical protocols	clinical protocols	clinical protocol	Clinical Protocols	7	singular
clothing	clothing	clothing	clothing	clothing	7	kept
Coercion	coercion	coercion	coercion	Coercion	7	kept
cohort study	cohort study	cohort study	cohort study	Cohort Studies	7	case_or_format
color	color	color	color	Color	7	case_or_format
color Doppler flowmetry	color doppler flowmetry	color doppler flowmetry	color doppler flowmetry	color Doppler flowmetry	7	kept
Colorectal Surgery	colorectal surgery	colorectal surgery	colorectal surgery	colorectal surgery	7	case_or_format
Colorimetry	colorimetry	colorimetry	colorimetry	Colorimetry	7	kept
comorbidities	comorbidities	comorbidities	comorbidity	comorbidity	7	singular

Compassionate care	compassionate care	compassionate care	compassionate care	Compassionate care	7	kept
compassionate care	compassionate care	compassionate care	compassionate care	Compassionate care	7	case_or_format
Complication	complication	complication	complication	complication	7	case_or_format
computer model	computer model	computer model	computer model	computer model	7	kept
computer security	computer security	computer security	computer security	computer security	7	kept
CONFIDENCE	confidence	confidence	confidence	Confidence	7	case_or_format
CONFLICT	conflict	conflict	conflict	conflict	7	case_or_format
Congenital heart surgery	congenital heart surgery	congenital heart surgery	congenital heart surgery	Congenital heart surgery	7	kept
congenital heart surgery	congenital heart surgery	congenital heart surgery	congenital heart surgery	Congenital heart surgery	7	case_or_format
congestive cardiomyopathy	congestive cardiomyopathy	congestive cardiomyopathy	congestive cardiomyopathy	congestive cardiomyopathy	7	kept
conjoined twins	conjoined twins	conjoined twins	conjoined twins	conjoined twins	7	kept
Conservative Treatment	conservative treatment	conservative treatment	conservative treatment	conservative treatment	7	case_or_format
continuous quality improvement	continuous quality improvement	continuous quality improvement	continuous quality improvement	continuous quality improvement	7	kept
continuous training	continuous training	continuous training	continuous training	continuous training	7	kept
Contracture	contracture	contracture	contracture	Contracture	7	kept
contraindication	contraindication	contraindication	contraindication	Contraindications	7	case_or_format

Control groups	control groups	control groups	control group	control group	7	singular
control strategy	control strategy	control strategy	control strategy	control strategy	7	kept
Coronary care units	coronary care units	coronary care units	coronary care unit	coronary care unit	7	singular
coronavirus infections	coronavirus infections	coronavirus infections	coronavirus infection	Coronavirus Infections	7	singular
Cortisol	cortisol	cortisol	cortisol	Cortisol	7	kept
cortisol	cortisol	cortisol	cortisol	Cortisol	7	case_or_format
Counselling	counselling	counselling	counselling	Counselling	7	kept
Courage	courage	courage	courage	Courage	7	kept
court	court	court	court	court	7	kept
COVID-19 vaccine	covid 19 vaccine	covid 19 vaccine	covid 19 vaccine	COVID-19 Vaccines	7	case_or_format
CPOE	cpoe	cpoe	cpoe	CPOE	7	kept
critical	critical	critical	critical	critical	7	kept
Critically ill children	critically ill children	critically ill children	critically ill children	Critically ill children	7	kept
cross clamp time	cross clamp time	cross clamp time	cross clamp time	cross clamp time	7	kept
cross‐sectional study	cross sectional study	cross sectional study	cross sectional study	Cross-Sectional Studies	7	case_or_format
crush trauma	crush trauma	crush trauma	crush trauma	crush trauma	7	kept

cryotherapy	cryotherapy	cryotherapy	cryotherapy	Cryotherapy	7	case_or_format
Cuidados de Enfermagem	cuidados de enfermagem	cuidados de enfermagem	cuidados de enfermagem	Cuidados de Enfermagem	7	kept
Cultural sensitivity	cultural sensitivity	cultural sensitivity	cultural sensitivity	cultural sensitivity	7	case_or_format
cultural value	cultural value	cultural value	cultural value	cultural value	7	kept
cutaneous T cell lymphoma	cutaneous t cell lymphoma	cutaneous t cell lymphoma	cutaneous t cell lymphoma	cutaneous T cell lymphoma	7	kept
data collection	data collection	data collection	data collection	Data Collection	7	case_or_format
Deception	deception	deception	deception	Deception	7	kept
DECISIONS	decisions	decisions	decision	DECISIONS	7	singular
Deep Learning	deep learning	deep learning	deep learning	deep learning	7	case_or_format
Deep vein thrombosis	deep vein thrombosis	deep vein thrombosis	deep vein thrombosis	deep vein thrombosis	7	case_or_format
Delaware	delaware	delaware	delaware	Delaware	7	kept
delayed cord clamping	delayed cord clamping	delayed cord clamping	delayed cord clamping	delayed cord clamping	7	kept
Deliberate practice	deliberate practice	deliberate practice	deliberate practice	Deliberate practice	7	kept
DELIVERY	delivery	delivery	delivery	delivery	7	case_or_format
dental hygienist	dental hygienist	dental hygienist	dental hygienist	dental hygienist	7	kept
Dermatitis, Allergic Contact	dermatitis allergic contact	dermatitis allergic contact	dermatitis allergic contact	Dermatitis, Allergic Contact	7	kept

device shortage	device shortage	device shortage	device shortage	device shortage	7	kept
Diabetic ketoacidosis	diabetic ketoacidosis	diabetic ketoacidosis	diabetic ketoacidosis	diabetic ketoacidosis	7	case_or_format
diabetic nephropathy	diabetic nephropathy	diabetic nephropathy	diabetic nephropathy	diabetic nephropathy	7	kept
diamorphine	diamorphine	diamorphine	diamorphine	diamorphine	7	kept
diaphragm hernia	diaphragm hernia	diaphragm hernia	diaphragm hernia	diaphragm hernia	7	kept
diaries	diaries	diaries	diary	Diary	7	singular
diastolic dysfunction	diastolic dysfunction	diastolic dysfunction	diastolic dysfunction	diastolic dysfunction	7	kept
digestive system	digestive system	digestive system	digestive system	digestive system	7	kept
digital imaging	digital imaging	digital imaging	digital imaging	digital imaging	7	kept
Digital Technology	digital technology	digital technology	digital technology	digital technology	7	case_or_format
digitalization	digitalization	digitalization	digitalization	digitalization	7	kept
Disabled Children	disabled children	disabled children	disabled children	Disabled Children	7	kept
Disabled Persons	disabled persons	disabled persons	disabled person	disabled person	7	singular
Disaster Medicine	disaster medicine	disaster medicine	disaster medicine	disaster medicine	7	case_or_format
DISCHARGE	discharge	discharge	discharge	Discharge	7	case_or_format
Discourse analysis	discourse analysis	discourse analysis	discourse analysis	Discourse analysis	7	kept

discourse analysis	discourse analysis	discourse analysis	discourse analysis	Discourse analysis	7	case_or_format
discriminant validity	discriminant validity	discriminant validity	discriminant validity	discriminant validity	7	kept
distilled water	distilled water	distilled water	distilled water	distilled water	7	kept
dog disease	dog disease	dog disease	dog disease	Dog Diseases	7	case_or_format
domperidone	domperidone	domperidone	domperidone	domperidone	7	kept
Donor Selection	donor selection	donor selection	donor selection	Donor Selection	7	kept
drinking	drinking	drinking	drinking	drinking	7	kept
drug bioavailability	drug bioavailability	drug bioavailability	drug bioavailability	drug bioavailability	7	kept
drug dosage form	drug dosage form	drug dosage form	drug dosage form	drug dosage form	7	kept
drug packaging	drug packaging	drug packaging	drug packaging	drug packaging	7	kept
drug response	drug response	drug response	drug response	drug response	7	kept
drug sensitivity	drug sensitivity	drug sensitivity	drug sensitivity	drug sensitivity	7	kept
drug stability	drug stability	drug stability	drug stability	drug stability	7	kept
dual antiplatelet therapy	dual antiplatelet therapy	dual antiplatelet therapy	dual antiplatelet therapy	dual antiplatelet therapy	7	kept
ear protective device	ear protective device	ear protective device	ear protective device	Ear Protective Devices	7	case_or_format
Early discharge	early discharge	early discharge	early discharge	Early discharge	7	kept

Early rehabilitation	early rehabilitation	early rehabilitation	early rehabilitation	early rehabilitation	7	case_or_format
EARLY WARNING SCORE	early warning score	early warning score	early warning score	early warning score	7	case_or_format
Early warning system	early warning system	early warning system	early warning system	early warning system	7	case_or_format
ECG	ecg	ecg	ecg	ECG	7	kept
ECG interpretation	ecg interpretation	ecg interpretation	ecg interpretation	ECG interpretation	7	kept
economic status	economic status	economic status	economic status	economic status	7	kept
Ecuador	ecuador	ecuador	ecuador	Ecuador	7	kept
educational intervention	educational intervention	educational intervention	educational intervention	Educational intervention	7	case_or_format
electrolyte blood level	electrolyte blood level	electrolyte blood level	electrolyte blood level	electrolyte blood level	7	kept
Electronic Prescribing	electronic prescribing	electronic prescribing	electronic prescribing	electronic prescribing	7	case_or_format
Electrophysiology	electrophysiology	electrophysiology	electrophysiology	electrophysiology	7	case_or_format
Eligibility Determination	eligibility determination	eligibility determination	eligibility determination	Eligibility Determination	7	kept
emergency management	emergency management	emergency management	emergency management	emergency management	7	kept
emollient agent	emollient agent	emollient agent	emollient agent	emollient agent	7	kept
empirical approaches	empirical approaches	empirical approaches	empirical approach	Empirical Approach	7	singular
enalapril	enalapril	enalapril	enalapril	enalapril	7	kept

END-OF-LIFE	end of life	end of life	end of life	End of life	7	case_or_format
Endemic Diseases	endemic diseases	endemic diseases	endemic disease	endemic disease	7	singular
Endocarditis	endocarditis	endocarditis	endocarditis	endocarditis	7	case_or_format
endoscopic surgery	endoscopic surgery	endoscopic surgery	endoscopic surgery	endoscopic surgery	7	kept
Enfermería de cuidados críticos	enfermer a de cuidados cr ticos	enfermer a de cuidados cr ticos	enfermer a de cuidados cr ticos	Enfermería de cuidados críticos	7	kept
environmental health	environmental health	environmental health	environmental health	environmental health	7	kept
environmental sanitation	environmental sanitation	environmental sanitation	environmental sanitation	environmental sanitation	7	kept
Epidemiologic Studies	epidemiologic studies	epidemiologic studies	epidemiologic studies	Epidemiologic Studies	7	kept
Epistaxis	epistaxis	epistaxis	epistaxis	epistaxis	7	case_or_format
errors	errors	errors	error	error	7	singular
Escalation of care	escalation of care	escalation of care	escalation of care	escalation of care	7	case_or_format
etoposide	etoposide	etoposide	etoposide	etoposide	7	kept
Evidence-based guidelines	evidence based guidelines	evidence based guidelines	evidence based guideline	Evidence-based guidelines	7	singular
excision	excision	excision	excision	excision	7	kept
exercise tolerance	exercise tolerance	exercise tolerance	exercise tolerance	exercise tolerance	7	kept
experimental model	experimental model	experimental model	experimental model	experimental model	7	kept

extended spectrum beta lactamase producing Klebsiella pneumoniae	extended spectrum beta lactamase producing klebsiella pneumoniae	extended spectrum beta lactamase producing klebsiella pneumoniae	extended spectrum beta lactamase producing klebsiella pneumoniae	extended spectrum beta lactamase producing Klebsiella pneumoniae	7	kept
Extracorporeal Circulation	extracorporeal circulation	extracorporeal circulation	extracorporeal circulation	extracorporeal circulation	7	case_or_format
Extracorporeal life support	extracorporeal life support	extracorporeal life support	extracorporeal life support	Extracorporeal life support	7	kept
Extraction, Obstetrical	extraction obstetrical	extraction obstetrical	extraction obstetrical	Extraction, Obstetrical	7	kept
extrapyramidal symptom	extrapyramidal symptom	extrapyramidal symptom	extrapyramidal symptom	extrapyramidal symptom	7	kept
eye protective device	eye protective device	eye protective device	eye protective device	Eye Protective Devices	7	case_or_format
face edema	face edema	face edema	face edema	face edema	7	kept
Facilitated tucking	facilitated tucking	facilitated tucking	facilitated tucking	facilitated tucking	7	case_or_format
Factor Xa Inhibitors	factor xa inhibitors	factor xa inhibitors	factor xa inhibitor	Factor Xa Inhibitors	7	singular
family care	family care	family care	family care	Family care	7	case_or_format
Family Characteristics	family characteristics	family characteristics	family characteristics	Family Characteristics	7	kept
Family member	family member	family member	family member	Family members	7	case_or_format
Fees and Charges	fees and charges	fees and charges	fees and charges	Fees and Charges	7	kept
Feminism	feminism	feminism	feminism	Feminism	7	kept
Femoral Fractures	femoral fractures	femoral fractures	femoral fractures	Femoral Fractures	7	kept
Femoral Vein	femoral vein	femoral vein	femoral vein	femoral vein	7	case_or_format

fetus hydrops	fetus hydrops	fetus hydrops	fetus hydrops	fetus hydrops	7	kept
Fibrinolysis	fibrinolysis	fibrinolysis	fibrinolysis	fibrinolysis	7	case_or_format
filter	filter	filter	filter	filter	7	kept
flow cytometry	flow cytometry	flow cytometry	flow cytometry	flow cytometry	7	kept
flucytosine	flucytosine	flucytosine	flucytosine	flucytosine	7	kept
Fluid resuscitation	fluid resuscitation	fluid resuscitation	fluid resuscitation	fluid resuscitation	7	case_or_format
Fluid therapy	fluid therapy	fluid therapy	fluid therapy	Fluid Therapy	7	case_or_format
focal epilepsy	focal epilepsy	focal epilepsy	focal epilepsy	focal epilepsy	7	kept
folinic acid	folinic acid	folinic acid	folinic acid	folinic acid	7	kept
Fontan Procedure	fontan procedure	fontan procedure	fontan procedure	Fontan Procedure	7	kept
forehead	forehead	forehead	forehead	forehead	7	kept
Formative Feedback	formative feedback	formative feedback	formative feedback	Formative Feedback	7	kept
fracture nonunion	fracture nonunion	fracture nonunion	fracture nonunion	fracture nonunion	7	kept
FREQUENCY	frequency	frequency	frequency	frequency	7	case_or_format
frontotemporal dementia	frontotemporal dementia	frontotemporal dementia	frontotemporal dementia	frontotemporal dementia	7	kept
Functional Status	functional status	functional status	functional status	functional status	7	case_or_format

Fundamentals of care	fundamentals of care	fundamentals of care	fundamentals of care	Fundamentals of care	7	kept
fungemia	fungemia	fungemia	fungemia	Fungemia	7	case_or_format
Fungi	fungi	fungi	fungi	Fungi	7	kept
fungus isolation	fungus isolation	fungus isolation	fungus isolation	fungus isolation	7	kept
Futility	futility	futility	futility	futility	7	case_or_format
Gastrectomy	gastrectomy	gastrectomy	gastrectomy	gastrectomy	7	case_or_format
Gastroenterology	gastroenterology	gastroenterology	gastroenterology	gastroenterology	7	case_or_format
gender identity	gender identity	gender identity	gender identity	Gender Identity	7	case_or_format
General Practitioners	general practitioners	general practitioners	general practitioner	general practitioner	7	singular
General ward	general ward	general ward	general ward	general ward	7	case_or_format
genetic susceptibility	genetic susceptibility	genetic susceptibility	genetic susceptibility	genetic susceptibility	7	kept
Genital Neoplasms, Female	genital neoplasms female	genital neoplasms female	genital neoplasms female	Genital Neoplasms, Female	7	kept
gestation period	gestation period	gestation period	gestation period	gestation period	7	kept
Glomerular Filtration Rate	glomerular filtration rate	glomerular filtration rate	glomerular filtration rate	Glomerular Filtration Rate	7	kept
glycopyrronium bromide	glycopyrronium bromide	glycopyrronium bromide	glycopyrronium bromide	glycopyrronium bromide	7	kept
Good death	good death	good death	good death	Good death	7	kept

good death	good death	good death	good death	Good death	7	case_or_format
graft versus host reaction	graft versus host reaction	graft versus host reaction	graft versus host reaction	graft versus host reaction	7	kept
guideline adherence	guideline adherence	guideline adherence	guideline adherence	Guideline Adherence	7	case_or_format
Haemodynamics	haemodynamics	haemodynamics	haemodynamic	Haemodynamics	7	singular
half life time	half life time	half life time	half life time	half life time	7	kept
HAND HYGIENE	hand hygiene	hand hygiene	hand hygiene	Hand Hygiene	7	case_or_format
handicapped child	handicapped child	handicapped child	handicapped child	handicapped child	7	kept
Handoffs	handoffs	handoffs	handoff	handoff	7	singular
Handwriting	handwriting	handwriting	handwriting	Handwriting	7	kept
handwriting	handwriting	handwriting	handwriting	Handwriting	7	case_or_format
Hawthorne effect	hawthorne effect	hawthorne effect	hawthorne effect	Hawthorne effect	7	kept
HCWs	hcws	hcws	hcw	HCWs	7	singular
Head Injuries, Closed	head injuries closed	head injuries closed	head injuries closed	Head Injuries, Closed	7	kept
health auxiliary	health auxiliary	health auxiliary	health auxiliary	health auxiliary	7	kept
Health care utilization	health care utilization	health care utilization	health care utilization	health care utilization	7	case_or_format
Health economics	health economics	health economics	health economics	health economics	7	case_or_format

Health equity	health equity	health equity	health equity	health equity	7	case_or_format
health information technology	health information technology	health information technology	health information technology	Health information technology	7	case_or_format
Health Insurance Portability and Accountability Act	health insurance portability and accountability act	health insurance portability and accountability act	health insurance portability and accountability act	Health Insurance Portability and Accountability Act	7	kept
Health literacy	health literacy	health literacy	health literacy	health literacy	7	case_or_format
health outcomes	health outcomes	health outcomes	health outcome	health outcome	7	singular
Health promotion	health promotion	health promotion	health promotion	Health Promotion	7	case_or_format
health services administration & management	health services administration and management	health services administration and management	health services administration and management	health services administration & management	7	kept
health status indicator	health status indicator	health status indicator	health status indicator	Health Status Indicators	7	case_or_format
Health systems	health systems	health systems	health system	Health systems	7	singular
Health-care workers	health care workers	health care workers	health care worker	Health care workers	7	singular
Healthcare delivery	healthcare delivery	healthcare delivery	healthcare delivery	Healthcare delivery	7	kept
healthcare management	healthcare management	healthcare management	healthcare management	healthcare management	7	kept
healthcare quality	healthcare quality	healthcare quality	healthcare quality	Healthcare quality	7	case_or_format
Healthcare staff	healthcare staff	healthcare staff	healthcare staff	Healthcare staff	7	kept
heart auscultation	heart auscultation	heart auscultation	heart auscultation	heart auscultation	7	kept
heart index	heart index	heart index	heart index	heart index	7	kept

heart left ventricle hypertrophy	heart left ventricle hypertrophy	heart left ventricle hypertrophy	heart left ventricle hypertrophy	heart left ventricle hypertrophy	7	kept
heart muscle revascularization	heart muscle revascularization	heart muscle revascularization	heart muscle revascularization	heart muscle revascularization	7	kept
Heart transplantation	heart transplantation	heart transplantation	heart transplantation	heart transplantation	7	case_or_format
heart valve	heart valve	heart valve	heart valve	Heart Valves	7	case_or_format
heart valve prosthesis	heart valve prosthesis	heart valve prosthesis	heart valve prosthesis	heart valve prosthesis	7	kept
heart ventricle	heart ventricle	heart ventricle	heart ventricle	Heart Ventricles	7	case_or_format
heart ventricle extrasystole	heart ventricle extrasystole	heart ventricle extrasystole	heart ventricle extrasystole	heart ventricle extrasystole	7	kept
helmet	helmet	helmet	helmet	helmet	7	kept
hematemesis	hematemesis	hematemesis	hematemesis	hematemesis	7	kept
Hematoma, Subdural, Acute	hematoma subdural acute	hematoma subdural acute	hematoma subdural acute	Hematoma, Subdural, Acute	7	kept
Hemodialysis Units, Hospital	hemodialysis units hospital	hemodialysis units hospital	hemodialysis units hospital	Hemodialysis Units, Hospital	7	kept
hemoperitoneum	hemoperitoneum	hemoperitoneum	hemoperitoneum	hemoperitoneum	7	kept
hemorrhage	hemorrhage	hemorrhage	hemorrhage	Hemorrhage	7	case_or_format
Hemostatics	hemostatics	hemostatics	hemostatics	Hemostatics	7	kept
Hepatitis A	hepatitis a	hepatitis a	hepatitis a	Hepatitis A	7	kept
Hepatitis C, Chronic	hepatitis c chronic	hepatitis c chronic	hepatitis c chronic	Hepatitis C, Chronic	7	kept

heterozygote	heterozygote	heterozygote	heterozygote	heterozygote	7	kept
High-flow nasal cannula	high flow nasal cannula	high flow nasal cannula	high flow nasal cannula	High-flow nasal cannula	7	kept
HIP FRACTURE	hip fracture	hip fracture	hip fracture	hip fracture	7	case_or_format
Hispanic Americans	hispanic americans	hispanic americans	hispanic americans	Hispanic Americans	7	kept
Holistic care	holistic care	holistic care	holistic care	holistic care	7	case_or_format
Home mechanical ventilation	home mechanical ventilation	home mechanical ventilation	home mechanical ventilation	Home mechanical ventilation	7	kept
home monitoring	home monitoring	home monitoring	home monitoring	home monitoring	7	kept
Hospice care	hospice care	hospice care	hospice care	Hospice Care	7	case_or_format
Hospital Bed Capacity, 300 to 499	hospital bed capacity 300 to 499	hospital bed capacity 300 to 499	hospital bed capacity 300 to 499	Hospital Bed Capacity, 300 to 499	7	kept
Hospital management	hospital management	hospital management	hospital management	hospital management	7	case_or_format
Hospitalisation	hospitalisation	hospitalisation	hospitalisation	hospitalisation	7	case_or_format
hurricane	hurricane	hurricane	hurricane	hurricane	7	kept
Hydroxymethylglutaryl-CoA Reductase Inhibitors	hydroxymethylglutaryl coa reductase inhibitors	hydroxymethylglutaryl coa reductase inhibitors	hydroxymethylglutaryl coa reductase inhibitors	Hydroxymethylglutaryl-CoA Reductase Inhibitors	7	kept
hyperphosphatemia	hyperphosphatemia	hyperphosphatemia	hyperphosphatemia	hyperphosphatemia	7	kept
Hypertension, Pregnancy-Induced	hypertension pregnancy induced	hypertension pregnancy induced	hypertension pregnancy induced	Hypertension, Pregnancy-Induced	7	kept
Hypertriglyceridemia	hypertriglyceridemia	hypertriglyceridemia	hypertriglyceridemia	hypertriglyceridemia	7	case_or_format

hypochlorite sodium	hypochlorite sodium	hypochlorite sodium	hypochlorite sodium	hypochlorite sodium	7	kept
hypoproteinemia	hypoproteinemia	hypoproteinemia	hypoproteinemia	hypoproteinemia	7	kept
Hypotension, Orthostatic	hypotension orthostatic	hypotension orthostatic	hypotension orthostatic	Hypotension, Orthostatic	7	kept
Hypothalamo-Hypophyseal System	hypothalamo hypophyseal system	hypothalamo hypophyseal system	hypothalamo hypophyseal system	Hypothalamo-Hypophyseal System	7	kept
Iatrogenic withdrawal syndrome	iatrogenic withdrawal syndrome	iatrogenic withdrawal syndrome	iatrogenic withdrawal syndrome	Iatrogenic withdrawal syndrome	7	kept
iatrogenic withdrawal syndrome	iatrogenic withdrawal syndrome	iatrogenic withdrawal syndrome	iatrogenic withdrawal syndrome	Iatrogenic withdrawal syndrome	7	case_or_format
ICD-9-CM	icd 9 cm	icd 9 cm	icd 9 cm	ICD-9-CM	7	kept
Ichthyosis, Lamellar	ichthyosis lamellar	ichthyosis lamellar	ichthyosis lamellar	Ichthyosis, Lamellar	7	kept
image processing	image processing	image processing	image processing	image processing	7	kept
Image Processing, Computer-Assisted	image processing computer assisted	image processing computer assisted	image processing computer assisted	Image Processing, Computer-Assisted	7	kept
impact	impact	impact	impact	IMPACT	7	case_or_format
Incident reporting	incident reporting	incident reporting	incident reporting	Incident reporting	7	kept
incident reporting	incident reporting	incident reporting	incident reporting	Incident reporting	7	case_or_format
Indians, North American	indians north american	indians north american	indians north american	Indians, North American	7	kept
individuality	individuality	individuality	individuality	individuality	7	kept
industrial noise	industrial noise	industrial noise	industrial noise	industrial noise	7	kept

Infant Death	infant death	infant death	infant death	Infant Death	7	kept
Infant Health	infant health	infant health	infant health	Infant Health	7	kept
infections	infections	infections	infection	infection	7	singular
infliximab	infliximab	infliximab	infliximab	infliximab	7	kept
Information dissemination	information dissemination	information dissemination	information dissemination	Information Dissemination	7	case_or_format
infusion system	infusion system	infusion system	infusion system	infusion system	7	kept
INJURIES	injuries	injuries	injury	injury	7	singular
Insomnia	insomnia	insomnia	insomnia	insomnia	7	case_or_format
Integer programming	integer programming	integer programming	integer programming	Integer programming	7	kept
intelligence	intelligence	intelligence	intelligence	intelligence	7	kept
Intelligent systems	intelligent systems	intelligent systems	intelligent systems	Intelligent systems	7	kept
intention	intention	intention	intention	Intention	7	case_or_format
Intention to Treat Analysis	intention to treat analysis	intention to treat analysis	intention to treat analysis	intention to treat analysis	7	case_or_format
interdisciplinary care	interdisciplinary care	interdisciplinary care	interdisciplinary care	interdisciplinary care	7	kept
Interdisciplinary collaboration	interdisciplinary collaboration	interdisciplinary collaboration	interdisciplinary collaboration	Interdisciplinary collaboration	7	kept
interhospital cooperation	interhospital cooperation	interhospital cooperation	interhospital cooperation	interhospital cooperation	7	kept

interleukin 2	interleukin 2	interleukin 2	interleukin 2	interleukin 2	7	kept
intermittent catheterization	intermittent catheterization	intermittent catheterization	intermittent catheterization	intermittent catheterization	7	kept
Intermittent hemodialysis	intermittent hemodialysis	intermittent hemodialysis	intermittent hemodialysis	Intermittent hemodialysis	7	kept
International	international	international	international	International	7	kept
International Educational Exchange	international educational exchange	international educational exchange	international educational exchange	International Educational Exchange	7	kept
interpretative phenomenological analysis	interpretative phenomenological analysis	interpretative phenomenological analysis	interpretative phenomenological analysis	interpretative phenomenological analysis	7	kept
interruptions	interruptions	interruptions	interruption	Interruptions	7	singular
intestinal failure	intestinal failure	intestinal failure	intestinal failure	intestinal failure	7	kept
intestine resection	intestine resection	intestine resection	intestine resection	intestine resection	7	kept
Intestine, Small	intestine small	intestine small	intestine small	Intestine, Small	7	kept
intracranial hemorrhage	intracranial hemorrhage	intracranial hemorrhage	intracranial hemorrhage	Intracranial Hemorrhages	7	case_or_format
Intrahospital transport	intrahospital transport	intrahospital transport	intrahospital transport	Intrahospital transport	7	kept
intrahospital transport	intrahospital transport	intrahospital transport	intrahospital transport	Intrahospital transport	7	case_or_format
intraoperative monitoring	intraoperative monitoring	intraoperative monitoring	intraoperative monitoring	intraoperative monitoring	7	kept
intravenous anesthetic agent	intravenous anesthetic agent	intravenous anesthetic agent	intravenous anesthetic agent	intravenous anesthetic agent	7	kept
IoT	iot	iot	iot	IoT	7	kept

ISBAR	isbar	isbar	isbar	ISBAR	7	kept
Isolation	isolation	isolation	isolation	isolation	7	case_or_format
isophane insulin	isophane insulin	isophane insulin	isophane insulin	isophane insulin	7	kept
itraconazole	itraconazole	itraconazole	itraconazole	itraconazole	7	kept
Jamaica	jamaica	jamaica	jamaica	Jamaica	7	kept
jejunostomy	jejunostomy	jejunostomy	jejunostomy	Jejunostomy	7	case_or_format
Joanna Briggs Institute critical appraisal checklist	joanna briggs institute critical appraisal checklist	joanna briggs institute critical appraisal checklist	joanna briggs institute critical appraisal checklist	Joanna Briggs Institute critical appraisal checklist	7	kept
job characteristics	job characteristics	job characteristics	job characteristics	job characteristics	7	kept
jurisprudence	jurisprudence	jurisprudence	jurisprudence	jurisprudence	7	kept
Karnofsky Performance Status	karnofsky performance status	karnofsky performance status	karnofsky performance status	Karnofsky Performance Status	7	kept
Keratoconjunctivitis	keratoconjunctivitis	keratoconjunctivitis	keratoconjunctivitis	Keratoconjunctivitis	7	kept
Kernicterus	kernicterus	kernicterus	kernicterus	Kernicterus	7	kept
Knowledge level	knowledge level	knowledge level	knowledge level	Knowledge level	7	kept
Laboratories	laboratories	laboratories	laboratory	laboratory	7	singular
laminectomy	laminectomy	laminectomy	laminectomy	laminectomy	7	kept
larynx edema	larynx edema	larynx edema	larynx edema	larynx edema	7	kept

Lavandula angustifolia	lavandula angustifolia	lavandula angustifolia	lavandula angustifolia	Lavandula angustifolia	7	kept
Lean	lean	lean	lean	Lean	7	kept
Learning Curve	learning curve	learning curve	learning curve	learning curve	7	case_or_format
Legal Guardians	legal guardians	legal guardians	legal guardian	Legal Guardians	7	singular
leukapheresis	leukapheresis	leukapheresis	leukapheresis	leukapheresis	7	kept
Leukemia	leukemia	leukemia	leukemia	leukemia	7	case_or_format
leukoencephalopathy	leukoencephalopathy	leukoencephalopathy	leukoencephalopathy	leukoencephalopathy	7	kept
licence	licence	licence	licence	licence	7	kept
Life Support Systems	life support systems	life support systems	life support systems	Life Support Systems	7	kept
LIFE-SUPPORT	life support	life support	life support	LIFE-SUPPORT	7	kept
limit of detection	limit of detection	limit of detection	limit of detection	limit of detection	7	kept
Linezolid	linezolid	linezolid	linezolid	linezolid	7	case_or_format
liquid	liquid	liquid	liquid	liquid	7	kept
Lithuania	lithuania	lithuania	lithuania	Lithuania	7	kept
liver	liver	liver	liver	Liver	7	case_or_format
liver toxicity	liver toxicity	liver toxicity	liver toxicity	liver toxicity	7	kept

loperamide	loperamide	loperamide	loperamide	loperamide	7	kept
low density lipoprotein cholesterol	low density lipoprotein cholesterol	low density lipoprotein cholesterol	low density lipoprotein cholesterol	low density lipoprotein cholesterol	7	kept
Low- and middle-income countries	low and middle income countries	low and middle income countries	low and middle income country	low- and middle-income countries	7	singular
lower extremity deep vein thrombosis	lower extremity deep vein thrombosis	lower extremity deep vein thrombosis	lower extremity deep vein thrombosis	lower extremity deep vein thrombosis	7	kept
lubricating agent	lubricating agent	lubricating agent	lubricating agent	lubricating agent	7	kept
lung volume	lung volume	lung volume	lung volume	lung volume	7	kept
lung wedge pressure	lung wedge pressure	lung wedge pressure	lung wedge pressure	lung wedge pressure	7	kept
lymph node dissection	lymph node dissection	lymph node dissection	lymph node dissection	lymph node dissection	7	kept
Lymphocytes	lymphocytes	lymphocytes	lymphocyte	lymphocyte	7	singular
Magnesium	magnesium	magnesium	magnesium	magnesium	7	case_or_format
Magnesium Deficiency	magnesium deficiency	magnesium deficiency	magnesium deficiency	Magnesium Deficiency	7	kept
Maine	maine	maine	maine	Maine	7	kept
Maintenance	maintenance	maintenance	maintenance	Maintenance	7	kept
maintenance drug dose	maintenance drug dose	maintenance drug dose	maintenance drug dose	maintenance drug dose	7	kept
Malaria, Falciparum	malaria falciparum	malaria falciparum	malaria falciparum	Malaria, Falciparum	7	kept
Manpower	manpower	manpower	manpower	manpower	7	case_or_format

mass medium	mass medium	mass medium	mass medium	mass medium	7	kept
mastectomy	mastectomy	mastectomy	mastectomy	mastectomy	7	kept
mathematical analysis	mathematical analysis	mathematical analysis	mathematical analysis	mathematical analysis	7	kept
measurement precision	measurement precision	measurement precision	measurement precision	measurement precision	7	kept
Mechanical ventilator	mechanical ventilator	mechanical ventilator	mechanical ventilator	mechanical ventilator	7	case_or_format
Meconium	meconium	meconium	meconium	meconium	7	case_or_format
mediastinitis	mediastinitis	mediastinitis	mediastinitis	mediastinitis	7	kept
Mediation Analysis	mediation analysis	mediation analysis	mediation analysis	mediation analysis	7	case_or_format
Medical Devices	medical devices	medical devices	medical device	medical device	7	singular
MEDICAL ERRORS	medical errors	medical errors	medical error	Medical Errors	7	singular
Medical information systems	medical information systems	medical information systems	medical information system	medical information system	7	singular
Medical Missions	medical missions	medical missions	medical missions	Medical Missions	7	kept
medical resource shortage	medical resource shortage	medical resource shortage	medical resource shortage	medical resource shortage	7	kept
mental function	mental function	mental function	mental function	mental function	7	kept
mental function assessment	mental function assessment	mental function assessment	mental function assessment	mental function assessment	7	kept
MENTAL HEALTH	mental health	mental health	mental health	mental health	7	case_or_format

mental health center	mental health center	mental health center	mental health center	mental health center	7	kept
Mental health services	mental health services	mental health services	mental health service	mental health service	7	singular
Mesenteric Ischemia	mesenteric ischemia	mesenteric ischemia	mesenteric ischemia	Mesenteric Ischemia	7	kept
metabolic clearance rate	metabolic clearance rate	metabolic clearance rate	metabolic clearance rate	Metabolic Clearance Rate	7	case_or_format
Methicillin-resistant Staphylococcus aureus	methicillin resistant staphylococcus aureus	methicillin resistant staphylococcus aureus	methicillin resistant staphylococcus aureus	methicillin resistant Staphylococcus aureus	7	case_or_format
methicillin-resistant Staphylococcus aureus	methicillin resistant staphylococcus aureus	methicillin resistant staphylococcus aureus	methicillin resistant staphylococcus aureus	methicillin resistant Staphylococcus aureus	7	case_or_format
Methylprednisolone	methylprednisolone	methylprednisolone	methylprednisolone	methylprednisolone	7	case_or_format
Mice	mice	mice	mice	Mice	7	kept
microcirculation	microcirculation	microcirculation	microcirculation	Microcirculation	7	case_or_format
microcomputer	microcomputer	microcomputer	microcomputer	microcomputer	7	kept
Middle East respiratory syndrome	middle east respiratory syndrome	middle east respiratory syndrome	middle east respiratory syndrome	Middle East respiratory syndrome	7	kept
middle income group	middle income group	middle income group	middle income group	middle income group	7	kept
midwives	midwives	midwives	midwives	Midwives	7	case_or_format
milk production	milk production	milk production	milk production	milk production	7	kept
mirtazapine	mirtazapine	mirtazapine	mirtazapine	mirtazapine	7	kept
mitral valve	mitral valve	mitral valve	mitral valve	Mitral Valve	7	case_or_format

mitral valve surgery	mitral valve surgery	mitral valve surgery	mitral valve surgery	mitral valve surgery	7	kept
Mixed method	mixed method	mixed method	mixed method	Mixed methods	7	case_or_format
Model For End Stage Liver Disease Score	model for end stage liver disease score	model for end stage liver disease score	model for end stage liver disease score	Model For End Stage Liver Disease Score	7	kept
money	money	money	money	money	7	kept
Monte Carlo Method	monte carlo method	monte carlo method	monte carlo method	Monte Carlo Method	7	kept
Mood Disorders	mood disorders	mood disorders	mood disorder	mood disorder	7	singular
Moraxella catarrhalis	moraxella catarrhalis	moraxella catarrhalis	moraxella catarrhalis	Moraxella catarrhalis	7	kept
mortality prediction	mortality prediction	mortality prediction	mortality prediction	Mortality prediction	7	case_or_format
mortality risk score	mortality risk score	mortality risk score	mortality risk score	mortality risk score	7	kept
mouth mucosa	mouth mucosa	mouth mucosa	mouth mucosa	Mouth Mucosa	7	case_or_format
mouth ulcer	mouth ulcer	mouth ulcer	mouth ulcer	mouth ulcer	7	kept
movement	movement	movement	movement	Movement	7	case_or_format
Mucormycosis	mucormycosis	mucormycosis	mucormycosis	Mucormycosis	7	kept
Multidimensional Scale of Perceived Social Support	multidimensional scale of perceived social support	multidimensional scale of perceived social support	multidimensional scale of perceived social support	Multidimensional Scale of Perceived Social Support	7	kept
Multidrug resistance	multidrug resistance	multidrug resistance	multidrug resistance	multidrug resistance	7	case_or_format
multidrug resistant tuberculosis	multidrug resistant tuberculosis	multidrug resistant tuberculosis	multidrug resistant tuberculosis	multidrug resistant tuberculosis	7	kept

Multiple Birth Offspring	multiple birth offspring	multiple birth offspring	multiple birth offspring	Multiple Birth Offspring	7	kept
multiple sclerosis	multiple sclerosis	multiple sclerosis	multiple sclerosis	multiple sclerosis	7	kept
multivariate analysis of variance	multivariate analysis of variance	multivariate analysis of variance	multivariate analysis of variance	multivariate analysis of variance	7	kept
Munchausen Syndrome by Proxy	munchausen syndrome by proxy	munchausen syndrome by proxy	munchausen syndrome by proxy	Munchausen Syndrome by Proxy	7	kept
mupirocin	mupirocin	mupirocin	mupirocin	mupirocin	7	kept
Musculoskeletal disorders	musculoskeletal disorders	musculoskeletal disorders	musculoskeletal disorder	Musculoskeletal disorders	7	singular
musculoskeletal injury	musculoskeletal injury	musculoskeletal injury	musculoskeletal injury	musculoskeletal injury	7	kept
Muslim	muslim	muslim	muslim	Muslim	7	kept
Mutation	mutation	mutation	mutation	Mutation	7	kept
Mycoplasma pneumoniae	mycoplasma pneumoniae	mycoplasma pneumoniae	mycoplasma pneumoniae	Mycoplasma pneumoniae	7	kept
Myeloproliferative Disorders	myeloproliferative disorders	myeloproliferative disorders	myeloproliferative disorder	Myeloproliferative Disorders	7	singular
narratives	narratives	narratives	narrative	narrative	7	singular
National Academies of Science, Engineering, and Medicine, U.S	national academies of science engineering and medicine u s	national academies of science engineering and medicine u s	national academies of science engineering and medicine u s	National Academies of Science, Engineering, and Medicine, U.S	7	kept
National Institutes of Health	national institutes of health	national institutes of health	national institutes of health	National Institutes of Health	7	kept
National survey	national survey	national survey	national survey	National survey	7	kept
Natural Childbirth	natural childbirth	natural childbirth	natural childbirth	Natural Childbirth	7	kept

Near-infrared spectroscopy	near infrared spectroscopy	near infrared spectroscopy	near infrared spectroscopy	near infrared spectroscopy	7	case_or_format
neck	neck	neck	neck	neck	7	kept
Necrotizing fasciitis	necrotizing fasciitis	necrotizing fasciitis	necrotizing fasciitis	necrotizing fasciitis	7	case_or_format
NEECHAM Confusion Scale	neecham confusion scale	neecham confusion scale	neecham confusion scale	NEECHAM Confusion Scale	7	kept
Needles	needles	needles	needle	needle	7	singular
NEMS	nems	nems	nems	NEMS	7	kept
neonatal unit	neonatal unit	neonatal unit	neonatal unit	Neonatal unit	7	case_or_format
neoplasms	neoplasms	neoplasms	neoplasm	Neoplasms	7	singular
nephrolithiasis	nephrolithiasis	nephrolithiasis	nephrolithiasis	nephrolithiasis	7	kept
nervous system injury	nervous system injury	nervous system injury	nervous system injury	nervous system injury	7	kept
Neuroimaging	neuroimaging	neuroimaging	neuroimaging	neuroimaging	7	case_or_format
neuroleptic malignant syndrome	neuroleptic malignant syndrome	neuroleptic malignant syndrome	neuroleptic malignant syndrome	neuroleptic malignant syndrome	7	kept
neurologic disease assessment	neurologic disease assessment	neurologic disease assessment	neurologic disease assessment	neurologic disease assessment	7	kept
Neuromuscular Nondepolarizing Agents	neuromuscular nondepolarizing agents	neuromuscular nondepolarizing agents	neuromuscular nondepolarizing agents	Neuromuscular Nondepolarizing Agents	7	kept
neurophysiological monitoring	neurophysiological monitoring	neurophysiological monitoring	neurophysiological monitoring	Neurophysiological Monitoring	7	case_or_format
Neuroprotective Agents	neuroprotective agents	neuroprotective agents	neuroprotective agent	Neuroprotective Agents	7	singular

Neurosurgeons	neurosurgeons	neurosurgeons	neurosurgeon	neurosurgeon	7	singular
New Mexico	new mexico	new mexico	new mexico	New Mexico	7	kept
newborn assessment	newborn assessment	newborn assessment	newborn assessment	newborn assessment	7	kept
Night shift	night shift	night shift	night shift	night shift	7	case_or_format
nitrogen	nitrogen	nitrogen	nitrogen	nitrogen	7	kept
Nitroglycerin	nitroglycerin	nitroglycerin	nitroglycerin	Nitroglycerin	7	kept
Noise pollution	noise pollution	noise pollution	noise pollution	Noise pollution	7	kept
non-governmental organization	non governmental organization	non governmental organization	non governmental organization	non-governmental organization	7	kept
nonhodgkin lymphoma	nonhodgkin lymphoma	nonhodgkin lymphoma	nonhodgkin lymphoma	nonhodgkin lymphoma	7	kept
Nonpharmacological interventions	nonpharmacological interventions	nonpharmacological interventions	nonpharmacological intervention	Nonpharmacological interventions	7	singular
norepinephrine	norepinephrine	norepinephrine	norepinephrine	Norepinephrine	7	case_or_format
norfloxacin	norfloxacin	norfloxacin	norfloxacin	norfloxacin	7	kept
nosocomial pneumonia	nosocomial pneumonia	nosocomial pneumonia	nosocomial pneumonia	nosocomial pneumonia	7	kept
Nutritional risk	nutritional risk	nutritional risk	nutritional risk	Nutritional risk	7	kept
Occupations	occupations	occupations	occupation	occupation	7	singular
off pump coronary surgery	off pump coronary surgery	off pump coronary surgery	off pump coronary surgery	off pump coronary surgery	7	kept

ofloxacin	ofloxacin	ofloxacin	ofloxacin	ofloxacin	7	kept
Ointments	ointments	ointments	ointment	Ointments	7	singular
oligohydramnios	oligohydramnios	oligohydramnios	oligohydramnios	oligohydramnios	7	kept
Operating theatre	operating theatre	operating theatre	operating theatre	Operating theatre	7	kept
Ophthalmic Solutions	ophthalmic solutions	ophthalmic solutions	ophthalmic solutions	Ophthalmic Solutions	7	kept
oral contraceptive agent	oral contraceptive agent	oral contraceptive agent	oral contraceptive agent	oral contraceptive agent	7	kept
oral rehydration solution	oral rehydration solution	oral rehydration solution	oral rehydration solution	oral rehydration solution	7	kept
Organ procurement	organ procurement	organ procurement	organ procurement	Organ procurement	7	kept
ORGANIZATION	organization	organization	organization	organization	7	case_or_format
ORGANIZATIONS	organizations	organizations	organization	organization	7	singular
osmolarity	osmolarity	osmolarity	osmolarity	osmolarity	7	kept
osteoarthritis	osteoarthritis	osteoarthritis	osteoarthritis	osteoarthritis	7	kept
Ostomy	ostomy	ostomy	ostomy	ostomy	7	case_or_format
Out-of-hospital cardiac arrest	out of hospital cardiac arrest	out of hospital cardiac arrest	out of hospital cardiac arrest	out of hospital cardiac arrest	7	case_or_format
Outcomes research	outcomes research	outcomes research	outcomes research	outcomes research	7	case_or_format
ovary cancer	ovary cancer	ovary cancer	ovary cancer	ovary cancer	7	kept

Overcrowding	overcrowding	overcrowding	overcrowding	Overcrowding	7	kept
Overdose	overdose	overdose	overdose	Overdose	7	kept
Pain Clinics	pain clinics	pain clinics	pain clinics	Pain Clinics	7	kept
Palliative Medicine	palliative medicine	palliative medicine	palliative medicine	palliative medicine	7	case_or_format
pancuronium	pancuronium	pancuronium	pancuronium	pancuronium	7	kept
pandemic preparedness	pandemic preparedness	pandemic preparedness	pandemic preparedness	pandemic preparedness	7	kept
participatory management	participatory management	participatory management	participatory management	participatory management	7	kept
pastoral care	pastoral care	pastoral care	pastoral care	Pastoral Care	7	case_or_format
peak expiratory flow	peak expiratory flow	peak expiratory flow	peak expiratory flow	peak expiratory flow	7	kept
pedestrian	pedestrian	pedestrian	pedestrian	pedestrian	7	kept
Pediatric Emergency Medicine	pediatric emergency medicine	pediatric emergency medicine	pediatric emergency medicine	pediatric emergency medicine	7	case_or_format
pediatric multisystem inflammatory syndrome	pediatric multisystem inflammatory syndrome	pediatric multisystem inflammatory syndrome	pediatric multisystem inflammatory syndrome	pediatric multisystem inflammatory syndrome	7	kept
Pediatric Quality of Life Inventory	pediatric quality of life inventory	pediatric quality of life inventory	pediatric quality of life inventory	Pediatric Quality of Life Inventory	7	kept
peer support	peer support	peer support	peer support	peer support	7	kept
percussion	percussion	percussion	percussion	percussion	7	kept
Perinatal	perinatal	perinatal	perinatal	Perinatal	7	kept

perinatal morbidity	perinatal morbidity	perinatal morbidity	perinatal morbidity	perinatal morbidity	7	kept
Perioperative	perioperative	perioperative	perioperative	Perioperative	7	kept
perioperative	perioperative	perioperative	perioperative	Perioperative	7	case_or_format
Perioperative Period	perioperative period	perioperative period	perioperative period	perioperative period	7	case_or_format
Peripheral Arterial Disease	peripheral arterial disease	peripheral arterial disease	peripheral arterial disease	Peripheral Arterial Disease	7	kept
peripheral neuropathy	peripheral neuropathy	peripheral neuropathy	peripheral neuropathy	peripheral neuropathy	7	kept
peripheral vein	peripheral vein	peripheral vein	peripheral vein	peripheral vein	7	kept
periprosthetic fracture	periprosthetic fracture	periprosthetic fracture	periprosthetic fracture	periprosthetic fracture	7	kept
personal digital assistant	personal digital assistant	personal digital assistant	personal digital assistant	personal digital assistant	7	kept
Personnel staffing and scheduling	personnel staffing and scheduling	personnel staffing and scheduling	personnel staffing and scheduling	Personnel Staffing and Scheduling	7	case_or_format
personnel turnover	personnel turnover	personnel turnover	personnel turnover	Personnel Turnover	7	case_or_format
pharyngitis	pharyngitis	pharyngitis	pharyngitis	pharyngitis	7	kept
phosphate	phosphate	phosphate	phosphate	phosphate	7	kept
phosphate blood level	phosphate blood level	phosphate blood level	phosphate blood level	phosphate blood level	7	kept
Physical Exertion	physical exertion	physical exertion	physical exertion	Physical Exertion	7	kept
Physical Functional Performance	physical functional performance	physical functional performance	physical functional performance	Physical Functional Performance	7	kept

physical stimulation	physical stimulation	physical stimulation	physical stimulation	Physical Stimulation	7	case_or_format
PHYSICIAN	physician	physician	physician	physician	7	case_or_format
physiotherapist attitude	physiotherapist attitude	physiotherapist attitude	physiotherapist attitude	physiotherapist attitude	7	kept
Placenta Accreta	placenta accreta	placenta accreta	placenta accreta	Placenta Accreta	7	kept
Platelet Count	platelet count	platelet count	platelet count	platelet count	7	case_or_format
pleura fluid	pleura fluid	pleura fluid	pleura fluid	pleura fluid	7	kept
Pleural Effusion	pleural effusion	pleural effusion	pleural effusion	Pleural Effusion	7	kept
pneumomediastinum	pneumomediastinum	pneumomediastinum	pneumomediastinum	pneumomediastinum	7	kept
Pneumonia Severity Index	pneumonia severity index	pneumonia severity index	pneumonia severity index	Pneumonia Severity Index	7	kept
posaconazole	posaconazole	posaconazole	posaconazole	posaconazole	7	kept
Positioning	positioning	positioning	positioning	Positioning	7	kept
positive feedback	positive feedback	positive feedback	positive feedback	positive feedback	7	kept
Post-acute care	post acute care	post acute care	post acute care	Post-acute care	7	kept
Post-traumatic	post traumatic	post traumatic	post traumatic	Post-traumatic	7	kept
Post-traumatic stress	post traumatic stress	post traumatic stress	post traumatic stress	Post-traumatic stress	7	kept
Postpartum depression	postpartum depression	postpartum depression	postpartum depression	Postpartum depression	7	kept

postpartum depression	postpartum depression	postpartum depression	postpartum depression	Postpartum depression	7	case_or_format
POSTTRAUMATIC STRESS	posttraumatic stress	posttraumatic stress	posttraumatic stress	POSTTRAUMATIC STRESS	7	kept
practice gap	practice gap	practice gap	practice gap	practice gap	7	kept
preceptorship	preceptorship	preceptorship	preceptorship	Preceptorship	7	case_or_format
Pregnancy Trimester, Third	pregnancy trimester third	pregnancy trimester third	pregnancy trimester third	Pregnancy Trimester, Third	7	kept
premature fetus membrane rupture	premature fetus membrane rupture	premature fetus membrane rupture	premature fetus membrane rupture	premature fetus membrane rupture	7	kept
Prescribing	prescribing	prescribing	prescribing	Prescribing	7	kept
Pressure injury prevention	pressure injury prevention	pressure injury prevention	pressure injury prevention	pressure injury prevention	7	case_or_format
primigravida	primigravida	primigravida	primigravida	primigravida	7	kept
Process evaluation	process evaluation	process evaluation	process evaluation	Process evaluation	7	kept
product design	product design	product design	product design	product design	7	kept
professional delegation	professional delegation	professional delegation	professional delegation	professional delegation	7	kept
professional role	professional role	professional role	professional role	Professional Role	7	case_or_format
professional-family relations	professional family relations	professional family relations	professional family relations	Professional-Family Relations	7	case_or_format
program sustainability	program sustainability	program sustainability	program sustainability	program sustainability	7	kept
Prone positioning	prone positioning	prone positioning	prone positioning	prone positioning	7	case_or_format

Prosthesis Implantation	prosthesis implantation	prosthesis implantation	prosthesis implantation	Prosthesis Implantation	7	kept
Protective Factors	protective factors	protective factors	protective factors	Protective Factors	7	kept
Protestantism	protestantism	protestantism	protestantism	Protestantism	7	kept
protocols	protocols	protocols	protocol	protocol	7	singular
Psychological adaptation	psychological adaptation	psychological adaptation	psychological adaptation	Psychological adaptation	7	kept
psychological resilience scale	psychological resilience scale	psychological resilience scale	psychological resilience scale	psychological resilience scale	7	kept
psychological theory	psychological theory	psychological theory	psychological theory	Psychological Theory	7	case_or_format
psychometric testing	psychometric testing	psychometric testing	psychometric testing	psychometric testing	7	kept
Psychomotor agitation	psychomotor agitation	psychomotor agitation	psychomotor agitation	Psychomotor Agitation	7	case_or_format
psychomotor disorder	psychomotor disorder	psychomotor disorder	psychomotor disorder	psychomotor disorder	7	kept
Psychosocial	psychosocial	psychosocial	psychosocial	Psychosocial	7	kept
psychotherapist	psychotherapist	psychotherapist	psychotherapist	psychotherapist	7	kept
public health problem	public health problem	public health problem	public health problem	public health problem	7	kept
publication bias	publication bias	publication bias	publication bias	publication bias	7	kept
Publications	publications	publications	publication	publication	7	singular
Pulmonary Emphysema	pulmonary emphysema	pulmonary emphysema	pulmonary emphysema	Pulmonary Emphysema	7	kept

Pulmonary Ventilation	pulmonary ventilation	pulmonary ventilation	pulmonary ventilation	Pulmonary Ventilation	7	kept
quality of sleep	quality of sleep	quality of sleep	quality of sleep	quality of sleep	7	kept
quality of working life	quality of working life	quality of working life	quality of working life	quality of working life	7	kept
Ramsay Sedation Scale	ramsay sedation scale	ramsay sedation scale	ramsay sedation scale	Ramsay Sedation Scale	7	kept
Randomized Controlled Trials	randomized controlled trials	randomized controlled trials	randomized controlled trial	randomized controlled trial	7	singular
Rapid response	rapid response	rapid response	rapid response	Rapid response	7	kept
Rapid Response System	rapid response system	rapid response system	rapid response system	Rapid response system	7	case_or_format
Rasch analysis	rasch analysis	rasch analysis	rasch analysis	Rasch analysis	7	kept
reactive oxygen metabolite	reactive oxygen metabolite	reactive oxygen metabolite	reactive oxygen metabolite	reactive oxygen metabolite	7	kept
Real time	real time	real time	real time	Real time	7	kept
RECOMMENDATIONS	recommendations	recommendations	recommendation	Recommendations	7	singular
Recruitment	recruitment	recruitment	recruitment	Recruitment	7	kept
referral	referral	referral	referral	referral	7	kept
reflexology	reflexology	reflexology	reflexology	reflexology	7	kept
reinfection	reinfection	reinfection	reinfection	reinfection	7	kept
relationship	relationship	relationship	relationship	relationships	7	case_or_format

Relief Work	relief work	relief work	relief work	Relief Work	7	kept
remote monitoring	remote monitoring	remote monitoring	remote monitoring	Remote monitoring	7	case_or_format
Remote Sensing Technology	remote sensing technology	remote sensing technology	remote sensing technology	Remote Sensing Technology	7	kept
repeat procedure	repeat procedure	repeat procedure	repeat procedure	repeat procedure	7	kept
Repositioning	repositioning	repositioning	repositioning	Repositioning	7	kept
Research Report	research report	research report	research report	Research Report	7	kept
Research Subjects	research subjects	research subjects	research subject	Research Subjects	7	singular
Resource-limited settings	resource limited settings	resource limited settings	resource limited setting	resource limited setting	7	singular
respiratory insufficiency	respiratory insufficiency	respiratory insufficiency	respiratory insufficiency	Respiratory Insufficiency	7	case_or_format
Respiratory rate	respiratory rate	respiratory rate	respiratory rate	Respiratory Rate	7	case_or_format
revascularization	revascularization	revascularization	revascularization	revascularization	7	kept
Rhabdomyolysis	rhabdomyolysis	rhabdomyolysis	rhabdomyolysis	rhabdomyolysis	7	case_or_format
Rhode Island	rhode island	rhode island	rhode island	Rhode Island	7	kept
Richmond Agitation-Sedation Scale	richmond agitation sedation scale	richmond agitation sedation scale	richmond agitation sedation scale	Richmond Agitation Sedation Scale	7	case_or_format
Risk prediction model	risk prediction model	risk prediction model	risk prediction model	Risk prediction model	7	kept
risk prediction model	risk prediction model	risk prediction model	risk prediction model	Risk prediction model	7	case_or_format

RNA 16S	rna 16s	rna 16s	rna 16s	RNA 16S	7	kept
Roy Adaptation Model	roy adaptation model	roy adaptation model	roy adaptation model	Roy Adaptation Model	7	kept
rural hospital	rural hospital	rural hospital	rural hospital	rural hospital	7	kept
Sacrum	sacrum	sacrum	sacrum	sacrum	7	case_or_format
salary and fringe benefit	salary and fringe benefit	salary and fringe benefit	salary and fringe benefit	salary and fringe benefit	7	kept
saliva	saliva	saliva	saliva	Saliva	7	case_or_format
SARS	sars	sars	sars	SARS	7	kept
SARS-CoV-2 Delta	sars cov 2 delta	sars cov 2 delta	sars cov 2 delta	SARS-CoV-2 Delta	7	kept
scale up	scale up	scale up	scale up	scale up	7	kept
Scales	scales	scales	scale	SCALE	7	singular
science	science	science	science	Science	7	case_or_format
SEER Program	seer program	seer program	seer program	SEER Program	7	kept
Self-management	self management	self management	self management	Self-Management	7	case_or_format
Sensitivity	sensitivity	sensitivity	sensitivity	SENSITIVITY	7	case_or_format
SEPTIC SHOCK	septic shock	septic shock	septic shock	septic shock	7	case_or_format
Seroprevalence	seroprevalence	seroprevalence	seroprevalence	seroprevalence	7	case_or_format

Severe sepsis	severe sepsis	severe sepsis	severe sepsis	severe sepsis	7	case_or_format
sexual dysfunction	sexual dysfunction	sexual dysfunction	sexual dysfunction	sexual dysfunction	7	kept
Shaken Baby Syndrome	shaken baby syndrome	shaken baby syndrome	shaken baby syndrome	Shaken Baby Syndrome	7	kept
Ships	ships	ships	ship	Ships	7	singular
singing	singing	singing	singing	singing	7	kept
skin graft	skin graft	skin graft	skin graft	skin graft	7	kept
skull fracture	skull fracture	skull fracture	skull fracture	skull fracture	7	kept
Sleep Disorders	sleep disorders	sleep disorders	sleep disorder	sleep disorder	7	singular
sleep disorders	sleep disorders	sleep disorders	sleep disorder	sleep disorder	7	singular
sleep disturbance	sleep disturbance	sleep disturbance	sleep disturbance	Sleep disturbance	7	case_or_format
sleep efficiency	sleep efficiency	sleep efficiency	sleep efficiency	sleep efficiency	7	kept
sleep latency	sleep latency	sleep latency	sleep latency	sleep latency	7	kept
sleep medicine	sleep medicine	sleep medicine	sleep medicine	sleep medicine	7	kept
social adaptation	social adaptation	social adaptation	social adaptation	social adaptation	7	kept
social network analysis	social network analysis	social network analysis	social network analysis	Social network analysis	7	case_or_format
social norm	social norm	social norm	social norm	social norm	7	kept

social stress	social stress	social stress	social stress	social stress	7	kept
society	society	society	society	SOCIETY	7	case_or_format
Sodium Bicarbonate	sodium bicarbonate	sodium bicarbonate	sodium bicarbonate	Sodium Bicarbonate	7	kept
sodium dihydrogen phosphate	sodium dihydrogen phosphate	sodium dihydrogen phosphate	sodium dihydrogen phosphate	sodium dihydrogen phosphate	7	kept
Specificity	specificity	specificity	specificity	specificity	7	case_or_format
Spinal Cord Neoplasms	spinal cord neoplasms	spinal cord neoplasms	spinal cord neoplasms	Spinal Cord Neoplasms	7	kept
spinal muscular atrophy	spinal muscular atrophy	spinal muscular atrophy	spinal muscular atrophy	spinal muscular atrophy	7	kept
Spine	spine	spine	spine	Spine	7	kept
spine fusion	spine fusion	spine fusion	spine fusion	spine fusion	7	kept
standard operating procedure	standard operating procedure	standard operating procedure	standard operating procedure	standard operating procedure	7	kept
standardized mortality ratio	standardized mortality ratio	standardized mortality ratio	standardized mortality ratio	standardized mortality ratio	7	kept
stent	stent	stent	stent	Stents	7	case_or_format
stereotypy	stereotypy	stereotypy	stereotypy	stereotypy	7	kept
Steroids	steroids	steroids	steroid	steroid	7	singular
stochastic model	stochastic model	stochastic model	stochastic model	stochastic model	7	kept
Stochastic Processes	stochastic processes	stochastic processes	stochastic processes	Stochastic Processes	7	kept

stomach cancer	stomach cancer	stomach cancer	stomach cancer	stomach cancer	7	kept
storage	storage	storage	storage	storage	7	kept
strategies	strategies	strategies	strategy	STRATEGIES	7	singular
Streptococcus pneumonia	streptococcus pneumonia	streptococcus pneumonia	streptococcus pneumonia	Streptococcus pneumonia	7	kept
Student	student	student	student	Students	7	case_or_format
Students, Health Occupations	students health occupations	students health occupations	students health occupations	Students, Health Occupations	7	kept
Suctioning	suctioning	suctioning	suctioning	Suctioning	7	kept
sudden infant death syndrome	sudden infant death syndrome	sudden infant death syndrome	sudden infant death syndrome	sudden infant death syndrome	7	kept
SupplMeshName: Chinese people, UI: C000724312, Type:	supplmeshname chinese people ui c000724312 type population	supplmeshname chinese people ui c000724312 type population	supplmeshname chinese people ui c000724312 type population	SupplMeshName: Chinese people, UI: C000724312, Type:	7	kept
supportive care	supportive care	supportive care	supportive care	supportive care	7	kept
supportive care need	supportive care need	supportive care need	supportive care need	supportive care need	7	kept
Surge capacity	surge capacity	surge capacity	surge capacity	surge capacity	7	case_or_format
Surgical	surgical	surgical	surgical	Surgical	7	kept
surgical	surgical	surgical	surgical	Surgical	7	case_or_format
surgical wound	surgical wound	surgical wound	surgical wound	surgical wound	7	kept
Surgicenters	surgicenters	surgicenters	surgicenters	Surgicenters	7	kept

surrogate decision-making	surrogate decision making	surrogate decision making	surrogate decision making	surrogate decision-making	7	kept
Symptom management	symptom management	symptom management	symptom management	symptom management	7	case_or_format
syphilis	syphilis	syphilis	syphilis	syphilis	7	kept
systemic disease	systemic disease	systemic disease	systemic disease	systemic disease	7	kept
Systems analysis	systems analysis	systems analysis	systems analysis	Systems Analysis	7	case_or_format
Systolic blood pressure	systolic blood pressure	systolic blood pressure	systolic blood pressure	systolic blood pressure	7	case_or_format
T-Lymphocytes	t lymphocytes	t lymphocytes	t lymphocyte	T-Lymphocytes	7	singular
T2 weighted imaging	t2 weighted imaging	t2 weighted imaging	t2 weighted imaging	T2 weighted imaging	7	kept
tablet	tablet	tablet	tablet	tablet	7	kept
Targeted temperature management	targeted temperature management	targeted temperature management	targeted temperature management	Targeted temperature management	7	kept
taste disorder	taste disorder	taste disorder	taste disorder	taste disorder	7	kept
television	television	television	television	television	7	kept
temazepam	temazepam	temazepam	temazepam	temazepam	7	kept
TERM	term	term	term	TERM	7	kept
Thanatology	thanatology	thanatology	thanatology	Thanatology	7	kept
Theory of Planned Behavior	theory of planned behavior	theory of planned behavior	theory of planned behavior	Theory of Planned Behavior	7	kept

Theory of planned behavior	theory of planned behavior	theory of planned behavior	theory of planned behavior	Theory of Planned Behavior	7	case_or_format
thermometer	thermometer	thermometer	thermometer	Thermometers	7	case_or_format
throat swab	throat swab	throat swab	throat swab	throat swab	7	kept
Time management	time management	time management	time management	Time Management	7	case_or_format
timeliness	timeliness	timeliness	timeliness	timeliness	7	kept
Tissue Distribution	tissue distribution	tissue distribution	tissue distribution	Tissue Distribution	7	kept
titrimetry	titrimetry	titrimetry	titrimetry	titrimetry	7	kept
tobacco dependence	tobacco dependence	tobacco dependence	tobacco dependence	tobacco dependence	7	kept
tobacco use	tobacco use	tobacco use	tobacco use	tobacco use	7	kept
Torsades de Pointes	torsades de pointes	torsades de pointes	torsades de pointes	Torsades de Pointes	7	kept
toxicity	toxicity	toxicity	toxicity	toxicity	7	kept
Tracheoesophageal Fistula	tracheoesophageal fistula	tracheoesophageal fistula	tracheoesophageal fistula	tracheoesophageal fistula	7	case_or_format
Transducers	transducers	transducers	transducer	Transducers	7	singular
Transducers, Pressure	transducers pressure	transducers pressure	transducers pressure	Transducers, Pressure	7	kept
transferrin	transferrin	transferrin	transferrin	transferrin	7	kept
Transitions of care	transitions of care	transitions of care	transitions of care	transitions of care	7	case_or_format

Translational Medical Research	translational medical research	translational medical research	translational medical research	Translational Medical Research	7	kept
Transplantation Conditioning	transplantation conditioning	transplantation conditioning	transplantation conditioning	Transplantation Conditioning	7	kept
TRENDS	trends	trends	trends	trends	7	case_or_format
Triglycerides	triglycerides	triglycerides	triglycerides	Triglycerides	7	kept
Trisomy	trisomy	trisomy	trisomy	Trisomy	7	kept
tumor volume	tumor volume	tumor volume	tumor volume	tumor volume	7	kept
turnover time	turnover time	turnover time	turnover time	turnover time	7	kept
ULCER PREVENTION	ulcer prevention	ulcer prevention	ulcer prevention	ULCER PREVENTION	7	kept
umbilical vein	umbilical vein	umbilical vein	umbilical vein	Umbilical Veins	7	case_or_format
United Nations	united nations	united nations	united nations	United Nations	7	kept
urban population	urban population	urban population	urban population	Urban Population	7	case_or_format
urethral catheterization	urethral catheterization	urethral catheterization	urethral catheterization	urethral catheterization	7	kept
User embracement	user embracement	user embracement	user embracement	User embracement	7	kept
User-centered design	user centered design	user centered design	user centered design	user-centered design	7	case_or_format
Uterine Neoplasms	uterine neoplasms	uterine neoplasms	uterine neoplasms	Uterine Neoplasms	7	kept
uterus contraction	uterus contraction	uterus contraction	uterus contraction	uterus contraction	7	kept

uterus rupture	uterus rupture	uterus rupture	uterus rupture	uterus rupture	7	kept
vagina bleeding	vagina bleeding	vagina bleeding	vagina bleeding	vagina bleeding	7	kept
Validation Studies	validation studies	validation studies	validation study	validation study	7	singular
Valproic Acid	valproic acid	valproic acid	valproic acid	valproic acid	7	case_or_format
Varicella zoster virus	varicella zoster virus	varicella zoster virus	varicella zoster virus	Varicella zoster virus	7	kept
Vascular Resistance	vascular resistance	vascular resistance	vascular resistance	vascular resistance	7	case_or_format
Vascular System Injuries	vascular system injuries	vascular system injuries	vascular system injuries	Vascular System Injuries	7	kept
venous blood	venous blood	venous blood	venous blood	venous blood	7	kept
Ventilator bundle	ventilator bundle	ventilator bundle	ventilator bundle	Ventilator bundle	7	kept
ventilator-associated	ventilator associated	ventilator associated	ventilator associated	Ventilator-associated	7	case_or_format
veterans health	veterans health	veterans health	veterans health	veterans health	7	kept
video recording	video recording	video recording	video recording	Video Recording	7	case_or_format
virulence factor	virulence factor	virulence factor	virulence factor	virulence factor	7	kept
virus replication	virus replication	virus replication	virus replication	virus replication	7	kept
visual stimulation	visual stimulation	visual stimulation	visual stimulation	visual stimulation	7	kept
vocal cord paralysis	vocal cord paralysis	vocal cord paralysis	vocal cord paralysis	vocal cord paralysis	7	kept

walking speed	walking speed	walking speed	walking speed	walking speed	7	kept
waste	waste	waste	waste	waste	7	kept
white matter	white matter	white matter	white matter	white matter	7	kept
White noise	white noise	white noise	white noise	white noise	7	case_or_format
wife	wife	wife	wife	wife	7	kept
Wireless sensor networks	wireless sensor networks	wireless sensor networks	wireless sensor network	Wireless sensor networks	7	singular
Withdrawal of treatment	withdrawal of treatment	withdrawal of treatment	withdrawal of treatment	Withdrawal of treatment	7	kept
Withholding	withholding	withholding	withholding	Withholding	7	kept
work of breathing	work of breathing	work of breathing	work of breathing	Work of Breathing	7	case_or_format
Wound	wound	wound	wound	wound	7	case_or_format
written communication	written communication	written communication	written communication	written communication	7	kept
zanamivir	zanamivir	zanamivir	zanamivir	zanamivir	7	kept
Abbreviations	abbreviations	abbreviations	abbreviations	Abbreviations	6	kept
abdominal abscess	abdominal abscess	abdominal abscess	abdominal abscess	abdominal abscess	6	kept
abdominal pressure	abdominal pressure	abdominal pressure	abdominal pressure	abdominal pressure	6	kept
Abscess	abscess	abscess	abscess	abscess	6	case_or_format

absolute neutrophil count	absolute neutrophil count	absolute neutrophil count	absolute neutrophil count	absolute neutrophil count	6	kept
Accident Prevention	accident prevention	accident prevention	accident prevention	accident prevention	6	case_or_format
accidental injury	accidental injury	accidental injury	accidental injury	accidental injury	6	kept
acidemia	acidemia	acidemia	acidemia	acidemia	6	kept
Acoustic environment	acoustic environment	acoustic environment	acoustic environment	Acoustic environment	6	kept
Acoustic variables control	acoustic variables control	acoustic variables control	acoustic variables control	Acoustic variables control	6	kept
acupressure	acupressure	acupressure	acupressure	Acupressure	6	case_or_format
acute care hospital	acute care hospital	acute care hospital	acute care hospital	acute care hospital	6	kept
Adenoviridae	adenoviridae	adenoviridae	adenoviridae	Adenoviridae	6	kept
Administration, Topical	administration topical	administration topical	administration topical	Administration, Topical	6	kept
administrative health data	administrative health data	administrative health data	administrative health data	administrative health data	6	kept
Adolescent Behavior	adolescent behavior	adolescent behavior	adolescent behavior	Adolescent Behavior	6	kept
Advanced cardiac life support	advanced cardiac life support	advanced cardiac life support	advanced cardiac life support	Advanced Cardiac Life Support	6	case_or_format
Adverse drug reactions	adverse drug reactions	adverse drug reactions	adverse drug reaction	adverse drug reaction	6	singular
Adverse outcomes	adverse outcomes	adverse outcomes	adverse outcome	adverse outcome	6	singular
aerobic capacity	aerobic capacity	aerobic capacity	aerobic capacity	aerobic capacity	6	kept

Afferent limb failure	afferent limb failure	afferent limb failure	afferent limb failure	Afferent limb failure	6	kept
Age of Onset	age of onset	age of onset	age of onset	Age of Onset	6	kept
AGITATION	agitation	agitation	agitation	agitation	6	case_or_format
air pollution	air pollution	air pollution	air pollution	air pollution	6	kept
aircraft accident	aircraft accident	aircraft accident	aircraft accident	aircraft accident	6	kept
airflow	airflow	airflow	airflow	airflow	6	kept
airplane pilot	airplane pilot	airplane pilot	airplane pilot	airplane pilot	6	kept
airway emergency	airway emergency	airway emergency	airway emergency	airway emergency	6	kept
airway extubation	airway extubation	airway extubation	airway extubation	Airway Extubation	6	case_or_format
Airway obstruction	airway obstruction	airway obstruction	airway obstruction	airway obstruction	6	case_or_format
Alarm	alarm	alarm	alarm	Alarms	6	case_or_format
Alcohol hand rub	alcohol hand rub	alcohol hand rub	alcohol hand rub	Alcohol hand rub	6	kept
Allergens	allergens	allergens	allergen	Allergens	6	singular
Allied health	allied health	allied health	allied health	Allied health	6	kept
allogeneic hematopoietic stem cell transplantation	allogeneic hematopoietic stem cell transplantation	allogeneic hematopoietic stem cell transplantation	allogeneic hematopoietic stem cell transplantation	allogeneic hematopoietic stem cell transplantation	6	kept
alpha tocopherol	alpha tocopherol	alpha tocopherol	alpha tocopherol	alpha tocopherol	6	kept

Aluminum	aluminum	aluminum	aluminum	Aluminum	6	kept
American Spinal Injury Association impairment scale	american spinal injury association impairment scale	american spinal injury association impairment scale	american spinal injury association impairment scale	American Spinal Injury Association impairment scale	6	kept
aminocaproic acid	aminocaproic acid	aminocaproic acid	aminocaproic acid	aminocaproic acid	6	kept
aminopenicillin	aminopenicillin	aminopenicillin	aminopenicillin	aminopenicillin	6	kept
Amphetamine-Related Disorders	amphetamine related disorders	amphetamine related disorders	amphetamine related disorders	Amphetamine-Related Disorders	6	kept
Analgesia, Obstetrical	analgesia obstetrical	analgesia obstetrical	analgesia obstetrical	Analgesia, Obstetrical	6	kept
anatomic landmark	anatomic landmark	anatomic landmark	anatomic landmark	anatomic landmark	6	kept
Anecdotes	anecdotes	anecdotes	anecdotes	Anecdotes	6	kept
anesthesia complication	anesthesia complication	anesthesia complication	anesthesia complication	anesthesia complication	6	kept
Anesthesia Department, Hospital	anesthesia department hospital	anesthesia department hospital	anesthesia department hospital	Anesthesia Department, Hospital	6	kept
animal care	animal care	animal care	animal care	animal care	6	kept
Anniversaries and Special Events	anniversaries and special events	anniversaries and special events	anniversaries and special events	Anniversaries and Special Events	6	kept
antibiotic	antibiotic	antibiotic	antibiotic	Antibiotics	6	case_or_format
anticipation	anticipation	anticipation	anticipation	anticipation	6	kept
Antineoplastic Combined Chemotherapy Protocols	antineoplastic combined chemotherapy protocols	antineoplastic combined chemotherapy protocols	antineoplastic combined chemotherapy protocols	Antineoplastic Combined Chemotherapy Protocols	6	kept
Anus, Imperforate	anus imperforate	anus imperforate	anus imperforate	Anus, Imperforate	6	kept

aorta stenosis	aorta stenosis	aorta stenosis	aorta stenosis	aorta stenosis	6	kept
aortic rupture	aortic rupture	aortic rupture	aortic rupture	Aortic Rupture	6	case_or_format
aortic surgery	aortic surgery	aortic surgery	aortic surgery	aortic surgery	6	kept
Aortic Valve Insufficiency	aortic valve insufficiency	aortic valve insufficiency	aortic valve insufficiency	Aortic Valve Insufficiency	6	kept
appetite	appetite	appetite	appetite	appetite	6	kept
Application effect	application effect	application effect	application effect	Application effect	6	kept
Arduino	arduino	arduino	arduino	Arduino	6	kept
argatroban	argatroban	argatroban	argatroban	argatroban	6	kept
arginine	arginine	arginine	arginine	arginine	6	kept
Armenia	armenia	armenia	armenia	Armenia	6	kept
Arterial blood pressure	arterial blood pressure	arterial blood pressure	arterial blood pressure	Arterial blood pressure	6	kept
Arterial catheter	arterial catheter	arterial catheter	arterial catheter	Arterial catheter	6	kept
arterial embolization	arterial embolization	arterial embolization	arterial embolization	arterial embolization	6	kept
artery occlusion	artery occlusion	artery occlusion	artery occlusion	artery occlusion	6	kept
arthroplasty	arthroplasty	arthroplasty	arthroplasty	arthroplasty	6	kept
Asian continental ancestry group	asian continental ancestry group	asian continental ancestry group	asian continental ancestry group	Asian Continental Ancestry Group	6	case_or_format

Asphyxia	asphyxia	asphyxia	asphyxia	asphyxia	6	case_or_format
Aspirin	aspirin	aspirin	aspirin	Aspirin	6	kept
assertiveness	assertiveness	assertiveness	assertiveness	Assertiveness	6	case_or_format
assisted suicide	assisted suicide	assisted suicide	assisted suicide	assisted suicide	6	kept
Associated factors	associated factors	associated factors	associated factor	Associated factors	6	singular
associated factors	associated factors	associated factors	associated factor	Associated factors	6	singular
asthenia	asthenia	asthenia	asthenia	asthenia	6	kept
atherosclerosis	atherosclerosis	atherosclerosis	atherosclerosis	atherosclerosis	6	kept
atopic dermatitis	atopic dermatitis	atopic dermatitis	atopic dermatitis	atopic dermatitis	6	kept
atopy	atopy	atopy	atopy	atopy	6	kept
Auditory Fatigue	auditory fatigue	auditory fatigue	auditory fatigue	Auditory Fatigue	6	kept
authority	authority	authority	authority	authority	6	kept
Authorship	authorship	authorship	authorship	Authorship	6	kept
autonomic dysfunction	autonomic dysfunction	autonomic dysfunction	autonomic dysfunction	autonomic dysfunction	6	kept
Avoidance Learning	avoidance learning	avoidance learning	avoidance learning	Avoidance Learning	6	kept
axilla temperature	axilla temperature	axilla temperature	axilla temperature	axilla temperature	6	kept

azathioprine	azathioprine	azathioprine	azathioprine	azathioprine	6	kept
back	back	back	back	back	6	kept
Back Injuries	back injuries	back injuries	back injury	Back Injuries	6	singular
Bacteroides fragilis	bacteroides fragilis	bacteroides fragilis	bacteroides fragilis	Bacteroides fragilis	6	kept
Bahrain	bahrain	bahrain	bahrain	Bahrain	6	kept
Barbiturates	barbiturates	barbiturates	barbiturates	Barbiturates	6	kept
Bathing	bathing	bathing	bathing	Bathing	6	kept
Beck Anxiety Inventory	beck anxiety inventory	beck anxiety inventory	beck anxiety inventory	Beck Anxiety Inventory	6	kept
Bed Conversion	bed conversion	bed conversion	bed conversion	Bed Conversion	6	kept
Bed occupancy	bed occupancy	bed occupancy	bed occupancy	Bed Occupancy	6	case_or_format
Bedridden Persons	bedridden persons	bedridden persons	bedridden persons	Bedridden Persons	6	kept
behavioral observation	behavioral observation	behavioral observation	behavioral observation	behavioral observation	6	kept
behavioral pain scale	behavioral pain scale	behavioral pain scale	behavioral pain scale	Behavioral Pain Scale	6	case_or_format
Beneficence	beneficence	beneficence	beneficence	beneficence	6	case_or_format
biocompatibility	biocompatibility	biocompatibility	biocompatibility	biocompatibility	6	kept
biomarkers	biomarkers	biomarkers	biomarker	Biomarkers	6	singular

Birth	birth	birth	birth	birth	6	case_or_format
bisphosphonic acid derivative	bisphosphonic acid derivative	bisphosphonic acid derivative	bisphosphonic acid derivative	bisphosphonic acid derivative	6	kept
Blood Banks	blood banks	blood banks	blood bank	blood bank	6	singular
blood chemistry	blood chemistry	blood chemistry	blood chemistry	blood chemistry	6	kept
blood clot	blood clot	blood clot	blood clot	blood clot	6	kept
Blood culture	blood culture	blood culture	blood culture	blood culture	6	case_or_format
blood donor	blood donor	blood donor	blood donor	blood donor	6	kept
Bloodstream infections	bloodstream infections	bloodstream infections	bloodstream infection	bloodstream infection	6	singular
BMI	bmi	bmi	bmi	BMI	6	kept
Body Image	body image	body image	body image	Body Image	6	kept
body temperature measurement	body temperature measurement	body temperature measurement	body temperature measurement	body temperature measurement	6	kept
body temperature monitoring	body temperature monitoring	body temperature monitoring	body temperature monitoring	body temperature monitoring	6	kept
bonding	bonding	bonding	bonding	Bonding	6	case_or_format
Botswana	botswana	botswana	botswana	Botswana	6	kept
BPS	bps	bps	bps	BPS	6	kept
Braden score	braden score	braden score	braden score	Braden score	6	kept

brain abscess	brain abscess	brain abscess	brain abscess	brain abscess	6	kept
brain artery aneurysm	brain artery aneurysm	brain artery aneurysm	brain artery aneurysm	brain artery aneurysm	6	kept
brain injuries	brain injuries	brain injuries	brain injury	Brain Injuries	6	singular
brain metastasis	brain metastasis	brain metastasis	brain metastasis	brain metastasis	6	kept
brain radiography	brain radiography	brain radiography	brain radiography	brain radiography	6	kept
BRAIN-INJURY	brain injury	brain injury	brain injury	Brain Injuries	6	case_or_format
breaking bad news	breaking bad news	breaking bad news	breaking bad news	breaking bad news	6	kept
breakthrough infection	breakthrough infection	breakthrough infection	breakthrough infection	breakthrough infection	6	kept
Brief Psychiatric Rating Scale	brief psychiatric rating scale	brief psychiatric rating scale	brief psychiatric rating scale	Brief Psychiatric Rating Scale	6	kept
bronchopleural fistula	bronchopleural fistula	bronchopleural fistula	bronchopleural fistula	bronchopleural fistula	6	kept
bronchopneumonia	bronchopneumonia	bronchopneumonia	bronchopneumonia	bronchopneumonia	6	kept
broth dilution	broth dilution	broth dilution	broth dilution	broth dilution	6	kept
Bundles	bundles	bundles	bundle	BUNDLE	6	singular
Buprenorphine	buprenorphine	buprenorphine	buprenorphine	buprenorphine	6	case_or_format
Burkholderia cepacia	burkholderia cepacia	burkholderia cepacia	burkholderia cepacia	Burkholderia cepacia	6	kept
buspirone	buspirone	buspirone	buspirone	buspirone	6	kept

CABG	cabg	cabg	cabg	CABG	6	kept
Caffeine	caffeine	caffeine	caffeine	caffeine	6	case_or_format
caffeine citrate	caffeine citrate	caffeine citrate	caffeine citrate	caffeine citrate	6	kept
calcineurin inhibitor	calcineurin inhibitor	calcineurin inhibitor	calcineurin inhibitor	calcineurin inhibitor	6	kept
calcium ion	calcium ion	calcium ion	calcium ion	calcium ion	6	kept
cancer adjuvant therapy	cancer adjuvant therapy	cancer adjuvant therapy	cancer adjuvant therapy	cancer adjuvant therapy	6	kept
cancer growth	cancer growth	cancer growth	cancer growth	cancer growth	6	kept
CAPD	capd	capd	capd	CAPD	6	kept
capillary	capillary	capillary	capillary	Capillaries	6	case_or_format
carbapenem resistant Acinetobacter baumannii	carbapenem resistant acinetobacter baumannii	carbapenem resistant acinetobacter baumannii	carbapenem resistant acinetobacter baumannii	carbapenem resistant Acinetobacter baumannii	6	kept
Carbapenem-Resistant Enterobacteriaceae	carbapenem resistant enterobacteriaceae	carbapenem resistant enterobacteriaceae	carbapenem resistant enterobacteriaceae	carbapenem-resistant Enterobacteriaceae	6	case_or_format
carbon monoxide intoxication	carbon monoxide intoxication	carbon monoxide intoxication	carbon monoxide intoxication	carbon monoxide intoxication	6	kept
carboplatin	carboplatin	carboplatin	carboplatin	carboplatin	6	kept
cardiac resynchronization therapy	cardiac resynchronization therapy	cardiac resynchronization therapy	cardiac resynchronization therapy	cardiac resynchronization therapy	6	kept
Cardiac tamponade	cardiac tamponade	cardiac tamponade	cardiac tamponade	Cardiac Tamponade	6	case_or_format
Cardiologists	cardiologists	cardiologists	cardiologist	cardiologist	6	singular

cardiopulmonary hemodynamics	cardiopulmonary hemodynamics	cardiopulmonary hemodynamics	cardiopulmonary hemodynamics	cardiopulmonary hemodynamics	6	kept
cardiovascular parameters	cardiovascular parameters	cardiovascular parameters	cardiovascular parameters	cardiovascular parameters	6	kept
Caregiver Burden	caregiver burden	caregiver burden	caregiver burden	caregiver burden	6	case_or_format
Caribbean	caribbean	caribbean	caribbean	Caribbean	6	kept
carnitine	carnitine	carnitine	carnitine	carnitine	6	kept
Case management	case management	case management	case management	Case Management	6	case_or_format
Case reports	case reports	case reports	case report	case report	6	singular
Case-studies	case studies	case studies	case study	case study	6	singular
Catecholamines	catecholamines	catecholamines	catecholamine	catecholamine	6	singular
catheter migration	catheter migration	catheter migration	catheter migration	catheter migration	6	kept
catheter thrombosis	catheter thrombosis	catheter thrombosis	catheter thrombosis	catheter thrombosis	6	kept
causality	causality	causality	causality	Causality	6	case_or_format
cefixime	cefixime	cefixime	cefixime	cefixime	6	kept
cefoperazone plus sulbactam	cefoperazone plus sulbactam	cefoperazone plus sulbactam	cefoperazone plus sulbactam	cefoperazone plus sulbactam	6	kept
cefpodoxime	cefpodoxime	cefpodoxime	cefpodoxime	cefpodoxime	6	kept
ceftolozane plus tazobactam	ceftolozane plus tazobactam	ceftolozane plus tazobactam	ceftolozane plus tazobactam	ceftolozane plus tazobactam	6	kept

Ceftriaxone	ceftriaxone	ceftriaxone	ceftriaxone	ceftriaxone	6	case_or_format
Cell phone	cell phone	cell phone	cell phone	Cell Phone	6	case_or_format
central nervous system agents	central nervous system agents	central nervous system agents	central nervous system agents	central nervous system agents	6	kept
CENTRAL VENOUS CATHETERS	central venous catheters	central venous catheters	central venous catheter	central venous catheter	6	singular
centrifugation	centrifugation	centrifugation	centrifugation	centrifugation	6	kept
Cerebral hemorrhage	cerebral hemorrhage	cerebral hemorrhage	cerebral hemorrhage	Cerebral Hemorrhage	6	case_or_format
Cerebral infarction	cerebral infarction	cerebral infarction	cerebral infarction	Cerebral Infarction	6	case_or_format
cetirizine	cetirizine	cetirizine	cetirizine	cetirizine	6	kept
CFIR	cfir	cfir	cfir	CFIR	6	kept
Chemistry, Pharmaceutical	chemistry pharmaceutical	chemistry pharmaceutical	chemistry pharmaceutical	Chemistry, Pharmaceutical	6	kept
chest pain	chest pain	chest pain	chest pain	Chest Pain	6	case_or_format
chickenpox vaccine	chickenpox vaccine	chickenpox vaccine	chickenpox vaccine	chickenpox vaccine	6	kept
Chinese medicine	chinese medicine	chinese medicine	chinese medicine	Chinese medicine	6	kept
cholesterol blood level	cholesterol blood level	cholesterol blood level	cholesterol blood level	cholesterol blood level	6	kept
cholinergic system	cholinergic system	cholinergic system	cholinergic system	cholinergic system	6	kept
chronic illness	chronic illness	chronic illness	chronic illness	chronic illness	6	kept

Chronic lung disease	chronic lung disease	chronic lung disease	chronic lung disease	chronic lung disease	6	case_or_format
Chronotype	chronotype	chronotype	chronotype	Chronotype	6	kept
chronotype	chronotype	chronotype	chronotype	Chronotype	6	case_or_format
cilastatin	cilastatin	cilastatin	cilastatin	cilastatin	6	kept
cimetidine	cimetidine	cimetidine	cimetidine	cimetidine	6	kept
Circumcision, Female	circumcision female	circumcision female	circumcision female	Circumcision, Female	6	kept
Citrates	citrates	citrates	citrate	Citrates	6	singular
Cleft Lip	cleft lip	cleft lip	cleft lip	cleft lip	6	case_or_format
clinical competency	clinical competency	clinical competency	clinical competency	Clinical competency	6	case_or_format
Clinical governance	clinical governance	clinical governance	clinical governance	Clinical governance	6	kept
clinical judgement	clinical judgement	clinical judgement	clinical judgement	clinical judgement	6	kept
clinical placement	clinical placement	clinical placement	clinical placement	clinical placement	6	kept
Clinical practices	clinical practices	clinical practices	clinical practice	clinical practice	6	singular
Clinical skills	clinical skills	clinical skills	clinical skill	clinical skills	6	singular
Clinical Trials	clinical trials	clinical trials	clinical trial	clinical trial	6	singular
CLINICAL-PRACTICE GUIDELINES	clinical practice guidelines	clinical practice guidelines	clinical practice guideline	clinical practice guideline	6	singular

Clostridium infection	clostridium infection	clostridium infection	clostridium infection	Clostridium Infections	6	case_or_format
Clothing	clothing	clothing	clothing	clothing	6	case_or_format
clozapine	clozapine	clozapine	clozapine	clozapine	6	kept
CNKI database	cnki database	cnki database	cnki database	CNKI database	6	kept
Cocaine	cocaine	cocaine	cocaine	cocaine	6	case_or_format
cognition assessment	cognition assessment	cognition assessment	cognition assessment	cognition assessment	6	kept
cognitive function	cognitive function	cognitive function	cognitive function	cognitive function	6	kept
cognitive load	cognitive load	cognitive load	cognitive load	cognitive load	6	kept
Cohen Mansfield Agitation Inventory	cohen mansfield agitation inventory	cohen mansfield agitation inventory	cohen mansfield agitation inventory	Cohen Mansfield Agitation Inventory	6	kept
Cold Temperature	cold temperature	cold temperature	cold temperature	Cold Temperature	6	kept
collaborative	collaborative	collaborative	collaborative	collaborative	6	kept
Collaborative practice	collaborative practice	collaborative practice	collaborative practice	Collaborative practice	6	kept
college student	college student	college student	college student	college student	6	kept
Colloids	colloids	colloids	colloid	colloid	6	singular
Colonic Diseases	colonic diseases	colonic diseases	colonic diseases	Colonic Diseases	6	kept
Colonoscopy	colonoscopy	colonoscopy	colonoscopy	colonoscopy	6	case_or_format

colorimetry	colorimetry	colorimetry	colorimetry	Colorimetry	6	case_or_format
Comfort care	comfort care	comfort care	comfort care	Comfort care	6	kept
comfort care	comfort care	comfort care	comfort care	Comfort care	6	case_or_format
Communication Aids for Disabled	communication aids for disabled	communication aids for disabled	communication aids for disabled	Communication Aids for Disabled	6	kept
communication software	communication software	communication software	communication software	communication software	6	kept
Community	community	community	community	community	6	case_or_format
community living	community living	community living	community living	community living	6	kept
community transmission	community transmission	community transmission	community transmission	community transmission	6	kept
comorbidity assessment	comorbidity assessment	comorbidity assessment	comorbidity assessment	comorbidity assessment	6	kept
Comparative Effectiveness Research	comparative effectiveness research	comparative effectiveness research	comparative effectiveness research	Comparative Effectiveness Research	6	kept
Competency-based education	competency based education	competency based education	competency based education	Competency-Based Education	6	case_or_format
compression therapy	compression therapy	compression therapy	compression therapy	compression therapy	6	kept
Computed Tomography Angiography	computed tomography angiography	computed tomography angiography	computed tomography angiography	Computed Tomography Angiography	6	kept
Computer	computer	computer	computer	Computers	6	case_or_format
Computer Graphics	computer graphics	computer graphics	computer graphics	Computer Graphics	6	kept
Comunicación	comunicaci n	comunicaci n	comunicaci n	Comunicación	6	kept

concentration	concentration	concentration	concentration	concentration	6	kept
Concept formation	concept formation	concept formation	concept formation	Concept Formation	6	case_or_format
conception	conception	conception	conception	conception	6	kept
congenital heart defect	congenital heart defect	congenital heart defect	congenital heart defect	congenital heart defect	6	kept
Conjunctivitis	conjunctivitis	conjunctivitis	conjunctivitis	conjunctivitis	6	case_or_format
connective tissue disease	connective tissue disease	connective tissue disease	connective tissue disease	connective tissue disease	6	kept
Connor Davidson resilience scale	connor davidson resilience scale	connor davidson resilience scale	connor davidson resilience scale	Connor Davidson resilience scale	6	kept
construction work and architectural phenomena	construction work and architectural phenomena	construction work and architectural phenomena	construction work and architectural phenomena	construction work and architectural phenomena	6	kept
contact dermatitis	contact dermatitis	contact dermatitis	contact dermatitis	contact dermatitis	6	kept
Contact precautions	contact precautions	contact precautions	contact precaution	Contact precautions	6	singular
CONTENT VALIDITY	content validity	content validity	content validity	content validity	6	case_or_format
continuity of care	continuity of care	continuity of care	continuity of care	Continuity of care	6	case_or_format
Continuous	continuous	continuous	continuous	Continuous	6	kept
contraception	contraception	contraception	contraception	contraception	6	kept
Contract Services	contract services	contract services	contract services	Contract Services	6	kept
contrast enhancement	contrast enhancement	contrast enhancement	contrast enhancement	contrast enhancement	6	kept

contrast induced nephropathy	contrast induced nephropathy	contrast induced nephropathy	contrast induced nephropathy	contrast induced nephropathy	6	kept
Controlled Clinical Trials as Topic	controlled clinical trials as topic	controlled clinical trials as topic	controlled clinical trials as topic	Controlled Clinical Trials as Topic	6	kept
convalescent plasma	convalescent plasma	convalescent plasma	convalescent plasma	convalescent plasma	6	kept
Convection	convection	convection	convection	Convection	6	kept
convolutional neural network	convolutional neural network	convolutional neural network	convolutional neural network	convolutional neural network	6	kept
Copper	copper	copper	copper	copper	6	case_or_format
coronary artery blood flow	coronary artery blood flow	coronary artery blood flow	coronary artery blood flow	coronary artery blood flow	6	kept
coronary artery bypass	coronary artery bypass	coronary artery bypass	coronary artery bypass	Coronary Artery Bypass	6	case_or_format
Coronary artery bypass grafting	coronary artery bypass grafting	coronary artery bypass grafting	coronary artery bypass grafting	Coronary artery bypass grafting	6	kept
Coronary artery disease	coronary artery disease	coronary artery disease	coronary artery disease	coronary artery disease	6	case_or_format
Coronary Circulation	coronary circulation	coronary circulation	coronary circulation	Coronary Circulation	6	kept
Coronary heart disease	coronary heart disease	coronary heart disease	coronary heart disease	Coronary heart disease	6	kept
Coronavirus disease	coronavirus disease	coronavirus disease	coronavirus disease	Coronavirus disease	6	kept
Corynebacterium diphtheriae	corynebacterium diphtheriae	corynebacterium diphtheriae	corynebacterium diphtheriae	Corynebacterium diphtheriae	6	kept
cost-effectiveness	cost effectiveness	cost effectiveness	cost effectiveness	Cost-effectiveness	6	case_or_format
Cost-Effectiveness Analysis	cost effectiveness analysis	cost effectiveness analysis	cost effectiveness analysis	cost effectiveness analysis	6	case_or_format

counselling	counselling	counselling	counselling	Counselling	6	case_or_format
cranial nerve	cranial nerve	cranial nerve	cranial nerve	cranial nerve	6	kept
crew member	crew member	crew member	crew member	crew member	6	kept
Cricoid Cartilage	cricoid cartilage	cricoid cartilage	cricoid cartilage	Cricoid Cartilage	6	kept
crime	crime	crime	crime	crime	6	kept
crisis management	crisis management	crisis management	crisis management	crisis management	6	kept
Crisis resource management	crisis resource management	crisis resource management	crisis resource management	Crisis resource management	6	kept
Critical	critical	critical	critical	critical	6	case_or_format
Critically ills	critically ills	critically ills	critically ill	Critically ill	6	singular
Cross-infection	cross infection	cross infection	cross infection	Cross Infection	6	case_or_format
Cross-transmission	cross transmission	cross transmission	cross transmission	Cross-transmission	6	kept
Crystalloid Solutions	crystalloid solutions	crystalloid solutions	crystalloid solutions	Crystalloid Solutions	6	kept
Cuff pressure	cuff pressure	cuff pressure	cuff pressure	Cuff pressure	6	kept
Cultural competency	cultural competency	cultural competency	cultural competency	Cultural Competency	6	case_or_format
cyanocobalamin	cyanocobalamin	cyanocobalamin	cyanocobalamin	cyanocobalamin	6	kept
cycle threshold value	cycle threshold value	cycle threshold value	cycle threshold value	cycle threshold value	6	kept

Cyclonic Storms	cyclonic storms	cyclonic storms	cyclonic storms	Cyclonic Storms	6	kept
cyclosporin A	cyclosporin a	cyclosporin a	cyclosporin a	cyclosporin A	6	kept
cytarabine	cytarabine	cytarabine	cytarabine	cytarabine	6	kept
dalteparin	dalteparin	dalteparin	dalteparin	dalteparin	6	kept
dantrolene	dantrolene	dantrolene	dantrolene	dantrolene	6	kept
Databases as Topic	databases as topic	databases as topic	databases as topic	Databases as Topic	6	kept
Death, Sudden	death sudden	death sudden	death sudden	Death, Sudden	6	kept
Decolonization	decolonization	decolonization	decolonization	Decolonization	6	kept
decompensated liver cirrhosis	decompensated liver cirrhosis	decompensated liver cirrhosis	decompensated liver cirrhosis	decompensated liver cirrhosis	6	kept
Deep Brain Stimulation	deep brain stimulation	deep brain stimulation	deep brain stimulation	Deep Brain Stimulation	6	kept
Delirium Observation Screening	delirium observation screening	delirium observation screening	delirium observation screening	Delirium Observation Screening	6	kept
delivery of health care	delivery of health care	delivery of health care	delivery of health care	Delivery of Health Care	6	case_or_format
Delivery room	delivery room	delivery room	delivery room	delivery room	6	case_or_format
dental health	dental health	dental health	dental health	dental health	6	kept
deployment	deployment	deployment	deployment	deployment	6	kept
desmopressin	desmopressin	desmopressin	desmopressin	desmopressin	6	kept

DEXMEDETOMIDINE	dexmedetomidine	dexmedetomidine	dexmedetomidine	dexmedetomidine	6	case_or_format
Diagnostic Self Evaluation	diagnostic self evaluation	diagnostic self evaluation	diagnostic self evaluation	Diagnostic Self Evaluation	6	kept
dialysis catheter	dialysis catheter	dialysis catheter	dialysis catheter	dialysis catheter	6	kept
diary	diary	diary	diary	Diary	6	case_or_format
dietary supplement	dietary supplement	dietary supplement	dietary supplement	Dietary Supplements	6	case_or_format
Digital health	digital health	digital health	digital health	digital health	6	case_or_format
directive counseling	directive counseling	directive counseling	directive counseling	directive counseling	6	kept
disability-adjusted life year	disability adjusted life year	disability adjusted life year	disability adjusted life year	disability-adjusted life year	6	kept
Disaster planning	disaster planning	disaster planning	disaster planning	Disaster Planning	6	case_or_format
disaster response	disaster response	disaster response	disaster response	disaster response	6	kept
discharge disposition	discharge disposition	discharge disposition	discharge disposition	discharge disposition	6	kept
disease free survival	disease free survival	disease free survival	disease free survival	disease free survival	6	kept
DISORDERS	disorders	disorders	disorder	DISORDERS	6	singular
DISRUPTION	disruption	disruption	disruption	DISRUPTION	6	kept
Distress	distress	distress	distress	DISTRESS	6	case_or_format
diuretic therapy	diuretic therapy	diuretic therapy	diuretic therapy	diuretic therapy	6	kept

Do not resuscitate	do not resuscitate	do not resuscitate	do not resuscitate	do-not-resuscitate	6	case_or_format
docusate sodium	docusate sodium	docusate sodium	docusate sodium	docusate sodium	6	kept
Dolor	dolor	dolor	dolor	Dolor	6	kept
donation after circulatory death	donation after circulatory death	donation after circulatory death	donation after circulatory death	donation after circulatory death	6	kept
donepezil	donepezil	donepezil	donepezil	donepezil	6	kept
donor milk	donor milk	donor milk	donor milk	donor milk	6	kept
doripenem	doripenem	doripenem	doripenem	doripenem	6	kept
drinking behavior	drinking behavior	drinking behavior	drinking behavior	drinking behavior	6	kept
drowning	drowning	drowning	drowning	drowning	6	kept
Drug Approval	drug approval	drug approval	drug approval	Drug Approval	6	kept
Drug Combinations	drug combinations	drug combinations	drug combination	drug combination	6	singular
Drug Delivery Systems	drug delivery systems	drug delivery systems	drug delivery system	drug delivery system	6	singular
drug extravasation	drug extravasation	drug extravasation	drug extravasation	drug extravasation	6	kept
drug fatality	drug fatality	drug fatality	drug fatality	drug fatality	6	kept
Drug Information Services	drug information services	drug information services	drug information services	Drug Information Services	6	kept
Drug resistance	drug resistance	drug resistance	drug resistance	drug resistance	6	case_or_format

drug tolerance	drug tolerance	drug tolerance	drug tolerance	Drug Tolerance	6	case_or_format
drug treatment failure	drug treatment failure	drug treatment failure	drug treatment failure	drug treatment failure	6	kept
DURATION	duration	duration	duration	DURATION	6	kept
duration	duration	duration	duration	DURATION	6	case_or_format
dynamics	dynamics	dynamics	dynamic	dynamics	6	singular
dysarthria	dysarthria	dysarthria	dysarthria	dysarthria	6	kept
dystonia	dystonia	dystonia	dystonia	dystonia	6	kept
early warning scores	early warning scores	early warning scores	early warning score	early warning score	6	singular
Ebolavirus	ebolavirus	ebolavirus	ebolavirus	Ebolavirus	6	kept
economic burden	economic burden	economic burden	economic burden	economic burden	6	kept
economic development	economic development	economic development	economic development	economic development	6	kept
Education, Professional	education professional	education professional	education professional	Education, Professional	6	kept
Educational needs	educational needs	educational needs	educational need	Educational needs	6	singular
Effect Modifier, Epidemiologic	effect modifier epidemiologic	effect modifier epidemiologic	effect modifier epidemiologic	Effect Modifier, Epidemiologic	6	kept
eHealth	ehealth	ehealth	ehealth	eHealth	6	kept
El Salvador	el salvador	el salvador	el salvador	El Salvador	6	kept

Electric Power Supplies	electric power supplies	electric power supplies	electric power supplies	Electric Power Supplies	6	kept
electromagnetic field	electromagnetic field	electromagnetic field	electromagnetic field	electromagnetic field	6	kept
Electromagnetic Fields	electromagnetic fields	electromagnetic fields	electromagnetic field	electromagnetic field	6	singular
electromagnetism	electromagnetism	electromagnetism	electromagnetism	electromagnetism	6	kept
Electronic Mail	electronic mail	electronic mail	electronic mail	Electronic Mail	6	kept
electronic medical records	electronic medical records	electronic medical records	electronic medical record	electronic medical record	6	singular
emergencies	emergencies	emergencies	emergency	Emergencies	6	singular
Emergency preparedness	emergency preparedness	emergency preparedness	emergency preparedness	Emergency preparedness	6	kept
Emergency response	emergency response	emergency response	emergency response	Emergency response	6	kept
Emergency room	emergency room	emergency room	emergency room	emergency room	6	case_or_format
Emotional Exhaustion	emotional exhaustion	emotional exhaustion	emotional exhaustion	emotional exhaustion	6	case_or_format
emphysema	emphysema	emphysema	emphysema	emphysema	6	kept
empirical research	empirical research	empirical research	empirical research	Empirical Research	6	case_or_format
Enablers	enablers	enablers	enablers	Enablers	6	kept
encephalomalacia	encephalomalacia	encephalomalacia	encephalomalacia	encephalomalacia	6	kept
End-of-life decision	end of life decision	end of life decision	end of life decision	End-of-life decisions	6	case_or_format

end-of-life decision-making	end of life decision making	end of life decision making	end of life decision making	end-of-life decision-making	6	kept
Endocrine System Diseases	endocrine system diseases	endocrine system diseases	endocrine system diseases	Endocrine System Diseases	6	kept
Endoscopy, Digestive System	endoscopy digestive system	endoscopy digestive system	endoscopy digestive system	Endoscopy, Digestive System	6	kept
Endotracheal	endotracheal	endotracheal	endotracheal	Endotracheal	6	kept
end－of－life care	end of life care	end of life care	end of life care	end-of-life care	6	case_or_format
Energy expenditure	energy expenditure	energy expenditure	energy expenditure	energy expenditure	6	case_or_format
Enfermagem	enfermagem	enfermagem	enfermagem	Enfermagem	6	kept
Enfermeras	enfermeras	enfermeras	enfermera	Enfermera	6	singular
enteropathy	enteropathy	enteropathy	enteropathy	enteropathy	6	kept
Enzyme-Linked Immunosorbent Assay	enzyme linked immunosorbent assay	enzyme linked immunosorbent assay	enzyme linked immunosorbent assay	enzyme linked immunosorbent assay	6	case_or_format
Epidemic	epidemic	epidemic	epidemic	epidemic	6	case_or_format
Episode of Care	episode of care	episode of care	episode of care	Episode of Care	6	kept
equipment and supplies	equipment and supplies	equipment and supplies	equipment and supplies	Equipment and Supplies	6	case_or_format
equipment contamination	equipment contamination	equipment contamination	equipment contamination	Equipment Contamination	6	case_or_format
Equipment design	equipment design	equipment design	equipment design	Equipment Design	6	case_or_format
equity	equity	equity	equity	equity	6	kept

Escape room	escape room	escape room	escape room	Escape room	6	kept
ESCHERICHIA-COLI	escherichia coli	escherichia coli	escherichia coli	Escherichia coli	6	case_or_format
esomeprazole	esomeprazole	esomeprazole	esomeprazole	esomeprazole	6	kept
Esophageal and Gastric Varices	esophageal and gastric varices	esophageal and gastric varices	esophageal and gastric varices	Esophageal and Gastric Varices	6	kept
esophagogastroduodenoscopy	esophagogastroduodenoscopy	esophagogastroduodenoscopy	esophagogastroduodenoscopy	esophagogastroduodenoscopy	6	kept
esophagus	esophagus	esophagus	esophagus	Esophagus	6	case_or_format
esophagus atresia	esophagus atresia	esophagus atresia	esophagus atresia	esophagus atresia	6	kept
esophagus varices	esophagus varices	esophagus varices	esophagus varices	esophagus varices	6	kept
Estonia	estonia	estonia	estonia	Estonia	6	kept
ethical dilemmas	ethical dilemmas	ethical dilemmas	ethical dilemma	ethical dilemma	6	singular
Ethical sensitivity	ethical sensitivity	ethical sensitivity	ethical sensitivity	ethical sensitivity	6	case_or_format
Ethical Theory	ethical theory	ethical theory	ethical theory	Ethical Theory	6	kept
Ethics Committees, Clinical	ethics committees clinical	ethics committees clinical	ethics committees clinical	Ethics Committees, Clinical	6	kept
ethics of care	ethics of care	ethics of care	ethics of care	ethics of care	6	kept
European Continental Ancestry Group	european continental ancestry group	european continental ancestry group	european continental ancestry group	European Continental Ancestry Group	6	kept
Evaluation Studies	evaluation studies	evaluation studies	evaluation study	evaluation study	6	singular

evening dosage	evening dosage	evening dosage	evening dosage	evening dosage	6	kept
evidence-based	evidence based	evidence based	evidence based	Evidence-based	6	case_or_format
Evidence-based practices	evidence based practices	evidence based practices	evidence based practice	evidence based practice	6	singular
Exercise Test	exercise test	exercise test	exercise test	exercise test	6	case_or_format
EXPOSURE	exposure	exposure	exposure	exposure	6	case_or_format
exudate	exudate	exudate	exudate	exudate	6	kept
Eye	eye	eye	eye	Eye	6	kept
eye infection	eye infection	eye infection	eye infection	eye infection	6	kept
eye movement	eye movement	eye movement	eye movement	Eye Movements	6	case_or_format
Faces Pain Scale	faces pain scale	faces pain scale	faces pain scale	Faces Pain Scale	6	kept
Failure to Rescue, Health Care	failure to rescue health care	failure to rescue health care	failure to rescue health care	Failure to Rescue, Health Care	6	kept
FALLS	falls	falls	fall	falls	6	singular
family caregiver	family caregiver	family caregiver	family caregiver	Family caregivers	6	case_or_format
family conflict	family conflict	family conflict	family conflict	family conflict	6	kept
family meeting	family meeting	family meeting	family meeting	family meeting	6	kept
Family presence during resuscitation	family presence during resuscitation	family presence during resuscitation	family presence during resuscitation	Family presence during resuscitation	6	kept

Family-integrated care	family integrated care	family integrated care	family integrated care	Family integrated care	6	case_or_format
Feasibility study	feasibility study	feasibility study	feasibility study	Feasibility Studies	6	case_or_format
feature selection	feature selection	feature selection	feature selection	feature selection	6	kept
FEEDBACK	feedback	feedback	feedback	Feedback	6	case_or_format
feeding disorder	feeding disorder	feeding disorder	feeding disorder	feeding disorder	6	kept
Feeding intolerance	feeding intolerance	feeding intolerance	feeding intolerance	feeding intolerance	6	case_or_format
ferritin blood level	ferritin blood level	ferritin blood level	ferritin blood level	ferritin blood level	6	kept
Fetal Growth Retardation	fetal growth retardation	fetal growth retardation	fetal growth retardation	Fetal Growth Retardation	6	kept
Fetal Macrosomia	fetal macrosomia	fetal macrosomia	fetal macrosomia	Fetal Macrosomia	6	kept
field study	field study	field study	field study	field study	6	kept
filtration	filtration	filtration	filtration	Filtration	6	case_or_format
Financial Management, Hospital	financial management hospital	financial management hospital	financial management hospital	Financial Management, Hospital	6	kept
firearm	firearm	firearm	firearm	firearm	6	kept
first-line treatment	first line treatment	first line treatment	first line treatment	first-line treatment	6	kept
fondaparinux	fondaparinux	fondaparinux	fondaparinux	fondaparinux	6	kept
Food	food	food	food	food	6	case_or_format

food allergy	food allergy	food allergy	food allergy	food allergy	6	kept
Food and Drug Administration	food and drug administration	food and drug administration	food and drug administration	food and drug administration	6	case_or_format
Food Contamination	food contamination	food contamination	food contamination	Food Contamination	6	kept
Food Handling	food handling	food handling	food handling	Food Handling	6	kept
food safety	food safety	food safety	food safety	food safety	6	kept
foot	foot	foot	foot	Foot	6	case_or_format
Foramen Ovale, Patent	foramen ovale patent	foramen ovale patent	foramen ovale patent	Foramen Ovale, Patent	6	kept
forceps delivery	forceps delivery	forceps delivery	forceps delivery	forceps delivery	6	kept
foreign body aspiration	foreign body aspiration	foreign body aspiration	foreign body aspiration	foreign body aspiration	6	kept
Forensic Medicine	forensic medicine	forensic medicine	forensic medicine	forensic medicine	6	case_or_format
fracture reduction	fracture reduction	fracture reduction	fracture reduction	fracture reduction	6	kept
Freedom	freedom	freedom	freedom	Freedom	6	kept
Friction	friction	friction	friction	friction	6	case_or_format
fungus	fungus	fungus	fungus	fungus	6	kept
fungus culture	fungus culture	fungus culture	fungus culture	fungus culture	6	kept
Furosemide	furosemide	furosemide	furosemide	furosemide	6	case_or_format

fusidic acid	fusidic acid	fusidic acid	fusidic acid	fusidic acid	6	kept
futile treatment	futile treatment	futile treatment	futile treatment	futile treatment	6	kept
futurology	futurology	futurology	futurology	futurology	6	kept
Galvanic Skin Response	galvanic skin response	galvanic skin response	galvanic skin response	Galvanic Skin Response	6	kept
ganciclovir	ganciclovir	ganciclovir	ganciclovir	ganciclovir	6	kept
Gastric Mucosa	gastric mucosa	gastric mucosa	gastric mucosa	Gastric Mucosa	6	kept
Gastrointestinal bleeding	gastrointestinal bleeding	gastrointestinal bleeding	gastrointestinal bleeding	Gastrointestinal bleeding	6	kept
gastrointestinal cancer	gastrointestinal cancer	gastrointestinal cancer	gastrointestinal cancer	gastrointestinal cancer	6	kept
Gastrointestinal Neoplasms	gastrointestinal neoplasms	gastrointestinal neoplasms	gastrointestinal neoplasms	Gastrointestinal Neoplasms	6	kept
Gastroplasty	gastroplasty	gastroplasty	gastroplasty	Gastroplasty	6	kept
gaze	gaze	gaze	gaze	gaze	6	kept
GENDER	gender	gender	gender	gender	6	case_or_format
General Health Questionnaire	general health questionnaire	general health questionnaire	general health questionnaire	General Health Questionnaire	6	kept
general medicine	general medicine	general medicine	general medicine	general medicine	6	kept
General Self-Efficacy Scale	general self efficacy scale	general self efficacy scale	general self efficacy scale	General Self-Efficacy Scale	6	kept
Generative artificial intelligence	generative artificial intelligence	generative artificial intelligence	generative artificial intelligence	Generative artificial intelligence	6	kept

genetic predisposition	genetic predisposition	genetic predisposition	genetic predisposition	genetic predisposition	6	kept
geriatric ward	geriatric ward	geriatric ward	geriatric ward	geriatric ward	6	kept
gerontopsychiatry	gerontopsychiatry	gerontopsychiatry	gerontopsychiatry	gerontopsychiatry	6	kept
gingivitis	gingivitis	gingivitis	gingivitis	gingivitis	6	kept
Gloves, Surgical	gloves surgical	gloves surgical	gloves surgical	Gloves, Surgical	6	kept
Glucose Metabolism Disorders	glucose metabolism disorders	glucose metabolism disorders	glucose metabolism disorders	Glucose Metabolism Disorders	6	kept
Glycaemic control	glycaemic control	glycaemic control	glycaemic control	Glycaemic control	6	kept
GLYCEMIC CONTROL	glycemic control	glycemic control	glycemic control	glycemic control	6	case_or_format
glycemic index	glycemic index	glycemic index	glycemic index	Glycemic Index	6	case_or_format
gout	gout	gout	gout	gout	6	kept
Governance	governance	governance	governance	Governance	6	kept
Government	government	government	government	government	6	case_or_format
Gram positive cocci	gram positive cocci	gram positive cocci	gram positive cocci	Gram positive cocci	6	kept
Gram-Positive Bacteria	gram positive bacteria	gram positive bacteria	gram positive bacteria	Gram-Positive Bacteria	6	kept
Grandparents	grandparents	grandparents	grandparent	Grandparents	6	singular
granulocyte colony stimulating factor	granulocyte colony stimulating factor	granulocyte colony stimulating factor	granulocyte colony stimulating factor	granulocyte colony stimulating factor	6	kept

group therapy	group therapy	group therapy	group therapy	group therapy	6	kept
growth hormone	growth hormone	growth hormone	growth hormone	growth hormone	6	kept
gynecologic surgery	gynecologic surgery	gynecologic surgery	gynecologic surgery	gynecologic surgery	6	kept
habit	habit	habit	habit	habit	6	kept
halothane	halothane	halothane	halothane	halothane	6	kept
HANDOVER	handover	handover	handover	Handover	6	case_or_format
Head Protective Devices	head protective devices	head protective devices	head protective devices	Head Protective Devices	6	kept
Health facility environment	health facility environment	health facility environment	health facility environment	Health Facility Environment	6	case_or_format
Health informatics	health informatics	health informatics	health informatics	health informatics	6	case_or_format
Health Information Systems	health information systems	health information systems	health information system	Health Information Systems	6	singular
Health information technologies	health information technologies	health information technologies	health information technology	Health information technology	6	singular
health infrastructure	health infrastructure	health infrastructure	health infrastructure	health infrastructure	6	kept
Health profession	health profession	health profession	health profession	Health profession	6	kept
health science	health science	health science	health science	health science	6	kept
Healthcare associated infections	healthcare associated infections	healthcare associated infections	healthcare associated infection	healthcare associated infection	6	singular
healthcare education	healthcare education	healthcare education	healthcare education	healthcare education	6	kept

Healthcare systems	healthcare systems	healthcare systems	healthcare system	healthcare system	6	singular
Hearing Disorders	hearing disorders	hearing disorders	hearing disorder	Hearing Disorders	6	singular
HEART	heart	heart	heart	Heart	6	case_or_format
Heart Atria	heart atria	heart atria	heart atria	Heart Atria	6	kept
Heart Catheterization	heart catheterization	heart catheterization	heart catheterization	heart catheterization	6	case_or_format
heart left ventricle function	heart left ventricle function	heart left ventricle function	heart left ventricle function	heart left ventricle function	6	kept
Heart Massage	heart massage	heart massage	heart massage	Heart Massage	6	kept
heart massage	heart massage	heart massage	heart massage	Heart Massage	6	case_or_format
heart muscle injury	heart muscle injury	heart muscle injury	heart muscle injury	heart muscle injury	6	kept
Heart Rate, Fetal	heart rate fetal	heart rate fetal	heart rate fetal	Heart Rate, Fetal	6	kept
Heart Septal Defects, Atrial	heart septal defects atrial	heart septal defects atrial	heart septal defects atrial	Heart Septal Defects, Atrial	6	kept
heart single ventricle	heart single ventricle	heart single ventricle	heart single ventricle	heart single ventricle	6	kept
heart ventricle function	heart ventricle function	heart ventricle function	heart ventricle function	heart ventricle function	6	kept
HEART-FAILURE	heart failure	heart failure	heart failure	heart failure	6	case_or_format
Heat Stroke	heat stroke	heat stroke	heat stroke	heat stroke	6	case_or_format
height	height	height	height	height	6	kept

hematologist	hematologist	hematologist	hematologist	hematologist	6	kept
Hematoma, Subdural	hematoma subdural	hematoma subdural	hematoma subdural	Hematoma, Subdural	6	kept
hemodilution	hemodilution	hemodilution	hemodilution	hemodilution	6	kept
hemolytic anemia	hemolytic anemia	hemolytic anemia	hemolytic anemia	hemolytic anemia	6	kept
Hemolytic-Uremic Syndrome	hemolytic uremic syndrome	hemolytic uremic syndrome	hemolytic uremic syndrome	Hemolytic-Uremic Syndrome	6	kept
Hepatic Encephalopathy	hepatic encephalopathy	hepatic encephalopathy	hepatic encephalopathy	hepatic encephalopathy	6	case_or_format
Hepatorenal Syndrome	hepatorenal syndrome	hepatorenal syndrome	hepatorenal syndrome	Hepatorenal Syndrome	6	kept
Hermeneutic phenomenology	hermeneutic phenomenology	hermeneutic phenomenology	hermeneutic phenomenology	Hermeneutic phenomenology	6	kept
hernioplasty	hernioplasty	hernioplasty	hernioplasty	hernioplasty	6	kept
Herniorrhaphy	herniorrhaphy	herniorrhaphy	herniorrhaphy	Herniorrhaphy	6	kept
herpes simplex	herpes simplex	herpes simplex	herpes simplex	herpes simplex	6	kept
herpes zoster	herpes zoster	herpes zoster	herpes zoster	herpes zoster	6	kept
hetastarch	hetastarch	hetastarch	hetastarch	hetastarch	6	kept
hierarchical clustering	hierarchical clustering	hierarchical clustering	hierarchical clustering	hierarchical clustering	6	kept
High fidelity simulation	high fidelity simulation	high fidelity simulation	high fidelity simulation	high-fidelity simulation	6	case_or_format
High risk	high risk	high risk	high risk	High risk	6	kept

high risk behavior	high risk behavior	high risk behavior	high risk behavior	high risk behavior	6	kept
Hip fracture	hip fracture	hip fracture	hip fracture	hip fracture	6	case_or_format
hip pain	hip pain	hip pain	hip pain	hip pain	6	kept
Histamine H2 Antagonists	histamine h2 antagonists	histamine h2 antagonists	histamine h2 antagonists	Histamine H2 Antagonists	6	kept
histogram	histogram	histogram	histogram	histogram	6	kept
home delivery	home delivery	home delivery	home delivery	home delivery	6	kept
homelessness	homelessness	homelessness	homelessness	homelessness	6	kept
Honduras	honduras	honduras	honduras	Honduras	6	kept
hospital finance	hospital finance	hospital finance	hospital finance	hospital finance	6	kept
hospital purchasing	hospital purchasing	hospital purchasing	hospital purchasing	hospital purchasing	6	kept
hospital rapid response team	hospital rapid response team	hospital rapid response team	hospital rapid response team	Hospital Rapid Response Team	6	case_or_format
Hospital setting	hospital setting	hospital setting	hospital setting	Hospital settings	6	case_or_format
Hospital Volunteers	hospital volunteers	hospital volunteers	hospital volunteer	Hospital Volunteers	6	singular
HOSPITALIZATION	hospitalization	hospitalization	hospitalization	hospitalization	6	case_or_format
Hospitalized child	hospitalized child	hospitalized child	hospitalized child	hospitalized child	6	case_or_format
Hospitals, Low-Volume	hospitals low volume	hospitals low volume	hospitals low volume	Hospitals, Low-Volume	6	kept

Hospitals, Packaged	hospitals packaged	hospitals packaged	hospitals packaged	Hospitals, Packaged	6	kept
hostility	hostility	hostility	hostility	Hostility	6	case_or_format
Hydrotherapy	hydrotherapy	hydrotherapy	hydrotherapy	Hydrotherapy	6	kept
hydrotherapy	hydrotherapy	hydrotherapy	hydrotherapy	Hydrotherapy	6	case_or_format
hyperactive delirium	hyperactive delirium	hyperactive delirium	hyperactive delirium	hyperactive delirium	6	kept
Hyperbaric Oxygenation	hyperbaric oxygenation	hyperbaric oxygenation	hyperbaric oxygenation	Hyperbaric Oxygenation	6	kept
Hyperglycemic Hyperosmolar Nonketotic Coma	hyperglycemic hyperosmolar nonketotic coma	hyperglycemic hyperosmolar nonketotic coma	hyperglycemic hyperosmolar nonketotic coma	Hyperglycemic Hyperosmolar Nonketotic Coma	6	kept
hyperinflation	hyperinflation	hyperinflation	hyperinflation	hyperinflation	6	kept
hyperreflexia	hyperreflexia	hyperreflexia	hyperreflexia	hyperreflexia	6	kept
hypersalivation	hypersalivation	hypersalivation	hypersalivation	hypersalivation	6	kept
Hypersensitivity	hypersensitivity	hypersensitivity	hypersensitivity	hypersensitivity	6	case_or_format
Hypertension, Portal	hypertension portal	hypertension portal	hypertension portal	Hypertension, Portal	6	kept
Hypocapnia	hypocapnia	hypocapnia	hypocapnia	hypocapnia	6	case_or_format
Hypophosphatemia	hypophosphatemia	hypophosphatemia	hypophosphatemia	hypophosphatemia	6	case_or_format
hypoventilation	hypoventilation	hypoventilation	hypoventilation	hypoventilation	6	kept
Ill-Housed Persons	ill housed persons	ill housed persons	ill housed persons	Ill-Housed Persons	6	kept

immunopathology	immunopathology	immunopathology	immunopathology	immunopathology	6	kept
improvement	improvement	improvement	improvement	IMPROVEMENT	6	case_or_format
in hospital cardiac arrest	in hospital cardiac arrest	in hospital cardiac arrest	in hospital cardiac arrest	In-hospital cardiac arrest	6	case_or_format
in-hospital cardiac arrest	in hospital cardiac arrest	in hospital cardiac arrest	in hospital cardiac arrest	In-hospital cardiac arrest	6	case_or_format
inappropriate vasopressin secretion	inappropriate vasopressin secretion	inappropriate vasopressin secretion	inappropriate vasopressin secretion	inappropriate vasopressin secretion	6	kept
incision	incision	incision	incision	incision	6	kept
incivility	incivility	incivility	incivility	Incivility	6	case_or_format
Incubator	incubator	incubator	incubator	incubator	6	case_or_format
INDEX	index	index	index	INDEX	6	kept
INDICATORS	indicators	indicators	indicator	INDICATORS	6	singular
induction chemotherapy	induction chemotherapy	induction chemotherapy	induction chemotherapy	induction chemotherapy	6	kept
infant disease	infant disease	infant disease	infant disease	infant disease	6	kept
Infant newborn	infant newborn	infant newborn	infant newborn	Infant, Newborn	6	case_or_format
infantile spasm	infantile spasm	infantile spasm	infantile spasm	infantile spasm	6	kept
Infarction, Middle Cerebral Artery	infarction middle cerebral artery	infarction middle cerebral artery	infarction middle cerebral artery	Infarction, Middle Cerebral Artery	6	kept
infection complication	infection complication	infection complication	infection complication	infection complication	6	kept

Infectious diseases	infectious diseases	infectious diseases	infectious disease	infectious disease	6	singular
inflammatory response	inflammatory response	inflammatory response	inflammatory response	inflammatory response	6	kept
influenza B	influenza b	influenza b	influenza b	influenza B	6	kept
information seeking	information seeking	information seeking	information seeking	information seeking	6	kept
Infrared Rays	infrared rays	infrared rays	infrared rays	Infrared Rays	6	kept
Infusion	infusion	infusion	infusion	infusion	6	case_or_format
ingestion	ingestion	ingestion	ingestion	ingestion	6	kept
Inhalation injury	inhalation injury	inhalation injury	inhalation injury	Inhalation injury	6	kept
inspiratory capacity	inspiratory capacity	inspiratory capacity	inspiratory capacity	inspiratory capacity	6	kept
INSTRUMENT	instrument	instrument	instrument	instrument	6	case_or_format
Instrument	instrument	instrument	instrument	instrument	6	case_or_format
insulin lispro	insulin lispro	insulin lispro	insulin lispro	insulin lispro	6	kept
Integration	integration	integration	integration	integration	6	case_or_format
interleukin 1	interleukin 1	interleukin 1	interleukin 1	interleukin 1	6	kept
intermediate care	intermediate care	intermediate care	intermediate care	Intermediate care	6	case_or_format
Intermediate care unit	intermediate care unit	intermediate care unit	intermediate care unit	intermediate care unit	6	case_or_format

international	international	international	international	International	6	case_or_format
Interpersonal and Communication Skills	interpersonal and communication skills	interpersonal and communication skills	interpersonal and communication skills	Interpersonal and Communication Skills	6	kept
intraaortic balloon pump	intraaortic balloon pump	intraaortic balloon pump	intraaortic balloon pump	intraaortic balloon pump	6	kept
Intractable Pain	intractable pain	intractable pain	intractable pain	Intractable Pain	6	kept
intradermal drug administration	intradermal drug administration	intradermal drug administration	intradermal drug administration	intradermal drug administration	6	kept
intramedullary nailing	intramedullary nailing	intramedullary nailing	intramedullary nailing	intramedullary nailing	6	kept
Intratracheal	intratracheal	intratracheal	intratracheal	intratracheal	6	case_or_format
intravenous infusions	intravenous infusions	intravenous infusions	intravenous infusion	intravenous infusions	6	singular
Investigación cualitativa	investigaci n cualitativa	investigaci n cualitativa	investigaci n cualitativa	Investigación cualitativa	6	kept
INVOLVEMENT	involvement	involvement	involvement	INVOLVEMENT	6	kept
Iranian people	iranian people	iranian people	iranian people	Iranian people	6	kept
joint	joint	joint	joint	joint	6	kept
keratopathy	keratopathy	keratopathy	keratopathy	keratopathy	6	kept
kidney biopsy	kidney biopsy	kidney biopsy	kidney biopsy	kidney biopsy	6	kept
kidney function test	kidney function test	kidney function test	kidney function test	kidney function test	6	kept
Klebsiella infection	klebsiella infection	klebsiella infection	klebsiella infection	Klebsiella Infections	6	case_or_format

knee replacement	knee replacement	knee replacement	knee replacement	knee replacement	6	kept
Knowledge acquisition	knowledge acquisition	knowledge acquisition	knowledge acquisition	Knowledge acquisition	6	kept
Kolmogorov Smirnov test	kolmogorov smirnov test	kolmogorov smirnov test	kolmogorov smirnov test	Kolmogorov Smirnov test	6	kept
Lactobacillus	lactobacillus	lactobacillus	lactobacillus	Lactobacillus	6	kept
Laos	laos	laos	laos	Laos	6	kept
laryngectomy	laryngectomy	laryngectomy	laryngectomy	laryngectomy	6	kept
latent profile analysis	latent profile analysis	latent profile analysis	latent profile analysis	latent profile analysis	6	kept
Latent Tuberculosis	latent tuberculosis	latent tuberculosis	latent tuberculosis	latent tuberculosis	6	case_or_format
lavage	lavage	lavage	lavage	lavage	6	kept
Lavandula	lavandula	lavandula	lavandula	Lavandula	6	kept
Law Enforcement	law enforcement	law enforcement	law enforcement	law enforcement	6	case_or_format
leg amputation	leg amputation	leg amputation	leg amputation	leg amputation	6	kept
leg edema	leg edema	leg edema	leg edema	leg edema	6	kept
Leisure Activities	leisure activities	leisure activities	leisure activities	Leisure Activities	6	kept
LENGTH	length	length	length	LENGTH	6	kept
Leukemia, Myeloid, Acute	leukemia myeloid acute	leukemia myeloid acute	leukemia myeloid acute	Leukemia, Myeloid, Acute	6	kept

Leukocytosis	leukocytosis	leukocytosis	leukocytosis	leukocytosis	6	case_or_format
Life support	life support	life support	life support	LIFE-SUPPORT	6	case_or_format
lobectomy	lobectomy	lobectomy	lobectomy	lobectomy	6	kept
Long COVID	long covid	long covid	long covid	long COVID	6	case_or_format
long-term care facilities	long term care facilities	long term care facilities	long term care facility	long-term care facilities	6	singular
low level laser therapy	low level laser therapy	low level laser therapy	low level laser therapy	low level laser therapy	6	kept
lung contusion	lung contusion	lung contusion	lung contusion	lung contusion	6	kept
Lung Diseases, Interstitial	lung diseases interstitial	lung diseases interstitial	lung diseases interstitial	Lung Diseases, Interstitial	6	kept
lung lobectomy	lung lobectomy	lung lobectomy	lung lobectomy	lung lobectomy	6	kept
lung mechanics	lung mechanics	lung mechanics	lung mechanics	lung mechanics	6	kept
lung minute volume	lung minute volume	lung minute volume	lung minute volume	lung minute volume	6	kept
lung perfusion	lung perfusion	lung perfusion	lung perfusion	lung perfusion	6	kept
lung tuberculosis	lung tuberculosis	lung tuberculosis	lung tuberculosis	lung tuberculosis	6	kept
lung vascular resistance	lung vascular resistance	lung vascular resistance	lung vascular resistance	lung vascular resistance	6	kept
lung vein drainage anomaly	lung vein drainage anomaly	lung vein drainage anomaly	lung vein drainage anomaly	lung vein drainage anomaly	6	kept
macronutrient	macronutrient	macronutrient	macronutrient	macronutrient	6	kept

MALNUTRITION	malnutrition	malnutrition	malnutrition	malnutrition	6	case_or_format
Managed Care Programs	managed care programs	managed care programs	managed care programs	Managed Care Programs	6	kept
Manikin	manikin	manikin	manikin	Manikins	6	case_or_format
Manuals as Topic	manuals as topic	manuals as topic	manuals as topic	Manuals as Topic	6	kept
materials management	materials management	materials management	materials management	materials management	6	kept
maternal child health care	maternal child health care	maternal child health care	maternal child health care	maternal child health care	6	kept
maternal diabetes mellitus	maternal diabetes mellitus	maternal diabetes mellitus	maternal diabetes mellitus	maternal diabetes mellitus	6	kept
maternal disease	maternal disease	maternal disease	maternal disease	maternal disease	6	kept
Maternal Health	maternal health	maternal health	maternal health	Maternal health	6	case_or_format
Maternity	maternity	maternity	maternity	Maternity	6	kept
Mathematics	mathematics	mathematics	mathematics	Mathematics	6	kept
meal	meal	meal	meal	meal	6	kept
Mean arterial pressure	mean arterial pressure	mean arterial pressure	mean arterial pressure	mean arterial pressure	6	case_or_format
meaningful recognition	meaningful recognition	meaningful recognition	meaningful recognition	meaningful recognition	6	kept
mechanical circulatory support	mechanical circulatory support	mechanical circulatory support	mechanical circulatory support	Mechanical circulatory support	6	case_or_format
Mechanical ventilators	mechanical ventilators	mechanical ventilators	mechanical ventilator	mechanical ventilator	6	singular

mediating effect	mediating effect	mediating effect	mediating effect	mediating effect	6	kept
Medical devices	medical devices	medical devices	medical device	medical device	6	singular
Medical Overuse	medical overuse	medical overuse	medical overuse	Medical Overuse	6	kept
Medical record	medical record	medical record	medical record	medical record	6	case_or_format
medical secretary	medical secretary	medical secretary	medical secretary	medical secretary	6	kept
Medical services	medical services	medical services	medical service	medical service	6	singular
medical terminology	medical terminology	medical terminology	medical terminology	medical terminology	6	kept
Medication administration	medication administration	medication administration	medication administration	medication administration	6	case_or_format
Medication systems	medication systems	medication systems	medication systems	Medication Systems	6	case_or_format
medication systems	medication systems	medication systems	medication systems	Medication Systems	6	case_or_format
Meningioma	meningioma	meningioma	meningioma	Meningioma	6	kept
mental health recovery	mental health recovery	mental health recovery	mental health recovery	mental health recovery	6	kept
mental performance	mental performance	mental performance	mental performance	mental performance	6	kept
Mental Status and Dementia Tests	mental status and dementia tests	mental status and dementia tests	mental status and dementia tests	Mental Status and Dementia Tests	6	kept
Mental Status Schedule	mental status schedule	mental status schedule	mental status schedule	Mental Status Schedule	6	kept
Meta-Analysis	meta analysis	meta analysis	meta analysis	meta analysis	6	case_or_format

metabolic regulation	metabolic regulation	metabolic regulation	metabolic regulation	metabolic regulation	6	kept
metallo beta lactamase	metallo beta lactamase	metallo beta lactamase	metallo beta lactamase	metallo beta lactamase	6	kept
metasynthesis	metasynthesis	metasynthesis	metasynthesis	metasynthesis	6	kept
methyldopa	methyldopa	methyldopa	methyldopa	methyldopa	6	kept
methylprednisolone sodium succinate	methylprednisolone sodium succinate	methylprednisolone sodium succinate	methylprednisolone sodium succinate	methylprednisolone sodium succinate	6	kept
metropolitan area	metropolitan area	metropolitan area	metropolitan area	metropolitan area	6	kept
Microbiological Techniques	microbiological techniques	microbiological techniques	microbiological techniques	Microbiological Techniques	6	kept
microflora	microflora	microflora	microflora	microflora	6	kept
Micronutrients	micronutrients	micronutrients	micronutrients	Micronutrients	6	kept
microsurgery	microsurgery	microsurgery	microsurgery	Microsurgery	6	case_or_format
milk bank	milk bank	milk bank	milk bank	Milk Banks	6	case_or_format
Minocycline	minocycline	minocycline	minocycline	minocycline	6	case_or_format
minority group	minority group	minority group	minority group	Minority Groups	6	case_or_format
mobile app	mobile app	mobile app	mobile app	mobile app	6	kept
Mobile phone	mobile phone	mobile phone	mobile phone	mobile phone	6	case_or_format
Mobilization	mobilization	mobilization	mobilization	mobilization	6	case_or_format

Model	model	model	model	model	6	case_or_format
Monitoring system	monitoring system	monitoring system	monitoring system	Monitoring system	6	kept
Monte Carlo method	monte carlo method	monte carlo method	monte carlo method	Monte Carlo Method	6	case_or_format
montelukast	montelukast	montelukast	montelukast	montelukast	6	kept
Morganella morganii	morganella morganii	morganella morganii	morganella morganii	Morganella morganii	6	kept
motion	motion	motion	motion	motion	6	kept
motivational interviewing	motivational interviewing	motivational interviewing	motivational interviewing	motivational interviewing	6	kept
Mozambique	mozambique	mozambique	mozambique	Mozambique	6	kept
MRI	mri	mri	mri	MRI	6	kept
mucormycosis	mucormycosis	mucormycosis	mucormycosis	Mucormycosis	6	case_or_format
mucosa	mucosa	mucosa	mucosa	mucosa	6	kept
mucosa inflammation	mucosa inflammation	mucosa inflammation	mucosa inflammation	mucosa inflammation	6	kept
multimedia	multimedia	multimedia	multimedia	Multimedia	6	case_or_format
Munchausen syndrome	munchausen syndrome	munchausen syndrome	munchausen syndrome	Munchausen syndrome	6	kept
Mupirocin	mupirocin	mupirocin	mupirocin	mupirocin	6	case_or_format
muscle contraction	muscle contraction	muscle contraction	muscle contraction	muscle contraction	6	kept

Muscle Tonus	muscle tonus	muscle tonus	muscle tonus	Muscle Tonus	6	kept
muscle training	muscle training	muscle training	muscle training	muscle training	6	kept
Musculoskeletal System	musculoskeletal system	musculoskeletal system	musculoskeletal system	Musculoskeletal System	6	kept
musculoskeletal system	musculoskeletal system	musculoskeletal system	musculoskeletal system	Musculoskeletal System	6	case_or_format
mutation	mutation	mutation	mutation	Mutation	6	case_or_format
myasthenia gravis	myasthenia gravis	myasthenia gravis	myasthenia gravis	myasthenia gravis	6	kept
myoclonus	myoclonus	myoclonus	myoclonus	myoclonus	6	kept
N-PASS	n pass	n pass	n pass	N-PASS	6	kept
Names	names	names	names	Names	6	kept
Nasal Cavity	nasal cavity	nasal cavity	nasal cavity	Nasal Cavity	6	kept
nasotracheal intubation	nasotracheal intubation	nasotracheal intubation	nasotracheal intubation	nasotracheal intubation	6	kept
natural disaster	natural disaster	natural disaster	natural disaster	natural disaster	6	kept
Neck Injuries	neck injuries	neck injuries	neck injury	Neck Injuries	6	singular
neighborhood	neighborhood	neighborhood	neighborhood	neighborhood	6	kept
Neisseria meningitidis	neisseria meningitidis	neisseria meningitidis	neisseria meningitidis	Neisseria meningitidis	6	kept
neonatal incubator	neonatal incubator	neonatal incubator	neonatal incubator	neonatal incubator	6	kept

Neonatal Infant Pain Scale	neonatal infant pain scale	neonatal infant pain scale	neonatal infant pain scale	Neonatal Infant Pain Scale	6	kept
neonatal outcome	neonatal outcome	neonatal outcome	neonatal outcome	neonatal outcome	6	kept
neonatal outcomes	neonatal outcomes	neonatal outcomes	neonatal outcome	neonatal outcome	6	singular
nephrotic syndrome	nephrotic syndrome	nephrotic syndrome	nephrotic syndrome	nephrotic syndrome	6	kept
nerve injury	nerve injury	nerve injury	nerve injury	nerve injury	6	kept
Nervous System	nervous system	nervous system	nervous system	nervous system	6	case_or_format
nervous system parameters	nervous system parameters	nervous system parameters	nervous system parameters	nervous system parameters	6	kept
Network	network	network	network	Network	6	kept
Network meta-analysis	network meta analysis	network meta analysis	network meta analysis	Network meta-analysis	6	kept
Neuromuscular blocking agents	neuromuscular blocking agents	neuromuscular blocking agents	neuromuscular blocking agent	neuromuscular blocking agent	6	singular
neurosis	neurosis	neurosis	neurosis	neurosis	6	kept
new devices	new devices	new devices	new devices	new devices	6	kept
Nicotine	nicotine	nicotine	nicotine	Nicotine	6	kept
nicotine	nicotine	nicotine	nicotine	Nicotine	6	case_or_format
night work	night work	night work	night work	night work	6	kept
noise pollution	noise pollution	noise pollution	noise pollution	Noise pollution	6	case_or_format

non heart beating donor	non heart beating donor	non heart beating donor	non heart beating donor	non heart beating donor	6	kept
Non-pharmacological interventions	non pharmacological interventions	non pharmacological interventions	non pharmacological intervention	Non-pharmacological interventions	6	singular
non-pharmacological interventions	non pharmacological interventions	non pharmacological interventions	non pharmacological intervention	Non-pharmacological interventions	6	singular
Normal Distribution	normal distribution	normal distribution	normal distribution	Normal Distribution	6	kept
Normal saline instillation	normal saline instillation	normal saline instillation	normal saline instillation	Normal saline instillation	6	kept
Normothermia	normothermia	normothermia	normothermia	Normothermia	6	kept
Norovirus	norovirus	norovirus	norovirus	Norovirus	6	kept
Northern Territory	northern territory	northern territory	northern territory	Northern Territory	6	kept
Nutrition assessment	nutrition assessment	nutrition assessment	nutrition assessment	Nutrition Assessment	6	case_or_format
nutrition education	nutrition education	nutrition education	nutrition education	nutrition education	6	kept
nutritional disorder	nutritional disorder	nutritional disorder	nutritional disorder	nutritional disorder	6	kept
nutritional health	nutritional health	nutritional health	nutritional health	nutritional health	6	kept
nutritional intolerance	nutritional intolerance	nutritional intolerance	nutritional intolerance	nutritional intolerance	6	kept
Nutritional status	nutritional status	nutritional status	nutritional status	nutritional status	6	case_or_format
OBSERVATION TOOL	observation tool	observation tool	observation tool	OBSERVATION TOOL	6	kept
Obsessive-Compulsive Disorder	obsessive compulsive disorder	obsessive compulsive disorder	obsessive compulsive disorder	obsessive compulsive disorder	6	case_or_format

obstetric analgesia	obstetric analgesia	obstetric analgesia	obstetric analgesia	obstetric analgesia	6	kept
obstruction	obstruction	obstruction	obstruction	obstruction	6	kept
obstructive airway disease	obstructive airway disease	obstructive airway disease	obstructive airway disease	obstructive airway disease	6	kept
Occupational burnout	occupational burnout	occupational burnout	occupational burnout	occupational burnout	6	case_or_format
Occupational Health Services	occupational health services	occupational health services	occupational health service	occupational health service	6	singular
occupational risks	occupational risks	occupational risks	occupational risk	Occupational risks	6	singular
Occupational Therapists	occupational therapists	occupational therapists	occupational therapist	occupational therapist	6	singular
OF-THE-LITERATURE	of the literature	of the literature	of the literature	OF-THE-LITERATURE	6	kept
off label drug use	off label drug use	off label drug use	off label drug use	off label drug use	6	kept
older adult	older adult	older adult	older adult	Older adults	6	case_or_format
oncology access	oncology access	oncology access	oncology access	oncology access	6	kept
Online Systems	online systems	online systems	online system	online system	6	singular
onset age	onset age	onset age	onset age	onset age	6	kept
Operations research	operations research	operations research	operations research	Operations research	6	kept
Opiate Substitution Treatment	opiate substitution treatment	opiate substitution treatment	opiate substitution treatment	Opiate Substitution Treatment	6	kept
optical coherence tomography	optical coherence tomography	optical coherence tomography	optical coherence tomography	optical coherence tomography	6	kept

oral mucositis	oral mucositis	oral mucositis	oral mucositis	oral mucositis	6	kept
ORGANIZATIONAL COMMITMENT	organizational commitment	organizational commitment	organizational commitment	Organizational commitment	6	case_or_format
organizational factors	organizational factors	organizational factors	organizational factors	organizational factors	6	kept
Organizations	organizations	organizations	organization	organization	6	singular
Oseltamivir	oseltamivir	oseltamivir	oseltamivir	oseltamivir	6	case_or_format
overnutrition	overnutrition	overnutrition	overnutrition	overnutrition	6	kept
oxaliplatin	oxaliplatin	oxaliplatin	oxaliplatin	oxaliplatin	6	kept
oxazepam	oxazepam	oxazepam	oxazepam	oxazepam	6	kept
oxygen toxicity	oxygen toxicity	oxygen toxicity	oxygen toxicity	oxygen toxicity	6	kept
Pacific islands	pacific islands	pacific islands	pacific islands	Pacific islands	6	kept
pacifier	pacifier	pacifier	pacifier	Pacifiers	6	case_or_format
paclitaxel	paclitaxel	paclitaxel	paclitaxel	paclitaxel	6	kept
Paediatric intensive	paediatric intensive	paediatric intensive	paediatric intensive	Paediatric intensive	6	kept
pain threshold	pain threshold	pain threshold	pain threshold	pain threshold	6	kept
Palivizumab	palivizumab	palivizumab	palivizumab	palivizumab	6	case_or_format
Panic Disorder	panic disorder	panic disorder	panic disorder	Panic Disorder	6	kept

Panton Valentine leukocidin	panton valentine leukocidin	panton valentine leukocidin	panton valentine leukocidin	Panton Valentine leukocidin	6	kept
Paracentesis	paracentesis	paracentesis	paracentesis	paracentesis	6	case_or_format
Paracetamol	paracetamol	paracetamol	paracetamol	paracetamol	6	case_or_format
Paralysis	paralysis	paralysis	paralysis	paralysis	6	case_or_format
Paramedic	paramedic	paramedic	paramedic	Paramedic	6	kept
paramedical profession	paramedical profession	paramedical profession	paramedical profession	paramedical profession	6	kept
Paramedics	paramedics	paramedics	paramedic	Paramedic	6	singular
partial nephrectomy	partial nephrectomy	partial nephrectomy	partial nephrectomy	partial nephrectomy	6	kept
participatory action research	participatory action research	participatory action research	participatory action research	participatory action research	6	kept
particle size	particle size	particle size	particle size	particle size	6	kept
partnership	partnership	partnership	partnership	partnership	6	kept
Parvoviridae Infections	parvoviridae infections	parvoviridae infections	parvoviridae infections	Parvoviridae Infections	6	kept
patience	patience	patience	patience	patience	6	kept
peak inspiratory flow	peak inspiratory flow	peak inspiratory flow	peak inspiratory flow	peak inspiratory flow	6	kept
pediatric care	pediatric care	pediatric care	pediatric care	pediatric care	6	kept
Pediatric delirium	pediatric delirium	pediatric delirium	pediatric delirium	pediatric delirium	6	case_or_format

Pediatric emergency medicine	pediatric emergency medicine	pediatric emergency medicine	pediatric emergency medicine	pediatric emergency medicine	6	case_or_format
Pediatric palliative care	pediatric palliative care	pediatric palliative care	pediatric palliative care	pediatric palliative care	6	case_or_format
Pedigree	pedigree	pedigree	pedigree	Pedigree	6	kept
PEEP	peep	peep	peep	PEEP	6	kept
peer pressure	peer pressure	peer pressure	peer pressure	peer pressure	6	kept
Peer Review, Health Care	peer review health care	peer review health care	peer review health care	Peer Review, Health Care	6	kept
Peer support	peer support	peer support	peer support	peer support	6	case_or_format
PERCEPTION	perception	perception	perception	perception	6	case_or_format
perinatal	perinatal	perinatal	perinatal	Perinatal	6	case_or_format
Peritonitis	peritonitis	peritonitis	peritonitis	peritonitis	6	case_or_format
personality test	personality test	personality test	personality test	personality test	6	kept
personnel	personnel	personnel	personnel	Personnel	6	case_or_format
Personnel downsizing	personnel downsizing	personnel downsizing	personnel downsizing	Personnel downsizing	6	kept
Personnel selection	personnel selection	personnel selection	personnel selection	Personnel Selection	6	case_or_format
perspective	perspective	perspective	perspective	PERSPECTIVES	6	case_or_format
pertussis vaccine	pertussis vaccine	pertussis vaccine	pertussis vaccine	pertussis vaccine	6	kept

petrolatum	petrolatum	petrolatum	petrolatum	petrolatum	6	kept
Phenobarbital	phenobarbital	phenobarbital	phenobarbital	phenobarbital	6	case_or_format
Philosophy, Medical	philosophy medical	philosophy medical	philosophy medical	Philosophy, Medical	6	kept
phobia	phobia	phobia	phobia	phobia	6	kept
phosphodiesterase inhibitor	phosphodiesterase inhibitor	phosphodiesterase inhibitor	phosphodiesterase inhibitor	phosphodiesterase inhibitor	6	kept
phylogenetic tree	phylogenetic tree	phylogenetic tree	phylogenetic tree	phylogenetic tree	6	kept
physical abuse	physical abuse	physical abuse	physical abuse	physical abuse	6	kept
Physical function	physical function	physical function	physical function	physical function	6	case_or_format
Physical therapy modalities	physical therapy modalities	physical therapy modalities	physical therapy modalities	Physical Therapy Modalities	6	case_or_format
Piperazines	piperazines	piperazines	piperazines	Piperazines	6	kept
placenta accreta	placenta accreta	placenta accreta	placenta accreta	Placenta Accreta	6	case_or_format
Planning	planning	planning	planning	planning	6	case_or_format
plasmid	plasmid	plasmid	plasmid	plasmid	6	kept
Play and Playthings	play and playthings	play and playthings	play and playthings	Play and Playthings	6	kept
pneumonectomy	pneumonectomy	pneumonectomy	pneumonectomy	Pneumonectomy	6	case_or_format
Pneumonia, Pneumococcal	pneumonia pneumococcal	pneumonia pneumococcal	pneumonia pneumococcal	Pneumonia, Pneumococcal	6	kept

Pneumonia, Pneumocystis	pneumonia pneumocystis	pneumonia pneumocystis	pneumonia pneumocystis	Pneumonia, Pneumocystis	6	kept
Pneumoperitoneum	pneumoperitoneum	pneumoperitoneum	pneumoperitoneum	pneumoperitoneum	6	case_or_format
Point-of-care ultrasound	point of care ultrasound	point of care ultrasound	point of care ultrasound	point of care ultrasound	6	case_or_format
poison center	poison center	poison center	poison center	poison center	6	kept
Polymerase chain reaction	polymerase chain reaction	polymerase chain reaction	polymerase chain reaction	polymerase chain reaction	6	case_or_format
polyvinylchloride	polyvinylchloride	polyvinylchloride	polyvinylchloride	polyvinylchloride	6	kept
population structure	population structure	population structure	population structure	population structure	6	kept
Post-Exposure Prophylaxis	post exposure prophylaxis	post exposure prophylaxis	post exposure prophylaxis	post exposure prophylaxis	6	case_or_format
Postgraduate	postgraduate	postgraduate	postgraduate	Postgraduate	6	kept
postoperative monitoring	postoperative monitoring	postoperative monitoring	postoperative monitoring	postoperative monitoring	6	kept
POSTOPERATIVE PAIN	postoperative pain	postoperative pain	postoperative pain	Postoperative Pain	6	case_or_format
Postpartum	postpartum	postpartum	postpartum	Postpartum	6	kept
POSTPARTUM DEPRESSION	postpartum depression	postpartum depression	postpartum depression	Postpartum depression	6	case_or_format
posttraumatic stress disorder checklist	posttraumatic stress disorder checklist	posttraumatic stress disorder checklist	posttraumatic stress disorder checklist	posttraumatic stress disorder checklist	6	kept
POSTTRAUMATIC-STRESS	posttraumatic stress	posttraumatic stress	posttraumatic stress	POSTTRAUMATIC STRESS	6	case_or_format
potentially inappropriate medication	potentially inappropriate medication	potentially inappropriate medication	potentially inappropriate medication	potentially inappropriate medication	6	kept

Povidone-Iodine	povidone iodine	povidone iodine	povidone iodine	povidone iodine	6	case_or_format
power	power	power	power	Power	6	case_or_format
Practice development	practice development	practice development	practice development	Practice development	6	kept
Practice guideline	practice guideline	practice guideline	practice guideline	practice guideline	6	case_or_format
PRACTITIONERS	practitioners	practitioners	practitioner	PRACTITIONERS	6	singular
PREDICTION	prediction	prediction	prediction	prediction	6	case_or_format
Prediction model	prediction model	prediction model	prediction model	prediction model	6	case_or_format
predictive factors	predictive factors	predictive factors	predictive factors	predictive factors	6	kept
preferences	preferences	preferences	preference	PREFERENCES	6	singular
prehabilitation	prehabilitation	prehabilitation	prehabilitation	prehabilitation	6	kept
Prehospital care	prehospital care	prehospital care	prehospital care	Prehospital care	6	kept
premature baby	premature baby	premature baby	premature baby	Premature baby	6	case_or_format
Premature neonate	premature neonate	premature neonate	premature neonate	Premature neonate	6	kept
PRESCRIBING ERRORS	prescribing errors	prescribing errors	prescribing error	prescribing error	6	singular
Prescription	prescription	prescription	prescription	prescription	6	case_or_format
PRESSURE ULCERS	pressure ulcers	pressure ulcers	pressure ulcer	Pressure Ulcer	6	singular

Presumed Consent	presumed consent	presumed consent	presumed consent	Presumed Consent	6	kept
Preventive Health Services	preventive health services	preventive health services	preventive health service	preventive health service	6	singular
preventive measures	preventive measures	preventive measures	preventive measures	preventive measures	6	kept
prison	prison	prison	prison	prison	6	kept
Prisoners	prisoners	prisoners	prisoner	Prisoners	6	singular
Professional role	professional role	professional role	professional role	Professional Role	6	case_or_format
professional well-being	professional well being	professional well being	professional well being	professional well-being	6	kept
Prognostication	prognostication	prognostication	prognostication	prognostication	6	case_or_format
prostaglandin E1	prostaglandin e1	prostaglandin e1	prostaglandin e1	prostaglandin E1	6	kept
protamine	protamine	protamine	protamine	protamine	6	kept
Protective clothing	protective clothing	protective clothing	protective clothing	Protective Clothing	6	case_or_format
Protein-Energy Malnutrition	protein energy malnutrition	protein energy malnutrition	protein energy malnutrition	Protein-Energy Malnutrition	6	kept
Proteins	proteins	proteins	protein	protein	6	singular
proteinuria	proteinuria	proteinuria	proteinuria	proteinuria	6	kept
Proteus	proteus	proteus	proteus	Proteus	6	kept
psychiatric	psychiatric	psychiatric	psychiatric	psychiatric	6	kept

psychological intervention	psychological intervention	psychological intervention	psychological intervention	psychological intervention	6	kept
psychological support	psychological support	psychological support	psychological support	psychological support	6	kept
psychological well being	psychological well being	psychological well being	psychological well being	psychological well-being	6	case_or_format
Psychometric	psychometric	psychometric	psychometric	Psychometrics	6	case_or_format
PSYCHOMETRIC EVALUATION	psychometric evaluation	psychometric evaluation	psychometric evaluation	PSYCHOMETRIC EVALUATION	6	kept
PSYCHOMETRIC PROPERTIES	psychometric properties	psychometric properties	psychometric properties	Psychometric properties	6	case_or_format
psychomotor performance	psychomotor performance	psychomotor performance	psychomotor performance	Psychomotor Performance	6	case_or_format
puerperal depression	puerperal depression	puerperal depression	puerperal depression	puerperal depression	6	kept
pulmonary artery catheterization	pulmonary artery catheterization	pulmonary artery catheterization	pulmonary artery catheterization	pulmonary artery catheterization	6	kept
pulmonary artery occlusion pressure	pulmonary artery occlusion pressure	pulmonary artery occlusion pressure	pulmonary artery occlusion pressure	pulmonary artery occlusion pressure	6	kept
Pulmonary embolism	pulmonary embolism	pulmonary embolism	pulmonary embolism	Pulmonary Embolism	6	case_or_format
pulse pressure	pulse pressure	pulse pressure	pulse pressure	pulse pressure	6	kept
pupillometry	pupillometry	pupillometry	pupillometry	pupillometry	6	kept
pylorus	pylorus	pylorus	pylorus	Pylorus	6	case_or_format
pyridoxine	pyridoxine	pyridoxine	pyridoxine	pyridoxine	6	kept
QTc interval	qtc interval	qtc interval	qtc interval	QTc interval	6	kept

QUALITY IMPROVEMENT	quality improvement	quality improvement	quality improvement	Quality Improvement	6	case_or_format
quality measures	quality measures	quality measures	quality measure	quality measures	6	singular
questionnaire design	questionnaire design	questionnaire design	questionnaire design	questionnaire design	6	kept
Questionnaire survey	questionnaire survey	questionnaire survey	questionnaire survey	questionnaire survey	6	case_or_format
quick Sequential Organ Failure Assessment Score	quick sequential organ failure assessment score	quick sequential organ failure assessment score	quick sequential organ failure assessment score	quick Sequential Organ Failure Assessment Score	6	kept
Radiation Exposure	radiation exposure	radiation exposure	radiation exposure	radiation exposure	6	case_or_format
radiographer	radiographer	radiographer	radiographer	radiographer	6	kept
Radiology Information Systems	radiology information systems	radiology information systems	radiology information systems	Radiology Information Systems	6	kept
Randomized controlled trials	randomized controlled trials	randomized controlled trials	randomized controlled trial	randomized controlled trial	6	singular
rapamycin	rapamycin	rapamycin	rapamycin	rapamycin	6	kept
Rape	rape	rape	rape	Rape	6	kept
rapid sequence induction	rapid sequence induction	rapid sequence induction	rapid sequence induction	rapid sequence induction	6	kept
RCT	rct	rct	rct	RCT	6	kept
Real-Time Polymerase Chain Reaction	real time polymerase chain reaction	real time polymerase chain reaction	real time polymerase chain reaction	real time polymerase chain reaction	6	case_or_format
recipient	recipient	recipient	recipient	recipient	6	kept
recommendations	recommendations	recommendations	recommendation	Recommendations	6	singular

Records	records	records	record	Records	6	singular
rectum hemorrhage	rectum hemorrhage	rectum hemorrhage	rectum hemorrhage	rectum hemorrhage	6	kept
recumbency	recumbency	recumbency	recumbency	recumbency	6	kept
red blood cell distribution width	red blood cell distribution width	red blood cell distribution width	red blood cell distribution width	red blood cell distribution width	6	kept
Reflex	reflex	reflex	reflex	Reflex	6	kept
Regional Health Planning	regional health planning	regional health planning	regional health planning	Regional Health Planning	6	kept
Reimbursement	reimbursement	reimbursement	reimbursement	reimbursement	6	case_or_format
relaxation	relaxation	relaxation	relaxation	relaxation	6	kept
reliability and validity	reliability and validity	reliability and validity	reliability and validity	reliability and validity	6	kept
Relocation stress	relocation stress	relocation stress	relocation stress	Relocation stress	6	kept
remuneration	remuneration	remuneration	remuneration	remuneration	6	kept
Renal dialysis	renal dialysis	renal dialysis	renal dialysis	Renal Dialysis	6	case_or_format
Reproducibility of results	reproducibility of results	reproducibility of results	reproducibility of results	Reproducibility of Results	6	case_or_format
research in practice	research in practice	research in practice	research in practice	research in practice	6	kept
Research priorities	research priorities	research priorities	research priority	Research priorities	6	singular
Residual Volume	residual volume	residual volume	residual volume	residual volume	6	case_or_format

Resource use	resource use	resource use	resource use	resource use	6	case_or_format
RESOURCES	resources	resources	resource	Resources	6	singular
respiratory alkalosis	respiratory alkalosis	respiratory alkalosis	respiratory alkalosis	respiratory alkalosis	6	kept
respiratory infections	respiratory infections	respiratory infections	respiratory infection	respiratory infections	6	singular
Respiratory Muscles	respiratory muscles	respiratory muscles	respiratory muscle	Respiratory Muscles	6	singular
Respiratory support	respiratory support	respiratory support	respiratory support	Respiratory support	6	kept
respiratory support	respiratory support	respiratory support	respiratory support	Respiratory support	6	case_or_format
Respiratory system	respiratory system	respiratory system	respiratory system	respiratory system	6	case_or_format
Respite Care	respite care	respite care	respite care	Respite Care	6	kept
Restraints	restraints	restraints	restraint	Restraint	6	singular
restraints	restraints	restraints	restraint	Restraint	6	singular
resuscitation orders	resuscitation orders	resuscitation orders	resuscitation orders	Resuscitation Orders	6	case_or_format
Rhinovirus	rhinovirus	rhinovirus	rhinovirus	Rhinovirus	6	kept
Rib fractures	rib fractures	rib fractures	rib fracture	rib fracture	6	singular
risk stratification	risk stratification	risk stratification	risk stratification	Risk stratification	6	case_or_format
Risk-Taking	risk taking	risk taking	risk taking	Risk-Taking	6	kept

RNA	rna	rna	rna	RNA	6	kept
role	role	role	role	Role	6	case_or_format
Root cause analysis	root cause analysis	root cause analysis	root cause analysis	root cause analysis	6	case_or_format
Rural Health	rural health	rural health	rural health	rural health	6	case_or_format
SARS-CoV-2 infection	sars cov 2 infection	sars cov 2 infection	sars cov 2 infection	SARS-CoV-2 infection	6	kept
Saudi	saudi	saudi	saudi	Saudi	6	kept
SBAR	sbar	sbar	sbar	SBAR	6	kept
scale development	scale development	scale development	scale development	Scale development	6	case_or_format
scar formation	scar formation	scar formation	scar formation	scar formation	6	kept
Scheduling	scheduling	scheduling	scheduling	Scheduling	6	kept
schizoaffective psychosis	schizoaffective psychosis	schizoaffective psychosis	schizoaffective psychosis	schizoaffective psychosis	6	kept
school	school	school	school	school	6	kept
Schools, Medical	schools medical	schools medical	schools medical	Schools, Medical	6	kept
seclusion	seclusion	seclusion	seclusion	seclusion	6	kept
secondary infection	secondary infection	secondary infection	secondary infection	secondary infection	6	kept
secretion	secretion	secretion	secretion	secretion	6	kept

Security	security	security	security	Security	6	kept
Sedation assessment	sedation assessment	sedation assessment	sedation assessment	Sedation assessment	6	kept
Seizure	seizure	seizure	seizure	seizure	6	case_or_format
Selection Bias	selection bias	selection bias	selection bias	Selection Bias	6	kept
self disclosure	self disclosure	self disclosure	self disclosure	self disclosure	6	kept
Self-assessment	self assessment	self assessment	self assessment	Self-Assessment	6	case_or_format
self-directed learning	self directed learning	self directed learning	self directed learning	self-directed learning	6	kept
Self-extubation	self extubation	self extubation	self extubation	Self-extubation	6	kept
Semantic Web	semantic web	semantic web	semantic web	Semantic Web	6	kept
Sense of Coherence	sense of coherence	sense of coherence	sense of coherence	Sense of Coherence	6	kept
Sensors	sensors	sensors	sensor	sensor	6	singular
sequence analysis	sequence analysis	sequence analysis	sequence analysis	sequence analysis	6	kept
sertraline	sertraline	sertraline	sertraline	sertraline	6	kept
severe acute pancreatitis	severe acute pancreatitis	severe acute pancreatitis	severe acute pancreatitis	severe acute pancreatitis	6	kept
severe asthma	severe asthma	severe asthma	severe asthma	severe asthma	6	kept
SEVERE SEPSIS	severe sepsis	severe sepsis	severe sepsis	severe sepsis	6	case_or_format

Severity	severity	severity	severity	SEVERITY	6	case_or_format
severity	severity	severity	severity	SEVERITY	6	case_or_format
severity of illness	severity of illness	severity of illness	severity of illness	Severity of illness	6	case_or_format
Sex	sex	sex	sex	sex	6	case_or_format
Sexism	sexism	sexism	sexism	Sexism	6	kept
sexual health	sexual health	sexual health	sexual health	sexual health	6	kept
Shared decision making	shared decision making	shared decision making	shared decision making	shared decision making	6	case_or_format
shoulder dystocia	shoulder dystocia	shoulder dystocia	shoulder dystocia	shoulder dystocia	6	kept
shoulder pain	shoulder pain	shoulder pain	shoulder pain	shoulder pain	6	kept
sickle cell anemia	sickle cell anemia	sickle cell anemia	sickle cell anemia	sickle cell anemia	6	kept
silicone derivative	silicone derivative	silicone derivative	silicone derivative	silicone derivative	6	kept
SIRS	sirs	sirs	sirs	SIRS	6	kept
Six Sigma	six sigma	six sigma	six sigma	Six Sigma	6	kept
SKIN	skin	skin	skin	Skin	6	case_or_format
skin flora	skin flora	skin flora	skin flora	skin flora	6	kept
skin incision	skin incision	skin incision	skin incision	skin incision	6	kept

skin pruritus	skin pruritus	skin pruritus	skin pruritus	skin pruritus	6	kept
skin test	skin test	skin test	skin test	Skin Tests	6	case_or_format
Skin Ulcer	skin ulcer	skin ulcer	skin ulcer	skin ulcer	6	case_or_format
Skin-to-skin	skin to skin	skin to skin	skin to skin	skin-to-skin	6	case_or_format
Sleep Apnea, Central	sleep apnea central	sleep apnea central	sleep apnea central	Sleep Apnea, Central	6	kept
Sleep disorders	sleep disorders	sleep disorders	sleep disorder	sleep disorder	6	singular
sleep disturbances	sleep disturbances	sleep disturbances	sleep disturbance	Sleep disturbance	6	singular
small intestine resection	small intestine resection	small intestine resection	small intestine resection	small intestine resection	6	kept
sneezing	sneezing	sneezing	sneezing	sneezing	6	kept
social justice	social justice	social justice	social justice	Social Justice	6	case_or_format
social needs	social needs	social needs	social needs	social needs	6	kept
sodium urine level	sodium urine level	sodium urine level	sodium urine level	sodium urine level	6	kept
solid tumor	solid tumor	solid tumor	solid tumor	solid tumor	6	kept
South America	south america	south america	south america	South America	6	kept
specimen handling	specimen handling	specimen handling	specimen handling	Specimen Handling	6	case_or_format
Spectrophotometry	spectrophotometry	spectrophotometry	spectrophotometry	Spectrophotometry	6	kept

Spinal Neoplasms	spinal neoplasms	spinal neoplasms	spinal neoplasms	Spinal Neoplasms	6	kept
spiritual healing	spiritual healing	spiritual healing	spiritual healing	spiritual healing	6	kept
spiritual well-being	spiritual well being	spiritual well being	spiritual well being	spiritual well-being	6	kept
Splenectomy	splenectomy	splenectomy	splenectomy	Splenectomy	6	kept
spring	spring	spring	spring	spring	6	kept
sputum analysis	sputum analysis	sputum analysis	sputum analysis	sputum analysis	6	kept
ST segment depression	st segment depression	st segment depression	st segment depression	ST segment depression	6	kept
stab wound	stab wound	stab wound	stab wound	stab wound	6	kept
Staff development	staff development	staff development	staff development	Staff Development	6	case_or_format
Staphylococcus aureus infection	staphylococcus aureus infection	staphylococcus aureus infection	staphylococcus aureus infection	Staphylococcus aureus infection	6	kept
statistically significant result	statistically significant result	statistically significant result	statistically significant result	statistically significant result	6	kept
Sternum	sternum	sternum	sternum	Sternum	6	kept
Stethoscope	stethoscope	stethoscope	stethoscope	Stethoscopes	6	case_or_format
Stomach Neoplasms	stomach neoplasms	stomach neoplasms	stomach neoplasms	Stomach Neoplasms	6	kept
stomach ulcer	stomach ulcer	stomach ulcer	stomach ulcer	stomach ulcer	6	kept
STRATEGY	strategy	strategy	strategy	STRATEGIES	6	case_or_format

Stress, psychological	stress psychological	stress psychological	stress psychological	Stress, Psychological	6	case_or_format
stressors	stressors	stressors	stressor	Stressors	6	singular
subcutaneous drug administration	subcutaneous drug administration	subcutaneous drug administration	subcutaneous drug administration	subcutaneous drug administration	6	kept
suburban area	suburban area	suburban area	suburban area	suburban area	6	kept
Suffering	suffering	suffering	suffering	suffering	6	case_or_format
Suicide Prevention	suicide prevention	suicide prevention	suicide prevention	Suicide Prevention	6	kept
Support vector machines	support vector machines	support vector machines	support vector machine	support vector machine	6	singular
Supportive care	supportive care	supportive care	supportive care	supportive care	6	case_or_format
suppository	suppository	suppository	suppository	suppository	6	kept
Surface-Active Agents	surface active agents	surface active agents	surface active agents	Surface-Active Agents	6	kept
surgical equipment	surgical equipment	surgical equipment	surgical equipment	surgical equipment	6	kept
surgical glove	surgical glove	surgical glove	surgical glove	surgical glove	6	kept
surgical mask	surgical mask	surgical mask	surgical mask	surgical mask	6	kept
surveys	surveys	surveys	survey	Survey	6	singular
Surviving Sepsis Campaign	surviving sepsis campaign	surviving sepsis campaign	surviving sepsis campaign	Surviving Sepsis Campaign	6	kept
SURVIVORS	survivors	survivors	survivor	Survivors	6	singular

suspension	suspension	suspension	suspension	suspension	6	kept
sustainable development	sustainable development	sustainable development	sustainable development	sustainable development	6	kept
Swiss	swiss	swiss	swiss	Swiss	6	kept
symbolism	symbolism	symbolism	symbolism	Symbolism	6	case_or_format
symposium	symposium	symposium	symposium	symposium	6	kept
Symptom Checklist 90	symptom checklist 90	symptom checklist 90	symptom checklist 90	Symptom Checklist 90	6	kept
Syria	syria	syria	syria	Syria	6	kept
Takotsubo cardiomyopathy	takotsubo cardiomyopathy	takotsubo cardiomyopathy	takotsubo cardiomyopathy	takotsubo cardiomyopathy	6	case_or_format
targeted temperature management	targeted temperature management	targeted temperature management	targeted temperature management	Targeted temperature management	6	case_or_format
taxonomy	taxonomy	taxonomy	taxonomy	taxonomy	6	kept
TBI	tbi	tbi	tbi	TBI	6	kept
team	team	team	team	Team	6	case_or_format
TEAMS	teams	teams	team	Team	6	singular
Technology Transfer	technology transfer	technology transfer	technology transfer	Technology Transfer	6	kept
Technology, Medical	technology medical	technology medical	technology medical	Technology, Medical	6	kept
telecommuting	telecommuting	telecommuting	telecommuting	telecommuting	6	kept

terbutaline	terbutaline	terbutaline	terbutaline	terbutaline	6	kept
term birth	term birth	term birth	term birth	term birth	6	kept
Terminology	terminology	terminology	terminology	Terminology	6	kept
tetracycline derivative	tetracycline derivative	tetracycline derivative	tetracycline derivative	tetracycline derivative	6	kept
Theophylline	theophylline	theophylline	theophylline	theophylline	6	case_or_format
Therapeutic communication	therapeutic communication	therapeutic communication	therapeutic communication	Therapeutic communication	6	kept
therapeutic error	therapeutic error	therapeutic error	therapeutic error	therapeutic error	6	kept
thoracic aorta	thoracic aorta	thoracic aorta	thoracic aorta	thoracic aorta	6	kept
ticlopidine	ticlopidine	ticlopidine	ticlopidine	ticlopidine	6	kept
Time Perception	time perception	time perception	time perception	time perception	6	case_or_format
TISS-28	tiss 28	tiss 28	tiss 28	TISS-28	6	kept
Tissue plasminogen activator	tissue plasminogen activator	tissue plasminogen activator	tissue plasminogen activator	tissue plasminogen activator	6	case_or_format
Tolerance	tolerance	tolerance	tolerance	Tolerance	6	kept
tongue	tongue	tongue	tongue	tongue	6	kept
total hip replacement	total hip replacement	total hip replacement	total hip replacement	total hip replacement	6	kept
tourniquet	tourniquet	tourniquet	tourniquet	tourniquet	6	kept

Tracheobronchomalacia	tracheobronchomalacia	tracheobronchomalacia	tracheobronchomalacia	Tracheobronchomalacia	6	kept
tracheomalacia	tracheomalacia	tracheomalacia	tracheomalacia	tracheomalacia	6	kept
Training program	training program	training program	training program	Training program	6	kept
training program	training program	training program	training program	Training program	6	case_or_format
TRANSITION	transition	transition	transition	Transition	6	case_or_format
TRANSMISSION	transmission	transmission	transmission	transmission	6	case_or_format
Transplants	transplants	transplants	transplant	Transplants	6	singular
Trauma, Nervous System	trauma nervous system	trauma nervous system	trauma nervous system	Trauma, Nervous System	6	kept
traumatic	traumatic	traumatic	traumatic	traumatic	6	kept
Traumatic brain injuries	traumatic brain injuries	traumatic brain injuries	traumatic brain injury	traumatic brain injury	6	singular
trisomy 18	trisomy 18	trisomy 18	trisomy 18	trisomy 18	6	kept
Tuberculin Test	tuberculin test	tuberculin test	tuberculin test	tuberculin test	6	case_or_format
TURNOVER	turnover	turnover	turnover	turnover	6	case_or_format
type A aortic dissection	type a aortic dissection	type a aortic dissection	type a aortic dissection	type A aortic dissection	6	kept
Uremia	uremia	uremia	uremia	uremia	6	case_or_format
uric acid	uric acid	uric acid	uric acid	uric acid	6	kept

Urinalysis	urinalysis	urinalysis	urinalysis	urinalysis	6	case_or_format
urinary catheterization	urinary catheterization	urinary catheterization	urinary catheterization	Urinary Catheterization	6	case_or_format
Urinary Retention	urinary retention	urinary retention	urinary retention	Urinary Retention	6	kept
Urinary tract infections	urinary tract infections	urinary tract infections	urinary tract infection	urinary tract infection	6	singular
Usability engineering	usability engineering	usability engineering	usability engineering	Usability engineering	6	kept
User centered design	user centered design	user centered design	user centered design	user-centered design	6	case_or_format
User interfaces	user interfaces	user interfaces	user interface	User interfaces	6	singular
Uterine Hemorrhage	uterine hemorrhage	uterine hemorrhage	uterine hemorrhage	Uterine Hemorrhage	6	kept
Vaginal Birth after Cesarean	vaginal birth after cesarean	vaginal birth after cesarean	vaginal birth after cesarean	Vaginal Birth after Cesarean	6	kept
validation studies	validation studies	validation studies	validation study	validation study	6	singular
Validation Studies as Topic	validation studies as topic	validation studies as topic	validation studies as topic	Validation Studies as Topic	6	kept
value	value	value	value	value	6	kept
Value of Life	value of life	value of life	value of life	Value of Life	6	kept
Vascular Patency	vascular patency	vascular patency	vascular patency	vascular patency	6	case_or_format
vasculitis	vasculitis	vasculitis	vasculitis	vasculitis	6	kept
vaxzevria	vaxzevria	vaxzevria	vaxzevria	vaxzevria	6	kept

venous access	venous access	venous access	venous access	venous access	6	kept
Ventilator-associated events	ventilator associated events	ventilator associated events	ventilator associated event	Ventilator-associated events	6	singular
Ventilator-Associated Pneumonia	ventilator associated pneumonia	ventilator associated pneumonia	ventilator associated pneumonia	ventilator associated pneumonia	6	case_or_format
verbal behavior	verbal behavior	verbal behavior	verbal behavior	Verbal Behavior	6	case_or_format
Very low birth weight infant	very low birth weight infant	very low birth weight infant	very low birth weight infant	Very low birth weight infant	6	kept
Very preterm infants	very preterm infants	very preterm infants	very preterm infant	Very preterm infants	6	singular
veterans	veterans	veterans	veteran	Veterans	6	singular
Video	video	video	video	Video	6	kept
vincristine	vincristine	vincristine	vincristine	vincristine	6	kept
viral bronchiolitis	viral bronchiolitis	viral bronchiolitis	viral bronchiolitis	viral bronchiolitis	6	kept
viral contamination	viral contamination	viral contamination	viral contamination	viral contamination	6	kept
virus encephalitis	virus encephalitis	virus encephalitis	virus encephalitis	virus encephalitis	6	kept
Vision Screening	vision screening	vision screening	vision screening	Vision Screening	6	kept
visitation	visitation	visitation	visitation	Visitation	6	case_or_format
visiting	visiting	visiting	visiting	Visiting	6	case_or_format
Visiting policy	visiting policy	visiting policy	visiting policy	Visiting policy	6	kept

visual acuity	visual acuity	visual acuity	visual acuity	visual acuity	6	kept
Vitamin D Deficiency	vitamin d deficiency	vitamin d deficiency	vitamin d deficiency	vitamin D deficiency	6	case_or_format
vocal cord	vocal cord	vocal cord	vocal cord	vocal cord	6	kept
VOLUME	volume	volume	volume	VOLUME	6	kept
waiting room	waiting room	waiting room	waiting room	waiting room	6	kept
water supply	water supply	water supply	water supply	water supply	6	kept
Wearable technology	wearable technology	wearable technology	wearable technology	Wearable technology	6	kept
web-based	web based	web based	web based	web-based	6	kept
Wilcoxon signed ranks test	wilcoxon signed ranks test	wilcoxon signed ranks test	wilcoxon signed ranks test	Wilcoxon signed ranks test	6	kept
winter	winter	winter	winter	winter	6	kept
Withdrawal symptoms	withdrawal symptoms	withdrawal symptoms	withdrawal symptoms	Withdrawal symptoms	6	kept
withholding treatment	withholding treatment	withholding treatment	withholding treatment	Withholding Treatment	6	case_or_format
Work hours	work hours	work hours	work hour	Work hours	6	singular
work satisfaction	work satisfaction	work satisfaction	work satisfaction	work satisfaction	6	kept
work stress	work stress	work stress	work stress	Work stress	6	case_or_format
Workers' Compensation	workers compensation	workers compensation	workers compensation	Workers' Compensation	6	kept

working memory	working memory	working memory	working memory	working memory	6	kept
WORKPLACE	workplace	workplace	workplace	Workplace	6	case_or_format
work‐life balance	work life balance	work life balance	work life balance	work-life balance	6	case_or_format
Wound management	wound management	wound management	wound management	Wound management	6	kept
Yoga	yoga	yoga	yoga	yoga	6	case_or_format
Zika Virus Infection	zika virus infection	zika virus infection	zika virus infection	Zika Virus Infection	6	kept
zolpidem	zolpidem	zolpidem	zolpidem	zolpidem	6	kept
4 methylpyrazole	4 methylpyrazole	4 methylpyrazole	4 methylpyrazole	4 methylpyrazole	5	kept
abdominal aorta	abdominal aorta	abdominal aorta	abdominal aorta	abdominal aorta	5	kept
abdominal aortic aneurysm	abdominal aortic aneurysm	abdominal aortic aneurysm	abdominal aortic aneurysm	abdominal aortic aneurysm	5	kept
Abdominal Wound Closure Techniques	abdominal wound closure techniques	abdominal wound closure techniques	abdominal wound closure techniques	Abdominal Wound Closure Techniques	5	kept
Abortion, Spontaneous	abortion spontaneous	abortion spontaneous	abortion spontaneous	Abortion, Spontaneous	5	kept
Accelerometry	accelerometry	accelerometry	accelerometry	Accelerometry	5	kept
accelerometry	accelerometry	accelerometry	accelerometry	Accelerometry	5	case_or_format
ACCESS	access	access	access	access	5	case_or_format
access to care	access to care	access to care	access to care	access to care	5	kept

accounting	accounting	accounting	accounting	accounting	5	kept
acepromazine	acepromazine	acepromazine	acepromazine	acepromazine	5	kept
acetazolamide	acetazolamide	acetazolamide	acetazolamide	acetazolamide	5	kept
acetic acid	acetic acid	acetic acid	acetic acid	acetic acid	5	kept
Acid-Base Imbalance	acid base imbalance	acid base imbalance	acid base imbalance	Acid-Base Imbalance	5	kept
Acidosis, Respiratory	acidosis respiratory	acidosis respiratory	acidosis respiratory	Acidosis, Respiratory	5	kept
acne	acne	acne	acne	acne	5	kept
acoustics	acoustics	acoustics	acoustics	Acoustics	5	case_or_format
activated carbon	activated carbon	activated carbon	activated carbon	activated carbon	5	kept
active listening	active listening	active listening	active listening	active listening	5	kept
Acuity	acuity	acuity	acuity	Acuity	5	kept
acute cholecystitis	acute cholecystitis	acute cholecystitis	acute cholecystitis	acute cholecystitis	5	kept
acute confusion	acute confusion	acute confusion	acute confusion	acute confusion	5	kept
ACUTE KIDNEY INJURY	acute kidney injury	acute kidney injury	acute kidney injury	Acute Kidney Injury	5	case_or_format
acute on chronic liver failure	acute on chronic liver failure	acute on chronic liver failure	acute on chronic liver failure	acute on chronic liver failure	5	kept
acute phase protein	acute phase protein	acute phase protein	acute phase protein	acute phase protein	5	kept

ACUTE RESPIRATORY-FAILURE	acute respiratory failure	acute respiratory failure	acute respiratory failure	acute respiratory failure	5	case_or_format
acute stress	acute stress	acute stress	acute stress	acute stress	5	kept
add on therapy	add on therapy	add on therapy	add on therapy	add on therapy	5	kept
Addiction	addiction	addiction	addiction	addiction	5	case_or_format
Adenocarcinoma	adenocarcinoma	adenocarcinoma	adenocarcinoma	Adenocarcinoma	5	kept
adenocarcinoma	adenocarcinoma	adenocarcinoma	adenocarcinoma	Adenocarcinoma	5	case_or_format
Adenosine Triphosphate	adenosine triphosphate	adenosine triphosphate	adenosine triphosphate	adenosine triphosphate	5	case_or_format
Adjuvants, Anesthesia	adjuvants anesthesia	adjuvants anesthesia	adjuvants anesthesia	Adjuvants, Anesthesia	5	kept
ADMINISTRATION ERRORS	administration errors	administration errors	administration error	ADMINISTRATION ERRORS	5	singular
Administration, Intranasal	administration intranasal	administration intranasal	administration intranasal	Administration, Intranasal	5	kept
Administrative Personnel	administrative personnel	administrative personnel	administrative personnel	administrative personnel	5	case_or_format
Admission	admission	admission	admission	ADMISSION	5	case_or_format
admission	admission	admission	admission	ADMISSION	5	case_or_format
Adrenal Gland Neoplasms	adrenal gland neoplasms	adrenal gland neoplasms	adrenal gland neoplasms	Adrenal Gland Neoplasms	5	kept
Adrenal Hyperplasia, Congenital	adrenal hyperplasia congenital	adrenal hyperplasia congenital	adrenal hyperplasia congenital	Adrenal Hyperplasia, Congenital	5	kept
Adrenal Insufficiency	adrenal insufficiency	adrenal insufficiency	adrenal insufficiency	Adrenal Insufficiency	5	kept

Adrenergic alpha-2 Receptor Agonists	adrenergic alpha 2 receptor agonists	adrenergic alpha 2 receptor agonists	adrenergic alpha 2 receptor agonists	Adrenergic alpha-2 Receptor Agonists	5	kept
adult palliative care	adult palliative care	adult palliative care	adult palliative care	adult palliative care	5	kept
ADVANCED CANCER	advanced cancer	advanced cancer	advanced cancer	advanced cancer	5	case_or_format
advanced directives	advanced directives	advanced directives	advanced directives	advanced directives	5	kept
Advanced heart failure	advanced heart failure	advanced heart failure	advanced heart failure	Advanced heart failure	5	kept
adverse drug events	adverse drug events	adverse drug events	adverse drug event	ADVERSE DRUG EVENTS	5	singular
Aeromedical evacuation	aeromedical evacuation	aeromedical evacuation	aeromedical evacuation	Aeromedical evacuation	5	kept
Africa, Western	africa western	africa western	africa western	Africa, Western	5	kept
Agent-based modeling	agent based modeling	agent based modeling	agent based modeling	Agent-based modeling	5	kept
agitation assessment	agitation assessment	agitation assessment	agitation assessment	agitation assessment	5	kept
AGREEMENT	agreement	agreement	agreement	AGREEMENT	5	kept
AHP	ahp	ahp	ahp	AHP	5	kept
air quality	air quality	air quality	air quality	air quality	5	kept
air transportation	air transportation	air transportation	air transportation	air transportation	5	kept
Airborne transmission	airborne transmission	airborne transmission	airborne transmission	airborne transmission	5	case_or_format
Airway Resistance	airway resistance	airway resistance	airway resistance	Airway Resistance	5	kept

airway resistance	airway resistance	airway resistance	airway resistance	Airway Resistance	5	case_or_format
alarm	alarm	alarm	alarm	Alarms	5	case_or_format
alcohol liver cirrhosis	alcohol liver cirrhosis	alcohol liver cirrhosis	alcohol liver cirrhosis	alcohol liver cirrhosis	5	kept
aldosterone antagonist	aldosterone antagonist	aldosterone antagonist	aldosterone antagonist	aldosterone antagonist	5	kept
Algeria	algeria	algeria	algeria	Algeria	5	kept
allergen	allergen	allergen	allergen	Allergens	5	case_or_format
allergic rhinitis	allergic rhinitis	allergic rhinitis	allergic rhinitis	allergic rhinitis	5	kept
alpha adrenergic receptor stimulating agent	alpha adrenergic receptor stimulating agent	alpha adrenergic receptor stimulating agent	alpha adrenergic receptor stimulating agent	alpha adrenergic receptor stimulating agent	5	kept
Alzheimer’ s disease	alzheimer s disease	alzheimer s disease	alzheimer s disease	Alzheimer’s disease	5	case_or_format
ambiguity	ambiguity	ambiguity	ambiguity	ambiguity	5	kept
American	american	american	american	American	5	kept
amino acid	amino acid	amino acid	amino acid	amino acid	5	kept
Amino Acid Metabolism, Inborn Errors	amino acid metabolism inborn errors	amino acid metabolism inborn errors	amino acid metabolism inborn errors	Amino Acid Metabolism, Inborn Errors	5	kept
Amino Acids	amino acids	amino acids	amino acid	amino acid	5	singular
aminotransferase	aminotransferase	aminotransferase	aminotransferase	aminotransferase	5	kept
amnion fluid	amnion fluid	amnion fluid	amnion fluid	amnion fluid	5	kept

amphotericin B lipid complex	amphotericin b lipid complex	amphotericin b lipid complex	amphotericin b lipid complex	amphotericin B lipid complex	5	kept
Amputation	amputation	amputation	amputation	amputation	5	case_or_format
Amputation, Surgical	amputation surgical	amputation surgical	amputation surgical	Amputation, Surgical	5	kept
anakinra	anakinra	anakinra	anakinra	anakinra	5	kept
analysis of covariance	analysis of covariance	analysis of covariance	analysis of covariance	analysis of covariance	5	kept
Anatomic Landmarks	anatomic landmarks	anatomic landmarks	anatomic landmark	anatomic landmark	5	singular
anatomy and histology	anatomy and histology	anatomy and histology	anatomy and histology	anatomy and histology	5	kept
Anemia, Neonatal	anemia neonatal	anemia neonatal	anemia neonatal	Anemia, Neonatal	5	kept
anesthesiological techniques	anesthesiological techniques	anesthesiological techniques	anesthesiological techniques	anesthesiological techniques	5	kept
Anesthetics, Dissociative	anesthetics dissociative	anesthetics dissociative	anesthetics dissociative	Anesthetics, Dissociative	5	kept
Aneurysmal subarachnoid hemorrhage	aneurysmal subarachnoid hemorrhage	aneurysmal subarachnoid hemorrhage	aneurysmal subarachnoid hemorrhage	Aneurysmal subarachnoid hemorrhage	5	kept
Angioplasty, Transluminal, Percutaneous Coronary	angioplasty transluminal percutaneous coronary	angioplasty transluminal percutaneous coronary	angioplasty transluminal percutaneous coronary	Angioplasty, Transluminal, Percutaneous Coronary	5	kept
Anorexia	anorexia	anorexia	anorexia	anorexia	5	case_or_format
anthropometric parameters	anthropometric parameters	anthropometric parameters	anthropometric parameters	anthropometric parameters	5	kept
Anti-HIV Agents	anti hiv agents	anti hiv agents	anti hiv agents	Anti-HIV Agents	5	kept
Antibacterial agents	antibacterial agents	antibacterial agents	antibacterial agents	Antibacterial agents	5	kept

antibiotic management	antibiotic management	antibiotic management	antibiotic management	antibiotic management	5	kept
Antibiotic prophylaxis	antibiotic prophylaxis	antibiotic prophylaxis	antibiotic prophylaxis	antibiotic prophylaxis	5	case_or_format
Antibodies	antibodies	antibodies	antibody	Antibodies	5	singular
Antibodies, Bacterial	antibodies bacterial	antibodies bacterial	antibodies bacterial	Antibodies, Bacterial	5	kept
anticipatory grief	anticipatory grief	anticipatory grief	anticipatory grief	anticipatory grief	5	kept
anticoagulants	anticoagulants	anticoagulants	anticoagulant	Anticoagulants	5	singular
anticoagulation reversal	anticoagulation reversal	anticoagulation reversal	anticoagulation reversal	anticoagulation reversal	5	kept
antifibrinolytic agent	antifibrinolytic agent	antifibrinolytic agent	antifibrinolytic agent	antifibrinolytic agent	5	kept
antimalarial agent	antimalarial agent	antimalarial agent	antimalarial agent	antimalarial agent	5	kept
ANTIMICROBIAL RESISTANCE	antimicrobial resistance	antimicrobial resistance	antimicrobial resistance	Antimicrobial resistance	5	case_or_format
antithrombin	antithrombin	antithrombin	antithrombin	antithrombin	5	kept
antithrombin III	antithrombin iii	antithrombin iii	antithrombin iii	antithrombin III	5	kept
Antitubercular Agents	antitubercular agents	antitubercular agents	antitubercular agents	Antitubercular Agents	5	kept
antiulcer agent	antiulcer agent	antiulcer agent	antiulcer agent	antiulcer agent	5	kept
aorta coarctation	aorta coarctation	aorta coarctation	aorta coarctation	aorta coarctation	5	kept
Aortic Aneurysm	aortic aneurysm	aortic aneurysm	aortic aneurysm	Aortic Aneurysm	5	kept

aortic regurgitation	aortic regurgitation	aortic regurgitation	aortic regurgitation	aortic regurgitation	5	kept
appendectomy	appendectomy	appendectomy	appendectomy	appendectomy	5	kept
application	application	application	application	application	5	kept
apps	apps	apps	app	apps	5	singular
Areas of practice	areas of practice	areas of practice	areas of practice	Areas of practice	5	kept
arm pain	arm pain	arm pain	arm pain	arm pain	5	kept
Armed Conflicts	armed conflicts	armed conflicts	armed conflicts	Armed Conflicts	5	kept
Arnold-Chiari Malformation	arnold chiari malformation	arnold chiari malformation	arnold chiari malformation	Arnold-Chiari Malformation	5	kept
art therapy	art therapy	art therapy	art therapy	art therapy	5	kept
Arterial blood gases	arterial blood gases	arterial blood gases	arterial blood gases	Arterial blood gases	5	kept
arteriovenous malformation	arteriovenous malformation	arteriovenous malformation	arteriovenous malformation	arteriovenous malformation	5	kept
artery dissection	artery dissection	artery dissection	artery dissection	artery dissection	5	kept
artery injury	artery injury	artery injury	artery injury	artery injury	5	kept
artery ligation	artery ligation	artery ligation	artery ligation	artery ligation	5	kept
artery thrombosis	artery thrombosis	artery thrombosis	artery thrombosis	artery thrombosis	5	kept
Arthroplasty	arthroplasty	arthroplasty	arthroplasty	arthroplasty	5	case_or_format

Aspergillus niger	aspergillus niger	aspergillus niger	aspergillus niger	Aspergillus niger	5	kept
assessment tools	assessment tools	assessment tools	assessment tool	Assessment tool	5	singular
asthmatic state	asthmatic state	asthmatic state	asthmatic state	asthmatic state	5	kept
asymptomatic carrier	asymptomatic carrier	asymptomatic carrier	asymptomatic carrier	asymptomatic carrier	5	kept
Atelectasis	atelectasis	atelectasis	atelectasis	atelectasis	5	case_or_format
Atención de Enfermería	atenci n de enfermer a	atenci n de enfermer a	atenci n de enfermer a	Atención de enfermería	5	case_or_format
Atracurium	atracurium	atracurium	atracurium	Atracurium	5	kept
atracurium	atracurium	atracurium	atracurium	Atracurium	5	case_or_format
Attention Deficit Disorder with Hyperactivity	attention deficit disorder with hyperactivity	attention deficit disorder with hyperactivity	attention deficit disorder with hyperactivity	Attention Deficit Disorder with Hyperactivity	5	kept
attention deficit hyperactivity disorder	attention deficit hyperactivity disorder	attention deficit hyperactivity disorder	attention deficit hyperactivity disorder	attention deficit hyperactivity disorder	5	kept
AUDIT	audit	audit	audit	Audit	5	case_or_format
augmentative and alternative communication	augmentative and alternative communication	augmentative and alternative communication	augmentative and alternative communication	Augmentative and alternative communication	5	case_or_format
Augmented Reality	augmented reality	augmented reality	augmented reality	augmented reality	5	case_or_format
Australasia	australasia	australasia	australasia	Australasia	5	kept
authentic leadership	authentic leadership	authentic leadership	authentic leadership	authentic leadership	5	kept
Autistic Disorder	autistic disorder	autistic disorder	autistic disorder	Autistic Disorder	5	kept

autologous hematopoietic stem cell transplantation	autologous hematopoietic stem cell transplantation	autologous hematopoietic stem cell transplantation	autologous hematopoietic stem cell transplantation	autologous hematopoietic stem cell transplantation	5	kept
AVIATION	aviation	aviation	aviation	aviation	5	case_or_format
axilla	axilla	axilla	axilla	Axilla	5	case_or_format
Axillary Vein	axillary vein	axillary vein	axillary vein	Axillary Vein	5	kept
Babesiosis	babesiosis	babesiosis	babesiosis	Babesiosis	5	kept
babies	babies	babies	baby	baby	5	singular
Bacillus subtilis	bacillus subtilis	bacillus subtilis	bacillus subtilis	Bacillus subtilis	5	kept
Bacteraemia	bacteraemia	bacteraemia	bacteraemia	Bacteraemia	5	kept
bacterial DNA	bacterial dna	bacterial dna	bacterial dna	bacterial DNA	5	kept
bacterial peritonitis	bacterial peritonitis	bacterial peritonitis	bacterial peritonitis	bacterial peritonitis	5	kept
bacterial superinfection	bacterial superinfection	bacterial superinfection	bacterial superinfection	bacterial superinfection	5	kept
bacterium antibody	bacterium antibody	bacterium antibody	bacterium antibody	bacterium antibody	5	kept
Bacteriuria	bacteriuria	bacteriuria	bacteriuria	Bacteriuria	5	kept
bacteriuria	bacteriuria	bacteriuria	bacteriuria	Bacteriuria	5	case_or_format
Bariatric	bariatric	bariatric	bariatric	Bariatric	5	kept
barriers and facilitators	barriers and facilitators	barriers and facilitators	barriers and facilitators	Barriers and facilitators	5	case_or_format

basal ganglion	basal ganglion	basal ganglion	basal ganglion	basal ganglion	5	kept
Bayesian learning	bayesian learning	bayesian learning	bayesian learning	Bayesian learning	5	kept
Bayesian network	bayesian network	bayesian network	bayesian network	Bayesian network	5	kept
Bayesian networks	bayesian networks	bayesian networks	bayesian network	Bayesian network	5	singular
BEDSIDE	bedside	bedside	bedside	BEDSIDE	5	kept
behavior modification	behavior modification	behavior modification	behavior modification	behavior modification	5	kept
Beijing	beijing	beijing	beijing	Beijing	5	kept
Berlin	berlin	berlin	berlin	Berlin	5	kept
bevacizumab	bevacizumab	bevacizumab	bevacizumab	bevacizumab	5	kept
Bicarbonates	bicarbonates	bicarbonates	bicarbonate	bicarbonate	5	singular
Bifidobacterium	bifidobacterium	bifidobacterium	bifidobacterium	Bifidobacterium	5	kept
Biological Products	biological products	biological products	biological product	Biological Products	5	singular
Birth asphyxia	birth asphyxia	birth asphyxia	birth asphyxia	Birth asphyxia	5	kept
birth center	birth center	birth center	birth center	birth center	5	kept
birth rate	birth rate	birth rate	birth rate	birth rate	5	kept
bites and stings	bites and stings	bites and stings	bites and stings	bites and stings	5	kept

Blogging	blogging	blogging	blogging	Blogging	5	kept
blood autotransfusion	blood autotransfusion	blood autotransfusion	blood autotransfusion	blood autotransfusion	5	kept
Blood Cell Count	blood cell count	blood cell count	blood cell count	blood cell count	5	case_or_format
blood clotting factor 9	blood clotting factor 9	blood clotting factor 9	blood clotting factor 9	blood clotting factor 9	5	kept
blood component	blood component	blood component	blood component	blood component	5	kept
blood conservation	blood conservation	blood conservation	blood conservation	blood conservation	5	kept
Blood Donors	blood donors	blood donors	blood donor	blood donor	5	singular
Blood Pressure Monitoring, Ambulatory	blood pressure monitoring ambulatory	blood pressure monitoring ambulatory	blood pressure monitoring ambulatory	Blood Pressure Monitoring, Ambulatory	5	kept
Blood sampling	blood sampling	blood sampling	blood sampling	blood sampling	5	case_or_format
Blood Transfusion, Autologous	blood transfusion autologous	blood transfusion autologous	blood transfusion autologous	Blood Transfusion, Autologous	5	kept
blood vessel transplantation	blood vessel transplantation	blood vessel transplantation	blood vessel transplantation	blood vessel transplantation	5	kept
bone marrow suppression	bone marrow suppression	bone marrow suppression	bone marrow suppression	bone marrow suppression	5	kept
Bosnia and Herzegovina	bosnia and herzegovina	bosnia and herzegovina	bosnia and herzegovina	Bosnia and Herzegovina	5	kept
brain derived neurotrophic factor	brain derived neurotrophic factor	brain derived neurotrophic factor	brain derived neurotrophic factor	brain derived neurotrophic factor	5	kept
brain infection	brain infection	brain infection	brain infection	brain infection	5	kept
brain metabolism	brain metabolism	brain metabolism	brain metabolism	brain metabolism	5	kept

Brain Stem	brain stem	brain stem	brain stem	brain stem	5	case_or_format
brain surgery	brain surgery	brain surgery	brain surgery	brain surgery	5	kept
breast pump	breast pump	breast pump	breast pump	breast pump	5	kept
Breathing exercises	breathing exercises	breathing exercises	breathing exercise	breathing exercise	5	singular
Bronchiolitis Obliterans	bronchiolitis obliterans	bronchiolitis obliterans	bronchiolitis obliterans	Bronchiolitis Obliterans	5	kept
Bronchitis	bronchitis	bronchitis	bronchitis	bronchitis	5	case_or_format
Bronchoalveolar Lavage	bronchoalveolar lavage	bronchoalveolar lavage	bronchoalveolar lavage	Bronchoalveolar Lavage	5	kept
Bronchoalveolar lavage	bronchoalveolar lavage	bronchoalveolar lavage	bronchoalveolar lavage	Bronchoalveolar Lavage	5	case_or_format
bronchoscope	bronchoscope	bronchoscope	bronchoscope	bronchoscope	5	kept
bronchus secretion	bronchus secretion	bronchus secretion	bronchus secretion	bronchus secretion	5	kept
budesonide	budesonide	budesonide	budesonide	budesonide	5	kept
bumetanide	bumetanide	bumetanide	bumetanide	bumetanide	5	kept
Burden	burden	burden	burden	Burden	5	kept
Burn units	burn units	burn units	burn unit	Burn Units	5	singular
burnout, professional	burnout professional	burnout professional	burnout professional	Burnout, Professional	5	case_or_format
busulfan	busulfan	busulfan	busulfan	busulfan	5	kept

C peptide	c peptide	c peptide	c peptide	C peptide	5	kept
calcium antagonist	calcium antagonist	calcium antagonist	calcium antagonist	calcium antagonist	5	kept
calcium carbonate	calcium carbonate	calcium carbonate	calcium carbonate	calcium carbonate	5	kept
camera	camera	camera	camera	camera	5	kept
cancer immunotherapy	cancer immunotherapy	cancer immunotherapy	cancer immunotherapy	cancer immunotherapy	5	kept
Candida glabrata	candida glabrata	candida glabrata	candida glabrata	Candida glabrata	5	kept
Capacity	capacity	capacity	capacity	Capacity	5	kept
Carbon Monoxide Poisoning	carbon monoxide poisoning	carbon monoxide poisoning	carbon monoxide poisoning	Carbon Monoxide Poisoning	5	kept
Carcinoma, Squamous Cell	carcinoma squamous cell	carcinoma squamous cell	carcinoma squamous cell	Carcinoma, Squamous Cell	5	kept
cardiac care unit	cardiac care unit	cardiac care unit	cardiac care unit	cardiac care unit	5	kept
Cardiac index	cardiac index	cardiac index	cardiac index	Cardiac index	5	kept
Cardiac Surgery	cardiac surgery	cardiac surgery	cardiac surgery	Cardiac surgery	5	case_or_format
Cardiomyopathy, Hypertrophic	cardiomyopathy hypertrophic	cardiomyopathy hypertrophic	cardiomyopathy hypertrophic	Cardiomyopathy, Hypertrophic	5	kept
Cardiopulmonary bypass	cardiopulmonary bypass	cardiopulmonary bypass	cardiopulmonary bypass	cardiopulmonary bypass	5	case_or_format
Cardiotocography	cardiotocography	cardiotocography	cardiotocography	Cardiotocography	5	kept
cardiotocography	cardiotocography	cardiotocography	cardiotocography	Cardiotocography	5	case_or_format

cardiovascular diseases	cardiovascular diseases	cardiovascular diseases	cardiovascular disease	cardiovascular disease	5	singular
care and caring	care and caring	care and caring	care and caring	care and caring	5	kept
care bundles	care bundles	care bundles	care bundle	care bundle	5	singular
care pathways	care pathways	care pathways	care pathway	care pathways	5	singular
care quality	care quality	care quality	care quality	care quality	5	kept
Care transition	care transition	care transition	care transition	care transitions	5	case_or_format
Carga de trabajo	carga de trabajo	carga de trabajo	carga de trabajo	Carga de trabajo	5	kept
Caring behaviors	caring behaviors	caring behaviors	caring behavior	Caring behavior	5	singular
CARRIAGE	carriage	carriage	carriage	CARRIAGE	5	kept
catalase	catalase	catalase	catalase	catalase	5	kept
Catalonia	catalonia	catalonia	catalonia	Catalonia	5	kept
cataract	cataract	cataract	cataract	cataract	5	kept
catering service	catering service	catering service	catering service	catering service	5	kept
Catheter associated urinary tract infection	catheter associated urinary tract infection	catheter associated urinary tract infection	catheter associated urinary tract infection	catheter associated urinary tract infection	5	case_or_format
Catheter-related infection	catheter related infection	catheter related infection	catheter related infection	Catheter-Related Infections	5	case_or_format
catheters	catheters	catheters	catheter	Catheters	5	singular

cefoperazone	cefoperazone	cefoperazone	cefoperazone	cefoperazone	5	kept
Cellulitis	cellulitis	cellulitis	cellulitis	cellulitis	5	case_or_format
Centers for Medicare and Medicaid Services, U.S.	centers for medicare and medicaid services u.s.	centers for medicare and medicaid services u.s.	centers for medicare and medicaid services u.s.	Centers for Medicare and Medicaid Services, U.S.	5	kept
central line	central line	central line	central line	Central line	5	case_or_format
central line-associated bloodstream infections	central line associated bloodstream infections	central line associated bloodstream infections	central line associated bloodstream infection	Central line-associated bloodstream infections	5	singular
central venous	central venous	central venous	central venous	Central venous	5	case_or_format
Cephalometry	cephalometry	cephalometry	cephalometry	Cephalometry	5	kept
Cerebellar Diseases	cerebellar diseases	cerebellar diseases	cerebellar diseases	Cerebellar Diseases	5	kept
Cesarean Section, Repeat	cesarean section repeat	cesarean section repeat	cesarean section repeat	Cesarean Section, Repeat	5	kept
change of shift report	change of shift report	change of shift report	change of shift report	change of shift report	5	kept
Chaplain	chaplain	chaplain	chaplain	Chaplain	5	kept
Charcoal	charcoal	charcoal	charcoal	Charcoal	5	kept
chemiluminescence immunoassay	chemiluminescence immunoassay	chemiluminescence immunoassay	chemiluminescence immunoassay	chemiluminescence immunoassay	5	kept
Chest Wall Oscillation	chest wall oscillation	chest wall oscillation	chest wall oscillation	Chest Wall Oscillation	5	kept
Child abuse	child abuse	child abuse	child abuse	Child Abuse	5	case_or_format
Child Behavior Disorders	child behavior disorders	child behavior disorders	child behavior disorders	Child Behavior Disorders	5	kept

Child Care	child care	child care	child care	child care	5	case_or_format
Child care	child care	child care	child care	child care	5	case_or_format
child psychology	child psychology	child psychology	child psychology	child psychology	5	kept
child welfare	child welfare	child welfare	child welfare	Child Welfare	5	case_or_format
Childbirth	childbirth	childbirth	childbirth	childbirth	5	case_or_format
childbirth education	childbirth education	childbirth education	childbirth education	childbirth education	5	kept
childhood adversity	childhood adversity	childhood adversity	childhood adversity	childhood adversity	5	kept
Chinese version	chinese version	chinese version	chinese version	Chinese version	5	kept
Chlamydophila pneumoniae	chlamydophila pneumoniae	chlamydophila pneumoniae	chlamydophila pneumoniae	Chlamydophila pneumoniae	5	kept
chlorine	chlorine	chlorine	chlorine	chlorine	5	kept
Cholecystectomy, Laparoscopic	cholecystectomy laparoscopic	cholecystectomy laparoscopic	cholecystectomy laparoscopic	Cholecystectomy, Laparoscopic	5	kept
cholinesterase	cholinesterase	cholinesterase	cholinesterase	cholinesterase	5	kept
chronic inflammation	chronic inflammation	chronic inflammation	chronic inflammation	chronic inflammation	5	kept
chronic wound	chronic wound	chronic wound	chronic wound	chronic wound	5	kept
chronically critically ill	chronically critically ill	chronically critically ill	chronically critically ill	chronically critically ill	5	kept
chronobiology	chronobiology	chronobiology	chronobiology	chronobiology	5	kept

circadian rhythm sleep disorder	circadian rhythm sleep disorder	circadian rhythm sleep disorder	circadian rhythm sleep disorder	circadian rhythm sleep disorder	5	kept
cirrhosis	cirrhosis	cirrhosis	cirrhosis	Cirrhosis	5	case_or_format
Citrobacter freundii	citrobacter freundii	citrobacter freundii	citrobacter freundii	Citrobacter freundii	5	kept
classical music	classical music	classical music	classical music	classical music	5	kept
clavicle fracture	clavicle fracture	clavicle fracture	clavicle fracture	clavicle fracture	5	kept
Climate Change	climate change	climate change	climate change	climate change	5	case_or_format
clinical indicator	clinical indicator	clinical indicator	clinical indicator	clinical indicator	5	kept
Clinical information system	clinical information system	clinical information system	clinical information system	Clinical information system	5	kept
Clinical notes	clinical notes	clinical notes	clinical notes	Clinical notes	5	kept
clinical performance	clinical performance	clinical performance	clinical performance	clinical performance	5	kept
Clinical pharmacist	clinical pharmacist	clinical pharmacist	clinical pharmacist	clinical pharmacist	5	case_or_format
Clinical pharmacy	clinical pharmacy	clinical pharmacy	clinical pharmacy	clinical pharmacy	5	case_or_format
Clinical training	clinical training	clinical training	clinical training	Clinical training	5	kept
Clostridioides difficile infection	clostridioides difficile infection	clostridioides difficile infection	clostridioides difficile infection	Clostridioides difficile infection	5	kept
Coccidioidomycosis	coccidioidomycosis	coccidioidomycosis	coccidioidomycosis	Coccidioidomycosis	5	kept
coccidioidomycosis	coccidioidomycosis	coccidioidomycosis	coccidioidomycosis	Coccidioidomycosis	5	case_or_format

code status	code status	code status	code status	code status	5	kept
Codes	codes	codes	codes	Codes	5	kept
Cognitive dysfunction	cognitive dysfunction	cognitive dysfunction	cognitive dysfunction	Cognitive Dysfunction	5	case_or_format
cognitive dysfunction	cognitive dysfunction	cognitive dysfunction	cognitive dysfunction	Cognitive Dysfunction	5	case_or_format
cognitive function test	cognitive function test	cognitive function test	cognitive function test	cognitive function test	5	kept
Cohort studies	cohort studies	cohort studies	cohort study	Cohort Studies	5	singular
cold	cold	cold	cold	cold	5	kept
Colistin	colistin	colistin	colistin	colistin	5	case_or_format
colitis	colitis	colitis	colitis	colitis	5	kept
collaborative care	collaborative care	collaborative care	collaborative care	collaborative care	5	kept
collapse	collapse	collapse	collapse	collapse	5	kept
Colon	colon	colon	colon	Colon	5	kept
Colostomy	colostomy	colostomy	colostomy	colostomy	5	case_or_format
combination chemotherapy	combination chemotherapy	combination chemotherapy	combination chemotherapy	combination chemotherapy	5	kept
COMFORT	comfort	comfort	comfort	comfort	5	case_or_format
communication protocol	communication protocol	communication protocol	communication protocol	communication protocol	5	kept

community dwelling person	community dwelling person	community dwelling person	community dwelling person	community dwelling person	5	kept
competencies	competencies	competencies	competency	Competency	5	singular
competition	competition	competition	competition	competition	5	kept
Complementary medicine	complementary medicine	complementary medicine	complementary medicine	Complementary medicine	5	kept
compulsion	compulsion	compulsion	compulsion	compulsion	5	kept
computer assisted drug therapy	computer assisted drug therapy	computer assisted drug therapy	computer assisted drug therapy	computer assisted drug therapy	5	kept
computer assisted impedance tomography	computer assisted impedance tomography	computer assisted impedance tomography	computer assisted impedance tomography	computer assisted impedance tomography	5	kept
computer language	computer language	computer language	computer language	computer language	5	kept
Condition	condition	condition	condition	Condition	5	kept
Confirmatory factor analysis	confirmatory factor analysis	confirmatory factor analysis	confirmatory factor analysis	confirmatory factor analysis	5	case_or_format
Confounding Factors, Epidemiologic	confounding factors epidemiologic	confounding factors epidemiologic	confounding factors epidemiologic	Confounding Factors, Epidemiologic	5	kept
Congenital	congenital	congenital	congenital	Congenital	5	kept
Congenital heart defects	congenital heart defects	congenital heart defects	congenital heart defect	congenital heart defect	5	singular
consanguinity	consanguinity	consanguinity	consanguinity	consanguinity	5	kept
Consciousness disorders	consciousness disorders	consciousness disorders	consciousness disorder	consciousness disorder	5	singular
Consensus methods	consensus methods	consensus methods	consensus methods	Consensus methods	5	kept

constructivist grounded theory	constructivist grounded theory	constructivist grounded theory	constructivist grounded theory	constructivist grounded theory	5	kept
consumer attitude	consumer attitude	consumer attitude	consumer attitude	consumer attitude	5	kept
Continental Population Groups	continental population groups	continental population groups	continental population groups	Continental Population Groups	5	kept
continuous hemodialysis	continuous hemodialysis	continuous hemodialysis	continuous hemodialysis	continuous hemodialysis	5	kept
contracture	contracture	contracture	contracture	Contracture	5	case_or_format
convalescent plasma therapy	convalescent plasma therapy	convalescent plasma therapy	convalescent plasma therapy	convalescent plasma therapy	5	kept
convergent validity	convergent validity	convergent validity	convergent validity	convergent validity	5	kept
cornea disease	cornea disease	cornea disease	cornea disease	cornea disease	5	kept
Cornell Assessment of Pediatric Delirium	cornell assessment of pediatric delirium	cornell assessment of pediatric delirium	cornell assessment of pediatric delirium	Cornell Assessment of Pediatric Delirium	5	kept
coronary artery	coronary artery	coronary artery	coronary artery	coronary artery	5	kept
Coronary artery bypass graft	coronary artery bypass graft	coronary artery bypass graft	coronary artery bypass graft	coronary artery bypass graft	5	case_or_format
Coronary artery bypass surgery	coronary artery bypass surgery	coronary artery bypass surgery	coronary artery bypass surgery	coronary artery bypass surgery	5	case_or_format
Coronary Artery Bypass, Off-Pump	coronary artery bypass off pump	coronary artery bypass off pump	coronary artery bypass off pump	Coronary Artery Bypass, Off-Pump	5	kept
Coronary Stenosis	coronary stenosis	coronary stenosis	coronary stenosis	Coronary Stenosis	5	kept
coronavac	coronavac	coronavac	coronavac	coronavac	5	kept
Coronaviruses	coronaviruses	coronaviruses	coronaviruses	Coronaviruses	5	kept

cost-benefit analysis	cost benefit analysis	cost benefit analysis	cost benefit analysis	Cost-Benefit Analysis	5	case_or_format
Cost-effectiveness analysis	cost effectiveness analysis	cost effectiveness analysis	cost effectiveness analysis	cost effectiveness analysis	5	case_or_format
course evaluation	course evaluation	course evaluation	course evaluation	course evaluation	5	kept
COVID-19 Nucleic Acid Testing	covid 19 nucleic acid testing	covid 19 nucleic acid testing	covid 19 nucleic acid testing	COVID-19 nucleic acid testing	5	case_or_format
COVID19	covid19	covid 19	covid 19	COVID-19	5	alias
CPR manikin	cpr manikin	cpr manikin	cpr manikin	CPR manikin	5	kept
craniopharyngioma	craniopharyngioma	craniopharyngioma	craniopharyngioma	craniopharyngioma	5	kept
Craniosynostosis	craniosynostosis	craniosynostosis	craniosynostosis	Craniosynostosis	5	kept
creatine kinase MB	creatine kinase mb	creatine kinase mb	creatine kinase mb	creatine kinase MB	5	kept
crew resource management	crew resource management	crew resource management	crew resource management	Crew resource management	5	case_or_format
Crime	crime	crime	crime	crime	5	case_or_format
crime victim	crime victim	crime victim	crime victim	crime victim	5	kept
Crimean Congo hemorrhagic fever	crimean congo hemorrhagic fever	crimean congo hemorrhagic fever	crimean congo hemorrhagic fever	Crimean Congo hemorrhagic fever	5	kept
critical access	critical access	critical access	critical access	critical access	5	kept
critical illness myopathy	critical illness myopathy	critical illness myopathy	critical illness myopathy	critical illness myopathy	5	kept
critically ill children	critically ill children	critically ill children	critically ill children	Critically ill children	5	case_or_format

croup	croup	croup	croup	croup	5	kept
Cuidados de enfermería	cuidados de enfermer a	cuidados de enfermer a	cuidados de enfermer a	Cuidados de enfermería	5	kept
CVC	cvc	cvc	cvc	CVC	5	kept
Cytochrome P-450 Enzyme System	cytochrome p 450 enzyme system	cytochrome p 450 enzyme system	cytochrome p 450 enzyme system	Cytochrome P-450 Enzyme System	5	kept
cytokines	cytokines	cytokines	cytokine	Cytokines	5	singular
Dabigatran	dabigatran	dabigatran	dabigatran	dabigatran	5	case_or_format
DAILY GOALS	daily goals	daily goals	daily goals	DAILY GOALS	5	kept
data processing	data processing	data processing	data processing	data processing	5	kept
death education	death education	death education	death education	death education	5	kept
decision support systems	decision support systems	decision support systems	decision support system	decision support system	5	singular
Decision supports	decision supports	decision supports	decision support	Decision support	5	singular
Deep sedation	deep sedation	deep sedation	deep sedation	Deep Sedation	5	case_or_format
deformity	deformity	deformity	deformity	deformity	5	kept
deglutition disorders	deglutition disorders	deglutition disorders	deglutition disorder	Deglutition Disorders	5	singular
Delayed-Action Preparations	delayed action preparations	delayed action preparations	delayed action preparations	Delayed-Action Preparations	5	kept
delirium prevention	delirium prevention	delirium prevention	delirium prevention	delirium prevention	5	kept

dementia assessment	dementia assessment	dementia assessment	dementia assessment	dementia assessment	5	kept
Dengue	dengue	dengue	dengue	dengue	5	case_or_format
Dermatologic Agents	dermatologic agents	dermatologic agents	dermatologic agents	Dermatologic Agents	5	kept
Desensitization, Immunologic	desensitization immunologic	desensitization immunologic	desensitization immunologic	Desensitization, Immunologic	5	kept
detection algorithm	detection algorithm	detection algorithm	detection algorithm	detection algorithm	5	kept
Developing	developing	developing	developing	Developing	5	kept
developing countries	developing countries	developing countries	developing country	Developing Countries	5	singular
Developing country	developing country	developing country	developing country	Developing Countries	5	case_or_format
developmental stage	developmental stage	developmental stage	developmental stage	developmental stage	5	kept
Diabetes Insipidus	diabetes insipidus	diabetes insipidus	diabetes insipidus	Diabetes Insipidus	5	kept
diabetes insipidus	diabetes insipidus	diabetes insipidus	diabetes insipidus	Diabetes Insipidus	5	case_or_format
diagnostic and statistical manual of mental disorders	diagnostic and statistical manual of mental disorders	diagnostic and statistical manual of mental disorders	diagnostic and statistical manual of mental disorder	Diagnostic and Statistical Manual of Mental Disorders	5	singular
Diagnostic Techniques, Cardiovascular	diagnostic techniques cardiovascular	diagnostic techniques cardiovascular	diagnostic techniques cardiovascular	Diagnostic Techniques, Cardiovascular	5	kept
diaper dermatitis	diaper dermatitis	diaper dermatitis	diaper dermatitis	diaper dermatitis	5	kept
Diapers, Infant	diapers infant	diapers infant	diapers infant	Diapers, Infant	5	kept
diaphragm paralysis	diaphragm paralysis	diaphragm paralysis	diaphragm paralysis	diaphragm paralysis	5	kept

Diet	diet	diet	diet	diet	5	case_or_format
dietetics	dietetics	dietetics	dietetics	Dietetics	5	case_or_format
Diethylhexyl Phthalate	diethylhexyl phthalate	diethylhexyl phthalate	diethylhexyl phthalate	Diethylhexyl Phthalate	5	kept
digestive system function disorder	digestive system function disorder	digestive system function disorder	digestive system function disorder	digestive system function disorder	5	kept
Digital technology	digital technology	digital technology	digital technology	digital technology	5	case_or_format
Digoxin	digoxin	digoxin	digoxin	digoxin	5	case_or_format
Disability	disability	disability	disability	disability	5	case_or_format
disasters	disasters	disasters	disaster	Disasters	5	singular
Discomfort	discomfort	discomfort	discomfort	discomfort	5	case_or_format
Discrete event simulation	discrete event simulation	discrete event simulation	discrete event simulation	Discrete event simulation	5	kept
discriminant analysis	discriminant analysis	discriminant analysis	discriminant analysis	discriminant analysis	5	kept
Disease outbreak	disease outbreak	disease outbreak	disease outbreak	Disease Outbreaks	5	case_or_format
Disease outbreaks	disease outbreaks	disease outbreaks	disease outbreak	Disease Outbreaks	5	singular
Disease Susceptibility	disease susceptibility	disease susceptibility	disease susceptibility	Disease Susceptibility	5	kept
disease treatment	disease treatment	disease treatment	disease treatment	disease treatment	5	kept
diseases	diseases	diseases	disease	Diseases	5	singular

disorders of acid base balance	disorders of acid base balance	disorders of acid base balance	disorders of acid base balance	disorders of acid base balance	5	kept
disorders of consciousness	disorders of consciousness	disorders of consciousness	disorders of consciousness	disorders of consciousness	5	kept
disorders of mitochondrial functions	disorders of mitochondrial functions	disorders of mitochondrial functions	disorders of mitochondrial functions	disorders of mitochondrial functions	5	kept
Diuresis	diuresis	diuresis	diuresis	diuresis	5	case_or_format
diversity	diversity	diversity	diversity	diversity	5	kept
DNA determination	dna determination	dna determination	dna determination	DNA determination	5	kept
DNA, Viral	dna viral	dna viral	dna viral	DNA, Viral	5	kept
DNR	dnr	dnr	dnr	DNR	5	kept
docosahexaenoic acid	docosahexaenoic acid	docosahexaenoic acid	docosahexaenoic acid	docosahexaenoic acid	5	kept
Doctor	doctor	doctor	doctor	Doctors	5	case_or_format
domestic violence	domestic violence	domestic violence	domestic violence	domestic violence	5	kept
doula	doula	doula	doula	doula	5	kept
dressing	dressing	dressing	dressing	dressing	5	kept
Dressings	dressings	dressings	dressing	dressing	5	singular
driver	driver	driver	driver	driver	5	kept
drug antagonism	drug antagonism	drug antagonism	drug antagonism	drug antagonism	5	kept

drug legislation	drug legislation	drug legislation	drug legislation	drug legislation	5	kept
drug misuse	drug misuse	drug misuse	drug misuse	drug misuse	5	kept
drug screening	drug screening	drug screening	drug screening	drug screening	5	kept
drug self administration	drug self administration	drug self administration	drug self administration	drug self administration	5	kept
Durable Medical Equipment	durable medical equipment	durable medical equipment	durable medical equipment	Durable Medical Equipment	5	kept
dyskinesia	dyskinesia	dyskinesia	dyskinesia	dyskinesia	5	kept
dyspepsia	dyspepsia	dyspepsia	dyspepsia	dyspepsia	5	kept
dysphonia	dysphonia	dysphonia	dysphonia	dysphonia	5	kept
dysthymia	dysthymia	dysthymia	dysthymia	dysthymia	5	kept
dystocia	dystocia	dystocia	dystocia	dystocia	5	kept
Early ambulation	early ambulation	early ambulation	early ambulation	Early Ambulation	5	case_or_format
Early diagnosis	early diagnosis	early diagnosis	early diagnosis	Early Diagnosis	5	case_or_format
EARLY MOBILIZATION	early mobilization	early mobilization	early mobilization	Early mobilization	5	case_or_format
Early recognition	early recognition	early recognition	early recognition	Early recognition	5	kept
Earthquake	earthquake	earthquake	earthquake	Earthquakes	5	case_or_format
Eating Disorders	eating disorders	eating disorders	eating disorder	eating disorder	5	singular

Ecchymosis	ecchymosis	ecchymosis	ecchymosis	ecchymosis	5	case_or_format
ECG monitoring	ecg monitoring	ecg monitoring	ecg monitoring	ECG monitoring	5	kept
ECLS	ecls	ecls	ecls	ECLS	5	kept
Eczema	eczema	eczema	eczema	eczema	5	case_or_format
EDUCATION & TRAINING	education and training	education and training	education and training	education and training	5	case_or_format
Education program	education program	education program	education program	education program	5	case_or_format
Education, Professional, Retraining	education professional retraining	education professional retraining	education professional retraining	Education, Professional, Retraining	5	kept
educational	educational	educational	educational	educational	5	kept
educational needs	educational needs	educational needs	educational need	Educational needs	5	singular
educational program	educational program	educational program	educational program	Educational program	5	case_or_format
Effective communication	effective communication	effective communication	effective communication	Effective communication	5	kept
effectiveness	effectiveness	effectiveness	effectiveness	Effectiveness	5	case_or_format
Elder Abuse	elder abuse	elder abuse	elder abuse	Elder Abuse	5	kept
electric shock	electric shock	electric shock	electric shock	electric shock	5	kept
Electric Stimulation	electric stimulation	electric stimulation	electric stimulation	Electric Stimulation	5	kept
electronic spreadsheet	electronic spreadsheet	electronic spreadsheet	electronic spreadsheet	electronic spreadsheet	5	kept

Electrotherapeutics	electrotherapeutics	electrotherapeutics	electrotherapeutics	Electrotherapeutics	5	kept
elevated blood pressure	elevated blood pressure	elevated blood pressure	elevated blood pressure	elevated blood pressure	5	kept
Emergency rooms	emergency rooms	emergency rooms	emergency room	emergency room	5	singular
Emergency services	emergency services	emergency services	emergency service	Emergency service	5	singular
emergency shelter	emergency shelter	emergency shelter	emergency shelter	emergency shelter	5	kept
emotional distress	emotional distress	emotional distress	emotional distress	emotional distress	5	kept
EMPATHY	empathy	empathy	empathy	Empathy	5	case_or_format
EMPOWERMENT	empowerment	empowerment	empowerment	empowerment	5	case_or_format
EMR	emr	emr	emr	EMR	5	kept
encephalopathy	encephalopathy	encephalopathy	encephalopathy	Encephalopathy	5	case_or_format
End-of-life decision-making	end of life decision making	end of life decision making	end of life decision making	end-of-life decision-making	5	case_or_format
end-of-life issues	end of life issues	end of life issues	end of life issues	end-of-life issues	5	kept
endocrinologist	endocrinologist	endocrinologist	endocrinologist	endocrinologist	5	kept
endophthalmitis	endophthalmitis	endophthalmitis	endophthalmitis	endophthalmitis	5	kept
endotracheal suction	endotracheal suction	endotracheal suction	endotracheal suction	Endotracheal suction	5	case_or_format
endotracheal tube cuff	endotracheal tube cuff	endotracheal tube cuff	endotracheal tube cuff	endotracheal tube cuff	5	kept

Enfermagem Neonatal	enfermagem neonatal	enfermagem neonatal	enfermagem neonatal	Enfermagem Neonatal	5	kept
Enfermería Neonatal	enfermer a neonatal	enfermer a neonatal	enfermer a neonatal	Enfermería Neonatal	5	kept
Enhanced recovery after surgery	enhanced recovery after surgery	enhanced recovery after surgery	enhanced recovery after surgery	enhanced recovery after surgery	5	case_or_format
Enterobacter infection	enterobacter infection	enterobacter infection	enterobacter infection	Enterobacter infection	5	kept
Enterovirus	enterovirus	enterovirus	enterovirus	Enterovirus	5	kept
environmental change	environmental change	environmental change	environmental change	environmental change	5	kept
environmental stress	environmental stress	environmental stress	environmental stress	environmental stress	5	kept
ENVIRONMENTS	environments	environments	environment	Environment	5	singular
enzyme immunoassay	enzyme immunoassay	enzyme immunoassay	enzyme immunoassay	enzyme immunoassay	5	kept
epidemiologist	epidemiologist	epidemiologist	epidemiologist	epidemiologist	5	kept
Epidermolysis Bullosa	epidermolysis bullosa	epidermolysis bullosa	epidermolysis bullosa	Epidermolysis Bullosa	5	kept
epistemology	epistemology	epistemology	epistemology	epistemology	5	kept
Epoprostenol	epoprostenol	epoprostenol	epoprostenol	Epoprostenol	5	kept
Equipment contamination	equipment contamination	equipment contamination	equipment contamination	Equipment Contamination	5	case_or_format
Equipment Reuse	equipment reuse	equipment reuse	equipment reuse	Equipment Reuse	5	kept
erosion	erosion	erosion	erosion	erosion	5	kept

Erratum	erratum	erratum	erratum	erratum	5	case_or_format
Errors, medication	errors medication	errors medication	errors medication	Errors, medication	5	kept
Esophageal Diseases	esophageal diseases	esophageal diseases	esophageal diseases	Esophageal Diseases	5	kept
essential care	essential care	essential care	essential care	essential care	5	kept
ethical awareness	ethical awareness	ethical awareness	ethical awareness	ethical awareness	5	kept
ETHICAL CLIMATE	ethical climate	ethical climate	ethical climate	Ethical climate	5	case_or_format
Ethical dilemmas	ethical dilemmas	ethical dilemmas	ethical dilemma	ethical dilemma	5	singular
ethicist	ethicist	ethicist	ethicist	ethicist	5	kept
Ethicists	ethicists	ethicists	ethicist	ethicist	5	singular
EuroSCORE	euroscore	euroscore	euroscore	EuroSCORE	5	kept
Euthanasia, Active	euthanasia active	euthanasia active	euthanasia active	Euthanasia, Active	5	kept
Evacuation	evacuation	evacuation	evacuation	Evacuation	5	kept
Evidence	evidence	evidence	evidence	Evidence	5	kept
evidence-based design	evidence based design	evidence based design	evidence based design	evidence-based design	5	kept
exchange blood transfusion	exchange blood transfusion	exchange blood transfusion	exchange blood transfusion	exchange blood transfusion	5	kept
exertional dyspnea	exertional dyspnea	exertional dyspnea	exertional dyspnea	exertional dyspnea	5	kept

EXPRESSION	expression	expression	expression	EXPRESSION	5	kept
extended spectrum beta lactamase producing Escherichia coli	extended spectrum beta lactamase producing escherichia coli	extended spectrum beta lactamase producing escherichia coli	extended spectrum beta lactamase producing escherichia coli	extended spectrum beta lactamase producing Escherichia coli	5	kept
external validity	external validity	external validity	external validity	external validity	5	kept
extraction	extraction	extraction	extraction	extraction	5	kept
Extravasation	extravasation	extravasation	extravasation	extravasation	5	case_or_format
extreme gradient boosting	extreme gradient boosting	extreme gradient boosting	extreme gradient boosting	extreme gradient boosting	5	kept
Extremely preterm infants	extremely preterm infants	extremely preterm infants	extremely preterm infant	Extremely preterm infants	5	singular
eye injury	eye injury	eye injury	eye injury	Eye Injuries	5	case_or_format
eye pain	eye pain	eye pain	eye pain	eye pain	5	kept
eye surgery	eye surgery	eye surgery	eye surgery	eye surgery	5	kept
eye-tracking technology	eye tracking technology	eye tracking technology	eye tracking technology	Eye-Tracking Technology	5	case_or_format
Face	face	face	face	face	5	case_or_format
Face recognition	face recognition	face recognition	face recognition	Face recognition	5	kept
Facilitation	facilitation	facilitation	facilitation	facilitation	5	case_or_format
facilitator	facilitator	facilitator	facilitator	Facilitators	5	case_or_format
Facilities and Services Utilization	facilities and services utilization	facilities and services utilization	facilities and services utilization	Facilities and Services Utilization	5	kept

failure to thrive	failure to thrive	failure to thrive	failure to thrive	failure to thrive	5	kept
family assessment	family assessment	family assessment	family assessment	family assessment	5	kept
family communication	family communication	family communication	family communication	family communication	5	kept
Family Conflict	family conflict	family conflict	family conflict	family conflict	5	case_or_format
Family experience	family experience	family experience	family experience	Family experience	5	kept
family income	family income	family income	family income	family income	5	kept
Family Therapy	family therapy	family therapy	family therapy	family therapy	5	case_or_format
family-integrated care	family integrated care	family integrated care	family integrated care	Family integrated care	5	case_or_format
fat intake	fat intake	fat intake	fat intake	fat intake	5	kept
fat mass	fat mass	fat mass	fat mass	fat mass	5	kept
FATHERS	fathers	fathers	father	Fathers	5	singular
Fatty Liver	fatty liver	fatty liver	fatty liver	Fatty Liver	5	kept
feasibility studies	feasibility studies	feasibility studies	feasibility study	Feasibility Studies	5	singular
feature extraction	feature extraction	feature extraction	feature extraction	feature extraction	5	kept
febrile convulsion	febrile convulsion	febrile convulsion	febrile convulsion	febrile convulsion	5	kept
feedback	feedback	feedback	feedback	Feedback	5	case_or_format

Feeding and Eating Disorders of Childhood	feeding and eating disorders of childhood	feeding and eating disorders of childhood	feeding and eating disorders of childhood	Feeding and Eating Disorders of Childhood	5	kept
Feeding methods	feeding methods	feeding methods	feeding method	Feeding Methods	5	singular
Feeding tube	feeding tube	feeding tube	feeding tube	Feeding tube	5	kept
female genital tract cancer	female genital tract cancer	female genital tract cancer	female genital tract cancer	female genital tract cancer	5	kept
female physician	female physician	female physician	female physician	female physician	5	kept
Femur	femur	femur	femur	Femur	5	kept
femur	femur	femur	femur	Femur	5	case_or_format
femur intertrochanteric fracture	femur intertrochanteric fracture	femur intertrochanteric fracture	femur intertrochanteric fracture	femur intertrochanteric fracture	5	kept
fentanyl citrate	fentanyl citrate	fentanyl citrate	fentanyl citrate	fentanyl citrate	5	kept
Fetal Blood	fetal blood	fetal blood	fetal blood	Fetal Blood	5	kept
Fetal Hypoxia	fetal hypoxia	fetal hypoxia	fetal hypoxia	Fetal Hypoxia	5	kept
fiber optics	fiber optics	fiber optics	fiber optics	fiber optics	5	kept
Fibula	fibula	fibula	fibula	Fibula	5	kept
FICare	ficare	ficare	ficare	FICare	5	kept
Fingers	fingers	fingers	finger	finger	5	singular
fire fighter	fire fighter	fire fighter	fire fighter	fire fighter	5	kept

flail chest	flail chest	flail chest	flail chest	flail chest	5	kept
Flavobacteriaceae Infections	flavobacteriaceae infections	flavobacteriaceae infections	flavobacteriaceae infections	Flavobacteriaceae Infections	5	kept
flexible bronchoscopy	flexible bronchoscopy	flexible bronchoscopy	flexible bronchoscopy	flexible bronchoscopy	5	kept
flucloxacillin	flucloxacillin	flucloxacillin	flucloxacillin	flucloxacillin	5	kept
Fluconazole	fluconazole	fluconazole	fluconazole	fluconazole	5	case_or_format
Fluid balance	fluid balance	fluid balance	fluid balance	fluid balance	5	case_or_format
Fluid management	fluid management	fluid management	fluid management	Fluid management	5	kept
fluid overload	fluid overload	fluid overload	fluid overload	Fluid overload	5	case_or_format
fluorouracil	fluorouracil	fluorouracil	fluorouracil	fluorouracil	5	kept
fluoxetine	fluoxetine	fluoxetine	fluoxetine	fluoxetine	5	kept
Flushing	flushing	flushing	flushing	flushing	5	case_or_format
Follow-up studies	follow up studies	follow up studies	follow up studies	Follow-Up Studies	5	case_or_format
food drug interaction	food drug interaction	food drug interaction	food drug interaction	food drug interaction	5	kept
Food Microbiology	food microbiology	food microbiology	food microbiology	Food Microbiology	5	kept
Food Service, Hospital	food service hospital	food service hospital	food service hospital	Food Service, Hospital	5	kept
Food, Fortified	food fortified	food fortified	food fortified	Food, Fortified	5	kept

Foreign Professional Personnel	foreign professional personnel	foreign professional personnel	foreign professional personnel	Foreign Professional Personnel	5	kept
fragility fracture	fragility fracture	fragility fracture	fragility fracture	fragility fracture	5	kept
Frail elderly	frail elderly	frail elderly	frail elderly	Frail Elderly	5	case_or_format
free radical	free radical	free radical	free radical	free radical	5	kept
Full Outline of Unresponsiveness score	full outline of unresponsiveness score	full outline of unresponsiveness score	full outline of unresponsiveness score	Full Outline of Unresponsiveness score	5	kept
functional residual capacity	functional residual capacity	functional residual capacity	functional residual capacity	functional residual capacity	5	kept
Fungal	fungal	fungal	fungal	Fungal	5	kept
fungal contamination	fungal contamination	fungal contamination	fungal contamination	fungal contamination	5	kept
fungus transmission	fungus transmission	fungus transmission	fungus transmission	fungus transmission	5	kept
futile care	futile care	futile care	futile care	Futile care	5	case_or_format
Fuzzy Logic	fuzzy logic	fuzzy logic	fuzzy logic	Fuzzy Logic	5	kept
Fuzzy logic	fuzzy logic	fuzzy logic	fuzzy logic	Fuzzy Logic	5	case_or_format
gait	gait	gait	gait	gait	5	kept
gamma glutamyltransferase	gamma glutamyltransferase	gamma glutamyltransferase	gamma glutamyltransferase	gamma glutamyltransferase	5	kept
Gammaproteobacteria	gammaproteobacteria	gammaproteobacteria	gammaproteobacteria	Gammaproteobacteria	5	kept
gangrene	gangrene	gangrene	gangrene	gangrene	5	kept

Gases	gases	gases	gases	Gases	5	kept
gastritis	gastritis	gastritis	gastritis	gastritis	5	kept
Gastrointestinal	gastrointestinal	gastrointestinal	gastrointestinal	Gastrointestinal	5	kept
gastrointestinal	gastrointestinal	gastrointestinal	gastrointestinal	Gastrointestinal	5	case_or_format
Gastrointestinal intubation	gastrointestinal intubation	gastrointestinal intubation	gastrointestinal intubation	Gastrointestinal intubation	5	kept
gastrointestinal tract function	gastrointestinal tract function	gastrointestinal tract function	gastrointestinal tract function	gastrointestinal tract function	5	kept
GCS	gcs	gcs	gcs	GCS	5	kept
gender bias	gender bias	gender bias	gender bias	gender bias	5	kept
general condition improvement	general condition improvement	general condition improvement	general condition improvement	general condition improvement	5	kept
Genetic Variation	genetic variation	genetic variation	genetic variation	Genetic Variation	5	kept
Genome, Bacterial	genome bacterial	genome bacterial	genome bacterial	Genome, Bacterial	5	kept
genomic DNA	genomic dna	genomic dna	genomic dna	genomic DNA	5	kept
geriatric hospital	geriatric hospital	geriatric hospital	geriatric hospital	geriatric hospital	5	kept
Glasgow Outcome Scale	glasgow outcome scale	glasgow outcome scale	glasgow outcome scale	Glasgow outcome scale	5	case_or_format
glaucoma	glaucoma	glaucoma	glaucoma	glaucoma	5	kept
Global	global	global	global	Global	5	kept

glucose control	glucose control	glucose control	glucose control	glucose control	5	kept
glucose homeostasis	glucose homeostasis	glucose homeostasis	glucose homeostasis	glucose homeostasis	5	kept
glutaraldehyde	glutaraldehyde	glutaraldehyde	glutaraldehyde	glutaraldehyde	5	kept
Glycated Hemoglobin	glycated hemoglobin	glycated hemoglobin	glycated hemoglobin	Glycated Hemoglobin	5	kept
glycosylated hemoglobin	glycosylated hemoglobin	glycosylated hemoglobin	glycosylated hemoglobin	glycosylated hemoglobin	5	kept
graduate	graduate	graduate	graduate	graduate	5	kept
group cohesion	group cohesion	group cohesion	group cohesion	group cohesion	5	kept
GROWTH	growth	growth	growth	Growth	5	case_or_format
Growth and development	growth and development	growth and development	growth and development	Growth and development	5	kept
growth disorder	growth disorder	growth disorder	growth disorder	Growth Disorders	5	case_or_format
guided imagery	guided imagery	guided imagery	guided imagery	guided imagery	5	kept
Habits	habits	habits	habit	habit	5	singular
Haemodynamic monitoring	haemodynamic monitoring	haemodynamic monitoring	haemodynamic monitoring	Haemodynamic monitoring	5	kept
hand disease	hand disease	hand disease	hand disease	hand disease	5	kept
hand-off	hand off	hand off	hand off	hand-off	5	kept
handoffs	handoffs	handoffs	handoff	handoff	5	singular

HAPI	hapi	hapi	hapi	HAPI	5	kept
hardiness	hardiness	hardiness	hardiness	hardiness	5	kept
Hazardous Substances	hazardous substances	hazardous substances	hazardous substances	Hazardous Substances	5	kept
HEAD	head	head	head	Head	5	case_or_format
head and neck tumor	head and neck tumor	head and neck tumor	head and neck tumor	head and neck tumor	5	kept
Head mounted displays	head mounted displays	head mounted displays	head mounted display	Head-mounted display	5	singular
Head trauma	head trauma	head trauma	head trauma	Head trauma	5	kept
Head-mounted display	head mounted display	head mounted display	head mounted display	Head-mounted display	5	kept
Health Belief Model	health belief model	health belief model	health belief model	Health Belief Model	5	kept
Health care associated infection	health care associated infection	health care associated infection	health care associated infection	Health care-associated infections	5	case_or_format
health care economics	health care economics	health care economics	health care economics	health care economics	5	kept
health care professional	health care professional	health care professional	health care professional	Health care professionals	5	case_or_format
Health Care Sector	health care sector	health care sector	health care sector	Health Care Sector	5	kept
Health disparities	health disparities	health disparities	health disparity	health disparity	5	singular
Health Facility Closure	health facility closure	health facility closure	health facility closure	Health Facility Closure	5	kept
Health Information Technology	health information technology	health information technology	health information technology	Health information technology	5	case_or_format

Health Manpower	health manpower	health manpower	health manpower	Health Manpower	5	kept
health services administration	health services administration	health services administration	health services administration	health services administration	5	kept
Health services for the aged	health services for the aged	health services for the aged	health services for the aged	Health Services for the Aged	5	case_or_format
health visitor	health visitor	health visitor	health visitor	health visitor	5	kept
healthcare costs	healthcare costs	healthcare costs	healthcare cost	Healthcare costs	5	singular
Healthcare provider	healthcare provider	healthcare provider	healthcare provider	Healthcare providers	5	case_or_format
healthcare provider	healthcare provider	healthcare provider	healthcare provider	Healthcare providers	5	case_or_format
Healthcare services	healthcare services	healthcare services	healthcare services	Healthcare services	5	kept
healthcare-associated infection	healthcare associated infection	healthcare associated infection	healthcare associated infection	healthcare associated infection	5	case_or_format
Healthy Volunteers	healthy volunteers	healthy volunteers	healthy volunteers	Healthy Volunteers	5	kept
healthy work environments	healthy work environments	healthy work environments	healthy work environment	healthy work environment	5	singular
hearing loss	hearing loss	hearing loss	hearing loss	Hearing Loss	5	case_or_format
heart beat	heart beat	heart beat	heart beat	heart beat	5	kept
heart block	heart block	heart block	heart block	heart block	5	kept
Heart Conduction System	heart conduction system	heart conduction system	heart conduction system	Heart Conduction System	5	kept
Heart defects	heart defects	heart defects	heart defects	Heart defects	5	kept

Heart Failure, Diastolic	heart failure diastolic	heart failure diastolic	heart failure diastolic	Heart Failure, Diastolic	5	kept
Heart Function Tests	heart function tests	heart function tests	heart function test	Heart Function Tests	5	singular
heart left ventricle failure	heart left ventricle failure	heart left ventricle failure	heart left ventricle failure	heart left ventricle failure	5	kept
heart muscle conduction system	heart muscle conduction system	heart muscle conduction system	heart muscle conduction system	heart muscle conduction system	5	kept
heart preload	heart preload	heart preload	heart preload	heart preload	5	kept
Heart rates	heart rates	heart rates	heart rate	heart rate	5	singular
heart right ventricle double outlet	heart right ventricle double outlet	heart right ventricle double outlet	heart right ventricle double outlet	heart right ventricle double outlet	5	kept
heart right ventricle function	heart right ventricle function	heart right ventricle function	heart right ventricle function	heart right ventricle function	5	kept
heart sound	heart sound	heart sound	heart sound	heart sound	5	kept
heat loss	heat loss	heat loss	heat loss	heat loss	5	kept
Heating	heating	heating	heating	Heating	5	kept
Heidegger	heidegger	heidegger	heidegger	Heidegger	5	kept
Helmet mounted displays	helmet mounted displays	helmet mounted displays	helmet mounted displays	Helmet mounted displays	5	kept
hemiarthroplasty	hemiarthroplasty	hemiarthroplasty	hemiarthroplasty	hemiarthroplasty	5	kept
Hemorrhagic Fever, Crimean	hemorrhagic fever crimean	hemorrhagic fever crimean	hemorrhagic fever crimean	Hemorrhagic Fever, Crimean	5	kept
Hemorrhagic shock	hemorrhagic shock	hemorrhagic shock	hemorrhagic shock	hemorrhagic shock	5	case_or_format

hepatitis A	hepatitis a	hepatitis a	hepatitis a	Hepatitis A	5	case_or_format
Hepatitis B Vaccines	hepatitis b vaccines	hepatitis b vaccines	hepatitis b vaccine	hepatitis B vaccine	5	singular
herbal medicine	herbal medicine	herbal medicine	herbal medicine	herbal medicine	5	kept
Hernia, Inguinal	hernia inguinal	hernia inguinal	hernia inguinal	Hernia, Inguinal	5	kept
heroin dependence	heroin dependence	heroin dependence	heroin dependence	heroin dependence	5	kept
High fidelity simulation training	high fidelity simulation training	high fidelity simulation training	high fidelity simulation training	High Fidelity Simulation Training	5	case_or_format
high risk	high risk	high risk	high risk	High risk	5	case_or_format
high throughput sequencing	high throughput sequencing	high throughput sequencing	high throughput sequencing	high throughput sequencing	5	kept
high-fidelity simulation training	high fidelity simulation training	high fidelity simulation training	high fidelity simulation training	High Fidelity Simulation Training	5	case_or_format
HIGH-RISK	high risk	high risk	high risk	High risk	5	case_or_format
High-risk	high risk	high risk	high risk	High risk	5	case_or_format
high-risk pregnancy	high risk pregnancy	high risk pregnancy	high risk pregnancy	high risk pregnancy	5	case_or_format
highest income group	highest income group	highest income group	highest income group	highest income group	5	kept
hip	hip	hip	hip	hip	5	kept
hip arthroplasty	hip arthroplasty	hip arthroplasty	hip arthroplasty	hip arthroplasty	5	kept
hip surgery	hip surgery	hip surgery	hip surgery	hip surgery	5	kept

Hirschsprung disease	hirschsprung disease	hirschsprung disease	hirschsprung disease	Hirschsprung Disease	5	case_or_format
Historically Controlled Study	historically controlled study	historically controlled study	historically controlled study	Historically Controlled Study	5	kept
History, 18th Century	history 18th century	history 18th century	history 18th century	History, 18th Century	5	kept
Holidays	holidays	holidays	holidays	Holidays	5	kept
home birth	home birth	home birth	home birth	home birth	5	kept
home dialysis	home dialysis	home dialysis	home dialysis	home dialysis	5	kept
home health agency	home health agency	home health agency	home health agency	home health agency	5	kept
honesty	honesty	honesty	honesty	honesty	5	kept
Honey	honey	honey	honey	Honey	5	kept
hormone	hormone	hormone	hormone	hormone	5	kept
hospital at home	hospital at home	hospital at home	hospital at home	hospital at home	5	kept
Hospital pharmacy	hospital pharmacy	hospital pharmacy	hospital pharmacy	hospital pharmacy	5	case_or_format
Hospital readmission	hospital readmission	hospital readmission	hospital readmission	hospital readmission	5	case_or_format
hospital running cost	hospital running cost	hospital running cost	hospital running cost	hospital running cost	5	kept
hospital volunteer	hospital volunteer	hospital volunteer	hospital volunteer	Hospital Volunteers	5	case_or_format
Hospital wards	hospital wards	hospital wards	hospital ward	Hospital wards	5	singular

hospital-acquired pressure injury	hospital acquired pressure injury	hospital acquired pressure injury	hospital acquired pressure injury	hospital-acquired pressure injury	5	kept
hospitalized	hospitalized	hospitalized	hospitalized	Hospitalized	5	case_or_format
host resistance	host resistance	host resistance	host resistance	host resistance	5	kept
humidified high flow nasal cannula therapy	humidified high flow nasal cannula therapy	humidified high flow nasal cannula therapy	humidified high flow nasal cannula therapy	humidified high flow nasal cannula therapy	5	kept
hydrocodone	hydrocodone	hydrocodone	hydrocodone	hydrocodone	5	kept
hydrocortisone blood level	hydrocortisone blood level	hydrocortisone blood level	hydrocortisone blood level	hydrocortisone blood level	5	kept
Hydrogen Peroxide	hydrogen peroxide	hydrogen peroxide	hydrogen peroxide	hydrogen peroxide	5	case_or_format
Hydroxyzine	hydroxyzine	hydroxyzine	hydroxyzine	Hydroxyzine	5	kept
hydroxyzine	hydroxyzine	hydroxyzine	hydroxyzine	Hydroxyzine	5	case_or_format
hyperhidrosis	hyperhidrosis	hyperhidrosis	hyperhidrosis	hyperhidrosis	5	kept
hyperlactatemia	hyperlactatemia	hyperlactatemia	hyperlactatemia	hyperlactatemia	5	kept
Hyperphosphatemia	hyperphosphatemia	hyperphosphatemia	hyperphosphatemia	hyperphosphatemia	5	case_or_format
Hyperthermic Intraperitoneal Chemotherapy	hyperthermic intraperitoneal chemotherapy	hyperthermic intraperitoneal chemotherapy	hyperthermic intraperitoneal chemotherapy	hyperthermic intraperitoneal chemotherapy	5	case_or_format
hyperthyroidism	hyperthyroidism	hyperthyroidism	hyperthyroidism	hyperthyroidism	5	kept
hypertonic solution	hypertonic solution	hypertonic solution	hypertonic solution	hypertonic solution	5	kept
Hyperuricemia	hyperuricemia	hyperuricemia	hyperuricemia	Hyperuricemia	5	kept

Hyperventilation	hyperventilation	hyperventilation	hyperventilation	hyperventilation	5	case_or_format
Hypnosis	hypnosis	hypnosis	hypnosis	hypnosis	5	case_or_format
Hypoglycaemia	hypoglycaemia	hypoglycaemia	hypoglycaemia	Hypoglycaemia	5	kept
hypothalamus hypophysis adrenal system	hypothalamus hypophysis adrenal system	hypothalamus hypophysis adrenal system	hypothalamus hypophysis adrenal system	hypothalamus hypophysis adrenal system	5	kept
Hypotonic Solutions	hypotonic solutions	hypotonic solutions	hypotonic solution	Hypotonic Solutions	5	singular
hypovolemic shock	hypovolemic shock	hypovolemic shock	hypovolemic shock	hypovolemic shock	5	kept
Hypoxemia	hypoxemia	hypoxemia	hypoxemia	hypoxemia	5	case_or_format
hypoxic-ischemic encephalopathy	hypoxic ischemic encephalopathy	hypoxic ischemic encephalopathy	hypoxic ischemic encephalopathy	hypoxic ischemic encephalopathy	5	case_or_format
Ileus	ileus	ileus	ileus	ileus	5	case_or_format
ILL	ill	ill	ill	ILL	5	kept
Illicit Drugs	illicit drugs	illicit drugs	illicit drug	illicit drug	5	singular
Imaging, Three-Dimensional	imaging three dimensional	imaging three dimensional	imaging three dimensional	Imaging, Three-Dimensional	5	kept
Imipenem	imipenem	imipenem	imipenem	imipenem	5	case_or_format
Immunocompromised Host	immunocompromised host	immunocompromised host	immunocompromised host	Immunocompromised Host	5	kept
Immunoglobulin A	immunoglobulin a	immunoglobulin a	immunoglobulin a	immunoglobulin A	5	case_or_format
immunoglobulin M antibody	immunoglobulin m antibody	immunoglobulin m antibody	immunoglobulin m antibody	immunoglobulin M antibody	5	kept

immunohistochemistry	immunohistochemistry	immunohistochemistry	immunohistochemistry	immunohistochemistry	5	kept
immunomodulation	immunomodulation	immunomodulation	immunomodulation	immunomodulation	5	kept
Impact	impact	impact	impact	IMPACT	5	case_or_format
implantable cardioverter defibrillator	implantable cardioverter defibrillator	implantable cardioverter defibrillator	implantable cardioverter defibrillator	implantable cardioverter defibrillator	5	kept
implicit bias	implicit bias	implicit bias	implicit bias	implicit bias	5	kept
IMPROVE	improve	improve	improve	IMPROVE	5	kept
impulsiveness	impulsiveness	impulsiveness	impulsiveness	impulsiveness	5	kept
in vitro fertilization	in vitro fertilization	in vitro fertilization	in vitro fertilization	in vitro fertilization	5	kept
IN-HOSPITAL MORTALITY	in hospital mortality	in hospital mortality	in hospital mortality	in-hospital mortality	5	case_or_format
Incontinence	incontinence	incontinence	incontinence	incontinence	5	case_or_format
independence	independence	independence	independence	independence	5	kept
indigenous people	indigenous people	indigenous people	indigenous people	indigenous people	5	kept
Individuality	individuality	individuality	individuality	individuality	5	case_or_format
Induced hypothermia	induced hypothermia	induced hypothermia	induced hypothermia	induced hypothermia	5	case_or_format
Infant feeding	infant feeding	infant feeding	infant feeding	infant feeding	5	case_or_format
Infant mortality	infant mortality	infant mortality	infant mortality	Infant Mortality	5	case_or_format

Infant premature	infant premature	infant premature	infant premature	Infant, Premature	5	case_or_format
INFECTION-CONTROL	infection control	infection control	infection control	Infection Control	5	case_or_format
Infectious disease	infectious disease	infectious disease	infectious disease	infectious disease	5	case_or_format
infective endocarditis	infective endocarditis	infective endocarditis	infective endocarditis	infective endocarditis	5	kept
inferior cava vein	inferior cava vein	inferior cava vein	inferior cava vein	inferior cava vein	5	kept
INFORMAL CAREGIVERS	informal caregivers	informal caregivers	informal caregiver	INFORMAL CAREGIVERS	5	singular
Informatics	informatics	informatics	informatics	informatics	5	case_or_format
Information exchange	information exchange	information exchange	information exchange	Information exchange	5	kept
Information literacy	information literacy	information literacy	information literacy	Information literacy	5	kept
information storage	information storage	information storage	information storage	information storage	5	kept
Information system	information system	information system	information system	information system	5	case_or_format
infrared radiation	infrared radiation	infrared radiation	infrared radiation	infrared radiation	5	kept
Inhalation	inhalation	inhalation	inhalation	inhalation	5	case_or_format
inotropism	inotropism	inotropism	inotropism	inotropism	5	kept
Institutional Management Teams	institutional management teams	institutional management teams	institutional management teams	Institutional Management Teams	5	kept
instrumental delivery	instrumental delivery	instrumental delivery	instrumental delivery	instrumental delivery	5	kept

insulin hypoglycemia	insulin hypoglycemia	insulin hypoglycemia	insulin hypoglycemia	insulin hypoglycemia	5	kept
insulin pump	insulin pump	insulin pump	insulin pump	insulin pump	5	kept
Insurance Claim Reporting	insurance claim reporting	insurance claim reporting	insurance claim reporting	Insurance Claim Reporting	5	kept
integrated care	integrated care	integrated care	integrated care	integrated care	5	kept
intensive insulin therapy	intensive insulin therapy	intensive insulin therapy	intensive insulin therapy	Intensive insulin therapy	5	case_or_format
Intensivists	intensivists	intensivists	intensivist	intensivist	5	singular
Inter-professional collaboration	inter professional collaboration	inter professional collaboration	inter professional collaboration	Inter-professional collaboration	5	kept
Interdisciplinary care	interdisciplinary care	interdisciplinary care	interdisciplinary care	interdisciplinary care	5	case_or_format
interdisciplinary collaboration	interdisciplinary collaboration	interdisciplinary collaboration	interdisciplinary collaboration	Interdisciplinary collaboration	5	case_or_format
Interdisciplinary team	interdisciplinary team	interdisciplinary team	interdisciplinary team	interdisciplinary team	5	case_or_format
interfacility transfer	interfacility transfer	interfacility transfer	interfacility transfer	interfacility transfer	5	kept
interleukin 8	interleukin 8	interleukin 8	interleukin 8	interleukin 8	5	kept
intermittent mandatory ventilation	intermittent mandatory ventilation	intermittent mandatory ventilation	intermittent mandatory ventilation	intermittent mandatory ventilation	5	kept
INTERRUPTIONS	interruptions	interruptions	interruption	Interruptions	5	singular
Intervention study	intervention study	intervention study	intervention study	intervention study	5	case_or_format
Intestinal failure	intestinal failure	intestinal failure	intestinal failure	intestinal failure	5	case_or_format

Intracranial hemorrhage	intracranial hemorrhage	intracranial hemorrhage	intracranial hemorrhage	Intracranial Hemorrhages	5	case_or_format
intrahepatic cholestasis	intrahepatic cholestasis	intrahepatic cholestasis	intrahepatic cholestasis	intrahepatic cholestasis	5	kept
intramuscular drug administration	intramuscular drug administration	intramuscular drug administration	intramuscular drug administration	intramuscular drug administration	5	kept
intravenous anesthesia	intravenous anesthesia	intravenous anesthesia	intravenous anesthesia	intravenous anesthesia	5	kept
intravenous feeding	intravenous feeding	intravenous feeding	intravenous feeding	intravenous feeding	5	kept
intrinsic motivation	intrinsic motivation	intrinsic motivation	intrinsic motivation	intrinsic motivation	5	kept
invasive aspergillosis	invasive aspergillosis	invasive aspergillosis	invasive aspergillosis	invasive aspergillosis	5	kept
invasive candidiasis	invasive candidiasis	invasive candidiasis	invasive candidiasis	invasive candidiasis	5	kept
Invasive mechanical ventilation	invasive mechanical ventilation	invasive mechanical ventilation	invasive mechanical ventilation	invasive mechanical ventilation	5	case_or_format
Invasive procedures	invasive procedures	invasive procedures	invasive procedure	invasive procedure	5	singular
Iranian	iranian	iranian	iranian	Iranian	5	kept
Iron	iron	iron	iron	iron	5	case_or_format
isolation procedure	isolation procedure	isolation procedure	isolation procedure	isolation procedure	5	kept
Jaundice	jaundice	jaundice	jaundice	jaundice	5	case_or_format
Jehovah's witness	jehovah s witness	jehovah s witness	jehovah s witness	Jehovah's witness	5	kept
Jehovah's Witnesses	jehovah s witnesses	jehovah s witnesses	jehovah s witnesses	Jehovah's Witnesses	5	kept

job adaptation	job adaptation	job adaptation	job adaptation	job adaptation	5	kept
job burnout	job burnout	job burnout	job burnout	job burnout	5	kept
JUDGMENT	judgment	judgment	judgment	Judgment	5	case_or_format
Karyotyping	karyotyping	karyotyping	karyotyping	Karyotyping	5	kept
Katz index	katz index	katz index	katz index	Katz index	5	kept
ketoacidosis	ketoacidosis	ketoacidosis	ketoacidosis	ketoacidosis	5	kept
ketobemidone	ketobemidone	ketobemidone	ketobemidone	ketobemidone	5	kept
KLEBSIELLA-PNEUMONIAE	klebsiella pneumoniae	klebsiella pneumoniae	klebsiella pneumoniae	Klebsiella pneumoniae	5	case_or_format
knee	knee	knee	knee	knee	5	kept
knee arthroplasty	knee arthroplasty	knee arthroplasty	knee arthroplasty	knee arthroplasty	5	kept
labor pain	labor pain	labor pain	labor pain	labor pain	5	kept
lactate dehydrogenase blood level	lactate dehydrogenase blood level	lactate dehydrogenase blood level	lactate dehydrogenase blood level	lactate dehydrogenase blood level	5	kept
lamotrigine	lamotrigine	lamotrigine	lamotrigine	lamotrigine	5	kept
language barriers	language barriers	language barriers	language barrier	language barriers	5	singular
language processing	language processing	language processing	language processing	language processing	5	kept
laparoscopic cholecystectomy	laparoscopic cholecystectomy	laparoscopic cholecystectomy	laparoscopic cholecystectomy	laparoscopic cholecystectomy	5	kept

latent class analysis	latent class analysis	latent class analysis	latent class analysis	Latent Class Analysis	5	case_or_format
lavender	lavender	lavender	lavender	lavender	5	kept
learning disorder	learning disorder	learning disorder	learning disorder	learning disorder	5	kept
Least-Squares Analysis	least squares analysis	least squares analysis	least squares analysis	Least-Squares Analysis	5	kept
Legal Approach	legal approach	legal approach	legal approach	Legal Approach	5	kept
legal guardian	legal guardian	legal guardian	legal guardian	Legal Guardians	5	case_or_format
leukomalacia	leukomalacia	leukomalacia	leukomalacia	leukomalacia	5	kept
level of care	level of care	level of care	level of care	level of care	5	kept
levodopa	levodopa	levodopa	levodopa	levodopa	5	kept
levomepromazine	levomepromazine	levomepromazine	levomepromazine	levomepromazine	5	kept
Licensure	licensure	licensure	licensure	Licensure	5	kept
life support equipment	life support equipment	life support equipment	life support equipment	life support equipment	5	kept
Life-sustaining treatment	life sustaining treatment	life sustaining treatment	life sustaining treatment	life sustaining treatment	5	case_or_format
life-sustaining treatment	life sustaining treatment	life sustaining treatment	life sustaining treatment	life sustaining treatment	5	case_or_format
life-sustaining treatments	life sustaining treatments	life sustaining treatments	life sustaining treatment	life sustaining treatment	5	singular
limb amputation	limb amputation	limb amputation	limb amputation	limb amputation	5	kept

linguistics	linguistics	linguistics	linguistics	linguistics	5	kept
lithium	lithium	lithium	lithium	lithium	5	kept
Live Birth	live birth	live birth	live birth	live birth	5	case_or_format
LIVED EXPERIENCE	lived experience	lived experience	lived experience	lived experience	5	case_or_format
Liver Function Tests	liver function tests	liver function tests	liver function test	liver function test	5	singular
liver resection	liver resection	liver resection	liver resection	liver resection	5	kept
living donor	living donor	living donor	living donor	Living Donors	5	case_or_format
Living Wills	living wills	living wills	living will	living will	5	singular
locus of control	locus of control	locus of control	locus of control	locus of control	5	kept
long term exposure	long term exposure	long term exposure	long term exposure	long term exposure	5	kept
lopinavir	lopinavir	lopinavir	lopinavir	lopinavir	5	kept
lung aspergillosis	lung aspergillosis	lung aspergillosis	lung aspergillosis	lung aspergillosis	5	kept
lung blood flow	lung blood flow	lung blood flow	lung blood flow	lung blood flow	5	kept
lung circulation	lung circulation	lung circulation	lung circulation	lung circulation	5	kept
magnesium blood level	magnesium blood level	magnesium blood level	magnesium blood level	magnesium blood level	5	kept
Magnetic Resonance Angiography	magnetic resonance angiography	magnetic resonance angiography	magnetic resonance angiography	magnetic resonance angiography	5	case_or_format

malabsorption	malabsorption	malabsorption	malabsorption	malabsorption	5	kept
Malta	malta	malta	malta	Malta	5	kept
Mammaplasty	mammaplasty	mammaplasty	mammaplasty	Mammaplasty	5	kept
mandatory program	mandatory program	mandatory program	mandatory program	mandatory program	5	kept
Marfan Syndrome	marfan syndrome	marfan syndrome	marfan syndrome	Marfan Syndrome	5	kept
Marketing of Health Services	marketing of health services	marketing of health services	marketing of health services	Marketing of Health Services	5	kept
Markov Chains	markov chains	markov chains	markov chain	Markov Chains	5	singular
mask ventilation	mask ventilation	mask ventilation	mask ventilation	mask ventilation	5	kept
Mass casualty incident	mass casualty incident	mass casualty incident	mass casualty incident	Mass Casualty Incidents	5	case_or_format
Mass Media	mass media	mass media	mass media	Mass Media	5	kept
masters education	masters education	masters education	masters education	masters education	5	kept
Materials Management, Hospital	materials management hospital	materials management hospital	materials management hospital	Materials Management, Hospital	5	kept
maternal	maternal	maternal	maternal	Maternal	5	case_or_format
Maternal Death	maternal death	maternal death	maternal death	maternal death	5	case_or_format
Maternal morbidity	maternal morbidity	maternal morbidity	maternal morbidity	maternal morbidity	5	case_or_format
Maternal-Child Health Services	maternal child health services	maternal child health services	maternal child health services	Maternal-Child Health Services	5	kept

maximal inspiratory pressure	maximal inspiratory pressure	maximal inspiratory pressure	maximal inspiratory pressure	maximal inspiratory pressure	5	kept
mean corpuscular volume	mean corpuscular volume	mean corpuscular volume	mean corpuscular volume	mean corpuscular volume	5	kept
meaningful use criteria	meaningful use criteria	meaningful use criteria	meaningful use criteria	meaningful use criteria	5	kept
MECHANISMS	mechanisms	mechanisms	mechanisms	MECHANISMS	5	kept
median survival time	median survival time	median survival time	median survival time	median survival time	5	kept
Mediastinitis	mediastinitis	mediastinitis	mediastinitis	mediastinitis	5	case_or_format
Medical applications	medical applications	medical applications	medical applications	Medical applications	5	kept
Medical decision-making	medical decision making	medical decision making	medical decision making	medical decision making	5	case_or_format
Medical device-related pressure injury	medical device related pressure injury	medical device related pressure injury	medical device related pressure injury	medical device-related pressure injury	5	case_or_format
Medical emergency teams	medical emergency teams	medical emergency teams	medical emergency team	Medical emergency team	5	singular
Medical Missions, Official	medical missions official	medical missions official	medical missions official	Medical Missions, Official	5	kept
Medical problems	medical problems	medical problems	medical problems	Medical problems	5	kept
Medical professionals	medical professionals	medical professionals	medical professionals	Medical professionals	5	kept
medical registrar	medical registrar	medical registrar	medical registrar	medical registrar	5	kept
Medical simulation	medical simulation	medical simulation	medical simulation	Medical simulation	5	kept
medical technologist	medical technologist	medical technologist	medical technologist	medical technologist	5	kept

MEDICAL-EDUCATION	medical education	medical education	medical education	medical education	5	case_or_format
Medicare Part A	medicare part a	medicare part a	medicare part a	Medicare Part A	5	kept
MEDICATION	medication	medication	medication	Medication	5	case_or_format
Medication administration errors	medication administration errors	medication administration errors	medication administration error	medication administration errors	5	singular
Medications	medications	medications	medication	Medication	5	singular
Melanoma	melanoma	melanoma	melanoma	melanoma	5	case_or_format
Men	men	men	men	Men	5	kept
meningoencephalitis	meningoencephalitis	meningoencephalitis	meningoencephalitis	meningoencephalitis	5	kept
mental development	mental development	mental development	mental development	mental development	5	kept
Meropenem	meropenem	meropenem	meropenem	meropenem	5	case_or_format
metabolic syndrome X	metabolic syndrome x	metabolic syndrome x	metabolic syndrome x	metabolic syndrome X	5	kept
Metabolism, Inborn Errors	metabolism inborn errors	metabolism inborn errors	metabolism inborn errors	Metabolism, Inborn Errors	5	kept
methemoglobinemia	methemoglobinemia	methemoglobinemia	methemoglobinemia	methemoglobinemia	5	kept
METHICILLIN-RESISTANT	methicillin resistant	methicillin resistant	methicillin resistant	METHICILLIN-RESISTANT	5	kept
Methicillin-resistant staphylococcus aureus	methicillin resistant staphylococcus aureus	methicillin resistant staphylococcus aureus	methicillin resistant staphylococcus aureus	methicillin resistant Staphylococcus aureus	5	case_or_format
Methodology	methodology	methodology	methodology	methodology	5	case_or_format

Methyl Ethers	methyl ethers	methyl ethers	methyl ethers	Methyl Ethers	5	kept
Microbiome	microbiome	microbiome	microbiome	microbiome	5	case_or_format
microorganism detection	microorganism detection	microorganism detection	microorganism detection	microorganism detection	5	kept
MIDAZOLAM	midazolam	midazolam	midazolam	midazolam	5	case_or_format
military hospital	military hospital	military hospital	military hospital	military hospital	5	kept
MIMIC-III	mimic iii	mimic iii	mimic iii	MIMIC-III	5	kept
Minas Gerais	minas gerais	minas gerais	minas gerais	Minas Gerais	5	kept
minimally conscious state	minimally conscious state	minimally conscious state	minimally conscious state	minimally conscious state	5	kept
minor surgery	minor surgery	minor surgery	minor surgery	minor surgery	5	kept
Mitral Valve Prolapse	mitral valve prolapse	mitral valve prolapse	mitral valve prolapse	Mitral Valve Prolapse	5	kept
mitral valve replacement	mitral valve replacement	mitral valve replacement	mitral valve replacement	mitral valve replacement	5	kept
mobile applications	mobile applications	mobile applications	mobile application	Mobile Applications	5	singular
Modelling	modelling	modelling	modelling	Modelling	5	kept
Models	models	models	model	model	5	singular
Models, Cardiovascular	models cardiovascular	models cardiovascular	models cardiovascular	Models, Cardiovascular	5	kept
Models, Economic	models economic	models economic	models economic	Models, Economic	5	kept

molecular diagnosis	molecular diagnosis	molecular diagnosis	molecular diagnosis	molecular diagnosis	5	kept
Molecular Typing	molecular typing	molecular typing	molecular typing	Molecular Typing	5	kept
molecular typing	molecular typing	molecular typing	molecular typing	Molecular Typing	5	case_or_format
mood change	mood change	mood change	mood change	mood change	5	kept
moral	moral	moral	moral	Morals	5	case_or_format
moral courage	moral courage	moral courage	moral courage	moral courage	5	kept
moral disengagement	moral disengagement	moral disengagement	moral disengagement	moral disengagement	5	kept
morals	morals	morals	moral	Morals	5	singular
morgue	morgue	morgue	morgue	morgue	5	kept
morphine equivalent dose	morphine equivalent dose	morphine equivalent dose	morphine equivalent dose	morphine equivalent dose	5	kept
Motor Skills Disorders	motor skills disorders	motor skills disorders	motor skills disorders	Motor Skills Disorders	5	kept
mouse	mouse	mouse	mouse	mouse	5	kept
Mucous Membrane	mucous membrane	mucous membrane	mucous membrane	Mucous Membrane	5	kept
Mucus	mucus	mucus	mucus	Mucus	5	kept
Multidisciplinary care	multidisciplinary care	multidisciplinary care	multidisciplinary care	multidisciplinary care	5	case_or_format
Multidisciplinary teams	multidisciplinary teams	multidisciplinary teams	multidisciplinary team	multidisciplinary team	5	singular

Multidrug-resistant organisms	multidrug resistant organisms	multidrug resistant organisms	multidrug resistant organism	Multidrug-resistant organisms	5	singular
multiinfarct dementia	multiinfarct dementia	multiinfarct dementia	multiinfarct dementia	multiinfarct dementia	5	kept
Multilevel Analysis	multilevel analysis	multilevel analysis	multilevel analysis	multilevel analysis	5	case_or_format
multimodal	multimodal	multimodal	multimodal	multimodal	5	kept
multimorbidity	multimorbidity	multimorbidity	multimorbidity	Multimorbidity	5	case_or_format
multinomial logistic regression	multinomial logistic regression	multinomial logistic regression	multinomial logistic regression	multinomial logistic regression	5	kept
Multiple Myeloma	multiple myeloma	multiple myeloma	multiple myeloma	multiple myeloma	5	case_or_format
Multiple organ failure	multiple organ failure	multiple organ failure	multiple organ failure	multiple organ failure	5	case_or_format
muscle cramp	muscle cramp	muscle cramp	muscle cramp	muscle cramp	5	kept
muscle function	muscle function	muscle function	muscle function	muscle function	5	kept
muscular dystrophy	muscular dystrophy	muscular dystrophy	muscular dystrophy	muscular dystrophy	5	kept
Musculoskeletal system	musculoskeletal system	musculoskeletal system	musculoskeletal system	Musculoskeletal System	5	case_or_format
mutism	mutism	mutism	mutism	mutism	5	kept
mycophenolic acid	mycophenolic acid	mycophenolic acid	mycophenolic acid	mycophenolic acid	5	kept
mydriasis	mydriasis	mydriasis	mydriasis	mydriasis	5	kept
myelodysplastic syndrome	myelodysplastic syndrome	myelodysplastic syndrome	myelodysplastic syndrome	myelodysplastic syndrome	5	kept

Myocardial Revascularization	myocardial revascularization	myocardial revascularization	myocardial revascularization	Myocardial Revascularization	5	kept
MYOCARDIAL-INFARCTION	myocardial infarction	myocardial infarction	myocardial infarction	Myocardial Infarction	5	case_or_format
Myocarditis	myocarditis	myocarditis	myocarditis	myocarditis	5	case_or_format
N-Methyl-3,4-methylenedioxyamphetamine	n methyl 3 4 methylenedioxyamphetamine	n methyl 3 4 methylenedioxyamphetamine	n methyl 3 4 methylenedioxyamphetamine	N-Methyl-3,4-methylenedioxyamphetamine	5	kept
naltrexone	naltrexone	naltrexone	naltrexone	naltrexone	5	kept
Narcotic Antagonists	narcotic antagonists	narcotic antagonists	narcotic antagonists	Narcotic Antagonists	5	kept
narrative inquiry	narrative inquiry	narrative inquiry	narrative inquiry	narrative inquiry	5	kept
NASA	nasa	nasa	nasa	NASA	5	kept
nasal cannula therapy	nasal cannula therapy	nasal cannula therapy	nasal cannula therapy	nasal cannula therapy	5	kept
nasopharynx	nasopharynx	nasopharynx	nasopharynx	nasopharynx	5	kept
NATIONAL-SURVEY	national survey	national survey	national survey	National survey	5	case_or_format
Near miss	near miss	near miss	near miss	near miss	5	case_or_format
near-infrared spectroscopy	near infrared spectroscopy	near infrared spectroscopy	near infrared spectroscopy	near infrared spectroscopy	5	case_or_format
neck injury	neck injury	neck injury	neck injury	Neck Injuries	5	case_or_format
necrotizing pneumonia	necrotizing pneumonia	necrotizing pneumonia	necrotizing pneumonia	necrotizing pneumonia	5	kept
nefopam	nefopam	nefopam	nefopam	nefopam	5	kept

Negativism	negativism	negativism	negativism	Negativism	5	kept
neglect	neglect	neglect	neglect	neglect	5	kept
neonatal death	neonatal death	neonatal death	neonatal death	neonatal death	5	kept
NEONATAL PAIN	neonatal pain	neonatal pain	neonatal pain	Neonatal pain	5	case_or_format
Neonatal Resuscitation Program	neonatal resuscitation program	neonatal resuscitation program	neonatal resuscitation program	Neonatal Resuscitation Program	5	kept
neonatal transport	neonatal transport	neonatal transport	neonatal transport	Neonatal transport	5	case_or_format
Nephrotic Syndrome	nephrotic syndrome	nephrotic syndrome	nephrotic syndrome	nephrotic syndrome	5	case_or_format
nerve cell plasticity	nerve cell plasticity	nerve cell plasticity	nerve cell plasticity	nerve cell plasticity	5	kept
nerve conduction	nerve conduction	nerve conduction	nerve conduction	nerve conduction	5	kept
Nervous System Malformations	nervous system malformations	nervous system malformations	nervous system malformation	Nervous System Malformations	5	singular
network meta-analysis	network meta analysis	network meta analysis	network meta analysis	Network meta-analysis	5	case_or_format
Neural networks	neural networks	neural networks	neural network	Neural networks	5	singular
neuroblastoma	neuroblastoma	neuroblastoma	neuroblastoma	neuroblastoma	5	kept
Neurologists	neurologists	neurologists	neurologist	neurologist	5	singular
Neuromonitoring	neuromonitoring	neuromonitoring	neuromonitoring	neuromonitoring	5	case_or_format
neuromuscular blockade	neuromuscular blockade	neuromuscular blockade	neuromuscular blockade	Neuromuscular Blockade	5	case_or_format

neuropalliative care	neuropalliative care	neuropalliative care	neuropalliative care	neuropalliative care	5	kept
neuroprotective agent	neuroprotective agent	neuroprotective agent	neuroprotective agent	Neuroprotective Agents	5	case_or_format
Neurosecretory Systems	neurosecretory systems	neurosecretory systems	neurosecretory systems	Neurosecretory Systems	5	kept
neutrophilia	neutrophilia	neutrophilia	neutrophilia	neutrophilia	5	kept
NICSS	nicss	nicss	nicss	NICSS	5	kept
Nitroprusside	nitroprusside	nitroprusside	nitroprusside	Nitroprusside	5	kept
Nitrous Oxide	nitrous oxide	nitrous oxide	nitrous oxide	nitrous oxide	5	case_or_format
nociceptive stimulation	nociceptive stimulation	nociceptive stimulation	nociceptive stimulation	nociceptive stimulation	5	kept
Noise levels	noise levels	noise levels	noise level	Noise levels	5	singular
Noise reduction	noise reduction	noise reduction	noise reduction	noise reduction	5	case_or_format
nomograms	nomograms	nomograms	nomogram	nomogram	5	singular
non-fermenting Gram-negative bacterium	non fermenting gram negative bacterium	non fermenting gram negative bacterium	non fermenting gram negative bacterium	non-fermenting Gram-negative bacterium	5	kept
Non-nutritive sucking	non nutritive sucking	non nutritive sucking	non nutritive sucking	Non-nutritive sucking	5	kept
Non-technical skills	non technical skills	non technical skills	non technical skill	Non-technical skills	5	singular
non-technical skills	non technical skills	non technical skills	non technical skill	Non-technical skills	5	singular
Noninvasive mechanical ventilation	noninvasive mechanical ventilation	noninvasive mechanical ventilation	noninvasive mechanical ventilation	Noninvasive mechanical ventilation	5	kept

Noninvasive medical procedures	noninvasive medical procedures	noninvasive medical procedures	noninvasive medical procedures	Noninvasive medical procedures	5	kept
nonREM sleep	nonrem sleep	nonrem sleep	nonrem sleep	nonREM sleep	5	kept
nootropic agent	nootropic agent	nootropic agent	nootropic agent	nootropic agent	5	kept
Northwestern United States	northwestern united states	northwestern united states	northwestern united states	Northwestern United States	5	kept
Norwood procedure	norwood procedure	norwood procedure	norwood procedure	Norwood procedure	5	kept
nose cavity	nose cavity	nose cavity	nose cavity	nose cavity	5	kept
nose mucosa	nose mucosa	nose mucosa	nose mucosa	nose mucosa	5	kept
Nosocomial Infection	nosocomial infection	nosocomial infection	nosocomial infection	Nosocomial infection	5	case_or_format
Nosocomial pneumonia	nosocomial pneumonia	nosocomial pneumonia	nosocomial pneumonia	nosocomial pneumonia	5	case_or_format
Nu-DESC	nu desc	nu desc	nu desc	Nu-DESC	5	kept
Observational	observational	observational	observational	Observational	5	kept
Obstacles	obstacles	obstacles	obstacles	Obstacles	5	kept
occlusive cerebrovascular disease	occlusive cerebrovascular disease	occlusive cerebrovascular disease	occlusive cerebrovascular disease	occlusive cerebrovascular disease	5	kept
OCCUPANCY	occupancy	occupancy	occupancy	OCCUPANCY	5	kept
occupational injuries	occupational injuries	occupational injuries	occupational injury	Occupational Injuries	5	singular
Occupational safety	occupational safety	occupational safety	occupational safety	occupational safety	5	case_or_format

odor	odor	odor	odor	odor	5	kept
odynophagia	odynophagia	odynophagia	odynophagia	odynophagia	5	kept
ointment	ointment	ointment	ointment	Ointments	5	case_or_format
OLDER	older	older	older	OLDER	5	kept
Oncology Service, Hospital	oncology service hospital	oncology service hospital	oncology service hospital	Oncology Service, Hospital	5	kept
Online learning	online learning	online learning	online learning	Online learning	5	kept
Ontology	ontology	ontology	ontology	Ontology	5	kept
open access publishing	open access publishing	open access publishing	open access publishing	open access publishing	5	kept
open fracture	open fracture	open fracture	open fracture	open fracture	5	kept
open reduction	open reduction	open reduction	open reduction	open reduction	5	kept
Open-heart surgery	open heart surgery	open heart surgery	open heart surgery	open heart surgery	5	case_or_format
optic nerve disease	optic nerve disease	optic nerve disease	optic nerve disease	optic nerve disease	5	kept
optic nerve sheath diameter	optic nerve sheath diameter	optic nerve sheath diameter	optic nerve sheath diameter	optic nerve sheath diameter	5	kept
Optimism	optimism	optimism	optimism	optimism	5	case_or_format
oral glucose tolerance test	oral glucose tolerance test	oral glucose tolerance test	oral glucose tolerance test	oral glucose tolerance test	5	kept
oral rehydration therapy	oral rehydration therapy	oral rehydration therapy	oral rehydration therapy	oral rehydration therapy	5	kept

Organ failure	organ failure	organ failure	organ failure	Organ failure	5	kept
organisation	organisation	organisation	organisation	Organisation	5	case_or_format
organizational support	organizational support	organizational support	organizational support	organizational support	5	kept
organophosphate	organophosphate	organophosphate	organophosphate	organophosphate	5	kept
Orthomyxoviridae	orthomyxoviridae	orthomyxoviridae	orthomyxoviridae	Orthomyxoviridae	5	kept
OSCE	osce	osce	osce	OSCE	5	kept
Osmolar Concentration	osmolar concentration	osmolar concentration	osmolar concentration	Osmolar Concentration	5	kept
Osteoarthritis	osteoarthritis	osteoarthritis	osteoarthritis	osteoarthritis	5	case_or_format
outcome of education	outcome of education	outcome of education	outcome of education	outcome of education	5	kept
overweight	overweight	overweight	overweight	Overweight	5	case_or_format
Oxygen inhalation therapy	oxygen inhalation therapy	oxygen inhalation therapy	oxygen inhalation therapy	Oxygen Inhalation Therapy	5	case_or_format
oxygen transport	oxygen transport	oxygen transport	oxygen transport	oxygen transport	5	kept
oxytocic agent	oxytocic agent	oxytocic agent	oxytocic agent	oxytocic agent	5	kept
pacemaker	pacemaker	pacemaker	pacemaker	pacemaker	5	kept
Pacific Islander	pacific islander	pacific islander	pacific islander	Pacific Islander	5	kept
pain control	pain control	pain control	pain control	pain control	5	kept

PAIN MANAGEMENT	pain management	pain management	pain management	Pain Management	5	case_or_format
Pain scales	pain scales	pain scales	pain scale	Pain scales	5	singular
Palestinian	palestinian	palestinian	palestinian	Palestinian	5	kept
Palliative	palliative	palliative	palliative	palliative	5	case_or_format
pancreas cancer	pancreas cancer	pancreas cancer	pancreas cancer	pancreas cancer	5	kept
pancreas resection	pancreas resection	pancreas resection	pancreas resection	pancreas resection	5	kept
Pancreaticoduodenectomy	pancreaticoduodenectomy	pancreaticoduodenectomy	pancreaticoduodenectomy	Pancreaticoduodenectomy	5	kept
Paraplegia	paraplegia	paraplegia	paraplegia	paraplegia	5	case_or_format
Parent education	parent education	parent education	parent education	Parent education	5	kept
parental experiences	parental experiences	parental experiences	parental experience	parental experiences	5	singular
parental involvement	parental involvement	parental involvement	parental involvement	parental involvement	5	kept
Parental participation	parental participation	parental participation	parental participation	Parental participation	5	kept
Parental satisfaction	parental satisfaction	parental satisfaction	parental satisfaction	Parental satisfaction	5	kept
parental satisfaction	parental satisfaction	parental satisfaction	parental satisfaction	Parental satisfaction	5	case_or_format
parenteral	parenteral	parenteral	parenteral	parenteral	5	kept
parenteral drug administration	parenteral drug administration	parenteral drug administration	parenteral drug administration	parenteral drug administration	5	kept

parenting education	parenting education	parenting education	parenting education	parenting education	5	kept
paresis	paresis	paresis	paresis	paresis	5	kept
partial pressure	partial pressure	partial pressure	partial pressure	Partial Pressure	5	case_or_format
Participatory action research	participatory action research	participatory action research	participatory action research	participatory action research	5	case_or_format
Partnership	partnership	partnership	partnership	partnership	5	case_or_format
passive euthanasia	passive euthanasia	passive euthanasia	passive euthanasia	passive euthanasia	5	kept
patent foramen ovale	patent foramen ovale	patent foramen ovale	patent foramen ovale	patent foramen ovale	5	kept
paternal behavior	paternal behavior	paternal behavior	paternal behavior	Paternal Behavior	5	case_or_format
pathogen load	pathogen load	pathogen load	pathogen load	pathogen load	5	kept
Pediatric Early Warning Score	pediatric early warning score	pediatric early warning score	pediatric early warning score	Pediatric Early Warning Score	5	kept
Pediatric Index of Mortality	pediatric index of mortality	pediatric index of mortality	pediatric index of mortality	Pediatric Index of Mortality	5	kept
Pediatric surgery	pediatric surgery	pediatric surgery	pediatric surgery	pediatric surgery	5	case_or_format
pelvic girdle	pelvic girdle	pelvic girdle	pelvic girdle	pelvic girdle	5	kept
Pelvis	pelvis	pelvis	pelvis	pelvis	5	case_or_format
penicillin binding protein 2a	penicillin binding protein 2a	penicillin binding protein 2a	penicillin binding protein 2a	penicillin binding protein 2a	5	kept
Penicillins	penicillins	penicillins	penicillins	Penicillins	5	kept

Peptoclostridium difficile	peptoclostridium difficile	peptoclostridium difficile	peptoclostridium difficile	Peptoclostridium difficile	5	kept
perceived social support	perceived social support	perceived social support	perceived social support	perceived social support	5	kept
perception disorder	perception disorder	perception disorder	perception disorder	perception disorder	5	kept
perfectionism	perfectionism	perfectionism	perfectionism	perfectionism	5	kept
Pericardial Effusion	pericardial effusion	pericardial effusion	pericardial effusion	pericardial effusion	5	case_or_format
pericarditis	pericarditis	pericarditis	pericarditis	pericarditis	5	kept
peripheral arterial disease	peripheral arterial disease	peripheral arterial disease	peripheral arterial disease	Peripheral Arterial Disease	5	case_or_format
Peripheral Nervous System Diseases	peripheral nervous system diseases	peripheral nervous system diseases	peripheral nervous system diseases	Peripheral Nervous System Diseases	5	kept
Peripheral Vascular Diseases	peripheral vascular diseases	peripheral vascular diseases	peripheral vascular disease	peripheral vascular disease	5	singular
periprosthetic joint infection	periprosthetic joint infection	periprosthetic joint infection	periprosthetic joint infection	periprosthetic joint infection	5	kept
Personal accomplishment	personal accomplishment	personal accomplishment	personal accomplishment	Personal accomplishment	5	kept
personal accomplishment	personal accomplishment	personal accomplishment	personal accomplishment	Personal accomplishment	5	case_or_format
personal value	personal value	personal value	personal value	personal value	5	kept
personality assessment	personality assessment	personality assessment	personality assessment	personality assessment	5	kept
Personnel Delegation	personnel delegation	personnel delegation	personnel delegation	Personnel Delegation	5	kept
personnel staffing	personnel staffing	personnel staffing	personnel staffing	personnel staffing	5	kept

Personnel Staffing and Scheduling Information Systems	personnel staffing and scheduling information systems	personnel staffing and scheduling information systems	personnel staffing and scheduling information systems	Personnel Staffing and Scheduling Information Systems	5	kept
Personnel turnover	personnel turnover	personnel turnover	personnel turnover	Personnel Turnover	5	case_or_format
Perspectives	perspectives	perspectives	perspective	PERSPECTIVES	5	singular
Pharmaceutical care	pharmaceutical care	pharmaceutical care	pharmaceutical care	pharmaceutical care	5	case_or_format
pharmaceutics	pharmaceutics	pharmaceutics	pharmaceutics	pharmaceutics	5	kept
Pharmacotherapy	pharmacotherapy	pharmacotherapy	pharmacotherapy	Pharmacotherapy	5	kept
pharmacy student	pharmacy student	pharmacy student	pharmacy student	pharmacy student	5	kept
Phenomenological research	phenomenological research	phenomenological research	phenomenological research	Phenomenological research	5	kept
Phosphodiesterase Inhibitors	phosphodiesterase inhibitors	phosphodiesterase inhibitors	phosphodiesterase inhibitor	phosphodiesterase inhibitor	5	singular
Photic Stimulation	photic stimulation	photic stimulation	photic stimulation	Photic Stimulation	5	kept
Photochemotherapy	photochemotherapy	photochemotherapy	photochemotherapy	Photochemotherapy	5	kept
physiatrist	physiatrist	physiatrist	physiatrist	physiatrist	5	kept
physical medicine	physical medicine	physical medicine	physical medicine	physical medicine	5	kept
physical parameters	physical parameters	physical parameters	physical parameters	physical parameters	5	kept
PHYSICIAN COLLABORATION	physician collaboration	physician collaboration	physician collaboration	PHYSICIAN COLLABORATION	5	kept
physician order entry system	physician order entry system	physician order entry system	physician order entry system	physician order entry system	5	kept

Physiological signals	physiological signals	physiological signals	physiological signals	Physiological signals	5	kept
Pilot trial	pilot trial	pilot trial	pilot trial	Pilot trial	5	kept
Pituitary Neoplasms	pituitary neoplasms	pituitary neoplasms	pituitary neoplasms	Pituitary Neoplasms	5	kept
Pituitary-Adrenal System	pituitary adrenal system	pituitary adrenal system	pituitary adrenal system	Pituitary-Adrenal System	5	kept
PLACEMENT	placement	placement	placement	PLACEMENT	5	kept
plasma	plasma	plasma	plasma	Plasma	5	case_or_format
Plasma exchange	plasma exchange	plasma exchange	plasma exchange	plasma exchange	5	case_or_format
Plasmids	plasmids	plasmids	plasmid	plasmid	5	singular
plastic	plastic	plastic	plastic	plastic	5	kept
plastic surgeon	plastic surgeon	plastic surgeon	plastic surgeon	plastic surgeon	5	kept
Plasticizers	plasticizers	plasticizers	plasticizer	Plasticizers	5	singular
Plastics	plastics	plastics	plastic	plastic	5	singular
policy making	policy making	policy making	policy making	Policy Making	5	case_or_format
polypropylene	polypropylene	polypropylene	polypropylene	polypropylene	5	kept
Population Dynamics	population dynamics	population dynamics	population dynamics	Population Dynamics	5	kept
population parameters	population parameters	population parameters	population parameters	population parameters	5	kept

population size	population size	population size	population size	population size	5	kept
Population statistics	population statistics	population statistics	population statistics	Population statistics	5	kept
Portal Vein	portal vein	portal vein	portal vein	Portal Vein	5	kept
Positive and Negative Syndrome Scale	positive and negative syndrome scale	positive and negative syndrome scale	positive and negative syndrome scale	Positive and Negative Syndrome Scale	5	kept
positivity rate	positivity rate	positivity rate	positivity rate	positivity rate	5	kept
Post-Acute COVID-19 Syndrome	post acute covid 19 syndrome	post acute covid 19 syndrome	post acute covid 19 syndrome	Post-Acute COVID-19 Syndrome	5	kept
Post-discharge	post discharge	post discharge	post discharge	Post-discharge	5	kept
post-operative care	post operative care	post operative care	post operative care	post-operative care	5	kept
Post-traumatic growth	post traumatic growth	post traumatic growth	post traumatic growth	post-traumatic growth	5	case_or_format
postal mail	postal mail	postal mail	postal mail	postal mail	5	kept
posterior reversible encephalopathy syndrome	posterior reversible encephalopathy syndrome	posterior reversible encephalopathy syndrome	posterior reversible encephalopathy syndrome	posterior reversible encephalopathy syndrome	5	kept
Postoperative period	postoperative period	postoperative period	postoperative period	postoperative period	5	case_or_format
postpartum	postpartum	postpartum	postpartum	Postpartum	5	case_or_format
Posttraumatic growth	posttraumatic growth	posttraumatic growth	posttraumatic growth	posttraumatic growth	5	case_or_format
postural drainage	postural drainage	postural drainage	postural drainage	postural drainage	5	kept
Potassium Chloride	potassium chloride	potassium chloride	potassium chloride	potassium chloride	5	case_or_format

Potentially Inappropriate Medication List	potentially inappropriate medication list	potentially inappropriate medication list	potentially inappropriate medication list	Potentially Inappropriate Medication List	5	kept
Practice change	practice change	practice change	practice change	Practice change	5	kept
PRACTICE GUIDELINES	practice guidelines	practice guidelines	practice guideline	practice guideline	5	singular
prealbumin	prealbumin	prealbumin	prealbumin	prealbumin	5	kept
predictive analytics	predictive analytics	predictive analytics	predictive analytics	predictive analytics	5	kept
Preeclampsia	preeclampsia	preeclampsia	preeclampsia	preeclampsia	5	case_or_format
pregnancy complications	pregnancy complications	pregnancy complications	pregnancy complication	Pregnancy Complications	5	singular
Prehospital	prehospital	prehospital	prehospital	Prehospital	5	kept
prehospital care	prehospital care	prehospital care	prehospital care	Prehospital care	5	case_or_format
Premature Infant Pain Profile	premature infant pain profile	premature infant pain profile	premature infant pain profile	Premature Infant Pain Profile	5	kept
preoperative treatment	preoperative treatment	preoperative treatment	preoperative treatment	preoperative treatment	5	kept
Prescriptions, Drug	prescriptions drug	prescriptions drug	prescriptions drug	Prescriptions, Drug	5	kept
pressure controlled ventilation	pressure controlled ventilation	pressure controlled ventilation	pressure controlled ventilation	pressure controlled ventilation	5	kept
preterm neonate	preterm neonate	preterm neonate	preterm neonate	Preterm neonates	5	case_or_format
Preventive measures	preventive measures	preventive measures	preventive measures	preventive measures	5	case_or_format
prick test	prick test	prick test	prick test	prick test	5	kept

primary palliative care	primary palliative care	primary palliative care	primary palliative care	primary palliative care	5	kept
prisoner	prisoner	prisoner	prisoner	Prisoners	5	case_or_format
problem behavior	problem behavior	problem behavior	problem behavior	problem behavior	5	kept
Problem solving	problem solving	problem solving	problem solving	Problem Solving	5	case_or_format
procedural sedation	procedural sedation	procedural sedation	procedural sedation	Procedural Sedation	5	case_or_format
process assessment	process assessment	process assessment	process assessment	Process Assessment	5	case_or_format
Process improvement	process improvement	process improvement	process improvement	Process improvement	5	kept
process improvement	process improvement	process improvement	process improvement	Process improvement	5	case_or_format
Professional development	professional development	professional development	professional development	professional development	5	case_or_format
Professional education	professional education	professional education	professional education	Professional education	5	kept
Program development	program development	program development	program development	Program Development	5	case_or_format
Programmed Instructions as Topic	programmed instructions as topic	programmed instructions as topic	programmed instructions as topic	Programmed Instructions as Topic	5	kept
prone position ventilation	prone position ventilation	prone position ventilation	prone position ventilation	prone position ventilation	5	kept
Propionibacterium	propionibacterium	propionibacterium	propionibacterium	Propionibacterium	5	kept
prosthesis infection	prosthesis infection	prosthesis infection	prosthesis infection	prosthesis infection	5	kept
prosthesis loosening	prosthesis loosening	prosthesis loosening	prosthesis loosening	prosthesis loosening	5	kept

protein degradation	protein degradation	protein degradation	protein degradation	protein degradation	5	kept
Protein Precursors	protein precursors	protein precursors	protein precursor	Protein Precursors	5	singular
protein S100B	protein s100b	protein s100b	protein s100b	protein S100B	5	kept
Proteomics	proteomics	proteomics	proteomic	Proteomics	5	singular
Prothrombin Time	prothrombin time	prothrombin time	prothrombin time	prothrombin time	5	case_or_format
PROVIDERS	providers	providers	provider	PROVIDERS	5	singular
proximal humerus fracture	proximal humerus fracture	proximal humerus fracture	proximal humerus fracture	proximal humerus fracture	5	kept
pseudomembranous colitis	pseudomembranous colitis	pseudomembranous colitis	pseudomembranous colitis	pseudomembranous colitis	5	kept
PSEUDOMONAS-AERUGINOSA	pseudomonas aeruginosa	pseudomonas aeruginosa	pseudomonas aeruginosa	Pseudomonas aeruginosa	5	case_or_format
psychiatric diagnosis	psychiatric diagnosis	psychiatric diagnosis	psychiatric diagnosis	psychiatric diagnosis	5	kept
psychological capital	psychological capital	psychological capital	psychological capital	psychological capital	5	kept
PSYCHOLOGICAL DISTRESS	psychological distress	psychological distress	psychological distress	Psychological Distress	5	case_or_format
psychological distress assessment	psychological distress assessment	psychological distress assessment	psychological distress assessment	psychological distress assessment	5	kept
psychological empowerment	psychological empowerment	psychological empowerment	psychological empowerment	Psychological empowerment	5	case_or_format
psychological health	psychological health	psychological health	psychological health	psychological health	5	kept
Psychological Well-Being	psychological well being	psychological well being	psychological well being	psychological well-being	5	case_or_format

Psychometric evaluation	psychometric evaluation	psychometric evaluation	psychometric evaluation	PSYCHOMETRIC EVALUATION	5	case_or_format
Psychosis	psychosis	psychosis	psychosis	psychosis	5	case_or_format
psychosocial	psychosocial	psychosocial	psychosocial	Psychosocial	5	case_or_format
Psychotropic drugs	psychotropic drugs	psychotropic drugs	psychotropic drugs	Psychotropic Drugs	5	case_or_format
public health emergencies	public health emergencies	public health emergencies	public health emergency	public health emergencies	5	singular
Public hospitals	public hospitals	public hospitals	public hospital	public hospital	5	singular
Pulmonary	pulmonary	pulmonary	pulmonary	Pulmonary	5	kept
pulmonary	pulmonary	pulmonary	pulmonary	Pulmonary	5	case_or_format
Pulmonary artery catheter	pulmonary artery catheter	pulmonary artery catheter	pulmonary artery catheter	pulmonary artery catheter	5	case_or_format
Pulmonary diseases	pulmonary diseases	pulmonary diseases	pulmonary disease	Pulmonary diseases	5	singular
Pulmonary infection	pulmonary infection	pulmonary infection	pulmonary infection	Pulmonary infection	5	kept
pulsatility index	pulsatility index	pulsatility index	pulsatility index	pulsatility index	5	kept
punishment	punishment	punishment	punishment	punishment	5	kept
pupil	pupil	pupil	pupil	Pupil	5	case_or_format
Pupillometer	pupillometer	pupillometer	pupillometer	Pupillometer	5	kept
pupillometer	pupillometer	pupillometer	pupillometer	Pupillometer	5	case_or_format

pyrazole derivative	pyrazole derivative	pyrazole derivative	pyrazole derivative	pyrazole derivative	5	kept
Pyridines	pyridines	pyridines	pyridines	Pyridines	5	kept
pyrrole derivative	pyrrole derivative	pyrrole derivative	pyrrole derivative	pyrrole derivative	5	kept
Qualitative analysis	qualitative analysis	qualitative analysis	qualitative analysis	qualitative analysis	5	case_or_format
quality assessment tool	quality assessment tool	quality assessment tool	quality assessment tool	quality assessment tool	5	kept
quality care	quality care	quality care	quality care	Quality care	5	case_or_format
Quality improvement methodologies	quality improvement methodologies	quality improvement methodologies	quality improvement methodologies	Quality improvement methodologies	5	kept
quality of dying	quality of dying	quality of dying	quality of dying	quality of dying	5	kept
Quality of service	quality of service	quality of service	quality of service	Quality of service	5	kept
Quality of work life	quality of work life	quality of work life	quality of work life	Quality of work life	5	kept
quantitative	quantitative	quantitative	quantitative	quantitative	5	kept
quaternary ammonium derivative	quaternary ammonium derivative	quaternary ammonium derivative	quaternary ammonium derivative	quaternary ammonium derivative	5	kept
rabies	rabies	rabies	rabies	rabies	5	kept
Radiation protection	radiation protection	radiation protection	radiation protection	Radiation protection	5	kept
radical gastrectomy	radical gastrectomy	radical gastrectomy	radical gastrectomy	radical gastrectomy	5	kept
Radiology Department, Hospital	radiology department hospital	radiology department hospital	radiology department hospital	Radiology Department, Hospital	5	kept

Random Forest	random forest	random forest	random forest	random forest	5	case_or_format
randomized trial	randomized trial	randomized trial	randomized trial	RANDOMIZED-TRIAL	5	case_or_format
rapid response	rapid response	rapid response	rapid response	Rapid response	5	case_or_format
RAPID RESPONSE SYSTEM	rapid response system	rapid response system	rapid response system	Rapid response system	5	case_or_format
Readiness	readiness	readiness	readiness	Readiness	5	kept
Readmissions	readmissions	readmissions	readmission	Readmission	5	singular
reasoning	reasoning	reasoning	reasoning	reasoning	5	kept
REBOA	reboa	reboa	reboa	REBOA	5	kept
RECORD	record	record	record	Records	5	case_or_format
recreation	recreation	recreation	recreation	recreation	5	kept
rectum anterior resection	rectum anterior resection	rectum anterior resection	rectum anterior resection	rectum anterior resection	5	kept
rectum tumor	rectum tumor	rectum tumor	rectum tumor	rectum tumor	5	kept
Reflexology	reflexology	reflexology	reflexology	reflexology	5	case_or_format
Refusal to Treat	refusal to treat	refusal to treat	refusal to treat	Refusal to Treat	5	kept
Regional Blood Flow	regional blood flow	regional blood flow	regional blood flow	Regional Blood Flow	5	kept
Reinfection	reinfection	reinfection	reinfection	reinfection	5	case_or_format

Reinforcement, Psychology	reinforcement psychology	reinforcement psychology	reinforcement psychology	Reinforcement, Psychology	5	kept
reintubation	reintubation	reintubation	reintubation	Reintubation	5	case_or_format
Related factors	related factors	related factors	related factors	Related factors	5	kept
Renal replacement therapies	renal replacement therapies	renal replacement therapies	renal replacement therapy	renal replacement therapy	5	singular
reperfusion	reperfusion	reperfusion	reperfusion	Reperfusion	5	case_or_format
reproductive health	reproductive health	reproductive health	reproductive health	reproductive health	5	kept
RESCUE	rescue	rescue	rescue	RESCUE	5	kept
research priority	research priority	research priority	research priority	Research priorities	5	case_or_format
research subject	research subject	research subject	research subject	Research Subjects	5	case_or_format
Research utilization	research utilization	research utilization	research utilization	Research utilization	5	kept
Resident	resident	resident	resident	resident	5	case_or_format
residential area	residential area	residential area	residential area	residential area	5	kept
residents	residents	residents	resident	resident	5	singular
Resilience Scale for Adults	resilience scale for adults	resilience scale for adults	resilience scale for adults	Resilience Scale for Adults	5	kept
resistance	resistance	resistance	resistance	RESISTANCE	5	case_or_format
resources	resources	resources	resource	Resources	5	singular

Respiratory diseases	respiratory diseases	respiratory diseases	respiratory disease	Respiratory diseases	5	singular
respiratory tract agent	respiratory tract agent	respiratory tract agent	respiratory tract agent	respiratory tract agent	5	kept
respiratory tract examination	respiratory tract examination	respiratory tract examination	respiratory tract examination	respiratory tract examination	5	kept
Responsibility	responsibility	responsibility	responsibility	responsibility	5	case_or_format
resting energy expenditure	resting energy expenditure	resting energy expenditure	resting energy expenditure	resting energy expenditure	5	kept
Restraint physical	restraint physical	restraint physical	restraint physical	Restraint, Physical	5	case_or_format
RETENTION	retention	retention	retention	Retention	5	case_or_format
rheumatic disease	rheumatic disease	rheumatic disease	rheumatic disease	rheumatic disease	5	kept
rheumatology	rheumatology	rheumatology	rheumatology	rheumatology	5	kept
rhinitis	rhinitis	rhinitis	rhinitis	rhinitis	5	kept
Richards Campbell Sleep Questionnaire	richards campbell sleep questionnaire	richards campbell sleep questionnaire	richards campbell sleep questionnaire	Richards Campbell Sleep Questionnaire	5	kept
rigor	rigor	rigor	rigor	rigor	5	kept
rimantadine	rimantadine	rimantadine	rimantadine	rimantadine	5	kept
Risk prediction	risk prediction	risk prediction	risk prediction	risk prediction	5	case_or_format
ritual	ritual	ritual	ritual	ritual	5	kept
Rocuronium	rocuronium	rocuronium	rocuronium	rocuronium	5	case_or_format

Role ambiguity	role ambiguity	role ambiguity	role ambiguity	Role ambiguity	5	kept
roles	roles	roles	role	Role	5	singular
room ventilation	room ventilation	room ventilation	room ventilation	room ventilation	5	kept
Ropivacaine	ropivacaine	ropivacaine	ropivacaine	ropivacaine	5	case_or_format
rosuvastatin	rosuvastatin	rosuvastatin	rosuvastatin	rosuvastatin	5	kept
Rotavirus	rotavirus	rotavirus	rotavirus	Rotavirus	5	kept
rounds	rounds	rounds	rounds	rounds	5	kept
Roux Y anastomosis	roux y anastomosis	roux y anastomosis	roux y anastomosis	Roux Y anastomosis	5	kept
RSV	rsv	rsv	rsv	RSV	5	kept
Rumination	rumination	rumination	rumination	Rumination	5	kept
running	running	running	running	running	5	kept
Rupture, Spontaneous	rupture spontaneous	rupture spontaneous	rupture spontaneous	Rupture, Spontaneous	5	kept
rural	rural	rural	rural	Rural	5	case_or_format
Russia	russia	russia	russia	Russia	5	kept
safety attitudes questionnaire	safety attitudes questionnaire	safety attitudes questionnaire	safety attitudes questionnaire	safety attitudes questionnaire	5	kept
safety management	safety management	safety management	safety management	Safety Management	5	case_or_format

SARS-CoV-2 variants	sars cov 2 variants	sars cov 2 variants	sars cov 2 variants	SARS-CoV-2 variants	5	kept
Scoliosis	scoliosis	scoliosis	scoliosis	scoliosis	5	case_or_format
Scope of Practice	scope of practice	scope of practice	scope of practice	scope of practice	5	case_or_format
SECLUSION	seclusion	seclusion	seclusion	seclusion	5	case_or_format
Second victim syndrome	second victim syndrome	second victim syndrome	second victim syndrome	Second victim syndrome	5	kept
second victim syndrome	second victim syndrome	second victim syndrome	second victim syndrome	Second victim syndrome	5	case_or_format
security	security	security	security	Security	5	case_or_format
self control	self control	self control	self control	self control	5	kept
Self-compassion	self compassion	self compassion	self compassion	Self-compassion	5	kept
self-confidence	self confidence	self confidence	self confidence	Self-confidence	5	case_or_format
Self-esteem	self esteem	self esteem	self esteem	self esteem	5	case_or_format
self-leadership	self leadership	self leadership	self leadership	self-leadership	5	kept
self－efficacy	self efficacy	self efficacy	self efficacy	Self Efficacy	5	case_or_format
semantics	semantics	semantics	semantics	Semantics	5	case_or_format
Semi structured interviews	semi structured interviews	semi structured interviews	semi structured interview	semi structured interview	5	singular
senescence	senescence	senescence	senescence	senescence	5	kept

sensitization	sensitization	sensitization	sensitization	sensitization	5	kept
SEPSIS	sepsis	sepsis	sepsis	Sepsis	5	case_or_format
Septicemia	septicemia	septicemia	septicemia	septicemia	5	case_or_format
Serbia	serbia	serbia	serbia	Serbia	5	kept
serious illness	serious illness	serious illness	serious illness	serious illness	5	kept
serum osmolality	serum osmolality	serum osmolality	serum osmolality	serum osmolality	5	kept
SERVICE	service	service	service	SERVICES	5	case_or_format
Severe acute pancreatitis	severe acute pancreatitis	severe acute pancreatitis	severe acute pancreatitis	severe acute pancreatitis	5	case_or_format
Severe Combined Immunodeficiency	severe combined immunodeficiency	severe combined immunodeficiency	severe combined immunodeficiency	Severe Combined Immunodeficiency	5	kept
Severity of illness index	severity of illness index	severity of illness index	severity of illness index	Severity of Illness Index	5	case_or_format
sexual function	sexual function	sexual function	sexual function	sexual function	5	kept
Sexual Harassment	sexual harassment	sexual harassment	sexual harassment	Sexual Harassment	5	kept
shaken baby syndrome	shaken baby syndrome	shaken baby syndrome	shaken baby syndrome	Shaken Baby Syndrome	5	case_or_format
Sharp injuries	sharp injuries	sharp injuries	sharp injury	Sharp injuries	5	singular
sharps disposal	sharps disposal	sharps disposal	sharps disposal	sharps disposal	5	kept
Shift work schedule	shift work schedule	shift work schedule	shift work schedule	Shift Work Schedule	5	case_or_format

short course therapy	short course therapy	short course therapy	short course therapy	short course therapy	5	kept
Short Form 12	short form 12	short form 12	short form 12	Short Form 12	5	kept
Simulation-based education	simulation based education	simulation based education	simulation based education	Simulation-based education	5	kept
simulation-based learning	simulation based learning	simulation based learning	simulation based learning	simulation-based learning	5	kept
Simulators	simulators	simulators	simulator	simulator	5	singular
single nucleotide polymorphism	single nucleotide polymorphism	single nucleotide polymorphism	single nucleotide polymorphism	single nucleotide polymorphism	5	kept
Sirolimus	sirolimus	sirolimus	sirolimus	Sirolimus	5	kept
Situational awareness	situational awareness	situational awareness	situational awareness	Situational awareness	5	kept
skin discoloration	skin discoloration	skin discoloration	skin discoloration	skin discoloration	5	kept
Skin integrity	skin integrity	skin integrity	skin integrity	Skin integrity	5	kept
skin redness	skin redness	skin redness	skin redness	skin redness	5	kept
sleep apnea syndromes	sleep apnea syndromes	sleep apnea syndromes	sleep apnea syndrome	sleep apnea syndrome	5	singular
sleep disorder assessment	sleep disorder assessment	sleep disorder assessment	sleep disorder assessment	sleep disorder assessment	5	kept
Sleep Duration	sleep duration	sleep duration	sleep duration	Sleep Duration	5	kept
Sleep Hygiene	sleep hygiene	sleep hygiene	sleep hygiene	sleep hygiene	5	case_or_format
Smoking cessation	smoking cessation	smoking cessation	smoking cessation	smoking cessation	5	case_or_format

social capital	social capital	social capital	social capital	social capital	5	kept
social control	social control	social control	social control	social control	5	kept
Social networking	social networking	social networking	social networking	Social Networking	5	case_or_format
Social psychology	social psychology	social psychology	social psychology	social psychology	5	case_or_format
Societies, Scientific	societies scientific	societies scientific	societies scientific	Societies, Scientific	5	kept
SOFA score	sofa score	sofa score	sofa score	SOFA score	5	kept
sotalol	sotalol	sotalol	sotalol	sotalol	5	kept
sound level measurement	sound level measurement	sound level measurement	sound level measurement	sound level measurement	5	kept
South Asia	south asia	south asia	south asia	South Asia	5	kept
South Dakota	south dakota	south dakota	south dakota	South Dakota	5	kept
Space syntax	space syntax	space syntax	space syntax	Space syntax	5	kept
spatial analysis	spatial analysis	spatial analysis	spatial analysis	spatial analysis	5	kept
spatiotemporal analysis	spatiotemporal analysis	spatiotemporal analysis	spatiotemporal analysis	spatiotemporal analysis	5	kept
Special care units	special care units	special care units	special care unit	Special care units	5	singular
Specialties, Medical	specialties medical	specialties medical	specialties medical	Specialties, Medical	5	kept
specialty	specialty	specialty	specialty	specialty	5	kept

speech and language rehabilitation	speech and language rehabilitation	speech and language rehabilitation	speech and language rehabilitation	speech and language rehabilitation	5	kept
Speech Disorders	speech disorders	speech disorders	speech disorder	speech disorder	5	singular
Speech-Language Pathology	speech language pathology	speech language pathology	speech language pathology	Speech-Language Pathology	5	kept
Spiritual climate	spiritual climate	spiritual climate	spiritual climate	Spiritual climate	5	kept
splinting	splinting	splinting	splinting	splinting	5	kept
sputum smear	sputum smear	sputum smear	sputum smear	sputum smear	5	kept
Standard care	standard care	standard care	standard care	Standard care	5	kept
Standardization	standardization	standardization	standardization	standardization	5	case_or_format
Standards of care	standards of care	standards of care	standards of care	Standards of care	5	kept
Stars	stars	stars	stars	Stars	5	kept
State Government	state government	state government	state government	State Government	5	kept
STATES	states	states	state	STATES	5	singular
Statistical tests	statistical tests	statistical tests	statistical tests	Statistical tests	5	kept
Statistics	statistics	statistics	statistics	statistics	5	case_or_format
sterile water	sterile water	sterile water	sterile water	sterile water	5	kept
Stevens-Johnson syndrome	stevens johnson syndrome	stevens johnson syndrome	stevens johnson syndrome	Stevens-Johnson Syndrome	5	case_or_format

stewardship	stewardship	stewardship	stewardship	stewardship	5	kept
Stockings, Compression	stockings compression	stockings compression	stockings compression	Stockings, Compression	5	kept
stomach bypass	stomach bypass	stomach bypass	stomach bypass	stomach bypass	5	kept
stomach content	stomach content	stomach content	stomach content	stomach content	5	kept
stomach hemorrhage	stomach hemorrhage	stomach hemorrhage	stomach hemorrhage	stomach hemorrhage	5	kept
stomach intubation	stomach intubation	stomach intubation	stomach intubation	stomach intubation	5	kept
stomach pain	stomach pain	stomach pain	stomach pain	stomach pain	5	kept
Stomach Ulcer	stomach ulcer	stomach ulcer	stomach ulcer	stomach ulcer	5	case_or_format
Strain	strain	strain	strain	Strain	5	kept
Streptococcus group A	streptococcus group a	streptococcus group a	streptococcus group a	Streptococcus group A	5	kept
Stress management	stress management	stress management	stress management	stress management	5	case_or_format
Stress psychological	stress psychological	stress psychological	stress psychological	Stress, Psychological	5	case_or_format
stress ulcer	stress ulcer	stress ulcer	stress ulcer	stress ulcer	5	kept
stroke survivor	stroke survivor	stroke survivor	stroke survivor	stroke survivor	5	kept
Structural empowerment	structural empowerment	structural empowerment	structural empowerment	Structural empowerment	5	kept
study skills	study skills	study skills	study skills	study skills	5	kept

Sturge-Weber Syndrome	sturge weber syndrome	sturge weber syndrome	sturge weber syndrome	Sturge-Weber Syndrome	5	kept
Subarachnoid haemorrhage	subarachnoid haemorrhage	subarachnoid haemorrhage	subarachnoid haemorrhage	Subarachnoid haemorrhage	5	kept
subcutaneous tissue	subcutaneous tissue	subcutaneous tissue	subcutaneous tissue	subcutaneous tissue	5	kept
substance use disorder	substance use disorder	substance use disorder	substance use disorder	substance use disorder	5	kept
substance-related disorders	substance related disorders	substance related disorders	substance related disorders	Substance-Related Disorders	5	case_or_format
sulfamethoxazole	sulfamethoxazole	sulfamethoxazole	sulfamethoxazole	sulfamethoxazole	5	kept
sulfonylurea	sulfonylurea	sulfonylurea	sulfonylurea	sulfonylurea	5	kept
superior cava vein	superior cava vein	superior cava vein	superior cava vein	superior cava vein	5	kept
supervision	supervision	supervision	supervision	supervision	5	kept
SupplMeshName: SARS-CoV-2 variants, UI: C000711908, Type:	supplmeshname sars cov 2 variants ui c000711908 type	supplmeshname sars cov 2 variants ui c000711908 type	supplmeshname sars cov 2 variants ui c000711908 type	SupplMeshName: SARS-CoV-2 variants, UI: C000711908, Type:	5	kept
Suppuration	suppuration	suppuration	suppuration	Suppuration	5	kept
surgical injury	surgical injury	surgical injury	surgical injury	surgical injury	5	kept
surgical stress	surgical stress	surgical stress	surgical stress	surgical stress	5	kept
surgical tape	surgical tape	surgical tape	surgical tape	Surgical Tape	5	case_or_format
survival factor	survival factor	survival factor	survival factor	survival factor	5	kept
susceptibility weighted imaging	susceptibility weighted imaging	susceptibility weighted imaging	susceptibility weighted imaging	susceptibility weighted imaging	5	kept

Sustainability	sustainability	sustainability	sustainability	sustainability	5	case_or_format
Sustainable Development	sustainable development	sustainable development	sustainable development	sustainable development	5	case_or_format
SUSTAINING TREATMENTS	sustaining treatments	sustaining treatments	sustaining treatment	SUSTAINING TREATMENT	5	singular
suvorexant	suvorexant	suvorexant	suvorexant	suvorexant	5	kept
Swallowing	swallowing	swallowing	swallowing	swallowing	5	case_or_format
Sweetening Agents	sweetening agents	sweetening agents	sweetening agent	Sweetening Agents	5	singular
symptom burden	symptom burden	symptom burden	symptom burden	symptom burden	5	kept
Syncope	syncope	syncope	syncope	Syncope	5	kept
syncope	syncope	syncope	syncope	Syncope	5	case_or_format
Syntactics	syntactics	syntactics	syntactics	Syntactics	5	kept
Syrian Arab Republic	syrian arab republic	syrian arab republic	syrian arab republic	Syrian Arab Republic	5	kept
Systemic inflammatory response syndrome	systemic inflammatory response syndrome	systemic inflammatory response syndrome	systemic inflammatory response syndrome	Systemic Inflammatory Response Syndrome	5	case_or_format
Systole	systole	systole	systole	Systole	5	kept
systolic heart failure	systolic heart failure	systolic heart failure	systolic heart failure	systolic heart failure	5	kept
Tachycardia, Ectopic Junctional	tachycardia ectopic junctional	tachycardia ectopic junctional	tachycardia ectopic junctional	Tachycardia, Ectopic Junctional	5	kept
Takotsubo Cardiomyopathy	takotsubo cardiomyopathy	takotsubo cardiomyopathy	takotsubo cardiomyopathy	takotsubo cardiomyopathy	5	case_or_format

tap water	tap water	tap water	tap water	tap water	5	kept
team dynamics	team dynamics	team dynamics	team dynamics	team dynamics	5	kept
Team performance	team performance	team performance	team performance	Team performance	5	kept
Technology, High-Cost	technology high cost	technology high cost	technology high cost	Technology, High-Cost	5	kept
telerehabilitation	telerehabilitation	telerehabilitation	telerehabilitation	telerehabilitation	5	kept
TEMPERATURE	temperature	temperature	temperature	temperature	5	case_or_format
tension pneumothorax	tension pneumothorax	tension pneumothorax	tension pneumothorax	tension pneumothorax	5	kept
Test Taking Skills	test taking skills	test taking skills	test taking skills	Test Taking Skills	5	kept
testosterone	testosterone	testosterone	testosterone	testosterone	5	kept
Thematic content analysis	thematic content analysis	thematic content analysis	thematic content analysis	Thematic content analysis	5	kept
Thermometry	thermometry	thermometry	thermometry	Thermometry	5	kept
thiamine deficiency	thiamine deficiency	thiamine deficiency	thiamine deficiency	thiamine deficiency	5	kept
Thoracic Surgery, Video-Assisted	thoracic surgery video assisted	thoracic surgery video assisted	thoracic surgery video assisted	Thoracic Surgery, Video-Assisted	5	kept
thoracic vertebra	thoracic vertebra	thoracic vertebra	thoracic vertebra	thoracic vertebra	5	kept
Thoracic Vertebrae	thoracic vertebrae	thoracic vertebrae	thoracic vertebrae	Thoracic Vertebrae	5	kept
Thoracic Wall	thoracic wall	thoracic wall	thoracic wall	Thoracic Wall	5	kept

three-dimensional imaging	three dimensional imaging	three dimensional imaging	three dimensional imaging	three-dimensional imaging	5	kept
Thriving at work	thriving at work	thriving at work	thriving at work	Thriving at work	5	kept
thrombin	thrombin	thrombin	thrombin	thrombin	5	kept
tight glycemic control	tight glycemic control	tight glycemic control	tight glycemic control	tight glycemic control	5	kept
time pressure	time pressure	time pressure	time pressure	time pressure	5	kept
timentin	timentin	timentin	timentin	timentin	5	kept
Tissue Adhesives	tissue adhesives	tissue adhesives	tissue adhesive	Tissue Adhesives	5	singular
Tissue donors	tissue donors	tissue donors	tissue donor	Tissue Donors	5	singular
tissue donors	tissue donors	tissue donors	tissue donor	Tissue Donors	5	singular
Tobacco Use Cessation	tobacco use cessation	tobacco use cessation	tobacco use cessation	Tobacco Use Cessation	5	kept
Tomography	tomography	tomography	tomography	Tomography	5	kept
Tongue	tongue	tongue	tongue	tongue	5	case_or_format
tooth injury	tooth injury	tooth injury	tooth injury	tooth injury	5	kept
toothpaste	toothpaste	toothpaste	toothpaste	toothpaste	5	kept
topical treatment	topical treatment	topical treatment	topical treatment	topical treatment	5	kept
Toxic epidermal necrolysis	toxic epidermal necrolysis	toxic epidermal necrolysis	toxic epidermal necrolysis	toxic epidermal necrolysis	5	case_or_format

Toxicity	toxicity	toxicity	toxicity	toxicity	5	case_or_format
trachea injury	trachea injury	trachea injury	trachea injury	trachea injury	5	kept
tracheal intubation	tracheal intubation	tracheal intubation	tracheal intubation	Tracheal intubation	5	case_or_format
tracheostomy decannulation	tracheostomy decannulation	tracheostomy decannulation	tracheostomy decannulation	tracheostomy decannulation	5	kept
tranquilizer	tranquilizer	tranquilizer	tranquilizer	tranquilizer	5	kept
tranquilizing activity	tranquilizing activity	tranquilizing activity	tranquilizing activity	tranquilizing activity	5	kept
transbronchial biopsy	transbronchial biopsy	transbronchial biopsy	transbronchial biopsy	transbronchial biopsy	5	kept
transcutaneous oxygen monitoring	transcutaneous oxygen monitoring	transcutaneous oxygen monitoring	transcutaneous oxygen monitoring	transcutaneous oxygen monitoring	5	kept
Transfer anxiety	transfer anxiety	transfer anxiety	transfer anxiety	Transfer anxiety	5	kept
Transformational leadership	transformational leadership	transformational leadership	transformational leadership	Transformational leadership	5	kept
Transition to Adult Care	transition to adult care	transition to adult care	transition to adult care	transition to adult care	5	case_or_format
TRANSITIONS	transitions	transitions	transition	Transition	5	singular
Transitions	transitions	transitions	transition	Transition	5	singular
TRANSLATION	translation	translation	translation	Translations	5	case_or_format
translation	translation	translation	translation	Translations	5	case_or_format
trauma care	trauma care	trauma care	trauma care	Trauma care	5	case_or_format

TRAUMATIC BRAIN-INJURY	traumatic brain injury	traumatic brain injury	traumatic brain injury	traumatic brain injury	5	case_or_format
TRIAGE	triage	triage	triage	Triage	5	case_or_format
tricyclic antidepressant agent	tricyclic antidepressant agent	tricyclic antidepressant agent	tricyclic antidepressant agent	tricyclic antidepressant agent	5	kept
trimethoprim	trimethoprim	trimethoprim	trimethoprim	trimethoprim	5	kept
trismus	trismus	trismus	trismus	trismus	5	kept
troponin T	troponin t	troponin t	troponin t	troponin T	5	kept
trunk	trunk	trunk	trunk	trunk	5	kept
turning	turning	turning	turning	turning	5	kept
Tympanic Membrane	tympanic membrane	tympanic membrane	tympanic membrane	Tympanic Membrane	5	kept
Türkiye	t rkiye	t rkiye	t rkiye	Türkiye	5	kept
Ulcer	ulcer	ulcer	ulcer	ulcer	5	case_or_format
Undergraduate	undergraduate	undergraduate	undergraduate	Undergraduate	5	kept
undergraduate education	undergraduate education	undergraduate education	undergraduate education	undergraduate education	5	kept
universal health care	universal health care	universal health care	universal health care	universal health care	5	kept
Upper Extremity Deep Vein Thrombosis	upper extremity deep vein thrombosis	upper extremity deep vein thrombosis	upper extremity deep vein thrombosis	Upper Extremity Deep Vein Thrombosis	5	kept
urinary tract infections	urinary tract infections	urinary tract infections	urinary tract infection	urinary tract infection	5	singular

urogenital tract disease	urogenital tract disease	urogenital tract disease	urogenital tract disease	urogenital tract disease	5	kept
urologic surgery	urologic surgery	urologic surgery	urologic surgery	urologic surgery	5	kept
User-Centered Design	user centered design	user centered design	user centered design	user-centered design	5	case_or_format
Utilization	utilization	utilization	utilization	utilization	5	case_or_format
vaginal birth after cesarean	vaginal birth after cesarean	vaginal birth after cesarean	vaginal birth after cesarean	Vaginal Birth after Cesarean	5	case_or_format
Vagus Nerve	vagus nerve	vagus nerve	vagus nerve	Vagus Nerve	5	kept
VAP prevention	vap prevention	vap prevention	vap prevention	VAP prevention	5	kept
VARIABILITY	variability	variability	variability	VARIABILITY	5	kept
Vasopressor	vasopressor	vasopressor	vasopressor	Vasopressor	5	kept
vegetable oil	vegetable oil	vegetable oil	vegetable oil	vegetable oil	5	kept
Vegetative state	vegetative state	vegetative state	vegetative state	Vegetative state	5	kept
velocity	velocity	velocity	velocity	velocity	5	kept
Venipuncture	venipuncture	venipuncture	venipuncture	Venipuncture	5	kept
venipuncture	venipuncture	venipuncture	venipuncture	Venipuncture	5	case_or_format
Venous thrombosis	venous thrombosis	venous thrombosis	venous thrombosis	Venous Thrombosis	5	case_or_format
ventilator‐associated pneumonia	ventilator associated pneumonia	ventilator associated pneumonia	ventilator associated pneumonia	ventilator associated pneumonia	5	case_or_format

VERSION	version	version	version	VERSION	5	kept
vertigo	vertigo	vertigo	vertigo	vertigo	5	kept
video consultation	video consultation	video consultation	video consultation	video consultation	5	kept
VIOLENCE	violence	violence	violence	Violence	5	case_or_format
Virtual education	virtual education	virtual education	virtual education	Virtual education	5	kept
virtual learning environment	virtual learning environment	virtual learning environment	virtual learning environment	virtual learning environment	5	kept
Virulence	virulence	virulence	virulence	Virulence	5	kept
virus strain	virus strain	virus strain	virus strain	virus strain	5	kept
virus virulence	virus virulence	virus virulence	virus virulence	virus virulence	5	kept
visual hallucination	visual hallucination	visual hallucination	visual hallucination	visual hallucination	5	kept
visualization	visualization	visualization	visualization	visualization	5	kept
Vital sign monitoring	vital sign monitoring	vital sign monitoring	vital sign monitoring	Vital sign monitoring	5	kept
Vital signs monitoring	vital signs monitoring	vital signs monitoring	vital signs monitoring	Vital signs monitoring	5	kept
VOSviewer	vosviewer	vosviewer	vosviewer	VOSviewer	5	kept
VRE	vre	vre	vre	VRE	5	kept
Wards	wards	wards	ward	ward	5	singular

Water Intoxication	water intoxication	water intoxication	water intoxication	Water Intoxication	5	kept
Water Microbiology	water microbiology	water microbiology	water microbiology	Water Microbiology	5	kept
Weaning from mechanical ventilation	weaning from mechanical ventilation	weaning from mechanical ventilation	weaning from mechanical ventilation	Weaning from mechanical ventilation	5	kept
Webcasts as Topic	webcasts as topic	webcasts as topic	webcasts as topic	Webcasts as Topic	5	kept
Weight gain	weight gain	weight gain	weight gain	Weight Gain	5	case_or_format
West Nile Fever	west nile fever	west nile fever	west nile fever	West Nile Fever	5	kept
WHOQOL-BREF	whoqol bref	whoqol bref	whoqol bref	WHOQOL-BREF	5	kept
work performance	work performance	work performance	work performance	Work Performance	5	case_or_format
Work system	work system	work system	work system	Work system	5	kept
workforce issues	workforce issues	workforce issues	workforce issues	workforce issues	5	kept
working environment	working environment	working environment	working environment	Working environment	5	case_or_format
workplace culture	workplace culture	workplace culture	workplace culture	workplace culture	5	kept
Wound care	wound care	wound care	wound care	wound care	5	case_or_format
wound complication	wound complication	wound complication	wound complication	wound complication	5	kept
X-ray	x ray	x ray	x ray	X ray	5	case_or_format
yeast	yeast	yeast	yeast	yeast	5	kept

Zinc	zinc	zinc	zinc	zinc	5	case_or_format
zinc oxide	zinc oxide	zinc oxide	zinc oxide	zinc oxide	5	kept
ziprasidone	ziprasidone	ziprasidone	ziprasidone	ziprasidone	5	kept
1p36 deletion syndrome	1p36 deletion syndrome	1p36 deletion syndrome	1p36 deletion syndrome	1p36 deletion syndrome	4	kept
4 aminobutyric acid	4 aminobutyric acid	4 aminobutyric acid	4 aminobutyric acid	4 aminobutyric acid	4	kept
Abdominal Cavity	abdominal cavity	abdominal cavity	abdominal cavity	abdominal cavity	4	case_or_format
abdominal discomfort	abdominal discomfort	abdominal discomfort	abdominal discomfort	abdominal discomfort	4	kept
Abdominal surgery	abdominal surgery	abdominal surgery	abdominal surgery	abdominal surgery	4	case_or_format
Abdominal Wall	abdominal wall	abdominal wall	abdominal wall	Abdominal Wall	4	kept
ablation therapy	ablation therapy	ablation therapy	ablation therapy	ablation therapy	4	kept
abortion	abortion	abortion	abortion	abortion	4	kept
Abusive head trauma	abusive head trauma	abusive head trauma	abusive head trauma	Abusive head trauma	4	kept
academic	academic	academic	academic	academic	4	kept
Academic medical centers	academic medical centers	academic medical centers	academic medical center	Academic Medical Centers	4	singular
access to medication	access to medication	access to medication	access to medication	access to medication	4	kept
ACCIDENT & EMERGENCY MEDICINE	accident and emergency medicine	accident and emergency medicine	accident and emergency medicine	accident & emergency medicine	4	case_or_format

Accident vasculaire cérébral	accident vasculaire c r bral	accident vasculaire c r bral	accident vasculaire c r bral	Accident vasculaire cérébral	4	kept
Accidents, Home	accidents home	accidents home	accidents home	Accidents, Home	4	kept
Accountable Care Organizations	accountable care organizations	accountable care organizations	accountable care organizations	Accountable Care Organizations	4	kept
acetaminophen	acetaminophen	acetaminophen	acetaminophen	Acetaminophen	4	case_or_format
ACINETOBACTER-BAUMANNII	acinetobacter baumannii	acinetobacter baumannii	acinetobacter baumannii	Acinetobacter baumannii	4	case_or_format
acquired brain injury	acquired brain injury	acquired brain injury	acquired brain injury	acquired brain injury	4	kept
ACQUIRED PNEUMONIA	acquired pneumonia	acquired pneumonia	acquired pneumonia	ACQUIRED PNEUMONIA	4	kept
activated protein C	activated protein c	activated protein c	activated protein c	activated protein C	4	kept
Acute	acute	acute	acute	acute	4	case_or_format
Acute Care Surgery	acute care surgery	acute care surgery	acute care surgery	acute care surgery	4	case_or_format
Acute care surgery	acute care surgery	acute care surgery	acute care surgery	acute care surgery	4	case_or_format
Acute coronary syndromes	acute coronary syndromes	acute coronary syndromes	acute coronary syndrome	acute coronary syndrome	4	singular
acute graft rejection	acute graft rejection	acute graft rejection	acute graft rejection	acute graft rejection	4	kept
Acute hypoxemic respiratory failure	acute hypoxemic respiratory failure	acute hypoxemic respiratory failure	acute hypoxemic respiratory failure	acute hypoxemic respiratory failure	4	case_or_format
acute kidney tubule necrosis	acute kidney tubule necrosis	acute kidney tubule necrosis	acute kidney tubule necrosis	acute kidney tubule necrosis	4	kept
ACUTE LUNG INJURY	acute lung injury	acute lung injury	acute lung injury	acute lung injury	4	case_or_format

acute respiratory tract disease	acute respiratory tract disease	acute respiratory tract disease	acute respiratory tract disease	acute respiratory tract disease	4	kept
Acute skin failure	acute skin failure	acute skin failure	acute skin failure	Acute skin failure	4	kept
acute skin failure	acute skin failure	acute skin failure	acute skin failure	Acute skin failure	4	case_or_format
Acute stress disorder	acute stress disorder	acute stress disorder	acute stress disorder	acute stress disorder	4	case_or_format
ACUTE STROKE	acute stroke	acute stroke	acute stroke	ACUTE STROKE	4	kept
Acute stroke	acute stroke	acute stroke	acute stroke	ACUTE STROKE	4	case_or_format
ACUTE-RENAL-FAILURE	acute renal failure	acute renal failure	acute renal failure	Acute renal failure	4	case_or_format
adenoidectomy	adenoidectomy	adenoidectomy	adenoidectomy	Adenoidectomy	4	case_or_format
Adenovirus	adenovirus	adenovirus	adenovirus	Adenovirus	4	kept
ADMISSIONS	admissions	admissions	admission	ADMISSION	4	singular
Admissions	admissions	admissions	admission	ADMISSION	4	singular
Adolescent, Hospitalized	adolescent hospitalized	adolescent hospitalized	adolescent hospitalized	Adolescent, Hospitalized	4	kept
adrenergic receptor stimulating agent	adrenergic receptor stimulating agent	adrenergic receptor stimulating agent	adrenergic receptor stimulating agent	adrenergic receptor stimulating agent	4	kept
adult disease	adult disease	adult disease	adult disease	adult disease	4	kept
adult health	adult health	adult health	adult health	adult health	4	kept
Advance directive	advance directive	advance directive	advance directive	Advance Directives	4	case_or_format

Advanced cancer	advanced cancer	advanced cancer	advanced cancer	advanced cancer	4	case_or_format
advanced trauma life support	advanced trauma life support	advanced trauma life support	advanced trauma life support	advanced trauma life support	4	kept
advanced trauma life support care	advanced trauma life support care	advanced trauma life support care	advanced trauma life support care	advanced trauma life support care	4	kept
Adverse Events	adverse events	adverse events	adverse event	adverse event	4	singular
aEEG	aeeg	aeeg	aeeg	aEEG	4	kept
Aeromedical transport	aeromedical transport	aeromedical transport	aeromedical transport	Aeromedical transport	4	kept
AFTER-DISCHARGE	after discharge	after discharge	after discharge	AFTER-DISCHARGE	4	kept
AGGRESSION	aggression	aggression	aggression	aggression	4	case_or_format
Air ambulances	air ambulances	air ambulances	air ambulance	Air Ambulances	4	singular
Air Conditioning	air conditioning	air conditioning	air conditioning	air conditioning	4	case_or_format
Air Microbiology	air microbiology	air microbiology	air microbiology	Air Microbiology	4	kept
Airway assessment	airway assessment	airway assessment	airway assessment	Airway assessment	4	kept
airway dynamics	airway dynamics	airway dynamics	airway dynamics	airway dynamics	4	kept
Alagoas	alagoas	alagoas	alagoas	Alagoas	4	kept
Albania	albania	albania	albania	Albania	4	kept
albuminuria	albuminuria	albuminuria	albuminuria	albuminuria	4	kept

Albuterol	albuterol	albuterol	albuterol	Albuterol	4	kept
Alcohol	alcohol	alcohol	alcohol	alcohol	4	case_or_format
alcohol abstinence	alcohol abstinence	alcohol abstinence	alcohol abstinence	alcohol abstinence	4	kept
Alcohol withdrawal	alcohol withdrawal	alcohol withdrawal	alcohol withdrawal	alcohol withdrawal	4	case_or_format
Alcohol-based hand rub	alcohol based hand rub	alcohol based hand rub	alcohol based hand rub	Alcohol-based hand rub	4	kept
alcoholic hepatitis	alcoholic hepatitis	alcoholic hepatitis	alcoholic hepatitis	alcoholic hepatitis	4	kept
alemtuzumab	alemtuzumab	alemtuzumab	alemtuzumab	alemtuzumab	4	kept
alkaline phosphatase blood level	alkaline phosphatase blood level	alkaline phosphatase blood level	alkaline phosphatase blood level	alkaline phosphatase blood level	4	kept
allied health education	allied health education	allied health education	allied health education	allied health education	4	kept
alopecia	alopecia	alopecia	alopecia	alopecia	4	kept
Altitude	altitude	altitude	altitude	Altitude	4	kept
altitude	altitude	altitude	altitude	Altitude	4	case_or_format
aluminum	aluminum	aluminum	aluminum	Aluminum	4	case_or_format
ALZHEIMERS-DISEASE	alzheimers disease	alzheimers disease	alzheimers disease	ALZHEIMERS-DISEASE	4	kept
ambulance response time	ambulance response time	ambulance response time	ambulance response time	ambulance response time	4	kept
ambulation	ambulation	ambulation	ambulation	ambulation	4	kept

AMERICAN SOCIETY	american society	american society	american society	AMERICAN SOCIETY	4	kept
amfebutamone	amfebutamone	amfebutamone	amfebutamone	amfebutamone	4	kept
Aminoglycosides	aminoglycosides	aminoglycosides	aminoglycoside	aminoglycoside	4	singular
Amish	amish	amish	amish	Amish	4	kept
Amnesia	amnesia	amnesia	amnesia	amnesia	4	case_or_format
Amniotic Fluid	amniotic fluid	amniotic fluid	amniotic fluid	Amniotic Fluid	4	kept
amphetamine	amphetamine	amphetamine	amphetamine	amphetamine	4	kept
amplitude integrated electroencephalography	amplitude integrated electroencephalography	amplitude integrated electroencephalography	amplitude integrated electroencephalography	amplitude integrated electroencephalography	4	kept
ampoule	ampoule	ampoule	ampoule	ampoule	4	kept
Amputation, Traumatic	amputation traumatic	amputation traumatic	amputation traumatic	Amputation, Traumatic	4	kept
Anal Canal	anal canal	anal canal	anal canal	Anal Canal	4	kept
Analgesic drugs	analgesic drugs	analgesic drugs	analgesic drugs	Analgesic drugs	4	kept
anatomic model	anatomic model	anatomic model	anatomic model	anatomic model	4	kept
ancestry group	ancestry group	ancestry group	ancestry group	ancestry group	4	kept
Androstanols	androstanols	androstanols	androstanols	Androstanols	4	kept
Anesthesia, Local	anesthesia local	anesthesia local	anesthesia local	Anesthesia, Local	4	kept

anesthesiological procedure	anesthesiological procedure	anesthesiological procedure	anesthesiological procedure	anesthesiological procedure	4	kept
Anesthetics, Combined	anesthetics combined	anesthetics combined	anesthetics combined	Anesthetics, Combined	4	kept
aneurysm rupture	aneurysm rupture	aneurysm rupture	aneurysm rupture	aneurysm rupture	4	kept
Angioedemas, Hereditary	angioedemas hereditary	angioedemas hereditary	angioedemas hereditary	Angioedemas, Hereditary	4	kept
Animal Assisted Therapy	animal assisted therapy	animal assisted therapy	animal assisted therapy	Animal Assisted Therapy	4	kept
Animals, Domestic	animals domestic	animals domestic	animals domestic	Animals, Domestic	4	kept
Animals, Newborn	animals newborn	animals newborn	animals newborn	Animals, Newborn	4	kept
ankle brachial index	ankle brachial index	ankle brachial index	ankle brachial index	ankle brachial index	4	kept
ankle pain	ankle pain	ankle pain	ankle pain	ankle pain	4	kept
ankyloglossia	ankyloglossia	ankyloglossia	ankyloglossia	ankyloglossia	4	kept
anonymised data	anonymised data	anonymised data	anonymised data	anonymised data	4	kept
anorectal malformation	anorectal malformation	anorectal malformation	anorectal malformation	anorectal malformation	4	kept
Anoxemia	anoxemia	anoxemia	anoxemia	Anoxemia	4	kept
Anti-N-Methyl-D-Aspartate Receptor Encephalitis	anti n methyl d aspartate receptor encephalitis	anti n methyl d aspartate receptor encephalitis	anti n methyl d aspartate receptor encephalitis	Anti-N-Methyl-D-Aspartate Receptor Encephalitis	4	kept
Antibiotic stewardship	antibiotic stewardship	antibiotic stewardship	antibiotic stewardship	antibiotic stewardship	4	case_or_format
ANTIBIOTIC USE	antibiotic use	antibiotic use	antibiotic use	ANTIBIOTIC USE	4	kept

Antibiotic use	antibiotic use	antibiotic use	antibiotic use	ANTIBIOTIC USE	4	case_or_format
antibody detection	antibody detection	antibody detection	antibody detection	antibody detection	4	kept
Antimicrobials	antimicrobials	antimicrobials	antimicrobial	Antimicrobials	4	singular
antiphospholipid syndrome	antiphospholipid syndrome	antiphospholipid syndrome	antiphospholipid syndrome	antiphospholipid syndrome	4	kept
antipsychotics	antipsychotics	antipsychotics	antipsychotic	Antipsychotics	4	singular
antiretrovirus agent	antiretrovirus agent	antiretrovirus agent	antiretrovirus agent	antiretrovirus agent	4	kept
antitussive agent	antitussive agent	antitussive agent	antitussive agent	antitussive agent	4	kept
Antivenins	antivenins	antivenins	antivenins	Antivenins	4	kept
antiviral resistance	antiviral resistance	antiviral resistance	antiviral resistance	antiviral resistance	4	kept
aorta	aorta	aorta	aorta	Aorta	4	case_or_format
aorta valve regurgitation	aorta valve regurgitation	aorta valve regurgitation	aorta valve regurgitation	aorta valve regurgitation	4	kept
aortic aneurysm	aortic aneurysm	aortic aneurysm	aortic aneurysm	Aortic Aneurysm	4	case_or_format
aortic valve stenosis	aortic valve stenosis	aortic valve stenosis	aortic valve stenosis	Aortic Valve Stenosis	4	case_or_format
APACHE II	apache ii	apache ii	apache ii	APACHE II	4	kept
Apathy	apathy	apathy	apathy	apathy	4	case_or_format
apnoea of prematurity	apnoea of prematurity	apnoea of prematurity	apnoea of prematurity	apnoea of prematurity	4	kept

apolipoprotein E4	apolipoprotein e4	apolipoprotein e4	apolipoprotein e4	apolipoprotein E4	4	kept
Apoptosis	apoptosis	apoptosis	apoptosis	apoptosis	4	case_or_format
app	app	app	app	apps	4	case_or_format
apparatus, equipment and supplies	apparatus equipment and supplies	apparatus equipment and supplies	apparatus equipment and supplies	apparatus, equipment and supplies	4	kept
application effect	application effect	application effect	application effect	Application effect	4	case_or_format
Application programs	application programs	application programs	application programs	Application programs	4	kept
applied research	applied research	applied research	applied research	applied research	4	kept
apraxia	apraxia	apraxia	apraxia	apraxia	4	kept
Arabic	arabic	arabic	arabic	Arabic	4	kept
Arginine	arginine	arginine	arginine	arginine	4	case_or_format
Art	art	art	art	art	4	case_or_format
Art Therapy	art therapy	art therapy	art therapy	art therapy	4	case_or_format
artesunate	artesunate	artesunate	artesunate	artesunate	4	kept
Arthroplasty, Replacement	arthroplasty replacement	arthroplasty replacement	arthroplasty replacement	Arthroplasty, Replacement	4	kept
Artificial airway	artificial airway	artificial airway	artificial airway	Artificial airway	4	kept
arytenoid cartilage	arytenoid cartilage	arytenoid cartilage	arytenoid cartilage	arytenoid cartilage	4	kept

ascending aorta	ascending aorta	ascending aorta	ascending aorta	ascending aorta	4	kept
aspergillosis	aspergillosis	aspergillosis	aspergillosis	Aspergillosis	4	case_or_format
assessment tool	assessment tool	assessment tool	assessment tool	Assessment tool	4	case_or_format
Assessment tools	assessment tools	assessment tools	assessment tool	Assessment tool	4	singular
asymptomatic bacteriuria	asymptomatic bacteriuria	asymptomatic bacteriuria	asymptomatic bacteriuria	asymptomatic bacteriuria	4	kept
asymptomatic coronavirus disease 2019	asymptomatic coronavirus disease 2019	asymptomatic coronavirus disease 2019	asymptomatic coronavirus disease 2019	asymptomatic coronavirus disease 2019	4	kept
asymptomatic disease	asymptomatic disease	asymptomatic disease	asymptomatic disease	asymptomatic disease	4	kept
Asymptomatic Infections	asymptomatic infections	asymptomatic infections	asymptomatic infection	asymptomatic infection	4	singular
atenolol	atenolol	atenolol	atenolol	atenolol	4	kept
ATIC terminology	atic terminology	atic terminology	atic terminology	ATIC terminology	4	kept
Atropine	atropine	atropine	atropine	atropine	4	case_or_format
attributable risk	attributable risk	attributable risk	attributable risk	attributable risk	4	kept
atypical antipsychotic agent	atypical antipsychotic agent	atypical antipsychotic agent	atypical antipsychotic agent	atypical antipsychotic agent	4	kept
audit and feedback	audit and feedback	audit and feedback	audit and feedback	audit and feedback	4	kept
auditory hallucination	auditory hallucination	auditory hallucination	auditory hallucination	auditory hallucination	4	kept
Auditory stimuli	auditory stimuli	auditory stimuli	auditory stimuli	Auditory stimuli	4	kept

authenticity	authenticity	authenticity	authenticity	authenticity	4	kept
Authoritarianism	authoritarianism	authoritarianism	authoritarianism	Authoritarianism	4	kept
Autism Spectrum Disorder	autism spectrum disorder	autism spectrum disorder	autism spectrum disorder	Autism Spectrum Disorder	4	kept
autonomic neuropathy	autonomic neuropathy	autonomic neuropathy	autonomic neuropathy	autonomic neuropathy	4	kept
autophagy	autophagy	autophagy	autophagy	autophagy	4	kept
autotransplantation	autotransplantation	autotransplantation	autotransplantation	autotransplantation	4	kept
axicabtagene ciloleucel	axicabtagene ciloleucel	axicabtagene ciloleucel	axicabtagene ciloleucel	axicabtagene ciloleucel	4	kept
axillary vein	axillary vein	axillary vein	axillary vein	Axillary Vein	4	case_or_format
Azithromycin	azithromycin	azithromycin	azithromycin	azithromycin	4	case_or_format
B lymphocyte	b lymphocyte	b lymphocyte	b lymphocyte	B lymphocyte	4	kept
Bacilli	bacilli	bacilli	bacilli	Bacilli	4	kept
bacitracin	bacitracin	bacitracin	bacitracin	bacitracin	4	kept
Bacterial contamination	bacterial contamination	bacterial contamination	bacterial contamination	Bacterial contamination	4	kept
Bacteriological Techniques	bacteriological techniques	bacteriological techniques	bacteriological techniques	Bacteriological Techniques	4	kept
bamlanivimab	bamlanivimab	bamlanivimab	bamlanivimab	bamlanivimab	4	kept
BAR-CODE TECHNOLOGY	bar code technology	bar code technology	bar code technology	BAR-CODE TECHNOLOGY	4	kept

basic needs	basic needs	basic needs	basic needs	basic needs	4	kept
basiliximab	basiliximab	basiliximab	basiliximab	basiliximab	4	kept
Bayley Scales of Infant Development	bayley scales of infant development	bayley scales of infant development	bayley scales of infant development	Bayley Scales of Infant Development	4	kept
bedside	bedside	bedside	bedside	BEDSIDE	4	case_or_format
Behavior Control	behavior control	behavior control	behavior control	Behavior Control	4	kept
behavior control	behavior control	behavior control	behavior control	Behavior Control	4	case_or_format
behavioral science	behavioral science	behavioral science	behavioral science	behavioral science	4	kept
BEHAVIORS	behaviors	behaviors	behavior	behavior	4	singular
Behaviors	behaviors	behaviors	behavior	behavior	4	singular
Best practice	best practice	best practice	best practice	best practice	4	case_or_format
beta lactamase CTX M	beta lactamase ctx m	beta lactamase ctx m	beta lactamase ctx m	beta lactamase CTX M	4	kept
beta-lactam resistance	beta lactam resistance	beta lactam resistance	beta lactam resistance	beta-Lactam Resistance	4	case_or_format
Bhutan	bhutan	bhutan	bhutan	Bhutan	4	kept
Bibliometric analysis	bibliometric analysis	bibliometric analysis	bibliometric analysis	bibliometric analysis	4	case_or_format
Big Data	big data	big data	big data	big data	4	case_or_format
bilateral pneumonia	bilateral pneumonia	bilateral pneumonia	bilateral pneumonia	bilateral pneumonia	4	kept

bile duct atresia	bile duct atresia	bile duct atresia	bile duct atresia	bile duct atresia	4	kept
biological warfare	biological warfare	biological warfare	biological warfare	biological warfare	4	kept
Biomedical signal processing	biomedical signal processing	biomedical signal processing	biomedical signal processing	Biomedical signal processing	4	kept
biomedicine	biomedicine	biomedicine	biomedicine	biomedicine	4	kept
Bioterrorism	bioterrorism	bioterrorism	bioterrorism	Bioterrorism	4	kept
Bipolar Disorder	bipolar disorder	bipolar disorder	bipolar disorder	bipolar disorder	4	case_or_format
birth injury	birth injury	birth injury	birth injury	Birth Injuries	4	case_or_format
bisoprolol	bisoprolol	bisoprolol	bisoprolol	bisoprolol	4	kept
Black People	black people	black people	black people	Black People	4	kept
bladder injury	bladder injury	bladder injury	bladder injury	bladder injury	4	kept
Blalock Taussig shunt	blalock taussig shunt	blalock taussig shunt	blalock taussig shunt	Blalock Taussig shunt	4	kept
blame	blame	blame	blame	blame	4	kept
Blended learning	blended learning	blended learning	blended learning	Blended learning	4	kept
blended learning	blended learning	blended learning	blended learning	Blended learning	4	case_or_format
bleomycin	bleomycin	bleomycin	bleomycin	bleomycin	4	kept
blood brain barrier	blood brain barrier	blood brain barrier	blood brain barrier	blood brain barrier	4	kept

Blood gas analysis	blood gas analysis	blood gas analysis	blood gas analysis	Blood Gas Analysis	4	case_or_format
Blood glucose measurements	blood glucose measurements	blood glucose measurements	blood glucose measurements	Blood glucose measurements	4	kept
blood pressure cuff	blood pressure cuff	blood pressure cuff	blood pressure cuff	blood pressure cuff	4	kept
blood safety	blood safety	blood safety	blood safety	blood safety	4	kept
blood smear	blood smear	blood smear	blood smear	blood smear	4	kept
Blood specimen collection	blood specimen collection	blood specimen collection	blood specimen collection	Blood Specimen Collection	4	case_or_format
Blood stream infection	blood stream infection	blood stream infection	blood stream infection	BLOOD-STREAM INFECTIONS	4	case_or_format
blood vessel	blood vessel	blood vessel	blood vessel	blood vessel	4	kept
bloodstream infections	bloodstream infections	bloodstream infections	bloodstream infection	bloodstream infection	4	singular
board of trustees	board of trustees	board of trustees	board of trustees	board of trustees	4	kept
bodily secretions	bodily secretions	bodily secretions	bodily secretions	bodily secretions	4	kept
Body Size	body size	body size	body size	Body Size	4	kept
Body weight	body weight	body weight	body weight	body weight	4	case_or_format
body weight change	body weight change	body weight change	body weight change	body weight change	4	kept
BODY-MASS INDEX	body mass index	body mass index	body mass index	Body Mass Index	4	case_or_format
bone	bone	bone	bone	bone	4	kept

bone metastasis	bone metastasis	bone metastasis	bone metastasis	bone metastasis	4	kept
Brain Abscess	brain abscess	brain abscess	brain abscess	brain abscess	4	case_or_format
brain arteriovenous malformation	brain arteriovenous malformation	brain arteriovenous malformation	brain arteriovenous malformation	brain arteriovenous malformation	4	kept
brain cortex	brain cortex	brain cortex	brain cortex	brain cortex	4	kept
brain level	brain level	brain level	brain level	brain level	4	kept
brain region	brain region	brain region	brain region	brain region	4	kept
BRAIN-DEATH	brain death	brain death	brain death	brain death	4	case_or_format
BRAIN-DEVELOPMENT	brain development	brain development	brain development	brain development	4	case_or_format
brainstorming	brainstorming	brainstorming	brainstorming	brainstorming	4	kept
breast pumping	breast pumping	breast pumping	breast pumping	breast pumping	4	kept
breastfeeding peer counselors	breastfeeding peer counselors	breastfeeding peer counselors	breastfeeding peer counselors	breastfeeding peer counselors	4	kept
breastfeeding support	breastfeeding support	breastfeeding support	breastfeeding support	breastfeeding support	4	kept
breastmilk	breastmilk	breastmilk	breastmilk	breastmilk	4	kept
Brief Symptom Inventory	brief symptom inventory	brief symptom inventory	brief symptom inventory	Brief Symptom Inventory	4	kept
Bronchi	bronchi	bronchi	bronchi	Bronchi	4	kept
bronchoalveolar lavage fluid	bronchoalveolar lavage fluid	bronchoalveolar lavage fluid	bronchoalveolar lavage fluid	bronchoalveolar lavage fluid	4	kept

building	building	building	building	building	4	kept
BURDEN	burden	burden	burden	Burden	4	case_or_format
Burkholderia cepacia complex	burkholderia cepacia complex	burkholderia cepacia complex	burkholderia cepacia complex	Burkholderia cepacia complex	4	kept
Burkitt lymphoma	burkitt lymphoma	burkitt lymphoma	burkitt lymphoma	Burkitt lymphoma	4	kept
burn care	burn care	burn care	burn care	Burn care	4	case_or_format
Burn out	burn out	burn out	burn out	Burn out	4	kept
burning sensation	burning sensation	burning sensation	burning sensation	burning sensation	4	kept
Burnout, professional	burnout professional	burnout professional	burnout professional	Burnout, Professional	4	case_or_format
Butyrylcholinesterase	butyrylcholinesterase	butyrylcholinesterase	butyrylcholinesterase	Butyrylcholinesterase	4	kept
C-REACTIVE PROTEIN	c reactive protein	c reactive protein	c reactive protein	C reactive protein	4	case_or_format
C-reactive protein	c reactive protein	c reactive protein	c reactive protein	C reactive protein	4	case_or_format
cachexia	cachexia	cachexia	cachexia	cachexia	4	kept
calcitonin	calcitonin	calcitonin	calcitonin	Calcitonin	4	case_or_format
Calcium Chloride	calcium chloride	calcium chloride	calcium chloride	calcium chloride	4	case_or_format
Calcium Gluconate	calcium gluconate	calcium gluconate	calcium gluconate	Calcium Gluconate	4	kept
calorie	calorie	calorie	calorie	calorie	4	kept

CAM	cam	cam	cam	CAM	4	kept
Candida auris infection	candida auris infection	candida auris infection	candida auris infection	Candida auris infection	4	kept
Candidiasis, Cutaneous	candidiasis cutaneous	candidiasis cutaneous	candidiasis cutaneous	Candidiasis, Cutaneous	4	kept
Canis familiaris	canis familiaris	canis familiaris	canis familiaris	Canis familiaris	4	kept
cannabidiol	cannabidiol	cannabidiol	cannabidiol	cannabidiol	4	kept
Cannabis	cannabis	cannabis	cannabis	cannabis	4	case_or_format
cannabis use	cannabis use	cannabis use	cannabis use	cannabis use	4	kept
capecitabine	capecitabine	capecitabine	capecitabine	capecitabine	4	kept
carbapenem resistant Pseudomonas aeruginosa	carbapenem resistant pseudomonas aeruginosa	carbapenem resistant pseudomonas aeruginosa	carbapenem resistant pseudomonas aeruginosa	carbapenem resistant Pseudomonas aeruginosa	4	kept
Carbapenem-resistant Klebsiella pneumoniae	carbapenem resistant klebsiella pneumoniae	carbapenem resistant klebsiella pneumoniae	carbapenem resistant klebsiella pneumoniae	carbapenem resistant Klebsiella pneumoniae	4	case_or_format
Carbapenemase	carbapenemase	carbapenemase	carbapenemase	carbapenemase	4	case_or_format
carbohydrate intake	carbohydrate intake	carbohydrate intake	carbohydrate intake	carbohydrate intake	4	kept
carbon dioxide equivalent	carbon dioxide equivalent	carbon dioxide equivalent	carbon dioxide equivalent	carbon dioxide equivalent	4	kept
Carcinoma, Hepatocellular	carcinoma hepatocellular	carcinoma hepatocellular	carcinoma hepatocellular	Carcinoma, Hepatocellular	4	kept
CARDIAC ARRESTS	cardiac arrests	cardiac arrests	cardiac arrest	Cardiac arrest	4	singular
Cardiac arrhythmia	cardiac arrhythmia	cardiac arrhythmia	cardiac arrhythmia	Cardiac arrhythmia	4	kept

cardiac care	cardiac care	cardiac care	cardiac care	cardiac care	4	kept
cardiac index	cardiac index	cardiac index	cardiac index	Cardiac index	4	case_or_format
Cardiac rehabilitation	cardiac rehabilitation	cardiac rehabilitation	cardiac rehabilitation	Cardiac Rehabilitation	4	case_or_format
Cardiography, Impedance	cardiography impedance	cardiography impedance	cardiography impedance	Cardiography, Impedance	4	kept
Cardiomyopathy	cardiomyopathy	cardiomyopathy	cardiomyopathy	cardiomyopathy	4	case_or_format
cardiopulmonary bypass time	cardiopulmonary bypass time	cardiopulmonary bypass time	cardiopulmonary bypass time	cardiopulmonary bypass time	4	kept
Cardiorenal syndrome	cardiorenal syndrome	cardiorenal syndrome	cardiorenal syndrome	cardiorenal syndrome	4	case_or_format
cardiorespiratory	cardiorespiratory	cardiorespiratory	cardiorespiratory	cardiorespiratory	4	kept
Cardiotoxicity	cardiotoxicity	cardiotoxicity	cardiotoxicity	cardiotoxicity	4	case_or_format
cardiovascular magnetic resonance	cardiovascular magnetic resonance	cardiovascular magnetic resonance	cardiovascular magnetic resonance	cardiovascular magnetic resonance	4	kept
Cardiovascular Physiological Phenomena	cardiovascular physiological phenomena	cardiovascular physiological phenomena	cardiovascular physiological phenomena	Cardiovascular Physiological Phenomena	4	kept
care delivery system	care delivery system	care delivery system	care delivery system	care delivery system	4	kept
Care quality	care quality	care quality	care quality	care quality	4	case_or_format
Care transitions	care transitions	care transitions	care transition	care transitions	4	singular
career planning	career planning	career planning	career planning	career planning	4	kept
Caregiver burden	caregiver burden	caregiver burden	caregiver burden	caregiver burden	4	case_or_format

caregiver burnout	caregiver burnout	caregiver burnout	caregiver burnout	caregiver burnout	4	kept
caring behavior	caring behavior	caring behavior	caring behavior	Caring behavior	4	case_or_format
carotid artery	carotid artery	carotid artery	carotid artery	carotid artery	4	kept
Carotid Artery Diseases	carotid artery diseases	carotid artery diseases	carotid artery diseases	Carotid Artery Diseases	4	kept
case series	case series	case series	case series	case series	4	kept
casirivimab	casirivimab	casirivimab	casirivimab	casirivimab	4	kept
cat disease	cat disease	cat disease	cat disease	Cat Diseases	4	case_or_format
catchment area	catchment area	catchment area	catchment area	catchment area	4	kept
Catchment Area, Health	catchment area health	catchment area health	catchment area health	Catchment Area, Health	4	kept
Cateterismo Periférico	cateterismo perif rico	cateterismo perif rico	cateterismo perif rico	Cateterismo Periférico	4	kept
catheter leakage	catheter leakage	catheter leakage	catheter leakage	catheter leakage	4	kept
catheter obstruction	catheter obstruction	catheter obstruction	catheter obstruction	Catheter Obstruction	4	case_or_format
Catheterization, central venous	catheterization central venous	catheterization central venous	catheterization central venous	Catheterization, Central Venous	4	case_or_format
Causal inference	causal inference	causal inference	causal inference	Causal inference	4	kept
CD8+ T lymphocyte	cd8 t lymphocyte	cd8 t lymphocyte	cd8 t lymphocyte	CD8+ T lymphocyte	4	kept
cefiderocol	cefiderocol	cefiderocol	cefiderocol	cefiderocol	4	kept

Center for Epidemiological Studies Depression	center for epidemiological studies depression	center for epidemiological studies depression	center for epidemiological studies depression	Center for Epidemiological Studies Depression	4	kept
Central Nervous System Stimulants	central nervous system stimulants	central nervous system stimulants	central nervous system stimulants	Central Nervous System Stimulants	4	kept
central nervous system tumor	central nervous system tumor	central nervous system tumor	central nervous system tumor	central nervous system tumor	4	kept
Central venous access	central venous access	central venous access	central venous access	Central venous access	4	kept
central venous access device	central venous access device	central venous access device	central venous access device	central venous access device	4	kept
CENTRAL VENOUS CATHETER	central venous catheter	central venous catheter	central venous catheter	central venous catheter	4	case_or_format
Cerebral Cortex	cerebral cortex	cerebral cortex	cerebral cortex	Cerebral Cortex	4	kept
cerebral hemorrhage	cerebral hemorrhage	cerebral hemorrhage	cerebral hemorrhage	Cerebral Hemorrhage	4	case_or_format
cerebral oxygenation	cerebral oxygenation	cerebral oxygenation	cerebral oxygenation	cerebral oxygenation	4	kept
Cerebrospinal Fluid Shunts	cerebrospinal fluid shunts	cerebrospinal fluid shunts	cerebrospinal fluid shunts	Cerebrospinal Fluid Shunts	4	kept
cervical spinal cord	cervical spinal cord	cervical spinal cord	cervical spinal cord	cervical spinal cord	4	kept
cervical spine injury	cervical spine injury	cervical spine injury	cervical spine injury	cervical spine injury	4	kept
cesarean birth	cesarean birth	cesarean birth	cesarean birth	cesarean birth	4	kept
Change Management	change management	change management	change management	change management	4	case_or_format
character	character	character	character	character	4	kept
Checklists	checklists	checklists	checklist	checklist	4	singular

Chemical and Drug Induced Liver Injury	chemical and drug induced liver injury	chemical and drug induced liver injury	chemical and drug induced liver injury	Chemical and Drug Induced Liver Injury	4	kept
chemical burn	chemical burn	chemical burn	chemical burn	chemical burn	4	kept
chemoembolization	chemoembolization	chemoembolization	chemoembolization	chemoembolization	4	kept
Chemokines	chemokines	chemokines	chemokines	Chemokines	4	kept
chemoradiotherapy	chemoradiotherapy	chemoradiotherapy	chemoradiotherapy	chemoradiotherapy	4	kept
chemosis	chemosis	chemosis	chemosis	chemosis	4	kept
Chest compression	chest compression	chest compression	chest compression	Chest compression	4	kept
Chest pain	chest pain	chest pain	chest pain	Chest Pain	4	case_or_format
Chest physiotherapy	chest physiotherapy	chest physiotherapy	chest physiotherapy	Chest physiotherapy	4	kept
chest physiotherapy	chest physiotherapy	chest physiotherapy	chest physiotherapy	Chest physiotherapy	4	case_or_format
Chest tube	chest tube	chest tube	chest tube	Chest Tubes	4	case_or_format
Chickenpox	chickenpox	chickenpox	chickenpox	chickenpox	4	case_or_format
Child Mortality	child mortality	child mortality	child mortality	Child Mortality	4	kept
child protection	child protection	child protection	child protection	child protection	4	kept
Child Restraint Systems	child restraint systems	child restraint systems	child restraint system	Child Restraint Systems	4	singular
childhood obesity	childhood obesity	childhood obesity	childhood obesity	childhood obesity	4	kept

chimeric antigen receptor T-cell immunotherapy	chimeric antigen receptor t cell immunotherapy	chimeric antigen receptor t cell immunotherapy	chimeric antigen receptor t cell immunotherapy	chimeric antigen receptor T-cell immunotherapy	4	kept
chlordiazepoxide	chlordiazepoxide	chlordiazepoxide	chlordiazepoxide	chlordiazepoxide	4	kept
chlorpheniramine	chlorpheniramine	chlorpheniramine	chlorpheniramine	chlorpheniramine	4	kept
choice	choice	choice	choice	choice	4	kept
cholelithiasis	cholelithiasis	cholelithiasis	cholelithiasis	cholelithiasis	4	kept
Cholestasis	cholestasis	cholestasis	cholestasis	cholestasis	4	case_or_format
Cholesterol, LDL	cholesterol ldl	cholesterol ldl	cholesterol ldl	Cholesterol, LDL	4	kept
cholinesterase inhibitor	cholinesterase inhibitor	cholinesterase inhibitor	cholinesterase inhibitor	cholinesterase inhibitor	4	kept
chromosome disorder	chromosome disorder	chromosome disorder	chromosome disorder	chromosome disorder	4	kept
chronic diseases	chronic diseases	chronic diseases	chronic disease	Chronic Disease	4	singular
Chronic kidney disease	chronic kidney disease	chronic kidney disease	chronic kidney disease	chronic kidney disease	4	case_or_format
chronic lymphatic leukemia	chronic lymphatic leukemia	chronic lymphatic leukemia	chronic lymphatic leukemia	chronic lymphatic leukemia	4	kept
chronic stress	chronic stress	chronic stress	chronic stress	chronic stress	4	kept
Chronically critically ill	chronically critically ill	chronically critically ill	chronically critically ill	chronically critically ill	4	case_or_format
circadian rhythms	circadian rhythms	circadian rhythms	circadian rhythm	circadian rhythm	4	singular
citalopram	citalopram	citalopram	citalopram	citalopram	4	kept

Citespace	citespace	citespace	citespace	Citespace	4	kept
Citrate	citrate	citrate	citrate	Citrates	4	case_or_format
citrate sodium	citrate sodium	citrate sodium	citrate sodium	citrate sodium	4	kept
Citrobacter	citrobacter	citrobacter	citrobacter	Citrobacter	4	kept
cladistics	cladistics	cladistics	cladistics	cladistics	4	kept
claustrophobia	claustrophobia	claustrophobia	claustrophobia	claustrophobia	4	kept
clearance	clearance	clearance	clearance	clearance	4	kept
cleft lip palate	cleft lip palate	cleft lip palate	cleft lip palate	cleft lip palate	4	kept
Clinical alarm	clinical alarm	clinical alarm	clinical alarm	Clinical Alarms	4	case_or_format
Clinical care	clinical care	clinical care	clinical care	Clinical care	4	kept
clinical care	clinical care	clinical care	clinical care	Clinical care	4	case_or_format
Clinical data	clinical data	clinical data	clinical data	Clinical data	4	kept
clinical decision rules	clinical decision rules	clinical decision rules	clinical decision rule	clinical decision rules	4	singular
clinical decision-making	clinical decision making	clinical decision making	clinical decision making	clinical decision making	4	case_or_format
Clinical evolution	clinical evolution	clinical evolution	clinical evolution	Clinical evolution	4	kept
Clinical experience	clinical experience	clinical experience	clinical experience	Clinical experience	4	kept

Clinical features	clinical features	clinical features	clinical feature	clinical feature	4	singular
Clinical Governance	clinical governance	clinical governance	clinical governance	Clinical governance	4	case_or_format
CLINICAL HANDOVER	clinical handover	clinical handover	clinical handover	clinical handover	4	case_or_format
clinical informatics	clinical informatics	clinical informatics	clinical informatics	clinical informatics	4	kept
Clinical pathway	clinical pathway	clinical pathway	clinical pathway	clinical pathway	4	case_or_format
Clinical protocol	clinical protocol	clinical protocol	clinical protocol	Clinical Protocols	4	case_or_format
clinical research coordinator	clinical research coordinator	clinical research coordinator	clinical research coordinator	clinical research coordinator	4	kept
Clinical settings	clinical settings	clinical settings	clinical setting	Clinical settings	4	singular
Clinical study	clinical study	clinical study	clinical study	clinical study	4	case_or_format
Clinical supervision	clinical supervision	clinical supervision	clinical supervision	clinical supervision	4	case_or_format
clinical teaching	clinical teaching	clinical teaching	clinical teaching	clinical teaching	4	kept
clinical training	clinical training	clinical training	clinical training	Clinical training	4	case_or_format
clinical trial protocol	clinical trial protocol	clinical trial protocol	clinical trial protocol	clinical trial protocol	4	kept
Clustering	clustering	clustering	clustering	Clustering	4	kept
Co-design	co design	co design	co design	Co-design	4	kept
coagulation	coagulation	coagulation	coagulation	coagulation	4	kept

Cocaine-Related Disorders	cocaine related disorders	cocaine related disorders	cocaine related disorders	Cocaine-Related Disorders	4	kept
Code blue	code blue	code blue	code blue	Code blue	4	kept
coercion	coercion	coercion	coercion	Coercion	4	case_or_format
Cognitive decline	cognitive decline	cognitive decline	cognitive decline	Cognitive decline	4	kept
Cognitive Test for Delirium	cognitive test for delirium	cognitive test for delirium	cognitive test for delirium	Cognitive Test for Delirium	4	kept
COHORT	cohort	cohort	cohort	COHORT	4	kept
Cohort	cohort	cohort	cohort	COHORT	4	case_or_format
colecalciferol	colecalciferol	colecalciferol	colecalciferol	colecalciferol	4	kept
collaborative practice	collaborative practice	collaborative practice	collaborative practice	Collaborative practice	4	case_or_format
colon cancer	colon cancer	colon cancer	colon cancer	colon cancer	4	kept
colon perforation	colon perforation	colon perforation	colon perforation	colon perforation	4	kept
color density spectral array	color density spectral array	color density spectral array	color density spectral array	color density spectral array	4	kept
colorectal tumor	colorectal tumor	colorectal tumor	colorectal tumor	colorectal tumor	4	kept
Commerce	commerce	commerce	commerce	Commerce	4	kept
common cold	common cold	common cold	common cold	common cold	4	kept
communication aids for disabled	communication aids for disabled	communication aids for disabled	communication aids for disabled	Communication Aids for Disabled	4	case_or_format

Communication board	communication board	communication board	communication board	Communication board	4	kept
Communication in ITU	communication in itu	communication in itu	communication in itu	Communication in ITU	4	kept
Communication Skills	communication skills	communication skills	communication skill	communication skill	4	singular
communication strategies	communication strategies	communication strategies	communication strategies	communication strategies	4	kept
Community acquired pneumonia	community acquired pneumonia	community acquired pneumonia	community acquired pneumonia	community acquired pneumonia	4	case_or_format
Community Health Planning	community health planning	community health planning	community health planning	Community Health Planning	4	kept
Community hospital	community hospital	community hospital	community hospital	community hospital	4	case_or_format
Comorbidities	comorbidities	comorbidities	comorbidity	comorbidity	4	singular
Comparative study	comparative study	comparative study	comparative study	comparative study	4	case_or_format
Compensation and Redress	compensation and redress	compensation and redress	compensation and redress	Compensation and Redress	4	kept
Computer Security	computer security	computer security	computer security	computer security	4	case_or_format
Computer vision	computer vision	computer vision	computer vision	Computer vision	4	kept
Computerized physician order entry	computerized physician order entry	computerized physician order entry	computerized physician order entry	Computerized physician order entry	4	kept
computers	computers	computers	computer	Computers	4	singular
Conjoined twins	conjoined twins	conjoined twins	conjoined twins	conjoined twins	4	case_or_format
CONSENSUS	consensus	consensus	consensus	consensus	4	case_or_format

CONSENSUS CONFERENCE	consensus conference	consensus conference	consensus conference	CONSENSUS CONFERENCE	4	kept
Consensus Development Conferences as Topic	consensus development conferences as topic	consensus development conferences as topic	consensus development conferences as topic	Consensus Development Conferences as Topic	4	kept
Constriction	constriction	constriction	constriction	Constriction	4	kept
Consultation	consultation	consultation	consultation	consultation	4	case_or_format
consumer health information	consumer health information	consumer health information	consumer health information	Consumer Health Information	4	case_or_format
Consumer participation	consumer participation	consumer participation	consumer participation	Consumer participation	4	kept
contact isolation	contact isolation	contact isolation	contact isolation	contact isolation	4	kept
contact precautions	contact precautions	contact precautions	contact precaution	Contact precautions	4	singular
Containment of Biohazards	containment of biohazards	containment of biohazards	containment of biohazards	Containment of Biohazards	4	kept
CONTEXT	context	context	context	CONTEXT	4	kept
Continuing	continuing	continuing	continuing	Continuing	4	kept
Continuing professional development	continuing professional development	continuing professional development	continuing professional development	Continuing professional development	4	kept
continuing professional development	continuing professional development	continuing professional development	continuing professional development	Continuing professional development	4	case_or_format
continuous glucose monitor	continuous glucose monitor	continuous glucose monitor	continuous glucose monitor	continuous glucose monitor	4	kept
Continuous quality improvement	continuous quality improvement	continuous quality improvement	continuous quality improvement	continuous quality improvement	4	case_or_format
Continuum of care	continuum of care	continuum of care	continuum of care	Continuum of care	4	kept

contract	contract	contract	contract	contract	4	kept
Contracts	contracts	contracts	contract	contract	4	singular
contributing factors	contributing factors	contributing factors	contributing factor	contributing factors	4	singular
Control	control	control	control	control	4	case_or_format
conversion to open surgery	conversion to open surgery	conversion to open surgery	conversion to open surgery	conversion to open surgery	4	kept
copeptin	copeptin	copeptin	copeptin	copeptin	4	kept
coping skills	coping skills	coping skills	coping skills	Coping Skills	4	case_or_format
Coping Strategy Questionnaire	coping strategy questionnaire	coping strategy questionnaire	coping strategy questionnaire	Coping Strategy Questionnaire	4	kept
Core competencies	core competencies	core competencies	core competency	Core competency	4	singular
Core competency	core competency	core competency	core competency	Core competency	4	kept
core competency	core competency	core competency	core competency	Core competency	4	case_or_format
core outcome set	core outcome set	core outcome set	core outcome set	core outcome set	4	kept
Cornea	cornea	cornea	cornea	Cornea	4	kept
corneal injuries	corneal injuries	corneal injuries	corneal injury	Corneal Injuries	4	singular
coronary artery atherosclerosis	coronary artery atherosclerosis	coronary artery atherosclerosis	coronary artery atherosclerosis	coronary artery atherosclerosis	4	kept
Coronary artery bypass	coronary artery bypass	coronary artery bypass	coronary artery bypass	Coronary Artery Bypass	4	case_or_format

coronary artery bypass grafting	coronary artery bypass grafting	coronary artery bypass grafting	coronary artery bypass grafting	Coronary artery bypass grafting	4	case_or_format
Coronary Care Unit	coronary care unit	coronary care unit	coronary care unit	coronary care unit	4	case_or_format
coronary heart disease	coronary heart disease	coronary heart disease	coronary heart disease	Coronary heart disease	4	case_or_format
coronavirus 2019	coronavirus 2019	coronavirus 2019	coronavirus 2019	coronavirus 2019	4	kept
coronavirus disease	coronavirus disease	coronavirus disease	coronavirus disease	Coronavirus disease	4	case_or_format
Coronavirus Disease 2019	coronavirus disease 2019	covid 19	covid 19	COVID-19	4	alias
Coronavirus disease-2019	coronavirus disease 2019	covid 19	covid 19	COVID-19	4	alias
coroner	coroner	coroner	coroner	coroner	4	kept
corticosteroids	corticosteroids	corticosteroids	corticosteroid	corticosteroid	4	singular
cost minimization analysis	cost minimization analysis	cost minimization analysis	cost minimization analysis	cost minimization analysis	4	kept
cost savings	cost savings	cost savings	cost saving	Cost Savings	4	singular
costs and cost analysis	costs and cost analysis	costs and cost analysis	costs and cost analysis	Costs and Cost Analysis	4	case_or_format
Counterpulsation	counterpulsation	counterpulsation	counterpulsation	Counterpulsation	4	kept
country economic status	country economic status	country economic status	country economic status	country economic status	4	kept
Covid	covid	covid	covid	COVID	4	case_or_format
COVID-19 Pandemic	covid 19 pandemic	covid 19 pandemic	covid 19 pandemic	COVID-19 pandemic	4	case_or_format

Covid-19 pandemic	covid 19 pandemic	covid 19 pandemic	covid 19 pandemic	COVID-19 pandemic	4	case_or_format
COVID-19 serological testing	covid 19 serological testing	covid 19 serological testing	covid 19 serological testing	COVID-19 serological testing	4	kept
COVID-19 vaccines	covid 19 vaccines	covid 19 vaccines	covid 19 vaccine	COVID-19 Vaccines	4	singular
Craniocerebral injury	craniocerebral injury	craniocerebral injury	craniocerebral injury	Craniocerebral injury	4	kept
Craniosynostoses	craniosynostoses	craniosynostoses	craniosynostoses	Craniosynostoses	4	kept
CRBSI	crbsi	crbsi	crbsi	CRBSI	4	kept
Creatine Kinase	creatine kinase	creatine kinase	creatine kinase	creatine kinase	4	case_or_format
creatinine urine level	creatinine urine level	creatinine urine level	creatinine urine level	creatinine urine level	4	kept
CRISIS	crisis	crisis	crisis	CRISIS	4	kept
CRITERIA	criteria	criteria	criteria	CRITERIA	4	kept
Critical condition	critical condition	critical condition	critical condition	Critical condition	4	kept
critical incident stress	critical incident stress	critical incident stress	critical incident stress	critical incident stress	4	kept
critical incident technique	critical incident technique	critical incident technique	critical incident technique	critical incident technique	4	kept
critical incidents	critical incidents	critical incidents	critical incident	critical incidents	4	singular
CRITICALLY-ILL CHILDREN	critically ill children	critically ill children	critically ill children	Critically ill children	4	case_or_format
CRM	crm	crm	crm	CRM	4	kept

CRNA	crna	crna	crna	CRNA	4	kept
cross-cultural comparison	cross cultural comparison	cross cultural comparison	cross cultural comparison	Cross-Cultural Comparison	4	case_or_format
cross-infection	cross infection	cross infection	cross infection	Cross Infection	4	case_or_format
crude mortality rate	crude mortality rate	crude mortality rate	crude mortality rate	crude mortality rate	4	kept
cuff pressure management	cuff pressure management	cuff pressure management	cuff pressure management	cuff pressure management	4	kept
cultural competency	cultural competency	cultural competency	cultural competency	Cultural Competency	4	case_or_format
Cystectomy	cystectomy	cystectomy	cystectomy	cystectomy	4	case_or_format
cytokine production	cytokine production	cytokine production	cytokine production	cytokine production	4	kept
cytokine release	cytokine release	cytokine release	cytokine release	cytokine release	4	kept
cytology	cytology	cytology	cytology	cytology	4	kept
dabigatran etexilate	dabigatran etexilate	dabigatran etexilate	dabigatran etexilate	dabigatran etexilate	4	kept
Damage control resuscitation	damage control resuscitation	damage control resuscitation	damage control resuscitation	Damage control resuscitation	4	kept
Data Analysis	data analysis	data analysis	data analysis	data analysis	4	case_or_format
data completeness	data completeness	data completeness	data completeness	data completeness	4	kept
data consistency	data consistency	data consistency	data consistency	data consistency	4	kept
Data envelopment analysis	data envelopment analysis	data envelopment analysis	data envelopment analysis	Data envelopment analysis	4	kept

Data handling	data handling	data handling	data handling	Data handling	4	kept
data science	data science	data science	data science	data science	4	kept
data source	data source	data source	data source	data source	4	kept
data visualization	data visualization	data visualization	data visualization	data visualization	4	kept
database	database	database	database	database	4	kept
Death Anxiety Scale	death anxiety scale	death anxiety scale	death anxiety scale	Death Anxiety Scale	4	kept
Decannulation	decannulation	decannulation	decannulation	decannulation	4	case_or_format
Decision support system	decision support system	decision support system	decision support system	decision support system	4	case_or_format
Decision Support Systems, Management	decision support systems management	decision support systems management	decision support systems management	Decision Support Systems, Management	4	kept
Decision support techniques	decision support techniques	decision support techniques	decision support technique	Decision Support Techniques	4	singular
Decision support tools	decision support tools	decision support tools	decision support tool	Decision support tools	4	singular
decision‑making	decision making	decision making	decision making	Decision Making	4	case_or_format
deconditioning	deconditioning	deconditioning	deconditioning	deconditioning	4	kept
Deep venous thrombosis	deep venous thrombosis	deep venous thrombosis	deep venous thrombosis	Deep venous thrombosis	4	kept
Defense Mechanisms	defense mechanisms	defense mechanisms	defense mechanism	defense mechanism	4	singular
Definitions	definitions	definitions	definition	Definitions	4	singular

Delayed Emergence from Anesthesia	delayed emergence from anesthesia	delayed emergence from anesthesia	delayed emergence from anesthesia	Delayed Emergence from Anesthesia	4	kept
delirium detection score	delirium detection score	delirium detection score	delirium detection score	delirium detection score	4	kept
delirium management	delirium management	delirium management	delirium management	Delirium management	4	case_or_format
Delirium prevention	delirium prevention	delirium prevention	delirium prevention	delirium prevention	4	case_or_format
Delirium Rating Scale	delirium rating scale	delirium rating scale	delirium rating scale	Delirium Rating Scale	4	kept
Delirium screening tools	delirium screening tools	delirium screening tools	delirium screening tool	Delirium screening tools	4	singular
DEMANDS	demands	demands	demand	DEMANDS	4	singular
dental assistant	dental assistant	dental assistant	dental assistant	dental assistant	4	kept
dental care	dental care	dental care	dental care	Dental Care	4	case_or_format
Dental Caries	dental caries	dental caries	dental caries	dental caries	4	case_or_format
Dental hygiene	dental hygiene	dental hygiene	dental hygiene	Dental hygiene	4	kept
DENTAL PLAQUE	dental plaque	dental plaque	dental plaque	Dental Plaque	4	case_or_format
Dental plaque	dental plaque	dental plaque	dental plaque	Dental Plaque	4	case_or_format
Dentistry	dentistry	dentistry	dentistry	Dentistry	4	kept
dentistry	dentistry	dentistry	dentistry	Dentistry	4	case_or_format
dependency	dependency	dependency	dependency	dependency	4	kept

Depression Anxiety Stress Scale	depression anxiety stress scale	depression anxiety stress scale	depression anxiety stress scale	Depression Anxiety Stress Scale	4	kept
Deprivation	deprivation	deprivation	deprivation	Deprivation	4	kept
depth perception	depth perception	depth perception	depth perception	depth perception	4	kept
Dermatitis, Contact	dermatitis contact	dermatitis contact	dermatitis contact	Dermatitis, Contact	4	kept
dermatological agent	dermatological agent	dermatological agent	dermatological agent	dermatological agent	4	kept
dermatologist	dermatologist	dermatologist	dermatologist	dermatologist	4	kept
Dermatology	dermatology	dermatology	dermatology	Dermatology	4	kept
dermatology	dermatology	dermatology	dermatology	Dermatology	4	case_or_format
Descriptors	descriptors	descriptors	descriptors	Descriptors	4	kept
detection	detection	detection	detection	detection	4	kept
Detergents	detergents	detergents	detergent	Detergents	4	singular
DETERIORATION	deterioration	deterioration	deterioration	deterioration	4	case_or_format
determinants	determinants	determinants	determinant	DETERMINANTS	4	singular
Device Approval	device approval	device approval	device approval	Device Approval	4	kept
device malposition	device malposition	device malposition	device malposition	device malposition	4	kept
Devices	devices	devices	device	devices	4	singular

diabetes education	diabetes education	diabetes education	diabetes education	diabetes education	4	kept
Diagnostic accuracy	diagnostic accuracy	diagnostic accuracy	diagnostic accuracy	diagnostic accuracy	4	case_or_format
diagnostic agent	diagnostic agent	diagnostic agent	diagnostic agent	diagnostic agent	4	kept
diagnostics	diagnostics	diagnostics	diagnostic	diagnostics	4	singular
dialysis fluid	dialysis fluid	dialysis fluid	dialysis fluid	dialysis fluid	4	kept
Dialysis Solutions	dialysis solutions	dialysis solutions	dialysis solutions	Dialysis Solutions	4	kept
diarrhoea	diarrhoea	diarrhoea	diarrhoea	diarrhoea	4	kept
diary study	diary study	diary study	diary study	diary study	4	kept
Diet, Diabetic	diet diabetic	diet diabetic	diet diabetic	Diet, Diabetic	4	kept
Differential diagnosis	differential diagnosis	differential diagnosis	differential diagnosis	differential diagnosis	4	case_or_format
diffuse Lewy body disease	diffuse lewy body disease	diffuse lewy body disease	diffuse lewy body disease	diffuse Lewy body disease	4	kept
Diffusion	diffusion	diffusion	diffusion	Diffusion	4	kept
diffusion	diffusion	diffusion	diffusion	Diffusion	4	case_or_format
digestion	digestion	digestion	digestion	digestion	4	kept
digestive system perforation	digestive system perforation	digestive system perforation	digestive system perforation	digestive system perforation	4	kept
digital learning	digital learning	digital learning	digital learning	digital learning	4	kept

digital retinal imaging	digital retinal imaging	digital retinal imaging	digital retinal imaging	digital retinal imaging	4	kept
Digital storage	digital storage	digital storage	digital storage	Digital storage	4	kept
digital subtraction angiography	digital subtraction angiography	digital subtraction angiography	digital subtraction angiography	digital subtraction angiography	4	kept
diluent	diluent	diluent	diluent	diluent	4	kept
Dinoprostone	dinoprostone	dinoprostone	dinoprostone	Dinoprostone	4	kept
dipeptidyl carboxypeptidase	dipeptidyl carboxypeptidase	dipeptidyl carboxypeptidase	dipeptidyl carboxypeptidase	dipeptidyl carboxypeptidase	4	kept
dipeptidyl peptidase IV inhibitor	dipeptidyl peptidase iv inhibitor	dipeptidyl peptidase iv inhibitor	dipeptidyl peptidase iv inhibitor	dipeptidyl peptidase IV inhibitor	4	kept
diphtheria	diphtheria	diphtheria	diphtheria	diphtheria	4	kept
Directive Counseling	directive counseling	directive counseling	directive counseling	directive counseling	4	case_or_format
directory	directory	directory	directory	directory	4	kept
Discriminant Analysis	discriminant analysis	discriminant analysis	discriminant analysis	discriminant analysis	4	case_or_format
DISCUSSIONS	discussions	discussions	discussion	DISCUSSIONS	4	singular
Disease management	disease management	disease management	disease management	Disease Management	4	case_or_format
disease prevalence	disease prevalence	disease prevalence	disease prevalence	disease prevalence	4	kept
Disease Transmission, Horizontal	disease transmission horizontal	disease transmission horizontal	disease transmission horizontal	Disease Transmission, Horizontal	4	kept
Disorders of consciousness	disorders of consciousness	disorders of consciousness	disorders of consciousness	disorders of consciousness	4	case_or_format

Disorders of Excessive Somnolence	disorders of excessive somnolence	disorders of excessive somnolence	disorders of excessive somnolence	Disorders of Excessive Somnolence	4	kept
dispersity	dispersity	dispersity	dispersity	dispersity	4	kept
Dispositivos de acceso vascular	dispositivos de acceso vascular	dispositivos de acceso vascular	dispositivos de acceso vascular	Dispositivos de acceso vascular	4	kept
disruptive behavior	disruptive behavior	disruptive behavior	disruptive behavior	disruptive behavior	4	kept
Diversity	diversity	diversity	diversity	diversity	4	case_or_format
Dizziness	dizziness	dizziness	dizziness	dizziness	4	case_or_format
DNR orders	dnr orders	dnr orders	dnr orders	DNR orders	4	kept
do not intubate order	do not intubate order	do not intubate order	do not intubate order	do not intubate order	4	kept
doctoral education	doctoral education	doctoral education	doctoral education	doctoral education	4	kept
doctors	doctors	doctors	doctor	Doctors	4	singular
dofetilide	dofetilide	dofetilide	dofetilide	dofetilide	4	kept
Donation after circulatory death	donation after circulatory death	donation after circulatory death	donation after circulatory death	donation after circulatory death	4	case_or_format
donor selection	donor selection	donor selection	donor selection	Donor Selection	4	case_or_format
dosage schedule comparison	dosage schedule comparison	dosage schedule comparison	dosage schedule comparison	dosage schedule comparison	4	kept
DOSING ERRORS	dosing errors	dosing errors	dosing error	DOSING ERRORS	4	singular
doxepin	doxepin	doxepin	doxepin	doxepin	4	kept

drainage	drainage	drainage	drainage	Drainage	4	case_or_format
dream	dream	dream	dream	Dreams	4	case_or_format
Drinking	drinking	drinking	drinking	drinking	4	case_or_format
droperidol	droperidol	droperidol	droperidol	droperidol	4	kept
Drowning	drowning	drowning	drowning	drowning	4	case_or_format
drug approval	drug approval	drug approval	drug approval	Drug Approval	4	case_or_format
Drug compounding	drug compounding	drug compounding	drug compounding	Drug Compounding	4	case_or_format
drug dose comparison	drug dose comparison	drug dose comparison	drug dose comparison	drug dose comparison	4	kept
drug excretion	drug excretion	drug excretion	drug excretion	drug excretion	4	kept
drug fever	drug fever	drug fever	drug fever	drug fever	4	kept
Drug infusion	drug infusion	drug infusion	drug infusion	drug infusion	4	case_or_format
Drug interactions	drug interactions	drug interactions	drug interaction	Drug Interactions	4	singular
drug manufacture	drug manufacture	drug manufacture	drug manufacture	drug manufacture	4	kept
Drug Packaging	drug packaging	drug packaging	drug packaging	drug packaging	4	case_or_format
Drug Resistance	drug resistance	drug resistance	drug resistance	drug resistance	4	case_or_format
Drug safety	drug safety	drug safety	drug safety	drug safety	4	case_or_format

drug solution	drug solution	drug solution	drug solution	drug solution	4	kept
Drug therapy	drug therapy	drug therapy	drug therapy	drug therapy	4	case_or_format
drug underdose	drug underdose	drug underdose	drug underdose	drug underdose	4	kept
drug utilization review	drug utilization review	drug utilization review	drug utilization review	Drug Utilization Review	4	case_or_format
drugs	drugs	drugs	drug	drug	4	singular
duodenum ulcer	duodenum ulcer	duodenum ulcer	duodenum ulcer	duodenum ulcer	4	kept
Duration	duration	duration	duration	DURATION	4	case_or_format
Duration of Therapy	duration of therapy	duration of therapy	duration of therapy	Duration of Therapy	4	kept
Dust	dust	dust	dust	Dust	4	kept
dysautonomia	dysautonomia	dysautonomia	dysautonomia	dysautonomia	4	kept
dysgeusia	dysgeusia	dysgeusia	dysgeusia	dysgeusia	4	kept
dysphoria	dysphoria	dysphoria	dysphoria	dysphoria	4	kept
dysuria	dysuria	dysuria	dysuria	dysuria	4	kept
ear infection	ear infection	ear infection	ear infection	ear infection	4	kept
early detection	early detection	early detection	early detection	early detection	4	kept
early goal-directed therapy	early goal directed therapy	early goal directed therapy	early goal directed therapy	early goal-directed therapy	4	kept

Early Mobilization	early mobilization	early mobilization	early mobilization	Early mobilization	4	case_or_format
early onset sepsis	early onset sepsis	early onset sepsis	early onset sepsis	early onset sepsis	4	kept
early recognition	early recognition	early recognition	early recognition	Early recognition	4	case_or_format
Early warning systems	early warning systems	early warning systems	early warning system	early warning system	4	singular
EARPLUGS	earplugs	earplugs	earplug	EARPLUGS	4	singular
earplugs	earplugs	earplugs	earplug	EARPLUGS	4	singular
eating habit	eating habit	eating habit	eating habit	eating habit	4	kept
Echocardiography, Doppler	echocardiography doppler	echocardiography doppler	echocardiography doppler	Echocardiography, Doppler	4	kept
echolalia	echolalia	echolalia	echolalia	echolalia	4	kept
ECMO specialist	ecmo specialist	ecmo specialist	ecmo specialist	ECMO specialist	4	kept
economic analysis	economic analysis	economic analysis	economic analysis	economic analysis	4	kept
economic inequality	economic inequality	economic inequality	economic inequality	economic inequality	4	kept
Ectodermal Dysplasia	ectodermal dysplasia	ectodermal dysplasia	ectodermal dysplasia	Ectodermal Dysplasia	4	kept
Educación	educaci n	educaci n	educaci n	Educación	4	kept
Education computing	education computing	education computing	education computing	Education computing	4	kept
Education Program	education program	education program	education program	education program	4	case_or_format

Educational Program	educational program	educational program	educational program	Educational program	4	case_or_format
Educational technology	educational technology	educational technology	educational technology	educational technology	4	case_or_format
effort-reward imbalance	effort reward imbalance	effort reward imbalance	effort reward imbalance	effort-reward imbalance	4	kept
ego development	ego development	ego development	ego development	ego development	4	kept
elasomeran	elasomeran	elasomeran	elasomeran	elasomeran	4	kept
Elective surgeries	elective surgeries	elective surgeries	elective surgery	elective surgery	4	singular
Elective surgery	elective surgery	elective surgery	elective surgery	elective surgery	4	case_or_format
Electric Impedance	electric impedance	electric impedance	electric impedance	Electric Impedance	4	kept
electroconvulsive therapy	electroconvulsive therapy	electroconvulsive therapy	electroconvulsive therapy	electroconvulsive therapy	4	kept
Electroencephalogram	electroencephalogram	electroencephalogram	electroencephalogram	electroencephalogram	4	case_or_format
electrolytes	electrolytes	electrolytes	electrolyte	electrolyte	4	singular
electron microscopy	electron microscopy	electron microscopy	electron microscopy	electron microscopy	4	kept
electronic device	electronic device	electronic device	electronic device	electronic device	4	kept
Electronic medical records	electronic medical records	electronic medical records	electronic medical record	electronic medical record	4	singular
electrotherapy	electrotherapy	electrotherapy	electrotherapy	electrotherapy	4	kept
eligibility	eligibility	eligibility	eligibility	eligibility	4	kept

embarrassment	embarrassment	embarrassment	embarrassment	embarrassment	4	kept
embolectomy	embolectomy	embolectomy	embolectomy	embolectomy	4	kept
Embolism	embolism	embolism	embolism	Embolism	4	kept
Emergency Departments	emergency departments	emergency departments	emergency department	Emergency department	4	singular
Emergency management	emergency management	emergency management	emergency management	emergency management	4	case_or_format
Emergency Responders	emergency responders	emergency responders	emergency responders	Emergency Responders	4	kept
emergency visit	emergency visit	emergency visit	emergency visit	emergency visit	4	kept
EMERGENCY-DEPARTMENTS	emergency departments	emergency departments	emergency department	Emergency department	4	singular
Emollients	emollients	emollients	emollient	Emollients	4	singular
Emotion	emotion	emotion	emotion	Emotions	4	case_or_format
Emotional Adjustment	emotional adjustment	emotional adjustment	emotional adjustment	Emotional Adjustment	4	kept
emotional eating	emotional eating	emotional eating	emotional eating	emotional eating	4	kept
EMOTIONAL EXHAUSTION	emotional exhaustion	emotional exhaustion	emotional exhaustion	emotional exhaustion	4	case_or_format
Emotional labor	emotional labor	emotional labor	emotional labor	Emotional labor	4	kept
Emotional support	emotional support	emotional support	emotional support	emotional support	4	case_or_format
empathy training	empathy training	empathy training	empathy training	empathy training	4	kept

Empirical research report	empirical research report	empirical research report	empirical research report	Empirical research report	4	kept
end stage kidney disease	end stage kidney disease	end stage kidney disease	end stage kidney disease	end stage kidney disease	4	kept
endothelium cell	endothelium cell	endothelium cell	endothelium cell	endothelium cell	4	kept
Endothelium-Dependent Relaxing Factors	endothelium dependent relaxing factors	endothelium dependent relaxing factors	endothelium dependent relaxing factors	Endothelium-Dependent Relaxing Factors	4	kept
endotracheal suctioning	endotracheal suctioning	endotracheal suctioning	endotracheal suctioning	Endotracheal suctioning	4	case_or_format
endotracheal tube suctioning	endotracheal tube suctioning	endotracheal tube suctioning	endotracheal tube suctioning	endotracheal tube suctioning	4	kept
Enfermeras clínicas	enfermeras cl nicas	enfermeras cl nicas	enfermeras cl nicas	Enfermeras clínicas	4	kept
Enfermeros	enfermeros	enfermeros	enfermero	Enfermeros	4	singular
Enfermería de cuidados intensivos	enfermer a de cuidados intensivos	enfermer a de cuidados intensivos	enfermer a de cuidados intensivos	Enfermería de cuidados intensivos	4	kept
Engineering	engineering	engineering	engineering	Engineering	4	kept
Engineering education	engineering education	engineering education	engineering education	Engineering education	4	kept
enhanced recovery	enhanced recovery	enhanced recovery	enhanced recovery	enhanced recovery	4	kept
Enteral	enteral	enteral	enteral	Enteral	4	kept
enteral	enteral	enteral	enteral	Enteral	4	case_or_format
ENTEROBACTERIACEAE	enterobacteriaceae	enterobacteriaceae	enterobacteriaceae	Enterobacteriaceae	4	case_or_format
enterococcal infection	enterococcal infection	enterococcal infection	enterococcal infection	enterococcal infection	4	kept

ENTEROCOCCI	enterococci	enterococci	enterococci	ENTEROCOCCI	4	kept
Enterostomy	enterostomy	enterostomy	enterostomy	Enterostomy	4	kept
enterostomy	enterostomy	enterostomy	enterostomy	Enterostomy	4	case_or_format
Enterovirus infection	enterovirus infection	enterovirus infection	enterovirus infection	Enterovirus infection	4	kept
entropy	entropy	entropy	entropy	entropy	4	kept
environmental protection	environmental protection	environmental protection	environmental protection	environmental protection	4	kept
Environmental sustainability	environmental sustainability	environmental sustainability	environmental sustainability	environmental sustainability	4	case_or_format
enzyme activity	enzyme activity	enzyme activity	enzyme activity	enzyme activity	4	kept
enzyme synthesis	enzyme synthesis	enzyme synthesis	enzyme synthesis	enzyme synthesis	4	kept
eosinophil	eosinophil	eosinophil	eosinophil	eosinophil	4	kept
epidermis	epidermis	epidermis	epidermis	epidermis	4	kept
epidural	epidural	epidural	epidural	epidural	4	kept
Epidural Abscess	epidural abscess	epidural abscess	epidural abscess	Epidural Abscess	4	kept
epidural catheter	epidural catheter	epidural catheter	epidural catheter	epidural catheter	4	kept
epidural drug administration	epidural drug administration	epidural drug administration	epidural drug administration	epidural drug administration	4	kept
epigastric pain	epigastric pain	epigastric pain	epigastric pain	epigastric pain	4	kept

epsilometer test	epsilometer test	epsilometer test	epsilometer test	epsilometer test	4	kept
Epworth sleepiness scale	epworth sleepiness scale	epworth sleepiness scale	epworth sleepiness scale	Epworth sleepiness scale	4	kept
Equidae	equidae	equidae	equidae	Equidae	4	kept
equipment and supplies utilization	equipment and supplies utilization	equipment and supplies utilization	equipment and supplies utilization	equipment and supplies utilization	4	kept
Equity	equity	equity	equity	equity	4	case_or_format
eradication therapy	eradication therapy	eradication therapy	eradication therapy	eradication therapy	4	kept
ERROR	error	error	error	error	4	case_or_format
error reporting	error reporting	error reporting	error reporting	error reporting	4	kept
Erythroblastosis, Fetal	erythroblastosis fetal	erythroblastosis fetal	erythroblastosis fetal	Erythroblastosis, Fetal	4	kept
Erythrocytes	erythrocytes	erythrocytes	erythrocyte	erythrocyte	4	singular
erythroderma	erythroderma	erythroderma	erythroderma	erythroderma	4	kept
Erythropoietin	erythropoietin	erythropoietin	erythropoietin	erythropoietin	4	case_or_format
escitalopram	escitalopram	escitalopram	escitalopram	escitalopram	4	kept
Esophageal Perforation	esophageal perforation	esophageal perforation	esophageal perforation	Esophageal Perforation	4	kept
esophagus tumor	esophagus tumor	esophagus tumor	esophagus tumor	esophagus tumor	4	kept
etanercept	etanercept	etanercept	etanercept	etanercept	4	kept

ethical challenges	ethical challenges	ethical challenges	ethical challenge	ethical challenges	4	singular
ethical decision-making	ethical decision making	ethical decision making	ethical decision making	ethical decision making	4	case_or_format
Ethical dilemma	ethical dilemma	ethical dilemma	ethical dilemma	ethical dilemma	4	case_or_format
ethical leadership	ethical leadership	ethical leadership	ethical leadership	ethical leadership	4	kept
ethics consultation	ethics consultation	ethics consultation	ethics consultation	Ethics Consultation	4	case_or_format
ethnographic research	ethnographic research	ethnographic research	ethnographic research	ethnographic research	4	kept
European Quality of Life 5 Dimensions 3 Level questionnaire	european quality of life 5 dimensions 3 level questionnaire	european quality of life 5 dimensions 3 level questionnaire	european quality of life 5 dimensions 3 level questionnaire	European Quality of Life 5 Dimensions 3 Level questionnaire	4	kept
evacuation	evacuation	evacuation	evacuation	Evacuation	4	case_or_format
evaluation and follow up	evaluation and follow up	evaluation and follow up	evaluation and follow up	evaluation and follow up	4	kept
evaluation studies	evaluation studies	evaluation studies	evaluation study	evaluation study	4	singular
evidence summary	evidence summary	evidence summary	evidence summary	evidence summary	4	kept
Evidence-based design	evidence based design	evidence based design	evidence based design	evidence-based design	4	case_or_format
evidence-based practices	evidence based practices	evidence based practices	evidence based practice	evidence based practice	4	singular
EXCELLENCE	excellence	excellence	excellence	EXCELLENCE	4	kept
Exclusive breastfeeding	exclusive breastfeeding	exclusive breastfeeding	exclusive breastfeeding	Exclusive breastfeeding	4	kept
Exercise therapy	exercise therapy	exercise therapy	exercise therapy	Exercise Therapy	4	case_or_format

experimental therapy	experimental therapy	experimental therapy	experimental therapy	experimental therapy	4	kept
EXPERT	expert	expert	expert	EXPERT	4	kept
Expert systems	expert systems	expert systems	expert system	expert system	4	singular
exposure variable	exposure variable	exposure variable	exposure variable	exposure variable	4	kept
extended daily dialysis	extended daily dialysis	extended daily dialysis	extended daily dialysis	extended daily dialysis	4	kept
Extended family	extended family	extended family	extended family	Extended family	4	kept
external fixator	external fixator	external fixator	external fixator	external fixator	4	kept
extracellular fluid	extracellular fluid	extracellular fluid	extracellular fluid	extracellular fluid	4	kept
extracorporeal therapy	extracorporeal therapy	extracorporeal therapy	extracorporeal therapy	extracorporeal therapy	4	kept
Extreme prematurity	extreme prematurity	extreme prematurity	extreme prematurity	extreme prematurity	4	case_or_format
Extremely low birth weight	extremely low birth weight	extremely low birth weight	extremely low birth weight	extremely low birth weight	4	case_or_format
extremely premature	extremely premature	extremely premature	extremely premature	extremely premature	4	kept
extremely premature infants	extremely premature infants	extremely premature infants	extremely premature infant	extremely premature infants	4	singular
EXTUBATION	extubation	extubation	extubation	extubation	4	case_or_format
eye	eye	eye	eye	Eye	4	case_or_format
EYE MASKS	eye masks	eye masks	eye mask	EYE MASKS	4	singular

eye masks	eye masks	eye masks	eye mask	EYE MASKS	4	singular
Eye tracking	eye tracking	eye tracking	eye tracking	eye tracking	4	case_or_format
eye-tracking	eye tracking	eye tracking	eye tracking	eye tracking	4	case_or_format
face pain	face pain	face pain	face pain	face pain	4	kept
facial nerve paralysis	facial nerve paralysis	facial nerve paralysis	facial nerve paralysis	facial nerve paralysis	4	kept
Facilitator	facilitator	facilitator	facilitator	Facilitators	4	case_or_format
FACILITATORS	facilitators	facilitators	facilitator	Facilitators	4	singular
Factor Xa	factor xa	factor xa	factor xa	Factor Xa	4	kept
Failure to Thrive	failure to thrive	failure to thrive	failure to thrive	failure to thrive	4	case_or_format
Fall	fall	fall	fall	falls	4	case_or_format
FALSE ALARMS	false alarms	false alarms	false alarm	False alarms	4	singular
familia	familia	familia	familia	Familia	4	case_or_format
family conference	family conference	family conference	family conference	family conference	4	kept
family experience	family experience	family experience	family experience	Family experience	4	case_or_format
Family Integrated Care	family integrated care	family integrated care	family integrated care	Family integrated care	4	case_or_format
family member	family member	family member	family member	Family members	4	case_or_format

Family participation	family participation	family participation	family participation	family participation	4	case_or_format
Family witnessed resuscitation	family witnessed resuscitation	family witnessed resuscitation	family witnessed resuscitation	Family-witnessed resuscitation	4	case_or_format
fatty liver	fatty liver	fatty liver	fatty liver	Fatty Liver	4	case_or_format
Feature extraction	feature extraction	feature extraction	feature extraction	feature extraction	4	case_or_format
Febrile Neutropenia	febrile neutropenia	febrile neutropenia	febrile neutropenia	febrile neutropenia	4	case_or_format
fecal incontinence	fecal incontinence	fecal incontinence	fecal incontinence	Fecal Incontinence	4	case_or_format
feces culture	feces culture	feces culture	feces culture	feces culture	4	kept
Feeding protocol	feeding protocol	feeding protocol	feeding protocol	Feeding protocol	4	kept
Fellowship	fellowship	fellowship	fellowship	Fellowship	4	kept
fellowship	fellowship	fellowship	fellowship	Fellowship	4	case_or_format
female worker	female worker	female worker	female worker	female worker	4	kept
Fetal Development	fetal development	fetal development	fetal development	Fetal Development	4	kept
fetoplacental unit	fetoplacental unit	fetoplacental unit	fetoplacental unit	fetoplacental unit	4	kept
fetus blood	fetus blood	fetus blood	fetus blood	fetus blood	4	kept
fetus malformation	fetus malformation	fetus malformation	fetus malformation	fetus malformation	4	kept
fibrin degradation product	fibrin degradation product	fibrin degradation product	fibrin degradation product	fibrin degradation product	4	kept

Fibrin Fibrinogen Degradation Products	fibrin fibrinogen degradation products	fibrin fibrinogen degradation products	fibrin fibrinogen degradation products	Fibrin Fibrinogen Degradation Products	4	kept
Fibrinogen	fibrinogen	fibrinogen	fibrinogen	fibrinogen	4	case_or_format
fibrosing alveolitis	fibrosing alveolitis	fibrosing alveolitis	fibrosing alveolitis	fibrosing alveolitis	4	kept
fidaxomicin	fidaxomicin	fidaxomicin	fidaxomicin	fidaxomicin	4	kept
FIDELITY	fidelity	fidelity	fidelity	FIDELITY	4	kept
fidelity	fidelity	fidelity	fidelity	FIDELITY	4	case_or_format
Field studies	field studies	field studies	field study	field study	4	singular
Financial Stress	financial stress	financial stress	financial stress	financial stress	4	case_or_format
fire protection	fire protection	fire protection	fire protection	fire protection	4	kept
Firearms	firearms	firearms	firearm	firearm	4	singular
Fistula	fistula	fistula	fistula	fistula	4	case_or_format
flaccid paralysis	flaccid paralysis	flaccid paralysis	flaccid paralysis	flaccid paralysis	4	kept
flatulence	flatulence	flatulence	flatulence	flatulence	4	kept
flecainide	flecainide	flecainide	flecainide	flecainide	4	kept
Flexible bronchoscopy	flexible bronchoscopy	flexible bronchoscopy	flexible bronchoscopy	flexible bronchoscopy	4	case_or_format
Fluid responsiveness	fluid responsiveness	fluid responsiveness	fluid responsiveness	Fluid responsiveness	4	kept

fluid retention	fluid retention	fluid retention	fluid retention	fluid retention	4	kept
Fluid Shifts	fluid shifts	fluid shifts	fluid shift	Fluid Shifts	4	singular
fluid-attenuated inversion recovery imaging	fluid attenuated inversion recovery imaging	fluid attenuated inversion recovery imaging	fluid attenuated inversion recovery imaging	fluid-attenuated inversion recovery imaging	4	kept
Fluorescein	fluorescein	fluorescein	fluorescein	Fluorescein	4	kept
Fluoroscopy	fluoroscopy	fluoroscopy	fluoroscopy	fluoroscopy	4	case_or_format
Focus group interviews	focus group interviews	focus group interviews	focus group interview	Focus group interviews	4	singular
focused assessment with sonography for trauma	focused assessment with sonography for trauma	focused assessment with sonography for trauma	focused assessment with sonography for trauma	focused assessment with sonography for trauma	4	kept
Foley catheter	foley catheter	foley catheter	foley catheter	Foley catheter	4	kept
Follow up	follow up	follow up	follow up	follow up	4	case_or_format
food insecurity	food insecurity	food insecurity	food insecurity	food insecurity	4	kept
food quality	food quality	food quality	food quality	food quality	4	kept
foot pain	foot pain	foot pain	foot pain	foot pain	4	kept
Foot reflexology	foot reflexology	foot reflexology	foot reflexology	Foot reflexology	4	kept
Foundations	foundations	foundations	foundation	Foundations	4	singular
fourth cervical vertebra	fourth cervical vertebra	fourth cervical vertebra	fourth cervical vertebra	fourth cervical vertebra	4	kept
fracture external fixation	fracture external fixation	fracture external fixation	fracture external fixation	fracture external fixation	4	kept

Fracture Fixation, Intramedullary	fracture fixation intramedullary	fracture fixation intramedullary	fracture fixation intramedullary	Fracture Fixation, Intramedullary	4	kept
fracture healing	fracture healing	fracture healing	fracture healing	fracture healing	4	kept
frailty index	frailty index	frailty index	frailty index	frailty index	4	kept
FRAMEWORK	framework	framework	framework	FRAMEWORK	4	kept
Free Radicals	free radicals	free radicals	free radical	free radical	4	singular
freedom	freedom	freedom	freedom	Freedom	4	case_or_format
French	french	french	french	French	4	kept
friendship	friendship	friendship	friendship	friendship	4	kept
Functional outcome	functional outcome	functional outcome	functional outcome	Functional outcome	4	kept
Functional recovery	functional recovery	functional recovery	functional recovery	Functional recovery	4	kept
fundamental care	fundamental care	fundamental care	fundamental care	fundamental care	4	kept
Fusobacterium necrophorum	fusobacterium necrophorum	fusobacterium necrophorum	fusobacterium necrophorum	Fusobacterium necrophorum	4	kept
Gabapentin	gabapentin	gabapentin	gabapentin	gabapentin	4	case_or_format
gas flow	gas flow	gas flow	gas flow	gas flow	4	kept
Gastric Acidity Determination	gastric acidity determination	gastric acidity determination	gastric acidity determination	Gastric Acidity Determination	4	kept
Gastric Lavage	gastric lavage	gastric lavage	gastric lavage	Gastric Lavage	4	kept

Gastroenteritis	gastroenteritis	gastroenteritis	gastroenteritis	gastroenteritis	4	case_or_format
Gastroesophageal reflux	gastroesophageal reflux	gastroesophageal reflux	gastroesophageal reflux	gastroesophageal reflux	4	case_or_format
gastrointestinal discomfort	gastrointestinal discomfort	gastrointestinal discomfort	gastrointestinal discomfort	gastrointestinal discomfort	4	kept
gastrointestinal intubation	gastrointestinal intubation	gastrointestinal intubation	gastrointestinal intubation	Gastrointestinal intubation	4	case_or_format
Gastroparesis	gastroparesis	gastroparesis	gastroparesis	Gastroparesis	4	kept
gastroscopy	gastroscopy	gastroscopy	gastroscopy	gastroscopy	4	kept
general	general	general	general	general	4	kept
General anesthesia	general anesthesia	general anesthesia	general anesthesia	general anesthesia	4	case_or_format
General hospital	general hospital	general hospital	general hospital	general hospital	4	case_or_format
General surgery	general surgery	general surgery	general surgery	general surgery	4	case_or_format
generative artificial intelligence	generative artificial intelligence	generative artificial intelligence	generative artificial intelligence	Generative artificial intelligence	4	case_or_format
genetic algorithm	genetic algorithm	genetic algorithm	genetic algorithm	genetic algorithm	4	kept
genetic variability	genetic variability	genetic variability	genetic variability	genetic variability	4	kept
genome	genome	genome	genome	genome	4	kept
Gentamicins	gentamicins	gentamicins	gentamicin	gentamicin	4	singular
geographic and geological parameters	geographic and geological parameters	geographic and geological parameters	geographic and geological parameters	geographic and geological parameters	4	kept

geriatric oncology	geriatric oncology	geriatric oncology	geriatric oncology	geriatric oncology	4	kept
Geriatric Psychiatry	geriatric psychiatry	geriatric psychiatry	geriatric psychiatry	Geriatric Psychiatry	4	kept
geriatric surgery	geriatric surgery	geriatric surgery	geriatric surgery	geriatric surgery	4	kept
Geriatric trauma	geriatric trauma	geriatric trauma	geriatric trauma	geriatric trauma	4	case_or_format
Geriatricians	geriatricians	geriatricians	geriatrician	geriatrician	4	singular
Glaucoma	glaucoma	glaucoma	glaucoma	glaucoma	4	case_or_format
glibenclamide	glibenclamide	glibenclamide	glibenclamide	glibenclamide	4	kept
glomerulopathy	glomerulopathy	glomerulopathy	glomerulopathy	glomerulopathy	4	kept
Gloves	gloves	gloves	glove	glove	4	singular
Glucose control	glucose control	glucose control	glucose control	glucose control	4	case_or_format
glucose sensor	glucose sensor	glucose sensor	glucose sensor	glucose sensor	4	kept
glycaemic control	glycaemic control	glycaemic control	glycaemic control	Glycaemic control	4	case_or_format
Glycerol	glycerol	glycerol	glycerol	glycerol	4	case_or_format
Goal-directed therapy	goal directed therapy	goal directed therapy	goal directed therapy	Goal-directed therapy	4	kept
governmental organization	governmental organization	governmental organization	governmental organization	governmental organization	4	kept
graft infection	graft infection	graft infection	graft infection	graft infection	4	kept

Graft vs Host Disease	graft vs host disease	graft vs host disease	graft vs host disease	Graft vs Host Disease	4	kept
Gram-negative	gram negative	gram negative	gram negative	Gram-negative	4	kept
grandparent	grandparent	grandparent	grandparent	Grandparents	4	case_or_format
gratitude	gratitude	gratitude	gratitude	gratitude	4	kept
gravity	gravity	gravity	gravity	gravity	4	kept
gray matter	gray matter	gray matter	gray matter	gray matter	4	kept
greenhouse gas emission	greenhouse gas emission	greenhouse gas emission	greenhouse gas emission	greenhouse gas emission	4	kept
Group Practice	group practice	group practice	group practice	Group Practice	4	kept
Group processes	group processes	group processes	group processes	Group Processes	4	case_or_format
grunting	grunting	grunting	grunting	grunting	4	kept
haematology	haematology	haematology	haematology	haematology	4	kept
haemodynamics	haemodynamics	haemodynamics	haemodynamic	Haemodynamics	4	singular
Hamilton Anxiety Scale	hamilton anxiety scale	hamilton anxiety scale	hamilton anxiety scale	Hamilton Anxiety Scale	4	kept
hand dermatitis	hand dermatitis	hand dermatitis	hand dermatitis	hand dermatitis	4	kept
hand injury	hand injury	hand injury	hand injury	hand injury	4	kept
Hand-off	hand off	hand off	hand off	hand-off	4	case_or_format

handheld device	handheld device	handheld device	handheld device	handheld device	4	kept
HANDOFFS	handoffs	handoffs	handoff	handoff	4	singular
Harm	harm	harm	harm	Harm	4	kept
Hazardous Waste	hazardous waste	hazardous waste	hazardous waste	Hazardous Waste	4	kept
hazardous waste	hazardous waste	hazardous waste	hazardous waste	Hazardous Waste	4	case_or_format
head and neck injury	head and neck injury	head and neck injury	head and neck injury	head and neck injury	4	kept
Head Injuries, Penetrating	head injuries penetrating	head injuries penetrating	head injuries penetrating	Head Injuries, Penetrating	4	kept
Head injury	head injury	head injury	head injury	head injury	4	case_or_format
head movement	head movement	head movement	head movement	head movement	4	kept
head of bed elevation	head of bed elevation	head of bed elevation	head of bed elevation	head of bed elevation	4	kept
healing environment	healing environment	healing environment	healing environment	healing environment	4	kept
Health belief model	health belief model	health belief model	health belief model	Health Belief Model	4	case_or_format
Health Care	health care	health care	health care	health care	4	case_or_format
Health care delivery	health care delivery	health care delivery	health care delivery	health care delivery	4	case_or_format
health care distribution	health care distribution	health care distribution	health care distribution	health care distribution	4	kept
Health care facilities	health care facilities	health care facilities	health care facility	health care facility	4	singular

health care facilities and services	health care facilities and services	health care facilities and services	health care facilities and services	health care facilities and services	4	kept
Health care professional	health care professional	health care professional	health care professional	Health care professionals	4	case_or_format
health care surveillance	health care surveillance	health care surveillance	health care surveillance	health care surveillance	4	kept
health care technology	health care technology	health care technology	health care technology	health care technology	4	kept
Health care-associated infection	health care associated infection	health care associated infection	health care associated infection	Health care-associated infections	4	case_or_format
health data	health data	health data	health data	health data	4	kept
health educator	health educator	health educator	health educator	health educator	4	kept
Health facilities	health facilities	health facilities	health facilities	Health Facilities	4	case_or_format
Health Facility Merger	health facility merger	health facility merger	health facility merger	Health Facility Merger	4	kept
Health Facility Moving	health facility moving	health facility moving	health facility moving	Health Facility Moving	4	kept
Health Facility Planning	health facility planning	health facility planning	health facility planning	Health Facility Planning	4	kept
Health Maintenance Organizations	health maintenance organizations	health maintenance organizations	health maintenance organization	Health Maintenance Organizations	4	singular
Health management	health management	health management	health management	Health management	4	kept
Health Services Administration	health services administration	health services administration	health services administration	health services administration	4	case_or_format
Health status	health status	health status	health status	health status	4	case_or_format
Health Status Disparities	health status disparities	health status disparities	health status disparities	Health Status Disparities	4	kept

health workers	health workers	health workers	health worker	health worker	4	singular
Health workforce	health workforce	health workforce	health workforce	health workforce	4	case_or_format
HEALTH-CARE WORKERS	health care workers	health care workers	health care worker	Health care workers	4	singular
health-care workers	health care workers	health care workers	health care worker	Health care workers	4	singular
Healthcare Failure Mode and Effect Analysis	healthcare failure mode and effect analysis	healthcare failure mode and effect analysis	healthcare failure mode and effect analysis	Healthcare Failure Mode and Effect Analysis	4	kept
Healthcare management	healthcare management	healthcare management	healthcare management	healthcare management	4	case_or_format
healthcare personnel	healthcare personnel	healthcare personnel	healthcare personnel	Healthcare personnel	4	case_or_format
Healthcare safety	healthcare safety	healthcare safety	healthcare safety	Healthcare safety	4	kept
Healthcare technology	healthcare technology	healthcare technology	healthcare technology	Healthcare technology	4	kept
healthcare utilization	healthcare utilization	healthcare utilization	healthcare utilization	Healthcare utilization	4	case_or_format
healthy lifestyle	healthy lifestyle	healthy lifestyle	healthy lifestyle	healthy lifestyle	4	kept
Healthy work environments	healthy work environments	healthy work environments	healthy work environment	healthy work environment	4	singular
hearing aid	hearing aid	hearing aid	hearing aid	hearing aid	4	kept
heart afterload	heart afterload	heart afterload	heart afterload	heart afterload	4	kept
Heart Auscultation	heart auscultation	heart auscultation	heart auscultation	heart auscultation	4	case_or_format
heart bundle branch block	heart bundle branch block	heart bundle branch block	heart bundle branch block	heart bundle branch block	4	kept

heart catheter	heart catheter	heart catheter	heart catheter	heart catheter	4	kept
heart electrophysiology	heart electrophysiology	heart electrophysiology	heart electrophysiology	heart electrophysiology	4	kept
heart failure with preserved ejection fraction	heart failure with preserved ejection fraction	heart failure with preserved ejection fraction	heart failure with preserved ejection fraction	heart failure with preserved ejection fraction	4	kept
heart function test	heart function test	heart function test	heart function test	Heart Function Tests	4	case_or_format
heart murmur	heart murmur	heart murmur	heart murmur	heart murmur	4	kept
heart muscle conduction disturbance	heart muscle conduction disturbance	heart muscle conduction disturbance	heart muscle conduction disturbance	heart muscle conduction disturbance	4	kept
heart muscle reperfusion	heart muscle reperfusion	heart muscle reperfusion	heart muscle reperfusion	heart muscle reperfusion	4	kept
heart performance	heart performance	heart performance	heart performance	heart performance	4	kept
heart protection	heart protection	heart protection	heart protection	heart protection	4	kept
heart right atrium	heart right atrium	heart right atrium	heart right atrium	heart right atrium	4	kept
Heart surgery	heart surgery	heart surgery	heart surgery	heart surgery	4	case_or_format
Heart transplant	heart transplant	heart transplant	heart transplant	Heart transplant	4	kept
HEART-DISEASE	heart disease	heart disease	heart disease	Heart Diseases	4	case_or_format
HeartWeek	heartweek	heartweek	heartweek	HeartWeek	4	kept
heating	heating	heating	heating	Heating	4	case_or_format
Helicobacter infection	helicobacter infection	helicobacter infection	helicobacter infection	Helicobacter infection	4	kept

HELLP Syndrome	hellp syndrome	hellp syndrome	hellp syndrome	HELLP syndrome	4	case_or_format
Helplessness, Learned	helplessness learned	helplessness learned	helplessness learned	Helplessness, Learned	4	kept
hematochezia	hematochezia	hematochezia	hematochezia	hematochezia	4	kept
hematological parameters	hematological parameters	hematological parameters	hematological parameters	hematological parameters	4	kept
Hematopoietic cell transplantation	hematopoietic cell transplantation	hematopoietic cell transplantation	hematopoietic cell transplantation	Hematopoietic cell transplantation	4	kept
hemisphere	hemisphere	hemisphere	hemisphere	hemisphere	4	kept
Hemodialysis Solutions	hemodialysis solutions	hemodialysis solutions	hemodialysis solutions	Hemodialysis Solutions	4	kept
Hemodynamic instability	hemodynamic instability	hemodynamic instability	hemodynamic instability	Hemodynamic instability	4	kept
Hemodynamic monitoring	hemodynamic monitoring	hemodynamic monitoring	hemodynamic monitoring	hemodynamic monitoring	4	case_or_format
hemophagocytic syndrome	hemophagocytic syndrome	hemophagocytic syndrome	hemophagocytic syndrome	hemophagocytic syndrome	4	kept
hemorrhagic stroke	hemorrhagic stroke	hemorrhagic stroke	hemorrhagic stroke	hemorrhagic stroke	4	kept
Hepatitis A virus	hepatitis a virus	hepatitis a virus	hepatitis a virus	Hepatitis A virus	4	kept
hepatobiliary disease	hepatobiliary disease	hepatobiliary disease	hepatobiliary disease	hepatobiliary disease	4	kept
Hepatomegaly	hepatomegaly	hepatomegaly	hepatomegaly	hepatomegaly	4	case_or_format
Hernia	hernia	hernia	hernia	Hernia	4	kept
hernia	hernia	hernia	hernia	Hernia	4	case_or_format

Herpes simplex virus	herpes simplex virus	herpes simplex virus	herpes simplex virus	Herpes simplex virus	4	kept
Heuristics	heuristics	heuristics	heuristic	Heuristics	4	singular
hiatus hernia	hiatus hernia	hiatus hernia	hiatus hernia	hiatus hernia	4	kept
Hierarchical task analysis	hierarchical task analysis	hierarchical task analysis	hierarchical task analysis	Hierarchical task analysis	4	kept
high density lipoprotein cholesterol	high density lipoprotein cholesterol	high density lipoprotein cholesterol	high density lipoprotein cholesterol	high density lipoprotein cholesterol	4	kept
High dependency care	high dependency care	high dependency care	high dependency care	High dependency care	4	kept
high dependency care	high dependency care	high dependency care	high dependency care	High dependency care	4	case_or_format
high fidelity simulation	high fidelity simulation	high fidelity simulation	high fidelity simulation	high-fidelity simulation	4	case_or_format
High flow nasal cannula	high flow nasal cannula	high flow nasal cannula	high flow nasal cannula	High-flow nasal cannula	4	case_or_format
high frequency oscillation	high frequency oscillation	high frequency oscillation	high frequency oscillation	high frequency oscillation	4	kept
high school	high school	high school	high school	high school	4	kept
High-fidelity simulations	high fidelity simulations	high fidelity simulations	high fidelity simulation	high-fidelity simulation	4	singular
High-risk infants	high risk infants	high risk infants	high risk infant	high risk infant	4	singular
hippocampus	hippocampus	hippocampus	hippocampus	hippocampus	4	kept
HLA Antigens	hla antigens	hla antigens	hla antigens	HLA Antigens	4	kept
holistic	holistic	holistic	holistic	holistic	4	kept

home health care	home health care	home health care	home health care	home health care	4	kept
home mechanical ventilation	home mechanical ventilation	home mechanical ventilation	home mechanical ventilation	Home mechanical ventilation	4	case_or_format
homeless person	homeless person	homeless person	homeless person	homeless person	4	kept
homocysteine	homocysteine	homocysteine	homocysteine	homocysteine	4	kept
honey	honey	honey	honey	Honey	4	case_or_format
hormone substitution	hormone substitution	hormone substitution	hormone substitution	hormone substitution	4	kept
hospital acquired	hospital acquired	hospital acquired	hospital acquired	hospital acquired	4	kept
Hospital air	hospital air	hospital air	hospital air	Hospital air	4	kept
Hospital bed capacity	hospital bed capacity	hospital bed capacity	hospital bed capacity	hospital bed capacity	4	case_or_format
Hospital Bed Capacity, 100 to 299	hospital bed capacity 100 to 299	hospital bed capacity 100 to 299	hospital bed capacity 100 to 299	Hospital Bed Capacity, 100 to 299	4	kept
hospital capacity	hospital capacity	hospital capacity	hospital capacity	hospital capacity	4	kept
Hospital Emergency Service	hospital emergency service	hospital emergency service	hospital emergency service	hospital emergency service	4	case_or_format
Hospital infection	hospital infection	hospital infection	hospital infection	hospital infection	4	case_or_format
Hospital units	hospital units	hospital units	hospital units	Hospital Units	4	case_or_format
hospital visit	hospital visit	hospital visit	hospital visit	hospital visit	4	kept
Hospital-acquired pneumonia	hospital acquired pneumonia	hospital acquired pneumonia	hospital acquired pneumonia	hospital acquired pneumonia	4	case_or_format

hospitalist	hospitalist	hospitalist	hospitalist	Hospitalists	4	case_or_format
Hospitals, Convalescent	hospitals convalescent	hospitals convalescent	hospitals convalescent	Hospitals, Convalescent	4	kept
Hotlines	hotlines	hotlines	hotline	Hotlines	4	singular
Housing	housing	housing	housing	housing	4	case_or_format
Housing for the Elderly	housing for the elderly	housing for the elderly	housing for the elderly	Housing for the Elderly	4	kept
Hyaluronoglucosaminidase	hyaluronoglucosaminidase	hyaluronoglucosaminidase	hyaluronoglucosaminidase	Hyaluronoglucosaminidase	4	kept
hydration status	hydration status	hydration status	hydration status	hydration status	4	kept
hydrocodone bitartrate plus paracetamol	hydrocodone bitartrate plus paracetamol	hydrocodone bitartrate plus paracetamol	hydrocodone bitartrate plus paracetamol	hydrocodone bitartrate plus paracetamol	4	kept
hydrocolloid	hydrocolloid	hydrocolloid	hydrocolloid	hydrocolloid	4	kept
hydrostatic pressure	hydrostatic pressure	hydrostatic pressure	hydrostatic pressure	hydrostatic pressure	4	kept
hydroxyurea	hydroxyurea	hydroxyurea	hydroxyurea	hydroxyurea	4	kept
HYGIENE	hygiene	hygiene	hygiene	hygiene	4	case_or_format
hyperactive	hyperactive	hyperactive	hyperactive	hyperactive	4	kept
hyperactivity	hyperactivity	hyperactivity	hyperactivity	hyperactivity	4	kept
hyperalgesia	hyperalgesia	hyperalgesia	hyperalgesia	hyperalgesia	4	kept
hyperchloremia	hyperchloremia	hyperchloremia	hyperchloremia	hyperchloremia	4	kept

hyperchloremic acidosis	hyperchloremic acidosis	hyperchloremic acidosis	hyperchloremic acidosis	hyperchloremic acidosis	4	kept
hyperglycaemia	hyperglycaemia	hyperglycaemia	hyperglycaemia	Hyperglycaemia	4	case_or_format
Hyperkinesis	hyperkinesis	hyperkinesis	hyperkinesis	Hyperkinesis	4	kept
hyperosmolarity	hyperosmolarity	hyperosmolarity	hyperosmolarity	hyperosmolarity	4	kept
hyperpnea	hyperpnea	hyperpnea	hyperpnea	hyperpnea	4	kept
hypersensitivity reaction	hypersensitivity reaction	hypersensitivity reaction	hypersensitivity reaction	hypersensitivity reaction	4	kept
hypertensive crisis	hypertensive crisis	hypertensive crisis	hypertensive crisis	hypertensive crisis	4	kept
Hypertensive Encephalopathy	hypertensive encephalopathy	hypertensive encephalopathy	hypertensive encephalopathy	Hypertensive Encephalopathy	4	kept
hyperthermic therapy	hyperthermic therapy	hyperthermic therapy	hyperthermic therapy	hyperthermic therapy	4	kept
Hyperthyroidism	hyperthyroidism	hyperthyroidism	hyperthyroidism	hyperthyroidism	4	case_or_format
hyperuricemia	hyperuricemia	hyperuricemia	hyperuricemia	Hyperuricemia	4	case_or_format
Hypoactive delirium	hypoactive delirium	hypoactive delirium	hypoactive delirium	Hypoactive delirium	4	kept
Hypoalbuminemia	hypoalbuminemia	hypoalbuminemia	hypoalbuminemia	hypoalbuminemia	4	case_or_format
hypochloremia	hypochloremia	hypochloremia	hypochloremia	hypochloremia	4	kept
hypochlorite	hypochlorite	hypochlorite	hypochlorite	hypochlorite	4	kept
hypokinesia	hypokinesia	hypokinesia	hypokinesia	hypokinesia	4	kept

hypospadias	hypospadias	hypospadias	hypospadias	hypospadias	4	kept
hypothalamus hypophysis system	hypothalamus hypophysis system	hypothalamus hypophysis system	hypothalamus hypophysis system	hypothalamus hypophysis system	4	kept
Hypoxic-ischemic encephalopathy	hypoxic ischemic encephalopathy	hypoxic ischemic encephalopathy	hypoxic ischemic encephalopathy	hypoxic ischemic encephalopathy	4	case_or_format
Hémorragie cérébrale	h morragie c r brale	h morragie c r brale	h morragie c r brale	Hémorragie cérébrale	4	kept
Ice	ice	ice	ice	Ice	4	kept
icosapentaenoic acid	icosapentaenoic acid	icosapentaenoic acid	icosapentaenoic acid	icosapentaenoic acid	4	kept
identification	identification	identification	identification	identification	4	kept
Idiopathic pulmonary fibrosis	idiopathic pulmonary fibrosis	idiopathic pulmonary fibrosis	idiopathic pulmonary fibrosis	Idiopathic pulmonary fibrosis	4	kept
illness perception	illness perception	illness perception	illness perception	illness perception	4	kept
Image Enhancement	image enhancement	image enhancement	image enhancement	Image Enhancement	4	kept
Imaging	imaging	imaging	imaging	imaging	4	case_or_format
immigrant	immigrant	immigrant	immigrant	immigrant	4	kept
immigration	immigration	immigration	immigration	immigration	4	kept
Immunization Schedule	immunization schedule	immunization schedule	immunization schedule	Immunization Schedule	4	kept
immunogenicity	immunogenicity	immunogenicity	immunogenicity	immunogenicity	4	kept
immunoglobulin enhancer binding protein	immunoglobulin enhancer binding protein	immunoglobulin enhancer binding protein	immunoglobulin enhancer binding protein	immunoglobulin enhancer binding protein	4	kept

Immunoglobulin M	immunoglobulin m	immunoglobulin m	immunoglobulin m	immunoglobulin M	4	case_or_format
Immunologic Factors	immunologic factors	immunologic factors	immunologic factor	Immunologic Factors	4	singular
immunologist	immunologist	immunologist	immunologist	immunologist	4	kept
immunomodulating agent	immunomodulating agent	immunomodulating agent	immunomodulating agent	immunomodulating agent	4	kept
impact of event scale-revised	impact of event scale revised	impact of event scale revised	impact of event scale revised	impact of event scale-revised	4	kept
impaired glucose tolerance	impaired glucose tolerance	impaired glucose tolerance	impaired glucose tolerance	impaired glucose tolerance	4	kept
IMPAIRMENT	impairment	impairment	impairment	IMPAIRMENT	4	kept
impedance	impedance	impedance	impedance	impedance	4	kept
Impella	impella	impella	impella	Impella	4	kept
impetigo	impetigo	impetigo	impetigo	impetigo	4	kept
In situ simulation	in situ simulation	in situ simulation	in situ simulation	in situ simulation	4	case_or_format
Inappropriate ADH Syndrome	inappropriate adh syndrome	inappropriate adh syndrome	inappropriate adh syndrome	Inappropriate ADH Syndrome	4	kept
INCIDENTS	incidents	incidents	incident	INCIDENTS	4	singular
Incubators	incubators	incubators	incubator	incubator	4	singular
incubators	incubators	incubators	incubator	incubator	4	singular
Indicators	indicators	indicators	indicator	INDICATORS	4	singular

Indomethacin	indomethacin	indomethacin	indomethacin	Indomethacin	4	kept
Indonesian	indonesian	indonesian	indonesian	Indonesian	4	kept
indoor air pollution	indoor air pollution	indoor air pollution	indoor air pollution	indoor air pollution	4	kept
Indwelling urinary catheter	indwelling urinary catheter	indwelling urinary catheter	indwelling urinary catheter	indwelling urinary catheter	4	case_or_format
infancy	infancy	infancy	infancy	infancy	4	kept
Infant care	infant care	infant care	infant care	Infant Care	4	case_or_format
Infant mental health	infant mental health	infant mental health	infant mental health	Infant mental health	4	kept
infant, premature	infant premature	infant premature	infant premature	Infant, Premature	4	case_or_format
Infant, Small for Gestational Age	infant small for gestational age	infant small for gestational age	infant small for gestational age	Infant, Small for Gestational Age	4	kept
Infarctus cérébral	infarctus c r bral	infarctus c r bral	infarctus c r bral	Infarctus cérébral	4	kept
infection severity	infection severity	infection severity	infection severity	infection severity	4	kept
infectious arthritis	infectious arthritis	infectious arthritis	infectious arthritis	infectious arthritis	4	kept
INFECTIOUS-DISEASES-SOCIETY	infectious diseases society	infectious diseases society	infectious diseases society	INFECTIOUS-DISEASES-SOCIETY	4	kept
Influencing factor	influencing factor	influencing factor	influencing factor	influencing factors	4	case_or_format
information literacy	information literacy	information literacy	information literacy	Information literacy	4	case_or_format
Information needs	information needs	information needs	information need	Information needs	4	singular

Information retrieval	information retrieval	information retrieval	information retrieval	information retrieval	4	case_or_format
information service	information service	information service	information service	Information Services	4	case_or_format
information source	information source	information source	information source	information source	4	kept
Infusion pump	infusion pump	infusion pump	infusion pump	Infusion Pumps	4	case_or_format
INFUSIONS	infusions	infusions	infusion	infusion	4	singular
inhalation anesthesia	inhalation anesthesia	inhalation anesthesia	inhalation anesthesia	inhalation anesthesia	4	kept
injection site	injection site	injection site	injection site	injection site	4	kept
Injections, Intramuscular	injections intramuscular	injections intramuscular	injections intramuscular	Injections, Intramuscular	4	kept
INSERTION	insertion	insertion	insertion	INSERTION	4	kept
Inservice training	inservice training	inservice training	inservice training	Inservice Training	4	case_or_format
Institutional Practice	institutional practice	institutional practice	institutional practice	Institutional Practice	4	kept
insulin autoimmune syndrome	insulin autoimmune syndrome	insulin autoimmune syndrome	insulin autoimmune syndrome	insulin autoimmune syndrome	4	kept
insulin blood level	insulin blood level	insulin blood level	insulin blood level	insulin blood level	4	kept
insulin detemir	insulin detemir	insulin detemir	insulin detemir	insulin detemir	4	kept
Insulins	insulins	insulins	insulin	Insulin	4	singular
Insurance	insurance	insurance	insurance	insurance	4	case_or_format

Integrative Medicine	integrative medicine	integrative medicine	integrative medicine	integrative medicine	4	case_or_format
Intelligence	intelligence	intelligence	intelligence	intelligence	4	case_or_format
Intensivstation	intensivstation	intensivstation	intensivstation	Intensivstation	4	kept
Intent to leave	intent to leave	intent to leave	intent to leave	Intent to leave	4	kept
inter-hospital transfer	inter hospital transfer	inter hospital transfer	inter hospital transfer	inter-hospital transfer	4	kept
Interactive Ventilatory Support	interactive ventilatory support	interactive ventilatory support	interactive ventilatory support	Interactive Ventilatory Support	4	kept
interactive ventilatory support	interactive ventilatory support	interactive ventilatory support	interactive ventilatory support	Interactive Ventilatory Support	4	case_or_format
Interdisciplinary health team	interdisciplinary health team	interdisciplinary health team	interdisciplinary health team	Interdisciplinary health team	4	kept
interdisciplinary teamwork	interdisciplinary teamwork	interdisciplinary teamwork	interdisciplinary teamwork	interdisciplinary teamwork	4	kept
Interfacility transport	interfacility transport	interfacility transport	interfacility transport	Interfacility transport	4	kept
interfacility transport	interfacility transport	interfacility transport	interfacility transport	Interfacility transport	4	case_or_format
interferon gamma release assay	interferon gamma release assay	interferon gamma release assay	interferon gamma release assay	interferon gamma release assay	4	kept
Interhospital transfer	interhospital transfer	interhospital transfer	interhospital transfer	Interhospital transfer	4	kept
interhospital transfer	interhospital transfer	interhospital transfer	interhospital transfer	Interhospital transfer	4	case_or_format
Interhospital transport	interhospital transport	interhospital transport	interhospital transport	Interhospital transport	4	kept
Interleukin-2	interleukin 2	interleukin 2	interleukin 2	interleukin 2	4	case_or_format

intermittent hemodialysis	intermittent hemodialysis	intermittent hemodialysis	intermittent hemodialysis	Intermittent hemodialysis	4	case_or_format
intermittent pneumatic compression device	intermittent pneumatic compression device	intermittent pneumatic compression device	intermittent pneumatic compression device	Intermittent Pneumatic Compression Devices	4	case_or_format
International Agencies	international agencies	international agencies	international agencies	International Agencies	4	kept
International Classification of Functioning	international classification of functioning disability	international classification of functioning disability	international classification of functioning disability	International Classification of Functioning	4	kept
International Normalized Ratio	international normalized ratio	international normalized ratio	international normalized ratio	international normalized ratio	4	case_or_format
international organization	international organization	international organization	international organization	international organization	4	kept
internet access	internet access	internet access	internet access	internet access	4	kept
Internet-Based Intervention	internet based intervention	internet based intervention	internet based intervention	Internet-Based Intervention	4	kept
Internship	internship	internship	internship	Internship	4	kept
Interpretive description	interpretive description	interpretive description	interpretive description	Interpretive description	4	kept
Interpretive phenomenology	interpretive phenomenology	interpretive phenomenology	interpretive phenomenology	Interpretive phenomenology	4	kept
interpretive phenomenology	interpretive phenomenology	interpretive phenomenology	interpretive phenomenology	Interpretive phenomenology	4	case_or_format
Interprofessional care	interprofessional care	interprofessional care	interprofessional care	interprofessional care	4	case_or_format
interprofessional practice	interprofessional practice	interprofessional practice	interprofessional practice	interprofessional practice	4	kept
interprofessional team	interprofessional team	interprofessional team	interprofessional team	interprofessional team	4	kept
Interprofessional teamwork	interprofessional teamwork	interprofessional teamwork	interprofessional teamwork	Interprofessional teamwork	4	kept

intervention development	intervention development	intervention development	intervention development	intervention development	4	kept
Interventional radiology	interventional radiology	interventional radiology	interventional radiology	interventional radiology	4	case_or_format
Intestinal Absorption	intestinal absorption	intestinal absorption	intestinal absorption	Intestinal Absorption	4	kept
intestine	intestine	intestine	intestine	Intestines	4	case_or_format
intestine fistula	intestine fistula	intestine fistula	intestine fistula	intestine fistula	4	kept
intestine flora	intestine flora	intestine flora	intestine flora	intestine flora	4	kept
intestine function	intestine function	intestine function	intestine function	intestine function	4	kept
intestine volvulus	intestine volvulus	intestine volvulus	intestine volvulus	intestine volvulus	4	kept
INTRACEREBRAL HEMORRHAGE	intracerebral hemorrhage	intracerebral hemorrhage	intracerebral hemorrhage	Intracerebral hemorrhage	4	case_or_format
Intracranial hypertension	intracranial hypertension	intracranial hypertension	intracranial hypertension	Intracranial Hypertension	4	case_or_format
Intravenous infusion	intravenous infusion	intravenous infusion	intravenous infusion	intravenous infusions	4	case_or_format
intravenous infusion	intravenous infusion	intravenous infusion	intravenous infusion	intravenous infusions	4	case_or_format
Intravenous infusions	intravenous infusions	intravenous infusions	intravenous infusion	intravenous infusions	4	singular
Intravenous thrombolysis	intravenous thrombolysis	intravenous thrombolysis	intravenous thrombolysis	Intravenous thrombolysis	4	kept
introspection	introspection	introspection	introspection	introspection	4	kept
INTUBATION	intubation	intubation	intubation	intubation	4	case_or_format

invasive procedures	invasive procedures	invasive procedures	invasive procedure	invasive procedure	4	singular
involvement	involvement	involvement	involvement	INVOLVEMENT	4	case_or_format
Iraq War, 2003 -	iraq war 2003	iraq war 2003	iraq war 2003	Iraq War, 2003 -	4	kept
Irish	irish	irish	irish	Irish	4	kept
irritant contact dermatitis	irritant contact dermatitis	irritant contact dermatitis	irritant contact dermatitis	irritant contact dermatitis	4	kept
irritant dermatitis	irritant dermatitis	irritant dermatitis	irritant dermatitis	irritant dermatitis	4	kept
Irritants	irritants	irritants	irritants	Irritants	4	kept
ischemic cardiomyopathy	ischemic cardiomyopathy	ischemic cardiomyopathy	ischemic cardiomyopathy	ischemic cardiomyopathy	4	kept
isolation room	isolation room	isolation room	isolation room	isolation room	4	kept
Iterative methods	iterative methods	iterative methods	iterative methods	Iterative methods	4	kept
JD-R model	jd r model	jd r model	jd r model	JD-R model	4	kept
Jewelry	jewelry	jewelry	jewelry	Jewelry	4	kept
Job analysis	job analysis	job analysis	job analysis	Job analysis	4	kept
job analysis	job analysis	job analysis	job analysis	Job analysis	4	case_or_format
Job Application	job application	job application	job application	Job Application	4	kept
job market	job market	job market	job market	job market	4	kept

Judaism	judaism	judaism	judaism	Judaism	4	kept
Jurisprudence	jurisprudence	jurisprudence	jurisprudence	jurisprudence	4	case_or_format
kangaroo	kangaroo	kangaroo	kangaroo	kangaroo	4	kept
Kangaroo Care	kangaroo care	kangaroo care	kangaroo care	kangaroo care	4	case_or_format
KAP	kap	kap	kap	KAP	4	kept
ketoconazole	ketoconazole	ketoconazole	ketoconazole	ketoconazole	4	kept
kidney blood flow	kidney blood flow	kidney blood flow	kidney blood flow	kidney blood flow	4	kept
kidney cancer	kidney cancer	kidney cancer	kidney cancer	kidney cancer	4	kept
Kinesics	kinesics	kinesics	kinesics	Kinesics	4	kept
knee pain	knee pain	knee pain	knee pain	knee pain	4	kept
knowledge management	knowledge management	knowledge management	knowledge management	knowledge management	4	kept
knowledge test	knowledge test	knowledge test	knowledge test	knowledge test	4	kept
Korean	korean	korean	korean	Korean	4	kept
laboratory information system	laboratory information system	laboratory information system	laboratory information system	laboratory information system	4	kept
labour	labour	labour	labour	labour	4	kept
lacosamide	lacosamide	lacosamide	lacosamide	lacosamide	4	kept

lactate sodium	lactate sodium	lactate sodium	lactate sodium	lactate sodium	4	kept
lactoferrin	lactoferrin	lactoferrin	lactoferrin	lactoferrin	4	kept
lactose	lactose	lactose	lactose	lactose	4	kept
laminar flow	laminar flow	laminar flow	laminar flow	laminar flow	4	kept
language disability	language disability	language disability	language disability	language disability	4	kept
Laryngeal Diseases	laryngeal diseases	laryngeal diseases	laryngeal diseases	Laryngeal Diseases	4	kept
Laryngostenosis	laryngostenosis	laryngostenosis	laryngostenosis	Laryngostenosis	4	kept
larynx	larynx	larynx	larynx	Larynx	4	case_or_format
Laser Coagulation	laser coagulation	laser coagulation	laser coagulation	Laser Coagulation	4	kept
laser coagulation	laser coagulation	laser coagulation	laser coagulation	Laser Coagulation	4	case_or_format
late preterm infant	late preterm infant	late preterm infant	late preterm infant	late preterm infant	4	kept
LATE-ONSET SEPSIS	late onset sepsis	late onset sepsis	late onset sepsis	LATE-ONSET SEPSIS	4	kept
Latent profile analysis	latent profile analysis	latent profile analysis	latent profile analysis	latent profile analysis	4	case_or_format
lawyer	lawyer	lawyer	lawyer	lawyer	4	kept
Lean methodology	lean methodology	lean methodology	lean methodology	Lean methodology	4	kept
learning algorithm	learning algorithm	learning algorithm	learning algorithm	learning algorithm	4	kept

Learning algorithms	learning algorithms	learning algorithms	learning algorithm	learning algorithm	4	singular
Learning Disabilities	learning disabilities	learning disabilities	learning disability	Learning Disabilities	4	singular
Learning models	learning models	learning models	learning model	Learning models	4	singular
LEAVE	leave	leave	leave	LEAVE	4	kept
left anterior descending coronary artery	left anterior descending coronary artery	left anterior descending coronary artery	left anterior descending coronary artery	left anterior descending coronary artery	4	kept
leg ischemia	leg ischemia	leg ischemia	leg ischemia	leg ischemia	4	kept
leg pain	leg pain	leg pain	leg pain	leg pain	4	kept
leg swelling	leg swelling	leg swelling	leg swelling	leg swelling	4	kept
leg thrombosis	leg thrombosis	leg thrombosis	leg thrombosis	leg thrombosis	4	kept
Leiomyoma	leiomyoma	leiomyoma	leiomyoma	Leiomyoma	4	kept
Length of hospital stay	length of hospital stay	length of hospital stay	length of hospital stay	Length of hospital stay	4	kept
lepirudin	lepirudin	lepirudin	lepirudin	lepirudin	4	kept
letter	letter	letter	letter	Letter	4	case_or_format
Levetiracetam	levetiracetam	levetiracetam	levetiracetam	levetiracetam	4	case_or_format
levothyroxine	levothyroxine	levothyroxine	levothyroxine	levothyroxine	4	kept
liaison psychiatry	liaison psychiatry	liaison psychiatry	liaison psychiatry	liaison psychiatry	4	kept

life history	life history	life history	life history	life history	4	kept
life support care	life support care	life support care	life support care	Life Support Care	4	case_or_format
Life-sustaining treatments	life sustaining treatments	life sustaining treatments	life sustaining treatment	life sustaining treatment	4	singular
Lifting equipment	lifting equipment	lifting equipment	lifting equipment	Lifting equipment	4	kept
ligation	ligation	ligation	ligation	ligation	4	kept
Light sedation	light sedation	light sedation	light sedation	Light sedation	4	kept
limb fracture	limb fracture	limb fracture	limb fracture	limb fracture	4	kept
limb salvage	limb salvage	limb salvage	limb salvage	limb salvage	4	kept
Limited English proficiency	limited english proficiency	limited english proficiency	limited english proficiency	Limited English Proficiency	4	case_or_format
limited mobility	limited mobility	limited mobility	limited mobility	limited mobility	4	kept
Linguistics	linguistics	linguistics	linguistics	linguistics	4	case_or_format
lipoprotein	lipoprotein	lipoprotein	lipoprotein	lipoprotein	4	kept
Listeria monocytogenes	listeria monocytogenes	listeria monocytogenes	listeria monocytogenes	Listeria monocytogenes	4	kept
liver cancer	liver cancer	liver cancer	liver cancer	liver cancer	4	kept
liver metastasis	liver metastasis	liver metastasis	liver metastasis	liver metastasis	4	kept
liver tumor	liver tumor	liver tumor	liver tumor	liver tumor	4	kept

living wills	living wills	living wills	living will	living will	4	singular
lobar pneumonia	lobar pneumonia	lobar pneumonia	lobar pneumonia	lobar pneumonia	4	kept
Logistic regression	logistic regression	logistic regression	logistic regression	Logistic regression	4	kept
logistic regression	logistic regression	logistic regression	logistic regression	Logistic regression	4	case_or_format
long acting drug	long acting drug	long acting drug	long acting drug	long acting drug	4	kept
long short term memory network	long short term memory network	long short term memory network	long short term memory network	long short term memory network	4	kept
long term memory	long term memory	long term memory	long term memory	long term memory	4	kept
LONG-TERM	long term	long term	long term	LONG-TERM	4	kept
Long-term outcomes	long term outcomes	long term outcomes	long term outcome	Long-term outcomes	4	singular
long-term outcomes	long term outcomes	long term outcomes	long term outcome	Long-term outcomes	4	singular
longevity	longevity	longevity	longevity	longevity	4	kept
longitudinal	longitudinal	longitudinal	longitudinal	longitudinal	4	kept
loop diuretic agent	loop diuretic agent	loop diuretic agent	loop diuretic agent	loop diuretic agent	4	kept
loratadine	loratadine	loratadine	loratadine	loratadine	4	kept
LOS	los	los	los	LOS	4	kept
Loss	loss	loss	loss	Loss	4	kept

Love	love	love	love	Love	4	kept
love	love	love	love	Love	4	case_or_format
low calory diet	low calory diet	low calory diet	low calory diet	low calory diet	4	kept
low tidal volume ventilation	low tidal volume ventilation	low tidal volume ventilation	low tidal volume ventilation	low tidal volume ventilation	4	kept
low volume hospital	low volume hospital	low volume hospital	low volume hospital	low volume hospital	4	kept
Low-resource settings	low resource settings	low resource settings	low resource setting	Low-resource settings	4	singular
Low-value care	low value care	low value care	low value care	Low-value care	4	kept
low-value care	low value care	low value care	low value care	Low-value care	4	case_or_format
lubrication	lubrication	lubrication	lubrication	lubrication	4	kept
Lullaby	lullaby	lullaby	lullaby	Lullaby	4	kept
Luminescence	luminescence	luminescence	luminescence	Luminescence	4	kept
lung angiography	lung angiography	lung angiography	lung angiography	lung angiography	4	kept
lung blood vessel	lung blood vessel	lung blood vessel	lung blood vessel	lung blood vessel	4	kept
Lung Compliance	lung compliance	lung compliance	lung compliance	lung compliance	4	case_or_format
lung diseases	lung diseases	lung diseases	lung disease	Lung Diseases	4	singular
lung emphysema	lung emphysema	lung emphysema	lung emphysema	lung emphysema	4	kept

lung hemodynamics	lung hemodynamics	lung hemodynamics	lung hemodynamics	lung hemodynamics	4	kept
lung nodule	lung nodule	lung nodule	lung nodule	lung nodule	4	kept
lung resistance	lung resistance	lung resistance	lung resistance	lung resistance	4	kept
lung ultrasound	lung ultrasound	lung ultrasound	lung ultrasound	lung ultrasound	4	kept
lupus erythematosus nephritis	lupus erythematosus nephritis	lupus erythematosus nephritis	lupus erythematosus nephritis	lupus erythematosus nephritis	4	kept
Lymph Node Excision	lymph node excision	lymph node excision	lymph node excision	Lymph Node Excision	4	kept
lymph node metastasis	lymph node metastasis	lymph node metastasis	lymph node metastasis	lymph node metastasis	4	kept
Lymphoma, Non-Hodgkin	lymphoma non hodgkin	lymphoma non hodgkin	lymphoma non hodgkin	Lymphoma, Non-Hodgkin	4	kept
Lymphopenia	lymphopenia	lymphopenia	lymphopenia	Lymphopenia	4	kept
lymphopenia	lymphopenia	lymphopenia	lymphopenia	Lymphopenia	4	case_or_format
Machine learning techniques	machine learning techniques	machine learning techniques	machine learning techniques	Machine learning techniques	4	kept
Machine-learning	machine learning	machine learning	machine learning	machine learning	4	case_or_format
Macroglossia	macroglossia	macroglossia	macroglossia	Macroglossia	4	kept
macrogol	macrogol	macrogol	macrogol	macrogol	4	kept
Macrolides	macrolides	macrolides	macrolide	macrolide	4	singular
macrophage	macrophage	macrophage	macrophage	macrophage	4	kept

magnetic field	magnetic field	magnetic field	magnetic field	magnetic field	4	kept
Magnetic resonance imaging	magnetic resonance imaging	magnetic resonance imaging	magnetic resonance imaging	Magnetic Resonance Imaging	4	case_or_format
Maharashtra	maharashtra	maharashtra	maharashtra	Maharashtra	4	kept
Major trauma	major trauma	major trauma	major trauma	Major trauma	4	kept
Malassezia	malassezia	malassezia	malassezia	Malassezia	4	kept
Manchester triage system	manchester triage system	manchester triage system	manchester triage system	Manchester triage system	4	kept
marketing	marketing	marketing	marketing	marketing	4	kept
maropitant	maropitant	maropitant	maropitant	maropitant	4	kept
Marriage	marriage	marriage	marriage	marriage	4	case_or_format
Masculinity	masculinity	masculinity	masculinity	Masculinity	4	kept
masculinity	masculinity	masculinity	masculinity	Masculinity	4	case_or_format
mass gathering	mass gathering	mass gathering	mass gathering	mass gathering	4	kept
materials handling	materials handling	materials handling	materials handling	materials handling	4	kept
maternal mental health	maternal mental health	maternal mental health	maternal mental health	maternal mental health	4	kept
Maternal voice	maternal voice	maternal voice	maternal voice	Maternal voice	4	kept
Maternal-Child Health Centers	maternal child health centers	maternal child health centers	maternal child health centers	Maternal-Child Health Centers	4	kept

Mathematical model	mathematical model	mathematical model	mathematical model	mathematical model	4	case_or_format
maxillofacial surgery	maxillofacial surgery	maxillofacial surgery	maxillofacial surgery	maxillofacial surgery	4	kept
maximum plasma concentration	maximum plasma concentration	maximum plasma concentration	maximum plasma concentration	maximum plasma concentration	4	kept
McGill Pain Questionnaire	mcgill pain questionnaire	mcgill pain questionnaire	mcgill pain questionnaire	McGill Pain Questionnaire	4	kept
McNemar test	mcnemar test	mcnemar test	mcnemar test	McNemar test	4	kept
meaning	meaning	meaning	meaning	meaning	4	kept
Measles	measles	measles	measles	measles	4	case_or_format
measles vaccine	measles vaccine	measles vaccine	measles vaccine	measles vaccine	4	kept
Measles virus	measles virus	measles virus	measles virus	Measles virus	4	kept
mechanical ventilators	mechanical ventilators	mechanical ventilators	mechanical ventilator	mechanical ventilator	4	singular
Mediastinum	mediastinum	mediastinum	mediastinum	Mediastinum	4	kept
mediastinum	mediastinum	mediastinum	mediastinum	Mediastinum	4	case_or_format
medical care need	medical care need	medical care need	medical care need	medical care need	4	kept
Medical conditions	medical conditions	medical conditions	medical conditions	Medical conditions	4	kept
medical decision-making	medical decision making	medical decision making	medical decision making	medical decision making	4	case_or_format
Medical doctors	medical doctors	medical doctors	medical doctors	Medical doctors	4	kept

medical education & training	medical education and training	medical education and training	medical education and training	medical education & training	4	kept
Medical Emergency Teams	medical emergency teams	medical emergency teams	medical emergency team	Medical emergency team	4	singular
Medical ethics	medical ethics	medical ethics	medical ethics	medical ethics	4	case_or_format
medical geography	medical geography	medical geography	medical geography	medical geography	4	kept
Medical information	medical information	medical information	medical information	medical information	4	case_or_format
Medical Knowledge	medical knowledge	medical knowledge	medical knowledge	Medical Knowledge	4	kept
medical liability	medical liability	medical liability	medical liability	medical liability	4	kept
Medical order entry systems	medical order entry systems	medical order entry systems	medical order entry system	Medical Order Entry Systems	4	singular
medical order entry systems	medical order entry systems	medical order entry systems	medical order entry system	Medical Order Entry Systems	4	singular
Medical Record Linkage	medical record linkage	medical record linkage	medical record linkage	Medical Record Linkage	4	kept
medical simulation	medical simulation	medical simulation	medical simulation	Medical simulation	4	case_or_format
Medical systems	medical systems	medical systems	medical systems	Medical systems	4	kept
Medical technology	medical technology	medical technology	medical technology	medical technology	4	case_or_format
Medication Reconciliation	medication reconciliation	medication reconciliation	medication reconciliation	Medication Reconciliation	4	kept
Medication reconciliation	medication reconciliation	medication reconciliation	medication reconciliation	Medication Reconciliation	4	case_or_format
medication reconciliation	medication reconciliation	medication reconciliation	medication reconciliation	Medication Reconciliation	4	case_or_format

melphalan	melphalan	melphalan	melphalan	melphalan	4	kept
memories	memories	memories	memory	memory	4	singular
Memory making	memory making	memory making	memory making	Memory making	4	kept
Meningitis, Meningococcal	meningitis meningococcal	meningitis meningococcal	meningitis meningococcal	Meningitis, Meningococcal	4	kept
Meningomyelocele	meningomyelocele	meningomyelocele	meningomyelocele	Meningomyelocele	4	kept
menstrual cycle	menstrual cycle	menstrual cycle	menstrual cycle	menstrual cycle	4	kept
mental fatigue	mental fatigue	mental fatigue	mental fatigue	Mental Fatigue	4	case_or_format
Mental Processes	mental processes	mental processes	mental processes	Mental Processes	4	kept
Menthol	menthol	menthol	menthol	Menthol	4	kept
Meperidine	meperidine	meperidine	meperidine	Meperidine	4	kept
mesenteric ischemia	mesenteric ischemia	mesenteric ischemia	mesenteric ischemia	Mesenteric Ischemia	4	case_or_format
metabolic alkalosis	metabolic alkalosis	metabolic alkalosis	metabolic alkalosis	metabolic alkalosis	4	kept
Metabolic Diseases	metabolic diseases	metabolic diseases	metabolic diseases	Metabolic Diseases	4	kept
Metabolic diseases	metabolic diseases	metabolic diseases	metabolic diseases	Metabolic Diseases	4	case_or_format
metagenomics	metagenomics	metagenomics	metagenomics	metagenomics	4	kept
methamphetamine	methamphetamine	methamphetamine	methamphetamine	methamphetamine	4	kept

methylene blue	methylene blue	methylene blue	methylene blue	methylene blue	4	kept
methylphenidate	methylphenidate	methylphenidate	methylphenidate	methylphenidate	4	kept
Metoclopramide	metoclopramide	metoclopramide	metoclopramide	metoclopramide	4	case_or_format
mevinolin	mevinolin	mevinolin	mevinolin	mevinolin	4	kept
MEWS	mews	mews	mews	MEWS	4	kept
microbial identification	microbial identification	microbial identification	microbial identification	microbial identification	4	kept
microbiologist	microbiologist	microbiologist	microbiologist	microbiologist	4	kept
micrognathia	micrognathia	micrognathia	micrognathia	micrognathia	4	kept
Microvascular Angina	microvascular angina	microvascular angina	microvascular angina	Microvascular Angina	4	kept
migraine	migraine	migraine	migraine	migraine	4	kept
migrant	migrant	migrant	migrant	migrant	4	kept
military	military	military	military	military	4	kept
Milk banks	milk banks	milk banks	milk bank	Milk Banks	4	singular
Mindfulness-based interventions	mindfulness based interventions	mindfulness based interventions	mindfulness based intervention	Mindfulness-based interventions	4	singular
MINI-MENTAL-STATE	mini mental state	mini mental state	mini mental state	MINI-MENTAL-STATE	4	kept
mining	mining	mining	mining	mining	4	kept

Mississippi	mississippi	mississippi	mississippi	Mississippi	4	kept
mitral valve disease	mitral valve disease	mitral valve disease	mitral valve disease	mitral valve disease	4	kept
mixed method	mixed method	mixed method	mixed method	Mixed methods	4	case_or_format
Mixed-methods research	mixed methods research	mixed methods research	mixed methods research	Mixed-methods research	4	kept
Mobile application	mobile application	mobile application	mobile application	Mobile Applications	4	case_or_format
Mobile applications	mobile applications	mobile applications	mobile application	Mobile Applications	4	singular
mobile health	mobile health	mobile health	mobile health	mobile health	4	kept
Model-based OPC	model based opc	model based opc	model based opc	Model-based OPC	4	kept
modeling	modeling	modeling	modeling	modeling	4	kept
models	models	models	model	model	4	singular
Models of care	models of care	models of care	models of care	Models of care	4	kept
models of care	models of care	models of care	models of care	Models of care	4	case_or_format
Models, Econometric	models econometric	models econometric	models econometric	Models, Econometric	4	kept
modified frailty index	modified frailty index	modified frailty index	modified frailty index	modified frailty index	4	kept
MODS	mods	mods	mods	MODS	4	kept
molecular biology	molecular biology	molecular biology	molecular biology	molecular biology	4	kept

moonlighting	moonlighting	moonlighting	moonlighting	moonlighting	4	kept
Moral courage	moral courage	moral courage	moral courage	moral courage	4	case_or_format
Moral Distress Scale	moral distress scale	moral distress scale	moral distress scale	Moral Distress Scale	4	kept
morning dosage	morning dosage	morning dosage	morning dosage	morning dosage	4	kept
MORPHINE	morphine	morphine	morphine	morphine	4	case_or_format
Mother-child relations	mother child relations	mother child relations	mother child relation	Mother-Child Relations	4	singular
Motion Therapy, Continuous Passive	motion therapy continuous passive	motion therapy continuous passive	motion therapy continuous passive	Motion Therapy, Continuous Passive	4	kept
motor development	motor development	motor development	motor development	motor development	4	kept
mouth examination	mouth examination	mouth examination	mouth examination	mouth examination	4	kept
mouth flora	mouth flora	mouth flora	mouth flora	mouth flora	4	kept
mouth tumor	mouth tumor	mouth tumor	mouth tumor	mouth tumor	4	kept
movement therapy	movement therapy	movement therapy	movement therapy	movement therapy	4	kept
mucolytic agent	mucolytic agent	mucolytic agent	mucolytic agent	mucolytic agent	4	kept
mucosal membrane pressure injury	mucosal membrane pressure injury	mucosal membrane pressure injury	mucosal membrane pressure injury	mucosal membrane pressure injury	4	kept
Muerte	muerte	muerte	muerte	Muerte	4	kept
Multi-disciplinary	multi disciplinary	multi disciplinary	multi disciplinary	Multi-disciplinary	4	kept

Multicenter study	multicenter study	multicenter study	multicenter study	multicenter study	4	case_or_format
Multidimensional approach	multidimensional approach	multidimensional approach	multidimensional approach	Multidimensional approach	4	kept
Multidisciplinary rounds	multidisciplinary rounds	multidisciplinary rounds	multidisciplinary round	Multidisciplinary rounds	4	singular
multidisciplinary teamwork	multidisciplinary teamwork	multidisciplinary teamwork	multidisciplinary teamwork	multidisciplinary teamwork	4	kept
multidrug resistant infection	multidrug resistant infection	multidrug resistant infection	multidrug resistant infection	multidrug resistant infection	4	kept
Multidrug-resistant bacteria	multidrug resistant bacteria	multidrug resistant bacteria	multidrug resistant bacteria	Multidrug-resistant bacteria	4	kept
multidrug-resistant organisms	multidrug resistant organisms	multidrug resistant organisms	multidrug resistant organism	Multidrug-resistant organisms	4	singular
Multimodal	multimodal	multimodal	multimodal	multimodal	4	case_or_format
multipara	multipara	multipara	multipara	multipara	4	kept
Multiple Sclerosis	multiple sclerosis	multiple sclerosis	multiple sclerosis	multiple sclerosis	4	case_or_format
mumps	mumps	mumps	mumps	mumps	4	kept
Munchausen syndrome by proxy	munchausen syndrome by proxy	munchausen syndrome by proxy	munchausen syndrome by proxy	Munchausen Syndrome by Proxy	4	case_or_format
muscarinic receptor blocking agent	muscarinic receptor blocking agent	muscarinic receptor blocking agent	muscarinic receptor blocking agent	muscarinic receptor blocking agent	4	kept
Muscle Hypotonia	muscle hypotonia	muscle hypotonia	muscle hypotonia	muscle hypotonia	4	case_or_format
Muscle Relaxation	muscle relaxation	muscle relaxation	muscle relaxation	muscle relaxation	4	case_or_format
muscle spasm	muscle spasm	muscle spasm	muscle spasm	muscle spasm	4	kept

Muscle strength	muscle strength	muscle strength	muscle strength	muscle strength	4	case_or_format
muscle thickness	muscle thickness	muscle thickness	muscle thickness	muscle thickness	4	kept
Musculoskeletal Pain	musculoskeletal pain	musculoskeletal pain	musculoskeletal pain	musculoskeletal pain	4	case_or_format
Musculoskeletal pain	musculoskeletal pain	musculoskeletal pain	musculoskeletal pain	musculoskeletal pain	4	case_or_format
MUSIC	music	music	music	Music	4	case_or_format
music intervention	music intervention	music intervention	music intervention	music intervention	4	kept
Mycological Typing Techniques	mycological typing techniques	mycological typing techniques	mycological typing techniques	Mycological Typing Techniques	4	kept
Myxedema	myxedema	myxedema	myxedema	Myxedema	4	kept
n methyl dextro aspartic acid receptor	n methyl dextro aspartic acid receptor	n methyl dextro aspartic acid receptor	n methyl dextro aspartic acid receptor	n methyl dextro aspartic acid receptor	4	kept
N95 Respirators	n95 respirators	n95 respirators	n95 respirator	N95 Respirators	4	singular
nafcillin	nafcillin	nafcillin	nafcillin	nafcillin	4	kept
Nails	nails	nails	nail	Nails	4	singular
Namibia	namibia	namibia	namibia	Namibia	4	kept
naproxen	naproxen	naproxen	naproxen	naproxen	4	kept
narrative medicine	narrative medicine	narrative medicine	narrative medicine	narrative medicine	4	kept
NASA-TLX	nasa tlx	nasa tlx	nasa tlx	NASA-TLX	4	kept

Nasal injury	nasal injury	nasal injury	nasal injury	Nasal injury	4	kept
nasal intermittent positive pressure ventilation	nasal intermittent positive pressure ventilation	nasal intermittent positive pressure ventilation	nasal intermittent positive pressure ventilation	nasal intermittent positive pressure ventilation	4	kept
Nasal Mucosa	nasal mucosa	nasal mucosa	nasal mucosa	Nasal Mucosa	4	kept
Nasopharynx	nasopharynx	nasopharynx	nasopharynx	nasopharynx	4	case_or_format
National Early Warning Score 2	national early warning score 2	national early warning score 2	national early warning score 2	National Early Warning Score 2	4	kept
NAtural language processing	natural language processing	natural language processing	natural language processing	natural language processing	4	case_or_format
naturalistic study	naturalistic study	naturalistic study	naturalistic study	naturalistic study	4	kept
near-death experiences	near death experiences	near death experiences	near death experience	near-death experiences	4	singular
NEC	nec	nec	nec	NEC	4	kept
Neck	neck	neck	neck	neck	4	case_or_format
Neck Pain	neck pain	neck pain	neck pain	neck pain	4	case_or_format
Needle stick injuries	needle stick injuries	needle stick injuries	needle stick injury	Needle stick injuries	4	singular
Negative pressure wound therapy	negative pressure wound therapy	negative pressure wound therapy	negative pressure wound therapy	Negative-Pressure Wound Therapy	4	case_or_format
negotiation	negotiation	negotiation	negotiation	negotiation	4	kept
Neonatal encephalopathy	neonatal encephalopathy	neonatal encephalopathy	neonatal encephalopathy	Neonatal encephalopathy	4	kept
Neonatal health	neonatal health	neonatal health	neonatal health	Neonatal health	4	kept

neonatal hyperbilirubinemia	neonatal hyperbilirubinemia	neonatal hyperbilirubinemia	neonatal hyperbilirubinemia	neonatal hyperbilirubinemia	4	kept
neonatal hypoglycemia	neonatal hypoglycemia	neonatal hypoglycemia	neonatal hypoglycemia	neonatal hypoglycemia	4	kept
neonatal opioid withdrawal syndrome	neonatal opioid withdrawal syndrome	neonatal opioid withdrawal syndrome	neonatal opioid withdrawal syndrome	neonatal opioid withdrawal syndrome	4	kept
neonatal pain assessment	neonatal pain assessment	neonatal pain assessment	neonatal pain assessment	neonatal pain assessment	4	kept
Neonatal sepsis	neonatal sepsis	neonatal sepsis	neonatal sepsis	Neonatal Sepsis	4	case_or_format
nephrectomy	nephrectomy	nephrectomy	nephrectomy	nephrectomy	4	kept
nephritis	nephritis	nephritis	nephritis	nephritis	4	kept
neurodisability	neurodisability	neurodisability	neurodisability	neurodisability	4	kept
Neuroleptic Malignant Syndrome	neuroleptic malignant syndrome	neuroleptic malignant syndrome	neuroleptic malignant syndrome	neuroleptic malignant syndrome	4	case_or_format
Neurological examination	neurological examination	neurological examination	neurological examination	Neurological examination	4	kept
neuron specific enolase	neuron specific enolase	neuron specific enolase	neuron specific enolase	neuron specific enolase	4	kept
neuropathology	neuropathology	neuropathology	neuropathology	neuropathology	4	kept
Neurophysiology	neurophysiology	neurophysiology	neurophysiology	neurophysiology	4	case_or_format
neuropsychological assessment	neuropsychological assessment	neuropsychological assessment	neuropsychological assessment	neuropsychological assessment	4	kept
neuroradiologist	neuroradiologist	neuroradiologist	neuroradiologist	neuroradiologist	4	kept
Neuroscience	neuroscience	neuroscience	neuroscience	neuroscience	4	case_or_format

neurosciences	neurosciences	neurosciences	neuroscience	neuroscience	4	singular
Neurotransmitter Agents	neurotransmitter agents	neurotransmitter agents	neurotransmitter agents	Neurotransmitter Agents	4	kept
Neurovascular unit	neurovascular unit	neurovascular unit	neurovascular unit	Neurovascular unit	4	kept
New Hampshire	new hampshire	new hampshire	new hampshire	New Hampshire	4	kept
newborn resuscitation	newborn resuscitation	newborn resuscitation	newborn resuscitation	newborn resuscitation	4	kept
Newfoundland and Labrador	newfoundland and labrador	newfoundland and labrador	newfoundland and labrador	Newfoundland and Labrador	4	kept
NEWS	news	news	news	NEWS	4	kept
Nicotinic Agonists	nicotinic agonists	nicotinic agonists	nicotinic agonists	Nicotinic Agonists	4	kept
night care	night care	night care	night care	Night Care	4	case_or_format
night sweat	night sweat	night sweat	night sweat	night sweat	4	kept
Nimodipine	nimodipine	nimodipine	nimodipine	nimodipine	4	case_or_format
NIPS	nips	nips	nips	NIPS	4	kept
NIRS	nirs	nirs	nirs	NIRS	4	kept
nitrate	nitrate	nitrate	nitrate	nitrate	4	kept
nitric acid derivative	nitric acid derivative	nitric acid derivative	nitric acid derivative	nitric acid derivative	4	kept
nitrofurantoin	nitrofurantoin	nitrofurantoin	nitrofurantoin	nitrofurantoin	4	kept

nivolumab	nivolumab	nivolumab	nivolumab	nivolumab	4	kept
NOISE	noise	noise	noise	Noise	4	case_or_format
Noise source	noise source	noise source	noise source	Noise source	4	kept
Noisy environment	noisy environment	noisy environment	noisy environment	Noisy environment	4	kept
Nomogram	nomogram	nomogram	nomogram	nomogram	4	case_or_format
non participant observation	non participant observation	non participant observation	non participant observation	non participant observation	4	kept
non prescription drug	non prescription drug	non prescription drug	non prescription drug	non prescription drug	4	kept
non profit hospital	non profit hospital	non profit hospital	non profit hospital	non profit hospital	4	kept
Non-compliance	non compliance	non compliance	non compliance	Non-compliance	4	kept
Non-invasive	non invasive	non invasive	non invasive	Non-invasive	4	kept
non-verbal communication	non verbal communication	non verbal communication	non verbal communication	non-verbal communication	4	kept
normal distribution	normal distribution	normal distribution	normal distribution	Normal Distribution	4	case_or_format
North American	north american	north american	north american	North American	4	kept
North Dakota	north dakota	north dakota	north dakota	North Dakota	4	kept
nortriptyline	nortriptyline	nortriptyline	nortriptyline	nortriptyline	4	kept
nose septum	nose septum	nose septum	nose septum	nose septum	4	kept

Notification	notification	notification	notification	Notification	4	kept
NPC	npc	npc	npc	NPC	4	kept
null result	null result	null result	null result	null result	4	kept
nutrient	nutrient	nutrient	nutrient	nutrient	4	kept
Nutrition Policy	nutrition policy	nutrition policy	nutrition policy	Nutrition Policy	4	kept
Nutrition Surveys	nutrition surveys	nutrition surveys	nutrition surveys	Nutrition Surveys	4	kept
Nutritional care	nutritional care	nutritional care	nutritional care	Nutritional care	4	kept
nutritional value	nutritional value	nutritional value	nutritional value	nutritional value	4	kept
Nutritionists	nutritionists	nutritionists	nutritionists	Nutritionists	4	kept
obesity hypoventilation syndrome	obesity hypoventilation syndrome	obesity hypoventilation syndrome	obesity hypoventilation syndrome	obesity hypoventilation syndrome	4	kept
Object detection	object detection	object detection	object detection	Object detection	4	kept
Observational studies	observational studies	observational studies	observational study	observational study	4	singular
OBSTACLES	obstacles	obstacles	obstacles	Obstacles	4	case_or_format
obstetric care	obstetric care	obstetric care	obstetric care	obstetric care	4	kept
occupational & industrial medicine	occupational and industrial medicine	occupational and industrial medicine	occupational and industrial medicine	occupational & industrial medicine	4	kept
occupational medicine	occupational medicine	occupational medicine	occupational medicine	occupational medicine	4	kept

OF-CARE	of care	of care	of care	OF-CARE	4	kept
Oldenburg Burnout Inventory	oldenburg burnout inventory	oldenburg burnout inventory	oldenburg burnout inventory	Oldenburg Burnout Inventory	4	kept
omalizumab	omalizumab	omalizumab	omalizumab	omalizumab	4	kept
Omicron	omicron	omicron	omicron	Omicron	4	kept
Omicron variant	omicron variant	omicron variant	omicron variant	Omicron variant	4	kept
omphalocele	omphalocele	omphalocele	omphalocele	omphalocele	4	kept
ONCOLOGY	oncology	oncology	oncology	oncology	4	case_or_format
oncotic pressure	oncotic pressure	oncotic pressure	oncotic pressure	oncotic pressure	4	kept
online education	online education	online education	online education	online education	4	kept
open abdomen	open abdomen	open abdomen	open abdomen	open abdomen	4	kept
open access	open access	open access	open access	open access	4	kept
Operating Room Information Systems	operating room information systems	operating room information systems	operating room information systems	Operating Room Information Systems	4	kept
operations research	operations research	operations research	operations research	Operations research	4	case_or_format
Ophthalmology	ophthalmology	ophthalmology	ophthalmology	ophthalmology	4	case_or_format
opiate derivative	opiate derivative	opiate derivative	opiate derivative	opiate derivative	4	kept
Opioid use disorder	opioid use disorder	opioid use disorder	opioid use disorder	Opioid use disorder	4	kept

Opportunistic Infections	opportunistic infections	opportunistic infections	opportunistic infection	opportunistic infection	4	singular
optimal drug dose	optimal drug dose	optimal drug dose	optimal drug dose	optimal drug dose	4	kept
oral surgery	oral surgery	oral surgery	oral surgery	oral surgery	4	kept
ORGAN DONATION	organ donation	organ donation	organ donation	Organ donation	4	case_or_format
organ injury	organ injury	organ injury	organ injury	organ injury	4	kept
organ perfusion	organ perfusion	organ perfusion	organ perfusion	organ perfusion	4	kept
organ systems	organ systems	organ systems	organ systems	organ systems	4	kept
Organisational culture	organisational culture	organisational culture	organisational culture	Organisational culture	4	kept
Organizational	organizational	organizational	organizational	Organizational	4	kept
Organophosphate Poisoning	organophosphate poisoning	organophosphate poisoning	organophosphate poisoning	Organophosphate Poisoning	4	kept
osteopenia	osteopenia	osteopenia	osteopenia	osteopenia	4	kept
Osteoporotic Fractures	osteoporotic fractures	osteoporotic fractures	osteoporotic fractures	Osteoporotic Fractures	4	kept
Oswestry Disability Index	oswestry disability index	oswestry disability index	oswestry disability index	Oswestry Disability Index	4	kept
otitis media	otitis media	otitis media	otitis media	otitis media	4	kept
otolaryngology	otolaryngology	otolaryngology	otolaryngology	Otolaryngology	4	case_or_format
outcome measures	outcome measures	outcome measures	outcome measure	outcome measures	4	singular

outreach	outreach	outreach	outreach	outreach	4	kept
overcrowding	overcrowding	overcrowding	overcrowding	Overcrowding	4	case_or_format
overtime	overtime	overtime	overtime	overtime	4	kept
Oxigenación por membrana extracorpórea	oxigenaci n por membrana extracorp rea	oxigenaci n por membrana extracorp rea	oxigenaci n por membrana extracorp rea	Oxigenación por membrana extracorpórea	4	kept
Oxycodone	oxycodone	oxycodone	oxycodone	oxycodone	4	case_or_format
oxygen delivery device	oxygen delivery device	oxygen delivery device	oxygen delivery device	oxygen delivery device	4	kept
Oxygenators, Membrane	oxygenators membrane	oxygenators membrane	oxygenators membrane	Oxygenators, Membrane	4	kept
P wave	p wave	p wave	p wave	P wave	4	kept
Paediatric surgery	paediatric surgery	paediatric surgery	paediatric surgery	Paediatric surgery	4	kept
PAEDIATRICS	paediatrics	paediatrics	paediatric	Paediatric	4	singular
Pain Assessment	pain assessment	pain assessment	pain assessment	pain assessment	4	case_or_format
Pain Threshold	pain threshold	pain threshold	pain threshold	pain threshold	4	case_or_format
Palliative sedation	palliative sedation	palliative sedation	palliative sedation	Palliative sedation	4	kept
pamidronic acid	pamidronic acid	pamidronic acid	pamidronic acid	pamidronic acid	4	kept
pancreatectomy	pancreatectomy	pancreatectomy	pancreatectomy	Pancreatectomy	4	case_or_format
pancreaticoduodenectomy	pancreaticoduodenectomy	pancreaticoduodenectomy	pancreaticoduodenectomy	Pancreaticoduodenectomy	4	case_or_format

Pancreatitis, Acute Necrotizing	pancreatitis acute necrotizing	pancreatitis acute necrotizing	pancreatitis acute necrotizing	Pancreatitis, Acute Necrotizing	4	kept
pancytopenia	pancytopenia	pancytopenia	pancytopenia	pancytopenia	4	kept
pandemic influenza	pandemic influenza	pandemic influenza	pandemic influenza	pandemic influenza	4	kept
Papua New Guinea	papua new guinea	papua new guinea	papua new guinea	Papua New Guinea	4	kept
papule	papule	papule	papule	papule	4	kept
parameter	parameter	parameter	parameter	parameters	4	case_or_format
Parent satisfaction	parent satisfaction	parent satisfaction	parent satisfaction	Parent satisfaction	4	kept
parent satisfaction	parent satisfaction	parent satisfaction	parent satisfaction	Parent satisfaction	4	case_or_format
Parental support	parental support	parental support	parental support	Parental support	4	kept
Parenthood	parenthood	parenthood	parenthood	parenthood	4	case_or_format
parietal lobe	parietal lobe	parietal lobe	parietal lobe	parietal lobe	4	kept
Parkinson's disease	parkinson s disease	parkinson s disease	parkinson s disease	Parkinson's disease	4	kept
parkinsonism	parkinsonism	parkinsonism	parkinsonism	parkinsonism	4	kept
Parkinson' s disease	parkinson s disease	parkinson s disease	parkinson s disease	Parkinson's disease	4	case_or_format
participant	participant	participant	participant	participant	4	kept
Participant observation	participant observation	participant observation	participant observation	participant observation	4	case_or_format

Participation	participation	participation	participation	participation	4	case_or_format
parttime employment	parttime employment	parttime employment	parttime employment	parttime employment	4	kept
passive movement	passive movement	passive movement	passive movement	passive movement	4	kept
pasteurization	pasteurization	pasteurization	pasteurization	pasteurization	4	kept
pattern recognition	pattern recognition	pattern recognition	pattern recognition	pattern recognition	4	kept
Pattern Recognition, Automated	pattern recognition automated	pattern recognition automated	pattern recognition automated	Pattern Recognition, Automated	4	kept
pediatric early warning score	pediatric early warning score	pediatric early warning score	pediatric early warning score	Pediatric Early Warning Score	4	case_or_format
Peer Review, Research	peer review research	peer review research	peer review research	Peer Review, Research	4	kept
pelvic examination	pelvic examination	pelvic examination	pelvic examination	pelvic examination	4	kept
Pelvic Inflammatory Disease	pelvic inflammatory disease	pelvic inflammatory disease	pelvic inflammatory disease	Pelvic Inflammatory Disease	4	kept
pelvis abscess	pelvis abscess	pelvis abscess	pelvis abscess	pelvis abscess	4	kept
pelvis radiography	pelvis radiography	pelvis radiography	pelvis radiography	pelvis radiography	4	kept
penicillin allergy	penicillin allergy	penicillin allergy	penicillin allergy	penicillin allergy	4	kept
pension	pension	pension	pension	pension	4	kept
Pentobarbital	pentobarbital	pentobarbital	pentobarbital	pentobarbital	4	case_or_format
Perceived organizational support	perceived organizational support	perceived organizational support	perceived organizational support	Perceived organizational support	4	kept

Percepción	percepci n	percepci n	percepci n	Percepción	4	kept
PERCUTANEOUS ENDOSCOPIC GASTROSTOMY	percutaneous endoscopic gastrostomy	percutaneous endoscopic gastrostomy	percutaneous endoscopic gastrostomy	percutaneous endoscopic gastrostomy	4	case_or_format
percutaneous transluminal angioplasty	percutaneous transluminal angioplasty	percutaneous transluminal angioplasty	percutaneous transluminal angioplasty	percutaneous transluminal angioplasty	4	kept
perforation	perforation	perforation	perforation	perforation	4	kept
performance assessment	performance assessment	performance assessment	performance assessment	performance assessment	4	kept
Performance improvement	performance improvement	performance improvement	performance improvement	Performance improvement	4	kept
performance measures	performance measures	performance measures	performance measure	performance measures	4	singular
perianesthesia	perianesthesia	perianesthesia	perianesthesia	perianesthesia	4	kept
Pericardiocentesis	pericardiocentesis	pericardiocentesis	pericardiocentesis	pericardiocentesis	4	case_or_format
Perinatal care	perinatal care	perinatal care	perinatal care	Perinatal Care	4	case_or_format
Perinatal death	perinatal death	perinatal death	perinatal death	Perinatal Death	4	case_or_format
perinatal development	perinatal development	perinatal development	perinatal development	perinatal development	4	kept
Perinatal palliative care	perinatal palliative care	perinatal palliative care	perinatal palliative care	Perinatal palliative care	4	kept
Periodontal Diseases	periodontal diseases	periodontal diseases	periodontal disease	Periodontal Diseases	4	singular
periodontitis	periodontitis	periodontitis	periodontitis	periodontitis	4	kept
Perioperative period	perioperative period	perioperative period	perioperative period	perioperative period	4	case_or_format

peripheral	peripheral	peripheral	peripheral	Peripheral	4	case_or_format
Peripheral intravenous catheter	peripheral intravenous catheter	peripheral intravenous catheter	peripheral intravenous catheter	Peripheral intravenous catheter	4	kept
Peripheral intravenous catheters	peripheral intravenous catheters	peripheral intravenous catheters	peripheral intravenous catheter	Peripheral intravenous catheter	4	singular
peripheral venous catheterization	peripheral venous catheterization	peripheral venous catheterization	peripheral venous catheterization	peripheral venous catheterization	4	kept
periviability	periviability	periviability	periviability	periviability	4	kept
personal needs	personal needs	personal needs	personal needs	personal needs	4	kept
Personal Space	personal space	personal space	personal space	Personal Space	4	kept
PERSONALITY	personality	personality	personality	personality	4	case_or_format
PERSONNEL	personnel	personnel	personnel	Personnel	4	case_or_format
Personnel staffing	personnel staffing	personnel staffing	personnel staffing	personnel staffing	4	case_or_format
Persuasive Communication	persuasive communication	persuasive communication	persuasive communication	persuasive communication	4	case_or_format
Pertussis Vaccine	pertussis vaccine	pertussis vaccine	pertussis vaccine	pertussis vaccine	4	case_or_format
petechia	petechia	petechia	petechia	petechia	4	kept
Petrolatum	petrolatum	petrolatum	petrolatum	petrolatum	4	case_or_format
Pharmaceutical services	pharmaceutical services	pharmaceutical services	pharmaceutical service	Pharmaceutical services	4	singular
pharmacoepidemiology	pharmacoepidemiology	pharmacoepidemiology	pharmacoepidemiology	pharmacoepidemiology	4	kept

Pharmacogenetics	pharmacogenetics	pharmacogenetics	pharmacogenetics	Pharmacogenetics	4	kept
pharmacogenomics	pharmacogenomics	pharmacogenomics	pharmacogenomics	pharmacogenomics	4	kept
pharmacy practice	pharmacy practice	pharmacy practice	pharmacy practice	pharmacy practice	4	kept
Pharmacy Technicians	pharmacy technicians	pharmacy technicians	pharmacy technician	pharmacy technician	4	singular
Pharmacy, institutional, hospital	pharmacy institutional hospital	pharmacy institutional hospital	pharmacy institutional hospital	Pharmacy, institutional, hospital	4	kept
pharynx	pharynx	pharynx	pharynx	Pharynx	4	case_or_format
Phenomenological study	phenomenological study	phenomenological study	phenomenological study	Phenomenological study	4	kept
phenomenological study	phenomenological study	phenomenological study	phenomenological study	Phenomenological study	4	case_or_format
Phenylephrine	phenylephrine	phenylephrine	phenylephrine	phenylephrine	4	case_or_format
pheochromocytoma	pheochromocytoma	pheochromocytoma	pheochromocytoma	pheochromocytoma	4	kept
Phonation	phonation	phonation	phonation	Phonation	4	kept
photoelectric plethysmography	photoelectric plethysmography	photoelectric plethysmography	photoelectric plethysmography	photoelectric plethysmography	4	kept
Photoperiod	photoperiod	photoperiod	photoperiod	Photoperiod	4	kept
photoplethysmography	photoplethysmography	photoplethysmography	photoplethysmography	photoplethysmography	4	kept
physical appearance	physical appearance	physical appearance	physical appearance	physical appearance	4	kept
physical environment	physical environment	physical environment	physical environment	physical environment	4	kept

physical functioning	physical functioning	physical functioning	physical functioning	physical functioning	4	kept
physical health	physical health	physical health	physical health	physical health	4	kept
physical therapy modalities	physical therapy modalities	physical therapy modalities	physical therapy modalities	Physical Therapy Modalities	4	case_or_format
physical therapy specialty	physical therapy specialty	physical therapy specialty	physical therapy specialty	Physical Therapy Specialty	4	case_or_format
PHYSICAL-ACTIVITY	physical activity	physical activity	physical activity	physical activity	4	case_or_format
Physician assistants	physician assistants	physician assistants	physician assistant	physician assistant	4	singular
PHYSICIAN-ASSISTED SUICIDE	physician assisted suicide	physician assisted suicide	physician assisted suicide	PHYSICIAN-ASSISTED SUICIDE	4	kept
physiologic	physiologic	physiologic	physiologic	Physiologic	4	case_or_format
physiological parameters	physiological parameters	physiological parameters	physiological parameter	Physiological parameters	4	singular
physiological process	physiological process	physiological process	physiological process	physiological process	4	kept
phytomenadione	phytomenadione	phytomenadione	phytomenadione	phytomenadione	4	kept
piracetam	piracetam	piracetam	piracetam	piracetam	4	kept
piritramide	piritramide	piritramide	piritramide	piritramide	4	kept
place of birth	place of birth	place of birth	place of birth	place of birth	4	kept
Place of death	place of death	place of death	place of death	place of death	4	case_or_format
placental transfer	placental transfer	placental transfer	placental transfer	placental transfer	4	kept

plague	plague	plague	plague	plague	4	kept
Plant Extracts	plant extracts	plant extracts	plant extract	Plant Extracts	4	singular
Plasma Substitutes	plasma substitutes	plasma substitutes	plasma substitute	Plasma Substitutes	4	singular
plastic waste	plastic waste	plastic waste	plastic waste	plastic waste	4	kept
pleura cavity	pleura cavity	pleura cavity	pleura cavity	pleura cavity	4	kept
Pneumocystis pneumonia	pneumocystis pneumonia	pneumocystis pneumonia	pneumocystis pneumonia	Pneumocystis pneumonia	4	kept
Pneumonia, ventilator-associated	pneumonia ventilator associated	pneumonia ventilator associated	pneumonia ventilator associated	Pneumonia, Ventilator-Associated	4	case_or_format
point of care	point of care	point of care	point of care	point of care	4	kept
point-of-care	point of care	point of care	point of care	point of care	4	case_or_format
poisoning	poisoning	poisoning	poisoning	Poisoning	4	case_or_format
Poisson regression	poisson regression	poisson regression	poisson regression	Poisson regression	4	kept
POLICIES	policies	policies	policy	policy	4	singular
pollution	pollution	pollution	pollution	pollution	4	kept
polyethylene	polyethylene	polyethylene	polyethylene	Polyethylene	4	case_or_format
polyethylene derivative	polyethylene derivative	polyethylene derivative	polyethylene derivative	polyethylene derivative	4	kept
Polyethylenes	polyethylenes	polyethylenes	polyethylene	Polyethylene	4	singular

polytrauma	polytrauma	polytrauma	polytrauma	polytrauma	4	kept
portal vein thrombosis	portal vein thrombosis	portal vein thrombosis	portal vein thrombosis	portal vein thrombosis	4	kept
Portuguese	portuguese	portuguese	portuguese	Portuguese	4	kept
position	position	position	position	position	4	kept
POSITIVE AIRWAY PRESSURE	positive airway pressure	positive airway pressure	positive airway pressure	POSITIVE AIRWAY PRESSURE	4	kept
Positive correlations	positive correlations	positive correlations	positive correlations	Positive correlations	4	kept
post-operative	post operative	post operative	post operative	post-operative	4	kept
post-traumatic	post traumatic	post traumatic	post traumatic	Post-traumatic	4	case_or_format
Postgraduate education	postgraduate education	postgraduate education	postgraduate education	postgraduate education	4	case_or_format
postmaturity	postmaturity	postmaturity	postmaturity	postmaturity	4	kept
Postmodernism	postmodernism	postmodernism	postmodernism	Postmodernism	4	kept
postoperative ileus	postoperative ileus	postoperative ileus	postoperative ileus	postoperative ileus	4	kept
postoperative outcomes	postoperative outcomes	postoperative outcomes	postoperative outcome	postoperative outcomes	4	singular
postpartum care	postpartum care	postpartum care	postpartum care	postpartum care	4	kept
postpartum period	postpartum period	postpartum period	postpartum period	Postpartum Period	4	case_or_format
power analysis	power analysis	power analysis	power analysis	power analysis	4	kept

power relations	power relations	power relations	power relations	power relations	4	kept
Practice Management, Medical	practice management medical	practice management medical	practice management medical	Practice Management, Medical	4	kept
practice patterns	practice patterns	practice patterns	practice pattern	practice patterns	4	singular
prasugrel	prasugrel	prasugrel	prasugrel	prasugrel	4	kept
Pre-term	pre term	pre term	pre term	Pre-term	4	kept
Prealbumin	prealbumin	prealbumin	prealbumin	prealbumin	4	case_or_format
precision medicine	precision medicine	precision medicine	precision medicine	Precision Medicine	4	case_or_format
Predictive analytics	predictive analytics	predictive analytics	predictive analytics	predictive analytics	4	case_or_format
PREDICTIVE-VALIDITY	predictive validity	predictive validity	predictive validity	predictive validity	4	case_or_format
Pregnant Women	pregnant women	pregnant women	pregnant women	Pregnant Women	4	kept
prehospital	prehospital	prehospital	prehospital	Prehospital	4	case_or_format
Prehospital emergency care	prehospital emergency care	prehospital emergency care	prehospital emergency care	Prehospital emergency care	4	kept
prejudice	prejudice	prejudice	prejudice	Prejudice	4	case_or_format
Premature Infant	premature infant	premature infant	premature infant	Premature infant	4	case_or_format
premature rupture of membranes	premature rupture of membranes	premature rupture of membranes	premature rupture of membranes	premature rupture of membranes	4	kept
prenatal drug exposure	prenatal drug exposure	prenatal drug exposure	prenatal drug exposure	prenatal drug exposure	4	kept

prenatal exposure	prenatal exposure	prenatal exposure	prenatal exposure	prenatal exposure	4	kept
prenatal screening	prenatal screening	prenatal screening	prenatal screening	prenatal screening	4	kept
preoperative education	preoperative education	preoperative education	preoperative education	preoperative education	4	kept
PRESCRIPTION ERRORS	prescription errors	prescription errors	prescription error	PRESCRIPTION ERRORS	4	singular
Prescriptions	prescriptions	prescriptions	prescription	prescription	4	singular
pressure transducer	pressure transducer	pressure transducer	pressure transducer	pressure transducer	4	kept
Preterm neonate	preterm neonate	preterm neonate	preterm neonate	Preterm neonates	4	case_or_format
Preterm newborn	preterm newborn	preterm newborn	preterm newborn	Preterm newborn	4	kept
Prevention guidelines	prevention guidelines	prevention guidelines	prevention guidelines	Prevention guidelines	4	kept
Prevention measures	prevention measures	prevention measures	prevention measure	Prevention measures	4	singular
Primary health care	primary health care	primary health care	primary health care	Primary Health Care	4	case_or_format
Prioritization	prioritization	prioritization	prioritization	Prioritization	4	kept
proadrenomedullin	proadrenomedullin	proadrenomedullin	proadrenomedullin	proadrenomedullin	4	kept
procainamide	procainamide	procainamide	procainamide	procainamide	4	kept
Procalcitonin	procalcitonin	procalcitonin	procalcitonin	procalcitonin	4	case_or_format
procedure	procedure	procedure	procedure	procedures	4	case_or_format

Procedures	procedures	procedures	procedure	procedures	4	singular
Process	process	process	process	Process	4	kept
process design	process design	process design	process design	process design	4	kept
process evaluation	process evaluation	process evaluation	process evaluation	Process evaluation	4	case_or_format
process mapping	process mapping	process mapping	process mapping	process mapping	4	kept
Procrastination	procrastination	procrastination	procrastination	Procrastination	4	kept
PROFESSION	profession	profession	profession	PROFESSION	4	kept
Professional boundaries	professional boundaries	professional boundaries	professional boundaries	Professional boundaries	4	kept
professional commitment	professional commitment	professional commitment	professional commitment	professional commitment	4	kept
Professional competency	professional competency	professional competency	professional competency	Professional competency	4	kept
professional quality of life scale	professional quality of life scale	professional quality of life scale	professional quality of life scale	professional quality of life scale	4	kept
progesterone	progesterone	progesterone	progesterone	progesterone	4	kept
prognostic factors	prognostic factors	prognostic factors	prognostic factor	prognostic factors	4	singular
program appropriateness	program appropriateness	program appropriateness	program appropriateness	program appropriateness	4	kept
Program evaluation	program evaluation	program evaluation	program evaluation	Program Evaluation	4	case_or_format
progression free survival	progression free survival	progression free survival	progression free survival	progression free survival	4	kept

Prolonged weaning	prolonged weaning	prolonged weaning	prolonged weaning	Prolonged weaning	4	kept
proof of concept	proof of concept	proof of concept	proof of concept	proof of concept	4	kept
Proof of Concept Study	proof of concept study	proof of concept study	proof of concept study	Proof of Concept Study	4	kept
propensity score matching	propensity score matching	propensity score matching	propensity score matching	propensity score matching	4	kept
proprioception	proprioception	proprioception	proprioception	proprioception	4	kept
Prospective	prospective	prospective	prospective	Prospective	4	kept
Prospective study	prospective study	prospective study	prospective study	Prospective Studies	4	case_or_format
Prostatic Neoplasms	prostatic neoplasms	prostatic neoplasms	prostatic neoplasm	Prostatic Neoplasms	4	singular
prosthesis complication	prosthesis complication	prosthesis complication	prosthesis complication	prosthesis complication	4	kept
Prosthesis Failure	prosthesis failure	prosthesis failure	prosthesis failure	Prosthesis Failure	4	kept
Protection	protection	protection	protection	protection	4	case_or_format
protein synthesis	protein synthesis	protein synthesis	protein synthesis	protein synthesis	4	kept
protein urine level	protein urine level	protein urine level	protein urine level	protein urine level	4	kept
prothrombin	prothrombin	prothrombin	prothrombin	prothrombin	4	kept
provider	provider	provider	provider	PROVIDERS	4	case_or_format
proxymetacaine	proxymetacaine	proxymetacaine	proxymetacaine	proxymetacaine	4	kept

psoas muscle	psoas muscle	psoas muscle	psoas muscle	psoas muscle	4	kept
Psoriasis	psoriasis	psoriasis	psoriasis	psoriasis	4	case_or_format
psychoeducation	psychoeducation	psychoeducation	psychoeducation	psychoeducation	4	kept
psychological experiences	psychological experiences	psychological experiences	psychological experience	psychological experiences	4	singular
psychological impact	psychological impact	psychological impact	psychological impact	psychological impact	4	kept
psychological outcomes	psychological outcomes	psychological outcomes	psychological outcome	psychological outcomes	4	singular
Psychological status	psychological status	psychological status	psychological status	Psychological status	4	kept
Psychological well-being	psychological well being	psychological well being	psychological well being	psychological well-being	4	case_or_format
Psychologist	psychologist	psychologist	psychologist	psychologist	4	case_or_format
psychometric evaluation	psychometric evaluation	psychometric evaluation	psychometric evaluation	PSYCHOMETRIC EVALUATION	4	case_or_format
Psychosocial care	psychosocial care	psychosocial care	psychosocial care	psychosocial care	4	case_or_format
psychosocial disorder	psychosocial disorder	psychosocial disorder	psychosocial disorder	psychosocial disorder	4	kept
psychosocial factors	psychosocial factors	psychosocial factors	psychosocial factors	psychosocial factors	4	kept
psychosocial impact	psychosocial impact	psychosocial impact	psychosocial impact	psychosocial impact	4	kept
Psychosocial Intervention	psychosocial intervention	psychosocial intervention	psychosocial intervention	psychosocial intervention	4	case_or_format
psychosocial support	psychosocial support	psychosocial support	psychosocial support	psychosocial support	4	kept

psychosocial withdrawal	psychosocial withdrawal	psychosocial withdrawal	psychosocial withdrawal	psychosocial withdrawal	4	kept
psychosomatic disorder	psychosomatic disorder	psychosomatic disorder	psychosomatic disorder	psychosomatic disorder	4	kept
public figure	public figure	public figure	public figure	public figure	4	kept
PUBLIC HEALTH	public health	public health	public health	public health	4	case_or_format
Public Health Surveillance	public health surveillance	public health surveillance	public health surveillance	Public Health Surveillance	4	kept
Puerto Rico	puerto rico	puerto rico	puerto rico	Puerto Rico	4	kept
Pulmonary Alveolar Proteinosis	pulmonary alveolar proteinosis	pulmonary alveolar proteinosis	pulmonary alveolar proteinosis	Pulmonary Alveolar Proteinosis	4	kept
Pulmonary Circulation	pulmonary circulation	pulmonary circulation	pulmonary circulation	Pulmonary Circulation	4	kept
Pulmonary hypertension	pulmonary hypertension	pulmonary hypertension	pulmonary hypertension	pulmonary hypertension	4	case_or_format
Pulmonary rehabilitation	pulmonary rehabilitation	pulmonary rehabilitation	pulmonary rehabilitation	pulmonary rehabilitation	4	case_or_format
pulmonary valve atresia	pulmonary valve atresia	pulmonary valve atresia	pulmonary valve atresia	pulmonary valve atresia	4	kept
pulmonary valve stenosis	pulmonary valve stenosis	pulmonary valve stenosis	pulmonary valve stenosis	pulmonary valve stenosis	4	kept
Pulmonary ventilation	pulmonary ventilation	pulmonary ventilation	pulmonary ventilation	Pulmonary Ventilation	4	case_or_format
pupil diameter	pupil diameter	pupil diameter	pupil diameter	pupil diameter	4	kept
Purines	purines	purines	purines	Purines	4	kept
qSOFA	qsofa	qsofa	qsofa	qSOFA	4	kept

Qualitative approach	qualitative approach	qualitative approach	qualitative approach	qualitative approaches	4	case_or_format
qualitative approach	qualitative approach	qualitative approach	qualitative approach	qualitative approaches	4	case_or_format
Qualitative approaches	qualitative approaches	qualitative approaches	qualitative approach	qualitative approaches	4	singular
Qualitative design	qualitative design	qualitative design	qualitative design	qualitative design	4	case_or_format
Qualitative synthesis	qualitative synthesis	qualitative synthesis	qualitative synthesis	Qualitative synthesis	4	kept
qualitative synthesis	qualitative synthesis	qualitative synthesis	qualitative synthesis	Qualitative synthesis	4	case_or_format
quality assessment	quality assessment	quality assessment	quality assessment	Quality assessment	4	case_or_format
Quality improvement project	quality improvement project	quality improvement project	quality improvement project	quality improvement project	4	case_or_format
quality In health care	quality in health care	quality in health care	quality in health care	quality in health care	4	case_or_format
Quality indicator	quality indicator	quality indicator	quality indicator	Quality indicators	4	case_or_format
quality management	quality management	quality management	quality management	Quality management	4	case_or_format
Quality of Care	quality of care	quality of care	quality of care	Quality of care	4	case_or_format
quality of work life	quality of work life	quality of work life	quality of work life	Quality of work life	4	case_or_format
Quantitative	quantitative	quantitative	quantitative	quantitative	4	case_or_format
Race	race	race	race	race	4	case_or_format
Radiation	radiation	radiation	radiation	Radiation	4	kept

radiation	radiation	radiation	radiation	Radiation	4	case_or_format
Radiation Dosage	radiation dosage	radiation dosage	radiation dosage	Radiation Dosage	4	kept
radiofrequency ablation	radiofrequency ablation	radiofrequency ablation	radiofrequency ablation	radiofrequency ablation	4	kept
Radiography, Interventional	radiography interventional	radiography interventional	radiography interventional	Radiography, Interventional	4	kept
radiological procedures	radiological procedures	radiological procedures	radiological procedures	radiological procedures	4	kept
Radiologists	radiologists	radiologists	radiologist	radiologist	4	singular
radiopharmaceutical agent	radiopharmaceutical agent	radiopharmaceutical agent	radiopharmaceutical agent	radiopharmaceutical agent	4	kept
Rajasthan	rajasthan	rajasthan	rajasthan	Rajasthan	4	kept
Random Amplified Polymorphic DNA Technique	random amplified polymorphic dna technique	random amplified polymorphic dna technique	random amplified polymorphic dna technique	Random Amplified Polymorphic DNA Technique	4	kept
Randomized	randomized	randomized	randomized	Randomized	4	kept
Rapid Response Team	rapid response team	rapid response team	rapid response team	rapid response team	4	case_or_format
rapid review	rapid review	rapid review	rapid review	rapid review	4	kept
Rapid Sequence Induction and Intubation	rapid sequence induction and intubation	rapid sequence induction and intubation	rapid sequence induction and intubation	Rapid Sequence Induction and Intubation	4	kept
Rationing	rationing	rationing	rationing	Rationing	4	kept
RCSQ	rcsq	rcsq	rcsq	RCSQ	4	kept
readiness	readiness	readiness	readiness	Readiness	4	case_or_format

Receptors, Antigen, T-Cell	receptors antigen t cell	receptors antigen t cell	receptors antigen t cell	Receptors, Antigen, T-Cell	4	kept
recertification	recertification	recertification	recertification	recertification	4	kept
Records as Topic	records as topic	records as topic	records as topic	Records as Topic	4	kept
rectal temperature	rectal temperature	rectal temperature	rectal temperature	rectal temperature	4	kept
recurrent neural network	recurrent neural network	recurrent neural network	recurrent neural network	recurrent neural network	4	kept
Recycling	recycling	recycling	recycling	recycling	4	case_or_format
Redeployment	redeployment	redeployment	redeployment	redeployment	4	case_or_format
Referral	referral	referral	referral	referral	4	case_or_format
referral and consultation	referral and consultation	referral and consultation	referral and consultation	Referral and Consultation	4	case_or_format
reflection	reflection	reflection	reflection	Reflection	4	case_or_format
Reflective practice	reflective practice	reflective practice	reflective practice	Reflective practice	4	kept
reflective practice	reflective practice	reflective practice	reflective practice	Reflective practice	4	case_or_format
refresher course	refresher course	refresher course	refresher course	refresher course	4	kept
Refugees	refugees	refugees	refugee	Refugees	4	singular
Regression analysis	regression analysis	regression analysis	regression analysis	Regression Analysis	4	case_or_format
regulatory mechanism	regulatory mechanism	regulatory mechanism	regulatory mechanism	regulatory mechanism	4	kept

Relaciones profesional-familia	relaciones profesional familia	relaciones profesional familia	relaciones profesional familia	Relaciones profesional-familia	4	kept
RELATIONAL COORDINATION	relational coordination	relational coordination	relational coordination	RELATIONAL COORDINATION	4	kept
relative humidity	relative humidity	relative humidity	relative humidity	relative humidity	4	kept
Relaxation	relaxation	relaxation	relaxation	relaxation	4	case_or_format
relebactam	relebactam	relebactam	relebactam	relebactam	4	kept
Reliability and validity	reliability and validity	reliability and validity	reliability and validity	reliability and validity	4	case_or_format
Religiosity	religiosity	religiosity	religiosity	Religiosity	4	kept
religious group	religious group	religious group	religious group	religious group	4	kept
renal cell carcinoma	renal cell carcinoma	renal cell carcinoma	renal cell carcinoma	renal cell carcinoma	4	kept
Renal failure	renal failure	renal failure	renal failure	Renal failure	4	kept
renin	renin	renin	renin	renin	4	kept
repeated drug dose	repeated drug dose	repeated drug dose	repeated drug dose	repeated drug dose	4	kept
replacement arthroplasty	replacement arthroplasty	replacement arthroplasty	replacement arthroplasty	replacement arthroplasty	4	kept
repositioning	repositioning	repositioning	repositioning	Repositioning	4	case_or_format
rescue work	rescue work	rescue work	rescue work	Rescue Work	4	case_or_format
research and diseases	research and diseases	research and diseases	research and diseases	research and diseases	4	kept

Research ethics	research ethics	research ethics	research ethics	research ethics	4	case_or_format
Research methodology	research methodology	research methodology	research methodology	Research methodology	4	kept
Researcher-Subject Relations	researcher subject relations	researcher subject relations	researcher subject relations	Researcher-Subject Relations	4	kept
resident education	resident education	resident education	resident education	Resident education	4	case_or_format
Residents	residents	residents	resident	resident	4	singular
Resistance	resistance	resistance	resistance	RESISTANCE	4	case_or_format
resource utilization	resource utilization	resource utilization	resource utilization	Resource utilization	4	case_or_format
Respiration artificial	respiration artificial	artificial ventilation	artificial ventilation	Artificial Ventilation	4	alias
Respirators	respirators	respirators	respirators	Respirators	4	kept
respiratory care practice	respiratory care practice	respiratory care practice	respiratory care practice	respiratory care practice	4	kept
respiratory gas humidifier	respiratory gas humidifier	respiratory gas humidifier	respiratory gas humidifier	respiratory gas humidifier	4	kept
respiratory infection	respiratory infection	respiratory infection	respiratory infection	respiratory infections	4	case_or_format
RESPIRATORY RATE	respiratory rate	respiratory rate	respiratory rate	Respiratory Rate	4	case_or_format
respiratory syncytial virus vaccine	respiratory syncytial virus vaccine	respiratory syncytial virus vaccine	respiratory syncytial virus vaccine	respiratory syncytial virus vaccine	4	kept
Respiratory therapists	respiratory therapists	respiratory therapists	respiratory therapist	respiratory therapist	4	singular
respiratory therapists	respiratory therapists	respiratory therapists	respiratory therapist	respiratory therapist	4	singular

Respiratory Therapy Department, Hospital	respiratory therapy department hospital	respiratory therapy department hospital	respiratory therapy department hospital	Respiratory Therapy Department, Hospital	4	kept
respiratory tract disease assessment	respiratory tract disease assessment	respiratory tract disease assessment	respiratory tract disease assessment	respiratory tract disease assessment	4	kept
respiratory tract hemorrhage	respiratory tract hemorrhage	respiratory tract hemorrhage	respiratory tract hemorrhage	respiratory tract hemorrhage	4	kept
Respiratory tract infections	respiratory tract infections	respiratory tract infections	respiratory tract infection	respiratory tract infection	4	singular
respiratory virus	respiratory virus	respiratory virus	respiratory virus	respiratory virus	4	kept
Respirovirus Infections	respirovirus infections	respirovirus infections	respirovirus infections	Respirovirus Infections	4	kept
respite care	respite care	respite care	respite care	Respite Care	4	case_or_format
Responsibilities	responsibilities	responsibilities	responsibility	responsibility	4	singular
RESTRAINT	restraint	restraint	restraint	Restraint	4	case_or_format
Resuscitation orders	resuscitation orders	resuscitation orders	resuscitation orders	Resuscitation Orders	4	case_or_format
Retirement	retirement	retirement	retirement	retirement	4	case_or_format
retrieval	retrieval	retrieval	retrieval	retrieval	4	kept
retrognathia	retrognathia	retrognathia	retrognathia	retrognathia	4	kept
Retrospective study	retrospective study	retrospective study	retrospective study	Retrospective Studies	4	case_or_format
Reverse Transcriptase Polymerase Chain Reaction	reverse transcriptase polymerase chain reaction	reverse transcriptase polymerase chain reaction	reverse transcriptase polymerase chain reaction	Reverse Transcriptase Polymerase Chain Reaction	4	kept
rhabdomyosarcoma	rhabdomyosarcoma	rhabdomyosarcoma	rhabdomyosarcoma	rhabdomyosarcoma	4	kept

rheumatologist	rheumatologist	rheumatologist	rheumatologist	rheumatologist	4	kept
rib	rib	rib	rib	rib	4	kept
Ribs	ribs	ribs	rib	rib	4	singular
Richmond Agitation and Sedation Scale	richmond agitation and sedation scale	richmond agitation and sedation scale	richmond agitation and sedation scale	Richmond Agitation and Sedation Scale	4	kept
Robotic surgery	robotic surgery	robotic surgery	robotic surgery	Robotic surgery	4	kept
Role transition	role transition	role transition	role transition	Role transition	4	kept
Roles	roles	roles	role	Role	4	singular
rooming in	rooming in	rooming in	rooming in	rooming in	4	kept
rotating shift	rotating shift	rotating shift	rotating shift	rotating shift	4	kept
Rothman Index	rothman index	rothman index	rothman index	Rothman Index	4	kept
Rounds	rounds	rounds	rounds	rounds	4	case_or_format
routinely collected health data	routinely collected health data	routinely collected health data	routinely collected health data	routinely collected health data	4	kept
Roux-en-Y gastric bypass	roux en y gastric bypass	roux en y gastric bypass	roux en y gastric bypass	Roux-en-Y gastric bypass	4	kept
rubella	rubella	rubella	rubella	rubella	4	kept
Rupture	rupture	rupture	rupture	Rupture	4	kept
Rural health services	rural health services	rural health services	rural health services	Rural Health Services	4	case_or_format

Russian Federation	russian federation	russian federation	russian federation	Russian Federation	4	kept
Réanimation	r animation	r animation	r animation	réanimation	4	case_or_format
sacrococcygeal region	sacrococcygeal region	sacrococcygeal region	sacrococcygeal region	Sacrococcygeal Region	4	case_or_format
Safety attitudes	safety attitudes	safety attitudes	safety attitude	Safety attitudes	4	singular
Safety Attitudes Questionnaire	safety attitudes questionnaire	safety attitudes questionnaire	safety attitudes questionnaire	safety attitudes questionnaire	4	case_or_format
Safety compliance	safety compliance	safety compliance	safety compliance	Safety compliance	4	kept
Salicylates	salicylates	salicylates	salicylates	Salicylates	4	kept
salivary cortisol	salivary cortisol	salivary cortisol	salivary cortisol	salivary cortisol	4	kept
sarcoidosis	sarcoidosis	sarcoidosis	sarcoidosis	sarcoidosis	4	kept
SARS-CoV-2 Alpha	sars cov 2 alpha	sars cov 2 alpha	sars cov 2 alpha	SARS-CoV-2 Alpha	4	kept
SARS-CoV-2 convalescent plasma	sars cov 2 convalescent plasma	sars cov 2 convalescent plasma	sars cov 2 convalescent plasma	SARS-CoV-2 convalescent plasma	4	kept
SARS-CoV-2	sars cov 2	sars cov 2	sars cov 2	SARS-CoV-2	4	case_or_format
Saskatchewan	saskatchewan	saskatchewan	saskatchewan	Saskatchewan	4	kept
scald	scald	scald	scald	scald	4	kept
Scalp	scalp	scalp	scalp	Scalp	4	kept
School Admission Criteria	school admission criteria	school admission criteria	school admission criteria	School Admission Criteria	4	kept

Schools	schools	schools	school	school	4	singular
SCIENCE	science	science	science	Science	4	case_or_format
scopolamine	scopolamine	scopolamine	scopolamine	scopolamine	4	kept
Screening tool	screening tool	screening tool	screening tool	Screening tool	4	kept
SDOH	sdoh	sdoh	sdoh	SDOH	4	kept
seatbelt	seatbelt	seatbelt	seatbelt	seatbelt	4	kept
second victim	second victim	second victim	second victim	Second victim	4	case_or_format
secondary trauma	secondary trauma	secondary trauma	secondary trauma	secondary trauma	4	kept
sedation protocol	sedation protocol	sedation protocol	sedation protocol	sedation protocol	4	kept
Sedation scales	sedation scales	sedation scales	sedation scale	Sedation scales	4	singular
sedatives	sedatives	sedatives	sedative	Sedatives	4	singular
Selenium	selenium	selenium	selenium	selenium	4	case_or_format
Self Administration	self administration	self administration	self administration	Self Administration	4	kept
self blame	self blame	self blame	self blame	self blame	4	kept
self monitoring	self monitoring	self monitoring	self monitoring	self monitoring	4	kept
self-assessment	self assessment	self assessment	self assessment	Self-Assessment	4	case_or_format

SELF-COMPASSION	self compassion	self compassion	self compassion	Self-compassion	4	case_or_format
self-compassion	self compassion	self compassion	self compassion	Self-compassion	4	case_or_format
SELF-EFFICACY	self efficacy	self efficacy	self efficacy	Self Efficacy	4	case_or_format
Self-regulation	self regulation	self regulation	self regulation	Self-regulation	4	kept
Sense of coherence	sense of coherence	sense of coherence	sense of coherence	Sense of Coherence	4	case_or_format
Sensing	sensing	sensing	sensing	Sensing	4	kept
Sensor nodes	sensor nodes	sensor nodes	sensor nodes	Sensor nodes	4	kept
Sensory Deprivation	sensory deprivation	sensory deprivation	sensory deprivation	Sensory Deprivation	4	kept
Sensory deprivation	sensory deprivation	sensory deprivation	sensory deprivation	Sensory Deprivation	4	case_or_format
Sensory stimulation	sensory stimulation	sensory stimulation	sensory stimulation	sensory stimulation	4	case_or_format
sentinel surveillance	sentinel surveillance	sentinel surveillance	sentinel surveillance	Sentinel Surveillance	4	case_or_format
Seoul	seoul	seoul	seoul	Seoul	4	kept
Separation	separation	separation	separation	Separation	4	kept
Sequential Organ Failure Assessment	sequential organ failure assessment	sequential organ failure assessment	sequential organ failure assessment	Sequential Organ Failure Assessment	4	kept
Serious game	serious game	serious game	serious game	Serious game	4	kept
Serious illness	serious illness	serious illness	serious illness	serious illness	4	case_or_format

Seroconversion	seroconversion	seroconversion	seroconversion	seroconversion	4	case_or_format
serodiagnosis	serodiagnosis	serodiagnosis	serodiagnosis	serodiagnosis	4	kept
Serology	serology	serology	serology	serology	4	case_or_format
Serratia infection	serratia infection	serratia infection	serratia infection	Serratia Infections	4	case_or_format
Service delivery	service delivery	service delivery	service delivery	Service delivery	4	kept
Services	services	services	service	SERVICES	4	singular
severe	severe	severe	severe	severe	4	kept
Severe acute respiratory syndrome	severe acute respiratory syndrome	severe acute respiratory syndrome	severe acute respiratory syndrome	severe acute respiratory syndrome	4	case_or_format
Severe pneumonia	severe pneumonia	severe pneumonia	severe pneumonia	severe pneumonia	4	case_or_format
severe traumatic brain injury	severe traumatic brain injury	severe traumatic brain injury	severe traumatic brain injury	severe traumatic brain injury	4	kept
sexism	sexism	sexism	sexism	Sexism	4	case_or_format
sexual education	sexual education	sexual education	sexual education	sexual education	4	kept
Sexuality	sexuality	sexuality	sexuality	Sexuality	4	kept
sexuality	sexuality	sexuality	sexuality	Sexuality	4	case_or_format
Shift	shift	shift	shift	Shift	4	kept
shift	shift	shift	shift	Shift	4	case_or_format

Shift Work	shift work	shift work	shift work	shift work	4	case_or_format
Shoes	shoes	shoes	shoes	Shoes	4	kept
Short Bowel Syndrome	short bowel syndrome	short bowel syndrome	short bowel syndrome	short bowel syndrome	4	case_or_format
sialidase inhibitor	sialidase inhibitor	sialidase inhibitor	sialidase inhibitor	sialidase inhibitor	4	kept
Sibling Relations	sibling relations	sibling relations	sibling relations	Sibling Relations	4	kept
Sick Role	sick role	sick role	sick role	Sick Role	4	kept
SIGN-OUT	sign out	sign out	sign out	SIGN-OUT	4	kept
signal detection	signal detection	signal detection	signal detection	signal detection	4	kept
signal noise ratio	signal noise ratio	signal noise ratio	signal noise ratio	signal noise ratio	4	kept
Signal processing	signal processing	signal processing	signal processing	signal processing	4	case_or_format
Signs and symptoms	signs and symptoms	signs and symptoms	signs and symptoms	signs and symptoms	4	case_or_format
Sildenafil Citrate	sildenafil citrate	sildenafil citrate	sildenafil citrate	Sildenafil Citrate	4	kept
silicone	silicone	silicone	silicone	Silicones	4	case_or_format
Silver	silver	silver	silver	Silver	4	kept
Simulation-based learning	simulation based learning	simulation based learning	simulation based learning	simulation-based learning	4	case_or_format
single photon emission computed tomography	single photon emission computed tomography	single photon emission computed tomography	single photon emission computed tomography	single photon emission computed tomography	4	kept

sinus bradycardia	sinus bradycardia	sinus bradycardia	sinus bradycardia	sinus bradycardia	4	kept
sinusitis	sinusitis	sinusitis	sinusitis	sinusitis	4	kept
SITE	site	site	site	SITE	4	kept
SITUATIONS	situations	situations	situation	SITUATIONS	4	singular
Skill mix	skill mix	skill mix	skill mix	skill mix	4	case_or_format
Skin Abnormalities	skin abnormalities	skin abnormalities	skin abnormalities	Skin Abnormalities	4	kept
SKIN CARE	skin care	skin care	skin care	Skin Care	4	case_or_format
Skin Cream	skin cream	skin cream	skin cream	Skin Cream	4	kept
skin cream	skin cream	skin cream	skin cream	Skin Cream	4	case_or_format
Skin Diseases, Infectious	skin diseases infectious	skin diseases infectious	skin diseases infectious	Skin Diseases, Infectious	4	kept
skin exfoliation	skin exfoliation	skin exfoliation	skin exfoliation	skin exfoliation	4	kept
skin failure	skin failure	skin failure	skin failure	skin failure	4	kept
Skin injury	skin injury	skin injury	skin injury	skin injury	4	case_or_format
Skin lesions	skin lesions	skin lesions	skin lesions	Skin lesions	4	kept
Skin Pigmentation	skin pigmentation	skin pigmentation	skin pigmentation	Skin Pigmentation	4	kept
skin pigmentation	skin pigmentation	skin pigmentation	skin pigmentation	Skin Pigmentation	4	case_or_format

skin surface	skin surface	skin surface	skin surface	skin surface	4	kept
Skin tears	skin tears	skin tears	skin tear	Skin tears	4	singular
skin tingling	skin tingling	skin tingling	skin tingling	skin tingling	4	kept
Skull Fractures	skull fractures	skull fractures	skull fracture	skull fracture	4	singular
Sleep Aids, Pharmaceutical	sleep aids pharmaceutical	sleep aids pharmaceutical	sleep aids pharmaceutical	Sleep Aids, Pharmaceutical	4	kept
Sleep assessment	sleep assessment	sleep assessment	sleep assessment	Sleep assessment	4	kept
sleep debt	sleep debt	sleep debt	sleep debt	sleep debt	4	kept
Sleep disturbances	sleep disturbances	sleep disturbances	sleep disturbance	Sleep disturbance	4	singular
Sleep research	sleep research	sleep research	sleep research	Sleep research	4	kept
Smallpox	smallpox	smallpox	smallpox	Smallpox	4	kept
smartphones	smartphones	smartphones	smartphone	Smartphone	4	singular
smoke	smoke	smoke	smoke	smoke	4	kept
smoking cessation program	smoking cessation program	smoking cessation program	smoking cessation program	smoking cessation program	4	kept
smoking habit	smoking habit	smoking habit	smoking habit	smoking habit	4	kept
snowball sample	snowball sample	snowball sample	snowball sample	snowball sample	4	kept
Social Adjustment	social adjustment	social adjustment	social adjustment	Social Adjustment	4	kept

social belief	social belief	social belief	social belief	social belief	4	kept
Social capital	social capital	social capital	social capital	social capital	4	case_or_format
social competence	social competence	social competence	social competence	social competence	4	kept
social connectedness	social connectedness	social connectedness	social connectedness	social connectedness	4	kept
social discrimination	social discrimination	social discrimination	social discrimination	social discrimination	4	kept
Social Dominance	social dominance	social dominance	social dominance	Social Dominance	4	kept
social inequality	social inequality	social inequality	social inequality	social inequality	4	kept
Social Network Analysis	social network analysis	social network analysis	social network analysis	Social network analysis	4	case_or_format
social problem	social problem	social problem	social problem	social problem	4	kept
social responsibility	social responsibility	social responsibility	social responsibility	Social Responsibility	4	case_or_format
social well-being	social well being	social well being	social well being	social well-being	4	kept
Social work	social work	social work	social work	social work	4	case_or_format
Societies	societies	societies	society	SOCIETY	4	singular
socioeconomic factors	socioeconomic factors	socioeconomic factors	socioeconomic factors	Socioeconomic Factors	4	case_or_format
socioeconomic status	socioeconomic status	socioeconomic status	socioeconomic status	socioeconomic status	4	kept
sodium thiosulfate	sodium thiosulfate	sodium thiosulfate	sodium thiosulfate	sodium thiosulfate	4	kept

Soft skills	soft skills	soft skills	soft skills	Soft skills	4	kept
soft tissue	soft tissue	soft tissue	soft tissue	soft tissue	4	kept
Somalia	somalia	somalia	somalia	Somalia	4	kept
somatization	somatization	somatization	somatization	somatization	4	kept
sonographer	sonographer	sonographer	sonographer	sonographer	4	kept
Sound environment	sound environment	sound environment	sound environment	Sound environment	4	kept
Sound source	sound source	sound source	sound source	Sound source	4	kept
Soundscapes	soundscapes	soundscapes	soundscape	Soundscapes	4	singular
Southern Europe	southern europe	southern europe	southern europe	Southern Europe	4	kept
Space Perception	space perception	space perception	space perception	Space Perception	4	kept
Special care unit	special care unit	special care unit	special care unit	Special care units	4	case_or_format
species difference	species difference	species difference	species difference	species difference	4	kept
species distribution	species distribution	species distribution	species distribution	species distribution	4	kept
Species Specificity	species specificity	species specificity	species specificity	Species Specificity	4	kept
Spinal Cord Compression	spinal cord compression	spinal cord compression	spinal cord compression	spinal cord compression	4	case_or_format
Spinal cord injury	spinal cord injury	spinal cord injury	spinal cord injury	Spinal Cord Injuries	4	case_or_format

spine radiography	spine radiography	spine radiography	spine radiography	spine radiography	4	kept
Spiritual health	spiritual health	spiritual health	spiritual health	Spiritual health	4	kept
spiritual therapies	spiritual therapies	spiritual therapies	spiritual therapies	Spiritual Therapies	4	case_or_format
Splanchnic Circulation	splanchnic circulation	splanchnic circulation	splanchnic circulation	Splanchnic Circulation	4	kept
splenectomy	splenectomy	splenectomy	splenectomy	Splenectomy	4	case_or_format
Splenic Artery	splenic artery	splenic artery	splenic artery	Splenic Artery	4	kept
Spontaneous breathing trial	spontaneous breathing trial	spontaneous breathing trial	spontaneous breathing trial	Spontaneous breathing trial	4	kept
spontaneous breathing trial	spontaneous breathing trial	spontaneous breathing trial	spontaneous breathing trial	Spontaneous breathing trial	4	case_or_format
sport	sport	sport	sport	sport	4	kept
squamous cell carcinoma	squamous cell carcinoma	squamous cell carcinoma	squamous cell carcinoma	squamous cell carcinoma	4	kept
ST segment	st segment	st segment	st segment	ST segment	4	kept
staff safety	staff safety	staff safety	staff safety	staff safety	4	kept
staff welfare	staff welfare	staff welfare	staff welfare	staff welfare	4	kept
Staff wellbeing	staff wellbeing	staff wellbeing	staff wellbeing	Staff wellbeing	4	kept
staining	staining	staining	staining	staining	4	kept
staphylococcal bacteremia	staphylococcal bacteremia	staphylococcal bacteremia	staphylococcal bacteremia	staphylococcal bacteremia	4	kept

staphylococcal pneumonia	staphylococcal pneumonia	staphylococcal pneumonia	staphylococcal pneumonia	staphylococcal pneumonia	4	kept
Staphylococcus hominis	staphylococcus hominis	staphylococcus hominis	staphylococcus hominis	Staphylococcus hominis	4	kept
starvation	starvation	starvation	starvation	starvation	4	kept
Statistical	statistical	statistical	statistical	Statistical	4	kept
statistical parameters	statistical parameters	statistical parameters	statistical parameters	statistical parameters	4	kept
STATISTICS & RESEARCH METHODS	statistics and research methods	statistics and research methods	statistics and research methods	STATISTICS & RESEARCH METHODS	4	kept
sternum	sternum	sternum	sternum	Sternum	4	case_or_format
Stigma	stigma	stigma	stigma	stigma	4	case_or_format
stimulus	stimulus	stimulus	stimulus	stimulus	4	kept
stomach lavage	stomach lavage	stomach lavage	stomach lavage	stomach lavage	4	kept
stomach tumor	stomach tumor	stomach tumor	stomach tumor	stomach tumor	4	kept
stomatitis	stomatitis	stomatitis	stomatitis	Stomatitis	4	case_or_format
stratified sample	stratified sample	stratified sample	stratified sample	stratified sample	4	kept
strength	strength	strength	strength	strength	4	kept
Stress Disorders, Traumatic	stress disorders traumatic	stress disorders traumatic	stress disorders traumatic	Stress Disorders, Traumatic	4	kept
stress hyperglycemia	stress hyperglycemia	stress hyperglycemia	stress hyperglycemia	stress hyperglycemia	4	kept

Stress, Mechanical	stress mechanical	stress mechanical	stress mechanical	Stress, Mechanical	4	kept
Stressor	stressor	stressor	stressor	Stressors	4	case_or_format
STRESSORS	stressors	stressors	stressor	Stressors	4	singular
STROKE	stroke	stroke	stroke	Stroke	4	case_or_format
Stroke unit	stroke unit	stroke unit	stroke unit	stroke unit	4	case_or_format
Stroke volume	stroke volume	stroke volume	stroke volume	Stroke Volume	4	case_or_format
structural equation model	structural equation model	structural equation model	structural equation model	structural equation model	4	kept
Structure	structure	structure	structure	Structure	4	kept
structure	structure	structure	structure	Structure	4	case_or_format
student attitude	student attitude	student attitude	student attitude	student attitude	4	kept
stupor	stupor	stupor	stupor	stupor	4	kept
Subcutaneous	subcutaneous	subcutaneous	subcutaneous	Subcutaneous	4	kept
Subjective well-being	subjective well being	subjective well being	subjective well being	Subjective well-being	4	kept
substitution therapy	substitution therapy	substitution therapy	substitution therapy	substitution therapy	4	kept
Subsyndromal delirium	subsyndromal delirium	subsyndromal delirium	subsyndromal delirium	Subsyndromal delirium	4	kept
suctioning	suctioning	suctioning	suctioning	Suctioning	4	case_or_format

Sudden infant death syndrome	sudden infant death syndrome	sudden infant death syndrome	sudden infant death syndrome	sudden infant death syndrome	4	case_or_format
suffocation	suffocation	suffocation	suffocation	suffocation	4	kept
suicide prevention	suicide prevention	suicide prevention	suicide prevention	Suicide Prevention	4	case_or_format
Sulfones	sulfones	sulfones	sulfones	Sulfones	4	kept
superoxide dismutase	superoxide dismutase	superoxide dismutase	superoxide dismutase	superoxide dismutase	4	kept
suppuration	suppuration	suppuration	suppuration	Suppuration	4	case_or_format
surfactant replacement therapy	surfactant replacement therapy	surfactant replacement therapy	surfactant replacement therapy	surfactant replacement therapy	4	kept
surge	surge	surge	surge	surge	4	kept
surgical care	surgical care	surgical care	surgical care	surgical care	4	kept
surgical error	surgical error	surgical error	surgical error	surgical error	4	kept
Surgical Mesh	surgical mesh	surgical mesh	surgical mesh	Surgical Mesh	4	kept
Surgical procedures	surgical procedures	surgical procedures	surgical procedures	Surgical procedures	4	kept
Surgical Stomas	surgical stomas	surgical stomas	surgical stomas	Surgical Stomas	4	kept
Surgical Wound	surgical wound	surgical wound	surgical wound	surgical wound	4	case_or_format
Surrogate	surrogate	surrogate	surrogate	Surrogate	4	kept
Surrogate decision making	surrogate decision making	surrogate decision making	surrogate decision making	surrogate decision-making	4	case_or_format

surrogate decision making	surrogate decision making	surrogate decision making	surrogate decision making	surrogate decision-making	4	case_or_format
Survival rate	survival rate	survival rate	survival rate	Survival Rate	4	case_or_format
Sustainable healthcare	sustainable healthcare	sustainable healthcare	sustainable healthcare	Sustainable healthcare	4	kept
Swine	swine	swine	swine	Swine	4	kept
Synergy	synergy	synergy	synergy	Synergy	4	kept
System	system	system	system	SYSTEM	4	case_or_format
Systems engineering	systems engineering	systems engineering	systems engineering	Systems engineering	4	kept
Systems Theory	systems theory	systems theory	systems theory	Systems Theory	4	kept
Systems-Based Practice	systems based practice	systems based practice	systems based practice	Systems-Based Practice	4	kept
T wave	t wave	t wave	t wave	T wave	4	kept
Tablets	tablets	tablets	tablet	tablet	4	singular
Tachypnea	tachypnea	tachypnea	tachypnea	tachypnea	4	case_or_format
TAIWAN	taiwan	taiwan	taiwan	Taiwan	4	case_or_format
Taiwanese	taiwanese	taiwanese	taiwanese	Taiwanese	4	kept
task shifting	task shifting	task shifting	task shifting	task shifting	4	kept
tasks	tasks	tasks	task	tasks	4	singular

TAVI	tavi	tavi	tavi	TAVI	4	kept
TAVR	tavr	tavr	tavr	TAVR	4	kept
Team communication	team communication	team communication	team communication	Team communication	4	kept
Team work	team work	team work	team work	Team work	4	kept
Team working	team working	team working	team working	Team working	4	kept
team-based care	team based care	team based care	team based care	team-based care	4	kept
technological innovation	technological innovation	technological innovation	technological innovation	technological innovation	4	kept
technology assessment	technology assessment	technology assessment	technology assessment	technology assessment	4	kept
Telangiectasia, Hereditary Hemorrhagic	telangiectasia hereditary hemorrhagic	telangiectasia hereditary hemorrhagic	telangiectasia hereditary hemorrhagic	Telangiectasia, Hereditary Hemorrhagic	4	kept
Television	television	television	television	television	4	case_or_format
temperament	temperament	temperament	temperament	temperament	4	kept
temporal lobe	temporal lobe	temporal lobe	temporal lobe	temporal lobe	4	kept
tenecteplase	tenecteplase	tenecteplase	tenecteplase	tenecteplase	4	kept
Terbutaline	terbutaline	terbutaline	terbutaline	terbutaline	4	case_or_format
Term Birth	term birth	term birth	term birth	term birth	4	case_or_format
terminally ill	terminally ill	terminally ill	terminally ill	Terminally Ill	4	case_or_format

Tertiary cares	tertiary cares	tertiary cares	tertiary care	Tertiary cares	4	singular
tetanus toxoid	tetanus toxoid	tetanus toxoid	tetanus toxoid	tetanus toxoid	4	kept
Thai	thai	thai	thai	Thai	4	kept
thalamus	thalamus	thalamus	thalamus	thalamus	4	kept
Theoretical domains framework	theoretical domains framework	theoretical domains framework	theoretical domains framework	Theoretical domains framework	4	kept
theory-practice relationship	theory practice relationship	theory practice relationship	theory practice relationship	theory-practice relationship	4	kept
Therapeutic	therapeutic	therapeutic	therapeutic	Therapeutics	4	case_or_format
therapeutic intervention scoring system	therapeutic intervention scoring system	therapeutic intervention scoring system	therapeutic intervention scoring system	therapeutic intervention scoring system	4	kept
therapeutic touch	therapeutic touch	therapeutic touch	therapeutic touch	Therapeutic Touch	4	case_or_format
Therapies, Investigational	therapies investigational	therapies investigational	therapies investigational	Therapies, Investigational	4	kept
thermal injury	thermal injury	thermal injury	thermal injury	thermal injury	4	kept
thermography	thermography	thermography	thermography	Thermography	4	case_or_format
thermometry	thermometry	thermometry	thermometry	Thermometry	4	case_or_format
thoracic aorta aneurysm	thoracic aorta aneurysm	thoracic aorta aneurysm	thoracic aorta aneurysm	thoracic aorta aneurysm	4	kept
thorax disease	thorax disease	thorax disease	thorax disease	thorax disease	4	kept
Thrombectomie mé canique	thrombectomie m canique	thrombectomie m canique	thrombectomie m canique	Thrombectomie mé canique	4	kept

Thrombelastography	thrombelastography	thrombelastography	thrombelastography	Thrombelastography	4	kept
thrombocyte activation	thrombocyte activation	thrombocyte activation	thrombocyte activation	thrombocyte activation	4	kept
thrombocytosis	thrombocytosis	thrombocytosis	thrombocytosis	thrombocytosis	4	kept
Thrombolyse intraveineuse	thrombolyse intraveineuse	thrombolyse intraveineuse	thrombolyse intraveineuse	Thrombolyse intraveineuse	4	kept
thrombophilia	thrombophilia	thrombophilia	thrombophilia	thrombophilia	4	kept
Thrombotic Microangiopathies	thrombotic microangiopathies	thrombotic microangiopathies	thrombotic microangiopathy	Thrombotic Microangiopathies	4	singular
thrush	thrush	thrush	thrush	thrush	4	kept
Thyroid Gland	thyroid gland	thyroid gland	thyroid gland	Thyroid Gland	4	kept
Thyroid Hormones	thyroid hormones	thyroid hormones	thyroid hormone	thyroid hormone	4	singular
Thyroidectomy	thyroidectomy	thyroidectomy	thyroidectomy	Thyroidectomy	4	kept
thyrotropin	thyrotropin	thyrotropin	thyrotropin	thyrotropin	4	kept
Tight glycemic control	tight glycemic control	tight glycemic control	tight glycemic control	tight glycemic control	4	case_or_format
time and motion studies	time and motion studies	time and motion studies	time and motion study	Time and Motion Studies	4	singular
time and motion study	time and motion study	time and motion study	time and motion study	Time and Motion Studies	4	case_or_format
time interval	time interval	time interval	time interval	time interval	4	kept
Time series	time series	time series	time series	Time series	4	kept

Time series analysis	time series analysis	time series analysis	time series analysis	time series analysis	4	case_or_format
tiotropium bromide	tiotropium bromide	tiotropium bromide	tiotropium bromide	tiotropium bromide	4	kept
tisagenlecleucel T	tisagenlecleucel t	tisagenlecleucel t	tisagenlecleucel t	tisagenlecleucel T	4	kept
tissue distribution	tissue distribution	tissue distribution	tissue distribution	Tissue Distribution	4	case_or_format
tissue necrosis	tissue necrosis	tissue necrosis	tissue necrosis	tissue necrosis	4	kept
tocolysis	tocolysis	tocolysis	tocolysis	tocolysis	4	kept
toddler	toddler	toddler	toddler	toddler	4	kept
Tokyo	tokyo	tokyo	tokyo	Tokyo	4	kept
Tomography Scanners, X-Ray Computed	tomography scanners x ray computed	tomography scanners x ray computed	tomography scanners x ray computed	Tomography Scanners, X-Ray Computed	4	kept
Tool	tool	tool	tool	TOOL	4	case_or_format
tool development	tool development	tool development	tool development	tool development	4	kept
tool use	tool use	tool use	tool use	tool use	4	kept
Tooth brushing	tooth brushing	tooth brushing	tooth brushing	tooth brushing	4	case_or_format
tooth disease	tooth disease	tooth disease	tooth disease	tooth disease	4	kept
topical anesthesia	topical anesthesia	topical anesthesia	topical anesthesia	topical anesthesia	4	kept
toxic megacolon	toxic megacolon	toxic megacolon	toxic megacolon	toxic megacolon	4	kept

Toxicology	toxicology	toxicology	toxicology	toxicology	4	case_or_format
trachea obstruction	trachea obstruction	trachea obstruction	trachea obstruction	trachea obstruction	4	kept
TRACHEAL INTUBATION	tracheal intubation	tracheal intubation	tracheal intubation	Tracheal intubation	4	case_or_format
tracheitis	tracheitis	tracheitis	tracheitis	tracheitis	4	kept
Tracheostomy care	tracheostomy care	tracheostomy care	tracheostomy care	Tracheostomy care	4	kept
TRACHEOTOMY	tracheotomy	tracheotomy	tracheotomy	tracheotomy	4	case_or_format
Tracking	tracking	tracking	tracking	Tracking	4	kept
Training Program	training program	training program	training program	Training program	4	case_or_format
Trajectory	trajectory	trajectory	trajectory	Trajectory	4	kept
Transfer, Psychology	transfer psychology	transfer psychology	transfer psychology	Transfer, Psychology	4	kept
transformational leadership	transformational leadership	transformational leadership	transformational leadership	Transformational leadership	4	case_or_format
transfusion medicine	transfusion medicine	transfusion medicine	transfusion medicine	transfusion medicine	4	kept
Transgender Persons	transgender persons	transgender persons	transgender persons	Transgender Persons	4	kept
transient tachypnea of the newborn	transient tachypnea of the newborn	transient tachypnea of the newborn	transient tachypnea of the newborn	transient tachypnea of the newborn	4	kept
transluminal valvuloplasty	transluminal valvuloplasty	transluminal valvuloplasty	transluminal valvuloplasty	transluminal valvuloplasty	4	kept
Transplant	transplant	transplant	transplant	Transplants	4	case_or_format

Transplant Recipients	transplant recipients	transplant recipients	transplant recipients	Transplant Recipients	4	kept
Transplantation Immunology	transplantation immunology	transplantation immunology	transplantation immunology	Transplantation Immunology	4	kept
transurethral resection	transurethral resection	transurethral resection	transurethral resection	transurethral resection	4	kept
Trauma centers	trauma centers	trauma centers	trauma center	Trauma Centers	4	singular
trauma centers	trauma centers	trauma centers	trauma center	Trauma Centers	4	singular
Trauma severity indices	trauma severity indices	trauma severity indices	trauma severity indices	Trauma Severity Indices	4	case_or_format
Trauma systems	trauma systems	trauma systems	trauma system	Trauma systems	4	singular
treprostinil	treprostinil	treprostinil	treprostinil	treprostinil	4	kept
Triazoles	triazoles	triazoles	triazoles	Triazoles	4	kept
trisomy 13	trisomy 13	trisomy 13	trisomy 13	trisomy 13	4	kept
Trisomy 13 Syndrome	trisomy 13 syndrome	trisomy 13 syndrome	trisomy 13 syndrome	Trisomy 13 Syndrome	4	kept
Trisomy 18 Syndrome	trisomy 18 syndrome	trisomy 18 syndrome	trisomy 18 syndrome	Trisomy 18 Syndrome	4	kept
Troponin I	troponin i	troponin i	troponin i	troponin I	4	case_or_format
trustworthiness	trustworthiness	trustworthiness	trustworthiness	trustworthiness	4	kept
tryptase	tryptase	tryptase	tryptase	tryptase	4	kept
Tuberculosis, Multidrug-Resistant	tuberculosis multidrug resistant	tuberculosis multidrug resistant	tuberculosis multidrug resistant	Tuberculosis, Multidrug-Resistant	4	kept

TURKEY	turkey	turkey	turkey	Turkey	4	case_or_format
Turner syndrome	turner syndrome	turner syndrome	turner syndrome	Turner syndrome	4	kept
TURNOVER INTENTION	turnover intention	turnover intention	turnover intention	Turnover intention	4	case_or_format
Ultrasonic Therapy	ultrasonic therapy	ultrasonic therapy	ultrasonic therapy	Ultrasonic Therapy	4	kept
Ultrasonics	ultrasonics	ultrasonics	ultrasonics	Ultrasonics	4	kept
ultrasound guidance	ultrasound guidance	ultrasound guidance	ultrasound guidance	ultrasound guidance	4	kept
understanding	understanding	understanding	understanding	understanding	4	kept
Unemployment	unemployment	unemployment	unemployment	unemployment	4	case_or_format
Unidade de Terapia Intensiva	unidade de terapia intensiva	unidade de terapia intensiva	unidade de terapia intensiva	Unidade de Terapia Intensiva	4	kept
Unité neurovasculaire	unit neurovasculaire	unit neurovasculaire	unit neurovasculaire	Unité neurovasculaire	4	kept
universal design	universal design	universal design	universal design	universal design	4	kept
upper respiratory tract	upper respiratory tract	upper respiratory tract	upper respiratory tract	upper respiratory tract	4	kept
upregulation	upregulation	upregulation	upregulation	upregulation	4	kept
Urban Health	urban health	urban health	urban health	Urban Health	4	kept
urban health	urban health	urban health	urban health	Urban Health	4	case_or_format
Urea	urea	urea	urea	urea	4	case_or_format

urgent care	urgent care	urgent care	urgent care	urgent care	4	kept
Urinary catheterization	urinary catheterization	urinary catheterization	urinary catheterization	Urinary Catheterization	4	case_or_format
urinary tract disease	urinary tract disease	urinary tract disease	urinary tract disease	urinary tract disease	4	kept
URINARY-TRACT-INFECTION	urinary tract infection	urinary tract infection	urinary tract infection	urinary tract infection	4	case_or_format
Urination Disorders	urination disorders	urination disorders	urination disorders	Urination Disorders	4	kept
urine catheter	urine catheter	urine catheter	urine catheter	urine catheter	4	kept
urine osmolality	urine osmolality	urine osmolality	urine osmolality	urine osmolality	4	kept
Urine output	urine output	urine output	urine output	Urine output	4	kept
Urine Specimen Collection	urine specimen collection	urine specimen collection	urine specimen collection	Urine Specimen Collection	4	kept
urodynamics	urodynamics	urodynamics	urodynamics	Urodynamics	4	case_or_format
urogenital tract cancer	urogenital tract cancer	urogenital tract cancer	urogenital tract cancer	urogenital tract cancer	4	kept
urologist	urologist	urologist	urologist	urologist	4	kept
Uruguay	uruguay	uruguay	uruguay	Uruguay	4	kept
User acceptance	user acceptance	user acceptance	user acceptance	User acceptance	4	kept
uterine atony	uterine atony	uterine atony	uterine atony	uterine atony	4	kept
uterine cervix cancer	uterine cervix cancer	uterine cervix cancer	uterine cervix cancer	uterine cervix cancer	4	kept

uterus myoma	uterus myoma	uterus myoma	uterus myoma	uterus myoma	4	kept
Vaccine Efficacy	vaccine efficacy	vaccine efficacy	vaccine efficacy	Vaccine Efficacy	4	kept
vaccinee	vaccinee	vaccinee	vaccinee	vaccinee	4	kept
vacuum extraction	vacuum extraction	vacuum extraction	vacuum extraction	vacuum extraction	4	kept
vagus nerve	vagus nerve	vagus nerve	vagus nerve	Vagus Nerve	4	case_or_format
valganciclovir	valganciclovir	valganciclovir	valganciclovir	valganciclovir	4	kept
Validation Study	validation study	validation study	validation study	validation study	4	case_or_format
Value	value	value	value	value	4	case_or_format
varicosis	varicosis	varicosis	varicosis	varicosis	4	kept
vascular surgeon	vascular surgeon	vascular surgeon	vascular surgeon	vascular surgeon	4	kept
vasculotropin	vasculotropin	vasculotropin	vasculotropin	vasculotropin	4	kept
vasculotropin inhibitor	vasculotropin inhibitor	vasculotropin inhibitor	vasculotropin inhibitor	vasculotropin inhibitor	4	kept
Vasoactive drugs	vasoactive drugs	vasoactive drugs	vasoactive drug	Vasoactive drugs	4	singular
Vasoconstriction	vasoconstriction	vasoconstriction	vasoconstriction	vasoconstriction	4	case_or_format
vasopressin derivative	vasopressin derivative	vasopressin derivative	vasopressin derivative	vasopressin derivative	4	kept
Vasopressors	vasopressors	vasopressors	vasopressor	Vasopressor	4	singular

vasopressors	vasopressors	vasopressors	vasopressor	Vasopressor	4	singular
Vena Cava Filters	vena cava filters	vena cava filters	vena cava filter	Vena Cava Filters	4	singular
Vena Cava, Superior	vena cava superior	vena cava superior	vena cava superior	Vena Cava, Superior	4	kept
venous thrombosis	venous thrombosis	venous thrombosis	venous thrombosis	Venous Thrombosis	4	case_or_format
ventilator-induced lung injury	ventilator induced lung injury	ventilator induced lung injury	ventilator induced lung injury	Ventilator-Induced Lung Injury	4	case_or_format
Ventilatory support	ventilatory support	ventilatory support	ventilatory support	Ventilatory support	4	kept
Very low birth weight infants	very low birth weight infants	very low birth weight infants	very low birth weight infant	Very low birth weight infant	4	singular
Veterans Health	veterans health	veterans health	veterans health	veterans health	4	case_or_format
veterans health service	veterans health service	veterans health service	veterans health service	veterans health service	4	kept
Veterinarians	veterinarians	veterinarians	veterinarian	veterinarian	4	singular
veterinary	veterinary	veterinary	veterinary	veterinary	4	kept
Veterinary Medicine	veterinary medicine	veterinary medicine	veterinary medicine	veterinary medicine	4	case_or_format
Viral infections	viral infections	viral infections	viral infections	Viral infections	4	kept
Viral Load	viral load	viral load	viral load	Viral Load	4	kept
viral respiratory tract infection	viral respiratory tract infection	viral respiratory tract infection	viral respiratory tract infection	viral respiratory tract infection	4	kept
virulence	virulence	virulence	virulence	Virulence	4	case_or_format

virus antigen	virus antigen	virus antigen	virus antigen	virus antigen	4	kept
virus particle	virus particle	virus particle	virus particle	virus particle	4	kept
Visitation policy	visitation policy	visitation policy	visitation policy	Visitation policy	4	kept
VISITS	visits	visits	visit	VISITS	4	singular
Visual	visual	visual	visual	Visual	4	kept
Visual cues	visual cues	visual cues	visual cues	Visual cues	4	kept
visual feedback	visual feedback	visual feedback	visual feedback	visual feedback	4	kept
visual system examination	visual system examination	visual system examination	visual system examination	visual system examination	4	kept
Visualization	visualization	visualization	visualization	visualization	4	case_or_format
vital signs monitoring	vital signs monitoring	vital signs monitoring	vital signs monitoring	Vital signs monitoring	4	case_or_format
vitamin deficiency	vitamin deficiency	vitamin deficiency	vitamin deficiency	vitamin deficiency	4	kept
Vocal Cord Paralysis	vocal cord paralysis	vocal cord paralysis	vocal cord paralysis	vocal cord paralysis	4	case_or_format
vocation	vocation	vocation	vocation	vocation	4	kept
vocational guidance	vocational guidance	vocational guidance	vocational guidance	vocational guidance	4	kept
Voice Disorders	voice disorders	voice disorders	voice disorder	Voice Disorders	4	singular
volume of distribution	volume of distribution	volume of distribution	volume of distribution	volume of distribution	4	kept

voluntary worker	voluntary worker	voluntary worker	voluntary worker	voluntary worker	4	kept
Voluntary Workers	voluntary workers	voluntary workers	voluntary worker	voluntary worker	4	singular
WARD	ward	ward	ward	ward	4	case_or_format
Ward	ward	ward	ward	ward	4	case_or_format
Ward rounds	ward rounds	ward rounds	ward round	Ward rounds	4	singular
wards	wards	wards	ward	ward	4	singular
water loss	water loss	water loss	water loss	water loss	4	kept
Water Purification	water purification	water purification	water purification	Water Purification	4	kept
Weaning protocol	weaning protocol	weaning protocol	weaning protocol	Weaning protocol	4	kept
weaning protocol	weaning protocol	weaning protocol	weaning protocol	Weaning protocol	4	case_or_format
wearable electronic devices	wearable electronic devices	wearable electronic devices	wearable electronic device	Wearable Electronic Devices	4	singular
webinar	webinar	webinar	webinar	webinar	4	kept
welfare	welfare	welfare	welfare	welfare	4	kept
Well being	well being	well being	well being	well-being	4	case_or_format
wellness	wellness	wellness	wellness	wellness	4	kept
whistleblowing	whistleblowing	whistleblowing	whistleblowing	whistleblowing	4	kept

Wireless telecommunication systems	wireless telecommunication systems	wireless telecommunication systems	wireless telecommunication systems	Wireless telecommunication systems	4	kept
withdrawal of life-sustaining treatment	withdrawal of life sustaining treatment	withdrawal of life sustaining treatment	withdrawal of life sustaining treatment	withdrawal of life-sustaining treatment	4	kept
Work Capacity Evaluation	work capacity evaluation	work capacity evaluation	work capacity evaluation	Work Capacity Evaluation	4	kept
work disability	work disability	work disability	work disability	work disability	4	kept
work from home	work from home	work from home	work from home	work from home	4	kept
Work Satisfaction	work satisfaction	work satisfaction	work satisfaction	work satisfaction	4	case_or_format
Work satisfaction	work satisfaction	work satisfaction	work satisfaction	work satisfaction	4	case_or_format
Work-life balance	work life balance	work life balance	work life balance	work-life balance	4	case_or_format
WORKFORCE	workforce	workforce	workforce	Workforce	4	case_or_format
workload management	workload management	workload management	workload management	workload management	4	kept
Workshop	workshop	workshop	workshop	workshop	4	case_or_format
work–family conflict	work family conflict	work family conflict	work family conflict	work–family conflict	4	kept
World health organization	world health organization	world health organization	world health organization	World Health Organization	4	case_or_format
wound healing impairment	wound healing impairment	wound healing impairment	wound healing impairment	wound healing impairment	4	kept
Wound infection	wound infection	wound infection	wound infection	wound infection	4	case_or_format
wounds	wounds	wounds	wound	wound	4	singular

Wounds, Stab	wounds stab	wounds stab	wounds stab	Wounds, Stab	4	kept
X ray film	x ray film	x ray film	x ray film	X ray film	4	kept
zidovudine	zidovudine	zidovudine	zidovudine	zidovudine	4	kept
zoledronic acid	zoledronic acid	zoledronic acid	zoledronic acid	zoledronic acid	4	kept
zone of inhibition	zone of inhibition	zone of inhibition	zone of inhibition	zone of inhibition	4	kept
zoonosis	zoonosis	zoonosis	zoonosis	zoonosis	4	kept
% reductions	reductions	reductions	reduction	REDUCTION	3	singular
’current	current	current	current	’current	3	kept
3,4 methylenedioxymethamph etamine	3 4 methylenedioxymethamph etamine	3 4 methylenedioxymethamph etamine	3 4 methylenedioxymethamph etamine	3,4 methylenedioxymethamph etamine	3	kept
abdominal cramp	abdominal cramp	abdominal cramp	abdominal cramp	abdominal cramp	3	kept
Abdominal hypertension	abdominal hypertension	abdominal hypertension	abdominal hypertension	Abdominal hypertension	3	kept
abdominal hysterectomy	abdominal hysterectomy	abdominal hysterectomy	abdominal hysterectomy	abdominal hysterectomy	3	kept
abdominal wall hernia	abdominal wall hernia	abdominal wall hernia	abdominal wall hernia	abdominal wall hernia	3	kept
abnormal laboratory result	abnormal laboratory result	abnormal laboratory result	abnormal laboratory result	abnormal laboratory result	3	kept
abnormalities	abnormalities	abnormalities	abnormalities	abnormalities	3	kept
abnormally high substrate concentration in blood	abnormally high substrate concentration in blood	abnormally high substrate concentration in blood	abnormally high substrate concentration in blood	abnormally high substrate concentration in blood	3	kept

abrasion	abrasion	abrasion	abrasion	abrasion	3	kept
abscess drainage	abscess drainage	abscess drainage	abscess drainage	abscess drainage	3	kept
absolute lymphocyte count	absolute lymphocyte count	absolute lymphocyte count	absolute lymphocyte count	absolute lymphocyte count	3	kept
absorption	absorption	absorption	absorption	absorption	3	kept
Abstracting and Indexing	abstracting and indexing	abstracting and indexing	abstracting and indexing	Abstracting and Indexing	3	kept
abuse	abuse	abuse	abuse	abuse	3	kept
Acceptance	acceptance	acceptance	acceptance	Acceptance	3	kept
acceptance	acceptance	acceptance	acceptance	Acceptance	3	case_or_format
Access to care	access to care	access to care	access to care	access to care	3	case_or_format
access to treatment	access to treatment	access to treatment	access to treatment	access to treatment	3	kept
Accidental extubation	accidental extubation	accidental extubation	accidental extubation	Accidental extubation	3	kept
accidental hypothermia	accidental hypothermia	accidental hypothermia	accidental hypothermia	accidental hypothermia	3	kept
Accounting	accounting	accounting	accounting	accounting	3	case_or_format
Accreditation Council for Graduate Medical Education	accreditation council for graduate medical education	accreditation council for graduate medical education	accreditation council for graduate medical education	Accreditation Council for Graduate Medical Education	3	kept
Acetamides	acetamides	acetamides	acetamides	Acetamides	3	kept
Acoustic fields	acoustic fields	acoustic fields	acoustic fields	Acoustic fields	3	kept

acoustic neuroma	acoustic neuroma	acoustic neuroma	acoustic neuroma	acoustic neuroma	3	kept
Acoustic variables measurement	acoustic variables measurement	acoustic variables measurement	acoustic variables measurement	Acoustic variables measurement	3	kept
Acoustic wave transmission	acoustic wave transmission	acoustic wave transmission	acoustic wave transmission	Acoustic wave transmission	3	kept
Acrylates	acrylates	acrylates	acrylates	Acrylates	3	kept
ACS	acs	acs	acs	ACS	3	kept
actigraphy	actigraphy	actigraphy	actigraphy	Actigraphy	3	case_or_format
Activation criteria	activation criteria	activation criteria	activation criteria	Activation criteria	3	kept
ACUITY	acuity	acuity	acuity	Acuity	3	case_or_format
acute brain disease	acute brain disease	acute brain disease	acute brain disease	acute brain disease	3	kept
acute brain injury	acute brain injury	acute brain injury	acute brain injury	acute brain injury	3	kept
Acute cardiac care	acute cardiac care	acute cardiac care	acute cardiac care	Acute cardiac care	3	kept
Acute Care	acute care	acute care	acute care	acute care	3	case_or_format
acute care settings	acute care settings	acute care settings	acute care setting	acute care setting	3	singular
acute chest syndrome	acute chest syndrome	acute chest syndrome	acute chest syndrome	acute chest syndrome	3	kept
Acute exacerbation of chronic obstructive pulmonary disease	acute exacerbation of chronic obstructive pulmonary disease	acute exacerbation of chronic obstructive pulmonary disease	acute exacerbation of chronic obstructive pulmonary disease	Acute exacerbation of chronic obstructive pulmonary disease	3	kept
acute gastroenteritis	acute gastroenteritis	acute gastroenteritis	acute gastroenteritis	acute gastroenteritis	3	kept

acute graft versus host disease	acute graft versus host disease	acute graft versus host disease	acute graft versus host disease	acute graft versus host disease	3	kept
acute granulocytic leukemia	acute granulocytic leukemia	acute granulocytic leukemia	acute granulocytic leukemia	acute granulocytic leukemia	3	kept
Acute heart failure	acute heart failure	acute heart failure	acute heart failure	acute heart failure	3	case_or_format
Acute hospitals	acute hospitals	acute hospitals	acute hospital	Acute hospitals	3	singular
Acute Hypotensive Episodes	acute hypotensive episodes	acute hypotensive episodes	acute hypotensive episodes	Acute Hypotensive Episodes	3	kept
Acute illness	acute illness	acute illness	acute illness	Acute illness	3	kept
ACUTE ISCHEMIC-STROKE	acute ischemic stroke	acute ischemic stroke	acute ischemic stroke	acute ischemic stroke	3	case_or_format
Acute kidney failure	acute kidney failure	acute kidney failure	acute kidney failure	acute kidney failure	3	case_or_format
Acute medicine	acute medicine	acute medicine	acute medicine	Acute medicine	3	kept
Acute pancreatitis	acute pancreatitis	acute pancreatitis	acute pancreatitis	acute pancreatitis	3	case_or_format
ACUTE PHYSIOLOGY	acute physiology	acute physiology	acute physiology	ACUTE PHYSIOLOGY	3	kept
Acute-Phase Proteins	acute phase proteins	acute phase proteins	acute phase protein	acute phase protein	3	singular
adalimumab	adalimumab	adalimumab	adalimumab	adalimumab	3	kept
ADAPTATION	adaptation	adaptation	adaptation	adaptation	3	case_or_format
adaptive immunity	adaptive immunity	adaptive immunity	adaptive immunity	adaptive immunity	3	kept
Adaptive support ventilation	adaptive support ventilation	adaptive support ventilation	adaptive support ventilation	Adaptive support ventilation	3	kept

ADDIE model	addie model	addie model	addie model	ADDIE model	3	kept
adenotonsillectomy	adenotonsillectomy	adenotonsillectomy	adenotonsillectomy	adenotonsillectomy	3	kept
adenovirus infection	adenovirus infection	adenovirus infection	adenovirus infection	adenovirus infection	3	kept
Adjunctive corticosteroids therapy	adjunctive corticosteroids therapy	adjunctive corticosteroids therapy	adjunctive corticosteroids therapy	Adjunctive corticosteroids therapy	3	kept
Adjustment Disorders	adjustment disorders	adjustment disorders	adjustment disorder	Adjustment Disorders	3	singular
Administration, Intravaginal	administration intravaginal	administration intravaginal	administration intravaginal	Administration, Intravaginal	3	kept
Administrative Claims, Healthcare	administrative claims healthcare	administrative claims healthcare	administrative claims healthcare	Administrative Claims, Healthcare	3	kept
ADMINISTRATIVE DATA	administrative data	administrative data	administrative data	ADMINISTRATIVE DATA	3	kept
administrative data	administrative data	administrative data	administrative data	ADMINISTRATIVE DATA	3	case_or_format
administrative database	administrative database	administrative database	administrative database	administrative database	3	kept
Adolescent Development	adolescent development	adolescent development	adolescent development	Adolescent Development	3	kept
Adolescente	adolescente	adolescente	adolescente	Adolescente	3	kept
Adoption	adoption	adoption	adoption	adoption	3	case_or_format
Adrenal insufficiency	adrenal insufficiency	adrenal insufficiency	adrenal insufficiency	Adrenal Insufficiency	3	case_or_format
adrenergic system	adrenergic system	adrenergic system	adrenergic system	adrenergic system	3	kept
Adrenergic Uptake Inhibitors	adrenergic uptake inhibitors	adrenergic uptake inhibitors	adrenergic uptake inhibitors	Adrenergic Uptake Inhibitors	3	kept

adult congenital heart disease	adult congenital heart disease	adult congenital heart disease	adult congenital heart disease	adult congenital heart disease	3	kept
Adult palliative care	adult palliative care	adult palliative care	adult palliative care	adult palliative care	3	case_or_format
adulthood	adulthood	adulthood	adulthood	adulthood	3	kept
advance directive	advance directive	advance directive	advance directive	Advance Directives	3	case_or_format
Advance practice providers	advance practice providers	advance practice providers	advance practice provider	Advance practice providers	3	singular
Advanced care planning	advanced care planning	advanced care planning	advanced care planning	Advanced care planning	3	kept
advanced care planning	advanced care planning	advanced care planning	advanced care planning	Advanced care planning	3	case_or_format
Advanced clinical practitioner	advanced clinical practitioner	advanced clinical practitioner	advanced clinical practitioner	Advanced clinical practitioner	3	kept
advanced dementia	advanced dementia	advanced dementia	advanced dementia	ADVANCED DEMENTIA	3	case_or_format
Advanced Trauma Life Support Care	advanced trauma life support care	advanced trauma life support care	advanced trauma life support care	advanced trauma life support care	3	case_or_format
Adverse Childhood Experiences	adverse childhood experiences	adverse childhood experiences	adverse childhood experiences	Adverse Childhood Experiences	3	kept
advocacy	advocacy	advocacy	advocacy	Advocacy	3	case_or_format
advocacy group	advocacy group	advocacy group	advocacy group	advocacy group	3	kept
aerobic exercise	aerobic exercise	aerobic exercise	aerobic exercise	aerobic exercise	3	kept
Aeromedical	aeromedical	aeromedical	aeromedical	Aeromedical	3	kept
aerospace medicine	aerospace medicine	aerospace medicine	aerospace medicine	Aerospace Medicine	3	case_or_format

affective psychosis	affective psychosis	affective psychosis	affective psychosis	affective psychosis	3	kept
affects	affects	affects	affect	Affect	3	singular
Affordable Care Act	affordable care act	affordable care act	affordable care act	Affordable Care Act	3	kept
AFRICAN-AMERICAN	african american	african american	african american	African American	3	case_or_format
Afzelin	afzelin	afzelin	afzelin	Afzelin	3	kept
Age groups	age groups	age groups	age groups	Age groups	3	kept
agents interacting with transmitter, hormone or drug	agents interacting with transmitter hormone or drug	agents interacting with transmitter hormone or drug	agents interacting with transmitter hormone or drug	agents interacting with transmitter, hormone or drug	3	kept
aggressiveness	aggressiveness	aggressiveness	aggressiveness	aggressiveness	3	kept
Agreement	agreement	agreement	agreement	AGREEMENT	3	case_or_format
air ambulance	air ambulance	air ambulance	air ambulance	Air Ambulances	3	case_or_format
air force	air force	air force	air force	air force	3	kept
air pollutant	air pollutant	air pollutant	air pollutant	air pollutant	3	kept
Airborne fungi	airborne fungi	airborne fungi	airborne fungi	Airborne fungi	3	kept
airborne infection	airborne infection	airborne infection	airborne infection	airborne infection	3	kept
airport	airport	airport	airport	airport	3	kept
airway conductance	airway conductance	airway conductance	airway conductance	airway conductance	3	kept

akinesia	akinesia	akinesia	akinesia	akinesia	3	kept
Alanine Transaminase	alanine transaminase	alanine transaminase	alanine transaminase	Alanine Transaminase	3	kept
Alarm handling	alarm handling	alarm handling	alarm handling	Alarm handling	3	kept
Alarm limits	alarm limits	alarm limits	alarm limits	Alarm limits	3	kept
ALARMS	alarms	alarms	alarm	Alarms	3	singular
Albumin	albumin	albumin	albumin	albumin	3	case_or_format
albumin dialysis	albumin dialysis	albumin dialysis	albumin dialysis	albumin dialysis	3	kept
Alcohol abuse	alcohol abuse	alcohol abuse	alcohol abuse	alcohol abuse	3	case_or_format
alcohol blood level	alcohol blood level	alcohol blood level	alcohol blood level	alcohol blood level	3	kept
alcohol derivative	alcohol derivative	alcohol derivative	alcohol derivative	alcohol derivative	3	kept
alcohol liver disease	alcohol liver disease	alcohol liver disease	alcohol liver disease	alcohol liver disease	3	kept
Alcohol withdrawal syndrome	alcohol withdrawal syndrome	alcohol withdrawal syndrome	alcohol withdrawal syndrome	alcohol withdrawal syndrome	3	case_or_format
Aldosterone	aldosterone	aldosterone	aldosterone	Aldosterone	3	kept
Aldrete score	aldrete score	aldrete score	aldrete score	Aldrete score	3	kept
allergic contact dermatitis	allergic contact dermatitis	allergic contact dermatitis	allergic contact dermatitis	allergic contact dermatitis	3	kept
allied health	allied health	allied health	allied health	Allied health	3	case_or_format

allogeneic stem cell transplantation	allogeneic stem cell transplantation	allogeneic stem cell transplantation	allogeneic stem cell transplantation	allogeneic stem cell transplantation	3	kept
alpha hemolytic Streptococcus	alpha hemolytic streptococcus	alpha hemolytic streptococcus	alpha hemolytic streptococcus	alpha hemolytic Streptococcus	3	kept
alpha2b interferon	alpha2b interferon	alpha2b interferon	alpha2b interferon	alpha2b interferon	3	kept
Alternative communication	alternative communication	alternative communication	alternative communication	Alternative communication	3	kept
ambient noise	ambient noise	ambient noise	ambient noise	ambient noise	3	kept
ambroxol	ambroxol	ambroxol	ambroxol	ambroxol	3	kept
ambulance diversion	ambulance diversion	ambulance diversion	ambulance diversion	ambulance diversion	3	kept
Amnion	amnion	amnion	amnion	Amnion	3	kept
amnion fluid embolism	amnion fluid embolism	amnion fluid embolism	amnion fluid embolism	amnion fluid embolism	3	kept
amniotomy	amniotomy	amniotomy	amniotomy	amniotomy	3	kept
Amphetamines	amphetamines	amphetamines	amphetamine	amphetamine	3	singular
Amplitude-integrated electroencephalography	amplitude integrated electroencephalography	amplitude integrated electroencephalography	amplitude integrated electroencephalography	amplitude integrated electroencephalography	3	case_or_format
Amylases	amylases	amylases	amylase	Amylases	3	singular
Amyotrophic Lateral Sclerosis	amyotrophic lateral sclerosis	amyotrophic lateral sclerosis	amyotrophic lateral sclerosis	amyotrophic lateral sclerosis	3	case_or_format
ANAESTHETICS	anaesthetics	anaesthetics	anaesthetics	ANAESTHETICS	3	kept
anal canal	anal canal	anal canal	anal canal	Anal Canal	3	case_or_format

Analgesic	analgesic	analgesic	analgesic	Analgesics	3	case_or_format
ANALGESICS	analgesics	analgesics	analgesic	Analgesics	3	singular
analgosedation	analgosedation	analgosedation	analgosedation	analgosedation	3	kept
anaphylactic shock	anaphylactic shock	anaphylactic shock	anaphylactic shock	anaphylactic shock	3	kept
Anastomosis, Surgical	anastomosis surgical	anastomosis surgical	anastomosis surgical	Anastomosis, Surgical	3	kept
Ancillary Services, Hospital	ancillary services hospital	ancillary services hospital	ancillary services hospital	Ancillary Services, Hospital	3	kept
and services	and services	and services	and services	and services	3	kept
anesthesia and analgesia	anesthesia and analgesia	anesthesia and analgesia	anesthesia and analgesia	anesthesia and analgesia	3	kept
anesthesia recovery period	anesthesia recovery period	anesthesia recovery period	anesthesia recovery period	Anesthesia Recovery Period	3	case_or_format
Anesthesia, Intravenous	anesthesia intravenous	anesthesia intravenous	anesthesia intravenous	Anesthesia, Intravenous	3	kept
Anesthetics, General	anesthetics general	anesthetics general	anesthetics general	Anesthetics, General	3	kept
Aneurysm	aneurysm	aneurysm	aneurysm	aneurysm	3	case_or_format
aneurysm surgery	aneurysm surgery	aneurysm surgery	aneurysm surgery	aneurysm surgery	3	kept
Aneurysm, Dissecting	aneurysm dissecting	aneurysm dissecting	aneurysm dissecting	Aneurysm, Dissecting	3	kept
Angehörige	angeh rige	angeh rige	angeh rige	Angehörige	3	kept
angiotensin 2 receptor antagonist	angiotensin 2 receptor antagonist	angiotensin 2 receptor antagonist	angiotensin 2 receptor antagonist	angiotensin 2 receptor antagonist	3	kept

Angiotensin Receptor Antagonists	angiotensin receptor antagonists	angiotensin receptor antagonists	angiotensin receptor antagonist	angiotensin receptor antagonist	3	singular
Angola	angola	angola	angola	Angola	3	kept
animal assisted therapy	animal assisted therapy	animal assisted therapy	animal assisted therapy	Animal Assisted Therapy	3	case_or_format
Animal Experimentation	animal experimentation	animal experimentation	animal experimentation	Animal Experimentation	3	kept
Animal Technicians	animal technicians	animal technicians	animal technicians	Animal Technicians	3	kept
Anisocoria	anisocoria	anisocoria	anisocoria	Anisocoria	3	kept
Ankle Joint	ankle joint	ankle joint	ankle joint	Ankle Joint	3	kept
anoxic brain injury	anoxic brain injury	anoxic brain injury	anoxic brain injury	anoxic brain injury	3	kept
antecedent variable	antecedent variable	antecedent variable	antecedent variable	antecedent variable	3	kept
Anthropology	anthropology	anthropology	anthropology	Anthropology	3	kept
anti n methyl d aspartate receptor encephalitis	anti n methyl d aspartate receptor encephalitis	anti n methyl d aspartate receptor encephalitis	anti n methyl d aspartate receptor encephalitis	Anti-N-Methyl-D-Aspartate Receptor Encephalitis	3	case_or_format
antiasthmatic agent	antiasthmatic agent	antiasthmatic agent	antiasthmatic agent	antiasthmatic agent	3	kept
antibacterial agents	antibacterial agents	antibacterial agents	antibacterial agents	Antibacterial agents	3	case_or_format
antibiotic use	antibiotic use	antibiotic use	antibiotic use	ANTIBIOTIC USE	3	case_or_format
ANTIBIOTIC-PROPHYLAXIS	antibiotic prophylaxis	antibiotic prophylaxis	antibiotic prophylaxis	antibiotic prophylaxis	3	case_or_format
ANTIBIOTIC-THERAPY	antibiotic therapy	antibiotic therapy	antibiotic therapy	antibiotic therapy	3	case_or_format

Antibody	antibody	antibody	antibody	Antibodies	3	case_or_format
antibody blood level	antibody blood level	antibody blood level	antibody blood level	antibody blood level	3	kept
antibody response	antibody response	antibody response	antibody response	antibody response	3	kept
anticholinergic effect	anticholinergic effect	anticholinergic effect	anticholinergic effect	anticholinergic effect	3	kept
Antidiuretic Agents	antidiuretic agents	antidiuretic agents	antidiuretic agents	Antidiuretic Agents	3	kept
antifungal activity	antifungal activity	antifungal activity	antifungal activity	antifungal activity	3	kept
antigen	antigen	antigen	antigen	antigen	3	kept
antiinflammatory activity	antiinflammatory activity	antiinflammatory activity	antiinflammatory activity	antiinflammatory activity	3	kept
Antimalarials	antimalarials	antimalarials	antimalarials	Antimalarials	3	kept
antimicrobial	antimicrobial	antimicrobial	antimicrobial	Antimicrobials	3	case_or_format
antioxidant activity	antioxidant activity	antioxidant activity	antioxidant activity	antioxidant activity	3	kept
Antiplatelet	antiplatelet	antiplatelet	antiplatelet	Antiplatelet	3	kept
antipsychotic medications	antipsychotic medications	antipsychotic medications	antipsychotic medications	antipsychotic medications	3	kept
Antiretroviral Therapy, Highly Active	antiretroviral therapy highly active	antiretroviral therapy highly active	antiretroviral therapy highly active	Antiretroviral Therapy, Highly Active	3	kept
antiseptics	antiseptics	antiseptics	antiseptic	antiseptics	3	singular
Anuria	anuria	anuria	anuria	anuria	3	case_or_format

Anxiety and depression	anxiety and depression	anxiety and depression	anxiety and depression	Anxiety and depression	3	kept
Anxiety disorders	anxiety disorders	anxiety disorders	anxiety disorder	anxiety disorder	3	singular
aorta clamping	aorta clamping	aorta clamping	aorta clamping	aorta clamping	3	kept
aorta valve	aorta valve	aorta valve	aorta valve	aorta valve	3	kept
aortic clamping	aortic clamping	aortic clamping	aortic clamping	aortic clamping	3	kept
Aortic Coarctation	aortic coarctation	aortic coarctation	aortic coarctation	Aortic Coarctation	3	kept
aortic reconstruction	aortic reconstruction	aortic reconstruction	aortic reconstruction	aortic reconstruction	3	kept
APACHE II score	apache ii score	apache ii score	apache ii score	APACHE II score	3	kept
apparent diffusion coefficient	apparent diffusion coefficient	apparent diffusion coefficient	apparent diffusion coefficient	apparent diffusion coefficient	3	kept
Appendicitis	appendicitis	appendicitis	appendicitis	appendicitis	3	case_or_format
Appetite	appetite	appetite	appetite	appetite	3	case_or_format
Appreciative inquiry	appreciative inquiry	appreciative inquiry	appreciative inquiry	Appreciative inquiry	3	kept
APPROPRIATENESS	appropriateness	appropriateness	appropriateness	APPROPRIATENESS	3	kept
Aprotinin	aprotinin	aprotinin	aprotinin	aprotinin	3	case_or_format
Architecture	architecture	architecture	architecture	architecture	3	case_or_format
arm circumference	arm circumference	arm circumference	arm circumference	arm circumference	3	kept

Arm Injuries	arm injuries	arm injuries	arm injury	arm injury	3	singular
arm injury	arm injury	arm injury	arm injury	arm injury	3	kept
AROMATHERAPY	aromatherapy	aromatherapy	aromatherapy	Aromatherapy	3	case_or_format
Arterial blood gas	arterial blood gas	arterial blood gas	arterial blood gas	Arterial blood gas	3	kept
Arterial line	arterial line	arterial line	arterial line	arterial line	3	case_or_format
Arterial Occlusive Diseases	arterial occlusive diseases	arterial occlusive diseases	arterial occlusive diseases	Arterial Occlusive Diseases	3	kept
Arterial pressure	arterial pressure	arterial pressure	arterial pressure	arterial pressure	3	case_or_format
Arterial pressures	arterial pressures	arterial pressures	arterial pressure	arterial pressure	3	singular
arteriography	arteriography	arteriography	arteriography	arteriography	3	kept
Arteriovenous malformation	arteriovenous malformation	arteriovenous malformation	arteriovenous malformation	arteriovenous malformation	3	case_or_format
Artesunate	artesunate	artesunate	artesunate	artesunate	3	case_or_format
arthrodesis	arthrodesis	arthrodesis	arthrodesis	arthrodesis	3	kept
artificial airway	artificial airway	artificial airway	artificial airway	Artificial airway	3	case_or_format
artificial embolism	artificial embolism	artificial embolism	artificial embolism	artificial embolism	3	kept
ascending aorta surgery	ascending aorta surgery	ascending aorta surgery	ascending aorta surgery	ascending aorta surgery	3	kept
Assessment scale	assessment scale	assessment scale	assessment scale	Assessment scale	3	kept

Assessment system	assessment system	assessment system	assessment system	Assessment system	3	kept
ASSESSMENT-TOOL	assessment tool	assessment tool	assessment tool	Assessment tool	3	case_or_format
Assisted suicide	assisted suicide	assisted suicide	assisted suicide	assisted suicide	3	case_or_format
assistive technology	assistive technology	assistive technology	assistive technology	assistive technology	3	kept
ASSOCIATIONS	associations	associations	association	ASSOCIATION	3	singular
Astrocytoma	astrocytoma	astrocytoma	astrocytoma	Astrocytoma	3	kept
Asymptomatic Diseases	asymptomatic diseases	asymptomatic diseases	asymptomatic disease	asymptomatic disease	3	singular
Atheist	atheist	atheist	atheist	Atheist	3	kept
atmospheric pressure	atmospheric pressure	atmospheric pressure	atmospheric pressure	atmospheric pressure	3	kept
ATTACHMENT	attachment	attachment	attachment	Attachment	3	case_or_format
attack rate	attack rate	attack rate	attack rate	attack rate	3	kept
Attitude to health	attitude to health	attitude to health	attitude to health	attitude to health	3	case_or_format
attitude toward death	attitude toward death	attitude toward death	attitude toward death	attitude toward death	3	kept
ATTITUDES QUESTIONNAIRE	attitudes questionnaire	attitudes questionnaire	attitudes questionnaire	ATTITUDES QUESTIONNAIRE	3	kept
audiologist	audiologist	audiologist	audiologist	audiologist	3	kept
audiology	audiology	audiology	audiology	audiology	3	kept

audiovisual aid	audiovisual aid	audiovisual aid	audiovisual aid	Audiovisual Aids	3	case_or_format
audiovisual recording	audiovisual recording	audiovisual recording	audiovisual recording	audiovisual recording	3	kept
Audit and feedback	audit and feedback	audit and feedback	audit and feedback	audit and feedback	3	case_or_format
auditory fatigue	auditory fatigue	auditory fatigue	auditory fatigue	Auditory Fatigue	3	case_or_format
Auditory stimulation	auditory stimulation	auditory stimulation	auditory stimulation	auditory stimulation	3	case_or_format
augmentation	augmentation	augmentation	augmentation	augmentation	3	kept
AUSTRALIAN HEALTH-CARE	australian health care	australian health care	australian health care	AUSTRALIAN HEALTH-CARE	3	kept
AUSTRALIAN MEDICAL SHEEPSKIN	australian medical sheepskin	australian medical sheepskin	australian medical sheepskin	AUSTRALIAN MEDICAL SHEEPSKIN	3	kept
autacoid	autacoid	autacoid	autacoid	autacoid	3	kept
Autogenic Training	autogenic training	autogenic training	autogenic training	Autogenic Training	3	kept
Autoimmune Diseases	autoimmune diseases	autoimmune diseases	autoimmune disease	autoimmune disease	3	singular
autoimmune hemolytic anemia	autoimmune hemolytic anemia	autoimmune hemolytic anemia	autoimmune hemolytic anemia	autoimmune hemolytic anemia	3	kept
autoimmune hepatitis	autoimmune hepatitis	autoimmune hepatitis	autoimmune hepatitis	autoimmune hepatitis	3	kept
autologous stem cell transplantation	autologous stem cell transplantation	autologous stem cell transplantation	autologous stem cell transplantation	autologous stem cell transplantation	3	kept
Automated external defibrillator	automated external defibrillator	automated external defibrillator	automated external defibrillator	Automated external defibrillator	3	kept
Automated generation	automated generation	automated generation	automated generation	Automated generation	3	kept

automated pain recognition	automated pain recognition	automated pain recognition	automated pain recognition	automated pain recognition	3	kept
Automated systems	automated systems	automated systems	automated system	Automated systems	3	singular
automatic	automatic	automatic	automatic	automatic	3	kept
automatic positive airway pressure	automatic positive airway pressure	automatic positive airway pressure	automatic positive airway pressure	automatic positive airway pressure	3	kept
autonomic dysreflexia	autonomic dysreflexia	autonomic dysreflexia	autonomic dysreflexia	autonomic dysreflexia	3	kept
autumn	autumn	autumn	autumn	autumn	3	kept
averaging	averaging	averaging	averaging	averaging	3	kept
avian influenza	avian influenza	avian influenza	avian influenza	avian influenza	3	kept
Aviation	aviation	aviation	aviation	aviation	3	case_or_format
avulsion injury	avulsion injury	avulsion injury	avulsion injury	avulsion injury	3	kept
awake craniotomy	awake craniotomy	awake craniotomy	awake craniotomy	awake craniotomy	3	kept
B cell lymphoma	b cell lymphoma	b cell lymphoma	b cell lymphoma	B cell lymphoma	3	kept
B-lines	b lines	b lines	b lines	B-lines	3	kept
Back	back	back	back	back	3	case_or_format
Back injuries	back injuries	back injuries	back injury	Back Injuries	3	singular
BACTERIA	bacteria	bacteria	bacteria	Bacteria	3	case_or_format

bacteria	bacteria	bacteria	bacteria	Bacteria	3	case_or_format
bacterial antigen	bacterial antigen	bacterial antigen	bacterial antigen	bacterial antigen	3	kept
bacterial arthritis	bacterial arthritis	bacterial arthritis	bacterial arthritis	bacterial arthritis	3	kept
Bacterial Outer Membrane Proteins	bacterial outer membrane proteins	bacterial outer membrane proteins	bacterial outer membrane proteins	Bacterial Outer Membrane Proteins	3	kept
Bacterial resistance	bacterial resistance	bacterial resistance	bacterial resistance	Bacterial resistance	3	kept
bacterial skin disease	bacterial skin disease	bacterial skin disease	bacterial skin disease	bacterial skin disease	3	kept
bacterial toxin	bacterial toxin	bacterial toxin	bacterial toxin	bacterial toxin	3	kept
Bacterial Toxins	bacterial toxins	bacterial toxins	bacterial toxin	bacterial toxin	3	singular
bacterial translocation	bacterial translocation	bacterial translocation	bacterial translocation	bacterial translocation	3	kept
bacterial viability	bacterial viability	bacterial viability	bacterial viability	bacterial viability	3	kept
Bahia	bahia	bahia	bahia	Bahia	3	kept
balance impairment	balance impairment	balance impairment	balance impairment	balance impairment	3	kept
balloon	balloon	balloon	balloon	balloon	3	kept
balloon catheter	balloon catheter	balloon catheter	balloon catheter	balloon catheter	3	kept
Balloon Dilatation	balloon dilatation	balloon dilatation	balloon dilatation	balloon dilatation	3	case_or_format
Balloon Occlusion	balloon occlusion	balloon occlusion	balloon occlusion	Balloon Occlusion	3	kept

balloon occlusion	balloon occlusion	balloon occlusion	balloon occlusion	Balloon Occlusion	3	case_or_format
bandaging technique	bandaging technique	bandaging technique	bandaging technique	bandaging technique	3	kept
Bariatric surgery	bariatric surgery	bariatric surgery	bariatric surgery	bariatric surgery	3	case_or_format
Bariatrics	bariatrics	bariatrics	bariatric	Bariatric	3	singular
bariatrics	bariatrics	bariatrics	bariatric	Bariatric	3	singular
barriers to care	barriers to care	barriers to care	barriers to care	barriers to care	3	kept
basal metabolic rate	basal metabolic rate	basal metabolic rate	basal metabolic rate	basal metabolic rate	3	kept
basic reproduction number	basic reproduction number	basic reproduction number	basic reproduction number	basic reproduction number	3	kept
Basque Country	basque country	basque country	basque country	Basque Country	3	kept
BCG vaccination	bcg vaccination	bcg vaccination	bcg vaccination	BCG vaccination	3	kept
Bedside monitoring	bedside monitoring	bedside monitoring	bedside monitoring	Bedside monitoring	3	kept
BEDSIDE ROUNDS	bedside rounds	bedside rounds	bedside rounds	BEDSIDE ROUNDS	3	kept
Bedside rounds	bedside rounds	bedside rounds	bedside rounds	BEDSIDE ROUNDS	3	case_or_format
Bedside teaching	bedside teaching	bedside teaching	bedside teaching	Bedside teaching	3	kept
bedsore	bedsore	bedsore	bedsore	bedsore	3	kept
Bedsores	bedsores	bedsores	bedsore	bedsore	3	singular

Behavior, Addictive	behavior addictive	behavior addictive	behavior addictive	Behavior, Addictive	3	kept
behavioral	behavioral	behavioral	behavioral	behavioral	3	kept
Behavioral health	behavioral health	behavioral health	behavioral health	Behavioral health	3	kept
Behavioral research	behavioral research	behavioral research	behavioral research	Behavioral research	3	kept
Belief	belief	belief	belief	beliefs	3	case_or_format
benefits	benefits	benefits	benefit	benefits	3	singular
benzalkonium chloride	benzalkonium chloride	benzalkonium chloride	benzalkonium chloride	benzalkonium chloride	3	kept
bereavement counseling	bereavement counseling	bereavement counseling	bereavement counseling	bereavement counseling	3	kept
beta 2 adrenergic receptor stimulating agent	beta 2 adrenergic receptor stimulating agent	beta 2 adrenergic receptor stimulating agent	beta 2 adrenergic receptor stimulating agent	beta 2 adrenergic receptor stimulating agent	3	kept
beta-Lactamase Inhibitors	beta lactamase inhibitors	beta lactamase inhibitors	beta lactamase inhibitor	beta lactamase inhibitor	3	singular
betamethasone dipropionate	betamethasone dipropionate	betamethasone dipropionate	betamethasone dipropionate	betamethasone dipropionate	3	kept
betamethasone valerate	betamethasone valerate	betamethasone valerate	betamethasone valerate	betamethasone valerate	3	kept
Bevacizumab	bevacizumab	bevacizumab	bevacizumab	bevacizumab	3	case_or_format
beverage	beverage	beverage	beverage	beverage	3	kept
biapenem	biapenem	biapenem	biapenem	biapenem	3	kept
bile acid	bile acid	bile acid	bile acid	bile acid	3	kept

bile duct carcinoma	bile duct carcinoma	bile duct carcinoma	bile duct carcinoma	bile duct carcinoma	3	kept
Biliary Atresia	biliary atresia	biliary atresia	biliary atresia	Biliary Atresia	3	kept
biliary tract infection	biliary tract infection	biliary tract infection	biliary tract infection	biliary tract infection	3	kept
bioavailability	bioavailability	bioavailability	bioavailability	bioavailability	3	kept
Bioinformatics	bioinformatics	bioinformatics	bioinformatics	Bioinformatics	3	kept
bioinformatics	bioinformatics	bioinformatics	bioinformatics	Bioinformatics	3	case_or_format
Biological organs	biological organs	biological organs	biological organs	Biological organs	3	kept
Biomedical imaging	biomedical imaging	biomedical imaging	biomedical imaging	Biomedical imaging	3	kept
biometry	biometry	biometry	biometry	biometry	3	kept
biosensor	biosensor	biosensor	biosensor	biosensor	3	kept
BiPAP	bipap	bipap	bipap	BiPAP	3	kept
birth order	birth order	birth order	birth order	birth order	3	kept
Birth weight	birth weight	birth weight	birth weight	birth weight	3	case_or_format
bisacodyl	bisacodyl	bisacodyl	bisacodyl	bisacodyl	3	kept
Bismuth	bismuth	bismuth	bismuth	Bismuth	3	kept
Bites and Stings	bites and stings	bites and stings	bites and stings	bites and stings	3	case_or_format

bladder carcinoma	bladder carcinoma	bladder carcinoma	bladder carcinoma	bladder carcinoma	3	kept
bladder disease	bladder disease	bladder disease	bladder disease	bladder disease	3	kept
bladder perforation	bladder perforation	bladder perforation	bladder perforation	bladder perforation	3	kept
Blalock-Taussig Procedure	blalock taussig procedure	blalock taussig procedure	blalock taussig procedure	Blalock-Taussig Procedure	3	kept
Blindness	blindness	blindness	blindness	blindness	3	case_or_format
BLOOD	blood	blood	blood	blood	3	case_or_format
blood biochemistry	blood biochemistry	blood biochemistry	blood biochemistry	blood biochemistry	3	kept
blood cell	blood cell	blood cell	blood cell	blood cell	3	kept
blood clotting factor 7a	blood clotting factor 7a	blood clotting factor 7a	blood clotting factor 7a	blood clotting factor 7a	3	kept
Blood coagulation	blood coagulation	blood coagulation	blood coagulation	Blood Coagulation	3	case_or_format
Blood component transfusion	blood component transfusion	blood component transfusion	blood component transfusion	Blood Component Transfusion	3	case_or_format
Blood conservation	blood conservation	blood conservation	blood conservation	blood conservation	3	case_or_format
blood group ABO incompatibility	blood group abo incompatibility	blood group abo incompatibility	blood group abo incompatibility	blood group ABO incompatibility	3	kept
Blood loss	blood loss	blood loss	blood loss	Blood loss	3	kept
blood management	blood management	blood management	blood management	blood management	3	kept
Blood oxygen saturation	blood oxygen saturation	blood oxygen saturation	blood oxygen saturation	Blood oxygen saturation	3	kept

blood oxygenation	blood oxygenation	blood oxygenation	blood oxygenation	blood oxygenation	3	kept
Blood Platelets	blood platelets	blood platelets	blood platelets	Blood Platelets	3	kept
blood pressure monitor	blood pressure monitor	blood pressure monitor	blood pressure monitor	Blood Pressure Monitors	3	case_or_format
Blood pressure monitoring	blood pressure monitoring	blood pressure monitoring	blood pressure monitoring	blood pressure monitoring	3	case_or_format
Blood Safety	blood safety	blood safety	blood safety	blood safety	3	case_or_format
blood vessel catheterization	blood vessel catheterization	blood vessel catheterization	blood vessel catheterization	blood vessel catheterization	3	kept
blood vessel occlusion	blood vessel occlusion	blood vessel occlusion	blood vessel occlusion	blood vessel occlusion	3	kept
Blood Vessel Prosthesis	blood vessel prosthesis	blood vessel prosthesis	blood vessel prosthesis	Blood Vessel Prosthesis	3	kept
Blood Volume Determination	blood volume determination	blood volume determination	blood volume determination	Blood Volume Determination	3	kept
bnt 162b 2	bnt 162b 2	bnt 162b 2	bnt 162b 2	bnt 162b 2	3	kept
Bochdalek hernia	bochdalek hernia	bochdalek hernia	bochdalek hernia	Bochdalek hernia	3	kept
body fluid compartment	body fluid compartment	body fluid compartment	body fluid compartment	body fluid compartment	3	kept
body mass index	body mass index	body mass index	body mass index	Body Mass Index	3	case_or_format
body regions	body regions	body regions	body regions	body regions	3	kept
bomb	bomb	bomb	bomb	bomb	3	kept
bone injury	bone injury	bone injury	bone injury	bone injury	3	kept

Bone marrow transplant	bone marrow transplant	bone marrow transplant	bone marrow transplant	Bone marrow transplant	3	kept
Bone Transplantation	bone transplantation	bone transplantation	bone transplantation	Bone Transplantation	3	kept
boredom	boredom	boredom	boredom	boredom	3	kept
Borg dyspnea scale	borg dyspnea scale	borg dyspnea scale	borg dyspnea scale	Borg dyspnea scale	3	kept
Bowel management	bowel management	bowel management	bowel management	Bowel management	3	kept
BPD	bpd	bpd	bpd	BPD	3	kept
Braden Q scale	braden q scale	braden q scale	braden q scale	Braden Q scale	3	kept
Braden QD scale	braden qd scale	braden qd scale	braden qd scale	Braden QD scale	3	kept
BRAIN	brain	brain	brain	Brain	3	case_or_format
brain artery aneurysm rupture	brain artery aneurysm rupture	brain artery aneurysm rupture	brain artery aneurysm rupture	brain artery aneurysm rupture	3	kept
brain injury assessment	brain injury assessment	brain injury assessment	brain injury assessment	brain injury assessment	3	kept
brain stem injury	brain stem injury	brain stem injury	brain stem injury	brain stem injury	3	kept
brain tissue	brain tissue	brain tissue	brain tissue	brain tissue	3	kept
Breast cancer	breast cancer	breast cancer	breast cancer	breast cancer	3	case_or_format
breast reconstruction	breast reconstruction	breast reconstruction	breast reconstruction	breast reconstruction	3	kept
Brochure	brochure	brochure	brochure	Brochure	3	kept

bronchiolitis obliterans	bronchiolitis obliterans	bronchiolitis obliterans	bronchiolitis obliterans	Bronchiolitis Obliterans	3	case_or_format
bronchiolitis obliterans organizing pneumonia	bronchiolitis obliterans organizing pneumonia	bronchiolitis obliterans organizing pneumonia	bronchiolitis obliterans organizing pneumonia	bronchiolitis obliterans organizing pneumonia	3	kept
Brussels Capital Region	brussels capital region	brussels capital region	brussels capital region	Brussels Capital Region	3	kept
buccal mucosa	buccal mucosa	buccal mucosa	buccal mucosa	buccal mucosa	3	kept
Built Environment	built environment	built environment	built environment	Built Environment	3	kept
Bulimia	bulimia	bulimia	bulimia	Bulimia	3	kept
bullous skin disease	bullous skin disease	bullous skin disease	bullous skin disease	bullous skin disease	3	kept
Bundle compliance	bundle compliance	bundle compliance	bundle compliance	Bundle compliance	3	kept
Bundle-Branch Block	bundle branch block	bundle branch block	bundle branch block	Bundle-Branch Block	3	kept
Burden of disease	burden of disease	burden of disease	burden of disease	Burden of disease	3	kept
Burden of illness	burden of illness	burden of illness	burden of illness	Burden of illness	3	kept
Burkholderia	burkholderia	burkholderia	burkholderia	Burkholderia	3	kept
Burkholderia Infections	burkholderia infections	burkholderia infections	burkholderia infection	Burkholderia Infections	3	singular
Burkina Faso	burkina faso	burkina faso	burkina faso	Burkina Faso	3	kept
burn excision	burn excision	burn excision	burn excision	burn excision	3	kept
burn shock	burn shock	burn shock	burn shock	burn shock	3	kept

burn-out	burn out	burn out	burn out	Burn out	3	case_or_format
Burnout professional	burnout professional	burnout professional	burnout professional	Burnout, Professional	3	case_or_format
Burnout Syndrome	burnout syndrome	burnout syndrome	burnout syndrome	burnout syndrome	3	case_or_format
Burns, Chemical	burns chemical	burns chemical	burns chemical	Burns, Chemical	3	kept
buttock	buttock	buttock	buttock	buttock	3	kept
Cachexia	cachexia	cachexia	cachexia	cachexia	3	case_or_format
Calciphylaxis	calciphylaxis	calciphylaxis	calciphylaxis	Calciphylaxis	3	kept
calculator	calculator	calculator	calculator	calculator	3	kept
Calidad del sueño	calidad del sue o	calidad del sue o	calidad del sue o	Calidad del sueño	3	kept
call center	call center	call center	call center	call center	3	kept
Calorimetry	calorimetry	calorimetry	calorimetry	calorimetry	3	case_or_format
CANCER CARE	cancer care	cancer care	cancer care	CANCER CARE	3	kept
cancer incidence	cancer incidence	cancer incidence	cancer incidence	cancer incidence	3	kept
Cancer Pain	cancer pain	cancer pain	cancer pain	cancer pain	3	case_or_format
cancer rehabilitation	cancer rehabilitation	cancer rehabilitation	cancer rehabilitation	cancer rehabilitation	3	kept
cancer research	cancer research	cancer research	cancer research	cancer research	3	kept

cancer screening	cancer screening	cancer screening	cancer screening	cancer screening	3	kept
cannabinoid	cannabinoid	cannabinoid	cannabinoid	cannabinoid	3	kept
capillary flow	capillary flow	capillary flow	capillary flow	capillary flow	3	kept
capillary permeability	capillary permeability	capillary permeability	capillary permeability	capillary permeability	3	kept
Capillary refill time	capillary refill time	capillary refill time	capillary refill time	Capillary refill time	3	kept
car	car	car	car	car	3	kept
Carbamazepine	carbamazepine	carbamazepine	carbamazepine	carbamazepine	3	case_or_format
carbapenem resistant Enterobacteriaceae	carbapenem resistant enterobacteriaceae	carbapenem resistant enterobacteriaceae	carbapenem resistant enterobacteriaceae	carbapenem-resistant Enterobacteriaceae	3	case_or_format
Carbon dioxide	carbon dioxide	carbon dioxide	carbon dioxide	carbon dioxide	3	case_or_format
Carbon Footprint	carbon footprint	carbon footprint	carbon footprint	carbon footprint	3	case_or_format
Carbon footprint	carbon footprint	carbon footprint	carbon footprint	carbon footprint	3	case_or_format
carbon monoxide	carbon monoxide	carbon monoxide	carbon monoxide	carbon monoxide	3	kept
Carboxymethylcellulose Sodium	carboxymethylcellulose sodium	carboxymethylcellulose sodium	carboxymethylcellulose sodium	Carboxymethylcellulose Sodium	3	kept
carcinomatous peritonitis	carcinomatous peritonitis	carcinomatous peritonitis	carcinomatous peritonitis	carcinomatous peritonitis	3	kept
cardiac agent	cardiac agent	cardiac agent	cardiac agent	cardiac agent	3	kept
cardiac arrest team	cardiac arrest team	cardiac arrest team	cardiac arrest team	cardiac arrest team	3	kept

Cardiac arrests	cardiac arrests	cardiac arrests	cardiac arrest	Cardiac arrest	3	singular
Cardiac care	cardiac care	cardiac care	cardiac care	cardiac care	3	case_or_format
Cardiac Catheters	cardiac catheters	cardiac catheters	cardiac catheters	Cardiac Catheters	3	kept
Cardiac disease	cardiac disease	cardiac disease	cardiac disease	Cardiac disease	3	kept
cardiac failure	cardiac failure	cardiac failure	cardiac failure	cardiac failure	3	kept
cardiac glycoside	cardiac glycoside	cardiac glycoside	cardiac glycoside	cardiac glycoside	3	kept
Cardiac monitoring	cardiac monitoring	cardiac monitoring	cardiac monitoring	Cardiac monitoring	3	kept
cardiac output monitoring	cardiac output monitoring	cardiac output monitoring	cardiac output monitoring	cardiac output monitoring	3	kept
Cardiogenic oscillations	cardiogenic oscillations	cardiogenic oscillations	cardiogenic oscillations	Cardiogenic oscillations	3	kept
cardioplegia	cardioplegia	cardioplegia	cardioplegia	cardioplegia	3	kept
cardiopulmonary disease	cardiopulmonary disease	cardiopulmonary disease	cardiopulmonary disease	cardiopulmonary disease	3	kept
cardiopulmonary exercise test	cardiopulmonary exercise test	cardiopulmonary exercise test	cardiopulmonary exercise test	cardiopulmonary exercise test	3	kept
cardiorespiratory fitness	cardiorespiratory fitness	cardiorespiratory fitness	cardiorespiratory fitness	cardiorespiratory fitness	3	kept
cardiothoracic	cardiothoracic	cardiothoracic	cardiothoracic	cardiothoracic	3	kept
cardiothoracic surgery	cardiothoracic surgery	cardiothoracic surgery	cardiothoracic surgery	Cardiothoracic surgery	3	case_or_format
Cardiovascular agents	cardiovascular agents	cardiovascular agents	cardiovascular agent	cardiovascular agent	3	singular

cardiovascular risk factor	cardiovascular risk factor	cardiovascular risk factor	cardiovascular risk factor	cardiovascular risk factor	3	kept
Care ethics	care ethics	care ethics	care ethics	Care ethics	3	kept
care model	care model	care model	care model	care model	3	kept
Care pathway	care pathway	care pathway	care pathway	care pathways	3	case_or_format
Care plan	care plan	care plan	care plan	Care plan	3	kept
Care rationing	care rationing	care rationing	care rationing	Care rationing	3	kept
care rationing	care rationing	care rationing	care rationing	Care rationing	3	case_or_format
care transition	care transition	care transition	care transition	care transitions	3	case_or_format
CARE-UNIT	care unit	care unit	care unit	CARE-UNIT	3	kept
Carers	carers	carers	carer	Carers	3	singular
Carga de Trabalho	carga de trabalho	carga de trabalho	carga de trabalho	Carga de Trabalho	3	kept
Caribbean Region	caribbean region	caribbean region	caribbean region	Caribbean Region	3	kept
caring behaviors	caring behaviors	caring behaviors	caring behavior	Caring behavior	3	singular
caring behaviour	caring behaviour	caring behaviour	caring behaviour	caring behaviour	3	kept
Caring behaviours	caring behaviours	caring behaviours	caring behaviour	caring behaviour	3	singular
Carnitine	carnitine	carnitine	carnitine	carnitine	3	case_or_format

carotid artery pulse	carotid artery pulse	carotid artery pulse	carotid artery pulse	carotid artery pulse	3	kept
carotid endarterectomy	carotid endarterectomy	carotid endarterectomy	carotid endarterectomy	carotid endarterectomy	3	kept
Carpal Tunnel Syndrome	carpal tunnel syndrome	carpal tunnel syndrome	carpal tunnel syndrome	Carpal Tunnel Syndrome	3	kept
carpal tunnel syndrome	carpal tunnel syndrome	carpal tunnel syndrome	carpal tunnel syndrome	Carpal Tunnel Syndrome	3	case_or_format
Cartilage Diseases	cartilage diseases	cartilage diseases	cartilage diseases	Cartilage Diseases	3	kept
Case fatality	case fatality	case fatality	case fatality	Case fatality	3	kept
Case fatality rate	case fatality rate	case fatality rate	case fatality rate	case fatality rate	3	case_or_format
Case Managers	case managers	case managers	case manager	case manager	3	singular
case reports	case reports	case reports	case report	case report	3	singular
Case-Based Learning	case based learning	case based learning	case based learning	Case-Based Learning	3	kept
Case-control study	case control study	case control study	case control study	Case-Control Studies	3	case_or_format
CASE-MANAGEMENT	case management	case management	case management	Case Management	3	case_or_format
Catastrophic Illness	catastrophic illness	catastrophic illness	catastrophic illness	Catastrophic Illness	3	kept
Cathartics	cathartics	cathartics	cathartics	Cathartics	3	kept
Catheter-associated urinary tract infections	catheter associated urinary tract infections	catheter associated urinary tract infections	catheter associated urinary tract infection	catheter associated urinary tract infection	3	singular
Catheter-related bloodstream infections	catheter related bloodstream infections	catheter related bloodstream infections	catheter related bloodstream infection	catheter related bloodstream infection	3	singular

catheter-related infection	catheter related infection	catheter related infection	catheter related infection	Catheter-Related Infections	3	case_or_format
CATHETER-RELATED INFECTIONS	catheter related infections	catheter related infections	catheter related infection	Catheter-Related Infections	3	singular
Catholicism	catholicism	catholicism	catholicism	Catholicism	3	kept
caudate nucleus	caudate nucleus	caudate nucleus	caudate nucleus	caudate nucleus	3	kept
Causes	causes	causes	causes	Causes	3	kept
CCFNI	ccfni	ccfni	ccfni	CCFNI	3	kept
CCHF	cchf	cchf	cchf	CCHF	3	kept
CD19 antigen	cd19 antigen	cd19 antigen	cd19 antigen	CD19 antigen	3	kept
CD3+ T lymphocyte	cd3 t lymphocyte	cd3 t lymphocyte	cd3 t lymphocyte	CD3+ T lymphocyte	3	kept
cefdinir	cefdinir	cefdinir	cefdinir	cefdinir	3	kept
cefmetazole	cefmetazole	cefmetazole	cefmetazole	cefmetazole	3	kept
Cefotaxime	cefotaxime	cefotaxime	cefotaxime	cefotaxime	3	case_or_format
ceftaroline	ceftaroline	ceftaroline	ceftaroline	ceftaroline	3	kept
Ceftazidime	ceftazidime	ceftazidime	ceftazidime	ceftazidime	3	case_or_format
ceftolozane	ceftolozane	ceftolozane	ceftolozane	ceftolozane	3	kept
cell culture	cell culture	cell culture	cell culture	cell culture	3	kept

cell infiltration	cell infiltration	cell infiltration	cell infiltration	cell infiltration	3	kept
Cell Phones	cell phones	cell phones	cell phone	Cell Phone	3	singular
Cells, Cultured	cells cultured	cells cultured	cells cultured	Cells, Cultured	3	kept
Cellular Senescence	cellular senescence	cellular senescence	cellular senescence	Cellular Senescence	3	kept
Centers for Medicare and Medicaid Services	centers for medicare and medicaid services	centers for medicare and medicaid services	centers for medicare and medicaid services	Centers for Medicare and Medicaid Services	3	kept
central depressant agent	central depressant agent	central depressant agent	central depressant agent	central depressant agent	3	kept
Central line associated bloodstream infection	central line associated bloodstream infection	central line associated bloodstream infection	central line associated bloodstream infection	Central line-associated bloodstream infections	3	case_or_format
central line associated bloodstream infection	central line associated bloodstream infection	central line associated bloodstream infection	central line associated bloodstream infection	Central line-associated bloodstream infections	3	case_or_format
central line bundle	central line bundle	central line bundle	central line bundle	central line bundle	3	kept
central line maintenance	central line maintenance	central line maintenance	central line maintenance	central line maintenance	3	kept
central line - associated bloodstream infection	central line associated bloodstream infection	central line associated bloodstream infection	central line associated bloodstream infection	Central line-associated bloodstream infections	3	case_or_format
central nervous system depression	central nervous system depression	central nervous system depression	central nervous system depression	central nervous system depression	3	kept
Central Nervous System Infections	central nervous system infections	central nervous system infections	central nervous system infection	central nervous system infection	3	singular
Central nervous system infections	central nervous system infections	central nervous system infections	central nervous system infection	central nervous system infection	3	singular
central pontine myelinolysis	central pontine myelinolysis	central pontine myelinolysis	central pontine myelinolysis	central pontine myelinolysis	3	kept
central retina artery occlusion	central retina artery occlusion	central retina artery occlusion	central retina artery occlusion	central retina artery occlusion	3	kept

central sleep apnea syndrome	central sleep apnea syndrome	central sleep apnea syndrome	central sleep apnea syndrome	central sleep apnea syndrome	3	kept
central stimulant agent	central stimulant agent	central stimulant agent	central stimulant agent	central stimulant agent	3	kept
Central venous access device	central venous access device	central venous access device	central venous access device	central venous access device	3	case_or_format
Central venous catheterisation	central venous catheterisation	central venous catheterisation	central venous catheterisation	Central venous catheterisation	3	kept
Central venous line	central venous line	central venous line	central venous line	Central venous line	3	kept
central venous oxygen saturation	central venous oxygen saturation	central venous oxygen saturation	central venous oxygen saturation	central venous oxygen saturation	3	kept
Central-line-associated bloodstream infection	central line associated bloodstream infection	central line associated bloodstream infection	central line associated bloodstream infection	Central line-associated bloodstream infections	3	case_or_format
Cerebellum	cerebellum	cerebellum	cerebellum	Cerebellum	3	kept
cerebellum	cerebellum	cerebellum	cerebellum	Cerebellum	3	case_or_format
cerebral artery disease	cerebral artery disease	cerebral artery disease	cerebral artery disease	cerebral artery disease	3	kept
Cerebral palsy	cerebral palsy	cerebral palsy	cerebral palsy	cerebral palsy	3	case_or_format
cerebral performance category scale	cerebral performance category scale	cerebral performance category scale	cerebral performance category scale	cerebral performance category scale	3	kept
Cerebral perfusion pressure	cerebral perfusion pressure	cerebral perfusion pressure	cerebral perfusion pressure	cerebral perfusion pressure	3	case_or_format
Cerebral spinal fluid	cerebral spinal fluid	cerebral spinal fluid	cerebral spinal fluid	Cerebral spinal fluid	3	kept
cerebrospinal fluid culture	cerebrospinal fluid culture	cerebrospinal fluid culture	cerebrospinal fluid culture	cerebrospinal fluid culture	3	kept
Cerebrospinal Fluid Proteins	cerebrospinal fluid proteins	cerebrospinal fluid proteins	cerebrospinal fluid proteins	Cerebrospinal Fluid Proteins	3	kept

cerebrospinal fluid shunting	cerebrospinal fluid shunting	cerebrospinal fluid shunting	cerebrospinal fluid shunting	cerebrospinal fluid shunting	3	kept
Cervical	cervical	cervical	cervical	Cervical	3	kept
Cervical Cord	cervical cord	cervical cord	cervical cord	Cervical Cord	3	kept
cervical spinal cord injury	cervical spinal cord injury	cervical spinal cord injury	cervical spinal cord injury	cervical spinal cord injury	3	kept
Cetuximab	cetuximab	cetuximab	cetuximab	Cetuximab	3	kept
CGM	cgm	cgm	cgm	CGM	3	kept
CHA2DS2-VASc score	cha2ds2 vasc score	cha2ds2 vasc score	cha2ds2 vasc score	CHA2DS2-VASc score	3	kept
chalazion	chalazion	chalazion	chalazion	chalazion	3	kept
Challenge	challenge	challenge	challenge	CHALLENGES	3	case_or_format
challenge	challenge	challenge	challenge	CHALLENGES	3	case_or_format
Change	change	change	change	Change	3	kept
Change of shift report	change of shift report	change of shift report	change of shift report	change of shift report	3	case_or_format
chaplaincy	chaplaincy	chaplaincy	chaplaincy	chaplaincy	3	kept
Characteristics	characteristics	characteristics	characteristic	Characteristics	3	singular
Charities	charities	charities	charity	Charities	3	singular
Charting	charting	charting	charting	Charting	3	kept

chest infection	chest infection	chest infection	chest infection	chest infection	3	kept
Child health services	child health services	child health services	child health services	Child Health Services	3	case_or_format
Child Nutrition Sciences	child nutrition sciences	child nutrition sciences	child nutrition sciences	Child Nutrition Sciences	3	kept
child psychiatry	child psychiatry	child psychiatry	child psychiatry	child psychiatry	3	kept
Child Psychology	child psychology	child psychology	child psychology	child psychology	3	case_or_format
child restraint system	child restraint system	child restraint system	child restraint system	Child Restraint Systems	3	case_or_format
CHILDBIRTH	childbirth	childbirth	childbirth	childbirth	3	case_or_format
CHILDREN BORN	children born	children born	children born	CHILDREN BORN	3	kept
Children with medical complexity	children with medical complexity	children with medical complexity	children with medical complexity	Children with medical complexity	3	kept
children with medical complexity	children with medical complexity	children with medical complexity	children with medical complexity	Children with medical complexity	3	case_or_format
Chlamydia pneumoniae	chlamydia pneumoniae	chlamydia pneumoniae	chlamydia pneumoniae	Chlamydia pneumoniae	3	kept
Chlamydophila psittaci	chlamydophila psittaci	chlamydophila psittaci	chlamydophila psittaci	Chlamydophila psittaci	3	kept
CHLORHEXIDINE GLUCONATE	chlorhexidine gluconate	chlorhexidine gluconate	chlorhexidine gluconate	chlorhexidine gluconate	3	case_or_format
chlormethine	chlormethine	chlormethine	chlormethine	chlormethine	3	kept
Chloroquine	chloroquine	chloroquine	chloroquine	chloroquine	3	case_or_format
cholangitis	cholangitis	cholangitis	cholangitis	cholangitis	3	kept

Cholecystitis, Acute	cholecystitis acute	cholecystitis acute	cholecystitis acute	Cholecystitis, Acute	3	kept
cholinergic receptor stimulating agent	cholinergic receptor stimulating agent	cholinergic receptor stimulating agent	cholinergic receptor stimulating agent	cholinergic receptor stimulating agent	3	kept
Christian	christian	christian	christian	Christian	3	kept
Chromatography, High Pressure Liquid	chromatography high pressure liquid	chromatography high pressure liquid	chromatography high pressure liquid	Chromatography, High Pressure Liquid	3	kept
chromium	chromium	chromium	chromium	chromium	3	kept
Chromosome Mapping	chromosome mapping	chromosome mapping	chromosome mapping	Chromosome Mapping	3	kept
Chronic	chronic	chronic	chronic	Chronic	3	kept
chronic care	chronic care	chronic care	chronic care	chronic care	3	kept
CHRONIC CRITICAL ILLNESS	chronic critical illness	chronic critical illness	chronic critical illness	chronic critical illness	3	case_or_format
Chronic disease management	chronic disease management	chronic disease management	chronic disease management	Chronic disease management	3	kept
chronic hepatitis C	chronic hepatitis c	chronic hepatitis c	chronic hepatitis c	chronic hepatitis C	3	kept
Chronic illness	chronic illness	chronic illness	chronic illness	chronic illness	3	case_or_format
Chronic Limb-Threatening Ischemia	chronic limb threatening ischemia	chronic limb threatening ischemia	chronic limb threatening ischemia	Chronic Limb-Threatening Ischemia	3	kept
chronicity	chronicity	chronicity	chronicity	chronicity	3	kept
chylous effusion	chylous effusion	chylous effusion	chylous effusion	chylous effusion	3	kept
Cicatrix	cicatrix	cicatrix	cicatrix	Cicatrix	3	kept

cidofovir	cidofovir	cidofovir	cidofovir	cidofovir	3	kept
CINAHL	cinahl	cinahl	cinahl	Cinahl	3	case_or_format
Circulatory support	circulatory support	circulatory support	circulatory support	Circulatory support	3	kept
Cisatracurium	cisatracurium	cisatracurium	cisatracurium	cisatracurium	3	case_or_format
CiteSpace	citespace	citespace	citespace	Citespace	3	case_or_format
citrate	citrate	citrate	citrate	Citrates	3	case_or_format
CLAIMS DATA	claims data	claims data	claims data	CLAIMS DATA	3	kept
Claims data	claims data	claims data	claims data	CLAIMS DATA	3	case_or_format
Clavicle	clavicle	clavicle	clavicle	Clavicle	3	kept
Cleaning	cleaning	cleaning	cleaning	cleaning	3	case_or_format
clinic	clinic	clinic	clinic	clinic	3	kept
clinical characteristics	clinical characteristics	clinical characteristics	clinical characteristics	clinical characteristics	3	kept
Clinical Chemistry Tests	clinical chemistry tests	clinical chemistry tests	clinical chemistry tests	Clinical Chemistry Tests	3	kept
Clinical decision rules	clinical decision rules	clinical decision rules	clinical decision rule	clinical decision rules	3	singular
Clinical Decision Support Systems	clinical decision support systems	clinical decision support systems	clinical decision support system	clinical decision support system	3	singular
CLINICAL DECISION-SUPPORT	clinical decision support	clinical decision support	clinical decision support	Clinical decision support	3	case_or_format

Clinical decisions	clinical decisions	clinical decisions	clinical decision	Clinical decisions	3	singular
Clinical effectiveness	clinical effectiveness	clinical effectiveness	clinical effectiveness	clinical effectiveness	3	case_or_format
Clinical environment	clinical environment	clinical environment	clinical environment	Clinical environment	3	kept
clinical experience	clinical experience	clinical experience	clinical experience	Clinical experience	3	case_or_format
Clinical frailty scale	clinical frailty scale	clinical frailty scale	clinical frailty scale	Clinical Frailty Scale	3	case_or_format
clinical governance	clinical governance	clinical governance	clinical governance	Clinical governance	3	case_or_format
clinical indicators	clinical indicators	clinical indicators	clinical indicator	clinical indicator	3	singular
Clinical informatics	clinical informatics	clinical informatics	clinical informatics	clinical informatics	3	case_or_format
Clinical information	clinical information	clinical information	clinical information	Clinical information	3	kept
clinical information systems	clinical information systems	clinical information systems	clinical information system	Clinical information system	3	singular
Clinical judgement	clinical judgement	clinical judgement	clinical judgement	clinical judgement	3	case_or_format
clinical laboratory personnel	clinical laboratory personnel	clinical laboratory personnel	clinical laboratory personnel	clinical laboratory personnel	3	kept
clinical leadership	clinical leadership	clinical leadership	clinical leadership	clinical leadership	3	kept
Clinical learning environment	clinical learning environment	clinical learning environment	clinical learning environment	Clinical learning environment	3	kept
clinical nutrition	clinical nutrition	clinical nutrition	clinical nutrition	clinical nutrition	3	kept
Clinical Opioid Withdrawal Scale	clinical opioid withdrawal scale	clinical opioid withdrawal scale	clinical opioid withdrawal scale	Clinical Opioid Withdrawal Scale	3	kept

Clinical practice improvement	clinical practice improvement	clinical practice improvement	clinical practice improvement	Clinical practice improvement	3	kept
clinical prediction	clinical prediction	clinical prediction	clinical prediction	clinical prediction	3	kept
Clinical procedure	clinical procedure	clinical procedure	clinical procedure	clinical procedures	3	case_or_format
Clinical rotation	clinical rotation	clinical rotation	clinical rotation	Clinical rotation	3	kept
clinical safety	clinical safety	clinical safety	clinical safety	clinical safety	3	kept
Clinical sessions	clinical sessions	clinical sessions	clinical sessions	Clinical sessions	3	kept
Clinical Trials, Phase II as Topic	clinical trials phase ii as topic	clinical trials phase ii as topic	clinical trials phase ii as topic	Clinical Trials, Phase II as Topic	3	kept
clinical workflow	clinical workflow	clinical workflow	clinical workflow	clinical workflow	3	kept
CLINICAL-OUTCOMES	clinical outcomes	clinical outcomes	clinical outcome	clinical outcome	3	singular
CLINICAL-TRIALS	clinical trials	clinical trials	clinical trial	clinical trial	3	singular
clinician confidence	clinician confidence	clinician confidence	clinician confidence	clinician confidence	3	kept
clobazam	clobazam	clobazam	clobazam	clobazam	3	kept
Closed-loop	closed loop	closed loop	closed loop	Closed-loop	3	kept
Closed-room air	closed room air	closed room air	closed room air	Closed-room air	3	kept
cloud computing	cloud computing	cloud computing	cloud computing	cloud computing	3	kept
Clubfoot	clubfoot	clubfoot	clubfoot	Clubfoot	3	kept

Coaching	coaching	coaching	coaching	Coaching	3	kept
coaching	coaching	coaching	coaching	Coaching	3	case_or_format
Coagulants	coagulants	coagulants	coagulants	Coagulants	3	kept
Coagulase	coagulase	coagulase	coagulase	Coagulase	3	kept
coagulation function	coagulation function	coagulation function	coagulation function	coagulation function	3	kept
coated tongue	coated tongue	coated tongue	coated tongue	coated tongue	3	kept
cocaine dependence	cocaine dependence	cocaine dependence	cocaine dependence	cocaine dependence	3	kept
Coccidioides	coccidioides	coccidioides	coccidioides	Coccidioides	3	kept
code blue	code blue	code blue	code blue	Code blue	3	case_or_format
codeine phosphate	codeine phosphate	codeine phosphate	codeine phosphate	codeine phosphate	3	kept
Codes of ethics	codes of ethics	codes of ethics	codes of ethics	Codes of Ethics	3	case_or_format
coffee	coffee	coffee	coffee	coffee	3	kept
coffee consumption	coffee consumption	coffee consumption	coffee consumption	coffee consumption	3	kept
Cognitive aids	cognitive aids	cognitive aids	cognitive aid	Cognitive aids	3	singular
cognitive control	cognitive control	cognitive control	cognitive control	cognitive control	3	kept
cognitive development	cognitive development	cognitive development	cognitive development	cognitive development	3	kept

Cognitive Dissonance	cognitive dissonance	cognitive dissonance	cognitive dissonance	Cognitive Dissonance	3	kept
cognitive flexibility	cognitive flexibility	cognitive flexibility	cognitive flexibility	cognitive flexibility	3	kept
cognitive fusion	cognitive fusion	cognitive fusion	cognitive fusion	cognitive fusion	3	kept
cognitive rehabilitation	cognitive rehabilitation	cognitive rehabilitation	cognitive rehabilitation	cognitive rehabilitation	3	kept
Cohorting	cohorting	cohorting	cohorting	Cohorting	3	kept
colectomy	colectomy	colectomy	colectomy	Colectomy	3	case_or_format
colistin resistance	colistin resistance	colistin resistance	colistin resistance	colistin resistance	3	kept
colon tumor	colon tumor	colon tumor	colon tumor	colon tumor	3	kept
Colonic Neoplasms	colonic neoplasms	colonic neoplasms	colonic neoplasms	Colonic Neoplasms	3	kept
Colonic Pseudo-Obstruction	colonic pseudo obstruction	colonic pseudo obstruction	colonic pseudo obstruction	Colonic Pseudo-Obstruction	3	kept
colonisation	colonisation	colonisation	colonisation	colonisation	3	kept
COLOR	color	color	color	Color	3	case_or_format
color ultrasound flowmetry	color ultrasound flowmetry	color ultrasound flowmetry	color ultrasound flowmetry	color ultrasound flowmetry	3	kept
Colorectal surgery	colorectal surgery	colorectal surgery	colorectal surgery	colorectal surgery	3	case_or_format
COMA	coma	coma	coma	Coma	3	case_or_format
Coma scale	coma scale	coma scale	coma scale	Coma scale	3	kept

comfort measures only order	comfort measures only order	comfort measures only order	comfort measures only order	comfort measures only order	3	kept
COMHON Index	comhon index	comhon index	comhon index	COMHON Index	3	kept
COMHON index	comhon index	comhon index	comhon index	COMHON Index	3	case_or_format
common bile duct stone	common bile duct stone	common bile duct stone	common bile duct stone	common bile duct stone	3	kept
COMMON PROCEDURES	common procedures	common procedures	common procedures	COMMON PROCEDURES	3	kept
Communication gap	communication gap	communication gap	communication gap	Communication gap	3	kept
communication skills	communication skills	communication skills	communication skill	communication skill	3	singular
Communication skills training	communication skills training	communication skills training	communication skills training	Communication skills training	3	kept
Communication systems	communication systems	communication systems	communication systems	Communication systems	3	kept
communication technology	communication technology	communication technology	communication technology	communication technology	3	kept
communications	communications	communications	communication	Communication	3	singular
Community care	community care	community care	community care	community care	3	case_or_format
Community Health Centers	community health centers	community health centers	community health centers	Community Health Centers	3	kept
Community Mental Health Services	community mental health services	community mental health services	community mental health service	Community Mental Health Services	3	singular
comparative analysis	comparative analysis	comparative analysis	comparative analysis	comparative analysis	3	kept
Comparison	comparison	comparison	comparison	Comparison	3	kept

compassionate use	compassionate use	compassionate use	compassionate use	compassionate use	3	kept
complementary therapies	complementary therapies	complementary therapies	complementary therapy	Complementary Therapies	3	singular
COMPLEX CHRONIC CONDITIONS	complex chronic conditions	complex chronic conditions	complex chronic conditions	COMPLEX CHRONIC CONDITIONS	3	kept
Complexity of care	complexity of care	complexity of care	complexity of care	Complexity of care	3	kept
Compliance control	compliance control	compliance control	compliance control	Compliance control	3	kept
complication rate	complication rate	complication rate	complication rate	complication rate	3	kept
compression stocking	compression stocking	compression stocking	compression stocking	compression stocking	3	kept
Compulsive Personality Disorder	compulsive personality disorder	compulsive personality disorder	compulsive personality disorder	Compulsive Personality Disorder	3	kept
computer aided design	computer aided design	computer aided design	computer aided design	computer aided design	3	kept
Computer aided instruction	computer aided instruction	computer aided instruction	computer aided instruction	Computer aided instruction	3	kept
Computer circuits	computer circuits	computer circuits	computer circuits	Computer circuits	3	kept
computer graphics	computer graphics	computer graphics	computer graphics	Computer Graphics	3	case_or_format
computer network	computer network	computer network	computer network	computer network	3	kept
Computerized	computerized	computerized	computerized	Computerized	3	kept
compétence	comp tence	comp tence	comp tence	compétence	3	kept
concentration loss	concentration loss	concentration loss	concentration loss	concentration loss	3	kept

concentration response	concentration response	concentration response	concentration response	concentration response	3	kept
Concept Analysis	concept analysis	concept analysis	concept analysis	concept analysis	3	case_or_format
concept development	concept development	concept development	concept development	concept development	3	kept
Conceptual model	conceptual model	conceptual model	conceptual model	conceptual model	3	case_or_format
concurrent infection	concurrent infection	concurrent infection	concurrent infection	concurrent infection	3	kept
conditioning	conditioning	conditioning	conditioning	conditioning	3	kept
Conflict of Interest	conflict of interest	conflict of interest	conflict of interest	conflict of interest	3	case_or_format
Conflict resolution	conflict resolution	conflict resolution	conflict resolution	Conflict resolution	3	kept
conflicts	conflicts	conflicts	conflict	conflict	3	singular
Confounding Factors	confounding factors	confounding factors	confounding factors	Confounding Factors	3	kept
CONFUSION	confusion	confusion	confusion	confusion	3	case_or_format
Confusion assessment method	confusion assessment method	confusion assessment method	confusion assessment method	Confusion Assessment Method	3	case_or_format
Congenital heart defect	congenital heart defect	congenital heart defect	congenital heart defect	congenital heart defect	3	case_or_format
CONGENITAL HEART-DISEASE	congenital heart disease	congenital heart disease	congenital heart disease	congenital heart disease	3	case_or_format
congenital hypothyroidism	congenital hypothyroidism	congenital hypothyroidism	congenital hypothyroidism	congenital hypothyroidism	3	kept
congenital infection	congenital infection	congenital infection	congenital infection	congenital infection	3	kept

congenital syphilis	congenital syphilis	congenital syphilis	congenital syphilis	congenital syphilis	3	kept
conjunctival hemorrhage	conjunctival hemorrhage	conjunctival hemorrhage	conjunctival hemorrhage	conjunctival hemorrhage	3	kept
CONNOR-DAVIDSON RESILIENCE	connor davidson resilience	connor davidson resilience	connor davidson resilience	CONNOR-DAVIDSON RESILIENCE	3	kept
Conocimientos	conocimientos	conocimientos	conocimientos	Conocimientos	3	kept
conscience	conscience	conscience	conscience	Conscience	3	case_or_format
CONSCIOUSNESS	consciousness	consciousness	consciousness	consciousness	3	case_or_format
Consciousness level	consciousness level	consciousness level	consciousness level	consciousness level	3	case_or_format
consent forms	consent forms	consent forms	consent forms	consent forms	3	kept
Consequences	consequences	consequences	consequence	CONSEQUENCES	3	singular
Conservation of Natural Resources	conservation of natural resources	conservation of natural resources	conservation of natural resources	Conservation of Natural Resources	3	kept
Constructivist grounded theory	constructivist grounded theory	constructivist grounded theory	constructivist grounded theory	constructivist grounded theory	3	case_or_format
Consumer Participation	consumer participation	consumer participation	consumer participation	Consumer participation	3	case_or_format
Consumer Product Safety	consumer product safety	consumer product safety	consumer product safety	Consumer Product Safety	3	kept
Contact tracing	contact tracing	contact tracing	contact tracing	Contact Tracing	3	case_or_format
contact tracing	contact tracing	contact tracing	contact tracing	Contact Tracing	3	case_or_format
containment	containment	containment	containment	containment	3	kept

Content validity	content validity	content validity	content validity	content validity	3	case_or_format
Contextual factors	contextual factors	contextual factors	contextual factors	Contextual factors	3	kept
Continuing Education	continuing education	continuing education	continuing education	continuing education	3	case_or_format
Continuing professional education	continuing professional education	continuing professional education	continuing professional education	Continuing professional education	3	kept
Continuity	continuity	continuity	continuity	Continuity	3	kept
continuous ambulatory peritoneal dialysis	continuous ambulatory peritoneal dialysis	continuous ambulatory peritoneal dialysis	continuous ambulatory peritoneal dialysis	continuous ambulatory peritoneal dialysis	3	kept
Continuous cardiac output	continuous cardiac output	continuous cardiac output	continuous cardiac output	Continuous cardiac output	3	kept
Continuous infusion	continuous infusion	continuous infusion	continuous infusion	continuous infusion	3	case_or_format
Continuous kidney replacement therapy	continuous kidney replacement therapy	continuous kidney replacement therapy	continuous kidney replacement therapy	Continuous kidney replacement therapy	3	kept
continuous kidney replacement therapy	continuous kidney replacement therapy	continuous kidney replacement therapy	continuous kidney replacement therapy	Continuous kidney replacement therapy	3	case_or_format
continuous monitoring	continuous monitoring	continuous monitoring	continuous monitoring	Continuous monitoring	3	case_or_format
continuous professional development	continuous professional development	continuous professional development	continuous professional development	continuous professional development	3	kept
continuum of care	continuum of care	continuum of care	continuum of care	Continuum of care	3	case_or_format
Control charts	control charts	control charts	control chart	control charts	3	singular
Control Groups	control groups	control groups	control group	control group	3	singular
controlled substance	controlled substance	controlled substance	controlled substance	controlled substance	3	kept

CONTROLLED TRIAL	controlled trial	controlled trial	controlled trial	CONTROLLED-TRIAL	3	case_or_format
Contusions	contusions	contusions	contusion	contusion	3	singular
Conversational analysis	conversational analysis	conversational analysis	conversational analysis	Conversational analysis	3	kept
Convolutional neural network	convolutional neural network	convolutional neural network	convolutional neural network	convolutional neural network	3	case_or_format
cooking	cooking	cooking	cooking	cooking	3	kept
Cooperative behavior	cooperative behavior	cooperative behavior	cooperative behavior	Cooperative Behavior	3	case_or_format
COPD exacerbation	copd exacerbation	copd exacerbation	copd exacerbation	COPD exacerbation	3	kept
Coping behavior	coping behavior	coping behavior	coping behavior	coping behavior	3	case_or_format
coping mechanisms	coping mechanisms	coping mechanisms	coping mechanism	coping mechanisms	3	singular
Coping skills	coping skills	coping skills	coping skills	Coping Skills	3	case_or_format
Core measures	core measures	core measures	core measures	Core measures	3	kept
cornea	cornea	cornea	cornea	Cornea	3	case_or_format
cornea injury	cornea injury	cornea injury	cornea injury	cornea injury	3	kept
cornea reflex	cornea reflex	cornea reflex	cornea reflex	cornea reflex	3	kept
Corneal diseases	corneal diseases	corneal diseases	corneal diseases	Corneal Diseases	3	case_or_format
Corneal injuries	corneal injuries	corneal injuries	corneal injury	Corneal Injuries	3	singular

Corneal Ulcer	corneal ulcer	corneal ulcer	corneal ulcer	Corneal Ulcer	3	kept
Corneal ulcer	corneal ulcer	corneal ulcer	corneal ulcer	Corneal Ulcer	3	case_or_format
cornell assessment of pediatric delirium	cornell assessment of pediatric delirium	cornell assessment of pediatric delirium	cornell assessment of pediatric delirium	Cornell Assessment of Pediatric Delirium	3	case_or_format
Coronary artery bypass graft surgery	coronary artery bypass graft surgery	coronary artery bypass graft surgery	coronary artery bypass graft surgery	Coronary artery bypass graft surgery	3	kept
coronary artery bypass graft surgery	coronary artery bypass graft surgery	coronary artery bypass graft surgery	coronary artery bypass graft surgery	Coronary artery bypass graft surgery	3	case_or_format
coronary artery calcification	coronary artery calcification	coronary artery calcification	coronary artery calcification	coronary artery calcification	3	kept
coronary artery surgery	coronary artery surgery	coronary artery surgery	coronary artery surgery	coronary artery surgery	3	kept
coronavirus pandemic	coronavirus pandemic	coronavirus pandemic	coronavirus pandemic	coronavirus pandemic	3	kept
coronavirus spike glycoprotein	coronavirus spike glycoprotein	coronavirus spike glycoprotein	coronavirus spike glycoprotein	coronavirus spike glycoprotein	3	kept
Coroners and Medical Examiners	coroners and medical examiners	coroners and medical examiners	coroners and medical examiners	Coroners and Medical Examiners	3	kept
corpus callosum	corpus callosum	corpus callosum	corpus callosum	corpus callosum	3	kept
correlation	correlation	correlation	correlation	correlation	3	kept
corticobasal degeneration	corticobasal degeneration	corticobasal degeneration	corticobasal degeneration	corticobasal degeneration	3	kept
Cost Allocation	cost allocation	cost allocation	cost allocation	Cost Allocation	3	kept
Cost benefit analysis	cost benefit analysis	cost benefit analysis	cost benefit analysis	Cost-Benefit Analysis	3	case_or_format
Cost control	cost control	cost control	cost control	cost control	3	case_or_format

cost effectiveness	cost effectiveness	cost effectiveness	cost effectiveness	Cost-effectiveness	3	case_or_format
Cost effectiveness analysis	cost effectiveness analysis	cost effectiveness analysis	cost effectiveness analysis	cost effectiveness analysis	3	case_or_format
Cost of illness	cost of illness	cost of illness	cost of illness	Cost of Illness	3	case_or_format
cost of living	cost of living	cost of living	cost of living	cost of living	3	kept
Costing	costing	costing	costing	Costing	3	kept
cost‐benefit analysis	cost benefit analysis	cost benefit analysis	cost benefit analysis	Cost-Benefit Analysis	3	case_or_format
COUNTRIES	countries	countries	countries	COUNTRIES	3	kept
courage	courage	courage	courage	Courage	3	case_or_format
Covid 19	covid 19	covid 19	covid 19	COVID-19	3	case_or_format
COVID-19 pharmacotherapy	covid 19 pharmacotherapy	covid 19 pharmacotherapy	covid 19 pharmacotherapy	COVID-19 pharmacotherapy	3	kept
COVID-19 Serological Testing	covid 19 serological testing	covid 19 serological testing	covid 19 serological testing	COVID-19 serological testing	3	case_or_format
COVID-19 severity	covid 19 severity	covid 19 severity	covid 19 severity	COVID-19 severity	3	kept
COVID‐19 pandemic	covid 19 pandemic	covid 19 pandemic	covid 19 pandemic	COVID-19 pandemic	3	case_or_format
cranial nerve paralysis	cranial nerve paralysis	cranial nerve paralysis	cranial nerve paralysis	cranial nerve paralysis	3	kept
Cranial Nerves	cranial nerves	cranial nerves	cranial nerve	cranial nerve	3	singular
craniofacial synostosis	craniofacial synostosis	craniofacial synostosis	craniofacial synostosis	craniofacial synostosis	3	kept

Creatine	creatine	creatine	creatine	Creatine	3	kept
credentialing	credentialing	credentialing	credentialing	Credentialing	3	case_or_format
cricoid	cricoid	cricoid	cricoid	cricoid	3	kept
Crime Victims	crime victims	crime victims	crime victim	crime victim	3	singular
Crisis	crisis	crisis	crisis	CRISIS	3	case_or_format
Crisis intervention	crisis intervention	crisis intervention	crisis intervention	Crisis Intervention	3	case_or_format
crisis resource management	crisis resource management	crisis resource management	crisis resource management	Crisis resource management	3	case_or_format
criterion variable	criterion variable	criterion variable	criterion variable	criterion variable	3	kept
Critical ethnography	critical ethnography	critical ethnography	critical ethnography	Critical ethnography	3	kept
critical ill	critical ill	critical ill	critical ill	critical ill	3	kept
Critical incident	critical incident	critical incident	critical incident	critical incidents	3	case_or_format
critical pathway	critical pathway	critical pathway	critical pathway	Critical Pathways	3	case_or_format
Critical pathways	critical pathways	critical pathways	critical pathway	Critical Pathways	3	singular
Criticality	criticality	criticality	criticality	Criticality	3	kept
critically illness	critically illness	critically illness	critically illness	critically illness	3	kept
Crohn Disease	crohn disease	crohn disease	crohn disease	Crohn disease	3	case_or_format

Cronobacter sakazakii	cronobacter sakazakii	cronobacter sakazakii	cronobacter sakazakii	Cronobacter sakazakii	3	kept
Cross contamination	cross contamination	cross contamination	cross contamination	Cross contamination	3	kept
Cross-sectional	cross sectional	cross sectional	cross sectional	Cross-sectional	3	kept
cross-sectional	cross sectional	cross sectional	cross sectional	Cross-sectional	3	case_or_format
Crotalus	crotalus	crotalus	crotalus	Crotalus	3	kept
cryopreservation	cryopreservation	cryopreservation	cryopreservation	cryopreservation	3	kept
Cryptococcus	cryptococcus	cryptococcus	cryptococcus	Cryptococcus	3	kept
CT scan	ct scan	ct scan	ct scan	CT scan	3	kept
Cuba	cuba	cuba	cuba	Cuba	3	kept
cues	cues	cues	cues	Cues	3	case_or_format
CUFF PRESSURE	cuff pressure	cuff pressure	cuff pressure	Cuff pressure	3	case_or_format
Cuidados Críticos	cuidados cr ticos	cuidados cr ticos	cuidados cr ticos	Cuidados críticos	3	case_or_format
Cuidados Paliativos	cuidados paliativos	cuidados paliativos	cuidados paliativos	Cuidados Paliativos	3	kept
Cuidados paliativos	cuidados paliativos	cuidados paliativos	cuidados paliativos	Cuidados Paliativos	3	case_or_format
cultural adaptation	cultural adaptation	cultural adaptation	cultural adaptation	cultural adaptation	3	kept
cultural background	cultural background	cultural background	cultural background	cultural background	3	kept

cultural care	cultural care	cultural care	cultural care	cultural care	3	kept
cultural issues	cultural issues	cultural issues	cultural issue	cultural issues	3	singular
culture technique	culture technique	culture technique	culture technique	culture technique	3	kept
Current practices	current practices	current practices	current practice	Current practices	3	singular
Cycled light	cycled light	cycled light	cycled light	Cycled light	3	kept
cyclooxygenase 2 inhibitor	cyclooxygenase 2 inhibitor	cyclooxygenase 2 inhibitor	cyclooxygenase 2 inhibitor	cyclooxygenase 2 inhibitor	3	kept
cyst	cyst	cyst	cyst	cyst	3	kept
Cystic fibrosis	cystic fibrosis	cystic fibrosis	cystic fibrosis	cystic fibrosis	3	case_or_format
cytostatic agent	cytostatic agent	cytostatic agent	cytostatic agent	cytostatic agent	3	kept
Daily interruption	daily interruption	daily interruption	daily interruption	Daily interruption	3	kept
daily interruption of sedation	daily interruption of sedation	daily interruption of sedation	daily interruption of sedation	daily interruption of sedation	3	kept
Damage control surgery	damage control surgery	damage control surgery	damage control surgery	Damage control surgery	3	kept
damage control surgery	damage control surgery	damage control surgery	damage control surgery	Damage control surgery	3	case_or_format
dapsone	dapsone	dapsone	dapsone	dapsone	3	kept
Daptomycin	daptomycin	daptomycin	daptomycin	daptomycin	3	case_or_format
darkness	darkness	darkness	darkness	darkness	3	kept

dashboard	dashboard	dashboard	dashboard	dashboard	3	kept
Data analysis	data analysis	data analysis	data analysis	data analysis	3	case_or_format
data envelopment analysis	data envelopment analysis	data envelopment analysis	data envelopment analysis	Data envelopment analysis	3	case_or_format
Data management	data management	data management	data management	Data management	3	kept
Data Visualization	data visualization	data visualization	data visualization	data visualization	3	case_or_format
Datasets as Topic	datasets as topic	datasets as topic	datasets as topic	Datasets as Topic	3	kept
daughter	daughter	daughter	daughter	daughter	3	kept
daytime somnolence	daytime somnolence	daytime somnolence	daytime somnolence	daytime somnolence	3	kept
DCV model	dcv model	dcv model	dcv model	DCV model	3	kept
DDAVP	ddavp	ddavp	ddavp	DDAVP	3	kept
DE-ESCALATION	de escalation	de escalation	de escalation	DE-ESCALATION	3	kept
death toll	death toll	death toll	death toll	death toll	3	kept
deaths	deaths	deaths	death	death	3	singular
debrief	debrief	debrief	debrief	debrief	3	kept
decibels	decibels	decibels	decibel	decibels	3	singular
decision aids	decision aids	decision aids	decision aid	decision aids	3	singular

Decision making process	decision making process	decision making process	decision making process	Decision making process	3	kept
Decision-making process	decision making process	decision making process	decision making process	Decision making process	3	case_or_format
DECOLONIZATION	decolonization	decolonization	decolonization	Decolonization	3	case_or_format
Decubitus ulcer	decubitus ulcer	decubitus ulcer	decubitus ulcer	Decubitus ulcer	3	kept
Defecation	defecation	defecation	defecation	defecation	3	case_or_format
defensive behavior	defensive behavior	defensive behavior	defensive behavior	defensive behavior	3	kept
DEFINITION	definition	definition	definition	Definitions	3	case_or_format
Delayed cerebral ischemia	delayed cerebral ischemia	delayed cerebral ischemia	delayed cerebral ischemia	Delayed cerebral ischemia	3	kept
DELIBERATE PRACTICE	deliberate practice	deliberate practice	deliberate practice	Deliberate practice	3	case_or_format
Delirio	delirio	delirio	delirio	Delirio	3	kept
Delirium O Meter	delirium o meter	delirium o meter	delirium o meter	Delirium O Meter	3	kept
Delirium Observation Screening Scale	delirium observation screening scale	delirium observation screening scale	delirium observation screening scale	Delirium Observation Screening Scale	3	kept
Delirium Rating Scale-revised	delirium rating scale revised	delirium rating scale revised	delirium rating scale revised	Delirium Rating Scale-revised	3	kept
Delirium screening	delirium screening	delirium screening	delirium screening	Delirium screening	3	kept
delirium screening	delirium screening	delirium screening	delirium screening	Delirium screening	3	case_or_format
delirium screening tools	delirium screening tools	delirium screening tools	delirium screening tool	Delirium screening tools	3	singular

Delirium Symptom Interview	delirium symptom interview	delirium symptom interview	delirium symptom interview	Delirium Symptom Interview	3	kept
Delivery	delivery	delivery	delivery	delivery	3	case_or_format
DELPHI	delphi	delphi	delphi	Delphi	3	case_or_format
Demographic factors	demographic factors	demographic factors	demographic factor	Demographic factors	3	singular
demoralization	demoralization	demoralization	demoralization	demoralization	3	kept
demyelination	demyelination	demyelination	demyelination	demyelination	3	kept
dental clinic	dental clinic	dental clinic	dental clinic	dental clinic	3	kept
Dental Staff, Hospital	dental staff hospital	dental staff hospital	dental staff hospital	Dental Staff, Hospital	3	kept
Dentists	dentists	dentists	dentist	dentist	3	singular
Dependency	dependency	dependency	dependency	dependency	3	case_or_format
Dependency, Psychological	dependency psychological	dependency psychological	dependency psychological	Dependency, Psychological	3	kept
Depersonalisation	depersonalisation	depersonalisation	depersonalisation	Depersonalisation	3	kept
Depression, Anxiety and Stress Scale	depression anxiety and stress scale	depression anxiety and stress scale	depression anxiety and stress scale	Depression, Anxiety and Stress Scale	3	kept
Depressive symptoms	depressive symptoms	depressive symptoms	depressive symptom	Depressive symptoms	3	singular
DEPRIVATION	deprivation	deprivation	deprivation	Deprivation	3	case_or_format
Dermatitis, Atopic	dermatitis atopic	dermatitis atopic	dermatitis atopic	Dermatitis, Atopic	3	kept

descending aorta	descending aorta	descending aorta	descending aorta	descending aorta	3	kept
Detection	detection	detection	detection	detection	3	case_or_format
detergent	detergent	detergent	detergent	Detergents	3	case_or_format
Developing world	developing world	developing world	developing world	developing world	3	case_or_format
Developmental Biology	developmental biology	developmental biology	developmental biology	Developmental Biology	3	kept
Developmental supportive care	developmental supportive care	developmental supportive care	developmental supportive care	Developmental supportive care	3	kept
DEVICE	device	device	device	devices	3	case_or_format
device therapy	device therapy	device therapy	device therapy	device therapy	3	kept
dextropropoxyphene	dextropropoxyphene	dextropropoxyphene	dextropropoxyphene	dextropropoxyphene	3	kept
dextrose	dextrose	dextrose	dextrose	dextrose	3	kept
diabetes control	diabetes control	diabetes control	diabetes control	diabetes control	3	kept
diabetes educator	diabetes educator	diabetes educator	diabetes educator	diabetes educator	3	kept
Diabetes Insipidus, Neurogenic	diabetes insipidus neurogenic	diabetes insipidus neurogenic	diabetes insipidus neurogenic	Diabetes Insipidus, Neurogenic	3	kept
DIABETES-MELLITUS	diabetes mellitus	diabetes mellitus	diabetes mellitus	diabetes mellitus	3	case_or_format
diabetic complication	diabetic complication	diabetic complication	diabetic complication	diabetic complication	3	kept
Diagnosis-related groups	diagnosis related groups	diagnosis related groups	diagnosis related group	Diagnosis-Related Groups	3	singular

Diagnostic	diagnostic	diagnostic	diagnostic	diagnostics	3	case_or_format
diagnostic	diagnostic	diagnostic	diagnostic	diagnostics	3	case_or_format
Diagnostic criteria	diagnostic criteria	diagnostic criteria	diagnostic criteria	Diagnostic criteria	3	kept
diagnostic tests	diagnostic tests	diagnostic tests	diagnostic test	diagnostic test	3	singular
dialysate	dialysate	dialysate	dialysate	dialysate	3	kept
dialysis access	dialysis access	dialysis access	dialysis access	dialysis access	3	kept
diaper	diaper	diaper	diaper	diaper	3	kept
Diapers, Adult	diapers adult	diapers adult	diapers adult	Diapers, Adult	3	kept
Diarrhoea	diarrhoea	diarrhoea	diarrhoea	diarrhoea	3	case_or_format
Diary as Topic	diary as topic	diary as topic	diary as topic	Diary as Topic	3	kept
Diastole	diastole	diastole	diastole	Diastole	3	kept
diastolic heart failure	diastolic heart failure	diastolic heart failure	diastolic heart failure	diastolic heart failure	3	kept
Diazepam	diazepam	diazepam	diazepam	diazepam	3	case_or_format
diazoxide	diazoxide	diazoxide	diazoxide	diazoxide	3	kept
Dietary Carbohydrates	dietary carbohydrates	dietary carbohydrates	dietary carbohydrates	Dietary Carbohydrates	3	kept
Dietary Fats	dietary fats	dietary fats	dietary fats	Dietary Fats	3	kept

Differential privacy	differential privacy	differential privacy	differential privacy	Differential privacy	3	kept
diffuse axonal injury	diffuse axonal injury	diffuse axonal injury	diffuse axonal injury	diffuse axonal injury	3	kept
diffusion tensor imaging	diffusion tensor imaging	diffusion tensor imaging	diffusion tensor imaging	diffusion tensor imaging	3	kept
DiGeorge syndrome	digeorge syndrome	digeorge syndrome	digeorge syndrome	DiGeorge syndrome	3	kept
Digestive System	digestive system	digestive system	digestive system	digestive system	3	case_or_format
Digestive System Diseases	digestive system diseases	digestive system diseases	digestive system disease	Digestive System Diseases	3	singular
digestive system ulcer	digestive system ulcer	digestive system ulcer	digestive system ulcer	digestive system ulcer	3	kept
digestive tract agent	digestive tract agent	digestive tract agent	digestive tract agent	digestive tract agent	3	kept
digestive tract parameters	digestive tract parameters	digestive tract parameters	digestive tract parameters	digestive tract parameters	3	kept
Digital	digital	digital	digital	Digital	3	kept
Digital literacy	digital literacy	digital literacy	digital literacy	Digital literacy	3	kept
digital radiography	digital radiography	digital radiography	digital radiography	digital radiography	3	kept
Digital storytelling	digital storytelling	digital storytelling	digital storytelling	Digital storytelling	3	kept
digoxin antibody F fragment	digoxin antibody f fragment	digoxin antibody f fragment	digoxin antibody f fragment	digoxin antibody F fragment	3	kept
Dilatation, Pathologic	dilatation pathologic	dilatation pathologic	dilatation pathologic	Dilatation, Pathologic	3	kept
DILEMMAS	dilemmas	dilemmas	dilemmas	DILEMMAS	3	kept

dimethyl sulfoxide	dimethyl sulfoxide	dimethyl sulfoxide	dimethyl sulfoxide	dimethyl sulfoxide	3	kept
diphtheria pertussis tetanus vaccine	diphtheria pertussis tetanus vaccine	diphtheria pertussis tetanus vaccine	diphtheria pertussis tetanus vaccine	diphtheria pertussis tetanus vaccine	3	kept
directed acyclic graph	directed acyclic graph	directed acyclic graph	directed acyclic graph	directed acyclic graph	3	kept
disabled child	disabled child	disabled child	disabled child	disabled child	3	kept
disadvantaged population	disadvantaged population	disadvantaged population	disadvantaged population	disadvantaged population	3	kept
Disaster preparedness	disaster preparedness	disaster preparedness	disaster preparedness	disaster preparedness	3	case_or_format
disaster victim	disaster victim	disaster victim	disaster victim	disaster victim	3	kept
discharge criteria	discharge criteria	discharge criteria	discharge criteria	discharge criteria	3	kept
Discharge disposition	discharge disposition	discharge disposition	discharge disposition	discharge disposition	3	case_or_format
discharge instructions	discharge instructions	discharge instructions	discharge instructions	discharge instructions	3	kept
Discharge process	discharge process	discharge process	discharge process	Discharge process	3	kept
discharge process	discharge process	discharge process	discharge process	Discharge process	3	case_or_format
discharge readiness	discharge readiness	discharge readiness	discharge readiness	discharge readiness	3	kept
Discrete choice experiment	discrete choice experiment	discrete choice experiment	discrete choice experiment	Discrete choice experiment	3	kept
Discrimination	discrimination	discrimination	discrimination	Discrimination	3	kept
discrimination	discrimination	discrimination	discrimination	Discrimination	3	case_or_format

disease	disease	disease	disease	Diseases	3	case_or_format
disease activity score	disease activity score	disease activity score	disease activity score	disease activity score	3	kept
Disease burden	disease burden	disease burden	disease burden	disease burden	3	case_or_format
Disease control	disease control	disease control	disease control	disease control	3	case_or_format
disease model	disease model	disease model	disease model	disease model	3	kept
Disease Notification	disease notification	disease notification	disease notification	Disease Notification	3	kept
Disease prevention	disease prevention	disease prevention	disease prevention	Disease prevention	3	kept
Disease-Free Survival	disease free survival	disease free survival	disease free survival	disease free survival	3	case_or_format
DISEASES	diseases	diseases	disease	Diseases	3	singular
dislocation	dislocation	dislocation	dislocation	dislocation	3	kept
dissecting aneurysm	dissecting aneurysm	dissecting aneurysm	dissecting aneurysm	dissecting aneurysm	3	kept
disseminated intravascular coagulation	disseminated intravascular coagulation	disseminated intravascular coagulation	disseminated intravascular coagulation	Disseminated Intravascular Coagulation	3	case_or_format
distance learning	distance learning	distance learning	distance learning	distance learning	3	kept
distractibility	distractibility	distractibility	distractibility	distractibility	3	kept
distraction	distraction	distraction	distraction	distraction	3	kept
divorced person	divorced person	divorced person	divorced person	divorced person	3	kept

DNA Fingerprinting	dna fingerprinting	dna fingerprinting	dna fingerprinting	DNA Fingerprinting	3	kept
DNA sequence	dna sequence	dna sequence	dna sequence	DNA sequence	3	kept
do not resuscitate	do not resuscitate	do not resuscitate	do not resuscitate	do-not-resuscitate	3	case_or_format
Do-not-resuscitate	do not resuscitate	do not resuscitate	do not resuscitate	do-not-resuscitate	3	case_or_format
docetaxel	docetaxel	docetaxel	docetaxel	docetaxel	3	kept
document analysis	document analysis	document analysis	document analysis	document analysis	3	kept
documentation burden	documentation burden	documentation burden	documentation burden	documentation burden	3	kept
Dominican Republic	dominican republic	dominican republic	dominican republic	Dominican Republic	3	kept
Donation	donation	donation	donation	Donation	3	kept
donor management	donor management	donor management	donor management	donor management	3	kept
Donor milk	donor milk	donor milk	donor milk	donor milk	3	case_or_format
dopexamine	dopexamine	dopexamine	dopexamine	dopexamine	3	kept
dose time effect relation	dose time effect relation	dose time effect relation	dose time effect relation	dose time effect relation	3	kept
dosing error	dosing error	dosing error	dosing error	DOSING ERRORS	3	case_or_format
Double-check	double check	double check	double check	Double-check	3	kept
Downtime	downtime	downtime	downtime	Downtime	3	kept

Drainage, Postural	drainage postural	drainage postural	drainage postural	Drainage, Postural	3	kept
DRESS syndrome	dress syndrome	dress syndrome	dress syndrome	DRESS syndrome	3	kept
drinking water	drinking water	drinking water	drinking water	drinking water	3	kept
drug concentration	drug concentration	drug concentration	drug concentration	drug concentration	3	kept
drug contamination	drug contamination	drug contamination	drug contamination	drug contamination	3	kept
drug dependence treatment	drug dependence treatment	drug dependence treatment	drug dependence treatment	drug dependence treatment	3	kept
drug dosage form comparison	drug dosage form comparison	drug dosage form comparison	drug dosage form comparison	drug dosage form comparison	3	kept
Drug dosing	drug dosing	drug dosing	drug dosing	Drug dosing	3	kept
Drug Evaluation	drug evaluation	drug evaluation	drug evaluation	Drug Evaluation	3	kept
drug formulary	drug formulary	drug formulary	drug formulary	drug formulary	3	kept
Drug incompatibility	drug incompatibility	drug incompatibility	drug incompatibility	drug incompatibility	3	case_or_format
drug induced headache	drug induced headache	drug induced headache	drug induced headache	drug induced headache	3	kept
drug instillation	drug instillation	drug instillation	drug instillation	drug instillation	3	kept
drug potency	drug potency	drug potency	drug potency	drug potency	3	kept
drug quality	drug quality	drug quality	drug quality	drug quality	3	kept
drug research	drug research	drug research	drug research	drug research	3	kept

Drug Resistance, Fungal	drug resistance fungal	drug resistance fungal	drug resistance fungal	Drug Resistance, Fungal	3	kept
Drug Storage	drug storage	drug storage	drug storage	drug storage	3	case_or_format
Drug Substitution	drug substitution	drug substitution	drug substitution	drug substitution	3	case_or_format
drug targeting	drug targeting	drug targeting	drug targeting	drug targeting	3	kept
drug toxicity	drug toxicity	drug toxicity	drug toxicity	drug toxicity	3	kept
drug use process	drug use process	drug use process	drug use process	drug use process	3	kept
DRUG-THERAPY	drug therapy	drug therapy	drug therapy	drug therapy	3	case_or_format
Drugs	drugs	drugs	drug	drug	3	singular
dry eye	dry eye	dry eye	dry eye	dry eye	3	kept
duodenum	duodenum	duodenum	duodenum	duodenum	3	kept
dura mater	dura mater	dura mater	dura mater	dura mater	3	kept
dust	dust	dust	dust	Dust	3	case_or_format
duty of care	duty of care	duty of care	duty of care	duty of care	3	kept
DVT	dvt	dvt	dvt	DVT	3	kept
Dysgeusia	dysgeusia	dysgeusia	dysgeusia	dysgeusia	3	case_or_format
DYSPHAGIA	dysphagia	dysphagia	dysphagia	dysphagia	3	case_or_format

DYSPNEA	dyspnea	dyspnea	dyspnea	dyspnea	3	case_or_format
dyssomnias	dyssomnias	dyssomnias	dyssomnia	Dyssomnias	3	singular
early childhood intervention	early childhood intervention	early childhood intervention	early childhood intervention	early childhood intervention	3	kept
Early Detection of Cancer	early detection of cancer	early detection of cancer	early detection of cancer	Early Detection of Cancer	3	kept
Early Goal-Directed Therapy	early goal directed therapy	early goal directed therapy	early goal directed therapy	early goal-directed therapy	3	case_or_format
Early recognition skills	early recognition skills	early recognition skills	early recognition skills	Early recognition skills	3	kept
early warning systems	early warning systems	early warning systems	early warning system	early warning system	3	singular
Eastern Europe	eastern europe	eastern europe	eastern europe	Eastern Europe	3	kept
eat sleep console	eat sleep console	eat sleep console	eat sleep console	eat sleep console	3	kept
EBGs	ebgs	ebgs	ebgs	EBGs	3	kept
Ebola	ebola	ebola	ebola	Ebola	3	kept
Ebstein anomaly	ebstein anomaly	ebstein anomaly	ebstein anomaly	Ebstein anomaly	3	kept
eclampsia and preeclampsia	eclampsia and preeclampsia	eclampsia and preeclampsia	eclampsia and preeclampsia	eclampsia and preeclampsia	3	kept
Economic analysis	economic analysis	economic analysis	economic analysis	economic analysis	3	case_or_format
economic model	economic model	economic model	economic model	economic model	3	kept
Economic Recession	economic recession	economic recession	economic recession	Economic Recession	3	kept

ECPR	ecpr	ecpr	ecpr	ECPR	3	kept
eculizumab	eculizumab	eculizumab	eculizumab	eculizumab	3	kept
edetic acid	edetic acid	edetic acid	edetic acid	edetic acid	3	kept
education & training	education and training	education and training	education and training	education and training	3	case_or_format
education intervention	education intervention	education intervention	education intervention	education intervention	3	kept
education training	education training	education training	education training	education training	3	kept
Education, Pharmacy	education pharmacy	education pharmacy	education pharmacy	Education, Pharmacy	3	kept
Educational	educational	educational	educational	educational	3	case_or_format
Educational interventions	educational interventions	educational interventions	educational intervention	Educational intervention	3	singular
Educational programme	educational programme	educational programme	educational programme	Educational programme	3	kept
Effectiveness of health care intervention	effectiveness of health care intervention	effectiveness of health care intervention	effectiveness of health care intervention	Effectiveness of health care intervention	3	kept
Efficacy	efficacy	efficacy	efficacy	EFFICACY	3	case_or_format
efficacy parameters	efficacy parameters	efficacy parameters	efficacy parameters	efficacy parameters	3	kept
EFFICIENCY	efficiency	efficiency	efficiency	Efficiency	3	case_or_format
egg	egg	egg	egg	egg	3	kept
EHR data	ehr data	ehr data	ehr data	EHR data	3	kept

Elapidae	elapidae	elapidae	elapidae	Elapidae	3	kept
elasticity	elasticity	elasticity	elasticity	elasticity	3	kept
ELBW	elbw	elbw	elbw	ELBW	3	kept
Elderly people	elderly people	elderly people	elderly people	Elderly people	3	kept
elderly population	elderly population	elderly population	elderly population	elderly population	3	kept
eLearning	elearning	elearning	elearning	eLearning	3	kept
electric burn	electric burn	electric burn	electric burn	electric burn	3	kept
electric potential	electric potential	electric potential	electric potential	electric potential	3	kept
Electrical impedance tomography	electrical impedance tomography	electrical impedance tomography	electrical impedance tomography	Electrical impedance tomography	3	kept
Electrocardiography, Ambulatory	electrocardiography ambulatory	electrocardiography ambulatory	electrocardiography ambulatory	Electrocardiography, Ambulatory	3	kept
electrocorticography	electrocorticography	electrocorticography	electrocorticography	electrocorticography	3	kept
electrodiagnosis	electrodiagnosis	electrodiagnosis	electrodiagnosis	electrodiagnosis	3	kept
Electronic checklist	electronic checklist	electronic checklist	electronic checklist	Electronic checklist	3	kept
electronic consultation	electronic consultation	electronic consultation	electronic consultation	electronic consultation	3	kept
Electronic document exchange	electronic document exchange	electronic document exchange	electronic document exchange	Electronic document exchange	3	kept
Electronic health	electronic health	electronic health	electronic health	Electronic health	3	kept

Electrophysiologic Techniques, Cardiac	electrophysiologic techniques cardiac	electrophysiologic techniques cardiac	electrophysiologic techniques cardiac	Electrophysiologic Techniques, Cardiac	3	kept
elemental diet	elemental diet	elemental diet	elemental diet	elemental diet	3	kept
Elizabethkingia meningoseptica	elizabethkingia meningoseptica	elizabethkingia meningoseptica	elizabethkingia meningoseptica	Elizabethkingia meningoseptica	3	kept
Embolectomy	embolectomy	embolectomy	embolectomy	embolectomy	3	case_or_format
embolism	embolism	embolism	embolism	Embolism	3	case_or_format
Embolism, Paradoxical	embolism paradoxical	embolism paradoxical	embolism paradoxical	Embolism, Paradoxical	3	kept
embryo transfer	embryo transfer	embryo transfer	embryo transfer	embryo transfer	3	kept
Emergency department triage	emergency department triage	emergency department triage	emergency department triage	Emergency department triage	3	kept
Emergency medical care	emergency medical care	emergency medical care	emergency medical care	Emergency medical care	3	kept
emergency medical dispatcher	emergency medical dispatcher	emergency medical dispatcher	emergency medical dispatcher	emergency medical dispatcher	3	kept
emergency medical service	emergency medical service	emergency medical service	emergency medical service	Emergency Medical Services	3	case_or_format
Emergency medical technician	emergency medical technician	emergency medical technician	emergency medical technician	Emergency Medical Technicians	3	case_or_format
Emergency physician	emergency physician	emergency physician	emergency physician	emergency physician	3	case_or_format
emergency response	emergency response	emergency response	emergency response	Emergency response	3	case_or_format
emergency response time	emergency response time	emergency response time	emergency response time	emergency response time	3	kept
Emergency Room Visits	emergency room visits	emergency room visits	emergency room visits	Emergency Room Visits	3	kept

Emergency Services	emergency services	emergency services	emergency service	Emergency service	3	singular
Emergency Services, Psychiatric	emergency services psychiatric	emergency services psychiatric	emergency services psychiatric	Emergency Services, Psychiatric	3	kept
emergency severity index	emergency severity index	emergency severity index	emergency severity index	emergency severity index	3	kept
Emergency situation	emergency situation	emergency situation	emergency situation	Emergency situation	3	kept
Emotional Regulation	emotional regulation	emotional regulation	emotional regulation	Emotional Regulation	3	kept
emotional regulation	emotional regulation	emotional regulation	emotional regulation	Emotional Regulation	3	case_or_format
emotional state	emotional state	emotional state	emotional state	emotional state	3	kept
Emotional stress	emotional stress	emotional stress	emotional stress	emotional stress	3	case_or_format
Emotional well-being	emotional well being	emotional well being	emotional well being	emotional well-being	3	case_or_format
Empiric antimicrobial therapy	empiric antimicrobial therapy	empiric antimicrobial therapy	empiric antimicrobial therapy	Empiric antimicrobial therapy	3	kept
empirical bioethics	empirical bioethics	empirical bioethics	empirical bioethics	empirical bioethics	3	kept
Employee Incentive Plans	employee incentive plans	employee incentive plans	employee incentive plans	Employee Incentive Plans	3	kept
Empower	empower	empower	empower	Empower	3	kept
empower	empower	empower	empower	Empower	3	case_or_format
EMS	ems	ems	ems	EMS	3	kept
emtricitabine	emtricitabine	emtricitabine	emtricitabine	emtricitabine	3	kept

EN barriers	en barriers	en barriers	en barriers	EN barriers	3	kept
enablers	enablers	enablers	enablers	Enablers	3	case_or_format
encouragement	encouragement	encouragement	encouragement	encouragement	3	kept
Encuestas y cuestionarios	encuestas y cuestionarios	encuestas y cuestionarios	encuestas y cuestionarios	Encuestas y cuestionarios	3	kept
end to end anastomosis	end to end anastomosis	end to end anastomosis	end to end anastomosis	end to end anastomosis	3	kept
End-of-Life care	end of life care	end of life care	end of life care	end-of-life care	3	case_or_format
end-of-life decision making	end of life decision making	end of life decision making	end of life decision making	end-of-life decision-making	3	case_or_format
Endarterectomy	endarterectomy	endarterectomy	endarterectomy	Endarterectomy	3	kept
Endothelium, Vascular	endothelium vascular	endothelium vascular	endothelium vascular	Endothelium, Vascular	3	kept
endotracheal	endotracheal	endotracheal	endotracheal	Endotracheal	3	case_or_format
endotracheal extubation	endotracheal extubation	endotracheal extubation	endotracheal extubation	endotracheal extubation	3	kept
ENDOTRACHEAL INTUBATION	endotracheal intubation	endotracheal intubation	endotracheal intubation	endotracheal intubation	3	case_or_format
endotracheal tube cuff pressure management	endotracheal tube cuff pressure management	endotracheal tube cuff pressure management	endotracheal tube cuff pressure management	endotracheal tube cuff pressure management	3	kept
endotracheal tube holder	endotracheal tube holder	endotracheal tube holder	endotracheal tube holder	endotracheal tube holder	3	kept
Endovascular	endovascular	endovascular	endovascular	Endovascular	3	kept
Endovascular Aneurysm Repair	endovascular aneurysm repair	endovascular aneurysm repair	endovascular aneurysm repair	endovascular aneurysm repair	3	case_or_format

endurance	endurance	endurance	endurance	endurance	3	kept
end - of - life	end of life	end of life	end of life	End of life	3	case_or_format
Enema	enema	enema	enema	enema	3	case_or_format
energy balance	energy balance	energy balance	energy balance	energy balance	3	kept
energy consumption	energy consumption	energy consumption	energy consumption	energy consumption	3	kept
ENERGY-EXPENDITURE	energy expenditure	energy expenditure	energy expenditure	energy expenditure	3	case_or_format
Enfermería de Cuidados Críticos	enfermer a de cuidados cr ticos	enfermer a de cuidados cr ticos	enfermer a de cuidados cr ticos	Enfermería de cuidados críticos	3	case_or_format
Enfermería de la familia	enfermer a de la familia	enfermer a de la familia	enfermer a de la familia	Enfermería de la familia	3	kept
ENGAGEMENT	engagement	engagement	engagement	engagement	3	case_or_format
Engineers	engineers	engineers	engineers	Engineers	3	kept
English proficiency	english proficiency	english proficiency	english proficiency	English proficiency	3	kept
Enhanced recovery	enhanced recovery	enhanced recovery	enhanced recovery	enhanced recovery	3	case_or_format
Enoxaparin	enoxaparin	enoxaparin	enoxaparin	enoxaparin	3	case_or_format
enoximone	enoximone	enoximone	enoximone	enoximone	3	kept
enrofloxacin	enrofloxacin	enrofloxacin	enrofloxacin	enrofloxacin	3	kept
ensemble learning	ensemble learning	ensemble learning	ensemble learning	ensemble learning	3	kept

Enteral feeding intolerance	enteral feeding intolerance	enteral feeding intolerance	enteral feeding intolerance	Enteral feeding intolerance	3	kept
enteral feeding tube	enteral feeding tube	enteral feeding tube	enteral feeding tube	enteral feeding tube	3	kept
enteric coated tablet	enteric coated tablet	enteric coated tablet	enteric coated tablet	enteric coated tablet	3	kept
enterobacterales	enterobacterales	enterobacterales	enterobacterales	Enterobacterales	3	case_or_format
enterococci	enterococci	enterococci	enterococci	ENTEROCOCCI	3	case_or_format
ENTEROCOCCUS	enterococcus	enterococcus	enterococcus	Enterococcus	3	case_or_format
Enterocolitis	enterocolitis	enterocolitis	enterocolitis	Enterocolitis	3	kept
enterocolitis	enterocolitis	enterocolitis	enterocolitis	Enterocolitis	3	case_or_format
envenomation	envenomation	envenomation	envenomation	envenomation	3	kept
Environmental contamination	environmental contamination	environmental contamination	environmental contamination	Environmental contamination	3	kept
environmental footprint	environmental footprint	environmental footprint	environmental footprint	environmental footprint	3	kept
Environmental Health	environmental health	environmental health	environmental health	environmental health	3	case_or_format
environmental management	environmental management	environmental management	environmental management	environmental management	3	kept
environmental microbiology	environmental microbiology	environmental microbiology	environmental microbiology	Environmental Microbiology	3	case_or_format
Environmental noise	environmental noise	environmental noise	environmental noise	Environmental noise	3	kept
eosinophil count	eosinophil count	eosinophil count	eosinophil count	eosinophil count	3	kept

eosinophilia	eosinophilia	eosinophilia	eosinophilia	eosinophilia	3	kept
eosinophilic esophagitis	eosinophilic esophagitis	eosinophilic esophagitis	eosinophilic esophagitis	eosinophilic esophagitis	3	kept
Epic	epic	epic	epic	Epic	3	kept
EPIDEMIC	epidemic	epidemic	epidemic	epidemic	3	case_or_format
epidemiological surveillance	epidemiological surveillance	epidemiological surveillance	epidemiological surveillance	epidemiological surveillance	3	kept
Epidural	epidural	epidural	epidural	epidural	3	case_or_format
Epigenetics	epigenetics	epigenetics	epigenetics	Epigenetics	3	kept
epigenetics	epigenetics	epigenetics	epigenetics	Epigenetics	3	case_or_format
equipment failure	equipment failure	equipment failure	equipment failure	Equipment Failure	3	case_or_format
Ergometry	ergometry	ergometry	ergometry	Ergometry	3	kept
Ergonomic	ergonomic	ergonomic	ergonomic	Ergonomics	3	case_or_format
Erythema	erythema	erythema	erythema	erythema	3	case_or_format
Erythromycin	erythromycin	erythromycin	erythromycin	erythromycin	3	case_or_format
ESKAPE	eskape	eskape	eskape	ESKAPE	3	kept
Esophageal Atresia	esophageal atresia	esophageal atresia	esophageal atresia	Esophageal Atresia	3	kept
esophagectomy	esophagectomy	esophagectomy	esophagectomy	Esophagectomy	3	case_or_format

esophagus achalasia	esophagus achalasia	esophagus achalasia	esophagus achalasia	esophagus achalasia	3	kept
esophagus stenosis	esophagus stenosis	esophagus stenosis	esophagus stenosis	esophagus stenosis	3	kept
essential hypertension	essential hypertension	essential hypertension	essential hypertension	essential hypertension	3	kept
Esthetics	esthetics	esthetics	esthetics	Esthetics	3	kept
Estreñimiento	estre imiento	estre imiento	estre imiento	Estreñimiento	3	kept
estriol	estriol	estriol	estriol	estriol	3	kept
ethambutol	ethambutol	ethambutol	ethambutol	ethambutol	3	kept
ethical	ethical	ethical	ethical	ethical	3	kept
Ethical Analysis	ethical analysis	ethical analysis	ethical analysis	Ethical Analysis	3	kept
Ethical attitude	ethical attitude	ethical attitude	ethical attitude	Ethical attitude	3	kept
ethical care	ethical care	ethical care	ethical care	ethical care	3	kept
Ethical decision making	ethical decision making	ethical decision making	ethical decision making	ethical decision making	3	case_or_format
ethics and leadership	ethics and leadership	ethics and leadership	ethics and leadership	ethics and leadership	3	kept
Ethics Committees	ethics committees	ethics committees	ethics committee	Ethics Committees	3	singular
ethics education	ethics education	ethics education	ethics education	ethics education	3	kept
Ethics of care	ethics of care	ethics of care	ethics of care	ethics of care	3	case_or_format

Ethnographic study	ethnographic study	ethnographic study	ethnographic study	Ethnographic study	3	kept
Etomidate	etomidate	etomidate	etomidate	etomidate	3	case_or_format
European American	european american	european american	european american	European American	3	kept
EUTHANASIA	euthanasia	euthanasia	euthanasia	Euthanasia	3	case_or_format
evaluation research	evaluation research	evaluation research	evaluation research	evaluation research	3	kept
EVD	evd	evd	evd	EVD	3	kept
event free survival	event free survival	event free survival	event free survival	event free survival	3	kept
Evidence based guidelines	evidence based guidelines	evidence based guidelines	evidence based guideline	Evidence-based guidelines	3	singular
evidence implementation	evidence implementation	evidence implementation	evidence implementation	evidence implementation	3	kept
Evidence-based care	evidence based care	evidence based care	evidence based care	Evidence-based care	3	kept
evidence-based guideline	evidence based guideline	evidence based guideline	evidence based guideline	Evidence-based guidelines	3	case_or_format
evidence-based management	evidence based management	evidence based management	evidence based management	evidence-based management	3	kept
evidence‐based care	evidence based care	evidence based care	evidence based care	Evidence-based care	3	case_or_format
Evoked Potentials	evoked potentials	evoked potentials	evoked potentials	Evoked Potentials	3	kept
Evoked Potentials, Auditory, Brain Stem	evoked potentials auditory brain stem	evoked potentials auditory brain stem	evoked potentials auditory brain stem	Evoked Potentials, Auditory, Brain Stem	3	kept
EVOLUTION	evolution	evolution	evolution	EVOLUTION	3	kept

evolution	evolution	evolution	evolution	EVOLUTION	3	case_or_format
Ewing sarcoma	ewing sarcoma	ewing sarcoma	ewing sarcoma	Ewing sarcoma	3	kept
examination	examination	examination	examination	examination	3	kept
excess mortality	excess mortality	excess mortality	excess mortality	excess mortality	3	kept
Exchange Transfusion, Whole Blood	exchange transfusion whole blood	exchange transfusion whole blood	exchange transfusion whole blood	Exchange Transfusion, Whole Blood	3	kept
excitotoxicity	excitotoxicity	excitotoxicity	excitotoxicity	excitotoxicity	3	kept
exclusive breastfeeding	exclusive breastfeeding	exclusive breastfeeding	exclusive breastfeeding	Exclusive breastfeeding	3	case_or_format
excretion	excretion	excretion	excretion	excretion	3	kept
exercise intensity	exercise intensity	exercise intensity	exercise intensity	exercise intensity	3	kept
Exhalation	exhalation	exhalation	exhalation	Exhalation	3	kept
Exotoxins	exotoxins	exotoxins	exotoxin	Exotoxins	3	singular
expectations	expectations	expectations	expectation	expectation	3	singular
Experimental validations	experimental validations	experimental validations	experimental validations	Experimental validations	3	kept
Expert consensus	expert consensus	expert consensus	expert consensus	Expert consensus	3	kept
Expertise	expertise	expertise	expertise	Expertise	3	kept
expertise	expertise	expertise	expertise	Expertise	3	case_or_format

EXPLORATION	exploration	exploration	exploration	EXPLORATION	3	kept
exploratory behavior	exploratory behavior	exploratory behavior	exploratory behavior	exploratory behavior	3	kept
exploratory methods	exploratory methods	exploratory methods	exploratory methods	exploratory methods	3	kept
Exposure keratopathy	exposure keratopathy	exposure keratopathy	exposure keratopathy	Exposure keratopathy	3	kept
exposure keratopathy	exposure keratopathy	exposure keratopathy	exposure keratopathy	Exposure keratopathy	3	case_or_format
exposure therapy	exposure therapy	exposure therapy	exposure therapy	exposure therapy	3	kept
Expressed Emotion	expressed emotion	expressed emotion	expressed emotion	Expressed Emotion	3	kept
Expression of concern	expression of concern	expression of concern	expression of concern	Expression of concern	3	kept
extemporaneous compounding	extemporaneous compounding	extemporaneous compounding	extemporaneous compounding	extemporaneous compounding	3	kept
extended family	extended family	extended family	extended family	Extended family	3	case_or_format
Extended-spectrum beta-lactamase	extended spectrum beta lactamase	extended spectrum beta lactamase	extended spectrum beta lactamase	extended spectrum beta lactamase	3	case_or_format
External Fixators	external fixators	external fixators	external fixator	external fixator	3	singular
Extracorporeal cardiopulmonary resuscitation	extracorporeal cardiopulmonary resuscitation	extracorporeal cardiopulmonary resuscitation	extracorporeal cardiopulmonary resuscitation	Extracorporeal cardiopulmonary resuscitation	3	kept
Extracorporeal circulation	extracorporeal circulation	extracorporeal circulation	extracorporeal circulation	extracorporeal circulation	3	case_or_format
extracorporeal life support	extracorporeal life support	extracorporeal life support	extracorporeal life support	Extracorporeal life support	3	case_or_format
extracorporeal membrane oxygenation device	extracorporeal membrane oxygenation device	extracorporeal membrane oxygenation device	extracorporeal membrane oxygenation device	extracorporeal membrane oxygenation device	3	kept

extraversion	extraversion	extraversion	extraversion	extraversion	3	kept
extremely preterm infant	extremely preterm infant	extremely preterm infant	extremely preterm infant	Extremely preterm infants	3	case_or_format
extubation failure	extubation failure	extubation failure	extubation failure	extubation failure	3	kept
eye mask	eye mask	eye mask	eye mask	EYE MASKS	3	case_or_format
eye ointment	eye ointment	eye ointment	eye ointment	eye ointment	3	kept
eye redness	eye redness	eye redness	eye redness	eye redness	3	kept
face seal	face seal	face seal	face seal	face seal	3	kept
Facial Expressions	facial expressions	facial expressions	facial expression	facial expression	3	singular
facial recognition	facial recognition	facial recognition	facial recognition	facial recognition	3	kept
facies	facies	facies	facies	facies	3	kept
Facility Regulation and Control	facility regulation and control	facility regulation and control	facility regulation and control	Facility Regulation and Control	3	kept
factorial design	factorial design	factorial design	factorial design	factorial design	3	kept
faculty practice	faculty practice	faculty practice	faculty practice	faculty practice	3	kept
Failure mode and effects analysis	failure mode and effects analysis	failure mode and effects analysis	failure mode and effects analysis	failure mode and effects analysis	3	case_or_format
failure-to-rescue	failure to rescue	failure to rescue	failure to rescue	failure to rescue	3	case_or_format
False alarm	false alarm	false alarm	false alarm	False alarms	3	case_or_format

False Negative Reactions	false negative reactions	false negative reactions	false negative reactions	False Negative Reactions	3	kept
Family conference	family conference	family conference	family conference	family conference	3	case_or_format
Family need	family need	family need	family need	Family needs	3	case_or_format
FAMILY PRESENCE	family presence	family presence	family presence	Family presence	3	case_or_format
family service	family service	family service	family service	family service	3	kept
family violence	family violence	family violence	family violence	family violence	3	kept
Family-Centered Care	family centered care	family centered care	family centered care	family centered care	3	case_or_format
family-centered rounds	family centered rounds	family centered rounds	family centered rounds	family-centered rounds	3	kept
Family-Centred care	family centred care	family centred care	family centred care	Family-centred care	3	case_or_format
family-focused care	family focused care	family focused care	family focused care	family-focused care	3	kept
fast-tracking	fast tracking	fast tracking	fast tracking	fast-tracking	3	kept
fat	fat	fat	fat	fat	3	kept
Fat Necrosis	fat necrosis	fat necrosis	fat necrosis	Fat Necrosis	3	kept
Fatigue Syndrome, Chronic	fatigue syndrome chronic	fatigue syndrome chronic	fatigue syndrome chronic	Fatigue Syndrome, Chronic	3	kept
favipiravir	favipiravir	favipiravir	favipiravir	favipiravir	3	kept
FCC	fcc	fcc	fcc	FCC	3	kept

fear of COVID-19 scale	fear of covid 19 scale	fear of covid 19 scale	fear of covid 19 scale	fear of COVID-19 scale	3	kept
Feasibility studies	feasibility studies	feasibility studies	feasibility study	Feasibility Studies	3	singular
fecal microbiota transplantation	fecal microbiota transplantation	fecal microbiota transplantation	fecal microbiota transplantation	fecal microbiota transplantation	3	kept
Federal Government	federal government	federal government	federal government	Federal Government	3	kept
Feeding behavior	feeding behavior	feeding behavior	feeding behavior	Feeding Behavior	3	case_or_format
feeding tube	feeding tube	feeding tube	feeding tube	Feeding tube	3	case_or_format
Fees, Medical	fees medical	fees medical	fees medical	Fees, Medical	3	kept
felodipine	felodipine	felodipine	felodipine	felodipine	3	kept
Femoral Neck Fractures	femoral neck fractures	femoral neck fractures	femoral neck fracture	femoral neck fracture	3	singular
Fertilization in Vitro	fertilization in vitro	fertilization in vitro	fertilization in vitro	Fertilization in Vitro	3	kept
fertilization in vitro	fertilization in vitro	fertilization in vitro	fertilization in vitro	Fertilization in Vitro	3	case_or_format
fetal anomalies	fetal anomalies	fetal anomalies	fetal anomalies	fetal anomalies	3	kept
Fetal Membranes, Premature Rupture	fetal membranes premature rupture	fetal membranes premature rupture	fetal membranes premature rupture	Fetal Membranes, Premature Rupture	3	kept
Fetal monitoring	fetal monitoring	fetal monitoring	fetal monitoring	Fetal Monitoring	3	case_or_format
fetal monitoring	fetal monitoring	fetal monitoring	fetal monitoring	Fetal Monitoring	3	case_or_format
fetus electrocardiography	fetus electrocardiography	fetus electrocardiography	fetus electrocardiography	fetus electrocardiography	3	kept

fetus growth	fetus growth	fetus growth	fetus growth	fetus growth	3	kept
fetus mortality	fetus mortality	fetus mortality	fetus mortality	fetus mortality	3	kept
fetus movement	fetus movement	fetus movement	fetus movement	fetus movement	3	kept
Fiber Optic Technology	fiber optic technology	fiber optic technology	fiber optic technology	Fiber Optic Technology	3	kept
fiberscope endoscopy	fiberscope endoscopy	fiberscope endoscopy	fiberscope endoscopy	fiberscope endoscopy	3	kept
FIELD GEL-ELECTROPHORESIS	field gel electrophoresis	field gel electrophoresis	field gel electrophoresis	FIELD GEL-ELECTROPHORESIS	3	kept
fieldwork	fieldwork	fieldwork	fieldwork	fieldwork	3	kept
fifth cervical vertebra	fifth cervical vertebra	fifth cervical vertebra	fifth cervical vertebra	fifth cervical vertebra	3	kept
financial distress	financial distress	financial distress	financial distress	financial distress	3	kept
finasteride	finasteride	finasteride	finasteride	finasteride	3	kept
finite element analysis	finite element analysis	finite element analysis	finite element analysis	finite element analysis	3	kept
Finnegan Neonatal Abstinence Scoring System	finnegan neonatal abstinence scoring system	finnegan neonatal abstinence scoring system	finnegan neonatal abstinence scoring system	Finnegan Neonatal Abstinence Scoring System	3	kept
Fish Oils	fish oils	fish oils	fish oil	fish oil	3	singular
Fixation, Ocular	fixation ocular	fixation ocular	fixation ocular	Fixation, Ocular	3	kept
Flavobacteriaceae	flavobacteriaceae	flavobacteriaceae	flavobacteriaceae	Flavobacteriaceae	3	kept
flight	flight	flight	flight	flight	3	kept

Floors	floors	floors	floor	Floors	3	singular
Fluid	fluid	fluid	fluid	Fluid	3	kept
fluorescence	fluorescence	fluorescence	fluorescence	fluorescence	3	kept
fluorodeoxyglucose f 18	fluorodeoxyglucose f 18	fluorodeoxyglucose f 18	fluorodeoxyglucose f 18	fluorodeoxyglucose f 18	3	kept
Fluorodeoxyglucose F18	fluorodeoxyglucose f18	fluorodeoxyglucose f18	fluorodeoxyglucose f18	Fluorodeoxyglucose F18	3	kept
fluoroquinolone resistance	fluoroquinolone resistance	fluoroquinolone resistance	fluoroquinolone resistance	fluoroquinolone resistance	3	kept
fluticasone	fluticasone	fluticasone	fluticasone	fluticasone	3	kept
foal	foal	foal	foal	foal	3	kept
foam	foam	foam	foam	foam	3	kept
Focused ethnography	focused ethnography	focused ethnography	focused ethnography	focused ethnography	3	case_or_format
Foley balloon catheter	foley balloon catheter	foley balloon catheter	foley balloon catheter	Foley balloon catheter	3	kept
Follow-up care	follow up care	follow up care	follow up care	Follow-up care	3	kept
Follow-up clinic	follow up clinic	follow up clinic	follow up clinic	Follow-up clinic	3	kept
food allergen	food allergen	food allergen	food allergen	food allergen	3	kept
Food Hypersensitivity	food hypersensitivity	food hypersensitivity	food hypersensitivity	Food Hypersensitivity	3	kept
food preference	food preference	food preference	food preference	food preference	3	kept

Forced Expiratory Volume	forced expiratory volume	forced expiratory volume	forced expiratory volume	forced expiratory volume	3	case_or_format
forensic science	forensic science	forensic science	forensic science	forensic science	3	kept
Formularies, Hospital as Topic	formularies hospital as topic	formularies hospital as topic	formularies hospital as topic	Formularies, Hospital as Topic	3	kept
fortified food	fortified food	fortified food	fortified food	fortified food	3	kept
foster care	foster care	foster care	foster care	foster care	3	kept
FOUR score	four score	four score	four score	FOUR score	3	kept
fracture immobilization	fracture immobilization	fracture immobilization	fracture immobilization	fracture immobilization	3	kept
fracture treatment	fracture treatment	fracture treatment	fracture treatment	fracture treatment	3	kept
Fractures, Open	fractures open	fractures open	fractures open	Fractures, Open	3	kept
Frenchman	frenchman	frenchman	frenchman	Frenchman	3	kept
Frequency	frequency	frequency	frequency	frequency	3	case_or_format
frontline	frontline	frontline	frontline	frontline	3	kept
Full outline of unresponsiveness score	full outline of unresponsiveness score	full outline of unresponsiveness score	full outline of unresponsiveness score	Full Outline of Unresponsiveness score	3	case_or_format
full time employment	full time employment	full time employment	full time employment	full time employment	3	kept
Functional assessment	functional assessment	functional assessment	functional assessment	functional assessment	3	case_or_format
FUNCTIONAL DISABILITY	functional disability	functional disability	functional disability	FUNCTIONAL DISABILITY	3	kept

functional impairment	functional impairment	functional impairment	functional impairment	functional impairment	3	kept
functional outcome	functional outcome	functional outcome	functional outcome	Functional outcome	3	case_or_format
Functionality	functionality	functionality	functionality	Functionality	3	kept
fundamentals of care	fundamentals of care	fundamentals of care	fundamentals of care	Fundamentals of care	3	case_or_format
fungal infection	fungal infection	fungal infection	fungal infection	fungal infection	3	kept
fungus growth	fungus growth	fungus growth	fungus growth	fungus growth	3	kept
fuzzy logic	fuzzy logic	fuzzy logic	fuzzy logic	Fuzzy Logic	3	case_or_format
gadolinium	gadolinium	gadolinium	gadolinium	gadolinium	3	kept
gait disorder	gait disorder	gait disorder	gait disorder	gait disorder	3	kept
galactomannan	galactomannan	galactomannan	galactomannan	galactomannan	3	kept
GAME	game	game	game	GAME	3	kept
game-based learning	game based learning	game based learning	game based learning	game-based learning	3	kept
gamification	gamification	gamification	gamification	Gamification	3	case_or_format
gas	gas	gas	gas	gas	3	kept
Gas gangrene	gas gangrene	gas gangrene	gas gangrene	Gas gangrene	3	kept
Gas Poisoning	gas poisoning	gas poisoning	gas poisoning	Gas Poisoning	3	kept

Gas Scavengers	gas scavengers	gas scavengers	gas scavengers	Gas Scavengers	3	kept
Gastric Juice	gastric juice	gastric juice	gastric juice	Gastric Juice	3	kept
gastric suction	gastric suction	gastric suction	gastric suction	gastric suction	3	kept
Gastrointestinal Agents	gastrointestinal agents	gastrointestinal agents	gastrointestinal agent	gastrointestinal agent	3	singular
Gastrointestinal disease	gastrointestinal disease	gastrointestinal disease	gastrointestinal disease	gastrointestinal disease	3	case_or_format
Gastrointestinal surgery	gastrointestinal surgery	gastrointestinal surgery	gastrointestinal surgery	gastrointestinal surgery	3	case_or_format
gastrointestinal transit	gastrointestinal transit	gastrointestinal transit	gastrointestinal transit	gastrointestinal transit	3	kept
gastrointestinal tumor	gastrointestinal tumor	gastrointestinal tumor	gastrointestinal tumor	gastrointestinal tumor	3	kept
gauze dressing	gauze dressing	gauze dressing	gauze dressing	gauze dressing	3	kept
Gaza Strip	gaza strip	gaza strip	gaza strip	Gaza Strip	3	kept
gelatinase B	gelatinase b	gelatinase b	gelatinase b	gelatinase B	3	kept
gemifloxacin	gemifloxacin	gemifloxacin	gemifloxacin	gemifloxacin	3	kept
gene amplification	gene amplification	gene amplification	gene amplification	gene amplification	3	kept
Gene Expression Profiling	gene expression profiling	gene expression profiling	gene expression profiling	Gene Expression Profiling	3	kept
gene therapy	gene therapy	gene therapy	gene therapy	gene therapy	3	kept
General Adaptation Syndrome	general adaptation syndrome	general adaptation syndrome	general adaptation syndrome	General Adaptation Syndrome	3	kept

general condition	general condition	general condition	general condition	general condition	3	kept
general device	general device	general device	general device	general device	3	kept
general health	general health	general health	general health	general health	3	kept
general health status assessment	general health status assessment	general health status assessment	general health status assessment	general health status assessment	3	kept
General hospitals	general hospitals	general hospitals	general hospital	general hospital	3	singular
GENERAL MEDICINE	general medicine	general medicine	general medicine	general medicine	3	case_or_format
general paediatrics	general paediatrics	general paediatrics	general paediatrics	general paediatrics	3	kept
General wards	general wards	general wards	general ward	general ward	3	singular
GENERAL-PRACTITIONERS	general practitioners	general practitioners	general practitioner	general practitioner	3	singular
generic drug	generic drug	generic drug	generic drug	generic drug	3	kept
Genes, Recessive	genes recessive	genes recessive	genes recessive	Genes, Recessive	3	kept
Genetics	genetics	genetics	genetics	genetics	3	case_or_format
genotyping technique	genotyping technique	genotyping technique	genotyping technique	genotyping technique	3	kept
geographic and geological phenomena	geographic and geological phenomena	geographic and geological phenomena	geographic and geological phenomena	geographic and geological phenomena	3	kept
Geriatric assessment	geriatric assessment	geriatric assessment	geriatric assessment	Geriatric Assessment	3	case_or_format
Geriatric medicine	geriatric medicine	geriatric medicine	geriatric medicine	geriatric medicine	3	case_or_format

gestational weight gain	gestational weight gain	gestational weight gain	gestational weight gain	gestational weight gain	3	kept
Glargine	glargine	glargine	glargine	Glargine	3	kept
Glass	glass	glass	glass	Glass	3	kept
glass	glass	glass	glass	Glass	3	case_or_format
Glenn shunt	glenn shunt	glenn shunt	glenn shunt	Glenn shunt	3	kept
glial fibrillary acidic protein	glial fibrillary acidic protein	glial fibrillary acidic protein	glial fibrillary acidic protein	glial fibrillary acidic protein	3	kept
Glioblastoma	glioblastoma	glioblastoma	glioblastoma	glioblastoma	3	case_or_format
Glioma	glioma	glioma	glioma	glioma	3	case_or_format
glipizide	glipizide	glipizide	glipizide	glipizide	3	kept
global	global	global	global	Global	3	case_or_format
global disease burden	global disease burden	global disease burden	global disease burden	global disease burden	3	kept
Global surgery	global surgery	global surgery	global surgery	Global surgery	3	kept
globulin	globulin	globulin	globulin	globulin	3	kept
globus pallidus	globus pallidus	globus pallidus	globus pallidus	globus pallidus	3	kept
glomerulus	glomerulus	glomerulus	glomerulus	glomerulus	3	kept
glottis	glottis	glottis	glottis	Glottis	3	case_or_format

glucose variability	glucose variability	glucose variability	glucose variability	glucose variability	3	kept
glutamic acid	glutamic acid	glutamic acid	glutamic acid	glutamic acid	3	kept
Glutamine	glutamine	glutamine	glutamine	glutamine	3	case_or_format
glycemia	glycemia	glycemia	glycemia	glycemia	3	kept
Glycogen Storage Disease Type II	glycogen storage disease type ii	glycogen storage disease type ii	glycogen storage disease type ii	Glycogen Storage Disease Type II	3	kept
Glycyrrhiza	glycyrrhiza	glycyrrhiza	glycyrrhiza	Glycyrrhiza	3	kept
GOAL-DIRECTED THERAPY	goal directed therapy	goal directed therapy	goal directed therapy	Goal-directed therapy	3	case_or_format
gold	gold	gold	gold	gold	3	kept
Government Agencies	government agencies	government agencies	government agencies	Government Agencies	3	kept
graduate education	graduate education	graduate education	graduate education	graduate education	3	kept
Graduate medical education	graduate medical education	graduate medical education	graduate medical education	graduate medical education	3	case_or_format
graft dysfunction	graft dysfunction	graft dysfunction	graft dysfunction	graft dysfunction	3	kept
gram-negative bacteria	gram negative bacteria	gram negative bacteria	gram negative bacteria	Gram-Negative Bacteria	3	case_or_format
Gram-Positive Cocci	gram positive cocci	gram positive cocci	gram positive cocci	Gram positive cocci	3	case_or_format
granuloma	granuloma	granuloma	granuloma	granuloma	3	kept
Graphical user interfaces	graphical user interfaces	graphical user interfaces	graphical user interfaces	Graphical user interfaces	3	kept

great vessels transposition	great vessels transposition	great vessels transposition	great vessels transposition	great vessels transposition	3	kept
Green Chemistry Technology	green chemistry technology	green chemistry technology	green chemistry technology	Green Chemistry Technology	3	kept
Gross Domestic Product	gross domestic product	gross domestic product	gross domestic product	Gross Domestic Product	3	kept
group dynamics	group dynamics	group dynamics	group dynamics	group dynamics	3	kept
group practice	group practice	group practice	group practice	Group Practice	3	case_or_format
growth rate	growth rate	growth rate	growth rate	growth rate	3	kept
growth retardation	growth retardation	growth retardation	growth retardation	growth retardation	3	kept
GSS	gss	gss	gss	GSS	3	kept
guide wire	guide wire	guide wire	guide wire	guide wire	3	kept
Guided participation	guided participation	guided participation	guided participation	Guided participation	3	kept
GUIDELINE	guideline	guideline	guideline	Guidelines	3	case_or_format
Guillain-Barré syndrome	guillain barr syndrome	guillain barr syndrome	guillain barr syndrome	Guillain-Barré syndrome	3	kept
Gynecology	gynecology	gynecology	gynecology	gynecology	3	case_or_format
H1N1	h1n1	h1n1	h1n1	H1N1	3	kept
Haematology	haematology	haematology	haematology	haematology	3	case_or_format
Haemodialysis	haemodialysis	haemodialysis	haemodialysis	Haemodialysis	3	kept

Haemophilus vaccine	haemophilus vaccine	haemophilus vaccine	haemophilus vaccine	Haemophilus vaccine	3	kept
Haemophilus Vaccines	haemophilus vaccines	haemophilus vaccines	haemophilus vaccine	Haemophilus vaccine	3	singular
Haemorrhagic shock	haemorrhagic shock	haemorrhagic shock	haemorrhagic shock	Haemorrhagic shock	3	kept
halitosis	halitosis	halitosis	halitosis	halitosis	3	kept
Hamilton Depression Rating Scale	hamilton depression rating scale	hamilton depression rating scale	hamilton depression rating scale	Hamilton Depression Rating Scale	3	kept
Hand antisepsis	hand antisepsis	hand antisepsis	hand antisepsis	Hand antisepsis	3	kept
hand hygiene compliance	hand hygiene compliance	hand hygiene compliance	hand hygiene compliance	Hand hygiene compliance	3	case_or_format
Hand Injuries	hand injuries	hand injuries	hand injury	hand injury	3	singular
hand movement	hand movement	hand movement	hand movement	hand movement	3	kept
Hand Strength	hand strength	hand strength	hand strength	Hand Strength	3	kept
HANDLING ACTIVITIES	handling activities	handling activities	handling activities	HANDLING ACTIVITIES	3	kept
handwashing	handwashing	handwashing	handwashing	Handwashing	3	case_or_format
HAP	hap	hap	hap	HAP	3	kept
haptoglobin	haptoglobin	haptoglobin	haptoglobin	haptoglobin	3	kept
Harar	harar	harar	harar	Harar	3	kept
HARM	harm	harm	harm	Harm	3	case_or_format

HCW	hcw	hcw	hcw	HCWs	3	case_or_format
Head injuries	head injuries	head injuries	head injury	head injury	3	singular
head trauma	head trauma	head trauma	head trauma	Head trauma	3	case_or_format
Head-Down Tilt	head down tilt	head down tilt	head down tilt	Head-Down Tilt	3	kept
health and safety	health and safety	health and safety	health and safety	health & safety	3	case_or_format
Health Care Coalitions	health care coalitions	health care coalitions	health care coalitions	Health Care Coalitions	3	kept
Health care personnel	health care personnel	health care personnel	health care personnel	health care personnel	3	case_or_format
Health care quality	health care quality	health care quality	health care quality	health care quality	3	case_or_format
health care rationing	health care rationing	health care rationing	health care rationing	Health Care Rationing	3	case_or_format
Health care services	health care services	health care services	health care services	Health care services	3	kept
health care staff	health care staff	health care staff	health care staff	health care staff	3	kept
health care surveys	health care surveys	health care surveys	health care survey	Health Care Surveys	3	singular
health communication	health communication	health communication	health communication	Health Communication	3	case_or_format
Health Equity	health equity	health equity	health equity	health equity	3	case_or_format
health impact	health impact	health impact	health impact	health impact	3	kept
health maintenance organization	health maintenance organization	health maintenance organization	health maintenance organization	Health Maintenance Organizations	3	case_or_format

Health Management	health management	health management	health management	Health management	3	case_or_format
Health manager	health manager	health manager	health manager	Health manager	3	kept
Health monitoring system	health monitoring system	health monitoring system	health monitoring system	Health monitoring system	3	kept
Health outcomes	health outcomes	health outcomes	health outcome	health outcome	3	singular
health professions education	health professions education	health professions education	health professions education	health professions education	3	kept
Health records	health records	health records	health record	Health records	3	singular
Health services accessibility	health services accessibility	health services accessibility	health services accessibility	Health Services Accessibility	3	case_or_format
HEALTH SERVICES ADMINISTRATION & MANAGEMENT	health services administration and management	health services administration and management	health services administration and management	health services administration & management	3	case_or_format
Health services evaluation	health services evaluation	health services evaluation	health services evaluation	Health services evaluation	3	kept
Health Spending	health spending	health spending	health spending	Health Spending	3	kept
health technology	health technology	health technology	health technology	health technology	3	kept
health-care quality	health care quality	health care quality	health care quality	health care quality	3	case_or_format
Health-care system	health care system	health care system	health care system	health care system	3	case_or_format
healthcare design	healthcare design	healthcare design	healthcare design	healthcare design	3	kept
Healthcare disparities	healthcare disparities	healthcare disparities	healthcare disparity	Healthcare Disparities	3	singular
Healthcare environments	healthcare environments	healthcare environments	healthcare environment	Healthcare environments	3	singular

Healthcare monitoring	healthcare monitoring	healthcare monitoring	healthcare monitoring	Healthcare monitoring	3	kept
healthcare operations	healthcare operations	healthcare operations	healthcare operations	healthcare operations	3	kept
Healthcare outcomes	healthcare outcomes	healthcare outcomes	healthcare outcomes	Healthcare outcomes	3	kept
healthcare policy	healthcare policy	healthcare policy	healthcare policy	healthcare policy	3	kept
healthcare professional	healthcare professional	healthcare professional	healthcare professional	healthcare professionals	3	case_or_format
Healthcare quality indicators	healthcare quality indicators	healthcare quality indicators	healthcare quality indicators	Healthcare quality indicators	3	kept
healthcare resource utilization	healthcare resource utilization	healthcare resource utilization	healthcare resource utilization	healthcare resource utilization	3	kept
Healthcare resources	healthcare resources	healthcare resources	healthcare resource	Healthcare resources	3	singular
healthcare services	healthcare services	healthcare services	healthcare services	Healthcare services	3	case_or_format
Healthcare system	healthcare system	healthcare system	healthcare system	healthcare system	3	case_or_format
healthcare workforce	healthcare workforce	healthcare workforce	healthcare workforce	healthcare workforce	3	kept
Healthy People Programs	healthy people programs	healthy people programs	healthy people programs	Healthy People Programs	3	kept
Hearing	hearing	hearing	hearing	hearing	3	case_or_format
hearing disorder	hearing disorder	hearing disorder	hearing disorder	Hearing Disorders	3	case_or_format
Hearing Loss, Sensorineural	hearing loss sensorineural	hearing loss sensorineural	hearing loss sensorineural	Hearing Loss, Sensorineural	3	kept
hearing test	hearing test	hearing test	hearing test	Hearing Tests	3	case_or_format

heart atrium arrhythmia	heart atrium arrhythmia	heart atrium arrhythmia	heart atrium arrhythmia	heart atrium arrhythmia	3	kept
Heart diseases	heart diseases	heart diseases	heart disease	Heart Diseases	3	singular
heart failure with reduced ejection fraction	heart failure with reduced ejection fraction	heart failure with reduced ejection fraction	heart failure with reduced ejection fraction	heart failure with reduced ejection fraction	3	kept
heart left right shunt	heart left right shunt	heart left right shunt	heart left right shunt	heart left right shunt	3	kept
heart left ventricle	heart left ventricle	heart left ventricle	heart left ventricle	heart left ventricle	3	kept
heart muscle biopsy	heart muscle biopsy	heart muscle biopsy	heart muscle biopsy	heart muscle biopsy	3	kept
heart perfusion	heart perfusion	heart perfusion	heart perfusion	heart perfusion	3	kept
heart rate measurement	heart rate measurement	heart rate measurement	heart rate measurement	heart rate measurement	3	kept
Heart Septal Defects	heart septal defects	heart septal defects	heart septal defects	Heart Septal Defects	3	kept
heart transplant	heart transplant	heart transplant	heart transplant	Heart transplant	3	case_or_format
Heart Valve Prosthesis	heart valve prosthesis	heart valve prosthesis	heart valve prosthesis	heart valve prosthesis	3	case_or_format
heart-assist devices	heart assist devices	heart assist devices	heart assist device	Heart-Assist Devices	3	singular
Heart-rate	heart rate	heart rate	heart rate	heart rate	3	case_or_format
heartburn	heartburn	heartburn	heartburn	heartburn	3	kept
Helicobacter Infections	helicobacter infections	helicobacter infections	helicobacter infection	Helicobacter infection	3	singular
Helicopter	helicopter	helicopter	helicopter	helicopter	3	case_or_format

hemadsorption	hemadsorption	hemadsorption	hemadsorption	hemadsorption	3	kept
hematological malignancy	hematological malignancy	hematological malignancy	hematological malignancy	hematological malignancy	3	kept
hematological procedure	hematological procedure	hematological procedure	hematological procedure	hematological procedure	3	kept
hematoma evacuation	hematoma evacuation	hematoma evacuation	hematoma evacuation	hematoma evacuation	3	kept
hematopneumothorax	hematopneumothorax	hematopneumothorax	hematopneumothorax	hematopneumothorax	3	kept
hematopoietic stem cell	hematopoietic stem cell	hematopoietic stem cell	hematopoietic stem cell	hematopoietic stem cell	3	kept
Hematopoietic stem cell transplant	hematopoietic stem cell transplant	hematopoietic stem cell transplant	hematopoietic stem cell transplant	Hematopoietic stem cell transplant	3	kept
hemicolectomy	hemicolectomy	hemicolectomy	hemicolectomy	hemicolectomy	3	kept
hemispheric dominance	hemispheric dominance	hemispheric dominance	hemispheric dominance	hemispheric dominance	3	kept
Hemochromatosis	hemochromatosis	hemochromatosis	hemochromatosis	Hemochromatosis	3	kept
Hemodilution	hemodilution	hemodilution	hemodilution	hemodilution	3	case_or_format
Hemodynamic	hemodynamic	hemodynamic	hemodynamic	hemodynamics	3	case_or_format
Hemodynamic changes	hemodynamic changes	hemodynamic changes	hemodynamic changes	Hemodynamic changes	3	kept
Hemoglobin A, Glycosylated	hemoglobin a glycosylated	hemoglobin a glycosylated	hemoglobin a glycosylated	Hemoglobin A, Glycosylated	3	kept
hemolytic uremic syndrome	hemolytic uremic syndrome	hemolytic uremic syndrome	hemolytic uremic syndrome	Hemolytic-Uremic Syndrome	3	case_or_format
hemophilia	hemophilia	hemophilia	hemophilia	hemophilia	3	kept

Hemorrhagic Fever Virus, Crimean-Congo	hemorrhagic fever virus crimean congo	hemorrhagic fever virus crimean congo	hemorrhagic fever virus crimean congo	Hemorrhagic Fever Virus, Crimean-Congo	3	kept
hemorrhoid	hemorrhoid	hemorrhoid	hemorrhoid	hemorrhoid	3	kept
Hemovigilance	hemovigilance	hemovigilance	hemovigilance	Hemovigilance	3	kept
HEMS	hems	hems	hem	HEMS	3	singular
hepatectomy	hepatectomy	hepatectomy	hepatectomy	Hepatectomy	3	case_or_format
hepatic artery thrombosis	hepatic artery thrombosis	hepatic artery thrombosis	hepatic artery thrombosis	hepatic artery thrombosis	3	kept
Hepatic Veno-Occlusive Disease	hepatic veno occlusive disease	hepatic veno occlusive disease	hepatic veno occlusive disease	Hepatic Veno-Occlusive Disease	3	kept
hepatitis B surface antigen	hepatitis b surface antigen	hepatitis b surface antigen	hepatitis b surface antigen	hepatitis B surface antigen	3	kept
hepatitis C antibody	hepatitis c antibody	hepatitis c antibody	hepatitis c antibody	hepatitis C antibody	3	kept
hepatologist	hepatologist	hepatologist	hepatologist	hepatologist	3	kept
hepatorenal syndrome	hepatorenal syndrome	hepatorenal syndrome	hepatorenal syndrome	Hepatorenal Syndrome	3	case_or_format
herbaceous agent	herbaceous agent	herbaceous agent	herbaceous agent	herbaceous agent	3	kept
herd immunity	herd immunity	herd immunity	herd immunity	herd immunity	3	kept
heredity	heredity	heredity	heredity	heredity	3	kept
hernia incarceration	hernia incarceration	hernia incarceration	hernia incarceration	hernia incarceration	3	kept
Hernia, Ventral	hernia ventral	hernia ventral	hernia ventral	Hernia, Ventral	3	kept

Herpes Simplex	herpes simplex	herpes simplex	herpes simplex	herpes simplex	3	case_or_format
Hetastarch	hetastarch	hetastarch	hetastarch	hetastarch	3	case_or_format
Heterometallic structure	heterometallic structure	heterometallic structure	heterometallic structure	Heterometallic structure	3	kept
hiccup	hiccup	hiccup	hiccup	hiccup	3	kept
High reliability organizations	high reliability organizations	high reliability organizations	high reliability organization	High reliability organizations	3	singular
high-dependency care	high dependency care	high dependency care	high dependency care	High dependency care	3	case_or_format
high-flow nasal cannula	high flow nasal cannula	high flow nasal cannula	high flow nasal cannula	High-flow nasal cannula	3	case_or_format
high-risk neonate	high risk neonate	high risk neonate	high risk neonate	high-risk neonate	3	kept
high-risk neonates	high risk neonates	high risk neonates	high risk neonate	high-risk neonate	3	singular
High-risk pregnancy	high risk pregnancy	high risk pregnancy	high risk pregnancy	high risk pregnancy	3	case_or_format
High-touch surfaces	high touch surfaces	high touch surfaces	high touch surfaces	High-touch surfaces	3	kept
higher education	higher education	higher education	higher education	higher education	3	kept
high‐risk pregnancy	high risk pregnancy	high risk pregnancy	high risk pregnancy	high risk pregnancy	3	case_or_format
Hip fractures	hip fractures	hip fractures	hip fracture	hip fracture	3	singular
hip hemiarthroplasty	hip hemiarthroplasty	hip hemiarthroplasty	hip hemiarthroplasty	hip hemiarthroplasty	3	kept
Hip Joint	hip joint	hip joint	hip joint	Hip Joint	3	kept

HIPEC	hipec	hipec	hipec	HIPEC	3	kept
histamine	histamine	histamine	histamine	histamine	3	kept
History, 16th Century	history 16th century	history 16th century	history 16th century	History, 16th Century	3	kept
History, 17th Century	history 17th century	history 17th century	history 17th century	History, 17th Century	3	kept
History, Ancient	history ancient	history ancient	history ancient	History, Ancient	3	kept
History, Medieval	history medieval	history medieval	history medieval	History, Medieval	3	kept
Hodgkin disease	hodgkin disease	hodgkin disease	hodgkin disease	Hodgkin disease	3	kept
Holter monitoring	holter monitoring	holter monitoring	holter monitoring	Holter monitoring	3	kept
Home	home	home	home	home	3	case_or_format
home care services	home care services	home care services	home care services	Home Care Services	3	case_or_format
Home healthcare	home healthcare	home healthcare	home healthcare	Home healthcare	3	kept
home immunization	home immunization	home immunization	home immunization	home immunization	3	kept
home respiratory care	home respiratory care	home respiratory care	home respiratory care	home respiratory care	3	kept
home safety	home safety	home safety	home safety	home safety	3	kept
Home ventilation	home ventilation	home ventilation	home ventilation	Home ventilation	3	kept
hormonal therapy	hormonal therapy	hormonal therapy	hormonal therapy	hormonal therapy	3	kept

hormone blood level	hormone blood level	hormone blood level	hormone blood level	hormone blood level	3	kept
Hormones	hormones	hormones	hormone	hormone	3	singular
Horses	horses	horses	horse	horse	3	singular
Hospital admission	hospital admission	hospital admission	hospital admission	hospital admission	3	case_or_format
HOSPITAL ANXIETY	hospital anxiety	hospital anxiety	hospital anxiety	HOSPITAL ANXIETY	3	kept
hospital based case control study	hospital based case control study	hospital based case control study	hospital based case control study	hospital based case control study	3	kept
Hospital Bed Capacity, under 100	hospital bed capacity under 100	hospital bed capacity under 100	hospital bed capacity under 100	Hospital Bed Capacity, under 100	3	kept
Hospital beds	hospital beds	hospital beds	hospital bed	hospital bed	3	singular
hospital costs	hospital costs	hospital costs	hospital cost	Hospital Costs	3	singular
Hospital design and construction	hospital design and construction	hospital design and construction	hospital design and construction	Hospital Design and Construction	3	case_or_format
hospital environment	hospital environment	hospital environment	hospital environment	Hospital environment	3	case_or_format
hospital food service	hospital food service	hospital food service	hospital food service	hospital food service	3	kept
Hospital hygiene	hospital hygiene	hospital hygiene	hospital hygiene	hospital hygiene	3	case_or_format
Hospital Information System	hospital information system	hospital information system	hospital information system	hospital information system	3	case_or_format
Hospital Medicine	hospital medicine	hospital medicine	hospital medicine	hospital medicine	3	case_or_format
Hospital noise	hospital noise	hospital noise	hospital noise	Hospital noise	3	kept

hospital operations	hospital operations	hospital operations	hospital operations	hospital operations	3	kept
HOSPITAL OUTBREAK	hospital outbreak	hospital outbreak	hospital outbreak	HOSPITAL OUTBREAK	3	kept
Hospital staff	hospital staff	hospital staff	hospital staff	Hospital staff	3	kept
hospital staff	hospital staff	hospital staff	hospital staff	Hospital staff	3	case_or_format
hospital staffing	hospital staffing	hospital staffing	hospital staffing	hospital staffing	3	kept
Hospital Stay	hospital stay	hospital stay	hospital stay	Hospital Stay	3	kept
HOSPITAL-ACQUIRED INFECTIONS	hospital acquired infections	hospital acquired infections	hospital acquired infection	Hospital-acquired infections	3	singular
hospital-acquired pneumonia	hospital acquired pneumonia	hospital acquired pneumonia	hospital acquired pneumonia	hospital acquired pneumonia	3	case_or_format
hospital-acquired pressure injuries	hospital acquired pressure injuries	hospital acquired pressure injuries	hospital acquired pressure injury	hospital-acquired pressure injury	3	singular
HOSPITALIZED CHILDREN	hospitalized children	hospitalized children	hospitalized children	HOSPITALIZED CHILDREN	3	kept
Hospitals, Animal	hospitals animal	hospitals animal	hospitals animal	Hospitals, Animal	3	kept
Hospitals, Municipal	hospitals municipal	hospitals municipal	hospitals municipal	Hospitals, Municipal	3	kept
hot water	hot water	hot water	hot water	hot water	3	kept
humerus fracture	humerus fracture	humerus fracture	humerus fracture	humerus fracture	3	kept
Hunger	hunger	hunger	hunger	hunger	3	case_or_format
Huntington chorea	huntington chorea	huntington chorea	huntington chorea	Huntington chorea	3	kept

husband	husband	husband	husband	husband	3	kept
hyaluronic acid	hyaluronic acid	hyaluronic acid	hyaluronic acid	hyaluronic acid	3	kept
hyaluronidase	hyaluronidase	hyaluronidase	hyaluronidase	hyaluronidase	3	kept
hybrid	hybrid	hybrid	hybrid	hybrid	3	kept
Hybrid surgery	hybrid surgery	hybrid surgery	hybrid surgery	Hybrid surgery	3	kept
Hydatidiform Mole	hydatidiform mole	hydatidiform mole	hydatidiform mole	Hydatidiform Mole	3	kept
hydramnios	hydramnios	hydramnios	hydramnios	hydramnios	3	kept
HYDRATION	hydration	hydration	hydration	hydration	3	case_or_format
hydrogel	hydrogel	hydrogel	hydrogel	hydrogel	3	kept
Hydromorphone	hydromorphone	hydromorphone	hydromorphone	hydromorphone	3	case_or_format
hydronephrosis	hydronephrosis	hydronephrosis	hydronephrosis	hydronephrosis	3	kept
Hydrostatic Pressure	hydrostatic pressure	hydrostatic pressure	hydrostatic pressure	hydrostatic pressure	3	case_or_format
hydroxychloroquine sulfate	hydroxychloroquine sulfate	hydroxychloroquine sulfate	hydroxychloroquine sulfate	hydroxychloroquine sulfate	3	kept
hygiene product	hygiene product	hygiene product	hygiene product	hygiene product	3	kept
Hyperactive delirium	hyperactive delirium	hyperactive delirium	hyperactive delirium	hyperactive delirium	3	case_or_format
hyperalimentation	hyperalimentation	hyperalimentation	hyperalimentation	hyperalimentation	3	kept

hyperammonemia	hyperammonemia	hyperammonemia	hyperammonemia	hyperammonemia	3	kept
Hyperbaric oxygen therapy	hyperbaric oxygen therapy	hyperbaric oxygen therapy	hyperbaric oxygen therapy	hyperbaric oxygen therapy	3	case_or_format
hyperbaric pressure	hyperbaric pressure	hyperbaric pressure	hyperbaric pressure	hyperbaric pressure	3	kept
Hypercholesterolemia	hypercholesterolemia	hypercholesterolemia	hypercholesterolemia	hypercholesterolemia	3	case_or_format
hyperinsulinism	hyperinsulinism	hyperinsulinism	hyperinsulinism	Hyperinsulinism	3	case_or_format
hyperosmolar hyperglycemic state	hyperosmolar hyperglycemic state	hyperosmolar hyperglycemic state	hyperosmolar hyperglycemic state	hyperosmolar hyperglycemic state	3	kept
hypertensive emergency	hypertensive emergency	hypertensive emergency	hypertensive emergency	hypertensive emergency	3	kept
hypertrophic pylorus stenosis	hypertrophic pylorus stenosis	hypertrophic pylorus stenosis	hypertrophic pylorus stenosis	hypertrophic pylorus stenosis	3	kept
Hypertrophy	hypertrophy	hypertrophy	hypertrophy	Hypertrophy	3	kept
hypoactive	hypoactive	hypoactive	hypoactive	hypoactive	3	kept
hypoactive delirium	hypoactive delirium	hypoactive delirium	hypoactive delirium	Hypoactive delirium	3	case_or_format
Hypopituitarism	hypopituitarism	hypopituitarism	hypopituitarism	Hypopituitarism	3	kept
HYPOTHERMIA	hypothermia	hypothermia	hypothermia	Hypothermia	3	case_or_format
hypotonic solution	hypotonic solution	hypotonic solution	hypotonic solution	Hypotonic Solutions	3	case_or_format
Hypoventilation	hypoventilation	hypoventilation	hypoventilation	hypoventilation	3	case_or_format
Hypovolemic shock	hypovolemic shock	hypovolemic shock	hypovolemic shock	hypovolemic shock	3	case_or_format

hypoxaemia	hypoxaemia	hypoxaemia	hypoxaemia	hypoxaemia	3	kept
Iatrogenesis	iatrogenesis	iatrogenesis	iatrogenesis	Iatrogenesis	3	kept
Iatrogenic	iatrogenic	iatrogenic	iatrogenic	Iatrogenic	3	kept
ibacovavec	ibacovavec	ibacovavec	ibacovavec	ibacovavec	3	kept
ibutilide	ibutilide	ibutilide	ibutilide	ibutilide	3	kept
ice	ice	ice	ice	Ice	3	case_or_format
ICF	icf	icf	icf	ICF	3	kept
ichthyosis	ichthyosis	ichthyosis	ichthyosis	ichthyosis	3	kept
ICP monitor placement	icp monitor placement	icp monitor placement	icp monitor placement	ICP monitor placement	3	kept
ICP monitoring	icp monitoring	icp monitoring	icp monitoring	ICP monitoring	3	kept
idarucizumab	idarucizumab	idarucizumab	idarucizumab	idarucizumab	3	kept
IDENTIFICATION	identification	identification	identification	identification	3	case_or_format
Identification	identification	identification	identification	identification	3	case_or_format
Idoso	idoso	idoso	idoso	Idoso	3	kept
ifosfamide	ifosfamide	ifosfamide	ifosfamide	ifosfamide	3	kept
Illness	illness	illness	illness	ILLNESS	3	case_or_format

Illness severity scores	illness severity scores	illness severity scores	illness severity scores	Illness severity scores	3	kept
iloprost	iloprost	iloprost	iloprost	iloprost	3	kept
Image	image	image	image	Image	3	kept
image artifact	image artifact	image artifact	image artifact	image artifact	3	kept
image segmentation	image segmentation	image segmentation	image segmentation	image segmentation	3	kept
imipramine	imipramine	imipramine	imipramine	imipramine	3	kept
immaturity	immaturity	immaturity	immaturity	immaturity	3	kept
immediate type hypersensitivity	immediate type hypersensitivity	immediate type hypersensitivity	immediate type hypersensitivity	immediate type hypersensitivity	3	kept
Immersive	immersive	immersive	immersive	Immersive	3	kept
Immersive learning	immersive learning	immersive learning	immersive learning	Immersive learning	3	kept
immune status	immune status	immune status	immune status	immune status	3	kept
Immunity	immunity	immunity	immunity	immunity	3	case_or_format
Immunity, Innate	immunity innate	immunity innate	immunity innate	Immunity, Innate	3	kept
immunocompetence	immunocompetence	immunocompetence	immunocompetence	immunocompetence	3	kept
immunoglobulin blood level	immunoglobulin blood level	immunoglobulin blood level	immunoglobulin blood level	immunoglobulin blood level	3	kept
Immunoglobulin E	immunoglobulin e	immunoglobulin e	immunoglobulin e	immunoglobulin E	3	case_or_format

Immunoglobulins	immunoglobulins	immunoglobulins	immunoglobulin	immunoglobulin	3	singular
immunophenotyping	immunophenotyping	immunophenotyping	immunophenotyping	immunophenotyping	3	kept
immunosenescence	immunosenescence	immunosenescence	immunosenescence	immunosenescence	3	kept
impact of event scale	impact of event scale	impact of event scale	impact of event scale	impact of event scale	3	kept
impedance cardiography	impedance cardiography	impedance cardiography	impedance cardiography	impedance cardiography	3	kept
Implementation strategies	implementation strategies	implementation strategies	implementation strategy	Implementation strategies	3	singular
implementation strategies	implementation strategies	implementation strategies	implementation strategy	Implementation strategies	3	singular
Improvement	improvement	improvement	improvement	IMPROVEMENT	3	case_or_format
impulse control disorder	impulse control disorder	impulse control disorder	impulse control disorder	impulse control disorder	3	kept
in vivo study	in vivo study	in vivo study	in vivo study	in vivo study	3	kept
In-depth interview	in depth interview	in depth interview	in depth interview	In-depth interview	3	kept
In-service education	in service education	in service education	in service education	In-service education	3	kept
In-service training	in service training	in service training	in service training	in service training	3	case_or_format
in-service training	in service training	in service training	in service training	in service training	3	case_or_format
INCIDENT	incident	incident	incident	INCIDENTS	3	case_or_format
Incident reports	incident reports	incident reports	incident report	incident report	3	singular

Inclusive leadership	inclusive leadership	inclusive leadership	inclusive leadership	Inclusive leadership	3	kept
Incontinence associated dermatitis	incontinence associated dermatitis	incontinence associated dermatitis	incontinence associated dermatitis	Incontinence-associated dermatitis	3	case_or_format
Incontinence dermatitis	incontinence dermatitis	incontinence dermatitis	incontinence dermatitis	Incontinence dermatitis	3	kept
Indicator	indicator	indicator	indicator	INDICATORS	3	case_or_format
indigenous health	indigenous health	indigenous health	indigenous health	indigenous health	3	kept
indirect costs	indirect costs	indirect costs	indirect cost	indirect costs	3	singular
Individual	individual	individual	individual	Individual	3	kept
Individualized care	individualized care	individualized care	individualized care	Individualized care	3	kept
individualized care	individualized care	individualized care	individualized care	Individualized care	3	case_or_format
INDIVIDUALS	individuals	individuals	individual	Individual	3	singular
Indoor air quality	indoor air quality	indoor air quality	indoor air quality	Indoor air quality	3	kept
induction of labor	induction of labor	induction of labor	induction of labor	induction of labor	3	kept
inequality	inequality	inequality	inequality	inequality	3	kept
Infant behavior	infant behavior	infant behavior	infant behavior	Infant Behavior	3	case_or_format
Infant death	infant death	infant death	infant death	Infant Death	3	case_or_format
infant equipment	infant equipment	infant equipment	infant equipment	Infant Equipment	3	case_or_format

infant mental health	infant mental health	infant mental health	infant mental health	Infant mental health	3	case_or_format
infant newborn	infant newborn	infant newborn	infant newborn	Infant, Newborn	3	case_or_format
Infant pain	infant pain	infant pain	infant pain	Infant pain	3	kept
infant stress	infant stress	infant stress	infant stress	infant stress	3	kept
infant, newborn	infant newborn	infant newborn	infant newborn	Infant, Newborn	3	case_or_format
Infarction	infarction	infarction	infarction	Infarction	3	kept
infection control and prevention	infection control and prevention	infection control and prevention	infection control and prevention	infection control and prevention	3	kept
infection control measures	infection control measures	infection control measures	infection control measures	infection control measures	3	kept
Infection control practices	infection control practices	infection control practices	infection control practices	Infection control practices	3	kept
Infection Prevention and Control	infection prevention and control	infection prevention and control	infection prevention and control	Infection prevention and control	3	case_or_format
infection sensitivity	infection sensitivity	infection sensitivity	infection sensitivity	infection sensitivity	3	kept
infectious pneumonia	infectious pneumonia	infectious pneumonia	infectious pneumonia	infectious pneumonia	3	kept
Infertility	infertility	infertility	infertility	Infertility	3	kept
infertility	infertility	infertility	infertility	Infertility	3	case_or_format
Infiltration	infiltration	infiltration	infiltration	Infiltration	3	kept
infiltration	infiltration	infiltration	infiltration	Infiltration	3	case_or_format

INFLAMMATION	inflammation	inflammation	inflammation	inflammation	3	case_or_format
inflammatory factors	inflammatory factors	inflammatory factors	inflammatory factors	inflammatory factors	3	kept
inflammatory infiltrate	inflammatory infiltrate	inflammatory infiltrate	inflammatory infiltrate	inflammatory infiltrate	3	kept
Influenza B virus	influenza b virus	influenza b virus	influenza b virus	Influenza B virus	3	kept
Influenza vaccine	influenza vaccine	influenza vaccine	influenza vaccine	influenza vaccine	3	case_or_format
information center	information center	information center	information center	information center	3	kept
Information contents	information contents	information contents	information contents	Information contents	3	kept
Information science	information science	information science	information science	information science	3	case_or_format
information security	information security	information security	information security	information security	3	kept
Information seeking	information seeking	information seeking	information seeking	information seeking	3	case_or_format
INFORMATION-TECHNOLOGY	information technology	information technology	information technology	information technology	3	case_or_format
Infrastructure	infrastructure	infrastructure	infrastructure	infrastructure	3	case_or_format
infusion related reaction	infusion related reaction	infusion related reaction	infusion related reaction	infusion related reaction	3	kept
Infusion therapy	infusion therapy	infusion therapy	infusion therapy	infusion therapy	3	case_or_format
infusions	infusions	infusions	infusion	infusion	3	singular
Inhalation sedation	inhalation sedation	inhalation sedation	inhalation sedation	Inhalation sedation	3	kept

Inheritance Patterns	inheritance patterns	inheritance patterns	inheritance patterns	Inheritance Patterns	3	kept
Injuries	injuries	injuries	injury	injury	3	singular
Inotropes	inotropes	inotropes	inotrope	Inotropes	3	singular
Insect Bites and Stings	insect bites and stings	insect bites and stings	insect bites and stings	Insect Bites and Stings	3	kept
instability	instability	instability	instability	instability	3	kept
institutional ethics	institutional ethics	institutional ethics	institutional ethics	institutional ethics	3	kept
institutionalized person	institutionalized person	institutionalized person	institutionalized person	institutionalized person	3	kept
Instrument validation	instrument validation	instrument validation	instrument validation	instrument validation	3	case_or_format
INSTRUMENTS	instruments	instruments	instrument	instrument	3	singular
insulin glulisine	insulin glulisine	insulin glulisine	insulin glulisine	insulin glulisine	3	kept
Insulin infusion	insulin infusion	insulin infusion	insulin infusion	insulin infusion	3	case_or_format
Insurance, Long-Term Care	insurance long term care	insurance long term care	insurance long term care	Insurance, Long-Term Care	3	kept
intellectual disability	intellectual disability	intellectual disability	intellectual disability	Intellectual Disability	3	case_or_format
intelligence quotient	intelligence quotient	intelligence quotient	intelligence quotient	intelligence quotient	3	kept
intelligent	intelligent	intelligent	intelligent	intelligent	3	kept
Intelligent medical system	intelligent medical system	intelligent medical system	intelligent medical system	Intelligent medical system	3	kept

Intensive	intensive	intensive	intensive	intensive	3	case_or_format
Intensive therapy	intensive therapy	intensive therapy	intensive therapy	Intensive therapy	3	kept
Intensivpflege	intensivpflege	intensivpflege	intensivpflege	Intensivpflege	3	kept
intent to leave	intent to leave	intent to leave	intent to leave	Intent to leave	3	case_or_format
Intent to stay	intent to stay	intent to stay	intent to stay	Intent to stay	3	kept
Inter professionals	inter professionals	inter professionals	inter professional	Inter professionals	3	singular
Inter-hospital transfer	inter hospital transfer	inter hospital transfer	inter hospital transfer	inter-hospital transfer	3	case_or_format
inter-rater reliability	inter rater reliability	inter rater reliability	inter rater reliability	inter-rater reliability	3	kept
interaction	interaction	interaction	interaction	Interaction	3	case_or_format
Interactions	interactions	interactions	interaction	Interaction	3	singular
intercostal muscle	intercostal muscle	intercostal muscle	intercostal muscle	intercostal muscle	3	kept
interdisciplinary health team	interdisciplinary health team	interdisciplinary health team	interdisciplinary health team	Interdisciplinary health team	3	case_or_format
Interdisciplinary Medicine	interdisciplinary medicine	interdisciplinary medicine	interdisciplinary medicine	Interdisciplinary Medicine	3	kept
Interdisciplinary Research	interdisciplinary research	interdisciplinary research	interdisciplinary research	interdisciplinary research	3	case_or_format
Interdisciplinary research	interdisciplinary research	interdisciplinary research	interdisciplinary research	interdisciplinary research	3	case_or_format
Interdisciplinary teams	interdisciplinary teams	interdisciplinary teams	interdisciplinary team	interdisciplinary team	3	singular

interdisciplinary teams	interdisciplinary teams	interdisciplinary teams	interdisciplinary team	interdisciplinary team	3	singular
Interface pressure	interface pressure	interface pressure	interface pressure	Interface pressure	3	kept
interferon	interferon	interferon	interferon	interferon	3	kept
Interleukin-10	interleukin 10	interleukin 10	interleukin 10	interleukin 10	3	case_or_format
Intermediate care facilities	intermediate care facilities	intermediate care facilities	intermediate care facility	Intermediate Care Facilities	3	singular
Intermediate Care Unit	intermediate care unit	intermediate care unit	intermediate care unit	intermediate care unit	3	case_or_format
Intermediate care units	intermediate care units	intermediate care units	intermediate care unit	intermediate care unit	3	singular
intermittent renal replacement therapy	intermittent renal replacement therapy	intermittent renal replacement therapy	intermittent renal replacement therapy	intermittent renal replacement therapy	3	kept
internal iliac artery	internal iliac artery	internal iliac artery	internal iliac artery	internal iliac artery	3	kept
internal mammary artery	internal mammary artery	internal mammary artery	internal mammary artery	internal mammary artery	3	kept
INTERNAL-MEDICINE	internal medicine	internal medicine	internal medicine	internal medicine	3	case_or_format
international child health	international child health	international child health	international child health	international child health	3	kept
International Nosocomial Infection Control Consortium	international nosocomial infection control consortium	international nosocomial infection control consortium	international nosocomial infection control consortium	International Nosocomial Infection Control Consortium	3	kept
international physical activity questionnaire	international physical activity questionnaire	international physical activity questionnaire	international physical activity questionnaire	international physical activity questionnaire	3	kept
Interoperability	interoperability	interoperability	interoperability	Interoperability	3	kept
Interpersonal relations	interpersonal relations	interpersonal relations	interpersonal relations	Interpersonal Relations	3	case_or_format

interpretation	interpretation	interpretation	interpretation	interpretation	3	kept
Interpretative phenomenological analysis	interpretative phenomenological analysis	interpretative phenomenological analysis	interpretative phenomenological analysis	interpretative phenomenological analysis	3	case_or_format
Interpreter	interpreter	interpreter	interpreter	Interpreter	3	kept
interpreter service	interpreter service	interpreter service	interpreter service	interpreter service	3	kept
interpretive description	interpretive description	interpretive description	interpretive description	Interpretive description	3	case_or_format
Interprofessional Collaboration	interprofessional collaboration	interprofessional collaboration	interprofessional collaboration	interprofessional collaboration	3	case_or_format
Interprofessional practice	interprofessional practice	interprofessional practice	interprofessional practice	interprofessional practice	3	case_or_format
Interprofessional teams	interprofessional teams	interprofessional teams	interprofessional team	interprofessional team	3	singular
interprofessional teams	interprofessional teams	interprofessional teams	interprofessional team	interprofessional team	3	singular
Interprofessional working	interprofessional working	interprofessional working	interprofessional working	Interprofessional working	3	kept
Interrater reliability	interrater reliability	interrater reliability	interrater reliability	interrater reliability	3	case_or_format
interruption	interruption	interruption	interruption	Interruptions	3	case_or_format
interstitial fluid	interstitial fluid	interstitial fluid	interstitial fluid	interstitial fluid	3	kept
intertrigo	intertrigo	intertrigo	intertrigo	intertrigo	3	kept
Intervention development	intervention development	intervention development	intervention development	intervention development	3	case_or_format
intervention program	intervention program	intervention program	intervention program	intervention program	3	kept

intervention research	intervention research	intervention research	intervention research	intervention research	3	kept
Intervention trials	intervention trials	intervention trials	intervention trials	Intervention trials	3	kept
interventional cardiovascular procedure	interventional cardiovascular procedure	interventional cardiovascular procedure	interventional cardiovascular procedure	interventional cardiovascular procedure	3	kept
interventional radiologist	interventional radiologist	interventional radiologist	interventional radiologist	interventional radiologist	3	kept
intestinal bleeding	intestinal bleeding	intestinal bleeding	intestinal bleeding	intestinal bleeding	3	kept
Intestinal Volvulus	intestinal volvulus	intestinal volvulus	intestinal volvulus	Intestinal Volvulus	3	kept
intestine absorption	intestine absorption	intestine absorption	intestine absorption	intestine absorption	3	kept
intestine atresia	intestine atresia	intestine atresia	intestine atresia	intestine atresia	3	kept
intestine motility	intestine motility	intestine motility	intestine motility	intestine motility	3	kept
intestine mucosa	intestine mucosa	intestine mucosa	intestine mucosa	intestine mucosa	3	kept
intestine necrosis	intestine necrosis	intestine necrosis	intestine necrosis	intestine necrosis	3	kept
intestine surgery	intestine surgery	intestine surgery	intestine surgery	intestine surgery	3	kept
Intra-abdominal pressure measurement	intra abdominal pressure measurement	intra abdominal pressure measurement	intra abdominal pressure measurement	Intra-abdominal pressure measurement	3	kept
intra-hospital transport	intra hospital transport	intra hospital transport	intra hospital transport	intra-hospital transport	3	kept
intracerebral hemorrhage	intracerebral hemorrhage	intracerebral hemorrhage	intracerebral hemorrhage	Intracerebral hemorrhage	3	case_or_format
Intracranial Arteriovenous Malformations	intracranial arteriovenous malformations	intracranial arteriovenous malformations	intracranial arteriovenous malformations	Intracranial Arteriovenous Malformations	3	kept

Intracranial Embolism	intracranial embolism	intracranial embolism	intracranial embolism	Intracranial Embolism	3	kept
Intracranial Hemorrhage, Traumatic	intracranial hemorrhage traumatic	intracranial hemorrhage traumatic	intracranial hemorrhage traumatic	Intracranial Hemorrhage, Traumatic	3	kept
Intraosseous	intraosseous	intraosseous	intraosseous	Intraosseous	3	kept
intraspinal drug administration	intraspinal drug administration	intraspinal drug administration	intraspinal drug administration	intraspinal drug administration	3	kept
intrauterine infection	intrauterine infection	intrauterine infection	intrauterine infection	intrauterine infection	3	kept
intravaginal drug administration	intravaginal drug administration	intravaginal drug administration	intravaginal drug administration	intravaginal drug administration	3	kept
Intravenous cannulation	intravenous cannulation	intravenous cannulation	intravenous cannulation	Intravenous cannulation	3	kept
intravenous insulin	intravenous insulin	intravenous insulin	intravenous insulin	intravenous insulin	3	kept
Intravenous therapy	intravenous therapy	intravenous therapy	intravenous therapy	Intravenous therapy	3	kept
intussusception	intussusception	intussusception	intussusception	intussusception	3	kept
Invasive Fungal Infections	invasive fungal infections	invasive fungal infections	invasive fungal infection	Invasive Fungal Infections	3	singular
Invasive ventilation	invasive ventilation	invasive ventilation	invasive ventilation	invasive ventilation	3	case_or_format
Involvement	involvement	involvement	involvement	INVOLVEMENT	3	case_or_format
ionizing radiation	ionizing radiation	ionizing radiation	ionizing radiation	ionizing radiation	3	kept
ipilimumab	ipilimumab	ipilimumab	ipilimumab	ipilimumab	3	kept
irinotecan	irinotecan	irinotecan	irinotecan	irinotecan	3	kept

iron deficiency anemia	iron deficiency anemia	iron deficiency anemia	iron deficiency anemia	iron deficiency anemia	3	kept
iron overload	iron overload	iron overload	iron overload	iron overload	3	kept
iron therapy	iron therapy	iron therapy	iron therapy	iron therapy	3	kept
Irrigation	irrigation	irrigation	irrigation	Irrigation	3	kept
ischaemic stroke	ischaemic stroke	ischaemic stroke	ischaemic stroke	ischaemic stroke	3	kept
ischemia time	ischemia time	ischemia time	ischemia time	ischemia time	3	kept
isolation hospital	isolation hospital	isolation hospital	isolation hospital	isolation hospital	3	kept
Isoxazoles	isoxazoles	isoxazoles	isoxazoles	Isoxazoles	3	kept
Israeli	israeli	israeli	israeli	Israeli	3	kept
ITU	itu	itu	itu	ITU	3	kept
Ixodes	ixodes	ixodes	ixodes	Ixodes	3	kept
Jejunum	jejunum	jejunum	jejunum	Jejunum	3	kept
jejunum	jejunum	jejunum	jejunum	Jejunum	3	case_or_format
jew	jew	jew	jew	jew	3	kept
Jews	jews	jews	jew	jew	3	singular
JOB STRESS	job stress	job stress	job stress	job stress	3	case_or_format

joint dislocation	joint dislocation	joint dislocation	joint dislocation	joint dislocation	3	kept
Joint Dislocations	joint dislocations	joint dislocations	joint dislocation	joint dislocation	3	singular
joint function	joint function	joint function	joint function	joint function	3	kept
Joint Instability	joint instability	joint instability	joint instability	Joint Instability	3	kept
joint mobility	joint mobility	joint mobility	joint mobility	joint mobility	3	kept
kanamycin	kanamycin	kanamycin	kanamycin	kanamycin	3	kept
Karnataka	karnataka	karnataka	karnataka	Karnataka	3	kept
ketogenic diet	ketogenic diet	ketogenic diet	ketogenic diet	ketogenic diet	3	kept
ketone	ketone	ketone	ketone	ketone	3	kept
kidney donor	kidney donor	kidney donor	kidney donor	kidney donor	3	kept
Kidney Failure	kidney failure	kidney failure	kidney failure	kidney failure	3	case_or_format
Kidney Function Tests	kidney function tests	kidney function tests	kidney function test	kidney function test	3	singular
Kidney Glomerulus	kidney glomerulus	kidney glomerulus	kidney glomerulus	Kidney Glomerulus	3	kept
Kidney transplantation	kidney transplantation	kidney transplantation	kidney transplantation	kidney transplantation	3	case_or_format
Killer Cells, Natural	killer cells natural	killer cells natural	killer cells natural	Killer Cells, Natural	3	kept
Kirkpatrick model	kirkpatrick model	kirkpatrick model	kirkpatrick model	Kirkpatrick model	3	kept

Knowledge Management	knowledge management	knowledge management	knowledge management	knowledge management	3	case_or_format
Kounis Syndrome	kounis syndrome	kounis syndrome	kounis syndrome	Kounis Syndrome	3	kept
KwaZulu-Natal	kwazulu natal	kwazulu natal	kwazulu natal	KwaZulu-Natal	3	kept
labor and delivery	labor and delivery	labor and delivery	labor and delivery	labor and delivery	3	kept
Labor induction	labor induction	labor induction	labor induction	labor induction	3	case_or_format
Labor Presentation	labor presentation	labor presentation	labor presentation	Labor Presentation	3	kept
Labor Stage, Second	labor stage second	labor stage second	labor stage second	Labor Stage, Second	3	kept
laboratory based surveillance	laboratory based surveillance	laboratory based surveillance	laboratory based surveillance	laboratory based surveillance	3	kept
Laboratory Techniques and Procedures	laboratory techniques and procedures	laboratory techniques and procedures	laboratory techniques and procedures	Laboratory Techniques and Procedures	3	kept
Lactates	lactates	lactates	lactate	Lactates	3	singular
lactation care	lactation care	lactation care	lactation care	lactation care	3	kept
Lactobacillus acidophilus	lactobacillus acidophilus	lactobacillus acidophilus	lactobacillus acidophilus	Lactobacillus acidophilus	3	kept
Lactobacillus casei	lactobacillus casei	lactobacillus casei	lactobacillus casei	Lactobacillus casei	3	kept
lagophthalmos	lagophthalmos	lagophthalmos	lagophthalmos	lagophthalmos	3	kept
Language Development	language development	language development	language development	language development	3	case_or_format
language therapy	language therapy	language therapy	language therapy	language therapy	3	kept

lansoprazole	lansoprazole	lansoprazole	lansoprazole	lansoprazole	3	kept
large for gestational age	large for gestational age	large for gestational age	large for gestational age	large for gestational age	3	kept
Laryngeal Edema	laryngeal edema	laryngeal edema	laryngeal edema	Laryngeal Edema	3	kept
Laryngectomy	laryngectomy	laryngectomy	laryngectomy	laryngectomy	3	case_or_format
Lasater Clinical Judgment Rubric	lasater clinical judgment rubric	lasater clinical judgment rubric	lasater clinical judgment rubric	Lasater Clinical Judgment Rubric	3	kept
Laser Therapy	laser therapy	laser therapy	laser therapy	Laser Therapy	3	kept
Laser-Doppler Flowmetry	laser doppler flowmetry	laser doppler flowmetry	laser doppler flowmetry	Laser-Doppler Flowmetry	3	kept
LASSO	lasso	lasso	lasso	LASSO	3	kept
late onset sepsis	late onset sepsis	late onset sepsis	late onset sepsis	LATE-ONSET SEPSIS	3	case_or_format
Late preterm	late preterm	late preterm	late preterm	Late preterm	3	kept
Late preterm infants	late preterm infants	late preterm infants	late preterm infant	late preterm infant	3	singular
lateral flow immunochromatography	lateral flow immunochromatography	lateral flow immunochromatography	lateral flow immunochromatography	lateral flow immunochromatography	3	kept
Latex Hypersensitivity	latex hypersensitivity	latex hypersensitivity	latex hypersensitivity	Latex Hypersensitivity	3	kept
Laughter Therapy	laughter therapy	laughter therapy	laughter therapy	Laughter Therapy	3	kept
Laundering	laundering	laundering	laundering	Laundering	3	kept
Lavender	lavender	lavender	lavender	lavender	3	case_or_format

lean methodology	lean methodology	lean methodology	lean methodology	Lean methodology	3	case_or_format
Learning disability	learning disability	learning disability	learning disability	Learning Disabilities	3	case_or_format
learning health system	learning health system	learning health system	learning health system	learning health system	3	kept
learning outcomes	learning outcomes	learning outcomes	learning outcome	learning outcomes	3	singular
Lecture	lecture	lecture	lecture	Lecture	3	kept
left hemisphere	left hemisphere	left hemisphere	left hemisphere	left hemisphere	3	kept
leg ulcer	leg ulcer	leg ulcer	leg ulcer	leg ulcer	3	kept
Legal	legal	legal	legal	Legal	3	kept
legal service	legal service	legal service	legal service	legal service	3	kept
Legislation	legislation	legislation	legislation	Legislation	3	kept
Legislation, Hospital	legislation hospital	legislation hospital	legislation hospital	Legislation, Hospital	3	kept
Legislation, Medical	legislation medical	legislation medical	legislation medical	Legislation, Medical	3	kept
leptin	leptin	leptin	leptin	leptin	3	kept
LESSONS	lessons	lessons	lessons	LESSONS	3	kept
lethality	lethality	lethality	lethality	lethality	3	kept
Leukemia, Lymphocytic, Chronic, B-Cell	leukemia lymphocytic chronic b cell	leukemia lymphocytic chronic b cell	leukemia lymphocytic chronic b cell	Leukemia, Lymphocytic, Chronic, B-Cell	3	kept

Leukocidins	leukocidins	leukocidins	leukocidin	Leukocidins	3	singular
Leukostasis	leukostasis	leukostasis	leukostasis	Leukostasis	3	kept
leukostasis	leukostasis	leukostasis	leukostasis	Leukostasis	3	case_or_format
leukotriene B4	leukotriene b4	leukotriene b4	leukotriene b4	leukotriene B4	3	kept
Liberia	liberia	liberia	liberia	Liberia	3	kept
library	library	library	library	library	3	kept
licensure	licensure	licensure	licensure	Licensure	3	case_or_format
Liderazgo	liderazgo	liderazgo	liderazgo	Liderazgo	3	kept
Life	life	life	life	LIFE	3	case_or_format
Life change events	life change events	life change events	life change events	Life Change Events	3	case_or_format
Life experiences	life experiences	life experiences	life experiences	Life experiences	3	kept
life experiences	life experiences	life experiences	life experiences	Life experiences	3	case_or_format
life support	life support	life support	life support	LIFE-SUPPORT	3	case_or_format
Life-changing events	life changing events	life changing events	life changing events	Life-changing events	3	kept
LIFE-SUSTAINING TREATMENT	life sustaining treatment	life sustaining treatment	life sustaining treatment	life sustaining treatment	3	case_or_format
LIFE-SUSTAINING TREATMENTS	life sustaining treatments	life sustaining treatments	life sustaining treatment	life sustaining treatment	3	singular

life-threatening diseases	life threatening diseases	life threatening diseases	life threatening diseases	life-threatening diseases	3	kept
light intensity	light intensity	light intensity	light intensity	light intensity	3	kept
Limb Deformities, Congenital	limb deformities congenital	limb deformities congenital	limb deformities congenital	Limb Deformities, Congenital	3	kept
limb movement	limb movement	limb movement	limb movement	limb movement	3	kept
Limb Salvage	limb salvage	limb salvage	limb salvage	limb salvage	3	case_or_format
lincosamide	lincosamide	lincosamide	lincosamide	lincosamide	3	kept
Linear programming	linear programming	linear programming	linear programming	Linear programming	3	kept
liothyronine	liothyronine	liothyronine	liothyronine	liothyronine	3	kept
lip	lip	lip	lip	lip	3	kept
lipid blood level	lipid blood level	lipid blood level	lipid blood level	lipid blood level	3	kept
liquid feces	liquid feces	liquid feces	liquid feces	liquid feces	3	kept
Literacy	literacy	literacy	literacy	Literacy	3	kept
literacy	literacy	literacy	literacy	Literacy	3	case_or_format
lithotripsy	lithotripsy	lithotripsy	lithotripsy	lithotripsy	3	kept
liver necrosis	liver necrosis	liver necrosis	liver necrosis	liver necrosis	3	kept
LMICs	lmics	lmics	lmic	LMIC	3	singular

Local anesthesia	local anesthesia	local anesthesia	local anesthesia	local anesthesia	3	case_or_format
Lockdown	lockdown	lockdown	lockdown	lockdown	3	case_or_format
Locus Coeruleus	locus coeruleus	locus coeruleus	locus coeruleus	Locus Coeruleus	3	kept
Locus of control	locus of control	locus of control	locus of control	locus of control	3	case_or_format
logic	logic	logic	logic	Logic	3	case_or_format
Logistics	logistics	logistics	logistics	Logistics	3	kept
Long short-term memory	long short term memory	long short term memory	long short term memory	Long short-term memory	3	kept
Long Term Adverse Effects	long term adverse effects	long term adverse effects	long term adverse effects	Long Term Adverse Effects	3	kept
Long term care	long term care	long term care	long term care	long term care	3	case_or_format
long-COVID	long covid	long covid	long covid	long COVID	3	case_or_format
LONG-TERM OUTCOMES	long term outcomes	long term outcomes	long term outcome	Long-term outcomes	3	singular
Long-term ventilation	long term ventilation	long term ventilation	long term ventilation	Long-term ventilation	3	kept
long-term ventilation	long term ventilation	long term ventilation	long term ventilation	Long-term ventilation	3	case_or_format
Longitudinal	longitudinal	longitudinal	longitudinal	longitudinal	3	case_or_format
losartan	losartan	losartan	losartan	losartan	3	kept
loudness	loudness	loudness	loudness	loudness	3	kept

low density lipoprotein	low density lipoprotein	low density lipoprotein	low density lipoprotein	low density lipoprotein	3	kept
Low resource setting	low resource setting	low resource setting	low resource setting	Low-resource settings	3	case_or_format
LOW-BACK-PAIN	low back pain	low back pain	low back pain	low back pain	3	case_or_format
Low-resource setting	low resource setting	low resource setting	low resource setting	Low-resource settings	3	case_or_format
Ludwig's Angina	ludwig s angina	ludwig s angina	ludwig s angina	Ludwig's Angina	3	kept
lullaby	lullaby	lullaby	lullaby	Lullaby	3	case_or_format
lumbar spine	lumbar spine	lumbar spine	lumbar spine	lumbar spine	3	kept
lumbar spine fracture	lumbar spine fracture	lumbar spine fracture	lumbar spine fracture	lumbar spine fracture	3	kept
lung aspiration	lung aspiration	lung aspiration	lung aspiration	lung aspiration	3	kept
lung biopsy	lung biopsy	lung biopsy	lung biopsy	lung biopsy	3	kept
lung cavitation	lung cavitation	lung cavitation	lung cavitation	lung cavitation	3	kept
lung infarction	lung infarction	lung infarction	lung infarction	lung infarction	3	kept
lung non small cell cancer	lung non small cell cancer	lung non small cell cancer	lung non small cell cancer	lung non small cell cancer	3	kept
lung pressure	lung pressure	lung pressure	lung pressure	lung pressure	3	kept
Lung recruitment	lung recruitment	lung recruitment	lung recruitment	Lung recruitment	3	kept
lung scintiscanning	lung scintiscanning	lung scintiscanning	lung scintiscanning	lung scintiscanning	3	kept

Lung transplant	lung transplant	lung transplant	lung transplant	Lung transplant	3	kept
Lung ultrasound	lung ultrasound	lung ultrasound	lung ultrasound	lung ultrasound	3	case_or_format
Lung Volume Measurements	lung volume measurements	lung volume measurements	lung volume measurements	Lung Volume Measurements	3	kept
lungs	lungs	lungs	lung	Lung	3	singular
Lyme Disease	lyme disease	lyme disease	lyme disease	Lyme Disease	3	kept
lymphedema	lymphedema	lymphedema	lymphedema	lymphedema	3	kept
lymphocyte antigen receptor	lymphocyte antigen receptor	lymphocyte antigen receptor	lymphocyte antigen receptor	lymphocyte antigen receptor	3	kept
Lymphoma	lymphoma	lymphoma	lymphoma	lymphoma	3	case_or_format
machine	machine	machine	machine	machine	3	kept
machine learning algorithm	machine learning algorithm	machine learning algorithm	machine learning algorithm	machine learning algorithm	3	kept
macrogol 3350	macrogol 3350	macrogol 3350	macrogol 3350	macrogol 3350	3	kept
maculopapular rash	maculopapular rash	maculopapular rash	maculopapular rash	maculopapular rash	3	kept
magnet	magnet	magnet	magnet	magnet	3	kept
magnetic resonance imaging	magnetic resonance imaging	magnetic resonance imaging	magnetic resonance imaging	Magnetic Resonance Imaging	3	case_or_format
malaria falciparum	malaria falciparum	malaria falciparum	malaria falciparum	Malaria, Falciparum	3	case_or_format
Malaria, Cerebral	malaria cerebral	malaria cerebral	malaria cerebral	Malaria, Cerebral	3	kept

malignant hypertension	malignant hypertension	malignant hypertension	malignant hypertension	malignant hypertension	3	kept
managed care organization	managed care organization	managed care organization	managed care organization	managed care organization	3	kept
Management Audit	management audit	management audit	management audit	Management Audit	3	kept
Management information systems	management information systems	management information systems	management information systems	Management information systems	3	kept
Management strategies	management strategies	management strategies	management strategies	Management strategies	3	kept
Mandatory Programs	mandatory programs	mandatory programs	mandatory program	mandatory program	3	singular
mania	mania	mania	mania	mania	3	kept
manipulative medicine	manipulative medicine	manipulative medicine	manipulative medicine	manipulative medicine	3	kept
Mantel Haenszel test	mantel haenszel test	mantel haenszel test	mantel haenszel test	Mantel Haenszel test	3	kept
marathon runner	marathon runner	marathon runner	marathon runner	marathon runner	3	kept
marjoram	marjoram	marjoram	marjoram	marjoram	3	kept
market	market	market	market	market	3	kept
MAS	mas	mas	mas	MAS	3	kept
masking	masking	masking	masking	masking	3	kept
Maslach burnout inventory	maslach burnout inventory	maslach burnout inventory	maslach burnout inventory	Maslach Burnout Inventory	3	case_or_format
Maslach Burnout Inventory-General Survey	maslach burnout inventory general survey	maslach burnout inventory general survey	maslach burnout inventory general survey	Maslach Burnout Inventory-General Survey	3	kept

Mass Behavior	mass behavior	mass behavior	mass behavior	Mass Behavior	3	kept
Mass spectrometry	mass spectrometry	mass spectrometry	mass spectrometry	Mass spectrometry	3	kept
MASSAGE	massage	massage	massage	Massage	3	case_or_format
Massage therapy	massage therapy	massage therapy	massage therapy	Massage therapy	3	kept
massage therapy	massage therapy	massage therapy	massage therapy	Massage therapy	3	case_or_format
Massive transfusion protocol	massive transfusion protocol	massive transfusion protocol	massive transfusion protocol	Massive transfusion protocol	3	kept
Master surgical plan	master surgical plan	master surgical plan	master surgical plan	Master surgical plan	3	kept
mastitis	mastitis	mastitis	mastitis	mastitis	3	kept
maternal health services	maternal health services	maternal health services	maternal health service	Maternal Health Services	3	singular
maternal nutrition	maternal nutrition	maternal nutrition	maternal nutrition	maternal nutrition	3	kept
maternal smoking	maternal smoking	maternal smoking	maternal smoking	maternal smoking	3	kept
Maternal stress	maternal stress	maternal stress	maternal stress	maternal stress	3	case_or_format
maternal transport	maternal transport	maternal transport	maternal transport	maternal transport	3	kept
mathematical parameters	mathematical parameters	mathematical parameters	mathematical parameters	mathematical parameters	3	kept
mathematics	mathematics	mathematics	mathematics	Mathematics	3	case_or_format
maturity	maturity	maturity	maturity	maturity	3	kept

maximal expiratory pressure	maximal expiratory pressure	maximal expiratory pressure	maximal expiratory pressure	maximal expiratory pressure	3	kept
maximal voluntary ventilation	maximal voluntary ventilation	maximal voluntary ventilation	maximal voluntary ventilation	maximal voluntary ventilation	3	kept
maximum likelihood method	maximum likelihood method	maximum likelihood method	maximum likelihood method	maximum likelihood method	3	kept
maximum permissible dose	maximum permissible dose	maximum permissible dose	maximum permissible dose	maximum permissible dose	3	kept
MDMA	mdma	mdma	mdma	MDMA	3	kept
Meals	meals	meals	meal	meal	3	singular
mean absolute relative difference	mean absolute relative difference	mean absolute relative difference	mean absolute relative difference	mean absolute relative difference	3	kept
mean platelet volume	mean platelet volume	mean platelet volume	mean platelet volume	mean platelet volume	3	kept
mean survival time	mean survival time	mean survival time	mean survival time	mean survival time	3	kept
Meaning	meaning	meaning	meaning	meaning	3	case_or_format
meaning-making	meaning making	meaning making	meaning making	meaning-making	3	kept
Meaningful Use	meaningful use	meaningful use	meaningful use	Meaningful Use	3	kept
measurements	measurements	measurements	measurement	measurement	3	singular
mechanics	mechanics	mechanics	mechanics	mechanics	3	kept
median sternotomy	median sternotomy	median sternotomy	median sternotomy	median sternotomy	3	kept
Mediation analysis	mediation analysis	mediation analysis	mediation analysis	mediation analysis	3	case_or_format

medical adhesive-related skin injury	medical adhesive related skin injury	medical adhesive related skin injury	medical adhesive related skin injury	medical adhesive-related skin injury	3	kept
Medical center	medical center	medical center	medical center	Medical center	3	kept
Medical decision making	medical decision making	medical decision making	medical decision making	medical decision making	3	case_or_format
medical device malfunction	medical device malfunction	medical device malfunction	medical device malfunction	medical device malfunction	3	kept
medical device related pressure injury	medical device related pressure injury	medical device related pressure injury	medical device related pressure injury	medical device-related pressure injury	3	case_or_format
Medical Education	medical education	medical education	medical education	medical education	3	case_or_format
MEDICAL EDUCATION & TRAINING	medical education and training	medical education and training	medical education and training	medical education & training	3	case_or_format
Medical equipment	medical equipment	medical equipment	medical equipment	Medical equipment	3	kept
Medical informatics applications	medical informatics applications	medical informatics applications	medical informatics applications	Medical Informatics Applications	3	case_or_format
Medical management	medical management	medical management	medical management	Medical management	3	kept
medical overuse	medical overuse	medical overuse	medical overuse	Medical Overuse	3	case_or_format
medical photography	medical photography	medical photography	medical photography	medical photography	3	kept
Medical procedures	medical procedures	medical procedures	medical procedures	medical procedures	3	case_or_format
medical professionals	medical professionals	medical professionals	medical professionals	Medical professionals	3	case_or_format
medical protocol	medical protocol	medical protocol	medical protocol	medical protocol	3	kept
medical receptionist	medical receptionist	medical receptionist	medical receptionist	medical receptionist	3	kept

Medical records	medical records	medical records	medical record	medical record	3	singular
medical records	medical records	medical records	medical record	medical record	3	singular
Medical records systems	medical records systems	medical records systems	medical records systems	Medical records systems	3	kept
medical records systems	medical records systems	medical records systems	medical records systems	Medical records systems	3	case_or_format
medical residency	medical residency	medical residency	medical residency	medical residency	3	kept
medical residents	medical residents	medical residents	medical resident	medical residents	3	singular
Medical resource utilization	medical resource utilization	medical resource utilization	medical resource utilization	Medical resource utilization	3	kept
Medical technologies	medical technologies	medical technologies	medical technology	medical technology	3	singular
Medical Waste Disposal	medical waste disposal	medical waste disposal	medical waste disposal	Medical Waste Disposal	3	kept
MEDICAL-CARE	medical care	medical care	medical care	medical care	3	case_or_format
Medication therapy management	medication therapy management	medication therapy management	medication therapy management	medication therapy management	3	case_or_format
Medicina intensiva	medicina intensiva	medicina intensiva	medicina intensiva	Medicina intensiva	3	kept
medicines	medicines	medicines	medicines	medicines	3	kept
medicines management	medicines management	medicines management	medicines management	medicines management	3	kept
Megacolon, Toxic	megacolon toxic	megacolon toxic	megacolon toxic	Megacolon, Toxic	3	kept
memantine	memantine	memantine	memantine	memantine	3	kept

Membrane Glycoproteins	membrane glycoproteins	membrane glycoproteins	membrane glycoproteins	Membrane Glycoproteins	3	kept
memory consolidation	memory consolidation	memory consolidation	memory consolidation	memory consolidation	3	kept
memory making	memory making	memory making	memory making	Memory making	3	case_or_format
Meningeal signs	meningeal signs	meningeal signs	meningeal signs	Meningeal signs	3	kept
meningism	meningism	meningism	meningism	meningism	3	kept
mental development assessment	mental development assessment	mental development assessment	mental development assessment	mental development assessment	3	kept
mental disorder	mental disorder	mental disorder	mental disorder	Mental Disorders	3	case_or_format
mental disorders	mental disorders	mental disorders	mental disorder	Mental Disorders	3	singular
mental health disorders	mental health disorders	mental health disorders	mental health disorders	mental health disorders	3	kept
mental instability	mental instability	mental instability	mental instability	mental instability	3	kept
mental test	mental test	mental test	mental test	mental test	3	kept
Mentorship	mentorship	mentorship	mentorship	Mentorship	3	kept
mesothelioma	mesothelioma	mesothelioma	mesothelioma	mesothelioma	3	kept
metabolic equivalent	metabolic equivalent	metabolic equivalent	metabolic equivalent	metabolic equivalent	3	kept
Metabolic Syndrome	metabolic syndrome	metabolic syndrome	metabolic syndrome	Metabolic Syndrome	3	kept
Metabolism	metabolism	metabolism	metabolism	metabolism	3	case_or_format

Metabolomics	metabolomics	metabolomics	metabolomics	Metabolomics	3	kept
Metacognition	metacognition	metacognition	metacognition	Metacognition	3	kept
Metaphor	metaphor	metaphor	metaphor	Metaphor	3	kept
meta‐synthesis	meta synthesis	meta synthesis	meta synthesis	meta-synthesis	3	case_or_format
methadone dose frequency	methadone dose frequency	methadone dose frequency	methadone dose frequency	methadone dose frequency	3	kept
Methamphetamine	methamphetamine	methamphetamine	methamphetamine	methamphetamine	3	case_or_format
Methanol	methanol	methanol	methanol	Methanol	3	kept
methanol poisoning	methanol poisoning	methanol poisoning	methanol poisoning	methanol poisoning	3	kept
Methemoglobinemia	methemoglobinemia	methemoglobinemia	methemoglobinemia	methemoglobinemia	3	case_or_format
Methicillin	methicillin	methicillin	methicillin	Methicillin	3	kept
methylxanthine	methylxanthine	methylxanthine	methylxanthine	methylxanthine	3	kept
metrics	metrics	metrics	metric	metrics	3	singular
Mice, Inbred C57BL	mice inbred c57bl	mice inbred c57bl	mice inbred c57bl	Mice, Inbred C57BL	3	kept
miconazole	miconazole	miconazole	miconazole	miconazole	3	kept
microangiopathy	microangiopathy	microangiopathy	microangiopathy	microangiopathy	3	kept
Microbial diversity	microbial diversity	microbial diversity	microbial diversity	Microbial diversity	3	kept

microbial diversity	microbial diversity	microbial diversity	microbial diversity	Microbial diversity	3	case_or_format
microbial growth	microbial growth	microbial growth	microbial growth	microbial growth	3	kept
Microbiology	microbiology	microbiology	microbiology	microbiology	3	case_or_format
Microcontroller	microcontroller	microcontroller	microcontroller	Microcontroller	3	kept
microorganisms	microorganisms	microorganisms	microorganism	microorganism	3	singular
Microphone arrays	microphone arrays	microphone arrays	microphone arrays	Microphone arrays	3	kept
Microphones	microphones	microphones	microphones	Microphones	3	kept
micturition	micturition	micturition	micturition	micturition	3	kept
middle cerebral artery occlusion	middle cerebral artery occlusion	middle cerebral artery occlusion	middle cerebral artery occlusion	middle cerebral artery occlusion	3	kept
Middle East Respiratory Syndrome Coronavirus	middle east respiratory syndrome coronavirus	middle east respiratory syndrome coronavirus	middle east respiratory syndrome coronavirus	Middle East respiratory syndrome coronavirus	3	case_or_format
midline catheter	midline catheter	midline catheter	midline catheter	midline catheter	3	kept
midwife attitude	midwife attitude	midwife attitude	midwife attitude	midwife attitude	3	kept
midwifery education	midwifery education	midwifery education	midwifery education	midwifery education	3	kept
miliary tuberculosis	miliary tuberculosis	miliary tuberculosis	miliary tuberculosis	miliary tuberculosis	3	kept
Military	military	military	military	military	3	case_or_format
Military medicine	military medicine	military medicine	military medicine	Military Medicine	3	case_or_format

MIMIC-IV	mimic iv	mimic iv	mimic iv	MIMIC-IV	3	kept
MIMIC-IV database	mimic iv database	mimic iv database	mimic iv database	MIMIC-IV database	3	kept
Mindfulness-based intervention	mindfulness based intervention	mindfulness based intervention	mindfulness based intervention	Mindfulness-based interventions	3	case_or_format
mineralocorticoid antagonist	mineralocorticoid antagonist	mineralocorticoid antagonist	mineralocorticoid antagonist	mineralocorticoid antagonist	3	kept
Mini-Cog assessment	mini cog assessment	mini cog assessment	mini cog assessment	Mini-Cog assessment	3	kept
Mini-Nutritional Assessment	mini nutritional assessment	mini nutritional assessment	mini nutritional assessment	Mini-Nutritional Assessment	3	kept
minimal clinically important difference	minimal clinically important difference	minimal clinically important difference	minimal clinically important difference	minimal clinically important difference	3	kept
MINIMUM DATA SET	minimum data set	minimum data set	minimum data set	MINIMUM DATA SET	3	kept
Mink	mink	mink	mink	Mink	3	kept
minoxidil	minoxidil	minoxidil	minoxidil	minoxidil	3	kept
misinformation	misinformation	misinformation	misinformation	misinformation	3	kept
Misoprostol	misoprostol	misoprostol	misoprostol	misoprostol	3	case_or_format
Mitral valve	mitral valve	mitral valve	mitral valve	Mitral Valve	3	case_or_format
Mixed methods research	mixed methods research	mixed methods research	mixed methods research	Mixed-methods research	3	case_or_format
mixed-integer programming	mixed integer programming	mixed integer programming	mixed integer programming	mixed-integer programming	3	kept
mixed-method research	mixed method research	mixed method research	mixed method research	mixed-method research	3	kept

Mixed-method study	mixed method study	mixed method study	mixed method study	Mixed-method study	3	kept
Mobile phones	mobile phones	mobile phones	mobile phone	mobile phone	3	singular
mobilisation	mobilisation	mobilisation	mobilisation	Mobilisation	3	case_or_format
model of care	model of care	model of care	model of care	model of care	3	kept
Model predictive control	model predictive control	model predictive control	model predictive control	Model predictive control	3	kept
Moderating effect	moderating effect	moderating effect	moderating effect	Moderating effect	3	kept
moisture-associated skin damage	moisture associated skin damage	moisture associated skin damage	moisture associated skin damage	moisture-associated skin damage	3	kept
Moldova	moldova	moldova	moldova	Moldova	3	kept
molecular weight	molecular weight	molecular weight	molecular weight	molecular weight	3	kept
Monitor	monitor	monitor	monitor	monitor	3	case_or_format
monkeypox	monkeypox	monkeypox	monkeypox	monkeypox	3	kept
monobactam derivative	monobactam derivative	monobactam derivative	monobactam derivative	monobactam derivative	3	kept
monoclonal antibody therapy	monoclonal antibody therapy	monoclonal antibody therapy	monoclonal antibody therapy	monoclonal antibody therapy	3	kept
monocyte	monocyte	monocyte	monocyte	monocyte	3	kept
Moral	moral	moral	moral	Morals	3	case_or_format
moral dilemma	moral dilemma	moral dilemma	moral dilemma	moral dilemma	3	kept

Moral injury	moral injury	moral injury	moral injury	moral injury	3	case_or_format
mortality awareness	mortality awareness	mortality awareness	mortality awareness	mortality awareness	3	kept
mortality rates	mortality rates	mortality rates	mortality rate	mortality rate	3	singular
moslem	moslem	moslem	moslem	moslem	3	kept
Mother-infant interaction	mother infant interaction	mother infant interaction	mother infant interaction	Mother-infant interaction	3	kept
mother-to-mother support	mother to mother support	mother to mother support	mother to mother support	mother-to-mother support	3	kept
Mothering	mothering	mothering	mothering	Mothering	3	kept
Motion	motion	motion	motion	motion	3	case_or_format
Mountaineering	mountaineering	mountaineering	mountaineering	Mountaineering	3	kept
mourning	mourning	mourning	mourning	mourning	3	kept
mouth infection	mouth infection	mouth infection	mouth infection	mouth infection	3	kept
mouth lesion	mouth lesion	mouth lesion	mouth lesion	mouth lesion	3	kept
mouth temperature	mouth temperature	mouth temperature	mouth temperature	mouth temperature	3	kept
Mouthwash	mouthwash	mouthwash	mouthwash	mouthwash	3	case_or_format
mucocutaneous lymph node syndrome	mucocutaneous lymph node syndrome	mucocutaneous lymph node syndrome	mucocutaneous lymph node syndrome	mucocutaneous lymph node syndrome	3	kept
mucous membrane pressure injury	mucous membrane pressure injury	mucous membrane pressure injury	mucous membrane pressure injury	mucous membrane pressure injury	3	kept

Multi-criteria decision making	multi criteria decision making	multi criteria decision making	multi criteria decision making	Multi-criteria decision making	3	kept
multi-disciplinary	multi disciplinary	multi disciplinary	multi disciplinary	Multi-disciplinary	3	case_or_format
Multi-disciplinary teams	multi disciplinary teams	multi disciplinary teams	multi disciplinary team	Multi-disciplinary teams	3	singular
Multi-sensory stimulation	multi sensory stimulation	multi sensory stimulation	multi sensory stimulation	Multi-sensory stimulation	3	kept
multicriteria decision analysis	multicriteria decision analysis	multicriteria decision analysis	multicriteria decision analysis	multicriteria decision analysis	3	kept
Multidisciplinary approach	multidisciplinary approach	multidisciplinary approach	multidisciplinary approach	Multidisciplinary approach	3	kept
Multidisciplinary collaboration	multidisciplinary collaboration	multidisciplinary collaboration	multidisciplinary collaboration	Multidisciplinary collaboration	3	kept
multidisciplinary collaboration	multidisciplinary collaboration	multidisciplinary collaboration	multidisciplinary collaboration	Multidisciplinary collaboration	3	case_or_format
multidisciplinary communication	multidisciplinary communication	multidisciplinary communication	multidisciplinary communication	multidisciplinary communication	3	kept
Multidisciplinary cooperation	multidisciplinary cooperation	multidisciplinary cooperation	multidisciplinary cooperation	Multidisciplinary cooperation	3	kept
multidrug resistant Pseudomonas aeruginosa	multidrug resistant pseudomonas aeruginosa	multidrug resistant pseudomonas aeruginosa	multidrug resistant pseudomonas aeruginosa	multidrug resistant Pseudomonas aeruginosa	3	kept
Multidrug-resistant	multidrug resistant	multidrug resistant	multidrug resistant	Multidrug-resistant	3	kept
multigravida	multigravida	multigravida	multigravida	multigravida	3	kept
multilayer perceptron	multilayer perceptron	multilayer perceptron	multilayer perceptron	multilayer perceptron	3	kept
Multilingualism	multilingualism	multilingualism	multilingualism	Multilingualism	3	kept
multimodal analgesia	multimodal analgesia	multimodal analgesia	multimodal analgesia	multimodal analgesia	3	kept

multimodal intervention	multimodal intervention	multimodal intervention	multimodal intervention	multimodal intervention	3	kept
Multimodal monitoring	multimodal monitoring	multimodal monitoring	multimodal monitoring	Multimodal monitoring	3	kept
Multiobjective optimization	multiobjective optimization	multiobjective optimization	multiobjective optimization	Multiobjective optimization	3	kept
multiorgan failure	multiorgan failure	multiorgan failure	multiorgan failure	multiorgan failure	3	kept
Multiple Endocrine Neoplasia	multiple endocrine neoplasia	multiple endocrine neoplasia	multiple endocrine neoplasia	Multiple Endocrine Neoplasia	3	kept
multiple fracture	multiple fracture	multiple fracture	multiple fracture	multiple fracture	3	kept
multiple organ dysfunction syndrome	multiple organ dysfunction syndrome	multiple organ dysfunction syndrome	multiple organ dysfunction syndrome	multiple organ dysfunction syndrome	3	kept
MUPIROCIN	mupirocin	mupirocin	mupirocin	mupirocin	3	case_or_format
Muscle	muscle	muscle	muscle	Muscle	3	kept
muscle	muscle	muscle	muscle	Muscle	3	case_or_format
muscle flap	muscle flap	muscle flap	muscle flap	muscle flap	3	kept
muscle hypertonia	muscle hypertonia	muscle hypertonia	muscle hypertonia	muscle hypertonia	3	kept
muscle injury	muscle injury	muscle injury	muscle injury	muscle injury	3	kept
Muscular Atrophy	muscular atrophy	muscular atrophy	muscular atrophy	Muscular Atrophy	3	kept
musculoskeletal	musculoskeletal	musculoskeletal	musculoskeletal	musculoskeletal	3	kept
musculoskeletal disorders	musculoskeletal disorders	musculoskeletal disorders	musculoskeletal disorder	Musculoskeletal disorders	3	singular

Musculoskeletal Manipulations	musculoskeletal manipulations	musculoskeletal manipulations	musculoskeletal manipulations	Musculoskeletal Manipulations	3	kept
music perception	music perception	music perception	music perception	music perception	3	kept
music therapist	music therapist	music therapist	music therapist	music therapist	3	kept
MUSIC-THERAPY	music therapy	music therapy	music therapy	Music Therapy	3	case_or_format
Myanmar	myanmar	myanmar	myanmar	Myanmar	3	kept
Mycoplasma pneumonia	mycoplasma pneumonia	mycoplasma pneumonia	mycoplasma pneumonia	Mycoplasma pneumonia	3	kept
myeloma	myeloma	myeloma	myeloma	myeloma	3	kept
myocardial disease	myocardial disease	myocardial disease	myocardial disease	myocardial disease	3	kept
Myocardial Reperfusion	myocardial reperfusion	myocardial reperfusion	myocardial reperfusion	Myocardial Reperfusion	3	kept
Myoglobin	myoglobin	myoglobin	myoglobin	Myoglobin	3	kept
myoglobin	myoglobin	myoglobin	myoglobin	Myoglobin	3	case_or_format
Médicos	m dicos	m dicos	m dico	Médicos	3	singular
N95 masks	n95 masks	n95 masks	n95 mask	N95 masks	3	singular
nadroparin	nadroparin	nadroparin	nadroparin	nadroparin	3	kept
nail bed	nail bed	nail bed	nail bed	nail bed	3	kept
Naloxone	naloxone	naloxone	naloxone	naloxone	3	case_or_format

NANDA International	nanda international	nanda international	nanda international	NANDA International	3	kept
NANDA international	nanda international	nanda international	nanda international	NANDA International	3	case_or_format
nanoparticle	nanoparticle	nanoparticle	nanoparticle	nanoparticle	3	kept
Nanoparticles	nanoparticles	nanoparticles	nanoparticle	nanoparticle	3	singular
napping	napping	napping	napping	napping	3	kept
Narcissism	narcissism	narcissism	narcissism	Narcissism	3	kept
narration	narration	narration	narration	Narration	3	case_or_format
Narrative	narrative	narrative	narrative	narrative	3	case_or_format
narrative research	narrative research	narrative research	narrative research	narrative research	3	kept
narrative therapy	narrative therapy	narrative therapy	narrative therapy	narrative therapy	3	kept
Narratives	narratives	narratives	narrative	narrative	3	singular
Nasogastric tube feeding	nasogastric tube feeding	nasogastric tube feeding	nasogastric tube feeding	Nasogastric tube feeding	3	kept
National early warning score	national early warning score	national early warning score	national early warning score	National Early Warning Score	3	case_or_format
NATIONAL-HEALTH	national health	national health	national health	NATIONAL-HEALTH	3	kept
nationality	nationality	nationality	nationality	nationality	3	kept
nationwide survey	nationwide survey	nationwide survey	nationwide survey	nationwide survey	3	kept

natriuresis	natriuresis	natriuresis	natriuresis	natriuresis	3	kept
natriuretic factor	natriuretic factor	natriuretic factor	natriuretic factor	natriuretic factor	3	kept
Natural childbirth	natural childbirth	natural childbirth	natural childbirth	Natural Childbirth	3	case_or_format
natural childbirth	natural childbirth	natural childbirth	natural childbirth	Natural Childbirth	3	case_or_format
naxitamab	naxitamab	naxitamab	naxitamab	naxitamab	3	kept
near drowning	near drowning	near drowning	near drowning	near drowning	3	kept
neck dissection	neck dissection	neck dissection	neck dissection	neck dissection	3	kept
NEED	need	need	need	NEEDS	3	case_or_format
needle stick	needle stick	needle stick	needle stick	needle stick	3	kept
Needle-stick injuries	needle stick injuries	needle stick injuries	needle stick injury	Needle stick injuries	3	singular
Needle-stick injury	needle stick injury	needle stick injury	needle stick injury	Needle stick injuries	3	case_or_format
Needlestick	needlestick	needlestick	needlestick	Needlestick	3	kept
NEEDLESTICK INJURIES	needlestick injuries	needlestick injuries	needlestick injury	needlestick injury	3	singular
needlestick injuries	needlestick injuries	needlestick injuries	needlestick injury	needlestick injury	3	singular
negative binomial regression	negative binomial regression	negative binomial regression	negative binomial regression	negative binomial regression	3	kept
negative syndrome	negative syndrome	negative syndrome	negative syndrome	negative syndrome	3	kept

neighborhood characteristic	neighborhood characteristic	neighborhood characteristic	neighborhood characteristic	neighborhood characteristic	3	kept
Neisseria gonorrhoeae	neisseria gonorrhoeae	neisseria gonorrhoeae	neisseria gonorrhoeae	Neisseria gonorrhoeae	3	kept
neoadjuvant chemotherapy	neoadjuvant chemotherapy	neoadjuvant chemotherapy	neoadjuvant chemotherapy	neoadjuvant chemotherapy	3	kept
neonatal care unit	neonatal care unit	neonatal care unit	neonatal care unit	neonatal care unit	3	kept
Neonatal death	neonatal death	neonatal death	neonatal death	neonatal death	3	case_or_format
neonatal encephalopathy	neonatal encephalopathy	neonatal encephalopathy	neonatal encephalopathy	Neonatal encephalopathy	3	case_or_format
Neonatal Extent of Work Rationing Instrument	neonatal extent of work rationing instrument	neonatal extent of work rationing instrument	neonatal extent of work rationing instrument	Neonatal Extent of Work Rationing Instrument	3	kept
Neonatal intensive	neonatal intensive	neonatal intensive	neonatal intensive	Neonatal intensive	3	kept
Neonatal morbidity	neonatal morbidity	neonatal morbidity	neonatal morbidity	Neonatal morbidity	3	kept
Neonatal outcomes	neonatal outcomes	neonatal outcomes	neonatal outcome	neonatal outcome	3	singular
neonatal pneumonia	neonatal pneumonia	neonatal pneumonia	neonatal pneumonia	neonatal pneumonia	3	kept
neonatal seizures	neonatal seizures	neonatal seizures	neonatal seizures	neonatal seizures	3	kept
neonatal sepsis	neonatal sepsis	neonatal sepsis	neonatal sepsis	Neonatal Sepsis	3	case_or_format
Neonatologist	neonatologist	neonatologist	neonatologist	neonatologist	3	case_or_format
Neoplasm Recurrence, Local	neoplasm recurrence local	neoplasm recurrence local	neoplasm recurrence local	Neoplasm Recurrence, Local	3	kept
Nephritis	nephritis	nephritis	nephritis	nephritis	3	case_or_format

Nephrologists	nephrologists	nephrologists	nephrologist	nephrologist	3	singular
Nerve block	nerve block	nerve block	nerve block	Nerve Block	3	case_or_format
nerve function	nerve function	nerve function	nerve function	nerve function	3	kept
nervous system inflammation	nervous system inflammation	nervous system inflammation	nervous system inflammation	nervous system inflammation	3	kept
nesiritide	nesiritide	nesiritide	nesiritide	nesiritide	3	kept
nesting	nesting	nesting	nesting	nesting	3	kept
Network Meta-Analysis	network meta analysis	network meta analysis	network meta analysis	Network meta-analysis	3	case_or_format
Networks	networks	networks	network	Network	3	singular
Neural Conduction	neural conduction	neural conduction	neural conduction	Neural Conduction	3	kept
neuroanatomy	neuroanatomy	neuroanatomy	neuroanatomy	neuroanatomy	3	kept
Neurobiology	neurobiology	neurobiology	neurobiology	Neurobiology	3	kept
neurocognitive disorders	neurocognitive disorders	neurocognitive disorders	neurocognitive disorders	neurocognitive disorders	3	kept
neuroendoscopy	neuroendoscopy	neuroendoscopy	neuroendoscopy	neuroendoscopy	3	kept
neurogenic bladder	neurogenic bladder	neurogenic bladder	neurogenic bladder	neurogenic bladder	3	kept
Neurological	neurological	neurological	neurological	Neurological	3	kept
Neurological assessment	neurological assessment	neurological assessment	neurological assessment	Neurological assessment	3	kept

neurological function	neurological function	neurological function	neurological function	neurological function	3	kept
neurological injury	neurological injury	neurological injury	neurological injury	neurological injury	3	kept
Neurological recovery	neurological recovery	neurological recovery	neurological recovery	Neurological recovery	3	kept
Neuroma, Acoustic	neuroma acoustic	neuroma acoustic	neuroma acoustic	Neuroma, Acoustic	3	kept
neuromodulation	neuromodulation	neuromodulation	neuromodulation	neuromodulation	3	kept
Neuromuscular blockade	neuromuscular blockade	neuromuscular blockade	neuromuscular blockade	Neuromuscular Blockade	3	case_or_format
Neuromuscular disease	neuromuscular disease	neuromuscular disease	neuromuscular disease	neuromuscular disease	3	case_or_format
Neuromuscular Junction	neuromuscular junction	neuromuscular junction	neuromuscular junction	Neuromuscular Junction	3	kept
neuropsychiatric symptoms	neuropsychiatric symptoms	neuropsychiatric symptoms	neuropsychiatric symptoms	neuropsychiatric symptoms	3	kept
neuroradiology	neuroradiology	neuroradiology	neuroradiology	neuroradiology	3	kept
Neuroticism	neuroticism	neuroticism	neuroticism	Neuroticism	3	kept
neurotransmitter	neurotransmitter	neurotransmitter	neurotransmitter	neurotransmitter	3	kept
neutrophil percentage	neutrophil percentage	neutrophil percentage	neutrophil percentage	neutrophil percentage	3	kept
Nevada	nevada	nevada	nevada	Nevada	3	kept
never smoker	never smoker	never smoker	never smoker	never smoker	3	kept
nevirapine	nevirapine	nevirapine	nevirapine	nevirapine	3	kept

New Brunswick	new brunswick	new brunswick	new brunswick	New Brunswick	3	kept
new-onset atrial fibrillation	new onset atrial fibrillation	new onset atrial fibrillation	new onset atrial fibrillation	new-onset atrial fibrillation	3	kept
newborn infant	newborn infant	newborn infant	newborn infant	Newborn infant	3	case_or_format
NEWBORNS	newborns	newborns	newborn	newborn	3	singular
NEWS2	news2	news2	news2	NEWS2	3	kept
NHPPD	nhppd	nhppd	nhppd	NHPPD	3	kept
NIC	nic	nic	nic	NIC	3	kept
nicotine replacement therapy	nicotine replacement therapy	nicotine replacement therapy	nicotine replacement therapy	nicotine replacement therapy	3	kept
night shift worker	night shift worker	night shift worker	night shift worker	night shift worker	3	kept
nirmatrelvir plus ritonavir	nirmatrelvir plus ritonavir	nirmatrelvir plus ritonavir	nirmatrelvir plus ritonavir	nirmatrelvir plus ritonavir	3	kept
nitrite	nitrite	nitrite	nitrite	nitrite	3	kept
NIV	niv	niv	niv	NIV	3	kept
No sedation	no sedation	no sedation	no sedation	No sedation	3	kept
NOC	noc	noc	noc	NOC	3	kept
Noise level	noise level	noise level	noise level	Noise levels	3	case_or_format
noise source	noise source	noise source	noise source	Noise source	3	case_or_format

Non-convulsive seizures	non convulsive seizures	non convulsive seizures	non convulsive seizures	Non-convulsive seizures	3	kept
non-Hodgkin lymphoma	non hodgkin lymphoma	non hodgkin lymphoma	non hodgkin lymphoma	non-Hodgkin lymphoma	3	kept
non-inferiority trial	non inferiority trial	non inferiority trial	non inferiority trial	non-inferiority trial	3	kept
non-invasive positive pressure ventilation	non invasive positive pressure ventilation	non invasive positive pressure ventilation	non invasive positive pressure ventilation	non-invasive positive pressure ventilation	3	kept
Non-invasive respiratory support	non invasive respiratory support	non invasive respiratory support	non invasive respiratory support	Non-invasive respiratory support	3	kept
Non-medical prescribing	non medical prescribing	non medical prescribing	non medical prescribing	Non-medical prescribing	3	kept
Non-nutritive suck	non nutritive suck	non nutritive suck	non nutritive suck	Non-nutritive suck	3	kept
Non-pharmacological	non pharmacological	non pharmacological	non pharmacological	Non-pharmacological	3	kept
non-pharmacological	non pharmacological	non pharmacological	non pharmacological	Non-pharmacological	3	case_or_format
Non-pharmacological intervention	non pharmacological intervention	non pharmacological intervention	non pharmacological intervention	Non-pharmacological interventions	3	case_or_format
Non-pharmacological pain management	non pharmacological pain management	non pharmacological pain management	non pharmacological pain management	Non-pharmacological pain management	3	kept
nonbeneficial care	nonbeneficial care	nonbeneficial care	nonbeneficial care	nonbeneficial care	3	kept
Noninvasive respiratory support	noninvasive respiratory support	noninvasive respiratory support	noninvasive respiratory support	Noninvasive respiratory support	3	kept
NONINVASIVE VENTILATION	noninvasive ventilation	noninvasive ventilation	noninvasive ventilation	noninvasive ventilation	3	case_or_format
nonketotic diabetic coma	nonketotic diabetic coma	nonketotic diabetic coma	nonketotic diabetic coma	nonketotic diabetic coma	3	kept
Nonpharmacologic	nonpharmacologic	nonpharmacologic	nonpharmacologic	Nonpharmacologic	3	kept

nonpharmacologic	nonpharmacologic	nonpharmacologic	nonpharmacologic	Nonpharmacologic	3	case_or_format
nonpharmacological interventions	nonpharmacological interventions	nonpharmacological interventions	nonpharmacological intervention	Nonpharmacological interventions	3	singular
nonsense mutation	nonsense mutation	nonsense mutation	nonsense mutation	nonsense mutation	3	kept
NONTECHNICAL SKILLS	nontechnical skills	nontechnical skills	nontechnical skills	NONTECHNICAL SKILLS	3	kept
nontechnical skills	nontechnical skills	nontechnical skills	nontechnical skills	NONTECHNICAL SKILLS	3	case_or_format
Nonverbal communication	nonverbal communication	nonverbal communication	nonverbal communication	Nonverbal Communication	3	case_or_format
Nordic Musculoskeletal Questionnaire	nordic musculoskeletal questionnaire	nordic musculoskeletal questionnaire	nordic musculoskeletal questionnaire	Nordic Musculoskeletal Questionnaire	3	kept
Normal saline	normal saline	normal saline	normal saline	Normal saline	3	kept
normalization process theory	normalization process theory	normalization process theory	normalization process theory	normalization process theory	3	kept
Norton Scale	norton scale	norton scale	norton scale	Norton Scale	3	kept
Norwood	norwood	norwood	norwood	Norwood	3	kept
nose injury	nose injury	nose injury	nose injury	nose injury	3	kept
nosocomial	nosocomial	nosocomial	nosocomial	Nosocomial	3	case_or_format
NOSOCOMIAL INFECTION	nosocomial infection	nosocomial infection	nosocomial infection	Nosocomial infection	3	case_or_format
Nosocomial transmission	nosocomial transmission	nosocomial transmission	nosocomial transmission	nosocomial transmission	3	case_or_format
NOT-RESUSCITATE ORDERS	not resuscitate orders	not resuscitate orders	not resuscitate orders	NOT-RESUSCITATE ORDERS	3	kept

Novel coronavirus	novel coronavirus	novel coronavirus	novel coronavirus	Novel coronavirus	3	kept
NRP	nrp	nrp	nrp	NRP	3	kept
nuclear magnetic resonance	nuclear magnetic resonance	nuclear magnetic resonance	nuclear magnetic resonance	nuclear magnetic resonance	3	kept
nucleic acid analysis	nucleic acid analysis	nucleic acid analysis	nucleic acid analysis	nucleic acid analysis	3	kept
nuisance alarms	nuisance alarms	nuisance alarms	nuisance alarms	nuisance alarms	3	kept
Numerical Analysis, Computer-Assisted	numerical analysis computer assisted	numerical analysis computer assisted	numerical analysis computer assisted	Numerical Analysis, Computer-Assisted	3	kept
nutrient availability	nutrient availability	nutrient availability	nutrient availability	nutrient availability	3	kept
nutrition support practice	nutrition support practice	nutrition support practice	nutrition support practice	nutrition support practice	3	kept
nutrition support teams	nutrition support teams	nutrition support teams	nutrition support team	nutrition support teams	3	singular
Nutrition therapy	nutrition therapy	nutrition therapy	nutrition therapy	Nutrition Therapy	3	case_or_format
Nutritional indicators	nutritional indicators	nutritional indicators	nutritional indicators	Nutritional indicators	3	kept
nutritional tolerance	nutritional tolerance	nutritional tolerance	nutritional tolerance	nutritional tolerance	3	kept
Nystatin	nystatin	nystatin	nystatin	nystatin	3	case_or_format
object attachment	object attachment	object attachment	object attachment	Object Attachment	3	case_or_format
Observation and response charts	observation and response charts	observation and response charts	observation and response charts	Observation and response charts	3	kept
observational method	observational method	observational method	observational method	observational method	3	kept

Observational Study	observational study	observational study	observational study	observational study	3	case_or_format
obstetric emergency	obstetric emergency	obstetric emergency	obstetric emergency	obstetric emergency	3	kept
obstetrics and gynecology	obstetrics and gynecology	obstetrics and gynecology	obstetrics and gynecology	obstetrics and gynecology	3	kept
obstructive lung disease	obstructive lung disease	obstructive lung disease	obstructive lung disease	obstructive lung disease	3	kept
OBSTRUCTIVE PULMONARY-DISEASE	obstructive pulmonary disease	obstructive pulmonary disease	obstructive pulmonary disease	OBSTRUCTIVE PULMONARY-DISEASE	3	kept
obstructive shock	obstructive shock	obstructive shock	obstructive shock	obstructive shock	3	kept
occlusive dressing	occlusive dressing	occlusive dressing	occlusive dressing	Occlusive Dressings	3	case_or_format
occult blood test	occult blood test	occult blood test	occult blood test	occult blood test	3	kept
occupation and occupation related phenomena	occupation and occupation related phenomena	occupation and occupation related phenomena	occupation and occupation related phenomena	occupation and occupation related phenomena	3	kept
Occupational	occupational	occupational	occupational	Occupational	3	kept
Occupational diseases	occupational diseases	occupational diseases	occupational disease	Occupational Diseases	3	singular
Occupational hazard	occupational hazard	occupational hazard	occupational hazard	occupational hazard	3	case_or_format
occupational hazards	occupational hazards	occupational hazards	occupational hazard	occupational hazard	3	singular
occupational risk factor	occupational risk factor	occupational risk factor	occupational risk factor	occupational risk factor	3	kept
occupational skin disease	occupational skin disease	occupational skin disease	occupational skin disease	occupational skin disease	3	kept
Octogenarians	octogenarians	octogenarians	octogenarian	Octogenarians	3	singular

Office Visits	office visits	office visits	office visits	Office Visits	3	kept
Ogilvie syndrome	ogilvie syndrome	ogilvie syndrome	ogilvie syndrome	Ogilvie syndrome	3	kept
Older	older	older	older	OLDER	3	case_or_format
older	older	older	older	OLDER	3	case_or_format
older person	older person	older person	older person	older person	3	kept
older persons	older persons	older persons	older person	older person	3	singular
Oligohydramnios	oligohydramnios	oligohydramnios	oligohydramnios	oligohydramnios	3	case_or_format
omphalitis	omphalitis	omphalitis	omphalitis	omphalitis	3	kept
one lung ventilation	one lung ventilation	one lung ventilation	one lung ventilation	one lung ventilation	3	kept
online	online	online	online	online	3	kept
online support group	online support group	online support group	online support group	online support group	3	kept
Online survey	online survey	online survey	online survey	Online survey	3	kept
Open abdomen	open abdomen	open abdomen	open abdomen	open abdomen	3	case_or_format
open fracture reduction	open fracture reduction	open fracture reduction	open fracture reduction	open fracture reduction	3	kept
Open visitation	open visitation	open visitation	open visitation	Open visitation	3	kept
open visitation	open visitation	open visitation	open visitation	Open visitation	3	case_or_format

Operating rooms	operating rooms	operating rooms	operating room	operating room	3	singular
Operational strategies	operational strategies	operational strategies	operational strategies	Operational strategies	3	kept
OPERATIONS	operations	operations	operation	OPERATIONS	3	singular
Operations management	operations management	operations management	operations management	Operations management	3	kept
Ophthalmologists	ophthalmologists	ophthalmologists	ophthalmologist	ophthalmologist	3	singular
Opinion	opinion	opinion	opinion	Opinion	3	kept
opioid analgesics	opioid analgesics	opioid analgesics	opioid analgesic	opioid analgesics	3	singular
opioid sparing	opioid sparing	opioid sparing	opioid sparing	opioid sparing	3	kept
opioid use	opioid use	opioid use	opioid use	opioid use	3	kept
Opportunities	opportunities	opportunities	opportunity	Opportunities	3	singular
optic nerve	optic nerve	optic nerve	optic nerve	optic nerve	3	kept
optimization	optimization	optimization	optimization	Optimization	3	case_or_format
oral bleeding	oral bleeding	oral bleeding	oral bleeding	oral bleeding	3	kept
Oral cancer	oral cancer	oral cancer	oral cancer	Oral cancer	3	kept
oral communication	oral communication	oral communication	oral communication	oral communication	3	kept
oral endotracheal tube	oral endotracheal tube	oral endotracheal tube	oral endotracheal tube	oral endotracheal tube	3	kept

oral frailty	oral frailty	oral frailty	oral frailty	oral frailty	3	kept
oral immunotherapy	oral immunotherapy	oral immunotherapy	oral immunotherapy	oral immunotherapy	3	kept
Oral intake	oral intake	oral intake	oral intake	Oral intake	3	kept
Oral stimulation	oral stimulation	oral stimulation	oral stimulation	Oral stimulation	3	kept
Oral Surgical Procedures	oral surgical procedures	oral surgical procedures	oral surgical procedures	Oral Surgical Procedures	3	kept
Organ donor	organ donor	organ donor	organ donor	organ donor	3	case_or_format
organ dysfunction	organ dysfunction	organ dysfunction	organ dysfunction	organ dysfunction	3	kept
organ dysfunction scores	organ dysfunction scores	organ dysfunction scores	organ dysfunction score	Organ Dysfunction Scores	3	singular
organisational development	organisational development	organisational development	organisational development	organisational development	3	kept
Organization-based self-esteem	organization based self esteem	organization based self esteem	organization based self esteem	Organization-based self-esteem	3	kept
Organizational Commitment	organizational commitment	organizational commitment	organizational commitment	Organizational commitment	3	case_or_format
ORGANIZATIONAL CULTURE	organizational culture	organizational culture	organizational culture	Organizational Culture	3	case_or_format
organizational downsizing	organizational downsizing	organizational downsizing	organizational downsizing	organizational downsizing	3	kept
Organizational factors	organizational factors	organizational factors	organizational factors	organizational factors	3	case_or_format
organizational intervention	organizational intervention	organizational intervention	organizational intervention	organizational intervention	3	kept
organizational restructuring	organizational restructuring	organizational restructuring	organizational restructuring	organizational restructuring	3	kept

Organometallic Compounds	organometallic compounds	organometallic compounds	organometallic compounds	Organometallic Compounds	3	kept
Orientia tsutsugamushi	orientia tsutsugamushi	orientia tsutsugamushi	orientia tsutsugamushi	Orientia tsutsugamushi	3	kept
orthosis	orthosis	orthosis	orthosis	orthosis	3	kept
oscillometry	oscillometry	oscillometry	oscillometry	oscillometry	3	kept
osmolality	osmolality	osmolality	osmolality	osmolality	3	kept
osteosarcoma	osteosarcoma	osteosarcoma	osteosarcoma	osteosarcoma	3	kept
otalgia	otalgia	otalgia	otalgia	otalgia	3	kept
Out-of-hospital	out of hospital	out of hospital	out of hospital	Out-of-hospital	3	kept
out-of-hospital cardiac arrest	out of hospital cardiac arrest	out of hospital cardiac arrest	out of hospital cardiac arrest	out of hospital cardiac arrest	3	case_or_format
Outcome and process assessment	outcome and process assessment	outcome and process assessment	outcome and process assessment	Outcome and Process Assessment	3	case_or_format
outcome and process assessment	outcome and process assessment	outcome and process assessment	outcome and process assessment	Outcome and Process Assessment	3	case_or_format
Outcome measures	outcome measures	outcome measures	outcome measure	outcome measures	3	singular
Outliers, DRG	outliers drg	outliers drg	outliers drg	Outliers, DRG	3	kept
Outreach	outreach	outreach	outreach	outreach	3	case_or_format
Outreach services	outreach services	outreach services	outreach service	Outreach services	3	singular
Ovarian cancer	ovarian cancer	ovarian cancer	ovarian cancer	Ovarian cancer	3	kept

Oxacillin	oxacillin	oxacillin	oxacillin	oxacillin	3	case_or_format
oxazolidinone derivative	oxazolidinone derivative	oxazolidinone derivative	oxazolidinone derivative	oxazolidinone derivative	3	kept
Oxazolidinones	oxazolidinones	oxazolidinones	oxazolidinones	Oxazolidinones	3	kept
Oxidative stress	oxidative stress	oxidative stress	oxidative stress	oxidative stress	3	case_or_format
Oximeters	oximeters	oximeters	oximeter	Oximeters	3	singular
Oxygen administration	oxygen administration	oxygen administration	oxygen administration	Oxygen administration	3	kept
Oxygen delivery	oxygen delivery	oxygen delivery	oxygen delivery	Oxygen delivery	3	kept
oxygenator	oxygenator	oxygenator	oxygenator	oxygenator	3	kept
Paclitaxel	paclitaxel	paclitaxel	paclitaxel	paclitaxel	3	case_or_format
Paediatric anaesthesia	paediatric anaesthesia	paediatric anaesthesia	paediatric anaesthesia	Paediatric anaesthesia	3	kept
paediatric delirium	paediatric delirium	paediatric delirium	paediatric delirium	paediatric delirium	3	kept
Paediatric oncology	paediatric oncology	paediatric oncology	paediatric oncology	Paediatric oncology	3	kept
Pain assessment tool	pain assessment tool	pain assessment tool	pain assessment tool	Pain assessment tool	3	kept
Painful procedures	painful procedures	painful procedures	painful procedure	Painful procedures	3	singular
palliative care consultation	palliative care consultation	palliative care consultation	palliative care consultation	palliative care consultation	3	kept
palliative care unit	palliative care unit	palliative care unit	palliative care unit	palliative care unit	3	kept

Palliative Performance Scale	palliative performance scale	palliative performance scale	palliative performance scale	Palliative Performance Scale	3	kept
Pancreas	pancreas	pancreas	pancreas	Pancreas	3	kept
pancreas adenocarcinoma	pancreas adenocarcinoma	pancreas adenocarcinoma	pancreas adenocarcinoma	pancreas adenocarcinoma	3	kept
pancreas fistula	pancreas fistula	pancreas fistula	pancreas fistula	pancreas fistula	3	kept
Pancreatic Neoplasms	pancreatic neoplasms	pancreatic neoplasms	pancreatic neoplasms	Pancreatic Neoplasms	3	kept
Pancreatic Pseudocyst	pancreatic pseudocyst	pancreatic pseudocyst	pancreatic pseudocyst	Pancreatic Pseudocyst	3	kept
Pantoprazole	pantoprazole	pantoprazole	pantoprazole	pantoprazole	3	case_or_format
Paper	paper	paper	paper	Paper	3	kept
paper	paper	paper	paper	Paper	3	case_or_format
Parainfluenza virus	parainfluenza virus	parainfluenza virus	parainfluenza virus	Parainfluenza virus	3	kept
paramedical disciplines	paramedical disciplines	paramedical disciplines	paramedical disciplines	paramedical disciplines	3	kept
parameters concerning the fetus, newborn and pregnancy	parameters concerning the fetus newborn and pregnancy	parameters concerning the fetus newborn and pregnancy	parameters concerning the fetus newborn and pregnancy	parameters concerning the fetus, newborn and pregnancy	3	kept
paranoia	paranoia	paranoia	paranoia	paranoia	3	kept
parent support	parent support	parent support	parent support	parent support	3	kept
parental	parental	parental	parental	parental	3	kept
Parental involvement	parental involvement	parental involvement	parental involvement	parental involvement	3	case_or_format

Parental presence	parental presence	parental presence	parental presence	Parental presence	3	kept
Parental self-efficacy	parental self efficacy	parental self efficacy	parental self efficacy	Parental self-efficacy	3	kept
Parenteral	parenteral	parenteral	parenteral	parenteral	3	case_or_format
Parenteral Nutrition Solutions	parenteral nutrition solutions	parenteral nutrition solutions	parenteral nutrition solutions	Parenteral Nutrition Solutions	3	kept
Parenteral Nutrition, Home	parenteral nutrition home	parenteral nutrition home	parenteral nutrition home	Parenteral Nutrition, Home	3	kept
parenteral solution	parenteral solution	parenteral solution	parenteral solution	parenteral solution	3	kept
PARENTERAL-NUTRITION	parenteral nutrition	parenteral nutrition	parenteral nutrition	parenteral nutrition	3	case_or_format
PARIHS	parihs	parihs	parihs	PARIHS	3	kept
paroxetine	paroxetine	paroxetine	paroxetine	paroxetine	3	kept
paroxysmal atrial fibrillation	paroxysmal atrial fibrillation	paroxysmal atrial fibrillation	paroxysmal atrial fibrillation	paroxysmal atrial fibrillation	3	kept
paroxysmal nocturnal hemoglobinuria	paroxysmal nocturnal hemoglobinuria	paroxysmal nocturnal hemoglobinuria	paroxysmal nocturnal hemoglobinuria	paroxysmal nocturnal hemoglobinuria	3	kept
participatory design	participatory design	participatory design	participatory design	Participatory design	3	case_or_format
Partnership practice	partnership practice	partnership practice	partnership practice	Partnership practice	3	kept
passive immunization	passive immunization	passive immunization	passive immunization	passive immunization	3	kept
passive leg raise	passive leg raise	passive leg raise	passive leg raise	passive leg raise	3	kept
Patent foramen ovale	patent foramen ovale	patent foramen ovale	patent foramen ovale	patent foramen ovale	3	case_or_format

Paternity	paternity	paternity	paternity	Paternity	3	kept
pathogen	pathogen	pathogen	pathogen	pathogen	3	kept
Pathogens	pathogens	pathogens	pathogen	pathogen	3	singular
Pathology	pathology	pathology	pathology	pathology	3	case_or_format
Pathway	pathway	pathway	pathway	Pathway	3	kept
pathway	pathway	pathway	pathway	Pathway	3	case_or_format
PCA	pca	pca	pca	PCA	3	kept
PDA	pda	pda	pda	PDA	3	kept
peanut	peanut	peanut	peanut	peanut	3	kept
Pediatric cancer	pediatric cancer	pediatric cancer	pediatric cancer	Pediatric cancer	3	kept
pediatric cancer	pediatric cancer	pediatric cancer	pediatric cancer	Pediatric cancer	3	case_or_format
pediatric emergency	pediatric emergency	pediatric emergency	pediatric emergency	pediatric emergency	3	kept
Pediatric Obesity	pediatric obesity	pediatric obesity	pediatric obesity	Pediatric Obesity	3	kept
pediatric oncologist	pediatric oncologist	pediatric oncologist	pediatric oncologist	pediatric oncologist	3	kept
Pediatric oncology	pediatric oncology	pediatric oncology	pediatric oncology	pediatric oncology	3	case_or_format
Peer Influence	peer influence	peer influence	peer influence	Peer Influence	3	kept

Peer learning	peer learning	peer learning	peer learning	Peer learning	3	kept
Peer review	peer review	peer review	peer review	peer review	3	case_or_format
PEG	peg	peg	peg	PEG	3	kept
pelvic pain	pelvic pain	pelvic pain	pelvic pain	pelvic pain	3	kept
pelvis injury	pelvis injury	pelvis injury	pelvis injury	pelvis injury	3	kept
Penicillanic Acid	penicillanic acid	penicillanic acid	penicillanic acid	Penicillanic Acid	3	kept
Penicillin-Binding Proteins	penicillin binding proteins	penicillin binding proteins	penicillin binding protein	Penicillin-Binding Proteins	3	singular
Pentalogy of Cantrell	pentalogy of cantrell	pentalogy of cantrell	pentalogy of cantrell	Pentalogy of Cantrell	3	kept
pentazocine	pentazocine	pentazocine	pentazocine	pentazocine	3	kept
people by vaccination status	people by vaccination status	people by vaccination status	people by vaccination status	people by vaccination status	3	kept
pepsin A	pepsin a	pepsin a	pepsin a	pepsin A	3	kept
perceived barriers	perceived barriers	perceived barriers	perceived barrier	Perceived barriers	3	singular
perception deafness	perception deafness	perception deafness	perception deafness	perception deafness	3	kept
percutaneous dilatational tracheostomy	percutaneous dilatational tracheostomy	percutaneous dilatational tracheostomy	percutaneous dilatational tracheostomy	percutaneous dilatational tracheostomy	3	kept
percutaneous nephrolithotomy	percutaneous nephrolithotomy	percutaneous nephrolithotomy	percutaneous nephrolithotomy	percutaneous nephrolithotomy	3	kept
performance feedback	performance feedback	performance feedback	performance feedback	performance feedback	3	kept

performance improvement	performance improvement	performance improvement	performance improvement	Performance improvement	3	case_or_format
Performance obstacles	performance obstacles	performance obstacles	performance obstacle	Performance obstacles	3	singular
pericardiotomy	pericardiotomy	pericardiotomy	pericardiotomy	pericardiotomy	3	kept
Perinatal loss	perinatal loss	perinatal loss	perinatal loss	Perinatal loss	3	kept
Perinatal mortality	perinatal mortality	perinatal mortality	perinatal mortality	Perinatal Mortality	3	case_or_format
perinatal outcome	perinatal outcome	perinatal outcome	perinatal outcome	perinatal outcome	3	kept
perineum injury	perineum injury	perineum injury	perineum injury	perineum injury	3	kept
periodic medical examination	periodic medical examination	periodic medical examination	periodic medical examination	periodic medical examination	3	kept
Periodicity	periodicity	periodicity	periodicity	Periodicity	3	kept
periodicity	periodicity	periodicity	periodicity	Periodicity	3	case_or_format
periodontal disease	periodontal disease	periodontal disease	periodontal disease	Periodontal Diseases	3	case_or_format
Perioperative management	perioperative management	perioperative management	perioperative management	Perioperative management	3	kept
perioperative medicine	perioperative medicine	perioperative medicine	perioperative medicine	perioperative medicine	3	kept
peripartum cardiomyopathy	peripartum cardiomyopathy	peripartum cardiomyopathy	peripartum cardiomyopathy	peripartum cardiomyopathy	3	kept
Peripheral catheterization	peripheral catheterization	peripheral catheterization	peripheral catheterization	Peripheral catheterization	3	kept
peripheral venous catheter	peripheral venous catheter	peripheral venous catheter	peripheral venous catheter	peripheral venous catheter	3	kept

peritoneum tumor	peritoneum tumor	peritoneum tumor	peritoneum tumor	peritoneum tumor	3	kept
permanent employment	permanent employment	permanent employment	permanent employment	permanent employment	3	kept
Person-centered care	person centered care	person centered care	person centered care	person centered care	3	case_or_format
person-centered care	person centered care	person centered care	person centered care	person centered care	3	case_or_format
person-centred	person centred	person centred	person centred	person-centred	3	kept
personal space	personal space	personal space	personal space	Personal Space	3	case_or_format
Personality traits	personality traits	personality traits	personality trait	Personality traits	3	singular
personalized care	personalized care	personalized care	personalized care	personalized care	3	kept
Persons with Psychiatric Disorders	persons with psychiatric disorders	persons with psychiatric disorders	persons with psychiatric disorders	Persons with Psychiatric Disorders	3	kept
Petechial rash	petechial rash	petechial rash	petechial rash	Petechial rash	3	kept
phage therapy	phage therapy	phage therapy	phage therapy	phage therapy	3	kept
Phantom Limb	phantom limb	phantom limb	phantom limb	Phantom Limb	3	kept
Pharmaceutical Services	pharmaceutical services	pharmaceutical services	pharmaceutical service	Pharmaceutical services	3	singular
Pharmaceutical Solutions	pharmaceutical solutions	pharmaceutical solutions	pharmaceutical solutions	Pharmaceutical Solutions	3	kept
pharmacist attitude	pharmacist attitude	pharmacist attitude	pharmacist attitude	pharmacist attitude	3	kept
Pharmacist intervention	pharmacist intervention	pharmacist intervention	pharmacist intervention	Pharmacist intervention	3	kept

pharmacogenetics	pharmacogenetics	pharmacogenetics	pharmacogenetics	Pharmacogenetics	3	case_or_format
pharmacokinetic parameters	pharmacokinetic parameters	pharmacokinetic parameters	pharmacokinetic parameters	pharmacokinetic parameters	3	kept
PHARMACOKINETICS	pharmacokinetics	pharmacokinetics	pharmacokinetic	pharmacokinetics	3	singular
Pharmacology, Clinical	pharmacology clinical	pharmacology clinical	pharmacology clinical	Pharmacology, Clinical	3	kept
Pharmacopoeias as Topic	pharmacopoeias as topic	pharmacopoeias as topic	pharmacopoeias as topic	Pharmacopoeias as Topic	3	kept
Pharmacovigilance	pharmacovigilance	pharmacovigilance	pharmacovigilance	pharmacovigilance	3	case_or_format
Pharmacy and Therapeutics Committee	pharmacy and therapeutics committee	pharmacy and therapeutics committee	pharmacy and therapeutics committee	Pharmacy and Therapeutics Committee	3	kept
phase 4 clinical trial	phase 4 clinical trial	phase 4 clinical trial	phase 4 clinical trial	phase 4 clinical trial	3	kept
Phenomenography	phenomenography	phenomenography	phenomenography	Phenomenography	3	kept
phenomenography	phenomenography	phenomenography	phenomenography	Phenomenography	3	case_or_format
Phenomenological hermeneutical	phenomenological hermeneutical	phenomenological hermeneutical	phenomenological hermeneutical	Phenomenological hermeneutical	3	kept
Pheochromocytoma	pheochromocytoma	pheochromocytoma	pheochromocytoma	pheochromocytoma	3	case_or_format
Philosophy	philosophy	philosophy	philosophy	philosophy	3	case_or_format
philosophy of medicine	philosophy of medicine	philosophy of medicine	philosophy of medicine	philosophy of medicine	3	kept
Phobic Disorders	phobic disorders	phobic disorders	phobic disorders	Phobic Disorders	3	kept
phonation	phonation	phonation	phonation	Phonation	3	case_or_format

phospholipid antibody	phospholipid antibody	phospholipid antibody	phospholipid antibody	phospholipid antibody	3	kept
Phosphorus	phosphorus	phosphorus	phosphorus	phosphorus	3	case_or_format
Physical distancing	physical distancing	physical distancing	physical distancing	Physical Distancing	3	case_or_format
Physical exertion	physical exertion	physical exertion	physical exertion	Physical Exertion	3	case_or_format
physical growth	physical growth	physical growth	physical growth	physical growth	3	kept
physical mobility	physical mobility	physical mobility	physical mobility	physical mobility	3	kept
Physical recovery	physical recovery	physical recovery	physical recovery	Physical recovery	3	kept
Physical Therapy	physical therapy	physical therapy	physical therapy	Physical therapy	3	case_or_format
physical tolerance	physical tolerance	physical tolerance	physical tolerance	physical tolerance	3	kept
Physical violence	physical violence	physical violence	physical violence	physical violence	3	case_or_format
physician assistant attitude	physician assistant attitude	physician assistant attitude	physician assistant attitude	physician assistant attitude	3	kept
physician associate	physician associate	physician associate	physician associate	physician associate	3	kept
physician shortage	physician shortage	physician shortage	physician shortage	physician shortage	3	kept
physician training	physician training	physician training	physician training	physician training	3	kept
Physicians, Women	physicians women	physicians women	physicians women	Physicians, Women	3	kept
Physiologic monitors	physiologic monitors	physiologic monitors	physiologic monitor	Physiologic monitors	3	singular

physiological	physiological	physiological	physiological	physiological	3	kept
Physiological condition	physiological condition	physiological condition	physiological condition	Physiological condition	3	kept
Physiological monitoring	physiological monitoring	physiological monitoring	physiological monitoring	Physiological monitoring	3	kept
physiological monitoring	physiological monitoring	physiological monitoring	physiological monitoring	Physiological monitoring	3	case_or_format
Physiological response	physiological response	physiological response	physiological response	Physiological response	3	kept
Physiological stress	physiological stress	physiological stress	physiological stress	physiological stress	3	case_or_format
physiological variable	physiological variable	physiological variable	physiological variable	physiological variable	3	kept
physiotherapy practice	physiotherapy practice	physiotherapy practice	physiotherapy practice	physiotherapy practice	3	kept
physostigmine	physostigmine	physostigmine	physostigmine	physostigmine	3	kept
Phytotherapy	phytotherapy	phytotherapy	phytotherapy	Phytotherapy	3	kept
PICS-F	pics f	pics f	pics f	PICS-F	3	kept
Pierre Robin Syndrome	pierre robin syndrome	pierre robin syndrome	pierre robin syndrome	Pierre Robin syndrome	3	case_or_format
pig	pig	pig	pig	pig	3	kept
pilot	pilot	pilot	pilot	pilot	3	kept
Pinealoma	pinealoma	pinealoma	pinealoma	Pinealoma	3	kept
Piperacillin, Tazobactam Drug Combination	piperacillin tazobactam drug combination	piperacillin tazobactam drug combination	piperacillin tazobactam drug combination	Piperacillin, Tazobactam Drug Combination	3	kept

PLACE	place	place	place	PLACE	3	kept
Placement	placement	placement	placement	PLACEMENT	3	case_or_format
placenta disorder	placenta disorder	placenta disorder	placenta disorder	placenta disorder	3	kept
plagiocephaly	plagiocephaly	plagiocephaly	plagiocephaly	plagiocephaly	3	kept
plan do study act	plan do study act	plan do study act	plan do study act	plan do study act	3	kept
PLANNED BEHAVIOR	planned behavior	planned behavior	planned behavior	PLANNED BEHAVIOR	3	kept
plant extract	plant extract	plant extract	plant extract	Plant Extracts	3	case_or_format
plant leaf	plant leaf	plant leaf	plant leaf	plant leaf	3	kept
PLAQUE ANTISEPTIC DECONTAMINATION	plaque antiseptic decontamination	plaque antiseptic decontamination	plaque antiseptic decontamination	PLAQUE ANTISEPTIC DECONTAMINATION	3	kept
Plasma Volume	plasma volume	plasma volume	plasma volume	Plasma Volume	3	kept
Platelet Transfusion	platelet transfusion	platelet transfusion	platelet transfusion	Platelet Transfusion	3	kept
Platelets	platelets	platelets	platelets	Platelets	3	kept
platinum	platinum	platinum	platinum	platinum	3	kept
Pleasure	pleasure	pleasure	pleasure	Pleasure	3	kept
pleura disease	pleura disease	pleura disease	pleura disease	pleura disease	3	kept
Pleural effusion	pleural effusion	pleural effusion	pleural effusion	Pleural Effusion	3	case_or_format

PLS-DA	pls da	pls da	pls da	PLS-DA	3	kept
pneumatosis intestinalis	pneumatosis intestinalis	pneumatosis intestinalis	pneumatosis intestinalis	pneumatosis intestinalis	3	kept
Pneumocystis jiroveci	pneumocystis jiroveci	pneumocystis jiroveci	pneumocystis jiroveci	Pneumocystis jiroveci	3	kept
Pneumopericardium	pneumopericardium	pneumopericardium	pneumopericardium	Pneumopericardium	3	kept
POCT	poct	poct	poct	POCT	3	kept
Point prevalence	point prevalence	point prevalence	point prevalence	point prevalence	3	case_or_format
point-of-care blood glucose	point of care blood glucose	point of care blood glucose	point of care blood glucose	point-of-care blood glucose	3	kept
point－of－care ultrasound	point of care ultrasound	point of care ultrasound	point of care ultrasound	point of care ultrasound	3	case_or_format
Poison Control Centers	poison control centers	poison control centers	poison control centers	Poison Control Centers	3	kept
Poland [Central Europe]	poland central europe	poland central europe	poland central europe	Poland [Central Europe]	3	kept
Police	police	police	police	police	3	case_or_format
POLICY	policy	policy	policy	policy	3	case_or_format
poliomyelitis vaccine	poliomyelitis vaccine	poliomyelitis vaccine	poliomyelitis vaccine	poliomyelitis vaccine	3	kept
Polish citizen	polish citizen	polish citizen	polish citizen	Polish citizen	3	kept
political system	political system	political system	political system	political system	3	kept
Pollution	pollution	pollution	pollution	pollution	3	case_or_format

POLST	polst	polst	polst	POLST	3	kept
polycythemia	polycythemia	polycythemia	polycythemia	polycythemia	3	kept
polydipsia	polydipsia	polydipsia	polydipsia	polydipsia	3	kept
Polymorphism, Single Nucleotide	polymorphism single nucleotide	polymorphism single nucleotide	polymorphism single nucleotide	Polymorphism, Single Nucleotide	3	kept
popliteal artery	popliteal artery	popliteal artery	popliteal artery	popliteal artery	3	kept
port protector	port protector	port protector	port protector	port protector	3	kept
POSITION	position	position	position	position	3	case_or_format
Position statement	position statement	position statement	position statement	Position statement	3	kept
positioning	positioning	positioning	positioning	Positioning	3	case_or_format
Positive-Pressure Respiration, Intrinsic	positive pressure respiration intrinsic	positive pressure respiration intrinsic	positive pressure respiration intrinsic	Positive-Pressure Respiration, Intrinsic	3	kept
positron emission tomography-computed tomography	positron emission tomography computed tomography	positron emission tomography computed tomography	positron emission tomography computed tomography	positron emission tomography-computed tomography	3	kept
Positron-Emission Tomography	positron emission tomography	positron emission tomography	positron emission tomography	positron emission tomography	3	case_or_format
Post-acute	post acute	post acute	post acute	Post-acute	3	kept
post-acute care	post acute care	post acute care	post acute care	Post-acute care	3	case_or_format
post-pandemic	post pandemic	post pandemic	post pandemic	post-pandemic	3	kept
post-registration education	post registration education	post registration education	post registration education	post-registration education	3	kept

Post-resuscitation care	post resuscitation care	post resuscitation care	post resuscitation care	Post-resuscitation care	3	kept
postacute care	postacute care	postacute care	postacute care	postacute care	3	kept
postextubation dysphagia	postextubation dysphagia	postextubation dysphagia	postextubation dysphagia	postextubation dysphagia	3	kept
postmarketing surveillance	postmarketing surveillance	postmarketing surveillance	postmarketing surveillance	postmarketing surveillance	3	kept
postmenopause	postmenopause	postmenopause	postmenopause	postmenopause	3	kept
postnatal development	postnatal development	postnatal development	postnatal development	postnatal development	3	kept
Postoperative analgesia	postoperative analgesia	postoperative analgesia	postoperative analgesia	postoperative analgesia	3	case_or_format
Postoperative bleeding	postoperative bleeding	postoperative bleeding	postoperative bleeding	Postoperative bleeding	3	kept
Postoperative monitoring	postoperative monitoring	postoperative monitoring	postoperative monitoring	postoperative monitoring	3	case_or_format
postoperative nausea	postoperative nausea	postoperative nausea	postoperative nausea	postoperative nausea	3	kept
Postoperative pain	postoperative pain	postoperative pain	postoperative pain	Postoperative Pain	3	case_or_format
Postoperative pain management	postoperative pain management	postoperative pain management	postoperative pain management	Postoperative pain management	3	kept
postoperative recovery	postoperative recovery	postoperative recovery	postoperative recovery	postoperative recovery	3	kept
postoperative thrombosis	postoperative thrombosis	postoperative thrombosis	postoperative thrombosis	postoperative thrombosis	3	kept
Postpartum period	postpartum period	postpartum period	postpartum period	Postpartum Period	3	case_or_format
Posttraumatic Growth Inventory	posttraumatic growth inventory	posttraumatic growth inventory	posttraumatic growth inventory	Posttraumatic Growth Inventory	3	kept

Posttraumatic stress	posttraumatic stress	posttraumatic stress	posttraumatic stress	POSTTRAUMATIC STRESS	3	case_or_format
posttraumatic stress	posttraumatic stress	posttraumatic stress	posttraumatic stress	POSTTRAUMATIC STRESS	3	case_or_format
postvoid residual urine volume	postvoid residual urine volume	postvoid residual urine volume	postvoid residual urine volume	postvoid residual urine volume	3	kept
post - traumatic stress disorder	post traumatic stress disorder	post traumatic stress disorder	post traumatic stress disorder	Post-traumatic stress disorder	3	case_or_format
potassium dihydrogen phosphate	potassium dihydrogen phosphate	potassium dihydrogen phosphate	potassium dihydrogen phosphate	potassium dihydrogen phosphate	3	kept
powder	powder	powder	powder	powder	3	kept
PPE, personal protective equipment	ppe personal protective equipment	ppe personal protective equipment	ppe personal protective equipment	PPE, personal protective equipment	3	kept
PRACTICE ENVIRONMENT	practice environment	practice environment	practice environment	practice environment	3	case_or_format
Practice environment	practice environment	practice environment	practice environment	practice environment	3	case_or_format
PRACTICE ENVIRONMENT SCALE	practice environment scale	practice environment scale	practice environment scale	PRACTICE ENVIRONMENT SCALE	3	kept
practice guidelines	practice guidelines	practice guidelines	practice guideline	practice guideline	3	singular
Practice Management	practice management	practice management	practice management	Practice Management	3	kept
Practice patterns	practice patterns	practice patterns	practice pattern	practice patterns	3	singular
practice readiness	practice readiness	practice readiness	practice readiness	practice readiness	3	kept
Practice standards	practice standards	practice standards	practice standards	Practice standards	3	kept
Practice-Based Learning and Improvement	practice based learning and improvement	practice based learning and improvement	practice based learning and improvement	Practice-Based Learning and Improvement	3	kept

pravastatin	pravastatin	pravastatin	pravastatin	pravastatin	3	kept
pre-analytical phase	pre analytical phase	pre analytical phase	pre analytical phase	pre-analytical phase	3	kept
Pre-eclampsia	pre eclampsia	pre eclampsia	pre eclampsia	Pre-Eclampsia	3	case_or_format
pre-exposure prophylaxis	pre exposure prophylaxis	pre exposure prophylaxis	pre exposure prophylaxis	pre-exposure prophylaxis	3	kept
Pre-hospital	pre hospital	pre hospital	pre hospital	Pre-hospital	3	kept
Preceptor	preceptor	preceptor	preceptor	Preceptor	3	kept
preceptor	preceptor	preceptor	preceptor	Preceptor	3	case_or_format
Preconception Care	preconception care	preconception care	preconception care	Preconception Care	3	kept
Prediction modelling	prediction modelling	prediction modelling	prediction modelling	Prediction modelling	3	kept
Prediction models	prediction models	prediction models	prediction model	prediction model	3	singular
prediction models	prediction models	prediction models	prediction model	prediction model	3	singular
Predictive model	predictive model	predictive model	predictive model	predictive model	3	case_or_format
Predictive value of tests	predictive value of tests	predictive value of tests	predictive value of tests	Predictive Value of Tests	3	case_or_format
PREGNANCY	pregnancy	pregnancy	pregnancy	Pregnancy	3	case_or_format
pregnancy disorder	pregnancy disorder	pregnancy disorder	pregnancy disorder	pregnancy disorder	3	kept
Pregnancy in Adolescence	pregnancy in adolescence	pregnancy in adolescence	pregnancy in adolescence	Pregnancy in Adolescence	3	kept

Pregnancy in Diabetics	pregnancy in diabetics	pregnancy in diabetics	pregnancy in diabetics	Pregnancy in Diabetics	3	kept
Pregnancy, Multiple	pregnancy multiple	pregnancy multiple	pregnancy multiple	Pregnancy, Multiple	3	kept
Pregnancy, Twin	pregnancy twin	pregnancy twin	pregnancy twin	Pregnancy, Twin	3	kept
Pregnant People	pregnant people	pregnant people	pregnant people	Pregnant People	3	kept
Premature Infant Pain Profile Revised	premature infant pain profile revised	premature infant pain profile revised	premature infant pain profile revised	Premature Infant Pain Profile Revised	3	kept
Premature Infants	premature infants	premature infants	premature infant	Premature infant	3	singular
Premature Rupture of Fetal Membranes	premature rupture of fetal membranes	premature rupture of fetal membranes	premature rupture of fetal membranes	Premature Rupture of Fetal Membranes	3	kept
Prenatal care	prenatal care	prenatal care	prenatal care	prenatal care	3	case_or_format
prenatal development	prenatal development	prenatal development	prenatal development	prenatal development	3	kept
Prenatal Education	prenatal education	prenatal education	prenatal education	Prenatal Education	3	kept
preoperative counseling	preoperative counseling	preoperative counseling	preoperative counseling	preoperative counseling	3	kept
preoperative exercise	preoperative exercise	preoperative exercise	preoperative exercise	preoperative exercise	3	kept
preoperative optimization	preoperative optimization	preoperative optimization	preoperative optimization	preoperative optimization	3	kept
Preparation	preparation	preparation	preparation	Preparation	3	kept
prescribing errors	prescribing errors	prescribing errors	prescribing error	prescribing error	3	singular
Prescription errors	prescription errors	prescription errors	prescription error	PRESCRIPTION ERRORS	3	singular

presence	presence	presence	presence	presence	3	kept
PRESSURE	pressure	pressure	pressure	Pressure	3	case_or_format
PRETERM	preterm	preterm	preterm	Preterm	3	case_or_format
Preterm baby	preterm baby	preterm baby	preterm baby	Preterm baby	3	kept
Preterm Birth	preterm birth	preterm birth	preterm birth	Preterm birth	3	case_or_format
Preterm born	preterm born	preterm born	preterm born	Preterm born	3	kept
Preterm Infants	preterm infants	preterm infants	preterm infant	preterm infant	3	singular
Preterm Newborn	preterm newborn	preterm newborn	preterm newborn	Preterm newborn	3	case_or_format
Preterm newborns	preterm newborns	preterm newborns	preterm newborn	Preterm newborn	3	singular
prevention strategies	prevention strategies	prevention strategies	prevention strategy	prevention strategies	3	singular
Preventive Medicine	preventive medicine	preventive medicine	preventive medicine	preventive medicine	3	case_or_format
Primary Graft Dysfunction	primary graft dysfunction	primary graft dysfunction	primary graft dysfunction	primary graft dysfunction	3	case_or_format
Primary graft dysfunction	primary graft dysfunction	primary graft dysfunction	primary graft dysfunction	primary graft dysfunction	3	case_or_format
Primary palliative care	primary palliative care	primary palliative care	primary palliative care	primary palliative care	3	case_or_format
Principal component analysis	principal component analysis	principal component analysis	principal component analysis	principal component analysis	3	case_or_format
Printing, Three-Dimensional	printing three dimensional	printing three dimensional	printing three dimensional	Printing, Three-Dimensional	3	kept

PRISMA	prisma	prisma	prisma	PRISMA	3	kept
Prisons	prisons	prisons	prison	prison	3	singular
Proactive	proactive	proactive	proactive	Proactive	3	kept
probiotics	probiotics	probiotics	probiotics	Probiotics	3	case_or_format
Problem Behavior	problem behavior	problem behavior	problem behavior	problem behavior	3	case_or_format
problem identification	problem identification	problem identification	problem identification	problem identification	3	kept
Problem-based learning	problem based learning	problem based learning	problem based learning	Problem-Based Learning	3	case_or_format
Procedure	procedure	procedure	procedure	procedures	3	case_or_format
Process mapping	process mapping	process mapping	process mapping	process mapping	3	case_or_format
process model	process model	process model	process model	process model	3	kept
process monitoring	process monitoring	process monitoring	process monitoring	process monitoring	3	kept
Product design	product design	product design	product design	product design	3	case_or_format
Product Labeling	product labeling	product labeling	product labeling	Product Labeling	3	kept
productive cough	productive cough	productive cough	productive cough	productive cough	3	kept
Professional aspects	professional aspects	professional aspects	professional aspects	Professional aspects	3	kept
Professional behavior	professional behavior	professional behavior	professional behavior	Professional behavior	3	kept

Professional commitment	professional commitment	professional commitment	professional commitment	professional commitment	3	case_or_format
professional education	professional education	professional education	professional education	Professional education	3	case_or_format
Professional exhaustion	professional exhaustion	professional exhaustion	professional exhaustion	Professional exhaustion	3	kept
Professional identity	professional identity	professional identity	professional identity	professional identity	3	case_or_format
Professional issues	professional issues	professional issues	professional issues	professional issues	3	case_or_format
Professional knowledge	professional knowledge	professional knowledge	professional knowledge	professional knowledge	3	case_or_format
Professional Misconduct	professional misconduct	professional misconduct	professional misconduct	Professional Misconduct	3	kept
Professional practice environment	professional practice environment	professional practice environment	professional practice environment	Professional practice environment	3	kept
Professional Practice Gaps	professional practice gaps	professional practice gaps	professional practice gaps	Professional Practice Gaps	3	kept
Professional Practice Location	professional practice location	professional practice location	professional practice location	Professional Practice Location	3	kept
professional secrecy	professional secrecy	professional secrecy	professional secrecy	professional secrecy	3	kept
professional self-efficacy	professional self efficacy	professional self efficacy	professional self efficacy	professional self-efficacy	3	kept
Professional stress	professional stress	professional stress	professional stress	Professional stress	3	kept
Program	program	program	program	PROGRAM	3	case_or_format
program efficacy	program efficacy	program efficacy	program efficacy	program efficacy	3	kept
Programming Languages	programming languages	programming languages	programming languages	Programming Languages	3	kept

progressive care	progressive care	progressive care	progressive care	progressive care	3	kept
Progressive muscle relaxation	progressive muscle relaxation	progressive muscle relaxation	progressive muscle relaxation	Progressive muscle relaxation	3	kept
progressive supranuclear palsy	progressive supranuclear palsy	progressive supranuclear palsy	progressive supranuclear palsy	progressive supranuclear palsy	3	kept
prokinetic agent	prokinetic agent	prokinetic agent	prokinetic agent	prokinetic agent	3	kept
Pronation	pronation	pronation	pronation	Pronation	3	kept
pronation	pronation	pronation	pronation	Pronation	3	case_or_format
proning	proning	proning	proning	proning	3	kept
Propensity score	propensity score	propensity score	propensity score	propensity score	3	case_or_format
Prosocial behavior	prosocial behavior	prosocial behavior	prosocial behavior	Prosocial behavior	3	kept
prospective	prospective	prospective	prospective	Prospective	3	case_or_format
prospective studies	prospective studies	prospective studies	prospective study	Prospective Studies	3	singular
prosthesis	prosthesis	prosthesis	prosthesis	prosthesis	3	kept
Prosthesis Design	prosthesis design	prosthesis design	prosthesis design	Prosthesis Design	3	kept
prosthetic joint infection	prosthetic joint infection	prosthetic joint infection	prosthetic joint infection	prosthetic joint infection	3	kept
protective ventilation	protective ventilation	protective ventilation	protective ventilation	protective ventilation	3	kept
protein cerebrospinal fluid level	protein cerebrospinal fluid level	protein cerebrospinal fluid level	protein cerebrospinal fluid level	protein cerebrospinal fluid level	3	kept

protein deficit	protein deficit	protein deficit	protein deficit	protein deficit	3	kept
protein diet	protein diet	protein diet	protein diet	protein diet	3	kept
proteinase	proteinase	proteinase	proteinase	proteinase	3	kept
Proteinuria	proteinuria	proteinuria	proteinuria	proteinuria	3	case_or_format
prothrombin complex concentrates	prothrombin complex concentrates	prothrombin complex concentrates	prothrombin complex concentrate	prothrombin complex concentrates	3	singular
Protocols & guidelines	protocols and guidelines	protocols and guidelines	protocols and guidelines	protocols & guidelines	3	case_or_format
Proton pump inhibitors	proton pump inhibitors	proton pump inhibitors	proton pump inhibitor	proton pump inhibitor	3	singular
pseudoephedrine	pseudoephedrine	pseudoephedrine	pseudoephedrine	pseudoephedrine	3	kept
Pseudomonas pneumonia	pseudomonas pneumonia	pseudomonas pneumonia	pseudomonas pneumonia	Pseudomonas pneumonia	3	kept
Pseudomonas stutzeri	pseudomonas stutzeri	pseudomonas stutzeri	pseudomonas stutzeri	Pseudomonas stutzeri	3	kept
PSQI	psqi	psqi	psqi	PSQI	3	kept
psychedelic agent	psychedelic agent	psychedelic agent	psychedelic agent	psychedelic agent	3	kept
psychological adaptation	psychological adaptation	psychological adaptation	psychological adaptation	Psychological adaptation	3	case_or_format
psychological burnout	psychological burnout	psychological burnout	psychological burnout	psychological burnout	3	kept
psychological contract	psychological contract	psychological contract	psychological contract	psychological contract	3	kept
psychological experience	psychological experience	psychological experience	psychological experience	psychological experiences	3	case_or_format

psychological flexibility	psychological flexibility	psychological flexibility	psychological flexibility	psychological flexibility	3	kept
PSYCHOLOGICAL IMPACT	psychological impact	psychological impact	psychological impact	psychological impact	3	case_or_format
psychological interview	psychological interview	psychological interview	psychological interview	psychological interview	3	kept
psychological symptoms	psychological symptoms	psychological symptoms	psychological symptoms	psychological symptoms	3	kept
Psychology, Adolescent	psychology adolescent	psychology adolescent	psychology adolescent	Psychology, Adolescent	3	kept
psychomotor activity	psychomotor activity	psychomotor activity	psychomotor activity	psychomotor activity	3	kept
psychomotor retardation	psychomotor retardation	psychomotor retardation	psychomotor retardation	psychomotor retardation	3	kept
Psychomotor skills	psychomotor skills	psychomotor skills	psychomotor skills	Psychomotor skills	3	kept
psychophysiology	psychophysiology	psychophysiology	psychophysiology	psychophysiology	3	kept
psychosocial environment	psychosocial environment	psychosocial environment	psychosocial environment	psychosocial environment	3	kept
Psychosocial factors	psychosocial factors	psychosocial factors	psychosocial factors	psychosocial factors	3	case_or_format
Psychosocial intervention	psychosocial intervention	psychosocial intervention	psychosocial intervention	psychosocial intervention	3	case_or_format
Psychosocial stress	psychosocial stress	psychosocial stress	psychosocial stress	Psychosocial stress	3	kept
psychotic disorder	psychotic disorder	psychotic disorder	psychotic disorder	Psychotic Disorders	3	case_or_format
PUBLIC HOSPITALS	public hospitals	public hospitals	public hospital	public hospital	3	singular
public hospitals	public hospitals	public hospitals	public hospital	public hospital	3	singular

Puerperal Infection	puerperal infection	puerperal infection	puerperal infection	Puerperal Infection	3	kept
puerperal infection	puerperal infection	puerperal infection	puerperal infection	Puerperal Infection	3	case_or_format
Pulmonary Alveoli	pulmonary alveoli	pulmonary alveoli	pulmonary alveoli	Pulmonary Alveoli	3	kept
Pulmonary Arterial Hypertension	pulmonary arterial hypertension	pulmonary arterial hypertension	pulmonary arterial hypertension	Pulmonary Arterial Hypertension	3	kept
pulmonary arterial hypertension	pulmonary arterial hypertension	pulmonary arterial hypertension	pulmonary arterial hypertension	Pulmonary Arterial Hypertension	3	case_or_format
pulmonary artery occlusion	pulmonary artery occlusion	pulmonary artery occlusion	pulmonary artery occlusion	pulmonary artery occlusion	3	kept
Pulmonary complications	pulmonary complications	pulmonary complications	pulmonary complications	Pulmonary complications	3	kept
pulmonary complications	pulmonary complications	pulmonary complications	pulmonary complications	Pulmonary complications	3	case_or_format
Pulmonary edema	pulmonary edema	pulmonary edema	pulmonary edema	Pulmonary Edema	3	case_or_format
Pulmonary embolus	pulmonary embolus	pulmonary embolus	pulmonary embolus	Pulmonary embolus	3	kept
pulmonary function	pulmonary function	pulmonary function	pulmonary function	pulmonary function	3	kept
Pulmonary medicine	pulmonary medicine	pulmonary medicine	pulmonary medicine	Pulmonary Medicine	3	case_or_format
pulmonary medicine	pulmonary medicine	pulmonary medicine	pulmonary medicine	Pulmonary Medicine	3	case_or_format
pulmonary vascular disease	pulmonary vascular disease	pulmonary vascular disease	pulmonary vascular disease	pulmonary vascular disease	3	kept
pulmonary vein stenosis	pulmonary vein stenosis	pulmonary vein stenosis	pulmonary vein stenosis	pulmonary vein stenosis	3	kept
Pulse oximeter	pulse oximeter	pulse oximeter	pulse oximeter	pulse oximeter	3	case_or_format

pulseless electrical activity	pulseless electrical activity	pulseless electrical activity	pulseless electrical activity	pulseless electrical activity	3	kept
purchasing	purchasing	purchasing	purchasing	purchasing	3	kept
purification	purification	purification	purification	purification	3	kept
pylorus stenosis	pylorus stenosis	pylorus stenosis	pylorus stenosis	pylorus stenosis	3	kept
pyrazinamide	pyrazinamide	pyrazinamide	pyrazinamide	pyrazinamide	3	kept
pyridine derivative	pyridine derivative	pyridine derivative	pyridine derivative	pyridine derivative	3	kept
pyridone derivative	pyridone derivative	pyridone derivative	pyridone derivative	pyridone derivative	3	kept
pyruvic acid	pyruvic acid	pyruvic acid	pyruvic acid	pyruvic acid	3	kept
pyuria	pyuria	pyuria	pyuria	pyuria	3	kept
Q methodology	q methodology	q methodology	q methodology	Q methodology	3	kept
Q-Sort	q sort	q sort	q sort	Q-Sort	3	kept
Qualitative description	qualitative description	qualitative description	qualitative description	Qualitative description	3	kept
qualitative Forschung	qualitative forschung	qualitative forschung	qualitative forschung	qualitative Forschung	3	kept
Qualitative interviews	qualitative interviews	qualitative interviews	qualitative interview	Qualitative interviews	3	singular
qualitative interviews	qualitative interviews	qualitative interviews	qualitative interview	Qualitative interviews	3	singular
Qualitative methodology	qualitative methodology	qualitative methodology	qualitative methodology	Qualitative methodology	3	kept

qualitative secondary analysis	qualitative secondary analysis	qualitative secondary analysis	qualitative secondary analysis	qualitative secondary analysis	3	kept
quality and safety of care	quality and safety of care	quality and safety of care	quality and safety of care	quality and safety of care	3	kept
quality in healthcare	quality in healthcare	quality in healthcare	quality in healthcare	quality in healthcare	3	kept
Quality indicators health care	quality indicators health care	quality indicators health care	quality indicators health care	Quality Indicators, Health Care	3	case_or_format
Quality indicators, health care	quality indicators health care	quality indicators health care	quality indicators health care	Quality Indicators, Health Care	3	case_or_format
Quality indicators, healthcare	quality indicators healthcare	quality indicators healthcare	quality indicators healthcare	Quality indicators, healthcare	3	kept
quality of death	quality of death	quality of death	quality of death	quality of death	3	kept
quality of healthcare	quality of healthcare	quality of healthcare	quality of healthcare	Quality of healthcare	3	case_or_format
quality of work	quality of work	quality of work	quality of work	quality of work	3	kept
Quality of working life	quality of working life	quality of working life	quality of working life	quality of working life	3	case_or_format
quantitative assay	quantitative assay	quantitative assay	quantitative assay	quantitative assay	3	kept
quantitative diagnosis	quantitative diagnosis	quantitative diagnosis	quantitative diagnosis	quantitative diagnosis	3	kept
quantitative electroencephalography	quantitative electroencephalography	quantitative electroencephalography	quantitative electroencephalography	quantitative electroencephalography	3	kept
Quantitative longitudinal study	quantitative longitudinal study	quantitative longitudinal study	quantitative longitudinal study	Quantitative longitudinal study	3	kept
Quantitative research	quantitative research	quantitative research	quantitative research	quantitative research	3	case_or_format
Quasi-experimental study	quasi experimental study	quasi experimental study	quasi experimental study	quasi experimental study	3	case_or_format

Questionnaire design	questionnaire design	questionnaire design	questionnaire design	questionnaire design	3	case_or_format
Questionnaire research	questionnaire research	questionnaire research	questionnaire research	Questionnaire research	3	kept
Questionnaire surveys	questionnaire surveys	questionnaire surveys	questionnaire survey	questionnaire survey	3	singular
Queueing theory	queueing theory	queueing theory	queueing theory	Queueing theory	3	kept
queueing theory	queueing theory	queueing theory	queueing theory	Queueing theory	3	case_or_format
Queuing	queuing	queuing	queuing	Queuing	3	kept
Quiet time	quiet time	quiet time	quiet time	Quiet time	3	kept
quinine	quinine	quinine	quinine	quinine	3	kept
Quit smoking	quit smoking	quit smoking	quit smoking	Quit smoking	3	kept
Rabies	rabies	rabies	rabies	rabies	3	case_or_format
Rabies virus	rabies virus	rabies virus	rabies virus	Rabies virus	3	kept
RACE	race	race	race	race	3	case_or_format
racial disparities	racial disparities	racial disparities	racial disparity	racial disparity	3	singular
Radial artery	radial artery	radial artery	radial artery	radial artery	3	case_or_format
radiation hazard	radiation hazard	radiation hazard	radiation hazard	radiation hazard	3	kept
radiation injury	radiation injury	radiation injury	radiation injury	radiation injury	3	kept

radiation oncologist	radiation oncologist	radiation oncologist	radiation oncologist	radiation oncologist	3	kept
radiation oncology	radiation oncology	radiation oncology	radiation oncology	radiation oncology	3	kept
Radiation Protection	radiation protection	radiation protection	radiation protection	Radiation protection	3	case_or_format
radiation protection	radiation protection	radiation protection	radiation protection	Radiation protection	3	case_or_format
radiation safety	radiation safety	radiation safety	radiation safety	radiation safety	3	kept
radical resection	radical resection	radical resection	radical resection	radical resection	3	kept
Radio frequency identification	radio frequency identification	radio frequency identification	radio frequency identification	Radio frequency identification	3	kept
radiofrequency identification	radiofrequency identification	radiofrequency identification	radiofrequency identification	radiofrequency identification	3	kept
Radiopharmaceuticals	radiopharmaceuticals	radiopharmaceuticals	radiopharmaceutical	Radiopharmaceuticals	3	singular
Ramsay scale	ramsay scale	ramsay scale	ramsay scale	Ramsay scale	3	kept
Random forest	random forest	random forest	random forest	random forest	3	case_or_format
Random forests	random forests	random forests	random forest	random forest	3	singular
randomised controlled trial	randomised controlled trial	randomised controlled trial	randomised controlled trial	Randomised controlled trial	3	case_or_format
Randomized clinical trial	randomized clinical trial	randomized clinical trial	randomized clinical trial	Randomized clinical trial	3	kept
randomized clinical trial	randomized clinical trial	randomized clinical trial	randomized clinical trial	Randomized clinical trial	3	case_or_format
RANDOMIZED CLINICAL-TRIAL	randomized clinical trial	randomized clinical trial	randomized clinical trial	Randomized clinical trial	3	case_or_format

randomized controlled trials	randomized controlled trials	randomized controlled trials	randomized controlled trial	randomized controlled trial	3	singular
Randomized controlled trials as topic	randomized controlled trials as topic	randomized controlled trials as topic	randomized controlled trials as topic	Randomized Controlled Trials as Topic	3	case_or_format
Randomized trial	randomized trial	randomized trial	randomized trial	RANDOMIZED-TRIAL	3	case_or_format
RANDOMIZED-CONTROLLED-TRIAL	randomized controlled trial	randomized controlled trial	randomized controlled trial	randomized controlled trial	3	case_or_format
rapid shallow breathing index	rapid shallow breathing index	rapid shallow breathing index	rapid shallow breathing index	rapid shallow breathing index	3	kept
Rasch model	rasch model	rasch model	rasch model	Rasch model	3	kept
rat	rat	rat	rat	rat	3	kept
Rational therapy	rational therapy	rational therapy	rational therapy	Rational therapy	3	kept
rationing	rationing	rationing	rationing	Rationing	3	case_or_format
Rats	rats	rats	rat	rat	3	singular
Raynaud phenomenon	raynaud phenomenon	raynaud phenomenon	raynaud phenomenon	Raynaud phenomenon	3	kept
Reactive Oxygen Species	reactive oxygen species	reactive oxygen species	reactive oxygen species	Reactive Oxygen Species	3	kept
readiness for discharge	readiness for discharge	readiness for discharge	readiness for discharge	readiness for discharge	3	kept
READMISSION	readmission	readmission	readmission	Readmission	3	case_or_format
READMISSIONS	readmissions	readmissions	readmission	Readmission	3	singular
Real time location systems	real time location systems	real time location systems	real time location systems	Real time location systems	3	kept

Real time monitoring	real time monitoring	real time monitoring	real time monitoring	Real time monitoring	3	kept
Real- time	real time	real time	real time	Real time	3	case_or_format
recanalization	recanalization	recanalization	recanalization	recanalization	3	kept
Receptors, Adrenergic, alpha-2	receptors adrenergic alpha 2	receptors adrenergic alpha 2	receptors adrenergic alpha 2	Receptors, Adrenergic, alpha-2	3	kept
Recién Nacido	reci n nacido	reci n nacido	reci n nacido	Recién Nacido	3	kept
Recién nacido	reci n nacido	reci n nacido	reci n nacido	Recién Nacido	3	case_or_format
recollections	recollections	recollections	recollection	recollections	3	singular
recombinant blood clotting factor 7a	recombinant blood clotting factor 7a	recombinant blood clotting factor 7a	recombinant blood clotting factor 7a	recombinant blood clotting factor 7a	3	kept
Recommendation	recommendation	recommendation	recommendation	Recommendations	3	case_or_format
Recovery room	recovery room	recovery room	recovery room	Recovery Room	3	case_or_format
Rectal Diseases	rectal diseases	rectal diseases	rectal diseases	Rectal Diseases	3	kept
rectum cancer	rectum cancer	rectum cancer	rectum cancer	rectum cancer	3	kept
recurrent aspiration	recurrent aspiration	recurrent aspiration	recurrent aspiration	recurrent aspiration	3	kept
Redesign	redesign	redesign	redesign	Redesign	3	kept
REDUCE	reduce	reduce	reduce	REDUCE	3	kept
reduction	reduction	reduction	reduction	REDUCTION	3	case_or_format

redundancy analysis	redundancy analysis	redundancy analysis	redundancy analysis	redundancy analysis	3	kept
Refeeding syndrome	refeeding syndrome	refeeding syndrome	refeeding syndrome	Refeeding Syndrome	3	case_or_format
Reference Books	reference books	reference books	reference books	Reference Books	3	kept
Referral and consultation	referral and consultation	referral and consultation	referral and consultation	Referral and Consultation	3	case_or_format
referral hospital	referral hospital	referral hospital	referral hospital	referral hospital	3	kept
reflex	reflex	reflex	reflex	Reflex	3	case_or_format
refugee	refugee	refugee	refugee	Refugees	3	case_or_format
refugee crisis	refugee crisis	refugee crisis	refugee crisis	refugee crisis	3	kept
Regional anesthesia	regional anesthesia	regional anesthesia	regional anesthesia	regional anesthesia	3	case_or_format
Regional citrate anticoagulation	regional citrate anticoagulation	regional citrate anticoagulation	regional citrate anticoagulation	Regional citrate anticoagulation	3	kept
regional citrate anticoagulation	regional citrate anticoagulation	regional citrate anticoagulation	regional citrate anticoagulation	Regional citrate anticoagulation	3	case_or_format
Registry	registry	registry	registry	Registries	3	case_or_format
registry	registry	registry	registry	Registries	3	case_or_format
Regulations	regulations	regulations	regulation	Regulations	3	singular
Regulatory compliance	regulatory compliance	regulatory compliance	regulatory compliance	Regulatory compliance	3	kept
Rehydration Solutions	rehydration solutions	rehydration solutions	rehydration solutions	Rehydration Solutions	3	kept

Reiki	reiki	reiki	reiki	Reiki	3	kept
Reimbursement, Incentive	reimbursement incentive	reimbursement incentive	reimbursement incentive	Reimbursement, Incentive	3	kept
related factors	related factors	related factors	related factors	Related factors	3	case_or_format
Relational coordination	relational coordination	relational coordination	relational coordination	RELATIONAL COORDINATION	3	case_or_format
Relationship	relationship	relationship	relationship	relationships	3	case_or_format
Relationships	relationships	relationships	relationship	relationships	3	singular
Relative	relative	relative	relative	relative	3	case_or_format
Relative Value Scales	relative value scales	relative value scales	relative value scales	Relative Value Scales	3	kept
relief work	relief work	relief work	relief work	Relief Work	3	case_or_format
RELIEVING SUPPORT SURFACES	relieving support surfaces	relieving support surfaces	relieving support surfaces	RELIEVING SUPPORT SURFACES	3	kept
Relocation	relocation	relocation	relocation	Relocation	3	kept
relocation	relocation	relocation	relocation	Relocation	3	case_or_format
remote consultation	remote consultation	remote consultation	remote consultation	Remote Consultation	3	case_or_format
renal artery	renal artery	renal artery	renal artery	renal artery	3	kept
renal clearance	renal clearance	renal clearance	renal clearance	renal clearance	3	kept
renal protection	renal protection	renal protection	renal protection	renal protection	3	kept

RENAL REPLACEMENT THERAPY	renal replacement therapy	renal replacement therapy	renal replacement therapy	renal replacement therapy	3	case_or_format
renfort	renfort	renfort	renfort	renfort	3	kept
Renin-Angiotensin System	renin angiotensin system	renin angiotensin system	renin angiotensin system	Renin-Angiotensin System	3	kept
Reperfusion Injury	reperfusion injury	reperfusion injury	reperfusion injury	reperfusion injury	3	case_or_format
replicon	replicon	replicon	replicon	replicon	3	kept
rescue	rescue	rescue	rescue	RESCUE	3	case_or_format
Research coordinator	research coordinator	research coordinator	research coordinator	Research coordinator	3	kept
research methodology	research methodology	research methodology	research methodology	Research methodology	3	case_or_format
research methods	research methods	research methods	research method	research methods	3	singular
Residency	residency	residency	residency	Residency	3	kept
Resident physician	resident physician	resident physician	resident physician	Resident physician	3	kept
resident training	resident training	resident training	resident training	resident training	3	kept
resiliency	resiliency	resiliency	resiliency	resiliency	3	kept
Resistance Training	resistance training	resistance training	resistance training	resistance training	3	case_or_format
RESISTANT	resistant	resistant	resistant	RESISTANT	3	kept
resource	resource	resource	resource	Resources	3	case_or_format

resource-limited setting	resource limited setting	resource limited setting	resource limited setting	resource limited setting	3	case_or_format
Respiratory arrest	respiratory arrest	respiratory arrest	respiratory arrest	respiratory arrest	3	case_or_format
respiratory disease	respiratory disease	respiratory disease	respiratory disease	Respiratory diseases	3	case_or_format
respiratory diseases	respiratory diseases	respiratory diseases	respiratory disease	Respiratory diseases	3	singular
respiratory drive	respiratory drive	respiratory drive	respiratory drive	respiratory drive	3	kept
Respiratory management	respiratory management	respiratory management	respiratory management	Respiratory management	3	kept
respiratory management	respiratory management	respiratory management	respiratory management	Respiratory management	3	case_or_format
RESPIRATORY PATHOGENS	respiratory pathogens	respiratory pathogens	respiratory pathogens	RESPIRATORY PATHOGENS	3	kept
respiratory rate	respiratory rate	respiratory rate	respiratory rate	Respiratory Rate	3	case_or_format
Respiratory Syncytial Viruses	respiratory syncytial viruses	respiratory syncytial viruses	respiratory syncytial viruses	Respiratory Syncytial Viruses	3	kept
Respiratory therapist	respiratory therapist	respiratory therapist	respiratory therapist	respiratory therapist	3	case_or_format
Respiratory tract diseases	respiratory tract diseases	respiratory tract diseases	respiratory tract disease	respiratory tract disease	3	singular
RESPIRATORY-FAILURE	respiratory failure	respiratory failure	respiratory failure	respiratory failure	3	case_or_format
Respite care	respite care	respite care	respite care	Respite Care	3	case_or_format
Response	response	response	response	RESPONSES	3	case_or_format
restaurant	restaurant	restaurant	restaurant	restaurant	3	kept

Restricción física	restricci n f sica	restricci n f sica	restricci n f sica	Restricción física	3	kept
Restructuring	restructuring	restructuring	restructuring	Restructuring	3	kept
Resuscitation medications	resuscitation medications	resuscitation medications	resuscitation medications	Resuscitation medications	3	kept
resuscitative endovascular balloon occlusion of the aorta	resuscitative endovascular balloon occlusion of the aorta	resuscitative endovascular balloon occlusion of the aorta	resuscitative endovascular balloon occlusion of the aorta	resuscitative endovascular balloon occlusion of the aorta	3	kept
Retention, Psychology	retention psychology	retention psychology	retention psychology	Retention, Psychology	3	kept
retina	retina	retina	retina	retina	3	kept
retina image	retina image	retina image	retina image	retina image	3	kept
Retreatment	retreatment	retreatment	retreatment	Retreatment	3	kept
retreatment	retreatment	retreatment	retreatment	Retreatment	3	case_or_format
retroperitoneal hematoma	retroperitoneal hematoma	retroperitoneal hematoma	retroperitoneal hematoma	retroperitoneal hematoma	3	kept
retroperitoneal hemorrhage	retroperitoneal hemorrhage	retroperitoneal hemorrhage	retroperitoneal hemorrhage	retroperitoneal hemorrhage	3	kept
retroperitoneum	retroperitoneum	retroperitoneum	retroperitoneum	retroperitoneum	3	kept
retrospective	retrospective	retrospective	retrospective	retrospective	3	kept
Retrospective studies	retrospective studies	retrospective studies	retrospective study	Retrospective Studies	3	singular
Return of Spontaneous Circulation	return of spontaneous circulation	return of spontaneous circulation	return of spontaneous circulation	return of spontaneous circulation	3	case_or_format
return to school	return to school	return to school	return to school	return to school	3	kept

Reviews	reviews	reviews	reviews	Reviews	3	kept
Rhinitis	rhinitis	rhinitis	rhinitis	rhinitis	3	case_or_format
rhythm	rhythm	rhythm	rhythm	rhythm	3	kept
Ribotyping	ribotyping	ribotyping	ribotyping	Ribotyping	3	kept
rifaximin	rifaximin	rifaximin	rifaximin	rifaximin	3	kept
right hemisphere	right hemisphere	right hemisphere	right hemisphere	right hemisphere	3	kept
Right to life	right to life	right to life	right to life	Right to life	3	kept
Rio Grande do Sul	rio grande do sul	rio grande do sul	rio grande do sul	Rio Grande do Sul	3	kept
Risk assessment scale	risk assessment scale	risk assessment scale	risk assessment scale	Risk assessment scale	3	kept
Risk score	risk score	risk score	risk score	Risk score	3	kept
RISK-FACTOR	risk factor	risk factor	risk factor	Risk Factors	3	case_or_format
Rituals	rituals	rituals	ritual	ritual	3	singular
rivastigmine	rivastigmine	rivastigmine	rivastigmine	rivastigmine	3	kept
RNA extraction	rna extraction	rna extraction	rna extraction	RNA extraction	3	kept
RNA, Complementary	rna complementary	rna complementary	rna complementary	RNA, Complementary	3	kept
RNA, Messenger	rna messenger	rna messenger	rna messenger	RNA, Messenger	3	kept

Robot-assisted	robot assisted	robot assisted	robot assisted	Robot-assisted	3	kept
Robotic arm motion control	robotic arm motion control	robotic arm motion control	robotic arm motion control	Robotic arm motion control	3	kept
roc curve	roc curve	roc curve	roc curve	ROC Curve	3	case_or_format
Role conflict	role conflict	role conflict	role conflict	Role conflict	3	kept
role-play	role play	role play	role play	role-play	3	kept
ROLES	roles	roles	role	Role	3	singular
roller pump	roller pump	roller pump	roller pump	roller pump	3	kept
ROOM	room	room	room	ROOM	3	kept
Roy adaptation model	roy adaptation model	roy adaptation model	roy adaptation model	Roy Adaptation Model	3	case_or_format
rumination	rumination	rumination	rumination	Rumination	3	case_or_format
Running	running	running	running	running	3	case_or_format
Rural health	rural health	rural health	rural health	rural health	3	case_or_format
Rural hospitals	rural hospitals	rural hospitals	rural hospital	rural hospital	3	singular
sacrococcyx teratoma	sacrococcyx teratoma	sacrococcyx teratoma	sacrococcyx teratoma	sacrococcyx teratoma	3	kept
Safety attitude	safety attitude	safety attitude	safety attitude	Safety attitudes	3	case_or_format
Safety Attitude Questionnaire	safety attitude questionnaire	safety attitude questionnaire	safety attitude questionnaire	Safety Attitude Questionnaire	3	kept

Safety critical systems	safety critical systems	safety critical systems	safety critical systems	Safety critical systems	3	kept
Safety-critical systems	safety critical systems	safety critical systems	safety critical systems	Safety critical systems	3	case_or_format
saliva analysis	saliva analysis	saliva analysis	saliva analysis	saliva analysis	3	kept
Saliva, Artificial	saliva artificial	saliva artificial	saliva artificial	Saliva, Artificial	3	kept
SALIVARY CORTISOL	salivary cortisol	salivary cortisol	salivary cortisol	salivary cortisol	3	case_or_format
Sao Paulo	sao paulo	sao paulo	sao paulo	Sao Paulo	3	kept
saphenous vein	saphenous vein	saphenous vein	saphenous vein	saphenous vein	3	kept
sarilumab	sarilumab	sarilumab	sarilumab	sarilumab	3	kept
SARS Virus	sars virus	sars virus	sars virus	SARS Virus	3	kept
SARS-CoV-19	sars cov 19	sars cov 19	sars cov 19	SARS-CoV-19	3	kept
SARS-cov-2	sars cov 2	sars cov 2	sars cov 2	SARS-CoV-2	3	case_or_format
SARS-CoV-2 pandemic	sars cov 2 pandemic	sars cov 2 pandemic	sars cov 2 pandemic	SARS-CoV-2 pandemic	3	kept
SARS-CoV2	sars cov2	sars cov2	sars cov2	SARS-CoV2	3	kept
SARS-related coronavirus	sars related coronavirus	sars related coronavirus	sars related coronavirus	SARS-related coronavirus	3	kept
Satisfaction with Life Scale	satisfaction with life scale	satisfaction with life scale	satisfaction with life scale	Satisfaction with Life Scale	3	kept
saudi arabia	saudi arabia	saudi arabia	saudi arabia	Saudi Arabia	3	case_or_format

SAUDI-ARABIA	saudi arabia	saudi arabia	saudi arabia	Saudi Arabia	3	case_or_format
scalp	scalp	scalp	scalp	Scalp	3	case_or_format
scapula fracture	scapula fracture	scapula fracture	scapula fracture	scapula fracture	3	kept
school attendance	school attendance	school attendance	school attendance	school attendance	3	kept
school health service	school health service	school health service	school health service	school health service	3	kept
SCI	sci	sci	sci	SCI	3	kept
Score	score	score	score	SCORE	3	case_or_format
score	score	score	score	SCORE	3	case_or_format
Screening tools	screening tools	screening tools	screening tool	Screening tool	3	singular
scrub	scrub	scrub	scrub	scrub	3	kept
scrub typhus	scrub typhus	scrub typhus	scrub typhus	scrub typhus	3	kept
second trimester pregnancy	second trimester pregnancy	second trimester pregnancy	second trimester pregnancy	second trimester pregnancy	3	kept
Secondary data analysis	secondary data analysis	secondary data analysis	secondary data analysis	Secondary data analysis	3	kept
secondary outcome	secondary outcome	secondary outcome	secondary outcome	secondary outcome	3	kept
Secondary prevention	secondary prevention	secondary prevention	secondary prevention	secondary prevention	3	case_or_format
SECONDARY TRAUMATIC STRESS	secondary traumatic stress	secondary traumatic stress	secondary traumatic stress	secondary traumatic stress	3	case_or_format

Security systems	security systems	security systems	security systems	Security systems	3	kept
sedation vacation	sedation vacation	sedation vacation	sedation vacation	sedation vacation	3	kept
Sedative	sedative	sedative	sedative	Sedatives	3	case_or_format
sedentary lifestyle	sedentary lifestyle	sedentary lifestyle	sedentary lifestyle	sedentary lifestyle	3	kept
selection bias	selection bias	selection bias	selection bias	Selection Bias	3	case_or_format
self administered questionnaire	self administered questionnaire	self administered questionnaire	self administered questionnaire	self administered questionnaire	3	kept
Self Disclosure	self disclosure	self disclosure	self disclosure	self disclosure	3	case_or_format
self efficacy	self efficacy	self efficacy	self efficacy	Self Efficacy	3	case_or_format
Self Mutilation	self mutilation	self mutilation	self mutilation	Self Mutilation	3	kept
self transcendence	self transcendence	self transcendence	self transcendence	self transcendence	3	kept
Self-control	self control	self control	self control	self control	3	case_or_format
self-esteem	self esteem	self esteem	self esteem	self esteem	3	case_or_format
Self-Evaluation Programs	self evaluation programs	self evaluation programs	self evaluation programs	Self-Evaluation Programs	3	kept
Self-Help Devices	self help devices	self help devices	self help devices	Self-Help Devices	3	kept
self-report	self report	self report	self report	self report	3	case_or_format
Semantic Differential	semantic differential	semantic differential	semantic differential	Semantic Differential	3	kept

Semisitting position	semisitting position	semisitting position	semisitting position	Semisitting position	3	kept
Senna extract	senna extract	senna extract	senna extract	Senna extract	3	kept
sense of belonging	sense of belonging	sense of belonging	sense of belonging	sense of belonging	3	kept
sense of coherence	sense of coherence	sense of coherence	sense of coherence	Sense of Coherence	3	case_or_format
Sensitivity analysis	sensitivity analysis	sensitivity analysis	sensitivity analysis	sensitivity analysis	3	case_or_format
sensorimotor function	sensorimotor function	sensorimotor function	sensorimotor function	sensorimotor function	3	kept
Sensory overload	sensory overload	sensory overload	sensory overload	Sensory overload	3	kept
separation	separation	separation	separation	Separation	3	case_or_format
separation anxiety	separation anxiety	separation anxiety	separation anxiety	separation anxiety	3	kept
Sepsis bundle	sepsis bundle	sepsis bundle	sepsis bundle	Sepsis bundle	3	kept
Sepsis education	sepsis education	sepsis education	sepsis education	Sepsis education	3	kept
Sepsis management	sepsis management	sepsis management	sepsis management	Sepsis management	3	kept
Sequence Analysis, DNA	sequence analysis dna	sequence analysis dna	sequence analysis dna	Sequence Analysis, DNA	3	kept
serious illness communication	serious illness communication	serious illness communication	serious illness communication	serious illness communication	3	kept
SERIOUSLY ILL	seriously ill	seriously ill	seriously ill	SERIOUSLY ILL	3	kept
serum	serum	serum	serum	serum	3	kept

Service evaluation	service evaluation	service evaluation	service evaluation	Service evaluation	3	kept
service evaluation	service evaluation	service evaluation	service evaluation	Service evaluation	3	case_or_format
Service provision	service provision	service provision	service provision	Service provision	3	kept
SETTINGS	settings	settings	settings	SETTINGS	3	kept
Severe acute respiratory infection	severe acute respiratory infection	severe acute respiratory infection	severe acute respiratory infection	Severe acute respiratory infection	3	kept
severe maternal morbidity	severe maternal morbidity	severe maternal morbidity	severe maternal morbidity	severe maternal morbidity	3	kept
Sevoflurano	sevoflurano	sevoflurano	sevoflurano	Sevoflurano	3	kept
sewage	sewage	sewage	sewage	sewage	3	kept
Sex Offenses	sex offenses	sex offenses	sex offenses	Sex Offenses	3	kept
sexual abuse	sexual abuse	sexual abuse	sexual abuse	sexual abuse	3	kept
Sexual and Gender Minorities	sexual and gender minorities	sexual and gender minorities	sexual and gender minority	Sexual and Gender Minorities	3	singular
sexual assault	sexual assault	sexual assault	sexual assault	sexual assault	3	kept
Sexual Dysfunctions, Psychological	sexual dysfunctions psychological	sexual dysfunctions psychological	sexual dysfunctions psychological	Sexual Dysfunctions, Psychological	3	kept
sexual harassment	sexual harassment	sexual harassment	sexual harassment	Sexual Harassment	3	case_or_format
sexual intercourse	sexual intercourse	sexual intercourse	sexual intercourse	sexual intercourse	3	kept
sexual violence	sexual violence	sexual violence	sexual violence	sexual violence	3	kept

Sexually Transmitted Diseases	sexually transmitted diseases	sexually transmitted diseases	sexually transmitted disease	sexually transmitted disease	3	singular
shared	shared	shared	shared	shared	3	kept
shared governance	shared governance	shared governance	shared governance	shared governance	3	kept
sharp injuries	sharp injuries	sharp injuries	sharp injury	Sharp injuries	3	singular
Sharps injuries	sharps injuries	sharps injuries	sharps injury	Sharps injuries	3	singular
sharps injuries	sharps injuries	sharps injuries	sharps injury	Sharps injuries	3	singular
SHIFT REPORT	shift report	shift report	shift report	SHIFT REPORT	3	kept
Shift scheduling	shift scheduling	shift scheduling	shift scheduling	Shift scheduling	3	kept
shift work schedule	shift work schedule	shift work schedule	shift work schedule	Shift Work Schedule	3	case_or_format
SHOCK	shock	shock	shock	shock	3	case_or_format
shock index	shock index	shock index	shock index	shock index	3	kept
Short bowel syndrome	short bowel syndrome	short bowel syndrome	short bowel syndrome	short bowel syndrome	3	case_or_format
short term survival	short term survival	short term survival	short term survival	short term survival	3	kept
sick building syndrome	sick building syndrome	sick building syndrome	sick building syndrome	sick building syndrome	3	kept
Sickness absence	sickness absence	sickness absence	sickness absence	Sickness absence	3	kept
SIDS	sids	sids	sids	SIDS	3	kept

Signal quality	signal quality	signal quality	signal quality	Signal quality	3	kept
Simulation studies	simulation studies	simulation studies	simulation study	Simulation studies	3	singular
simulation-based education	simulation based education	simulation based education	simulation based education	Simulation-based education	3	case_or_format
Simulator	simulator	simulator	simulator	simulator	3	case_or_format
single family room	single family room	single family room	single family room	single family room	3	kept
Single Question in Delirium	single question in delirium	single question in delirium	single question in delirium	Single Question in Delirium	3	kept
single-family room	single family room	single family room	single family room	single family room	3	case_or_format
sinus node	sinus node	sinus node	sinus node	sinus node	3	kept
Sinus Thrombosis, Intracranial	sinus thrombosis intracranial	sinus thrombosis intracranial	sinus thrombosis intracranial	Sinus Thrombosis, Intracranial	3	kept
SITUATION AWARENESS	situation awareness	situation awareness	situation awareness	situation awareness	3	case_or_format
Situation Awareness	situation awareness	situation awareness	situation awareness	situation awareness	3	case_or_format
Skill	skill	skill	skill	skill	3	case_or_format
Skills acquisition	skills acquisition	skills acquisition	skills acquisition	Skills acquisition	3	kept
skin abrasion	skin abrasion	skin abrasion	skin abrasion	skin abrasion	3	kept
skin abscess	skin abscess	skin abscess	skin abscess	skin abscess	3	kept
Skin Absorption	skin absorption	skin absorption	skin absorption	Skin Absorption	3	kept

skin allergy	skin allergy	skin allergy	skin allergy	skin allergy	3	kept
skin assessment	skin assessment	skin assessment	skin assessment	skin assessment	3	kept
skin barrier	skin barrier	skin barrier	skin barrier	skin barrier	3	kept
Skin breakdown	skin breakdown	skin breakdown	skin breakdown	Skin breakdown	3	kept
skin disease assessment	skin disease assessment	skin disease assessment	skin disease assessment	skin disease assessment	3	kept
skin function	skin function	skin function	skin function	skin function	3	kept
skin pain	skin pain	skin pain	skin pain	skin pain	3	kept
Skin temperature	skin temperature	skin temperature	skin temperature	Skin Temperature	3	case_or_format
skin to skin contact	skin to skin contact	skin to skin contact	skin to skin contact	Skin-to-skin contact	3	case_or_format
skin turgor	skin turgor	skin turgor	skin turgor	skin turgor	3	kept
Skull	skull	skull	skull	Skull	3	kept
skull	skull	skull	skull	Skull	3	case_or_format
sleep depth	sleep depth	sleep depth	sleep depth	sleep depth	3	kept
Sleep disorder	sleep disorder	sleep disorder	sleep disorder	sleep disorder	3	case_or_format
SLEEP DISRUPTION	sleep disruption	sleep disruption	sleep disruption	SLEEP DISRUPTION	3	kept
Sleep disruption	sleep disruption	sleep disruption	sleep disruption	SLEEP DISRUPTION	3	case_or_format

Sleep efficiency	sleep efficiency	sleep efficiency	sleep efficiency	sleep efficiency	3	case_or_format
Sleep hygiene	sleep hygiene	sleep hygiene	sleep hygiene	sleep hygiene	3	case_or_format
Sleep intervention	sleep intervention	sleep intervention	sleep intervention	Sleep intervention	3	kept
Sleep Medicine Specialty	sleep medicine specialty	sleep medicine specialty	sleep medicine specialty	Sleep Medicine Specialty	3	kept
sleep parameters	sleep parameters	sleep parameters	sleep parameters	sleep parameters	3	kept
SLEEP QUALITY	sleep quality	sleep quality	sleep quality	sleep quality	3	case_or_format
sleep questionnaire	sleep questionnaire	sleep questionnaire	sleep questionnaire	sleep questionnaire	3	kept
Sleep, REM	sleep rem	sleep rem	sleep rem	Sleep, REM	3	kept
SLEEP-DEPRIVATION	sleep deprivation	sleep deprivation	sleep deprivation	Sleep Deprivation	3	case_or_format
Sleep-wake cycle	sleep wake cycle	sleep wake cycle	sleep wake cycle	Sleep-wake cycle	3	kept
Sleep-wake disorders	sleep wake disorders	sleep wake disorders	sleep wake disorders	Sleep Wake Disorders	3	case_or_format
sleeve gastrectomy	sleeve gastrectomy	sleeve gastrectomy	sleeve gastrectomy	sleeve gastrectomy	3	kept
small for gestational age	small for gestational age	small for gestational age	small for gestational age	small for gestational age	3	kept
small intestine obstruction	small intestine obstruction	small intestine obstruction	small intestine obstruction	small intestine obstruction	3	kept
small intestine perforation	small intestine perforation	small intestine perforation	small intestine perforation	small intestine perforation	3	kept
smallpox	smallpox	smallpox	smallpox	Smallpox	3	case_or_format

smallpox vaccine	smallpox vaccine	smallpox vaccine	smallpox vaccine	smallpox vaccine	3	kept
Smartphones	smartphones	smartphones	smartphone	Smartphone	3	singular
smear	smear	smear	smear	smear	3	kept
Smith-Lemli-Opitz Syndrome	smith lemli opitz syndrome	smith lemli opitz syndrome	smith lemli opitz syndrome	Smith-Lemli-Opitz Syndrome	3	kept
Smoking Prevention	smoking prevention	smoking prevention	smoking prevention	Smoking Prevention	3	kept
snake venom	snake venom	snake venom	snake venom	snake venom	3	kept
Sneddon syndrome	sneddon syndrome	sneddon syndrome	sneddon syndrome	Sneddon syndrome	3	kept
Social Capital	social capital	social capital	social capital	social capital	3	case_or_format
social cognition	social cognition	social cognition	social cognition	social cognition	3	kept
Social Cognitive Theory	social cognitive theory	social cognitive theory	social cognitive theory	Social Cognitive Theory	3	kept
Social cognitive theory	social cognitive theory	social cognitive theory	social cognitive theory	Social Cognitive Theory	3	case_or_format
social cohesion	social cohesion	social cohesion	social cohesion	social cohesion	3	kept
Social determinants of health	social determinants of health	social determinants of health	social determinants of health	social determinants of health	3	case_or_format
Social Distance	social distance	social distance	social distance	social distance	3	case_or_format
social factors	social factors	social factors	social factors	social factors	3	kept
social identity	social identity	social identity	social identity	social identity	3	kept

Social interactions	social interactions	social interactions	social interaction	social interaction	3	singular
Social justice	social justice	social justice	social justice	Social Justice	3	case_or_format
social learning	social learning	social learning	social learning	social learning	3	kept
social phobia	social phobia	social phobia	social phobia	social phobia	3	kept
Social Skills	social skills	social skills	social skills	Social Skills	3	kept
social structure	social structure	social structure	social structure	social structure	3	kept
Social Vulnerability Index	social vulnerability index	social vulnerability index	social vulnerability index	Social Vulnerability Index	3	kept
social worker attitude	social worker attitude	social worker attitude	social worker attitude	social worker attitude	3	kept
Sociodemographic Factors	sociodemographic factors	sociodemographic factors	sociodemographic factors	Sociodemographic Factors	3	kept
Socioeconomic factors	socioeconomic factors	socioeconomic factors	socioeconomic factors	Socioeconomic Factors	3	case_or_format
Sociology, Medical	sociology medical	sociology medical	sociology medical	Sociology, Medical	3	kept
sociometric status	sociometric status	sociometric status	sociometric status	sociometric status	3	kept
Sociotechnical systems	sociotechnical systems	sociotechnical systems	sociotechnical system	Sociotechnical systems	3	singular
sodium carbonate	sodium carbonate	sodium carbonate	sodium carbonate	sodium carbonate	3	kept
sodium excretion	sodium excretion	sodium excretion	sodium excretion	sodium excretion	3	kept
sodium glucose cotransporter 2 inhibitor	sodium glucose cotransporter 2 inhibitor	sodium glucose cotransporter 2 inhibitor	sodium glucose cotransporter 2 inhibitor	sodium glucose cotransporter 2 inhibitor	3	kept

SOFA	sofa	sofa	sofa	SOFA	3	kept
software design	software design	software design	software design	Software Design	3	case_or_format
solidarity	solidarity	solidarity	solidarity	solidarity	3	kept
solvent	solvent	solvent	solvent	solvent	3	kept
somatomedin binding protein	somatomedin binding protein	somatomedin binding protein	somatomedin binding protein	somatomedin binding protein	3	kept
somatomedin C	somatomedin c	somatomedin c	somatomedin c	somatomedin C	3	kept
sophia observation withdrawal symptoms scale	sophia observation withdrawal symptoms scale	sophia observation withdrawal symptoms scale	sophia observation withdrawal symptoms scale	sophia observation withdrawal symptoms scale	3	kept
Sorbitol	sorbitol	sorbitol	sorbitol	sorbitol	3	case_or_format
Sotos syndrome	sotos syndrome	sotos syndrome	sotos syndrome	Sotos syndrome	3	kept
sotrovimab	sotrovimab	sotrovimab	sotrovimab	sotrovimab	3	kept
sound environment management	sound environment management	sound environment management	sound environment management	sound environment management	3	kept
sound pressure	sound pressure	sound pressure	sound pressure	sound pressure	3	kept
Sound pressure level	sound pressure level	sound pressure level	sound pressure level	Sound pressure level	3	kept
Sound pressure levels	sound pressure levels	sound pressure levels	sound pressure level	Sound pressure level	3	singular
South Asian	south asian	south asian	south asian	South Asian	3	kept
SOUTH-AFRICA	south africa	south africa	south africa	South Africa	3	case_or_format

soybean oil	soybean oil	soybean oil	soybean oil	soybean oil	3	kept
space flight	space flight	space flight	space flight	space flight	3	kept
Spatio-Temporal Analysis	spatio temporal analysis	spatio temporal analysis	spatio temporal analysis	Spatio-Temporal Analysis	3	kept
special care	special care	special care	special care	special care	3	kept
Speech communication	speech communication	speech communication	speech communication	Speech communication	3	kept
speech-language pathology	speech language pathology	speech language pathology	speech language pathology	Speech-Language Pathology	3	case_or_format
spinal cord decompression	spinal cord decompression	spinal cord decompression	spinal cord decompression	spinal cord decompression	3	kept
Spinal cord injuries	spinal cord injuries	spinal cord injuries	spinal cord injury	Spinal Cord Injuries	3	singular
spinal dysraphism	spinal dysraphism	spinal dysraphism	spinal dysraphism	Spinal Dysraphism	3	case_or_format
spinal hematoma	spinal hematoma	spinal hematoma	spinal hematoma	spinal hematoma	3	kept
Spinal Muscular Atrophies of Childhood	spinal muscular atrophies of childhood	spinal muscular atrophies of childhood	spinal muscular atrophies of childhood	Spinal Muscular Atrophies of Childhood	3	kept
SPINAL-CORD-INJURY	spinal cord injury	spinal cord injury	spinal cord injury	Spinal Cord Injuries	3	case_or_format
spine	spine	spine	spine	Spine	3	case_or_format
spine malformation	spine malformation	spine malformation	spine malformation	spine malformation	3	kept
spine tumor	spine tumor	spine tumor	spine tumor	spine tumor	3	kept
Spiritual	spiritual	spiritual	spiritual	Spiritual	3	kept

spiritual health	spiritual health	spiritual health	spiritual health	Spiritual health	3	case_or_format
Spiritual needs	spiritual needs	spiritual needs	spiritual needs	Spiritual needs	3	kept
Spiritual well-being	spiritual well being	spiritual well being	spiritual well being	spiritual well-being	3	case_or_format
Spiritualism	spiritualism	spiritualism	spiritualism	Spiritualism	3	kept
spleen injury	spleen injury	spleen injury	spleen injury	spleen injury	3	kept
splenomegaly	splenomegaly	splenomegaly	splenomegaly	splenomegaly	3	kept
split thickness skin graft	split thickness skin graft	split thickness skin graft	split thickness skin graft	split thickness skin graft	3	kept
SpO2	spo2	spo2	spo2	SpO2	3	kept
spontaneous awakening trial	spontaneous awakening trial	spontaneous awakening trial	spontaneous awakening trial	spontaneous awakening trial	3	kept
Spontaneous breathing	spontaneous breathing	spontaneous breathing	spontaneous breathing	Spontaneous breathing	3	kept
sport injury	sport injury	sport injury	sport injury	sport injury	3	kept
sports medicine	sports medicine	sports medicine	sports medicine	sports medicine	3	kept
Spray	spray	spray	spray	Spray	3	kept
Staff education	staff education	staff education	staff education	Staff education	3	kept
staff education	staff education	staff education	staff education	Staff education	3	case_or_format
Staff satisfaction	staff satisfaction	staff satisfaction	staff satisfaction	Staff satisfaction	3	kept

staff satisfaction	staff satisfaction	staff satisfaction	staff satisfaction	Staff satisfaction	3	case_or_format
Staff turnover	staff turnover	staff turnover	staff turnover	Staff turnover	3	kept
staff wellbeing	staff wellbeing	staff wellbeing	staff wellbeing	Staff wellbeing	3	case_or_format
STAFFING LEVELS	staffing levels	staffing levels	staffing level	STAFFING LEVELS	3	singular
staffing models	staffing models	staffing models	staffing model	staffing models	3	singular
STAFFING PATTERNS	staffing patterns	staffing patterns	staffing patterns	STAFFING PATTERNS	3	kept
Stakeholders	stakeholders	stakeholders	stakeholder	Stakeholders	3	singular
Standard deviation	standard deviation	standard deviation	standard deviation	Standard deviation	3	kept
Standard of care	standard of care	standard of care	standard of care	Standard of Care	3	case_or_format
standard precautions	standard precautions	standard precautions	standard precautions	Standard precautions	3	case_or_format
standardized incidence ratio	standardized incidence ratio	standardized incidence ratio	standardized incidence ratio	standardized incidence ratio	3	kept
Standardized training	standardized training	standardized training	standardized training	Standardized training	3	kept
Statins	statins	statins	statins	Statins	3	kept
STEMI	stemi	stemi	stemi	STEMI	3	kept
Stenotrophomonas	stenotrophomonas	stenotrophomonas	stenotrophomonas	Stenotrophomonas	3	kept
step down unit	step down unit	step down unit	step down unit	step down unit	3	kept

Stepped wedge	stepped wedge	stepped wedge	stepped wedge	Stepped wedge	3	kept
stereotaxic surgery	stereotaxic surgery	stereotaxic surgery	stereotaxic surgery	stereotaxic surgery	3	kept
sternum fracture	sternum fracture	sternum fracture	sternum fracture	sternum fracture	3	kept
steroids	steroids	steroids	steroid	steroid	3	singular
Stewardship	stewardship	stewardship	stewardship	stewardship	3	case_or_format
Stochastic systems	stochastic systems	stochastic systems	stochastic systems	Stochastic systems	3	kept
Stoma	stoma	stoma	stoma	stoma	3	case_or_format
stomach disease	stomach disease	stomach disease	stomach disease	stomach disease	3	kept
stomach fundoplication	stomach fundoplication	stomach fundoplication	stomach fundoplication	stomach fundoplication	3	kept
stomach motility	stomach motility	stomach motility	stomach motility	stomach motility	3	kept
stomach mucosa	stomach mucosa	stomach mucosa	stomach mucosa	stomach mucosa	3	kept
strangulation	strangulation	strangulation	strangulation	strangulation	3	kept
strategic approach	strategic approach	strategic approach	strategic approach	strategic approach	3	kept
strategy	strategy	strategy	strategy	STRATEGIES	3	case_or_format
stress reduction	stress reduction	stress reduction	stress reduction	stress reduction	3	kept
Stress response	stress response	stress response	stress response	Stress response	3	kept

stress response	stress response	stress response	stress response	Stress response	3	case_or_format
stressor	stressor	stressor	stressor	Stressors	3	case_or_format
structure activity relation	structure activity relation	structure activity relation	structure activity relation	structure activity relation	3	kept
structure analysis	structure analysis	structure analysis	structure analysis	structure analysis	3	kept
Students, Pharmacy	students pharmacy	students pharmacy	students pharmacy	Students, Pharmacy	3	kept
Subacute care	subacute care	subacute care	subacute care	Subacute Care	3	case_or_format
subcutaneous insulin	subcutaneous insulin	subcutaneous insulin	subcutaneous insulin	subcutaneous insulin	3	kept
Subdural hematoma	subdural hematoma	subdural hematoma	subdural hematoma	subdural hematoma	3	case_or_format
subglottic stenosis	subglottic stenosis	subglottic stenosis	subglottic stenosis	subglottic stenosis	3	kept
Substance Abuse Detection	substance abuse detection	substance abuse detection	substance abuse detection	Substance Abuse Detection	3	kept
Substance withdrawal syndrome	substance withdrawal syndrome	substance withdrawal syndrome	substance withdrawal syndrome	Substance Withdrawal Syndrome	3	case_or_format
subsyndromal delirium	subsyndromal delirium	subsyndromal delirium	subsyndromal delirium	Subsyndromal delirium	3	case_or_format
Succinylcholine	succinylcholine	succinylcholine	succinylcholine	Succinylcholine	3	kept
sucking reflex	sucking reflex	sucking reflex	sucking reflex	sucking reflex	3	kept
SUCROSE	sucrose	sucrose	sucrose	Sucrose	3	case_or_format
Sugammadex	sugammadex	sugammadex	sugammadex	sugammadex	3	case_or_format

Sunlight	sunlight	sunlight	sunlight	Sunlight	3	kept
Supervised machine learning	supervised machine learning	supervised machine learning	supervised machine learning	Supervised machine learning	3	kept
Supervisory	supervisory	supervisory	supervisory	Supervisory	3	kept
Supervisory leadership	supervisory leadership	supervisory leadership	supervisory leadership	Supervisory leadership	3	kept
SupplMeshName: pediatric multisystem inflammatory disease	supplmeshname pediatric multisystem inflammatory disease	supplmeshname pediatric multisystem inflammatory disease	supplmeshname pediatric multisystem inflammatory disease	SupplMeshName: pediatric multisystem inflammatory disease	3	kept
SupplMeshName: Respiratory Distress Syndrome In Premature	supplmeshname respiratory distress syndrome in premature	supplmeshname respiratory distress syndrome in premature	supplmeshname respiratory distress syndrome in premature	SupplMeshName: Respiratory Distress Syndrome In Premature	3	kept
Support surfaces	support surfaces	support surfaces	support surface	Support surfaces	3	singular
Supportive behaviors	supportive behaviors	supportive behaviors	supportive behaviors	Supportive behaviors	3	kept
Suppository	suppository	suppository	suppository	suppository	3	case_or_format
surface area	surface area	surface area	surface area	surface area	3	kept
Surfactant	surfactant	surfactant	surfactant	surfactant	3	case_or_format
Surgery, Computer-Assisted	surgery computer assisted	surgery computer assisted	surgery computer assisted	Surgery, Computer-Assisted	3	kept
Surgery, Plastic	surgery plastic	surgery plastic	surgery plastic	Surgery, Plastic	3	kept
surgical anatomy	surgical anatomy	surgical anatomy	surgical anatomy	surgical anatomy	3	kept
surgical dashboard	surgical dashboard	surgical dashboard	surgical dashboard	surgical dashboard	3	kept
surgical education	surgical education	surgical education	surgical education	surgical education	3	kept

surgical oncology	surgical oncology	surgical oncology	surgical oncology	surgical oncology	3	kept
surgical outcomes	surgical outcomes	surgical outcomes	surgical outcome	surgical outcomes	3	singular
surgical palliative care	surgical palliative care	surgical palliative care	surgical palliative care	surgical palliative care	3	kept
Surgical Procedures, Elective	surgical procedures elective	surgical procedures elective	surgical procedures elective	Surgical Procedures, Elective	3	kept
Surgical Procedures, Minimally Invasive	surgical procedures minimally invasive	surgical procedures minimally invasive	surgical procedures minimally invasive	Surgical Procedures, Minimally Invasive	3	kept
surgical site	surgical site	surgical site	surgical site	surgical site	3	kept
surgical volume	surgical volume	surgical volume	surgical volume	surgical volume	3	kept
Surgical Wound Dehiscence	surgical wound dehiscence	surgical wound dehiscence	surgical wound dehiscence	Surgical Wound Dehiscence	3	kept
Surgical wound infection	surgical wound infection	surgical wound infection	surgical wound infection	Surgical Wound Infection	3	case_or_format
SURROGATE DECISION-MAKING	surrogate decision making	surrogate decision making	surrogate decision making	surrogate decision-making	3	case_or_format
surrogates	surrogates	surrogates	surrogate	Surrogate	3	singular
Surveillance monitoring	surveillance monitoring	surveillance monitoring	surveillance monitoring	Surveillance monitoring	3	kept
Surveillance systems	surveillance systems	surveillance systems	surveillance systems	Surveillance systems	3	kept
Survival analysis	survival analysis	survival analysis	survival analysis	Survival Analysis	3	case_or_format
Surviving sepsis campaign	surviving sepsis campaign	surviving sepsis campaign	surviving sepsis campaign	Surviving Sepsis Campaign	3	case_or_format
Survivor	survivor	survivor	survivor	Survivors	3	case_or_format

Sus scrofa	sus scrofa	sus scrofa	sus scrofa	Sus scrofa	3	kept
SUSCEPTIBILITY	susceptibility	susceptibility	susceptibility	SUSCEPTIBILITY	3	kept
Suture Techniques	suture techniques	suture techniques	suture technique	Suture Techniques	3	singular
suturing method	suturing method	suturing method	suturing method	suturing method	3	kept
Swallowing disorder	swallowing disorder	swallowing disorder	swallowing disorder	Swallowing disorder	3	kept
Swallowing disorders	swallowing disorders	swallowing disorders	swallowing disorder	Swallowing disorder	3	singular
Swedish massage	swedish massage	swedish massage	swedish massage	Swedish massage	3	kept
symbolic interactionism	symbolic interactionism	symbolic interactionism	symbolic interactionism	symbolic interactionism	3	kept
Sympathetic Nervous System	sympathetic nervous system	sympathetic nervous system	sympathetic nervous system	Sympathetic Nervous System	3	kept
Symptom assessment	symptom assessment	symptom assessment	symptom assessment	symptom assessment	3	case_or_format
syringe pump	syringe pump	syringe pump	syringe pump	syringe pump	3	kept
systemic circulation	systemic circulation	systemic circulation	systemic circulation	systemic circulation	3	kept
systemic sclerosis	systemic sclerosis	systemic sclerosis	systemic sclerosis	systemic sclerosis	3	kept
Systems	systems	systems	system	SYSTEM	3	singular
systems	systems	systems	system	SYSTEM	3	singular
Systems Biology	systems biology	systems biology	systems biology	Systems Biology	3	kept

T lymphocyte	t lymphocyte	t lymphocyte	t lymphocyte	T-Lymphocytes	3	case_or_format
tablet formulation	tablet formulation	tablet formulation	tablet formulation	tablet formulation	3	kept
Tanzanian	tanzanian	tanzanian	tanzanian	Tanzanian	3	kept
Task analysis	task analysis	task analysis	task analysis	Task analysis	3	kept
Task severity	task severity	task severity	task severity	Task severity	3	kept
Task shifting	task shifting	task shifting	task shifting	task shifting	3	case_or_format
task-shifting	task shifting	task shifting	task shifting	task shifting	3	case_or_format
taste	taste	taste	taste	taste	3	kept
Tazobactam	tazobactam	tazobactam	tazobactam	tazobactam	3	case_or_format
Teaching hospital	teaching hospital	teaching hospital	teaching hospital	teaching hospital	3	case_or_format
teaching methods	teaching methods	teaching methods	teaching methods	teaching methods	3	kept
Teaching rounds	teaching rounds	teaching rounds	teaching round	Teaching Rounds	3	singular
team communication	team communication	team communication	team communication	Team communication	3	case_or_format
Team dynamics	team dynamics	team dynamics	team dynamics	team dynamics	3	case_or_format
Team-Based Learning	team based learning	team based learning	team based learning	Team-Based Learning	3	kept
TeamSTEPPS	teamstepps	teamstepps	teamstepps	TeamSTEPPS	3	kept

TeamSTEPPS®	teamstepps	teamstepps	teamstepps	TeamSTEPPS	3	case_or_format
technical efficiency	technical efficiency	technical efficiency	technical efficiency	technical efficiency	3	kept
techniques & procedures	techniques and procedures	techniques and procedures	techniques and procedure	techniques and procedures	3	singular
Technology acceptance model	technology acceptance model	technology acceptance model	technology acceptance model	Technology acceptance model	3	kept
Technology adoption	technology adoption	technology adoption	technology adoption	Technology adoption	3	kept
Tele-monitoring	tele monitoring	tele monitoring	tele monitoring	Tele-monitoring	3	kept
Telecommunication	telecommunication	telecommunication	telecommunication	telecommunication	3	case_or_format
telecommunications	telecommunications	telecommunications	telecommunication	telecommunication	3	singular
Teleconsultation	teleconsultation	teleconsultation	teleconsultation	teleconsultation	3	case_or_format
Telementoring	telementoring	telementoring	telementoring	Telementoring	3	kept
telepsychiatry	telepsychiatry	telepsychiatry	telepsychiatry	telepsychiatry	3	kept
teleradiology	teleradiology	teleradiology	teleradiology	teleradiology	3	kept
Telerehabilitation	telerehabilitation	telerehabilitation	telerehabilitation	telerehabilitation	3	case_or_format
telesimulation	telesimulation	telesimulation	telesimulation	telesimulation	3	kept
temozolomide	temozolomide	temozolomide	temozolomide	temozolomide	3	kept
Temperature measurement	temperature measurement	temperature measurement	temperature measurement	temperature measurement	3	case_or_format

temporary tracheostomy	temporary tracheostomy	temporary tracheostomy	temporary tracheostomy	temporary tracheostomy	3	kept
temporomandibular joint disorder	temporomandibular joint disorder	temporomandibular joint disorder	temporomandibular joint disorder	temporomandibular joint disorder	3	kept
tendinitis	tendinitis	tendinitis	tendinitis	tendinitis	3	kept
tendon reflex	tendon reflex	tendon reflex	tendon reflex	tendon reflex	3	kept
teratoma	teratoma	teratoma	teratoma	Teratoma	3	case_or_format
terlipressin	terlipressin	terlipressin	terlipressin	terlipressin	3	kept
terminal extubation	terminal extubation	terminal extubation	terminal extubation	terminal extubation	3	kept
Terminal weaning	terminal weaning	terminal weaning	terminal weaning	Terminal weaning	3	kept
tertiary care hospital	tertiary care hospital	tertiary care hospital	tertiary care hospital	tertiary care hospital	3	kept
Tertiary hospital	tertiary hospital	tertiary hospital	tertiary hospital	Tertiary hospital	3	kept
Testing	testing	testing	testing	Testing	3	kept
testing	testing	testing	testing	Testing	3	case_or_format
testing capacity	testing capacity	testing capacity	testing capacity	testing capacity	3	kept
tetracaine	tetracaine	tetracaine	tetracaine	tetracaine	3	kept
Text mining	text mining	text mining	text mining	Text mining	3	kept
TF-IDF	tf idf	tf idf	tf idf	TF-IDF	3	kept

thallium	thallium	thallium	thallium	thallium	3	kept
thanatophoric dwarfism	thanatophoric dwarfism	thanatophoric dwarfism	thanatophoric dwarfism	thanatophoric dwarfism	3	kept
The Netherlands	the netherlands	the netherlands	the netherlands	The Netherlands	3	kept
Theoretical Domains Framework	theoretical domains framework	theoretical domains framework	theoretical domains framework	Theoretical domains framework	3	case_or_format
theory of planned behavior	theory of planned behavior	theory of planned behavior	theory of planned behavior	Theory of Planned Behavior	3	case_or_format
therapeutic abortion	therapeutic abortion	therapeutic abortion	therapeutic abortion	therapeutic abortion	3	kept
therapeutic drug monitoring	therapeutic drug monitoring	therapeutic drug monitoring	therapeutic drug monitoring	therapeutic drug monitoring	3	kept
Therapeutic intervention scoring system	therapeutic intervention scoring system	therapeutic intervention scoring system	therapeutic intervention scoring system	therapeutic intervention scoring system	3	case_or_format
therapeutic relationship	therapeutic relationship	therapeutic relationship	therapeutic relationship	therapeutic relationship	3	kept
therapeutics	therapeutics	therapeutics	therapeutic	Therapeutics	3	singular
therapy escalation	therapy escalation	therapy escalation	therapy escalation	therapy escalation	3	kept
thermodynamics	thermodynamics	thermodynamics	thermodynamics	thermodynamics	3	kept
theta rhythm	theta rhythm	theta rhythm	theta rhythm	theta rhythm	3	kept
THINKING	thinking	thinking	thinking	Thinking	3	case_or_format
thirdhand smoke	thirdhand smoke	thirdhand smoke	thirdhand smoke	thirdhand smoke	3	kept
thoracic cavity	thoracic cavity	thoracic cavity	thoracic cavity	thoracic cavity	3	kept

thoracoscopy	thoracoscopy	thoracoscopy	thoracoscopy	thoracoscopy	3	kept
thorax blunt trauma	thorax blunt trauma	thorax blunt trauma	thorax blunt trauma	thorax blunt trauma	3	kept
thorax compression	thorax compression	thorax compression	thorax compression	thorax compression	3	kept
thorax epidural anesthesia	thorax epidural anesthesia	thorax epidural anesthesia	thorax epidural anesthesia	thorax epidural anesthesia	3	kept
thorax pressure	thorax pressure	thorax pressure	thorax pressure	thorax pressure	3	kept
threshold limit value	threshold limit value	threshold limit value	threshold limit value	threshold limit value	3	kept
thrombin inhibitor	thrombin inhibitor	thrombin inhibitor	thrombin inhibitor	thrombin inhibitor	3	kept
thrombomodulin	thrombomodulin	thrombomodulin	thrombomodulin	thrombomodulin	3	kept
Thrombophlebitis	thrombophlebitis	thrombophlebitis	thrombophlebitis	thrombophlebitis	3	case_or_format
thymocyte antibody	thymocyte antibody	thymocyte antibody	thymocyte antibody	thymocyte antibody	3	kept
thyroid cancer	thyroid cancer	thyroid cancer	thyroid cancer	thyroid cancer	3	kept
thyroid gland	thyroid gland	thyroid gland	thyroid gland	Thyroid Gland	3	case_or_format
Thyroid Neoplasms	thyroid neoplasms	thyroid neoplasms	thyroid neoplasms	Thyroid Neoplasms	3	kept
Thyrotoxicosis	thyrotoxicosis	thyrotoxicosis	thyrotoxicosis	Thyrotoxicosis	3	kept
Thyrotropin	thyrotropin	thyrotropin	thyrotropin	thyrotropin	3	case_or_format
Thyroxine	thyroxine	thyroxine	thyroxine	Thyroxine	3	kept

thyroxine	thyroxine	thyroxine	thyroxine	Thyroxine	3	case_or_format
Tibia	tibia	tibia	tibia	Tibia	3	kept
tibia	tibia	tibia	tibia	Tibia	3	case_or_format
Ticagrelor	ticagrelor	ticagrelor	ticagrelor	ticagrelor	3	case_or_format
tick paralysis	tick paralysis	tick paralysis	tick paralysis	tick paralysis	3	kept
Tidal volume	tidal volume	tidal volume	tidal volume	tidal volume	3	case_or_format
Time and motion study	time and motion study	time and motion study	time and motion study	Time and Motion Studies	3	case_or_format
Timing	timing	timing	timing	Timing	3	kept
timing	timing	timing	timing	Timing	3	case_or_format
tinnitus	tinnitus	tinnitus	tinnitus	tinnitus	3	kept
tissue culture	tissue culture	tissue culture	tissue culture	tissue culture	3	kept
tissue inhibitor of metalloproteinase 2	tissue inhibitor of metalloproteinase 2	tissue inhibitor of metalloproteinase 2	tissue inhibitor of metalloproteinase 2	tissue inhibitor of metalloproteinase 2	3	kept
Tocolytic Agents	tocolytic agents	tocolytic agents	tocolytic agents	Tocolytic Agents	3	kept
Toma de decisiones	toma de decisiones	toma de decisiones	toma de decisiones	Toma de decisiones	3	kept
tonic seizure	tonic seizure	tonic seizure	tonic seizure	tonic seizure	3	kept
Tools	tools	tools	tool	TOOL	3	singular

tooth extraction	tooth extraction	tooth extraction	tooth extraction	tooth extraction	3	kept
tooth infection	tooth infection	tooth infection	tooth infection	tooth infection	3	kept
topical agent	topical agent	topical agent	topical agent	topical agent	3	kept
topical drug administration	topical drug administration	topical drug administration	topical drug administration	topical drug administration	3	kept
Torsades de pointes	torsades de pointes	torsades de pointes	torsades de pointes	Torsades de Pointes	3	case_or_format
Total artificial heart	total artificial heart	total artificial heart	total artificial heart	Total artificial heart	3	kept
Total hip arthroplasty	total hip arthroplasty	total hip arthroplasty	total hip arthroplasty	Total hip arthroplasty	3	kept
total hip prosthesis	total hip prosthesis	total hip prosthesis	total hip prosthesis	total hip prosthesis	3	kept
total knee replacement	total knee replacement	total knee replacement	total knee replacement	total knee replacement	3	kept
total lung capacity	total lung capacity	total lung capacity	total lung capacity	total lung capacity	3	kept
Total parenteral nutrition	total parenteral nutrition	total parenteral nutrition	total parenteral nutrition	total parenteral nutrition	3	case_or_format
TOTAL PARENTERAL-NUTRITION	total parenteral nutrition	total parenteral nutrition	total parenteral nutrition	total parenteral nutrition	3	case_or_format
TPN	tpn	tpn	tpn	TPN	3	kept
Trabajo en equipo	trabajo en equipo	trabajo en equipo	trabajo en equipo	Trabajo en equipo	3	kept
Trace elements	trace elements	trace elements	trace element	trace element	3	singular
trachea stoma	trachea stoma	trachea stoma	trachea stoma	trachea stoma	3	kept

TRACK	track	track	track	TRACK	3	kept
Track and trigger	track and trigger	track and trigger	track and trigger	Track and trigger	3	kept
trade union	trade union	trade union	trade union	trade union	3	kept
train of four	train of four	train of four	train of four	train of four	3	kept
training and education	training and education	training and education	training and education	training and education	3	kept
training course	training course	training course	training course	training course	3	kept
Training programs	training programs	training programs	training program	Training program	3	singular
TRAINING-PROGRAM	training program	training program	training program	Training program	3	case_or_format
Transcatheter aortic valve implantation	transcatheter aortic valve implantation	transcatheter aortic valve implantation	transcatheter aortic valve implantation	transcatheter aortic valve implantation	3	case_or_format
Transcutaneous Electric Nerve Stimulation	transcutaneous electric nerve stimulation	transcutaneous electric nerve stimulation	transcutaneous electric nerve stimulation	Transcutaneous Electric Nerve Stimulation	3	kept
transcutaneous nerve stimulation	transcutaneous nerve stimulation	transcutaneous nerve stimulation	transcutaneous nerve stimulation	transcutaneous nerve stimulation	3	kept
transducer	transducer	transducer	transducer	Transducers	3	case_or_format
transfer of care	transfer of care	transfer of care	transfer of care	transfer of care	3	kept
transfer of learning	transfer of learning	transfer of learning	transfer of learning	transfer of learning	3	kept
Transfusion practices	transfusion practices	transfusion practices	transfusion practice	Transfusion practices	3	singular
Transients and Migrants	transients and migrants	transients and migrants	transients and migrants	Transients and Migrants	3	kept

Transition shock	transition shock	transition shock	transition shock	Transition shock	3	kept
transition to home	transition to home	transition to home	transition to home	transition to home	3	kept
transitions	transitions	transitions	transition	Transition	3	singular
transitions in care	transitions in care	transitions in care	transitions in care	transitions in care	3	kept
Translational research	translational research	translational research	translational research	translational research	3	case_or_format
Translational Science, Biomedical	translational science biomedical	translational science biomedical	translational science biomedical	Translational Science, Biomedical	3	kept
Transmissions	transmissions	transmissions	transmission	transmission	3	singular
Transplant Donor Site	transplant donor site	transplant donor site	transplant donor site	Transplant Donor Site	3	kept
Transplantation, Homologous	transplantation homologous	transplantation homologous	transplantation homologous	Transplantation, Homologous	3	kept
trastuzumab	trastuzumab	trastuzumab	trastuzumab	trastuzumab	3	kept
Tratamiento	tratamiento	tratamiento	tratamiento	Tratamiento	3	kept
Trauma center	trauma center	trauma center	trauma center	Trauma Centers	3	case_or_format
trauma center	trauma center	trauma center	trauma center	Trauma Centers	3	case_or_format
Trauma education	trauma education	trauma education	trauma education	Trauma education	3	kept
Trauma management	trauma management	trauma management	trauma management	Trauma management	3	kept
trauma management	trauma management	trauma management	trauma management	Trauma management	3	case_or_format

Trauma surgery	trauma surgery	trauma surgery	trauma surgery	trauma surgery	3	case_or_format
Traumatic	traumatic	traumatic	traumatic	traumatic	3	case_or_format
Traumatic Brain Injury	traumatic brain injury	traumatic brain injury	traumatic brain injury	traumatic brain injury	3	case_or_format
traumatic spinal cord injury	traumatic spinal cord injury	traumatic spinal cord injury	traumatic spinal cord injury	traumatic spinal cord injury	3	kept
Treatment interference	treatment interference	treatment interference	treatment interference	Treatment interference	3	kept
Treatment limitation	treatment limitation	treatment limitation	treatment limitation	Treatment limitation	3	kept
treatment response time	treatment response time	treatment response time	treatment response time	treatment response time	3	kept
Treatment withdrawal	treatment withdrawal	treatment withdrawal	treatment withdrawal	treatment withdrawal	3	case_or_format
Trees	trees	trees	tree	Trees	3	singular
Tremor	tremor	tremor	tremor	tremor	3	case_or_format
Treponema pallidum	treponema pallidum	treponema pallidum	treponema pallidum	Treponema pallidum	3	kept
triacylglycerol lipase	triacylglycerol lipase	triacylglycerol lipase	triacylglycerol lipase	triacylglycerol lipase	3	kept
Triazines	triazines	triazines	triazines	Triazines	3	kept
Triiodothyronine	triiodothyronine	triiodothyronine	triiodothyronine	Triiodothyronine	3	kept
triplets	triplets	triplets	triplets	triplets	3	kept
tropical disease	tropical disease	tropical disease	tropical disease	tropical disease	3	kept

Troponin	troponin	troponin	troponin	troponin	3	case_or_format
Tuberculin skin test	tuberculin skin test	tuberculin skin test	tuberculin skin test	Tuberculin skin test	3	kept
Tubos Torácicos	tubos tor cicos	tubos tor cicos	tubos tor cicos	Tubos Torácicos	3	kept
tumor necrosis factor receptor 1	tumor necrosis factor receptor 1	tumor necrosis factor receptor 1	tumor necrosis factor receptor 1	tumor necrosis factor receptor 1	3	kept
Turk	turk	turk	turk	Turk	3	kept
Turning	turning	turning	turning	turning	3	case_or_format
two dimensional echocardiography	two dimensional echocardiography	two dimensional echocardiography	two dimensional echocardiography	two dimensional echocardiography	3	kept
type 2 diabetes	type 2 diabetes	type 2 diabetes	type 2 diabetes	type 2 diabetes	3	kept
UAE	uae	uae	uae	UAE	3	kept
UCI	uci	uci	uci	UCI	3	kept
ulcer healing	ulcer healing	ulcer healing	ulcer healing	ulcer healing	3	kept
Ultrasound-guided	ultrasound guided	ultrasound guided	ultrasound guided	Ultrasound-guided	3	kept
Umbilical Arteries	umbilical arteries	umbilical arteries	umbilical artery	umbilical artery	3	singular
umbilical artery	umbilical artery	umbilical artery	umbilical artery	umbilical artery	3	kept
umbilical artery catheter	umbilical artery catheter	umbilical artery catheter	umbilical artery catheter	umbilical artery catheter	3	kept
umbilical cord blood	umbilical cord blood	umbilical cord blood	umbilical cord blood	umbilical cord blood	3	kept

umbilical cord clamping	umbilical cord clamping	umbilical cord clamping	umbilical cord clamping	umbilical cord clamping	3	kept
umbilical hernia	umbilical hernia	umbilical hernia	umbilical hernia	umbilical hernia	3	kept
Underfeeding	underfeeding	underfeeding	underfeeding	Underfeeding	3	kept
undernutrition	undernutrition	undernutrition	undernutrition	undernutrition	3	kept
Unidades de Cuidado Intensivo Neonatal	unidades de cuidado intensivo neonatal	unidades de cuidado intensivo neonatal	unidades de cuidado intensivo neonatal	Unidades de Cuidado Intensivo Neonatal	3	kept
Unidades de Cuidado Intensivo Pediátrico	unidades de cuidado intensivo pedi trico	unidades de cuidado intensivo pedi trico	unidades de cuidado intensivo pedi trico	Unidades de Cuidado Intensivo Pediátrico	3	kept
Unidades de terapia intensiva	unidades de terapia intensiva	unidades de terapia intensiva	unidades de terapia intensiva	Unidades de Terapia Intensiva	3	case_or_format
Unidades de Terapia Intensiva Neonatal	unidades de terapia intensiva neonatal	unidades de terapia intensiva neonatal	unidades de terapia intensiva neonatal	Unidades de Terapia Intensiva Neonatal	3	kept
Unified Parkinson Disease Rating Scale	unified parkinson disease rating scale	unified parkinson disease rating scale	unified parkinson disease rating scale	Unified Parkinson Disease Rating Scale	3	kept
Universal health coverage	universal health coverage	universal health coverage	universal health coverage	Universal health coverage	3	kept
Universal precautions	universal precautions	universal precautions	universal precaution	Universal Precautions	3	singular
unmet medical need	unmet medical need	unmet medical need	unmet medical need	unmet medical need	3	kept
UNPLANNED ADMISSION	unplanned admission	unplanned admission	unplanned admission	UNPLANNED ADMISSION	3	kept
upper abdominal pain	upper abdominal pain	upper abdominal pain	upper abdominal pain	upper abdominal pain	3	kept
Upper Gastrointestinal Tract	upper gastrointestinal tract	upper gastrointestinal tract	upper gastrointestinal tract	Upper Gastrointestinal Tract	3	kept
urapidil	urapidil	urapidil	urapidil	urapidil	3	kept

urban rural difference	urban rural difference	urban rural difference	urban rural difference	urban rural difference	3	kept
urbanization	urbanization	urbanization	urbanization	urbanization	3	kept
urethra	urethra	urethra	urethra	urethra	3	kept
Uric Acid	uric acid	uric acid	uric acid	uric acid	3	case_or_format
Urinary catheter	urinary catheter	urinary catheter	urinary catheter	urinary catheter	3	case_or_format
urinary tract	urinary tract	urinary tract	urinary tract	urinary tract	3	kept
urinary tract agent	urinary tract agent	urinary tract agent	urinary tract agent	urinary tract agent	3	kept
Urination	urination	urination	urination	Urination	3	kept
urogenital tract infection	urogenital tract infection	urogenital tract infection	urogenital tract infection	urogenital tract infection	3	kept
urokinase	urokinase	urokinase	urokinase	urokinase	3	kept
Urologic Surgical Procedures	urologic surgical procedures	urologic surgical procedures	urologic surgical procedures	Urologic Surgical Procedures	3	kept
ursodeoxycholic acid	ursodeoxycholic acid	ursodeoxycholic acid	ursodeoxycholic acid	ursodeoxycholic acid	3	kept
Urticaria	urticaria	urticaria	urticaria	urticaria	3	case_or_format
USABILITY	usability	usability	usability	usability	3	case_or_format
Usability testing	usability testing	usability testing	usability testing	usability testing	3	case_or_format
uterine artery embolization	uterine artery embolization	uterine artery embolization	uterine artery embolization	uterine artery embolization	3	kept

uterine cervix dilatation	uterine cervix dilatation	uterine cervix dilatation	uterine cervix dilatation	uterine cervix dilatation	3	kept
Utility	utility	utility	utility	Utility	3	kept
Uzbekistan	uzbekistan	uzbekistan	uzbekistan	Uzbekistan	3	kept
vaccination reaction	vaccination reaction	vaccination reaction	vaccination reaction	vaccination reaction	3	kept
vaccination refusal	vaccination refusal	vaccination refusal	vaccination refusal	vaccination refusal	3	kept
Vaccine hesitancy	vaccine hesitancy	vaccine hesitancy	vaccine hesitancy	vaccine hesitancy	3	case_or_format
Vaccines, Inactivated	vaccines inactivated	vaccines inactivated	vaccines inactivated	Vaccines, Inactivated	3	kept
Vacuum Extraction, Obstetrical	vacuum extraction obstetrical	vacuum extraction obstetrical	vacuum extraction obstetrical	Vacuum Extraction, Obstetrical	3	kept
Vacuum-assisted closure	vacuum assisted closure	vacuum assisted closure	vacuum assisted closure	vacuum assisted closure	3	case_or_format
Vagina	vagina	vagina	vagina	Vagina	3	kept
vagina	vagina	vagina	vagina	Vagina	3	case_or_format
vagus nerve stimulation	vagus nerve stimulation	vagus nerve stimulation	vagus nerve stimulation	vagus nerve stimulation	3	kept
valaciclovir	valaciclovir	valaciclovir	valaciclovir	valaciclovir	3	kept
valproate semisodium	valproate semisodium	valproate semisodium	valproate semisodium	valproate semisodium	3	kept
vancomycin resistance	vancomycin resistance	vancomycin resistance	vancomycin resistance	Vancomycin Resistance	3	case_or_format
VANCOMYCIN-RESISTANT ENTEROCOCCI	vancomycin resistant enterococci	vancomycin resistant enterococci	vancomycin resistant enterococci	Vancomycin-Resistant Enterococci	3	case_or_format

Vancomycin-resistant enterococci	vancomycin resistant enterococci	vancomycin resistant enterococci	vancomycin resistant enterococci	Vancomycin-Resistant Enterococci	3	case_or_format
Varicella	varicella	varicella	varicella	Varicella	3	kept
varimax rotation	varimax rotation	varimax rotation	varimax rotation	varimax rotation	3	kept
VASCULAR ACCESS	vascular access	vascular access	vascular access	vascular access	3	case_or_format
Vascular catheter	vascular catheter	vascular catheter	vascular catheter	Vascular catheter	3	kept
Vascular Malformations	vascular malformations	vascular malformations	vascular malformations	Vascular Malformations	3	kept
Vasospasm	vasospasm	vasospasm	vasospasm	vasospasm	3	case_or_format
vein embolism	vein embolism	vein embolism	vein embolism	vein embolism	3	kept
Vena Cava, Inferior	vena cava inferior	vena cava inferior	vena cava inferior	Vena Cava, Inferior	3	kept
venom antiserum	venom antiserum	venom antiserum	venom antiserum	venom antiserum	3	kept
Venous access	venous access	venous access	venous access	venous access	3	case_or_format
Venous air embolism	venous air embolism	venous air embolism	venous air embolism	Venous air embolism	3	kept
venous congestion	venous congestion	venous congestion	venous congestion	venous congestion	3	kept
venous oxygen tension	venous oxygen tension	venous oxygen tension	venous oxygen tension	venous oxygen tension	3	kept
venous pressure	venous pressure	venous pressure	venous pressure	venous pressure	3	kept
venous stasis	venous stasis	venous stasis	venous stasis	venous stasis	3	kept

venovenous extracorporeal membrane oxygenation	venovenous extracorporeal membrane oxygenation	venovenous extracorporeal membrane oxygenation	venovenous extracorporeal membrane oxygenation	venovenous extracorporeal membrane oxygenation	3	kept
Ventilated	ventilated	ventilated	ventilated	Ventilated	3	kept
Ventilator associated	ventilator associated	ventilator associated	ventilator associated	Ventilator-associated	3	case_or_format
Ventilator auto-cycling	ventilator auto cycling	ventilator auto cycling	ventilator auto cycling	Ventilator auto-cycling	3	kept
ventilator bundle	ventilator bundle	ventilator bundle	ventilator bundle	Ventilator bundle	3	case_or_format
Ventilator management	ventilator management	ventilator management	ventilator management	Ventilator management	3	kept
Ventilators, mechanical	ventilators mechanical	ventilators mechanical	ventilators mechanical	Ventilators, Mechanical	3	case_or_format
Vermont	vermont	vermont	vermont	Vermont	3	kept
Vertebral Artery	vertebral artery	vertebral artery	vertebral artery	Vertebral Artery	3	kept
vertebral artery	vertebral artery	vertebral artery	vertebral artery	Vertebral Artery	3	case_or_format
Vertebral Artery Dissection	vertebral artery dissection	vertebral artery dissection	vertebral artery dissection	Vertebral Artery Dissection	3	kept
very low birth-weight infant	very low birth weight infant	very low birth weight infant	very low birth weight infant	Very low birth weight infant	3	case_or_format
Very low birthweight	very low birthweight	very low birthweight	very low birthweight	Very low birthweight	3	kept
very premature birth	very premature birth	very premature birth	very premature birth	very premature birth	3	kept
Veterans Health Services	veterans health services	veterans health services	veterans health service	veterans health service	3	singular
veterinary emergency	veterinary emergency	veterinary emergency	veterinary emergency	veterinary emergency	3	kept

vicarious trauma	vicarious trauma	vicarious trauma	vicarious trauma	vicarious trauma	3	kept
video call	video call	video call	video call	video call	3	kept
Video recording	video recording	video recording	video recording	Video Recording	3	case_or_format
video surveillance	video surveillance	video surveillance	video surveillance	video surveillance	3	kept
Vietnamese	vietnamese	vietnamese	vietnamese	Vietnamese	3	kept
VIEWS	views	views	views	VIEWS	3	kept
Viral Vaccines	viral vaccines	viral vaccines	viral vaccines	Viral Vaccines	3	kept
viremia	viremia	viremia	viremia	viremia	3	kept
virtual care	virtual care	virtual care	virtual care	virtual care	3	kept
virtual reality simulation	virtual reality simulation	virtual reality simulation	virtual reality simulation	virtual reality simulation	3	kept
virtual training	virtual training	virtual training	virtual training	virtual training	3	kept
virtual visits	virtual visits	virtual visits	virtual visits	virtual visits	3	kept
Virulence Factors	virulence factors	virulence factors	virulence factor	virulence factor	3	singular
virus hepatitis	virus hepatitis	virus hepatitis	virus hepatitis	virus hepatitis	3	kept
virus isolation	virus isolation	virus isolation	virus isolation	virus isolation	3	kept
virus meningitis	virus meningitis	virus meningitis	virus meningitis	virus meningitis	3	kept

virus nucleocapsid	virus nucleocapsid	virus nucleocapsid	virus nucleocapsid	virus nucleocapsid	3	kept
virus viability	virus viability	virus viability	virus viability	virus viability	3	kept
Viruses	viruses	viruses	viruses	Viruses	3	kept
Vision	vision	vision	vision	vision	3	case_or_format
Visit	visit	visit	visit	VISITS	3	case_or_format
Visiting hours	visiting hours	visiting hours	visiting hour	Visiting hours	3	singular
Visiting policies	visiting policies	visiting policies	visiting policy	Visiting policy	3	singular
visiting policy	visiting policy	visiting policy	visiting policy	Visiting policy	3	case_or_format
visitors	visitors	visitors	visitors	visitors	3	kept
Visual analog scale	visual analog scale	visual analog scale	visual analog scale	visual analog scale	3	case_or_format
vital capacity	vital capacity	vital capacity	vital capacity	vital capacity	3	kept
vitality	vitality	vitality	vitality	vitality	3	kept
Vitamin A	vitamin a	vitamin a	vitamin a	Vitamin A	3	kept
vitamin blood level	vitamin blood level	vitamin blood level	vitamin blood level	vitamin blood level	3	kept
Vitamin K	vitamin k	vitamin k	vitamin k	Vitamin K	3	kept
VLBW	vlbw	vlbw	vlbw	VLBW	3	kept

vocational rehabilitation	vocational rehabilitation	vocational rehabilitation	vocational rehabilitation	vocational rehabilitation	3	kept
Volume status	volume status	volume status	volume status	Volume status	3	kept
Voluntary Health Agencies	voluntary health agencies	voluntary health agencies	voluntary health agencies	Voluntary Health Agencies	3	kept
vulnerable populations	vulnerable populations	vulnerable populations	vulnerable population	vulnerable population	3	singular
Waist Circumference	waist circumference	waist circumference	waist circumference	Waist Circumference	3	kept
waist circumference	waist circumference	waist circumference	waist circumference	Waist Circumference	3	case_or_format
ward round	ward round	ward round	ward round	Ward rounds	3	case_or_format
WARDS	wards	wards	ward	ward	3	singular
warfare	warfare	warfare	warfare	Warfare	3	case_or_format
Waste Management	waste management	waste management	waste management	waste management	3	case_or_format
Waste management	waste management	waste management	waste management	waste management	3	case_or_format
Waterlow scale	waterlow scale	waterlow scale	waterlow scale	Waterlow scale	3	kept
Watson's Theory of Caring	watson s theory of caring	watson s theory of caring	watson s theory of caring	Watson's Theory of Caring	3	kept
Weakness	weakness	weakness	weakness	weakness	3	case_or_format
Wearable alarm system	wearable alarm system	wearable alarm system	wearable alarm system	Wearable alarm system	3	kept
Wearable devices	wearable devices	wearable devices	wearable device	Wearable devices	3	singular

Wearable sensors	wearable sensors	wearable sensors	wearable sensor	Wearable sensors	3	singular
Web camera	web camera	web camera	web camera	Web camera	3	kept
Wechsler adult intelligence scale	wechsler adult intelligence scale	wechsler adult intelligence scale	wechsler adult intelligence scale	Wechsler adult intelligence scale	3	kept
weekend effect	weekend effect	weekend effect	weekend effect	weekend effect	3	kept
Weight	weight	weight	weight	Weight	3	kept
well‑being	well being	well being	well being	well-being	3	case_or_format
West Nile flavivirus	west nile flavivirus	west nile flavivirus	west nile flavivirus	West Nile flavivirus	3	kept
Western Hemisphere	western hemisphere	western hemisphere	western hemisphere	Western Hemisphere	3	kept
Whistleblowing	whistleblowing	whistleblowing	whistleblowing	whistleblowing	3	case_or_format
Whole-genome sequencing	whole genome sequencing	whole genome sequencing	whole genome sequencing	whole genome sequencing	3	case_or_format
widow	widow	widow	widow	widow	3	kept
wildlife	wildlife	wildlife	wildlife	wildlife	3	kept
willingness to work	willingness to work	willingness to work	willingness to work	willingness to work	3	kept
Wireless sensor	wireless sensor	wireless sensor	wireless sensor	Wireless sensor	3	kept
Wireless technologies	wireless technologies	wireless technologies	wireless technology	Wireless Technology	3	singular
Withdrawing	withdrawing	withdrawing	withdrawing	Withdrawing	3	kept

withholding	withholding	withholding	withholding	Withholding	3	case_or_format
WITNESSED RESUSCITATION	witnessed resuscitation	witnessed resuscitation	witnessed resuscitation	WITNESSED RESUSCITATION	3	kept
Wolff-Parkinson-White Syndrome	wolff parkinson white syndrome	wolff parkinson white syndrome	wolff parkinson white syndrome	Wolff-Parkinson-White Syndrome	3	kept
Women’ s health	women s health	women s health	women s health	women's health	3	case_or_format
women’ s health	women s health	women s health	women s health	women's health	3	case_or_format
work alienation	work alienation	work alienation	work alienation	work alienation	3	kept
Work efficiency	work efficiency	work efficiency	work efficiency	Work efficiency	3	kept
Work Environment	work environment	work environment	work environment	work environment	3	case_or_format
Work environments	work environments	work environments	work environment	work environment	3	singular
WORK HOURS	work hours	work hours	work hour	Work hours	3	singular
work hours	work hours	work hours	work hour	Work hours	3	singular
Work motivation	work motivation	work motivation	work motivation	Work motivation	3	kept
Work organization	work organization	work organization	work organization	Work organization	3	kept
Work patterns	work patterns	work patterns	work patterns	Work patterns	3	kept
Work profile	work profile	work profile	work profile	Work profile	3	kept
work related stress	work related stress	work related stress	work related stress	Work-related stress	3	case_or_format

Work shift	work shift	work shift	work shift	Work shift	3	kept
Work-flows	work flows	work flows	work flow	Work-flows	3	singular
Workers’	workers	workers	worker	worker	3	singular
Workflow patterns	workflow patterns	workflow patterns	workflow patterns	Workflow patterns	3	kept
workforce diversity	workforce diversity	workforce diversity	workforce diversity	workforce diversity	3	kept
workforce planning	workforce planning	workforce planning	workforce planning	workforce planning	3	kept
Workforce shortage	workforce shortage	workforce shortage	workforce shortage	Workforce shortage	3	kept
Working experience	working experience	working experience	working experience	Working experience	3	kept
workman compensation	workman compensation	workman compensation	workman compensation	workman compensation	3	kept
workplace distress	workplace distress	workplace distress	workplace distress	workplace distress	3	kept
workplace incivility	workplace incivility	workplace incivility	workplace incivility	workplace incivility	3	kept
workplace safety	workplace safety	workplace safety	workplace safety	workplace safety	3	kept
WORKPLACE VIOLENCE	workplace violence	workplace violence	workplace violence	workplace violence	3	case_or_format
Worry	worry	worry	worry	Worry	3	kept
wound assessment	wound assessment	wound assessment	wound assessment	wound assessment	3	kept
wound management	wound management	wound management	wound management	Wound management	3	case_or_format

wrist	wrist	wrist	wrist	wrist	3	kept
X ray analysis	x ray analysis	x ray analysis	x ray analysis	X ray analysis	3	kept
Xenon	xenon	xenon	xenon	Xenon	3	kept
xenon	xenon	xenon	xenon	Xenon	3	case_or_format
Yakson	yakson	yakson	yakson	Yakson	3	kept
yawning	yawning	yawning	yawning	yawning	3	kept
Zika fever	zika fever	zika fever	zika fever	Zika fever	3	kept
Zinc Oxide	zinc oxide	zinc oxide	zinc oxide	zinc oxide	3	case_or_format
zinc sulfate	zinc sulfate	zinc sulfate	zinc sulfate	zinc sulfate	3	kept
zopiclone	zopiclone	zopiclone	zopiclone	zopiclone	3	kept
Zung Self Rating Depression Scale	zung self rating depression scale	zung self rating depression scale	zung self rating depression scale	Zung Self Rating Depression Scale	3	kept
éthique	thique	thique	thique	éthique	3	kept
%Alcons	alcons	alcons	alcons	%Alcons	2	kept
1-Propanol	1 propanol	1 propanol	1 propanol	1-Propanol	2	kept
12-h shifts	12 h shifts	12 h shifts	12 h shift	12-h shifts	2	singular
16S rRNA sequencing	16s rrna sequencing	16s rrna sequencing	16s rrna sequencing	16S rRNA sequencing	2	kept

18-MONTH FOLLOW-UP	18 month follow up	18 month follow up	18 month follow up	18-MONTH FOLLOW-UP	2	kept
2019 novel coronavirus infection	2019 novel coronavirus infection	2019 novel coronavirus infection	2019 novel coronavirus infection	2019 novel coronavirus infection	2	kept
25 hydroxyvitamin D	25 hydroxyvitamin d	25 hydroxyvitamin d	25 hydroxyvitamin d	25 hydroxyvitamin D	2	kept
3 hydroxybutyric acid	3 hydroxybutyric acid	3 hydroxybutyric acid	3 hydroxybutyric acid	3 hydroxybutyric acid	2	kept
3 SCORES	3 scores	3 scores	3 scores	3 SCORES	2	kept
3 Wishes Project	3 wishes project	3 wishes project	3 wishes project	3 Wishes Project	2	kept
3-Hydroxybutyric Acid	3 hydroxybutyric acid	3 hydroxybutyric acid	3 hydroxybutyric acid	3 hydroxybutyric acid	2	case_or_format
360° virtual reality	360 virtual reality	360 virtual reality	360 virtual reality	360° virtual reality	2	kept
3D-CAM	3d cam	3d cam	3d cam	3D-CAM	2	kept
4 hydroxybutyric acid	4 hydroxybutyric acid	4 hydroxybutyric acid	4 hydroxybutyric acid	4 hydroxybutyric acid	2	kept
5E Learning Cycle	5e learning cycle	5e learning cycle	5e learning cycle	5E Learning Cycle	2	kept
9 EQUIVALENTS	9 equivalents	9 equivalents	9 equivalents	9 EQUIVALENTS	2	kept
A-lines	a lines	a lines	a lines	A-lines	2	kept
ABA	aba	aba	aba	ABA	2	kept
abbreviated cognitive test for delirium	abbreviated cognitive test for delirium	abbreviated cognitive test for delirium	abbreviated cognitive test for delirium	abbreviated cognitive test for delirium	2	kept
Abbreviated injury scale	abbreviated injury scale	abbreviated injury scale	abbreviated injury scale	Abbreviated Injury Scale	2	case_or_format

ABCDEF care bundle	abcdef care bundle	abcdef care bundle	abcdef care bundle	ABCDEF care bundle	2	kept
abciximab	abciximab	abciximab	abciximab	abciximab	2	kept
Abdominal aortic aneurysm	abdominal aortic aneurysm	abdominal aortic aneurysm	abdominal aortic aneurysm	abdominal aortic aneurysm	2	case_or_format
abdominal bleeding	abdominal bleeding	abdominal bleeding	abdominal bleeding	abdominal bleeding	2	kept
abdominal blunt trauma	abdominal blunt trauma	abdominal blunt trauma	abdominal blunt trauma	abdominal blunt trauma	2	kept
abdominal circumference	abdominal circumference	abdominal circumference	abdominal circumference	abdominal circumference	2	kept
Abdominal massage	abdominal massage	abdominal massage	abdominal massage	Abdominal massage	2	kept
Abdominal Muscles	abdominal muscles	abdominal muscles	abdominal muscles	Abdominal Muscles	2	kept
Abdominal pain	abdominal pain	abdominal pain	abdominal pain	abdominal pain	2	case_or_format
Abdominal pain management	abdominal pain management	abdominal pain management	abdominal pain management	Abdominal pain management	2	kept
abdominal wall	abdominal wall	abdominal wall	abdominal wall	Abdominal Wall	2	case_or_format
abdominal wound closure	abdominal wound closure	abdominal wound closure	abdominal wound closure	abdominal wound closure	2	kept
ABILITY	ability	ability	ability	ABILITY	2	kept
abnormal value	abnormal value	abnormal value	abnormal value	abnormal value	2	kept
Aborigine	aborigine	aborigine	aborigine	Aborigine	2	kept
Abortion, Illegal	abortion illegal	abortion illegal	abortion illegal	Abortion, Illegal	2	kept

absence of side effects	absence of side effects	absence of side effects	absence of side effects	absence of side effects	2	kept
abstinence	abstinence	abstinence	abstinence	abstinence	2	kept
Abuse	abuse	abuse	abuse	abuse	2	case_or_format
abusive head trauma	abusive head trauma	abusive head trauma	abusive head trauma	Abusive head trauma	2	case_or_format
Academic medical center	academic medical center	academic medical center	academic medical center	Academic Medical Centers	2	case_or_format
academic medical centers	academic medical centers	academic medical centers	academic medical center	Academic Medical Centers	2	singular
ACADEMIC MEDICAL-CENTERS	academic medical centers	academic medical centers	academic medical center	Academic Medical Centers	2	singular
academic research	academic research	academic research	academic research	academic research	2	kept
Academic success	academic success	academic success	academic success	Academic success	2	kept
academic success	academic success	academic success	academic success	Academic success	2	case_or_format
Acanthaceae	acanthaceae	acanthaceae	acanthaceae	Acanthaceae	2	kept
acarbose	acarbose	acarbose	acarbose	acarbose	2	kept
acceleration	acceleration	acceleration	acceleration	acceleration	2	kept
Accelerometer	accelerometer	accelerometer	accelerometer	Accelerometer	2	kept
accelerometers	accelerometers	accelerometers	accelerometer	Accelerometer	2	singular
acceptance and commitment therapy	acceptance and commitment therapy	acceptance and commitment therapy	acceptance and commitment therapy	acceptance and commitment therapy	2	kept

Access	access	access	access	access	2	case_or_format
Access to health care	access to health care	access to health care	access to health care	Access to health care	2	kept
Access to information	access to information	access to information	access to information	access to information	2	case_or_format
Accessibility	accessibility	accessibility	accessibility	Accessibility	2	kept
ACCIDENT	accident	accident	accident	accident	2	case_or_format
Accident and emergency	accident and emergency	accident and emergency	accident and emergency	Accident and emergency	2	kept
accidental death	accidental death	accidental death	accidental death	accidental death	2	kept
Accidental falls	accidental falls	accidental falls	accidental falls	Accidental Falls	2	case_or_format
accidental falls	accidental falls	accidental falls	accidental falls	Accidental Falls	2	case_or_format
Accidents, Aviation	accidents aviation	accidents aviation	accidents aviation	Accidents, Aviation	2	kept
accommodation	accommodation	accommodation	accommodation	accommodation	2	kept
Acculturation	acculturation	acculturation	acculturation	Acculturation	2	kept
acetabulum fracture	acetabulum fracture	acetabulum fracture	acetabulum fracture	acetabulum fracture	2	kept
acetic acid plus gluconate sodium plus magnesium chloride	acetic acid plus gluconate sodium plus magnesium chloride	acetic acid plus gluconate sodium plus magnesium chloride	acetic acid plus gluconate sodium plus magnesium chloride	acetic acid plus gluconate sodium plus magnesium chloride	2	kept
acetylcholine	acetylcholine	acetylcholine	acetylcholine	acetylcholine	2	kept
Acetylcholinesterase	acetylcholinesterase	acetylcholinesterase	acetylcholinesterase	Acetylcholinesterase	2	kept

Acetylcysteine	acetylcysteine	acetylcysteine	acetylcysteine	acetylcysteine	2	case_or_format
Achievement	achievement	achievement	achievement	achievement	2	case_or_format
Achievement motivation	achievement motivation	achievement motivation	achievement motivation	Achievement motivation	2	kept
acid fast bacterium	acid fast bacterium	acid fast bacterium	acid fast bacterium	acid fast bacterium	2	kept
Acinetobacter baumannii complex	acinetobacter baumannii complex	acinetobacter baumannii complex	acinetobacter baumannii complex	Acinetobacter baumannii complex	2	kept
Acinetobacter lwoffii	acinetobacter lwoffii	acinetobacter lwoffii	acinetobacter lwoffii	Acinetobacter lwoffii	2	kept
ACLS	acls	acls	acls	ACLS	2	kept
Acoustic alarms	acoustic alarms	acoustic alarms	acoustic alarms	Acoustic alarms	2	kept
Acoustic problems	acoustic problems	acoustic problems	acoustic problems	Acoustic problems	2	kept
acoustic stress	acoustic stress	acoustic stress	acoustic stress	acoustic stress	2	kept
Acquired weakness	acquired weakness	acquired weakness	acquired weakness	Acquired weakness	2	kept
acquisition	acquisition	acquisition	acquisition	ACQUISITION	2	case_or_format
Acrylic Resins	acrylic resins	acrylic resins	acrylic resins	Acrylic Resins	2	kept
Action learning	action learning	action learning	action learning	Action learning	2	kept
Active learning	active learning	active learning	active learning	Active learning	2	kept
active learning approach	active learning approach	active learning approach	active learning approach	active learning approach	2	kept

active mobilization	active mobilization	active mobilization	active mobilization	active mobilization	2	kept
ACTIVE SURVEILLANCE	active surveillance	active surveillance	active surveillance	active surveillance	2	case_or_format
Active surveillance	active surveillance	active surveillance	active surveillance	active surveillance	2	case_or_format
ACTIVE SURVEILLANCE CULTURES	active surveillance cultures	active surveillance cultures	active surveillance cultures	ACTIVE SURVEILLANCE CULTURES	2	kept
ACTIVITIES SCORE	activities score	activities score	activities score	ACTIVITIES SCORE	2	kept
ACTIVITIES SCORE NAS	activities score nas	activities score nas	activities score nas	ACTIVITIES SCORE NAS	2	kept
activity and mobility	activity and mobility	activity and mobility	activity and mobility	activity and mobility	2	kept
activity of daily living	activity of daily living	activity of daily living	activity of daily living	activity of daily living	2	kept
activity of daily living assessment	activity of daily living assessment	activity of daily living assessment	activity of daily living assessment	activity of daily living assessment	2	kept
Activity recognition	activity recognition	activity recognition	activity recognition	Activity recognition	2	kept
actual body weight	actual body weight	actual body weight	actual body weight	actual body weight	2	kept
Acuity assessment	acuity assessment	acuity assessment	acuity assessment	Acuity assessment	2	kept
Acuity score	acuity score	acuity score	acuity score	Acuity score	2	kept
ACUPRESSURE	acupressure	acupressure	acupressure	Acupressure	2	case_or_format
acute abdominal pain	acute abdominal pain	acute abdominal pain	acute abdominal pain	acute abdominal pain	2	kept
Acute cardiovascular care	acute cardiovascular care	acute cardiovascular care	acute cardiovascular care	Acute cardiovascular care	2	kept

Acute care hospital	acute care hospital	acute care hospital	acute care hospital	acute care hospital	2	case_or_format
acute care NP	acute care np	acute care np	acute care np	acute care NP	2	kept
Acute care setting	acute care setting	acute care setting	acute care setting	acute care setting	2	case_or_format
Acute care units	acute care units	acute care units	acute care units	Acute care units	2	kept
acute care units	acute care units	acute care units	acute care units	Acute care units	2	case_or_format
Acute confusion	acute confusion	acute confusion	acute confusion	acute confusion	2	case_or_format
Acute confusional state	acute confusional state	acute confusional state	acute confusional state	Acute confusional state	2	kept
acute confusional state	acute confusional state	acute confusional state	acute confusional state	Acute confusional state	2	case_or_format
Acute decompensated heart failure	acute decompensated heart failure	acute decompensated heart failure	acute decompensated heart failure	Acute decompensated heart failure	2	kept
acute diarrhea	acute diarrhea	acute diarrhea	acute diarrhea	acute diarrhea	2	kept
acute disease management	acute disease management	acute disease management	acute disease management	acute disease management	2	kept
acute disseminated encephalomyelitis	acute disseminated encephalomyelitis	acute disseminated encephalomyelitis	acute disseminated encephalomyelitis	acute disseminated encephalomyelitis	2	kept
acute encephalopathy	acute encephalopathy	acute encephalopathy	acute encephalopathy	acute encephalopathy	2	kept
acute exacerbation of chronic obstructive pulmonary disease	acute exacerbation of chronic obstructive pulmonary disease	acute exacerbation of chronic obstructive pulmonary disease	acute exacerbation of chronic obstructive pulmonary disease	Acute exacerbation of chronic obstructive pulmonary disease	2	case_or_format
Acute exacerbations	acute exacerbations	acute exacerbations	acute exacerbations	Acute exacerbations	2	kept
acute fatigue	acute fatigue	acute fatigue	acute fatigue	acute fatigue	2	kept

acute fatty liver of pregnancy	acute fatty liver of pregnancy	acute fatty liver of pregnancy	acute fatty liver of pregnancy	acute fatty liver of pregnancy	2	kept
Acute fulminant myocarditis	acute fulminant myocarditis	acute fulminant myocarditis	acute fulminant myocarditis	Acute fulminant myocarditis	2	kept
acute generalized exanthematous pustulosis	acute generalized exanthematous pustulosis	acute generalized exanthematous pustulosis	acute generalized exanthematous pustulosis	acute generalized exanthematous pustulosis	2	kept
acute hepatitis	acute hepatitis	acute hepatitis	acute hepatitis	acute hepatitis	2	kept
Acute hospital	acute hospital	acute hospital	acute hospital	Acute hospitals	2	case_or_format
Acute hospital setting	acute hospital setting	acute hospital setting	acute hospital setting	Acute hospital setting	2	kept
Acute kidney disease	acute kidney disease	acute kidney disease	acute kidney disease	Acute kidney disease	2	kept
Acute limb ischemia	acute limb ischemia	acute limb ischemia	acute limb ischemia	Acute limb ischemia	2	kept
acute lymphocytic leukemia	acute lymphocytic leukemia	acute lymphocytic leukemia	acute lymphocytic leukemia	acute lymphocytic leukemia	2	kept
Acute Mania	acute mania	acute mania	acute mania	Acute Mania	2	kept
acute medicine	acute medicine	acute medicine	acute medicine	Acute medicine	2	case_or_format
Acute Myocardial Infarction	acute myocardial infarction	acute myocardial infarction	acute myocardial infarction	acute myocardial infarction	2	case_or_format
acute phase response	acute phase response	acute phase response	acute phase response	acute phase response	2	kept
acute phosphate nephropathy	acute phosphate nephropathy	acute phosphate nephropathy	acute phosphate nephropathy	acute phosphate nephropathy	2	kept
Acute Physiology and Chronic Health evaluation	acute physiology and chronic health evaluation	acute physiology and chronic health evaluation	acute physiology and chronic health evaluation	Acute Physiology and Chronic Health evaluation	2	kept
acute poisoning management	acute poisoning management	acute poisoning management	acute poisoning management	acute poisoning management	2	kept

Acute Respiratory Failure	acute respiratory failure	acute respiratory failure	acute respiratory failure	acute respiratory failure	2	case_or_format
Acute Respiratory failure	acute respiratory failure	acute respiratory failure	acute respiratory failure	acute respiratory failure	2	case_or_format
acute setting	acute setting	acute setting	acute setting	acute setting	2	kept
Acute stress	acute stress	acute stress	acute stress	acute stress	2	case_or_format
acute stroke	acute stroke	acute stroke	acute stroke	ACUTE STROKE	2	case_or_format
Acute tubular necrosis	acute tubular necrosis	acute tubular necrosis	acute tubular necrosis	Acute tubular necrosis	2	kept
Acute-care hospital	acute care hospital	acute care hospital	acute care hospital	acute care hospital	2	case_or_format
acute-on-chronic liver failure	acute on chronic liver failure	acute on chronic liver failure	acute on chronic liver failure	acute on chronic liver failure	2	case_or_format
Acyclovir	acyclovir	acyclovir	acyclovir	Acyclovir	2	kept
adaptation of an instrument	adaptation of an instrument	adaptation of an instrument	adaptation of an instrument	adaptation of an instrument	2	kept
Adaptive algorithms	adaptive algorithms	adaptive algorithms	adaptive algorithm	Adaptive algorithms	2	singular
adaptive capacity	adaptive capacity	adaptive capacity	adaptive capacity	adaptive capacity	2	kept
Adaptor Proteins, Signal Transducing	adaptor proteins signal transducing	adaptor proteins signal transducing	adaptor proteins signal transducing	Adaptor Proteins, Signal Transducing	2	kept
Adenoma	adenoma	adenoma	adenoma	Adenoma	2	kept
Adenosine triphosphate	adenosine triphosphate	adenosine triphosphate	adenosine triphosphate	adenosine triphosphate	2	case_or_format
Adherence rate	adherence rate	adherence rate	adherence rate	Adherence rate	2	kept

adhesion	adhesion	adhesion	adhesion	adhesion	2	kept
adhesive	adhesive	adhesive	adhesive	Adhesives	2	case_or_format
adhesives	adhesives	adhesives	adhesive	Adhesives	2	singular
Adiposity	adiposity	adiposity	adiposity	Adiposity	2	kept
Adjustment	adjustment	adjustment	adjustment	Adjustment	2	kept
adjustment	adjustment	adjustment	adjustment	Adjustment	2	case_or_format
adjustment disorder	adjustment disorder	adjustment disorder	adjustment disorder	Adjustment Disorders	2	case_or_format
ADMINISTERED PROPOFOL SEDATION	administered propofol sedation	administered propofol sedation	administered propofol sedation	ADMINISTERED PROPOFOL SEDATION	2	kept
Administration and dosage	administration and dosage	administration and dosage	administration and dosage	administration and dosage	2	case_or_format
administration error	administration error	administration error	administration error	ADMINISTRATION ERRORS	2	case_or_format
Administrative data	administrative data	administrative data	administrative data	ADMINISTRATIVE DATA	2	case_or_format
administrative databases	administrative databases	administrative databases	administrative database	administrative database	2	singular
Administrative models	administrative models	administrative models	administrative models	Administrative models	2	kept
Administrative staff	administrative staff	administrative staff	administrative staff	Administrative staff	2	kept
admissions	admissions	admissions	admission	ADMISSION	2	singular
Admitting Department, Hospital	admitting department hospital	admitting department hospital	admitting department hospital	Admitting Department, Hospital	2	kept

adolescent health	adolescent health	adolescent health	adolescent health	adolescent health	2	kept
Adolescent Health Services	adolescent health services	adolescent health services	adolescent health services	Adolescent Health Services	2	kept
adolescent mother	adolescent mother	adolescent mother	adolescent mother	adolescent mother	2	kept
adolescent pregnancy	adolescent pregnancy	adolescent pregnancy	adolescent pregnancy	adolescent pregnancy	2	kept
adrenal gland	adrenal gland	adrenal gland	adrenal gland	adrenal gland	2	kept
adrenal insufficiency	adrenal insufficiency	adrenal insufficiency	adrenal insufficiency	Adrenal Insufficiency	2	case_or_format
Adrenergic Agonists	adrenergic agonists	adrenergic agonists	adrenergic agonists	Adrenergic Agonists	2	kept
Adrenergic alpha-Antagonists	adrenergic alpha antagonists	adrenergic alpha antagonists	adrenergic alpha antagonists	Adrenergic alpha-Antagonists	2	kept
adrenergic receptor	adrenergic receptor	adrenergic receptor	adrenergic receptor	adrenergic receptor	2	kept
adrenergic stimulation	adrenergic stimulation	adrenergic stimulation	adrenergic stimulation	adrenergic stimulation	2	kept
Adrenocorticotropic Hormone	adrenocorticotropic hormone	adrenocorticotropic hormone	adrenocorticotropic hormone	Adrenocorticotropic Hormone	2	kept
Adsorption	adsorption	adsorption	adsorption	Adsorption	2	kept
Adult anaesthesia	adult anaesthesia	adult anaesthesia	adult anaesthesia	Adult anaesthesia	2	kept
adult child	adult child	adult child	adult child	adult child	2	kept
adult learner	adult learner	adult learner	adult learner	adult learner	2	kept
adult medicine	adult medicine	adult medicine	adult medicine	adult medicine	2	kept

adult psychiatry	adult psychiatry	adult psychiatry	adult psychiatry	adult psychiatry	2	kept
Adultos	adultos	adultos	adulto	Adultos	2	singular
advanced	advanced	advanced	advanced	advanced	2	kept
advanced age	advanced age	advanced age	advanced age	advanced age	2	kept
advanced lung cancer	advanced lung cancer	advanced lung cancer	advanced lung cancer	advanced lung cancer	2	kept
Advanced practice providers	advanced practice providers	advanced practice providers	advanced practice provider	advanced practice provider	2	singular
Advanced practitioner	advanced practitioner	advanced practitioner	advanced practitioner	Advanced practitioner	2	kept
Advanced providers	advanced providers	advanced providers	advanced providers	Advanced providers	2	kept
adverse discharge	adverse discharge	adverse discharge	adverse discharge	adverse discharge	2	kept
Adverse drug event	adverse drug event	adverse drug event	adverse drug event	ADVERSE DRUG EVENTS	2	case_or_format
Adverse drug reaction	adverse drug reaction	adverse drug reaction	adverse drug reaction	adverse drug reaction	2	case_or_format
Adverse effect	adverse effect	adverse effect	adverse effect	adverse effects	2	case_or_format
adverse effect	adverse effect	adverse effect	adverse effect	adverse effects	2	case_or_format
Adverse local tissue reactions	adverse local tissue reactions	adverse local tissue reactions	adverse local tissue reactions	Adverse local tissue reactions	2	kept
adverse neonatal outcome	adverse neonatal outcome	adverse neonatal outcome	adverse neonatal outcome	adverse neonatal outcome	2	kept
adversity quotient	adversity quotient	adversity quotient	adversity quotient	adversity quotient	2	kept

aerobic bacterium	aerobic bacterium	aerobic bacterium	aerobic bacterium	aerobic bacterium	2	kept
aeromedical	aeromedical	aeromedical	aeromedical	Aeromedical	2	case_or_format
Aerosol	aerosol	aerosol	aerosol	aerosol	2	case_or_format
Aerosol and surface transmission	aerosol and surface transmission	aerosol and surface transmission	aerosol and surface transmission	Aerosol and surface transmission	2	kept
Aerosol generating procedure	aerosol generating procedure	aerosol generating procedure	aerosol generating procedure	aerosol generating procedure	2	case_or_format
aerosols	aerosols	aerosols	aerosol	aerosol	2	singular
affective responses	affective responses	affective responses	affective responses	affective responses	2	kept
Afrontamiento	afrontamiento	afrontamiento	afrontamiento	Afrontamiento	2	kept
After-hours care	after hours care	after hours care	after hours care	After-Hours Care	2	case_or_format
afternoon shift	afternoon shift	afternoon shift	afternoon shift	afternoon shift	2	kept
age determination	age determination	age determination	age determination	age determination	2	kept
Age factors	age factors	age factors	age factors	Age Factors	2	case_or_format
age-appropriate care	age appropriate care	age appropriate care	age appropriate care	age-appropriate care	2	kept
Ageing population	ageing population	ageing population	ageing population	Ageing population	2	kept
agents affecting metabolism	agents affecting metabolism	agents affecting metabolism	agents affecting metabolism	agents affecting metabolism	2	kept
agglutination test	agglutination test	agglutination test	agglutination test	agglutination test	2	kept

Aggressive care	aggressive care	aggressive care	aggressive care	Aggressive care	2	kept
aggressive care	aggressive care	aggressive care	aggressive care	Aggressive care	2	case_or_format
Aggressiveness	aggressiveness	aggressiveness	aggressiveness	aggressiveness	2	case_or_format
Agile	agile	agile	agile	Agile	2	kept
Agitation and Sedation Scale	agitation and sedation scale	agitation and sedation scale	agitation and sedation scale	Agitation and Sedation Scale	2	kept
AGITATION SCALE	agitation scale	agitation scale	agitation scale	AGITATION SCALE	2	kept
Agonistic Behavior	agonistic behavior	agonistic behavior	agonistic behavior	Agonistic Behavior	2	kept
agranulocytosis	agranulocytosis	agranulocytosis	agranulocytosis	agranulocytosis	2	kept
AGREE II	agree ii	agree ii	agree ii	AGREE II	2	kept
agreement	agreement	agreement	agreement	AGREEMENT	2	case_or_format
agreement analysis	agreement analysis	agreement analysis	agreement analysis	agreement analysis	2	kept
agyria	agyria	agyria	agyria	agyria	2	kept
AI attitudes	ai attitudes	ai attitudes	ai attitudes	AI attitudes	2	kept
AI implementation	ai implementation	ai implementation	ai implementation	AI implementation	2	kept
AIDS	aids	aids	aid	AIDS	2	singular
Air ambulance	air ambulance	air ambulance	air ambulance	Air Ambulances	2	case_or_format

air filter	air filter	air filter	air filter	air filter	2	kept
air monitoring	air monitoring	air monitoring	air monitoring	air monitoring	2	kept
Air Movements	air movements	air movements	air movements	Air Movements	2	kept
Air Pressure	air pressure	air pressure	air pressure	Air Pressure	2	kept
air pressure	air pressure	air pressure	air pressure	Air Pressure	2	case_or_format
Air pressures	air pressures	air pressures	air pressure	Air Pressure	2	singular
Air transport	air transport	air transport	air transport	Air transport	2	kept
Airborne	airborne	airborne	airborne	Airborne	2	kept
airborne	airborne	airborne	airborne	Airborne	2	case_or_format
Airborne bacteria	airborne bacteria	airborne bacteria	airborne bacteria	Airborne bacteria	2	kept
AIRBORNE TRANSMISSION	airborne transmission	airborne transmission	airborne transmission	airborne transmission	2	case_or_format
Airplane	airplane	airplane	airplane	Airplane	2	kept
AIRWAY	airway	airway	airway	airway	2	case_or_format
Airway care	airway care	airway care	airway care	Airway care	2	kept
airway care interventions	airway care interventions	airway care interventions	airway care interventions	airway care interventions	2	kept
Airway clearance	airway clearance	airway clearance	airway clearance	Airway clearance	2	kept

Airway clearance techniques	airway clearance techniques	airway clearance techniques	airway clearance technique	Airway clearance techniques	2	singular
airway humidification	airway humidification	airway humidification	airway humidification	airway humidification	2	kept
AIRWAY MANAGEMENT	airway management	airway management	airway management	Airway Management	2	case_or_format
airway pressure release ventilation	airway pressure release ventilation	airway pressure release ventilation	airway pressure release ventilation	airway pressure release ventilation	2	kept
airway suction device	airway suction device	airway suction device	airway suction device	airway suction device	2	kept
AL amyloidosis	al amyloidosis	al amyloidosis	al amyloidosis	AL amyloidosis	2	kept
Alagille syndrome	alagille syndrome	alagille syndrome	alagille syndrome	Alagille syndrome	2	kept
alanine aminotransferase level	alanine aminotransferase level	alanine aminotransferase level	alanine aminotransferase level	alanine aminotransferase level	2	kept
ALARM FATIGUE	alarm fatigue	alarm fatigue	alarm fatigue	Alarm fatigue	2	case_or_format
Alarm Fatigue	alarm fatigue	alarm fatigue	alarm fatigue	Alarm fatigue	2	case_or_format
Alarm masking	alarm masking	alarm masking	alarm masking	Alarm masking	2	kept
alarm reduction	alarm reduction	alarm reduction	alarm reduction	alarm reduction	2	kept
Alarm response	alarm response	alarm response	alarm response	Alarm response	2	kept
alarm system	alarm system	alarm system	alarm system	Alarm systems	2	case_or_format
alarm system quality	alarm system quality	alarm system quality	alarm system quality	alarm system quality	2	kept
Alaska	alaska	alaska	alaska	Alaska	2	kept

Alaska Native	alaska native	alaska native	alaska native	Alaska Native	2	kept
Albuminuria	albuminuria	albuminuria	albuminuria	albuminuria	2	case_or_format
alcohol withdrawal seizure	alcohol withdrawal seizure	alcohol withdrawal seizure	alcohol withdrawal seizure	alcohol withdrawal seizure	2	kept
Alcohol Withdrawal Seizures	alcohol withdrawal seizures	alcohol withdrawal seizures	alcohol withdrawal seizure	alcohol withdrawal seizure	2	singular
alcohol-based hand rub	alcohol based hand rub	alcohol based hand rub	alcohol based hand rub	Alcohol-based hand rub	2	case_or_format
aldosterone	aldosterone	aldosterone	aldosterone	Aldosterone	2	case_or_format
Aldosterone Antagonists	aldosterone antagonists	aldosterone antagonists	aldosterone antagonist	aldosterone antagonist	2	singular
Alemtuzumab	alemtuzumab	alemtuzumab	alemtuzumab	alemtuzumab	2	case_or_format
alert fatigue health personnel	alert fatigue health personnel	alert fatigue health personnel	alert fatigue health personnel	Alert Fatigue, Health Personnel	2	case_or_format
alert systems	alert systems	alert systems	alert systems	alert systems	2	kept
Alertness	alertness	alertness	alertness	alertness	2	case_or_format
alexithymia	alexithymia	alexithymia	alexithymia	alexithymia	2	kept
ALGORITHM	algorithm	algorithm	algorithm	Algorithms	2	case_or_format
alienation	alienation	alienation	alienation	alienation	2	kept
Alkaline Phosphatase	alkaline phosphatase	alkaline phosphatase	alkaline phosphatase	alkaline phosphatase	2	case_or_format
Alkalosis, Respiratory	alkalosis respiratory	alkalosis respiratory	alkalosis respiratory	Alkalosis, Respiratory	2	kept

alkylating agent	alkylating agent	alkylating agent	alkylating agent	alkylating agent	2	kept
all cause readmission	all cause readmission	all cause readmission	all cause readmission	all cause readmission	2	kept
All-cause mortality	all cause mortality	all cause mortality	all cause mortality	all cause mortality	2	case_or_format
allele	allele	allele	allele	allele	2	kept
allergic asthma	allergic asthma	allergic asthma	allergic asthma	allergic asthma	2	kept
allergic disease	allergic disease	allergic disease	allergic disease	allergic disease	2	kept
allergic pneumonitis	allergic pneumonitis	allergic pneumonitis	allergic pneumonitis	allergic pneumonitis	2	kept
Allergy	allergy	allergy	allergy	allergy	2	case_or_format
allergy test	allergy test	allergy test	allergy test	allergy test	2	kept
Allied Health Occupations	allied health occupations	allied health occupations	allied health occupations	Allied Health Occupations	2	kept
allied health workers	allied health workers	allied health workers	allied health workers	allied health workers	2	kept
allocation of health care resources	allocation of health care resources	allocation of health care resources	allocation of health care resources	allocation of health care resources	2	kept
allocation of scarce resources	allocation of scarce resources	allocation of scarce resources	allocation of scarce resources	allocation of scarce resources	2	kept
allotransplantation	allotransplantation	allotransplantation	allotransplantation	allotransplantation	2	kept
Alloys	alloys	alloys	alloys	Alloys	2	kept
Aloe vera	aloe vera	aloe vera	aloe vera	Aloe vera	2	kept

Aloe vera extract	aloe vera extract	aloe vera extract	aloe vera extract	Aloe vera extract	2	kept
alpha 2 adrenergic receptor	alpha 2 adrenergic receptor	alpha 2 adrenergic receptor	alpha 2 adrenergic receptor	alpha 2 adrenergic receptor	2	kept
alpha adrenergic receptor blocking agent	alpha adrenergic receptor blocking agent	alpha adrenergic receptor blocking agent	alpha adrenergic receptor blocking agent	alpha adrenergic receptor blocking agent	2	kept
alpha fetoprotein	alpha fetoprotein	alpha fetoprotein	alpha fetoprotein	alpha fetoprotein	2	kept
Alteplase	alteplase	alteplase	alteplase	alteplase	2	case_or_format
Altered level of consciousness	altered level of consciousness	altered level of consciousness	altered level of consciousness	Altered level of consciousness	2	kept
Alternative medicine	alternative medicine	alternative medicine	alternative medicine	alternative medicine	2	case_or_format
Alternative resource	alternative resource	alternative resource	alternative resource	Alternative resource	2	kept
alternative therapies	alternative therapies	alternative therapies	alternative therapies	alternative therapies	2	kept
Alzheimer's disease and related dementias	alzheimer s disease and related dementias	alzheimer s disease and related dementias	alzheimer s disease and related dementia	Alzheimer's disease and related dementias	2	singular
ALZHEIMER-DISEASE	alzheimer disease	alzheimer disease	alzheimer disease	Alzheimer disease	2	case_or_format
Amantadine	amantadine	amantadine	amantadine	amantadine	2	case_or_format
AMBIGUITY	ambiguity	ambiguity	ambiguity	ambiguity	2	case_or_format
ambrisentan	ambrisentan	ambrisentan	ambrisentan	ambrisentan	2	kept
Ambroxol	ambroxol	ambroxol	ambroxol	ambroxol	2	case_or_format
Ambulance diversion	ambulance diversion	ambulance diversion	ambulance diversion	ambulance diversion	2	case_or_format

Ambulatory care	ambulatory care	ambulatory care	ambulatory care	Ambulatory Care	2	case_or_format
ambulatory disease management	ambulatory disease management	ambulatory disease management	ambulatory disease management	ambulatory disease management	2	kept
ambulatory monitoring	ambulatory monitoring	ambulatory monitoring	ambulatory monitoring	ambulatory monitoring	2	kept
amenorrhea	amenorrhea	amenorrhea	amenorrhea	amenorrhea	2	kept
American Burn Association	american burn association	american burn association	american burn association	American Burn Association	2	kept
american society for parenteral and enteral nutrition	american society for parenteral and enteral nutrition	american society for parenteral and enteral nutrition	american society for parenteral and enteral nutrition	american society for parenteral and enteral nutrition	2	kept
Amifostine	amifostine	amifostine	amifostine	Amifostine	2	kept
Amines	amines	amines	amine	Amines	2	singular
aminoglycoside derivative	aminoglycoside derivative	aminoglycoside derivative	aminoglycoside derivative	aminoglycoside derivative	2	kept
aminotransferase blood level	aminotransferase blood level	aminotransferase blood level	aminotransferase blood level	aminotransferase blood level	2	kept
amlodipine besylate	amlodipine besylate	amlodipine besylate	amlodipine besylate	amlodipine besylate	2	kept
ammonia	ammonia	ammonia	ammonia	ammonia	2	kept
ammonia blood level	ammonia blood level	ammonia blood level	ammonia blood level	ammonia blood level	2	kept
Amnestic	amnestic	amnestic	amnestic	Amnestic	2	kept
amniocentesis	amniocentesis	amniocentesis	amniocentesis	amniocentesis	2	kept
amnion	amnion	amnion	amnion	Amnion	2	case_or_format

amniotic fluid embolism	amniotic fluid embolism	amniotic fluid embolism	amniotic fluid embolism	amniotic fluid embolism	2	kept
amphetamine derivative	amphetamine derivative	amphetamine derivative	amphetamine derivative	amphetamine derivative	2	kept
Amphibia	amphibia	amphibia	amphibia	Amphibia	2	kept
amphotericin B deoxycholate	amphotericin b deoxycholate	amphotericin b deoxycholate	amphotericin b deoxycholate	amphotericin B deoxycholate	2	kept
Ampicillin	ampicillin	ampicillin	ampicillin	ampicillin	2	case_or_format
Amplitude-integrated EEG	amplitude integrated eeg	amplitude integrated eeg	amplitude integrated eeg	Amplitude-integrated EEG	2	kept
amplitude-integrated electroencephalography	amplitude integrated electroencephalography	amplitude integrated electroencephalography	amplitude integrated electroencephalography	amplitude integrated electroencephalography	2	case_or_format
Amputations	amputations	amputations	amputation	amputation	2	singular
Amputees	amputees	amputees	amputees	Amputees	2	kept
amrinone	amrinone	amrinone	amrinone	amrinone	2	kept
amylase	amylase	amylase	amylase	Amylases	2	case_or_format
Amyloidosis	amyloidosis	amyloidosis	amyloidosis	Amyloidosis	2	kept
AnaConDa	anaconda	anaconda	anaconda	AnaConDa	2	kept
anaerobe	anaerobe	anaerobe	anaerobe	anaerobe	2	kept
Anaesthesiologist	anaesthesiologist	anaesthesiologist	anaesthesiologist	Anaesthesiologist	2	kept
anaesthesiology	anaesthesiology	anaesthesiology	anaesthesiology	Anaesthesiology	2	case_or_format

anaesthetics	anaesthetics	anaesthetics	anaesthetics	ANAESTHETICS	2	case_or_format
Anafilaxia	anafilaxia	anafilaxia	anafilaxia	Anafilaxia	2	kept
Analgesic administration	analgesic administration	analgesic administration	analgesic administration	Analgesic administration	2	kept
Analgesic use	analgesic use	analgesic use	analgesic use	Analgesic use	2	kept
analgesics	analgesics	analgesics	analgesic	Analgesics	2	singular
analog digital converter	analog digital converter	analog digital converter	analog digital converter	analog digital converter	2	kept
Analysis method	analysis method	analysis method	analysis method	Analysis method	2	kept
analysis of influencing factor	analysis of influencing factor	analysis of influencing factor	analysis of influencing factor	analysis of influencing factor	2	kept
analytic autoethnography	analytic autoethnography	analytic autoethnography	analytic autoethnography	analytic autoethnography	2	kept
Analytic hierarchy process	analytic hierarchy process	analytic hierarchy process	analytic hierarchy process	Analytic hierarchy process	2	kept
analytic hierarchy process	analytic hierarchy process	analytic hierarchy process	analytic hierarchy process	Analytic hierarchy process	2	case_or_format
Analytical Approach	analytical approach	analytical approach	analytical approach	Analytical Approach	2	kept
Analytical Hierarchy Process	analytical hierarchy process	analytical hierarchy process	analytical hierarchy process	Analytical Hierarchy Process	2	kept
anaplasma	anaplasma	anaplasma	anaplasma	anaplasma	2	kept
Anaplasmosis	anaplasmosis	anaplasmosis	anaplasmosis	Anaplasmosis	2	kept
anasarca	anasarca	anasarca	anasarca	anasarca	2	kept

anatomical region	anatomical region	anatomical region	anatomical region	anatomical region	2	kept
anatomical variation	anatomical variation	anatomical variation	anatomical variation	anatomical variation	2	kept
anatomy	anatomy	anatomy	anatomy	anatomy	2	kept
Anciano	anciano	anciano	anciano	Anciano	2	kept
and early exercise	and early exercise	and early exercise	and early exercise	and early exercise	2	kept
and evaluation	and evaluation	and evaluation	and evaluation	and evaluation	2	kept
And practice	and practice	and practice	and practice	And practice	2	kept
And umbrella review	and umbrella review	and umbrella review	and umbrella review	And umbrella review	2	kept
and workplace violence	and workplace violence	and workplace violence	and workplace violence	and workplace violence	2	kept
Andexanet alfa	andexanet alfa	andexanet alfa	andexanet alfa	andexanet alfa	2	case_or_format
androstane derivative	androstane derivative	androstane derivative	androstane derivative	androstane derivative	2	kept
Anemia, Hemolytic	anemia hemolytic	anemia hemolytic	anemia hemolytic	Anemia, Hemolytic	2	kept
Anemia, Iron-Deficiency	anemia iron deficiency	anemia iron deficiency	anemia iron deficiency	Anemia, Iron-Deficiency	2	kept
Anemia, Pernicious	anemia pernicious	anemia pernicious	anemia pernicious	Anemia, Pernicious	2	kept
Anesthesia, Cardiac Procedures	anesthesia cardiac procedures	anesthesia cardiac procedures	anesthesia cardiac procedures	Anesthesia, Cardiac Procedures	2	kept
Anesthesia - general	anesthesia general	anesthesia general	anesthesia general	Anesthesia, General	2	case_or_format

Anesthesiologist	anesthesiologist	anesthesiologist	anesthesiologist	anesthesiologist	2	case_or_format
anesthesiology monitoring device	anesthesiology monitoring device	anesthesiology monitoring device	anesthesiology monitoring device	anesthesiology monitoring device	2	kept
Anesthetic agents	anesthetic agents	anesthetic agents	anesthetic agent	anesthetic agent	2	singular
anesthetic equipment	anesthetic equipment	anesthetic equipment	anesthetic equipment	anesthetic equipment	2	kept
anesthetic gas	anesthetic gas	anesthetic gas	anesthetic gas	anesthetic gas	2	kept
aneurysm size	aneurysm size	aneurysm size	aneurysm size	aneurysm size	2	kept
angiogenesis	angiogenesis	angiogenesis	angiogenesis	angiogenesis	2	kept
Angiography, Digital Subtraction	angiography digital subtraction	angiography digital subtraction	angiography digital subtraction	Angiography, Digital Subtraction	2	kept
Angiotensin II	angiotensin ii	angiotensin ii	angiotensin ii	Angiotensin II	2	kept
angiotensin II	angiotensin ii	angiotensin ii	angiotensin ii	Angiotensin II	2	case_or_format
animal behavior	animal behavior	animal behavior	animal behavior	animal behavior	2	kept
animal cell	animal cell	animal cell	animal cell	animal cell	2	kept
animal welfare	animal welfare	animal welfare	animal welfare	animal welfare	2	kept
Animal-assisted activity	animal assisted activity	animal assisted activity	animal assisted activity	Animal-assisted activity	2	kept
anisocoria	anisocoria	anisocoria	anisocoria	Anisocoria	2	case_or_format
Ankle Brachial Index	ankle brachial index	ankle brachial index	ankle brachial index	ankle brachial index	2	case_or_format

ankle injury	ankle injury	ankle injury	ankle injury	ankle injury	2	kept
annoyance	annoyance	annoyance	annoyance	annoyance	2	kept
anorexia nervosa	anorexia nervosa	anorexia nervosa	anorexia nervosa	anorexia nervosa	2	kept
Anosmia	anosmia	anosmia	anosmia	anosmia	2	case_or_format
Ansiedad	ansiedad	ansiedad	ansiedad	Ansiedad	2	kept
Antecedents	antecedents	antecedents	antecedents	ANTECEDENTS	2	case_or_format
antecubital fossa	antecubital fossa	antecubital fossa	antecubital fossa	antecubital fossa	2	kept
antenatal	antenatal	antenatal	antenatal	antenatal	2	kept
antenatal care	antenatal care	antenatal care	antenatal care	antenatal care	2	kept
ANTENATAL CORTICOSTEROIDS	antenatal corticosteroids	antenatal corticosteroids	antenatal corticosteroids	ANTENATAL CORTICOSTEROIDS	2	kept
antepartum	antepartum	antepartum	antepartum	antepartum	2	kept
anterior communicating artery aneurysm	anterior communicating artery aneurysm	anterior communicating artery aneurysm	anterior communicating artery aneurysm	anterior communicating artery aneurysm	2	kept
anterior fontanel	anterior fontanel	anterior fontanel	anterior fontanel	anterior fontanel	2	kept
anthropology	anthropology	anthropology	anthropology	Anthropology	2	case_or_format
anthropometric shoes	anthropometric shoes	anthropometric shoes	anthropometric shoes	anthropometric shoes	2	kept
Anti-infective agents	anti infective agents	anti infective agents	anti infective agents	Anti-Infective Agents	2	case_or_format

anti-infective agents	anti infective agents	anti infective agents	anti infective agents	Anti-Infective Agents	2	case_or_format
Anti-infectives	anti infectives	anti infectives	anti infectives	Anti-infectives	2	kept
Anti-inflammatory	anti inflammatory	anti inflammatory	anti inflammatory	Anti-inflammatory	2	kept
Anti-N-methyl-D-aspartate receptor encephalitis	anti n methyl d aspartate receptor encephalitis	anti n methyl d aspartate receptor encephalitis	anti n methyl d aspartate receptor encephalitis	Anti-N-Methyl-D-Aspartate Receptor Encephalitis	2	case_or_format
Anti-oxidative	anti oxidative	anti oxidative	anti oxidative	Anti-oxidative	2	kept
anti-PU bed	anti pu bed	anti pu bed	anti pu bed	anti-PU bed	2	kept
antianemic agent	antianemic agent	antianemic agent	antianemic agent	antianemic agent	2	kept
antibacterial drug resistance	antibacterial drug resistance	antibacterial drug resistance	antibacterial drug resistance	antibacterial drug resistance	2	kept
Antibiogram	antibiogram	antibiogram	antibiogram	Antibiogram	2	kept
Antibiotic consumption	antibiotic consumption	antibiotic consumption	antibiotic consumption	Antibiotic consumption	2	kept
antibiotic prescribing	antibiotic prescribing	antibiotic prescribing	antibiotic prescribing	antibiotic prescribing	2	kept
antibiotic resistome	antibiotic resistome	antibiotic resistome	antibiotic resistome	antibiotic resistome	2	kept
Antibodies, Neutralizing	antibodies neutralizing	antibodies neutralizing	antibodies neutralizing	Antibodies, Neutralizing	2	kept
antibody	antibody	antibody	antibody	Antibodies	2	case_or_format
anticipated turnover	anticipated turnover	anticipated turnover	anticipated turnover	anticipated turnover	2	kept
Antidepressants	antidepressants	antidepressants	antidepressants	Antidepressants	2	kept

antidiarrheal agent	antidiarrheal agent	antidiarrheal agent	antidiarrheal agent	antidiarrheal agent	2	kept
Antidiarrheals	antidiarrheals	antidiarrheals	antidiarrheals	Antidiarrheals	2	kept
Antidote	antidote	antidote	antidote	antidote	2	case_or_format
Antiepileptic drugs	antiepileptic drugs	antiepileptic drugs	antiepileptic drugs	Antiepileptic drugs	2	kept
Antigens, CD19	antigens cd19	antigens cd19	antigens cd19	Antigens, CD19	2	kept
Antihypertensives	antihypertensives	antihypertensives	antihypertensives	Antihypertensives	2	kept
Antimicrobial	antimicrobial	antimicrobial	antimicrobial	Antimicrobials	2	case_or_format
Antimicrobial copper	antimicrobial copper	antimicrobial copper	antimicrobial copper	Antimicrobial copper	2	kept
Antimicrobial infusions	antimicrobial infusions	antimicrobial infusions	antimicrobial infusions	Antimicrobial infusions	2	kept
antimicrobial susceptibility	antimicrobial susceptibility	antimicrobial susceptibility	antimicrobial susceptibility	antimicrobial susceptibility	2	kept
Antimicrobial use	antimicrobial use	antimicrobial use	antimicrobial use	Antimicrobial use	2	kept
Antineoplastic agents	antineoplastic agents	antineoplastic agents	antineoplastic agent	antineoplastic agent	2	singular
antinociception	antinociception	antinociception	antinociception	antinociception	2	kept
antinuclear antibody	antinuclear antibody	antinuclear antibody	antinuclear antibody	antinuclear antibody	2	kept
Antiparkinson Agents	antiparkinson agents	antiparkinson agents	antiparkinson agent	antiparkinson agent	2	singular
Antipruritics	antipruritics	antipruritics	antipruritics	Antipruritics	2	kept

Antipsychotic	antipsychotic	antipsychotic	antipsychotic	Antipsychotics	2	case_or_format
Antipsychotic agents	antipsychotic agents	antipsychotic agents	antipsychotic agents	Antipsychotic Agents	2	case_or_format
antipsychotic agents	antipsychotic agents	antipsychotic agents	antipsychotic agents	Antipsychotic Agents	2	case_or_format
antirheumatic agent	antirheumatic agent	antirheumatic agent	antirheumatic agent	antirheumatic agent	2	kept
Antirheumatic Agents	antirheumatic agents	antirheumatic agents	antirheumatic agent	antirheumatic agent	2	singular
ANTISEPTIC-IMPREGNATED CATHETER	antiseptic impregnated catheter	antiseptic impregnated catheter	antiseptic impregnated catheter	ANTISEPTIC-IMPREGNATED CATHETER	2	kept
antiserum	antiserum	antiserum	antiserum	antiserum	2	kept
Antithrombin III	antithrombin iii	antithrombin iii	antithrombin iii	antithrombin III	2	case_or_format
Antithrombins	antithrombins	antithrombins	antithrombin	antithrombin	2	singular
antithrombotic activity	antithrombotic activity	antithrombotic activity	antithrombotic activity	antithrombotic activity	2	kept
Antivenom	antivenom	antivenom	antivenom	Antivenom	2	kept
antiviral activity	antiviral activity	antiviral activity	antiviral activity	antiviral activity	2	kept
anus	anus	anus	anus	anus	2	kept
anus atresia	anus atresia	anus atresia	anus atresia	anus atresia	2	kept
anxiety management	anxiety management	anxiety management	anxiety management	anxiety management	2	kept
aorta dissection	aorta dissection	aorta dissection	aorta dissection	aorta dissection	2	kept

aorta valve prosthesis	aorta valve prosthesis	aorta valve prosthesis	aorta valve prosthesis	aorta valve prosthesis	2	kept
aortic arch surgery	aortic arch surgery	aortic arch surgery	aortic arch surgery	aortic arch surgery	2	kept
Aortic dissection	aortic dissection	aortic dissection	aortic dissection	Aortic Dissection	2	case_or_format
aortic trauma	aortic trauma	aortic trauma	aortic trauma	aortic trauma	2	kept
aortography	aortography	aortography	aortography	aortography	2	kept
Apallic syndrome	apallic syndrome	apallic syndrome	apallic syndrome	Apallic syndrome	2	kept
Aphasia	aphasia	aphasia	aphasia	aphasia	2	case_or_format
Aphonia	aphonia	aphonia	aphonia	Aphonia	2	kept
Apixaban	apixaban	apixaban	apixaban	apixaban	2	case_or_format
aplastic anemia	aplastic anemia	aplastic anemia	aplastic anemia	aplastic anemia	2	kept
APN score	apn score	apn score	apn score	APN score	2	kept
apnea hypopnea index	apnea hypopnea index	apnea hypopnea index	apnea hypopnea index	apnea hypopnea index	2	kept
Apnoea	apnoea	apnoea	apnoea	Apnoea	2	kept
Apolipoprotein E4	apolipoprotein e4	apolipoprotein e4	apolipoprotein e4	apolipoprotein E4	2	case_or_format
apolipoprotein L1	apolipoprotein l1	apolipoprotein l1	apolipoprotein l1	apolipoprotein L1	2	kept
apomorphine	apomorphine	apomorphine	apomorphine	apomorphine	2	kept

APP	app	app	app	apps	2	case_or_format
Appendectomy	appendectomy	appendectomy	appendectomy	appendectomy	2	case_or_format
appendix cancer	appendix cancer	appendix cancer	appendix cancer	appendix cancer	2	kept
appendix tumor	appendix tumor	appendix tumor	appendix tumor	appendix tumor	2	kept
Application	application	application	application	application	2	case_or_format
application of evidence	application of evidence	application of evidence	application of evidence	application of evidence	2	kept
applications	applications	applications	application	application	2	singular
applied narratology	applied narratology	applied narratology	applied narratology	applied narratology	2	kept
appreciative inquiry	appreciative inquiry	appreciative inquiry	appreciative inquiry	Appreciative inquiry	2	case_or_format
apprenticeship	apprenticeship	apprenticeship	apprenticeship	apprenticeship	2	kept
approach	approach	approach	approach	approach	2	kept
appropriate	appropriate	appropriate	appropriate	appropriate	2	kept
Appropriateness	appropriateness	appropriateness	appropriateness	APPROPRIATENESS	2	case_or_format
APR-DRG	apr drg	apr drg	apr drg	APR-DRG	2	kept
APRV	aprv	aprv	aprv	APRV	2	kept
aptitude	aptitude	aptitude	aptitude	aptitude	2	kept

aqueous solution	aqueous solution	aqueous solution	aqueous solution	aqueous solution	2	kept
ARABIA	arabia	arabia	arabia	ARABIA	2	kept
arachidonic acid	arachidonic acid	arachidonic acid	arachidonic acid	arachidonic acid	2	kept
Architectural acoustics	architectural acoustics	architectural acoustics	architectural acoustics	Architectural acoustics	2	kept
Architectural layout	architectural layout	architectural layout	architectural layout	Architectural layout	2	kept
Arduino Uno	arduino uno	arduino uno	arduino uno	Arduino Uno	2	kept
Area Deprivation Index	area deprivation index	area deprivation index	area deprivation index	Area Deprivation Index	2	kept
areflexia	areflexia	areflexia	areflexia	areflexia	2	kept
Arginine Vasopressin	arginine vasopressin	arginine vasopressin	arginine vasopressin	Arginine Vasopressin	2	kept
argon plasma coagulation	argon plasma coagulation	argon plasma coagulation	argon plasma coagulation	argon plasma coagulation	2	kept
ARIMA	arima	arima	arima	ARIMA	2	kept
arithmetic	arithmetic	arithmetic	arithmetic	arithmetic	2	kept
arketamine	arketamine	arketamine	arketamine	arketamine	2	kept
arm swelling	arm swelling	arm swelling	arm swelling	arm swelling	2	kept
arm weakness	arm weakness	arm weakness	arm weakness	arm weakness	2	kept
Arnold Chiari malformation	arnold chiari malformation	arnold chiari malformation	arnold chiari malformation	Arnold-Chiari Malformation	2	case_or_format

arrhythmia detector	arrhythmia detector	arrhythmia detector	arrhythmia detector	arrhythmia detector	2	kept
arrhythmias	arrhythmias	arrhythmias	arrhythmia	Arrhythmia	2	singular
Arsenicals	arsenicals	arsenicals	arsenicals	Arsenicals	2	kept
artemether	artemether	artemether	artemether	artemether	2	kept
arterial access	arterial access	arterial access	arterial access	arterial access	2	kept
arterial blood gas	arterial blood gas	arterial blood gas	arterial blood gas	Arterial blood gas	2	case_or_format
arterial thromboembolism	arterial thromboembolism	arterial thromboembolism	arterial thromboembolism	arterial thromboembolism	2	kept
arteriovenous graft	arteriovenous graft	arteriovenous graft	arteriovenous graft	arteriovenous graft	2	kept
artery bypass	artery bypass	artery bypass	artery bypass	artery bypass	2	kept
artificial heart	artificial heart	artificial heart	artificial heart	artificial heart	2	kept
artificial hydration and nutrition	artificial hydration and nutrition	artificial hydration and nutrition	artificial hydration and nutrition	artificial hydration and nutrition	2	kept
Artificial intelligence technologies	artificial intelligence technologies	artificial intelligence technologies	artificial intelligence technology	Artificial intelligence technologies	2	singular
Artificial intelligent	artificial intelligent	artificial intelligent	artificial intelligent	Artificial intelligent	2	kept
artificial liver support system	artificial liver support system	artificial liver support system	artificial liver support system	artificial liver support system	2	kept
Artificial neural network	artificial neural network	artificial neural network	artificial neural network	artificial neural network	2	case_or_format
ARTIFICIAL NUTRITION	artificial nutrition	artificial nutrition	artificial nutrition	ARTIFICIAL NUTRITION	2	kept

artificial nutrition	artificial nutrition	artificial nutrition	artificial nutrition	ARTIFICIAL NUTRITION	2	case_or_format
ARTIFICIAL PANCREAS	artificial pancreas	artificial pancreas	artificial pancreas	ARTIFICIAL PANCREAS	2	kept
Artificial Respiration	artificial respiration	artificial respiration	artificial respiration	Artificial respiration	2	case_or_format
artificial tear	artificial tear	artificial tear	artificial tear	artificial tear	2	kept
Artificial ventilation	artificial ventilation	artificial ventilation	artificial ventilation	Artificial Ventilation	2	case_or_format
artistic awareness	artistic awareness	artistic awareness	artistic awareness	artistic awareness	2	kept
Aryl Hydrocarbon Hydroxylases	aryl hydrocarbon hydroxylases	aryl hydrocarbon hydroxylases	aryl hydrocarbon hydroxylases	Aryl Hydrocarbon Hydroxylases	2	kept
ASA = American Society of Anesthesiologists	asa american society of anesthesiologists	asa american society of anesthesiologists	asa american society of anesthesiologists	ASA = American Society of Anesthesiologists	2	kept
Ascites	ascites	ascites	ascites	ascites	2	case_or_format
Ascorbic Acid	ascorbic acid	ascorbic acid	ascorbic acid	ascorbic acid	2	case_or_format
Asian American	asian american	asian american	asian american	Asian American	2	kept
asparaginase	asparaginase	asparaginase	asparaginase	asparaginase	2	kept
Aspartate Aminotransferases	aspartate aminotransferases	aspartate aminotransferases	aspartate aminotransferase	aspartate aminotransferase	2	singular
Aspartic Acid	aspartic acid	aspartic acid	aspartic acid	Aspartic Acid	2	kept
ASPERGILLOSIS	aspergillosis	aspergillosis	aspergillosis	Aspergillosis	2	case_or_format
Aspergillus flavus	aspergillus flavus	aspergillus flavus	aspergillus flavus	Aspergillus flavus	2	kept

Aspergillus fumigatus	aspergillus fumigatus	aspergillus fumigatus	aspergillus fumigatus	Aspergillus fumigatus	2	kept
asphyxia neonatorum	asphyxia neonatorum	asphyxia neonatorum	asphyxia neonatorum	Asphyxia Neonatorum	2	case_or_format
ASPIRATION	aspiration	aspiration	aspiration	aspiration	2	case_or_format
assay	assay	assay	assay	assay	2	kept
Assessment documents	assessment documents	assessment documents	assessment documents	Assessment documents	2	kept
assessment frequency	assessment frequency	assessment frequency	assessment frequency	assessment frequency	2	kept
ASSESSMENT TOOLS	assessment tools	assessment tools	assessment tool	Assessment tool	2	singular
assistance	assistance	assistance	assistance	assistance	2	kept
Assistant device	assistant device	assistant device	assistant device	Assistant device	2	kept
assisted living	assisted living	assisted living	assisted living	assisted living	2	kept
Assistive devices	assistive devices	assistive devices	assistive devices	Assistive devices	2	kept
assistive technology device	assistive technology device	assistive technology device	assistive technology device	assistive technology device	2	kept
associated factor	associated factor	associated factor	associated factor	Associated factors	2	case_or_format
Associated pneumonia	associated pneumonia	associated pneumonia	associated pneumonia	Associated pneumonia	2	kept
associated pneumonia	associated pneumonia	associated pneumonia	associated pneumonia	Associated pneumonia	2	case_or_format
ASSOCIATION GUIDELINES UPDATE	association guidelines update	association guidelines update	association guidelines update	ASSOCIATION GUIDELINES UPDATE	2	kept

asthma action plan	asthma action plan	asthma action plan	asthma action plan	asthma action plan	2	kept
Asthma Control Test	asthma control test	asthma control test	asthma control test	Asthma Control Test	2	kept
Astringents	astringents	astringents	astringents	Astringents	2	kept
asymptomatic transmission	asymptomatic transmission	asymptomatic transmission	asymptomatic transmission	asymptomatic transmission	2	kept
At-risk	at risk	at risk	at risk	At-risk	2	kept
ATCN	atcn	atcn	atcn	ATCN	2	kept
Athens Insomnia Scale	athens insomnia scale	athens insomnia scale	athens insomnia scale	Athens Insomnia Scale	2	kept
Atherosclerosis	atherosclerosis	atherosclerosis	atherosclerosis	atherosclerosis	2	case_or_format
athlete	athlete	athlete	athlete	athlete	2	kept
Athletic Injuries	athletic injuries	athletic injuries	athletic injuries	Athletic Injuries	2	kept
atlantooccipital joint	atlantooccipital joint	atlantooccipital joint	atlantooccipital joint	atlantooccipital joint	2	kept
Atmosphere	atmosphere	atmosphere	atmosphere	atmosphere	2	case_or_format
Atmospheric pressure	atmospheric pressure	atmospheric pressure	atmospheric pressure	atmospheric pressure	2	case_or_format
Atopobium minutum	atopobium minutum	atopobium minutum	atopobium minutum	Atopobium minutum	2	kept
atraumatic restorative treatment	atraumatic restorative treatment	atraumatic restorative treatment	atraumatic restorative treatment	atraumatic restorative treatment	2	kept
atresia	atresia	atresia	atresia	atresia	2	kept

Atrial Flutter	atrial flutter	atrial flutter	atrial flutter	Atrial Flutter	2	kept
Atrial isomerism	atrial isomerism	atrial isomerism	atrial isomerism	Atrial isomerism	2	kept
Atrial Natriuretic Factor	atrial natriuretic factor	atrial natriuretic factor	atrial natriuretic factor	Atrial Natriuretic Factor	2	kept
atrial natriuretic factor	atrial natriuretic factor	atrial natriuretic factor	atrial natriuretic factor	Atrial Natriuretic Factor	2	case_or_format
AtriAmp	atriamp	atriamp	atriamp	AtriAmp	2	kept
Atrophy	atrophy	atrophy	atrophy	Atrophy	2	kept
atrophy	atrophy	atrophy	atrophy	Atrophy	2	case_or_format
Attachment theory	attachment theory	attachment theory	attachment theory	Attachment theory	2	kept
Attack rate	attack rate	attack rate	attack rate	attack rate	2	case_or_format
attendance	attendance	attendance	attendance	attendance	2	kept
ATTENDING PHYSICIAN WORKLOAD	attending physician workload	attending physician workload	attending physician workload	ATTENDING PHYSICIAN WORKLOAD	2	kept
ATTENTION	attention	attention	attention	attention	2	case_or_format
ATTITUDE	attitude	attitude	attitude	attitude	2	case_or_format
attitude and practice	attitude and practice	attitude and practice	attitude and practice	attitude and practice	2	kept
Attitude scale	attitude scale	attitude scale	attitude scale	attitude scale	2	case_or_format
attitude to life	attitude to life	attitude to life	attitude to life	attitude to life	2	kept

Attitude toward death	attitude toward death	attitude toward death	attitude toward death	attitude toward death	2	case_or_format
attitude towards death	attitude towards death	attitude towards death	attitude towards death	attitude towards death	2	kept
attitudes towards death	attitudes towards death	attitudes towards death	attitudes towards death	attitudes towards death	2	kept
atypical mycobacteriosis	atypical mycobacteriosis	atypical mycobacteriosis	atypical mycobacteriosis	atypical mycobacteriosis	2	kept
atypical sleep	atypical sleep	atypical sleep	atypical sleep	atypical sleep	2	kept
Audio alarm	audio alarm	audio alarm	audio alarm	Audio alarm	2	kept
audiometry	audiometry	audiometry	audiometry	audiometry	2	kept
audit log	audit log	audit log	audit log	audit log	2	kept
Audition	audition	audition	audition	Audition	2	kept
auditory	auditory	auditory	auditory	auditory	2	kept
Auditory alarm	auditory alarm	auditory alarm	auditory alarm	Auditory alarm	2	kept
auditory evoked potential	auditory evoked potential	auditory evoked potential	auditory evoked potential	auditory evoked potential	2	kept
auditory neuropathy	auditory neuropathy	auditory neuropathy	auditory neuropathy	auditory neuropathy	2	kept
auditory screening	auditory screening	auditory screening	auditory screening	auditory screening	2	kept
AUREUS	aureus	aureus	aureus	AUREUS	2	kept
Auscultation skills	auscultation skills	auscultation skills	auscultation skills	Auscultation skills	2	kept

AUSTRALIA	australia	australia	australia	Australia	2	case_or_format
AUTHENTIC LEADERSHIP	authentic leadership	authentic leadership	authentic leadership	authentic leadership	2	case_or_format
Authentic leadership	authentic leadership	authentic leadership	authentic leadership	authentic leadership	2	case_or_format
author	author	author	author	author	2	kept
Authority	authority	authority	authority	authority	2	case_or_format
Authority delegation	authority delegation	authority delegation	authority delegation	Authority delegation	2	kept
authorization	authorization	authorization	authorization	authorization	2	kept
Auto-documentation	auto documentation	auto documentation	auto documentation	Auto-documentation	2	kept
Autoanalysis	autoanalysis	autoanalysis	autoanalysis	Autoanalysis	2	kept
autoanalysis	autoanalysis	autoanalysis	autoanalysis	Autoanalysis	2	case_or_format
autoanalyzer	autoanalyzer	autoanalyzer	autoanalyzer	autoanalyzer	2	kept
autoantibody	autoantibody	autoantibody	autoantibody	autoantibody	2	kept
autocorrelation	autocorrelation	autocorrelation	autocorrelation	autocorrelation	2	kept
autoimmune liver disease	autoimmune liver disease	autoimmune liver disease	autoimmune liver disease	autoimmune liver disease	2	kept
autoimmune thrombocytopenia	autoimmune thrombocytopenia	autoimmune thrombocytopenia	autoimmune thrombocytopenia	autoimmune thrombocytopenia	2	kept
Automated dispensing cabinet, ADC	automated dispensing cabinet adc	automated dispensing cabinet adc	automated dispensing cabinet adc	Automated dispensing cabinet, ADC	2	kept

Automated execution	automated execution	automated execution	automated execution	Automated execution	2	kept
automated external defibrillator	automated external defibrillator	automated external defibrillator	automated external defibrillator	Automated external defibrillator	2	case_or_format
Automated external defibrillator training	automated external defibrillator training	automated external defibrillator training	automated external defibrillator training	Automated external defibrillator training	2	kept
Automated monitoring	automated monitoring	automated monitoring	automated monitoring	Automated monitoring	2	kept
Automated pupillometry	automated pupillometry	automated pupillometry	automated pupillometry	Automated pupillometry	2	kept
automated pupillometry	automated pupillometry	automated pupillometry	automated pupillometry	Automated pupillometry	2	case_or_format
automated sedation	automated sedation	automated sedation	automated sedation	automated sedation	2	kept
Automatic pain assessment	automatic pain assessment	automatic pain assessment	automatic pain assessment	Automatic pain assessment	2	kept
Automobile Driving	automobile driving	automobile driving	automobile driving	Automobile Driving	2	kept
autonomous motivation	autonomous motivation	autonomous motivation	autonomous motivation	autonomous motivation	2	kept
avascular necrosis	avascular necrosis	avascular necrosis	avascular necrosis	avascular necrosis	2	kept
Average life expectancy	average life expectancy	average life expectancy	average life expectancy	Average life expectancy	2	kept
aversion	aversion	aversion	aversion	aversion	2	kept
avian influenza virus	avian influenza virus	avian influenza virus	avian influenza virus	avian influenza virus	2	kept
avibactam	avibactam	avibactam	avibactam	avibactam	2	kept
AVPU scale	avpu scale	avpu scale	avpu scale	AVPU scale	2	kept

awakening and breathing coordination	awakening and breathing coordination	awakening and breathing coordination	awakening and breathing coordination	awakening and breathing coordination	2	kept
axillary lymph node	axillary lymph node	axillary lymph node	axillary lymph node	axillary lymph node	2	kept
Azepines	azepines	azepines	azepines	Azepines	2	kept
Azygos Vein	azygos vein	azygos vein	azygos vein	Azygos Vein	2	kept
B-Lymphocytes	b lymphocytes	b lymphocytes	b lymphocyte	B lymphocyte	2	singular
Babies	babies	babies	baby	baby	2	singular
Baby	baby	baby	baby	baby	2	case_or_format
baby boomer	baby boomer	baby boomer	baby boomer	baby boomer	2	kept
baby food	baby food	baby food	baby food	baby food	2	kept
Baby Friendly Hospital Initiative	baby friendly hospital initiative	baby friendly hospital initiative	baby friendly hospital initiative	Baby Friendly Hospital Initiative	2	kept
Baby-Friendly Hospital Initiative	baby friendly hospital initiative	baby friendly hospital initiative	baby friendly hospital initiative	Baby Friendly Hospital Initiative	2	case_or_format
back pain	back pain	back pain	back pain	Back Pain	2	case_or_format
Back school	back school	back school	back school	Back school	2	kept
background noise	background noise	background noise	background noise	background noise	2	kept
BACKREST ELEVATION	backrest elevation	backrest elevation	backrest elevation	BACKREST ELEVATION	2	kept
bacteraemia	bacteraemia	bacteraemia	bacteraemia	Bacteraemia	2	case_or_format

bacterial contamination	bacterial contamination	bacterial contamination	bacterial contamination	Bacterial contamination	2	case_or_format
bacterial disease	bacterial disease	bacterial disease	bacterial disease	bacterial disease	2	kept
bacterial flora	bacterial flora	bacterial flora	bacterial flora	bacterial flora	2	kept
bacterial genetic phenomena and functions	bacterial genetic phenomena and functions	bacterial genetic phenomena and functions	bacterial genetic phenomena and functions	bacterial genetic phenomena and functions	2	kept
bacterial infections	bacterial infections	bacterial infections	bacterial infection	Bacterial Infections	2	singular
Bacterial meningitis	bacterial meningitis	bacterial meningitis	bacterial meningitis	bacterial meningitis	2	case_or_format
Bacterial translocation	bacterial translocation	bacterial translocation	bacterial translocation	bacterial translocation	2	case_or_format
bacterial vaccine	bacterial vaccine	bacterial vaccine	bacterial vaccine	bacterial vaccine	2	kept
bacteriology	bacteriology	bacteriology	bacteriology	bacteriology	2	kept
BAD-NEWS	bad news	bad news	bad news	BAD-NEWS	2	kept
balance disorder	balance disorder	balance disorder	balance disorder	balance disorder	2	kept
Balint group	balint group	balint group	balint group	Balint group	2	kept
baloxavir	baloxavir	baloxavir	baloxavir	baloxavir	2	kept
Baltic States	baltic states	baltic states	baltic states	Baltic States	2	kept
bar code medication administration	bar code medication administration	bar code medication administration	bar code medication administration	bar code medication administration	2	kept
Bar coded medication administration	bar coded medication administration	bar coded medication administration	bar coded medication administration	Bar coded medication administration	2	kept

bar coded medication administration	bar coded medication administration	bar coded medication administration	bar coded medication administration	Bar coded medication administration	2	case_or_format
barcode medication administration	barcode medication administration	barcode medication administration	barcode medication administration	barcode medication administration	2	kept
bariatric	bariatric	bariatric	bariatric	Bariatric	2	case_or_format
Barotrauma	barotrauma	barotrauma	barotrauma	barotrauma	2	case_or_format
Barreras	barreras	barreras	barreras	Barreras	2	kept
Barrett Esophagus	barrett esophagus	barrett esophagus	barrett esophagus	Barrett Esophagus	2	kept
Barrett esophagus	barrett esophagus	barrett esophagus	barrett esophagus	Barrett Esophagus	2	case_or_format
Barriers to implement non-pharmacological pain methods	barriers to implement non pharmacological pain methods	barriers to implement non pharmacological pain methods	barriers to implement non pharmacological pain methods	Barriers to implement non-pharmacological pain methods	2	kept
barriers to palliative care	barriers to palliative care	barriers to palliative care	barriers to palliative care	barriers to palliative care	2	kept
barriers to translation	barriers to translation	barriers to translation	barriers to translation	barriers to translation	2	kept
Bartter syndrome	bartter syndrome	bartter syndrome	bartter syndrome	Bartter syndrome	2	kept
Base-line performance	base line performance	base line performance	base line performance	Base-line performance	2	kept
basic research	basic research	basic research	basic research	basic research	2	kept
bathing	bathing	bathing	bathing	Bathing	2	case_or_format
Baumannii	baumannii	baumannii	baumannii	Baumannii	2	kept
BAVIQ	baviq	baviq	baviq	BAVIQ	2	kept

Bayesian	bayesian	bayesian	bayesian	Bayesian	2	kept
Bayesian analysis	bayesian analysis	bayesian analysis	bayesian analysis	Bayesian analysis	2	kept
BCG Vaccine	bcg vaccine	bcg vaccine	bcg vaccine	BCG vaccine	2	case_or_format
Beckwith-Wiedemann Syndrome	beckwith wiedemann syndrome	beckwith wiedemann syndrome	beckwith wiedemann syndrome	Beckwith-Wiedemann Syndrome	2	kept
Bed bath	bed bath	bed bath	bed bath	Bed bath	2	kept
BED NUMBERS	bed numbers	bed numbers	bed numbers	BED NUMBERS	2	kept
BED OCCUPANCY	bed occupancy	bed occupancy	bed occupancy	Bed Occupancy	2	case_or_format
Bed pooling	bed pooling	bed pooling	bed pooling	Bed pooling	2	kept
Bed sore	bed sore	bed sore	bed sore	Bed sore	2	kept
bed sores	bed sores	bed sores	bed sore	Bed sore	2	singular
Bedbugs	bedbugs	bedbugs	bedbugs	Bedbugs	2	kept
beds	beds	beds	bed	Beds	2	singular
bedside endoscopy	bedside endoscopy	bedside endoscopy	bedside endoscopy	bedside endoscopy	2	kept
Bedside handover	bedside handover	bedside handover	bedside handover	Bedside handover	2	kept
Bedside laparotomy	bedside laparotomy	bedside laparotomy	bedside laparotomy	Bedside laparotomy	2	kept
bedside milk expression	bedside milk expression	bedside milk expression	bedside milk expression	bedside milk expression	2	kept

bedside monitoring	bedside monitoring	bedside monitoring	bedside monitoring	Bedside monitoring	2	case_or_format
BEDSIDE PLACEMENT	bedside placement	bedside placement	bedside placement	BEDSIDE PLACEMENT	2	kept
bedside rationing	bedside rationing	bedside rationing	bedside rationing	bedside rationing	2	kept
Bedside report	bedside report	bedside report	bedside report	Bedside report	2	kept
bedside training	bedside training	bedside training	bedside training	bedside training	2	kept
Behavior control	behavior control	behavior control	behavior control	Behavior Control	2	case_or_format
behavior monitoring	behavior monitoring	behavior monitoring	behavior monitoring	behavior monitoring	2	kept
Behavior, Animal	behavior animal	behavior animal	behavior animal	Behavior, Animal	2	kept
Behavioral and psychological symptoms of dementia	behavioral and psychological symptoms of dementia	behavioral and psychological symptoms of dementia	behavioral and psychological symptoms of dementia	Behavioral and psychological symptoms of dementia	2	kept
behavioral and psychological symptoms of dementia	behavioral and psychological symptoms of dementia	behavioral and psychological symptoms of dementia	behavioral and psychological symptoms of dementia	Behavioral and psychological symptoms of dementia	2	case_or_format
Behavioral assessment	behavioral assessment	behavioral assessment	behavioral assessment	Behavioral assessment	2	kept
Behavioral mapping	behavioral mapping	behavioral mapping	behavioral mapping	Behavioral mapping	2	kept
Behavioral pain scale	behavioral pain scale	behavioral pain scale	behavioral pain scale	Behavioral Pain Scale	2	case_or_format
Behavioral rating scale	behavioral rating scale	behavioral rating scale	behavioral rating scale	Behavioral rating scale	2	kept
behavioral response	behavioral response	behavioral response	behavioral response	behavioral response	2	kept
behavioral stress	behavioral stress	behavioral stress	behavioral stress	behavioral stress	2	kept

Behavioral Symptoms	behavioral symptoms	behavioral symptoms	behavioral symptoms	Behavioral Symptoms	2	kept
Behavioral system	behavioral system	behavioral system	behavioral system	Behavioral system	2	kept
BEHAVIORAL-ASSESSMENT	behavioral assessment	behavioral assessment	behavioral assessment	Behavioral assessment	2	case_or_format
Behaviour change	behaviour change	behaviour change	behaviour change	Behaviour change	2	kept
Behaviour Rating Scale	behaviour rating scale	behaviour rating scale	behaviour rating scale	Behaviour Rating Scale	2	kept
Behavioural competence	behavioural competence	behavioural competence	behavioural competence	Behavioural competence	2	kept
behavioural consequences	behavioural consequences	behavioural consequences	behavioural consequences	behavioural consequences	2	kept
Behavioural research	behavioural research	behavioural research	behavioural research	Behavioural research	2	kept
Behavioural responses	behavioural responses	behavioural responses	behavioural response	Behavioural responses	2	singular
Belgian	belgian	belgian	belgian	Belgian	2	kept
belief	belief	belief	belief	beliefs	2	case_or_format
below knee amputation	below knee amputation	below knee amputation	below knee amputation	below knee amputation	2	kept
benazepril	benazepril	benazepril	benazepril	benazepril	2	kept
benefit	benefit	benefit	benefit	benefits	2	case_or_format
Benner's Novice to Expert model	benner s novice to expert model	benner s novice to expert model	benner s novice to expert model	Benner's Novice to Expert model	2	kept
benzathine penicillin	benzathine penicillin	benzathine penicillin	benzathine penicillin	benzathine penicillin	2	kept

benzatropine	benzatropine	benzatropine	benzatropine	benzatropine	2	kept
Benzethonium	benzethonium	benzethonium	benzethonium	Benzethonium	2	kept
benzimidazole derivative	benzimidazole derivative	benzimidazole derivative	benzimidazole derivative	benzimidazole derivative	2	kept
Benzimidazoles	benzimidazoles	benzimidazoles	benzimidazoles	Benzimidazoles	2	kept
beractant	beractant	beractant	beractant	beractant	2	kept
bereaved	bereaved	bereaved	bereaved	bereaved	2	kept
Bereaved parents	bereaved parents	bereaved parents	bereaved parent	Bereaved parents	2	singular
bereaved parents	bereaved parents	bereaved parents	bereaved parent	Bereaved parents	2	singular
Bereaved relatives	bereaved relatives	bereaved relatives	bereaved relatives	Bereaved relatives	2	kept
Bereavement care	bereavement care	bereavement care	bereavement care	Bereavement care	2	kept
bereavement care	bereavement care	bereavement care	bereavement care	Bereavement care	2	case_or_format
Best practice guidelines	best practice guidelines	best practice guidelines	best practice guidelines	Best practice guidelines	2	kept
beta 2 adrenergic receptor	beta 2 adrenergic receptor	beta 2 adrenergic receptor	beta 2 adrenergic receptor	beta 2 adrenergic receptor	2	kept
beta 2 microglobulin	beta 2 microglobulin	beta 2 microglobulin	beta 2 microglobulin	beta 2 microglobulin	2	kept
beta carotene	beta carotene	beta carotene	beta carotene	beta carotene	2	kept
beta Lactam Antibiotics	beta lactam antibiotics	beta lactam antibiotics	beta lactam antibiotic	beta lactam antibiotic	2	singular

beta lactamase AmpC	beta lactamase ampc	beta lactamase ampc	beta lactamase ampc	beta lactamase AmpC	2	kept
beta1b interferon	beta1b interferon	beta1b interferon	beta1b interferon	beta1b interferon	2	kept
betrayal	betrayal	betrayal	betrayal	betrayal	2	kept
betweenness centrality	betweenness centrality	betweenness centrality	betweenness centrality	betweenness centrality	2	kept
Bezugspflege	bezugspflege	bezugspflege	bezugspflege	Bezugspflege	2	kept
Bias, Implicit	bias implicit	bias implicit	bias implicit	Bias, Implicit	2	kept
bibliometric	bibliometric	bibliometric	bibliometric	Bibliometrics	2	case_or_format
Bibliometric study	bibliometric study	bibliometric study	bibliometric study	Bibliometric study	2	kept
Bicycle	bicycle	bicycle	bicycle	Bicycle	2	kept
Bidirectional Glenn procedure	bidirectional glenn procedure	bidirectional glenn procedure	bidirectional glenn procedure	Bidirectional Glenn procedure	2	kept
BIFIDOBACTERIUM-BREVE	bifidobacterium breve	bifidobacterium breve	bifidobacterium breve	BIFIDOBACTERIUM-BREVE	2	kept
Big Five Inventory	big five inventory	big five inventory	big five inventory	Big Five Inventory	2	kept
Big five teamwork	big five teamwork	big five teamwork	big five teamwork	Big five teamwork	2	kept
biguanide derivative	biguanide derivative	biguanide derivative	biguanide derivative	biguanide derivative	2	kept
biliary tract disease	biliary tract disease	biliary tract disease	biliary tract disease	biliary tract disease	2	kept
Biliary Tract Diseases	biliary tract diseases	biliary tract diseases	biliary tract disease	biliary tract disease	2	singular

biliopancreatic bypass	biliopancreatic bypass	biliopancreatic bypass	biliopancreatic bypass	biliopancreatic bypass	2	kept
bilious vomiting	bilious vomiting	bilious vomiting	bilious vomiting	bilious vomiting	2	kept
bilirubin glucuronide	bilirubin glucuronide	bilirubin glucuronide	bilirubin glucuronide	bilirubin glucuronide	2	kept
Billing and coding	billing and coding	billing and coding	billing and coding	Billing and coding	2	kept
Bio-psycho-pharmaco-social approach	bio psycho pharmaco social approach	bio psycho pharmaco social approach	bio psycho pharmaco social approach	Bio-psycho-pharmaco-social approach	2	kept
bioassay	bioassay	bioassay	bioassay	bioassay	2	kept
biobrane	biobrane	biobrane	biobrane	biobrane	2	kept
biochemical marker	biochemical marker	biochemical marker	biochemical marker	biochemical marker	2	kept
Bioengineering	bioengineering	bioengineering	bioengineering	bioengineering	2	case_or_format
biofeedback	biofeedback	biofeedback	biofeedback	biofeedback	2	kept
biological accident	biological accident	biological accident	biological accident	biological accident	2	kept
Biological Availability	biological availability	biological availability	biological availability	Biological Availability	2	kept
biological monitoring	biological monitoring	biological monitoring	biological monitoring	biological monitoring	2	kept
biological product	biological product	biological product	biological product	Biological Products	2	case_or_format
biological rhythm	biological rhythm	biological rhythm	biological rhythm	biological rhythm	2	kept
biological therapy	biological therapy	biological therapy	biological therapy	biological therapy	2	kept

biomarker	biomarker	biomarker	biomarker	Biomarkers	2	case_or_format
BIOMARKERS	biomarkers	biomarkers	biomarker	Biomarkers	2	singular
biomaterial	biomaterial	biomaterial	biomaterial	biomaterial	2	kept
biomaterials	biomaterials	biomaterials	biomaterial	biomaterial	2	singular
Biomechanics	biomechanics	biomechanics	biomechanics	biomechanics	2	case_or_format
Biometry	biometry	biometry	biometry	biometry	2	case_or_format
Bioprosthesis	bioprosthesis	bioprosthesis	bioprosthesis	Bioprosthesis	2	kept
bioprosthesis	bioprosthesis	bioprosthesis	bioprosthesis	Bioprosthesis	2	case_or_format
biopsy technique	biopsy technique	biopsy technique	biopsy technique	biopsy technique	2	kept
biopsychosocial model	biopsychosocial model	biopsychosocial model	biopsychosocial model	biopsychosocial model	2	kept
Biosensing	biosensing	biosensing	biosensing	Biosensing	2	kept
Biosensing Techniques	biosensing techniques	biosensing techniques	biosensing techniques	Biosensing Techniques	2	kept
biostatistician	biostatistician	biostatistician	biostatistician	biostatistician	2	kept
biosynthesis	biosynthesis	biosynthesis	biosynthesis	biosynthesis	2	kept
biovigilance	biovigilance	biovigilance	biovigilance	biovigilance	2	kept
Birjand	birjand	birjand	birjand	Birjand	2	kept

birth certificate	birth certificate	birth certificate	birth certificate	birth certificate	2	kept
Birth defects	birth defects	birth defects	birth defect	Birth defects	2	singular
birth length	birth length	birth length	birth length	birth length	2	kept
birth outcomes	birth outcomes	birth outcomes	birth outcomes	birth outcomes	2	kept
Birth Setting	birth setting	birth setting	birth setting	birth setting	2	case_or_format
BIRTH-WEIGHT	birth weight	birth weight	birth weight	birth weight	2	case_or_format
BIRTH-WEIGHT NEWBORNS	birth weight newborns	birth weight newborns	birth weight newborns	BIRTH-WEIGHT NEWBORNS	2	kept
Bispectral Index	bispectral index	bispectral index	bispectral index	bispectral index	2	case_or_format
BLACK-AND-WHITE	black and white	black and white	black and white	BLACK-AND-WHITE	2	kept
Blacks	blacks	blacks	blacks	Blacks	2	kept
bladder distension	bladder distension	bladder distension	bladder distension	bladder distension	2	kept
bladder exstrophy	bladder exstrophy	bladder exstrophy	bladder exstrophy	Bladder Exstrophy	2	case_or_format
bladder function	bladder function	bladder function	bladder function	bladder function	2	kept
bladder irrigation	bladder irrigation	bladder irrigation	bladder irrigation	bladder irrigation	2	kept
bladder pressure	bladder pressure	bladder pressure	bladder pressure	bladder pressure	2	kept
bladder rupture	bladder rupture	bladder rupture	bladder rupture	bladder rupture	2	kept

bladder scanner	bladder scanner	bladder scanner	bladder scanner	bladder scanner	2	kept
Blakemore tube placement	blakemore tube placement	blakemore tube placement	blakemore tube placement	Blakemore tube placement	2	kept
Blastomyces	blastomyces	blastomyces	blastomyces	Blastomyces	2	kept
Blastomycosis	blastomycosis	blastomycosis	blastomycosis	Blastomycosis	2	kept
bleaching	bleaching	bleaching	bleaching	bleaching	2	kept
Blinking	blinking	blinking	blinking	Blinking	2	kept
blinking	blinking	blinking	blinking	Blinking	2	case_or_format
bloating	bloating	bloating	bloating	bloating	2	kept
blogging	blogging	blogging	blogging	Blogging	2	case_or_format
Blood chemistry tests	blood chemistry tests	blood chemistry tests	blood chemistry tests	Blood chemistry tests	2	kept
blood clotting factor 5 Leiden	blood clotting factor 5 leiden	blood clotting factor 5 leiden	blood clotting factor 5 leiden	blood clotting factor 5 Leiden	2	kept
blood clotting factor 7	blood clotting factor 7	blood clotting factor 7	blood clotting factor 7	blood clotting factor 7	2	kept
blood clotting factor 8	blood clotting factor 8	blood clotting factor 8	blood clotting factor 8	blood clotting factor 8	2	kept
blood coagulation	blood coagulation	blood coagulation	blood coagulation	Blood Coagulation	2	case_or_format
blood collection	blood collection	blood collection	blood collection	blood collection	2	kept
Blood components	blood components	blood components	blood component	blood component	2	singular

Blood cultures	blood cultures	blood cultures	blood culture	blood culture	2	singular
blood gas analyzer	blood gas analyzer	blood gas analyzer	blood gas analyzer	blood gas analyzer	2	kept
blood gases	blood gases	blood gases	blood gases	blood gases	2	kept
Blood glucose level	blood glucose level	blood glucose level	blood glucose level	Blood glucose level	2	kept
Blood glucose management	blood glucose management	blood glucose management	blood glucose management	Blood glucose management	2	kept
Blood glucose monitoring	blood glucose monitoring	blood glucose monitoring	blood glucose monitoring	blood glucose monitoring	2	case_or_format
blood group typing	blood group typing	blood group typing	blood group typing	blood group typing	2	kept
Blood Grouping and Crossmatching	blood grouping and crossmatching	blood grouping and crossmatching	blood grouping and crossmatching	Blood Grouping and Crossmatching	2	kept
blood oxygen saturation	blood oxygen saturation	blood oxygen saturation	blood oxygen saturation	Blood oxygen saturation	2	case_or_format
Blood pressure determination	blood pressure determination	blood pressure determination	blood pressure determination	Blood Pressure Determination	2	case_or_format
blood pressure variability	blood pressure variability	blood pressure variability	blood pressure variability	blood pressure variability	2	kept
Blood products	blood products	blood products	blood products	Blood products	2	kept
blood salvage	blood salvage	blood salvage	blood salvage	blood salvage	2	kept
Blood sample collection	blood sample collection	blood sample collection	blood sample collection	Blood sample collection	2	kept
blood storage	blood storage	blood storage	blood storage	blood storage	2	kept
blood toxicity	blood toxicity	blood toxicity	blood toxicity	blood toxicity	2	kept

Blood Urea Nitrogen	blood urea nitrogen	blood urea nitrogen	blood urea nitrogen	Blood Urea Nitrogen	2	kept
blood vessel prosthesis	blood vessel prosthesis	blood vessel prosthesis	blood vessel prosthesis	Blood Vessel Prosthesis	2	case_or_format
Blood vessels	blood vessels	blood vessels	blood vessel	blood vessel	2	singular
Blood Viscosity	blood viscosity	blood viscosity	blood viscosity	Blood Viscosity	2	kept
Blood volume	blood volume	blood volume	blood volume	blood volume	2	case_or_format
BLOOD-FLOW	blood flow	blood flow	blood flow	blood flow	2	case_or_format
BLOOD-PRESSURE	blood pressure	blood pressure	blood pressure	blood pressure	2	case_or_format
Blood-pressure monitoring	blood pressure monitoring	blood pressure monitoring	blood pressure monitoring	blood pressure monitoring	2	case_or_format
Blood-sparing techniques	blood sparing techniques	blood sparing techniques	blood sparing techniques	Blood-sparing techniques	2	kept
Bloodless Medical and Surgical Procedures	bloodless medical and surgical procedures	bloodless medical and surgical procedures	bloodless medical and surgical procedures	Bloodless Medical and Surgical Procedures	2	kept
Bloodless medicine	bloodless medicine	bloodless medicine	bloodless medicine	Bloodless medicine	2	kept
Bluetooth-based	bluetooth based	bluetooth based	bluetooth based	Bluetooth-based	2	kept
blunt	blunt	blunt	blunt	blunt	2	kept
BNT162 Vaccine	bnt162 vaccine	bnt162 vaccine	bnt162 vaccine	BNT162 Vaccine	2	kept
Board games	board games	board games	board games	Board games	2	kept
Boarding	boarding	boarding	boarding	Boarding	2	kept

Bodily Secretions	bodily secretions	bodily secretions	bodily secretions	bodily secretions	2	case_or_format
BODY	body	body	body	BODY	2	kept
Body Fluid Compartments	body fluid compartments	body fluid compartments	body fluid compartment	body fluid compartment	2	singular
Body height	body height	body height	body height	body height	2	case_or_format
Body mechanics	body mechanics	body mechanics	body mechanics	Body mechanics	2	kept
BODY POSITION	body position	body position	body position	body position	2	case_or_format
body size	body size	body size	body size	Body Size	2	case_or_format
Body Weights and Measures	body weights and measures	body weights and measures	body weights and measures	Body Weights and Measures	2	kept
BODY-TEMPERATURE	body temperature	body temperature	body temperature	body temperature	2	case_or_format
Boerhaave syndrome	boerhaave syndrome	boerhaave syndrome	boerhaave syndrome	Boerhaave syndrome	2	kept
Bone and Bones	bone and bones	bone and bones	bone and bones	Bone and Bones	2	kept
bone and joint infections	bone and joint infections	bone and joint infections	bone and joint infections	bone and joint infections	2	kept
bone biopsy	bone biopsy	bone biopsy	bone biopsy	bone biopsy	2	kept
Bone Diseases, Metabolic	bone diseases metabolic	bone diseases metabolic	bone diseases metabolic	Bone Diseases, Metabolic	2	kept
bone marrow aplasia	bone marrow aplasia	bone marrow aplasia	bone marrow aplasia	bone marrow aplasia	2	kept
bone marrow transplant	bone marrow transplant	bone marrow transplant	bone marrow transplant	Bone marrow transplant	2	case_or_format

bone metabolism	bone metabolism	bone metabolism	bone metabolism	bone metabolism	2	kept
bone mineral density	bone mineral density	bone mineral density	bone mineral density	bone mineral density	2	kept
Bone Nails	bone nails	bone nails	bone nail	Bone Nails	2	singular
bone pain	bone pain	bone pain	bone pain	bone pain	2	kept
bone radiography	bone radiography	bone radiography	bone radiography	bone radiography	2	kept
BoNT-A	bont a	bont a	bont a	BoNT-A	2	kept
boosting machine learning algorithm	boosting machine learning algorithm	boosting machine learning algorithm	boosting machine learning algorithm	boosting machine learning algorithm	2	kept
borderline state	borderline state	borderline state	borderline state	borderline state	2	kept
botulinum antiserum	botulinum antiserum	botulinum antiserum	botulinum antiserum	botulinum antiserum	2	kept
Botulinum Antitoxin	botulinum antitoxin	botulinum antitoxin	botulinum antitoxin	Botulinum Antitoxin	2	kept
Botulinum Toxin Type A	botulinum toxin type a	botulinum toxin type a	botulinum toxin type a	Botulinum Toxin Type A	2	kept
Boundaries	boundaries	boundaries	boundaries	Boundaries	2	kept
BoW	bow	bow	bow	BoW	2	kept
Bowel sounds	bowel sounds	bowel sounds	bowel sound	Bowel sounds	2	singular
Boxing	boxing	boxing	boxing	Boxing	2	kept
Braces	braces	braces	brace	Braces	2	singular

brachial artery	brachial artery	brachial artery	brachial artery	brachial artery	2	kept
brachial plexus anesthesia	brachial plexus anesthesia	brachial plexus anesthesia	brachial plexus anesthesia	brachial plexus anesthesia	2	kept
Brachial Plexus Block	brachial plexus block	brachial plexus block	brachial plexus block	Brachial Plexus Block	2	kept
Brachycephaly	brachycephaly	brachycephaly	brachycephaly	Brachycephaly	2	kept
brachytherapy	brachytherapy	brachytherapy	brachytherapy	brachytherapy	2	kept
braden index	braden index	braden index	braden index	braden index	2	kept
braden qd scale	braden qd scale	braden qd scale	braden qd scale	Braden QD scale	2	case_or_format
bradypnea	bradypnea	bradypnea	bradypnea	bradypnea	2	kept
brain blood volume	brain blood volume	brain blood volume	brain blood volume	brain blood volume	2	kept
brain cancer	brain cancer	brain cancer	brain cancer	brain cancer	2	kept
brain decompression	brain decompression	brain decompression	brain decompression	brain decompression	2	kept
Brain edema	brain edema	brain edema	brain edema	brain edema	2	case_or_format
brain growth	brain growth	brain growth	brain growth	brain growth	2	kept
Brain Infarction	brain infarction	brain infarction	brain infarction	brain infarction	2	case_or_format
Brain infection	brain infection	brain infection	brain infection	brain infection	2	case_or_format
brain injury recovery	brain injury recovery	brain injury recovery	brain injury recovery	brain injury recovery	2	kept

Brain monitoring	brain monitoring	brain monitoring	brain monitoring	Brain monitoring	2	kept
brain protection	brain protection	brain protection	brain protection	brain protection	2	kept
brain radiation	brain radiation	brain radiation	brain radiation	brain radiation	2	kept
brain size	brain size	brain size	brain size	brain size	2	kept
Brain Stem Neoplasms	brain stem neoplasms	brain stem neoplasms	brain stem neoplasms	Brain Stem Neoplasms	2	kept
brain stem response	brain stem response	brain stem response	brain stem response	brain stem response	2	kept
Brain surgery	brain surgery	brain surgery	brain surgery	brain surgery	2	case_or_format
brain temperature	brain temperature	brain temperature	brain temperature	brain temperature	2	kept
Brain tissue oxygenation	brain tissue oxygenation	brain tissue oxygenation	brain tissue oxygenation	Brain tissue oxygenation	2	kept
Brain trauma	brain trauma	brain trauma	brain trauma	Brain trauma	2	kept
Brain tumor	brain tumor	brain tumor	brain tumor	brain tumor	2	case_or_format
Brain tumour	brain tumour	brain tumour	brain tumour	Brain tumour	2	kept
brain vascular resistance	brain vascular resistance	brain vascular resistance	brain vascular resistance	brain vascular resistance	2	kept
brain ventricle	brain ventricle	brain ventricle	brain ventricle	brain ventricle	2	kept
Brain Waves	brain waves	brain waves	brain waves	Brain Waves	2	kept
Brain-Derived Neurotrophic Factor	brain derived neurotrophic factor	brain derived neurotrophic factor	brain derived neurotrophic factor	brain derived neurotrophic factor	2	case_or_format

Braun and Clarke	braun and clarke	braun and clarke	braun and clarke	Braun and Clarke	2	kept
Brazilian Unified Public Health System	brazilian unified public health system	brazilian unified public health system	brazilian unified public health system	Brazilian Unified Public Health System	2	kept
breakthrough groups	breakthrough groups	breakthrough groups	breakthrough groups	breakthrough groups	2	kept
breakthrough pain	breakthrough pain	breakthrough pain	breakthrough pain	breakthrough pain	2	kept
breast adenocarcinoma	breast adenocarcinoma	breast adenocarcinoma	breast adenocarcinoma	breast adenocarcinoma	2	kept
breast augmentation	breast augmentation	breast augmentation	breast augmentation	breast augmentation	2	kept
breast surgery	breast surgery	breast surgery	breast surgery	breast surgery	2	kept
Breastfeeding initiation	breastfeeding initiation	breastfeeding initiation	breastfeeding initiation	Breastfeeding initiation	2	kept
Breath Tests	breath tests	breath tests	breath tests	Breath Tests	2	kept
breech extraction	breech extraction	breech extraction	breech extraction	breech extraction	2	kept
Breech Presentation	breech presentation	breech presentation	breech presentation	Breech Presentation	2	kept
breech presentation	breech presentation	breech presentation	breech presentation	Breech Presentation	2	case_or_format
Brief COPE Inventory	brief cope inventory	brief cope inventory	brief cope inventory	Brief COPE Inventory	2	kept
Brief intervention	brief intervention	brief intervention	brief intervention	Brief intervention	2	kept
brief resilient coping scale	brief resilient coping scale	brief resilient coping scale	brief resilient coping scale	brief resilient coping scale	2	kept
BRIGHT LIGHT	bright light	bright light	bright light	BRIGHT LIGHT	2	kept

bristol stool scale	bristol stool scale	bristol stool scale	bristol stool scale	bristol stool scale	2	kept
bronchial aspiration procedure	bronchial aspiration procedure	bronchial aspiration procedure	bronchial aspiration procedure	bronchial aspiration procedure	2	kept
Bronchial brushing	bronchial brushing	bronchial brushing	bronchial brushing	Bronchial brushing	2	kept
bronchialoccluder	bronchialoccluder	bronchialoccluder	bronchialoccluder	bronchialoccluder	2	kept
Bronchodilator	bronchodilator	bronchodilator	bronchodilator	Bronchodilator	2	kept
bronchomalacia	bronchomalacia	bronchomalacia	bronchomalacia	bronchomalacia	2	kept
Bronchoscope examination	bronchoscope examination	bronchoscope examination	bronchoscope examination	Bronchoscope examination	2	kept
Bronchoscopes	bronchoscopes	bronchoscopes	bronchoscope	bronchoscope	2	singular
bronchus	bronchus	bronchus	bronchus	bronchus	2	kept
bronchus obstruction	bronchus obstruction	bronchus obstruction	bronchus obstruction	bronchus obstruction	2	kept
Broset Violence Checklist	broset violence checklist	broset violence checklist	broset violence checklist	Broset Violence Checklist	2	kept
brother	brother	brother	brother	brother	2	kept
bruising	bruising	bruising	bruising	bruising	2	kept
BUBBLE CPAP	bubble cpap	bubble cpap	bubble cpap	BUBBLE CPAP	2	kept
Bubble humidification	bubble humidification	bubble humidification	bubble humidification	Bubble humidification	2	kept
Buddhist	buddhist	buddhist	buddhist	Buddhist	2	kept

Budget	budget	budget	budget	budget	2	case_or_format
Budget control	budget control	budget control	budget control	Budget control	2	kept
built environment	built environment	built environment	built environment	Built Environment	2	case_or_format
bulbar paralysis	bulbar paralysis	bulbar paralysis	bulbar paralysis	bulbar paralysis	2	kept
bulimia	bulimia	bulimia	bulimia	Bulimia	2	case_or_format
Bunching strategy	bunching strategy	bunching strategy	bunching strategy	Bunching strategy	2	kept
Bundle care	bundle care	bundle care	bundle care	Bundle care	2	kept
bundle care	bundle care	bundle care	bundle care	Bundle care	2	case_or_format
Bundle management	bundle management	bundle management	bundle management	Bundle management	2	kept
Bundled payments	bundled payments	bundled payments	bundled payment	Bundled payments	2	singular
Bundles of changes	bundles of changes	bundles of changes	bundles of changes	Bundles of changes	2	kept
burden of disease	burden of disease	burden of disease	burden of disease	Burden of disease	2	case_or_format
burial	burial	burial	burial	burial	2	kept
Burkholderia infection	burkholderia infection	burkholderia infection	burkholderia infection	Burkholderia Infections	2	case_or_format
Burkholderia pseudomallei	burkholderia pseudomallei	burkholderia pseudomallei	burkholderia pseudomallei	Burkholderia pseudomallei	2	kept
Burkholderiaceae	burkholderiaceae	burkholderiaceae	burkholderiaceae	Burkholderiaceae	2	kept

Burn center	burn center	burn center	burn center	Burn center	2	kept
burn contracture	burn contracture	burn contracture	burn contracture	burn contracture	2	kept
burn infection	burn infection	burn infection	burn infection	burn infection	2	kept
Burn injury	burn injury	burn injury	burn injury	Burn injury	2	kept
Burn rehabilitation	burn rehabilitation	burn rehabilitation	burn rehabilitation	Burn rehabilitation	2	kept
burn survivor	burn survivor	burn survivor	burn survivor	burn survivor	2	kept
Burn wound care	burn wound care	burn wound care	burn wound care	Burn wound care	2	kept
Burn wounds	burn wounds	burn wounds	burn wounds	Burn wounds	2	kept
Burnout assessment	burnout assessment	burnout assessment	burnout assessment	Burnout assessment	2	kept
burnout prevention	burnout prevention	burnout prevention	burnout prevention	burnout prevention	2	kept
burnout, psychological	burnout psychological	burnout psychological	burnout psychological	Burnout, Psychological	2	case_or_format
burnouts	burnouts	burnouts	burnout	burnout	2	singular
burnout—professional	burnout professional	burnout professional	burnout professional	Burnout, Professional	2	case_or_format
business concepts	business concepts	business concepts	business concepts	business concepts	2	kept
Buttocks	buttocks	buttocks	buttock	buttock	2	singular
Bypass	bypass	bypass	bypass	Bypass	2	kept

C-CHEWS score	c chews score	c chews score	c chews score	C-CHEWS score	2	kept
C-Peptide	c peptide	c peptide	c peptide	C peptide	2	case_or_format
C57BL mouse	c57bl mouse	c57bl mouse	c57bl mouse	C57BL mouse	2	kept
Cabg	cabg	cabg	cabg	CABG	2	case_or_format
cabotegravir	cabotegravir	cabotegravir	cabotegravir	cabotegravir	2	kept
caesarean section	caesarean section	caesarean section	caesarean section	caesarean section	2	kept
calcification	calcification	calcification	calcification	calcification	2	kept
Calcinosis	calcinosis	calcinosis	calcinosis	Calcinosis	2	kept
calcinosis	calcinosis	calcinosis	calcinosis	Calcinosis	2	case_or_format
calcium acetate	calcium acetate	calcium acetate	calcium acetate	calcium acetate	2	kept
Calcium channel blocker	calcium channel blocker	calcium channel blocker	calcium channel blocker	Calcium Channel Blockers	2	case_or_format
Calcium Metabolism Disorders	calcium metabolism disorders	calcium metabolism disorders	calcium metabolism disorders	Calcium Metabolism Disorders	2	kept
calculi	calculi	calculi	calculi	calculi	2	kept
Calf-compresses	calf compresses	calf compresses	calf compresses	Calf-compresses	2	kept
Caliciviridae Infections	caliciviridae infections	caliciviridae infections	caliciviridae infections	Caliciviridae Infections	2	kept
Calidad	calidad	calidad	calidad	Calidad	2	kept

CALIFORNIA	california	california	california	California	2	case_or_format
Call	call	call	call	Call	2	kept
calling	calling	calling	calling	calling	2	kept
camera monitoring	camera monitoring	camera monitoring	camera monitoring	camera monitoring	2	kept
Campaign	campaign	campaign	campaign	Campaign	2	kept
Campylobacter	campylobacter	campylobacter	campylobacter	Campylobacter	2	kept
CANADA	canada	canada	canada	Canada	2	case_or_format
Cancer care facilities	cancer care facilities	cancer care facilities	cancer care facilities	Cancer Care Facilities	2	case_or_format
cancer combination chemotherapy	cancer combination chemotherapy	cancer combination chemotherapy	cancer combination chemotherapy	cancer combination chemotherapy	2	kept
cancer control	cancer control	cancer control	cancer control	cancer control	2	kept
cancer localization	cancer localization	cancer localization	cancer localization	cancer localization	2	kept
cancer model	cancer model	cancer model	cancer model	cancer model	2	kept
Candida albicans germ tube antibody	candida albicans germ tube antibody	candida albicans germ tube antibody	candida albicans germ tube antibody	Candida albicans germ tube antibody	2	kept
Candida krusei	candida krusei	candida krusei	candida krusei	Candida krusei	2	kept
Candida species	candida species	candida species	candida species	Candida species	2	kept
canine	canine	canine	canine	canine	2	kept

Canis	canis	canis	canis	Canis	2	kept
Cannabinoid Receptor Agonists	cannabinoid receptor agonists	cannabinoid receptor agonists	cannabinoid receptor agonist	Cannabinoid Receptor Agonists	2	singular
Cannabis abuse	cannabis abuse	cannabis abuse	cannabis abuse	Cannabis abuse	2	kept
cannabis addiction	cannabis addiction	cannabis addiction	cannabis addiction	cannabis addiction	2	kept
Cannulations	cannulations	cannulations	cannulation	cannulation	2	singular
CAPABILITY	capability	capability	capability	CAPABILITY	2	kept
Capability	capability	capability	capability	CAPABILITY	2	case_or_format
capacity boosting	capacity boosting	capacity boosting	capacity boosting	capacity boosting	2	kept
Capacity development	capacity development	capacity development	capacity development	Capacity development	2	kept
capacity development	capacity development	capacity development	capacity development	Capacity development	2	case_or_format
capillary refill time	capillary refill time	capillary refill time	capillary refill time	Capillary refill time	2	case_or_format
Capital Expenditures	capital expenditures	capital expenditures	capital expenditures	Capital Expenditures	2	kept
Capital Financing	capital financing	capital financing	capital financing	Capital Financing	2	kept
Capnocytophaga	capnocytophaga	capnocytophaga	capnocytophaga	Capnocytophaga	2	kept
capsaicin	capsaicin	capsaicin	capsaicin	capsaicin	2	kept
capsule endoscopy	capsule endoscopy	capsule endoscopy	capsule endoscopy	capsule endoscopy	2	kept

car driving	car driving	car driving	car driving	car driving	2	kept
carbapenem antibiotics	carbapenem antibiotics	carbapenem antibiotics	carbapenem antibiotics	carbapenem antibiotics	2	kept
Carbapenem resistance	carbapenem resistance	carbapenem resistance	carbapenem resistance	carbapenem resistance	2	case_or_format
carbapenem resistant Escherichia coli	carbapenem resistant escherichia coli	carbapenem resistant escherichia coli	carbapenem resistant escherichia coli	carbapenem resistant Escherichia coli	2	kept
Carbapenem-Resistant Enterobacterales	carbapenem resistant enterobacterales	carbapenem resistant enterobacterales	carbapenem resistant enterobacterale	Carbapenem-Resistant Enterobacterales	2	singular
Carbapenem-resistant Enterobacteriaceae	carbapenem resistant enterobacteriaceae	carbapenem resistant enterobacteriaceae	carbapenem resistant enterobacteriaceae	carbapenem-resistant Enterobacteriaceae	2	case_or_format
carbapenemase gene	carbapenemase gene	carbapenemase gene	carbapenemase gene	carbapenemase gene	2	kept
Carbapenemase-producing Enterobacterales	carbapenemase producing enterobacterales	carbapenemase producing enterobacterales	carbapenemase producing enterobacterale	Carbapenemase-producing Enterobacterales	2	singular
carbapenemases	carbapenemases	carbapenemases	carbapenemase	carbapenemase	2	singular
carbapenems	carbapenems	carbapenems	carbapenem	carbapenem	2	singular
carbidopa plus levodopa	carbidopa plus levodopa	carbidopa plus levodopa	carbidopa plus levodopa	carbidopa plus levodopa	2	kept
carbomer	carbomer	carbomer	carbomer	carbomer	2	kept
carbon emission	carbon emission	carbon emission	carbon emission	carbon emission	2	kept
Carbon monoxide	carbon monoxide	carbon monoxide	carbon monoxide	carbon monoxide	2	case_or_format
Carboplatin	carboplatin	carboplatin	carboplatin	carboplatin	2	case_or_format
carboxy terminal telopeptide	carboxy terminal telopeptide	carboxy terminal telopeptide	carboxy terminal telopeptide	carboxy terminal telopeptide	2	kept

carboxymethylcellulose	carboxymethylcellulose	carboxymethylcellulose	carboxymethylcellulose	carboxymethylcellulose	2	kept
carcinoembryonic antigen	carcinoembryonic antigen	carcinoembryonic antigen	carcinoembryonic antigen	carcinoembryonic antigen	2	kept
carcinogenesis	carcinogenesis	carcinogenesis	carcinogenesis	carcinogenesis	2	kept
Carcinoma, Large Cell	carcinoma large cell	carcinoma large cell	carcinoma large cell	Carcinoma, Large Cell	2	kept
Cardiac Arrest	cardiac arrest	cardiac arrest	cardiac arrest	Cardiac arrest	2	case_or_format
cardiac care facilities	cardiac care facilities	cardiac care facilities	cardiac care facilities	Cardiac Care Facilities	2	case_or_format
Cardiac catheterization laboratory	cardiac catheterization laboratory	cardiac catheterization laboratory	cardiac catheterization laboratory	Cardiac catheterization laboratory	2	kept
cardiac cycle	cardiac cycle	cardiac cycle	cardiac cycle	cardiac cycle	2	kept
Cardiac failure	cardiac failure	cardiac failure	cardiac failure	cardiac failure	2	case_or_format
Cardiac Intensivist	cardiac intensivist	cardiac intensivist	cardiac intensivist	Cardiac Intensivist	2	kept
Cardiac medications	cardiac medications	cardiac medications	cardiac medications	Cardiac medications	2	kept
cardiac monitoring	cardiac monitoring	cardiac monitoring	cardiac monitoring	Cardiac monitoring	2	case_or_format
cardiac muscle cell	cardiac muscle cell	cardiac muscle cell	cardiac muscle cell	cardiac muscle cell	2	kept
cardiac output monitor	cardiac output monitor	cardiac output monitor	cardiac output monitor	cardiac output monitor	2	kept
CARDIAC REHABILITATION	cardiac rehabilitation	cardiac rehabilitation	cardiac rehabilitation	Cardiac Rehabilitation	2	case_or_format
Cardiac Resynchronization Therapy Devices	cardiac resynchronization therapy devices	cardiac resynchronization therapy devices	cardiac resynchronization therapy device	Cardiac Resynchronization Therapy Devices	2	singular

Cardiac surgical procedure	cardiac surgical procedure	cardiac surgical procedure	cardiac surgical procedure	Cardiac Surgical Procedures	2	case_or_format
CARDIAC-CATHETERIZATION	cardiac catheterization	cardiac catheterization	cardiac catheterization	Cardiac Catheterization	2	case_or_format
cardio	cardio	cardio	cardio	cardio	2	kept
Cardio-oncology	cardio oncology	cardio oncology	cardio oncology	Cardio-oncology	2	kept
Cardio-Renal Syndrome	cardio renal syndrome	cardio renal syndrome	cardio renal syndrome	Cardio-Renal Syndrome	2	kept
cardiogenic pulmonary edema	cardiogenic pulmonary edema	cardiogenic pulmonary edema	cardiogenic pulmonary edema	cardiogenic pulmonary edema	2	kept
Cardiopulmonary	cardiopulmonary	cardiopulmonary	cardiopulmonary	Cardiopulmonary	2	kept
cardiopulmonary	cardiopulmonary	cardiopulmonary	cardiopulmonary	Cardiopulmonary	2	case_or_format
cardiopulmonary comorbidities	cardiopulmonary comorbidities	cardiopulmonary comorbidities	cardiopulmonary comorbidities	cardiopulmonary comorbidities	2	kept
cardiopulmonary management	cardiopulmonary management	cardiopulmonary management	cardiopulmonary management	cardiopulmonary management	2	kept
Cardiorespiratory arrest	cardiorespiratory arrest	cardiorespiratory arrest	cardiorespiratory arrest	Cardiorespiratory arrest	2	kept
cardiovascular care unit	cardiovascular care unit	cardiovascular care unit	cardiovascular care unit	cardiovascular care unit	2	kept
Cardiovascular Disease	cardiovascular disease	cardiovascular disease	cardiovascular disease	cardiovascular disease	2	case_or_format
cardiovascular disease assessment	cardiovascular disease assessment	cardiovascular disease assessment	cardiovascular disease assessment	cardiovascular disease assessment	2	kept
cardiovascular equipment	cardiovascular equipment	cardiovascular equipment	cardiovascular equipment	cardiovascular equipment	2	kept
cardiovascular medicine	cardiovascular medicine	cardiovascular medicine	cardiovascular medicine	cardiovascular medicine	2	kept

Cardiovascular monitoring	cardiovascular monitoring	cardiovascular monitoring	cardiovascular monitoring	Cardiovascular monitoring	2	kept
cardiovascular procedure	cardiovascular procedure	cardiovascular procedure	cardiovascular procedure	cardiovascular procedure	2	kept
Cardiovascular system	cardiovascular system	cardiovascular system	cardiovascular system	cardiovascular system	2	case_or_format
care complexity index	care complexity index	care complexity index	care complexity index	care complexity index	2	kept
care continuity	care continuity	care continuity	care continuity	care continuity	2	kept
Care delivery models	care delivery models	care delivery models	care delivery models	Care delivery models	2	kept
care dependency	care dependency	care dependency	care dependency	care dependency	2	kept
Care encounter	care encounter	care encounter	care encounter	Care encounter	2	kept
care ethics	care ethics	care ethics	care ethics	Care ethics	2	case_or_format
Care givers	care givers	care givers	care givers	Care givers	2	kept
care goal	care goal	care goal	care goal	care goal	2	kept
Care homes	care homes	care homes	care homes	Care homes	2	kept
Care limitation	care limitation	care limitation	care limitation	Care limitation	2	kept
Care package	care package	care package	care package	Care package	2	kept
care process	care process	care process	care process	care process	2	kept
Care protocol	care protocol	care protocol	care protocol	Care protocol	2	kept

Care provision	care provision	care provision	care provision	Care provision	2	kept
Care settings	care settings	care settings	care setting	Care settings	2	singular
Care transition outcomes	care transition outcomes	care transition outcomes	care transition outcomes	Care transition outcomes	2	kept
care utilization	care utilization	care utilization	care utilization	care utilization	2	kept
Care worker	care worker	care worker	care worker	Care worker	2	kept
care workload	care workload	care workload	care workload	care workload	2	kept
career calling	career calling	career calling	career calling	career calling	2	kept
Career development	career development	career development	career development	Career development	2	kept
career development	career development	career development	career development	Career development	2	case_or_format
career ladder	career ladder	career ladder	career ladder	career ladder	2	kept
Career motivation	career motivation	career motivation	career motivation	Career motivation	2	kept
Career success	career success	career success	career success	Career success	2	kept
CAREGIVER BURDEN	caregiver burden	caregiver burden	caregiver burden	caregiver burden	2	case_or_format
caregiver experience	caregiver experience	caregiver experience	caregiver experience	caregiver experience	2	kept
caregiver involvement	caregiver involvement	caregiver involvement	caregiver involvement	caregiver involvement	2	kept
Caregiver support	caregiver support	caregiver support	caregiver support	caregiver support	2	case_or_format

Caregiving	caregiving	caregiving	caregiving	caregiving	2	case_or_format
Caregiving burden	caregiving burden	caregiving burden	caregiving burden	Caregiving burden	2	kept
Carer	carer	carer	carer	Carers	2	case_or_format
Carga de Trabajo	carga de trabajo	carga de trabajo	carga de trabajo	Carga de trabajo	2	case_or_format
Carga de trabalho	carga de trabalho	carga de trabalho	carga de trabalho	Carga de Trabalho	2	case_or_format
Caring behaviour	caring behaviour	caring behaviour	caring behaviour	caring behaviour	2	case_or_format
caring behaviours	caring behaviours	caring behaviours	caring behaviour	caring behaviour	2	singular
caring efficacy	caring efficacy	caring efficacy	caring efficacy	caring efficacy	2	kept
caring practice	caring practice	caring practice	caring practice	caring practice	2	kept
Caring science	caring science	caring science	caring science	Caring science	2	kept
carotid artery obstruction	carotid artery obstruction	carotid artery obstruction	carotid artery obstruction	carotid artery obstruction	2	kept
carotid stenosis	carotid stenosis	carotid stenosis	carotid stenosis	Carotid Stenosis	2	case_or_format
Case design	case design	case design	case design	Case design	2	kept
Case Report	case report	case report	case report	case report	2	case_or_format
Case Reports	case reports	case reports	case report	case report	2	singular
Case-based learning	case based learning	case based learning	case based learning	Case-Based Learning	2	case_or_format

case-control study	case control study	case control study	case control study	Case-Control Studies	2	case_or_format
casirivimab plus imdevimab	casirivimab plus imdevimab	casirivimab plus imdevimab	casirivimab plus imdevimab	casirivimab plus imdevimab	2	kept
catabolism	catabolism	catabolism	catabolism	catabolism	2	kept
catalepsy	catalepsy	catalepsy	catalepsy	catalepsy	2	kept
catalyst	catalyst	catalyst	catalyst	catalyst	2	kept
catecholamine derivative	catecholamine derivative	catecholamine derivative	catecholamine derivative	catecholamine derivative	2	kept
catecholamines	catecholamines	catecholamines	catecholamine	catecholamine	2	singular
Cateterismo Venoso Central	cateterismo venoso central	cateterismo venoso central	cateterismo venoso central	Cateterismo Venoso Central	2	kept
Cateterismo Venoso Central.	cateterismo venoso central	cateterismo venoso central	cateterismo venoso central	Cateterismo Venoso Central	2	case_or_format
catharsis	catharsis	catharsis	catharsis	catharsis	2	kept
CATHETER	catheter	catheter	catheter	Catheters	2	case_or_format
catheter breakage	catheter breakage	catheter breakage	catheter breakage	catheter breakage	2	kept
catheter dysfunction	catheter dysfunction	catheter dysfunction	catheter dysfunction	catheter dysfunction	2	kept
catheter fracture	catheter fracture	catheter fracture	catheter fracture	catheter fracture	2	kept
catheter insertion	catheter insertion	catheter insertion	catheter insertion	catheter insertion	2	kept
Catheter placement	catheter placement	catheter placement	catheter placement	Catheter placement	2	kept

Catheter related bloodstream infection	catheter related bloodstream infection	catheter related bloodstream infection	catheter related bloodstream infection	catheter related bloodstream infection	2	case_or_format
Catheter securement	catheter securement	catheter securement	catheter securement	Catheter securement	2	kept
catheter-related bacteremia	catheter related bacteremia	catheter related bacteremia	catheter related bacteremia	catheter-related bacteremia	2	kept
Catheter-related urinary tract infection	catheter related urinary tract infection	catheter related urinary tract infection	catheter related urinary tract infection	Catheter-related urinary tract infection	2	kept
Catheterisation	catheterisation	catheterisation	catheterisation	Catheterisation	2	kept
Catheterization peripheral	catheterization peripheral	catheterization peripheral	catheterization peripheral	Catheterization, Peripheral	2	case_or_format
CATHETERS	catheters	catheters	catheter	Catheters	2	singular
Catholic	catholic	catholic	catholic	Catholic	2	kept
causal attribution	causal attribution	causal attribution	causal attribution	causal attribution	2	kept
Causal random forest	causal random forest	causal random forest	causal random forest	Causal random forest	2	kept
causative microorganisms	causative microorganisms	causative microorganisms	causative microorganisms	causative microorganisms	2	kept
Cause of death	cause of death	cause of death	cause of death	cause of death	2	case_or_format
CBT	cbt	cbt	cbt	CBT	2	kept
CCC	ccc	ccc	ccc	CCC	2	kept
CCNs	ccns	ccns	ccns	CCNs	2	kept
CCRN	ccrn	ccrn	ccrn	CCRN	2	kept

CD-ROM	cd rom	cd rom	cd rom	CD-ROM	2	kept
CD8 lymphocyte count	cd8 lymphocyte count	cd8 lymphocyte count	cd8 lymphocyte count	CD8 lymphocyte count	2	kept
CD8-Positive T-Lymphocytes	cd8 positive t lymphocytes	cd8 positive t lymphocytes	cd8 positive t lymphocytes	CD8-Positive T-Lymphocytes	2	kept
CDAD	cdad	cdad	cdad	CDAD	2	kept
CDIO model	cdio model	cdio model	cdio model	CDIO model	2	kept
cefaclor	cefaclor	cefaclor	cefaclor	cefaclor	2	kept
cefadroxil	cefadroxil	cefadroxil	cefadroxil	cefadroxil	2	kept
cefalotin	cefalotin	cefalotin	cefalotin	cefalotin	2	kept
cefotetan	cefotetan	cefotetan	cefotetan	cefotetan	2	kept
cefprozil	cefprozil	cefprozil	cefprozil	cefprozil	2	kept
ceftibuten	ceftibuten	ceftibuten	ceftibuten	ceftibuten	2	kept
ceftriaxone plus sulbactam	ceftriaxone plus sulbactam	ceftriaxone plus sulbactam	ceftriaxone plus sulbactam	ceftriaxone plus sulbactam	2	kept
celecoxib	celecoxib	celecoxib	celecoxib	celecoxib	2	kept
cell activity	cell activity	cell activity	cell activity	cell activity	2	kept
cell cycle arrest	cell cycle arrest	cell cycle arrest	cell cycle arrest	cell cycle arrest	2	kept
cell death	cell death	cell death	cell death	cell death	2	kept

cell heterogeneity	cell heterogeneity	cell heterogeneity	cell heterogeneity	cell heterogeneity	2	kept
Cell Hypoxia	cell hypoxia	cell hypoxia	cell hypoxia	Cell Hypoxia	2	kept
cell proliferation	cell proliferation	cell proliferation	cell proliferation	cell proliferation	2	kept
cell structure	cell structure	cell structure	cell structure	cell structure	2	kept
cell transplantation	cell transplantation	cell transplantation	cell transplantation	cell transplantation	2	kept
Cell- and Tissue-Based Therapy	cell and tissue based therapy	cell and tissue based therapy	cell and tissue based therapy	Cell- and Tissue-Based Therapy	2	kept
Cellular Phone	cellular phone	cellular phone	cellular phone	Cellular Phone	2	kept
center of coordination	center of coordination	center of coordination	center of coordination	center of coordination	2	kept
Central America	central america	central america	central america	Central America	2	kept
Central apneas	central apneas	central apneas	central apneas	Central apneas	2	kept
Central line associated blood stream infection	central line associated blood stream infection	central line associated blood stream infection	central line associated blood stream infection	Central line associated blood stream infection	2	kept
central line associated blood stream infection	central line associated blood stream infection	central line associated blood stream infection	central line associated blood stream infection	Central line associated blood stream infection	2	case_or_format
Central line associated bloodstream infections	central line associated bloodstream infections	central line associated bloodstream infections	central line associated bloodstream infection	Central line-associated bloodstream infections	2	singular
Central line infections	central line infections	central line infections	central line infection	Central line infections	2	singular
Central line-associated bloodstream infection	central line associated bloodstream infection	central line associated bloodstream infection	central line associated bloodstream infection	Central line-associated bloodstream infections	2	case_or_format
central line - associated bloodstream infection	central line associated bloodstream infection	central line associated bloodstream infection	central line associated bloodstream infection	Central line-associated bloodstream infections	2	case_or_format

central muscle relaxant	central muscle relaxant	central muscle relaxant	central muscle relaxant	central muscle relaxant	2	kept
central nervous system cancer	central nervous system cancer	central nervous system cancer	central nervous system cancer	central nervous system cancer	2	kept
central nervous system function	central nervous system function	central nervous system function	central nervous system function	central nervous system function	2	kept
central vascular catheter	central vascular catheter	central vascular catheter	central vascular catheter	central vascular catheter	2	kept
central vein	central vein	central vein	central vein	central vein	2	kept
central venous access	central venous access	central venous access	central venous access	Central venous access	2	case_or_format
central venous cannulation	central venous cannulation	central venous cannulation	central venous cannulation	central venous cannulation	2	kept
Central Venous Catheter	central venous catheter	central venous catheter	central venous catheter	central venous catheter	2	case_or_format
central venous catheter infection	central venous catheter infection	central venous catheter infection	central venous catheter infection	central venous catheter infection	2	kept
Central Venous Catheterization	central venous catheterization	central venous catheterization	central venous catheterization	central venous catheterization	2	case_or_format
central venous line	central venous line	central venous line	central venous line	Central venous line	2	case_or_format
central venous occlusion	central venous occlusion	central venous occlusion	central venous occlusion	central venous occlusion	2	kept
Central venous oxygen saturation	central venous oxygen saturation	central venous oxygen saturation	central venous oxygen saturation	central venous oxygen saturation	2	case_or_format
Central-line associated bloodstream infection	central line associated bloodstream infection	central line associated bloodstream infection	central line associated bloodstream infection	Central line-associated bloodstream infections	2	case_or_format
Central-line bundle	central line bundle	central line bundle	central line bundle	central line bundle	2	case_or_format
Central-line-associated bloodstream infections	central line associated bloodstream infections	central line associated bloodstream infections	central line associated bloodstream infection	Central line-associated bloodstream infections	2	singular

Centralized Hospital Services	centralized hospital services	centralized hospital services	centralized hospital services	Centralized Hospital Services	2	kept
centralized remote fetal monitoring	centralized remote fetal monitoring	centralized remote fetal monitoring	centralized remote fetal monitoring	centralized remote fetal monitoring	2	kept
centrifugal pump	centrifugal pump	centrifugal pump	centrifugal pump	centrifugal pump	2	kept
cephalic vein	cephalic vein	cephalic vein	cephalic vein	cephalic vein	2	kept
cephalometry	cephalometry	cephalometry	cephalometry	Cephalometry	2	case_or_format
Cephalosporin Resistance	cephalosporin resistance	cephalosporin resistance	cephalosporin resistance	cephalosporin resistance	2	case_or_format
cephamycin derivative	cephamycin derivative	cephamycin derivative	cephamycin derivative	cephamycin derivative	2	kept
cerebellum infarction	cerebellum infarction	cerebellum infarction	cerebellum infarction	cerebellum infarction	2	kept
Cerebral aneurysm	cerebral aneurysm	cerebral aneurysm	cerebral aneurysm	Cerebral aneurysm	2	kept
Cerebral blood flow	cerebral blood flow	cerebral blood flow	cerebral blood flow	Cerebral blood flow	2	kept
cerebral blood flow	cerebral blood flow	cerebral blood flow	cerebral blood flow	Cerebral blood flow	2	case_or_format
Cerebral oxygenation	cerebral oxygenation	cerebral oxygenation	cerebral oxygenation	cerebral oxygenation	2	case_or_format
Cerebral perfusion	cerebral perfusion	cerebral perfusion	cerebral perfusion	Cerebral perfusion	2	kept
cerebral salt wasting	cerebral salt wasting	cerebral salt wasting	cerebral salt wasting	cerebral salt wasting	2	kept
cerebral sinus thrombosis	cerebral sinus thrombosis	cerebral sinus thrombosis	cerebral sinus thrombosis	cerebral sinus thrombosis	2	kept
Cerebrospinal Fluid Pressure	cerebrospinal fluid pressure	cerebrospinal fluid pressure	cerebrospinal fluid pressure	Cerebrospinal Fluid Pressure	2	kept

cerebrospinal fluid shunts	cerebrospinal fluid shunts	cerebrospinal fluid shunts	cerebrospinal fluid shunts	Cerebrospinal Fluid Shunts	2	case_or_format
Cerebrovascular disease	cerebrovascular disease	cerebrovascular disease	cerebrovascular disease	cerebrovascular disease	2	case_or_format
Cerebrovascular diseases	cerebrovascular diseases	cerebrovascular diseases	cerebrovascular disease	cerebrovascular disease	2	singular
Cerebrovascular Trauma	cerebrovascular trauma	cerebrovascular trauma	cerebrovascular trauma	Cerebrovascular Trauma	2	kept
CERTIFICATION	certification	certification	certification	Certification	2	case_or_format
cervical spine radiography	cervical spine radiography	cervical spine radiography	cervical spine radiography	cervical spine radiography	2	kept
cervical spondylosis	cervical spondylosis	cervical spondylosis	cervical spondylosis	cervical spondylosis	2	kept
cesarean	cesarean	cesarean	cesarean	cesarean	2	kept
Cesarean delivery	cesarean delivery	cesarean delivery	cesarean delivery	Cesarean delivery	2	kept
cetuximab	cetuximab	cetuximab	cetuximab	Cetuximab	2	case_or_format
cetylpyridinium salt	cetylpyridinium salt	cetylpyridinium salt	cetylpyridinium salt	cetylpyridinium salt	2	kept
cgMLST	cgmlst	cgmlst	cgmlst	cgMLST	2	kept
chain mediating effect	chain mediating effect	chain mediating effect	chain mediating effect	chain mediating effect	2	kept
Chain mediation effect	chain mediation effect	chain mediation effect	chain mediation effect	Chain mediation effect	2	kept
Chalazion	chalazion	chalazion	chalazion	chalazion	2	case_or_format
Change process	change process	change process	change process	Change process	2	kept

Chaplains	chaplains	chaplains	chaplain	Chaplain	2	singular
chaplains	chaplains	chaplains	chaplain	Chaplain	2	singular
Character	character	character	character	character	2	case_or_format
CHARLSON COMORBIDITY INDEX	charlson comorbidity index	charlson comorbidity index	charlson comorbidity index	Charlson Comorbidity Index	2	case_or_format
Chart-field completion	chart field completion	chart field completion	chart field completion	Chart-field completion	2	kept
Checking	checking	checking	checking	Checking	2	kept
cheek	cheek	cheek	cheek	cheek	2	kept
Chemical	chemical	chemical	chemical	Chemical	2	kept
chemical binding	chemical binding	chemical binding	chemical binding	chemical binding	2	kept
Chemokine CXCL10	chemokine cxcl10	chemokine cxcl10	chemokine cxcl10	Chemokine CXCL10	2	kept
Chemoprophylaxis	chemoprophylaxis	chemoprophylaxis	chemoprophylaxis	chemoprophylaxis	2	case_or_format
CHEMOTHERAPY	chemotherapy	chemotherapy	chemotherapy	chemotherapy	2	case_or_format
Chemotherapy, Adjuvant	chemotherapy adjuvant	chemotherapy adjuvant	chemotherapy adjuvant	Chemotherapy, Adjuvant	2	kept
chest compression	chest compression	chest compression	chest compression	Chest compression	2	case_or_format
chest compression quality	chest compression quality	chest compression quality	chest compression quality	chest compression quality	2	kept
Chest drain	chest drain	chest drain	chest drain	Chest drain	2	kept

Chest drains	chest drains	chest drains	chest drain	Chest drain	2	singular
chest drains	chest drains	chest drains	chest drain	Chest drain	2	singular
Chest imaging	chest imaging	chest imaging	chest imaging	Chest imaging	2	kept
chest wall oscillation	chest wall oscillation	chest wall oscillation	chest wall oscillation	Chest Wall Oscillation	2	case_or_format
chest X-ray	chest x ray	chest x ray	chest x ray	chest X-ray	2	kept
chest X-rays	chest x rays	chest x rays	chest x ray	chest X-ray	2	singular
chewing gum	chewing gum	chewing gum	chewing gum	chewing gum	2	kept
chikungunya	chikungunya	chikungunya	chikungunya	chikungunya	2	kept
Child advocacy	child advocacy	child advocacy	child advocacy	Child Advocacy	2	case_or_format
Child Behavior Checklist	child behavior checklist	child behavior checklist	child behavior checklist	Child Behavior Checklist	2	kept
Child death	child death	child death	child death	child death	2	case_or_format
child neglect	child neglect	child neglect	child neglect	child neglect	2	kept
child opportunity index	child opportunity index	child opportunity index	child opportunity index	child opportunity index	2	kept
Child, hospitalized	child hospitalized	child hospitalized	child hospitalized	Child, Hospitalized	2	case_or_format
Child-Pugh score	child pugh score	child pugh score	child pugh score	Child Pugh score	2	case_or_format
childbirth hospital services	childbirth hospital services	childbirth hospital services	childbirth hospital services	childbirth hospital services	2	kept

Childcare	childcare	childcare	childcare	Childcare	2	kept
childhood	childhood	childhood	childhood	childhood	2	kept
Chills	chills	chills	chill	chill	2	singular
chimeric antigen receptor	chimeric antigen receptor	chimeric antigen receptor	chimeric antigen receptor	chimeric antigen receptor	2	kept
Chinese drug	chinese drug	chinese drug	chinese drug	Chinese drug	2	kept
Chinese hospital	chinese hospital	chinese hospital	chinese hospital	Chinese hospital	2	kept
chiropractic	chiropractic	chiropractic	chiropractic	chiropractic	2	kept
chiropractor	chiropractor	chiropractor	chiropractor	chiropractor	2	kept
Chlamydia	chlamydia	chlamydia	chlamydia	Chlamydia	2	kept
chlamydial pneumonia	chlamydial pneumonia	chlamydial pneumonia	chlamydial pneumonia	chlamydial pneumonia	2	kept
chlamydiasis	chlamydiasis	chlamydiasis	chlamydiasis	chlamydiasis	2	kept
chlorhexidine bathing	chlorhexidine bathing	chlorhexidine bathing	chlorhexidine bathing	chlorhexidine bathing	2	kept
Chlorhexidine gluconate wipes	chlorhexidine gluconate wipes	chlorhexidine gluconate wipes	chlorhexidine gluconate wipes	Chlorhexidine gluconate wipes	2	kept
Chlorides	chlorides	chlorides	chloride	chloride	2	singular
Chlorine	chlorine	chlorine	chlorine	chlorine	2	case_or_format
Chlorocebus aethiops	chlorocebus aethiops	chlorocebus aethiops	chlorocebus aethiops	Chlorocebus aethiops	2	kept

chlorpropamide	chlorpropamide	chlorpropamide	chlorpropamide	chlorpropamide	2	kept
chlortalidone	chlortalidone	chlortalidone	chlortalidone	chlortalidone	2	kept
chocolate	chocolate	chocolate	chocolate	chocolate	2	kept
Choice	choice	choice	choice	choice	2	case_or_format
Cholangiocarcinoma	cholangiocarcinoma	cholangiocarcinoma	cholangiocarcinoma	Cholangiocarcinoma	2	kept
Cholangitis, Sclerosing	cholangitis sclerosing	cholangitis sclerosing	cholangitis sclerosing	Cholangitis, Sclerosing	2	kept
Cholecalciferol	cholecalciferol	cholecalciferol	cholecalciferol	Cholecalciferol	2	kept
Cholecystectomy	cholecystectomy	cholecystectomy	cholecystectomy	cholecystectomy	2	case_or_format
Cholecystostomy	cholecystostomy	cholecystostomy	cholecystostomy	Cholecystostomy	2	kept
cholecystostomy	cholecystostomy	cholecystostomy	cholecystostomy	Cholecystostomy	2	case_or_format
cholera	cholera	cholera	cholera	cholera	2	kept
Cholestasis, Intrahepatic	cholestasis intrahepatic	cholestasis intrahepatic	cholestasis intrahepatic	Cholestasis, Intrahepatic	2	kept
cholestatic hepatitis	cholestatic hepatitis	cholestatic hepatitis	cholestatic hepatitis	cholestatic hepatitis	2	kept
Choline	choline	choline	choline	Choline	2	kept
choline	choline	choline	choline	Choline	2	case_or_format
Cholinergic Agents	cholinergic agents	cholinergic agents	cholinergic agents	Cholinergic Agents	2	kept

Cholinergic Antagonists	cholinergic antagonists	cholinergic antagonists	cholinergic antagonists	Cholinergic Antagonists	2	kept
cholinesterase blood level	cholinesterase blood level	cholinesterase blood level	cholinesterase blood level	cholinesterase blood level	2	kept
choreography	choreography	choreography	choreography	choreography	2	kept
chorionic gonadotropin	chorionic gonadotropin	chorionic gonadotropin	chorionic gonadotropin	chorionic gonadotropin	2	kept
chorionic gonadotropin beta subunit	chorionic gonadotropin beta subunit	chorionic gonadotropin beta subunit	chorionic gonadotropin beta subunit	chorionic gonadotropin beta subunit	2	kept
choroid	choroid	choroid	choroid	choroid	2	kept
Chromatography, Liquid	chromatography liquid	chromatography liquid	chromatography liquid	Chromatography, Liquid	2	kept
chromosome	chromosome	chromosome	chromosome	chromosome	2	kept
chromosome 1	chromosome 1	chromosome 1	chromosome 1	chromosome 1	2	kept
chromosome aberration	chromosome aberration	chromosome aberration	chromosome aberration	chromosome aberration	2	kept
Chromosome Deletion	chromosome deletion	chromosome deletion	chromosome deletion	Chromosome Deletion	2	kept
chromosome deletion	chromosome deletion	chromosome deletion	chromosome deletion	Chromosome Deletion	2	case_or_format
chromosome deletion 22q11	chromosome deletion 22q11	chromosome deletion 22q11	chromosome deletion 22q11	chromosome deletion 22q11	2	kept
Chromosomes	chromosomes	chromosomes	chromosome	chromosome	2	singular
chronic bronchitis	chronic bronchitis	chronic bronchitis	chronic bronchitis	chronic bronchitis	2	kept
Chronic conditions	chronic conditions	chronic conditions	chronic conditions	Chronic conditions	2	kept

chronic conditions	chronic conditions	chronic conditions	chronic conditions	Chronic conditions	2	case_or_format
chronic disease management	chronic disease management	chronic disease management	chronic disease management	Chronic disease management	2	case_or_format
chronic fatigue	chronic fatigue	chronic fatigue	chronic fatigue	chronic fatigue	2	kept
chronic hepatitis B	chronic hepatitis b	chronic hepatitis b	chronic hepatitis b	chronic hepatitis B	2	kept
chronic myeloid leukemia	chronic myeloid leukemia	chronic myeloid leukemia	chronic myeloid leukemia	chronic myeloid leukemia	2	kept
Chronic or prolonged mechanical ventilation	chronic or prolonged mechanical ventilation	chronic or prolonged mechanical ventilation	chronic or prolonged mechanical ventilation	Chronic or prolonged mechanical ventilation	2	kept
chronic osteomyelitis	chronic osteomyelitis	chronic osteomyelitis	chronic osteomyelitis	chronic osteomyelitis	2	kept
chronic pancreatitis	chronic pancreatitis	chronic pancreatitis	chronic pancreatitis	chronic pancreatitis	2	kept
Chronic Renal Failure	chronic renal failure	chronic renal failure	chronic renal failure	Chronic Renal Failure	2	kept
Chronic respiratory failure	chronic respiratory failure	chronic respiratory failure	chronic respiratory failure	chronic respiratory failure	2	case_or_format
Chryseobacterium	chryseobacterium	chryseobacterium	chryseobacterium	Chryseobacterium	2	kept
chylous ascites	chylous ascites	chylous ascites	chylous ascites	chylous ascites	2	kept
chyme reinfusion	chyme reinfusion	chyme reinfusion	chyme reinfusion	chyme reinfusion	2	kept
Cimex	cimex	cimex	cimex	Cimex	2	kept
Circadian activity rhythms	circadian activity rhythms	circadian activity rhythms	circadian activity rhythms	Circadian activity rhythms	2	kept
Circadian variation	circadian variation	circadian variation	circadian variation	Circadian variation	2	kept

CIRCADIAN-RHYTHMS	circadian rhythms	circadian rhythms	circadian rhythm	circadian rhythm	2	singular
circulatory shock	circulatory shock	circulatory shock	circulatory shock	circulatory shock	2	kept
Circumcision, Male	circumcision male	circumcision male	circumcision male	Circumcision, Male	2	kept
CIS	cis	cis	cis	CIS	2	kept
Cisplatin	cisplatin	cisplatin	cisplatin	cisplatin	2	case_or_format
citation analysis	citation analysis	citation analysis	citation analysis	citation analysis	2	kept
citizenship	citizenship	citizenship	citizenship	citizenship	2	kept
citrate anticoagulation	citrate anticoagulation	citrate anticoagulation	citrate anticoagulation	citrate anticoagulation	2	kept
Citrus	citrus	citrus	citrus	Citrus	2	kept
CITU	citu	citu	citu	CITU	2	kept
civil disorder	civil disorder	civil disorder	civil disorder	civil disorder	2	kept
CKRT	ckrt	ckrt	ckrt	CKRT	2	kept
Classical test theory	classical test theory	classical test theory	classical test theory	Classical test theory	2	kept
Classification and regression trees	classification and regression trees	classification and regression trees	classification and regression trees	Classification and regression trees	2	kept
Classification models	classification models	classification models	classification models	Classification models	2	kept
classifier	classifier	classifier	classifier	classifier	2	kept

clavicle	clavicle	clavicle	clavicle	Clavicle	2	case_or_format
CLD	cld	cld	cld	CLD	2	kept
CLEAN INTERMITTENT CATHETERIZATION	clean intermittent catheterization	clean intermittent catheterization	clean intermittent catheterization	CLEAN INTERMITTENT CATHETERIZATION	2	kept
cleaner	cleaner	cleaner	cleaner	cleaner	2	kept
Cleft lip	cleft lip	cleft lip	cleft lip	cleft lip	2	case_or_format
Clerkship	clerkship	clerkship	clerkship	Clerkship	2	kept
client centered therapy	client centered therapy	client centered therapy	client centered therapy	client centered therapy	2	kept
Climate	climate	climate	climate	climate	2	case_or_format
clinical academic pathway	clinical academic pathway	clinical academic pathway	clinical academic pathway	clinical academic pathway	2	kept
Clinical adaptation	clinical adaptation	clinical adaptation	clinical adaptation	Clinical adaptation	2	kept
clinical alarm	clinical alarm	clinical alarm	clinical alarm	Clinical Alarms	2	case_or_format
clinical application	clinical application	clinical application	clinical application	clinical application	2	kept
Clinical assessment	clinical assessment	clinical assessment	clinical assessment	clinical assessment	2	case_or_format
Clinical belongingness	clinical belongingness	clinical belongingness	clinical belongingness	Clinical belongingness	2	kept
clinical benefit	clinical benefit	clinical benefit	clinical benefit	clinical benefit	2	kept
Clinical Care Quality	clinical care quality	clinical care quality	clinical care quality	Clinical Care Quality	2	kept

clinical challenges	clinical challenges	clinical challenges	clinical challenges	clinical challenges	2	kept
clinical chemistry	clinical chemistry	clinical chemistry	clinical chemistry	clinical chemistry	2	kept
Clinical database	clinical database	clinical database	clinical database	Clinical database	2	kept
clinical decision rule	clinical decision rule	clinical decision rule	clinical decision rule	clinical decision rules	2	case_or_format
Clinical Decision Support System	clinical decision support system	clinical decision support system	clinical decision support system	clinical decision support system	2	case_or_format
clinical decision-making confidence	clinical decision making confidence	clinical decision making confidence	clinical decision making confidence	clinical decision-making confidence	2	kept
Clinical Dementia Rating	clinical dementia rating	clinical dementia rating	clinical dementia rating	Clinical Dementia Rating	2	kept
clinical dementia rating scale	clinical dementia rating scale	clinical dementia rating scale	clinical dementia rating scale	clinical dementia rating scale	2	kept
Clinical determinants	clinical determinants	clinical determinants	clinical determinants	Clinical determinants	2	kept
clinical documentation	clinical documentation	clinical documentation	clinical documentation	clinical documentation	2	kept
clinical early warning	clinical early warning	clinical early warning	clinical early warning	clinical early warning	2	kept
Clinical education	clinical education	clinical education	clinical education	clinical education	2	case_or_format
clinical environment	clinical environment	clinical environment	clinical environment	Clinical environment	2	case_or_format
clinical epidemiology	clinical epidemiology	clinical epidemiology	clinical epidemiology	clinical epidemiology	2	kept
clinical ethics consultation	clinical ethics consultation	clinical ethics consultation	clinical ethics consultation	clinical ethics consultation	2	kept
Clinical event debriefing	clinical event debriefing	clinical event debriefing	clinical event debriefing	Clinical event debriefing	2	kept

Clinical Global Impression scale	clinical global impression scale	clinical global impression scale	clinical global impression scale	Clinical Global Impression scale	2	kept
Clinical guideline	clinical guideline	clinical guideline	clinical guideline	clinical guidelines	2	case_or_format
clinical guideline	clinical guideline	clinical guideline	clinical guideline	clinical guidelines	2	case_or_format
Clinical handovers	clinical handovers	clinical handovers	clinical handover	clinical handover	2	singular
clinical health	clinical health	clinical health	clinical health	clinical health	2	kept
Clinical improvement	clinical improvement	clinical improvement	clinical improvement	Clinical improvement	2	kept
clinical information system	clinical information system	clinical information system	clinical information system	Clinical information system	2	case_or_format
Clinical Laboratory Information Systems	clinical laboratory information systems	clinical laboratory information systems	clinical laboratory information systems	Clinical Laboratory Information Systems	2	kept
Clinical laboratory tests	clinical laboratory tests	clinical laboratory tests	clinical laboratory tests	Clinical laboratory tests	2	kept
clinical ladder	clinical ladder	clinical ladder	clinical ladder	clinical ladder	2	kept
Clinical Medicine	clinical medicine	clinical medicine	clinical medicine	clinical medicine	2	case_or_format
Clinical microsystems	clinical microsystems	clinical microsystems	clinical microsystems	Clinical microsystems	2	kept
Clinical observation	clinical observation	clinical observation	clinical observation	clinical observation	2	case_or_format
CLINICAL PATHWAY	clinical pathway	clinical pathway	clinical pathway	clinical pathway	2	case_or_format
Clinical pathway teaching	clinical pathway teaching	clinical pathway teaching	clinical pathway teaching	Clinical pathway teaching	2	kept
Clinical Pharmacist	clinical pharmacist	clinical pharmacist	clinical pharmacist	clinical pharmacist	2	case_or_format

Clinical placement	clinical placement	clinical placement	clinical placement	clinical placement	2	case_or_format
Clinical placements	clinical placements	clinical placements	clinical placement	clinical placement	2	singular
Clinical Pulmonary infection Score	clinical pulmonary infection score	clinical pulmonary infection score	clinical pulmonary infection score	Clinical Pulmonary infection Score	2	kept
clinical questions	clinical questions	clinical questions	clinical questions	clinical questions	2	kept
Clinical reasoning competency	clinical reasoning competency	clinical reasoning competency	clinical reasoning competency	Clinical reasoning competency	2	kept
Clinical resources	clinical resources	clinical resources	clinical resources	Clinical resources	2	kept
Clinical scenario	clinical scenario	clinical scenario	clinical scenario	Clinical scenario	2	kept
clinical score	clinical score	clinical score	clinical score	clinical score	2	kept
Clinical self-efficacy	clinical self efficacy	clinical self efficacy	clinical self efficacy	Clinical self-efficacy	2	kept
Clinical shift	clinical shift	clinical shift	clinical shift	Clinical shift	2	kept
Clinical simulation	clinical simulation	clinical simulation	clinical simulation	Clinical simulation	2	kept
clinical simulation	clinical simulation	clinical simulation	clinical simulation	Clinical simulation	2	case_or_format
clinical skills education	clinical skills education	clinical skills education	clinical skills education	clinical skills education	2	kept
Clinical staff	clinical staff	clinical staff	clinical staff	Clinical staff	2	kept
clinical staff	clinical staff	clinical staff	clinical staff	Clinical staff	2	case_or_format
Clinical Studies as Topic	clinical studies as topic	clinical studies as topic	clinical studies as topic	Clinical Studies as Topic	2	kept

Clinical teaching	clinical teaching	clinical teaching	clinical teaching	clinical teaching	2	case_or_format
clinical treatment	clinical treatment	clinical treatment	clinical treatment	clinical treatment	2	kept
clinical trial registration	clinical trial registration	clinical trial registration	clinical trial registration	clinical trial registration	2	kept
clinical trial registry	clinical trial registry	clinical trial registry	clinical trial registry	clinical trial registry	2	kept
Clinical Trials, Phase III as Topic	clinical trials phase iii as topic	clinical trials phase iii as topic	clinical trials phase iii as topic	Clinical Trials, Phase III as Topic	2	kept
Clinical utility	clinical utility	clinical utility	clinical utility	Clinical utility	2	kept
clinical utility	clinical utility	clinical utility	clinical utility	Clinical utility	2	case_or_format
Clinical ward management	clinical ward management	clinical ward management	clinical ward management	Clinical ward management	2	kept
CLINICAL-ASSESSMENT	clinical assessment	clinical assessment	clinical assessment	clinical assessment	2	case_or_format
CLINICIAN	clinician	clinician	clinician	clinician	2	case_or_format
clinician well-being	clinician well being	clinician well being	clinician well being	clinician well-being	2	kept
clinicians	clinicians	clinicians	clinician	clinician	2	singular
clinimetrics	clinimetrics	clinimetrics	clinimetrics	clinimetrics	2	kept
Cloaca	cloaca	cloaca	cloaca	Cloaca	2	kept
clock	clock	clock	clock	clock	2	kept
clock drawing test	clock drawing test	clock drawing test	clock drawing test	clock drawing test	2	kept

clomethiazole	clomethiazole	clomethiazole	clomethiazole	clomethiazole	2	kept
Clopidogrel	clopidogrel	clopidogrel	clopidogrel	clopidogrel	2	case_or_format
Closed blood sampling devices	closed blood sampling devices	closed blood sampling devices	closed blood sampling devices	Closed blood sampling devices	2	kept
Closed loop systems	closed loop systems	closed loop systems	closed loop system	Closed-loop system	2	singular
closed loop ventilation	closed loop ventilation	closed loop ventilation	closed loop ventilation	closed loop ventilation	2	kept
closed reduction	closed reduction	closed reduction	closed reduction	closed reduction	2	kept
Closed system suctioning	closed system suctioning	closed system suctioning	closed system suctioning	Closed system suctioning	2	kept
Closed tracheal suctioning	closed tracheal suctioning	closed tracheal suctioning	closed tracheal suctioning	Closed tracheal suctioning	2	kept
Closed-circuit television	closed circuit television	closed circuit television	closed circuit television	Closed-circuit television	2	kept
Closed-loop glycemic control	closed loop glycemic control	closed loop glycemic control	closed loop glycemic control	Closed-loop glycemic control	2	kept
Closed-loop management mode	closed loop management mode	closed loop management mode	closed loop management mode	Closed-loop management mode	2	kept
Closed-loop system	closed loop system	closed loop system	closed loop system	Closed-loop system	2	kept
Closed-loop ventilation	closed loop ventilation	closed loop ventilation	closed loop ventilation	closed loop ventilation	2	case_or_format
Closeness	closeness	closeness	closeness	Closeness	2	kept
closeness centrality	closeness centrality	closeness centrality	closeness centrality	closeness centrality	2	kept
clostridioides difficile infection	clostridioides difficile infection	clostridioides difficile infection	clostridioides difficile infection	Clostridioides difficile infection	2	case_or_format

Clostridium tetani	clostridium tetani	clostridium tetani	clostridium tetani	Clostridium tetani	2	kept
clotrimazole	clotrimazole	clotrimazole	clotrimazole	clotrimazole	2	kept
Cloud Computing	cloud computing	cloud computing	cloud computing	cloud computing	2	case_or_format
Cloud-based	cloud based	cloud based	cloud based	Cloud-based	2	kept
cloxacillin	cloxacillin	cloxacillin	cloxacillin	cloxacillin	2	kept
cluster	cluster	cluster	cluster	cluster	2	kept
Cluster care	cluster care	cluster care	cluster care	Cluster care	2	kept
cluster care	cluster care	cluster care	cluster care	Cluster care	2	case_or_format
cluster randomized clinical trial	cluster randomized clinical trial	cluster randomized clinical trial	cluster randomized clinical trial	cluster randomized clinical trial	2	kept
Clustering analysis	clustering analysis	clustering analysis	clustering analysis	Clustering analysis	2	kept
Clusters	clusters	clusters	cluster	cluster	2	singular
CMI	cmi	cmi	cmi	CMI	2	kept
Co-management	co management	co management	co management	Co-management	2	kept
CO<sub>2<	co sub 2	co sub 2	co sub 2	CO<sub>2<	2	kept
coaching needs	coaching needs	coaching needs	coaching needs	coaching needs	2	kept
coagulase	coagulase	coagulase	coagulase	Coagulase	2	case_or_format

Coagulase-Negative Staphylococci	coagulase negative staphylococci	coagulase negative staphylococci	coagulase negative staphylococci	Coagulase-Negative Staphylococci	2	kept
Coagulase-negative staphylococci	coagulase negative staphylococci	coagulase negative staphylococci	coagulase negative staphylococci	Coagulase-Negative Staphylococci	2	case_or_format
coagulating agent	coagulating agent	coagulating agent	coagulating agent	coagulating agent	2	kept
coagulopathy	coagulopathy	coagulopathy	coagulopathy	Coagulopathy	2	case_or_format
Coated Materials, Biocompatible	coated materials biocompatible	coated materials biocompatible	coated materials biocompatible	Coated Materials, Biocompatible	2	kept
Cobalt toxicity	cobalt toxicity	cobalt toxicity	cobalt toxicity	Cobalt toxicity	2	kept
coccygectomy	coccygectomy	coccygectomy	coccygectomy	coccygectomy	2	kept
cochlear implantation	cochlear implantation	cochlear implantation	cochlear implantation	cochlear implantation	2	kept
COCKPIT MANAGEMENT ATTITUDES	cockpit management attitudes	cockpit management attitudes	cockpit management attitudes	COCKPIT MANAGEMENT ATTITUDES	2	kept
coconut	coconut	coconut	coconut	coconut	2	kept
Coconut oil	coconut oil	coconut oil	coconut oil	Coconut oil	2	kept
coconut oil	coconut oil	coconut oil	coconut oil	Coconut oil	2	case_or_format
cod liver oil	cod liver oil	cod liver oil	cod liver oil	cod liver oil	2	kept
Code Blue	code blue	code blue	code blue	Code blue	2	case_or_format
Code blue teams	code blue teams	code blue teams	code blue team	Code blue teams	2	singular
Code Cart	code cart	code cart	code cart	Code Cart	2	kept

code documentation	code documentation	code documentation	code documentation	code documentation	2	kept
Code status	code status	code status	code status	code status	2	case_or_format
Codeine	codeine	codeine	codeine	codeine	2	case_or_format
cognitive	cognitive	cognitive	cognitive	cognitive	2	kept
Cognitive aid	cognitive aid	cognitive aid	cognitive aid	Cognitive aids	2	case_or_format
cognitive aid	cognitive aid	cognitive aid	cognitive aid	Cognitive aids	2	case_or_format
Cognitive artifacts	cognitive artifacts	cognitive artifacts	cognitive artifacts	Cognitive artifacts	2	kept
cognitive assessment	cognitive assessment	cognitive assessment	cognitive assessment	cognitive assessment	2	kept
cognitive assessment method	cognitive assessment method	cognitive assessment method	cognitive assessment method	cognitive assessment method	2	kept
Cognitive behavioral therapy	cognitive behavioral therapy	cognitive behavioral therapy	cognitive behavioral therapy	cognitive behavioral therapy	2	case_or_format
Cognitive bias	cognitive bias	cognitive bias	cognitive bias	Cognitive bias	2	kept
cognitive decline	cognitive decline	cognitive decline	cognitive decline	Cognitive decline	2	case_or_format
Cognitive deficits	cognitive deficits	cognitive deficits	cognitive deficits	Cognitive deficits	2	kept
cognitive differences	cognitive differences	cognitive differences	cognitive differences	cognitive differences	2	kept
Cognitive disorders	cognitive disorders	cognitive disorders	cognitive disorders	Cognitive disorders	2	kept
cognitive failure	cognitive failure	cognitive failure	cognitive failure	cognitive failure	2	kept

Cognitive function	cognitive function	cognitive function	cognitive function	cognitive function	2	case_or_format
Cognitive fusion	cognitive fusion	cognitive fusion	cognitive fusion	cognitive fusion	2	case_or_format
cognitive impairment assessment	cognitive impairment assessment	cognitive impairment assessment	cognitive impairment assessment	cognitive impairment assessment	2	kept
Cognitive impairments	cognitive impairments	cognitive impairments	cognitive impairment	Cognitive impairment	2	singular
cognitive impairments	cognitive impairments	cognitive impairments	cognitive impairment	Cognitive impairment	2	singular
cognitive interview	cognitive interview	cognitive interview	cognitive interview	cognitive interview	2	kept
Cognitive performance	cognitive performance	cognitive performance	cognitive performance	Cognitive performance	2	kept
Cognitive Reflection	cognitive reflection	cognitive reflection	cognitive reflection	Cognitive Reflection	2	kept
cognitive reflection	cognitive reflection	cognitive reflection	cognitive reflection	Cognitive Reflection	2	case_or_format
cognitive remediation therapy	cognitive remediation therapy	cognitive remediation therapy	cognitive remediation therapy	cognitive remediation therapy	2	kept
cognitive rumination	cognitive rumination	cognitive rumination	cognitive rumination	cognitive rumination	2	kept
Cognitive systems	cognitive systems	cognitive systems	cognitive systems	Cognitive systems	2	kept
cognitive test for delirium	cognitive test for delirium	cognitive test for delirium	cognitive test for delirium	Cognitive Test for Delirium	2	case_or_format
Cognitive therapy	cognitive therapy	cognitive therapy	cognitive therapy	cognitive therapy	2	case_or_format
Cognitive training	cognitive training	cognitive training	cognitive training	Cognitive training	2	kept
cognitive work analysis	cognitive work analysis	cognitive work analysis	cognitive work analysis	cognitive work analysis	2	kept

cohort	cohort	cohort	cohort	COHORT	2	case_or_format
cohort studies	cohort studies	cohort studies	cohort study	Cohort Studies	2	singular
Colaboración interprofesional	colaboraci n interprofesional	colaboraci n interprofesional	colaboraci n interprofesional	Colaboración interprofesional	2	kept
cold application	cold application	cold application	cold application	cold application	2	kept
Cold Ischemia	cold ischemia	cold ischemia	cold ischemia	Cold Ischemia	2	kept
cold treatment	cold treatment	cold treatment	cold treatment	cold treatment	2	kept
colestyramine	colestyramine	colestyramine	colestyramine	colestyramine	2	kept
Colic	colic	colic	colic	Colic	2	kept
Collaborating	collaborating	collaborating	collaborating	Collaborating	2	kept
collaborative development	collaborative development	collaborative development	collaborative development	collaborative development	2	kept
collaborative work	collaborative work	collaborative work	collaborative work	collaborative work	2	kept
collaborative working	collaborative working	collaborative working	collaborative working	collaborative working	2	kept
Collective experience	collective experience	collective experience	collective experience	Collective experience	2	kept
Colombian	colombian	colombian	colombian	Colombian	2	kept
colon	colon	colon	colon	Colon	2	case_or_format
Colon cancer	colon cancer	colon cancer	colon cancer	colon cancer	2	case_or_format

colon disease	colon disease	colon disease	colon disease	colon disease	2	kept
Colonization surveillance	colonization surveillance	colonization surveillance	colonization surveillance	Colonization surveillance	2	kept
color density spectral array electroencephalography	color density spectral array electroencephalography	color density spectral array electroencephalography	color density spectral array electroencephalography	color density spectral array electroencephalography	2	kept
coloring agent	coloring agent	coloring agent	coloring agent	coloring agent	2	kept
Coloring Agents	coloring agents	coloring agents	coloring agent	coloring agent	2	singular
COM-B model	com b model	com b model	com b model	COM-B model	2	kept
coma recovery scale revised	coma recovery scale revised	coma recovery scale revised	coma recovery scale revised	coma recovery scale revised	2	kept
Combat casualty care	combat casualty care	combat casualty care	combat casualty care	Combat casualty care	2	kept
Combat Disorders	combat disorders	combat disorders	combat disorders	Combat Disorders	2	kept
Combat support hospital	combat support hospital	combat support hospital	combat support hospital	Combat support hospital	2	kept
Combinatorial optimization	combinatorial optimization	combinatorial optimization	combinatorial optimization	Combinatorial optimization	2	kept
Combined modality therapy	combined modality therapy	combined modality therapy	combined modality therapy	Combined Modality Therapy	2	case_or_format
Comfort Behavior Scale	comfort behavior scale	comfort behavior scale	comfort behavior scale	Comfort Behavior Scale	2	kept
Comfort behavioral scale	comfort behavioral scale	comfort behavioral scale	comfort behavioral scale	Comfort behavioral scale	2	kept
Comfort Theory	comfort theory	comfort theory	comfort theory	Comfort Theory	2	kept
comfort with touch	comfort with touch	comfort with touch	comfort with touch	comfort with touch	2	kept

COMFORT-B score	comfort b score	comfort b score	comfort b score	COMFORT-B score	2	kept
COMFORTneo	comfortneo	comfortneo	comfortneo	COMFORTneo	2	kept
COMMITMENT	commitment	commitment	commitment	COMMITMENT	2	kept
Commitment of Persons with Psychiatric Disorders	commitment of persons with psychiatric disorders	commitment of persons with psychiatric disorders	commitment of persons with psychiatric disorders	Commitment of Persons with Psychiatric Disorders	2	kept
Committee Membership	committee membership	committee membership	committee membership	Committee Membership	2	kept
common arterial trunk	common arterial trunk	common arterial trunk	common arterial trunk	common arterial trunk	2	kept
common bile duct	common bile duct	common bile duct	common bile duct	common bile duct	2	kept
communicating hydrocephalus	communicating hydrocephalus	communicating hydrocephalus	communicating hydrocephalus	communicating hydrocephalus	2	kept
Communication and coordination	communication and coordination	communication and coordination	communication and coordination	Communication and coordination	2	kept
communication and knowledge exchange	communication and knowledge exchange	communication and knowledge exchange	communication and knowledge exchange	communication and knowledge exchange	2	kept
Communication barrier	communication barrier	communication barrier	communication barrier	Communication Barriers	2	case_or_format
communication board	communication board	communication board	communication board	Communication board	2	case_or_format
Communication methods	communication methods	communication methods	communication methods	Communication methods	2	kept
Communication networks	communication networks	communication networks	communication networks	Communication networks	2	kept
Communication pattern	communication pattern	communication pattern	communication pattern	Communication pattern	2	kept
Communication satisfaction	communication satisfaction	communication satisfaction	communication satisfaction	Communication satisfaction	2	kept

communication skill training	communication skill training	communication skill training	communication skill training	communication skill training	2	kept
Communication technologies	communication technologies	communication technologies	communication technology	communication technology	2	singular
communication tool	communication tool	communication tool	communication tool	communication tool	2	kept
Communication tools	communication tools	communication tools	communication tool	communication tool	2	singular
communication vulnerable	communication vulnerable	communication vulnerable	communication vulnerable	communication vulnerable	2	kept
Communication workshops	communication workshops	communication workshops	communication workshops	Communication workshops	2	kept
Communications Media	communications media	communications media	communications media	Communications Media	2	kept
communities of practice	communities of practice	communities of practice	communities of practice	communities of practice	2	kept
Community health	community health	community health	community health	Community health	2	kept
community health status	community health status	community health status	community health status	community health status	2	kept
community health worker	community health worker	community health worker	community health worker	community health worker	2	kept
community pharmacist	community pharmacist	community pharmacist	community pharmacist	community pharmacist	2	kept
community reintegration	community reintegration	community reintegration	community reintegration	community reintegration	2	kept
community risk factor	community risk factor	community risk factor	community risk factor	community risk factor	2	kept
Community-Acquired Pneumonia	community acquired pneumonia	community acquired pneumonia	community acquired pneumonia	community acquired pneumonia	2	case_or_format
community-based neurorehabilitation	community based neurorehabilitation	community based neurorehabilitation	community based neurorehabilitation	community-based neurorehabilitation	2	kept

Comorbidities in trauma	comorbidities in trauma	comorbidities in trauma	comorbidities in trauma	Comorbidities in trauma	2	kept
COMORBIDITY	comorbidity	comorbidity	comorbidity	comorbidity	2	case_or_format
Comparative effectiveness	comparative effectiveness	comparative effectiveness	comparative effectiveness	comparative effectiveness	2	case_or_format
Comparative effectiveness research	comparative effectiveness research	comparative effectiveness research	comparative effectiveness research	Comparative Effectiveness Research	2	case_or_format
comparison	comparison	comparison	comparison	Comparison	2	case_or_format
compatibility	compatibility	compatibility	compatibility	compatibility	2	kept
compensated liver cirrhosis	compensated liver cirrhosis	compensated liver cirrhosis	compensated liver cirrhosis	compensated liver cirrhosis	2	kept
Competence-based education	competence based education	competence based education	competence based education	Competence-based education	2	kept
competency assessment	competency assessment	competency assessment	competency assessment	competency assessment	2	kept
competency training	competency training	competency training	competency training	competency training	2	kept
competency-based assessment	competency based assessment	competency based assessment	competency based assessment	competency-based assessment	2	kept
Competitive Behavior	competitive behavior	competitive behavior	competitive behavior	Competitive Behavior	2	kept
complement	complement	complement	complement	complement	2	kept
complement activation	complement activation	complement activation	complement activation	complement activation	2	kept
complementary	complementary	complementary	complementary	complementary	2	kept
complementary and alternative medicine	complementary and alternative medicine	complementary and alternative medicine	complementary and alternative medicine	complementary and alternative medicine	2	kept

complementary medicine	complementary medicine	complementary medicine	complementary medicine	Complementary medicine	2	case_or_format
complementary RNA	complementary rna	complementary rna	complementary rna	complementary RNA	2	kept
Complementary therapy	complementary therapy	complementary therapy	complementary therapy	Complementary Therapies	2	case_or_format
complete AV block	complete av block	complete av block	complete av block	complete AV block	2	kept
complex cardiac care	complex cardiac care	complex cardiac care	complex cardiac care	complex cardiac care	2	kept
Complex care	complex care	complex care	complex care	Complex care	2	kept
Complex environments	complex environments	complex environments	complex environments	Complex environments	2	kept
Complex intervention	complex intervention	complex intervention	complex intervention	Complex interventions	2	case_or_format
complex interventions	complex interventions	complex interventions	complex intervention	Complex interventions	2	singular
complexity	complexity	complexity	complexity	Complexity	2	case_or_format
complicated grief	complicated grief	complicated grief	complicated grief	complicated grief	2	kept
Compound chlorhexidine gargle	compound chlorhexidine gargle	compound chlorhexidine gargle	compound chlorhexidine gargle	Compound chlorhexidine gargle	2	kept
Comprehensive	comprehensive	comprehensive	comprehensive	Comprehensive	2	kept
Comprehensive care	comprehensive care	comprehensive care	comprehensive care	Comprehensive care	2	kept
comprehensive geriatric assessment	comprehensive geriatric assessment	comprehensive geriatric assessment	comprehensive geriatric assessment	comprehensive geriatric assessment	2	kept
COMPRESSION	compression	compression	compression	compression	2	case_or_format

Computational results	computational results	computational results	computational results	Computational results	2	kept
Computed tomography	computed tomography	computed tomography	computed tomography	Computed tomography	2	kept
Computer aided design	computer aided design	computer aided design	computer aided design	computer aided design	2	case_or_format
computer analysis	computer analysis	computer analysis	computer analysis	computer analysis	2	kept
computer assisted therapy	computer assisted therapy	computer assisted therapy	computer assisted therapy	computer assisted therapy	2	kept
Computer graphics	computer graphics	computer graphics	computer graphics	Computer Graphics	2	case_or_format
Computer networks	computer networks	computer networks	computer network	computer network	2	singular
Computer programming languages	computer programming languages	computer programming languages	computer programming languages	Computer programming languages	2	kept
computer protocol	computer protocol	computer protocol	computer protocol	computer protocol	2	kept
Computer Terminals	computer terminals	computer terminals	computer terminals	Computer Terminals	2	kept
computer vision	computer vision	computer vision	computer vision	Computer vision	2	case_or_format
Computer-aided design	computer aided design	computer aided design	computer aided design	computer aided design	2	case_or_format
computer-aided design	computer aided design	computer aided design	computer aided design	computer aided design	2	case_or_format
Computer-based simulation	computer based simulation	computer based simulation	computer based simulation	Computer-based simulation	2	kept
Computerised decision support	computerised decision support	computerised decision support	computerised decision support	Computerised decision support	2	kept
Computerised equipment	computerised equipment	computerised equipment	computerised equipment	Computerised equipment	2	kept

Computerised monitoring	computerised monitoring	computerised monitoring	computerised monitoring	Computerised monitoring	2	kept
computerized	computerized	computerized	computerized	Computerized	2	case_or_format
computerized decision support	computerized decision support	computerized decision support	computerized decision support	computerized decision support	2	kept
Computerized provider order entry	computerized provider order entry	computerized provider order entry	computerized provider order entry	computerized provider order entry	2	case_or_format
Computerized tomography	computerized tomography	computerized tomography	computerized tomography	Computerized tomography	2	kept
compétence infirmière	comp tence infirmi re	comp tence infirmi re	comp tence infirmi re	compétence infirmière	2	kept
Conceptual framework	conceptual framework	conceptual framework	conceptual framework	conceptual framework	2	case_or_format
Conceptual frameworks	conceptual frameworks	conceptual frameworks	conceptual framework	conceptual framework	2	singular
concordance	concordance	concordance	concordance	concordance	2	kept
concussion	concussion	concussion	concussion	concussion	2	kept
Conditioning, Psychological	conditioning psychological	conditioning psychological	conditioning psychological	Conditioning, Psychological	2	kept
Conducta alimentaria Infant	conducta alimentaria infant	conducta alimentaria infant	conducta alimentaria infant	Conducta alimentaria Infant	2	kept
cone beam computed tomography	cone beam computed tomography	cone beam computed tomography	cone beam computed tomography	cone beam computed tomography	2	kept
conference abstract	conference abstract	conference abstract	conference abstract	conference abstract	2	kept
Confidence interval	confidence interval	confidence interval	confidence interval	confidence interval	2	case_or_format
Confidence intervals	confidence intervals	confidence intervals	confidence interval	confidence interval	2	singular

Confidence-based marking	confidence based marking	confidence based marking	confidence based marking	Confidence-based marking	2	kept
Confinement	confinement	confinement	confinement	Confinement	2	kept
Conflict Resolution	conflict resolution	conflict resolution	conflict resolution	Conflict resolution	2	case_or_format
CONFLICTS	conflicts	conflicts	conflict	conflict	2	singular
Conflicts	conflicts	conflicts	conflict	conflict	2	singular
Confusional State Evaluation	confusional state evaluation	confusional state evaluation	confusional state evaluation	Confusional State Evaluation	2	kept
congenital	congenital	congenital	congenital	Congenital	2	case_or_format
congenital abnorm	congenital abnorm	congenital abnorm	congenital abnorm	congenital abnorm	2	kept
Congenital abnormalities	congenital abnormalities	congenital abnormalities	congenital abnormality	Congenital Abnormalities	2	singular
Congenital anomalies	congenital anomalies	congenital anomalies	congenital anomaly	Congenital anomaly	2	singular
Congenital anomaly	congenital anomaly	congenital anomaly	congenital anomaly	Congenital anomaly	2	kept
congenital anomaly	congenital anomaly	congenital anomaly	congenital anomaly	Congenital anomaly	2	case_or_format
congenital blood vessel malformation	congenital blood vessel malformation	congenital blood vessel malformation	congenital blood vessel malformation	congenital blood vessel malformation	2	kept
Congenital diaphragmatic hernia	congenital diaphragmatic hernia	congenital diaphragmatic hernia	congenital diaphragmatic hernia	Congenital diaphragmatic hernia	2	kept
Congenital Heart Disease	congenital heart disease	congenital heart disease	congenital heart disease	congenital heart disease	2	case_or_format
congestion	congestion	congestion	congestion	congestion	2	kept

Congestive heart failure	congestive heart failure	congestive heart failure	congestive heart failure	congestive heart failure	2	case_or_format
Conjoint analysis	conjoint analysis	conjoint analysis	conjoint analysis	Conjoint analysis	2	kept
Conjunctiva	conjunctiva	conjunctiva	conjunctiva	Conjunctiva	2	kept
conjunctiva disease	conjunctiva disease	conjunctiva disease	conjunctiva disease	conjunctiva disease	2	kept
Conscious	conscious	conscious	conscious	Conscious	2	kept
consecutive working days	consecutive working days	consecutive working days	consecutive working day	consecutive working days	2	singular
Consensus conference	consensus conference	consensus conference	consensus conference	CONSENSUS CONFERENCE	2	case_or_format
Consensus Development Conferences	consensus development conferences	consensus development conferences	consensus development conference	Consensus Development Conferences	2	singular
consensus model	consensus model	consensus model	consensus model	consensus model	2	kept
CONSENSUS STATEMENT	consensus statement	consensus statement	consensus statement	CONSENSUS STATEMENT	2	kept
consensus statement	consensus statement	consensus statement	consensus statement	CONSENSUS STATEMENT	2	case_or_format
Consent	consent	consent	consent	Consent	2	kept
consent	consent	consent	consent	Consent	2	case_or_format
Constriction, Pathologic	constriction pathologic	constriction pathologic	constriction pathologic	Constriction, Pathologic	2	kept
Constructive criticism	constructive criticism	constructive criticism	constructive criticism	Constructive criticism	2	kept
Consultant training	consultant training	consultant training	consultant training	Consultant training	2	kept

consultant work pattern	consultant work pattern	consultant work pattern	consultant work pattern	consultant work pattern	2	kept
consultant-level	consultant level	consultant level	consultant level	consultant-level	2	kept
CONSULTATION	consultation	consultation	consultation	consultation	2	case_or_format
Consumer quality index	consumer quality index	consumer quality index	consumer quality index	Consumer quality index	2	kept
CONSUMPTION	consumption	consumption	consumption	CONSUMPTION	2	kept
Contact Lenses	contact lenses	contact lenses	contact lenses	Contact Lenses	2	kept
contact transmission	contact transmission	contact transmission	contact transmission	contact transmission	2	kept
Containment	containment	containment	containment	containment	2	case_or_format
Contención mecánica	contenci n mec nica	contenci n mec nica	contenci n mec nica	Contención mecánica	2	kept
Content Analysis	content analysis	content analysis	content analysis	content analysis	2	case_or_format
content text analysis	content text analysis	content text analysis	content text analysis	content text analysis	2	kept
Content validation	content validation	content validation	content validation	Content validation	2	kept
content validity index	content validity index	content validity index	content validity index	content validity index	2	kept
Context	context	context	context	CONTEXT	2	case_or_format
context	context	context	context	CONTEXT	2	case_or_format
Contextual design	contextual design	contextual design	contextual design	Contextual design	2	kept

Contextual inquiry	contextual inquiry	contextual inquiry	contextual inquiry	Contextual inquiry	2	kept
continence	continence	continence	continence	continence	2	kept
Contingency management	contingency management	contingency management	contingency management	Contingency management	2	kept
contingency table	contingency table	contingency table	contingency table	contingency table	2	kept
continuing care	continuing care	continuing care	continuing care	continuing care	2	kept
Continuing medical education	continuing medical education	continuing medical education	continuing medical education	Continuing medical education	2	kept
continuing medical education	continuing medical education	continuing medical education	continuing medical education	Continuing medical education	2	case_or_format
Continuous blood pressure	continuous blood pressure	continuous blood pressure	continuous blood pressure	Continuous blood pressure	2	kept
continuous blood purification	continuous blood purification	continuous blood purification	continuous blood purification	continuous blood purification	2	kept
Continuous blood purification therapy	continuous blood purification therapy	continuous blood purification therapy	continuous blood purification therapy	Continuous blood purification therapy	2	kept
continuous chest compression	continuous chest compression	continuous chest compression	continuous chest compression	continuous chest compression	2	kept
continuous education	continuous education	continuous education	continuous education	continuous education	2	kept
Continuous EEG	continuous eeg	continuous eeg	continuous eeg	Continuous EEG	2	kept
Continuous glucosemonitoring	continuous glucosemonitoring	continuous glucosemonitoring	continuous glucosemonitoring	Continuous glucosemonitoring	2	kept
Continuous monitoring systems	continuous monitoring systems	continuous monitoring systems	continuous monitoring systems	Continuous monitoring systems	2	kept
Continuous predictive analytics monitoring	continuous predictive analytics monitoring	continuous predictive analytics monitoring	continuous predictive analytics monitoring	Continuous predictive analytics monitoring	2	kept

continuous process	continuous process	continuous process	continuous process	continuous process	2	kept
Continuous professional development	continuous professional development	continuous professional development	continuous professional development	continuous professional development	2	case_or_format
Continuous renal replacement rate	continuous renal replacement rate	continuous renal replacement rate	continuous renal replacement rate	Continuous renal replacement rate	2	kept
continuous time Markov chain	continuous time markov chain	continuous time markov chain	continuous time markov chain	continuous time Markov chain	2	kept
Continuous venovenous hemofiltration	continuous venovenous hemofiltration	continuous venovenous hemofiltration	continuous venovenous hemofiltration	Continuous venovenous hemofiltration	2	kept
continuous vital sign monitoring	continuous vital sign monitoring	continuous vital sign monitoring	continuous vital sign monitoring	continuous vital sign monitoring	2	kept
Contraception Behavior	contraception behavior	contraception behavior	contraception behavior	Contraception Behavior	2	kept
contraceptive behavior	contraceptive behavior	contraceptive behavior	contraceptive behavior	contraceptive behavior	2	kept
Contrast media	contrast media	contrast media	contrast media	Contrast Media	2	case_or_format
Contributing factor	contributing factor	contributing factor	contributing factor	contributing factors	2	case_or_format
CONTRIBUTING FACTORS	contributing factors	contributing factors	contributing factor	contributing factors	2	singular
Control protocols	control protocols	control protocols	control protocols	Control protocols	2	kept
Controlled drug delivery	controlled drug delivery	controlled drug delivery	controlled drug delivery	Controlled drug delivery	2	kept
controlled motivation	controlled motivation	controlled motivation	controlled motivation	controlled motivation	2	kept
controlled vocabulary	controlled vocabulary	controlled vocabulary	controlled vocabulary	controlled vocabulary	2	kept
Conventional methods	conventional methods	conventional methods	conventional methods	Conventional methods	2	kept

conversion disorder	conversion disorder	conversion disorder	conversion disorder	conversion disorder	2	kept
Conversion factor	conversion factor	conversion factor	conversion factor	Conversion factor	2	kept
cooling tower	cooling tower	cooling tower	cooling tower	cooling tower	2	kept
Coombs test	coombs test	coombs test	coombs test	Coombs test	2	kept
Cooperation	cooperation	cooperation	cooperation	cooperation	2	case_or_format
COOPERATIVE CARDIOVASCULAR PROJECT	cooperative cardiovascular project	cooperative cardiovascular project	cooperative cardiovascular project	COOPERATIVE CARDIOVASCULAR PROJECT	2	kept
cooperative learning	cooperative learning	cooperative learning	cooperative learning	cooperative learning	2	kept
COORDINATION	coordination	coordination	coordination	coordination	2	case_or_format
Copenhagen Burnout Inventory	copenhagen burnout inventory	copenhagen burnout inventory	copenhagen burnout inventory	Copenhagen Burnout Inventory	2	kept
Coping behaviors	coping behaviors	coping behaviors	coping behavior	coping behavior	2	singular
Coping mechanism	coping mechanism	coping mechanism	coping mechanism	coping mechanisms	2	case_or_format
Coping mechanisms	coping mechanisms	coping mechanisms	coping mechanism	coping mechanisms	2	singular
coping self-efficacy	coping self efficacy	coping self efficacy	coping self efficacy	coping self-efficacy	2	kept
Coping Strategies	coping strategies	coping strategies	coping strategy	Coping strategies	2	singular
Coping strategy	coping strategy	coping strategy	coping strategy	Coping strategies	2	case_or_format
coping strategy	coping strategy	coping strategy	coping strategy	Coping strategies	2	case_or_format

coping style	coping style	coping style	coping style	coping style	2	kept
Coping styles	coping styles	coping styles	coping style	coping style	2	singular
Coping with death competence	coping with death competence	coping with death competence	coping with death competence	Coping with death competence	2	kept
Copula	copula	copula	copula	Copula	2	kept
cor pulmonale	cor pulmonale	cor pulmonale	cor pulmonale	cor pulmonale	2	kept
Core Competency	core competency	core competency	core competency	Core competency	2	case_or_format
CORE TEMPERATURE	core temperature	core temperature	core temperature	core temperature	2	case_or_format
cornea transplantation	cornea transplantation	cornea transplantation	cornea transplantation	cornea transplantation	2	kept
cornea ulcer	cornea ulcer	cornea ulcer	cornea ulcer	cornea ulcer	2	kept
Corneal complications	corneal complications	corneal complications	corneal complications	Corneal complications	2	kept
Corona pandemic	corona pandemic	corona pandemic	corona pandemic	Corona pandemic	2	kept
Corona virus	corona virus	corona virus	corona virus	Corona virus	2	kept
Coronary angioplasty	coronary angioplasty	coronary angioplasty	coronary angioplasty	Coronary angioplasty	2	kept
coronary artery anomaly	coronary artery anomaly	coronary artery anomaly	coronary artery anomaly	coronary artery anomaly	2	kept
coronary atherosclerosis	coronary atherosclerosis	coronary atherosclerosis	coronary atherosclerosis	coronary atherosclerosis	2	kept
coronary care	coronary care	coronary care	coronary care	coronary care	2	kept

coronary diseases	coronary diseases	coronary diseases	coronary disease	Coronary Disease	2	singular
CORONARY-HEART-DISEASE	coronary heart disease	coronary heart disease	coronary heart disease	Coronary heart disease	2	case_or_format
coronavirus anxiety scale	coronavirus anxiety scale	coronavirus anxiety scale	coronavirus anxiety scale	coronavirus anxiety scale	2	kept
CORONAVIRUS DISEASE 2019	coronavirus disease 2019	covid 19	covid 19	COVID-19	2	alias
coronavirus disease 2019 pneumonia	coronavirus disease 2019 pneumonia	coronavirus disease 2019 pneumonia	coronavirus disease 2019 pneumonia	coronavirus disease 2019 pneumonia	2	kept
Coronavirus disease warriors	coronavirus disease warriors	coronavirus disease warriors	coronavirus disease warriors	Coronavirus disease warriors	2	kept
Correlation	correlation	correlation	correlation	correlation	2	case_or_format
correlation function	correlation function	correlation function	correlation function	correlation function	2	kept
correspondence analysis	correspondence analysis	correspondence analysis	correspondence analysis	correspondence analysis	2	kept
Correspondence as Topic	correspondence as topic	correspondence as topic	correspondence as topic	Correspondence as Topic	2	kept
corticotropin	corticotropin	corticotropin	corticotropin	corticotropin	2	kept
COSMIN checklist	cosmin checklist	cosmin checklist	cosmin checklist	COSMIN checklist	2	kept
Cost and cost analysis	cost and cost analysis	cost and cost analysis	cost and cost analysis	Cost and cost analysis	2	kept
Cost comparison	cost comparison	cost comparison	cost comparison	Cost comparison	2	kept
cost of care	cost of care	cost of care	cost of care	cost of care	2	kept
Cost Of Health Care	cost of health care	cost of health care	cost of health care	Cost Of Health Care	2	kept

Cost savings	cost savings	cost savings	cost saving	Cost Savings	2	singular
cost-analysis	cost analysis	cost analysis	cost analysis	Cost analysis	2	case_or_format
Cost-benefit analysis	cost benefit analysis	cost benefit analysis	cost benefit analysis	Cost-Benefit Analysis	2	case_or_format
cost-effectiveness analysis	cost effectiveness analysis	cost effectiveness analysis	cost effectiveness analysis	cost effectiveness analysis	2	case_or_format
Cost-saving	cost saving	cost saving	cost saving	Cost Savings	2	case_or_format
Costs and costs analysis	costs and costs analysis	costs and costs analysis	costs and costs analysis	Costs and costs analysis	2	kept
Cost－benefit analysis	cost benefit analysis	cost benefit analysis	cost benefit analysis	Cost-Benefit Analysis	2	case_or_format
Cough Assist	cough assist	cough assist	cough assist	Cough Assist	2	kept
COUNCIL	council	council	council	COUNCIL	2	kept
counterpulsation	counterpulsation	counterpulsation	counterpulsation	Counterpulsation	2	case_or_format
couplet care	couplet care	couplet care	couplet care	couplet care	2	kept
COVID- 19	covid 19	covid 19	covid 19	COVID-19	2	case_or_format
cOVID-19	covid 19	covid 19	covid 19	COVID-19	2	case_or_format
COVID-19 hospitalization	covid 19 hospitalization	covid 19 hospitalization	covid 19 hospitalization	COVID-19 hospitalization	2	kept
COVID-19 infection	covid 19 infection	covid 19 infection	covid 19 infection	COVID-19 infection	2	kept
covid-19 pandemic	covid 19 pandemic	covid 19 pandemic	covid 19 pandemic	COVID-19 pandemic	2	case_or_format

COVID-19 public health policy	covid 19 public health policy	covid 19 public health policy	covid 19 public health policy	COVID-19 public health policy	2	kept
COVID-19, coronavirus disease 2019	covid 19 coronavirus disease 2019	covid 19 coronavirus disease 2019	covid 19 coronavirus disease 2019	COVID-19, coronavirus disease 2019	2	kept
Covid19	covid19	covid 19	covid 19	COVID-19	2	alias
Coxiella burnetii	coxiella burnetii	coxiella burnetii	coxiella burnetii	Coxiella burnetii	2	kept
Coûts	co ts	co ts	co ts	Coûts	2	kept
CPE	cpe	cpe	cpe	CPE	2	kept
CPR—cardiopulmonary resuscitation	cpr cardiopulmonary resuscitation	cpr cardiopulmonary resuscitation	cpr cardiopulmonary resuscitation	CPR—cardiopulmonary resuscitation	2	kept
CPSS	cpss	cpss	cpss	CPSS	2	kept
CQI	cqi	cqi	cqi	CQI	2	kept
cranial nerves	cranial nerves	cranial nerves	cranial nerve	cranial nerve	2	singular
cranial sinus	cranial sinus	cranial sinus	cranial sinus	cranial sinus	2	kept
craniofacial malformation	craniofacial malformation	craniofacial malformation	craniofacial malformation	craniofacial malformation	2	kept
Craniopharyngioma	craniopharyngioma	craniopharyngioma	craniopharyngioma	craniopharyngioma	2	case_or_format
cranioplasty	cranioplasty	cranioplasty	cranioplasty	cranioplasty	2	kept
craving	craving	craving	craving	craving	2	kept
CRE	cre	cre	cre	CRE	2	kept

creatine	creatine	creatine	creatine	Creatine	2	case_or_format
creatine kinase blood level	creatine kinase blood level	creatine kinase blood level	creatine kinase blood level	creatine kinase blood level	2	kept
Creative Self-efficacy	creative self efficacy	creative self efficacy	creative self efficacy	Creative Self-efficacy	2	kept
creative self-efficacy	creative self efficacy	creative self efficacy	creative self efficacy	Creative Self-efficacy	2	case_or_format
Creutzfeldt Jakob disease	creutzfeldt jakob disease	creutzfeldt jakob disease	creutzfeldt jakob disease	Creutzfeldt Jakob disease	2	kept
cricoid cartilage	cricoid cartilage	cricoid cartilage	cricoid cartilage	Cricoid Cartilage	2	case_or_format
cricothyrotomy	cricothyrotomy	cricothyrotomy	cricothyrotomy	cricothyrotomy	2	kept
criminal justice	criminal justice	criminal justice	criminal justice	criminal justice	2	kept
Criminal Law	criminal law	criminal law	criminal law	Criminal Law	2	kept
criminal law	criminal law	criminal law	criminal law	Criminal Law	2	case_or_format
crisis	crisis	crisis	crisis	CRISIS	2	case_or_format
Crisis checklists	crisis checklists	crisis checklists	crisis checklist	Crisis checklists	2	singular
Critical caring	critical caring	critical caring	critical caring	Critical caring	2	kept
Critical data	critical data	critical data	critical data	Critical data	2	kept
Critical environment	critical environment	critical environment	critical environment	Critical environment	2	kept
critical ethnography	critical ethnography	critical ethnography	critical ethnography	Critical ethnography	2	case_or_format

Critical ill	critical ill	critical ill	critical ill	critical ill	2	case_or_format
Critical illness myopathy	critical illness myopathy	critical illness myopathy	critical illness myopathy	critical illness myopathy	2	case_or_format
critical illness polyneuropathy	critical illness polyneuropathy	critical illness polyneuropathy	critical illness polyneuropathy	critical illness polyneuropathy	2	kept
critical illness survivorship	critical illness survivorship	critical illness survivorship	critical illness survivorship	critical illness survivorship	2	kept
critical incident	critical incident	critical incident	critical incident	critical incidents	2	case_or_format
Critical incident reporting	critical incident reporting	critical incident reporting	critical incident reporting	Critical incident reporting	2	kept
Critical incident technique	critical incident technique	critical incident technique	critical incident technique	critical incident technique	2	case_or_format
Critical incidents	critical incidents	critical incidents	critical incident	critical incidents	2	singular
critical limb ischemia	critical limb ischemia	critical limb ischemia	critical limb ischemia	critical limb ischemia	2	kept
Critical skills assessment	critical skills assessment	critical skills assessment	critical skills assessment	Critical skills assessment	2	kept
critical theory	critical theory	critical theory	critical theory	critical theory	2	kept
critical value	critical value	critical value	critical value	critical value	2	kept
Critical values	critical values	critical values	critical value	critical value	2	singular
critically ill neonates	critically ill neonates	critically ill neonates	critically ill neonates	critically ill neonates	2	kept
Critically-ill	critically ill	critically ill	critically ill	Critically ill	2	case_or_format
cross cultural comparison	cross cultural comparison	cross cultural comparison	cross cultural comparison	Cross-Cultural Comparison	2	case_or_format

cross reaction	cross reaction	cross reaction	cross reaction	cross reaction	2	kept
cross section	cross section	cross section	cross section	cross section	2	kept
cross sectional study	cross sectional study	cross sectional study	cross sectional study	Cross-Sectional Studies	2	case_or_format
Cross-cultural adaptation	cross cultural adaptation	cross cultural adaptation	cross cultural adaptation	Cross-cultural adaptation	2	kept
Cross-cultural comparison	cross cultural comparison	cross cultural comparison	cross cultural comparison	Cross-Cultural Comparison	2	case_or_format
cross-lagged panel analysis	cross lagged panel analysis	cross lagged panel analysis	cross lagged panel analysis	cross-lagged panel analysis	2	kept
cross-sectional analysis	cross sectional analysis	cross sectional analysis	cross sectional analysis	cross-sectional analysis	2	kept
Cross-sectional surveys	cross sectional surveys	cross sectional surveys	cross sectional survey	cross-sectional survey	2	singular
cross‐sectional studies	cross sectional studies	cross sectional studies	cross sectional study	Cross-Sectional Studies	2	singular
cross‐sectional survey	cross sectional survey	cross sectional survey	cross sectional survey	cross-sectional survey	2	case_or_format
Croup	croup	croup	croup	croup	2	case_or_format
Crowdsourcing	crowdsourcing	crowdsourcing	crowdsourcing	Crowdsourcing	2	kept
crowdsourcing	crowdsourcing	crowdsourcing	crowdsourcing	Crowdsourcing	2	case_or_format
CRRT dose	crrt dose	crrt dose	crrt dose	CRRT dose	2	kept
Crushing	crushing	crushing	crushing	Crushing	2	kept
crushing	crushing	crushing	crushing	Crushing	2	case_or_format

CRY	cry	cry	cry	CRY	2	kept
cryoablation	cryoablation	cryoablation	cryoablation	cryoablation	2	kept
Cryptogenic Organizing Pneumonia	cryptogenic organizing pneumonia	cryptogenic organizing pneumonia	cryptogenic organizing pneumonia	Cryptogenic Organizing Pneumonia	2	kept
crystalloid solutions	crystalloid solutions	crystalloid solutions	crystalloid solutions	Crystalloid Solutions	2	case_or_format
crystalloids	crystalloids	crystalloids	crystalloid	crystalloid	2	singular
Cue-based feeding	cue based feeding	cue based feeding	cue based feeding	Cue-based feeding	2	kept
cuff pressure	cuff pressure	cuff pressure	cuff pressure	Cuff pressure	2	case_or_format
cuff pressure monitoring	cuff pressure monitoring	cuff pressure monitoring	cuff pressure monitoring	cuff pressure monitoring	2	kept
Cuidado de enfermería	cuidado de enfermer a	cuidado de enfermer a	cuidado de enfermer a	Cuidado de enfermería	2	kept
Cuidados al final de la vida	cuidados al final de la vida	cuidados al final de la vida	cuidados al final de la vida	Cuidados al final de la vida	2	kept
Cuidados de enfermagem	cuidados de enfermagem	cuidados de enfermagem	cuidados de enfermagem	Cuidados de Enfermagem	2	case_or_format
Cuidados de Enfermería	cuidados de enfermer a	cuidados de enfermer a	cuidados de enfermer a	Cuidados de enfermería	2	case_or_format
Cuidados Intensivos	cuidados intensivos	cuidados intensivos	cuidados intensivos	Cuidados intensivos	2	case_or_format
Cultural	cultural	cultural	cultural	Cultural	2	kept
Cultural adaptation	cultural adaptation	cultural adaptation	cultural adaptation	cultural adaptation	2	case_or_format
cultural care competencies	cultural care competencies	cultural care competencies	cultural care competencies	cultural care competencies	2	kept

CULTURAL COMPETENCE	cultural competence	cultural competence	cultural competence	cultural competence	2	case_or_format
Cultural context	cultural context	cultural context	cultural context	Cultural context	2	kept
Cultural issues	cultural issues	cultural issues	cultural issue	cultural issues	2	singular
Cultural safety	cultural safety	cultural safety	cultural safety	cultural safety	2	case_or_format
culturally competent care	culturally competent care	culturally competent care	culturally competent care	Culturally Competent Care	2	case_or_format
culture medium	culture medium	culture medium	culture medium	culture medium	2	kept
culture of excellence	culture of excellence	culture of excellence	culture of excellence	culture of excellence	2	kept
Culture of safety	culture of safety	culture of safety	culture of safety	Culture of safety	2	kept
culture of safety	culture of safety	culture of safety	culture of safety	Culture of safety	2	case_or_format
Cumulative Illness Rating Scale	cumulative illness rating scale	cumulative illness rating scale	cumulative illness rating scale	Cumulative Illness Rating Scale	2	kept
cumulative meta-analysis	cumulative meta analysis	cumulative meta analysis	cumulative meta analysis	cumulative meta-analysis	2	kept
cumulative trauma disorders	cumulative trauma disorders	cumulative trauma disorders	cumulative trauma disorders	cumulative trauma disorders	2	kept
Cunninghamella	cunninghamella	cunninghamella	cunninghamella	Cunninghamella	2	kept
cup feeding	cup feeding	cup feeding	cup feeding	cup feeding	2	kept
Cupping Therapy	cupping therapy	cupping therapy	cupping therapy	Cupping Therapy	2	kept
CURB-65	curb 65	curb 65	curb 65	CURB-65	2	kept

CURB-65 score	curb 65 score	curb 65 score	curb 65 score	CURB-65 score	2	kept
custodial care	custodial care	custodial care	custodial care	custodial care	2	kept
custodial grandparent	custodial grandparent	custodial grandparent	custodial grandparent	custodial grandparent	2	kept
customer experience	customer experience	customer experience	customer experience	customer experience	2	kept
Customisation	customisation	customisation	customisation	Customisation	2	kept
Customizable	customizable	customizable	customizable	Customizable	2	kept
CVC maintenance care bundle	cvc maintenance care bundle	cvc maintenance care bundle	cvc maintenance care bundle	CVC maintenance care bundle	2	kept
CVVH	cvvh	cvvh	cvvh	CVVH	2	kept
cyanide	cyanide	cyanide	cyanide	cyanide	2	kept
cyanoacrylate	cyanoacrylate	cyanoacrylate	cyanoacrylate	cyanoacrylate	2	kept
cyanotic heart disease	cyanotic heart disease	cyanotic heart disease	cyanotic heart disease	cyanotic heart disease	2	kept
Cyclin-Dependent Kinase Inhibitor p16	cyclin dependent kinase inhibitor p16	cyclin dependent kinase inhibitor p16	cyclin dependent kinase inhibitor p16	Cyclin-Dependent Kinase Inhibitor p16	2	kept
cycling	cycling	cycling	cycling	cycling	2	kept
Cyclohexanecarboxylic Acids	cyclohexanecarboxylic acids	cyclohexanecarboxylic acids	cyclohexanecarboxylic acids	Cyclohexanecarboxylic Acids	2	kept
cycloserine	cycloserine	cycloserine	cycloserine	cycloserine	2	kept
cystatin C	cystatin c	cystatin c	cystatin c	cystatin C	2	kept

cystic lymphangioma	cystic lymphangioma	cystic lymphangioma	cystic lymphangioma	cystic lymphangioma	2	kept
cystitis	cystitis	cystitis	cystitis	cystitis	2	kept
cystostomy	cystostomy	cystostomy	cystostomy	cystostomy	2	kept
Cytochrome P-450 CYP1A2	cytochrome p 450 cyp1a2	cytochrome p 450 cyp1a2	cytochrome p 450 cyp1a2	Cytochrome P-450 CYP1A2	2	kept
cytochrome P450	cytochrome p450	cytochrome p450	cytochrome p450	cytochrome P450	2	kept
cytochrome P450 1A2	cytochrome p450 1a2	cytochrome p450 1a2	cytochrome p450 1a2	cytochrome P450 1A2	2	kept
cytogenetics	cytogenetics	cytogenetics	cytogenetics	cytogenetics	2	kept
cytolysis	cytolysis	cytolysis	cytolysis	cytolysis	2	kept
Cytomegalovirus Infections	cytomegalovirus infections	cytomegalovirus infections	cytomegalovirus infection	cytomegalovirus infection	2	singular
cytopenia	cytopenia	cytopenia	cytopenia	cytopenia	2	kept
Cytoreductive surgery	cytoreductive surgery	cytoreductive surgery	cytoreductive surgery	cytoreductive surgery	2	case_or_format
cytotoxic agent	cytotoxic agent	cytotoxic agent	cytotoxic agent	cytotoxic agent	2	kept
cytotoxic T lymphocyte	cytotoxic t lymphocyte	cytotoxic t lymphocyte	cytotoxic t lymphocyte	cytotoxic T lymphocyte	2	kept
cytotoxicity	cytotoxicity	cytotoxicity	cytotoxicity	cytotoxicity	2	kept
Czech	czech	czech	czech	Czech	2	kept
D-dimer	d dimer	d dimer	d dimer	D dimer	2	case_or_format

daclizumab	daclizumab	daclizumab	daclizumab	daclizumab	2	kept
daily goals	daily goals	daily goals	daily goals	DAILY GOALS	2	case_or_format
dalfopristin plus quinupristin	dalfopristin plus quinupristin	dalfopristin plus quinupristin	dalfopristin plus quinupristin	dalfopristin plus quinupristin	2	kept
damage control	damage control	damage control	damage control	damage control	2	kept
damage control resuscitation	damage control resuscitation	damage control resuscitation	damage control resuscitation	Damage control resuscitation	2	case_or_format
dancing	dancing	dancing	dancing	dancing	2	kept
Danish citizen	danish citizen	danish citizen	danish citizen	Danish citizen	2	kept
daptomycin resistance	daptomycin resistance	daptomycin resistance	daptomycin resistance	daptomycin resistance	2	kept
Dar es Salaam	dar es salaam	dar es salaam	dar es salaam	Dar es Salaam	2	kept
Dasein	dasein	dasein	dasein	Dasein	2	kept
Dashboard	dashboard	dashboard	dashboard	dashboard	2	case_or_format
DASS 21	dass 21	dass 21	dass 21	DASS 21	2	kept
Data acquisition	data acquisition	data acquisition	data acquisition	Data acquisition	2	kept
Data analytics	data analytics	data analytics	data analytics	Data analytics	2	kept
data availability	data availability	data availability	data availability	data availability	2	kept
data classification	data classification	data classification	data classification	data classification	2	kept

data collection methods	data collection methods	data collection methods	data collection method	data collection method	2	singular
Data communication systems	data communication systems	data communication systems	data communication systems	Data communication systems	2	kept
Data Compression	data compression	data compression	data compression	Data Compression	2	kept
Data flow analysis	data flow analysis	data flow analysis	data flow analysis	Data flow analysis	2	kept
data representation	data representation	data representation	data representation	data representation	2	kept
Data Science	data science	data science	data science	data science	2	case_or_format
Data science	data science	data science	data science	data science	2	case_or_format
Data visualization	data visualization	data visualization	data visualization	data visualization	2	case_or_format
Data-driven control method	data driven control method	data driven control method	data driven control method	Data-driven control method	2	kept
Database software	database software	database software	database software	Database software	2	kept
Database systems	database systems	database systems	database systems	Database systems	2	kept
Day Care, Medical	day care medical	day care medical	day care medical	Day Care, Medical	2	kept
day surgery	day surgery	day surgery	day surgery	day surgery	2	kept
daytime sleepiness	daytime sleepiness	daytime sleepiness	daytime sleepiness	daytime sleepiness	2	kept
DCD procedures	dcd procedures	dcd procedures	dcd procedures	DCD procedures	2	kept
de-escalation	de escalation	de escalation	de escalation	DE-ESCALATION	2	case_or_format

de-escalation techniques	de escalation techniques	de escalation techniques	de escalation techniques	de-escalation techniques	2	kept
de-implementation	de implementation	de implementation	de implementation	de-implementation	2	kept
Deactivation	deactivation	deactivation	deactivation	Deactivation	2	kept
Death Anxiety	death anxiety	death anxiety	death anxiety	death anxiety	2	case_or_format
Death anxiety	death anxiety	death anxiety	death anxiety	death anxiety	2	case_or_format
Death Certificates	death certificates	death certificates	death certificate	death certificate	2	singular
death experiences	death experiences	death experiences	death experiences	death experiences	2	kept
death rattles	death rattles	death rattles	death rattle	death rattles	2	singular
Debilidad muscular	debilidad muscular	debilidad muscular	debilidad muscular	Debilidad muscular	2	kept
Debre Berhan	debre berhan	debre berhan	debre berhan	Debre Berhan	2	kept
Debriefing structure	debriefing structure	debriefing structure	debriefing structure	Debriefing structure	2	kept
Decent work perception	decent work perception	decent work perception	decent work perception	Decent work perception	2	kept
decentralization	decentralization	decentralization	decentralization	decentralization	2	kept
deception	deception	deception	deception	Deception	2	case_or_format
deciduous tooth	deciduous tooth	deciduous tooth	deciduous tooth	deciduous tooth	2	kept
DECISION	decision	decision	decision	DECISIONS	2	case_or_format

decision aid	decision aid	decision aid	decision aid	decision aids	2	case_or_format
Decision analysis	decision analysis	decision analysis	decision analysis	Decision analysis	2	kept
Decision making models	decision making models	decision making models	decision making models	Decision making models	2	kept
decision making task	decision making task	decision making task	decision making task	decision making task	2	kept
Decision theory	decision theory	decision theory	decision theory	Decision theory	2	kept
decision trees	decision trees	decision trees	decision tree	Decision Trees	2	singular
decision-making dilemma	decision making dilemma	decision making dilemma	decision making dilemma	decision-making dilemma	2	kept
decision-making process	decision making process	decision making process	decision making process	Decision making process	2	case_or_format
DECISION-SUPPORT	decision support	decision support	decision support	Decision support	2	case_or_format
Decisional conflict	decisional conflict	decisional conflict	decisional conflict	Decisional conflict	2	kept
decitabine	decitabine	decitabine	decitabine	decitabine	2	kept
Decompressive craniectomy	decompressive craniectomy	decompressive craniectomy	decompressive craniectomy	decompressive craniectomy	2	case_or_format
decompressive laparotomy	decompressive laparotomy	decompressive laparotomy	decompressive laparotomy	decompressive laparotomy	2	kept
DECONTAMINATION	decontamination	decontamination	decontamination	decontamination	2	case_or_format
decortication	decortication	decortication	decortication	decortication	2	kept
decubitus ulcer	decubitus ulcer	decubitus ulcer	decubitus ulcer	Decubitus ulcer	2	case_or_format

Decubitus ulcers	decubitus ulcers	decubitus ulcers	decubitus ulcer	Decubitus ulcer	2	singular
Dedicated team	dedicated team	dedicated team	dedicated team	Dedicated team	2	kept
deductive reasoning	deductive reasoning	deductive reasoning	deductive reasoning	deductive reasoning	2	kept
deep venous thrombosis	deep venous thrombosis	deep venous thrombosis	deep venous thrombosis	Deep venous thrombosis	2	case_or_format
DEEP-VEIN THROMBOSIS	deep vein thrombosis	deep vein thrombosis	deep vein thrombosis	deep vein thrombosis	2	case_or_format
DEFECTS	defects	defects	defects	DEFECTS	2	kept
Defibrillation	defibrillation	defibrillation	defibrillation	defibrillation	2	case_or_format
deficiency	deficiency	deficiency	deficiency	deficiency	2	kept
Defined daily dose	defined daily dose	defined daily dose	defined daily dose	Defined daily dose	2	kept
Definition	definition	definition	definition	Definitions	2	case_or_format
DEFINITIONS	definitions	definitions	definition	Definitions	2	singular
deflazacort	deflazacort	deflazacort	deflazacort	deflazacort	2	kept
Deformational plagiocephaly	deformational plagiocephaly	deformational plagiocephaly	deformational plagiocephaly	Deformational plagiocephaly	2	kept
degree of centrality	degree of centrality	degree of centrality	degree of centrality	degree of centrality	2	kept
delay	delay	delay	delay	delay	2	kept
delay time	delay time	delay time	delay time	delay time	2	kept

delayed release formulation	delayed release formulation	delayed release formulation	delayed release formulation	delayed release formulation	2	kept
Delayed response time	delayed response time	delayed response time	delayed response time	Delayed response time	2	kept
delayed surgery	delayed surgery	delayed surgery	delayed surgery	delayed surgery	2	kept
DELEGATION	delegation	delegation	delegation	DELEGATION	2	kept
Delirium & cognitive disorders	delirium and cognitive disorders	delirium and cognitive disorders	delirium and cognitive disorders	Delirium & cognitive disorders	2	kept
delirium & cognitive disorders	delirium and cognitive disorders	delirium and cognitive disorders	delirium and cognitive disorders	Delirium & cognitive disorders	2	case_or_format
Delirium Index	delirium index	delirium index	delirium index	Delirium Index	2	kept
Delirium knowledge	delirium knowledge	delirium knowledge	delirium knowledge	Delirium knowledge	2	kept
delirium knowledge	delirium knowledge	delirium knowledge	delirium knowledge	Delirium knowledge	2	case_or_format
Delirium observation screening Scale	delirium observation screening scale	delirium observation screening scale	delirium observation screening scale	Delirium Observation Screening Scale	2	case_or_format
delirium rating scale	delirium rating scale	delirium rating scale	delirium rating scale	Delirium Rating Scale	2	case_or_format
Delirium Rating Scale Revised 98	delirium rating scale revised 98	delirium rating scale revised 98	delirium rating scale revised 98	Delirium Rating Scale Revised 98	2	kept
Delirium screening tool	delirium screening tool	delirium screening tool	delirium screening tool	Delirium screening tools	2	case_or_format
Delirium Severity Scale	delirium severity scale	delirium severity scale	delirium severity scale	Delirium Severity Scale	2	kept
Delirium subtype	delirium subtype	delirium subtype	delirium subtype	Delirium subtype	2	kept
delirium subtypes	delirium subtypes	delirium subtypes	delirium subtype	Delirium subtype	2	singular

Delirium tremens	delirium tremens	delirium tremens	delirium tremens	delirium tremens	2	case_or_format
delivery of health care, integrated	delivery of health care integrated	delivery of health care integrated	delivery of health care integrated	Delivery of Health Care, Integrated	2	case_or_format
Delivery room management	delivery room management	delivery room management	delivery room management	Delivery room management	2	kept
delivery rooms	delivery rooms	delivery rooms	delivery room	delivery room	2	singular
Delphi evaluation	delphi evaluation	delphi evaluation	delphi evaluation	Delphi evaluation	2	kept
delta rhythm	delta rhythm	delta rhythm	delta rhythm	delta rhythm	2	kept
DEMAND	demand	demand	demand	DEMANDS	2	case_or_format
demands	demands	demands	demand	DEMANDS	2	singular
Dementia care	dementia care	dementia care	dementia care	Dementia care	2	kept
dementia care	dementia care	dementia care	dementia care	Dementia care	2	case_or_format
Dementia special care units	dementia special care units	dementia special care units	dementia special care units	Dementia special care units	2	kept
dementia specialty care unit	dementia specialty care unit	dementia specialty care unit	dementia specialty care unit	dementia specialty care unit	2	kept
DEMOGRAPHIC CHARACTERISTICS	demographic characteristics	demographic characteristics	demographic characteristics	DEMOGRAPHIC CHARACTERISTICS	2	kept
Demographics	demographics	demographics	demographics	demographics	2	case_or_format
Dempster Practice Behavior Scale	dempster practice behavior scale	dempster practice behavior scale	dempster practice behavior scale	Dempster Practice Behavior Scale	2	kept
demyelinating disease	demyelinating disease	demyelinating disease	demyelinating disease	demyelinating disease	2	kept

Dendrograms	dendrograms	dendrograms	dendrograms	Dendrograms	2	kept
Denervated heart	denervated heart	denervated heart	denervated heart	Denervated heart	2	kept
Denial, Psychological	denial psychological	denial psychological	denial psychological	Denial, Psychological	2	kept
density	density	density	density	density	2	kept
dental care for aged	dental care for aged	dental care for aged	dental care for aged	dental care for aged	2	kept
dental care for chronically ill	dental care for chronically ill	dental care for chronically ill	dental care for chronically ill	dental care for chronically ill	2	kept
Dental Hygienists	dental hygienists	dental hygienists	dental hygienist	dental hygienist	2	singular
dental personnel	dental personnel	dental personnel	dental personnel	dental personnel	2	kept
Dental prosthesis	dental prosthesis	dental prosthesis	dental prosthesis	Dental prosthesis	2	kept
dental restoration	dental restoration	dental restoration	dental restoration	dental restoration	2	kept
Dental Restoration, Permanent	dental restoration permanent	dental restoration permanent	dental restoration permanent	Dental Restoration, Permanent	2	kept
dental student	dental student	dental student	dental student	dental student	2	kept
denture	denture	denture	denture	denture	2	kept
Department of Gastrointestinal Surgery	department of gastrointestinal surgery	department of gastrointestinal surgery	department of gastrointestinal surgery	Department of Gastrointestinal Surgery	2	kept
Dependency structures	dependency structures	dependency structures	dependency structures	Dependency structures	2	kept
deprescription	deprescription	deprescription	deprescription	deprescription	2	kept

depressed blood pressure	depressed blood pressure	depressed blood pressure	depressed blood pressure	depressed blood pressure	2	kept
depression assessment	depression assessment	depression assessment	depression assessment	depression assessment	2	kept
Depressive disorders	depressive disorders	depressive disorders	depressive disorder	Depressive Disorder	2	singular
depressive symptoms	depressive symptoms	depressive symptoms	depressive symptom	Depressive symptoms	2	singular
Depth of hypnosis	depth of hypnosis	depth of hypnosis	depth of hypnosis	Depth of hypnosis	2	kept
DERMATITIS	dermatitis	dermatitis	dermatitis	Dermatitis	2	case_or_format
dermatological procedure	dermatological procedure	dermatological procedure	dermatological procedure	dermatological procedure	2	kept
dermatomycosis	dermatomycosis	dermatomycosis	dermatomycosis	dermatomycosis	2	kept
desaturation	desaturation	desaturation	desaturation	desaturation	2	kept
Descriptive	descriptive	descriptive	descriptive	Descriptive	2	kept
descriptive	descriptive	descriptive	descriptive	Descriptive	2	case_or_format
Descriptive statistics	descriptive statistics	descriptive statistics	descriptive statistics	Descriptive statistics	2	kept
Descriptive studies	descriptive studies	descriptive studies	descriptive study	Descriptive studies	2	singular
Desensitization	desensitization	desensitization	desensitization	desensitization	2	case_or_format
Desflurane	desflurane	desflurane	desflurane	desflurane	2	case_or_format
Design guidelines	design guidelines	design guidelines	design guideline	Design guidelines	2	singular

Designated hospital	designated hospital	designated hospital	designated hospital	Designated hospital	2	kept
designated hospital	designated hospital	designated hospital	designated hospital	Designated hospital	2	case_or_format
designing simulation scenarios	designing simulation scenarios	designing simulation scenarios	designing simulation scenarios	designing simulation scenarios	2	kept
desirability function	desirability function	desirability function	desirability function	desirability function	2	kept
desorption	desorption	desorption	desorption	desorption	2	kept
desquamation	desquamation	desquamation	desquamation	desquamation	2	kept
destination therapy	destination therapy	destination therapy	destination therapy	destination therapy	2	kept
detachment	detachment	detachment	detachment	detachment	2	kept
detergents	detergents	detergents	detergent	Detergents	2	singular
Determinant factors	determinant factors	determinant factors	determinant factors	Determinant factors	2	kept
Determination of death	determination of death	determination of death	determination of death	Determination of death	2	kept
developmental biology	developmental biology	developmental biology	developmental biology	Developmental Biology	2	case_or_format
developmental disabilities	developmental disabilities	developmental disabilities	developmental disabilities	Developmental Disabilities	2	case_or_format
Developmental support	developmental support	developmental support	developmental support	Developmental support	2	kept
developmental supportive care	developmental supportive care	developmental supportive care	developmental supportive care	Developmental supportive care	2	case_or_format
developmentally supportive care	developmentally supportive care	developmentally supportive care	developmentally supportive care	developmentally supportive care	2	kept

device migration	device migration	device migration	device migration	device migration	2	kept
Device utilization	device utilization	device utilization	device utilization	Device utilization	2	kept
Device-associated infections	device associated infections	device associated infections	device associated infection	Device-associated infections	2	singular
dextran	dextran	dextran	dextran	dextran	2	kept
dezocine	dezocine	dezocine	dezocine	dezocine	2	kept
Diabetes Insipidus, Nephrogenic	diabetes insipidus nephrogenic	diabetes insipidus nephrogenic	diabetes insipidus nephrogenic	Diabetes Insipidus, Nephrogenic	2	kept
diabetes management	diabetes management	diabetes management	diabetes management	diabetes management	2	kept
diabetes technology	diabetes technology	diabetes technology	diabetes technology	diabetes technology	2	kept
diabetic diet	diabetic diet	diabetic diet	diabetic diet	diabetic diet	2	kept
diabetic foot	diabetic foot	diabetic foot	diabetic foot	diabetic foot	2	kept
Diabetic Nephropathies	diabetic nephropathies	diabetic nephropathies	diabetic nephropathy	diabetic nephropathy	2	singular
diagnosis time	diagnosis time	diagnosis time	diagnosis time	diagnosis time	2	kept
Diagnostic accuracy test	diagnostic accuracy test	diagnostic accuracy test	diagnostic accuracy test	Diagnostic accuracy test	2	kept
diagnostic approach route	diagnostic approach route	diagnostic approach route	diagnostic approach route	diagnostic approach route	2	kept
Diagnostic bronchoscopy	diagnostic bronchoscopy	diagnostic bronchoscopy	diagnostic bronchoscopy	Diagnostic bronchoscopy	2	kept
Diagnostic characteristics	diagnostic characteristics	diagnostic characteristics	diagnostic characteristics	Diagnostic characteristics	2	kept

Diagnostic error	diagnostic error	diagnostic error	diagnostic error	Diagnostic Errors	2	case_or_format
diagnostic reasoning	diagnostic reasoning	diagnostic reasoning	diagnostic reasoning	diagnostic reasoning	2	kept
Diagnostic tools	diagnostic tools	diagnostic tools	diagnostic tools	Diagnostic tools	2	kept
dialysis-related disease	dialysis related disease	dialysis related disease	dialysis related disease	dialysis-related disease	2	kept
diaphragm disease	diaphragm disease	diaphragm disease	diaphragm disease	diaphragm disease	2	kept
diastole	diastole	diastole	diastole	Diastole	2	case_or_format
Diastolic dysfunction	diastolic dysfunction	diastolic dysfunction	diastolic dysfunction	diastolic dysfunction	2	case_or_format
dietary compliance	dietary compliance	dietary compliance	dietary compliance	dietary compliance	2	kept
Dietary Fiber	dietary fiber	dietary fiber	dietary fiber	Dietary Fiber	2	kept
dietary reference intake	dietary reference intake	dietary reference intake	dietary reference intake	dietary reference intake	2	kept
dietary service	dietary service	dietary service	dietary service	dietary service	2	kept
Dietitian	dietitian	dietitian	dietitian	dietitian	2	case_or_format
Differential item functioning	differential item functioning	differential item functioning	differential item functioning	Differential item functioning	2	kept
Diffuse Intrinsic Pontine Glioma	diffuse intrinsic pontine glioma	diffuse intrinsic pontine glioma	diffuse intrinsic pontine glioma	Diffuse Intrinsic Pontine Glioma	2	kept
diffuse large B cell lymphoma	diffuse large b cell lymphoma	diffuse large b cell lymphoma	diffuse large b cell lymphoma	diffuse large B cell lymphoma	2	kept
Diffusion of innovation	diffusion of innovation	diffusion of innovation	diffusion of innovation	Diffusion of Innovation	2	case_or_format

diffusion of innovation	diffusion of innovation	diffusion of innovation	diffusion of innovation	Diffusion of Innovation	2	case_or_format
Digestion	digestion	digestion	digestion	digestion	2	case_or_format
Digestive System Abnormalities	digestive system abnormalities	digestive system abnormalities	digestive system abnormality	Digestive System Abnormalities	2	singular
digestive system disease assessment	digestive system disease assessment	digestive system disease assessment	digestive system disease assessment	digestive system disease assessment	2	kept
Digestive System Physiological Phenomena	digestive system physiological phenomena	digestive system physiological phenomena	digestive system physiological phenomena	Digestive System Physiological Phenomena	2	kept
digital	digital	digital	digital	Digital	2	case_or_format
digital assessment tool	digital assessment tool	digital assessment tool	digital assessment tool	digital assessment tool	2	kept
digital competence	digital competence	digital competence	digital competence	digital competence	2	kept
digital culture	digital culture	digital culture	digital culture	digital culture	2	kept
digital health intervention	digital health intervention	digital health intervention	digital health intervention	digital health intervention	2	kept
Digital hospital visits	digital hospital visits	digital hospital visits	digital hospital visits	Digital hospital visits	2	kept
Digital medicine	digital medicine	digital medicine	digital medicine	Digital medicine	2	kept
digital medicine	digital medicine	digital medicine	digital medicine	Digital medicine	2	case_or_format
Digital penandpaper	digital penandpaper	digital penandpaper	digital penandpaper	Digital penandpaper	2	kept
digital rectal examination	digital rectal examination	digital rectal examination	digital rectal examination	digital rectal examination	2	kept
digital transformation	digital transformation	digital transformation	digital transformation	digital transformation	2	kept

digitalis intoxication	digitalis intoxication	digitalis intoxication	digitalis intoxication	digitalis intoxication	2	kept
digitalisation	digitalisation	digitalisation	digitalisation	digitalisation	2	kept
dignified care	dignified care	dignified care	dignified care	dignified care	2	kept
dignified death	dignified death	dignified death	dignified death	dignified death	2	kept
DIGNITY	dignity	dignity	dignity	dignity	2	case_or_format
Dilatation and Curettage	dilatation and curettage	dilatation and curettage	dilatation and curettage	Dilatation and Curettage	2	kept
Dilemmas	dilemmas	dilemmas	dilemmas	DILEMMAS	2	case_or_format
dimenhydrinate	dimenhydrinate	dimenhydrinate	dimenhydrinate	dimenhydrinate	2	kept
Dimensional analysis	dimensional analysis	dimensional analysis	dimensional analysis	Dimensional analysis	2	kept
dimensional analysis	dimensional analysis	dimensional analysis	dimensional analysis	Dimensional analysis	2	case_or_format
Dimensional Measurement Accuracy	dimensional measurement accuracy	dimensional measurement accuracy	dimensional measurement accuracy	Dimensional Measurement Accuracy	2	kept
dimensional measurement accuracy	dimensional measurement accuracy	dimensional measurement accuracy	dimensional measurement accuracy	Dimensional Measurement Accuracy	2	case_or_format
Diphtheria	diphtheria	diphtheria	diphtheria	diphtheria	2	case_or_format
diplopia	diplopia	diplopia	diplopia	diplopia	2	kept
Diptera	diptera	diptera	diptera	Diptera	2	kept
direct contact transmission	direct contact transmission	direct contact transmission	direct contact transmission	direct contact transmission	2	kept

DIRECT LARYNGOSCOPY	direct laryngoscopy	direct laryngoscopy	direct laryngoscopy	direct laryngoscopy	2	case_or_format
Direct observation	direct observation	direct observation	direct observation	Direct observation	2	kept
Direct oral anticoagulants	direct oral anticoagulants	direct oral anticoagulants	direct oral anticoagulant	Direct oral anticoagulants	2	singular
directed content analysis	directed content analysis	directed content analysis	directed content analysis	directed content analysis	2	kept
Directories	directories	directories	directory	directory	2	singular
Directories as Topic	directories as topic	directories as topic	directories as topic	Directories as Topic	2	kept
DISABILITY	disability	disability	disability	disability	2	case_or_format
disability discrimination	disability discrimination	disability discrimination	disability discrimination	disability discrimination	2	kept
disability rating scale	disability rating scale	disability rating scale	disability rating scale	disability rating scale	2	kept
disabled children	disabled children	disabled children	disabled children	Disabled Children	2	case_or_format
Disaster management	disaster management	disaster management	disaster management	disaster management	2	case_or_format
disaster mitigation	disaster mitigation	disaster mitigation	disaster mitigation	disaster mitigation	2	kept
Disaster plans	disaster plans	disaster plans	disaster plan	Disaster plans	2	singular
Disaster relief	disaster relief	disaster relief	disaster relief	Disaster relief	2	kept
Disaster response	disaster response	disaster response	disaster response	disaster response	2	case_or_format
disaster response resources	disaster response resources	disaster response resources	disaster response resources	disaster response resources	2	kept

discectomy	discectomy	discectomy	discectomy	discectomy	2	kept
discharge against medical advice	discharge against medical advice	discharge against medical advice	discharge against medical advice	discharge against medical advice	2	kept
discharge barriers	discharge barriers	discharge barriers	discharge barriers	discharge barriers	2	kept
discharge delay	discharge delay	discharge delay	discharge delay	discharge delay	2	kept
Discharge location	discharge location	discharge location	discharge location	Discharge location	2	kept
discharge location	discharge location	discharge location	discharge location	Discharge location	2	case_or_format
discharge planner	discharge planner	discharge planner	discharge planner	discharge planner	2	kept
discharge preparing service	discharge preparing service	discharge preparing service	discharge preparing service	discharge preparing service	2	kept
Discharge readiness	discharge readiness	discharge readiness	discharge readiness	discharge readiness	2	case_or_format
discharge tools	discharge tools	discharge tools	discharge tools	discharge tools	2	kept
Discitis	discitis	discitis	discitis	Discitis	2	kept
DISCLOSURE	disclosure	disclosure	disclosure	Disclosure	2	case_or_format
Discourse	discourse	discourse	discourse	Discourse	2	kept
discourse	discourse	discourse	discourse	Discourse	2	case_or_format
DISCOURSE ANALYSIS	discourse analysis	discourse analysis	discourse analysis	Discourse analysis	2	case_or_format
Disease	disease	disease	disease	Diseases	2	case_or_format

disease diagnosis	disease diagnosis	disease diagnosis	disease diagnosis	disease diagnosis	2	kept
disease modifying antirheumatic drug	disease modifying antirheumatic drug	disease modifying antirheumatic drug	disease modifying antirheumatic drug	disease modifying antirheumatic drug	2	kept
disease notification	disease notification	disease notification	disease notification	Disease Notification	2	case_or_format
disease outbreaks	disease outbreaks	disease outbreaks	disease outbreak	Disease Outbreaks	2	singular
Disease Prevention	disease prevention	disease prevention	disease prevention	Disease prevention	2	case_or_format
disease prevention	disease prevention	disease prevention	disease prevention	Disease prevention	2	case_or_format
disease simulation	disease simulation	disease simulation	disease simulation	disease simulation	2	kept
Disinfectant resistance	disinfectant resistance	disinfectant resistance	disinfectant resistance	Disinfectant resistance	2	kept
disinfection cap	disinfection cap	disinfection cap	disinfection cap	disinfection cap	2	kept
disinfection system	disinfection system	disinfection system	disinfection system	disinfection system	2	kept
diskitis	diskitis	diskitis	diskitis	diskitis	2	kept
Disorders	disorders	disorders	disorder	DISORDERS	2	singular
disorders of amino acid and protein metabolism	disorders of amino acid and protein metabolism	disorders of amino acid and protein metabolism	disorders of amino acid and protein metabolism	disorders of amino acid and protein metabolism	2	kept
DISPARITIES	disparities	disparities	disparity	disparities	2	singular
Disparities	disparities	disparities	disparity	disparities	2	singular
Dispensing	dispensing	dispensing	dispensing	Dispensing	2	kept

dispensing error	dispensing error	dispensing error	dispensing error	dispensing error	2	kept
DISPENSING ERRORS	dispensing errors	dispensing errors	dispensing error	dispensing error	2	singular
dispersion	dispersion	dispersion	dispersion	dispersion	2	kept
Display devices	display devices	display devices	display devices	Display devices	2	kept
DISPLAYS	displays	displays	displays	DISPLAYS	2	kept
Disposition planning	disposition planning	disposition planning	disposition planning	Disposition planning	2	kept
Dissection	dissection	dissection	dissection	Dissection	2	kept
Disseminated intravascular coagulation	disseminated intravascular coagulation	disseminated intravascular coagulation	disseminated intravascular coagulation	Disseminated Intravascular Coagulation	2	case_or_format
DISSEMINATION	dissemination	dissemination	dissemination	DISSEMINATION	2	kept
dissemination	dissemination	dissemination	dissemination	DISSEMINATION	2	case_or_format
Distal feeding	distal feeding	distal feeding	distal feeding	Distal feeding	2	kept
distal femur fracture	distal femur fracture	distal femur fracture	distal femur fracture	distal femur fracture	2	kept
distal radius fracture	distal radius fracture	distal radius fracture	distal radius fracture	distal radius fracture	2	kept
Distance	distance	distance	distance	Distance	2	kept
Distance Education	distance education	distance education	distance education	Distance Education	2	kept
Distance learning	distance learning	distance learning	distance learning	distance learning	2	case_or_format

Distance to hospital	distance to hospital	distance to hospital	distance to hospital	Distance to hospital	2	kept
Distraction	distraction	distraction	distraction	distraction	2	case_or_format
Distraction distance	distraction distance	distraction distance	distraction distance	Distraction distance	2	kept
Distress tolerance	distress tolerance	distress tolerance	distress tolerance	Distress tolerance	2	kept
distribution characteristics	distribution characteristics	distribution characteristics	distribution characteristics	distribution characteristics	2	kept
District hospitals	district hospitals	district hospitals	district hospital	District hospitals	2	singular
DISTURBANCE	disturbance	disturbance	disturbance	DISTURBANCE	2	kept
disulfiram	disulfiram	disulfiram	disulfiram	disulfiram	2	kept
diuretics	diuretics	diuretics	diuretics	Diuretics	2	case_or_format
Diurnal variation	diurnal variation	diurnal variation	diurnal variation	Diurnal variation	2	kept
DNA 16S	dna 16s	dna 16s	dna 16s	DNA 16S	2	kept
DNA damage	dna damage	dna damage	dna damage	DNA damage	2	kept
DNA fingerprinting	dna fingerprinting	dna fingerprinting	dna fingerprinting	DNA Fingerprinting	2	case_or_format
DNA isolation	dna isolation	dna isolation	dna isolation	DNA isolation	2	kept
DNA methylation	dna methylation	dna methylation	dna methylation	DNA methylation	2	kept
DNA, Fungal	dna fungal	dna fungal	dna fungal	DNA, Fungal	2	kept

DNAR	dnar	dnar	dnar	DNAR	2	kept
Do Not Resuscitate order	do not resuscitate order	do not resuscitate order	do not resuscitate order	do not resuscitate order	2	case_or_format
do-not-resuscitate orders	do not resuscitate orders	do not resuscitate orders	do not resuscitate order	do not resuscitate order	2	singular
doctor	doctor	doctor	doctor	Doctors	2	case_or_format
document examination	document examination	document examination	document examination	document examination	2	kept
Documentation accuracy	documentation accuracy	documentation accuracy	documentation accuracy	Documentation accuracy	2	kept
documentations	documentations	documentations	documentation	Documentation	2	singular
Doffing	doffing	doffing	doffing	Doffing	2	kept
DOGS	dogs	dogs	dog	Dogs	2	singular
Dolichocephaly	dolichocephaly	dolichocephaly	dolichocephaly	Dolichocephaly	2	kept
domestic animal	domestic animal	domestic animal	domestic animal	domestic animal	2	kept
domestic chemical	domestic chemical	domestic chemical	domestic chemical	domestic chemical	2	kept
domestic partner	domestic partner	domestic partner	domestic partner	domestic partner	2	kept
Dominance, Cerebral	dominance cerebral	dominance cerebral	dominance cerebral	Dominance, Cerebral	2	kept
Dominance-Subordination	dominance subordination	dominance subordination	dominance subordination	Dominance-Subordination	2	kept
Donabedian	donabedian	donabedian	donabedian	Donabedian	2	kept

DONATION	donation	donation	donation	Donation	2	case_or_format
DONOR	donor	donor	donor	donor	2	case_or_format
donor breast milk	donor breast milk	donor breast milk	donor breast milk	donor breast milk	2	kept
donor relatives	donor relatives	donor relatives	donor relatives	donor relatives	2	kept
donor site	donor site	donor site	donor site	donor site	2	kept
Dopamine Antagonists	dopamine antagonists	dopamine antagonists	dopamine antagonists	Dopamine Antagonists	2	kept
Doppler echocardiography	doppler echocardiography	doppler echocardiography	doppler echocardiography	Doppler echocardiography	2	kept
DOS	dos	dos	dos	DOS	2	kept
Dosage	dosage	dosage	dosage	Dosage	2	kept
Dosage Forms	dosage forms	dosage forms	dosage form	Dosage Forms	2	singular
dose-response	dose response	dose response	dose response	dose response	2	case_or_format
dosimeter	dosimeter	dosimeter	dosimeter	dosimeter	2	kept
dosimetry	dosimetry	dosimetry	dosimetry	dosimetry	2	kept
Dosing	dosing	dosing	dosing	Dosing	2	kept
Double dilution	double dilution	double dilution	double dilution	Double dilution	2	kept
double lumen catheter	double lumen catheter	double lumen catheter	double lumen catheter	double lumen catheter	2	kept

Doulas	doulas	doulas	doula	doula	2	singular
downes’ score	downes score	downes score	downes score	downes’ score	2	kept
downtime	downtime	downtime	downtime	Downtime	2	case_or_format
doxapram	doxapram	doxapram	doxapram	doxapram	2	kept
doxazosin	doxazosin	doxazosin	doxazosin	doxazosin	2	kept
Drawing	drawing	drawing	drawing	drawing	2	case_or_format
Dressing	dressing	dressing	dressing	dressing	2	case_or_format
Dressing change	dressing change	dressing change	dressing change	Dressing change	2	kept
DRESSINGS	dressings	dressings	dressing	dressing	2	singular
DRG	drg	drg	drg	DRG	2	kept
droplet transmission	droplet transmission	droplet transmission	droplet transmission	droplet transmission	2	kept
Drug	drug	drug	drug	drug	2	case_or_format
Drug abuse	drug abuse	drug abuse	drug abuse	drug abuse	2	case_or_format
DRUG ADMINISTRATION ERRORS	drug administration errors	drug administration errors	drug administration errors	DRUG ADMINISTRATION ERRORS	2	kept
Drug and Narcotic Control	drug and narcotic control	drug and narcotic control	drug and narcotic control	Drug and Narcotic Control	2	kept
drug classification	drug classification	drug classification	drug classification	drug classification	2	kept

drug compounding	drug compounding	drug compounding	drug compounding	Drug Compounding	2	case_or_format
Drug Contamination	drug contamination	drug contamination	drug contamination	drug contamination	2	case_or_format
Drug delivery	drug delivery	drug delivery	drug delivery	Drug delivery	2	kept
drug desensitization	drug desensitization	drug desensitization	drug desensitization	drug desensitization	2	kept
drug design	drug design	drug design	drug design	drug design	2	kept
drug development	drug development	drug development	drug development	drug development	2	kept
Drug dosage	drug dosage	drug dosage	drug dosage	Drug dosage	2	kept
drug dose calculator	drug dose calculator	drug dose calculator	drug dose calculator	drug dose calculator	2	kept
drug dose sequence	drug dose sequence	drug dose sequence	drug dose sequence	drug dose sequence	2	kept
drug dosing	drug dosing	drug dosing	drug dosing	Drug dosing	2	case_or_format
Drug eluting stent	drug eluting stent	drug eluting stent	drug eluting stent	Drug eluting stent	2	kept
Drug Eruptions	drug eruptions	drug eruptions	drug eruption	drug eruption	2	singular
drug granule	drug granule	drug granule	drug granule	drug granule	2	kept
Drug Industry	drug industry	drug industry	drug industry	drug industry	2	case_or_format
Drug library	drug library	drug library	drug library	Drug library	2	kept
drug library	drug library	drug library	drug library	Drug library	2	case_or_format

drug metabolite	drug metabolite	drug metabolite	drug metabolite	drug metabolite	2	kept
Drug monitoring	drug monitoring	drug monitoring	drug monitoring	Drug Monitoring	2	case_or_format
Drug preparation	drug preparation	drug preparation	drug preparation	Drug preparation	2	kept
drug preparation	drug preparation	drug preparation	drug preparation	Drug preparation	2	case_or_format
drug protein binding	drug protein binding	drug protein binding	drug protein binding	drug protein binding	2	kept
drug pulse therapy	drug pulse therapy	drug pulse therapy	drug pulse therapy	drug pulse therapy	2	kept
drug reaction	drug reaction	drug reaction	drug reaction	drug reaction	2	kept
drug repositioning	drug repositioning	drug repositioning	drug repositioning	drug repositioning	2	kept
Drug resistance, microbial	drug resistance microbial	drug resistance microbial	drug resistance microbial	Drug Resistance, Microbial	2	case_or_format
Drug Stability	drug stability	drug stability	drug stability	drug stability	2	case_or_format
Drug stability	drug stability	drug stability	drug stability	drug stability	2	case_or_format
Drug storage and distribution system	drug storage and distribution system	drug storage and distribution system	drug storage and distribution system	Drug storage and distribution system	2	kept
Drug Synergism	drug synergism	drug synergism	drug synergism	Drug Synergism	2	kept
drug tissue level	drug tissue level	drug tissue level	drug tissue level	drug tissue level	2	kept
Drug use evaluation	drug use evaluation	drug use evaluation	drug use evaluation	Drug use evaluation	2	kept
Drug utilization	drug utilization	drug utilization	drug utilization	Drug Utilization	2	case_or_format

drug vehicle	drug vehicle	drug vehicle	drug vehicle	drug vehicle	2	kept
Drugs, Investigational	drugs investigational	drugs investigational	drugs investigational	Drugs, Investigational	2	kept
drunken driving	drunken driving	drunken driving	drunken driving	drunken driving	2	kept
Dry eye syndrome	dry eye syndrome	dry eye syndrome	dry eye syndrome	Dry Eye Syndromes	2	case_or_format
Dry mouth	dry mouth	dry mouth	dry mouth	Dry mouth	2	kept
DSM-IV-TR	dsm iv tr	dsm iv tr	dsm iv tr	DSM-IV-TR	2	kept
dual diagnosis	dual diagnosis	dual diagnosis	dual diagnosis	dual diagnosis	2	kept
dual eligibility	dual eligibility	dual eligibility	dual eligibility	dual eligibility	2	kept
dual energy X ray absorptiometry	dual energy x ray absorptiometry	dual energy x ray absorptiometry	dual energy x ray absorptiometry	dual energy X ray absorptiometry	2	kept
dual role	dual role	dual role	dual role	dual role	2	kept
duloxetine	duloxetine	duloxetine	duloxetine	duloxetine	2	kept
Duodenum	duodenum	duodenum	duodenum	duodenum	2	case_or_format
duration of mechanical ventilation	duration of mechanical ventilation	duration of mechanical ventilation	duration of mechanical ventilation	duration of mechanical ventilation	2	kept
Dutchman	dutchman	dutchman	dutchman	Dutchman	2	kept
Duty hours	duty hours	duty hours	duty hours	Duty hours	2	kept
duty hours	duty hours	duty hours	duty hours	Duty hours	2	case_or_format

Dynamic composition	dynamic composition	dynamic composition	dynamic composition	Dynamic composition	2	kept
Dynamic threshold	dynamic threshold	dynamic threshold	dynamic threshold	Dynamic threshold	2	kept
Dynamic uncertainty	dynamic uncertainty	dynamic uncertainty	dynamic uncertainty	Dynamic uncertainty	2	kept
DYNAMICS	dynamics	dynamics	dynamic	dynamics	2	singular
Dysarthria	dysarthria	dysarthria	dysarthria	dysarthria	2	case_or_format
Dysbiosis	dysbiosis	dysbiosis	dysbiosis	Dysbiosis	2	kept
dysesthesia	dysesthesia	dysesthesia	dysesthesia	dysesthesia	2	kept
DYSFUNCTION	dysfunction	dysfunction	dysfunction	DYSFUNCTION	2	kept
Dysfunction	dysfunction	dysfunction	dysfunction	DYSFUNCTION	2	case_or_format
dysglycemia	dysglycemia	dysglycemia	dysglycemia	dysglycemia	2	kept
Dyslipidemias	dyslipidemias	dyslipidemias	dyslipidemia	dyslipidemia	2	singular
dysphasia	dysphasia	dysphasia	dysphasia	dysphasia	2	kept
Dysphonia	dysphonia	dysphonia	dysphonia	dysphonia	2	case_or_format
dysplasia	dysplasia	dysplasia	dysplasia	dysplasia	2	kept
Dyspnoea	dyspnoea	dyspnoea	dyspnoea	Dyspnoea	2	kept
dysrhythmia	dysrhythmia	dysrhythmia	dysrhythmia	dysrhythmia	2	kept

Dysrhythmias	dysrhythmias	dysrhythmias	dysrhythmia	dysrhythmia	2	singular
Dystocia	dystocia	dystocia	dystocia	dystocia	2	case_or_format
E-bike	e bike	e bike	e bike	E-bike	2	kept
E-Delphi	e delphi	e delphi	e delphi	E-Delphi	2	kept
E-health	e health	e health	e health	E-health	2	kept
E. coli	e coli	e coli	e coli	E. coli	2	kept
Ear	ear	ear	ear	Ear	2	kept
ear	ear	ear	ear	Ear	2	case_or_format
Ear surgery noise	ear surgery noise	ear surgery noise	ear surgery noise	Ear surgery noise	2	kept
early activity	early activity	early activity	early activity	early activity	2	kept
early cancer diagnosis	early cancer diagnosis	early cancer diagnosis	early cancer diagnosis	early cancer diagnosis	2	kept
EARLY DISCHARGE	early discharge	early discharge	early discharge	Early discharge	2	case_or_format
early discharge	early discharge	early discharge	early discharge	Early discharge	2	case_or_format
early enteral nutrition	early enteral nutrition	early enteral nutrition	early enteral nutrition	early enteral nutrition	2	kept
Early goals of care	early goals of care	early goals of care	early goals of care	Early goals of care	2	kept
early identification	early identification	early identification	early identification	early identification	2	kept

Early Intervention	early intervention	early intervention	early intervention	early intervention	2	case_or_format
Early Mobilisation	early mobilisation	early mobilisation	early mobilisation	Early mobilisation	2	case_or_format
Early nasojejunal enteral nutrition	early nasojejunal enteral nutrition	early nasojejunal enteral nutrition	early nasojejunal enteral nutrition	Early nasojejunal enteral nutrition	2	kept
EARLY PALLIATIVE CARE	early palliative care	early palliative care	early palliative care	EARLY PALLIATIVE CARE	2	kept
early psychological support	early psychological support	early psychological support	early psychological support	early psychological support	2	kept
early rehabilitation activity	early rehabilitation activity	early rehabilitation activity	early rehabilitation activity	early rehabilitation activity	2	kept
Early rehabilitation training	early rehabilitation training	early rehabilitation training	early rehabilitation training	Early rehabilitation training	2	kept
Early tracheal extubation	early tracheal extubation	early tracheal extubation	early tracheal extubation	Early tracheal extubation	2	kept
Early warning	early warning	early warning	early warning	Early warning	2	kept
early warning	early warning	early warning	early warning	Early warning	2	case_or_format
EARLY WARNING SCORES	early warning scores	early warning scores	early warning score	early warning score	2	singular
Early warning scoring system	early warning scoring system	early warning scoring system	early warning scoring system	Early warning scoring system	2	kept
Early-warning score	early warning score	early warning score	early warning score	early warning score	2	case_or_format
earplug	earplug	earplug	earplug	EARPLUGS	2	case_or_format
Earplugs	earplugs	earplugs	earplug	EARPLUGS	2	singular
East Asian	east asian	east asian	east asian	East Asian	2	kept

Eastern Cape	eastern cape	eastern cape	eastern cape	Eastern Cape	2	kept
eastern Ethiopia	eastern ethiopia	eastern ethiopia	eastern ethiopia	eastern Ethiopia	2	kept
Eastern European	eastern european	eastern european	eastern european	Eastern European	2	kept
Eastern Mediterranean region	eastern mediterranean region	eastern mediterranean region	eastern mediterranean region	Eastern Mediterranean region	2	kept
eastern Mediterranean region	eastern mediterranean region	eastern mediterranean region	eastern mediterranean region	Eastern Mediterranean region	2	case_or_format
Eat	eat	eat	eat	Eat	2	kept
Ebola vaccine	ebola vaccine	ebola vaccine	ebola vaccine	Ebola vaccine	2	kept
Ebolavirus infection	ebolavirus infection	ebolavirus infection	ebolavirus infection	Ebolavirus infection	2	kept
EBP	ebp	ebp	ebp	EBP	2	kept
EBP experiences	ebp experiences	ebp experiences	ebp experiences	EBP experiences	2	kept
ECG abnormality	ecg abnormality	ecg abnormality	ecg abnormality	ECG abnormality	2	kept
ECG transmission	ecg transmission	ecg transmission	ecg transmission	ECG transmission	2	kept
ECG, electrocardiogram	ecg electrocardiogram	ecg electrocardiogram	ecg electrocardiogram	ECG, electrocardiogram	2	kept
Echinocandins	echinocandins	echinocandins	echinocandin	echinocandin	2	singular
echocardiogram	echocardiogram	echocardiogram	echocardiogram	echocardiogram	2	kept
ecological model	ecological model	ecological model	ecological model	ecological model	2	kept

Ecological Momentary Assessment	ecological momentary assessment	ecological momentary assessment	ecological momentary assessment	Ecological Momentary Assessment	2	kept
ecological systems theory	ecological systems theory	ecological systems theory	ecological systems theory	ecological systems theory	2	kept
Economic and social effects	economic and social effects	economic and social effects	economic and social effects	Economic and social effects	2	kept
Economic Competition	economic competition	economic competition	economic competition	Economic Competition	2	kept
Economic crisis	economic crisis	economic crisis	economic crisis	Economic crisis	2	kept
Economic evaluation	economic evaluation	economic evaluation	economic evaluation	economic evaluation	2	case_or_format
economic recession	economic recession	economic recession	economic recession	Economic Recession	2	case_or_format
economic well-being	economic well being	economic well being	economic well being	economic well-being	2	kept
ECONOMIC-IMPACT	economic impact	economic impact	economic impact	ECONOMIC-IMPACT	2	kept
Economics, Behavioral	economics behavioral	economics behavioral	economics behavioral	Economics, Behavioral	2	kept
Economics, Medical	economics medical	economics medical	economics medical	Economics, Medical	2	kept
Ecstasy	ecstasy	ecstasy	ecstasy	Ecstasy	2	kept
ectodermal dysplasia	ectodermal dysplasia	ectodermal dysplasia	ectodermal dysplasia	Ectodermal Dysplasia	2	case_or_format
eDelphi	edelphi	edelphi	edelphi	eDelphi	2	kept
Edema, Cardiac	edema cardiac	edema cardiac	edema cardiac	Edema, Cardiac	2	kept
editor	editor	editor	editor	editor	2	kept

Editorial Policies	editorial policies	editorial policies	editorial policies	Editorial Policies	2	kept
Edmonton Symptom Assessment System	edmonton symptom assessment system	edmonton symptom assessment system	edmonton symptom assessment system	Edmonton Symptom Assessment System	2	kept
educate	educate	educate	educate	educate	2	kept
Education and training	education and training	education and training	education and training	education and training	2	case_or_format
education research	education research	education research	education research	education research	2	kept
Education, distance	education distance	education distance	education distance	Education, Distance	2	case_or_format
Education, evidence-based practice	education evidence based practice	education evidence based practice	education evidence based practice	Education, evidence-based practice	2	kept
Education, higher	education higher	education higher	education higher	Education, higher	2	kept
Education, Pharmacy, Graduate	education pharmacy graduate	education pharmacy graduate	education pharmacy graduate	Education, Pharmacy, Graduate	2	kept
Education, professional	education professional	education professional	education professional	Education, Professional	2	case_or_format
Education, Special	education special	education special	education special	Education, Special	2	kept
Educational assessment	educational assessment	educational assessment	educational assessment	Educational assessment	2	kept
Educational escape room	educational escape room	educational escape room	educational escape room	Educational escape room	2	kept
educational exchange	educational exchange	educational exchange	educational exchange	educational exchange	2	kept
Educational Interventions	educational interventions	educational interventions	educational intervention	Educational intervention	2	singular
Educational issues	educational issues	educational issues	educational issues	Educational issues	2	kept

educational models	educational models	educational models	educational model	educational model	2	singular
Educational module	educational module	educational module	educational module	Educational module	2	kept
educational need	educational need	educational need	educational need	Educational needs	2	case_or_format
educational programme	educational programme	educational programme	educational programme	Educational programme	2	case_or_format
Educational status	educational status	educational status	educational status	educational status	2	case_or_format
educational theory	educational theory	educational theory	educational theory	educational theory	2	kept
educational video	educational video	educational video	educational video	educational video	2	kept
educational workshops	educational workshops	educational workshops	educational workshop	educational workshops	2	singular
efavirenz	efavirenz	efavirenz	efavirenz	efavirenz	2	kept
Effect modification	effect modification	effect modification	effect modification	Effect modification	2	kept
effective reproduction number	effective reproduction number	effective reproduction number	effective reproduction number	effective reproduction number	2	kept
effectiveness of simulation training	effectiveness of simulation training	effectiveness of simulation training	effectiveness of simulation training	effectiveness of simulation training	2	kept
Efficiency-thoroughness trade-off	efficiency thoroughness trade off	efficiency thoroughness trade off	efficiency thoroughness trade off	Efficiency-thoroughness trade-off	2	kept
Eficiencia	eficiencia	eficiencia	eficiencia	Eficiencia	2	kept
ego	ego	ego	ego	ego	2	kept
EHR systems	ehr systems	ehr systems	ehr systems	EHR systems	2	kept

Eicosapentaenoic Acid	eicosapentaenoic acid	eicosapentaenoic acid	eicosapentaenoic acid	Eicosapentaenoic Acid	2	kept
EIT	eit	eit	eit	EIT	2	kept
Elapid Venoms	elapid venoms	elapid venoms	elapid venoms	Elapid Venoms	2	kept
elbow	elbow	elbow	elbow	elbow	2	kept
Elder	elder	elder	elder	Elder	2	kept
elder	elder	elder	elder	Elder	2	case_or_format
Elder care	elder care	elder care	elder care	Elder care	2	kept
Elderly care	elderly care	elderly care	elderly care	elderly care	2	case_or_format
elderly suicides	elderly suicides	elderly suicides	elderly suicide	elderly suicides	2	singular
elderly trauma	elderly trauma	elderly trauma	elderly trauma	elderly trauma	2	kept
Elders	elders	elders	elder	Elder	2	singular
elective	elective	elective	elective	elective	2	kept
Elective delivery	elective delivery	elective delivery	elective delivery	Elective delivery	2	kept
Electric impedance	electric impedance	electric impedance	electric impedance	Electric Impedance	2	case_or_format
Electric impedance measurement	electric impedance measurement	electric impedance measurement	electric impedance measurement	Electric impedance measurement	2	kept
Electric impedance tomography	electric impedance tomography	electric impedance tomography	electric impedance tomography	Electric impedance tomography	2	kept

Electric Wiring	electric wiring	electric wiring	electric wiring	Electric Wiring	2	kept
Electrical impe dance tomography	electrical impe dance tomography	electrical impe dance tomography	electrical impe dance tomography	Electrical impe dance tomography	2	kept
electrocadiography	electrocadiography	electrocadiography	electrocadiography	electrocadiography	2	kept
Electrocardiogram interpretation	electrocardiogram interpretation	electrocardiogram interpretation	electrocardiogram interpretation	Electrocardiogram interpretation	2	kept
electrocardiogram monitoring	electrocardiogram monitoring	electrocardiogram monitoring	electrocardiogram monitoring	electrocardiogram monitoring	2	kept
electrocardiogram timing	electrocardiogram timing	electrocardiogram timing	electrocardiogram timing	electrocardiogram timing	2	kept
electrocardiograph	electrocardiograph	electrocardiograph	electrocardiograph	electrocardiograph	2	kept
electrocardiographic monitoring	electrocardiographic monitoring	electrocardiographic monitoring	electrocardiographic monitoring	electrocardiographic monitoring	2	kept
Electrocardiography interpretation	electrocardiography interpretation	electrocardiography interpretation	electrocardiography interpretation	Electrocardiography interpretation	2	kept
electrodermal activity	electrodermal activity	electrodermal activity	electrodermal activity	electrodermal activity	2	kept
Electrodiagnosis	electrodiagnosis	electrodiagnosis	electrodiagnosis	electrodiagnosis	2	case_or_format
electrographic seizure	electrographic seizure	electrographic seizure	electrographic seizure	electrographic seizure	2	kept
electrography	electrography	electrography	electrography	electrography	2	kept
Electrolyte imbalance	electrolyte imbalance	electrolyte imbalance	electrolyte imbalance	Electrolyte imbalance	2	kept
electrolyte solution	electrolyte solution	electrolyte solution	electrolyte solution	electrolyte solution	2	kept
electromagnetic guidance	electromagnetic guidance	electromagnetic guidance	electromagnetic guidance	electromagnetic guidance	2	kept

electromyogram	electromyogram	electromyogram	electromyogram	electromyogram	2	kept
electronic alert	electronic alert	electronic alert	electronic alert	electronic alert	2	kept
ELECTRONIC HEALTH RECORD	electronic health record	electronic health record	electronic health record	Electronic Health Records	2	case_or_format
Electronic health records and systems	electronic health records and systems	electronic health records and systems	electronic health records and systems	Electronic health records and systems	2	kept
Electronic medical record system	electronic medical record system	electronic medical record system	electronic medical record system	electronic medical record system	2	case_or_format
Electronic medication management systems	electronic medication management systems	electronic medication management systems	electronic medication management system	Electronic medication management systems	2	singular
Electronic monitoring	electronic monitoring	electronic monitoring	electronic monitoring	Electronic monitoring	2	kept
electrophoresis	electrophoresis	electrophoresis	electrophoresis	electrophoresis	2	kept
Electrophysiological monitoring	electrophysiological monitoring	electrophysiological monitoring	electrophysiological monitoring	Electrophysiological monitoring	2	kept
electrophysiological procedures	electrophysiological procedures	electrophysiological procedures	electrophysiological procedures	electrophysiological procedures	2	kept
Elizabethkingia spp.	elizabethkingia spp	elizabethkingia spp	elizabethkingia spp	Elizabethkingia spp.	2	kept
ELNEC	elnec	elnec	elnec	ELNEC	2	kept
EMBASE	embase	embase	embase	Embase	2	case_or_format
Embedded systems	embedded systems	embedded systems	embedded systems	Embedded systems	2	kept
EMBEDDEDNESS	embeddedness	embeddedness	embeddedness	EMBEDDEDNESS	2	kept
embedding	embedding	embedding	embedding	embedding	2	kept

Embeddings	embeddings	embeddings	embedding	embedding	2	singular
Embodiment	embodiment	embodiment	embodiment	Embodiment	2	kept
Embolization	embolization	embolization	embolization	Embolization	2	kept
Embryo Transfer	embryo transfer	embryo transfer	embryo transfer	embryo transfer	2	case_or_format
EMERGENCE	emergence	emergence	emergence	EMERGENCE	2	kept
Emergence delirium	emergence delirium	emergence delirium	emergence delirium	Emergence Delirium	2	case_or_format
emergence delirium	emergence delirium	emergence delirium	emergence delirium	Emergence Delirium	2	case_or_format
Emergency care systems	emergency care systems	emergency care systems	emergency care systems	Emergency care systems	2	kept
emergency care systems	emergency care systems	emergency care systems	emergency care systems	Emergency care systems	2	case_or_format
Emergency department overcrowding	emergency department overcrowding	emergency department overcrowding	emergency department overcrowding	Emergency department overcrowding	2	kept
emergency department presenting sepsis	emergency department presenting sepsis	emergency department presenting sepsis	emergency department presenting sepsis	emergency department presenting sepsis	2	kept
Emergency department visits	emergency department visits	emergency department visits	emergency department visit	emergency department visit	2	singular
emergency departments	emergency departments	emergency departments	emergency department	Emergency department	2	singular
Emergency general surgery	emergency general surgery	emergency general surgery	emergency general surgery	Emergency general surgery	2	kept
Emergency General Surgery Specific Frailty Index	emergency general surgery specific frailty index	emergency general surgery specific frailty index	emergency general surgery specific frailty index	Emergency General Surgery Specific Frailty Index	2	kept
Emergency health services	emergency health services	emergency health services	emergency health service	emergency health service	2	singular

emergency medical dispatch	emergency medical dispatch	emergency medical dispatch	emergency medical dispatch	emergency medical dispatch	2	kept
Emergency Medical Dispatcher	emergency medical dispatcher	emergency medical dispatcher	emergency medical dispatcher	emergency medical dispatcher	2	case_or_format
Emergency medical service	emergency medical service	emergency medical service	emergency medical service	Emergency Medical Services	2	case_or_format
Emergency medical services for children	emergency medical services for children	emergency medical services for children	emergency medical services for children	Emergency medical services for children	2	kept
emergency medical system	emergency medical system	emergency medical system	emergency medical system	emergency medical system	2	kept
emergency pediatrics	emergency pediatrics	emergency pediatrics	emergency pediatrics	emergency pediatrics	2	kept
Emergency planning	emergency planning	emergency planning	emergency planning	Emergency planning	2	kept
emergency preparedness	emergency preparedness	emergency preparedness	emergency preparedness	Emergency preparedness	2	case_or_format
Emergency Response Performance Tool	emergency response performance tool	emergency response performance tool	emergency response performance tool	Emergency Response Performance Tool	2	kept
Emergency service, hospital	emergency service hospital	emergency service hospital	emergency service hospital	Emergency Service, Hospital	2	case_or_format
Emergency Severity Index	emergency severity index	emergency severity index	emergency severity index	emergency severity index	2	case_or_format
Emergency severity index	emergency severity index	emergency severity index	emergency severity index	emergency severity index	2	case_or_format
emergency transport	emergency transport	emergency transport	emergency transport	emergency transport	2	kept
Emergency treatment	emergency treatment	emergency treatment	emergency treatment	Emergency Treatment	2	case_or_format
Emergency ward	emergency ward	emergency ward	emergency ward	emergency ward	2	case_or_format
EMERGENCY-DEPARTMENT VISITS	emergency department visits	emergency department visits	emergency department visit	emergency department visit	2	singular

Emerging economy	emerging economy	emerging economy	emerging economy	Emerging economy	2	kept
Emetics	emetics	emetics	emetics	Emetics	2	kept
emigration and immigration	emigration and immigration	emigration and immigration	emigration and immigration	Emigration and Immigration	2	case_or_format
EMLA cream	emla cream	emla cream	emla cream	EMLA cream	2	kept
EMOTION	emotion	emotion	emotion	Emotions	2	case_or_format
emotion assessment	emotion assessment	emotion assessment	emotion assessment	emotion assessment	2	kept
Emotion regulation skills	emotion regulation skills	emotion regulation skills	emotion regulation skills	Emotion regulation skills	2	kept
emotion-focused coping	emotion focused coping	emotion focused coping	emotion focused coping	emotion-focused coping	2	kept
emotional consequences	emotional consequences	emotional consequences	emotional consequences	emotional consequences	2	kept
Emotional distress	emotional distress	emotional distress	emotional distress	emotional distress	2	case_or_format
emotional effort	emotional effort	emotional effort	emotional effort	emotional effort	2	kept
emotional experiences	emotional experiences	emotional experiences	emotional experiences	emotional experiences	2	kept
emotional labor	emotional labor	emotional labor	emotional labor	Emotional labor	2	case_or_format
emotional outcomes	emotional outcomes	emotional outcomes	emotional outcomes	emotional outcomes	2	kept
Emotional reflexivity	emotional reflexivity	emotional reflexivity	emotional reflexivity	Emotional reflexivity	2	kept
EMPATHIC	empathic	empathic	empathic	EMPATHIC	2	kept

empathic communication	empathic communication	empathic communication	empathic communication	empathic communication	2	kept
Empathic tendency	empathic tendency	empathic tendency	empathic tendency	Empathic tendency	2	kept
empathy fatigue	empathy fatigue	empathy fatigue	empathy fatigue	empathy fatigue	2	kept
empathy skills	empathy skills	empathy skills	empathy skills	empathy skills	2	kept
empiric therapy	empiric therapy	empiric therapy	empiric therapy	empiric therapy	2	kept
Empirical approaches	empirical approaches	empirical approaches	empirical approach	Empirical Approach	2	singular
Empirical distributions	empirical distributions	empirical distributions	empirical distributions	Empirical distributions	2	kept
Employee Discipline	employee discipline	employee discipline	employee discipline	Employee Discipline	2	kept
employee heath	employee heath	employee heath	employee heath	employee heath	2	kept
Employee performance appraisal	employee performance appraisal	employee performance appraisal	employee performance appraisal	Employee Performance Appraisal	2	case_or_format
employee relationships	employee relationships	employee relationships	employee relationships	employee relationships	2	kept
Employee retention	employee retention	employee retention	employee retention	Employee retention	2	kept
EMPLOYMENT	employment	employment	employment	employment	2	case_or_format
empowerment program	empowerment program	empowerment program	empowerment program	empowerment program	2	kept
enacting death	enacting death	enacting death	enacting death	enacting death	2	kept
enalapril maleate	enalapril maleate	enalapril maleate	enalapril maleate	enalapril maleate	2	kept

enalaprilat	enalaprilat	enalaprilat	enalaprilat	enalaprilat	2	kept
Encephalitis, Herpes Simplex	encephalitis herpes simplex	encephalitis herpes simplex	encephalitis herpes simplex	Encephalitis, Herpes Simplex	2	kept
Encephalitis, Viral	encephalitis viral	encephalitis viral	encephalitis viral	Encephalitis, Viral	2	kept
encephalomyelitis	encephalomyelitis	encephalomyelitis	encephalomyelitis	encephalomyelitis	2	kept
end of life issues	end of life issues	end of life issues	end of life issues	end-of-life issues	2	case_or_format
End-of-Life Care	end of life care	end of life care	end of life care	end-of-life care	2	case_or_format
End-of-life communication	end of life communication	end of life communication	end of life communication	End-of-life communication	2	kept
end-of-life conversation	end of life conversation	end of life conversation	end of life conversation	end-of-life conversation	2	kept
end-of-life decision	end of life decision	end of life decision	end of life decision	End-of-life decisions	2	case_or_format
End-of-life decision making	end of life decision making	end of life decision making	end of life decision making	end-of-life decision-making	2	case_or_format
end-of-life illnesses	end of life illnesses	end of life illnesses	end of life illnesses	end-of-life illnesses	2	kept
End-of-life issues	end of life issues	end of life issues	end of life issues	end-of-life issues	2	case_or_format
End-stage heart disease	end stage heart disease	end stage heart disease	end stage heart disease	End-stage heart disease	2	kept
End-stage kidney disease	end stage kidney disease	end stage kidney disease	end stage kidney disease	end stage kidney disease	2	case_or_format
End-stage lung disease	end stage lung disease	end stage lung disease	end stage lung disease	End-stage lung disease	2	kept
Endocrinology	endocrinology	endocrinology	endocrinology	endocrinology	2	case_or_format

endometriosis	endometriosis	endometriosis	endometriosis	endometriosis	2	kept
endometritis	endometritis	endometritis	endometritis	endometritis	2	kept
endometrium	endometrium	endometrium	endometrium	endometrium	2	kept
Endophthalmitis	endophthalmitis	endophthalmitis	endophthalmitis	endophthalmitis	2	case_or_format
Endoscopes, Gastrointestinal	endoscopes gastrointestinal	endoscopes gastrointestinal	endoscopes gastrointestinal	Endoscopes, Gastrointestinal	2	kept
endoscopic echography	endoscopic echography	endoscopic echography	endoscopic echography	endoscopic echography	2	kept
endoscopist	endoscopist	endoscopist	endoscopist	endoscopist	2	kept
endothelial dysfunction	endothelial dysfunction	endothelial dysfunction	endothelial dysfunction	endothelial dysfunction	2	kept
endothelin 1	endothelin 1	endothelin 1	endothelin 1	endothelin 1	2	kept
endothelin receptor antagonist	endothelin receptor antagonist	endothelin receptor antagonist	endothelin receptor antagonist	endothelin receptor antagonist	2	kept
endothelium	endothelium	endothelium	endothelium	endothelium	2	kept
endotracheal cuff	endotracheal cuff	endotracheal cuff	endotracheal cuff	endotracheal cuff	2	kept
Endotracheal Suctioning	endotracheal suctioning	endotracheal suctioning	endotracheal suctioning	Endotracheal suctioning	2	case_or_format
Endotracheal Tube	endotracheal tube	endotracheal tube	endotracheal tube	endotracheal tube	2	case_or_format
endovascular	endovascular	endovascular	endovascular	Endovascular	2	case_or_format
Endovascular thrombectomy	endovascular thrombectomy	endovascular thrombectomy	endovascular thrombectomy	Endovascular thrombectomy	2	kept

Endpoint Determination	endpoint determination	endpoint determination	endpoint determination	Endpoint Determination	2	kept
endurance training	endurance training	endurance training	endurance training	endurance training	2	kept
ENERGY	energy	energy	energy	energy	2	case_or_format
Energy	energy	energy	energy	energy	2	case_or_format
Energy balance	energy balance	energy balance	energy balance	energy balance	2	case_or_format
energy deficit	energy deficit	energy deficit	energy deficit	energy deficit	2	kept
Energy efficiency	energy efficiency	energy efficiency	energy efficiency	Energy efficiency	2	kept
Energy efficient	energy efficient	energy efficient	energy efficient	Energy efficient	2	kept
Energy intake	energy intake	energy intake	energy intake	Energy Intake	2	case_or_format
energy intake	energy intake	energy intake	energy intake	Energy Intake	2	case_or_format
energy resource	energy resource	energy resource	energy resource	energy resource	2	kept
Enfermedad crítica	enfermedad cr tica	enfermedad cr tica	enfermedad cr tica	Enfermedad crítica	2	kept
Enfermera de práctica avanzada	enfermera de pr ctica avanzada	enfermera de pr ctica avanzada	enfermera de pr ctica avanzada	Enfermera de práctica avanzada	2	kept
Enfermería de práctica avanzada	enfermer a de pr ctica avanzada	enfermer a de pr ctica avanzada	enfermer a de pr ctica avanzada	Enfermería de práctica avanzada	2	kept
Enfermería neonatal	enfermer a neonatal	enfermer a neonatal	enfermer a neonatal	Enfermería Neonatal	2	case_or_format
Enfermería obstétrica	enfermer a obst trica	enfermer a obst trica	enfermer a obst trica	Enfermería obstétrica	2	kept

Enfermería Pediátrica	enfermer a pedi trica	enfermer a pedi trica	enfermer a pedi trica	Enfermería Pediátrica	2	kept
Enfermería pediátrica	enfermer a pedi trica	enfermer a pedi trica	enfermer a pedi trica	Enfermería Pediátrica	2	case_or_format
engineering	engineering	engineering	engineering	Engineering	2	case_or_format
ENGLAND	england	england	england	England	2	case_or_format
English as a second language	english as a second language	english as a second language	english as a second language	English as a second language	2	kept
ENHANCED RECOVERY	enhanced recovery	enhanced recovery	enhanced recovery	enhanced recovery	2	case_or_format
enkephalinase inhibitor	enkephalinase inhibitor	enkephalinase inhibitor	enkephalinase inhibitor	enkephalinase inhibitor	2	kept
enrichment culture	enrichment culture	enrichment culture	enrichment culture	enrichment culture	2	kept
Ensemble learning	ensemble learning	ensemble learning	ensemble learning	ensemble learning	2	case_or_format
ensemble model	ensemble model	ensemble model	ensemble model	ensemble model	2	kept
enteral drug administration	enteral drug administration	enteral drug administration	enteral drug administration	enteral drug administration	2	kept
Enteral Feeding	enteral feeding	enteral feeding	enteral feeding	Enteral feeding	2	case_or_format
enteral feeding intolerance	enteral feeding intolerance	enteral feeding intolerance	enteral feeding intolerance	Enteral feeding intolerance	2	case_or_format
Enteral feeding tubes	enteral feeding tubes	enteral feeding tubes	enteral feeding tube	enteral feeding tube	2	singular
enteral formulas	enteral formulas	enteral formulas	enteral formulas	enteral formulas	2	kept
Enteral nutrition interruption	enteral nutrition interruption	enteral nutrition interruption	enteral nutrition interruption	Enteral nutrition interruption	2	kept

enteral nutrition support	enteral nutrition support	enteral nutrition support	enteral nutrition support	enteral nutrition support	2	kept
enteral nutrition-associated diarrhea	enteral nutrition associated diarrhea	enteral nutrition associated diarrhea	enteral nutrition associated diarrhea	enteral nutrition-associated diarrhea	2	kept
enteral nutritional support	enteral nutritional support	enteral nutritional support	enteral nutritional support	enteral nutritional support	2	kept
enteritis	enteritis	enteritis	enteritis	enteritis	2	kept
enterobacteria	enterobacteria	enterobacteria	enterobacteria	enterobacteria	2	kept
enterobacterial repetitive intergenic consensus polymerase	enterobacterial repetitive intergenic consensus polymerase	enterobacterial repetitive intergenic consensus polymerase	enterobacterial repetitive intergenic consensus polymerase	enterobacterial repetitive intergenic consensus polymerase	2	kept
Enterocolitis, Neutropenic	enterocolitis neutropenic	enterocolitis neutropenic	enterocolitis neutropenic	Enterocolitis, Neutropenic	2	kept
enterocutaneous	enterocutaneous	enterocutaneous	enterocutaneous	enterocutaneous	2	kept
Entrustable professional activity	entrustable professional activity	entrustable professional activity	entrustable professional activity	Entrustable professional activity	2	kept
environmental challenges	environmental challenges	environmental challenges	environmental challenges	environmental challenges	2	kept
environmental cleaning	environmental cleaning	environmental cleaning	environmental cleaning	environmental cleaning	2	kept
environmental contamination	environmental contamination	environmental contamination	environmental contamination	Environmental contamination	2	case_or_format
environmental design	environmental design	environmental design	environmental design	environmental design	2	kept
environmental disinfection	environmental disinfection	environmental disinfection	environmental disinfection	environmental disinfection	2	kept
Environmental impact	environmental impact	environmental impact	environmental impact	environmental impact	2	case_or_format
ENVIRONMENTAL NOISE	environmental noise	environmental noise	environmental noise	Environmental noise	2	case_or_format

environmental noise	environmental noise	environmental noise	environmental noise	Environmental noise	2	case_or_format
Environmental noise levels	environmental noise levels	environmental noise levels	environmental noise levels	Environmental noise levels	2	kept
environmental parameters	environmental parameters	environmental parameters	environmental parameters	environmental parameters	2	kept
Environmental performance	environmental performance	environmental performance	environmental performance	Environmental performance	2	kept
environmental planning	environmental planning	environmental planning	environmental planning	environmental planning	2	kept
Environmental Pollutants	environmental pollutants	environmental pollutants	environmental pollutants	Environmental Pollutants	2	kept
Environmental Pollution	environmental pollution	environmental pollution	environmental pollution	Environmental Pollution	2	kept
environmental resilience	environmental resilience	environmental resilience	environmental resilience	environmental resilience	2	kept
Environmental situation	environmental situation	environmental situation	environmental situation	Environmental situation	2	kept
Environmental stressor	environmental stressor	environmental stressor	environmental stressor	Environmental stressor	2	kept
enzyme inhibitor	enzyme inhibitor	enzyme inhibitor	enzyme inhibitor	enzyme inhibitor	2	kept
Enzyme Inhibitors	enzyme inhibitors	enzyme inhibitors	enzyme inhibitor	enzyme inhibitor	2	singular
Eosinophils	eosinophils	eosinophils	eosinophil	eosinophil	2	singular
epidemic curve	epidemic curve	epidemic curve	epidemic curve	epidemic curve	2	kept
Epidemiologic studies	epidemiologic studies	epidemiologic studies	epidemiologic studies	Epidemiologic Studies	2	case_or_format
Epidemiological investigation	epidemiological investigation	epidemiological investigation	epidemiological investigation	Epidemiological investigation	2	kept

Epidermal Necrolysis, Toxic	epidermal necrolysis toxic	epidermal necrolysis toxic	epidermal necrolysis toxic	Epidermal Necrolysis, Toxic	2	kept
epidermal stripping	epidermal stripping	epidermal stripping	epidermal stripping	epidermal stripping	2	kept
Epidermis	epidermis	epidermis	epidermis	epidermis	2	case_or_format
epidermolysis	epidermolysis	epidermolysis	epidermolysis	epidermolysis	2	kept
Epidural anesthesia	epidural anesthesia	epidural anesthesia	epidural anesthesia	epidural anesthesia	2	case_or_format
Epigenomics	epigenomics	epigenomics	epigenomics	Epigenomics	2	kept
epiglottis	epiglottis	epiglottis	epiglottis	epiglottis	2	kept
epiglottitis	epiglottitis	epiglottitis	epiglottitis	epiglottitis	2	kept
epilepsy monitoring unit	epilepsy monitoring unit	epilepsy monitoring unit	epilepsy monitoring unit	epilepsy monitoring unit	2	kept
epileptologist	epileptologist	epileptologist	epileptologist	epileptologist	2	kept
Epinet	epinet	epinet	epinet	Epinet	2	kept
epistemic injustice	epistemic injustice	epistemic injustice	epistemic injustice	epistemic injustice	2	kept
Epoetin Alfa	epoetin alfa	epoetin alfa	epoetin alfa	Epoetin Alfa	2	kept
EPR	epr	epr	epr	EPR	2	kept
Epstein Barr virus	epstein barr virus	epstein barr virus	epstein barr virus	Epstein Barr virus	2	kept
Epstein Barr virus infection	epstein barr virus infection	epstein barr virus infection	epstein barr virus infection	Epstein Barr virus infection	2	kept

equipoise	equipoise	equipoise	equipoise	equipoise	2	kept
equivalence trial	equivalence trial	equivalence trial	equivalence trial	equivalence trial	2	kept
Equus	equus	equus	equus	Equus	2	kept
Equus caballus	equus caballus	equus caballus	equus caballus	Equus caballus	2	kept
ER noise	er noise	er noise	er noise	ER noise	2	kept
ERADICATION	eradication	eradication	eradication	ERADICATION	2	kept
ERAS protocol	eras protocol	eras protocol	eras protocol	ERAS protocol	2	kept
erectile dysfunction	erectile dysfunction	erectile dysfunction	erectile dysfunction	erectile dysfunction	2	kept
Erfahrungen	erfahrungen	erfahrungen	erfahrungen	Erfahrungen	2	kept
ergocalciferol	ergocalciferol	ergocalciferol	ergocalciferol	ergocalciferol	2	kept
ergometrine	ergometrine	ergometrine	ergometrine	ergometrine	2	kept
Ergonomic risk	ergonomic risk	ergonomic risk	ergonomic risk	Ergonomic risk	2	kept
Ergonomic Risks	ergonomic risks	ergonomic risks	ergonomic risk	Ergonomic risk	2	singular
ERGONOMICS	ergonomics	ergonomics	ergonomic	Ergonomics	2	singular
Ericsson	ericsson	ericsson	ericsson	Ericsson	2	kept
Eritrea	eritrea	eritrea	eritrea	Eritrea	2	kept

Erlang loss model	erlang loss model	erlang loss model	erlang loss model	Erlang loss model	2	kept
Error analysis	error analysis	error analysis	error analysis	Error analysis	2	kept
Error detection	error detection	error detection	error detection	Error detection	2	kept
Error prediction	error prediction	error prediction	error prediction	Error prediction	2	kept
Error reporting	error reporting	error reporting	error reporting	error reporting	2	case_or_format
Error reporting impediments	error reporting impediments	error reporting impediments	error reporting impediments	Error reporting impediments	2	kept
Ertapenem	ertapenem	ertapenem	ertapenem	ertapenem	2	case_or_format
erythema infectiosum	erythema infectiosum	erythema infectiosum	erythema infectiosum	erythema infectiosum	2	kept
Erythema Multiforme	erythema multiforme	erythema multiforme	erythema multiforme	Erythema Multiforme	2	kept
erythema multiforme	erythema multiforme	erythema multiforme	erythema multiforme	Erythema Multiforme	2	case_or_format
Erythrocyte Indices	erythrocyte indices	erythrocyte indices	erythrocyte indices	Erythrocyte Indices	2	kept
erythrocyte volume	erythrocyte volume	erythrocyte volume	erythrocyte volume	erythrocyte volume	2	kept
erythromycin ethylsuccinate	erythromycin ethylsuccinate	erythromycin ethylsuccinate	erythromycin ethylsuccinate	erythromycin ethylsuccinate	2	kept
erythromycin lactobionate	erythromycin lactobionate	erythromycin lactobionate	erythromycin lactobionate	erythromycin lactobionate	2	kept
Escala de Evaluación de la Conducta	escala de evaluaci n de la conducta	escala de evaluaci n de la conducta	escala de evaluaci n de la conducta	Escala de Evaluación de la Conducta	2	kept
ESCALATION	escalation	escalation	escalation	ESCALATION	2	kept

escalation	escalation	escalation	escalation	ESCALATION	2	case_or_format
escape behavior	escape behavior	escape behavior	escape behavior	escape behavior	2	kept
Escharotomy	escharotomy	escharotomy	escharotomy	Escharotomy	2	kept
esophageal adenocarcinoma	esophageal adenocarcinoma	esophageal adenocarcinoma	esophageal adenocarcinoma	esophageal adenocarcinoma	2	kept
Esophageal cancer	esophageal cancer	esophageal cancer	esophageal cancer	Esophageal cancer	2	kept
esophagitis	esophagitis	esophagitis	esophagitis	esophagitis	2	kept
esophagus hemorrhage	esophagus hemorrhage	esophagus hemorrhage	esophagus hemorrhage	esophagus hemorrhage	2	kept
esophagus injury	esophagus injury	esophagus injury	esophagus injury	esophagus injury	2	kept
esophagus perforation	esophagus perforation	esophagus perforation	esophagus perforation	esophagus perforation	2	kept
esophagus pressure	esophagus pressure	esophagus pressure	esophagus pressure	esophagus pressure	2	kept
esophagus ulcer	esophagus ulcer	esophagus ulcer	esophagus ulcer	esophagus ulcer	2	kept
Especialidades de enfermería	especialidades de enfermer a	especialidades de enfermer a	especialidades de enfermer a	Especialidades de enfermería	2	kept
ESRD	esrd	esrd	esrd	ESRD	2	kept
Essays	essays	essays	essays	Essays	2	kept
Essential care	essential care	essential care	essential care	essential care	2	case_or_format
Essential oil	essential oil	essential oil	essential oil	essential oil	2	case_or_format

Essential oils	essential oils	essential oils	essential oil	essential oil	2	singular
Establishing new surgical program	establishing new surgical program	establishing new surgical program	establishing new surgical program	Establishing new surgical program	2	kept
estradiol	estradiol	estradiol	estradiol	estradiol	2	kept
estrogen	estrogen	estrogen	estrogen	estrogen	2	kept
Estrés psicológico	estr s psicol gico	estr s psicol gico	estr s psicol gico	Estrés psicológico	2	kept
Estudio de validación	estudio de validaci n	estudio de validaci n	estudio de validaci n	Estudio de validación	2	kept
Estándares de referencia	est ndares de referencia	est ndares de referencia	est ndares de referencia	Estándares de referencia	2	kept
EtCO<sub>2<	etco sub 2	etco sub 2	etco sub 2	EtCO<sub>2<	2	kept
ethic	ethic	ethic	ethic	ethics	2	case_or_format
Ethical	ethical	ethical	ethical	ethical	2	case_or_format
Ethical analytic	ethical analytic	ethical analytic	ethical analytic	Ethical analytic	2	kept
ethical challenge	ethical challenge	ethical challenge	ethical challenge	ethical challenges	2	case_or_format
Ethical challenges	ethical challenges	ethical challenges	ethical challenge	ethical challenges	2	singular
ethical characteristics	ethical characteristics	ethical characteristics	ethical characteristics	ethical characteristics	2	kept
ethical confrontation	ethical confrontation	ethical confrontation	ethical confrontation	ethical confrontation	2	kept
ETHICAL DILEMMAS	ethical dilemmas	ethical dilemmas	ethical dilemma	ethical dilemma	2	singular

ethical inquiry	ethical inquiry	ethical inquiry	ethical inquiry	ethical inquiry	2	kept
ethical issues in practice	ethical issues in practice	ethical issues in practice	ethical issues in practice	ethical issues in practice	2	kept
ethical practice	ethical practice	ethical practice	ethical practice	ethical practice	2	kept
Ethical principles	ethical principles	ethical principles	ethical principles	Ethical principles	2	kept
ethical principles	ethical principles	ethical principles	ethical principles	Ethical principles	2	case_or_format
ethical theory	ethical theory	ethical theory	ethical theory	Ethical Theory	2	case_or_format
Ethics courage	ethics courage	ethics courage	ethics courage	Ethics courage	2	kept
Ethics, medical	ethics medical	ethics medical	ethics medical	Ethics, Medical	2	case_or_format
Ethics, Professional	ethics professional	ethics professional	ethics professional	Ethics, Professional	2	kept
Ethnic and racial minorities	ethnic and racial minorities	ethnic and racial minorities	ethnic and racial minorities	Ethnic and racial minorities	2	kept
ethnic conflict	ethnic conflict	ethnic conflict	ethnic conflict	ethnic conflict	2	kept
ETHNOGRAPHY	ethnography	ethnography	ethnography	ethnography	2	case_or_format
ethnomethodology	ethnomethodology	ethnomethodology	ethnomethodology	ethnomethodology	2	kept
ethylene glycol	ethylene glycol	ethylene glycol	ethylene glycol	ethylene glycol	2	kept
Etiology	etiology	etiology	etiology	etiology	2	case_or_format
euglycemic diabetic ketoacidosis	euglycemic diabetic ketoacidosis	euglycemic diabetic ketoacidosis	euglycemic diabetic ketoacidosis	euglycemic diabetic ketoacidosis	2	kept

euphoria	euphoria	euphoria	euphoria	euphoria	2	kept
European Working Time Directive	european working time directive	european working time directive	european working time directive	European Working Time Directive	2	kept
evacuation safety	evacuation safety	evacuation safety	evacuation safety	evacuation safety	2	kept
Evaluación de necesidades	evaluaci n de necesidades	evaluaci n de necesidades	evaluaci n de necesidades	Evaluación de necesidades	2	kept
evaluation and assessment	evaluation and assessment	evaluation and assessment	evaluation and assessment	evaluation and assessment	2	kept
Evaluation methodology	evaluation methodology	evaluation methodology	evaluation methodology	Evaluation methodology	2	kept
Evaluation research	evaluation research	evaluation research	evaluation research	evaluation research	2	case_or_format
event related potential	event related potential	event related potential	event related potential	event related potential	2	kept
evidence	evidence	evidence	evidence	Evidence	2	case_or_format
Evidence based	evidence based	evidence based	evidence based	Evidence-based	2	case_or_format
Evidence Based Medicine	evidence based medicine	evidence based medicine	evidence based medicine	Evidence-Based Medicine	2	case_or_format
evidence gap	evidence gap	evidence gap	evidence gap	evidence gap	2	kept
evidence needs	evidence needs	evidence needs	evidence needs	evidence needs	2	kept
Evidence summary	evidence summary	evidence summary	evidence summary	evidence summary	2	case_or_format
evidence synthesis	evidence synthesis	evidence synthesis	evidence synthesis	evidence synthesis	2	kept
evidence-based care	evidence based care	evidence based care	evidence based care	Evidence-based care	2	case_or_format

evidence-based guidelines	evidence based guidelines	evidence based guidelines	evidence based guideline	Evidence-based guidelines	2	singular
Evidence-based practices module	evidence based practices module	evidence based practices module	evidence based practices module	Evidence-based practices module	2	kept
evidence-based quality improvement	evidence based quality improvement	evidence based quality improvement	evidence based quality improvement	evidence-based quality improvement	2	kept
evidence-based rehabilitation medicine	evidence based rehabilitation medicine	evidence based rehabilitation medicine	evidence based rehabilitation medicine	evidence-based rehabilitation medicine	2	kept
evidencebased practice	evidencebased practice	evidencebased practice	evidencebased practice	evidencebased practice	2	kept
evoked auditory response	evoked auditory response	evoked auditory response	evoked auditory response	evoked auditory response	2	kept
evoked brain stem auditory response	evoked brain stem auditory response	evoked brain stem auditory response	evoked brain stem auditory response	evoked brain stem auditory response	2	kept
Evoked Potentials, Auditory	evoked potentials auditory	evoked potentials auditory	evoked potentials auditory	Evoked Potentials, Auditory	2	kept
evolutionary adaptation	evolutionary adaptation	evolutionary adaptation	evolutionary adaptation	evolutionary adaptation	2	kept
EVPI	evpi	evpi	evpi	EVPI	2	kept
EVPPI	evppi	evppi	evppi	EVPPI	2	kept
EWTD	ewtd	ewtd	ewtd	EWTD	2	kept
Ex Utero Intrapartum Treatment	ex utero intrapartum treatment	ex utero intrapartum treatment	ex utero intrapartum treatment	Ex Utero Intrapartum Treatment	2	kept
ex vivo study	ex vivo study	ex vivo study	ex vivo study	ex vivo study	2	kept
Exanthema	exanthema	exanthema	exanthema	Exanthema	2	kept
Excess mortality	excess mortality	excess mortality	excess mortality	excess mortality	2	case_or_format

Exchange of information	exchange of information	exchange of information	exchange of information	Exchange of information	2	kept
Excision and grafting	excision and grafting	excision and grafting	excision and grafting	Excision and grafting	2	kept
excitement	excitement	excitement	excitement	excitement	2	kept
Executive Function	executive function	executive function	executive function	executive function	2	case_or_format
Exercise capacity	exercise capacity	exercise capacity	exercise capacity	Exercise capacity	2	kept
EXHAUSTION	exhaustion	exhaustion	exhaustion	exhaustion	2	case_or_format
Exhaustion	exhaustion	exhaustion	exhaustion	exhaustion	2	case_or_format
existentialia	existentialia	existentialia	existentialia	existentialia	2	kept
EXIT	exit	exit	exit	EXIT	2	kept
exon	exon	exon	exon	exon	2	kept
Exons	exons	exons	exon	exon	2	singular
exophthalmos	exophthalmos	exophthalmos	exophthalmos	exophthalmos	2	kept
exotoxin	exotoxin	exotoxin	exotoxin	Exotoxins	2	case_or_format
expectancy	expectancy	expectancy	expectancy	expectancy	2	kept
Expectation	expectation	expectation	expectation	expectation	2	case_or_format
EXPECTATIONS	expectations	expectations	expectation	expectation	2	singular

Expectations	expectations	expectations	expectation	expectation	2	singular
Expectorants	expectorants	expectorants	expectorants	Expectorants	2	kept
experience sampling method	experience sampling method	experience sampling method	experience sampling method	experience sampling method	2	kept
Experiencing coronavirus disease-2019	experiencing coronavirus disease 2019	experiencing coronavirus disease 2019	experiencing coronavirus disease 2019	Experiencing coronavirus disease-2019	2	kept
Experiential learning	experiential learning	experiential learning	experiential learning	experiential learning	2	case_or_format
Experiential training	experiential training	experiential training	experiential training	Experiential training	2	kept
experiment	experiment	experiment	experiment	experiment	2	kept
Experimental	experimental	experimental	experimental	Experimental	2	kept
experimentation	experimentation	experimentation	experimentation	experimentation	2	kept
EXPERT PERFORMANCE	expert performance	expert performance	expert performance	EXPERT PERFORMANCE	2	kept
EXPERTISE	expertise	expertise	expertise	Expertise	2	case_or_format
expiratory reserve volume	expiratory reserve volume	expiratory reserve volume	expiratory reserve volume	expiratory reserve volume	2	kept
Explainable artificial intelligence	explainable artificial intelligence	explainable artificial intelligence	explainable artificial intelligence	Explainable artificial intelligence	2	kept
explainable artificial intelligence	explainable artificial intelligence	explainable artificial intelligence	explainable artificial intelligence	Explainable artificial intelligence	2	case_or_format
explanatory analysis	explanatory analysis	explanatory analysis	explanatory analysis	explanatory analysis	2	kept
Exploratory factor analysis	exploratory factor analysis	exploratory factor analysis	exploratory factor analysis	exploratory factor analysis	2	case_or_format

Exposed to	exposed to	exposed to	exposed to	Exposed to	2	kept
Exposure to biological agents	exposure to biological agents	exposure to biological agents	exposure to biological agents	Exposure to biological agents	2	kept
exposure to violence	exposure to violence	exposure to violence	exposure to violence	exposure to violence	2	kept
Expressing errors	expressing errors	expressing errors	expressing errors	Expressing errors	2	kept
extended shift patterns	extended shift patterns	extended shift patterns	extended shift patterns	extended shift patterns	2	kept
extended working lives	extended working lives	extended working lives	extended working lives	extended working lives	2	kept
EXTENSION	extension	extension	extension	EXTENSION	2	kept
extensive subdural hematoma	extensive subdural hematoma	extensive subdural hematoma	extensive subdural hematoma	extensive subdural hematoma	2	kept
external jugular vein	external jugular vein	external jugular vein	external jugular vein	external jugular vein	2	kept
extra corporeal membrane oxygenation	extra corporeal membrane oxygenation	extra corporeal membrane oxygenation	extra corporeal membrane oxygenation	extra corporeal membrane oxygenation	2	kept
extracellular matrix	extracellular matrix	extracellular matrix	extracellular matrix	extracellular matrix	2	kept
extracellular vesicle	extracellular vesicle	extracellular vesicle	extracellular vesicle	extracellular vesicle	2	kept
extracorporeal cardiopulmonary resuscitation	extracorporeal cardiopulmonary resuscitation	extracorporeal cardiopulmonary resuscitation	extracorporeal cardiopulmonary resuscitation	Extracorporeal cardiopulmonary resuscitation	2	case_or_format
Extracorporeal life supports	extracorporeal life supports	extracorporeal life supports	extracorporeal life support	Extracorporeal life support	2	singular
extracorporeal lung support	extracorporeal lung support	extracorporeal lung support	extracorporeal lung support	extracorporeal lung support	2	kept
Extracorporeal membrane oxygen	extracorporeal membrane oxygen	extracorporeal membrane oxygen	extracorporeal membrane oxygen	Extracorporeal membrane oxygen	2	kept

Extracorporeal renal replacement therapy	extracorporeal renal replacement therapy	extracorporeal renal replacement therapy	extracorporeal renal replacement therapy	Extracorporeal renal replacement therapy	2	kept
Extrasystoles	extrasystoles	extrasystoles	extrasystoles	Extrasystoles	2	kept
Extreme learning machine	extreme learning machine	extreme learning machine	extreme learning machine	Extreme learning machine	2	kept
extremely low birth weight infants	extremely low birth weight infants	extremely low birth weight infants	extremely low birth weight infant	extremely low birth-weight infant	2	singular
extremely low birth-weight infant	extremely low birth weight infant	extremely low birth weight infant	extremely low birth weight infant	extremely low birth-weight infant	2	kept
extremely premature infant	extremely premature infant	extremely premature infant	extremely premature infant	extremely premature infants	2	case_or_format
Extremely preterm infant	extremely preterm infant	extremely preterm infant	extremely preterm infant	Extremely preterm infants	2	case_or_format
extremely preterm infants	extremely preterm infants	extremely preterm infants	extremely preterm infant	Extremely preterm infants	2	singular
Extremities	extremities	extremities	extremity	Extremity	2	singular
Extremity	extremity	extremity	extremity	Extremity	2	kept
Extubation protocol	extubation protocol	extubation protocol	extubation protocol	Extubation protocol	2	kept
EYE CARE	eye care	eye care	eye care	eye care	2	case_or_format
Eye Care	eye care	eye care	eye care	eye care	2	case_or_format
Eye care clinical guidelines	eye care clinical guidelines	eye care clinical guidelines	eye care clinical guidelines	Eye care clinical guidelines	2	kept
eye fundus	eye fundus	eye fundus	eye fundus	eye fundus	2	kept
Eye Health	eye health	eye health	eye health	Eye Health	2	kept

Eye Infections, Bacterial	eye infections bacterial	eye infections bacterial	eye infections bacterial	Eye Infections, Bacterial	2	kept
eye photography	eye photography	eye photography	eye photography	eye photography	2	kept
eye swelling	eye swelling	eye swelling	eye swelling	eye swelling	2	kept
Eyeglasses	eyeglasses	eyeglasses	eyeglasses	Eyeglasses	2	kept
eyelid disease	eyelid disease	eyelid disease	eyelid disease	eyelid disease	2	kept
Eyelid Diseases	eyelid diseases	eyelid diseases	eyelid disease	eyelid disease	2	singular
eyelid edema	eyelid edema	eyelid edema	eyelid edema	eyelid edema	2	kept
eyelid reflex	eyelid reflex	eyelid reflex	eyelid reflex	eyelid reflex	2	kept
face deformity	face deformity	face deformity	face deformity	face deformity	2	kept
face fracture	face fracture	face fracture	face fracture	face fracture	2	kept
face to face support group	face to face support group	face to face support group	face to face support group	face to face support group	2	kept
facial pressure injury	facial pressure injury	facial pressure injury	facial pressure injury	facial pressure injury	2	kept
facilitated communication	facilitated communication	facilitated communication	facilitated communication	facilitated communication	2	kept
Facilitated tucking by parents	facilitated tucking by parents	facilitated tucking by parents	facilitated tucking by parents	Facilitated tucking by parents	2	kept
facilitators and barriers	facilitators and barriers	facilitators and barriers	facilitators and barriers	facilitators and barriers	2	kept
facilities and services utilization	facilities and services utilization	facilities and services utilization	facilities and services utilization	Facilities and Services Utilization	2	case_or_format

Facility design and construction	facility design and construction	facility design and construction	facility design and construction	Facility Design and Construction	2	case_or_format
facility design and construction	facility design and construction	facility design and construction	facility design and construction	Facility Design and Construction	2	case_or_format
factitious disease	factitious disease	factitious disease	factitious disease	factitious disease	2	kept
factor	factor	factor	factor	Factors	2	case_or_format
Factor Analysis	factor analysis	factor analysis	factor analysis	factor analysis	2	case_or_format
Factor IX	factor ix	factor ix	factor ix	Factor IX	2	kept
Factors associated	factors associated	factors associated	factors associated	Factors associated	2	kept
Faecal incontinence	faecal incontinence	faecal incontinence	faecal incontinence	Faecal incontinence	2	kept
Faecal management system	faecal management system	faecal management system	faecal management system	Faecal management system	2	kept
Failure assessment	failure assessment	failure assessment	failure assessment	Failure assessment	2	kept
FAILURE MODE	failure mode	failure mode	failure mode	FAILURE MODE	2	kept
Failure mode and effect analysis	failure mode and effect analysis	failure mode and effect analysis	failure mode and effect analysis	Failure mode and effect analysis	2	kept
FAILURES	failures	failures	failure	FAILURE	2	singular
Fairness in resource allocation	fairness in resource allocation	fairness in resource allocation	fairness in resource allocation	Fairness in resource allocation	2	kept
Faith Healing	faith healing	faith healing	faith healing	Faith Healing	2	kept
fall	fall	fall	fall	falls	2	case_or_format

fall injury	fall injury	fall injury	fall injury	fall injury	2	kept
false alarm	false alarm	false alarm	false alarm	False alarms	2	case_or_format
false alarms	false alarms	false alarms	false alarm	False alarms	2	singular
False positive	false positive	false positive	false positive	False positive	2	kept
famciclovir	famciclovir	famciclovir	famciclovir	famciclovir	2	kept
familial Mediterranean fever	familial mediterranean fever	familial mediterranean fever	familial mediterranean fever	familial Mediterranean fever	2	kept
FAMILY CAREGIVERS	family caregivers	family caregivers	family caregiver	Family caregivers	2	singular
family communications	family communications	family communications	family communication	family communication	2	singular
Family Confusion Assessment Method	family confusion assessment method	family confusion assessment method	family confusion assessment method	Family Confusion Assessment Method	2	kept
Family crisis	family crisis	family crisis	family crisis	Family crisis	2	kept
family experiences	family experiences	family experiences	family experience	Family experience	2	singular
Family function	family function	family function	family function	Family function	2	kept
Family functioning	family functioning	family functioning	family functioning	family functioning	2	case_or_format
Family health	family health	family health	family health	Family Health	2	case_or_format
Family information meeting	family information meeting	family information meeting	family information meeting	Family information meeting	2	kept
Family intervention	family intervention	family intervention	family intervention	Family intervention	2	kept

family management	family management	family management	family management	family management	2	kept
Family medicine	family medicine	family medicine	family medicine	family medicine	2	case_or_format
family meetings	family meetings	family meetings	family meeting	family meeting	2	singular
Family Members	family members	family members	family member	Family members	2	singular
Family partnership	family partnership	family partnership	family partnership	Family partnership	2	kept
Family perspectives	family perspectives	family perspectives	family perspectives	Family perspectives	2	kept
Family Planning Services	family planning services	family planning services	family planning services	Family Planning Services	2	kept
family resilience	family resilience	family resilience	family resilience	family resilience	2	kept
family separation	family separation	family separation	family separation	family separation	2	kept
family size	family size	family size	family size	family size	2	kept
Family stress	family stress	family stress	family stress	family stress	2	case_or_format
family structure	family structure	family structure	family structure	family structure	2	kept
Family suffering	family suffering	family suffering	family suffering	Family suffering	2	kept
Family visit	family visit	family visit	family visit	Family visit	2	kept
family visit	family visit	family visit	family visit	Family visit	2	case_or_format
Family visitors	family visitors	family visitors	family visitors	Family visitors	2	kept

family visits	family visits	family visits	family visit	Family visit	2	singular
family wellbeing	family wellbeing	family wellbeing	family wellbeing	family wellbeing	2	kept
Family-centered	family centered	family centered	family centered	Family-centered	2	kept
family-centered	family centered	family centered	family centered	Family-centered	2	case_or_format
Family-centered rounds	family centered rounds	family centered rounds	family centered rounds	family-centered rounds	2	case_or_format
family-witnessed resuscitation	family witnessed resuscitation	family witnessed resuscitation	family witnessed resuscitation	Family-witnessed resuscitation	2	case_or_format
family‐centered care	family centered care	family centered care	family centered care	family centered care	2	case_or_format
family‐centred care	family centred care	family centred care	family centred care	Family-centred care	2	case_or_format
Famotidine	famotidine	famotidine	famotidine	famotidine	2	case_or_format
fan therapy	fan therapy	fan therapy	fan therapy	fan therapy	2	kept
Fanconi renotubular syndrome	fanconi renotubular syndrome	fanconi renotubular syndrome	fanconi renotubular syndrome	Fanconi renotubular syndrome	2	kept
fantasy	fantasy	fantasy	fantasy	fantasy	2	kept
fast track protocol	fast track protocol	fast track protocol	fast track protocol	fast track protocol	2	kept
Fast track surgery	fast track surgery	fast track surgery	fast track surgery	Fast track surgery	2	kept
Fast-track	fast track	fast track	fast track	Fast-track	2	kept
Fast-tracking pediatric cardiac surgery	fast tracking pediatric cardiac surgery	fast tracking pediatric cardiac surgery	fast tracking pediatric cardiac surgery	Fast-tracking pediatric cardiac surgery	2	kept

Father-child relations	father child relations	father child relations	father child relation	Father-Child Relations	2	singular
father－child relations	father child relations	father child relations	father child relation	Father-Child Relations	2	singular
fatigue management	fatigue management	fatigue management	fatigue management	fatigue management	2	kept
Fatigue Severity Scale	fatigue severity scale	fatigue severity scale	fatigue severity scale	Fatigue Severity Scale	2	kept
fatty acid	fatty acid	fatty acid	fatty acid	fatty acid	2	kept
fault detection	fault detection	fault detection	fault detection	fault detection	2	kept
fault-tolerant systems	fault tolerant systems	fault tolerant systems	fault tolerant systems	fault-tolerant systems	2	kept
FDS	fds	fds	fds	FDS	2	kept
fear of childbirth	fear of childbirth	fear of childbirth	fear of childbirth	fear of childbirth	2	kept
Fear of movement	fear of movement	fear of movement	fear of movement	Fear of movement	2	kept
FEASIBILITY	feasibility	feasibility	feasibility	Feasibility	2	case_or_format
feature importance	feature importance	feature importance	feature importance	feature importance	2	kept
Feature selection	feature selection	feature selection	feature selection	feature selection	2	case_or_format
Febrile neutropenia	febrile neutropenia	febrile neutropenia	febrile neutropenia	febrile neutropenia	2	case_or_format
feces impaction	feces impaction	feces impaction	feces impaction	feces impaction	2	kept
feed intolerance	feed intolerance	feed intolerance	feed intolerance	feed intolerance	2	kept

Feeding algorithm	feeding algorithm	feeding algorithm	feeding algorithm	Feeding algorithm	2	kept
feeding methods	feeding methods	feeding methods	feeding method	Feeding Methods	2	singular
feeding protocol	feeding protocol	feeding protocol	feeding protocol	Feeding protocol	2	case_or_format
Feeding stimulation	feeding stimulation	feeding stimulation	feeding stimulation	Feeding stimulation	2	kept
feeding tube placement	feeding tube placement	feeding tube placement	feeding tube placement	feeding tube placement	2	kept
FEEDING TUBES	feeding tubes	feeding tubes	feeding tube	Feeding tube	2	singular
Feeding tubes	feeding tubes	feeding tubes	feeding tube	Feeding tube	2	singular
feeling safe	feeling safe	feeling safe	feeling safe	feeling safe	2	kept
feelings	feelings	feelings	feelings	feelings	2	kept
Fellow	fellow	fellow	fellow	Fellow	2	kept
female infertility	female infertility	female infertility	female infertility	female infertility	2	kept
feminist ethics	feminist ethics	feminist ethics	feminist ethics	feminist ethics	2	kept
femoral angiography	femoral angiography	femoral angiography	femoral angiography	femoral angiography	2	kept
femorally inserted central catheter	femorally inserted central catheter	femorally inserted central catheter	femorally inserted central catheter	femorally inserted central catheter	2	kept
Femur fracture	femur fracture	femur fracture	femur fracture	femur fracture	2	case_or_format
femur neck fracture	femur neck fracture	femur neck fracture	femur neck fracture	femur neck fracture	2	kept

femur pertrochanteric fracture	femur pertrochanteric fracture	femur pertrochanteric fracture	femur pertrochanteric fracture	femur pertrochanteric fracture	2	kept
femur subtrochanteric fracture	femur subtrochanteric fracture	femur subtrochanteric fracture	femur subtrochanteric fracture	femur subtrochanteric fracture	2	kept
fenestration	fenestration	fenestration	fenestration	fenestration	2	kept
Ferimentos e Lesões	ferimentos e les es	ferimentos e les es	ferimentos e les es	Ferimentos e Lesões	2	kept
Ferritins	ferritins	ferritins	ferritin	ferritin	2	singular
ferrous sulfate	ferrous sulfate	ferrous sulfate	ferrous sulfate	ferrous sulfate	2	kept
fertility	fertility	fertility	fertility	fertility	2	kept
FETAL	fetal	fetal	fetal	FETAL	2	kept
fetal	fetal	fetal	fetal	FETAL	2	case_or_format
fetal arrhythmia	fetal arrhythmia	fetal arrhythmia	fetal arrhythmia	fetal arrhythmia	2	kept
Fetal death	fetal death	fetal death	fetal death	Fetal Death	2	case_or_format
Fetal Distress	fetal distress	fetal distress	fetal distress	Fetal Distress	2	kept
Fetal Therapies	fetal therapies	fetal therapies	fetal therapies	Fetal Therapies	2	kept
fetal viability	fetal viability	fetal viability	fetal viability	Fetal Viability	2	case_or_format
Fetal Weight	fetal weight	fetal weight	fetal weight	Fetal Weight	2	kept
fetus hypoxia	fetus hypoxia	fetus hypoxia	fetus hypoxia	fetus hypoxia	2	kept

fetus surgery	fetus surgery	fetus surgery	fetus surgery	fetus surgery	2	kept
FEVER	fever	fever	fever	fever	2	case_or_format
Fever management	fever management	fever management	fever management	Fever management	2	kept
Fever-phases	fever phases	fever phases	fever phases	Fever-phases	2	kept
FFP	ffp	ffp	ffp	FFP	2	kept
FFP3	ffp3	ffp3	ffp3	FFP3	2	kept
FGD	fgd	fgd	fgd	FGD	2	kept
FI-LAB	fi lab	fi lab	fi lab	FI-LAB	2	kept
fiberoptic tracheal intubation	fiberoptic tracheal intubation	fiberoptic tracheal intubation	fiberoptic tracheal intubation	fiberoptic tracheal intubation	2	kept
fibrin fragment D	fibrin fragment d	fibrin fragment d	fibrin fragment d	fibrin fragment D	2	kept
fibrinogen concentrate	fibrinogen concentrate	fibrinogen concentrate	fibrinogen concentrate	fibrinogen concentrate	2	kept
fibromyalgia	fibromyalgia	fibromyalgia	fibromyalgia	fibromyalgia	2	kept
Fibrosis	fibrosis	fibrosis	fibrosis	Fibrosis	2	kept
fibrosis	fibrosis	fibrosis	fibrosis	Fibrosis	2	case_or_format
fibula	fibula	fibula	fibula	Fibula	2	case_or_format
fibula graft	fibula graft	fibula graft	fibula graft	fibula graft	2	kept

field expertise	field expertise	field expertise	field expertise	field expertise	2	kept
field programmable gate array	field programmable gate array	field programmable gate array	field programmable gate array	field programmable gate array	2	kept
Fieldwork	fieldwork	fieldwork	fieldwork	fieldwork	2	case_or_format
fight or flight response	fight or flight response	fight or flight response	fight or flight response	fight or flight response	2	kept
fighting	fighting	fighting	fighting	fighting	2	kept
Fiji	fiji	fiji	fiji	Fiji	2	kept
Filter life	filter life	filter life	filter life	Filter life	2	kept
Fin de vie	fin de vie	fin de vie	fin de vie	Fin de vie	2	kept
Financial management	financial management	financial management	financial management	financial management	2	case_or_format
Financing, Government	financing government	financing government	financing government	Financing, Government	2	kept
Financing, Organized	financing organized	financing organized	financing organized	Financing, Organized	2	kept
Financing, Personal	financing personal	financing personal	financing personal	Financing, Personal	2	kept
fine needle aspiration biopsy	fine needle aspiration biopsy	fine needle aspiration biopsy	fine needle aspiration biopsy	fine needle aspiration biopsy	2	kept
finger injury	finger injury	finger injury	finger injury	finger injury	2	kept
finger nail	finger nail	finger nail	finger nail	finger nail	2	kept
Finite element method	finite element method	finite element method	finite element method	Finite element method	2	kept

Finite element modeling	finite element modeling	finite element modeling	finite element modeling	Finite element modeling	2	kept
Finnegan scoring	finnegan scoring	finnegan scoring	finnegan scoring	Finnegan scoring	2	kept
FIO<sub>2<	fio sub 2	fio sub 2	fio sub 2	FIO<sub>2<	2	kept
fire risk assessment	fire risk assessment	fire risk assessment	fire risk assessment	fire risk assessment	2	kept
first responder	first responder	first responder	first responder	first responder	2	kept
first trimester pregnancy	first trimester pregnancy	first trimester pregnancy	first trimester pregnancy	first trimester pregnancy	2	kept
First wave	first wave	first wave	first wave	First wave	2	kept
first wave	first wave	first wave	first wave	First wave	2	case_or_format
fish	fish	fish	fish	fish	2	kept
Fish oil	fish oil	fish oil	fish oil	fish oil	2	case_or_format
fitness	fitness	fitness	fitness	fitness	2	kept
Five moments	five moments	five moments	five moments	Five moments	2	kept
flame	flame	flame	flame	flame	2	kept
FLANDERS	flanders	flanders	flanders	FLANDERS	2	kept
Flanders	flanders	flanders	flanders	FLANDERS	2	case_or_format
FLANDERS BELGIUM	flanders belgium	flanders belgium	flanders belgium	FLANDERS BELGIUM	2	kept

flank pain	flank pain	flank pain	flank pain	flank pain	2	kept
Flexibility	flexibility	flexibility	flexibility	Flexibility	2	kept
flexible laryngoscopy	flexible laryngoscopy	flexible laryngoscopy	flexible laryngoscopy	flexible laryngoscopy	2	kept
Flexsim healthcare	flexsim healthcare	flexsim healthcare	flexsim healthcare	Flexsim healthcare	2	kept
Flipped classroom	flipped classroom	flipped classroom	flipped classroom	Flipped classroom	2	kept
floorings	floorings	floorings	flooring	floorings	2	singular
Florence Nightingale	florence nightingale	florence nightingale	florence nightingale	Florence Nightingale	2	kept
FLOW	flow	flow	flow	FLOW	2	kept
flow chart	flow chart	flow chart	flow chart	flow chart	2	kept
Flow Cytometry	flow cytometry	flow cytometry	flow cytometry	flow cytometry	2	case_or_format
Flow diverter	flow diverter	flow diverter	flow diverter	Flow diverter	2	kept
Flow of fluids	flow of fluids	flow of fluids	flow of fluids	Flow of fluids	2	kept
Flow rate	flow rate	flow rate	flow rate	flow rate	2	case_or_format
Flower framework	flower framework	flower framework	flower framework	Flower framework	2	kept
Fludrocortisone	fludrocortisone	fludrocortisone	fludrocortisone	fludrocortisone	2	case_or_format
fluid	fluid	fluid	fluid	Fluid	2	case_or_format

Fluid administration	fluid administration	fluid administration	fluid administration	Fluid administration	2	kept
fluid flow	fluid flow	fluid flow	fluid flow	fluid flow	2	kept
Fluid protocol	fluid protocol	fluid protocol	fluid protocol	Fluid protocol	2	kept
FLUID RESUSCITATION	fluid resuscitation	fluid resuscitation	fluid resuscitation	fluid resuscitation	2	case_or_format
fluid shift	fluid shift	fluid shift	fluid shift	Fluid Shifts	2	case_or_format
flunitrazepam	flunitrazepam	flunitrazepam	flunitrazepam	flunitrazepam	2	kept
fluorescein	fluorescein	fluorescein	fluorescein	Fluorescein	2	case_or_format
Fluorescence	fluorescence	fluorescence	fluorescence	fluorescence	2	case_or_format
Fluorescence Polarization Immunoassay	fluorescence polarization immunoassay	fluorescence polarization immunoassay	fluorescence polarization immunoassay	Fluorescence Polarization Immunoassay	2	kept
Fluorescent Dyes	fluorescent dyes	fluorescent dyes	fluorescent dyes	Fluorescent Dyes	2	kept
fluorescent lighting	fluorescent lighting	fluorescent lighting	fluorescent lighting	fluorescent lighting	2	kept
fluoride	fluoride	fluoride	fluoride	fluoride	2	kept
FMEA	fmea	fmea	fmea	FMEA	2	kept
focal glomerulosclerosis	focal glomerulosclerosis	focal glomerulosclerosis	focal glomerulosclerosis	focal glomerulosclerosis	2	kept
focal seizures	focal seizures	focal seizures	focal seizures	focal seizures	2	kept
FOCUS	focus	focus	focus	FOCUS	2	kept

focus group discussion	focus group discussion	focus group discussion	focus group discussion	focus group discussion	2	kept
Focus group discussions	focus group discussions	focus group discussions	focus group discussion	focus group discussion	2	singular
Focused ultrasound	focused ultrasound	focused ultrasound	focused ultrasound	Focused ultrasound	2	kept
follow-up service	follow up service	follow up service	follow up service	follow-up service	2	kept
Follow-up services	follow up services	follow up services	follow up service	follow-up service	2	singular
fomepizole	fomepizole	fomepizole	fomepizole	fomepizole	2	kept
fomites	fomites	fomites	fomite	Fomites	2	singular
Fontan	fontan	fontan	fontan	Fontan	2	kept
Food additives	food additives	food additives	food additives	Food additives	2	kept
food aspiration	food aspiration	food aspiration	food aspiration	food aspiration	2	kept
food assistance	food assistance	food assistance	food assistance	food assistance	2	kept
food aversion	food aversion	food aversion	food aversion	food aversion	2	kept
food composition	food composition	food composition	food composition	food composition	2	kept
food contamination	food contamination	food contamination	food contamination	Food Contamination	2	case_or_format
food handling	food handling	food handling	food handling	Food Handling	2	case_or_format
Food Industry	food industry	food industry	food industry	Food Industry	2	kept

Food Insecurity	food insecurity	food insecurity	food insecurity	food insecurity	2	case_or_format
food regurgitation	food regurgitation	food regurgitation	food regurgitation	food regurgitation	2	kept
Food Storage	food storage	food storage	food storage	Food Storage	2	kept
Food-Drug Interactions	food drug interactions	food drug interactions	food drug interaction	food drug interaction	2	singular
Foot Diseases	foot diseases	foot diseases	foot disease	Foot Diseases	2	singular
foot injury	foot injury	foot injury	foot injury	foot injury	2	kept
Foot Ulcer	foot ulcer	foot ulcer	foot ulcer	Foot Ulcer	2	kept
foot ulcer	foot ulcer	foot ulcer	foot ulcer	Foot Ulcer	2	case_or_format
Football	football	football	football	Football	2	kept
Footsteps	footsteps	footsteps	footstep	Footsteps	2	singular
footwear	footwear	footwear	footwear	footwear	2	kept
force	force	force	force	force	2	kept
force-sensing array	force sensing array	force sensing array	force sensing array	force-sensing array	2	kept
forceps	forceps	forceps	forceps	forceps	2	kept
Forehead	forehead	forehead	forehead	forehead	2	case_or_format
foreigner	foreigner	foreigner	foreigner	foreigner	2	kept

Forensic medicine	forensic medicine	forensic medicine	forensic medicine	forensic medicine	2	case_or_format
Forensic Psychiatry	forensic psychiatry	forensic psychiatry	forensic psychiatry	Forensic Psychiatry	2	kept
Forensic Sciences	forensic sciences	forensic sciences	forensic science	forensic science	2	singular
forest	forest	forest	forest	forest	2	kept
formaldehyde	formaldehyde	formaldehyde	formaldehyde	formaldehyde	2	kept
Formative evaluation	formative evaluation	formative evaluation	formative evaluation	Formative evaluation	2	kept
foscarnet	foscarnet	foscarnet	foscarnet	foscarnet	2	kept
fosinopril	fosinopril	fosinopril	fosinopril	fosinopril	2	kept
FOUR Coma Scale	four coma scale	four coma scale	four coma scale	FOUR Coma Scale	2	kept
Fournier gangrene	fournier gangrene	fournier gangrene	fournier gangrene	Fournier gangrene	2	kept
FPDR	fpdr	fpdr	fpdr	FPDR	2	kept
fracking	fracking	fracking	fracking	fracking	2	kept
fracture dislocation	fracture dislocation	fracture dislocation	fracture dislocation	fracture dislocation	2	kept
Fractures, Multiple	fractures multiple	fractures multiple	fractures multiple	Fractures, Multiple	2	kept
fragility fractures	fragility fractures	fragility fractures	fragility fracture	fragility fracture	2	singular
fragrance	fragrance	fragrance	fragrance	fragrance	2	kept

FRAM	fram	fram	fram	FRAM	2	kept
Framework analysis	framework analysis	framework analysis	framework analysis	Framework analysis	2	kept
framework approach	framework approach	framework approach	framework approach	framework approach	2	kept
Free flap	free flap	free flap	free flap	Free flap	2	kept
Free flap reconstruction	free flap reconstruction	free flap reconstruction	free flap reconstruction	Free flap reconstruction	2	kept
Free Radical Scavengers	free radical scavengers	free radical scavengers	free radical scavengers	Free Radical Scavengers	2	kept
Free radicals	free radicals	free radicals	free radical	free radical	2	singular
free tissue transfer	free tissue transfer	free tissue transfer	free tissue transfer	free tissue transfer	2	kept
freeze drying	freeze drying	freeze drying	freeze drying	freeze drying	2	kept
FREQUENCY OSCILLATORY VENTILATION	frequency oscillatory ventilation	frequency oscillatory ventilation	frequency oscillatory ventilation	FREQUENCY OSCILLATORY VENTILATION	2	kept
Fresh frozen plasma	fresh frozen plasma	fresh frozen plasma	fresh frozen plasma	fresh frozen plasma	2	case_or_format
FRICTION	friction	friction	friction	friction	2	case_or_format
Friedman test	friedman test	friedman test	friedman test	Friedman test	2	kept
Frontal Lobe	frontal lobe	frontal lobe	frontal lobe	Frontal Lobe	2	kept
frontal lobe	frontal lobe	frontal lobe	frontal lobe	Frontal Lobe	2	case_or_format
frontal sinus	frontal sinus	frontal sinus	frontal sinus	frontal sinus	2	kept

Frostbite	frostbite	frostbite	frostbite	Frostbite	2	kept
frostbite	frostbite	frostbite	frostbite	Frostbite	2	case_or_format
fruit juice	fruit juice	fruit juice	fruit juice	fruit juice	2	kept
Full Outline of UnResponsiveness	full outline of unresponsiveness	full outline of unresponsiveness	full outline of unresponsiveness	Full Outline of UnResponsiveness	2	kept
fulminant hepatic failure	fulminant hepatic failure	fulminant hepatic failure	fulminant hepatic failure	fulminant hepatic failure	2	kept
fulminating purpura	fulminating purpura	fulminating purpura	fulminating purpura	fulminating purpura	2	kept
functional connectivity	functional connectivity	functional connectivity	functional connectivity	functional connectivity	2	kept
Functional decline	functional decline	functional decline	functional decline	Functional decline	2	kept
functional decline	functional decline	functional decline	functional decline	Functional decline	2	case_or_format
functional dyspepsia	functional dyspepsia	functional dyspepsia	functional dyspepsia	functional dyspepsia	2	kept
functional electrical stimulation	functional electrical stimulation	functional electrical stimulation	functional electrical stimulation	functional electrical stimulation	2	kept
Functional impairment	functional impairment	functional impairment	functional impairment	functional impairment	2	case_or_format
functional neuroimaging	functional neuroimaging	functional neuroimaging	functional neuroimaging	functional neuroimaging	2	kept
Functional requirements	functional requirements	functional requirements	functional requirement	Functional requirements	2	singular
Functional Residual Capacity	functional residual capacity	functional residual capacity	functional residual capacity	functional residual capacity	2	case_or_format
functional training	functional training	functional training	functional training	functional training	2	kept

functions of the skin and its appendages	functions of the skin and its appendages	functions of the skin and its appendages	functions of the skin and its appendages	functions of the skin and its appendages	2	kept
Funeral arrangements	funeral arrangements	funeral arrangements	funeral arrangements	Funeral arrangements	2	kept
fungal cell culture	fungal cell culture	fungal cell culture	fungal cell culture	fungal cell culture	2	kept
fungal strain	fungal strain	fungal strain	fungal strain	fungal strain	2	kept
fungal virulence	fungal virulence	fungal virulence	fungal virulence	fungal virulence	2	kept
Fusobacterium Infections	fusobacterium infections	fusobacterium infections	fusobacterium infection	Fusobacterium Infections	2	singular
FUTILITY	futility	futility	futility	futility	2	case_or_format
Fuzzy constraint satisfaction problems	fuzzy constraint satisfaction problems	fuzzy constraint satisfaction problems	fuzzy constraint satisfaction problems	Fuzzy constraint satisfaction problems	2	kept
Fuzzy fingerprints	fuzzy fingerprints	fuzzy fingerprints	fuzzy fingerprints	Fuzzy fingerprints	2	kept
Fuzzy inference	fuzzy inference	fuzzy inference	fuzzy inference	Fuzzy inference	2	kept
Fuzzy rule in routing	fuzzy rule in routing	fuzzy rule in routing	fuzzy rule in routing	Fuzzy rule in routing	2	kept
GABA Modulators	gaba modulators	gaba modulators	gaba modulators	GABA Modulators	2	kept
Gadamer	gadamer	gadamer	gadamer	Gadamer	2	kept
galactogogue	galactogogue	galactogogue	galactogogue	galactogogue	2	kept
Galactogogues	galactogogues	galactogogues	galactogogue	galactogogue	2	singular
gallstone	gallstone	gallstone	gallstone	gallstone	2	kept

gallstone ileus	gallstone ileus	gallstone ileus	gallstone ileus	gallstone ileus	2	kept
game	game	game	game	GAME	2	case_or_format
Game Theory	game theory	game theory	game theory	Game Theory	2	kept
gamma glutamyl transferase blood level	gamma glutamyl transferase blood level	gamma glutamyl transferase blood level	gamma glutamyl transferase blood level	gamma glutamyl transferase blood level	2	kept
gamma-Aminobutyric Acid	gamma aminobutyric acid	gamma aminobutyric acid	gamma aminobutyric acid	gamma-Aminobutyric Acid	2	kept
gargle	gargle	gargle	gargle	gargle	2	kept
gas chromatography	gas chromatography	gas chromatography	gas chromatography	gas chromatography	2	kept
gas gangrene	gas gangrene	gas gangrene	gas gangrene	Gas gangrene	2	case_or_format
gas mask	gas mask	gas mask	gas mask	gas mask	2	kept
gas poisoning	gas poisoning	gas poisoning	gas poisoning	Gas Poisoning	2	case_or_format
Gasoline	gasoline	gasoline	gasoline	Gasoline	2	kept
Gastric cancer	gastric cancer	gastric cancer	gastric cancer	Gastric cancer	2	kept
gastric cancer	gastric cancer	gastric cancer	gastric cancer	Gastric cancer	2	case_or_format
gastric feeding tube	gastric feeding tube	gastric feeding tube	gastric feeding tube	gastric feeding tube	2	kept
Gastric Outlet Obstruction	gastric outlet obstruction	gastric outlet obstruction	gastric outlet obstruction	Gastric Outlet Obstruction	2	kept
gastrocnemius muscle	gastrocnemius muscle	gastrocnemius muscle	gastrocnemius muscle	gastrocnemius muscle	2	kept

gastroenterologist	gastroenterologist	gastroenterologist	gastroenterologist	gastroenterologist	2	kept
Gastroenterostomy	gastroenterostomy	gastroenterostomy	gastroenterostomy	Gastroenterostomy	2	kept
gastrointestinal function	gastrointestinal function	gastrointestinal function	gastrointestinal function	gastrointestinal function	2	kept
Gastrointestinal problems	gastrointestinal problems	gastrointestinal problems	gastrointestinal problems	Gastrointestinal problems	2	kept
gastrointestinal reflux	gastrointestinal reflux	gastrointestinal reflux	gastrointestinal reflux	gastrointestinal reflux	2	kept
gastrointestinal stromal tumor	gastrointestinal stromal tumor	gastrointestinal stromal tumor	gastrointestinal stromal tumor	gastrointestinal stromal tumor	2	kept
Gastrointestinal symptoms	gastrointestinal symptoms	gastrointestinal symptoms	gastrointestinal symptom	gastrointestinal symptom	2	singular
gastrointestinal toxicity	gastrointestinal toxicity	gastrointestinal toxicity	gastrointestinal toxicity	gastrointestinal toxicity	2	kept
gastrointestinal tract examination	gastrointestinal tract examination	gastrointestinal tract examination	gastrointestinal tract examination	gastrointestinal tract examination	2	kept
gastrojejunostomy	gastrojejunostomy	gastrojejunostomy	gastrojejunostomy	gastrojejunostomy	2	kept
gastropexy	gastropexy	gastropexy	gastropexy	gastropexy	2	kept
gastroplasty	gastroplasty	gastroplasty	gastroplasty	Gastroplasty	2	case_or_format
Gastroscopy	gastroscopy	gastroscopy	gastroscopy	gastroscopy	2	case_or_format
gastrotomy	gastrotomy	gastrotomy	gastrotomy	gastrotomy	2	kept
gatifloxacin	gatifloxacin	gatifloxacin	gatifloxacin	gatifloxacin	2	kept
gemcitabine	gemcitabine	gemcitabine	gemcitabine	gemcitabine	2	kept

gender and health	gender and health	gender and health	gender and health	gender and health	2	kept
Gender disparity	gender disparity	gender disparity	gender disparity	Gender disparity	2	kept
Gender Equity	gender equity	gender equity	gender equity	Gender Equity	2	kept
gender equity	gender equity	gender equity	gender equity	Gender Equity	2	case_or_format
gene identification	gene identification	gene identification	gene identification	gene identification	2	kept
gene mapping	gene mapping	gene mapping	gene mapping	gene mapping	2	kept
general aspects of disease	general aspects of disease	general aspects of disease	general aspects of disease	general aspects of disease	2	kept
general cardiology	general cardiology	general cardiology	general cardiology	general cardiology	2	kept
General care monitoring	general care monitoring	general care monitoring	general care monitoring	General care monitoring	2	kept
GENERAL HEALTH	general health	general health	general health	general health	2	case_or_format
general internal medicine	general internal medicine	general internal medicine	general internal medicine	general internal medicine	2	kept
general medical device	general medical device	general medical device	general medical device	general medical device	2	kept
General population	general population	general population	general population	General population	2	kept
General Practice	general practice	general practice	general practice	general practice	2	case_or_format
General practice	general practice	general practice	general practice	general practice	2	case_or_format
GENERAL WARD	general ward	general ward	general ward	general ward	2	case_or_format

general wards	general wards	general wards	general ward	general ward	2	singular
GENERAL-ANESTHESIA	general anesthesia	general anesthesia	general anesthesia	general anesthesia	2	case_or_format
Generalized Anxiety Disorder Scale	generalized anxiety disorder scale	generalized anxiety disorder scale	generalized anxiety disorder scale	Generalized Anxiety Disorder Scale	2	kept
Generalized Anxiety Disorder-2	generalized anxiety disorder 2	generalized anxiety disorder 2	generalized anxiety disorder 2	Generalized Anxiety Disorder-2	2	kept
generalized edema	generalized edema	generalized edema	generalized edema	generalized edema	2	kept
Generation Z	generation z	generation z	generation z	Generation Z	2	kept
generative adversarial network	generative adversarial network	generative adversarial network	generative adversarial network	generative adversarial network	2	kept
Genes, Bacterial	genes bacterial	genes bacterial	genes bacterial	Genes, Bacterial	2	kept
Genes, MDR	genes mdr	genes mdr	genes mdr	Genes, MDR	2	kept
Genetic algorithms	genetic algorithms	genetic algorithms	genetic algorithm	genetic algorithm	2	singular
genetic association	genetic association	genetic association	genetic association	genetic association	2	kept
Genetic Privacy	genetic privacy	genetic privacy	genetic privacy	Genetic Privacy	2	kept
genetic risk	genetic risk	genetic risk	genetic risk	genetic risk	2	kept
Genetic Screening	genetic screening	genetic screening	genetic screening	genetic screening	2	case_or_format
Genetic Techniques	genetic techniques	genetic techniques	genetic techniques	Genetic Techniques	2	kept
genetic testing	genetic testing	genetic testing	genetic testing	Genetic Testing	2	case_or_format

genetic variation	genetic variation	genetic variation	genetic variation	Genetic Variation	2	case_or_format
genome-wide association study	genome wide association study	genome wide association study	genome wide association study	genome-wide association study	2	kept
genotyping	genotyping	genotyping	genotyping	genotyping	2	kept
Geographic information systems	geographic information systems	geographic information systems	geographic information system	Geographic information systems	2	singular
geriatric attitude	geriatric attitude	geriatric attitude	geriatric attitude	geriatric attitude	2	kept
Geriatric fracture	geriatric fracture	geriatric fracture	geriatric fracture	Geriatric fracture	2	kept
Geriatric Nutritional Risk Index	geriatric nutritional risk index	geriatric nutritional risk index	geriatric nutritional risk index	Geriatric Nutritional Risk Index	2	kept
Geriatric traumatology	geriatric traumatology	geriatric traumatology	geriatric traumatology	Geriatric traumatology	2	kept
German translation	german translation	german translation	german translation	German translation	2	kept
GESTATION	gestation	gestation	gestation	GESTATION	2	kept
Gestational diabetes mellitus	gestational diabetes mellitus	gestational diabetes mellitus	gestational diabetes mellitus	Gestational diabetes mellitus	2	kept
GESTATIONAL-AGE	gestational age	gestational age	gestational age	gestational age	2	case_or_format
GFR	gfr	gfr	gfr	GFR	2	kept
gift giving	gift giving	gift giving	gift giving	gift giving	2	kept
Gingivitis	gingivitis	gingivitis	gingivitis	gingivitis	2	case_or_format
Gini coefficient	gini coefficient	gini coefficient	gini coefficient	Gini coefficient	2	kept

Glamorgan scale	glamorgan scale	glamorgan scale	glamorgan scale	Glamorgan scale	2	kept
global neonatology	global neonatology	global neonatology	global neonatology	global neonatology	2	kept
global perspective	global perspective	global perspective	global perspective	global perspective	2	kept
global south	global south	global south	global south	global south	2	kept
global surgery	global surgery	global surgery	global surgery	Global surgery	2	case_or_format
glomerulosclerosis	glomerulosclerosis	glomerulosclerosis	glomerulosclerosis	glomerulosclerosis	2	kept
glomerulus filtration	glomerulus filtration	glomerulus filtration	glomerulus filtration	glomerulus filtration	2	kept
Glove juice	glove juice	glove juice	glove juice	Glove juice	2	kept
GLOVES	gloves	gloves	glove	glove	2	singular
Glucagon	glucagon	glucagon	glucagon	glucagon	2	case_or_format
glucagon like peptide 1 receptor agonist	glucagon like peptide 1 receptor agonist	glucagon like peptide 1 receptor agonist	glucagon like peptide 1 receptor agonist	glucagon like peptide 1 receptor agonist	2	kept
glucocorticoid replacement	glucocorticoid replacement	glucocorticoid replacement	glucocorticoid replacement	glucocorticoid replacement	2	kept
glucocorticoids	glucocorticoids	glucocorticoids	glucocorticoid	glucocorticoid	2	singular
Glucosamine	glucosamine	glucosamine	glucosamine	Glucosamine	2	kept
glucose 6 phosphate dehydrogenase	glucose 6 phosphate dehydrogenase	glucose 6 phosphate dehydrogenase	glucose 6 phosphate dehydrogenase	glucose 6 phosphate dehydrogenase	2	kept
glucose 6 phosphate dehydrogenase deficiency	glucose 6 phosphate dehydrogenase deficiency	glucose 6 phosphate dehydrogenase deficiency	glucose 6 phosphate dehydrogenase deficiency	glucose 6 phosphate dehydrogenase deficiency	2	kept

GLUCOSE CONTROL	glucose control	glucose control	glucose control	glucose control	2	case_or_format
glucose dehydrogenase	glucose dehydrogenase	glucose dehydrogenase	glucose dehydrogenase	glucose dehydrogenase	2	kept
glucose infusion	glucose infusion	glucose infusion	glucose infusion	glucose infusion	2	kept
glucose level	glucose level	glucose level	glucose level	glucose level	2	kept
glucose metabolism	glucose metabolism	glucose metabolism	glucose metabolism	glucose metabolism	2	kept
Glucose monitors	glucose monitors	glucose monitors	glucose monitors	Glucose monitors	2	kept
glucose oxidase	glucose oxidase	glucose oxidase	glucose oxidase	glucose oxidase	2	kept
glucose regulation	glucose regulation	glucose regulation	glucose regulation	glucose regulation	2	kept
Glucose Solution, Hypertonic	glucose solution hypertonic	glucose solution hypertonic	glucose solution hypertonic	Glucose Solution, Hypertonic	2	kept
Glucose Tolerance Test	glucose tolerance test	glucose tolerance test	glucose tolerance test	Glucose Tolerance Test	2	kept
glucose tolerance test	glucose tolerance test	glucose tolerance test	glucose tolerance test	Glucose Tolerance Test	2	case_or_format
glutamate dehydrogenase	glutamate dehydrogenase	glutamate dehydrogenase	glutamate dehydrogenase	glutamate dehydrogenase	2	kept
Glycaemic variability	glycaemic variability	glycaemic variability	glycaemic variability	Glycaemic variability	2	kept
glycated hemoglobin	glycated hemoglobin	glycated hemoglobin	glycated hemoglobin	Glycated Hemoglobin	2	case_or_format
glycemic protocol	glycemic protocol	glycemic protocol	glycemic protocol	glycemic protocol	2	kept
glycemic variability	glycemic variability	glycemic variability	glycemic variability	glycemic variability	2	kept

Glycine max	glycine max	glycine max	glycine max	Glycine max	2	kept
glycolysis	glycolysis	glycolysis	glycolysis	glycolysis	2	kept
Goal Concordant Care	goal concordant care	goal concordant care	goal concordant care	Goal Concordant Care	2	kept
Goal-aligned care	goal aligned care	goal aligned care	goal aligned care	Goal-aligned care	2	kept
goal-concordant care	goal concordant care	goal concordant care	goal concordant care	Goal Concordant Care	2	case_or_format
goal-directed lung physiotherapy	goal directed lung physiotherapy	goal directed lung physiotherapy	goal directed lung physiotherapy	goal-directed lung physiotherapy	2	kept
GOALS	goals	goals	goals	Goals	2	case_or_format
goals-of-care	goals of care	goals of care	goals of care	Goals of care	2	case_or_format
Goiter	goiter	goiter	goiter	Goiter	2	kept
Gold	gold	gold	gold	gold	2	case_or_format
Golden Hour	golden hour	golden hour	golden hour	Golden Hour	2	kept
Golden hour	golden hour	golden hour	golden hour	Golden Hour	2	case_or_format
golden hour	golden hour	golden hour	golden hour	Golden Hour	2	case_or_format
gonadotropin	gonadotropin	gonadotropin	gonadotropin	gonadotropin	2	kept
GOOD DEATH	good death	good death	good death	Good death	2	case_or_format
good health and well-being	good health and well being	good health and well being	good health and well being	good health and well-being	2	kept

good practice standard	good practice standard	good practice standard	good practice standard	good practice standard	2	kept
governance	governance	governance	governance	Governance	2	case_or_format
governance approach	governance approach	governance approach	governance approach	governance approach	2	kept
GRADUATE MEDICAL-EDUCATION	graduate medical education	graduate medical education	graduate medical education	graduate medical education	2	case_or_format
graduate student	graduate student	graduate student	graduate student	graduate student	2	kept
Graft	graft	graft	graft	Graft	2	kept
Graft dysfunction	graft dysfunction	graft dysfunction	graft dysfunction	graft dysfunction	2	case_or_format
graft patency	graft patency	graft patency	graft patency	graft patency	2	kept
gram-negative	gram negative	gram negative	gram negative	Gram-negative	2	case_or_format
Gram-negative bacilli	gram negative bacilli	gram negative bacilli	gram negative bacilli	Gram-negative bacilli	2	kept
GRAM-NEGATIVE BACTERIA	gram negative bacteria	gram negative bacteria	gram negative bacteria	Gram-Negative Bacteria	2	case_or_format
Gram-negative bacteria	gram negative bacteria	gram negative bacteria	gram negative bacteria	Gram-Negative Bacteria	2	case_or_format
Gram-negative microorganisms	gram negative microorganisms	gram negative microorganisms	gram negative microorganisms	Gram-negative microorganisms	2	kept
grandfather	grandfather	grandfather	grandfather	grandfather	2	kept
grandiose delusion	grandiose delusion	grandiose delusion	grandiose delusion	grandiose delusion	2	kept
grandmother	grandmother	grandmother	grandmother	grandmother	2	kept

grandparenting	grandparenting	grandparenting	grandparenting	grandparenting	2	kept
granular corneal dystrophy type 2	granular corneal dystrophy type 2	granular corneal dystrophy type 2	granular corneal dystrophy type 2	granular corneal dystrophy type 2	2	kept
granulocyte	granulocyte	granulocyte	granulocyte	granulocyte	2	kept
granulocyte macrophage colony stimulating factor	granulocyte macrophage colony stimulating factor	granulocyte macrophage colony stimulating factor	granulocyte macrophage colony stimulating factor	granulocyte macrophage colony stimulating factor	2	kept
granulomatous inflammation	granulomatous inflammation	granulomatous inflammation	granulomatous inflammation	granulomatous inflammation	2	kept
Graphic facilitation	graphic facilitation	graphic facilitation	graphic facilitation	Graphic facilitation	2	kept
Graves Disease	graves disease	graves disease	graves disease	Graves Disease	2	kept
Gravitation	gravitation	gravitation	gravitation	Gravitation	2	kept
greater trochanter	greater trochanter	greater trochanter	greater trochanter	greater trochanter	2	kept
Green	green	green	green	Green	2	kept
greenhouse gas	greenhouse gas	greenhouse gas	greenhouse gas	greenhouse gas	2	kept
GRIEF EXPERIENCES	grief experiences	grief experiences	grief experiences	GRIEF EXPERIENCES	2	kept
Grieving	grieving	grieving	grieving	Grieving	2	kept
grocery store	grocery store	grocery store	grocery store	grocery store	2	kept
Groin	groin	groin	groin	Groin	2	kept
grooming	grooming	grooming	grooming	grooming	2	kept

GROUNDED THEORY	grounded theory	grounded theory	grounded theory	grounded theory	2	case_or_format
grounded theory approach	grounded theory approach	grounded theory approach	grounded theory approach	grounded theory approach	2	kept
grounded theory method	grounded theory method	grounded theory method	grounded theory method	grounded theory method	2	kept
group	group	group	group	group	2	kept
group A streptococcal infection	group a streptococcal infection	group a streptococcal infection	group a streptococcal infection	group A streptococcal infection	2	kept
group B streptococcal infection	group b streptococcal infection	group b streptococcal infection	group b streptococcal infection	group B streptococcal infection	2	kept
Group clinical supervision	group clinical supervision	group clinical supervision	group clinical supervision	Group clinical supervision	2	kept
Group discussion	group discussion	group discussion	group discussion	Group discussion	2	kept
group prenatal care	group prenatal care	group prenatal care	group prenatal care	group prenatal care	2	kept
growth and development	growth and development	growth and development	growth and development	Growth and development	2	case_or_format
growth hormone deficiency	growth hormone deficiency	growth hormone deficiency	growth hormone deficiency	growth hormone deficiency	2	kept
growth trajectory	growth trajectory	growth trajectory	growth trajectory	growth trajectory	2	kept
guanidine	guanidine	guanidine	guanidine	guanidine	2	kept
GUIDANCE	guidance	guidance	guidance	GUIDANCE	2	kept
Guidance	guidance	guidance	guidance	GUIDANCE	2	case_or_format
guidance	guidance	guidance	guidance	GUIDANCE	2	case_or_format

GUIDE	guide	guide	guide	GUIDE	2	kept
Guideline implementation	guideline implementation	guideline implementation	guideline implementation	Guideline implementation	2	kept
guideline implementation	guideline implementation	guideline implementation	guideline implementation	Guideline implementation	2	case_or_format
guideline usage	guideline usage	guideline usage	guideline usage	guideline usage	2	kept
guidelines as topic	guidelines as topic	guidelines as topic	guidelines as topic	Guidelines as Topic	2	case_or_format
Gulf War	gulf war	gulf war	gulf war	Gulf War	2	kept
gun violence	gun violence	gun violence	gun violence	gun violence	2	kept
gustatory	gustatory	gustatory	gustatory	gustatory	2	kept
gynecologic oncologist	gynecologic oncologist	gynecologic oncologist	gynecologic oncologist	gynecologic oncologist	2	kept
Gynecologic Surgical Procedures	gynecologic surgical procedures	gynecologic surgical procedures	gynecologic surgical procedures	Gynecologic Surgical Procedures	2	kept
Habituation, Psychophysiologic	habituation psychophysiologic	habituation psychophysiologic	habituation psychophysiologic	Habituation, Psychophysiologic	2	kept
Haemato-oncology	haemato oncology	haemato oncology	haemato oncology	Haemato-oncology	2	kept
Haemodiafiltration	haemodiafiltration	haemodiafiltration	haemodiafiltration	Haemodiafiltration	2	kept
haemodynamic monitoring	haemodynamic monitoring	haemodynamic monitoring	haemodynamic monitoring	Haemodynamic monitoring	2	case_or_format
haemodynamic parameters	haemodynamic parameters	haemodynamic parameters	haemodynamic parameters	haemodynamic parameters	2	kept
Haemofiltration	haemofiltration	haemofiltration	haemofiltration	Haemofiltration	2	kept

haemolysis	haemolysis	haemolysis	haemolysis	haemolysis	2	kept
Haemorrhage	haemorrhage	haemorrhage	haemorrhage	Haemorrhage	2	kept
HAI prevention	hai prevention	hai prevention	hai prevention	HAI prevention	2	kept
Hailey Hailey disease	hailey hailey disease	hailey hailey disease	hailey hailey disease	Hailey Hailey disease	2	kept
Hair	hair	hair	hair	Hair	2	kept
hair	hair	hair	hair	Hair	2	case_or_format
hair loss	hair loss	hair loss	hair loss	hair loss	2	kept
Halitosis	halitosis	halitosis	halitosis	halitosis	2	case_or_format
HALOPERIDOL	haloperidol	haloperidol	haloperidol	haloperidol	2	case_or_format
Hammersmith Neonatal Neurological Examination	hammersmith neonatal neurological examination	hammersmith neonatal neurological examination	hammersmith neonatal neurological examination	Hammersmith Neonatal Neurological Examination	2	kept
Hand decontamination	hand decontamination	hand decontamination	hand decontamination	Hand decontamination	2	kept
Hand dermatitis	hand dermatitis	hand dermatitis	hand dermatitis	hand dermatitis	2	case_or_format
Hand drying	hand drying	hand drying	hand drying	Hand drying	2	kept
hand foot and mouth disease	hand foot and mouth disease	hand foot and mouth disease	hand foot and mouth disease	hand foot and mouth disease	2	kept
Hand hygiene adherence rate	hand hygiene adherence rate	hand hygiene adherence rate	hand hygiene adherence rate	Hand hygiene adherence rate	2	kept
HAND HYGIENE COMPLIANCE	hand hygiene compliance	hand hygiene compliance	hand hygiene compliance	Hand hygiene compliance	2	case_or_format

hand pain	hand pain	hand pain	hand pain	hand pain	2	kept
Hand rub	hand rub	hand rub	hand rub	Hand rub	2	kept
hand tremor	hand tremor	hand tremor	hand tremor	hand tremor	2	kept
Hand-over	hand over	hand over	hand over	Hand-over	2	kept
Handgrip strength	handgrip strength	handgrip strength	handgrip strength	Handgrip strength	2	kept
handheld	handheld	handheld	handheld	handheld	2	kept
handheld [L01.224.230.260.550.500]	handheld 101 224 230 260 550 500	handheld 101 224 230 260 550 500	handheld 101 224 230 260 550 500	handheld [L01.224.230.260.550.500]	2	kept
Handling, Psychological	handling psychological	handling psychological	handling psychological	Handling, Psychological	2	kept
Handover quality	handover quality	handover quality	handover quality	Handover quality	2	kept
HANDWASHING PRACTICES	handwashing practices	handwashing practices	handwashing practices	HANDWASHING PRACTICES	2	kept
Hanging	hanging	hanging	hanging	Hanging	2	kept
hangover	hangover	hangover	hangover	hangover	2	kept
harassment	harassment	harassment	harassment	harassment	2	kept
harm	harm	harm	harm	Harm	2	case_or_format
Hazard	hazard	hazard	hazard	Hazard	2	kept
hazard	hazard	hazard	hazard	Hazard	2	case_or_format

HbSC	hbsc	hbsc	hbsc	HbSC	2	kept
HCAHPS	hcahps	hcahps	hcahps	HCAHPS	2	kept
HCAP	hcap	hcap	hcap	HCAP	2	kept
HCID	hcid	hcid	hcid	HCID	2	kept
HCUP NIS	hcup nis	hcup nis	hcup nis	HCUP NIS	2	kept
Head and neck cancer	head and neck cancer	head and neck cancer	head and neck cancer	head and neck cancer	2	case_or_format
head and neck disease	head and neck disease	head and neck disease	head and neck disease	head and neck disease	2	kept
head and neck surgery	head and neck surgery	head and neck surgery	head and neck surgery	head and neck surgery	2	kept
Head-of-bed elevation	head of bed elevation	head of bed elevation	head of bed elevation	head of bed elevation	2	case_or_format
headaches	headaches	headaches	headaches	headaches	2	kept
Healing environment	healing environment	healing environment	healing environment	healing environment	2	case_or_format
Health & safety	health and safety	health and safety	health and safety	health & safety	2	case_or_format
Health and safety	health and safety	health and safety	health and safety	health & safety	2	case_or_format
Health assessment	health assessment	health assessment	health assessment	Health assessment	2	kept
Health Assessment Questionnaire	health assessment questionnaire	health assessment questionnaire	health assessment questionnaire	Health Assessment Questionnaire	2	kept
health care assistants	health care assistants	health care assistants	health care assistants	health care assistants	2	kept

Health care associated infections	health care associated infections	health care associated infections	health care associated infection	Health care-associated infections	2	singular
health care challenges	health care challenges	health care challenges	health care challenges	health care challenges	2	kept
Health care disparities	health care disparities	health care disparities	health care disparity	health care disparity	2	singular
Health care economics	health care economics	health care economics	health care economics	health care economics	2	case_or_format
health care equipment	health care equipment	health care equipment	health care equipment	health care equipment	2	kept
Health care inequalities	health care inequalities	health care inequalities	health care inequalities	Health care inequalities	2	kept
Health care management	health care management	health care management	health care management	health care management	2	case_or_format
health care personnel perspective	health care personnel perspective	health care personnel perspective	health care personnel perspective	health care personnel perspective	2	kept
Health care planning	health care planning	health care planning	health care planning	health care planning	2	case_or_format
Health Care Professionals	health care professionals	health care professionals	health care professional	Health care professionals	2	singular
health care provider	health care provider	health care provider	health care provider	health care providers	2	case_or_format
health care proxy	health care proxy	health care proxy	health care proxy	health care proxy	2	kept
health care resources	health care resources	health care resources	health care resources	health care resources	2	kept
Health care survey	health care survey	health care survey	health care survey	Health Care Surveys	2	case_or_format
Health care system	health care system	health care system	health care system	health care system	2	case_or_format
health care team	health care team	health care team	health care team	health care team	2	kept

health care training	health care training	health care training	health care training	health care training	2	kept
Health care-acquired infections	health care acquired infections	health care acquired infections	health care acquired infection	Health care-acquired infections	2	singular
health care - associated infections	health care associated infections	health care associated infections	health care associated infection	Health care-associated infections	2	singular
Health complaints	health complaints	health complaints	health complaints	Health complaints	2	kept
health economic analysis	health economic analysis	health economic analysis	health economic analysis	health economic analysis	2	kept
Health Educators	health educators	health educators	health educator	health educator	2	singular
Health Evaluation	health evaluation	health evaluation	health evaluation	Health Evaluation	2	kept
health expenditures	health expenditures	health expenditures	health expenditure	Health Expenditures	2	singular
Health Facility Administration	health facility administration	health facility administration	health facility administration	Health Facility Administration	2	kept
Health Information Interoperability	health information interoperability	health information interoperability	health information interoperability	Health Information Interoperability	2	kept
health information interoperability	health information interoperability	health information interoperability	health information interoperability	Health Information Interoperability	2	case_or_format
Health Information Management	health information management	health information management	health information management	Health Information Management	2	kept
Health information systems	health information systems	health information systems	health information system	Health Information Systems	2	singular
Health insurance	health insurance	health insurance	health insurance	health insurance	2	case_or_format
health intervention	health intervention	health intervention	health intervention	health intervention	2	kept
health legislation	health legislation	health legislation	health legislation	health legislation	2	kept

Health monitoring	health monitoring	health monitoring	health monitoring	Health monitoring	2	kept
Health plan implementation	health plan implementation	health plan implementation	health plan implementation	Health Plan Implementation	2	case_or_format
Health planning	health planning	health planning	health planning	Health Planning	2	case_or_format
Health policies	health policies	health policies	health policy	Health Policy	2	singular
Health professional	health professional	health professional	health professional	Health professionals	2	case_or_format
Health professions education	health professions education	health professions education	health professions education	health professions education	2	case_or_format
Health Profile	health profile	health profile	health profile	Health Profile	2	kept
health profile	health profile	health profile	health profile	Health Profile	2	case_or_format
health psychosis	health psychosis	health psychosis	health psychosis	health psychosis	2	kept
health risk assessment	health risk assessment	health risk assessment	health risk assessment	health risk assessment	2	kept
health sciences education	health sciences education	health sciences education	health sciences education	health sciences education	2	kept
Health service research	health service research	health service research	health service research	Health service research	2	kept
health services accessibility	health services accessibility	health services accessibility	health services accessibility	Health Services Accessibility	2	case_or_format
health services and outcomes research	health services and outcomes research	health services and outcomes research	health services and outcomes research	health services and outcomes research	2	kept
health student	health student	health student	health student	health student	2	kept
Health surveys	health surveys	health surveys	health survey	health survey	2	singular

health system reorganization	health system reorganization	health system reorganization	health system reorganization	health system reorganization	2	kept
health technologies	health technologies	health technologies	health technology	health technology	2	singular
Health technology	health technology	health technology	health technology	health technology	2	case_or_format
Health Workers	health workers	health workers	health worker	health worker	2	singular
health-care facilities	health care facilities	health care facilities	health care facility	health care facility	2	singular
Health-care worker	health care worker	health care worker	health care worker	Health care workers	2	case_or_format
HEALTH-STATUS	health status	health status	health status	health status	2	case_or_format
Healthcare analytics	healthcare analytics	healthcare analytics	healthcare analytics	Healthcare analytics	2	kept
Healthcare architecture	healthcare architecture	healthcare architecture	healthcare architecture	Healthcare architecture	2	kept
Healthcare associated infection	healthcare associated infection	healthcare associated infection	healthcare associated infection	healthcare associated infection	2	case_or_format
healthcare associated infections	healthcare associated infections	healthcare associated infections	healthcare associated infection	healthcare associated infection	2	singular
Healthcare barriers	healthcare barriers	healthcare barriers	healthcare barriers	Healthcare barriers	2	kept
Healthcare Common Procedure Coding System	healthcare common procedure coding system	healthcare common procedure coding system	healthcare common procedure coding system	Healthcare Common Procedure Coding System	2	kept
healthcare consumption	healthcare consumption	healthcare consumption	healthcare consumption	healthcare consumption	2	kept
healthcare delivery	healthcare delivery	healthcare delivery	healthcare delivery	Healthcare delivery	2	case_or_format
healthcare disparities	healthcare disparities	healthcare disparities	healthcare disparity	Healthcare Disparities	2	singular

healthcare economic perspective	healthcare economic perspective	healthcare economic perspective	healthcare economic perspective	healthcare economic perspective	2	kept
Healthcare education	healthcare education	healthcare education	healthcare education	healthcare education	2	case_or_format
healthcare environment	healthcare environment	healthcare environment	healthcare environment	Healthcare environments	2	case_or_format
healthcare equity	healthcare equity	healthcare equity	healthcare equity	healthcare equity	2	kept
healthcare failure mode and effect analysis	healthcare failure mode and effect analysis	healthcare failure mode and effect analysis	healthcare failure mode and effect analysis	Healthcare Failure Mode and Effect Analysis	2	case_or_format
healthcare improvement	healthcare improvement	healthcare improvement	healthcare improvement	healthcare improvement	2	kept
Healthcare industry	healthcare industry	healthcare industry	healthcare industry	Healthcare industry	2	kept
Healthcare network design	healthcare network design	healthcare network design	healthcare network design	Healthcare network design	2	kept
healthcare operations management	healthcare operations management	healthcare operations management	healthcare operations management	healthcare operations management	2	kept
Healthcare organization	healthcare organization	healthcare organization	healthcare organization	Healthcare organization	2	kept
healthcare professional-led follow-up care	healthcare professional led follow up care	healthcare professional led follow up care	healthcare professional led follow up care	healthcare professional-led follow-up care	2	kept
Healthcare Professionals	healthcare professionals	healthcare professionals	healthcare professional	healthcare professionals	2	singular
healthcare provider outcomes	healthcare provider outcomes	healthcare provider outcomes	healthcare provider outcomes	healthcare provider outcomes	2	kept
Healthcare Quality Indicators	healthcare quality indicators	healthcare quality indicators	healthcare quality indicators	Healthcare quality indicators	2	case_or_format
Healthcare rationing	healthcare rationing	healthcare rationing	healthcare rationing	Healthcare rationing	2	kept
Healthcare reform	healthcare reform	healthcare reform	healthcare reform	Healthcare reform	2	kept

healthcare resources	healthcare resources	healthcare resources	healthcare resource	Healthcare resources	2	singular
healthcare survey	healthcare survey	healthcare survey	healthcare survey	healthcare survey	2	kept
Healthcare team	healthcare team	healthcare team	healthcare team	Healthcare team	2	kept
healthcare team	healthcare team	healthcare team	healthcare team	Healthcare team	2	case_or_format
healthcare utilisation	healthcare utilisation	healthcare utilisation	healthcare utilisation	healthcare utilisation	2	kept
Healthcare Worker	healthcare worker	healthcare worker	healthcare worker	Healthcare workers	2	case_or_format
Healthcare Workers	healthcare workers	healthcare workers	healthcare worker	Healthcare workers	2	singular
Healthcare-associated pneumonia	healthcare associated pneumonia	healthcare associated pneumonia	healthcare associated pneumonia	Healthcare-Associated Pneumonia	2	case_or_format
healthcare‐associated infections	healthcare associated infections	healthcare associated infections	healthcare associated infection	healthcare associated infection	2	singular
Healthy People 2030	healthy people 2030	healthy people 2030	healthy people 2030	Healthy People 2030	2	kept
Hearing Aids	hearing aids	hearing aids	hearing aid	hearing aid	2	singular
Hearing Loss, Noise-Induced	hearing loss noise induced	hearing loss noise induced	hearing loss noise induced	Hearing Loss, Noise-Induced	2	kept
Hearing Loss, Unilateral	hearing loss unilateral	hearing loss unilateral	hearing loss unilateral	Hearing Loss, Unilateral	2	kept
heart abscess	heart abscess	heart abscess	heart abscess	heart abscess	2	kept
heart atrium	heart atrium	heart atrium	heart atrium	heart atrium	2	kept
heart atrium contraction	heart atrium contraction	heart atrium contraction	heart atrium contraction	heart atrium contraction	2	kept

heart atrium thrombosis	heart atrium thrombosis	heart atrium thrombosis	heart atrium thrombosis	heart atrium thrombosis	2	kept
heart cycle	heart cycle	heart cycle	heart cycle	heart cycle	2	kept
heart defects	heart defects	heart defects	heart defects	Heart defects	2	case_or_format
heart disease risk factors	heart disease risk factors	heart disease risk factors	heart disease risk factors	heart disease risk factors	2	kept
heart enzyme	heart enzyme	heart enzyme	heart enzyme	heart enzyme	2	kept
heart injury	heart injury	heart injury	heart injury	heart injury	2	kept
heart left atrium	heart left atrium	heart left atrium	heart left atrium	heart left atrium	2	kept
heart muscle	heart muscle	heart muscle	heart muscle	heart muscle	2	kept
heart muscle blood flow	heart muscle blood flow	heart muscle blood flow	heart muscle blood flow	heart muscle blood flow	2	kept
heart muscle cell	heart muscle cell	heart muscle cell	heart muscle cell	heart muscle cell	2	kept
heart muscle fibrosis	heart muscle fibrosis	heart muscle fibrosis	heart muscle fibrosis	heart muscle fibrosis	2	kept
Heart Neoplasms	heart neoplasms	heart neoplasms	heart neoplasms	Heart Neoplasms	2	kept
Heart rate and blood pressure	heart rate and blood pressure	heart rate and blood pressure	heart rate and blood pressure	Heart rate and blood pressure	2	kept
heart right atrium pressure	heart right atrium pressure	heart right atrium pressure	heart right atrium pressure	heart right atrium pressure	2	kept
heart right bundle branch block	heart right bundle branch block	heart right bundle branch block	heart right bundle branch block	heart right bundle branch block	2	kept
heart right left shunt	heart right left shunt	heart right left shunt	heart right left shunt	heart right left shunt	2	kept

heart right ventricle	heart right ventricle	heart right ventricle	heart right ventricle	heart right ventricle	2	kept
heart right ventricle outflow tract	heart right ventricle outflow tract	heart right ventricle outflow tract	heart right ventricle outflow tract	heart right ventricle outflow tract	2	kept
heart scintiscanning	heart scintiscanning	heart scintiscanning	heart scintiscanning	heart scintiscanning	2	kept
Heart Septum	heart septum	heart septum	heart septum	Heart Septum	2	kept
Heart Sounds	heart sounds	heart sounds	heart sound	heart sound	2	singular
Heart surgical theater	heart surgical theater	heart surgical theater	heart surgical theater	Heart surgical theater	2	kept
Heart valve prosthesis implantation	heart valve prosthesis implantation	heart valve prosthesis implantation	heart valve prosthesis implantation	Heart Valve Prosthesis Implantation	2	case_or_format
heart ventricle hypertrophy	heart ventricle hypertrophy	heart ventricle hypertrophy	heart ventricle hypertrophy	heart ventricle hypertrophy	2	kept
heart ventricle pressure	heart ventricle pressure	heart ventricle pressure	heart ventricle pressure	heart ventricle pressure	2	kept
heart work	heart work	heart work	heart work	heart work	2	kept
Heart, Artificial	heart artificial	heart artificial	heart artificial	Heart, Artificial	2	kept
HEART-ASSOCIATION GUIDELINES	heart association guidelines	heart association guidelines	heart association guidelines	HEART-ASSOCIATION GUIDELINES	2	kept
Heart-Lung Transplantation	heart lung transplantation	heart lung transplantation	heart lung transplantation	Heart-Lung Transplantation	2	kept
Heat	heat	heat	heat	heat	2	case_or_format
heat exchange	heat exchange	heat exchange	heat exchange	heat exchange	2	kept
heat wave	heat wave	heat wave	heat wave	heat wave	2	kept

Heavy workloads	heavy workloads	heavy workloads	heavy workloads	Heavy workloads	2	kept
Hebron hospitals	hebron hospitals	hebron hospitals	hebron hospitals	Hebron hospitals	2	kept
Heel lance	heel lance	heel lance	heel lance	Heel lance	2	kept
heel lance	heel lance	heel lance	heel lance	Heel lance	2	case_or_format
heel lancing	heel lancing	heel lancing	heel lancing	heel lancing	2	kept
heel pressure injuries	heel pressure injuries	heel pressure injuries	heel pressure injury	heel pressure injuries	2	singular
heel prick	heel prick	heel prick	heel prick	heel prick	2	kept
heel stick	heel stick	heel stick	heel stick	heel stick	2	kept
Heelstick	heelstick	heelstick	heelstick	Heelstick	2	kept
Heideggerian phenomenology	heideggerian phenomenology	heideggerian phenomenology	heideggerian phenomenology	Heideggerian phenomenology	2	kept
Height	height	height	height	height	2	case_or_format
Heilongjiang province	heilongjiang province	heilongjiang province	heilongjiang province	Heilongjiang province	2	kept
Heimlich valve catheter placement	heimlich valve catheter placement	heimlich valve catheter placement	heimlich valve catheter placement	Heimlich valve catheter placement	2	kept
helicopter emergency medical service	helicopter emergency medical service	helicopter emergency medical service	helicopter emergency medical service	helicopter emergency medical service	2	kept
Helicopter Emergency Medical Services	helicopter emergency medical services	helicopter emergency medical services	helicopter emergency medical service	helicopter emergency medical service	2	singular
helicopter pilot	helicopter pilot	helicopter pilot	helicopter pilot	helicopter pilot	2	kept

helicopter transport	helicopter transport	helicopter transport	helicopter transport	helicopter transport	2	kept
Helmet	helmet	helmet	helmet	helmet	2	case_or_format
Help-Seeking Behavior	help seeking behavior	help seeking behavior	help seeking behavior	help seeking behavior	2	case_or_format
Hemangioma	hemangioma	hemangioma	hemangioma	Hemangioma	2	kept
hemangioma	hemangioma	hemangioma	hemangioma	Hemangioma	2	case_or_format
Hematemesis	hematemesis	hematemesis	hematemesis	hematemesis	2	case_or_format
Hematinics	hematinics	hematinics	hematinics	Hematinics	2	kept
hematologic agent	hematologic agent	hematologic agent	hematologic agent	hematologic agent	2	kept
Hematological malignant cancer	hematological malignant cancer	hematological malignant cancer	hematological malignant cancer	Hematological malignant cancer	2	kept
Hematoma, Epidural, Cranial	hematoma epidural cranial	hematoma epidural cranial	hematoma epidural cranial	Hematoma, Epidural, Cranial	2	kept
Hematopoietic stem cell transplantation	hematopoietic stem cell transplantation	hematopoietic stem cell transplantation	hematopoietic stem cell transplantation	Hematopoietic Stem Cell Transplantation	2	case_or_format
Hematuria	hematuria	hematuria	hematuria	hematuria	2	case_or_format
heme oxygenase 1	heme oxygenase 1	heme oxygenase 1	heme oxygenase 1	heme oxygenase 1	2	kept
hemicraniectomy	hemicraniectomy	hemicraniectomy	hemicraniectomy	hemicraniectomy	2	kept
Hemiplegia	hemiplegia	hemiplegia	hemiplegia	hemiplegia	2	case_or_format
hemodynamic stability	hemodynamic stability	hemodynamic stability	hemodynamic stability	hemodynamic stability	2	kept

hemoglobin A	hemoglobin a	hemoglobin a	hemoglobin a	hemoglobin A	2	kept
hemoglobin F	hemoglobin f	hemoglobin f	hemoglobin f	hemoglobin F	2	kept
Hemoglobinuria, Paroxysmal	hemoglobinuria paroxysmal	hemoglobinuria paroxysmal	hemoglobinuria paroxysmal	Hemoglobinuria, Paroxysmal	2	kept
Hemolytic uremic syndrome	hemolytic uremic syndrome	hemolytic uremic syndrome	hemolytic uremic syndrome	Hemolytic-Uremic Syndrome	2	case_or_format
Hemoperitoneum	hemoperitoneum	hemoperitoneum	hemoperitoneum	hemoperitoneum	2	case_or_format
Hemophagocytic lymphohistiocytosis	hemophagocytic lymphohistiocytosis	hemophagocytic lymphohistiocytosis	hemophagocytic lymphohistiocytosis	Hemophagocytic lymphohistiocytosis	2	kept
hemophilia A	hemophilia a	hemophilia a	hemophilia a	hemophilia A	2	kept
hemophilia B	hemophilia b	hemophilia b	hemophilia b	hemophilia B	2	kept
Hemoptysis	hemoptysis	hemoptysis	hemoptysis	hemoptysis	2	case_or_format
hemorrhagic fever	hemorrhagic fever	hemorrhagic fever	hemorrhagic fever	hemorrhagic fever	2	kept
Hemorrhagic Fevers, Viral	hemorrhagic fevers viral	hemorrhagic fevers viral	hemorrhagic fevers viral	Hemorrhagic Fevers, Viral	2	kept
Hemostasis, Surgical	hemostasis surgical	hemostasis surgical	hemostasis surgical	Hemostasis, Surgical	2	kept
Hemostatic Techniques	hemostatic techniques	hemostatic techniques	hemostatic technique	Hemostatic Techniques	2	singular
hemotherapy	hemotherapy	hemotherapy	hemotherapy	hemotherapy	2	kept
Hemothorax	hemothorax	hemothorax	hemothorax	Hemothorax	2	kept
Hepacivirus	hepacivirus	hepacivirus	hepacivirus	Hepacivirus	2	kept

HEPARIN	heparin	heparin	heparin	heparin	2	case_or_format
heparinoid	heparinoid	heparinoid	heparinoid	heparinoid	2	kept
Hepatic encephalopathy	hepatic encephalopathy	hepatic encephalopathy	hepatic encephalopathy	hepatic encephalopathy	2	case_or_format
Hepatic Veins	hepatic veins	hepatic veins	hepatic veins	Hepatic Veins	2	kept
Hepatitis	hepatitis	hepatitis	hepatitis	hepatitis	2	case_or_format
hepatitis A antibody	hepatitis a antibody	hepatitis a antibody	hepatitis a antibody	hepatitis A antibody	2	kept
hepatitis A vaccine	hepatitis a vaccine	hepatitis a vaccine	hepatitis a vaccine	hepatitis A vaccine	2	kept
hepatitis B antibody	hepatitis b antibody	hepatitis b antibody	hepatitis b antibody	hepatitis B antibody	2	kept
hepatitis B surface antibody	hepatitis b surface antibody	hepatitis b surface antibody	hepatitis b surface antibody	hepatitis B surface antibody	2	kept
Hepatitis, Alcoholic	hepatitis alcoholic	hepatitis alcoholic	hepatitis alcoholic	Hepatitis, Alcoholic	2	kept
hepatology	hepatology	hepatology	hepatology	hepatology	2	kept
herbal dressing	herbal dressing	herbal dressing	herbal dressing	herbal dressing	2	kept
Herbal Medicine	herbal medicine	herbal medicine	herbal medicine	herbal medicine	2	case_or_format
Hernia, Hiatal	hernia hiatal	hernia hiatal	hernia hiatal	Hernia, Hiatal	2	kept
Heroin	heroin	heroin	heroin	Heroin	2	kept
Heroin Dependence	heroin dependence	heroin dependence	heroin dependence	heroin dependence	2	case_or_format

herpes simplex encephalitis	herpes simplex encephalitis	herpes simplex encephalitis	herpes simplex encephalitis	herpes simplex encephalitis	2	kept
herpes virus infection	herpes virus infection	herpes virus infection	herpes virus infection	herpes virus infection	2	kept
herpetic stomatitis	herpetic stomatitis	herpetic stomatitis	herpetic stomatitis	herpetic stomatitis	2	kept
heuristic method	heuristic method	heuristic method	heuristic method	heuristic method	2	kept
heuristics	heuristics	heuristics	heuristic	Heuristics	2	singular
HFNC	hfnc	hfnc	hfnc	HFNC	2	kept
HFOV	hfov	hfov	hfov	HFOV	2	kept
hiatal hernia	hiatal hernia	hiatal hernia	hiatal hernia	hiatal hernia	2	kept
Hierarchical systems	hierarchical systems	hierarchical systems	hierarchical systems	Hierarchical systems	2	kept
High acuity	high acuity	high acuity	high acuity	High acuity	2	kept
High acuity settings	high acuity settings	high acuity settings	high acuity setting	High acuity settings	2	singular
high alert medication	high alert medication	high alert medication	high alert medication	high alert medication	2	kept
High alert medications	high alert medications	high alert medications	high alert medication	high alert medication	2	singular
High Dependency Unit	high dependency unit	high dependency unit	high dependency unit	high dependency unit	2	case_or_format
high flow nasal cannula	high flow nasal cannula	high flow nasal cannula	high flow nasal cannula	High-flow nasal cannula	2	case_or_format
High frequency HF	high frequency hf	high frequency hf	high frequency hf	High frequency HF	2	kept

high frequency jet ventilation	high frequency jet ventilation	high frequency jet ventilation	high frequency jet ventilation	high frequency jet ventilation	2	kept
high frequency ultrasound	high frequency ultrasound	high frequency ultrasound	high frequency ultrasound	high frequency ultrasound	2	kept
High intensity	high intensity	high intensity	high intensity	High intensity	2	kept
high intensity exercise	high intensity exercise	high intensity exercise	high intensity exercise	high intensity exercise	2	kept
High reliability	high reliability	high reliability	high reliability	High reliability	2	kept
high temperature	high temperature	high temperature	high temperature	high temperature	2	kept
high-alert medications	high alert medications	high alert medications	high alert medication	high alert medication	2	singular
high-fidelity	high fidelity	high fidelity	high fidelity	high-fidelity	2	kept
high-flow nasal cannula therapy	high flow nasal cannula therapy	high flow nasal cannula therapy	high flow nasal cannula therapy	high flow nasal cannula therapy	2	case_or_format
High-flow nasal oxygen	high flow nasal oxygen	high flow nasal oxygen	high flow nasal oxygen	High-flow nasal oxygen	2	kept
High-Frequency Jet Ventilation	high frequency jet ventilation	high frequency jet ventilation	high frequency jet ventilation	high frequency jet ventilation	2	case_or_format
high-income countries	high income countries	high income countries	high income country	high income country	2	singular
High-Intensity Interval Training	high intensity interval training	high intensity interval training	high intensity interval training	High-Intensity Interval Training	2	kept
High-risk infant	high risk infant	high risk infant	high risk infant	high risk infant	2	case_or_format
high-risk infants	high risk infants	high risk infants	high risk infant	high risk infant	2	singular
high-risk neonate’s mother	high risk neonate s mother	high risk neonate s mother	high risk neonate s mother	high-risk neonate’s mother	2	kept

High-volume centre	high volume centre	high volume centre	high volume centre	High-volume centre	2	kept
higher music education	higher music education	higher music education	higher music education	higher music education	2	kept
Hinduism	hinduism	hinduism	hinduism	Hinduism	2	kept
hip circumference	hip circumference	hip circumference	hip circumference	hip circumference	2	kept
hip dislocation	hip dislocation	hip dislocation	hip dislocation	hip dislocation	2	kept
hip fracture surgery	hip fracture surgery	hip fracture surgery	hip fracture surgery	hip fracture surgery	2	kept
hip osteoarthritis	hip osteoarthritis	hip osteoarthritis	hip osteoarthritis	hip osteoarthritis	2	kept
hip radiography	hip radiography	hip radiography	hip radiography	hip radiography	2	kept
hiring congruency	hiring congruency	hiring congruency	hiring congruency	hiring congruency	2	kept
Hirudins	hirudins	hirudins	hirudin	Hirudins	2	singular
Histamine	histamine	histamine	histamine	histamine	2	case_or_format
Histamine H1 Antagonists	histamine h1 antagonists	histamine h1 antagonists	histamine h1 antagonists	Histamine H1 Antagonists	2	kept
Histoplasmosis	histoplasmosis	histoplasmosis	histoplasmosis	Histoplasmosis	2	kept
Holding	holding	holding	holding	Holding	2	kept
Holistic health	holistic health	holistic health	holistic health	Holistic Health	2	case_or_format
HoloLens	hololens	hololens	hololens	HoloLens	2	kept

holoprosencephaly	holoprosencephaly	holoprosencephaly	holoprosencephaly	holoprosencephaly	2	kept
Home Based Functional Electrical Stimulation	home based functional electrical stimulation	home based functional electrical stimulation	home based functional electrical stimulation	Home Based Functional Electrical Stimulation	2	kept
Home Care Agencies	home care agencies	home care agencies	home care agencies	Home Care Agencies	2	kept
Home childbirth	home childbirth	home childbirth	home childbirth	Home Childbirth	2	case_or_format
home health	home health	home health	home health	home health	2	kept
Home health care	home health care	home health care	home health care	home health care	2	case_or_format
HOME HEALTH-CARE	home health care	home health care	home health care	home health care	2	case_or_format
home hospital	home hospital	home hospital	home hospital	home hospital	2	kept
home invasive mechanical ventilation	home invasive mechanical ventilation	home invasive mechanical ventilation	home invasive mechanical ventilation	home invasive mechanical ventilation	2	kept
home mental health care	home mental health care	home mental health care	home mental health care	home mental health care	2	kept
home oxygen	home oxygen	home oxygen	home oxygen	home oxygen	2	kept
home palliative care	home palliative care	home palliative care	home palliative care	home palliative care	2	kept
Home return	home return	home return	home return	Home return	2	kept
home ventilation	home ventilation	home ventilation	home ventilation	Home ventilation	2	case_or_format
home-based care	home based care	home based care	home based care	home-based care	2	kept
homebound	homebound	homebound	homebound	homebound	2	kept

Homelessness	homelessness	homelessness	homelessness	homelessness	2	case_or_format
Homogeneous management	homogeneous management	homogeneous management	homogeneous management	Homogeneous management	2	kept
HOPE	hope	hope	hope	Hope	2	case_or_format
Hopelessness	hopelessness	hopelessness	hopelessness	hopelessness	2	case_or_format
Hopkins verbal learning test	hopkins verbal learning test	hopkins verbal learning test	hopkins verbal learning test	Hopkins verbal learning test	2	kept
horizontal violence	horizontal violence	horizontal violence	horizontal violence	horizontal violence	2	kept
Hosmer and Lemeshow goodness-of-fit test	hosmer and lemeshow goodness of fit test	hosmer and lemeshow goodness of fit test	hosmer and lemeshow goodness of fit test	Hosmer and Lemeshow goodness-of-fit test	2	kept
hospices	hospices	hospices	hospice	hospice	2	singular
hospital acquired infection	hospital acquired infection	hospital acquired infection	hospital acquired infection	Hospital-acquired infections	2	case_or_format
hospital acquired infections	hospital acquired infections	hospital acquired infections	hospital acquired infection	Hospital-acquired infections	2	singular
hospital administration	hospital administration	hospital administration	hospital administration	Hospital Administration	2	case_or_format
HOSPITAL ADMISSION	hospital admission	hospital admission	hospital admission	hospital admission	2	case_or_format
HOSPITAL ADMISSIONS	hospital admissions	hospital admissions	hospital admission	hospital admission	2	singular
hospital bed numbers	hospital bed numbers	hospital bed numbers	hospital bed numbers	hospital bed numbers	2	kept
Hospital buildings	hospital buildings	hospital buildings	hospital building	hospital building	2	singular
hospital construction	hospital construction	hospital construction	hospital construction	hospital construction	2	kept

hospital dental services	hospital dental services	hospital dental services	hospital dental services	hospital dental services	2	kept
hospital departments	hospital departments	hospital departments	hospital department	hospital department	2	singular
hospital design and construction	hospital design and construction	hospital design and construction	hospital design and construction	Hospital Design and Construction	2	case_or_format
Hospital emergency service	hospital emergency service	hospital emergency service	hospital emergency service	hospital emergency service	2	case_or_format
Hospital emergency services	hospital emergency services	hospital emergency services	hospital emergency service	hospital emergency service	2	singular
hospital engineering	hospital engineering	hospital engineering	hospital engineering	hospital engineering	2	kept
Hospital errors	hospital errors	hospital errors	hospital errors	Hospital errors	2	kept
hospital gown	hospital gown	hospital gown	hospital gown	hospital gown	2	kept
Hospital incident reporting	hospital incident reporting	hospital incident reporting	hospital incident reporting	Hospital incident reporting	2	kept
Hospital infection control	hospital infection control	hospital infection control	hospital infection control	Hospital infection control	2	kept
Hospital information systems	hospital information systems	hospital information systems	hospital information system	hospital information system	2	singular
Hospital length of stay	hospital length of stay	hospital length of stay	hospital length of stay	Hospital length of stay	2	kept
hospital length of stay	hospital length of stay	hospital length of stay	hospital length of stay	Hospital length of stay	2	case_or_format
Hospital levels	hospital levels	hospital levels	hospital levels	Hospital levels	2	kept
Hospital medical staff	hospital medical staff	hospital medical staff	hospital medical staff	Hospital medical staff	2	kept
Hospital personnel	hospital personnel	hospital personnel	hospital personnel	hospital personnel	2	case_or_format

hospital pharmacists	hospital pharmacists	hospital pharmacists	hospital pharmacist	hospital pharmacist	2	singular
Hospital planning	hospital planning	hospital planning	hospital planning	hospital planning	2	case_or_format
hospital presenting sepsis	hospital presenting sepsis	hospital presenting sepsis	hospital presenting sepsis	hospital presenting sepsis	2	kept
hospital quality	hospital quality	hospital quality	hospital quality	hospital quality	2	kept
Hospital reorganization	hospital reorganization	hospital reorganization	hospital reorganization	Hospital reorganization	2	kept
Hospital response	hospital response	hospital response	hospital response	Hospital response	2	kept
HOSPITAL RESTRUCTURING EFFORTS	hospital restructuring efforts	hospital restructuring efforts	hospital restructuring efforts	HOSPITAL RESTRUCTURING EFFORTS	2	kept
hospital settings	hospital settings	hospital settings	hospital setting	Hospital settings	2	singular
HOSPITAL STAY	hospital stay	hospital stay	hospital stay	Hospital Stay	2	case_or_format
Hospital stay	hospital stay	hospital stay	hospital stay	Hospital Stay	2	case_or_format
hospital stay	hospital stay	hospital stay	hospital stay	Hospital Stay	2	case_or_format
Hospital sustainability	hospital sustainability	hospital sustainability	hospital sustainability	Hospital sustainability	2	kept
hospital units	hospital units	hospital units	hospital units	Hospital Units	2	case_or_format
Hospital visiting	hospital visiting	hospital visiting	hospital visiting	Hospital visiting	2	kept
HOSPITAL VOLUME	hospital volume	hospital volume	hospital volume	hospital volume	2	case_or_format
Hospital ward layout	hospital ward layout	hospital ward layout	hospital ward layout	Hospital ward layout	2	kept

hospital worker	hospital worker	hospital worker	hospital worker	hospital worker	2	kept
Hospital-acquired	hospital acquired	hospital acquired	hospital acquired	hospital acquired	2	case_or_format
hospital-acquired conditions	hospital acquired conditions	hospital acquired conditions	hospital acquired condition	hospital-acquired conditions	2	singular
Hospital-acquired pressure ulcer	hospital acquired pressure ulcer	hospital acquired pressure ulcer	hospital acquired pressure ulcer	Hospital-acquired pressure ulcers	2	case_or_format
hospital-acquired pressure ulcer	hospital acquired pressure ulcer	hospital acquired pressure ulcer	hospital acquired pressure ulcer	Hospital-acquired pressure ulcers	2	case_or_format
hospital-acquired venous thromboembolism	hospital acquired venous thromboembolism	hospital acquired venous thromboembolism	hospital acquired venous thromboembolism	hospital-acquired venous thromboembolism	2	kept
hospital-based maternity unit	hospital based maternity unit	hospital based maternity unit	hospital based maternity unit	hospital-based maternity unit	2	kept
HOSPITALIST HANDOFFS	hospitalist handoffs	hospitalist handoffs	hospitalist handoffs	HOSPITALIST HANDOFFS	2	kept
hospitalization area pediatrics	hospitalization area pediatrics	hospitalization area pediatrics	hospitalization area pediatrics	hospitalization area pediatrics	2	kept
hospitalizations	hospitalizations	hospitalizations	hospitalization	hospitalization	2	singular
hospitalized children	hospitalized children	hospitalized children	hospitalized children	HOSPITALIZED CHILDREN	2	case_or_format
Hospitals, Federal	hospitals federal	hospitals federal	hospitals federal	Hospitals, Federal	2	kept
Hospitals, Rehabilitation	hospitals rehabilitation	hospitals rehabilitation	hospitals rehabilitation	Hospitals, Rehabilitation	2	kept
Hospitals-acquired pressure sores	hospitals acquired pressure sores	hospitals acquired pressure sores	hospitals acquired pressure sores	Hospitals-acquired pressure sores	2	kept
hot flush	hot flush	hot flush	hot flush	hot flush	2	kept
hotline	hotline	hotline	hotline	Hotlines	2	case_or_format

hotspots	hotspots	hotspots	hotspots	hotspots	2	kept
House officers	house officers	house officers	house officer	House officers	2	singular
household economic status	household economic status	household economic status	household economic status	household economic status	2	kept
housestaff	housestaff	housestaff	housestaff	housestaff	2	kept
HRQoL	hrqol	hrqol	hrqol	HRQoL	2	kept
HSOPSC	hsopsc	hsopsc	hsopsc	HSOPSC	2	kept
HTA	hta	hta	hta	HTA	2	kept
humidification	humidification	humidification	humidification	humidification	2	kept
Humidifiers	humidifiers	humidifiers	humidifier	humidifier	2	singular
humor	humor	humor	humor	humor	2	kept
Hutterite	hutterite	hutterite	hutterite	Hutterite	2	kept
hyaline membrane disease	hyaline membrane disease	hyaline membrane disease	hyaline membrane disease	hyaline membrane disease	2	kept
hybrid CGM-POC monitor	hybrid cgm poc monitor	hybrid cgm poc monitor	hybrid cgm poc monitor	hybrid CGM-POC monitor	2	kept
hybrid model	hybrid model	hybrid model	hybrid model	hybrid model	2	kept
hybrid protocol	hybrid protocol	hybrid protocol	hybrid protocol	hybrid protocol	2	kept
Hydration	hydration	hydration	hydration	hydration	2	case_or_format

hydrocarbon	hydrocarbon	hydrocarbon	hydrocarbon	hydrocarbon	2	kept
hydrocortisone release	hydrocortisone release	hydrocortisone release	hydrocortisone release	hydrocortisone release	2	kept
hydrocortisone sodium succinate	hydrocortisone sodium succinate	hydrocortisone sodium succinate	hydrocortisone sodium succinate	hydrocortisone sodium succinate	2	kept
Hydrodynamics	hydrodynamics	hydrodynamics	hydrodynamics	Hydrodynamics	2	kept
hydrolase	hydrolase	hydrolase	hydrolase	hydrolase	2	kept
hydrophilicity	hydrophilicity	hydrophilicity	hydrophilicity	hydrophilicity	2	kept
Hydrops Fetalis	hydrops fetalis	hydrops fetalis	hydrops fetalis	Hydrops Fetalis	2	kept
Hydrothorax	hydrothorax	hydrothorax	hydrothorax	Hydrothorax	2	kept
hydrothorax	hydrothorax	hydrothorax	hydrothorax	Hydrothorax	2	case_or_format
hydroxocobalamin	hydroxocobalamin	hydroxocobalamin	hydroxocobalamin	hydroxocobalamin	2	kept
hydroxypropylmethylcellulose	hydroxypropylmethylcellulose	hydroxypropylmethylcellulose	hydroxypropylmethylcellulose	hydroxypropylmethylcellulose	2	kept
hyperacute graft rejection	hyperacute graft rejection	hyperacute graft rejection	hyperacute graft rejection	hyperacute graft rejection	2	kept
Hyperbaric oxygen treatment	hyperbaric oxygen treatment	hyperbaric oxygen treatment	hyperbaric oxygen treatment	Hyperbaric oxygen treatment	2	kept
Hypercalcemia	hypercalcemia	hypercalcemia	hypercalcemia	hypercalcemia	2	case_or_format
hypercapnic respiratory failure	hypercapnic respiratory failure	hypercapnic respiratory failure	hypercapnic respiratory failure	hypercapnic respiratory failure	2	kept
hypercoagulability	hypercoagulability	hypercoagulability	hypercoagulability	hypercoagulability	2	kept

hyperemesis gravidarum	hyperemesis gravidarum	hyperemesis gravidarum	hyperemesis gravidarum	hyperemesis gravidarum	2	kept
Hyperemia	hyperemia	hyperemia	hyperemia	Hyperemia	2	kept
hyperemia	hyperemia	hyperemia	hyperemia	Hyperemia	2	case_or_format
Hyperesthesia	hyperesthesia	hyperesthesia	hyperesthesia	Hyperesthesia	2	kept
Hyperglycemia during COVID-19 hospitalizations	hyperglycemia during covid 19 hospitalizations	hyperglycemia during covid 19 hospitalizations	hyperglycemia during covid 19 hospitalizations	Hyperglycemia during COVID-19 hospitalizations	2	kept
hyperimmune globulin	hyperimmune globulin	hyperimmune globulin	hyperimmune globulin	hyperimmune globulin	2	kept
hyperinflammation	hyperinflammation	hyperinflammation	hyperinflammation	hyperinflammation	2	kept
hyperinsulinemia	hyperinsulinemia	hyperinsulinemia	hyperinsulinemia	hyperinsulinemia	2	kept
Hyperlipidemias	hyperlipidemias	hyperlipidemias	hyperlipidemia	hyperlipidemia	2	singular
hypermagnesemia	hypermagnesemia	hypermagnesemia	hypermagnesemia	hypermagnesemia	2	kept
hyperosmolar therapy	hyperosmolar therapy	hyperosmolar therapy	hyperosmolar therapy	hyperosmolar therapy	2	kept
hyperoxemia	hyperoxemia	hyperoxemia	hyperoxemia	hyperoxemia	2	kept
hyperpigmentation	hyperpigmentation	hyperpigmentation	hyperpigmentation	hyperpigmentation	2	kept
hyperplasia	hyperplasia	hyperplasia	hyperplasia	hyperplasia	2	kept
HYPERTHERMIA	hyperthermia	hyperthermia	hyperthermia	hyperthermia	2	case_or_format
Hyperthermia	hyperthermia	hyperthermia	hyperthermia	hyperthermia	2	case_or_format

HYPERTONIC SALINE	hypertonic saline	hypertonic saline	hypertonic saline	HYPERTONIC SALINE	2	kept
hypertrophic scar	hypertrophic scar	hypertrophic scar	hypertrophic scar	hypertrophic scar	2	kept
Hypervirulent	hypervirulent	hypervirulent	hypervirulent	Hypervirulent	2	kept
hypesthesia	hypesthesia	hypesthesia	hypesthesia	hypesthesia	2	kept
hypnotics and sedatives	hypnotics and sedatives	hypnotics and sedatives	hypnotics and sedatives	Hypnotics and Sedatives	2	case_or_format
hypoglycaemia	hypoglycaemia	hypoglycaemia	hypoglycaemia	Hypoglycaemia	2	case_or_format
Hypohidrosis	hypohidrosis	hypohidrosis	hypohidrosis	Hypohidrosis	2	kept
hypophysis adrenal system	hypophysis adrenal system	hypophysis adrenal system	hypophysis adrenal system	hypophysis adrenal system	2	kept
hypoplasia	hypoplasia	hypoplasia	hypoplasia	hypoplasia	2	kept
Hypoplastic left heart syndrome	hypoplastic left heart syndrome	hypoplastic left heart syndrome	hypoplastic left heart syndrome	Hypoplastic Left Heart Syndrome	2	case_or_format
Hypoproteinemia	hypoproteinemia	hypoproteinemia	hypoproteinemia	hypoproteinemia	2	case_or_format
hyposalivation	hyposalivation	hyposalivation	hyposalivation	hyposalivation	2	kept
Hypothermia treatment	hypothermia treatment	hypothermia treatment	hypothermia treatment	Hypothermia treatment	2	kept
Hypoxaemia	hypoxaemia	hypoxaemia	hypoxaemia	hypoxaemia	2	case_or_format
Hypoxemic respiratory failure	hypoxemic respiratory failure	hypoxemic respiratory failure	hypoxemic respiratory failure	hypoxemic respiratory failure	2	case_or_format
hypoxic brain injury	hypoxic brain injury	hypoxic brain injury	hypoxic brain injury	hypoxic brain injury	2	kept

hôpital	h pital	h pital	h pital	hôpital	2	kept
Iade	iade	iade	iade	Iade	2	kept
Iatrogenic disease	iatrogenic disease	iatrogenic disease	iatrogenic disease	Iatrogenic Disease	2	case_or_format
ibalizumab	ibalizumab	ibalizumab	ibalizumab	ibalizumab	2	kept
ICD-11	icd 11	icd 11	icd 11	ICD-11	2	kept
ICNP	icnp	icnp	icnp	ICNP	2	kept
ICOCFAS score	icocfas score	icocfas score	icocfas score	ICOCFAS score	2	kept
Icon	icon	icon	icon	Icon	2	kept
ICP monitor	icp monitor	icp monitor	icp monitor	ICP monitor	2	kept
Ideal Body Weight	ideal body weight	ideal body weight	ideal body weight	Ideal Body Weight	2	kept
ideal body weight	ideal body weight	ideal body weight	ideal body weight	Ideal Body Weight	2	case_or_format
idiopathic disease	idiopathic disease	idiopathic disease	idiopathic disease	idiopathic disease	2	kept
Idiopathic Pulmonary Fibrosis	idiopathic pulmonary fibrosis	idiopathic pulmonary fibrosis	idiopathic pulmonary fibrosis	Idiopathic pulmonary fibrosis	2	case_or_format
idiopathic thrombocytopenic purpura	idiopathic thrombocytopenic purpura	idiopathic thrombocytopenic purpura	idiopathic thrombocytopenic purpura	idiopathic thrombocytopenic purpura	2	kept
Idle days	idle days	idle days	idle days	Idle days	2	kept
IEC 60601-1-8	iec 60601 1 8	iec 60601 1 8	iec 60601 1 8	IEC 60601-1-8	2	kept

IGF-1	igf 1	igf 1	igf 1	IGF-1	2	kept
ILCOR, International Liaison Committee on Resuscitation	ilcor international liaison committee on resuscitation	ilcor international liaison committee on resuscitation	ilcor international liaison committee on resuscitation	ILCOR, International Liaison Committee on Resuscitation	2	kept
ileal conduit	ileal conduit	ileal conduit	ileal conduit	ileal conduit	2	kept
iliac artery	iliac artery	iliac artery	iliac artery	iliac artery	2	kept
illness behavior	illness behavior	illness behavior	illness behavior	illness behavior	2	kept
Image Interpretation, Computer-Assisted	image interpretation computer assisted	image interpretation computer assisted	image interpretation computer assisted	Image Interpretation, Computer-Assisted	2	kept
Image reconstruction	image reconstruction	image reconstruction	image reconstruction	Image reconstruction	2	kept
Imagery	imagery	imagery	imagery	Imagery	2	kept
imagery	imagery	imagery	imagery	Imagery	2	case_or_format
imagination	imagination	imagination	imagination	imagination	2	kept
imaging system	imaging system	imaging system	imaging system	imaging system	2	kept
imdevimab	imdevimab	imdevimab	imdevimab	imdevimab	2	kept
Immersion	immersion	immersion	immersion	Immersion	2	kept
immersion	immersion	immersion	immersion	Immersion	2	case_or_format
Immune Reconstitution Inflammatory Syndrome	immune reconstitution inflammatory syndrome	immune reconstitution inflammatory syndrome	immune reconstitution inflammatory syndrome	Immune Reconstitution Inflammatory Syndrome	2	kept
Immune system	immune system	immune system	immune system	immune system	2	case_or_format

Immune-mediated inflammatory disease	immune mediated inflammatory disease	immune mediated inflammatory disease	immune mediated inflammatory disease	Immune-mediated inflammatory disease	2	kept
Immunity, Cellular	immunity cellular	immunity cellular	immunity cellular	Immunity, Cellular	2	kept
Immunization, Secondary	immunization secondary	immunization secondary	immunization secondary	Immunization, Secondary	2	kept
Immunizations	immunizations	immunizations	immunization	immunization	2	singular
Immunocompromised	immunocompromised	immunocompromised	immunocompromised	Immunocompromised	2	kept
immunoglobulin deficiency	immunoglobulin deficiency	immunoglobulin deficiency	immunoglobulin deficiency	immunoglobulin deficiency	2	kept
immunologic factor	immunologic factor	immunologic factor	immunologic factor	Immunologic Factors	2	case_or_format
immunological tolerance	immunological tolerance	immunological tolerance	immunological tolerance	immunological tolerance	2	kept
Immunomodulation	immunomodulation	immunomodulation	immunomodulation	immunomodulation	2	case_or_format
immunopathogenesis	immunopathogenesis	immunopathogenesis	immunopathogenesis	immunopathogenesis	2	kept
immunoprophylaxis	immunoprophylaxis	immunoprophylaxis	immunoprophylaxis	immunoprophylaxis	2	kept
immunoregulation	immunoregulation	immunoregulation	immunoregulation	immunoregulation	2	kept
immunostimulation	immunostimulation	immunostimulation	immunostimulation	immunostimulation	2	kept
IMMUNOSUPPRESSION	immunosuppression	immunosuppression	immunosuppression	Immunosuppression	2	case_or_format
immunosuppression	immunosuppression	immunosuppression	immunosuppression	Immunosuppression	2	case_or_format
Immunotherapy, Adoptive	immunotherapy adoptive	immunotherapy adoptive	immunotherapy adoptive	Immunotherapy, Adoptive	2	kept

impact evaluation	impact evaluation	impact evaluation	impact evaluation	impact evaluation	2	kept
implant complication	implant complication	implant complication	implant complication	implant complication	2	kept
implant costs	implant costs	implant costs	implant costs	implant costs	2	kept
implantable [E07.505.254]	implantable e07 505 254	implantable e07 505 254	implantable e07 505 254	implantable [E07.505.254]	2	kept
Implantable cardiac defibrillators	implantable cardiac defibrillators	implantable cardiac defibrillators	implantable cardiac defibrillators	Implantable cardiac defibrillators	2	kept
implementation and deployment	implementation and deployment	implementation and deployment	implementation and deployment	implementation and deployment	2	kept
Implementation plan	implementation plan	implementation plan	implementation plan	Implementation plan	2	kept
implementation program	implementation program	implementation program	implementation program	implementation program	2	kept
implementation project	implementation project	implementation project	implementation project	implementation project	2	kept
implementation research	implementation research	implementation research	implementation research	implementation research	2	kept
implementing simulation training	implementing simulation training	implementing simulation training	implementing simulation training	implementing simulation training	2	kept
implications	implications	implications	implications	implications	2	kept
importance-performance analysis	importance performance analysis	importance performance analysis	importance performance analysis	importance-performance analysis	2	kept
Improve	improve	improve	improve	IMPROVE	2	case_or_format
Improved outcomes	improved outcomes	improved outcomes	improved outcomes	Improved outcomes	2	kept
IMPROVES	improves	improves	improve	IMPROVE	2	singular

Improving Pediatric Sepsis Outcomes collaborative	improving pediatric sepsis outcomes collaborative	improving pediatric sepsis outcomes collaborative	improving pediatric sepsis outcomes collaborative	Improving Pediatric Sepsis Outcomes collaborative	2	kept
IMPROVING TEAMWORK	improving teamwork	improving teamwork	improving teamwork	IMPROVING TEAMWORK	2	kept
impulsive noise	impulsive noise	impulsive noise	impulsive noise	impulsive noise	2	kept
impulsivity	impulsivity	impulsivity	impulsivity	impulsivity	2	kept
in silico validation	in silico validation	in silico validation	in silico validation	in silico validation	2	kept
In situ	in situ	in situ	in situ	In situ	2	kept
In vivo mouse model	in vivo mouse model	in vivo mouse model	in vivo mouse model	In vivo mouse model	2	kept
In-bed sensor	in bed sensor	in bed sensor	in bed sensor	In-bed sensor	2	kept
in-dwelling catheters	in dwelling catheters	in dwelling catheters	in dwelling catheters	in-dwelling catheters	2	kept
In-hospital	in hospital	in hospital	in hospital	In-hospital	2	kept
in-service	in service	in service	in service	in-service	2	kept
in-situ simulation	in situ simulation	in situ simulation	in situ simulation	in situ simulation	2	case_or_format
IN-VITRO ACTIVITY	in vitro activity	in vitro activity	in vitro activity	IN-VITRO ACTIVITY	2	kept
inadvertent perioperative hypothermia	inadvertent perioperative hypothermia	inadvertent perioperative hypothermia	inadvertent perioperative hypothermia	inadvertent perioperative hypothermia	2	kept
inappropriate care	inappropriate care	inappropriate care	inappropriate care	inappropriate care	2	kept
INAPPROPRIATE MEDICATION USE	inappropriate medication use	inappropriate medication use	inappropriate medication use	INAPPROPRIATE MEDICATION USE	2	kept

Inappropriate prescribing	inappropriate prescribing	inappropriate prescribing	inappropriate prescribing	inappropriate prescribing	2	case_or_format
Inappropriate transfer	inappropriate transfer	inappropriate transfer	inappropriate transfer	Inappropriate transfer	2	kept
Inattention	inattention	inattention	inattention	Inattention	2	kept
inborn error of metabolism	inborn error of metabolism	inborn error of metabolism	inborn error of metabolism	inborn error of metabolism	2	kept
incentive spirometer	incentive spirometer	incentive spirometer	incentive spirometer	incentive spirometer	2	kept
Incentive spirometry	incentive spirometry	incentive spirometry	incentive spirometry	Incentive spirometry	2	kept
incentive spirometry	incentive spirometry	incentive spirometry	incentive spirometry	Incentive spirometry	2	case_or_format
Incidence of adverse reactions	incidence of adverse reactions	incidence of adverse reactions	incidence of adverse reactions	Incidence of adverse reactions	2	kept
Incidence rates	incidence rates	incidence rates	incidence rate	Incidence rates	2	singular
Incidencia	incidencia	incidencia	incidencia	Incidencia	2	kept
incident	incident	incident	incident	INCIDENTS	2	case_or_format
Incident Reporting	incident reporting	incident reporting	incident reporting	Incident reporting	2	case_or_format
Incident reporting culture	incident reporting culture	incident reporting culture	incident reporting culture	Incident reporting culture	2	kept
Incident reporting system	incident reporting system	incident reporting system	incident reporting system	Incident reporting system	2	kept
incident reports	incident reports	incident reports	incident report	incident report	2	singular
incidental finding	incidental finding	incidental finding	incidental finding	incidental finding	2	kept

inclusion	inclusion	inclusion	inclusion	inclusion	2	kept
INCONTINENCE	incontinence	incontinence	incontinence	incontinence	2	case_or_format
INCREASE	increase	increase	increase	INCREASE	2	kept
INCREASED MORTALITY	increased mortality	increased mortality	increased mortality	INCREASED MORTALITY	2	kept
Indenes	indenes	indenes	indenes	Indenes	2	kept
Independent sector	independent sector	independent sector	independent sector	Independent sector	2	kept
Indian Ocean	indian ocean	indian ocean	indian ocean	Indian Ocean	2	kept
indication	indication	indication	indication	indication	2	kept
indicator	indicator	indicator	indicator	INDICATORS	2	case_or_format
indicators	indicators	indicators	indicator	INDICATORS	2	singular
indicators and metrics	indicators and metrics	indicators and metrics	indicators and metrics	indicators and metrics	2	kept
Indigenous health	indigenous health	indigenous health	indigenous health	indigenous health	2	case_or_format
Indigenous Peoples	indigenous peoples	indigenous peoples	indigenous people	indigenous people	2	singular
Indigenous peoples	indigenous peoples	indigenous peoples	indigenous people	indigenous people	2	singular
Indirect calorimetry	indirect calorimetry	indirect calorimetry	indirect calorimetry	indirect calorimetry	2	case_or_format
indirect contact transmission	indirect contact transmission	indirect contact transmission	indirect contact transmission	indirect contact transmission	2	kept

indirect laryngoscopy	indirect laryngoscopy	indirect laryngoscopy	indirect laryngoscopy	indirect laryngoscopy	2	kept
individual coping	individual coping	individual coping	individual coping	individual coping	2	kept
Individualised developmental care	individualised developmental care	individualised developmental care	individualised developmental care	Individualised developmental care	2	kept
individualized developmental care	individualized developmental care	individualized developmental care	individualized developmental care	individualized developmental care	2	kept
Individualized Medicine	individualized medicine	individualized medicine	individualized medicine	Individualized Medicine	2	kept
Indoor air	indoor air	indoor air	indoor air	Indoor air	2	kept
indoor environment	indoor environment	indoor environment	indoor environment	indoor environment	2	kept
Indoor localization	indoor localization	indoor localization	indoor localization	Indoor localization	2	kept
Indoor positioning systems	indoor positioning systems	indoor positioning systems	indoor positioning systems	Indoor positioning systems	2	kept
indoor positioning systems	indoor positioning systems	indoor positioning systems	indoor positioning systems	Indoor positioning systems	2	case_or_format
Induced	induced	induced	induced	Induced	2	kept
induced abortion	induced abortion	induced abortion	induced abortion	induced abortion	2	kept
induction	induction	induction	induction	induction	2	kept
Industrial hygiene	industrial hygiene	industrial hygiene	industrial hygiene	Industrial hygiene	2	kept
Industrial research	industrial research	industrial research	industrial research	Industrial research	2	kept
Indwelling	indwelling	indwelling	indwelling	Indwelling	2	kept

Indwelling Catheter	indwelling catheter	indwelling catheter	indwelling catheter	indwelling catheter	2	case_or_format
Indwelling catheter	indwelling catheter	indwelling catheter	indwelling catheter	indwelling catheter	2	case_or_format
Inequality	inequality	inequality	inequality	inequality	2	case_or_format
Infant development	infant development	infant development	infant development	Infant development	2	kept
infant health	infant health	infant health	infant health	Infant Health	2	case_or_format
Infant neonates	infant neonates	infant neonates	infant neonates	Infant neonates	2	kept
Infant Nutrition Physiology	infant nutrition physiology	infant nutrition physiology	infant nutrition physiology	Infant Nutrition Physiology	2	kept
infant parent psychotherapy	infant parent psychotherapy	infant parent psychotherapy	infant parent psychotherapy	infant parent psychotherapy	2	kept
infant premature	infant premature	infant premature	infant premature	Infant, Premature	2	case_or_format
Infant welfare	infant welfare	infant welfare	infant welfare	Infant Welfare	2	case_or_format
Infant, Postmature	infant postmature	infant postmature	infant postmature	Infant, Postmature	2	kept
Infant, very low birth weight	infant very low birth weight	infant very low birth weight	infant very low birth weight	Infant, Very Low Birth Weight	2	case_or_format
Infant-newborn	infant newborn	infant newborn	infant newborn	Infant, Newborn	2	case_or_format
Infant-premature	infant premature	infant premature	infant premature	Infant, Premature	2	case_or_format
infarction	infarction	infarction	infarction	Infarction	2	case_or_format
Infecciones por coronavirus	infecciones por coronavirus	infecciones por coronavirus	infecciones por coronavirus	Infecciones por coronavirus	2	kept

Infection control and prevention	infection control and prevention	infection control and prevention	infection control and prevention	infection control and prevention	2	case_or_format
infection control assessment tool	infection control assessment tool	infection control assessment tool	infection control assessment tool	infection control assessment tool	2	kept
Infection control guidelines	infection control guidelines	infection control guidelines	infection control guidelines	Infection control guidelines	2	kept
infection control performance	infection control performance	infection control performance	infection control performance	infection control performance	2	kept
Infection control program	infection control program	infection control program	infection control program	Infection control program	2	kept
INFECTION PREVENTION	infection prevention	infection prevention	infection prevention	infection prevention	2	case_or_format
Infection rate	infection rate	infection rate	infection rate	infection rate	2	case_or_format
Infection rates	infection rates	infection rates	infection rate	infection rate	2	singular
infectious colitis	infectious colitis	infectious colitis	infectious colitis	infectious colitis	2	kept
Infectious disease control	infectious disease control	infectious disease control	infectious disease control	Infectious disease control	2	kept
infectious disease medicine	infectious disease medicine	infectious disease medicine	infectious disease medicine	infectious disease medicine	2	kept
Infectious disease transmission	infectious disease transmission	infectious disease transmission	infectious disease transmission	Infectious disease transmission	2	kept
Infectious Diseases	infectious diseases	infectious diseases	infectious disease	infectious disease	2	singular
infectious waste	infectious waste	infectious waste	infectious waste	infectious waste	2	kept
INFECTIOUS-DISEASES SOCIETY	infectious diseases society	infectious diseases society	infectious diseases society	INFECTIOUS-DISEASES-SOCIETY	2	case_or_format
Infecções por Coronaví rus	infec es por coronav rus	infec es por coronav rus	infec es por coronav rus	Infecções por Coronaví rus	2	kept

inferior mesenteric artery	inferior mesenteric artery	inferior mesenteric artery	inferior mesenteric artery	inferior mesenteric artery	2	kept
infertility therapy	infertility therapy	infertility therapy	infertility therapy	infertility therapy	2	kept
infestation	infestation	infestation	infestation	infestation	2	kept
Inflammatory bowel disease	inflammatory bowel disease	inflammatory bowel disease	inflammatory bowel disease	inflammatory bowel disease	2	case_or_format
Inflammatory Bowel Diseases	inflammatory bowel diseases	inflammatory bowel diseases	inflammatory bowel disease	inflammatory bowel disease	2	singular
inflammatory cell	inflammatory cell	inflammatory cell	inflammatory cell	inflammatory cell	2	kept
inflammatory pain	inflammatory pain	inflammatory pain	inflammatory pain	inflammatory pain	2	kept
inflation pressure	inflation pressure	inflation pressure	inflation pressure	inflation pressure	2	kept
Influence	influence	influence	influence	Influence	2	kept
influence	influence	influence	influence	Influence	2	case_or_format
influence factors	influence factors	influence factors	influence factor	influence factors	2	singular
INFLUENZA	influenza	influenza	influenza	influenza	2	case_or_format
influenza A virus	influenza a virus	influenza a virus	influenza a virus	Influenza A virus	2	case_or_format
Influenza virus A	influenza virus a	influenza virus a	influenza virus a	Influenza virus A	2	kept
Influenza virus B	influenza virus b	influenza virus b	influenza virus b	Influenza virus B	2	kept
informal care	informal care	informal care	informal care	informal care	2	kept

informal payment	informal payment	informal payment	informal payment	informal payment	2	kept
Informant Questionnaire on [illegible]	informant questionnaire on [illegible]	informant questionnaire on [illegible]	informant questionnaire on [illegible]	Informant Questionnaire on [illegible]	2	kept
informatician	informatician	informatician	informatician	informatician	2	kept
Information and Communication [illegible]	information and communication [illegible]	information and communication [illegible]	information and communication [illegible]	Information and Communication [illegible]	2	kept
Information Display	information display	information display	information display	Information Display	2	kept
Information display	information display	information display	information display	Information Display	2	case_or_format
Information exchanges	information exchanges	information exchanges	information exchange	Information exchange	2	singular
information gap	information gap	information gap	information gap	information gap	2	kept
Information requirement	information requirement	information requirement	information requirement	Information requirement	2	kept
Information services	information services	information services	information service	Information Services	2	singular
information sharing	information sharing	information sharing	information sharing	information sharing	2	kept
Information to family	information to family	information to family	information to family	Information to family	2	kept
Information transfer	information transfer	information transfer	information transfer	Information transfer	2	kept
information transfer	information transfer	information transfer	information transfer	Information transfer	2	case_or_format
Infrared devices	infrared devices	infrared devices	infrared devices	Infrared devices	2	kept
infrared spectroscopy	infrared spectroscopy	infrared spectroscopy	infrared spectroscopy	infrared spectroscopy	2	kept

Infusions, Subcutaneous	infusions subcutaneous	infusions subcutaneous	infusions subcutaneous	Infusions, Subcutaneous	2	kept
inguinal hernia	inguinal hernia	inguinal hernia	inguinal hernia	inguinal hernia	2	kept
inhalant abuse	inhalant abuse	inhalant abuse	inhalant abuse	inhalant abuse	2	kept
Inhalation Exposure	inhalation exposure	inhalation exposure	inhalation exposure	Inhalation Exposure	2	kept
INHALATION INJURY	inhalation injury	inhalation injury	inhalation injury	Inhalation injury	2	case_or_format
inhalation injury	inhalation injury	inhalation injury	inhalation injury	Inhalation injury	2	case_or_format
inhibition	inhibition	inhibition	inhibition	inhibition	2	kept
inhibitors	inhibitors	inhibitors	inhibitors	inhibitors	2	kept
inhibitory factors	inhibitory factors	inhibitory factors	inhibitory factors	inhibitory factors	2	kept
injectable antiseizure medication	injectable antiseizure medication	injectable antiseizure medication	injectable antiseizure medication	injectable antiseizure medication	2	kept
injection drug user	injection drug user	injection drug user	injection drug user	injection drug user	2	kept
injection duration	injection duration	injection duration	injection duration	injection duration	2	kept
injection pain	injection pain	injection pain	injection pain	injection pain	2	kept
injection site extravasation	injection site extravasation	injection site extravasation	injection site extravasation	injection site extravasation	2	kept
injection site pain	injection site pain	injection site pain	injection site pain	injection site pain	2	kept
injection site reaction	injection site reaction	injection site reaction	injection site reaction	injection site reaction	2	kept

Injections, Epidural	injections epidural	injections epidural	injections epidural	Injections, Epidural	2	kept
Injections, Intradermal	injections intradermal	injections intradermal	injections intradermal	Injections, Intradermal	2	kept
Injury prevention	injury prevention	injury prevention	injury prevention	Injury prevention	2	kept
Injury severity score	injury severity score	injury severity score	injury severity score	Injury Severity Score	2	case_or_format
innovative	innovative	innovative	innovative	innovative	2	kept
Innovative behaviour	innovative behaviour	innovative behaviour	innovative behaviour	Innovative behaviour	2	kept
inoculation	inoculation	inoculation	inoculation	inoculation	2	kept
inotropes	inotropes	inotropes	inotrope	Inotropes	2	singular
insect bite	insect bite	insect bite	insect bite	insect bite	2	kept
Insecticides	insecticides	insecticides	insecticide	Insecticides	2	singular
INSERTED CENTRAL CATHETERS	inserted central catheters	inserted central catheters	inserted central catheters	INSERTED CENTRAL CATHETERS	2	kept
INSIGHTS	insights	insights	insights	INSIGHTS	2	kept
Insomnia severity	insomnia severity	insomnia severity	insomnia severity	Insomnia severity	2	kept
Instituciones oncoló gicas	instituciones oncol gicas	instituciones oncol gicas	instituciones oncol gicas	Instituciones oncoló gicas	2	kept
Institute of Medicine	institute of medicine	institute of medicine	institute of medicine	Institute of Medicine	2	kept
institutional	institutional	institutional	institutional	institutional	2	kept

institutional case volume	institutional case volume	institutional case volume	institutional case volume	institutional case volume	2	kept
institutional climate	institutional climate	institutional climate	institutional climate	institutional climate	2	kept
Institutional ethnography	institutional ethnography	institutional ethnography	institutional ethnography	Institutional ethnography	2	kept
institutional ethnography	institutional ethnography	institutional ethnography	institutional ethnography	Institutional ethnography	2	case_or_format
Institutional models	institutional models	institutional models	institutional models	Institutional models	2	kept
institutional practice	institutional practice	institutional practice	institutional practice	Institutional Practice	2	case_or_format
INSTITUTIONS	institutions	institutions	institution	INSTITUTIONS	2	singular
instrument adaptation	instrument adaptation	instrument adaptation	instrument adaptation	instrument adaptation	2	kept
Instrument design	instrument design	instrument design	instrument design	Instrument design	2	kept
Instruments	instruments	instruments	instrument	instrument	2	singular
Insulin Aspart	insulin aspart	insulin aspart	insulin aspart	insulin aspart	2	case_or_format
Insulin aspart	insulin aspart	insulin aspart	insulin aspart	insulin aspart	2	case_or_format
insulin derivative	insulin derivative	insulin derivative	insulin derivative	insulin derivative	2	kept
insulin disposal	insulin disposal	insulin disposal	insulin disposal	insulin disposal	2	kept
Insulin Glargine	insulin glargine	insulin glargine	insulin glargine	insulin glargine	2	case_or_format
Insulin infusion systems	insulin infusion systems	insulin infusion systems	insulin infusion system	Insulin Infusion Systems	2	singular

Insulin therapy	insulin therapy	insulin therapy	insulin therapy	Insulin therapy	2	kept
insulin therapy	insulin therapy	insulin therapy	insulin therapy	Insulin therapy	2	case_or_format
Insulin, Long-Acting	insulin long acting	insulin long acting	insulin long acting	Insulin, Long-Acting	2	kept
INSURE	insure	insure	insure	INSURE	2	kept
Intcare	intcare	intcare	intcare	Intcare	2	kept
INTCare system	intcare system	intcare system	intcare system	INTCare system	2	kept
Integer Linear Programming	integer linear programming	integer linear programming	integer linear programming	Integer Linear Programming	2	kept
Integral healthcare practice	integral healthcare practice	integral healthcare practice	integral healthcare practice	Integral healthcare practice	2	kept
integrated	integrated	integrated	integrated	integrated	2	kept
integrated healthcare	integrated healthcare	integrated healthcare	integrated healthcare	integrated healthcare	2	kept
integrated management mode	integrated management mode	integrated management mode	integrated management mode	integrated management mode	2	kept
Integrated medical care	integrated medical care	integrated medical care	integrated medical care	Integrated medical care	2	kept
integrated monitoring system	integrated monitoring system	integrated monitoring system	integrated monitoring system	integrated monitoring system	2	kept
integrated palliative care	integrated palliative care	integrated palliative care	integrated palliative care	integrated palliative care	2	kept
integrated system	integrated system	integrated system	integrated system	integrated system	2	kept
Integrative care	integrative care	integrative care	integrative care	Integrative care	2	kept

Integrative health care	integrative health care	integrative health care	integrative health care	Integrative health care	2	kept
integrative literature review	integrative literature review	integrative literature review	integrative literature review	integrative literature review	2	kept
Integrative Oncology	integrative oncology	integrative oncology	integrative oncology	Integrative Oncology	2	kept
integrative oncology	integrative oncology	integrative oncology	integrative oncology	Integrative Oncology	2	case_or_format
Integrative therapies	integrative therapies	integrative therapies	integrative therapies	Integrative therapies	2	kept
Integrity	integrity	integrity	integrity	Integrity	2	kept
Inteligencia emocional	inteligencia emocional	inteligencia emocional	inteligencia emocional	Inteligencia emocional	2	kept
intelligence test	intelligence test	intelligence test	intelligence test	intelligence test	2	kept
Intelligence Tests	intelligence tests	intelligence tests	intelligence test	intelligence test	2	singular
Intelligent alarms	intelligent alarms	intelligent alarms	intelligent alarms	Intelligent alarms	2	kept
Intelligent control	intelligent control	intelligent control	intelligent control	Intelligent control	2	kept
Intelligent decision support systems	intelligent decision support systems	intelligent decision support systems	intelligent decision support systems	Intelligent decision support systems	2	kept
Intelligent robot	intelligent robot	intelligent robot	intelligent robot	Intelligent robot	2	kept
Intensity of treatment	intensity of treatment	intensity of treatment	intensity of treatment	Intensity of treatment	2	kept
Intensive Cardiac Care Unit	intensive cardiac care unit	intensive cardiac care unit	intensive cardiac care unit	Intensive Cardiac Care Unit	2	kept
Intensive insulin therapies	intensive insulin therapies	intensive insulin therapies	intensive insulin therapy	Intensive insulin therapy	2	singular

intensive neonatal care	intensive neonatal care	intensive neonatal care	intensive neonatal care	intensive neonatal care	2	kept
Intensive therapy units	intensive therapy units	intensive therapy units	intensive therapy unit	Intensive therapy units	2	singular
INTENTION	intention	intention	intention	Intention	2	case_or_format
Intention to leave career	intention to leave career	intention to leave career	intention to leave career	Intention to leave career	2	kept
Intention to quit	intention to quit	intention to quit	intention to quit	Intention to quit	2	kept
Intention to stay	intention to stay	intention to stay	intention to stay	Intention to stay	2	kept
Inter quartile ranges	inter quartile ranges	inter quartile ranges	inter quartile ranges	Inter quartile ranges	2	kept
inter-hospital transport	inter hospital transport	inter hospital transport	inter hospital transport	inter-hospital transport	2	kept
Inter-observer variability	inter observer variability	inter observer variability	inter observer variability	Inter-observer variability	2	kept
inter-rater variability	inter rater variability	inter rater variability	inter rater variability	inter-rater variability	2	kept
Interaction design	interaction design	interaction design	interaction design	Interaction design	2	kept
Interactional justice	interactional justice	interactional justice	interactional justice	Interactional justice	2	kept
interactional psychology	interactional psychology	interactional psychology	interactional psychology	interactional psychology	2	kept
Interactive computer systems	interactive computer systems	interactive computer systems	interactive computer systems	Interactive computer systems	2	kept
interactive display	interactive display	interactive display	interactive display	interactive display	2	kept
Interactive learning	interactive learning	interactive learning	interactive learning	Interactive learning	2	kept

interactive learning	interactive learning	interactive learning	interactive learning	Interactive learning	2	case_or_format
Interactive ventilatory support	interactive ventilatory support	interactive ventilatory support	interactive ventilatory support	Interactive Ventilatory Support	2	case_or_format
interactive voice response system	interactive voice response system	interactive voice response system	interactive voice response system	interactive voice response system	2	kept
interatrial septum	interatrial septum	interatrial septum	interatrial septum	interatrial septum	2	kept
intercellular adhesion molecule 1	intercellular adhesion molecule 1	intercellular adhesion molecule 1	intercellular adhesion molecule 1	intercellular adhesion molecule 1	2	kept
intercostal nerve	intercostal nerve	intercostal nerve	intercostal nerve	intercostal nerve	2	kept
intercostal nerve block	intercostal nerve block	intercostal nerve block	intercostal nerve block	intercostal nerve block	2	kept
INTERDEPENDENCE	interdependence	interdependence	interdependence	INTERDEPENDENCE	2	kept
Interdisciplinarity	interdisciplinarity	interdisciplinarity	interdisciplinarity	Interdisciplinarity	2	kept
Interdisciplinary cooperation	interdisciplinary cooperation	interdisciplinary cooperation	interdisciplinary cooperation	Interdisciplinary cooperation	2	kept
Interdisciplinary Placement	interdisciplinary placement	interdisciplinary placement	interdisciplinary placement	Interdisciplinary Placement	2	kept
interdisciplinary rounds	interdisciplinary rounds	interdisciplinary rounds	interdisciplinary rounds	interdisciplinary rounds	2	kept
interface pressure	interface pressure	interface pressure	interface pressure	Interface pressure	2	case_or_format
interfacility	interfacility	interfacility	interfacility	interfacility	2	kept
Interference	interference	interference	interference	Interference	2	kept
Interferon-gamma	interferon gamma	interferon gamma	interferon gamma	Interferon-gamma	2	kept

interhospital transfers	interhospital transfers	interhospital transfers	interhospital transfer	Interhospital transfer	2	singular
INTERHOSPITAL TRANSPORT	interhospital transport	interhospital transport	interhospital transport	Interhospital transport	2	case_or_format
interior design	interior design	interior design	interior design	interior design	2	kept
interleukin 15	interleukin 15	interleukin 15	interleukin 15	interleukin 15	2	kept
interleukin 4	interleukin 4	interleukin 4	interleukin 4	interleukin 4	2	kept
interleukin 6 receptor	interleukin 6 receptor	interleukin 6 receptor	interleukin 6 receptor	interleukin 6 receptor	2	kept
intermediate care units	intermediate care units	intermediate care units	intermediate care unit	intermediate care unit	2	singular
intermediate respiratory care unit	intermediate respiratory care unit	intermediate respiratory care unit	intermediate respiratory care unit	intermediate respiratory care unit	2	kept
intermediate risk population	intermediate risk population	intermediate risk population	intermediate risk population	intermediate risk population	2	kept
INTERMITTENT	intermittent	intermittent	intermittent	INTERMITTENT	2	kept
Intermittent Claudication	intermittent claudication	intermittent claudication	intermittent claudication	Intermittent Claudication	2	kept
intermittent claudication	intermittent claudication	intermittent claudication	intermittent claudication	Intermittent Claudication	2	case_or_format
Intermittent Renal Replacement Therapy	intermittent renal replacement therapy	intermittent renal replacement therapy	intermittent renal replacement therapy	intermittent renal replacement therapy	2	case_or_format
internal carotid artery occlusion	internal carotid artery occlusion	internal carotid artery occlusion	internal carotid artery occlusion	internal carotid artery occlusion	2	kept
Internal Fixators	internal fixators	internal fixators	internal fixator	Internal Fixators	2	singular
internal medicine unit	internal medicine unit	internal medicine unit	internal medicine unit	internal medicine unit	2	kept

internal transcribed spacer	internal transcribed spacer	internal transcribed spacer	internal transcribed spacer	internal transcribed spacer	2	kept
INTERNATIONAL CONSENSUS DEFINITIONS	international consensus definitions	international consensus definitions	international consensus definitions	INTERNATIONAL CONSENSUS DEFINITIONS	2	kept
international health services	international health services	international health services	international health services	international health services	2	kept
International Issues	international issues	international issues	international issues	International Issues	2	kept
International standards	international standards	international standards	international standard	International standards	2	singular
internet	internet	internet	internet	Internet	2	case_or_format
internet literacy	internet literacy	internet literacy	internet literacy	internet literacy	2	kept
Internet of Things devices	internet of things devices	internet of things devices	internet of things devices	Internet of Things devices	2	kept
Internship and residency	internship and residency	internship and residency	internship and residency	Internship and Residency	2	case_or_format
Internship year	internship year	internship year	internship year	Internship year	2	kept
Interpersonal communication	interpersonal communication	interpersonal communication	interpersonal communication	interpersonal communication	2	case_or_format
interpersonal dynamics	interpersonal dynamics	interpersonal dynamics	interpersonal dynamics	interpersonal dynamics	2	kept
interpersonal relationship	interpersonal relationship	interpersonal relationship	interpersonal relationship	interpersonal relationship	2	kept
interpersonal relationships	interpersonal relationships	interpersonal relationships	interpersonal relationship	interpersonal relationship	2	singular
Interpretability	interpretability	interpretability	interpretability	Interpretability	2	kept
Interpretation	interpretation	interpretation	interpretation	interpretation	2	case_or_format

interpretive research	interpretive research	interpretive research	interpretive research	interpretive research	2	kept
INTERPROFESSIONAL COLLABORATION	interprofessional collaboration	interprofessional collaboration	interprofessional collaboration	interprofessional collaboration	2	case_or_format
Interprofessional relationships	interprofessional relationships	interprofessional relationships	interprofessional relationship	Interprofessional relationships	2	singular
interprofessional simulation	interprofessional simulation	interprofessional simulation	interprofessional simulation	interprofessional simulation	2	kept
interprofessional teamwork	interprofessional teamwork	interprofessional teamwork	interprofessional teamwork	Interprofessional teamwork	2	case_or_format
Interrupted time series	interrupted time series	interrupted time series	interrupted time series	Interrupted time series	2	kept
Interrupted time series analysis	interrupted time series analysis	interrupted time series analysis	interrupted time series analysis	Interrupted Time Series Analysis	2	case_or_format
interruption events	interruption events	interruption events	interruption events	interruption events	2	kept
Interruption management	interruption management	interruption management	interruption management	Interruption management	2	kept
intershift recovery	intershift recovery	intershift recovery	intershift recovery	intershift recovery	2	kept
Interstitial	interstitial	interstitial	interstitial	Interstitial	2	kept
Intertrigo	intertrigo	intertrigo	intertrigo	intertrigo	2	case_or_format
intertrochanteric femoral fracture	intertrochanteric femoral fracture	intertrochanteric femoral fracture	intertrochanteric femoral fracture	intertrochanteric femoral fracture	2	kept
intertrochanteric fracture	intertrochanteric fracture	intertrochanteric fracture	intertrochanteric fracture	intertrochanteric fracture	2	kept
Intervenciones no farmacológicas	intervenciones no farmacol gicas	intervenciones no farmacol gicas	intervenciones no farmacol gicas	Intervenciones no farmacológicas	2	kept
Intervention implementation	intervention implementation	intervention implementation	intervention implementation	Intervention implementation	2	kept

Intervention measures	intervention measures	intervention measures	intervention measures	Intervention measures	2	kept
INTERVENTION SCORING SYSTEM	intervention scoring system	intervention scoring system	intervention scoring system	INTERVENTION SCORING SYSTEM	2	kept
Intervention studies	intervention studies	intervention studies	intervention study	intervention study	2	singular
Interventional	interventional	interventional	interventional	Interventional	2	kept
interventional pulmonology	interventional pulmonology	interventional pulmonology	interventional pulmonology	interventional pulmonology	2	kept
Interventional study	interventional study	interventional study	interventional study	Interventional study	2	kept
INTERVIEW	interview	interview	interview	interview	2	case_or_format
Intestinal Failure	intestinal failure	intestinal failure	intestinal failure	intestinal failure	2	case_or_format
intestinal intussusception	intestinal intussusception	intestinal intussusception	intestinal intussusception	intestinal intussusception	2	kept
Intestinal Mucosa	intestinal mucosa	intestinal mucosa	intestinal mucosa	Intestinal Mucosa	2	kept
Intestinal obstruction	intestinal obstruction	intestinal obstruction	intestinal obstruction	Intestinal Obstruction	2	case_or_format
intestine duplication	intestine duplication	intestine duplication	intestine duplication	intestine duplication	2	kept
intestine injury	intestine injury	intestine injury	intestine injury	intestine injury	2	kept
Intoxication	intoxication	intoxication	intoxication	intoxication	2	case_or_format
intra-amniotic infection	intra amniotic infection	intra amniotic infection	intra amniotic infection	intra-amniotic infection	2	kept
Intra-aortic balloon	intra aortic balloon	intra aortic balloon	intra aortic balloon	Intra-aortic balloon	2	kept

Intra-Aortic Balloon Pump	intra aortic balloon pump	intra aortic balloon pump	intra aortic balloon pump	Intra-Aortic Balloon Pump	2	kept
intra-aortic balloon pump	intra aortic balloon pump	intra aortic balloon pump	intra aortic balloon pump	Intra-Aortic Balloon Pump	2	case_or_format
Intra-hospital transport	intra hospital transport	intra hospital transport	intra hospital transport	intra-hospital transport	2	case_or_format
Intraabdominal Infections	intraabdominal infections	intraabdominal infections	intraabdominal infections	Intraabdominal Infections	2	kept
intracardiac echocardiography	intracardiac echocardiography	intracardiac echocardiography	intracardiac echocardiography	intracardiac echocardiography	2	kept
intracardiac thrombosis	intracardiac thrombosis	intracardiac thrombosis	intracardiac thrombosis	intracardiac thrombosis	2	kept
Intracellular Fluid	intracellular fluid	intracellular fluid	intracellular fluid	Intracellular Fluid	2	kept
intracellular fluid	intracellular fluid	intracellular fluid	intracellular fluid	Intracellular Fluid	2	case_or_format
Intracerebral haemorrhage	intracerebral haemorrhage	intracerebral haemorrhage	intracerebral haemorrhage	Intracerebral haemorrhage	2	kept
Intracranial aneurysm	intracranial aneurysm	intracranial aneurysm	intracranial aneurysm	Intracranial Aneurysm	2	case_or_format
Intracranial pressure monitoring	intracranial pressure monitoring	intracranial pressure monitoring	intracranial pressure monitoring	intracranial pressure monitoring	2	case_or_format
Intracranial Thrombosis	intracranial thrombosis	intracranial thrombosis	intracranial thrombosis	Intracranial Thrombosis	2	kept
intractable epilepsy	intractable epilepsy	intractable epilepsy	intractable epilepsy	intractable epilepsy	2	kept
intractable pain	intractable pain	intractable pain	intractable pain	Intractable Pain	2	case_or_format
intrahepatic cholestasis of pregnancy	intrahepatic cholestasis of pregnancy	intrahepatic cholestasis of pregnancy	intrahepatic cholestasis of pregnancy	intrahepatic cholestasis of pregnancy	2	kept
intrahospital transfer	intrahospital transfer	intrahospital transfer	intrahospital transfer	intrahospital transfer	2	kept

intramedullary nail	intramedullary nail	intramedullary nail	intramedullary nail	intramedullary nail	2	kept
INTRANASAL MUPIROCIN	intranasal mupirocin	intranasal mupirocin	intranasal mupirocin	INTRANASAL MUPIROCIN	2	kept
intraocular pressure	intraocular pressure	intraocular pressure	intraocular pressure	intraocular pressure	2	kept
Intraoperative management	intraoperative management	intraoperative management	intraoperative management	Intraoperative management	2	kept
Intraoperative monitoring	intraoperative monitoring	intraoperative monitoring	intraoperative monitoring	intraoperative monitoring	2	case_or_format
Intraoperative Neurophysiological Monitoring	intraoperative neurophysiological monitoring	intraoperative neurophysiological monitoring	intraoperative neurophysiological monitoring	Intraoperative Neurophysiological Monitoring	2	kept
intraoperative neurophysiological monitoring	intraoperative neurophysiological monitoring	intraoperative neurophysiological monitoring	intraoperative neurophysiological monitoring	Intraoperative Neurophysiological Monitoring	2	case_or_format
Intraoperative Period	intraoperative period	intraoperative period	intraoperative period	intraoperative period	2	case_or_format
intraoperatively acquired pressure injuries	intraoperatively acquired pressure injuries	intraoperatively acquired pressure injuries	intraoperatively acquired pressure injuries	intraoperatively acquired pressure injuries	2	kept
intraosseous drug administration	intraosseous drug administration	intraosseous drug administration	intraosseous drug administration	intraosseous drug administration	2	kept
intraosseous infusion	intraosseous infusion	intraosseous infusion	intraosseous infusion	intraosseous infusion	2	kept
intrapartum	intrapartum	intrapartum	intrapartum	intrapartum	2	kept
Intratracheal intubation	intratracheal intubation	intratracheal intubation	intratracheal intubation	Intratracheal intubation	2	kept
intratracheal intubation	intratracheal intubation	intratracheal intubation	intratracheal intubation	Intratracheal intubation	2	case_or_format
intrauterine growth retardation, mortality rate	intrauterine growth retardation mortality rate	intrauterine growth retardation mortality rate	intrauterine growth retardation mortality rate	intrauterine growth retardation, mortality rate	2	kept
Intravenous access	intravenous access	intravenous access	intravenous access	Intravenous access	2	kept

Intravenous Administration	intravenous administration	intravenous administration	intravenous administration	intravenous administration	2	case_or_format
Intravenous administration	intravenous administration	intravenous administration	intravenous administration	intravenous administration	2	case_or_format
intravenous bolus	intravenous bolus	intravenous bolus	intravenous bolus	intravenous bolus	2	kept
Intravenous Drug Administration	intravenous drug administration	intravenous drug administration	intravenous drug administration	intravenous drug administration	2	case_or_format
Intravenous fluid	intravenous fluid	intravenous fluid	intravenous fluid	Intravenous fluid	2	kept
Intravenous fluids	intravenous fluids	intravenous fluids	intravenous fluid	Intravenous fluid	2	singular
Intravenous immunoglobulin	intravenous immunoglobulin	intravenous immunoglobulin	intravenous immunoglobulin	Intravenous immunoglobulin	2	kept
intravenous injection	intravenous injection	intravenous injection	intravenous injection	intravenous injection	2	kept
intravenous levetiracetam	intravenous levetiracetam	intravenous levetiracetam	intravenous levetiracetam	intravenous levetiracetam	2	kept
INTRAVENOUS MEDICATION	intravenous medication	intravenous medication	intravenous medication	INTRAVENOUS MEDICATION	2	kept
intravenous medication	intravenous medication	intravenous medication	intravenous medication	INTRAVENOUS MEDICATION	2	case_or_format
intravenous medication errors	intravenous medication errors	intravenous medication errors	intravenous medication errors	intravenous medication errors	2	kept
Intravenous system	intravenous system	intravenous system	intravenous system	Intravenous system	2	kept
INTRAVENOUS THERAPY TEAM	intravenous therapy team	intravenous therapy team	intravenous therapy team	INTRAVENOUS THERAPY TEAM	2	kept
intravenous treatment safety	intravenous treatment safety	intravenous treatment safety	intravenous treatment safety	intravenous treatment safety	2	kept
intravenous tubing	intravenous tubing	intravenous tubing	intravenous tubing	intravenous tubing	2	kept

intrinsic influences	intrinsic influences	intrinsic influences	intrinsic influences	intrinsic influences	2	kept
Introduction	introduction	introduction	introduction	Introduction	2	kept
intron	intron	intron	intron	intron	2	kept
introversion	introversion	introversion	introversion	introversion	2	kept
Intubated	intubated	intubated	intubated	Intubated	2	kept
intubated	intubated	intubated	intubated	Intubated	2	case_or_format
intubated children	intubated children	intubated children	intubated children	intubated children	2	kept
intubation attempt	intubation attempt	intubation attempt	intubation attempt	intubation attempt	2	kept
intubation gastrointestinal	intubation gastrointestinal	intubation gastrointestinal	intubation gastrointestinal	Intubation, Gastrointestinal	2	case_or_format
Intubation, gastrointestinal	intubation gastrointestinal	intubation gastrointestinal	intubation gastrointestinal	Intubation, Gastrointestinal	2	case_or_format
Intuitive	intuitive	intuitive	intuitive	Intuitive	2	kept
Invariance	invariance	invariance	invariance	Invariance	2	kept
Invasive	invasive	invasive	invasive	Invasive	2	kept
Invasive blood pressure monitoring	invasive blood pressure monitoring	invasive blood pressure monitoring	invasive blood pressure monitoring	Invasive blood pressure monitoring	2	kept
Invasive E. coli disease	invasive e coli disease	invasive e coli disease	invasive e coli disease	Invasive E. coli disease	2	kept
Invasive fungal infection	invasive fungal infection	invasive fungal infection	invasive fungal infection	Invasive Fungal Infections	2	case_or_format

invasive monitoring	invasive monitoring	invasive monitoring	invasive monitoring	invasive monitoring	2	kept
Invasive pneumococcal infection	invasive pneumococcal infection	invasive pneumococcal infection	invasive pneumococcal infection	Invasive pneumococcal infection	2	kept
invasive Prozeduren	invasive prozeduren	invasive prozeduren	invasive prozeduren	invasive Prozeduren	2	kept
Invasive surgery	invasive surgery	invasive surgery	invasive surgery	Invasive surgery	2	kept
Invasive ventilation, lung protection	invasive ventilation lung protection	invasive ventilation lung protection	invasive ventilation lung protection	Invasive ventilation, lung protection	2	kept
INVENTORY	inventory	inventory	inventory	INVENTORY	2	kept
inventory time	inventory time	inventory time	inventory time	inventory time	2	kept
Investments	investments	investments	investment	investment	2	singular
IoT device	iot device	iot device	iot device	IoT device	2	kept
IPAQ	ipaq	ipaq	ipaq	IPAQ	2	kept
IPC	ipc	ipc	ipc	IPC	2	kept
IPC core components	ipc core components	ipc core components	ipc core components	IPC core components	2	kept
Ipecac	ipecac	ipecac	ipecac	Ipecac	2	kept
ipratropium bromide plus salbutamol	ipratropium bromide plus salbutamol	ipratropium bromide plus salbutamol	ipratropium bromide plus salbutamol	ipratropium bromide plus salbutamol	2	kept
IPS	ips	ips	ips	IPS	2	kept
iron blood level	iron blood level	iron blood level	iron blood level	iron blood level	2	kept

Irritable Mood	irritable mood	irritable mood	irritable mood	Irritable Mood	2	kept
irritant agent	irritant agent	irritant agent	irritant agent	irritant agent	2	kept
ischemic colitis	ischemic colitis	ischemic colitis	ischemic colitis	ischemic colitis	2	kept
Ischemic disease	ischemic disease	ischemic disease	ischemic disease	Ischemic disease	2	kept
ISO Standards	iso standards	iso standards	iso standards	ISO Standards	2	kept
isoelectric focusing	isoelectric focusing	isoelectric focusing	isoelectric focusing	isoelectric focusing	2	kept
Isoflurano	isoflurano	isoflurano	isoflurano	Isoflurano	2	kept
isolation and standard precautions	isolation and standard precautions	isolation and standard precautions	isolation and standard precautions	isolation and standard precautions	2	kept
Isolation education	isolation education	isolation education	isolation education	Isolation education	2	kept
ISOLATION PRECAUTIONS	isolation precautions	isolation precautions	isolation precaution	ISOLATION PRECAUTIONS	2	singular
Isolation precautions	isolation precautions	isolation precautions	isolation precaution	ISOLATION PRECAUTIONS	2	singular
isolation precautions	isolation precautions	isolation precautions	isolation precaution	ISOLATION PRECAUTIONS	2	singular
Isolation wards	isolation wards	isolation wards	isolation ward	Isolation wards	2	singular
Isoniazid	isoniazid	isoniazid	isoniazid	isoniazid	2	case_or_format
isotretinoin	isotretinoin	isotretinoin	isotretinoin	isotretinoin	2	kept
Isovist	isovist	isovist	isovist	Isovist	2	kept

ispagula	ispagula	ispagula	ispagula	ispagula	2	kept
Item response theory	item response theory	item response theory	item response theory	Item response theory	2	kept
item total correlation	item total correlation	item total correlation	item total correlation	item total correlation	2	kept
IV escalation pathway	iv escalation pathway	iv escalation pathway	iv escalation pathway	IV escalation pathway	2	kept
IV infusion	iv infusion	iv infusion	iv infusion	IV infusion	2	kept
IV smart pump	iv smart pump	iv smart pump	iv smart pump	IV smart pump	2	kept
IV, intravenous	iv intravenous	iv intravenous	iv intravenous	IV, intravenous	2	kept
IVC filter placement	ivc filter placement	ivc filter placement	ivc filter placement	IVC filter placement	2	kept
ivermectin	ivermectin	ivermectin	ivermectin	ivermectin	2	kept
Ixodes holocyclus	ixodes holocyclus	ixodes holocyclus	ixodes holocyclus	Ixodes holocyclus	2	kept
Jails	jails	jails	jails	Jails	2	kept
JAPAN	japan	japan	japan	Japan	2	case_or_format
Japanese version	japanese version	japanese version	japanese version	Japanese version	2	kept
JCI	jci	jci	jci	JCI	2	kept
Jefferson Scale of Physician Empathy	jefferson scale of physician empathy	jefferson scale of physician empathy	jefferson scale of physician empathy	Jefferson Scale of Physician Empathy	2	kept
Jeffries' simulation model	jeffries simulation model	jeffries simulation model	jeffries simulation model	Jeffries' simulation model	2	kept

Jerk	jerk	jerk	jerk	Jerk	2	kept
jigsaw	jigsaw	jigsaw	jigsaw	jigsaw	2	kept
Job	job	job	job	Job	2	kept
Job burnout	job burnout	job burnout	job burnout	job burnout	2	case_or_format
Job conflict	job conflict	job conflict	job conflict	Job conflict	2	kept
Job crafting	job crafting	job crafting	job crafting	Job crafting	2	kept
job crafting	job crafting	job crafting	job crafting	Job crafting	2	case_or_format
job demands-resources	job demands resources	job demands resources	job demands resources	job demands-resources	2	kept
Job demands‐resources model	job demands resources model	job demands resources model	job demands resources model	Job demands‐resources model	2	kept
job dissatisfaction	job dissatisfaction	job dissatisfaction	job dissatisfaction	job dissatisfaction	2	kept
job embeddedness	job embeddedness	job embeddedness	job embeddedness	job embeddedness	2	kept
JOB OUTCOMES	job outcomes	job outcomes	job outcomes	JOB OUTCOMES	2	kept
Job strain	job strain	job strain	job strain	Job strain	2	kept
job strain	job strain	job strain	job strain	Job strain	2	case_or_format
Job Stress	job stress	job stress	job stress	job stress	2	case_or_format
Job stressors	job stressors	job stressors	job stressors	Job stressors	2	kept

Job turnover intention	job turnover intention	job turnover intention	job turnover intention	Job turnover intention	2	kept
Johnson's model	johnson s model	johnson s model	johnson s model	Johnson's model	2	kept
joint characteristics and functions	joint characteristics and functions	joint characteristics and functions	joint characteristics and functions	joint characteristics and functions	2	kept
Joint flexibility	joint flexibility	joint flexibility	joint flexibility	Joint flexibility	2	kept
Joint Prosthesis	joint prosthesis	joint prosthesis	joint prosthesis	Joint Prosthesis	2	kept
joint prosthesis	joint prosthesis	joint prosthesis	joint prosthesis	Joint Prosthesis	2	case_or_format
joint stiffness	joint stiffness	joint stiffness	joint stiffness	joint stiffness	2	kept
Joints	joints	joints	joint	joint	2	singular
Jordanian healthcare	jordanian healthcare	jordanian healthcare	jordanian healthcare	Jordanian healthcare	2	kept
journal	journal	journal	journal	journal	2	kept
Journal Impact Factor	journal impact factor	journal impact factor	journal impact factor	Journal Impact Factor	2	kept
journal impact factor	journal impact factor	journal impact factor	journal impact factor	Journal Impact Factor	2	case_or_format
July Effect	july effect	july effect	july effect	July Effect	2	kept
JUNIOR DOCTORS	junior doctors	junior doctors	junior doctor	JUNIOR DOCTORS	2	singular
JUST-IN-TIME	just in time	just in time	just in time	JUST-IN-TIME	2	kept
Justice	justice	justice	justice	justice	2	case_or_format

juvenile	juvenile	juvenile	juvenile	juvenile	2	kept
juvenile systemic sclerosis	juvenile systemic sclerosis	juvenile systemic sclerosis	juvenile systemic sclerosis	juvenile systemic sclerosis	2	kept
k means clustering	k means clustering	k means clustering	k means clustering	k means clustering	2	kept
k nearest neighbor	k nearest neighbor	k nearest neighbor	k nearest neighbor	k nearest neighbor	2	kept
Kaiser Permanente early onset sepsis calculator	kaiser permanente early onset sepsis calculator	kaiser permanente early onset sepsis calculator	kaiser permanente early onset sepsis calculator	Kaiser Permanente early onset sepsis calculator	2	kept
KANGAROO CARE	kangaroo care	kangaroo care	kangaroo care	kangaroo care	2	case_or_format
KANGAROO MOTHER CARE	kangaroo mother care	kangaroo mother care	kangaroo mother care	Kangaroo mother care	2	case_or_format
Kangaroo mother care method	kangaroo mother care method	kangaroo mother care method	kangaroo mother care method	Kangaroo-Mother Care Method	2	case_or_format
kangaroo-mother care method	kangaroo mother care method	kangaroo mother care method	kangaroo mother care method	Kangaroo-Mother Care Method	2	case_or_format
karyotype	karyotype	karyotype	karyotype	karyotype	2	kept
Kasabach Merritt syndrome	kasabach merritt syndrome	kasabach merritt syndrome	kasabach merritt syndrome	Kasabach Merritt syndrome	2	kept
KASRP	kasrp	kasrp	kasrp	KASRP	2	kept
Kaze features	kaze features	kaze features	kaze features	Kaze features	2	kept
Keepsake	keepsake	keepsake	keepsake	Keepsake	2	kept
keepsake	keepsake	keepsake	keepsake	Keepsake	2	case_or_format
Kendall plot	kendall plot	kendall plot	kendall plot	Kendall plot	2	kept

Kennedy terminal ulcer	kennedy terminal ulcer	kennedy terminal ulcer	kennedy terminal ulcer	Kennedy terminal ulcer	2	kept
keratoconjunctivitis	keratoconjunctivitis	keratoconjunctivitis	keratoconjunctivitis	Keratoconjunctivitis	2	case_or_format
Kerman	kerman	kerman	kerman	Kerman	2	kept
kernicterus	kernicterus	kernicterus	kernicterus	Kernicterus	2	case_or_format
Kessler Psychological Distress Scale	kessler psychological distress scale	kessler psychological distress scale	kessler psychological distress scale	Kessler Psychological Distress Scale	2	kept
Ketogenic diet	ketogenic diet	ketogenic diet	ketogenic diet	ketogenic diet	2	case_or_format
Ketones	ketones	ketones	ketone	ketone	2	singular
ketonuria	ketonuria	ketonuria	ketonuria	ketonuria	2	kept
ketotifen	ketotifen	ketotifen	ketotifen	ketotifen	2	kept
key performance indicator	key performance indicator	key performance indicator	key performance indicator	key performance indicator	2	kept
Kidney Calculi	kidney calculi	kidney calculi	kidney calculi	Kidney Calculi	2	kept
Kidney cancer	kidney cancer	kidney cancer	kidney cancer	kidney cancer	2	case_or_format
kidney infarction	kidney infarction	kidney infarction	kidney infarction	kidney infarction	2	kept
Kidney Neoplasms	kidney neoplasms	kidney neoplasms	kidney neoplasms	Kidney Neoplasms	2	kept
kidney perfusion	kidney perfusion	kidney perfusion	kidney perfusion	kidney perfusion	2	kept
kidney polycystic disease	kidney polycystic disease	kidney polycystic disease	kidney polycystic disease	kidney polycystic disease	2	kept

kidney replacement therapy	kidney replacement therapy	kidney replacement therapy	kidney replacement therapy	kidney replacement therapy	2	kept
Kidney Tubular Necrosis, Acute	kidney tubular necrosis acute	kidney tubular necrosis acute	kidney tubular necrosis acute	Kidney Tubular Necrosis, Acute	2	kept
Killer Cells, Lymphokine-Activated	killer cells lymphokine activated	killer cells lymphokine activated	killer cells lymphokine activated	Killer Cells, Lymphokine-Activated	2	kept
kinesthesia	kinesthesia	kinesthesia	kinesthesia	kinesthesia	2	kept
kinesthetic	kinesthetic	kinesthetic	kinesthetic	kinesthetic	2	kept
Kinetic therapy	kinetic therapy	kinetic therapy	kinetic therapy	Kinetic therapy	2	kept
Kinetics	kinetics	kinetics	kinetics	Kinetics	2	kept
Kingdom of Saudi Arabia	kingdom of saudi arabia	kingdom of saudi arabia	kingdom of saudi arabia	Kingdom of Saudi Arabia	2	kept
kinship care	kinship care	kinship care	kinship care	kinship care	2	kept
Kirkpatrick	kirkpatrick	kirkpatrick	kirkpatrick	Kirkpatrick	2	kept
Klebsiella Pneumoniae	klebsiella pneumoniae	klebsiella pneumoniae	klebsiella pneumoniae	Klebsiella pneumoniae	2	case_or_format
KMC	kmc	kmc	kmc	KMC	2	kept
Knee	knee	knee	knee	knee	2	case_or_format
knee function	knee function	knee function	knee function	knee function	2	kept
knee injury	knee injury	knee injury	knee injury	knee injury	2	kept
knowledge and attitude	knowledge and attitude	knowledge and attitude	knowledge and attitude	knowledge and attitude	2	kept

Knowledge and attitudes	knowledge and attitudes	knowledge and attitudes	knowledge and attitude	knowledge and attitude	2	singular
knowledge and skills acquisition	knowledge and skills acquisition	knowledge and skills acquisition	knowledge and skills acquisition	knowledge and skills acquisition	2	kept
Knowledge assessment	knowledge assessment	knowledge assessment	knowledge assessment	Knowledge assessment	2	kept
Knowledge Attitudes	knowledge attitudes	knowledge attitudes	knowledge attitudes	Knowledge Attitudes	2	kept
knowledge attitudes and practices	knowledge attitudes and practices	knowledge attitudes and practices	knowledge attitudes and practices	knowledge attitudes and practices	2	kept
Knowledge based systems	knowledge based systems	knowledge based systems	knowledge based systems	Knowledge based systems	2	kept
Knowledge level of delirium	knowledge level of delirium	knowledge level of delirium	knowledge level of delirium	Knowledge level of delirium	2	kept
Knowledge transfer	knowledge transfer	knowledge transfer	knowledge transfer	Knowledge transfer	2	kept
KNOWLEDGE TRANSLATION	knowledge translation	knowledge translation	knowledge translation	Knowledge translation	2	case_or_format
Knowledge use in pain care	knowledge use in pain care	knowledge use in pain care	knowledge use in pain care	Knowledge use in pain care	2	kept
knowledge, attitude, and practice	knowledge attitude and practice	knowledge attitude and practice	knowledge attitude and practice	knowledge, attitude, and practice	2	kept
Knowledge-attitude-practice	knowledge attitude practice	knowledge attitude practice	knowledge attitude practice	Knowledge-attitude-practice	2	kept
knowledge-attitude-practice	knowledge attitude practice	knowledge attitude practice	knowledge attitude practice	Knowledge-attitude-practice	2	case_or_format
knowledge－attitude－practice	knowledge attitude practice	knowledge attitude practice	knowledge attitude practice	Knowledge-attitude-practice	2	case_or_format
Kolcaba	kolcaba	kolcaba	kolcaba	Kolcaba	2	kept
Kyphosis	kyphosis	kyphosis	kyphosis	Kyphosis	2	kept

Labetalol	labetalol	labetalol	labetalol	labetalol	2	case_or_format
LABOR	labor	labor	labor	labor	2	case_or_format
Labor	labor	labor	labor	labor	2	case_or_format
labor analgesia	labor analgesia	labor analgesia	labor analgesia	labor analgesia	2	kept
Labor and delivery	labor and delivery	labor and delivery	labor and delivery	labor and delivery	2	case_or_format
labor management	labor management	labor management	labor management	labor management	2	kept
Labor Onset	labor onset	labor onset	labor onset	Labor Onset	2	kept
labor onset	labor onset	labor onset	labor onset	Labor Onset	2	case_or_format
Labor Pain	labor pain	labor pain	labor pain	labor pain	2	case_or_format
Labor pain	labor pain	labor pain	labor pain	labor pain	2	case_or_format
labor stage 2	labor stage 2	labor stage 2	labor stage 2	labor stage 2	2	kept
labor strike	labor strike	labor strike	labor strike	labor strike	2	kept
Labov’s model of narrative analysis	labov s model of narrative analysis	labov s model of narrative analysis	labov s model of narrative analysis	Labov’s model of narrative analysis	2	kept
LabVIEW	labview	labview	labview	LabVIEW	2	kept
LabViEW	labview	labview	labview	LabVIEW	2	case_or_format
lacrimation	lacrimation	lacrimation	lacrimation	lacrimation	2	kept

Lactams	lactams	lactams	lactams	Lactams	2	kept
Lactate	lactate	lactate	lactate	Lactates	2	case_or_format
lactate	lactate	lactate	lactate	Lactates	2	case_or_format
Lactate dehydrogenase	lactate dehydrogenase	lactate dehydrogenase	lactate dehydrogenase	lactate dehydrogenase	2	case_or_format
lactation disorder	lactation disorder	lactation disorder	lactation disorder	Lactation Disorders	2	case_or_format
Lactation support	lactation support	lactation support	lactation support	Lactation support	2	kept
Lactobacillus plantarum	lactobacillus plantarum	lactobacillus plantarum	lactobacillus plantarum	Lactobacillus plantarum	2	kept
Lactobacillus rhamnosus	lactobacillus rhamnosus	lactobacillus rhamnosus	lactobacillus rhamnosus	Lactobacillus rhamnosus	2	kept
Lactoferrin	lactoferrin	lactoferrin	lactoferrin	lactoferrin	2	case_or_format
lactogenesis	lactogenesis	lactogenesis	lactogenesis	lactogenesis	2	kept
Lactulose	lactulose	lactulose	lactulose	lactulose	2	case_or_format
lacunar infarction	lacunar infarction	lacunar infarction	lacunar infarction	lacunar infarction	2	kept
Laminar flow	laminar flow	laminar flow	laminar flow	laminar flow	2	case_or_format
laminoplasty	laminoplasty	laminoplasty	laminoplasty	laminoplasty	2	kept
lamivudine	lamivudine	lamivudine	lamivudine	lamivudine	2	kept
Lamotrigine	lamotrigine	lamotrigine	lamotrigine	lamotrigine	2	case_or_format

landscape	landscape	landscape	landscape	landscape	2	kept
language ability	language ability	language ability	language ability	language ability	2	kept
Language barriers	language barriers	language barriers	language barrier	language barriers	2	singular
language concordant care	language concordant care	language concordant care	language concordant care	language concordant care	2	kept
Language development	language development	language development	language development	language development	2	case_or_format
Language processing	language processing	language processing	language processing	language processing	2	case_or_format
language proficiency	language proficiency	language proficiency	language proficiency	language proficiency	2	kept
laninamivir	laninamivir	laninamivir	laninamivir	laninamivir	2	kept
Lanolin	lanolin	lanolin	lanolin	Lanolin	2	kept
lanolin	lanolin	lanolin	lanolin	Lanolin	2	case_or_format
laparoscopic colectomy	laparoscopic colectomy	laparoscopic colectomy	laparoscopic colectomy	laparoscopic colectomy	2	kept
large language models	large language models	large language models	large language model	large language models	2	singular
laryngitis	laryngitis	laryngitis	laryngitis	laryngitis	2	kept
laryngology	laryngology	laryngology	laryngology	laryngology	2	kept
Laryngomalacia	laryngomalacia	laryngomalacia	laryngomalacia	Laryngomalacia	2	kept
laryngopharyngeal reflux	laryngopharyngeal reflux	laryngopharyngeal reflux	laryngopharyngeal reflux	laryngopharyngeal reflux	2	kept

Laryngoplasty	laryngoplasty	laryngoplasty	laryngoplasty	Laryngoplasty	2	kept
laryngoplasty	laryngoplasty	laryngoplasty	laryngoplasty	Laryngoplasty	2	case_or_format
laryngotracheal reconstruction	laryngotracheal reconstruction	laryngotracheal reconstruction	laryngotracheal reconstruction	laryngotracheal reconstruction	2	kept
laryngotracheal stenosis	laryngotracheal stenosis	laryngotracheal stenosis	laryngotracheal stenosis	laryngotracheal stenosis	2	kept
larynx cancer	larynx cancer	larynx cancer	larynx cancer	larynx cancer	2	kept
larynx disorder	larynx disorder	larynx disorder	larynx disorder	larynx disorder	2	kept
larynx injury	larynx injury	larynx injury	larynx injury	larynx injury	2	kept
larynx stenosis	larynx stenosis	larynx stenosis	larynx stenosis	larynx stenosis	2	kept
laser surgery	laser surgery	laser surgery	laser surgery	laser surgery	2	kept
laser therapy	laser therapy	laser therapy	laser therapy	Laser Therapy	2	case_or_format
Lasso	lasso	lasso	lasso	LASSO	2	case_or_format
late life	late life	late life	late life	late life	2	kept
late onset disorder	late onset disorder	late onset disorder	late onset disorder	late onset disorder	2	kept
late preterm	late preterm	late preterm	late preterm	Late preterm	2	case_or_format
Late-onset sepsis	late onset sepsis	late onset sepsis	late onset sepsis	LATE-ONSET SEPSIS	2	case_or_format
late-onset sepsis	late onset sepsis	late onset sepsis	late onset sepsis	LATE-ONSET SEPSIS	2	case_or_format

Late-preterm	late preterm	late preterm	late preterm	Late preterm	2	case_or_format
latent growth mixture modeling	latent growth mixture modeling	latent growth mixture modeling	latent growth mixture modeling	latent growth mixture modeling	2	kept
latent profile	latent profile	latent profile	latent profile	latent profile	2	kept
Latent tuberculosis	latent tuberculosis	latent tuberculosis	latent tuberculosis	latent tuberculosis	2	case_or_format
latex	latex	latex	latex	latex	2	kept
Laughter	laughter	laughter	laughter	Laughter	2	kept
Laundry Service, Hospital	laundry service hospital	laundry service hospital	laundry service hospital	Laundry Service, Hospital	2	kept
Law	law	law	law	law	2	case_or_format
Lawsonia plant	lawsonia plant	lawsonia plant	lawsonia plant	Lawsonia plant	2	kept
laxatives	laxatives	laxatives	laxative	laxative	2	singular
Layout	layout	layout	layout	Layout	2	kept
layout	layout	layout	layout	Layout	2	case_or_format
layperson	layperson	layperson	layperson	layperson	2	kept
LEADER-MEMBER EXCHANGE	leader member exchange	leader member exchange	leader member exchange	LEADER-MEMBER EXCHANGE	2	kept
Leadership coaching behavior	leadership coaching behavior	leadership coaching behavior	leadership coaching behavior	Leadership coaching behavior	2	kept
Leadership development	leadership development	leadership development	leadership development	Leadership development	2	kept

leadership styles	leadership styles	leadership styles	leadership styles	leadership styles	2	kept
lean management	lean management	lean management	lean management	lean management	2	kept
Learning and memory	learning and memory	learning and memory	learning and memory	Learning and memory	2	kept
learning attitudes	learning attitudes	learning attitudes	learning attitudes	learning attitudes	2	kept
Learning Disorders	learning disorders	learning disorders	learning disorder	learning disorder	2	singular
Learning engagement	learning engagement	learning engagement	learning engagement	Learning engagement	2	kept
Learning health system	learning health system	learning health system	learning health system	learning health system	2	case_or_format
learning immersion	learning immersion	learning immersion	learning immersion	learning immersion	2	kept
learning model	learning model	learning model	learning model	Learning models	2	case_or_format
learning motivation	learning motivation	learning motivation	learning motivation	learning motivation	2	kept
Learning outcomes	learning outcomes	learning outcomes	learning outcome	learning outcomes	2	singular
learning platform	learning platform	learning platform	learning platform	learning platform	2	kept
Learning process	learning process	learning process	learning process	Learning process	2	kept
Learning satisfaction	learning satisfaction	learning satisfaction	learning satisfaction	Learning satisfaction	2	kept
Learning styles	learning styles	learning styles	learning style	learning style	2	singular
Least squares approximations	least squares approximations	least squares approximations	least squares approximations	Least squares approximations	2	kept

lecture method	lecture method	lecture method	lecture method	lecture method	2	kept
Lecture-based learning	lecture based learning	lecture based learning	lecture based learning	Lecture-based learning	2	kept
Leeching	leeching	leeching	leeching	Leeching	2	kept
leeching	leeching	leeching	leeching	Leeching	2	case_or_format
left coronary artery	left coronary artery	left coronary artery	left coronary artery	left coronary artery	2	kept
leg muscle	leg muscle	leg muscle	leg muscle	leg muscle	2	kept
Legal guardian	legal guardian	legal guardian	legal guardian	Legal Guardians	2	case_or_format
Legionella longbeachae	legionella longbeachae	legionella longbeachae	legionella longbeachae	Legionella longbeachae	2	kept
Legionellaceae	legionellaceae	legionellaceae	legionellaceae	Legionellaceae	2	kept
legionnaire disease	legionnaire disease	legionnaire disease	legionnaire disease	legionnaire disease	2	kept
legislation	legislation	legislation	legislation	Legislation	2	case_or_format
leiomyoma	leiomyoma	leiomyoma	leiomyoma	Leiomyoma	2	case_or_format
leiomyosarcoma	leiomyosarcoma	leiomyosarcoma	leiomyosarcoma	leiomyosarcoma	2	kept
lemborexant	lemborexant	lemborexant	lemborexant	lemborexant	2	kept
Lemierre Syndrome	lemierre syndrome	lemierre syndrome	lemierre syndrome	Lemierre Syndrome	2	kept
Lemierre syndrome	lemierre syndrome	lemierre syndrome	lemierre syndrome	Lemierre Syndrome	2	case_or_format

lemon	lemon	lemon	lemon	lemon	2	kept
lenacapavir	lenacapavir	lenacapavir	lenacapavir	lenacapavir	2	kept
lenalidomide	lenalidomide	lenalidomide	lenalidomide	lenalidomide	2	kept
length of hospital stay	length of hospital stay	length of hospital stay	length of hospital stay	Length of hospital stay	2	case_or_format
lens	lens	lens	lens	lens	2	kept
leprosy	leprosy	leprosy	leprosy	leprosy	2	kept
Leptospira	leptospira	leptospira	leptospira	Leptospira	2	kept
leptospirosis	leptospirosis	leptospirosis	leptospirosis	leptospirosis	2	kept
Lesotho	lesotho	lesotho	lesotho	Lesotho	2	kept
less invasive surfactant administration	less invasive surfactant administration	less invasive surfactant administration	less invasive surfactant administration	less invasive surfactant administration	2	kept
lessons learned	lessons learned	lessons learned	lessons learned	lessons learned	2	kept
letter inquiry	letter inquiry	letter inquiry	letter inquiry	letter inquiry	2	kept
Leukapheresis	leukapheresis	leukapheresis	leukapheresis	leukapheresis	2	case_or_format
Leukemia, Promyelocytic, Acute	leukemia promyelocytic acute	leukemia promyelocytic acute	leukemia promyelocytic acute	Leukemia, Promyelocytic, Acute	2	kept
leukocidin	leukocidin	leukocidin	leukocidin	Leukocidins	2	case_or_format
leukocyte differential count	leukocyte differential count	leukocyte differential count	leukocyte differential count	leukocyte differential count	2	kept

leukoreduction	leukoreduction	leukoreduction	leukoreduction	leukoreduction	2	kept
leukotriene	leukotriene	leukotriene	leukotriene	leukotriene	2	kept
levalbuterol	levalbuterol	levalbuterol	levalbuterol	levalbuterol	2	kept
level of knowledge	level of knowledge	level of knowledge	level of knowledge	level of knowledge	2	kept
Level of sedation	level of sedation	level of sedation	level of sedation	Level of sedation	2	kept
levels of self-concept	levels of self concept	levels of self concept	levels of self concept	levels of self-concept	2	kept
levothyroxine sodium	levothyroxine sodium	levothyroxine sodium	levothyroxine sodium	levothyroxine sodium	2	kept
liaison	liaison	liaison	liaison	liaison	2	kept
Liberation	liberation	liberation	liberation	Liberation	2	kept
Liberation bundle	liberation bundle	liberation bundle	liberation bundle	Liberation bundle	2	kept
liberation bundle	liberation bundle	liberation bundle	liberation bundle	Liberation bundle	2	case_or_format
Librarians	librarians	librarians	librarian	Librarians	2	singular
Lidocaine chloride	lidocaine chloride	lidocaine chloride	lidocaine chloride	Lidocaine chloride	2	kept
LIFE CARE	life care	life care	life care	LIFE CARE	2	kept
life change events	life change events	life change events	life change events	Life Change Events	2	case_or_format
life cycle assessment	life cycle assessment	life cycle assessment	life cycle assessment	life cycle assessment	2	kept

Life quality	life quality	life quality	life quality	Life quality	2	kept
Life satisfaction	life satisfaction	life satisfaction	life satisfaction	life satisfaction	2	case_or_format
Life supports	life supports	life supports	life support	LIFE-SUPPORT	2	singular
Life threatening conditions	life threatening conditions	life threatening conditions	life threatening condition	Life threatening conditions	2	singular
life-limiting illness	life limiting illness	life limiting illness	life limiting illness	life-limiting illness	2	kept
life-supporting treatment limitation	life supporting treatment limitation	life supporting treatment limitation	life supporting treatment limitation	life-supporting treatment limitation	2	kept
life-threatening conditions	life threatening conditions	life threatening conditions	life threatening condition	Life threatening conditions	2	singular
Life-threatening illness	life threatening illness	life threatening illness	life threatening illness	Life-threatening illness	2	kept
lifestyles	lifestyles	lifestyles	lifestyle	lifestyle	2	singular
light emitting diode	light emitting diode	light emitting diode	light emitting diode	light emitting diode	2	kept
Light environment	light environment	light environment	light environment	Light environment	2	kept
light sedation	light sedation	light sedation	light sedation	Light sedation	2	case_or_format
light-dark cycles	light dark cycles	light dark cycles	light dark cycle	light-dark cycles	2	singular
lighting	lighting	lighting	lighting	Lighting	2	case_or_format
limb pain	limb pain	limb pain	limb pain	limb pain	2	kept
Limb salvage	limb salvage	limb salvage	limb salvage	limb salvage	2	case_or_format

Liminality	liminality	liminality	liminality	Liminality	2	kept
limit of quantitation	limit of quantitation	limit of quantitation	limit of quantitation	limit of quantitation	2	kept
limitation of therapeutic effort	limitation of therapeutic effort	limitation of therapeutic effort	limitation of therapeutic effort	limitation of therapeutic effort	2	kept
limited resources	limited resources	limited resources	limited resource	limited resources	2	singular
limiting factors	limiting factors	limiting factors	limiting factors	limiting factors	2	kept
lincomycin	lincomycin	lincomycin	lincomycin	lincomycin	2	kept
line management	line management	line management	line management	line management	2	kept
line of therapy	line of therapy	line of therapy	line of therapy	line of therapy	2	kept
linoleic acid	linoleic acid	linoleic acid	linoleic acid	linoleic acid	2	kept
lip disease	lip disease	lip disease	lip disease	lip disease	2	kept
lipid fingerprinting	lipid fingerprinting	lipid fingerprinting	lipid fingerprinting	lipid fingerprinting	2	kept
lipid metabolism	lipid metabolism	lipid metabolism	lipid metabolism	lipid metabolism	2	kept
lipocalin	lipocalin	lipocalin	lipocalin	lipocalin	2	kept
Lipocalins	lipocalins	lipocalins	lipocalin	lipocalin	2	singular
lipophilicity	lipophilicity	lipophilicity	lipophilicity	lipophilicity	2	kept
Lipopolysaccharide	lipopolysaccharide	lipopolysaccharide	lipopolysaccharide	Lipopolysaccharide	2	kept

Lipoproteins	lipoproteins	lipoproteins	lipoprotein	lipoprotein	2	singular
liposome	liposome	liposome	liposome	liposome	2	kept
liquid chromatography-mass spectrometry	liquid chromatography mass spectrometry	liquid chromatography mass spectrometry	liquid chromatography mass spectrometry	liquid chromatography-mass spectrometry	2	kept
Liquid Ventilation	liquid ventilation	liquid ventilation	liquid ventilation	Liquid Ventilation	2	kept
LISA	lisa	lisa	lisa	LISA	2	kept
Listening Visits	listening visits	listening visits	listening visit	Listening Visits	2	singular
Literature reviews	literature reviews	literature reviews	literature review	literature review	2	singular
Literature studies	literature studies	literature studies	literature studies	Literature studies	2	kept
Lithotripsy	lithotripsy	lithotripsy	lithotripsy	lithotripsy	2	case_or_format
Lived Experience Advisory Group	lived experience advisory group	lived experience advisory group	lived experience advisory group	Lived Experience Advisory Group	2	kept
liver cell	liver cell	liver cell	liver cell	liver cell	2	kept
Liver Cirrhosis, Biliary	liver cirrhosis biliary	liver cirrhosis biliary	liver cirrhosis biliary	Liver Cirrhosis, Biliary	2	kept
Liver Diseases, Alcoholic	liver diseases alcoholic	liver diseases alcoholic	liver diseases alcoholic	Liver Diseases, Alcoholic	2	kept
liver donor	liver donor	liver donor	liver donor	liver donor	2	kept
liver support	liver support	liver support	liver support	liver support	2	kept
Liver Transplant	liver transplant	liver transplant	liver transplant	Liver Transplant	2	kept

Liver transplant	liver transplant	liver transplant	liver transplant	Liver Transplant	2	case_or_format
liver venoocclusive disease	liver venoocclusive disease	liver venoocclusive disease	liver venoocclusive disease	liver venoocclusive disease	2	kept
Living will	living will	living will	living will	living will	2	case_or_format
Living wills	living wills	living wills	living will	living will	2	singular
LMS	lms	lms	lms	LMS	2	kept
LOAD	load	load	load	LOAD	2	kept
Lobbying	lobbying	lobbying	lobbying	Lobbying	2	kept
Local anaesthesia	local anaesthesia	local anaesthesia	local anaesthesia	Local anaesthesia	2	kept
locked in syndrome	locked in syndrome	locked in syndrome	locked in syndrome	locked in syndrome	2	kept
Locomotion	locomotion	locomotion	locomotion	locomotion	2	case_or_format
Logistic regressions	logistic regressions	logistic regressions	logistic regression	Logistic regression	2	singular
LONG	long	long	long	LONG	2	kept
Long and short term memory neural network	long and short term memory neural network	long and short term memory neural network	long and short term memory neural network	Long and short term memory neural network	2	kept
long of stay	long of stay	long of stay	long of stay	long of stay	2	kept
long term acute care hospital	long term acute care hospital	long term acute care hospital	long term acute care hospital	long term acute care hospital	2	kept
Long term care facility	long term care facility	long term care facility	long term care facility	long-term care facilities	2	case_or_format

long term care insurance	long term care insurance	long term care insurance	long term care insurance	long term care insurance	2	kept
long-term acute care hospitals	long term acute care hospitals	long term acute care hospitals	long term acute care hospital	long term acute care hospital	2	singular
Long-term care costs	long term care costs	long term care costs	long term care costs	Long-term care costs	2	kept
Long-term care facilities	long term care facilities	long term care facilities	long term care facility	long-term care facilities	2	singular
long-term care facility	long term care facility	long term care facility	long term care facility	long-term care facilities	2	case_or_format
Long-term mechanical ventilation	long term mechanical ventilation	long term mechanical ventilation	long term mechanical ventilation	Long-term mechanical ventilation	2	kept
long-term neurological conditions	long term neurological conditions	long term neurological conditions	long term neurological conditions	long-term neurological conditions	2	kept
Long-term outcome	long term outcome	long term outcome	long term outcome	Long-term outcomes	2	case_or_format
long-term outcome	long term outcome	long term outcome	long term outcome	Long-term outcomes	2	case_or_format
long-term sequelae	long term sequelae	long term sequelae	long term sequelae	long-term sequelae	2	kept
long-term survival	long term survival	long term survival	long term survival	long term survival	2	case_or_format
Longevity	longevity	longevity	longevity	longevity	2	case_or_format
Longitudinal studies	longitudinal studies	longitudinal studies	longitudinal study	Longitudinal Studies	2	singular
longitudinal studies	longitudinal studies	longitudinal studies	longitudinal study	Longitudinal Studies	2	singular
Longitudinal study	longitudinal study	longitudinal study	longitudinal study	Longitudinal Studies	2	case_or_format
long - term care	long term care	long term care	long term care	long term care	2	case_or_format

LOS = length of stay	los length of stay	los length of stay	los length of stay	LOS = length of stay	2	kept
losartan potassium	losartan potassium	losartan potassium	losartan potassium	losartan potassium	2	kept
Loudness Perception	loudness perception	loudness perception	loudness perception	Loudness Perception	2	kept
Low and middle income countries	low and middle income countries	low and middle income countries	low and middle income country	low- and middle-income countries	2	singular
Low and middle-income countries	low and middle income countries	low and middle income countries	low and middle income country	low- and middle-income countries	2	singular
Low birth weight infant	low birth weight infant	low birth weight infant	low birth weight infant	Low birth weight infant	2	kept
low carbohydrate diet	low carbohydrate diet	low carbohydrate diet	low carbohydrate diet	low carbohydrate diet	2	kept
Low cardiac output syndrome	low cardiac output syndrome	low cardiac output syndrome	low cardiac output syndrome	Low cardiac output syndrome	2	kept
low fat diet	low fat diet	low fat diet	low fat diet	low fat diet	2	kept
low fidelity simulation	low fidelity simulation	low fidelity simulation	low fidelity simulation	low fidelity simulation	2	kept
Low frequency	low frequency	low frequency	low frequency	Low frequency	2	kept
low resource	low resource	low resource	low resource	low resource	2	kept
low resource settings	low resource settings	low resource settings	low resource setting	Low-resource settings	2	singular
Low resourced countries	low resourced countries	low resourced countries	low resourced countries	Low resourced countries	2	kept
low resources settings	low resources settings	low resources settings	low resources settings	low resources settings	2	kept
low temperature	low temperature	low temperature	low temperature	low temperature	2	kept

Low-and middle-income countries	low and middle income countries	low and middle income countries	low and middle income country	low- and middle-income countries	2	singular
low-and middleincome countries	low and middleincome countries	low and middleincome countries	low and middleincome countries	low-and middleincome countries	2	kept
low-dose high-frequency training	low dose high frequency training	low dose high frequency training	low dose high frequency training	low-dose high-frequency training	2	kept
Low-income countries	low income countries	low income countries	low income country	low income country	2	singular
Low-income country	low income country	low income country	low income country	low income country	2	case_or_format
low-resource settings	low resource settings	low resource settings	low resource setting	Low-resource settings	2	singular
low-to-middle-income country	low to middle income country	low to middle income country	low to middle income country	low-to-middle-income country	2	kept
Low-volume centre	low volume centre	low volume centre	low volume centre	Low-volume centre	2	kept
lower abdominal pain	lower abdominal pain	lower abdominal pain	lower abdominal pain	lower abdominal pain	2	kept
lower esophagus sphincter	lower esophagus sphincter	lower esophagus sphincter	lower esophagus sphincter	lower esophagus sphincter	2	kept
Lower middle-income country	lower middle income country	lower middle income country	lower middle income country	Lower middle-income country	2	kept
lower middle-income country	lower middle income country	lower middle income country	lower middle income country	Lower middle-income country	2	case_or_format
lumbar spinal stenosis	lumbar spinal stenosis	lumbar spinal stenosis	lumbar spinal stenosis	lumbar spinal stenosis	2	kept
lumbar vertebra	lumbar vertebra	lumbar vertebra	lumbar vertebra	lumbar vertebra	2	kept
Lumbar Vertebrae	lumbar vertebrae	lumbar vertebrae	lumbar vertebrae	Lumbar Vertebrae	2	kept
Lunar New Year holiday	lunar new year holiday	lunar new year holiday	lunar new year holiday	Lunar New Year holiday	2	kept

lung alveolus	lung alveolus	lung alveolus	lung alveolus	lung alveolus	2	kept
lung alveolus proteinosis	lung alveolus proteinosis	lung alveolus proteinosis	lung alveolus proteinosis	lung alveolus proteinosis	2	kept
lung carcinoma	lung carcinoma	lung carcinoma	lung carcinoma	lung carcinoma	2	kept
lung cyst	lung cyst	lung cyst	lung cyst	lung cyst	2	kept
lung dead space	lung dead space	lung dead space	lung dead space	lung dead space	2	kept
lung development	lung development	lung development	lung development	lung development	2	kept
lung diffusion capacity	lung diffusion capacity	lung diffusion capacity	lung diffusion capacity	lung diffusion capacity	2	kept
Lung Diseases, Fungal	lung diseases fungal	lung diseases fungal	lung diseases fungal	Lung Diseases, Fungal	2	kept
lung extravascular fluid	lung extravascular fluid	lung extravascular fluid	lung extravascular fluid	lung extravascular fluid	2	kept
lung hypoplasia	lung hypoplasia	lung hypoplasia	lung hypoplasia	lung hypoplasia	2	kept
lung lesion	lung lesion	lung lesion	lung lesion	lung lesion	2	kept
lung lobe	lung lobe	lung lobe	lung lobe	lung lobe	2	kept
lung model	lung model	lung model	lung model	lung model	2	kept
Lung protective ventilation	lung protective ventilation	lung protective ventilation	lung protective ventilation	Lung protective ventilation	2	kept
lung surgery	lung surgery	lung surgery	lung surgery	lung surgery	2	kept
lung toxicity	lung toxicity	lung toxicity	lung toxicity	lung toxicity	2	kept

lung ultrasound score	lung ultrasound score	lung ultrasound score	lung ultrasound score	lung ultrasound score	2	kept
Lungs	lungs	lungs	lung	Lung	2	singular
lupus anticoagulant	lupus anticoagulant	lupus anticoagulant	lupus anticoagulant	lupus anticoagulant	2	kept
Lupus Nephritis	lupus nephritis	lupus nephritis	lupus nephritis	Lupus Nephritis	2	kept
LVAD	lvad	lvad	lvad	LVAD	2	kept
lying	lying	lying	lying	lying	2	kept
lymph node biopsy	lymph node biopsy	lymph node biopsy	lymph node biopsy	lymph node biopsy	2	kept
Lymphangiectasis, Intestinal	lymphangiectasis intestinal	lymphangiectasis intestinal	lymphangiectasis intestinal	Lymphangiectasis, Intestinal	2	kept
lymphangioma	lymphangioma	lymphangioma	lymphangioma	lymphangioma	2	kept
lymphocytosis	lymphocytosis	lymphocytosis	lymphocytosis	lymphocytosis	2	kept
Lymphohistiocytosis, Hemophagocytic	lymphohistiocytosis hemophagocytic	lymphohistiocytosis hemophagocytic	lymphohistiocytosis hemophagocytic	Lymphohistiocytosis, Hemophagocytic	2	kept
Lymphoma, T-Cell, Cutaneous	lymphoma t cell cutaneous	lymphoma t cell cutaneous	lymphoma t cell cutaneous	Lymphoma, T-Cell, Cutaneous	2	kept
Lypressin	lypressin	lypressin	lypressin	Lypressin	2	kept
lysosome storage disease	lysosome storage disease	lysosome storage disease	lysosome storage disease	lysosome storage disease	2	kept
M-learning	m learning	m learning	m learning	M-learning	2	kept
Maastricht classification	maastricht classification	maastricht classification	maastricht classification	Maastricht classification	2	kept

Macedonia	macedonia	macedonia	macedonia	Macedonia	2	kept
Machine design	machine design	machine design	machine design	Machine design	2	kept
Machine learning methods	machine learning methods	machine learning methods	machine learning methods	Machine learning methods	2	kept
Macro-ergonomics	macro ergonomics	macro ergonomics	macro ergonomics	Macro-ergonomics	2	kept
Macroergonomics	macroergonomics	macroergonomics	macroergonomics	Macroergonomics	2	kept
macrolide resistance	macrolide resistance	macrolide resistance	macrolide resistance	macrolide resistance	2	kept
Macronutrients	macronutrients	macronutrients	macronutrient	macronutrient	2	singular
Macrophage Migration-Inhibitory Factors	macrophage migration inhibitory factors	macrophage migration inhibitory factors	macrophage migration inhibitory factors	Macrophage Migration-Inhibitory Factors	2	kept
Macrophages	macrophages	macrophages	macrophage	macrophage	2	singular
magnesium hydroxide	magnesium hydroxide	magnesium hydroxide	magnesium hydroxide	magnesium hydroxide	2	kept
Magnesium sulfate	magnesium sulfate	magnesium sulfate	magnesium sulfate	magnesium sulfate	2	case_or_format
MAGNET	magnet	magnet	magnet	magnet	2	case_or_format
MAGNET HOSPITAL CHARACTERISTICS	magnet hospital characteristics	magnet hospital characteristics	magnet hospital characteristics	MAGNET HOSPITAL CHARACTERISTICS	2	kept
MAGNET HOSPITALS	magnet hospitals	magnet hospitals	magnet hospital	MAGNET HOSPITALS	2	singular
Magnet recognition	magnet recognition	magnet recognition	magnet recognition	Magnet recognition	2	kept
Magnetic fields	magnetic fields	magnetic fields	magnetic field	magnetic field	2	singular

Magnetic Resonance Imaging, Cine	magnetic resonance imaging cine	magnetic resonance imaging cine	magnetic resonance imaging cine	Magnetic Resonance Imaging, Cine	2	kept
Magnets	magnets	magnets	magnet	magnet	2	singular
Major adverse events	major adverse events	major adverse events	major adverse events	Major adverse events	2	kept
major injury	major injury	major injury	major injury	major injury	2	kept
Major surgery	major surgery	major surgery	major surgery	major surgery	2	case_or_format
MAJOR TRAUMA	major trauma	major trauma	major trauma	Major trauma	2	case_or_format
Malabsorption Syndromes	malabsorption syndromes	malabsorption syndromes	malabsorption syndromes	Malabsorption Syndromes	2	kept
Malaysians	malaysians	malaysians	malaysian	Malaysians	2	singular
Malignant	malignant	malignant	malignant	Malignant	2	kept
malnutrition assessment	malnutrition assessment	malnutrition assessment	malnutrition assessment	malnutrition assessment	2	kept
malonaldehyde	malonaldehyde	malonaldehyde	malonaldehyde	malonaldehyde	2	kept
man machine interaction	man machine interaction	man machine interaction	man machine interaction	man machine interaction	2	kept
Man-Machine Systems	man machine systems	man machine systems	man machine systems	Man-Machine Systems	2	kept
Management by objectives	management by objectives	management by objectives	management by objectives	Management by objectives	2	kept
Management guideline	management guideline	management guideline	management guideline	Management guideline	2	kept
Management Information Systems	management information systems	management information systems	management information systems	Management information systems	2	case_or_format

Management Innovation	management innovation	management innovation	management innovation	Management Innovation	2	kept
Management of crisis	management of crisis	management of crisis	management of crisis	Management of crisis	2	kept
Management Quality Circles	management quality circles	management quality circles	management quality circles	Management Quality Circles	2	kept
Management system	management system	management system	management system	Management system	2	kept
Management systems	management systems	management systems	management system	Management system	2	singular
Managers	managers	managers	manager	manager	2	singular
Mandatory testing	mandatory testing	mandatory testing	mandatory testing	Mandatory testing	2	kept
mandible	mandible	mandible	mandible	mandible	2	kept
mandible injury	mandible injury	mandible injury	mandible injury	mandible injury	2	kept
mandibular distraction osteogenesis	mandibular distraction osteogenesis	mandibular distraction osteogenesis	mandibular distraction osteogenesis	mandibular distraction osteogenesis	2	kept
Manmade disaster	manmade disaster	manmade disaster	manmade disaster	Manmade disaster	2	kept
mannequin	mannequin	mannequin	mannequin	mannequin	2	kept
Mannikin simulator	mannikin simulator	mannikin simulator	mannikin simulator	Mannikin simulator	2	kept
manometry	manometry	manometry	manometry	Manometry	2	case_or_format
MANPOWER USE SCORE	manpower use score	manpower use score	manpower use score	MANPOWER USE SCORE	2	kept
manual emergency ventilator	manual emergency ventilator	manual emergency ventilator	manual emergency ventilator	manual emergency ventilator	2	kept

Maori	maori	maori	maori	Maori	2	kept
MAPK signaling	mapk signaling	mapk signaling	mapk signaling	MAPK signaling	2	kept
marbofloxacin	marbofloxacin	marbofloxacin	marbofloxacin	marbofloxacin	2	kept
Marburg virus	marburg virus	marburg virus	marburg virus	Marburg virus	2	kept
MARD	mard	mard	mard	MARD	2	kept
marijuana	marijuana	marijuana	marijuana	marijuana	2	kept
Marketing	marketing	marketing	marketing	marketing	2	case_or_format
Markov model	markov model	markov model	markov model	Markov model	2	kept
Markov processes	markov processes	markov processes	markov processes	Markov processes	2	kept
married woman	married woman	married woman	married woman	married woman	2	kept
Mask	mask	mask	mask	Masks	2	case_or_format
Mask ventilation	mask ventilation	mask ventilation	mask ventilation	mask ventilation	2	case_or_format
maslach burnout inventory	maslach burnout inventory	maslach burnout inventory	maslach burnout inventory	Maslach Burnout Inventory	2	case_or_format
Mass burns	mass burns	mass burns	mass burns	Mass burns	2	kept
Mass casualty	mass casualty	mass casualty	mass casualty	Mass casualty	2	kept
mass casualty incidents	mass casualty incidents	mass casualty incidents	mass casualty incident	Mass Casualty Incidents	2	singular

mass fragmentography	mass fragmentography	mass fragmentography	mass fragmentography	mass fragmentography	2	kept
mass immunization	mass immunization	mass immunization	mass immunization	mass immunization	2	kept
mass spectrometry	mass spectrometry	mass spectrometry	mass spectrometry	Mass spectrometry	2	case_or_format
Mass Vaccination	mass vaccination	mass vaccination	mass vaccination	Mass Vaccination	2	kept
Massive transfusion	massive transfusion	massive transfusion	massive transfusion	Massive transfusion	2	kept
Mastectomy	mastectomy	mastectomy	mastectomy	mastectomy	2	case_or_format
material coating	material coating	material coating	material coating	material coating	2	kept
maternal and child health	maternal and child health	maternal and child health	maternal and child health	maternal and child health	2	kept
Maternal and neonatal outcome	maternal and neonatal outcome	maternal and neonatal outcome	maternal and neonatal outcome	Maternal and neonatal outcome	2	kept
Maternal behavior	maternal behavior	maternal behavior	maternal behavior	Maternal Behavior	2	case_or_format
maternal behaviour	maternal behaviour	maternal behaviour	maternal behaviour	maternal behaviour	2	kept
maternal blood	maternal blood	maternal blood	maternal blood	maternal blood	2	kept
Maternal death	maternal death	maternal death	maternal death	maternal death	2	case_or_format
maternal emotional distress	maternal emotional distress	maternal emotional distress	maternal emotional distress	maternal emotional distress	2	kept
Maternal engagement	maternal engagement	maternal engagement	maternal engagement	Maternal engagement	2	kept
Maternal Exposure	maternal exposure	maternal exposure	maternal exposure	Maternal Exposure	2	kept

Maternal involvement	maternal involvement	maternal involvement	maternal involvement	Maternal involvement	2	kept
maternal levels of care	maternal levels of care	maternal levels of care	maternal levels of care	maternal levels of care	2	kept
maternal medicine	maternal medicine	maternal medicine	maternal medicine	maternal medicine	2	kept
Maternal near miss	maternal near miss	maternal near miss	maternal near miss	Maternal near miss	2	kept
Maternal outcomes	maternal outcomes	maternal outcomes	maternal outcome	Maternal outcomes	2	singular
maternal role attainment	maternal role attainment	maternal role attainment	maternal role attainment	maternal role attainment	2	kept
maternal satisfaction	maternal satisfaction	maternal satisfaction	maternal satisfaction	maternal satisfaction	2	kept
Maternal self-efficacy	maternal self efficacy	maternal self efficacy	maternal self efficacy	Maternal self-efficacy	2	kept
Maternal sensitivity	maternal sensitivity	maternal sensitivity	maternal sensitivity	Maternal sensitivity	2	kept
Maternity care	maternity care	maternity care	maternity care	Maternity care	2	kept
maternity care	maternity care	maternity care	maternity care	Maternity care	2	case_or_format
Mathematical models	mathematical models	mathematical models	mathematical model	mathematical model	2	singular
Mathematical programming	mathematical programming	mathematical programming	mathematical programming	Mathematical programming	2	kept
mathematical proposition	mathematical proposition	mathematical proposition	mathematical proposition	mathematical proposition	2	kept
matrix-assisted laser desorption-ionization mass spectrometry	matrix assisted laser desorption ionization mass spectrometry	matrix assisted laser desorption ionization mass spectrometry	matrix assisted laser desorption ionization mass spectrometry	matrix-assisted laser desorption-ionization mass spectrometry	2	kept
Mattering	mattering	mattering	mattering	Mattering	2	kept

Mature	mature	mature	mature	Mature	2	kept
Max-min fairness	max min fairness	max min fairness	max min fairness	Max-min fairness	2	kept
Maximum Allowable Concentration	maximum allowable concentration	maximum allowable concentration	maximum allowable concentration	Maximum Allowable Concentration	2	kept
MCDM	mcdm	mcdm	mcdm	MCDM	2	kept
MDR	mdr	mdr	mdr	MDR	2	kept
MDROs	mdros	mdros	mdro	MDROs	2	singular
Mean arterial blood pressures	mean arterial blood pressures	mean arterial blood pressures	mean arterial blood pressures	Mean arterial blood pressures	2	kept
Mean errors	mean errors	mean errors	mean errors	Mean errors	2	kept
Mean platelet volume	mean platelet volume	mean platelet volume	mean platelet volume	mean platelet volume	2	case_or_format
Mean pressures	mean pressures	mean pressures	mean pressures	Mean pressures	2	kept
meaning in life	meaning in life	meaning in life	meaning in life	meaning in life	2	kept
Meaningful use	meaningful use	meaningful use	meaningful use	Meaningful Use	2	case_or_format
measles mumps rubella vaccine	measles mumps rubella vaccine	measles mumps rubella vaccine	measles mumps rubella vaccine	measles mumps rubella vaccine	2	kept
measles vaccination	measles vaccination	measles vaccination	measles vaccination	measles vaccination	2	kept
Measurement instrument	measurement instrument	measurement instrument	measurement instrument	Measurement instrument	2	kept
Measurement Noise	measurement noise	measurement noise	measurement noise	Measurement Noise	2	kept

Measurement system	measurement system	measurement system	measurement system	Measurement system	2	kept
Measurement techniques	measurement techniques	measurement techniques	measurement techniques	Measurement techniques	2	kept
MecA gene	meca gene	meca gene	meca gene	MecA gene	2	kept
mechanical	mechanical	mechanical	mechanical	Mechanical	2	case_or_format
Mechanical Insufflator-Exsufflator device	mechanical insufflator exsufflator device	mechanical insufflator exsufflator device	mechanical insufflator exsufflator device	Mechanical Insufflator-Exsufflator device	2	kept
mechanical stress	mechanical stress	mechanical stress	mechanical stress	mechanical stress	2	kept
mechanical ventilation education	mechanical ventilation education	mechanical ventilation education	mechanical ventilation education	mechanical ventilation education	2	kept
mechanical ventilation in old	mechanical ventilation in old	mechanical ventilation in old	mechanical ventilation in old	mechanical ventilation in old	2	kept
Mechanical ventilation interactive ventilatory support	mechanical ventilation interactive ventilatory support	mechanical ventilation interactive ventilatory support	mechanical ventilation interactive ventilatory support	Mechanical ventilation interactive ventilatory support	2	kept
Mechanical Ventilator	mechanical ventilator	mechanical ventilator	mechanical ventilator	mechanical ventilator	2	case_or_format
Mechanically assisted crevice corrosion	mechanically assisted crevice corrosion	mechanically assisted crevice corrosion	mechanically assisted crevice corrosion	Mechanically assisted crevice corrosion	2	kept
Mechanically ventilated	mechanically ventilated	mechanically ventilated	mechanically ventilated	Mechanically ventilated	2	kept
mechanoreceptor	mechanoreceptor	mechanoreceptor	mechanoreceptor	mechanoreceptor	2	kept
Mechanoreceptors	mechanoreceptors	mechanoreceptors	mechanoreceptor	mechanoreceptor	2	singular
Meconium aspiration syndrome	meconium aspiration syndrome	meconium aspiration syndrome	meconium aspiration syndrome	Meconium Aspiration Syndrome	2	case_or_format
meconium ileus	meconium ileus	meconium ileus	meconium ileus	meconium ileus	2	kept

Media	media	media	media	Media	2	kept
mediating and moderating variables	mediating and moderating variables	mediating and moderating variables	mediating and moderating variables	mediating and moderating variables	2	kept
Mediating role	mediating role	mediating role	mediating role	Mediating role	2	kept
mediating role	mediating role	mediating role	mediating role	Mediating role	2	case_or_format
mediation	mediation	mediation	mediation	mediation	2	kept
Mediation effect	mediation effect	mediation effect	mediation effect	Mediation effect	2	kept
Mediation role	mediation role	mediation role	mediation role	Mediation role	2	kept
mediator	mediator	mediator	mediator	mediator	2	kept
Medicaid spending	medicaid spending	medicaid spending	medicaid spending	Medicaid spending	2	kept
medical adhesive	medical adhesive	medical adhesive	medical adhesive	medical adhesive	2	kept
Medical air transportation	medical air transportation	medical air transportation	medical air transportation	Medical air transportation	2	kept
Medical Assistance	medical assistance	medical assistance	medical assistance	Medical Assistance	2	kept
medical cannabis	medical cannabis	medical cannabis	medical cannabis	medical cannabis	2	kept
Medical care	medical care	medical care	medical care	medical care	2	case_or_format
medical clowning	medical clowning	medical clowning	medical clowning	medical clowning	2	kept
Medical communication	medical communication	medical communication	medical communication	Medical communication	2	kept

MEDICAL COMPLEXITY	medical complexity	medical complexity	medical complexity	MEDICAL COMPLEXITY	2	kept
Medical Costs	medical costs	medical costs	medical cost	Medical Costs	2	singular
medical countermeasure	medical countermeasure	medical countermeasure	medical countermeasure	medical countermeasure	2	kept
Medical crises	medical crises	medical crises	medical crises	Medical crises	2	kept
MEDICAL DECISION-MAKING	medical decision making	medical decision making	medical decision making	medical decision making	2	case_or_format
Medical device related pressure injury	medical device related pressure injury	medical device related pressure injury	medical device related pressure injury	medical device-related pressure injury	2	case_or_format
Medical device-related pressure ulcer	medical device related pressure ulcer	medical device related pressure ulcer	medical device related pressure ulcer	medical device related pressure ulcer	2	case_or_format
medical devices and technologies	medical devices and technologies	medical devices and technologies	medical devices and technologies	medical devices and technologies	2	kept
Medical emergency	medical emergency	medical emergency	medical emergency	Medical emergency	2	kept
medical emergency	medical emergency	medical emergency	medical emergency	Medical emergency	2	case_or_format
Medical emergency response	medical emergency response	medical emergency response	medical emergency response	Medical emergency response	2	kept
medical high dependency unit	medical high dependency unit	medical high dependency unit	medical high dependency unit	medical high dependency unit	2	kept
Medical image	medical image	medical image	medical image	Medical image	2	kept
medical informatics applications	medical informatics applications	medical informatics applications	medical informatics applications	Medical Informatics Applications	2	case_or_format
Medical instrumentation	medical instrumentation	medical instrumentation	medical instrumentation	medical instrumentation	2	case_or_format
medical interpretation	medical interpretation	medical interpretation	medical interpretation	medical interpretation	2	kept

Medical Laboratory Personnel	medical laboratory personnel	medical laboratory personnel	medical laboratory personnel	Medical Laboratory Personnel	2	kept
Medical malpractice	medical malpractice	medical malpractice	medical malpractice	Medical malpractice	2	kept
Medical material management	medical material management	medical material management	medical material management	Medical material management	2	kept
Medical NLP	medical nlp	medical nlp	medical nlp	Medical NLP	2	kept
medical nutrition therapy	medical nutrition therapy	medical nutrition therapy	medical nutrition therapy	medical nutrition therapy	2	kept
medical oncologist	medical oncologist	medical oncologist	medical oncologist	medical oncologist	2	kept
medical or surgical	medical or surgical	medical or surgical	medical or surgical	medical or surgical	2	kept
Medical Practice	medical practice	medical practice	medical practice	medical practice	2	case_or_format
Medical residents	medical residents	medical residents	medical resident	medical residents	2	singular
Medical simulations	medical simulations	medical simulations	medical simulation	Medical simulation	2	singular
Medical specialists	medical specialists	medical specialists	medical specialist	medical specialist	2	singular
medical staff visits	medical staff visits	medical staff visits	medical staff visits	medical staff visits	2	kept
medical staffing	medical staffing	medical staffing	medical staffing	medical staffing	2	kept
Medical training	medical training	medical training	medical training	Medical training	2	kept
medical training	medical training	medical training	medical training	Medical training	2	case_or_format
medical units	medical units	medical units	medical units	medical units	2	kept

medical ward	medical ward	medical ward	medical ward	medical ward	2	kept
medical wards	medical wards	medical wards	medical ward	medical ward	2	singular
Medical Waste	medical waste	medical waste	medical waste	Medical Waste	2	kept
Medical workers	medical workers	medical workers	medical worker	Medical workers	2	singular
MEDICAL-STUDENTS	medical students	medical students	medical student	medical student	2	singular
medical-surgical unit	medical surgical unit	medical surgical unit	medical surgical unit	medical-surgical unit	2	kept
medically fragile infant	medically fragile infant	medically fragile infant	medically fragile infant	medically fragile infant	2	kept
Medicare Assignment	medicare assignment	medicare assignment	medicare assignment	Medicare Assignment	2	kept
Medicare Part B	medicare part b	medicare part b	medicare part b	Medicare Part B	2	kept
Medicare Part C	medicare part c	medicare part c	medicare part c	Medicare Part C	2	kept
Medicare spending	medicare spending	medicare spending	medicare spending	Medicare spending	2	kept
medication [N04.452.515.360.500]	medication n04 452 515 360 500	medication n04 452 515 360 500	medication n04 452 515 360 500	medication [N04.452.515.360.500]	2	kept
Medication adherence	medication adherence	medication adherence	medication adherence	Medication Adherence	2	case_or_format
medication adherence	medication adherence	medication adherence	medication adherence	Medication Adherence	2	case_or_format
Medication administration error	medication administration error	medication administration error	medication administration error	medication administration errors	2	case_or_format
medication administration error survey	medication administration error survey	medication administration error survey	medication administration error survey	medication administration error survey	2	kept

MEDICATION ADMINISTRATION ERRORS	medication administration errors	medication administration errors	medication administration error	medication administration errors	2	singular
medication errors [E02.319.529]	medication errors e02 319 529	medication errors e02 319 529	medication errors e02 319 529	medication errors [E02.319.529]	2	kept
Medication Management	medication management	medication management	medication management	medication management	2	case_or_format
Medication orders	medication orders	medication orders	medication orders	Medication orders	2	kept
medication transition	medication transition	medication transition	medication transition	medication transition	2	kept
medications	medications	medications	medication	Medication	2	singular
medications errors	medications errors	medications errors	medications errors	medications errors	2	kept
Medicine in Literature	medicine in literature	medicine in literature	medicine in literature	Medicine in Literature	2	kept
medicine in the arts	medicine in the arts	medicine in the arts	medicine in the arts	medicine in the arts	2	kept
Medicine, Chinese Traditional	medicine chinese traditional	medicine chinese traditional	medicine chinese traditional	Medicine, Chinese Traditional	2	kept
Medicine, Traditional	medicine traditional	medicine traditional	medicine traditional	Medicine, Traditional	2	kept
MEDICINES	medicines	medicines	medicines	medicines	2	case_or_format
medicolegal	medicolegal	medicolegal	medicolegal	medicolegal	2	kept
medium chain triacylglycerol	medium chain triacylglycerol	medium chain triacylglycerol	medium chain triacylglycerol	medium chain triacylglycerol	2	kept
meglitinide	meglitinide	meglitinide	meglitinide	meglitinide	2	kept
melena	melena	melena	melena	melena	2	kept

Melissa officinalis	melissa officinalis	melissa officinalis	melissa officinalis	Melissa officinalis	2	kept
Melphalan	melphalan	melphalan	melphalan	melphalan	2	case_or_format
MEMS microphones	mems microphones	mems microphones	mems microphone	MEMS microphones	2	singular
MEN	men	men	men	Men	2	case_or_format
Mendelian randomization analysis	mendelian randomization analysis	mendelian randomization analysis	mendelian randomization analysis	Mendelian randomization analysis	2	kept
Mendelian randomization study	mendelian randomization study	mendelian randomization study	mendelian randomization study	Mendelian randomization study	2	kept
meningioma	meningioma	meningioma	meningioma	Meningioma	2	case_or_format
meningococcosis	meningococcosis	meningococcosis	meningococcosis	meningococcosis	2	kept
Meningoencephalitis	meningoencephalitis	meningoencephalitis	meningoencephalitis	meningoencephalitis	2	case_or_format
menorrhagia	menorrhagia	menorrhagia	menorrhagia	menorrhagia	2	kept
menstruation	menstruation	menstruation	menstruation	menstruation	2	kept
mental abnormality	mental abnormality	mental abnormality	mental abnormality	mental abnormality	2	kept
mental demand	mental demand	mental demand	mental demand	mental demand	2	kept
Mental health outcomes	mental health outcomes	mental health outcomes	mental health outcome	Mental health outcomes	2	singular
mental health problem	mental health problem	mental health problem	mental health problem	mental health problem	2	kept
Mental health problems	mental health problems	mental health problems	mental health problem	mental health problem	2	singular

mental health promotion	mental health promotion	mental health promotion	mental health promotion	mental health promotion	2	kept
mental health screening	mental health screening	mental health screening	mental health screening	mental health screening	2	kept
mental health status	mental health status	mental health status	mental health status	mental health status	2	kept
Mental illness	mental illness	mental illness	mental illness	Mental illness	2	kept
mental load	mental load	mental load	mental load	mental load	2	kept
Mental models	mental models	mental models	mental model	Mental models	2	singular
Mental Workload	mental workload	mental workload	mental workload	mental workload	2	case_or_format
mentalization	mentalization	mentalization	mentalization	mentalization	2	kept
Mentor	mentor	mentor	mentor	Mentors	2	case_or_format
mepivacaine	mepivacaine	mepivacaine	mepivacaine	mepivacaine	2	kept
Mercury	mercury	mercury	mercury	Mercury	2	kept
Mercury compounds	mercury compounds	mercury compounds	mercury compounds	Mercury compounds	2	kept
meropenem plus vaborbactam	meropenem plus vaborbactam	meropenem plus vaborbactam	meropenem plus vaborbactam	meropenem plus vaborbactam	2	kept
MERS	mers	mers	mers	MERS	2	kept
MERS-CoV complications	mers cov complications	mers cov complications	mers cov complications	MERS-CoV complications	2	kept
mesenchymal stem cell	mesenchymal stem cell	mesenchymal stem cell	mesenchymal stem cell	mesenchymal stem cell	2	kept

mesenteric artery	mesenteric artery	mesenteric artery	mesenteric artery	mesenteric artery	2	kept
MeSH Terms	mesh terms	mesh terms	mesh terms	MeSH Terms	2	kept
MeSH terms	mesh terms	mesh terms	mesh terms	MeSH Terms	2	case_or_format
MESH Terms: COVID-19	mesh terms covid 19	mesh terms covid 19	mesh terms covid 19	MESH Terms: COVID-19	2	kept
mesna	mesna	mesna	mesna	mesna	2	kept
messenger RNA	messenger rna	messenger rna	messenger rna	messenger RNA	2	kept
MET	met	met	met	MET	2	kept
Meta analysis	meta analysis	meta analysis	meta analysis	meta analysis	2	case_or_format
Meta synthesis	meta synthesis	meta synthesis	meta synthesis	meta-synthesis	2	case_or_format
metabolic bone disease	metabolic bone disease	metabolic bone disease	metabolic bone disease	metabolic bone disease	2	kept
metabolic parameters	metabolic parameters	metabolic parameters	metabolic parameters	metabolic parameters	2	kept
metabolic rate	metabolic rate	metabolic rate	metabolic rate	metabolic rate	2	kept
metabolic stability	metabolic stability	metabolic stability	metabolic stability	metabolic stability	2	kept
metachromatic leukodystrophy	metachromatic leukodystrophy	metachromatic leukodystrophy	metachromatic leukodystrophy	metachromatic leukodystrophy	2	kept
Metaethnography	metaethnography	metaethnography	metaethnography	Metaethnography	2	kept
metamorphosis	metamorphosis	metamorphosis	metamorphosis	metamorphosis	2	kept

metastatic cancer	metastatic cancer	metastatic cancer	metastatic cancer	metastatic cancer	2	kept
metastatic colon cancer	metastatic colon cancer	metastatic colon cancer	metastatic colon cancer	metastatic colon cancer	2	kept
Metaverse	metaverse	metaverse	metaverse	Metaverse	2	kept
metered dose inhaler	metered dose inhaler	metered dose inhaler	metered dose inhaler	metered dose inhaler	2	kept
Metered Dose Inhalers	metered dose inhalers	metered dose inhalers	metered dose inhaler	metered dose inhaler	2	singular
Metformin	metformin	metformin	metformin	metformin	2	case_or_format
methanol	methanol	methanol	methanol	Methanol	2	case_or_format
methemoglobin	methemoglobin	methemoglobin	methemoglobin	methemoglobin	2	kept
methicillin susceptible Staphylococcus aureus	methicillin susceptible staphylococcus aureus	methicillin susceptible staphylococcus aureus	methicillin susceptible staphylococcus aureus	methicillin susceptible Staphylococcus aureus	2	kept
methimazole	methimazole	methimazole	methimazole	methimazole	2	kept
methocarbamol	methocarbamol	methocarbamol	methocarbamol	methocarbamol	2	kept
Methodological overview	methodological overview	methodological overview	methodological overview	Methodological overview	2	kept
METHODOLOGY	methodology	methodology	methodology	methodology	2	case_or_format
Methylene Blue	methylene blue	methylene blue	methylene blue	methylene blue	2	case_or_format
Methylmalonic Acid	methylmalonic acid	methylmalonic acid	methylmalonic acid	Methylmalonic Acid	2	kept
methylmalonic aciduria	methylmalonic aciduria	methylmalonic aciduria	methylmalonic aciduria	methylmalonic aciduria	2	kept

Meticillin-resistant Staphylococcus aureus	meticillin resistant staphylococcus aureus	meticillin resistant staphylococcus aureus	meticillin resistant staphylococcus aureus	Meticillin-resistant Staphylococcus aureus	2	kept
metoprolol succinate	metoprolol succinate	metoprolol succinate	metoprolol succinate	metoprolol succinate	2	kept
Metric	metric	metric	metric	metrics	2	case_or_format
metric system	metric system	metric system	metric system	metric system	2	kept
Metronidazole	metronidazole	metronidazole	metronidazole	metronidazole	2	case_or_format
Metropolitan and regional hospitals	metropolitan and regional hospitals	metropolitan and regional hospitals	metropolitan and regional hospitals	Metropolitan and regional hospitals	2	kept
Meyerozyma guilliermondii	meyerozyma guilliermondii	meyerozyma guilliermondii	meyerozyma guilliermondii	Meyerozyma guilliermondii	2	kept
MHD	mhd	mhd	mhd	MHD	2	kept
Micro-costing	micro costing	micro costing	micro costing	Micro-costing	2	kept
Micro-Electrical-Mechanical Systems	micro electrical mechanical systems	micro electrical mechanical systems	micro electrical mechanical systems	Micro-Electrical-Mechanical Systems	2	kept
Micro-learning	micro learning	micro learning	micro learning	Micro-learning	2	kept
microarray analysis	microarray analysis	microarray analysis	microarray analysis	microarray analysis	2	kept
Microaspiration	microaspiration	microaspiration	microaspiration	Microaspiration	2	kept
microbial community	microbial community	microbial community	microbial community	microbial community	2	kept
Microbial contamination	microbial contamination	microbial contamination	microbial contamination	microbial contamination	2	case_or_format
microbial keratitis	microbial keratitis	microbial keratitis	microbial keratitis	microbial keratitis	2	kept

microbial morphology	microbial morphology	microbial morphology	microbial morphology	microbial morphology	2	kept
Microbial Viability	microbial viability	microbial viability	microbial viability	Microbial Viability	2	kept
microcephaly	microcephaly	microcephaly	microcephaly	microcephaly	2	kept
microcirculatory shock	microcirculatory shock	microcirculatory shock	microcirculatory shock	microcirculatory shock	2	kept
Micrococcus luteus	micrococcus luteus	micrococcus luteus	micrococcus luteus	Micrococcus luteus	2	kept
microcontroller	microcontroller	microcontroller	microcontroller	Microcontroller	2	case_or_format
microcounseling skills	microcounseling skills	microcounseling skills	microcounseling skills	microcounseling skills	2	kept
microenvironment	microenvironment	microenvironment	microenvironment	microenvironment	2	kept
Microfilaria	microfilaria	microfilaria	microfilaria	Microfilaria	2	kept
microglia	microglia	microglia	microglia	microglia	2	kept
microlaryngoscopy	microlaryngoscopy	microlaryngoscopy	microlaryngoscopy	microlaryngoscopy	2	kept
MicroNIR	micronir	micronir	micronir	MicroNIR	2	kept
Microorganism	microorganism	microorganism	microorganism	microorganism	2	case_or_format
Microscopy, Electron, Scanning	microscopy electron scanning	microscopy electron scanning	microscopy electron scanning	Microscopy, Electron, Scanning	2	kept
microvascular	microvascular	microvascular	microvascular	microvascular	2	kept
micturition disorder	micturition disorder	micturition disorder	micturition disorder	micturition disorder	2	kept

midazolam maleate	midazolam maleate	midazolam maleate	midazolam maleate	midazolam maleate	2	kept
Middle Eastern People	middle eastern people	middle eastern people	middle eastern people	Middle Eastern People	2	kept
MIDDLE-INCOME COUNTRIES	middle income countries	middle income countries	middle income country	middle income country	2	singular
midomafetamine	midomafetamine	midomafetamine	midomafetamine	midomafetamine	2	kept
midregional proatrial natriuretic peptide	midregional proatrial natriuretic peptide	midregional proatrial natriuretic peptide	midregional proatrial natriuretic peptide	midregional proatrial natriuretic peptide	2	kept
Midwife-led care	midwife led care	midwife led care	midwife led care	Midwife-led care	2	kept
midwifery student	midwifery student	midwifery student	midwifery student	midwifery student	2	kept
MILD HYPOTHERMIA	mild hypothermia	mild hypothermia	mild hypothermia	MILD HYPOTHERMIA	2	kept
mild renal impairment	mild renal impairment	mild renal impairment	mild renal impairment	mild renal impairment	2	kept
MILK	milk	milk	milk	milk	2	case_or_format
milk allergy	milk allergy	milk allergy	milk allergy	milk allergy	2	kept
milk donor	milk donor	milk donor	milk donor	milk donor	2	kept
Milk Ejection	milk ejection	milk ejection	milk ejection	Milk Ejection	2	kept
Milk expression	milk expression	milk expression	milk expression	Milk expression	2	kept
milk expression	milk expression	milk expression	milk expression	Milk expression	2	case_or_format
Miller pyramid model	miller pyramid model	miller pyramid model	miller pyramid model	Miller pyramid model	2	kept

MIMIC database	mimic database	mimic database	mimic database	MIMIC database	2	kept
MIMIC III	mimic iii	mimic iii	mimic iii	MIMIC-III	2	case_or_format
Mind-Body Relations, Metaphysical	mind body relations metaphysical	mind body relations metaphysical	mind body relations metaphysical	Mind-Body Relations, Metaphysical	2	kept
Mind-Body Therapies	mind body therapies	mind body therapies	mind body therapies	Mind-Body Therapies	2	kept
MINDFULNESS	mindfulness	mindfulness	mindfulness	Mindfulness	2	case_or_format
MINDFULNESS-BASED INTERVENTION	mindfulness based intervention	mindfulness based intervention	mindfulness based intervention	Mindfulness-based interventions	2	case_or_format
mindfulness-based stress reduction	mindfulness based stress reduction	mindfulness based stress reduction	mindfulness based stress reduction	mindfulness-based stress reduction	2	kept
mineral	mineral	mineral	mineral	mineral	2	kept
mineral deficiency	mineral deficiency	mineral deficiency	mineral deficiency	mineral deficiency	2	kept
Minerals	minerals	minerals	mineral	mineral	2	singular
MINI-NUTRITIONAL ASSESSMENT	mini nutritional assessment	mini nutritional assessment	mini nutritional assessment	Mini-Nutritional Assessment	2	case_or_format
Mini-pig	mini pig	mini pig	mini pig	Mini-pig	2	kept
Minimally invasive	minimally invasive	minimally invasive	minimally invasive	Minimally invasive	2	kept
Minimally invasive surgery	minimally invasive surgery	minimally invasive surgery	minimally invasive surgery	minimally invasive surgery	2	case_or_format
Minimum dataset	minimum dataset	minimum dataset	minimum dataset	Minimum dataset	2	kept
minnesota satisfaction questionnaire	minnesota satisfaction questionnaire	minnesota satisfaction questionnaire	minnesota satisfaction questionnaire	minnesota satisfaction questionnaire	2	kept

MIS-C	mis c	mis c	mis c	MIS-C	2	kept
miscellaneous named groups	miscellaneous named groups	miscellaneous named groups	miscellaneous named groups	miscellaneous named groups	2	kept
missed diagnosis	missed diagnosis	missed diagnosis	missed diagnosis	missed diagnosis	2	kept
missense mutation	missense mutation	missense mutation	missense mutation	missense mutation	2	kept
mistake	mistake	mistake	mistake	mistake	2	kept
mitochondrial DNA	mitochondrial dna	mitochondrial dna	mitochondrial dna	mitochondrial DNA	2	kept
mitogen activated protein kinase kinase 2	mitogen activated protein kinase kinase 2	mitogen activated protein kinase kinase 2	mitogen activated protein kinase kinase 2	mitogen activated protein kinase kinase 2	2	kept
Mitral valve regurgitation	mitral valve regurgitation	mitral valve regurgitation	mitral valve regurgitation	mitral valve regurgitation	2	case_or_format
Mitral Valve Stenosis	mitral valve stenosis	mitral valve stenosis	mitral valve stenosis	Mitral Valve Stenosis	2	kept
mitral valve stenosis	mitral valve stenosis	mitral valve stenosis	mitral valve stenosis	Mitral Valve Stenosis	2	case_or_format
mivacurium	mivacurium	mivacurium	mivacurium	mivacurium	2	kept
Mixed effects	mixed effects	mixed effects	mixed effects	Mixed effects	2	kept
Mixed Function Oxygenases	mixed function oxygenases	mixed function oxygenases	mixed function oxygenases	Mixed Function Oxygenases	2	kept
Mixed reality	mixed reality	mixed reality	mixed reality	Mixed reality	2	kept
Mixed-method	mixed method	mixed method	mixed method	Mixed methods	2	case_or_format
mixed-method design	mixed method design	mixed method design	mixed method design	mixed-method design	2	kept

Mixed-methods	mixed methods	mixed methods	mixed method	Mixed methods	2	singular
MMSE	mmse	mmse	mmse	MMSE	2	kept
Mobile apps	mobile apps	mobile apps	mobile app	mobile app	2	singular
mobile apps	mobile apps	mobile apps	mobile app	mobile app	2	singular
Mobile device	mobile device	mobile device	mobile device	Mobile device	2	kept
Mobile devices	mobile devices	mobile devices	mobile device	Mobile device	2	singular
Mobile e-learning	mobile e learning	mobile e learning	mobile e learning	Mobile e-learning	2	kept
mobile ECMO	mobile ecmo	mobile ecmo	mobile ecmo	mobile ECMO	2	kept
mobile genetic element	mobile genetic element	mobile genetic element	mobile genetic element	mobile genetic element	2	kept
Mobile Health	mobile health	mobile health	mobile health	mobile health	2	case_or_format
Mobile health application	mobile health application	mobile health application	mobile health application	Mobile health application	2	kept
Mobile learning	mobile learning	mobile learning	mobile learning	Mobile learning	2	kept
mobile x ray unit	mobile x ray unit	mobile x ray unit	mobile x ray unit	mobile x ray unit	2	kept
MOBILITY	mobility	mobility	mobility	Mobility	2	case_or_format
Mobility assessment	mobility assessment	mobility assessment	mobility assessment	Mobility assessment	2	kept
Mobility limitation	mobility limitation	mobility limitation	mobility limitation	Mobility Limitation	2	case_or_format

Mobility monitoring	mobility monitoring	mobility monitoring	mobility monitoring	Mobility monitoring	2	kept
Mobility team	mobility team	mobility team	mobility team	Mobility team	2	kept
mobility test	mobility test	mobility test	mobility test	mobility test	2	kept
Mode of birth	mode of birth	mode of birth	mode of birth	Mode of birth	2	kept
Model development	model development	model development	model development	Model development	2	kept
Model of care	model of care	model of care	model of care	model of care	2	case_or_format
Model-based protocol	model based protocol	model based protocol	model based protocol	Model-based protocol	2	kept
Modelcomputer simulation	modelcomputer simulation	modelcomputer simulation	modelcomputer simulation	Modelcomputer simulation	2	kept
Models, Animal	models animal	models animal	models animal	Models, Animal	2	kept
Models, Structural	models structural	models structural	models structural	Models, Structural	2	kept
moderate renal impairment	moderate renal impairment	moderate renal impairment	moderate renal impairment	moderate renal impairment	2	kept
Moderate sedation	moderate sedation	moderate sedation	moderate sedation	Moderate sedation	2	kept
moderated moderation analysis	moderated moderation analysis	moderated moderation analysis	moderated moderation analysis	moderated moderation analysis	2	kept
modified Delphi	modified delphi	modified delphi	modified delphi	modified Delphi	2	kept
modified Delphi consensus process	modified delphi consensus process	modified delphi consensus process	modified delphi consensus process	modified Delphi consensus process	2	kept
modified early warning score	modified early warning score	modified early warning score	modified early warning score	Modified Early Warning Score	2	case_or_format

Moisture associated skin damage	moisture associated skin damage	moisture associated skin damage	moisture associated skin damage	moisture-associated skin damage	2	case_or_format
moisture lesion	moisture lesion	moisture lesion	moisture lesion	moisture lesion	2	kept
Moisture lesions	moisture lesions	moisture lesions	moisture lesion	moisture lesion	2	singular
molar tooth	molar tooth	molar tooth	molar tooth	molar tooth	2	kept
molecular mechanics	molecular mechanics	molecular mechanics	molecular mechanics	molecular mechanics	2	kept
molecular recognition	molecular recognition	molecular recognition	molecular recognition	molecular recognition	2	kept
monitored anaesthesia care	monitored anaesthesia care	monitored anaesthesia care	monitored anaesthesia care	monitored anaesthesia care	2	kept
monitoring and assessment	monitoring and assessment	monitoring and assessment	monitoring and assessment	monitoring and assessment	2	kept
Monitoring device	monitoring device	monitoring device	monitoring device	Monitoring device	2	kept
Monitoring physiologic	monitoring physiologic	monitoring physiologic	monitoring physiologic	Monitoring, Physiologic	2	case_or_format
Monitoring tools	monitoring tools	monitoring tools	monitoring tools	Monitoring tools	2	kept
Monitors	monitors	monitors	monitor	monitor	2	singular
Monoclonal antibodies	monoclonal antibodies	monoclonal antibodies	monoclonal antibody	monoclonal antibody	2	singular
monoclonal immunoglobulinemia	monoclonal immunoglobulinemia	monoclonal immunoglobulinemia	monoclonal immunoglobulinemia	monoclonal immunoglobulinemia	2	kept
monocyte chemotactic protein 1	monocyte chemotactic protein 1	monocyte chemotactic protein 1	monocyte chemotactic protein 1	monocyte chemotactic protein 1	2	kept
Monocytes	monocytes	monocytes	monocyte	monocyte	2	singular

MOOD	mood	mood	mood	mood	2	case_or_format
Mood states	mood states	mood states	mood state	Mood states	2	singular
Moonlighting	moonlighting	moonlighting	moonlighting	moonlighting	2	case_or_format
moral agency	moral agency	moral agency	moral agency	moral agency	2	kept
moral comfort	moral comfort	moral comfort	moral comfort	moral comfort	2	kept
Moral community	moral community	moral community	moral community	Moral community	2	kept
Moral Development	moral development	moral development	moral development	Moral Development	2	kept
Moral disengagement	moral disengagement	moral disengagement	moral disengagement	moral disengagement	2	case_or_format
moral distress frequency	moral distress frequency	moral distress frequency	moral distress frequency	moral distress frequency	2	kept
moral distress intensity	moral distress intensity	moral distress intensity	moral distress intensity	moral distress intensity	2	kept
moral distress scale	moral distress scale	moral distress scale	moral distress scale	Moral Distress Scale	2	case_or_format
Moral Distress Scale Neonatal Pediatric Version	moral distress scale neonatal pediatric version	moral distress scale neonatal pediatric version	moral distress scale neonatal pediatric version	Moral Distress Scale Neonatal Pediatric Version	2	kept
Moral emotions	moral emotions	moral emotions	moral emotions	Moral emotions	2	kept
moral identity	moral identity	moral identity	moral identity	moral identity	2	kept
Moral Sensitivity	moral sensitivity	moral sensitivity	moral sensitivity	moral sensitivity	2	case_or_format
moral stress	moral stress	moral stress	moral stress	moral stress	2	kept

Moraxella	moraxella	moraxella	moraxella	Moraxella	2	kept
Morbid obesity	morbid obesity	morbid obesity	morbid obesity	morbid obesity	2	case_or_format
Morbidity and mortality	morbidity and mortality	morbidity and mortality	morbidity and mortality	Morbidity and mortality	2	kept
morbidity and mortality conference	morbidity and mortality conference	morbidity and mortality conference	morbidity and mortality conference	morbidity and mortality conference	2	kept
Morbidity and mortality conferences	morbidity and mortality conferences	morbidity and mortality conferences	morbidity and mortality conference	morbidity and mortality conference	2	singular
Morganella	morganella	morganella	morganella	Morganella	2	kept
Morning care	morning care	morning care	morning care	Morning care	2	kept
Morphine Derivatives	morphine derivatives	morphine derivatives	morphine derivative	Morphine Derivatives	2	singular
morpholine derivative	morpholine derivative	morpholine derivative	morpholine derivative	morpholine derivative	2	kept
Morpholines	morpholines	morpholines	morpholines	Morpholines	2	kept
morphology	morphology	morphology	morphology	morphology	2	kept
Morse Fall Scale	morse fall scale	morse fall scale	morse fall scale	Morse Fall Scale	2	kept
Mortalidad	mortalidad	mortalidad	mortalidad	Mortalidad	2	kept
MORTALITY PREDICTION	mortality prediction	mortality prediction	mortality prediction	Mortality prediction	2	case_or_format
Mortality risk	mortality risk	mortality risk	mortality risk	mortality risk	2	case_or_format
mosapride	mosapride	mosapride	mosapride	mosapride	2	kept

mother of preterm newborn	mother of preterm newborn	mother of preterm newborn	mother of preterm newborn	mother of preterm newborn	2	kept
mother to child transmission	mother to child transmission	mother to child transmission	mother to child transmission	mother to child transmission	2	kept
Mother's voice	mother s voice	mother s voice	mother s voice	Mother's voice	2	kept
Mother-child relation	mother child relation	mother child relation	mother child relation	Mother-Child Relations	2	case_or_format
mother-child relations	mother child relations	mother child relations	mother child relation	Mother-Child Relations	2	singular
Mother-infant attachment	mother infant attachment	mother infant attachment	mother infant attachment	Mother-infant attachment	2	kept
mother-infant interaction	mother infant interaction	mother infant interaction	mother infant interaction	Mother-infant interaction	2	case_or_format
mother-infant separation	mother infant separation	mother infant separation	mother infant separation	mother-infant separation	2	kept
Motherhood	motherhood	motherhood	motherhood	Motherhood	2	kept
motherhood experiences	motherhood experiences	motherhood experiences	motherhood experiences	motherhood experiences	2	kept
mothering	mothering	mothering	mothering	Mothering	2	case_or_format
mothers of premature infants	mothers of premature infants	mothers of premature infants	mothers of premature infant	mothers of premature infants	2	singular
mothers of preterm infants	mothers of preterm infants	mothers of preterm infants	mothers of preterm infants	mothers of preterm infants	2	kept
mothers' experiences	mothers experiences	mothers experiences	mothers experience	mothers' experiences	2	singular
Motion capture	motion capture	motion capture	motion capture	Motion capture	2	kept
motion detector	motion detector	motion detector	motion detector	motion detector	2	kept

Motion study	motion study	motion study	motion study	Motion study	2	kept
Motivational Interviewing	motivational interviewing	motivational interviewing	motivational interviewing	motivational interviewing	2	case_or_format
Motor Disorders	motor disorders	motor disorders	motor disorders	Motor Disorders	2	kept
Motor Neuron Disease	motor neuron disease	motor neuron disease	motor neuron disease	Motor Neuron Disease	2	kept
motor neuron disease	motor neuron disease	motor neuron disease	motor neuron disease	Motor Neuron Disease	2	case_or_format
Motor subtypes of delirium	motor subtypes of delirium	motor subtypes of delirium	motor subtypes of delirium	Motor subtypes of delirium	2	kept
Motor Vehicles	motor vehicles	motor vehicles	motor vehicle	motor vehicle	2	singular
Motorcycles	motorcycles	motorcycles	motorcycle	Motorcycles	2	singular
mountain	mountain	mountain	mountain	mountain	2	kept
mountaineering	mountaineering	mountaineering	mountaineering	Mountaineering	2	case_or_format
mouth cancer	mouth cancer	mouth cancer	mouth cancer	mouth cancer	2	kept
Mouth care	mouth care	mouth care	mouth care	Mouth care	2	kept
mouth care	mouth care	mouth care	mouth care	Mouth care	2	case_or_format
Mouth Floor	mouth floor	mouth floor	mouth floor	Mouth Floor	2	kept
mouth injury	mouth injury	mouth injury	mouth injury	mouth injury	2	kept
Movement pattern	movement pattern	movement pattern	movement pattern	Movement pattern	2	kept

Movilización precoz	movilizaci n precoz	movilizaci n precoz	movilizaci n precoz	Movilización precoz	2	kept
Moving average	moving average	moving average	moving average	Moving average	2	kept
MSD prevalence	msd prevalence	msd prevalence	msd prevalence	MSD prevalence	2	kept
MSSA	mssa	mssa	mssa	MSSA	2	kept
Mucociliary Clearance	mucociliary clearance	mucociliary clearance	mucociliary clearance	Mucociliary Clearance	2	kept
mucociliary clearance	mucociliary clearance	mucociliary clearance	mucociliary clearance	Mucociliary Clearance	2	case_or_format
Mucocutaneous Lymph Node Syndrome	mucocutaneous lymph node syndrome	mucocutaneous lymph node syndrome	mucocutaneous lymph node syndrome	mucocutaneous lymph node syndrome	2	case_or_format
mucosal bleeding	mucosal bleeding	mucosal bleeding	mucosal bleeding	mucosal bleeding	2	kept
Mucosal pressure injury	mucosal pressure injury	mucosal pressure injury	mucosal pressure injury	Mucosal pressure injury	2	kept
mucus	mucus	mucus	mucus	Mucus	2	case_or_format
mukbang videos	mukbang videos	mukbang videos	mukbang videos	mukbang videos	2	kept
multi-criteria decision analysis	multi criteria decision analysis	multi criteria decision analysis	multi criteria decision analysis	multi-criteria decision analysis	2	kept
multi-disciplinary team	multi disciplinary team	multi disciplinary team	multi disciplinary team	Multi-disciplinary teams	2	case_or_format
Multi-drug resistance	multi drug resistance	multi drug resistance	multi drug resistance	Multi-drug resistance	2	kept
Multi-hazard	multi hazard	multi hazard	multi hazard	Multi-hazard	2	kept
Multi-modal	multi modal	multi modal	multi modal	Multi-modal	2	kept

Multi-organ failure	multi organ failure	multi organ failure	multi organ failure	Multi-organ failure	2	kept
multi-organ failure	multi organ failure	multi organ failure	multi organ failure	Multi-organ failure	2	case_or_format
Multicentre study	multicentre study	multicentre study	multicentre study	Multicentre study	2	kept
Multicomponent intervention	multicomponent intervention	multicomponent intervention	multicomponent intervention	Multicomponent intervention	2	kept
Multidetector Computed Tomography	multidetector computed tomography	multidetector computed tomography	multidetector computed tomography	Multidetector Computed Tomography	2	kept
multidetector computed tomography	multidetector computed tomography	multidetector computed tomography	multidetector computed tomography	Multidetector Computed Tomography	2	case_or_format
multidimensional approach	multidimensional approach	multidimensional approach	multidimensional approach	Multidimensional approach	2	case_or_format
multidimensional framework	multidimensional framework	multidimensional framework	multidimensional framework	multidimensional framework	2	kept
multidimensional scaling	multidimensional scaling	multidimensional scaling	multidimensional scaling	multidimensional scaling	2	kept
multidisciplinary approach	multidisciplinary approach	multidisciplinary approach	multidisciplinary approach	Multidisciplinary approach	2	case_or_format
Multidisciplinary handover communication	multidisciplinary handover communication	multidisciplinary handover communication	multidisciplinary handover communication	Multidisciplinary handover communication	2	kept
multidisciplinary management	multidisciplinary management	multidisciplinary management	multidisciplinary management	multidisciplinary management	2	kept
Multidisciplinary neurotraumatology	multidisciplinary neurotraumatology	multidisciplinary neurotraumatology	multidisciplinary neurotraumatology	Multidisciplinary neurotraumatology	2	kept
MULTIDISCIPLINARY ROUNDS	multidisciplinary rounds	multidisciplinary rounds	multidisciplinary round	Multidisciplinary rounds	2	singular
multidisciplinary team approach	multidisciplinary team approach	multidisciplinary team approach	multidisciplinary team approach	multidisciplinary team approach	2	kept
multidisciplinary teams	multidisciplinary teams	multidisciplinary teams	multidisciplinary team	multidisciplinary team	2	singular

Multidisciplinary teamwork	multidisciplinary teamwork	multidisciplinary teamwork	multidisciplinary teamwork	multidisciplinary teamwork	2	case_or_format
multidrug resistant Enterobacteriaceae	multidrug resistant enterobacteriaceae	multidrug resistant enterobacteriaceae	multidrug resistant enterobacteriaceae	multidrug resistant Enterobacteriaceae	2	kept
multidrug resistant organism	multidrug resistant organism	multidrug resistant organism	multidrug resistant organism	Multidrug-resistant organisms	2	case_or_format
multidrug-resistant organism	multidrug resistant organism	multidrug resistant organism	multidrug resistant organism	Multidrug-resistant organisms	2	case_or_format
Multifactorial analysis	multifactorial analysis	multifactorial analysis	multifactorial analysis	Multifactorial analysis	2	kept
Multilayer network	multilayer network	multilayer network	multilayer network	Multilayer network	2	kept
MULTILEVEL ANALYSIS	multilevel analysis	multilevel analysis	multilevel analysis	multilevel analysis	2	case_or_format
Multilevel modelling	multilevel modelling	multilevel modelling	multilevel modelling	Multilevel modelling	2	kept
Multimedia tool	multimedia tool	multimedia tool	multimedia tool	Multimedia tool	2	kept
multimodal models	multimodal models	multimodal models	multimodal models	multimodal models	2	kept
multimodal monitoring	multimodal monitoring	multimodal monitoring	multimodal monitoring	Multimodal monitoring	2	case_or_format
multimodal sensor systems	multimodal sensor systems	multimodal sensor systems	multimodal sensor systems	multimodal sensor systems	2	kept
multimodality chest physiotherapy	multimodality chest physiotherapy	multimodality chest physiotherapy	multimodality chest physiotherapy	multimodality chest physiotherapy	2	kept
Multiple births	multiple births	multiple births	multiple birth	Multiple births	2	singular
Multiple Chronic Conditions	multiple chronic conditions	multiple chronic conditions	multiple chronic conditions	multiple chronic conditions	2	case_or_format
Multiple chronic conditions	multiple chronic conditions	multiple chronic conditions	multiple chronic conditions	multiple chronic conditions	2	case_or_format

multiple comorbidities	multiple comorbidities	multiple comorbidities	multiple comorbidities	multiple comorbidities	2	kept
multiple malformation syndrome	multiple malformation syndrome	multiple malformation syndrome	multiple malformation syndrome	multiple malformation syndrome	2	kept
multiple ontologies	multiple ontologies	multiple ontologies	multiple ontologies	multiple ontologies	2	kept
Multiple trauma	multiple trauma	multiple trauma	multiple trauma	Multiple Trauma	2	case_or_format
Multiple-dose vial	multiple dose vial	multiple dose vial	multiple dose vial	Multiple-dose vial	2	kept
Multiplex Polymerase Chain Reaction	multiplex polymerase chain reaction	multiplex polymerase chain reaction	multiplex polymerase chain reaction	multiplex polymerase chain reaction	2	case_or_format
multiprofessional	multiprofessional	multiprofessional	multiprofessional	multiprofessional	2	kept
Multiprofessional care	multiprofessional care	multiprofessional care	multiprofessional care	Multiprofessional care	2	kept
Multiprofessional education	multiprofessional education	multiprofessional education	multiprofessional education	Multiprofessional education	2	kept
Multiresistant bacteria	multiresistant bacteria	multiresistant bacteria	multiresistant bacteria	Multiresistant bacteria	2	kept
multisensory	multisensory	multisensory	multisensory	multisensory	2	kept
multisource multitask learning	multisource multitask learning	multisource multitask learning	multisource multitask learning	multisource multitask learning	2	kept
Multivariate matching	multivariate matching	multivariate matching	multivariate matching	Multivariate matching	2	kept
Munchausen Syndrome	munchausen syndrome	munchausen syndrome	munchausen syndrome	Munchausen syndrome	2	case_or_format
Muscarinic Antagonists	muscarinic antagonists	muscarinic antagonists	muscarinic antagonists	Muscarinic Antagonists	2	kept
muscle biopsy	muscle biopsy	muscle biopsy	muscle biopsy	muscle biopsy	2	kept

muscle contractility	muscle contractility	muscle contractility	muscle contractility	muscle contractility	2	kept
muscle excitation	muscle excitation	muscle excitation	muscle excitation	muscle excitation	2	kept
Muscle Fatigue	muscle fatigue	muscle fatigue	muscle fatigue	Muscle Fatigue	2	kept
muscle fatigue	muscle fatigue	muscle fatigue	muscle fatigue	Muscle Fatigue	2	case_or_format
Muscle Relaxants, Central	muscle relaxants central	muscle relaxants central	muscle relaxants central	Muscle Relaxants, Central	2	kept
Muscle Rigidity	muscle rigidity	muscle rigidity	muscle rigidity	muscle rigidity	2	case_or_format
Muscle Spasticity	muscle spasticity	muscle spasticity	muscle spasticity	Muscle Spasticity	2	kept
Muscles	muscles	muscles	muscle	Muscle	2	singular
Muscular Disorders, Atrophic	muscular disorders atrophic	muscular disorders atrophic	muscular disorders atrophic	Muscular Disorders, Atrophic	2	kept
Muscular Dystrophies	muscular dystrophies	muscular dystrophies	muscular dystrophy	muscular dystrophy	2	singular
Muscular Dystrophy, Duchenne	muscular dystrophy duchenne	muscular dystrophy duchenne	muscular dystrophy duchenne	Muscular Dystrophy, Duchenne	2	kept
musculoskeletal disease assessment	musculoskeletal disease assessment	musculoskeletal disease assessment	musculoskeletal disease assessment	musculoskeletal disease assessment	2	kept
Musculoskeletal disorder	musculoskeletal disorder	musculoskeletal disorder	musculoskeletal disorder	Musculoskeletal disorders	2	case_or_format
musculoskeletal function	musculoskeletal function	musculoskeletal function	musculoskeletal function	musculoskeletal function	2	kept
musculoskeletal system examination	musculoskeletal system examination	musculoskeletal system examination	musculoskeletal system examination	musculoskeletal system examination	2	kept
Mushroom Poisoning	mushroom poisoning	mushroom poisoning	mushroom poisoning	Mushroom Poisoning	2	kept

MUSIC INTERVENTION	music intervention	music intervention	music intervention	music intervention	2	case_or_format
Music intervention	music intervention	music intervention	music intervention	music intervention	2	case_or_format
Music listening	music listening	music listening	music listening	Music listening	2	kept
music medicine	music medicine	music medicine	music medicine	music medicine	2	kept
musical identity	musical identity	musical identity	musical identity	musical identity	2	kept
musician	musician	musician	musician	musician	2	kept
Musicoterapia	musicoterapia	musicoterapia	musicoterapia	Musicoterapia	2	kept
Mutism	mutism	mutism	mutism	mutism	2	case_or_format
mutual support	mutual support	mutual support	mutual support	mutual support	2	kept
Myasthenia Gravis	myasthenia gravis	myasthenia gravis	myasthenia gravis	myasthenia gravis	2	case_or_format
Mycobacterium Infections, Nontuberculous	mycobacterium infections nontuberculous	mycobacterium infections nontuberculous	mycobacterium infections nontuberculous	Mycobacterium Infections, Nontuberculous	2	kept
mycosis fungoides	mycosis fungoides	mycosis fungoides	mycosis fungoides	mycosis fungoides	2	kept
Myeloablative Agonists	myeloablative agonists	myeloablative agonists	myeloablative agonists	Myeloablative Agonists	2	kept
myocardial infarction with non-obstructive coronary arteries	myocardial infarction with non obstructive coronary arteries	myocardial infarction with non obstructive coronary arteries	myocardial infarction with non obstructive coronary arteries	myocardial infarction with non-obstructive coronary arteries	2	kept
myoclonus seizure	myoclonus seizure	myoclonus seizure	myoclonus seizure	myoclonus seizure	2	kept
Myokines	myokines	myokines	myokines	Myokines	2	kept

myomectomy	myomectomy	myomectomy	myomectomy	myomectomy	2	kept
Myonecrosis	myonecrosis	myonecrosis	myonecrosis	Myonecrosis	2	kept
myopathies nemaline	myopathies nemaline	myopathies nemaline	myopathies nemaline	myopathies nemaline	2	kept
Myristates	myristates	myristates	myristates	Myristates	2	kept
Mythology	mythology	mythology	mythology	Mythology	2	kept
myxedema	myxedema	myxedema	myxedema	Myxedema	2	case_or_format
médicament	m dicament	m dicament	m dicament	médicament	2	kept
N95 respirators	n95 respirators	n95 respirators	n95 respirator	N95 Respirators	2	singular
nacubactam	nacubactam	nacubactam	nacubactam	nacubactam	2	kept
nafamstat	nafamstat	nafamstat	nafamstat	nafamstat	2	kept
nail	nail	nail	nail	Nails	2	case_or_format
Nairovirus	nairovirus	nairovirus	nairovirus	Nairovirus	2	kept
Najran	najran	najran	najran	Najran	2	kept
Nalbuphine	nalbuphine	nalbuphine	nalbuphine	nalbuphine	2	case_or_format
NANDA	nanda	nanda	nanda	NANDA	2	kept
nanopore sequencing	nanopore sequencing	nanopore sequencing	nanopore sequencing	nanopore sequencing	2	kept

narrative interviews	narrative interviews	narrative interviews	narrative interviews	narrative interviews	2	kept
narrative synthesis	narrative synthesis	narrative synthesis	narrative synthesis	narrative synthesis	2	kept
Nasal continuous positive airway pressure	nasal continuous positive airway pressure	nasal continuous positive airway pressure	nasal continuous positive airway pressure	Nasal continuous positive airway pressure	2	kept
nasal continuous positive airway pressure	nasal continuous positive airway pressure	nasal continuous positive airway pressure	nasal continuous positive airway pressure	Nasal continuous positive airway pressure	2	case_or_format
nasal high flow therapy	nasal high flow therapy	nasal high flow therapy	nasal high flow therapy	nasal high flow therapy	2	kept
Nasal Microorganism	nasal microorganism	nasal microorganism	nasal microorganism	Nasal Microorganism	2	kept
Nasogastric feeding	nasogastric feeding	nasogastric feeding	nasogastric feeding	Nasogastric feeding	2	kept
nasointestinal tube	nasointestinal tube	nasointestinal tube	nasointestinal tube	nasointestinal tube	2	kept
nasopharyngeal aspiration	nasopharyngeal aspiration	nasopharyngeal aspiration	nasopharyngeal aspiration	nasopharyngeal aspiration	2	kept
National Aeronautics and Space Administration Task	national aeronautics and space administration task	national aeronautics and space administration task	national aeronautics and space administration task	National Aeronautics and Space Administration Task	2	kept
national framework	national framework	national framework	national framework	national framework	2	kept
National Health Insurance	national health insurance	national health insurance	national health insurance	national health insurance	2	case_or_format
National Health Service	national health service	national health service	national health service	national health service	2	case_or_format
National health service	national health service	national health service	national health service	national health service	2	case_or_format
national healthcare plans	national healthcare plans	national healthcare plans	national healthcare plans	national healthcare plans	2	kept
National Institute for Occupational Safety and Health, U.S	national institute for occupational safety and health u s	national institute for occupational safety and health u s	national institute for occupational safety and health u s	National Institute for Occupational Safety and Health, U.S	2	kept

Nationwide questionnaire in Japan	nationwide questionnaire in japan	nationwide questionnaire in japan	nationwide questionnaire in japan	Nationwide questionnaire in Japan	2	kept
Native Hawaiian	native hawaiian	native hawaiian	native hawaiian	Native Hawaiian	2	kept
Natriuretic Agents	natriuretic agents	natriuretic agents	natriuretic agents	Natriuretic Agents	2	kept
Natriuretic peptides	natriuretic peptides	natriuretic peptides	natriuretic peptide	Natriuretic peptides	2	singular
Natural disaster	natural disaster	natural disaster	natural disaster	natural disaster	2	case_or_format
natural disasters	natural disasters	natural disasters	natural disaster	natural disaster	2	singular
Natural hazard	natural hazard	natural hazard	natural hazard	Natural hazard	2	kept
natural killer cell	natural killer cell	natural killer cell	natural killer cell	natural killer cell	2	kept
natural killer T cell	natural killer t cell	natural killer t cell	natural killer t cell	natural killer T cell	2	kept
Natural languages	natural languages	natural languages	natural languages	Natural languages	2	kept
natural orifice transluminal endoscopic surgery	natural orifice transluminal endoscopic surgery	natural orifice transluminal endoscopic surgery	natural orifice transluminal endoscopic surgery	natural orifice transluminal endoscopic surgery	2	kept
NAVA	nava	nava	nava	NAVA	2	kept
Navarre	navarre	navarre	navarre	Navarre	2	kept
NCPAP	ncpap	ncpap	ncpap	NCPAP	2	kept
NDNQI	ndnqi	ndnqi	ndnqi	NDNQI	2	kept
Near Infrared Spectroscopy Technology	near infrared spectroscopy technology	near infrared spectroscopy technology	near infrared spectroscopy technology	Near Infrared Spectroscopy Technology	2	kept

near-death experience	near death experience	near death experience	near death experience	near-death experiences	2	case_or_format
near-infrared	near infrared	near infrared	near infrared	near-infrared	2	kept
NEAR-INFRARED SPECTROSCOPY	near infrared spectroscopy	near infrared spectroscopy	near infrared spectroscopy	near infrared spectroscopy	2	case_or_format
Nebulization	nebulization	nebulization	nebulization	nebulization	2	case_or_format
neck swelling	neck swelling	neck swelling	neck swelling	neck swelling	2	kept
NECROSIS-FACTOR-ALPHA	necrosis factor alpha	necrosis factor alpha	necrosis factor alpha	NECROSIS-FACTOR-ALPHA	2	kept
Necrotising fasciitis	necrotising fasciitis	necrotising fasciitis	necrotising fasciitis	Necrotising fasciitis	2	kept
necrotizing	necrotizing	necrotizing	necrotizing	necrotizing	2	kept
NECROTIZING ENTEROCOLITIS	necrotizing enterocolitis	necrotizing enterocolitis	necrotizing enterocolitis	necrotizing enterocolitis	2	case_or_format
NEECHAM confusion scale	neecham confusion scale	neecham confusion scale	neecham confusion scale	NEECHAM Confusion Scale	2	case_or_format
Need	need	need	need	NEEDS	2	case_or_format
need	need	need	need	NEEDS	2	case_or_format
need for oxygenation	need for oxygenation	need for oxygenation	need for oxygenation	need for oxygenation	2	kept
Needle insertion	needle insertion	needle insertion	needle insertion	Needle insertion	2	kept
needle recapping	needle recapping	needle recapping	needle recapping	needle recapping	2	kept
Needle stick injury	needle stick injury	needle stick injury	needle stick injury	Needle stick injuries	2	case_or_format

Needle sticks	needle sticks	needle sticks	needle stick	needle stick	2	singular
needle-free	needle free	needle free	needle free	needle-free	2	kept
needle-stick injuries	needle stick injuries	needle stick injuries	needle stick injury	Needle stick injuries	2	singular
Needleless connector	needleless connector	needleless connector	needleless connector	Needleless connector	2	kept
needleless connector	needleless connector	needleless connector	needleless connector	Needleless connector	2	case_or_format
Needleless connectors	needleless connectors	needleless connectors	needleless connector	Needleless connector	2	singular
Needlestick injury	needlestick injury	needlestick injury	needlestick injury	needlestick injury	2	case_or_format
Needlesticks	needlesticks	needlesticks	needlestick	Needlestick	2	singular
Needs of the family	needs of the family	needs of the family	needs of the family	Needs of the family	2	kept
Negative attitude	negative attitude	negative attitude	negative attitude	Negative attitude	2	kept
negative emotion	negative emotion	negative emotion	negative emotion	negative emotion	2	kept
Negative pressure ward	negative pressure ward	negative pressure ward	negative pressure ward	Negative pressure ward	2	kept
negative pressure wound therapy	negative pressure wound therapy	negative pressure wound therapy	negative pressure wound therapy	Negative-Pressure Wound Therapy	2	case_or_format
Negative-pressure ward	negative pressure ward	negative pressure ward	negative pressure ward	Negative pressure ward	2	case_or_format
Negligence	negligence	negligence	negligence	negligence	2	case_or_format
Neisseria	neisseria	neisseria	neisseria	Neisseria	2	kept

neoadjuvant chemoradiotherapy	neoadjuvant chemoradiotherapy	neoadjuvant chemoradiotherapy	neoadjuvant chemoradiotherapy	neoadjuvant chemoradiotherapy	2	kept
neomycin	neomycin	neomycin	neomycin	neomycin	2	kept
Neonatal brain imaging	neonatal brain imaging	neonatal brain imaging	neonatal brain imaging	Neonatal brain imaging	2	kept
neonatal care bundle	neonatal care bundle	neonatal care bundle	neonatal care bundle	neonatal care bundle	2	kept
Neonatal emergency care	neonatal emergency care	neonatal emergency care	neonatal emergency care	Neonatal emergency care	2	kept
Neonatal epidemiology	neonatal epidemiology	neonatal epidemiology	neonatal epidemiology	Neonatal epidemiology	2	kept
Neonatal ethics	neonatal ethics	neonatal ethics	neonatal ethics	Neonatal ethics	2	kept
Neonatal hypoglycemia	neonatal hypoglycemia	neonatal hypoglycemia	neonatal hypoglycemia	neonatal hypoglycemia	2	case_or_format
neonatal hypothermia	neonatal hypothermia	neonatal hypothermia	neonatal hypothermia	neonatal hypothermia	2	kept
Neonatal infection	neonatal infection	neonatal infection	neonatal infection	Neonatal infection	2	kept
neonatal infection	neonatal infection	neonatal infection	neonatal infection	Neonatal infection	2	case_or_format
Neonatal jaundice	neonatal jaundice	neonatal jaundice	neonatal jaundice	Neonatal jaundice	2	kept
neonatal jaundice	neonatal jaundice	neonatal jaundice	neonatal jaundice	Neonatal jaundice	2	case_or_format
Neonatal networking	neonatal networking	neonatal networking	neonatal networking	Neonatal networking	2	kept
Neonatal nutrition	neonatal nutrition	neonatal nutrition	neonatal nutrition	Neonatal nutrition	2	kept
neonatal pain agitation and sedation scale	neonatal pain agitation and sedation scale	neonatal pain agitation and sedation scale	neonatal pain agitation and sedation scale	neonatal pain agitation and sedation scale	2	kept

Neonatal pain management	neonatal pain management	neonatal pain management	neonatal pain management	Neonatal pain management	2	kept
Neonatal Palliative care	neonatal palliative care	neonatal palliative care	neonatal palliative care	neonatal palliative care	2	case_or_format
neonatal respiratory failure	neonatal respiratory failure	neonatal respiratory failure	neonatal respiratory failure	neonatal respiratory failure	2	kept
neonatal respiratory support	neonatal respiratory support	neonatal respiratory support	neonatal respiratory support	neonatal respiratory support	2	kept
neonatal resuscitation program	neonatal resuscitation program	neonatal resuscitation program	neonatal resuscitation program	Neonatal Resuscitation Program	2	case_or_format
neonatal resuscitation program algorithm	neonatal resuscitation program algorithm	neonatal resuscitation program algorithm	neonatal resuscitation program algorithm	neonatal resuscitation program algorithm	2	kept
neonatal retrieval	neonatal retrieval	neonatal retrieval	neonatal retrieval	neonatal retrieval	2	kept
Neonatal screening	neonatal screening	neonatal screening	neonatal screening	Neonatal Screening	2	case_or_format
neonatal screening	neonatal screening	neonatal screening	neonatal screening	Neonatal Screening	2	case_or_format
Neonatal seizures	neonatal seizures	neonatal seizures	neonatal seizures	neonatal seizures	2	case_or_format
neonatal sleep	neonatal sleep	neonatal sleep	neonatal sleep	neonatal sleep	2	kept
neonatal standards	neonatal standards	neonatal standards	neonatal standard	neonatal standards	2	singular
neonatal stress	neonatal stress	neonatal stress	neonatal stress	neonatal stress	2	kept
Neonatal surgery	neonatal surgery	neonatal surgery	neonatal surgery	Neonatal surgery	2	kept
NEONATAL UNIT	neonatal unit	neonatal unit	neonatal unit	Neonatal unit	2	case_or_format
Neonatal unit designation	neonatal unit designation	neonatal unit designation	neonatal unit designation	Neonatal unit designation	2	kept

Neonatalogy	neonatalogy	neonatalogy	neonatalogy	Neonatalogy	2	kept
neonate care	neonate care	neonate care	neonate care	neonate care	2	kept
Neoplasm Invasiveness	neoplasm invasiveness	neoplasm invasiveness	neoplasm invasiveness	Neoplasm Invasiveness	2	kept
Neostigmine	neostigmine	neostigmine	neostigmine	neostigmine	2	case_or_format
nephroblastoma	nephroblastoma	nephroblastoma	nephroblastoma	nephroblastoma	2	kept
nephrologists	nephrologists	nephrologists	nephrologist	nephrologist	2	singular
Nephrotoxicity	nephrotoxicity	nephrotoxicity	nephrotoxicity	nephrotoxicity	2	case_or_format
nephroureterectomy	nephroureterectomy	nephroureterectomy	nephroureterectomy	nephroureterectomy	2	kept
nero-linguistic programming	nero linguistic programming	nero linguistic programming	nero linguistic programming	nero-linguistic programming	2	kept
nerve cell network	nerve cell network	nerve cell network	nerve cell network	nerve cell network	2	kept
Nerve conduction studies	nerve conduction studies	nerve conduction studies	nerve conduction study	Nerve conduction studies	2	singular
nervous system function	nervous system function	nervous system function	nervous system function	nervous system function	2	kept
nervous system malformation	nervous system malformation	nervous system malformation	nervous system malformation	Nervous System Malformations	2	case_or_format
Net ultrafiltration	net ultrafiltration	net ultrafiltration	net ultrafiltration	Net ultrafiltration	2	kept
net ultrafiltration	net ultrafiltration	net ultrafiltration	net ultrafiltration	Net ultrafiltration	2	case_or_format
NETWORK	network	network	network	Network	2	case_or_format

network science	network science	network science	network science	network science	2	kept
Neural network model	neural network model	neural network model	neural network model	Neural network model	2	kept
neural networks	neural networks	neural networks	neural network	Neural networks	2	singular
Neural Pathways	neural pathways	neural pathways	neural pathways	Neural Pathways	2	kept
Neuro-Development Treatment	neuro development treatment	neuro development treatment	neuro development treatment	Neuro-Development Treatment	2	kept
Neurobehavior	neurobehavior	neurobehavior	neurobehavior	Neurobehavior	2	kept
Neurobehavioral development	neurobehavioral development	neurobehavioral development	neurobehavioral development	Neurobehavioral development	2	kept
Neuroblastoma	neuroblastoma	neuroblastoma	neuroblastoma	neuroblastoma	2	case_or_format
Neurocognitive Disorders	neurocognitive disorders	neurocognitive disorders	neurocognitive disorders	neurocognitive disorders	2	case_or_format
Neurocognitive disorders	neurocognitive disorders	neurocognitive disorders	neurocognitive disorders	neurocognitive disorders	2	case_or_format
neurocritical	neurocritical	neurocritical	neurocritical	neurocritical	2	kept
neurocysticercosis	neurocysticercosis	neurocysticercosis	neurocysticercosis	neurocysticercosis	2	kept
Neurodegenerative diseases	neurodegenerative diseases	neurodegenerative diseases	neurodegenerative disease	Neurodegenerative diseases	2	singular
neurodegenerative diseases	neurodegenerative diseases	neurodegenerative diseases	neurodegenerative disease	Neurodegenerative diseases	2	singular
neurodevelopmental outcome	neurodevelopmental outcome	neurodevelopmental outcome	neurodevelopmental outcome	neurodevelopmental outcome	2	kept
Neurodevelopmental outcomes	neurodevelopmental outcomes	neurodevelopmental outcomes	neurodevelopmental outcome	neurodevelopmental outcome	2	singular

Neurodevelopmental treatment	neurodevelopmental treatment	neurodevelopmental treatment	neurodevelopmental treatment	Neurodevelopmental treatment	2	kept
Neuroendoscopy	neuroendoscopy	neuroendoscopy	neuroendoscopy	neuroendoscopy	2	case_or_format
neurofibromatosis	neurofibromatosis	neurofibromatosis	neurofibromatosis	neurofibromatosis	2	kept
Neurofunction monitoring	neurofunction monitoring	neurofunction monitoring	neurofunction monitoring	Neurofunction monitoring	2	kept
neurogenic diabetes insipidus	neurogenic diabetes insipidus	neurogenic diabetes insipidus	neurogenic diabetes insipidus	neurogenic diabetes insipidus	2	kept
Neurologic assessment	neurologic assessment	neurologic assessment	neurologic assessment	Neurologic assessment	2	kept
Neurologic dysfunction	neurologic dysfunction	neurologic dysfunction	neurologic dysfunction	Neurologic dysfunction	2	kept
Neurologic examination	neurologic examination	neurologic examination	neurologic examination	neurologic examination	2	case_or_format
Neurologic outcome	neurologic outcome	neurologic outcome	neurologic outcome	Neurologic outcome	2	kept
neurologic outcome	neurologic outcome	neurologic outcome	neurologic outcome	Neurologic outcome	2	case_or_format
Neurologic prognosis	neurologic prognosis	neurologic prognosis	neurologic prognosis	Neurologic prognosis	2	kept
neurological complications	neurological complications	neurological complications	neurological complication	neurological complication	2	singular
Neurological disorders	neurological disorders	neurological disorders	neurological disorders	Neurological disorders	2	kept
Neurological function	neurological function	neurological function	neurological function	neurological function	2	case_or_format
Neurological problems	neurological problems	neurological problems	neurological problems	Neurological problems	2	kept
Neurological prognosis	neurological prognosis	neurological prognosis	neurological prognosis	Neurological prognosis	2	kept

neurological pupillary index	neurological pupillary index	neurological pupillary index	neurological pupillary index	neurological pupillary index	2	kept
NEUROLOGY	neurology	neurology	neurology	neurology	2	case_or_format
Neuromuscular	neuromuscular	neuromuscular	neuromuscular	Neuromuscular	2	kept
Neuromuscular Agents	neuromuscular agents	neuromuscular agents	neuromuscular agents	Neuromuscular Agents	2	kept
Neuromuscular blockers	neuromuscular blockers	neuromuscular blockers	neuromuscular blockers	Neuromuscular blockers	2	kept
neuromuscular blocking agents	neuromuscular blocking agents	neuromuscular blocking agents	neuromuscular blocking agent	neuromuscular blocking agent	2	singular
Neuromuscular diseases	neuromuscular diseases	neuromuscular diseases	neuromuscular disease	neuromuscular disease	2	singular
neuromuscular junction	neuromuscular junction	neuromuscular junction	neuromuscular junction	Neuromuscular Junction	2	case_or_format
Neuronal Plasticity	neuronal plasticity	neuronal plasticity	neuronal plasticity	Neuronal Plasticity	2	kept
Neurons, Afferent	neurons afferent	neurons afferent	neurons afferent	Neurons, Afferent	2	kept
Neuroprotective	neuroprotective	neuroprotective	neuroprotective	Neuroprotective	2	kept
neuroprotective care	neuroprotective care	neuroprotective care	neuroprotective care	neuroprotective care	2	kept
Neuropsychiatric Inventory	neuropsychiatric inventory	neuropsychiatric inventory	neuropsychiatric inventory	neuropsychiatric inventory	2	case_or_format
neuropsychiatry	neuropsychiatry	neuropsychiatry	neuropsychiatry	neuropsychiatry	2	kept
Neuropsychology	neuropsychology	neuropsychology	neuropsychology	neuropsychology	2	case_or_format
neurosensory disability	neurosensory disability	neurosensory disability	neurosensory disability	neurosensory disability	2	kept

neurosurgical procedure	neurosurgical procedure	neurosurgical procedure	neurosurgical procedure	Neurosurgical Procedures	2	case_or_format
neurosyphilis	neurosyphilis	neurosyphilis	neurosyphilis	neurosyphilis	2	kept
Neurotoxicity Syndromes	neurotoxicity syndromes	neurotoxicity syndromes	neurotoxicity syndromes	Neurotoxicity Syndromes	2	kept
neurotoxin	neurotoxin	neurotoxin	neurotoxin	neurotoxin	2	kept
neurotransmission	neurotransmission	neurotransmission	neurotransmission	neurotransmission	2	kept
Neurotrauma	neurotrauma	neurotrauma	neurotrauma	Neurotrauma	2	kept
neurotrauma	neurotrauma	neurotrauma	neurotrauma	Neurotrauma	2	case_or_format
neutral insulin	neutral insulin	neutral insulin	neutral insulin	neutral insulin	2	kept
neutralizing antibody	neutralizing antibody	neutralizing antibody	neutralizing antibody	neutralizing antibody	2	kept
neutrophil collagenase	neutrophil collagenase	neutrophil collagenase	neutrophil collagenase	neutrophil collagenase	2	kept
neutrophil gelatinase associated lipocalin	neutrophil gelatinase associated lipocalin	neutrophil gelatinase associated lipocalin	neutrophil gelatinase associated lipocalin	neutrophil gelatinase associated lipocalin	2	kept
Never events	never events	never events	never events	Never events	2	kept
New Zealander	new zealander	new zealander	new zealander	New Zealander	2	kept
New-born	new born	new born	new born	New-born	2	kept
NEW-ZEALAND	new zealand	new zealand	new zealand	New Zealand	2	case_or_format
NEWBORN	newborn	newborn	newborn	newborn	2	case_or_format

newborn anemia	newborn anemia	newborn anemia	newborn anemia	newborn anemia	2	kept
newborn hemolytic disease	newborn hemolytic disease	newborn hemolytic disease	newborn hemolytic disease	newborn hemolytic disease	2	kept
Newborn Infants	newborn infants	newborn infants	newborn infant	Newborn infant	2	singular
Newborn unit	newborn unit	newborn unit	newborn unit	Newborn unit	2	kept
newsvendor model	newsvendor model	newsvendor model	newsvendor model	newsvendor model	2	kept
next of kin	next of kin	next of kin	next of kin	next of kin	2	kept
NHS HOSPITALS	nhs hospitals	nhs hospitals	nhs hospitals	NHS HOSPITALS	2	kept
NHS Safety Thermometer	nhs safety thermometer	nhs safety thermometer	nhs safety thermometer	NHS Safety Thermometer	2	kept
nicotine patch	nicotine patch	nicotine patch	nicotine patch	nicotine patch	2	kept
nicotinic acid	nicotinic acid	nicotinic acid	nicotinic acid	nicotinic acid	2	kept
Nifedipine	nifedipine	nifedipine	nifedipine	nifedipine	2	case_or_format
nirsevimab	nirsevimab	nirsevimab	nirsevimab	nirsevimab	2	kept
nitrazepam	nitrazepam	nitrazepam	nitrazepam	nitrazepam	2	kept
Nitric oxide	nitric oxide	nitric oxide	nitric oxide	nitric oxide	2	case_or_format
nitrogen balance	nitrogen balance	nitrogen balance	nitrogen balance	nitrogen balance	2	kept
Nitrogen Dioxide	nitrogen dioxide	nitrogen dioxide	nitrogen dioxide	Nitrogen Dioxide	2	kept

nitrogen dioxide	nitrogen dioxide	nitrogen dioxide	nitrogen dioxide	Nitrogen Dioxide	2	case_or_format
nitrous oxide plus oxygen	nitrous oxide plus oxygen	nitrous oxide plus oxygen	nitrous oxide plus oxygen	nitrous oxide plus oxygen	2	kept
Niño	ni o	ni o	ni o	Niño	2	kept
NLCM	nlcm	nlcm	nlcm	NLCM	2	kept
No visitors policy	no visitors policy	no visitors policy	no visitors policy	No visitors policy	2	kept
Nociceptive Pain	nociceptive pain	nociceptive pain	nociceptive pain	Nociceptive Pain	2	kept
nociceptive pain	nociceptive pain	nociceptive pain	nociceptive pain	Nociceptive Pain	2	case_or_format
nociceptive receptor	nociceptive receptor	nociceptive receptor	nociceptive receptor	nociceptive receptor	2	kept
Noise abatement	noise abatement	noise abatement	noise abatement	Noise abatement	2	kept
noise control	noise control	noise control	noise control	noise control	2	kept
Noise management	noise management	noise management	noise management	Noise management	2	kept
noise metrics	noise metrics	noise metrics	noise metrics	noise metrics	2	kept
Noise reduction system	noise reduction system	noise reduction system	noise reduction system	Noise reduction system	2	kept
Noise source localization	noise source localization	noise source localization	noise source localization	Noise source localization	2	kept
NOISE-LEVELS	noise levels	noise levels	noise level	Noise levels	2	singular
Nominal group technique	nominal group technique	nominal group technique	nominal group technique	Nominal group technique	2	kept

nomogram model	nomogram model	nomogram model	nomogram model	nomogram model	2	kept
non anion gap metabolic acidemia	non anion gap metabolic acidemia	non anion gap metabolic acidemia	non anion gap metabolic acidemia	non anion gap metabolic acidemia	2	kept
non cardiac surgery	non cardiac surgery	non cardiac surgery	non cardiac surgery	non cardiac surgery	2	kept
non communicable disease	non communicable disease	non communicable disease	non communicable disease	non communicable disease	2	kept
non invasive blood pressure monitor	non invasive blood pressure monitor	non invasive blood pressure monitor	non invasive blood pressure monitor	non invasive blood pressure monitor	2	kept
non profit organization	non profit organization	non profit organization	non profit organization	non profit organization	2	kept
non-artificial airway	non artificial airway	non artificial airway	non artificial airway	non-artificial airway	2	kept
Non-communicative	non communicative	non communicative	non communicative	Non-communicative	2	kept
non-home discharge	non home discharge	non home discharge	non home discharge	non-home discharge	2	kept
Non-inferiority	non inferiority	non inferiority	non inferiority	Non-inferiority	2	kept
Non-invasive mechanical ventilation	non invasive mechanical ventilation	non invasive mechanical ventilation	non invasive mechanical ventilation	Non-invasive mechanical ventilation	2	kept
non-invasive ward	non invasive ward	non invasive ward	non invasive ward	non-invasive ward	2	kept
non-pharmaceutical interventions	non pharmaceutical interventions	non pharmaceutical interventions	non pharmaceutical interventions	non-pharmaceutical interventions	2	kept
non-pharmacologic interventions	non pharmacologic interventions	non pharmacologic interventions	non pharmacologic interventions	non-pharmacologic interventions	2	kept
Non-pharmacological approach	non pharmacological approach	non pharmacological approach	non pharmacological approach	Non-pharmacological approach	2	kept
Non-pharmacological pain methods	non pharmacological pain methods	non pharmacological pain methods	non pharmacological pain methods	Non-pharmacological pain methods	2	kept

Non-pharmacological treatment	non pharmacological treatment	non pharmacological treatment	non pharmacological treatment	Non-pharmacological treatment	2	kept
Non-randomized controlled trial	non randomized controlled trial	non randomized controlled trial	non randomized controlled trial	Non-randomized controlled trial	2	kept
non-smoker	non smoker	non smoker	non smoker	non-smoker	2	kept
Non-traumatic subarachnoid hemorrhage	non traumatic subarachnoid hemorrhage	non traumatic subarachnoid hemorrhage	non traumatic subarachnoid hemorrhage	Non-traumatic subarachnoid hemorrhage	2	kept
nonattendance	nonattendance	nonattendance	nonattendance	nonattendance	2	kept
noncommunicable disease	noncommunicable disease	noncommunicable disease	noncommunicable disease	noncommunicable disease	2	kept
Noncompliance	noncompliance	noncompliance	noncompliance	Noncompliance	2	kept
noncontact monitoring	noncontact monitoring	noncontact monitoring	noncontact monitoring	noncontact monitoring	2	kept
Nonconvulsive seizures	nonconvulsive seizures	nonconvulsive seizures	nonconvulsive seizures	Nonconvulsive seizures	2	kept
nonhome discharge	nonhome discharge	nonhome discharge	nonhome discharge	nonhome discharge	2	kept
noninvasive	noninvasive	noninvasive	noninvasive	noninvasive	2	kept
noninvasive blood pressure	noninvasive blood pressure	noninvasive blood pressure	noninvasive blood pressure	noninvasive blood pressure	2	kept
noninvasive ventilator	noninvasive ventilator	noninvasive ventilator	noninvasive ventilator	noninvasive ventilator	2	kept
nonischemic cardiomyopathy	nonischemic cardiomyopathy	nonischemic cardiomyopathy	nonischemic cardiomyopathy	nonischemic cardiomyopathy	2	kept
Nonlinear dependencies	nonlinear dependencies	nonlinear dependencies	nonlinear dependencies	Nonlinear dependencies	2	kept
Nonlinear Dynamics	nonlinear dynamics	nonlinear dynamics	nonlinear dynamics	Nonlinear Dynamics	2	kept

Nonlinear Mapping	nonlinear mapping	nonlinear mapping	nonlinear mapping	Nonlinear Mapping	2	kept
nonmaleficence	nonmaleficence	nonmaleficence	nonmaleficence	nonmaleficence	2	kept
nonmedical occupations	nonmedical occupations	nonmedical occupations	nonmedical occupations	nonmedical occupations	2	kept
Nonnutritive sucking	nonnutritive sucking	nonnutritive sucking	nonnutritive sucking	Nonnutritive sucking	2	kept
nonnutritive sucking	nonnutritive sucking	nonnutritive sucking	nonnutritive sucking	Nonnutritive sucking	2	case_or_format
nonpharmacologic intervention	nonpharmacologic intervention	nonpharmacologic intervention	nonpharmacologic intervention	nonpharmacologic intervention	2	kept
Nonpharmacological	nonpharmacological	nonpharmacological	nonpharmacological	Nonpharmacological	2	kept
Nonpharmacological intervention	nonpharmacological intervention	nonpharmacological intervention	nonpharmacological intervention	Nonpharmacological interventions	2	case_or_format
nonpharmacological pain care	nonpharmacological pain care	nonpharmacological pain care	nonpharmacological pain care	nonpharmacological pain care	2	kept
nonsteroidal anti-inflammatory drugs	nonsteroidal anti inflammatory drugs	nonsteroidal anti inflammatory drugs	nonsteroidal anti inflammatory drugs	nonsteroidal anti-inflammatory drugs	2	kept
Nontechnical skills	nontechnical skills	nontechnical skills	nontechnical skills	NONTECHNICAL SKILLS	2	case_or_format
Nonverbal pain scale	nonverbal pain scale	nonverbal pain scale	nonverbal pain scale	Nonverbal pain scale	2	kept
Noradrenaline	noradrenaline	noradrenaline	noradrenaline	Noradrenaline	2	kept
noradrénaline	noradr naline	noradr naline	noradr naline	noradrénaline	2	kept
normal saline	normal saline	normal saline	normal saline	Normal saline	2	case_or_format
normal saline solution	normal saline solution	normal saline solution	normal saline solution	normal saline solution	2	kept

Northwest Ethiopia	northwest ethiopia	northwest ethiopia	northwest ethiopia	Northwest Ethiopia	2	kept
Norwegian	norwegian	norwegian	norwegian	Norwegian	2	kept
Norwood Procedures	norwood procedures	norwood procedures	norwood procedure	Norwood procedure	2	singular
Nose Diseases	nose diseases	nose diseases	nose diseases	Nose Diseases	2	kept
nose infection	nose infection	nose infection	nose infection	nose infection	2	kept
nose spray	nose spray	nose spray	nose spray	nose spray	2	kept
nose ulcer	nose ulcer	nose ulcer	nose ulcer	nose ulcer	2	kept
nosocomial infection density	nosocomial infection density	nosocomial infection density	nosocomial infection density	nosocomial infection density	2	kept
Nosocomial infection prevention	nosocomial infection prevention	nosocomial infection prevention	nosocomial infection prevention	Nosocomial infection prevention	2	kept
Nosocomial Infections	nosocomial infections	nosocomial infections	nosocomial infection	Nosocomial infection	2	singular
Nosocomial infectious diarrhea	nosocomial infectious diarrhea	nosocomial infectious diarrhea	nosocomial infectious diarrhea	Nosocomial infectious diarrhea	2	kept
novel Coronavirus	novel coronavirus	novel coronavirus	novel coronavirus	Novel coronavirus	2	case_or_format
Novel coronavirus disease 2019	novel coronavirus disease 2019	novel coronavirus disease 2019	novel coronavirus disease 2019	Novel coronavirus disease 2019	2	kept
novel coronavirus infection	novel coronavirus infection	novel coronavirus infection	novel coronavirus infection	novel coronavirus infection	2	kept
Novel methods	novel methods	novel methods	novel methods	Novel methods	2	kept
novice	novice	novice	novice	novice	2	kept

Noxae	noxae	noxae	noxae	Noxae	2	kept
Noxious stimulus	noxious stimulus	noxious stimulus	noxious stimulus	Noxious stimulus	2	kept
NPi	npi	npi	npi	NPi	2	kept
NPSIRS	npsirs	npsirs	npsirs	NPSIRS	2	kept
NSE	nse	nse	nse	NSE	2	kept
NTS	nts	nts	nts	NTS	2	kept
Nuclear Family	nuclear family	nuclear family	nuclear family	Nuclear Family	2	kept
nuclear family	nuclear family	nuclear family	nuclear family	Nuclear Family	2	case_or_format
nuclear medicine	nuclear medicine	nuclear medicine	nuclear medicine	nuclear medicine	2	kept
nucleic acid amplification	nucleic acid amplification	nucleic acid amplification	nucleic acid amplification	nucleic acid amplification	2	kept
nucleic acid amplification techniques	nucleic acid amplification techniques	nucleic acid amplification techniques	nucleic acid amplification techniques	nucleic acid amplification techniques	2	kept
nucleocapsid protein	nucleocapsid protein	nucleocapsid protein	nucleocapsid protein	nucleocapsid protein	2	kept
NuDesc	nudesc	nudesc	nudesc	NuDesc	2	kept
Nudesc	nudesc	nudesc	nudesc	NuDesc	2	case_or_format
null hypothesis	null hypothesis	null hypothesis	null hypothesis	null hypothesis	2	kept
Numeric rating scale	numeric rating scale	numeric rating scale	numeric rating scale	numeric rating scale	2	case_or_format

Nunavut	nunavut	nunavut	nunavut	Nunavut	2	kept
Nurture	nurture	nurture	nurture	Nurture	2	kept
nutrient content	nutrient content	nutrient content	nutrient content	nutrient content	2	kept
nutrient intake	nutrient intake	nutrient intake	nutrient intake	nutrient intake	2	kept
nutrient uptake	nutrient uptake	nutrient uptake	nutrient uptake	nutrient uptake	2	kept
NUTRITION	nutrition	nutrition	nutrition	nutrition	2	case_or_format
NUTRITION & DIETETICS	nutrition and dietetics	nutrition and dietetics	nutrition and dietetics	NUTRITION & DIETETICS	2	kept
nutrition risk	nutrition risk	nutrition risk	nutrition risk	nutrition risk	2	kept
nutrition screening	nutrition screening	nutrition screening	nutrition screening	nutrition screening	2	kept
Nutrition status	nutrition status	nutrition status	nutrition status	Nutrition status	2	kept
Nutrition support	nutrition support	nutrition support	nutrition support	nutrition support	2	case_or_format
Nutrition support practices	nutrition support practices	nutrition support practices	nutrition support practice	nutrition support practice	2	singular
nutrition therapy	nutrition therapy	nutrition therapy	nutrition therapy	Nutrition Therapy	2	case_or_format
nutritional counseling	nutritional counseling	nutritional counseling	nutritional counseling	nutritional counseling	2	kept
nutritional myodegeneration	nutritional myodegeneration	nutritional myodegeneration	nutritional myodegeneration	nutritional myodegeneration	2	kept
Nutritional Physiological Phenomena	nutritional physiological phenomena	nutritional physiological phenomena	nutritional physiological phenomena	Nutritional Physiological Phenomena	2	kept

nutritional risk	nutritional risk	nutritional risk	nutritional risk	Nutritional risk	2	case_or_format
Nutritional Risk Index	nutritional risk index	nutritional risk index	nutritional risk index	Nutritional Risk Index	2	kept
nutritional risk score	nutritional risk score	nutritional risk score	nutritional risk score	nutritional risk score	2	kept
Nutritional state	nutritional state	nutritional state	nutritional state	Nutritional state	2	kept
Nutritional therapy	nutritional therapy	nutritional therapy	nutritional therapy	Nutritional therapy	2	kept
nutritionDay	nutritionday	nutritionday	nutritionday	nutritionDay	2	kept
NWI-R	nwi r	nwi r	nwi r	NWI-R	2	kept
Object attachment	object attachment	object attachment	object attachment	Object Attachment	2	case_or_format
objective structured clinical exam	objective structured clinical exam	objective structured clinical exam	objective structured clinical exam	objective structured clinical exam	2	kept
Observation data	observation data	observation data	observation data	Observation data	2	kept
observation, participant	observation participant	observation participant	observation participant	observation, participant	2	kept
observational and multicentre study	observational and multicentre study	observational and multicentre study	observational and multicentre study	observational and multicentre study	2	kept
Observational data	observational data	observational data	observational data	Observational data	2	kept
Observations	observations	observations	observation	Observation	2	singular
observations	observations	observations	observation	Observation	2	singular
obsession	obsession	obsession	obsession	obsession	2	kept

Obsessive Behavior	obsessive behavior	obsessive behavior	obsessive behavior	Obsessive Behavior	2	kept
obstacle analysis	obstacle analysis	obstacle analysis	obstacle analysis	obstacle analysis	2	kept
obstetric emergencies	obstetric emergencies	obstetric emergencies	obstetric emergency	obstetric emergency	2	singular
Obstetric high dependency care	obstetric high dependency care	obstetric high dependency care	obstetric high dependency care	Obstetric high dependency care	2	kept
Obstetric high dependency unit	obstetric high dependency unit	obstetric high dependency unit	obstetric high dependency unit	Obstetric high dependency unit	2	kept
obstetrical	obstetrical	obstetrical	obstetrical	obstetrical	2	kept
obstetrical hemorrhage	obstetrical hemorrhage	obstetrical hemorrhage	obstetrical hemorrhage	obstetrical hemorrhage	2	kept
OBSTETRICS	obstetrics	obstetrics	obstetric	Obstetrics	2	singular
Obstetrics and gynaecology	obstetrics and gynaecology	obstetrics and gynaecology	obstetrics and gynaecology	Obstetrics and gynaecology	2	kept
Obstetrics and gynecology	obstetrics and gynecology	obstetrics and gynecology	obstetrics and gynecology	obstetrics and gynecology	2	case_or_format
Obstructive Sleep Apnea	obstructive sleep apnea	obstructive sleep apnea	obstructive sleep apnea	Obstructive sleep apnea	2	case_or_format
Obtención de tejidos y órganos	obtenci n de tejidos y rganos	obtenci n de tejidos y rganos	obtenci n de tejidos y rganos	Obtención de tejidos y órganos	2	kept
occipital lobe	occipital lobe	occipital lobe	occipital lobe	occipital lobe	2	kept
occiput	occiput	occiput	occiput	occiput	2	kept
Occlusion	occlusion	occlusion	occlusion	occlusion	2	case_or_format
Occlusive dressings	occlusive dressings	occlusive dressings	occlusive dressing	Occlusive Dressings	2	singular

Occult Blood	occult blood	occult blood	occult blood	Occult Blood	2	kept
occult blood	occult blood	occult blood	occult blood	Occult Blood	2	case_or_format
Occupancy	occupancy	occupancy	occupancy	OCCUPANCY	2	case_or_format
Occupation	occupation	occupation	occupation	occupation	2	case_or_format
occupational benefit perception	occupational benefit perception	occupational benefit perception	occupational benefit perception	occupational benefit perception	2	kept
OCCUPATIONAL BURNOUT	occupational burnout	occupational burnout	occupational burnout	occupational burnout	2	case_or_format
occupational burnout syndrome	occupational burnout syndrome	occupational burnout syndrome	occupational burnout syndrome	occupational burnout syndrome	2	kept
Occupational distress	occupational distress	occupational distress	occupational distress	Occupational distress	2	kept
Occupational Ethics	occupational ethics	occupational ethics	occupational ethics	Occupational Ethics	2	kept
occupational fatigue	occupational fatigue	occupational fatigue	occupational fatigue	occupational fatigue	2	kept
occupational health issues	occupational health issues	occupational health issues	occupational health issues	occupational health issues	2	kept
Occupational infections	occupational infections	occupational infections	occupational infections	Occupational infections	2	kept
Occupational noise	occupational noise	occupational noise	occupational noise	Occupational noise	2	kept
Occupational risk	occupational risk	occupational risk	occupational risk	Occupational risks	2	case_or_format
occupational therapist attitude	occupational therapist attitude	occupational therapist attitude	occupational therapist attitude	occupational therapist attitude	2	kept
Occupational well-being	occupational well being	occupational well being	occupational well being	Occupational well-being	2	kept

occupational well-being	occupational well being	occupational well being	occupational well being	Occupational well-being	2	case_or_format
Oceania	oceania	oceania	oceania	Oceania	2	kept
Oceanic Ancestry Group	oceanic ancestry group	oceanic ancestry group	oceanic ancestry group	Oceanic Ancestry Group	2	kept
Oceanic ancestry group	oceanic ancestry group	oceanic ancestry group	oceanic ancestry group	Oceanic Ancestry Group	2	case_or_format
octogenarians	octogenarians	octogenarians	octogenarian	Octogenarians	2	singular
ocular health	ocular health	ocular health	ocular health	ocular health	2	kept
ocular surface disorder	ocular surface disorder	ocular surface disorder	ocular surface disorder	ocular surface disorder	2	kept
Odisha	odisha	odisha	odisha	Odisha	2	kept
odontoid process fracture	odontoid process fracture	odontoid process fracture	odontoid process fracture	odontoid process fracture	2	kept
Odors	odors	odors	odor	odor	2	singular
off pump surgery	off pump surgery	off pump surgery	off pump surgery	off pump surgery	2	kept
offline	offline	offline	offline	offline	2	kept
Ofloxacin	ofloxacin	ofloxacin	ofloxacin	ofloxacin	2	case_or_format
OKT 3	okt 3	okt 3	okt 3	OKT 3	2	kept
OLDER PERSONS	older persons	older persons	older person	older person	2	singular
olfactory	olfactory	olfactory	olfactory	olfactory	2	kept

omadacycline	omadacycline	omadacycline	omadacycline	omadacycline	2	kept
omega-3 polyunsaturated fatty acids	omega 3 polyunsaturated fatty acids	omega 3 polyunsaturated fatty acids	omega 3 polyunsaturated fatty acids	omega-3 polyunsaturated fatty acids	2	kept
omission errors	omission errors	omission errors	omission error	omission errors	2	singular
on-table extubation protocol	on table extubation protocol	on table extubation protocol	on table extubation protocol	on-table extubation protocol	2	kept
Oncologists	oncologists	oncologists	oncologist	oncologist	2	singular
oncoprotein	oncoprotein	oncoprotein	oncoprotein	oncoprotein	2	kept
One Health	one health	one health	one health	One Health	2	kept
Online	online	online	online	online	2	case_or_format
online survey	online survey	online survey	online survey	Online survey	2	case_or_format
ONSET	onset	onset	onset	ONSET	2	kept
ontology	ontology	ontology	ontology	Ontology	2	case_or_format
oocyte donation	oocyte donation	oocyte donation	oocyte donation	oocyte donation	2	kept
Open abdomen complications	open abdomen complications	open abdomen complications	open abdomen complications	Open abdomen complications	2	kept
open bedside tracheostomy	open bedside tracheostomy	open bedside tracheostomy	open bedside tracheostomy	open bedside tracheostomy	2	kept
open cardiac surgery	open cardiac surgery	open cardiac surgery	open cardiac surgery	open cardiac surgery	2	kept
open visitation policy	open visitation policy	open visitation policy	open visitation policy	open visitation policy	2	kept

Open visiting	open visiting	open visiting	open visiting	Open visiting	2	kept
open-heart surgery	open heart surgery	open heart surgery	open heart surgery	open heart surgery	2	case_or_format
Operating Room Technicians	operating room technicians	operating room technicians	operating room technicians	Operating Room Technicians	2	kept
operating theater	operating theater	operating theater	operating theater	operating theater	2	kept
Operating theatre planning	operating theatre planning	operating theatre planning	operating theatre planning	Operating theatre planning	2	kept
operation	operation	operation	operation	OPERATIONS	2	case_or_format
operations management	operations management	operations management	operations management	Operations management	2	case_or_format
Operations Research	operations research	operations research	operations research	Operations research	2	case_or_format
Ophthalmologic Surgical Procedures	ophthalmologic surgical procedures	ophthalmologic surgical procedures	ophthalmologic surgical procedures	Ophthalmologic Surgical Procedures	2	kept
ophthalmoplegia	ophthalmoplegia	ophthalmoplegia	ophthalmoplegia	ophthalmoplegia	2	kept
opiates	opiates	opiates	opiate	opiate	2	singular
Opinions	opinions	opinions	opinion	Opinion	2	singular
Opioid administration	opioid administration	opioid administration	opioid administration	Opioid administration	2	kept
opioid induced constipation	opioid induced constipation	opioid induced constipation	opioid induced constipation	opioid induced constipation	2	kept
opioid infusion	opioid infusion	opioid infusion	opioid infusion	opioid infusion	2	kept
opioid use disorder	opioid use disorder	opioid use disorder	opioid use disorder	Opioid use disorder	2	case_or_format

opioid withdrawal	opioid withdrawal	opioid withdrawal	opioid withdrawal	opioid withdrawal	2	kept
opioid-induced respiratory depression	opioid induced respiratory depression	opioid induced respiratory depression	opioid induced respiratory depression	opioid-induced respiratory depression	2	kept
OPPORTUNITIES	opportunities	opportunities	opportunity	Opportunities	2	singular
opportunity cost	opportunity cost	opportunity cost	opportunity cost	opportunity cost	2	kept
Optic nerve sheath diameter	optic nerve sheath diameter	optic nerve sheath diameter	optic nerve sheath diameter	optic nerve sheath diameter	2	case_or_format
Optical Devices	optical devices	optical devices	optical devices	Optical Devices	2	kept
Optimisations	optimisations	optimisations	optimisation	Optimisations	2	singular
Optimization algorithms	optimization algorithms	optimization algorithms	optimization algorithms	Optimization algorithms	2	kept
Optimization modeling	optimization modeling	optimization modeling	optimization modeling	Optimization modeling	2	kept
oral administration	oral administration	oral administration	oral administration	oral administration	2	kept
ORAL CARE	oral care	oral care	oral care	Oral care	2	case_or_format
oral care hygiene	oral care hygiene	oral care hygiene	oral care hygiene	oral care hygiene	2	kept
Oral contraceptive pills	oral contraceptive pills	oral contraceptive pills	oral contraceptive pills	Oral contraceptive pills	2	kept
oral endotracheal intubation	oral endotracheal intubation	oral endotracheal intubation	oral endotracheal intubation	oral endotracheal intubation	2	kept
Oral Feeding Function	oral feeding function	oral feeding function	oral feeding function	Oral Feeding Function	2	kept
oral feeding practices	oral feeding practices	oral feeding practices	oral feeding practices	oral feeding practices	2	kept

oral feeding readiness	oral feeding readiness	oral feeding readiness	oral feeding readiness	oral feeding readiness	2	kept
ORAL GLUCOSE	oral glucose	oral glucose	oral glucose	ORAL GLUCOSE	2	kept
Oral hygiene care	oral hygiene care	oral hygiene care	oral hygiene care	Oral hygiene care	2	kept
oral lesion	oral lesion	oral lesion	oral lesion	oral lesion	2	kept
oral microbiome	oral microbiome	oral microbiome	oral microbiome	oral microbiome	2	kept
Oral motor function	oral motor function	oral motor function	oral motor function	Oral motor function	2	kept
oral motor stimulation	oral motor stimulation	oral motor stimulation	oral motor stimulation	oral motor stimulation	2	kept
Oral mucosa	oral mucosa	oral mucosa	oral mucosa	Oral mucosa	2	kept
oral mucosal disease	oral mucosal disease	oral mucosal disease	oral mucosal disease	oral mucosal disease	2	kept
Oral mucositis	oral mucositis	oral mucositis	oral mucositis	oral mucositis	2	case_or_format
Oral nutrition	oral nutrition	oral nutrition	oral nutrition	Oral nutrition	2	kept
ORAL SUPPLEMENTATION	oral supplementation	oral supplementation	oral supplementation	ORAL SUPPLEMENTATION	2	kept
orbit fracture	orbit fracture	orbit fracture	orbit fracture	orbit fracture	2	kept
Orbital Diseases	orbital diseases	orbital diseases	orbital diseases	Orbital Diseases	2	kept
ORDER ENTRY	order entry	order entry	order entry	ORDER ENTRY	2	kept
Ordinal logistic regression	ordinal logistic regression	ordinal logistic regression	ordinal logistic regression	Ordinal logistic regression	2	kept

Orem self-care model	orem self care model	orem self care model	orem self care model	Orem self-care model	2	kept
organ	organ	organ	organ	organ	2	kept
Organ and tissue donation	organ and tissue donation	organ and tissue donation	organ and tissue donation	Organ and tissue donation	2	kept
Organ Donation	organ donation	organ donation	organ donation	Organ donation	2	case_or_format
Organ donation process	organ donation process	organ donation process	organ donation process	Organ donation process	2	kept
Organ donations	organ donations	organ donations	organ donation	Organ donation	2	singular
Organ donors	organ donors	organ donors	organ donor	organ donor	2	singular
ORGAN DYSFUNCTION	organ dysfunction	organ dysfunction	organ dysfunction	organ dysfunction	2	case_or_format
Organ dysfunction scores	organ dysfunction scores	organ dysfunction scores	organ dysfunction score	Organ Dysfunction Scores	2	singular
organ preservation	organ preservation	organ preservation	organ preservation	organ preservation	2	kept
organ procurement	organ procurement	organ procurement	organ procurement	Organ procurement	2	case_or_format
organ procurement organization	organ procurement organization	organ procurement organization	organ procurement organization	organ procurement organization	2	kept
Organ retrieval	organ retrieval	organ retrieval	organ retrieval	Organ retrieval	2	kept
Organ Size	organ size	organ size	organ size	Organ Size	2	kept
organic psychosyndrome	organic psychosyndrome	organic psychosyndrome	organic psychosyndrome	organic psychosyndrome	2	kept
Organisation for Economic Co-operation and Development	organisation for economic co operation and development	organisation for economic co operation and development	organisation for economic co operation and development	Organisation for Economic Co-operation and Development	2	kept

organisational adaptation	organisational adaptation	organisational adaptation	organisational adaptation	organisational adaptation	2	kept
organisational factors	organisational factors	organisational factors	organisational factors	organisational factors	2	kept
organisational learning	organisational learning	organisational learning	organisational learning	organisational learning	2	kept
Organisational strategies	organisational strategies	organisational strategies	organisational strategies	Organisational strategies	2	kept
organisational support	organisational support	organisational support	organisational support	organisational support	2	kept
organisms	organisms	organisms	organisms	organisms	2	kept
Organization and Administration	organization and administration	organization and administration	organization and administration	Organization and Administration	2	kept
organization and administration	organization and administration	organization and administration	organization and administration	Organization and Administration	2	case_or_format
Organization of care	organization of care	organization of care	organization of care	Organization of care	2	kept
Organizational Affiliation	organizational affiliation	organizational affiliation	organizational affiliation	Organizational Affiliation	2	kept
organizational behavior	organizational behavior	organizational behavior	organizational behavior	organizational behavior	2	kept
organizational change	organizational change	organizational change	organizational change	organizational change	2	kept
ORGANIZATIONAL CHARACTERISTICS	organizational characteristics	organizational characteristics	organizational characteristics	ORGANIZATIONAL CHARACTERISTICS	2	kept
Organizational characteristics	organizational characteristics	organizational characteristics	organizational characteristics	ORGANIZATIONAL CHARACTERISTICS	2	case_or_format
organizational characteristics	organizational characteristics	organizational characteristics	organizational characteristics	ORGANIZATIONAL CHARACTERISTICS	2	case_or_format
ORGANIZATIONAL CITIZENSHIP BEHAVIOR	organizational citizenship behavior	organizational citizenship behavior	organizational citizenship behavior	ORGANIZATIONAL CITIZENSHIP BEHAVIOR	2	kept

organizational ethics	organizational ethics	organizational ethics	organizational ethics	organizational ethics	2	kept
Organizational Intelligence	organizational intelligence	organizational intelligence	organizational intelligence	Organizational Intelligence	2	kept
Organizational justice	organizational justice	organizational justice	organizational justice	Organizational justice	2	kept
Organizational politics	organizational politics	organizational politics	organizational politics	Organizational politics	2	kept
Organizational psychology	organizational psychology	organizational psychology	organizational psychology	Organizational psychology	2	kept
Organizational support	organizational support	organizational support	organizational support	organizational support	2	case_or_format
organizational theory	organizational theory	organizational theory	organizational theory	organizational theory	2	kept
Organizational-based self-esteem	organizational based self esteem	organizational based self esteem	organizational based self esteem	Organizational-based self-esteem	2	kept
ORGANIZATIONAL-CLIMATE	organizational climate	organizational climate	organizational climate	Organizational climate	2	case_or_format
Organizations, Nonprofit	organizations nonprofit	organizations nonprofit	organizations nonprofit	Organizations, Nonprofit	2	kept
organophosphate insecticide	organophosphate insecticide	organophosphate insecticide	organophosphate insecticide	organophosphate insecticide	2	kept
organophosphate poisoning	organophosphate poisoning	organophosphate poisoning	organophosphate poisoning	Organophosphate Poisoning	2	case_or_format
Organophosphates	organophosphates	organophosphates	organophosphate	organophosphate	2	singular
oriental medicine	oriental medicine	oriental medicine	oriental medicine	oriental medicine	2	kept
ornithosis	ornithosis	ornithosis	ornithosis	ornithosis	2	kept
Orogastric tube	orogastric tube	orogastric tube	orogastric tube	Orogastric tube	2	kept

orogastric tube	orogastric tube	orogastric tube	orogastric tube	Orogastric tube	2	case_or_format
Oromia	oromia	oromia	oromia	Oromia	2	kept
oropharyngeal dysphagia	oropharyngeal dysphagia	oropharyngeal dysphagia	oropharyngeal dysphagia	oropharyngeal dysphagia	2	kept
Orotracheal intubation	orotracheal intubation	orotracheal intubation	orotracheal intubation	Orotracheal intubation	2	kept
Orthopaedic	orthopaedic	orthopaedic	orthopaedic	Orthopaedic	2	kept
Orthopedic surgery	orthopedic surgery	orthopedic surgery	orthopedic surgery	orthopedic surgery	2	case_or_format
orthotics	orthotics	orthotics	orthotics	orthotics	2	kept
orthotopic liver transplantation	orthotopic liver transplantation	orthotopic liver transplantation	orthotopic liver transplantation	orthotopic liver transplantation	2	kept
osmosis	osmosis	osmosis	osmosis	osmosis	2	kept
Osmotherapy	osmotherapy	osmotherapy	osmotherapy	Osmotherapy	2	kept
osmotic diuretic agent	osmotic diuretic agent	osmotic diuretic agent	osmotic diuretic agent	osmotic diuretic agent	2	kept
osmotic pressure	osmotic pressure	osmotic pressure	osmotic pressure	osmotic pressure	2	kept
osseointegration	osseointegration	osseointegration	osseointegration	osseointegration	2	kept
osteogenesis imperfecta	osteogenesis imperfecta	osteogenesis imperfecta	osteogenesis imperfecta	osteogenesis imperfecta	2	kept
osteogenesis imperfecta type II	osteogenesis imperfecta type ii	osteogenesis imperfecta type ii	osteogenesis imperfecta type ii	osteogenesis imperfecta type II	2	kept
osteolysis	osteolysis	osteolysis	osteolysis	osteolysis	2	kept

Osteomyelitis	osteomyelitis	osteomyelitis	osteomyelitis	osteomyelitis	2	case_or_format
osteopathic medicine	osteopathic medicine	osteopathic medicine	osteopathic medicine	osteopathic medicine	2	kept
Osteosarcoma	osteosarcoma	osteosarcoma	osteosarcoma	osteosarcoma	2	case_or_format
Oswestry questionnaire	oswestry questionnaire	oswestry questionnaire	oswestry questionnaire	Oswestry questionnaire	2	kept
ototoxicity	ototoxicity	ototoxicity	ototoxicity	ototoxicity	2	kept
out-of-hospital mortality	out of hospital mortality	out of hospital mortality	out of hospital mortality	out-of-hospital mortality	2	kept
outborn	outborn	outborn	outborn	outborn	2	kept
Outbreak setting	outbreak setting	outbreak setting	outbreak setting	Outbreak setting	2	kept
Outbreaks	outbreaks	outbreaks	outbreak	Outbreak	2	singular
outbreaks	outbreaks	outbreaks	outbreak	Outbreak	2	singular
Outcome following severe brain damage	outcome following severe brain damage	outcome following severe brain damage	outcome following severe brain damage	Outcome following severe brain damage	2	kept
Outcome prediction	outcome prediction	outcome prediction	outcome prediction	Outcome prediction	2	kept
Outcomes assessment	outcomes assessment	outcomes assessment	outcomes assessment	Outcomes assessment	2	kept
Outer membrane protein	outer membrane protein	outer membrane protein	outer membrane protein	Outer membrane protein	2	kept
Outreach service	outreach service	outreach service	outreach service	Outreach services	2	case_or_format
outreach services	outreach services	outreach services	outreach service	Outreach services	2	singular

Ovako working posture analyzing system	ovako working posture analyzing system	ovako working posture analyzing system	ovako working posture analyzing system	Ovako working posture analyzing system	2	kept
Ovarian Neoplasms	ovarian neoplasms	ovarian neoplasms	ovarian neoplasms	Ovarian Neoplasms	2	kept
Overall health	overall health	overall health	overall health	Overall health	2	kept
overdose	overdose	overdose	overdose	Overdose	2	case_or_format
overlapping gene	overlapping gene	overlapping gene	overlapping gene	overlapping gene	2	kept
overnight	overnight	overnight	overnight	overnight	2	kept
overnight shift	overnight shift	overnight shift	overnight shift	overnight shift	2	kept
Overtime	overtime	overtime	overtime	overtime	2	case_or_format
Overtriage	overtriage	overtriage	overtriage	Overtriage	2	kept
ovulation induction	ovulation induction	ovulation induction	ovulation induction	ovulation induction	2	kept
OWL-S	owl s	owl s	owl s	OWL-S	2	kept
OXA-48	oxa 48	oxa 48	oxa 48	OXA-48	2	kept
oxandrolone	oxandrolone	oxandrolone	oxandrolone	oxandrolone	2	kept
OXIDATIVE STRESS	oxidative stress	oxidative stress	oxidative stress	oxidative stress	2	case_or_format
Oxides	oxides	oxides	oxide	Oxides	2	singular
oximeter	oximeter	oximeter	oximeter	Oximeters	2	case_or_format

oxygen blood level	oxygen blood level	oxygen blood level	oxygen blood level	oxygen blood level	2	kept
oxygen breathing	oxygen breathing	oxygen breathing	oxygen breathing	oxygen breathing	2	kept
oxygen delivery	oxygen delivery	oxygen delivery	oxygen delivery	Oxygen delivery	2	case_or_format
oxygen extraction fraction	oxygen extraction fraction	oxygen extraction fraction	oxygen extraction fraction	oxygen extraction fraction	2	kept
oxygen inhalation therapy	oxygen inhalation therapy	oxygen inhalation therapy	oxygen inhalation therapy	Oxygen Inhalation Therapy	2	case_or_format
oxygen saturation monitoring	oxygen saturation monitoring	oxygen saturation monitoring	oxygen saturation monitoring	oxygen saturation monitoring	2	kept
Oxygen supplementation	oxygen supplementation	oxygen supplementation	oxygen supplementation	Oxygen supplementation	2	kept
oxygenation status	oxygenation status	oxygenation status	oxygenation status	oxygenation status	2	kept
Ozone	ozone	ozone	ozone	Ozone	2	kept
P-ASTERISK MODELS	p asterisk models	p asterisk models	p asterisk models	P-ASTERISK MODELS	2	kept
packaging	packaging	packaging	packaging	packaging	2	kept
PAD guidelines	pad guidelines	pad guidelines	pad guidelines	PAD guidelines	2	kept
PADIS	padis	padis	padis	PADIS	2	kept
paedaitrics	paedaitrics	paedaitrics	paedaitrics	paedaitrics	2	kept
paediatric care	paediatric care	paediatric care	paediatric care	paediatric care	2	kept
Paediatric delirium	paediatric delirium	paediatric delirium	paediatric delirium	paediatric delirium	2	case_or_format

Paediatric Disorder of Consciousness	paediatric disorder of consciousness	paediatric disorder of consciousness	paediatric disorder of consciousness	Paediatric Disorder of Consciousness	2	kept
Paediatric gastroenterology	paediatric gastroenterology	paediatric gastroenterology	paediatric gastroenterology	Paediatric gastroenterology	2	kept
paediatric hospitals	paediatric hospitals	paediatric hospitals	paediatric hospital	paediatric hospitals	2	singular
paediatric palliative care	paediatric palliative care	paediatric palliative care	paediatric palliative care	paediatric palliative care	2	kept
Paediatric Practice	paediatric practice	paediatric practice	paediatric practice	Paediatric Practice	2	kept
paediatric staffing	paediatric staffing	paediatric staffing	paediatric staffing	paediatric staffing	2	kept
paediatric surgery	paediatric surgery	paediatric surgery	paediatric surgery	Paediatric surgery	2	case_or_format
Paediatric transport	paediatric transport	paediatric transport	paediatric transport	Paediatric transport	2	kept
pager	pager	pager	pager	pager	2	kept
Pain control	pain control	pain control	pain control	pain control	2	case_or_format
pain education	pain education	pain education	pain education	pain education	2	kept
Pain management algorithm	pain management algorithm	pain management algorithm	pain management algorithm	Pain management algorithm	2	kept
Pain monitoring	pain monitoring	pain monitoring	pain monitoring	Pain monitoring	2	kept
pain perception	pain perception	pain perception	pain perception	Pain Perception	2	case_or_format
Pain practice	pain practice	pain practice	pain practice	Pain practice	2	kept
pain prevention	pain prevention	pain prevention	pain prevention	pain prevention	2	kept

Pain reduction	pain reduction	pain reduction	pain reduction	Pain reduction	2	kept
Pain scale assessment	pain scale assessment	pain scale assessment	pain scale assessment	Pain scale assessment	2	kept
pain severity score	pain severity score	pain severity score	pain severity score	pain severity score	2	kept
pain terms	pain terms	pain terms	pain terms	pain terms	2	kept
pain treatment	pain treatment	pain treatment	pain treatment	pain treatment	2	kept
Pain, Intractable	pain intractable	pain intractable	pain intractable	Pain, Intractable	2	kept
Painful procedure	painful procedure	painful procedure	painful procedure	Painful procedures	2	case_or_format
Painful vaso-occlusive crises	painful vaso occlusive crises	painful vaso occlusive crises	painful vaso occlusive crises	Painful vaso-occlusive crises	2	kept
Paired comparison	paired comparison	paired comparison	paired comparison	Paired comparison	2	kept
Pais	pais	pais	pais	Pais	2	kept
paladai feeding	paladai feeding	paladai feeding	paladai feeding	paladai feeding	2	kept
palady feeding	palady feeding	palady feeding	palady feeding	palady feeding	2	kept
palate	palate	palate	palate	palate	2	kept
palatoplasty	palatoplasty	palatoplasty	palatoplasty	palatoplasty	2	kept
paliperidone	paliperidone	paliperidone	paliperidone	paliperidone	2	kept
palliative care consultation team	palliative care consultation team	palliative care consultation team	palliative care consultation team	palliative care consultation team	2	kept

palliative care research	palliative care research	palliative care research	palliative care research	palliative care research	2	kept
palliative communication	palliative communication	palliative communication	palliative communication	palliative communication	2	kept
palliative extubation	palliative extubation	palliative extubation	palliative extubation	palliative extubation	2	kept
palliative treatment	palliative treatment	palliative treatment	palliative treatment	palliative treatment	2	kept
palonosetron	palonosetron	palonosetron	palonosetron	palonosetron	2	kept
palpitations	palpitations	palpitations	palpitations	palpitations	2	kept
pancreas	pancreas	pancreas	pancreas	Pancreas	2	case_or_format
pancreas and biliary tract	pancreas and biliary tract	pancreas and biliary tract	pancreas and biliary tract	pancreas and biliary tract	2	kept
pancreas tumor	pancreas tumor	pancreas tumor	pancreas tumor	pancreas tumor	2	kept
Pancreas, Artificial	pancreas artificial	pancreas artificial	pancreas artificial	Pancreas, Artificial	2	kept
Pancreatic cancer	pancreatic cancer	pancreatic cancer	pancreatic cancer	Pancreatic cancer	2	kept
pancreatic disease	pancreatic disease	pancreatic disease	pancreatic disease	pancreatic disease	2	kept
pancreatic pseudocyst	pancreatic pseudocyst	pancreatic pseudocyst	pancreatic pseudocyst	Pancreatic Pseudocyst	2	case_or_format
Pancreatic resection	pancreatic resection	pancreatic resection	pancreatic resection	Pancreatic resection	2	kept
Pancreatic surgery	pancreatic surgery	pancreatic surgery	pancreatic surgery	Pancreatic surgery	2	kept
Pandemic control policies	pandemic control policies	pandemic control policies	pandemic control policies	Pandemic control policies	2	kept

pandemic hospitals	pandemic hospitals	pandemic hospitals	pandemic hospitals	pandemic hospitals	2	kept
pandemic planning	pandemic planning	pandemic planning	pandemic planning	pandemic planning	2	kept
pandemic response	pandemic response	pandemic response	pandemic response	pandemic response	2	kept
Pandemic response experiences	pandemic response experiences	pandemic response experiences	pandemic response experiences	Pandemic response experiences	2	kept
panel data analysis	panel data analysis	panel data analysis	panel data analysis	panel data analysis	2	kept
PANTON-VALENTINE LEUKOCIDIN	panton valentine leukocidin	panton valentine leukocidin	panton valentine leukocidin	Panton Valentine leukocidin	2	case_or_format
Panton-Valentine leukocidin	panton valentine leukocidin	panton valentine leukocidin	panton valentine leukocidin	Panton Valentine leukocidin	2	case_or_format
PaO2	pao2	pao2	pao2	PaO2	2	kept
PaO<sub>2<	pao sub 2	pao sub 2	pao sub 2	PaO<sub>2<	2	kept
Papaverine	papaverine	papaverine	papaverine	Papaverine	2	kept
papillomatosis	papillomatosis	papillomatosis	papillomatosis	papillomatosis	2	kept
paradoxical embolism	paradoxical embolism	paradoxical embolism	paradoxical embolism	paradoxical embolism	2	kept
Parainfluenza virus infection	parainfluenza virus infection	parainfluenza virus infection	parainfluenza virus infection	Parainfluenza virus infection	2	kept
paralanguage	paralanguage	paralanguage	paralanguage	paralanguage	2	kept
paralytic ileus	paralytic ileus	paralytic ileus	paralytic ileus	paralytic ileus	2	kept
paramedic	paramedic	paramedic	paramedic	Paramedic	2	case_or_format

paramedical student	paramedical student	paramedical student	paramedical student	paramedical student	2	kept
parametric test	parametric test	parametric test	parametric test	parametric test	2	kept
Paranasal Sinus Diseases	paranasal sinus diseases	paranasal sinus diseases	paranasal sinus diseases	Paranasal Sinus Diseases	2	kept
paraproteinemia	paraproteinemia	paraproteinemia	paraproteinemia	paraproteinemia	2	kept
parasite control	parasite control	parasite control	parasite control	parasite control	2	kept
parasitosis	parasitosis	parasitosis	parasitosis	parasitosis	2	kept
Parasympathetic Nervous System	parasympathetic nervous system	parasympathetic nervous system	parasympathetic nervous system	Parasympathetic Nervous System	2	kept
parent education	parent education	parent education	parent education	Parent education	2	case_or_format
Parent engagement	parent engagement	parent engagement	parent engagement	Parent engagement	2	kept
Parent experience	parent experience	parent experience	parent experience	Parent experience	2	kept
parent perspective	parent perspective	parent perspective	parent perspective	parent perspective	2	kept
Parent support	parent support	parent support	parent support	parent support	2	case_or_format
Parent support group	parent support group	parent support group	parent support group	Parent support group	2	kept
parent-child relationship	parent child relationship	parent child relationship	parent child relationship	parent-child relationship	2	kept
parent-infant bonding	parent infant bonding	parent infant bonding	parent infant bonding	parent-infant bonding	2	kept
Parent-infant closeness	parent infant closeness	parent infant closeness	parent infant closeness	Parent-infant closeness	2	kept

Parent-staff communication	parent staff communication	parent staff communication	parent staff communication	Parent-staff communication	2	kept
parental anxiety	parental anxiety	parental anxiety	parental anxiety	parental anxiety	2	kept
Parental competence	parental competence	parental competence	parental competence	Parental competence	2	kept
parental education	parental education	parental education	parental education	parental education	2	kept
parental experience	parental experience	parental experience	parental experience	parental experiences	2	case_or_format
Parental mental health	parental mental health	parental mental health	parental mental health	Parental mental health	2	kept
parental mental health	parental mental health	parental mental health	parental mental health	Parental mental health	2	case_or_format
Parental perceptions	parental perceptions	parental perceptions	parental perception	Parental perceptions	2	singular
parental perspectives	parental perspectives	parental perspectives	parental perspectives	parental perspectives	2	kept
PARENTAL PRESENCE	parental presence	parental presence	parental presence	Parental presence	2	case_or_format
parental presence	parental presence	parental presence	parental presence	Parental presence	2	case_or_format
parental survey	parental survey	parental survey	parental survey	parental survey	2	kept
Parenteral feeding	parenteral feeding	parenteral feeding	parenteral feeding	Parenteral feeding	2	kept
Parenteral Nutrition, Home Total	parenteral nutrition home total	parenteral nutrition home total	parenteral nutrition home total	Parenteral Nutrition, Home Total	2	kept
PARENTING STRESS	parenting stress	parenting stress	parenting stress	PARENTING STRESS	2	kept
parents’ preferences	parents preferences	parents preferences	parents preferences	parents’ preferences	2	kept

Parents’ satisfaction	parents satisfaction	parents satisfaction	parents satisfaction	Parents’ satisfaction	2	kept
parent－infant bonding	parent infant bonding	parent infant bonding	parent infant bonding	parent-infant bonding	2	case_or_format
Paresis	paresis	paresis	paresis	paresis	2	case_or_format
Partial least square	partial least square	partial least square	partial least square	Partial least square	2	kept
partial pressure of oxygen	partial pressure of oxygen	partial pressure of oxygen	partial pressure of oxygen	partial pressure of oxygen	2	kept
participant recruitment	participant recruitment	participant recruitment	participant recruitment	participant recruitment	2	kept
participatory system dynamics modeling	participatory system dynamics modeling	participatory system dynamics modeling	participatory system dynamics modeling	participatory system dynamics modeling	2	kept
Particle Size	particle size	particle size	particle size	particle size	2	case_or_format
partner violence	partner violence	partner violence	partner violence	partner violence	2	kept
Partners	partners	partners	partners	Partners	2	kept
Partnership Practice	partnership practice	partnership practice	partnership practice	Partnership practice	2	case_or_format
partnership practice	partnership practice	partnership practice	partnership practice	Partnership practice	2	case_or_format
Passion	passion	passion	passion	Passion	2	kept
Passive data	passive data	passive data	passive data	Passive data	2	kept
Pasteurization	pasteurization	pasteurization	pasteurization	pasteurization	2	case_or_format
Path analysis	path analysis	path analysis	path analysis	path analysis	2	case_or_format

Pathogenic bacteria	pathogenic bacteria	pathogenic bacteria	pathogenic bacteria	Pathogenic bacteria	2	kept
pathologic fracture	pathologic fracture	pathologic fracture	pathologic fracture	pathologic fracture	2	kept
Pathological conditions	pathological conditions	pathological conditions	pathological conditions	Pathological conditions	2	kept
pathological conditions	pathological conditions	pathological conditions	pathological conditions	Pathological conditions	2	case_or_format
Pathology, Clinical	pathology clinical	pathology clinical	pathology clinical	Pathology, Clinical	2	kept
pathway analysis	pathway analysis	pathway analysis	pathway analysis	pathway analysis	2	kept
Patterns of knowing	patterns of knowing	patterns of knowing	patterns of knowing	Patterns of knowing	2	kept
payment reform	payment reform	payment reform	payment reform	payment reform	2	kept
PCA-EHR interoperability	pca ehr interoperability	pca ehr interoperability	pca ehr interoperability	PCA-EHR interoperability	2	kept
PCR	pcr	pcr	pcr	PCR	2	kept
PDCA	pdca	pdca	pdca	PDCA	2	kept
PDMS	pdms	pdms	pdms	PDMS	2	kept
PDSA cycle	pdsa cycle	pdsa cycle	pdsa cycle	PDSA cycle	2	kept
PDSA cycles	pdsa cycles	pdsa cycles	pdsa cycle	PDSA cycle	2	singular
pedagogics	pedagogics	pedagogics	pedagogics	pedagogics	2	kept
Pediatric acute-onset neuropsychiatric syndrome	pediatric acute onset neuropsychiatric syndrome	pediatric acute onset neuropsychiatric syndrome	pediatric acute onset neuropsychiatric syndrome	Pediatric acute-onset neuropsychiatric syndrome	2	kept

Pediatric advanced life support	pediatric advanced life support	pediatric advanced life support	pediatric advanced life support	pediatric advanced life support	2	case_or_format
pediatric anesthesia emergence delirium scale	pediatric anesthesia emergence delirium scale	pediatric anesthesia emergence delirium scale	pediatric anesthesia emergence delirium scale	pediatric anesthesia emergence delirium scale	2	kept
Pediatric cardiac surgery	pediatric cardiac surgery	pediatric cardiac surgery	pediatric cardiac surgery	Pediatric cardiac surgery	2	kept
pediatric cardiac surgery	pediatric cardiac surgery	pediatric cardiac surgery	pediatric cardiac surgery	Pediatric cardiac surgery	2	case_or_format
Pediatric care	pediatric care	pediatric care	pediatric care	pediatric care	2	case_or_format
Pediatric Early Warning System	pediatric early warning system	pediatric early warning system	pediatric early warning system	Pediatric Early Warning System	2	kept
pediatric early warning system	pediatric early warning system	pediatric early warning system	pediatric early warning system	Pediatric Early Warning System	2	case_or_format
Pediatric emergency department	pediatric emergency department	pediatric emergency department	pediatric emergency department	Pediatric emergency department	2	kept
Pediatric hospital	pediatric hospital	pediatric hospital	pediatric hospital	pediatric hospital	2	case_or_format
Pediatric hospital medicine	pediatric hospital medicine	pediatric hospital medicine	pediatric hospital medicine	Pediatric hospital medicine	2	kept
Pediatric liver failure	pediatric liver failure	pediatric liver failure	pediatric liver failure	Pediatric liver failure	2	kept
pediatric medical device	pediatric medical device	pediatric medical device	pediatric medical device	pediatric medical device	2	kept
pediatric multisystem inflammatory syndrome temporally associated	pediatric multisystem inflammatory syndrome temporally associated	pediatric multisystem inflammatory syndrome temporally associated	pediatric multisystem inflammatory syndrome temporally associated	pediatric multisystem inflammatory syndrome temporally associated	2	kept
pediatric nephrology	pediatric nephrology	pediatric nephrology	pediatric nephrology	pediatric nephrology	2	kept
Pediatric pain	pediatric pain	pediatric pain	pediatric pain	Pediatric pain	2	kept
pediatric radiologist	pediatric radiologist	pediatric radiologist	pediatric radiologist	pediatric radiologist	2	kept

pediatric radiology	pediatric radiology	pediatric radiology	pediatric radiology	pediatric radiology	2	kept
pediatric rehabilitation	pediatric rehabilitation	pediatric rehabilitation	pediatric rehabilitation	pediatric rehabilitation	2	kept
Pediatric resuscitation	pediatric resuscitation	pediatric resuscitation	pediatric resuscitation	Pediatric resuscitation	2	kept
Pediatric Risk of Mortality	pediatric risk of mortality	pediatric risk of mortality	pediatric risk of mortality	Pediatric Risk of Mortality	2	kept
Pediatric sedation	pediatric sedation	pediatric sedation	pediatric sedation	Pediatric sedation	2	kept
pediatric sedation	pediatric sedation	pediatric sedation	pediatric sedation	Pediatric sedation	2	case_or_format
Pediatric tracheostomy	pediatric tracheostomy	pediatric tracheostomy	pediatric tracheostomy	Pediatric tracheostomy	2	kept
Pediatric wards	pediatric wards	pediatric wards	pediatric ward	pediatric ward	2	singular
pedigree	pedigree	pedigree	pedigree	Pedigree	2	case_or_format
pedometer	pedometer	pedometer	pedometer	pedometer	2	kept
PEDro	pedro	pedro	pedro	PEDro	2	kept
peer counseling	peer counseling	peer counseling	peer counseling	peer counseling	2	kept
Peer mentoring	peer mentoring	peer mentoring	peer mentoring	Peer mentoring	2	kept
pelvic fracture	pelvic fracture	pelvic fracture	pelvic fracture	pelvic fracture	2	kept
pelvic fractures	pelvic fractures	pelvic fractures	pelvic fracture	pelvic fracture	2	singular
pemphigus	pemphigus	pemphigus	pemphigus	pemphigus	2	kept

penicillanic acid	penicillanic acid	penicillanic acid	penicillanic acid	Penicillanic Acid	2	case_or_format
Penicillin allergy	penicillin allergy	penicillin allergy	penicillin allergy	penicillin allergy	2	case_or_format
pentamidine	pentamidine	pentamidine	pentamidine	pentamidine	2	kept
Penumonia	penumonia	penumonia	penumonia	Penumonia	2	kept
people by smoking status	people by smoking status	people by smoking status	people by smoking status	people by smoking status	2	kept
Peptic Ulcer Hemorrhage	peptic ulcer hemorrhage	peptic ulcer hemorrhage	peptic ulcer hemorrhage	Peptic Ulcer Hemorrhage	2	kept
Peptide Fragments	peptide fragments	peptide fragments	peptide fragments	Peptide Fragments	2	kept
PERCEIVED BARRIERS	perceived barriers	perceived barriers	perceived barrier	Perceived barriers	2	singular
Perceived management commitment to safety	perceived management commitment to safety	perceived management commitment to safety	perceived management commitment to safety	Perceived management commitment to safety	2	kept
Perceived needs	perceived needs	perceived needs	perceived need	Perceived needs	2	singular
Perceived quality	perceived quality	perceived quality	perceived quality	Perceived quality	2	kept
Perceived social support	perceived social support	perceived social support	perceived social support	perceived social support	2	case_or_format
Perception of autonomy	perception of autonomy	perception of autonomy	perception of autonomy	Perception of autonomy	2	kept
Perceptual Masking	perceptual masking	perceptual masking	perceptual masking	Perceptual Masking	2	kept
percocet	percocet	percocet	percocet	percocet	2	kept
PERCUTANEOUS CORONARY INTERVENTION	percutaneous coronary intervention	percutaneous coronary intervention	percutaneous coronary intervention	percutaneous coronary intervention	2	case_or_format

Percutaneous dilatational tracheostomy	percutaneous dilatational tracheostomy	percutaneous dilatational tracheostomy	percutaneous dilatational tracheostomy	percutaneous dilatational tracheostomy	2	case_or_format
Percutaneous dilatative tracheostomy	percutaneous dilatative tracheostomy	percutaneous dilatative tracheostomy	percutaneous dilatative tracheostomy	Percutaneous dilatative tracheostomy	2	kept
percutaneous dilational tracheostomy	percutaneous dilational tracheostomy	percutaneous dilational tracheostomy	percutaneous dilational tracheostomy	percutaneous dilational tracheostomy	2	kept
percutaneous drainage	percutaneous drainage	percutaneous drainage	percutaneous drainage	percutaneous drainage	2	kept
Percutaneous gastrostomy	percutaneous gastrostomy	percutaneous gastrostomy	percutaneous gastrostomy	Percutaneous gastrostomy	2	kept
Percutaneous injuries	percutaneous injuries	percutaneous injuries	percutaneous injury	percutaneous injury	2	singular
percutaneous injury	percutaneous injury	percutaneous injury	percutaneous injury	percutaneous injury	2	kept
percutaneous thrombectomy	percutaneous thrombectomy	percutaneous thrombectomy	percutaneous thrombectomy	percutaneous thrombectomy	2	kept
percutaneous tracheostomy	percutaneous tracheostomy	percutaneous tracheostomy	percutaneous tracheostomy	percutaneous tracheostomy	2	kept
performance appraisal	performance appraisal	performance appraisal	performance appraisal	performance appraisal	2	kept
Performance management	performance management	performance management	performance management	Performance management	2	kept
Performance measures	performance measures	performance measures	performance measure	performance measures	2	singular
Performance monitoring	performance monitoring	performance monitoring	performance monitoring	Performance monitoring	2	kept
Performance prediction	performance prediction	performance prediction	performance prediction	Performance prediction	2	kept
performance score	performance score	performance score	performance score	performance score	2	kept
performance status	performance status	performance status	performance status	performance status	2	kept

Perfusion pressure	perfusion pressure	perfusion pressure	perfusion pressure	perfusion pressure	2	case_or_format
Perinatal grief	perinatal grief	perinatal grief	perinatal grief	Perinatal grief	2	kept
perinatal infection	perinatal infection	perinatal infection	perinatal infection	perinatal infection	2	kept
Perinatal substance use	perinatal substance use	perinatal substance use	perinatal substance use	Perinatal substance use	2	kept
PERINATAL-CARE	perinatal care	perinatal care	perinatal care	Perinatal Care	2	case_or_format
perindopril	perindopril	perindopril	perindopril	perindopril	2	kept
Perineal Assessment Tool	perineal assessment tool	perineal assessment tool	perineal assessment tool	Perineal Assessment Tool	2	kept
perineural invasion	perineural invasion	perineural invasion	perineural invasion	perineural invasion	2	kept
PERIODONTITIS	periodontitis	periodontitis	periodontitis	periodontitis	2	case_or_format
Periodontitis	periodontitis	periodontitis	periodontitis	periodontitis	2	case_or_format
perioperative complication	perioperative complication	perioperative complication	perioperative complication	perioperative complication	2	kept
perioperative management	perioperative management	perioperative management	perioperative management	Perioperative management	2	case_or_format
Perioperative medicine	perioperative medicine	perioperative medicine	perioperative medicine	perioperative medicine	2	case_or_format
perioperative monitoring	perioperative monitoring	perioperative monitoring	perioperative monitoring	perioperative monitoring	2	kept
perioperative outcomes	perioperative outcomes	perioperative outcomes	perioperative outcomes	perioperative outcomes	2	kept
Perioperative practice	perioperative practice	perioperative practice	perioperative practice	Perioperative practice	2	kept

Peripartum	peripartum	peripartum	peripartum	Peripartum	2	kept
Peripartum cardiomyopathy	peripartum cardiomyopathy	peripartum cardiomyopathy	peripartum cardiomyopathy	peripartum cardiomyopathy	2	case_or_format
peripheral capillary oxygen saturation	peripheral capillary oxygen saturation	peripheral capillary oxygen saturation	peripheral capillary oxygen saturation	peripheral capillary oxygen saturation	2	kept
Peripheral catheterisation	peripheral catheterisation	peripheral catheterisation	peripheral catheterisation	Peripheral catheterisation	2	kept
Peripheral Catheterization	peripheral catheterization	peripheral catheterization	peripheral catheterization	Peripheral catheterization	2	case_or_format
peripheral catheterization	peripheral catheterization	peripheral catheterization	peripheral catheterization	Peripheral catheterization	2	case_or_format
Peripheral interaction	peripheral interaction	peripheral interaction	peripheral interaction	Peripheral interaction	2	kept
Peripheral Nerves	peripheral nerves	peripheral nerves	peripheral nerve	Peripheral Nerves	2	singular
peripheral oxygen saturation	peripheral oxygen saturation	peripheral oxygen saturation	peripheral oxygen saturation	peripheral oxygen saturation	2	kept
peripheral parenteral nutrition	peripheral parenteral nutrition	peripheral parenteral nutrition	peripheral parenteral nutrition	peripheral parenteral nutrition	2	kept
Peripherally inserted central catheters	peripherally inserted central catheters	peripherally inserted central catheters	peripherally inserted central catheter	peripherally inserted central catheter	2	singular
peripherally inserted central catheters	peripherally inserted central catheters	peripherally inserted central catheters	peripherally inserted central catheter	peripherally inserted central catheter	2	singular
Periprosthetic Fractures	periprosthetic fractures	periprosthetic fractures	periprosthetic fracture	periprosthetic fracture	2	singular
perirectal swab	perirectal swab	perirectal swab	perirectal swab	perirectal swab	2	kept
peritoneal fluid	peritoneal fluid	peritoneal fluid	peritoneal fluid	peritoneal fluid	2	kept
Peritoneal malignancy	peritoneal malignancy	peritoneal malignancy	peritoneal malignancy	Peritoneal malignancy	2	kept

Peritoneal surface malignancies	peritoneal surface malignancies	peritoneal surface malignancies	peritoneal surface malignancies	Peritoneal surface malignancies	2	kept
peritoneum adhesion	peritoneum adhesion	peritoneum adhesion	peritoneum adhesion	peritoneum adhesion	2	kept
peritoneum mesothelioma	peritoneum mesothelioma	peritoneum mesothelioma	peritoneum mesothelioma	peritoneum mesothelioma	2	kept
Persian Weaning Tool	persian weaning tool	persian weaning tool	persian weaning tool	Persian Weaning Tool	2	kept
persistent critical illness	persistent critical illness	persistent critical illness	persistent critical illness	persistent critical illness	2	kept
PERSISTENT INFLAMMATION	persistent inflammation	persistent inflammation	persistent inflammation	PERSISTENT INFLAMMATION	2	kept
person-centeredness	person centeredness	person centeredness	person centeredness	person-centeredness	2	kept
person-centred communication	person centred communication	person centred communication	person centred communication	person-centred communication	2	kept
person-centredness	person centredness	person centredness	person centredness	person-centredness	2	kept
Personal characteristics	personal characteristics	personal characteristics	personal characteristics	Personal characteristics	2	kept
Personal control	personal control	personal control	personal control	Personal control	2	kept
Personal digital assistant	personal digital assistant	personal digital assistant	personal digital assistant	personal digital assistant	2	case_or_format
Personal monitoring	personal monitoring	personal monitoring	personal monitoring	Personal monitoring	2	kept
personal monitoring	personal monitoring	personal monitoring	personal monitoring	Personal monitoring	2	case_or_format
Personal narratives	personal narratives	personal narratives	personal narratives	Personal narratives	2	kept
Personal Narratives as Topic	personal narratives as topic	personal narratives as topic	personal narratives as topic	Personal Narratives as Topic	2	kept

Personal satisfaction	personal satisfaction	personal satisfaction	personal satisfaction	Personal Satisfaction	2	case_or_format
Personality traits& safety attitude	personality traits and safety attitude	personality traits and safety attitude	personality traits and safety attitude	Personality traits& safety attitude	2	kept
personality traits	personality traits	personality traits	personality trait	Personality traits	2	singular
personality type	personality type	personality type	personality type	personality type	2	kept
Personalized medicine	personalized medicine	personalized medicine	personalized medicine	personalized medicine	2	case_or_format
Personalized stigma	personalized stigma	personalized stigma	personalized stigma	Personalized stigma	2	kept
personnel downsizing	personnel downsizing	personnel downsizing	personnel downsizing	Personnel downsizing	2	case_or_format
Personnel, pharmacy	personnel pharmacy	personnel pharmacy	personnel pharmacy	Personnel, pharmacy	2	kept
Perspective	perspective	perspective	perspective	PERSPECTIVES	2	case_or_format
Pertussis	pertussis	pertussis	pertussis	pertussis	2	case_or_format
PES-NWI	pes nwi	pes nwi	pes nwi	PES-NWI	2	kept
pessimism	pessimism	pessimism	pessimism	pessimism	2	kept
Pesticides	pesticides	pesticides	pesticide	Pesticides	2	singular
pet animal	pet animal	pet animal	pet animal	pet animal	2	kept
pews	pews	pews	pew	PEWS	2	singular
PFCC	pfcc	pfcc	pfcc	PFCC	2	kept

PFGE	pfge	pfge	pfge	PFGE	2	kept
Pflegesystem	pflegesystem	pflegesystem	pflegesystem	Pflegesystem	2	kept
pH measurement	ph measurement	ph measurement	ph measurement	pH measurement	2	kept
Phantoms	phantoms	phantoms	phantom	Phantoms	2	singular
Phantoms, Imaging	phantoms imaging	phantoms imaging	phantoms imaging	Phantoms, Imaging	2	kept
pharmaceutical preparations	pharmaceutical preparations	pharmaceutical preparations	pharmaceutical preparations	Pharmaceutical Preparations	2	case_or_format
Pharmacists, hospital	pharmacists hospital	pharmacists hospital	pharmacists hospital	Pharmacists, hospital	2	kept
Pharmacodynamics	pharmacodynamics	pharmacodynamics	pharmacodynamics	pharmacodynamics	2	case_or_format
Pharmacoeconomics	pharmacoeconomics	pharmacoeconomics	pharmacoeconomics	pharmacoeconomics	2	case_or_format
Pharmacologic therapy	pharmacologic therapy	pharmacologic therapy	pharmacologic therapy	Pharmacologic therapy	2	kept
pharmacologic treatment	pharmacologic treatment	pharmacologic treatment	pharmacologic treatment	pharmacologic treatment	2	kept
pharmacotherapy	pharmacotherapy	pharmacotherapy	pharmacotherapy	Pharmacotherapy	2	case_or_format
Pharmacy service	pharmacy service	pharmacy service	pharmacy service	Pharmacy service	2	kept
pharmacy service	pharmacy service	pharmacy service	pharmacy service	Pharmacy service	2	case_or_format
Pharyngitis	pharyngitis	pharyngitis	pharyngitis	pharyngitis	2	case_or_format
pharynx disease	pharynx disease	pharynx disease	pharynx disease	pharynx disease	2	kept

Phase 1	phase 1	phase 1	phase 1	Phase 1	2	kept
pheniramine	pheniramine	pheniramine	pheniramine	pheniramine	2	kept
Phenolic compounds	phenolic compounds	phenolic compounds	phenolic compounds	Phenolic compounds	2	kept
Phenomenological hermeneutic	phenomenological hermeneutic	phenomenological hermeneutic	phenomenological hermeneutic	Phenomenological hermeneutic	2	kept
phenomenological hermeneutic interpretation	phenomenological hermeneutic interpretation	phenomenological hermeneutic interpretation	phenomenological hermeneutic interpretation	phenomenological hermeneutic interpretation	2	kept
phenomenological research	phenomenological research	phenomenological research	phenomenological research	Phenomenological research	2	case_or_format
Phentolamine	phentolamine	phentolamine	phentolamine	Phentolamine	2	kept
phentolamine	phentolamine	phentolamine	phentolamine	Phentolamine	2	case_or_format
Phenylpropanolamine	phenylpropanolamine	phenylpropanolamine	phenylpropanolamine	Phenylpropanolamine	2	kept
Phlebography	phlebography	phlebography	phlebography	Phlebography	2	kept
phlebography	phlebography	phlebography	phlebography	Phlebography	2	case_or_format
phone app	phone app	phone app	phone app	phone app	2	kept
phosphine	phosphine	phosphine	phosphine	phosphine	2	kept
phospholipid	phospholipid	phospholipid	phospholipid	phospholipid	2	kept
Phosphorus Metabolism Disorders	phosphorus metabolism disorders	phosphorus metabolism disorders	phosphorus metabolism disorders	Phosphorus Metabolism Disorders	2	kept
photochemotherapy	photochemotherapy	photochemotherapy	photochemotherapy	Photochemotherapy	2	case_or_format

photodynamic therapy	photodynamic therapy	photodynamic therapy	photodynamic therapy	photodynamic therapy	2	kept
Photograph	photograph	photograph	photograph	Photograph	2	kept
Photographs	photographs	photographs	photograph	Photograph	2	singular
photophobia	photophobia	photophobia	photophobia	photophobia	2	kept
photoplethysmogram	photoplethysmogram	photoplethysmogram	photoplethysmogram	photoplethysmogram	2	kept
Photoplythesmography	photoplythesmography	photoplythesmography	photoplythesmography	Photoplythesmography	2	kept
photosensitivity	photosensitivity	photosensitivity	photosensitivity	photosensitivity	2	kept
Physical and Rehabilitation Medicine	physical and rehabilitation medicine	physical and rehabilitation medicine	physical and rehabilitation medicine	Physical and Rehabilitation Medicine	2	kept
PHYSICAL ASSESSMENT	physical assessment	physical assessment	physical assessment	PHYSICAL ASSESSMENT	2	kept
physical assessment	physical assessment	physical assessment	physical assessment	PHYSICAL ASSESSMENT	2	case_or_format
physical assessment skills	physical assessment skills	physical assessment skills	physical assessment skills	physical assessment skills	2	kept
physical complications	physical complications	physical complications	physical complications	physical complications	2	kept
Physical cooling	physical cooling	physical cooling	physical cooling	Physical cooling	2	kept
physical discomfort	physical discomfort	physical discomfort	physical discomfort	physical discomfort	2	kept
Physical disorders	physical disorders	physical disorders	physical disorders	Physical disorders	2	kept
Physical Endurance	physical endurance	physical endurance	physical endurance	Physical Endurance	2	kept

Physical environment	physical environment	physical environment	physical environment	physical environment	2	case_or_format
Physical exam	physical exam	physical exam	physical exam	Physical exam	2	kept
Physical exercise	physical exercise	physical exercise	physical exercise	Physical exercise	2	kept
Physical health	physical health	physical health	physical health	physical health	2	case_or_format
Physical impairments	physical impairments	physical impairments	physical impairment	Physical impairments	2	singular
physical inactivity	physical inactivity	physical inactivity	physical inactivity	physical inactivity	2	kept
Physical Medicine	physical medicine	physical medicine	physical medicine	physical medicine	2	case_or_format
physical rehabilitation	physical rehabilitation	physical rehabilitation	physical rehabilitation	physical rehabilitation	2	kept
physical restraining	physical restraining	physical restraining	physical restraining	physical restraining	2	kept
Physical Restraint	physical restraint	physical restraint	physical restraint	physical restraint	2	case_or_format
PHYSICAL RESTRAINTS	physical restraints	physical restraints	physical restraint	physical restraint	2	singular
Physical therapists	physical therapists	physical therapists	physical therapist	Physical Therapists	2	singular
physical therapists	physical therapists	physical therapists	physical therapist	Physical Therapists	2	singular
PHYSICAL TOUCH	physical touch	physical touch	physical touch	PHYSICAL TOUCH	2	kept
Physician Assistant	physician assistant	physician assistant	physician assistant	physician assistant	2	case_or_format
Physician documentation	physician documentation	physician documentation	physician documentation	Physician documentation	2	kept

physician education	physician education	physician education	physician education	physician education	2	kept
physician efficiency	physician efficiency	physician efficiency	physician efficiency	physician efficiency	2	kept
Physician Impairment	physician impairment	physician impairment	physician impairment	Physician Impairment	2	kept
physician income	physician income	physician income	physician income	physician income	2	kept
physician knowledge	physician knowledge	physician knowledge	physician knowledge	physician knowledge	2	kept
physician practice patterns	physician practice patterns	physician practice patterns	physician practice patterns	physician practice patterns	2	kept
physicians and students	physicians and students	physicians and students	physicians and students	physicians and students	2	kept
Physicians, Primary Care	physicians primary care	physicians primary care	physicians primary care	Physicians, Primary Care	2	kept
Physiologic monitor	physiologic monitor	physiologic monitor	physiologic monitor	Physiologic monitors	2	case_or_format
physiologic monitors	physiologic monitors	physiologic monitors	physiologic monitor	Physiologic monitors	2	singular
physiological adaptation	physiological adaptation	physiological adaptation	physiological adaptation	physiological adaptation	2	kept
PHYSIOLOGICAL INDICATORS	physiological indicators	physiological indicators	physiological indicators	PHYSIOLOGICAL INDICATORS	2	kept
physiological needs	physiological needs	physiological needs	physiological needs	physiological needs	2	kept
Physiological parameter	physiological parameter	physiological parameter	physiological parameter	Physiological parameters	2	case_or_format
Physiological Processes	physiological processes	physiological processes	physiological processes	Physiological Processes	2	kept
Physiological responses	physiological responses	physiological responses	physiological response	Physiological response	2	singular

physiological responses	physiological responses	physiological responses	physiological response	Physiological response	2	singular
Physiological states	physiological states	physiological states	physiological states	Physiological states	2	kept
physiopathology	physiopathology	physiopathology	physiopathology	physiopathology	2	kept
Physiotherapist	physiotherapist	physiotherapist	physiotherapist	physiotherapist	2	case_or_format
Physiotherapists	physiotherapists	physiotherapists	physiotherapist	physiotherapist	2	singular
PICC-peripherally inserted central catheter	picc peripherally inserted central catheter	picc peripherally inserted central catheter	picc peripherally inserted central catheter	PICC-peripherally inserted central catheter	2	kept
PICCs	piccs	piccs	picc	PICC	2	singular
picosulfate sodium	picosulfate sodium	picosulfate sodium	picosulfate sodium	picosulfate sodium	2	kept
Pierre Robin sequence	pierre robin sequence	pierre robin sequence	pierre robin sequence	Pierre Robin sequence	2	kept
PILOT	pilot	pilot	pilot	pilot	2	case_or_format
Pilot projects	pilot projects	pilot projects	pilot project	Pilot Projects	2	singular
PIMS-TS	pims ts	pims ts	pims ts	PIMS-TS	2	kept
pindolol	pindolol	pindolol	pindolol	pindolol	2	kept
pioglitazone	pioglitazone	pioglitazone	pioglitazone	pioglitazone	2	kept
PIPERACILLIN-TAZOBACTAM	piperacillin tazobactam	piperacillin tazobactam	piperacillin tazobactam	PIPERACILLIN-TAZOBACTAM	2	kept
Piperacillin-tazobactam	piperacillin tazobactam	piperacillin tazobactam	piperacillin tazobactam	PIPERACILLIN-TAZOBACTAM	2	case_or_format

piperazine derivative	piperazine derivative	piperazine derivative	piperazine derivative	piperazine derivative	2	kept
Piracetam	piracetam	piracetam	piracetam	piracetam	2	case_or_format
PIRRT	pirrt	pirrt	pirrt	PIRRT	2	kept
Pitfalls	pitfalls	pitfalls	pitfalls	Pitfalls	2	kept
PIV	piv	piv	piv	PIV	2	kept
pivmecillinam	pivmecillinam	pivmecillinam	pivmecillinam	pivmecillinam	2	kept
Placenta accreta spectrum	placenta accreta spectrum	placenta accreta spectrum	placenta accreta spectrum	Placenta accreta spectrum	2	kept
placenta accreta spectrum	placenta accreta spectrum	placenta accreta spectrum	placenta accreta spectrum	Placenta accreta spectrum	2	case_or_format
Placental Circulation	placental circulation	placental circulation	placental circulation	Placental Circulation	2	kept
placental transfusion	placental transfusion	placental transfusion	placental transfusion	placental transfusion	2	kept
Plagiocephaly	plagiocephaly	plagiocephaly	plagiocephaly	plagiocephaly	2	case_or_format
plan of care	plan of care	plan of care	plan of care	plan of care	2	kept
planned home birth	planned home birth	planned home birth	planned home birth	planned home birth	2	kept
plaque index	plaque index	plaque index	plaque index	plaque index	2	kept
plasma osmolality	plasma osmolality	plasma osmolality	plasma osmolality	plasma osmolality	2	kept
plasma substitute	plasma substitute	plasma substitute	plasma substitute	Plasma Substitutes	2	case_or_format

plasma volume	plasma volume	plasma volume	plasma volume	Plasma Volume	2	case_or_format
plasmacytoma	plasmacytoma	plasmacytoma	plasmacytoma	plasmacytoma	2	kept
plasminogen activator	plasminogen activator	plasminogen activator	plasminogen activator	plasminogen activator	2	kept
Plasmodium falciparum	plasmodium falciparum	plasmodium falciparum	plasmodium falciparum	Plasmodium falciparum	2	kept
plate fixation	plate fixation	plate fixation	plate fixation	plate fixation	2	kept
platelet lymphocyte ratio	platelet lymphocyte ratio	platelet lymphocyte ratio	platelet lymphocyte ratio	platelet lymphocyte ratio	2	kept
Play	play	play	play	Play	2	kept
pleocytosis	pleocytosis	pleocytosis	pleocytosis	pleocytosis	2	kept
Pleura	pleura	pleura	pleura	Pleura	2	kept
pleura empyema	pleura empyema	pleura empyema	pleura empyema	pleura empyema	2	kept
Pleural Effusion, Malignant	pleural effusion malignant	pleural effusion malignant	pleural effusion malignant	Pleural Effusion, Malignant	2	kept
pleurodesis	pleurodesis	pleurodesis	pleurodesis	pleurodesis	2	kept
PLISSIT model	plissit model	plissit model	plissit model	PLISSIT model	2	kept
Pneumatosis Cystoides Intestinalis	pneumatosis cystoides intestinalis	pneumatosis cystoides intestinalis	pneumatosis cystoides intestinalis	Pneumatosis Cystoides Intestinalis	2	kept
pneumococcal meningitis	pneumococcal meningitis	pneumococcal meningitis	pneumococcal meningitis	pneumococcal meningitis	2	kept
pneumococcal vaccine	pneumococcal vaccine	pneumococcal vaccine	pneumococcal vaccine	Pneumococcal Vaccines	2	case_or_format

pneumococcal vaccines	pneumococcal vaccines	pneumococcal vaccines	pneumococcal vaccine	Pneumococcal Vaccines	2	singular
Pneumocystis	pneumocystis	pneumocystis	pneumocystis	Pneumocystis	2	kept
Pneumocystis jirovecii	pneumocystis jirovecii	pneumocystis jirovecii	pneumocystis jirovecii	Pneumocystis jirovecii	2	kept
Pneumonia, Mycoplasma	pneumonia mycoplasma	pneumonia mycoplasma	pneumonia mycoplasma	Pneumonia, Mycoplasma	2	kept
POD	pod	pod	pod	POD	2	kept
podcast	podcast	podcast	podcast	podcast	2	kept
Poder	poder	poder	poder	Poder	2	kept
podiatrist	podiatrist	podiatrist	podiatrist	podiatrist	2	kept
podocyte	podocyte	podocyte	podocyte	podocyte	2	kept
Poetry	poetry	poetry	poetry	Poetry	2	kept
Poetry as Topic	poetry as topic	poetry as topic	poetry as topic	Poetry as Topic	2	kept
point mutation	point mutation	point mutation	point mutation	point mutation	2	kept
point of care technology	point of care technology	point of care technology	point of care technology	point of care technology	2	kept
Point of care test	point of care test	point of care test	point of care test	Point of care test	2	kept
POINT PREVALENCE	point prevalence	point prevalence	point prevalence	point prevalence	2	case_or_format
Point-of-care systems	point of care systems	point of care systems	point of care system	Point-of-Care Systems	2	singular

point-of-care systems	point of care systems	point of care systems	point of care system	Point-of-Care Systems	2	singular
point-of-care testing	point of care testing	point of care testing	point of care testing	point of care testing	2	case_or_format
Poiseuille flow	poiseuille flow	poiseuille flow	poiseuille flow	Poiseuille flow	2	kept
Poisson distribution	poisson distribution	poisson distribution	poisson distribution	Poisson Distribution	2	case_or_format
policy implementation	policy implementation	policy implementation	policy implementation	policy implementation	2	kept
Poliovirus Vaccine, Inactivated	poliovirus vaccine inactivated	poliovirus vaccine inactivated	poliovirus vaccine inactivated	Poliovirus Vaccine, Inactivated	2	kept
political action	political action	political action	political action	political action	2	kept
Polycythemia Vera	polycythemia vera	polycythemia vera	polycythemia vera	Polycythemia Vera	2	kept
polyene antibiotic agent	polyene antibiotic agent	polyene antibiotic agent	polyene antibiotic agent	polyene antibiotic agent	2	kept
polyester	polyester	polyester	polyester	polyester	2	kept
Polyethylene Glycols	polyethylene glycols	polyethylene glycols	polyethylene glycols	Polyethylene Glycols	2	kept
polyhydramnios	polyhydramnios	polyhydramnios	polyhydramnios	polyhydramnios	2	kept
polymer	polymer	polymer	polymer	polymer	2	kept
Polymyxin	polymyxin	polymyxin	polymyxin	polymyxin	2	case_or_format
Polyneuropathy	polyneuropathy	polyneuropathy	polyneuropathy	Polyneuropathies	2	case_or_format
polystyrenesulfonate sodium	polystyrenesulfonate sodium	polystyrenesulfonate sodium	polystyrenesulfonate sodium	polystyrenesulfonate sodium	2	kept

polyurethan foam	polyurethan foam	polyurethan foam	polyurethan foam	polyurethan foam	2	kept
polyvagal theory	polyvagal theory	polyvagal theory	polyvagal theory	polyvagal theory	2	kept
Poor working posture	poor working posture	poor working posture	poor working posture	Poor working posture	2	kept
popliteal vein	popliteal vein	popliteal vein	popliteal vein	popliteal vein	2	kept
population based case control study	population based case control study	population based case control study	population based case control study	population based case control study	2	kept
Population Density	population density	population density	population density	population density	2	case_or_format
population focus	population focus	population focus	population focus	population focus	2	kept
population growth	population growth	population growth	population growth	population growth	2	kept
population study	population study	population study	population study	population study	2	kept
population surveillance	population surveillance	population surveillance	population surveillance	Population Surveillance	2	case_or_format
Population-based	population based	population based	population based	Population-based	2	kept
poractant	poractant	poractant	poractant	poractant	2	kept
portable equipment	portable equipment	portable equipment	portable equipment	portable equipment	2	kept
Position	position	position	position	position	2	case_or_format
Positional drainage	positional drainage	positional drainage	positional drainage	Positional drainage	2	kept
Positive end expiratory pressure	positive end expiratory pressure	positive end expiratory pressure	positive end expiratory pressure	positive end expiratory pressure	2	case_or_format

positive mental health	positive mental health	positive mental health	positive mental health	positive mental health	2	kept
Positive predictive value	positive predictive value	positive predictive value	positive predictive value	Positive predictive value	2	kept
Positive pressure ventilation	positive pressure ventilation	positive pressure ventilation	positive pressure ventilation	positive pressure ventilation	2	case_or_format
Positive psychology	positive psychology	positive psychology	positive psychology	Positive psychology	2	kept
positive reinforcement	positive reinforcement	positive reinforcement	positive reinforcement	positive reinforcement	2	kept
Positive religious coping	positive religious coping	positive religious coping	positive religious coping	Positive religious coping	2	kept
positive suggestion	positive suggestion	positive suggestion	positive suggestion	positive suggestion	2	kept
Positive-pressure respiration	positive pressure respiration	positive pressure respiration	positive pressure respiration	Positive-Pressure Respiration	2	case_or_format
post anaesthesia care unit	post anaesthesia care unit	post anaesthesia care unit	post anaesthesia care unit	post anaesthesia care unit	2	kept
post neonatal tetanus	post neonatal tetanus	post neonatal tetanus	post neonatal tetanus	post neonatal tetanus	2	kept
Post occupancy evaluation	post occupancy evaluation	post occupancy evaluation	post occupancy evaluation	Post occupancy evaluation	2	kept
Post surgery	post surgery	post surgery	post surgery	Post surgery	2	kept
post-anaesthesia care unit	post anaesthesia care unit	post anaesthesia care unit	post anaesthesia care unit	post anaesthesia care unit	2	case_or_format
post-cardiac arrest care	post cardiac arrest care	post cardiac arrest care	post cardiac arrest care	post-cardiac arrest care	2	kept
Post-code stress	post code stress	post code stress	post code stress	Post-code stress	2	kept
Post-discharge care	post discharge care	post discharge care	post discharge care	Post-discharge care	2	kept

Post-mortem care	post mortem care	post mortem care	post mortem care	Post-mortem care	2	kept
Post-Occupancy Evaluation	post occupancy evaluation	post occupancy evaluation	post occupancy evaluation	Post occupancy evaluation	2	case_or_format
Post-operative	post operative	post operative	post operative	post-operative	2	case_or_format
Post-operative care	post operative care	post operative care	post operative care	post-operative care	2	case_or_format
Post-resuscitation	post resuscitation	post resuscitation	post resuscitation	Post-resuscitation	2	kept
post-secondary	post secondary	post secondary	post secondary	post-secondary	2	kept
Post-Sepsis-Syndrome	post sepsis syndrome	post sepsis syndrome	post sepsis syndrome	Post-Sepsis-Syndrome	2	kept
post-sepsis-syndrome	post sepsis syndrome	post sepsis syndrome	post sepsis syndrome	Post-Sepsis-Syndrome	2	case_or_format
post-traumatic stress	post traumatic stress	post traumatic stress	post traumatic stress	Post-traumatic stress	2	case_or_format
post-traumatic stress disorders	post traumatic stress disorders	post traumatic stress disorders	post traumatic stress disorder	Post-traumatic stress disorder	2	singular
Post-traumatic stress symptoms	post traumatic stress symptoms	post traumatic stress symptoms	post traumatic stress symptoms	Post-traumatic stress symptoms	2	kept
post-traumatic stress syndrome	post traumatic stress syndrome	post traumatic stress syndrome	post traumatic stress syndrome	post-traumatic stress syndrome	2	kept
Postal Service	postal service	postal service	postal service	Postal Service	2	kept
postanesthesia recovery	postanesthesia recovery	postanesthesia recovery	postanesthesia recovery	postanesthesia recovery	2	kept
postcolonial	postcolonial	postcolonial	postcolonial	postcolonial	2	kept
posterior fossa	posterior fossa	posterior fossa	posterior fossa	posterior fossa	2	kept

Posterior Leukoencephalopathy Syndrome	posterior leukoencephalopathy syndrome	posterior leukoencephalopathy syndrome	posterior leukoencephalopathy syndrome	Posterior Leukoencephalopathy Syndrome	2	kept
Posterior reversible encephalopathy syndrome	posterior reversible encephalopathy syndrome	posterior reversible encephalopathy syndrome	posterior reversible encephalopathy syndrome	posterior reversible encephalopathy syndrome	2	case_or_format
posterior spine fusion	posterior spine fusion	posterior spine fusion	posterior spine fusion	posterior spine fusion	2	kept
Posters as Topic	posters as topic	posters as topic	posters as topic	Posters as Topic	2	kept
postgraduate medical education	postgraduate medical education	postgraduate medical education	postgraduate medical education	postgraduate medical education	2	kept
Postgraduate students	postgraduate students	postgraduate students	postgraduate student	postgraduate student	2	singular
Postgraduate training	postgraduate training	postgraduate training	postgraduate training	Postgraduate training	2	kept
POSTMENOPAUSAL WOMEN	postmenopausal women	postmenopausal women	postmenopausal women	POSTMENOPAUSAL WOMEN	2	kept
Postmenopause	postmenopause	postmenopause	postmenopause	postmenopause	2	case_or_format
postnatal	postnatal	postnatal	postnatal	postnatal	2	kept
postnatal growth	postnatal growth	postnatal growth	postnatal growth	postnatal growth	2	kept
postnatal growth failure, very low birth weight	postnatal growth failure very low birth weight	postnatal growth failure very low birth weight	postnatal growth failure very low birth weight	postnatal growth failure, very low birth weight	2	kept
POSTOPERATIVE ANALGESIA	postoperative analgesia	postoperative analgesia	postoperative analgesia	postoperative analgesia	2	case_or_format
Postoperative assessment	postoperative assessment	postoperative assessment	postoperative assessment	Postoperative assessment	2	kept
postoperative assessment	postoperative assessment	postoperative assessment	postoperative assessment	Postoperative assessment	2	case_or_format
postoperative cognitive complications	postoperative cognitive complications	postoperative cognitive complications	postoperative cognitive complications	Postoperative Cognitive Complications	2	case_or_format

Postoperative cognitive dysfunction	postoperative cognitive dysfunction	postoperative cognitive dysfunction	postoperative cognitive dysfunction	postoperative cognitive dysfunction	2	case_or_format
Postoperative Delirium	postoperative delirium	postoperative delirium	postoperative delirium	postoperative delirium	2	case_or_format
Postoperative handover	postoperative handover	postoperative handover	postoperative handover	Postoperative handover	2	kept
postoperative handover	postoperative handover	postoperative handover	postoperative handover	Postoperative handover	2	case_or_format
postoperative management	postoperative management	postoperative management	postoperative management	postoperative management	2	kept
Postoperative pain management model	postoperative pain management model	postoperative pain management model	postoperative pain management model	Postoperative pain management model	2	kept
Postoperative pulmonary complications	postoperative pulmonary complications	postoperative pulmonary complications	postoperative pulmonary complication	Postoperative pulmonary complications	2	singular
Postoperative recovery	postoperative recovery	postoperative recovery	postoperative recovery	postoperative recovery	2	case_or_format
postoperative vomiting	postoperative vomiting	postoperative vomiting	postoperative vomiting	postoperative vomiting	2	kept
postpartum depression screening	postpartum depression screening	postpartum depression screening	postpartum depression screening	postpartum depression screening	2	kept
Postphlebitic Syndrome	postphlebitic syndrome	postphlebitic syndrome	postphlebitic syndrome	Postphlebitic Syndrome	2	kept
Posttraumatic	posttraumatic	posttraumatic	posttraumatic	Posttraumatic	2	kept
posttraumatic	posttraumatic	posttraumatic	posttraumatic	Posttraumatic	2	case_or_format
posttraumatic stress disorder checklist-civilian version	posttraumatic stress disorder checklist civilian version	posttraumatic stress disorder checklist civilian version	posttraumatic stress disorder checklist civilian version	posttraumatic stress disorder checklist-civilian version	2	kept
POSTTRAUMATIC STRESS SYMPTOMS	posttraumatic stress symptoms	posttraumatic stress symptoms	posttraumatic stress symptoms	POSTTRAUMATIC STRESS SYMPTOMS	2	kept
posttraumatic stress symptoms	posttraumatic stress symptoms	posttraumatic stress symptoms	posttraumatic stress symptoms	POSTTRAUMATIC STRESS SYMPTOMS	2	case_or_format

potassium cell level	potassium cell level	potassium cell level	potassium cell level	potassium cell level	2	kept
potato	potato	potato	potato	potato	2	kept
potential difference	potential difference	potential difference	potential difference	potential difference	2	kept
potential organ donor	potential organ donor	potential organ donor	potential organ donor	potential organ donor	2	kept
POTENTIAL SOURCE	potential source	potential source	potential source	POTENTIAL SOURCE	2	kept
potentially inappropriate treatment	potentially inappropriate treatment	potentially inappropriate treatment	potentially inappropriate treatment	potentially inappropriate treatment	2	kept
Poverty Areas	poverty areas	poverty areas	poverty areas	Poverty Areas	2	kept
poverty level	poverty level	poverty level	poverty level	poverty level	2	kept
Povidone-iodine	povidone iodine	povidone iodine	povidone iodine	povidone iodine	2	case_or_format
power of attorney	power of attorney	power of attorney	power of attorney	power of attorney	2	kept
PowerPoint training intervention	powerpoint training intervention	powerpoint training intervention	powerpoint training intervention	PowerPoint training intervention	2	kept
PPD test	ppd test	ppd test	ppd test	PPD test	2	kept
PPG	ppg	ppg	ppg	PPG	2	kept
PPH	pph	pph	pph	PPH	2	kept
PPI	ppi	ppi	ppi	PPI	2	kept
Practic	practic	practic	practic	Practic	2	kept

Practical	practical	practical	practical	Practical	2	kept
Practice and sepsis	practice and sepsis	practice and sepsis	practice and sepsis	Practice and sepsis	2	kept
practice change	practice change	practice change	practice change	Practice change	2	case_or_format
practice development	practice development	practice development	practice development	Practice development	2	case_or_format
practice guidelines as topic	practice guidelines as topic	practice guidelines as topic	practice guidelines as topic	Practice Guidelines as Topic	2	case_or_format
practice implications	practice implications	practice implications	practice implications	practice implications	2	kept
Practice management	practice management	practice management	practice management	Practice Management	2	case_or_format
Practice readiness	practice readiness	practice readiness	practice readiness	practice readiness	2	case_or_format
Practice variation	practice variation	practice variation	practice variation	Practice variation	2	kept
Prader-Willi Syndrome	prader willi syndrome	prader willi syndrome	prader willi syndrome	Prader-Willi Syndrome	2	kept
Pragmatic clinical trial	pragmatic clinical trial	pragmatic clinical trial	pragmatic clinical trial	Pragmatic clinical trial	2	kept
pragmatic clinical trial	pragmatic clinical trial	pragmatic clinical trial	pragmatic clinical trial	Pragmatic clinical trial	2	case_or_format
Pragmatic clinical trials	pragmatic clinical trials	pragmatic clinical trials	pragmatic clinical trial	Pragmatic clinical trial	2	singular
pralidoxime	pralidoxime	pralidoxime	pralidoxime	pralidoxime	2	kept
Pralidoxime Compounds	pralidoxime compounds	pralidoxime compounds	pralidoxime compounds	Pralidoxime Compounds	2	kept
PRAM	pram	pram	pram	PRAM	2	kept

PRAMS	prams	prams	pram	PRAM	2	singular
prazosin	prazosin	prazosin	prazosin	prazosin	2	kept
Pre-anesthesia evaluation	pre anesthesia evaluation	pre anesthesia evaluation	pre anesthesia evaluation	Pre-anesthesia evaluation	2	kept
Pre-Exposure Prophylaxis	pre exposure prophylaxis	pre exposure prophylaxis	pre exposure prophylaxis	pre-exposure prophylaxis	2	case_or_format
pre-operative care	pre operative care	pre operative care	pre operative care	pre-operative care	2	kept
preanalytical error	preanalytical error	preanalytical error	preanalytical error	preanalytical error	2	kept
prebiotic agent	prebiotic agent	prebiotic agent	prebiotic agent	prebiotic agent	2	kept
prebriefing	prebriefing	prebriefing	prebriefing	prebriefing	2	kept
Precipitating factors	precipitating factors	precipitating factors	precipitating factors	Precipitating Factors	2	case_or_format
precipitation	precipitation	precipitation	precipitation	precipitation	2	kept
Precision medicine	precision medicine	precision medicine	precision medicine	Precision Medicine	2	case_or_format
Precision surveillance	precision surveillance	precision surveillance	precision surveillance	Precision surveillance	2	kept
preclinical study	preclinical study	preclinical study	preclinical study	preclinical study	2	kept
PREDICT	predict	predict	predict	PREDICT	2	kept
Prediction of influenza-like virus pathogen	prediction of influenza like virus pathogen	prediction of influenza like virus pathogen	prediction of influenza like virus pathogen	Prediction of influenza-like virus pathogen	2	kept
PREDICTIONS	predictions	predictions	prediction	prediction	2	singular

predictive medicine	predictive medicine	predictive medicine	predictive medicine	predictive medicine	2	kept
Predictive modeling	predictive modeling	predictive modeling	predictive modeling	predictive modeling	2	case_or_format
PREDICTOR	predictor	predictor	predictor	PREDICTORS	2	case_or_format
Predictor	predictor	predictor	predictor	PREDICTORS	2	case_or_format
predictor	predictor	predictor	predictor	PREDICTORS	2	case_or_format
Prednisone	prednisone	prednisone	prednisone	prednisone	2	case_or_format
preemie	preemie	preemie	preemie	preemie	2	kept
preemployment medical examination	preemployment medical examination	preemployment medical examination	preemployment medical examination	preemployment medical examination	2	kept
preemptive analgesia	preemptive analgesia	preemptive analgesia	preemptive analgesia	preemptive analgesia	2	kept
Preferences	preferences	preferences	preference	PREFERENCES	2	singular
prefrontal cortex	prefrontal cortex	prefrontal cortex	prefrontal cortex	prefrontal cortex	2	kept
Pregnancy burn injury	pregnancy burn injury	pregnancy burn injury	pregnancy burn injury	Pregnancy burn injury	2	kept
Pregnancy complications	pregnancy complications	pregnancy complications	pregnancy complication	Pregnancy Complications	2	singular
Pregnancy high-risk	pregnancy high risk	pregnancy high risk	pregnancy high risk	Pregnancy, High-Risk	2	case_or_format
pregnancy induced hypertension	pregnancy induced hypertension	pregnancy induced hypertension	pregnancy induced hypertension	pregnancy induced hypertension	2	kept
pregnancy outcomes	pregnancy outcomes	pregnancy outcomes	pregnancy outcome	Pregnancy Outcome	2	singular

Pregnancy, Ectopic	pregnancy ectopic	pregnancy ectopic	pregnancy ectopic	Pregnancy, Ectopic	2	kept
Pregnant	pregnant	pregnant	pregnant	Pregnant	2	kept
pregnant laboring women	pregnant laboring women	pregnant laboring women	pregnant laboring women	pregnant laboring women	2	kept
pregnant labouring women delivery and breastfeeding	pregnant labouring women delivery and breastfeeding	pregnant labouring women delivery and breastfeeding	pregnant labouring women delivery and breastfeeding	pregnant labouring women delivery and breastfeeding	2	kept
pregnant women	pregnant women	pregnant women	pregnant women	Pregnant Women	2	case_or_format
Prelicensure	prelicensure	prelicensure	prelicensure	Prelicensure	2	kept
PREMATURE	premature	premature	premature	Premature	2	case_or_format
premature infant care	premature infant care	premature infant care	premature infant care	premature infant care	2	kept
Premature infant pain profile	premature infant pain profile	premature infant pain profile	premature infant pain profile	Premature Infant Pain Profile	2	case_or_format
premature infant pain profile	premature infant pain profile	premature infant pain profile	premature infant pain profile	Premature Infant Pain Profile	2	case_or_format
Premature neonates	premature neonates	premature neonates	premature neonate	Premature neonate	2	singular
premature neonates	premature neonates	premature neonates	premature neonate	Premature neonate	2	singular
prenatal	prenatal	prenatal	prenatal	prenatal	2	kept
Prenatal diagnosis	prenatal diagnosis	prenatal diagnosis	prenatal diagnosis	Prenatal Diagnosis	2	case_or_format
Prenatal Injuries	prenatal injuries	prenatal injuries	prenatal injury	prenatal injury	2	singular
prenatal injury	prenatal injury	prenatal injury	prenatal injury	prenatal injury	2	kept

preoperative	preoperative	preoperative	preoperative	preoperative	2	kept
preoperative assessment	preoperative assessment	preoperative assessment	preoperative assessment	preoperative assessment	2	kept
Preoperative Exercise	preoperative exercise	preoperative exercise	preoperative exercise	preoperative exercise	2	case_or_format
Preparation errors	preparation errors	preparation errors	preparation error	Preparation errors	2	singular
PREPAREDNESS	preparedness	preparedness	preparedness	Preparedness	2	case_or_format
prepregnancy care	prepregnancy care	prepregnancy care	prepregnancy care	prepregnancy care	2	kept
preschool	preschool	preschool	preschool	preschool	2	kept
prescribing	prescribing	prescribing	prescribing	Prescribing	2	case_or_format
prescribing practices	prescribing practices	prescribing practices	prescribing practice	prescribing practice	2	singular
Prescription Drug Misuse	prescription drug misuse	prescription drug misuse	prescription drug misuse	Prescription Drug Misuse	2	kept
PRESCRIPTIONS	prescriptions	prescriptions	prescription	prescription	2	singular
prescriptions	prescriptions	prescriptions	prescription	prescription	2	singular
pressure injures	pressure injures	pressure injures	pressure injures	pressure injures	2	kept
Pressure measurement	pressure measurement	pressure measurement	pressure measurement	pressure measurement	2	case_or_format
Pressure risk assessment scales	pressure risk assessment scales	pressure risk assessment scales	pressure risk assessment scales	Pressure risk assessment scales	2	kept
pressure sore	pressure sore	pressure sore	pressure sore	pressure sore	2	kept

Pressure sores	pressure sores	pressure sores	pressure sore	pressure sore	2	singular
pressure ulcer care	pressure ulcer care	pressure ulcer care	pressure ulcer care	pressure ulcer care	2	kept
pressure ulcer medical	pressure ulcer medical	pressure ulcer medical	pressure ulcer medical	pressure ulcer medical	2	kept
pressure ulceration	pressure ulceration	pressure ulceration	pressure ulceration	pressure ulceration	2	kept
pressure volume curve	pressure volume curve	pressure volume curve	pressure volume curve	pressure volume curve	2	kept
presumed consent	presumed consent	presumed consent	presumed consent	Presumed Consent	2	case_or_format
presyncope	presyncope	presyncope	presyncope	presyncope	2	kept
preterm baby	preterm baby	preterm baby	preterm baby	Preterm baby	2	case_or_format
preterm baby care	preterm baby care	preterm baby care	preterm baby care	preterm baby care	2	kept
PRETERM BIRTH	preterm birth	preterm birth	preterm birth	Preterm birth	2	case_or_format
PRETERM INFANT	preterm infant	preterm infant	preterm infant	preterm infant	2	case_or_format
Preterm infant development	preterm infant development	preterm infant development	preterm infant development	Preterm infant development	2	kept
Preterm mothers	preterm mothers	preterm mothers	preterm mothers	Preterm mothers	2	kept
preterm newborn	preterm newborn	preterm newborn	preterm newborn	Preterm newborn	2	case_or_format
preterm rupture of membranes	preterm rupture of membranes	preterm rupture of membranes	preterm rupture of membranes	preterm rupture of membranes	2	kept
pretest-posttest study	pretest posttest study	pretest posttest study	pretest posttest study	pretest-posttest study	2	kept

Prethrombotic state	prethrombotic state	prethrombotic state	prethrombotic state	Prethrombotic state	2	kept
prevalence rate	prevalence rate	prevalence rate	prevalence rate	prevalence rate	2	kept
PREVENT DELIRIUM	prevent delirium	prevent delirium	prevent delirium	PREVENT DELIRIUM	2	kept
Preventable complications	preventable complications	preventable complications	preventable complications	Preventable complications	2	kept
Prevention & control	prevention and control	prevention and control	prevention and control	prevention and control	2	case_or_format
Prevention and control of COVID-19 pandemic	prevention and control of covid 19 pandemic	prevention and control of covid 19 pandemic	prevention and control of covid 19 pandemic	Prevention and control of COVID-19 pandemic	2	kept
PREVENTION KNOWLEDGE	prevention knowledge	prevention knowledge	prevention knowledge	PREVENTION KNOWLEDGE	2	kept
Prevention of pressure ulcers	prevention of pressure ulcers	prevention of pressure ulcers	prevention of pressure ulcers	Prevention of pressure ulcers	2	kept
Prevention practice	prevention practice	prevention practice	prevention practice	Prevention practice	2	kept
Prevention strategies	prevention strategies	prevention strategies	prevention strategy	prevention strategies	2	singular
preventive dentistry	preventive dentistry	preventive dentistry	preventive dentistry	preventive dentistry	2	kept
Preventive interventions	preventive interventions	preventive interventions	preventive interventions	Preventive interventions	2	kept
Prilocaine	prilocaine	prilocaine	prilocaine	Prilocaine	2	kept
primary culture	primary culture	primary culture	primary culture	primary culture	2	kept
primary infection	primary infection	primary infection	primary infection	primary infection	2	kept
primary outcome	primary outcome	primary outcome	primary outcome	primary outcome	2	kept

Primary prevention	primary prevention	primary prevention	primary prevention	Primary Prevention	2	case_or_format
primary sclerosing cholangitis	primary sclerosing cholangitis	primary sclerosing cholangitis	primary sclerosing cholangitis	primary sclerosing cholangitis	2	kept
printing	printing	printing	printing	printing	2	kept
prioritization matrix	prioritization matrix	prioritization matrix	prioritization matrix	prioritization matrix	2	kept
pristinamycin	pristinamycin	pristinamycin	pristinamycin	pristinamycin	2	kept
Privacy preserving	privacy preserving	privacy preserving	privacy preserving	Privacy preserving	2	kept
Private hospital	private hospital	private hospital	private hospital	private hospital	2	case_or_format
Private Practice	private practice	private practice	private practice	private practice	2	case_or_format
Private room	private room	private room	private room	Private room	2	kept
Private sector	private sector	private sector	private sector	private sector	2	case_or_format
Privileging	privileging	privileging	privileging	Privileging	2	kept
Proactive personality	proactive personality	proactive personality	proactive personality	Proactive personality	2	kept
Proactive round	proactive round	proactive round	proactive round	Proactive round	2	kept
Proactivity	proactivity	proactivity	proactivity	Proactivity	2	kept
Probability Distribution Pattern Analysis	probability distribution pattern analysis	probability distribution pattern analysis	probability distribution pattern analysis	Probability Distribution Pattern Analysis	2	kept
Problem based learning	problem based learning	problem based learning	problem based learning	Problem-Based Learning	2	case_or_format

procarbazine	procarbazine	procarbazine	procarbazine	procarbazine	2	kept
Procedural	procedural	procedural	procedural	Procedural	2	kept
procedural pain management	procedural pain management	procedural pain management	procedural pain management	procedural pain management	2	kept
PROCEDURAL SEDATION	procedural sedation	procedural sedation	procedural sedation	Procedural Sedation	2	case_or_format
Proceduralization	proceduralization	proceduralization	proceduralization	Proceduralization	2	kept
Procedures and Techniques Utilization	procedures and techniques utilization	procedures and techniques utilization	procedures and techniques utilization	Procedures and Techniques Utilization	2	kept
process	process	process	process	Process	2	case_or_format
Process assessment	process assessment	process assessment	process assessment	Process Assessment	2	case_or_format
Process mining	process mining	process mining	process mining	Process mining	2	kept
process mining	process mining	process mining	process mining	Process mining	2	case_or_format
Process quality	process quality	process quality	process quality	Process quality	2	kept
processing speed	processing speed	processing speed	processing speed	processing speed	2	kept
procrastination	procrastination	procrastination	procrastination	Procrastination	2	case_or_format
PROCUREMENT	procurement	procurement	procurement	PROCUREMENT	2	kept
prodromal symptom	prodromal symptom	prodromal symptom	prodromal symptom	prodromal symptom	2	kept
Product development	product development	product development	product development	Product development	2	kept

product development	product development	product development	product development	Product development	2	case_or_format
Product Line Management	product line management	product line management	product line management	Product Line Management	2	kept
PRODUCTIVITY	productivity	productivity	productivity	productivity	2	case_or_format
Productivity	productivity	productivity	productivity	productivity	2	case_or_format
profession selection	profession selection	profession selection	profession selection	profession selection	2	kept
Profession turnover intention	profession turnover intention	profession turnover intention	profession turnover intention	Profession turnover intention	2	kept
Professional activities	professional activities	professional activities	professional activities	Professional activities	2	kept
professional characteristics	professional characteristics	professional characteristics	professional characteristics	professional characteristics	2	kept
Professional experience	professional experience	professional experience	professional experience	Professional experience	2	kept
Professional exposure	professional exposure	professional exposure	professional exposure	Professional exposure	2	kept
professional focus	professional focus	professional focus	professional focus	professional focus	2	kept
professional fulfillment	professional fulfillment	professional fulfillment	professional fulfillment	professional fulfillment	2	kept
professional growth	professional growth	professional growth	professional growth	professional growth	2	kept
Professional Impairment	professional impairment	professional impairment	professional impairment	Professional Impairment	2	kept
Professional Issues	professional issues	professional issues	professional issues	professional issues	2	case_or_format
professional practice environment	professional practice environment	professional practice environment	professional practice environment	Professional practice environment	2	case_or_format

Professional practice environment work passion	professional practice environment work passion	professional practice environment work passion	professional practice environment work passion	Professional practice environment work passion	2	kept
Professional training	professional training	professional training	professional training	Professional training	2	kept
professional training	professional training	professional training	professional training	Professional training	2	case_or_format
Professional value	professional value	professional value	professional value	Professional value	2	kept
Professional values	professional values	professional values	professional value	Professional value	2	singular
PROFESSIONALISM	professionalism	professionalism	professionalism	professionalism	2	case_or_format
professional－family relations	professional family relations	professional family relations	professional family relations	Professional-Family Relations	2	case_or_format
Professions	professions	professions	profession	PROFESSION	2	singular
progeny	progeny	progeny	progeny	progeny	2	kept
PROGNOSIS	prognosis	prognosis	prognosis	Prognosis	2	case_or_format
Prognosis, quality of health care	prognosis quality of health care	prognosis quality of health care	prognosis quality of health care	Prognosis, quality of health care	2	kept
Prognostic factors	prognostic factors	prognostic factors	prognostic factor	prognostic factors	2	singular
Prognostic value	prognostic value	prognostic value	prognostic value	Prognostic value	2	kept
program	program	program	program	PROGRAM	2	case_or_format
program implementation	program implementation	program implementation	program implementation	program implementation	2	kept
program usability	program usability	program usability	program usability	program usability	2	kept

Programme	programme	programme	programme	Programme	2	kept
Programmed analgesia and sedation	programmed analgesia and sedation	programmed analgesia and sedation	programmed analgesia and sedation	Programmed analgesia and sedation	2	kept
programmed death 1 ligand 1	programmed death 1 ligand 1	programmed death 1 ligand 1	programmed death 1 ligand 1	programmed death 1 ligand 1	2	kept
Programming, Linear	programming linear	programming linear	programming linear	Programming, Linear	2	kept
PROGRAMS	programs	programs	program	PROGRAM	2	singular
Progress records	progress records	progress records	progress records	Progress records	2	kept
progressive care unit	progressive care unit	progressive care unit	progressive care unit	progressive care unit	2	kept
Progressive mobility	progressive mobility	progressive mobility	progressive mobility	Progressive mobility	2	kept
Progressive mobilization	progressive mobilization	progressive mobilization	progressive mobilization	Progressive mobilization	2	kept
progressive muscle relaxation	progressive muscle relaxation	progressive muscle relaxation	progressive muscle relaxation	Progressive muscle relaxation	2	case_or_format
PROJECT	project	project	project	PROJECT	2	kept
Project management	project management	project management	project management	Project management	2	kept
project-based learning	project based learning	project based learning	project based learning	project-based learning	2	kept
prolactin	prolactin	prolactin	prolactin	prolactin	2	kept
prolapse	prolapse	prolapse	prolapse	prolapse	2	kept
prolonged	prolonged	prolonged	prolonged	prolonged	2	kept

Prolonged illness	prolonged illness	prolonged illness	prolonged illness	Prolonged illness	2	kept
PROLONGED MECHANICAL VENTILATION	prolonged mechanical ventilation	prolonged mechanical ventilation	prolonged mechanical ventilation	prolonged mechanical ventilation	2	case_or_format
PROLONGED PAIN	prolonged pain	prolonged pain	prolonged pain	PROLONGED PAIN	2	kept
Prone	prone	prone	prone	Prone	2	kept
prone	prone	prone	prone	Prone	2	case_or_format
Prone Positioning	prone positioning	prone positioning	prone positioning	prone positioning	2	case_or_format
PROPHYLAXIS	prophylaxis	prophylaxis	prophylaxis	prophylaxis	2	case_or_format
Propionibacterium acnes	propionibacterium acnes	propionibacterium acnes	propionibacterium acnes	Propionibacterium acnes	2	kept
propionic acidemia	propionic acidemia	propionic acidemia	propionic acidemia	propionic acidemia	2	kept
propofol infusion syndrome	propofol infusion syndrome	propofol infusion syndrome	propofol infusion syndrome	propofol infusion syndrome	2	kept
Propoxycaine	propoxycaine	propoxycaine	propoxycaine	Propoxycaine	2	kept
Prosopagnosia	prosopagnosia	prosopagnosia	prosopagnosia	Prosopagnosia	2	kept
Prospective studies	prospective studies	prospective studies	prospective study	Prospective Studies	2	singular
prostacyclin derivative	prostacyclin derivative	prostacyclin derivative	prostacyclin derivative	prostacyclin derivative	2	kept
prostaglandin synthase inhibitor	prostaglandin synthase inhibitor	prostaglandin synthase inhibitor	prostaglandin synthase inhibitor	prostaglandin synthase inhibitor	2	kept
prostate biopsy	prostate biopsy	prostate biopsy	prostate biopsy	prostate biopsy	2	kept

prostate surgery	prostate surgery	prostate surgery	prostate surgery	prostate surgery	2	kept
prostatectomy	prostatectomy	prostatectomy	prostatectomy	prostatectomy	2	kept
prosthesis dislocation	prosthesis dislocation	prosthesis dislocation	prosthesis dislocation	prosthesis dislocation	2	kept
prosthesis failure	prosthesis failure	prosthesis failure	prosthesis failure	Prosthesis Failure	2	case_or_format
prosthesis implantation	prosthesis implantation	prosthesis implantation	prosthesis implantation	Prosthesis Implantation	2	case_or_format
prosthetic valve thrombosis	prosthetic valve thrombosis	prosthetic valve thrombosis	prosthetic valve thrombosis	prosthetic valve thrombosis	2	kept
protamine sulfate	protamine sulfate	protamine sulfate	protamine sulfate	protamine sulfate	2	kept
protective agent	protective agent	protective agent	protective agent	protective agent	2	kept
Protective Agents	protective agents	protective agents	protective agent	protective agent	2	singular
protective factors	protective factors	protective factors	protective factors	Protective Factors	2	case_or_format
Protective lung strategies	protective lung strategies	protective lung strategies	protective lung strategy	Protective lung strategies	2	singular
Protective reactions	protective reactions	protective reactions	protective reactions	Protective reactions	2	kept
PROTEIN	protein	protein	protein	protein	2	case_or_format
Protein	protein	protein	protein	protein	2	case_or_format
protein C deficiency	protein c deficiency	protein c deficiency	protein c deficiency	protein C deficiency	2	kept
protein calorie malnutrition	protein calorie malnutrition	protein calorie malnutrition	protein calorie malnutrition	protein calorie malnutrition	2	kept

protein p53	protein p53	protein p53	protein p53	protein p53	2	kept
protein precursor	protein precursor	protein precursor	protein precursor	Protein Precursors	2	case_or_format
protein S	protein s	protein s	protein s	protein S	2	kept
Protein Synthesis Inhibitors	protein synthesis inhibitors	protein synthesis inhibitors	protein synthesis inhibitors	Protein Synthesis Inhibitors	2	kept
Protein-Losing Enteropathies	protein losing enteropathies	protein losing enteropathies	protein losing enteropathies	Protein-Losing Enteropathies	2	kept
proteinase inhibitor	proteinase inhibitor	proteinase inhibitor	proteinase inhibitor	proteinase inhibitor	2	kept
proteins	proteins	proteins	protein	protein	2	singular
proteomics	proteomics	proteomics	proteomic	Proteomics	2	singular
Proto-Oncogene Proteins	proto oncogene proteins	proto oncogene proteins	proto oncogene proteins	Proto-Oncogene Proteins	2	kept
Protocol-based care	protocol based care	protocol based care	protocol based care	Protocol-based care	2	kept
prototype	prototype	prototype	prototype	prototype	2	kept
Providencia	providencia	providencia	providencia	Providencia	2	kept
provider costs	provider costs	provider costs	provider costs	provider costs	2	kept
providers	providers	providers	provider	PROVIDERS	2	singular
PROVISION	provision	provision	provision	PROVISION	2	kept
provocation test	provocation test	provocation test	provocation test	provocation test	2	kept

proximal femur	proximal femur	proximal femur	proximal femur	proximal femur	2	kept
proximal femur fracture	proximal femur fracture	proximal femur fracture	proximal femur fracture	proximal femur fracture	2	kept
proximal interphalangeal joint	proximal interphalangeal joint	proximal interphalangeal joint	proximal interphalangeal joint	proximal interphalangeal joint	2	kept
proximity	proximity	proximity	proximity	proximity	2	kept
Proximity sensing	proximity sensing	proximity sensing	proximity sensing	Proximity sensing	2	kept
Práctica de enfermería	pr ctica de enfermer a	pr ctica de enfermer a	pr ctica de enfermer a	Práctica de enfermería	2	kept
Pseudoaneurysm	pseudoaneurysm	pseudoaneurysm	pseudoaneurysm	Pseudoaneurysm	2	kept
Pseudomonadaceae	pseudomonadaceae	pseudomonadaceae	pseudomonadaceae	Pseudomonadaceae	2	kept
Pseudomonas Aureginosa	pseudomonas aureginosa	pseudomonas aureginosa	pseudomonas aureginosa	Pseudomonas Aureginosa	2	kept
Psicometría	psicometr a	psicometr a	psicometr a	Psicometría	2	kept
Psittacosis	psittacosis	psittacosis	psittacosis	Psittacosis	2	kept
Psoas Muscles	psoas muscles	psoas muscles	psoas muscle	psoas muscle	2	singular
psoriatic arthritis	psoriatic arthritis	psoriatic arthritis	psoriatic arthritis	psoriatic arthritis	2	kept
psychedelic drugs	psychedelic drugs	psychedelic drugs	psychedelic drugs	psychedelic drugs	2	kept
Psychiatric	psychiatric	psychiatric	psychiatric	psychiatric	2	case_or_format
psychiatric complication	psychiatric complication	psychiatric complication	psychiatric complication	psychiatric complication	2	kept

psychiatric hospitals	psychiatric hospitals	psychiatric hospitals	psychiatric hospital	psychiatric hospitals	2	singular
Psychiatric symptoms	psychiatric symptoms	psychiatric symptoms	psychiatric symptoms	Psychiatric symptoms	2	kept
psychiatric ward	psychiatric ward	psychiatric ward	psychiatric ward	psychiatric ward	2	kept
PSYCHIATRY	psychiatry	psychiatry	psychiatry	psychiatry	2	case_or_format
psycho-educational program	psycho educational program	psycho educational program	psycho educational program	psycho-educational program	2	kept
psycho-oncology	psycho oncology	psycho oncology	psycho oncology	psycho-oncology	2	kept
psychoanalysis	psychoanalysis	psychoanalysis	psychoanalysis	psychoanalysis	2	kept
Psychoanalytic Theory	psychoanalytic theory	psychoanalytic theory	psychoanalytic theory	Psychoanalytic Theory	2	kept
psychoeducational program	psychoeducational program	psychoeducational program	psychoeducational program	psychoeducational program	2	kept
Psycholinguistics	psycholinguistics	psycholinguistics	psycholinguistics	Psycholinguistics	2	kept
Psychological and behavioral factors	psychological and behavioral factors	psychological and behavioral factors	psychological and behavioral factors	Psychological and behavioral factors	2	kept
Psychological burden	psychological burden	psychological burden	psychological burden	Psychological burden	2	kept
Psychological capital	psychological capital	psychological capital	psychological capital	psychological capital	2	case_or_format
psychological comfort	psychological comfort	psychological comfort	psychological comfort	psychological comfort	2	kept
PSYCHOLOGICAL EMPOWERMENT	psychological empowerment	psychological empowerment	psychological empowerment	Psychological empowerment	2	case_or_format
psychological first aid	psychological first aid	psychological first aid	psychological first aid	psychological first aid	2	kept

Psychological flexibility	psychological flexibility	psychological flexibility	psychological flexibility	psychological flexibility	2	case_or_format
Psychological health	psychological health	psychological health	psychological health	psychological health	2	case_or_format
Psychological impact	psychological impact	psychological impact	psychological impact	psychological impact	2	case_or_format
Psychological intervention	psychological intervention	psychological intervention	psychological intervention	psychological intervention	2	case_or_format
Psychological needs	psychological needs	psychological needs	psychological needs	Psychological needs	2	kept
Psychological outcome	psychological outcome	psychological outcome	psychological outcome	psychological outcomes	2	case_or_format
Psychological recovery	psychological recovery	psychological recovery	psychological recovery	Psychological recovery	2	kept
Psychological Safety	psychological safety	psychological safety	psychological safety	psychological safety	2	case_or_format
Psychological safety	psychological safety	psychological safety	psychological safety	psychological safety	2	case_or_format
psychological status	psychological status	psychological status	psychological status	Psychological status	2	case_or_format
Psychological Stress	psychological stress	psychological stress	psychological stress	Psychological stress	2	case_or_format
Psychological support	psychological support	psychological support	psychological support	psychological support	2	case_or_format
Psychological symptoms	psychological symptoms	psychological symptoms	psychological symptoms	psychological symptoms	2	case_or_format
psychological trauma	psychological trauma	psychological trauma	psychological trauma	Psychological Trauma	2	case_or_format
psychological wellbeing	psychological wellbeing	psychological wellbeing	psychological wellbeing	psychological wellbeing	2	kept
Psychology, Industrial	psychology industrial	psychology industrial	psychology industrial	Psychology, Industrial	2	kept

psychometric assessment	psychometric assessment	psychometric assessment	psychometric assessment	psychometric assessment	2	kept
psychometric studies	psychometric studies	psychometric studies	psychometric studies	psychometric studies	2	kept
Psychometric testing	psychometric testing	psychometric testing	psychometric testing	psychometric testing	2	case_or_format
PSYCHOMETRIC VALIDATION	psychometric validation	psychometric validation	psychometric validation	PSYCHOMETRIC VALIDATION	2	kept
psychometric validation	psychometric validation	psychometric validation	psychometric validation	PSYCHOMETRIC VALIDATION	2	case_or_format
Psychometrics assessment	psychometrics assessment	psychometrics assessment	psychometrics assessment	Psychometrics assessment	2	kept
Psychometry	psychometry	psychometry	psychometry	psychometry	2	case_or_format
psychomotor agitation	psychomotor agitation	psychomotor agitation	psychomotor agitation	Psychomotor Agitation	2	case_or_format
psychomotor development	psychomotor development	psychomotor development	psychomotor development	psychomotor development	2	kept
Psychomotor Disorders	psychomotor disorders	psychomotor disorders	psychomotor disorder	psychomotor disorder	2	singular
Psychoneuroimmunology	psychoneuroimmunology	psychoneuroimmunology	psychoneuroimmunology	Psychoneuroimmunology	2	kept
psychopathology	psychopathology	psychopathology	psychopathology	psychopathology	2	kept
psychopharmacotherapy	psychopharmacotherapy	psychopharmacotherapy	psychopharmacotherapy	psychopharmacotherapy	2	kept
Psychophysiology	psychophysiology	psychophysiology	psychophysiology	psychophysiology	2	case_or_format
psychosocial adjustment	psychosocial adjustment	psychosocial adjustment	psychosocial adjustment	psychosocial adjustment	2	kept
psychosocial issues	psychosocial issues	psychosocial issues	psychosocial issues	psychosocial issues	2	kept

psychosocial needs	psychosocial needs	psychosocial needs	psychosocial needs	psychosocial needs	2	kept
psychosocial risks	psychosocial risks	psychosocial risks	psychosocial risks	psychosocial risks	2	kept
Psychosocial support	psychosocial support	psychosocial support	psychosocial support	psychosocial support	2	case_or_format
Psychosocial support post-burn	psychosocial support post burn	psychosocial support post burn	psychosocial support post burn	Psychosocial support post-burn	2	kept
Psychosocial support systems	psychosocial support systems	psychosocial support systems	psychosocial support systems	Psychosocial Support Systems	2	case_or_format
psychosocial well-being	psychosocial well being	psychosocial well being	psychosocial well being	psychosocial well-being	2	kept
Psychosocial work environment	psychosocial work environment	psychosocial work environment	psychosocial work environment	Psychosocial work environment	2	kept
psychosomatic symptom	psychosomatic symptom	psychosomatic symptom	psychosomatic symptom	psychosomatic symptom	2	kept
Psychosomatic symptoms	psychosomatic symptoms	psychosomatic symptoms	psychosomatic symptom	psychosomatic symptom	2	singular
psychostimulant agent	psychostimulant agent	psychostimulant agent	psychostimulant agent	psychostimulant agent	2	kept
Psychotherapy, Group	psychotherapy group	psychotherapy group	psychotherapy group	Psychotherapy, Group	2	kept
PTS	pts	pts	pts	PTS	2	kept
Public	public	public	public	Public	2	kept
public attitude	public attitude	public attitude	public attitude	public attitude	2	kept
public crisis	public crisis	public crisis	public crisis	public crisis	2	kept
public finance	public finance	public finance	public finance	public finance	2	kept

public health campaign	public health campaign	public health campaign	public health campaign	public health campaign	2	kept
Public health emergencies	public health emergencies	public health emergencies	public health emergency	public health emergencies	2	singular
public health ethics	public health ethics	public health ethics	public health ethics	public health ethics	2	kept
Public Health Practice	public health practice	public health practice	public health practice	Public Health Practice	2	kept
Public health preparedness	public health preparedness	public health preparedness	public health preparedness	Public health preparedness	2	kept
public health surveillance	public health surveillance	public health surveillance	public health surveillance	Public Health Surveillance	2	case_or_format
Public hospital	public hospital	public hospital	public hospital	public hospital	2	case_or_format
Public Hospitals	public hospitals	public hospitals	public hospital	public hospital	2	singular
public reporting	public reporting	public reporting	public reporting	public reporting	2	kept
Public Sector	public sector	public sector	public sector	public sector	2	case_or_format
Public sector	public sector	public sector	public sector	public sector	2	case_or_format
Public tertiary hospitals	public tertiary hospitals	public tertiary hospitals	public tertiary hospitals	Public tertiary hospitals	2	kept
Public-Private Sector Partnerships	public private sector partnerships	public private sector partnerships	public private sector partnerships	Public-Private Sector Partnerships	2	kept
Publication Bias	publication bias	publication bias	publication bias	publication bias	2	case_or_format
pull through operation	pull through operation	pull through operation	pull through operation	pull through operation	2	kept
Pulmonary artery	pulmonary artery	pulmonary artery	pulmonary artery	Pulmonary Artery	2	case_or_format

pulmonary artery systolic pressure	pulmonary artery systolic pressure	pulmonary artery systolic pressure	pulmonary artery systolic pressure	pulmonary artery systolic pressure	2	kept
PULMONARY ASPIRATION	pulmonary aspiration	pulmonary aspiration	pulmonary aspiration	pulmonary aspiration	2	case_or_format
Pulmonary Atresia	pulmonary atresia	pulmonary atresia	pulmonary atresia	Pulmonary Atresia	2	kept
pulmonary atresia	pulmonary atresia	pulmonary atresia	pulmonary atresia	Pulmonary Atresia	2	case_or_format
Pulmonary Disease	pulmonary disease	pulmonary disease	pulmonary disease	Pulmonary diseases	2	case_or_format
pulmonary edema	pulmonary edema	pulmonary edema	pulmonary edema	Pulmonary Edema	2	case_or_format
pulmonary endarterectomy	pulmonary endarterectomy	pulmonary endarterectomy	pulmonary endarterectomy	pulmonary endarterectomy	2	kept
Pulmonary Fibrosis	pulmonary fibrosis	pulmonary fibrosis	pulmonary fibrosis	Pulmonary Fibrosis	2	kept
Pulmonary Heart Disease	pulmonary heart disease	pulmonary heart disease	pulmonary heart disease	Pulmonary Heart Disease	2	kept
pulmonary infection	pulmonary infection	pulmonary infection	pulmonary infection	Pulmonary infection	2	case_or_format
pulmonary infections	pulmonary infections	pulmonary infections	pulmonary infection	Pulmonary infection	2	singular
pulmonary valve	pulmonary valve	pulmonary valve	pulmonary valve	pulmonary valve	2	kept
PULMONARY-ARTERY	pulmonary artery	pulmonary artery	pulmonary artery	Pulmonary Artery	2	case_or_format
Pulmonology	pulmonology	pulmonology	pulmonology	pulmonology	2	case_or_format
Pulse contour cardiac output	pulse contour cardiac output	pulse contour cardiac output	pulse contour cardiac output	Pulse contour cardiac output	2	kept
Pulse oximeters	pulse oximeters	pulse oximeters	pulse oximeter	pulse oximeter	2	singular

Pulse transit time	pulse transit time	pulse transit time	pulse transit time	Pulse transit time	2	kept
pulse wave	pulse wave	pulse wave	pulse wave	pulse wave	2	kept
Pulse-induced contour cardiac output	pulse induced contour cardiac output	pulse induced contour cardiac output	pulse induced contour cardiac output	Pulse-induced contour cardiac output	2	kept
pump	pump	pump	pump	pump	2	kept
Pumps	pumps	pumps	pump	pump	2	singular
punch biopsy	punch biopsy	punch biopsy	punch biopsy	punch biopsy	2	kept
Punjab	punjab	punjab	punjab	Punjab	2	kept
pupil assessment	pupil assessment	pupil assessment	pupil assessment	pupil assessment	2	kept
Pupillary	pupillary	pupillary	pupillary	Pupillary	2	kept
puppy	puppy	puppy	puppy	puppy	2	kept
Purchasing, Hospital	purchasing hospital	purchasing hospital	purchasing hospital	Purchasing, Hospital	2	kept
purinergic P2Y receptor antagonist	purinergic p2y receptor antagonist	purinergic p2y receptor antagonist	purinergic p2y receptor antagonist	purinergic P2Y receptor antagonist	2	kept
purinergic P2Y12 receptor	purinergic p2y12 receptor	purinergic p2y12 receptor	purinergic p2y12 receptor	purinergic P2Y12 receptor	2	kept
purpura	purpura	purpura	purpura	purpura	2	kept
Purpura, Thrombocytopenic, Idiopathic	purpura thrombocytopenic idiopathic	purpura thrombocytopenic idiopathic	purpura thrombocytopenic idiopathic	Purpura, Thrombocytopenic, Idiopathic	2	kept
purpuric rash	purpuric rash	purpuric rash	purpuric rash	purpuric rash	2	kept

putamen	putamen	putamen	putamen	putamen	2	kept
Pyelonephritis	pyelonephritis	pyelonephritis	pyelonephritis	pyelonephritis	2	case_or_format
Q set	q set	q set	q set	Q set	2	kept
QEEG	qeeg	qeeg	qeeg	QEEG	2	kept
QoL	qol	qol	qol	QoL	2	kept
QoS algorithms	qos algorithms	qos algorithms	qos algorithms	QoS algorithms	2	kept
QoS in WBMS	qos in wbms	qos in wbms	qos in wbms	QoS in WBMS	2	kept
QTc prolongation	qtc prolongation	qtc prolongation	qtc prolongation	QTc prolongation	2	kept
quadriceps femoris muscle	quadriceps femoris muscle	quadriceps femoris muscle	quadriceps femoris muscle	quadriceps femoris muscle	2	kept
Quadriceps Muscle	quadriceps muscle	quadriceps muscle	quadriceps muscle	Quadriceps Muscle	2	kept
qualified rate	qualified rate	qualified rate	qualified rate	qualified rate	2	kept
qualitative descriptive	qualitative descriptive	qualitative descriptive	qualitative descriptive	qualitative descriptive	2	kept
Qualitative evaluation	qualitative evaluation	qualitative evaluation	qualitative evaluation	Qualitative evaluation	2	kept
Qualitative Methods	qualitative methods	qualitative methods	qualitative methods	Qualitative methods	2	case_or_format
qualitative review	qualitative review	qualitative review	qualitative review	qualitative review	2	kept
Qualitative thematic content analysis	qualitative thematic content analysis	qualitative thematic content analysis	qualitative thematic content analysis	Qualitative thematic content analysis	2	kept

quality accreditation	quality accreditation	quality accreditation	quality accreditation	quality accreditation	2	kept
Quality and safety	quality and safety	quality and safety	quality and safety	quality and safety	2	case_or_format
quality control procedures	quality control procedures	quality control procedures	quality control procedures	quality control procedures	2	kept
Quality health care	quality health care	quality health care	quality health care	Quality health care	2	kept
Quality Health Outcomes Model	quality health outcomes model	quality health outcomes model	quality health outcomes model	Quality Health Outcomes Model	2	kept
quality improvement methodologies	quality improvement methodologies	quality improvement methodologies	quality improvement methodologies	Quality improvement methodologies	2	case_or_format
Quality improvement program	quality improvement program	quality improvement program	quality improvement program	Quality improvement program	2	kept
QUALITY INDICATORS	quality indicators	quality indicators	quality indicator	Quality indicators	2	singular
Quality Indicators	quality indicators	quality indicators	quality indicator	Quality indicators	2	singular
quality initiative	quality initiative	quality initiative	quality initiative	quality initiative	2	kept
Quality of death	quality of death	quality of death	quality of death	quality of death	2	case_or_format
quality of dying and death	quality of dying and death	quality of dying and death	quality of dying and death	quality of dying and death	2	kept
quality of handover	quality of handover	quality of handover	quality of handover	quality of handover	2	kept
Quality of newborn care	quality of newborn care	quality of newborn care	quality of newborn care	Quality of newborn care	2	kept
Quality of recovery	quality of recovery	quality of recovery	quality of recovery	Quality of recovery	2	kept
quality of work-life	quality of work life	quality of work life	quality of work life	Quality of work life	2	case_or_format

quality standards	quality standards	quality standards	quality standards	quality standards	2	kept
Quality-improvement	quality improvement	quality improvement	quality improvement	Quality Improvement	2	case_or_format
quantitative approaches	quantitative approaches	quantitative approaches	quantitative approaches	quantitative approaches	2	kept
Quantitative EEG	quantitative eeg	quantitative eeg	quantitative eeg	Quantitative EEG	2	kept
Quantitative method	quantitative method	quantitative method	quantitative method	Quantitative method	2	kept
Quantitative study	quantitative study	quantitative study	quantitative study	quantitative study	2	case_or_format
quasi-experimental	quasi experimental	quasi experimental	quasi experimental	quasi-experimental	2	kept
quasi-experimental study	quasi experimental study	quasi experimental study	quasi experimental study	quasi experimental study	2	case_or_format
Quebec Back Pain Disability Scale	quebec back pain disability scale	quebec back pain disability scale	quebec back pain disability scale	Quebec Back Pain Disability Scale	2	kept
Questionnaire development	questionnaire development	questionnaire development	questionnaire development	Questionnaire development	2	kept
QUESTIONNAIRE SURVEY	questionnaire survey	questionnaire survey	questionnaire survey	questionnaire survey	2	case_or_format
Quetiapine Fumarate	quetiapine fumarate	quetiapine fumarate	quetiapine fumarate	Quetiapine Fumarate	2	kept
queueing	queueing	queueing	queueing	queueing	2	kept
Queuing models	queuing models	queuing models	queuing models	Queuing models	2	kept
quick COVID-19 severity index	quick covid 19 severity index	quick covid 19 severity index	quick covid 19 severity index	quick COVID-19 severity index	2	kept
quick Sequential Organ Failure Assessment	quick sequential organ failure assessment	quick sequential organ failure assessment	quick sequential organ failure assessment	quick Sequential Organ Failure Assessment	2	kept

quiet time	quiet time	quiet time	quiet time	Quiet time	2	case_or_format
quinapril	quinapril	quinapril	quinapril	quinapril	2	kept
Quinine	quinine	quinine	quinine	quinine	2	case_or_format
Quinolones	quinolones	quinolones	quinolone	quinolone	2	singular
R-bibliometrix	r bibliometrix	r bibliometrix	r bibliometrix	R-bibliometrix	2	kept
rabeprazole	rabeprazole	rabeprazole	rabeprazole	rabeprazole	2	kept
rabies vaccine	rabies vaccine	rabies vaccine	rabies vaccine	rabies vaccine	2	kept
racetrack layout	racetrack layout	racetrack layout	racetrack layout	racetrack layout	2	kept
racial differences	racial differences	racial differences	racial differences	racial differences	2	kept
Racial disparities	racial disparities	racial disparities	racial disparity	racial disparity	2	singular
Radiant warmer	radiant warmer	radiant warmer	radiant warmer	Radiant warmer	2	kept
Radiation Injuries	radiation injuries	radiation injuries	radiation injury	radiation injury	2	singular
radiation therapy	radiation therapy	radiation therapy	radiation therapy	radiation therapy	2	kept
radical cystectomy	radical cystectomy	radical cystectomy	radical cystectomy	radical cystectomy	2	kept
radical nephrectomy	radical nephrectomy	radical nephrectomy	radical nephrectomy	radical nephrectomy	2	kept
Radio Frequency Identification Device	radio frequency identification device	radio frequency identification device	radio frequency identification device	Radio Frequency Identification Device	2	kept

radioactive iodine	radioactive iodine	radioactive iodine	radioactive iodine	radioactive iodine	2	kept
radiofrequency	radiofrequency	radiofrequency	radiofrequency	radiofrequency	2	kept
Radiosurgery	radiosurgery	radiosurgery	radiosurgery	Radiosurgery	2	kept
radiosurgery	radiosurgery	radiosurgery	radiosurgery	Radiosurgery	2	case_or_format
Radiotherapy	radiotherapy	radiotherapy	radiotherapy	Radiotherapy	2	kept
radiotherapy	radiotherapy	radiotherapy	radiotherapy	Radiotherapy	2	case_or_format
Radiotherapy, Adjuvant	radiotherapy adjuvant	radiotherapy adjuvant	radiotherapy adjuvant	Radiotherapy, Adjuvant	2	kept
ramipril	ramipril	ramipril	ramipril	ramipril	2	kept
Rancho Los Amigos scale	rancho los amigos scale	rancho los amigos scale	rancho los amigos scale	Rancho Los Amigos scale	2	kept
random amplified polymorphic DNA	random amplified polymorphic dna	random amplified polymorphic dna	random amplified polymorphic dna	random amplified polymorphic DNA	2	kept
Random forest classifier	random forest classifier	random forest classifier	random forest classifier	Random forest classifier	2	kept
Random forest model	random forest model	random forest model	random forest model	Random forest model	2	kept
Randomised clinical trial	randomised clinical trial	randomised clinical trial	randomised clinical trial	Randomised clinical trial	2	kept
Randomised controlled clinical trial	randomised controlled clinical trial	randomised controlled clinical trial	randomised controlled clinical trial	Randomised controlled clinical trial	2	kept
Randomised controlled trials	randomised controlled trials	randomised controlled trials	randomised controlled trial	Randomised controlled trial	2	singular
randomised controlled trials	randomised controlled trials	randomised controlled trials	randomised controlled trial	Randomised controlled trial	2	singular

Randomization	randomization	randomization	randomization	randomization	2	case_or_format
randomized	randomized	randomized	randomized	Randomized	2	case_or_format
randomized controlled	randomized controlled	randomized controlled	randomized controlled	randomized controlled	2	kept
Range of motion	range of motion	range of motion	range of motion	range of motion	2	case_or_format
ranibizumab	ranibizumab	ranibizumab	ranibizumab	ranibizumab	2	kept
Ranitidine	ranitidine	ranitidine	ranitidine	ranitidine	2	case_or_format
Raoultella planticola	raoultella planticola	raoultella planticola	raoultella planticola	Raoultella planticola	2	kept
rapid cycle deliberate practice	rapid cycle deliberate practice	rapid cycle deliberate practice	rapid cycle deliberate practice	rapid cycle deliberate practice	2	kept
Rapid diagnostics	rapid diagnostics	rapid diagnostics	rapid diagnostics	Rapid diagnostics	2	kept
RAPID RESPONSE	rapid response	rapid response	rapid response	Rapid response	2	case_or_format
RAPID RESPONSE SYSTEMS	rapid response systems	rapid response systems	rapid response system	Rapid response system	2	singular
Rapid Response Systems	rapid response systems	rapid response systems	rapid response system	Rapid response system	2	singular
Rapid Upper Limb Assessment	rapid upper limb assessment	rapid upper limb assessment	rapid upper limb assessment	Rapid Upper Limb Assessment	2	kept
RAPID-RESPONSE SYSTEMS	rapid response systems	rapid response systems	rapid response system	Rapid response system	2	singular
RATING-SCALE	rating scale	rating scale	rating scale	rating scale	2	case_or_format
RATINGS	ratings	ratings	ratings	RATINGS	2	kept

Ratio	ratio	ratio	ratio	Ratio	2	kept
Rationed care	rationed care	rationed care	rationed care	Rationed care	2	kept
ratios	ratios	ratios	ratio	Ratio	2	singular
RATS	rats	rats	rat	rat	2	singular
ravulizumab	ravulizumab	ravulizumab	ravulizumab	ravulizumab	2	kept
RCT, randomized controlled trial	rct randomized controlled trial	rct randomized controlled trial	rct randomized controlled trial	RCT, randomized controlled trial	2	kept
RE-AIM	re aim	re aim	re aim	RE-AIM	2	kept
reaction	reaction	reaction	reaction	reaction	2	kept
reactivate	reactivate	reactivate	reactivate	reactivate	2	kept
Readiness for hospital discharge	readiness for hospital discharge	readiness for hospital discharge	readiness for hospital discharge	Readiness for hospital discharge	2	kept
readmissions	readmissions	readmissions	readmission	Readmission	2	singular
reaginic antibody	reaginic antibody	reaginic antibody	reaginic antibody	reaginic antibody	2	kept
Real time measurements	real time measurements	real time measurements	real time measurements	Real time measurements	2	kept
Real time safety audits	real time safety audits	real time safety audits	real time safety audits	Real time safety audits	2	kept
Real time systems	real time systems	real time systems	real time systems	Real time systems	2	kept
Real World Evidence	real world evidence	real world evidence	real world evidence	Real World Evidence	2	kept

real-time data	real time data	real time data	real time data	real-time data	2	kept
real-time noncontact visit program	real time noncontact visit program	real time noncontact visit program	real time noncontact visit program	real-time noncontact visit program	2	kept
Real-time safety audits	real time safety audits	real time safety audits	real time safety audits	Real time safety audits	2	case_or_format
Real-world	real world	real world	real world	Real-world	2	kept
realism	realism	realism	realism	realism	2	kept
Realist evaluation	realist evaluation	realist evaluation	realist evaluation	Realist evaluation	2	kept
realist review	realist review	realist review	realist review	realist review	2	kept
Reasonable adjustments	reasonable adjustments	reasonable adjustments	reasonable adjustments	Reasonable adjustments	2	kept
Reasoning competency	reasoning competency	reasoning competency	reasoning competency	Reasoning competency	2	kept
REBA	reba	reba	reba	REBA	2	kept
rebound	rebound	rebound	rebound	rebound	2	kept
Recall	recall	recall	recall	recall	2	case_or_format
Receptors, Vasopressin	receptors vasopressin	receptors vasopressin	receptors vasopressin	Receptors, Vasopressin	2	kept
recidivism	recidivism	recidivism	recidivism	recidivism	2	kept
Recién Nacido Prematuro	reci n nacido prematuro	reci n nacido prematuro	reci n nacido prematuro	Recién Nacido Prematuro	2	kept
Recognising	recognising	recognising	recognising	Recognising	2	kept

recombinant erythropoietin	recombinant erythropoietin	recombinant erythropoietin	recombinant erythropoietin	recombinant erythropoietin	2	kept
Recombinant factor VIIa	recombinant factor viia	recombinant factor viia	recombinant factor viia	Recombinant factor VIIa	2	kept
recombinant FVIIa	recombinant fviia	recombinant fviia	recombinant fviia	recombinant FVIIa	2	kept
Recommended vaccinations	recommended vaccinations	recommended vaccinations	recommended vaccinations	Recommended vaccinations	2	kept
reconnaissance	reconnaissance	reconnaissance	reconnaissance	reconnaissance	2	kept
Recording data	recording data	recording data	recording data	Recording data	2	kept
Records management	records management	records management	records management	Records management	2	kept
Recovery and rehabilitation	recovery and rehabilitation	recovery and rehabilitation	recovery and rehabilitation	Recovery and rehabilitation	2	kept
Recovery center	recovery center	recovery center	recovery center	Recovery center	2	kept
Recovery of external-work contractility	recovery of external work contractility	recovery of external work contractility	recovery of external work contractility	Recovery of external-work contractility	2	kept
Recovery of function	recovery of function	recovery of function	recovery of function	Recovery of Function	2	case_or_format
Recovery process	recovery process	recovery process	recovery process	Recovery process	2	kept
Recovery questionnaire	recovery questionnaire	recovery questionnaire	recovery questionnaire	Recovery questionnaire	2	kept
Recreation	recreation	recreation	recreation	recreation	2	case_or_format
recreational drug	recreational drug	recreational drug	recreational drug	recreational drug	2	kept
Recreational drugs	recreational drugs	recreational drugs	recreational drug	recreational drug	2	singular

Rectal	rectal	rectal	rectal	Rectal	2	kept
rectal drug administration	rectal drug administration	rectal drug administration	rectal drug administration	rectal drug administration	2	kept
Rectal perforation	rectal perforation	rectal perforation	rectal perforation	Rectal perforation	2	kept
rectum perforation	rectum perforation	rectum perforation	rectum perforation	rectum perforation	2	kept
rectum surgery	rectum surgery	rectum surgery	rectum surgery	rectum surgery	2	kept
rectum temperature	rectum temperature	rectum temperature	rectum temperature	rectum temperature	2	kept
rectum ulcer	rectum ulcer	rectum ulcer	rectum ulcer	rectum ulcer	2	kept
rectus sheath hematoma	rectus sheath hematoma	rectus sheath hematoma	rectus sheath hematoma	rectus sheath hematoma	2	kept
recurrent laryngeal nerve palsy	recurrent laryngeal nerve palsy	recurrent laryngeal nerve palsy	recurrent laryngeal nerve palsy	recurrent laryngeal nerve palsy	2	kept
Recurrent neural networks	recurrent neural networks	recurrent neural networks	recurrent neural network	recurrent neural network	2	singular
Recém-Nascido	rec m nascido	rec m nascido	rec m nascido	Recém-Nascido	2	kept
Red blood cell transfusion	red blood cell transfusion	red blood cell transfusion	red blood cell transfusion	Red blood cell transfusion	2	kept
red blood cell transfusion	red blood cell transfusion	red blood cell transfusion	red blood cell transfusion	Red blood cell transfusion	2	case_or_format
reentry tachycardia	reentry tachycardia	reentry tachycardia	reentry tachycardia	reentry tachycardia	2	kept
reference database	reference database	reference database	reference database	reference database	2	kept
Reference standards	reference standards	reference standards	reference standard	Reference Standards	2	singular

REFLECTIONS	reflections	reflections	reflection	Reflection	2	singular
reflective case study	reflective case study	reflective case study	reflective case study	reflective case study	2	kept
Reflective lifeworld research	reflective lifeworld research	reflective lifeworld research	reflective lifeworld research	Reflective lifeworld research	2	kept
Reflective supervision	reflective supervision	reflective supervision	reflective supervision	Reflective supervision	2	kept
reflexivity	reflexivity	reflexivity	reflexivity	reflexivity	2	kept
refractory disease	refractory disease	refractory disease	refractory disease	refractory disease	2	kept
refractory period	refractory period	refractory period	refractory period	refractory period	2	kept
refusal to participate	refusal to participate	refusal to participate	refusal to participate	refusal to participate	2	kept
Refused admissions	refused admissions	refused admissions	refused admissions	Refused admissions	2	kept
regenerative medicine	regenerative medicine	regenerative medicine	regenerative medicine	regenerative medicine	2	kept
regional	regional	regional	regional	regional	2	kept
regional anaesthesia	regional anaesthesia	regional anaesthesia	regional anaesthesia	regional anaesthesia	2	kept
Regional oxygen saturation	regional oxygen saturation	regional oxygen saturation	regional oxygen saturation	Regional oxygen saturation	2	kept
regional perfusion	regional perfusion	regional perfusion	regional perfusion	regional perfusion	2	kept
regional trauma centre	regional trauma centre	regional trauma centre	regional trauma centre	regional trauma centre	2	kept
Regional variation	regional variation	regional variation	regional variation	Regional variation	2	kept

Regionalization	regionalization	regionalization	regionalization	regionalization	2	case_or_format
Registered respiratory therapists	registered respiratory therapists	registered respiratory therapists	registered respiratory therapist	Registered respiratory therapists	2	singular
regulation	regulation	regulation	regulation	Regulations	2	case_or_format
regulatory T lymphocyte	regulatory t lymphocyte	regulatory t lymphocyte	regulatory t lymphocyte	regulatory T lymphocyte	2	kept
Rehabilitación	rehabilitaci n	rehabilitaci n	rehabilitaci n	Rehabilitación	2	kept
REHABILITATION	rehabilitation	rehabilitation	rehabilitation	rehabilitation	2	case_or_format
REHABILITATION MEDICINE	rehabilitation medicine	rehabilitation medicine	rehabilitation medicine	rehabilitation medicine	2	case_or_format
Rehabilitation outcome	rehabilitation outcome	rehabilitation outcome	rehabilitation outcome	Rehabilitation outcome	2	kept
Rehabilitation programs	rehabilitation programs	rehabilitation programs	rehabilitation program	Rehabilitation programs	2	singular
rehabilitation protocol	rehabilitation protocol	rehabilitation protocol	rehabilitation protocol	rehabilitation protocol	2	kept
rehospitalization	rehospitalization	rehospitalization	rehospitalization	rehospitalization	2	kept
Rehospitalizations	rehospitalizations	rehospitalizations	rehospitalization	rehospitalization	2	singular
reintroduction	reintroduction	reintroduction	reintroduction	reintroduction	2	kept
Rejection	rejection	rejection	rejection	Rejection	2	kept
Relaciones familiares	relaciones familiares	relaciones familiares	relaciones familiares	Relaciones familiares	2	kept
Relaciones Médico-Enfermero	relaciones m dico enfermero	relaciones m dico enfermero	relaciones m dico enfermero	Relaciones Médico-Enfermero	2	kept

relaparotomy	relaparotomy	relaparotomy	relaparotomy	relaparotomy	2	kept
Relapse	relapse	relapse	relapse	relapse	2	case_or_format
relational coordination	relational coordination	relational coordination	relational coordination	RELATIONAL COORDINATION	2	case_or_format
relational ethics	relational ethics	relational ethics	relational ethics	relational ethics	2	kept
Relatives needs	relatives needs	relatives needs	relatives needs	Relatives needs	2	kept
Relações Médico-Enfermeiro	rela es m dico enfermeiro	rela es m dico enfermeiro	rela es m dico enfermeiro	Relações Médico-Enfermeiro	2	kept
RELIEF	relief	relief	relief	RELIEF	2	kept
Religious	religious	religious	religious	Religious	2	kept
Religious Approach	religious approach	religious approach	religious approach	Religious Approach	2	kept
religiousness	religiousness	religiousness	religiousness	religiousness	2	kept
relocation stress	relocation stress	relocation stress	relocation stress	Relocation stress	2	case_or_format
Remimazolam Besylate	remimazolam besylate	remimazolam besylate	remimazolam besylate	Remimazolam Besylate	2	kept
Reminder systems	reminder systems	reminder systems	reminder system	Reminder Systems	2	singular
Remote	remote	remote	remote	Remote	2	kept
remote	remote	remote	remote	Remote	2	case_or_format
remote education	remote education	remote education	remote education	remote education	2	kept

REMOVAL	removal	removal	removal	REMOVAL	2	kept
renal	renal	renal	renal	renal	2	kept
renal dialysis	renal dialysis	renal dialysis	renal dialysis	Renal Dialysis	2	case_or_format
renal failure	renal failure	renal failure	renal failure	Renal failure	2	case_or_format
Renal insufficiency, acute	renal insufficiency acute	renal insufficiency acute	renal insufficiency acute	Renal insufficiency, acute	2	kept
Renal leiomyosarcoma	renal leiomyosarcoma	renal leiomyosarcoma	renal leiomyosarcoma	Renal leiomyosarcoma	2	kept
Renal recovery	renal recovery	renal recovery	renal recovery	Renal recovery	2	kept
renal system parameters	renal system parameters	renal system parameters	renal system parameters	renal system parameters	2	kept
repeated measures analysis	repeated measures analysis	repeated measures analysis	repeated measures analysis	repeated measures analysis	2	kept
Repetitive simulation	repetitive simulation	repetitive simulation	repetitive simulation	Repetitive simulation	2	kept
replicability	replicability	replicability	replicability	replicability	2	kept
reporting and data system	reporting and data system	reporting and data system	reporting and data system	reporting and data system	2	kept
Reporting systems	reporting systems	reporting systems	reporting systems	Reporting systems	2	kept
Representation	representation	representation	representation	Representation	2	kept
Repressor Proteins	repressor proteins	repressor proteins	repressor proteins	Repressor Proteins	2	kept
reproduction	reproduction	reproduction	reproduction	reproduction	2	kept

Requirements	requirements	requirements	requirements	Requirements	2	kept
Rescue	rescue	rescue	rescue	RESCUE	2	case_or_format
Rescue missions	rescue missions	rescue missions	rescue missions	Rescue missions	2	kept
rescue personnel attitude	rescue personnel attitude	rescue personnel attitude	rescue personnel attitude	rescue personnel attitude	2	kept
Rescue success rate	rescue success rate	rescue success rate	rescue success rate	Rescue success rate	2	kept
rescue treatment	rescue treatment	rescue treatment	rescue treatment	rescue treatment	2	kept
Research designs	research designs	research designs	research designs	Research designs	2	kept
research findings	research findings	research findings	research findings	research findings	2	kept
Research personnel	research personnel	research personnel	research personnel	Research Personnel	2	case_or_format
research priorities	research priorities	research priorities	research priority	Research priorities	2	singular
Research programs	research programs	research programs	research programs	Research programs	2	kept
research protocol	research protocol	research protocol	research protocol	research protocol	2	kept
Research report	research report	research report	research report	Research Report	2	case_or_format
Research utilisation	research utilisation	research utilisation	research utilisation	Research utilisation	2	kept
research work	research work	research work	research work	research work	2	kept
reservoir	reservoir	reservoir	reservoir	reservoir	2	kept

residency	residency	residency	residency	Residency	2	case_or_format
Residency training	residency training	residency training	residency training	Residency training	2	kept
Residual dense block	residual dense block	residual dense block	residual dense block	Residual dense block	2	kept
Resilience engineering	resilience engineering	resilience engineering	resilience engineering	Resilience engineering	2	kept
Resiliency	resiliency	resiliency	resiliency	resiliency	2	case_or_format
resilient	resilient	resilient	resilient	resilient	2	kept
resilient coping	resilient coping	resilient coping	resilient coping	resilient coping	2	kept
Resilient healthcare	resilient healthcare	resilient healthcare	resilient healthcare	Resilient healthcare	2	kept
Resistance training	resistance training	resistance training	resistance training	resistance training	2	case_or_format
Resource limited setting	resource limited setting	resource limited setting	resource limited setting	resource limited setting	2	case_or_format
resource-constrained health systems	resource constrained health systems	resource constrained health systems	resource constrained health systems	resource-constrained health systems	2	kept
resource-limited settings	resource limited settings	resource limited settings	resource limited setting	resource limited setting	2	singular
RESOURCE-MANAGEMENT	resource management	resource management	resource management	resource management	2	case_or_format
respiration center	respiration center	respiration center	respiration center	respiration center	2	kept
respiration load	respiration load	respiration load	respiration load	respiration load	2	kept
respiratory airflow	respiratory airflow	respiratory airflow	respiratory airflow	respiratory airflow	2	kept

Respiratory care practitioner	respiratory care practitioner	respiratory care practitioner	respiratory care practitioner	Respiratory care practitioner	2	kept
respiratory care skills	respiratory care skills	respiratory care skills	respiratory care skills	respiratory care skills	2	kept
respiratory depression	respiratory depression	respiratory depression	respiratory depression	respiratory depression	2	kept
Respiratory function	respiratory function	respiratory function	respiratory function	respiratory function	2	case_or_format
Respiratory functional reeducation	respiratory functional reeducation	respiratory functional reeducation	respiratory functional reeducation	Respiratory functional reeducation	2	kept
Respiratory infections	respiratory infections	respiratory infections	respiratory infection	respiratory infections	2	singular
RESPIRATORY MEDICINE	respiratory medicine	respiratory medicine	respiratory medicine	RESPIRATORY MEDICINE	2	kept
respiratory medicine	respiratory medicine	respiratory medicine	respiratory medicine	RESPIRATORY MEDICINE	2	case_or_format
Respiratory Mucosa	respiratory mucosa	respiratory mucosa	respiratory mucosa	Respiratory Mucosa	2	kept
Respiratory muscles	respiratory muscles	respiratory muscles	respiratory muscle	Respiratory Muscles	2	singular
Respiratory Physiology	respiratory physiology	respiratory physiology	respiratory physiology	Respiratory Physiology	2	kept
respiratory Rate	respiratory rate	respiratory rate	respiratory rate	Respiratory Rate	2	case_or_format
Respiratory rehabilitation	respiratory rehabilitation	respiratory rehabilitation	respiratory rehabilitation	Respiratory rehabilitation	2	kept
respiratory severity score	respiratory severity score	respiratory severity score	respiratory severity score	respiratory severity score	2	kept
Respiratory simulation scenario	respiratory simulation scenario	respiratory simulation scenario	respiratory simulation scenario	Respiratory simulation scenario	2	kept
respiratory therapeutic device	respiratory therapeutic device	respiratory therapeutic device	respiratory therapeutic device	respiratory therapeutic device	2	kept

Respiratory therapies	respiratory therapies	respiratory therapies	respiratory therapy	Respiratory Therapy	2	singular
Respiratory tract infection	respiratory tract infection	respiratory tract infection	respiratory tract infection	respiratory tract infection	2	case_or_format
respiratory tract infections	respiratory tract infections	respiratory tract infections	respiratory tract infection	respiratory tract infection	2	singular
respiratory tract injury	respiratory tract injury	respiratory tract injury	respiratory tract injury	respiratory tract injury	2	kept
respiratory tract mucosa	respiratory tract mucosa	respiratory tract mucosa	respiratory tract mucosa	respiratory tract mucosa	2	kept
Responding	responding	responding	responding	Responding	2	kept
response	response	response	response	RESPONSES	2	case_or_format
Response rate	response rate	response rate	response rate	Response rate	2	kept
responsive bassinet	responsive bassinet	responsive bassinet	responsive bassinet	responsive bassinet	2	kept
Restless legs syndrome	restless legs syndrome	restless legs syndrome	restless legs syndrome	Restless legs syndrome	2	kept
restless legs syndrome	restless legs syndrome	restless legs syndrome	restless legs syndrome	Restless legs syndrome	2	case_or_format
restoration	restoration	restoration	restoration	restoration	2	kept
Restraint alternatives	restraint alternatives	restraint alternatives	restraint alternatives	Restraint alternatives	2	kept
restraint physical	restraint physical	restraint physical	restraint physical	Restraint, Physical	2	case_or_format
Restraint, physical	restraint physical	restraint physical	restraint physical	Restraint, Physical	2	case_or_format
restraint, physical	restraint physical	restraint physical	restraint physical	Restraint, Physical	2	case_or_format

RESTRICTION	restriction	restriction	restriction	RESTRICTION	2	kept
restrictive practice	restrictive practice	restrictive practice	restrictive practice	restrictive practice	2	kept
Resuscitation decision	resuscitation decision	resuscitation decision	resuscitation decision	Resuscitation decision	2	kept
Resuscitative thoracotomy	resuscitative thoracotomy	resuscitative thoracotomy	resuscitative thoracotomy	Resuscitative thoracotomy	2	kept
retained placenta	retained placenta	retained placenta	retained placenta	retained placenta	2	kept
retained surgical item	retained surgical item	retained surgical item	retained surgical item	retained surgical item	2	kept
reteplase	reteplase	reteplase	reteplase	reteplase	2	kept
Retina	retina	retina	retina	retina	2	case_or_format
retina blood vessel	retina blood vessel	retina blood vessel	retina blood vessel	retina blood vessel	2	kept
retinal nerve fiber layer	retinal nerve fiber layer	retinal nerve fiber layer	retinal nerve fiber layer	retinal nerve fiber layer	2	kept
Retinitis	retinitis	retinitis	retinitis	Retinitis	2	kept
retinitis	retinitis	retinitis	retinitis	Retinitis	2	case_or_format
retirement decisions	retirement decisions	retirement decisions	retirement decisions	retirement decisions	2	kept
Retrieval	retrieval	retrieval	retrieval	retrieval	2	case_or_format
Retrieval medicine	retrieval medicine	retrieval medicine	retrieval medicine	Retrieval medicine	2	kept
Retrieval time	retrieval time	retrieval time	retrieval time	Retrieval time	2	kept

retrobulbar hemorrhage	retrobulbar hemorrhage	retrobulbar hemorrhage	retrobulbar hemorrhage	retrobulbar hemorrhage	2	kept
retrosigmoid	retrosigmoid	retrosigmoid	retrosigmoid	retrosigmoid	2	kept
Retrospective	retrospective	retrospective	retrospective	retrospective	2	case_or_format
Rett syndrome	rett syndrome	rett syndrome	rett syndrome	Rett syndrome	2	kept
Return mapping	return mapping	return mapping	return mapping	Return mapping	2	kept
Return visit	return visit	return visit	return visit	Return visit	2	kept
Reverberation time	reverberation time	reverberation time	reverberation time	Reverberation time	2	kept
Reversal	reversal	reversal	reversal	Reversal	2	kept
reverse osmosis	reverse osmosis	reverse osmosis	reverse osmosis	reverse osmosis	2	kept
Review literature	review literature	review literature	review literature	Review literature	2	kept
Review literature as topic	review literature as topic	review literature as topic	review literature as topic	Review Literature as Topic	2	case_or_format
revision arthroplasty	revision arthroplasty	revision arthroplasty	revision arthroplasty	revision arthroplasty	2	kept
Revisit	revisit	revisit	revisit	Revisit	2	kept
Revues de morbi-mortalité	revues de morbi mortalit	revues de morbi mortalit	revues de morbi mortalit	Revues de morbi-mortalité	2	kept
rewriting	rewriting	rewriting	rewriting	rewriting	2	kept
RFID	rfid	rfid	rfid	RFID	2	kept

Rhabdomyosarcoma	rhabdomyosarcoma	rhabdomyosarcoma	rhabdomyosarcoma	rhabdomyosarcoma	2	case_or_format
Rhabdomyosarcoma, Embryonal	rhabdomyosarcoma embryonal	rhabdomyosarcoma embryonal	rhabdomyosarcoma embryonal	Rhabdomyosarcoma, Embryonal	2	kept
rhesus D antibody	rhesus d antibody	rhesus d antibody	rhesus d antibody	rhesus D antibody	2	kept
rheumatic heart disease	rheumatic heart disease	rheumatic heart disease	rheumatic heart disease	rheumatic heart disease	2	kept
Rhinitis, Allergic	rhinitis allergic	rhinitis allergic	rhinitis allergic	Rhinitis, Allergic	2	kept
Rhizotomy	rhizotomy	rhizotomy	rhizotomy	Rhizotomy	2	kept
rib fractures	rib fractures	rib fractures	rib fracture	rib fracture	2	singular
riboflavin	riboflavin	riboflavin	riboflavin	riboflavin	2	kept
ribosome DNA	ribosome dna	ribosome dna	ribosome dna	ribosome DNA	2	kept
RibScore	ribscore	ribscore	ribscore	RibScore	2	kept
Richards-Campbell Sleep Questionnaire	richards campbell sleep questionnaire	richards campbell sleep questionnaire	richards campbell sleep questionnaire	Richards Campbell Sleep Questionnaire	2	case_or_format
Richmond agitation-sedation scale	richmond agitation sedation scale	richmond agitation sedation scale	richmond agitation sedation scale	Richmond Agitation Sedation Scale	2	case_or_format
Rickettsia	rickettsia	rickettsia	rickettsia	Rickettsia	2	kept
Rickettsiaceae infection	rickettsiaceae infection	rickettsiaceae infection	rickettsiaceae infection	Rickettsiaceae infection	2	kept
Rifampin	rifampin	rifampin	rifampin	Rifampin	2	kept
Rift Valley fever	rift valley fever	rift valley fever	rift valley fever	Rift Valley fever	2	kept

right coronary artery	right coronary artery	right coronary artery	right coronary artery	right coronary artery	2	kept
right to healthcare	right to healthcare	right to healthcare	right to healthcare	right to healthcare	2	kept
Right ventricle	right ventricle	right ventricle	right ventricle	Right ventricle	2	kept
Rigid bronchoscopy	rigid bronchoscopy	rigid bronchoscopy	rigid bronchoscopy	Rigid bronchoscopy	2	kept
rigidity	rigidity	rigidity	rigidity	rigidity	2	kept
Riker sedation agitation scale	riker sedation agitation scale	riker sedation agitation scale	riker sedation agitation scale	Riker sedation agitation scale	2	kept
Rimantadine	rimantadine	rimantadine	rimantadine	rimantadine	2	case_or_format
Ringer's Lactate	ringer s lactate	ringer s lactate	ringer s lactate	Ringer's Lactate	2	kept
RIPLS	ripls	ripls	ripls	RIPLS	2	kept
RISK ADJUSTMENT	risk adjustment	risk adjustment	risk adjustment	Risk Adjustment	2	case_or_format
Risk Analysis Index	risk analysis index	risk analysis index	risk analysis index	Risk Analysis Index	2	kept
risk and protective factors	risk and protective factors	risk and protective factors	risk and protective factors	risk and protective factors	2	kept
risk evaluation and mitigation	risk evaluation and mitigation	risk evaluation and mitigation	risk evaluation and mitigation	risk evaluation and mitigation	2	kept
risk evaluation and mitigation strategy	risk evaluation and mitigation strategy	risk evaluation and mitigation strategy	risk evaluation and mitigation strategy	risk evaluation and mitigation strategy	2	kept
Risk index	risk index	risk index	risk index	Risk index	2	kept
risk perception	risk perception	risk perception	risk perception	Risk perception	2	case_or_format

Risk plot	risk plot	risk plot	risk plot	Risk plot	2	kept
risk scale	risk scale	risk scale	risk scale	risk scale	2	kept
risk-appropriate care	risk appropriate care	risk appropriate care	risk appropriate care	risk-appropriate care	2	kept
RISK-ASSESSMENT	risk assessment	risk assessment	risk assessment	Risk Assessment	2	case_or_format
RISK-ASSESSMENT SCALES	risk assessment scales	risk assessment scales	risk assessment scale	Risk assessment scale	2	singular
RISK-STRATIFICATION	risk stratification	risk stratification	risk stratification	Risk stratification	2	case_or_format
Risks	risks	risks	risk	risk	2	singular
Risks assessments	risks assessments	risks assessments	risks assessments	Risks assessments	2	kept
ritonavir	ritonavir	ritonavir	ritonavir	ritonavir	2	kept
Riyadh	riyadh	riyadh	riyadh	Riyadh	2	kept
RNA Splice Sites	rna splice sites	rna splice sites	rna splice site	RNA Splice Sites	2	singular
RNA splicing	rna splicing	rna splicing	rna splicing	RNA splicing	2	kept
RNs	rns	rns	rns	RNs	2	kept
road traffic injuries	road traffic injuries	road traffic injuries	road traffic injury	road traffic injuries	2	singular
Robot	robot	robot	robot	Robot	2	kept
robot-assisted prostatectomy	robot assisted prostatectomy	robot assisted prostatectomy	robot assisted prostatectomy	robot-assisted prostatectomy	2	kept

robot-assisted surgery	robot assisted surgery	robot assisted surgery	robot assisted surgery	robot assisted surgery	2	case_or_format
robotic cardiac surgery	robotic cardiac surgery	robotic cardiac surgery	robotic cardiac surgery	robotic cardiac surgery	2	kept
Robotic simulation	robotic simulation	robotic simulation	robotic simulation	Robotic simulation	2	kept
Robotics surgery	robotics surgery	robotics surgery	robotics surgery	Robotics surgery	2	kept
robust algorithm	robust algorithm	robust algorithm	robust algorithm	robust algorithm	2	kept
robust newsvendor optimization	robust newsvendor optimization	robust newsvendor optimization	robust newsvendor optimization	robust newsvendor optimization	2	kept
Robust optimization	robust optimization	robust optimization	robust optimization	Robust optimization	2	kept
ROC curve	roc curve	roc curve	roc curve	ROC Curve	2	case_or_format
Role and scope	role and scope	role and scope	role and scope	Role and scope	2	kept
Role Conflict	role conflict	role conflict	role conflict	Role conflict	2	case_or_format
ROLE DELINEATION	role delineation	role delineation	role delineation	ROLE DELINEATION	2	kept
Role development	role development	role development	role development	Role development	2	kept
role expansion	role expansion	role expansion	role expansion	role expansion	2	kept
Role extension	role extension	role extension	role extension	Role extension	2	kept
Role of health care professionals	role of health care professionals	role of health care professionals	role of health care professionals	Role of health care professionals	2	kept
role theory	role theory	role theory	role theory	role theory	2	kept

role transition	role transition	role transition	role transition	Role transition	2	case_or_format
roles and responsibilities	roles and responsibilities	roles and responsibilities	roles and responsibilities	roles and responsibilities	2	kept
Rolling Refresher	rolling refresher	rolling refresher	rolling refresher	Rolling Refresher	2	kept
ROM	rom	rom	rom	ROM	2	kept
Room temperature	room temperature	room temperature	room temperature	room temperature	2	case_or_format
Rooming-in	rooming in	rooming in	rooming in	rooming in	2	case_or_format
Root cause	root cause	root cause	root cause	Root cause	2	kept
root mean squared error	root mean squared error	root mean squared error	root mean squared error	root mean squared error	2	kept
Rostering	rostering	rostering	rostering	Rostering	2	kept
rostering	rostering	rostering	rostering	Rostering	2	case_or_format
Rotational therapy	rotational therapy	rotational therapy	rotational therapy	Rotational therapy	2	kept
Rotavirus infection	rotavirus infection	rotavirus infection	rotavirus infection	Rotavirus infection	2	kept
Rotavirus Infections	rotavirus infections	rotavirus infections	rotavirus infection	Rotavirus infection	2	singular
Rotavirus vaccine	rotavirus vaccine	rotavirus vaccine	rotavirus vaccine	Rotavirus vaccine	2	kept
ROTEM	rotem	rotem	rotem	ROTEM	2	kept
Rounding	rounding	rounding	rounding	Rounding	2	kept

ROUNDS	rounds	rounds	rounds	rounds	2	case_or_format
Routine care	routine care	routine care	routine care	Routine care	2	kept
routine data	routine data	routine data	routine data	routine data	2	kept
roxithromycin	roxithromycin	roxithromycin	roxithromycin	roxithromycin	2	kept
Roy’s adaptation model	roy s adaptation model	roy s adaptation model	roy s adaptation model	Roy’s adaptation model	2	kept
RR interval	rr interval	rr interval	rr interval	RR interval	2	kept
RRT	rrt	rrt	rrt	RRT	2	kept
RSV vaccine	rsv vaccine	rsv vaccine	rsv vaccine	RSV vaccine	2	kept
RT-PCR	rt pcr	rt pcr	rt pcr	RT-PCR	2	kept
RTLS	rtls	rtls	rtls	RTLS	2	kept
RUB	rub	rub	rub	RUB	2	kept
RULA	rula	rula	rula	RULA	2	kept
Rule generation	rule generation	rule generation	rule generation	Rule generation	2	kept
rule-based expert system	rule based expert system	rule based expert system	rule based expert system	rule-based expert system	2	kept
run charts	run charts	run charts	run charts	run charts	2	kept
rupture	rupture	rupture	rupture	Rupture	2	case_or_format

Rural and remote	rural and remote	rural and remote	rural and remote	Rural and remote	2	kept
Rural and remote areas	rural and remote areas	rural and remote areas	rural and remote areas	Rural and remote areas	2	kept
rural hospitals	rural hospitals	rural hospitals	rural hospital	rural hospital	2	singular
réanimation néonatale	r animation n onatale	r animation n onatale	r animation n onatale	réanimation néonatale	2	kept
réanimation pé diatrique	r animation p diatrique	r animation p diatrique	r animation p diatrique	réanimation pé diatrique	2	kept
rôle infirmier	r le infirmier	r le infirmier	r le infirmier	rôle infirmier	2	kept
S aureaus	s aureaus	s aureaus	s aureaus	S aureaus	2	kept
S. aureus	s aureus	s aureus	s aureus	S. aureus	2	kept
S100β	s100	s100	s100	S100β	2	kept
sacrococcygeal teratoma	sacrococcygeal teratoma	sacrococcygeal teratoma	sacrococcygeal teratoma	sacrococcygeal teratoma	2	kept
sacrum fracture	sacrum fracture	sacrum fracture	sacrum fracture	sacrum fracture	2	kept
SAFE	safe	safe	safe	SAFE	2	kept
Safe managements	safe managements	safe managements	safe management	Safe managements	2	singular
Safe medication administration	safe medication administration	safe medication administration	safe medication administration	Safe medication administration	2	kept
safe sleep	safe sleep	safe sleep	safe sleep	safe sleep	2	kept
safe sleep practices	safe sleep practices	safe sleep practices	safe sleep practice	safe sleep practices	2	singular

safety analytics	safety analytics	safety analytics	safety analytics	safety analytics	2	kept
safety attitude questionnaire	safety attitude questionnaire	safety attitude questionnaire	safety attitude questionnaire	Safety Attitude Questionnaire	2	case_or_format
safety attitudes	safety attitudes	safety attitudes	safety attitude	Safety attitudes	2	singular
Safety behavior	safety behavior	safety behavior	safety behavior	Safety behavior	2	kept
safety behavior	safety behavior	safety behavior	safety behavior	Safety behavior	2	case_or_format
Safety Culture	safety culture	safety culture	safety culture	safety culture	2	case_or_format
Safety issues	safety issues	safety issues	safety issue	Safety issues	2	singular
safety net health care	safety net health care	safety net health care	safety net health care	safety net health care	2	kept
Safety net hospitals	safety net hospitals	safety net hospitals	safety net hospital	safety net hospital	2	singular
Safety participation	safety participation	safety participation	safety participation	Safety participation	2	kept
safety performance	safety performance	safety performance	safety performance	safety performance	2	kept
safety planning	safety planning	safety planning	safety planning	safety planning	2	kept
Safety-oriented organization	safety oriented organization	safety oriented organization	safety oriented organization	Safety-oriented organization	2	kept
salazosulfapyridine	salazosulfapyridine	salazosulfapyridine	salazosulfapyridine	salazosulfapyridine	2	kept
salbutamol sulfate	salbutamol sulfate	salbutamol sulfate	salbutamol sulfate	salbutamol sulfate	2	kept
saliva level	saliva level	saliva level	saliva level	saliva level	2	kept

Salivary cortisol	salivary cortisol	salivary cortisol	salivary cortisol	salivary cortisol	2	case_or_format
salivation	salivation	salivation	salivation	salivation	2	kept
Salmonella enterica	salmonella enterica	salmonella enterica	salmonella enterica	Salmonella enterica	2	kept
Salmonella enterica serovar Typhi	salmonella enterica serovar typhi	salmonella enterica serovar typhi	salmonella enterica serovar typhi	Salmonella enterica serovar Typhi	2	kept
Salmonella Infections	salmonella infections	salmonella infections	salmonella infections	Salmonella Infections	2	kept
salmonellosis	salmonellosis	salmonellosis	salmonellosis	salmonellosis	2	kept
salpingooophorectomy	salpingooophorectomy	salpingooophorectomy	salpingooophorectomy	salpingooophorectomy	2	kept
salt wasting	salt wasting	salt wasting	salt wasting	salt wasting	2	kept
Salts	salts	salts	salts	Salts	2	kept
Salvia	salvia	salvia	salvia	Salvia	2	kept
SAMPLE	sample	sample	sample	sample	2	case_or_format
SAMPLE-SIZE	sample size	sample size	sample size	sample size	2	case_or_format
Sampling	sampling	sampling	sampling	sampling	2	case_or_format
Sanger sequencing	sanger sequencing	sanger sequencing	sanger sequencing	Sanger sequencing	2	kept
Sanitation	sanitation	sanitation	sanitation	sanitation	2	case_or_format
SAQ	saq	saq	saq	SAQ	2	kept

SARS-CoV	sars cov	sars cov	sars cov	SARS-CoV	2	kept
SARS-CoV-2 antibodies	sars cov 2 antibodies	sars cov 2 antibodies	sars cov 2 antibody	SARS-CoV-2 antibody	2	singular
SARS-CoV-2 breakthrough infections	sars cov 2 breakthrough infections	sars cov 2 breakthrough infections	sars cov 2 breakthrough infections	SARS-CoV-2 breakthrough infections	2	kept
SARS - CoV - 2	sars cov 2	sars cov 2	sars cov 2	SARS-CoV-2	2	case_or_format
satisfaction rate	satisfaction rate	satisfaction rate	satisfaction rate	satisfaction rate	2	kept
satisfaction with care	satisfaction with care	satisfaction with care	satisfaction with care	satisfaction with care	2	kept
SATURATION	saturation	saturation	saturation	SATURATION	2	kept
scabies	scabies	scabies	scabies	scabies	2	kept
SCALES	scales	scales	scale	SCALE	2	singular
scanning electron microscopy	scanning electron microscopy	scanning electron microscopy	scanning electron microscopy	scanning electron microscopy	2	kept
scavenging system	scavenging system	scavenging system	scavenging system	scavenging system	2	kept
SCBU	scbu	scbu	scbu	SCBU	2	kept
Scenario	scenario	scenario	scenario	Scenario	2	kept
Scenario-based training	scenario based training	scenario based training	scenario based training	Scenario-based training	2	kept
scheduling	scheduling	scheduling	scheduling	Scheduling	2	case_or_format
Scheduling models	scheduling models	scheduling models	scheduling models	Scheduling models	2	kept

Scheduling strategic behavior	scheduling strategic behavior	scheduling strategic behavior	scheduling strategic behavior	Scheduling strategic behavior	2	kept
SCHIZOPHRENIA	schizophrenia	schizophrenia	schizophrenia	schizophrenia	2	case_or_format
schizophrenia spectrum disorder	schizophrenia spectrum disorder	schizophrenia spectrum disorder	schizophrenia spectrum disorder	schizophrenia spectrum disorder	2	kept
Schmerzassessment	schmerzassessment	schmerzassessment	schmerzassessment	Schmerzassessment	2	kept
School	school	school	school	school	2	case_or_format
school admission	school admission	school admission	school admission	school admission	2	kept
School Health Services	school health services	school health services	school health service	school health service	2	singular
sciatic nerve	sciatic nerve	sciatic nerve	sciatic nerve	sciatic nerve	2	kept
Scientific publications	scientific publications	scientific publications	scientific publications	Scientific publications	2	kept
scintiscanning	scintiscanning	scintiscanning	scintiscanning	scintiscanning	2	kept
sclerosing cholangitis	sclerosing cholangitis	sclerosing cholangitis	sclerosing cholangitis	sclerosing cholangitis	2	kept
sclerosis	sclerosis	sclerosis	sclerosis	sclerosis	2	kept
scopolamine butyl bromide	scopolamine butyl bromide	scopolamine butyl bromide	scopolamine butyl bromide	scopolamine butyl bromide	2	kept
Scoring	scoring	scoring	scoring	Scoring	2	kept
SCORING SYSTEM	scoring system	scoring system	scoring system	scoring system	2	case_or_format
Scoring systems	scoring systems	scoring systems	scoring system	scoring system	2	singular

screening symptoms	screening symptoms	screening symptoms	screening symptoms	screening symptoms	2	kept
screening tool	screening tool	screening tool	screening tool	Screening tool	2	case_or_format
SDGs	sdgs	sdgs	sdgs	SDGs	2	kept
SDU	sdu	sdu	sdu	SDU	2	kept
seashore	seashore	seashore	seashore	seashore	2	kept
Seat Belts	seat belts	seat belts	seat belts	Seat Belts	2	kept
Seclusion	seclusion	seclusion	seclusion	seclusion	2	case_or_format
second victim phenomenon	second victim phenomenon	second victim phenomenon	second victim phenomenon	second victim phenomenon	2	kept
second-person narration	second person narration	second person narration	second person narration	second-person narration	2	kept
secondary	secondary	secondary	secondary	secondary	2	kept
secondary attack rate	secondary attack rate	secondary attack rate	secondary attack rate	secondary attack rate	2	kept
secondary data analysis	secondary data analysis	secondary data analysis	secondary data analysis	Secondary data analysis	2	case_or_format
secondary education	secondary education	secondary education	secondary education	secondary education	2	kept
secondary immunization	secondary immunization	secondary immunization	secondary immunization	secondary immunization	2	kept
Secondary trauma	secondary trauma	secondary trauma	secondary trauma	secondary trauma	2	case_or_format
Secondary Traumatic Stress Scale	secondary traumatic stress scale	secondary traumatic stress scale	secondary traumatic stress scale	Secondary Traumatic Stress Scale	2	kept

Securement	securement	securement	securement	Securement	2	kept
Security measures	security measures	security measures	security measures	Security Measures	2	case_or_format
security personnel	security personnel	security personnel	security personnel	security personnel	2	kept
Sedación	sedaci n	sedaci n	sedaci n	Sedación	2	kept
sedated	sedated	sedated	sedated	sedated	2	kept
Sedation and analgesia	sedation and analgesia	sedation and analgesia	sedation and analgesia	Sedation and analgesia	2	kept
sedation assessment	sedation assessment	sedation assessment	sedation assessment	Sedation assessment	2	case_or_format
Sedation management	sedation management	sedation management	sedation management	Sedation management	2	kept
Sedation monitoring	sedation monitoring	sedation monitoring	sedation monitoring	Sedation monitoring	2	kept
Sedation protocols	sedation protocols	sedation protocols	sedation protocol	sedation protocol	2	singular
sedation scale	sedation scale	sedation scale	sedation scale	Sedation scales	2	case_or_format
Sedentary Behavior	sedentary behavior	sedentary behavior	sedentary behavior	Sedentary Behavior	2	kept
Sedentary behavior	sedentary behavior	sedentary behavior	sedentary behavior	Sedentary Behavior	2	case_or_format
Segurança do Paciente	seguran a do paciente	seguran a do paciente	seguran a do paciente	Segurança do Paciente	2	kept
Seguridad del Paciente	seguridad del paciente	seguridad del paciente	seguridad del paciente	Seguridad del paciente	2	case_or_format
SEIPS	seips	seips	seips	SEIPS	2	kept

SEIZURES	seizures	seizures	seizure	seizure	2	singular
Selective decontamination of the digestive tract	selective decontamination of the digestive tract	selective decontamination of the digestive tract	selective decontamination of the digestive tract	Selective decontamination of the digestive tract	2	kept
selective decontamination of the digestive tract	selective decontamination of the digestive tract	selective decontamination of the digestive tract	selective decontamination of the digestive tract	Selective decontamination of the digestive tract	2	case_or_format
selective digestive tract decontamination	selective digestive tract decontamination	selective digestive tract decontamination	selective digestive tract decontamination	selective digestive tract decontamination	2	kept
Selective serotonin reuptake inhibitors	selective serotonin reuptake inhibitors	selective serotonin reuptake inhibitors	selective serotonin reuptake inhibitors	Selective serotonin reuptake inhibitors	2	kept
self	self	self	self	self	2	kept
self advocacy	self advocacy	self advocacy	self advocacy	self advocacy	2	kept
Self Assessment	self assessment	self assessment	self assessment	Self-Assessment	2	case_or_format
Self care	self care	self care	self care	self care	2	case_or_format
Self confidence	self confidence	self confidence	self confidence	Self-confidence	2	case_or_format
self examination	self examination	self examination	self examination	self examination	2	kept
self extubation	self extubation	self extubation	self extubation	Self-extubation	2	case_or_format
self injection	self injection	self injection	self injection	self injection	2	kept
self poisoning	self poisoning	self poisoning	self poisoning	self poisoning	2	kept
Self Rating Anxiety Scale	self rating anxiety scale	self rating anxiety scale	self rating anxiety scale	Self-rating Anxiety Scale	2	case_or_format
Self-administration	self administration	self administration	self administration	Self Administration	2	case_or_format

self-awareness	self awareness	self awareness	self awareness	self-awareness	2	kept
self-concept	self concept	self concept	self concept	self concept	2	case_or_format
Self-harm	self harm	self harm	self harm	Self-harm	2	kept
self-monitoring blood glucose	self monitoring blood glucose	self monitoring blood glucose	self monitoring blood glucose	self-monitoring blood glucose	2	kept
self-reflection	self reflection	self reflection	self reflection	self-reflection	2	kept
Self-report	self report	self report	self report	self report	2	case_or_format
semaglutide	semaglutide	semaglutide	semaglutide	semaglutide	2	kept
semantic web	semantic web	semantic web	semantic web	Semantic Web	2	case_or_format
semi-solid feeds	semi solid feeds	semi solid feeds	semi solid feeds	semi-solid feeds	2	kept
Semi-structured	semi structured	semi structured	semi structured	Semi-structured	2	kept
Senegal	senegal	senegal	senegal	Senegal	2	kept
Senior leadership	senior leadership	senior leadership	senior leadership	Senior leadership	2	kept
Sensation Disorders	sensation disorders	sensation disorders	sensation disorders	Sensation Disorders	2	kept
SENSE	sense	sense	sense	SENSE	2	kept
Sense of professional mission	sense of professional mission	sense of professional mission	sense of professional mission	Sense of professional mission	2	kept
sense of self	sense of self	sense of self	sense of self	sense of self	2	kept

sensibility	sensibility	sensibility	sensibility	sensibility	2	kept
sensitive indicators	sensitive indicators	sensitive indicators	sensitive indicators	sensitive indicators	2	kept
sensor rate of change	sensor rate of change	sensor rate of change	sensor rate of change	sensor rate of change	2	kept
Sensory	sensory	sensory	sensory	Sensory	2	kept
sensory aid	sensory aid	sensory aid	sensory aid	sensory aid	2	kept
sensory cortex	sensory cortex	sensory cortex	sensory cortex	sensory cortex	2	kept
sensory deprivation	sensory deprivation	sensory deprivation	sensory deprivation	Sensory Deprivation	2	case_or_format
sensory feedback	sensory feedback	sensory feedback	sensory feedback	sensory feedback	2	kept
sensory integration	sensory integration	sensory integration	sensory integration	sensory integration	2	kept
sensory system	sensory system	sensory system	sensory system	sensory system	2	kept
Sensory Thresholds	sensory thresholds	sensory thresholds	sensory thresholds	Sensory Thresholds	2	kept
sensory-based interventions	sensory based interventions	sensory based interventions	sensory based interventions	sensory-based interventions	2	kept
sentiment	sentiment	sentiment	sentiment	sentiment	2	kept
Sentiment Analysis	sentiment analysis	sentiment analysis	sentiment analysis	Sentiment Analysis	2	kept
Sentiment analysis	sentiment analysis	sentiment analysis	sentiment analysis	Sentiment Analysis	2	case_or_format
Sepsis bundles	sepsis bundles	sepsis bundles	sepsis bundle	Sepsis bundle	2	singular

sepsis management	sepsis management	sepsis management	sepsis management	Sepsis management	2	case_or_format
sepsis six performance	sepsis six performance	sepsis six performance	sepsis six performance	sepsis six performance	2	kept
sepsis workup	sepsis workup	sepsis workup	sepsis workup	sepsis workup	2	kept
Septic	septic	septic	septic	Septic	2	kept
septic	septic	septic	septic	Septic	2	case_or_format
Sequelae	sequelae	sequelae	sequelae	Sequelae	2	kept
sequelae	sequelae	sequelae	sequelae	Sequelae	2	case_or_format
Sergipe	sergipe	sergipe	sergipe	Sergipe	2	kept
serial-multiple mediation model	serial multiple mediation model	serial multiple mediation model	serial multiple mediation model	serial-multiple mediation model	2	kept
serious game	serious game	serious game	serious game	Serious game	2	case_or_format
serotonin	serotonin	serotonin	serotonin	serotonin	2	kept
serotonin noradrenalin reuptake inhibitor	serotonin noradrenalin reuptake inhibitor	serotonin noradrenalin reuptake inhibitor	serotonin noradrenalin reuptake inhibitor	serotonin noradrenalin reuptake inhibitor	2	kept
Serotonin Plasma Membrane Transport Proteins	serotonin plasma membrane transport proteins	serotonin plasma membrane transport proteins	serotonin plasma membrane transport proteins	Serotonin Plasma Membrane Transport Proteins	2	kept
Serotonin Syndrome	serotonin syndrome	serotonin syndrome	serotonin syndrome	Serotonin Syndrome	2	kept
Serotonin syndrome	serotonin syndrome	serotonin syndrome	serotonin syndrome	Serotonin Syndrome	2	case_or_format
serotype	serotype	serotype	serotype	serotype	2	kept

Serotyping	serotyping	serotyping	serotyping	Serotyping	2	kept
Serratia	serratia	serratia	serratia	Serratia	2	kept
Service development	service development	service development	service development	Service development	2	kept
Service improvement	service improvement	service improvement	service improvement	Service improvement	2	kept
service improvement	service improvement	service improvement	service improvement	Service improvement	2	case_or_format
Service Quality	service quality	service quality	service quality	Service Quality	2	kept
service quality	service quality	service quality	service quality	Service Quality	2	case_or_format
services	services	services	service	SERVICES	2	singular
Sesame Oil	sesame oil	sesame oil	sesame oil	Sesame Oil	2	kept
Sesiones clínicas	sesiones cl nicas	sesiones cl nicas	sesiones cl nicas	Sesiones clínicas	2	kept
SET	set	set	set	SET	2	kept
setting of care	setting of care	setting of care	setting of care	setting of care	2	kept
sevelamer	sevelamer	sevelamer	sevelamer	sevelamer	2	kept
severe acute asthma	severe acute asthma	severe acute asthma	severe acute asthma	severe acute asthma	2	kept
Severe acute respiratory syndrome coronavirus	severe acute respiratory syndrome coronavirus	severe acute respiratory syndrome coronavirus	severe acute respiratory syndrome coronavirus	Severe acute respiratory syndrome coronavirus	2	kept
severe acute respiratory syndrome-associated coronavirus	severe acute respiratory syndrome associated coronavirus	severe acute respiratory syndrome associated coronavirus	severe acute respiratory syndrome associated coronavirus	severe acute respiratory syndrome-associated coronavirus	2	kept

Severe Burns	severe burns	severe burns	severe burns	Severe Burns	2	kept
Severe cutaneous adverse reactions	severe cutaneous adverse reactions	severe cutaneous adverse reactions	severe cutaneous adverse reaction	Severe cutaneous adverse reactions	2	singular
SEVERE HEAD-INJURY	severe head injury	severe head injury	severe head injury	SEVERE HEAD-INJURY	2	kept
Severe maternal morbidity	severe maternal morbidity	severe maternal morbidity	severe maternal morbidity	severe maternal morbidity	2	case_or_format
severe neurologic impairment	severe neurologic impairment	severe neurologic impairment	severe neurologic impairment	severe neurologic impairment	2	kept
severe pancreatitis	severe pancreatitis	severe pancreatitis	severe pancreatitis	severe pancreatitis	2	kept
Severe stroke	severe stroke	severe stroke	severe stroke	Severe stroke	2	kept
Severe traumatic brain injury	severe traumatic brain injury	severe traumatic brain injury	severe traumatic brain injury	severe traumatic brain injury	2	case_or_format
Severity scores	severity scores	severity scores	severity score	Severity scores	2	singular
sex hormone	sex hormone	sex hormone	sex hormone	sex hormone	2	kept
sex role	sex role	sex role	sex role	sex role	2	kept
sexual and gender identity disorders	sexual and gender identity disorders	sexual and gender identity disorders	sexual and gender identity disorders	sexual and gender identity disorders	2	kept
sexual development	sexual development	sexual development	sexual development	sexual development	2	kept
Sexual harassment	sexual harassment	sexual harassment	sexual harassment	Sexual Harassment	2	case_or_format
SF-36	sf 36	sf 36	sf 36	SF-36	2	kept
Shaken baby syndrome	shaken baby syndrome	shaken baby syndrome	shaken baby syndrome	Shaken Baby Syndrome	2	case_or_format

Shapley Value	shapley value	shapley value	shapley value	Shapley Value	2	kept
Shared Decision Making	shared decision making	shared decision making	shared decision making	shared decision making	2	case_or_format
Shared governance	shared governance	shared governance	shared governance	shared governance	2	case_or_format
Shared leadership	shared leadership	shared leadership	shared leadership	Shared leadership	2	kept
shared leadership	shared leadership	shared leadership	shared leadership	Shared leadership	2	case_or_format
shared resource iteration	shared resource iteration	shared resource iteration	shared resource iteration	shared resource iteration	2	kept
sharp injury	sharp injury	sharp injury	sharp injury	Sharp injuries	2	case_or_format
sharps injury	sharps injury	sharps injury	sharps injury	Sharps injuries	2	case_or_format
shear stress	shear stress	shear stress	shear stress	shear stress	2	kept
Sheehan Disability Scale	sheehan disability scale	sheehan disability scale	sheehan disability scale	Sheehan Disability Scale	2	kept
Sheep	sheep	sheep	sheep	Sheep	2	kept
SHEL model	shel model	shel model	shel model	SHEL model	2	kept
Shift handover	shift handover	shift handover	shift handover	Shift handover	2	kept
Shift pattern	shift pattern	shift pattern	shift pattern	Shift pattern	2	kept
Shift report	shift report	shift report	shift report	SHIFT REPORT	2	case_or_format
shift reports	shift reports	shift reports	shift report	SHIFT REPORT	2	singular

SHIFT WORK	shift work	shift work	shift work	shift work	2	case_or_format
Shift work sleep disorder	shift work sleep disorder	shift work sleep disorder	shift work sleep disorder	Shift work sleep disorder	2	kept
shiftwork	shiftwork	shiftwork	shiftwork	shiftwork	2	kept
ship	ship	ship	ship	Ships	2	case_or_format
Shirley Graves	shirley graves	shirley graves	shirley graves	Shirley Graves	2	kept
Shock, septic	shock septic	shock septic	shock septic	Shock, Septic	2	case_or_format
short acting drug	short acting drug	short acting drug	short acting drug	short acting drug	2	kept
Short Portable Mental Status Questionnaire	short portable mental status questionnaire	short portable mental status questionnaire	short portable mental status questionnaire	Short Portable Mental Status Questionnaire	2	kept
short stature	short stature	short stature	short stature	short stature	2	kept
short term	short term	short term	short term	short term	2	kept
short term memory	short term memory	short term memory	short term memory	short term memory	2	kept
short term psychotherapy	short term psychotherapy	short term psychotherapy	short term psychotherapy	short term psychotherapy	2	kept
Shortage	shortage	shortage	shortage	Shortage	2	kept
shortages	shortages	shortages	shortage	Shortage	2	singular
Shortness of breath	shortness of breath	shortness of breath	shortness of breath	Shortness of breath	2	kept
Shoulder	shoulder	shoulder	shoulder	Shoulder	2	kept

shoulder	shoulder	shoulder	shoulder	Shoulder	2	case_or_format
shoulder injury	shoulder injury	shoulder injury	shoulder injury	shoulder injury	2	kept
Shunt	shunt	shunt	shunt	Shunt	2	kept
Sialorrhea	sialorrhea	sialorrhea	sialorrhea	Sialorrhea	2	kept
sialorrhea	sialorrhea	sialorrhea	sialorrhea	Sialorrhea	2	case_or_format
siblings	siblings	siblings	sibling	sibling	2	singular
Sickle cell	sickle cell	sickle cell	sickle cell	Sickle cell	2	kept
Sickle cell anemia	sickle cell anemia	sickle cell anemia	sickle cell anemia	sickle cell anemia	2	case_or_format
sickle cell disease	sickle cell disease	sickle cell disease	sickle cell disease	sickle cell disease	2	kept
sickle cell trait	sickle cell trait	sickle cell trait	sickle cell trait	sickle cell trait	2	kept
Side effects	side effects	side effects	side effect	side effect	2	singular
sigmoid	sigmoid	sigmoid	sigmoid	sigmoid	2	kept
sigmoid volvulus	sigmoid volvulus	sigmoid volvulus	sigmoid volvulus	sigmoid volvulus	2	kept
sigmoidoscopy	sigmoidoscopy	sigmoidoscopy	sigmoidoscopy	sigmoidoscopy	2	kept
Sign Language	sign language	sign language	sign language	Sign Language	2	kept
sign language	sign language	sign language	sign language	Sign Language	2	case_or_format

sign-out	sign out	sign out	sign out	SIGN-OUT	2	case_or_format
Signal abnormality index	signal abnormality index	signal abnormality index	signal abnormality index	Signal abnormality index	2	kept
SIGNS	signs	signs	sign	SIGNS	2	singular
silica gel	silica gel	silica gel	silica gel	silica gel	2	kept
silicon	silicon	silicon	silicon	silicon	2	kept
silicone gel	silicone gel	silicone gel	silicone gel	silicone gel	2	kept
silver	silver	silver	silver	Silver	2	case_or_format
silver dressing	silver dressing	silver dressing	silver dressing	silver dressing	2	kept
silver nitrate	silver nitrate	silver nitrate	silver nitrate	silver nitrate	2	kept
silymarin	silymarin	silymarin	silymarin	silymarin	2	kept
Simple++	simple	simple	simple	Simple++	2	kept
Simulated cough	simulated cough	simulated cough	simulated cough	Simulated cough	2	kept
Simulation based learning experiences	simulation based learning experiences	simulation based learning experiences	simulation based learning experience	Simulation based learning experiences	2	singular
Simulation based training	simulation based training	simulation based training	simulation based training	Simulation-based training	2	case_or_format
simulation evaluation	simulation evaluation	simulation evaluation	simulation evaluation	simulation evaluation	2	kept
simulation hurdle	simulation hurdle	simulation hurdle	simulation hurdle	simulation hurdle	2	kept

simulation in healthcare	simulation in healthcare	simulation in healthcare	simulation in healthcare	simulation in healthcare	2	kept
simulation learning	simulation learning	simulation learning	simulation learning	simulation learning	2	kept
simulation mastery	simulation mastery	simulation mastery	simulation mastery	simulation mastery	2	kept
simulation-based mastery learning	simulation based mastery learning	simulation based mastery learning	simulation based mastery learning	simulation-based mastery learning	2	kept
Simulation-based Teaching	simulation based teaching	simulation based teaching	simulation based teaching	Simulation-based Teaching	2	kept
simulation-based team training	simulation based team training	simulation based team training	simulation based team training	simulation-based team training	2	kept
simulations	simulations	simulations	simulation	simulation	2	singular
Simultaneous pancreas-kidney transplantation	simultaneous pancreas kidney transplantation	simultaneous pancreas kidney transplantation	simultaneous pancreas kidney transplantation	Simultaneous pancreas-kidney transplantation	2	kept
single photon emission computer tomography	single photon emission computer tomography	single photon emission computer tomography	single photon emission computer tomography	single photon emission computer tomography	2	kept
Single room	single room	single room	single room	Single room	2	kept
Single-dose vial	single dose vial	single dose vial	single dose vial	Single-dose vial	2	kept
Single-family rooms	single family rooms	single family rooms	single family room	single family room	2	singular
Sistemas de apoyo psicosocial	sistemas de apoyo psicosocial	sistemas de apoyo psicosocial	sistemas de apoyo psicosocial	Sistemas de apoyo psicosocial	2	kept
sister	sister	sister	sister	sister	2	kept
Situated learning	situated learning	situated learning	situated learning	Situated learning	2	kept
SITUATION	situation	situation	situation	SITUATIONS	2	case_or_format

situation	situation	situation	situation	SITUATIONS	2	case_or_format
Situation-Background-Assessment-Recommendation	situation background assessment recommendation	situation background assessment recommendation	situation background assessment recommendation	Situation-Background-Assessment-Recommendation	2	kept
situational awareness	situational awareness	situational awareness	situational awareness	Situational awareness	2	case_or_format
size	size	size	size	size	2	kept
size reduction	size reduction	size reduction	size reduction	size reduction	2	kept
skepticism	skepticism	skepticism	skepticism	skepticism	2	kept
Skewed distribution	skewed distribution	skewed distribution	skewed distribution	Skewed distribution	2	kept
Ski patrol	ski patrol	ski patrol	ski patrol	Ski patrol	2	kept
skill retention	skill retention	skill retention	skill retention	skill retention	2	kept
skill test	skill test	skill test	skill test	skill test	2	kept
Skill-mix	skill mix	skill mix	skill mix	skill mix	2	case_or_format
skills	skills	skills	skill	skill	2	singular
Skills development	skills development	skills development	skills development	Skills development	2	kept
skin allograft	skin allograft	skin allograft	skin allograft	skin allograft	2	kept
skin bruising	skin bruising	skin bruising	skin bruising	skin bruising	2	kept
skin calcification	skin calcification	skin calcification	skin calcification	skin calcification	2	kept

skin culture	skin culture	skin culture	skin culture	skin culture	2	kept
Skin Diseases, Bacterial	skin diseases bacterial	skin diseases bacterial	skin diseases bacterial	Skin Diseases, Bacterial	2	kept
Skin failure	skin failure	skin failure	skin failure	skin failure	2	case_or_format
Skin injuries	skin injuries	skin injuries	skin injury	skin injury	2	singular
Skin Neoplasms	skin neoplasms	skin neoplasms	skin neoplasms	Skin Neoplasms	2	kept
Skin physiology	skin physiology	skin physiology	skin physiology	Skin physiology	2	kept
Skin risk assessment tool	skin risk assessment tool	skin risk assessment tool	skin risk assessment tool	Skin risk assessment tool	2	kept
skin tear	skin tear	skin tear	skin tear	Skin tears	2	case_or_format
skin tears	skin tears	skin tears	skin tear	Skin tears	2	singular
Skin to skin contact	skin to skin contact	skin to skin contact	skin to skin contact	Skin-to-skin contact	2	case_or_format
skin tumor	skin tumor	skin tumor	skin tumor	skin tumor	2	kept
Skin-to-skin Contact	skin to skin contact	skin to skin contact	skin to skin contact	Skin-to-skin contact	2	case_or_format
Skincare	skincare	skincare	skincare	Skincare	2	kept
Skinfold Thickness	skinfold thickness	skinfold thickness	skinfold thickness	Skinfold Thickness	2	kept
skinfold thickness	skinfold thickness	skinfold thickness	skinfold thickness	Skinfold Thickness	2	case_or_format
skull base surgery	skull base surgery	skull base surgery	skull base surgery	skull base surgery	2	kept

skull defect	skull defect	skull defect	skull defect	skull defect	2	kept
skull surgery	skull surgery	skull surgery	skull surgery	skull surgery	2	kept
Sleep apnea	sleep apnea	sleep apnea	sleep apnea	Sleep apnea	2	kept
sleep disruption	sleep disruption	sleep disruption	sleep disruption	SLEEP DISRUPTION	2	case_or_format
sleep induction	sleep induction	sleep induction	sleep induction	sleep induction	2	kept
Sleep medicine	sleep medicine	sleep medicine	sleep medicine	sleep medicine	2	case_or_format
Sleep monitoring	sleep monitoring	sleep monitoring	sleep monitoring	Sleep monitoring	2	kept
sleep monitoring	sleep monitoring	sleep monitoring	sleep monitoring	Sleep monitoring	2	case_or_format
Sleep promotion	sleep promotion	sleep promotion	sleep promotion	Sleep promotion	2	kept
sleep promotion	sleep promotion	sleep promotion	sleep promotion	Sleep promotion	2	case_or_format
Sleep protection	sleep protection	sleep protection	sleep protection	Sleep protection	2	kept
Sleep questionnaire	sleep questionnaire	sleep questionnaire	sleep questionnaire	sleep questionnaire	2	case_or_format
sleep study	sleep study	sleep study	sleep study	sleep study	2	kept
Sleep wake disorders	sleep wake disorders	sleep wake disorders	sleep wake disorders	Sleep Wake Disorders	2	case_or_format
sleep wake-disorders	sleep wake disorders	sleep wake disorders	sleep wake disorders	Sleep Wake Disorders	2	case_or_format
SLEEP-APNEA	sleep apnea	sleep apnea	sleep apnea	Sleep apnea	2	case_or_format

Sleep-wake cycles	sleep wake cycles	sleep wake cycles	sleep wake cycle	Sleep-wake cycle	2	singular
Sleep-wake transition disorders	sleep wake transition disorders	sleep wake transition disorders	sleep wake transition disorders	Sleep-wake transition disorders	2	kept
sleepiness	sleepiness	sleepiness	sleepiness	Sleepiness	2	case_or_format
Sleeping	sleeping	sleeping	sleeping	Sleeping	2	kept
sliding mode control	sliding mode control	sliding mode control	sliding mode control	sliding mode control	2	kept
slips	slips	slips	slip	slips	2	singular
slit lamp	slit lamp	slit lamp	slit lamp	slit lamp	2	kept
slit lamp microscopy	slit lamp microscopy	slit lamp microscopy	slit lamp microscopy	slit lamp microscopy	2	kept
slow code	slow code	slow code	slow code	slow code	2	kept
Small and sick newborns	small and sick newborns	small and sick newborns	small and sick newborns	Small and sick newborns	2	kept
Small baby	small baby	small baby	small baby	Small baby	2	kept
small bowel obstruction	small bowel obstruction	small bowel obstruction	small bowel obstruction	small bowel obstruction	2	kept
small cell lung cancer	small cell lung cancer	small cell lung cancer	small cell lung cancer	small cell lung cancer	2	kept
Small inestine	small inestine	small inestine	small inestine	Small inestine	2	kept
small intestine	small intestine	small intestine	small intestine	small intestine	2	kept
Small volume tubes	small volume tubes	small volume tubes	small volume tubes	Small volume tubes	2	kept

Smallpox Vaccine	smallpox vaccine	smallpox vaccine	smallpox vaccine	smallpox vaccine	2	case_or_format
Smallpox virus	smallpox virus	smallpox virus	smallpox virus	Smallpox virus	2	kept
Smart Glasses	smart glasses	smart glasses	smart glasses	Smart Glasses	2	kept
Smart pump	smart pump	smart pump	smart pump	Smart pump	2	kept
smart pumps	smart pumps	smart pumps	smart pump	Smart pump	2	singular
Smart pump—electronic health record interoperability	smart pump electronic health record interoperability	smart pump electronic health record interoperability	smart pump electronic health record interoperability	Smart pump—electronic health record interoperability	2	kept
SMCR communication model	smcr communication model	smcr communication model	smcr communication model	SMCR communication model	2	kept
Smoke inhalation	smoke inhalation	smoke inhalation	smoke inhalation	Smoke inhalation	2	kept
smoking ban	smoking ban	smoking ban	smoking ban	smoking ban	2	kept
SNOMED CT	snomed ct	snomed ct	snomed ct	SNOMED CT	2	kept
Snow	snow	snow	snow	Snow	2	kept
snowball sampling	snowball sampling	snowball sampling	snowball sampling	snowball sampling	2	kept
SOAP	soap	soap	soap	soap	2	case_or_format
social aspects and related phenomena	social aspects and related phenomena	social aspects and related phenomena	social aspects and related phenomena	social aspects and related phenomena	2	kept
social bonding	social bonding	social bonding	social bonding	social bonding	2	kept
social context	social context	social context	social context	social context	2	kept

Social Control, Formal	social control formal	social control formal	social control formal	Social Control, Formal	2	kept
social embeddedness	social embeddedness	social embeddedness	social embeddedness	social embeddedness	2	kept
social engagement	social engagement	social engagement	social engagement	social engagement	2	kept
Social environment	social environment	social environment	social environment	social environment	2	case_or_format
social evolution	social evolution	social evolution	social evolution	social evolution	2	kept
social exclusion	social exclusion	social exclusion	social exclusion	social exclusion	2	kept
Social Factors	social factors	social factors	social factors	social factors	2	case_or_format
social hierarchy	social hierarchy	social hierarchy	social hierarchy	social hierarchy	2	kept
Social isolation	social isolation	social isolation	social isolation	social isolation	2	case_or_format
social medicine	social medicine	social medicine	social medicine	social medicine	2	kept
Social pressure	social pressure	social pressure	social pressure	Social pressure	2	kept
Social Problems	social problems	social problems	social problem	social problem	2	singular
Social representation	social representation	social representation	social representation	Social representation	2	kept
social resilience	social resilience	social resilience	social resilience	social resilience	2	kept
Social skills	social skills	social skills	social skills	Social Skills	2	case_or_format
social support assessment	social support assessment	social support assessment	social support assessment	social support assessment	2	kept

social theory	social theory	social theory	social theory	social theory	2	kept
Social undermining	social undermining	social undermining	social undermining	Social undermining	2	kept
SOCIAL-WORKERS	social workers	social workers	social worker	social worker	2	singular
Societies, Hospital	societies hospital	societies hospital	societies hospital	Societies, Hospital	2	kept
Socio-demographic	socio demographic	socio demographic	socio demographic	Socio-demographic	2	kept
socio-demographic factors	socio demographic factors	socio demographic factors	socio demographic factors	socio-demographic factors	2	kept
Sociocultural	sociocultural	sociocultural	sociocultural	Sociocultural	2	kept
sociodemographic factors	sociodemographic factors	sociodemographic factors	sociodemographic factors	Sociodemographic Factors	2	case_or_format
socioeconomic vulnerability	socioeconomic vulnerability	socioeconomic vulnerability	socioeconomic vulnerability	socioeconomic vulnerability	2	kept
sociologist	sociologist	sociologist	sociologist	sociologist	2	kept
Sociometric sensors	sociometric sensors	sociometric sensors	sociometric sensors	Sociometric sensors	2	kept
sociopathy	sociopathy	sociopathy	sociopathy	sociopathy	2	kept
sociotechnical	sociotechnical	sociotechnical	sociotechnical	sociotechnical	2	kept
sodium fluoride	sodium fluoride	sodium fluoride	sodium fluoride	sodium fluoride	2	kept
sodium glucose cotransporter 2	sodium glucose cotransporter 2	sodium glucose cotransporter 2	sodium glucose cotransporter 2	sodium glucose cotransporter 2	2	kept
Sodium Hypochlorite	sodium hypochlorite	sodium hypochlorite	sodium hypochlorite	Sodium Hypochlorite	2	kept

sodium retention	sodium retention	sodium retention	sodium retention	sodium retention	2	kept
SOFA SCORE	sofa score	sofa score	sofa score	SOFA score	2	case_or_format
SOFFI	soffi	soffi	soffi	SOFFI	2	kept
soft tissue disease	soft tissue disease	soft tissue disease	soft tissue disease	soft tissue disease	2	kept
Soft Tissue Injuries	soft tissue injuries	soft tissue injuries	soft tissue injury	soft tissue injury	2	singular
soft tissue sarcoma	soft tissue sarcoma	soft tissue sarcoma	soft tissue sarcoma	soft tissue sarcoma	2	kept
Soft-tissue infection	soft tissue infection	soft tissue infection	soft tissue infection	soft tissue infection	2	case_or_format
software engineering	software engineering	software engineering	software engineering	software engineering	2	kept
Software testing	software testing	software testing	software testing	Software testing	2	kept
SOI	soi	soi	soi	SOI	2	kept
Solid organ transplant	solid organ transplant	solid organ transplant	solid organ transplant	Solid organ transplant	2	kept
solid tumour	solid tumour	solid tumour	solid tumour	solid tumour	2	kept
solitary tract nucleus	solitary tract nucleus	solitary tract nucleus	solitary tract nucleus	solitary tract nucleus	2	kept
solution and solubility	solution and solubility	solution and solubility	solution and solubility	solution and solubility	2	kept
Somali region	somali region	somali region	somali region	Somali region	2	kept
Somaliland	somaliland	somaliland	somaliland	Somaliland	2	kept

somatomedin	somatomedin	somatomedin	somatomedin	somatomedin	2	kept
somatosensory evoked potential	somatosensory evoked potential	somatosensory evoked potential	somatosensory evoked potential	somatosensory evoked potential	2	kept
somatostatin	somatostatin	somatostatin	somatostatin	somatostatin	2	kept
somatostatin derivative	somatostatin derivative	somatostatin derivative	somatostatin derivative	somatostatin derivative	2	kept
sorafenib	sorafenib	sorafenib	sorafenib	sorafenib	2	kept
SORES	sores	sores	sores	SORES	2	kept
SOUND	sound	sound	sound	Sound	2	case_or_format
Sound level	sound level	sound level	sound level	Sound level	2	kept
Sound levels	sound levels	sound levels	sound level	Sound level	2	singular
Sound pressures level	sound pressures level	sound pressures level	sound pressures level	Sound pressures level	2	kept
Sound sources	sound sources	sound sources	sound source	Sound source	2	singular
Sound's levels	sound s levels	sound s levels	sound s levels	Sound's levels	2	kept
Soundscape	soundscape	soundscape	soundscape	Soundscapes	2	case_or_format
Sour Seven Questionnaire	sour seven questionnaire	sour seven questionnaire	sour seven questionnaire	Sour Seven Questionnaire	2	kept
Source tracking	source tracking	source tracking	source tracking	Source tracking	2	kept
sources	sources	sources	sources	sources	2	kept

SOUTH	south	south	south	SOUTH	2	kept
South African	south african	south african	south african	South African	2	kept
South Korean	south korean	south korean	south korean	South Korean	2	kept
Southeast Asia	southeast asia	southeast asia	southeast asia	Southeast Asia	2	kept
Southeast Asian People	southeast asian people	southeast asian people	southeast asian people	Southeast Asian People	2	kept
Southeast Iran	southeast iran	southeast iran	southeast iran	Southeast Iran	2	kept
Spaces arrangement	spaces arrangement	spaces arrangement	spaces arrangement	Spaces arrangement	2	kept
spacing effect	spacing effect	spacing effect	spacing effect	spacing effect	2	kept
Spanish influenza	spanish influenza	spanish influenza	spanish influenza	Spanish influenza	2	kept
sparse recovery	sparse recovery	sparse recovery	sparse recovery	sparse recovery	2	kept
Spasticity	spasticity	spasticity	spasticity	spasticity	2	case_or_format
Spatial Analysis	spatial analysis	spatial analysis	spatial analysis	spatial analysis	2	case_or_format
Spatial configuration	spatial configuration	spatial configuration	spatial configuration	Spatial configuration	2	kept
Speaking up	speaking up	speaking up	speaking up	Speaking up	2	kept
speaking up	speaking up	speaking up	speaking up	Speaking up	2	case_or_format
special care unit	special care unit	special care unit	special care unit	Special care units	2	case_or_format

special care units	special care units	special care units	special care unit	Special care units	2	singular
Specialist	specialist	specialist	specialist	Specialist	2	kept
specialist palliative care	specialist palliative care	specialist palliative care	specialist palliative care	specialist palliative care	2	kept
specialist registrar	specialist registrar	specialist registrar	specialist registrar	specialist registrar	2	kept
Specialities	specialities	specialities	specialities	Specialities	2	kept
specialized palliative care	specialized palliative care	specialized palliative care	specialized palliative care	specialized palliative care	2	kept
specialized weaning units	specialized weaning units	specialized weaning units	specialized weaning units	specialized weaning units	2	kept
Specialties	specialties	specialties	specialty	specialty	2	singular
Specialty Boards	specialty boards	specialty boards	specialty boards	Specialty Boards	2	kept
specialty certification	specialty certification	specialty certification	specialty certification	specialty certification	2	kept
specialty palliative care	specialty palliative care	specialty palliative care	specialty palliative care	specialty palliative care	2	kept
species identification	species identification	species identification	species identification	species identification	2	kept
specificity of training	specificity of training	specificity of training	specificity of training	specificity of training	2	kept
spectacles	spectacles	spectacles	spectacles	spectacles	2	kept
spectrophotometry	spectrophotometry	spectrophotometry	spectrophotometry	Spectrophotometry	2	case_or_format
Spectrophotometry, Infrared	spectrophotometry infrared	spectrophotometry infrared	spectrophotometry infrared	Spectrophotometry, Infrared	2	kept

spectroscopy	spectroscopy	spectroscopy	spectroscopy	spectroscopy	2	kept
SPECTRUM BETA-LACTAMASE	spectrum beta lactamase	spectrum beta lactamase	spectrum beta lactamase	SPECTRUM BETA-LACTAMASE	2	kept
speech and language	speech and language	speech and language	speech and language	speech and language	2	kept
speech discrimination	speech discrimination	speech discrimination	speech discrimination	speech discrimination	2	kept
Speech Intelligibility	speech intelligibility	speech intelligibility	speech intelligibility	Speech Intelligibility	2	kept
speech intelligibility	speech intelligibility	speech intelligibility	speech intelligibility	Speech Intelligibility	2	case_or_format
Speech Perception	speech perception	speech perception	speech perception	Speech Perception	2	kept
speech recognition system	speech recognition system	speech recognition system	speech recognition system	speech recognition system	2	kept
Speech-language pathology	speech language pathology	speech language pathology	speech language pathology	Speech-Language Pathology	2	case_or_format
sphygmomanometer	sphygmomanometer	sphygmomanometer	sphygmomanometer	sphygmomanometer	2	kept
Spinal Cord	spinal cord	spinal cord	spinal cord	Spinal Cord	2	kept
spinal cord	spinal cord	spinal cord	spinal cord	Spinal Cord	2	case_or_format
spinal cord ischemia	spinal cord ischemia	spinal cord ischemia	spinal cord ischemia	spinal cord ischemia	2	kept
spinal cord surgery	spinal cord surgery	spinal cord surgery	spinal cord surgery	spinal cord surgery	2	kept
Spinal Diseases	spinal diseases	spinal diseases	spinal diseases	Spinal Diseases	2	kept
spinal reflex	spinal reflex	spinal reflex	spinal reflex	spinal reflex	2	kept

spindle cell	spindle cell	spindle cell	spindle cell	spindle cell	2	kept
spine stabilization	spine stabilization	spine stabilization	spine stabilization	spine stabilization	2	kept
spiritual care competencies	spiritual care competencies	spiritual care competencies	spiritual care competency	spiritual care competencies	2	singular
Spiritual Intelligence	spiritual intelligence	spiritual intelligence	spiritual intelligence	Spiritual Intelligence	2	kept
Spiritual self-care	spiritual self care	spiritual self care	spiritual self care	Spiritual self-care	2	kept
Spiritual support	spiritual support	spiritual support	spiritual support	Spiritual support	2	kept
Spiritual wellbeing	spiritual wellbeing	spiritual wellbeing	spiritual wellbeing	Spiritual wellbeing	2	kept
splanchnic blood flow	splanchnic blood flow	splanchnic blood flow	splanchnic blood flow	splanchnic blood flow	2	kept
Spleen	spleen	spleen	spleen	Spleen	2	kept
spleen artery aneurysm	spleen artery aneurysm	spleen artery aneurysm	spleen artery aneurysm	spleen artery aneurysm	2	kept
spleen rupture	spleen rupture	spleen rupture	spleen rupture	spleen rupture	2	kept
Splenic Diseases	splenic diseases	splenic diseases	splenic diseases	Splenic Diseases	2	kept
Splints	splints	splints	splints	Splints	2	kept
SPO2	spo2	spo2	spo2	SpO2	2	case_or_format
SPOC	spoc	spoc	spoc	SPOC	2	kept
Spondylolisthesis	spondylolisthesis	spondylolisthesis	spondylolisthesis	Spondylolisthesis	2	kept

Spondylosis	spondylosis	spondylosis	spondylosis	Spondylosis	2	kept
Spontaneous Awakening Trials	spontaneous awakening trials	spontaneous awakening trials	spontaneous awakening trial	spontaneous awakening trial	2	singular
Spontaneous Breathing Trials	spontaneous breathing trials	spontaneous breathing trials	spontaneous breathing trial	Spontaneous breathing trial	2	singular
spontaneous breathing trials	spontaneous breathing trials	spontaneous breathing trials	spontaneous breathing trial	Spontaneous breathing trial	2	singular
spontaneous placental delivery	spontaneous placental delivery	spontaneous placental delivery	spontaneous placental delivery	spontaneous placental delivery	2	kept
Spontaneously breathing	spontaneously breathing	spontaneously breathing	spontaneously breathing	Spontaneously breathing	2	kept
Sports	sports	sports	sport	sport	2	singular
Spot measurements	spot measurements	spot measurements	spot measurements	Spot measurements	2	kept
spouses	spouses	spouses	spouse	Spouses	2	singular
SSC	ssc	ssc	ssc	SSC	2	kept
ST-elevation myocardial infarction	st elevation myocardial infarction	st elevation myocardial infarction	st elevation myocardial infarction	ST Elevation Myocardial Infarction	2	case_or_format
STABILITY	stability	stability	stability	STABILITY	2	kept
Stabilization	stabilization	stabilization	stabilization	Stabilization	2	kept
staff allocation	staff allocation	staff allocation	staff allocation	staff allocation	2	kept
staff attitudes	staff attitudes	staff attitudes	staff attitude	staff attitudes	2	singular
staff awareness	staff awareness	staff awareness	staff awareness	staff awareness	2	kept

staff development	staff development	staff development	staff development	Staff Development	2	case_or_format
staff empowerment	staff empowerment	staff empowerment	staff empowerment	staff empowerment	2	kept
staff health	staff health	staff health	staff health	staff health	2	kept
Staff members	staff members	staff members	staff members	Staff members	2	kept
Staff perception	staff perception	staff perception	staff perception	Staff perception	2	kept
Staff perceptions	staff perceptions	staff perceptions	staff perception	Staff perception	2	singular
staff perspectives	staff perspectives	staff perspectives	staff perspectives	staff perspectives	2	kept
staff retention	staff retention	staff retention	staff retention	staff retention	2	kept
Staff safety	staff safety	staff safety	staff safety	staff safety	2	case_or_format
Staff support	staff support	staff support	staff support	Staff support	2	kept
Staff well-being	staff well being	staff well being	staff well being	Staff well-being	2	kept
STAFF WORKLOAD	staff workload	staff workload	staff workload	STAFF WORKLOAD	2	kept
staffing adequacy	staffing adequacy	staffing adequacy	staffing adequacy	staffing adequacy	2	kept
Staffing costs	staffing costs	staffing costs	staffing costs	Staffing costs	2	kept
Staffing levels	staffing levels	staffing levels	staffing level	STAFFING LEVELS	2	singular
staffing levels	staffing levels	staffing levels	staffing level	STAFFING LEVELS	2	singular

staffing ratio	staffing ratio	staffing ratio	staffing ratio	staffing ratio	2	kept
Staffing ratios	staffing ratios	staffing ratios	staffing ratio	staffing ratio	2	singular
STAGE	stage	stage	stage	STAGE	2	kept
Staging	staging	staging	staging	Staging	2	kept
staging	staging	staging	staging	Staging	2	case_or_format
Standard	standard	standard	standard	standards	2	case_or_format
standard of care	standard of care	standard of care	standard of care	Standard of Care	2	case_or_format
Standard of cares	standard of cares	standard of cares	standard of care	Standard of Care	2	singular
standard operating procedures	standard operating procedures	standard operating procedures	standard operating procedure	standard operating procedure	2	singular
Standard operational procedure	standard operational procedure	standard operational procedure	standard operational procedure	Standard operational procedure	2	kept
standardized management pathway	standardized management pathway	standardized management pathway	standardized management pathway	standardized management pathway	2	kept
Standardized mortality ratios	standardized mortality ratios	standardized mortality ratios	standardized mortality ratio	standardized mortality ratio	2	singular
STANDARDS	standards	standards	standard	standards	2	singular
standards of care	standards of care	standards of care	standards of care	Standards of care	2	case_or_format
Staphylococci Aureus	staphylococci aureus	staphylococci aureus	staphylococci aureus	Staphylococci Aureus	2	kept
Staphylococcus aureus MRSA	staphylococcus aureus mrsa	staphylococcus aureus mrsa	staphylococcus aureus mrsa	Staphylococcus aureus MRSA	2	kept

Staphylococcus vaccine	staphylococcus vaccine	staphylococcus vaccine	staphylococcus vaccine	Staphylococcus vaccine	2	kept
STAPHYLOCOCCUS-AUREUS BACTEREMIA	staphylococcus aureus bacteremia	staphylococcus aureus bacteremia	staphylococcus aureus bacteremia	STAPHYLOCOCCUS-AUREUS BACTEREMIA	2	kept
STAPHYLOCOCCUS-AUREUS INFECTIONS	staphylococcus aureus infections	staphylococcus aureus infections	staphylococcus aureus infection	Staphylococcus aureus infection	2	singular
STAR Protocol	star protocol	star protocol	star protocol	STAR Protocol	2	kept
Starling law	starling law	starling law	starling law	Starling law	2	kept
STATE	state	state	state	STATES	2	case_or_format
State Behavioral Scale	state behavioral scale	state behavioral scale	state behavioral scale	State Behavioral Scale	2	kept
state behavioral scale	state behavioral scale	state behavioral scale	state behavioral scale	State Behavioral Scale	2	case_or_format
State of the art	state of the art	state of the art	state of the art	State of the art	2	kept
State-Trait Anxiety Trait	state trait anxiety trait	state trait anxiety trait	state trait anxiety trait	State-Trait Anxiety Trait	2	kept
Statistical significance	statistical significance	statistical significance	statistical significance	statistical significance	2	case_or_format
Status	status	status	status	Status	2	kept
steady state	steady state	steady state	steady state	steady state	2	kept
Stem Cell Transplantation	stem cell transplantation	stem cell transplantation	stem cell transplantation	stem cell transplantation	2	case_or_format
Stem cell transplantation	stem cell transplantation	stem cell transplantation	stem cell transplantation	stem cell transplantation	2	case_or_format
STENOSIS	stenosis	stenosis	stenosis	STENOSIS	2	kept

stenosis	stenosis	stenosis	stenosis	STENOSIS	2	case_or_format
stent dislodgement	stent dislodgement	stent dislodgement	stent dislodgement	stent dislodgement	2	kept
Step-down unit	step down unit	step down unit	step down unit	step down unit	2	case_or_format
step-down units	step down units	step down units	step down unit	step down unit	2	singular
Stepped-wedge cluster-randomised trial	stepped wedge cluster randomised trial	stepped wedge cluster randomised trial	stepped wedge cluster randomised trial	Stepped-wedge cluster-randomised trial	2	kept
stereotactic body radiation therapy	stereotactic body radiation therapy	stereotactic body radiation therapy	stereotactic body radiation therapy	stereotactic body radiation therapy	2	kept
stereotyping	stereotyping	stereotyping	stereotyping	Stereotyping	2	case_or_format
Stevens Johnson	stevens johnson	stevens johnson	stevens johnson	Stevens Johnson	2	kept
Stickler syndrome	stickler syndrome	stickler syndrome	stickler syndrome	Stickler syndrome	2	kept
STIGMA	stigma	stigma	stigma	stigma	2	case_or_format
stimulus response	stimulus response	stimulus response	stimulus response	stimulus response	2	kept
stomach antrum	stomach antrum	stomach antrum	stomach antrum	stomach antrum	2	kept
stomach carcinoma	stomach carcinoma	stomach carcinoma	stomach carcinoma	stomach carcinoma	2	kept
stomach erosion	stomach erosion	stomach erosion	stomach erosion	stomach erosion	2	kept
stomach obstruction	stomach obstruction	stomach obstruction	stomach obstruction	stomach obstruction	2	kept
stomach parameters	stomach parameters	stomach parameters	stomach parameters	stomach parameters	2	kept

stomach pH	stomach ph	stomach ph	stomach ph	stomach pH	2	kept
stomach radiography	stomach radiography	stomach radiography	stomach radiography	stomach radiography	2	kept
stomach surgery	stomach surgery	stomach surgery	stomach surgery	stomach surgery	2	kept
stomach volume	stomach volume	stomach volume	stomach volume	stomach volume	2	kept
stomatology	stomatology	stomatology	stomatology	stomatology	2	kept
storage condition	storage condition	storage condition	storage condition	storage condition	2	kept
strain of care	strain of care	strain of care	strain of care	strain of care	2	kept
STRAINS	strains	strains	strain	Strain	2	singular
stratification	stratification	stratification	stratification	stratification	2	kept
street drug	street drug	street drug	street drug	street drug	2	kept
street medicine	street medicine	street medicine	street medicine	street medicine	2	kept
Streptococcus anginosus	streptococcus anginosus	streptococcus anginosus	streptococcus anginosus	Streptococcus anginosus	2	kept
Streptococcus mitis	streptococcus mitis	streptococcus mitis	streptococcus mitis	Streptococcus mitis	2	kept
Streptococcus mutans	streptococcus mutans	streptococcus mutans	streptococcus mutans	Streptococcus mutans	2	kept
Streptococcus thermophilus	streptococcus thermophilus	streptococcus thermophilus	streptococcus thermophilus	Streptococcus thermophilus	2	kept
STREPTOCOCCUS-PNEUMONIAE	streptococcus pneumoniae	streptococcus pneumoniae	streptococcus pneumoniae	Streptococcus pneumoniae	2	case_or_format

streptokinase	streptokinase	streptokinase	streptokinase	streptokinase	2	kept
streptomycin	streptomycin	streptomycin	streptomycin	streptomycin	2	kept
stress and coping	stress and coping	stress and coping	stress and coping	stress and coping	2	kept
Stress at work	stress at work	stress at work	stress at work	Stress at work	2	kept
Stress cardiomyopathy	stress cardiomyopathy	stress cardiomyopathy	stress cardiomyopathy	Stress cardiomyopathy	2	kept
Stress disorder	stress disorder	stress disorder	stress disorder	Stress disorders	2	case_or_format
stress echocardiography	stress echocardiography	stress echocardiography	stress echocardiography	stress echocardiography	2	kept
Stress hyperglycemia	stress hyperglycemia	stress hyperglycemia	stress hyperglycemia	stress hyperglycemia	2	case_or_format
stress mitigation	stress mitigation	stress mitigation	stress mitigation	stress mitigation	2	kept
Stress of conscience	stress of conscience	stress of conscience	stress of conscience	Stress of conscience	2	kept
STRESS REDUCTION	stress reduction	stress reduction	stress reduction	stress reduction	2	case_or_format
Stress ulcer prophylaxis	stress ulcer prophylaxis	stress ulcer prophylaxis	stress ulcer prophylaxis	Stress ulcer prophylaxis	2	kept
stress, physiological	stress physiological	stress physiological	stress physiological	Stress, Physiological	2	case_or_format
Stress-induced	stress induced	stress induced	stress induced	Stress-induced	2	kept
Stretcher	stretcher	stretcher	stretcher	Stretcher	2	kept
stretcher	stretcher	stretcher	stretcher	Stretcher	2	case_or_format

Stretchers	stretchers	stretchers	stretcher	Stretcher	2	singular
stretching	stretching	stretching	stretching	stretching	2	kept
stretching exercise	stretching exercise	stretching exercise	stretching exercise	stretching exercise	2	kept
Strikes, Employee	strikes employee	strikes employee	strikes employee	Strikes, Employee	2	kept
Stroke care transitions	stroke care transitions	stroke care transitions	stroke care transitions	Stroke care transitions	2	kept
Stroke management	stroke management	stroke management	stroke management	Stroke management	2	kept
Stroke outcomes	stroke outcomes	stroke outcomes	stroke outcome	Stroke outcomes	2	singular
Stroke Tracheotomy with Dysphagia	stroke tracheotomy with dysphagia	stroke tracheotomy with dysphagia	stroke tracheotomy with dysphagia	Stroke Tracheotomy with Dysphagia	2	kept
Stroke units	stroke units	stroke units	stroke unit	stroke unit	2	singular
STRONG MODEL	strong model	strong model	strong model	STRONG MODEL	2	kept
structural empowerment	structural empowerment	structural empowerment	structural empowerment	Structural empowerment	2	case_or_format
Structural equation modelling	structural equation modelling	structural equation modelling	structural equation modelling	Structural equation modelling	2	kept
structural equation modelling	structural equation modelling	structural equation modelling	structural equation modelling	Structural equation modelling	2	case_or_format
Structural nutritional care management model	structural nutritional care management model	structural nutritional care management model	structural nutritional care management model	Structural nutritional care management model	2	kept
structural racism	structural racism	structural racism	structural racism	structural racism	2	kept
STRUCTURED INTERDISCIPLINARY ROUNDS	structured interdisciplinary rounds	structured interdisciplinary rounds	structured interdisciplinary rounds	STRUCTURED INTERDISCIPLINARY ROUNDS	2	kept

STS	sts	sts	sts	STS	2	kept
Student education	student education	student education	student education	Student education	2	kept
student satisfaction	student satisfaction	student satisfaction	student satisfaction	student satisfaction	2	kept
student-debriefer interaction	student debriefer interaction	student debriefer interaction	student debriefer interaction	student-debriefer interaction	2	kept
Students, medical	students medical	students medical	students medical	Students, Medical	2	case_or_format
Study design	study design	study design	study design	study design	2	case_or_format
Study protocol	study protocol	study protocol	study protocol	Study protocol	2	kept
study recruitment	study recruitment	study recruitment	study recruitment	study recruitment	2	kept
stunned heart muscle	stunned heart muscle	stunned heart muscle	stunned heart muscle	stunned heart muscle	2	kept
STYLE	style	style	style	STYLE	2	kept
SUB-SAHARAN AFRICA	sub saharan africa	sub saharan africa	sub saharan africa	Sub-Saharan Africa	2	case_or_format
SUBARACHNOID HEMORRHAGE	subarachnoid hemorrhage	subarachnoid hemorrhage	subarachnoid hemorrhage	subarachnoid hemorrhage	2	case_or_format
subclavian artery	subclavian artery	subclavian artery	subclavian artery	subclavian artery	2	kept
subclavian vein catheter	subclavian vein catheter	subclavian vein catheter	subclavian vein catheter	subclavian vein catheter	2	kept
subclavian vein catheterization	subclavian vein catheterization	subclavian vein catheterization	subclavian vein catheterization	subclavian vein catheterization	2	kept
Subcutaneous Tissue	subcutaneous tissue	subcutaneous tissue	subcutaneous tissue	subcutaneous tissue	2	case_or_format

Subglottic secretion drainage	subglottic secretion drainage	subglottic secretion drainage	subglottic secretion drainage	Subglottic secretion drainage	2	kept
subglottic secretion drainage	subglottic secretion drainage	subglottic secretion drainage	subglottic secretion drainage	Subglottic secretion drainage	2	case_or_format
Subintensive geriatric care unit	subintensive geriatric care unit	subintensive geriatric care unit	subintensive geriatric care unit	Subintensive geriatric care unit	2	kept
Subjective assessments	subjective assessments	subjective assessments	subjective assessments	Subjective assessments	2	kept
subjective fatigue	subjective fatigue	subjective fatigue	subjective fatigue	subjective fatigue	2	kept
subjective perception	subjective perception	subjective perception	subjective perception	subjective perception	2	kept
Subjective perceptions	subjective perceptions	subjective perceptions	subjective perception	subjective perception	2	singular
Subjective workload	subjective workload	subjective workload	subjective workload	Subjective workload	2	kept
subjective workload assessment technique	subjective workload assessment technique	subjective workload assessment technique	subjective workload assessment technique	subjective workload assessment technique	2	kept
suboccipital	suboccipital	suboccipital	suboccipital	suboccipital	2	kept
SUBSEQUENT DISABILITY	subsequent disability	subsequent disability	subsequent disability	SUBSEQUENT DISABILITY	2	kept
Substance	substance	substance	substance	Substance	2	kept
Substance Abuse, Intravenous	substance abuse intravenous	substance abuse intravenous	substance abuse intravenous	Substance Abuse, Intravenous	2	kept
SUBSTANCE USE	substance use	substance use	substance use	substance use	2	case_or_format
Substance use	substance use	substance use	substance use	substance use	2	case_or_format
Substance use disorder	substance use disorder	substance use disorder	substance use disorder	substance use disorder	2	case_or_format

substance use disorders	substance use disorders	substance use disorders	substance use disorder	substance use disorder	2	singular
substance-exposed pregnancy	substance exposed pregnancy	substance exposed pregnancy	substance exposed pregnancy	substance-exposed pregnancy	2	kept
substitute decision-making	substitute decision making	substitute decision making	substitute decision making	substitute decision-making	2	kept
Subthalamic Nucleus	subthalamic nucleus	subthalamic nucleus	subthalamic nucleus	Subthalamic Nucleus	2	kept
Success rate of rescue	success rate of rescue	success rate of rescue	success rate of rescue	Success rate of rescue	2	kept
Sucking	sucking	sucking	sucking	sucking	2	case_or_format
Sucking success	sucking success	sucking success	sucking success	Sucking success	2	kept
Sucralfate	sucralfate	sucralfate	sucralfate	sucralfate	2	case_or_format
suction drainage	suction drainage	suction drainage	suction drainage	suction drainage	2	kept
Sudden death	sudden death	sudden death	sudden death	sudden death	2	case_or_format
Sueño	sue o	sue o	sue o	Sueño	2	kept
sugar-free cola	sugar free cola	sugar free cola	sugar free cola	sugar-free cola	2	kept
Suicidal Ideation	suicidal ideation	suicidal ideation	suicidal ideation	suicidal ideation	2	case_or_format
Suicidal ideation	suicidal ideation	suicidal ideation	suicidal ideation	suicidal ideation	2	case_or_format
Suicide prevention	suicide prevention	suicide prevention	suicide prevention	Suicide Prevention	2	case_or_format
Sulfonamides	sulfonamides	sulfonamides	sulfonamide	sulfonamide	2	singular

sulfonylurea derivative	sulfonylurea derivative	sulfonylurea derivative	sulfonylurea derivative	sulfonylurea derivative	2	kept
sulperazon	sulperazon	sulperazon	sulperazon	sulperazon	2	kept
summary of evidence	summary of evidence	summary of evidence	summary of evidence	summary of evidence	2	kept
Sunflower Oil	sunflower oil	sunflower oil	sunflower oil	Sunflower Oil	2	kept
Superinfection	superinfection	superinfection	superinfection	superinfection	2	case_or_format
superior cava vein syndrome	superior cava vein syndrome	superior cava vein syndrome	superior cava vein syndrome	superior cava vein syndrome	2	kept
Superior Vena Cava Syndrome	superior vena cava syndrome	superior vena cava syndrome	superior vena cava syndrome	Superior Vena Cava Syndrome	2	kept
supervised machine learning	supervised machine learning	supervised machine learning	supervised machine learning	Supervised machine learning	2	case_or_format
Supine position	supine position	supine position	supine position	supine position	2	case_or_format
SupplMeshName: Maslach Burnout Inventory, UI: C000728151, Type:	supplmeshname maslach burnout inventory ui c000728151 type	supplmeshname maslach burnout inventory ui c000728151 type	supplmeshname maslach burnout inventory ui c000728151 type	SupplMeshName: Maslach Burnout Inventory, UI: C000728151, Type:	2	kept
SupplMeshName: Necrophobia, UI: C000719207, Type:	supplmeshname necrophobia ui c000719207 type	supplmeshname necrophobia ui c000719207 type	supplmeshname necrophobia ui c000719207 type	SupplMeshName: Necrophobia, UI: C000719207, Type:	2	kept
supply chain	supply chain	supply chain	supply chain	supply chain	2	kept
Supply chain management	supply chain management	supply chain management	supply chain management	Supply chain management	2	kept
Support needs	support needs	support needs	support needs	Support needs	2	kept
Support services	support services	support services	support service	Support services	2	singular
Support system	support system	support system	support system	Support system	2	kept

Supporting	supporting	supporting	supporting	Supporting	2	kept
Supportive therapy	supportive therapy	supportive therapy	supportive therapy	Supportive therapy	2	kept
suppression ratio	suppression ratio	suppression ratio	suppression ratio	suppression ratio	2	kept
suprapubic catheter	suprapubic catheter	suprapubic catheter	suprapubic catheter	suprapubic catheter	2	kept
Surface Properties	surface properties	surface properties	surface property	surface property	2	singular
surfactant therapy	surfactant therapy	surfactant therapy	surfactant therapy	surfactant therapy	2	kept
surge planning	surge planning	surge planning	surge planning	surge planning	2	kept
Surgeon	surgeon	surgeon	surgeon	surgeon	2	case_or_format
surgeons transfer	surgeons transfer	surgeons transfer	surgeons transfer	surgeons transfer	2	kept
surgery unit	surgery unit	surgery unit	surgery unit	surgery unit	2	kept
Surgery wards	surgery wards	surgery wards	surgery wards	Surgery wards	2	kept
Surgery, Oral	surgery oral	surgery oral	surgery oral	Surgery, Oral	2	kept
surgical areas	surgical areas	surgical areas	surgical areas	surgical areas	2	kept
Surgical complication	surgical complication	surgical complication	surgical complication	Surgical complication	2	kept
Surgical education	surgical education	surgical education	surgical education	surgical education	2	case_or_format
Surgical error	surgical error	surgical error	surgical error	surgical error	2	case_or_format

surgical flaps	surgical flaps	surgical flaps	surgical flaps	Surgical Flaps	2	case_or_format
surgical gown	surgical gown	surgical gown	surgical gown	surgical gown	2	kept
Surgical Instruments	surgical instruments	surgical instruments	surgical instrument	Surgical Instruments	2	singular
Surgical operation	surgical operation	surgical operation	surgical operation	Surgical operation	2	kept
SURGICAL SITE INFECTION	surgical site infection	surgical site infection	surgical site infection	SURGICAL SITE INFECTION	2	kept
Surgical site infection	surgical site infection	surgical site infection	surgical site infection	SURGICAL SITE INFECTION	2	case_or_format
surgical site infection	surgical site infection	surgical site infection	surgical site infection	SURGICAL SITE INFECTION	2	case_or_format
Surgical stomas	surgical stomas	surgical stomas	surgical stomas	Surgical Stomas	2	case_or_format
surgical tracheostomy	surgical tracheostomy	surgical tracheostomy	surgical tracheostomy	surgical tracheostomy	2	kept
Suriname	suriname	suriname	suriname	Suriname	2	kept
surrogate	surrogate	surrogate	surrogate	Surrogate	2	case_or_format
Surrogate decision maker	surrogate decision maker	surrogate decision maker	surrogate decision maker	Surrogate decision maker	2	kept
surrogate decision maker	surrogate decision maker	surrogate decision maker	surrogate decision maker	Surrogate decision maker	2	case_or_format
SURVEILLANCE CULTURES	surveillance cultures	surveillance cultures	surveillance cultures	SURVEILLANCE CULTURES	2	kept
Surveillance video observation	surveillance video observation	surveillance video observation	surveillance video observation	Surveillance video observation	2	kept
Survey and questionnaires	survey and questionnaires	survey and questionnaires	survey and questionnaire	Survey and questionnaires	2	singular

survey methodology	survey methodology	survey methodology	survey methodology	survey methodology	2	kept
survey methods	survey methods	survey methods	survey method	survey methods	2	singular
Survey research	survey research	survey research	survey research	Survey research	2	kept
Survey study	survey study	survey study	survey study	Survey study	2	kept
Survival machine learning algorithm	survival machine learning algorithm	survival machine learning algorithm	survival machine learning algorithm	Survival machine learning algorithm	2	kept
Survival prediction	survival prediction	survival prediction	survival prediction	survival prediction	2	case_or_format
surviving sepsis campaign	surviving sepsis campaign	surviving sepsis campaign	surviving sepsis campaign	Surviving Sepsis Campaign	2	case_or_format
Suspension	suspension	suspension	suspension	suspension	2	case_or_format
Sustainable development	sustainable development	sustainable development	sustainable development	sustainable development	2	case_or_format
sustainable development goal	sustainable development goal	sustainable development goal	sustainable development goal	sustainable development goal	2	kept
Sustainable development goals	sustainable development goals	sustainable development goals	sustainable development goal	sustainable development goal	2	singular
Sustainable employment	sustainable employment	sustainable employment	sustainable employment	Sustainable employment	2	kept
sustainable healthcare	sustainable healthcare	sustainable healthcare	sustainable healthcare	Sustainable healthcare	2	case_or_format
Sustained low-efficiency dialysis	sustained low efficiency dialysis	sustained low efficiency dialysis	sustained low efficiency dialysis	Sustained low-efficiency dialysis	2	kept
sustained release preparation	sustained release preparation	sustained release preparation	sustained release preparation	sustained release preparation	2	kept
suture technique	suture technique	suture technique	suture technique	Suture Techniques	2	case_or_format

swabbing	swabbing	swabbing	swabbing	swabbing	2	kept
Swaddling	swaddling	swaddling	swaddling	Swaddling	2	kept
SwAge model	swage model	swage model	swage model	SwAge model	2	kept
swallow	swallow	swallow	swallow	swallow	2	kept
swallowing disorders	swallowing disorders	swallowing disorders	swallowing disorder	Swallowing disorder	2	singular
Sweating	sweating	sweating	sweating	sweating	2	case_or_format
sweetening agent	sweetening agent	sweetening agent	sweetening agent	Sweetening Agents	2	case_or_format
swimming pool	swimming pool	swimming pool	swimming pool	swimming pool	2	kept
sympathectomy	sympathectomy	sympathectomy	sympathectomy	sympathectomy	2	kept
sympathetic tone	sympathetic tone	sympathetic tone	sympathetic tone	sympathetic tone	2	kept
Sympathomimetics	sympathomimetics	sympathomimetics	sympathomimetics	Sympathomimetics	2	kept
symptom control	symptom control	symptom control	symptom control	symptom control	2	kept
symptom rebound	symptom rebound	symptom rebound	symptom rebound	symptom rebound	2	kept
Symptoms and symptom management	symptoms and symptom management	symptoms and symptom management	symptoms and symptom management	Symptoms and symptom management	2	kept
Synaptic Transmission	synaptic transmission	synaptic transmission	synaptic transmission	Synaptic Transmission	2	kept
synaptic transmission	synaptic transmission	synaptic transmission	synaptic transmission	Synaptic Transmission	2	case_or_format

syndrome CHARGE	syndrome charge	syndrome charge	syndrome charge	syndrome CHARGE	2	kept
Synergy Model	synergy model	synergy model	synergy model	Synergy Model	2	kept
Synergy model	synergy model	synergy model	synergy model	Synergy Model	2	case_or_format
Syphilis, Congenital	syphilis congenital	syphilis congenital	syphilis congenital	Syphilis, Congenital	2	kept
syrup	syrup	syrup	syrup	syrup	2	kept
System design	system design	system design	system design	System design	2	kept
System development	system development	system development	system development	System development	2	kept
system evaluation	system evaluation	system evaluation	system evaluation	system evaluation	2	kept
System thinking	system thinking	system thinking	system thinking	System thinking	2	kept
system usability scale	system usability scale	system usability scale	system usability scale	system usability scale	2	kept
System use	system use	system use	system use	System use	2	kept
System-on-chip	system on chip	system on chip	system on chip	System-on-chip	2	kept
Systematic challenges	systematic challenges	systematic challenges	systematic challenges	Systematic challenges	2	kept
systematic error	systematic error	systematic error	systematic error	systematic error	2	kept
Systematic literature review	systematic literature review	systematic literature review	systematic literature review	Systematic literature review	2	kept
Systematized Nomenclature of Medicine	systematized nomenclature of medicine	systematized nomenclature of medicine	systematized nomenclature of medicine	Systematized Nomenclature of Medicine	2	kept

Systematized review	systematized review	systematized review	systematized review	Systematized review	2	kept
systemic	systemic	systemic	systemic	systemic	2	kept
systems biology	systems biology	systems biology	systems biology	Systems Biology	2	case_or_format
Systems development	systems development	systems development	systems development	Systems development	2	kept
systems engineering	systems engineering	systems engineering	systems engineering	Systems engineering	2	case_or_format
systole	systole	systole	systole	Systole	2	case_or_format
systolic dysfunction	systolic dysfunction	systolic dysfunction	systolic dysfunction	systolic dysfunction	2	kept
systolic heart murmur	systolic heart murmur	systolic heart murmur	systolic heart murmur	systolic heart murmur	2	kept
sécurité	s curit	s curit	s curit	sécurité	2	kept
Sézary syndrome	s zary syndrome	s zary syndrome	s zary syndrome	Sézary syndrome	2	kept
Síndrome de distrés respiratorio agudo	s ndrome de distr s respiratorio agudo	s ndrome de distr s respiratorio agudo	s ndrome de distr s respiratorio agudo	Síndrome de distrés respiratorio agudo	2	kept
T lymphocyte subpopulation	t lymphocyte subpopulation	t lymphocyte subpopulation	t lymphocyte subpopulation	T lymphocyte subpopulation	2	kept
T lymphocyte subsets	t lymphocyte subsets	t lymphocyte subsets	t lymphocyte subsets	T lymphocyte subsets	2	kept
T wave inversion	t wave inversion	t wave inversion	t wave inversion	T wave inversion	2	kept
T1 weighted imaging	t1 weighted imaging	t1 weighted imaging	t1 weighted imaging	T1 weighted imaging	2	kept
Tablet applications	tablet applications	tablet applications	tablet applications	Tablet applications	2	kept

Tachycardia, Ectopic Atrial	tachycardia ectopic atrial	tachycardia ectopic atrial	tachycardia ectopic atrial	Tachycardia, Ectopic Atrial	2	kept
tachyphylaxis	tachyphylaxis	tachyphylaxis	tachyphylaxis	tachyphylaxis	2	kept
tachysystole	tachysystole	tachysystole	tachysystole	tachysystole	2	kept
tactile	tactile	tactile	tactile	tactile	2	kept
Tactile stimulation	tactile stimulation	tactile stimulation	tactile stimulation	tactile stimulation	2	case_or_format
tadalafil	tadalafil	tadalafil	tadalafil	tadalafil	2	kept
Tags	tags	tags	tags	Tags	2	kept
Tai Chi	tai chi	tai chi	tai chi	Tai Chi	2	kept
Tail dependence	tail dependence	tail dependence	tail dependence	Tail dependence	2	kept
tamoxifen	tamoxifen	tamoxifen	tamoxifen	tamoxifen	2	kept
tamsulosin	tamsulosin	tamsulosin	tamsulosin	tamsulosin	2	kept
Tandem Mass Spectrometry	tandem mass spectrometry	tandem mass spectrometry	tandem mass spectrometry	Tandem Mass Spectrometry	2	kept
Tanner Clinical Judgment Model	tanner clinical judgment model	tanner clinical judgment model	tanner clinical judgment model	Tanner Clinical Judgment Model	2	kept
Target population	target population	target population	target population	Target population	2	kept
Targeted drug delivery	targeted drug delivery	targeted drug delivery	targeted drug delivery	Targeted drug delivery	2	kept
Task Completion Time	task completion time	task completion time	task completion time	Task Completion Time	2	kept

TASK COMPLEXITY	task complexity	task complexity	task complexity	TASK COMPLEXITY	2	kept
Task performance	task performance	task performance	task performance	task performance	2	case_or_format
TASK-FORCE	task force	task force	task force	TASK-FORCE	2	kept
Tasmania	tasmania	tasmania	tasmania	Tasmania	2	kept
Taste	taste	taste	taste	taste	2	case_or_format
tax	tax	tax	tax	tax	2	kept
taxane derivative	taxane derivative	taxane derivative	taxane derivative	taxane derivative	2	kept
TBI = traumatic brain injury	tbi traumatic brain injury	tbi traumatic brain injury	tbi traumatic brain injury	TBI = traumatic brain injury	2	kept
TDABC	tdabc	tdabc	tdabc	TDABC	2	kept
TDM	tdm	tdm	tdm	TDM	2	kept
tea	tea	tea	tea	tea	2	kept
Teaching and learning	teaching and learning	teaching and learning	teaching and learning	Teaching and learning	2	kept
teaching design	teaching design	teaching design	teaching design	teaching design	2	kept
TEACHING HOSPITALS	teaching hospitals	teaching hospitals	teaching hospital	teaching hospital	2	singular
Teaching methods	teaching methods	teaching methods	teaching methods	teaching methods	2	case_or_format
teaching rounds	teaching rounds	teaching rounds	teaching round	Teaching Rounds	2	singular

Teaching strategy	teaching strategy	teaching strategy	teaching strategy	Teaching strategy	2	kept
TEACHING UNIT	teaching unit	teaching unit	teaching unit	TEACHING UNIT	2	kept
Team cognition	team cognition	team cognition	team cognition	Team cognition	2	kept
team cognition	team cognition	team cognition	team cognition	Team cognition	2	case_or_format
Team commitment	team commitment	team commitment	team commitment	Team commitment	2	kept
team culture	team culture	team culture	team culture	team culture	2	kept
team effectiveness	team effectiveness	team effectiveness	team effectiveness	team effectiveness	2	kept
Team efficacy	team efficacy	team efficacy	team efficacy	Team efficacy	2	kept
Team Emergency Assessment Measure	team emergency assessment measure	team emergency assessment measure	team emergency assessment measure	Team Emergency Assessment Measure	2	kept
Team interaction	team interaction	team interaction	team interaction	Team interaction	2	kept
Team interactions	team interactions	team interactions	team interaction	Team interaction	2	singular
team work	team work	team work	team work	Team work	2	case_or_format
Teams	teams	teams	team	Team	2	singular
Tears	tears	tears	tears	Tears	2	kept
Techniques and procedures	techniques and procedures	techniques and procedures	techniques and procedure	techniques and procedures	2	singular
Technology acceptance	technology acceptance	technology acceptance	technology acceptance	Technology acceptance	2	kept

Technology Acceptance Model	technology acceptance model	technology acceptance model	technology acceptance model	Technology acceptance model	2	case_or_format
technology-enhanced learning	technology enhanced learning	technology enhanced learning	technology enhanced learning	technology-enhanced learning	2	kept
Technostress	technostress	technostress	technostress	Technostress	2	kept
tedizolid	tedizolid	tedizolid	tedizolid	tedizolid	2	kept
Tele-intensivist	tele intensivist	tele intensivist	tele intensivist	Tele-intensivist	2	kept
telecardiology	telecardiology	telecardiology	telecardiology	telecardiology	2	kept
telediagnosis	telediagnosis	telediagnosis	telediagnosis	telediagnosis	2	kept
telemedicine and drones	telemedicine and drones	telemedicine and drones	telemedicine and drones	telemedicine and drones	2	kept
teleneonatology	teleneonatology	teleneonatology	teleneonatology	teleneonatology	2	kept
telepharmacy	telepharmacy	telepharmacy	telepharmacy	telepharmacy	2	kept
telephone telemetry	telephone telemetry	telephone telemetry	telephone telemetry	telephone telemetry	2	kept
Telepresence	telepresence	telepresence	telepresence	Telepresence	2	kept
television viewing	television viewing	television viewing	television viewing	television viewing	2	kept
telling rights	telling rights	telling rights	telling rights	telling rights	2	kept
Temperament	temperament	temperament	temperament	temperament	2	case_or_format
temperature sensitivity	temperature sensitivity	temperature sensitivity	temperature sensitivity	temperature sensitivity	2	kept

Template matching	template matching	template matching	template matching	Template matching	2	kept
Temporal Arteries	temporal arteries	temporal arteries	temporal artery	temporal artery	2	singular
temporal artery	temporal artery	temporal artery	temporal artery	temporal artery	2	kept
Temporary pacing	temporary pacing	temporary pacing	temporary pacing	Temporary pacing	2	kept
Temporomandibular joint	temporomandibular joint	temporomandibular joint	temporomandibular joint	Temporomandibular joint	2	kept
tendon reconstruction	tendon reconstruction	tendon reconstruction	tendon reconstruction	tendon reconstruction	2	kept
tendon rupture	tendon rupture	tendon rupture	tendon rupture	tendon rupture	2	kept
tenofovir	tenofovir	tenofovir	tenofovir	tenofovir	2	kept
tenosynovitis	tenosynovitis	tenosynovitis	tenosynovitis	tenosynovitis	2	kept
tension headache	tension headache	tension headache	tension headache	tension headache	2	kept
tensive adaptation	tensive adaptation	tensive adaptation	tensive adaptation	tensive adaptation	2	kept
Term	term	term	term	TERM	2	case_or_format
term	term	term	term	TERM	2	case_or_format
TERM COGNITIVE IMPAIRMENT	term cognitive impairment	term cognitive impairment	term cognitive impairment	TERM COGNITIVE IMPAIRMENT	2	kept
Term infant	term infant	term infant	term infant	Term infant	2	kept
term pregnancy	term pregnancy	term pregnancy	term pregnancy	term pregnancy	2	kept

TERM-CARE FACILITIES	term care facilities	term care facilities	term care facilities	TERM-CARE FACILITIES	2	kept
terminal cleaning	terminal cleaning	terminal cleaning	terminal cleaning	terminal cleaning	2	kept
Terminal extubation	terminal extubation	terminal extubation	terminal extubation	terminal extubation	2	case_or_format
Terminal illness	terminal illness	terminal illness	terminal illness	Terminal illness	2	kept
terminal illness	terminal illness	terminal illness	terminal illness	Terminal illness	2	case_or_format
tertiary	tertiary	tertiary	tertiary	tertiary	2	kept
Tertiary care	tertiary care	tertiary care	tertiary care	Tertiary cares	2	case_or_format
tertiary care	tertiary care	tertiary care	tertiary care	Tertiary cares	2	case_or_format
Tertiary Care Hospital	tertiary care hospital	tertiary care hospital	tertiary care hospital	tertiary care hospital	2	case_or_format
Tertiary care hospital	tertiary care hospital	tertiary care hospital	tertiary care hospital	tertiary care hospital	2	case_or_format
Tertiary hospitals	tertiary hospitals	tertiary hospitals	tertiary hospital	Tertiary hospital	2	singular
Test turnaround time	test turnaround time	test turnaround time	test turnaround time	Test turnaround time	2	kept
testis cancer	testis cancer	testis cancer	testis cancer	testis cancer	2	kept
tetanus antibody	tetanus antibody	tetanus antibody	tetanus antibody	tetanus antibody	2	kept
Tetany	tetany	tetany	tetany	Tetany	2	kept
tethered cord syndrome	tethered cord syndrome	tethered cord syndrome	tethered cord syndrome	tethered cord syndrome	2	kept

tetrahydrocannabinol	tetrahydrocannabinol	tetrahydrocannabinol	tetrahydrocannabinol	tetrahydrocannabinol	2	kept
tetralogy of fallot	tetralogy of fallot	tetralogy of fallot	tetralogy of fallot	Tetralogy of Fallot	2	case_or_format
TEVAR	tevar	tevar	tevar	TEVAR	2	kept
TEXT	text	text	text	TEXT	2	kept
Text analytics	text analytics	text analytics	text analytics	Text analytics	2	kept
Text messaging	text messaging	text messaging	text messaging	Text Messaging	2	case_or_format
Textiles	textiles	textiles	textiles	Textiles	2	kept
Thalamus	thalamus	thalamus	thalamus	thalamus	2	case_or_format
thalidomide	thalidomide	thalidomide	thalidomide	thalidomide	2	kept
Thanatophobia	thanatophobia	thanatophobia	thanatophobia	Thanatophobia	2	kept
thawing	thawing	thawing	thawing	thawing	2	kept
the stability and workload index for transfer	the stability and workload index for transfer	the stability and workload index for transfer	the stability and workload index for transfer	the stability and workload index for transfer	2	kept
the theory of planned behavior	the theory of planned behavior	the theory of planned behavior	the theory of planned behavior	the theory of planned behavior	2	kept
THE-LITERATURE	the literature	the literature	the literature	THE-LITERATURE	2	kept
theme analysis	theme analysis	theme analysis	theme analysis	theme analysis	2	kept
theoretical domains framework	theoretical domains framework	theoretical domains framework	theoretical domains framework	Theoretical domains framework	2	case_or_format

Theoretical framework	theoretical framework	theoretical framework	theoretical framework	Theoretical framework	2	kept
Theoretical framework of acceptability	theoretical framework of acceptability	theoretical framework of acceptability	theoretical framework of acceptability	Theoretical framework of acceptability	2	kept
Theoretical modeling	theoretical modeling	theoretical modeling	theoretical modeling	Theoretical modeling	2	kept
theoretical models	theoretical models	theoretical models	theoretical model	theoretical model	2	singular
Theory of planned behaviour	theory of planned behaviour	theory of planned behaviour	theory of planned behaviour	Theory of planned behaviour	2	kept
theory of planned behaviour	theory of planned behaviour	theory of planned behaviour	theory of planned behaviour	Theory of planned behaviour	2	case_or_format
theory testing	theory testing	theory testing	theory testing	theory testing	2	kept
theory-practice gap	theory practice gap	theory practice gap	theory practice gap	theory-practice gap	2	kept
therapeutic communication	therapeutic communication	therapeutic communication	therapeutic communication	Therapeutic communication	2	case_or_format
therapeutic misconception	therapeutic misconception	therapeutic misconception	therapeutic misconception	therapeutic misconception	2	kept
therapeutic music listening	therapeutic music listening	therapeutic music listening	therapeutic music listening	therapeutic music listening	2	kept
Therapeutic plasmapheresis	therapeutic plasmapheresis	therapeutic plasmapheresis	therapeutic plasmapheresis	Therapeutic plasmapheresis	2	kept
Therapeutic touch	therapeutic touch	therapeutic touch	therapeutic touch	Therapeutic Touch	2	case_or_format
therapeutic use	therapeutic use	therapeutic use	therapeutic use	therapeutic use	2	kept
Therapeutics, treatment outcome	therapeutics treatment outcome	therapeutics treatment outcome	therapeutics treatment outcome	Therapeutics, treatment outcome	2	kept
therapies	therapies	therapies	therapy	therapy	2	singular

therapist	therapist	therapist	therapist	therapist	2	kept
therapy]	therapy	therapy	therapy	therapy	2	case_or_format
thermotherapy	thermotherapy	thermotherapy	thermotherapy	thermotherapy	2	kept
thiamazole	thiamazole	thiamazole	thiamazole	thiamazole	2	kept
Thiamine	thiamine	thiamine	thiamine	thiamine	2	case_or_format
Thiamine Deficiency	thiamine deficiency	thiamine deficiency	thiamine deficiency	thiamine deficiency	2	case_or_format
thiazide diuretic agent	thiazide diuretic agent	thiazide diuretic agent	thiazide diuretic agent	thiazide diuretic agent	2	kept
thiophene derivative	thiophene derivative	thiophene derivative	thiophene derivative	thiophene derivative	2	kept
Thiophenes	thiophenes	thiophenes	thiophenes	Thiophenes	2	kept
thioridazine	thioridazine	thioridazine	thioridazine	thioridazine	2	kept
Third International Consensus Definitions for Sepsis and Septic	third international consensus definitions for sepsis and septic	third international consensus definitions for sepsis and septic	third international consensus definitions for sepsis and septic	Third International Consensus Definitions for Sepsis and Septic	2	kept
thirst management	thirst management	thirst management	thirst management	thirst management	2	kept
thoracic aorta surgery	thoracic aorta surgery	thoracic aorta surgery	thoracic aorta surgery	thoracic aorta surgery	2	kept
thoracic aortic aneurysm	thoracic aortic aneurysm	thoracic aortic aneurysm	thoracic aortic aneurysm	thoracic aortic aneurysm	2	kept
thoracic aortic dissection	thoracic aortic dissection	thoracic aortic dissection	thoracic aortic dissection	thoracic aortic dissection	2	kept
Thoracic aortic endograft	thoracic aortic endograft	thoracic aortic endograft	thoracic aortic endograft	Thoracic aortic endograft	2	kept

thoracic aortic surgery	thoracic aortic surgery	thoracic aortic surgery	thoracic aortic surgery	thoracic aortic surgery	2	kept
thoracic medicine	thoracic medicine	thoracic medicine	thoracic medicine	thoracic medicine	2	kept
thoracic procedures	thoracic procedures	thoracic procedures	thoracic procedures	thoracic procedures	2	kept
Thoracic trauma	thoracic trauma	thoracic trauma	thoracic trauma	Thoracic trauma	2	kept
thoracolumbar fracture	thoracolumbar fracture	thoracolumbar fracture	thoracolumbar fracture	thoracolumbar fracture	2	kept
thorax wall defect	thorax wall defect	thorax wall defect	thorax wall defect	thorax wall defect	2	kept
thought disorder	thought disorder	thought disorder	thought disorder	thought disorder	2	kept
Threats	threats	threats	threat	threat	2	singular
Thriving	thriving	thriving	thriving	Thriving	2	kept
thriving at work	thriving at work	thriving at work	thriving at work	Thriving at work	2	case_or_format
throat disease	throat disease	throat disease	throat disease	throat disease	2	kept
thrombin time	thrombin time	thrombin time	thrombin time	thrombin time	2	kept
THROMBOLYSIS	thrombolysis	thrombolysis	thrombolysis	Thrombolysis	2	case_or_format
Thrombolytic	thrombolytic	thrombolytic	thrombolytic	Thrombolytic	2	kept
Thrombophilia	thrombophilia	thrombophilia	thrombophilia	thrombophilia	2	case_or_format
Thromboplastin	thromboplastin	thromboplastin	thromboplastin	Thromboplastin	2	kept

Thromboprophylaxis	thromboprophylaxis	thromboprophylaxis	thromboprophylaxis	Thromboprophylaxis	2	kept
Thrombotic microangiopathy	thrombotic microangiopathy	thrombotic microangiopathy	thrombotic microangiopathy	Thrombotic Microangiopathies	2	case_or_format
thrombus	thrombus	thrombus	thrombus	thrombus	2	kept
Throughput	throughput	throughput	throughput	Throughput	2	kept
thyroid crisis	thyroid crisis	thyroid crisis	thyroid crisis	Thyroid Crisis	2	case_or_format
thyroid disease	thyroid disease	thyroid disease	thyroid disease	thyroid disease	2	kept
thyroid function	thyroid function	thyroid function	thyroid function	thyroid function	2	kept
tiapride	tiapride	tiapride	tiapride	tiapride	2	kept
tibia fracture	tibia fracture	tibia fracture	tibia fracture	tibia fracture	2	kept
tibial artery	tibial artery	tibial artery	tibial artery	tibial artery	2	kept
tibial plateau fracture	tibial plateau fracture	tibial plateau fracture	tibial plateau fracture	tibial plateau fracture	2	kept
tick bite	tick bite	tick bite	tick bite	tick bite	2	kept
tick borne encephalitis	tick borne encephalitis	tick borne encephalitis	tick borne encephalitis	tick borne encephalitis	2	kept
Tick-Borne Diseases	tick borne diseases	tick borne diseases	tick borne diseases	Tick-Borne Diseases	2	kept
Ticlopidine	ticlopidine	ticlopidine	ticlopidine	ticlopidine	2	case_or_format
Tidal volume, low tidal volume ventilation	tidal volume low tidal volume ventilation	tidal volume low tidal volume ventilation	tidal volume low tidal volume ventilation	Tidal volume, low tidal volume ventilation	2	kept

Tigecycline	tigecycline	tigecycline	tigecycline	tigecycline	2	case_or_format
tight glycaemic control	tight glycaemic control	tight glycaemic control	tight glycaemic control	tight glycaemic control	2	kept
Tilapia	tilapia	tilapia	tilapia	Tilapia	2	kept
tilt table test	tilt table test	tilt table test	tilt table test	tilt table test	2	kept
tilting	tilting	tilting	tilting	tilting	2	kept
Time and motion studies	time and motion studies	time and motion studies	time and motion study	Time and Motion Studies	2	singular
Time domain analysis	time domain analysis	time domain analysis	time domain analysis	Time domain analysis	2	kept
Time factors	time factors	time factors	time factor	Time Factors	2	singular
time in bed	time in bed	time in bed	time in bed	time in bed	2	kept
Time motions	time motions	time motions	time motions	Time motions	2	kept
time of admission	time of admission	time of admission	time of admission	time of admission	2	kept
time of birth	time of birth	time of birth	time of birth	time of birth	2	kept
time out	time out	time out	time out	time out	2	kept
time series	time series	time series	time series	Time series	2	case_or_format
Time spent	time spent	time spent	time spent	Time spent	2	kept
TIME TRENDS	time trends	time trends	time trend	TIME TRENDS	2	singular

time-efficiency	time efficiency	time efficiency	time efficiency	time-efficiency	2	kept
Time-motion study	time motion study	time motion study	time motion study	Time-motion study	2	kept
time-motion study	time motion study	time motion study	time motion study	Time-motion study	2	case_or_format
Timeliness	timeliness	timeliness	timeliness	timeliness	2	case_or_format
timolol maleate	timolol maleate	timolol maleate	timolol maleate	timolol maleate	2	kept
tirilazad	tirilazad	tirilazad	tirilazad	tirilazad	2	kept
tirofiban	tirofiban	tirofiban	tirofiban	tirofiban	2	kept
tisagenlecleucel	tisagenlecleucel	tisagenlecleucel	tisagenlecleucel	tisagenlecleucel	2	kept
Tissue	tissue	tissue	tissue	Tissue	2	kept
Tissue Adhesions	tissue adhesions	tissue adhesions	tissue adhesions	Tissue Adhesions	2	kept
Tissue donation	tissue donation	tissue donation	tissue donation	Tissue donation	2	kept
Tissue donor	tissue donor	tissue donor	tissue donor	Tissue Donors	2	case_or_format
tissue reaction	tissue reaction	tissue reaction	tissue reaction	tissue reaction	2	kept
tissue regeneration	tissue regeneration	tissue regeneration	tissue regeneration	tissue regeneration	2	kept
tissue survival	tissue survival	tissue survival	tissue survival	tissue survival	2	kept
tissue viability	tissue viability	tissue viability	tissue viability	tissue viability	2	kept

Titration	titration	titration	titration	Titration	2	kept
Titrimetry	titrimetry	titrimetry	titrimetry	titrimetry	2	case_or_format
TO-SKIN CONTACT	to skin contact	to skin contact	to skin contact	TO-SKIN CONTACT	2	kept
tobacco	tobacco	tobacco	tobacco	tobacco	2	kept
tobacco consumption	tobacco consumption	tobacco consumption	tobacco consumption	tobacco consumption	2	kept
Tobacco control	tobacco control	tobacco control	tobacco control	Tobacco control	2	kept
Tobacco Use Cessation Devices	tobacco use cessation devices	tobacco use cessation devices	tobacco use cessation devices	Tobacco Use Cessation Devices	2	kept
Tobacco-control	tobacco control	tobacco control	tobacco control	Tobacco control	2	case_or_format
toileting	toileting	toileting	toileting	toileting	2	kept
Tomography, Emission-Computed, Single-Photon	tomography emission computed single photon	tomography emission computed single photon	tomography emission computed single photon	Tomography, Emission-Computed, Single-Photon	2	kept
Tool development	tool development	tool development	tool development	tool development	2	case_or_format
TOOLS	tools	tools	tool	TOOL	2	singular
tooth calculus	tooth calculus	tooth calculus	tooth calculus	tooth calculus	2	kept
tooth crown	tooth crown	tooth crown	tooth crown	tooth crown	2	kept
Toothpastes	toothpastes	toothpastes	toothpaste	toothpaste	2	singular
Topic Modeling	topic modeling	topic modeling	topic modeling	Topic Modeling	2	kept

topiramate	topiramate	topiramate	topiramate	topiramate	2	kept
TOPSIS	topsis	topsis	topsis	TOPSIS	2	kept
TORCH	torch	torch	torch	TORCH	2	kept
Torticollis	torticollis	torticollis	torticollis	Torticollis	2	kept
total arthroplasty	total arthroplasty	total arthroplasty	total arthroplasty	total arthroplasty	2	kept
total artificial heart	total artificial heart	total artificial heart	total artificial heart	Total artificial heart	2	case_or_format
total body water	total body water	total body water	total body water	total body water	2	kept
total distance traveled	total distance traveled	total distance traveled	total distance traveled	total distance traveled	2	kept
total hip arthroplasty	total hip arthroplasty	total hip arthroplasty	total hip arthroplasty	Total hip arthroplasty	2	case_or_format
total joint replacement	total joint replacement	total joint replacement	total joint replacement	total joint replacement	2	kept
toxic hepatitis	toxic hepatitis	toxic hepatitis	toxic hepatitis	toxic hepatitis	2	kept
Toxic megacolon	toxic megacolon	toxic megacolon	toxic megacolon	toxic megacolon	2	case_or_format
toxic shock syndrome toxin 1	toxic shock syndrome toxin 1	toxic shock syndrome toxin 1	toxic shock syndrome toxin 1	toxic shock syndrome toxin 1	2	kept
toxic stress	toxic stress	toxic stress	toxic stress	toxic stress	2	kept
toxic substance	toxic substance	toxic substance	toxic substance	toxic substance	2	kept
toxicity testing	toxicity testing	toxicity testing	toxicity testing	toxicity testing	2	kept

toxin	toxin	toxin	toxin	toxin	2	kept
tPA	tpa	tpa	tpa	tPA	2	kept
TQIP	tqip	tqip	tqip	TQIP	2	kept
Trace Elements	trace elements	trace elements	trace element	trace element	2	singular
tracer	tracer	tracer	tracer	tracer	2	kept
Tracheal cannula	tracheal cannula	tracheal cannula	tracheal cannula	Tracheal cannula	2	kept
tracheal intubation and extubation	tracheal intubation and extubation	tracheal intubation and extubation	tracheal intubation and extubation	tracheal intubation and extubation	2	kept
Tracheal secretions	tracheal secretions	tracheal secretions	tracheal secretions	Tracheal secretions	2	kept
Tracheal suctioning	tracheal suctioning	tracheal suctioning	tracheal suctioning	Tracheal suctioning	2	kept
Tracheoesophageal fistula	tracheoesophageal fistula	tracheoesophageal fistula	tracheoesophageal fistula	tracheoesophageal fistula	2	case_or_format
TRACHEOSTOMY	tracheostomy	tracheostomy	tracheostomy	tracheostomy	2	case_or_format
tracheostomy care	tracheostomy care	tracheostomy care	tracheostomy care	Tracheostomy care	2	case_or_format
Tracheostomy complications	tracheostomy complications	tracheostomy complications	tracheostomy complications	Tracheostomy complications	2	kept
track and trigger system	track and trigger system	track and trigger system	track and trigger system	track and trigger system	2	kept
traction therapy	traction therapy	traction therapy	traction therapy	traction therapy	2	kept
traditional Chinese culture	traditional chinese culture	traditional chinese culture	traditional chinese culture	traditional Chinese culture	2	kept

Traditional Chinese Medicine	traditional chinese medicine	traditional chinese medicine	traditional chinese medicine	Traditional Chinese Medicine	2	kept
traditional medicine	traditional medicine	traditional medicine	traditional medicine	traditional medicine	2	kept
trail making test	trail making test	trail making test	trail making test	trail making test	2	kept
Train-of-four	train of four	train of four	train of four	train of four	2	case_or_format
Trainee	trainee	trainee	trainee	Trainee	2	kept
trainees	trainees	trainees	trainee	Trainee	2	singular
Training and education	training and education	training and education	training and education	training and education	2	case_or_format
Training demands	training demands	training demands	training demand	Training demands	2	singular
Training improvement	training improvement	training improvement	training improvement	Training improvement	2	kept
Training reflections	training reflections	training reflections	training reflections	Training reflections	2	kept
trajectory	trajectory	trajectory	trajectory	Trajectory	2	case_or_format
Tranexamic acid	tranexamic acid	tranexamic acid	tranexamic acid	tranexamic acid	2	case_or_format
trans-sectoral cooperation	trans sectoral cooperation	trans sectoral cooperation	trans sectoral cooperation	trans-sectoral cooperation	2	kept
transactional leadership	transactional leadership	transactional leadership	transactional leadership	transactional leadership	2	kept
Transcatheter aortic valve replacement	transcatheter aortic valve replacement	transcatheter aortic valve replacement	transcatheter aortic valve replacement	Transcatheter Aortic Valve Replacement	2	case_or_format
Transcranial Direct Current Stimulation	transcranial direct current stimulation	transcranial direct current stimulation	transcranial direct current stimulation	Transcranial Direct Current Stimulation	2	kept

transcranial direct current stimulation	transcranial direct current stimulation	transcranial direct current stimulation	transcranial direct current stimulation	Transcranial Direct Current Stimulation	2	case_or_format
Transcranial Magnetic Stimulation	transcranial magnetic stimulation	transcranial magnetic stimulation	transcranial magnetic stimulation	Transcranial Magnetic Stimulation	2	kept
transcription initiation	transcription initiation	transcription initiation	transcription initiation	transcription initiation	2	kept
transcriptomics	transcriptomics	transcriptomics	transcriptomics	transcriptomics	2	kept
transcutaneous carbon dioxide monitoring	transcutaneous carbon dioxide monitoring	transcutaneous carbon dioxide monitoring	transcutaneous carbon dioxide monitoring	transcutaneous carbon dioxide monitoring	2	kept
transcutaneous electrical nerve stimulation	transcutaneous electrical nerve stimulation	transcutaneous electrical nerve stimulation	transcutaneous electrical nerve stimulation	transcutaneous electrical nerve stimulation	2	kept
transdisciplinary research	transdisciplinary research	transdisciplinary research	transdisciplinary research	transdisciplinary research	2	kept
transepidermal water loss	transepidermal water loss	transepidermal water loss	transepidermal water loss	transepidermal water loss	2	kept
Transfer Agreement	transfer agreement	transfer agreement	transfer agreement	Transfer Agreement	2	kept
transfer anxiety	transfer anxiety	transfer anxiety	transfer anxiety	Transfer anxiety	2	case_or_format
transfer home	transfer home	transfer home	transfer home	transfer home	2	kept
Transfer of care	transfer of care	transfer of care	transfer of care	transfer of care	2	case_or_format
Transfer time	transfer time	transfer time	transfer time	Transfer time	2	kept
transfers	transfers	transfers	transfer	Transfer	2	singular
Transformation	transformation	transformation	transformation	Transformation	2	kept
TRANSFORMATIONAL LEADERSHIP	transformational leadership	transformational leadership	transformational leadership	Transformational leadership	2	case_or_format

TRANSFUSION	transfusion	transfusion	transfusion	transfusion	2	case_or_format
Transfusion practice	transfusion practice	transfusion practice	transfusion practice	Transfusion practices	2	case_or_format
transfusion practices	transfusion practices	transfusion practices	transfusion practice	Transfusion practices	2	singular
transfusion reaction	transfusion reaction	transfusion reaction	transfusion reaction	Transfusion Reaction	2	case_or_format
transfusion related acute lung injury	transfusion related acute lung injury	transfusion related acute lung injury	transfusion related acute lung injury	transfusion related acute lung injury	2	kept
Transfusion safety	transfusion safety	transfusion safety	transfusion safety	Transfusion safety	2	kept
transfusion-associated circulatory overload	transfusion associated circulatory overload	transfusion associated circulatory overload	transfusion associated circulatory overload	transfusion-associated circulatory overload	2	kept
Transillumination	transillumination	transillumination	transillumination	Transillumination	2	kept
Transition after birth	transition after birth	transition after birth	transition after birth	Transition after birth	2	kept
transition into practice	transition into practice	transition into practice	transition into practice	transition into practice	2	kept
transition of care	transition of care	transition of care	transition of care	transition of care	2	kept
transition process	transition process	transition process	transition process	transition process	2	kept
Transitional object	transitional object	transitional object	transitional object	Transitional object	2	kept
transitional object	transitional object	transitional object	transitional object	Transitional object	2	case_or_format
Transitions in care	transitions in care	transitions in care	transitions in care	transitions in care	2	case_or_format
Transitions theory	transitions theory	transitions theory	transitions theory	Transitions theory	2	kept

translabyrinthine	translabyrinthine	translabyrinthine	translabyrinthine	translabyrinthine	2	kept
Translation and cross-cultural adaptation	translation and cross cultural adaptation	translation and cross cultural adaptation	translation and cross cultural adaptation	Translation and cross-cultural adaptation	2	kept
translational medical research	translational medical research	translational medical research	translational medical research	Translational Medical Research	2	case_or_format
Translocation, Genetic	translocation genetic	translocation genetic	translocation genetic	Translocation, Genetic	2	kept
transmembrane pressure	transmembrane pressure	transmembrane pressure	transmembrane pressure	transmembrane pressure	2	kept
TRANSMISSION DYNAMICS	transmission dynamics	transmission dynamics	transmission dynamics	TRANSMISSION DYNAMICS	2	kept
transmission electron microscopy	transmission electron microscopy	transmission electron microscopy	transmission electron microscopy	transmission electron microscopy	2	kept
transmission risk	transmission risk	transmission risk	transmission risk	transmission risk	2	kept
transnational supervision	transnational supervision	transnational supervision	transnational supervision	transnational supervision	2	kept
transparent dressing	transparent dressing	transparent dressing	transparent dressing	transparent dressing	2	kept
transplant surgeon	transplant surgeon	transplant surgeon	transplant surgeon	transplant surgeon	2	kept
TRANSPLANTATION	transplantation	transplantation	transplantation	transplantation	2	case_or_format
transplantation conditioning	transplantation conditioning	transplantation conditioning	transplantation conditioning	Transplantation Conditioning	2	case_or_format
TRANSPORT	transport	transport	transport	Transport	2	case_or_format
Transport medicine	transport medicine	transport medicine	transport medicine	Transport medicine	2	kept
transport medicine	transport medicine	transport medicine	transport medicine	Transport medicine	2	case_or_format

transportation	transportation	transportation	transportation	Transportation	2	case_or_format
transpulmonary thermodilution	transpulmonary thermodilution	transpulmonary thermodilution	transpulmonary thermodilution	transpulmonary thermodilution	2	kept
transsphenoidal hypophysectomy	transsphenoidal hypophysectomy	transsphenoidal hypophysectomy	transsphenoidal hypophysectomy	transsphenoidal hypophysectomy	2	kept
transvaginal echography	transvaginal echography	transvaginal echography	transvaginal echography	transvaginal echography	2	kept
Transversal Care Unit	transversal care unit	transversal care unit	transversal care unit	Transversal Care Unit	2	kept
transverse colon	transverse colon	transverse colon	transverse colon	transverse colon	2	kept
Trauma discharge	trauma discharge	trauma discharge	trauma discharge	Trauma discharge	2	kept
Trauma disposition	trauma disposition	trauma disposition	trauma disposition	Trauma disposition	2	kept
Trauma medicine	trauma medicine	trauma medicine	trauma medicine	Trauma medicine	2	kept
Trauma mortality	trauma mortality	trauma mortality	trauma mortality	Trauma mortality	2	kept
Trauma registry	trauma registry	trauma registry	trauma registry	Trauma registry	2	kept
Trauma surgeon	trauma surgeon	trauma surgeon	trauma surgeon	trauma surgeon	2	case_or_format
Trauma team	trauma team	trauma team	trauma team	Trauma team	2	kept
trauma-focused psychotherapy	trauma focused psychotherapy	trauma focused psychotherapy	trauma focused psychotherapy	trauma-focused psychotherapy	2	kept
Trauma-informed care	trauma informed care	trauma informed care	trauma informed care	trauma-informed care	2	case_or_format
Traumatic amputation	traumatic amputation	traumatic amputation	traumatic amputation	Traumatic amputation	2	kept

traumatic amputation	traumatic amputation	traumatic amputation	traumatic amputation	Traumatic amputation	2	case_or_format
Traumatic Brain Injuries	traumatic brain injuries	traumatic brain injuries	traumatic brain injury	traumatic brain injury	2	singular
Traumatic catheter insertion	traumatic catheter insertion	traumatic catheter insertion	traumatic catheter insertion	Traumatic catheter insertion	2	kept
Traumatic grief	traumatic grief	traumatic grief	traumatic grief	Traumatic grief	2	kept
Traumatic injury	traumatic injury	traumatic injury	traumatic injury	Traumatic injury	2	kept
traumatic injury	traumatic injury	traumatic injury	traumatic injury	Traumatic injury	2	case_or_format
TRAUMATIC STRESS	traumatic stress	traumatic stress	traumatic stress	TRAUMATIC STRESS	2	kept
traumatic stress	traumatic stress	traumatic stress	traumatic stress	TRAUMATIC STRESS	2	case_or_format
Treatment adherence	treatment adherence	treatment adherence	treatment adherence	Treatment adherence	2	kept
Treatment fidelity	treatment fidelity	treatment fidelity	treatment fidelity	Treatment fidelity	2	kept
treatment guideline	treatment guideline	treatment guideline	treatment guideline	treatment guideline	2	kept
treatment interference	treatment interference	treatment interference	treatment interference	Treatment interference	2	case_or_format
treatment limitation decision	treatment limitation decision	treatment limitation decision	treatment limitation decision	treatment limitation decision	2	kept
Treatment limitations	treatment limitations	treatment limitations	treatment limitation	Treatment limitation	2	singular
Treatment protocols	treatment protocols	treatment protocols	treatment protocol	treatment protocol	2	singular
Trends	trends	trends	trends	trends	2	case_or_format

triacylglycerol blood level	triacylglycerol blood level	triacylglycerol blood level	triacylglycerol blood level	triacylglycerol blood level	2	kept
triacylglycerol lipase blood level	triacylglycerol lipase blood level	triacylglycerol lipase blood level	triacylglycerol lipase blood level	triacylglycerol lipase blood level	2	kept
Triage decision-making	triage decision making	triage decision making	triage decision making	Triage decision-making	2	kept
triage protocol	triage protocol	triage protocol	triage protocol	triage protocol	2	kept
triage system	triage system	triage system	triage system	triage system	2	kept
trial of labor	trial of labor	trial of labor	trial of labor	trial of labor	2	kept
TRIALS	trials	trials	trial	TRIAL	2	singular
Trials	trials	trials	trial	TRIAL	2	singular
triamcinolone	triamcinolone	triamcinolone	triamcinolone	triamcinolone	2	kept
triazine derivative	triazine derivative	triazine derivative	triazine derivative	triazine derivative	2	kept
triazole derivative	triazole derivative	triazole derivative	triazole derivative	triazole derivative	2	kept
triclosan	triclosan	triclosan	triclosan	triclosan	2	kept
trigger	trigger	trigger	trigger	trigger	2	kept
Trigger tools	trigger tools	trigger tools	trigger tool	Trigger tools	2	singular
triggers	triggers	triggers	trigger	trigger	2	singular
Trimethoprim, Sulfamethoxazole Drug Combination	trimethoprim sulfamethoxazole drug combination	trimethoprim sulfamethoxazole drug combination	trimethoprim sulfamethoxazole drug combination	Trimethoprim, Sulfamethoxazole Drug Combination	2	kept

triplet pregnancy	triplet pregnancy	triplet pregnancy	triplet pregnancy	triplet pregnancy	2	kept
Triplets	triplets	triplets	triplets	triplets	2	case_or_format
Trismus	trismus	trismus	trismus	trismus	2	case_or_format
trisomy	trisomy	trisomy	trisomy	Trisomy	2	case_or_format
Trisomy 21	trisomy 21	trisomy 21	trisomy 21	Trisomy 21	2	kept
trisomy 21	trisomy 21	trisomy 21	trisomy 21	Trisomy 21	2	case_or_format
trophic feeding	trophic feeding	trophic feeding	trophic feeding	trophic feeding	2	kept
tropic climate	tropic climate	tropic climate	tropic climate	tropic climate	2	kept
Tropical Climate	tropical climate	tropical climate	tropical climate	Tropical Climate	2	kept
Tropical Medicine	tropical medicine	tropical medicine	tropical medicine	Tropical Medicine	2	kept
tropicamide	tropicamide	tropicamide	tropicamide	tropicamide	2	kept
trough concentration	trough concentration	trough concentration	trough concentration	trough concentration	2	kept
Truncated product of P-values	truncated product of p values	truncated product of p values	truncated product of p values	Truncated product of P-values	2	kept
Truncus Arteriosus, Persistent	truncus arteriosus persistent	truncus arteriosus persistent	truncus arteriosus persistent	Truncus Arteriosus, Persistent	2	kept
TRUST	trust	trust	trust	Trust	2	case_or_format
truth disclosure	truth disclosure	truth disclosure	truth disclosure	Truth Disclosure	2	case_or_format

TST	tst	tst	tst	TST	2	kept
TUBE	tube	tube	tube	tube	2	case_or_format
Tube feedings	tube feedings	tube feedings	tube feeding	Tube feeding	2	singular
tuberculin	tuberculin	tuberculin	tuberculin	tuberculin	2	kept
Tuberculin test	tuberculin test	tuberculin test	tuberculin test	tuberculin test	2	case_or_format
tuberculous meningitis	tuberculous meningitis	tuberculous meningitis	tuberculous meningitis	tuberculous meningitis	2	kept
Tubes	tubes	tubes	tube	tube	2	singular
tubes	tubes	tubes	tube	tube	2	singular
tularemia	tularemia	tularemia	tularemia	tularemia	2	kept
tumor	tumor	tumor	tumor	tumor	2	kept
tumor bleeding	tumor bleeding	tumor bleeding	tumor bleeding	tumor bleeding	2	kept
Tumor lysis syndrome	tumor lysis syndrome	tumor lysis syndrome	tumor lysis syndrome	Tumor Lysis Syndrome	2	case_or_format
tumor lysis syndrome	tumor lysis syndrome	tumor lysis syndrome	tumor lysis syndrome	Tumor Lysis Syndrome	2	case_or_format
tumor necrosis factor inhibitor	tumor necrosis factor inhibitor	tumor necrosis factor inhibitor	tumor necrosis factor inhibitor	tumor necrosis factor inhibitor	2	kept
tumor regression	tumor regression	tumor regression	tumor regression	tumor regression	2	kept
Tunisian	tunisian	tunisian	tunisian	Tunisian	2	kept

TURNOVER INTENTIONS	turnover intentions	turnover intentions	turnover intention	Turnover intention	2	singular
Turnover intentions	turnover intentions	turnover intentions	turnover intention	Turnover intention	2	singular
turnover intentions	turnover intentions	turnover intentions	turnover intention	Turnover intention	2	singular
Twin	twin	twin	twin	Twins	2	case_or_format
Twins, Monozygotic	twins monozygotic	twins monozygotic	twins monozygotic	Twins, Monozygotic	2	kept
Two-step principal component analysis	two step principal component analysis	two step principal component analysis	two step principal component analysis	Two-step principal component analysis	2	kept
tympanic temperature	tympanic temperature	tympanic temperature	tympanic temperature	tympanic temperature	2	kept
tympanometry	tympanometry	tympanometry	tympanometry	tympanometry	2	kept
Type A aortic dissection	type a aortic dissection	type a aortic dissection	type a aortic dissection	type A aortic dissection	2	case_or_format
Typhoid Fever	typhoid fever	typhoid fever	typhoid fever	Typhoid Fever	2	kept
typhoid fever	typhoid fever	typhoid fever	typhoid fever	Typhoid Fever	2	case_or_format
ubidecarenone	ubidecarenone	ubidecarenone	ubidecarenone	ubidecarenone	2	kept
UDI-6	udi 6	udi 6	udi 6	UDI-6	2	kept
ULCER	ulcer	ulcer	ulcer	ulcer	2	case_or_format
ULCER DEVELOPMENT	ulcer development	ulcer development	ulcer development	ULCER DEVELOPMENT	2	kept
ULCER PREVALENCE	ulcer prevalence	ulcer prevalence	ulcer prevalence	ULCER PREVALENCE	2	kept

ulnar nerve	ulnar nerve	ulnar nerve	ulnar nerve	ulnar nerve	2	kept
ultra-wideband	ultra wideband	ultra wideband	ultra wideband	ultra-wideband	2	kept
Ultrasonography, Doppler, Duplex	ultrasonography doppler duplex	ultrasonography doppler duplex	ultrasonography doppler duplex	Ultrasonography, Doppler, Duplex	2	kept
Ultrasound-guided regional anestheia	ultrasound guided regional anestheia	ultrasound guided regional anestheia	ultrasound guided regional anestheia	Ultrasound-guided regional anestheia	2	kept
ultrastructure	ultrastructure	ultrastructure	ultrastructure	ultrastructure	2	kept
ultraviolet C radiation	ultraviolet c radiation	ultraviolet c radiation	ultraviolet c radiation	ultraviolet C radiation	2	kept
ultraviolet disinfection	ultraviolet disinfection	ultraviolet disinfection	ultraviolet disinfection	ultraviolet disinfection	2	kept
ultraviolet radiation	ultraviolet radiation	ultraviolet radiation	ultraviolet radiation	ultraviolet radiation	2	kept
Ultraviolet Rays	ultraviolet rays	ultraviolet rays	ultraviolet rays	Ultraviolet Rays	2	kept
umbilical artery catheterization	umbilical artery catheterization	umbilical artery catheterization	umbilical artery catheterization	umbilical artery catheterization	2	kept
Umbrella review	umbrella review	umbrella review	umbrella review	umbrella review	2	case_or_format
umbrella reviews	umbrella reviews	umbrella reviews	umbrella review	umbrella review	2	singular
uncertain prognosis	uncertain prognosis	uncertain prognosis	uncertain prognosis	uncertain prognosis	2	kept
UNCERTAINTY	uncertainty	uncertainty	uncertainty	Uncertainty	2	case_or_format
Uncompensated Care	uncompensated care	uncompensated care	uncompensated care	Uncompensated Care	2	kept
underdiagnosis	underdiagnosis	underdiagnosis	underdiagnosis	underdiagnosis	2	kept

Undergraduate education	undergraduate education	undergraduate education	undergraduate education	undergraduate education	2	case_or_format
underreporting	underreporting	underreporting	underreporting	underreporting	2	kept
Underserved	underserved	underserved	underserved	Underserved	2	kept
Understaffing	understaffing	understaffing	understaffing	Understaffing	2	kept
understaffing	understaffing	understaffing	understaffing	Understaffing	2	case_or_format
Undertriage	undertriage	undertriage	undertriage	Undertriage	2	kept
Unidad de cuidados críticos	unidad de cuidados cr ticos	unidad de cuidados cr ticos	unidad de cuidados cr ticos	Unidad de cuidados críticos	2	kept
Unidade de Terapia Intensiva Pediátrica	unidade de terapia intensiva pedi trica	unidade de terapia intensiva pedi trica	unidade de terapia intensiva pedi trica	Unidade de Terapia Intensiva Pediátrica	2	kept
Unidades de cuidado intensivo neonatal	unidades de cuidado intensivo neonatal	unidades de cuidado intensivo neonatal	unidades de cuidado intensivo neonatal	Unidades de Cuidado Intensivo Neonatal	2	case_or_format
Unidades de cuidado intensivo pediátrico	unidades de cuidado intensivo pedi trico	unidades de cuidado intensivo pedi trico	unidades de cuidado intensivo pedi trico	Unidades de Cuidado Intensivo Pediátrico	2	case_or_format
UNINTENDED CONSEQUENCES	unintended consequences	unintended consequences	unintended consequences	UNINTENDED CONSEQUENCES	2	kept
uniprost	uniprost	uniprost	uniprost	uniprost	2	kept
Unit operation decisions	unit operation decisions	unit operation decisions	unit operation decisions	Unit operation decisions	2	kept
United kingdom	united kingdom	united kingdom	united kingdom	United Kingdom	2	case_or_format
United States Agency for Healthcare Research and Quality	united states agency for healthcare research and quality	united states agency for healthcare research and quality	united states agency for healthcare research and quality	United States Agency for Healthcare Research and Quality	2	kept
UNITED-KINGDOM	united kingdom	united kingdom	united kingdom	United Kingdom	2	case_or_format

UNITED-STATES HOSPITALS	united states hospitals	united states hospitals	united states hospitals	UNITED-STATES HOSPITALS	2	kept
unité de soins intensifs	unit de soins intensifs	unit de soins intensifs	unit de soins intensifs	unité de soins intensifs	2	kept
Universal Health Care	universal health care	universal health care	universal health care	universal health care	2	case_or_format
universal health insurance	universal health insurance	universal health insurance	universal health insurance	universal health insurance	2	kept
universal newborn hearing screening	universal newborn hearing screening	universal newborn hearing screening	universal newborn hearing screening	universal newborn hearing screening	2	kept
UNIVERSITY	university	university	university	university	2	case_or_format
University of Missouri	university of missouri	university of missouri	university of missouri	University of Missouri	2	kept
Unobtrusive measure	unobtrusive measure	unobtrusive measure	unobtrusive measure	Unobtrusive measure	2	kept
Unplanned interruption rate	unplanned interruption rate	unplanned interruption rate	unplanned interruption rate	Unplanned interruption rate	2	kept
unplanned weaning	unplanned weaning	unplanned weaning	unplanned weaning	unplanned weaning	2	kept
Unstable angina	unstable angina	unstable angina	unstable angina	Unstable angina	2	kept
Unsupervised Machine Learning	unsupervised machine learning	unsupervised machine learning	unsupervised machine learning	Unsupervised Machine Learning	2	kept
unwillingness	unwillingness	unwillingness	unwillingness	unwillingness	2	kept
UPDATED METAANALYSIS	updated metaanalysis	updated metaanalysis	updated metaanalysis	UPDATED METAANALYSIS	2	kept
upper back pain	upper back pain	upper back pain	upper back pain	upper back pain	2	kept
upper extremity deep vein thrombosis	upper extremity deep vein thrombosis	upper extremity deep vein thrombosis	upper extremity deep vein thrombosis	Upper Extremity Deep Vein Thrombosis	2	case_or_format

upper gastrointestinal tract	upper gastrointestinal tract	upper gastrointestinal tract	upper gastrointestinal tract	Upper Gastrointestinal Tract	2	case_or_format
upper lip	upper lip	upper lip	upper lip	upper lip	2	kept
URBAN	urban	urban	urban	URBAN	2	kept
ureter	ureter	ureter	ureter	ureter	2	kept
urethra injury	urethra injury	urethra injury	urethra injury	urethra injury	2	kept
Urethral catheterization	urethral catheterization	urethral catheterization	urethral catheterization	urethral catheterization	2	case_or_format
urgence	urgence	urgence	urgence	urgence	2	kept
Urgencias	urgencias	urgencias	urgencias	Urgencias	2	kept
Urgency	urgency	urgency	urgency	Urgency	2	kept
Urgently field hospital	urgently field hospital	urgently field hospital	urgently field hospital	Urgently field hospital	2	kept
uric acid blood level	uric acid blood level	uric acid blood level	uric acid blood level	uric acid blood level	2	kept
Urinary catheters	urinary catheters	urinary catheters	urinary catheter	urinary catheter	2	singular
urinary dysfunction	urinary dysfunction	urinary dysfunction	urinary dysfunction	urinary dysfunction	2	kept
urinary incontinence	urinary incontinence	urinary incontinence	urinary incontinence	Urinary Incontinence	2	case_or_format
Urinary retention	urinary retention	urinary retention	urinary retention	Urinary Retention	2	case_or_format
urinary tract cancer	urinary tract cancer	urinary tract cancer	urinary tract cancer	urinary tract cancer	2	kept

urinary tract function	urinary tract function	urinary tract function	urinary tract function	urinary tract function	2	kept
URINARY-TRACT-INFECTIONS	urinary tract infections	urinary tract infections	urinary tract infection	urinary tract infection	2	singular
urine color	urine color	urine color	urine color	urine color	2	kept
urine level	urine level	urine level	urine level	urine level	2	kept
urine sediment	urine sediment	urine sediment	urine sediment	urine sediment	2	kept
Urochordata	urochordata	urochordata	urochordata	Urochordata	2	kept
urological care	urological care	urological care	urological care	urological care	2	kept
US HOSPITALS	us hospitals	us hospitals	us hospitals	US HOSPITALS	2	kept
US-guided pericardiocentesis	us guided pericardiocentesis	us guided pericardiocentesis	us guided pericardiocentesis	US-guided pericardiocentesis	2	kept
usability questionnaire	usability questionnaire	usability questionnaire	usability questionnaire	usability questionnaire	2	kept
usability test	usability test	usability test	usability test	usability test	2	kept
usefulness	usefulness	usefulness	usefulness	usefulness	2	kept
User computer interface	user computer interface	user computer interface	user computer interface	User-Computer Interface	2	case_or_format
User engagement	user engagement	user engagement	user engagement	User engagement	2	kept
user interface	user interface	user interface	user interface	User interfaces	2	case_or_format
USER INTERFACES	user interfaces	user interfaces	user interface	User interfaces	2	singular

user interfaces	user interfaces	user interfaces	user interface	User interfaces	2	singular
user perception	user perception	user perception	user perception	user perception	2	kept
User requirement analysis	user requirement analysis	user requirement analysis	user requirement analysis	User requirement analysis	2	kept
User test	user test	user test	user test	User test	2	kept
User-computer interfaces	user computer interfaces	user computer interfaces	user computer interface	User-Computer Interface	2	singular
User-friendliness	user friendliness	user friendliness	user friendliness	User-friendliness	2	kept
uterine cervix	uterine cervix	uterine cervix	uterine cervix	uterine cervix	2	kept
uterine cervix ripening	uterine cervix ripening	uterine cervix ripening	uterine cervix ripening	uterine cervix ripening	2	kept
Uterine Rupture	uterine rupture	uterine rupture	uterine rupture	Uterine Rupture	2	kept
Uterus	uterus	uterus	uterus	Uterus	2	kept
uterus	uterus	uterus	uterus	Uterus	2	case_or_format
uterus blood flow	uterus blood flow	uterus blood flow	uterus blood flow	uterus blood flow	2	kept
uterus cancer	uterus cancer	uterus cancer	uterus cancer	uterus cancer	2	kept
uterus inversion	uterus inversion	uterus inversion	uterus inversion	uterus inversion	2	kept
uterus spasmolytic agent	uterus spasmolytic agent	uterus spasmolytic agent	uterus spasmolytic agent	uterus spasmolytic agent	2	kept
UTI	uti	uti	uti	UTI	2	kept

Utrecht Work Engagement Scale	utrecht work engagement scale	utrecht work engagement scale	utrecht work engagement scale	Utrecht Work Engagement Scale	2	kept
Uttarakhand	uttarakhand	uttarakhand	uttarakhand	Uttarakhand	2	kept
Uveitis	uveitis	uveitis	uveitis	Uveitis	2	kept
Uvula	uvula	uvula	uvula	Uvula	2	kept
uvulopalatopharyngoplasty	uvulopalatopharyngoplasty	uvulopalatopharyngoplasty	uvulopalatopharyngoplasty	uvulopalatopharyngoplasty	2	kept
UWB	uwb	uwb	uwb	UWB	2	kept
Vaccination coverage	vaccination coverage	vaccination coverage	vaccination coverage	vaccination coverage	2	case_or_format
Vaccination Refusal	vaccination refusal	vaccination refusal	vaccination refusal	vaccination refusal	2	case_or_format
Vaccination strategy	vaccination strategy	vaccination strategy	vaccination strategy	Vaccination strategy	2	kept
vaccine effectiveness	vaccine effectiveness	vaccine effectiveness	vaccine effectiveness	vaccine effectiveness	2	kept
vaccine preventable disease	vaccine preventable disease	vaccine preventable disease	vaccine preventable disease	vaccine preventable disease	2	kept
Vaccine uptake	vaccine uptake	vaccine uptake	vaccine uptake	Vaccine uptake	2	kept
vaccine uptake	vaccine uptake	vaccine uptake	vaccine uptake	Vaccine uptake	2	case_or_format
VACTE	vacte	vacte	vacte	VACTE	2	kept
vacuum	vacuum	vacuum	vacuum	vacuum	2	kept
VAGINAL PROLAPSE SURGERY	vaginal prolapse surgery	vaginal prolapse surgery	vaginal prolapse surgery	VAGINAL PROLAPSE SURGERY	2	kept

Validation studies as topic	validation studies as topic	validation studies as topic	validation studies as topic	Validation Studies as Topic	2	case_or_format
validation studies as topic	validation studies as topic	validation studies as topic	validation studies as topic	Validation Studies as Topic	2	case_or_format
Validez	validez	validez	validez	Validez	2	kept
Validity and reliability	validity and reliability	validity and reliability	validity and reliability	Validity and reliability	2	kept
Validity evidence	validity evidence	validity evidence	validity evidence	Validity evidence	2	kept
valorization	valorization	valorization	valorization	valorization	2	kept
Valproic acid	valproic acid	valproic acid	valproic acid	valproic acid	2	case_or_format
valsartan	valsartan	valsartan	valsartan	valsartan	2	kept
value congruence	value congruence	value congruence	value congruence	value congruence	2	kept
Value of information analysis	value of information analysis	value of information analysis	value of information analysis	Value of information analysis	2	kept
valued	valued	valued	valued	valued	2	kept
Values	values	values	value	value	2	singular
Values affecting end-of-life care	values affecting end of life care	values affecting end of life care	values affecting end of life care	Values affecting end-of-life care	2	kept
valvuloplasty	valvuloplasty	valvuloplasty	valvuloplasty	valvuloplasty	2	kept
Vancomycin-resistant Enterococcus	vancomycin resistant enterococcus	vancomycin resistant enterococcus	vancomycin resistant enterococcus	vancomycin resistant Enterococcus	2	case_or_format
VAP bundle	vap bundle	vap bundle	vap bundle	VAP bundle	2	kept

vaping	vaping	vaping	vaping	vaping	2	kept
Variant of concern	variant of concern	variant of concern	variant of concern	Variant of concern	2	kept
variant of concern	variant of concern	variant of concern	variant of concern	Variant of concern	2	case_or_format
Variations	variations	variations	variation	Variations	2	singular
varicella	varicella	varicella	varicella	Varicella	2	case_or_format
VAS	vas	vas	vas	VAS	2	kept
vasa previa	vasa previa	vasa previa	vasa previa	vasa previa	2	kept
Vascular	vascular	vascular	vascular	Vascular	2	kept
Vascular Access Team	vascular access team	vascular access team	vascular access team	Vascular Access Team	2	kept
vascular access team	vascular access team	vascular access team	vascular access team	Vascular Access Team	2	case_or_format
vascular complication	vascular complication	vascular complication	vascular complication	vascular complication	2	kept
Vascular Devices	vascular devices	vascular devices	vascular device	Vascular Devices	2	singular
vascular endothelial function	vascular endothelial function	vascular endothelial function	vascular endothelial function	vascular endothelial function	2	kept
Vascular Endothelial Growth Factor A	vascular endothelial growth factor a	vascular endothelial growth factor a	vascular endothelial growth factor a	Vascular Endothelial Growth Factor A	2	kept
vascular endothelium	vascular endothelium	vascular endothelium	vascular endothelium	vascular endothelium	2	kept
Vasculitis, Leukocytoclastic, Cutaneous	vasculitis leukocytoclastic cutaneous	vasculitis leukocytoclastic cutaneous	vasculitis leukocytoclastic cutaneous	Vasculitis, Leukocytoclastic, Cutaneous	2	kept

Vasoactive drug	vasoactive drug	vasoactive drug	vasoactive drug	Vasoactive drugs	2	case_or_format
vasoactive inotropic score	vasoactive inotropic score	vasoactive inotropic score	vasoactive inotropic score	vasoactive inotropic score	2	kept
Vasomotor System	vasomotor system	vasomotor system	vasomotor system	Vasomotor System	2	kept
vasoplegia	vasoplegia	vasoplegia	vasoplegia	vasoplegia	2	kept
vasopressin receptor	vasopressin receptor	vasopressin receptor	vasopressin receptor	vasopressin receptor	2	kept
vasopressin release	vasopressin release	vasopressin release	vasopressin release	vasopressin release	2	kept
vasopressor	vasopressor	vasopressor	vasopressor	Vasopressor	2	case_or_format
VATS	vats	vats	vat	VATS	2	singular
Vecuronium Bromide	vecuronium bromide	vecuronium bromide	vecuronium bromide	Vecuronium Bromide	2	kept
vegetable	vegetable	vegetable	vegetable	vegetable	2	kept
vegetative state	vegetative state	vegetative state	vegetative state	Vegetative state	2	case_or_format
vein perforation	vein perforation	vein perforation	vein perforation	vein perforation	2	kept
vein surgery	vein surgery	vein surgery	vein surgery	vein surgery	2	kept
vena cava filter	vena cava filter	vena cava filter	vena cava filter	Vena Cava Filters	2	case_or_format
Venezuela	venezuela	venezuela	venezuela	Venezuela	2	kept
Venezuelan	venezuelan	venezuelan	venezuelan	Venezuelan	2	kept

Veno-arterial extracorporeal membrane oxygenation	veno arterial extracorporeal membrane oxygenation	veno arterial extracorporeal membrane oxygenation	veno arterial extracorporeal membrane oxygenation	Veno-arterial extracorporeal membrane oxygenation	2	kept
venous access team	venous access team	venous access team	venous access team	venous access team	2	kept
Venous catheterization	venous catheterization	venous catheterization	venous catheterization	Venous catheterization	2	kept
Venous oxygen saturation	venous oxygen saturation	venous oxygen saturation	venous oxygen saturation	Venous oxygen saturation	2	kept
venous return	venous return	venous return	venous return	venous return	2	kept
Venous thromboembolic disease	venous thromboembolic disease	venous thromboembolic disease	venous thromboembolic disease	Venous thromboembolic disease	2	kept
Venovenous extracorporeal membrane oxygenation	venovenous extracorporeal membrane oxygenation	venovenous extracorporeal membrane oxygenation	venovenous extracorporeal membrane oxygenation	venovenous extracorporeal membrane oxygenation	2	case_or_format
Ventilación mecánica	ventilaci n mec nica	ventilaci n mec nica	ventilaci n mec nica	Ventilación mecánica	2	kept
ventilated	ventilated	ventilated	ventilated	Ventilated	2	case_or_format
Ventilation system	ventilation system	ventilation system	ventilation system	Ventilation system	2	kept
Ventilation-Perfusion Ratio	ventilation perfusion ratio	ventilation perfusion ratio	ventilation perfusion ratio	Ventilation-Perfusion Ratio	2	kept
Ventilator care bundle	ventilator care bundle	ventilator care bundle	ventilator care bundle	Ventilator care bundle	2	kept
Ventilator days	ventilator days	ventilator days	ventilator days	Ventilator days	2	kept
Ventilator liberation	ventilator liberation	ventilator liberation	ventilator liberation	Ventilator liberation	2	kept
ventilator waveform	ventilator waveform	ventilator waveform	ventilator waveform	ventilator waveform	2	kept
Ventilator-associated event	ventilator associated event	ventilator associated event	ventilator associated event	Ventilator-associated events	2	case_or_format

ventilator-associated events	ventilator associated events	ventilator associated events	ventilator associated event	Ventilator-associated events	2	singular
Ventilator-Associated pneumonia	ventilator associated pneumonia	ventilator associated pneumonia	ventilator associated pneumonia	ventilator associated pneumonia	2	case_or_format
ventilator-Associated pneumonia	ventilator associated pneumonia	ventilator associated pneumonia	ventilator associated pneumonia	ventilator associated pneumonia	2	case_or_format
Ventilator-associated pneumonia bundle	ventilator associated pneumonia bundle	ventilator associated pneumonia bundle	ventilator associated pneumonia bundle	Ventilator-associated pneumonia bundle	2	kept
ventilators, mechanical	ventilators mechanical	ventilators mechanical	ventilators mechanical	Ventilators, Mechanical	2	case_or_format
Ventilatory performance	ventilatory performance	ventilatory performance	ventilatory performance	Ventilatory performance	2	kept
Ventilatory weaning	ventilatory weaning	ventilatory weaning	ventilatory weaning	Ventilatory weaning	2	kept
verbal abuse	verbal abuse	verbal abuse	verbal abuse	verbal abuse	2	kept
Verbal communication	verbal communication	verbal communication	verbal communication	verbal communication	2	case_or_format
Verbal communications	verbal communications	verbal communications	verbal communication	verbal communication	2	singular
Verbal rating scale	verbal rating scale	verbal rating scale	verbal rating scale	Verbal rating scale	2	kept
Verbal violence	verbal violence	verbal violence	verbal violence	Verbal violence	2	kept
verbalization	verbalization	verbalization	verbalization	verbalization	2	kept
very low birth weight infant	very low birth weight infant	very low birth weight infant	very low birth weight infant	Very low birth weight infant	2	case_or_format
very low birth weight infants	very low birth weight infants	very low birth weight infants	very low birth weight infant	Very low birth weight infant	2	singular
very low birthweight infant	very low birthweight infant	very low birthweight infant	very low birthweight infant	very low birthweight infant	2	kept

very old	very old	very old	very old	very old	2	kept
Very preterm birth	very preterm birth	very preterm birth	very preterm birth	Very preterm birth	2	kept
vestibular	vestibular	vestibular	vestibular	vestibular	2	kept
vestibular schwannoma	vestibular schwannoma	vestibular schwannoma	vestibular schwannoma	vestibular schwannoma	2	kept
vestibular stimulation	vestibular stimulation	vestibular stimulation	vestibular stimulation	vestibular stimulation	2	kept
Veteran	veteran	veteran	veteran	Veterans	2	case_or_format
veterinary technicians	veterinary technicians	veterinary technicians	veterinary technician	veterinary technicians	2	singular
Vial contamination	vial contamination	vial contamination	vial contamination	Vial contamination	2	kept
Vibration technique	vibration technique	vibration technique	vibration technique	Vibration technique	2	kept
Vibrotactile display	vibrotactile display	vibrotactile display	vibrotactile display	Vibrotactile display	2	kept
Vibrotactile displays	vibrotactile displays	vibrotactile displays	vibrotactile display	Vibrotactile display	2	singular
vidarabine	vidarabine	vidarabine	vidarabine	vidarabine	2	kept
video	video	video	video	Video	2	case_or_format
video game	video game	video game	video game	Video Games	2	case_or_format
video telemedicine	video telemedicine	video telemedicine	video telemedicine	video telemedicine	2	kept
video-assisted techniques	video assisted techniques	video assisted techniques	video assisted techniques	video-assisted techniques	2	kept

video-messaging service	video messaging service	video messaging service	video messaging service	video-messaging service	2	kept
Video-recording	video recording	video recording	video recording	Video Recording	2	case_or_format
Videodisc Recording	videodisc recording	videodisc recording	videodisc recording	Videodisc Recording	2	kept
videolaryngoscope	videolaryngoscope	videolaryngoscope	videolaryngoscope	videolaryngoscope	2	kept
videorecorder	videorecorder	videorecorder	videorecorder	videorecorder	2	kept
Vienna Protocol for Neonatal Pain and Sedation	vienna protocol for neonatal pain and sedation	vienna protocol for neonatal pain and sedation	vienna protocol for neonatal pain and sedation	Vienna Protocol for Neonatal Pain and Sedation	2	kept
Views	views	views	views	VIEWS	2	case_or_format
VIGILANCE	vigilance	vigilance	vigilance	VIGILANCE	2	kept
Vigilance	vigilance	vigilance	vigilance	VIGILANCE	2	case_or_format
Vignettes	vignettes	vignettes	vignette	vignette	2	singular
vinblastine	vinblastine	vinblastine	vinblastine	vinblastine	2	kept
Vinyl Compounds	vinyl compounds	vinyl compounds	vinyl compounds	Vinyl Compounds	2	kept
Viral Envelope Proteins	viral envelope proteins	viral envelope proteins	viral envelope proteins	Viral Envelope Proteins	2	kept
Viral load	viral load	viral load	viral load	Viral Load	2	case_or_format
Virtual	virtual	virtual	virtual	Virtual	2	kept
virtual	virtual	virtual	virtual	Virtual	2	case_or_format

Virtual bronchoscopy	virtual bronchoscopy	virtual bronchoscopy	virtual bronchoscopy	Virtual bronchoscopy	2	kept
virtual education	virtual education	virtual education	virtual education	Virtual education	2	case_or_format
virtual environments	virtual environments	virtual environments	virtual environment	virtual environments	2	singular
Virtual glasses	virtual glasses	virtual glasses	virtual glasses	Virtual glasses	2	kept
virtual medicine	virtual medicine	virtual medicine	virtual medicine	virtual medicine	2	kept
Virtual Reality Exposure Therapy	virtual reality exposure therapy	virtual reality exposure therapy	virtual reality exposure therapy	Virtual Reality Exposure Therapy	2	kept
Virtual reality simulation	virtual reality simulation	virtual reality simulation	virtual reality simulation	virtual reality simulation	2	case_or_format
virtual reflection group	virtual reflection group	virtual reflection group	virtual reflection group	virtual reflection group	2	kept
virtual resus room	virtual resus room	virtual resus room	virtual resus room	virtual resus room	2	kept
Virtual Trial	virtual trial	virtual trial	virtual trial	Virtual Trial	2	kept
Virtues	virtues	virtues	virtues	Virtues	2	kept
virtues	virtues	virtues	virtues	Virtues	2	case_or_format
Virus	virus	virus	virus	Virus	2	kept
virus	virus	virus	virus	Virus	2	case_or_format
virus culture	virus culture	virus culture	virus culture	virus culture	2	kept
virus diagnosis	virus diagnosis	virus diagnosis	virus diagnosis	virus diagnosis	2	kept

virus DNA	virus dna	virus dna	virus dna	virus DNA	2	kept
virus genome	virus genome	virus genome	virus genome	virus genome	2	kept
virus mutation	virus mutation	virus mutation	virus mutation	virus mutation	2	kept
virus myocarditis	virus myocarditis	virus myocarditis	virus myocarditis	virus myocarditis	2	kept
virus pathogenesis	virus pathogenesis	virus pathogenesis	virus pathogenesis	virus pathogenesis	2	kept
virus reactivation	virus reactivation	virus reactivation	virus reactivation	virus reactivation	2	kept
virus shedding	virus shedding	virus shedding	virus shedding	virus shedding	2	kept
Viscosity	viscosity	viscosity	viscosity	Viscosity	2	kept
viscosity	viscosity	viscosity	viscosity	Viscosity	2	case_or_format
Vision, Ocular	vision ocular	vision ocular	vision ocular	Vision, Ocular	2	kept
Visitas a pacientes	visitas a pacientes	visitas a pacientes	visitas a pacientes	Visitas a pacientes	2	kept
VISITATION	visitation	visitation	visitation	Visitation	2	case_or_format
visitation policy	visitation policy	visitation policy	visitation policy	Visitation policy	2	case_or_format
visiting policies	visiting policies	visiting policies	visiting policy	Visiting policy	2	singular
visiting restriction	visiting restriction	visiting restriction	visiting restriction	visiting restriction	2	kept
Visiting restrictions	visiting restrictions	visiting restrictions	visiting restriction	visiting restriction	2	singular

visual	visual	visual	visual	Visual	2	case_or_format
Visual analogue scale	visual analogue scale	visual analogue scale	visual analogue scale	Visual analogue scale	2	kept
Visual attention	visual attention	visual attention	visual attention	visual attention	2	case_or_format
visual evoked potential	visual evoked potential	visual evoked potential	visual evoked potential	visual evoked potential	2	kept
visual field	visual field	visual field	visual field	visual field	2	kept
visual information	visual information	visual information	visual information	visual information	2	kept
visual management	visual management	visual management	visual management	visual management	2	kept
Visualization of infection risk assessment	visualization of infection risk assessment	visualization of infection risk assessment	visualization of infection risk assessment	Visualization of infection risk assessment	2	kept
Vital Capacity	vital capacity	vital capacity	vital capacity	vital capacity	2	case_or_format
vital parameter	vital parameter	vital parameter	vital parameter	vital parameter	2	kept
Vital Sings	vital sings	vital sings	vital sings	Vital Sings	2	kept
Vitamin B 12 Deficiency	vitamin b 12 deficiency	vitamin b 12 deficiency	vitamin b 12 deficiency	Vitamin B 12 Deficiency	2	kept
Vitamin E	vitamin e	vitamin e	vitamin e	Vitamin E	2	kept
Vitis	vitis	vitis	vitis	Vitis	2	kept
VLBW infants	vlbw infants	vlbw infants	vlbw infant	VLBW infants	2	singular
vocabulary	vocabulary	vocabulary	vocabulary	vocabulary	2	kept

vocal cord disorder	vocal cord disorder	vocal cord disorder	vocal cord disorder	vocal cord disorder	2	kept
vocal cord immobility	vocal cord immobility	vocal cord immobility	vocal cord immobility	vocal cord immobility	2	kept
Vocal Cords	vocal cords	vocal cords	vocal cord	vocal cord	2	singular
Vocal soothing	vocal soothing	vocal soothing	vocal soothing	Vocal soothing	2	kept
voice behavior	voice behavior	voice behavior	voice behavior	voice behavior	2	kept
voice disorder	voice disorder	voice disorder	voice disorder	Voice Disorders	2	case_or_format
Voice disorders	voice disorders	voice disorders	voice disorder	Voice Disorders	2	singular
volatile agent	volatile agent	volatile agent	volatile agent	volatile agent	2	kept
Volume	volume	volume	volume	VOLUME	2	case_or_format
volume	volume	volume	volume	VOLUME	2	case_or_format
volume controlled ventilation	volume controlled ventilation	volume controlled ventilation	volume controlled ventilation	volume controlled ventilation	2	kept
volume flexibility strategies	volume flexibility strategies	volume flexibility strategies	volume flexibility strategies	volume flexibility strategies	2	kept
volume status	volume status	volume status	volume status	Volume status	2	case_or_format
volumetry	volumetry	volumetry	volumetry	volumetry	2	kept
von Willebrand factor	von willebrand factor	von willebrand factor	von willebrand factor	von Willebrand factor	2	kept
VR sickness symptoms	vr sickness symptoms	vr sickness symptoms	vr sickness symptoms	VR sickness symptoms	2	kept

VSD	vsd	vsd	vsd	VSD	2	kept
Vulnerable populations	vulnerable populations	vulnerable populations	vulnerable population	vulnerable population	2	singular
vulva	vulva	vulva	vulva	vulva	2	kept
waist hip ratio	waist hip ratio	waist hip ratio	waist hip ratio	waist hip ratio	2	kept
Waist-Hip Ratio	waist hip ratio	waist hip ratio	waist hip ratio	waist hip ratio	2	case_or_format
Waiting	waiting	waiting	waiting	Waiting	2	kept
Waiting list	waiting list	waiting list	waiting list	Waiting Lists	2	case_or_format
Wake	wake	wake	wake	Wake	2	kept
Walker and Avant's model	walker and avant s model	walker and avant s model	walker and avant s model	Walker and Avant's model	2	kept
walking aid	walking aid	walking aid	walking aid	walking aid	2	kept
walking distance	walking distance	walking distance	walking distance	walking distance	2	kept
Walking Speed	walking speed	walking speed	walking speed	walking speed	2	case_or_format
ward environment	ward environment	ward environment	ward environment	ward environment	2	kept
ward rounds	ward rounds	ward rounds	ward round	Ward rounds	2	singular
Warning indicator	warning indicator	warning indicator	warning indicator	Warning indicator	2	kept
Wart virus	wart virus	wart virus	wart virus	Wart virus	2	kept

Washing	washing	washing	washing	Washing	2	kept
Waste	waste	waste	waste	waste	2	case_or_format
wasting syndrome	wasting syndrome	wasting syndrome	wasting syndrome	wasting syndrome	2	kept
Watchful Waiting	watchful waiting	watchful waiting	watchful waiting	Watchful Waiting	2	kept
WATER	water	water	water	water	2	case_or_format
water contamination	water contamination	water contamination	water contamination	water contamination	2	kept
Water immersion	water immersion	water immersion	water immersion	Water immersion	2	kept
water intoxication	water intoxication	water intoxication	water intoxication	Water Intoxication	2	case_or_format
Water Loss, Insensible	water loss insensible	water loss insensible	water loss insensible	Water Loss, Insensible	2	kept
Water temperature	water temperature	water temperature	water temperature	Water temperature	2	kept
water vapor	water vapor	water vapor	water vapor	water vapor	2	kept
Water-electrolyte balance	water electrolyte balance	water electrolyte balance	water electrolyte balance	Water-Electrolyte Balance	2	case_or_format
waterbirth	waterbirth	waterbirth	waterbirth	waterbirth	2	kept
Waterborne transmission	waterborne transmission	waterborne transmission	waterborne transmission	Waterborne transmission	2	kept
Waterlow Scale	waterlow scale	waterlow scale	waterlow scale	Waterlow scale	2	case_or_format
Waterlow score	waterlow score	waterlow score	waterlow score	Waterlow score	2	kept

watery stool	watery stool	watery stool	watery stool	watery stool	2	kept
Watson's theory	watson s theory	watson s theory	watson s theory	Watson's theory	2	kept
Waveform analysis	waveform analysis	waveform analysis	waveform analysis	Waveform analysis	2	kept
Wean	wean	wean	wean	Wean	2	kept
wean	wean	wean	wean	Wean	2	case_or_format
Weaning failure	weaning failure	weaning failure	weaning failure	Weaning failure	2	kept
weaning failure	weaning failure	weaning failure	weaning failure	Weaning failure	2	case_or_format
Wear of materials	wear of materials	wear of materials	wear of materials	Wear of materials	2	kept
wearable	wearable	wearable	wearable	Wearable	2	case_or_format
Wearable computers	wearable computers	wearable computers	wearable computers	Wearable computers	2	kept
wearable devices	wearable devices	wearable devices	wearable device	Wearable devices	2	singular
wearable monitoring	wearable monitoring	wearable monitoring	wearable monitoring	wearable monitoring	2	kept
wearable sensor	wearable sensor	wearable sensor	wearable sensor	Wearable sensors	2	case_or_format
weather	weather	weather	weather	weather	2	kept
web browser	web browser	web browser	web browser	web browser	2	kept
Web services	web services	web services	web services	Web services	2	kept

web-based intervention	web based intervention	web based intervention	web based intervention	web-based intervention	2	kept
web-based survey	web based survey	web based survey	web based survey	web-based survey	2	kept
Web-based training	web based training	web based training	web based training	Web-based training	2	kept
web-based training	web based training	web based training	web based training	Web-based training	2	case_or_format
Webcam	webcam	webcam	webcam	Webcam	2	kept
Webcams	webcams	webcams	webcam	Webcam	2	singular
WEEKEND ADMISSION	weekend admission	weekend admission	weekend admission	WEEKEND ADMISSION	2	kept
Wegener granulomatosis	wegener granulomatosis	wegener granulomatosis	wegener granulomatosis	Wegener granulomatosis	2	kept
weight	weight	weight	weight	Weight	2	case_or_format
weight bias	weight bias	weight bias	weight bias	weight bias	2	kept
weight control	weight control	weight control	weight control	weight control	2	kept
weight lifting	weight lifting	weight lifting	weight lifting	weight lifting	2	kept
Weight loss	weight loss	weight loss	weight loss	Weight Loss	2	case_or_format
Weight-Bearing	weight bearing	weight bearing	weight bearing	weight bearing	2	case_or_format
weights and measures	weights and measures	weights and measures	weights and measures	Weights and Measures	2	case_or_format
Weka	weka	weka	weka	Weka	2	kept

Wells score	wells score	wells score	wells score	Wells score	2	kept
Wernicke encephalopathy	wernicke encephalopathy	wernicke encephalopathy	wernicke encephalopathy	Wernicke encephalopathy	2	kept
West Nile virus	west nile virus	west nile virus	west nile virus	West Nile virus	2	kept
West Shoa	west shoa	west shoa	west shoa	West Shoa	2	kept
Western Ethiopia	western ethiopia	western ethiopia	western ethiopia	Western Ethiopia	2	kept
wet work	wet work	wet work	wet work	wet work	2	kept
WGS	wgs	wgs	wgs	WGS	2	kept
wheelchair user	wheelchair user	wheelchair user	wheelchair user	wheelchair user	2	kept
Wheelchairs	wheelchairs	wheelchairs	wheelchair	Wheelchairs	2	singular
White	white	white	white	White	2	kept
white card test	white card test	white card test	white card test	white card test	2	kept
white matter lesion	white matter lesion	white matter lesion	white matter lesion	white matter lesion	2	kept
white muscle disease	white muscle disease	white muscle disease	white muscle disease	white muscle disease	2	kept
WHO, World Health Organization	who world health organization	who world health organization	who world health organization	WHO, World Health Organization	2	kept
whole body CT	whole body ct	whole body ct	whole body ct	whole body CT	2	kept
Whole genome sequencing	whole genome sequencing	whole genome sequencing	whole genome sequencing	whole genome sequencing	2	case_or_format

wilderness medicine	wilderness medicine	wilderness medicine	wilderness medicine	wilderness medicine	2	kept
willingness	willingness	willingness	willingness	willingness	2	kept
Willingness to participate	willingness to participate	willingness to participate	willingness to participate	Willingness to participate	2	kept
Willingness To Pay	willingness to pay	willingness to pay	willingness to pay	Willingness To Pay	2	kept
Willis-Ekbom syndrome	willis ekbom syndrome	willis ekbom syndrome	willis ekbom syndrome	Willis-Ekbom syndrome	2	kept
willpower	willpower	willpower	willpower	willpower	2	kept
wing	wing	wing	wing	wing	2	kept
Wireless body area network	wireless body area network	wireless body area network	wireless body area network	Wireless body area network	2	kept
Wireless local area networks	wireless local area networks	wireless local area networks	wireless local area networks	Wireless local area networks	2	kept
wireless sensor	wireless sensor	wireless sensor	wireless sensor	Wireless sensor	2	case_or_format
wireless technologies	wireless technologies	wireless technologies	wireless technology	Wireless Technology	2	singular
Wireless technology	wireless technology	wireless technology	wireless technology	Wireless Technology	2	case_or_format
Withdraw	withdraw	withdraw	withdraw	Withdraw	2	kept
Withdrawal and withholding of treatment	withdrawal and withholding of treatment	withdrawal and withholding of treatment	withdrawal and withholding of treatment	Withdrawal and withholding of treatment	2	kept
withdrawal assessment tool 1	withdrawal assessment tool 1	withdrawal assessment tool 1	withdrawal assessment tool 1	withdrawal assessment tool 1	2	kept
withdrawal of life-sustaining therapies	withdrawal of life sustaining therapies	withdrawal of life sustaining therapies	withdrawal of life sustaining therapy	Withdrawal of life-sustaining therapy	2	singular

Withdrawal of life-sustaining therapy	withdrawal of life sustaining therapy	withdrawal of life sustaining therapy	withdrawal of life sustaining therapy	Withdrawal of life-sustaining therapy	2	kept
withdrawal of life-sustaining therapy	withdrawal of life sustaining therapy	withdrawal of life sustaining therapy	withdrawal of life sustaining therapy	Withdrawal of life-sustaining therapy	2	case_or_format
Withdrawal of life-sustaining treatment	withdrawal of life sustaining treatment	withdrawal of life sustaining treatment	withdrawal of life sustaining treatment	withdrawal of life-sustaining treatment	2	case_or_format
Withdrawal of nutrition and hydration	withdrawal of nutrition and hydration	withdrawal of nutrition and hydration	withdrawal of nutrition and hydration	Withdrawal of nutrition and hydration	2	kept
withdrawal of treatment	withdrawal of treatment	withdrawal of treatment	withdrawal of treatment	Withdrawal of treatment	2	case_or_format
Withdrawal syndrome	withdrawal syndrome	withdrawal syndrome	withdrawal syndrome	withdrawal syndrome	2	case_or_format
withdrawing life-sustaining treatment	withdrawing life sustaining treatment	withdrawing life sustaining treatment	withdrawing life sustaining treatment	withdrawing life-sustaining treatment	2	kept
Withdrawing treatment	withdrawing treatment	withdrawing treatment	withdrawing treatment	Withdrawing treatment	2	kept
withholding life-sustaining treatment	withholding life sustaining treatment	withholding life sustaining treatment	withholding life sustaining treatment	withholding life-sustaining treatment	2	kept
Witnessed resuscitation	witnessed resuscitation	witnessed resuscitation	witnessed resuscitation	WITNESSED RESUSCITATION	2	case_or_format
Women's Health Services	women s health services	women s health services	women s health services	Women's Health Services	2	kept
Word Embedding	word embedding	word embedding	word embedding	Word Embedding	2	kept
WORK ABILITY	work ability	work ability	work ability	WORK ABILITY	2	kept
Work ability	work ability	work ability	work ability	WORK ABILITY	2	case_or_format
WORK ENGAGEMENT	work engagement	work engagement	work engagement	work engagement	2	case_or_format
Work flow	work flow	work flow	work flow	Work-flows	2	case_or_format

WORK INDEX	work index	work index	work index	WORK INDEX	2	kept
Work Motivation	work motivation	work motivation	work motivation	Work motivation	2	case_or_format
work organisation	work organisation	work organisation	work organisation	work organisation	2	kept
work overload	work overload	work overload	work overload	work overload	2	kept
Work process	work process	work process	work process	Work process	2	kept
Work processes	work processes	work processes	work processes	Work processes	2	kept
Work productivity	work productivity	work productivity	work productivity	Work productivity	2	kept
Work Productivity and Activity Impairment Questionnaire	work productivity and activity impairment questionnaire	work productivity and activity impairment questionnaire	work productivity and activity impairment questionnaire	Work Productivity and Activity Impairment Questionnaire	2	kept
Work related stress	work related stress	work related stress	work related stress	Work-related stress	2	case_or_format
Work sampling	work sampling	work sampling	work sampling	Work sampling	2	kept
work values	work values	work values	work values	work values	2	kept
Work-family conflict	work family conflict	work family conflict	work family conflict	work－family conflict	2	case_or_format
work-family conflict	work family conflict	work family conflict	work family conflict	work－family conflict	2	case_or_format
work-family interaction	work family interaction	work family interaction	work family interaction	work-family interaction	2	kept
Work-related demands	work related demands	work related demands	work related demands	Work-related demands	2	kept
WORK-RELATED FATIGUE	work related fatigue	work related fatigue	work related fatigue	WORK-RELATED FATIGUE	2	kept

Work-related musculoskeletal disorders	work related musculoskeletal disorders	work related musculoskeletal disorders	work related musculoskeletal disorders	Work-related musculoskeletal disorders	2	kept
Work-Related stress	work related stress	work related stress	work related stress	Work-related stress	2	case_or_format
work-related well-being	work related well being	work related well being	work related well being	work-related well-being	2	kept
work-resources	work resources	work resources	work resources	work-resources	2	kept
Work-stress	work stress	work stress	work stress	Work stress	2	case_or_format
Workaholism	workaholism	workaholism	workaholism	Workaholism	2	kept
workers	workers	workers	worker	worker	2	singular
Workers' health	workers health	workers health	workers health	Workers' health	2	kept
workflow efficiency	workflow efficiency	workflow efficiency	workflow efficiency	workflow efficiency	2	kept
workforce development	workforce development	workforce development	workforce development	workforce development	2	kept
Workforce planning	workforce planning	workforce planning	workforce planning	workforce planning	2	case_or_format
workforce shortage	workforce shortage	workforce shortage	workforce shortage	Workforce shortage	2	case_or_format
working hours	working hours	working hours	working hour	working hours	2	singular
Working memory	working memory	working memory	working memory	working memory	2	case_or_format
Working time	working time	working time	working time	working time	2	case_or_format
working women	working women	working women	working women	working women	2	kept

WORKING-CONDITIONS	working conditions	working conditions	working conditions	Working Conditions	2	case_or_format
Workload balance	workload balance	workload balance	workload balance	Workload balance	2	kept
Workload management	workload management	workload management	workload management	workload management	2	case_or_format
Workload measurement	workload measurement	workload measurement	workload measurement	Workload measurement	2	kept
workplace challenges	workplace challenges	workplace challenges	workplace challenges	workplace challenges	2	kept
WORKPLACE INTERRUPTIONS	workplace interruptions	workplace interruptions	workplace interruptions	WORKPLACE INTERRUPTIONS	2	kept
workplace ostracism	workplace ostracism	workplace ostracism	workplace ostracism	workplace ostracism	2	kept
Workplace physical environment	workplace physical environment	workplace physical environment	workplace physical environment	Workplace physical environment	2	kept
Workplace stress	workplace stress	workplace stress	workplace stress	Workplace stress	2	kept
workplace stress	workplace stress	workplace stress	workplace stress	Workplace stress	2	case_or_format
WORKPLACE STRESSORS	workplace stressors	workplace stressors	workplace stressors	WORKPLACE STRESSORS	2	kept
Workplace stressors	workplace stressors	workplace stressors	workplace stressors	WORKPLACE STRESSORS	2	case_or_format
Workplace well-being	workplace well being	workplace well being	workplace well being	Workplace well-being	2	kept
World Café methodology	world caf methodology	world caf methodology	world caf methodology	World Café methodology	2	kept
World War II	world war ii	world war ii	world war ii	World War II	2	kept
WOUND INFECTIONS	wound infections	wound infections	wound infection	wound infection	2	singular

wound irrigation	wound irrigation	wound irrigation	wound irrigation	wound irrigation	2	kept
wound treatment	wound treatment	wound treatment	wound treatment	wound treatment	2	kept
WOUNDS	wounds	wounds	wound	wound	2	singular
Wounds	wounds	wounds	wound	wound	2	singular
Wrist	wrist	wrist	wrist	wrist	2	case_or_format
wrist fracture	wrist fracture	wrist fracture	wrist fracture	wrist fracture	2	kept
Wyoming	wyoming	wyoming	wyoming	Wyoming	2	kept
X-Rays	x rays	x rays	x ray	X ray	2	singular
X-rays	x rays	x rays	x ray	X ray	2	singular
XGBoost	xgboost	xgboost	xgboost	XGBoost	2	kept
Xiphoid Bone	xiphoid bone	xiphoid bone	xiphoid bone	Xiphoid Bone	2	kept
xiphoid bone	xiphoid bone	xiphoid bone	xiphoid bone	Xiphoid Bone	2	case_or_format
xylazine	xylazine	xylazine	xylazine	xylazine	2	kept
Young adults	young adults	young adults	young adults	Young adults	2	kept
young onset dementia	young onset dementia	young onset dementia	young onset dementia	young onset dementia	2	kept
Young people	young people	young people	young people	Young people	2	kept

Young-onset dementia	young onset dementia	young onset dementia	young onset dementia	young onset dementia	2	case_or_format
Yugoslavia	yugoslavia	yugoslavia	yugoslavia	Yugoslavia	2	kept
Zanamivir	zanamivir	zanamivir	zanamivir	zanamivir	2	case_or_format
zidebactam	zidebactam	zidebactam	zidebactam	zidebactam	2	kept
ZigBee	zigbee	zigbee	zigbee	ZigBee	2	kept
Zika Virus	zika virus	zika virus	zika virus	Zika Virus	2	kept
zinc deficiency	zinc deficiency	zinc deficiency	zinc deficiency	zinc deficiency	2	kept
Zinc Sulfate	zinc sulfate	zinc sulfate	zinc sulfate	zinc sulfate	2	case_or_format
zinc supplement	zinc supplement	zinc supplement	zinc supplement	zinc supplement	2	kept
Zoom clinical rounds	zoom clinical rounds	zoom clinical rounds	zoom clinical rounds	Zoom clinical rounds	2	kept
zuclopenthixol	zuclopenthixol	zuclopenthixol	zuclopenthixol	zuclopenthixol	2	kept
Zung Self Rating Anxiety Scale	zung self rating anxiety scale	zung self rating anxiety scale	zung self rating anxiety scale	Zung Self Rating Anxiety Scale	2	kept
épidémie	pid mie	pid mie	pid mie	épidémie	2	kept
“6S” management	6s management	6s management	6s management	“6S” management	2	kept
″good death″	good death	good death	good death	Good death	1	case_or_format
˝Gram-negative pathogens ’susceptibility″	gram negative pathogens susceptibility	gram negative pathogens susceptibility	gram negative pathogens susceptibility	˝Gram-negative pathogens ’susceptibility″	1	kept

"just-in-time" training	just in time training	just in time training	just in time training	"just-in-time" training	1	kept
"Mechanical ventilation"	mechanical ventilation	mechanical ventilation	mechanical ventilation	Mechanical ventilation	1	case_or_format
"No lift" policy	no lift policy	no lift policy	no lift policy	"No lift" policy	1	kept
"Ventilator-associated pneumonia"	ventilator associated pneumonia	ventilator associated pneumonia	ventilator associated pneumonia	ventilator associated pneumonia	1	case_or_format
'Low flow' and 'high flow' oxygen therapy	low flow and high flow oxygen therapy	low flow and high flow oxygen therapy	low flow and high flow oxygen therapy	'Low flow' and 'high flow' oxygen therapy	1	kept
-β-D-glucan	d glucan	d glucan	d glucan	-β-D-glucan	1	kept
. Infection	infection	infection	infection	infection	1	case_or_format
00A69	00a69	00a69	00a69	00A69	1	kept
1,1 dichloro 2,2 bis ethane	1 1 dichloro 2 2 bis ethane	1 1 dichloro 2 2 bis ethane	1 1 dichloro 2 2 bis ethane	1,1 dichloro 2,2 bis ethane	1	kept
1,4 dichlorobenzene	1 4 dichlorobenzene	1 4 dichlorobenzene	1 4 dichlorobenzene	1,4 dichlorobenzene	1	kept
1,4-dichlorobenzene	1 4 dichlorobenzene	1 4 dichlorobenzene	1 4 dichlorobenzene	1,4 dichlorobenzene	1	case_or_format
1-hour bundle	1 hour bundle	1 hour bundle	1 hour bundle	1-hour bundle	1	kept
1-piece drainable pouch	1 piece drainable pouch	1 piece drainable pouch	1 piece drainable pouch	1-piece drainable pouch	1	kept
10 EUROPEAN COUNTRIES	10 european countries	10 european countries	10 european countries	10 EUROPEAN COUNTRIES	1	kept
10 STEPS	10 steps	10 steps	10 steps	10 STEPS	1	kept
100,000 LIVES CAMPAIGN	100 000 lives campaign	100 000 lives campaign	100 000 lives campaign	100,000 LIVES CAMPAIGN	1	kept

105 year old and alteplase	105 year old and alteplase	105 year old and alteplase	105 year old and alteplase	105 year old and alteplase	1	kept
12 item Kansas City Cardiomyopathy Questionnaire	12 item kansas city cardiomyopathy questionnaire	12 item kansas city cardiomyopathy questionnaire	12 item kansas city cardiomyopathy questionnaire	12 item Kansas City Cardiomyopathy Questionnaire	1	kept
12-h shift	12 h shift	12 h shift	12 h shift	12-h shifts	1	case_or_format
12-HOUR SHIFT	12 hour shift	12 hour shift	12 hour shift	12-HOUR SHIFT	1	kept
12-hour shifts	12 hour shifts	12 hour shifts	12 hour shift	12-HOUR SHIFT	1	singular
12-hr shifts	12 hr shifts	12 hr shifts	12 hr shifts	12-hr shifts	1	kept
12-lead	12 lead	12 lead	12 lead	12-lead	1	kept
12-lead ECG	12 lead ecg	12 lead ecg	12 lead ecg	12-lead ECG	1	kept
12-month mortality	12 month mortality	12 month mortality	12 month mortality	12-month mortality	1	kept
12h shifts	12h shifts	12h shifts	12h shifts	12h shifts	1	kept
13-valent pneumococcal conjugate vaccine recommendation	13 valent pneumococcal conjugate vaccine recommendation	13 valent pneumococcal conjugate vaccine recommendation	13 valent pneumococcal conjugate vaccine recommendation	13-valent pneumococcal conjugate vaccine recommendation	1	kept
130-273-11555	130 273 11555	130 273 11555	130 273 11555	130-273-11555	1	kept
130-273-572	130 273 572	130 273 572	130 273 572	130-273-572	1	kept
130-3814-5488	130 3814 5488	130 3814 5488	130 3814 5488	130-3814-5488	1	kept
16S rRNA	16s rrna	16s rrna	16s rrna	16S rRNA	1	kept
16S rRNA amplicon sequencing	16s rrna amplicon sequencing	16s rrna amplicon sequencing	16s rrna amplicon sequencing	16S rRNA amplicon sequencing	1	kept

16S rRNA sequence analysis	16s rrna sequence analysis	16s rrna sequence analysis	16s rrna sequence analysis	16S rRNA sequence analysis	1	kept
16s rRNA sequencing	16s rrna sequencing	16s rrna sequencing	16s rrna sequencing	16S rRNA sequencing	1	case_or_format
16sribosomal RNA sequencing	16sribosomal rna sequencing	16sribosomal rna sequencing	16sribosomal rna sequencing	16sribosomal RNA sequencing	1	kept
17 methylnaltrexone	17 methylnaltrexone	17 methylnaltrexone	17 methylnaltrexone	17 methylnaltrexone	1	kept
19th century	19th century	19th century	19th century	19th century	1	kept
1:1-Care	1 1 care	1 1 care	1 1 care	1:1-Care	1	kept
1q4 deletion	1q4 deletion	1q4 deletion	1q4 deletion	1q4 deletion	1	kept
1ST YEAR	1st year	1st year	1st year	1ST YEAR	1	kept
2 ⌊⌊ methyl]sulfinyl]benzimidazole derivative	2 methyl sulfinyl benzimidazole derivative	2 methyl sulfinyl benzimidazole derivative	2 methyl sulfinyl benzimidazole derivative	2 ⌊⌊ methyl]sulfinyl]benzimidazole derivative	1	kept
2 PARTS	2 parts	2 parts	2 parts	2 PARTS	1	kept
2,4 dinitrophenylhydrazine	2 4 dinitrophenylhydrazine	2 4 dinitrophenylhydrazine	2 4 dinitrophenylhydrazine	2,4 dinitrophenylhydrazine	1	kept
2,4 thiazolidinedione derivative	2 4 thiazolidinedione derivative	2 4 thiazolidinedione derivative	2 4 thiazolidinedione derivative	2,4 thiazolidinedione derivative	1	kept
2-Pyridinylmethylsulfinylbenzimidazoles	2 pyridinylmethylsulfinylbenzimidazoles	2 pyridinylmethylsulfinylbenzimidazoles	2 pyridinylmethylsulfinylbenzimidazoles	2-Pyridinylmethylsulfinylbenzimidazoles	1	kept
2008 UPDATE	2008 update	2008 update	2008 update	2008 UPDATE	1	kept
2010 INTERNATIONAL CONSENSUS	2010 international consensus	2010 international consensus	2010 international consensus	2010 INTERNATIONAL CONSENSUS	1	kept
2019 coronavirus disease	2019 coronavirus disease	2019 coronavirus disease	2019 coronavirus disease	2019 coronavirus disease	1	kept

2019 novel coronavirus	2019 novel coronavirus	2019 novel coronavirus	2019 novel coronavirus	2019 novel coronavirus	1	kept
20th Cent. Medicine	20th cent medicine	20th cent medicine	20th cent medicine	20th Cent. Medicine	1	kept
20th century	20th century	20th century	20th century	20th century	1	kept
21st Century Cures Act	21st century cures act	21st century cures act	21st century cures act	21st Century Cures Act	1	kept
21st century cures act	21st century cures act	21st century cures act	21st century cures act	21st Century Cures Act	1	case_or_format
23 item preterm birth risk assessment scale	23 item preterm birth risk assessment scale	23 item preterm birth risk assessment scale	23 item preterm birth risk assessment scale	23 item preterm birth risk assessment scale	1	kept
24-h rhythm	24 h rhythm	24 h rhythm	24 h rhythm	24-h rhythm	1	kept
24-h shifts	24 h shifts	24 h shifts	24 h shifts	24-h shifts	1	kept
24-hour in-house coverage	24 hour in house coverage	24 hour in house coverage	24 hour in house coverage	24-hour in-house coverage	1	kept
25 hydroxyergocalciferol	25 hydroxyergocalciferol	25 hydroxyergocalciferol	25 hydroxyergocalciferol	25 hydroxyergocalciferol	1	kept
25-hydroxyvitamin D	25 hydroxyvitamin d	25 hydroxyvitamin d	25 hydroxyvitamin d	25 hydroxyvitamin D	1	case_or_format
261-492-574	261 492 574	261 492 574	261 492 574	261-492-574	1	kept
28-Day mortality	28 day mortality	28 day mortality	28 day mortality	28-Day mortality	1	kept
28-day mortality	28 day mortality	28 day mortality	28 day mortality	28-Day mortality	1	case_or_format
283-217-7597	283 217 7597	283 217 7597	283 217 7597	283-217-7597	1	kept
283-239-5059	283 239 5059	283 239 5059	283 239 5059	283-239-5059	1	kept

2HELPS2B score	2helps2b score	2helps2b score	2helps2b score	2HELPS2B score	1	kept
2helps2b score	2helps2b score	2helps2b score	2helps2b score	2HELPS2B score	1	case_or_format
2ND VICTIM	2nd victim	2nd victim	2nd victim	2ND VICTIM	1	kept
2ND VICTIMS	2nd victims	2nd victims	2nd victim	2ND VICTIM	1	singular
3 CITIES	3 cities	3 cities	3 cities	3 CITIES	1	kept
3 dimensional Cognitive Assessment Method	3 dimensional cognitive assessment method	3 dimensional cognitive assessment method	3 dimensional cognitive assessment method	3 dimensional Cognitive Assessment Method	1	kept
3 minute Diagnostic Interview for CAM defined Delirium	3 minute diagnostic interview for cam defined delirium	3 minute diagnostic interview for cam defined delirium	3 minute diagnostic interview for cam defined delirium	3 minute Diagnostic Interview for CAM defined Delirium	1	kept
3 wishes project	3 wishes project	3 wishes project	3 wishes project	3 Wishes Project	1	case_or_format
3,4 methylenedioxyamphetamine	3 4 methylenedioxyamphetamine	3 4 methylenedioxyamphetamine	3 4 methylenedioxyamphetamine	3,4 methylenedioxyamphetamine	1	kept
3-axis accelerometer	3 axis accelerometer	3 axis accelerometer	3 axis accelerometer	3-axis accelerometer	1	kept
3-compartment bag system	3 compartment bag system	3 compartment bag system	3 compartment bag system	3-compartment bag system	1	kept
3-Iodobenzylguanidine	3 iodobenzylguanidine	3 iodobenzylguanidine	3 iodobenzylguanidine	3-Iodobenzylguanidine	1	kept
3-midnight rule	3 midnight rule	3 midnight rule	3 midnight rule	3-midnight rule	1	kept
3-MINUTE DIAGNOSTIC INTERVIEW	3 minute diagnostic interview	3 minute diagnostic interview	3 minute diagnostic interview	3-MINUTE DIAGNOSTIC INTERVIEW	1	kept
3-MONTH-OLD	3 month old	3 month old	3 month old	3-MONTH-OLD	1	kept
30 day readmission	30 day readmission	30 day readmission	30 day readmission	30 day readmission	1	kept

30 day thromboischemic event rates	30 day thromboischemic event rates	30 day thromboischemic event rates	30 day thromboischemic event rates	30 day thromboischemic event rates	1	kept
30-day mortality	30 day mortality	30 day mortality	30 day mortality	30-day mortality	1	kept
30-day readmissions	30 day readmissions	30 day readmissions	30 day readmission	30 day readmission	1	singular
329-475-488-3831-3629	329 475 488 3831 3629	329 475 488 3831 3629	329 475 488 3831 3629	329-475-488-3831-3629	1	kept
33 item Preterm Birth Knowledge Scale	33 item preterm birth knowledge scale	33 item preterm birth knowledge scale	33 item preterm birth knowledge scale	33 item Preterm Birth Knowledge Scale	1	kept
39-Week rule	39 week rule	39 week rule	39 week rule	39-Week rule	1	kept
3D game	3d game	3d game	3d game	3D game	1	kept
3D imaging sensor	3d imaging sensor	3d imaging sensor	3d imaging sensor	3D imaging sensor	1	kept
3D point cloud	3d point cloud	3d point cloud	3d point cloud	3D point cloud	1	kept
3D printing	3d printing	3d printing	3d printing	3D printing	1	kept
3D scanning and measuring	3d scanning and measuring	3d scanning and measuring	3d scanning and measuring	3D scanning and measuring	1	kept
3MS	3ms	3ms	3ms	3MS	1	kept
4 aminobutyric acid derivative	4 aminobutyric acid derivative	4 aminobutyric acid derivative	4 aminobutyric acid derivative	4 aminobutyric acid derivative	1	kept
4 aminobutyric acid receptor	4 aminobutyric acid receptor	4 aminobutyric acid receptor	4 aminobutyric acid receptor	4 aminobutyric acid receptor	1	kept
4 A’s Test	4 a s test	4 a s test	4 a s test	4 A’s Test	1	kept
4 disciplines of execution	4 disciplines of execution	4 disciplines of execution	4 disciplines of execution	4 disciplines of execution	1	kept

4 factor prothombin complex concentrate	4 factor prothombin complex concentrate	4 factor prothombin complex concentrate	4 factor prothombin complex concentrate	4 factor prothombin complex concentrate	1	kept
4 factor prothrombin complex concentrate	4 factor prothrombin complex concentrate	4 factor prothrombin complex concentrate	4 factor prothrombin complex concentrate	4 factor prothrombin complex concentrate	1	kept
4 hydroxycoumarin derivative	4 hydroxycoumarin derivative	4 hydroxycoumarin derivative	4 hydroxycoumarin derivative	4 hydroxycoumarin derivative	1	kept
4-dichlorobenzene	4 dichlorobenzene	4 dichlorobenzene	4 dichlorobenzene	4-dichlorobenzene	1	kept
4-hydroxybutyric acid	4 hydroxybutyric acid	4 hydroxybutyric acid	4 hydroxybutyric acid	4 hydroxybutyric acid	1	case_or_format
4-Hydroxycoumarins	4 hydroxycoumarins	4 hydroxycoumarins	4 hydroxycoumarins	4-Hydroxycoumarins	1	kept
4-methylenedioxyamphetam ine	4 methylenedioxyamphetam ine	4 methylenedioxyamphetam ine	4 methylenedioxyamphetam ine	4-methylenedioxyamphetam ine	1	kept
40% dextrose gel	40 dextrose gel	40 dextrose gel	40 dextrose gel	40% dextrose gel	1	kept
46, XX Disorders of Sex Development	46 xx disorders of sex development	46 xx disorders of sex development	46 xx disorders of sex development	46, XX Disorders of Sex Development	1	kept
48-hour	48 hour	48 hour	48 hour	48-hour	1	kept
4AT	4at	4at	4at	4AT	1	kept
4F-PCC	4f pcc	4f pcc	4f pcc	4F-PCC	1	kept
5 [1 1,2,3,4 tetrahydro 6 quinolyl] 3 6 dihydro 6 methyl	5 1 1 2 3 4 tetrahydro 6 quinolyl 3 6 dihydro 6 methyl 2h 1 3 4	5 1 1 2 3 4 tetrahydro 6 quinolyl 3 6 dihydro 6 methyl 2h 1 3 4	5 1 1 2 3 4 tetrahydro 6 quinolyl 3 6 dihydro 6 methyl 2h 1 3 4	5 [1 1,2,3,4 tetrahydro 6 quinolyl] 3 6 dihydro 6 methyl	1	kept
5 item modified frailty index	5 item modified frailty index	5 item modified frailty index	5 item modified frailty index	5 item modified frailty index	1	kept
5 moments	5 moments	5 moments	5 moments	5 moments	1	kept
5 point scale	5 point scale	5 point scale	5 point scale	5 point scale	1	kept

5-YEAR FOLLOW-UP	5 year follow up	5 year follow up	5 year follow up	5-YEAR FOLLOW-UP	1	kept
50% nitrous oxide	50 nitrous oxide	50 nitrous oxide	50 nitrous oxide	50% nitrous oxide	1	kept
5PFQ Questionnaire	5pfq questionnaire	5pfq questionnaire	5pfq questionnaire	5PFQ Questionnaire	1	kept
6 Cs	6 cs	6 cs	6 cs	6 Cs	1	kept
6 EUROPEAN COUNTRIES	6 european countries	6 european countries	6 european countries	6 EUROPEAN COUNTRIES	1	kept
6 hydroxymelatonin o sulfate	6 hydroxymelatonin o sulfate	6 hydroxymelatonin o sulfate	6 hydroxymelatonin o sulfate	6 hydroxymelatonin o sulfate	1	kept
6-Item Cognitive Impairment Test	6 item cognitive impairment test	6 item cognitive impairment test	6 item cognitive impairment test	6-Item Cognitive Impairment Test	1	kept
6-minute walk test	6 minute walk test	6 minute walk test	6 minute walk test	6-minute walk test	1	kept
6-MONTH MORTALITY	6 month mortality	6 month mortality	6 month mortality	6-MONTH MORTALITY	1	kept
6-sulphatoxymelatonin	6 sulphatoxymelatonin	6 sulphatoxymelatonin	6 sulphatoxymelatonin	6-sulphatoxymelatonin	1	kept
60-day survival	60 day survival	60 day survival	60 day survival	60-day survival	1	kept
7 : 417-421	7 417 421	7 417 421	7 417 421	7 : 417-421	1	kept
7 catecholamines	7 catecholamines	7 catecholamines	7 catecholamines	7 catecholamines	1	kept
7 point ordinal scale	7 point ordinal scale	7 point ordinal scale	7 point ordinal scale	7 point ordinal scale	1	kept
8A model	8a model	8a model	8a model	8A model	1	kept
9 point GRADE scale	9 point grade scale	9 point grade scale	9 point grade scale	9 point GRADE scale	1	kept

9 point scale	9 point scale	9 point scale	9 point scale	9 point scale	1	kept
91B30	91b30	91b30	91b30	91B30	1	kept
92C50	92c50	92c50	92c50	92C50	1	kept
93C41	93c41	93c41	93c41	93C41	1	kept
[Secondary] brain injury	secondary brain injury	secondary brain injury	secondary brain injury	secondary brain injury	1	case_or_format
a brief mindfulness-based intervention	a brief mindfulness based intervention	a brief mindfulness based intervention	a brief mindfulness based intervention	a brief mindfulness-based intervention	1	kept
A care bundle protocol	a care bundle protocol	a care bundle protocol	a care bundle protocol	A care bundle protocol	1	kept
A case report	a case report	a case report	a case report	A case report	1	kept
a creative option to maintain staffing ratios	a creative option to maintain staffing ratios	a creative option to maintain staffing ratios	a creative option to maintain staffing ratios	a creative option to maintain staffing ratios	1	kept
A H3N2	a h3n2	a h3n2	a h3n2	A H3N2	1	kept
A meta-analysis	a meta analysis	a meta analysis	a meta analysis	A meta-analysis	1	kept
a meta-analysis	a meta analysis	a meta analysis	a meta analysis	A meta-analysis	1	case_or_format
a methapred	a methapred	a methapred	a methapred	a methapred	1	kept
a phenomenological study	a phenomenological study	a phenomenological study	a phenomenological study	a phenomenological study	1	kept
A Quick Test of Cognitive Speed	a quick test of cognitive speed	a quick test of cognitive speed	a quick test of cognitive speed	A Quick Test of Cognitive Speed	1	kept
A-EEG	a eeg	a eeg	a eeg	A-EEG	1	kept

A. baumannii XDR pneumonia	a baumannii xdr pneumonia	a baumannii xdr pneumonia	a baumannii xdr pneumonia	A. baumannii XDR pneumonia	1	kept
AA = anesthesiologist assistant	aa anesthesiologist assistant	aa anesthesiologist assistant	aa anesthesiologist assistant	AA = anesthesiologist assistant	1	kept
aac bacterial gene	aac bacterial gene	aac bacterial gene	aac bacterial gene	aac bacterial gene	1	kept
AACN	aacn	aacn	aacn	AACN	1	kept
AACN´s standards for establishing and sustaining healthy	aacn s standards for establishing and sustaining healthy	aacn s standards for establishing and sustaining healthy	aacn s standards for establishing and sustaining healthy	AACN´s standards for establishing and sustaining healthy	1	kept
AAPD = Association of Academic Program Directors	aapd association of academic program directors	aapd association of academic program directors	aapd association of academic program directors	AAPD = Association of Academic Program Directors	1	kept
AAST	aast	aast	aast	AAST	1	kept
AAU	aau	aau	aau	AAU	1	kept
ABA = American Board of Anesthesiology	aba american board of anesthesiology	aba american board of anesthesiology	aba american board of anesthesiology	ABA = American Board of Anesthesiology	1	kept
ABA-American Burn Association	aba american burn association	aba american burn association	aba american burn association	ABA-American Burn Association	1	kept
abacavir	abacavir	abacavir	abacavir	abacavir	1	kept
Abadan	abadan	abadan	abadan	Abadan	1	kept
Abandoned babies	abandoned babies	abandoned babies	abandoned babies	Abandoned babies	1	kept
abandonment	abandonment	abandonment	abandonment	abandonment	1	kept
Abandono de la profesi ón	abandono de la profesi n	abandono de la profesi n	abandono de la profesi n	Abandono de la profesi ón	1	kept
ABAQUS	abaqus	abaqus	abaqus	ABAQUS	1	kept

Abbott	abbott	abbott	abbott	Abbott	1	kept
abbreviated burn severity index	abbreviated burn severity index	abbreviated burn severity index	abbreviated burn severity index	abbreviated burn severity index	1	kept
Abbreviated Injury Scale score for the head	abbreviated injury scale score for the head	abbreviated injury scale score for the head	abbreviated injury scale score for the head	Abbreviated Injury Scale score for the head	1	kept
Abbreviated Injury Score	abbreviated injury score	abbreviated injury score	abbreviated injury score	Abbreviated Injury Score	1	kept
abbreviated mental test	abbreviated mental test	abbreviated mental test	abbreviated mental test	abbreviated mental test	1	kept
ABCD classification tool	abcd classification tool	abcd classification tool	abcd classification tool	ABCD classification tool	1	kept
ABCDE	abcde	abcde	abcde	ABCDE	1	kept
ABCDEF	abcdef	abcdef	abcdef	ABCDEF	1	kept
ABCDEF BUNDLE	abcdef bundle	abcdef bundle	abcdef bundle	ABCDEF bundle	1	case_or_format
ABCDEF Bundle	abcdef bundle	abcdef bundle	abcdef bundle	ABCDEF bundle	1	case_or_format
abcdef bundle	abcdef bundle	abcdef bundle	abcdef bundle	ABCDEF bundle	1	case_or_format
ABCDEFGH bundle	abcdefgh bundle	abcdefgh bundle	abcdefgh bundle	ABCDEFGH bundle	1	kept
ABCDF bundle	abcdf bundle	abcdf bundle	abcdf bundle	ABCDF bundle	1	kept
Abdomen, Acute	abdomen acute	abdomen acute	abdomen acute	Abdomen, Acute	1	kept
Abdominal	abdominal	abdominal	abdominal	Abdominal	1	kept
abdominal adhesions	abdominal adhesions	abdominal adhesions	abdominal adhesions	abdominal adhesions	1	kept

abdominal angiography	abdominal angiography	abdominal angiography	abdominal angiography	abdominal angiography	1	kept
Abdominal aortic aneurysm surgery	abdominal aortic aneurysm surgery	abdominal aortic aneurysm surgery	abdominal aortic aneurysm surgery	Abdominal aortic aneurysm surgery	1	kept
abdominal assessment	abdominal assessment	abdominal assessment	abdominal assessment	abdominal assessment	1	kept
Abdominal decompression	abdominal decompression	abdominal decompression	abdominal decompression	Abdominal decompression	1	kept
abdominal decompression	abdominal decompression	abdominal decompression	abdominal decompression	Abdominal decompression	1	case_or_format
Abdominal infection	abdominal infection	abdominal infection	abdominal infection	abdominal infection	1	case_or_format
abdominal injuries	abdominal injuries	abdominal injuries	abdominal injury	Abdominal Injuries	1	singular
abdominal mass	abdominal mass	abdominal mass	abdominal mass	abdominal mass	1	kept
ABDOMINAL MASSAGE	abdominal massage	abdominal massage	abdominal massage	Abdominal massage	1	case_or_format
abdominal massage	abdominal massage	abdominal massage	abdominal massage	Abdominal massage	1	case_or_format
abdominal pelvic ultrasound	abdominal pelvic ultrasound	abdominal pelvic ultrasound	abdominal pelvic ultrasound	abdominal pelvic ultrasound	1	kept
Abdominal perfusion pressure	abdominal perfusion pressure	abdominal perfusion pressure	abdominal perfusion pressure	Abdominal perfusion pressure	1	kept
abdominal perineal injury	abdominal perineal injury	abdominal perineal injury	abdominal perineal injury	abdominal perineal injury	1	kept
abdominal pregnancy	abdominal pregnancy	abdominal pregnancy	abdominal pregnancy	abdominal pregnancy	1	kept
Abdominal pressure	abdominal pressure	abdominal pressure	abdominal pressure	abdominal pressure	1	case_or_format
Abdominal sepsis	abdominal sepsis	abdominal sepsis	abdominal sepsis	Abdominal sepsis	1	kept

abdominal signs	abdominal signs	abdominal signs	abdominal signs	abdominal signs	1	kept
abdominal swelling	abdominal swelling	abdominal swelling	abdominal swelling	abdominal swelling	1	kept
Abdominal trauma	abdominal trauma	abdominal trauma	abdominal trauma	Abdominal trauma	1	kept
Abdominal ultrasound	abdominal ultrasound	abdominal ultrasound	abdominal ultrasound	Abdominal ultrasound	1	kept
abdominal wall closure	abdominal wall closure	abdominal wall closure	abdominal wall closure	abdominal wall closure	1	kept
abdominal wall defect	abdominal wall defect	abdominal wall defect	abdominal wall defect	abdominal wall defect	1	kept
abdominoplasty	abdominoplasty	abdominoplasty	abdominoplasty	abdominoplasty	1	kept
abducens nerve paralysis	abducens nerve paralysis	abducens nerve paralysis	abducens nerve paralysis	abducens nerve paralysis	1	kept
abductor muscle	abductor muscle	abductor muscle	abductor muscle	abductor muscle	1	kept
Abdul-Qadir-Jilani	abdul qadir jilani	abdul qadir jilani	abdul qadir jilani	Abdul-Qadir-Jilani	1	kept
aberrant motor behavior	aberrant motor behavior	aberrant motor behavior	aberrant motor behavior	aberrant motor behavior	1	kept
Aberrant splice site	aberrant splice site	aberrant splice site	aberrant splice site	Aberrant splice site	1	kept
ABG	abg	abg	abg	ABG	1	kept
Abha	abha	abha	abha	Abha	1	kept
Ability	ability	ability	ability	ABILITY	1	case_or_format
ability	ability	ability	ability	ABILITY	1	case_or_format

Ability evaluation	ability evaluation	ability evaluation	ability evaluation	Ability evaluation	1	kept
ability level principle	ability level principle	ability level principle	ability level principle	ability level principle	1	kept
ABIM	abim	abim	abim	ABIM	1	kept
ablation	ablation	ablation	ablation	ablation	1	kept
ABLS	abls	abls	abls	ABLS	1	kept
ABLS provider course	abls provider course	abls provider course	abls provider course	ABLS provider course	1	kept
Abnormal conditions	abnormal conditions	abnormal conditions	abnormal conditions	Abnormal conditions	1	kept
abnormal feces	abnormal feces	abnormal feces	abnormal feces	abnormal feces	1	kept
Abnormal Involuntary Movement Scale	abnormal involuntary movement scale	abnormal involuntary movement scale	abnormal involuntary movement scale	Abnormal Involuntary Movement Scale	1	kept
abnormal placenta adherence	abnormal placenta adherence	abnormal placenta adherence	abnormal placenta adherence	abnormal placenta adherence	1	kept
abnormal posture	abnormal posture	abnormal posture	abnormal posture	abnormal posture	1	kept
abnormal sensation	abnormal sensation	abnormal sensation	abnormal sensation	abnormal sensation	1	kept
abnormal vision	abnormal vision	abnormal vision	abnormal vision	abnormal vision	1	kept
Abnormalities	abnormalities	abnormalities	abnormalities	abnormalities	1	case_or_format
Abnormality index	abnormality index	abnormality index	abnormality index	Abnormality index	1	kept
ABO Blood-Group System	abo blood group system	abo blood group system	abo blood group system	ABO Blood-Group System	1	kept

ABO incompatibility	abo incompatibility	abo incompatibility	abo incompatibility	ABO incompatibility	1	kept
aboriginal	aboriginal	aboriginal	aboriginal	aboriginal	1	kept
Aboriginal people, North America	aboriginal people north america	aboriginal people north america	aboriginal people north america	Aboriginal people, North America	1	kept
Abortion, Therapeutic	abortion therapeutic	abortion therapeutic	abortion therapeutic	Abortion, Therapeutic	1	kept
Abortion, Threatened	abortion threatened	abortion threatened	abortion threatened	Abortion, Threatened	1	kept
above cuff vocalization	above cuff vocalization	above cuff vocalization	above cuff vocalization	above cuff vocalization	1	kept
above knee amputation	above knee amputation	above knee amputation	above knee amputation	above knee amputation	1	kept
abruptio placentae	abruptio placentae	abruptio placentae	abruptio placentae	abruptio placentae	1	kept
abscess formation	abscess formation	abscess formation	abscess formation	abscess formation	1	kept
absconding	absconding	absconding	absconding	absconding	1	kept
absent basal cistern	absent basal cistern	absent basal cistern	absent basal cistern	absent basal cistern	1	kept
Absolute difference	absolute difference	absolute difference	absolute difference	Absolute difference	1	kept
absolute risk prediction	absolute risk prediction	absolute risk prediction	absolute risk prediction	absolute risk prediction	1	kept
Absorbent pad	absorbent pad	absorbent pad	absorbent pad	Absorbent pad	1	kept
absorbent pad	absorbent pad	absorbent pad	absorbent pad	Absorbent pad	1	case_or_format
ABSORPTION	absorption	absorption	absorption	absorption	1	case_or_format

Absorption	absorption	absorption	absorption	absorption	1	case_or_format
Absorption spectroscopy	absorption spectroscopy	absorption spectroscopy	absorption spectroscopy	Absorption spectroscopy	1	kept
Abstinence	abstinence	abstinence	abstinence	abstinence	1	case_or_format
Abstinence syndrome	abstinence syndrome	abstinence syndrome	abstinence syndrome	Abstinence syndrome	1	kept
abstinence syndrome	abstinence syndrome	abstinence syndrome	abstinence syndrome	Abstinence syndrome	1	case_or_format
Abstract concept	abstract concept	abstract concept	abstract concept	Abstract concept	1	kept
Abuelos	abuelos	abuelos	abuelos	Abuelos	1	kept
abus des aines	abus des aines	abus des aines	abus des aines	abus des aines	1	kept
ABUSE	abuse	abuse	abuse	abuse	1	case_or_format
ABW	abw	abw	abw	ABW	1	kept
ac = antibiotic-coated	ac antibiotic coated	ac antibiotic coated	ac antibiotic coated	ac = antibiotic-coated	1	kept
Academic development	academic development	academic development	academic development	Academic development	1	kept
Academic Dissertations	academic dissertations	academic dissertations	academic dissertations	Academic Dissertations	1	kept
Academic Dissertations as Topic	academic dissertations as topic	academic dissertations as topic	academic dissertations as topic	Academic Dissertations as Topic	1	kept
academic field	academic field	academic field	academic field	academic field	1	kept
academic health network	academic health network	academic health network	academic health network	academic health network	1	kept

academic hospital	academic hospital	academic hospital	academic hospital	academic hospital	1	kept
Academic literature	academic literature	academic literature	academic literature	Academic literature	1	kept
Academic medical centre	academic medical centre	academic medical centre	academic medical centre	Academic medical centre	1	kept
academic medicine	academic medicine	academic medicine	academic medicine	academic medicine	1	kept
Academic motivation	academic motivation	academic motivation	academic motivation	Academic motivation	1	kept
Academic Motivation Scale	academic motivation scale	academic motivation scale	academic motivation scale	Academic Motivation Scale	1	kept
Academic partnership	academic partnership	academic partnership	academic partnership	Academic partnership	1	kept
Academic promotion	academic promotion	academic promotion	academic promotion	Academic promotion	1	kept
Academic radiology	academic radiology	academic radiology	academic radiology	Academic radiology	1	kept
Academic Success	academic success	academic success	academic success	Academic success	1	case_or_format
Academic surgery	academic surgery	academic surgery	academic surgery	Academic surgery	1	kept
Academic Training	academic training	academic training	academic training	Academic Training	1	kept
ACADEMIC-MEDICAL-CENTER	academic medical center	academic medical center	academic medical center	Academic Medical Centers	1	case_or_format
Academic-practice partners	academic practice partners	academic practice partners	academic practice partners	Academic-practice partners	1	kept
Academic‐Practice partnership	academic practice partnership	academic practice partnership	academic practice partnership	Academic‐Practice partnership	1	kept
acanthocytosis	acanthocytosis	acanthocytosis	acanthocytosis	acanthocytosis	1	kept

acantholysis	acantholysis	acantholysis	acantholysis	acantholysis	1	kept
acantholytic dyskeratosis	acantholytic dyskeratosis	acantholytic dyskeratosis	acantholytic dyskeratosis	acantholytic dyskeratosis	1	kept
acanthosis	acanthosis	acanthosis	acanthosis	acanthosis	1	kept
ACC	acc	acc	acc	ACC	1	kept
acc	acc	acc	acc	ACC	1	case_or_format
ACC Competency Statement	acc competency statement	acc competency statement	acc competency statement	ACC Competency Statement	1	kept
accelerate time to death	accelerate time to death	accelerate time to death	accelerate time to death	accelerate time to death	1	kept
accelerometer	accelerometer	accelerometer	accelerometer	Accelerometer	1	case_or_format
Accelerometers	accelerometers	accelerometers	accelerometer	Accelerometer	1	singular
Accelerometers or wearable sensors	accelerometers or wearable sensors	accelerometers or wearable sensors	accelerometers or wearable sensors	Accelerometers or wearable sensors	1	kept
acceptability intervention measure	acceptability intervention measure	acceptability intervention measure	acceptability intervention measure	acceptability intervention measure	1	kept
Acceptable limit	acceptable limit	acceptable limit	acceptable limit	Acceptable limit	1	kept
ACCEPTANCE	acceptance	acceptance	acceptance	Acceptance	1	case_or_format
acceptance and action questionnaire	acceptance and action questionnaire	acceptance and action questionnaire	acceptance and action questionnaire	acceptance and action questionnaire	1	kept
acceptance and action questionnaire II	acceptance and action questionnaire ii	acceptance and action questionnaire ii	acceptance and action questionnaire ii	acceptance and action questionnaire II	1	kept
Acceptance and Commitment Therapy	acceptance and commitment therapy	acceptance and commitment therapy	acceptance and commitment therapy	acceptance and commitment therapy	1	case_or_format

Acceptance and commitment therapy	acceptance and commitment therapy	acceptance and commitment therapy	acceptance and commitment therapy	acceptance and commitment therapy	1	case_or_format
Acceptance and commitment training Depression Stress	acceptance and commitment training depression stress	acceptance and commitment training depression stress	acceptance and commitment training depression stress	Acceptance and commitment training Depression Stress	1	kept
Acceptance tests	acceptance tests	acceptance tests	acceptance tests	Acceptance tests	1	kept
Access block	access block	access block	access block	Access block	1	kept
access to health care	access to health care	access to health care	access to health care	Access to health care	1	case_or_format
access to nature	access to nature	access to nature	access to nature	access to nature	1	kept
access to palliative care	access to palliative care	access to palliative care	access to palliative care	access to palliative care	1	kept
access to research	access to research	access to research	access to research	access to research	1	kept
access to services	access to services	access to services	access to services	access to services	1	kept
access to staff	access to staff	access to staff	access to staff	access to staff	1	kept
ACCESS, WATCH and RESERVE	access watch and reserve	access watch and reserve	access watch and reserve	ACCESS, WATCH and RESERVE	1	kept
Access-block	access block	access block	access block	Access block	1	case_or_format
Accident	accident	accident	accident	accident	1	case_or_format
Accident & Emergency	accident and emergency	accident and emergency	accident and emergency	Accident and emergency	1	case_or_format
Accident and Emergency Department	accident and emergency department	accident and emergency department	accident and emergency department	Accident and Emergency Department	1	kept
accident and emergency medicine	accident and emergency medicine	accident and emergency medicine	accident and emergency medicine	accident & emergency medicine	1	case_or_format

Accident prevention	accident prevention	accident prevention	accident prevention	accident prevention	1	case_or_format
accident vasculaire cérébral	accident vasculaire c r bral	accident vasculaire c r bral	accident vasculaire c r bral	Accident vasculaire cérébral	1	case_or_format
Accidental	accidental	accidental	accidental	Accidental	1	kept
Accidental catheter removal	accidental catheter removal	accidental catheter removal	accidental catheter removal	Accidental catheter removal	1	kept
accidental catheter removal	accidental catheter removal	accidental catheter removal	accidental catheter removal	Accidental catheter removal	1	case_or_format
accidental decannulation	accidental decannulation	accidental decannulation	accidental decannulation	accidental decannulation	1	kept
ACCIDENTAL DISLODGMENT	accidental dislodgment	accidental dislodgment	accidental dislodgment	ACCIDENTAL DISLODGMENT	1	kept
Accidental exposure	accidental exposure	accidental exposure	accidental exposure	Accidental exposure	1	kept
accidental exposure	accidental exposure	accidental exposure	accidental exposure	Accidental exposure	1	case_or_format
accidental extubation	accidental extubation	accidental extubation	accidental extubation	Accidental extubation	1	case_or_format
Accidental ingestion	accidental ingestion	accidental ingestion	accidental ingestion	Accidental ingestion	1	kept
accidental PN bolus	accidental pn bolus	accidental pn bolus	accidental pn bolus	accidental PN bolus	1	kept
ACCIDENTAL PNEUMOTHORAX	accidental pneumothorax	accidental pneumothorax	accidental pneumothorax	ACCIDENTAL PNEUMOTHORAX	1	kept
accidental poisoning	accidental poisoning	accidental poisoning	accidental poisoning	accidental poisoning	1	kept
accidental removal	accidental removal	accidental removal	accidental removal	accidental removal	1	kept
ACCIDENTS	accidents	accidents	accident	accident	1	singular

Accidents, occupational	accidents occupational	accidents occupational	accidents occupational	Accidents, Occupational	1	case_or_format
accidents, occupational	accidents occupational	accidents occupational	accidents occupational	Accidents, Occupational	1	case_or_format
Accountability	accountability	accountability	accountability	Accountability	1	kept
accountable care organizations	accountable care organizations	accountable care organizations	accountable care organizations	Accountable Care Organizations	1	case_or_format
Accounts Payable and Receivable	accounts payable and receivable	accounts payable and receivable	accounts payable and receivable	Accounts Payable and Receivable	1	kept
ACCREDITATION	accreditation	accreditation	accreditation	accreditation	1	case_or_format
accreta	accreta	accreta	accreta	accreta	1	kept
acculturation	acculturation	acculturation	acculturation	Acculturation	1	case_or_format
Accuracy of decision makings	accuracy of decision makings	accuracy of decision makings	accuracy of decision makings	Accuracy of decision makings	1	kept
accuracy of documentation	accuracy of documentation	accuracy of documentation	accuracy of documentation	accuracy of documentation	1	kept
accuracy rate	accuracy rate	accuracy rate	accuracy rate	accuracy rate	1	kept
AccuVein AV400	accuvein av400	accuvein av400	accuvein av400	AccuVein AV400	1	kept
ACDU scale	acdu scale	acdu scale	acdu scale	ACDU scale	1	kept
ACE inhibitor	ace inhibitor	ace inhibitor	ace inhibitor	ACE inhibitor	1	kept
ACE Star model	ace star model	ace star model	ace star model	ACE Star model	1	kept
ACE-2	ace 2	ace 2	ace 2	ACE-2	1	kept

acecainide	acecainide	acecainide	acecainide	acecainide	1	kept
Aceh	aceh	aceh	aceh	Aceh	1	kept
acellular dermal matrix	acellular dermal matrix	acellular dermal matrix	acellular dermal matrix	acellular dermal matrix	1	kept
Acellular Dermis	acellular dermis	acellular dermis	acellular dermis	Acellular Dermis	1	kept
acenocoumarol	acenocoumarol	acenocoumarol	acenocoumarol	acenocoumarol	1	kept
ACES	aces	aces	aces	ACES	1	kept
ACEs	aces	aces	aces	ACES	1	case_or_format
acetabular osteomyelitis	acetabular osteomyelitis	acetabular osteomyelitis	acetabular osteomyelitis	acetabular osteomyelitis	1	kept
Acetabulum	acetabulum	acetabulum	acetabulum	Acetabulum	1	kept
acetazolamide side effects	acetazolamide side effects	acetazolamide side effects	acetazolamide side effects	acetazolamide side effects	1	kept
Acetic Acid	acetic acid	acetic acid	acetic acid	acetic acid	1	case_or_format
acetylcholinesterase	acetylcholinesterase	acetylcholinesterase	acetylcholinesterase	Acetylcholinesterase	1	case_or_format
acetylsalicylic acid plus dipyridamole	acetylsalicylic acid plus dipyridamole	acetylsalicylic acid plus dipyridamole	acetylsalicylic acid plus dipyridamole	acetylsalicylic acid plus dipyridamole	1	kept
ACGME	acgme	acgme	acgme	ACGME	1	kept
ACGME = Accreditation Council for Graduate Medical Education	acgme accreditation council for graduate medical education	acgme accreditation council for graduate medical education	acgme accreditation council for graduate medical education	ACGME = Accreditation Council for Graduate Medical Education	1	kept
ACGME CASE LOGS	acgme case logs	acgme case logs	acgme case logs	ACGME CASE LOGS	1	kept

ACGME competencies	acgme competencies	acgme competencies	acgme competencies	ACGME competencies	1	kept
ACHD	achd	achd	achd	ACHD	1	kept
achievement test	achievement test	achievement test	achievement test	achievement test	1	kept
Achieving best care	achieving best care	achieving best care	achieving best care	Achieving best care	1	kept
ACHIEVING CONSENSUS	achieving consensus	achieving consensus	achieving consensus	ACHIEVING CONSENSUS	1	kept
achilles tendon	achilles tendon	achilles tendon	achilles tendon	achilles tendon	1	kept
Achromobacter denitrificans	achromobacter denitrificans	achromobacter denitrificans	achromobacter denitrificans	Achromobacter denitrificans	1	kept
ACID	acid	acid	acid	ACID	1	kept
ACID ASPIRATION	acid aspiration	acid aspiration	acid aspiration	acid aspiration	1	case_or_format
Acid base balance	acid base balance	acid base balance	acid base balance	acid base balance	1	case_or_format
Acid base disturbances	acid base disturbances	acid base disturbances	acid base disturbances	Acid base disturbances	1	kept
acid citrate dextrose	acid citrate dextrose	acid citrate dextrose	acid citrate dextrose	acid citrate dextrose	1	kept
acid citrate dextrose solution A	acid citrate dextrose solution a	acid citrate dextrose solution a	acid citrate dextrose solution a	acid citrate dextrose solution A	1	kept
acid ice stimulation	acid ice stimulation	acid ice stimulation	acid ice stimulation	acid ice stimulation	1	kept
acid lipase	acid lipase	acid lipase	acid lipase	acid lipase	1	kept
Acid suppression	acid suppression	acid suppression	acid suppression	Acid suppression	1	kept

acid suppressive agent	acid suppressive agent	acid suppressive agent	acid suppressive agent	acid suppressive agent	1	kept
Acid suppressive therapy	acid suppressive therapy	acid suppressive therapy	acid suppressive therapy	Acid suppressive therapy	1	kept
acid-base disorder	acid base disorder	acid base disorder	acid base disorder	acid-base disorder	1	kept
acidity	acidity	acidity	acidity	acidity	1	kept
acidosis acute	acidosis acute	acidosis acute	acidosis acute	acidosis acute	1	kept
Acidosis, Lactic	acidosis lactic	acidosis lactic	acidosis lactic	Acidosis, Lactic	1	kept
Acidosis, Renal Tubular	acidosis renal tubular	acidosis renal tubular	acidosis renal tubular	Acidosis, Renal Tubular	1	kept
Acids	acids	acids	acid	ACID	1	singular
Acinetobacter baumanii	acinetobacter baumanii	acinetobacter baumanii	acinetobacter baumanii	Acinetobacter baumanii	1	kept
Acinetobacter baylyi	acinetobacter baylyi	acinetobacter baylyi	acinetobacter baylyi	Acinetobacter baylyi	1	kept
Acinetobacter pittii	acinetobacter pittii	acinetobacter pittii	acinetobacter pittii	Acinetobacter pittii	1	kept
ACINETOBACTER-BAUMANNII INFECTION	acinetobacter baumannii infection	acinetobacter baumannii infection	acinetobacter baumannii infection	ACINETOBACTER-BAUMANNII INFECTION	1	kept
acitretin	acitretin	acitretin	acitretin	acitretin	1	kept
ACLF	aclf	aclf	aclf	ACLF	1	kept
acmeological approach	acmeological approach	acmeological approach	acmeological approach	acmeological approach	1	kept
ACNP	acnp	acnp	acnp	ACNP	1	kept

ACNP scope of practice	acnp scope of practice	acnp scope of practice	acnp scope of practice	ACNP scope of practice	1	kept
ACO	aco	aco	aco	ACO	1	kept
Acolhimento	acolhimento	acolhimento	acolhimento	Acolhimento	1	kept
aconite oil	aconite oil	aconite oil	aconite oil	aconite oil	1	kept
Acoustic conditions	acoustic conditions	acoustic conditions	acoustic conditions	Acoustic conditions	1	kept
ACOUSTIC ENVIRONMENT	acoustic environment	acoustic environment	acoustic environment	Acoustic environment	1	case_or_format
acoustic environment	acoustic environment	acoustic environment	acoustic environment	Acoustic environment	1	case_or_format
acoustic environment evaluation	acoustic environment evaluation	acoustic environment evaluation	acoustic environment evaluation	acoustic environment evaluation	1	kept
Acoustic experiments	acoustic experiments	acoustic experiments	acoustic experiments	Acoustic experiments	1	kept
Acoustic generators	acoustic generators	acoustic generators	acoustic generators	Acoustic generators	1	kept
Acoustic measurements	acoustic measurements	acoustic measurements	acoustic measurements	Acoustic measurements	1	kept
Acoustic noise measurement	acoustic noise measurement	acoustic noise measurement	acoustic noise measurement	Acoustic noise measurement	1	kept
Acoustic preferences	acoustic preferences	acoustic preferences	acoustic preferences	Acoustic preferences	1	kept
Acoustic respiratory rate monitoring	acoustic respiratory rate monitoring	acoustic respiratory rate monitoring	acoustic respiratory rate monitoring	Acoustic respiratory rate monitoring	1	kept
Acoustic signal processing	acoustic signal processing	acoustic signal processing	acoustic signal processing	Acoustic signal processing	1	kept
Acoustic source localization	acoustic source localization	acoustic source localization	acoustic source localization	Acoustic source localization	1	kept

Acoustic sources	acoustic sources	acoustic sources	acoustic sources	Acoustic sources	1	kept
Acoustic stimulation	acoustic stimulation	acoustic stimulation	acoustic stimulation	Acoustic Stimulation	1	case_or_format
Acoustic streaming	acoustic streaming	acoustic streaming	acoustic streaming	Acoustic streaming	1	kept
Acoustic treatment	acoustic treatment	acoustic treatment	acoustic treatment	Acoustic treatment	1	kept
Acoustic wave absorption	acoustic wave absorption	acoustic wave absorption	acoustic wave absorption	Acoustic wave absorption	1	kept
Acoustical treatment	acoustical treatment	acoustical treatment	acoustical treatment	Acoustical treatment	1	kept
ACP	acp	acp	acp	ACP	1	kept
Acquired brain injury	acquired brain injury	acquired brain injury	acquired brain injury	acquired brain injury	1	case_or_format
Acquired coagulopathy	acquired coagulopathy	acquired coagulopathy	acquired coagulopathy	Acquired coagulopathy	1	kept
acquired heart disease	acquired heart disease	acquired heart disease	acquired heart disease	acquired heart disease	1	kept
Acquired Infection	acquired infection	acquired infection	acquired infection	Acquired Infection	1	kept
ACQUIRED INFECTIONS	acquired infections	acquired infections	acquired infection	Acquired Infection	1	singular
ACQUIRED PRESSURE ULCERS	acquired pressure ulcers	acquired pressure ulcers	acquired pressure ulcers	ACQUIRED PRESSURE ULCERS	1	kept
ACRM	acrm	acrm	acrm	ACRM	1	kept
Acrocallosal Syndrome	acrocallosal syndrome	acrocallosal syndrome	acrocallosal syndrome	Acrocallosal Syndrome	1	kept
acrocallosal syndrome	acrocallosal syndrome	acrocallosal syndrome	acrocallosal syndrome	Acrocallosal Syndrome	1	case_or_format

acrocephalosyndactyly	acrocephalosyndactyly	acrocephalosyndactyly	acrocephalosyndactyly	acrocephalosyndactyly	1	kept
acromegaly	acromegaly	acromegaly	acromegaly	acromegaly	1	kept
Acromion	acromion	acromion	acromion	Acromion	1	kept
acromion	acromion	acromion	acromion	Acromion	1	case_or_format
acronym syndrome	acronym syndrome	acronym syndrome	acronym syndrome	acronym syndrome	1	kept
Acrylamides	acrylamides	acrylamides	acrylamides	Acrylamides	1	kept
acrylic acid	acrylic acid	acrylic acid	acrylic acid	acrylic acid	1	kept
acrylic acid derivative	acrylic acid derivative	acrylic acid derivative	acrylic acid derivative	acrylic acid derivative	1	kept
acrylic acid resin	acrylic acid resin	acrylic acid resin	acrylic acid resin	acrylic acid resin	1	kept
act in case of depression	act in case of depression	act in case of depression	act in case of depression	act in case of depression	1	kept
Actaea	actaea	actaea	actaea	Actaea	1	kept
acte exclusif	acte exclusif	acte exclusif	acte exclusif	acte exclusif	1	kept
Acticoat	acticoat	acticoat	acticoat	Acticoat	1	kept
ACTIGRAPHY	actigraphy	actigraphy	actigraphy	Actigraphy	1	case_or_format
actin	actin	actin	actin	actin	1	kept
actin myosin interaction	actin myosin interaction	actin myosin interaction	actin myosin interaction	actin myosin interaction	1	kept

Actinobacteria	actinobacteria	actinobacteria	actinobacteria	Actinobacteria	1	kept
Actinomyces	actinomyces	actinomyces	actinomyces	Actinomyces	1	kept
Actins	actins	actins	actin	actin	1	singular
Action accountability	action accountability	action accountability	action accountability	Action accountability	1	kept
Action controls	action controls	action controls	action controls	Action controls	1	kept
action maze	action maze	action maze	action maze	action maze	1	kept
action plan	action plan	action plan	action plan	action plan	1	kept
Action planning	action planning	action planning	action planning	Action planning	1	kept
Action potential	action potential	action potential	action potential	Action potential	1	kept
action potential	action potential	action potential	action potential	Action potential	1	case_or_format
Action research model	action research model	action research model	action research model	Action research model	1	kept
Action Unit	action unit	action unit	action unit	Action Unit	1	kept
action units guided network	action units guided network	action units guided network	action units guided network	action units guided network	1	kept
actionable feedback model	actionable feedback model	actionable feedback model	actionable feedback model	actionable feedback model	1	kept
actionable processes	actionable processes	actionable processes	actionable processes	actionable processes	1	kept
Actions at the scene	actions at the scene	actions at the scene	actions at the scene	Actions at the scene	1	kept

Actitudes y Práctica en Salud	actitudes y pr ctica en salud	actitudes y pr ctica en salud	actitudes y pr ctica en salud	Actitudes y Práctica en Salud	1	kept
Activated clotting time	activated clotting time	activated clotting time	activated clotting time	Activated clotting time	1	kept
activated clotting time	activated clotting time	activated clotting time	activated clotting time	Activated clotting time	1	case_or_format
ACTIVATED PROTEIN-C	activated protein c	activated protein c	activated protein c	activated protein C	1	case_or_format
activated prothrombin complex	activated prothrombin complex	activated prothrombin complex	activated prothrombin complex	activated prothrombin complex	1	kept
Activated prothrombin complex concentrates	activated prothrombin complex concentrates	activated prothrombin complex concentrates	activated prothrombin complex concentrates	Activated prothrombin complex concentrates	1	kept
Activation	activation	activation	activation	ACTIVATION	1	case_or_format
Activation barriers	activation barriers	activation barriers	activation barriers	Activation barriers	1	kept
Active and Passive Components	active and passive components	active and passive components	active and passive components	Active and Passive Components	1	kept
ACTIVE ANTIRETROVIRAL THERAPY	active antiretroviral therapy	active antiretroviral therapy	active antiretroviral therapy	ACTIVE ANTIRETROVIRAL THERAPY	1	kept
active hyperemia	active hyperemia	active hyperemia	active hyperemia	active hyperemia	1	kept
active involvement	active involvement	active involvement	active involvement	active involvement	1	kept
active learning	active learning	active learning	active learning	Active learning	1	case_or_format
ACTIVE LIFE EXPECTANCY	active life expectancy	active life expectancy	active life expectancy	ACTIVE LIFE EXPECTANCY	1	kept
Active mobilization	active mobilization	active mobilization	active mobilization	active mobilization	1	case_or_format
Active noise control	active noise control	active noise control	active noise control	Active noise control	1	kept

Active radio frequency identification	active radio frequency identification	active radio frequency identification	active radio frequency identification	Active radio frequency identification	1	kept
active rewarming	active rewarming	active rewarming	active rewarming	active rewarming	1	kept
active shooter	active shooter	active shooter	active shooter	active shooter	1	kept
active shortening of the dying process	active shortening of the dying process	active shortening of the dying process	active shortening of the dying process	active shortening of the dying process	1	kept
active surveillance culturing	active surveillance culturing	active surveillance culturing	active surveillance culturing	active surveillance culturing	1	kept
active transport	active transport	active transport	active transport	active transport	1	kept
ACTIVE VOLUNTARY EUTHANASIA	active voluntary euthanasia	active voluntary euthanasia	active voluntary euthanasia	ACTIVE VOLUNTARY EUTHANASIA	1	kept
active warming	active warming	active warming	active warming	active warming	1	kept
active weight bearing	active weight bearing	active weight bearing	active weight bearing	active weight bearing	1	kept
Actividades	actividades	actividades	actividades	Actividades	1	kept
activism	activism	activism	activism	activism	1	kept
Activities	activities	activities	activity	Activity	1	singular
Activity	activity	activity	activity	Activity	1	kept
Activity Cycles	activity cycles	activity cycles	activity cycles	Activity Cycles	1	kept
Activity index	activity index	activity index	activity index	Activity index	1	kept
Activity intensities	activity intensities	activity intensities	activity intensities	Activity intensities	1	kept

Activity levels	activity levels	activity levels	activity levels	Activity levels	1	kept
Activity logs	activity logs	activity logs	activity logs	Activity logs	1	kept
Activity of daily lives	activity of daily lives	activity of daily lives	activity of daily lives	Activity of daily lives	1	kept
Activity of daily living	activity of daily living	activity of daily living	activity of daily living	activity of daily living	1	case_or_format
Activity patterns	activity patterns	activity patterns	activity patterns	Activity patterns	1	kept
activity recognition	activity recognition	activity recognition	activity recognition	Activity recognition	1	case_or_format
Activity register	activity register	activity register	activity register	Activity register	1	kept
activity theory	activity theory	activity theory	activity theory	activity theory	1	kept
activity timing	activity timing	activity timing	activity timing	activity timing	1	kept
Activity tracker	activity tracker	activity tracker	activity tracker	Activity tracker	1	kept
activity tracker	activity tracker	activity tracker	activity tracker	Activity tracker	1	case_or_format
activity-based costing	activity based costing	activity based costing	activity based costing	activity-based costing	1	kept
activity-based funding	activity based funding	activity based funding	activity based funding	activity-based funding	1	kept
activity-based therapy	activity based therapy	activity based therapy	activity based therapy	activity-based therapy	1	kept
Actiwatch	actiwatch	actiwatch	actiwatch	Actiwatch	1	kept
actron	actron	actron	actron	actron	1	kept

actual blood loss	actual blood loss	actual blood loss	actual blood loss	actual blood loss	1	kept
Actual stress	actual stress	actual stress	actual stress	Actual stress	1	kept
actual work condition	actual work condition	actual work condition	actual work condition	actual work condition	1	kept
ACTUARIAL	actuarial	actuarial	actuarial	ACTUARIAL	1	kept
Acuity adaptable	acuity adaptable	acuity adaptable	acuity adaptable	Acuity adaptable	1	kept
acuity adaptable model	acuity adaptable model	acuity adaptable model	acuity adaptable model	acuity adaptable model	1	kept
acuity management	acuity management	acuity management	acuity management	acuity management	1	kept
acuity scale level	acuity scale level	acuity scale level	acuity scale level	acuity scale level	1	kept
Acuity system	acuity system	acuity system	acuity system	Acuity system	1	kept
acuity system	acuity system	acuity system	acuity system	Acuity system	1	case_or_format
Acuity systems	acuity systems	acuity systems	acuity system	Acuity system	1	singular
acuity systems	acuity systems	acuity systems	acuity system	Acuity system	1	singular
Acuity-adaptable	acuity adaptable	acuity adaptable	acuity adaptable	Acuity adaptable	1	case_or_format
acuity-adaptable	acuity adaptable	acuity adaptable	acuity adaptable	Acuity adaptable	1	case_or_format
ACUPUNCTURE	acupuncture	acupuncture	acupuncture	acupuncture	1	case_or_format
Acupuncture Analgesia	acupuncture analgesia	acupuncture analgesia	acupuncture analgesia	Acupuncture Analgesia	1	kept

acupuncture point	acupuncture point	acupuncture point	acupuncture point	acupuncture point	1	kept
Acupuncture Points	acupuncture points	acupuncture points	acupuncture point	acupuncture point	1	singular
acupuncturist	acupuncturist	acupuncturist	acupuncturist	acupuncturist	1	kept
Acute abdomen	acute abdomen	acute abdomen	acute abdomen	acute abdomen	1	case_or_format
Acute abdominal pain	acute abdominal pain	acute abdominal pain	acute abdominal pain	acute abdominal pain	1	case_or_format
acute adult wards	acute adult wards	acute adult wards	acute adult wards	acute adult wards	1	kept
acute alithiasic pancreatitis	acute alithiasic pancreatitis	acute alithiasic pancreatitis	acute alithiasic pancreatitis	acute alithiasic pancreatitis	1	kept
Acute and critical illness	acute and critical illness	acute and critical illness	acute and critical illness	Acute and critical illness	1	kept
Acute and high dependency	acute and high dependency	acute and high dependency	acute and high dependency	Acute and high dependency	1	kept
acute aortic dissection	acute aortic dissection	acute aortic dissection	acute aortic dissection	acute aortic dissection	1	kept
acute aortic syndrome	acute aortic syndrome	acute aortic syndrome	acute aortic syndrome	acute aortic syndrome	1	kept
acute aortic syndromes	acute aortic syndromes	acute aortic syndromes	acute aortic syndrome	acute aortic syndrome	1	singular
acute aplastic crisis	acute aplastic crisis	acute aplastic crisis	acute aplastic crisis	acute aplastic crisis	1	kept
Acute asthma	acute asthma	acute asthma	acute asthma	Acute asthma	1	kept
Acute attacks	acute attacks	acute attacks	acute attacks	Acute attacks	1	kept
Acute bereavement	acute bereavement	acute bereavement	acute bereavement	Acute bereavement	1	kept

Acute bereavement care	acute bereavement care	acute bereavement care	acute bereavement care	Acute bereavement care	1	kept
Acute brain failure	acute brain failure	acute brain failure	acute brain failure	Acute brain failure	1	kept
Acute brain injury	acute brain injury	acute brain injury	acute brain injury	acute brain injury	1	case_or_format
Acute bronchiolitis	acute bronchiolitis	acute bronchiolitis	acute bronchiolitis	Acute bronchiolitis	1	kept
Acute cardiac tamponade	acute cardiac tamponade	acute cardiac tamponade	acute cardiac tamponade	Acute cardiac tamponade	1	kept
acute cardiovascular disease	acute cardiovascular disease	acute cardiovascular disease	acute cardiovascular disease	acute cardiovascular disease	1	kept
ACUTE CARE	acute care	acute care	acute care	acute care	1	case_or_format
acute care center	acute care center	acute care center	acute care center	acute care center	1	kept
acute care common stem	acute care common stem	acute care common stem	acute care common stem	acute care common stem	1	kept
Acute care education	acute care education	acute care education	acute care education	Acute care education	1	kept
Acute care for elders	acute care for elders	acute care for elders	acute care for elders	Acute care for elders	1	kept
Acute care general wards	acute care general wards	acute care general wards	acute care general wards	Acute care general wards	1	kept
acute care hospitals	acute care hospitals	acute care hospitals	acute care hospital	acute care hospital	1	singular
Acute care imaging	acute care imaging	acute care imaging	acute care imaging	Acute care imaging	1	kept
acute care needs	acute care needs	acute care needs	acute care needs	acute care needs	1	kept
Acute care of at risk newborn	acute care of at risk newborn	acute care of at risk newborn	acute care of at risk newborn	Acute care of at risk newborn	1	kept

Acute care provider	acute care provider	acute care provider	acute care provider	Acute care provider	1	kept
acute care rehabilitation	acute care rehabilitation	acute care rehabilitation	acute care rehabilitation	acute care rehabilitation	1	kept
Acute care services	acute care services	acute care services	acute care services	Acute care services	1	kept
Acute care work environment	acute care work environment	acute care work environment	acute care work environment	Acute care work environment	1	kept
Acute cerebral infarction	acute cerebral infarction	acute cerebral infarction	acute cerebral infarction	Acute cerebral infarction	1	kept
Acute Chest Syndrome	acute chest syndrome	acute chest syndrome	acute chest syndrome	acute chest syndrome	1	case_or_format
Acute children’s ward	acute children s ward	acute children s ward	acute children s ward	Acute children’s ward	1	kept
Acute clinical deterioration	acute clinical deterioration	acute clinical deterioration	acute clinical deterioration	Acute clinical deterioration	1	kept
acute cognitive impairment	acute cognitive impairment	acute cognitive impairment	acute cognitive impairment	acute cognitive impairment	1	kept
Acute Complex Care Model	acute complex care model	acute complex care model	acute complex care model	Acute Complex Care Model	1	kept
ACUTE CONFUSION	acute confusion	acute confusion	acute confusion	acute confusion	1	case_or_format
Acute confusional state cognitive function	acute confusional state cognitive function	acute confusional state cognitive function	acute confusional state cognitive function	Acute confusional state cognitive function	1	kept
ACUTE CORONARY SYNDROMES	acute coronary syndromes	acute coronary syndromes	acute coronary syndrome	acute coronary syndrome	1	singular
acute coronary syndromes	acute coronary syndromes	acute coronary syndromes	acute coronary syndrome	acute coronary syndrome	1	singular
acute decompensated heart failure	acute decompensated heart failure	acute decompensated heart failure	acute decompensated heart failure	Acute decompensated heart failure	1	case_or_format
Acute deterioration	acute deterioration	acute deterioration	acute deterioration	Acute deterioration	1	kept

acute deterioration	acute deterioration	acute deterioration	acute deterioration	Acute deterioration	1	case_or_format
acute deteriorations	acute deteriorations	acute deteriorations	acute deterioration	Acute deterioration	1	singular
Acute diarrhea	acute diarrhea	acute diarrhea	acute diarrhea	acute diarrhea	1	case_or_format
Acute disease	acute disease	acute disease	acute disease	Acute Disease	1	case_or_format
Acute exacerbation period	acute exacerbation period	acute exacerbation period	acute exacerbation period	Acute exacerbation period	1	kept
Acute faecal incontinence	acute faecal incontinence	acute faecal incontinence	acute faecal incontinence	Acute faecal incontinence	1	kept
Acute fatty liver of pregnancy	acute fatty liver of pregnancy	acute fatty liver of pregnancy	acute fatty liver of pregnancy	acute fatty liver of pregnancy	1	case_or_format
acute fatty liver of pregnancy, liver failure in pregnancy	acute fatty liver of pregnancy liver failure in pregnancy	acute fatty liver of pregnancy liver failure in pregnancy	acute fatty liver of pregnancy liver failure in pregnancy	acute fatty liver of pregnancy, liver failure in pregnancy	1	kept
acute fulminant myocarditis	acute fulminant myocarditis	acute fulminant myocarditis	acute fulminant myocarditis	Acute fulminant myocarditis	1	case_or_format
ACUTE GASTROENTERITIS	acute gastroenteritis	acute gastroenteritis	acute gastroenteritis	acute gastroenteritis	1	case_or_format
Acute gastrointestinal bleeding	acute gastrointestinal bleeding	acute gastrointestinal bleeding	acute gastrointestinal bleeding	Acute gastrointestinal bleeding	1	kept
Acute Generalized Exanthematous Pustulosis	acute generalized exanthematous pustulosis	acute generalized exanthematous pustulosis	acute generalized exanthematous pustulosis	acute generalized exanthematous pustulosis	1	case_or_format
Acute heart failure care	acute heart failure care	acute heart failure care	acute heart failure care	Acute heart failure care	1	kept
Acute heart failure syndrome	acute heart failure syndrome	acute heart failure syndrome	acute heart failure syndrome	Acute heart failure syndrome	1	kept
ACUTE HEART-FAILURE	acute heart failure	acute heart failure	acute heart failure	acute heart failure	1	case_or_format
acute hemorrhagic pancreatitis	acute hemorrhagic pancreatitis	acute hemorrhagic pancreatitis	acute hemorrhagic pancreatitis	acute hemorrhagic pancreatitis	1	kept

acute hospital	acute hospital	acute hospital	acute hospital	Acute hospitals	1	case_or_format
acute hyperglycemic emergencies	acute hyperglycemic emergencies	acute hyperglycemic emergencies	acute hyperglycemic emergencies	acute hyperglycemic emergencies	1	kept
Acute Hypoxemic Respiratory Failure	acute hypoxemic respiratory failure	acute hypoxemic respiratory failure	acute hypoxemic respiratory failure	acute hypoxemic respiratory failure	1	case_or_format
acute hypoxic respiratory failure	acute hypoxic respiratory failure	acute hypoxic respiratory failure	acute hypoxic respiratory failure	acute hypoxic respiratory failure	1	kept
Acute Illness Management	acute illness management	acute illness management	acute illness management	Acute Illness Management	1	kept
acute illnesses	acute illnesses	acute illnesses	acute illnesses	acute illnesses	1	kept
acute inflammation	acute inflammation	acute inflammation	acute inflammation	acute inflammation	1	kept
acute intensive trauma rehabilitation	acute intensive trauma rehabilitation	acute intensive trauma rehabilitation	acute intensive trauma rehabilitation	acute intensive trauma rehabilitation	1	kept
Acute intermittent porphyria	acute intermittent porphyria	acute intermittent porphyria	acute intermittent porphyria	Acute intermittent porphyria	1	kept
acute intermittent porphyria	acute intermittent porphyria	acute intermittent porphyria	acute intermittent porphyria	Acute intermittent porphyria	1	case_or_format
Acute intestinal failure	acute intestinal failure	acute intestinal failure	acute intestinal failure	Acute intestinal failure	1	kept
acute intestinal failure	acute intestinal failure	acute intestinal failure	acute intestinal failure	Acute intestinal failure	1	case_or_format
Acute ischaemic stroke	acute ischaemic stroke	acute ischaemic stroke	acute ischaemic stroke	Acute ischaemic stroke	1	kept
Acute Kidney injury	acute kidney injury	acute kidney injury	acute kidney injury	Acute Kidney Injury	1	case_or_format
Acute kidney Injury	acute kidney injury	acute kidney injury	acute kidney injury	Acute Kidney Injury	1	case_or_format
Acute Kidney Injury Network classification	acute kidney injury network classification	acute kidney injury network classification	acute kidney injury network classification	Acute Kidney Injury Network classification	1	kept

Acute kidney insufficiency	acute kidney insufficiency	acute kidney insufficiency	acute kidney insufficiency	Acute kidney insufficiency	1	kept
Acute leukemia	acute leukemia	acute leukemia	acute leukemia	acute leukemia	1	case_or_format
Acute life threatening event	acute life threatening event	acute life threatening event	acute life threatening event	Acute life threatening event	1	kept
Acute Life-threatening Events - Recognition and Treatment	acute life threatening events recognition and treatment	acute life threatening events recognition and treatment	acute life threatening events recognition and treatment	Acute Life-threatening Events - Recognition and Treatment	1	kept
acute limb ischemia	acute limb ischemia	acute limb ischemia	acute limb ischemia	Acute limb ischemia	1	case_or_format
Acute Liver Failure	acute liver failure	acute liver failure	acute liver failure	acute liver failure	1	case_or_format
Acute Lung Injury [mortality	acute lung injury mortality	acute lung injury mortality	acute lung injury mortality	Acute Lung Injury [mortality	1	kept
Acute maternal illness	acute maternal illness	acute maternal illness	acute maternal illness	Acute maternal illness	1	kept
ACUTE MECHANICAL VENTILATION	acute mechanical ventilation	acute mechanical ventilation	acute mechanical ventilation	ACUTE MECHANICAL VENTILATION	1	kept
acute medical care	acute medical care	acute medical care	acute medical care	acute medical care	1	kept
Acute medical units	acute medical units	acute medical units	acute medical units	Acute medical units	1	kept
Acute Medicine	acute medicine	acute medicine	acute medicine	Acute medicine	1	case_or_format
Acute mesenteric ischemia	acute mesenteric ischemia	acute mesenteric ischemia	acute mesenteric ischemia	Acute mesenteric ischemia	1	kept
Acute muscle wasting	acute muscle wasting	acute muscle wasting	acute muscle wasting	Acute muscle wasting	1	kept
Acute myeloid leukemia	acute myeloid leukemia	acute myeloid leukemia	acute myeloid leukemia	acute myeloid leukemia	1	case_or_format
acute neurology	acute neurology	acute neurology	acute neurology	acute neurology	1	kept

Acute nontraumatic weakness	acute nontraumatic weakness	acute nontraumatic weakness	acute nontraumatic weakness	Acute nontraumatic weakness	1	kept
Acute normovolemic hemodilution	acute normovolemic hemodilution	acute normovolemic hemodilution	acute normovolemic hemodilution	Acute normovolemic hemodilution	1	kept
acute normovolemic hemodilution	acute normovolemic hemodilution	acute normovolemic hemodilution	acute normovolemic hemodilution	Acute normovolemic hemodilution	1	case_or_format
acute on chronic inflammation	acute on chronic inflammation	acute on chronic inflammation	acute on chronic inflammation	acute on chronic inflammation	1	kept
Acute on chronic respiratory failure	acute on chronic respiratory failure	acute on chronic respiratory failure	acute on chronic respiratory failure	Acute on chronic respiratory failure	1	kept
acute otitis media	acute otitis media	acute otitis media	acute otitis media	acute otitis media	1	kept
acute oxalate nephropathy	acute oxalate nephropathy	acute oxalate nephropathy	acute oxalate nephropathy	acute oxalate nephropathy	1	kept
acute paediatric care	acute paediatric care	acute paediatric care	acute paediatric care	acute paediatric care	1	kept
Acute pain guidelines	acute pain guidelines	acute pain guidelines	acute pain guidelines	Acute pain guidelines	1	kept
acute pain management guidelines	acute pain management guidelines	acute pain management guidelines	acute pain management guidelines	acute pain management guidelines	1	kept
Acute Pain Program	acute pain program	acute pain program	acute pain program	Acute Pain Program	1	kept
acute pain service	acute pain service	acute pain service	acute pain service	acute pain service	1	kept
acute palliative care unit	acute palliative care unit	acute palliative care unit	acute palliative care unit	acute palliative care unit	1	kept
Acute peritoneal dialysis	acute peritoneal dialysis	acute peritoneal dialysis	acute peritoneal dialysis	Acute peritoneal dialysis	1	kept
Acute phase	acute phase	acute phase	acute phase	Acute phase	1	kept
Acute phase cardiac rehabilitation	acute phase cardiac rehabilitation	acute phase cardiac rehabilitation	acute phase cardiac rehabilitation	Acute phase cardiac rehabilitation	1	kept

Acute physiologic and chronic health evaluation II	acute physiologic and chronic health evaluation ii	acute physiologic and chronic health evaluation ii	acute physiologic and chronic health evaluation ii	Acute physiologic and chronic health evaluation II	1	kept
Acute physiological deterioration	acute physiological deterioration	acute physiological deterioration	acute physiological deterioration	Acute physiological deterioration	1	kept
Acute Physiology and Chronic Health Evaluation II	acute physiology and chronic health evaluation ii	acute physiology and chronic health evaluation ii	acute physiology and chronic health evaluation ii	Acute Physiology and Chronic Health Evaluation II	1	kept
Acute physiology and chronic health evaluation II	acute physiology and chronic health evaluation ii	acute physiology and chronic health evaluation ii	acute physiology and chronic health evaluation ii	Acute Physiology and Chronic Health Evaluation II	1	case_or_format
Acute physiology and chronic health evaluation II score	acute physiology and chronic health evaluation ii score	acute physiology and chronic health evaluation ii score	acute physiology and chronic health evaluation ii score	Acute physiology and chronic health evaluation II score	1	kept
Acute physiology and chronic health evaluation score	acute physiology and chronic health evaluation score	acute physiology and chronic health evaluation score	acute physiology and chronic health evaluation score	Acute physiology and chronic health evaluation score	1	kept
Acute Physiology and Chronic Health Evaluation-II	acute physiology and chronic health evaluation ii	acute physiology and chronic health evaluation ii	acute physiology and chronic health evaluation ii	Acute Physiology and Chronic Health Evaluation II	1	case_or_format
ACUTE PHYSIOLOGY SCORE	acute physiology score	acute physiology score	acute physiology score	ACUTE PHYSIOLOGY SCORE	1	kept
acute postoperative emergencies	acute postoperative emergencies	acute postoperative emergencies	acute postoperative emergencies	acute postoperative emergencies	1	kept
acute postoperative pain	acute postoperative pain	acute postoperative pain	acute postoperative pain	acute postoperative pain	1	kept
Acute posttraumatic stress disorder	acute posttraumatic stress disorder	acute posttraumatic stress disorder	acute posttraumatic stress disorder	Acute posttraumatic stress disorder	1	kept
acute psychiatric care	acute psychiatric care	acute psychiatric care	acute psychiatric care	acute psychiatric care	1	kept
Acute Psychiatric Unit	acute psychiatric unit	acute psychiatric unit	acute psychiatric unit	Acute Psychiatric Unit	1	kept
Acute psychiatric wards	acute psychiatric wards	acute psychiatric wards	acute psychiatric wards	Acute psychiatric wards	1	kept
Acute pulmonary disorders	acute pulmonary disorders	acute pulmonary disorders	acute pulmonary disorders	Acute pulmonary disorders	1	kept
acute pulmonary embolism	acute pulmonary embolism	acute pulmonary embolism	acute pulmonary embolism	acute pulmonary embolism	1	kept

Acute pulmonary thromboembolism	acute pulmonary thromboembolism	acute pulmonary thromboembolism	acute pulmonary thromboembolism	Acute pulmonary thromboembolism	1	kept
acute pyelonephritis	acute pyelonephritis	acute pyelonephritis	acute pyelonephritis	acute pyelonephritis	1	kept
Acute rehabilitation	acute rehabilitation	acute rehabilitation	acute rehabilitation	Acute rehabilitation	1	kept
acute rehabilitation practice	acute rehabilitation practice	acute rehabilitation practice	acute rehabilitation practice	acute rehabilitation practice	1	kept
Acute Renal Injury	acute renal injury	acute renal injury	acute renal injury	Acute Renal Injury	1	kept
acute respiratory care unit	acute respiratory care unit	acute respiratory care unit	acute respiratory care unit	acute respiratory care unit	1	kept
Acute respiratory distress	acute respiratory distress	acute respiratory distress	acute respiratory distress	Acute respiratory distress	1	kept
acute respiratory distress	acute respiratory distress	acute respiratory distress	acute respiratory distress	Acute respiratory distress	1	case_or_format
Acute respiratory distress syndrome, ARDS	acute respiratory distress syndrome ards	acute respiratory distress syndrome ards	acute respiratory distress syndrome ards	Acute respiratory distress syndrome, ARDS	1	kept
acute respiratory illness	acute respiratory illness	acute respiratory illness	acute respiratory illness	acute respiratory illness	1	kept
ACUTE RESPIRATORY-DISTRESS	acute respiratory distress	acute respiratory distress	acute respiratory distress	Acute respiratory distress	1	case_or_format
Acute services	acute services	acute services	acute services	Acute services	1	kept
ACUTE STRESS DISORDER	acute stress disorder	acute stress disorder	acute stress disorder	acute stress disorder	1	case_or_format
Acute stress scale ± anxiety	acute stress scale anxiety	acute stress scale anxiety	acute stress scale anxiety	Acute stress scale ± anxiety	1	kept
acute stroke care	acute stroke care	acute stroke care	acute stroke care	acute stroke care	1	kept
Acute stroke intervention	acute stroke intervention	acute stroke intervention	acute stroke intervention	Acute stroke intervention	1	kept

Acute stroke therapy	acute stroke therapy	acute stroke therapy	acute stroke therapy	Acute stroke therapy	1	kept
acute stroke therapy	acute stroke therapy	acute stroke therapy	acute stroke therapy	Acute stroke therapy	1	case_or_format
Acute subarachnoid hemorrhage	acute subarachnoid hemorrhage	acute subarachnoid hemorrhage	acute subarachnoid hemorrhage	Acute subarachnoid hemorrhage	1	kept
acute subdural	acute subdural	acute subdural	acute subdural	acute subdural	1	kept
acute tongue dystonia	acute tongue dystonia	acute tongue dystonia	acute tongue dystonia	acute tongue dystonia	1	kept
acute toxicity	acute toxicity	acute toxicity	acute toxicity	acute toxicity	1	kept
Acute trauma care	acute trauma care	acute trauma care	acute trauma care	Acute trauma care	1	kept
Acute viral bronchiolitis	acute viral bronchiolitis	acute viral bronchiolitis	acute viral bronchiolitis	Acute viral bronchiolitis	1	kept
ACUTE-CARE	acute care	acute care	acute care	acute care	1	case_or_format
acute-care	acute care	acute care	acute care	acute care	1	case_or_format
Acute-care facilities	acute care facilities	acute care facilities	acute care facilities	Acute-care facilities	1	kept
ACUTE-CARE HOSPITALS	acute care hospitals	acute care hospitals	acute care hospital	acute care hospital	1	singular
Acute-on-chronic liver failure	acute on chronic liver failure	acute on chronic liver failure	acute on chronic liver failure	acute on chronic liver failure	1	case_or_format
acute-on-chronic respiratory failure	acute on chronic respiratory failure	acute on chronic respiratory failure	acute on chronic respiratory failure	Acute on chronic respiratory failure	1	case_or_format
acute-surgical-unit	acute surgical unit	acute surgical unit	acute surgical unit	acute-surgical-unit	1	kept
Acute-to-chronic	acute to chronic	acute to chronic	acute to chronic	Acute-to-chronic	1	kept

Acutely unwell	acutely unwell	acutely unwell	acutely unwell	Acutely unwell	1	kept
acylcarnitine	acylcarnitine	acylcarnitine	acylcarnitine	acylcarnitine	1	kept
ad hoc team	ad hoc team	ad hoc team	ad hoc team	ad hoc team	1	kept
ad libitum feeding	ad libitum feeding	ad libitum feeding	ad libitum feeding	ad libitum feeding	1	kept
AD8 screening	ad8 screening	ad8 screening	ad8 screening	AD8 screening	1	kept
Adapt	adapt	adapt	adapt	Adapt	1	kept
Adaptación	adaptaci n	adaptaci n	adaptaci n	Adaptación	1	kept
Adaptation Strategies	adaptation strategies	adaptation strategies	adaptation strategies	Adaptation Strategies	1	kept
adaptation syndrome	adaptation syndrome	adaptation syndrome	adaptation syndrome	adaptation syndrome	1	kept
adaptations	adaptations	adaptations	adaptation	adaptation	1	singular
Adapted physical activityCoronavirus	adapted physical activitycoronavirus	adapted physical activitycoronavirus	adapted physical activitycoronavirus	Adapted physical activityCoronavirus	1	kept
Adaptive algorithm	adaptive algorithm	adaptive algorithm	adaptive algorithm	Adaptive algorithms	1	case_or_format
adaptive boosting	adaptive boosting	adaptive boosting	adaptive boosting	adaptive boosting	1	kept
adaptive clinical trial	adaptive clinical trial	adaptive clinical trial	adaptive clinical trial	adaptive clinical trial	1	kept
Adaptive Clinical Trials as Topic	adaptive clinical trials as topic	adaptive clinical trials as topic	adaptive clinical trials as topic	Adaptive Clinical Trials as Topic	1	kept
Adaptive control	adaptive control	adaptive control	adaptive control	Adaptive control	1	kept

adaptive control	adaptive control	adaptive control	adaptive control	Adaptive control	1	case_or_format
Adaptive control systems	adaptive control systems	adaptive control systems	adaptive control systems	Adaptive control systems	1	kept
adaptive education program	adaptive education program	adaptive education program	adaptive education program	adaptive education program	1	kept
Adaptive filters	adaptive filters	adaptive filters	adaptive filters	Adaptive filters	1	kept
Adaptive Immunity	adaptive immunity	adaptive immunity	adaptive immunity	adaptive immunity	1	case_or_format
Adaptive Leadership	adaptive leadership	adaptive leadership	adaptive leadership	Adaptive Leadership	1	kept
adaptive performance	adaptive performance	adaptive performance	adaptive performance	adaptive performance	1	kept
adaptive responses	adaptive responses	adaptive responses	adaptive responses	adaptive responses	1	kept
adaptive reuse	adaptive reuse	adaptive reuse	adaptive reuse	adaptive reuse	1	kept
adaptive servo ventilation	adaptive servo ventilation	adaptive servo ventilation	adaptive servo ventilation	adaptive servo ventilation	1	kept
Adaptive systems	adaptive systems	adaptive systems	adaptive systems	Adaptive systems	1	kept
Adaptive touch	adaptive touch	adaptive touch	adaptive touch	Adaptive touch	1	kept
addell	addell	addell	addell	addell	1	kept
Addicted	addicted	addicted	addicted	Addicted	1	kept
ADDICTION	addiction	addiction	addiction	addiction	1	case_or_format
addiction treatment	addiction treatment	addiction treatment	addiction treatment	addiction treatment	1	kept

Addictions to alcohol	addictions to alcohol	addictions to alcohol	addictions to alcohol	Addictions to alcohol	1	kept
Addison Disease	addison disease	addison disease	addison disease	Addison Disease	1	kept
Addison disease	addison disease	addison disease	addison disease	Addison Disease	1	case_or_format
ADDITION trial	addition trial	addition trial	addition trial	ADDITION trial	1	kept
additional barrier methods	additional barrier methods	additional barrier methods	additional barrier methods	additional barrier methods	1	kept
additional training	additional training	additional training	additional training	additional training	1	kept
ADDRESSING MORAL DISTRESS	addressing moral distress	addressing moral distress	addressing moral distress	ADDRESSING MORAL DISTRESS	1	kept
ADE - adverse drug events	ade adverse drug events	ade adverse drug events	ade adverse drug events	ADE - adverse drug events	1	kept
Adecuación	adecuaci n	adecuaci n	adecuaci n	Adecuación	1	kept
adefovir	adefovir	adefovir	adefovir	adefovir	1	kept
adenine	adenine	adenine	adenine	adenine	1	kept
Adennotonsillectomy	adennotonsillectomy	adennotonsillectomy	adennotonsillectomy	Adennotonsillectomy	1	kept
adenoid	adenoid	adenoid	adenoid	adenoid	1	kept
adenoma	adenoma	adenoma	adenoma	Adenoma	1	case_or_format
adenomyosis	adenomyosis	adenomyosis	adenomyosis	adenomyosis	1	kept
Adenosine	adenosine	adenosine	adenosine	adenosine	1	case_or_format

adenosine triphosphatase	adenosine triphosphatase	adenosine triphosphatase	adenosine triphosphatase	adenosine triphosphatase	1	kept
Adenosine triphosphate bioluminescence	adenosine triphosphate bioluminescence	adenosine triphosphate bioluminescence	adenosine triphosphate bioluminescence	Adenosine triphosphate bioluminescence	1	kept
adenosine triphosphate test	adenosine triphosphate test	adenosine triphosphate test	adenosine triphosphate test	adenosine triphosphate test	1	kept
adenotonsillar hypertrophy	adenotonsillar hypertrophy	adenotonsillar hypertrophy	adenotonsillar hypertrophy	adenotonsillar hypertrophy	1	kept
Adenotonsillectomy	adenotonsillectomy	adenotonsillectomy	adenotonsillectomy	adenotonsillectomy	1	case_or_format
Adenovirus type 8	adenovirus type 8	adenovirus type 8	adenovirus type 8	Adenovirus type 8	1	kept
adenovirus vector	adenovirus vector	adenovirus vector	adenovirus vector	adenovirus vector	1	kept
Adequacy of treatment of therapeutic effort	adequacy of treatment of therapeutic effort	adequacy of treatment of therapeutic effort	adequacy of treatment of therapeutic effort	Adequacy of treatment of therapeutic effort	1	kept
Adesivos Teciduais	adesivos teciduais	adesivos teciduais	adesivos teciduais	Adesivos Teciduais	1	kept
adherence intervention	adherence intervention	adherence intervention	adherence intervention	adherence intervention	1	kept
Adherence to guidelines	adherence to guidelines	adherence to guidelines	adherence to guidelines	Adherence to guidelines	1	kept
adherence to guidelines	adherence to guidelines	adherence to guidelines	adherence to guidelines	Adherence to guidelines	1	case_or_format
Adherence to hand washing	adherence to hand washing	adherence to hand washing	adherence to hand washing	Adherence to hand washing	1	kept
Adherence to interventions	adherence to interventions	adherence to interventions	adherence to interventions	Adherence to interventions	1	kept
adhesin	adhesin	adhesin	adhesin	adhesin	1	kept
adhesiolysis	adhesiolysis	adhesiolysis	adhesiolysis	adhesiolysis	1	kept

Adhesion molecules	adhesion molecules	adhesion molecules	adhesion molecules	Adhesion molecules	1	kept
adhesive electrodes	adhesive electrodes	adhesive electrodes	adhesive electrodes	adhesive electrodes	1	kept
adhesive sensors	adhesive sensors	adhesive sensors	adhesive sensors	adhesive sensors	1	kept
adhesive tape	adhesive tape	adhesive tape	adhesive tape	adhesive tape	1	kept
Adhesiveness	adhesiveness	adhesiveness	adhesiveness	Adhesiveness	1	kept
Adhesivos Tisulares	adhesivos tisulares	adhesivos tisulares	adhesivos tisulares	Adhesivos Tisulares	1	kept
adipocytokine	adipocytokine	adipocytokine	adipocytokine	adipocytokine	1	kept
adipose	adipose	adipose	adipose	adipose	1	kept
adipose tissue	adipose tissue	adipose tissue	adipose tissue	adipose tissue	1	kept
ADIPOSE-TISSUE	adipose tissue	adipose tissue	adipose tissue	adipose tissue	1	case_or_format
Adjudication	adjudication	adjudication	adjudication	Adjudication	1	kept
Adjustable stabilizing set	adjustable stabilizing set	adjustable stabilizing set	adjustable stabilizing set	Adjustable stabilizing set	1	kept
Adjustment disorders	adjustment disorders	adjustment disorders	adjustment disorder	Adjustment Disorders	1	singular
adjuvant analgesics	adjuvant analgesics	adjuvant analgesics	adjuvant analgesics	adjuvant analgesics	1	kept
adjuvant chemoradiotherapy	adjuvant chemoradiotherapy	adjuvant chemoradiotherapy	adjuvant chemoradiotherapy	adjuvant chemoradiotherapy	1	kept
adjuvant chemotherapy	adjuvant chemotherapy	adjuvant chemotherapy	adjuvant chemotherapy	adjuvant chemotherapy	1	kept

adjuvant radiotherapy	adjuvant radiotherapy	adjuvant radiotherapy	adjuvant radiotherapy	adjuvant radiotherapy	1	kept
ADL	adl	adl	adl	ADL	1	kept
Adl	adl	adl	adl	ADL	1	case_or_format
ADL = activities of daily living	adl activities of daily living	adl activities of daily living	adl activities of daily living	ADL = activities of daily living	1	kept
Administración	administraci n	administraci n	administraci n	Administración	1	kept
Administración de los Servicios de Salud	administraci n de los servicios de salud	administraci n de los servicios de salud	administraci n de los servicios de salud	Administración de los Servicios de Salud	1	kept
Administración de Personal	administraci n de personal	administraci n de personal	administraci n de personal	Administración de Personal	1	kept
Administración de servicios sanitarios	administraci n de servicios sanitarios	administraci n de servicios sanitarios	administraci n de servicios sanitarios	Administración de servicios sanitarios	1	kept
Administración Intravenosa	administraci n intravenosa	administraci n intravenosa	administraci n intravenosa	Administración Intravenosa	1	kept
Administración y dosificación	administraci n y dosificaci n	administraci n y dosificaci n	administraci n y dosificaci n	Administración y dosificación	1	kept
Administration and health services	administration and health services	administration and health services	administration and health services	Administration and health services	1	kept
Administration and organization	administration and organization	administration and organization	administration and organization	Administration and organization	1	kept
administration changeover	administration changeover	administration changeover	administration changeover	administration changeover	1	kept
Administration error	administration error	administration error	administration error	ADMINISTRATION ERRORS	1	case_or_format
Administration Errors	administration errors	administration errors	administration error	ADMINISTRATION ERRORS	1	singular
Administration errors	administration errors	administration errors	administration error	ADMINISTRATION ERRORS	1	singular

ADMINISTRATION SYSTEM	administration system	administration system	administration system	ADMINISTRATION SYSTEM	1	kept
administration times	administration times	administration times	administration times	administration times	1	kept
Administration, Buccal	administration buccal	administration buccal	administration buccal	Administration, Buccal	1	kept
administration, hospital	administration hospital	administration hospital	administration hospital	administration, hospital	1	kept
Administration, inhalation [MeSH]	administration inhalation mesh	administration inhalation mesh	administration inhalation mesh	Administration, inhalation [MeSH]	1	kept
Administration, intravenous	administration intravenous	administration intravenous	administration intravenous	Administration, Intravenous	1	case_or_format
administration, intravenous	administration intravenous	administration intravenous	administration intravenous	Administration, Intravenous	1	case_or_format
administrative assistant	administrative assistant	administrative assistant	administrative assistant	administrative assistant	1	kept
Administrative database	administrative database	administrative database	administrative database	administrative database	1	case_or_format
administrative director	administrative director	administrative director	administrative director	administrative director	1	kept
Administrative pharmacy	administrative pharmacy	administrative pharmacy	administrative pharmacy	Administrative pharmacy	1	kept
Administrative principles	administrative principles	administrative principles	administrative principles	Administrative principles	1	kept
administrative quality indicators	administrative quality indicators	administrative quality indicators	administrative quality indicators	administrative quality indicators	1	kept
Administrative structure	administrative structure	administrative structure	administrative structure	Administrative structure	1	kept
Administrative structures	administrative structures	administrative structures	administrative structure	Administrative structure	1	singular
administrative work	administrative work	administrative work	administrative work	administrative work	1	kept

ADMINISTRATOR TURNOVER	administrator turnover	administrator turnover	administrator turnover	ADMINISTRATOR TURNOVER	1	kept
Administrators	administrators	administrators	administrators	Administrators	1	kept
Administração de Serviços de Saúde	administra o de servios de sa de	administra o de servios de sa de	administra o de servios de sa de	Administração de Serviços de Saúde	1	kept
Admission and treatment process	admission and treatment process	admission and treatment process	admission and treatment process	Admission and treatment process	1	kept
Admission avoidance	admission avoidance	admission avoidance	admission avoidance	Admission avoidance	1	kept
Admission cost	admission cost	admission cost	admission cost	Admission cost	1	kept
Admission criteria	admission criteria	admission criteria	admission criteria	Admission criteria	1	kept
Admission evaluation	admission evaluation	admission evaluation	admission evaluation	Admission evaluation	1	kept
ADMISSION HYPERGLYCEMIA	admission hyperglycemia	admission hyperglycemia	admission hyperglycemia	ADMISSION HYPERGLYCEMIA	1	kept
ADMISSION HYPONATREMIA	admission hyponatremia	admission hyponatremia	admission hyponatremia	ADMISSION HYPONATREMIA	1	kept
admission planning discharge	admission planning discharge	admission planning discharge	admission planning discharge	admission planning discharge	1	kept
admission process	admission process	admission process	admission process	admission process	1	kept
admission rates	admission rates	admission rates	admission rates	admission rates	1	kept
Admission selection criteria	admission selection criteria	admission selection criteria	admission selection criteria	Admission selection criteria	1	kept
Admission time	admission time	admission time	admission time	Admission time	1	kept
ADMISSION UNIT	admission unit	admission unit	admission unit	ADMISSION UNIT	1	kept

admitting department	admitting department	admitting department	admitting department	admitting department	1	kept
adolescence	adolescence	adolescence	adolescence	adolescence	1	kept
adolescent behavior	adolescent behavior	adolescent behavior	adolescent behavior	Adolescent Behavior	1	case_or_format
adolescent development	adolescent development	adolescent development	adolescent development	Adolescent Development	1	case_or_format
adolescent idiopathic scoliosis	adolescent idiopathic scoliosis	adolescent idiopathic scoliosis	adolescent idiopathic scoliosis	adolescent idiopathic scoliosis	1	kept
Adolescent medicine	adolescent medicine	adolescent medicine	adolescent medicine	Adolescent medicine	1	kept
ADOLESCENT MOTHERS	adolescent mothers	adolescent mothers	adolescent mother	adolescent mother	1	singular
Adolescent Mothers	adolescent mothers	adolescent mothers	adolescent mother	adolescent mother	1	singular
adolescent obesity	adolescent obesity	adolescent obesity	adolescent obesity	adolescent obesity	1	kept
Adolescent parents	adolescent parents	adolescent parents	adolescent parents	Adolescent parents	1	kept
Adolescent Psychology	adolescent psychology	adolescent psychology	adolescent psychology	Adolescent Psychology	1	kept
adoptive immunotherapy	adoptive immunotherapy	adoptive immunotherapy	adoptive immunotherapy	adoptive immunotherapy	1	kept
adrenal disease	adrenal disease	adrenal disease	adrenal disease	adrenal disease	1	kept
Adrenal Glands	adrenal glands	adrenal glands	adrenal gland	adrenal gland	1	singular
Adrenal hypofunction	adrenal hypofunction	adrenal hypofunction	adrenal hypofunction	Adrenal hypofunction	1	kept
ADRENAL INSUFFICIENCY	adrenal insufficiency	adrenal insufficiency	adrenal insufficiency	Adrenal Insufficiency	1	case_or_format

adrenal metastasis	adrenal metastasis	adrenal metastasis	adrenal metastasis	adrenal metastasis	1	kept
adrenal tumor	adrenal tumor	adrenal tumor	adrenal tumor	adrenal tumor	1	kept
adrenalectomy	adrenalectomy	adrenalectomy	adrenalectomy	adrenalectomy	1	kept
Adrenaline	adrenaline	adrenaline	adrenaline	Adrenaline	1	kept
adrenaline	adrenaline	adrenaline	adrenaline	Adrenaline	1	case_or_format
adrenergic 2-receptor agonists	adrenergic 2 receptor agonists	adrenergic 2 receptor agonists	adrenergic 2 receptor agonists	adrenergic 2-receptor agonists	1	kept
Adrenergic Antagonists	adrenergic antagonists	adrenergic antagonists	adrenergic antagonists	Adrenergic Antagonists	1	kept
Adrenergic beta-1 Receptor Antagonists	adrenergic beta 1 receptor antagonists	adrenergic beta 1 receptor antagonists	adrenergic beta 1 receptor antagonists	Adrenergic beta-1 Receptor Antagonists	1	kept
Adrenergic beta-Agonists	adrenergic beta agonists	adrenergic beta agonists	adrenergic beta agonists	Adrenergic beta-Agonists	1	kept
adrenergic receptor blocking agent	adrenergic receptor blocking agent	adrenergic receptor blocking agent	adrenergic receptor blocking agent	adrenergic receptor blocking agent	1	kept
adrenergic transmission	adrenergic transmission	adrenergic transmission	adrenergic transmission	adrenergic transmission	1	kept
adrenochrome	adrenochrome	adrenochrome	adrenochrome	adrenochrome	1	kept
Adrenocorticotropic hormone	adrenocorticotropic hormone	adrenocorticotropic hormone	adrenocorticotropic hormone	Adrenocorticotropic Hormone	1	case_or_format
Adrenomedullin	adrenomedullin	adrenomedullin	adrenomedullin	Adrenomedullin	1	kept
adrenomedullin	adrenomedullin	adrenomedullin	adrenomedullin	Adrenomedullin	1	case_or_format
adsorption	adsorption	adsorption	adsorption	Adsorption	1	case_or_format

ADT work intensity	adt work intensity	adt work intensity	adt work intensity	ADT work intensity	1	kept
Adult airway management	adult airway management	adult airway management	adult airway management	Adult airway management	1	kept
adult anaesthesia	adult anaesthesia	adult anaesthesia	adult anaesthesia	Adult anaesthesia	1	case_or_format
Adult Attention Deficit Hyperactivity Disorder Self Report	adult attention deficit hyperactivity disorder self report	adult attention deficit hyperactivity disorder self report	adult attention deficit hyperactivity disorder self report	Adult Attention Deficit Hyperactivity Disorder Self Report	1	kept
adult brain injury	adult brain injury	adult brain injury	adult brain injury	adult brain injury	1	kept
Adult cardiology	adult cardiology	adult cardiology	adult cardiology	Adult cardiology	1	kept
adult cardiology	adult cardiology	adult cardiology	adult cardiology	Adult cardiology	1	case_or_format
adult carer quality of life questionnaire	adult carer quality of life questionnaire	adult carer quality of life questionnaire	adult carer quality of life questionnaire	adult carer quality of life questionnaire	1	kept
Adult Children	adult children	adult children	adult children	Adult Children	1	kept
ADULT COMATOSE SURVIVORS	adult comatose survivors	adult comatose survivors	adult comatose survivors	ADULT COMATOSE SURVIVORS	1	kept
Adult Congenital Heart Disease	adult congenital heart disease	adult congenital heart disease	adult congenital heart disease	adult congenital heart disease	1	case_or_format
adult day care	adult day care	adult day care	adult day care	adult day care	1	kept
adult day services	adult day services	adult day services	adult day services	adult day services	1	kept
Adult diseases, personality disorders, and prenatal	adult diseases personality disorders and prenatal	adult diseases personality disorders and prenatal	adult diseases personality disorders and prenatal	Adult diseases, personality disorders, and prenatal	1	kept
Adult ECMO	adult ecmo	adult ecmo	adult ecmo	Adult ECMO	1	kept
Adult education	adult education	adult education	adult education	Adult education	1	kept

adult education	adult education	adult education	adult education	Adult education	1	case_or_format
Adult fever	adult fever	adult fever	adult fever	Adult fever	1	kept
Adult intussusceptions	adult intussusceptions	adult intussusceptions	adult intussusceptions	Adult intussusceptions	1	kept
adult learners	adult learners	adult learners	adult learner	adult learner	1	singular
Adult learning	adult learning	adult learning	adult learning	Adult learning	1	kept
adult learning theories	adult learning theories	adult learning theories	adult learning theory	Adult learning theory	1	singular
Adult learning theory	adult learning theory	adult learning theory	adult learning theory	Adult learning theory	1	kept
adult management	adult management	adult management	adult management	adult management	1	kept
Adult neurology	adult neurology	adult neurology	adult neurology	Adult neurology	1	kept
adult oncology	adult oncology	adult oncology	adult oncology	adult oncology	1	kept
adult orphan	adult orphan	adult orphan	adult orphan	adult orphan	1	kept
Adult surgery	adult surgery	adult surgery	adult surgery	Adult surgery	1	kept
Adult tetanus	adult tetanus	adult tetanus	adult tetanus	Adult tetanus	1	kept
Adult thoracic medicine	adult thoracic medicine	adult thoracic medicine	adult thoracic medicine	Adult thoracic medicine	1	kept
adult thoracic medicine	adult thoracic medicine	adult thoracic medicine	adult thoracic medicine	Adult thoracic medicine	1	case_or_format
adultism	adultism	adultism	adultism	adultism	1	kept

Adulto	adulto	adulto	adulto	Adultos	1	case_or_format
Advance care directives	advance care directives	advance care directives	advance care directives	Advance care directives	1	kept
advance care directives	advance care directives	advance care directives	advance care directives	Advance care directives	1	case_or_format
advance care discussion	advance care discussion	advance care discussion	advance care discussion	advance care discussion	1	kept
Advance health directive	advance health directive	advance health directive	advance health directive	Advance health directive	1	kept
advance health directive	advance health directive	advance health directive	advance health directive	Advance health directive	1	case_or_format
advance limitation	advance limitation	advance limitation	advance limitation	advance limitation	1	kept
Advance practice provider	advance practice provider	advance practice provider	advance practice provider	Advance practice providers	1	case_or_format
Advance treatment	advance treatment	advance treatment	advance treatment	Advance treatment	1	kept
Advanced	advanced	advanced	advanced	advanced	1	case_or_format
Advanced age	advanced age	advanced age	advanced age	advanced age	1	case_or_format
Advanced assessment	advanced assessment	advanced assessment	advanced assessment	Advanced assessment	1	kept
Advanced Cardiac Life Support [E02 365 647 110 500]	advanced cardiac life support e02 365 647 110 500	advanced cardiac life support e02 365 647 110 500	advanced cardiac life support e02 365 647 110 500	Advanced Cardiac Life Support [E02 365 647 110 500]	1	kept
Advanced Cardiac Life Support in pregnancy	advanced cardiac life support in pregnancy	advanced cardiac life support in pregnancy	advanced cardiac life support in pregnancy	Advanced Cardiac Life Support in pregnancy	1	kept
Advanced cardiorenal clinics	advanced cardiorenal clinics	advanced cardiorenal clinics	advanced cardiorenal clinics	Advanced cardiorenal clinics	1	kept
advanced cardiovascular life support	advanced cardiovascular life support	advanced cardiovascular life support	advanced cardiovascular life support	advanced cardiovascular life support	1	kept

Advanced care	advanced care	advanced care	advanced care	Advanced care	1	kept
Advanced care practitioner	advanced care practitioner	advanced care practitioner	advanced care practitioner	Advanced care practitioner	1	kept
Advanced care provider	advanced care provider	advanced care provider	advanced care provider	Advanced care provider	1	kept
Advanced clinical practice	advanced clinical practice	advanced clinical practice	advanced clinical practice	Advanced clinical practice	1	kept
advanced clinical practitioner	advanced clinical practitioner	advanced clinical practitioner	advanced clinical practitioner	Advanced clinical practitioner	1	case_or_format
Advanced clinical practitioners	advanced clinical practitioners	advanced clinical practitioners	advanced clinical practitioner	Advanced clinical practitioner	1	singular
ADVANCED COGNITIVE IMPAIRMENT	advanced cognitive impairment	advanced cognitive impairment	advanced cognitive impairment	ADVANCED COGNITIVE IMPAIRMENT	1	kept
Advanced controller	advanced controller	advanced controller	advanced controller	Advanced controller	1	kept
Advanced dementia	advanced dementia	advanced dementia	advanced dementia	ADVANCED DEMENTIA	1	case_or_format
advanced disease	advanced disease	advanced disease	advanced disease	advanced disease	1	kept
ADVANCED HEAD	advanced head	advanced head	advanced head	ADVANCED HEAD	1	kept
Advanced home care	advanced home care	advanced home care	advanced home care	Advanced home care	1	kept
advanced life support in obstetrics	advanced life support in obstetrics	advanced life support in obstetrics	advanced life support in obstetrics	advanced life support in obstetrics	1	kept
Advanced lung cancer	advanced lung cancer	advanced lung cancer	advanced lung cancer	advanced lung cancer	1	case_or_format
Advanced maternal age	advanced maternal age	advanced maternal age	advanced maternal age	Advanced maternal age	1	kept
advanced planning	advanced planning	advanced planning	advanced planning	advanced planning	1	kept

advanced practice professional	advanced practice professional	advanced practice professional	advanced practice professional	advanced practice professional	1	kept
advanced practice professionals	advanced practice professionals	advanced practice professionals	advanced practice professional	advanced practice professional	1	singular
Advanced Practice Provider Models	advanced practice provider models	advanced practice provider models	advanced practice provider models	Advanced Practice Provider Models	1	kept
ADVANCED PRACTICE PROVIDERS	advanced practice providers	advanced practice providers	advanced practice provider	advanced practice provider	1	singular
Advanced Practice Providers	advanced practice providers	advanced practice providers	advanced practice provider	advanced practice provider	1	singular
Advanced practices	advanced practices	advanced practices	advanced practice	Advanced practice	1	singular
advanced practitioners	advanced practitioners	advanced practitioners	advanced practitioner	Advanced practitioner	1	singular
Advanced respiratory disease	advanced respiratory disease	advanced respiratory disease	advanced respiratory disease	Advanced respiratory disease	1	kept
Advanced respiratory monitoring	advanced respiratory monitoring	advanced respiratory monitoring	advanced respiratory monitoring	Advanced respiratory monitoring	1	kept
Advanced role	advanced role	advanced role	advanced role	Advanced role	1	kept
Advanced roles	advanced roles	advanced roles	advanced role	Advanced role	1	singular
Advanced technology	advanced technology	advanced technology	advanced technology	Advanced technology	1	kept
advanced technology	advanced technology	advanced technology	advanced technology	Advanced technology	1	case_or_format
Advanced Trauma Life Support	advanced trauma life support	advanced trauma life support	advanced trauma life support	advanced trauma life support	1	case_or_format
Advanced trauma life support	advanced trauma life support	advanced trauma life support	advanced trauma life support	advanced trauma life support	1	case_or_format
Advancing practice	advancing practice	advancing practice	advancing practice	Advancing practice	1	kept

advancing practice	advancing practice	advancing practice	advancing practice	Advancing practice	1	case_or_format
Advantages	advantages	advantages	advantages	Advantages	1	kept
adverse childhood experiences	adverse childhood experiences	adverse childhood experiences	adverse childhood experiences	Adverse Childhood Experiences	1	case_or_format
adverse discharge disposition	adverse discharge disposition	adverse discharge disposition	adverse discharge disposition	adverse discharge disposition	1	kept
adverse drug event	adverse drug event	adverse drug event	adverse drug event	ADVERSE DRUG EVENTS	1	case_or_format
Adverse drug events prevention	adverse drug events prevention	adverse drug events prevention	adverse drug events prevention	Adverse drug events prevention	1	kept
Adverse Drug Reaction	adverse drug reaction	adverse drug reaction	adverse drug reaction	adverse drug reaction	1	case_or_format
Adverse Drug Reactions	adverse drug reactions	adverse drug reactions	adverse drug reaction	adverse drug reaction	1	singular
adverse drug reactions	adverse drug reactions	adverse drug reactions	adverse drug reaction	adverse drug reaction	1	singular
ADVERSE DRUG-REACTIONS	adverse drug reactions	adverse drug reactions	adverse drug reaction	adverse drug reaction	1	singular
adverse event detection	adverse event detection	adverse event detection	adverse event detection	adverse event detection	1	kept
adverse event monitoring	adverse event monitoring	adverse event monitoring	adverse event monitoring	adverse event monitoring	1	kept
Adverse events analgesia related	adverse events analgesia related	adverse events analgesia related	adverse events analgesia related	Adverse events analgesia related	1	kept
Adverse events in hospitals	adverse events in hospitals	adverse events in hospitals	adverse events in hospitals	Adverse events in hospitals	1	kept
adverse events outcome	adverse events outcome	adverse events outcome	adverse events outcome	adverse events outcome	1	kept
Adverse events, epidemiology and detection	adverse events epidemiology and detection	adverse events epidemiology and detection	adverse events epidemiology and detection	Adverse events, epidemiology and detection	1	kept

Adverse incident	adverse incident	adverse incident	adverse incident	Adverse incident	1	kept
adverse incident	adverse incident	adverse incident	adverse incident	Adverse incident	1	case_or_format
adverse outcome pathway	adverse outcome pathway	adverse outcome pathway	adverse outcome pathway	adverse outcome pathway	1	kept
Adverse Outcome Pathways	adverse outcome pathways	adverse outcome pathways	adverse outcome pathway	adverse outcome pathway	1	singular
adverse outcomes	adverse outcomes	adverse outcomes	adverse outcome	adverse outcome	1	singular
Adverse perinatal outcomes	adverse perinatal outcomes	adverse perinatal outcomes	adverse perinatal outcomes	Adverse perinatal outcomes	1	kept
adverse perinatal outcomes	adverse perinatal outcomes	adverse perinatal outcomes	adverse perinatal outcomes	Adverse perinatal outcomes	1	case_or_format
Adverse postoperative outcomes	adverse postoperative outcomes	adverse postoperative outcomes	adverse postoperative outcomes	Adverse postoperative outcomes	1	kept
adverse prognosis	adverse prognosis	adverse prognosis	adverse prognosis	adverse prognosis	1	kept
Adverse reaction	adverse reaction	adverse reaction	adverse reaction	Adverse reaction	1	kept
adverse reactions	adverse reactions	adverse reactions	adverse reaction	Adverse reaction	1	singular
adverse reports	adverse reports	adverse reports	adverse reports	adverse reports	1	kept
Adverse skin reactions	adverse skin reactions	adverse skin reactions	adverse skin reactions	Adverse skin reactions	1	kept
Adverse transfusion reactions	adverse transfusion reactions	adverse transfusion reactions	adverse transfusion reactions	Adverse transfusion reactions	1	kept
Adverse-drug-reactions	adverse drug reactions	adverse drug reactions	adverse drug reaction	adverse drug reaction	1	singular
ADVERSITY	adversity	adversity	adversity	ADVERSITY	1	kept

Adversity quotient	adversity quotient	adversity quotient	adversity quotient	adversity quotient	1	case_or_format
Advertising	advertising	advertising	advertising	Advertising	1	kept
advertising	advertising	advertising	advertising	Advertising	1	case_or_format
ADVICE	advice	advice	advice	ADVICE	1	kept
Advisory Committees[MeSH]	advisory committees mesh	advisory committees mesh	advisory committees mesh	Advisory Committees[MeSH]	1	kept
Advisory systems	advisory systems	advisory systems	advisory systems	Advisory systems	1	kept
advocacy and child health	advocacy and child health	advocacy and child health	advocacy and child health	advocacy and child health	1	kept
Advocacy in health	advocacy in health	advocacy in health	advocacy in health	Advocacy in health	1	kept
Advocate	advocate	advocate	advocate	Advocate	1	kept
AE - adverse event	ae adverse event	ae adverse event	ae adverse event	AE - adverse event	1	kept
AECOPD	aecopd	aecopd	aecopd	AECOPD	1	kept
AEEG teaching	aeeg teaching	aeeg teaching	aeeg teaching	AEEG teaching	1	kept
aeration and oxygenation	aeration and oxygenation	aeration and oxygenation	aeration and oxygenation	aeration and oxygenation	1	kept
aerius oral solution	aerius oral solution	aerius oral solution	aerius oral solution	aerius oral solution	1	kept
aerobe	aerobe	aerobe	aerobe	aerobe	1	kept
aerobic glycolysis	aerobic glycolysis	aerobic glycolysis	aerobic glycolysis	aerobic glycolysis	1	kept

Aerococcus	aerococcus	aerococcus	aerococcus	Aerococcus	1	kept
Aeromedical Transport	aeromedical transport	aeromedical transport	aeromedical transport	Aeromedical transport	1	case_or_format
aeromedical transport	aeromedical transport	aeromedical transport	aeromedical transport	Aeromedical transport	1	case_or_format
aerophagia	aerophagia	aerophagia	aerophagia	aerophagia	1	kept
Aerosol generating	aerosol generating	aerosol generating	aerosol generating	Aerosol generating	1	kept
aerosol generating	aerosol generating	aerosol generating	aerosol generating	Aerosol generating	1	case_or_format
aerosol transmission	aerosol transmission	aerosol transmission	aerosol transmission	aerosol transmission	1	kept
aerosol-generating procedure	aerosol generating procedure	aerosol generating procedure	aerosol generating procedure	aerosol generating procedure	1	case_or_format
Aerosolisation	aerosolisation	aerosolisation	aerosolisation	Aerosolisation	1	kept
Aerosolization	aerosolization	aerosolization	aerosolization	Aerosolization	1	kept
aerosolized particles and droplets	aerosolized particles and droplets	aerosolized particles and droplets	aerosolized particles and droplets	aerosolized particles and droplets	1	kept
AERUGINOSA BACTEREMIA	aeruginosa bacteremia	aeruginosa bacteremia	aeruginosa bacteremia	AERUGINOSA BACTEREMIA	1	kept
aesthetics	aesthetics	aesthetics	aesthetics	aesthetics	1	kept
Aetiology	aetiology	aetiology	aetiology	Aetiology	1	kept
Affective disorder	affective disorder	affective disorder	affective disorder	Affective disorder	1	kept
Affective Disorders, Psychotic	affective disorders psychotic	affective disorders psychotic	affective disorders psychotic	Affective Disorders, Psychotic	1	kept

Affects	affects	affects	affect	Affect	1	singular
AFFERENT LIMB	afferent limb	afferent limb	afferent limb	AFFERENT LIMB	1	kept
afferent limb failure	afferent limb failure	afferent limb failure	afferent limb failure	Afferent limb failure	1	case_or_format
afferent pupillary defect	afferent pupillary defect	afferent pupillary defect	afferent pupillary defect	afferent pupillary defect	1	kept
Affinity chromatography	affinity chromatography	affinity chromatography	affinity chromatography	Affinity chromatography	1	kept
afflux de victimes	afflux de victimes	afflux de victimes	afflux de victimes	afflux de victimes	1	kept
AFGHANISTAN	afghanistan	afghanistan	afghanistan	Afghanistan	1	case_or_format
Afibrinogenemia	afibrinogenemia	afibrinogenemia	afibrinogenemia	Afibrinogenemia	1	kept
afibrinogenemia	afibrinogenemia	afibrinogenemia	afibrinogenemia	Afibrinogenemia	1	case_or_format
aflatoxin	aflatoxin	aflatoxin	aflatoxin	aflatoxin	1	kept
AFLP	aflp	aflp	aflp	AFLP	1	kept
AFM and KPC	afm and kpc	afm and kpc	afm and kpc	AFM and KPC	1	kept
afoxolaner	afoxolaner	afoxolaner	afoxolaner	afoxolaner	1	kept
Africa, Eastern	africa eastern	africa eastern	africa eastern	Africa, Eastern	1	kept
African	african	african	african	African	1	kept
African Continental Ancestry Group	african continental ancestry group	african continental ancestry group	african continental ancestry group	African Continental Ancestry Group	1	kept

AFRICAN-AMERICANS	african americans	african americans	african american	African American	1	singular
After death care	after death care	after death care	after death care	After death care	1	kept
after hours	after hours	after hours	after hours	after hours	1	kept
after hours care	after hours care	after hours care	after hours care	After-Hours Care	1	case_or_format
After-death care	after death care	after death care	after death care	After death care	1	case_or_format
After-hours	after hours	after hours	after hours	after hours	1	case_or_format
after-hours care	after hours care	after hours care	after hours care	After-Hours Care	1	case_or_format
AFTER-HOURS DISCHARGE	after hours discharge	after hours discharge	after hours discharge	AFTER-HOURS DISCHARGE	1	kept
after-hours discharge	after hours discharge	after hours discharge	after hours discharge	AFTER-HOURS DISCHARGE	1	case_or_format
after-hours transfers	after hours transfers	after hours transfers	after hours transfers	after-hours transfers	1	kept
Aftercare paths	aftercare paths	aftercare paths	aftercare paths	Aftercare paths	1	kept
Afterload	afterload	afterload	afterload	Afterload	1	kept
after‑death care	after death care	after death care	after death care	After death care	1	case_or_format
Ag NPs	ag nps	ag nps	ag nps	Ag NPs	1	kept
Agar	agar	agar	agar	agar	1	case_or_format
age difference	age difference	age difference	age difference	age difference	1	kept

Age related malnutrition	age related malnutrition	age related malnutrition	age related malnutrition	Age related malnutrition	1	kept
age specific care	age specific care	age specific care	age specific care	age specific care	1	kept
age-friendly health services	age friendly health services	age friendly health services	age friendly health services	age-friendly health services	1	kept
age-related hearing loss	age related hearing loss	age related hearing loss	age related hearing loss	age-related hearing loss	1	kept
age-specific risk factors	age specific risk factors	age specific risk factors	age specific risk factors	age-specific risk factors	1	kept
Aged 80	aged 80	aged 80	aged 80	Aged 80	1	kept
aged 80-years and over	aged 80 years and over	aged 80 years and over	aged 80 years and over	aged 80-years and over	1	kept
Aged and terminally ill	aged and terminally ill	aged and terminally ill	aged and terminally ill	Aged and terminally ill	1	kept
aged care	aged care	aged care	aged care	aged care	1	kept
aged, 80 and over	aged 80 and over	aged 80 and over	aged 80 and over	Aged, 80 and over	1	case_or_format
ageing	ageing	ageing	ageing	ageing	1	kept
Ageism Attitude Scale	ageism attitude scale	ageism attitude scale	ageism attitude scale	Ageism Attitude Scale	1	kept
Agency	agency	agency	agency	Agency	1	kept
agency	agency	agency	agency	Agency	1	case_or_format
Agency workers	agency workers	agency workers	agency workers	Agency workers	1	kept
agenesis	agenesis	agenesis	agenesis	agenesis	1	kept

AGENT	agent	agent	agent	AGENT	1	kept
Agent-based model	agent based model	agent based model	agent based model	Agent-based model	1	kept
Agent-based models	agent based models	agent based models	agent based model	Agent-based model	1	singular
Agent-based simulation	agent based simulation	agent based simulation	agent based simulation	Agent-based simulation	1	kept
Agentes bloqueantes neuromusculares	agentes bloqueantes neuromusculares	agentes bloqueantes neuromusculares	agentes bloqueantes neuromusculares	Agentes bloqueantes neuromusculares	1	kept
agents acting on the eye	agents acting on the eye	agents acting on the eye	agents acting on the eye	agents acting on the eye	1	kept
agents acting on the genital system	agents acting on the genital system	agents acting on the genital system	agents acting on the genital system	agents acting on the genital system	1	kept
Agesim	agesim	agesim	agesim	Agesim	1	kept
age‐adjusted Charlson comorbidity index	age adjusted charlson comorbidity index	age adjusted charlson comorbidity index	age adjusted charlson comorbidity index	age‐adjusted Charlson comorbidity index	1	kept
aggregate national early warning score	aggregate national early warning score	aggregate national early warning score	aggregate national early warning score	aggregate national early warning score	1	kept
Aggregatibacter actinomycetemcomitans	aggregatibacter actinomycetemcomitans	aggregatibacter actinomycetemcomitans	aggregatibacter actinomycetemcomitans	Aggregatibacter actinomycetemcomitans	1	kept
aggression assessment	aggression assessment	aggression assessment	aggression assessment	aggression assessment	1	kept
aggression incidents	aggression incidents	aggression incidents	aggression incidents	aggression incidents	1	kept
Aggression management	aggression management	aggression management	aggression management	Aggression management	1	kept
Aggressive behavior	aggressive behavior	aggressive behavior	aggressive behavior	Aggressive behavior	1	kept
aggressive behaviors	aggressive behaviors	aggressive behaviors	aggressive behavior	Aggressive behavior	1	singular

Aggressive behaviour	aggressive behaviour	aggressive behaviour	aggressive behaviour	Aggressive behaviour	1	kept
aggressive end-of-life care	aggressive end of life care	aggressive end of life care	aggressive end of life care	aggressive end-of-life care	1	kept
AGGRESSIVENESS	aggressiveness	aggressiveness	aggressiveness	aggressiveness	1	case_or_format
Agile manufacturing systems	agile manufacturing systems	agile manufacturing systems	agile manufacturing systems	Agile manufacturing systems	1	kept
Agility	agility	agility	agility	Agility	1	kept
Aging biomarker	aging biomarker	aging biomarker	aging biomarker	Aging biomarker	1	kept
aging decay	aging decay	aging decay	aging decay	aging decay	1	kept
Aging medicine	aging medicine	aging medicine	aging medicine	Aging medicine	1	kept
Aging of the skin	aging of the skin	aging of the skin	aging of the skin	Aging of the skin	1	kept
Aging population	aging population	aging population	aging population	Aging population	1	kept
aging population	aging population	aging population	aging population	Aging population	1	case_or_format
Aging skin	aging skin	aging skin	aging skin	Aging skin	1	kept
Aging society	aging society	aging society	aging society	Aging society	1	kept
aging workforce	aging workforce	aging workforce	aging workforce	aging workforce	1	kept
Agitated	agitated	agitated	agitated	Agitated	1	kept
Agitated Behavior Scale	agitated behavior scale	agitated behavior scale	agitated behavior scale	Agitated Behavior Scale	1	kept

agitated behaviors	agitated behaviors	agitated behaviors	agitated behaviors	agitated behaviors	1	kept
Agitation intensity	agitation intensity	agitation intensity	agitation intensity	Agitation intensity	1	kept
AGITATION SEDATION SCALE	agitation sedation scale	agitation sedation scale	agitation sedation scale	AGITATION SEDATION SCALE	1	kept
Agitation-sedation assessment	agitation sedation assessment	agitation sedation assessment	agitation sedation assessment	Agitation-sedation assessment	1	kept
Agnostic	agnostic	agnostic	agnostic	Agnostic	1	kept
Agotamiento Emocional	agotamiento emocional	agotamiento emocional	agotamiento emocional	Agotamiento Emocional	1	kept
Agotamiento profesional	agotamiento profesional	agotamiento profesional	agotamiento profesional	Agotamiento profesional	1	kept
agotamiento profesional	agotamiento profesional	agotamiento profesional	agotamiento profesional	Agotamiento profesional	1	case_or_format
AGP	agp	agp	agp	AGP	1	kept
agpar score	agpar score	agpar score	agpar score	agpar score	1	kept
AGREE instrument	agree instrument	agree instrument	agree instrument	AGREE instrument	1	kept
AGREE-II	agree ii	agree ii	agree ii	AGREE II	1	case_or_format
AGREE-REX	agree rex	agree rex	agree rex	AGREE-REX	1	kept
Agressive behavior	agressive behavior	agressive behavior	agressive behavior	Agressive behavior	1	kept
Agua	agua	agua	agua	Agua	1	kept
AHA	aha	aha	aha	AHA	1	kept

aHUS	ahus	ahus	ahus	aHUS	1	kept
Ahvaz	ahvaz	ahvaz	ahvaz	Ahvaz	1	kept
AI adoption	ai adoption	ai adoption	ai adoption	AI adoption	1	kept
AI in health care	ai in health care	ai in health care	ai in health care	AI in health care	1	kept
AID	aid	aid	aid	AIDS	1	case_or_format
aide-soignant	aide soignant	aide soignant	aide soignant	aide-soignant	1	kept
Aids	aids	aids	aid	AIDS	1	singular
AIDS CARE	aids care	aids care	aids care	AIDS CARE	1	kept
AIDS related complex	aids related complex	aids related complex	aids related complex	AIDS related complex	1	kept
AIP	aip	aip	aip	AIP	1	kept
AIR	air	air	air	Air	1	case_or_format
air	air	air	air	Air	1	case_or_format
air ambulances	air ambulances	air ambulances	air ambulance	Air Ambulances	1	singular
Air Bags	air bags	air bags	air bags	Air Bags	1	kept
air bubble detector	air bubble detector	air bubble detector	air bubble detector	air bubble detector	1	kept
Air changes per hour	air changes per hour	air changes per hour	air changes per hour	Air changes per hour	1	kept

air changes per hour	air changes per hour	air changes per hour	air changes per hour	Air changes per hour	1	case_or_format
Air changes per hours	air changes per hours	air changes per hours	air changes per hour	Air changes per hour	1	singular
Air cleaners	air cleaners	air cleaners	air cleaners	Air cleaners	1	kept
Air conditioning	air conditioning	air conditioning	air conditioning	air conditioning	1	case_or_format
Air filtration	air filtration	air filtration	air filtration	Air filtration	1	kept
Air leak	air leak	air leak	air leak	Air leak	1	kept
air leak	air leak	air leak	air leak	Air leak	1	case_or_format
air leak syndrome	air leak syndrome	air leak syndrome	air leak syndrome	air leak syndrome	1	kept
air leakage	air leakage	air leakage	air leakage	air leakage	1	kept
air mattress	air mattress	air mattress	air mattress	air mattress	1	kept
air medical	air medical	air medical	air medical	air medical	1	kept
Air Pollutants	air pollutants	air pollutants	air pollutant	air pollutant	1	singular
Air pollution	air pollution	air pollution	air pollution	air pollution	1	case_or_format
Air purification	air purification	air purification	air purification	Air purification	1	kept
air purification	air purification	air purification	air purification	Air purification	1	case_or_format
air sampler	air sampler	air sampler	air sampler	air sampler	1	kept

Air sampling	air sampling	air sampling	air sampling	air sampling	1	case_or_format
Air temperature	air temperature	air temperature	air temperature	Air temperature	1	kept
air temperature	air temperature	air temperature	air temperature	Air temperature	1	case_or_format
air transport	air transport	air transport	air transport	Air transport	1	case_or_format
air-conditioning	air conditioning	air conditioning	air conditioning	air conditioning	1	case_or_format
AIR-LOSS BEDS	air loss beds	air loss beds	air loss beds	AIR-LOSS BEDS	1	kept
Airbag management	airbag management	airbag management	airbag management	Airbag management	1	kept
AIRBORNE	airborne	airborne	airborne	Airborne	1	case_or_format
AIRBORNE INFECTION	airborne infection	airborne infection	airborne infection	airborne infection	1	case_or_format
Airborne latex particles	airborne latex particles	airborne latex particles	airborne latex particles	Airborne latex particles	1	kept
airborne microorganisms	airborne microorganisms	airborne microorganisms	airborne microorganisms	airborne microorganisms	1	kept
airborne particle	airborne particle	airborne particle	airborne particle	airborne particle	1	kept
airborne precautions	airborne precautions	airborne precautions	airborne precautions	airborne precautions	1	kept
Airborne spread	airborne spread	airborne spread	airborne spread	Airborne spread	1	kept
airborne spread	airborne spread	airborne spread	airborne spread	Airborne spread	1	case_or_format
airborne virus	airborne virus	airborne virus	airborne virus	airborne virus	1	kept

Airlocks	airlocks	airlocks	airlocks	Airlocks	1	kept
Airports	airports	airports	airport	airport	1	singular
Airtraq®	airtraq	airtraq	airtraq	Airtraq®	1	kept
airway assessment	airway assessment	airway assessment	airway assessment	Airway assessment	1	case_or_format
airway assessment: co-existing disease	airway assessment co existing disease	airway assessment co existing disease	airway assessment co existing disease	airway assessment: co-existing disease	1	kept
Airway assistant	airway assistant	airway assistant	airway assistant	Airway assistant	1	kept
airway based educational intervention	airway based educational intervention	airway based educational intervention	airway based educational intervention	airway based educational intervention	1	kept
Airway basics	airway basics	airway basics	airway basics	Airway basics	1	kept
airway breathing circulation disability and exposure approach	airway breathing circulation disability and exposure approach	airway breathing circulation disability and exposure approach	airway breathing circulation disability and exposure approach	airway breathing circulation disability and exposure approach	1	kept
airway bundle	airway bundle	airway bundle	airway bundle	airway bundle	1	kept
airway care	airway care	airway care	airway care	Airway care	1	case_or_format
airway cart	airway cart	airway cart	airway cart	airway cart	1	kept
Airway clearance technique	airway clearance technique	airway clearance technique	airway clearance technique	Airway clearance techniques	1	case_or_format
airway closure	airway closure	airway closure	airway closure	airway closure	1	kept
airway connector	airway connector	airway connector	airway connector	airway connector	1	kept
Airway emergency	airway emergency	airway emergency	airway emergency	airway emergency	1	case_or_format

airway equipment	airway equipment	airway equipment	airway equipment	airway equipment	1	kept
Airway guidelines	airway guidelines	airway guidelines	airway guidelines	Airway guidelines	1	kept
Airway patency	airway patency	airway patency	airway patency	Airway patency	1	kept
airway safety	airway safety	airway safety	airway safety	airway safety	1	kept
Airway score	airway score	airway score	airway score	Airway score	1	kept
Airway secretion clearance	airway secretion clearance	airway secretion clearance	airway secretion clearance	Airway secretion clearance	1	kept
Airway secretions	airway secretions	airway secretions	airway secretions	Airway secretions	1	kept
airway specialist	airway specialist	airway specialist	airway specialist	airway specialist	1	kept
Airway Suctioning	airway suctioning	airway suctioning	airway suctioning	Airway Suctioning	1	kept
airway suctioning	airway suctioning	airway suctioning	airway suctioning	Airway Suctioning	1	case_or_format
Airway team	airway team	airway team	airway team	Airway team	1	kept
airway trolley	airway trolley	airway trolley	airway trolley	airway trolley	1	kept
airway-obstructing tumor	airway obstructing tumor	airway obstructing tumor	airway obstructing tumor	airway-obstructing tumor	1	kept
airway: neonate	airway neonate	airway neonate	airway neonate	airway: neonate	1	kept
airway: paediatric	airway paediatric	airway paediatric	airway paediatric	airway: paediatric	1	kept
Airways Breathing Circulation Disability Exposure	airways breathing circulation disability exposure	airways breathing circulation disability exposure	airways breathing circulation disability exposure	Airways Breathing Circulation Disability Exposure	1	kept

AIS	ais	ais	ais	AIS	1	kept
AIS = American Spinal Injury Association Impairment Scale	ais american spinal injury association impairment scale	ais american spinal injury association impairment scale	ais american spinal injury association impairment scale	AIS = American Spinal Injury Association Impairment Scale	1	kept
AIS-H = Abbreviated Injury Score for the head	ais h abbreviated injury score for the head	ais h abbreviated injury score for the head	ais h abbreviated injury score for the head	AIS-H = Abbreviated Injury Score for the head	1	kept
Aislamiento preventivo	aislamiento preventivo	aislamiento preventivo	aislamiento preventivo	Aislamiento preventivo	1	kept
Ajellomyces dermatitidis	ajellomyces dermatitidis	ajellomyces dermatitidis	ajellomyces dermatitidis	Ajellomyces dermatitidis	1	kept
Ajuste emocional	ajuste emocional	ajuste emocional	ajuste emocional	Ajuste emocional	1	kept
Akaike information criteria	akaike information criteria	akaike information criteria	akaike information criteria	Akaike information criteria	1	kept
akathisia	akathisia	akathisia	akathisia	akathisia	1	kept
AKI = acute kidney injury	aki acute kidney injury	aki acute kidney injury	aki acute kidney injury	AKI, acute kidney injury	1	case_or_format
AKI definition	aki definition	aki definition	aki definition	AKI definition	1	kept
AKI epidemiology	aki epidemiology	aki epidemiology	aki epidemiology	AKI epidemiology	1	kept
AKI survivors	aki survivors	aki survivors	aki survivors	AKI survivors	1	kept
AKI, acute kidney injury	aki acute kidney injury	aki acute kidney injury	aki acute kidney injury	AKI, acute kidney injury	1	kept
Akinesia	akinesia	akinesia	akinesia	akinesia	1	case_or_format
akinetic mutism	akinetic mutism	akinetic mutism	akinetic mutism	akinetic mutism	1	kept
Aktion Saubere Hände	aktion saubere h nde	aktion saubere h nde	aktion saubere h nde	Aktion Saubere Hände	1	kept

Alanine	alanine	alanine	alanine	Alanine	1	kept
alanine	alanine	alanine	alanine	Alanine	1	case_or_format
alarm avoidance	alarm avoidance	alarm avoidance	alarm avoidance	alarm avoidance	1	kept
Alarm Customization	alarm customization	alarm customization	alarm customization	Alarm Customization	1	kept
Alarm desensitisation	alarm desensitisation	alarm desensitisation	alarm desensitisation	Alarm desensitisation	1	kept
alarm desensitization	alarm desensitization	alarm desensitization	alarm desensitization	alarm desensitization	1	kept
Alarm Fatigue Questionnaire	alarm fatigue questionnaire	alarm fatigue questionnaire	alarm fatigue questionnaire	Alarm Fatigue Questionnaire	1	kept
Alarm Hazards	alarm hazards	alarm hazards	alarm hazards	Alarm Hazards	1	kept
Alarm message	alarm message	alarm message	alarm message	Alarm message	1	kept
alarm pressure	alarm pressure	alarm pressure	alarm pressure	alarm pressure	1	kept
Alarm rates	alarm rates	alarm rates	alarm rates	Alarm rates	1	kept
Alarm safety	alarm safety	alarm safety	alarm safety	Alarm safety	1	kept
Alarm signals	alarm signals	alarm signals	alarm signals	Alarm signals	1	kept
Alarm thresholds	alarm thresholds	alarm thresholds	alarm thresholds	Alarm thresholds	1	kept
Alaska Natives	alaska natives	alaska natives	alaska native	Alaska Native	1	singular
albendazole	albendazole	albendazole	albendazole	albendazole	1	kept

Alberta Motor Infant scale	alberta motor infant scale	alberta motor infant scale	alberta motor infant scale	Alberta Motor Infant scale	1	kept
Alberta polio epidemic	alberta polio epidemic	alberta polio epidemic	alberta polio epidemic	Alberta polio epidemic	1	kept
ALBUMIN	albumin	albumin	albumin	albumin	1	case_or_format
Albumin dialysis	albumin dialysis	albumin dialysis	albumin dialysis	albumin dialysis	1	case_or_format
ALBUMIN LEVELS	albumin levels	albumin levels	albumin level	albumin level	1	singular
ALBUMIN SYNTHESIS	albumin synthesis	albumin synthesis	albumin synthesis	ALBUMIN SYNTHESIS	1	kept
Albumin-corrected anion gap	albumin corrected anion gap	albumin corrected anion gap	albumin corrected anion gap	Albumin-corrected anion gap	1	kept
albuminoid	albuminoid	albuminoid	albuminoid	albuminoid	1	kept
albuterol	albuterol	albuterol	albuterol	Albuterol	1	case_or_format
Alcance de práctica	alcance de pr ctica	alcance de pr ctica	alcance de pr ctica	Alcance de práctica	1	kept
ALCOHOL	alcohol	alcohol	alcohol	alcohol	1	case_or_format
Alcohol Amnestic Disorder	alcohol amnestic disorder	alcohol amnestic disorder	alcohol amnestic disorder	Alcohol Amnestic Disorder	1	kept
alcohol based handrub	alcohol based handrub	alcohol based handrub	alcohol based handrub	alcohol based handrub	1	kept
alcohol dehydrogenase inhibition	alcohol dehydrogenase inhibition	alcohol dehydrogenase inhibition	alcohol dehydrogenase inhibition	alcohol dehydrogenase inhibition	1	kept
Alcohol Deterrents	alcohol deterrents	alcohol deterrents	alcohol deterrents	Alcohol Deterrents	1	kept
alcohol drinking	alcohol drinking	alcohol drinking	alcohol drinking	Alcohol Drinking	1	case_or_format

alcohol hand rub	alcohol hand rub	alcohol hand rub	alcohol hand rub	Alcohol hand rub	1	case_or_format
Alcohol rub	alcohol rub	alcohol rub	alcohol rub	Alcohol rub	1	kept
alcohol septal ablation	alcohol septal ablation	alcohol septal ablation	alcohol septal ablation	alcohol septal ablation	1	kept
alcohol symptoms	alcohol symptoms	alcohol symptoms	alcohol symptoms	alcohol symptoms	1	kept
Alcohol use disorder	alcohol use disorder	alcohol use disorder	alcohol use disorder	Alcohol use disorder	1	kept
alcohol use disorder	alcohol use disorder	alcohol use disorder	alcohol use disorder	Alcohol use disorder	1	case_or_format
Alcohol Use Disorders Identification Test	alcohol use disorders identification test	alcohol use disorders identification test	alcohol use disorders identification test	Alcohol Use Disorders Identification Test	1	kept
Alcohol withdrawal delirium	alcohol withdrawal delirium	alcohol withdrawal delirium	alcohol withdrawal delirium	Alcohol Withdrawal Delirium	1	case_or_format
Alcohol withdrawal protocol	alcohol withdrawal protocol	alcohol withdrawal protocol	alcohol withdrawal protocol	Alcohol withdrawal protocol	1	kept
alcohol withdrawal seizures	alcohol withdrawal seizures	alcohol withdrawal seizures	alcohol withdrawal seizure	alcohol withdrawal seizure	1	singular
Alcohol withdrawal symptoms	alcohol withdrawal symptoms	alcohol withdrawal symptoms	alcohol withdrawal symptoms	Alcohol withdrawal symptoms	1	kept
Alcohol-based cleanser	alcohol based cleanser	alcohol based cleanser	alcohol based cleanser	Alcohol-based cleanser	1	kept
alcohol-based hand rub dispenser	alcohol based hand rub dispenser	alcohol based hand rub dispenser	alcohol based hand rub dispenser	alcohol-based hand rub dispenser	1	kept
Alcohol-based hand rub dispensers	alcohol based hand rub dispensers	alcohol based hand rub dispensers	alcohol based hand rub dispenser	alcohol-based hand rub dispenser	1	singular
Alcohol-based hand rubs	alcohol based hand rubs	alcohol based hand rubs	alcohol based hand rub	Alcohol-based hand rub	1	singular
alcohol-based handrub	alcohol based handrub	alcohol based handrub	alcohol based handrub	alcohol based handrub	1	case_or_format

Alcohol-based solution	alcohol based solution	alcohol based solution	alcohol based solution	Alcohol-based solution	1	kept
Alcohol-Induced Disorders	alcohol induced disorders	alcohol induced disorders	alcohol induced disorders	Alcohol-Induced Disorders	1	kept
Alcohol-Induced Disorders, Nervous System	alcohol induced disorders nervous system	alcohol induced disorders nervous system	alcohol induced disorders nervous system	Alcohol-Induced Disorders, Nervous System	1	kept
Alcohol-related disorders	alcohol related disorders	alcohol related disorders	alcohol related disorders	Alcohol-Related Disorders	1	case_or_format
Alcoholic based handrub	alcoholic based handrub	alcoholic based handrub	alcoholic based handrub	Alcoholic based handrub	1	kept
alcoholic beverage	alcoholic beverage	alcoholic beverage	alcoholic beverage	alcoholic beverage	1	kept
Alcoholic beverages	alcoholic beverages	alcoholic beverages	alcoholic beverage	alcoholic beverage	1	singular
alcoholic delirium	alcoholic delirium	alcoholic delirium	alcoholic delirium	alcoholic delirium	1	kept
alcoholic fatty liver	alcoholic fatty liver	alcoholic fatty liver	alcoholic fatty liver	alcoholic fatty liver	1	kept
Alcoholic hand rub	alcoholic hand rub	alcoholic hand rub	alcoholic hand rub	Alcoholic hand rub	1	kept
Alcoholic hepatitis	alcoholic hepatitis	alcoholic hepatitis	alcoholic hepatitis	alcoholic hepatitis	1	case_or_format
Alcoholic Intoxication	alcoholic intoxication	alcoholic intoxication	alcoholic intoxication	Alcoholic Intoxication	1	kept
alcoholic pancreatitis	alcoholic pancreatitis	alcoholic pancreatitis	alcoholic pancreatitis	alcoholic pancreatitis	1	kept
Alergy: latex	alergy latex	alergy latex	alergy latex	Alergy: latex	1	kept
ALERT	alert	alert	alert	ALERT	1	kept
Alert	alert	alert	alert	ALERT	1	case_or_format

alert	alert	alert	alert	ALERT	1	case_or_format
ALERT DOCTORS	alert doctors	alert doctors	alert doctors	ALERT DOCTORS	1	kept
Alert fatigue	alert fatigue	alert fatigue	alert fatigue	alert fatigue	1	case_or_format
alert responds to voice responds to pain unresponsive	alert responds to voice responds to pain unresponsive	alert responds to voice responds to pain unresponsive	alert responds to voice responds to pain unresponsive	alert responds to voice responds to pain unresponsive	1	kept
Alert systems	alert systems	alert systems	alert systems	alert systems	1	case_or_format
Alert Voice Pain Unresponsive scale	alert voice pain unresponsive scale	alert voice pain unresponsive scale	alert voice pain unresponsive scale	Alert Voice Pain Unresponsive scale	1	kept
Alerts	alerts	alerts	alert	ALERT	1	singular
ALF	alf	alf	alf	ALF	1	kept
Alfabetización en Salud	alfabetizaci n en salud	alfabetizaci n en salud	alfabetizaci n en salud	Alfabetización en Salud	1	kept
alfaxalone	alfaxalone	alfaxalone	alfaxalone	alfaxalone	1	kept
alfaxan	alfaxan	alfaxan	alfaxan	alfaxan	1	kept
alginic acid	alginic acid	alginic acid	alginic acid	alginic acid	1	kept
Algorithm design	algorithm design	algorithm design	algorithm design	Algorithm design	1	kept
Algorithmic Approach	algorithmic approach	algorithmic approach	algorithmic approach	Algorithmic Approach	1	kept
Algorithms development	algorithms development	algorithms development	algorithms development	Algorithms development	1	kept
ALI	ali	ali	ali	ALI	1	kept

Alias	alias	alias	alias	Alias	1	kept
ALIF = anterior lumbar interbody fusion	alif anterior lumbar interbody fusion	alif anterior lumbar interbody fusion	alif anterior lumbar interbody fusion	ALIF = anterior lumbar interbody fusion	1	kept
alimemazine	alimemazine	alimemazine	alimemazine	alimemazine	1	kept
alimemazine tartrate	alimemazine tartrate	alimemazine tartrate	alimemazine tartrate	alimemazine tartrate	1	kept
Alkali therapy	alkali therapy	alkali therapy	alkali therapy	Alkali therapy	1	kept
Alkaline phosphatase	alkaline phosphatase	alkaline phosphatase	alkaline phosphatase	alkaline phosphatase	1	case_or_format
All India Institute of Medical Science	all india institute of medical science	all india institute of medical science	all india institute of medical science	All India Institute of Medical Science	1	kept
All phase	all phase	all phase	all phase	All phase	1	kept
ALL-CAUSE MORTALITY	all cause mortality	all cause mortality	all cause mortality	all cause mortality	1	case_or_format
all-cause mortality	all cause mortality	all cause mortality	all cause mortality	all cause mortality	1	case_or_format
allantoin	allantoin	allantoin	allantoin	allantoin	1	kept
Allen's test	allen s test	allen s test	allen s test	Allen's test	1	kept
Allergic disease	allergic disease	allergic disease	allergic disease	allergic disease	1	case_or_format
Allergic emergencies	allergic emergencies	allergic emergencies	allergic emergencies	Allergic emergencies	1	kept
allergic encephalitis	allergic encephalitis	allergic encephalitis	allergic encephalitis	allergic encephalitis	1	kept
Allergic rhinitis	allergic rhinitis	allergic rhinitis	allergic rhinitis	allergic rhinitis	1	case_or_format

Allergist care	allergist care	allergist care	allergist care	Allergist care	1	kept
Allergological study	allergological study	allergological study	allergological study	Allergological study	1	kept
Allergy clinic	allergy clinic	allergy clinic	allergy clinic	Allergy clinic	1	kept
alleviation	alleviation	alleviation	alleviation	alleviation	1	kept
Alleviation of pain	alleviation of pain	alleviation of pain	alleviation of pain	Alleviation of pain	1	kept
Allied health personnel	allied health personnel	allied health personnel	allied health personnel	Allied Health Personnel	1	case_or_format
allied health professiona	allied health professiona	allied health professiona	allied health professiona	allied health professiona	1	kept
Allied Health Professionals	allied health professionals	allied health professionals	allied health professionals	Allied Health Professionals	1	kept
allied health professionals	allied health professionals	allied health professionals	allied health professionals	Allied Health Professionals	1	case_or_format
Allied health professions	allied health professions	allied health professions	allied health professions	Allied health professions	1	kept
allied healthcare professionals	allied healthcare professionals	allied healthcare professionals	allied healthcare professionals	allied healthcare professionals	1	kept
Allocating time	allocating time	allocating time	allocating time	Allocating time	1	kept
ALLOCATION	allocation	allocation	allocation	ALLOCATION	1	kept
Allocation of resources	allocation of resources	allocation of resources	allocation of resources	Allocation of resources	1	kept
allodynia	allodynia	allodynia	allodynia	allodynia	1	kept
Allogeneic transplantation	allogeneic transplantation	allogeneic transplantation	allogeneic transplantation	Allogeneic transplantation	1	kept

Allostasis	allostasis	allostasis	allostasis	Allostasis	1	kept
allostasis	allostasis	allostasis	allostasis	Allostasis	1	case_or_format
allostatic load	allostatic load	allostatic load	allostatic load	allostatic load	1	kept
allosterism	allosterism	allosterism	allosterism	allosterism	1	kept
Allow natural death	allow natural death	allow natural death	allow natural death	Allow natural death	1	kept
almond milk	almond milk	almond milk	almond milk	almond milk	1	kept
almond oil	almond oil	almond oil	almond oil	almond oil	1	kept
Almost adverse events	almost adverse events	almost adverse events	almost adverse events	Almost adverse events	1	kept
almotriptan	almotriptan	almotriptan	almotriptan	almotriptan	1	kept
Aloe	aloe	aloe	aloe	Aloe	1	kept
Alopecia	alopecia	alopecia	alopecia	alopecia	1	case_or_format
Alopecia areata	alopecia areata	alopecia areata	alopecia areata	Alopecia areata	1	kept
alores syrup	alores syrup	alores syrup	alores syrup	alores syrup	1	kept
alosetron	alosetron	alosetron	alosetron	alosetron	1	kept
alpha 1 antitrypsin	alpha 1 antitrypsin	alpha 1 antitrypsin	alpha 1 antitrypsin	alpha 1 antitrypsin	1	kept
alpha 1 antitrypsin deficiency	alpha 1 antitrypsin deficiency	alpha 1 antitrypsin deficiency	alpha 1 antitrypsin deficiency	alpha 1 antitrypsin deficiency	1	kept

alpha 1-Antitrypsin	alpha 1 antitrypsin	alpha 1 antitrypsin	alpha 1 antitrypsin	alpha 1 antitrypsin	1	case_or_format
alpha 1-Antitrypsin Deficiency	alpha 1 antitrypsin deficiency	alpha 1 antitrypsin deficiency	alpha 1 antitrypsin deficiency	alpha 1 antitrypsin deficiency	1	case_or_format
alpha adrenergic receptor	alpha adrenergic receptor	alpha adrenergic receptor	alpha adrenergic receptor	alpha adrenergic receptor	1	kept
alpha amanitin	alpha amanitin	alpha amanitin	alpha amanitin	alpha amanitin	1	kept
alpha defensin	alpha defensin	alpha defensin	alpha defensin	alpha defensin	1	kept
alpha interferon	alpha interferon	alpha interferon	alpha interferon	alpha interferon	1	kept
alpha tocopherol deficiency	alpha tocopherol deficiency	alpha tocopherol deficiency	alpha tocopherol deficiency	alpha tocopherol deficiency	1	kept
alpha tumor necrosis factor	alpha tumor necrosis factor	alpha tumor necrosis factor	alpha tumor necrosis factor	alpha tumor necrosis factor	1	kept
alpha-2 adrenergic agonist	alpha 2 adrenergic agonist	alpha 2 adrenergic agonist	alpha 2 adrenergic agonist	alpha-2 adrenergic agonist	1	kept
Alpha-2 agonists	alpha 2 agonists	alpha 2 agonists	alpha 2 agonists	Alpha-2 agonists	1	kept
alpha-amylase	alpha amylase	alpha amylase	alpha amylase	alpha-amylase	1	kept
alpha-Fetoproteins	alpha fetoproteins	alpha fetoproteins	alpha fetoprotein	alpha fetoprotein	1	singular
alpha-Glucosidases	alpha glucosidases	alpha glucosidases	alpha glucosidases	alpha-Glucosidases	1	kept
Alphavirus Infections	alphavirus infections	alphavirus infections	alphavirus infections	Alphavirus Infections	1	kept
Alprostadil	alprostadil	alprostadil	alprostadil	Alprostadil	1	kept
ALS	als	als	als	ALS	1	kept

Alta de la UCI	alta de la uci	alta de la uci	alta de la uci	Alta de la UCI	1	kept
Alta del paciente	alta del paciente	alta del paciente	alta del paciente	Alta del paciente	1	kept
Alta hospitalaria	alta hospitalaria	alta hospitalaria	alta hospitalaria	Alta hospitalaria	1	kept
Alta precoz prematuros	alta precoz prematuros	alta precoz prematuros	alta precoz prematuros	Alta precoz prematuros	1	kept
alteplase in elderly	alteplase in elderly	alteplase in elderly	alteplase in elderly	alteplase in elderly	1	kept
Alterancy	alterancy	alterancy	alterancy	Alterancy	1	kept
altered mental status	altered mental status	altered mental status	altered mental status	altered mental status	1	kept
Alternaria	alternaria	alternaria	alternaria	Alternaria	1	kept
alternate care site	alternate care site	alternate care site	alternate care site	alternate care site	1	kept
Alternate level of care	alternate level of care	alternate level of care	alternate level of care	Alternate level of care	1	kept
Alternate therapy	alternate therapy	alternate therapy	alternate therapy	Alternate therapy	1	kept
Alternating pressure	alternating pressure	alternating pressure	alternating pressure	Alternating pressure	1	kept
alternative	alternative	alternative	alternative	alternative	1	kept
alternative access	alternative access	alternative access	alternative access	alternative access	1	kept
Alternative analgesia	alternative analgesia	alternative analgesia	alternative analgesia	Alternative analgesia	1	kept
Alternative and augmentative communication	alternative and augmentative communication	alternative and augmentative communication	alternative and augmentative communication	Alternative and augmentative communication	1	kept

alternative and augmentative communication	alternative and augmentative communication	alternative and augmentative communication	alternative and augmentative communication	Alternative and augmentative communication	1	case_or_format
Alternative augmented communication	alternative augmented communication	alternative augmented communication	alternative augmented communication	Alternative augmented communication	1	kept
alternative care site	alternative care site	alternative care site	alternative care site	alternative care site	1	kept
Alternative communication methods	alternative communication methods	alternative communication methods	alternative communication methods	Alternative communication methods	1	kept
Alternative Diversion Programs	alternative diversion programs	alternative diversion programs	alternative diversion programs	Alternative Diversion Programs	1	kept
alternative diversion programs	alternative diversion programs	alternative diversion programs	alternative diversion programs	Alternative Diversion Programs	1	case_or_format
alternative RNA splicing	alternative rna splicing	alternative rna splicing	alternative rna splicing	alternative RNA splicing	1	kept
Alternative Splicing	alternative splicing	alternative splicing	alternative splicing	Alternative Splicing	1	kept
Alternative strategies	alternative strategies	alternative strategies	alternative strategies	Alternative strategies	1	kept
Alternative technologies	alternative technologies	alternative technologies	alternative technologies	Alternative technologies	1	kept
Alternative therapies	alternative therapies	alternative therapies	alternative therapies	alternative therapies	1	case_or_format
Alternative waters	alternative waters	alternative waters	alternative waters	Alternative waters	1	kept
ALTERNATIVES	alternatives	alternatives	alternative	alternative	1	singular
alternatives	alternatives	alternatives	alternative	alternative	1	singular
Alternatives to neonatal autopsy	alternatives to neonatal autopsy	alternatives to neonatal autopsy	alternatives to neonatal autopsy	Alternatives to neonatal autopsy	1	kept
altitude disease	altitude disease	altitude disease	altitude disease	altitude disease	1	kept

altizide plus spironolactone	altizide plus spironolactone	altizide plus spironolactone	altizide plus spironolactone	altizide plus spironolactone	1	kept
Altruistic behavior	altruistic behavior	altruistic behavior	altruistic behavior	Altruistic behavior	1	kept
aluminum acetate	aluminum acetate	aluminum acetate	aluminum acetate	aluminum acetate	1	kept
aluminum hydroxide	aluminum hydroxide	aluminum hydroxide	aluminum hydroxide	aluminum hydroxide	1	kept
aluminum magnesium hydroxide	aluminum magnesium hydroxide	aluminum magnesium hydroxide	aluminum magnesium hydroxide	aluminum magnesium hydroxide	1	kept
alumnus	alumnus	alumnus	alumnus	alumnus	1	kept
alveolar bone loss	alveolar bone loss	alveolar bone loss	alveolar bone loss	alveolar bone loss	1	kept
alveolar consolidation	alveolar consolidation	alveolar consolidation	alveolar consolidation	alveolar consolidation	1	kept
ALVEOLAR MACROPHAGES	alveolar macrophages	alveolar macrophages	alveolar macrophages	ALVEOLAR MACROPHAGES	1	kept
Alveolar recruitment maneuvers	alveolar recruitment maneuvers	alveolar recruitment maneuvers	alveolar recruitment maneuvers	Alveolar recruitment maneuvers	1	kept
alveolar ridge	alveolar ridge	alveolar ridge	alveolar ridge	alveolar ridge	1	kept
Alzheimer disease [psychology]	alzheimer disease psychology	alzheimer disease psychology	alzheimer disease psychology	Alzheimer disease [psychology]	1	kept
alzheimer disease and related dementia care	alzheimer disease and related dementia care	alzheimer disease and related dementia care	alzheimer disease and related dementia care	alzheimer disease and related dementia care	1	kept
Alzheimer's	alzheimer s	alzheimer s	alzheimer s	Alzheimer's	1	kept
Alzheimer's dementia	alzheimer s dementia	alzheimer s dementia	alzheimer s dementia	Alzheimer's dementia	1	kept
Alzheimer's disease and related dementia	alzheimer s disease and related dementia	alzheimer s disease and related dementia	alzheimer s disease and related dementia	Alzheimer's disease and related dementias	1	case_or_format

Alzheimer's special care units	alzheimer s special care units	alzheimer s special care units	alzheimer s special care units	Alzheimer's special care units	1	kept
AMA = American Medical Association	ama american medical association	ama american medical association	ama american medical association	AMA = American Medical Association	1	kept
amanita	amanita	amanita	amanita	amanita	1	kept
Amazonas	amazonas	amazonas	amazonas	Amazonas	1	kept
amber	amber	amber	amber	amber	1	kept
Ambidextrous leadership	ambidextrous leadership	ambidextrous leadership	ambidextrous leadership	Ambidextrous leadership	1	kept
ambidextrous leadership	ambidextrous leadership	ambidextrous leadership	ambidextrous leadership	Ambidextrous leadership	1	case_or_format
Ambient air	ambient air	ambient air	ambient air	ambient air	1	case_or_format
Ambient displays for device status	ambient displays for device status	ambient displays for device status	ambient displays for device status	Ambient displays for device status	1	kept
AMBIENT INTELLIGENCE	ambient intelligence	ambient intelligence	ambient intelligence	AMBIENT INTELLIGENCE	1	kept
ambient light	ambient light	ambient light	ambient light	ambient light	1	kept
Ambient noise	ambient noise	ambient noise	ambient noise	ambient noise	1	case_or_format
Ambiente de la prá ctica	ambiente de la pr ctica	ambiente de la pr ctica	ambiente de la pr ctica	Ambiente de la prá ctica	1	kept
Ambiente de Trabajo	ambiente de trabajo	ambiente de trabajo	ambiente de trabajo	Ambiente de Trabajo	1	kept
Ambiente de Trabalho	ambiente de trabalho	ambiente de trabalho	ambiente de trabalho	Ambiente de Trabalho	1	kept
ambulance care service	ambulance care service	ambulance care service	ambulance care service	ambulance care service	1	kept

Ambulance Diversion	ambulance diversion	ambulance diversion	ambulance diversion	ambulance diversion	1	case_or_format
Ambulance diversions	ambulance diversions	ambulance diversions	ambulance diversion	ambulance diversion	1	singular
ambulance service	ambulance service	ambulance service	ambulance service	ambulance service	1	kept
Ambulance services	ambulance services	ambulance services	ambulance service	ambulance service	1	singular
Ambulance transport	ambulance transport	ambulance transport	ambulance transport	Ambulance transport	1	kept
ambulances	ambulances	ambulances	ambulance	ambulance	1	singular
Ambulate	ambulate	ambulate	ambulate	Ambulate	1	kept
AMBULATION	ambulation	ambulation	ambulation	ambulation	1	case_or_format
Ambulation	ambulation	ambulation	ambulation	ambulation	1	case_or_format
Ambulation, early	ambulation early	ambulation early	ambulation early	Ambulation, early	1	kept
Ambulatory anaesthesia	ambulatory anaesthesia	ambulatory anaesthesia	ambulatory anaesthesia	Ambulatory anaesthesia	1	kept
Ambulatory blood pressure	ambulatory blood pressure	ambulatory blood pressure	ambulatory blood pressure	Ambulatory blood pressure	1	kept
ambulatory blood pressure monitoring	ambulatory blood pressure monitoring	ambulatory blood pressure monitoring	ambulatory blood pressure monitoring	ambulatory blood pressure monitoring	1	kept
ambulatory bloodstream infections	ambulatory bloodstream infections	ambulatory bloodstream infections	ambulatory bloodstream infections	ambulatory bloodstream infections	1	kept
ambulatory care facilities	ambulatory care facilities	ambulatory care facilities	ambulatory care facilities	Ambulatory Care Facilities	1	case_or_format
Ambulatory Care Information Systems	ambulatory care information systems	ambulatory care information systems	ambulatory care information systems	Ambulatory Care Information Systems	1	kept

Ambulatory care sensitive conditions	ambulatory care sensitive conditions	ambulatory care sensitive conditions	ambulatory care sensitive conditions	Ambulatory care sensitive conditions	1	kept
Ambulatory care-sensitive conditions	ambulatory care sensitive conditions	ambulatory care sensitive conditions	ambulatory care sensitive conditions	Ambulatory care sensitive conditions	1	case_or_format
ambulatory clinic	ambulatory clinic	ambulatory clinic	ambulatory clinic	ambulatory clinic	1	kept
ambulatory healthcare associated infection	ambulatory healthcare associated infection	ambulatory healthcare associated infection	ambulatory healthcare associated infection	ambulatory healthcare associated infection	1	kept
Ambulatory monitoring	ambulatory monitoring	ambulatory monitoring	ambulatory monitoring	ambulatory monitoring	1	case_or_format
Ambulatory setting	ambulatory setting	ambulatory setting	ambulatory setting	Ambulatory setting	1	kept
Ambulatory surgery	ambulatory surgery	ambulatory surgery	ambulatory surgery	ambulatory surgery	1	case_or_format
ambulatory training	ambulatory training	ambulatory training	ambulatory training	ambulatory training	1	kept
Ambulatory ventilation	ambulatory ventilation	ambulatory ventilation	ambulatory ventilation	Ambulatory ventilation	1	kept
amcinonide	amcinonide	amcinonide	amcinonide	amcinonide	1	kept
Amebiasis	amebiasis	amebiasis	amebiasis	Amebiasis	1	kept
amenity	amenity	amenity	amenity	amenity	1	kept
AMERICAN ASSOCIATION	american association	american association	american association	AMERICAN ASSOCIATION	1	kept
American Association for the Surgery of Trauma	american association for the surgery of trauma	american association for the surgery of trauma	american association for the surgery of trauma	American Association for the Surgery of Trauma	1	kept
American College of Obstetricians and Gynecologists	american college of obstetricians and gynecologists	american college of obstetricians and gynecologists	american college of obstetricians and gynecologists	American College of Obstetricians and Gynecologists	1	kept
American College of Surgeons	american college of surgeons	american college of surgeons	american college of surgeons	American College of Surgeons	1	kept

American College of Surgeons National Surgical Quality	american college of surgeons national surgical quality	american college of surgeons national surgical quality	american college of surgeons national surgical quality	American College of Surgeons National Surgical Quality	1	kept
American college of surgeons trauma quality improvement	american college of surgeons trauma quality improvement	american college of surgeons trauma quality improvement	american college of surgeons trauma quality improvement	American college of surgeons trauma quality improvement	1	kept
American Congress of Rehabilitation Medicine	american congress of rehabilitation medicine	american congress of rehabilitation medicine	american congress of rehabilitation medicine	American Congress of Rehabilitation Medicine	1	kept
American Diabetic Association	american diabetic association	american diabetic association	american diabetic association	American Diabetic Association	1	kept
american gastroenterological association	american gastroenterological association	american gastroenterological association	american gastroenterological association	american gastroenterological association	1	kept
American Hospital Association	american hospital association	american hospital association	american hospital association	American Hospital Association	1	kept
American Indian or Alaska Native	american indian or alaska native	american indian or alaska native	american indian or alaska native	American Indian or Alaska Native	1	kept
American Indians	american indians	american indians	american indian	American Indian	1	singular
American Knee Society score	american knee society score	american knee society score	american knee society score	American Knee Society score	1	kept
American Recovery and Reinvestment Act	american recovery and reinvestment act	american recovery and reinvestment act	american recovery and reinvestment act	American Recovery and Reinvestment Act	1	kept
American Society of Anesthesiologists physical status	american society of anesthesiologists physical status	american society of anesthesiologists physical status	american society of anesthesiologists physical status	American Society of Anesthesiologists physical status	1	kept
american society of nutrition	american society of nutrition	american society of nutrition	american society of nutrition	american society of nutrition	1	kept
American Spinal Injury Association motor and sensory scales	american spinal injury association motor and sensory scales	american spinal injury association motor and sensory scales	american spinal injury association motor and sensory scales	American Spinal Injury Association motor and sensory scales	1	kept
AMERICAN-COLLEGE	american college	american college	american college	AMERICAN-COLLEGE	1	kept
AMERICAN-HEART-ASSOCIATION	american heart association	american heart association	american heart association	American Heart Association	1	case_or_format
Americas	americas	americas	americas	Americas	1	kept

AMG = American medical graduate	amg american medical graduate	amg american medical graduate	amg american medical graduate	AMG = American medical graduate	1	kept
AMI Standard Clinical Pathway	ami standard clinical pathway	ami standard clinical pathway	ami standard clinical pathway	AMI Standard Clinical Pathway	1	kept
Amides	amides	amides	amides	Amides	1	kept
amifostine	amifostine	amifostine	amifostine	Amifostine	1	case_or_format
Amigurumi toy	amigurumi toy	amigurumi toy	amigurumi toy	Amigurumi toy	1	kept
Amikacin bladder irrigation	amikacin bladder irrigation	amikacin bladder irrigation	amikacin bladder irrigation	Amikacin bladder irrigation	1	kept
amiloride	amiloride	amiloride	amiloride	amiloride	1	kept
amine	amine	amine	amine	Amines	1	case_or_format
Amino acids	amino acids	amino acids	amino acid	amino acid	1	singular
AMINO-ACID ENRICHMENT	amino acid enrichment	amino acid enrichment	amino acid enrichment	AMINO-ACID ENRICHMENT	1	kept
AMINO-ACIDS	amino acids	amino acids	amino acid	amino acid	1	singular
aminoacidemia	aminoacidemia	aminoacidemia	aminoacidemia	aminoacidemia	1	kept
aminobromine	aminobromine	aminobromine	aminobromine	aminobromine	1	kept
aminoglycoside resistance	aminoglycoside resistance	aminoglycoside resistance	aminoglycoside resistance	aminoglycoside resistance	1	kept
aminomethylcycline	aminomethylcycline	aminomethylcycline	aminomethylcycline	aminomethylcycline	1	kept
Aminophylline	aminophylline	aminophylline	aminophylline	aminophylline	1	case_or_format

Amish heritage	amish heritage	amish heritage	amish heritage	Amish heritage	1	kept
Amish mutual networks	amish mutual networks	amish mutual networks	amish mutual networks	Amish mutual networks	1	kept
amisulpride	amisulpride	amisulpride	amisulpride	amisulpride	1	kept
Amlodipine	amlodipine	amlodipine	amlodipine	amlodipine	1	case_or_format
Ammonia	ammonia	ammonia	ammonia	ammonia	1	case_or_format
ammonium chloride	ammonium chloride	ammonium chloride	ammonium chloride	ammonium chloride	1	kept
ammonium compound	ammonium compound	ammonium compound	ammonium compound	ammonium compound	1	kept
Amnesia, Retrograde	amnesia retrograde	amnesia retrograde	amnesia retrograde	Amnesia, Retrograde	1	kept
Amniocentesis	amniocentesis	amniocentesis	amniocentesis	amniocentesis	1	case_or_format
amnioinfusion	amnioinfusion	amnioinfusion	amnioinfusion	amnioinfusion	1	kept
amnion infection syndrome	amnion infection syndrome	amnion infection syndrome	amnion infection syndrome	amnion infection syndrome	1	kept
amnionitis	amnionitis	amnionitis	amnionitis	amnionitis	1	kept
Amniota	amniota	amniota	amniota	Amniota	1	kept
amniote	amniote	amniote	amniote	amniote	1	kept
Amount of information	amount of information	amount of information	amount of information	Amount of information	1	kept
Amount of time	amount of time	amount of time	amount of time	Amount of time	1	kept

Amoxicillin	amoxicillin	amoxicillin	amoxicillin	amoxicillin	1	case_or_format
AmpC beta-lactamase	ampc beta lactamase	ampc beta lactamase	ampc beta lactamase	AmpC beta-lactamase	1	kept
AmpC beta-lactamases	ampc beta lactamases	ampc beta lactamases	ampc beta lactamase	AmpC beta-lactamase	1	singular
AmpC gene	ampc gene	ampc gene	ampc gene	AmpC gene	1	kept
amphotericin B cholesterol sulfate	amphotericin b cholesterol sulfate	amphotericin b cholesterol sulfate	amphotericin b cholesterol sulfate	amphotericin B cholesterol sulfate	1	kept
Amplifiers	amplifiers	amplifiers	amplifiers	Amplifiers	1	kept
amplitude integrated electroencephalogram	amplitude integrated electroencephalogram	amplitude integrated electroencephalogram	amplitude integrated electroencephalogram	amplitude integrated electroencephalogram	1	kept
Amplitude motions	amplitude motions	amplitude motions	amplitude motions	Amplitude motions	1	kept
amplitude-integrated electroencephalogram	amplitude integrated electroencephalogram	amplitude integrated electroencephalogram	amplitude integrated electroencephalogram	amplitude integrated electroencephalogram	1	case_or_format
AMPLITUDE-INTEGRATED ELECTROENCEPHALOGRAPHY	amplitude integrated electroencephalography	amplitude integrated electroencephalography	amplitude integrated electroencephalography	amplitude integrated electroencephalography	1	case_or_format
amputation stump	amputation stump	amputation stump	amputation stump	amputation stump	1	kept
AMR	amr	amr	amr	AMR	1	kept
amrizole	amrizole	amrizole	amrizole	amrizole	1	kept
AMS	ams	ams	ams	AMS	1	kept
AMSU	amsu	amsu	amsu	AMSU	1	kept
amubarvimab	amubarvimab	amubarvimab	amubarvimab	amubarvimab	1	kept

amygdaloid nucleus	amygdaloid nucleus	amygdaloid nucleus	amygdaloid nucleus	amygdaloid nucleus	1	kept
Amylase	amylase	amylase	amylase	Amylases	1	case_or_format
amylase blood level	amylase blood level	amylase blood level	amylase blood level	amylase blood level	1	kept
AMYLASE LEVEL	amylase level	amylase level	amylase level	AMYLASE LEVEL	1	kept
Amyloid	amyloid	amyloid	amyloid	Amyloid	1	kept
amyloid beta protein	amyloid beta protein	amyloid beta protein	amyloid beta protein	amyloid beta protein	1	kept
Amyloid light chain amyloidosis	amyloid light chain amyloidosis	amyloid light chain amyloidosis	amyloid light chain amyloidosis	Amyloid light chain amyloidosis	1	kept
Amyloid Neuropathies, Familial	amyloid neuropathies familial	amyloid neuropathies familial	amyloid neuropathies familial	Amyloid Neuropathies, Familial	1	kept
amyloidosis	amyloidosis	amyloidosis	amyloidosis	Amyloidosis	1	case_or_format
Amyloidosis, Hereditary, Transthyretin-Related	amyloidosis hereditary transthyretin related	amyloidosis hereditary transthyretin related	amyloidosis hereditary transthyretin related	Amyloidosis, Hereditary, Transthyretin-Related	1	kept
AMYOTROPHIC-LATERAL-SCLEROSIS	amyotrophic lateral sclerosis	amyotrophic lateral sclerosis	amyotrophic lateral sclerosis	amyotrophic lateral sclerosis	1	case_or_format
amélioration de la qualité	am lioration de la qualit	am lioration de la qualit	am lioration de la qualit	amélioration de la qualité	1	kept
América Latina	am rica latina	am rica latina	am rica latina	América Latina	1	kept
anabolic agent	anabolic agent	anabolic agent	anabolic agent	anabolic agent	1	kept
anaemia prevention	anaemia prevention	anaemia prevention	anaemia prevention	anaemia prevention	1	kept
anaerobic bacterium	anaerobic bacterium	anaerobic bacterium	anaerobic bacterium	anaerobic bacterium	1	kept

anaerobic cell culture	anaerobic cell culture	anaerobic cell culture	anaerobic cell culture	anaerobic cell culture	1	kept
anaerobic infection	anaerobic infection	anaerobic infection	anaerobic infection	anaerobic infection	1	kept
anaerobic metabolism	anaerobic metabolism	anaerobic metabolism	anaerobic metabolism	anaerobic metabolism	1	kept
Anaesthesia care	anaesthesia care	anaesthesia care	anaesthesia care	Anaesthesia care	1	kept
Anaesthesia in cardiology	anaesthesia in cardiology	anaesthesia in cardiology	anaesthesia in cardiology	Anaesthesia in cardiology	1	kept
Anaesthesia in obstetrics	anaesthesia in obstetrics	anaesthesia in obstetrics	anaesthesia in obstetrics	Anaesthesia in obstetrics	1	kept
anaesthesia machine turnover	anaesthesia machine turnover	anaesthesia machine turnover	anaesthesia machine turnover	anaesthesia machine turnover	1	kept
Anaesthesia monitoring	anaesthesia monitoring	anaesthesia monitoring	anaesthesia monitoring	Anaesthesia monitoring	1	kept
Anaesthesia, audit	anaesthesia audit	anaesthesia audit	anaesthesia audit	Anaesthesia, audit	1	kept
anaesthesia, team	anaesthesia team	anaesthesia team	anaesthesia team	anaesthesia, team	1	kept
anaesthesiologist	anaesthesiologist	anaesthesiologist	anaesthesiologist	Anaesthesiologist	1	case_or_format
Anaesthesiologists	anaesthesiologists	anaesthesiologists	anaesthesiologist	Anaesthesiologist	1	singular
anaesthesiology and perioperative care	anaesthesiology and perioperative care	anaesthesiology and perioperative care	anaesthesiology and perioperative care	anaesthesiology and perioperative care	1	kept
anaesthesiology care providers	anaesthesiology care providers	anaesthesiology care providers	anaesthesiology care providers	anaesthesiology care providers	1	kept
Anaesthetic agents	anaesthetic agents	anaesthetic agents	anaesthetic agents	Anaesthetic agents	1	kept
Anaesthetic clinic	anaesthetic clinic	anaesthetic clinic	anaesthetic clinic	Anaesthetic clinic	1	kept

Anaesthetic emergence	anaesthetic emergence	anaesthetic emergence	anaesthetic emergence	Anaesthetic emergence	1	kept
Anaesthetic emergence agitation	anaesthetic emergence agitation	anaesthetic emergence agitation	anaesthetic emergence agitation	Anaesthetic emergence agitation	1	kept
Anaesthetics	anaesthetics	anaesthetics	anaesthetics	ANAESTHETICS	1	case_or_format
Anaesthetics: management and care	anaesthetics management and care	anaesthetics management and care	anaesthetics management and care	Anaesthetics: management and care	1	kept
Anaesthetist	anaesthetist	anaesthetist	anaesthetist	Anaesthetist	1	kept
anaesthetist	anaesthetist	anaesthetist	anaesthetist	Anaesthetist	1	case_or_format
anaesthetist non technical skill	anaesthetist non technical skill	anaesthetist non technical skill	anaesthetist non technical skill	anaesthetist non technical skill	1	kept
analgesia and sedation	analgesia and sedation	analgesia and sedation	analgesia and sedation	analgesia and sedation	1	kept
analgesia and sedation protocol	analgesia and sedation protocol	analgesia and sedation protocol	analgesia and sedation protocol	analgesia and sedation protocol	1	kept
analgesia nociception index	analgesia nociception index	analgesia nociception index	analgesia nociception index	analgesia nociception index	1	kept
analgesia scale	analgesia scale	analgesia scale	analgesia scale	analgesia scale	1	kept
Analgesia, epidural	analgesia epidural	analgesia epidural	analgesia epidural	Analgesia, Epidural	1	case_or_format
analgesic administration	analgesic administration	analgesic administration	analgesic administration	Analgesic administration	1	case_or_format
Analgesic delivery	analgesic delivery	analgesic delivery	analgesic delivery	Analgesic delivery	1	kept
analgesic delivery	analgesic delivery	analgesic delivery	analgesic delivery	Analgesic delivery	1	case_or_format
analgesics and sedatives	analgesics and sedatives	analgesics and sedatives	analgesics and sedatives	analgesics and sedatives	1	kept

Analgesics, opioid	analgesics opioid	analgesics opioid	analgesics opioid	Analgesics, Opioid	1	case_or_format
Analgosedation	analgosedation	analgosedation	analgosedation	analgosedation	1	case_or_format
Analogue-to-digital conversion	analogue to digital conversion	analogue to digital conversion	analogue to digital conversion	Analogue-to-digital conversion	1	kept
analyse qualitative	analyse qualitative	analyse qualitative	analyse qualitative	analyse qualitative	1	kept
Analyseur social	analyseur social	analyseur social	analyseur social	Analyseur social	1	kept
Analysis of influencing factors	analysis of influencing factors	analysis of influencing factors	analysis of influencing factor	analysis of influencing factor	1	singular
analysis protocol	analysis protocol	analysis protocol	analysis protocol	analysis protocol	1	kept
analytical	analytical	analytical	analytical	analytical	1	kept
Analytical analysis	analytical analysis	analytical analysis	analytical analysis	Analytical analysis	1	kept
Analytical approach	analytical approach	analytical approach	analytical approach	Analytical Approach	1	case_or_format
analytical equipment	analytical equipment	analytical equipment	analytical equipment	analytical equipment	1	kept
Analytical hierarchical process	analytical hierarchical process	analytical hierarchical process	analytical hierarchical process	Analytical hierarchical process	1	kept
analytical method	analytical method	analytical method	analytical method	analytical method	1	kept
analytical phase	analytical phase	analytical phase	analytical phase	analytical phase	1	kept
analyzer	analyzer	analyzer	analyzer	analyzer	1	kept
Anaphylaxis management	anaphylaxis management	anaphylaxis management	anaphylaxis management	Anaphylaxis management	1	kept

Anaplasma phagocytophilum	anaplasma phagocytophilum	anaplasma phagocytophilum	anaplasma phagocytophilum	Anaplasma phagocytophilum	1	kept
anaplasmosis	anaplasmosis	anaplasmosis	anaplasmosis	Anaplasmosis	1	case_or_format
Anaplastic	anaplastic	anaplastic	anaplastic	Anaplastic	1	kept
anaplastic lymphoma kinase	anaplastic lymphoma kinase	anaplastic lymphoma kinase	anaplastic lymphoma kinase	anaplastic lymphoma kinase	1	kept
Anasarca	anasarca	anasarca	anasarca	anasarca	1	case_or_format
Anastomotic Leak	anastomotic leak	anastomotic leak	anastomotic leak	Anastomotic Leak	1	kept
Anastomotic leak	anastomotic leak	anastomotic leak	anastomotic leak	Anastomotic Leak	1	case_or_format
Anastomotic leakage	anastomotic leakage	anastomotic leakage	anastomotic leakage	Anastomotic leakage	1	kept
anastrozole	anastrozole	anastrozole	anastrozole	anastrozole	1	kept
anatomical concepts	anatomical concepts	anatomical concepts	anatomical concepts	anatomical concepts	1	kept
anatomical landmark	anatomical landmark	anatomical landmark	anatomical landmark	anatomical landmark	1	kept
anatomical location	anatomical location	anatomical location	anatomical location	anatomical location	1	kept
Anatomical Variation	anatomical variation	anatomical variation	anatomical variation	anatomical variation	1	case_or_format
Anatomy	anatomy	anatomy	anatomy	anatomy	1	case_or_format
ANC	anc	anc	anc	ANC	1	kept
ANC, absolute neutrophil count	anc absolute neutrophil count	anc absolute neutrophil count	anc absolute neutrophil count	ANC, absolute neutrophil count	1	kept

ANCA associated vasculitis	anca associated vasculitis	anca associated vasculitis	anca associated vasculitis	ANCA associated vasculitis	1	kept
ANCHORED RATING-SCALES	anchored rating scales	anchored rating scales	anchored rating scales	ANCHORED RATING-SCALES	1	kept
AnchorFast	anchorfast	anchorfast	anchorfast	AnchorFast	1	kept
Ancianos	ancianos	ancianos	anciano	Anciano	1	singular
Ancillary services	ancillary services	ancillary services	ancillary services	Ancillary services	1	kept
Ancillary services, hospital	ancillary services hospital	ancillary services hospital	ancillary services hospital	Ancillary Services, Hospital	1	case_or_format
And acute exacerbation	and acute exacerbation	and acute exacerbation	and acute exacerbation	And acute exacerbation	1	kept
and care report	and care report	and care report	and care report	and care report	1	kept
and communication	and communication	and communication	and communication	and communication	1	kept
And debriefing	and debriefing	and debriefing	and debriefing	And debriefing	1	kept
and descriptive phenomenology	and descriptive phenomenology	and descriptive phenomenology	and descriptive phenomenology	and descriptive phenomenology	1	kept
And healthcare policy	and healthcare policy	and healthcare policy	and healthcare policy	And healthcare policy	1	kept
And healthcare workers	and healthcare workers	and healthcare workers	and healthcare workers	And healthcare workers	1	kept
And Jordan	and jordan	and jordan	and jordan	And Jordan	1	kept
And management	and management	and management	and management	And management	1	kept
and nutrition	and nutrition	and nutrition	and nutrition	and nutrition	1	kept

and practice	and practice	and practice	and practice	And practice	1	case_or_format
And premature skin	and premature skin	and premature skin	and premature skin	And premature skin	1	kept
And psychometric evaluation	and psychometric evaluation	and psychometric evaluation	and psychometric evaluation	And psychometric evaluation	1	kept
and subcutaneous insulin	and subcutaneous insulin	and subcutaneous insulin	and subcutaneous insulin	and subcutaneous insulin	1	kept
and witnessed resuscitation	and witnessed resuscitation	and witnessed resuscitation	and witnessed resuscitation	and witnessed resuscitation	1	kept
and workshop	and workshop	and workshop	and workshop	and workshop	1	kept
Andersen	andersen	andersen	andersen	Andersen	1	kept
anderson behavioural state scale	anderson behavioural state scale	anderson behavioural state scale	anderson behavioural state scale	anderson behavioural state scale	1	kept
androgen	androgen	androgen	androgen	androgen	1	kept
androgen deprivation therapy	androgen deprivation therapy	androgen deprivation therapy	androgen deprivation therapy	androgen deprivation therapy	1	kept
Androgens	androgens	androgens	androgen	androgen	1	singular
Android	android	android	android	Android	1	kept
Android Application	android application	android application	android application	Android Application	1	kept
Android application	android application	android application	android application	Android Application	1	case_or_format
Android applications	android applications	android applications	android application	Android Application	1	singular
androstenedione	androstenedione	androstenedione	androstenedione	androstenedione	1	kept

Anemia without bleeding	anemia without bleeding	anemia without bleeding	anemia without bleeding	Anemia without bleeding	1	kept
Anemia, Aplastic	anemia aplastic	anemia aplastic	anemia aplastic	Anemia, Aplastic	1	kept
Anestesiología	anestesiolog a	anestesiolog a	anestesiolog a	Anestesiología	1	kept
Anesthesia and analgesia	anesthesia and analgesia	anesthesia and analgesia	anesthesia and analgesia	anesthesia and analgesia	1	case_or_format
Anesthesia and reanimation	anesthesia and reanimation	anesthesia and reanimation	anesthesia and reanimation	Anesthesia and reanimation	1	kept
anesthesia and surgery	anesthesia and surgery	anesthesia and surgery	anesthesia and surgery	anesthesia and surgery	1	kept
Anesthesia Assistant	anesthesia assistant	anesthesia assistant	anesthesia assistant	Anesthesia Assistant	1	kept
anesthesia assistant	anesthesia assistant	anesthesia assistant	anesthesia assistant	Anesthesia Assistant	1	case_or_format
Anesthesia care unit	anesthesia care unit	anesthesia care unit	anesthesia care unit	Anesthesia care unit	1	kept
anesthesia complications	anesthesia complications	anesthesia complications	anesthesia complication	anesthesia complication	1	singular
anesthesia consideration	anesthesia consideration	anesthesia consideration	anesthesia consideration	anesthesia consideration	1	kept
anesthesia end time	anesthesia end time	anesthesia end time	anesthesia end time	anesthesia end time	1	kept
anesthesia information	anesthesia information	anesthesia information	anesthesia information	anesthesia information	1	kept
Anesthesia inhalation	anesthesia inhalation	anesthesia inhalation	anesthesia inhalation	Anesthesia, Inhalation	1	case_or_format
Anesthesia provider	anesthesia provider	anesthesia provider	anesthesia provider	Anesthesia provider	1	kept
Anesthesia Record Keeping	anesthesia record keeping	anesthesia record keeping	anesthesia record keeping	Anesthesia Record Keeping	1	kept

Anesthesia recovery period	anesthesia recovery period	anesthesia recovery period	anesthesia recovery period	Anesthesia Recovery Period	1	case_or_format
anesthesia recovery postanesthesia care unit	anesthesia recovery postanesthesia care unit	anesthesia recovery postanesthesia care unit	anesthesia recovery postanesthesia care unit	anesthesia recovery postanesthesia care unit	1	kept
Anesthesia Sedation	anesthesia sedation	anesthesia sedation	anesthesia sedation	Anesthesia Sedation	1	kept
ANESTHESIA SOCIETY-SOCIETY	anesthesia society society	anesthesia society society	anesthesia society society	ANESTHESIA SOCIETY-SOCIETY	1	kept
Anesthesia team	anesthesia team	anesthesia team	anesthesia team	Anesthesia team	1	kept
anesthesia team	anesthesia team	anesthesia team	anesthesia team	Anesthesia team	1	case_or_format
anesthesia technician	anesthesia technician	anesthesia technician	anesthesia technician	anesthesia technician	1	kept
Anesthesia technicians	anesthesia technicians	anesthesia technicians	anesthesia technician	anesthesia technician	1	singular
anesthesia technicians	anesthesia technicians	anesthesia technicians	anesthesia technician	anesthesia technician	1	singular
Anesthesia type	anesthesia type	anesthesia type	anesthesia type	Anesthesia type	1	kept
anesthesia ventilator	anesthesia ventilator	anesthesia ventilator	anesthesia ventilator	anesthesia ventilator	1	kept
anesthesia workforce	anesthesia workforce	anesthesia workforce	anesthesia workforce	anesthesia workforce	1	kept
Anesthesia, Caudal	anesthesia caudal	anesthesia caudal	anesthesia caudal	Anesthesia, Caudal	1	kept
Anesthesia, Endotracheal	anesthesia endotracheal	anesthesia endotracheal	anesthesia endotracheal	Anesthesia, Endotracheal	1	kept
Anesthesiologist subspecialty	anesthesiologist subspecialty	anesthesiologist subspecialty	anesthesiologist subspecialty	Anesthesiologist subspecialty	1	kept
ANESTHESIOLOGISTS	anesthesiologists	anesthesiologists	anesthesiologist	anesthesiologist	1	singular

anesthesiologists	anesthesiologists	anesthesiologists	anesthesiologist	anesthesiologist	1	singular
ANESTHESIOLOGY	anesthesiology	anesthesiology	anesthesiology	Anesthesiology	1	case_or_format
anesthesiology department	anesthesiology department	anesthesiology department	anesthesiology department	anesthesiology department	1	kept
anesthesiology diagnostic device	anesthesiology diagnostic device	anesthesiology diagnostic device	anesthesiology diagnostic device	anesthesiology diagnostic device	1	kept
anesthesiology general device	anesthesiology general device	anesthesiology general device	anesthesiology general device	anesthesiology general device	1	kept
anesthesiology room general equipment	anesthesiology room general equipment	anesthesiology room general equipment	anesthesiology room general equipment	anesthesiology room general equipment	1	kept
anesthesiology therapeutic device	anesthesiology therapeutic device	anesthesiology therapeutic device	anesthesiology therapeutic device	anesthesiology therapeutic device	1	kept
anesthesiology unit	anesthesiology unit	anesthesiology unit	anesthesiology unit	anesthesiology unit	1	kept
anesthetic complexity	anesthetic complexity	anesthetic complexity	anesthetic complexity	anesthetic complexity	1	kept
Anesthetic drugs	anesthetic drugs	anesthetic drugs	anesthetic drugs	Anesthetic drugs	1	kept
anesthetic management	anesthetic management	anesthetic management	anesthetic management	anesthetic management	1	kept
Anesthetic team model	anesthetic team model	anesthetic team model	anesthetic team model	Anesthetic team model	1	kept
Anesthetic techniques	anesthetic techniques	anesthetic techniques	anesthetic techniques	Anesthetic techniques	1	kept
anesthetic vaporizer	anesthetic vaporizer	anesthetic vaporizer	anesthetic vaporizer	anesthetic vaporizer	1	kept
Anesthetist	anesthetist	anesthetist	anesthetist	Anesthetists	1	case_or_format
anesthetists	anesthetists	anesthetists	anesthetist	Anesthetists	1	singular

ANESTHETIZED CHILDREN	anesthetized children	anesthetized children	anesthetized children	ANESTHETIZED CHILDREN	1	kept
anesthésie	anesth sie	anesth sie	anesth sie	anesthésie	1	kept
anesthésie-réanimation	anesth sie r animation	anesth sie r animation	anesth sie r animation	anesthésie-réanimation	1	kept
Anestésicos	anest sicos	anest sicos	anest sicos	Anestésicos	1	kept
anetoderma of prematurity	anetoderma of prematurity	anetoderma of prematurity	anetoderma of prematurity	anetoderma of prematurity	1	kept
aneurysm clipping	aneurysm clipping	aneurysm clipping	aneurysm clipping	aneurysm clipping	1	kept
Aneurysm surgery	aneurysm surgery	aneurysm surgery	aneurysm surgery	aneurysm surgery	1	case_or_format
Aneurysm, False	aneurysm false	aneurysm false	aneurysm false	Aneurysm, False	1	kept
Aneurysm, Infected	aneurysm infected	aneurysm infected	aneurysm infected	Aneurysm, Infected	1	kept
ANEURYSMAL SUBARACHNOID HEMORRHAGE	aneurysmal subarachnoid hemorrhage	aneurysmal subarachnoid hemorrhage	aneurysmal subarachnoid hemorrhage	Aneurysmal subarachnoid hemorrhage	1	case_or_format
Anger Expression Scale	anger expression scale	anger expression scale	anger expression scale	Anger Expression Scale	1	kept
Anger management	anger management	anger management	anger management	Anger management	1	kept
Anger Management Therapy	anger management therapy	anger management therapy	anger management therapy	Anger Management Therapy	1	kept
angina	angina	angina	angina	angina	1	kept
Angiodysplasia	angiodysplasia	angiodysplasia	angiodysplasia	Angiodysplasia	1	kept
Angiogram	angiogram	angiogram	angiogram	Angiogram	1	kept

Angiographic evaluation of the cerebral and carotid	angiographic evaluation of the cerebral and carotid	angiographic evaluation of the cerebral and carotid	angiographic evaluation of the cerebral and carotid	Angiographic evaluation of the cerebral and carotid	1	kept
angioma	angioma	angioma	angioma	angioma	1	kept
angiomyolipoma	angiomyolipoma	angiomyolipoma	angiomyolipoma	angiomyolipoma	1	kept
ANGIOPLASTY	angioplasty	angioplasty	angioplasty	Angioplasty	1	case_or_format
Angioscopy	angioscopy	angioscopy	angioscopy	Angioscopy	1	kept
angiotensin	angiotensin	angiotensin	angiotensin	angiotensin	1	kept
angiotensin converting enzyme inhibitors	angiotensin converting enzyme inhibitors	angiotensin converting enzyme inhibitors	angiotensin converting enzyme inhibitor	Angiotensin-Converting Enzyme Inhibitors	1	singular
angiotensin II receptor blockers	angiotensin ii receptor blockers	angiotensin ii receptor blockers	angiotensin ii receptor blockers	angiotensin II receptor blockers	1	kept
Angiotensin II Type 1 Receptor Blockers	angiotensin ii type 1 receptor blockers	angiotensin ii type 1 receptor blockers	angiotensin ii type 1 receptor blockers	Angiotensin II Type 1 Receptor Blockers	1	kept
Angiotensin receptor blocker	angiotensin receptor blocker	angiotensin receptor blocker	angiotensin receptor blocker	Angiotensin receptor blocker	1	kept
angiotensin receptor blockers	angiotensin receptor blockers	angiotensin receptor blockers	angiotensin receptor blocker	Angiotensin receptor blocker	1	singular
angiotensin-converting enzyme inhibitor	angiotensin converting enzyme inhibitor	angiotensin converting enzyme inhibitor	angiotensin converting enzyme inhibitor	Angiotensin-Converting Enzyme Inhibitors	1	case_or_format
Anglophone	anglophone	anglophone	anglophone	Anglophone	1	kept
anhedonia	anhedonia	anhedonia	anhedonia	anhedonia	1	kept
Anhui province	anhui province	anhui province	anhui province	Anhui province	1	kept
Animal assisted intervention	animal assisted intervention	animal assisted intervention	animal assisted intervention	Animal assisted intervention	1	kept

animal assisted intervention	animal assisted intervention	animal assisted intervention	animal assisted intervention	Animal assisted intervention	1	case_or_format
Animal assisted therapy	animal assisted therapy	animal assisted therapy	animal assisted therapy	Animal Assisted Therapy	1	case_or_format
animal disease	animal disease	animal disease	animal disease	animal disease	1	kept
Animal Diseases	animal diseases	animal diseases	animal disease	animal disease	1	singular
Animal experimentation	animal experimentation	animal experimentation	animal experimentation	Animal Experimentation	1	case_or_format
animal food	animal food	animal food	animal food	animal food	1	kept
Animal foods	animal foods	animal foods	animal food	animal food	1	singular
Animal husbandry	animal husbandry	animal husbandry	animal husbandry	Animal husbandry	1	kept
animal husbandry	animal husbandry	animal husbandry	animal husbandry	Animal husbandry	1	case_or_format
Animal model	animal model	animal model	animal model	animal model	1	case_or_format
animal science	animal science	animal science	animal science	animal science	1	kept
Animal welfare	animal welfare	animal welfare	animal welfare	animal welfare	1	case_or_format
animal-assisted activity	animal assisted activity	animal assisted activity	animal assisted activity	Animal-assisted activity	1	case_or_format
Animal-assisted intervention	animal assisted intervention	animal assisted intervention	animal assisted intervention	Animal assisted intervention	1	case_or_format
Animal-assisted interventions	animal assisted interventions	animal assisted interventions	animal assisted intervention	Animal assisted intervention	1	singular
animal-assisted interventions	animal assisted interventions	animal assisted interventions	animal assisted intervention	Animal assisted intervention	1	singular

Animal-assisted therapy	animal assisted therapy	animal assisted therapy	animal assisted therapy	Animal Assisted Therapy	1	case_or_format
animal-assisted therapy	animal assisted therapy	animal assisted therapy	animal assisted therapy	Animal Assisted Therapy	1	case_or_format
ANIMAL-MODEL	animal model	animal model	animal model	animal model	1	case_or_format
anion exchange	anion exchange	anion exchange	anion exchange	anion exchange	1	kept
Ankle	ankle	ankle	ankle	Ankle	1	kept
ankle	ankle	ankle	ankle	Ankle	1	case_or_format
Ankle and foot pain	ankle and foot pain	ankle and foot pain	ankle and foot pain	Ankle and foot pain	1	kept
ankle and foot pain	ankle and foot pain	ankle and foot pain	ankle and foot pain	Ankle and foot pain	1	case_or_format
ankle circumference	ankle circumference	ankle circumference	ankle circumference	ankle circumference	1	kept
ankle dislocation	ankle dislocation	ankle dislocation	ankle dislocation	ankle dislocation	1	kept
ankle dorsiflexion angle	ankle dorsiflexion angle	ankle dorsiflexion angle	ankle dorsiflexion angle	ankle dorsiflexion angle	1	kept
ankle fracture	ankle fracture	ankle fracture	ankle fracture	ankle fracture	1	kept
Ankle Injuries	ankle injuries	ankle injuries	ankle injury	ankle injury	1	singular
ankle plantarflexion angle	ankle plantarflexion angle	ankle plantarflexion angle	ankle plantarflexion angle	ankle plantarflexion angle	1	kept
Ankle-brachial index	ankle brachial index	ankle brachial index	ankle brachial index	ankle brachial index	1	case_or_format
Ankyloglossia	ankyloglossia	ankyloglossia	ankyloglossia	ankyloglossia	1	case_or_format

annotation	annotation	annotation	annotation	annotation	1	kept
annotation protocol	annotation protocol	annotation protocol	annotation protocol	annotation protocol	1	kept
Annotation scheme	annotation scheme	annotation scheme	annotation scheme	Annotation scheme	1	kept
ANNOTeM	annotem	annotem	annotem	ANNOTeM	1	kept
ANNOYANCE	annoyance	annoyance	annoyance	annoyance	1	case_or_format
Annual Reports as Topic	annual reports as topic	annual reports as topic	annual reports as topic	Annual Reports as Topic	1	kept
annuloplasty	annuloplasty	annuloplasty	annuloplasty	annuloplasty	1	kept
Anomaly detection	anomaly detection	anomaly detection	anomaly detection	Anomaly detection	1	kept
anomaly detection	anomaly detection	anomaly detection	anomaly detection	Anomaly detection	1	case_or_format
anonymization	anonymization	anonymization	anonymization	anonymization	1	kept
anonymous	anonymous	anonymous	anonymous	anonymous	1	kept
Anonyms and Pseudonyms	anonyms and pseudonyms	anonyms and pseudonyms	anonyms and pseudonyms	Anonyms and Pseudonyms	1	kept
anoplasty	anoplasty	anoplasty	anoplasty	anoplasty	1	kept
anorectal disease	anorectal disease	anorectal disease	anorectal disease	anorectal disease	1	kept
Anorexia Nervosa	anorexia nervosa	anorexia nervosa	anorexia nervosa	anorexia nervosa	1	case_or_format
anoxic myoclonus	anoxic myoclonus	anoxic myoclonus	anoxic myoclonus	anoxic myoclonus	1	kept

ansamycin derivative	ansamycin derivative	ansamycin derivative	ansamycin derivative	ansamycin derivative	1	kept
Answer set programming	answer set programming	answer set programming	answer set programming	Answer set programming	1	kept
ant bacterial gene	ant bacterial gene	ant bacterial gene	ant bacterial gene	ant bacterial gene	1	kept
Antagonism	antagonism	antagonism	antagonism	Antagonism	1	kept
Antagonists	antagonists	antagonists	antagonists	Antagonists	1	kept
antagonists and inhibitors	antagonists and inhibitors	antagonists and inhibitors	antagonists and inhibitors	antagonists and inhibitors	1	kept
antecedents	antecedents	antecedents	antecedents	ANTECEDENTS	1	case_or_format
Antenatal Care	antenatal care	antenatal care	antenatal care	antenatal care	1	case_or_format
Antenatal care	antenatal care	antenatal care	antenatal care	antenatal care	1	case_or_format
antenatal corticosteroids	antenatal corticosteroids	antenatal corticosteroids	antenatal corticosteroids	ANTENATAL CORTICOSTEROIDS	1	case_or_format
antenatal counseling	antenatal counseling	antenatal counseling	antenatal counseling	antenatal counseling	1	kept
antenatal depression	antenatal depression	antenatal depression	antenatal depression	antenatal depression	1	kept
Antenatal palliative care	antenatal palliative care	antenatal palliative care	antenatal palliative care	Antenatal palliative care	1	kept
Antepartum surveillance	antepartum surveillance	antepartum surveillance	antepartum surveillance	Antepartum surveillance	1	kept
anterior	anterior	anterior	anterior	anterior	1	kept
anterior approach	anterior approach	anterior approach	anterior approach	anterior approach	1	kept

anterior cerebral artery	anterior cerebral artery	anterior cerebral artery	anterior cerebral artery	anterior cerebral artery	1	kept
anterior cervical decompression and fusion	anterior cervical decompression and fusion	anterior cervical decompression and fusion	anterior cervical decompression and fusion	anterior cervical decompression and fusion	1	kept
ANTERIOR CIRCULATION ANEURYSMS	anterior circulation aneurysms	anterior circulation aneurysms	anterior circulation aneurysms	ANTERIOR CIRCULATION ANEURYSMS	1	kept
ANTERIOR COLPORRHAPHY	anterior colporrhaphy	anterior colporrhaphy	anterior colporrhaphy	ANTERIOR COLPORRHAPHY	1	kept
anterior communication artery aneurysm	anterior communication artery aneurysm	anterior communication artery aneurysm	anterior communication artery aneurysm	anterior communication artery aneurysm	1	kept
anterior myocardial infarction	anterior myocardial infarction	anterior myocardial infarction	anterior myocardial infarction	anterior myocardial infarction	1	kept
anterior pelvic ring fracture	anterior pelvic ring fracture	anterior pelvic ring fracture	anterior pelvic ring fracture	anterior pelvic ring fracture	1	kept
anterior spine fusion	anterior spine fusion	anterior spine fusion	anterior spine fusion	anterior spine fusion	1	kept
anterior surgical approach	anterior surgical approach	anterior surgical approach	anterior surgical approach	anterior surgical approach	1	kept
anterior thoracolumbar fusion	anterior thoracolumbar fusion	anterior thoracolumbar fusion	anterior thoracolumbar fusion	anterior thoracolumbar fusion	1	kept
Anterior wall defects	anterior wall defects	anterior wall defects	anterior wall defects	Anterior wall defects	1	kept
anteverted nostril	anteverted nostril	anteverted nostril	anteverted nostril	anteverted nostril	1	kept
anthelmintic agent	anthelmintic agent	anthelmintic agent	anthelmintic agent	anthelmintic agent	1	kept
anthocyanin	anthocyanin	anthocyanin	anthocyanin	anthocyanin	1	kept
anthracycline	anthracycline	anthracycline	anthracycline	anthracycline	1	kept
anthracycline derivative	anthracycline derivative	anthracycline derivative	anthracycline derivative	anthracycline derivative	1	kept

anthrax	anthrax	anthrax	anthrax	anthrax	1	kept
anthropology of health	anthropology of health	anthropology of health	anthropology of health	anthropology of health	1	kept
Anthropology, Medical	anthropology medical	anthropology medical	anthropology medical	Anthropology, Medical	1	kept
anthropometric measurements	anthropometric measurements	anthropometric measurements	anthropometric measurements	anthropometric measurements	1	kept
Anthropometric measures	anthropometric measures	anthropometric measures	anthropometric measures	Anthropometric measures	1	kept
anthroposophic medicine	anthroposophic medicine	anthroposophic medicine	anthroposophic medicine	anthroposophic medicine	1	kept
Anthroposophical medicine	anthroposophical medicine	anthroposophical medicine	anthroposophical medicine	Anthroposophical medicine	1	kept
Anthroposophy	anthroposophy	anthroposophy	anthroposophy	Anthroposophy	1	kept
Anti-bacterial activity	anti bacterial activity	anti bacterial activity	anti bacterial activity	Anti-bacterial activity	1	kept
Anti-bacterial agents	anti bacterial agents	anti bacterial agents	anti bacterial agents	Anti-Bacterial Agents	1	case_or_format
Anti-bacterial agents [MeSH]	anti bacterial agents mesh	anti bacterial agents mesh	anti bacterial agents mesh	Anti-bacterial agents [MeSH]	1	kept
Anti-Factor Xa	anti factor xa	anti factor xa	anti factor xa	Anti-Factor Xa	1	kept
Anti-infection	anti infection	anti infection	anti infection	Anti-infection	1	kept
Anti-Infective Agents, Urinary [therapeutic use]	anti infective agents urinary therapeutic use	anti infective agents urinary therapeutic use	anti infective agents urinary therapeutic use	Anti-Infective Agents, Urinary [therapeutic use]	1	kept
Anti-infective drugs	anti infective drugs	anti infective drugs	anti infective drugs	Anti-infective drugs	1	kept
anti-infective therapy	anti infective therapy	anti infective therapy	anti infective therapy	anti-infective therapy	1	kept

Anti-inflammator	anti inflammator	anti inflammator	anti inflammator	Anti-inflammator	1	kept
Anti-inflammatory Drugs	anti inflammatory drugs	anti inflammatory drugs	anti inflammatory drugs	Anti-inflammatory Drugs	1	kept
Anti-mannan antibody	anti mannan antibody	anti mannan antibody	anti mannan antibody	Anti-mannan antibody	1	kept
anti-maskers	anti maskers	anti maskers	anti maskers	anti-maskers	1	kept
Anti-Mullerian Hormone	anti mullerian hormone	anti mullerian hormone	anti mullerian hormone	Anti-Mullerian Hormone	1	kept
Anti-Mullerian hormone	anti mullerian hormone	anti mullerian hormone	anti mullerian hormone	Anti-Mullerian Hormone	1	case_or_format
Anti-NMDA encephalitis	anti nmda encephalitis	anti nmda encephalitis	anti nmda encephalitis	Anti-NMDA encephalitis	1	kept
Anti-NMDA receptor encephalitis	anti nmda receptor encephalitis	anti nmda receptor encephalitis	anti nmda receptor encephalitis	Anti-NMDA receptor encephalitis	1	kept
Anti-pressure	anti pressure	anti pressure	anti pressure	Anti-pressure	1	kept
Anti-pressure ulcer bed	anti pressure ulcer bed	anti pressure ulcer bed	anti pressure ulcer bed	Anti-pressure ulcer bed	1	kept
Anti-Retroviral Agents	anti retroviral agents	anti retroviral agents	anti retroviral agents	Anti-Retroviral Agents	1	kept
anti-SARS-CoV-2 agent	anti sars cov 2 agent	anti sars cov 2 agent	anti sars cov 2 agent	anti-SARS-CoV-2 agent	1	kept
Anti-seizure medications	anti seizure medications	anti seizure medications	anti seizure medications	Anti-seizure medications	1	kept
Anti-TNF agents	anti tnf agents	anti tnf agents	anti tnf agents	Anti-TNF agents	1	kept
anti-TNF-α	anti tnf	anti tnf	anti tnf	anti-TNF-α	1	kept
Anti-ulcer agent	anti ulcer agent	anti ulcer agent	anti ulcer agent	Anti-Ulcer Agents	1	case_or_format

anti-vaccination movement	anti vaccination movement	anti vaccination movement	anti vaccination movement	anti-vaccination movement	1	kept
anti-vaccine	anti vaccine	anti vaccine	anti vaccine	anti-vaccine	1	kept
anti-VEGF	anti vegf	anti vegf	anti vegf	anti-VEGF	1	kept
Anti-VEGF agents	anti vegf agents	anti vegf agents	anti vegf agents	Anti-VEGF agents	1	kept
antiangina pectoris agent	antiangina pectoris agent	antiangina pectoris agent	antiangina pectoris agent	antiangina pectoris agent	1	kept
antiapoptotic activity	antiapoptotic activity	antiapoptotic activity	antiapoptotic activity	antiapoptotic activity	1	kept
antiarrhythmic drugs	antiarrhythmic drugs	antiarrhythmic drugs	antiarrhythmic drugs	antiarrhythmic drugs	1	kept
Antibacterial activity	antibacterial activity	antibacterial activity	antibacterial activity	antibacterial activity	1	case_or_format
Antibacterial efficacy	antibacterial efficacy	antibacterial efficacy	antibacterial efficacy	Antibacterial efficacy	1	kept
Antibacterials	antibacterials	antibacterials	antibacterials	Antibacterials	1	kept
antibiorésistance	antibior sistance	antibior sistance	antibior sistance	antibiorésistance	1	kept
antibiothérapie	antibioth rapie	antibioth rapie	antibioth rapie	antibiothérapie	1	kept
antibiotic associated diarrhea	antibiotic associated diarrhea	antibiotic associated diarrhea	antibiotic associated diarrhea	antibiotic associated diarrhea	1	kept
Antibiotic coated catheter	antibiotic coated catheter	antibiotic coated catheter	antibiotic coated catheter	Antibiotic coated catheter	1	kept
ANTIBIOTIC CONSUMPTION	antibiotic consumption	antibiotic consumption	antibiotic consumption	Antibiotic consumption	1	case_or_format
antibiotic duration	antibiotic duration	antibiotic duration	antibiotic duration	antibiotic duration	1	kept

antibiotic exposure	antibiotic exposure	antibiotic exposure	antibiotic exposure	antibiotic exposure	1	kept
Antibiotic prescribing	antibiotic prescribing	antibiotic prescribing	antibiotic prescribing	antibiotic prescribing	1	case_or_format
antibiotic reduction	antibiotic reduction	antibiotic reduction	antibiotic reduction	antibiotic reduction	1	kept
antibiotic regimen modification behavior	antibiotic regimen modification behavior	antibiotic regimen modification behavior	antibiotic regimen modification behavior	antibiotic regimen modification behavior	1	kept
Antibiotic resistance genes	antibiotic resistance genes	antibiotic resistance genes	antibiotic resistance genes	Antibiotic resistance genes	1	kept
Antibiotic resistance, microbial	antibiotic resistance microbial	antibiotic resistance microbial	antibiotic resistance microbial	Antibiotic resistance, microbial	1	kept
ANTIBIOTIC RESTRICTION POLICY	antibiotic restriction policy	antibiotic restriction policy	antibiotic restriction policy	ANTIBIOTIC RESTRICTION POLICY	1	kept
Antibiotic stewardship programs	antibiotic stewardship programs	antibiotic stewardship programs	antibiotic stewardship programs	Antibiotic stewardship programs	1	kept
antibiotic stewardship programs	antibiotic stewardship programs	antibiotic stewardship programs	antibiotic stewardship programs	Antibiotic stewardship programs	1	case_or_format
ANTIBIOTIC SUSCEPTIBILITY	antibiotic susceptibility	antibiotic susceptibility	antibiotic susceptibility	ANTIBIOTIC SUSCEPTIBILITY	1	kept
antibiotic susceptibility testing	antibiotic susceptibility testing	antibiotic susceptibility testing	antibiotic susceptibility testing	antibiotic susceptibility testing	1	kept
Antibiotic therapy	antibiotic therapy	antibiotic therapy	antibiotic therapy	antibiotic therapy	1	case_or_format
Antibiotic time	antibiotic time	antibiotic time	antibiotic time	Antibiotic time	1	kept
antibiotic utilization rate	antibiotic utilization rate	antibiotic utilization rate	antibiotic utilization rate	antibiotic utilization rate	1	kept
antibiotic wait time	antibiotic wait time	antibiotic wait time	antibiotic wait time	antibiotic wait time	1	kept
ANTIBIOTIC-PRESCRIBING PRACTICES	antibiotic prescribing practices	antibiotic prescribing practices	antibiotic prescribing practices	ANTIBIOTIC-PRESCRIBING PRACTICES	1	kept

ANTIBIOTIC-RESISTANCE	antibiotic resistance	antibiotic resistance	antibiotic resistance	antibiotic resistance	1	case_or_format
antibiotic-resistant bacteria	antibiotic resistant bacteria	antibiotic resistant bacteria	antibiotic resistant bacteria	antibiotic-resistant bacteria	1	kept
Antibiotic-resistant Gram-negative bacteria	antibiotic resistant gram negative bacteria	antibiotic resistant gram negative bacteria	antibiotic resistant gram negative bacteria	Antibiotic-resistant Gram-negative bacteria	1	kept
ANTIBIOTIC-TREATMENT	antibiotic treatment	antibiotic treatment	antibiotic treatment	ANTIBIOTIC-TREATMENT	1	kept
Antibiotics stewardship	antibiotics stewardship	antibiotics stewardship	antibiotics stewardship	Antibiotics stewardship	1	kept
Antibodies monoclonal	antibodies monoclonal	antibodies monoclonal	antibodies monoclonal	Antibodies, Monoclonal	1	case_or_format
Antibodies, Fungal	antibodies fungal	antibodies fungal	antibodies fungal	Antibodies, Fungal	1	kept
Antibody Formation	antibody formation	antibody formation	antibody formation	Antibody Formation	1	kept
antibody mediated rejection	antibody mediated rejection	antibody mediated rejection	antibody mediated rejection	antibody mediated rejection	1	kept
ANTICHOLINERGIC ACTIVITY	anticholinergic activity	anticholinergic activity	anticholinergic activity	ANTICHOLINERGIC ACTIVITY	1	kept
Anticholinergic drugs	anticholinergic drugs	anticholinergic drugs	anticholinergic drugs	Anticholinergic drugs	1	kept
Anticholinergic properties	anticholinergic properties	anticholinergic properties	anticholinergic properties	Anticholinergic properties	1	kept
Anticholinergic Syndrome	anticholinergic syndrome	anticholinergic syndrome	anticholinergic syndrome	Anticholinergic Syndrome	1	kept
Anticholinergics	anticholinergics	anticholinergics	anticholinergics	Anticholinergics	1	kept
Anticipated experiences	anticipated experiences	anticipated experiences	anticipated experiences	Anticipated experiences	1	kept
anticipated loss	anticipated loss	anticipated loss	anticipated loss	anticipated loss	1	kept

Anticipation, Psychological	anticipation psychological	anticipation psychological	anticipation psychological	Anticipation, Psychological	1	kept
Anticipatory grief	anticipatory grief	anticipatory grief	anticipatory grief	anticipatory grief	1	case_or_format
anticipatory grief scale	anticipatory grief scale	anticipatory grief scale	anticipatory grief scale	anticipatory grief scale	1	kept
Anticipatory guidance	anticipatory guidance	anticipatory guidance	anticipatory guidance	Anticipatory guidance	1	kept
anticipatory guidance	anticipatory guidance	anticipatory guidance	anticipatory guidance	Anticipatory guidance	1	case_or_format
anticipatory medical care decisions	anticipatory medical care decisions	anticipatory medical care decisions	anticipatory medical care decisions	anticipatory medical care decisions	1	kept
Anticoagulant	anticoagulant	anticoagulant	anticoagulant	Anticoagulants	1	case_or_format
anticoagulant citrate dextrose A	anticoagulant citrate dextrose a	anticoagulant citrate dextrose a	anticoagulant citrate dextrose a	anticoagulant citrate dextrose A	1	kept
anticoagulant reversal	anticoagulant reversal	anticoagulant reversal	anticoagulant reversal	anticoagulant reversal	1	kept
ANTICOAGULATION	anticoagulation	anticoagulation	anticoagulation	anticoagulation	1	case_or_format
Anticoagulation complication	anticoagulation complication	anticoagulation complication	anticoagulation complication	Anticoagulation complication	1	kept
Anticoagulation complications	anticoagulation complications	anticoagulation complications	anticoagulation complication	Anticoagulation complication	1	singular
anticoagulation reversal agents	anticoagulation reversal agents	anticoagulation reversal agents	anticoagulation reversal agents	anticoagulation reversal agents	1	kept
ANTICOAGULATION SERVICE	anticoagulation service	anticoagulation service	anticoagulation service	ANTICOAGULATION SERVICE	1	kept
Antidepressive Agents, Second-Generation	antidepressive agents second generation	antidepressive agents second generation	antidepressive agents second generation	Antidepressive Agents, Second-Generation	1	kept
antidiarrheal activity	antidiarrheal activity	antidiarrheal activity	antidiarrheal activity	antidiarrheal activity	1	kept

Antifungal	antifungal	antifungal	antifungal	Antifungal	1	kept
antifungal drug resistance	antifungal drug resistance	antifungal drug resistance	antifungal drug resistance	antifungal drug resistance	1	kept
Antifungal drugs	antifungal drugs	antifungal drugs	antifungal drugs	Antifungal drugs	1	kept
antifungal prophylaxis	antifungal prophylaxis	antifungal prophylaxis	antifungal prophylaxis	antifungal prophylaxis	1	kept
Antifungal stewardship	antifungal stewardship	antifungal stewardship	antifungal stewardship	Antifungal stewardship	1	kept
Antifungal therapy	antifungal therapy	antifungal therapy	antifungal therapy	antifungal therapy	1	case_or_format
antifungal treatment	antifungal treatment	antifungal treatment	antifungal treatment	antifungal treatment	1	kept
antigen detection	antigen detection	antigen detection	antigen detection	antigen detection	1	kept
antigen presentation	antigen presentation	antigen presentation	antigen presentation	antigen presentation	1	kept
antigen recognition	antigen recognition	antigen recognition	antigen recognition	antigen recognition	1	kept
Antigenome technology	antigenome technology	antigenome technology	antigenome technology	Antigenome technology	1	kept
Antigens	antigens	antigens	antigen	antigen	1	singular
Antigens, Bacterial	antigens bacterial	antigens bacterial	antigens bacterial	Antigens, Bacterial	1	kept
Antigens, Neoplasm	antigens neoplasm	antigens neoplasm	antigens neoplasm	Antigens, Neoplasm	1	kept
antigout agent	antigout agent	antigout agent	antigout agent	antigout agent	1	kept
Antihistamine	antihistamine	antihistamine	antihistamine	Antihistamine	1	kept

Antihistamines	antihistamines	antihistamines	antihistamine	Antihistamine	1	singular
antihistamines	antihistamines	antihistamines	antihistamine	Antihistamine	1	singular
antihyperglycemics	antihyperglycemics	antihyperglycemics	antihyperglycemics	antihyperglycemics	1	kept
antihypertensive medications	antihypertensive medications	antihypertensive medications	antihypertensive medications	antihypertensive medications	1	kept
Antiinfecciosos	antiinfecciosos	antiinfecciosos	antiinfecciosos	Antiinfecciosos	1	kept
Antiinflammatory agents	antiinflammatory agents	antiinflammatory agents	antiinflammatory agent	antiinflammatory agent	1	singular
antileukemic agent	antileukemic agent	antileukemic agent	antileukemic agent	antileukemic agent	1	kept
antimalarial activity	antimalarial activity	antimalarial activity	antimalarial activity	antimalarial activity	1	kept
Antimanic Agents	antimanic agents	antimanic agents	antimanic agents	Antimanic Agents	1	kept
Antimetabolites, Antineoplastic	antimetabolites antineoplastic	antimetabolites antineoplastic	antimetabolites antineoplastic	Antimetabolites, Antineoplastic	1	kept
Antimicrobial agents	antimicrobial agents	antimicrobial agents	antimicrobial agents	Antimicrobial agents	1	kept
Antimicrobial Cationic Peptides	antimicrobial cationic peptides	antimicrobial cationic peptides	antimicrobial cationic peptides	Antimicrobial Cationic Peptides	1	kept
Antimicrobial coated catheter	antimicrobial coated catheter	antimicrobial coated catheter	antimicrobial coated catheter	Antimicrobial coated catheter	1	kept
Antimicrobial failure	antimicrobial failure	antimicrobial failure	antimicrobial failure	Antimicrobial failure	1	kept
antimicrobial guideline	antimicrobial guideline	antimicrobial guideline	antimicrobial guideline	antimicrobial guideline	1	kept
Antimicrobial indication	antimicrobial indication	antimicrobial indication	antimicrobial indication	Antimicrobial indication	1	kept

antimicrobial management	antimicrobial management	antimicrobial management	antimicrobial management	antimicrobial management	1	kept
Antimicrobial optimisation	antimicrobial optimisation	antimicrobial optimisation	antimicrobial optimisation	Antimicrobial optimisation	1	kept
antimicrobial prescribing	antimicrobial prescribing	antimicrobial prescribing	antimicrobial prescribing	antimicrobial prescribing	1	kept
ANTIMICROBIAL STEWARDSHIP	antimicrobial stewardship	antimicrobial stewardship	antimicrobial stewardship	antimicrobial stewardship	1	case_or_format
Antimicrobial Stewardship Program	antimicrobial stewardship program	antimicrobial stewardship program	antimicrobial stewardship program	Antimicrobial Stewardship Program	1	kept
antimicrobial stewardship program	antimicrobial stewardship program	antimicrobial stewardship program	antimicrobial stewardship program	Antimicrobial Stewardship Program	1	case_or_format
Antimicrobial stewardship programme	antimicrobial stewardship programme	antimicrobial stewardship programme	antimicrobial stewardship programme	Antimicrobial stewardship programme	1	kept
antimicrobial stewardship programs	antimicrobial stewardship programs	antimicrobial stewardship programs	antimicrobial stewardship program	Antimicrobial Stewardship Program	1	singular
Antimicrobial susceptibility	antimicrobial susceptibility	antimicrobial susceptibility	antimicrobial susceptibility	antimicrobial susceptibility	1	case_or_format
ANTIMICROBIAL THERAPY	antimicrobial therapy	antimicrobial therapy	antimicrobial therapy	antimicrobial therapy	1	case_or_format
ANTIMICROBIAL TREATMENT	antimicrobial treatment	antimicrobial treatment	antimicrobial treatment	ANTIMICROBIAL TREATMENT	1	kept
Antimicrobial treatment	antimicrobial treatment	antimicrobial treatment	antimicrobial treatment	ANTIMICROBIAL TREATMENT	1	case_or_format
Antimicrobial use. elderly facility	antimicrobial use elderly facility	antimicrobial use elderly facility	antimicrobial use elderly facility	Antimicrobial use. elderly facility	1	kept
ANTIMICROBIAL UTILIZATION	antimicrobial utilization	antimicrobial utilization	antimicrobial utilization	ANTIMICROBIAL UTILIZATION	1	kept
antimicrobial-impregnated central venous catheters	antimicrobial impregnated central venous catheters	antimicrobial impregnated central venous catheters	antimicrobial impregnated central venous catheters	antimicrobial-impregnated central venous catheters	1	kept
antimicrobial-resistant infection	antimicrobial resistant infection	antimicrobial resistant infection	antimicrobial resistant infection	antimicrobial-resistant infection	1	kept

antimicrobial-resistant infections	antimicrobial resistant infections	antimicrobial resistant infections	antimicrobial resistant infection	antimicrobial-resistant infection	1	singular
antinematodal agent	antinematodal agent	antinematodal agent	antinematodal agent	antinematodal agent	1	kept
Antinematodal Agents	antinematodal agents	antinematodal agents	antinematodal agent	antinematodal agent	1	singular
antineoplastic agents	antineoplastic agents	antineoplastic agents	antineoplastic agent	antineoplastic agent	1	singular
Antineoplastic Agents, Alkylating	antineoplastic agents alkylating	antineoplastic agents alkylating	antineoplastic agents alkylating	Antineoplastic Agents, Alkylating	1	kept
Antineoplastic Agents, Phytogenic	antineoplastic agents phytogenic	antineoplastic agents phytogenic	antineoplastic agents phytogenic	Antineoplastic Agents, Phytogenic	1	kept
antineoplastic antimetabolite	antineoplastic antimetabolite	antineoplastic antimetabolite	antineoplastic antimetabolite	antineoplastic antimetabolite	1	kept
antineoplastic hormone agonists and antagonists	antineoplastic hormone agonists and antagonists	antineoplastic hormone agonists and antagonists	antineoplastic hormone agonists and antagonists	antineoplastic hormone agonists and antagonists	1	kept
Antioxidant enzymes	antioxidant enzymes	antioxidant enzymes	antioxidant enzymes	Antioxidant enzymes	1	kept
antioxidants	antioxidants	antioxidants	antioxidant	Antioxidants	1	singular
antioxidative	antioxidative	antioxidative	antioxidative	antioxidative	1	kept
antiparasitic agent	antiparasitic agent	antiparasitic agent	antiparasitic agent	antiparasitic agent	1	kept
antiplatelet	antiplatelet	antiplatelet	antiplatelet	Antiplatelet	1	case_or_format
Antiplatelet drugs	antiplatelet drugs	antiplatelet drugs	antiplatelet drugs	Antiplatelet drugs	1	kept
antiplatelet medications	antiplatelet medications	antiplatelet medications	antiplatelet medications	antiplatelet medications	1	kept
antiproliferative activity	antiproliferative activity	antiproliferative activity	antiproliferative activity	antiproliferative activity	1	kept

ANTIPSYCHOTIC MEDICATIONS	antipsychotic medications	antipsychotic medications	antipsychotic medications	antipsychotic medications	1	case_or_format
antipyresis	antipyresis	antipyresis	antipyresis	antipyresis	1	kept
ANTIPYRETIC THERAPY	antipyretic therapy	antipyretic therapy	antipyretic therapy	ANTIPYRETIC THERAPY	1	kept
Antipyretic treatment	antipyretic treatment	antipyretic treatment	antipyretic treatment	Antipyretic treatment	1	kept
antipyretics	antipyretics	antipyretics	antipyretics	Antipyretics	1	case_or_format
antiseizure medication	antiseizure medication	antiseizure medication	antiseizure medication	antiseizure medication	1	kept
Antiseizure medications	antiseizure medications	antiseizure medications	antiseizure medication	antiseizure medication	1	singular
Antiseptic	antiseptic	antiseptic	antiseptic	antiseptics	1	case_or_format
antiseptic impregnated catheter	antiseptic impregnated catheter	antiseptic impregnated catheter	antiseptic impregnated catheter	ANTISEPTIC-IMPREGNATED CATHETER	1	case_or_format
Antiseptics	antiseptics	antiseptics	antiseptic	antiseptics	1	singular
antisocial behavior	antisocial behavior	antisocial behavior	antisocial behavior	antisocial behavior	1	kept
antistreptolysin	antistreptolysin	antistreptolysin	antistreptolysin	antistreptolysin	1	kept
Antithrombotic	antithrombotic	antithrombotic	antithrombotic	Antithrombotic	1	kept
Antithrombotic drugs	antithrombotic drugs	antithrombotic drugs	antithrombotic drugs	Antithrombotic drugs	1	kept
antithrombotic therapy	antithrombotic therapy	antithrombotic therapy	antithrombotic therapy	antithrombotic therapy	1	kept
antithyroid agent	antithyroid agent	antithyroid agent	antithyroid agent	antithyroid agent	1	kept

antitoxin	antitoxin	antitoxin	antitoxin	antitoxin	1	kept
antitussive activity	antitussive activity	antitussive activity	antitussive activity	antitussive activity	1	kept
antivenom	antivenom	antivenom	antivenom	Antivenom	1	case_or_format
Antiviral	antiviral	antiviral	antiviral	Antiviral	1	kept
antiviral agents	antiviral agents	antiviral agents	antiviral agents	Antiviral Agents	1	case_or_format
Antivirus protocol	antivirus protocol	antivirus protocol	antivirus protocol	Antivirus protocol	1	kept
ANTS system	ants system	ants system	ants system	ANTS system	1	kept
anus continence	anus continence	anus continence	anus continence	anus continence	1	kept
Anus Diseases	anus diseases	anus diseases	anus diseases	Anus Diseases	1	kept
anus fissure	anus fissure	anus fissure	anus fissure	anus fissure	1	kept
anus injury	anus injury	anus injury	anus injury	anus injury	1	kept
anus surgery	anus surgery	anus surgery	anus surgery	anus surgery	1	kept
anxiety and depression	anxiety and depression	anxiety and depression	anxiety and depression	Anxiety and depression	1	case_or_format
Anxiety and PTSS	anxiety and ptss	anxiety and ptss	anxiety and ptss	Anxiety and PTSS	1	kept
anxiety COVID-19	anxiety covid 19	anxiety covid 19	anxiety covid 19	anxiety COVID-19	1	kept
anxiety depression	anxiety depression	anxiety depression	anxiety depression	anxiety depression	1	kept

Anxiety disorder	anxiety disorder	anxiety disorder	anxiety disorder	anxiety disorder	1	case_or_format
anxiety disorders	anxiety disorders	anxiety disorders	anxiety disorder	anxiety disorder	1	singular
anxiety factors	anxiety factors	anxiety factors	anxiety factors	anxiety factors	1	kept
anxiety level	anxiety level	anxiety level	anxiety level	anxiety level	1	kept
anxiety neurosis	anxiety neurosis	anxiety neurosis	anxiety neurosis	anxiety neurosis	1	kept
anxiety of mothers	anxiety of mothers	anxiety of mothers	anxiety of mothers	anxiety of mothers	1	kept
anxiety relief	anxiety relief	anxiety relief	anxiety relief	anxiety relief	1	kept
Anxiety Sensitivity Index	anxiety sensitivity index	anxiety sensitivity index	anxiety sensitivity index	Anxiety Sensitivity Index	1	kept
Anxiety symptom	anxiety symptom	anxiety symptom	anxiety symptom	Anxiety symptom	1	kept
ANXIETY-LIKE BEHAVIORS	anxiety like behaviors	anxiety like behaviors	anxiety like behaviors	ANXIETY-LIKE BEHAVIORS	1	kept
Anxiolytics	anxiolytics	anxiolytics	anxiolytics	Anxiolytics	1	kept
ANZICS	anzics	anzics	anzics	ANZICS	1	kept
ANZSCTS	anzscts	anzscts	anzscts	ANZSCTS	1	kept
Análisis bibliométrico	an lisis bibliom trico	an lisis bibliom trico	an lisis bibliom trico	Análisis bibliométrico	1	kept
Análisis de contenido	an lisis de contenido	an lisis de contenido	an lisis de contenido	Análisis de contenido	1	kept
Análisis de coste-efectividad	an lisis de coste efectividad	an lisis de coste efectividad	an lisis de coste efectividad	Análisis de coste-efectividad	1	kept

Análisis de datos secundarios	an lisis de datos secundarios	an lisis de datos secundarios	an lisis de datos secundarios	Análisis de datos secundarios	1	kept
Análisis factorial	an lisis factorial	an lisis factorial	an lisis factorial	Análisis factorial	1	kept
Análisis químico de la sangre	an lisis qu mico de la sangre	an lisis qu mico de la sangre	an lisis qu mico de la sangre	Análisis químico de la sangre	1	kept
AOR, Adjusted odds ratio	aor adjusted odds ratio	aor adjusted odds ratio	aor adjusted odds ratio	AOR, Adjusted odds ratio	1	kept
aorta arch	aorta arch	aorta arch	aorta arch	aorta arch	1	kept
aorta arch anomaly	aorta arch anomaly	aorta arch anomaly	aorta arch anomaly	aorta arch anomaly	1	kept
aorta bifurcation	aorta bifurcation	aorta bifurcation	aorta bifurcation	aorta bifurcation	1	kept
aorta flow	aorta flow	aorta flow	aorta flow	aorta flow	1	kept
aorta subvalvular stenosis	aorta subvalvular stenosis	aorta subvalvular stenosis	aorta subvalvular stenosis	aorta subvalvular stenosis	1	kept
aorta surgery	aorta surgery	aorta surgery	aorta surgery	aorta surgery	1	kept
aorta thrombosis	aorta thrombosis	aorta thrombosis	aorta thrombosis	aorta thrombosis	1	kept
aorta valve disease	aorta valve disease	aorta valve disease	aorta valve disease	aorta valve disease	1	kept
aortic	aortic	aortic	aortic	aortic	1	kept
aortic annulus	aortic annulus	aortic annulus	aortic annulus	aortic annulus	1	kept
aortic arch	aortic arch	aortic arch	aortic arch	aortic arch	1	kept
aortic arch syndrome	aortic arch syndrome	aortic arch syndrome	aortic arch syndrome	aortic arch syndrome	1	kept

aortic atherosclerosis	aortic atherosclerosis	aortic atherosclerosis	aortic atherosclerosis	aortic atherosclerosis	1	kept
aortic bifurcation	aortic bifurcation	aortic bifurcation	aortic bifurcation	aortic bifurcation	1	kept
aortic coarctation	aortic coarctation	aortic coarctation	aortic coarctation	Aortic Coarctation	1	case_or_format
aortic disease	aortic disease	aortic disease	aortic disease	Aortic Diseases	1	case_or_format
aortic graft	aortic graft	aortic graft	aortic graft	aortic graft	1	kept
Aortic occlusion	aortic occlusion	aortic occlusion	aortic occlusion	Aortic occlusion	1	kept
aortic occlusion	aortic occlusion	aortic occlusion	aortic occlusion	Aortic occlusion	1	case_or_format
Aortic regurgitation	aortic regurgitation	aortic regurgitation	aortic regurgitation	aortic regurgitation	1	case_or_format
aortic root	aortic root	aortic root	aortic root	aortic root	1	kept
aortic root replacement	aortic root replacement	aortic root replacement	aortic root replacement	aortic root replacement	1	kept
aortic root surgery	aortic root surgery	aortic root surgery	aortic root surgery	aortic root surgery	1	kept
aortic sinus	aortic sinus	aortic sinus	aortic sinus	aortic sinus	1	kept
aortic tissue	aortic tissue	aortic tissue	aortic tissue	aortic tissue	1	kept
aortic valve calcification	aortic valve calcification	aortic valve calcification	aortic valve calcification	aortic valve calcification	1	kept
Aortic valve operation	aortic valve operation	aortic valve operation	aortic valve operation	Aortic valve operation	1	kept
aortic valve reintervention	aortic valve reintervention	aortic valve reintervention	aortic valve reintervention	aortic valve reintervention	1	kept

aortic valve repair	aortic valve repair	aortic valve repair	aortic valve repair	aortic valve repair	1	kept
Aortic valve stenosis	aortic valve stenosis	aortic valve stenosis	aortic valve stenosis	Aortic Valve Stenosis	1	case_or_format
aortic valve surgery	aortic valve surgery	aortic valve surgery	aortic valve surgery	aortic valve surgery	1	kept
Aortobronchial	aortobronchial	aortobronchial	aortobronchial	Aortobronchial	1	kept
Aortoesophageal fistula	aortoesophageal fistula	aortoesophageal fistula	aortoesophageal fistula	Aortoesophageal fistula	1	kept
aortoesophageal fistula	aortoesophageal fistula	aortoesophageal fistula	aortoesophageal fistula	Aortoesophageal fistula	1	case_or_format
Aortography	aortography	aortography	aortography	aortography	1	case_or_format
aortoplasty	aortoplasty	aortoplasty	aortoplasty	aortoplasty	1	kept
aortopulmonary shunt	aortopulmonary shunt	aortopulmonary shunt	aortopulmonary shunt	aortopulmonary shunt	1	kept
ap 001	ap 001	ap 001	ap 001	ap 001	1	kept
Apache	apache	apache	apache	APACHE	1	case_or_format
APACHE III score	apache iii score	apache iii score	apache iii score	APACHE III score	1	kept
APACHE Score	apache score	apache score	apache score	APACHE Score	1	kept
APACHE-II	apache ii	apache ii	apache ii	APACHE II	1	case_or_format
APACHE2	apache2	apache2	apache2	APACHE2	1	kept
apartheid	apartheid	apartheid	apartheid	apartheid	1	kept

aPCC	apcc	apcc	apcc	aPCC	1	kept
Apfel score	apfel score	apfel score	apfel score	Apfel score	1	kept
aph bacterial gene	aph bacterial gene	aph bacterial gene	aph bacterial gene	aph bacterial gene	1	kept
Apheresis	apheresis	apheresis	apheresis	apheresis	1	case_or_format
APIB	apib	apib	apib	APIB	1	kept
apireks pediatric suspension	apireks pediatric suspension	apireks pediatric suspension	apireks pediatric suspension	apireks pediatric suspension	1	kept
aplastic crisis	aplastic crisis	aplastic crisis	aplastic crisis	aplastic crisis	1	kept
Aplicaciones móviles	aplicaciones m viles	aplicaciones m viles	aplicaciones m viles	Aplicaciones móviles	1	kept
APN	apn	apn	apn	APN	1	kept
APNEA	apnea	apnea	apnea	apnea	1	case_or_format
Apnea detection	apnea detection	apnea detection	apnea detection	Apnea detection	1	kept
apnea of prematurity	apnea of prematurity	apnea of prematurity	apnea of prematurity	apnea of prematurity	1	kept
apnea prevention device	apnea prevention device	apnea prevention device	apnea prevention device	apnea prevention device	1	kept
apnea test	apnea test	apnea test	apnea test	apnea test	1	kept
apnoea	apnoea	apnoea	apnoea	Apnoea	1	case_or_format
Apogon lineatus	apogon lineatus	apogon lineatus	apogon lineatus	Apogon lineatus	1	kept

Apolipoprotein L1	apolipoprotein l1	apolipoprotein l1	apolipoprotein l1	apolipoprotein L1	1	case_or_format
APP role	app role	app role	app role	APP role	1	kept
Appalachia	appalachia	appalachia	appalachia	Appalachia	1	kept
Appalachian	appalachian	appalachian	appalachian	Appalachian	1	kept
Appearance	appearance	appearance	appearance	Appearance	1	kept
Appendiceal Neoplasms	appendiceal neoplasms	appendiceal neoplasms	appendiceal neoplasms	Appendiceal Neoplasms	1	kept
appendix	appendix	appendix	appendix	appendix	1	kept
appendix perforation	appendix perforation	appendix perforation	appendix perforation	appendix perforation	1	kept
appetite stimulant	appetite stimulant	appetite stimulant	appetite stimulant	appetite stimulant	1	kept
Appetite Stimulants	appetite stimulants	appetite stimulants	appetite stimulant	appetite stimulant	1	singular
apple	apple	apple	apple	apple	1	kept
Application development	application development	application development	application development	Application development	1	kept
application effects	application effects	application effects	application effect	Application effect	1	singular
application of advanced cardiac life support	application of advanced cardiac life support	application of advanced cardiac life support	application of advanced cardiac life support	application of advanced cardiac life support	1	kept
Application of evidence-based practice	application of evidence based practice	application of evidence based practice	application of evidence based practice	Application of evidence-based practice	1	kept
Application of iot	application of iot	application of iot	application of iot	Application of iot	1	kept

Application performance	application performance	application performance	application performance	Application performance	1	kept
Application programming interfaces	application programming interfaces	application programming interfaces	application programming interfaces	Application programming interfaces	1	kept
application site bleeding	application site bleeding	application site bleeding	application site bleeding	application site bleeding	1	kept
Application strategies	application strategies	application strategies	application strategy	Application strategy	1	singular
Application strategy	application strategy	application strategy	application strategy	Application strategy	1	kept
application study	application study	application study	application study	application study	1	kept
Application value	application value	application value	application value	Application value	1	kept
application value	application value	application value	application value	Application value	1	case_or_format
Applied and Professional Ethics	applied and professional ethics	applied and professional ethics	applied and professional ethics	Applied and Professional Ethics	1	kept
Appraisal form development	appraisal form development	appraisal form development	appraisal form development	Appraisal form development	1	kept
Appraisal of Guidelines for Research and	appraisal of guidelines for research and	appraisal of guidelines for research and	appraisal of guidelines for research and	Appraisal of Guidelines for Research and	1	kept
appreciate	appreciate	appreciate	appreciate	appreciate	1	kept
Appreciation	appreciation	appreciation	appreciation	Appreciation	1	kept
Apprenticeship	apprenticeship	apprenticeship	apprenticeship	apprenticeship	1	case_or_format
Appropriate care	appropriate care	appropriate care	appropriate care	Appropriate care	1	kept
appropriate transfer	appropriate transfer	appropriate transfer	appropriate transfer	appropriate transfer	1	kept

Appropriate treatment	appropriate treatment	appropriate treatment	appropriate treatment	Appropriate treatment	1	kept
APPROPRIATE USE	appropriate use	appropriate use	appropriate use	APPROPRIATE USE	1	kept
appropriateness	appropriateness	appropriateness	appropriateness	APPROPRIATENESS	1	case_or_format
Appropriateness evaluation protocol	appropriateness evaluation protocol	appropriateness evaluation protocol	appropriateness evaluation protocol	Appropriateness evaluation protocol	1	kept
appropriateness of transfer	appropriateness of transfer	appropriateness of transfer	appropriateness of transfer	appropriateness of transfer	1	kept
Appropriation	appropriation	appropriation	appropriation	Appropriation	1	kept
APPs	apps	apps	app	apps	1	singular
Aprendizaje	aprendizaje	aprendizaje	aprendizaje	Aprendizaje	1	kept
aprepitant	aprepitant	aprepitant	aprepitant	aprepitant	1	kept
APRN	aprn	aprn	aprn	APRN	1	kept
APRN Consensus Model	aprn consensus model	aprn consensus model	aprn consensus model	APRN Consensus Model	1	kept
APRN productivity tracking	aprn productivity tracking	aprn productivity tracking	aprn productivity tracking	APRN productivity tracking	1	kept
APSIII score	apsiii score	apsiii score	apsiii score	APSIII score	1	kept
Aptamer	aptamer	aptamer	aptamer	Aptamer	1	kept
aptamer	aptamer	aptamer	aptamer	Aptamer	1	case_or_format
Aptamers	aptamers	aptamers	aptamer	Aptamer	1	singular

Aptitude	aptitude	aptitude	aptitude	aptitude	1	case_or_format
aptitude test	aptitude test	aptitude test	aptitude test	aptitude test	1	kept
aPTT	aptt	aptt	aptt	aPTT	1	kept
Aquatic exercise	aquatic exercise	aquatic exercise	aquatic exercise	Aquatic exercise	1	kept
aquatic exercise	aquatic exercise	aquatic exercise	aquatic exercise	Aquatic exercise	1	case_or_format
Aquatic Therapy	aquatic therapy	aquatic therapy	aquatic therapy	Aquatic Therapy	1	kept
Arab spring	arab spring	arab spring	arab spring	Arab spring	1	kept
Arab women	arab women	arab women	arab women	Arab women	1	kept
Arab World	arab world	arab world	arab world	Arab World	1	kept
Arab world	arab world	arab world	arab world	Arab World	1	case_or_format
Arabic culture	arabic culture	arabic culture	arabic culture	Arabic culture	1	kept
Arabidopsis Proteins	arabidopsis proteins	arabidopsis proteins	arabidopsis proteins	Arabidopsis Proteins	1	kept
Arachidonic Acid	arachidonic acid	arachidonic acid	arachidonic acid	arachidonic acid	1	case_or_format
Arachnidism	arachnidism	arachnidism	arachnidism	Arachnidism	1	kept
arachnoid cyst	arachnoid cyst	arachnoid cyst	arachnoid cyst	arachnoid cyst	1	kept
Arbeitsbelastung	arbeitsbelastung	arbeitsbelastung	arbeitsbelastung	Arbeitsbelastung	1	kept

archeology	archeology	archeology	archeology	archeology	1	kept
architect	architect	architect	architect	architect	1	kept
Architectural Accessibility	architectural accessibility	architectural accessibility	architectural accessibility	Architectural Accessibility	1	kept
Archive	archive	archive	archive	Archive	1	kept
Archived data	archived data	archived data	archived data	Archived data	1	kept
Archives	archives	archives	archive	Archive	1	singular
ARCS Motivational Design Model	arcs motivational design model	arcs motivational design model	arcs motivational design model	ARCS Motivational Design Model	1	kept
Arctic Regions	arctic regions	arctic regions	arctic regions	Arctic Regions	1	kept
Ardabil	ardabil	ardabil	ardabil	Ardabil	1	kept
Arden Syntax	arden syntax	arden syntax	arden syntax	Arden Syntax	1	kept
Arden syntax	arden syntax	arden syntax	arden syntax	Arden Syntax	1	case_or_format
ards	ards	ards	ards	ARDS	1	case_or_format
ARDS definition	ards definition	ards definition	ards definition	ARDS definition	1	kept
ARDS survival outcomes	ards survival outcomes	ards survival outcomes	ards survival outcomes	ARDS survival outcomes	1	kept
ARDS, acute respiratory distress syndrome	ards acute respiratory distress syndrome	ards acute respiratory distress syndrome	ards acute respiratory distress syndrome	ARDS, acute respiratory distress syndrome	1	kept
Arduino kit	arduino kit	arduino kit	arduino kit	Arduino kit	1	kept

Area under curve	area under curve	area under curve	area under curve	Area Under Curve	1	case_or_format
area under curve	area under curve	area under curve	area under curve	Area Under Curve	1	case_or_format
Area under the curve	area under the curve	area under the curve	area under the curve	area under the curve	1	case_or_format
area under the curve ratio	area under the curve ratio	area under the curve ratio	area under the curve ratio	area under the curve ratio	1	kept
Area under the curves	area under the curves	area under the curves	area under the curve	area under the curve	1	singular
areas of practice	areas of practice	areas of practice	areas of practice	Areas of practice	1	case_or_format
Areas of Worklife Scale	areas of worklife scale	areas of worklife scale	areas of worklife scale	Areas of Worklife Scale	1	kept
arformoterol	arformoterol	arformoterol	arformoterol	arformoterol	1	kept
ARG	arg	arg	arg	ARG	1	kept
Argatroban	argatroban	argatroban	argatroban	argatroban	1	case_or_format
Argentina kagoshimae	argentina kagoshimae	argentina kagoshimae	argentina kagoshimae	Argentina kagoshimae	1	kept
Argentina-health personnel-psychiatric disorders	argentina health personnel psychiatric disorders	argentina health personnel psychiatric disorders	argentina health personnel psychiatric disorders	Argentina-health personnel-psychiatric disorders	1	kept
Argentinian	argentinian	argentinian	argentinian	Argentinian	1	kept
arginine vasopressin	arginine vasopressin	arginine vasopressin	arginine vasopressin	Arginine Vasopressin	1	case_or_format
argipressin[1 deamino]	argipressin 1 deamino	argipressin 1 deamino	argipressin 1 deamino	argipressin[1 deamino]	1	kept
Argumentation	argumentation	argumentation	argumentation	Argumentation	1	kept

Argyrosomus argentatus	argyrosomus argentatus	argyrosomus argentatus	argyrosomus argentatus	Argyrosomus argentatus	1	kept
Aristotle	aristotle	aristotle	aristotle	Aristotle	1	kept
ARM CIRCUMFERENCE	arm circumference	arm circumference	arm circumference	arm circumference	1	case_or_format
arm curl test	arm curl test	arm curl test	arm curl test	arm curl test	1	kept
arm edema	arm edema	arm edema	arm edema	arm edema	1	kept
Arm exercise	arm exercise	arm exercise	arm exercise	Arm exercise	1	kept
arm exercise	arm exercise	arm exercise	arm exercise	Arm exercise	1	case_or_format
Arm motions	arm motions	arm motions	arm motions	Arm motions	1	kept
arm movement	arm movement	arm movement	arm movement	arm movement	1	kept
arm position	arm position	arm position	arm position	arm position	1	kept
ARM processor	arm processor	arm processor	arm processor	ARM processor	1	kept
ARM processors	arm processors	arm processors	arm processor	ARM processor	1	singular
Arm span measurement	arm span measurement	arm span measurement	arm span measurement	Arm span measurement	1	kept
Arnica montana extract	arnica montana extract	arnica montana extract	arnica montana extract	Arnica montana extract	1	kept
Aroma therapy	aroma therapy	aroma therapy	aroma therapy	Aroma therapy	1	kept
Aromatherapy massage	aromatherapy massage	aromatherapy massage	aromatherapy massage	Aromatherapy massage	1	kept

ARREST	arrest	arrest	arrest	ARREST	1	kept
ARRESTS	arrests	arrests	arrest	ARREST	1	singular
arrhythmia < cardiovascular	arrhythmia cardiovascular	arrhythmia cardiovascular	arrhythmia cardiovascular	arrhythmia < cardiovascular	1	kept
ARRHYTHMIA ALARMS	arrhythmia alarms	arrhythmia alarms	arrhythmia alarms	ARRHYTHMIA ALARMS	1	kept
arrhythmia interpreting	arrhythmia interpreting	arrhythmia interpreting	arrhythmia interpreting	arrhythmia interpreting	1	kept
Arrhythmias, cardiac	arrhythmias cardiac	arrhythmias cardiac	arrhythmias cardiac	Arrhythmias, Cardiac	1	case_or_format
arrhythmogenesis	arrhythmogenesis	arrhythmogenesis	arrhythmogenesis	arrhythmogenesis	1	kept
arrhythmogenic cardiomyopathy	arrhythmogenic cardiomyopathy	arrhythmogenic cardiomyopathy	arrhythmogenic cardiomyopathy	arrhythmogenic cardiomyopathy	1	kept
Arrival rates	arrival rates	arrival rates	arrival rates	Arrival rates	1	kept
Arsenic	arsenic	arsenic	arsenic	Arsenic	1	kept
Arsenic Poisoning	arsenic poisoning	arsenic poisoning	arsenic poisoning	Arsenic Poisoning	1	kept
Arsenic Trioxide	arsenic trioxide	arsenic trioxide	arsenic trioxide	Arsenic Trioxide	1	kept
arsenic trioxide	arsenic trioxide	arsenic trioxide	arsenic trioxide	Arsenic Trioxide	1	case_or_format
arson	arson	arson	arson	arson	1	kept
art therapist	art therapist	art therapist	art therapist	art therapist	1	kept
Art therapy and pregnancy	art therapy and pregnancy	art therapy and pregnancy	art therapy and pregnancy	Art therapy and pregnancy	1	kept

Artefact	artefact	artefact	artefact	Artefact	1	kept
ArtekMed	artekmed	artekmed	artekmed	ArtekMed	1	kept
Artemether, Lumefantrine Drug Combination	artemether lumefantrine drug combination	artemether lumefantrine drug combination	artemether lumefantrine drug combination	Artemether, Lumefantrine Drug Combination	1	kept
ARTEMIS	artemis	artemis	artemis	ARTEMIS	1	kept
artemisinin	artemisinin	artemisinin	artemisinin	artemisinin	1	kept
Artemisinins	artemisinins	artemisinins	artemisinin	artemisinin	1	singular
Arterial Blood Gas	arterial blood gas	arterial blood gas	arterial blood gas	Arterial blood gas	1	case_or_format
Arterial blood gas sampling	arterial blood gas sampling	arterial blood gas sampling	arterial blood gas sampling	Arterial blood gas sampling	1	kept
Arterial blood oxygen saturation	arterial blood oxygen saturation	arterial blood oxygen saturation	arterial blood oxygen saturation	Arterial blood oxygen saturation	1	kept
arterial blood pressure	arterial blood pressure	arterial blood pressure	arterial blood pressure	Arterial blood pressure	1	case_or_format
ARTERIAL CARBON-DIOXIDE	arterial carbon dioxide	arterial carbon dioxide	arterial carbon dioxide	ARTERIAL CARBON-DIOXIDE	1	kept
arterial catheter	arterial catheter	arterial catheter	arterial catheter	Arterial catheter	1	case_or_format
arterial catheterization	arterial catheterization	arterial catheterization	arterial catheterization	arterial catheterization	1	kept
arterial embolectomy	arterial embolectomy	arterial embolectomy	arterial embolectomy	arterial embolectomy	1	kept
Arterial embolization	arterial embolization	arterial embolization	arterial embolization	arterial embolization	1	case_or_format
arterial grafts	arterial grafts	arterial grafts	arterial grafts	arterial grafts	1	kept

Arterial hypertension	arterial hypertension	arterial hypertension	arterial hypertension	Arterial hypertension	1	kept
Arterial ligation	arterial ligation	arterial ligation	arterial ligation	Arterial ligation	1	kept
arterial line placements	arterial line placements	arterial line placements	arterial line placements	arterial line placements	1	kept
Arterial puncture	arterial puncture	arterial puncture	arterial puncture	Arterial puncture	1	kept
Arterial Switch Operation	arterial switch operation	arterial switch operation	arterial switch operation	Arterial Switch Operation	1	kept
arterial trunk	arterial trunk	arterial trunk	arterial trunk	arterial trunk	1	kept
arteries	arteries	arteries	artery	Arteries	1	singular
arterio-venous ECMO	arterio venous ecmo	arterio venous ecmo	arterio venous ecmo	arterio-venous ECMO	1	kept
arteriosclerosis	arteriosclerosis	arteriosclerosis	arteriosclerosis	arteriosclerosis	1	kept
Arteriovenous Fistula	arteriovenous fistula	arteriovenous fistula	arteriovenous fistula	arteriovenous fistula	1	case_or_format
Arteriovenous fistula	arteriovenous fistula	arteriovenous fistula	arteriovenous fistula	arteriovenous fistula	1	case_or_format
Arteriovenous Malformations	arteriovenous malformations	arteriovenous malformations	arteriovenous malformation	arteriovenous malformation	1	singular
arteriovenous shunt	arteriovenous shunt	arteriovenous shunt	arteriovenous shunt	arteriovenous shunt	1	kept
arteritis	arteritis	arteritis	arteritis	arteritis	1	kept
Artery blood flow	artery blood flow	artery blood flow	artery blood flow	Artery blood flow	1	kept
artery blood flow	artery blood flow	artery blood flow	artery blood flow	Artery blood flow	1	case_or_format

artery clamp	artery clamp	artery clamp	artery clamp	artery clamp	1	kept
artery compression	artery compression	artery compression	artery compression	artery compression	1	kept
artery embolism	artery embolism	artery embolism	artery embolism	artery embolism	1	kept
artery intima	artery intima	artery intima	artery intima	artery intima	1	kept
artery media	artery media	artery media	artery media	artery media	1	kept
Artery of Percheron	artery of percheron	artery of percheron	artery of percheron	Artery of Percheron	1	kept
artery of Percheron	artery of percheron	artery of percheron	artery of percheron	Artery of Percheron	1	case_or_format
artery perforation	artery perforation	artery perforation	artery perforation	artery perforation	1	kept
ARTERY-BYPASS GRAFT	artery bypass graft	artery bypass graft	artery bypass graft	ARTERY-BYPASS GRAFT	1	kept
Arthralgias	arthralgias	arthralgias	arthralgia	arthralgia	1	singular
Arthritis	arthritis	arthritis	arthritis	arthritis	1	case_or_format
Arthritis, Infectious	arthritis infectious	arthritis infectious	arthritis infectious	Arthritis, Infectious	1	kept
Arthritis, Rheumatoid	arthritis rheumatoid	arthritis rheumatoid	arthritis rheumatoid	Arthritis, Rheumatoid	1	kept
arthrocentesis	arthrocentesis	arthrocentesis	arthrocentesis	arthrocentesis	1	kept
Arthrodesis	arthrodesis	arthrodesis	arthrodesis	arthrodesis	1	case_or_format
arthropathy	arthropathy	arthropathy	arthropathy	arthropathy	1	kept

Arthroplasty Risk Score	arthroplasty risk score	arthroplasty risk score	arthroplasty risk score	Arthroplasty Risk Score	1	kept
Arthroscopy	arthroscopy	arthroscopy	arthroscopy	Arthroscopy	1	kept
arthroscopy	arthroscopy	arthroscopy	arthroscopy	Arthroscopy	1	case_or_format
Article critique	article critique	article critique	article critique	Article critique	1	kept
artifact reduction	artifact reduction	artifact reduction	artifact reduction	artifact reduction	1	kept
Artificial airway management	artificial airway management	artificial airway management	artificial airway management	Artificial airway management	1	kept
Artificial breathing	artificial breathing	artificial breathing	artificial breathing	Artificial breathing	1	kept
Artificial cardiac pacing	artificial cardiac pacing	artificial cardiac pacing	artificial cardiac pacing	Artificial cardiac pacing	1	kept
artificial cardiac pacing	artificial cardiac pacing	artificial cardiac pacing	artificial cardiac pacing	Artificial cardiac pacing	1	case_or_format
artificial cough	artificial cough	artificial cough	artificial cough	artificial cough	1	kept
artificial fetal reduction	artificial fetal reduction	artificial fetal reduction	artificial fetal reduction	artificial fetal reduction	1	kept
ARTIFICIAL FINGERNAILS WORN	artificial fingernails worn	artificial fingernails worn	artificial fingernails worn	ARTIFICIAL FINGERNAILS WORN	1	kept
artificial insemination	artificial insemination	artificial insemination	artificial insemination	artificial insemination	1	kept
artificial Intelligence	artificial intelligence	artificial intelligence	artificial intelligence	artificial intelligence	1	case_or_format
artificial intelligence and machine learning	artificial intelligence and machine learning	artificial intelligence and machine learning	artificial intelligence and machine learning	artificial intelligence and machine learning	1	kept
Artificial intelligence technology	artificial intelligence technology	artificial intelligence technology	artificial intelligence technology	Artificial intelligence technologies	1	case_or_format

artificial kidney	artificial kidney	artificial kidney	artificial kidney	artificial kidney	1	kept
artificial light	artificial light	artificial light	artificial light	artificial light	1	kept
artificial liver	artificial liver	artificial liver	artificial liver	artificial liver	1	kept
artificial liver support	artificial liver support	artificial liver support	artificial liver support	artificial liver support	1	kept
artificial lung	artificial lung	artificial lung	artificial lung	artificial lung	1	kept
artificial membrane	artificial membrane	artificial membrane	artificial membrane	artificial membrane	1	kept
Artificial Neural Network	artificial neural network	artificial neural network	artificial neural network	artificial neural network	1	case_or_format
Artificial neural networks	artificial neural networks	artificial neural networks	artificial neural network	artificial neural network	1	singular
ARTIFICIAL NEURAL-NETWORK	artificial neural network	artificial neural network	artificial neural network	artificial neural network	1	case_or_format
Artificial nutrition	artificial nutrition	artificial nutrition	artificial nutrition	ARTIFICIAL NUTRITION	1	case_or_format
artificial nutrition and hydration	artificial nutrition and hydration	artificial nutrition and hydration	artificial nutrition and hydration	artificial nutrition and hydration	1	kept
artificial organ	artificial organ	artificial organ	artificial organ	artificial organ	1	kept
Artificial Organs	artificial organs	artificial organs	artificial organ	artificial organ	1	singular
Artificial pancreas	artificial pancreas	artificial pancreas	artificial pancreas	ARTIFICIAL PANCREAS	1	case_or_format
artificial pancreas	artificial pancreas	artificial pancreas	artificial pancreas	ARTIFICIAL PANCREAS	1	case_or_format
Artificial saliva	artificial saliva	artificial saliva	artificial saliva	Artificial saliva	1	kept

Artificial uterus	artificial uterus	artificial uterus	artificial uterus	Artificial uterus	1	kept
Artificial Ventilation	artificial ventilation	artificial ventilation	artificial ventilation	Artificial Ventilation	1	kept
Arytenoid Cartilage	arytenoid cartilage	arytenoid cartilage	arytenoid cartilage	arytenoid cartilage	1	case_or_format
Arytenoid cartilage dislocation	arytenoid cartilage dislocation	arytenoid cartilage dislocation	arytenoid cartilage dislocation	Arytenoid cartilage dislocation	1	kept
Arytenoid dislocation	arytenoid dislocation	arytenoid dislocation	arytenoid dislocation	Arytenoid dislocation	1	kept
arytenoid dislocation	arytenoid dislocation	arytenoid dislocation	arytenoid dislocation	Arytenoid dislocation	1	case_or_format
ASA	asa	asa	asa	ASA	1	kept
ASAMD = absolute standardized average mean differences	asamd absolute standardized average mean differences	asamd absolute standardized average mean differences	asamd absolute standardized average mean differences	ASAMD = absolute standardized average mean differences	1	kept
asbestos	asbestos	asbestos	asbestos	asbestos	1	kept
ascending aortic aneurysm	ascending aortic aneurysm	ascending aortic aneurysm	ascending aortic aneurysm	ascending aortic aneurysm	1	kept
Ascending arousal system	ascending arousal system	ascending arousal system	ascending arousal system	Ascending arousal system	1	kept
ascitis	ascitis	ascitis	ascitis	ascitis	1	kept
ASD occluder	asd occluder	asd occluder	asd occluder	ASD occluder	1	kept
Aseer Central hospital	aseer central hospital	aseer central hospital	aseer central hospital	Aseer Central hospital	1	kept
aseptic meningitis	aseptic meningitis	aseptic meningitis	aseptic meningitis	aseptic meningitis	1	kept
aseptic non touch technique	aseptic non touch technique	aseptic non touch technique	aseptic non touch technique	aseptic non touch technique	1	kept

aseptic non-touch technique	aseptic non touch technique	aseptic non touch technique	aseptic non touch technique	aseptic non touch technique	1	case_or_format
aseptic revision arthroplasty	aseptic revision arthroplasty	aseptic revision arthroplasty	aseptic revision arthroplasty	aseptic revision arthroplasty	1	kept
Aseptic Technique	aseptic technique	aseptic technique	aseptic technique	Aseptic Technique	1	kept
aseptic techniques	aseptic techniques	aseptic techniques	aseptic technique	Aseptic Technique	1	singular
ASF	asf	asf	asf	ASF	1	kept
ASHEVILLE-PROJECT	asheville project	asheville project	asheville project	ASHEVILLE-PROJECT	1	kept
ASIA Impairment Scale	asia impairment scale	asia impairment scale	asia impairment scale	ASIA Impairment Scale	1	kept
Asian Americans	asian americans	asian americans	asian american	Asian American	1	singular
Asian children	asian children	asian children	asian children	Asian children	1	kept
Asia‑Pacific	asia pacific	asia pacific	asia pacific	Asia‑Pacific	1	kept
ASM chart	asm chart	asm chart	asm chart	ASM chart	1	kept
aspartame	aspartame	aspartame	aspartame	aspartame	1	kept
aspartate aminotransferase level	aspartate aminotransferase level	aspartate aminotransferase level	aspartate aminotransferase level	aspartate aminotransferase level	1	kept
aspartic acid	aspartic acid	aspartic acid	aspartic acid	Aspartic Acid	1	case_or_format
aspects of sleep	aspects of sleep	aspects of sleep	aspects of sleep	aspects of sleep	1	kept
ASPEN	aspen	aspen	aspen	ASPEN	1	kept

Asphyxia neonatorum	asphyxia neonatorum	asphyxia neonatorum	asphyxia neonatorum	Asphyxia Neonatorum	1	case_or_format
asphyxiation	asphyxiation	asphyxiation	asphyxiation	asphyxiation	1	kept
aspiration biomarker	aspiration biomarker	aspiration biomarker	aspiration biomarker	aspiration biomarker	1	kept
aspiration biopsy	aspiration biopsy	aspiration biopsy	aspiration biopsy	aspiration biopsy	1	kept
Aspiration mechanical	aspiration mechanical	aspiration mechanical	aspiration mechanical	Aspiration mechanical	1	kept
ASPIRATION PNEUMONIA	aspiration pneumonia	aspiration pneumonia	aspiration pneumonia	aspiration pneumonia	1	case_or_format
aspiration pneumonia, acute lung injury	aspiration pneumonia acute lung injury	aspiration pneumonia acute lung injury	aspiration pneumonia acute lung injury	aspiration pneumonia, acute lung injury	1	kept
ASPIRATION PNEUMONITIS	aspiration pneumonitis	aspiration pneumonitis	aspiration pneumonitis	ASPIRATION PNEUMONITIS	1	kept
aspiration pneumonitis	aspiration pneumonitis	aspiration pneumonitis	aspiration pneumonitis	ASPIRATION PNEUMONITIS	1	case_or_format
aspirin exacerbated respiratory disease	aspirin exacerbated respiratory disease	aspirin exacerbated respiratory disease	aspirin exacerbated respiratory disease	aspirin exacerbated respiratory disease	1	kept
Assault	assault	assault	assault	assault	1	case_or_format
assembly of sleep and respiratory neurobiology of the	assembly of sleep and respiratory neurobiology of the	assembly of sleep and respiratory neurobiology of the	assembly of sleep and respiratory neurobiology of the	assembly of sleep and respiratory neurobiology of the	1	kept
Assertive communication	assertive communication	assertive communication	assertive communication	Assertive communication	1	kept
assessement tool	assessement tool	assessement tool	assessement tool	assessement tool	1	kept
assessing	assessing	assessing	assessing	assessing	1	kept
Assessing Pain Both Spontaneous Awakening and Breathing Trials	assessing pain both spontaneous awakening and breathing trials	assessing pain both spontaneous awakening and breathing trials	assessing pain both spontaneous awakening and breathing trials	Assessing Pain Both Spontaneous Awakening and Breathing Trials	1	kept

Assessing sedation	assessing sedation	assessing sedation	assessing sedation	Assessing sedation	1	kept
Assessment - Ventilated	assessment ventilated	assessment ventilated	assessment ventilated	Assessment - Ventilated	1	kept
ASSESSMENT CHECKLIST	assessment checklist	assessment checklist	assessment checklist	ASSESSMENT CHECKLIST	1	kept
Assessment Electrocardiogram	assessment electrocardiogram	assessment electrocardiogram	assessment electrocardiogram	Assessment Electrocardiogram	1	kept
Assessment for palliative care	assessment for palliative care	assessment for palliative care	assessment for palliative care	Assessment for palliative care	1	kept
Assessment Indicator	assessment indicator	assessment indicator	assessment indicator	Assessment Indicator	1	kept
Assessment instruments	assessment instruments	assessment instruments	assessment instruments	Assessment instruments	1	kept
assessment method	assessment method	assessment method	assessment method	assessment method	1	kept
assessment methods	assessment methods	assessment methods	assessment method	assessment method	1	singular
assessment model	assessment model	assessment model	assessment model	assessment model	1	kept
Assessment models	assessment models	assessment models	assessment model	assessment model	1	singular
Assessment of pain	assessment of pain	assessment of pain	assessment of pain	Assessment of pain	1	kept
ASSESSMENT PROGRAM	assessment program	assessment program	assessment program	ASSESSMENT PROGRAM	1	kept
ASSESSMENT RETROSPECTIVE ANALYSIS	assessment retrospective analysis	assessment retrospective analysis	assessment retrospective analysis	ASSESSMENT RETROSPECTIVE ANALYSIS	1	kept
ASSESSMENT SCALE	assessment scale	assessment scale	assessment scale	Assessment scale	1	case_or_format
ASSESSMENT SCALES	assessment scales	assessment scales	assessment scale	Assessment scale	1	singular

Assessment scales	assessment scales	assessment scales	assessment scale	Assessment scale	1	singular
Assessments	assessments	assessments	assessment	Assessment	1	singular
Assessments scale	assessments scale	assessments scale	assessments scale	Assessments scale	1	kept
ASSIGNMENT	assignment	assignment	assignment	ASSIGNMENT	1	kept
Assignment problems	assignment problems	assignment problems	assignment problems	Assignment problems	1	kept
Assimilation	assimilation	assimilation	assimilation	Assimilation	1	kept
Assistance	assistance	assistance	assistance	assistance	1	case_or_format
Assistance in dying	assistance in dying	assistance in dying	assistance in dying	Assistance in dying	1	kept
assisted care facility	assisted care facility	assisted care facility	assisted care facility	assisted care facility	1	kept
Assisted conception	assisted conception	assisted conception	assisted conception	Assisted conception	1	kept
Assisted death	assisted death	assisted death	assisted death	Assisted death	1	kept
Assisted dying	assisted dying	assisted dying	assisted dying	Assisted dying	1	kept
Assisted Invasive ventilation	assisted invasive ventilation	assisted invasive ventilation	assisted invasive ventilation	Assisted Invasive ventilation	1	kept
Assisted Living	assisted living	assisted living	assisted living	assisted living	1	case_or_format
Assisted living	assisted living	assisted living	assisted living	assisted living	1	case_or_format
ASSISTED SUICIDE	assisted suicide	assisted suicide	assisted suicide	assisted suicide	1	case_or_format

Assisted ventilator modes	assisted ventilator modes	assisted ventilator modes	assisted ventilator modes	Assisted ventilator modes	1	kept
assistive	assistive	assistive	assistive	assistive	1	kept
Assistive Technology	assistive technology	assistive technology	assistive technology	assistive technology	1	case_or_format
Assistive technology	assistive technology	assistive technology	assistive technology	assistive technology	1	case_or_format
Assistência Ambulatorial	assist ncia ambulatorial	assist ncia ambulatorial	assist ncia ambulatorial	Assistência Ambulatorial	1	kept
Associated Factors	associated factors	associated factors	associated factor	Associated factors	1	singular
Associated risk factors	associated risk factors	associated risk factors	associated risk factors	Associated risk factors	1	kept
Association	association	association	association	ASSOCIATION	1	case_or_format
Associative group analysis	associative group analysis	associative group analysis	associative group analysis	Associative group analysis	1	kept
AST, aspartate aminotransferase	ast aspartate aminotransferase	ast aspartate aminotransferase	ast aspartate aminotransferase	AST, aspartate aminotransferase	1	kept
Asthma Control Questionnaire	asthma control questionnaire	asthma control questionnaire	asthma control questionnaire	Asthma Control Questionnaire	1	kept
Asthma education	asthma education	asthma education	asthma education	Asthma education	1	kept
asthma education	asthma education	asthma education	asthma education	Asthma education	1	case_or_format
Asthma exacerbation	asthma exacerbation	asthma exacerbation	asthma exacerbation	Asthma exacerbation	1	kept
Asthma management	asthma management	asthma management	asthma management	Asthma management	1	kept
Asthma specialist care	asthma specialist care	asthma specialist care	asthma specialist care	Asthma specialist care	1	kept

asthma specialty care	asthma specialty care	asthma specialty care	asthma specialty care	asthma specialty care	1	kept
ASTHMA-CARE	asthma care	asthma care	asthma care	ASTHMA-CARE	1	kept
astrocytoma	astrocytoma	astrocytoma	astrocytoma	Astrocytoma	1	case_or_format
ASV	asv	asv	asv	ASV	1	kept
Asylum	asylum	asylum	asylum	Asylum	1	kept
asylum seeker	asylum seeker	asylum seeker	asylum seeker	asylum seeker	1	kept
Asymmetric cost functions	asymmetric cost functions	asymmetric cost functions	asymmetric cost functions	Asymmetric cost functions	1	kept
Asymptomatic bacteriuria	asymptomatic bacteriuria	asymptomatic bacteriuria	asymptomatic bacteriuria	asymptomatic bacteriuria	1	case_or_format
asymptomatic cases	asymptomatic cases	asymptomatic cases	asymptomatic cases	asymptomatic cases	1	kept
Asymptomatic infection	asymptomatic infection	asymptomatic infection	asymptomatic infection	asymptomatic infection	1	case_or_format
asynchronous	asynchronous	asynchronous	asynchronous	asynchronous	1	kept
Asynchronous Learning	asynchronous learning	asynchronous learning	asynchronous learning	Asynchronous Learning	1	kept
Asynchrony	asynchrony	asynchrony	asynchrony	Asynchrony	1	kept
asynchrony	asynchrony	asynchrony	asynchrony	Asynchrony	1	case_or_format
Asystole	asystole	asystole	asystole	asystole	1	case_or_format
At home	at home	at home	at home	At home	1	kept

at home mortality	at home mortality	at home mortality	at home mortality	at home mortality	1	kept
at-breast supplementer	at breast supplementer	at breast supplementer	at breast supplementer	at-breast supplementer	1	kept
at-homeness	at homeness	at homeness	at homeness	at-homeness	1	kept
at-risk infants	at risk infants	at risk infants	at risk infants	at-risk infants	1	kept
At-risk population	at risk population	at risk population	at risk population	At-risk population	1	kept
Ataxia	ataxia	ataxia	ataxia	ataxia	1	case_or_format
ataxic aphasia	ataxic aphasia	ataxic aphasia	ataxic aphasia	ataxic aphasia	1	kept
ATELECTASIS	atelectasis	atelectasis	atelectasis	atelectasis	1	case_or_format
atelosteogenesis type 3	atelosteogenesis type 3	atelosteogenesis type 3	atelosteogenesis type 3	atelosteogenesis type 3	1	kept
Atención Ambulatoria	atenci n ambulatoria	atenci n ambulatoria	atenci n ambulatoria	Atención Ambulatoria	1	kept
Atención de bajo valor	atenci n de bajo valor	atenci n de bajo valor	atenci n de bajo valor	Atención de bajo valor	1	kept
Atención de Enfermeria	atenci n de enfermeria	atenci n de enfermeria	atenci n de enfermeria	Atención de Enfermeria	1	kept
atención de enfermería	atenci n de enfermer a	atenci n de enfermer a	atenci n de enfermer a	Atención de enfermería	1	case_or_format
Atención domiciliaria de salud	atenci n domiciliaria de salud	atenci n domiciliaria de salud	atenci n domiciliaria de salud	Atención domiciliaria de salud	1	kept
Atención farmacéutica	atenci n farmac utica	atenci n farmac utica	atenci n farmac utica	Atención farmacéutica	1	kept
Atención integral de la salud	atenci n integral de la salud	atenci n integral de la salud	atenci n integral de la salud	Atención integral de la salud	1	kept

Atención plena	atenci n plena	atenci n plena	atenci n plena	Atención plena	1	kept
atenolol plus chlortalidone	atenolol plus chlortalidone	atenolol plus chlortalidone	atenolol plus chlortalidone	atenolol plus chlortalidone	1	kept
atheroma	atheroma	atheroma	atheroma	atheroma	1	kept
athletic trainer	athletic trainer	athletic trainer	athletic trainer	athletic trainer	1	kept
Atient safety	atient safety	atient safety	atient safety	Atient safety	1	kept
atient safety	atient safety	atient safety	atient safety	Atient safety	1	case_or_format
Atitudes e Prática em Saúde	atitudes e pr tica em sa de	atitudes e pr tica em sa de	atitudes e pr tica em sa de	Atitudes e Prática em Saúde	1	kept
atlanto occipital dissociation	atlanto occipital dissociation	atlanto occipital dissociation	atlanto occipital dissociation	atlanto occipital dissociation	1	kept
Atlanto-Axial Joint	atlanto axial joint	atlanto axial joint	atlanto axial joint	Atlanto-Axial Joint	1	kept
atlanto-occipital dislocation	atlanto occipital dislocation	atlanto occipital dislocation	atlanto occipital dislocation	atlanto-occipital dislocation	1	kept
atlanto-occipital dissociation	atlanto occipital dissociation	atlanto occipital dissociation	atlanto occipital dissociation	atlanto occipital dissociation	1	case_or_format
Atlanto-Occipital Joint	atlanto occipital joint	atlanto occipital joint	atlanto occipital joint	Atlanto-Occipital Joint	1	kept
atlantoaxial dislocation	atlantoaxial dislocation	atlantoaxial dislocation	atlantoaxial dislocation	atlantoaxial dislocation	1	kept
Atlantoaxial fixation	atlantoaxial fixation	atlantoaxial fixation	atlantoaxial fixation	Atlantoaxial fixation	1	kept
atlantoaxial joint	atlantoaxial joint	atlantoaxial joint	atlantoaxial joint	atlantoaxial joint	1	kept
atlas fracture	atlas fracture	atlas fracture	atlas fracture	atlas fracture	1	kept

Atmospheric Pressure	atmospheric pressure	atmospheric pressure	atmospheric pressure	atmospheric pressure	1	case_or_format
Atomizing nasal cleaning method	atomizing nasal cleaning method	atomizing nasal cleaning method	atomizing nasal cleaning method	Atomizing nasal cleaning method	1	kept
Atopobium	atopobium	atopobium	atopobium	Atopobium	1	kept
Atopobium minutum infection	atopobium minutum infection	atopobium minutum infection	atopobium minutum infection	Atopobium minutum infection	1	kept
Atorvastatin	atorvastatin	atorvastatin	atorvastatin	atorvastatin	1	case_or_format
atovaquone	atovaquone	atovaquone	atovaquone	atovaquone	1	kept
ATP bioluminescence	atp bioluminescence	atp bioluminescence	atp bioluminescence	ATP bioluminescence	1	kept
ATP2C1 gene	atp2c1 gene	atp2c1 gene	atp2c1 gene	ATP2C1 gene	1	kept
Atresia choanae	atresia choanae	atresia choanae	atresia choanae	Atresia choanae	1	kept
Atrial Appendage	atrial appendage	atrial appendage	atrial appendage	Atrial Appendage	1	kept
atrial appendage atriotomy	atrial appendage atriotomy	atrial appendage atriotomy	atrial appendage atriotomy	atrial appendage atriotomy	1	kept
Atrial Fibrilation	atrial fibrilation	atrial fibrilation	atrial fibrilation	Atrial Fibrilation	1	kept
Atrial flutter	atrial flutter	atrial flutter	atrial flutter	Atrial Flutter	1	case_or_format
atrial natriuretic factor alpha	atrial natriuretic factor alpha	atrial natriuretic factor alpha	atrial natriuretic factor alpha	atrial natriuretic factor alpha	1	kept
atrial natriuretic factor derivative	atrial natriuretic factor derivative	atrial natriuretic factor derivative	atrial natriuretic factor derivative	atrial natriuretic factor derivative	1	kept
atrial septostomy	atrial septostomy	atrial septostomy	atrial septostomy	atrial septostomy	1	kept

atrial stent placement	atrial stent placement	atrial stent placement	atrial stent placement	atrial stent placement	1	kept
Atrial-femoral bypass	atrial femoral bypass	atrial femoral bypass	atrial femoral bypass	Atrial-femoral bypass	1	kept
ATROPHY	atrophy	atrophy	atrophy	Atrophy	1	case_or_format
attack on doctors	attack on doctors	attack on doctors	attack on doctors	attack on doctors	1	kept
Attacks	attacks	attacks	attacks	Attacks	1	kept
Attacks on healthcare	attacks on healthcare	attacks on healthcare	attacks on healthcare	Attacks on healthcare	1	kept
Attempted suicide	attempted suicide	attempted suicide	attempted suicide	Attempted suicide	1	kept
ATTEND WORK	attend work	attend work	attend work	ATTEND WORK	1	kept
attendants	attendants	attendants	attendants	attendants	1	kept
attending physician	attending physician	attending physician	attending physician	attending physician	1	kept
Attention mechanisms	attention mechanisms	attention mechanisms	attention mechanisms	Attention mechanisms	1	kept
attention performance	attention performance	attention performance	attention performance	attention performance	1	kept
Attentional load	attentional load	attentional load	attentional load	Attentional load	1	kept
attenuation	attenuation	attenuation	attenuation	attenuation	1	kept
Attenuation tendency	attenuation tendency	attenuation tendency	attenuation tendency	Attenuation tendency	1	kept
attire	attire	attire	attire	attire	1	kept

attitude and behaviour	attitude and behaviour	attitude and behaviour	attitude and behaviour	attitude and behaviour	1	kept
Attitude and health personnel	attitude and health personnel	attitude and health personnel	attitude and health personnel	Attitude and health personnel	1	kept
Attitude of health professional	attitude of health professional	attitude of health professional	attitude of health professional	Attitude of health professional	1	kept
Attitude questionnaire	attitude questionnaire	attitude questionnaire	attitude questionnaire	Attitude questionnaire	1	kept
Attitude scales	attitude scales	attitude scales	attitude scale	attitude scale	1	singular
attitude scales	attitude scales	attitude scales	attitude scale	attitude scale	1	singular
Attitude toward euthanasia	attitude toward euthanasia	attitude toward euthanasia	attitude toward euthanasia	Attitude toward euthanasia	1	kept
Attitude toward Pressure Ulcer Prevention Instrument	attitude toward pressure ulcer prevention instrument	attitude toward pressure ulcer prevention instrument	attitude toward pressure ulcer prevention instrument	Attitude toward Pressure Ulcer Prevention Instrument	1	kept
Attitude toward Pressure Ulcer tool	attitude toward pressure ulcer tool	attitude toward pressure ulcer tool	attitude toward pressure ulcer tool	Attitude toward Pressure Ulcer tool	1	kept
attitude, and practice	attitude and practice	attitude and practice	attitude and practice	attitude and practice	1	case_or_format
Attitudes and beliefs	attitudes and beliefs	attitudes and beliefs	attitudes and beliefs	Attitudes and beliefs	1	kept
Attitudes and perceptions	attitudes and perceptions	attitudes and perceptions	attitudes and perceptions	Attitudes and perceptions	1	kept
Attitudes of health personnel	attitudes of health personnel	attitudes of health personnel	attitudes of health personnel	Attitudes of health personnel	1	kept
Attitudes of healthcare personnel	attitudes of healthcare personnel	attitudes of healthcare personnel	attitudes of healthcare personnel	Attitudes of healthcare personnel	1	kept
Attitudes Related to Trauma Informed Care Scale	attitudes related to trauma informed care scale	attitudes related to trauma informed care scale	attitudes related to trauma informed care scale	Attitudes Related to Trauma Informed Care Scale	1	kept
attitudes to death	attitudes to death	attitudes to death	attitudes to death	attitudes to death	1	kept

Attitudes to fever	attitudes to fever	attitudes to fever	attitudes to fever	Attitudes to fever	1	kept
Attitudes to organ donation	attitudes to organ donation	attitudes to organ donation	attitudes to organ donation	Attitudes to organ donation	1	kept
Attitudes to organ or tissue donations	attitudes to organ or tissue donations	attitudes to organ or tissue donations	attitudes to organ or tissue donations	Attitudes to organ or tissue donations	1	kept
attitudes toward implicit bias instrument	attitudes toward implicit bias instrument	attitudes toward implicit bias instrument	attitudes toward implicit bias instrument	attitudes toward implicit bias instrument	1	kept
attitudes toward palliative care	attitudes toward palliative care	attitudes toward palliative care	attitudes toward palliative care	attitudes toward palliative care	1	kept
Attitudes toward Principles about Dying with Dignity Scale	attitudes toward principles about dying with dignity scale	attitudes toward principles about dying with dignity scale	attitudes toward principles about dying with dignity scale	Attitudes toward Principles about Dying with Dignity Scale	1	kept
Attitudes towards death	attitudes towards death	attitudes towards death	attitudes towards death	attitudes towards death	1	case_or_format
Attractive strategies	attractive strategies	attractive strategies	attractive strategies	Attractive strategies	1	kept
Attributable costs	attributable costs	attributable costs	attributable costs	Attributable costs	1	kept
ATTRIBUTABLE MORTALITY	attributable mortality	attributable mortality	attributable mortality	ATTRIBUTABLE MORTALITY	1	kept
ATTRIBUTES	attributes	attributes	attributes	ATTRIBUTES	1	kept
attribution	attribution	attribution	attribution	attribution	1	kept
Attrition	attrition	attrition	attrition	Attrition	1	kept
attrition	attrition	attrition	attrition	Attrition	1	case_or_format
Attrition rate	attrition rate	attrition rate	attrition rate	Attrition rate	1	kept
atypical antipsychotic	atypical antipsychotic	atypical antipsychotic	atypical antipsychotic	atypical antipsychotic	1	kept

Atypical antipsychotics	atypical antipsychotics	atypical antipsychotics	atypical antipsychotic	atypical antipsychotic	1	singular
Atypical bacterial forms	atypical bacterial forms	atypical bacterial forms	atypical bacterial forms	Atypical bacterial forms	1	kept
Atypical hemolytic uremic syndrome	atypical hemolytic uremic syndrome	atypical hemolytic uremic syndrome	atypical hemolytic uremic syndrome	Atypical hemolytic uremic syndrome	1	kept
atypical Mycobacterium	atypical mycobacterium	atypical mycobacterium	atypical mycobacterium	atypical Mycobacterium	1	kept
ATYPICAL SYMPTOMS	atypical symptoms	atypical symptoms	atypical symptoms	ATYPICAL SYMPTOMS	1	kept
Au NPs	au nps	au nps	au nps	Au NPs	1	kept
AUC	auc	auc	auc	AUC	1	kept
Audience response system	audience response system	audience response system	audience response system	Audience response system	1	kept
audio	audio	audio	audio	audio	1	kept
Audio acoustics	audio acoustics	audio acoustics	audio acoustics	Audio acoustics	1	kept
Audio book	audio book	audio book	audio book	Audio book	1	kept
Audio intervention	audio intervention	audio intervention	audio intervention	Audio intervention	1	kept
audio interview	audio interview	audio interview	audio interview	audio interview	1	kept
Audio recordings	audio recordings	audio recordings	audio recording	audio recording	1	singular
Audio signal processing	audio signal processing	audio signal processing	audio signal processing	Audio signal processing	1	kept
Audio therapy	audio therapy	audio therapy	audio therapy	Audio therapy	1	kept

audio therapy	audio therapy	audio therapy	audio therapy	Audio therapy	1	case_or_format
Audio-visual perceptions	audio visual perceptions	audio visual perceptions	audio visual perceptions	Audio-visual perceptions	1	kept
audiobook	audiobook	audiobook	audiobook	audiobook	1	kept
Audiology	audiology	audiology	audiology	audiology	1	case_or_format
Audiometry	audiometry	audiometry	audiometry	audiometry	1	case_or_format
Audiometry, Evoked Response	audiometry evoked response	audiometry evoked response	audiometry evoked response	Audiometry, Evoked Response	1	kept
Audiometry, Pure-Tone	audiometry pure tone	audiometry pure tone	audiometry pure tone	Audiometry, Pure-Tone	1	kept
Audiovisual	audiovisual	audiovisual	audiovisual	Audiovisual	1	kept
audiovisual research	audiovisual research	audiovisual research	audiovisual research	audiovisual research	1	kept
Audit and Feedback	audit and feedback	audit and feedback	audit and feedback	audit and feedback	1	case_or_format
Audit and feedback intervention	audit and feedback intervention	audit and feedback intervention	audit and feedback intervention	Audit and feedback intervention	1	kept
Audit log	audit log	audit log	audit log	audit log	1	case_or_format
Audit logs	audit logs	audit logs	audit log	audit log	1	singular
audit logs	audit logs	audit logs	audit log	audit log	1	singular
Audit steps	audit steps	audit steps	audit steps	Audit steps	1	kept
auditory brainstem response	auditory brainstem response	auditory brainstem response	auditory brainstem response	auditory brainstem response	1	kept

Auditory brainstem responses	auditory brainstem responses	auditory brainstem responses	auditory brainstem response	auditory brainstem response	1	singular
auditory development	auditory development	auditory development	auditory development	auditory development	1	kept
Auditory Diseases, Central	auditory diseases central	auditory diseases central	auditory diseases central	Auditory Diseases, Central	1	kept
auditory displays	auditory displays	auditory displays	auditory displays	auditory displays	1	kept
auditory dys-synchrony	auditory dys synchrony	auditory dys synchrony	auditory dys synchrony	auditory dys-synchrony	1	kept
auditory environment	auditory environment	auditory environment	auditory environment	auditory environment	1	kept
Auditory fatigues	auditory fatigues	auditory fatigues	auditory fatigue	Auditory Fatigue	1	singular
auditory hallucinations	auditory hallucinations	auditory hallucinations	auditory hallucination	auditory hallucination	1	singular
Auditory interventions	auditory interventions	auditory interventions	auditory interventions	Auditory interventions	1	kept
auditory masking	auditory masking	auditory masking	auditory masking	auditory masking	1	kept
auditory nervous system	auditory nervous system	auditory nervous system	auditory nervous system	auditory nervous system	1	kept
Auditory perception	auditory perception	auditory perception	auditory perception	Auditory Perception	1	case_or_format
auditory prompt	auditory prompt	auditory prompt	auditory prompt	auditory prompt	1	kept
Auditory streaming	auditory streaming	auditory streaming	auditory streaming	Auditory streaming	1	kept
Auditory Threshold	auditory threshold	auditory threshold	auditory threshold	Auditory Threshold	1	kept
Auditory virtual environment	auditory virtual environment	auditory virtual environment	auditory virtual environment	Auditory virtual environment	1	kept

Auditory-evoked potential	auditory evoked potential	auditory evoked potential	auditory evoked potential	auditory evoked potential	1	case_or_format
Auditorías	auditor as	auditor as	auditor as	Auditorías	1	kept
Auditorías en tiempo real	auditor as en tiempo real	auditor as en tiempo real	auditor as en tiempo real	Auditorías en tiempo real	1	kept
Augmentative alternative communication	augmentative alternative communication	augmentative alternative communication	augmentative alternative communication	Augmentative alternative communication	1	kept
Augmentative alternative communications systems	augmentative alternative communications systems	augmentative alternative communications systems	augmentative alternative communications systems	Augmentative alternative communications systems	1	kept
augmentative and alternative communication	augmentative and alternative communication	augmentative and alternative communication	augmentative and alternative communication	augmentative and alternative communication	1	kept
Augmentative and Alternative Communication Systems	augmentative and alternative communication systems	augmentative and alternative communication systems	augmentative and alternative communication systems	Augmentative and Alternative Communication Systems	1	kept
Augmentative and alternative communications systems	augmentative and alternative communications systems	augmentative and alternative communications systems	augmentative and alternative communications systems	Augmentative and alternative communications systems	1	kept
Augmentative-and-alternative communication	augmentative and alternative communication	augmentative and alternative communication	augmentative and alternative communication	Augmentative and alternative communication	1	case_or_format
Augmented alternative communication	augmented alternative communication	augmented alternative communication	augmented alternative communication	Augmented alternative communication	1	kept
augmented reality assisted video laryngoscopy	augmented reality assisted video laryngoscopy	augmented reality assisted video laryngoscopy	augmented reality assisted video laryngoscopy	augmented reality assisted video laryngoscopy	1	kept
Augmented reality systems	augmented reality systems	augmented reality systems	augmented reality systems	Augmented reality systems	1	kept
augmented renal clearance	augmented renal clearance	augmented renal clearance	augmented renal clearance	augmented renal clearance	1	kept
aureus	aureus	aureus	aureus	AUREUS	1	case_or_format
Auscultatory sounds	auscultatory sounds	auscultatory sounds	auscultatory sounds	Auscultatory sounds	1	kept
Aussie Heart Guide Program	aussie heart guide program	aussie heart guide program	aussie heart guide program	Aussie Heart Guide Program	1	kept

Austere environment	austere environment	austere environment	austere environment	Austere environment	1	kept
Austere environment surgery	austere environment surgery	austere environment surgery	austere environment surgery	Austere environment surgery	1	kept
austerity	austerity	austerity	austerity	austerity	1	kept
Australian Aboriginal and Torres Strait Islander Peoples	australian aboriginal and torres strait islander peoples	australian aboriginal and torres strait islander peoples	australian aboriginal and torres strait islander peoples	Australian Aboriginal and Torres Strait Islander Peoples	1	kept
Australian Capital Territory	australian capital territory	australian capital territory	australian capital territory	Australian Capital Territory	1	kept
AUTHENTICATION	authentication	authentication	authentication	AUTHENTICATION	1	kept
Authentication	authentication	authentication	authentication	AUTHENTICATION	1	case_or_format
Authorized users	authorized users	authorized users	authorized users	Authorized users	1	kept
authorship	authorship	authorship	authorship	Authorship	1	case_or_format
Authors’ medical education	authors medical education	authors medical education	authors medical education	Authors’ medical education	1	kept
AUTHORS’ Postgraduation	authors postgraduation	authors postgraduation	authors postgraduation	AUTHORS’ Postgraduation	1	kept
Autism	autism	autism	autism	autism	1	case_or_format
Autism spectrum disorder	autism spectrum disorder	autism spectrum disorder	autism spectrum disorder	Autism Spectrum Disorder	1	case_or_format
Auto regressive models	auto regressive models	auto regressive models	auto regressive models	Auto regressive models	1	kept
auto-communication	auto communication	auto communication	auto communication	auto-communication	1	kept
auto-documentation	auto documentation	auto documentation	auto documentation	Auto-documentation	1	case_or_format

auto-programming	auto programming	auto programming	auto programming	auto-programming	1	kept
Autoantibodies	autoantibodies	autoantibodies	autoantibody	autoantibody	1	singular
Autobiographies as Topic	autobiographies as topic	autobiographies as topic	autobiographies as topic	Autobiographies as Topic	1	kept
Autobiography	autobiography	autobiography	autobiography	Autobiography	1	kept
Autobiography as Topic	autobiography as topic	autobiography as topic	autobiography as topic	Autobiography as Topic	1	kept
Autoeficacia materna	autoeficacia materna	autoeficacia materna	autoeficacia materna	Autoeficacia materna	1	kept
autograft	autograft	autograft	autograft	autograft	1	kept
autoimmune bullous skin diseases	autoimmune bullous skin diseases	autoimmune bullous skin diseases	autoimmune bullous skin diseases	autoimmune bullous skin diseases	1	kept
autoimmune cytopenia	autoimmune cytopenia	autoimmune cytopenia	autoimmune cytopenia	autoimmune cytopenia	1	kept
Autoimmune disease	autoimmune disease	autoimmune disease	autoimmune disease	autoimmune disease	1	case_or_format
autoimmune diseases	autoimmune diseases	autoimmune diseases	autoimmune disease	autoimmune disease	1	singular
Autoimmune disorder	autoimmune disorder	autoimmune disorder	autoimmune disorder	Autoimmune disorder	1	kept
autoimmune inflammation	autoimmune inflammation	autoimmune inflammation	autoimmune inflammation	autoimmune inflammation	1	kept
autoimmune pancreatitis	autoimmune pancreatitis	autoimmune pancreatitis	autoimmune pancreatitis	autoimmune pancreatitis	1	kept
autoimmune skin disease	autoimmune skin disease	autoimmune skin disease	autoimmune skin disease	autoimmune skin disease	1	kept
Autoimmunity	autoimmunity	autoimmunity	autoimmunity	Autoimmunity	1	kept

autoinflammatory disease	autoinflammatory disease	autoinflammatory disease	autoinflammatory disease	autoinflammatory disease	1	kept
autologous	autologous	autologous	autologous	autologous	1	kept
Autologous blood	autologous blood	autologous blood	autologous blood	Autologous blood	1	kept
Autologous bone graft	autologous bone graft	autologous bone graft	autologous bone graft	Autologous bone graft	1	kept
Autologous platelet-derived gel	autologous platelet derived gel	autologous platelet derived gel	autologous platelet derived gel	Autologous platelet-derived gel	1	kept
AUTOLOGOUS TRANSFUSION	autologous transfusion	autologous transfusion	autologous transfusion	AUTOLOGOUS TRANSFUSION	1	kept
Autologous-salvaged blood	autologous salvaged blood	autologous salvaged blood	autologous salvaged blood	Autologous-salvaged blood	1	kept
autolytic debridement	autolytic debridement	autolytic debridement	autolytic debridement	autolytic debridement	1	kept
Automatable	automatable	automatable	automatable	Automatable	1	kept
automated	automated	automated	automated	automated	1	kept
automated alert system	automated alert system	automated alert system	automated alert system	automated alert system	1	kept
Automated classification	automated classification	automated classification	automated classification	Automated classification	1	kept
Automated deduction	automated deduction	automated deduction	automated deduction	Automated deduction	1	kept
AUTOMATED DETECTION	automated detection	automated detection	automated detection	AUTOMATED DETECTION	1	kept
Automated detection	automated detection	automated detection	automated detection	AUTOMATED DETECTION	1	case_or_format
automated dispensing cabinet	automated dispensing cabinet	automated dispensing cabinet	automated dispensing cabinet	automated dispensing cabinet	1	kept

automated dispensing cabinets	automated dispensing cabinets	automated dispensing cabinets	automated dispensing cabinet	automated dispensing cabinet	1	singular
Automated drug-dispensing systems	automated drug dispensing systems	automated drug dispensing systems	automated drug dispensing systems	Automated drug-dispensing systems	1	kept
automated early warning system	automated early warning system	automated early warning system	automated early warning system	automated early warning system	1	kept
Automated external defibrillators	automated external defibrillators	automated external defibrillators	automated external defibrillator	Automated external defibrillator	1	singular
automated glucose monitoring	automated glucose monitoring	automated glucose monitoring	automated glucose monitoring	automated glucose monitoring	1	kept
automated infrared pupillometry	automated infrared pupillometry	automated infrared pupillometry	automated infrared pupillometry	automated infrared pupillometry	1	kept
Automated Insulin Delivery	automated insulin delivery	automated insulin delivery	automated insulin delivery	Automated Insulin Delivery	1	kept
Automated insulin delivery	automated insulin delivery	automated insulin delivery	automated insulin delivery	Automated Insulin Delivery	1	case_or_format
automated insulin dosing	automated insulin dosing	automated insulin dosing	automated insulin dosing	automated insulin dosing	1	kept
automated measurement	automated measurement	automated measurement	automated measurement	automated measurement	1	kept
Automated methods	automated methods	automated methods	automated methods	Automated methods	1	kept
automated monitoring	automated monitoring	automated monitoring	automated monitoring	Automated monitoring	1	case_or_format
Automated monitoring system	automated monitoring system	automated monitoring system	automated monitoring system	Automated monitoring system	1	kept
automated notification system	automated notification system	automated notification system	automated notification system	automated notification system	1	kept
Automated process	automated process	automated process	automated process	Automated process	1	kept
AUTOMATED PUPILLOMETRY	automated pupillometry	automated pupillometry	automated pupillometry	Automated pupillometry	1	case_or_format

Automated QT measurement	automated qt measurement	automated qt measurement	automated qt measurement	Automated QT measurement	1	kept
automated resuscitation recorder	automated resuscitation recorder	automated resuscitation recorder	automated resuscitation recorder	automated resuscitation recorder	1	kept
Automated risk assessment system	automated risk assessment system	automated risk assessment system	automated risk assessment system	Automated risk assessment system	1	kept
automated risk assessment system for multidrug resistant	automated risk assessment system for multidrug resistant	automated risk assessment system for multidrug resistant	automated risk assessment system for multidrug resistant	automated risk assessment system for multidrug resistant	1	kept
Automated scoring	automated scoring	automated scoring	automated scoring	Automated scoring	1	kept
automated scoring system	automated scoring system	automated scoring system	automated scoring system	automated scoring system	1	kept
automated screening	automated screening	automated screening	automated screening	automated screening	1	kept
automated self adjusting subcutaneous insulin algorithm	automated self adjusting subcutaneous insulin algorithm	automated self adjusting subcutaneous insulin algorithm	automated self adjusting subcutaneous insulin algorithm	automated self adjusting subcutaneous insulin algorithm	1	kept
automated sepsis screening and alert system	automated sepsis screening and alert system	automated sepsis screening and alert system	automated sepsis screening and alert system	automated sepsis screening and alert system	1	kept
Automated surveillance system	automated surveillance system	automated surveillance system	automated surveillance system	Automated surveillance system	1	kept
Automated system	automated system	automated system	automated system	Automated systems	1	case_or_format
Automated tracking	automated tracking	automated tracking	automated tracking	Automated tracking	1	kept
Automated ventilation	automated ventilation	automated ventilation	automated ventilation	Automated ventilation	1	kept
automated vigilance	automated vigilance	automated vigilance	automated vigilance	automated vigilance	1	kept
Automated weaning modes	automated weaning modes	automated weaning modes	automated weaning modes	Automated weaning modes	1	kept
Automatic classification	automatic classification	automatic classification	automatic classification	Automatic classification	1	kept

Automatic composition	automatic composition	automatic composition	automatic composition	Automatic composition	1	kept
Automatic detection	automatic detection	automatic detection	automatic detection	Automatic detection	1	kept
Automatic evaluation	automatic evaluation	automatic evaluation	automatic evaluation	Automatic evaluation	1	kept
Automatic FIO<sub>2<	automatic fio sub 2	automatic fio sub 2	automatic fio sub 2	Automatic FIO<sub>2<	1	kept
Automatic recognition	automatic recognition	automatic recognition	automatic recognition	Automatic recognition	1	kept
automatic speech recognition	automatic speech recognition	automatic speech recognition	automatic speech recognition	automatic speech recognition	1	kept
Automatic syntactic analysis	automatic syntactic analysis	automatic syntactic analysis	automatic syntactic analysis	Automatic syntactic analysis	1	kept
Automatic transcription	automatic transcription	automatic transcription	automatic transcription	Automatic transcription	1	kept
Automatic wheel chair	automatic wheel chair	automatic wheel chair	automatic wheel chair	Automatic wheel chair	1	kept
Automatically generated	automatically generated	automatically generated	automatically generated	Automatically generated	1	kept
Automaticity	automaticity	automaticity	automaticity	Automaticity	1	kept
automation, health care policy and regulatory foresight	automation health care policy and regulatory foresight	automation health care policy and regulatory foresight	automation health care policy and regulatory foresight	automation, health care policy and regulatory foresight	1	kept
Automation, Laboratory	automation laboratory	automation laboratory	automation laboratory	Automation, Laboratory	1	kept
Automation.	automation	automation	automation	automation	1	case_or_format
Autonomic dysfunction	autonomic dysfunction	autonomic dysfunction	autonomic dysfunction	autonomic dysfunction	1	case_or_format
Autonomic Dysreflexia	autonomic dysreflexia	autonomic dysreflexia	autonomic dysreflexia	autonomic dysreflexia	1	case_or_format

Autonomic functions	autonomic functions	autonomic functions	autonomic functions	Autonomic functions	1	kept
Autonomic instability	autonomic instability	autonomic instability	autonomic instability	Autonomic instability	1	kept
autonomic measurement	autonomic measurement	autonomic measurement	autonomic measurement	autonomic measurement	1	kept
Autonomic storming	autonomic storming	autonomic storming	autonomic storming	Autonomic storming	1	kept
Autonomous agents	autonomous agents	autonomous agents	autonomous agents	Autonomous agents	1	kept
autonomous clinician	autonomous clinician	autonomous clinician	autonomous clinician	autonomous clinician	1	kept
Autonomous community of Madrid	autonomous community of madrid	autonomous community of madrid	autonomous community of madrid	Autonomous community of Madrid	1	kept
Autonomous detection	autonomous detection	autonomous detection	autonomous detection	Autonomous detection	1	kept
autonomous learning	autonomous learning	autonomous learning	autonomous learning	autonomous learning	1	kept
Autonomous monitoring	autonomous monitoring	autonomous monitoring	autonomous monitoring	Autonomous monitoring	1	kept
Autonomous Robots	autonomous robots	autonomous robots	autonomous robots	Autonomous Robots	1	kept
Autonomy score	autonomy score	autonomy score	autonomy score	Autonomy score	1	kept
autonomy score	autonomy score	autonomy score	autonomy score	Autonomy score	1	case_or_format
Autopsies	autopsies	autopsies	autopsy	autopsy	1	singular
AUTOREGULATION	autoregulation	autoregulation	autoregulation	autoregulation	1	case_or_format
Autoresuscitation	autoresuscitation	autoresuscitation	autoresuscitation	Autoresuscitation	1	kept

autoresuscitation	autoresuscitation	autoresuscitation	autoresuscitation	Autoresuscitation	1	case_or_format
autosomal dominant hypoparathyroidism 1	autosomal dominant hypoparathyroidism 1	autosomal dominant hypoparathyroidism 1	autosomal dominant hypoparathyroidism 1	autosomal dominant hypoparathyroidism 1	1	kept
autosomal dominant inheritance	autosomal dominant inheritance	autosomal dominant inheritance	autosomal dominant inheritance	autosomal dominant inheritance	1	kept
autosomal inheritance	autosomal inheritance	autosomal inheritance	autosomal inheritance	autosomal inheritance	1	kept
Auxiliares de enfermería	auxiliares de enfermer a	auxiliares de enfermer a	auxiliares de enfermer a	Auxiliares de enfermería	1	kept
AV canal	av canal	av canal	av canal	AV canal	1	kept
AV nodal active tachycardia	av nodal active tachycardia	av nodal active tachycardia	av nodal active tachycardia	AV nodal active tachycardia	1	kept
AV nodal passive	av nodal passive	av nodal passive	av nodal passive	AV nodal passive	1	kept
Availability	availability	availability	availability	Availability	1	kept
availability	availability	availability	availability	Availability	1	case_or_format
Avaliação de necessidades	avalia o de necessidades	avalia o de necessidades	avalia o de necessidades	Avaliação de necessidades	1	kept
Avaliação em saúde	avalia o em sa de	avalia o em sa de	avalia o em sa de	Avaliação em saúde	1	kept
Avalon	avalon	avalon	avalon	Avalon	1	kept
avatar-based monitoring	avatar based monitoring	avatar based monitoring	avatar based monitoring	avatar-based monitoring	1	kept
AVC	avc	avc	avc	AVC	1	kept
Average Distance	average distance	average distance	average distance	Average Distance	1	kept

Average numbers	average numbers	average numbers	average numbers	Average numbers	1	kept
Average rates	average rates	average rates	average rates	Average rates	1	kept
Average treatment effects	average treatment effects	average treatment effects	average treatment effects	Average treatment effects	1	kept
aversive behavior	aversive behavior	aversive behavior	aversive behavior	aversive behavior	1	kept
Aves	aves	aves	aves	Aves	1	kept
avi 7537	avi 7537	avi 7537	avi 7537	avi 7537	1	kept
Avoidance behavior	avoidance behavior	avoidance behavior	avoidance behavior	avoidance behavior	1	case_or_format
avoidance behaviour	avoidance behaviour	avoidance behaviour	avoidance behaviour	avoidance behaviour	1	kept
avoidant coping	avoidant coping	avoidant coping	avoidant coping	avoidant coping	1	kept
Avoidant restrictive food intake disorder	avoidant restrictive food intake disorder	avoidant restrictive food intake disorder	avoidant restrictive food intake disorder	Avoidant restrictive food intake disorder	1	kept
AVP receptor antagonists	avp receptor antagonists	avp receptor antagonists	avp receptor antagonists	AVP receptor antagonists	1	kept
AVSD	avsd	avsd	avsd	AVSD	1	kept
avulsion fracture	avulsion fracture	avulsion fracture	avulsion fracture	avulsion fracture	1	kept
Avós	av s	av s	av s	Avós	1	kept
Awake	awake	awake	awake	Awake	1	kept
awake	awake	awake	awake	Awake	1	case_or_format

AWAKE FIBEROPTIC INTUBATION	awake fiberoptic intubation	awake fiberoptic intubation	awake fiberoptic intubation	AWAKE FIBEROPTIC INTUBATION	1	kept
awake-prone positioning	awake prone positioning	awake prone positioning	awake prone positioning	awake-prone positioning	1	kept
Awakening and Breathing Coordination, Delirium	awakening and breathing coordination delirium monitoring	awakening and breathing coordination delirium monitoring	awakening and breathing coordination delirium monitoring	Awakening and Breathing Coordination, Delirium	1	kept
Awakening and breathing trials	awakening and breathing trials	awakening and breathing trials	awakening and breathing trials	Awakening and breathing trials	1	kept
Awards	awards	awards	awards	Awards	1	kept
AWARENESS	awareness	awareness	awareness	awareness	1	case_or_format
Awareness campaign	awareness campaign	awareness campaign	awareness campaign	Awareness campaign	1	kept
Awareness displays	awareness displays	awareness displays	awareness displays	Awareness displays	1	kept
awareness level	awareness level	awareness level	awareness level	awareness level	1	kept
awareness of mortality	awareness of mortality	awareness of mortality	awareness of mortality	awareness of mortality	1	kept
Awareness raising	awareness raising	awareness raising	awareness raising	Awareness raising	1	kept
axial spondyloarthritis	axial spondyloarthritis	axial spondyloarthritis	axial spondyloarthritis	axial spondyloarthritis	1	kept
axid ar	axid ar	axid ar	axid ar	axid ar	1	kept
AXILLARY	axillary	axillary	axillary	AXILLARY	1	kept
axillary	axillary	axillary	axillary	AXILLARY	1	case_or_format
Axillary Artery	axillary artery	axillary artery	axillary artery	Axillary Artery	1	kept

axillary artery	axillary artery	axillary artery	axillary artery	Axillary Artery	1	case_or_format
Axillary lymph node	axillary lymph node	axillary lymph node	axillary lymph node	axillary lymph node	1	case_or_format
axillary lymph node dissection	axillary lymph node dissection	axillary lymph node dissection	axillary lymph node dissection	axillary lymph node dissection	1	kept
axillary region	axillary region	axillary region	axillary region	axillary region	1	kept
Axillary temperature	axillary temperature	axillary temperature	axillary temperature	Axillary temperature	1	kept
axillary temperature	axillary temperature	axillary temperature	axillary temperature	Axillary temperature	1	case_or_format
axillary thermometer	axillary thermometer	axillary thermometer	axillary thermometer	axillary thermometer	1	kept
Ayder comprehensive specialized hospital	ayder comprehensive specialized hospital	ayder comprehensive specialized hospital	ayder comprehensive specialized hospital	Ayder comprehensive specialized hospital	1	kept
Ayurveda	ayurveda	ayurveda	ayurveda	Ayurveda	1	kept
ayurvedic drug	ayurvedic drug	ayurvedic drug	ayurvedic drug	ayurvedic drug	1	kept
Aza Compounds	aza compounds	aza compounds	aza compounds	Aza Compounds	1	kept
azacitidine	azacitidine	azacitidine	azacitidine	azacitidine	1	kept
Azerbaijan	azerbaijan	azerbaijan	azerbaijan	Azerbaijan	1	kept
Azoles	azoles	azoles	azoles	Azoles	1	kept
azoles	azoles	azoles	azoles	Azoles	1	case_or_format
azotemia	azotemia	azotemia	azotemia	azotemia	1	kept

B Raf kinase	b raf kinase	b raf kinase	b raf kinase	B Raf kinase	1	kept
B scan	b scan	b scan	b scan	B scan	1	kept
B-cell lymphoma	b cell lymphoma	b cell lymphoma	b cell lymphoma	B cell lymphoma	1	case_or_format
B-natriuretic peptides	b natriuretic peptides	b natriuretic peptides	b natriuretic peptides	B-natriuretic peptides	1	kept
b-Type natriuretic peptide	b type natriuretic peptide	b type natriuretic peptide	b type natriuretic peptide	b-Type natriuretic peptide	1	kept
BA9 genotype	ba9 genotype	ba9 genotype	ba9 genotype	BA9 genotype	1	kept
Babesia	babesia	babesia	babesia	Babesia	1	kept
BABIES	babies	babies	baby	baby	1	singular
babies-understanding the pain	babies understanding the pain	babies understanding the pain	babies understanding the pain	babies-understanding the pain	1	kept
Babinski reflex	babinski reflex	babinski reflex	babinski reflex	Babinski reflex	1	kept
Baby body language and psychotherapy	baby body language and psychotherapy	baby body language and psychotherapy	baby body language and psychotherapy	Baby body language and psychotherapy	1	kept
Baby bottle	baby bottle	baby bottle	baby bottle	Baby bottle	1	kept
Baby Friendly Health Initiative	baby friendly health initiative	baby friendly health initiative	baby friendly health initiative	Baby Friendly Health Initiative	1	kept
baby friendly hospital initiative	baby friendly hospital initiative	baby friendly hospital initiative	baby friendly hospital initiative	Baby Friendly Hospital Initiative	1	case_or_format
Baby Incubator	baby incubator	baby incubator	baby incubator	Baby Incubator	1	kept
Baby incubator	baby incubator	baby incubator	baby incubator	Baby Incubator	1	case_or_format

Baby-Friendly	baby friendly	baby friendly	baby friendly	Baby-Friendly	1	kept
Baby-friendly	baby friendly	baby friendly	baby friendly	Baby-Friendly	1	case_or_format
baby-friendly	baby friendly	baby friendly	baby friendly	Baby-Friendly	1	case_or_format
Baby-friendly hospital	baby friendly hospital	baby friendly hospital	baby friendly hospital	Baby-friendly hospital	1	kept
Baby-MONITOR	baby monitor	baby monitor	baby monitor	Baby-MONITOR	1	kept
Babylon-Iraq	babylon iraq	babylon iraq	babylon iraq	Babylon-Iraq	1	kept
BabySteps	babysteps	babysteps	babysteps	BabySteps	1	kept
babywearing	babywearing	babywearing	babywearing	babywearing	1	kept
Baccalaureate	baccalaureate	baccalaureate	baccalaureate	Baccalaureate	1	kept
Baccalaureate education	baccalaureate education	baccalaureate education	baccalaureate education	Baccalaureate education	1	kept
Baccalaureate program	baccalaureate program	baccalaureate program	baccalaureate program	Baccalaureate program	1	kept
Bachelor's degree	bachelor s degree	bachelor s degree	bachelor s degree	Bachelor's degree	1	kept
BACILLI	bacilli	bacilli	bacilli	Bacilli	1	case_or_format
Bacillus cereus infection	bacillus cereus infection	bacillus cereus infection	bacillus cereus infection	Bacillus cereus infection	1	kept
Bacillus paranthracis	bacillus paranthracis	bacillus paranthracis	bacillus paranthracis	Bacillus paranthracis	1	kept
Bacillus spp	bacillus spp	bacillus spp	bacillus spp	Bacillus spp	1	kept

Bacillus thuringiensis	bacillus thuringiensis	bacillus thuringiensis	bacillus thuringiensis	Bacillus thuringiensis	1	kept
Bacitracin	bacitracin	bacitracin	bacitracin	bacitracin	1	case_or_format
back college	back college	back college	back college	back college	1	kept
Back Depression Scale	back depression scale	back depression scale	back depression scale	Back Depression Scale	1	kept
back injury	back injury	back injury	back injury	Back Injuries	1	case_or_format
Back massage	back massage	back massage	back massage	Back massage	1	kept
Back pain	back pain	back pain	back pain	Back Pain	1	case_or_format
Back pain frequency	back pain frequency	back pain frequency	back pain frequency	Back pain frequency	1	kept
back scratch test	back scratch test	back scratch test	back scratch test	back scratch test	1	kept
back to sleep	back to sleep	back to sleep	back to sleep	back to sleep	1	kept
back transfer	back transfer	back transfer	back transfer	back transfer	1	kept
Back transport	back transport	back transport	back transport	Back transport	1	kept
Back-referral	back referral	back referral	back referral	Back-referral	1	kept
Background noise levels	background noise levels	background noise levels	background noise levels	Background noise levels	1	kept
Backrest elevation	backrest elevation	backrest elevation	backrest elevation	BACKREST ELEVATION	1	case_or_format
backward translation	backward translation	backward translation	backward translation	backward translation	1	kept

backward upward and rightward pressure maneuver	backward upward and rightward pressure maneuver	backward upward and rightward pressure maneuver	backward upward and rightward pressure maneuver	backward upward and rightward pressure maneuver	1	kept
bacteremic nosocomial pneumonia	bacteremic nosocomial pneumonia	bacteremic nosocomial pneumonia	bacteremic nosocomial pneumonia	bacteremic nosocomial pneumonia	1	kept
Bacteria care	bacteria care	bacteria care	bacteria care	Bacteria care	1	kept
bacteria, cerebrospinal fluid, encephalitis	bacteria cerebrospinal fluid encephalitis inflammation virus	bacteria cerebrospinal fluid encephalitis inflammation virus	bacteria cerebrospinal fluid encephalitis inflammation virus	bacteria, cerebrospinal fluid, encephalitis	1	kept
BACTERIAL	bacterial	bacterial	bacterial	BACTERIAL	1	kept
Bacterial	bacterial	bacterial	bacterial	BACTERIAL	1	case_or_format
bacterial	bacterial	bacterial	bacterial	BACTERIAL	1	case_or_format
BACTERIAL BIOFILMS	bacterial biofilms	bacterial biofilms	bacterial biofilms	BACTERIAL BIOFILMS	1	kept
bacterial burden	bacterial burden	bacterial burden	bacterial burden	bacterial burden	1	kept
Bacterial Capsules	bacterial capsules	bacterial capsules	bacterial capsules	Bacterial Capsules	1	kept
bacterial cell wall	bacterial cell wall	bacterial cell wall	bacterial cell wall	bacterial cell wall	1	kept
bacterial clearance	bacterial clearance	bacterial clearance	bacterial clearance	bacterial clearance	1	kept
Bacterial colonisation	bacterial colonisation	bacterial colonisation	bacterial colonisation	Bacterial colonisation	1	kept
Bacterial community	bacterial community	bacterial community	bacterial community	Bacterial community	1	kept
Bacterial Contamination	bacterial contamination	bacterial contamination	bacterial contamination	Bacterial contamination	1	case_or_format
bacterial decolonization	bacterial decolonization	bacterial decolonization	bacterial decolonization	bacterial decolonization	1	kept

BACTERIAL DIVERSITY	bacterial diversity	bacterial diversity	bacterial diversity	BACTERIAL DIVERSITY	1	kept
Bacterial drug resistance	bacterial drug resistance	bacterial drug resistance	bacterial drug resistance	Bacterial drug resistance	1	kept
Bacterial flora	bacterial flora	bacterial flora	bacterial flora	bacterial flora	1	case_or_format
bacterial genetics	bacterial genetics	bacterial genetics	bacterial genetics	bacterial genetics	1	kept
Bacterial isolates	bacterial isolates	bacterial isolates	bacterial isolates	Bacterial isolates	1	kept
Bacterial pathogens	bacterial pathogens	bacterial pathogens	bacterial pathogens	Bacterial pathogens	1	kept
bacterial protein femB	bacterial protein femb	bacterial protein femb	bacterial protein femb	bacterial protein femB	1	kept
BACTERIAL SEPSIS	bacterial sepsis	bacterial sepsis	bacterial sepsis	BACTERIAL SEPSIS	1	kept
Bacterial Sepsis	bacterial sepsis	bacterial sepsis	bacterial sepsis	BACTERIAL SEPSIS	1	case_or_format
BACTERIAL TRANSLOCATION	bacterial translocation	bacterial translocation	bacterial translocation	bacterial translocation	1	case_or_format
Bacterial Translocation	bacterial translocation	bacterial translocation	bacterial translocation	bacterial translocation	1	case_or_format
bacterial vector	bacterial vector	bacterial vector	bacterial vector	bacterial vector	1	kept
BACTERIAL-COLONIZATION	bacterial colonization	bacterial colonization	bacterial colonization	bacterial colonization	1	case_or_format
BACTERIAL-CONTAMINATION	bacterial contamination	bacterial contamination	bacterial contamination	Bacterial contamination	1	case_or_format
Bacterias multirresistentes	bacterias multirresistentes	bacterias multirresistentes	bacterias multirresistentes	Bacterias multirresistentes	1	kept
Bactericides	bactericides	bactericides	bactericides	Bactericides	1	kept

Bacteriemia	bacteriemia	bacteriemia	bacteriemia	Bacteriemia	1	kept
Bacteriology	bacteriology	bacteriology	bacteriology	bacteriology	1	case_or_format
bacteriophage	bacteriophage	bacteriophage	bacteriophage	bacteriophage	1	kept
bacterium conjugation	bacterium conjugation	bacterium conjugation	bacterium conjugation	bacterium conjugation	1	kept
bacterium examination	bacterium examination	bacterium examination	bacterium examination	bacterium examination	1	kept
Bacteroides	bacteroides	bacteroides	bacteroides	Bacteroides	1	kept
Bacteroides infection	bacteroides infection	bacteroides infection	bacteroides infection	Bacteroides infection	1	kept
Bacteroidetes	bacteroidetes	bacteroidetes	bacteroidetes	Bacteroidetes	1	kept
bactrim suspension	bactrim suspension	bactrim suspension	bactrim suspension	bactrim suspension	1	kept
Bactérias multirresistentes	bact rias multirresistentes	bact rias multirresistentes	bact rias multirresistentes	Bactérias multirresistentes	1	kept
bactérie multiré sistante	bact rie multir sistante	bact rie multir sistante	bact rie multir sistante	bactérie multiré sistante	1	kept
bactérie émergente	bact rie mergente	bact rie mergente	bact rie mergente	bactérie émergente	1	kept
Bactéries multiré sistantes	bact ries multir sistantes	bact ries multir sistantes	bact ries multir sistantes	Bactéries multiré sistantes	1	kept
bad death	bad death	bad death	bad death	bad death	1	kept
BAD methods	bad methods	bad methods	bad methods	BAD methods	1	kept
Bad news	bad news	bad news	bad news	BAD-NEWS	1	case_or_format

Baden-Baden	baden baden	baden baden	baden baden	Baden-Baden	1	kept
Baden-Wurttemberg	baden wurttemberg	baden wurttemberg	baden wurttemberg	Baden-Wurttemberg	1	kept
Baduanjin	baduanjin	baduanjin	baduanjin	Baduanjin	1	kept
Bag and mask	bag and mask	bag and mask	bag and mask	Bag and mask	1	kept
bag and mask ventilation	bag and mask ventilation	bag and mask ventilation	bag and mask ventilation	bag and mask ventilation	1	kept
Bag valve mask	bag valve mask	bag valve mask	bag valve mask	Bag valve mask	1	kept
bag-mask ventilation	bag mask ventilation	bag mask ventilation	bag mask ventilation	bag mask ventilation	1	case_or_format
Bag-valve ventilation	bag valve ventilation	bag valve ventilation	bag valve ventilation	Bag-valve ventilation	1	kept
Bagg albino mouse	bagg albino mouse	bagg albino mouse	bagg albino mouse	Bagg albino mouse	1	kept
Baghdad Teaching Hospitals	baghdad teaching hospitals	baghdad teaching hospitals	baghdad teaching hospitals	Baghdad Teaching Hospitals	1	kept
Baja California	baja california	baja california	baja california	Baja California	1	kept
Baker’s health literacy model	baker s health literacy model	baker s health literacy model	baker s health literacy model	Baker’s health literacy model	1	kept
Balamuthia mandrillaris	balamuthia mandrillaris	balamuthia mandrillaris	balamuthia mandrillaris	Balamuthia mandrillaris	1	kept
BALANCE	balance	balance	balance	BALANCE	1	kept
balance	balance	balance	balance	BALANCE	1	case_or_format
balanced	balanced	balanced	balanced	balanced	1	kept

Balanced diet	balanced diet	balanced diet	balanced diet	Balanced diet	1	kept
Balanced propofol solution	balanced propofol solution	balanced propofol solution	balanced propofol solution	Balanced propofol solution	1	kept
balanced propofol solution	balanced propofol solution	balanced propofol solution	balanced propofol solution	Balanced propofol solution	1	case_or_format
Balanced solutions	balanced solutions	balanced solutions	balanced solutions	Balanced solutions	1	kept
Balanced translocation	balanced translocation	balanced translocation	balanced translocation	Balanced translocation	1	kept
Balancing	balancing	balancing	balancing	Balancing	1	kept
ballistics	ballistics	ballistics	ballistics	ballistics	1	kept
Balloon diameter	balloon diameter	balloon diameter	balloon diameter	Balloon diameter	1	kept
Balloon dilatational tracheostomy	balloon dilatational tracheostomy	balloon dilatational tracheostomy	balloon dilatational tracheostomy	Balloon dilatational tracheostomy	1	kept
balloon migration	balloon migration	balloon migration	balloon migration	balloon migration	1	kept
balm oil	balm oil	balm oil	balm oil	balm oil	1	kept
Baltic Sea	baltic sea	baltic sea	baltic sea	Baltic Sea	1	kept
Bamlanivimab	bamlanivimab	bamlanivimab	bamlanivimab	bamlanivimab	1	case_or_format
banana	banana	banana	banana	banana	1	kept
Bancroftian filariasis	bancroftian filariasis	bancroftian filariasis	bancroftian filariasis	Bancroftian filariasis	1	kept
Bandage	bandage	bandage	bandage	Bandages	1	case_or_format

bandages and dressings	bandages and dressings	bandages and dressings	bandages and dressings	bandages and dressings	1	kept
Bandages, hydrocolloid	bandages hydrocolloid	bandages hydrocolloid	bandages hydrocolloid	Bandages, Hydrocolloid	1	case_or_format
Bandaging materials	bandaging materials	bandaging materials	bandaging materials	Bandaging materials	1	kept
bandemia	bandemia	bandemia	bandemia	bandemia	1	kept
bandwidth	bandwidth	bandwidth	bandwidth	bandwidth	1	kept
Banhos	banhos	banhos	banhos	Banhos	1	kept
Bankrupt	bankrupt	bankrupt	bankrupt	Bankrupt	1	kept
Bantane Hospital	bantane hospital	bantane hospital	bantane hospital	Bantane Hospital	1	kept
Bar code medication administration	bar code medication administration	bar code medication administration	bar code medication administration	bar code medication administration	1	case_or_format
bar code medication system	bar code medication system	bar code medication system	bar code medication system	bar code medication system	1	kept
Bar codes	bar codes	bar codes	bar codes	Bar codes	1	kept
Bar-code medication administration	bar code medication administration	bar code medication administration	bar code medication administration	bar code medication administration	1	case_or_format
Barbados	barbados	barbados	barbados	Barbados	1	kept
barbiturates	barbiturates	barbiturates	barbiturates	Barbiturates	1	case_or_format
Barcoding	barcoding	barcoding	barcoding	Barcoding	1	kept
barcoding	barcoding	barcoding	barcoding	Barcoding	1	case_or_format

Bardet Biedl syndrome	bardet biedl syndrome	bardet biedl syndrome	bardet biedl syndrome	Bardet Biedl syndrome	1	kept
bare below the elbow	bare below the elbow	bare below the elbow	bare below the elbow	bare below the elbow	1	kept
bare below the elbows	bare below the elbows	bare below the elbows	bare below the elbow	bare below the elbow	1	singular
Bariatric Medicine	bariatric medicine	bariatric medicine	bariatric medicine	Bariatric Medicine	1	kept
barium	barium	barium	barium	barium	1	kept
barium enema	barium enema	barium enema	barium enema	barium enema	1	kept
barium sulfate	barium sulfate	barium sulfate	barium sulfate	barium sulfate	1	kept
Baroreflex	baroreflex	baroreflex	baroreflex	Baroreflex	1	kept
Barratt Impulsiveness Scale	barratt impulsiveness scale	barratt impulsiveness scale	barratt impulsiveness scale	Barratt Impulsiveness Scale	1	kept
barrier cream	barrier cream	barrier cream	barrier cream	barrier cream	1	kept
barrier factors	barrier factors	barrier factors	barrier factors	barrier factors	1	kept
barrier film	barrier film	barrier film	barrier film	barrier film	1	kept
barrier function	barrier function	barrier function	barrier function	barrier function	1	kept
barrier to early mobilization	barrier to early mobilization	barrier to early mobilization	barrier to early mobilization	barrier to early mobilization	1	kept
Barriers of EBP	barriers of ebp	barriers of ebp	barriers of ebp	Barriers of EBP	1	kept
barriers of EBP	barriers of ebp	barriers of ebp	barriers of ebp	Barriers of EBP	1	case_or_format

Barriers to activation	barriers to activation	barriers to activation	barriers to activation	Barriers to activation	1	kept
Barriers to and Facilitators of Implementing the	barriers to and facilitators of implementing the	barriers to and facilitators of implementing the	barriers to and facilitators of implementing the	Barriers to and Facilitators of Implementing the	1	kept
Barriers to care	barriers to care	barriers to care	barriers to care	barriers to care	1	case_or_format
barriers to compliance	barriers to compliance	barriers to compliance	barriers to compliance	barriers to compliance	1	kept
Barriers to effective communication	barriers to effective communication	barriers to effective communication	barriers to effective communication	Barriers to effective communication	1	kept
barriers to enteral nutrition	barriers to enteral nutrition	barriers to enteral nutrition	barriers to enteral nutrition	barriers to enteral nutrition	1	kept
barriers to FCC	barriers to fcc	barriers to fcc	barriers to fcc	barriers to FCC	1	kept
barriers to recovery	barriers to recovery	barriers to recovery	barriers to recovery	barriers to recovery	1	kept
Barriers to reporting errors	barriers to reporting errors	barriers to reporting errors	barriers to reporting errors	Barriers to reporting errors	1	kept
Barriers to restraints	barriers to restraints	barriers to restraints	barriers to restraints	Barriers to restraints	1	kept
Barriers to treatment	barriers to treatment	barriers to treatment	barriers to treatment	Barriers to treatment	1	kept
Barriers, hand hygiene surveillance	barriers hand hygiene surveillance	barriers hand hygiene surveillance	barriers hand hygiene surveillance	Barriers, hand hygiene surveillance	1	kept
Barrow oral care protocol	barrow oral care protocol	barrow oral care protocol	barrow oral care protocol	Barrow oral care protocol	1	kept
Barthel score	barthel score	barthel score	barthel score	Barthel score	1	kept
basal bolus insulin	basal bolus insulin	basal bolus insulin	basal bolus insulin	basal bolus insulin	1	kept
basal cistern compression	basal cistern compression	basal cistern compression	basal cistern compression	basal cistern compression	1	kept

basal forebrain	basal forebrain	basal forebrain	basal forebrain	basal forebrain	1	kept
basal nucleus of Meynert	basal nucleus of meynert	basal nucleus of meynert	basal nucleus of meynert	basal nucleus of Meynert	1	kept
Basal stimulation	basal stimulation	basal stimulation	basal stimulation	Basal stimulation	1	kept
basal vein	basal vein	basal vein	basal vein	basal vein	1	kept
basal-bolus	basal bolus	basal bolus	basal bolus	basal-bolus	1	kept
Base deficit	base deficit	base deficit	base deficit	Base deficit	1	kept
base of skull surgery	base of skull surgery	base of skull surgery	base of skull surgery	base of skull surgery	1	kept
Base stations	base stations	base stations	base stations	Base stations	1	kept
BASE-LINE	base line	base line	base line	BASE-LINE	1	kept
Based modelling	based modelling	based modelling	based modelling	Based modelling	1	kept
Baseline assessment	baseline assessment	baseline assessment	baseline assessment	Baseline assessment	1	kept
Baseline covariates	baseline covariates	baseline covariates	baseline covariates	Baseline covariates	1	kept
Baseline survey	baseline survey	baseline survey	baseline survey	Baseline survey	1	kept
baseline survey	baseline survey	baseline survey	baseline survey	Baseline survey	1	case_or_format
BASIC = Brain and Spinal Injury Center	basic brain and spinal injury center	basic brain and spinal injury center	basic brain and spinal injury center	BASIC = Brain and Spinal Injury Center	1	kept
Basic airway management	basic airway management	basic airway management	basic airway management	Basic airway management	1	kept

Basic Cardiac Life Support	basic cardiac life support	basic cardiac life support	basic cardiac life support	Basic Cardiac Life Support	1	kept
Basic cardiac life support	basic cardiac life support	basic cardiac life support	basic cardiac life support	Basic Cardiac Life Support	1	case_or_format
basic cognitive sensory recovery	basic cognitive sensory recovery	basic cognitive sensory recovery	basic cognitive sensory recovery	basic cognitive sensory recovery	1	kept
basic echocardiography	basic echocardiography	basic echocardiography	basic echocardiography	basic echocardiography	1	kept
Basic Life Support	basic life support	basic life support	basic life support	basic life support	1	case_or_format
basic need satisfaction	basic need satisfaction	basic need satisfaction	basic need satisfaction	basic need satisfaction	1	kept
Basic Newborn Resuscitation	basic newborn resuscitation	basic newborn resuscitation	basic newborn resuscitation	Basic Newborn Resuscitation	1	kept
Basic structure	basic structure	basic structure	basic structure	Basic structure	1	kept
BASICS	basics	basics	basics	BASICS	1	kept
basics	basics	basics	basics	BASICS	1	case_or_format
Basidiomycota	basidiomycota	basidiomycota	basidiomycota	Basidiomycota	1	kept
basilar artery	basilar artery	basilar artery	basilar artery	basilar artery	1	kept
Basilic vein	basilic vein	basilic vein	basilic vein	Basilic vein	1	kept
BASIS model	basis model	basis model	basis model	BASIS model	1	kept
basophilia	basophilia	basophilia	basophilia	basophilia	1	kept
Basra	basra	basra	basra	Basra	1	kept

Batch frequency	batch frequency	batch frequency	batch frequency	Batch frequency	1	kept
Batch treatment	batch treatment	batch treatment	batch treatment	Batch treatment	1	kept
BATDOK	batdok	batdok	batdok	BATDOK	1	kept
Bath	bath	bath	bath	Baths	1	case_or_format
Bathing and honoring	bathing and honoring	bathing and honoring	bathing and honoring	Bathing and honoring	1	kept
Bathing practice	bathing practice	bathing practice	bathing practice	Bathing practice	1	kept
baths	baths	baths	bath	Baths	1	singular
Battered Women	battered women	battered women	battered women	Battered Women	1	kept
battery life	battery life	battery life	battery life	battery life	1	kept
Baux score	baux score	baux score	baux score	Baux score	1	kept
Bayesian information criterion	bayesian information criterion	bayesian information criterion	bayesian information criterion	Bayesian information criterion	1	kept
Bayesian prediction	bayesian prediction	bayesian prediction	bayesian prediction	Bayesian prediction	1	kept
Bays	bays	bays	bays	Bays	1	kept
Baños	ba os	ba os	ba os	Baños	1	kept
bbibp corv	bbibp corv	bbibp corv	bbibp corv	bbibp corv	1	kept
bbibp-corv vaccine	bbibp corv vaccine	bbibp corv vaccine	bbibp corv vaccine	bbibp-corv vaccine	1	kept

BCG revaccination	bcg revaccination	bcg revaccination	bcg revaccination	BCG revaccination	1	kept
BCVI	bcvi	bcvi	bcvi	BCVI	1	kept
BCVI = blunt cerebrovascular injury	bcvi blunt cerebrovascular injury	bcvi blunt cerebrovascular injury	bcvi blunt cerebrovascular injury	BCVI = blunt cerebrovascular injury	1	kept
BD GeneOhm methicillin resistant Staphylococcus aureus	bd geneohm methicillin resistant staphylococcus aureus	bd geneohm methicillin resistant staphylococcus aureus	bd geneohm methicillin resistant staphylococcus aureus	BD GeneOhm methicillin resistant Staphylococcus aureus	1	kept
BD Max methicillin resistant Staphylococcus aureus	bd max methicillin resistant staphylococcus aureus	bd max methicillin resistant staphylococcus aureus	bd max methicillin resistant staphylococcus aureus	BD Max methicillin resistant Staphylococcus aureus	1	kept
Beacon award	beacon award	beacon award	beacon award	Beacon award	1	kept
beagle	beagle	beagle	beagle	beagle	1	kept
Beam width artefact	beam width artefact	beam width artefact	beam width artefact	Beam width artefact	1	kept
beam width artefact	beam width artefact	beam width artefact	beam width artefact	Beam width artefact	1	case_or_format
Beamforming	beamforming	beamforming	beamforming	Beamforming	1	kept
bear	bear	bear	bear	bear	1	kept
Bearing witness	bearing witness	bearing witness	bearing witness	Bearing witness	1	kept
Bebé prematuro	beb prematuro	beb prematuro	beb prematuro	Bebé prematuro	1	kept
Beck checklist	beck checklist	beck checklist	beck checklist	Beck checklist	1	kept
Beck Depression Scale	beck depression scale	beck depression scale	beck depression scale	Beck Depression Scale	1	kept
Beck Hopelessness Scale	beck hopelessness scale	beck hopelessness scale	beck hopelessness scale	Beck Hopelessness Scale	1	kept

Beck oral assessment scale	beck oral assessment scale	beck oral assessment scale	beck oral assessment scale	Beck oral assessment scale	1	kept
Beck oral scoring scale	beck oral scoring scale	beck oral scoring scale	beck oral scoring scale	Beck oral scoring scale	1	kept
Becker nevus	becker nevus	becker nevus	becker nevus	Becker nevus	1	kept
Beckwith Wiedemann syndrome	beckwith wiedemann syndrome	beckwith wiedemann syndrome	beckwith wiedemann syndrome	Beckwith-Wiedemann Syndrome	1	case_or_format
beclometasone	beclometasone	beclometasone	beclometasone	beclometasone	1	kept
beclometasone dipropionate	beclometasone dipropionate	beclometasone dipropionate	beclometasone dipropionate	beclometasone dipropionate	1	kept
Becoming a mother	becoming a mother	becoming a mother	becoming a mother	Becoming a mother	1	kept
becoming mother	becoming mother	becoming mother	becoming mother	becoming mother	1	kept
BED	bed	bed	bed	Beds	1	case_or_format
Bed allocation	bed allocation	bed allocation	bed allocation	Bed allocation	1	kept
bed assignment standardization	bed assignment standardization	bed assignment standardization	bed assignment standardization	bed assignment standardization	1	kept
Bed availability	bed availability	bed availability	bed availability	Bed availability	1	kept
bed availability	bed availability	bed availability	bed availability	Bed availability	1	case_or_format
bed bath	bed bath	bed bath	bed bath	Bed bath	1	case_or_format
Bed bathing	bed bathing	bed bathing	bed bathing	Bed bathing	1	kept
Bed bathing practice	bed bathing practice	bed bathing practice	bed bathing practice	Bed bathing practice	1	kept

Bed capacity	bed capacity	bed capacity	bed capacity	Bed capacity	1	kept
Bed capacity planning	bed capacity planning	bed capacity planning	bed capacity planning	Bed capacity planning	1	kept
Bed control	bed control	bed control	bed control	Bed control	1	kept
bed control	bed control	bed control	bed control	Bed control	1	case_or_format
bed head angle	bed head angle	bed head angle	bed head angle	bed head angle	1	kept
Bed manager	bed manager	bed manager	bed manager	Bed manager	1	kept
bed models	bed models	bed models	bed models	bed models	1	kept
bed occupancy	bed occupancy	bed occupancy	bed occupancy	Bed Occupancy	1	case_or_format
Bed occupancy rate	bed occupancy rate	bed occupancy rate	bed occupancy rate	Bed occupancy rate	1	kept
Bed pads	bed pads	bed pads	bed pads	Bed pads	1	kept
bed rail	bed rail	bed rail	bed rail	bed rail	1	kept
Bed sores	bed sores	bed sores	bed sore	Bed sore	1	singular
Bed spaces	bed spaces	bed spaces	bed spaces	Bed spaces	1	kept
Bed surface	bed surface	bed surface	bed surface	Bed surface	1	kept
BED THERAPY	bed therapy	bed therapy	bed therapy	BED THERAPY	1	kept
bedbug bite	bedbug bite	bedbug bite	bedbug bite	bedbug bite	1	kept

bedrails	bedrails	bedrails	bedrails	bedrails	1	kept
Bedrest	bedrest	bedrest	bedrest	Bedrest	1	kept
bedridden persons	bedridden persons	bedridden persons	bedridden persons	Bedridden Persons	1	case_or_format
BEDS	beds	beds	bed	Beds	1	singular
Bedside	bedside	bedside	bedside	BEDSIDE	1	case_or_format
Bedside barcode medication administration	bedside barcode medication administration	bedside barcode medication administration	bedside barcode medication administration	Bedside barcode medication administration	1	kept
bedside barcode medication administration	bedside barcode medication administration	bedside barcode medication administration	bedside barcode medication administration	Bedside barcode medication administration	1	case_or_format
bedside blind insertion	bedside blind insertion	bedside blind insertion	bedside blind insertion	bedside blind insertion	1	kept
bedside calculation	bedside calculation	bedside calculation	bedside calculation	bedside calculation	1	kept
Bedside caregiver	bedside caregiver	bedside caregiver	bedside caregiver	Bedside caregiver	1	kept
Bedside Confusion Scale	bedside confusion scale	bedside confusion scale	bedside confusion scale	Bedside Confusion Scale	1	kept
bedside device	bedside device	bedside device	bedside device	bedside device	1	kept
bedside discussion	bedside discussion	bedside discussion	bedside discussion	bedside discussion	1	kept
Bedside flowsheet	bedside flowsheet	bedside flowsheet	bedside flowsheet	Bedside flowsheet	1	kept
Bedside follow-up	bedside follow up	bedside follow up	bedside follow up	Bedside follow-up	1	kept
Bedside handovers	bedside handovers	bedside handovers	bedside handover	Bedside handover	1	singular

Bedside interdisciplinary rounds	bedside interdisciplinary rounds	bedside interdisciplinary rounds	bedside interdisciplinary rounds	Bedside interdisciplinary rounds	1	kept
bedside mobility assessment tool	bedside mobility assessment tool	bedside mobility assessment tool	bedside mobility assessment tool	bedside mobility assessment tool	1	kept
bedside oral exam	bedside oral exam	bedside oral exam	bedside oral exam	bedside oral exam	1	kept
bedside placement	bedside placement	bedside placement	bedside placement	BEDSIDE PLACEMENT	1	case_or_format
Bedside rationing	bedside rationing	bedside rationing	bedside rationing	bedside rationing	1	case_or_format
bedside report	bedside report	bedside report	bedside report	Bedside report	1	case_or_format
Bedside rounding	bedside rounding	bedside rounding	bedside rounding	Bedside rounding	1	kept
bedside rounds	bedside rounds	bedside rounds	bedside rounds	BEDSIDE ROUNDS	1	case_or_format
Bedside shift report	bedside shift report	bedside shift report	bedside shift report	Bedside shift report	1	kept
Bedside simulation	bedside simulation	bedside simulation	bedside simulation	Bedside simulation	1	kept
Bedside surgery	bedside surgery	bedside surgery	bedside surgery	Bedside surgery	1	kept
Bedside swallowing examinations	bedside swallowing examinations	bedside swallowing examinations	bedside swallowing examinations	Bedside swallowing examinations	1	kept
bedside testing	bedside testing	bedside testing	bedside testing	bedside testing	1	kept
bedside tool	bedside tool	bedside tool	bedside tool	bedside tool	1	kept
Bedside ultrasonography	bedside ultrasonography	bedside ultrasonography	bedside ultrasonography	Bedside ultrasonography	1	kept
Bedside ultrasound	bedside ultrasound	bedside ultrasound	bedside ultrasound	Bedside ultrasound	1	kept

bedside visualisation tool	bedside visualisation tool	bedside visualisation tool	bedside visualisation tool	bedside visualisation tool	1	kept
bedside X-ray	bedside x ray	bedside x ray	bedside x ray	bedside X-ray	1	kept
bedside-PEWS	bedside pews	bedside pews	bedside pews	bedside-PEWS	1	kept
BedsidePEWS	bedsidepews	bedsidepews	bedsidepews	BedsidePEWS	1	kept
Bedspace	bedspace	bedspace	bedspace	Bedspace	1	kept
bee	bee	bee	bee	bee	1	kept
bee venom	bee venom	bee venom	bee venom	bee venom	1	kept
Bee Venoms	bee venoms	bee venoms	bee venom	bee venom	1	singular
BEERS CRITERIA	beers criteria	beers criteria	beers criteria	BEERS CRITERIA	1	kept
Beers criteria	beers criteria	beers criteria	beers criteria	BEERS CRITERIA	1	case_or_format
Bees	bees	bees	bee	bee	1	singular
BEFIP questionnaire	befip questionnaire	befip questionnaire	befip questionnaire	BEFIP questionnaire	1	kept
Before and after	before and after	before and after	before and after	Before and after	1	kept
before and after design	before and after design	before and after design	before and after design	before and after design	1	kept
before-after	before after	before after	before after	before-after	1	kept
Behavior and attitude	behavior and attitude	behavior and attitude	behavior and attitude	Behavior and attitude	1	kept

behavior and social drivers	behavior and social drivers	behavior and social drivers	behavior and social drivers	behavior and social drivers	1	kept
Behavior change	behavior change	behavior change	behavior change	behavior change	1	case_or_format
Behavior change theory	behavior change theory	behavior change theory	behavior change theory	Behavior change theory	1	kept
Behavior Pain Assessment Tool	behavior pain assessment tool	behavior pain assessment tool	behavior pain assessment tool	Behavior Pain Assessment Tool	1	kept
Behavior Pain Scale	behavior pain scale	behavior pain scale	behavior pain scale	Behavior Pain Scale	1	kept
behavior rating inventory of executive function	behavior rating inventory of executive function	behavior rating inventory of executive function	behavior rating inventory of executive function	behavior rating inventory of executive function	1	kept
Behavior Rating Scale	behavior rating scale	behavior rating scale	behavior rating scale	Behavior Rating Scale	1	kept
behavior response team	behavior response team	behavior response team	behavior response team	behavior response team	1	kept
Behavioral	behavioral	behavioral	behavioral	behavioral	1	case_or_format
behavioral and psychiatric symptoms of dementia	behavioral and psychiatric symptoms of dementia	behavioral and psychiatric symptoms of dementia	behavioral and psychiatric symptoms of dementia	behavioral and psychiatric symptoms of dementia	1	kept
behavioral and psychological symptom of dementia	behavioral and psychological symptom of dementia	behavioral and psychological symptom of dementia	behavioral and psychological symptom of dementia	behavioral and psychological symptom of dementia	1	kept
Behavioral and psychological symptoms associated with	behavioral and psychological symptoms associated with	behavioral and psychological symptoms associated with	behavioral and psychological symptoms associated with	Behavioral and psychological symptoms associated with	1	kept
Behavioral change	behavioral change	behavioral change	behavioral change	Behavioral change	1	kept
behavioral change	behavioral change	behavioral change	behavioral change	Behavioral change	1	case_or_format
behavioral cue based oral feeding	behavioral cue based oral feeding	behavioral cue based oral feeding	behavioral cue based oral feeding	behavioral cue based oral feeding	1	kept
Behavioral development	behavioral development	behavioral development	behavioral development	Behavioral development	1	kept

behavioral disinhibition	behavioral disinhibition	behavioral disinhibition	behavioral disinhibition	behavioral disinhibition	1	kept
BEHAVIORAL DISTRESS	behavioral distress	behavioral distress	behavioral distress	BEHAVIORAL DISTRESS	1	kept
Behavioral disturbances	behavioral disturbances	behavioral disturbances	behavioral disturbances	Behavioral disturbances	1	kept
behavioral economics	behavioral economics	behavioral economics	behavioral economics	behavioral economics	1	kept
behavioral epigenetics	behavioral epigenetics	behavioral epigenetics	behavioral epigenetics	behavioral epigenetics	1	kept
behavioral health	behavioral health	behavioral health	behavioral health	Behavioral health	1	case_or_format
Behavioral Indicators of Infant Pain score	behavioral indicators of infant pain score	behavioral indicators of infant pain score	behavioral indicators of infant pain score	Behavioral Indicators of Infant Pain score	1	kept
Behavioral Intention	behavioral intention	behavioral intention	behavioral intention	Behavioral Intention	1	kept
behavioral intentions	behavioral intentions	behavioral intentions	behavioral intention	Behavioral Intention	1	singular
behavioral intervention	behavioral intervention	behavioral intervention	behavioral intervention	behavioral intervention	1	kept
behavioral mapping study	behavioral mapping study	behavioral mapping study	behavioral mapping study	behavioral mapping study	1	kept
behavioral model	behavioral model	behavioral model	behavioral model	behavioral model	1	kept
Behavioral modification	behavioral modification	behavioral modification	behavioral modification	Behavioral modification	1	kept
Behavioral observation	behavioral observation	behavioral observation	behavioral observation	behavioral observation	1	case_or_format
Behavioral outcomes for at-risk infants	behavioral outcomes for at risk infants	behavioral outcomes for at risk infants	behavioral outcomes for at risk infants	Behavioral outcomes for at-risk infants	1	kept
behavioral pain scale non intubated	behavioral pain scale non intubated	behavioral pain scale non intubated	behavioral pain scale non intubated	behavioral pain scale non intubated	1	kept

Behavioral response	behavioral response	behavioral response	behavioral response	behavioral response	1	case_or_format
Behavioral responses	behavioral responses	behavioral responses	behavioral response	behavioral response	1	singular
behavioral risk factor surveillance system	behavioral risk factor surveillance system	behavioral risk factor surveillance system	behavioral risk factor surveillance system	behavioral risk factor surveillance system	1	kept
Behavioral scale	behavioral scale	behavioral scale	behavioral scale	Behavioral scale	1	kept
Behavioral scale of pain monitoring	behavioral scale of pain monitoring	behavioral scale of pain monitoring	behavioral scale of pain monitoring	Behavioral scale of pain monitoring	1	kept
Behavioral scales	behavioral scales	behavioral scales	behavioral scale	Behavioral scale	1	singular
Behavioral science	behavioral science	behavioral science	behavioral science	behavioral science	1	case_or_format
Behavioral sciences	behavioral sciences	behavioral sciences	behavioral science	behavioral science	1	singular
Behavioral states	behavioral states	behavioral states	behavioral states	Behavioral states	1	kept
behavioral states	behavioral states	behavioral states	behavioral states	Behavioral states	1	case_or_format
Behavioral status	behavioral status	behavioral status	behavioral status	Behavioral status	1	kept
Behavioral symptoms	behavioral symptoms	behavioral symptoms	behavioral symptoms	Behavioral Symptoms	1	case_or_format
BEHAVIORAL-MODEL	behavioral model	behavioral model	behavioral model	behavioral model	1	case_or_format
behaviors	behaviors	behaviors	behavior	behavior	1	singular
Behaviour Change Wheel	behaviour change wheel	behaviour change wheel	behaviour change wheel	Behaviour Change Wheel	1	kept
Behaviour changing	behaviour changing	behaviour changing	behaviour changing	Behaviour changing	1	kept

behaviour pain scale	behaviour pain scale	behaviour pain scale	behaviour pain scale	behaviour pain scale	1	kept
Behaviour research	behaviour research	behaviour research	behaviour research	Behaviour research	1	kept
Behaviour state	behaviour state	behaviour state	behaviour state	Behaviour state	1	kept
behavioural and psychological symptoms of dementia	behavioural and psychological symptoms of dementia	behavioural and psychological symptoms of dementia	behavioural and psychological symptoms of dementia	behavioural and psychological symptoms of dementia	1	kept
Behavioural assessment	behavioural assessment	behavioural assessment	behavioural assessment	Behavioural assessment	1	kept
Behavioural change	behavioural change	behavioural change	behavioural change	Behavioural change	1	kept
Behavioural cues	behavioural cues	behavioural cues	behavioural cues	Behavioural cues	1	kept
behavioural economics	behavioural economics	behavioural economics	behavioural economics	behavioural economics	1	kept
behavioural intentions	behavioural intentions	behavioural intentions	behavioural intentions	behavioural intentions	1	kept
Behavioural intervention	behavioural intervention	behavioural intervention	behavioural intervention	Behavioural intervention	1	kept
Behavioural interventions	behavioural interventions	behavioural interventions	behavioural intervention	Behavioural intervention	1	singular
Behavioural Marker Risk Index	behavioural marker risk index	behavioural marker risk index	behavioural marker risk index	Behavioural Marker Risk Index	1	kept
Behavioural Observational Pain Scale	behavioural observational pain scale	behavioural observational pain scale	behavioural observational pain scale	Behavioural Observational Pain Scale	1	kept
behavioural observational pain scale	behavioural observational pain scale	behavioural observational pain scale	behavioural observational pain scale	Behavioural Observational Pain Scale	1	case_or_format
Behavioural outcomes	behavioural outcomes	behavioural outcomes	behavioural outcomes	Behavioural outcomes	1	kept
Behavioural pain assessment tool	behavioural pain assessment tool	behavioural pain assessment tool	behavioural pain assessment tool	Behavioural pain assessment tool	1	kept

Behavioural Pain Scale	behavioural pain scale	behavioural pain scale	behavioural pain scale	Behavioural Pain Scale	1	kept
Behavioural Research	behavioural research	behavioural research	behavioural research	Behavioural research	1	case_or_format
Behavioural response	behavioural response	behavioural response	behavioural response	Behavioural responses	1	case_or_format
behavioural responses	behavioural responses	behavioural responses	behavioural response	Behavioural responses	1	singular
Behavioural scales	behavioural scales	behavioural scales	behavioural scales	Behavioural scales	1	kept
behavioural scales	behavioural scales	behavioural scales	behavioural scales	Behavioural scales	1	case_or_format
Behavioural symptoms	behavioural symptoms	behavioural symptoms	behavioural symptoms	Behavioural symptoms	1	kept
Behaviours	behaviours	behaviours	behaviour	Behaviour	1	singular
behaviours	behaviours	behaviours	behaviour	Behaviour	1	singular
behaviours and psychological symptoms of dementia	behaviours and psychological symptoms of dementia	behaviours and psychological symptoms of dementia	behaviours and psychological symptoms of dementia	behaviours and psychological symptoms of dementia	1	kept
Behcet disease	behcet disease	behcet disease	behcet disease	Behcet disease	1	kept
Being	being	being	being	Being	1	kept
Being voiceless	being voiceless	being voiceless	being voiceless	Being voiceless	1	kept
belatacept	belatacept	belatacept	belatacept	belatacept	1	kept
Belgian settings	belgian settings	belgian settings	belgian settings	Belgian settings	1	kept
belief practice	belief practice	belief practice	belief practice	belief practice	1	kept

beliefs and attitudes	beliefs and attitudes	beliefs and attitudes	beliefs and attitudes	beliefs and attitudes	1	kept
beliefs and attitudes visitation questionnaire	beliefs and attitudes visitation questionnaire	beliefs and attitudes visitation questionnaire	beliefs and attitudes visitation questionnaire	beliefs and attitudes visitation questionnaire	1	kept
Belize	belize	belize	belize	Belize	1	kept
Bell palsy	bell palsy	bell palsy	bell palsy	Bell palsy	1	kept
Bell's criteria	bell s criteria	bell s criteria	bell s criteria	Bell's criteria	1	kept
Bellwether	bellwether	bellwether	bellwether	Bellwether	1	kept
Belonging	belonging	belonging	belonging	Belonging	1	kept
BELONGINGNESS	belongingness	belongingness	belongingness	BELONGINGNESS	1	kept
Belongingness	belongingness	belongingness	belongingness	BELONGINGNESS	1	case_or_format
belt	belt	belt	belt	belt	1	kept
bemiparin	bemiparin	bemiparin	bemiparin	bemiparin	1	kept
Bence Jones protein	bence jones protein	bence jones protein	bence jones protein	Bence Jones protein	1	kept
Bench-mark problems	bench mark problems	bench mark problems	bench mark problems	Bench-mark problems	1	kept
Benchmark	benchmark	benchmark	benchmark	Benchmark	1	kept
Benchmark datasets	benchmark datasets	benchmark datasets	benchmark datasets	Benchmark datasets	1	kept
Beneficial	beneficial	beneficial	beneficial	Beneficial	1	kept

BENEFICIARIES	beneficiaries	beneficiaries	beneficiaries	BENEFICIARIES	1	kept
beneficiaries	beneficiaries	beneficiaries	beneficiaries	BENEFICIARIES	1	case_or_format
BENEFIT	benefit	benefit	benefit	benefits	1	case_or_format
Benefit cost	benefit cost	benefit cost	benefit cost	Benefit cost	1	kept
Benefit rehabilitation coverage	benefit rehabilitation coverage	benefit rehabilitation coverage	benefit rehabilitation coverage	Benefit rehabilitation coverage	1	kept
benefit-cost	benefit cost	benefit cost	benefit cost	Benefit cost	1	case_or_format
BENEFITS	benefits	benefits	benefit	benefits	1	singular
benefits and burdens	benefits and burdens	benefits and burdens	benefits and burdens	benefits and burdens	1	kept
Benefits of certification	benefits of certification	benefits of certification	benefits of certification	Benefits of certification	1	kept
Benefits of early mobilization	benefits of early mobilization	benefits of early mobilization	benefits of early mobilization	Benefits of early mobilization	1	kept
benevolence	benevolence	benevolence	benevolence	benevolence	1	kept
benflumetol	benflumetol	benflumetol	benflumetol	benflumetol	1	kept
benign childhood epilepsy	benign childhood epilepsy	benign childhood epilepsy	benign childhood epilepsy	benign childhood epilepsy	1	kept
benign head and neck tumor	benign head and neck tumor	benign head and neck tumor	benign head and neck tumor	benign head and neck tumor	1	kept
benign paroxysmal positional vertigo	benign paroxysmal positional vertigo	benign paroxysmal positional vertigo	benign paroxysmal positional vertigo	benign paroxysmal positional vertigo	1	kept
Benner	benner	benner	benner	Benner	1	kept

Benner’ s model	benner s model	benner s model	benner s model	Benner’ s model	1	kept
benralizumab	benralizumab	benralizumab	benralizumab	benralizumab	1	kept
benserazide	benserazide	benserazide	benserazide	benserazide	1	kept
benserazide plus levodopa	benserazide plus levodopa	benserazide plus levodopa	benserazide plus levodopa	benserazide plus levodopa	1	kept
Benthosema pterotum	benthosema pterotum	benthosema pterotum	benthosema pterotum	Benthosema pterotum	1	kept
benzalkonium	benzalkonium	benzalkonium	benzalkonium	benzalkonium	1	kept
Benzalkonium chloride	benzalkonium chloride	benzalkonium chloride	benzalkonium chloride	benzalkonium chloride	1	case_or_format
Benzalkonium Compounds	benzalkonium compounds	benzalkonium compounds	benzalkonium compounds	Benzalkonium Compounds	1	kept
benzatropine mesilate	benzatropine mesilate	benzatropine mesilate	benzatropine mesilate	benzatropine mesilate	1	kept
benzazepine derivative	benzazepine derivative	benzazepine derivative	benzazepine derivative	benzazepine derivative	1	kept
Benzazepines	benzazepines	benzazepines	benzazepines	Benzazepines	1	kept
benzene	benzene	benzene	benzene	benzene	1	kept
Benzenesulfonates	benzenesulfonates	benzenesulfonates	benzenesulfonates	Benzenesulfonates	1	kept
benzethonium chloride	benzethonium chloride	benzethonium chloride	benzethonium chloride	benzethonium chloride	1	kept
Benzhydryl Compounds	benzhydryl compounds	benzhydryl compounds	benzhydryl compounds	Benzhydryl Compounds	1	kept
Benzocaine	benzocaine	benzocaine	benzocaine	Benzocaine	1	kept

benzocaine	benzocaine	benzocaine	benzocaine	Benzocaine	1	case_or_format
BENZODIAZEPINE	benzodiazepine	benzodiazepine	benzodiazepine	benzodiazepine	1	case_or_format
benzodiazepine receptor	benzodiazepine receptor	benzodiazepine receptor	benzodiazepine receptor	benzodiazepine receptor	1	kept
BENZODIAZEPINE WITHDRAWAL	benzodiazepine withdrawal	benzodiazepine withdrawal	benzodiazepine withdrawal	BENZODIAZEPINE WITHDRAWAL	1	kept
Benzydamine	benzydamine	benzydamine	benzydamine	Benzydamine	1	kept
benzydamine	benzydamine	benzydamine	benzydamine	Benzydamine	1	case_or_format
BENZYDAMINE HYDROCHLORIDE	benzydamine hydrochloride	benzydamine hydrochloride	benzydamine hydrochloride	BENZYDAMINE HYDROCHLORIDE	1	kept
benzylcetyldimethylammonium	benzylcetyldimethylammonium	benzylcetyldimethylammonium	benzylcetyldimethylammonium	benzylcetyldimethylammonium	1	kept
bepridil	bepridil	bepridil	bepridil	bepridil	1	kept
Bereaved family members’	bereaved family members	bereaved family members	bereaved family members	Bereaved family members’	1	kept
bereaved parent	bereaved parent	bereaved parent	bereaved parent	Bereaved parents	1	case_or_format
BEREAVED PARENTS	bereaved parents	bereaved parents	bereaved parent	Bereaved parents	1	singular
BEREAVEMENT	bereavement	bereavement	bereavement	Bereavement	1	case_or_format
BEREAVEMENT CARE	bereavement care	bereavement care	bereavement care	Bereavement care	1	case_or_format
Bereavement programs	bereavement programs	bereavement programs	bereavement programs	Bereavement programs	1	kept
bereavement programs	bereavement programs	bereavement programs	bereavement programs	Bereavement programs	1	case_or_format

Bereavement support	bereavement support	bereavement support	bereavement support	bereavement support	1	case_or_format
bereavement.	bereavement	bereavement	bereavement	Bereavement	1	case_or_format
bergamot	bergamot	bergamot	bergamot	bergamot	1	kept
Bergen Insomnia Scale	bergen insomnia scale	bergen insomnia scale	bergen insomnia scale	Bergen Insomnia Scale	1	kept
Berger situational anxiety questionnaire	berger situational anxiety questionnaire	berger situational anxiety questionnaire	berger situational anxiety questionnaire	Berger situational anxiety questionnaire	1	kept
Berner Schmerzscore für Neugeborene	berner schmerzscore f r neugeborene	berner schmerzscore f r neugeborene	berner schmerzscore f r neugeborene	Berner Schmerzscore für Neugeborene	1	kept
Bernese pain scale	bernese pain scale	bernese pain scale	bernese pain scale	Bernese pain scale	1	kept
Bernese Pain Scale for Neonate	bernese pain scale for neonate	bernese pain scale for neonate	bernese pain scale for neonate	Bernese Pain Scale for Neonate	1	kept
Bernese Pain Scale for Neonates	bernese pain scale for neonates	bernese pain scale for neonates	bernese pain scale for neonate	Bernese Pain Scale for Neonate	1	singular
Bernese pain scale for neonates	bernese pain scale for neonates	bernese pain scale for neonates	bernese pain scale for neonate	Bernese Pain Scale for Neonate	1	singular
berufliche Praxis	berufliche praxis	berufliche praxis	berufliche praxis	berufliche Praxis	1	kept
Beryllium compounds	beryllium compounds	beryllium compounds	beryllium compounds	Beryllium compounds	1	kept
Best choice	best choice	best choice	best choice	Best choice	1	kept
best corrected visual acuity	best corrected visual acuity	best corrected visual acuity	best corrected visual acuity	best corrected visual acuity	1	kept
Best designs	best designs	best designs	best designs	Best designs	1	kept
Best evidence	best evidence	best evidence	best evidence	Best evidence	1	kept

best evidence	best evidence	best evidence	best evidence	Best evidence	1	case_or_format
best interest principle	best interest principle	best interest principle	best interest principle	best interest principle	1	kept
Best interests	best interests	best interests	best interests	Best interests	1	kept
Best position	best position	best position	best position	Best position	1	kept
Best possible medication history	best possible medication history	best possible medication history	best possible medication history	Best possible medication history	1	kept
best possible medication history	best possible medication history	best possible medication history	best possible medication history	Best possible medication history	1	case_or_format
Best practice advisory	best practice advisory	best practice advisory	best practice advisory	Best practice advisory	1	kept
Best practice standards	best practice standards	best practice standards	best practice standards	Best practice standards	1	kept
Best-bad method	best bad method	best bad method	best bad method	Best-bad method	1	kept
beta 1 adrenergic receptor	beta 1 adrenergic receptor	beta 1 adrenergic receptor	beta 1 adrenergic receptor	beta 1 adrenergic receptor	1	kept
beta 1 adrenergic receptor blocking agent	beta 1 adrenergic receptor blocking agent	beta 1 adrenergic receptor blocking agent	beta 1 adrenergic receptor blocking agent	beta 1 adrenergic receptor blocking agent	1	kept
beta 1,3 glucan	beta 1 3 glucan	beta 1 3 glucan	beta 1 3 glucan	beta 1,3 glucan	1	kept
beta adrenergic receptor blocking	beta adrenergic receptor blocking	beta adrenergic receptor blocking	beta adrenergic receptor blocking	beta adrenergic receptor blocking	1	kept
beta chemokine	beta chemokine	beta chemokine	beta chemokine	beta chemokine	1	kept
beta glucan	beta glucan	beta glucan	beta glucan	beta glucan	1	kept
beta interferon	beta interferon	beta interferon	beta interferon	beta interferon	1	kept

beta lactam derivative	beta lactam derivative	beta lactam derivative	beta lactam derivative	beta lactam derivative	1	kept
beta lactamase CTX M 15	beta lactamase ctx m 15	beta lactamase ctx m 15	beta lactamase ctx m 15	beta lactamase CTX M 15	1	kept
beta lactamase CTX M 3	beta lactamase ctx m 3	beta lactamase ctx m 3	beta lactamase ctx m 3	beta lactamase CTX M 3	1	kept
Beta lactamase inhibitors	beta lactamase inhibitors	beta lactamase inhibitors	beta lactamase inhibitor	beta lactamase inhibitor	1	singular
beta lactamase KPC 3	beta lactamase kpc 3	beta lactamase kpc 3	beta lactamase kpc 3	beta lactamase KPC 3	1	kept
beta lactamase OXA 48	beta lactamase oxa 48	beta lactamase oxa 48	beta lactamase oxa 48	beta lactamase OXA 48	1	kept
beta lactamase SHV 12	beta lactamase shv 12	beta lactamase shv 12	beta lactamase shv 12	beta lactamase SHV 12	1	kept
beta lactamase TEM 1	beta lactamase tem 1	beta lactamase tem 1	beta lactamase tem 1	beta lactamase TEM 1	1	kept
beta lactamase VIM 1	beta lactamase vim 1	beta lactamase vim 1	beta lactamase vim 1	beta lactamase VIM 1	1	kept
beta radiation	beta radiation	beta radiation	beta radiation	beta radiation	1	kept
beta rhythm	beta rhythm	beta rhythm	beta rhythm	beta rhythm	1	kept
beta-blocker	beta blocker	beta blocker	beta blocker	beta-blocker	1	kept
BETA-ENDORPHIN	beta endorphin	beta endorphin	beta endorphin	BETA-ENDORPHIN	1	kept
Beta-lactam	beta lactam	beta lactam	beta lactam	beta lactam	1	case_or_format
Beta-lactam allergy	beta lactam allergy	beta lactam allergy	beta lactam allergy	Beta-lactam allergy	1	kept
BETA-LACTAM INFUSION	beta lactam infusion	beta lactam infusion	beta lactam infusion	BETA-LACTAM INFUSION	1	kept

BETA-LACTAMASE	beta lactamase	beta lactamase	beta lactamase	beta-Lactamases	1	case_or_format
beta-lactamase	beta lactamase	beta lactamase	beta lactamase	beta-Lactamases	1	case_or_format
beta-lactamase NDM-1	beta lactamase ndm 1	beta lactamase ndm 1	beta lactamase ndm 1	beta-lactamase NDM-1	1	kept
beta-lactamases	beta lactamases	beta lactamases	beta lactamase	beta-Lactamases	1	singular
beta-lactams	beta lactams	beta lactams	beta lactam	beta lactam	1	singular
beta2 glycoprotein 1	beta2 glycoprotein 1	beta2 glycoprotein 1	beta2 glycoprotein 1	beta2 glycoprotein 1	1	kept
Betamethasone Valerate	betamethasone valerate	betamethasone valerate	betamethasone valerate	betamethasone valerate	1	case_or_format
Betrayal	betrayal	betrayal	betrayal	betrayal	1	case_or_format
betrixaban	betrixaban	betrixaban	betrixaban	betrixaban	1	kept
Betty neuman model	betty neuman model	betty neuman model	betty neuman model	Betty neuman model	1	kept
Bevel orientation	bevel orientation	bevel orientation	bevel orientation	Bevel orientation	1	kept
beyond	beyond	beyond	beyond	beyond	1	kept
BFHI	bfhi	bfhi	bfhi	BFHI	1	kept
bi-directional learning	bi directional learning	bi directional learning	bi directional learning	bi-directional learning	1	kept
Biapenem	biapenem	biapenem	biapenem	biapenem	1	case_or_format
BIAS	bias	bias	bias	Bias	1	case_or_format

bias	bias	bias	bias	Bias	1	case_or_format
bias and stigma	bias and stigma	bias and stigma	bias and stigma	bias and stigma	1	kept
Bias reduction	bias reduction	bias reduction	bias reduction	Bias reduction	1	kept
Bibliographies as Topic	bibliographies as topic	bibliographies as topic	bibliographies as topic	Bibliographies as Topic	1	kept
Bibliography	bibliography	bibliography	bibliography	Bibliography	1	kept
Bibliometric	bibliometric	bibliometric	bibliometric	Bibliometrics	1	case_or_format
BIBLIOMETRIC ANALYSIS	bibliometric analysis	bibliometric analysis	bibliometric analysis	bibliometric analysis	1	case_or_format
Bibliotherapy	bibliotherapy	bibliotherapy	bibliotherapy	Bibliotherapy	1	kept
Bicarbonate	bicarbonate	bicarbonate	bicarbonate	bicarbonate	1	case_or_format
bictegravir plus emtricitabine plus tenofovir alafenamide	bictegravir plus emtricitabine plus tenofovir alafenamide	bictegravir plus emtricitabine plus tenofovir alafenamide	bictegravir plus emtricitabine plus tenofovir alafenamide	bictegravir plus emtricitabine plus tenofovir alafenamide	1	kept
bicycle ergometry	bicycle ergometry	bicycle ergometry	bicycle ergometry	bicycle ergometry	1	kept
Bicycle trauma	bicycle trauma	bicycle trauma	bicycle trauma	Bicycle trauma	1	kept
Bicycling	bicycling	bicycling	bicycling	Bicycling	1	kept
bidirectional automatic interfacing	bidirectional automatic interfacing	bidirectional automatic interfacing	bidirectional automatic interfacing	bidirectional automatic interfacing	1	kept
bidirectional communication	bidirectional communication	bidirectional communication	bidirectional communication	bidirectional communication	1	kept
Bidirectional Encoder Representations from Transformers	bidirectional encoder representations from transformers	bidirectional encoder representations from transformers	bidirectional encoder representations from transformers	Bidirectional Encoder Representations from Transformers	1	kept

Bienestar	bienestar	bienestar	bienestar	Bienestar	1	kept
Bifidobacterium longum	bifidobacterium longum	bifidobacterium longum	bifidobacterium longum	Bifidobacterium longum	1	kept
Bifrontal contusions	bifrontal contusions	bifrontal contusions	bifrontal contusions	Bifrontal contusions	1	kept
Big data analysis	big data analysis	big data analysis	big data analysis	Big data analysis	1	kept
big data and analytics	big data and analytics	big data and analytics	big data and analytics	big data and analytics	1	kept
Big Five	big five	big five	big five	Big Five	1	kept
Biguanides	biguanides	biguanides	biguanides	Biguanides	1	kept
Bihar	bihar	bihar	bihar	Bihar	1	kept
Bilateral asymmetric	bilateral asymmetric	bilateral asymmetric	bilateral asymmetric	Bilateral asymmetric	1	kept
Bilateral cleft	bilateral cleft	bilateral cleft	bilateral cleft	Bilateral cleft	1	kept
Bilateral cleft palate	bilateral cleft palate	bilateral cleft palate	bilateral cleft palate	Bilateral cleft palate	1	kept
Bilateral distraction	bilateral distraction	bilateral distraction	bilateral distraction	Bilateral distraction	1	kept
bilateral lung transplantation	bilateral lung transplantation	bilateral lung transplantation	bilateral lung transplantation	bilateral lung transplantation	1	kept
Bilateral thalamic infarct	bilateral thalamic infarct	bilateral thalamic infarct	bilateral thalamic infarct	Bilateral thalamic infarct	1	kept
bilateral thalamic infarct	bilateral thalamic infarct	bilateral thalamic infarct	bilateral thalamic infarct	Bilateral thalamic infarct	1	case_or_format
bile	bile	bile	bile	bile	1	kept

bile acid blood level	bile acid blood level	bile acid blood level	bile acid blood level	bile acid blood level	1	kept
Bile Acids and Salts	bile acids and salts	bile acids and salts	bile acids and salts	Bile Acids and Salts	1	kept
bile acids and salts	bile acids and salts	bile acids and salts	bile acids and salts	Bile Acids and Salts	1	case_or_format
bile duct dilatation	bile duct dilatation	bile duct dilatation	bile duct dilatation	bile duct dilatation	1	kept
bile duct injury	bile duct injury	bile duct injury	bile duct injury	bile duct injury	1	kept
Bile Ducts, Intrahepatic	bile ducts intrahepatic	bile ducts intrahepatic	bile ducts intrahepatic	Bile Ducts, Intrahepatic	1	kept
Bile leak	bile leak	bile leak	bile leak	Bile leak	1	kept
bile leak	bile leak	bile leak	bile leak	Bile leak	1	case_or_format
bile leakage	bile leakage	bile leakage	bile leakage	bile leakage	1	kept
bile salt	bile salt	bile salt	bile salt	bile salt	1	kept
Biliary atresia	biliary atresia	biliary atresia	biliary atresia	Biliary Atresia	1	case_or_format
biliary atresia	biliary atresia	biliary atresia	biliary atresia	Biliary Atresia	1	case_or_format
biliary intervention	biliary intervention	biliary intervention	biliary intervention	biliary intervention	1	kept
biliary tract drainage	biliary tract drainage	biliary tract drainage	biliary tract drainage	biliary tract drainage	1	kept
biliary tract endoscopy	biliary tract endoscopy	biliary tract endoscopy	biliary tract endoscopy	biliary tract endoscopy	1	kept
biliary tract surgery	biliary tract surgery	biliary tract surgery	biliary tract surgery	biliary tract surgery	1	kept

Biliary Tract Surgical Procedures	biliary tract surgical procedures	biliary tract surgical procedures	biliary tract surgical procedures	Biliary Tract Surgical Procedures	1	kept
bilingualism	bilingualism	bilingualism	bilingualism	bilingualism	1	kept
Bilirubin level	bilirubin level	bilirubin level	bilirubin level	Bilirubin level	1	kept
bilirubinuria	bilirubinuria	bilirubinuria	bilirubinuria	bilirubinuria	1	kept
Bilisphere phototherapy	bilisphere phototherapy	bilisphere phototherapy	bilisphere phototherapy	Bilisphere phototherapy	1	kept
billing	billing	billing	billing	billing	1	kept
billing practices	billing practices	billing practices	billing practices	billing practices	1	kept
bimatoprost	bimatoprost	bimatoprost	bimatoprost	bimatoprost	1	kept
Bin packing problem	bin packing problem	bin packing problem	bin packing problem	Bin packing problem	1	kept
Binary alloys	binary alloys	binary alloys	binary alloys	Binary alloys	1	kept
Binary classification problems	binary classification problems	binary classification problems	binary classification problems	Binary classification problems	1	kept
binary logistic regression	binary logistic regression	binary logistic regression	binary logistic regression	binary logistic regression	1	kept
binary logistic regression analysis	binary logistic regression analysis	binary logistic regression analysis	binary logistic regression analysis	binary logistic regression analysis	1	kept
binary pain assessment	binary pain assessment	binary pain assessment	binary pain assessment	binary pain assessment	1	kept
binder jetting	binder jetting	binder jetting	binder jetting	binder jetting	1	kept
binding affinity	binding affinity	binding affinity	binding affinity	binding affinity	1	kept

Binge Drinking	binge drinking	binge drinking	binge drinking	Binge Drinking	1	kept
binge drinking	binge drinking	binge drinking	binge drinking	Binge Drinking	1	case_or_format
binge eating disorder	binge eating disorder	binge eating disorder	binge eating disorder	binge eating disorder	1	kept
binocular indirect ophthalmoscopy	binocular indirect ophthalmoscopy	binocular indirect ophthalmoscopy	binocular indirect ophthalmoscopy	binocular indirect ophthalmoscopy	1	kept
binomial distribution	binomial distribution	binomial distribution	binomial distribution	binomial distribution	1	kept
Bio-aerosol	bio aerosol	bio aerosol	bio aerosol	Bio-aerosol	1	kept
bio-aerosol	bio aerosol	bio aerosol	bio aerosol	Bio-aerosol	1	case_or_format
bio-debridement	bio debridement	bio debridement	bio debridement	bio-debridement	1	kept
Bio-monitoring system	bio monitoring system	bio monitoring system	bio monitoring system	Bio-monitoring system	1	kept
BIOAEROSOLS	bioaerosols	bioaerosols	bioaerosols	BIOAEROSOLS	1	kept
Bioaerosols	bioaerosols	bioaerosols	bioaerosols	BIOAEROSOLS	1	case_or_format
Bioartificial liver support	bioartificial liver support	bioartificial liver support	bioartificial liver support	Bioartificial liver support	1	kept
BIOAVAILABILITY	bioavailability	bioavailability	bioavailability	bioavailability	1	case_or_format
biobehavioral	biobehavioral	biobehavioral	biobehavioral	biobehavioral	1	kept
biobehavioural model	biobehavioural model	biobehavioural model	biobehavioural model	biobehavioural model	1	kept
biochemical composition	biochemical composition	biochemical composition	biochemical composition	biochemical composition	1	kept

Biochemistry	biochemistry	biochemistry	biochemistry	biochemistry	1	case_or_format
biocide	biocide	biocide	biocide	biocide	1	kept
Biocontainment	biocontainment	biocontainment	biocontainment	Biocontainment	1	kept
Biocontainment unit	biocontainment unit	biocontainment unit	biocontainment unit	Biocontainment unit	1	kept
biocontainment unit	biocontainment unit	biocontainment unit	biocontainment unit	Biocontainment unit	1	case_or_format
biocontainment units	biocontainment units	biocontainment units	biocontainment unit	Biocontainment unit	1	singular
biodegradability	biodegradability	biodegradability	biodegradability	biodegradability	1	kept
biodiversity	biodiversity	biodiversity	biodiversity	biodiversity	1	kept
bioethical dilemmas	bioethical dilemmas	bioethical dilemmas	bioethical dilemmas	bioethical dilemmas	1	kept
Bioethical issues	bioethical issues	bioethical issues	bioethical issues	Bioethical Issues	1	case_or_format
bioethicist	bioethicist	bioethicist	bioethicist	bioethicist	1	kept
Biofeedback	biofeedback	biofeedback	biofeedback	biofeedback	1	case_or_format
Biofeedback, Psychology	biofeedback psychology	biofeedback psychology	biofeedback psychology	Biofeedback, Psychology	1	kept
Biofilm	biofilm	biofilm	biofilm	biofilm	1	case_or_format
Biofilm-associated disease	biofilm associated disease	biofilm associated disease	biofilm associated disease	Biofilm-associated disease	1	kept
biofilms	biofilms	biofilms	biofilm	biofilm	1	singular

Biographies as Topic	biographies as topic	biographies as topic	biographies as topic	Biographies as Topic	1	kept
Biography intervention	biography intervention	biography intervention	biography intervention	Biography intervention	1	kept
bioinformatician	bioinformatician	bioinformatician	bioinformatician	bioinformatician	1	kept
Biologic mesh	biologic mesh	biologic mesh	biologic mesh	Biologic mesh	1	kept
biological activity	biological activity	biological activity	biological activity	biological activity	1	kept
Biological Evolution	biological evolution	biological evolution	biological evolution	Biological Evolution	1	kept
biological functions	biological functions	biological functions	biological functions	biological functions	1	kept
Biological Hazards	biological hazards	biological hazards	biological hazards	Biological Hazards	1	kept
Biological membranes	biological membranes	biological membranes	biological membranes	Biological membranes	1	kept
Biological Monitoring	biological monitoring	biological monitoring	biological monitoring	biological monitoring	1	case_or_format
Biological risk prevention	biological risk prevention	biological risk prevention	biological risk prevention	Biological risk prevention	1	kept
Biological Science Disciplines	biological science disciplines	biological science disciplines	biological science disciplines	Biological Science Disciplines	1	kept
biological sex	biological sex	biological sex	biological sex	biological sex	1	kept
Biological signal processing	biological signal processing	biological signal processing	biological signal processing	Biological signal processing	1	kept
Biological signals	biological signals	biological signals	biological signals	Biological signals	1	kept
Biological Specimen Banks	biological specimen banks	biological specimen banks	biological specimen banks	Biological Specimen Banks	1	kept

Biological Warfare	biological warfare	biological warfare	biological warfare	biological warfare	1	case_or_format
Biological waste	biological waste	biological waste	biological waste	Biological waste	1	kept
Biology	biology	biology	biology	Biology	1	kept
biology	biology	biology	biology	Biology	1	case_or_format
bioluminescence	bioluminescence	bioluminescence	bioluminescence	bioluminescence	1	kept
Biomarker testing experience	biomarker testing experience	biomarker testing experience	biomarker testing experience	Biomarker testing experience	1	kept
Biomarkers, Tumor	biomarkers tumor	biomarkers tumor	biomarkers tumor	Biomarkers, Tumor	1	kept
Biomaterials	biomaterials	biomaterials	biomaterial	biomaterial	1	singular
Biomechanical phenomena	biomechanical phenomena	biomechanical phenomena	biomechanical phenomena	Biomechanical Phenomena	1	case_or_format
Biomedical	biomedical	biomedical	biomedical	Biomedical	1	kept
biomedical equipment	biomedical equipment	biomedical equipment	biomedical equipment	Biomedical equipment	1	case_or_format
Biomedical ethics	biomedical ethics	biomedical ethics	biomedical ethics	Biomedical ethics	1	kept
biomedical ethics	biomedical ethics	biomedical ethics	biomedical ethics	Biomedical ethics	1	case_or_format
Biomedical instrumentation	biomedical instrumentation	biomedical instrumentation	biomedical instrumentation	Biomedical instrumentation	1	kept
Biomedical monitoring	biomedical monitoring	biomedical monitoring	biomedical monitoring	Biomedical monitoring	1	kept
biomedical optical imaging	biomedical optical imaging	biomedical optical imaging	biomedical optical imaging	biomedical optical imaging	1	kept

Biomedical research	biomedical research	biomedical research	biomedical research	Biomedical Research	1	case_or_format
Biomedical technologies	biomedical technologies	biomedical technologies	biomedical technology	Biomedical Technology	1	singular
Biomedical Technology Assessment	biomedical technology assessment	biomedical technology assessment	biomedical technology assessment	biomedical technology assessment	1	case_or_format
Biomedical Waste	biomedical waste	biomedical waste	biomedical waste	Biomedical Waste	1	kept
biomonitoring	biomonitoring	biomonitoring	biomonitoring	biomonitoring	1	kept
Bionecteur®	bionecteur	bionecteur	bionecteur	Bionecteur®	1	kept
Biopharmaceutical justification	biopharmaceutical justification	biopharmaceutical justification	biopharmaceutical justification	Biopharmaceutical justification	1	kept
Biopharmaceutics	biopharmaceutics	biopharmaceutics	biopharmaceutics	Biopharmaceutics	1	kept
biophilia hypothesis	biophilia hypothesis	biophilia hypothesis	biophilia hypothesis	biophilia hypothesis	1	kept
Biophysical profile	biophysical profile	biophysical profile	biophysical profile	Biophysical profile	1	kept
Biophysics	biophysics	biophysics	biophysics	Biophysics	1	kept
Biophysiologic stress	biophysiologic stress	biophysiologic stress	biophysiologic stress	Biophysiologic stress	1	kept
biopolitics	biopolitics	biopolitics	biopolitics	biopolitics	1	kept
biopower	biopower	biopower	biopower	biopower	1	kept
bioprosthetic heart valve prosthesis	bioprosthetic heart valve prosthesis	bioprosthetic heart valve prosthesis	bioprosthetic heart valve prosthesis	bioprosthetic heart valve prosthesis	1	kept
Biopsy, Needle	biopsy needle	biopsy needle	biopsy needle	Biopsy, Needle	1	kept

Biopsychosocial-spiritual model	biopsychosocial spiritual model	biopsychosocial spiritual model	biopsychosocial spiritual model	Biopsychosocial-spiritual model	1	kept
Bioreactance	bioreactance	bioreactance	bioreactance	Bioreactance	1	kept
Biosafety incident response competence	biosafety incident response competence	biosafety incident response competence	biosafety incident response competence	Biosafety incident response competence	1	kept
biosafety level 2	biosafety level 2	biosafety level 2	biosafety level 2	biosafety level 2	1	kept
Biosecurity	biosecurity	biosecurity	biosecurity	Biosecurity	1	kept
Biosensors	biosensors	biosensors	biosensor	biosensor	1	singular
biosensors	biosensors	biosensors	biosensor	biosensor	1	singular
biosimilar agent	biosimilar agent	biosimilar agent	biosimilar agent	biosimilar agent	1	kept
Biostatistics	biostatistics	biostatistics	biostatistics	Biostatistics	1	kept
biostatistics	biostatistics	biostatistics	biostatistics	Biostatistics	1	case_or_format
biosurfactant	biosurfactant	biosurfactant	biosurfactant	biosurfactant	1	kept
biosurveillance	biosurveillance	biosurveillance	biosurveillance	biosurveillance	1	kept
Biotechnology	biotechnology	biotechnology	biotechnology	Biotechnology	1	kept
biotechnology	biotechnology	biotechnology	biotechnology	Biotechnology	1	case_or_format
biotherapy	biotherapy	biotherapy	biotherapy	biotherapy	1	kept
Biothreats	biothreats	biothreats	biothreats	Biothreats	1	kept

biotin deficiency	biotin deficiency	biotin deficiency	biotin deficiency	biotin deficiency	1	kept
Bioética	bio tica	bio tica	bio tica	Bioética	1	kept
BIPAP	bipap	bipap	bipap	BiPAP	1	case_or_format
Biphasic positive airway pressure	biphasic positive airway pressure	biphasic positive airway pressure	biphasic positive airway pressure	Biphasic positive airway pressure	1	kept
biplane imaging	biplane imaging	biplane imaging	biplane imaging	biplane imaging	1	kept
Bipolar disorder	bipolar disorder	bipolar disorder	bipolar disorder	bipolar disorder	1	case_or_format
bipolar I disorder	bipolar i disorder	bipolar i disorder	bipolar i disorder	bipolar I disorder	1	kept
bird	bird	bird	bird	bird	1	kept
Bird vacuum extractor	bird vacuum extractor	bird vacuum extractor	bird vacuum extractor	Bird vacuum extractor	1	kept
Birds	birds	birds	bird	bird	1	singular
Birmingham	birmingham	birmingham	birmingham	Birmingham	1	kept
birth asphyxia	birth asphyxia	birth asphyxia	birth asphyxia	Birth asphyxia	1	case_or_format
Birth center	birth center	birth center	birth center	birth center	1	case_or_format
Birth centers	birth centers	birth centers	birth center	birth center	1	singular
Birth centre	birth centre	birth centre	birth centre	Birth centre	1	kept
Birth centres	birth centres	birth centres	birth centre	Birth centre	1	singular

Birth Certificates	birth certificates	birth certificates	birth certificate	birth certificate	1	singular
birth cohort	birth cohort	birth cohort	birth cohort	birth cohort	1	kept
birth control	birth control	birth control	birth control	birth control	1	kept
birth defect	birth defect	birth defect	birth defect	Birth defects	1	case_or_format
Birth injuries	birth injuries	birth injuries	birth injury	Birth Injuries	1	singular
birth interval	birth interval	birth interval	birth interval	birth interval	1	kept
Birth Intervals	birth intervals	birth intervals	birth interval	birth interval	1	singular
Birth interventions	birth interventions	birth interventions	birth interventions	Birth interventions	1	kept
Birth modes	birth modes	birth modes	birth modes	Birth modes	1	kept
Birth order	birth order	birth order	birth order	birth order	1	case_or_format
Birth trauma	birth trauma	birth trauma	birth trauma	Birth trauma	1	kept
Birth trauma outcomes	birth trauma outcomes	birth trauma outcomes	birth trauma outcomes	Birth trauma outcomes	1	kept
BIRTH-WEIGHT INFANT	birth weight infant	birth weight infant	birth weight infant	BIRTH-WEIGHT INFANTS	1	case_or_format
birthing ball exercise	birthing ball exercise	birthing ball exercise	birthing ball exercise	birthing ball exercise	1	kept
birthing center	birthing center	birthing center	birthing center	Birthing Centers	1	case_or_format
birthing centers	birthing centers	birthing centers	birthing center	Birthing Centers	1	singular

Birthing centre	birthing centre	birthing centre	birthing centre	Birthing centre	1	kept
Birthing centres, free-standing	birthing centres free standing	birthing centres free standing	birthing centres free standing	Birthing centres, free-standing	1	kept
Birthweight	birthweight	birthweight	birthweight	Birthweight	1	kept
birthweight weighing scale	birthweight weighing scale	birthweight weighing scale	birthweight weighing scale	birthweight weighing scale	1	kept
BIS Monitoring	bis monitoring	bis monitoring	bis monitoring	BIS Monitoring	1	kept
BIS XP	bis xp	bis xp	bis xp	BIS XP	1	kept
bisexual female	bisexual female	bisexual female	bisexual female	bisexual female	1	kept
bismuth	bismuth	bismuth	bismuth	Bismuth	1	case_or_format
Bisoprolol	bisoprolol	bisoprolol	bisoprolol	bisoprolol	1	case_or_format
BISPECTRAL INDEX	bispectral index	bispectral index	bispectral index	bispectral index	1	case_or_format
Bispectral index monitoring system	bispectral index monitoring system	bispectral index monitoring system	bispectral index monitoring system	Bispectral index monitoring system	1	kept
bispectral index monitoring system	bispectral index monitoring system	bispectral index monitoring system	bispectral index monitoring system	Bispectral index monitoring system	1	case_or_format
Bisphenol A Compounds	bisphenol a compounds	bisphenol a compounds	bisphenol a compounds	Bisphenol A Compounds	1	kept
Bisphosphonates	bisphosphonates	bisphosphonates	bisphosphonates	Bisphosphonates	1	kept
bite	bite	bite	bite	bite	1	kept
Bite-block	bite block	bite block	bite block	Bite-block	1	kept

Bitter taste	bitter taste	bitter taste	bitter taste	Bitter taste	1	kept
bitter taste	bitter taste	bitter taste	bitter taste	Bitter taste	1	case_or_format
bivalent vaccine	bivalent vaccine	bivalent vaccine	bivalent vaccine	bivalent vaccine	1	kept
bivalirudin	bivalirudin	bivalirudin	bivalirudin	bivalirudin	1	kept
bivariate logistic regression	bivariate logistic regression	bivariate logistic regression	bivariate logistic regression	bivariate logistic regression	1	kept
bla KPC 2 gene	bla kpc 2 gene	bla kpc 2 gene	bla kpc 2 gene	bla KPC 2 gene	1	kept
bla ndm 1 gene	bla ndm 1 gene	bla ndm 1 gene	bla ndm 1 gene	bla ndm 1 gene	1	kept
bla oxa 23 gene	bla oxa 23 gene	bla oxa 23 gene	bla oxa 23 gene	bla oxa 23 gene	1	kept
bla OXA 48 gene	bla oxa 48 gene	bla oxa 48 gene	bla oxa 48 gene	bla OXA 48 gene	1	kept
bla oxa 91 gene	bla oxa 91 gene	bla oxa 91 gene	bla oxa 91 gene	bla oxa 91 gene	1	kept
Black Americans	black americans	black americans	black americans	Black Americans	1	kept
Black Feminist Theory	black feminist theory	black feminist theory	black feminist theory	Black Feminist Theory	1	kept
Black mothers	black mothers	black mothers	black mothers	Black mothers	1	kept
Black participant enrollment	black participant enrollment	black participant enrollment	black participant enrollment	Black participant enrollment	1	kept
Black tar heroin	black tar heroin	black tar heroin	black tar heroin	Black tar heroin	1	kept
Black Widow Spider	black widow spider	black widow spider	black widow spider	Black Widow Spider	1	kept

Black widow spider bites	black widow spider bites	black widow spider bites	black widow spider bites	Black widow spider bites	1	kept
Black women	black women	black women	black women	Black women	1	kept
black women health study	black women health study	black women health study	black women health study	black women health study	1	kept
Bladder	bladder	bladder	bladder	bladder	1	case_or_format
Bladder cancer	bladder cancer	bladder cancer	bladder cancer	bladder cancer	1	case_or_format
bladder capacity	bladder capacity	bladder capacity	bladder capacity	bladder capacity	1	kept
BLADDER CATHETERIZATION	bladder catheterization	bladder catheterization	bladder catheterization	bladder catheterization	1	case_or_format
bladder emptying	bladder emptying	bladder emptying	bladder emptying	bladder emptying	1	kept
Bladder pressure	bladder pressure	bladder pressure	bladder pressure	bladder pressure	1	case_or_format
bladder pressure measurement	bladder pressure measurement	bladder pressure measurement	bladder pressure measurement	bladder pressure measurement	1	kept
Bladder pressures	bladder pressures	bladder pressures	bladder pressure	bladder pressure	1	singular
bladder puncture	bladder puncture	bladder puncture	bladder puncture	bladder puncture	1	kept
bladder spasm	bladder spasm	bladder spasm	bladder spasm	bladder spasm	1	kept
bladder surgery	bladder surgery	bladder surgery	bladder surgery	bladder surgery	1	kept
bladder thermometer	bladder thermometer	bladder thermometer	bladder thermometer	bladder thermometer	1	kept
bladder tumor	bladder tumor	bladder tumor	bladder tumor	bladder tumor	1	kept

Bladder wash	bladder wash	bladder wash	bladder wash	Bladder wash	1	kept
Blame culture	blame culture	blame culture	blame culture	Blame culture	1	kept
blame related distress	blame related distress	blame related distress	blame related distress	blame related distress	1	kept
blanket restrictions	blanket restrictions	blanket restrictions	blanket restrictions	blanket restrictions	1	kept
blast cell	blast cell	blast cell	blast cell	blast cell	1	kept
Blast injury	blast injury	blast injury	blast injury	blast injury	1	case_or_format
Blastomyces dermatitidis	blastomyces dermatitidis	blastomyces dermatitidis	blastomyces dermatitidis	Blastomyces dermatitidis	1	kept
blastomycosis	blastomycosis	blastomycosis	blastomycosis	Blastomycosis	1	case_or_format
bleaching agent	bleaching agent	bleaching agent	bleaching agent	bleaching agent	1	kept
Bleed	bleed	bleed	bleed	Bleed	1	kept
bleeding at the peripheral catheter injection site	bleeding at the peripheral catheter injection site	bleeding at the peripheral catheter injection site	bleeding at the peripheral catheter injection site	bleeding at the peripheral catheter injection site	1	kept
Bleeding complication	bleeding complication	bleeding complication	bleeding complication	Bleeding complication	1	kept
Bleeding complications	bleeding complications	bleeding complications	bleeding complication	Bleeding complication	1	singular
Bleeding control	bleeding control	bleeding control	bleeding control	Bleeding control	1	kept
bleeding disorder	bleeding disorder	bleeding disorder	bleeding disorder	bleeding disorder	1	kept
bleeding management	bleeding management	bleeding management	bleeding management	bleeding management	1	kept

bleeding prophylaxis	bleeding prophylaxis	bleeding prophylaxis	bleeding prophylaxis	bleeding prophylaxis	1	kept
Bleeding risk	bleeding risk	bleeding risk	bleeding risk	Bleeding risk	1	kept
bleeding tendency	bleeding tendency	bleeding tendency	bleeding tendency	bleeding tendency	1	kept
bleep filtering	bleep filtering	bleep filtering	bleep filtering	bleep filtering	1	kept
blended interprofessional education program	blended interprofessional education program	blended interprofessional education program	blended interprofessional education program	blended interprofessional education program	1	kept
blended online learning	blended online learning	blended online learning	blended online learning	blended online learning	1	kept
blended-learning	blended learning	blended learning	blended learning	Blended learning	1	case_or_format
Blenderized diet	blenderized diet	blenderized diet	blenderized diet	Blenderized diet	1	kept
blenderized tube-feeding	blenderized tube feeding	blenderized tube feeding	blenderized tube feeding	blenderized tube-feeding	1	kept
Blending-learning	blending learning	blending learning	blending learning	Blending-learning	1	kept
Blind bedside	blind bedside	blind bedside	blind bedside	Blind bedside	1	kept
blind bedside	blind bedside	blind bedside	blind bedside	Blind bedside	1	case_or_format
blind bronchial sampling	blind bronchial sampling	blind bronchial sampling	blind bronchial sampling	blind bronchial sampling	1	kept
blind guidance	blind guidance	blind guidance	blind guidance	blind guidance	1	kept
blinded randomized clinical trial	blinded randomized clinical trial	blinded randomized clinical trial	blinded randomized clinical trial	blinded randomized clinical trial	1	kept
BLISS-1 questionnaire	bliss 1 questionnaire	bliss 1 questionnaire	bliss 1 questionnaire	BLISS-1 questionnaire	1	kept

Blister	blister	blister	blister	blister	1	case_or_format
blizzard sign	blizzard sign	blizzard sign	blizzard sign	blizzard sign	1	kept
Bloc opératoire	bloc op ratoire	bloc op ratoire	bloc op ratoire	Bloc opératoire	1	kept
BLOCKCHAIN	blockchain	blockchain	blockchain	BLOCKCHAIN	1	kept
Blockchain	blockchain	blockchain	blockchain	BLOCKCHAIN	1	case_or_format
Blocking	blocking	blocking	blocking	Blocking	1	kept
Blog	blog	blog	blog	Blog	1	kept
blog analysis	blog analysis	blog analysis	blog analysis	blog analysis	1	kept
blog posts	blog posts	blog posts	blog posts	blog posts	1	kept
blood administration	blood administration	blood administration	blood administration	blood administration	1	kept
blood alcohol level	blood alcohol level	blood alcohol level	blood alcohol level	blood alcohol level	1	kept
blood and body fluids	blood and body fluids	blood and body fluids	blood and body fluids	blood and body fluids	1	kept
Blood and urine samples	blood and urine samples	blood and urine samples	blood and urine samples	Blood and urine samples	1	kept
Blood bank & transfusion medicine	blood bank and transfusion medicine	blood bank and transfusion medicine	blood bank and transfusion medicine	Blood bank & transfusion medicine	1	kept
blood banking	blood banking	blood banking	blood banking	blood banking	1	kept
Blood borne pathogens	blood borne pathogens	blood borne pathogens	blood borne pathogens	Blood-Borne Pathogens	1	case_or_format

blood cancer	blood cancer	blood cancer	blood cancer	blood cancer	1	kept
blood cancers	blood cancers	blood cancers	blood cancer	blood cancer	1	singular
blood clotting factor 10	blood clotting factor 10	blood clotting factor 10	blood clotting factor 10	blood clotting factor 10	1	kept
blood clotting factor 8 cryoprecipitate	blood clotting factor 8 cryoprecipitate	blood clotting factor 8 cryoprecipitate	blood clotting factor 8 cryoprecipitate	blood clotting factor 8 cryoprecipitate	1	kept
blood clotting factor 8 inhibitor	blood clotting factor 8 inhibitor	blood clotting factor 8 inhibitor	blood clotting factor 8 inhibitor	blood clotting factor 8 inhibitor	1	kept
blood clotting factor 8a	blood clotting factor 8a	blood clotting factor 8a	blood clotting factor 8a	blood clotting factor 8a	1	kept
blood clotting factor 9 inhibitor	blood clotting factor 9 inhibitor	blood clotting factor 9 inhibitor	blood clotting factor 9 inhibitor	blood clotting factor 9 inhibitor	1	kept
blood clotting inhibitor	blood clotting inhibitor	blood clotting inhibitor	blood clotting inhibitor	blood clotting inhibitor	1	kept
Blood collection	blood collection	blood collection	blood collection	blood collection	1	case_or_format
blood component removal	blood component removal	blood component removal	blood component removal	Blood Component Removal	1	case_or_format
Blood concentration monitoring	blood concentration monitoring	blood concentration monitoring	blood concentration monitoring	Blood concentration monitoring	1	kept
Blood conservation strategies	blood conservation strategies	blood conservation strategies	blood conservation strategies	Blood conservation strategies	1	kept
Blood culture contamination	blood culture contamination	blood culture contamination	blood culture contamination	Blood culture contamination	1	kept
blood culture contamination	blood culture contamination	blood culture contamination	blood culture contamination	Blood culture contamination	1	case_or_format
Blood culture sample	blood culture sample	blood culture sample	blood culture sample	Blood culture sample	1	kept
Blood delivery	blood delivery	blood delivery	blood delivery	Blood delivery	1	kept

blood derivative	blood derivative	blood derivative	blood derivative	blood derivative	1	kept
Blood discard volume	blood discard volume	blood discard volume	blood discard volume	Blood discard volume	1	kept
blood disease	blood disease	blood disease	blood disease	blood disease	1	kept
Blood donation	blood donation	blood donation	blood donation	Blood donation	1	kept
Blood draw practice	blood draw practice	blood draw practice	blood draw practice	Blood draw practice	1	kept
Blood draws	blood draws	blood draws	blood draws	Blood draws	1	kept
blood filter	blood filter	blood filter	blood filter	blood filter	1	kept
blood filtration	blood filtration	blood filtration	blood filtration	blood filtration	1	kept
Blood flow velocity	blood flow velocity	blood flow velocity	blood flow velocity	blood flow velocity	1	case_or_format
blood flowmetry	blood flowmetry	blood flowmetry	blood flowmetry	blood flowmetry	1	kept
Blood Gas	blood gas	blood gas	blood gas	blood gas	1	case_or_format
Blood gas	blood gas	blood gas	blood gas	blood gas	1	case_or_format
Blood gas analysis indicators	blood gas analysis indicators	blood gas analysis indicators	blood gas analysis indicators	Blood gas analysis indicators	1	kept
blood gas analysis, coagulation tests	blood gas analysis coagulation tests	blood gas analysis coagulation tests	blood gas analysis coagulation tests	blood gas analysis, coagulation tests	1	kept
blood gas monitoring	blood gas monitoring	blood gas monitoring	blood gas monitoring	blood gas monitoring	1	kept
Blood gas values	blood gas values	blood gas values	blood gas values	Blood gas values	1	kept

Blood gases	blood gases	blood gases	blood gases	blood gases	1	case_or_format
Blood gasses	blood gasses	blood gasses	blood gasses	Blood gasses	1	kept
Blood glucose control	blood glucose control	blood glucose control	blood glucose control	Blood glucose control	1	kept
blood glucose control	blood glucose control	blood glucose control	blood glucose control	Blood glucose control	1	case_or_format
blood glucose meter	blood glucose meter	blood glucose meter	blood glucose meter	blood glucose meter	1	kept
blood glucose monitoring device	blood glucose monitoring device	blood glucose monitoring device	blood glucose monitoring device	blood glucose monitoring device	1	kept
Blood glucose regulations	blood glucose regulations	blood glucose regulations	blood glucose regulations	Blood glucose regulations	1	kept
Blood glucose target range	blood glucose target range	blood glucose target range	blood glucose target range	Blood glucose target range	1	kept
blood glucose variability	blood glucose variability	blood glucose variability	blood glucose variability	blood glucose variability	1	kept
blood group	blood group	blood group	blood group	blood group	1	kept
blood group A	blood group a	blood group a	blood group a	blood group A	1	kept
blood group ABO system	blood group abo system	blood group abo system	blood group abo system	blood group ABO system	1	kept
Blood Group Incompatibility	blood group incompatibility	blood group incompatibility	blood group incompatibility	Blood Group Incompatibility	1	kept
blood group incompatibility	blood group incompatibility	blood group incompatibility	blood group incompatibility	Blood Group Incompatibility	1	case_or_format
blood group Rh	blood group rh	blood group rh	blood group rh	blood group Rh	1	kept
Blood indexes	blood indexes	blood indexes	blood indexes	Blood indexes	1	kept

blood infection	blood infection	blood infection	blood infection	blood infection	1	kept
blood loss estimation	blood loss estimation	blood loss estimation	blood loss estimation	blood loss estimation	1	kept
Blood management	blood management	blood management	blood management	blood management	1	case_or_format
Blood Preservation	blood preservation	blood preservation	blood preservation	Blood Preservation	1	kept
Blood pressure augmentation	blood pressure augmentation	blood pressure augmentation	blood pressure augmentation	Blood pressure augmentation	1	kept
Blood pressure cuff	blood pressure cuff	blood pressure cuff	blood pressure cuff	blood pressure cuff	1	case_or_format
blood pressure fluctuation	blood pressure fluctuation	blood pressure fluctuation	blood pressure fluctuation	blood pressure fluctuation	1	kept
blood pressure location	blood pressure location	blood pressure location	blood pressure location	blood pressure location	1	kept
blood pressure monitors	blood pressure monitors	blood pressure monitors	blood pressure monitor	Blood Pressure Monitors	1	singular
blood pressure transducer	blood pressure transducer	blood pressure transducer	blood pressure transducer	blood pressure transducer	1	kept
blood pump	blood pump	blood pump	blood pump	blood pump	1	kept
Blood purification	blood purification	blood purification	blood purification	blood purification	1	case_or_format
blood relative	blood relative	blood relative	blood relative	blood relative	1	kept
Blood safety	blood safety	blood safety	blood safety	blood safety	1	case_or_format
blood sample collection	blood sample collection	blood sample collection	blood sample collection	Blood sample collection	1	case_or_format
Blood sampling technique	blood sampling technique	blood sampling technique	blood sampling technique	Blood sampling technique	1	kept

blood specimen collection	blood specimen collection	blood specimen collection	blood specimen collection	Blood Specimen Collection	1	case_or_format
blood stream infection	blood stream infection	blood stream infection	blood stream infection	BLOOD-STREAM INFECTIONS	1	case_or_format
blood stream infections	blood stream infections	blood stream infections	blood stream infection	BLOOD-STREAM INFECTIONS	1	singular
Blood sugar	blood sugar	blood sugar	blood sugar	Blood sugar	1	kept
Blood supply	blood supply	blood supply	blood supply	Blood supply	1	kept
blood test	blood test	blood test	blood test	blood test	1	kept
blood testis barrier	blood testis barrier	blood testis barrier	blood testis barrier	blood testis barrier	1	kept
blood tinged emesis	blood tinged emesis	blood tinged emesis	blood tinged emesis	blood tinged emesis	1	kept
Blood Transfusion Procedure	blood transfusion procedure	blood transfusion procedure	blood transfusion procedure	Blood Transfusion Procedure	1	kept
blood urea nitrogen	blood urea nitrogen	blood urea nitrogen	blood urea nitrogen	Blood Urea Nitrogen	1	case_or_format
blood vessel calcification	blood vessel calcification	blood vessel calcification	blood vessel calcification	blood vessel calcification	1	kept
blood vessel fistula	blood vessel fistula	blood vessel fistula	blood vessel fistula	blood vessel fistula	1	kept
blood vessel wall	blood vessel wall	blood vessel wall	blood vessel wall	blood vessel wall	1	kept
Blood Vessels	blood vessels	blood vessels	blood vessel	blood vessel	1	singular
blood vessels	blood vessels	blood vessels	blood vessel	blood vessel	1	singular
blood viscosity	blood viscosity	blood viscosity	blood viscosity	Blood Viscosity	1	case_or_format

Blood wastage	blood wastage	blood wastage	blood wastage	Blood wastage	1	kept
Blood-Air Barrier	blood air barrier	blood air barrier	blood air barrier	Blood-Air Barrier	1	kept
blood-borne infection	blood borne infection	blood borne infection	blood borne infection	blood-borne infection	1	kept
Blood-Borne Infections	blood borne infections	blood borne infections	blood borne infection	blood-borne infection	1	singular
Blood-borne pathogens	blood borne pathogens	blood borne pathogens	blood borne pathogens	Blood-Borne Pathogens	1	case_or_format
Blood-borne virus	blood borne virus	blood borne virus	blood borne virus	Blood-borne virus	1	kept
Blood-Brain Barrier	blood brain barrier	blood brain barrier	blood brain barrier	blood brain barrier	1	case_or_format
BLOOD-GAS	blood gas	blood gas	blood gas	blood gas	1	case_or_format
BLOOD-GLUCOSE	blood glucose	blood glucose	blood glucose	Blood Glucose	1	case_or_format
BLOOD-GLUCOSE CONTROL	blood glucose control	blood glucose control	blood glucose control	Blood glucose control	1	case_or_format
blood-glucose control	blood glucose control	blood glucose control	blood glucose control	Blood glucose control	1	case_or_format
BLOOD-LOSS	blood loss	blood loss	blood loss	Blood loss	1	case_or_format
BLOOD-PRESSURE CONTROL	blood pressure control	blood pressure control	blood pressure control	BLOOD-PRESSURE CONTROL	1	kept
bloodless care	bloodless care	bloodless care	bloodless care	bloodless care	1	kept
Bloodless transplantation	bloodless transplantation	bloodless transplantation	bloodless transplantation	Bloodless transplantation	1	kept
Bloodletting	bloodletting	bloodletting	bloodletting	Bloodletting	1	kept

bloodletting	bloodletting	bloodletting	bloodletting	Bloodletting	1	case_or_format
Bloodstream Infection	bloodstream infection	bloodstream infection	bloodstream infection	bloodstream infection	1	case_or_format
bloody	bloody	bloody	bloody	bloody	1	kept
blood‑sparing techniques	blood sparing techniques	blood sparing techniques	blood sparing techniques	Blood-sparing techniques	1	case_or_format
Bloqueo nervioso	bloqueo nervioso	bloqueo nervioso	bloqueo nervioso	Bloqueo nervioso	1	kept
BLS training	bls training	bls training	bls training	BLS training	1	kept
BLSD	blsd	blsd	blsd	BLSD	1	kept
Blue code	blue code	blue code	blue code	Blue code	1	kept
blue code	blue code	blue code	blue code	Blue code	1	case_or_format
Blue Cross Blue Shield Insurance Plans	blue cross blue shield insurance plans	blue cross blue shield insurance plans	blue cross blue shield insurance plans	Blue Cross Blue Shield Insurance Plans	1	kept
Blue dye swallow test	blue dye swallow test	blue dye swallow test	blue dye swallow test	Blue dye swallow test	1	kept
BlueBERT	bluebert	bluebert	bluebert	BlueBERT	1	kept
Bluetooth connectivity	bluetooth connectivity	bluetooth connectivity	bluetooth connectivity	Bluetooth connectivity	1	kept
Bluetooth Low Energy	bluetooth low energy	bluetooth low energy	bluetooth low energy	Bluetooth Low Energy	1	kept
Bluetooth scatternets	bluetooth scatternets	bluetooth scatternets	bluetooth scatternets	Bluetooth scatternets	1	kept
Bluewater Health	bluewater health	bluewater health	bluewater health	Bluewater Health	1	kept

Blunt abdominal trauma	blunt abdominal trauma	blunt abdominal trauma	blunt abdominal trauma	Blunt abdominal trauma	1	kept
blunt aortic injury	blunt aortic injury	blunt aortic injury	blunt aortic injury	blunt aortic injury	1	kept
blunt cerebrovascular injury	blunt cerebrovascular injury	blunt cerebrovascular injury	blunt cerebrovascular injury	blunt cerebrovascular injury	1	kept
Blunt chest trauma	blunt chest trauma	blunt chest trauma	blunt chest trauma	Blunt chest trauma	1	kept
Blunt chest wall injury	blunt chest wall injury	blunt chest wall injury	blunt chest wall injury	Blunt chest wall injury	1	kept
Blunt chest wall trauma	blunt chest wall trauma	blunt chest wall trauma	blunt chest wall trauma	Blunt chest wall trauma	1	kept
Blunt thoracic trauma	blunt thoracic trauma	blunt thoracic trauma	blunt thoracic trauma	Blunt thoracic trauma	1	kept
BLUNT TRAUMA	blunt trauma	blunt trauma	blunt trauma	blunt trauma	1	case_or_format
blunted affect	blunted affect	blunted affect	blunted affect	blunted affect	1	kept
BMI = body mass index	bmi body mass index	bmi body mass index	bmi body mass index	BMI, body mass index	1	case_or_format
BMI, body mass index	bmi body mass index	bmi body mass index	bmi body mass index	BMI, body mass index	1	kept
BNP	bnp	bnp	bnp	BNP	1	kept
bnt 162 vaccine	bnt 162 vaccine	bnt 162 vaccine	bnt 162 vaccine	bnt 162 vaccine	1	kept
BNT162b2	bnt162b2	bnt162b2	bnt162b2	BNT162b2	1	kept
Bocavirus	bocavirus	bocavirus	bocavirus	Bocavirus	1	kept
bocavirus	bocavirus	bocavirus	bocavirus	Bocavirus	1	case_or_format

BODIES	bodies	bodies	body	BODY	1	singular
Bodily functions	bodily functions	bodily functions	bodily functions	Bodily functions	1	kept
Bodily Objectification	bodily objectification	bodily objectification	bodily objectification	Bodily Objectification	1	kept
Body	body	body	body	BODY	1	case_or_format
body adiposity index	body adiposity index	body adiposity index	body adiposity index	body adiposity index	1	kept
Body and self	body and self	body and self	body and self	Body and self	1	kept
Body Burden	body burden	body burden	body burden	Body Burden	1	kept
body burden	body burden	body burden	body burden	Body Burden	1	case_or_format
body condition score	body condition score	body condition score	body condition score	body condition score	1	kept
body contouring	body contouring	body contouring	body contouring	body contouring	1	kept
Body Fat	body fat	body fat	body fat	Body Fat	1	kept
Body fat	body fat	body fat	body fat	Body Fat	1	case_or_format
Body fluid compartments	body fluid compartments	body fluid compartments	body fluid compartment	body fluid compartment	1	singular
Body fluid exposure	body fluid exposure	body fluid exposure	body fluid exposure	Body fluid exposure	1	kept
Body fluids	body fluids	body fluids	body fluid	body fluid	1	singular
Body heat exchange	body heat exchange	body heat exchange	body heat exchange	Body heat exchange	1	kept

body heat exchange	body heat exchange	body heat exchange	body heat exchange	Body heat exchange	1	case_or_format
Body image	body image	body image	body image	Body Image	1	case_or_format
body image	body image	body image	body image	Body Image	1	case_or_format
Body language	body language	body language	body language	Body language	1	kept
Body mapping	body mapping	body mapping	body mapping	Body mapping	1	kept
Body mass	body mass	body mass	body mass	body mass	1	case_or_format
body plethysmography	body plethysmography	body plethysmography	body plethysmography	body plethysmography	1	kept
Body position change	body position change	body position change	body position change	Body position change	1	kept
Body Position Measurement	body position measurement	body position measurement	body position measurement	Body Position Measurement	1	kept
Body positioning	body positioning	body positioning	body positioning	Body positioning	1	kept
Body positions	body positions	body positions	body position	body position	1	singular
body posture change	body posture change	body posture change	body posture change	body posture change	1	kept
body procedure technique	body procedure technique	body procedure technique	body procedure technique	body procedure technique	1	kept
body rash	body rash	body rash	body rash	body rash	1	kept
BODY REGIONS	body regions	body regions	body regions	body regions	1	case_or_format
Body sensor networks	body sensor networks	body sensor networks	body sensor networks	Body sensor networks	1	kept

Body surface area	body surface area	body surface area	body surface area	Body Surface Area	1	case_or_format
Body Surface Potential Mapping	body surface potential mapping	body surface potential mapping	body surface potential mapping	Body Surface Potential Mapping	1	kept
Body Temperature Measurement	body temperature measurement	body temperature measurement	body temperature measurement	body temperature measurement	1	case_or_format
Body temperature measurements	body temperature measurements	body temperature measurements	body temperature measurement	body temperature measurement	1	singular
Body temperature regulation	body temperature regulation	body temperature regulation	body temperature regulation	Body Temperature Regulation	1	case_or_format
body temperature regulation	body temperature regulation	body temperature regulation	body temperature regulation	Body Temperature Regulation	1	case_or_format
Body temperature sensor	body temperature sensor	body temperature sensor	body temperature sensor	Body temperature sensor	1	kept
Body Water	body water	body water	body water	Body Water	1	kept
body water	body water	body water	body water	Body Water	1	case_or_format
body weight disorder	body weight disorder	body weight disorder	body weight disorder	body weight disorder	1	kept
Body worn cameras	body worn cameras	body worn cameras	body worn cameras	Body worn cameras	1	kept
BODY-FLUIDS	body fluids	body fluids	body fluid	body fluid	1	singular
bodyweight measurements	bodyweight measurements	bodyweight measurements	bodyweight measurements	bodyweight measurements	1	kept
boiling liquid expanding vapor explosion	boiling liquid expanding vapor explosion	boiling liquid expanding vapor explosion	boiling liquid expanding vapor explosion	boiling liquid expanding vapor explosion	1	kept
Bolus	bolus	bolus	bolus	Bolus	1	kept
Bolus dose	bolus dose	bolus dose	bolus dose	Bolus dose	1	kept

bolus enteral feeding	bolus enteral feeding	bolus enteral feeding	bolus enteral feeding	bolus enteral feeding	1	kept
Bolus fluid therapy	bolus fluid therapy	bolus fluid therapy	bolus fluid therapy	Bolus fluid therapy	1	kept
bolus infusion	bolus infusion	bolus infusion	bolus infusion	bolus infusion	1	kept
Bombs	bombs	bombs	bomb	bomb	1	singular
bon usage	bon usage	bon usage	bon usage	bon usage	1	kept
Bond	bond	bond	bond	Bond	1	kept
Bonding process	bonding process	bonding process	bonding process	Bonding process	1	kept
Bone	bone	bone	bone	bone	1	case_or_format
Bone and joint infections	bone and joint infections	bone and joint infections	bone and joint infections	bone and joint infections	1	case_or_format
bone atrophy	bone atrophy	bone atrophy	bone atrophy	bone atrophy	1	kept
Bone conduction	bone conduction	bone conduction	bone conduction	Bone conduction	1	kept
bone densitometry	bone densitometry	bone densitometry	bone densitometry	bone densitometry	1	kept
Bone Density	bone density	bone density	bone density	bone density	1	case_or_format
bone disease	bone disease	bone disease	bone disease	bone disease	1	kept
bone dysplasia	bone dysplasia	bone dysplasia	bone dysplasia	bone dysplasia	1	kept
bone erosion	bone erosion	bone erosion	bone erosion	bone erosion	1	kept

bone fragility	bone fragility	bone fragility	bone fragility	bone fragility	1	kept
bone growth	bone growth	bone growth	bone growth	bone growth	1	kept
bone marrow	bone marrow	bone marrow	bone marrow	bone marrow	1	kept
bone marrow cell	bone marrow cell	bone marrow cell	bone marrow cell	bone marrow cell	1	kept
bone marrow depression	bone marrow depression	bone marrow depression	bone marrow depression	bone marrow depression	1	kept
bone marrow metastasis	bone marrow metastasis	bone marrow metastasis	bone marrow metastasis	bone marrow metastasis	1	kept
bone marrow toxicity	bone marrow toxicity	bone marrow toxicity	bone marrow toxicity	bone marrow toxicity	1	kept
bone maturation	bone maturation	bone maturation	bone maturation	bone maturation	1	kept
bone mineralization	bone mineralization	bone mineralization	bone mineralization	bone mineralization	1	kept
bone nail	bone nail	bone nail	bone nail	Bone Nails	1	case_or_format
bone necrosis	bone necrosis	bone necrosis	bone necrosis	bone necrosis	1	kept
bone plate	bone plate	bone plate	bone plate	bone plate	1	kept
Bone Plates	bone plates	bone plates	bone plate	bone plate	1	singular
bone remodeling	bone remodeling	bone remodeling	bone remodeling	bone remodeling	1	kept
bone retroversion	bone retroversion	bone retroversion	bone retroversion	bone retroversion	1	kept
Bone traction	bone traction	bone traction	bone traction	Bone traction	1	kept

bone transplantation	bone transplantation	bone transplantation	bone transplantation	Bone Transplantation	1	case_or_format
bone tumor	bone tumor	bone tumor	bone tumor	bone tumor	1	kept
Bone-Conductive Sound	bone conductive sound	bone conductive sound	bone conductive sound	Bone-Conductive Sound	1	kept
Bonferroni correction	bonferroni correction	bonferroni correction	bonferroni correction	Bonferroni correction	1	kept
book club	book club	book club	book club	book club	1	kept
Books	books	books	book	book	1	singular
Boolean function simplification	boolean function simplification	boolean function simplification	boolean function simplification	Boolean function simplification	1	kept
BOOP	boop	boop	boop	BOOP	1	kept
booster dose	booster dose	booster dose	booster dose	booster dose	1	kept
booster vaccine	booster vaccine	booster vaccine	booster vaccine	booster vaccine	1	kept
boot camp	boot camp	boot camp	boot camp	boot camp	1	kept
bootcamp	bootcamp	bootcamp	bootcamp	bootcamp	1	kept
BOPPPS	boppps	boppps	boppps	BOPPPS	1	kept
Borderline Personality Disorder	borderline personality disorder	borderline personality disorder	borderline personality disorder	Borderline Personality Disorder	1	kept
borderline personality disorder	borderline personality disorder	borderline personality disorder	borderline personality disorder	Borderline Personality Disorder	1	case_or_format
Borderline viability	borderline viability	borderline viability	borderline viability	Borderline viability	1	kept

Bordetella	bordetella	bordetella	bordetella	Bordetella	1	kept
Bordetella parapertussis	bordetella parapertussis	bordetella parapertussis	bordetella parapertussis	Bordetella parapertussis	1	kept
bordetellosis	bordetellosis	bordetellosis	bordetellosis	bordetellosis	1	kept
Boredom	boredom	boredom	boredom	boredom	1	case_or_format
BORN	born	born	born	BORN	1	kept
BORN EXTREMELY PRETERM	born extremely preterm	born extremely preterm	born extremely preterm	BORN EXTREMELY PRETERM	1	kept
bortezomib	bortezomib	bortezomib	bortezomib	bortezomib	1	kept
BOS	bos	bos	bos	BOS	1	kept
bosentan	bosentan	bosentan	bosentan	bosentan	1	kept
Boswellia	boswellia	boswellia	boswellia	Boswellia	1	kept
bottle	bottle	bottle	bottle	bottle	1	kept
Bottle gourd	bottle gourd	bottle gourd	bottle gourd	Bottle gourd	1	kept
bottle-feeding	bottle feeding	bottle feeding	bottle feeding	Bottle Feeding	1	case_or_format
Bottles	bottles	bottles	bottle	bottle	1	singular
botulinum toxin	botulinum toxin	botulinum toxin	botulinum toxin	botulinum toxin	1	kept
botulinum toxin A	botulinum toxin a	botulinum toxin a	botulinum toxin a	botulinum toxin A	1	kept

Botulismo	botulismo	botulismo	botulismo	Botulismo	1	kept
Bouc émissaire	bouc missaire	bouc missaire	bouc missaire	Bouc émissaire	1	kept
bougie	bougie	bougie	bougie	bougie	1	kept
boundaries	boundaries	boundaries	boundaries	Boundaries	1	case_or_format
bounded rationality	bounded rationality	bounded rationality	bounded rationality	bounded rationality	1	kept
bovine serum albumin	bovine serum albumin	bovine serum albumin	bovine serum albumin	bovine serum albumin	1	kept
bow-tie method	bow tie method	bow tie method	bow tie method	bow-tie method	1	kept
Bowel	bowel	bowel	bowel	Bowel	1	kept
Bowel atresia	bowel atresia	bowel atresia	bowel atresia	Bowel atresia	1	kept
bowel evacuation	bowel evacuation	bowel evacuation	bowel evacuation	bowel evacuation	1	kept
Bowel function	bowel function	bowel function	bowel function	Bowel function	1	kept
bowel management	bowel management	bowel management	bowel management	Bowel management	1	case_or_format
bowel movement	bowel movement	bowel movement	bowel movement	bowel movement	1	kept
Bowel obstruction	bowel obstruction	bowel obstruction	bowel obstruction	Bowel obstruction	1	kept
bowel rest	bowel rest	bowel rest	bowel rest	bowel rest	1	kept
Bowel sound	bowel sound	bowel sound	bowel sound	Bowel sounds	1	case_or_format

Bowels	bowels	bowels	bowel	Bowel	1	singular
bowling	bowling	bowling	bowling	bowling	1	kept
Bowman Birk inhibitor	bowman birk inhibitor	bowman birk inhibitor	bowman birk inhibitor	Bowman Birk inhibitor	1	kept
Bowman Capsule	bowman capsule	bowman capsule	bowman capsule	Bowman Capsule	1	kept
Bowman capsule	bowman capsule	bowman capsule	bowman capsule	Bowman Capsule	1	case_or_format
BPSD	bpsd	bpsd	bpsd	BPSD	1	kept
brace	brace	brace	brace	Braces	1	case_or_format
brachial plexus injury	brachial plexus injury	brachial plexus injury	brachial plexus injury	brachial plexus injury	1	kept
Brachial plexus neuropathies	brachial plexus neuropathies	brachial plexus neuropathies	brachial plexus neuropathies	Brachial plexus neuropathies	1	kept
brachial plexus palsy	brachial plexus palsy	brachial plexus palsy	brachial plexus palsy	brachial plexus palsy	1	kept
brachial vein deep vein thrombosis	brachial vein deep vein thrombosis	brachial vein deep vein thrombosis	brachial vein deep vein thrombosis	brachial vein deep vein thrombosis	1	kept
brachiocephalic trunk	brachiocephalic trunk	brachiocephalic trunk	brachiocephalic trunk	brachiocephalic trunk	1	kept
brachiocephalic vein	brachiocephalic vein	brachiocephalic vein	brachiocephalic vein	brachiocephalic vein	1	kept
Brachiocephalic Veins	brachiocephalic veins	brachiocephalic veins	brachiocephalic vein	brachiocephalic vein	1	singular
brachycephaly	brachycephaly	brachycephaly	brachycephaly	Brachycephaly	1	case_or_format
Brachytherapy	brachytherapy	brachytherapy	brachytherapy	brachytherapy	1	case_or_format

Bracing	bracing	bracing	bracing	Bracing	1	kept
BRADEN	braden	braden	braden	BRADEN	1	kept
Braden	braden	braden	braden	BRADEN	1	case_or_format
Braden Q	braden q	braden q	braden q	Braden Q	1	kept
braden q scale	braden q scale	braden q scale	braden q scale	Braden Q scale	1	case_or_format
Braden QD Scale	braden qd scale	braden qd scale	braden qd scale	Braden QD scale	1	case_or_format
Braden risk assessment scale	braden risk assessment scale	braden risk assessment scale	braden risk assessment scale	Braden risk assessment scale	1	kept
Braden risk score	braden risk score	braden risk score	braden risk score	Braden risk score	1	kept
braden scale	braden scale	braden scale	braden scale	Braden Scale	1	case_or_format
Braden tool	braden tool	braden tool	braden tool	Braden tool	1	kept
BRADEN-SCALE	braden scale	braden scale	braden scale	Braden Scale	1	case_or_format
bradycardia identification	bradycardia identification	bradycardia identification	bradycardia identification	bradycardia identification	1	kept
bradykinesia	bradykinesia	bradykinesia	bradykinesia	bradykinesia	1	kept
bradykinin	bradykinin	bradykinin	bradykinin	bradykinin	1	kept
bradypnoea	bradypnoea	bradypnoea	bradypnoea	bradypnoea	1	kept
BRAF	braf	braf	braf	BRAF	1	kept

Brain abscess	brain abscess	brain abscess	brain abscess	brain abscess	1	case_or_format
brain aqueduct	brain aqueduct	brain aqueduct	brain aqueduct	brain aqueduct	1	kept
brain aqueduct stenosis	brain aqueduct stenosis	brain aqueduct stenosis	brain aqueduct stenosis	brain aqueduct stenosis	1	kept
brain artery	brain artery	brain artery	brain artery	brain artery	1	kept
brain asymmetry	brain asymmetry	brain asymmetry	brain asymmetry	brain asymmetry	1	kept
brain atherosclerosis	brain atherosclerosis	brain atherosclerosis	brain atherosclerosis	brain atherosclerosis	1	kept
brain biopsy	brain biopsy	brain biopsy	brain biopsy	brain biopsy	1	kept
brain blood vessel	brain blood vessel	brain blood vessel	brain blood vessel	brain blood vessel	1	kept
Brain computer interface	brain computer interface	brain computer interface	brain computer interface	Brain computer interface	1	kept
Brain Concussion	brain concussion	brain concussion	brain concussion	Brain Concussion	1	kept
brain concussion	brain concussion	brain concussion	brain concussion	Brain Concussion	1	case_or_format
Brain Contusion	brain contusion	brain contusion	brain contusion	brain contusion	1	case_or_format
brain cortex lesion	brain cortex lesion	brain cortex lesion	brain cortex lesion	brain cortex lesion	1	kept
brain damage of preterm infants	brain damage of preterm infants	brain damage of preterm infants	brain damage of preterm infants	brain damage of preterm infants	1	kept
Brain dead	brain dead	brain dead	brain dead	Brain dead	1	kept
Brain dead donor	brain dead donor	brain dead donor	brain dead donor	Brain dead donor	1	kept

brain dead donor	brain dead donor	brain dead donor	brain dead donor	Brain dead donor	1	case_or_format
brain death knowledge	brain death knowledge	brain death knowledge	brain death knowledge	brain death knowledge	1	kept
Brain development	brain development	brain development	brain development	brain development	1	case_or_format
Brain Diseases, Metabolic	brain diseases metabolic	brain diseases metabolic	brain diseases metabolic	Brain Diseases, Metabolic	1	kept
brain drain	brain drain	brain drain	brain drain	brain drain	1	kept
brain fourth ventricle	brain fourth ventricle	brain fourth ventricle	brain fourth ventricle	brain fourth ventricle	1	kept
Brain function	brain function	brain function	brain function	brain function	1	case_or_format
brain heart infusion broth	brain heart infusion broth	brain heart infusion broth	brain heart infusion broth	brain heart infusion broth	1	kept
Brain herniation	brain herniation	brain herniation	brain herniation	Brain herniation	1	kept
brain herniation	brain herniation	brain herniation	brain herniation	Brain herniation	1	case_or_format
brain histology	brain histology	brain histology	brain histology	brain histology	1	kept
Brain Injury Guidelines	brain injury guidelines	brain injury guidelines	brain injury guidelines	Brain Injury Guidelines	1	kept
brain injury medicine	brain injury medicine	brain injury medicine	brain injury medicine	brain injury medicine	1	kept
Brain insult	brain insult	brain insult	brain insult	Brain insult	1	kept
brain interstitial fluid	brain interstitial fluid	brain interstitial fluid	brain interstitial fluid	brain interstitial fluid	1	kept
brain malaria	brain malaria	brain malaria	brain malaria	brain malaria	1	kept

brain mass	brain mass	brain mass	brain mass	brain mass	1	kept
Brain metabolism	brain metabolism	brain metabolism	brain metabolism	brain metabolism	1	case_or_format
brain microcirculation	brain microcirculation	brain microcirculation	brain microcirculation	brain microcirculation	1	kept
brain mineralization	brain mineralization	brain mineralization	brain mineralization	brain mineralization	1	kept
BRAIN NATRIURETIC PEPTIDE	brain natriuretic peptide	brain natriuretic peptide	brain natriuretic peptide	brain natriuretic peptide	1	case_or_format
Brain natriuretic peptide	brain natriuretic peptide	brain natriuretic peptide	brain natriuretic peptide	brain natriuretic peptide	1	case_or_format
brain necrosis	brain necrosis	brain necrosis	brain necrosis	brain necrosis	1	kept
Brain oxygen	brain oxygen	brain oxygen	brain oxygen	Brain oxygen	1	kept
brain oxygen consumption	brain oxygen consumption	brain oxygen consumption	brain oxygen consumption	brain oxygen consumption	1	kept
brain oxygen tension	brain oxygen tension	brain oxygen tension	brain oxygen tension	brain oxygen tension	1	kept
Brain physiology	brain physiology	brain physiology	brain physiology	Brain physiology	1	kept
Brain protection	brain protection	brain protection	brain protection	brain protection	1	case_or_format
brain pseudotumor	brain pseudotumor	brain pseudotumor	brain pseudotumor	brain pseudotumor	1	kept
brain regeneration	brain regeneration	brain regeneration	brain regeneration	brain regeneration	1	kept
Brain Roadmap	brain roadmap	brain roadmap	brain roadmap	Brain Roadmap	1	kept
Brain stem death	brain stem death	brain stem death	brain stem death	Brain stem death	1	kept

Brain Stem Infarctions	brain stem infarctions	brain stem infarctions	brain stem infarctions	Brain Stem Infarctions	1	kept
brain swelling	brain swelling	brain swelling	brain swelling	brain swelling	1	kept
brain third ventricle	brain third ventricle	brain third ventricle	brain third ventricle	brain third ventricle	1	kept
brain tomography	brain tomography	brain tomography	brain tomography	brain tomography	1	kept
brain trauma injury	brain trauma injury	brain trauma injury	brain trauma injury	brain trauma injury	1	kept
brain tumour	brain tumour	brain tumour	brain tumour	Brain tumour	1	case_or_format
brain vein	brain vein	brain vein	brain vein	brain vein	1	kept
brain ventricle pressure	brain ventricle pressure	brain ventricle pressure	brain ventricle pressure	brain ventricle pressure	1	kept
brain weight	brain weight	brain weight	brain weight	brain weight	1	kept
brain-dead	brain dead	brain dead	brain dead	Brain dead	1	case_or_format
BrainPath	brainpath	brainpath	brainpath	BrainPath	1	kept
brainstem death	brainstem death	brainstem death	brainstem death	brainstem death	1	kept
brainstem death testing	brainstem death testing	brainstem death testing	brainstem death testing	brainstem death testing	1	kept
brainstem lesion	brainstem lesion	brainstem lesion	brainstem lesion	brainstem lesion	1	kept
brainstem reflex	brainstem reflex	brainstem reflex	brainstem reflex	brainstem reflex	1	kept
brave	brave	brave	brave	brave	1	kept

BRAZILIAN VERSION	brazilian version	brazilian version	brazilian version	BRAZILIAN VERSION	1	kept
breach	breach	breach	breach	breach	1	kept
Break-up	break up	break up	break up	Break-up	1	kept
Breaking bad news	breaking bad news	breaking bad news	breaking bad news	breaking bad news	1	case_or_format
Breakthrough infection	breakthrough infection	breakthrough infection	breakthrough infection	breakthrough infection	1	case_or_format
Breakthrough Infections	breakthrough infections	breakthrough infections	breakthrough infection	breakthrough infection	1	singular
BREAST	breast	breast	breast	BREAST	1	kept
Breast	breast	breast	breast	BREAST	1	case_or_format
breast	breast	breast	breast	BREAST	1	case_or_format
Breast augmentation	breast augmentation	breast augmentation	breast augmentation	breast augmentation	1	case_or_format
Breast cancer surgery	breast cancer surgery	breast cancer surgery	breast cancer surgery	Breast cancer surgery	1	kept
breast endoprosthesis	breast endoprosthesis	breast endoprosthesis	breast endoprosthesis	breast endoprosthesis	1	kept
breast engorgement	breast engorgement	breast engorgement	breast engorgement	breast engorgement	1	kept
Breast Implantation	breast implantation	breast implantation	breast implantation	Breast Implantation	1	kept
breast milk donation	breast milk donation	breast milk donation	breast milk donation	breast milk donation	1	kept
Breast milk expression	breast milk expression	breast milk expression	breast milk expression	Breast Milk Expression	1	case_or_format

Breast milk feeding	breast milk feeding	breast milk feeding	breast milk feeding	Breast milk feeding	1	kept
breast milk jaundice	breast milk jaundice	breast milk jaundice	breast milk jaundice	breast milk jaundice	1	kept
Breast milk odor	breast milk odor	breast milk odor	breast milk odor	Breast milk odor	1	kept
breast milk smell	breast milk smell	breast milk smell	breast milk smell	breast milk smell	1	kept
Breast neoplasms	breast neoplasms	breast neoplasms	breast neoplasms	Breast Neoplasms	1	case_or_format
Breast reconstruction	breast reconstruction	breast reconstruction	breast reconstruction	breast reconstruction	1	case_or_format
breast reduction	breast reduction	breast reduction	breast reduction	breast reduction	1	kept
Breast-feeding	breast feeding	breast feeding	breast feeding	Breast Feeding	1	case_or_format
BREAST-FEEDING OUTCOMES	breast feeding outcomes	breast feeding outcomes	breast feeding outcomes	BREAST-FEEDING OUTCOMES	1	kept
BREAST-MILK	breast milk	breast milk	breast milk	breast milk	1	case_or_format
BREAST-MILK USE	breast milk use	breast milk use	breast milk use	BREAST-MILK USE	1	kept
breastfeeding - psychosocial factors	breastfeeding psychosocial factors	breastfeeding psychosocial factors	breastfeeding psychosocial factors	breastfeeding - psychosocial factors	1	kept
breastfeeding at discharge	breastfeeding at discharge	breastfeeding at discharge	breastfeeding at discharge	breastfeeding at discharge	1	kept
breastfeeding attitude	breastfeeding attitude	breastfeeding attitude	breastfeeding attitude	breastfeeding attitude	1	kept
Breastfeeding duration	breastfeeding duration	breastfeeding duration	breastfeeding duration	Breastfeeding duration	1	kept
breastfeeding experience	breastfeeding experience	breastfeeding experience	breastfeeding experience	breastfeeding experience	1	kept

breastfeeding initiation	breastfeeding initiation	breastfeeding initiation	breastfeeding initiation	Breastfeeding initiation	1	case_or_format
breastfeeding interventions	breastfeeding interventions	breastfeeding interventions	breastfeeding interventions	breastfeeding interventions	1	kept
Breastfeeding performance	breastfeeding performance	breastfeeding performance	breastfeeding performance	Breastfeeding performance	1	kept
breastfeeding promotion	breastfeeding promotion	breastfeeding promotion	breastfeeding promotion	breastfeeding promotion	1	kept
breastfeeding quality improvement	breastfeeding quality improvement	breastfeeding quality improvement	breastfeeding quality improvement	breastfeeding quality improvement	1	kept
breastfeeding self efficacy scale short form	breastfeeding self efficacy scale short form	breastfeeding self efficacy scale short form	breastfeeding self efficacy scale short form	breastfeeding self efficacy scale short form	1	kept
Breastfeeding Self-Efficacy	breastfeeding self efficacy	breastfeeding self efficacy	breastfeeding self efficacy	Breastfeeding Self-Efficacy	1	kept
Breastfeeding self-efficacy	breastfeeding self efficacy	breastfeeding self efficacy	breastfeeding self efficacy	Breastfeeding Self-Efficacy	1	case_or_format
breastfeeding self-efficacy	breastfeeding self efficacy	breastfeeding self efficacy	breastfeeding self efficacy	Breastfeeding Self-Efficacy	1	case_or_format
Breastfeeding success	breastfeeding success	breastfeeding success	breastfeeding success	Breastfeeding success	1	kept
breastfeeding success	breastfeeding success	breastfeeding success	breastfeeding success	Breastfeeding success	1	case_or_format
breastfeeding transmission	breastfeeding transmission	breastfeeding transmission	breastfeeding transmission	breastfeeding transmission	1	kept
Breastfeeding.	breastfeeding	breastfeeding	breastfeeding	breastfeeding	1	case_or_format
Breath representation	breath representation	breath representation	breath representation	Breath representation	1	kept
Breathing	breathing	breathing	breathing	breathing	1	case_or_format
breathing apparatus	breathing apparatus	breathing apparatus	breathing apparatus	breathing apparatus	1	kept

breathing issues	breathing issues	breathing issues	breathing issues	breathing issues	1	kept
breathing rate estimation	breathing rate estimation	breathing rate estimation	breathing rate estimation	breathing rate estimation	1	kept
Breathing trials	breathing trials	breathing trials	breathing trials	Breathing trials	1	kept
breathing trials	breathing trials	breathing trials	breathing trials	Breathing trials	1	case_or_format
BREATHLESSNESS	breathlessness	breathlessness	breathlessness	BREATHLESSNESS	1	kept
brentuximab vedotin	brentuximab vedotin	brentuximab vedotin	brentuximab vedotin	brentuximab vedotin	1	kept
bretylium	bretylium	bretylium	bretylium	bretylium	1	kept
Brevundimonas	brevundimonas	brevundimonas	brevundimonas	Brevundimonas	1	kept
Bricolage	bricolage	bricolage	bricolage	Bricolage	1	kept
Bridge symptoms	bridge symptoms	bridge symptoms	bridge symptoms	Bridge symptoms	1	kept
bridge to decision	bridge to decision	bridge to decision	bridge to decision	bridge to decision	1	kept
Bridging	bridging	bridging	bridging	Bridging	1	kept
Brief Confusion Assessment Method	brief confusion assessment method	brief confusion assessment method	brief confusion assessment method	Brief Confusion Assessment Method	1	kept
Brief Confusion Assessment Method questionnaire	brief confusion assessment method questionnaire	brief confusion assessment method questionnaire	brief confusion assessment method questionnaire	Brief Confusion Assessment Method questionnaire	1	kept
Brief Fatigue Inventory	brief fatigue inventory	brief fatigue inventory	brief fatigue inventory	Brief Fatigue Inventory	1	kept
brief intervention	brief intervention	brief intervention	brief intervention	Brief intervention	1	case_or_format

brief psychotic disorder	brief psychotic disorder	brief psychotic disorder	brief psychotic disorder	brief psychotic disorder	1	kept
Brief Resilient Coping Scale	brief resilient coping scale	brief resilient coping scale	brief resilient coping scale	brief resilient coping scale	1	case_or_format
Brief text messages	brief text messages	brief text messages	brief text messages	Brief text messages	1	kept
briefing	briefing	briefing	briefing	briefing	1	kept
BRIGHT LIGHT THERAPY	bright light therapy	bright light therapy	bright light therapy	BRIGHT LIGHT THERAPY	1	kept
brimonidine	brimonidine	brimonidine	brimonidine	brimonidine	1	kept
brincidofovir	brincidofovir	brincidofovir	brincidofovir	brincidofovir	1	kept
Bristol Breastfeeding Assessment Scale	bristol breastfeeding assessment scale	bristol breastfeeding assessment scale	bristol breastfeeding assessment scale	Bristol Breastfeeding Assessment Scale	1	kept
British citizen	british citizen	british citizen	british citizen	British citizen	1	kept
British Thoracic Society	british thoracic society	british thoracic society	british thoracic society	British Thoracic Society	1	kept
brivaracetam	brivaracetam	brivaracetam	brivaracetam	brivaracetam	1	kept
Broad range 16S DNA PCR	broad range 16s dna pcr	broad range 16s dna pcr	broad range 16s dna pcr	Broad range 16S DNA PCR	1	kept
Broad spectrum antibiotics	broad spectrum antibiotics	broad spectrum antibiotics	broad spectrum antibiotics	Broad spectrum antibiotics	1	kept
Broad-spectr um antibiotics	broad spectr um antibiotics	broad spectr um antibiotics	broad spectr um antibiotics	Broad-spectr um antibiotics	1	kept
broadly neutralizing antibody	broadly neutralizing antibody	broadly neutralizing antibody	broadly neutralizing antibody	broadly neutralizing antibody	1	kept
brodifacoum	brodifacoum	brodifacoum	brodifacoum	brodifacoum	1	kept

Broken heart syndrome	broken heart syndrome	broken heart syndrome	broken heart syndrome	Broken heart syndrome	1	kept
bromide	bromide	bromide	bromide	bromide	1	kept
bronchial artery embolization	bronchial artery embolization	bronchial artery embolization	bronchial artery embolization	bronchial artery embolization	1	kept
Bronchial asthma	bronchial asthma	bronchial asthma	bronchial asthma	Bronchial asthma	1	kept
bronchial asthma	bronchial asthma	bronchial asthma	bronchial asthma	Bronchial asthma	1	case_or_format
Bronchial disease	bronchial disease	bronchial disease	bronchial disease	Bronchial disease	1	kept
Bronchial Diseases	bronchial diseases	bronchial diseases	bronchial disease	Bronchial disease	1	singular
Bronchiectasis	bronchiectasis	bronchiectasis	bronchiectasis	bronchiectasis	1	case_or_format
Bronchiolitis obliterans organizing pneumonia	bronchiolitis obliterans organizing pneumonia	bronchiolitis obliterans organizing pneumonia	bronchiolitis obliterans organizing pneumonia	bronchiolitis obliterans organizing pneumonia	1	case_or_format
Bronchiolitis Obliterans Syndrome	bronchiolitis obliterans syndrome	bronchiolitis obliterans syndrome	bronchiolitis obliterans syndrome	Bronchiolitis Obliterans Syndrome	1	kept
bronchiolitis, mechanical ventilation	bronchiolitis mechanical ventilation	bronchiolitis mechanical ventilation	bronchiolitis mechanical ventilation	bronchiolitis, mechanical ventilation	1	kept
broncho-aspiration	broncho aspiration	broncho aspiration	broncho aspiration	broncho-aspiration	1	kept
BRONCHOALVEOLAR LAVAGE	bronchoalveolar lavage	bronchoalveolar lavage	bronchoalveolar lavage	Bronchoalveolar Lavage	1	case_or_format
bronchoalveolar lavage	bronchoalveolar lavage	bronchoalveolar lavage	bronchoalveolar lavage	Bronchoalveolar Lavage	1	case_or_format
Bronchoalveolar Lavage Fluid	bronchoalveolar lavage fluid	bronchoalveolar lavage fluid	bronchoalveolar lavage fluid	bronchoalveolar lavage fluid	1	case_or_format
Bronchodilator agents [MeSH]	bronchodilator agents mesh	bronchodilator agents mesh	bronchodilator agents mesh	Bronchodilator agents [MeSH]	1	kept

bronchodilators	bronchodilators	bronchodilators	bronchodilator	Bronchodilator	1	singular
bronchodysplasia	bronchodysplasia	bronchodysplasia	bronchodysplasia	bronchodysplasia	1	kept
Bronchogenic Cyst	bronchogenic cyst	bronchogenic cyst	bronchogenic cyst	Bronchogenic Cyst	1	kept
Bronchogenic cyst	bronchogenic cyst	bronchogenic cyst	bronchogenic cyst	Bronchogenic Cyst	1	case_or_format
bronchopulmonary fistula	bronchopulmonary fistula	bronchopulmonary fistula	bronchopulmonary fistula	bronchopulmonary fistula	1	kept
BRONCHOSCOPE	bronchoscope	bronchoscope	bronchoscope	bronchoscope	1	case_or_format
bronchoscopist	bronchoscopist	bronchoscopist	bronchoscopist	bronchoscopist	1	kept
bronchus fistula	bronchus fistula	bronchus fistula	bronchus fistula	bronchus fistula	1	kept
bronchus injury	bronchus injury	bronchus injury	bronchus injury	bronchus injury	1	kept
bronchyolitis	bronchyolitis	bronchyolitis	bronchyolitis	bronchyolitis	1	kept
Bronx	bronx	bronx	bronx	Bronx	1	kept
brother-in-law	brother in law	brother in law	brother in law	brother-in-law	1	kept
brotizolam	brotizolam	brotizolam	brotizolam	brotizolam	1	kept
Brown Sequard syndrome	brown sequard syndrome	brown sequard syndrome	brown sequard syndrome	Brown Sequard syndrome	1	kept
Brown-Séquard syndrome	brown s quard syndrome	brown s quard syndrome	brown s quard syndrome	Brown-Séquard syndrome	1	kept
brownout	brownout	brownout	brownout	brownout	1	kept

Brucella	brucella	brucella	brucella	Brucella	1	kept
Brucella melitensis	brucella melitensis	brucella melitensis	brucella melitensis	Brucella melitensis	1	kept
brucellosis	brucellosis	brucellosis	brucellosis	brucellosis	1	kept
Brugada Syndrome	brugada syndrome	brugada syndrome	brugada syndrome	Brugada Syndrome	1	kept
Brugada syndrome	brugada syndrome	brugada syndrome	brugada syndrome	Brugada Syndrome	1	case_or_format
Brunswik	brunswik	brunswik	brunswik	Brunswik	1	kept
Brøset Violence Checklist	br set violence checklist	br set violence checklist	br set violence checklist	Brøset Violence Checklist	1	kept
brûlure	br lure	br lure	br lure	brûlure	1	kept
BSI = bloodstream infection	bsi bloodstream infection	bsi bloodstream infection	bsi bloodstream infection	BSI = bloodstream infection	1	kept
BSN	bsn	bsn	bsn	BSN	1	kept
BSN students	bsn students	bsn students	bsn students	BSN students	1	kept
Bubble continuous positive airway pressure	bubble continuous positive airway pressure	bubble continuous positive airway pressure	bubble continuous positive airway pressure	bubble continuous positive airway pressure	1	case_or_format
bubble continuous positive airway pressure disconnection	bubble continuous positive airway pressure disconnection	bubble continuous positive airway pressure disconnection	bubble continuous positive airway pressure disconnection	bubble continuous positive airway pressure disconnection	1	kept
bubble CPAP	bubble cpap	bubble cpap	bubble cpap	BUBBLE CPAP	1	case_or_format
Bubble formation	bubble formation	bubble formation	bubble formation	Bubble formation	1	kept
bubble humidified oxygen	bubble humidified oxygen	bubble humidified oxygen	bubble humidified oxygen	bubble humidified oxygen	1	kept

bubble noninvasive positive pressure ventilation	bubble noninvasive positive pressure ventilation	bubble noninvasive positive pressure ventilation	bubble noninvasive positive pressure ventilation	bubble noninvasive positive pressure ventilation	1	kept
Buckman protocol	buckman protocol	buckman protocol	buckman protocol	Buckman protocol	1	kept
budding	budding	budding	budding	budding	1	kept
Budesonide	budesonide	budesonide	budesonide	budesonide	1	case_or_format
budesonide plus formoterol	budesonide plus formoterol	budesonide plus formoterol	budesonide plus formoterol	budesonide plus formoterol	1	kept
Budget allocation	budget allocation	budget allocation	budget allocation	Budget allocation	1	kept
Budget impact	budget impact	budget impact	budget impact	Budget impact	1	kept
Buerger-Allen exercises	buerger allen exercises	buerger allen exercises	buerger allen exercises	Buerger-Allen exercises	1	kept
buffer	buffer	buffer	buffer	buffer	1	kept
Buffering	buffering	buffering	buffering	Buffering	1	kept
Buffers	buffers	buffers	buffer	buffer	1	singular
Bugis philosophy	bugis philosophy	bugis philosophy	bugis philosophy	Bugis philosophy	1	kept
Building blockes	building blockes	building blockes	building blockes	Building blockes	1	kept
Building layout	building layout	building layout	building layout	Building layout	1	kept
Building trust	building trust	building trust	building trust	Building trust	1	kept
Buildings layout	buildings layout	buildings layout	buildings layout	Buildings layout	1	kept

BULB OXYGEN-SATURATION	bulb oxygen saturation	bulb oxygen saturation	bulb oxygen saturation	BULB OXYGEN-SATURATION	1	kept
Bulgaria	bulgaria	bulgaria	bulgaria	Bulgaria	1	kept
bullet	bullet	bullet	bullet	bullet	1	kept
bullock	bullock	bullock	bullock	bullock	1	kept
bullous dermatitis	bullous dermatitis	bullous dermatitis	bullous dermatitis	bullous dermatitis	1	kept
bullous pemphigoid	bullous pemphigoid	bullous pemphigoid	bullous pemphigoid	bullous pemphigoid	1	kept
bulsuphan	bulsuphan	bulsuphan	bulsuphan	bulsuphan	1	kept
Bumetanide	bumetanide	bumetanide	bumetanide	bumetanide	1	case_or_format
Bundle care approach	bundle care approach	bundle care approach	bundle care approach	Bundle care approach	1	kept
Bundle of His	bundle of his	bundle of his	bundle of his	Bundle of His	1	kept
Bundle protocol	bundle protocol	bundle protocol	bundle protocol	Bundle protocol	1	kept
Bundle set intervention	bundle set intervention	bundle set intervention	bundle set intervention	Bundle set intervention	1	kept
Bundle therapy	bundle therapy	bundle therapy	bundle therapy	Bundle therapy	1	kept
bundled care model	bundled care model	bundled care model	bundled care model	bundled care model	1	kept
bundled informed consent	bundled informed consent	bundled informed consent	bundled informed consent	bundled informed consent	1	kept
bundled payment	bundled payment	bundled payment	bundled payment	Bundled payments	1	case_or_format

BUNDLES	bundles	bundles	bundle	BUNDLE	1	singular
bundles	bundles	bundles	bundle	BUNDLE	1	singular
Bundles of Care	bundles of care	bundles of care	bundles of care	Bundles of Care	1	kept
Bundles of care	bundles of care	bundles of care	bundles of care	Bundles of Care	1	case_or_format
Buprenorphine maintenance therapy	buprenorphine maintenance therapy	buprenorphine maintenance therapy	buprenorphine maintenance therapy	Buprenorphine maintenance therapy	1	kept
buprenorphine plus naloxone	buprenorphine plus naloxone	buprenorphine plus naloxone	buprenorphine plus naloxone	buprenorphine plus naloxone	1	kept
Buprenorphine, Naloxone Drug Combination	buprenorphine naloxone drug combination	buprenorphine naloxone drug combination	buprenorphine naloxone drug combination	Buprenorphine, Naloxone Drug Combination	1	kept
Bupropion	bupropion	bupropion	bupropion	Bupropion	1	kept
burden	burden	burden	burden	Burden	1	case_or_format
burden of care	burden of care	burden of care	burden of care	burden of care	1	kept
Burden of Clostridioides difficile infection	burden of clostridioides difficile infection	burden of clostridioides difficile infection	burden of clostridioides difficile infection	Burden of Clostridioides difficile infection	1	kept
Burden of treatment	burden of treatment	burden of treatment	burden of treatment	Burden of treatment	1	kept
burdensome transitions	burdensome transitions	burdensome transitions	burdensome transitions	burdensome transitions	1	kept
Burkholderia ambifaria	burkholderia ambifaria	burkholderia ambifaria	burkholderia ambifaria	Burkholderia ambifaria	1	kept
Burkholderia contaminans	burkholderia contaminans	burkholderia contaminans	burkholderia contaminans	Burkholderia contaminans	1	kept
Burkholderia stabilis	burkholderia stabilis	burkholderia stabilis	burkholderia stabilis	Burkholderia stabilis	1	kept

burn care hospital	burn care hospital	burn care hospital	burn care hospital	burn care hospital	1	kept
Burn care team	burn care team	burn care team	burn care team	Burn care team	1	kept
burn care team	burn care team	burn care team	burn care team	Burn care team	1	case_or_format
Burn care unit	burn care unit	burn care unit	burn care unit	Burn care unit	1	kept
Burn Center	burn center	burn center	burn center	Burn center	1	case_or_format
Burn centers	burn centers	burn centers	burn center	Burn center	1	singular
Burn centre	burn centre	burn centre	burn centre	Burn centre	1	kept
Burn centres	burn centres	burn centres	burn centre	Burn centre	1	singular
burn clinician	burn clinician	burn clinician	burn clinician	burn clinician	1	kept
burn dressing	burn dressing	burn dressing	burn dressing	burn dressing	1	kept
Burn injuries	burn injuries	burn injuries	burn injury	Burn injury	1	singular
Burn intervention score	burn intervention score	burn intervention score	burn intervention score	Burn intervention score	1	kept
Burn Low income country Standardized training Essential	burn low income country standardized training essential	burn low income country standardized training essential	burn low income country standardized training essential	Burn Low income country Standardized training Essential	1	kept
burn out	burn out	burn out	burn out	Burn out	1	case_or_format
Burn outcomes	burn outcomes	burn outcomes	burn outcomes	Burn outcomes	1	kept
Burn pain	burn pain	burn pain	burn pain	Burn pain	1	kept

burn pain	burn pain	burn pain	burn pain	Burn pain	1	case_or_format
Burn prevention	burn prevention	burn prevention	burn prevention	Burn prevention	1	kept
Burn resuscitation	burn resuscitation	burn resuscitation	burn resuscitation	Burn resuscitation	1	kept
burn scar	burn scar	burn scar	burn scar	burn scar	1	kept
burn units	burn units	burn units	burn unit	Burn Units	1	singular
Burn victims	burn victims	burn victims	burn victims	Burn victims	1	kept
Burn wound infection	burn wound infection	burn wound infection	burn wound infection	Burn wound infection	1	kept
Burn wound sepsis	burn wound sepsis	burn wound sepsis	burn wound sepsis	Burn wound sepsis	1	kept
burn-associated mortalities	burn associated mortalities	burn associated mortalities	burn associated mortalities	burn-associated mortalities	1	kept
burning	burning	burning	burning	burning	1	kept
burning feet syndrome	burning feet syndrome	burning feet syndrome	burning feet syndrome	burning feet syndrome	1	kept
burning pain	burning pain	burning pain	burning pain	burning pain	1	kept
Burnout and compassion fatigue	burnout and compassion fatigue	burnout and compassion fatigue	burnout and compassion fatigue	Burnout and compassion fatigue	1	kept
BURNOUT LEVELS	burnout levels	burnout levels	burnout levels	BURNOUT LEVELS	1	kept
Burnout Mitigation	burnout mitigation	burnout mitigation	burnout mitigation	Burnout Mitigation	1	kept
Burnout Professional	burnout professional	burnout professional	burnout professional	Burnout, Professional	1	case_or_format

burnout professional	burnout professional	burnout professional	burnout professional	Burnout, Professional	1	case_or_format
Burnout psychological	burnout psychological	burnout psychological	burnout psychological	Burnout, Psychological	1	case_or_format
Burnout symptoms	burnout symptoms	burnout symptoms	burnout symptoms	Burnout symptoms	1	kept
BURNOUT SYNDROME	burnout syndrome	burnout syndrome	burnout syndrome	burnout syndrome	1	case_or_format
Burnout, professional, prevention and control	burnout professional prevention and control	burnout professional prevention and control	burnout professional prevention and control	Burnout, professional, prevention and control	1	kept
Burnout, psychological	burnout psychological	burnout psychological	burnout psychological	Burnout, Psychological	1	case_or_format
BURNS UNIT	burns unit	burns unit	burns unit	BURNS UNIT	1	kept
Burns Weaning Assessment Protocol	burns weaning assessment protocol	burns weaning assessment protocol	burns weaning assessment protocol	Burns Weaning Assessment Protocol	1	kept
Burns, inhalation	burns inhalation	burns inhalation	burns inhalation	Burns, Inhalation	1	case_or_format
Burn’s wean assessment program	burn s wean assessment program	burn s wean assessment program	burn s wean assessment program	Burn’s wean assessment program	1	kept
burr hole drainage	burr hole drainage	burr hole drainage	burr hole drainage	burr hole drainage	1	kept
burst abdomen	burst abdomen	burst abdomen	burst abdomen	burst abdomen	1	kept
burst fracture	burst fracture	burst fracture	burst fracture	burst fracture	1	kept
Bushfire trauma	bushfire trauma	bushfire trauma	bushfire trauma	Bushfire trauma	1	kept
business	business	business	business	business	1	kept
Business efficiency	business efficiency	business efficiency	business efficiency	Business efficiency	1	kept

business ethics	business ethics	business ethics	business ethics	business ethics	1	kept
business intelligence	business intelligence	business intelligence	business intelligence	business intelligence	1	kept
business process management	business process management	business process management	business process management	business process management	1	kept
BUSINESS-UNIT	business unit	business unit	business unit	BUSINESS-UNIT	1	kept
Buss Perry Aggression Questionnaire	buss perry aggression questionnaire	buss perry aggression questionnaire	buss perry aggression questionnaire	Buss Perry Aggression Questionnaire	1	kept
Busulfan	busulfan	busulfan	busulfan	busulfan	1	case_or_format
butamirate citrate	butamirate citrate	butamirate citrate	butamirate citrate	butamirate citrate	1	kept
butorphanol	butorphanol	butorphanol	butorphanol	butorphanol	1	kept
butorphanol tartrate	butorphanol tartrate	butorphanol tartrate	butorphanol tartrate	butorphanol tartrate	1	kept
butterfly	butterfly	butterfly	butterfly	butterfly	1	kept
butterfly flap fixation	butterfly flap fixation	butterfly flap fixation	butterfly flap fixation	butterfly flap fixation	1	kept
Buttock care	buttock care	buttock care	buttock care	Buttock care	1	kept
Buttock lifting angle	buttock lifting angle	buttock lifting angle	buttock lifting angle	Buttock lifting angle	1	kept
butyrophenone derivative	butyrophenone derivative	butyrophenone derivative	butyrophenone derivative	butyrophenone derivative	1	kept
butyrophenones	butyrophenones	butyrophenones	butyrophenones	butyrophenones	1	kept
Buzzer	buzzer	buzzer	buzzer	Buzzer	1	kept

BW-based	bw based	bw based	bw based	BW-based	1	kept
BWM	bwm	bwm	bwm	BWM	1	kept
BYPASS	bypass	bypass	bypass	Bypass	1	case_or_format
BYPASS GRAFT-SURGERY	bypass graft surgery	bypass graft surgery	bypass graft surgery	BYPASS GRAFT-SURGERY	1	kept
Bypass surgery	bypass surgery	bypass surgery	bypass surgery	bypass surgery	1	case_or_format
B - lines	b lines	b lines	b lines	B-lines	1	case_or_format
C difficile	c difficile	c difficile	c difficile	C difficile	1	kept
c ritical care	c ritical care	c ritical care	c ritical care	c ritical care	1	kept
C-CHEWS	c chews	c chews	c chews	C-CHEWS	1	kept
C-clamp	c clamp	c clamp	c clamp	C-clamp	1	kept
C-PICSQ	c picsq	c picsq	c picsq	C-PICSQ	1	kept
C-POT scale	c pot scale	c pot scale	c pot scale	C-POT scale	1	kept
C-section	c section	c section	c section	C-section	1	kept
C-trend	c trend	c trend	c trend	C-trend	1	kept
C. difficile	c difficile	c difficile	c difficile	C difficile	1	case_or_format
C. Lindau extract	c lindau extract	c lindau extract	c lindau extract	C. Lindau extract	1	kept

C57BL 6 mouse	c57bl 6 mouse	c57bl 6 mouse	c57bl 6 mouse	C57BL 6 mouse	1	kept
CABG SURGERY	cabg surgery	cabg surgery	cabg surgery	CABG SURGERY	1	kept
cabotegravir plus rilpivirine	cabotegravir plus rilpivirine	cabotegravir plus rilpivirine	cabotegravir plus rilpivirine	cabotegravir plus rilpivirine	1	kept
cabozantinib	cabozantinib	cabozantinib	cabozantinib	cabozantinib	1	kept
Cacao	cacao	cacao	cacao	Cacao	1	kept
CAD	cad	cad	cad	CAD	1	kept
cadaver-based course	cadaver based course	cadaver based course	cadaver based course	cadaver-based course	1	kept
cadherin 23	cadherin 23	cadherin 23	cadherin 23	cadherin 23	1	kept
Cadmium compounds	cadmium compounds	cadmium compounds	cadmium compounds	Cadmium compounds	1	kept
Caesarean	caesarean	caesarean	caesarean	Caesarean	1	kept
caesarean	caesarean	caesarean	caesarean	Caesarean	1	case_or_format
Caesarean delivery	caesarean delivery	caesarean delivery	caesarean delivery	Caesarean delivery	1	kept
Caesarean Section	caesarean section	caesarean section	caesarean section	caesarean section	1	case_or_format
Caesarean section	caesarean section	caesarean section	caesarean section	caesarean section	1	case_or_format
CAFFEINE	caffeine	caffeine	caffeine	caffeine	1	case_or_format
caffeine use disorder	caffeine use disorder	caffeine use disorder	caffeine use disorder	caffeine use disorder	1	kept

café	caf	caf	caf	café	1	kept
CAG repeat	cag repeat	cag repeat	cag repeat	CAG repeat	1	kept
CAGE questionnaire	cage questionnaire	cage questionnaire	cage questionnaire	CAGE questionnaire	1	kept
Cairo [Egypt]	cairo egypt	cairo egypt	cairo egypt	Cairo [Egypt]	1	kept
cake	cake	cake	cake	cake	1	kept
Caking	caking	caking	caking	Caking	1	kept
Calabash	calabash	calabash	calabash	Calabash	1	kept
Calcific uremic arteriolopathy	calcific uremic arteriolopathy	calcific uremic arteriolopathy	calcific uremic arteriolopathy	Calcific uremic arteriolopathy	1	kept
Calcineurin inhibitors	calcineurin inhibitors	calcineurin inhibitors	calcineurin inhibitor	calcineurin inhibitor	1	singular
Calcinosis Cutis	calcinosis cutis	calcinosis cutis	calcinosis cutis	Calcinosis Cutis	1	kept
calcipotriol	calcipotriol	calcipotriol	calcipotriol	calcipotriol	1	kept
calcitonin blood level	calcitonin blood level	calcitonin blood level	calcitonin blood level	calcitonin blood level	1	kept
calcitonin gene related peptide	calcitonin gene related peptide	calcitonin gene related peptide	calcitonin gene related peptide	calcitonin gene related peptide	1	kept
Calcitonin Gene-Related Peptide	calcitonin gene related peptide	calcitonin gene related peptide	calcitonin gene related peptide	calcitonin gene related peptide	1	case_or_format
calcitriol	calcitriol	calcitriol	calcitriol	calcitriol	1	kept
CALCIUM	calcium	calcium	calcium	calcium	1	case_or_format

calcium alginate	calcium alginate	calcium alginate	calcium alginate	calcium alginate	1	kept
Calcium Carbonate	calcium carbonate	calcium carbonate	calcium carbonate	calcium carbonate	1	case_or_format
calcium cell level	calcium cell level	calcium cell level	calcium cell level	calcium cell level	1	kept
calcium channel blocker	calcium channel blocker	calcium channel blocker	calcium channel blocker	Calcium Channel Blockers	1	case_or_format
calcium channel stimulating agent	calcium channel stimulating agent	calcium channel stimulating agent	calcium channel stimulating agent	calcium channel stimulating agent	1	kept
calcium glubionate	calcium glubionate	calcium glubionate	calcium glubionate	calcium glubionate	1	kept
calcium gluconate	calcium gluconate	calcium gluconate	calcium gluconate	Calcium Gluconate	1	case_or_format
calcium hydroxide	calcium hydroxide	calcium hydroxide	calcium hydroxide	calcium hydroxide	1	kept
calcium phosphate	calcium phosphate	calcium phosphate	calcium phosphate	calcium phosphate	1	kept
Calcium Phosphates	calcium phosphates	calcium phosphates	calcium phosphate	calcium phosphate	1	singular
calcium salt	calcium salt	calcium salt	calcium salt	calcium salt	1	kept
calcium stone	calcium stone	calcium stone	calcium stone	calcium stone	1	kept
calcium urine level	calcium urine level	calcium urine level	calcium urine level	calcium urine level	1	kept
Calcium-Transporting ATPases	calcium transporting atpases	calcium transporting atpases	calcium transporting atpases	Calcium-Transporting ATPases	1	kept
Calculation skill	calculation skill	calculation skill	calculation skill	Calculation skill	1	kept
CALCULATION SKILLS	calculation skills	calculation skills	calculation skill	Calculation skill	1	singular

calendula oil	calendula oil	calendula oil	calendula oil	calendula oil	1	kept
calf	calf	calf	calf	calf	1	kept
CALF CIRCUMFERENCE	calf circumference	calf circumference	calf circumference	CALF CIRCUMFERENCE	1	kept
calf circumference	calf circumference	calf circumference	calf circumference	CALF CIRCUMFERENCE	1	case_or_format
Calf management	calf management	calf management	calf management	Calf management	1	kept
calfactant	calfactant	calfactant	calfactant	calfactant	1	kept
calgranulin	calgranulin	calgranulin	calgranulin	calgranulin	1	kept
calicivirus infection	calicivirus infection	calicivirus infection	calicivirus infection	calicivirus infection	1	kept
Calidad de la atención de salud	calidad de la atenci n de salud	calidad de la atenci n de salud	calidad de la atenci n de salud	Calidad de la atención de salud	1	kept
Calidad de vida	calidad de vida	calidad de vida	calidad de vida	Calidad de vida	1	kept
Calidad del sue?o	calidad del sue o	calidad del sue o	calidad del sue o	Calidad del sueño	1	case_or_format
Calidad en salud	calidad en salud	calidad en salud	calidad en salud	Calidad en salud	1	kept
Califia perfusion simulator system	califia perfusion simulator system	califia perfusion simulator system	califia perfusion simulator system	Califia perfusion simulator system	1	kept
California hospitals	california hospitals	california hospitals	california hospitals	California hospitals	1	kept
California verbal learning test	california verbal learning test	california verbal learning test	california verbal learning test	California verbal learning test	1	kept
CALL	call	call	call	Call	1	case_or_format

CALL CENTERS	call centers	call centers	call center	call center	1	singular
call intake approach	call intake approach	call intake approach	call intake approach	call intake approach	1	kept
Calling	calling	calling	calling	calling	1	case_or_format
Calling criteria	calling criteria	calling criteria	calling criteria	Calling criteria	1	kept
calls for action	calls for action	calls for action	calls for action	calls for action	1	kept
Calm Technology	calm technology	calm technology	calm technology	Calm Technology	1	kept
Calm technology	calm technology	calm technology	calm technology	Calm Technology	1	case_or_format
calmness	calmness	calmness	calmness	calmness	1	kept
Caloric agents	caloric agents	caloric agents	caloric agents	Caloric agents	1	kept
Caloric intake	caloric intake	caloric intake	caloric intake	caloric intake	1	case_or_format
caloric requirements	caloric requirements	caloric requirements	caloric requirements	caloric requirements	1	kept
Calorie	calorie	calorie	calorie	calorie	1	case_or_format
calorimeter	calorimeter	calorimeter	calorimeter	calorimeter	1	kept
CALS	cals	cals	cals	CALS	1	kept
Calves	calves	calves	calves	Calves	1	kept
cam	cam	cam	cam	CAM	1	case_or_format

CAM-DEFINED DELIRIUM	cam defined delirium	cam defined delirium	cam defined delirium	CAM-DEFINED DELIRIUM	1	kept
CAM-S	cam s	cam s	cam s	CAM-S	1	kept
Cambridge	cambridge	cambridge	cambridge	Cambridge	1	kept
camellia oil	camellia oil	camellia oil	camellia oil	camellia oil	1	kept
Camera	camera	camera	camera	camera	1	case_or_format
Camera-based	camera based	camera based	camera based	Camera-based	1	kept
camera-derived estimates	camera derived estimates	camera derived estimates	camera derived estimates	camera-derived estimates	1	kept
Cameras	cameras	cameras	camera	camera	1	singular
Cameras monitoring	cameras monitoring	cameras monitoring	cameras monitoring	Cameras monitoring	1	kept
CAMPAIGN	campaign	campaign	campaign	Campaign	1	case_or_format
Campbell sleep questionnaire	campbell sleep questionnaire	campbell sleep questionnaire	campbell sleep questionnaire	Campbell sleep questionnaire	1	kept
Campylobacter enteritis	campylobacter enteritis	campylobacter enteritis	campylobacter enteritis	Campylobacter enteritis	1	kept
Campylobacter jejuni	campylobacter jejuni	campylobacter jejuni	campylobacter jejuni	Campylobacter jejuni	1	kept
CAMPYLOBACTER-JEJUNI INFECTIONS	campylobacter jejuni infections	campylobacter jejuni infections	campylobacter jejuni infections	CAMPYLOBACTER-JEJUNI INFECTIONS	1	kept
Cams	cams	cams	cam	CAM	1	singular
Can design	can design	can design	can design	Can design	1	kept

Can-cer	can cer	can cer	can cer	Can-cer	1	kept
CAN-IMPLEMENT©	can implement	can implement	can implement	CAN-IMPLEMENT©	1	kept
canagliflozin	canagliflozin	canagliflozin	canagliflozin	canagliflozin	1	kept
Cancellation	cancellation	cancellation	cancellation	Cancellation	1	kept
cancer associated fibroblast	cancer associated fibroblast	cancer associated fibroblast	cancer associated fibroblast	cancer associated fibroblast	1	kept
Cancer care	cancer care	cancer care	cancer care	CANCER CARE	1	case_or_format
cancer care	cancer care	cancer care	cancer care	CANCER CARE	1	case_or_format
cancer cell	cancer cell	cancer cell	cancer cell	cancer cell	1	kept
cancer classification	cancer classification	cancer classification	cancer classification	cancer classification	1	kept
Cancer communication	cancer communication	cancer communication	cancer communication	Cancer communication	1	kept
Cancer de la prostate	cancer de la prostate	cancer de la prostate	cancer de la prostate	Cancer de la prostate	1	kept
cancer drugs	cancer drugs	cancer drugs	cancer drugs	cancer drugs	1	kept
Cancer du poumon	cancer du poumon	cancer du poumon	cancer du poumon	Cancer du poumon	1	kept
cancer fatigue	cancer fatigue	cancer fatigue	cancer fatigue	cancer fatigue	1	kept
cancer grading	cancer grading	cancer grading	cancer grading	cancer grading	1	kept
cancer hormone therapy	cancer hormone therapy	cancer hormone therapy	cancer hormone therapy	cancer hormone therapy	1	kept

cancer of unknown primary site	cancer of unknown primary site	cancer of unknown primary site	cancer of unknown primary site	cancer of unknown primary site	1	kept
CANCER PAIN MANAGEMENT	cancer pain management	cancer pain management	cancer pain management	CANCER PAIN MANAGEMENT	1	kept
cancer regression	cancer regression	cancer regression	cancer regression	cancer regression	1	kept
cancer specific survival	cancer specific survival	cancer specific survival	cancer specific survival	cancer specific survival	1	kept
CANCER STATISTICS	cancer statistics	cancer statistics	cancer statistics	CANCER STATISTICS	1	kept
Cancer surgery	cancer surgery	cancer surgery	cancer surgery	cancer surgery	1	case_or_format
cancer survivor	cancer survivor	cancer survivor	cancer survivor	cancer survivor	1	kept
Cancer therapy	cancer therapy	cancer therapy	cancer therapy	cancer therapy	1	case_or_format
cancer treatments	cancer treatments	cancer treatments	cancer treatments	cancer treatments	1	kept
Cancer-directed therapy	cancer directed therapy	cancer directed therapy	cancer directed therapy	Cancer-directed therapy	1	kept
CANCER-RELATED FATIGUE	cancer related fatigue	cancer related fatigue	cancer related fatigue	CANCER-RELATED FATIGUE	1	kept
candesartan	candesartan	candesartan	candesartan	candesartan	1	kept
Candida auris admission screening health care facility	candida auris admission screening health care facility	candida auris admission screening health care facility	candida auris admission screening health care facility	Candida auris admission screening health care facility	1	kept
Candida hemulonii	candida hemulonii	candida hemulonii	candida hemulonii	Candida hemulonii	1	kept
candida infection	candida infection	candida infection	candida infection	candida infection	1	kept
Candida paraspilosis	candida paraspilosis	candida paraspilosis	candida paraspilosis	Candida paraspilosis	1	kept

Candida PCR	candida pcr	candida pcr	candida pcr	Candida PCR	1	kept
Candida prophylaxis	candida prophylaxis	candida prophylaxis	candida prophylaxis	Candida prophylaxis	1	kept
Candida spp. colonization	candida spp colonization	candida spp colonization	candida spp colonization	Candida spp. colonization	1	kept
Candida tropiana	candida tropiana	candida tropiana	candida tropiana	Candida tropiana	1	kept
CANDIDA-ALBICANS	candida albicans	candida albicans	candida albicans	Candida albicans	1	case_or_format
Candidaemia	candidaemia	candidaemia	candidaemia	Candidaemia	1	kept
Candidate evaluation, cardiac transplantation	candidate evaluation cardiac transplantation	candidate evaluation cardiac transplantation	candidate evaluation cardiac transplantation	Candidate evaluation, cardiac transplantation	1	kept
Candiduria	candiduria	candiduria	candiduria	Candiduria	1	kept
cane	cane	cane	cane	cane	1	kept
Canes	canes	canes	cane	cane	1	singular
Canidae	canidae	canidae	canidae	Canidae	1	kept
canine model	canine model	canine model	canine model	canine model	1	kept
Cannabidiol	cannabidiol	cannabidiol	cannabidiol	cannabidiol	1	case_or_format
cannabinoid receptor agonist	cannabinoid receptor agonist	cannabinoid receptor agonist	cannabinoid receptor agonist	Cannabinoid Receptor Agonists	1	case_or_format
cannabinoids	cannabinoids	cannabinoids	cannabinoid	cannabinoid	1	singular
cannot intubate cannot ventilate	cannot intubate cannot ventilate	cannot intubate cannot ventilate	cannot intubate cannot ventilate	cannot intubate cannot ventilate	1	kept

cannula dislodgement	cannula dislodgement	cannula dislodgement	cannula dislodgement	cannula dislodgement	1	kept
cannula obstruction	cannula obstruction	cannula obstruction	cannula obstruction	cannula obstruction	1	kept
CANNULA OXYGEN	cannula oxygen	cannula oxygen	cannula oxygen	CANNULA OXYGEN	1	kept
CANNULA OXYGEN-THERAPY	cannula oxygen therapy	cannula oxygen therapy	cannula oxygen therapy	CANNULA OXYGEN-THERAPY	1	kept
cannulating physician	cannulating physician	cannulating physician	cannulating physician	cannulating physician	1	kept
CANNULATION	cannulation	cannulation	cannulation	cannulation	1	case_or_format
Cannulation	cannulation	cannulation	cannulation	cannulation	1	case_or_format
canrenoate potassium	canrenoate potassium	canrenoate potassium	canrenoate potassium	canrenoate potassium	1	kept
CAPA	capa	capa	capa	CAPA	1	kept
capability well-being	capability well being	capability well being	capability well being	capability well-being	1	kept
Capacitación en servicio	capacitaci n en servicio	capacitaci n en servicio	capacitaci n en servicio	Capacitación en servicio	1	kept
Capacitive sensors	capacitive sensors	capacitive sensors	capacitive sensors	Capacitive sensors	1	kept
CAPACITY	capacity	capacity	capacity	Capacity	1	case_or_format
capacity	capacity	capacity	capacity	Capacity	1	case_or_format
capacity assessment	capacity assessment	capacity assessment	capacity assessment	capacity assessment	1	kept
Capacity building program	capacity building program	capacity building program	capacity building program	Capacity building program	1	kept

Capacity Development	capacity development	capacity development	capacity development	Capacity development	1	case_or_format
Capacity hospice	capacity hospice	capacity hospice	capacity hospice	Capacity hospice	1	kept
Capacity management	capacity management	capacity management	capacity management	Capacity management	1	kept
Capacity planning	capacity planning	capacity planning	capacity planning	Capacity planning	1	kept
capacity planning	capacity planning	capacity planning	capacity planning	Capacity planning	1	case_or_format
capacity-building programme	capacity building programme	capacity building programme	capacity building programme	capacity-building programme	1	kept
cape triage scoring	cape triage scoring	cape triage scoring	cape triage scoring	cape triage scoring	1	kept
Capecitabine	capecitabine	capecitabine	capecitabine	capecitabine	1	case_or_format
Capillary closing pressure	capillary closing pressure	capillary closing pressure	capillary closing pressure	Capillary closing pressure	1	kept
capillary density	capillary density	capillary density	capillary density	capillary density	1	kept
capillary filling time	capillary filling time	capillary filling time	capillary filling time	capillary filling time	1	kept
Capillary glucose testing	capillary glucose testing	capillary glucose testing	capillary glucose testing	Capillary glucose testing	1	kept
capillary heel stick	capillary heel stick	capillary heel stick	capillary heel stick	capillary heel stick	1	kept
CAPILLARY LEAK SYNDROME	capillary leak syndrome	capillary leak syndrome	capillary leak syndrome	capillary leak syndrome	1	case_or_format
Capillary Leak Syndrome	capillary leak syndrome	capillary leak syndrome	capillary leak syndrome	capillary leak syndrome	1	case_or_format
Capillary refill	capillary refill	capillary refill	capillary refill	Capillary refill	1	kept

capillary refill test	capillary refill test	capillary refill test	capillary refill test	capillary refill test	1	kept
capillary resistance	capillary resistance	capillary resistance	capillary resistance	capillary resistance	1	kept
capillary wall	capillary wall	capillary wall	capillary wall	capillary wall	1	kept
capital	capital	capital	capital	capital	1	kept
Capital Punishment	capital punishment	capital punishment	capital punishment	Capital Punishment	1	kept
Capitation Fee	capitation fee	capitation fee	capitation fee	Capitation Fee	1	kept
capitation fee	capitation fee	capitation fee	capitation fee	Capitation Fee	1	case_or_format
Capnocytophaga canimorsus	capnocytophaga canimorsus	capnocytophaga canimorsus	capnocytophaga canimorsus	Capnocytophaga canimorsus	1	kept
capnometer	capnometer	capnometer	capnometer	capnometer	1	kept
CAPS	caps	caps	caps	CAPS	1	kept
Capsule Endoscopy	capsule endoscopy	capsule endoscopy	capsule endoscopy	capsule endoscopy	1	case_or_format
Capsule endoscopy	capsule endoscopy	capsule endoscopy	capsule endoscopy	capsule endoscopy	1	case_or_format
capture ELISA	capture elisa	capture elisa	capture elisa	capture ELISA	1	kept
Car seats	car seats	car seats	car seats	Car seats	1	kept
CAR T cells	car t cells	car t cells	car t cells	CAR T cells	1	kept
CAR T-cell therapy	car t cell therapy	car t cell therapy	car t cell therapy	CAR T-cell therapy	1	kept

CAR T-cell-related encephalopathy syndrome	car t cell related encephalopathy syndrome	car t cell related encephalopathy syndrome	car t cell related encephalopathy syndrome	CAR T-cell-related encephalopathy syndrome	1	kept
Carative	carative	carative	carative	Carative	1	kept
Carbapenem	carbapenem	carbapenem	carbapenem	carbapenem	1	case_or_format
carbapenem resistant	carbapenem resistant	carbapenem resistant	carbapenem resistant	carbapenem resistant	1	kept
Carbapenem resistant Enterobacterales	carbapenem resistant enterobacterales	carbapenem resistant enterobacterales	carbapenem resistant enterobacterale	Carbapenem-Resistant Enterobacterales	1	singular
Carbapenem resistant Enterobacteriaceae	carbapenem resistant enterobacteriaceae	carbapenem resistant enterobacteriaceae	carbapenem resistant enterobacteriaceae	carbapenem-resistant Enterobacteriaceae	1	case_or_format
carbapenem resistant gram-negative bacteria	carbapenem resistant gram negative bacteria	carbapenem resistant gram negative bacteria	carbapenem resistant gram negative bacteria	carbapenem resistant gram-negative bacteria	1	kept
carbapenem resistant organism	carbapenem resistant organism	carbapenem resistant organism	carbapenem resistant organism	carbapenem resistant organism	1	kept
carbapenem-resistant	carbapenem resistant	carbapenem resistant	carbapenem resistant	carbapenem resistant	1	case_or_format
Carbapenem-resistant A	carbapenem resistant a	carbapenem resistant a	carbapenem resistant a	Carbapenem-resistant A	1	kept
Carbapenem-resistant Acinetobacter baumannii	carbapenem resistant acinetobacter baumannii	carbapenem resistant acinetobacter baumannii	carbapenem resistant acinetobacter baumannii	carbapenem resistant Acinetobacter baumannii	1	case_or_format
carbapenem-resistant Acinetobacter baumannii	carbapenem resistant acinetobacter baumannii	carbapenem resistant acinetobacter baumannii	carbapenem resistant acinetobacter baumannii	carbapenem resistant Acinetobacter baumannii	1	case_or_format
Carbapenem-resistant enterobacterale	carbapenem resistant enterobacterale	carbapenem resistant enterobacterale	carbapenem resistant enterobacterale	Carbapenem-Resistant Enterobacterales	1	case_or_format
Carbapenem-resistant Enterobacterales	carbapenem resistant enterobacterales	carbapenem resistant enterobacterales	carbapenem resistant enterobacterale	Carbapenem-Resistant Enterobacterales	1	singular
Carbapenem-resistant Enterobacteriaceae colonization	carbapenem resistant enterobacteriaceae colonization	carbapenem resistant enterobacteriaceae colonization	carbapenem resistant enterobacteriaceae colonization	Carbapenem-resistant Enterobacteriaceae colonization	1	kept
Carbapenem-resistant Klebsiella pneumonia	carbapenem resistant klebsiella pneumonia	carbapenem resistant klebsiella pneumonia	carbapenem resistant klebsiella pneumonia	Carbapenem-resistant Klebsiella pneumonia	1	kept

carbapenem-resistant Klebsiella pneumoniae	carbapenem resistant klebsiella pneumoniae	carbapenem resistant klebsiella pneumoniae	carbapenem resistant klebsiella pneumoniae	carbapenem resistant Klebsiella pneumoniae	1	case_or_format
carbapenem-resistant organisms	carbapenem resistant organisms	carbapenem resistant organisms	carbapenem resistant organism	carbapenem resistant organism	1	singular
Carbapenem-resistant P. aeruginosa	carbapenem resistant p aeruginosa	carbapenem resistant p aeruginosa	carbapenem resistant p aeruginosa	Carbapenem-resistant P. aeruginosa	1	kept
carbapenem-resistant Pseudomonas aeruginosa	carbapenem resistant pseudomonas aeruginosa	carbapenem resistant pseudomonas aeruginosa	carbapenem resistant pseudomonas aeruginosa	carbapenem resistant Pseudomonas aeruginosa	1	case_or_format
Carbapenem-sparing regimen	carbapenem sparing regimen	carbapenem sparing regimen	carbapenem sparing regimen	Carbapenem-sparing regimen	1	kept
carbapenemase OXA 48	carbapenemase oxa 48	carbapenemase oxa 48	carbapenemase oxa 48	carbapenemase OXA 48	1	kept
Carbapenemase producing enterobacterales	carbapenemase producing enterobacterales	carbapenemase producing enterobacterales	carbapenemase producing enterobacterale	Carbapenemase-producing Enterobacterales	1	singular
carbapenemase producing Escherichia coli	carbapenemase producing escherichia coli	carbapenemase producing escherichia coli	carbapenemase producing escherichia coli	carbapenemase producing Escherichia coli	1	kept
carbapenemase producing Klebsiella pneumoniae	carbapenemase producing klebsiella pneumoniae	carbapenemase producing klebsiella pneumoniae	carbapenemase producing klebsiella pneumoniae	carbapenemase producing Klebsiella pneumoniae	1	kept
carbapenemase VIM	carbapenemase vim	carbapenemase vim	carbapenemase vim	carbapenemase VIM	1	kept
Carbapenemase-producing enterobacterale	carbapenemase producing enterobacterale	carbapenemase producing enterobacterale	carbapenemase producing enterobacterale	Carbapenemase-producing Enterobacterales	1	case_or_format
Carbapenemase-Producing Enterobacterales	carbapenemase producing enterobacterales	carbapenemase producing enterobacterales	carbapenemase producing enterobacterale	Carbapenemase-producing Enterobacterales	1	singular
Carbapenemase-producing enterobacterales	carbapenemase producing enterobacterales	carbapenemase producing enterobacterales	carbapenemase producing enterobacterale	Carbapenemase-producing Enterobacterales	1	singular
Carbapenemases	carbapenemases	carbapenemases	carbapenemase	carbapenemase	1	singular
Carbapenms	carbapenms	carbapenms	carbapenms	Carbapenms	1	kept
carbenicillin	carbenicillin	carbenicillin	carbenicillin	carbenicillin	1	kept

carbetocin	carbetocin	carbetocin	carbetocin	carbetocin	1	kept
carbidopa	carbidopa	carbidopa	carbidopa	carbidopa	1	kept
carbohydrate antigen	carbohydrate antigen	carbohydrate antigen	carbohydrate antigen	carbohydrate antigen	1	kept
carbohydrate diet	carbohydrate diet	carbohydrate diet	carbohydrate diet	carbohydrate diet	1	kept
Carbohydrate supply	carbohydrate supply	carbohydrate supply	carbohydrate supply	Carbohydrate supply	1	kept
carbohydrate supply	carbohydrate supply	carbohydrate supply	carbohydrate supply	Carbohydrate supply	1	case_or_format
carbohydrate to sodium ratio	carbohydrate to sodium ratio	carbohydrate to sodium ratio	carbohydrate to sodium ratio	carbohydrate to sodium ratio	1	kept
carbon dioxide blood level	carbon dioxide blood level	carbon dioxide blood level	carbon dioxide blood level	carbon dioxide blood level	1	kept
carbon dioxide emission	carbon dioxide emission	carbon dioxide emission	carbon dioxide emission	carbon dioxide emission	1	kept
carbon dioxide excretion	carbon dioxide excretion	carbon dioxide excretion	carbon dioxide excretion	carbon dioxide excretion	1	kept
carboprost	carboprost	carboprost	carboprost	carboprost	1	kept
carboxyhemoglobin	carboxyhemoglobin	carboxyhemoglobin	carboxyhemoglobin	carboxyhemoglobin	1	kept
Carboxylation	carboxylation	carboxylation	carboxylation	Carboxylation	1	kept
Carboxymethylcellulose	carboxymethylcellulose	carboxymethylcellulose	carboxymethylcellulose	carboxymethylcellulose	1	case_or_format
carboxypeptidase G2	carboxypeptidase g2	carboxypeptidase g2	carboxypeptidase g2	carboxypeptidase G2	1	kept
carboxypeptidase transpeptidase	carboxypeptidase transpeptidase	carboxypeptidase transpeptidase	carboxypeptidase transpeptidase	carboxypeptidase transpeptidase	1	kept

carcinogen	carcinogen	carcinogen	carcinogen	carcinogen	1	kept
Carcinoma	carcinoma	carcinoma	carcinoma	Carcinoma	1	kept
carcinoma	carcinoma	carcinoma	carcinoma	Carcinoma	1	case_or_format
Carcinoma, Medullary	carcinoma medullary	carcinoma medullary	carcinoma medullary	Carcinoma, Medullary	1	kept
Carcinoma, Ovarian Epithelial	carcinoma ovarian epithelial	carcinoma ovarian epithelial	carcinoma ovarian epithelial	Carcinoma, Ovarian Epithelial	1	kept
cardiac action potential generation	cardiac action potential generation	cardiac action potential generation	cardiac action potential generation	cardiac action potential generation	1	kept
cardiac advanced life support	cardiac advanced life support	cardiac advanced life support	cardiac advanced life support	cardiac advanced life support	1	kept
cardiac allograft vasculopathy	cardiac allograft vasculopathy	cardiac allograft vasculopathy	cardiac allograft vasculopathy	cardiac allograft vasculopathy	1	kept
cardiac anatomy	cardiac anatomy	cardiac anatomy	cardiac anatomy	cardiac anatomy	1	kept
cardiac arrest and brain injuries	cardiac arrest and brain injuries	cardiac arrest and brain injuries	cardiac arrest and brain injuries	cardiac arrest and brain injuries	1	kept
Cardiac arrest centre	cardiac arrest centre	cardiac arrest centre	cardiac arrest centre	Cardiac arrest centre	1	kept
Cardiac Arrest Team	cardiac arrest team	cardiac arrest team	cardiac arrest team	cardiac arrest team	1	case_or_format
cardiac arrests	cardiac arrests	cardiac arrests	cardiac arrest	Cardiac arrest	1	singular
Cardiac arrhythmias	cardiac arrhythmias	cardiac arrhythmias	cardiac arrhythmia	Cardiac arrhythmia	1	singular
Cardiac artery bypass graft	cardiac artery bypass graft	cardiac artery bypass graft	cardiac artery bypass graft	Cardiac artery bypass graft	1	kept
Cardiac assessment	cardiac assessment	cardiac assessment	cardiac assessment	Cardiac assessment	1	kept

Cardiac assist	cardiac assist	cardiac assist	cardiac assist	Cardiac assist	1	kept
cardiac beriberi	cardiac beriberi	cardiac beriberi	cardiac beriberi	cardiac beriberi	1	kept
Cardiac care facilities	cardiac care facilities	cardiac care facilities	cardiac care facilities	Cardiac Care Facilities	1	case_or_format
Cardiac care unit	cardiac care unit	cardiac care unit	cardiac care unit	cardiac care unit	1	case_or_format
Cardiac catheterisation	cardiac catheterisation	cardiac catheterisation	cardiac catheterisation	Cardiac catheterisation	1	kept
Cardiac catheterisation. (JPMA 75: 70	cardiac catheterisation jpma 75 70	cardiac catheterisation jpma 75 70	cardiac catheterisation jpma 75 70	Cardiac catheterisation. (JPMA 75: 70	1	kept
Cardiac catheterization	cardiac catheterization	cardiac catheterization	cardiac catheterization	Cardiac Catheterization	1	case_or_format
cardiac catheterization laboratory	cardiac catheterization laboratory	cardiac catheterization laboratory	cardiac catheterization laboratory	Cardiac catheterization laboratory	1	case_or_format
Cardiac conduction system	cardiac conduction system	cardiac conduction system	cardiac conduction system	Cardiac conduction system	1	kept
Cardiac Conduction System Disease	cardiac conduction system disease	cardiac conduction system disease	cardiac conduction system disease	Cardiac Conduction System Disease	1	kept
cardiac death organ donation program	cardiac death organ donation program	cardiac death organ donation program	cardiac death organ donation program	cardiac death organ donation program	1	kept
cardiac disease	cardiac disease	cardiac disease	cardiac disease	Cardiac disease	1	case_or_format
Cardiac disorders	cardiac disorders	cardiac disorders	cardiac disorders	Cardiac disorders	1	kept
CARDIAC DYSFUNCTION	cardiac dysfunction	cardiac dysfunction	cardiac dysfunction	CARDIAC DYSFUNCTION	1	kept
Cardiac dysrhythmias	cardiac dysrhythmias	cardiac dysrhythmias	cardiac dysrhythmias	Cardiac dysrhythmias	1	kept
cardiac dysrhythmias	cardiac dysrhythmias	cardiac dysrhythmias	cardiac dysrhythmias	Cardiac dysrhythmias	1	case_or_format

Cardiac Electrophysiology	cardiac electrophysiology	cardiac electrophysiology	cardiac electrophysiology	Cardiac Electrophysiology	1	kept
Cardiac electrophysiology	cardiac electrophysiology	cardiac electrophysiology	cardiac electrophysiology	Cardiac Electrophysiology	1	case_or_format
cardiac electrophysiology	cardiac electrophysiology	cardiac electrophysiology	cardiac electrophysiology	Cardiac Electrophysiology	1	case_or_format
cardiac emergencies	cardiac emergencies	cardiac emergencies	cardiac emergencies	cardiac emergencies	1	kept
cardiac fluid responsiveness	cardiac fluid responsiveness	cardiac fluid responsiveness	cardiac fluid responsiveness	cardiac fluid responsiveness	1	kept
Cardiac function	cardiac function	cardiac function	cardiac function	Cardiac function	1	kept
cardiac function	cardiac function	cardiac function	cardiac function	Cardiac function	1	case_or_format
cardiac gated imaging	cardiac gated imaging	cardiac gated imaging	cardiac gated imaging	cardiac gated imaging	1	kept
Cardiac genetics	cardiac genetics	cardiac genetics	cardiac genetics	Cardiac genetics	1	kept
cardiac genetics	cardiac genetics	cardiac genetics	cardiac genetics	Cardiac genetics	1	case_or_format
cardiac graft rejection	cardiac graft rejection	cardiac graft rejection	cardiac graft rejection	cardiac graft rejection	1	kept
Cardiac imaging	cardiac imaging	cardiac imaging	cardiac imaging	Cardiac imaging	1	kept
cardiac imaging	cardiac imaging	cardiac imaging	cardiac imaging	Cardiac imaging	1	case_or_format
Cardiac implanted electronic device extraction	cardiac implanted electronic device extraction	cardiac implanted electronic device extraction	cardiac implanted electronic device extraction	Cardiac implanted electronic device extraction	1	kept
Cardiac implanted electronic device system infection	cardiac implanted electronic device system infection	cardiac implanted electronic device system infection	cardiac implanted electronic device system infection	Cardiac implanted electronic device system infection	1	kept
Cardiac impulse	cardiac impulse	cardiac impulse	cardiac impulse	Cardiac impulse	1	kept

Cardiac markers	cardiac markers	cardiac markers	cardiac markers	Cardiac markers	1	kept
Cardiac monitors	cardiac monitors	cardiac monitors	cardiac monitors	Cardiac monitors	1	kept
cardiac monitors	cardiac monitors	cardiac monitors	cardiac monitors	Cardiac monitors	1	case_or_format
cardiac muscle	cardiac muscle	cardiac muscle	cardiac muscle	cardiac muscle	1	kept
Cardiac neurodevelopment	cardiac neurodevelopment	cardiac neurodevelopment	cardiac neurodevelopment	Cardiac neurodevelopment	1	kept
cardiac neurodevelopment	cardiac neurodevelopment	cardiac neurodevelopment	cardiac neurodevelopment	Cardiac neurodevelopment	1	case_or_format
cardiac output	cardiac output	cardiac output	cardiac output	Cardiac Output	1	case_or_format
Cardiac output optimization	cardiac output optimization	cardiac output optimization	cardiac output optimization	Cardiac output optimization	1	kept
cardiac rehabilitation	cardiac rehabilitation	cardiac rehabilitation	cardiac rehabilitation	Cardiac Rehabilitation	1	case_or_format
Cardiac research	cardiac research	cardiac research	cardiac research	Cardiac research	1	kept
cardiac resternotomy	cardiac resternotomy	cardiac resternotomy	cardiac resternotomy	cardiac resternotomy	1	kept
cardiac resuscitation	cardiac resuscitation	cardiac resuscitation	cardiac resuscitation	cardiac resuscitation	1	kept
cardiac resuscitator	cardiac resuscitator	cardiac resuscitator	cardiac resuscitator	cardiac resuscitator	1	kept
cardiac resynchronization therapy device	cardiac resynchronization therapy device	cardiac resynchronization therapy device	cardiac resynchronization therapy device	Cardiac Resynchronization Therapy Devices	1	case_or_format
cardiac revascularization	cardiac revascularization	cardiac revascularization	cardiac revascularization	cardiac revascularization	1	kept
Cardiac rhythm	cardiac rhythm	cardiac rhythm	cardiac rhythm	Cardiac rhythm	1	kept

Cardiac rhythm interpretation	cardiac rhythm interpretation	cardiac rhythm interpretation	cardiac rhythm interpretation	Cardiac rhythm interpretation	1	kept
Cardiac services	cardiac services	cardiac services	cardiac services	Cardiac services	1	kept
cardiac support	cardiac support	cardiac support	cardiac support	cardiac support	1	kept
cardiac surgery associated acute kidney injury	cardiac surgery associated acute kidney injury	cardiac surgery associated acute kidney injury	cardiac surgery associated acute kidney injury	cardiac surgery associated acute kidney injury	1	kept
Cardiac surgery simulation	cardiac surgery simulation	cardiac surgery simulation	cardiac surgery simulation	Cardiac surgery simulation	1	kept
cardiac tamponade	cardiac tamponade	cardiac tamponade	cardiac tamponade	Cardiac Tamponade	1	case_or_format
cardiac telemetry	cardiac telemetry	cardiac telemetry	cardiac telemetry	cardiac telemetry	1	kept
cardiac telemetry alarms	cardiac telemetry alarms	cardiac telemetry alarms	cardiac telemetry alarms	cardiac telemetry alarms	1	kept
cardiac transplant	cardiac transplant	cardiac transplant	cardiac transplant	cardiac transplant	1	kept
Cardiac transplantation	cardiac transplantation	cardiac transplantation	cardiac transplantation	Cardiac transplantation	1	kept
Cardiac transplants	cardiac transplants	cardiac transplants	cardiac transplant	cardiac transplant	1	singular
CARDIAC TROPONIN	cardiac troponin	cardiac troponin	cardiac troponin	CARDIAC TROPONIN	1	kept
cardiac troponin I and T	cardiac troponin i and t	cardiac troponin i and t	cardiac troponin i and t	cardiac troponin I and T	1	kept
cardiac ultrasound	cardiac ultrasound	cardiac ultrasound	cardiac ultrasound	cardiac ultrasound	1	kept
cardiac valvular surgery	cardiac valvular surgery	cardiac valvular surgery	cardiac valvular surgery	cardiac valvular surgery	1	kept
CARDIAC-OUTPUT	cardiac output	cardiac output	cardiac output	Cardiac Output	1	case_or_format

cardio gastroenterology unit	cardio gastroenterology unit	cardio gastroenterology unit	cardio gastroenterology unit	cardio gastroenterology unit	1	kept
Cardio-embolism	cardio embolism	cardio embolism	cardio embolism	Cardio-embolism	1	kept
cardio-oncology	cardio oncology	cardio oncology	cardio oncology	Cardio-oncology	1	case_or_format
Cardio-pulmonary resuscitation	cardio pulmonary resuscitation	cardio pulmonary resuscitation	cardio pulmonary resuscitation	Cardio-pulmonary resuscitation	1	kept
Cardio-surgery	cardio surgery	cardio surgery	cardio surgery	Cardio-surgery	1	kept
Cardio-thoracic unit	cardio thoracic unit	cardio thoracic unit	cardio thoracic unit	Cardio-thoracic unit	1	kept
Cardio-vascular disease	cardio vascular disease	cardio vascular disease	cardio vascular disease	Cardio-vascular disease	1	kept
cardiocirculatory determination of death	cardiocirculatory determination of death	cardiocirculatory determination of death	cardiocirculatory determination of death	cardiocirculatory determination of death	1	kept
cardioembolic stroke	cardioembolic stroke	cardioembolic stroke	cardioembolic stroke	cardioembolic stroke	1	kept
cardioesophagomyotomy	cardioesophagomyotomy	cardioesophagomyotomy	cardioesophagomyotomy	cardioesophagomyotomy	1	kept
Cardiofaciocutaneous syndrome	cardiofaciocutaneous syndrome	cardiofaciocutaneous syndrome	cardiofaciocutaneous syndrome	Cardiofaciocutaneous syndrome	1	kept
cardiofaciocutaneous syndrome	cardiofaciocutaneous syndrome	cardiofaciocutaneous syndrome	cardiofaciocutaneous syndrome	Cardiofaciocutaneous syndrome	1	case_or_format
CARDIOGENIC PULMONARY-EDEMA	cardiogenic pulmonary edema	cardiogenic pulmonary edema	cardiogenic pulmonary edema	cardiogenic pulmonary edema	1	case_or_format
Cardiogenic shock Extracorporeal Membrane Oxygenation	cardiogenic shock extracorporeal membrane oxygenation	cardiogenic shock extracorporeal membrane oxygenation	cardiogenic shock extracorporeal membrane oxygenation	Cardiogenic shock Extracorporeal Membrane Oxygenation	1	kept
Cardiography	cardiography	cardiography	cardiography	Cardiography	1	kept
cardiolipin	cardiolipin	cardiolipin	cardiolipin	cardiolipin	1	kept

cardiologie	cardiologie	cardiologie	cardiologie	cardiologie	1	kept
Cardiologist	cardiologist	cardiologist	cardiologist	cardiologist	1	case_or_format
CARDIOLOGY	cardiology	cardiology	cardiology	Cardiology	1	case_or_format
cardiology fellow in training	cardiology fellow in training	cardiology fellow in training	cardiology fellow in training	cardiology fellow in training	1	kept
CardioMEMS™ HF-System	cardiomems hf system	cardiomems hf system	cardiomems hf system	CardioMEMS™ HF-System	1	kept
Cardionephrology	cardionephrology	cardionephrology	cardionephrology	Cardionephrology	1	kept
Cardiopatía congénita	cardiopat a cong nita	cardiopat a cong nita	cardiopat a cong nita	Cardiopatía congénita	1	kept
Cardiopul monary resuscitation	cardiopul monary resuscitation	cardiopul monary resuscitation	cardiopul monary resuscitation	Cardiopul monary resuscitation	1	kept
CARDIOPULMONARY	cardiopulmonary	cardiopulmonary	cardiopulmonary	Cardiopulmonary	1	case_or_format
Cardiopulmonary Arrest	cardiopulmonary arrest	cardiopulmonary arrest	cardiopulmonary arrest	cardiopulmonary arrest	1	case_or_format
CARDIOPULMONARY ARRESTS	cardiopulmonary arrests	cardiopulmonary arrests	cardiopulmonary arrest	cardiopulmonary arrest	1	singular
CARDIOPULMONARY BYPASS	cardiopulmonary bypass	cardiopulmonary bypass	cardiopulmonary bypass	cardiopulmonary bypass	1	case_or_format
Cardiopulmonary bypass system	cardiopulmonary bypass system	cardiopulmonary bypass system	cardiopulmonary bypass system	Cardiopulmonary bypass system	1	kept
cardiopulmonary instability	cardiopulmonary instability	cardiopulmonary instability	cardiopulmonary instability	cardiopulmonary instability	1	kept
Cardiopulmonary reanimation	cardiopulmonary reanimation	cardiopulmonary reanimation	cardiopulmonary reanimation	Cardiopulmonary reanimation	1	kept
cardiopulmonary rehabilitation	cardiopulmonary rehabilitation	cardiopulmonary rehabilitation	cardiopulmonary rehabilitation	cardiopulmonary rehabilitation	1	kept

Cardiopulmonary Resuscitation [E02 365 647 110]	cardiopulmonary resuscitation e02 365 647 110	cardiopulmonary resuscitation e02 365 647 110	cardiopulmonary resuscitation e02 365 647 110	Cardiopulmonary Resuscitation [E02 365 647 110]	1	kept
Cardiopulmonary resuscitation CPR training	cardiopulmonary resuscitation cpr training	cardiopulmonary resuscitation cpr training	cardiopulmonary resuscitation cpr training	Cardiopulmonary resuscitation CPR training	1	kept
Cardiopulmonary resuscitation decision making	cardiopulmonary resuscitation decision making	cardiopulmonary resuscitation decision making	cardiopulmonary resuscitation decision making	Cardiopulmonary resuscitation decision making	1	kept
Cardiopulmonary resuscitation priorities	cardiopulmonary resuscitation priorities	cardiopulmonary resuscitation priorities	cardiopulmonary resuscitation priorities	Cardiopulmonary resuscitation priorities	1	kept
Cardiopulmonary support	cardiopulmonary support	cardiopulmonary support	cardiopulmonary support	Cardiopulmonary support	1	kept
cardiopulmonary transplantation	cardiopulmonary transplantation	cardiopulmonary transplantation	cardiopulmonary transplantation	cardiopulmonary transplantation	1	kept
CARDIOPULMONARY VARIABLES	cardiopulmonary variables	cardiopulmonary variables	cardiopulmonary variables	CARDIOPULMONARY VARIABLES	1	kept
Cardiopulmonay resuscitation	cardiopulmonay resuscitation	cardiopulmonay resuscitation	cardiopulmonay resuscitation	Cardiopulmonay resuscitation	1	kept
cardiorenal axis	cardiorenal axis	cardiorenal axis	cardiorenal axis	cardiorenal axis	1	kept
Cardiorenal interactions	cardiorenal interactions	cardiorenal interactions	cardiorenal interactions	Cardiorenal interactions	1	kept
cardiorenal syndrome type 1	cardiorenal syndrome type 1	cardiorenal syndrome type 1	cardiorenal syndrome type 1	cardiorenal syndrome type 1	1	kept
cardiorenal syndrome type 2	cardiorenal syndrome type 2	cardiorenal syndrome type 2	cardiorenal syndrome type 2	cardiorenal syndrome type 2	1	kept
cardiorenal syndrome type 3	cardiorenal syndrome type 3	cardiorenal syndrome type 3	cardiorenal syndrome type 3	cardiorenal syndrome type 3	1	kept
cardiorenal syndrome type 4	cardiorenal syndrome type 4	cardiorenal syndrome type 4	cardiorenal syndrome type 4	cardiorenal syndrome type 4	1	kept
cardiorenal syndrome type 5	cardiorenal syndrome type 5	cardiorenal syndrome type 5	cardiorenal syndrome type 5	cardiorenal syndrome type 5	1	kept
Cardiorenal unit	cardiorenal unit	cardiorenal unit	cardiorenal unit	Cardiorenal unit	1	kept

Cardiorespiratory instability	cardiorespiratory instability	cardiorespiratory instability	cardiorespiratory instability	Cardiorespiratory instability	1	kept
Cardiorespiratory physiotherapy	cardiorespiratory physiotherapy	cardiorespiratory physiotherapy	cardiorespiratory physiotherapy	Cardiorespiratory physiotherapy	1	kept
cardiorespiratory physiotherapy	cardiorespiratory physiotherapy	cardiorespiratory physiotherapy	cardiorespiratory physiotherapy	Cardiorespiratory physiotherapy	1	case_or_format
cardioscopy	cardioscopy	cardioscopy	cardioscopy	cardioscopy	1	kept
Cardiothoracic	cardiothoracic	cardiothoracic	cardiothoracic	cardiothoracic	1	case_or_format
cardiothoracic and vascular surgery	cardiothoracic and vascular surgery	cardiothoracic and vascular surgery	cardiothoracic and vascular surgery	cardiothoracic and vascular surgery	1	kept
Cardiothoracic Surgery	cardiothoracic surgery	cardiothoracic surgery	cardiothoracic surgery	Cardiothoracic surgery	1	case_or_format
Cardiothoracic training	cardiothoracic training	cardiothoracic training	cardiothoracic training	Cardiothoracic training	1	kept
cardiothoracic training	cardiothoracic training	cardiothoracic training	cardiothoracic training	Cardiothoracic training	1	case_or_format
Cardiothoracics	cardiothoracics	cardiothoracics	cardiothoracic	cardiothoracic	1	singular
cardiotocogram	cardiotocogram	cardiotocogram	cardiotocogram	cardiotocogram	1	kept
Cardiotoxins	cardiotoxins	cardiotoxins	cardiotoxins	Cardiotoxins	1	kept
Cardiovascular Abnormalities	cardiovascular abnormalities	cardiovascular abnormalities	cardiovascular abnormalities	Cardiovascular Abnormalities	1	kept
cardiovascular agents	cardiovascular agents	cardiovascular agents	cardiovascular agent	cardiovascular agent	1	singular
cardiovascular assessment	cardiovascular assessment	cardiovascular assessment	cardiovascular assessment	cardiovascular assessment	1	kept
Cardiovascular care	cardiovascular care	cardiovascular care	cardiovascular care	Cardiovascular care	1	kept

cardiovascular care and outcomes	cardiovascular care and outcomes	cardiovascular care and outcomes	cardiovascular care and outcomes	cardiovascular care and outcomes	1	kept
cardiovascular care coordination	cardiovascular care coordination	cardiovascular care coordination	cardiovascular care coordination	cardiovascular care coordination	1	kept
CARDIOVASCULAR CARE SCIENCE	cardiovascular care science	cardiovascular care science	cardiovascular care science	CARDIOVASCULAR CARE SCIENCE	1	kept
cardiovascular changes in pregnancy	cardiovascular changes in pregnancy	cardiovascular changes in pregnancy	cardiovascular changes in pregnancy	cardiovascular changes in pregnancy	1	kept
Cardiovascular complications	cardiovascular complications	cardiovascular complications	cardiovascular complications	Cardiovascular complications	1	kept
CARDIOVASCULAR COMPUTED-TOMOGRAPHY	cardiovascular computed tomography	cardiovascular computed tomography	cardiovascular computed tomography	CARDIOVASCULAR COMPUTED-TOMOGRAPHY	1	kept
cardiovascular drugs	cardiovascular drugs	cardiovascular drugs	cardiovascular drugs	cardiovascular drugs	1	kept
cardiovascular effect	cardiovascular effect	cardiovascular effect	cardiovascular effect	cardiovascular effect	1	kept
cardiovascular emergencies	cardiovascular emergencies	cardiovascular emergencies	cardiovascular emergencies	cardiovascular emergencies	1	kept
CARDIOVASCULAR EVENTS	cardiovascular events	cardiovascular events	cardiovascular events	CARDIOVASCULAR EVENTS	1	kept
Cardiovascular function	cardiovascular function	cardiovascular function	cardiovascular function	cardiovascular function	1	case_or_format
Cardiovascular Health Study Frailty Index criteria	cardiovascular health study frailty index criteria	cardiovascular health study frailty index criteria	cardiovascular health study frailty index criteria	Cardiovascular Health Study Frailty Index criteria	1	kept
cardiovascular hospital	cardiovascular hospital	cardiovascular hospital	cardiovascular hospital	cardiovascular hospital	1	kept
cardiovascular infection	cardiovascular infection	cardiovascular infection	cardiovascular infection	cardiovascular infection	1	kept
cardiovascular instability	cardiovascular instability	cardiovascular instability	cardiovascular instability	cardiovascular instability	1	kept
cardiovascular life support	cardiovascular life support	cardiovascular life support	cardiovascular life support	cardiovascular life support	1	kept

cardiovascular metabolism	cardiovascular metabolism	cardiovascular metabolism	cardiovascular metabolism	cardiovascular metabolism	1	kept
cardiovascular monitoring device	cardiovascular monitoring device	cardiovascular monitoring device	cardiovascular monitoring device	cardiovascular monitoring device	1	kept
cardiovascular obstetrics	cardiovascular obstetrics	cardiovascular obstetrics	cardiovascular obstetrics	cardiovascular obstetrics	1	kept
cardiovascular physiological phenomena	cardiovascular physiological phenomena	cardiovascular physiological phenomena	cardiovascular physiological phenomena	Cardiovascular Physiological Phenomena	1	case_or_format
Cardiovascular Physiology	cardiovascular physiology	cardiovascular physiology	cardiovascular physiology	Cardiovascular Physiology	1	kept
cardiovascular pregnancy complication	cardiovascular pregnancy complication	cardiovascular pregnancy complication	cardiovascular pregnancy complication	cardiovascular pregnancy complication	1	kept
cardiovascular procedures	cardiovascular procedures	cardiovascular procedures	cardiovascular procedure	cardiovascular procedure	1	singular
cardiovascular response	cardiovascular response	cardiovascular response	cardiovascular response	cardiovascular response	1	kept
Cardiovascular specialty	cardiovascular specialty	cardiovascular specialty	cardiovascular specialty	Cardiovascular specialty	1	kept
Cardiovascular support	cardiovascular support	cardiovascular support	cardiovascular support	Cardiovascular support	1	kept
Cardiovascular Surgery	cardiovascular surgery	cardiovascular surgery	cardiovascular surgery	cardiovascular surgery	1	case_or_format
cardiovascular surgery training	cardiovascular surgery training	cardiovascular surgery training	cardiovascular surgery training	cardiovascular surgery training	1	kept
cardiovascular surgical equipment	cardiovascular surgical equipment	cardiovascular surgical equipment	cardiovascular surgical equipment	cardiovascular surgical equipment	1	kept
Cardiovascular surgical procedures	cardiovascular surgical procedures	cardiovascular surgical procedures	cardiovascular surgical procedures	Cardiovascular Surgical Procedures	1	case_or_format
cardiovascular surgical procedures	cardiovascular surgical procedures	cardiovascular surgical procedures	cardiovascular surgical procedures	Cardiovascular Surgical Procedures	1	case_or_format
cardiovascular symptom	cardiovascular symptom	cardiovascular symptom	cardiovascular symptom	cardiovascular symptom	1	kept

cardiovascular unit	cardiovascular unit	cardiovascular unit	cardiovascular unit	cardiovascular unit	1	kept
CARDIOVASCULAR-DISEASE	cardiovascular disease	cardiovascular disease	cardiovascular disease	cardiovascular disease	1	case_or_format
carditis	carditis	carditis	carditis	carditis	1	kept
Care activities	care activities	care activities	care activities	Care activities	1	kept
care activities	care activities	care activities	care activities	Care activities	1	case_or_format
Care and maintenance	care and maintenance	care and maintenance	care and maintenance	Care and maintenance	1	kept
Care assistants	care assistants	care assistants	care assistants	Care assistants	1	kept
Care at the end of life	care at the end of life	care at the end of life	care at the end of life	Care at the end of life	1	kept
CARE BUNDLE	care bundle	care bundle	care bundle	care bundle	1	case_or_format
CARE BUNDLES	care bundles	care bundles	care bundle	care bundle	1	singular
Care Bundles	care bundles	care bundles	care bundle	care bundle	1	singular
Care burden of primary caregivers	care burden of primary caregivers	care burden of primary caregivers	care burden of primary caregivers	Care burden of primary caregivers	1	kept
care clinicians	care clinicians	care clinicians	care clinicians	care clinicians	1	kept
Care complexity	care complexity	care complexity	care complexity	Care complexity	1	kept
care complexity	care complexity	care complexity	care complexity	Care complexity	1	case_or_format
Care continuity	care continuity	care continuity	care continuity	care continuity	1	case_or_format

Care coordination competency	care coordination competency	care coordination competency	care coordination competency	Care coordination competency	1	kept
Care Coordination Competency Scale	care coordination competency scale	care coordination competency scale	care coordination competency scale	Care Coordination Competency Scale	1	kept
Care coordinator	care coordinator	care coordinator	care coordinator	care coordinator	1	case_or_format
Care culture	care culture	care culture	care culture	Care culture	1	kept
CARE DELIVERY	care delivery	care delivery	care delivery	care delivery	1	case_or_format
Care delivery	care delivery	care delivery	care delivery	care delivery	1	case_or_format
Care demand	care demand	care demand	care demand	Care demand	1	kept
Care dependency	care dependency	care dependency	care dependency	care dependency	1	case_or_format
Care dependent	care dependent	care dependent	care dependent	Care dependent	1	kept
Care economics	care economics	care economics	care economics	Care economics	1	kept
care environment	care environment	care environment	care environment	care environment	1	kept
care erosion	care erosion	care erosion	care erosion	care erosion	1	kept
Care escalation barriers	care escalation barriers	care escalation barriers	care escalation barriers	Care escalation barriers	1	kept
care experiences	care experiences	care experiences	care experiences	care experiences	1	kept
care first fund later	care first fund later	care first fund later	care first fund later	care first fund later	1	kept
care for caregiver	care for caregiver	care for caregiver	care for caregiver	care for caregiver	1	kept

Care for the Caregiver	care for the caregiver	care for the caregiver	care for the caregiver	Care for the Caregiver	1	kept
Care for the caregiver	care for the caregiver	care for the caregiver	care for the caregiver	Care for the Caregiver	1	case_or_format
Care for the elderly	care for the elderly	care for the elderly	care for the elderly	Care for the elderly	1	kept
care goals	care goals	care goals	care goal	care goal	1	singular
care homes	care homes	care homes	care homes	Care homes	1	case_or_format
care in pediatric therapy units	care in pediatric therapy units	care in pediatric therapy units	care in pediatric therapy units	care in pediatric therapy units	1	kept
Care intensity	care intensity	care intensity	care intensity	Care intensity	1	kept
care intensity	care intensity	care intensity	care intensity	Care intensity	1	case_or_format
care management needs	care management needs	care management needs	care management needs	care management needs	1	kept
Care model	care model	care model	care model	care model	1	case_or_format
Care needs	care needs	care needs	care needs	Care needs	1	kept
care needs	care needs	care needs	care needs	Care needs	1	case_or_format
Care network design	care network design	care network design	care network design	Care network design	1	kept
care of quality	care of quality	care of quality	care of quality	care of quality	1	kept
Care of the suicidal client	care of the suicidal client	care of the suicidal client	care of the suicidal client	Care of the suicidal client	1	kept
care omission	care omission	care omission	care omission	care omission	1	kept

care organisation	care organisation	care organisation	care organisation	care organisation	1	kept
Care Outcome	care outcome	care outcome	care outcome	Care Outcome	1	kept
CARE OUTREACH TEAM	care outreach team	care outreach team	care outreach team	CARE OUTREACH TEAM	1	kept
care package	care package	care package	care package	Care package	1	case_or_format
Care pain observation tool	care pain observation tool	care pain observation tool	care pain observation tool	Care pain observation tool	1	kept
care pathway	care pathway	care pathway	care pathway	care pathways	1	case_or_format
care performance	care performance	care performance	care performance	care performance	1	kept
Care plan Continuous renal replacement [illegible]	care plan continuous renal replacement [illegible]	care plan continuous renal replacement [illegible]	care plan continuous renal replacement [illegible]	Care plan Continuous renal replacement [illegible]	1	kept
Care planning	care planning	care planning	care planning	Care planning	1	kept
care planning	care planning	care planning	care planning	Care planning	1	case_or_format
Care preferences	care preferences	care preferences	care preferences	Care preferences	1	kept
Care processes	care processes	care processes	care processes	Care processes	1	kept
care processes	care processes	care processes	care processes	Care processes	1	case_or_format
care protocol	care protocol	care protocol	care protocol	Care protocol	1	case_or_format
care provider	care provider	care provider	care provider	care provider	1	kept
CARE RECORDING-SYSTEM	care recording system	care recording system	care recording system	CARE RECORDING-SYSTEM	1	kept

Care relationships	care relationships	care relationships	care relationships	Care relationships	1	kept
CARE RESOURCE BURDEN	care resource burden	care resource burden	care resource burden	CARE RESOURCE BURDEN	1	kept
care safety	care safety	care safety	care safety	care safety	1	kept
Care satisfaction	care satisfaction	care satisfaction	care satisfaction	Care satisfaction	1	kept
care setting	care setting	care setting	care setting	Care settings	1	case_or_format
Care standard	care standard	care standard	care standard	Care standard	1	kept
care standards	care standards	care standards	care standard	Care standard	1	singular
care strategies	care strategies	care strategies	care strategies	care strategies	1	kept
Care supply	care supply	care supply	care supply	Care supply	1	kept
Care systems	care systems	care systems	care systems	Care systems	1	kept
Care team	care team	care team	care team	Care team	1	kept
care team perspectives	care team perspectives	care team perspectives	care team perspectives	care team perspectives	1	kept
care teams	care teams	care teams	care team	Care team	1	singular
Care transition and handoff	care transition and handoff	care transition and handoff	care transition and handoff	Care transition and handoff	1	kept
care transitions and handoffs	care transitions and handoffs	care transitions and handoffs	care transitions and handoffs	care transitions and handoffs	1	kept
care triad	care triad	care triad	care triad	care triad	1	kept

Care unit	care unit	care unit	care unit	CARE-UNIT	1	case_or_format
Care unit, intensive	care unit intensive	care unit intensive	care unit intensive	Care unit, intensive	1	kept
CARE-ASSOCIATED INFECTIONS	care associated infections	care associated infections	care associated infections	CARE-ASSOCIATED INFECTIONS	1	kept
Care-by-parent	care by parent	care by parent	care by parent	Care-by-parent	1	kept
Care-giving practices	care giving practices	care giving practices	care giving practices	Care-giving practices	1	kept
care-related pain	care related pain	care related pain	care related pain	care-related pain	1	kept
Career	career	career	career	career	1	case_or_format
Career advancement	career advancement	career advancement	career advancement	Career advancement	1	kept
Career Aspirations	career aspirations	career aspirations	career aspirations	Career Aspirations	1	kept
Career commitment	career commitment	career commitment	career commitment	Career commitment	1	kept
Career growth	career growth	career growth	career growth	Career growth	1	kept
career options	career options	career options	career options	career options	1	kept
Career preferences	career preferences	career preferences	career preferences	Career preferences	1	kept
career resilience	career resilience	career resilience	career resilience	career resilience	1	kept
CAREGIVER	caregiver	caregiver	caregiver	caregiver	1	case_or_format
Caregiver Burden Inventory	caregiver burden inventory	caregiver burden inventory	caregiver burden inventory	Caregiver Burden Inventory	1	kept

Caregiver engagement	caregiver engagement	caregiver engagement	caregiver engagement	Caregiver engagement	1	kept
caregiver functioning	caregiver functioning	caregiver functioning	caregiver functioning	caregiver functioning	1	kept
Caregiver help-seeking behavior	caregiver help seeking behavior	caregiver help seeking behavior	caregiver help seeking behavior	Caregiver help-seeking behavior	1	kept
Caregiver preparation	caregiver preparation	caregiver preparation	caregiver preparation	Caregiver preparation	1	kept
caregiver role strain	caregiver role strain	caregiver role strain	caregiver role strain	caregiver role strain	1	kept
caregiver satisfaction score	caregiver satisfaction score	caregiver satisfaction score	caregiver satisfaction score	caregiver satisfaction score	1	kept
caregiver services	caregiver services	caregiver services	caregiver services	caregiver services	1	kept
caregiver stress	caregiver stress	caregiver stress	caregiver stress	caregiver stress	1	kept
caregiver willingness	caregiver willingness	caregiver willingness	caregiver willingness	caregiver willingness	1	kept
Caregiver-infant interaction	caregiver infant interaction	caregiver infant interaction	caregiver infant interaction	Caregiver-infant interaction	1	kept
CAREGIVERS EXPERIENCES	caregivers experiences	caregivers experiences	caregivers experiences	CAREGIVERS EXPERIENCES	1	kept
Caregivers’ burden	caregivers burden	caregivers burden	caregivers burden	Caregivers’ burden	1	kept
Caregivers’ help-seeking behavior	caregivers help seeking behavior	caregivers help seeking behavior	caregivers help seeking behavior	Caregivers’ help-seeking behavior	1	kept
caregiving experience	caregiving experience	caregiving experience	caregiving experience	caregiving experience	1	kept
caribbean	caribbean	caribbean	caribbean	Caribbean	1	case_or_format
Caribbean region	caribbean region	caribbean region	caribbean region	Caribbean Region	1	case_or_format

Caring Ability	caring ability	caring ability	caring ability	Caring Ability	1	kept
Caring ability	caring ability	caring ability	caring ability	Caring Ability	1	case_or_format
Caring attributes	caring attributes	caring attributes	caring attributes	Caring attributes	1	kept
CARING BEHAVIOR	caring behavior	caring behavior	caring behavior	Caring behavior	1	case_or_format
Caring Behavior Assessment scale	caring behavior assessment scale	caring behavior assessment scale	caring behavior assessment scale	Caring Behavior Assessment scale	1	kept
Caring Behaviors	caring behaviors	caring behaviors	caring behavior	Caring behavior	1	singular
caring behaviors inventory 24	caring behaviors inventory 24	caring behaviors inventory 24	caring behaviors inventory 24	caring behaviors inventory 24	1	kept
Caring challenges	caring challenges	caring challenges	caring challenges	Caring challenges	1	kept
Caring ethics, ethical demand	caring ethics ethical demand	caring ethics ethical demand	caring ethics ethical demand	Caring ethics, ethical demand	1	kept
Caring Experience	caring experience	caring experience	caring experience	Caring Experience	1	kept
caring for parents of sick newborns	caring for parents of sick newborns	caring for parents of sick newborns	caring for parents of sick newborns	caring for parents of sick newborns	1	kept
caring healing environment	caring healing environment	caring healing environment	caring healing environment	caring healing environment	1	kept
Caring intervention	caring intervention	caring intervention	caring intervention	Caring intervention	1	kept
caring intervention	caring intervention	caring intervention	caring intervention	Caring intervention	1	case_or_format
caring performance	caring performance	caring performance	caring performance	caring performance	1	kept
Caring practice	caring practice	caring practice	caring practice	caring practice	1	case_or_format

Caring practices	caring practices	caring practices	caring practice	caring practice	1	singular
caring science	caring science	caring science	caring science	Caring science	1	case_or_format
caring touch	caring touch	caring touch	caring touch	caring touch	1	kept
caring values	caring values	caring values	caring values	caring values	1	kept
Caring, General units	caring general units	caring general units	caring general units	Caring, General units	1	kept
Caring-efficacy	caring efficacy	caring efficacy	caring efficacy	caring efficacy	1	case_or_format
Caritas Processes®	caritas processes	caritas processes	caritas processes	Caritas Processes®	1	kept
carmustine	carmustine	carmustine	carmustine	carmustine	1	kept
carotene	carotene	carotene	carotene	carotene	1	kept
Carotid	carotid	carotid	carotid	Carotid	1	kept
carotid artery aneurysm	carotid artery aneurysm	carotid artery aneurysm	carotid artery aneurysm	carotid artery aneurysm	1	kept
carotid artery endarterectomy	carotid artery endarterectomy	carotid artery endarterectomy	carotid artery endarterectomy	carotid artery endarterectomy	1	kept
Carotid Artery Injuries	carotid artery injuries	carotid artery injuries	carotid artery injury	carotid artery injury	1	singular
carotid artery injury	carotid artery injury	carotid artery injury	carotid artery injury	carotid artery injury	1	kept
carotid artery surgery	carotid artery surgery	carotid artery surgery	carotid artery surgery	carotid artery surgery	1	kept
Carotid Artery, Internal	carotid artery internal	carotid artery internal	carotid artery internal	Carotid Artery, Internal	1	kept

Carotid blowout	carotid blowout	carotid blowout	carotid blowout	Carotid blowout	1	kept
Carotid stenosis	carotid stenosis	carotid stenosis	carotid stenosis	Carotid Stenosis	1	case_or_format
Carotid stenting	carotid stenting	carotid stenting	carotid stenting	Carotid stenting	1	kept
carpal tunnel release	carpal tunnel release	carpal tunnel release	carpal tunnel release	carpal tunnel release	1	kept
carpenter	carpenter	carpenter	carpenter	carpenter	1	kept
Carriage	carriage	carriage	carriage	CARRIAGE	1	case_or_format
carriage	carriage	carriage	carriage	CARRIAGE	1	case_or_format
carriage system	carriage system	carriage system	carriage system	carriage system	1	kept
carrier protein	carrier protein	carrier protein	carrier protein	carrier protein	1	kept
Carrier Proteins	carrier proteins	carrier proteins	carrier protein	carrier protein	1	singular
carrier risk	carrier risk	carrier risk	carrier risk	carrier risk	1	kept
Carriers	carriers	carriers	carriers	Carriers	1	kept
carrot	carrot	carrot	carrot	carrot	1	kept
Carspecken	carspecken	carspecken	carspecken	Carspecken	1	kept
cartilage degeneration	cartilage degeneration	cartilage degeneration	cartilage degeneration	cartilage degeneration	1	kept
cartilage graft	cartilage graft	cartilage graft	cartilage graft	cartilage graft	1	kept

case approach	case approach	case approach	case approach	case approach	1	kept
case based learning	case based learning	case based learning	case based learning	Case-Based Learning	1	case_or_format
Case conferences	case conferences	case conferences	case conferences	Case conferences	1	kept
Case control	case control	case control	case control	Case control	1	kept
case control	case control	case control	case control	Case control	1	case_or_format
case fatality	case fatality	case fatality	case fatality	Case fatality	1	case_or_format
Case finding	case finding	case finding	case finding	Case finding	1	kept
case finding	case finding	case finding	case finding	Case finding	1	case_or_format
Case history	case history	case history	case history	Case history	1	kept
Case history of the basal stimulation	case history of the basal stimulation	case history of the basal stimulation	case history of the basal stimulation	Case history of the basal stimulation	1	kept
case identification	case identification	case identification	case identification	case identification	1	kept
Case method	case method	case method	case method	Case method	1	kept
case review	case review	case review	case review	case review	1	kept
Case scenario assessment	case scenario assessment	case scenario assessment	case scenario assessment	Case scenario assessment	1	kept
Case series	case series	case series	case series	case series	1	case_or_format
CASE STUDIES	case studies	case studies	case study	case study	1	singular

Case studies	case studies	case studies	case study	case study	1	singular
case studies	case studies	case studies	case study	case study	1	singular
case Study	case study	case study	case study	case study	1	case_or_format
case study research	case study research	case study research	case study research	case study research	1	kept
Case-based approach	case based approach	case based approach	case based approach	Case-based approach	1	kept
Case-based Learning	case based learning	case based learning	case based learning	Case-Based Learning	1	case_or_format
case-based learning	case based learning	case based learning	case based learning	Case-Based Learning	1	case_or_format
case-based rounds	case based rounds	case based rounds	case based rounds	case-based rounds	1	kept
Case-based teaching	case based teaching	case based teaching	case based teaching	Case-based teaching	1	kept
case-control	case control	case control	case control	Case control	1	case_or_format
Case-control studies	case control studies	case control studies	case control study	Case-Control Studies	1	singular
Case-control studyl	case control studyl	case control studyl	case control studyl	Case-control studyl	1	kept
Caseload	caseload	caseload	caseload	Caseload	1	kept
Caseload midwifery	caseload midwifery	caseload midwifery	caseload midwifery	Caseload midwifery	1	kept
case‐control	case control	case control	case control	Case control	1	case_or_format
Case‐control study	case control study	case control study	case control study	Case-Control Studies	1	case_or_format

cashew nut	cashew nut	cashew nut	cashew nut	cashew nut	1	kept
Casirivimab	casirivimab	casirivimab	casirivimab	casirivimab	1	case_or_format
caspase inhibitor	caspase inhibitor	caspase inhibitor	caspase inhibitor	caspase inhibitor	1	kept
Casts, Surgical	casts surgical	casts surgical	casts surgical	Casts, Surgical	1	kept
Cat scratch disease	cat scratch disease	cat scratch disease	cat scratch disease	Cat scratch disease	1	kept
CAT use	cat use	cat use	cat use	CAT use	1	kept
Cataract Disease Analysis	cataract disease analysis	cataract disease analysis	cataract disease analysis	Cataract Disease Analysis	1	kept
cataract extraction	cataract extraction	cataract extraction	cataract extraction	cataract extraction	1	kept
catastrophe	catastrophe	catastrophe	catastrophe	catastrophe	1	kept
catastrophic case management	catastrophic case management	catastrophic case management	catastrophic case management	catastrophic case management	1	kept
CATASTROPHIC ILLNESS	catastrophic illness	catastrophic illness	catastrophic illness	Catastrophic Illness	1	case_or_format
Catastrophization	catastrophization	catastrophization	catastrophization	Catastrophization	1	kept
catastrophizing	catastrophizing	catastrophizing	catastrophizing	catastrophizing	1	kept
catatonic schizophrenia	catatonic schizophrenia	catatonic schizophrenia	catatonic schizophrenia	catatonic schizophrenia	1	kept
catchment	catchment	catchment	catchment	catchment	1	kept
Catchment Area	catchment area	catchment area	catchment area	catchment area	1	case_or_format

Catchment area	catchment area	catchment area	catchment area	catchment area	1	case_or_format
Catecholamine	catecholamine	catecholamine	catecholamine	catecholamine	1	case_or_format
catecholamine blood level	catecholamine blood level	catecholamine blood level	catecholamine blood level	catecholamine blood level	1	kept
catecholamine release	catecholamine release	catecholamine release	catecholamine release	catecholamine release	1	kept
Categories of care	categories of care	categories of care	categories of care	Categories of care	1	kept
categories of care	categories of care	categories of care	categories of care	Categories of care	1	case_or_format
categories of complexity	categories of complexity	categories of complexity	categories of complexity	categories of complexity	1	kept
categories of healthcare professionals	categories of healthcare professionals	categories of healthcare professionals	categories of healthcare professionals	categories of healthcare professionals	1	kept
category	category	category	category	category	1	kept
category boosting	category boosting	category boosting	category boosting	category boosting	1	kept
Cateteres Venosos Centrais	cateteres venosos centrais	cateteres venosos centrais	cateteres venosos centrais	Cateteres Venosos Centrais	1	kept
Cateterismo periférico	cateterismo perif rico	cateterismo perif rico	cateterismo perif rico	Cateterismo Periférico	1	case_or_format
Cateterismo venoso periférico	cateterismo venoso perif rico	cateterismo venoso perif rico	cateterismo venoso perif rico	Cateterismo venoso periférico	1	kept
Cath lab	cath lab	cath lab	cath lab	Cath lab	1	kept
Catherer-associated urinary tract infection	catherer associated urinary tract infection	catherer associated urinary tract infection	catherer associated urinary tract infection	Catherer-associated urinary tract infection	1	kept
Catheter ablation	catheter ablation	catheter ablation	catheter ablation	Catheter Ablation	1	case_or_format

Catheter anchor	catheter anchor	catheter anchor	catheter anchor	Catheter anchor	1	kept
catheter associated bloodstream infection	catheter associated bloodstream infection	catheter associated bloodstream infection	catheter associated bloodstream infection	catheter associated bloodstream infection	1	kept
catheter associated meatal pressure injury	catheter associated meatal pressure injury	catheter associated meatal pressure injury	catheter associated meatal pressure injury	catheter associated meatal pressure injury	1	kept
Catheter Associated Urinary Tract Infection	catheter associated urinary tract infection	catheter associated urinary tract infection	catheter associated urinary tract infection	catheter associated urinary tract infection	1	case_or_format
Catheter associated urinary tract infections	catheter associated urinary tract infections	catheter associated urinary tract infections	catheter associated urinary tract infection	catheter associated urinary tract infection	1	singular
catheter cleaning	catheter cleaning	catheter cleaning	catheter cleaning	catheter cleaning	1	kept
catheter clearance	catheter clearance	catheter clearance	catheter clearance	catheter clearance	1	kept
catheter colonisation	catheter colonisation	catheter colonisation	catheter colonisation	catheter colonisation	1	kept
Catheter colonization	catheter colonization	catheter colonization	catheter colonization	Catheter colonization	1	kept
Catheter complications	catheter complications	catheter complications	catheter complication	catheter complication	1	singular
catheter complications	catheter complications	catheter complications	catheter complication	catheter complication	1	singular
catheter dialysate leakage	catheter dialysate leakage	catheter dialysate leakage	catheter dialysate leakage	catheter dialysate leakage	1	kept
catheter directed thrombolysis	catheter directed thrombolysis	catheter directed thrombolysis	catheter directed thrombolysis	catheter directed thrombolysis	1	kept
catheter dislocation	catheter dislocation	catheter dislocation	catheter dislocation	catheter dislocation	1	kept
catheter dislodgment	catheter dislodgment	catheter dislodgment	catheter dislodgment	catheter dislodgment	1	kept
catheter displacement	catheter displacement	catheter displacement	catheter displacement	catheter displacement	1	kept

catheter dressing	catheter dressing	catheter dressing	catheter dressing	catheter dressing	1	kept
catheter dwell time	catheter dwell time	catheter dwell time	catheter dwell time	catheter dwell time	1	kept
Catheter dysfunction	catheter dysfunction	catheter dysfunction	catheter dysfunction	catheter dysfunction	1	case_or_format
catheter exchange	catheter exchange	catheter exchange	catheter exchange	catheter exchange	1	kept
catheter indwelling block	catheter indwelling block	catheter indwelling block	catheter indwelling block	catheter indwelling block	1	kept
catheter indwelling pneumonia	catheter indwelling pneumonia	catheter indwelling pneumonia	catheter indwelling pneumonia	catheter indwelling pneumonia	1	kept
catheter leaking	catheter leaking	catheter leaking	catheter leaking	catheter leaking	1	kept
Catheter location	catheter location	catheter location	catheter location	Catheter location	1	kept
catheter malfunction	catheter malfunction	catheter malfunction	catheter malfunction	catheter malfunction	1	kept
catheter malposition	catheter malposition	catheter malposition	catheter malposition	catheter malposition	1	kept
Catheter migration	catheter migration	catheter migration	catheter migration	catheter migration	1	case_or_format
catheter misplacement	catheter misplacement	catheter misplacement	catheter misplacement	catheter misplacement	1	kept
Catheter patency	catheter patency	catheter patency	catheter patency	Catheter patency	1	kept
catheter prolapse	catheter prolapse	catheter prolapse	catheter prolapse	catheter prolapse	1	kept
catheter reinsertion	catheter reinsertion	catheter reinsertion	catheter reinsertion	catheter reinsertion	1	kept
catheter related bacteremia	catheter related bacteremia	catheter related bacteremia	catheter related bacteremia	catheter-related bacteremia	1	case_or_format

catheter related bloodstream infection reduction bundle	catheter related bloodstream infection reduction bundle	catheter related bloodstream infection reduction bundle	catheter related bloodstream infection reduction bundle	catheter related bloodstream infection reduction bundle	1	kept
catheter related bloodstream infections	catheter related bloodstream infections	catheter related bloodstream infections	catheter related bloodstream infection	catheter related bloodstream infection	1	singular
Catheter related urinary tract infection	catheter related urinary tract infection	catheter related urinary tract infection	catheter related urinary tract infection	Catheter-related urinary tract infection	1	case_or_format
catheter related urinary tract infection	catheter related urinary tract infection	catheter related urinary tract infection	catheter related urinary tract infection	Catheter-related urinary tract infection	1	case_or_format
catheter rupture	catheter rupture	catheter rupture	catheter rupture	catheter rupture	1	kept
CATHETER SELF-MANAGEMENT	catheter self management	catheter self management	catheter self management	CATHETER SELF-MANAGEMENT	1	kept
CATHETER SEPSIS	catheter sepsis	catheter sepsis	catheter sepsis	CATHETER SEPSIS	1	kept
Catheter thrombosis	catheter thrombosis	catheter thrombosis	catheter thrombosis	catheter thrombosis	1	case_or_format
CATHETER USE	catheter use	catheter use	catheter use	CATHETER USE	1	kept
catheter valve	catheter valve	catheter valve	catheter valve	catheter valve	1	kept
Catheter-associated	catheter associated	catheter associated	catheter associated	Catheter-associated	1	kept
CATHETER-ASSOCIATED BACTERIURIA	catheter associated bacteriuria	catheter associated bacteriuria	catheter associated bacteriuria	CATHETER-ASSOCIATED BACTERIURIA	1	kept
Catheter-associated Candida bloodstream infection	catheter associated candida bloodstream infection	catheter associated candida bloodstream infection	catheter associated candida bloodstream infection	Catheter-associated Candida bloodstream infection	1	kept
Catheter-associated infection	catheter associated infection	catheter associated infection	catheter associated infection	Catheter-associated infection	1	kept
Catheter-associated infections	catheter associated infections	catheter associated infections	catheter associated infection	Catheter-associated infection	1	singular
Catheter-related bacteremia	catheter related bacteremia	catheter related bacteremia	catheter related bacteremia	catheter-related bacteremia	1	case_or_format

catheter-related bloodstream infection	catheter related bloodstream infection	catheter related bloodstream infection	catheter related bloodstream infection	catheter related bloodstream infection	1	case_or_format
catheter-related complication	catheter related complication	catheter related complication	catheter related complication	catheter-related complication	1	kept
Catheter-related complications	catheter related complications	catheter related complications	catheter related complication	catheter-related complication	1	singular
Catheter-Related Infection	catheter related infection	catheter related infection	catheter related infection	Catheter-Related Infections	1	case_or_format
catheter-related ı nfections	catheter related nfections	catheter related nfections	catheter related nfections	catheter-related ı nfections	1	kept
catheterisation laboratory	catheterisation laboratory	catheterisation laboratory	catheterisation laboratory	catheterisation laboratory	1	kept
Catheterization central venous	catheterization central venous	catheterization central venous	catheterization central venous	Catheterization, Central Venous	1	case_or_format
Catheterization Peripheral	catheterization peripheral	catheterization peripheral	catheterization peripheral	Catheterization, Peripheral	1	case_or_format
catheterization, central venous	catheterization central venous	catheterization central venous	catheterization central venous	Catheterization, Central Venous	1	case_or_format
Catheters, Indwelling [adverse effects]	catheters indwelling adverse effects	catheters indwelling adverse effects	catheters indwelling adverse effects	Catheters, Indwelling [adverse effects]	1	kept
Catheters. RESUMO	catheters resumo	catheters resumo	catheters resumo	Catheters. RESUMO	1	kept
catheter‐associated thrombus	catheter associated thrombus	catheter associated thrombus	catheter associated thrombus	catheter‐associated thrombus	1	kept
catheter‐related infections	catheter related infections	catheter related infections	catheter related infection	Catheter-Related Infections	1	singular
cation exchange	cation exchange	cation exchange	cation exchange	cation exchange	1	kept
CATS	cats	cats	cat	Cats	1	singular
cats	cats	cats	cat	Cats	1	singular

Cattle	cattle	cattle	cattle	Cattle	1	kept
Catéter	cat ter	cat ter	cat ter	Catéter	1	kept
Catéteres	cat teres	cat teres	cat teres	Catéteres	1	kept
Catéteres Venosos Centrales	cat teres venosos centrales	cat teres venosos centrales	cat teres venosos centrales	Catéteres Venosos Centrales	1	kept
Catéteres venosos centrales	cat teres venosos centrales	cat teres venosos centrales	cat teres venosos centrales	Catéteres Venosos Centrales	1	case_or_format
Cauda Equina	cauda equina	cauda equina	cauda equina	Cauda Equina	1	kept
cauda equina	cauda equina	cauda equina	cauda equina	Cauda Equina	1	case_or_format
cauda equina syndrome	cauda equina syndrome	cauda equina syndrome	cauda equina syndrome	cauda equina syndrome	1	kept
caudal	caudal	caudal	caudal	caudal	1	kept
Causal attributions	causal attributions	causal attributions	causal attribution	causal attribution	1	singular
Causal factors	causal factors	causal factors	causal factors	Causal factors	1	kept
causal inference	causal inference	causal inference	causal inference	Causal inference	1	case_or_format
Causal inferences	causal inferences	causal inferences	causal inference	Causal inference	1	singular
causal mediation analysis	causal mediation analysis	causal mediation analysis	causal mediation analysis	causal mediation analysis	1	kept
Causal pathway	causal pathway	causal pathway	causal pathway	Causal pathway	1	kept
causal reasoning	causal reasoning	causal reasoning	causal reasoning	causal reasoning	1	kept

causes	causes	causes	causes	Causes	1	case_or_format
causes and management	causes and management	causes and management	causes and management	causes and management	1	kept
Causes of death	causes of death	causes of death	causes of death	Causes of death	1	kept
Causes of Errors	causes of errors	causes of errors	causes of errors	Causes of Errors	1	kept
causing incidents at work	causing incidents at work	causing incidents at work	causing incidents at work	causing incidents at work	1	kept
Caustics	caustics	caustics	caustics	Caustics	1	kept
CAUTI rate	cauti rate	cauti rate	cauti rate	CAUTI rate	1	kept
Cavalier King Charles spaniel	cavalier king charles spaniel	cavalier king charles spaniel	cavalier king charles spaniel	Cavalier King Charles spaniel	1	kept
cavernous sinus	cavernous sinus	cavernous sinus	cavernous sinus	cavernous sinus	1	kept
cavitary lesion	cavitary lesion	cavitary lesion	cavitary lesion	cavitary lesion	1	kept
cavopulmonary connection	cavopulmonary connection	cavopulmonary connection	cavopulmonary connection	cavopulmonary connection	1	kept
Caídas	ca das	ca das	ca das	Caídas	1	kept
CBC	cbc	cbc	cbc	CBC	1	kept
CBI	cbi	cbi	cbi	CBI	1	kept
CBL	cbl	cbl	cbl	CBL	1	kept
CBRN	cbrn	cbrn	cbrn	CBRN	1	kept

CCATT	ccatt	ccatt	ccatt	CCATT	1	kept
CCC Complex chronic condition	ccc complex chronic condition	ccc complex chronic condition	ccc complex chronic condition	CCC Complex chronic condition	1	kept
CCFNI instrument	ccfni instrument	ccfni instrument	ccfni instrument	CCFNI instrument	1	kept
CCFNI-ED	ccfni ed	ccfni ed	ccfni ed	CCFNI-ED	1	kept
CCHD screening in developing countries	cchd screening in developing countries	cchd screening in developing countries	cchd screening in developing countries	CCHD screening in developing countries	1	kept
CCHS	cchs	cchs	cchs	CCHS	1	kept
CCI	cci	cci	cci	CCI	1	kept
CCPOT	ccpot	ccpot	ccpot	CCPOT	1	kept
CCU limited resource	ccu limited resource	ccu limited resource	ccu limited resource	CCU limited resource	1	kept
CCU readmission	ccu readmission	ccu readmission	ccu readmission	CCU readmission	1	kept
CCU, Coronary care unit	ccu coronary care unit	ccu coronary care unit	ccu coronary care unit	CCU, Coronary care unit	1	kept
CCUs	ccus	ccus	ccus	CCUs	1	kept
CD-based BLS	cd based bls	cd based bls	cd based bls	CD-based BLS	1	kept
CD-I	cd i	cd i	cd i	CD-I	1	kept
CD135 antigen	cd135 antigen	cd135 antigen	cd135 antigen	CD135 antigen	1	kept
CD34 antigen	cd34 antigen	cd34 antigen	cd34 antigen	CD34 antigen	1	kept

CD4-Positive T-Lymphocytes	cd4 positive t lymphocytes	cd4 positive t lymphocytes	cd4 positive t lymphocytes	CD4-Positive T-Lymphocytes	1	kept
CD8 antigen	cd8 antigen	cd8 antigen	cd8 antigen	CD8 antigen	1	kept
CDC	cdc	cdc	cdc	CDC	1	kept
cdc	cdc	cdc	cdc	CDC	1	case_or_format
CDC = Centers for Disease Control and Prevention	cdc centers for disease control and prevention	cdc centers for disease control and prevention	cdc centers for disease control and prevention	CDC = Centers for Disease Control and Prevention	1	kept
CDI	cdi	cdi	cdi	CDI	1	kept
CEEG	ceeg	ceeg	ceeg	CEEG	1	kept
CEFEPIME	cefepime	cefepime	cefepime	cefepime	1	case_or_format
CEFEPIME THERAPY	cefepime therapy	cefepime therapy	cefepime therapy	CEFEPIME THERAPY	1	kept
cefodizime	cefodizime	cefodizime	cefodizime	cefodizime	1	kept
cefovecin	cefovecin	cefovecin	cefovecin	cefovecin	1	kept
ceftazidime resistance	ceftazidime resistance	ceftazidime resistance	ceftazidime resistance	ceftazidime resistance	1	kept
ceftazidime-avibactam	ceftazidime avibactam	ceftazidime avibactam	ceftazidime avibactam	ceftazidime-avibactam	1	kept
ceftazidime－avibactam	ceftazidime avibactam	ceftazidime avibactam	ceftazidime avibactam	ceftazidime-avibactam	1	case_or_format
ceftiofur	ceftiofur	ceftiofur	ceftiofur	ceftiofur	1	kept
ceftizoxime	ceftizoxime	ceftizoxime	ceftizoxime	ceftizoxime	1	kept

Cefuroxime	cefuroxime	cefuroxime	cefuroxime	cefuroxime	1	case_or_format
celiac disease	celiac disease	celiac disease	celiac disease	celiac disease	1	kept
cell activation	cell activation	cell activation	cell activation	cell activation	1	kept
cell adhesion molecule	cell adhesion molecule	cell adhesion molecule	cell adhesion molecule	cell adhesion molecule	1	kept
cell aging	cell aging	cell aging	cell aging	cell aging	1	kept
Cell Count	cell count	cell count	cell count	Cell Count	1	kept
cell count	cell count	cell count	cell count	Cell Count	1	case_or_format
cell culture cytotoxicity neutralization assay	cell culture cytotoxicity neutralization assay	cell culture cytotoxicity neutralization assay	cell culture cytotoxicity neutralization assay	cell culture cytotoxicity neutralization assay	1	kept
cell culture technique	cell culture technique	cell culture technique	cell culture technique	cell culture technique	1	kept
Cell Culture Techniques	cell culture techniques	cell culture techniques	cell culture technique	cell culture technique	1	singular
cell cycle checkpoint	cell cycle checkpoint	cell cycle checkpoint	cell cycle checkpoint	cell cycle checkpoint	1	kept
Cell Cycle Checkpoints	cell cycle checkpoints	cell cycle checkpoints	cell cycle checkpoint	cell cycle checkpoint	1	singular
cell cycle regulation	cell cycle regulation	cell cycle regulation	cell cycle regulation	cell cycle regulation	1	kept
Cell Death	cell death	cell death	cell death	cell death	1	case_or_format
cell differentiation	cell differentiation	cell differentiation	cell differentiation	cell differentiation	1	kept
cell granule	cell granule	cell granule	cell granule	cell granule	1	kept

cell hyperplasia	cell hyperplasia	cell hyperplasia	cell hyperplasia	cell hyperplasia	1	kept
cell hypoxia	cell hypoxia	cell hypoxia	cell hypoxia	Cell Hypoxia	1	case_or_format
cell maturation	cell maturation	cell maturation	cell maturation	cell maturation	1	kept
Cell Membrane Permeability	cell membrane permeability	cell membrane permeability	cell membrane permeability	Cell Membrane Permeability	1	kept
cell membrane permeability	cell membrane permeability	cell membrane permeability	cell membrane permeability	Cell Membrane Permeability	1	case_or_format
cell organelle	cell organelle	cell organelle	cell organelle	cell organelle	1	kept
Cell phones	cell phones	cell phones	cell phone	Cell Phone	1	singular
cell phones	cell phones	cell phones	cell phone	Cell Phone	1	singular
cell polarity	cell polarity	cell polarity	cell polarity	cell polarity	1	kept
cell regeneration	cell regeneration	cell regeneration	cell regeneration	cell regeneration	1	kept
CELL SALVAGE	cell salvage	cell salvage	cell salvage	CELL SALVAGE	1	kept
Cell saver	cell saver	cell saver	cell saver	Cell saver	1	kept
Cell saver waste	cell saver waste	cell saver waste	cell saver waste	Cell saver waste	1	kept
cell surface protein	cell surface protein	cell surface protein	cell surface protein	cell surface protein	1	kept
Cell Transplantation	cell transplantation	cell transplantation	cell transplantation	cell transplantation	1	case_or_format
cell vacuole	cell vacuole	cell vacuole	cell vacuole	cell vacuole	1	kept

cell viability	cell viability	cell viability	cell viability	cell viability	1	kept
cell wall	cell wall	cell wall	cell wall	cell wall	1	kept
CELL-ACTIVITY	cell activity	cell activity	cell activity	cell activity	1	case_or_format
Cell-Derived Microparticles	cell derived microparticles	cell derived microparticles	cell derived microparticles	Cell-Derived Microparticles	1	kept
Cellphone	cellphone	cellphone	cellphone	Cellphone	1	kept
cellular distribution	cellular distribution	cellular distribution	cellular distribution	cellular distribution	1	kept
cellular immunity	cellular immunity	cellular immunity	cellular immunity	cellular immunity	1	kept
Cellular system	cellular system	cellular system	cellular system	Cellular system	1	kept
Cellular telephone systems	cellular telephone systems	cellular telephone systems	cellular telephone systems	Cellular telephone systems	1	kept
cellulose	cellulose	cellulose	cellulose	cellulose	1	kept
cement	cement	cement	cement	cement	1	kept
Centella asiatica	centella asiatica	centella asiatica	centella asiatica	Centella asiatica	1	kept
Centella asiatica extract	centella asiatica extract	centella asiatica extract	centella asiatica extract	Centella asiatica extract	1	kept
Centenarians	centenarians	centenarians	centenarians	Centenarians	1	kept
centenarians	centenarians	centenarians	centenarians	Centenarians	1	case_or_format
Center for Clinical Research in Animal Envenomations	center for clinical research in animal envenomations	center for clinical research in animal envenomations	center for clinical research in animal envenomations	Center for Clinical Research in Animal Envenomations	1	kept

center of excellence	center of excellence	center of excellence	center of excellence	center of excellence	1	kept
CENTERED CARE INTERVENTIONS	centered care interventions	centered care interventions	centered care interventions	CENTERED CARE INTERVENTIONS	1	kept
CenteringPregnancy	centeringpregnancy	centeringpregnancy	centeringpregnancy	CenteringPregnancy	1	kept
CENTERS	centers	centers	centers	CENTERS	1	kept
CENTERS EXPERIENCE	centers experience	centers experience	centers experience	CENTERS EXPERIENCE	1	kept
Centers for Disease Control	centers for disease control	centers for disease control	centers for disease control	Centers for Disease Control	1	kept
Centers for Disease Control and Prevention CLABSI	centers for disease control and prevention clabsi	centers for disease control and prevention clabsi	centers for disease control and prevention clabsi	Centers for Disease Control and Prevention CLABSI	1	kept
Centers for diseases control and preventions	centers for diseases control and preventions	centers for diseases control and preventions	centers for diseases control and preventions	Centers for diseases control and preventions	1	kept
Centers for medicare and medicaid services	centers for medicare and medicaid services	centers for medicare and medicaid services	centers for medicare and medicaid services	Centers for Medicare and Medicaid Services	1	case_or_format
centile charts	centile charts	centile charts	centile charts	centile charts	1	kept
Central access	central access	central access	central access	Central access	1	kept
central alveolar hypoventilation	central alveolar hypoventilation	central alveolar hypoventilation	central alveolar hypoventilation	central alveolar hypoventilation	1	kept
Central bed registry	central bed registry	central bed registry	central bed registry	Central bed registry	1	kept
central call center	central call center	central call center	central call center	central call center	1	kept
central catheter access	central catheter access	central catheter access	central catheter access	central catheter access	1	kept
central catheter infections	central catheter infections	central catheter infections	central catheter infections	central catheter infections	1	kept

Central dispatch communication center	central dispatch communication center	central dispatch communication center	central dispatch communication center	Central dispatch communication center	1	kept
Central European	central european	central european	central european	Central European	1	kept
central gray matter	central gray matter	central gray matter	central gray matter	central gray matter	1	kept
central hypoventilation syndrome	central hypoventilation syndrome	central hypoventilation syndrome	central hypoventilation syndrome	central hypoventilation syndrome	1	kept
central line associated blood stream infections	central line associated blood stream infections	central line associated blood stream infections	central line associated blood stream infection	Central line associated blood stream infection	1	singular
central line associated bloodstream infections	central line associated bloodstream infections	central line associated bloodstream infections	central line associated bloodstream infection	Central line-associated bloodstream infections	1	singular
Central line associated infections	central line associated infections	central line associated infections	central line associated infections	Central line associated infections	1	kept
Central line associated sepsis	central line associated sepsis	central line associated sepsis	central line associated sepsis	Central line associated sepsis	1	kept
Central line bundle	central line bundle	central line bundle	central line bundle	central line bundle	1	case_or_format
Central line care	central line care	central line care	central line care	Central line care	1	kept
central line care	central line care	central line care	central line care	Central line care	1	case_or_format
Central line checklist	central line checklist	central line checklist	central line checklist	Central line checklist	1	kept
Central line complications	central line complications	central line complications	central line complications	Central line complications	1	kept
central line dressing kits	central line dressing kits	central line dressing kits	central line dressing kits	central line dressing kits	1	kept
central line infection	central line infection	central line infection	central line infection	Central line infections	1	case_or_format
central line insertion checklist	central line insertion checklist	central line insertion checklist	central line insertion checklist	central line insertion checklist	1	kept

Central Line-Associated blood stream infection	central line associated blood stream infection	central line associated blood stream infection	central line associated blood stream infection	Central line associated blood stream infection	1	case_or_format
Central line-associated blood stream infections	central line associated blood stream infections	central line associated blood stream infections	central line associated blood stream infection	Central line associated blood stream infection	1	singular
Central line-associated bloodstream	central line associated bloodstream	central line associated bloodstream	central line associated bloodstream	Central line-associated bloodstream	1	kept
Central line-Associated bloodstream infection	central line associated bloodstream infection	central line associated bloodstream infection	central line associated bloodstream infection	Central line-associated bloodstream infections	1	case_or_format
central line-Associated bloodstream infection	central line associated bloodstream infection	central line associated bloodstream infection	central line associated bloodstream infection	Central line-associated bloodstream infections	1	case_or_format
Central Line-Associated Bloodstream Infections	central line associated bloodstream infections	central line associated bloodstream infections	central line associated bloodstream infection	Central line-associated bloodstream infections	1	singular
CENTRAL LINES	central lines	central lines	central line	Central line	1	singular
Central lines	central lines	central lines	central line	Central line	1	singular
central lines	central lines	central lines	central line	Central line	1	singular
Central line -associated blood stream infections	central line associated blood stream infections	central line associated blood stream infections	central line associated blood stream infection	Central line associated blood stream infection	1	singular
central line -associated blood stream infections	central line associated blood stream infections	central line associated blood stream infections	central line associated blood stream infection	Central line associated blood stream infection	1	singular
Central line -associated bloodstream infection	central line associated bloodstream infection	central line associated bloodstream infection	central line associated bloodstream infection	Central line-associated bloodstream infections	1	case_or_format
Central line -associated bloodstream infections	central line associated bloodstream infections	central line associated bloodstream infections	central line associated bloodstream infection	Central line-associated bloodstream infections	1	singular
central line -associated bloodstream infections	central line associated bloodstream infections	central line associated bloodstream infections	central line associated bloodstream infection	Central line-associated bloodstream infections	1	singular
Central Nervous System	central nervous system	central nervous system	central nervous system	central nervous system	1	case_or_format
Central nervous system	central nervous system	central nervous system	central nervous system	central nervous system	1	case_or_format

Central nervous system drugs	central nervous system drugs	central nervous system drugs	central nervous system drugs	Central nervous system drugs	1	kept
central nervous system infections	central nervous system infections	central nervous system infections	central nervous system infection	central nervous system infection	1	singular
central nervous system malformation	central nervous system malformation	central nervous system malformation	central nervous system malformation	central nervous system malformation	1	kept
central nervous system sensitization	central nervous system sensitization	central nervous system sensitization	central nervous system sensitization	central nervous system sensitization	1	kept
Central Ohio Trauma System	central ohio trauma system	central ohio trauma system	central ohio trauma system	Central Ohio Trauma System	1	kept
Central pontine myelinolysis	central pontine myelinolysis	central pontine myelinolysis	central pontine myelinolysis	central pontine myelinolysis	1	case_or_format
central retina vein occlusion	central retina vein occlusion	central retina vein occlusion	central retina vein occlusion	central retina vein occlusion	1	kept
Central sensitization	central sensitization	central sensitization	central sensitization	Central sensitization	1	kept
Central vascular catheter	central vascular catheter	central vascular catheter	central vascular catheter	central vascular catheter	1	case_or_format
central vascular catheterization	central vascular catheterization	central vascular catheterization	central vascular catheterization	central vascular catheterization	1	kept
Central vein	central vein	central vein	central vein	central vein	1	case_or_format
central venous access devices	central venous access devices	central venous access devices	central venous access device	central venous access device	1	singular
central venous blood sampling	central venous blood sampling	central venous blood sampling	central venous blood sampling	central venous blood sampling	1	kept
Central venous cannulae	central venous cannulae	central venous cannulae	central venous cannulae	Central venous cannulae	1	kept
CENTRAL VENOUS CANNULATION	central venous cannulation	central venous cannulation	central venous cannulation	central venous cannulation	1	case_or_format
Central Venous Cathete	central venous cathete	central venous cathete	central venous cathete	Central Venous Cathete	1	kept

Central venous catheter bundle	central venous catheter bundle	central venous catheter bundle	central venous catheter bundle	Central venous catheter bundle	1	kept
central venous catheter complications	central venous catheter complications	central venous catheter complications	central venous catheter complications	central venous catheter complications	1	kept
central venous catheter related bloodstream infection	central venous catheter related bloodstream infection	central venous catheter related bloodstream infection	central venous catheter related bloodstream infection	central venous catheter related bloodstream infection	1	kept
Central venous catheter-related infection	central venous catheter related infection	central venous catheter related infection	central venous catheter related infection	Central venous catheter-related infection	1	kept
central venous catheter-related infection	central venous catheter related infection	central venous catheter related infection	central venous catheter related infection	Central venous catheter-related infection	1	case_or_format
Central venous catheters associated bloodstream infections	central venous catheters associated bloodstream infections	central venous catheters associated bloodstream infections	central venous catheters associated bloodstream infections	Central venous catheters associated bloodstream infections	1	kept
Central Venous Catheters, Treatment Failure	central venous catheters treatment failure	central venous catheters treatment failure	central venous catheters treatment failure	Central Venous Catheters, Treatment Failure	1	kept
Central Venous Catheters. Infant	central venous catheters infant	central venous catheters infant	central venous catheters infant	Central Venous Catheters. Infant	1	kept
Central Venous Infant	central venous infant	central venous infant	central venous infant	Central Venous Infant	1	kept
central venous line maintenance bundle	central venous line maintenance bundle	central venous line maintenance bundle	central venous line maintenance bundle	central venous line maintenance bundle	1	kept
Central-line associated	central line associated	central line associated	central line associated	Central-line associated	1	kept
Central-line - associated bloodstream infection	central line associated bloodstream infection	central line associated bloodstream infection	central line associated bloodstream infection	Central line- associated bloodstream infections	1	case_or_format
CENTRAL-VENOUS-PRESSURE	central venous pressure	central venous pressure	central venous pressure	central venous pressure	1	case_or_format
Centralised	centralised	centralised	centralised	Centralised	1	kept
Centralised control	centralised control	centralised control	centralised control	Centralised control	1	kept
Centrality	centrality	centrality	centrality	Centrality	1	kept

centralized	centralized	centralized	centralized	centralized	1	kept
centralized control interface	centralized control interface	centralized control interface	centralized control interface	centralized control interface	1	kept
Centralized management	centralized management	centralized management	centralized management	Centralized management	1	kept
centre size	centre size	centre size	centre size	centre size	1	kept
centrifugal blood pump	centrifugal blood pump	centrifugal blood pump	centrifugal blood pump	centrifugal blood pump	1	kept
centrifugal blood pumps	centrifugal blood pumps	centrifugal blood pumps	centrifugal blood pump	centrifugal blood pump	1	singular
Centrifugation	centrifugation	centrifugation	centrifugation	centrifugation	1	case_or_format
Cephalic vein	cephalic vein	cephalic vein	cephalic vein	cephalic vein	1	case_or_format
cephalopelvic disproportion	cephalopelvic disproportion	cephalopelvic disproportion	cephalopelvic disproportion	cephalopelvic disproportion	1	kept
CEPHALOSPORINS	cephalosporins	cephalosporins	cephalosporin	cephalosporin	1	singular
cephamycin	cephamycin	cephamycin	cephamycin	cephamycin	1	kept
ceproject	ceproject	ceproject	ceproject	ceproject	1	kept
ceramics	ceramics	ceramics	ceramics	ceramics	1	kept
cerebellar ataxia	cerebellar ataxia	cerebellar ataxia	cerebellar ataxia	cerebellar ataxia	1	kept
Cerebellar stroke	cerebellar stroke	cerebellar stroke	cerebellar stroke	Cerebellar stroke	1	kept
cerebellum atrophy	cerebellum atrophy	cerebellum atrophy	cerebellum atrophy	cerebellum atrophy	1	kept

cerebellum cortex	cerebellum cortex	cerebellum cortex	cerebellum cortex	cerebellum cortex	1	kept
cerebellum hemorrhage	cerebellum hemorrhage	cerebellum hemorrhage	cerebellum hemorrhage	cerebellum hemorrhage	1	kept
cerebellum injury	cerebellum injury	cerebellum injury	cerebellum injury	cerebellum injury	1	kept
Cerebelo-pontine angle	cerebelo pontine angle	cerebelo pontine angle	cerebelo pontine angle	Cerebelo-pontine angle	1	kept
Cerebral air embolism	cerebral air embolism	cerebral air embolism	cerebral air embolism	Cerebral air embolism	1	kept
Cerebral Amyloid Angiopathy	cerebral amyloid angiopathy	cerebral amyloid angiopathy	cerebral amyloid angiopathy	Cerebral Amyloid Angiopathy	1	kept
cerebral amyloid angiopathy	cerebral amyloid angiopathy	cerebral amyloid angiopathy	cerebral amyloid angiopathy	Cerebral Amyloid Angiopathy	1	case_or_format
cerebral aneurysm	cerebral aneurysm	cerebral aneurysm	cerebral aneurysm	Cerebral aneurysm	1	case_or_format
Cerebral Aqueduct	cerebral aqueduct	cerebral aqueduct	cerebral aqueduct	Cerebral Aqueduct	1	kept
cerebral cortex	cerebral cortex	cerebral cortex	cerebral cortex	Cerebral Cortex	1	case_or_format
Cerebral edema	cerebral edema	cerebral edema	cerebral edema	Cerebral edema	1	kept
cerebral edema	cerebral edema	cerebral edema	cerebral edema	Cerebral edema	1	case_or_format
Cerebral function monitoring	cerebral function monitoring	cerebral function monitoring	cerebral function monitoring	Cerebral function monitoring	1	kept
Cerebral haemodynamics and oxygenation	cerebral haemodynamics and oxygenation	cerebral haemodynamics and oxygenation	cerebral haemodynamics and oxygenation	Cerebral haemodynamics and oxygenation	1	kept
cerebral hemiatrophy	cerebral hemiatrophy	cerebral hemiatrophy	cerebral hemiatrophy	cerebral hemiatrophy	1	kept
cerebral hemodynamics	cerebral hemodynamics	cerebral hemodynamics	cerebral hemodynamics	cerebral hemodynamics	1	kept

cerebral hyperperfusion	cerebral hyperperfusion	cerebral hyperperfusion	cerebral hyperperfusion	cerebral hyperperfusion	1	kept
CEREBRAL INFARCTION	cerebral infarction	cerebral infarction	cerebral infarction	Cerebral Infarction	1	case_or_format
Cerebral infraction	cerebral infraction	cerebral infraction	cerebral infraction	Cerebral infraction	1	kept
cerebral malaria	cerebral malaria	cerebral malaria	cerebral malaria	cerebral malaria	1	kept
Cerebral metastases	cerebral metastases	cerebral metastases	cerebral metastases	Cerebral metastases	1	kept
Cerebral microdialysis	cerebral microdialysis	cerebral microdialysis	cerebral microdialysis	Cerebral microdialysis	1	kept
Cerebral neoplasms	cerebral neoplasms	cerebral neoplasms	cerebral neoplasms	Cerebral neoplasms	1	kept
Cerebral oedema	cerebral oedema	cerebral oedema	cerebral oedema	Cerebral oedema	1	kept
cerebral oximetry	cerebral oximetry	cerebral oximetry	cerebral oximetry	cerebral oximetry	1	kept
Cerebral oxygen delivery	cerebral oxygen delivery	cerebral oxygen delivery	cerebral oxygen delivery	Cerebral oxygen delivery	1	kept
Cerebral Palsy [prevention & control]	cerebral palsy prevention and control	cerebral palsy prevention and control	cerebral palsy prevention and control	Cerebral Palsy [prevention & control]	1	kept
Cerebral palsy and developmental follow-up for at-risk infants	cerebral palsy and developmental follow up for at risk infants	cerebral palsy and developmental follow up for at risk infants	cerebral palsy and developmental follow up for at risk infants	Cerebral palsy and developmental follow-up for at-risk infants	1	kept
Cerebral performance category	cerebral performance category	cerebral performance category	cerebral performance category	Cerebral performance category	1	kept
CEREBRAL PERFUSION-PRESSURE	cerebral perfusion pressure	cerebral perfusion pressure	cerebral perfusion pressure	cerebral perfusion pressure	1	case_or_format
cerebral revascularization	cerebral revascularization	cerebral revascularization	cerebral revascularization	cerebral revascularization	1	kept
Cerebral sinus thrombosis	cerebral sinus thrombosis	cerebral sinus thrombosis	cerebral sinus thrombosis	cerebral sinus thrombosis	1	case_or_format

Cerebral Small Vessel Diseases	cerebral small vessel diseases	cerebral small vessel diseases	cerebral small vessel diseases	Cerebral Small Vessel Diseases	1	kept
cerebral toxoplasmosis	cerebral toxoplasmosis	cerebral toxoplasmosis	cerebral toxoplasmosis	cerebral toxoplasmosis	1	kept
Cerebral vasospasm	cerebral vasospasm	cerebral vasospasm	cerebral vasospasm	Cerebral vasospasm	1	kept
Cerebral venous and sinus thrombosis	cerebral venous and sinus thrombosis	cerebral venous and sinus thrombosis	cerebral venous and sinus thrombosis	Cerebral venous and sinus thrombosis	1	kept
Cerebral venous thrombosis	cerebral venous thrombosis	cerebral venous thrombosis	cerebral venous thrombosis	Cerebral venous thrombosis	1	kept
CEREBRAL-PALSY	cerebral palsy	cerebral palsy	cerebral palsy	cerebral palsy	1	case_or_format
cerebrocentric model of consciousness	cerebrocentric model of consciousness	cerebrocentric model of consciousness	cerebrocentric model of consciousness	cerebrocentric model of consciousness	1	kept
cerebrolysin	cerebrolysin	cerebrolysin	cerebrolysin	cerebrolysin	1	kept
cerebroside sulfatase	cerebroside sulfatase	cerebroside sulfatase	cerebroside sulfatase	cerebroside sulfatase	1	kept
Cerebrospinal fluid	cerebrospinal fluid	cerebrospinal fluid	cerebrospinal fluid	cerebrospinal fluid	1	case_or_format
Cerebrospinal fluid analysis	cerebrospinal fluid analysis	cerebrospinal fluid analysis	cerebrospinal fluid analysis	cerebrospinal fluid analysis	1	case_or_format
cerebrospinal fluid cytology	cerebrospinal fluid cytology	cerebrospinal fluid cytology	cerebrospinal fluid cytology	cerebrospinal fluid cytology	1	kept
Cerebrospinal Fluid Leak	cerebrospinal fluid leak	cerebrospinal fluid leak	cerebrospinal fluid leak	Cerebrospinal Fluid Leak	1	kept
cerebrospinal fluid level	cerebrospinal fluid level	cerebrospinal fluid level	cerebrospinal fluid level	cerebrospinal fluid level	1	kept
cerebrospinal fluid pressure	cerebrospinal fluid pressure	cerebrospinal fluid pressure	cerebrospinal fluid pressure	Cerebrospinal Fluid Pressure	1	case_or_format
CEREBROSPINAL-FLUID	cerebrospinal fluid	cerebrospinal fluid	cerebrospinal fluid	cerebrospinal fluid	1	case_or_format

Cerebrovascular accident	cerebrovascular accident	cerebrovascular accident	cerebrovascular accident	cerebrovascular accident	1	case_or_format
Cerebrovascular disorders	cerebrovascular disorders	cerebrovascular disorders	cerebrovascular disorders	Cerebrovascular Disorders	1	case_or_format
cerebrovascular disorders	cerebrovascular disorders	cerebrovascular disorders	cerebrovascular disorders	Cerebrovascular Disorders	1	case_or_format
Cerebrovascular disorders - therapy	cerebrovascular disorders therapy	cerebrovascular disorders therapy	cerebrovascular disorders therapy	Cerebrovascular disorders - therapy	1	kept
Cerner software	cerner software	cerner software	cerner software	Cerner software	1	kept
cernevit	cernevit	cernevit	cernevit	cernevit	1	kept
Certificate of Need	certificate of need	certificate of need	certificate of need	Certificate of Need	1	kept
Certification examination	certification examination	certification examination	certification examination	Certification examination	1	kept
Certified child life specialists	certified child life specialists	certified child life specialists	certified child life specialists	Certified child life specialists	1	kept
Certified health training	certified health training	certified health training	certified health training	Certified health training	1	kept
certified therapeutic recreation specialist	certified therapeutic recreation specialist	certified therapeutic recreation specialist	certified therapeutic recreation specialist	certified therapeutic recreation specialist	1	kept
certolizumab pegol	certolizumab pegol	certolizumab pegol	certolizumab pegol	certolizumab pegol	1	kept
Cervical collar	cervical collar	cervical collar	cervical collar	Cervical collar	1	kept
cervical disc arthroplasty	cervical disc arthroplasty	cervical disc arthroplasty	cervical disc arthroplasty	cervical disc arthroplasty	1	kept
cervical flattening	cervical flattening	cervical flattening	cervical flattening	cervical flattening	1	kept
cervical intervertebral disk hernia	cervical intervertebral disk hernia	cervical intervertebral disk hernia	cervical intervertebral disk hernia	cervical intervertebral disk hernia	1	kept

cervical laminectomy	cervical laminectomy	cervical laminectomy	cervical laminectomy	cervical laminectomy	1	kept
cervical lymphadenopathy	cervical lymphadenopathy	cervical lymphadenopathy	cervical lymphadenopathy	cervical lymphadenopathy	1	kept
cervical lymphangioma	cervical lymphangioma	cervical lymphangioma	cervical lymphangioma	cervical lymphangioma	1	kept
Cervical Rib Syndrome	cervical rib syndrome	cervical rib syndrome	cervical rib syndrome	Cervical Rib Syndrome	1	kept
cervical vertebra	cervical vertebra	cervical vertebra	cervical vertebra	cervical vertebra	1	kept
Cervical vertebrae	cervical vertebrae	cervical vertebrae	cervical vertebrae	Cervical Vertebrae	1	case_or_format
CES-D	ces d	ces d	ces d	CES-D	1	kept
Cesarean	cesarean	cesarean	cesarean	cesarean	1	case_or_format
cesarean delivery	cesarean delivery	cesarean delivery	cesarean delivery	Cesarean delivery	1	case_or_format
cesarean reduction	cesarean reduction	cesarean reduction	cesarean reduction	cesarean reduction	1	kept
Cesarean section	cesarean section	cesarean section	cesarean section	cesarean section	1	case_or_format
cesarean surgery	cesarean surgery	cesarean surgery	cesarean surgery	cesarean surgery	1	kept
Cessation	cessation	cessation	cessation	Cessation	1	kept
Cessation of active treatment	cessation of active treatment	cessation of active treatment	cessation of active treatment	Cessation of active treatment	1	kept
ceterizine	ceterizine	ceterizine	ceterizine	ceterizine	1	kept
cethrin	cethrin	cethrin	cethrin	cethrin	1	kept

cetrimide	cetrimide	cetrimide	cetrimide	cetrimide	1	kept
cetryn syrup	cetryn syrup	cetryn syrup	cetryn syrup	cetryn syrup	1	kept
Cetylpyridinium	cetylpyridinium	cetylpyridinium	cetylpyridinium	Cetylpyridinium	1	kept
Cetylpyridinium chloride	cetylpyridinium chloride	cetylpyridinium chloride	cetylpyridinium chloride	Cetylpyridinium chloride	1	kept
CEWT	cewt	cewt	cewt	CEWT	1	kept
CFAR	cfar	cfar	cfar	CFAR	1	kept
CGM accuracy	cgm accuracy	cgm accuracy	cgm accuracy	CGM accuracy	1	kept
CGM SYSTEM	cgm system	cgm system	cgm system	CGM SYSTEM	1	kept
CHA	cha	cha	cha	CHA	1	kept
ChAdOx1 nCoV-19	chadox1 ncov 19	chadox1 ncov 19	chadox1 ncov 19	ChAdOx1 nCoV-19	1	kept
CHADS2 score	chads2 score	chads2 score	chads2 score	CHADS2 score	1	kept
Chagas Cardiomyopathy	chagas cardiomyopathy	chagas cardiomyopathy	chagas cardiomyopathy	Chagas Cardiomyopathy	1	kept
Chagas Disease	chagas disease	chagas disease	chagas disease	Chagas Disease	1	kept
Chagas disease	chagas disease	chagas disease	chagas disease	Chagas Disease	1	case_or_format
CHAID	chaid	chaid	chaid	CHAID	1	kept
chain mediation	chain mediation	chain mediation	chain mediation	chain mediation	1	kept

Chains of infection	chains of infection	chains of infection	chains of infection	Chains of infection	1	kept
chair sit and reach test	chair sit and reach test	chair sit and reach test	chair sit and reach test	chair sit and reach test	1	kept
chair sitting exercise	chair sitting exercise	chair sitting exercise	chair sitting exercise	chair sitting exercise	1	kept
chair stand test	chair stand test	chair stand test	chair stand test	chair stand test	1	kept
Chair-sitting	chair sitting	chair sitting	chair sitting	Chair-sitting	1	kept
challenges back home	challenges back home	challenges back home	challenges back home	challenges back home	1	kept
challenges in ECMO care	challenges in ecmo care	challenges in ecmo care	challenges in ecmo care	challenges in ECMO care	1	kept
challenging behavior	challenging behavior	challenging behavior	challenging behavior	challenging behavior	1	kept
Challenging behaviour	challenging behaviour	challenging behaviour	challenging behaviour	Challenging behaviour	1	kept
chamomile	chamomile	chamomile	chamomile	chamomile	1	kept
Champion	champion	champion	champion	Champion	1	kept
Champions	champions	champions	champion	Champion	1	singular
Champsodon snyderi	champsodon snyderi	champsodon snyderi	champsodon snyderi	Champsodon snyderi	1	kept
Change agent	change agent	change agent	change agent	Change agent	1	kept
Change detection	change detection	change detection	change detection	Change detection	1	kept
change fatigue	change fatigue	change fatigue	change fatigue	change fatigue	1	kept

Change implementation	change implementation	change implementation	change implementation	Change implementation	1	kept
change implementation	change implementation	change implementation	change implementation	Change implementation	1	case_or_format
Change of shift	change of shift	change of shift	change of shift	Change of shift	1	kept
change of shift	change of shift	change of shift	change of shift	Change of shift	1	case_or_format
change of therapy	change of therapy	change of therapy	change of therapy	change of therapy	1	kept
change process	change process	change process	change process	Change process	1	case_or_format
change team	change team	change team	change team	change team	1	kept
Change Theory	change theory	change theory	change theory	Change Theory	1	kept
Change theory	change theory	change theory	change theory	Change Theory	1	case_or_format
change theory	change theory	change theory	change theory	Change Theory	1	case_or_format
changeover	changeover	changeover	changeover	changeover	1	kept
Changes	changes	changes	change	Change	1	singular
changes in Health, End Stage Disease and Symptoms and Signs	changes in health end stage disease and symptoms and signs	changes in health end stage disease and symptoms and signs	changes in health end stage disease and symptoms and signs	changes in Health, End Stage Disease and Symptoms and Signs	1	kept
Changes in Health, End-Stage Disease, Signs, and Symptoms	changes in health end stage disease signs and symptoms scale	changes in health end stage disease signs and symptoms scale	changes in health end stage disease signs and symptoms scale	Changes in Health, End-Stage Disease, Signs, and Symptoms	1	kept
Changes of psychological status	changes of psychological status	changes of psychological status	changes of psychological status	Changes of psychological status	1	kept
changing KAP	changing kap	changing kap	changing kap	changing KAP	1	kept

CHANGING PATTERN	changing pattern	changing pattern	changing pattern	CHANGING PATTERN	1	kept
changing position	changing position	changing position	changing position	changing position	1	kept
Channel Characterization	channel characterization	channel characterization	channel characterization	Channel Characterization	1	kept
CHAOS	chaos	chaos	chaos	CHAOS	1	kept
chaotic environment	chaotic environment	chaotic environment	chaotic environment	chaotic environment	1	kept
chaplain	chaplain	chaplain	chaplain	Chaplain	1	case_or_format
Chaplaincy	chaplaincy	chaplaincy	chaplaincy	chaplaincy	1	case_or_format
chapped lips	chapped lips	chapped lips	chapped lips	chapped lips	1	kept
characteristic	characteristic	characteristic	characteristic	Characteristics	1	case_or_format
Characteristic difference	characteristic difference	characteristic difference	characteristic difference	Characteristic difference	1	kept
characteristics of workplace	characteristics of workplace	characteristics of workplace	characteristics of workplace	characteristics of workplace	1	kept
Characterization	characterization	characterization	characterization	Characterization	1	kept
CHARGE	charge	charge	charge	CHARGE	1	kept
CHARGE syndrome	charge syndrome	charge syndrome	charge syndrome	CHARGE syndrome	1	kept
Charges	charges	charges	charge	CHARGE	1	singular
charges	charges	charges	charge	CHARGE	1	singular

charity	charity	charity	charity	Charities	1	case_or_format
Charles Bonnet syndrome	charles bonnet syndrome	charles bonnet syndrome	charles bonnet syndrome	Charles Bonnet syndrome	1	kept
Charlson Index	charlson index	charlson index	charlson index	Charlson Index	1	kept
Charlson-Quan comorbidity index	charlson quan comorbidity index	charlson quan comorbidity index	charlson quan comorbidity index	Charlson-Quan comorbidity index	1	kept
Chatbot	chatbot	chatbot	chatbot	Chatbot	1	kept
chatbot	chatbot	chatbot	chatbot	Chatbot	1	case_or_format
Check-List	check list	check list	check list	Check-List	1	kept
Checklist for communication	checklist for communication	checklist for communication	checklist for communication	Checklist for communication	1	kept
Checklist for Early Recognition and Treatment of Acute [illegible]	checklist for early recognition and treatment of acute [illegible]	checklist for early recognition and treatment of acute [illegible]	checklist for early recognition and treatment of acute [illegible]	Checklist for Early Recognition and Treatment of Acute [illegible]	1	kept
Checklist for Reporting Results of Internet E Survey [illegible]	checklist for reporting results of internet e survey [illegible]	checklist for reporting results of internet e survey [illegible]	checklist for reporting results of internet e survey [illegible]	Checklist for Reporting Results of Internet E Survey [illegible]	1	kept
CHECKLISTS	checklists	checklists	checklist	checklist	1	singular
checkpoint inhibitor therapy	checkpoint inhibitor therapy	checkpoint inhibitor therapy	checkpoint inhibitor therapy	checkpoint inhibitor therapy	1	kept
cheek mucosa	cheek mucosa	cheek mucosa	cheek mucosa	cheek mucosa	1	kept
cheerfulness	cheerfulness	cheerfulness	cheerfulness	cheerfulness	1	kept
Chelation Therapy	chelation therapy	chelation therapy	chelation therapy	Chelation Therapy	1	kept
chemexfoliation	chemexfoliation	chemexfoliation	chemexfoliation	chemexfoliation	1	kept

chemical	chemical	chemical	chemical	Chemical	1	case_or_format
Chemical activation	chemical activation	chemical activation	chemical activation	Chemical activation	1	kept
Chemical burns	chemical burns	chemical burns	chemical burn	chemical burn	1	singular
Chemical casualties	chemical casualties	chemical casualties	chemical casualties	Chemical casualties	1	kept
chemical composition	chemical composition	chemical composition	chemical composition	chemical composition	1	kept
Chemical compounds	chemical compounds	chemical compounds	chemical compounds	Chemical compounds	1	kept
chemical debridement	chemical debridement	chemical debridement	chemical debridement	chemical debridement	1	kept
CHEMICAL INDUSTRIES	chemical industries	chemical industries	chemical industries	CHEMICAL INDUSTRIES	1	kept
chemical injury	chemical injury	chemical injury	chemical injury	chemical injury	1	kept
CHEMICAL PATHOLOGY	chemical pathology	chemical pathology	chemical pathology	CHEMICAL PATHOLOGY	1	kept
Chemical Precipitation	chemical precipitation	chemical precipitation	chemical precipitation	Chemical Precipitation	1	kept
chemical reaction	chemical reaction	chemical reaction	chemical reaction	chemical reaction	1	kept
Chemical Terrorism	chemical terrorism	chemical terrorism	chemical terrorism	Chemical Terrorism	1	kept
Chemical terrorism	chemical terrorism	chemical terrorism	chemical terrorism	Chemical Terrorism	1	case_or_format
Chemical Warfare	chemical warfare	chemical warfare	chemical warfare	Chemical Warfare	1	kept
chemical warfare	chemical warfare	chemical warfare	chemical warfare	Chemical Warfare	1	case_or_format

chemical warfare agent	chemical warfare agent	chemical warfare agent	chemical warfare agent	chemical warfare agent	1	kept
Chemical Warfare Agents	chemical warfare agents	chemical warfare agents	chemical warfare agent	chemical warfare agent	1	singular
Chemical weapons	chemical weapons	chemical weapons	chemical weapons	Chemical weapons	1	kept
chemiluminescent microparticle immunoassay	chemiluminescent microparticle immunoassay	chemiluminescent microparticle immunoassay	chemiluminescent microparticle immunoassay	chemiluminescent microparticle immunoassay	1	kept
Chemokines, CC	chemokines cc	chemokines cc	chemokines cc	Chemokines, CC	1	kept
chemoreceptor	chemoreceptor	chemoreceptor	chemoreceptor	chemoreceptor	1	kept
Chemoreceptors	chemoreceptors	chemoreceptors	chemoreceptor	chemoreceptor	1	singular
CHEMOSENSOR	chemosensor	chemosensor	chemosensor	CHEMOSENSOR	1	kept
Chemosis	chemosis	chemosis	chemosis	chemosis	1	case_or_format
chemotaxis	chemotaxis	chemotaxis	chemotaxis	chemotaxis	1	kept
Chemotherapy agents	chemotherapy agents	chemotherapy agents	chemotherapy agents	Chemotherapy agents	1	kept
Chemotherapy biotherapy	chemotherapy biotherapy	chemotherapy biotherapy	chemotherapy biotherapy	Chemotherapy biotherapy	1	kept
chemotherapy induced emesis	chemotherapy induced emesis	chemotherapy induced emesis	chemotherapy induced emesis	chemotherapy induced emesis	1	kept
chemotherapy induced nausea and vomiting	chemotherapy induced nausea and vomiting	chemotherapy induced nausea and vomiting	chemotherapy induced nausea and vomiting	chemotherapy induced nausea and vomiting	1	kept
Chemotherapy, Cancer, Regional Perfusion	chemotherapy cancer regional perfusion	chemotherapy cancer regional perfusion	chemotherapy cancer regional perfusion	Chemotherapy, Cancer, Regional Perfusion	1	kept
Chemotherapy-Induced Febrile Neutropenia	chemotherapy induced febrile neutropenia	chemotherapy induced febrile neutropenia	chemotherapy induced febrile neutropenia	Chemotherapy-Induced Febrile Neutropenia	1	kept

cherry	cherry	cherry	cherry	cherry	1	kept
CHESS II	chess ii	chess ii	chess ii	CHESS II	1	kept
chest	chest	chest	chest	chest	1	kept
Chest clearance	chest clearance	chest clearance	chest clearance	Chest clearance	1	kept
chest compression interruption time	chest compression interruption time	chest compression interruption time	chest compression interruption time	chest compression interruption time	1	kept
Chest compressions	chest compressions	chest compressions	chest compression	Chest compression	1	singular
Chest CT	chest ct	chest ct	chest ct	Chest CT	1	kept
Chest drainage	chest drainage	chest drainage	chest drainage	Chest drainage	1	kept
chest drainage	chest drainage	chest drainage	chest drainage	Chest drainage	1	case_or_format
Chest excursion	chest excursion	chest excursion	chest excursion	Chest excursion	1	kept
Chest image interpretation	chest image interpretation	chest image interpretation	chest image interpretation	Chest image interpretation	1	kept
chest movement	chest movement	chest movement	chest movement	chest movement	1	kept
Chest physical therapy	chest physical therapy	chest physical therapy	chest physical therapy	Chest physical therapy	1	kept
Chest radiograph	chest radiograph	chest radiograph	chest radiograph	Chest radiograph	1	kept
Chest radiograph evaluation	chest radiograph evaluation	chest radiograph evaluation	chest radiograph evaluation	Chest radiograph evaluation	1	kept
Chest radiograph interpretation	chest radiograph interpretation	chest radiograph interpretation	chest radiograph interpretation	Chest radiograph interpretation	1	kept

Chest radiographs	chest radiographs	chest radiographs	chest radiograph	Chest radiograph	1	singular
chest radiographs	chest radiographs	chest radiographs	chest radiograph	Chest radiograph	1	singular
CHEST RADIOGRAPHY	chest radiography	chest radiography	chest radiography	CHEST RADIOGRAPHY	1	kept
Chest radiography	chest radiography	chest radiography	chest radiography	CHEST RADIOGRAPHY	1	case_or_format
Chest symptoms	chest symptoms	chest symptoms	chest symptoms	Chest symptoms	1	kept
chest trauma	chest trauma	chest trauma	chest trauma	chest trauma	1	kept
Chest tube bleeding	chest tube bleeding	chest tube bleeding	chest tube bleeding	Chest tube bleeding	1	kept
Chest tube discomfort	chest tube discomfort	chest tube discomfort	chest tube discomfort	Chest tube discomfort	1	kept
Chest tube dressing	chest tube dressing	chest tube dressing	chest tube dressing	Chest tube dressing	1	kept
chest tube management	chest tube management	chest tube management	chest tube management	chest tube management	1	kept
Chest tube patency	chest tube patency	chest tube patency	chest tube patency	Chest tube patency	1	kept
CHEST TUBE REMOVAL	chest tube removal	chest tube removal	chest tube removal	CHEST TUBE REMOVAL	1	kept
Chest tube removal	chest tube removal	chest tube removal	chest tube removal	CHEST TUBE REMOVAL	1	case_or_format
chest tube thoracostomy	chest tube thoracostomy	chest tube thoracostomy	chest tube thoracostomy	chest tube thoracostomy	1	kept
Chest tubes	chest tubes	chest tubes	chest tube	Chest Tubes	1	singular
chest tubes	chest tubes	chest tubes	chest tube	Chest Tubes	1	singular

chest wall compression	chest wall compression	chest wall compression	chest wall compression	chest wall compression	1	kept
chest wall stiffness	chest wall stiffness	chest wall stiffness	chest wall stiffness	chest wall stiffness	1	kept
Chest X-ray	chest x ray	chest x ray	chest x ray	chest X-ray	1	case_or_format
CHEST-PAIN	chest pain	chest pain	chest pain	Chest Pain	1	case_or_format
chest-tube placement simulation	chest tube placement simulation	chest tube placement simulation	chest tube placement simulation	chest-tube placement simulation	1	kept
chestnut	chestnut	chestnut	chestnut	chestnut	1	kept
chewable tablet	chewable tablet	chewable tablet	chewable tablet	chewable tablet	1	kept
Chewing Gum	chewing gum	chewing gum	chewing gum	chewing gum	1	case_or_format
Cheyne Stokes breathing	cheyne stokes breathing	cheyne stokes breathing	cheyne stokes breathing	Cheyne Stokes breathing	1	kept
Chez-soi	chez soi	chez soi	chez soi	Chez-soi	1	kept
CHG	chg	chg	chg	CHG	1	kept
Chhattisgarh	chhattisgarh	chhattisgarh	chhattisgarh	Chhattisgarh	1	kept
Chhattisgarh India	chhattisgarh india	chhattisgarh india	chhattisgarh india	Chhattisgarh India	1	kept
chi inner harmony	chi inner harmony	chi inner harmony	chi inner harmony	chi inner harmony	1	kept
Chi square Automatic Interaction Detector decision tree model	chi square automatic interaction detector decision tree model	chi square automatic interaction detector decision tree model	chi square automatic interaction detector decision tree model	Chi square Automatic Interaction Detector decision tree model	1	kept
Chi square test	chi square test	chi square test	chi square test	chi square test	1	case_or_format

Chiari malformation	chiari malformation	chiari malformation	chiari malformation	Chiari malformation	1	kept
Chicken-pox	chicken pox	chicken pox	chicken pox	Chicken-pox	1	kept
Chickenpox outbreak	chickenpox outbreak	chickenpox outbreak	chickenpox outbreak	Chickenpox outbreak	1	kept
ChiCTR2200059093	chictr2200059093	chictr2200059093	chictr2200059093	ChiCTR2200059093	1	kept
chief complain	chief complain	chief complain	chief complain	chief complain	1	kept
Chief Doctor	chief doctor	chief doctor	chief doctor	Chief Doctor	1	kept
Chief Executive Officers, Hospital	chief executive officers hospital	chief executive officers hospital	chief executive officers hospital	Chief Executive Officers, Hospital	1	kept
Chihuahua	chihuahua	chihuahua	chihuahua	Chihuahua	1	kept
Chikungunya	chikungunya	chikungunya	chikungunya	chikungunya	1	case_or_format
Chikungunya virus	chikungunya virus	chikungunya virus	chikungunya virus	Chikungunya virus	1	kept
Child behavior	child behavior	child behavior	child behavior	child behavior	1	case_or_format
Child Custody	child custody	child custody	child custody	Child Custody	1	kept
child custody	child custody	child custody	child custody	Child Custody	1	case_or_format
Child Day Care Centers	child day care centers	child day care centers	child day care centers	Child Day Care Centers	1	kept
child deaths	child deaths	child deaths	child death	child death	1	singular
Child delirium assessment scale	child delirium assessment scale	child delirium assessment scale	child delirium assessment scale	Child delirium assessment scale	1	kept

Child Development Disorders, Pervasive	child development disorders pervasive	child development disorders pervasive	child development disorders pervasive	Child Development Disorders, Pervasive	1	kept
child experience	child experience	child experience	child experience	child experience	1	kept
Child guidance clinics	child guidance clinics	child guidance clinics	child guidance clinics	Child guidance clinics	1	kept
child health insurance	child health insurance	child health insurance	child health insurance	child health insurance	1	kept
child health workforce	child health workforce	child health workforce	child health workforce	child health workforce	1	kept
Child Hospitalized	child hospitalized	child hospitalized	child hospitalized	Child, Hospitalized	1	case_or_format
Child hospitalized	child hospitalized	child hospitalized	child hospitalized	Child, Hospitalized	1	case_or_format
child hospitalized	child hospitalized	child hospitalized	child hospitalized	Child, Hospitalized	1	case_or_format
Child maltreatment	child maltreatment	child maltreatment	child maltreatment	Child maltreatment	1	kept
Child mortality	child mortality	child mortality	child mortality	Child Mortality	1	case_or_format
Child Nutritional Physiological Phenomena	child nutritional physiological phenomena	child nutritional physiological phenomena	child nutritional physiological phenomena	Child Nutritional Physiological Phenomena	1	kept
Child of Impaired Parents	child of impaired parents	child of impaired parents	child of impaired parents	Child of Impaired Parents	1	kept
Child passenger safety	child passenger safety	child passenger safety	child passenger safety	Child passenger safety	1	kept
Child physical abuse	child physical abuse	child physical abuse	child physical abuse	Child physical abuse	1	kept
child physical abuse	child physical abuse	child physical abuse	child physical abuse	Child physical abuse	1	case_or_format
child post traumatic stress disorder symptom scale	child post traumatic stress disorder symptom scale	child post traumatic stress disorder symptom scale	child post traumatic stress disorder symptom scale	child post traumatic stress disorder symptom scale	1	kept

child posttraumatic stress disorder symptom score	child posttraumatic stress disorder symptom score	child posttraumatic stress disorder symptom score	child posttraumatic stress disorder symptom score	child posttraumatic stress disorder symptom score	1	kept
Child Poverty	child poverty	child poverty	child poverty	Child Poverty	1	kept
Child Psychiatry	child psychiatry	child psychiatry	child psychiatry	child psychiatry	1	case_or_format
Child psychiatry	child psychiatry	child psychiatry	child psychiatry	child psychiatry	1	case_or_format
Child restraint systems	child restraint systems	child restraint systems	child restraint system	Child Restraint Systems	1	singular
child rights	child rights	child rights	child rights	child rights	1	kept
child sexual abuse	child sexual abuse	child sexual abuse	child sexual abuse	child sexual abuse	1	kept
Child survival	child survival	child survival	child survival	Child survival	1	kept
child urology	child urology	child urology	child urology	child urology	1	kept
Child visitation	child visitation	child visitation	child visitation	Child visitation	1	kept
Child visitors	child visitors	child visitors	child visitors	Child visitors	1	kept
CHILD VULNERABILITY	child vulnerability	child vulnerability	child vulnerability	CHILD VULNERABILITY	1	kept
child vulnerability	child vulnerability	child vulnerability	child vulnerability	CHILD VULNERABILITY	1	case_or_format
child well being	child well being	child well being	child well being	child well being	1	kept
Child with a disability	child with a disability	child with a disability	child with a disability	Child with a disability	1	kept
Child • Visit	child visit	child visit	child visit	Child • Visit	1	kept

Child, preschool	child preschool	child preschool	child preschool	Child, Preschool	1	case_or_format
Child-Centered Care	child centered care	child centered care	child centered care	Child-Centered Care	1	kept
CHILD-MORTALITY	child mortality	child mortality	child mortality	Child Mortality	1	case_or_format
Child-Pugh	child pugh	child pugh	child pugh	Child-Pugh	1	kept
child-rearing	child rearing	child rearing	child rearing	child-rearing	1	kept
Childbearing culture	childbearing culture	childbearing culture	childbearing culture	Childbearing culture	1	kept
Childbirth and Health	childbirth and health	childbirth and health	childbirth and health	Childbirth and Health	1	kept
childbirth educator	childbirth educator	childbirth educator	childbirth educator	childbirth educator	1	kept
childbirth hospital	childbirth hospital	childbirth hospital	childbirth hospital	childbirth hospital	1	kept
childbirth hospital types	childbirth hospital types	childbirth hospital types	childbirth hospital types	childbirth hospital types	1	kept
childbirth satisfaction	childbirth satisfaction	childbirth satisfaction	childbirth satisfaction	childbirth satisfaction	1	kept
Childbirth trauma	childbirth trauma	childbirth trauma	childbirth trauma	Childbirth trauma	1	kept
Childbirth-related post-traumatic stress disorder	childbirth related post traumatic stress disorder	childbirth related post traumatic stress disorder	childbirth related post traumatic stress disorder	Childbirth-related post-traumatic stress disorder	1	kept
childcare	childcare	childcare	childcare	Childcare	1	case_or_format
CHILDHOOD	childhood	childhood	childhood	childhood	1	case_or_format
Childhood blindness	childhood blindness	childhood blindness	childhood blindness	Childhood blindness	1	kept

childhood blindness prevention	childhood blindness prevention	childhood blindness prevention	childhood blindness prevention	childhood blindness prevention	1	kept
Childhood death	childhood death	childhood death	childhood death	Childhood death	1	kept
childhood epilepsy	childhood epilepsy	childhood epilepsy	childhood epilepsy	childhood epilepsy	1	kept
childhood leukemia	childhood leukemia	childhood leukemia	childhood leukemia	childhood leukemia	1	kept
childhood neurodevelopmental disorders	childhood neurodevelopmental disorders	childhood neurodevelopmental disorders	childhood neurodevelopmental disorders	childhood neurodevelopmental disorders	1	kept
CHILDHOOD OBESITY	childhood obesity	childhood obesity	childhood obesity	childhood obesity	1	case_or_format
childhood stroke	childhood stroke	childhood stroke	childhood stroke	childhood stroke	1	kept
Childhood TB diagnostics	childhood tb diagnostics	childhood tb diagnostics	childhood tb diagnostics	Childhood TB diagnostics	1	kept
CHILDHOOD-CANCER	childhood cancer	childhood cancer	childhood cancer	childhood cancer	1	case_or_format
children and infant care	children and infant care	children and infant care	children and infant care	children and infant care	1	kept
Children Cardiovascular Early Warning Scale	children cardiovascular early warning scale	children cardiovascular early warning scale	children cardiovascular early warning scale	Children Cardiovascular Early Warning Scale	1	kept
Children visiting	children visiting	children visiting	children visiting	Children visiting	1	kept
Children with complex chronic conditions	children with complex chronic conditions	children with complex chronic conditions	children with complex chronic conditions	Children with complex chronic conditions	1	kept
children with pneumonia	children with pneumonia	children with pneumonia	children with pneumonia	children with pneumonia	1	kept
Children with special health care needs	children with special health care needs	children with special health care needs	children with special health care needs	Children with special health care needs	1	kept
children with special healthcare needs	children with special healthcare needs	children with special healthcare needs	children with special healthcare needs	children with special healthcare needs	1	kept

Children Yale Brown Obsessive Compulsive Scale	children yale brown obsessive compulsive scale	children yale brown obsessive compulsive scale	children yale brown obsessive compulsive scale	Children Yale Brown Obsessive Compulsive Scale	1	kept
Children's Early Warning Tool	children s early warning tool	children s early warning tool	children s early warning tool	Children's Early Warning Tool	1	kept
Children's Health	children s health	children s health	children s health	Children's Health	1	kept
Children's hospital	children s hospital	children s hospital	children s hospital	Children's hospital	1	kept
children's hospital	children s hospital	children s hospital	children s hospital	Children's hospital	1	case_or_format
Children's Hospital Association	children s hospital association	children s hospital association	children s hospital association	Children's Hospital Association	1	kept
Children's hospital neonatal database	children s hospital neonatal database	children s hospital neonatal database	children s hospital neonatal database	Children's hospital neonatal database	1	kept
Children's pain	children s pain	children s pain	children s pain	Children's pain	1	kept
children's rights	children s rights	children s rights	children s rights	children's rights	1	kept
Children's surgical services	children s surgical services	children s surgical services	children s surgical services	Children's surgical services	1	kept
Children's wards	children s wards	children s wards	children s wards	Children's wards	1	kept
Children’ s brain injury global health	children s brain injury global health	children s brain injury global health	children s brain injury global health	Children’ s brain injury global health	1	kept
children’ s brain injury global health	children s brain injury global health	children s brain injury global health	children s brain injury global health	Children’ s brain injury global health	1	case_or_format
children’ s deaths	children s deaths	children s deaths	children s deaths	children’ s deaths	1	kept
Children’ s health	children s health	children s health	children s health	Children's Health	1	case_or_format
children’ s health	children s health	children s health	children s health	Children's Health	1	case_or_format

Children’ s Hospital	children s hospital	children s hospital	children s hospital	Children’s hospital	1	case_or_format
children’ s visits	children s visits	children s visits	children s visits	children’ s visits	1	kept
child‑ and family‑ centred care	child and family centred care	child and family centred care	child and family centred care	child‑ and family‑ centred care	1	kept
chimeric antigen receptor T cell therapy	chimeric antigen receptor t cell therapy	chimeric antigen receptor t cell therapy	chimeric antigen receptor t cell therapy	chimeric antigen receptor T cell therapy	1	kept
chimeric antigen receptor T-cell therapy	chimeric antigen receptor t cell therapy	chimeric antigen receptor t cell therapy	chimeric antigen receptor t cell therapy	chimeric antigen receptor T cell therapy	1	case_or_format
CHINA	china	china	china	China	1	case_or_format
chinese essen trauma inventory	chinese essen trauma inventory	chinese essen trauma inventory	chinese essen trauma inventory	chinese essen trauma inventory	1	kept
CHINESE VERSION	chinese version	chinese version	chinese version	Chinese version	1	case_or_format
Chinese-Americans	chinese americans	chinese americans	chinese americans	Chinese-Americans	1	kept
chinstraps	chinstraps	chinstraps	chinstraps	chinstraps	1	kept
chirurgie	chirurgie	chirurgie	chirurgie	chirurgie	1	kept
Chitosan	chitosan	chitosan	chitosan	Chitosan	1	kept
Chlamydia trachomatis	chlamydia trachomatis	chlamydia trachomatis	chlamydia trachomatis	Chlamydia trachomatis	1	kept
Chlamydophila	chlamydophila	chlamydophila	chlamydophila	Chlamydophila	1	kept
CHLORAL HYDRATE	chloral hydrate	chloral hydrate	chloral hydrate	chloral hydrate	1	case_or_format
Chloramphenicol	chloramphenicol	chloramphenicol	chloramphenicol	chloramphenicol	1	case_or_format

Chlordiazepoxide	chlordiazepoxide	chlordiazepoxide	chlordiazepoxide	chlordiazepoxide	1	case_or_format
Chlorhexidine bath	chlorhexidine bath	chlorhexidine bath	chlorhexidine bath	Chlorhexidine bath	1	kept
Chlorhexidine bathing	chlorhexidine bathing	chlorhexidine bathing	chlorhexidine bathing	chlorhexidine bathing	1	case_or_format
chlorhexidine cloths	chlorhexidine cloths	chlorhexidine cloths	chlorhexidine cloths	chlorhexidine cloths	1	kept
Chlorhexidine dressing	chlorhexidine dressing	chlorhexidine dressing	chlorhexidine dressing	Chlorhexidine dressing	1	kept
Chlorhexidine Gluconate	chlorhexidine gluconate	chlorhexidine gluconate	chlorhexidine gluconate	chlorhexidine gluconate	1	case_or_format
Chlorhexidine gluconate solution	chlorhexidine gluconate solution	chlorhexidine gluconate solution	chlorhexidine gluconate solution	Chlorhexidine gluconate solution	1	kept
Chlorhexidine gluconate washcloths	chlorhexidine gluconate washcloths	chlorhexidine gluconate washcloths	chlorhexidine gluconate washcloths	Chlorhexidine gluconate washcloths	1	kept
Chlorhexidine securement dressing	chlorhexidine securement dressing	chlorhexidine securement dressing	chlorhexidine securement dressing	Chlorhexidine securement dressing	1	kept
Chlorhexidine-gluconate impregnated dressing	chlorhexidine gluconate impregnated dressing	chlorhexidine gluconate impregnated dressing	chlorhexidine gluconate impregnated dressing	Chlorhexidine-gluconate impregnated dressing	1	kept
Chlorhexidine-impregnated sponges	chlorhexidine impregnated sponges	chlorhexidine impregnated sponges	chlorhexidine impregnated sponges	Chlorhexidine-impregnated sponges	1	kept
chloride transport	chloride transport	chloride transport	chloride transport	chloride transport	1	kept
chlorinated water	chlorinated water	chlorinated water	chlorinated water	chlorinated water	1	kept
Chlorine compounds	chlorine compounds	chlorine compounds	chlorine compounds	Chlorine compounds	1	kept
chlorobenzene	chlorobenzene	chlorobenzene	chlorobenzene	chlorobenzene	1	kept
Chlorobenzenes	chlorobenzenes	chlorobenzenes	chlorobenzene	chlorobenzene	1	singular

Chlorobutanol	chlorobutanol	chlorobutanol	chlorobutanol	Chlorobutanol	1	kept
chlorothiazide	chlorothiazide	chlorothiazide	chlorothiazide	chlorothiazide	1	kept
chlorpheniramine maleate	chlorpheniramine maleate	chlorpheniramine maleate	chlorpheniramine maleate	chlorpheniramine maleate	1	kept
chlorpheniramine maleate plus ibuprofen plus pseudoephedrine	chlorpheniramine maleate plus ibuprofen plus pseudoephedrine	chlorpheniramine maleate plus ibuprofen plus pseudoephedrine	chlorpheniramine maleate plus ibuprofen plus pseudoephedrine	chlorpheniramine maleate plus ibuprofen plus pseudoephedrine	1	kept
chlorphenotane	chlorphenotane	chlorphenotane	chlorphenotane	chlorphenotane	1	kept
choana atresia	choana atresia	choana atresia	choana atresia	choana atresia	1	kept
choc septique	choc septique	choc septique	choc septique	choc septique	1	kept
Chocolate	chocolate	chocolate	chocolate	chocolate	1	case_or_format
CHOICE BEHAVIOUR	choice behaviour	choice behaviour	choice behaviour	CHOICE BEHAVIOUR	1	kept
choice of drugs	choice of drugs	choice of drugs	choice of drugs	choice of drugs	1	kept
choking	choking	choking	choking	choking	1	kept
cholangiography	cholangiography	cholangiography	cholangiography	cholangiography	1	kept
Cholangiopancreatography, Endoscopic Retrograde	cholangiopancreatography endoscopic retrograde	cholangiopancreatography endoscopic retrograde	cholangiopancreatography endoscopic retrograde	Cholangiopancreatography, Endoscopic Retrograde	1	kept
cholecystoduodenostomy	cholecystoduodenostomy	cholecystoduodenostomy	cholecystoduodenostomy	cholecystoduodenostomy	1	kept
choledocholithotomy	choledocholithotomy	choledocholithotomy	choledocholithotomy	choledocholithotomy	1	kept
cholera toxin	cholera toxin	cholera toxin	cholera toxin	cholera toxin	1	kept

cholera vaccine	cholera vaccine	cholera vaccine	cholera vaccine	cholera vaccine	1	kept
Cholesterol	cholesterol	cholesterol	cholesterol	cholesterol	1	case_or_format
Cholesterol, HDL	cholesterol hdl	cholesterol hdl	cholesterol hdl	Cholesterol, HDL	1	kept
cholestin	cholestin	cholestin	cholestin	cholestin	1	kept
choline magnesium trisalicylate	choline magnesium trisalicylate	choline magnesium trisalicylate	choline magnesium trisalicylate	choline magnesium trisalicylate	1	kept
Cholinergic transmission	cholinergic transmission	cholinergic transmission	cholinergic transmission	Cholinergic transmission	1	kept
Cholinesterase activity	cholinesterase activity	cholinesterase activity	cholinesterase activity	Cholinesterase activity	1	kept
Cholinesterase inhibitor	cholinesterase inhibitor	cholinesterase inhibitor	cholinesterase inhibitor	cholinesterase inhibitor	1	case_or_format
Cholinesterase Inhibitors	cholinesterase inhibitors	cholinesterase inhibitors	cholinesterase inhibitor	cholinesterase inhibitor	1	singular
Cholinesterase inhibitors	cholinesterase inhibitors	cholinesterase inhibitors	cholinesterase inhibitor	cholinesterase inhibitor	1	singular
Cholinesterases	cholinesterases	cholinesterases	cholinesterase	cholinesterase	1	singular
chondrodysplasia	chondrodysplasia	chondrodysplasia	chondrodysplasia	chondrodysplasia	1	kept
Choosing Wisely	choosing wisely	choosing wisely	choosing wisely	Choosing Wisely	1	kept
choosing wisely	choosing wisely	choosing wisely	choosing wisely	Choosing Wisely	1	case_or_format
chorion villus sampling	chorion villus sampling	chorion villus sampling	chorion villus sampling	chorion villus sampling	1	kept
Chorionic Gonadotropin	chorionic gonadotropin	chorionic gonadotropin	chorionic gonadotropin	chorionic gonadotropin	1	case_or_format

Chorionic Villi Sampling	chorionic villi sampling	chorionic villi sampling	chorionic villi sampling	Chorionic Villi Sampling	1	kept
Chorioretinitis	chorioretinitis	chorioretinitis	chorioretinitis	Chorioretinitis	1	kept
chorioretinopathy	chorioretinopathy	chorioretinopathy	chorioretinopathy	chorioretinopathy	1	kept
Chromatography, Gas	chromatography gas	chromatography gas	chromatography gas	Chromatography, Gas	1	kept
Chromium	chromium	chromium	chromium	chromium	1	case_or_format
chromium blood level	chromium blood level	chromium blood level	chromium blood level	chromium blood level	1	kept
chromoendoscopy	chromoendoscopy	chromoendoscopy	chromoendoscopy	chromoendoscopy	1	kept
CHROMOGENIC AGAR	chromogenic agar	chromogenic agar	chromogenic agar	CHROMOGENIC AGAR	1	kept
Chromophores	chromophores	chromophores	chromophores	Chromophores	1	kept
chromosomal	chromosomal	chromosomal	chromosomal	chromosomal	1	kept
chromosomal anomalies	chromosomal anomalies	chromosomal anomalies	chromosomal anomalies	chromosomal anomalies	1	kept
chromosome 3	chromosome 3	chromosome 3	chromosome 3	chromosome 3	1	kept
chromosome 4	chromosome 4	chromosome 4	chromosome 4	chromosome 4	1	kept
chromosome 6	chromosome 6	chromosome 6	chromosome 6	chromosome 6	1	kept
chromosome 8	chromosome 8	chromosome 8	chromosome 8	chromosome 8	1	kept
chromosome 9	chromosome 9	chromosome 9	chromosome 9	chromosome 9	1	kept

chromosome analysis	chromosome analysis	chromosome analysis	chromosome analysis	chromosome analysis	1	kept
Chromosome Disorders	chromosome disorders	chromosome disorders	chromosome disorder	chromosome disorder	1	singular
chromosome map	chromosome map	chromosome map	chromosome map	chromosome map	1	kept
chromosome mutation	chromosome mutation	chromosome mutation	chromosome mutation	chromosome mutation	1	kept
Chromosome Painting	chromosome painting	chromosome painting	chromosome painting	Chromosome Painting	1	kept
chromosome painting	chromosome painting	chromosome painting	chromosome painting	Chromosome Painting	1	case_or_format
Chromosomes, Bacterial	chromosomes bacterial	chromosomes bacterial	chromosomes bacterial	Chromosomes, Bacterial	1	kept
Chromosomes, Fungal	chromosomes fungal	chromosomes fungal	chromosomes fungal	Chromosomes, Fungal	1	kept
Chromotherapy	chromotherapy	chromotherapy	chromotherapy	Chromotherapy	1	kept
chronic	chronic	chronic	chronic	Chronic	1	case_or_format
chronic airways disease	chronic airways disease	chronic airways disease	chronic airways disease	chronic airways disease	1	kept
chronic brain disease	chronic brain disease	chronic brain disease	chronic brain disease	chronic brain disease	1	kept
Chronic care	chronic care	chronic care	chronic care	chronic care	1	case_or_format
chronic care facilities	chronic care facilities	chronic care facilities	chronic care facility	chronic care facility	1	singular
chronic care facility	chronic care facility	chronic care facility	chronic care facility	chronic care facility	1	kept
Chronic care model	chronic care model	chronic care model	chronic care model	Chronic care model	1	kept

Chronic childhood disease	chronic childhood disease	chronic childhood disease	chronic childhood disease	Chronic childhood disease	1	kept
chronic constipation	chronic constipation	chronic constipation	chronic constipation	chronic constipation	1	kept
chronic cough	chronic cough	chronic cough	chronic cough	chronic cough	1	kept
chronic critically ill	chronic critically ill	chronic critically ill	chronic critically ill	chronic critically ill	1	kept
chronic critically illness	chronic critically illness	chronic critically illness	chronic critically illness	chronic critically illness	1	kept
CHRONIC DISEASE	chronic disease	chronic disease	chronic disease	Chronic Disease	1	case_or_format
Chronic diseases	chronic diseases	chronic diseases	chronic disease	Chronic Disease	1	singular
chronic drug administration	chronic drug administration	chronic drug administration	chronic drug administration	chronic drug administration	1	kept
Chronic exposure	chronic exposure	chronic exposure	chronic exposure	Chronic exposure	1	kept
Chronic fatigue syndrome	chronic fatigue syndrome	chronic fatigue syndrome	chronic fatigue syndrome	chronic fatigue syndrome	1	case_or_format
chronic graft rejection	chronic graft rejection	chronic graft rejection	chronic graft rejection	chronic graft rejection	1	kept
CHRONIC HEALTH EVALUATION	chronic health evaluation	chronic health evaluation	chronic health evaluation	CHRONIC HEALTH EVALUATION	1	kept
chronic health evaluation II score	chronic health evaluation ii score	chronic health evaluation ii score	chronic health evaluation ii score	chronic health evaluation II score	1	kept
chronic heart disease	chronic heart disease	chronic heart disease	chronic heart disease	chronic heart disease	1	kept
Chronic heart failure	chronic heart failure	chronic heart failure	chronic heart failure	Chronic heart failure	1	kept
Chronic heart failures	chronic heart failures	chronic heart failures	chronic heart failure	Chronic heart failure	1	singular

CHRONIC HEART-FAILURE	chronic heart failure	chronic heart failure	chronic heart failure	Chronic heart failure	1	case_or_format
chronic hemodialysis	chronic hemodialysis	chronic hemodialysis	chronic hemodialysis	chronic hemodialysis	1	kept
Chronic hospitalization	chronic hospitalization	chronic hospitalization	chronic hospitalization	Chronic hospitalization	1	kept
chronic hypertension	chronic hypertension	chronic hypertension	chronic hypertension	chronic hypertension	1	kept
chronic hypoxemic respiratory failure	chronic hypoxemic respiratory failure	chronic hypoxemic respiratory failure	chronic hypoxemic respiratory failure	chronic hypoxemic respiratory failure	1	kept
chronic illnesses	chronic illnesses	chronic illnesses	chronic illnesses	chronic illnesses	1	kept
Chronic inflammation	chronic inflammation	chronic inflammation	chronic inflammation	chronic inflammation	1	case_or_format
chronic insomnia	chronic insomnia	chronic insomnia	chronic insomnia	chronic insomnia	1	kept
chronic leukemia	chronic leukemia	chronic leukemia	chronic leukemia	chronic leukemia	1	kept
Chronic limb-threatening	chronic limb threatening	chronic limb threatening	chronic limb threatening	Chronic limb-threatening	1	kept
Chronic limb-threatening ischemia	chronic limb threatening ischemia	chronic limb threatening ischemia	chronic limb threatening ischemia	Chronic Limb-Threatening Ischemia	1	case_or_format
chronic lung allograft dysfunction	chronic lung allograft dysfunction	chronic lung allograft dysfunction	chronic lung allograft dysfunction	chronic lung allograft dysfunction	1	kept
chronic lymphocytic leukemia	chronic lymphocytic leukemia	chronic lymphocytic leukemia	chronic lymphocytic leukemia	chronic lymphocytic leukemia	1	kept
Chronic medications	chronic medications	chronic medications	chronic medications	Chronic medications	1	kept
chronic obstructive pulmonary disease decision modeling	chronic obstructive pulmonary disease decision modeling	chronic obstructive pulmonary disease decision modeling	chronic obstructive pulmonary disease decision modeling	chronic obstructive pulmonary disease decision modeling	1	kept
chronic obstructive pulmonary disease exacerbations	chronic obstructive pulmonary disease exacerbations	chronic obstructive pulmonary disease exacerbations	chronic obstructive pulmonary disease exacerbations	chronic obstructive pulmonary disease exacerbations	1	kept

chronic obstructive pulmonary diseases	chronic obstructive pulmonary diseases	chronic obstructive pulmonary diseases	chronic obstructive pulmonary disease	Chronic obstructive pulmonary disease	1	singular
CHRONIC PAIN	chronic pain	chronic pain	chronic pain	chronic pain	1	case_or_format
chronic pain in pregnancy	chronic pain in pregnancy	chronic pain in pregnancy	chronic pain in pregnancy	chronic pain in pregnancy	1	kept
chronic pain post vaccination	chronic pain post vaccination	chronic pain post vaccination	chronic pain post vaccination	chronic pain post vaccination	1	kept
chronic post-COVID pain 2	chronic post covid pain 2	chronic post covid pain 2	chronic post covid pain 2	chronic post-COVID pain 2	1	kept
Chronic pulmonary obstructive disease	chronic pulmonary obstructive disease	chronic pulmonary obstructive disease	chronic pulmonary obstructive disease	Chronic pulmonary obstructive disease	1	kept
Chronic renal insufficiency	chronic renal insufficiency	chronic renal insufficiency	chronic renal insufficiency	Chronic renal insufficiency	1	kept
Chronic respiratory diseases	chronic respiratory diseases	chronic respiratory diseases	chronic respiratory diseases	Chronic respiratory diseases	1	kept
chronic sialorrhea	chronic sialorrhea	chronic sialorrhea	chronic sialorrhea	chronic sialorrhea	1	kept
chronic sinusitis	chronic sinusitis	chronic sinusitis	chronic sinusitis	chronic sinusitis	1	kept
Chronic sleep problems	chronic sleep problems	chronic sleep problems	chronic sleep problems	Chronic sleep problems	1	kept
Chronic sorrow	chronic sorrow	chronic sorrow	chronic sorrow	Chronic sorrow	1	kept
chronic stress syndrome	chronic stress syndrome	chronic stress syndrome	chronic stress syndrome	chronic stress syndrome	1	kept
chronic subdural	chronic subdural	chronic subdural	chronic subdural	chronic subdural	1	kept
Chronic thromboembolic pulmonary hypertension	chronic thromboembolic pulmonary hypertension	chronic thromboembolic pulmonary hypertension	chronic thromboembolic pulmonary hypertension	Chronic thromboembolic pulmonary hypertension	1	kept
chronic thromboembolic pulmonary hypertension	chronic thromboembolic pulmonary hypertension	chronic thromboembolic pulmonary hypertension	chronic thromboembolic pulmonary hypertension	Chronic thromboembolic pulmonary hypertension	1	case_or_format

chronic viral hepatitis	chronic viral hepatitis	chronic viral hepatitis	chronic viral hepatitis	chronic viral hepatitis	1	kept
Chronically critically III	chronically critically iii	chronically critically iii	chronically critically iii	Chronically critically III	1	kept
Chronically Critically Ill	chronically critically ill	chronically critically ill	chronically critically ill	chronically critically ill	1	case_or_format
Chronically ill	chronically ill	chronically ill	chronically ill	Chronically ill	1	kept
chronodisruption	chronodisruption	chronodisruption	chronodisruption	chronodisruption	1	kept
Chronological age	chronological age	chronological age	chronological age	Chronological age	1	kept
chronology	chronology	chronology	chronology	chronology	1	kept
Chronology as Topic	chronology as topic	chronology as topic	chronology as topic	Chronology as Topic	1	kept
Chronophobia	chronophobia	chronophobia	chronophobia	Chronophobia	1	kept
chronophobia	chronophobia	chronophobia	chronophobia	Chronophobia	1	case_or_format
chronotherapeutics	chronotherapeutics	chronotherapeutics	chronotherapeutics	chronotherapeutics	1	kept
chronotherapy	chronotherapy	chronotherapy	chronotherapy	chronotherapy	1	kept
Chryseobacterium indologenes	chryseobacterium indologenes	chryseobacterium indologenes	chryseobacterium indologenes	Chryseobacterium indologenes	1	kept
chyle	chyle	chyle	chyle	chyle	1	kept
chyluria	chyluria	chyluria	chyluria	chyluria	1	kept
Chyme recycling	chyme recycling	chyme recycling	chyme recycling	Chyme recycling	1	kept

CI - confidence interval	ci confidence interval	ci confidence interval	ci confidence interval	CI, Confidence interval	1	case_or_format
CI, Confidence interval	ci confidence interval	ci confidence interval	ci confidence interval	CI, Confidence interval	1	kept
CI, confidence interval	ci confidence interval	ci confidence interval	ci confidence interval	CI, Confidence interval	1	case_or_format
Ciaglia serial dilatational technique	ciaglia serial dilatational technique	ciaglia serial dilatational technique	ciaglia serial dilatational technique	Ciaglia serial dilatational technique	1	kept
Ciaglia single dilator tracheostomy	ciaglia single dilator tracheostomy	ciaglia single dilator tracheostomy	ciaglia single dilator tracheostomy	Ciaglia single dilator tracheostomy	1	kept
Cialgia technique	cialgia technique	cialgia technique	cialgia technique	Cialgia technique	1	kept
Cicatrix, Hypertrophic	cicatrix hypertrophic	cicatrix hypertrophic	cicatrix hypertrophic	Cicatrix, Hypertrophic	1	kept
Cidosporin	cidosporin	cidosporin	cidosporin	Cidosporin	1	kept
Ciencia de la implementación	ciencia de la implementaci n	ciencia de la implementaci n	ciencia de la implementaci n	Ciencia de la implementación	1	kept
cilastatin sodium plus imipenem plus relebactam	cilastatin sodium plus imipenem plus relebactam	cilastatin sodium plus imipenem plus relebactam	cilastatin sodium plus imipenem plus relebactam	cilastatin sodium plus imipenem plus relebactam	1	kept
Cilia	cilia	cilia	cilia	Cilia	1	kept
ciliary dyskinesia	ciliary dyskinesia	ciliary dyskinesia	ciliary dyskinesia	ciliary dyskinesia	1	kept
ciliary motility	ciliary motility	ciliary motility	ciliary motility	ciliary motility	1	kept
Cilium	cilium	cilium	cilium	Cilium	1	kept
Cimicifuga	cimicifuga	cimicifuga	cimicifuga	Cimicifuga	1	kept
cinacalcet	cinacalcet	cinacalcet	cinacalcet	cinacalcet	1	kept

cineole	cineole	cineole	cineole	cineole	1	kept
cinnamedrine	cinnamedrine	cinnamedrine	cinnamedrine	cinnamedrine	1	kept
CINV	cinv	cinv	cinv	CINV	1	kept
CIP	cip	cip	cip	CIP	1	kept
CIPROFLOXACIN	ciprofloxacin	ciprofloxacin	ciprofloxacin	ciprofloxacin	1	case_or_format
Ciprofol	ciprofol	ciprofol	ciprofol	Ciprofol	1	kept
ciraparantag	ciraparantag	ciraparantag	ciraparantag	ciraparantag	1	kept
Circadian	circadian	circadian	circadian	Circadian	1	kept
circadian	circadian	circadian	circadian	Circadian	1	case_or_format
circadian clock	circadian clock	circadian clock	circadian clock	circadian clock	1	kept
Circadian Clocks	circadian clocks	circadian clocks	circadian clock	circadian clock	1	singular
circadian misalignment	circadian misalignment	circadian misalignment	circadian misalignment	circadian misalignment	1	kept
circadian rhythm disorder	circadian rhythm disorder	circadian rhythm disorder	circadian rhythm disorder	circadian rhythm disorder	1	kept
Circadian Rhytm	circadian rhytm	circadian rhytm	circadian rhytm	Circadian Rhytm	1	kept
Circadian stimulus	circadian stimulus	circadian stimulus	circadian stimulus	Circadian stimulus	1	kept
circadian stimulus	circadian stimulus	circadian stimulus	circadian stimulus	Circadian stimulus	1	case_or_format

Circle of Willis	circle of willis	circle of willis	circle of willis	Circle of Willis	1	kept
Circuit	circuit	circuit	circuit	Circuit	1	kept
circuit lifespan	circuit lifespan	circuit lifespan	circuit lifespan	circuit lifespan	1	kept
Circuit survival	circuit survival	circuit survival	circuit survival	Circuit survival	1	kept
Circuit-Based Exercise	circuit based exercise	circuit based exercise	circuit based exercise	Circuit-Based Exercise	1	kept
Circulación Sanguínea	circulaci n sangu nea	circulaci n sangu nea	circulaci n sangu nea	Circulación Sanguínea	1	kept
Circulation	circulation	circulation	circulation	circulation	1	case_or_format
circulation spaces	circulation spaces	circulation spaces	circulation spaces	circulation spaces	1	kept
Circulatory Arrest, Deep Hypothermia Induced	circulatory arrest deep hypothermia induced	circulatory arrest deep hypothermia induced	circulatory arrest deep hypothermia induced	Circulatory Arrest, Deep Hypothermia Induced	1	kept
circulatory collapse	circulatory collapse	circulatory collapse	circulatory collapse	circulatory collapse	1	kept
circulatory death	circulatory death	circulatory death	circulatory death	circulatory death	1	kept
Circulatory death donor	circulatory death donor	circulatory death donor	circulatory death donor	Circulatory death donor	1	kept
Circulação Sanguínea	circula o sangu nea	circula o sangu nea	circula o sangu nea	Circulação Sanguínea	1	kept
circumcision	circumcision	circumcision	circumcision	circumcision	1	kept
circumoral cyanosis	circumoral cyanosis	circumoral cyanosis	circumoral cyanosis	circumoral cyanosis	1	kept
CIRCUMSTANCES	circumstances	circumstances	circumstances	CIRCUMSTANCES	1	kept

cirrhose	cirrhose	cirrhose	cirrhose	cirrhose	1	kept
CIRS analysis	cirs analysis	cirs analysis	cirs analysis	CIRS analysis	1	kept
Cirugía cardiaca	cirug a cardiaca	cirug a cardiaca	cirug a cardiaca	Cirugía cardiaca	1	kept
CITATION-CLASSICS	citation classics	citation classics	citation classics	CITATION-CLASSICS	1	kept
citations	citations	citations	citations	citations	1	kept
Citizenship	citizenship	citizenship	citizenship	citizenship	1	case_or_format
CITRATE	citrate	citrate	citrate	Citrates	1	case_or_format
Citrus limon	citrus limon	citrus limon	citrus limon	Citrus limon	1	kept
citrus oil	citrus oil	citrus oil	citrus oil	citrus oil	1	kept
CITY	city	city	city	city	1	case_or_format
City Hospitals	city hospitals	city hospitals	city hospitals	City Hospitals	1	kept
civil disobedience	civil disobedience	civil disobedience	civil disobedience	civil disobedience	1	kept
Civil disorder	civil disorder	civil disorder	civil disorder	civil disorder	1	case_or_format
Civil Rights	civil rights	civil rights	civil rights	Civil Rights	1	kept
civil rights	civil rights	civil rights	civil rights	Civil Rights	1	case_or_format
Civility	civility	civility	civility	Civility	1	kept

CIWA	ciwa	ciwa	ciwa	CIWA	1	kept
CIWA-Ar	ciwa ar	ciwa ar	ciwa ar	CIWA-Ar	1	kept
ck 2066260	ck 2066260	ck 2066260	ck 2066260	ck 2066260	1	kept
CK, creatinine kinase	ck creatinine kinase	ck creatinine kinase	ck creatinine kinase	CK, creatinine kinase	1	kept
CK-MB	ck mb	ck mb	ck mb	CK-MB	1	kept
CKRT dose	ckrt dose	ckrt dose	ckrt dose	CKRT dose	1	kept
CLA-BSI	cla bsi	cla bsi	cla bsi	CLA-BSI	1	kept
CLABSI = central line - associated BSI	clabsi central line associated bsi	clabsi central line associated bsi	clabsi central line associated bsi	CLABSI = central line - associated BSI	1	kept
CLABSI prevention	clabsi prevention	clabsi prevention	clabsi prevention	CLABSI prevention	1	kept
CLABSI rate	clabsi rate	clabsi rate	clabsi rate	CLABSI rate	1	kept
cladribine	cladribine	cladribine	cladribine	cladribine	1	kept
Claim data	claim data	claim data	claim data	Claim data	1	kept
CLAIMS	claims	claims	claims	CLAIMS	1	kept
claims	claims	claims	claims	CLAIMS	1	case_or_format
Claims-based Frailty Index	claims based frailty index	claims based frailty index	claims based frailty index	Claims-based Frailty Index	1	kept
Clarke Error Grid	clarke error grid	clarke error grid	clarke error grid	Clarke Error Grid	1	kept

Clasificación	clasificaci n	clasificaci n	clasificaci n	Clasificación	1	kept
Class hospital	class hospital	class hospital	class hospital	Class hospital	1	kept
class of recommendation	class of recommendation	class of recommendation	class of recommendation	class of recommendation	1	kept
Classic grounded theory	classic grounded theory	classic grounded theory	classic grounded theory	Classic grounded theory	1	kept
Classical Lissencephalies and Subcortical Band	classical lissencephalies and subcortical band	classical lissencephalies and subcortical band	classical lissencephalies and subcortical band	Classical Lissencephalies and Subcortical Band	1	kept
Classical methods	classical methods	classical methods	classical methods	Classical methods	1	kept
Classical teaching method	classical teaching method	classical teaching method	classical teaching method	Classical teaching method	1	kept
Classification algorithm	classification algorithm	classification algorithm	classification algorithm	Classification algorithm	1	kept
classification algorithm	classification algorithm	classification algorithm	classification algorithm	Classification algorithm	1	case_or_format
classification and regression trees	classification and regression trees	classification and regression trees	classification and regression trees	Classification and regression trees	1	case_or_format
Classification machine learning techniques	classification machine learning techniques	classification machine learning techniques	classification machine learning techniques	Classification machine learning techniques	1	kept
Classification methods	classification methods	classification methods	classification methods	Classification methods	1	kept
Classification precision	classification precision	classification precision	classification precision	Classification precision	1	kept
Classification results	classification results	classification results	classification results	Classification results	1	kept
Classification systems	classification systems	classification systems	classification system	CLASSIFICATION-SYSTEM	1	singular
Classification technique	classification technique	classification technique	classification technique	Classification technique	1	kept

CLASSIFICATION-SYSTEM	classification system	classification system	classification system	CLASSIFICATION-SYSTEM	1	kept
CLASSIFICATION-SYSTEM EVALUATION	classification system evaluation	classification system evaluation	classification system evaluation	CLASSIFICATION-SYSTEM EVALUATION	1	kept
Classifying	classifying	classifying	classifying	Classifying	1	kept
CLASSROOM ASSIGNMENT	classroom assignment	classroom assignment	classroom assignment	CLASSROOM ASSIGNMENT	1	kept
Claude	claude	claude	claude	Claude	1	kept
CLAVE	clave	clave	clave	CLAVE	1	kept
Clavien‑Dindo classification	clavien dindo classification	clavien dindo classification	clavien dindo classification	Clavien‑Dindo classification	1	kept
Clean supply room	clean supply room	clean supply room	clean supply room	Clean supply room	1	kept
cleaning equipment	cleaning equipment	cleaning equipment	cleaning equipment	cleaning equipment	1	kept
Cleaning of the airways	cleaning of the airways	cleaning of the airways	cleaning of the airways	Cleaning of the airways	1	kept
cleaning quality	cleaning quality	cleaning quality	cleaning quality	cleaning quality	1	kept
cleaning training intervention	cleaning training intervention	cleaning training intervention	cleaning training intervention	cleaning training intervention	1	kept
clear cell sarcoma	clear cell sarcoma	clear cell sarcoma	clear cell sarcoma	clear cell sarcoma	1	kept
Clearlink	clearlink	clearlink	clearlink	Clearlink	1	kept
cleft lip and	cleft lip and	cleft lip and	cleft lip and	cleft lip and	1	kept
Cleft lip and palate	cleft lip and palate	cleft lip and palate	cleft lip and palate	Cleft lip and palate	1	kept

Cleft palate	cleft palate	cleft palate	cleft palate	cleft palate	1	case_or_format
cleft palate surgery	cleft palate surgery	cleft palate surgery	cleft palate surgery	cleft palate surgery	1	kept
CLEFT-LIP	cleft lip	cleft lip	cleft lip	cleft lip	1	case_or_format
Cleisthenes pinetorum	cleisthenes pinetorum	cleisthenes pinetorum	cleisthenes pinetorum	Cleisthenes pinetorum	1	kept
Cleveland	cleveland	cleveland	cleveland	Cleveland	1	kept
clevidipine	clevidipine	clevidipine	clevidipine	clevidipine	1	kept
Client satisfaction	client satisfaction	client satisfaction	client satisfaction	Client satisfaction	1	kept
Clima ético	clima tico	clima tico	clima tico	Clima ético	1	kept
Climacteric	climacteric	climacteric	climacteric	Climacteric	1	kept
climate conditions	climate conditions	climate conditions	climate conditions	climate conditions	1	kept
Climate impact	climate impact	climate impact	climate impact	Climate impact	1	kept
climate resilience	climate resilience	climate resilience	climate resilience	climate resilience	1	kept
CLIN	clin	clin	clin	CLIN	1	kept
Clinacanthus nutans Lindau extract	clinacanthus nutans lindau extract	clinacanthus nutans lindau extract	clinacanthus nutans lindau extract	Clinacanthus nutans Lindau extract	1	kept
clinic evaluation	clinic evaluation	clinic evaluation	clinic evaluation	clinic evaluation	1	kept
Clinic internal emergency management	clinic internal emergency management	clinic internal emergency management	clinic internal emergency management	Clinic internal emergency management	1	kept

CLINIC VISITS	clinic visits	clinic visits	clinic visits	CLINIC VISITS	1	kept
clinic visits	clinic visits	clinic visits	clinic visits	CLINIC VISITS	1	case_or_format
clinical academic	clinical academic	clinical academic	clinical academic	clinical academic	1	kept
Clinical accompaniment	clinical accompaniment	clinical accompaniment	clinical accompaniment	Clinical accompaniment	1	kept
clinical activity	clinical activity	clinical activity	clinical activity	clinical activity	1	kept
Clinical adoption framework	clinical adoption framework	clinical adoption framework	clinical adoption framework	Clinical adoption framework	1	kept
clinical adoption framework	clinical adoption framework	clinical adoption framework	clinical adoption framework	Clinical adoption framework	1	case_or_format
clinical advantages	clinical advantages	clinical advantages	clinical advantages	clinical advantages	1	kept
Clinical Alarm	clinical alarm	clinical alarm	clinical alarm	Clinical Alarms	1	case_or_format
clinical alarm management	clinical alarm management	clinical alarm management	clinical alarm management	clinical alarm management	1	kept
Clinical alarm system	clinical alarm system	clinical alarm system	clinical alarm system	Clinical alarm system	1	kept
CLINICAL ALARMS	clinical alarms	clinical alarms	clinical alarm	Clinical Alarms	1	singular
Clinical application	clinical application	clinical application	clinical application	clinical application	1	case_or_format
Clinical applications	clinical applications	clinical applications	clinical application	clinical application	1	singular
Clinical area transitions	clinical area transitions	clinical area transitions	clinical area transitions	Clinical area transitions	1	kept
Clinical assessment tools	clinical assessment tools	clinical assessment tools	clinical assessment tool	clinical assessment tool	1	singular

clinical assistant	clinical assistant	clinical assistant	clinical assistant	clinical assistant	1	kept
clinical audit, health services administration and	clinical audit health services administration and	clinical audit health services administration and	clinical audit health services administration and	clinical audit, health services administration and	1	kept
Clinical Autonomy	clinical autonomy	clinical autonomy	clinical autonomy	Clinical Autonomy	1	kept
clinical cancer research	clinical cancer research	clinical cancer research	clinical cancer research	clinical cancer research	1	kept
Clinical capability and confidence	clinical capability and confidence	clinical capability and confidence	clinical capability and confidence	Clinical capability and confidence	1	kept
clinical capital	clinical capital	clinical capital	clinical capital	clinical capital	1	kept
clinical care classification	clinical care classification	clinical care classification	clinical care classification	clinical care classification	1	kept
Clinical Care Classification System	clinical care classification system	clinical care classification system	clinical care classification system	Clinical Care Classification System	1	kept
clinical care pathways	clinical care pathways	clinical care pathways	clinical care pathways	clinical care pathways	1	kept
clinical care strategy	clinical care strategy	clinical care strategy	clinical care strategy	clinical care strategy	1	kept
clinical care time	clinical care time	clinical care time	clinical care time	clinical care time	1	kept
CLINICAL CHARACTERISTICS	clinical characteristics	clinical characteristics	clinical characteristics	clinical characteristics	1	case_or_format
Clinical characteristics	clinical characteristics	clinical characteristics	clinical characteristics	clinical characteristics	1	case_or_format
clinical chorioamnionitis	clinical chorioamnionitis	clinical chorioamnionitis	clinical chorioamnionitis	clinical chorioamnionitis	1	kept
Clinical clerk-ship	clinical clerk ship	clinical clerk ship	clinical clerk ship	Clinical clerk-ship	1	kept
Clinical clerkship	clinical clerkship	clinical clerkship	clinical clerkship	Clinical Clerkship	1	case_or_format

Clinical coach support	clinical coach support	clinical coach support	clinical coach support	Clinical coach support	1	kept
clinical coding	clinical coding	clinical coding	clinical coding	Clinical Coding	1	case_or_format
Clinical communication	clinical communication	clinical communication	clinical communication	Clinical communication	1	kept
clinical communication	clinical communication	clinical communication	clinical communication	Clinical communication	1	case_or_format
CLINICAL COMORBIDITY INDEX	clinical comorbidity index	clinical comorbidity index	clinical comorbidity index	CLINICAL COMORBIDITY INDEX	1	kept
Clinical Competencies	clinical competencies	clinical competencies	clinical competency	Clinical competency	1	singular
clinical competencies	clinical competencies	clinical competencies	clinical competency	Clinical competency	1	singular
Clinical complexity	clinical complexity	clinical complexity	clinical complexity	Clinical complexity	1	kept
clinical complexity	clinical complexity	clinical complexity	clinical complexity	Clinical complexity	1	case_or_format
Clinical Conclusions	clinical conclusions	clinical conclusions	clinical conclusions	Clinical Conclusions	1	kept
clinical conference	clinical conference	clinical conference	clinical conference	clinical conference	1	kept
clinical confidence	clinical confidence	clinical confidence	clinical confidence	clinical confidence	1	kept
clinical costing	clinical costing	clinical costing	clinical costing	clinical costing	1	kept
clinical coverage scoring system	clinical coverage scoring system	clinical coverage scoring system	clinical coverage scoring system	clinical coverage scoring system	1	kept
Clinical criteria	clinical criteria	clinical criteria	clinical criteria	Clinical criteria	1	kept
Clinical Dashboard	clinical dashboard	clinical dashboard	clinical dashboard	Clinical Dashboard	1	kept

Clinical dashboard	clinical dashboard	clinical dashboard	clinical dashboard	Clinical Dashboard	1	case_or_format
clinical dashboard	clinical dashboard	clinical dashboard	clinical dashboard	Clinical Dashboard	1	case_or_format
clinical data repository	clinical data repository	clinical data repository	clinical data repository	clinical data repository	1	kept
Clinical Data Warehouse	clinical data warehouse	clinical data warehouse	clinical data warehouse	Clinical Data Warehouse	1	kept
Clinical databases	clinical databases	clinical databases	clinical database	Clinical database	1	singular
clinical death	clinical death	clinical death	clinical death	clinical death	1	kept
Clinical debriefing	clinical debriefing	clinical debriefing	clinical debriefing	Clinical debriefing	1	kept
Clinical decision	clinical decision	clinical decision	clinical decision	Clinical decisions	1	case_or_format
Clinical decision - making	clinical decision making	clinical decision making	clinical decision making	clinical decision making	1	case_or_format
Clinical decision assessment system	clinical decision assessment system	clinical decision assessment system	clinical decision assessment system	Clinical decision assessment system	1	kept
Clinical decision processes	clinical decision processes	clinical decision processes	clinical decision processes	Clinical decision processes	1	kept
Clinical Decision Rules	clinical decision rules	clinical decision rules	clinical decision rule	clinical decision rules	1	singular
Clinical decision rules COVID-19 Risk Scores NEWS	clinical decision rules covid 19 risk scores news	clinical decision rules covid 19 risk scores news	clinical decision rules covid 19 risk scores news	Clinical decision rules COVID-19 Risk Scores NEWS	1	kept
clinical decision support tool	clinical decision support tool	clinical decision support tool	clinical decision support tool	clinical decision support tool	1	kept
Clinical Decision Supporting System	clinical decision supporting system	clinical decision supporting system	clinical decision supporting system	Clinical Decision Supporting System	1	kept
Clinical decision supporting system	clinical decision supporting system	clinical decision supporting system	clinical decision supporting system	Clinical Decision Supporting System	1	case_or_format

Clinical Decision Tree	clinical decision tree	clinical decision tree	clinical decision tree	Clinical Decision Tree	1	kept
clinical decisions	clinical decisions	clinical decisions	clinical decision	Clinical decisions	1	singular
clinical decisions rules	clinical decisions rules	clinical decisions rules	clinical decisions rules	clinical decisions rules	1	kept
Clinical decisions support systems	clinical decisions support systems	clinical decisions support systems	clinical decisions support systems	Clinical decisions support systems	1	kept
clinical department effect	clinical department effect	clinical department effect	clinical department effect	clinical department effect	1	kept
clinical deteriorations	clinical deteriorations	clinical deteriorations	clinical deterioration	Clinical Deterioration	1	singular
Clinical device	clinical device	clinical device	clinical device	Clinical device	1	kept
clinical diagnosis and treatment	clinical diagnosis and treatment	clinical diagnosis and treatment	clinical diagnosis and treatment	clinical diagnosis and treatment	1	kept
Clinical dietitian	clinical dietitian	clinical dietitian	clinical dietitian	Clinical dietitian	1	kept
clinical documentation audit	clinical documentation audit	clinical documentation audit	clinical documentation audit	clinical documentation audit	1	kept
Clinical effect	clinical effect	clinical effect	clinical effect	Clinical effect	1	kept
clinical effects	clinical effects	clinical effects	clinical effect	Clinical effect	1	singular
Clinical elective	clinical elective	clinical elective	clinical elective	Clinical elective	1	kept
Clinical emergency situations	clinical emergency situations	clinical emergency situations	clinical emergency situations	Clinical emergency situations	1	kept
Clinical engineering	clinical engineering	clinical engineering	clinical engineering	Clinical engineering	1	kept
Clinical Engineers	clinical engineers	clinical engineers	clinical engineers	Clinical Engineers	1	kept

Clinical engineers	clinical engineers	clinical engineers	clinical engineers	Clinical Engineers	1	case_or_format
Clinical environments	clinical environments	clinical environments	clinical environment	Clinical environment	1	singular
Clinical epidemiology	clinical epidemiology	clinical epidemiology	clinical epidemiology	clinical epidemiology	1	case_or_format
clinical error	clinical error	clinical error	clinical error	clinical error	1	kept
Clinical errors	clinical errors	clinical errors	clinical error	clinical error	1	singular
clinical errors	clinical errors	clinical errors	clinical error	clinical error	1	singular
clinical Ethics	clinical ethics	clinical ethics	clinical ethics	clinical ethics	1	case_or_format
clinical ethics committee	clinical ethics committee	clinical ethics committee	clinical ethics committee	clinical ethics committee	1	kept
clinical ethics support	clinical ethics support	clinical ethics support	clinical ethics support	clinical ethics support	1	kept
clinical ethics support services	clinical ethics support services	clinical ethics support services	clinical ethics support services	clinical ethics support services	1	kept
Clinical evaluation	clinical evaluation	clinical evaluation	clinical evaluation	clinical evaluation	1	case_or_format
Clinical events	clinical events	clinical events	clinical events	Clinical events	1	kept
Clinical Evolution	clinical evolution	clinical evolution	clinical evolution	Clinical evolution	1	case_or_format
Clinical experience portal	clinical experience portal	clinical experience portal	clinical experience portal	Clinical experience portal	1	kept
Clinical experiences	clinical experiences	clinical experiences	clinical experience	Clinical experience	1	singular
clinical experiences	clinical experiences	clinical experiences	clinical experience	Clinical experience	1	singular

clinical features	clinical features	clinical features	clinical feature	clinical feature	1	singular
Clinical features changes	clinical features changes	clinical features changes	clinical features changes	Clinical features changes	1	kept
clinical frailty	clinical frailty	clinical frailty	clinical frailty	clinical frailty	1	kept
Clinical Frailty Scale Spain	clinical frailty scale spain	clinical frailty scale spain	clinical frailty scale spain	Clinical Frailty Scale Spain	1	kept
clinical frailty score	clinical frailty score	clinical frailty score	clinical frailty score	clinical frailty score	1	kept
CLINICAL GENETICS	clinical genetics	clinical genetics	clinical genetics	CLINICAL GENETICS	1	kept
clinical gestalt	clinical gestalt	clinical gestalt	clinical gestalt	clinical gestalt	1	kept
clinical governance: crisis intervention	clinical governance crisis intervention	clinical governance crisis intervention	clinical governance crisis intervention	clinical governance: crisis intervention	1	kept
CLINICAL GUIDELINES	clinical guidelines	clinical guidelines	clinical guideline	clinical guidelines	1	singular
Clinical guidelines, Hyperbilirubinaemia	clinical guidelines hyperbilirubinaemia	clinical guidelines hyperbilirubinaemia	clinical guidelines hyperbilirubinaemia	Clinical guidelines, Hyperbilirubinaemia	1	kept
clinical immunology	clinical immunology	clinical immunology	clinical immunology	clinical immunology	1	kept
Clinical Impact	clinical impact	clinical impact	clinical impact	Clinical Impact	1	kept
clinical incident	clinical incident	clinical incident	clinical incident	clinical incident	1	kept
CLINICAL INDICATORS	clinical indicators	clinical indicators	clinical indicator	clinical indicator	1	singular
Clinical indicators	clinical indicators	clinical indicators	clinical indicator	clinical indicator	1	singular
CLINICAL INFORMATION	clinical information	clinical information	clinical information	Clinical information	1	case_or_format

Clinical Information Systems	clinical information systems	clinical information systems	clinical information system	Clinical information system	1	singular
Clinical information systems design	clinical information systems design	clinical information systems design	clinical information systems design	Clinical information systems design	1	kept
Clinical Institute Withdrawal Assessment of Alcohol Scale	clinical institute withdrawal assessment of alcohol scale	clinical institute withdrawal assessment of alcohol scale	clinical institute withdrawal assessment of alcohol scale	Clinical Institute Withdrawal Assessment of Alcohol Scale	1	kept
Clinical Institute Withdrawal Assessment —Alcohol revised	clinical institute withdrawal assessment alcohol revised	clinical institute withdrawal assessment alcohol revised	clinical institute withdrawal assessment alcohol revised	Clinical Institute Withdrawal Assessment —Alcohol revised	1	kept
clinical instructor	clinical instructor	clinical instructor	clinical instructor	clinical instructor	1	kept
clinical internship	clinical internship	clinical internship	clinical internship	clinical internship	1	kept
Clinical intervention	clinical intervention	clinical intervention	clinical intervention	Clinical intervention	1	kept
Clinical interventions	clinical interventions	clinical interventions	clinical intervention	Clinical intervention	1	singular
clinical interventions	clinical interventions	clinical interventions	clinical intervention	Clinical intervention	1	singular
clinical investigator	clinical investigator	clinical investigator	clinical investigator	clinical investigator	1	kept
Clinical isolates	clinical isolates	clinical isolates	clinical isolates	Clinical isolates	1	kept
CLINICAL JUDGMENT	clinical judgment	clinical judgment	clinical judgment	Clinical judgment	1	case_or_format
Clinical laboratory science students	clinical laboratory science students	clinical laboratory science students	clinical laboratory science students	Clinical laboratory science students	1	kept
clinical laboratory science students	clinical laboratory science students	clinical laboratory science students	clinical laboratory science students	Clinical laboratory science students	1	case_or_format
clinical laboratory services	clinical laboratory services	clinical laboratory services	clinical laboratory services	clinical laboratory services	1	kept
Clinical laboratory techniques	clinical laboratory techniques	clinical laboratory techniques	clinical laboratory techniques	Clinical Laboratory Techniques	1	case_or_format

clinical laboratory techniques	clinical laboratory techniques	clinical laboratory techniques	clinical laboratory techniques	Clinical Laboratory Techniques	1	case_or_format
clinical lactation care	clinical lactation care	clinical lactation care	clinical lactation care	clinical lactation care	1	kept
Clinical ladder-3	clinical ladder 3	clinical ladder 3	clinical ladder 3	Clinical ladder-3	1	kept
Clinical language variants	clinical language variants	clinical language variants	clinical language variants	Clinical language variants	1	kept
Clinical Leadership	clinical leadership	clinical leadership	clinical leadership	clinical leadership	1	case_or_format
Clinical leadership	clinical leadership	clinical leadership	clinical leadership	clinical leadership	1	case_or_format
clinical learning environment	clinical learning environment	clinical learning environment	clinical learning environment	Clinical learning environment	1	case_or_format
CLINICAL LEARNING-ENVIRONMENT	clinical learning environment	clinical learning environment	clinical learning environment	Clinical learning environment	1	case_or_format
Clinical lessons	clinical lessons	clinical lessons	clinical lessons	Clinical lessons	1	kept
clinical letter	clinical letter	clinical letter	clinical letter	clinical letter	1	kept
Clinical librarian program	clinical librarian program	clinical librarian program	clinical librarian program	Clinical librarian program	1	kept
clinical management	clinical management	clinical management	clinical management	Clinical management	1	case_or_format
Clinical manifestation	clinical manifestation	clinical manifestation	clinical manifestation	Clinical manifestation	1	kept
clinical medical ethics	clinical medical ethics	clinical medical ethics	clinical medical ethics	clinical medical ethics	1	kept
CLINICAL MICROBIOLOGISTS	clinical microbiologists	clinical microbiologists	clinical microbiologists	CLINICAL MICROBIOLOGISTS	1	kept
Clinical microbiology	clinical microbiology	clinical microbiology	clinical microbiology	Clinical microbiology	1	kept

Clinical monitoring	clinical monitoring	clinical monitoring	clinical monitoring	clinical monitoring	1	case_or_format
CLINICAL NEUROLOGY	clinical neurology	clinical neurology	clinical neurology	CLINICAL NEUROLOGY	1	kept
Clinical nutrition	clinical nutrition	clinical nutrition	clinical nutrition	clinical nutrition	1	case_or_format
Clinical nutritionist	clinical nutritionist	clinical nutritionist	clinical nutritionist	Clinical nutritionist	1	kept
Clinical nutritionists	clinical nutritionists	clinical nutritionists	clinical nutritionist	Clinical nutritionist	1	singular
Clinical Observation Units	clinical observation units	clinical observation units	clinical observation units	Clinical Observation Units	1	kept
Clinical operations	clinical operations	clinical operations	clinical operations	Clinical operations	1	kept
clinical opioid withdrawal scale	clinical opioid withdrawal scale	clinical opioid withdrawal scale	clinical opioid withdrawal scale	Clinical Opioid Withdrawal Scale	1	case_or_format
Clinical Outcomes Research	clinical outcomes research	clinical outcomes research	clinical outcomes research	Clinical Outcomes Research	1	kept
clinical parameter	clinical parameter	clinical parameter	clinical parameter	clinical parameter	1	kept
clinical pathology	clinical pathology	clinical pathology	clinical pathology	clinical pathology	1	kept
clinical pathways	clinical pathways	clinical pathways	clinical pathway	clinical pathway	1	singular
Clinical performance	clinical performance	clinical performance	clinical performance	clinical performance	1	case_or_format
Clinical Performance Questionnaire	clinical performance questionnaire	clinical performance questionnaire	clinical performance questionnaire	Clinical Performance Questionnaire	1	kept
clinical perfusionist	clinical perfusionist	clinical perfusionist	clinical perfusionist	clinical perfusionist	1	kept
CLINICAL PHARMACIST INTERVENTIONS	clinical pharmacist interventions	clinical pharmacist interventions	clinical pharmacist interventions	CLINICAL PHARMACIST INTERVENTIONS	1	kept

Clinical pharmacists	clinical pharmacists	clinical pharmacists	clinical pharmacist	clinical pharmacist	1	singular
clinical pharmacists	clinical pharmacists	clinical pharmacists	clinical pharmacist	clinical pharmacist	1	singular
Clinical Pharmacy	clinical pharmacy	clinical pharmacy	clinical pharmacy	clinical pharmacy	1	case_or_format
clinical pharmacy information systems	clinical pharmacy information systems	clinical pharmacy information systems	clinical pharmacy information systems	Clinical Pharmacy Information Systems	1	case_or_format
CLINICAL PHARMACY SERVICES	clinical pharmacy services	clinical pharmacy services	clinical pharmacy services	CLINICAL PHARMACY SERVICES	1	kept
clinical pharmacy services	clinical pharmacy services	clinical pharmacy services	clinical pharmacy services	CLINICAL PHARMACY SERVICES	1	case_or_format
Clinical pharmacy, Computers, Economics, Health professions [illegible]	clinical pharmacy computers economics health professions [illegible]	clinical pharmacy computers economics health professions [illegible]	clinical pharmacy computers economics health professions [illegible]	Clinical pharmacy, Computers, Economics, Health professions [illegible]	1	kept
clinical phenotype	clinical phenotype	clinical phenotype	clinical phenotype	clinical phenotype	1	kept
CLINICAL PHYSIOLOGY	clinical physiology	clinical physiology	clinical physiology	CLINICAL PHYSIOLOGY	1	kept
Clinical picture	clinical picture	clinical picture	clinical picture	Clinical picture	1	kept
clinical placements	clinical placements	clinical placements	clinical placement	clinical placement	1	singular
Clinical Practice	clinical practice	clinical practice	clinical practice	clinical practice	1	case_or_format
Clinical practice based evidence	clinical practice based evidence	clinical practice based evidence	clinical practice based evidence	Clinical practice based evidence	1	kept
Clinical Practice Guidelines	clinical practice guidelines	clinical practice guidelines	clinical practice guideline	clinical practice guideline	1	singular
Clinical practice guidelines for quality palliative care	clinical practice guidelines for quality palliative care	clinical practice guidelines for quality palliative care	clinical practice guidelines for quality palliative care	Clinical practice guidelines for quality palliative care	1	kept
clinical practice improvement	clinical practice improvement	clinical practice improvement	clinical practice improvement	Clinical practice improvement	1	case_or_format

Clinical practice nutrition guidelines	clinical practice nutrition guidelines	clinical practice nutrition guidelines	clinical practice nutrition guidelines	Clinical practice nutrition guidelines	1	kept
clinical practice pattern	clinical practice pattern	clinical practice pattern	clinical practice pattern	clinical practice pattern	1	kept
Clinical practice patterns	clinical practice patterns	clinical practice patterns	clinical practice pattern	clinical practice pattern	1	singular
clinical practice patterns	clinical practice patterns	clinical practice patterns	clinical practice pattern	clinical practice pattern	1	singular
Clinical practice stressors	clinical practice stressors	clinical practice stressors	clinical practice stressors	Clinical practice stressors	1	kept
Clinical practice survey	clinical practice survey	clinical practice survey	clinical practice survey	Clinical practice survey	1	kept
Clinical practice variation	clinical practice variation	clinical practice variation	clinical practice variation	Clinical practice variation	1	kept
clinical preceptor	clinical preceptor	clinical preceptor	clinical preceptor	clinical preceptor	1	kept
Clinical prediction	clinical prediction	clinical prediction	clinical prediction	clinical prediction	1	case_or_format
Clinical prediction model	clinical prediction model	clinical prediction model	clinical prediction model	Clinical prediction model	1	kept
Clinical prediction models	clinical prediction models	clinical prediction models	clinical prediction model	Clinical prediction model	1	singular
Clinical prediction rule	clinical prediction rule	clinical prediction rule	clinical prediction rule	Clinical prediction rule	1	kept
clinical predictions	clinical predictions	clinical predictions	clinical prediction	clinical prediction	1	singular
CLINICAL PRESENTATION	clinical presentation	clinical presentation	clinical presentation	CLINICAL PRESENTATION	1	kept
Clinical presentation	clinical presentation	clinical presentation	clinical presentation	CLINICAL PRESENTATION	1	case_or_format
Clinical process	clinical process	clinical process	clinical process	Clinical process	1	kept

clinical profiles	clinical profiles	clinical profiles	clinical profiles	clinical profiles	1	kept
CLINICAL PSYCHOLOGY	clinical psychology	clinical psychology	clinical psychology	clinical psychology	1	case_or_format
Clinical public health	clinical public health	clinical public health	clinical public health	Clinical public health	1	kept
clinical pulmonary infection score	clinical pulmonary infection score	clinical pulmonary infection score	clinical pulmonary infection score	Clinical Pulmonary infection Score	1	case_or_format
clinical quality indicators	clinical quality indicators	clinical quality indicators	clinical quality indicators	clinical quality indicators	1	kept
Clinical questions	clinical questions	clinical questions	clinical questions	clinical questions	1	case_or_format
Clinical reasoning ability	clinical reasoning ability	clinical reasoning ability	clinical reasoning ability	Clinical reasoning ability	1	kept
Clinical reengineering	clinical reengineering	clinical reengineering	clinical reengineering	Clinical reengineering	1	kept
Clinical Relevance	clinical relevance	clinical relevance	clinical relevance	Clinical Relevance	1	kept
Clinical relevance	clinical relevance	clinical relevance	clinical relevance	Clinical Relevance	1	case_or_format
clinical research areas	clinical research areas	clinical research areas	clinical research areas	clinical research areas	1	kept
Clinical research organisation	clinical research organisation	clinical research organisation	clinical research organisation	Clinical research organisation	1	kept
Clinical research practitioners	clinical research practitioners	clinical research practitioners	clinical research practitioners	Clinical research practitioners	1	kept
clinical research prevention	clinical research prevention	clinical research prevention	clinical research prevention	clinical research prevention	1	kept
Clinical research professional	clinical research professional	clinical research professional	clinical research professional	Clinical research professional	1	kept
clinical research protocol	clinical research protocol	clinical research protocol	clinical research protocol	clinical research protocol	1	kept

clinical risk for babies	clinical risk for babies	clinical risk for babies	clinical risk for babies	clinical risk for babies	1	kept
clinical risk index for babies II	clinical risk index for babies ii	clinical risk index for babies ii	clinical risk index for babies ii	clinical risk index for babies II	1	kept
clinical risk prediction	clinical risk prediction	clinical risk prediction	clinical risk prediction	clinical risk prediction	1	kept
Clinical role	clinical role	clinical role	clinical role	Clinical role	1	kept
clinical rotation program	clinical rotation program	clinical rotation program	clinical rotation program	clinical rotation program	1	kept
Clinical rounds	clinical rounds	clinical rounds	clinical rounds	Clinical rounds	1	kept
clinical rounds	clinical rounds	clinical rounds	clinical rounds	Clinical rounds	1	case_or_format
clinical routine data reuse	clinical routine data reuse	clinical routine data reuse	clinical routine data reuse	clinical routine data reuse	1	kept
Clinical safety	clinical safety	clinical safety	clinical safety	clinical safety	1	case_or_format
Clinical scales	clinical scales	clinical scales	clinical scales	Clinical scales	1	kept
Clinical scenarios	clinical scenarios	clinical scenarios	clinical scenario	Clinical scenario	1	singular
clinical scenarios	clinical scenarios	clinical scenarios	clinical scenario	Clinical scenario	1	singular
Clinical Scene Investigator Academy	clinical scene investigator academy	clinical scene investigator academy	clinical scene investigator academy	Clinical Scene Investigator Academy	1	kept
Clinical scene investigator academy	clinical scene investigator academy	clinical scene investigator academy	clinical scene investigator academy	Clinical Scene Investigator Academy	1	case_or_format
Clinical scoring	clinical scoring	clinical scoring	clinical scoring	Clinical scoring	1	kept
clinical sequalae	clinical sequalae	clinical sequalae	clinical sequalae	clinical sequalae	1	kept

clinical service delivery	clinical service delivery	clinical service delivery	clinical service delivery	clinical service delivery	1	kept
Clinical setting	clinical setting	clinical setting	clinical setting	Clinical settings	1	case_or_format
CLINICAL SIMULATION	clinical simulation	clinical simulation	clinical simulation	Clinical simulation	1	case_or_format
Clinical simulation unit	clinical simulation unit	clinical simulation unit	clinical simulation unit	Clinical simulation unit	1	kept
Clinical site	clinical site	clinical site	clinical site	Clinical site	1	kept
Clinical situations	clinical situations	clinical situations	clinical situations	Clinical situations	1	kept
Clinical skill	clinical skill	clinical skill	clinical skill	clinical skills	1	case_or_format
clinical skills laboratory	clinical skills laboratory	clinical skills laboratory	clinical skills laboratory	clinical skills laboratory	1	kept
Clinical Specialist	clinical specialist	clinical specialist	clinical specialist	Clinical Specialist	1	kept
CLINICAL STAFF	clinical staff	clinical staff	clinical staff	Clinical staff	1	case_or_format
Clinical studies	clinical studies	clinical studies	clinical study	clinical study	1	singular
Clinical supervision model	clinical supervision model	clinical supervision model	clinical supervision model	Clinical supervision model	1	kept
Clinical support	clinical support	clinical support	clinical support	Clinical support	1	kept
Clinical surveillance	clinical surveillance	clinical surveillance	clinical surveillance	Clinical surveillance	1	kept
Clinical Sustainability Assessment Tool	clinical sustainability assessment tool	clinical sustainability assessment tool	clinical sustainability assessment tool	Clinical Sustainability Assessment Tool	1	kept
clinical teacher	clinical teacher	clinical teacher	clinical teacher	clinical teacher	1	kept

Clinical Teaching	clinical teaching	clinical teaching	clinical teaching	clinical teaching	1	case_or_format
clinical teaching behaviours	clinical teaching behaviours	clinical teaching behaviours	clinical teaching behaviours	clinical teaching behaviours	1	kept
clinical teaching unit	clinical teaching unit	clinical teaching unit	clinical teaching unit	clinical teaching unit	1	kept
Clinical teams	clinical teams	clinical teams	clinical teams	Clinical teams	1	kept
clinical technologies	clinical technologies	clinical technologies	clinical technologies	clinical technologies	1	kept
Clinical thinking	clinical thinking	clinical thinking	clinical thinking	Clinical thinking	1	kept
Clinical time series	clinical time series	clinical time series	clinical time series	Clinical time series	1	kept
clinical tools	clinical tools	clinical tools	clinical tools	clinical tools	1	kept
Clinical treatments	clinical treatments	clinical treatments	clinical treatment	clinical treatment	1	singular
Clinical trial design	clinical trial design	clinical trial design	clinical trial design	Clinical trial design	1	kept
Clinical trial management	clinical trial management	clinical trial management	clinical trial management	Clinical trial management	1	kept
clinical trial research	clinical trial research	clinical trial research	clinical trial research	clinical trial research	1	kept
Clinical Trials Data Monitoring Committees	clinical trials data monitoring committees	clinical trials data monitoring committees	clinical trials data monitoring committees	Clinical Trials Data Monitoring Committees	1	kept
Clinical trials priorities	clinical trials priorities	clinical trials priorities	clinical trials priorities	Clinical trials priorities	1	kept
Clinical Trials, Phase I as Topic	clinical trials phase i as topic	clinical trials phase i as topic	clinical trials phase i as topic	Clinical Trials, Phase I as Topic	1	kept
Clinical Trials, Phase IV as Topic	clinical trials phase iv as topic	clinical trials phase iv as topic	clinical trials phase iv as topic	Clinical Trials, Phase IV as Topic	1	kept

CLINICAL UTILITY	clinical utility	clinical utility	clinical utility	Clinical utility	1	case_or_format
Clinical validation	clinical validation	clinical validation	clinical validation	Clinical validation	1	kept
Clinical value	clinical value	clinical value	clinical value	Clinical value	1	kept
Clinical vignettes	clinical vignettes	clinical vignettes	clinical vignettes	Clinical vignettes	1	kept
clinical wisdom	clinical wisdom	clinical wisdom	clinical wisdom	clinical wisdom	1	kept
Clinical work	clinical work	clinical work	clinical work	Clinical work	1	kept
CLINICAL WORKFLOW	clinical workflow	clinical workflow	clinical workflow	clinical workflow	1	case_or_format
clinical-decision making	clinical decision making	clinical decision making	clinical decision making	clinical decision making	1	case_or_format
clinical-decision-making	clinical decision making	clinical decision making	clinical decision making	clinical decision making	1	case_or_format
CLINICAL-FEATURES	clinical features	clinical features	clinical feature	clinical feature	1	singular
CLINICAL-RESEARCH	clinical research	clinical research	clinical research	clinical research	1	case_or_format
CLINICAL-RESEARCH PRIORITIES	clinical research priorities	clinical research priorities	clinical research priorities	CLINICAL-RESEARCH PRIORITIES	1	kept
CLINICAL-SIGNIFICANCE	clinical significance	clinical significance	clinical significance	clinical significance	1	case_or_format
CLINICAL-TRIAL PARTICIPATION	clinical trial participation	clinical trial participation	clinical trial participation	CLINICAL-TRIAL PARTICIPATION	1	kept
CLINICAL-VARIABLES	clinical variables	clinical variables	clinical variables	CLINICAL-VARIABLES	1	kept
CLINICALLY ABNORMAL OBSERVATIONS	clinically abnormal observations	clinically abnormal observations	clinically abnormal observations	CLINICALLY ABNORMAL OBSERVATIONS	1	kept

Clinicial informatics	clinicial informatics	clinicial informatics	clinicial informatics	Clinicial informatics	1	kept
Clinician	clinician	clinician	clinician	clinician	1	case_or_format
clinician activities	clinician activities	clinician activities	clinician activities	clinician activities	1	kept
Clinician awareness	clinician awareness	clinician awareness	clinician awareness	Clinician awareness	1	kept
clinician behavior	clinician behavior	clinician behavior	clinician behavior	clinician behavior	1	kept
Clinician burnout	clinician burnout	clinician burnout	clinician burnout	Clinician burnout	1	kept
Clinician discomfort predictors	clinician discomfort predictors	clinician discomfort predictors	clinician discomfort predictors	Clinician discomfort predictors	1	kept
clinician distress	clinician distress	clinician distress	clinician distress	clinician distress	1	kept
clinician experience	clinician experience	clinician experience	clinician experience	clinician experience	1	kept
Clinician experiences	clinician experiences	clinician experiences	clinician experience	clinician experience	1	singular
Clinician Global Rating of Delirium Scale	clinician global rating of delirium scale	clinician global rating of delirium scale	clinician global rating of delirium scale	Clinician Global Rating of Delirium Scale	1	kept
clinician insight	clinician insight	clinician insight	clinician insight	clinician insight	1	kept
Clinician interaction	clinician interaction	clinician interaction	clinician interaction	Clinician interaction	1	kept
clinician opinion	clinician opinion	clinician opinion	clinician opinion	clinician opinion	1	kept
clinician perception	clinician perception	clinician perception	clinician perception	clinician perception	1	kept
Clinician perspectives	clinician perspectives	clinician perspectives	clinician perspectives	Clinician perspectives	1	kept

clinician survey	clinician survey	clinician survey	clinician survey	clinician survey	1	kept
Clinician training	clinician training	clinician training	clinician training	Clinician training	1	kept
clinician turnaround time	clinician turnaround time	clinician turnaround time	clinician turnaround time	clinician turnaround time	1	kept
Clinician-parent communication	clinician parent communication	clinician parent communication	clinician parent communication	Clinician-parent communication	1	kept
Clinician-reported outcome measure	clinician reported outcome measure	clinician reported outcome measure	clinician reported outcome measure	Clinician-reported outcome measure	1	kept
clinician-researcher	clinician researcher	clinician researcher	clinician researcher	clinician-researcher	1	kept
Clinicians	clinicians	clinicians	clinician	clinician	1	singular
clinicians’ disaster preparedness	clinicians disaster preparedness	clinicians disaster preparedness	clinicians disaster preparedness	clinicians’ disaster preparedness	1	kept
clinicians’ perception	clinicians perception	clinicians perception	clinicians perception	clinicians’ perception	1	kept
clinicians’ risk mitigation	clinicians risk mitigation	clinicians risk mitigation	clinicians risk mitigation	clinicians’ risk mitigation	1	kept
CLINICS	clinics	clinics	clinic	clinic	1	singular
Clinics	clinics	clinics	clinic	clinic	1	singular
Clinimetrics	clinimetrics	clinimetrics	clinimetrics	clinimetrics	1	case_or_format
CLIP	clip	clip	clip	CLIP	1	kept
clobetasol	clobetasol	clobetasol	clobetasol	clobetasol	1	kept
clofarabine	clofarabine	clofarabine	clofarabine	clofarabine	1	kept

Clogged chest tubes	clogged chest tubes	clogged chest tubes	clogged chest tubes	Clogged chest tubes	1	kept
Clogging	clogging	clogging	clogging	Clogging	1	kept
clomifene	clomifene	clomifene	clomifene	clomifene	1	kept
Clonazepam	clonazepam	clonazepam	clonazepam	clonazepam	1	case_or_format
clone	clone	clone	clone	clone	1	kept
clonic seizure	clonic seizure	clonic seizure	clonic seizure	clonic seizure	1	kept
Cloning	cloning	cloning	cloning	Cloning	1	kept
clonus	clonus	clonus	clonus	clonus	1	kept
clorazepate	clorazepate	clorazepate	clorazepate	clorazepate	1	kept
Close Collaboration with Parents	close collaboration with parents	close collaboration with parents	close collaboration with parents	Close Collaboration with Parents	1	kept
Close contact	close contact	close contact	close contact	Close contact	1	kept
Close loop IntelliVent ®-ASV mode	close loop intellivent asv mode	close loop intellivent asv mode	close loop intellivent asv mode	Close loop IntelliVent ®-ASV mode	1	kept
Close proximity	close proximity	close proximity	close proximity	Close proximity	1	kept
CLOSE RELATIVES	close relatives	close relatives	close relatives	CLOSE RELATIVES	1	kept
Close tracheal suction system	close tracheal suction system	close tracheal suction system	close tracheal suction system	Close tracheal suction system	1	kept
close-observation	close observation	close observation	close observation	close-observation	1	kept

closed angle glaucoma	closed angle glaucoma	closed angle glaucoma	closed angle glaucoma	closed angle glaucoma	1	kept
closed blood sampling devices	closed blood sampling devices	closed blood sampling devices	closed blood sampling devices	Closed blood sampling devices	1	case_or_format
Closed drainage system	closed drainage system	closed drainage system	closed drainage system	Closed drainage system	1	kept
Closed endotracheal suction	closed endotracheal suction	closed endotracheal suction	closed endotracheal suction	Closed endotracheal suction	1	kept
Closed head injury	closed head injury	closed head injury	closed head injury	Closed head injury	1	kept
closed loop automated oxygen control	closed loop automated oxygen control	closed loop automated oxygen control	closed loop automated oxygen control	closed loop automated oxygen control	1	kept
Closed loop control systems	closed loop control systems	closed loop control systems	closed loop control systems	Closed loop control systems	1	kept
closed loop glucose control	closed loop glucose control	closed loop glucose control	closed loop glucose control	closed loop glucose control	1	kept
Closed loop medication administration	closed loop medication administration	closed loop medication administration	closed loop medication administration	Closed loop medication administration	1	kept
Closed loop oxygen control	closed loop oxygen control	closed loop oxygen control	closed loop oxygen control	Closed loop oxygen control	1	kept
Closed medication system	closed medication system	closed medication system	closed medication system	Closed medication system	1	kept
Closed reduction	closed reduction	closed reduction	closed reduction	closed reduction	1	case_or_format
Closed sampling system	closed sampling system	closed sampling system	closed sampling system	Closed sampling system	1	kept
Closed suction	closed suction	closed suction	closed suction	Closed suction	1	kept
closed suction	closed suction	closed suction	closed suction	Closed suction	1	case_or_format
Closed suction system	closed suction system	closed suction system	closed suction system	Closed suction system	1	kept

Closed suctioning	closed suctioning	closed suctioning	closed suctioning	Closed suctioning	1	kept
Closed suctioning system	closed suctioning system	closed suctioning system	closed suctioning system	Closed suctioning system	1	kept
Closed system drug transfer device	closed system drug transfer device	closed system drug transfer device	closed system drug transfer device	Closed system drug transfer device	1	kept
closed system suctioning	closed system suctioning	closed system suctioning	closed system suctioning	Closed system suctioning	1	case_or_format
Closed-loop control	closed loop control	closed loop control	closed loop control	Closed-loop control	1	kept
Closed-loop FIO<sub>2<	closed loop fio sub 2	closed loop fio sub 2	closed loop fio sub 2	Closed-loop FIO<sub>2<	1	kept
closed-loop glucose control	closed loop glucose control	closed loop glucose control	closed loop glucose control	closed loop glucose control	1	case_or_format
Closed-loop glycemic control system	closed loop glycemic control system	closed loop glycemic control system	closed loop glycemic control system	Closed-loop glycemic control system	1	kept
closed-loop management	closed loop management	closed loop management	closed loop management	closed-loop management	1	kept
Closed-loop systems	closed loop systems	closed loop systems	closed loop system	Closed-loop system	1	singular
closed-loop ventilation	closed loop ventilation	closed loop ventilation	closed loop ventilation	closed loop ventilation	1	case_or_format
CLOSENESS	closeness	closeness	closeness	Closeness	1	case_or_format
closeness	closeness	closeness	closeness	Closeness	1	case_or_format
Clostridium	clostridium	clostridium	clostridium	Clostridium	1	kept
Clostridium botulinum	clostridium botulinum	clostridium botulinum	clostridium botulinum	Clostridium botulinum	1	kept
Clostridium difficile toxin A	clostridium difficile toxin a	clostridium difficile toxin a	clostridium difficile toxin a	Clostridium difficile toxin A	1	kept

Clostridium difficile toxin B	clostridium difficile toxin b	clostridium difficile toxin b	clostridium difficile toxin b	Clostridium difficile toxin B	1	kept
Clostridium toxin	clostridium toxin	clostridium toxin	clostridium toxin	Clostridium toxin	1	kept
CLOSTRIDIUM-DIFFICILE	clostridium difficile	clostridium difficile	clostridium difficile	Clostridium difficile	1	case_or_format
CLOSTRIDIUM-DIFFICILE INFECTION	clostridium difficile infection	clostridium difficile infection	clostridium difficile infection	Clostridium difficile infection	1	case_or_format
Clothing design	clothing design	clothing design	clothing design	Clothing design	1	kept
Clotting	clotting	clotting	clotting	Clotting	1	kept
Cloud	cloud	cloud	cloud	Cloud	1	kept
Cloud computing	cloud computing	cloud computing	cloud computing	cloud computing	1	case_or_format
Cloud platforms	cloud platforms	cloud platforms	cloud platforms	Cloud platforms	1	kept
cloud storage	cloud storage	cloud storage	cloud storage	cloud storage	1	kept
cloud-based	cloud based	cloud based	cloud based	Cloud-based	1	case_or_format
clouding of consciousness	clouding of consciousness	clouding of consciousness	clouding of consciousness	clouding of consciousness	1	kept
Clouds	clouds	clouds	cloud	Cloud	1	singular
Clown	clown	clown	clown	Clown	1	kept
CLRN	clrn	clrn	clrn	CLRN	1	kept
cluster headache	cluster headache	cluster headache	cluster headache	cluster headache	1	kept

Cluster randomization	cluster randomization	cluster randomization	cluster randomization	Cluster randomization	1	kept
cluster randomized controlled trial	cluster randomized controlled trial	cluster randomized controlled trial	cluster randomized controlled trial	cluster randomized controlled trial	1	kept
Cluster randomized design	cluster randomized design	cluster randomized design	cluster randomized design	Cluster randomized design	1	kept
cluster randomized trial	cluster randomized trial	cluster randomized trial	cluster randomized trial	cluster randomized trial	1	kept
Cluster treatment	cluster treatment	cluster treatment	cluster treatment	Cluster treatment	1	kept
cluster trials	cluster trials	cluster trials	cluster trials	cluster trials	1	kept
CLUSTER-ANALYSIS	cluster analysis	cluster analysis	cluster analysis	Cluster Analysis	1	case_or_format
Cluster-randomized controlled trial	cluster randomized controlled trial	cluster randomized controlled trial	cluster randomized controlled trial	cluster randomized controlled trial	1	case_or_format
Cluster-randomized trial	cluster randomized trial	cluster randomized trial	cluster randomized trial	cluster randomized trial	1	case_or_format
CLUSTER-RANDOMIZED-TRIAL	cluster randomized trial	cluster randomized trial	cluster randomized trial	cluster randomized trial	1	case_or_format
CLUSTER-RANDOMIZED-TRIALS	cluster randomized trials	cluster randomized trials	cluster randomized trial	cluster randomized trial	1	singular
Clustered care	clustered care	clustered care	clustered care	Clustered care	1	kept
clustered care	clustered care	clustered care	clustered care	Clustered care	1	case_or_format
clustering algorithm	clustering algorithm	clustering algorithm	clustering algorithm	clustering algorithm	1	kept
Clustering algorithms	clustering algorithms	clustering algorithms	clustering algorithm	clustering algorithm	1	singular
clustering analysis	clustering analysis	clustering analysis	clustering analysis	Clustering analysis	1	case_or_format

clustering care activities	clustering care activities	clustering care activities	clustering care activities	clustering care activities	1	kept
Clustering techniques	clustering techniques	clustering techniques	clustering techniques	Clustering techniques	1	kept
CME	cme	cme	cme	CME	1	kept
CNL	cnl	cnl	cnl	CNL	1	kept
CNN	cnn	cnn	cnn	CNN	1	kept
CNN models	cnn models	cnn models	cnn models	CNN models	1	kept
CNOR	cnor	cnor	cnor	CNOR	1	kept
CNS	cns	cns	cns	CNS	1	kept
CNS Complications	cns complications	cns complications	cns complications	CNS Complications	1	kept
CNS TOXICITY	cns toxicity	cns toxicity	cns toxicity	CNS TOXICITY	1	kept
co management	co management	co management	co management	Co-management	1	case_or_format
co-administration	co administration	co administration	co administration	co-administration	1	kept
Co-creation	co creation	co creation	co creation	Co-creation	1	kept
Co-Design	co design	co design	co design	Co-design	1	case_or_format
co-design	co design	co design	co design	Co-design	1	case_or_format
Co-designs	co designs	co designs	co design	Co-design	1	singular

co-determination	co determination	co determination	co determination	co-determination	1	kept
Co-development	co development	co development	co development	Co-development	1	kept
co-management	co management	co management	co management	Co-management	1	case_or_format
Co-morbidities	co morbidities	co morbidities	co morbidities	Co-morbidities	1	kept
co-operative Working	co operative working	co operative working	co operative working	co-operative Working	1	kept
co-ordinated care	co ordinated care	co ordinated care	co ordinated care	co-ordinated care	1	kept
co-ordination	co ordination	co ordination	co ordination	co-ordination	1	kept
Co-production	co production	co production	co production	Co-production	1	kept
Co-word analysis	co word analysis	co word analysis	co word analysis	Co-word analysis	1	kept
Co-workers	co workers	co workers	co workers	Co-workers	1	kept
CO2 level	co2 level	co2 level	co2 level	CO2 level	1	kept
coagulase negative staphylococci	coagulase negative staphylococci	coagulase negative staphylococci	coagulase negative staphylococci	Coagulase-Negative Staphylococci	1	case_or_format
coagulase negative staphylococci infection	coagulase negative staphylococci infection	coagulase negative staphylococci infection	coagulase negative staphylococci infection	coagulase negative staphylococci infection	1	kept
coagulase-negative staphylococci	coagulase negative staphylococci	coagulase negative staphylococci	coagulase negative staphylococci	Coagulase-Negative Staphylococci	1	case_or_format
Coagulasenegative staphylococci	coagulasenegative staphylococci	coagulasenegative staphylococci	coagulasenegative staphylococci	Coagulasenegative staphylococci	1	kept
Coagulated	coagulated	coagulated	coagulated	Coagulated	1	kept

Coagulation	coagulation	coagulation	coagulation	coagulation	1	case_or_format
Coagulation test	coagulation test	coagulation test	coagulation test	Coagulation test	1	kept
coal tar	coal tar	coal tar	coal tar	coal tar	1	kept
Coastal India	coastal india	coastal india	coastal india	Coastal India	1	kept
COATED LATEX	coated latex	coated latex	coated latex	COATED LATEX	1	kept
cobalamin	cobalamin	cobalamin	cobalamin	cobalamin	1	kept
cobalt	cobalt	cobalt	cobalt	cobalt	1	kept
cobalt blood level	cobalt blood level	cobalt blood level	cobalt blood level	cobalt blood level	1	kept
cobedding	cobedding	cobedding	cobedding	cobedding	1	kept
cobicistat	cobicistat	cobicistat	cobicistat	cobicistat	1	kept
cobicistat plus darunavir	cobicistat plus darunavir	cobicistat plus darunavir	cobicistat plus darunavir	cobicistat plus darunavir	1	kept
cobicistat plus darunavir plus emtricitabine plus [illegible]	cobicistat plus darunavir plus emtricitabine plus [illegible]	cobicistat plus darunavir plus emtricitabine plus [illegible]	cobicistat plus darunavir plus emtricitabine plus [illegible]	cobicistat plus darunavir plus emtricitabine plus [illegible]	1	kept
cobicistat plus elvitegravir plus emtricitabine plus [illegible]	cobicistat plus elvitegravir plus emtricitabine plus [illegible]	cobicistat plus elvitegravir plus emtricitabine plus [illegible]	cobicistat plus elvitegravir plus emtricitabine plus [illegible]	cobicistat plus elvitegravir plus emtricitabine plus [illegible]	1	kept
cocaína crack	coca na crack	coca na crack	coca na crack	cocaína crack	1	kept
Coccyx	coccyx	coccyx	coccyx	Coccyx	1	kept
coccyx	coccyx	coccyx	coccyx	Coccyx	1	case_or_format

cochlea prosthesis	cochlea prosthesis	cochlea prosthesis	cochlea prosthesis	cochlea prosthesis	1	kept
Cochlear Implantation	cochlear implantation	cochlear implantation	cochlear implantation	cochlear implantation	1	case_or_format
Cochlear Nerve	cochlear nerve	cochlear nerve	cochlear nerve	Cochlear Nerve	1	kept
Cochrane Review Protocol	cochrane review protocol	cochrane review protocol	cochrane review protocol	Cochrane Review Protocol	1	kept
cocodamol	cocodamol	cocodamol	cocodamol	cocodamol	1	kept
Coconut fall-related trauma	coconut fall related trauma	coconut fall related trauma	coconut fall related trauma	Coconut fall-related trauma	1	kept
Coconut falls	coconut falls	coconut falls	coconut falls	Coconut falls	1	kept
Cocos	cocos	cocos	cocos	Cocos	1	kept
Code barriers	code barriers	code barriers	code barriers	Code barriers	1	kept
Code blue events	code blue events	code blue events	code blue events	Code blue events	1	kept
code blue team	code blue team	code blue team	code blue team	Code blue teams	1	case_or_format
Code debriefing	code debriefing	code debriefing	code debriefing	Code debriefing	1	kept
Code grey	code grey	code grey	code grey	Code grey	1	kept
code of ethics	code of ethics	code of ethics	code of ethics	code of ethics	1	kept
Code of Hygiene	code of hygiene	code of hygiene	code of hygiene	Code of Hygiene	1	kept
Code reduction	code reduction	code reduction	code reduction	Code reduction	1	kept

Code selection	code selection	code selection	code selection	Code selection	1	kept
Coded signal	coded signal	coded signal	coded signal	Coded signal	1	kept
Codeine phosphate	codeine phosphate	codeine phosphate	codeine phosphate	codeine phosphate	1	case_or_format
Codependency	codependency	codependency	codependency	Codependency	1	kept
Coding errors	coding errors	coding errors	coding errors	Coding errors	1	kept
Coding Interactive Behavior	coding interactive behavior	coding interactive behavior	coding interactive behavior	Coding Interactive Behavior	1	kept
Coding process	coding process	coding process	coding process	Coding process	1	kept
Codon, Nonsense	codon nonsense	codon nonsense	codon nonsense	Codon, Nonsense	1	kept
Coelorinchus multispinulosus	coelorinchus multispinulosus	coelorinchus multispinulosus	coelorinchus multispinulosus	Coelorinchus multispinulosus	1	kept
coenrollment	coenrollment	coenrollment	coenrollment	coenrollment	1	kept
coenzyme A	coenzyme a	coenzyme a	coenzyme a	coenzyme A	1	kept
COERCION	coercion	coercion	coercion	Coercion	1	case_or_format
coercive measures	coercive measures	coercive measures	coercive measures	coercive measures	1	kept
CogCheck	cogcheck	cogcheck	cogcheck	CogCheck	1	kept
cogenital central hypoventilation syndrome	cogenital central hypoventilation syndrome	cogenital central hypoventilation syndrome	cogenital central hypoventilation syndrome	cogenital central hypoventilation syndrome	1	kept
cogmeas	cogmeas	cogmeas	cogmeas	cogmeas	1	kept

Cognition bias	cognition bias	cognition bias	cognition bias	Cognition bias	1	kept
cognition disorder	cognition disorder	cognition disorder	cognition disorder	Cognition Disorders	1	case_or_format
Cognition disorders	cognition disorders	cognition disorders	cognition disorder	Cognition Disorders	1	singular
Cognition Disorders [prevention & control]	cognition disorders prevention and control	cognition disorders prevention and control	cognition disorders prevention and control	Cognition Disorders [prevention & control]	1	kept
Cognitive	cognitive	cognitive	cognitive	cognitive	1	case_or_format
Cognitive ability	cognitive ability	cognitive ability	cognitive ability	Cognitive ability	1	kept
cognitive adaptation training	cognitive adaptation training	cognitive adaptation training	cognitive adaptation training	cognitive adaptation training	1	kept
cognitive AIDS	cognitive aids	cognitive aids	cognitive aid	Cognitive aids	1	singular
cognitive aspects of task execution and general incidents	cognitive aspects of task execution and general incidents	cognitive aspects of task execution and general incidents	cognitive aspects of task execution and general incidents	cognitive aspects of task execution and general incidents	1	kept
Cognitive assessment method	cognitive assessment method	cognitive assessment method	cognitive assessment method	cognitive assessment method	1	case_or_format
cognitive assessment screening instrument	cognitive assessment screening instrument	cognitive assessment screening instrument	cognitive assessment screening instrument	cognitive assessment screening instrument	1	kept
cognitive behavioral theory	cognitive behavioral theory	cognitive behavioral theory	cognitive behavioral theory	cognitive behavioral theory	1	kept
cognitive behavioural therapy	cognitive behavioural therapy	cognitive behavioural therapy	cognitive behavioural therapy	cognitive behavioural therapy	1	kept
cognitive bias	cognitive bias	cognitive bias	cognitive bias	Cognitive bias	1	case_or_format
cognitive coping	cognitive coping	cognitive coping	cognitive coping	cognitive coping	1	kept
COGNITIVE DECLINE	cognitive decline	cognitive decline	cognitive decline	Cognitive decline	1	case_or_format

Cognitive development	cognitive development	cognitive development	cognitive development	cognitive development	1	case_or_format
COGNITIVE DISABILITIES	cognitive disabilities	cognitive disabilities	cognitive disabilities	COGNITIVE DISABILITIES	1	kept
Cognitive domain	cognitive domain	cognitive domain	cognitive domain	Cognitive domain	1	kept
Cognitive domains	cognitive domains	cognitive domains	cognitive domain	Cognitive domain	1	singular
COGNITIVE DYSFUNCTION	cognitive dysfunction	cognitive dysfunction	cognitive dysfunction	Cognitive Dysfunction	1	case_or_format
cognitive effort	cognitive effort	cognitive effort	cognitive effort	cognitive effort	1	kept
Cognitive Ergonomics Components	cognitive ergonomics components	cognitive ergonomics components	cognitive ergonomics components	Cognitive Ergonomics Components	1	kept
cognitive errors	cognitive errors	cognitive errors	cognitive errors	cognitive errors	1	kept
Cognitive failure	cognitive failure	cognitive failure	cognitive failure	cognitive failure	1	case_or_format
Cognitive Function	cognitive function	cognitive function	cognitive function	cognitive function	1	case_or_format
cognitive function subscale consists of 10 item	cognitive function subscale consists of 10 item	cognitive function subscale consists of 10 item	cognitive function subscale consists of 10 item	cognitive function subscale consists of 10 item	1	kept
Cognitive function training	cognitive function training	cognitive function training	cognitive function training	Cognitive function training	1	kept
cognitive function training	cognitive function training	cognitive function training	cognitive function training	Cognitive function training	1	case_or_format
cognitive fusion questionaire	cognitive fusion questionaire	cognitive fusion questionaire	cognitive fusion questionaire	cognitive fusion questionaire	1	kept
Cognitive interview	cognitive interview	cognitive interview	cognitive interview	cognitive interview	1	case_or_format
cognitive interviewing	cognitive interviewing	cognitive interviewing	cognitive interviewing	cognitive interviewing	1	kept

cognitive load theory	cognitive load theory	cognitive load theory	cognitive load theory	cognitive load theory	1	kept
Cognitive outcomes	cognitive outcomes	cognitive outcomes	cognitive outcomes	Cognitive outcomes	1	kept
cognitive outcomes	cognitive outcomes	cognitive outcomes	cognitive outcomes	Cognitive outcomes	1	case_or_format
COGNITIVE PERSPECTIVE	cognitive perspective	cognitive perspective	cognitive perspective	COGNITIVE PERSPECTIVE	1	kept
cognitive preference	cognitive preference	cognitive preference	cognitive preference	cognitive preference	1	kept
cognitive process	cognitive process	cognitive process	cognitive process	cognitive process	1	kept
cognitive processing of trauma	cognitive processing of trauma	cognitive processing of trauma	cognitive processing of trauma	cognitive processing of trauma	1	kept
cognitive processing therapy	cognitive processing therapy	cognitive processing therapy	cognitive processing therapy	cognitive processing therapy	1	kept
Cognitive recovery	cognitive recovery	cognitive recovery	cognitive recovery	Cognitive recovery	1	kept
COGNITIVE REHABILITATION	cognitive rehabilitation	cognitive rehabilitation	cognitive rehabilitation	cognitive rehabilitation	1	case_or_format
Cognitive remediation	cognitive remediation	cognitive remediation	cognitive remediation	Cognitive remediation	1	kept
cognitive resilience	cognitive resilience	cognitive resilience	cognitive resilience	cognitive resilience	1	kept
Cognitive resources	cognitive resources	cognitive resources	cognitive resources	Cognitive resources	1	kept
cognitive restructuring	cognitive restructuring	cognitive restructuring	cognitive restructuring	cognitive restructuring	1	kept
Cognitive Schema	cognitive schema	cognitive schema	cognitive schema	Cognitive Schema	1	kept
Cognitive screening	cognitive screening	cognitive screening	cognitive screening	Cognitive screening	1	kept

Cognitive situation	cognitive situation	cognitive situation	cognitive situation	Cognitive situation	1	kept
Cognitive status	cognitive status	cognitive status	cognitive status	Cognitive status	1	kept
cognitive support	cognitive support	cognitive support	cognitive support	cognitive support	1	kept
COGNITIVE TEST	cognitive test	cognitive test	cognitive test	COGNITIVE TEST	1	kept
COGNITIVE THERAPY	cognitive therapy	cognitive therapy	cognitive therapy	cognitive therapy	1	case_or_format
Cognitive workload	cognitive workload	cognitive workload	cognitive workload	Cognitive workload	1	kept
cognitive workload	cognitive workload	cognitive workload	cognitive workload	Cognitive workload	1	case_or_format
Cognitive-behavior therapy	cognitive behavior therapy	cognitive behavior therapy	cognitive behavior therapy	Cognitive-behavior therapy	1	kept
cognitive-behavior therapy	cognitive behavior therapy	cognitive behavior therapy	cognitive behavior therapy	Cognitive-behavior therapy	1	case_or_format
Cognitive-behavioral	cognitive behavioral	cognitive behavioral	cognitive behavioral	Cognitive-behavioral	1	kept
COGNITIVE-BEHAVIORAL THERAPY	cognitive behavioral therapy	cognitive behavioral therapy	cognitive behavioral therapy	cognitive behavioral therapy	1	case_or_format
cognitive-behavioral therapy	cognitive behavioral therapy	cognitive behavioral therapy	cognitive behavioral therapy	cognitive behavioral therapy	1	case_or_format
Cognitive-behavioral training	cognitive behavioral training	cognitive behavioral training	cognitive behavioral training	Cognitive-behavioral training	1	kept
cognitive-behavioural intervention	cognitive behavioural intervention	cognitive behavioural intervention	cognitive behavioural intervention	cognitive-behavioural intervention	1	kept
cognitive-behavioural therapy	cognitive behavioural therapy	cognitive behavioural therapy	cognitive behavioural therapy	cognitive behavioural therapy	1	case_or_format
COGNITIVE-DEVELOPMENT	cognitive development	cognitive development	cognitive development	cognitive development	1	case_or_format

cohabitation	cohabitation	cohabitation	cohabitation	cohabitation	1	kept
cohabiting person	cohabiting person	cohabiting person	cohabiting person	cohabiting person	1	kept
cohen mans agitation inventory	cohen mans agitation inventory	cohen mans agitation inventory	cohen mans agitation inventory	cohen mans agitation inventory	1	kept
Cohort Effect	cohort effect	cohort effect	cohort effect	Cohort Effect	1	kept
cohort effect	cohort effect	cohort effect	cohort effect	Cohort Effect	1	case_or_format
cohort isolation	cohort isolation	cohort isolation	cohort isolation	cohort isolation	1	kept
cohorting	cohorting	cohorting	cohorting	Cohorting	1	case_or_format
coinfections	coinfections	coinfections	coinfection	coinfection	1	singular
col1a2 gene	col1a2 gene	col1a2 gene	col1a2 gene	col1a2 gene	1	kept
Cola	cola	cola	cola	Cola	1	kept
Colaboración	colaboraci n	colaboraci n	colaboraci n	Colaboración	1	kept
Colchicine	colchicine	colchicine	colchicine	colchicine	1	case_or_format
Cold	cold	cold	cold	cold	1	case_or_format
cold air	cold air	cold air	cold air	cold air	1	kept
Cold application	cold application	cold application	cold application	cold application	1	case_or_format
Cold chain	cold chain	cold chain	cold chain	Cold chain	1	kept

cold clammy skin	cold clammy skin	cold clammy skin	cold clammy skin	cold clammy skin	1	kept
cold exposure	cold exposure	cold exposure	cold exposure	cold exposure	1	kept
cold fluid	cold fluid	cold fluid	cold fluid	cold fluid	1	kept
cold ischemia time	cold ischemia time	cold ischemia time	cold ischemia time	cold ischemia time	1	kept
Cold stress	cold stress	cold stress	cold stress	Cold stress	1	kept
cold stress	cold stress	cold stress	cold stress	Cold stress	1	case_or_format
COLD THERAPY	cold therapy	cold therapy	cold therapy	COLD THERAPY	1	kept
colestipol	colestipol	colestipol	colestipol	colestipol	1	kept
colic	colic	colic	colic	Colic	1	case_or_format
coliform bacterium	coliform bacterium	coliform bacterium	coliform bacterium	coliform bacterium	1	kept
colistimethate	colistimethate	colistimethate	colistimethate	colistimethate	1	kept
Colitis	colitis	colitis	colitis	colitis	1	case_or_format
Colitis, Ischemic	colitis ischemic	colitis ischemic	colitis ischemic	Colitis, Ischemic	1	kept
Collaboration structure	collaboration structure	collaboration structure	collaboration structure	Collaboration structure	1	kept
collaboration structure	collaboration structure	collaboration structure	collaboration structure	Collaboration structure	1	case_or_format
Collaborative approach	collaborative approach	collaborative approach	collaborative approach	Collaborative approach	1	kept

collaborative behaviors	collaborative behaviors	collaborative behaviors	collaborative behaviors	collaborative behaviors	1	kept
collaborative care model	collaborative care model	collaborative care model	collaborative care model	collaborative care model	1	kept
Collaborative clinical reasoning	collaborative clinical reasoning	collaborative clinical reasoning	collaborative clinical reasoning	Collaborative clinical reasoning	1	kept
Collaborative clinical research trial	collaborative clinical research trial	collaborative clinical research trial	collaborative clinical research trial	Collaborative clinical research trial	1	kept
Collaborative decision making	collaborative decision making	collaborative decision making	collaborative decision making	Collaborative decision making	1	kept
Collaborative decision-making	collaborative decision making	collaborative decision making	collaborative decision making	Collaborative decision making	1	case_or_format
collaborative decision-making	collaborative decision making	collaborative decision making	collaborative decision making	Collaborative decision making	1	case_or_format
collaborative effort	collaborative effort	collaborative effort	collaborative effort	collaborative effort	1	kept
Collaborative health information technology	collaborative health information technology	collaborative health information technology	collaborative health information technology	Collaborative health information technology	1	kept
Collaborative Hyperbaric Medicine and Extreme [illegible]	collaborative hyperbaric medicine and extreme [illegible]	collaborative hyperbaric medicine and extreme [illegible]	collaborative hyperbaric medicine and extreme [illegible]	Collaborative Hyperbaric Medicine and Extreme [illegible]	1	kept
Collaborative leadership	collaborative leadership	collaborative leadership	collaborative leadership	Collaborative leadership	1	kept
Collaborative request	collaborative request	collaborative request	collaborative request	Collaborative request	1	kept
Collaborative Research	collaborative research	collaborative research	collaborative research	Collaborative Research	1	kept
collaborative response	collaborative response	collaborative response	collaborative response	collaborative response	1	kept
Collaborative teams	collaborative teams	collaborative teams	collaborative teams	Collaborative teams	1	kept
Collaborative work	collaborative work	collaborative work	collaborative work	collaborative work	1	case_or_format

Collaborative work environment	collaborative work environment	collaborative work environment	collaborative work environment	Collaborative work environment	1	kept
Collaborative, breakthrough groups	collaborative breakthrough groups	collaborative breakthrough groups	collaborative breakthrough groups	Collaborative, breakthrough groups	1	kept
collagen metabolism	collagen metabolism	collagen metabolism	collagen metabolism	collagen metabolism	1	kept
collagen synthesis	collagen synthesis	collagen synthesis	collagen synthesis	collagen synthesis	1	kept
Collapse	collapse	collapse	collapse	collapse	1	case_or_format
collapsing	collapsing	collapsing	collapsing	collapsing	1	kept
Collateral Circulation	collateral circulation	collateral circulation	collateral circulation	Collateral Circulation	1	kept
collateral circulation	collateral circulation	collateral circulation	collateral circulation	Collateral Circulation	1	case_or_format
Collateral damage	collateral damage	collateral damage	collateral damage	Collateral damage	1	kept
colleagues	colleagues	colleagues	colleagues	colleagues	1	kept
Collective Bargaining	collective bargaining	collective bargaining	collective bargaining	Collective Bargaining	1	kept
collective case studies	collective case studies	collective case studies	collective case studies	collective case studies	1	kept
Collective Efficacy	collective efficacy	collective efficacy	collective efficacy	Collective Efficacy	1	kept
collective efficacy	collective efficacy	collective efficacy	collective efficacy	Collective Efficacy	1	case_or_format
Collective subject discourse	collective subject discourse	collective subject discourse	collective subject discourse	Collective subject discourse	1	kept
COLLECTIVE TURNOVER	collective turnover	collective turnover	collective turnover	COLLECTIVE TURNOVER	1	kept

collectivist	collectivist	collectivist	collectivist	collectivist	1	kept
COLLEGE	college	college	college	college	1	case_or_format
College freshmen	college freshmen	college freshmen	college freshmen	College freshmen	1	kept
College of American Pathology	college of american pathology	college of american pathology	college of american pathology	College of American Pathology	1	kept
Collegial feedback	collegial feedback	collegial feedback	collegial feedback	Collegial feedback	1	kept
Collegiality	collegiality	collegiality	collegiality	Collegiality	1	kept
Collett-Lester Death Anxiety Scale	collett lester death anxiety scale	collett lester death anxiety scale	collett lester death anxiety scale	Collett-Lester Death Anxiety Scale	1	kept
Collett-Lester Fear of Death Scale	collett lester fear of death scale	collett lester fear of death scale	collett lester fear of death scale	Collett-Lester Fear of Death Scale	1	kept
collimator	collimator	collimator	collimator	collimator	1	kept
Colloid	colloid	colloid	colloid	colloid	1	case_or_format
Colloid solutions	colloid solutions	colloid solutions	colloid solutions	Colloid solutions	1	kept
colloids	colloids	colloids	colloid	colloid	1	singular
Colocación del paciente	colocaci n del paciente	colocaci n del paciente	colocaci n del paciente	Colocación del paciente	1	kept
colon adenocarcinoma	colon adenocarcinoma	colon adenocarcinoma	colon adenocarcinoma	colon adenocarcinoma	1	kept
colon biopsy	colon biopsy	colon biopsy	colon biopsy	colon biopsy	1	kept
colon lavage	colon lavage	colon lavage	colon lavage	colon lavage	1	kept

colon obstruction	colon obstruction	colon obstruction	colon obstruction	colon obstruction	1	kept
colon polyp	colon polyp	colon polyp	colon polyp	colon polyp	1	kept
colon surgery	colon surgery	colon surgery	colon surgery	colon surgery	1	kept
colon volvulus	colon volvulus	colon volvulus	colon volvulus	colon volvulus	1	kept
Colon, Sigmoid	colon sigmoid	colon sigmoid	colon sigmoid	Colon, Sigmoid	1	kept
colonialism	colonialism	colonialism	colonialism	colonialism	1	kept
colonic inertia	colonic inertia	colonic inertia	colonic inertia	colonic inertia	1	kept
Colonic perforation	colonic perforation	colonic perforation	colonic perforation	Colonic perforation	1	kept
Colonic volvulus	colonic volvulus	colonic volvulus	colonic volvulus	Colonic volvulus	1	kept
Colonisation	colonisation	colonisation	colonisation	colonisation	1	case_or_format
Colonization and decolonization	colonization and decolonization	colonization and decolonization	colonization and decolonization	Colonization and decolonization	1	kept
colonization prediction model	colonization prediction model	colonization prediction model	colonization prediction model	colonization prediction model	1	kept
COLONIZATION PRESSURE	colonization pressure	colonization pressure	colonization pressure	COLONIZATION PRESSURE	1	kept
Colonization rate	colonization rate	colonization rate	colonization rate	Colonization rate	1	kept
Colonization screening	colonization screening	colonization screening	colonization screening	Colonization screening	1	kept
colonoscope	colonoscope	colonoscope	colonoscope	colonoscope	1	kept

Colorectal Cancer	colorectal cancer	colorectal cancer	colorectal cancer	colorectal cancer	1	case_or_format
colorectal carcinoma	colorectal carcinoma	colorectal carcinoma	colorectal carcinoma	colorectal carcinoma	1	kept
colorectal neoplasms	colorectal neoplasms	colorectal neoplasms	colorectal neoplasms	Colorectal Neoplasms	1	case_or_format
colorectal obstruction	colorectal obstruction	colorectal obstruction	colorectal obstruction	colorectal obstruction	1	kept
colorectal peritoneal metastasis	colorectal peritoneal metastasis	colorectal peritoneal metastasis	colorectal peritoneal metastasis	colorectal peritoneal metastasis	1	kept
COLORECTAL-CANCER	colorectal cancer	colorectal cancer	colorectal cancer	colorectal cancer	1	case_or_format
Colorimeter	colorimeter	colorimeter	colorimeter	Colorimeter	1	kept
Colorimetric scale	colorimetric scale	colorimetric scale	colorimetric scale	Colorimetric scale	1	kept
Colorimétrie	colorim trie	colorim trie	colorim trie	Colorimétrie	1	kept
coloring	coloring	coloring	coloring	coloring	1	kept
COLOSTRUM	colostrum	colostrum	colostrum	Colostrum	1	case_or_format
colovaginal fistula	colovaginal fistula	colovaginal fistula	colovaginal fistula	colovaginal fistula	1	kept
colporrhaphy	colporrhaphy	colporrhaphy	colporrhaphy	colporrhaphy	1	kept
Columbidae	columbidae	columbidae	columbidae	Columbidae	1	kept
column-line diagram	column line diagram	column line diagram	column line diagram	column-line diagram	1	kept
COM-B	com b	com b	com b	COM-B	1	kept

coma recovery scale - revised	coma recovery scale revised	coma recovery scale revised	coma recovery scale revised	coma recovery scale revised	1	case_or_format
Coma Recovery Scale Revised	coma recovery scale revised	coma recovery scale revised	coma recovery scale revised	coma recovery scale revised	1	case_or_format
Coma Recovery Scale Revised Score	coma recovery scale revised score	coma recovery scale revised score	coma recovery scale revised score	Coma Recovery Scale Revised Score	1	kept
Coma Recovery Scale - Revised	coma recovery scale revised	coma recovery scale revised	coma recovery scale revised	coma recovery scale revised	1	case_or_format
COMA SCALE	coma scale	coma scale	coma scale	Coma scale	1	case_or_format
coma scale	coma scale	coma scale	coma scale	Coma scale	1	case_or_format
coma scale revised	coma scale revised	coma scale revised	coma scale revised	coma scale revised	1	kept
COMA-SCALE	coma scale	coma scale	coma scale	Coma scale	1	case_or_format
Comanagement	comanagement	comanagement	comanagement	Comanagement	1	kept
Comatose	comatose	comatose	comatose	Comatose	1	kept
COMATOSE SURVIVORS	comatose survivors	comatose survivors	comatose survivors	COMATOSE SURVIVORS	1	kept
Combat	combat	combat	combat	Combat	1	kept
Combat casualties	combat casualties	combat casualties	combat casualties	Combat casualties	1	kept
combat casualty care	combat casualty care	combat casualty care	combat casualty care	Combat casualty care	1	case_or_format
Combat hospital	combat hospital	combat hospital	combat hospital	Combat hospital	1	kept
combat wounds	combat wounds	combat wounds	combat wounds	combat wounds	1	kept

COMBINATION	combination	combination	combination	COMBINATION	1	kept
combination antibiotic therapy	combination antibiotic therapy	combination antibiotic therapy	combination antibiotic therapy	combination antibiotic therapy	1	kept
COMBINATION ANTIMICROBIAL THERAPY	combination antimicrobial therapy	combination antimicrobial therapy	combination antimicrobial therapy	COMBINATION ANTIMICROBIAL THERAPY	1	kept
Combination Rule	combination rule	combination rule	combination rule	Combination Rule	1	kept
Combinatorial complexity	combinatorial complexity	combinatorial complexity	combinatorial complexity	Combinatorial complexity	1	kept
Combinatorial mathematics	combinatorial mathematics	combinatorial mathematics	combinatorial mathematics	Combinatorial mathematics	1	kept
combined care	combined care	combined care	combined care	combined care	1	kept
combined immunodeficiency	combined immunodeficiency	combined immunodeficiency	combined immunodeficiency	combined immunodeficiency	1	kept
Combined kidney liver transplantation	combined kidney liver transplantation	combined kidney liver transplantation	combined kidney liver transplantation	Combined kidney liver transplantation	1	kept
combined online and offline learning	combined online and offline learning	combined online and offline learning	combined online and offline learning	combined online and offline learning	1	kept
combined spinal-epidural anesthesia	combined spinal epidural anesthesia	combined spinal epidural anesthesia	combined spinal epidural anesthesia	combined spinal-epidural anesthesia	1	kept
COMFORT B scale	comfort b scale	comfort b scale	comfort b scale	COMFORT B scale	1	kept
Comfort B scale	comfort b scale	comfort b scale	comfort b scale	COMFORT B scale	1	case_or_format
COMFORT Behavior Scale	comfort behavior scale	comfort behavior scale	comfort behavior scale	Comfort Behavior Scale	1	case_or_format
Comfort behavior scale	comfort behavior scale	comfort behavior scale	comfort behavior scale	Comfort Behavior Scale	1	case_or_format
comfort behavior scale	comfort behavior scale	comfort behavior scale	comfort behavior scale	Comfort Behavior Scale	1	case_or_format

COMFORT behavioral scale	comfort behavioral scale	comfort behavioral scale	comfort behavioral scale	Comfort behavioral scale	1	case_or_format
COMFORT CARE	comfort care	comfort care	comfort care	Comfort care	1	case_or_format
comfort measure	comfort measure	comfort measure	comfort measure	comfort measure	1	kept
Comfort measures	comfort measures	comfort measures	comfort measure	comfort measure	1	singular
comfort measures	comfort measures	comfort measures	comfort measure	comfort measure	1	singular
Comfort measures only	comfort measures only	comfort measures only	comfort measures only	Comfort measures only	1	kept
comfort measures only	comfort measures only	comfort measures only	comfort measures only	Comfort measures only	1	case_or_format
Comfort Scale	comfort scale	comfort scale	comfort scale	Comfort Scale	1	kept
Comfort Scale Score	comfort scale score	comfort scale score	comfort scale score	Comfort Scale Score	1	kept
Comfort theory	comfort theory	comfort theory	comfort theory	Comfort Theory	1	case_or_format
Comfort zone	comfort zone	comfort zone	comfort zone	Comfort zone	1	kept
COMFORT-B	comfort b	comfort b	comfort b	COMFORT-B	1	kept
COMFORT-BEHAVIOR SCALE	comfort behavior scale	comfort behavior scale	comfort behavior scale	Comfort Behavior Scale	1	case_or_format
comfort-focused care	comfort focused care	comfort focused care	comfort focused care	comfort-focused care	1	kept
COMFORT-Neo score	comfort neo score	comfort neo score	comfort neo score	COMFORT-Neo score	1	kept
Comforting touch	comforting touch	comforting touch	comforting touch	Comforting touch	1	kept

comics	comics	comics	comics	comics	1	kept
comirnaty	comirnaty	comirnaty	comirnaty	comirnaty	1	kept
Command center	command center	command center	command center	Command center	1	kept
commensal	commensal	commensal	commensal	commensal	1	kept
Commission on Professional and Hospital Activities	commission on professional and hospital activities	commission on professional and hospital activities	commission on professional and hospital activities	Commission on Professional and Hospital Activities	1	kept
commitment	commitment	commitment	commitment	COMMITMENT	1	case_or_format
Committee on Trauma	committee on trauma	committee on trauma	committee on trauma	Committee on Trauma	1	kept
committee translation	committee translation	committee translation	committee translation	committee translation	1	kept
commodification	commodification	commodification	commodification	commodification	1	kept
common bile duct stenosis	common bile duct stenosis	common bile duct stenosis	common bile duct stenosis	common bile duct stenosis	1	kept
common carotid artery	common carotid artery	common carotid artery	common carotid artery	common carotid artery	1	kept
Common Cold	common cold	common cold	common cold	common cold	1	case_or_format
Common Data Elements	common data elements	common data elements	common data elements	Common Data Elements	1	kept
Common data elements	common data elements	common data elements	common data elements	Common Data Elements	1	case_or_format
common data elements	common data elements	common data elements	common data elements	Common Data Elements	1	case_or_format
COMMON FACTOR-ANALYSIS	common factor analysis	common factor analysis	common factor analysis	COMMON FACTOR-ANALYSIS	1	kept

common iliac artery	common iliac artery	common iliac artery	common iliac artery	common iliac artery	1	kept
Common iliac artery aneurysm	common iliac artery aneurysm	common iliac artery aneurysm	common iliac artery aneurysm	Common iliac artery aneurysm	1	kept
Common pitfalls	common pitfalls	common pitfalls	common pitfalls	Common pitfalls	1	kept
Common Terminology Criteria for Adverse Events	common terminology criteria for adverse events	common terminology criteria for adverse events	common terminology criteria for adverse events	Common Terminology Criteria for Adverse Events	1	kept
common variable immunodeficiency	common variable immunodeficiency	common variable immunodeficiency	common variable immunodeficiency	common variable immunodeficiency	1	kept
common-trunk	common trunk	common trunk	common trunk	common-trunk	1	kept
commonsense precepts	commonsense precepts	commonsense precepts	commonsense precepts	commonsense precepts	1	kept
communal dining	communal dining	communal dining	communal dining	communal dining	1	kept
Communicable disease control	communicable disease control	communicable disease control	communicable disease control	Communicable Disease Control	1	case_or_format
Communicable diseases	communicable diseases	communicable diseases	communicable disease	communicable disease	1	singular
communicable diseases	communicable diseases	communicable diseases	communicable disease	communicable disease	1	singular
Communicable Diseases, Imported	communicable diseases imported	communicable diseases imported	communicable diseases imported	Communicable Diseases, Imported	1	kept
CommuniCARE Newborn questionnaire	communicare newborn questionnaire	communicare newborn questionnaire	communicare newborn questionnaire	CommuniCARE Newborn questionnaire	1	kept
Communicating bad news	communicating bad news	communicating bad news	communicating bad news	Communicating bad news	1	kept
Communication aid	communication aid	communication aid	communication aid	communication aid	1	case_or_format
Communication aids	communication aids	communication aids	communication aid	communication aid	1	singular

communication aids	communication aids	communication aids	communication aid	communication aid	1	singular
Communication aids for disabled	communication aids for disabled	communication aids for disabled	communication aids for disabled	Communication Aids for Disabled	1	case_or_format
Communication and collaboration	communication and collaboration	communication and collaboration	communication and collaboration	Communication and collaboration	1	kept
Communication and optimal resolution	communication and optimal resolution	communication and optimal resolution	communication and optimal resolution	Communication and optimal resolution	1	kept
Communication and teamwork in health care	communication and teamwork in health care	communication and teamwork in health care	communication and teamwork in health care	Communication and teamwork in health care	1	kept
communication and teamwork in health care	communication and teamwork in health care	communication and teamwork in health care	communication and teamwork in health care	Communication and teamwork in health care	1	case_or_format
Communication and Teamwork Skills Assessment	communication and teamwork skills assessment	communication and teamwork skills assessment	communication and teamwork skills assessment	Communication and Teamwork Skills Assessment	1	kept
COMMUNICATION BARRIERS	communication barriers	communication barriers	communication barrier	Communication Barriers	1	singular
communication behaviour	communication behaviour	communication behaviour	communication behaviour	communication behaviour	1	kept
communication center	communication center	communication center	communication center	communication center	1	kept
communication challenges	communication challenges	communication challenges	communication challenges	communication challenges	1	kept
Communication Competence	communication competence	communication competence	communication competence	Communication Competence	1	kept
communication competence	communication competence	communication competence	communication competence	Communication Competence	1	case_or_format
communication competencies	communication competencies	communication competencies	communication competencies	communication competencies	1	kept
Communication content	communication content	communication content	communication content	Communication content	1	kept
Communication device	communication device	communication device	communication device	Communication device	1	kept

Communication disorders	communication disorders	communication disorders	communication disorder	communication disorder	1	singular
Communication effectiveness	communication effectiveness	communication effectiveness	communication effectiveness	Communication effectiveness	1	kept
communication enablers	communication enablers	communication enablers	communication enablers	communication enablers	1	kept
communication error	communication error	communication error	communication error	communication error	1	kept
Communication errors	communication errors	communication errors	communication error	communication error	1	singular
communication errors	communication errors	communication errors	communication error	communication error	1	singular
COMMUNICATION EXPERIENCE	communication experience	communication experience	communication experience	COMMUNICATION EXPERIENCE	1	kept
communication experiences	communication experiences	communication experiences	communication experience	COMMUNICATION EXPERIENCE	1	singular
Communication facilitator	communication facilitator	communication facilitator	communication facilitator	Communication facilitator	1	kept
Communication functions	communication functions	communication functions	communication functions	Communication functions	1	kept
communication gap	communication gap	communication gap	communication gap	Communication gap	1	case_or_format
Communication improvement	communication improvement	communication improvement	communication improvement	Communication improvement	1	kept
communication in health care	communication in health care	communication in health care	communication in health care	communication in health care	1	kept
Communication interferences	communication interferences	communication interferences	communication interferences	Communication interferences	1	kept
Communication Management	communication management	communication management	communication management	Communication Management	1	kept
communication material	communication material	communication material	communication material	communication material	1	kept

Communication needs and gaps	communication needs and gaps	communication needs and gaps	communication needs and gaps	Communication needs and gaps	1	kept
communication networks	communication networks	communication networks	communication networks	Communication networks	1	case_or_format
communication observation method	communication observation method	communication observation method	communication observation method	communication observation method	1	kept
communication openness	communication openness	communication openness	communication openness	communication openness	1	kept
Communication partners	communication partners	communication partners	communication partners	Communication partners	1	kept
communication practices	communication practices	communication practices	communication practices	communication practices	1	kept
communication preferences	communication preferences	communication preferences	communication preferences	communication preferences	1	kept
Communication program	communication program	communication program	communication program	Communication program	1	kept
Communication services	communication services	communication services	communication services	Communication services	1	kept
Communication skill	communication skill	communication skill	communication skill	communication skill	1	case_or_format
Communication skills of health workers	communication skills of health workers	communication skills of health workers	communication skills of health workers	Communication skills of health workers	1	kept
Communication spaces	communication spaces	communication spaces	communication spaces	Communication spaces	1	kept
Communication strategies	communication strategies	communication strategies	communication strategies	communication strategies	1	case_or_format
Communication Structure	communication structure	communication structure	communication structure	Communication Structure	1	kept
Communication structure	communication structure	communication structure	communication structure	Communication Structure	1	case_or_format
communication systems	communication systems	communication systems	communication systems	Communication systems	1	case_or_format

COMMUNICATION TECHNOLOGIES	communication technologies	communication technologies	communication technology	communication technology	1	singular
Communication tool	communication tool	communication tool	communication tool	communication tool	1	case_or_format
Communication training	communication training	communication training	communication training	Communication training	1	kept
communication with medical providers	communication with medical providers	communication with medical providers	communication with medical providers	communication with medical providers	1	kept
Communication, handover	communication handover	communication handover	communication handover	Communication, handover	1	kept
COMMUNICATION-SKILLS	communication skills	communication skills	communication skill	communication skill	1	singular
communication-vulnerable	communication vulnerable	communication vulnerable	communication vulnerable	communication vulnerable	1	case_or_format
Communications	communications	communications	communication	Communication	1	singular
Communications skills	communications skills	communications skills	communications skills	Communications skills	1	kept
communicative dynamics	communicative dynamics	communicative dynamics	communicative dynamics	communicative dynamics	1	kept
communicative musicality	communicative musicality	communicative musicality	communicative musicality	communicative musicality	1	kept
Communicative technology	communicative technology	communicative technology	communicative technology	Communicative technology	1	kept
communitas	communitas	communitas	communitas	communitas	1	kept
community assessment	community assessment	community assessment	community assessment	community assessment	1	kept
Community based	community based	community based	community based	Community based	1	kept
community based rehabilitation	community based rehabilitation	community based rehabilitation	community based rehabilitation	community based rehabilitation	1	kept

community benefit	community benefit	community benefit	community benefit	community benefit	1	kept
community birth	community birth	community birth	community birth	community birth	1	kept
Community cancer care	community cancer care	community cancer care	community cancer care	Community cancer care	1	kept
COMMUNITY CARE	community care	community care	community care	community care	1	case_or_format
Community Care Networks	community care networks	community care networks	community care networks	Community Care Networks	1	kept
Community child health	community child health	community child health	community child health	Community child health	1	kept
community child health	community child health	community child health	community child health	Community child health	1	case_or_format
community college	community college	community college	community college	community college	1	kept
community discharge	community discharge	community discharge	community discharge	community discharge	1	kept
community ecology	community ecology	community ecology	community ecology	community ecology	1	kept
community emergency department overcrowding scale	community emergency department overcrowding scale	community emergency department overcrowding scale	community emergency department overcrowding scale	community emergency department overcrowding scale	1	kept
Community health care	community health care	community health care	community health care	Community health care	1	kept
Community health services	community health services	community health services	community health services	Community Health Services	1	case_or_format
community health services	community health services	community health services	community health services	Community Health Services	1	case_or_format
community healthyactivities model program for	community healthyactivities model program for	community healthyactivities model program for	community healthyactivities model program for	community healthyactivities model program for	1	kept
Community hospitals	community hospitals	community hospitals	community hospital	community hospital	1	singular

community hospitals	community hospitals	community hospitals	community hospital	community hospital	1	singular
Community Integration	community integration	community integration	community integration	Community Integration	1	kept
community integration	community integration	community integration	community integration	Community Integration	1	case_or_format
Community Medicine	community medicine	community medicine	community medicine	Community Medicine	1	kept
community medicine	community medicine	community medicine	community medicine	Community Medicine	1	case_or_format
community mental health center	community mental health center	community mental health center	community mental health center	community mental health center	1	kept
community mental health service	community mental health service	community mental health service	community mental health service	Community Mental Health Services	1	case_or_format
community midwifery	community midwifery	community midwifery	community midwifery	community midwifery	1	kept
community midwifery model	community midwifery model	community midwifery model	community midwifery model	community midwifery model	1	kept
Community of inquiry	community of inquiry	community of inquiry	community of inquiry	Community of inquiry	1	kept
community onset stroke	community onset stroke	community onset stroke	community onset stroke	community onset stroke	1	kept
Community outreach	community outreach	community outreach	community outreach	Community outreach	1	kept
COMMUNITY PHARMACY	community pharmacy	community pharmacy	community pharmacy	COMMUNITY PHARMACY	1	kept
Community Pharmacy Services	community pharmacy services	community pharmacy services	community pharmacy services	Community Pharmacy Services	1	kept
community program	community program	community program	community program	community program	1	kept
community resilience	community resilience	community resilience	community resilience	community resilience	1	kept

community resource	community resource	community resource	community resource	community resource	1	kept
Community Services Card	community services card	community services card	community services card	Community Services Card	1	kept
community stakeholders	community stakeholders	community stakeholders	community stakeholders	community stakeholders	1	kept
community structure	community structure	community structure	community structure	community structure	1	kept
Community transmission	community transmission	community transmission	community transmission	community transmission	1	case_or_format
community views	community views	community views	community views	community views	1	kept
community violence	community violence	community violence	community violence	community violence	1	kept
Community-acquired co-infection	community acquired co infection	community acquired co infection	community acquired co infection	Community-acquired co-infection	1	kept
community-acquired infections	community acquired infections	community acquired infections	community acquired infection	Community-Acquired Infections	1	singular
community-acquired pressure ulcer	community acquired pressure ulcer	community acquired pressure ulcer	community acquired pressure ulcer	community-acquired pressure ulcer	1	kept
Community-associated infection	community associated infection	community associated infection	community associated infection	Community-associated infection	1	kept
Community-associated infections	community associated infections	community associated infections	community associated infection	Community-associated infection	1	singular
Community-associated methicillin-resistant Staphylococcus aureus	community associated methicillin resistant staphylococcus aureus	community associated methicillin resistant staphylococcus aureus	community associated methicillin resistant staphylococcus aureus	Community-associated methicillin-resistant Staphylococcus aureus	1	kept
Community-based	community based	community based	community based	Community based	1	case_or_format
community-based	community based	community based	community based	Community based	1	case_or_format
community-based palliative care	community based palliative care	community based palliative care	community based palliative care	community-based palliative care	1	kept

Community-Based Participatory Research	community based participatory research	community based participatory research	community based participatory research	Community-Based Participatory Research	1	kept
community-dwelling	community dwelling	community dwelling	community dwelling	community-dwelling	1	kept
COMMUNITY-LIVING ELDERS	community living elders	community living elders	community living elders	COMMUNITY-LIVING ELDERS	1	kept
community-living older adults	community living older adults	community living older adults	community living older adults	community-living older adults	1	kept
Community-onset pneumonia	community onset pneumonia	community onset pneumonia	community onset pneumonia	Community-onset pneumonia	1	kept
Community-onset stroke	community onset stroke	community onset stroke	community onset stroke	community onset stroke	1	case_or_format
Comodidad	comodidad	comodidad	comodidad	Comodidad	1	kept
comorbidity index	comorbidity index	comorbidity index	comorbidity index	comorbidity index	1	kept
Comorbidity Polypharmacy Score	comorbidity polypharmacy score	comorbidity polypharmacy score	comorbidity polypharmacy score	Comorbidity Polypharmacy Score	1	kept
comorbidity polypharmacy score	comorbidity polypharmacy score	comorbidity polypharmacy score	comorbidity polypharmacy score	Comorbidity Polypharmacy Score	1	case_or_format
Compact disk	compact disk	compact disk	compact disk	Compact disk	1	kept
compact disk	compact disk	compact disk	compact disk	Compact disk	1	case_or_format
Compact Disks	compact disks	compact disks	compact disk	Compact disk	1	singular
Compact fluorescent light	compact fluorescent light	compact fluorescent light	compact fluorescent light	Compact fluorescent light	1	kept
companion animals	companion animals	companion animals	companion animals	companion animals	1	kept
Companionship	companionship	companionship	companionship	Companionship	1	kept

companionship	companionship	companionship	companionship	Companionship	1	case_or_format
Comparative analysis	comparative analysis	comparative analysis	comparative analysis	comparative analysis	1	case_or_format
Comparative effectiveness trial	comparative effectiveness trial	comparative effectiveness trial	comparative effectiveness trial	Comparative effectiveness trial	1	kept
Comparative performance	comparative performance	comparative performance	comparative performance	Comparative performance	1	kept
Comparatives studies	comparatives studies	comparatives studies	comparatives studies	Comparatives studies	1	kept
compare	compare	compare	compare	compare	1	kept
COMPARING IMMEDIATE	comparing immediate	comparing immediate	comparing immediate	COMPARING IMMEDIATE	1	kept
COMPARING SAFETY	comparing safety	comparing safety	comparing safety	COMPARING SAFETY	1	kept
comparison method	comparison method	comparison method	comparison method	comparison method	1	kept
Comparison of validity	comparison of validity	comparison of validity	comparison of validity	Comparison of validity	1	kept
compartment model	compartment model	compartment model	compartment model	compartment model	1	kept
compasion satisfaction	compasion satisfaction	compasion satisfaction	compasion satisfaction	compasion satisfaction	1	kept
compassion cafes	compassion cafes	compassion cafes	compassion cafes	compassion cafes	1	kept
compassion fatigue and personality traits	compassion fatigue and personality traits	compassion fatigue and personality traits	compassion fatigue and personality traits	compassion fatigue and personality traits	1	kept
compassion focused therapy	compassion focused therapy	compassion focused therapy	compassion focused therapy	compassion focused therapy	1	kept
compassion-fatigue	compassion fatigue	compassion fatigue	compassion fatigue	Compassion Fatigue	1	case_or_format

compassionate deactivation	compassionate deactivation	compassionate deactivation	compassionate deactivation	compassionate deactivation	1	kept
Compassionate extubation	compassionate extubation	compassionate extubation	compassionate extubation	Compassionate extubation	1	kept
compassionate extubation	compassionate extubation	compassionate extubation	compassionate extubation	Compassionate extubation	1	case_or_format
compassionate leadership	compassionate leadership	compassionate leadership	compassionate leadership	compassionate leadership	1	kept
compassionate love	compassionate love	compassionate love	compassionate love	compassionate love	1	kept
compatibility platelets	compatibility platelets	compatibility platelets	compatibility platelets	compatibility platelets	1	kept
Compensated occupational infectious diseases	compensated occupational infectious diseases	compensated occupational infectious diseases	compensated occupational infectious diseases	Compensated occupational infectious diseases	1	kept
Compensating wage differentials	compensating wage differentials	compensating wage differentials	compensating wage differentials	Compensating wage differentials	1	kept
Compensation and redress	compensation and redress	compensation and redress	compensation and redress	Compensation and Redress	1	case_or_format
compensation distance evaluation technique	compensation distance evaluation technique	compensation distance evaluation technique	compensation distance evaluation technique	compensation distance evaluation technique	1	kept
compensatory sleep	compensatory sleep	compensatory sleep	compensatory sleep	compensatory sleep	1	kept
Compentence	compentence	compentence	compentence	Compentence	1	kept
Competence development	competence development	competence development	competence development	Competence development	1	kept
competence-based education	competence based education	competence based education	competence based education	Competence-based education	1	case_or_format
COMPETENCES	competences	competences	competence	competence	1	singular
Competencia profesional	competencia profesional	competencia profesional	competencia profesional	Competencia profesional	1	kept

Competencias	competencias	competencias	competencias	Competencias	1	kept
Competency assessment	competency assessment	competency assessment	competency assessment	competency assessment	1	case_or_format
competency based education	competency based education	competency based education	competency based education	Competency-Based Education	1	case_or_format
Competency based training	competency based training	competency based training	competency based training	Competency based training	1	kept
competency based training	competency based training	competency based training	competency based training	Competency based training	1	case_or_format
Competency development	competency development	competency development	competency development	Competency development	1	kept
competency level	competency level	competency level	competency level	competency level	1	kept
Competency Outcomes and Performance Assessment	competency outcomes and performance assessment	competency outcomes and performance assessment	competency outcomes and performance assessment	Competency Outcomes and Performance Assessment	1	kept
Competency standards	competency standards	competency standards	competency standards	Competency standards	1	kept
Competency testing	competency testing	competency testing	competency testing	Competency testing	1	kept
Competing risk	competing risk	competing risk	competing risk	Competing risk	1	kept
Competing trials	competing trials	competing trials	competing trials	Competing trials	1	kept
Competition	competition	competition	competition	competition	1	case_or_format
Competitive behavior	competitive behavior	competitive behavior	competitive behavior	Competitive Behavior	1	case_or_format
Complainer	complainer	complainer	complainer	Complainer	1	kept
Complaint	complaint	complaint	complaint	Complaint	1	kept

COMPLAINTS	complaints	complaints	complaint	Complaint	1	singular
Complement	complement	complement	complement	complement	1	case_or_format
Complement Activation	complement activation	complement activation	complement activation	complement activation	1	case_or_format
Complement C5	complement c5	complement c5	complement c5	Complement C5	1	kept
complement component C1q	complement component c1q	complement component c1q	complement component c1q	complement component C1q	1	kept
complement component C1s inhibitor	complement component c1s inhibitor	complement component c1s inhibitor	complement component c1s inhibitor	complement component C1s inhibitor	1	kept
complement component C3b receptor	complement component c3b receptor	complement component c3b receptor	complement component c3b receptor	complement component C3b receptor	1	kept
complement component C4	complement component c4	complement component c4	complement component c4	complement component C4	1	kept
complement component C4d	complement component c4d	complement component c4d	complement component c4d	complement component C4d	1	kept
complement component C5	complement component c5	complement component c5	complement component c5	complement component C5	1	kept
Complement Inactivating Agents	complement inactivating agents	complement inactivating agents	complement inactivating agents	Complement Inactivating Agents	1	kept
complement inhibitor	complement inhibitor	complement inhibitor	complement inhibitor	complement inhibitor	1	kept
complement system	complement system	complement system	complement system	complement system	1	kept
Complement System Proteins	complement system proteins	complement system proteins	complement system proteins	Complement System Proteins	1	kept
COMPLEMENTARY	complementary	complementary	complementary	complementary	1	case_or_format
Complementary	complementary	complementary	complementary	complementary	1	case_or_format

Complementary and Alternative Therapies	complementary and alternative therapies	complementary and alternative therapies	complementary and alternative therapies	Complementary and Alternative Therapies	1	kept
Complementary andalternative medicine	complementary andalternative medicine	complementary andalternative medicine	complementary andalternative medicine	Complementary andalternative medicine	1	kept
COMPLEMENTARY FOODS	complementary foods	complementary foods	complementary foods	COMPLEMENTARY FOODS	1	kept
complementary foods	complementary foods	complementary foods	complementary foods	COMPLEMENTARY FOODS	1	case_or_format
Complementary health approaches	complementary health approaches	complementary health approaches	complementary health approaches	Complementary health approaches	1	kept
complementary health approaches	complementary health approaches	complementary health approaches	complementary health approaches	Complementary health approaches	1	case_or_format
Complementary interventions	complementary interventions	complementary interventions	complementary interventions	Complementary interventions	1	kept
COMPLEMENTARY MEDICINE	complementary medicine	complementary medicine	complementary medicine	Complementary medicine	1	case_or_format
complementary skill	complementary skill	complementary skill	complementary skill	complementary skill	1	kept
complementary therapy	complementary therapy	complementary therapy	complementary therapy	Complementary Therapies	1	case_or_format
complete diagnostic autopsy	complete diagnostic autopsy	complete diagnostic autopsy	complete diagnostic autopsy	complete diagnostic autopsy	1	kept
complete heart block	complete heart block	complete heart block	complete heart block	complete heart block	1	kept
Complete oral feeding	complete oral feeding	complete oral feeding	complete oral feeding	Complete oral feeding	1	kept
complete peritonectomy	complete peritonectomy	complete peritonectomy	complete peritonectomy	complete peritonectomy	1	kept
Complete response	complete response	complete response	complete response	Complete response	1	kept
COMPLETION	completion	completion	completion	COMPLETION	1	kept

Complex 1	complex 1	complex 1	complex 1	Complex 1	1	kept
Complex abdominal wall reconstruction	complex abdominal wall reconstruction	complex abdominal wall reconstruction	complex abdominal wall reconstruction	Complex abdominal wall reconstruction	1	kept
complex abdominal wall reconstruction	complex abdominal wall reconstruction	complex abdominal wall reconstruction	complex abdominal wall reconstruction	Complex abdominal wall reconstruction	1	case_or_format
Complex adaptive system	complex adaptive system	complex adaptive system	complex adaptive system	Complex adaptive system	1	kept
complex care	complex care	complex care	complex care	Complex care	1	case_or_format
Complex chronic conditions	complex chronic conditions	complex chronic conditions	complex chronic conditions	COMPLEX CHRONIC CONDITIONS	1	case_or_format
complex chronic conditions	complex chronic conditions	complex chronic conditions	complex chronic conditions	COMPLEX CHRONIC CONDITIONS	1	case_or_format
complex chronic disease	complex chronic disease	complex chronic disease	complex chronic disease	complex chronic disease	1	kept
Complex chronic illness	complex chronic illness	complex chronic illness	complex chronic illness	Complex chronic illness	1	kept
complex conditions	complex conditions	complex conditions	complex conditions	complex conditions	1	kept
Complex congenital heart disease	complex congenital heart disease	complex congenital heart disease	complex congenital heart disease	Complex congenital heart disease	1	kept
complex decisions	complex decisions	complex decisions	complex decisions	complex decisions	1	kept
Complex endovascular aortic repair	complex endovascular aortic repair	complex endovascular aortic repair	complex endovascular aortic repair	Complex endovascular aortic repair	1	kept
Complex family health interventions	complex family health interventions	complex family health interventions	complex family health interventions	Complex family health interventions	1	kept
complex intervention	complex intervention	complex intervention	complex intervention	Complex interventions	1	case_or_format
Complex lesion	complex lesion	complex lesion	complex lesion	Complex lesion	1	kept

Complex lesions	complex lesions	complex lesions	complex lesion	Complex lesion	1	singular
complex mandibular surgery	complex mandibular surgery	complex mandibular surgery	complex mandibular surgery	complex mandibular surgery	1	kept
complex medical needs	complex medical needs	complex medical needs	complex medical needs	complex medical needs	1	kept
complex needs	complex needs	complex needs	complex needs	complex needs	1	kept
Complex networks	complex networks	complex networks	complex networks	Complex networks	1	kept
Complex regional pain syndrome	complex regional pain syndrome	complex regional pain syndrome	complex regional pain syndrome	Complex regional pain syndrome	1	kept
complex regional pain syndrome	complex regional pain syndrome	complex regional pain syndrome	complex regional pain syndrome	Complex regional pain syndrome	1	case_or_format
Complex Regional Pain Syndromes	complex regional pain syndromes	complex regional pain syndromes	complex regional pain syndrome	Complex regional pain syndrome	1	singular
COMPLEX RELATIONSHIP	complex relationship	complex relationship	complex relationship	COMPLEX RELATIONSHIP	1	kept
Complex sociotechnical systems	complex sociotechnical systems	complex sociotechnical systems	complex sociotechnical systems	Complex sociotechnical systems	1	kept
complex technology	complex technology	complex technology	complex technology	complex technology	1	kept
Complex thoracoabdominal aortic aneurysm	complex thoracoabdominal aortic aneurysm	complex thoracoabdominal aortic aneurysm	complex thoracoabdominal aortic aneurysm	Complex thoracoabdominal aortic aneurysm	1	kept
Complex transformations	complex transformations	complex transformations	complex transformations	Complex transformations	1	kept
Complex ventral hernia repair	complex ventral hernia repair	complex ventral hernia repair	complex ventral hernia repair	Complex ventral hernia repair	1	kept
complexity compression	complexity compression	complexity compression	complexity compression	complexity compression	1	kept
complexity of care	complexity of care	complexity of care	complexity of care	Complexity of care	1	case_or_format

Compliance analysis	compliance analysis	compliance analysis	compliance analysis	Compliance analysis	1	kept
compliance and knowledge	compliance and knowledge	compliance and knowledge	compliance and knowledge	compliance and knowledge	1	kept
compliance barrier	compliance barrier	compliance barrier	compliance barrier	compliance barrier	1	kept
Compliance behavior	compliance behavior	compliance behavior	compliance behavior	Compliance behavior	1	kept
compliance error	compliance error	compliance error	compliance error	compliance error	1	kept
Compliance pathways	compliance pathways	compliance pathways	compliance pathways	Compliance pathways	1	kept
compliance rates	compliance rates	compliance rates	compliance rates	compliance rates	1	kept
Compliance with guidelines	compliance with guidelines	compliance with guidelines	compliance with guidelines	Compliance with guidelines	1	kept
Complicaciones	complicaciones	complicaciones	complicaciones	Complicaciones	1	kept
Complicated childbirth	complicated childbirth	complicated childbirth	complicated childbirth	Complicated childbirth	1	kept
complicated cholecystitis	complicated cholecystitis	complicated cholecystitis	complicated cholecystitis	complicated cholecystitis	1	kept
COMPLICATED GRIEF	complicated grief	complicated grief	complicated grief	complicated grief	1	case_or_format
Complicated Grief	complicated grief	complicated grief	complicated grief	complicated grief	1	case_or_format
Complicated grief	complicated grief	complicated grief	complicated grief	complicated grief	1	case_or_format
Complication of lung transplantation	complication of lung transplantation	complication of lung transplantation	complication of lung transplantation	Complication of lung transplantation	1	kept
COMPLICATION RATES	complication rates	complication rates	complication rate	complication rate	1	singular

Complication scoring systems	complication scoring systems	complication scoring systems	complication scoring systems	Complication scoring systems	1	kept
Complications of lung transplantation	complications of lung transplantation	complications of lung transplantation	complications of lung transplantation	Complications of lung transplantation	1	kept
complications of pediatric rheumatic disease	complications of pediatric rheumatic disease	complications of pediatric rheumatic disease	complications of pediatric rheumatic disease	complications of pediatric rheumatic disease	1	kept
Complications of portal hypertension	complications of portal hypertension	complications of portal hypertension	complications of portal hypertension	Complications of portal hypertension	1	kept
Complications of VADs	complications of vads	complications of vads	complications of vads	Complications of VADs	1	kept
Complications registration	complications registration	complications registration	complications registration	Complications registration	1	kept
COMPONENT ANALYSIS	component analysis	component analysis	component analysis	COMPONENT ANALYSIS	1	kept
Component network meta-analysis	component network meta analysis	component network meta analysis	component network meta analysis	Component network meta-analysis	1	kept
Component ratio	component ratio	component ratio	component ratio	Component ratio	1	kept
COMPONENTS	components	components	components	COMPONENTS	1	kept
composite measure	composite measure	composite measure	composite measure	composite measure	1	kept
composite outcomes	composite outcomes	composite outcomes	composite outcomes	composite outcomes	1	kept
Composite Resins	composite resins	composite resins	composite resins	Composite Resins	1	kept
Composite scores	composite scores	composite scores	composite scores	Composite scores	1	kept
Compound fracture	compound fracture	compound fracture	compound fracture	Compound fracture	1	kept
Compounded sterile products	compounded sterile products	compounded sterile products	compounded sterile products	Compounded sterile products	1	kept

compounding	compounding	compounding	compounding	compounding	1	kept
compounding center preparation	compounding center preparation	compounding center preparation	compounding center preparation	compounding center preparation	1	kept
Compounding errors	compounding errors	compounding errors	compounding errors	Compounding errors	1	kept
COMPREHENSION	comprehension	comprehension	comprehension	comprehension	1	case_or_format
Comprehensive airway management	comprehensive airway management	comprehensive airway management	comprehensive airway management	Comprehensive airway management	1	kept
comprehensive care	comprehensive care	comprehensive care	comprehensive care	Comprehensive care	1	case_or_format
Comprehensive error rate testing	comprehensive error rate testing	comprehensive error rate testing	comprehensive error rate testing	Comprehensive error rate testing	1	kept
Comprehensive geriatric treatment	comprehensive geriatric treatment	comprehensive geriatric treatment	comprehensive geriatric treatment	Comprehensive geriatric treatment	1	kept
Comprehensive health care	comprehensive health care	comprehensive health care	comprehensive health care	Comprehensive Health Care	1	case_or_format
comprehensive healthcare	comprehensive healthcare	comprehensive healthcare	comprehensive healthcare	comprehensive healthcare	1	kept
Comprehensive intervention	comprehensive intervention	comprehensive intervention	comprehensive intervention	Comprehensive intervention	1	kept
Comprehensive literature reviews	comprehensive literature reviews	comprehensive literature reviews	comprehensive literature reviews	Comprehensive literature reviews	1	kept
Comprehensive oxygen management	comprehensive oxygen management	comprehensive oxygen management	comprehensive oxygen management	Comprehensive oxygen management	1	kept
Comprehensive program	comprehensive program	comprehensive program	comprehensive program	Comprehensive program	1	kept
Comprehensive projects	comprehensive projects	comprehensive projects	comprehensive projects	Comprehensive projects	1	kept
comprehensive response strategies	comprehensive response strategies	comprehensive response strategies	comprehensive response strategies	comprehensive response strategies	1	kept

Comprehensive Safety Management	comprehensive safety management	comprehensive safety management	comprehensive safety management	Comprehensive Safety Management	1	kept
Comprehensive Severity Index	comprehensive severity index	comprehensive severity index	comprehensive severity index	Comprehensive Severity Index	1	kept
Comprehensive stroke center	comprehensive stroke center	comprehensive stroke center	comprehensive stroke center	Comprehensive stroke center	1	kept
Comprehensive Unit-based Safety Program	comprehensive unit based safety program	comprehensive unit based safety program	comprehensive unit based safety program	Comprehensive Unit-based Safety Program	1	kept
compressed density spectral array	compressed density spectral array	compressed density spectral array	compressed density spectral array	compressed density spectral array	1	kept
Compressed working week	compressed working week	compressed working week	compressed working week	Compressed working week	1	kept
compression bandage	compression bandage	compression bandage	compression bandage	compression bandage	1	kept
Compression Bandages	compression bandages	compression bandages	compression bandage	compression bandage	1	singular
compression garment	compression garment	compression garment	compression garment	compression garment	1	kept
compression instrument	compression instrument	compression instrument	compression instrument	compression instrument	1	kept
compression quality	compression quality	compression quality	compression quality	compression quality	1	kept
compression syndrome	compression syndrome	compression syndrome	compression syndrome	compression syndrome	1	kept
compression ultrasound	compression ultrasound	compression ultrasound	compression ultrasound	compression ultrasound	1	kept
Compression ultrasound, thrombosis	compression ultrasound thrombosis	compression ultrasound thrombosis	compression ultrasound thrombosis	Compression ultrasound, thrombosis	1	kept
Compressive Strength	compressive strength	compressive strength	compressive strength	Compressive Strength	1	kept
Compromised infant	compromised infant	compromised infant	compromised infant	Compromised infant	1	kept

Compulsive Behavior	compulsive behavior	compulsive behavior	compulsive behavior	Compulsive Behavior	1	kept
Computable biomarker	computable biomarker	computable biomarker	computable biomarker	Computable biomarker	1	kept
Computable phenotype	computable phenotype	computable phenotype	computable phenotype	Computable phenotype	1	kept
computable phenotype	computable phenotype	computable phenotype	computable phenotype	Computable phenotype	1	case_or_format
Computation theory	computation theory	computation theory	computation theory	Computation theory	1	kept
computational fluid dynamics	computational fluid dynamics	computational fluid dynamics	computational fluid dynamics	computational fluid dynamics	1	kept
Computational linguistics	computational linguistics	computational linguistics	computational linguistics	Computational linguistics	1	kept
Computational methods	computational methods	computational methods	computational methods	Computational methods	1	kept
COMPUTATIONAL MODEL	computational model	computational model	computational model	COMPUTATIONAL MODEL	1	kept
Computational modelling	computational modelling	computational modelling	computational modelling	Computational modelling	1	kept
Computational physiology	computational physiology	computational physiology	computational physiology	Computational physiology	1	kept
computed tomographic colonography	computed tomographic colonography	computed tomographic colonography	computed tomographic colonography	computed tomographic colonography	1	kept
computed tomographic venography	computed tomographic venography	computed tomographic venography	computed tomographic venography	computed tomographic venography	1	kept
Computed Tomography	computed tomography	computed tomography	computed tomography	Computed tomography	1	case_or_format
computed tomography	computed tomography	computed tomography	computed tomography	Computed tomography	1	case_or_format
Computed tomography angiography	computed tomography angiography	computed tomography angiography	computed tomography angiography	Computed Tomography Angiography	1	case_or_format

computed tomography pulmonary angiography	computed tomography pulmonary angiography	computed tomography pulmonary angiography	computed tomography pulmonary angiography	computed tomography pulmonary angiography	1	kept
computed tomography scanner	computed tomography scanner	computed tomography scanner	computed tomography scanner	computed tomography scanner	1	kept
COMPUTED-TOMOGRAPHY	computed tomography	computed tomography	computed tomography	Computed tomography	1	case_or_format
Computer Aided Design	computer aided design	computer aided design	computer aided design	computer aided design	1	case_or_format
Computer aided diagnosis	computer aided diagnosis	computer aided diagnosis	computer aided diagnosis	Computer aided diagnosis	1	kept
computer aided diagnosis	computer aided diagnosis	computer aided diagnosis	computer aided diagnosis	Computer aided diagnosis	1	case_or_format
Computer algorithm	computer algorithm	computer algorithm	computer algorithm	Computer algorithm	1	kept
Computer application	computer application	computer application	computer application	Computer application	1	kept
Computer Applications	computer applications	computer applications	computer application	Computer application	1	singular
computer applications	computer applications	computer applications	computer application	Computer application	1	singular
Computer assisted	computer assisted	computer assisted	computer assisted	Computer assisted	1	kept
computer assisted decision support system	computer assisted decision support system	computer assisted decision support system	computer assisted decision support system	computer assisted decision support system	1	kept
Computer assisted drug therapy	computer assisted drug therapy	computer assisted drug therapy	computer assisted drug therapy	computer assisted drug therapy	1	case_or_format
computer assisted emission tomography	computer assisted emission tomography	computer assisted emission tomography	computer assisted emission tomography	computer assisted emission tomography	1	kept
Computer assisted learning	computer assisted learning	computer assisted learning	computer assisted learning	Computer assisted learning	1	kept
Computer based	computer based	computer based	computer based	Computer based	1	kept

computer based	computer based	computer based	computer based	Computer based	1	case_or_format
computer communication networks	computer communication networks	computer communication networks	computer communication networks	Computer Communication Networks	1	case_or_format
Computer control systems	computer control systems	computer control systems	computer control systems	Computer control systems	1	kept
Computer Data Processing	computer data processing	computer data processing	computer data processing	Computer Data Processing	1	kept
Computer documentation	computer documentation	computer documentation	computer documentation	Computer documentation	1	kept
computer generated blood glucose alert	computer generated blood glucose alert	computer generated blood glucose alert	computer generated blood glucose alert	computer generated blood glucose alert	1	kept
COMPUTER HEURISTICS	computer heuristics	computer heuristics	computer heuristics	COMPUTER HEURISTICS	1	kept
Computer managements	computer managements	computer managements	computer managements	Computer managements	1	kept
Computer mediated communication	computer mediated communication	computer mediated communication	computer mediated communication	Computer mediated communication	1	kept
computer mediated communication	computer mediated communication	computer mediated communication	computer mediated communication	Computer mediated communication	1	case_or_format
computer numerical control router	computer numerical control router	computer numerical control router	computer numerical control router	computer numerical control router	1	kept
Computer Peripherals	computer peripherals	computer peripherals	computer peripherals	Computer Peripherals	1	kept
Computer Prescribing Order Entry	computer prescribing order entry	computer prescribing order entry	computer prescribing order entry	Computer Prescribing Order Entry	1	kept
Computer programming	computer programming	computer programming	computer programming	Computer programming	1	kept
Computer protocol	computer protocol	computer protocol	computer protocol	computer protocol	1	case_or_format
Computer science	computer science	computer science	computer science	Computer science	1	kept

COMPUTER SIMULATION	computer simulation	computer simulation	computer simulation	Computer Simulation	1	case_or_format
Computer software	computer software	computer software	computer software	Computer software	1	kept
Computer systems	computer systems	computer systems	computer system	computer system	1	singular
Computer technology	computer technology	computer technology	computer technology	Computer technology	1	kept
Computer usage	computer usage	computer usage	computer usage	Computer usage	1	kept
computer user	computer user	computer user	computer user	computer user	1	kept
Computer user training	computer user training	computer user training	computer user training	Computer User Training	1	case_or_format
Computer vision techniques	computer vision techniques	computer vision techniques	computer vision techniques	Computer vision techniques	1	kept
Computer-aided	computer aided	computer aided	computer aided	Computer-aided	1	kept
Computer-Aided Design	computer aided design	computer aided design	computer aided design	computer aided design	1	case_or_format
Computer-assisted decision making	computer assisted decision making	computer assisted decision making	computer assisted decision making	Computer-assisted decision making	1	kept
computer-assisted decision making	computer assisted decision making	computer assisted decision making	computer assisted decision making	Computer-assisted decision making	1	case_or_format
Computer-assisted decision support system	computer assisted decision support system	computer assisted decision support system	computer assisted decision support system	computer assisted decision support system	1	case_or_format
computer-assisted information flow	computer assisted information flow	computer assisted information flow	computer assisted information flow	computer-assisted information flow	1	kept
Computer-assisted instruction	computer assisted instruction	computer assisted instruction	computer assisted instruction	Computer-Assisted Instruction	1	case_or_format
computer-assisted instruction	computer assisted instruction	computer assisted instruction	computer assisted instruction	Computer-Assisted Instruction	1	case_or_format

COMPUTER-ASSISTED MANAGEMENT	computer assisted management	computer assisted management	computer assisted management	COMPUTER-ASSISTED MANAGEMENT	1	kept
Computer-base protocol	computer base protocol	computer base protocol	computer base protocol	Computer-base protocol	1	kept
computer-based communication tool	computer based communication tool	computer based communication tool	computer based communication tool	computer-based communication tool	1	kept
Computer-based self-instructional module	computer based self instructional module	computer based self instructional module	computer based self instructional module	Computer-based self-instructional module	1	kept
computer-based simulation	computer based simulation	computer based simulation	computer based simulation	Computer-based simulation	1	case_or_format
COMPUTER-PROGRAM	computer program	computer program	computer program	computer program	1	case_or_format
computerised	computerised	computerised	computerised	computerised	1	kept
computerised decision support systems	computerised decision support systems	computerised decision support systems	computerised decision support systems	computerised decision support systems	1	kept
computerised equipment	computerised equipment	computerised equipment	computerised equipment	Computerised equipment	1	case_or_format
Computerised physician order entry	computerised physician order entry	computerised physician order entry	computerised physician order entry	Computerised physician order entry	1	kept
Computerization	computerization	computerization	computerization	Computerization	1	kept
computerized clinical information systems	computerized clinical information systems	computerized clinical information systems	computerized clinical information systems	computerized clinical information systems	1	kept
Computerized decision	computerized decision	computerized decision	computerized decision	Computerized decision	1	kept
Computerized decision support	computerized decision support	computerized decision support	computerized decision support	computerized decision support	1	case_or_format
computerized information systems	computerized information systems	computerized information systems	computerized information systems	computerized information systems	1	kept
computerized insulin algorithm	computerized insulin algorithm	computerized insulin algorithm	computerized insulin algorithm	computerized insulin algorithm	1	kept

Computerized management	computerized management	computerized management	computerized management	Computerized management	1	kept
Computerized medical record systems	computerized medical record systems	computerized medical record systems	computerized medical record systems	Computerized medical record systems	1	kept
computerized order entry	computerized order entry	computerized order entry	computerized order entry	computerized order entry	1	kept
computerized parenteral nutrition worksheet	computerized parenteral nutrition worksheet	computerized parenteral nutrition worksheet	computerized parenteral nutrition worksheet	computerized parenteral nutrition worksheet	1	kept
computerized physician order entry	computerized physician order entry	computerized physician order entry	computerized physician order entry	Computerized physician order entry	1	case_or_format
computerized prescriber order entry	computerized prescriber order entry	computerized prescriber order entry	computerized prescriber order entry	computerized prescriber order entry	1	kept
Computerized protocol	computerized protocol	computerized protocol	computerized protocol	Computerized protocol	1	kept
Computerized shift report	computerized shift report	computerized shift report	computerized shift report	Computerized shift report	1	kept
Computing Methodologies	computing methodologies	computing methodologies	computing methodologies	Computing Methodologies	1	kept
Comunicación no verbal	comunicaci n no verbal	comunicaci n no verbal	comunicaci n no verbal	Comunicación no verbal	1	kept
Comunicação	comunica o	comunica o	comunica o	Comunicação	1	kept
Concentration Camps	concentration camps	concentration camps	concentration camps	Concentration Camps	1	kept
concentration camps	concentration camps	concentration camps	concentration camps	Concentration Camps	1	case_or_format
concentration process	concentration process	concentration process	concentration process	concentration process	1	kept
concentration ratio	concentration ratio	concentration ratio	concentration ratio	concentration ratio	1	kept
Concept building	concept building	concept building	concept building	Concept building	1	kept

concept clarification	concept clarification	concept clarification	concept clarification	concept clarification	1	kept
concept delineation	concept delineation	concept delineation	concept delineation	concept delineation	1	kept
Concept mapping	concept mapping	concept mapping	concept mapping	Concept mapping	1	kept
concept mapping	concept mapping	concept mapping	concept mapping	Concept mapping	1	case_or_format
concept of good death	concept of good death	concept of good death	concept of good death	concept of good death	1	kept
Concept structures	concept structures	concept structures	concept structures	Concept structures	1	kept
Concepts	concepts	concepts	concepts	Concepts	1	kept
conceptual	conceptual	conceptual	conceptual	conceptual	1	kept
Conceptual analyses	conceptual analyses	conceptual analyses	conceptual analyses	Conceptual analyses	1	kept
concern	concern	concern	concern	concern	1	kept
Concerns	concerns	concerns	concern	concern	1	singular
concerns	concerns	concerns	concern	concern	1	singular
Conciencia plena	conciencia plena	conciencia plena	conciencia plena	Conciencia plena	1	kept
Concordance	concordance	concordance	concordance	concordance	1	case_or_format
Concrete pavements	concrete pavements	concrete pavements	concrete pavements	Concrete pavements	1	kept
Concurrent engineering	concurrent engineering	concurrent engineering	concurrent engineering	Concurrent engineering	1	kept

concurrent hospice care	concurrent hospice care	concurrent hospice care	concurrent hospice care	concurrent hospice care	1	kept
Concurrent interaction	concurrent interaction	concurrent interaction	concurrent interaction	Concurrent interaction	1	kept
Concurrent Review	concurrent review	concurrent review	concurrent review	Concurrent Review	1	kept
Concurrent surgery	concurrent surgery	concurrent surgery	concurrent surgery	Concurrent surgery	1	kept
Concurrent tasks	concurrent tasks	concurrent tasks	concurrent tasks	Concurrent tasks	1	kept
Concussion	concussion	concussion	concussion	concussion	1	case_or_format
Condiciones de Trabajo	condiciones de trabajo	condiciones de trabajo	condiciones de trabajo	Condiciones de Trabajo	1	kept
Condiciones de trabajo	condiciones de trabajo	condiciones de trabajo	condiciones de trabajo	Condiciones de Trabajo	1	case_or_format
condiciones de trabajo	condiciones de trabajo	condiciones de trabajo	condiciones de trabajo	Condiciones de Trabajo	1	case_or_format
condition of work effectiveness questionnaire ii	condition of work effectiveness questionnaire ii	condition of work effectiveness questionnaire ii	condition of work effectiveness questionnaire ii	condition of work effectiveness questionnaire ii	1	kept
conditional alarm triggers	conditional alarm triggers	conditional alarm triggers	conditional alarm triggers	conditional alarm triggers	1	kept
Conditional average	conditional average	conditional average	conditional average	Conditional average	1	kept
Conditional survival	conditional survival	conditional survival	conditional survival	Conditional survival	1	kept
CONDITIONING REGIMENS	conditioning regimens	conditioning regimens	conditioning regimens	CONDITIONING REGIMENS	1	kept
Conditioning, Operant	conditioning operant	conditioning operant	conditioning operant	Conditioning, Operant	1	kept
Condições de Trabalho	condi es de trabalho	condi es de trabalho	condi es de trabalho	Condições de Trabalho	1	kept

condom catheter	condom catheter	condom catheter	condom catheter	condom catheter	1	kept
conduct disorder	conduct disorder	conduct disorder	conduct disorder	conduct disorder	1	kept
conductance	conductance	conductance	conductance	conductance	1	kept
conduction	conduction	conduction	conduction	conduction	1	kept
Cone-Beam Computed Tomography	cone beam computed tomography	cone beam computed tomography	cone beam computed tomography	cone beam computed tomography	1	case_or_format
Conference consensus	conference consensus	conference consensus	conference consensus	Conference consensus	1	kept
conference review	conference review	conference review	conference review	conference review	1	kept
CONFERENCES	conferences	conferences	conferences	CONFERENCES	1	kept
confidence intervals	confidence intervals	confidence intervals	confidence interval	confidence interval	1	singular
Confidence levels	confidence levels	confidence levels	confidence levels	Confidence levels	1	kept
Confidential enquiry maternal deaths	confidential enquiry maternal deaths	confidential enquiry maternal deaths	confidential enquiry maternal deaths	Confidential enquiry maternal deaths	1	kept
Confirmation	confirmation	confirmation	confirmation	Confirmation	1	kept
confirmation of diagnosis	confirmation of diagnosis	confirmation of diagnosis	confirmation of diagnosis	confirmation of diagnosis	1	kept
Confirmatory Factor Analysis	confirmatory factor analysis	confirmatory factor analysis	confirmatory factor analysis	confirmatory factor analysis	1	case_or_format
Confirming	confirming	confirming	confirming	Confirming	1	kept
Conflict handling styles	conflict handling styles	conflict handling styles	conflict handling styles	Conflict handling styles	1	kept

conflict management strategies	conflict management strategies	conflict management strategies	conflict management strategies	conflict management strategies	1	kept
conflict of conscience	conflict of conscience	conflict of conscience	conflict of conscience	conflict of conscience	1	kept
conflict resolution	conflict resolution	conflict resolution	conflict resolution	Conflict resolution	1	case_or_format
Conflict zone healthcare	conflict zone healthcare	conflict zone healthcare	conflict zone healthcare	Conflict zone healthcare	1	kept
Conflicto ético	conflicto tico	conflicto tico	conflicto tico	Conflicto ético	1	kept
conflicts and irritations with colleagues	conflicts and irritations with colleagues	conflicts and irritations with colleagues	conflicts and irritations with colleagues	conflicts and irritations with colleagues	1	kept
conflicts of conscience	conflicts of conscience	conflicts of conscience	conflicts of conscience	conflicts of conscience	1	kept
Conformational position	conformational position	conformational position	conformational position	Conformational position	1	kept
Conformational positioner	conformational positioner	conformational positioner	conformational positioner	Conformational positioner	1	kept
confounding	confounding	confounding	confounding	confounding	1	kept
Confrontation	confrontation	confrontation	confrontation	Confrontation	1	kept
confrontation	confrontation	confrontation	confrontation	Confrontation	1	case_or_format
Confusion Assessment Method Severity	confusion assessment method severity	confusion assessment method severity	confusion assessment method severity	Confusion Assessment Method Severity	1	kept
Confusion Assessment Method-Severity	confusion assessment method severity	confusion assessment method severity	confusion assessment method severity	Confusion Assessment Method Severity	1	case_or_format
confusion matrix	confusion matrix	confusion matrix	confusion matrix	confusion matrix	1	kept
confusion syndrome	confusion syndrome	confusion syndrome	confusion syndrome	confusion syndrome	1	kept

Confusional state	confusional state	confusional state	confusional state	Confusional state	1	kept
congenital abnormality	congenital abnormality	congenital abnormality	congenital abnormality	Congenital Abnormalities	1	case_or_format
Congenital adrenal hyperplasia	congenital adrenal hyperplasia	congenital adrenal hyperplasia	congenital adrenal hyperplasia	Congenital adrenal hyperplasia	1	kept
congenital adrenal hyperplasia	congenital adrenal hyperplasia	congenital adrenal hyperplasia	congenital adrenal hyperplasia	Congenital adrenal hyperplasia	1	case_or_format
congenital anomalies	congenital anomalies	congenital anomalies	congenital anomaly	Congenital anomaly	1	singular
Congenital anomalies and developmental follow-up for infants [illegible]	congenital anomalies and developmental follow up for infants [illegible]	congenital anomalies and developmental follow up for infants [illegible]	congenital anomalies and developmental follow up for infants [illegible]	Congenital anomalies and developmental follow-up for infants [illegible]	1	kept
Congenital cardiac condition	congenital cardiac condition	congenital cardiac condition	congenital cardiac condition	Congenital cardiac condition	1	kept
Congenital cardiac defects	congenital cardiac defects	congenital cardiac defects	congenital cardiac defects	Congenital cardiac defects	1	kept
congenital cardiac percutaneous interventions	congenital cardiac percutaneous interventions	congenital cardiac percutaneous interventions	congenital cardiac percutaneous interventions	congenital cardiac percutaneous interventions	1	kept
Congenital cardiac surgery	congenital cardiac surgery	congenital cardiac surgery	congenital cardiac surgery	Congenital cardiac surgery	1	kept
congenital cardiac surgery	congenital cardiac surgery	congenital cardiac surgery	congenital cardiac surgery	Congenital cardiac surgery	1	case_or_format
congenital central hypoventilation syndrome	congenital central hypoventilation syndrome	congenital central hypoventilation syndrome	congenital central hypoventilation syndrome	congenital central hypoventilation syndrome	1	kept
Congenital cutis aplasia	congenital cutis aplasia	congenital cutis aplasia	congenital cutis aplasia	Congenital cutis aplasia	1	kept
CONGENITAL CYTOMEGALOVIRUS-INFECTION	congenital cytomegalovirus infection	congenital cytomegalovirus infection	congenital cytomegalovirus infection	CONGENITAL CYTOMEGALOVIRUS-INFECTION	1	kept
congenital diaphragmatic hernia	congenital diaphragmatic hernia	congenital diaphragmatic hernia	congenital diaphragmatic hernia	Congenital diaphragmatic hernia	1	case_or_format
Congenital disorders	congenital disorders	congenital disorders	congenital disorder	congenital disorder	1	singular

congenital disorders	congenital disorders	congenital disorders	congenital disorder	congenital disorder	1	singular
CONGENITAL ELECTROPHYSIOLOGY	congenital electrophysiology	congenital electrophysiology	congenital electrophysiology	CONGENITAL ELECTROPHYSIOLOGY	1	kept
Congenital heart	congenital heart	congenital heart	congenital heart	Congenital heart	1	kept
congenital heart	congenital heart	congenital heart	congenital heart	Congenital heart	1	case_or_format
Congenital heart block	congenital heart block	congenital heart block	congenital heart block	Congenital heart block	1	kept
congenital heart block	congenital heart block	congenital heart block	congenital heart block	Congenital heart block	1	case_or_format
congenital heart defects	congenital heart defects	congenital heart defects	congenital heart defect	congenital heart defect	1	singular
Congenital heart defects, depression, cardiac surgery	congenital heart defects depression cardiac surgery	congenital heart defects depression cardiac surgery	congenital heart defects depression cardiac surgery	Congenital heart defects, depression, cardiac surgery	1	kept
Congenital heart diseases	congenital heart diseases	congenital heart diseases	congenital heart disease	congenital heart disease	1	singular
Congenital Heart Surgery	congenital heart surgery	congenital heart surgery	congenital heart surgery	Congenital heart surgery	1	case_or_format
congenital high airway obstruction syndrome	congenital high airway obstruction syndrome	congenital high airway obstruction syndrome	congenital high airway obstruction syndrome	congenital high airway obstruction syndrome	1	kept
Congenital High Airway Obstructive Syndrome	congenital high airway obstructive syndrome	congenital high airway obstructive syndrome	congenital high airway obstructive syndrome	Congenital High Airway Obstructive Syndrome	1	kept
Congenital Hypothyroidism	congenital hypothyroidism	congenital hypothyroidism	congenital hypothyroidism	congenital hypothyroidism	1	case_or_format
Congenital lobar emphysema	congenital lobar emphysema	congenital lobar emphysema	congenital lobar emphysema	Congenital lobar emphysema	1	kept
congenital lobar emphysema	congenital lobar emphysema	congenital lobar emphysema	congenital lobar emphysema	Congenital lobar emphysema	1	case_or_format
Congenital malformations	congenital malformations	congenital malformations	congenital malformation	congenital malformation	1	singular

congenital nephrotic syndrome	congenital nephrotic syndrome	congenital nephrotic syndrome	congenital nephrotic syndrome	congenital nephrotic syndrome	1	kept
congenital skin disease	congenital skin disease	congenital skin disease	congenital skin disease	congenital skin disease	1	kept
congenital tuberculosis	congenital tuberculosis	congenital tuberculosis	congenital tuberculosis	congenital tuberculosis	1	kept
Conger myriaster	conger myriaster	conger myriaster	conger myriaster	Conger myriaster	1	kept
Congestion	congestion	congestion	congestion	congestion	1	case_or_format
Congo	congo	congo	congo	Congo	1	kept
Congresses	congresses	congresses	congresses	Congresses	1	kept
Congruence	congruence	congruence	congruence	Congruence	1	kept
Conhecimentos	conhecimentos	conhecimentos	conhecimentos	Conhecimentos	1	kept
conivaptan	conivaptan	conivaptan	conivaptan	conivaptan	1	kept
Conjoin	conjoin	conjoin	conjoin	Conjoin	1	kept
Conjoined	conjoined	conjoined	conjoined	Conjoined	1	kept
conjoint analysis	conjoint analysis	conjoint analysis	conjoint analysis	Conjoint analysis	1	case_or_format
conjugate vaccine	conjugate vaccine	conjugate vaccine	conjugate vaccine	conjugate vaccine	1	kept
Conjunctival Diseases	conjunctival diseases	conjunctival diseases	conjunctival diseases	Conjunctival Diseases	1	kept
conjunctival hyperemia	conjunctival hyperemia	conjunctival hyperemia	conjunctival hyperemia	conjunctival hyperemia	1	kept

connaissance	connaissance	connaissance	connaissance	connaissance	1	kept
CONNECT	connect	connect	connect	CONNECT	1	kept
Connect, Introduce, Communicate, Ask, Respond, Exit	connect introduce communicate ask respond exit	connect introduce communicate ask respond exit	connect introduce communicate ask respond exit	Connect, Introduce, Communicate, Ask, Respond, Exit	1	kept
Connected Health Symposium	connected health symposium	connected health symposium	connected health symposium	Connected Health Symposium	1	kept
Connected healths	connected healths	connected healths	connected healths	Connected healths	1	kept
connections	connections	connections	connections	connections	1	kept
connective tissue	connective tissue	connective tissue	connective tissue	connective tissue	1	kept
Connectivism	connectivism	connectivism	connectivism	Connectivism	1	kept
Conner Davidson resilience scale	conner davidson resilience scale	conner davidson resilience scale	conner davidson resilience scale	Conner Davidson resilience scale	1	kept
Connor-Davidson Resilience Scale	connor davidson resilience scale	connor davidson resilience scale	connor davidson resilience scale	Connor Davidson resilience scale	1	case_or_format
Conocimiento administrativo	conocimiento administrativo	conocimiento administrativo	conocimiento administrativo	Conocimiento administrativo	1	kept
conscientious	conscientious	conscientious	conscientious	conscientious	1	kept
conscientious intelligence	conscientious intelligence	conscientious intelligence	conscientious intelligence	conscientious intelligence	1	kept
conscientious objection	conscientious objection	conscientious objection	conscientious objection	conscientious objection	1	kept
conscientisation	conscientisation	conscientisation	conscientisation	conscientisation	1	kept
conscious	conscious	conscious	conscious	Conscious	1	case_or_format

Conscious level	conscious level	conscious level	conscious level	Conscious level	1	kept
consciousness disorders	consciousness disorders	consciousness disorders	consciousness disorder	consciousness disorder	1	singular
Consciousness Level	consciousness level	consciousness level	consciousness level	consciousness level	1	case_or_format
consciousness monitor	consciousness monitor	consciousness monitor	consciousness monitor	Consciousness Monitors	1	case_or_format
Consciousness monitors	consciousness monitors	consciousness monitors	consciousness monitor	Consciousness Monitors	1	singular
Consciousness monitors neurophysiological	consciousness monitors neurophysiological	consciousness monitors neurophysiological	consciousness monitors neurophysiological	Consciousness monitors neurophysiological	1	kept
consciousness recovery	consciousness recovery	consciousness recovery	consciousness recovery	consciousness recovery	1	kept
Consecutive working day	consecutive working day	consecutive working day	consecutive working day	consecutive working days	1	case_or_format
consensus building	consensus building	consensus building	consensus building	consensus building	1	kept
Consensus development	consensus development	consensus development	consensus development	consensus development	1	case_or_format
Consensus development conference	consensus development conference	consensus development conference	consensus development conference	Consensus Development Conferences	1	case_or_format
Consensus document	consensus document	consensus document	consensus document	Consensus document	1	kept
consensus methods	consensus methods	consensus methods	consensus methods	Consensus methods	1	case_or_format
Consensus model	consensus model	consensus model	consensus model	consensus model	1	case_or_format
Consensus model licensure	consensus model licensure	consensus model licensure	consensus model licensure	Consensus model licensure	1	kept
Consensus statement	consensus statement	consensus statement	consensus statement	CONSENSUS STATEMENT	1	case_or_format

CONSENT	consent	consent	consent	Consent	1	case_or_format
Consent Forms	consent forms	consent forms	consent forms	consent forms	1	case_or_format
Consent rate	consent rate	consent rate	consent rate	Consent rate	1	kept
consent rate	consent rate	consent rate	consent rate	Consent rate	1	case_or_format
Consequence	consequence	consequence	consequence	CONSEQUENCES	1	case_or_format
CONSERVATION	conservation	conservation	conservation	CONSERVATION	1	kept
Conservation of Resources	conservation of resources	conservation of resources	conservation of resources	Conservation of Resources	1	kept
Conservation of resources theory	conservation of resources theory	conservation of resources theory	conservation of resources theory	Conservation of resources theory	1	kept
conservative care	conservative care	conservative care	conservative care	conservative care	1	kept
Conservative oxygenation	conservative oxygenation	conservative oxygenation	conservative oxygenation	Conservative oxygenation	1	kept
consistency	consistency	consistency	consistency	consistency	1	kept
Console	console	console	console	Console	1	kept
Consolidated Framework for Implementation Research 2.0 framework	consolidated framework for implementation research 2.0 framework	consolidated framework for implementation research 2.0 framework	consolidated framework for implementation research 2.0 framework	Consolidated Framework for Implementation Research 2.0 framework	1	kept
Consolidation	consolidation	consolidation	consolidation	Consolidation	1	kept
Constantes vitales	constantes vitales	constantes vitales	constantes vitales	Constantes vitales	1	kept
constants and coefficients	constants and coefficients	constants and coefficients	constants and coefficients	constants and coefficients	1	kept

Constellation	constellation	constellation	constellation	Constellation	1	kept
Constipation Severity Scale	constipation severity scale	constipation severity scale	constipation severity scale	Constipation Severity Scale	1	kept
Constraint programming	constraint programming	constraint programming	constraint programming	Constraint programming	1	kept
Constraint theory	constraint theory	constraint theory	constraint theory	Constraint theory	1	kept
Constraints	constraints	constraints	constraints	Constraints	1	kept
constraints	constraints	constraints	constraints	Constraints	1	case_or_format
Construct validity	construct validity	construct validity	construct validity	construct validity	1	case_or_format
CONSTRUCTION	construction	construction	construction	CONSTRUCTION	1	kept
Construction	construction	construction	construction	CONSTRUCTION	1	case_or_format
construction	construction	construction	construction	CONSTRUCTION	1	case_or_format
Construction noise	construction noise	construction noise	construction noise	Construction noise	1	kept
construction worker	construction worker	construction worker	construction worker	construction worker	1	kept
Constructivist Grounded Theory	constructivist grounded theory	constructivist grounded theory	constructivist grounded theory	constructivist grounded theory	1	case_or_format
Constructivist grounded theory design	constructivist grounded theory design	constructivist grounded theory design	constructivist grounded theory design	Constructivist grounded theory design	1	kept
constructivist GT	constructivist gt	constructivist gt	constructivist gt	constructivist GT	1	kept
Constructivist training	constructivist training	constructivist training	constructivist training	Constructivist training	1	kept

constructivist training	constructivist training	constructivist training	constructivist training	Constructivist training	1	case_or_format
Consult	consult	consult	consult	Consult	1	kept
consult	consult	consult	consult	Consult	1	case_or_format
consult team	consult team	consult team	consult team	consult team	1	kept
Consultancy services	consultancy services	consultancy services	consultancy services	Consultancy services	1	kept
consultation liaison psychiatry physician	consultation liaison psychiatry physician	consultation liaison psychiatry physician	consultation liaison psychiatry physician	consultation liaison psychiatry physician	1	kept
consultation-liaison psychiatry	consultation liaison psychiatry	consultation liaison psychiatry	consultation liaison psychiatry	consultation-liaison psychiatry	1	kept
Consultative	consultative	consultative	consultative	Consultative	1	kept
Consultative model	consultative model	consultative model	consultative model	Consultative model	1	kept
CONSULTEES CONCORDANCE	consultees concordance	consultees concordance	consultees concordance	CONSULTEES CONCORDANCE	1	kept
Consults	consults	consults	consult	Consult	1	singular
consults	consults	consults	consult	Consult	1	singular
consumable	consumable	consumable	consumable	consumable	1	kept
Consumer Advocacy	consumer advocacy	consumer advocacy	consumer advocacy	Consumer Advocacy	1	kept
consumer advocacy	consumer advocacy	consumer advocacy	consumer advocacy	Consumer Advocacy	1	case_or_format
consumer engagement	consumer engagement	consumer engagement	consumer engagement	consumer engagement	1	kept

Consumer health information	consumer health information	consumer health information	consumer health information	Consumer Health Information	1	case_or_format
Consumer products	consumer products	consumer products	consumer products	Consumer products	1	kept
Consumer satisfaction	consumer satisfaction	consumer satisfaction	consumer satisfaction	Consumer Satisfaction	1	case_or_format
consumers	consumers	consumers	consumer	consumer	1	singular
Consumption datum	consumption datum	consumption datum	consumption datum	Consumption datum	1	kept
Consumption of opiates	consumption of opiates	consumption of opiates	consumption of opiates	Consumption of opiates	1	kept
CONTACT	contact	contact	contact	CONTACT	1	kept
contact angle	contact angle	contact angle	contact angle	contact angle	1	kept
Contact behavior	contact behavior	contact behavior	contact behavior	Contact behavior	1	kept
Contact event	contact event	contact event	contact event	Contact event	1	kept
Contact heterogeneity	contact heterogeneity	contact heterogeneity	contact heterogeneity	Contact heterogeneity	1	kept
Contact isolation	contact isolation	contact isolation	contact isolation	contact isolation	1	case_or_format
contact lens	contact lens	contact lens	contact lens	contact lens	1	kept
Contact less	contact less	contact less	contact less	Contact less	1	kept
Contact networks	contact networks	contact networks	contact networks	Contact networks	1	kept
Contact precaution	contact precaution	contact precaution	contact precaution	Contact precautions	1	case_or_format

contact precaution	contact precaution	contact precaution	contact precaution	Contact precautions	1	case_or_format
CONTACT PRECAUTIONS	contact precautions	contact precautions	contact precaution	Contact precautions	1	singular
Contact Precautions	contact precautions	contact precautions	contact precaution	Contact precautions	1	singular
contact pressure	contact pressure	contact pressure	contact pressure	contact pressure	1	kept
Contact pressures	contact pressures	contact pressures	contact pressure	contact pressure	1	singular
contact time	contact time	contact time	contact time	contact time	1	kept
contacted surface	contacted surface	contacted surface	contacted surface	contacted surface	1	kept
Contactless care	contactless care	contactless care	contactless care	Contactless care	1	kept
CONTAINMENT	containment	containment	containment	containment	1	case_or_format
Containment methods	containment methods	containment methods	containment methods	Containment methods	1	kept
contaminated handwashing sink	contaminated handwashing sink	contaminated handwashing sink	contaminated handwashing sink	contaminated handwashing sink	1	kept
Contaminating accidents	contaminating accidents	contaminating accidents	contaminating accidents	Contaminating accidents	1	kept
Contamination injury	contamination injury	contamination injury	contamination injury	Contamination injury	1	kept
Contamination rates	contamination rates	contamination rates	contamination rates	Contamination rates	1	kept
CONTEMPORARY MOTION-PICTURES	contemporary motion pictures	contemporary motion pictures	contemporary motion pictures	CONTEMPORARY MOTION-PICTURES	1	kept
Contenciones mecánicas	contenciones mec nicas	contenciones mec nicas	contenciones mec nicas	Contenciones mecánicas	1	kept

Content	content	content	content	Content	1	kept
Content analysis study	content analysis study	content analysis study	content analysis study	Content analysis study	1	kept
content areas	content areas	content areas	content areas	content areas	1	kept
CONTENT OVERLAP	content overlap	content overlap	content overlap	CONTENT OVERLAP	1	kept
Content overlap	content overlap	content overlap	content overlap	CONTENT OVERLAP	1	case_or_format
content validation	content validation	content validation	content validation	Content validation	1	case_or_format
Content validity assessment	content validity assessment	content validity assessment	content validity assessment	Content validity assessment	1	kept
Content Validity Index	content validity index	content validity index	content validity index	content validity index	1	case_or_format
content validity ratio	content validity ratio	content validity ratio	content validity ratio	content validity ratio	1	kept
Context information	context information	context information	context information	Context information	1	kept
context mechanism outcome	context mechanism outcome	context mechanism outcome	context mechanism outcome	context mechanism outcome	1	kept
Context of care	context of care	context of care	context of care	Context of care	1	kept
Context-management systems	context management systems	context management systems	context management systems	Context-management systems	1	kept
Context-specific	context specific	context specific	context specific	Context-specific	1	kept
Contextual analysis	contextual analysis	contextual analysis	contextual analysis	Contextual analysis	1	kept
contextual determinants	contextual determinants	contextual determinants	contextual determinants	contextual determinants	1	kept

contextual factors	contextual factors	contextual factors	contextual factors	Contextual factors	1	case_or_format
Contextual inquiries	contextual inquiries	contextual inquiries	contextual inquiry	Contextual inquiry	1	singular
Contextualize	contextualize	contextualize	contextualize	Contextualize	1	kept
contingency	contingency	contingency	contingency	contingency	1	kept
Contingency approach	contingency approach	contingency approach	contingency approach	Contingency approach	1	kept
contingency level care	contingency level care	contingency level care	contingency level care	contingency level care	1	kept
contingency plan	contingency plan	contingency plan	contingency plan	contingency plan	1	kept
contingent labor	contingent labor	contingent labor	contingent labor	contingent labor	1	kept
Contingent music	contingent music	contingent music	contingent music	Contingent music	1	kept
contingent valuation analysis	contingent valuation analysis	contingent valuation analysis	contingent valuation analysis	contingent valuation analysis	1	kept
contingent valuation method	contingent valuation method	contingent valuation method	contingent valuation method	contingent valuation method	1	kept
Continual Education	continual education	continual education	continual education	Continual Education	1	kept
Continued education	continued education	continued education	continued education	Continued education	1	kept
Continuidad de la Atención al Paciente	continuidad de la atenci n al paciente	continuidad de la atenci n al paciente	continuidad de la atenci n al paciente	Continuidad de la Atención al Paciente	1	kept
Continuidad de la atención al paciente	continuidad de la atenci n al paciente	continuidad de la atenci n al paciente	continuidad de la atenci n al paciente	Continuidad de la Atención al Paciente	1	case_or_format
Continuidade da Assistência ao Paciente	continuidade da assist ncia ao paciente	continuidade da assist ncia ao paciente	continuidade da assist ncia ao paciente	Continuidade da Assistência ao Paciente	1	kept

continuing	continuing	continuing	continuing	Continuing	1	case_or_format
Continuing care	continuing care	continuing care	continuing care	continuing care	1	case_or_format
continuing education provider	continuing education provider	continuing education provider	continuing education provider	continuing education provider	1	kept
Continuing medical educations	continuing medical educations	continuing medical educations	continuing medical education	Continuing medical education	1	singular
CONTINUING MEDICAL-EDUCATION	continuing medical education	continuing medical education	continuing medical education	Continuing medical education	1	case_or_format
CONTINUING PROFESSIONAL-DEVELOPMENT	continuing professional development	continuing professional development	continuing professional development	Continuing professional development	1	case_or_format
CONTINUING-EDUCATION	continuing education	continuing education	continuing education	continuing education	1	case_or_format
CONTINUITY	continuity	continuity	continuity	Continuity	1	case_or_format
Continuity of cares	continuity of cares	continuity of cares	continuity of care	Continuity of care	1	singular
continuity of management	continuity of management	continuity of management	continuity of management	continuity of management	1	kept
Continuous and deep sedation until death	continuous and deep sedation until death	continuous and deep sedation until death	continuous and deep sedation until death	Continuous and deep sedation until death	1	kept
Continuous assessment	continuous assessment	continuous assessment	continuous assessment	Continuous assessment	1	kept
Continuous blood glucose monitoring devices	continuous blood glucose monitoring devices	continuous blood glucose monitoring devices	continuous blood glucose monitoring devices	Continuous blood glucose monitoring devices	1	kept
continuous cardiac monitoring	continuous cardiac monitoring	continuous cardiac monitoring	continuous cardiac monitoring	continuous cardiac monitoring	1	kept
Continuous cardiac output monitoring	continuous cardiac output monitoring	continuous cardiac output monitoring	continuous cardiac output monitoring	Continuous cardiac output monitoring	1	kept
continuous cardiac output monitoring	continuous cardiac output monitoring	continuous cardiac output monitoring	continuous cardiac output monitoring	Continuous cardiac output monitoring	1	case_or_format

continuous closed circle television	continuous closed circle television	continuous closed circle television	continuous closed circle television	continuous closed circle television	1	kept
Continuous cuff pressure monitoring	continuous cuff pressure monitoring	continuous cuff pressure monitoring	continuous cuff pressure monitoring	Continuous cuff pressure monitoring	1	kept
Continuous data acquisition	continuous data acquisition	continuous data acquisition	continuous data acquisition	Continuous data acquisition	1	kept
Continuous ECG monitoring	continuous ecg monitoring	continuous ecg monitoring	continuous ecg monitoring	Continuous ECG monitoring	1	kept
Continuous education	continuous education	continuous education	continuous education	continuous education	1	case_or_format
Continuous education programs	continuous education programs	continuous education programs	continuous education programs	Continuous education programs	1	kept
continuous EEG	continuous eeg	continuous eeg	continuous eeg	Continuous EEG	1	case_or_format
Continuous EEG monitoring	continuous eeg monitoring	continuous eeg monitoring	continuous eeg monitoring	Continuous EEG monitoring	1	kept
continuous electrocardiography monitoring	continuous electrocardiography monitoring	continuous electrocardiography monitoring	continuous electrocardiography monitoring	continuous electrocardiography monitoring	1	kept
continuous enteral feeding	continuous enteral feeding	continuous enteral feeding	continuous enteral feeding	continuous enteral feeding	1	kept
Continuous fecal drainage systems	continuous fecal drainage systems	continuous fecal drainage systems	continuous fecal drainage systems	Continuous fecal drainage systems	1	kept
Continuous feeding	continuous feeding	continuous feeding	continuous feeding	Continuous feeding	1	kept
continuous feeding	continuous feeding	continuous feeding	continuous feeding	Continuous feeding	1	case_or_format
continuous flow isotope ratio mass spectrometry	continuous flow isotope ratio mass spectrometry	continuous flow isotope ratio mass spectrometry	continuous flow isotope ratio mass spectrometry	continuous flow isotope ratio mass spectrometry	1	kept
continuous flush method	continuous flush method	continuous flush method	continuous flush method	continuous flush method	1	kept
Continuous glucose monitor	continuous glucose monitor	continuous glucose monitor	continuous glucose monitor	continuous glucose monitor	1	case_or_format

Continuous glucose monitoring sensors	continuous glucose monitoring sensors	continuous glucose monitoring sensors	continuous glucose monitoring sensors	Continuous glucose monitoring sensors	1	kept
continuous heart rate estimation	continuous heart rate estimation	continuous heart rate estimation	continuous heart rate estimation	continuous heart rate estimation	1	kept
Continuous hemofiltration	continuous hemofiltration	continuous hemofiltration	continuous hemofiltration	continuous hemofiltration	1	case_or_format
continuous improvement	continuous improvement	continuous improvement	continuous improvement	continuous improvement	1	kept
Continuous improvements	continuous improvements	continuous improvements	continuous improvement	continuous improvement	1	singular
continuous intrapartum fetal monitoring	continuous intrapartum fetal monitoring	continuous intrapartum fetal monitoring	continuous intrapartum fetal monitoring	continuous intrapartum fetal monitoring	1	kept
continuous intravenous treatment	continuous intravenous treatment	continuous intravenous treatment	continuous intravenous treatment	continuous intravenous treatment	1	kept
Continuous kidney replacement therapy dose	continuous kidney replacement therapy dose	continuous kidney replacement therapy dose	continuous kidney replacement therapy dose	Continuous kidney replacement therapy dose	1	kept
Continuous KRT	continuous krt	continuous krt	continuous krt	Continuous KRT	1	kept
Continuous learning	continuous learning	continuous learning	continuous learning	Continuous learning	1	kept
continuous noncontact vital sign monitoring	continuous noncontact vital sign monitoring	continuous noncontact vital sign monitoring	continuous noncontact vital sign monitoring	continuous noncontact vital sign monitoring	1	kept
Continuous observation	continuous observation	continuous observation	continuous observation	Continuous observation	1	kept
continuous oxygen saturation estimation	continuous oxygen saturation estimation	continuous oxygen saturation estimation	continuous oxygen saturation estimation	continuous oxygen saturation estimation	1	kept
continuous paravertebral block	continuous paravertebral block	continuous paravertebral block	continuous paravertebral block	continuous paravertebral block	1	kept
continuous passive movement device	continuous passive movement device	continuous passive movement device	continuous passive movement device	continuous passive movement device	1	kept
Continuous phototherapy	continuous phototherapy	continuous phototherapy	continuous phototherapy	Continuous phototherapy	1	kept

continuous phototherapy	continuous phototherapy	continuous phototherapy	continuous phototherapy	Continuous phototherapy	1	case_or_format
Continuous Positive Air Pressure	continuous positive air pressure	continuous positive air pressure	continuous positive air pressure	Continuous Positive Air Pressure	1	kept
CONTINUOUS POSITIVE AIRWAY PRESSURE	continuous positive airway pressure	continuous positive airway pressure	continuous positive airway pressure	Continuous Positive Airway Pressure	1	case_or_format
Continuous positive airway pressures	continuous positive airway pressures	continuous positive airway pressures	continuous positive airway pressure	Continuous Positive Airway Pressure	1	singular
continuous pre-term infant monitoring	continuous pre term infant monitoring	continuous pre term infant monitoring	continuous pre term infant monitoring	continuous pre-term infant monitoring	1	kept
continuous predictive monitoring	continuous predictive monitoring	continuous predictive monitoring	continuous predictive monitoring	continuous predictive monitoring	1	kept
continuous process improvement	continuous process improvement	continuous process improvement	continuous process improvement	continuous process improvement	1	kept
Continuous pulse oximetry	continuous pulse oximetry	continuous pulse oximetry	continuous pulse oximetry	Continuous pulse oximetry	1	kept
continuous pulse oximetry	continuous pulse oximetry	continuous pulse oximetry	continuous pulse oximetry	Continuous pulse oximetry	1	case_or_format
continuous renal replacement	continuous renal replacement	continuous renal replacement	continuous renal replacement	continuous renal replacement	1	kept
Continuous Renal Replacement Therapies	continuous renal replacement therapies	continuous renal replacement therapies	continuous renal replacement therapy	continuous renal replacement therapy	1	singular
Continuous renal replacement therapies	continuous renal replacement therapies	continuous renal replacement therapies	continuous renal replacement therapy	continuous renal replacement therapy	1	singular
Continuous renal replacement therapy device	continuous renal replacement therapy device	continuous renal replacement therapy device	continuous renal replacement therapy device	Continuous renal replacement therapy device	1	kept
continuous renal replacement therapy hemofiltration	continuous renal replacement therapy hemofiltration	continuous renal replacement therapy hemofiltration	continuous renal replacement therapy hemofiltration	continuous renal replacement therapy hemofiltration	1	kept
continuous renal replacement treatment	continuous renal replacement treatment	continuous renal replacement treatment	continuous renal replacement treatment	continuous renal replacement treatment	1	kept
continuous respiratory rate estimation	continuous respiratory rate estimation	continuous respiratory rate estimation	continuous respiratory rate estimation	continuous respiratory rate estimation	1	kept

Continuous sedation until death	continuous sedation until death	continuous sedation until death	continuous sedation until death	Continuous sedation until death	1	kept
continuous sedation until death	continuous sedation until death	continuous sedation until death	continuous sedation until death	Continuous sedation until death	1	case_or_format
continuous sleep time	continuous sleep time	continuous sleep time	continuous sleep time	continuous sleep time	1	kept
Continuous time Markov chain	continuous time markov chain	continuous time markov chain	continuous time markov chain	continuous time Markov chain	1	case_or_format
Continuous time systems	continuous time systems	continuous time systems	continuous time systems	Continuous time systems	1	kept
continuous veno venous hemodialysis	continuous veno venous hemodialysis	continuous veno venous hemodialysis	continuous veno venous hemodialysis	continuous veno venous hemodialysis	1	kept
Continuous veno-venous hemodialysis	continuous veno venous hemodialysis	continuous veno venous hemodialysis	continuous veno venous hemodialysis	continuous veno venous hemodialysis	1	case_or_format
Continuous veno-venous hemofiltration	continuous veno venous hemofiltration	continuous veno venous hemofiltration	continuous veno venous hemofiltration	Continuous veno-venous hemofiltration	1	kept
Continuous venovenous hemodiafiltration	continuous venovenous hemodiafiltration	continuous venovenous hemodiafiltration	continuous venovenous hemodiafiltration	Continuous venovenous hemodiafiltration	1	kept
continuous venovenous hemodialysis without filtration	continuous venovenous hemodialysis without filtration	continuous venovenous hemodialysis without filtration	continuous venovenous hemodialysis without filtration	continuous venovenous hemodialysis without filtration	1	kept
CONTINUOUS VENOVENOUS HEMOFILTRATION	continuous venovenous hemofiltration	continuous venovenous hemofiltration	continuous venovenous hemofiltration	Continuous venovenous hemofiltration	1	case_or_format
continuous ventilator	continuous ventilator	continuous ventilator	continuous ventilator	continuous ventilator	1	kept
continuous vital sign monitoring technologies	continuous vital sign monitoring technologies	continuous vital sign monitoring technologies	continuous vital sign monitoring technologies	continuous vital sign monitoring technologies	1	kept
Continuous-flow pumps	continuous flow pumps	continuous flow pumps	continuous flow pumps	Continuous-flow pumps	1	kept
CONTINUOUS-QUALITY-IMPROVEMENT	continuous quality improvement	continuous quality improvement	continuous quality improvement	continuous quality improvement	1	case_or_format
CONTINUUM	continuum	continuum	continuum	CONTINUUM	1	kept

continuum	continuum	continuum	continuum	CONTINUUM	1	case_or_format
Continuum supports	continuum supports	continuum supports	continuum supports	Continuum supports	1	kept
Contraception	contraception	contraception	contraception	contraception	1	case_or_format
contraceptive agent	contraceptive agent	contraceptive agent	contraceptive agent	contraceptive agent	1	kept
Contract services	contract services	contract services	contract services	Contract Services	1	case_or_format
Contractility	contractility	contractility	contractility	Contractility	1	kept
contractual staff	contractual staff	contractual staff	contractual staff	contractual staff	1	kept
contraindications	contraindications	contraindications	contraindication	Contraindications	1	singular
Contralateral	contralateral	contralateral	contralateral	Contralateral	1	kept
Contrast echocardiography	contrast echocardiography	contrast echocardiography	contrast echocardiography	Contrast echocardiography	1	kept
contrast echocardiography	contrast echocardiography	contrast echocardiography	contrast echocardiography	Contrast echocardiography	1	case_or_format
Contrast extravasation	contrast extravasation	contrast extravasation	contrast extravasation	Contrast extravasation	1	kept
Contrast media induced nephropathy scores	contrast media induced nephropathy scores	contrast media induced nephropathy scores	contrast media induced nephropathy scores	Contrast media induced nephropathy scores	1	kept
Contrast microbubbles	contrast microbubbles	contrast microbubbles	contrast microbubbles	Contrast microbubbles	1	kept
Contrast safety	contrast safety	contrast safety	contrast safety	Contrast safety	1	kept
Contributing or influential factors	contributing or influential factors	contributing or influential factors	contributing or influential factors	Contributing or influential factors	1	kept

control and restraint	control and restraint	control and restraint	control and restraint	control and restraint	1	kept
Control approach	control approach	control approach	control approach	Control approach	1	kept
control chart	control chart	control chart	control chart	control charts	1	case_or_format
Control chart patterns	control chart patterns	control chart patterns	control chart patterns	Control chart patterns	1	kept
control charts, run charts	control charts run charts	control charts run charts	control charts run charts	control charts, run charts	1	kept
Control glucémico	control gluc mico	control gluc mico	control gluc mico	Control glucémico	1	kept
Control group	control group	control group	control group	control group	1	case_or_format
Control of communicable diseases	control of communicable diseases	control of communicable diseases	control of communicable diseases	Control of communicable diseases	1	kept
Control performance	control performance	control performance	control performance	Control performance	1	kept
Control preference scale	control preference scale	control preference scale	control preference scale	Control preference scale	1	kept
CONTROL PROGRAMS	control programs	control programs	control programs	CONTROL PROGRAMS	1	kept
CONTROL ROOM	control room	control room	control room	CONTROL ROOM	1	kept
Control settings	control settings	control settings	control settings	Control settings	1	kept
Control strategies	control strategies	control strategies	control strategy	control strategy	1	singular
Control waveforms	control waveforms	control waveforms	control waveforms	Control waveforms	1	kept
Controle de Infecções	controle de infec es	controle de infec es	controle de infec es	Controle de Infecções	1	kept

controlled atmosphere	controlled atmosphere	controlled atmosphere	controlled atmosphere	controlled atmosphere	1	kept
controlled before - after studies	controlled before after studies	controlled before after studies	controlled before after studies	Controlled Before-After Studies	1	case_or_format
CONTROLLED CLINICAL-TRIAL	controlled clinical trial	controlled clinical trial	controlled clinical trial	controlled clinical trial	1	case_or_format
Controlled donation after circulatory death	controlled donation after circulatory death	controlled donation after circulatory death	controlled donation after circulatory death	Controlled donation after circulatory death	1	kept
controlled donation after circulatory death	controlled donation after circulatory death	controlled donation after circulatory death	controlled donation after circulatory death	Controlled donation after circulatory death	1	case_or_format
Controlled experiment	controlled experiment	controlled experiment	controlled experiment	Controlled experiment	1	kept
Controlled normothermia	controlled normothermia	controlled normothermia	controlled normothermia	Controlled normothermia	1	kept
Controlled parameter	controlled parameter	controlled parameter	controlled parameter	Controlled parameter	1	kept
Controlled study	controlled study	controlled study	controlled study	controlled study	1	case_or_format
Controlled Temperature Storage	controlled temperature storage	controlled temperature storage	controlled temperature storage	Controlled Temperature Storage	1	kept
Controlled trial	controlled trial	controlled trial	controlled trial	CONTROLLED-TRIAL	1	case_or_format
Controlled vocabulary	controlled vocabulary	controlled vocabulary	controlled vocabulary	controlled vocabulary	1	case_or_format
Controllers	controllers	controllers	controllers	Controllers	1	kept
controllers	controllers	controllers	controllers	Controllers	1	case_or_format
Controlling	controlling	controlling	controlling	Controlling	1	kept
Controlling Nutritional Status score	controlling nutritional status score	controlling nutritional status score	controlling nutritional status score	Controlling Nutritional Status score	1	kept

conus medullaris	conus medullaris	conus medullaris	conus medullaris	conus medullaris	1	kept
Convalescent plasma	convalescent plasma	convalescent plasma	convalescent plasma	convalescent plasma	1	case_or_format
Convection enhanced delivery	convection enhanced delivery	convection enhanced delivery	convection enhanced delivery	Convection enhanced delivery	1	kept
conventional angiography	conventional angiography	conventional angiography	conventional angiography	conventional angiography	1	kept
Conventional approach	conventional approach	conventional approach	conventional approach	Conventional approach	1	kept
Conventional blood culture	conventional blood culture	conventional blood culture	conventional blood culture	Conventional blood culture	1	kept
Conventional care	conventional care	conventional care	conventional care	Conventional care	1	kept
conventional content analysis	conventional content analysis	conventional content analysis	conventional content analysis	conventional content analysis	1	kept
conventional mechanical ventilation	conventional mechanical ventilation	conventional mechanical ventilation	conventional mechanical ventilation	conventional mechanical ventilation	1	kept
conventional stethoscope	conventional stethoscope	conventional stethoscope	conventional stethoscope	conventional stethoscope	1	kept
conventional surgery	conventional surgery	conventional surgery	conventional surgery	conventional surgery	1	kept
Conventional teaching method	conventional teaching method	conventional teaching method	conventional teaching method	Conventional teaching method	1	kept
conventional ventilation mode	conventional ventilation mode	conventional ventilation mode	conventional ventilation mode	conventional ventilation mode	1	kept
CONVERSATION	conversation	conversation	conversation	conversation	1	case_or_format
CONVERSATION ANALYSIS	conversation analysis	conversation analysis	conversation analysis	CONVERSATION ANALYSIS	1	kept
Conversation analysis	conversation analysis	conversation analysis	conversation analysis	CONVERSATION ANALYSIS	1	case_or_format

conversation guides	conversation guides	conversation guides	conversation guides	conversation guides	1	kept
CONVERSATIONS	conversations	conversations	conversation	conversation	1	singular
Conversations	conversations	conversations	conversation	conversation	1	singular
CONVEX DELAY COSTS	convex delay costs	convex delay costs	convex delay costs	CONVEX DELAY COSTS	1	kept
convidecia	convidecia	convidecia	convidecia	convidecia	1	kept
Convolution	convolution	convolution	convolution	Convolution	1	kept
Convolutional neural networks	convolutional neural networks	convolutional neural networks	convolutional neural network	convolutional neural network	1	singular
Convulsion	convulsion	convulsion	convulsion	convulsion	1	case_or_format
convulsions	convulsions	convulsions	convulsion	convulsion	1	singular
coolcuddle	coolcuddle	coolcuddle	coolcuddle	coolcuddle	1	kept
Cooling	cooling	cooling	cooling	cooling	1	case_or_format
cooling in middle-income countries	cooling in middle income countries	cooling in middle income countries	cooling in middle income countries	cooling in middle-income countries	1	kept
cooling survey	cooling survey	cooling survey	cooling survey	cooling survey	1	kept
cooperation protocol	cooperation protocol	cooperation protocol	cooperation protocol	cooperation protocol	1	kept
cooperative behavior	cooperative behavior	cooperative behavior	cooperative behavior	Cooperative Behavior	1	case_or_format
Cooperative behaviour	cooperative behaviour	cooperative behaviour	cooperative behaviour	Cooperative behaviour	1	kept

COOPERATIVE COMMUNICATION	cooperative communication	cooperative communication	cooperative communication	COOPERATIVE COMMUNICATION	1	kept
Coordinación	coordinaci n	coordinaci n	coordinaci n	Coordinación	1	kept
Coordinated care	coordinated care	coordinated care	coordinated care	Coordinated care	1	kept
coordinated transitional care	coordinated transitional care	coordinated transitional care	coordinated transitional care	coordinated transitional care	1	kept
Coordination of care	coordination of care	coordination of care	coordination of care	Coordination of care	1	kept
coordination of care	coordination of care	coordination of care	coordination of care	Coordination of care	1	case_or_format
Coordination team	coordination team	coordination team	coordination team	Coordination team	1	kept
COORDINATOR	coordinator	coordinator	coordinator	COORDINATOR	1	kept
Coordinator	coordinator	coordinator	coordinator	COORDINATOR	1	case_or_format
coordinator	coordinator	coordinator	coordinator	COORDINATOR	1	case_or_format
COP9 Signalosome Complex	cop9 signalosome complex	cop9 signalosome complex	cop9 signalosome complex	COP9 Signalosome Complex	1	kept
COPD assessment test	copd assessment test	copd assessment test	copd assessment test	COPD assessment test	1	kept
COPD Exacerbations	copd exacerbations	copd exacerbations	copd exacerbation	COPD exacerbation	1	singular
COPD--oral care	copd oral care	copd oral care	copd oral care	COPD--oral care	1	kept
COPD-exacerbation	copd exacerbation	copd exacerbation	copd exacerbation	COPD exacerbation	1	case_or_format
Copenhagen burnout inventory	copenhagen burnout inventory	copenhagen burnout inventory	copenhagen burnout inventory	Copenhagen Burnout Inventory	1	case_or_format

Copenhagen Burnout Scale	copenhagen burnout scale	copenhagen burnout scale	copenhagen burnout scale	Copenhagen Burnout Scale	1	kept
Copenhagen Psychosocial Questionnaire II	copenhagen psychosocial questionnaire ii	copenhagen psychosocial questionnaire ii	copenhagen psychosocial questionnaire ii	Copenhagen Psychosocial Questionnaire II	1	kept
Coping ability	coping ability	coping ability	coping ability	Coping ability	1	kept
Coping behaviour	coping behaviour	coping behaviour	coping behaviour	Coping behaviour	1	kept
Coping fear of recurrence	coping fear of recurrence	coping fear of recurrence	coping fear of recurrence	Coping fear of recurrence	1	kept
coping flexibility	coping flexibility	coping flexibility	coping flexibility	coping flexibility	1	kept
Coping Health Inventory for Parents	coping health inventory for parents	coping health inventory for parents	coping health inventory for parents	Coping Health Inventory for Parents	1	kept
coping mechanism	coping mechanism	coping mechanism	coping mechanism	coping mechanisms	1	case_or_format
coping methods	coping methods	coping methods	coping methods	coping methods	1	kept
Coping process	coping process	coping process	coping process	Coping process	1	kept
COPING SELF-EFFICACY	coping self efficacy	coping self efficacy	coping self efficacy	coping self-efficacy	1	case_or_format
Coping style	coping style	coping style	coping style	coping style	1	case_or_format
coping styles	coping styles	coping styles	coping style	coping style	1	singular
copper deficiency	copper deficiency	copper deficiency	copper deficiency	copper deficiency	1	kept
copper surface	copper surface	copper surface	copper surface	copper surface	1	kept
COPS2 score	cops2 score	cops2 score	cops2 score	COPS2 score	1	kept

Copula modelling	copula modelling	copula modelling	copula modelling	Copula modelling	1	kept
Copyrights	copyrights	copyrights	copyrights	Copyrights	1	kept
COR, Crude odds ratio	cor crude odds ratio	cor crude odds ratio	cor crude odds ratio	COR, Crude odds ratio	1	kept
Coral snakes	coral snakes	coral snakes	coral snakes	Coral snakes	1	kept
cord avulsion	cord avulsion	cord avulsion	cord avulsion	cord avulsion	1	kept
cord blood harvesting	cord blood harvesting	cord blood harvesting	cord blood harvesting	cord blood harvesting	1	kept
Cord Blood Stem Cell Transplantation	cord blood stem cell transplantation	cord blood stem cell transplantation	cord blood stem cell transplantation	Cord Blood Stem Cell Transplantation	1	kept
cord blood stem cell transplantation	cord blood stem cell transplantation	cord blood stem cell transplantation	cord blood stem cell transplantation	Cord Blood Stem Cell Transplantation	1	case_or_format
Cord clamping	cord clamping	cord clamping	cord clamping	Cord clamping	1	kept
cord lactate	cord lactate	cord lactate	cord lactate	cord lactate	1	kept
Cord milking	cord milking	cord milking	cord milking	Cord milking	1	kept
cord milking	cord milking	cord milking	cord milking	Cord milking	1	case_or_format
cord pH	cord ph	cord ph	cord ph	cord pH	1	kept
core competence	core competence	core competence	core competence	core competence	1	kept
CORE COMPETENCES	core competences	core competences	core competence	core competence	1	singular
core competences	core competences	core competences	core competence	core competence	1	singular

core competencies	core competencies	core competencies	core competency	Core competency	1	singular
core genome	core genome	core genome	core genome	core genome	1	kept
core genome analysis	core genome analysis	core genome analysis	core genome analysis	core genome analysis	1	kept
core laboratory	core laboratory	core laboratory	core laboratory	core laboratory	1	kept
core outcome measures in effectiveness trial	core outcome measures in effectiveness trial	core outcome measures in effectiveness trial	core outcome measures in effectiveness trial	core outcome measures in effectiveness trial	1	kept
Core Outcome Set	core outcome set	core outcome set	core outcome set	core outcome set	1	case_or_format
Core outcome set	core outcome set	core outcome set	core outcome set	core outcome set	1	case_or_format
Core outcome set development	core outcome set development	core outcome set development	core outcome set development	Core outcome set development	1	kept
core outcome set development	core outcome set development	core outcome set development	core outcome set development	Core outcome set development	1	case_or_format
core outcomes	core outcomes	core outcomes	core outcomes	core outcomes	1	kept
Core prevention and control measures	core prevention and control measures	core prevention and control measures	core prevention and control measures	Core prevention and control measures	1	kept
core qualities	core qualities	core qualities	core qualities	core qualities	1	kept
CORE SELF-EVALUATIONS	core self evaluations	core self evaluations	core self evaluations	CORE SELF-EVALUATIONS	1	kept
core staff	core staff	core staff	core staff	core staff	1	kept
core story creation	core story creation	core story creation	core story creation	core story creation	1	kept
Core temperature	core temperature	core temperature	core temperature	core temperature	1	case_or_format

Coregulation	coregulation	coregulation	coregulation	Coregulation	1	kept
corley moral distress scale	corley moral distress scale	corley moral distress scale	corley moral distress scale	corley moral distress scale	1	kept
Corleys Moral distress scale	corleys moral distress scale	corleys moral distress scale	corleys moral distress scale	Corleys Moral distress scale	1	kept
cormack and lehane grade	cormack and lehane grade	cormack and lehane grade	cormack and lehane grade	cormack and lehane grade	1	kept
cornea dryness	cornea dryness	cornea dryness	cornea dryness	cornea dryness	1	kept
cornea epithelium	cornea epithelium	cornea epithelium	cornea epithelium	cornea epithelium	1	kept
cornea haziness	cornea haziness	cornea haziness	cornea haziness	cornea haziness	1	kept
Corneal	corneal	corneal	corneal	Corneal	1	kept
Corneal abrasion	corneal abrasion	corneal abrasion	corneal abrasion	Corneal abrasion	1	kept
corneal abrasion	corneal abrasion	corneal abrasion	corneal abrasion	Corneal abrasion	1	case_or_format
corneal assessment	corneal assessment	corneal assessment	corneal assessment	corneal assessment	1	kept
corneal exposure	corneal exposure	corneal exposure	corneal exposure	corneal exposure	1	kept
corneal injury	corneal injury	corneal injury	corneal injury	Corneal Injuries	1	case_or_format
Corneal pathology	corneal pathology	corneal pathology	corneal pathology	Corneal pathology	1	kept
Corneal transplant	corneal transplant	corneal transplant	corneal transplant	Corneal transplant	1	kept
Corneal Transplantation	corneal transplantation	corneal transplantation	corneal transplantation	Corneal Transplantation	1	kept

Corneal transplantation	corneal transplantation	corneal transplantation	corneal transplantation	Corneal Transplantation	1	case_or_format
CORNELL ASSESSMENT	cornell assessment	cornell assessment	cornell assessment	CORNELL ASSESSMENT	1	kept
Cornell assessment of pediatric delirium	cornell assessment of pediatric delirium	cornell assessment of pediatric delirium	cornell assessment of pediatric delirium	Cornell Assessment of Pediatric Delirium	1	case_or_format
Cornell Assessment of Pediatric Delirium Scale	cornell assessment of pediatric delirium scale	cornell assessment of pediatric delirium scale	cornell assessment of pediatric delirium scale	Cornell Assessment of Pediatric Delirium Scale	1	kept
Cornell Assessment of Pediatric Delirium scale	cornell assessment of pediatric delirium scale	cornell assessment of pediatric delirium scale	cornell assessment of pediatric delirium scale	Cornell Assessment of Pediatric Delirium Scale	1	case_or_format
Cornell Assessment Pediatric Delirium	cornell assessment pediatric delirium	cornell assessment pediatric delirium	cornell assessment pediatric delirium	Cornell Assessment Pediatric Delirium	1	kept
Cornell Musculoskeletal Discomfort	cornell musculoskeletal discomfort	cornell musculoskeletal discomfort	cornell musculoskeletal discomfort	Cornell Musculoskeletal Discomfort	1	kept
Cornell musculoskeletal discomfort	cornell musculoskeletal discomfort	cornell musculoskeletal discomfort	cornell musculoskeletal discomfort	Cornell Musculoskeletal Discomfort	1	case_or_format
cornell musculoskeletal discomfort	cornell musculoskeletal discomfort	cornell musculoskeletal discomfort	cornell musculoskeletal discomfort	Cornell Musculoskeletal Discomfort	1	case_or_format
Cornell Scale for Depression in Dementia	cornell scale for depression in dementia	cornell scale for depression in dementia	cornell scale for depression in dementia	Cornell Scale for Depression in Dementia	1	kept
corona	corona	corona	corona	corona	1	kept
corona virus	corona virus	corona virus	corona virus	Corona virus	1	case_or_format
corona virus crisis management model	corona virus crisis management model	corona virus crisis management model	corona virus crisis management model	corona virus crisis management model	1	kept
Corona virus disease	corona virus disease	corona virus disease	corona virus disease	Corona virus disease	1	kept
Corona Virus Disease 2019	corona virus disease 2019	corona virus disease 2019	corona virus disease 2019	Corona Virus Disease 2019	1	kept
Coronary Aneurysm	coronary aneurysm	coronary aneurysm	coronary aneurysm	Coronary Aneurysm	1	kept

Coronary angiography	coronary angiography	coronary angiography	coronary angiography	coronary angiography	1	case_or_format
Coronary Artery	coronary artery	coronary artery	coronary artery	coronary artery	1	case_or_format
coronary artery aneurysm	coronary artery aneurysm	coronary artery aneurysm	coronary artery aneurysm	coronary artery aneurysm	1	kept
Coronary artery bypass graft Surgery	coronary artery bypass graft surgery	coronary artery bypass graft surgery	coronary artery bypass graft surgery	Coronary artery bypass graft surgery	1	case_or_format
coronary artery bypass grafts	coronary artery bypass grafts	coronary artery bypass grafts	coronary artery bypass graft	coronary artery bypass graft	1	singular
Coronary Artery Bypass Surgery	coronary artery bypass surgery	coronary artery bypass surgery	coronary artery bypass surgery	coronary artery bypass surgery	1	case_or_format
Coronary artery bypass, off-pump	coronary artery bypass off pump	coronary artery bypass off pump	coronary artery bypass off pump	Coronary Artery Bypass, Off-Pump	1	case_or_format
coronary artery circumflex branch	coronary artery circumflex branch	coronary artery circumflex branch	coronary artery circumflex branch	coronary artery circumflex branch	1	kept
coronary artery diseases	coronary artery diseases	coronary artery diseases	coronary artery disease	coronary artery disease	1	singular
coronary artery obstruction	coronary artery obstruction	coronary artery obstruction	coronary artery obstruction	coronary artery obstruction	1	kept
coronary artery thrombosis	coronary artery thrombosis	coronary artery thrombosis	coronary artery thrombosis	coronary artery thrombosis	1	kept
coronary blood vessel	coronary blood vessel	coronary blood vessel	coronary blood vessel	coronary blood vessel	1	kept
Coronary bypass	coronary bypass	coronary bypass	coronary bypass	Coronary bypass	1	kept
Coronary disease	coronary disease	coronary disease	coronary disease	Coronary Disease	1	case_or_format
Coronary intervention	coronary intervention	coronary intervention	coronary intervention	Coronary intervention	1	kept
coronary reperfusion	coronary reperfusion	coronary reperfusion	coronary reperfusion	coronary reperfusion	1	kept

Coronary Restenosis	coronary restenosis	coronary restenosis	coronary restenosis	Coronary Restenosis	1	kept
coronary risk	coronary risk	coronary risk	coronary risk	coronary risk	1	kept
coronary sinus	coronary sinus	coronary sinus	coronary sinus	coronary sinus	1	kept
coronary stenosis	coronary stenosis	coronary stenosis	coronary stenosis	Coronary Stenosis	1	case_or_format
coronary stent	coronary stent	coronary stent	coronary stent	coronary stent	1	kept
coronary syndromes	coronary syndromes	coronary syndromes	coronary syndromes	coronary syndromes	1	kept
Coronary units	coronary units	coronary units	coronary units	Coronary units	1	kept
Coronary Vessel Anomalies	coronary vessel anomalies	coronary vessel anomalies	coronary vessel anomalies	Coronary Vessel Anomalies	1	kept
coronary vessel malformation	coronary vessel malformation	coronary vessel malformation	coronary vessel malformation	coronary vessel malformation	1	kept
Coronary Vessels	coronary vessels	coronary vessels	coronary vessels	Coronary Vessels	1	kept
CORONARY-ARTERY-DISEASE	coronary artery disease	coronary artery disease	coronary artery disease	coronary artery disease	1	case_or_format
Coronavac	coronavac	coronavac	coronavac	coronavac	1	case_or_format
Coronaviridae	coronaviridae	coronaviridae	coronaviridae	Coronaviridae	1	kept
Coronaviridae infection	coronaviridae infection	coronaviridae infection	coronaviridae infection	Coronaviridae infection	1	kept
coronavirus 19 phobia scale	coronavirus 19 phobia scale	coronavirus 19 phobia scale	coronavirus 19 phobia scale	coronavirus 19 phobia scale	1	kept
Coronavirus 2019	coronavirus 2019	coronavirus 2019	coronavirus 2019	coronavirus 2019	1	case_or_format

Coronavirus Act 2020	coronavirus act 2020	coronavirus act 2020	coronavirus act 2020	Coronavirus Act 2020	1	kept
Coronavirus Anxiety Inventory	coronavirus anxiety inventory	coronavirus anxiety inventory	coronavirus anxiety inventory	Coronavirus Anxiety Inventory	1	kept
Coronavirus disease 19	coronavirus disease 19	coronavirus disease 19	coronavirus disease 19	Coronavirus disease 19	1	kept
coronavirus disease 2019 response	coronavirus disease 2019 response	coronavirus disease 2019 response	coronavirus disease 2019 response	coronavirus disease 2019 response	1	kept
coronavirus disease 2019-related acute respiratory distress [illegible]	coronavirus disease 2019 related acute respiratory distress [illegible]	coronavirus disease 2019 related acute respiratory distress [illegible]	coronavirus disease 2019 related acute respiratory distress [illegible]	coronavirus disease 2019-related acute respiratory distress [illegible]	1	kept
Coronavirus disease of 2019	coronavirus disease of 2019	coronavirus disease of 2019	coronavirus disease of 2019	Coronavirus disease of 2019	1	kept
Coronavirus disease-19	coronavirus disease 19	coronavirus disease 19	coronavirus disease 19	Coronavirus disease 19	1	case_or_format
Coronavirus disease-19 in pregnancy	coronavirus disease 19 in pregnancy	coronavirus disease 19 in pregnancy	coronavirus disease 19 in pregnancy	Coronavirus disease-19 in pregnancy	1	kept
coronavirus diseases-2019	coronavirus diseases 2019	coronavirus diseases 2019	coronavirus diseases 2019	coronavirus diseases-2019	1	kept
Coronavirus Envelope Proteins	coronavirus envelope proteins	coronavirus envelope proteins	coronavirus envelope proteins	Coronavirus Envelope Proteins	1	kept
coronavirus nucleocapsid protein	coronavirus nucleocapsid protein	coronavirus nucleocapsid protein	coronavirus nucleocapsid protein	coronavirus nucleocapsid protein	1	kept
CORONAVIRUS OUTBREAK	coronavirus outbreak	coronavirus outbreak	coronavirus outbreak	CORONAVIRUS OUTBREAK	1	kept
coronavirus outbreak	coronavirus outbreak	coronavirus outbreak	coronavirus outbreak	CORONAVIRUS OUTBREAK	1	case_or_format
Coronavirus pandemic	coronavirus pandemic	coronavirus pandemic	coronavirus pandemic	coronavirus pandemic	1	case_or_format
Coronavirus RNA-Dependent RNA Polymerase	coronavirus rna dependent rna polymerase	coronavirus rna dependent rna polymerase	coronavirus rna dependent rna polymerase	Coronavirus RNA-Dependent RNA Polymerase	1	kept
Coronavirus-19 phobia scale	coronavirus 19 phobia scale	coronavirus 19 phobia scale	coronavirus 19 phobia scale	coronavirus 19 phobia scale	1	case_or_format

coronavirus-2	coronavirus 2	coronavirus 2	coronavirus 2	coronavirus-2	1	kept
corporate social responsibility	corporate social responsibility	corporate social responsibility	corporate social responsibility	corporate social responsibility	1	kept
correct diagnosis	correct diagnosis	correct diagnosis	correct diagnosis	correct diagnosis	1	kept
Correct IV device	correct iv device	correct iv device	correct iv device	Correct IV device	1	kept
correctional health care	correctional health care	correctional health care	correctional health care	correctional health care	1	kept
corrective and palliative surgery	corrective and palliative surgery	corrective and palliative surgery	corrective and palliative surgery	corrective and palliative surgery	1	kept
Correlation features	correlation features	correlation features	correlation features	Correlation features	1	kept
corridors	corridors	corridors	corridors	corridors	1	kept
corrosion	corrosion	corrosion	corrosion	corrosion	1	kept
Corrosive esophageal injury	corrosive esophageal injury	corrosive esophageal injury	corrosive esophageal injury	Corrosive esophageal injury	1	kept
cortical excitability	cortical excitability	cortical excitability	cortical excitability	cortical excitability	1	kept
Cortical spreading depression	cortical spreading depression	cortical spreading depression	cortical spreading depression	Cortical spreading depression	1	kept
cortical stroke	cortical stroke	cortical stroke	cortical stroke	cortical stroke	1	kept
corticospinal tract	corticospinal tract	corticospinal tract	corticospinal tract	corticospinal tract	1	kept
Corticosteroid	corticosteroid	corticosteroid	corticosteroid	corticosteroid	1	case_or_format
Corticosteroid therapy	corticosteroid therapy	corticosteroid therapy	corticosteroid therapy	corticosteroid therapy	1	case_or_format

Corticosteroid use	corticosteroid use	corticosteroid use	corticosteroid use	Corticosteroid use	1	kept
Corticotropin	corticotropin	corticotropin	corticotropin	corticotropin	1	case_or_format
CORTISOL	cortisol	cortisol	cortisol	Cortisol	1	case_or_format
CORTISOL RESPONSES	cortisol responses	cortisol responses	cortisol responses	CORTISOL RESPONSES	1	kept
CORTISOL-LEVELS	cortisol levels	cortisol levels	cortisol levels	CORTISOL-LEVELS	1	kept
Cortisone	cortisone	cortisone	cortisone	Cortisone	1	kept
cortisone	cortisone	cortisone	cortisone	Cortisone	1	case_or_format
Cortrack	cortrack	cortrack	cortrack	Cortrack	1	kept
Cortrak	cortrak	cortrak	cortrak	Cortrak	1	kept
Corynebacterium amycolatum	corynebacterium amycolatum	corynebacterium amycolatum	corynebacterium amycolatum	Corynebacterium amycolatum	1	kept
Corynebacterium bovis	corynebacterium bovis	corynebacterium bovis	corynebacterium bovis	Corynebacterium bovis	1	kept
Corynebacterium casei	corynebacterium casei	corynebacterium casei	corynebacterium casei	Corynebacterium casei	1	kept
Corynebacterium jeikeium	corynebacterium jeikeium	corynebacterium jeikeium	corynebacterium jeikeium	Corynebacterium jeikeium	1	kept
Corynebacterium matruchoti	corynebacterium matruchoti	corynebacterium matruchoti	corynebacterium matruchoti	Corynebacterium matruchoti	1	kept
Corynebacterium minutissimum	corynebacterium minutissimum	corynebacterium minutissimum	corynebacterium minutissimum	Corynebacterium minutissimum	1	kept
Corynebacterium striatum	corynebacterium striatum	corynebacterium striatum	corynebacterium striatum	Corynebacterium striatum	1	kept

Corynebacterium ulcerans	corynebacterium ulcerans	corynebacterium ulcerans	corynebacterium ulcerans	Corynebacterium ulcerans	1	kept
Cosmetics	cosmetics	cosmetics	cosmetics	Cosmetics	1	kept
COSMIN	cosmin	cosmin	cosmin	COSMIN	1	kept
cosmological phenomena	cosmological phenomena	cosmological phenomena	cosmological phenomena	cosmological phenomena	1	kept
cost accounting	cost accounting	cost accounting	cost accounting	cost accounting	1	kept
Cost allocation	cost allocation	cost allocation	cost allocation	Cost Allocation	1	case_or_format
Cost Analysis	cost analysis	cost analysis	cost analysis	Cost analysis	1	case_or_format
cost and cost analysis	cost and cost analysis	cost and cost analysis	cost and cost analysis	Cost and cost analysis	1	case_or_format
COST AUDITS	cost audits	cost audits	cost audits	COST AUDITS	1	kept
cost avoidance	cost avoidance	cost avoidance	cost avoidance	cost avoidance	1	kept
Cost awareness	cost awareness	cost awareness	cost awareness	Cost awareness	1	kept
Cost benefit	cost benefit	cost benefit	cost benefit	Cost benefit	1	kept
Cost consciousness	cost consciousness	cost consciousness	cost consciousness	Cost consciousness	1	kept
cost controlling	cost controlling	cost controlling	cost controlling	cost controlling	1	kept
Cost driver	cost driver	cost driver	cost driver	Cost driver	1	kept
Cost effective	cost effective	cost effective	cost effective	Cost effective	1	kept

cost effective	cost effective	cost effective	cost effective	Cost effective	1	case_or_format
Cost effective health care	cost effective health care	cost effective health care	cost effective health care	Cost effective health care	1	kept
cost effective interventions	cost effective interventions	cost effective interventions	cost effective interventions	cost effective interventions	1	kept
Cost efficiency	cost efficiency	cost efficiency	cost efficiency	Cost efficiency	1	kept
cost inflation	cost inflation	cost inflation	cost inflation	cost inflation	1	kept
Cost minimisation analysis	cost minimisation analysis	cost minimisation analysis	cost minimisation analysis	Cost minimisation analysis	1	kept
Cost neutral	cost neutral	cost neutral	cost neutral	Cost neutral	1	kept
Cost of care	cost of care	cost of care	cost of care	cost of care	1	case_or_format
Cost of diseases OR economic burden of diseases	cost of diseases or economic burden of diseases	cost of diseases or economic burden of diseases	cost of diseases or economic burden of diseases	Cost of diseases OR economic burden of diseases	1	kept
Cost of hospitalization	cost of hospitalization	cost of hospitalization	cost of hospitalization	Cost of hospitalization	1	kept
cost of pressure ulcers	cost of pressure ulcers	cost of pressure ulcers	cost of pressure ulcers	cost of pressure ulcers	1	kept
cost of rehabilitation	cost of rehabilitation	cost of rehabilitation	cost of rehabilitation	cost of rehabilitation	1	kept
cost of stay	cost of stay	cost of stay	cost of stay	cost of stay	1	kept
COST REDUCTION	cost reduction	cost reduction	cost reduction	COST REDUCTION	1	kept
Cost reduction	cost reduction	cost reduction	cost reduction	COST REDUCTION	1	case_or_format
Cost saving	cost saving	cost saving	cost saving	Cost Savings	1	case_or_format

cost saving	cost saving	cost saving	cost saving	Cost Savings	1	case_or_format
cost shift	cost shift	cost shift	cost shift	cost shift	1	kept
Cost study	cost study	cost study	cost study	Cost study	1	kept
Cost, Healthcare utilization, Hospice, Palliative care	cost healthcare utilization hospice palliative care	cost healthcare utilization hospice palliative care	cost healthcare utilization hospice palliative care	Cost, Healthcare utilization, Hospice, Palliative care	1	kept
Cost-analysis	cost analysis	cost analysis	cost analysis	Cost analysis	1	case_or_format
Cost-consequence analysis	cost consequence analysis	cost consequence analysis	cost consequence analysis	Cost-consequence analysis	1	kept
Cost-containment	cost containment	cost containment	cost containment	Cost-containment	1	kept
cost-containment	cost containment	cost containment	cost containment	Cost-containment	1	case_or_format
Cost-effective	cost effective	cost effective	cost effective	Cost effective	1	case_or_format
cost-effective	cost effective	cost effective	cost effective	Cost effective	1	case_or_format
Cost-effective care	cost effective care	cost effective care	cost effective care	Cost-effective care	1	kept
cost-effective neonatal care	cost effective neonatal care	cost effective neonatal care	cost effective neonatal care	cost-effective neonatal care	1	kept
Cost-effectiveness evaluation	cost effectiveness evaluation	cost effectiveness evaluation	cost effectiveness evaluation	Cost-effectiveness evaluation	1	kept
Cost-effectiveness ratios	cost effectiveness ratios	cost effectiveness ratios	cost effectiveness ratios	Cost-effectiveness ratios	1	kept
Cost-effectiveness study	cost effectiveness study	cost effectiveness study	cost effectiveness study	Cost-effectiveness study	1	kept
Cost-efficacy analysis	cost efficacy analysis	cost efficacy analysis	cost efficacy analysis	Cost-efficacy analysis	1	kept

Cost-minimisation	cost minimisation	cost minimisation	cost minimisation	Cost-minimisation	1	kept
Cost-of-illness	cost of illness	cost of illness	cost of illness	Cost of Illness	1	case_or_format
cost-of-illness	cost of illness	cost of illness	cost of illness	Cost of Illness	1	case_or_format
cost-utility	cost utility	cost utility	cost utility	cost-utility	1	kept
Cost-utility analysis	cost utility analysis	cost utility analysis	cost utility analysis	cost utility analysis	1	case_or_format
Costa Rica	costa rica	costa rica	costa rica	Costa Rica	1	kept
costing	costing	costing	costing	Costing	1	case_or_format
Costs analysis	costs analysis	costs analysis	costs analysis	Costs analysis	1	kept
Costs and cost-analysis	costs and cost analysis	costs and cost analysis	costs and cost analysis	Costs and Cost Analysis	1	case_or_format
costs and costs analyses	costs and costs analyses	costs and costs analyses	costs and costs analysis	Costs and costs analysis	1	singular
Costs of trauma	costs of trauma	costs of trauma	costs of trauma	Costs of trauma	1	kept
cost‐effectiveness	cost effectiveness	cost effectiveness	cost effectiveness	Cost-effectiveness	1	case_or_format
Cosyntropin	cosyntropin	cosyntropin	cosyntropin	Cosyntropin	1	kept
COT	cot	cot	cot	COT	1	kept
Cot days	cot days	cot days	cot days	Cot days	1	kept
cotton	cotton	cotton	cotton	cotton	1	kept

COUGH	cough	cough	cough	Cough	1	case_or_format
Cough assist	cough assist	cough assist	cough assist	Cough Assist	1	case_or_format
Cough peak expiratory flow	cough peak expiratory flow	cough peak expiratory flow	cough peak expiratory flow	Cough peak expiratory flow	1	kept
cough reflex	cough reflex	cough reflex	cough reflex	cough reflex	1	kept
Coumadin	coumadin	coumadin	coumadin	Coumadin	1	kept
coumarin	coumarin	coumarin	coumarin	coumarin	1	kept
Counseling and supportive interventions	counseling and supportive interventions	counseling and supportive interventions	counseling and supportive interventions	Counseling and supportive interventions	1	kept
COUNSELORS	counselors	counselors	counselor	counselor	1	singular
Counselors	counselors	counselors	counselor	counselor	1	singular
counterproductive behavior	counterproductive behavior	counterproductive behavior	counterproductive behavior	counterproductive behavior	1	kept
counterterrorism	counterterrorism	counterterrorism	counterterrorism	counterterrorism	1	kept
Countertransference	countertransference	countertransference	countertransference	Countertransference	1	kept
COUNTS	counts	counts	counts	COUNTS	1	kept
County-level hospital	county level hospital	county level hospital	county level hospital	County-level hospital	1	kept
couple	couple	couple	couple	couple	1	kept
Couples	couples	couples	couple	couple	1	singular

Couplet care	couplet care	couplet care	couplet care	couplet care	1	case_or_format
court-appointed guardians	court appointed guardians	court appointed guardians	court appointed guardians	court-appointed guardians	1	kept
Couvelaire uterus	couvelaire uterus	couvelaire uterus	couvelaire uterus	Couvelaire uterus	1	kept
covariance	covariance	covariance	covariance	covariance	1	kept
COVERAGE	coverage	coverage	coverage	COVERAGE	1	kept
Coverage	coverage	coverage	coverage	COVERAGE	1	case_or_format
covert medication	covert medication	covert medication	covert medication	covert medication	1	kept
Covert observation	covert observation	covert observation	covert observation	Covert observation	1	kept
COVID -19	covid 19	covid 19	covid 19	COVID-19	1	case_or_format
covid 19	covid 19	covid 19	covid 19	COVID-19	1	case_or_format
COVID 19 delirium	covid 19 delirium	covid 19 delirium	covid 19 delirium	COVID 19 delirium	1	kept
COVID 19 encephalopathy	covid 19 encephalopathy	covid 19 encephalopathy	covid 19 encephalopathy	COVID 19 encephalopathy	1	kept
Covid 19 pandemic	covid 19 pandemic	covid 19 pandemic	covid 19 pandemic	COVID-19 pandemic	1	case_or_format
COVID 19 Stigma Scale	covid 19 stigma scale	covid 19 stigma scale	covid 19 stigma scale	COVID 19 Stigma Scale	1	kept
COVID 19 unit	covid 19 unit	covid 19 unit	covid 19 unit	COVID 19 unit	1	kept
covid 19 vulnerability index	covid 19 vulnerability index	covid 19 vulnerability index	covid 19 vulnerability index	covid 19 vulnerability index	1	kept

Covid cell	covid cell	covid cell	covid cell	Covid cell	1	kept
Covid circuit	covid circuit	covid circuit	covid circuit	Covid circuit	1	kept
covid coagulopathy	covid coagulopathy	covid coagulopathy	covid coagulopathy	covid coagulopathy	1	kept
COVID hospital	covid hospital	covid hospital	covid hospital	COVID hospital	1	kept
COVID management	covid management	covid management	covid management	COVID management	1	kept
COVID nephropathy	covid nephropathy	covid nephropathy	covid nephropathy	COVID nephropathy	1	kept
COVID-11	covid 11	covid 11	covid 11	COVID-11	1	kept
CoViD-19	covid 19	covid 19	covid 19	COVID-19	1	case_or_format
COVID-19 [supplementary concept]	covid 19 supplementary concept	covid 19 supplementary concept	covid 19 supplementary concept	COVID-19 [supplementary concept]	1	kept
COVID-19 antibody	covid 19 antibody	covid 19 antibody	covid 19 antibody	COVID-19 antibody	1	kept
COVID-19 attitude	covid 19 attitude	covid 19 attitude	covid 19 attitude	COVID-19 attitude	1	kept
COVID-19 breakthrough infections	covid 19 breakthrough infections	covid 19 breakthrough infections	covid 19 breakthrough infections	COVID-19 breakthrough infections	1	kept
COVID-19 burnout scale	covid 19 burnout scale	covid 19 burnout scale	covid 19 burnout scale	COVID-19 burnout scale	1	kept
covid-19 cases	covid 19 cases	covid 19 cases	covid 19 cases	covid-19 cases	1	kept
COVID-19 crisis	covid 19 crisis	covid 19 crisis	covid 19 crisis	COVID-19 crisis	1	kept
covid-19 crisis	covid 19 crisis	covid 19 crisis	covid 19 crisis	COVID-19 crisis	1	case_or_format

COVID-19 hospital	covid 19 hospital	covid 19 hospital	covid 19 hospital	COVID-19 hospital	1	kept
COVID-19 in Greece	covid 19 in greece	covid 19 in greece	covid 19 in greece	COVID-19 in Greece	1	kept
COVID-19 India	covid 19 india	covid 19 india	covid 19 india	COVID-19 India	1	kept
COVID-19 isolated hospitals	covid 19 isolated hospitals	covid 19 isolated hospitals	covid 19 isolated hospitals	COVID-19 isolated hospitals	1	kept
covid-19 mortality	covid 19 mortality	covid 19 mortality	covid 19 mortality	covid-19 mortality	1	kept
COVID-19 outbreak	covid 19 outbreak	covid 19 outbreak	covid 19 outbreak	COVID-19 outbreak	1	kept
covid-19 outbreak	covid 19 outbreak	covid 19 outbreak	covid 19 outbreak	COVID-19 outbreak	1	case_or_format
COVID-19 pandemic experience	covid 19 pandemic experience	covid 19 pandemic experience	covid 19 pandemic experience	COVID-19 pandemic experience	1	kept
CoVid-19 pneumonia	covid 19 pneumonia	covid 19 pneumonia	covid 19 pneumonia	COVID-19 pneumonia	1	case_or_format
COVID-19 prevention and control	covid 19 prevention and control	covid 19 prevention and control	covid 19 prevention and control	COVID-19 prevention and control	1	kept
COVID-19 Rapid Quantitative Assessment Tool	covid 19 rapid quantitative assessment tool	covid 19 rapid quantitative assessment tool	covid 19 rapid quantitative assessment tool	COVID-19 Rapid Quantitative Assessment Tool	1	kept
COVID-19 related psychological distress	covid 19 related psychological distress	covid 19 related psychological distress	covid 19 related psychological distress	COVID-19 related psychological distress	1	kept
COVID-19 survivors	covid 19 survivors	covid 19 survivors	covid 19 survivors	COVID-19 survivors	1	kept
COVID-19 symptoms	covid 19 symptoms	covid 19 symptoms	covid 19 symptoms	COVID-19 symptoms	1	kept
COVID-19 vaccination	covid 19 vaccination	covid 19 vaccination	covid 19 vaccination	COVID-19 vaccination	1	kept
COVID-19 vaccine hesitancy	covid 19 vaccine hesitancy	covid 19 vaccine hesitancy	covid 19 vaccine hesitancy	COVID-19 vaccine hesitancy	1	kept

COVID-19 virus	covid 19 virus	covid 19 virus	covid 19 virus	COVID-19 virus	1	kept
COVID-19 workforce	covid 19 workforce	covid 19 workforce	covid 19 workforce	COVID-19 workforce	1	kept
COVID-19, Coronavirus disease 2019	covid 19 coronavirus disease 2019	covid 19 coronavirus disease 2019	covid 19 coronavirus disease 2019	COVID-19, coronavirus disease 2019	1	case_or_format
COVID-19-associated coagulopathy	covid 19 associated coagulopathy	covid 19 associated coagulopathy	covid 19 associated coagulopathy	COVID-19-associated coagulopathy	1	kept
COVID-19-related ARDS	covid 19 related ards	covid 19 related ards	covid 19 related ards	COVID-19-related ARDS	1	kept
COVID-19.	covid 19	covid 19	covid 19	COVID-19	1	case_or_format
COVID-HCFs	covid hcfs	covid hcfs	covid hcfs	COVID-HCFs	1	kept
COVID-induced severe rhabdomyolysis	covid induced severe rhabdomyolysis	covid induced severe rhabdomyolysis	covid induced severe rhabdomyolysis	COVID-induced severe rhabdomyolysis	1	kept
covid19	covid19	covid 19	covid 19	COVID-19	1	alias
COVID<sup>-<	covid sup	covid sup	covid sup	COVID<sup>-<	1	kept
Covid‐19	covid 19	covid 19	covid 19	COVID-19	1	case_or_format
covilo	covilo	covilo	covilo	covilo	1	kept
COVIP-study	covip study	covip study	covip study	COVIP-study	1	kept
cow milk	cow milk	cow milk	cow milk	cow milk	1	kept
coworker support	coworker support	coworker support	coworker support	coworker support	1	kept
coworker support web-based survey	coworker support web based survey	coworker support web based survey	coworker support web based survey	coworker support web-based survey	1	kept

Cox proportional hazards models	cox proportional hazards models	cox proportional hazards models	cox proportional hazards models	Cox proportional hazards models	1	kept
Cox regression	cox regression	cox regression	cox regression	Cox regression	1	kept
Coxsackie virus B	coxsackie virus b	coxsackie virus b	coxsackie virus b	Coxsackie virus B	1	kept
Coxsackie virus infection	coxsackie virus infection	coxsackie virus infection	coxsackie virus infection	Coxsackie virus infection	1	kept
Coxsackievirus Infections	coxsackievirus infections	coxsackievirus infections	coxsackievirus infections	Coxsackievirus Infections	1	kept
CPAN	cpan	cpan	cpan	CPAN	1	kept
CPAP ventilation	cpap ventilation	cpap ventilation	cpap ventilation	CPAP ventilation	1	kept
CPAP, Continuous Positive Airway Pressure	cpap continuous positive airway pressure	cpap continuous positive airway pressure	cpap continuous positive airway pressure	CPAP, Continuous Positive Airway Pressure	1	kept
CPOT Evaluation	cpot evaluation	cpot evaluation	cpot evaluation	CPOT Evaluation	1	kept
CPQCC = California Perinatal Quality Care Collaborative	cpqcc california perinatal quality care collaborative	cpqcc california perinatal quality care collaborative	cpqcc california perinatal quality care collaborative	CPQCC = California Perinatal Quality Care Collaborative	1	kept
CPR educational package	cpr educational package	cpr educational package	cpr educational package	CPR educational package	1	kept
CPR experiences	cpr experiences	cpr experiences	cpr experiences	CPR experiences	1	kept
CPR FEEDBACK	cpr feedback	cpr feedback	cpr feedback	CPR FEEDBACK	1	kept
CPR guidelines	cpr guidelines	cpr guidelines	cpr guidelines	CPR guidelines	1	kept
CPR quality	cpr quality	cpr quality	cpr quality	CPR quality	1	kept
CPR training	cpr training	cpr training	cpr training	CPR training	1	kept

CR-BSI	cr bsi	cr bsi	cr bsi	CR-BSI	1	kept
Crack Cocaine	crack cocaine	crack cocaine	crack cocaine	Crack Cocaine	1	kept
cracker	cracker	cracker	cracker	cracker	1	kept
Cracks	cracks	cracks	cracks	Cracks	1	kept
Cramer's V	cramer s v	cramer s v	cramer s v	Cramer's V	1	kept
Cranial index	cranial index	cranial index	cranial index	Cranial index	1	kept
Cranial nerve assessment	cranial nerve assessment	cranial nerve assessment	cranial nerve assessment	Cranial nerve assessment	1	kept
Cranial Nerve Diseases	cranial nerve diseases	cranial nerve diseases	cranial nerve diseases	Cranial Nerve Diseases	1	kept
cranial suture	cranial suture	cranial suture	cranial suture	cranial suture	1	kept
Cranial sutures	cranial sutures	cranial sutures	cranial suture	cranial suture	1	singular
Cranial ultrasonography	cranial ultrasonography	cranial ultrasonography	cranial ultrasonography	Cranial ultrasonography	1	kept
cranial ultrasound	cranial ultrasound	cranial ultrasound	cranial ultrasound	cranial ultrasound	1	kept
cranial ultrasound scanning	cranial ultrasound scanning	cranial ultrasound scanning	cranial ultrasound scanning	cranial ultrasound scanning	1	kept
cranial volume	cranial volume	cranial volume	cranial volume	cranial volume	1	kept
Craniocerebral trauma	craniocerebral trauma	craniocerebral trauma	craniocerebral trauma	Craniocerebral Trauma	1	case_or_format
craniocervical distraction	craniocervical distraction	craniocervical distraction	craniocervical distraction	craniocervical distraction	1	kept

Craniofacial	craniofacial	craniofacial	craniofacial	Craniofacial	1	kept
craniofacial	craniofacial	craniofacial	craniofacial	Craniofacial	1	case_or_format
Craniofacial Abnormalities	craniofacial abnormalities	craniofacial abnormalities	craniofacial abnormalities	Craniofacial Abnormalities	1	kept
Craniofacial surgery	craniofacial surgery	craniofacial surgery	craniofacial surgery	Craniofacial surgery	1	kept
craniosacral therapy	craniosacral therapy	craniosacral therapy	craniosacral therapy	craniosacral therapy	1	kept
Craniotomy postop	craniotomy postop	craniotomy postop	craniotomy postop	Craniotomy postop	1	kept
CRB 65	crb 65	crb 65	crb 65	CRB 65	1	kept
CRB-65 Index	crb 65 index	crb 65 index	crb 65 index	CRB-65 Index	1	kept
crbsi	crbsi	crbsi	crbsi	CRBSI	1	case_or_format
CRBSI, Catheter Related Blood Stream Infection	crbsi catheter related blood stream infection	crbsi catheter related blood stream infection	crbsi catheter related blood stream infection	CRBSI, Catheter Related Blood Stream Infection	1	kept
CREATING OPPORTUNITIES	creating opportunities	creating opportunities	creating opportunities	CREATING OPPORTUNITIES	1	kept
Creating opportunities for parent empowerment	creating opportunities for parent empowerment	creating opportunities for parent empowerment	creating opportunities for parent empowerment	Creating opportunities for parent empowerment	1	kept
Creatinine enzymatic assay	creatinine enzymatic assay	creatinine enzymatic assay	creatinine enzymatic assay	Creatinine enzymatic assay	1	kept
creative arts therapy	creative arts therapy	creative arts therapy	creative arts therapy	creative arts therapy	1	kept
Creativeness	creativeness	creativeness	creativeness	Creativeness	1	kept
Credibility	credibility	credibility	credibility	Credibility	1	kept

credit scorecard model	credit scorecard model	credit scorecard model	credit scorecard model	credit scorecard model	1	kept
CREEP	creep	creep	creep	CREEP	1	kept
cremation	cremation	cremation	cremation	cremation	1	kept
cremophor	cremophor	cremophor	cremophor	cremophor	1	kept
Crescendo effect	crescendo effect	crescendo effect	crescendo effect	Crescendo effect	1	kept
Crew Resource Management	crew resource management	crew resource management	crew resource management	Crew resource management	1	case_or_format
Crew resources management	crew resources management	crew resources management	crew resources management	Crew resources management	1	kept
Crew scheduling	crew scheduling	crew scheduling	crew scheduling	Crew scheduling	1	kept
Criança Hospitalizada	crian a hospitalizada	crian a hospitalizada	crian a hospitalizada	Criança Hospitalizada	1	kept
CRIB	crib	crib	crib	CRIB	1	kept
cricoarytenoid joint fixation	cricoarytenoid joint fixation	cricoarytenoid joint fixation	cricoarytenoid joint fixation	cricoarytenoid joint fixation	1	kept
cricoid pressure	cricoid pressure	cricoid pressure	cricoid pressure	cricoid pressure	1	kept
CRIES pain scale	cries pain scale	cries pain scale	cries pain scale	CRIES pain scale	1	kept
criminal behavior	criminal behavior	criminal behavior	criminal behavior	criminal behavior	1	kept
Criminal justice	criminal justice	criminal justice	criminal justice	criminal justice	1	case_or_format
Criminal Police	criminal police	criminal police	criminal police	Criminal Police	1	kept

criminalistics	criminalistics	criminalistics	criminalistics	criminalistics	1	kept
crise	crise	crise	crise	crise	1	kept
Crise covid-19	crise covid 19	crise covid 19	crise covid 19	Crise covid-19	1	kept
crise sanitaire	crise sanitaire	crise sanitaire	crise sanitaire	crise sanitaire	1	kept
crises	crises	crises	crise	crise	1	singular
Crisis alarms	crisis alarms	crisis alarms	crisis alarms	Crisis alarms	1	kept
crisis care conditions	crisis care conditions	crisis care conditions	crisis care conditions	crisis care conditions	1	kept
crisis checklist	crisis checklist	crisis checklist	crisis checklist	Crisis checklists	1	case_or_format
crisis conditions	crisis conditions	crisis conditions	crisis conditions	crisis conditions	1	kept
Crisis driven innovation	crisis driven innovation	crisis driven innovation	crisis driven innovation	Crisis driven innovation	1	kept
Crisis management	crisis management	crisis management	crisis management	crisis management	1	case_or_format
crisis preparedness	crisis preparedness	crisis preparedness	crisis preparedness	crisis preparedness	1	kept
Crisis resolution	crisis resolution	crisis resolution	crisis resolution	Crisis resolution	1	kept
CRISIS RESOURCE-MANAGEMENT	crisis resource management	crisis resource management	crisis resource management	Crisis resource management	1	case_or_format
Crisis response	crisis response	crisis response	crisis response	Crisis response	1	kept
crisis situation	crisis situation	crisis situation	crisis situation	crisis situation	1	kept

Crisis standards of care	crisis standards of care	crisis standards of care	crisis standards of care	Crisis standards of care	1	kept
crisis standards of care	crisis standards of care	crisis standards of care	crisis standards of care	Crisis standards of care	1	case_or_format
Criteria	criteria	criteria	criteria	CRITERIA	1	case_or_format
Criterion validity	criterion validity	criterion validity	criterion validity	Criterion validity	1	kept
Criterion-related validity	criterion related validity	criterion related validity	criterion related validity	criterion related validity	1	case_or_format
critical access hospital	critical access hospital	critical access hospital	critical access hospital	critical access hospital	1	kept
Critical activities	critical activities	critical activities	critical activity	critical activity	1	singular
critical activity	critical activity	critical activity	critical activity	critical activity	1	kept
critical adults	critical adults	critical adults	critical adults	critical adults	1	kept
Critical airway occlusion	critical airway occlusion	critical airway occlusion	critical airway occlusion	Critical airway occlusion	1	kept
critical alarms	critical alarms	critical alarms	critical alarms	critical alarms	1	kept
Critical and emergency care	critical and emergency care	critical and emergency care	critical and emergency care	Critical and emergency care	1	kept
Critical and non-critical practice areas	critical and non critical practice areas	critical and non critical practice areas	critical and non critical practice areas	Critical and non-critical practice areas	1	kept
critical anemia	critical anemia	critical anemia	critical anemia	critical anemia	1	kept
Critical appraisal	critical appraisal	critical appraisal	critical appraisal	Critical appraisal	1	kept
Critical congenital heart defects	critical congenital heart defects	critical congenital heart defects	critical congenital heart defects	Critical congenital heart defects	1	kept

critical congenital heart disease	critical congenital heart disease	critical congenital heart disease	critical congenital heart disease	critical congenital heart disease	1	kept
Critical congenital heart disease screening	critical congenital heart disease screening	critical congenital heart disease screening	critical congenital heart disease screening	Critical congenital heart disease screening	1	kept
Critical deterioration	critical deterioration	critical deterioration	critical deterioration	Critical deterioration	1	kept
critical emergency management	critical emergency management	critical emergency management	critical emergency management	critical emergency management	1	kept
Critical event	critical event	critical event	critical event	Critical event	1	kept
critical event	critical event	critical event	critical event	Critical event	1	case_or_format
Critical events	critical events	critical events	critical event	Critical event	1	singular
Critical friend	critical friend	critical friend	critical friend	Critical friend	1	kept
critical heart disease	critical heart disease	critical heart disease	critical heart disease	critical heart disease	1	kept
Critical ill-ness	critical ill ness	critical ill ness	critical ill ness	Critical ill-ness	1	kept
critical illness education	critical illness education	critical illness education	critical illness education	critical illness education	1	kept
critical illness microbleeds	critical illness microbleeds	critical illness microbleeds	critical illness microbleeds	critical illness microbleeds	1	kept
Critical illness neuropathy	critical illness neuropathy	critical illness neuropathy	critical illness neuropathy	Critical illness neuropathy	1	kept
Critical illness polyneuromyopathy	critical illness polyneuromyopathy	critical illness polyneuromyopathy	critical illness polyneuromyopathy	Critical illness polyneuromyopathy	1	kept
Critical illness polyneuropathy	critical illness polyneuropathy	critical illness polyneuropathy	critical illness polyneuropathy	critical illness polyneuropathy	1	case_or_format
Critical illness recovery	critical illness recovery	critical illness recovery	critical illness recovery	Critical illness recovery	1	kept

critical illness survivors	critical illness survivors	critical illness survivors	critical illness survivors	critical illness survivors	1	kept
Critical illness: arterial catheterisation	critical illness arterial catheterisation	critical illness arterial catheterisation	critical illness arterial catheterisation	Critical illness: arterial catheterisation	1	kept
Critical illnesses	critical illnesses	critical illnesses	critical illnesses	Critical illnesses	1	kept
Critical Incident	critical incident	critical incident	critical incident	critical incidents	1	case_or_format
critical incident reporting	critical incident reporting	critical incident reporting	critical incident reporting	Critical incident reporting	1	case_or_format
Critical incident reporting system	critical incident reporting system	critical incident reporting system	critical incident reporting system	Critical incident reporting system	1	kept
critical incident reporting system	critical incident reporting system	critical incident reporting system	critical incident reporting system	Critical incident reporting system	1	case_or_format
Critical incident stress debriefing	critical incident stress debriefing	critical incident stress debriefing	critical incident stress debriefing	Critical incident stress debriefing	1	kept
critical incidents method	critical incidents method	critical incidents method	critical incidents method	critical incidents method	1	kept
Critical injury	critical injury	critical injury	critical injury	Critical injury	1	kept
critical intervention	critical intervention	critical intervention	critical intervention	critical intervention	1	kept
Critical Medicine	critical medicine	critical medicine	critical medicine	Critical Medicine	1	kept
Critical methods	critical methods	critical methods	critical methods	Critical methods	1	kept
critical methods	critical methods	critical methods	critical methods	Critical methods	1	case_or_format
Critical moment	critical moment	critical moment	critical moment	Critical moment	1	kept
critical neurological disease	critical neurological disease	critical neurological disease	critical neurological disease	critical neurological disease	1	kept

Critical newborn	critical newborn	critical newborn	critical newborn	Critical newborn	1	kept
Critical outcome	critical outcome	critical outcome	critical outcome	Critical outcome	1	kept
Critical outcomes	critical outcomes	critical outcomes	critical outcome	Critical outcome	1	singular
critical pathways	critical pathways	critical pathways	critical pathway	Critical Pathways	1	singular
critical points of care	critical points of care	critical points of care	critical points of care	critical points of care	1	kept
Critical practice guidelines	critical practice guidelines	critical practice guidelines	critical practice guidelines	Critical practice guidelines	1	kept
critical reflection	critical reflection	critical reflection	critical reflection	critical reflection	1	kept
CRITICAL REQUIREMENTS	critical requirements	critical requirements	critical requirements	CRITICAL REQUIREMENTS	1	kept
Critical respiratory care	critical respiratory care	critical respiratory care	critical respiratory care	Critical respiratory care	1	kept
Critical respiratory events	critical respiratory events	critical respiratory events	critical respiratory events	Critical respiratory events	1	kept
critical services	critical services	critical services	critical services	critical services	1	kept
Critical situation	critical situation	critical situation	critical situation	Critical situation	1	kept
critical situation	critical situation	critical situation	critical situation	Critical situation	1	case_or_format
critical skills scarcity	critical skills scarcity	critical skills scarcity	critical skills scarcity	critical skills scarcity	1	kept
Critical tasks	critical tasks	critical tasks	critical tasks	Critical tasks	1	kept
Critical technological environments	critical technological environments	critical technological environments	critical technological environments	Critical technological environments	1	kept

critical technological environments	critical technological environments	critical technological environments	critical technological environments	Critical technological environments	1	case_or_format
CRITICAL THINKING	critical thinking	critical thinking	critical thinking	critical thinking	1	case_or_format
critical thinking disposition	critical thinking disposition	critical thinking disposition	critical thinking disposition	critical thinking disposition	1	kept
CRITICAL THINKING DISPOSITIONS	critical thinking dispositions	critical thinking dispositions	critical thinking disposition	critical thinking disposition	1	singular
critical thinking skills	critical thinking skills	critical thinking skills	critical thinking skills	critical thinking skills	1	kept
Critical ultrasonography	critical ultrasonography	critical ultrasonography	critical ultrasonography	Critical ultrasonography	1	kept
Critical units	critical units	critical units	critical units	Critical units	1	kept
critical units	critical units	critical units	critical units	Critical units	1	case_or_format
critical vital signs	critical vital signs	critical vital signs	critical vital signs	critical vital signs	1	kept
critical ward	critical ward	critical ward	critical ward	critical ward	1	kept
Critical zones	critical zones	critical zones	critical zones	Critical zones	1	kept
Critical-incident technique	critical incident technique	critical incident technique	critical incident technique	critical incident technique	1	case_or_format
CriticalIllness	criticalillness	criticalillness	criticalillness	CriticalIllness	1	kept
Criticalities	criticalities	criticalities	criticality	Criticality	1	singular
Critically ill adults	critically ill adults	critically ill adults	critically ill adults	Critically ill adults	1	kept
critically ill animals	critically ill animals	critically ill animals	critically ill animals	critically ill animals	1	kept

critically ill aspiration pneumonia	critically ill aspiration pneumonia	critically ill aspiration pneumonia	critically ill aspiration pneumonia	critically ill aspiration pneumonia	1	kept
Critically ill child	critically ill child	critically ill child	critically ill child	Critically ill child	1	kept
critically ill child	critically ill child	critically ill child	critically ill child	Critically ill child	1	case_or_format
critically ill infants	critically ill infants	critically ill infants	critically ill infants	critically ill infants	1	kept
critically ill newborn	critically ill newborn	critically ill newborn	critically ill newborn	critically ill newborn	1	kept
Critically ill older adults	critically ill older adults	critically ill older adults	critically ill older adults	Critically ill older adults	1	kept
Critically Ill Person	critically ill person	critically ill person	critically ill person	Critically Ill Person	1	kept
Critically ill transport	critically ill transport	critically ill transport	critically ill transport	Critically ill transport	1	kept
Critically illness	critically illness	critically illness	critically illness	critically illness	1	case_or_format
critically-ill	critically ill	critically ill	critically ill	Critically ill	1	case_or_format
CRITICALLY-ILL ADULTS	critically ill adults	critically ill adults	critically ill adults	Critically ill adults	1	case_or_format
Critics	critics	critics	critics	Critics	1	kept
crizotinib	crizotinib	crizotinib	crizotinib	crizotinib	1	kept
CRN	crn	crn	crn	CRN	1	kept
CRNA leadership	crna leadership	crna leadership	crna leadership	CRNA leadership	1	kept
CRNA organizational climate	crna organizational climate	crna organizational climate	crna organizational climate	CRNA organizational climate	1	kept

CRNFA	crnfa	crnfa	crnfa	CRNFA	1	kept
crocheted octopus	crocheted octopus	crocheted octopus	crocheted octopus	crocheted octopus	1	kept
Crohn's disease	crohn s disease	crohn s disease	crohn s disease	Crohn's disease	1	kept
Crohn’ s disease	crohn s disease	crohn s disease	crohn s disease	Crohn's disease	1	case_or_format
croissance post-traumatique	croissance post traumatique	croissance post traumatique	croissance post traumatique	croissance post-traumatique	1	kept
Cronbach's alpha	cronbach s alpha	cronbach s alpha	cronbach s alpha	Cronbach's alpha	1	kept
Cronbach’ s alpha	cronbach s alpha	cronbach s alpha	cronbach s alpha	Cronbach's alpha	1	case_or_format
CROs	cros	cros	cros	CROs	1	kept
cross allergy	cross allergy	cross allergy	cross allergy	cross allergy	1	kept
Cross Infection [etiology]	cross infection etiology	cross infection etiology	cross infection etiology	Cross Infection [etiology]	1	kept
cross linking	cross linking	cross linking	cross linking	cross linking	1	kept
Cross Protection	cross protection	cross protection	cross protection	Cross Protection	1	kept
cross protection	cross protection	cross protection	cross protection	Cross Protection	1	case_or_format
Cross Reactions	cross reactions	cross reactions	cross reaction	cross reaction	1	singular
Cross sectional	cross sectional	cross sectional	cross sectional	Cross-sectional	1	case_or_format
Cross sectional study	cross sectional study	cross sectional study	cross sectional study	Cross-Sectional Studies	1	case_or_format

cross site working	cross site working	cross site working	cross site working	cross site working	1	kept
Cross transmission	cross transmission	cross transmission	cross transmission	Cross-transmission	1	case_or_format
Cross-contamination	cross contamination	cross contamination	cross contamination	Cross contamination	1	case_or_format
cross-country comparison	cross country comparison	cross country comparison	cross country comparison	cross-country comparison	1	kept
cross-cultural	cross cultural	cross cultural	cross cultural	cross-cultural	1	kept
Cross-cultural interaction	cross cultural interaction	cross cultural interaction	cross cultural interaction	Cross-cultural interaction	1	kept
Cross-cultural issues	cross cultural issues	cross cultural issues	cross cultural issues	Cross-cultural issues	1	kept
cross-cultural validity	cross cultural validity	cross cultural validity	cross cultural validity	cross-cultural validity	1	kept
Cross-functional teams	cross functional teams	cross functional teams	cross functional teams	Cross-functional teams	1	kept
cross-hospital contamination	cross hospital contamination	cross hospital contamination	cross hospital contamination	cross-hospital contamination	1	kept
Cross-lagged model	cross lagged model	cross lagged model	cross lagged model	Cross-lagged model	1	kept
Cross-matching	cross matching	cross matching	cross matching	Cross-matching	1	kept
cross-national	cross national	cross national	cross national	cross-national	1	kept
cross-protection	cross protection	cross protection	cross protection	Cross Protection	1	case_or_format
Cross-Sectional	cross sectional	cross sectional	cross sectional	Cross-sectional	1	case_or_format
CROSS-SECTIONAL ANALYSIS	cross sectional analysis	cross sectional analysis	cross sectional analysis	cross-sectional analysis	1	case_or_format

Cross-sectional design	cross sectional design	cross sectional design	cross sectional design	Cross-sectional design	1	kept
Cross-sectional Studies	cross sectional studies	cross sectional studies	cross sectional study	Cross-Sectional Studies	1	singular
cross-sectional studies.	cross sectional studies	cross sectional studies	cross sectional study	Cross-Sectional Studies	1	singular
Cross-sectional Study	cross sectional study	cross sectional study	cross sectional study	Cross-Sectional Studies	1	case_or_format
CROSS-SECTIONAL SURVEY	cross sectional survey	cross sectional survey	cross sectional survey	cross-sectional survey	1	case_or_format
Cross-Sectional Survey	cross sectional survey	cross sectional survey	cross sectional survey	cross-sectional survey	1	case_or_format
cross-sector collaboration	cross sector collaboration	cross sector collaboration	cross sector collaboration	cross-sector collaboration	1	kept
Cross-sectoral collaboration	cross sectoral collaboration	cross sectoral collaboration	cross sectoral collaboration	Cross-sectoral collaboration	1	kept
cross-temporal meta-analysis	cross temporal meta analysis	cross temporal meta analysis	cross temporal meta analysis	cross-temporal meta-analysis	1	kept
cross-transmission	cross transmission	cross transmission	cross transmission	Cross-transmission	1	case_or_format
CROSS-VALIDATION	cross validation	cross validation	cross validation	cross validation	1	case_or_format
Crossing traditional boundaries	crossing traditional boundaries	crossing traditional boundaries	crossing traditional boundaries	Crossing traditional boundaries	1	kept
crossover study	crossover study	crossover study	crossover study	crossover study	1	kept
cross‐cultural validation	cross cultural validation	cross cultural validation	cross cultural validation	cross‐cultural validation	1	kept
Cross‐sectional study	cross sectional study	cross sectional study	cross sectional study	Cross-Sectional Studies	1	case_or_format
Cross-sectional studies	cross sectional studies	cross sectional studies	cross sectional study	Cross-Sectional Studies	1	singular

cross-sectional study	cross sectional study	cross sectional study	cross sectional study	Cross-Sectional Studies	1	case_or_format
Cross-sectional study	cross sectional study	cross sectional study	cross sectional study	Cross-Sectional Studies	1	case_or_format
Crowns	crowns	crowns	crowns	Crowns	1	kept
CRP, C-reactive protein	crp c reactive protein	crp c reactive protein	crp c reactive protein	CRP, C-reactive protein	1	kept
CRRT circuit	crrt circuit	crrt circuit	crrt circuit	CRRT circuit	1	kept
CRRT device	crrt device	crrt device	crrt device	CRRT device	1	kept
CRS	crs	crs	crs	CRS	1	kept
CRS-R	crs r	crs r	crs r	CRS-R	1	kept
crush injury	crush injury	crush injury	crush injury	Crush Injuries	1	case_or_format
Crush Syndrome	crush syndrome	crush syndrome	crush syndrome	Crush Syndrome	1	kept
crush syndrome	crush syndrome	crush syndrome	crush syndrome	Crush Syndrome	1	case_or_format
Crying baby	crying baby	crying baby	crying baby	Crying baby	1	kept
crying require increased oxygen administration	crying require increased oxygen administration	crying require increased oxygen administration	crying require increased oxygen administration	crying require increased oxygen administration	1	kept
crying,Newborn	crying newborn	crying newborn	crying newborn	crying,Newborn	1	kept
Cryoprecipitate	cryoprecipitate	cryoprecipitate	cryoprecipitate	cryoprecipitate	1	case_or_format
Cryosurgery	cryosurgery	cryosurgery	cryosurgery	Cryosurgery	1	kept

cryptococcal meningitis	cryptococcal meningitis	cryptococcal meningitis	cryptococcal meningitis	cryptococcal meningitis	1	kept
cryptococcosis	cryptococcosis	cryptococcosis	cryptococcosis	cryptococcosis	1	kept
cryptorchism	cryptorchism	cryptorchism	cryptorchism	cryptorchism	1	kept
Crystalloid	crystalloid	crystalloid	crystalloid	crystalloid	1	case_or_format
Crystalloid solutions	crystalloid solutions	crystalloid solutions	crystalloid solutions	Crystalloid Solutions	1	case_or_format
Crystalloids	crystalloids	crystalloids	crystalloid	crystalloid	1	singular
crystalluria	crystalluria	crystalluria	crystalluria	crystalluria	1	kept
CSANZ	csanz	csanz	csanz	CSANZ	1	kept
csf	csf	csf	csf	csf	1	kept
CSF DIVERSION	csf diversion	csf diversion	csf diversion	CSF DIVERSION	1	kept
CSF drainage	csf drainage	csf drainage	csf drainage	CSF drainage	1	kept
CSN6	csn6	csn6	csn6	CSN6	1	kept
CSSD management	cssd management	cssd management	cssd management	CSSD management	1	kept
CT Image	ct image	ct image	ct image	CT Image	1	kept
CTL019 chimeric antigen receptor	ctl019 chimeric antigen receptor	ctl019 chimeric antigen receptor	ctl019 chimeric antigen receptor	CTL019 chimeric antigen receptor	1	kept
CTraC	ctrac	ctrac	ctrac	CTraC	1	kept

CTRS	ctrs	ctrs	ctrs	CTRS	1	kept
CTX-M-3	ctx m 3	ctx m 3	ctx m 3	CTX-M-3	1	kept
Cubbin & Jackson scale	cubbin and jackson scale	cubbin and jackson scale	cubbin and jackson scale	Cubbin & Jackson scale	1	kept
Cucumis	cucumis	cucumis	cucumis	Cucumis	1	kept
Cucurbitaceae	cucurbitaceae	cucurbitaceae	cucurbitaceae	Cucurbitaceae	1	kept
cucurbitacin	cucurbitacin	cucurbitacin	cucurbitacin	cucurbitacin	1	kept
Cucurbitacins	cucurbitacins	cucurbitacins	cucurbitacin	cucurbitacin	1	singular
cuddle	cuddle	cuddle	cuddle	cuddle	1	kept
cue based feeding	cue based feeding	cue based feeding	cue based feeding	Cue-based feeding	1	case_or_format
Cue utilisation	cue utilisation	cue utilisation	cue utilisation	Cue utilisation	1	kept
cue utilization	cue utilization	cue utilization	cue utilization	cue utilization	1	kept
cue-based	cue based	cue based	cue based	cue-based	1	kept
cue-based feeding	cue based feeding	cue based feeding	cue based feeding	Cue-based feeding	1	case_or_format
cue-based feedings	cue based feedings	cue based feedings	cue based feeding	Cue-based feeding	1	singular
Cuff	cuff	cuff	cuff	Cuff	1	kept
cuff	cuff	cuff	cuff	Cuff	1	case_or_format

cuff diameter	cuff diameter	cuff diameter	cuff diameter	cuff diameter	1	kept
Cuff Pressure	cuff pressure	cuff pressure	cuff pressure	Cuff pressure	1	case_or_format
Cuff pressure management	cuff pressure management	cuff pressure management	cuff pressure management	cuff pressure management	1	case_or_format
Cuff pressure monitoring	cuff pressure monitoring	cuff pressure monitoring	cuff pressure monitoring	cuff pressure monitoring	1	case_or_format
Cuffed endotracheal tube	cuffed endotracheal tube	cuffed endotracheal tube	cuffed endotracheal tube	Cuffed endotracheal tube	1	kept
Cuffed tracheostomy tube	cuffed tracheostomy tube	cuffed tracheostomy tube	cuffed tracheostomy tube	Cuffed tracheostomy tube	1	kept
Cuidado de Enfermagem	cuidado de enfermagem	cuidado de enfermagem	cuidado de enfermagem	Cuidado de Enfermagem	1	kept
Cuidado de transición	cuidado de transici n	cuidado de transici n	cuidado de transici n	Cuidado de transición	1	kept
Cuidado del Niño	cuidado del ni o	cuidado del ni o	cuidado del ni o	Cuidado del Niño	1	kept
Cuidado familiar	cuidado familiar	cuidado familiar	cuidado familiar	Cuidado familiar	1	kept
Cuidado Intensivo Neonatal	cuidado intensivo neonatal	cuidado intensivo neonatal	cuidado intensivo neonatal	Cuidado Intensivo Neonatal	1	kept
Cuidado neonatal	cuidado neonatal	cuidado neonatal	cuidado neonatal	Cuidado neonatal	1	kept
Cuidados agudos	cuidados agudos	cuidados agudos	cuidados agudos	Cuidados agudos	1	kept
Cuidados intensivos enfermería	cuidados intensivos enfermer a	cuidados intensivos enfermer a	cuidados intensivos enfermer a	Cuidados intensivos enfermería	1	kept
Cuidados intensivos neonatales	cuidados intensivos neonatales	cuidados intensivos neonatales	cuidados intensivos neonatales	Cuidados intensivos neonatales	1	kept
Cuidados intensivos postoperatorios	cuidados intensivos postoperatorios	cuidados intensivos postoperatorios	cuidados intensivos postoperatorios	Cuidados intensivos postoperatorios	1	kept

Cuidados terminales	cuidados terminales	cuidados terminales	cuidados terminales	Cuidados terminales	1	kept
Culex tritaeniorhynchus	culex tritaeniorhynchus	culex tritaeniorhynchus	culex tritaeniorhynchus	Culex tritaeniorhynchus	1	kept
Cultura de seguridad	cultura de seguridad	cultura de seguridad	cultura de seguridad	Cultura de seguridad	1	kept
Cultura organizacional	cultura organizacional	cultura organizacional	cultura organizacional	Cultura organizacional	1	kept
Cultural adaption	cultural adaption	cultural adaption	cultural adaption	Cultural adaption	1	kept
cultural and language barriers	cultural and language barriers	cultural and language barriers	cultural and language barriers	cultural and language barriers	1	kept
Cultural and organisational influences	cultural and organisational influences	cultural and organisational influences	cultural and organisational influences	Cultural and organisational influences	1	kept
Cultural and spiritual comfort	cultural and spiritual comfort	cultural and spiritual comfort	cultural and spiritual comfort	Cultural and spiritual comfort	1	kept
Cultural and spiritual needs	cultural and spiritual needs	cultural and spiritual needs	cultural and spiritual needs	Cultural and spiritual needs	1	kept
cultural awareness	cultural awareness	cultural awareness	cultural awareness	cultural awareness	1	kept
cultural barriers	cultural barriers	cultural barriers	cultural barriers	cultural barriers	1	kept
cultural change	cultural change	cultural change	cultural change	cultural change	1	kept
Cultural determinants	cultural determinants	cultural determinants	cultural determinants	Cultural determinants	1	kept
Cultural differences	cultural differences	cultural differences	cultural differences	Cultural differences	1	kept
cultural factors	cultural factors	cultural factors	cultural factor	cultural factor	1	singular
Cultural humility	cultural humility	cultural humility	cultural humility	Cultural humility	1	kept

cultural humility	cultural humility	cultural humility	cultural humility	Cultural humility	1	case_or_format
Cultural issue	cultural issue	cultural issue	cultural issue	cultural issues	1	case_or_format
cultural norms	cultural norms	cultural norms	cultural norms	cultural norms	1	kept
cultural practices	cultural practices	cultural practices	cultural practices	cultural practices	1	kept
Cultural psychology and prenatal experiences	cultural psychology and prenatal experiences	cultural psychology and prenatal experiences	cultural psychology and prenatal experiences	Cultural psychology and prenatal experiences	1	kept
cultural tradition	cultural tradition	cultural tradition	cultural tradition	cultural tradition	1	kept
Cultural values	cultural values	cultural values	cultural value	cultural value	1	singular
culturalehistorical activity theory	culturalehistorical activity theory	culturalehistorical activity theory	culturalehistorical activity theory	culturalehistorical activity theory	1	kept
Culturally and linguistically diverse	culturally and linguistically diverse	culturally and linguistically diverse	culturally and linguistically diverse	Culturally and linguistically diverse	1	kept
culturally and linguistically diverse	culturally and linguistically diverse	culturally and linguistically diverse	culturally and linguistically diverse	Culturally and linguistically diverse	1	case_or_format
Culturally competent care	culturally competent care	culturally competent care	culturally competent care	Culturally Competent Care	1	case_or_format
Culturally diverse	culturally diverse	culturally diverse	culturally diverse	Culturally diverse	1	kept
Culturally sensitive care	culturally sensitive care	culturally sensitive care	culturally sensitive care	Culturally sensitive care	1	kept
culturally-appropriate care	culturally appropriate care	culturally appropriate care	culturally appropriate care	culturally-appropriate care	1	kept
Culture and religion	culture and religion	culture and religion	culture and religion	Culture and religion	1	kept
Culture and sensitivity testing	culture and sensitivity testing	culture and sensitivity testing	culture and sensitivity testing	Culture and sensitivity testing	1	kept

Culture change	culture change	culture change	culture change	Culture change	1	kept
culture change	culture change	culture change	culture change	Culture change	1	case_or_format
culture contamination	culture contamination	culture contamination	culture contamination	culture contamination	1	kept
culture de sécurité	culture de s curit	culture de s curit	culture de s curit	culture de sécurité	1	kept
Culture Media	culture media	culture media	culture media	Culture Media	1	kept
Culture negative sepsis	culture negative sepsis	culture negative sepsis	culture negative sepsis	Culture negative sepsis	1	kept
culture of	culture of	culture of	culture of	culture of	1	kept
culture of health care	culture of health care	culture of health care	culture of health care	culture of health care	1	kept
culture shift	culture shift	culture shift	culture shift	culture shift	1	kept
Culture shock	culture shock	culture shock	culture shock	Culture shock	1	kept
Culture Techniques	culture techniques	culture techniques	culture technique	culture technique	1	singular
Culture techniques	culture techniques	culture techniques	culture technique	culture technique	1	singular
culture-specific palliative care	culture specific palliative care	culture specific palliative care	culture specific palliative care	culture-specific palliative care	1	kept
CULTURES	cultures	cultures	culture	Culture	1	singular
Cultures	cultures	cultures	culture	Culture	1	singular
Cumulative incidence	cumulative incidence	cumulative incidence	cumulative incidence	cumulative incidence	1	case_or_format

Cumulative risk	cumulative risk	cumulative risk	cumulative risk	Cumulative risk	1	kept
cumulative stress	cumulative stress	cumulative stress	cumulative stress	cumulative stress	1	kept
Cumulative Trauma Disorders	cumulative trauma disorders	cumulative trauma disorders	cumulative trauma disorders	cumulative trauma disorders	1	case_or_format
Cunninghamella bertholletiae	cunninghamella bertholletiae	cunninghamella bertholletiae	cunninghamella bertholletiae	Cunninghamella bertholletiae	1	kept
curare	curare	curare	curare	curare	1	kept
Curative	curative	curative	curative	Curative	1	kept
Curative care beds	curative care beds	curative care beds	curative care beds	Curative care beds	1	kept
CURB 65	curb 65	curb 65	curb 65	CURB-65	1	case_or_format
CURB 65 Score	curb 65 score	curb 65 score	curb 65 score	CURB-65 score	1	case_or_format
CURB score	curb score	curb score	curb score	CURB score	1	kept
curettage	curettage	curettage	curettage	curettage	1	kept
currant jelly stool	currant jelly stool	currant jelly stool	currant jelly stool	currant jelly stool	1	kept
CURRENT KNOWLEDGE	current knowledge	current knowledge	current knowledge	CURRENT KNOWLEDGE	1	kept
Current practice	current practice	current practice	current practice	Current practices	1	case_or_format
Current Procedural Terminology code	current procedural terminology code	current procedural terminology code	current procedural terminology code	Current Procedural Terminology code	1	kept
Current situation	current situation	current situation	current situation	Current situation	1	kept

Current situation investigation	current situation investigation	current situation investigation	current situation investigation	Current situation investigation	1	kept
current situation investigation	current situation investigation	current situation investigation	current situation investigation	Current situation investigation	1	case_or_format
Current status	current status	current status	current status	Current status	1	kept
Current status survey	current status survey	current status survey	current status survey	Current status survey	1	kept
Custodial Care	custodial care	custodial care	custodial care	custodial care	1	case_or_format
custodial unit	custodial unit	custodial unit	custodial unit	custodial unit	1	kept
CUSTOMER SATISFACTION	customer satisfaction	customer satisfaction	customer satisfaction	CUSTOMER SATISFACTION	1	kept
customization	customization	customization	customization	customization	1	kept
CUSUM	cusum	cusum	cusum	CUSUM	1	kept
cutaneous candidiasis	cutaneous candidiasis	cutaneous candidiasis	cutaneous candidiasis	cutaneous candidiasis	1	kept
cutaneous drug administration	cutaneous drug administration	cutaneous drug administration	cutaneous drug administration	cutaneous drug administration	1	kept
Cutaneous Fistula	cutaneous fistula	cutaneous fistula	cutaneous fistula	Cutaneous Fistula	1	kept
Cutaneous hyperpigmentation	cutaneous hyperpigmentation	cutaneous hyperpigmentation	cutaneous hyperpigmentation	Cutaneous hyperpigmentation	1	kept
Cutaneous necrosis	cutaneous necrosis	cutaneous necrosis	cutaneous necrosis	Cutaneous necrosis	1	kept
cutaneous parameters	cutaneous parameters	cutaneous parameters	cutaneous parameters	cutaneous parameters	1	kept
cutdown technique	cutdown technique	cutdown technique	cutdown technique	cutdown technique	1	kept

cutting corners	cutting corners	cutting corners	cutting corners	cutting corners	1	kept
CVAT, Central Venous Access Team	cvat central venous access team	cvat central venous access team	cvat central venous access team	CVAT, Central Venous Access Team	1	kept
CVC bundles	cvc bundles	cvc bundles	cvc bundles	CVC bundles	1	kept
CVC daily management	cvc daily management	cvc daily management	cvc daily management	CVC daily management	1	kept
CVC placement	cvc placement	cvc placement	cvc placement	CVC placement	1	kept
CVC, Central venous catheter	cvc central venous catheter	cvc central venous catheter	cvc central venous catheter	CVC, Central venous catheter	1	kept
CVI	cvi	cvi	cvi	CVI	1	kept
CVP	cvp	cvp	cvp	CVP	1	kept
CVS (chorionic villous sampling	cvs chorionic villous sampling	cvs chorionic villous sampling	cvs chorionic villous sampling	CVS (chorionic villous sampling	1	kept
CVVHD	cvvhd	cvvhd	cvvhd	CVVHD	1	kept
CVVHDF	cvvhdf	cvvhdf	cvvhdf	CVVHDF	1	kept
CXCL14 chemokine	cxcl14 chemokine	cxcl14 chemokine	cxcl14 chemokine	CXCL14 chemokine	1	kept
CXR	cxr	cxr	cxr	CXR	1	kept
CXR, chest radiograph	cxr chest radiograph	cxr chest radiograph	cxr chest radiograph	CXR, chest radiograph	1	kept
cyanide poisoning	cyanide poisoning	cyanide poisoning	cyanide poisoning	cyanide poisoning	1	kept
Cyanides	cyanides	cyanides	cyanide	cyanide	1	singular

cyanoacrylic acid octyl ester	cyanoacrylic acid octyl ester	cyanoacrylic acid octyl ester	cyanoacrylic acid octyl ester	cyanoacrylic acid octyl ester	1	kept
cyanocobalamin deficiency	cyanocobalamin deficiency	cyanocobalamin deficiency	cyanocobalamin deficiency	cyanocobalamin deficiency	1	kept
Cyber physicals	cyber physicals	cyber physicals	cyber physicals	Cyber physicals	1	kept
cyberattack	cyberattack	cyberattack	cyberattack	cyberattack	1	kept
Cybernetics	cybernetics	cybernetics	cybernetics	Cybernetics	1	kept
cybersickness	cybersickness	cybersickness	cybersickness	cybersickness	1	kept
cyborgs	cyborgs	cyborgs	cyborgs	cyborgs	1	kept
Cycle ergometry	cycle ergometry	cycle ergometry	cycle ergometry	Cycle ergometry	1	kept
cycline	cycline	cycline	cycline	cycline	1	kept
Cycling	cycling	cycling	cycling	cycling	1	case_or_format
cyclizine	cyclizine	cyclizine	cyclizine	cyclizine	1	kept
cyclobenzaprine	cyclobenzaprine	cyclobenzaprine	cyclobenzaprine	cyclobenzaprine	1	kept
cyclohexanecarboxylic acid derivative	cyclohexanecarboxylic acid derivative	cyclohexanecarboxylic acid derivative	cyclohexanecarboxylic acid derivative	cyclohexanecarboxylic acid derivative	1	kept
Cyclooxygenase Inhibitors	cyclooxygenase inhibitors	cyclooxygenase inhibitors	cyclooxygenase inhibitors	Cyclooxygenase Inhibitors	1	kept
cyclopentolate	cyclopentolate	cyclopentolate	cyclopentolate	cyclopentolate	1	kept
cyclopentolate plus phenylephrine	cyclopentolate plus phenylephrine	cyclopentolate plus phenylephrine	cyclopentolate plus phenylephrine	cyclopentolate plus phenylephrine	1	kept

Cyclophosphamide	cyclophosphamide	cyclophosphamide	cyclophosphamide	cyclophosphamide	1	case_or_format
cycloplegic agent	cycloplegic agent	cycloplegic agent	cycloplegic agent	cycloplegic agent	1	kept
cyclopropane	cyclopropane	cyclopropane	cyclopropane	cyclopropane	1	kept
Cyclosporine	cyclosporine	cyclosporine	cyclosporine	cyclosporine	1	case_or_format
cyproheptadine	cyproheptadine	cyproheptadine	cyproheptadine	cyproheptadine	1	kept
Cystatin C	cystatin c	cystatin c	cystatin c	cystatin C	1	case_or_format
Cysteine Endopeptidases	cysteine endopeptidases	cysteine endopeptidases	cysteine endopeptidases	Cysteine Endopeptidases	1	kept
Cystic Fibrosis Transmembrane Conductance Regulator	cystic fibrosis transmembrane conductance regulator	cystic fibrosis transmembrane conductance regulator	cystic fibrosis transmembrane conductance regulator	Cystic Fibrosis Transmembrane Conductance Regulator	1	kept
CYSTIC-FIBROSIS	cystic fibrosis	cystic fibrosis	cystic fibrosis	cystic fibrosis	1	case_or_format
Cystoscopy	cystoscopy	cystoscopy	cystoscopy	Cystoscopy	1	kept
cystoscopy	cystoscopy	cystoscopy	cystoscopy	Cystoscopy	1	case_or_format
cystourethrography	cystourethrography	cystourethrography	cystourethrography	cystourethrography	1	kept
cytapheresis	cytapheresis	cytapheresis	cytapheresis	cytapheresis	1	kept
Cytidine	cytidine	cytidine	cytidine	Cytidine	1	kept
Cytochrome P-450 CYP2C9	cytochrome p 450 cyp2c9	cytochrome p 450 cyp2c9	cytochrome p 450 cyp2c9	Cytochrome P-450 CYP2C9	1	kept
Cytochrome P-450 CYP3A	cytochrome p 450 cyp3a	cytochrome p 450 cyp3a	cytochrome p 450 cyp3a	Cytochrome P-450 CYP3A	1	kept

Cytochrome P-450 Enzyme Inhibitors	cytochrome p 450 enzyme inhibitors	cytochrome p 450 enzyme inhibitors	cytochrome p 450 enzyme inhibitors	Cytochrome P-450 Enzyme Inhibitors	1	kept
cytokeratin	cytokeratin	cytokeratin	cytokeratin	cytokeratin	1	kept
Cytokine	cytokine	cytokine	cytokine	Cytokines	1	case_or_format
cytokine receptor antagonist	cytokine receptor antagonist	cytokine receptor antagonist	cytokine receptor antagonist	cytokine receptor antagonist	1	kept
Cytokine Release Syndrome	cytokine release syndrome	cytokine release syndrome	cytokine release syndrome	cytokine release syndrome	1	case_or_format
Cytokine storm	cytokine storm	cytokine storm	cytokine storm	cytokine storm	1	case_or_format
cytokinerelease syndrome	cytokinerelease syndrome	cytokinerelease syndrome	cytokinerelease syndrome	cytokinerelease syndrome	1	kept
CYTOKINES	cytokines	cytokines	cytokine	Cytokines	1	singular
cytomegalovirus	cytomegalovirus	cytomegalovirus	cytomegalovirus	Cytomegalovirus	1	case_or_format
cytoreduction	cytoreduction	cytoreduction	cytoreduction	cytoreduction	1	kept
CYTOREDUCTIVE SURGERY	cytoreductive surgery	cytoreductive surgery	cytoreductive surgery	cytoreductive surgery	1	case_or_format
CytoSorb	cytosorb	cytosorb	cytosorb	CytoSorb	1	kept
Cytotastic drug	cytotastic drug	cytotastic drug	cytotastic drug	Cytotastic drug	1	kept
cytotoxic lymphocyte	cytotoxic lymphocyte	cytotoxic lymphocyte	cytotoxic lymphocyte	cytotoxic lymphocyte	1	kept
C-C motif chemokine ligand 14	c c motif chemokine ligand 14	c c motif chemokine ligand 14	c c motif chemokine ligand 14	C-C motif chemokine ligand 14	1	kept
D1, day 1	d1 day 1	d1 day 1	d1 day 1	D1, day 1	1	kept

D2B door-to-balloon	d2b door to balloon	d2b door to balloon	d2b door to balloon	D2B door-to-balloon	1	kept
D2D	d2d	d2d	d2d	D2D	1	kept
D2D communications	d2d communications	d2d communications	d2d communications	D2D communications	1	kept
D3 lymph node dissection	d3 lymph node dissection	d3 lymph node dissection	d3 lymph node dissection	D3 lymph node dissection	1	kept
Da Vinci robot	da vinci robot	da vinci robot	da vinci robot	Da Vinci robot	1	kept
Da vinci robot	da vinci robot	da vinci robot	da vinci robot	Da Vinci robot	1	case_or_format
dabrafenib	dabrafenib	dabrafenib	dabrafenib	dabrafenib	1	kept
Dacarbazine	dacarbazine	dacarbazine	dacarbazine	Dacarbazine	1	kept
dacarbazine	dacarbazine	dacarbazine	dacarbazine	Dacarbazine	1	case_or_format
Daily activities	daily activities	daily activities	daily activity	Daily activity	1	singular
daily activities of living	daily activities of living	daily activities of living	daily activities of living	daily activities of living	1	kept
Daily activity	daily activity	daily activity	daily activity	Daily activity	1	kept
daily awakening	daily awakening	daily awakening	daily awakening	daily awakening	1	kept
Daily checklist	daily checklist	daily checklist	daily checklist	Daily checklist	1	kept
daily goal sheet	daily goal sheet	daily goal sheet	daily goal sheet	daily goal sheet	1	kept
Daily goals	daily goals	daily goals	daily goals	DAILY GOALS	1	case_or_format

daily goals sheet	daily goals sheet	daily goals sheet	daily goals sheet	daily goals sheet	1	kept
daily interruption	daily interruption	daily interruption	daily interruption	Daily interruption	1	case_or_format
Daily interruption of sedation	daily interruption of sedation	daily interruption of sedation	daily interruption of sedation	daily interruption of sedation	1	case_or_format
daily life	daily life	daily life	daily life	daily life	1	kept
DAILY MEATAL CARE	daily meatal care	daily meatal care	daily meatal care	DAILY MEATAL CARE	1	kept
DAILY MULTIDISCIPLINARY ROUNDS	daily multidisciplinary rounds	daily multidisciplinary rounds	daily multidisciplinary rounds	DAILY MULTIDISCIPLINARY ROUNDS	1	kept
Daily routine Saudi Arabia	daily routine saudi arabia	daily routine saudi arabia	daily routine saudi arabia	Daily routine Saudi Arabia	1	kept
Daily sedation interruption	daily sedation interruption	daily sedation interruption	daily sedation interruption	Daily sedation interruption	1	kept
daily sedation interruption	daily sedation interruption	daily sedation interruption	daily sedation interruption	Daily sedation interruption	1	case_or_format
Daily sedation interruption and qualitative study	daily sedation interruption and qualitative study	daily sedation interruption and qualitative study	daily sedation interruption and qualitative study	Daily sedation interruption and qualitative study	1	kept
daily sedation interuption	daily sedation interuption	daily sedation interuption	daily sedation interuption	daily sedation interuption	1	kept
daily sedative interruption	daily sedative interruption	daily sedative interruption	daily sedative interruption	daily sedative interruption	1	kept
Daily tasks	daily tasks	daily tasks	daily tasks	Daily tasks	1	kept
Dairies	dairies	dairies	dairies	Dairies	1	kept
dairy cattle	dairy cattle	dairy cattle	dairy cattle	dairy cattle	1	kept
dalbavancin	dalbavancin	dalbavancin	dalbavancin	dalbavancin	1	kept

Dalteparin	dalteparin	dalteparin	dalteparin	dalteparin	1	case_or_format
Damage control	damage control	damage control	damage control	damage control	1	case_or_format
Damage control orthopaedic surgery	damage control orthopaedic surgery	damage control orthopaedic surgery	damage control orthopaedic surgery	Damage control orthopaedic surgery	1	kept
damage-associated molecular pattern-triggered immunity	damage associated molecular pattern triggered immunity	damage associated molecular pattern triggered immunity	damage associated molecular pattern triggered immunity	damage-associated molecular pattern-triggered immunity	1	kept
damage-control resuscitation	damage control resuscitation	damage control resuscitation	damage control resuscitation	Damage control resuscitation	1	case_or_format
danaparoid	danaparoid	danaparoid	danaparoid	danaparoid	1	kept
danazol	danazol	danazol	danazol	danazol	1	kept
Dance of death	dance of death	dance of death	dance of death	Dance of death	1	kept
Dandy Walker syndrome	dandy walker syndrome	dandy walker syndrome	dandy walker syndrome	Dandy Walker syndrome	1	kept
Dandy-Walker	dandy walker	dandy walker	dandy walker	Dandy-Walker	1	kept
Dandy-Walker Syndrome	dandy walker syndrome	dandy walker syndrome	dandy walker syndrome	Dandy Walker syndrome	1	case_or_format
dangerous goods	dangerous goods	dangerous goods	dangerous goods	dangerous goods	1	kept
Dangers of malnourishment	dangers of malnourishment	dangers of malnourishment	dangers of malnourishment	Dangers of malnourishment	1	kept
DANMAP	danmap	danmap	danmap	DANMAP	1	kept
Dantrolene	dantrolene	dantrolene	dantrolene	dantrolene	1	case_or_format
dantrolene sodium	dantrolene sodium	dantrolene sodium	dantrolene sodium	dantrolene sodium	1	kept

DAR-ES-SALAAM	dar es salaam	dar es salaam	dar es salaam	Dar es Salaam	1	case_or_format
daratumumab	daratumumab	daratumumab	daratumumab	daratumumab	1	kept
DARE, daytime activity ratio estimate	dare daytime activity ratio estimate	dare daytime activity ratio estimate	dare daytime activity ratio estimate	DARE, daytime activity ratio estimate	1	kept
Darkness	darkness	darkness	darkness	darkness	1	case_or_format
darunavir	darunavir	darunavir	darunavir	darunavir	1	kept
DAS28	das28	das28	das28	DAS28	1	kept
DASH	dash	dash	dash	DASH	1	kept
Dashboard system	dashboard system	dashboard system	dashboard system	Dashboard system	1	kept
DASS	dass	dass	dass	DASS	1	kept
DASS-21	dass 21	dass 21	dass 21	DASS 21	1	case_or_format
Data Aggregation	data aggregation	data aggregation	data aggregation	Data Aggregation	1	kept
data aggregation	data aggregation	data aggregation	data aggregation	Data Aggregation	1	case_or_format
Data Analytics	data analytics	data analytics	data analytics	Data analytics	1	case_or_format
Data capture system	data capture system	data capture system	data capture system	Data capture system	1	kept
data collection tool	data collection tool	data collection tool	data collection tool	data collection tool	1	kept
Data collections	data collections	data collections	data collection	Data Collection	1	singular

Data communication equipment	data communication equipment	data communication equipment	data communication equipment	Data communication equipment	1	kept
data correlation	data correlation	data correlation	data correlation	data correlation	1	kept
data dissemination	data dissemination	data dissemination	data dissemination	data dissemination	1	kept
Data documentation	data documentation	data documentation	data documentation	Data documentation	1	kept
Data driven	data driven	data driven	data driven	Data driven	1	kept
Data fusion	data fusion	data fusion	data fusion	Data fusion	1	kept
data infrastructure	data infrastructure	data infrastructure	data infrastructure	data infrastructure	1	kept
data interoperability	data interoperability	data interoperability	data interoperability	data interoperability	1	kept
Data linkage	data linkage	data linkage	data linkage	Data linkage	1	kept
Data Management	data management	data management	data management	Data management	1	case_or_format
DATA MANAGEMENT-SYSTEM	data management system	data management system	data management system	DATA MANAGEMENT-SYSTEM	1	kept
Data monitoring	data monitoring	data monitoring	data monitoring	Data monitoring	1	kept
data overload reduction	data overload reduction	data overload reduction	data overload reduction	data overload reduction	1	kept
data pooling	data pooling	data pooling	data pooling	data pooling	1	kept
Data preprocessing	data preprocessing	data preprocessing	data preprocessing	Data preprocessing	1	kept
data privacy	data privacy	data privacy	data privacy	data privacy	1	kept

data protection	data protection	data protection	data protection	data protection	1	kept
Data quality	data quality	data quality	data quality	data quality	1	case_or_format
Data retrieval	data retrieval	data retrieval	data retrieval	Data retrieval	1	kept
data retrieval	data retrieval	data retrieval	data retrieval	Data retrieval	1	case_or_format
DATA SET	data set	data set	data set	DATA SET	1	kept
data standards	data standards	data standards	data standards	data standards	1	kept
data stewardship	data stewardship	data stewardship	data stewardship	data stewardship	1	kept
data system	data system	data system	data system	data system	1	kept
data to text	data to text	data to text	data to text	data to text	1	kept
Data transfer	data transfer	data transfer	data transfer	Data transfer	1	kept
data validation	data validation	data validation	data validation	data validation	1	kept
data validity	data validity	data validity	data validity	data validity	1	kept
data warehouse	data warehouse	data warehouse	data warehouse	data warehouse	1	kept
data warehousing	data warehousing	data warehousing	data warehousing	data warehousing	1	kept
Data-driven algorithms	data driven algorithms	data driven algorithms	data driven algorithms	Data-driven algorithms	1	kept
Data-driven control	data driven control	data driven control	data driven control	Data-driven control	1	kept

Data-driven model	data driven model	data driven model	data driven model	Data-driven model	1	kept
Data-transmission	data transmission	data transmission	data transmission	Data-transmission	1	kept
Databanks	databanks	databanks	databanks	Databanks	1	kept
Database	database	database	database	database	1	case_or_format
Database management system	database management system	database management system	database management system	Database Management Systems	1	case_or_format
database management system	database management system	database management system	database management system	Database Management Systems	1	case_or_format
Database management systems	database management systems	database management systems	database management system	Database Management Systems	1	singular
DATABASES	databases	databases	database	database	1	singular
databases	databases	databases	database	database	1	singular
databases as topic	databases as topic	databases as topic	databases as topic	Databases as Topic	1	case_or_format
daunorubicin	daunorubicin	daunorubicin	daunorubicin	daunorubicin	1	kept
Davies-Bouldin index	davies bouldin index	davies bouldin index	davies bouldin index	Davies-Bouldin index	1	kept
day	day	day	day	day	1	kept
Day Care	day care	day care	day care	day care	1	case_or_format
Day care centers	day care centers	day care centers	day care centers	Day care centers	1	kept
day case	day case	day case	day case	day case	1	kept

Day clinic	day clinic	day clinic	day clinic	Day clinic	1	kept
Day hospital	day hospital	day hospital	day hospital	Day hospital	1	kept
day of death	day of death	day of death	day of death	day of death	1	kept
day shift	day shift	day shift	day shift	day shift	1	kept
day shift work	day shift work	day shift work	day shift work	day shift work	1	kept
Day surgery	day surgery	day surgery	day surgery	day surgery	1	case_or_format
Day-clinic	day clinic	day clinic	day clinic	Day clinic	1	case_or_format
Daylight	daylight	daylight	daylight	Daylight	1	kept
daylighting	daylighting	daylighting	daylighting	daylighting	1	kept
days at home	days at home	days at home	days at home	days at home	1	kept
daytime dysfunction	daytime dysfunction	daytime dysfunction	daytime dysfunction	daytime dysfunction	1	kept
Daytime rest	daytime rest	daytime rest	daytime rest	Daytime rest	1	kept
dCBTi	dcbti	dcbti	dcbti	dCBTi	1	kept
DCC, Data Coordinating Center	dcc data coordinating center	dcc data coordinating center	dcc data coordinating center	DCC, Data Coordinating Center	1	kept
DDD = degenerative disc disease	ddd degenerative disc disease	ddd degenerative disc disease	ddd degenerative disc disease	DDD = degenerative disc disease	1	kept
DDOS	ddos	ddos	ddos	DDOS	1	kept

de dépistage	de d pistage	de d pistage	de d pistage	de dépistage	1	kept
De-escalation	de escalation	de escalation	de escalation	DE-ESCALATION	1	case_or_format
De-escalation techniques	de escalation techniques	de escalation techniques	de escalation techniques	de-escalation techniques	1	case_or_format
DE-IMPLEMENTATION	de implementation	de implementation	de implementation	de-implementation	1	case_or_format
De-implementation	de implementation	de implementation	de implementation	de-implementation	1	case_or_format
de-motivation	de motivation	de motivation	de motivation	de-motivation	1	kept
DEA	dea	dea	dea	DEA	1	kept
Dead body	dead body	dead body	dead body	Dead body	1	kept
Deaf	deaf	deaf	deaf	Deaf	1	kept
Deafness	deafness	deafness	deafness	Deafness	1	kept
Deamino Arginine Vasopressin	deamino arginine vasopressin	deamino arginine vasopressin	deamino arginine vasopressin	Deamino Arginine Vasopressin	1	kept
death & dying	death and dying	death and dying	death and dying	death and dying	1	case_or_format
DEATH ANXIETY	death anxiety	death anxiety	death anxiety	death anxiety	1	case_or_format
death attitude	death attitude	death attitude	death attitude	death attitude	1	kept
Death attitudes	death attitudes	death attitudes	death attitude	death attitude	1	singular
death awareness	death awareness	death awareness	death awareness	death awareness	1	kept

Death Café	death caf	death caf	death caf	Death Café	1	kept
Death certificates	death certificates	death certificates	death certificate	death certificate	1	singular
Death competence	death competence	death competence	death competence	Death competence	1	kept
death coping ability	death coping ability	death coping ability	death coping ability	death coping ability	1	kept
Death coping self-efficacy	death coping self efficacy	death coping self efficacy	death coping self efficacy	Death coping self-efficacy	1	kept
death depression	death depression	death depression	death depression	death depression	1	kept
Death education	death education	death education	death education	death education	1	case_or_format
death in isolation	death in isolation	death in isolation	death in isolation	death in isolation	1	kept
Death notification	death notification	death notification	death notification	Death notification	1	kept
death notification	death notification	death notification	death notification	Death notification	1	case_or_format
death of a loved one	death of a loved one	death of a loved one	death of a loved one	death of a loved one	1	kept
Death rates	death rates	death rates	death rates	Death rates	1	kept
DEATH RATTLE	death rattle	death rattle	death rattle	death rattles	1	case_or_format
Death rattle	death rattle	death rattle	death rattle	death rattles	1	case_or_format
death receptor	death receptor	death receptor	death receptor	death receptor	1	kept
death reporting	death reporting	death reporting	death reporting	death reporting	1	kept

DEATH-SYNDROME	death syndrome	death syndrome	death syndrome	DEATH-SYNDROME	1	kept
Debaryomyces hansenii	debaryomyces hansenii	debaryomyces hansenii	debaryomyces hansenii	Debaryomyces hansenii	1	kept
Debilidad adquirida en la UCI	debilidad adquirida en la uci	debilidad adquirida en la uci	debilidad adquirida en la uci	Debilidad adquirida en la UCI	1	kept
debriefing models	debriefing models	debriefing models	debriefing models	debriefing models	1	kept
debt burden	debt burden	debt burden	debt burden	debt burden	1	kept
Decavac	decavac	decavac	decavac	Decavac	1	kept
deceased	deceased	deceased	deceased	deceased	1	kept
Deceased adults	deceased adults	deceased adults	deceased adults	Deceased adults	1	kept
deceased donation	deceased donation	deceased donation	deceased donation	deceased donation	1	kept
Deceased donors	deceased donors	deceased donors	deceased donor	deceased donor	1	singular
deceased organ donation	deceased organ donation	deceased organ donation	deceased organ donation	deceased organ donation	1	kept
deceased organ donor	deceased organ donor	deceased organ donor	deceased organ donor	deceased organ donor	1	kept
Deceleration	deceleration	deceleration	deceleration	Deceleration	1	kept
deceleration	deceleration	deceleration	deceleration	Deceleration	1	case_or_format
decentralized	decentralized	decentralized	decentralized	decentralized	1	kept
Decentralized management	decentralized management	decentralized management	decentralized management	Decentralized management	1	kept

decibel	decibel	decibel	decibel	decibels	1	case_or_format
Decision	decision	decision	decision	DECISIONS	1	case_or_format
decision	decision	decision	decision	DECISIONS	1	case_or_format
Decision aid	decision aid	decision aid	decision aid	decision aids	1	case_or_format
Decision aid tool	decision aid tool	decision aid tool	decision aid tool	Decision aid tool	1	kept
Decision aids	decision aids	decision aids	decision aid	decision aids	1	singular
decision AIDS	decision aids	decision aids	decision aid	decision aids	1	singular
Decision algorithm	decision algorithm	decision algorithm	decision algorithm	Decision algorithm	1	kept
decision analysis	decision analysis	decision analysis	decision analysis	Decision analysis	1	case_or_format
Decision coaching	decision coaching	decision coaching	decision coaching	Decision coaching	1	kept
decision control preferences	decision control preferences	decision control preferences	decision control preferences	decision control preferences	1	kept
decision curve analysis	decision curve analysis	decision curve analysis	decision curve analysis	decision curve analysis	1	kept
Decision fatigue	decision fatigue	decision fatigue	decision fatigue	Decision fatigue	1	kept
decision maker	decision maker	decision maker	decision maker	decision maker	1	kept
decision makers	decision makers	decision makers	decision maker	decision maker	1	singular
Decision making for end-of-life care	decision making for end of life care	decision making for end of life care	decision making for end of life care	Decision making for end-of-life care	1	kept

Decision Making: Organizational	decision making organizational	decision making organizational	decision making organizational	Decision Making, Organizational	1	case_or_format
decision model	decision model	decision model	decision model	decision model	1	kept
DECISION REGRET	decision regret	decision regret	decision regret	DECISION REGRET	1	kept
Decision regret	decision regret	decision regret	decision regret	DECISION REGRET	1	case_or_format
decision regret	decision regret	decision regret	decision regret	DECISION REGRET	1	case_or_format
Decision support systems, clinical	decision support systems clinical	decision support systems clinical	decision support systems clinical	Decision Support Systems, Clinical	1	case_or_format
decision support technique	decision support technique	decision support technique	decision support technique	Decision Support Techniques	1	case_or_format
decision support tool	decision support tool	decision support tool	decision support tool	Decision support tools	1	case_or_format
Decision support, clinical	decision support clinical	decision support clinical	decision support clinical	Decision support, clinical	1	kept
decision support, clinical	decision support clinical	decision support clinical	decision support clinical	Decision support, clinical	1	case_or_format
Decision support, computerized	decision support computerized	decision support computerized	decision support computerized	Decision support, computerized	1	kept
Decision Theory	decision theory	decision theory	decision theory	Decision theory	1	case_or_format
decision theory	decision theory	decision theory	decision theory	Decision theory	1	case_or_format
Decision tree	decision tree	decision tree	decision tree	Decision Trees	1	case_or_format
Decision-maker	decision maker	decision maker	decision maker	decision maker	1	case_or_format
Decision-Making	decision making	decision making	decision making	Decision Making	1	case_or_format

decision-making aid	decision making aid	decision making aid	decision making aid	decision-making aid	1	kept
DECISION-MAKING CAPACITY	decision making capacity	decision making capacity	decision making capacity	DECISION-MAKING CAPACITY	1	kept
decision-making capacity	decision making capacity	decision making capacity	decision making capacity	DECISION-MAKING CAPACITY	1	case_or_format
Decision-making climate	decision making climate	decision making climate	decision making climate	Decision-making climate	1	kept
decision-making processes	decision making processes	decision making processes	decision making processes	decision-making processes	1	kept
Decision-making skills	decision making skills	decision making skills	decision making skills	Decision-making skills	1	kept
decision-making skills	decision making skills	decision making skills	decision making skills	Decision-making skills	1	case_or_format
Decision-making strategies	decision making strategies	decision making strategies	decision making strategies	Decision-making strategies	1	kept
decision-making support	decision making support	decision making support	decision making support	decision-making support	1	kept
Decision-making systems	decision making systems	decision making systems	decision making systems	Decision-making systems	1	kept
Decision-making, shared	decision making shared	decision making shared	decision making shared	Decision Making, Shared	1	case_or_format
decision-support systems	decision support systems	decision support systems	decision support system	decision support system	1	singular
DECISION-SUPPORT-SYSTEM	decision support system	decision support system	decision support system	decision support system	1	case_or_format
decisional conflict	decisional conflict	decisional conflict	decisional conflict	Decisional conflict	1	case_or_format
decisional conflict scale score	decisional conflict scale score	decisional conflict scale score	decisional conflict scale score	decisional conflict scale score	1	kept
decisions	decisions	decisions	decision	DECISIONS	1	singular

Decisions makings	decisions makings	decisions makings	decisions makings	Decisions makings	1	kept
Decisions to foregolife sustaining treatment	decisions to foregolife sustaining treatment	decisions to foregolife sustaining treatment	decisions to foregolife sustaining treatment	Decisions to foregolife sustaining treatment	1	kept
decision-making and judgement	decision making and judgement	decision making and judgement	decision making and judgement	decision-making and judgement	1	kept
decision-making process	decision making process	decision making process	decision making process	Decision making process	1	case_or_format
decolonization	decolonization	decolonization	decolonization	Decolonization	1	case_or_format
decom-pensated heart failure	decom pensated heart failure	decom pensated heart failure	decom pensated heart failure	decom-pensated heart failure	1	kept
Decompensated	decompensated	decompensated	decompensated	Decompensated	1	kept
decompensation	decompensation	decompensation	decompensation	decompensation	1	kept
decomposition	decomposition	decomposition	decomposition	decomposition	1	kept
Decompressive laparotomy	decompressive laparotomy	decompressive laparotomy	decompressive laparotomy	decompressive laparotomy	1	case_or_format
Deconditioning	deconditioning	deconditioning	deconditioning	deconditioning	1	case_or_format
Decrease infections	decrease infections	decrease infections	decrease infections	Decrease infections	1	kept
Decrease risk of repeat preterm birth	decrease risk of repeat preterm birth	decrease risk of repeat preterm birth	decrease risk of repeat preterm birth	Decrease risk of repeat preterm birth	1	kept
Decreased consciousness	decreased consciousness	decreased consciousness	decreased consciousness	Decreased consciousness	1	kept
DECREASES COLLAPSIBILITY	decreases collapsibility	decreases collapsibility	decreases collapsibility	DECREASES COLLAPSIBILITY	1	kept
Decree of competences	decree of competences	decree of competences	decree of competences	Decree of competences	1	kept

DECUBITUS ULCERS	decubitus ulcers	decubitus ulcers	decubitus ulcer	Decubitus ulcer	1	singular
Decúbito prono	dec bito prono	dec bito prono	dec bito prono	Decúbito prono	1	kept
Dedicated education unit	dedicated education unit	dedicated education unit	dedicated education unit	Dedicated education unit	1	kept
Dedicated transport team	dedicated transport team	dedicated transport team	dedicated transport team	Dedicated transport team	1	kept
Deductive	deductive	deductive	deductive	Deductive	1	kept
Deductive analysis	deductive analysis	deductive analysis	deductive analysis	Deductive analysis	1	kept
Deemed consent	deemed consent	deemed consent	deemed consent	Deemed consent	1	kept
deemed consent	deemed consent	deemed consent	deemed consent	Deemed consent	1	case_or_format
Deep acting	deep acting	deep acting	deep acting	Deep acting	1	kept
deep brain stimulation	deep brain stimulation	deep brain stimulation	deep brain stimulation	Deep Brain Stimulation	1	case_or_format
deep breathing exercise	deep breathing exercise	deep breathing exercise	deep breathing exercise	deep breathing exercise	1	kept
Deep breathing relaxation exercise	deep breathing relaxation exercise	deep breathing relaxation exercise	deep breathing relaxation exercise	Deep breathing relaxation exercise	1	kept
deep breathing relaxation exercise	deep breathing relaxation exercise	deep breathing relaxation exercise	deep breathing relaxation exercise	Deep breathing relaxation exercise	1	case_or_format
deep breathing relaxation technique	deep breathing relaxation technique	deep breathing relaxation technique	deep breathing relaxation technique	deep breathing relaxation technique	1	kept
deep femoral artery	deep femoral artery	deep femoral artery	deep femoral artery	deep femoral artery	1	kept
deep hypothermic circulatory arrest	deep hypothermic circulatory arrest	deep hypothermic circulatory arrest	deep hypothermic circulatory arrest	deep hypothermic circulatory arrest	1	kept

deep inferior epigastric perforator flap	deep inferior epigastric perforator flap	deep inferior epigastric perforator flap	deep inferior epigastric perforator flap	deep inferior epigastric perforator flap	1	kept
Deep intronic mutation	deep intronic mutation	deep intronic mutation	deep intronic mutation	Deep intronic mutation	1	kept
deep learning neural network	deep learning neural network	deep learning neural network	deep learning neural network	deep learning neural network	1	kept
deep neural network	deep neural network	deep neural network	deep neural network	deep neural network	1	kept
DEEP SEDATION	deep sedation	deep sedation	deep sedation	Deep Sedation	1	case_or_format
deep surgical site infection	deep surgical site infection	deep surgical site infection	deep surgical site infection	deep surgical site infection	1	kept
Deep tissue injury	deep tissue injury	deep tissue injury	deep tissue injury	Deep tissue injury	1	kept
Deep tissue pressure injury	deep tissue pressure injury	deep tissue pressure injury	deep tissue pressure injury	Deep tissue pressure injury	1	kept
DEEP TISSUE-INJURY	deep tissue injury	deep tissue injury	deep tissue injury	Deep tissue injury	1	case_or_format
Deep tracheal suctioning	deep tracheal suctioning	deep tracheal suctioning	deep tracheal suctioning	Deep tracheal suctioning	1	kept
deep venous catheter infection	deep venous catheter infection	deep venous catheter infection	deep venous catheter infection	deep venous catheter infection	1	kept
deep-brain stimulation	deep brain stimulation	deep brain stimulation	deep brain stimulation	Deep Brain Stimulation	1	case_or_format
Deep-tissue injury	deep tissue injury	deep tissue injury	deep tissue injury	Deep tissue injury	1	case_or_format
deep-tissue pressure injuries	deep tissue pressure injuries	deep tissue pressure injuries	deep tissue pressure injury	Deep tissue pressure injury	1	singular
Deep-vein thrombosis	deep vein thrombosis	deep vein thrombosis	deep vein thrombosis	deep vein thrombosis	1	case_or_format
Default alarm settings	default alarm settings	default alarm settings	default alarm settings	Default alarm settings	1	kept

default alarm settings	default alarm settings	default alarm settings	default alarm settings	Default alarm settings	1	case_or_format
default mode network	default mode network	default mode network	default mode network	default mode network	1	kept
defecation disorder	defecation disorder	defecation disorder	defecation disorder	defecation disorder	1	kept
defecation habit	defecation habit	defecation habit	defecation habit	defecation habit	1	kept
defecation measurement	defecation measurement	defecation measurement	defecation measurement	defecation measurement	1	kept
Defensa del niño	defensa del ni o	defensa del ni o	defensa del ni o	Defensa del niño	1	kept
Defense mechanisms	defense mechanisms	defense mechanisms	defense mechanism	defense mechanism	1	singular
Defensins	defensins	defensins	defensins	Defensins	1	kept
Defensive Medicine	defensive medicine	defensive medicine	defensive medicine	Defensive Medicine	1	kept
defensive medicine	defensive medicine	defensive medicine	defensive medicine	Defensive Medicine	1	case_or_format
Defensive practice	defensive practice	defensive practice	defensive practice	Defensive practice	1	kept
deference	deference	deference	deference	deference	1	kept
Deferoxamine	deferoxamine	deferoxamine	deferoxamine	Deferoxamine	1	kept
deferoxamine	deferoxamine	deferoxamine	deferoxamine	Deferoxamine	1	case_or_format
deferoxamine mesylate	deferoxamine mesylate	deferoxamine mesylate	deferoxamine mesylate	deferoxamine mesylate	1	kept
DEFIBRILLATION	defibrillation	defibrillation	defibrillation	defibrillation	1	case_or_format

defibrotide	defibrotide	defibrotide	defibrotide	defibrotide	1	kept
DEFICIENCIES	deficiencies	deficiencies	deficiency	deficiency	1	singular
deficiencies	deficiencies	deficiencies	deficiency	deficiency	1	singular
defined daily dose	defined daily dose	defined daily dose	defined daily dose	Defined daily dose	1	case_or_format
DEFINING MEDICAL FUTILITY	defining medical futility	defining medical futility	defining medical futility	DEFINING MEDICAL FUTILITY	1	kept
definition	definition	definition	definition	Definitions	1	case_or_format
definition of death	definition of death	definition of death	definition of death	definition of death	1	kept
Deflation	deflation	deflation	deflation	Deflation	1	kept
defusing	defusing	defusing	defusing	defusing	1	kept
degenerative	degenerative	degenerative	degenerative	degenerative	1	kept
deglutition	deglutition	deglutition	deglutition	Deglutition	1	case_or_format
Deglutition disorder	deglutition disorder	deglutition disorder	deglutition disorder	Deglutition Disorders	1	case_or_format
Degree of complexity	degree of complexity	degree of complexity	degree of complexity	Degree of complexity	1	kept
Dehiscence	dehiscence	dehiscence	dehiscence	Dehiscence	1	kept
dehydrated tissue	dehydrated tissue	dehydrated tissue	dehydrated tissue	dehydrated tissue	1	kept
Dehydroepiandrosterone sulfate	dehydroepiandrosterone sulfate	dehydroepiandrosterone sulfate	dehydroepiandrosterone sulfate	Dehydroepiandrosterone sulfate	1	kept

DELAY	delay	delay	delay	delay	1	case_or_format
Delay	delay	delay	delay	delay	1	case_or_format
delay and interruption of nutritional support	delay and interruption of nutritional support	delay and interruption of nutritional support	delay and interruption of nutritional support	delay and interruption of nutritional support	1	kept
delay in discharge	delay in discharge	delay in discharge	delay in discharge	delay in discharge	1	kept
delayed	delayed	delayed	delayed	delayed	1	kept
delayed activation	delayed activation	delayed activation	delayed activation	delayed activation	1	kept
Delayed admission	delayed admission	delayed admission	delayed admission	Delayed admission	1	kept
delayed brain ischemia	delayed brain ischemia	delayed brain ischemia	delayed brain ischemia	delayed brain ischemia	1	kept
Delayed care	delayed care	delayed care	delayed care	Delayed care	1	kept
delayed cerebral ischemia	delayed cerebral ischemia	delayed cerebral ischemia	delayed cerebral ischemia	Delayed cerebral ischemia	1	case_or_format
Delayed closure of chest	delayed closure of chest	delayed closure of chest	delayed closure of chest	Delayed closure of chest	1	kept
DELAYED DIAGNOSIS	delayed diagnosis	delayed diagnosis	delayed diagnosis	delayed diagnosis	1	case_or_format
Delayed discharge	delayed discharge	delayed discharge	delayed discharge	Delayed discharge	1	kept
Delayed discharges	delayed discharges	delayed discharges	delayed discharge	Delayed discharge	1	singular
delayed emergence from anesthesia	delayed emergence from anesthesia	delayed emergence from anesthesia	delayed emergence from anesthesia	Delayed Emergence from Anesthesia	1	case_or_format
Delayed extubation	delayed extubation	delayed extubation	delayed extubation	Delayed extubation	1	kept

delayed first bath	delayed first bath	delayed first bath	delayed first bath	delayed first bath	1	kept
delayed gastric emptying	delayed gastric emptying	delayed gastric emptying	delayed gastric emptying	delayed gastric emptying	1	kept
delayed neurocognitive recovery	delayed neurocognitive recovery	delayed neurocognitive recovery	delayed neurocognitive recovery	delayed neurocognitive recovery	1	kept
Delayed recoveries	delayed recoveries	delayed recoveries	delayed recoveries	Delayed recoveries	1	kept
Delayed referral	delayed referral	delayed referral	delayed referral	Delayed referral	1	kept
delayed rib plating	delayed rib plating	delayed rib plating	delayed rib plating	delayed rib plating	1	kept
delayed umbilical cord clamping	delayed umbilical cord clamping	delayed umbilical cord clamping	delayed umbilical cord clamping	delayed umbilical cord clamping	1	kept
Delays	delays	delays	delay	delay	1	singular
Delegated tasks	delegated tasks	delegated tasks	delegated tasks	Delegated tasks	1	kept
delegation	delegation	delegation	delegation	DELEGATION	1	case_or_format
deliberate practice	deliberate practice	deliberate practice	deliberate practice	Deliberate practice	1	case_or_format
Deliberate rumination	deliberate rumination	deliberate rumination	deliberate rumination	Deliberate rumination	1	kept
DELIBERATE SELF-HARM	deliberate self harm	deliberate self harm	deliberate self harm	DELIBERATE SELF-HARM	1	kept
Deliriogenic	deliriogenic	deliriogenic	deliriogenic	Deliriogenic	1	kept
deliriogenic	deliriogenic	deliriogenic	deliriogenic	Deliriogenic	1	case_or_format
delirious behavior	delirious behavior	delirious behavior	delirious behavior	delirious behavior	1	kept

Delirium and ABCDE bundle	delirium and abcde bundle	delirium and abcde bundle	delirium and abcde bundle	Delirium and ABCDE bundle	1	kept
delirium and COVID	delirium and covid	delirium and covid	delirium and covid	delirium and COVID	1	kept
Delirium assessment	delirium assessment	delirium assessment	delirium assessment	delirium assessment	1	case_or_format
delirium assessment and management	delirium assessment and management	delirium assessment and management	delirium assessment and management	delirium assessment and management	1	kept
Delirium assessment tools	delirium assessment tools	delirium assessment tools	delirium assessment tools	Delirium assessment tools	1	kept
delirium assessment tools	delirium assessment tools	delirium assessment tools	delirium assessment tools	Delirium assessment tools	1	case_or_format
delirium biomarkers	delirium biomarkers	delirium biomarkers	delirium biomarkers	delirium biomarkers	1	kept
Delirium care	delirium care	delirium care	delirium care	Delirium care	1	kept
delirium care self-efficacy	delirium care self efficacy	delirium care self efficacy	delirium care self efficacy	delirium care self-efficacy	1	kept
Delirium Care Self-Efficacy Scale	delirium care self efficacy scale	delirium care self efficacy scale	delirium care self efficacy scale	Delirium Care Self-Efficacy Scale	1	kept
Delirium Detection Score	delirium detection score	delirium detection score	delirium detection score	delirium detection score	1	case_or_format
Delirium diagnostics	delirium diagnostics	delirium diagnostics	delirium diagnostics	Delirium diagnostics	1	kept
delirium duration	delirium duration	delirium duration	delirium duration	delirium duration	1	kept
delirium experience	delirium experience	delirium experience	delirium experience	delirium experience	1	kept
Delirium Experience Questionnaire	delirium experience questionnaire	delirium experience questionnaire	delirium experience questionnaire	Delirium Experience Questionnaire	1	kept
Delirium intervention	delirium intervention	delirium intervention	delirium intervention	Delirium intervention	1	kept

Delirium management strategies	delirium management strategies	delirium management strategies	delirium management strategies	Delirium management strategies	1	kept
Delirium monitoring	delirium monitoring	delirium monitoring	delirium monitoring	Delirium monitoring	1	kept
delirium monitoring	delirium monitoring	delirium monitoring	delirium monitoring	Delirium monitoring	1	case_or_format
Delirium Observation Scale	delirium observation scale	delirium observation scale	delirium observation scale	Delirium Observation Scale	1	kept
Delirium Observation screening scale	delirium observation screening scale	delirium observation screening scale	delirium observation screening scale	Delirium Observation Screening Scale	1	case_or_format
delirium observation screening scale	delirium observation screening scale	delirium observation screening scale	delirium observation screening scale	Delirium Observation Screening Scale	1	case_or_format
Delirium Observational Screening Scale	delirium observational screening scale	delirium observational screening scale	delirium observational screening scale	Delirium Observational Screening Scale	1	kept
Delirium prevention and management	delirium prevention and management	delirium prevention and management	delirium prevention and management	Delirium prevention and management	1	kept
Delirium prevention strategies	delirium prevention strategies	delirium prevention strategies	delirium prevention strategies	Delirium prevention strategies	1	kept
Delirium Rating scale	delirium rating scale	delirium rating scale	delirium rating scale	Delirium Rating Scale	1	case_or_format
delirium risk assessment	delirium risk assessment	delirium risk assessment	delirium risk assessment	delirium risk assessment	1	kept
Delirium Scale Observation	delirium scale observation	delirium scale observation	delirium scale observation	Delirium Scale Observation	1	kept
Delirium Screening	delirium screening	delirium screening	delirium screening	Delirium screening	1	case_or_format
Delirium severity	delirium severity	delirium severity	delirium severity	Delirium severity	1	kept
delirium toolbox	delirium toolbox	delirium toolbox	delirium toolbox	delirium toolbox	1	kept
delirium treatment	delirium treatment	delirium treatment	delirium treatment	delirium treatment	1	kept

Delirium • Children	delirium children	delirium children	delirium children	Delirium • Children	1	kept
delirium-superim-posed-on-dementia	delirium superim posed on dementia	delirium superim posed on dementia	delirium superim posed on dementia	delirium-superim-posed-on-dementia	1	kept
DELIRIUM-TREMENS	delirium tremens	delirium tremens	delirium tremens	delirium tremens	1	case_or_format
Delivery chain	delivery chain	delivery chain	delivery chain	Delivery chain	1	kept
Delivery experience	delivery experience	delivery experience	delivery experience	Delivery experience	1	kept
delivery model	delivery model	delivery model	delivery model	delivery model	1	kept
Delivery of healthcare	delivery of healthcare	delivery of healthcare	delivery of healthcare	Delivery of healthcare	1	kept
delivery outcomes	delivery outcomes	delivery outcomes	delivery outcomes	delivery outcomes	1	kept
delivery rate	delivery rate	delivery rate	delivery rate	delivery rate	1	kept
DELIVERY ROOM	delivery room	delivery room	delivery room	delivery room	1	case_or_format
Delivery Room	delivery room	delivery room	delivery room	delivery room	1	case_or_format
delivery room management	delivery room management	delivery room management	delivery room management	Delivery room management	1	case_or_format
Delivery systems	delivery systems	delivery systems	delivery systems	Delivery systems	1	kept
delivery time	delivery time	delivery time	delivery time	delivery time	1	kept
Delphi consensus	delphi consensus	delphi consensus	delphi consensus	Delphi consensus	1	kept
Delphi consensus survey	delphi consensus survey	delphi consensus survey	delphi consensus survey	Delphi consensus survey	1	kept

Delphi consultation	delphi consultation	delphi consultation	delphi consultation	Delphi consultation	1	kept
DELPHI METHOD	delphi method	delphi method	delphi method	Delphi method	1	case_or_format
delphi Method	delphi method	delphi method	delphi method	Delphi method	1	case_or_format
delphi method	delphi method	delphi method	delphi method	Delphi method	1	case_or_format
Delphi methodology	delphi methodology	delphi methodology	delphi methodology	Delphi methodology	1	kept
Delphi Study	delphi study	delphi study	delphi study	Delphi study	1	case_or_format
delphi study	delphi study	delphi study	delphi study	Delphi study	1	case_or_format
Delphi survey	delphi survey	delphi survey	delphi survey	Delphi survey	1	kept
Delphi-survey	delphi survey	delphi survey	delphi survey	Delphi survey	1	case_or_format
Delphi-technique	delphi technique	delphi technique	delphi technique	Delphi Technique	1	case_or_format
Deltacoronavirus	deltacoronavirus	deltacoronavirus	deltacoronavirus	Deltacoronavirus	1	kept
deltopectoral approach	deltopectoral approach	deltopectoral approach	deltopectoral approach	deltopectoral approach	1	kept
Delusion	delusion	delusion	delusion	delusion	1	case_or_format
DELUSIONAL MEMORIES	delusional memories	delusional memories	delusional memory	delusional memory	1	singular
Delusional Memories	delusional memories	delusional memories	delusional memory	delusional memory	1	singular
delusional memory	delusional memory	delusional memory	delusional memory	delusional memory	1	kept

demand	demand	demand	demand	DEMANDS	1	case_or_format
Demand distribution	demand distribution	demand distribution	demand distribution	Demand distribution	1	kept
demand feeding	demand feeding	demand feeding	demand feeding	demand feeding	1	kept
Demand for care	demand for care	demand for care	demand for care	Demand for care	1	kept
Demand forecasting	demand forecasting	demand forecasting	demand forecasting	Demand forecasting	1	kept
Demands	demands	demands	demand	DEMANDS	1	singular
Dementia [psychology]	dementia psychology	dementia psychology	dementia psychology	Dementia [psychology]	1	kept
dementia attitude scale	dementia attitude scale	dementia attitude scale	dementia attitude scale	dementia attitude scale	1	kept
Dementia Care	dementia care	dementia care	dementia care	Dementia care	1	case_or_format
dementia friendly communities environment assessment	dementia friendly communities environment assessment	dementia friendly communities environment assessment	dementia friendly communities environment assessment	dementia friendly communities environment assessment	1	kept
Dementia Prevention	dementia prevention	dementia prevention	dementia prevention	Dementia Prevention	1	kept
Dementia Screening Scale	dementia screening scale	dementia screening scale	dementia screening scale	Dementia Screening Scale	1	kept
Dementia unit	dementia unit	dementia unit	dementia unit	Dementia unit	1	kept
dementia with Lewy bodies	dementia with lewy bodies	dementia with lewy bodies	dementia with lewy bodies	dementia with Lewy bodies	1	kept
Dementia workforce	dementia workforce	dementia workforce	dementia workforce	Dementia workforce	1	kept
dementia worry	dementia worry	dementia worry	dementia worry	dementia worry	1	kept

dementia-enabling environment	dementia enabling environment	dementia enabling environment	dementia enabling environment	dementia-enabling environment	1	kept
dementia-enabling environments	dementia enabling environments	dementia enabling environments	dementia enabling environment	dementia-enabling environment	1	singular
Democratic Republic Congo	democratic republic congo	democratic republic congo	democratic republic congo	Democratic Republic Congo	1	kept
Democratic Republic of the Congo, DRC	democratic republic of the congo drc	democratic republic of the congo drc	democratic republic of the congo drc	Democratic Republic of the Congo, DRC	1	kept
Demographic characteristics	demographic characteristics	demographic characteristics	demographic characteristics	DEMOGRAPHIC CHARACTERISTICS	1	case_or_format
Demographic Data	demographic data	demographic data	demographic data	Demographic Data	1	kept
demographic disparity	demographic disparity	demographic disparity	demographic disparity	demographic disparity	1	kept
Demographic factor	demographic factor	demographic factor	demographic factor	Demographic factors	1	case_or_format
Demographic information	demographic information	demographic information	demographic information	Demographic information	1	kept
Demographic Information Questionnaire	demographic information questionnaire	demographic information questionnaire	demographic information questionnaire	Demographic Information Questionnaire	1	kept
Demographic profile	demographic profile	demographic profile	demographic profile	Demographic profile	1	kept
Demographic variables	demographic variables	demographic variables	demographic variables	Demographic variables	1	kept
Demoralisation	demoralisation	demoralisation	demoralisation	Demoralisation	1	kept
demoralisation syndrome	demoralisation syndrome	demoralisation syndrome	demoralisation syndrome	demoralisation syndrome	1	kept
Demoralization	demoralization	demoralization	demoralization	demoralization	1	case_or_format
DemTect	demtect	demtect	demtect	DemTect	1	kept

Demyelinating Diseases	demyelinating diseases	demyelinating diseases	demyelinating disease	demyelinating disease	1	singular
Denaturing Gradient Gel Electrophoresis	denaturing gradient gel electrophoresis	denaturing gradient gel electrophoresis	denaturing gradient gel electrophoresis	Denaturing Gradient Gel Electrophoresis	1	kept
dendritic cell	dendritic cell	dendritic cell	dendritic cell	dendritic cell	1	kept
denervation	denervation	denervation	denervation	denervation	1	kept
Denervation and type grouping	denervation and type grouping	denervation and type grouping	denervation and type grouping	Denervation and type grouping	1	kept
Dengue fever	dengue fever	dengue fever	dengue fever	Dengue fever	1	kept
Dengue Virus	dengue virus	dengue virus	dengue virus	Dengue Virus	1	kept
Dengue virus	dengue virus	dengue virus	dengue virus	Dengue Virus	1	case_or_format
denial	denial	denial	denial	denial	1	kept
Denials	denials	denials	denial	denial	1	singular
DENMARK	denmark	denmark	denmark	Denmark	1	case_or_format
denosumab	denosumab	denosumab	denosumab	denosumab	1	kept
Density	density	density	density	density	1	case_or_format
Dental	dental	dental	dental	Dental	1	kept
Dental Amalgam	dental amalgam	dental amalgam	dental amalgam	Dental Amalgam	1	kept
dental amalgam	dental amalgam	dental amalgam	dental amalgam	Dental Amalgam	1	case_or_format

dental anxiety	dental anxiety	dental anxiety	dental anxiety	dental anxiety	1	kept
Dental Atraumatic Restorative Treatment	dental atraumatic restorative treatment	dental atraumatic restorative treatment	dental atraumatic restorative treatment	Dental Atraumatic Restorative Treatment	1	kept
Dental atraumatic restorative treatment	dental atraumatic restorative treatment	dental atraumatic restorative treatment	dental atraumatic restorative treatment	Dental Atraumatic Restorative Treatment	1	case_or_format
Dental Care for Chronically Ill	dental care for chronically ill	dental care for chronically ill	dental care for chronically ill	dental care for chronically ill	1	case_or_format
Dental caries	dental caries	dental caries	dental caries	dental caries	1	case_or_format
Dental Clinics	dental clinics	dental clinics	dental clinic	dental clinic	1	singular
dental debridement	dental debridement	dental debridement	dental debridement	dental debridement	1	kept
dental disease	dental disease	dental disease	dental disease	dental disease	1	kept
dental education	dental education	dental education	dental education	dental education	1	kept
dental equipment	dental equipment	dental equipment	dental equipment	dental equipment	1	kept
dental examination	dental examination	dental examination	dental examination	dental examination	1	kept
dental facility	dental facility	dental facility	dental facility	dental facility	1	kept
Dental Implantation, Endosseous	dental implantation endosseous	dental implantation endosseous	dental implantation endosseous	Dental Implantation, Endosseous	1	kept
dental material	dental material	dental material	dental material	dental material	1	kept
Dental Materials	dental materials	dental materials	dental material	dental material	1	singular
Dental practice	dental practice	dental practice	dental practice	Dental practice	1	kept

dental practice	dental practice	dental practice	dental practice	Dental practice	1	case_or_format
dental prophylaxis	dental prophylaxis	dental prophylaxis	dental prophylaxis	dental prophylaxis	1	kept
Dental Restoration Failure	dental restoration failure	dental restoration failure	dental restoration failure	Dental Restoration Failure	1	kept
Dental Scaling	dental scaling	dental scaling	dental scaling	Dental Scaling	1	kept
dental surgery	dental surgery	dental surgery	dental surgery	dental surgery	1	kept
DENTAL-CARE	dental care	dental care	dental care	Dental Care	1	case_or_format
dentate gyrus	dentate gyrus	dentate gyrus	dentate gyrus	dentate gyrus	1	kept
DENTITION	dentition	dentition	dentition	DENTITION	1	kept
dentition	dentition	dentition	dentition	DENTITION	1	case_or_format
Dentures	dentures	dentures	denture	denture	1	singular
DENWIS	denwis	denwis	denwis	DENWIS	1	kept
deocal	deocal	deocal	deocal	deocal	1	kept
deodorizers	deodorizers	deodorizers	deodorizers	deodorizers	1	kept
deontological ethics	deontological ethics	deontological ethics	deontological ethics	deontological ethics	1	kept
deontology	deontology	deontology	deontology	deontology	1	kept
deoxyhemoglobin	deoxyhemoglobin	deoxyhemoglobin	deoxyhemoglobin	deoxyhemoglobin	1	kept

deoxypyridinoline	deoxypyridinoline	deoxypyridinoline	deoxypyridinoline	deoxypyridinoline	1	kept
Deoxyribonucleases, Type II Site-Specific	deoxyribonucleases type ii site specific	deoxyribonucleases type ii site specific	deoxyribonucleases type ii site specific	Deoxyribonucleases, Type II Site-Specific	1	kept
Department of Corrections	department of corrections	department of corrections	department of corrections	Department of Corrections	1	kept
Department of internal medicine	department of internal medicine	department of internal medicine	department of internal medicine	Department of internal medicine	1	kept
departmental culture	departmental culture	departmental culture	departmental culture	departmental culture	1	kept
Departmental evaluation	departmental evaluation	departmental evaluation	departmental evaluation	Departmental evaluation	1	kept
dependence	dependence	dependence	dependence	dependence	1	kept
Dependence measures	dependence measures	dependence measures	dependence measures	Dependence measures	1	kept
dependence related skin lesion	dependence related skin lesion	dependence related skin lesion	dependence related skin lesion	dependence related skin lesion	1	kept
dependence-related lesions	dependence related lesions	dependence related lesions	dependence related lesions	dependence-related lesions	1	kept
Dependence-related skin lesions	dependence related skin lesions	dependence related skin lesions	dependence related skin lesion	dependence related skin lesion	1	singular
DEPENDENCY	dependency	dependency	dependency	dependency	1	case_or_format
Dependency parsing	dependency parsing	dependency parsing	dependency parsing	Dependency parsing	1	kept
Dependent Ambulation	dependent ambulation	dependent ambulation	dependent ambulation	Dependent Ambulation	1	kept
depletion	depletion	depletion	depletion	depletion	1	kept
DEPLOYMENT	deployment	deployment	deployment	deployment	1	case_or_format

Deployment	deployment	deployment	deployment	deployment	1	case_or_format
deployment medicine	deployment medicine	deployment medicine	deployment medicine	deployment medicine	1	kept
Deployment stressors	deployment stressors	deployment stressors	deployment stressors	Deployment stressors	1	kept
depolarization	depolarization	depolarization	depolarization	depolarization	1	kept
Deprescribing	deprescribing	deprescribing	deprescribing	Deprescribing	1	kept
deprescribing	deprescribing	deprescribing	deprescribing	Deprescribing	1	case_or_format
Deprescriptions	deprescriptions	deprescriptions	deprescription	deprescription	1	singular
depressed skull fracture	depressed skull fracture	depressed skull fracture	depressed skull fracture	depressed skull fracture	1	kept
Depression & mood disorders	depression and mood disorders	depression and mood disorders	depression and mood disorders	Depression & mood disorders	1	kept
DEPRESSION SCALE	depression scale	depression scale	depression scale	DEPRESSION SCALE	1	kept
depression screening	depression screening	depression screening	depression screening	depression screening	1	kept
depressive disorder	depressive disorder	depressive disorder	depressive disorder	Depressive Disorder	1	case_or_format
Depressive disorder, major	depressive disorder major	depressive disorder major	depressive disorder major	Depressive Disorder, Major	1	case_or_format
Depressive disorder, major, epidemiology	depressive disorder major epidemiology	depressive disorder major epidemiology	depressive disorder major epidemiology	Depressive disorder, major, epidemiology	1	kept
Depressive symptom	depressive symptom	depressive symptom	depressive symptom	Depressive symptoms	1	case_or_format
Depressive symptomatology	depressive symptomatology	depressive symptomatology	depressive symptomatology	Depressive symptomatology	1	kept

DEPRESSIVE SYMPTOMS	depressive symptoms	depressive symptoms	depressive symptom	Depressive symptoms	1	singular
deprivation of liberty safeguards	deprivation of liberty safeguards	deprivation of liberty safeguards	deprivation of liberty safeguards	deprivation of liberty safeguards	1	kept
Depth electroencephalography	depth electroencephalography	depth electroencephalography	depth electroencephalography	Depth electroencephalography	1	kept
Depth of compressions	depth of compressions	depth of compressions	depth of compressions	Depth of compressions	1	kept
DERIVATION	derivation	derivation	derivation	DERIVATION	1	kept
Dermacentor	dermacentor	dermacentor	dermacentor	Dermacentor	1	kept
dermal burns	dermal burns	dermal burns	dermal burns	dermal burns	1	kept
Dermatitis asociada a la incontinencia	dermatitis asociada a la incontinencia	dermatitis asociada a la incontinencia	dermatitis asociada a la incontinencia	Dermatitis asociada a la incontinencia	1	kept
Dermatitis intertriginosa	dermatitis intertriginosa	dermatitis intertriginosa	dermatitis intertriginosa	Dermatitis intertriginosa	1	kept
dermatologic emergency	dermatologic emergency	dermatologic emergency	dermatologic emergency	dermatologic emergency	1	kept
Dermatological Diseases	dermatological diseases	dermatological diseases	dermatological diseases	Dermatological Diseases	1	kept
Dermatological emergencies	dermatological emergencies	dermatological emergencies	dermatological emergencies	Dermatological emergencies	1	kept
dermatology admissions	dermatology admissions	dermatology admissions	dermatology admissions	dermatology admissions	1	kept
dermatology consults	dermatology consults	dermatology consults	dermatology consults	dermatology consults	1	kept
dermatology diagnosis related groups	dermatology diagnosis related groups	dermatology diagnosis related groups	dermatology diagnosis related groups	dermatology diagnosis related groups	1	kept
Dermatology Life Quality Index	dermatology life quality index	dermatology life quality index	dermatology life quality index	Dermatology Life Quality Index	1	kept

dermatology specific and dermatology related diagnosis	dermatology specific and dermatology related diagnosis	dermatology specific and dermatology related diagnosis	dermatology specific and dermatology related diagnosis	dermatology specific and dermatology related diagnosis	1	kept
Dermatome	dermatome	dermatome	dermatome	Dermatome	1	kept
dermatomes	dermatomes	dermatomes	dermatome	Dermatome	1	singular
dermatomyositis	dermatomyositis	dermatomyositis	dermatomyositis	dermatomyositis	1	kept
dermatovenereologist	dermatovenereologist	dermatovenereologist	dermatovenereologist	dermatovenereologist	1	kept
Dermiplus Prevent	dermiplus prevent	dermiplus prevent	dermiplus prevent	Dermiplus Prevent	1	kept
dermis	dermis	dermis	dermis	dermis	1	kept
dermoepidermal junction	dermoepidermal junction	dermoepidermal junction	dermoepidermal junction	dermoepidermal junction	1	kept
Derriford Appearance Scale 24	derriford appearance scale 24	derriford appearance scale 24	derriford appearance scale 24	Derriford Appearance Scale 24	1	kept
Desasosiego moral	desasosiego moral	desasosiego moral	desasosiego moral	Desasosiego moral	1	kept
Descartes	descartes	descartes	descartes	Descartes	1	kept
descending aortic surgery	descending aortic surgery	descending aortic surgery	descending aortic surgery	descending aortic surgery	1	kept
Descending thoracic aortic aneurysm	descending thoracic aortic aneurysm	descending thoracic aortic aneurysm	descending thoracic aortic aneurysm	Descending thoracic aortic aneurysm	1	kept
Description	description	description	description	Description	1	kept
descriptive analysis	descriptive analysis	descriptive analysis	descriptive analysis	descriptive analysis	1	kept
Descriptive cross-sectional	descriptive cross sectional	descriptive cross sectional	descriptive cross sectional	Descriptive cross-sectional	1	kept

descriptive design	descriptive design	descriptive design	descriptive design	descriptive design	1	kept
Descriptive epidemiology	descriptive epidemiology	descriptive epidemiology	descriptive epidemiology	Descriptive epidemiology	1	kept
Descriptive exploratory	descriptive exploratory	descriptive exploratory	descriptive exploratory	Descriptive exploratory	1	kept
Descriptive exploratory study	descriptive exploratory study	descriptive exploratory study	descriptive exploratory study	Descriptive exploratory study	1	kept
Descriptive interpretative study	descriptive interpretative study	descriptive interpretative study	descriptive interpretative study	Descriptive interpretative study	1	kept
Descriptive methodology	descriptive methodology	descriptive methodology	descriptive methodology	Descriptive methodology	1	kept
descriptive methods	descriptive methods	descriptive methods	descriptive methods	descriptive methods	1	kept
Descriptive Model	descriptive model	descriptive model	descriptive model	Descriptive Model	1	kept
Descriptive phenomenology	descriptive phenomenology	descriptive phenomenology	descriptive phenomenology	Descriptive phenomenology	1	kept
descriptive qualitative design	descriptive qualitative design	descriptive qualitative design	descriptive qualitative design	descriptive qualitative design	1	kept
Descriptive qualitative method	descriptive qualitative method	descriptive qualitative method	descriptive qualitative method	Descriptive qualitative method	1	kept
Descriptive study	descriptive study	descriptive study	descriptive study	Descriptive studies	1	case_or_format
descriptive Study	descriptive study	descriptive study	descriptive study	Descriptive studies	1	case_or_format
descriptive study	descriptive study	descriptive study	descriptive study	Descriptive studies	1	case_or_format
Descriptive-exploratory study	descriptive exploratory study	descriptive exploratory study	descriptive exploratory study	Descriptive exploratory study	1	case_or_format
DESCRIPTORS: Enteral nutrition	descriptors enteral nutrition	descriptors enteral nutrition	descriptors enteral nutrition	DESCRIPTORS: Enteral nutrition	1	kept

Descriptors: enteral nutrition	descriptors enteral nutrition	descriptors enteral nutrition	descriptors enteral nutrition	DESCRIPTORS: Enteral nutrition	1	case_or_format
Descriptors: Infant	descriptors infant	descriptors infant	descriptors infant	Descriptors: Infant	1	kept
Descritores	descritores	descritores	descritores	Descritores	1	kept
Dese	dese	dese	dese	Dese	1	kept
Desenvolvimento tecnológico	desenvolvimento tecnol gico	desenvolvimento tecnol gico	desenvolvimento tecnol gico	Desenvolvimento tecnológico	1	kept
Desfibrilhação precoce	desfibrilha o precoce	desfibrilha o precoce	desfibrilha o precoce	Desfibrilhação precoce	1	kept
Desgaste profesional	desgaste profesional	desgaste profesional	desgaste profesional	Desgaste profesional	1	kept
Design and analysis	design and analysis	design and analysis	design and analysis	Design and analysis	1	kept
Design and Development	design and development	design and development	design and development	Design and Development	1	kept
Design and implementations	design and implementations	design and implementations	design and implementations	Design and implementations	1	kept
Design concept	design concept	design concept	design concept	Design concept	1	kept
Design considerations	design considerations	design considerations	design considerations	Design considerations	1	kept
Design data	design data	design data	design data	Design data	1	kept
Design direction	design direction	design direction	design direction	Design direction	1	kept
Design flaw	design flaw	design flaw	design flaw	Design flaw	1	kept
Design for Healthcare	design for healthcare	design for healthcare	design for healthcare	Design for Healthcare	1	kept

Design guideline	design guideline	design guideline	design guideline	Design guidelines	1	case_or_format
Design implications	design implications	design implications	design implications	Design implications	1	kept
Design intervention	design intervention	design intervention	design intervention	Design intervention	1	kept
Design optimization	design optimization	design optimization	design optimization	Design optimization	1	kept
Design performance	design performance	design performance	design performance	Design performance	1	kept
Design Principles	design principles	design principles	design principles	Design Principles	1	kept
Design programs	design programs	design programs	design programs	Design programs	1	kept
design propositions	design propositions	design propositions	design propositions	design propositions	1	kept
design research	design research	design research	design research	design research	1	kept
Design robustness	design robustness	design robustness	design robustness	Design robustness	1	kept
Design science research	design science research	design science research	design science research	Design science research	1	kept
DESIGN SENSITIVITY	design sensitivity	design sensitivity	design sensitivity	DESIGN SENSITIVITY	1	kept
Design thinking	design thinking	design thinking	design thinking	Design thinking	1	kept
designated health facility	designated health facility	designated health facility	designated health facility	designated health facility	1	kept
designated hospitals	designated hospitals	designated hospitals	designated hospital	Designated hospital	1	singular
designer drug	designer drug	designer drug	designer drug	designer drug	1	kept

desipramine	desipramine	desipramine	desipramine	desipramine	1	kept
Desirability function	desirability function	desirability function	desirability function	desirability function	1	case_or_format
desloratadine	desloratadine	desloratadine	desloratadine	desloratadine	1	kept
desmoglein 1 antibody	desmoglein 1 antibody	desmoglein 1 antibody	desmoglein 1 antibody	desmoglein 1 antibody	1	kept
desmoglein 3 antibody	desmoglein 3 antibody	desmoglein 3 antibody	desmoglein 3 antibody	desmoglein 3 antibody	1	kept
desmoid tumor	desmoid tumor	desmoid tumor	desmoid tumor	desmoid tumor	1	kept
Desmopressin	desmopressin	desmopressin	desmopressin	desmopressin	1	case_or_format
desmopressin acetate	desmopressin acetate	desmopressin acetate	desmopressin acetate	desmopressin acetate	1	kept
DESORPTION IONIZATION-TIME	desorption ionization time	desorption ionization time	desorption ionization time	DESORPTION IONIZATION-TIME	1	kept
Dessie Town	dessie town	dessie town	dessie town	Dessie Town	1	kept
DESTINATION	destination	destination	destination	DESTINATION	1	kept
Destination care	destination care	destination care	destination care	Destination care	1	kept
Details management	details management	details management	details management	Details management	1	kept
DETECT DELIRIUM	detect delirium	detect delirium	detect delirium	DETECT DELIRIUM	1	kept
Detecting	detecting	detecting	detecting	Detecting	1	kept
DETECTING CLINICAL DETERIORATION	detecting clinical deterioration	detecting clinical deterioration	detecting clinical deterioration	DETECTING CLINICAL DETERIORATION	1	kept

DETECTING CRITICAL ILLNESS	detecting critical illness	detecting critical illness	detecting critical illness	DETECTING CRITICAL ILLNESS	1	kept
Detection Algorithms	detection algorithms	detection algorithms	detection algorithm	detection algorithm	1	singular
detection method	detection method	detection method	detection method	detection method	1	kept
Detection methods	detection methods	detection methods	detection method	detection method	1	singular
detection model	detection model	detection model	detection model	detection model	1	kept
Detection models	detection models	detection models	detection model	detection model	1	singular
detention	detention	detention	detention	detention	1	kept
deteriorated drug error	deteriorated drug error	deteriorated drug error	deteriorated drug error	deteriorated drug error	1	kept
Deteriorating	deteriorating	deteriorating	deteriorating	Deteriorating	1	kept
Deteriorating condition	deteriorating condition	deteriorating condition	deteriorating condition	Deteriorating condition	1	kept
Deterioro clínico	deterioro cl nico	deterioro cl nico	deterioro cl nico	Deterioro clínico	1	kept
Determinant	determinant	determinant	determinant	DETERMINANTS	1	case_or_format
Determinants	determinants	determinants	determinant	DETERMINANTS	1	singular
determinants frameworks	determinants frameworks	determinants frameworks	determinants frameworks	determinants frameworks	1	kept
Determinants of stress	determinants of stress	determinants of stress	determinants of stress	Determinants of stress	1	kept
Determination	determination	determination	determination	Determination	1	kept

detoxification	detoxification	detoxification	detoxification	detoxification	1	kept
DEUTERIUM-LABELED DEHP	deuterium labeled dehp	deuterium labeled dehp	deuterium labeled dehp	DEUTERIUM-LABELED DEHP	1	kept
devascularization	devascularization	devascularization	devascularization	devascularization	1	kept
develop	develop	develop	develop	develop	1	kept
Developed	developed	developed	developed	Developed	1	kept
Developed countries	developed countries	developed countries	developed country	developed country	1	singular
Developing nations	developing nations	developing nations	developing nations	Developing nations	1	kept
DEVELOPING-COUNTRIES	developing countries	developing countries	developing country	Developing Countries	1	singular
DEVELOPING-WORLD	developing world	developing world	developing world	developing world	1	case_or_format
Development and applications	development and applications	development and applications	development and applications	Development and applications	1	kept
Development and construction	development and construction	development and construction	development and construction	Development and construction	1	kept
Development and growth	development and growth	development and growth	development and growth	Development and growth	1	kept
Development care	development care	development care	development care	Development care	1	kept
development care	development care	development care	development care	Development care	1	case_or_format
Development models	development models	development models	development models	Development models	1	kept
Development problems	development problems	development problems	development problems	Development problems	1	kept

Development process	development process	development process	development process	Development process	1	kept
development quotient	development quotient	development quotient	development quotient	development quotient	1	kept
Developmental anchor points	developmental anchor points	developmental anchor points	developmental anchor points	Developmental anchor points	1	kept
developmental anomalies	developmental anomalies	developmental anomalies	developmental anomalies	developmental anomalies	1	kept
DEVELOPMENTAL CARE	developmental care	developmental care	developmental care	Developmental care	1	case_or_format
Developmental Care	developmental care	developmental care	developmental care	Developmental care	1	case_or_format
Developmental Care Scale for Neonates with Congenital Heart	developmental care scale for neonates with congenital heart	developmental care scale for neonates with congenital heart	developmental care scale for neonates with congenital heart	Developmental Care Scale for Neonates with Congenital Heart	1	kept
Developmental delay and developmental follow-up for infants	developmental delay and developmental follow up for infants	developmental delay and developmental follow up for infants	developmental delay and developmental follow up for infants	Developmental delay and developmental follow-up for infants	1	kept
developmental delays	developmental delays	developmental delays	developmental delay	developmental delay	1	singular
developmental disorders	developmental disorders	developmental disorders	developmental disorder	developmental disorder	1	singular
developmental education	developmental education	developmental education	developmental education	developmental education	1	kept
developmental guidance	developmental guidance	developmental guidance	developmental guidance	developmental guidance	1	kept
developmental intervention	developmental intervention	developmental intervention	developmental intervention	developmental intervention	1	kept
developmental neurology & neurodisability	developmental neurology and neurodisability	developmental neurology and neurodisability	developmental neurology and neurodisability	developmental neurology & neurodisability	1	kept
developmental neuroscience	developmental neuroscience	developmental neuroscience	developmental neuroscience	developmental neuroscience	1	kept
developmental origins	developmental origins	developmental origins	developmental origins	developmental origins	1	kept

Developmental outcomes for at-risk infants	developmental outcomes for at risk infants	developmental outcomes for at risk infants	developmental outcomes for at risk infants	Developmental outcomes for at-risk infants	1	kept
Developmental paediatrics	developmental paediatrics	developmental paediatrics	developmental paediatrics	Developmental paediatrics	1	kept
developmental position	developmental position	developmental position	developmental position	developmental position	1	kept
Developmental positioning	developmental positioning	developmental positioning	developmental positioning	Developmental positioning	1	kept
developmental positioning	developmental positioning	developmental positioning	developmental positioning	Developmental positioning	1	case_or_format
developmental psychology	developmental psychology	developmental psychology	developmental psychology	developmental psychology	1	kept
Developmental support competency	developmental support competency	developmental support competency	developmental support competency	Developmental support competency	1	kept
Developmental support status	developmental support status	developmental support status	developmental support status	Developmental support status	1	kept
DEVELOPMENTAL-CARE	developmental care	developmental care	developmental care	Developmental care	1	case_or_format
Developmentally supportive care	developmentally supportive care	developmentally supportive care	developmentally supportive care	developmentally supportive care	1	case_or_format
developmentally supportive positioning	developmentally supportive positioning	developmentally supportive positioning	developmentally supportive positioning	developmentally supportive positioning	1	kept
DEVIANCE	deviance	deviance	deviance	DEVIANCE	1	kept
Device	device	device	device	devices	1	case_or_format
Device Aggregator	device aggregator	device aggregator	device aggregator	Device Aggregator	1	kept
device aggregator	device aggregator	device aggregator	device aggregator	Device Aggregator	1	case_or_format
device alarm	device alarm	device alarm	device alarm	device alarm	1	kept

Device alarms	device alarms	device alarms	device alarm	device alarm	1	singular
device approval	device approval	device approval	device approval	Device Approval	1	case_or_format
Device disruption	device disruption	device disruption	device disruption	Device disruption	1	kept
device economics	device economics	device economics	device economics	device economics	1	kept
Device failure	device failure	device failure	device failure	device failure	1	case_or_format
device failure analysis	device failure analysis	device failure analysis	device failure analysis	device failure analysis	1	kept
Device infection	device infection	device infection	device infection	device infection	1	case_or_format
device loosening	device loosening	device loosening	device loosening	device loosening	1	kept
device mishandling	device mishandling	device mishandling	device mishandling	device mishandling	1	kept
Device performance	device performance	device performance	device performance	Device performance	1	kept
device privacy	device privacy	device privacy	device privacy	device privacy	1	kept
Device removal	device removal	device removal	device removal	Device Removal	1	case_or_format
Device system	device system	device system	device system	Device system	1	kept
device thrombosis	device thrombosis	device thrombosis	device thrombosis	device thrombosis	1	kept
device utilization ratio	device utilization ratio	device utilization ratio	device utilization ratio	device utilization ratio	1	kept
device-associated infection	device associated infection	device associated infection	device associated infection	Device-associated infections	1	case_or_format

Device-related	device related	device related	device related	Device-related	1	kept
Device-related pressure injury	device related pressure injury	device related pressure injury	device related pressure injury	Device-related pressure injury	1	kept
Device-related pressure ulcer	device related pressure ulcer	device related pressure ulcer	device related pressure ulcer	Device-related pressure ulcer	1	kept
deviceassociated-hospital-acquired infections	deviceassociated hospital acquired infections	deviceassociated hospital acquired infections	deviceassociated hospital acquired infections	deviceassociated-hospital-acquired infections	1	kept
DEVICES	devices	devices	device	devices	1	singular
Device - object pairing	device object pairing	device object pairing	device object pairing	Device - object pairing	1	kept
Dewey	dewey	dewey	dewey	Dewey	1	kept
dexamethasone sodium phosphate	dexamethasone sodium phosphate	dexamethasone sodium phosphate	dexamethasone sodium phosphate	dexamethasone sodium phosphate	1	kept
dexpanthenol	dexpanthenol	dexpanthenol	dexpanthenol	dexpanthenol	1	kept
Dextrocardia	dextrocardia	dextrocardia	dextrocardia	Dextrocardia	1	kept
dextrocardia	dextrocardia	dextrocardia	dextrocardia	Dextrocardia	1	case_or_format
dextromethorphan	dextromethorphan	dextromethorphan	dextromethorphan	dextromethorphan	1	kept
dextropropoxyphene napsilate	dextropropoxyphene napsilate	dextropropoxyphene napsilate	dextropropoxyphene napsilate	dextropropoxyphene napsilate	1	kept
dextropropoxyphene plus paracetamol	dextropropoxyphene plus paracetamol	dextropropoxyphene plus paracetamol	dextropropoxyphene plus paracetamol	dextropropoxyphene plus paracetamol	1	kept
Dextrose	dextrose	dextrose	dextrose	dextrose	1	case_or_format
dextrose 40 percent oral gel	dextrose 40 percent oral gel	dextrose 40 percent oral gel	dextrose 40 percent oral gel	dextrose 40 percent oral gel	1	kept

dextrose gel	dextrose gel	dextrose gel	dextrose gel	dextrose gel	1	kept
DI PHTHALATE	di phthalate	di phthalate	di phthalate	DI PHTHALATE	1	kept
DI PHTHALATE DEHP	di phthalate dehp	di phthalate dehp	di phthalate dehp	DI PHTHALATE DEHP	1	kept
DI- PHTHALATE	di phthalate	di phthalate	di phthalate	DI PHTHALATE	1	case_or_format
Diabetes complications	diabetes complications	diabetes complications	diabetes complications	Diabetes Complications	1	case_or_format
Diabetes in pregnancy	diabetes in pregnancy	diabetes in pregnancy	diabetes in pregnancy	Diabetes in pregnancy	1	kept
Diabetes ketoacidosis	diabetes ketoacidosis	diabetes ketoacidosis	diabetes ketoacidosis	Diabetes ketoacidosis	1	kept
DIABETES MANAGEMENT	diabetes management	diabetes management	diabetes management	diabetes management	1	case_or_format
Diabetes mellitus, type 1	diabetes mellitus type 1	diabetes mellitus type 1	diabetes mellitus type 1	Diabetes Mellitus, Type 1	1	case_or_format
Diabetes mellitus, type 2	diabetes mellitus type 2	diabetes mellitus type 2	diabetes mellitus type 2	Diabetes Mellitus, Type 2	1	case_or_format
Diabetes Technology	diabetes technology	diabetes technology	diabetes technology	diabetes technology	1	case_or_format
Diabetic emergencies	diabetic emergencies	diabetic emergencies	diabetic emergencies	Diabetic emergencies	1	kept
Diabetic Foot	diabetic foot	diabetic foot	diabetic foot	diabetic foot	1	case_or_format
Diabetic foot	diabetic foot	diabetic foot	diabetic foot	diabetic foot	1	case_or_format
diabetic hypertension	diabetic hypertension	diabetic hypertension	diabetic hypertension	diabetic hypertension	1	kept
diabetic induced cognitive dysfunction	diabetic induced cognitive dysfunction	diabetic induced cognitive dysfunction	diabetic induced cognitive dysfunction	diabetic induced cognitive dysfunction	1	kept

Diabetic nephropathy	diabetic nephropathy	diabetic nephropathy	diabetic nephropathy	diabetic nephropathy	1	case_or_format
diabetologist	diabetologist	diabetologist	diabetologist	diabetologist	1	kept
diacritics	diacritics	diacritics	diacritics	diacritics	1	kept
DIAGNOSES	diagnoses	diagnoses	diagnoses	DIAGNOSES	1	kept
Diagnoses	diagnoses	diagnoses	diagnoses	DIAGNOSES	1	case_or_format
Diagnoses codes	diagnoses codes	diagnoses codes	diagnoses codes	Diagnoses codes	1	kept
Diagnosing	diagnosing	diagnosing	diagnosing	Diagnosing	1	kept
Diagnosis and procedure combination	diagnosis and procedure combination	diagnosis and procedure combination	diagnosis and procedure combination	Diagnosis and procedure combination	1	kept
diagnosis and procedure combination	diagnosis and procedure combination	diagnosis and procedure combination	diagnosis and procedure combination	Diagnosis and procedure combination	1	case_or_format
diagnosis codes	diagnosis codes	diagnosis codes	diagnosis codes	diagnosis codes	1	kept
Diagnosis methods	diagnosis methods	diagnosis methods	diagnosis methods	Diagnosis methods	1	kept
Diagnosis procedure combination	diagnosis procedure combination	diagnosis procedure combination	diagnosis procedure combination	Diagnosis procedure combination	1	kept
Diagnosis related groups	diagnosis related groups	diagnosis related groups	diagnosis related group	Diagnosis-Related Groups	1	singular
diagnosis related groups reimbursement	diagnosis related groups reimbursement	diagnosis related groups reimbursement	diagnosis related groups reimbursement	diagnosis related groups reimbursement	1	kept
Diagnosis, Dual	diagnosis dual	diagnosis dual	diagnosis dual	Diagnosis, Dual	1	kept
diagnosis, measurement and analysis	diagnosis measurement and analysis	diagnosis measurement and analysis	diagnosis measurement and analysis	diagnosis, measurement and analysis	1	kept

Diagnosis, Oral	diagnosis oral	diagnosis oral	diagnosis oral	Diagnosis, Oral	1	kept
Diagnostic algorithm	diagnostic algorithm	diagnostic algorithm	diagnostic algorithm	Diagnostic algorithm	1	kept
Diagnostic and Statistical Manual of Mental Disorder	diagnostic and statistical manual of mental disorder	diagnostic and statistical manual of mental disorder	diagnostic and statistical manual of mental disorder	Diagnostic and Statistical Manual of Mental Disorders	1	case_or_format
Diagnostic and statistical manual of mental disorders	diagnostic and statistical manual of mental disorders	diagnostic and statistical manual of mental disorders	diagnostic and statistical manual of mental disorder	Diagnostic and Statistical Manual of Mental Disorders	1	singular
diagnostic criteria	diagnostic criteria	diagnostic criteria	diagnostic criteria	Diagnostic criteria	1	case_or_format
Diagnostic Equipment	diagnostic equipment	diagnostic equipment	diagnostic equipment	Diagnostic Equipment	1	kept
Diagnostic errors	diagnostic errors	diagnostic errors	diagnostic error	Diagnostic Errors	1	singular
diagnostic errors	diagnostic errors	diagnostic errors	diagnostic error	Diagnostic Errors	1	singular
Diagnostic imaging	diagnostic imaging	diagnostic imaging	diagnostic imaging	diagnostic imaging	1	case_or_format
diagnostic imaging equipment	diagnostic imaging equipment	diagnostic imaging equipment	diagnostic imaging equipment	diagnostic imaging equipment	1	kept
Diagnostic instrument	diagnostic instrument	diagnostic instrument	diagnostic instrument	Diagnostic instrument	1	kept
diagnostic kit	diagnostic kit	diagnostic kit	diagnostic kit	diagnostic kit	1	kept
Diagnostic markers	diagnostic markers	diagnostic markers	diagnostic markers	Diagnostic markers	1	kept
diagnostic modalities	diagnostic modalities	diagnostic modalities	diagnostic modalities	diagnostic modalities	1	kept
Diagnostic NOTES	diagnostic notes	diagnostic notes	diagnostic notes	Diagnostic NOTES	1	kept
Diagnostic Procedure	diagnostic procedure	diagnostic procedure	diagnostic procedure	diagnostic procedure	1	case_or_format

Diagnostic radiology	diagnostic radiology	diagnostic radiology	diagnostic radiology	Diagnostic radiology	1	kept
diagnostic stewardship	diagnostic stewardship	diagnostic stewardship	diagnostic stewardship	diagnostic stewardship	1	kept
Diagnostic Techniques, Respiratory System	diagnostic techniques respiratory system	diagnostic techniques respiratory system	diagnostic techniques respiratory system	Diagnostic Techniques, Respiratory System	1	kept
Diagnostic test	diagnostic test	diagnostic test	diagnostic test	diagnostic test	1	case_or_format
diagnostic test accuracy	diagnostic test accuracy	diagnostic test accuracy	diagnostic test accuracy	diagnostic test accuracy	1	kept
Diagnostic testing	diagnostic testing	diagnostic testing	diagnostic testing	Diagnostic testing	1	kept
Diagnostic tests	diagnostic tests	diagnostic tests	diagnostic test	diagnostic test	1	singular
Diagnostic time-out	diagnostic time out	diagnostic time out	diagnostic time out	Diagnostic time-out	1	kept
Diagnostic use	diagnostic use	diagnostic use	diagnostic use	Diagnostic use	1	kept
DIAGNOSTIC-TESTS	diagnostic tests	diagnostic tests	diagnostic test	diagnostic test	1	singular
Diagnostics	diagnostics	diagnostics	diagnostic	diagnostics	1	singular
Diagnóstico de Enfermagem	diagn stico de enfermagem	diagn stico de enfermagem	diagn stico de enfermagem	Diagnóstico de Enfermagem	1	kept
Diagnóstico de Enfermería	diagn stico de enfermer a	diagn stico de enfermer a	diagn stico de enfermer a	Diagnóstico de Enfermería	1	kept
Diagnóstico de enfermería	diagn stico de enfermer a	diagn stico de enfermer a	diagn stico de enfermer a	Diagnóstico de Enfermería	1	case_or_format
Diagnósticos enfermeros	diagn sticos enfermeros	diagn sticos enfermeros	diagn sticos enfermeros	Diagnósticos enfermeros	1	kept
dialectical behavior therapy	dialectical behavior therapy	dialectical behavior therapy	dialectical behavior therapy	dialectical behavior therapy	1	kept

Dialogue	dialogue	dialogue	dialogue	Dialogue	1	kept
Dialysate	dialysate	dialysate	dialysate	dialysate	1	case_or_format
Dialysates	dialysates	dialysates	dialysate	dialysate	1	singular
dialysis dose	dialysis dose	dialysis dose	dialysis dose	dialysis dose	1	kept
Dialysis efficiency	dialysis efficiency	dialysis efficiency	dialysis efficiency	Dialysis efficiency	1	kept
dialysis efficiency	dialysis efficiency	dialysis efficiency	dialysis efficiency	Dialysis efficiency	1	case_or_format
dialysis equipment and supplies	dialysis equipment and supplies	dialysis equipment and supplies	dialysis equipment and supplies	dialysis equipment and supplies	1	kept
Dialysis machines	dialysis machines	dialysis machines	dialysis machines	Dialysis machines	1	kept
dialysis membrane	dialysis membrane	dialysis membrane	dialysis membrane	dialysis membrane	1	kept
Dialysis prescription	dialysis prescription	dialysis prescription	dialysis prescription	Dialysis prescription	1	kept
dialysis pump	dialysis pump	dialysis pump	dialysis pump	dialysis pump	1	kept
dialysis pump speed	dialysis pump speed	dialysis pump speed	dialysis pump speed	dialysis pump speed	1	kept
dialysis related amyloidosis	dialysis related amyloidosis	dialysis related amyloidosis	dialysis related amyloidosis	dialysis related amyloidosis	1	kept
dialysis room	dialysis room	dialysis room	dialysis room	dialysis room	1	kept
dialysis unit	dialysis unit	dialysis unit	dialysis unit	dialysis unit	1	kept
dialysis unit design	dialysis unit design	dialysis unit design	dialysis unit design	dialysis unit design	1	kept

Dialysis units	dialysis units	dialysis units	dialysis unit	dialysis unit	1	singular
Dialyzer	dialyzer	dialyzer	dialyzer	Dialyzer	1	kept
diamond	diamond	diamond	diamond	diamond	1	kept
Diaper	diaper	diaper	diaper	diaper	1	case_or_format
DIAPER CHANGE	diaper change	diaper change	diaper change	DIAPER CHANGE	1	kept
diaper cleansing	diaper cleansing	diaper cleansing	diaper cleansing	diaper cleansing	1	kept
Diaper rash	diaper rash	diaper rash	diaper rash	Diaper Rash	1	case_or_format
diaphragm fatigue	diaphragm fatigue	diaphragm fatigue	diaphragm fatigue	diaphragm fatigue	1	kept
Diaphragm function exercise	diaphragm function exercise	diaphragm function exercise	diaphragm function exercise	Diaphragm function exercise	1	kept
diaphragm injury	diaphragm injury	diaphragm injury	diaphragm injury	diaphragm injury	1	kept
diaphragm muscle	diaphragm muscle	diaphragm muscle	diaphragm muscle	diaphragm muscle	1	kept
diaphragm rupture	diaphragm rupture	diaphragm rupture	diaphragm rupture	diaphragm rupture	1	kept
diaphragm thickening fraction	diaphragm thickening fraction	diaphragm thickening fraction	diaphragm thickening fraction	diaphragm thickening fraction	1	kept
diaphragmatic function training	diaphragmatic function training	diaphragmatic function training	diaphragmatic function training	diaphragmatic function training	1	kept
Diaphragmatic-breathing	diaphragmatic breathing	diaphragmatic breathing	diaphragmatic breathing	Diaphragmatic-breathing	1	kept
Diaries as topic	diaries as topic	diaries as topic	diaries as topic	Diaries as Topic	1	case_or_format

Diario	diario	diario	diario	Diario	1	kept
diary number	diary number	diary number	diary number	diary number	1	kept
Diary or diaries	diary or diaries	diary or diaries	diary or diaries	Diary or diaries	1	kept
diastolic heart murmur	diastolic heart murmur	diastolic heart murmur	diastolic heart murmur	diastolic heart murmur	1	kept
diastolic hypertension	diastolic hypertension	diastolic hypertension	diastolic hypertension	diastolic hypertension	1	kept
diathermy	diathermy	diathermy	diathermy	diathermy	1	kept
diatrizoate	diatrizoate	diatrizoate	diatrizoate	diatrizoate	1	kept
Diazoxide	diazoxide	diazoxide	diazoxide	diazoxide	1	case_or_format
dibenzothiepin derivative	dibenzothiepin derivative	dibenzothiepin derivative	dibenzothiepin derivative	dibenzothiepin derivative	1	kept
Dibenzothiepins	dibenzothiepins	dibenzothiepins	dibenzothiepins	Dibenzothiepins	1	kept
DIC	dic	dic	dic	DIC	1	kept
Diclofenac	diclofenac	diclofenac	diclofenac	diclofenac	1	case_or_format
didanosine	didanosine	didanosine	didanosine	didanosine	1	kept
didecyldimethylammonium chloride	didecyldimethylammonium chloride	didecyldimethylammonium chloride	didecyldimethylammonium chloride	didecyldimethylammonium chloride	1	kept
DIEP	diep	diep	diep	DIEP	1	kept
diet composition	diet composition	diet composition	diet composition	diet composition	1	kept

diet history	diet history	diet history	diet history	diet history	1	kept
diet intervention	diet intervention	diet intervention	diet intervention	diet intervention	1	kept
Diet Records	diet records	diet records	diet records	Diet Records	1	kept
Diet records	diet records	diet records	diet records	Diet Records	1	case_or_format
diet soda	diet soda	diet soda	diet soda	diet soda	1	kept
diet technician	diet technician	diet technician	diet technician	diet technician	1	kept
Diet, Carbohydrate-Restricted	diet carbohydrate restricted	diet carbohydrate restricted	diet carbohydrate restricted	Diet, Carbohydrate-Restricted	1	kept
Diet, Fat-Restricted	diet fat restricted	diet fat restricted	diet fat restricted	Diet, Fat-Restricted	1	kept
Diet, Ketogenic	diet ketogenic	diet ketogenic	diet ketogenic	Diet, Ketogenic	1	kept
Diet, Protein-Restricted	diet protein restricted	diet protein restricted	diet protein restricted	Diet, Protein-Restricted	1	kept
Diet, Sodium-Restricted	diet sodium restricted	diet sodium restricted	diet sodium restricted	Diet, Sodium-Restricted	1	kept
dietary care	dietary care	dietary care	dietary care	dietary care	1	kept
dietary fiber	dietary fiber	dietary fiber	dietary fiber	Dietary Fiber	1	case_or_format
Dietary fibre	dietary fibre	dietary fibre	dietary fibre	Dietary fibre	1	kept
Dietary program	dietary program	dietary program	dietary program	Dietary program	1	kept
Dietary Services	dietary services	dietary services	dietary service	dietary service	1	singular

diethylcarbamazine	diethylcarbamazine	diethylcarbamazine	diethylcarbamazine	diethylcarbamazine	1	kept
diethylstilbestrol	diethylstilbestrol	diethylstilbestrol	diethylstilbestrol	diethylstilbestrol	1	kept
Dietician	dietician	dietician	dietician	Dietician	1	kept
dietitian attitude	dietitian attitude	dietitian attitude	dietitian attitude	dietitian attitude	1	kept
dietitians	dietitians	dietitians	dietitian	dietitian	1	singular
DIFFER	differ	differ	differ	DIFFER	1	kept
DIFFERENCE	difference	difference	difference	DIFFERENCE	1	kept
difference in differences	difference in differences	difference in differences	difference in differences	difference in differences	1	kept
difference-in-differences modeling	difference in differences modeling	difference in differences modeling	difference in differences modeling	difference-in-differences modeling	1	kept
Differences in perceptions	differences in perceptions	differences in perceptions	differences in perceptions	Differences in perceptions	1	kept
different transportation methods	different transportation methods	different transportation methods	different transportation methods	different transportation methods	1	kept
differential diagnostic ability	differential diagnostic ability	differential diagnostic ability	differential diagnostic ability	differential diagnostic ability	1	kept
Differential privacies	differential privacies	differential privacies	differential privacy	Differential privacy	1	singular
differential scanning calorimetry	differential scanning calorimetry	differential scanning calorimetry	differential scanning calorimetry	differential scanning calorimetry	1	kept
differentiation	differentiation	differentiation	differentiation	differentiation	1	kept
differentiation of self	differentiation of self	differentiation of self	differentiation of self	differentiation of self	1	kept

differentiation syndrome	differentiation syndrome	differentiation syndrome	differentiation syndrome	differentiation syndrome	1	kept
diffuse brain contusion	diffuse brain contusion	diffuse brain contusion	diffuse brain contusion	diffuse brain contusion	1	kept
diffuse noxious inhibitory control	diffuse noxious inhibitory control	diffuse noxious inhibitory control	diffuse noxious inhibitory control	diffuse noxious inhibitory control	1	kept
diffusing capacity for carbon monoxide	diffusing capacity for carbon monoxide	diffusing capacity for carbon monoxide	diffusing capacity for carbon monoxide	diffusing capacity for carbon monoxide	1	kept
DIFFUSION	diffusion	diffusion	diffusion	Diffusion	1	case_or_format
diffusion analysis	diffusion analysis	diffusion analysis	diffusion analysis	diffusion analysis	1	kept
Diffusion kurtosis imaging	diffusion kurtosis imaging	diffusion kurtosis imaging	diffusion kurtosis imaging	Diffusion kurtosis imaging	1	kept
Diffusion Magnetic Resonance Imaging	diffusion magnetic resonance imaging	diffusion magnetic resonance imaging	diffusion magnetic resonance imaging	Diffusion Magnetic Resonance Imaging	1	kept
Diffusion magnetic resonance imaging	diffusion magnetic resonance imaging	diffusion magnetic resonance imaging	diffusion magnetic resonance imaging	Diffusion Magnetic Resonance Imaging	1	case_or_format
Diffusion of innovation theory	diffusion of innovation theory	diffusion of innovation theory	diffusion of innovation theory	Diffusion of innovation theory	1	kept
diffusion of innovation theory	diffusion of innovation theory	diffusion of innovation theory	diffusion of innovation theory	Diffusion of innovation theory	1	case_or_format
Diffusion of Innovations	diffusion of innovations	diffusion of innovations	diffusion of innovation	Diffusion of Innovation	1	singular
Diffusion Tensor Imaging	diffusion tensor imaging	diffusion tensor imaging	diffusion tensor imaging	diffusion tensor imaging	1	case_or_format
Digastric	digastric	digastric	digastric	Digastric	1	kept
DiGeorge Syndrome	digeorge syndrome	digeorge syndrome	digeorge syndrome	DiGeorge syndrome	1	case_or_format
Digestive activities	digestive activities	digestive activities	digestive activities	Digestive activities	1	kept

digestive system abnormality	digestive system abnormality	digestive system abnormality	digestive system abnormality	Digestive System Abnormalities	1	case_or_format
digestive system disease	digestive system disease	digestive system disease	digestive system disease	Digestive System Diseases	1	case_or_format
digestive system examination	digestive system examination	digestive system examination	digestive system examination	digestive system examination	1	kept
digestive system fistula	digestive system fistula	digestive system fistula	digestive system fistula	digestive system fistula	1	kept
digestive system injury	digestive system injury	digestive system injury	digestive system injury	digestive system injury	1	kept
digestive system malformation	digestive system malformation	digestive system malformation	digestive system malformation	digestive system malformation	1	kept
Digestive System Neoplasms	digestive system neoplasms	digestive system neoplasms	digestive system neoplasms	Digestive System Neoplasms	1	kept
digestive tract endoscopy	digestive tract endoscopy	digestive tract endoscopy	digestive tract endoscopy	digestive tract endoscopy	1	kept
digestive tube	digestive tube	digestive tube	digestive tube	digestive tube	1	kept
digit span test	digit span test	digit span test	digit span test	digit span test	1	kept
digit symbol substitution test	digit symbol substitution test	digit symbol substitution test	digit symbol substitution test	digit symbol substitution test	1	kept
digital artery	digital artery	digital artery	digital artery	digital artery	1	kept
Digital assistants	digital assistants	digital assistants	digital assistants	Digital assistants	1	kept
digital Clock Drawing Test	digital clock drawing test	digital clock drawing test	digital clock drawing test	digital Clock Drawing Test	1	kept
digital clubbing	digital clubbing	digital clubbing	digital clubbing	digital clubbing	1	kept
Digital Communication Perceptions	digital communication perceptions	digital communication perceptions	digital communication perceptions	Digital Communication Perceptions	1	kept

Digital Communication Regulations	digital communication regulations	digital communication regulations	digital communication regulations	Digital Communication Regulations	1	kept
digital computer	digital computer	digital computer	digital computer	digital computer	1	kept
DIGITAL CONTINUITY	digital continuity	digital continuity	digital continuity	DIGITAL CONTINUITY	1	kept
digital evolution	digital evolution	digital evolution	digital evolution	digital evolution	1	kept
Digital form	digital form	digital form	digital form	Digital form	1	kept
Digital forms	digital forms	digital forms	digital form	Digital form	1	singular
digital fundus imaging	digital fundus imaging	digital fundus imaging	digital fundus imaging	digital fundus imaging	1	kept
Digital Health	digital health	digital health	digital health	digital health	1	case_or_format
digital health services	digital health services	digital health services	digital health services	digital health services	1	kept
Digital health technologies	digital health technologies	digital health technologies	digital health technologies	Digital health technologies	1	kept
digital health technologies	digital health technologies	digital health technologies	digital health technologies	Digital health technologies	1	case_or_format
Digital health tools	digital health tools	digital health tools	digital health tools	Digital health tools	1	kept
Digital healths	digital healths	digital healths	digital health	digital health	1	singular
Digital imaging	digital imaging	digital imaging	digital imaging	digital imaging	1	case_or_format
digital imaging and communications in medicine	digital imaging and communications in medicine	digital imaging and communications in medicine	digital imaging and communications in medicine	digital imaging and communications in medicine	1	kept
Digital innovation	digital innovation	digital innovation	digital innovation	Digital innovation	1	kept

digital innovation	digital innovation	digital innovation	digital innovation	Digital innovation	1	case_or_format
digital intervention	digital intervention	digital intervention	digital intervention	digital intervention	1	kept
Digital learning	digital learning	digital learning	digital learning	digital learning	1	case_or_format
digital literacy	digital literacy	digital literacy	digital literacy	Digital literacy	1	case_or_format
digital participation	digital participation	digital participation	digital participation	digital participation	1	kept
Digital program	digital program	digital program	digital program	Digital program	1	kept
Digital signal processing	digital signal processing	digital signal processing	digital signal processing	Digital signal processing	1	kept
digital simulation	digital simulation	digital simulation	digital simulation	digital simulation	1	kept
digital simulator	digital simulator	digital simulator	digital simulator	digital simulator	1	kept
digital storytelling	digital storytelling	digital storytelling	digital storytelling	Digital storytelling	1	case_or_format
Digital surviellance	digital surviellance	digital surviellance	digital surviellance	Digital surviellance	1	kept
Digital therapeutics	digital therapeutics	digital therapeutics	digital therapeutics	Digital therapeutics	1	kept
digital therapy	digital therapy	digital therapy	digital therapy	digital therapy	1	kept
Digital thermometers	digital thermometers	digital thermometers	digital thermometers	Digital thermometers	1	kept
digital tool	digital tool	digital tool	digital tool	digital tool	1	kept
Digital training tool	digital training tool	digital training tool	digital training tool	Digital training tool	1	kept

Digital Transformation	digital transformation	digital transformation	digital transformation	digital transformation	1	case_or_format
Digital twin	digital twin	digital twin	digital twin	Digital twin	1	kept
digital video camera	digital video camera	digital video camera	digital video camera	digital video camera	1	kept
DigitalHub	digitalhub	digitalhub	digitalhub	DigitalHub	1	kept
digitalis	digitalis	digitalis	digitalis	digitalis	1	kept
digitally enabled care pathway	digitally enabled care pathway	digitally enabled care pathway	digitally enabled care pathway	digitally enabled care pathway	1	kept
Digitisation	digitisation	digitisation	digitisation	Digitisation	1	kept
Dignified	dignified	dignified	dignified	Dignified	1	kept
Dignified care	dignified care	dignified care	dignified care	dignified care	1	case_or_format
DIGNIFIED DEATH	dignified death	dignified death	dignified death	dignified death	1	case_or_format
Dignified dying	dignified dying	dignified dying	dignified dying	Dignified dying	1	kept
dignity conserving palliative care	dignity conserving palliative care	dignity conserving palliative care	dignity conserving palliative care	dignity conserving palliative care	1	kept
dignity in care	dignity in care	dignity in care	dignity in care	dignity in care	1	kept
digoxin blood level	digoxin blood level	digoxin blood level	digoxin blood level	digoxin blood level	1	kept
digoxin immune fab	digoxin immune fab	digoxin immune fab	digoxin immune fab	digoxin immune fab	1	kept
DIHYDROCHLORIDE	dihydrochloride	dihydrochloride	dihydrochloride	DIHYDROCHLORIDE	1	kept

dihydrocodeine	dihydrocodeine	dihydrocodeine	dihydrocodeine	dihydrocodeine	1	kept
dihydroxyaluminum sodium carbonate	dihydroxyaluminum sodium carbonate	dihydroxyaluminum sodium carbonate	dihydroxyaluminum sodium carbonate	dihydroxyaluminum sodium carbonate	1	kept
Diketopiperazines	diketopiperazines	diketopiperazines	diketopiperazines	Diketopiperazines	1	kept
dilatation and curettage	dilatation and curettage	dilatation and curettage	dilatation and curettage	Dilatation and Curettage	1	case_or_format
dilatation tracheotomy	dilatation tracheotomy	dilatation tracheotomy	dilatation tracheotomy	dilatation tracheotomy	1	kept
Dilatational tracheotomy	dilatational tracheotomy	dilatational tracheotomy	dilatational tracheotomy	Dilatational tracheotomy	1	kept
Dilated cardiomyopathy	dilated cardiomyopathy	dilated cardiomyopathy	dilated cardiomyopathy	Dilated cardiomyopathy	1	kept
dilated cardiomyopathy	dilated cardiomyopathy	dilated cardiomyopathy	dilated cardiomyopathy	Dilated cardiomyopathy	1	case_or_format
Dimensionamento de pessoal	dimensionamento de pessoal	dimensionamento de pessoal	dimensionamento de pessoal	Dimensionamento de pessoal	1	kept
DIMENSIONS	dimensions	dimensions	dimensions	DIMENSIONS	1	kept
Dimensions	dimensions	dimensions	dimensions	DIMENSIONS	1	case_or_format
dimensions	dimensions	dimensions	dimensions	DIMENSIONS	1	case_or_format
Dimensions of stress	dimensions of stress	dimensions of stress	dimensions of stress	Dimensions of stress	1	kept
Dimethylpolysiloxanes	dimethylpolysiloxanes	dimethylpolysiloxanes	dimethylpolysiloxanes	Dimethylpolysiloxanes	1	kept
dimeticone	dimeticone	dimeticone	dimeticone	dimeticone	1	kept
dinoprostone	dinoprostone	dinoprostone	dinoprostone	Dinoprostone	1	case_or_format

DIPG	dipg	dipg	dipg	DIPG	1	kept
Diphosphonates	diphosphonates	diphosphonates	diphosphonates	Diphosphonates	1	kept
Diphtheria and Tetanus Toxoids	diphtheria and tetanus toxoids	diphtheria and tetanus toxoids	diphtheria and tetanus toxoids	Diphtheria and Tetanus Toxoids	1	kept
diphtheria pertussis poliomyelitis tetanus hepatitis B vaccine	diphtheria pertussis poliomyelitis tetanus hepatitis b vaccine	diphtheria pertussis poliomyelitis tetanus hepatitis b vaccine	diphtheria pertussis poliomyelitis tetanus hepatitis b vaccine	diphtheria pertussis poliomyelitis tetanus hepatitis B vaccine	1	kept
diphtheria pertussis tetanus Haemophilus influenzae type b	diphtheria pertussis tetanus haemophilus influenzae type b	diphtheria pertussis tetanus haemophilus influenzae type b	diphtheria pertussis tetanus haemophilus influenzae type b	diphtheria pertussis tetanus Haemophilus influenzae type b	1	kept
diphtheria tetanus vaccine	diphtheria tetanus vaccine	diphtheria tetanus vaccine	diphtheria tetanus vaccine	diphtheria tetanus vaccine	1	kept
diphtheria toxin	diphtheria toxin	diphtheria toxin	diphtheria toxin	diphtheria toxin	1	kept
Diphtheria Toxoid, Tetanus Toxoid, Acellular Pertussis	diphtheria toxoid tetanus toxoid acellular pertussis	diphtheria toxoid tetanus toxoid acellular pertussis	diphtheria toxoid tetanus toxoid acellular pertussis	Diphtheria Toxoid, Tetanus Toxoid, Acellular Pertussis	1	kept
diphtheria vaccine	diphtheria vaccine	diphtheria vaccine	diphtheria vaccine	diphtheria vaccine	1	kept
Diphtheria-Tetanus-acellular Pertussis Vaccines	diphtheria tetanus acellular pertussis vaccines	diphtheria tetanus acellular pertussis vaccines	diphtheria tetanus acellular pertussis vaccines	Diphtheria-Tetanus-acellular Pertussis Vaccines	1	kept
Diphtheria-Tetanus-Pertussis Vaccine	diphtheria tetanus pertussis vaccine	diphtheria tetanus pertussis vaccine	diphtheria tetanus pertussis vaccine	Diphtheria-Tetanus-Pertussis Vaccine	1	kept
Diphtheroid	diphtheroid	diphtheroid	diphtheroid	Diphtheroid	1	kept
diploidy	diploidy	diploidy	diploidy	diploidy	1	kept
Direct acting antivirals	direct acting antivirals	direct acting antivirals	direct acting antivirals	Direct acting antivirals	1	kept
direct admissions	direct admissions	direct admissions	direct admissions	direct admissions	1	kept
Direct breastfeeding	direct breastfeeding	direct breastfeeding	direct breastfeeding	Direct breastfeeding	1	kept

direct content analysis	direct content analysis	direct content analysis	direct content analysis	direct content analysis	1	kept
Direct cost	direct cost	direct cost	direct cost	Direct cost	1	kept
direct cost	direct cost	direct cost	direct cost	Direct cost	1	case_or_format
DIRECT COSTS	direct costs	direct costs	direct cost	Direct cost	1	singular
direct current shock	direct current shock	direct current shock	direct current shock	direct current shock	1	kept
Direct medical cost	direct medical cost	direct medical cost	direct medical cost	Direct medical cost	1	kept
direct medical cost	direct medical cost	direct medical cost	direct medical cost	Direct medical cost	1	case_or_format
direct medical cost of illness	direct medical cost of illness	direct medical cost of illness	direct medical cost of illness	direct medical cost of illness	1	kept
direct observation	direct observation	direct observation	direct observation	Direct observation	1	case_or_format
Direct observation method	direct observation method	direct observation method	direct observation method	Direct observation method	1	kept
direct observation of procedural skills	direct observation of procedural skills	direct observation of procedural skills	direct observation of procedural skills	direct observation of procedural skills	1	kept
Direct observations	direct observations	direct observations	direct observation	Direct observation	1	singular
direct observations	direct observations	direct observations	direct observation	Direct observation	1	singular
Direct oral anticoagulant	direct oral anticoagulant	direct oral anticoagulant	direct oral anticoagulant	Direct oral anticoagulants	1	case_or_format
DIRECT ORAL ANTICOAGULANTS	direct oral anticoagulants	direct oral anticoagulants	direct oral anticoagulant	Direct oral anticoagulants	1	singular
direct oral anticoagulants	direct oral anticoagulants	direct oral anticoagulants	direct oral anticoagulant	Direct oral anticoagulants	1	singular

Direct Peritoneal Resuscitation	direct peritoneal resuscitation	direct peritoneal resuscitation	direct peritoneal resuscitation	Direct Peritoneal Resuscitation	1	kept
Direct thrombin inhibitor	direct thrombin inhibitor	direct thrombin inhibitor	direct thrombin inhibitor	Direct thrombin inhibitor	1	kept
directed observers	directed observers	directed observers	directed observers	directed observers	1	kept
directed qualitative content analysis	directed qualitative content analysis	directed qualitative content analysis	directed qualitative content analysis	directed qualitative content analysis	1	kept
Directed Tissue Donation	directed tissue donation	directed tissue donation	directed tissue donation	Directed Tissue Donation	1	kept
directive anticipée	directive anticip e	directive anticip e	directive anticip e	directive anticipée	1	kept
directives and request	directives and request	directives and request	directives and request	directives and request	1	kept
Directly Observed Therapy	directly observed therapy	directly observed therapy	directly observed therapy	Directly Observed Therapy	1	kept
directly observed therapy	directly observed therapy	directly observed therapy	directly observed therapy	Directly Observed Therapy	1	case_or_format
director	director	director	director	director	1	kept
Directrices para la planificación en salud	directrices para la planificaci n en salud	directrices para la planificaci n en salud	directrices para la planificaci n en salud	Directrices para la planificación en salud	1	kept
disability advocacy	disability advocacy	disability advocacy	disability advocacy	disability advocacy	1	kept
disability and health	disability and health	disability and health	disability and health	disability and health	1	kept
disability assessment	disability assessment	disability assessment	disability assessment	disability assessment	1	kept
Disability Discrimination	disability discrimination	disability discrimination	disability discrimination	disability discrimination	1	case_or_format
Disability evaluation	disability evaluation	disability evaluation	disability evaluation	Disability Evaluation	1	case_or_format

Disability Rating Scale	disability rating scale	disability rating scale	disability rating scale	disability rating scale	1	case_or_format
DISABILITY RATING-SCALE	disability rating scale	disability rating scale	disability rating scale	disability rating scale	1	case_or_format
disability severity	disability severity	disability severity	disability severity	disability severity	1	kept
disability studies	disability studies	disability studies	disability studies	disability studies	1	kept
Disabled children	disabled children	disabled children	disabled children	Disabled Children	1	case_or_format
Disabled persons	disabled persons	disabled persons	disabled person	disabled person	1	singular
disaccharide	disaccharide	disaccharide	disaccharide	disaccharide	1	kept
Disaccharides	disaccharides	disaccharides	disaccharide	disaccharide	1	singular
Disadvantaged populations	disadvantaged populations	disadvantaged populations	disadvantaged population	disadvantaged population	1	singular
disaster evacuation	disaster evacuation	disaster evacuation	disaster evacuation	disaster evacuation	1	kept
Disaster management teams	disaster management teams	disaster management teams	disaster management teams	Disaster management teams	1	kept
Disaster Medicine education	disaster medicine education	disaster medicine education	disaster medicine education	Disaster Medicine education	1	kept
disaster plan	disaster plan	disaster plan	disaster plan	Disaster plans	1	case_or_format
disaster preparation	disaster preparation	disaster preparation	disaster preparation	disaster preparation	1	kept
Disaster triage	disaster triage	disaster triage	disaster triage	Disaster triage	1	kept
Disaster Victims	disaster victims	disaster victims	disaster victim	disaster victim	1	singular

DISASTERS	disasters	disasters	disaster	Disasters	1	singular
DISCERN	discern	discern	discern	DISCERN	1	kept
Discharg	discharg	discharg	discharg	Discharg	1	kept
Discharge before noon	discharge before noon	discharge before noon	discharge before noon	Discharge before noon	1	kept
discharge care plan	discharge care plan	discharge care plan	discharge care plan	discharge care plan	1	kept
Discharge checklist	discharge checklist	discharge checklist	discharge checklist	Discharge checklist	1	kept
Discharge Criteria Checklist	discharge criteria checklist	discharge criteria checklist	discharge criteria checklist	Discharge Criteria Checklist	1	kept
Discharge destination	discharge destination	discharge destination	discharge destination	Discharge destination	1	kept
discharge destination	discharge destination	discharge destination	discharge destination	Discharge destination	1	case_or_format
Discharge dispositions	discharge dispositions	discharge dispositions	discharge disposition	discharge disposition	1	singular
Discharge intervention	discharge intervention	discharge intervention	discharge intervention	Discharge intervention	1	kept
Discharge length of stay	discharge length of stay	discharge length of stay	discharge length of stay	Discharge length of stay	1	kept
Discharge letter	discharge letter	discharge letter	discharge letter	Discharge letter	1	kept
Discharge letters	discharge letters	discharge letters	discharge letter	Discharge letter	1	singular
Discharge outcome	discharge outcome	discharge outcome	discharge outcome	Discharge outcome	1	kept
discharge outcome	discharge outcome	discharge outcome	discharge outcome	Discharge outcome	1	case_or_format

Discharge outcomes	discharge outcomes	discharge outcomes	discharge outcome	Discharge outcome	1	singular
Discharge policy	discharge policy	discharge policy	discharge policy	Discharge policy	1	kept
Discharge practices	discharge practices	discharge practices	discharge practices	Discharge practices	1	kept
Discharge preparation	discharge preparation	discharge preparation	discharge preparation	Discharge preparation	1	kept
discharge preparation	discharge preparation	discharge preparation	discharge preparation	Discharge preparation	1	case_or_format
Discharge Preparation Service	discharge preparation service	discharge preparation service	discharge preparation service	Discharge Preparation Service	1	kept
Discharge preparedness	discharge preparedness	discharge preparedness	discharge preparedness	Discharge preparedness	1	kept
Discharge protocol	discharge protocol	discharge protocol	discharge protocol	Discharge protocol	1	kept
discharge setting	discharge setting	discharge setting	discharge setting	discharge setting	1	kept
Discharge status	discharge status	discharge status	discharge status	Discharge status	1	kept
Discharge summary	discharge summary	discharge summary	discharge summary	Discharge summary	1	kept
discharge teaching	discharge teaching	discharge teaching	discharge teaching	discharge teaching	1	kept
Discharge training	discharge training	discharge training	discharge training	Discharge training	1	kept
Discharge ward	discharge ward	discharge ward	discharge ward	Discharge ward	1	kept
Discharge-support program	discharge support program	discharge support program	discharge support program	Discharge-support program	1	kept
disciplinary power	disciplinary power	disciplinary power	disciplinary power	disciplinary power	1	kept

discipline	discipline	discipline	discipline	discipline	1	kept
Discipline cooperation	discipline cooperation	discipline cooperation	discipline cooperation	Discipline cooperation	1	kept
Discipline operation condition	discipline operation condition	discipline operation condition	discipline operation condition	Discipline operation condition	1	kept
Discipline scale	discipline scale	discipline scale	discipline scale	Discipline scale	1	kept
disclosure	disclosure	disclosure	disclosure	Disclosure	1	case_or_format
Disclosure and healthcare personnel	disclosure and healthcare personnel	disclosure and healthcare personnel	disclosure and healthcare personnel	Disclosure and healthcare personnel	1	kept
DISCOMFORT	discomfort	discomfort	discomfort	discomfort	1	case_or_format
Discomfort factors	discomfort factors	discomfort factors	discomfort factors	Discomfort factors	1	kept
Discomfort symptoms	discomfort symptoms	discomfort symptoms	discomfort symptoms	Discomfort symptoms	1	kept
DISCONTINUATION	discontinuation	discontinuation	discontinuation	DISCONTINUATION	1	kept
Discontinuation	discontinuation	discontinuation	discontinuation	DISCONTINUATION	1	case_or_format
discontinuation	discontinuation	discontinuation	discontinuation	DISCONTINUATION	1	case_or_format
discontinuities in care	discontinuities in care	discontinuities in care	discontinuities in care	discontinuities in care	1	kept
DISCONTINUITY	discontinuity	discontinuity	discontinuity	DISCONTINUITY	1	kept
Discordance	discordance	discordance	discordance	Discordance	1	kept
discordance	discordance	discordance	discordance	Discordance	1	case_or_format

discrete choice analysis	discrete choice analysis	discrete choice analysis	discrete choice analysis	discrete choice analysis	1	kept
discrete choice experiment	discrete choice experiment	discrete choice experiment	discrete choice experiment	Discrete choice experiment	1	case_or_format
discrete cosine transform	discrete cosine transform	discrete cosine transform	discrete cosine transform	discrete cosine transform	1	kept
discrete element analysis	discrete element analysis	discrete element analysis	discrete element analysis	discrete element analysis	1	kept
Discrete event simulation model	discrete event simulation model	discrete event simulation model	discrete event simulation model	Discrete event simulation model	1	kept
Discrete-event simulation	discrete event simulation	discrete event simulation	discrete event simulation	Discrete event simulation	1	case_or_format
discrete-event simulation	discrete event simulation	discrete event simulation	discrete event simulation	Discrete event simulation	1	case_or_format
Discrete-event simulation model	discrete event simulation model	discrete event simulation model	discrete event simulation model	Discrete event simulation model	1	case_or_format
Discrete-event simulations	discrete event simulations	discrete event simulations	discrete event simulation	Discrete event simulation	1	singular
Discretion	discretion	discretion	discretion	Discretion	1	kept
Discretization algorithms	discretization algorithms	discretization algorithms	discretization algorithms	Discretization algorithms	1	kept
Discretization technique	discretization technique	discretization technique	discretization technique	Discretization technique	1	kept
Discretizations	discretizations	discretizations	discretizations	Discretizations	1	kept
Discriminant analysis	discriminant analysis	discriminant analysis	discriminant analysis	discriminant analysis	1	case_or_format
Discriminant validity	discriminant validity	discriminant validity	discriminant validity	discriminant validity	1	case_or_format
Discrimination Learning	discrimination learning	discrimination learning	discrimination learning	Discrimination Learning	1	kept

Discrimination, Psychological	discrimination psychological	discrimination psychological	discrimination psychological	Discrimination, Psychological	1	kept
DISCUSSING RELIGION	discussing religion	discussing religion	discussing religion	DISCUSSING RELIGION	1	kept
Discussion	discussion	discussion	discussion	DISCUSSIONS	1	case_or_format
discussion	discussion	discussion	discussion	DISCUSSIONS	1	case_or_format
Discussions	discussions	discussions	discussion	DISCUSSIONS	1	singular
disease clearance	disease clearance	disease clearance	disease clearance	disease clearance	1	kept
Disease diagnosis	disease diagnosis	disease diagnosis	disease diagnosis	disease diagnosis	1	case_or_format
Disease Eradication	disease eradication	disease eradication	disease eradication	Disease Eradication	1	kept
disease eradication	disease eradication	disease eradication	disease eradication	Disease Eradication	1	case_or_format
Disease exacerbation	disease exacerbation	disease exacerbation	disease exacerbation	disease exacerbation	1	case_or_format
Disease experience	disease experience	disease experience	disease experience	Disease experience	1	kept
Disease group prediction	disease group prediction	disease group prediction	disease group prediction	Disease group prediction	1	kept
Disease Hotspot	disease hotspot	disease hotspot	disease hotspot	Disease Hotspot	1	kept
disease hotspot	disease hotspot	disease hotspot	disease hotspot	Disease Hotspot	1	case_or_format
disease incidence	disease incidence	disease incidence	disease incidence	disease incidence	1	kept
Disease management program	disease management program	disease management program	disease management program	Disease management program	1	kept

Disease Management programme	disease management programme	disease management programme	disease management programme	Disease Management programme	1	kept
Disease management programs	disease management programs	disease management programs	disease management program	Disease management program	1	singular
disease modifying therapy	disease modifying therapy	disease modifying therapy	disease modifying therapy	disease modifying therapy	1	kept
Disease notification	disease notification	disease notification	disease notification	Disease Notification	1	case_or_format
disease ontology	disease ontology	disease ontology	disease ontology	disease ontology	1	kept
Disease prediction	disease prediction	disease prediction	disease prediction	Disease prediction	1	kept
Disease prevention emergency room	disease prevention emergency room	disease prevention emergency room	disease prevention emergency room	Disease prevention emergency room	1	kept
disease progression monitoring	disease progression monitoring	disease progression monitoring	disease progression monitoring	disease progression monitoring	1	kept
disease related criteria	disease related criteria	disease related criteria	disease related criteria	disease related criteria	1	kept
Disease related malnutrition	disease related malnutrition	disease related malnutrition	disease related malnutrition	Disease related malnutrition	1	kept
Disease severity	disease severity	disease severity	disease severity	disease severity	1	case_or_format
Disease trans-mission, infectious	disease trans mission infectious	disease trans mission infectious	disease trans mission infectious	Disease trans-mission, infectious	1	kept
Disease transmission: infectious	disease transmission infectious	disease transmission infectious	disease transmission infectious	Disease Transmission, Infectious	1	case_or_format
Disease worsening	disease worsening	disease worsening	disease worsening	Disease worsening	1	kept
DISEASE-CONTROL	disease control	disease control	disease control	disease control	1	case_or_format
disease-modifying therapies	disease modifying therapies	disease modifying therapies	disease modifying therapy	disease modifying therapy	1	singular

DISEASE-RELATED MALNUTRITION	disease related malnutrition	disease related malnutrition	disease related malnutrition	Disease related malnutrition	1	case_or_format
Diseases [prevention and control]	diseases prevention and control	diseases prevention and control	diseases prevention and control	Diseases [prevention and control]	1	kept
Diseases, obesity: morbid	diseases obesity morbid	diseases obesity morbid	diseases obesity morbid	Diseases, obesity: morbid	1	kept
DISEASES-SOCIETY	diseases society	diseases society	diseases society	DISEASES-SOCIETY	1	kept
Diseases: occupational	diseases occupational	diseases occupational	diseases occupational	Diseases: occupational	1	kept
Disenfranchised Grief	disenfranchised grief	disenfranchised grief	disenfranchised grief	Disenfranchised Grief	1	kept
Disenfranchised grief	disenfranchised grief	disenfranchised grief	disenfranchised grief	Disenfranchised Grief	1	case_or_format
disenfranchised grief	disenfranchised grief	disenfranchised grief	disenfranchised grief	Disenfranchised Grief	1	case_or_format
disengagement	disengagement	disengagement	disengagement	disengagement	1	kept
Disfagia	disfagia	disfagia	disfagia	Disfagia	1	kept
Disfigurement	disfigurement	disfigurement	disfigurement	Disfigurement	1	kept
Disfunción cognitiva	disfunci n cognitiva	disfunci n cognitiva	disfunci n cognitiva	Disfunción cognitiva	1	kept
Disgust	disgust	disgust	disgust	Disgust	1	kept
disgust sensitivity	disgust sensitivity	disgust sensitivity	disgust sensitivity	disgust sensitivity	1	kept
dishonesty	dishonesty	dishonesty	dishonesty	dishonesty	1	kept
Disinfectant	disinfectant	disinfectant	disinfectant	Disinfectants	1	case_or_format

disinfectant contamination	disinfectant contamination	disinfectant contamination	disinfectant contamination	disinfectant contamination	1	kept
disinfectants	disinfectants	disinfectants	disinfectant	Disinfectants	1	singular
DISINFECTION	disinfection	disinfection	disinfection	disinfection	1	case_or_format
Disinfection cap	disinfection cap	disinfection cap	disinfection cap	disinfection cap	1	case_or_format
disinformation	disinformation	disinformation	disinformation	disinformation	1	kept
disinhibition	disinhibition	disinhibition	disinhibition	disinhibition	1	kept
disintegrin	disintegrin	disintegrin	disintegrin	disintegrin	1	kept
Disk Diffusion Antimicrobial Tests	disk diffusion antimicrobial tests	disk diffusion antimicrobial tests	disk diffusion antimicrobial tests	Disk Diffusion Antimicrobial Tests	1	kept
Dislocation	dislocation	dislocation	dislocation	dislocation	1	case_or_format
dislodgement	dislodgement	dislodgement	dislodgement	dislodgement	1	kept
dismissive attitude	dismissive attitude	dismissive attitude	dismissive attitude	dismissive attitude	1	kept
disobedience	disobedience	disobedience	disobedience	disobedience	1	kept
disopyramide	disopyramide	disopyramide	disopyramide	disopyramide	1	kept
disopyramide phosphate	disopyramide phosphate	disopyramide phosphate	disopyramide phosphate	disopyramide phosphate	1	kept
DISORDER	disorder	disorder	disorder	DISORDERS	1	case_or_format
disorder of sex development	disorder of sex development	disorder of sex development	disorder of sex development	disorder of sex development	1	kept

DISORDER-RELATED SYMPTOMS	disorder related symptoms	disorder related symptoms	disorder related symptoms	DISORDER-RELATED SYMPTOMS	1	kept
disorders	disorders	disorders	disorder	DISORDERS	1	singular
Disorders of consciousness program	disorders of consciousness program	disorders of consciousness program	disorders of consciousness program	Disorders of consciousness program	1	kept
Disorders of environmental origin	disorders of environmental origin	disorders of environmental origin	disorders of environmental origin	Disorders of environmental origin	1	kept
disorders of the gut-brain interaction	disorders of the gut brain interaction	disorders of the gut brain interaction	disorders of the gut brain interaction	disorders of the gut-brain interaction	1	kept
disparities health	disparities health	disparities health	disparities health	disparities health	1	kept
disparity	disparity	disparity	disparity	disparities	1	case_or_format
Dispatch	dispatch	dispatch	dispatch	Dispatch	1	kept
dispatcher call processing time	dispatcher call processing time	dispatcher call processing time	dispatcher call processing time	dispatcher call processing time	1	kept
dispensing extra dose	dispensing extra dose	dispensing extra dose	dispensing extra dose	dispensing extra dose	1	kept
dispersion analysis	dispersion analysis	dispersion analysis	dispersion analysis	dispersion analysis	1	kept
Dispersion quality	dispersion quality	dispersion quality	dispersion quality	Dispersion quality	1	kept
displacement	displacement	displacement	displacement	displacement	1	kept
Displacement process	displacement process	displacement process	displacement process	Displacement process	1	kept
Display designs	display designs	display designs	display designs	Display designs	1	kept
DISPLAY IMPROVES	display improves	display improves	display improves	DISPLAY IMPROVES	1	kept

display system	display system	display system	display system	display system	1	kept
disposable pre impregnated wipes	disposable pre impregnated wipes	disposable pre impregnated wipes	disposable pre impregnated wipes	disposable pre impregnated wipes	1	kept
disposal	disposal	disposal	disposal	disposal	1	kept
dispositif intraveineux longue durée	dispositif intraveineux longue dur e	dispositif intraveineux longue dur e	dispositif intraveineux longue dur e	dispositif intraveineux longue durée	1	kept
disposition	disposition	disposition	disposition	disposition	1	kept
Disposition based on total psoas volume	disposition based on total psoas volume	disposition based on total psoas volume	disposition based on total psoas volume	Disposition based on total psoas volume	1	kept
disposition outcome	disposition outcome	disposition outcome	disposition outcome	disposition outcome	1	kept
disproportionate care	disproportionate care	disproportionate care	disproportionate care	disproportionate care	1	kept
Disproportionate increase in intracranial pressure	disproportionate increase in intracranial pressure	disproportionate increase in intracranial pressure	disproportionate increase in intracranial pressure	Disproportionate increase in intracranial pressure	1	kept
Disrespect	disrespect	disrespect	disrespect	Disrespect	1	kept
disrespect	disrespect	disrespect	disrespect	Disrespect	1	case_or_format
Disruption	disruption	disruption	disruption	DISRUPTION	1	case_or_format
dissection	dissection	dissection	dissection	Dissection	1	case_or_format
Disseminated infection	disseminated infection	disseminated infection	disseminated infection	Disseminated infection	1	kept
dissemination and implementation	dissemination and implementation	dissemination and implementation	dissemination and implementation	dissemination and implementation	1	kept
dissemination of innovation	dissemination of innovation	dissemination of innovation	dissemination of innovation	dissemination of innovation	1	kept

dissociative disorder	dissociative disorder	dissociative disorder	dissociative disorder	dissociative disorder	1	kept
Dissociative Disorders	dissociative disorders	dissociative disorders	dissociative disorder	dissociative disorder	1	singular
distal femur	distal femur	distal femur	distal femur	distal femur	1	kept
Distal femur fracture	distal femur fracture	distal femur fracture	distal femur fracture	distal femur fracture	1	case_or_format
distal internal jugular vein	distal internal jugular vein	distal internal jugular vein	distal internal jugular vein	distal internal jugular vein	1	kept
DISTANCE	distance	distance	distance	Distance	1	case_or_format
Distance education	distance education	distance education	distance education	Distance Education	1	case_or_format
distance education	distance education	distance education	distance education	Distance Education	1	case_or_format
Distance from skin to hyoid bone	distance from skin to hyoid bone	distance from skin to hyoid bone	distance from skin to hyoid bone	Distance from skin to hyoid bone	1	kept
Distance from skin to thyroid isthmus	distance from skin to thyroid isthmus	distance from skin to thyroid isthmus	distance from skin to thyroid isthmus	Distance from skin to thyroid isthmus	1	kept
distance simulation	distance simulation	distance simulation	distance simulation	distance simulation	1	kept
distillation	distillation	distillation	distillation	distillation	1	kept
Distilled water	distilled water	distilled water	distilled water	distilled water	1	case_or_format
DISTRACTION	distraction	distraction	distraction	distraction	1	case_or_format
distraction osteogenesis	distraction osteogenesis	distraction osteogenesis	distraction osteogenesis	distraction osteogenesis	1	kept
Distractions	distractions	distractions	distraction	distraction	1	singular

Distractor	distractor	distractor	distractor	Distractor	1	kept
distress interolerance	distress interolerance	distress interolerance	distress interolerance	distress interolerance	1	kept
DISTRESS SCALE K10	distress scale k10	distress scale k10	distress scale k10	DISTRESS SCALE K10	1	kept
DISTRESS-SYNDROME	distress syndrome	distress syndrome	distress syndrome	distress syndrome	1	case_or_format
Distressed Communities Index	distressed communities index	distressed communities index	distressed communities index	Distressed Communities Index	1	kept
Distressing death	distressing death	distressing death	distressing death	Distressing death	1	kept
Distributed cognition	distributed cognition	distributed cognition	distributed cognition	Distributed cognition	1	kept
distributed cognition	distributed cognition	distributed cognition	distributed cognition	Distributed cognition	1	case_or_format
Distributed computer systems	distributed computer systems	distributed computer systems	distributed computer systems	Distributed computer systems	1	kept
Distributed leadership	distributed leadership	distributed leadership	distributed leadership	Distributed leadership	1	kept
Distributed situation awareness	distributed situation awareness	distributed situation awareness	distributed situation awareness	Distributed situation awareness	1	kept
Distributed systems	distributed systems	distributed systems	distributed systems	Distributed systems	1	kept
distribution	distribution	distribution	distribution	distribution	1	kept
Distribution factor	distribution factor	distribution factor	distribution factor	Distribution factor	1	kept
Distribution of disease	distribution of disease	distribution of disease	distribution of disease	Distribution of disease	1	kept
distribution parameters	distribution parameters	distribution parameters	distribution parameters	distribution parameters	1	kept

Distribution patterns	distribution patterns	distribution patterns	distribution patterns	Distribution patterns	1	kept
Distribution systems	distribution systems	distribution systems	distribution systems	Distribution systems	1	kept
Distributional semantics	distributional semantics	distributional semantics	distributional semantics	Distributional semantics	1	kept
Distributive justice	distributive justice	distributive justice	distributive justice	Distributive justice	1	kept
distributive justice	distributive justice	distributive justice	distributive justice	Distributive justice	1	case_or_format
district hospital	district hospital	district hospital	district hospital	District hospitals	1	case_or_format
distrust	distrust	distrust	distrust	distrust	1	kept
Distrés moral	distr s moral	distr s moral	distr s moral	Distrés moral	1	kept
Distrés psicológico	distr s psicol gico	distr s psicol gico	distr s psicol gico	Distrés psicológico	1	kept
Disturbance	disturbance	disturbance	disturbance	DISTURBANCE	1	case_or_format
disturbance	disturbance	disturbance	disturbance	DISTURBANCE	1	case_or_format
Disturbed sleep	disturbed sleep	disturbed sleep	disturbed sleep	Disturbed sleep	1	kept
dithiaden	dithiaden	dithiaden	dithiaden	dithiaden	1	kept
dithranol	dithranol	dithranol	dithranol	dithranol	1	kept
diurnal enuresis	diurnal enuresis	diurnal enuresis	diurnal enuresis	diurnal enuresis	1	kept
Diurnal patterns	diurnal patterns	diurnal patterns	diurnal patterns	Diurnal patterns	1	kept

Diurnal rhythm	diurnal rhythm	diurnal rhythm	diurnal rhythm	Diurnal rhythm	1	kept
diurnal variations	diurnal variations	diurnal variations	diurnal variation	Diurnal variation	1	singular
diverse teaching	diverse teaching	diverse teaching	diverse teaching	diverse teaching	1	kept
DIVERSION	diversion	diversion	diversion	DIVERSION	1	kept
DIVERSITY	diversity	diversity	diversity	diversity	1	case_or_format
Diversity and inclusion in healthcare	diversity and inclusion in healthcare	diversity and inclusion in healthcare	diversity and inclusion in healthcare	Diversity and inclusion in healthcare	1	kept
diversity, equity and inclusion	diversity equity and inclusion	diversity equity and inclusion	diversity equity and inclusion	diversity, equity and inclusion	1	kept
Division of labour	division of labour	division of labour	division of labour	Division of labour	1	kept
divorce	divorce	divorce	divorce	divorce	1	kept
DLCO	dlco	dlco	dlco	DLCO	1	kept
DLQI	dlqi	dlqi	dlqi	DLQI	1	kept
DMAIC	dmaic	dmaic	dmaic	DMAIC	1	kept
DMM	dmm	dmm	dmm	DMM	1	kept
DMS, data management system	dms data management system	dms data management system	dms data management system	DMS, data management system	1	kept
DNA 23S	dna 23s	dna 23s	dna 23s	DNA 23S	1	kept
DNA base composition	dna base composition	dna base composition	dna base composition	DNA base composition	1	kept

DNA Damage	dna damage	dna damage	dna damage	DNA damage	1	case_or_format
DNA ligase	dna ligase	dna ligase	dna ligase	DNA ligase	1	kept
DNA methyltransferase 3A	dna methyltransferase 3a	dna methyltransferase 3a	dna methyltransferase 3a	DNA methyltransferase 3A	1	kept
DNA microarray	dna microarray	dna microarray	dna microarray	DNA microarray	1	kept
DNA molecular detection	dna molecular detection	dna molecular detection	dna molecular detection	DNA molecular detection	1	kept
DNA polymerase	dna polymerase	dna polymerase	dna polymerase	DNA polymerase	1	kept
DNA polymorphism	dna polymorphism	dna polymorphism	dna polymorphism	DNA polymorphism	1	kept
DNA Primers	dna primers	dna primers	dna primers	DNA Primers	1	kept
DNA repair	dna repair	dna repair	dna repair	DNA repair	1	kept
DNA supercoiling	dna supercoiling	dna supercoiling	dna supercoiling	DNA supercoiling	1	kept
DNA template	dna template	dna template	dna template	DNA template	1	kept
DNA topoisomerase A	dna topoisomerase a	dna topoisomerase a	dna topoisomerase a	DNA topoisomerase A	1	kept
DNA topoisomerase B	dna topoisomerase b	dna topoisomerase b	dna topoisomerase b	DNA topoisomerase B	1	kept
DNA, Ribosomal	dna ribosomal	dna ribosomal	dna ribosomal	DNA, Ribosomal	1	kept
DNA-Binding Proteins	dna binding proteins	dna binding proteins	dna binding proteins	DNA-Binding Proteins	1	kept
DNA-Formamidopyrimidine Glycosylase	dna formamidopyrimidine glycosylase	dna formamidopyrimidine glycosylase	dna formamidopyrimidine glycosylase	DNA-Formamidopyrimidine Glycosylase	1	kept

DNACPR	dnacpr	dnacpr	dnacpr	DNACPR	1	kept
DNAemia	dnaemia	dnaemia	dnaemia	DNAemia	1	kept
DNAR order	dnar order	dnar order	dnar order	DNAR order	1	kept
dnr	dnr	dnr	dnr	DNR	1	case_or_format
do not attempt resuscitation	do not attempt resuscitation	do not attempt resuscitation	do not attempt resuscitation	do not attempt resuscitation	1	kept
do not hospitalize	do not hospitalize	do not hospitalize	do not hospitalize	do not hospitalize	1	kept
Do not intubate	do not intubate	do not intubate	do not intubate	Do not intubate	1	kept
do not resuscitate orders	do not resuscitate orders	do not resuscitate orders	do not resuscitate order	do not resuscitate order	1	singular
Do-not-intubate	do not intubate	do not intubate	do not intubate	Do not intubate	1	case_or_format
do-not-resuscitate order	do not resuscitate order	do not resuscitate order	do not resuscitate order	do not resuscitate order	1	case_or_format
Do-Not-Resuscitate orders	do not resuscitate orders	do not resuscitate orders	do not resuscitate order	do not resuscitate order	1	singular
Do-not-resuscitate orders	do not resuscitate orders	do not resuscitate orders	do not resuscitate order	do not resuscitate order	1	singular
DOACs	doacs	doacs	doacs	DOACs	1	kept
DOC	doc	doc	doc	DOC	1	kept
Docosahexaenoic Acids	docosahexaenoic acids	docosahexaenoic acids	docosahexaenoic acid	docosahexaenoic acid	1	singular
DOCTOR RELATIONSHIP	doctor relationship	doctor relationship	doctor relationship	DOCTOR RELATIONSHIP	1	kept

Doctor satisfaction	doctor satisfaction	doctor satisfaction	doctor satisfaction	Doctor satisfaction	1	kept
doctor satisfaction	doctor satisfaction	doctor satisfaction	doctor satisfaction	Doctor satisfaction	1	case_or_format
doctor to bed ratio	doctor to bed ratio	doctor to bed ratio	doctor to bed ratio	doctor to bed ratio	1	kept
doctor-managers	doctor managers	doctor managers	doctor managers	doctor-managers	1	kept
Document Classification	document classification	document classification	document classification	Document Classification	1	kept
Document classification	document classification	document classification	document classification	Document Classification	1	case_or_format
document review and approval	document review and approval	document review and approval	document review and approval	document review and approval	1	kept
Document templates	document templates	document templates	document templates	Document templates	1	kept
Documentaries and factual films	documentaries and factual films	documentaries and factual films	documentaries and factual films	Documentaries and factual films	1	kept
Documentary	documentary	documentary	documentary	Documentary	1	kept
DOCUMENTATION AND ARCHIVES	documentation and archives	documentation and archives	documentation and archives	DOCUMENTATION AND ARCHIVES	1	kept
Documentation compliance	documentation compliance	documentation compliance	documentation compliance	Documentation compliance	1	kept
documentation compliance	documentation compliance	documentation compliance	documentation compliance	Documentation compliance	1	case_or_format
Documentation errors	documentation errors	documentation errors	documentation errors	Documentation errors	1	kept
DOCUMENTATION TIME	documentation time	documentation time	documentation time	DOCUMENTATION TIME	1	kept
Documentation tools	documentation tools	documentation tools	documentation tools	Documentation tools	1	kept

docusate sodium plus Senna extract	docusate sodium plus senna extract	docusate sodium plus senna extract	docusate sodium plus senna extract	docusate sodium plus Senna extract	1	kept
Doederleinia berycoides	doederleinia berycoides	doederleinia berycoides	doederleinia berycoides	Doederleinia berycoides	1	kept
doffing	doffing	doffing	doffing	Doffing	1	case_or_format
Dog	dog	dog	dog	Dogs	1	case_or_format
Dog bite	dog bite	dog bite	dog bite	Dog bite	1	kept
dog bite	dog bite	dog bite	dog bite	Dog bite	1	case_or_format
dogs	dogs	dogs	dog	Dogs	1	singular
dolichocephaly	dolichocephaly	dolichocephaly	dolichocephaly	Dolichocephaly	1	case_or_format
Dolor posquirúrgico	dolor posquir rgico	dolor posquir rgico	dolor posquir rgico	Dolor posquirúrgico	1	kept
dolutegravir	dolutegravir	dolutegravir	dolutegravir	dolutegravir	1	kept
dolven ped syrup	dolven ped syrup	dolven ped syrup	dolven ped syrup	dolven ped syrup	1	kept
Domain-adaptation	domain adaptation	domain adaptation	domain adaptation	Domain-adaptation	1	kept
Domains	domains	domains	domains	Domains	1	kept
Domestic appliances	domestic appliances	domestic appliances	domestic appliances	Domestic appliances	1	kept
domestic cat	domestic cat	domestic cat	domestic cat	domestic cat	1	kept
domestic partnership	domestic partnership	domestic partnership	domestic partnership	domestic partnership	1	kept

Domestic setting	domestic setting	domestic setting	domestic setting	Domestic setting	1	kept
domestic sewage discharge behavior	domestic sewage discharge behavior	domestic sewage discharge behavior	domestic sewage discharge behavior	domestic sewage discharge behavior	1	kept
Domestic violence	domestic violence	domestic violence	domestic violence	domestic violence	1	case_or_format
dominance behavior	dominance behavior	dominance behavior	dominance behavior	dominance behavior	1	kept
Don d'organes	don d organes	don d organes	don d organes	Don d'organes	1	kept
don d'organes	don d organes	don d organes	don d organes	Don d'organes	1	case_or_format
Don d'organes après mort cardiaque	don d organes apr s mort cardiaque	don d organes apr s mort cardiaque	don d organes apr s mort cardiaque	Don d'organes après mort cardiaque	1	kept
Donabedian's quality care model	donabedian s quality care model	donabedian s quality care model	donabedian s quality care model	Donabedian's quality care model	1	kept
Donantes de tejidos	donantes de tejidos	donantes de tejidos	donantes de tejidos	Donantes de tejidos	1	kept
donation after brain death	donation after brain death	donation after brain death	donation after brain death	donation after brain death	1	kept
Donation After Cardiac Death	donation after cardiac death	donation after cardiac death	donation after cardiac death	Donation After Cardiac Death	1	kept
Donation after cardiac death	donation after cardiac death	donation after cardiac death	donation after cardiac death	Donation After Cardiac Death	1	case_or_format
donation after cardiac death	donation after cardiac death	donation after cardiac death	donation after cardiac death	Donation After Cardiac Death	1	case_or_format
donation after circulatory death donor	donation after circulatory death donor	donation after circulatory death donor	donation after circulatory death donor	donation after circulatory death donor	1	kept
donation after circulatory determination of death	donation after circulatory determination of death	donation after circulatory determination of death	donation after circulatory determination of death	donation after circulatory determination of death	1	kept
Donation and transplantation	donation and transplantation	donation and transplantation	donation and transplantation	Donation and transplantation	1	kept

donation and transplantation program	donation and transplantation program	donation and transplantation program	donation and transplantation program	donation and transplantation program	1	kept
donation process	donation process	donation process	donation process	donation process	1	kept
Donation rates	donation rates	donation rates	donation rates	Donation rates	1	kept
donation rates	donation rates	donation rates	donation rates	Donation rates	1	case_or_format
Donation request	donation request	donation request	donation request	Donation request	1	kept
donkey	donkey	donkey	donkey	donkey	1	kept
Donna Wright Competency Assessment	donna wright competency assessment	donna wright competency assessment	donna wright competency assessment	Donna Wright Competency Assessment	1	kept
donning and doffing	donning and doffing	donning and doffing	donning and doffing	donning and doffing	1	kept
Donohue syndrome	donohue syndrome	donohue syndrome	donohue syndrome	Donohue syndrome	1	kept
Donor Action<sup>®<	donor action sup	donor action sup	donor action sup	Donor Action<sup>®<	1	kept
Donor efficiency	donor efficiency	donor efficiency	donor efficiency	Donor efficiency	1	kept
Donor identification	donor identification	donor identification	donor identification	Donor identification	1	kept
Donor management	donor management	donor management	donor management	donor management	1	case_or_format
Donor oocyte recipients	donor oocyte recipients	donor oocyte recipients	donor oocyte recipients	Donor oocyte recipients	1	kept
DONOR ORGANS	donor organs	donor organs	donor organs	DONOR ORGANS	1	kept
Donor referral	donor referral	donor referral	donor referral	Donor referral	1	kept

Donor selection	donor selection	donor selection	donor selection	Donor Selection	1	case_or_format
Donor staffing	donor staffing	donor staffing	donor staffing	Donor staffing	1	kept
DONORS	donors	donors	donor	donor	1	singular
donors	donors	donors	donor	donor	1	singular
donors and donation: donor evaluation	donors and donation donor evaluation	donors and donation donor evaluation	donors and donation donor evaluation	donors and donation: donor evaluation	1	kept
donors, organ transplantation	donors organ transplantation	donors organ transplantation	donors organ transplantation	donors, organ transplantation	1	kept
Door to balloon time	door to balloon time	door to balloon time	door to balloon time	Door to balloon time	1	kept
door to balloon time	door to balloon time	door to balloon time	door to balloon time	Door to balloon time	1	case_or_format
door to needle time	door to needle time	door to needle time	door to needle time	door to needle time	1	kept
Door-testing	door testing	door testing	door testing	Door-testing	1	kept
Door-to-provider	door to provider	door to provider	door to provider	Door-to-provider	1	kept
Door-to-transfer	door to transfer	door to transfer	door to transfer	Door-to-transfer	1	kept
doormat	doormat	doormat	doormat	doormat	1	kept
Dopamine Agents	dopamine agents	dopamine agents	dopamine agents	Dopamine Agents	1	kept
dopamine agonist withdrawal syndrome	dopamine agonist withdrawal syndrome	dopamine agonist withdrawal syndrome	dopamine agonist withdrawal syndrome	dopamine agonist withdrawal syndrome	1	kept
dopamine receptor	dopamine receptor	dopamine receptor	dopamine receptor	dopamine receptor	1	kept

dopamine receptor blocking agent	dopamine receptor blocking agent	dopamine receptor blocking agent	dopamine receptor blocking agent	dopamine receptor blocking agent	1	kept
dopamine transporter	dopamine transporter	dopamine transporter	dopamine transporter	dopamine transporter	1	kept
dopaminergic system	dopaminergic system	dopaminergic system	dopaminergic system	dopaminergic system	1	kept
dopaminergic transmission	dopaminergic transmission	dopaminergic transmission	dopaminergic transmission	dopaminergic transmission	1	kept
Doppler flowmeter	doppler flowmeter	doppler flowmeter	doppler flowmeter	Doppler flowmeter	1	kept
Dormant MRSA	dormant mrsa	dormant mrsa	dormant mrsa	Dormant MRSA	1	kept
dormazolam	dormazolam	dormazolam	dormazolam	dormazolam	1	kept
dornase alfa	dornase alfa	dornase alfa	dornase alfa	dornase alfa	1	kept
dorsal region	dorsal region	dorsal region	dorsal region	dorsal region	1	kept
dorsolateral medulla	dorsolateral medulla	dorsolateral medulla	dorsolateral medulla	dorsolateral medulla	1	kept
dorsolateral prefrontal cortex	dorsolateral prefrontal cortex	dorsolateral prefrontal cortex	dorsolateral prefrontal cortex	dorsolateral prefrontal cortex	1	kept
dorzolamide plus timolol	dorzolamide plus timolol	dorzolamide plus timolol	dorzolamide plus timolol	dorzolamide plus timolol	1	kept
Dosage calculations	dosage calculations	dosage calculations	dosage calculations	Dosage calculations	1	kept
dosage form	dosage form	dosage form	dosage form	Dosage Forms	1	case_or_format
Dosage schedule	dosage schedule	dosage schedule	dosage schedule	Dosage schedule	1	kept
Dose	dose	dose	dose	Dose	1	kept

dose	dose	dose	dose	Dose	1	case_or_format
dose calculation algorithm	dose calculation algorithm	dose calculation algorithm	dose calculation algorithm	dose calculation algorithm	1	kept
dose capping	dose capping	dose capping	dose capping	dose capping	1	kept
DOSE DEXMEDETOMIDINE	dose dexmedetomidine	dose dexmedetomidine	dose dexmedetomidine	DOSE DEXMEDETOMIDINE	1	kept
Dose limits	dose limits	dose limits	dose limits	Dose limits	1	kept
dose omission	dose omission	dose omission	dose omission	dose omission	1	kept
Dose optimization	dose optimization	dose optimization	dose optimization	Dose optimization	1	kept
Dose range	dose range	dose range	dose range	Dose range	1	kept
dose regimen	dose regimen	dose regimen	dose regimen	dose regimen	1	kept
Dose tracking	dose tracking	dose tracking	dose tracking	Dose tracking	1	kept
dose tracking	dose tracking	dose tracking	dose tracking	Dose tracking	1	case_or_format
Dose-finding	dose finding	dose finding	dose finding	Dose-finding	1	kept
Dose-Response relationship	dose response relationship	dose response relationship	dose response relationship	Dose-Response relationship	1	kept
dose-response relationship	dose response relationship	dose response relationship	dose response relationship	Dose-Response relationship	1	case_or_format
Doses	doses	doses	dose	Dose	1	singular
dosing chart	dosing chart	dosing chart	dosing chart	dosing chart	1	kept

dosing errors	dosing errors	dosing errors	dosing error	DOSING ERRORS	1	singular
dosing sheets	dosing sheets	dosing sheets	dosing sheets	dosing sheets	1	kept
dosotequine	dosotequine	dosotequine	dosotequine	dosotequine	1	kept
double balloon enteroscopy	double balloon enteroscopy	double balloon enteroscopy	double balloon enteroscopy	double balloon enteroscopy	1	kept
Double Bind Interaction	double bind interaction	double bind interaction	double bind interaction	Double Bind Interaction	1	kept
Double Diamond	double diamond	double diamond	double diamond	Double Diamond	1	kept
double difference analysis	double difference analysis	double difference analysis	double difference analysis	double difference analysis	1	kept
Double Effect	double effect	double effect	double effect	Double Effect	1	kept
Double Effect Principle	double effect principle	double effect principle	double effect principle	Double Effect Principle	1	kept
double enterostomy	double enterostomy	double enterostomy	double enterostomy	double enterostomy	1	kept
double lumen tube	double lumen tube	double lumen tube	double lumen tube	double lumen tube	1	kept
Double lumen vascular catheter	double lumen vascular catheter	double lumen vascular catheter	double lumen vascular catheter	Double lumen vascular catheter	1	kept
Double Outlet Right Ventricle	double outlet right ventricle	double outlet right ventricle	double outlet right ventricle	Double Outlet Right Ventricle	1	kept
Double Pumping	double pumping	double pumping	double pumping	Double Pumping	1	kept
Double-balloon catheter	double balloon catheter	double balloon catheter	double balloon catheter	Double-balloon catheter	1	kept
Double-blind method	double blind method	double blind method	double blind method	Double-Blind Method	1	case_or_format

Double-capsule fecal catheter device	double capsule fecal catheter device	double capsule fecal catheter device	double capsule fecal catheter device	Double-capsule fecal catheter device	1	kept
DOUBLE-CHECKING	double checking	double checking	double checking	DOUBLE-CHECKING	1	kept
double-checking	double checking	double checking	double checking	DOUBLE-CHECKING	1	case_or_format
double-pocket fecal catheter system	double pocket fecal catheter system	double pocket fecal catheter system	double pocket fecal catheter system	double-pocket fecal catheter system	1	kept
douleur	douleur	douleur	douleur	douleur	1	kept
Douleur Aigue Nouveau ne Scale	douleur aigue nouveau ne scale	douleur aigue nouveau ne scale	douleur aigue nouveau ne scale	Douleur Aigue Nouveau ne Scale	1	kept
down regulation	down regulation	down regulation	down regulation	down regulation	1	kept
down syndrome	down syndrome	down syndrome	down syndrome	Down syndrome	1	case_or_format
Down-Regulation	down regulation	down regulation	down regulation	down regulation	1	case_or_format
downe score	downe score	downe score	downe score	downe score	1	kept
downes score	downes score	downes score	downes score	downes’ score	1	case_or_format
downstream outcome	downstream outcome	downstream outcome	downstream outcome	downstream outcome	1	kept
downton index	downton index	downton index	downton index	downton index	1	kept
downton scale	downton scale	downton scale	downton scale	downton scale	1	kept
downward tract adherence method	downward tract adherence method	downward tract adherence method	downward tract adherence method	downward tract adherence method	1	kept
doxafin syrup	doxafin syrup	doxafin syrup	doxafin syrup	doxafin syrup	1	kept

Doxorubicin	doxorubicin	doxorubicin	doxorubicin	doxorubicin	1	case_or_format
Doxycycline	doxycycline	doxycycline	doxycycline	doxycycline	1	case_or_format
DP, Deformational plagiocephaly	dp deformational plagiocephaly	dp deformational plagiocephaly	dp deformational plagiocephaly	DP, Deformational plagiocephaly	1	kept
drain	drain	drain	drain	drain	1	kept
drainage angle	drainage angle	drainage angle	drainage angle	drainage angle	1	kept
drainage catheter	drainage catheter	drainage catheter	drainage catheter	drainage catheter	1	kept
Drainage device	drainage device	drainage device	drainage device	Drainage device	1	kept
drainage procedure	drainage procedure	drainage procedure	drainage procedure	drainage procedure	1	kept
DRAINAGE SYSTEMS	drainage systems	drainage systems	drainage systems	DRAINAGE SYSTEMS	1	kept
Drainage, Sanitary	drainage sanitary	drainage sanitary	drainage sanitary	Drainage, Sanitary	1	kept
Drama	drama	drama	drama	Drama	1	kept
dreaming	dreaming	dreaming	dreaming	dreaming	1	kept
DRESS	dress	dress	dress	DRESS	1	kept
Dressing changes	dressing changes	dressing changes	dressing change	Dressing change	1	singular
Dressing material	dressing material	dressing material	dressing material	Dressing material	1	kept
Dressings and securement device methods	dressings and securement device methods	dressings and securement device methods	dressings and securement device methods	Dressings and securement device methods	1	kept

DRG system	drg system	drg system	drg system	DRG system	1	kept
DRG-system	drg system	drg system	drg system	DRG system	1	case_or_format
Drill	drill	drill	drill	Drill	1	kept
Drinking Behavior	drinking behavior	drinking behavior	drinking behavior	drinking behavior	1	case_or_format
Drinking Water	drinking water	drinking water	drinking water	drinking water	1	case_or_format
Drive	drive	drive	drive	Drive	1	kept
driver diagram	driver diagram	driver diagram	driver diagram	driver diagram	1	kept
driving ability	driving ability	driving ability	driving ability	driving ability	1	kept
Drotrecogin alfa	drotrecogin alfa	drotrecogin alfa	drotrecogin alfa	Drotrecogin alfa	1	kept
DRPU	drpu	drpu	drpu	DRPU	1	kept
drug accumulation	drug accumulation	drug accumulation	drug accumulation	drug accumulation	1	kept
drug activation	drug activation	drug activation	drug activation	drug activation	1	kept
drug activity	drug activity	drug activity	drug activity	drug activity	1	kept
drug administration delay	drug administration delay	drug administration delay	drug administration delay	drug administration delay	1	kept
drug administration errors	drug administration errors	drug administration errors	drug administration errors	DRUG ADMINISTRATION ERRORS	1	case_or_format
drug administration procedure steps	drug administration procedure steps	drug administration procedure steps	drug administration procedure steps	drug administration procedure steps	1	kept

Drug administration rate	drug administration rate	drug administration rate	drug administration rate	Drug administration rate	1	kept
Drug administration schedule	drug administration schedule	drug administration schedule	drug administration schedule	Drug Administration Schedule	1	case_or_format
drug administration schedule	drug administration schedule	drug administration schedule	drug administration schedule	Drug Administration Schedule	1	case_or_format
Drug administration systems	drug administration systems	drug administration systems	drug administration systems	Drug administration systems	1	kept
drug adsorption	drug adsorption	drug adsorption	drug adsorption	drug adsorption	1	kept
Drug allergy	drug allergy	drug allergy	drug allergy	Drug allergy	1	kept
drug analysis	drug analysis	drug analysis	drug analysis	drug analysis	1	kept
Drug and Device Safety	drug and device safety	drug and device safety	drug and device safety	Drug and Device Safety	1	kept
drug antibody	drug antibody	drug antibody	drug antibody	drug antibody	1	kept
drug binding	drug binding	drug binding	drug binding	drug binding	1	kept
drug capsule	drug capsule	drug capsule	drug capsule	drug capsule	1	kept
Drug Carriers	drug carriers	drug carriers	drug carriers	Drug Carriers	1	kept
Drug class	drug class	drug class	drug class	Drug class	1	kept
drug comparison	drug comparison	drug comparison	drug comparison	drug comparison	1	kept
Drug compatibility	drug compatibility	drug compatibility	drug compatibility	Drug compatibility	1	kept
drug compatibility	drug compatibility	drug compatibility	drug compatibility	Drug compatibility	1	case_or_format

Drug contamination	drug contamination	drug contamination	drug contamination	drug contamination	1	case_or_format
drug control	drug control	drug control	drug control	drug control	1	kept
drug delivery device	drug delivery device	drug delivery device	drug delivery device	drug delivery device	1	kept
Drug delivery systems	drug delivery systems	drug delivery systems	drug delivery system	drug delivery system	1	singular
drug delivery systems	drug delivery systems	drug delivery systems	drug delivery system	drug delivery system	1	singular
drug detoxification	drug detoxification	drug detoxification	drug detoxification	drug detoxification	1	kept
Drug discontinuation	drug discontinuation	drug discontinuation	drug discontinuation	Drug discontinuation	1	kept
Drug discovery	drug discovery	drug discovery	drug discovery	Drug discovery	1	kept
Drug distribution systems	drug distribution systems	drug distribution systems	drug distribution systems	Drug distribution systems	1	kept
Drug dosage calculations	drug dosage calculations	drug dosage calculations	drug dosage calculations	Drug Dosage Calculations	1	case_or_format
Drug error	drug error	drug error	drug error	Drug error	1	kept
drug error	drug error	drug error	drug error	Drug error	1	case_or_format
Drug errors	drug errors	drug errors	drug error	Drug error	1	singular
drug errors	drug errors	drug errors	drug error	Drug error	1	singular
DRUG EVENTS	drug events	drug events	drug events	DRUG EVENTS	1	kept
drug hydrolysis	drug hydrolysis	drug hydrolysis	drug hydrolysis	drug hydrolysis	1	kept

Drug hypersensitivity	drug hypersensitivity	drug hypersensitivity	drug hypersensitivity	drug hypersensitivity	1	case_or_format
drug identification	drug identification	drug identification	drug identification	drug identification	1	kept
Drug incompatibilities	drug incompatibilities	drug incompatibilities	drug incompatibility	drug incompatibility	1	singular
Drug induced pancreatitis	drug induced pancreatitis	drug induced pancreatitis	drug induced pancreatitis	Drug induced pancreatitis	1	kept
drug information offering	drug information offering	drug information offering	drug information offering	drug information offering	1	kept
Drug infusion safety	drug infusion safety	drug infusion safety	drug infusion safety	Drug infusion safety	1	kept
Drug Interaction	drug interaction	drug interaction	drug interaction	Drug Interactions	1	case_or_format
Drug interaction	drug interaction	drug interaction	drug interaction	Drug Interactions	1	case_or_format
drug interactions	drug interactions	drug interactions	drug interaction	Drug Interactions	1	singular
drug inventory control	drug inventory control	drug inventory control	drug inventory control	drug inventory control	1	kept
Drug inventory policy	drug inventory policy	drug inventory policy	drug inventory policy	Drug inventory policy	1	kept
drug labelling	drug labelling	drug labelling	drug labelling	drug labelling	1	kept
Drug libraries	drug libraries	drug libraries	drug library	Drug library	1	singular
Drug management system	drug management system	drug management system	drug management system	Drug management system	1	kept
DRUG MANUFACTURING	drug manufacturing	drug manufacturing	drug manufacturing	DRUG MANUFACTURING	1	kept
drug marketing	drug marketing	drug marketing	drug marketing	drug marketing	1	kept

drug mixture	drug mixture	drug mixture	drug mixture	drug mixture	1	kept
Drug overdosage	drug overdosage	drug overdosage	drug overdosage	Drug overdosage	1	kept
Drug overdoses	drug overdoses	drug overdoses	drug overdose	drug overdose	1	singular
drug penetration	drug penetration	drug penetration	drug penetration	drug penetration	1	kept
DRUG PREPARATION	drug preparation	drug preparation	drug preparation	Drug preparation	1	case_or_format
drug preparation error	drug preparation error	drug preparation error	drug preparation error	drug preparation error	1	kept
drug prescribing	drug prescribing	drug prescribing	drug prescribing	drug prescribing	1	kept
drug prescription methods	drug prescription methods	drug prescription methods	drug prescription methods	drug prescription methods	1	kept
Drug price	drug price	drug price	drug price	Drug price	1	kept
DRUG PROCUREMENT	drug procurement	drug procurement	drug procurement	DRUG PROCUREMENT	1	kept
Drug rash	drug rash	drug rash	drug rash	Drug rash	1	kept
drug recall	drug recall	drug recall	drug recall	drug recall	1	kept
drug release	drug release	drug release	drug release	drug release	1	kept
Drug resistance microbial	drug resistance microbial	drug resistance microbial	drug resistance microbial	Drug Resistance, Microbial	1	case_or_format
drug resistance, bacterial	drug resistance bacterial	drug resistance bacterial	drug resistance bacterial	Drug Resistance, Bacterial	1	case_or_format
Drug Resistance, Viral	drug resistance viral	drug resistance viral	drug resistance viral	Drug Resistance, Viral	1	kept

Drug resistant epilepsy	drug resistant epilepsy	drug resistant epilepsy	drug resistant epilepsy	Drug resistant epilepsy	1	kept
drug resistant infection	drug resistant infection	drug resistant infection	drug resistant infection	drug resistant infection	1	kept
Drug resistant pathogens	drug resistant pathogens	drug resistant pathogens	drug resistant pathogens	Drug resistant pathogens	1	kept
drug retention	drug retention	drug retention	drug retention	drug retention	1	kept
drug selectivity	drug selectivity	drug selectivity	drug selectivity	drug selectivity	1	kept
Drug sensitivity	drug sensitivity	drug sensitivity	drug sensitivity	drug sensitivity	1	case_or_format
drug sensitization	drug sensitization	drug sensitization	drug sensitization	drug sensitization	1	kept
Drug shortage	drug shortage	drug shortage	drug shortage	drug shortage	1	case_or_format
drug skin level	drug skin level	drug skin level	drug skin level	drug skin level	1	kept
Drug supply	drug supply	drug supply	drug supply	Drug supply	1	kept
Drug supply chain	drug supply chain	drug supply chain	drug supply chain	Drug supply chain	1	kept
drug tampering	drug tampering	drug tampering	drug tampering	drug tampering	1	kept
Drug tolerance	drug tolerance	drug tolerance	drug tolerance	Drug Tolerance	1	case_or_format
Drug Toxicity	drug toxicity	drug toxicity	drug toxicity	drug toxicity	1	case_or_format
Drug Trafficking	drug trafficking	drug trafficking	drug trafficking	Drug Trafficking	1	kept
drug urine level	drug urine level	drug urine level	drug urine level	drug urine level	1	kept

DRUG USE	drug use	drug use	drug use	drug use	1	case_or_format
Drug use	drug use	drug use	drug use	drug use	1	case_or_format
Drug utilization review	drug utilization review	drug utilization review	drug utilization review	Drug Utilization Review	1	case_or_format
drug waste	drug waste	drug waste	drug waste	drug waste	1	kept
Drug withdrawal	drug withdrawal	drug withdrawal	drug withdrawal	drug withdrawal	1	case_or_format
Drug withdrawal symptoms	drug withdrawal symptoms	drug withdrawal symptoms	drug withdrawal symptoms	Drug withdrawal symptoms	1	kept
DRUG-ABUSE	drug abuse	drug abuse	drug abuse	drug abuse	1	case_or_format
drug-compatibility	drug compatibility	drug compatibility	drug compatibility	Drug compatibility	1	case_or_format
Drug-drug interactions	drug drug interactions	drug drug interactions	drug drug interactions	Drug-drug interactions	1	kept
Drug-incompabilities	drug incompabilities	drug incompabilities	drug incompabilities	Drug-incompabilities	1	kept
Drug-Induced Liver Injury	drug induced liver injury	drug induced liver injury	drug induced liver injury	Drug-Induced Liver Injury	1	kept
drug-induced liver injury	drug induced liver injury	drug induced liver injury	drug induced liver injury	Drug-Induced Liver Injury	1	case_or_format
DRUG-INTERACTIONS	drug interactions	drug interactions	drug interaction	Drug Interactions	1	singular
DRUG-RELATED MORBIDITY	drug related morbidity	drug related morbidity	drug related morbidity	DRUG-RELATED MORBIDITY	1	kept
Drug-related problem	drug related problem	drug related problem	drug related problem	Drug-related problem	1	kept
drug-related side effects	drug related side effects	drug related side effects	drug related side effects	drug-related side effects	1	kept

drug-related side effects and adverse reactions	drug related side effects and adverse reactions	drug related side effects and adverse reactions	drug related side effects and adverse reactions	Drug-Related Side Effects and Adverse Reactions	1	case_or_format
Drug-Seeking Behavior	drug seeking behavior	drug seeking behavior	drug seeking behavior	Drug-Seeking Behavior	1	kept
DRUG-UTILIZATION EVALUATION	drug utilization evaluation	drug utilization evaluation	drug utilization evaluation	DRUG-UTILIZATION EVALUATION	1	kept
Drugs and equipment	drugs and equipment	drugs and equipment	drugs and equipment	Drugs and equipment	1	kept
Drugs inventory policies	drugs inventory policies	drugs inventory policies	drugs inventory policies	Drugs inventory policies	1	kept
Drugs of abuse	drugs of abuse	drugs of abuse	drugs of abuse	Drugs of abuse	1	kept
drugs used in the treatment of addiction	drugs used in the treatment of addiction	drugs used in the treatment of addiction	drugs used in the treatment of addiction	drugs used in the treatment of addiction	1	kept
Drugs, Essential	drugs essential	drugs essential	drugs essential	Drugs, Essential	1	kept
Drugs, Generic	drugs generic	drugs generic	drugs generic	Drugs, Generic	1	kept
DRY EYE	dry eye	dry eye	dry eye	dry eye	1	case_or_format
dry eye disease	dry eye disease	dry eye disease	dry eye disease	dry eye disease	1	kept
dry eye syndrome	dry eye syndrome	dry eye syndrome	dry eye syndrome	Dry Eye Syndromes	1	case_or_format
dry lip	dry lip	dry lip	dry lip	dry lip	1	kept
dry nose	dry nose	dry nose	dry nose	dry nose	1	kept
dry oxygen	dry oxygen	dry oxygen	dry oxygen	dry oxygen	1	kept
dry weight	dry weight	dry weight	dry weight	dry weight	1	kept

Drying time	drying time	drying time	drying time	Drying time	1	kept
Dryness	dryness	dryness	dryness	Dryness	1	kept
DSC, Developmental supportive care	dsc developmental supportive care	dsc developmental supportive care	dsc developmental supportive care	DSC, Developmental supportive care	1	kept
DTaP	dtap	dtap	dtap	DTaP	1	kept
DTI	dti	dti	dti	DTI	1	kept
DTP	dtp	dtp	dtp	DTP	1	kept
Dual agency	dual agency	dual agency	dual agency	Dual agency	1	kept
dual agency	dual agency	dual agency	dual agency	Dual agency	1	case_or_format
Dual Anti-Platelet Therapy	dual anti platelet therapy	dual anti platelet therapy	dual anti platelet therapy	Dual Anti-Platelet Therapy	1	kept
Dual Item Measure of Burnout	dual item measure of burnout	dual item measure of burnout	dual item measure of burnout	Dual Item Measure of Burnout	1	kept
Dual MEDICAID MEDICARE Eligibility	dual medicaid medicare eligibility	dual medicaid medicare eligibility	dual medicaid medicare eligibility	Dual MEDICAID MEDICARE Eligibility	1	kept
Dual mode	dual mode	dual mode	dual mode	Dual mode	1	kept
dual occupancy	dual occupancy	dual occupancy	dual occupancy	dual occupancy	1	kept
Dual process theory	dual process theory	dual process theory	dual process theory	Dual process theory	1	kept
Dual responsibility	dual responsibility	dual responsibility	dual responsibility	Dual responsibility	1	kept
Dual roles	dual roles	dual roles	dual role	dual role	1	singular

DUAL-CHAMBER	dual chamber	dual chamber	dual chamber	DUAL-CHAMBER	1	kept
DUAL-USE	dual use	dual use	dual use	DUAL-USE	1	kept
Ductus arteriosus	ductus arteriosus	ductus arteriosus	ductus arteriosus	Ductus arteriosus	1	kept
ductus arteriosus	ductus arteriosus	ductus arteriosus	ductus arteriosus	Ductus arteriosus	1	case_or_format
Duke Activity Status Index	duke activity status index	duke activity status index	duke activity status index	Duke Activity Status Index	1	kept
dulaglutide	dulaglutide	dulaglutide	dulaglutide	dulaglutide	1	kept
dummies	dummies	dummies	dummies	dummies	1	kept
dumping	dumping	dumping	dumping	dumping	1	kept
dumping syndrome	dumping syndrome	dumping syndrome	dumping syndrome	dumping syndrome	1	kept
Duodenal feeding tube	duodenal feeding tube	duodenal feeding tube	duodenal feeding tube	Duodenal feeding tube	1	kept
Duodenal Ulcer	duodenal ulcer	duodenal ulcer	duodenal ulcer	Duodenal Ulcer	1	kept
duodenoscopy	duodenoscopy	duodenoscopy	duodenoscopy	duodenoscopy	1	kept
Duodenostomy	duodenostomy	duodenostomy	duodenostomy	Duodenostomy	1	kept
duodenum atresia	duodenum atresia	duodenum atresia	duodenum atresia	duodenum atresia	1	kept
duoderm	duoderm	duoderm	duoderm	duoderm	1	kept
dupilumab	dupilumab	dupilumab	dupilumab	dupilumab	1	kept

Duplex ultrasonography	duplex ultrasonography	duplex ultrasonography	duplex ultrasonography	Duplex ultrasonography	1	kept
duplicate medication order	duplicate medication order	duplicate medication order	duplicate medication order	duplicate medication order	1	kept
dura	dura	dura	dura	dura	1	kept
dural arteriovenous fistula	dural arteriovenous fistula	dural arteriovenous fistula	dural arteriovenous fistula	dural arteriovenous fistula	1	kept
dural leak	dural leak	dural leak	dural leak	dural leak	1	kept
dural puncture	dural puncture	dural puncture	dural puncture	dural puncture	1	kept
dural tear	dural tear	dural tear	dural tear	dural tear	1	kept
duration of smoking	duration of smoking	duration of smoking	duration of smoking	duration of smoking	1	kept
Duration of therapy	duration of therapy	duration of therapy	duration of therapy	Duration of Therapy	1	case_or_format
duration of ventilator support	duration of ventilator support	duration of ventilator support	duration of ventilator support	duration of ventilator support	1	kept
during	during	during	during	during	1	kept
durlobactam	durlobactam	durlobactam	durlobactam	durlobactam	1	kept
duroplasty	duroplasty	duroplasty	duroplasty	duroplasty	1	kept
durotomy	durotomy	durotomy	durotomy	durotomy	1	kept
DUTCH	dutch	dutch	dutch	DUTCH	1	kept
Dutch healthcare system	dutch healthcare system	dutch healthcare system	dutch healthcare system	Dutch healthcare system	1	kept

Duty	duty	duty	duty	Duty	1	kept
Duty hour restrictions	duty hour restrictions	duty hour restrictions	duty hour restrictions	Duty hour restrictions	1	kept
Duty Hours	duty hours	duty hours	duty hours	Duty hours	1	case_or_format
Duty of care	duty of care	duty of care	duty of care	duty of care	1	case_or_format
DVT = deep vein thrombosis	dvt deep vein thrombosis	dvt deep vein thrombosis	dvt deep vein thrombosis	DVT = deep vein thrombosis	1	kept
DVT prevention	dvt prevention	dvt prevention	dvt prevention	DVT prevention	1	kept
DVT preventive measures	dvt preventive measures	dvt preventive measures	dvt preventive measures	DVT preventive measures	1	kept
DVT prophylaxis	dvt prophylaxis	dvt prophylaxis	dvt prophylaxis	DVT prophylaxis	1	kept
dwarfism	dwarfism	dwarfism	dwarfism	dwarfism	1	kept
DWELL TIME	dwell time	dwell time	dwell time	dwell time	1	case_or_format
dyad	dyad	dyad	dyad	dyad	1	kept
dyadic adjustment	dyadic adjustment	dyadic adjustment	dyadic adjustment	dyadic adjustment	1	kept
Dyadic Adjustment Scale	dyadic adjustment scale	dyadic adjustment scale	dyadic adjustment scale	Dyadic Adjustment Scale	1	kept
dyadic interviews	dyadic interviews	dyadic interviews	dyadic interviews	dyadic interviews	1	kept
dye	dye	dye	dye	dye	1	kept
dye dilution curve	dye dilution curve	dye dilution curve	dye dilution curve	dye dilution curve	1	kept

dyes, reagents, indicators, markers and buffers	dyes reagents indicators markers and buffers	dyes reagents indicators markers and buffers	dyes reagents indicators markers and buffers	dyes, reagents, indicators, markers and buffers	1	kept
dying alone	dying alone	dying alone	dying alone	dying alone	1	kept
dying and death	dying and death	dying and death	dying and death	dying and death	1	kept
Dying at home	dying at home	dying at home	dying at home	Dying at home	1	kept
dying baby	dying baby	dying baby	dying baby	dying baby	1	kept
dying child	dying child	dying child	dying child	dying child	1	kept
Dying children	dying children	dying children	dying children	Dying children	1	kept
Dying in hospital	dying in hospital	dying in hospital	dying in hospital	Dying in hospital	1	kept
DYING INFANTS	dying infants	dying infants	dying infants	DYING INFANTS	1	kept
Dying infants	dying infants	dying infants	dying infants	DYING INFANTS	1	case_or_format
dying phase	dying phase	dying phase	dying phase	dying phase	1	kept
Dying preferences	dying preferences	dying preferences	dying preferences	Dying preferences	1	kept
dying preterm newborns	dying preterm newborns	dying preterm newborns	dying preterm newborns	dying preterm newborns	1	kept
Dying process	dying process	dying process	dying process	Dying process	1	kept
Dying trajectories	dying trajectories	dying trajectories	dying trajectories	Dying trajectories	1	kept
Dying well	dying well	dying well	dying well	Dying well	1	kept

dying wish	dying wish	dying wish	dying wish	dying wish	1	kept
Dyna-Form Static Air HZ	dyna form static air hz	dyna form static air hz	dyna form static air hz	Dyna-Form Static Air HZ	1	kept
dynamic	dynamic	dynamic	dynamic	dynamics	1	case_or_format
Dynamic detection	dynamic detection	dynamic detection	dynamic detection	Dynamic detection	1	kept
dynamic detection	dynamic detection	dynamic detection	dynamic detection	Dynamic detection	1	case_or_format
Dynamic factors	dynamic factors	dynamic factors	dynamic factors	Dynamic factors	1	kept
dynamic fascial closure	dynamic fascial closure	dynamic fascial closure	dynamic fascial closure	dynamic fascial closure	1	kept
dynamic hip screw	dynamic hip screw	dynamic hip screw	dynamic hip screw	dynamic hip screw	1	kept
Dynamic insulin infusion protocol	dynamic insulin infusion protocol	dynamic insulin infusion protocol	dynamic insulin infusion protocol	Dynamic insulin infusion protocol	1	kept
dynamic insulin nomogram	dynamic insulin nomogram	dynamic insulin nomogram	dynamic insulin nomogram	dynamic insulin nomogram	1	kept
DYNAMIC LIGHT	dynamic light	dynamic light	dynamic light	DYNAMIC LIGHT	1	kept
dynamic model	dynamic model	dynamic model	dynamic model	dynamic model	1	kept
dynamic needle tip positioning	dynamic needle tip positioning	dynamic needle tip positioning	dynamic needle tip positioning	dynamic needle tip positioning	1	kept
dynamic nomogram	dynamic nomogram	dynamic nomogram	dynamic nomogram	dynamic nomogram	1	kept
Dynamic policy	dynamic policy	dynamic policy	dynamic policy	Dynamic policy	1	kept
Dynamic relationships	dynamic relationships	dynamic relationships	dynamic relationships	Dynamic relationships	1	kept

Dynamic response	dynamic response	dynamic response	dynamic response	Dynamic response	1	kept
Dynamic satisfaction function	dynamic satisfaction function	dynamic satisfaction function	dynamic satisfaction function	Dynamic satisfaction function	1	kept
Dynamic standing	dynamic standing	dynamic standing	dynamic standing	Dynamic standing	1	kept
dynamic standing	dynamic standing	dynamic standing	dynamic standing	Dynamic standing	1	case_or_format
dynamique d’équipe	dynamique d quipe	dynamique d quipe	dynamique d quipe	dynamique d’équipe	1	kept
dynamometer	dynamometer	dynamometer	dynamometer	dynamometer	1	kept
dynamometry	dynamometry	dynamometry	dynamometry	dynamometry	1	kept
dysbiosis	dysbiosis	dysbiosis	dysbiosis	Dysbiosis	1	case_or_format
Dysfunctional beliefs	dysfunctional beliefs	dysfunctional beliefs	dysfunctional beliefs	Dysfunctional beliefs	1	kept
dysgerminoma	dysgerminoma	dysgerminoma	dysgerminoma	dysgerminoma	1	kept
Dyslexia	dyslexia	dyslexia	dyslexia	Dyslexia	1	kept
Dyslipidemia	dyslipidemia	dyslipidemia	dyslipidemia	dyslipidemia	1	case_or_format
dysmenorrhea	dysmenorrhea	dysmenorrhea	dysmenorrhea	dysmenorrhea	1	kept
dyspareunia	dyspareunia	dyspareunia	dyspareunia	dyspareunia	1	kept
Dysphagia management	dysphagia management	dysphagia management	dysphagia management	Dysphagia management	1	kept
Dysphagia Rehabilitation	dysphagia rehabilitation	dysphagia rehabilitation	dysphagia rehabilitation	Dysphagia Rehabilitation	1	kept

dysphagia screening	dysphagia screening	dysphagia screening	dysphagia screening	dysphagia screening	1	kept
dysphagia team	dysphagia team	dysphagia team	dysphagia team	dysphagia team	1	kept
Dysphoria	dysphoria	dysphoria	dysphoria	dysphoria	1	case_or_format
Dysregulated immune system	dysregulated immune system	dysregulated immune system	dysregulated immune system	Dysregulated immune system	1	kept
Dysrhythmia	dysrhythmia	dysrhythmia	dysrhythmia	dysrhythmia	1	case_or_format
dyssomnia	dyssomnia	dyssomnia	dyssomnia	Dyssomnias	1	case_or_format
dysthanasia	dysthanasia	dysthanasia	dysthanasia	dysthanasia	1	kept
dystrophin	dystrophin	dystrophin	dystrophin	dystrophin	1	kept
dystrophy	dystrophy	dystrophy	dystrophy	dystrophy	1	kept
débriefing	d briefing	d briefing	d briefing	débriefing	1	kept
décision	d cision	d cision	d cision	décision	1	kept
décubitus ventral	d cubitus ventral	d cubitus ventral	d cubitus ventral	décubitus ventral	1	kept
Décès en éhpad	d c s en hpad	d c s en hpad	d c s en hpad	Décès en éhpad	1	kept
défense des personnes handicapées	d fense des personnes handicap es	d fense des personnes handicap es	d fense des personnes handicap es	défense des personnes handicapées	1	kept
E - learning	e learning	e learning	e learning	e-learning	1	case_or_format
E coli	e coli	e coli	e coli	E. coli	1	case_or_format

E hospital	e hospital	e hospital	e hospital	E hospital	1	kept
E-alert	e alert	e alert	e alert	E-alert	1	kept
e-book	e book	e book	e book	e-book	1	kept
E-books	e books	e books	e book	e-book	1	singular
e-cigarettes	e cigarettes	e cigarettes	e cigarettes	e-cigarettes	1	kept
E-consultation	e consultation	e consultation	e consultation	E-consultation	1	kept
E-CURB 65	e curb 65	e curb 65	e curb 65	E-CURB 65	1	kept
e-Delphi	e delphi	e delphi	e delphi	E-Delphi	1	case_or_format
e-Delphi technique	e delphi technique	e delphi technique	e delphi technique	e-Delphi technique	1	kept
e-Health	e health	e health	e health	E-health	1	case_or_format
e-health	e health	e health	e health	E-health	1	case_or_format
e-health surveillance systems	e health surveillance systems	e health surveillance systems	e health surveillance systems	e-health surveillance systems	1	kept
e-health technology	e health technology	e health technology	e health technology	e-health technology	1	kept
E-health-surveillance-systems	e health surveillance systems	e health surveillance systems	e health surveillance systems	e-health surveillance systems	1	case_or_format
E-learning system	e learning system	e learning system	e learning system	E-learning system	1	kept
e-learning tool	e learning tool	e learning tool	e learning tool	e-learning tool	1	kept

e-NEC Scoring System	e nec scoring system	e nec scoring system	e nec scoring system	e-NEC Scoring System	1	kept
e-Prescribing	e prescribing	e prescribing	e prescribing	e-Prescribing	1	kept
e-prescribing	e prescribing	e prescribing	e prescribing	e-Prescribing	1	case_or_format
E-Selectin	e selectin	e selectin	e selectin	E-Selectin	1	kept
E-selectin	e selectin	e selectin	e selectin	E-Selectin	1	case_or_format
E. faecalis	e faecalis	e faecalis	e faecalis	E. faecalis	1	kept
EAF management algorithm	eaf management algorithm	eaf management algorithm	eaf management algorithm	EAF management algorithm	1	kept
ear muff	ear muff	ear muff	ear muff	ear muff	1	kept
Ear protective devices	ear protective devices	ear protective devices	ear protective device	Ear Protective Devices	1	singular
ear surgery	ear surgery	ear surgery	ear surgery	ear surgery	1	kept
Ear, Middle	ear middle	ear middle	ear middle	Ear, Middle	1	kept
Ear-plugs	ear plugs	ear plugs	ear plugs	Ear-plugs	1	kept
ear-to-ear distance	ear to ear distance	ear to ear distance	ear to ear distance	ear-to-ear distance	1	kept
Early	early	early	early	Early	1	kept
early	early	early	early	Early	1	case_or_format
Early active mobilization	early active mobilization	early active mobilization	early active mobilization	Early active mobilization	1	kept

early active mobilization	early active mobilization	early active mobilization	early active mobilization	Early active mobilization	1	case_or_format
Early active movements	early active movements	early active movements	early active movements	Early active movements	1	kept
early active movements	early active movements	early active movements	early active movements	Early active movements	1	case_or_format
Early adopters	early adopters	early adopters	early adopters	Early adopters	1	kept
EARLY AMBULATION	early ambulation	early ambulation	early ambulation	Early Ambulation	1	case_or_format
Early child development and prenatal experiences	early child development and prenatal experiences	early child development and prenatal experiences	early child development and prenatal experiences	Early child development and prenatal experiences	1	kept
Early childhood development	early childhood development	early childhood development	early childhood development	Early childhood development	1	kept
early childhood development	early childhood development	early childhood development	early childhood development	Early childhood development	1	case_or_format
Early clinical deterioration	early clinical deterioration	early clinical deterioration	early clinical deterioration	Early clinical deterioration	1	kept
early communication	early communication	early communication	early communication	early communication	1	kept
Early death	early death	early death	early death	Early death	1	kept
Early defibrillation	early defibrillation	early defibrillation	early defibrillation	Early defibrillation	1	kept
early depression	early depression	early depression	early depression	early depression	1	kept
Early detection	early detection	early detection	early detection	early detection	1	case_or_format
Early Detection of Disease	early detection of disease	early detection of disease	early detection of disease	Early Detection of Disease	1	kept
Early discharge of preterm infants	early discharge of preterm infants	early discharge of preterm infants	early discharge of preterm infants	Early discharge of preterm infants	1	kept

EARLY DISCHARGE PROGRAM	early discharge program	early discharge program	early discharge program	EARLY DISCHARGE PROGRAM	1	kept
Early discharge programme	early discharge programme	early discharge programme	early discharge programme	Early discharge programme	1	kept
Early enteral nutrition	early enteral nutrition	early enteral nutrition	early enteral nutrition	early enteral nutrition	1	case_or_format
EARLY EXERCISE	early exercise	early exercise	early exercise	EARLY EXERCISE	1	kept
Early Exercise	early exercise	early exercise	early exercise	EARLY EXERCISE	1	case_or_format
Early Exercise and Progressive Mobility	early exercise and progressive mobility	early exercise and progressive mobility	early exercise and progressive mobility	Early Exercise and Progressive Mobility	1	kept
EARLY EXTUBATION	early extubation	early extubation	early extubation	EARLY EXTUBATION	1	kept
Early Extubation	early extubation	early extubation	early extubation	EARLY EXTUBATION	1	case_or_format
Early extubation	early extubation	early extubation	early extubation	EARLY EXTUBATION	1	case_or_format
Early feeding	early feeding	early feeding	early feeding	Early feeding	1	kept
early findings	early findings	early findings	early findings	early findings	1	kept
Early goal	early goal	early goal	early goal	Early goal	1	kept
Early Goal Directed Therapy	early goal directed therapy	early goal directed therapy	early goal directed therapy	early goal-directed therapy	1	case_or_format
Early goal-directed mobilization	early goal directed mobilization	early goal directed mobilization	early goal directed mobilization	Early goal-directed mobilization	1	kept
Early goal-directed resuscitation	early goal directed resuscitation	early goal directed resuscitation	early goal directed resuscitation	Early goal-directed resuscitation	1	kept
Early goal-directed therapy	early goal directed therapy	early goal directed therapy	early goal directed therapy	early goal-directed therapy	1	case_or_format

Early goal-oriented rehabilition exercise	early goal oriented rehabilition exercise	early goal oriented rehabilition exercise	early goal oriented rehabilition exercise	Early goal-oriented rehabilition exercise	1	kept
Early goaldirected therapy	early goaldirected therapy	early goaldirected therapy	early goaldirected therapy	Early goaldirected therapy	1	kept
EARLY HOME INTERVENTION	early home intervention	early home intervention	early home intervention	EARLY HOME INTERVENTION	1	kept
Early immobilizations	early immobilizations	early immobilizations	early immobilizations	Early immobilizations	1	kept
Early integration	early integration	early integration	early integration	Early integration	1	kept
early integration	early integration	early integration	early integration	Early integration	1	case_or_format
Early interactions with parents	early interactions with parents	early interactions with parents	early interactions with parents	Early interactions with parents	1	kept
Early Intervention [methods]	early intervention methods	early intervention methods	early intervention methods	Early Intervention [methods]	1	kept
Early intervention for at-risk infants	early intervention for at risk infants	early intervention for at risk infants	early intervention for at risk infants	Early intervention for at-risk infants	1	kept
Early intervention for preterm infants	early intervention for preterm infants	early intervention for preterm infants	early intervention for preterm infants	Early intervention for preterm infants	1	kept
Early intervention program	early intervention program	early intervention program	early intervention program	Early intervention program	1	kept
Early interventions	early interventions	early interventions	early intervention	early intervention	1	singular
early interventions	early interventions	early interventions	early intervention	early intervention	1	singular
Early interventions for preterm infants	early interventions for preterm infants	early interventions for preterm infants	early interventions for preterm infants	Early interventions for preterm infants	1	kept
early language and literacy	early language and literacy	early language and literacy	early language and literacy	early language and literacy	1	kept
Early medical intervention	early medical intervention	early medical intervention	early medical intervention	Early Medical Intervention	1	case_or_format

Early Mobility	early mobility	early mobility	early mobility	early mobility	1	case_or_format
early mobility protocol	early mobility protocol	early mobility protocol	early mobility protocol	early mobility protocol	1	kept
early mobilization and rehabilitation protocol	early mobilization and rehabilitation protocol	early mobilization and rehabilitation protocol	early mobilization and rehabilitation protocol	early mobilization and rehabilitation protocol	1	kept
Early mortality	early mortality	early mortality	early mortality	Early mortality	1	kept
EARLY MOTHERHOOD	early motherhood	early motherhood	early motherhood	EARLY MOTHERHOOD	1	kept
Early nasal-CPAP	early nasal cpap	early nasal cpap	early nasal cpap	Early nasal-CPAP	1	kept
early obstetric warning score	early obstetric warning score	early obstetric warning score	early obstetric warning score	early obstetric warning score	1	kept
Early onset pneumonia	early onset pneumonia	early onset pneumonia	early onset pneumonia	Early onset pneumonia	1	kept
early onset pneumonia	early onset pneumonia	early onset pneumonia	early onset pneumonia	Early onset pneumonia	1	case_or_format
Early onset sepsis	early onset sepsis	early onset sepsis	early onset sepsis	early onset sepsis	1	case_or_format
early oral feed	early oral feed	early oral feed	early oral feed	early oral feed	1	kept
early outbreaks	early outbreaks	early outbreaks	early outbreaks	early outbreaks	1	kept
early palliative care	early palliative care	early palliative care	early palliative care	EARLY PALLIATIVE CARE	1	case_or_format
Early parenthood	early parenthood	early parenthood	early parenthood	Early parenthood	1	kept
Early parenting strategies	early parenting strategies	early parenting strategies	early parenting strategies	Early parenting strategies	1	kept
Early Physical Activity	early physical activity	early physical activity	early physical activity	Early Physical Activity	1	kept

Early physiotherapy	early physiotherapy	early physiotherapy	early physiotherapy	Early physiotherapy	1	kept
EARLY PHYSIOTHERAPY INTERVENTION	early physiotherapy intervention	early physiotherapy intervention	early physiotherapy intervention	EARLY PHYSIOTHERAPY INTERVENTION	1	kept
early post-operative period	early post operative period	early post operative period	early post operative period	early post-operative period	1	kept
Early post-operative shower	early post operative shower	early post operative shower	early post operative shower	Early post-operative shower	1	kept
early postpartum follow-up	early postpartum follow up	early postpartum follow up	early postpartum follow up	early postpartum follow-up	1	kept
Early prediction	early prediction	early prediction	early prediction	Early prediction	1	kept
early prediction	early prediction	early prediction	early prediction	Early prediction	1	case_or_format
early predictive assessment	early predictive assessment	early predictive assessment	early predictive assessment	early predictive assessment	1	kept
early readmission	early readmission	early readmission	early readmission	early readmission	1	kept
EARLY RECOGNITION	early recognition	early recognition	early recognition	Early recognition	1	case_or_format
early recognition of delirium	early recognition of delirium	early recognition of delirium	early recognition of delirium	early recognition of delirium	1	kept
EARLY REHABILITATION	early rehabilitation	early rehabilitation	early rehabilitation	early rehabilitation	1	case_or_format
Early Rehabilitation	early rehabilitation	early rehabilitation	early rehabilitation	early rehabilitation	1	case_or_format
Early resuscitation	early resuscitation	early resuscitation	early resuscitation	Early resuscitation	1	kept
Early skin-to-skin	early skin to skin	early skin to skin	early skin to skin	Early skin-to-skin	1	kept
Early skin-to-skin contact	early skin to skin contact	early skin to skin contact	early skin to skin contact	Early skin-to-skin contact	1	kept

Early stage	early stage	early stage	early stage	Early stage	1	kept
early stage	early stage	early stage	early stage	Early stage	1	case_or_format
Early supported transfer	early supported transfer	early supported transfer	early supported transfer	Early supported transfer	1	kept
early surgery	early surgery	early surgery	early surgery	early surgery	1	kept
Early term delivery	early term delivery	early term delivery	early term delivery	Early term delivery	1	kept
EARLY TRACHEAL EXTUBATION	early tracheal extubation	early tracheal extubation	early tracheal extubation	Early tracheal extubation	1	case_or_format
Early tracheostomy	early tracheostomy	early tracheostomy	early tracheostomy	Early tracheostomy	1	kept
early tracheostomy	early tracheostomy	early tracheostomy	early tracheostomy	Early tracheostomy	1	case_or_format
Early treatment	early treatment	early treatment	early treatment	Early treatment	1	kept
Early vocal contact	early vocal contact	early vocal contact	early vocal contact	Early vocal contact	1	kept
Early Warning	early warning	early warning	early warning	Early warning	1	case_or_format
Early warning assessment tools	early warning assessment tools	early warning assessment tools	early warning assessment tools	Early warning assessment tools	1	kept
early warning assessment tools	early warning assessment tools	early warning assessment tools	early warning assessment tools	Early warning assessment tools	1	case_or_format
early warning criteria	early warning criteria	early warning criteria	early warning criteria	early warning criteria	1	kept
early warning measures	early warning measures	early warning measures	early warning measures	early warning measures	1	kept
early warning score system	early warning score system	early warning score system	early warning score system	early warning score system	1	kept

early warning score systems	early warning score systems	early warning score systems	early warning score system	early warning score system	1	singular
early warning scores, machine learning	early warning scores machine learning	early warning scores machine learning	early warning scores machine learning	early warning scores, machine learning	1	kept
Early Warning Scoring	early warning scoring	early warning scoring	early warning scoring	Early Warning Scoring	1	kept
Early warning scoring systems	early warning scoring systems	early warning scoring systems	early warning scoring system	Early warning scoring system	1	singular
Early warning signs	early warning signs	early warning signs	early warning signs	Early warning signs	1	kept
early warning signs	early warning signs	early warning signs	early warning signs	Early warning signs	1	case_or_format
EARLY WARNING SYSTEM	early warning system	early warning system	early warning system	early warning system	1	case_or_format
Early Warning System	early warning system	early warning system	early warning system	early warning system	1	case_or_format
Early Warning Systems	early warning systems	early warning systems	early warning system	early warning system	1	singular
Early warning tool	early warning tool	early warning tool	early warning tool	Early warning tool	1	kept
early warning tool	early warning tool	early warning tool	early warning tool	Early warning tool	1	case_or_format
EARLY-INTERVENTION PROGRAM	early intervention program	early intervention program	early intervention program	Early intervention program	1	case_or_format
early-onset sepsis	early onset sepsis	early onset sepsis	early onset sepsis	early onset sepsis	1	case_or_format
Early-term infant	early term infant	early term infant	early term infant	Early-term infant	1	kept
Earmuff	earmuff	earmuff	earmuff	Earmuff	1	kept
earmuff	earmuff	earmuff	earmuff	Earmuff	1	case_or_format

EARSS	earss	earss	earss	EARSS	1	kept
EASE COVID-19	ease covid 19	ease covid 19	ease covid 19	EASE COVID-19	1	kept
EASI lead system	easi lead system	easi lead system	easi lead system	EASI lead system	1	kept
EAST	east	east	east	EAST	1	kept
East Wollega	east wollega	east wollega	east wollega	East Wollega	1	kept
Eastern Association for the Surgery of Trauma	eastern association for the surgery of trauma	eastern association for the surgery of trauma	eastern association for the surgery of trauma	Eastern Association for the Surgery of Trauma	1	kept
Eastern Ethiopia	eastern ethiopia	eastern ethiopia	eastern ethiopia	eastern Ethiopia	1	case_or_format
Eastern Nepal	eastern nepal	eastern nepal	eastern nepal	Eastern Nepal	1	kept
Eastern Orthodox	eastern orthodox	eastern orthodox	eastern orthodox	Eastern Orthodox	1	kept
eastern province	eastern province	eastern province	eastern province	eastern province	1	kept
EASY-NET	easy net	easy net	easy net	EASY-NET	1	kept
Eat Sleep Console	eat sleep console	eat sleep console	eat sleep console	eat sleep console	1	case_or_format
Eating Attitudes Test	eating attitudes test	eating attitudes test	eating attitudes test	Eating Attitudes Test	1	kept
eating behaviours	eating behaviours	eating behaviours	eating behaviours	eating behaviours	1	kept
Eating disorder	eating disorder	eating disorder	eating disorder	eating disorder	1	case_or_format
Eating habits	eating habits	eating habits	eating habit	eating habit	1	singular

Eaton Lambert syndrome	eaton lambert syndrome	eaton lambert syndrome	eaton lambert syndrome	Eaton Lambert syndrome	1	kept
EBIIAL	ebiial	ebiial	ebiial	EBIIAL	1	kept
EBM	ebm	ebm	ebm	EBM	1	kept
EBM Reviews	ebm reviews	ebm reviews	ebm reviews	EBM Reviews	1	kept
Ebola Treatment Unit	ebola treatment unit	ebola treatment unit	ebola treatment unit	Ebola Treatment Unit	1	kept
Ebola Vaccines	ebola vaccines	ebola vaccines	ebola vaccine	Ebola vaccine	1	singular
Ebola virus	ebola virus	ebola virus	ebola virus	Ebola virus	1	kept
Ebola Virus Disease	ebola virus disease	ebola virus disease	ebola virus disease	Ebola Virus Disease	1	kept
EBP implementation	ebp implementation	ebp implementation	ebp implementation	EBP implementation	1	kept
EBP process	ebp process	ebp process	ebp process	EBP process	1	kept
EBP projects	ebp projects	ebp projects	ebp projects	EBP projects	1	kept
EBP strategies	ebp strategies	ebp strategies	ebp strategies	EBP strategies	1	kept
EBP, Evidence-based practice	ebp evidence based practice	ebp evidence based practice	ebp evidence based practice	EBP, Evidence-based practice	1	kept
Ebstein's anomaly	ebstein s anomaly	ebstein s anomaly	ebstein s anomaly	Ebstein's anomaly	1	kept
eccentricity	eccentricity	eccentricity	eccentricity	eccentricity	1	kept
ECDC point prevalence survey	ecdc point prevalence survey	ecdc point prevalence survey	ecdc point prevalence survey	ECDC point prevalence survey	1	kept

ECG alarm	ecg alarm	ecg alarm	ecg alarm	ECG alarm	1	kept
ECG electrocardiogram	ecg electrocardiogram	ecg electrocardiogram	ecg electrocardiogram	ECG, electrocardiogram	1	case_or_format
ECG features	ecg features	ecg features	ecg features	ECG features	1	kept
ecg interpretation	ecg interpretation	ecg interpretation	ecg interpretation	ECG interpretation	1	case_or_format
Ecg monitoring	ecg monitoring	ecg monitoring	ecg monitoring	ECG monitoring	1	case_or_format
ECG-derived value	ecg derived value	ecg derived value	ecg derived value	ECG-derived value	1	kept
Echinacea	echinacea	echinacea	echinacea	Echinacea	1	kept
Echinacea extract	echinacea extract	echinacea extract	echinacea extract	Echinacea extract	1	kept
echinococcosis	echinococcosis	echinococcosis	echinococcosis	echinococcosis	1	kept
echinoderm microtubule associated protein like 4	echinoderm microtubule associated protein like 4	echinoderm microtubule associated protein like 4	echinoderm microtubule associated protein like 4	echinoderm microtubule associated protein like 4	1	kept
ECHM - European Committee for Hyperbaric Medicine	echm european committee for hyperbaric medicine	echm european committee for hyperbaric medicine	echm european committee for hyperbaric medicine	ECHM - European Committee for Hyperbaric Medicine	1	kept
echo contrast medium	echo contrast medium	echo contrast medium	echo contrast medium	echo contrast medium	1	kept
Echo virus	echo virus	echo virus	echo virus	Echo virus	1	kept
Echocardiogram	echocardiogram	echocardiogram	echocardiogram	echocardiogram	1	case_or_format
ECHOCARDIOGRAPHY	echocardiography	echocardiography	echocardiography	echocardiography	1	case_or_format
Echocardiography, Stress	echocardiography stress	echocardiography stress	echocardiography stress	Echocardiography, Stress	1	kept

echoencephalography	echoencephalography	echoencephalography	echoencephalography	echoencephalography	1	kept
Echoguiding	echoguiding	echoguiding	echoguiding	Echoguiding	1	kept
Ecmo	ecmo	ecmo	ecmo	ECMO	1	case_or_format
ECMO care knowledge	ecmo care knowledge	ecmo care knowledge	ecmo care knowledge	ECMO care knowledge	1	kept
ECMO clinical specialists	ecmo clinical specialists	ecmo clinical specialists	ecmo clinical specialists	ECMO clinical specialists	1	kept
ECMO complications	ecmo complications	ecmo complications	ecmo complications	ECMO complications	1	kept
ECMO Education Magnet Research	ecmo education magnet research	ecmo education magnet research	ecmo education magnet research	ECMO Education Magnet Research	1	kept
ECMO program development	ecmo program development	ecmo program development	ecmo program development	ECMO program development	1	kept
ECMO specialists	ecmo specialists	ecmo specialists	ecmo specialist	ECMO specialist	1	singular
ECMO Transport	ecmo transport	ecmo transport	ecmo transport	ECMO Transport	1	kept
ECMO transport	ecmo transport	ecmo transport	ecmo transport	ECMO Transport	1	case_or_format
ECMO, extracorporeal membrane oxygenation	ecmo extracorporeal membrane oxygenation	ecmo extracorporeal membrane oxygenation	ecmo extracorporeal membrane oxygenation	ECMO, extracorporeal membrane oxygenation	1	kept
ECMO-VV	ecmo vv	ecmo vv	ecmo vv	ECMO-VV	1	kept
ecological civilization education	ecological civilization education	ecological civilization education	ecological civilization education	ecological civilization education	1	kept
Ecological display	ecological display	ecological display	ecological display	Ecological display	1	kept
Ecological Medicine Model	ecological medicine model	ecological medicine model	ecological medicine model	Ecological Medicine Model	1	kept

Ecological momentary assessment	ecological momentary assessment	ecological momentary assessment	ecological momentary assessment	Ecological Momentary Assessment	1	case_or_format
ecological momentary assessment	ecological momentary assessment	ecological momentary assessment	ecological momentary assessment	Ecological Momentary Assessment	1	case_or_format
ecological niche	ecological niche	ecological niche	ecological niche	ecological niche	1	kept
ecology	ecology	ecology	ecology	ecology	1	kept
Economic	economic	economic	economic	economics	1	case_or_format
ECONOMIC ANALYSIS	economic analysis	economic analysis	economic analysis	economic analysis	1	case_or_format
Economic Burden	economic burden	economic burden	economic burden	economic burden	1	case_or_format
Economic burden	economic burden	economic burden	economic burden	economic burden	1	case_or_format
Economic costs	economic costs	economic costs	economic costs	Economic costs	1	kept
economic costs	economic costs	economic costs	economic costs	Economic costs	1	case_or_format
economic crisis	economic crisis	economic crisis	economic crisis	Economic crisis	1	case_or_format
Economic development	economic development	economic development	economic development	economic development	1	case_or_format
Economic effects	economic effects	economic effects	economic effects	Economic effects	1	kept
economic efficiency	economic efficiency	economic efficiency	economic efficiency	economic efficiency	1	kept
Economic Factors	economic factors	economic factors	economic factors	Economic Factors	1	kept
economic incentive	economic incentive	economic incentive	economic incentive	economic incentive	1	kept

Economic indicators	economic indicators	economic indicators	economic indicators	Economic indicators	1	kept
Economic loss	economic loss	economic loss	economic loss	Economic loss	1	kept
economic outcome	economic outcome	economic outcome	economic outcome	economic outcome	1	kept
ECONOMIC OUTCOMES	economic outcomes	economic outcomes	economic outcome	economic outcome	1	singular
economic reason	economic reason	economic reason	economic reason	economic reason	1	kept
Economic resources	economic resources	economic resources	economic resources	Economic resources	1	kept
Economic Status	economic status	economic status	economic status	economic status	1	case_or_format
economics and health services	economics and health services	economics and health services	economics and health services	economics and health services	1	kept
economics, medical	economics medical	economics medical	economics medical	Economics, Medical	1	case_or_format
Economics, Pharmaceutical	economics pharmaceutical	economics pharmaceutical	economics pharmaceutical	Economics, Pharmaceutical	1	kept
eConsult	econsult	econsult	econsult	eConsult	1	kept
ECPELLA	ecpella	ecpella	ecpella	ECPELLA	1	kept
ECRS Method	ecrs method	ecrs method	ecrs method	ECRS Method	1	kept
ED = emergency department	ed emergency department	ed emergency department	ed emergency department	ED, emergency department	1	case_or_format
ED Boarding	ed boarding	ed boarding	ed boarding	ED Boarding	1	kept
ED child death	ed child death	ed child death	ed child death	ED child death	1	kept

ED Extubation	ed extubation	ed extubation	ed extubation	ED Extubation	1	kept
ED LOS	ed los	ed los	ed los	ED LOS	1	kept
ED management	ed management	ed management	ed management	ED management	1	kept
ED observation unit	ed observation unit	ed observation unit	ed observation unit	ED observation unit	1	kept
ED, emergency department	ed emergency department	ed emergency department	ed emergency department	ED, emergency department	1	kept
ED: emergency department	ed emergency department	ed emergency department	ed emergency department	ED, emergency department	1	case_or_format
edentulousness	edentulousness	edentulousness	edentulousness	edentulousness	1	kept
edetate disodium	edetate disodium	edetate disodium	edetate disodium	edetate disodium	1	kept
Edetic Acid	edetic acid	edetic acid	edetic acid	edetic acid	1	case_or_format
EDHEP	edhep	edhep	edhep	EDHEP	1	kept
Edmonton Frail Scale	edmonton frail scale	edmonton frail scale	edmonton frail scale	Edmonton Frail Scale	1	kept
Edmonton Symptom Assessment Scale	edmonton symptom assessment scale	edmonton symptom assessment scale	edmonton symptom assessment scale	Edmonton Symptom Assessment Scale	1	kept
edmonton symptom assessment system	edmonton symptom assessment system	edmonton symptom assessment system	edmonton symptom assessment system	Edmonton Symptom Assessment System	1	case_or_format
Edoxaban	edoxaban	edoxaban	edoxaban	edoxaban	1	case_or_format
Educación basada en competencias	educaci n basada en competencias	educaci n basada en competencias	educaci n basada en competencias	Educación basada en competencias	1	kept
Educación de postgrado en enfermería	educaci n de postgrado en enfermer a	educaci n de postgrado en enfermer a	educaci n de postgrado en enfermer a	Educación de postgrado en enfermería	1	kept

Educación en Enfermería	educaci n en enfermer a	educaci n en enfermer a	educaci n en enfermer a	Educación en Enfermería	1	kept
Educación en enfermería	educaci n en enfermer a	educaci n en enfermer a	educaci n en enfermer a	Educación en Enfermería	1	case_or_format
Educación en Salud	educaci n en salud	educaci n en salud	educaci n en salud	Educación en Salud	1	kept
Educación para la Salud	educaci n para la salud	educaci n para la salud	educaci n para la salud	Educación para la Salud	1	kept
education and counseling	education and counseling	education and counseling	education and counseling	education and counseling	1	kept
Education at a distance	education at a distance	education at a distance	education at a distance	Education at a distance	1	kept
Education continuing	education continuing	education continuing	education continuing	Education, Continuing	1	case_or_format
Education Department, Hospital	education department hospital	education department hospital	education department hospital	Education Department, Hospital	1	kept
Education evaluation	education evaluation	education evaluation	education evaluation	Education evaluation	1	kept
Education Health Program	education health program	education health program	education health program	Education Health Program	1	kept
Education in practice	education in practice	education in practice	education in practice	Education in practice	1	kept
Education innovation	education innovation	education innovation	education innovation	Education innovation	1	kept
Education intervention	education intervention	education intervention	education intervention	education intervention	1	case_or_format
Education needs	education needs	education needs	education needs	Education needs	1	kept
Education of Physicians on End-of-Life Care	education of physicians on end of life care	education of physicians on end of life care	education of physicians on end of life care	Education of Physicians on End-of-Life Care	1	kept
education of resuscitation providers	education of resuscitation providers	education of resuscitation providers	education of resuscitation providers	education of resuscitation providers	1	kept

Education professional	education professional	education professional	education professional	Education, Professional	1	case_or_format
education programme	education programme	education programme	education programme	education programme	1	kept
Education research	education research	education research	education research	education research	1	case_or_format
education standards	education standards	education standards	education standards	education standards	1	kept
Education&Higher	education and higher	education and higher	education and higher	Education&Higher	1	kept
Education, all levels	education all levels	education all levels	education all levels	Education, all levels	1	kept
Education, Dental	education dental	education dental	education dental	Education, Dental	1	kept
Education, Dental, Continuing	education dental continuing	education dental continuing	education dental continuing	Education, Dental, Continuing	1	kept
Education, medical	education medical	education medical	education medical	Education, Medical	1	case_or_format
EDUCATION-PROGRAM	education program	education program	education program	education program	1	case_or_format
educational achievement	educational achievement	educational achievement	educational achievement	educational achievement	1	kept
educational activities	educational activities	educational activities	educational activities	educational activities	1	kept
educational assessment	educational assessment	educational assessment	educational assessment	Educational assessment	1	case_or_format
educational attainment	educational attainment	educational attainment	educational attainment	educational attainment	1	kept
Educational background	educational background	educational background	educational background	Educational background	1	kept
educational contents	educational contents	educational contents	educational contents	educational contents	1	kept

Educational courses	educational courses	educational courses	educational courses	Educational courses	1	kept
educational early intervention	educational early intervention	educational early intervention	educational early intervention	educational early intervention	1	kept
Educational environment	educational environment	educational environment	educational environment	Educational environment	1	kept
Educational film	educational film	educational film	educational film	Educational film	1	kept
Educational game	educational game	educational game	educational game	Educational game	1	kept
Educational initiative	educational initiative	educational initiative	educational initiative	Educational initiative	1	kept
EDUCATIONAL INTERVENTION	educational intervention	educational intervention	educational intervention	Educational intervention	1	case_or_format
EDUCATIONAL INTERVENTIONS	educational interventions	educational interventions	educational intervention	Educational intervention	1	singular
educational interventions	educational interventions	educational interventions	educational intervention	Educational intervention	1	singular
Educational leadership	educational leadership	educational leadership	educational leadership	Educational leadership	1	kept
educational level	educational level	educational level	educational level	educational level	1	kept
Educational material	educational material	educational material	educational material	Educational material	1	kept
Educational measurement	educational measurement	educational measurement	educational measurement	Educational Measurement	1	case_or_format
educational measurement	educational measurement	educational measurement	educational measurement	Educational Measurement	1	case_or_format
educational methods	educational methods	educational methods	educational methods	educational methods	1	kept
Educational modelling	educational modelling	educational modelling	educational modelling	Educational modelling	1	kept

Educational models	educational models	educational models	educational model	educational model	1	singular
Educational need analysis	educational need analysis	educational need analysis	educational need analysis	Educational need analysis	1	kept
educational outcomes	educational outcomes	educational outcomes	educational outcomes	educational outcomes	1	kept
EDUCATIONAL OUTREACH	educational outreach	educational outreach	educational outreach	EDUCATIONAL OUTREACH	1	kept
Educational outreach	educational outreach	educational outreach	educational outreach	EDUCATIONAL OUTREACH	1	case_or_format
educational outreach	educational outreach	educational outreach	educational outreach	EDUCATIONAL OUTREACH	1	case_or_format
educational package	educational package	educational package	educational package	educational package	1	kept
educational pathway	educational pathway	educational pathway	educational pathway	educational pathway	1	kept
Educational Personnel	educational personnel	educational personnel	educational personnel	Educational Personnel	1	kept
Educational pre-internship package	educational pre internship package	educational pre internship package	educational pre internship package	Educational pre-internship package	1	kept
EDUCATIONAL PREPARATION	educational preparation	educational preparation	educational preparation	EDUCATIONAL PREPARATION	1	kept
Educational profile	educational profile	educational profile	educational profile	Educational profile	1	kept
Educational program: Performance	educational program performance	educational program performance	educational program performance	Educational program: Performance	1	kept
educational science	educational science	educational science	educational science	educational science	1	kept
educational strategies	educational strategies	educational strategies	educational strategy	Educational strategy	1	singular
Educational strategy	educational strategy	educational strategy	educational strategy	Educational strategy	1	kept

educational training concept	educational training concept	educational training concept	educational training concept	educational training concept	1	kept
educational workshop	educational workshop	educational workshop	educational workshop	educational workshops	1	case_or_format
Educational-supportive program	educational supportive program	educational supportive program	educational supportive program	Educational-supportive program	1	kept
EDUCATIONAL-LEVELS	educational levels	educational levels	educational level	educational level	1	singular
EDUCATIONAL-PROGRAM	educational program	educational program	educational program	Educational program	1	case_or_format
EDUCATOR	educator	educator	educator	EDUCATOR	1	kept
Educator role development	educator role development	educator role development	educator role development	Educator role development	1	kept
EDUCATORS	educators	educators	educator	EDUCATOR	1	singular
Educação	educa o	educa o	educa o	Educação	1	kept
Educação em Saúde	educa o em sa de	educa o em sa de	educa o em sa de	Educação em Saúde	1	kept
Edwards SAPIEN	edwards sapien	edwards sapien	edwards sapien	Edwards SAPIEN	1	kept
EEG abnormality	eeg abnormality	eeg abnormality	eeg abnormality	EEG abnormality	1	kept
EEG burst suppression	eeg burst suppression	eeg burst suppression	eeg burst suppression	EEG burst suppression	1	kept
EEG monitoring	eeg monitoring	eeg monitoring	eeg monitoring	EEG monitoring	1	kept
EEG signals	eeg signals	eeg signals	eeg signals	EEG signals	1	kept
EEG, electroencephalography	eeg electroencephalography	eeg electroencephalography	eeg electroencephalography	EEG, electroencephalography	1	kept

efavirenz plus emtricitabine plus tenofovir disoproxil	efavirenz plus emtricitabine plus tenofovir disoproxil	efavirenz plus emtricitabine plus tenofovir disoproxil	efavirenz plus emtricitabine plus tenofovir disoproxil	efavirenz plus emtricitabine plus tenofovir disoproxil	1	kept
EfCCNa	efccna	efccna	efccna	EfCCNa	1	kept
Effect	effect	effect	effect	Effect	1	kept
effect comparison	effect comparison	effect comparison	effect comparison	effect comparison	1	kept
Effect evaluation	effect evaluation	effect evaluation	effect evaluation	Effect evaluation	1	kept
Effect factors	effect factors	effect factors	effect factors	Effect factors	1	kept
Effect Modifiers	effect modifiers	effect modifiers	effect modifiers	Effect Modifiers	1	kept
Effect size	effect size	effect size	effect size	effect size	1	case_or_format
Effective approaches	effective approaches	effective approaches	effective approaches	Effective approaches	1	kept
EFFECTIVE COMMUNICATION	effective communication	effective communication	effective communication	Effective communication	1	case_or_format
effective cough	effective cough	effective cough	effective cough	effective cough	1	kept
effective handover	effective handover	effective handover	effective handover	effective handover	1	kept
Effective implementation	effective implementation	effective implementation	effective implementation	Effective implementation	1	kept
Effective Instructor	effective instructor	effective instructor	effective instructor	Effective Instructor	1	kept
Effective solution	effective solution	effective solution	effective solution	Effective solution	1	kept
EFFECTIVE TEAMWORK	effective teamwork	effective teamwork	effective teamwork	EFFECTIVE TEAMWORK	1	kept

Effective utilization of laboratory services	effective utilization of laboratory services	effective utilization of laboratory services	effective utilization of laboratory services	Effective utilization of laboratory services	1	kept
effectiveness in education	effectiveness in education	effectiveness in education	effectiveness in education	effectiveness in education	1	kept
Effectiveness instructional program	effectiveness instructional program	effectiveness instructional program	effectiveness instructional program	Effectiveness instructional program	1	kept
Effectiveness of Educational Program	effectiveness of educational program	effectiveness of educational program	effectiveness of educational program	Effectiveness of Educational Program	1	kept
effector cell	effector cell	effector cell	effector cell	effector cell	1	kept
effects	effects	effects	effect	Effect	1	singular
Effects of aging	effects of aging	effects of aging	effects of aging	Effects of aging	1	kept
effects of bullying	effects of bullying	effects of bullying	effects of bullying	effects of bullying	1	kept
Effects of education	effects of education	effects of education	effects of education	Effects of education	1	kept
Efferent limb	efferent limb	efferent limb	efferent limb	Efferent limb	1	kept
Efficacy beliefs	efficacy beliefs	efficacy beliefs	efficacy beliefs	Efficacy beliefs	1	kept
Efficacy evaluation	efficacy evaluation	efficacy evaluation	efficacy evaluation	Efficacy evaluation	1	kept
Efficacy of prevention	efficacy of prevention	efficacy of prevention	efficacy of prevention	Efficacy of prevention	1	kept
Efficiency and reliability	efficiency and reliability	efficiency and reliability	efficiency and reliability	Efficiency and reliability	1	kept
Efficiency ratio	efficiency ratio	efficiency ratio	efficiency ratio	Efficiency ratio	1	kept
efficiency, organizational	efficiency organizational	efficiency organizational	efficiency organizational	Efficiency, Organizational	1	case_or_format

Efficient monitoring	efficient monitoring	efficient monitoring	efficient monitoring	Efficient monitoring	1	kept
efficient triage	efficient triage	efficient triage	efficient triage	efficient triage	1	kept
effort vein thrombosis	effort vein thrombosis	effort vein thrombosis	effort vein thrombosis	effort vein thrombosis	1	kept
EFFORT-REWARD IMBALANCE	effort reward imbalance	effort reward imbalance	effort reward imbalance	effort-reward imbalance	1	case_or_format
Effort-reward imbalance	effort reward imbalance	effort reward imbalance	effort reward imbalance	effort-reward imbalance	1	case_or_format
effort‑reward imbalance model	effort reward imbalance model	effort reward imbalance model	effort reward imbalance model	effort‑reward imbalance model	1	kept
EGS	egs	egs	egs	EGS	1	kept
Egyptian	egyptian	egyptian	egyptian	Egyptian	1	kept
Egyptian Neonatal Safety Standards	egyptian neonatal safety standards	egyptian neonatal safety standards	egyptian neonatal safety standards	Egyptian Neonatal Safety Standards	1	kept
Egyptian uprising	egyptian uprising	egyptian uprising	egyptian uprising	Egyptian uprising	1	kept
Egyptians	egyptians	egyptians	egyptian	Egyptian	1	singular
EHealth	ehealth	ehealth	ehealth	eHealth	1	case_or_format
Ehealth	ehealth	ehealth	ehealth	eHealth	1	case_or_format
eHealth strategies	ehealth strategies	ehealth strategies	ehealth strategies	eHealth strategies	1	kept
EHR transition	ehr transition	ehr transition	ehr transition	EHR transition	1	kept
EHR, Electronic health records	ehr electronic health records	ehr electronic health records	ehr electronic health records	EHR, Electronic health records	1	kept

EHRs	ehrs	ehrs	ehr	EHR	1	singular
eicosapentaenoic acid	eicosapentaenoic acid	eicosapentaenoic acid	eicosapentaenoic acid	Eicosapentaenoic Acid	1	case_or_format
eigenvector centrality	eigenvector centrality	eigenvector centrality	eigenvector centrality	eigenvector centrality	1	kept
EIR, early interdisciplinary rehabilitation	eir early interdisciplinary rehabilitation	eir early interdisciplinary rehabilitation	eir early interdisciplinary rehabilitation	EIR, early interdisciplinary rehabilitation	1	kept
ejection fraction	ejection fraction	ejection fraction	ejection fraction	ejection fraction	1	kept
EKG artifact	ekg artifact	ekg artifact	ekg artifact	EKG artifact	1	kept
elastase ii	elastase ii	elastase ii	elastase ii	elastase ii	1	kept
Elastic net	elastic net	elastic net	elastic net	Elastic net	1	kept
Elasticity	elasticity	elasticity	elasticity	elasticity	1	case_or_format
elastomer	elastomer	elastomer	elastomer	elastomer	1	kept
elastomeric respirator	elastomeric respirator	elastomeric respirator	elastomeric respirator	elastomeric respirator	1	kept
elbow injury	elbow injury	elbow injury	elbow injury	elbow injury	1	kept
elbow pain	elbow pain	elbow pain	elbow pain	elbow pain	1	kept
ELBW infants	elbw infants	elbw infants	elbw infants	ELBW infants	1	kept
elder abuse	elder abuse	elder abuse	elder abuse	Elder Abuse	1	case_or_format
elder design	elder design	elder design	elder design	elder design	1	kept

elder risk assessment	elder risk assessment	elder risk assessment	elder risk assessment	elder risk assessment	1	kept
elderly < specific populations	elderly specific populations	elderly specific populations	elderly specific populations	elderly < specific populations	1	kept
ELDERLY CARE	elderly care	elderly care	elderly care	elderly care	1	case_or_format
elderly citizens	elderly citizens	elderly citizens	elderly citizens	elderly citizens	1	kept
elderly fall	elderly fall	elderly fall	elderly fall	elderly fall	1	kept
elderly health	elderly health	elderly health	elderly health	elderly health	1	kept
elderly hip fracture	elderly hip fracture	elderly hip fracture	elderly hip fracture	elderly hip fracture	1	kept
Elderly mobility scale	elderly mobility scale	elderly mobility scale	elderly mobility scale	Elderly mobility scale	1	kept
Elderly person	elderly person	elderly person	elderly person	Elderly person	1	kept
Elderly persons	elderly persons	elderly persons	elderly person	Elderly person	1	singular
elderly resident	elderly resident	elderly resident	elderly resident	elderly resident	1	kept
elderly suicide	elderly suicide	elderly suicide	elderly suicide	elderly suicides	1	case_or_format
ELDERLY-PEOPLE	elderly people	elderly people	elderly people	Elderly people	1	case_or_format
ELDERLY-WOMEN	elderly women	elderly women	elderly women	ELDERLY-WOMEN	1	kept
elders	elders	elders	elder	Elder	1	singular
Elearning	elearning	elearning	elearning	eLearning	1	case_or_format

Elective	elective	elective	elective	elective	1	case_or_format
Elective aortic abdominal aneurysm repair	elective aortic abdominal aneurysm repair	elective aortic abdominal aneurysm repair	elective aortic abdominal aneurysm repair	Elective aortic abdominal aneurysm repair	1	kept
ELECTIVE CRANIOTOMY	elective craniotomy	elective craniotomy	elective craniotomy	ELECTIVE CRANIOTOMY	1	kept
Elective induction	elective induction	elective induction	elective induction	Elective induction	1	kept
Elective neurosurgery	elective neurosurgery	elective neurosurgery	elective neurosurgery	Elective neurosurgery	1	kept
elective noncardiac surgery	elective noncardiac surgery	elective noncardiac surgery	elective noncardiac surgery	elective noncardiac surgery	1	kept
Elective repeat cesarean section	elective repeat cesarean section	elective repeat cesarean section	elective repeat cesarean section	Elective repeat cesarean section	1	kept
ELECTIVE SURGERY	elective surgery	elective surgery	elective surgery	elective surgery	1	case_or_format
Elective ventilation	elective ventilation	elective ventilation	elective ventilation	Elective ventilation	1	kept
electric battery	electric battery	electric battery	electric battery	electric battery	1	kept
Electric burns	electric burns	electric burns	electric burn	electric burn	1	singular
Electric defibrillation	electric defibrillation	electric defibrillation	electric defibrillation	Electric defibrillation	1	kept
Electric lighting	electric lighting	electric lighting	electric lighting	Electric lighting	1	kept
electric resistance	electric resistance	electric resistance	electric resistance	electric resistance	1	kept
electric stimulation therapy	electric stimulation therapy	electric stimulation therapy	electric stimulation therapy	Electric Stimulation Therapy	1	case_or_format
Electric syringe pump	electric syringe pump	electric syringe pump	electric syringe pump	Electric syringe pump	1	kept

Electric Wheelchair	electric wheelchair	electric wheelchair	electric wheelchair	Electric Wheelchair	1	kept
Electric wheelchair	electric wheelchair	electric wheelchair	electric wheelchair	Electric Wheelchair	1	case_or_format
Electrical activities	electrical activities	electrical activities	electrical activities	Electrical activities	1	kept
electrical activity of the diaphragm	electrical activity of the diaphragm	electrical activity of the diaphragm	electrical activity of the diaphragm	electrical activity of the diaphragm	1	kept
Electrical bioimpedance of the chest	electrical bioimpedance of the chest	electrical bioimpedance of the chest	electrical bioimpedance of the chest	Electrical bioimpedance of the chest	1	kept
electrical cardiometry	electrical cardiometry	electrical cardiometry	electrical cardiometry	electrical cardiometry	1	kept
electrical conductance	electrical conductance	electrical conductance	electrical conductance	electrical conductance	1	kept
electrical equipment	electrical equipment	electrical equipment	electrical equipment	electrical equipment	1	kept
electrical impedance	electrical impedance	electrical impedance	electrical impedance	electrical impedance	1	kept
Electrical Impedance Tomography	electrical impedance tomography	electrical impedance tomography	electrical impedance tomography	Electrical impedance tomography	1	case_or_format
ELECTRICAL NERVE-STIMULATION	electrical nerve stimulation	electrical nerve stimulation	electrical nerve stimulation	ELECTRICAL NERVE-STIMULATION	1	kept
Electrical resistances	electrical resistances	electrical resistances	electrical resistances	Electrical resistances	1	kept
Electro-encephalogram	electro encephalogram	electro encephalogram	electro encephalogram	Electro-encephalogram	1	kept
Electrocadiography	electrocadiography	electrocadiography	electrocadiography	electrocadiography	1	case_or_format
Electrocardiogram central line confirmation	electrocardiogram central line confirmation	electrocardiogram central line confirmation	electrocardiogram central line confirmation	Electrocardiogram central line confirmation	1	kept
electrocardiogram interpretation	electrocardiogram interpretation	electrocardiogram interpretation	electrocardiogram interpretation	Electrocardiogram interpretation	1	case_or_format

Electrocardiogram signal	electrocardiogram signal	electrocardiogram signal	electrocardiogram signal	Electrocardiogram signal	1	kept
electrocardiographic	electrocardiographic	electrocardiographic	electrocardiographic	electrocardiographic	1	kept
Electrocardiography Interpretation	electrocardiography interpretation	electrocardiography interpretation	electrocardiography interpretation	Electrocardiography interpretation	1	case_or_format
electrochemical analysis	electrochemical analysis	electrochemical analysis	electrochemical analysis	electrochemical analysis	1	kept
Electroconvulsive Therapy	electroconvulsive therapy	electroconvulsive therapy	electroconvulsive therapy	electroconvulsive therapy	1	case_or_format
Electroconvulsive therapy	electroconvulsive therapy	electroconvulsive therapy	electroconvulsive therapy	electroconvulsive therapy	1	case_or_format
Electrocorticography	electrocorticography	electrocorticography	electrocorticography	electrocorticography	1	case_or_format
electrodermal response	electrodermal response	electrodermal response	electrodermal response	electrodermal response	1	kept
Electrodes, Implanted	electrodes implanted	electrodes implanted	electrodes implanted	Electrodes, Implanted	1	kept
Electrodiagnostic	electrodiagnostic	electrodiagnostic	electrodiagnostic	Electrodiagnostic	1	kept
electrodiagnostic studies	electrodiagnostic studies	electrodiagnostic studies	electrodiagnostic studies	electrodiagnostic studies	1	kept
electrogastrography	electrogastrography	electrogastrography	electrogastrography	electrogastrography	1	kept
electrographic seizure burden	electrographic seizure burden	electrographic seizure burden	electrographic seizure burden	electrographic seizure burden	1	kept
ELECTROGRAPHIC SEIZURES	electrographic seizures	electrographic seizures	electrographic seizure	electrographic seizure	1	singular
Electrographic seizures	electrographic seizures	electrographic seizures	electrographic seizure	electrographic seizure	1	singular
electrographic seizures	electrographic seizures	electrographic seizures	electrographic seizure	electrographic seizure	1	singular

ELECTROLARYNX	electrolarynx	electrolarynx	electrolarynx	ELECTROLARYNX	1	kept
Electrolarynx	electrolarynx	electrolarynx	electrolarynx	ELECTROLARYNX	1	case_or_format
Electrolyte	electrolyte	electrolyte	electrolyte	electrolyte	1	case_or_format
electrolyte disorders	electrolyte disorders	electrolyte disorders	electrolyte disorders	electrolyte disorders	1	kept
Electrolyte disturbances	electrolyte disturbances	electrolyte disturbances	electrolyte disturbance	electrolyte disturbance	1	singular
electrolyte excretion	electrolyte excretion	electrolyte excretion	electrolyte excretion	electrolyte excretion	1	kept
electrolyte imbalance	electrolyte imbalance	electrolyte imbalance	electrolyte imbalance	Electrolyte imbalance	1	case_or_format
electrolyte transport	electrolyte transport	electrolyte transport	electrolyte transport	electrolyte transport	1	kept
electrolyte urine level	electrolyte urine level	electrolyte urine level	electrolyte urine level	electrolyte urine level	1	kept
Electromagnetic field	electromagnetic field	electromagnetic field	electromagnetic field	electromagnetic field	1	case_or_format
Electromagnetic guidance	electromagnetic guidance	electromagnetic guidance	electromagnetic guidance	electromagnetic guidance	1	case_or_format
electromagnetic guided feeding tube placement	electromagnetic guided feeding tube placement	electromagnetic guided feeding tube placement	electromagnetic guided feeding tube placement	electromagnetic guided feeding tube placement	1	kept
Electromagnetic pulse	electromagnetic pulse	electromagnetic pulse	electromagnetic pulse	Electromagnetic pulse	1	kept
Electromyogram	electromyogram	electromyogram	electromyogram	electromyogram	1	case_or_format
Electromyographic	electromyographic	electromyographic	electromyographic	Electromyographic	1	kept
electromyographic endotracheal tube	electromyographic endotracheal tube	electromyographic endotracheal tube	electromyographic endotracheal tube	electromyographic endotracheal tube	1	kept

Electronic	electronic	electronic	electronic	Electronics	1	case_or_format
ELECTRONIC BOOKS	electronic books	electronic books	electronic books	ELECTRONIC BOOKS	1	kept
electronic calculator	electronic calculator	electronic calculator	electronic calculator	electronic calculator	1	kept
Electronic charts	electronic charts	electronic charts	electronic charts	Electronic charts	1	kept
electronic checklist	electronic checklist	electronic checklist	electronic checklist	Electronic checklist	1	case_or_format
electronic cigarette smoking	electronic cigarette smoking	electronic cigarette smoking	electronic cigarette smoking	electronic cigarette smoking	1	kept
electronic circular dichroism	electronic circular dichroism	electronic circular dichroism	electronic circular dichroism	electronic circular dichroism	1	kept
Electronic communication	electronic communication	electronic communication	electronic communication	Electronic communication	1	kept
electronic dashboard	electronic dashboard	electronic dashboard	electronic dashboard	electronic dashboard	1	kept
Electronic dashboards	electronic dashboards	electronic dashboards	electronic dashboard	electronic dashboard	1	singular
Electronic data collection	electronic data collection	electronic data collection	electronic data collection	Electronic data collection	1	kept
Electronic data interchange	electronic data interchange	electronic data interchange	electronic data interchange	Electronic data interchange	1	kept
Electronic database	electronic database	electronic database	electronic database	Electronic database	1	kept
electronic decision support system	electronic decision support system	electronic decision support system	electronic decision support system	electronic decision support system	1	kept
Electronic digital signature	electronic digital signature	electronic digital signature	electronic digital signature	Electronic digital signature	1	kept
Electronic documentation	electronic documentation	electronic documentation	electronic documentation	Electronic documentation	1	kept

electronic documentation	electronic documentation	electronic documentation	electronic documentation	Electronic documentation	1	case_or_format
electronic drug administration	electronic drug administration	electronic drug administration	electronic drug administration	electronic drug administration	1	kept
Electronic equipment	electronic equipment	electronic equipment	electronic equipment	Electronic equipment	1	kept
Electronic feedback	electronic feedback	electronic feedback	electronic feedback	Electronic feedback	1	kept
Electronic fingerprint	electronic fingerprint	electronic fingerprint	electronic fingerprint	Electronic fingerprint	1	kept
electronic follow up system	electronic follow up system	electronic follow up system	electronic follow up system	electronic follow up system	1	kept
Electronic formats	electronic formats	electronic formats	electronic formats	Electronic formats	1	kept
electronic hand hygiene monitoring system	electronic hand hygiene monitoring system	electronic hand hygiene monitoring system	electronic hand hygiene monitoring system	electronic hand hygiene monitoring system	1	kept
Electronic handover	electronic handover	electronic handover	electronic handover	Electronic handover	1	kept
electronic handover tool	electronic handover tool	electronic handover tool	electronic handover tool	electronic handover tool	1	kept
Electronic handwash counter	electronic handwash counter	electronic handwash counter	electronic handwash counter	Electronic handwash counter	1	kept
Electronic Health Record	electronic health record	electronic health record	electronic health record	Electronic Health Records	1	case_or_format
ELECTRONIC HEALTH RECORDS	electronic health records	electronic health records	electronic health record	Electronic Health Records	1	singular
electronic health records and systems	electronic health records and systems	electronic health records and systems	electronic health records and systems	Electronic health records and systems	1	case_or_format
electronic healthcare record utilization	electronic healthcare record utilization	electronic healthcare record utilization	electronic healthcare record utilization	electronic healthcare record utilization	1	kept
Electronic heath record	electronic heath record	electronic heath record	electronic heath record	Electronic heath record	1	kept

Electronic HH system	electronic hh system	electronic hh system	electronic hh system	Electronic HH system	1	kept
Electronic information system	electronic information system	electronic information system	electronic information system	Electronic information system	1	kept
Electronic learning	electronic learning	electronic learning	electronic learning	Electronic learning	1	kept
electronic learning	electronic learning	electronic learning	electronic learning	Electronic learning	1	case_or_format
Electronic medical database	electronic medical database	electronic medical database	electronic medical database	Electronic medical database	1	kept
Electronic Medical Record	electronic medical record	electronic medical record	electronic medical record	electronic medical record	1	case_or_format
Electronic medical record systems	electronic medical record systems	electronic medical record systems	electronic medical record system	electronic medical record system	1	singular
electronic medication administering registration	electronic medication administering registration	electronic medication administering registration	electronic medication administering registration	electronic medication administering registration	1	kept
Electronic medication administration	electronic medication administration	electronic medication administration	electronic medication administration	Electronic medication administration	1	kept
Electronic medication administration records	electronic medication administration records	electronic medication administration records	electronic medication administration records	Electronic medication administration records	1	kept
electronic medication management system	electronic medication management system	electronic medication management system	electronic medication management system	Electronic medication management systems	1	case_or_format
electronic medication management systems	electronic medication management systems	electronic medication management systems	electronic medication management system	Electronic medication management systems	1	singular
Electronic method	electronic method	electronic method	electronic method	Electronic method	1	kept
Electronic monitoring systems	electronic monitoring systems	electronic monitoring systems	electronic monitoring systems	Electronic monitoring systems	1	kept
Electronic order set	electronic order set	electronic order set	electronic order set	Electronic order set	1	kept
electronic order set	electronic order set	electronic order set	electronic order set	Electronic order set	1	case_or_format

Electronic prescribing	electronic prescribing	electronic prescribing	electronic prescribing	electronic prescribing	1	case_or_format
electronic process of care checklist	electronic process of care checklist	electronic process of care checklist	electronic process of care checklist	electronic process of care checklist	1	kept
Electronic publishing	electronic publishing	electronic publishing	electronic publishing	Electronic publishing	1	kept
Electronic records	electronic records	electronic records	electronic records	Electronic records	1	kept
electronic records	electronic records	electronic records	electronic records	Electronic records	1	case_or_format
Electronic registries	electronic registries	electronic registries	electronic registries	Electronic registries	1	kept
Electronic reminder	electronic reminder	electronic reminder	electronic reminder	Electronic reminder	1	kept
Electronic reporting tool	electronic reporting tool	electronic reporting tool	electronic reporting tool	Electronic reporting tool	1	kept
electronic resuscitation record	electronic resuscitation record	electronic resuscitation record	electronic resuscitation record	electronic resuscitation record	1	kept
electronic rounding tool	electronic rounding tool	electronic rounding tool	electronic rounding tool	electronic rounding tool	1	kept
electronic sensor	electronic sensor	electronic sensor	electronic sensor	electronic sensor	1	kept
electronic stethoscope	electronic stethoscope	electronic stethoscope	electronic stethoscope	electronic stethoscope	1	kept
electronic surveillance	electronic surveillance	electronic surveillance	electronic surveillance	electronic surveillance	1	kept
Electronic surveillance systems	electronic surveillance systems	electronic surveillance systems	electronic surveillance systems	Electronic surveillance systems	1	kept
Electronic system	electronic system	electronic system	electronic system	Electronic system	1	kept
Electronic systems	electronic systems	electronic systems	electronic system	Electronic system	1	singular

Electronic transfusion verification	electronic transfusion verification	electronic transfusion verification	electronic transfusion verification	Electronic transfusion verification	1	kept
electronic triage	electronic triage	electronic triage	electronic triage	electronic triage	1	kept
Electronic versions	electronic versions	electronic versions	electronic versions	Electronic versions	1	kept
Electronics, Medical	electronics medical	electronics medical	electronics medical	Electronics, Medical	1	kept
electrooculogram	electrooculogram	electrooculogram	electrooculogram	electrooculogram	1	kept
ELECTROPHORESIS	electrophoresis	electrophoresis	electrophoresis	electrophoresis	1	case_or_format
Electrophysiology studies	electrophysiology studies	electrophysiology studies	electrophysiology studies	Electrophysiology studies	1	kept
electrospray mass spectrometry	electrospray mass spectrometry	electrospray mass spectrometry	electrospray mass spectrometry	electrospray mass spectrometry	1	kept
electrostatic adsorption	electrostatic adsorption	electrostatic adsorption	electrostatic adsorption	electrostatic adsorption	1	kept
electrostimulation therapy	electrostimulation therapy	electrostimulation therapy	electrostimulation therapy	electrostimulation therapy	1	kept
electrosurgery	electrosurgery	electrosurgery	electrosurgery	electrosurgery	1	kept
Element models	element models	element models	element models	Element models	1	kept
elephant	elephant	elephant	elephant	elephant	1	kept
Elevación del cabezal de la cama	elevaci n del cabezal de la cama	elevaci n del cabezal de la cama	elevaci n del cabezal de la cama	Elevación del cabezal de la cama	1	kept
Elevated intracranial pressure	elevated intracranial pressure	elevated intracranial pressure	elevated intracranial pressure	Elevated intracranial pressure	1	kept
Elevated liver enzymes	elevated liver enzymes	elevated liver enzymes	elevated liver enzymes	Elevated liver enzymes	1	kept

elevated temperature	elevated temperature	elevated temperature	elevated temperature	elevated temperature	1	kept
elevating tubing	elevating tubing	elevating tubing	elevating tubing	elevating tubing	1	kept
ELF	elf	elf	elf	ELF	1	kept
Elicitation study	elicitation study	elicitation study	elicitation study	Elicitation study	1	kept
eliciting consent	eliciting consent	eliciting consent	eliciting consent	eliciting consent	1	kept
eliglustat	eliglustat	eliglustat	eliglustat	eliglustat	1	kept
elimination care	elimination care	elimination care	elimination care	elimination care	1	kept
elimination rate constant	elimination rate constant	elimination rate constant	elimination rate constant	elimination rate constant	1	kept
ELISA	elisa	elisa	elisa	ELISA	1	kept
Elisabethkingia meningoseptica	elisabethkingia meningoseptica	elisabethkingia meningoseptica	elisabethkingia meningoseptica	Elisabethkingia meningoseptica	1	kept
elixhauser score	elixhauser score	elixhauser score	elixhauser score	elixhauser score	1	kept
elixir	elixir	elixir	elixir	elixir	1	kept
Elizabethkingia	elizabethkingia	elizabethkingia	elizabethkingia	Elizabethkingia	1	kept
Elizabethkingia anophelis	elizabethkingia anophelis	elizabethkingia anophelis	elizabethkingia anophelis	Elizabethkingia anophelis	1	kept
ellipsoid zone	ellipsoid zone	ellipsoid zone	ellipsoid zone	ellipsoid zone	1	kept
ELSO, Extracorporeal Life Support Organization	elso extracorporeal life support organization	elso extracorporeal life support organization	elso extracorporeal life support organization	ELSO, Extracorporeal Life Support Organization	1	kept

eltrombopag	eltrombopag	eltrombopag	eltrombopag	eltrombopag	1	kept
elvitegravir	elvitegravir	elvitegravir	elvitegravir	elvitegravir	1	kept
EM resident education	em resident education	em resident education	em resident education	EM resident education	1	kept
email	email	email	email	email	1	kept
Email communication	email communication	email communication	email communication	Email communication	1	kept
Emancipatory Model	emancipatory model	emancipatory model	emancipatory model	Emancipatory Model	1	kept
EMBASE: Interrater reliability	embase interrater reliability	embase interrater reliability	embase interrater reliability	EMBASE: Interrater reliability	1	kept
Embedded	embedded	embedded	embedded	Embedded	1	kept
Embedded Web Medical System	embedded web medical system	embedded web medical system	embedded web medical system	Embedded Web Medical System	1	kept
Embeddedness	embeddedness	embeddedness	embeddedness	EMBEDDEDNESS	1	case_or_format
embesin	embesin	embesin	embesin	embesin	1	kept
embodied	embodied	embodied	embodied	embodied	1	kept
Embodied voice and presence	embodied voice and presence	embodied voice and presence	embodied voice and presence	Embodied voice and presence	1	kept
Embolisation	embolisation	embolisation	embolisation	Embolisation	1	kept
Embolism, Fat	embolism fat	embolism fat	embolism fat	Embolism, Fat	1	kept
embolization coil	embolization coil	embolization coil	embolization coil	embolization coil	1	kept

Embrace care	embrace care	embrace care	embrace care	Embrace care	1	kept
embryo	embryo	embryo	embryo	embryo	1	kept
embryology	embryology	embryology	embryology	embryology	1	kept
Embryonic kidney	embryonic kidney	embryonic kidney	embryonic kidney	Embryonic kidney	1	kept
Emer-gency Medicine	emer gency medicine	emer gency medicine	emer gency medicine	Emer-gency Medicine	1	kept
Emergencies room	emergencies room	emergencies room	emergencies room	Emergencies room	1	kept
Emergency abdominal surgery	emergency abdominal surgery	emergency abdominal surgery	emergency abdominal surgery	Emergency abdominal surgery	1	kept
emergency abdominal surgery	emergency abdominal surgery	emergency abdominal surgery	emergency abdominal surgery	Emergency abdominal surgery	1	case_or_format
Emergency air medical transport	emergency air medical transport	emergency air medical transport	emergency air medical transport	Emergency air medical transport	1	kept
emergency ambulance systems	emergency ambulance systems	emergency ambulance systems	emergency ambulance systems	emergency ambulance systems	1	kept
EMERGENCY ASSISTANCE	emergency assistance	emergency assistance	emergency assistance	EMERGENCY ASSISTANCE	1	kept
Emergency caesarean section	emergency caesarean section	emergency caesarean section	emergency caesarean section	Emergency caesarean section	1	kept
emergency capability	emergency capability	emergency capability	emergency capability	emergency capability	1	kept
emergency cardiovascular care	emergency cardiovascular care	emergency cardiovascular care	emergency cardiovascular care	emergency cardiovascular care	1	kept
Emergency Care	emergency care	emergency care	emergency care	emergency care	1	case_or_format
emergency care brief alcohol intervention	emergency care brief alcohol intervention	emergency care brief alcohol intervention	emergency care brief alcohol intervention	emergency care brief alcohol intervention	1	kept

Emergency care research	emergency care research	emergency care research	emergency care research	Emergency care research	1	kept
emergency care systems, admission avoidance	emergency care systems admission avoidance	emergency care systems admission avoidance	emergency care systems admission avoidance	emergency care systems, admission avoidance	1	kept
emergency care systems, emergency departments	emergency care systems emergency departments	emergency care systems emergency departments	emergency care systems emergency departments	emergency care systems, emergency departments	1	kept
emergency care training	emergency care training	emergency care training	emergency care training	emergency care training	1	kept
emergency center or department	emergency center or department	emergency center or department	emergency center or department	emergency center or department	1	kept
EMERGENCY CESAREAN-SECTION	emergency cesarean section	emergency cesarean section	emergency cesarean section	EMERGENCY CESAREAN-SECTION	1	kept
Emergency conditions	emergency conditions	emergency conditions	emergency conditions	Emergency conditions	1	kept
emergency critical illness	emergency critical illness	emergency critical illness	emergency critical illness	emergency critical illness	1	kept
emergency decrees	emergency decrees	emergency decrees	emergency decrees	emergency decrees	1	kept
Emergency department boarding	emergency department boarding	emergency department boarding	emergency department boarding	Emergency department boarding	1	kept
emergency department end of life	emergency department end of life	emergency department end of life	emergency department end of life	emergency department end of life	1	kept
emergency department length of stay	emergency department length of stay	emergency department length of stay	emergency department length of stay	emergency department length of stay	1	kept
emergency department operations	emergency department operations	emergency department operations	emergency department operations	emergency department operations	1	kept
Emergency department preparedness	emergency department preparedness	emergency department preparedness	emergency department preparedness	Emergency department preparedness	1	kept
Emergency department thoracotomies	emergency department thoracotomies	emergency department thoracotomies	emergency department thoracotomy	Emergency department thoracotomy	1	singular
Emergency department thoracotomy	emergency department thoracotomy	emergency department thoracotomy	emergency department thoracotomy	Emergency department thoracotomy	1	kept

Emergency department transfer	emergency department transfer	emergency department transfer	emergency department transfer	Emergency department transfer	1	kept
emergency department transfer	emergency department transfer	emergency department transfer	emergency department transfer	Emergency department transfer	1	case_or_format
emergency department triage	emergency department triage	emergency department triage	emergency department triage	Emergency department triage	1	case_or_format
Emergency department visit	emergency department visit	emergency department visit	emergency department visit	emergency department visit	1	case_or_format
emergency endotracheal intubation	emergency endotracheal intubation	emergency endotracheal intubation	emergency endotracheal intubation	emergency endotracheal intubation	1	kept
Emergency equipment	emergency equipment	emergency equipment	emergency equipment	Emergency equipment	1	kept
emergency equipment and supply	emergency equipment and supply	emergency equipment and supply	emergency equipment and supply	emergency equipment and supply	1	kept
emergency evacuation	emergency evacuation	emergency evacuation	emergency evacuation	emergency evacuation	1	kept
Emergency evaluation	emergency evaluation	emergency evaluation	emergency evaluation	Emergency evaluation	1	kept
Emergency groups	emergency groups	emergency groups	emergency groups	Emergency groups	1	kept
Emergency handling	emergency handling	emergency handling	emergency handling	Emergency handling	1	kept
Emergency health care workers	emergency health care workers	emergency health care workers	emergency health care workers	Emergency health care workers	1	kept
emergency health care workers	emergency health care workers	emergency health care workers	emergency health care workers	Emergency health care workers	1	case_or_format
emergency hospital service	emergency hospital service	emergency hospital service	emergency hospital service	emergency hospital service	1	kept
Emergency information form	emergency information form	emergency information form	emergency information form	Emergency information form	1	kept
Emergency intubation	emergency intubation	emergency intubation	emergency intubation	Emergency intubation	1	kept

emergency intubation	emergency intubation	emergency intubation	emergency intubation	Emergency intubation	1	case_or_format
emergency laparotomy	emergency laparotomy	emergency laparotomy	emergency laparotomy	emergency laparotomy	1	kept
Emergency leadership	emergency leadership	emergency leadership	emergency leadership	Emergency leadership	1	kept
emergency leave	emergency leave	emergency leave	emergency leave	emergency leave	1	kept
Emergency medical call center	emergency medical call center	emergency medical call center	emergency medical call center	Emergency medical call center	1	kept
Emergency Medical Dispatch	emergency medical dispatch	emergency medical dispatch	emergency medical dispatch	emergency medical dispatch	1	case_or_format
Emergency medical dispatcher	emergency medical dispatcher	emergency medical dispatcher	emergency medical dispatcher	emergency medical dispatcher	1	case_or_format
Emergency medical schools	emergency medical schools	emergency medical schools	emergency medical schools	Emergency medical schools	1	kept
Emergency medical service system	emergency medical service system	emergency medical service system	emergency medical service system	Emergency medical service system	1	kept
Emergency medical services development	emergency medical services development	emergency medical services development	emergency medical services development	Emergency medical services development	1	kept
emergency medical services education	emergency medical services education	emergency medical services education	emergency medical services education	emergency medical services education	1	kept
emergency medical services systems	emergency medical services systems	emergency medical services systems	emergency medical services systems	emergency medical services systems	1	kept
Emergency medical technicians	emergency medical technicians	emergency medical technicians	emergency medical technician	Emergency Medical Technicians	1	singular
emergency medical technicians	emergency medical technicians	emergency medical technicians	emergency medical technician	Emergency Medical Technicians	1	singular
Emergency medical transportation	emergency medical transportation	emergency medical transportation	emergency medical transportation	Emergency medical transportation	1	kept
emergency medical treatment and active labor act	emergency medical treatment and active labor act	emergency medical treatment and active labor act	emergency medical treatment and active labor act	emergency medical treatment and active labor act	1	kept

emergency medications	emergency medications	emergency medications	emergency medications	emergency medications	1	kept
emergency medicine education	emergency medicine education	emergency medicine education	emergency medicine education	emergency medicine education	1	kept
Emergency medicine journals	emergency medicine journals	emergency medicine journals	emergency medicine journals	Emergency medicine journals	1	kept
Emergency medicine system	emergency medicine system	emergency medicine system	emergency medicine system	Emergency medicine system	1	kept
emergency medicine training	emergency medicine training	emergency medicine training	emergency medicine training	emergency medicine training	1	kept
emergency mobile units	emergency mobile units	emergency mobile units	emergency mobile units	emergency mobile units	1	kept
emergency multiple injury	emergency multiple injury	emergency multiple injury	emergency multiple injury	emergency multiple injury	1	kept
Emergency Neurological Life Support	emergency neurological life support	emergency neurological life support	emergency neurological life support	Emergency Neurological Life Support	1	kept
Emergency neurology	emergency neurology	emergency neurology	emergency neurology	Emergency neurology	1	kept
emergency neurology	emergency neurology	emergency neurology	emergency neurology	Emergency neurology	1	case_or_format
Emergency neurosurgery	emergency neurosurgery	emergency neurosurgery	emergency neurosurgery	Emergency neurosurgery	1	kept
emergency paediatric	emergency paediatric	emergency paediatric	emergency paediatric	emergency paediatric	1	kept
emergency palliative medicine	emergency palliative medicine	emergency palliative medicine	emergency palliative medicine	emergency palliative medicine	1	kept
emergency physicians	emergency physicians	emergency physicians	emergency physician	emergency physician	1	singular
Emergency pre-examination and triage	emergency pre examination and triage	emergency pre examination and triage	emergency pre examination and triage	Emergency pre-examination and triage	1	kept
EMERGENCY PREPAREDNESS	emergency preparedness	emergency preparedness	emergency preparedness	Emergency preparedness	1	case_or_format

Emergency Preparedness	emergency preparedness	emergency preparedness	emergency preparedness	Emergency preparedness	1	case_or_format
Emergency psychiatry	emergency psychiatry	emergency psychiatry	emergency psychiatry	Emergency psychiatry	1	kept
emergency public health response capacity	emergency public health response capacity	emergency public health response capacity	emergency public health response capacity	emergency public health response capacity	1	kept
emergency radiology	emergency radiology	emergency radiology	emergency radiology	emergency radiology	1	kept
Emergency readiness	emergency readiness	emergency readiness	emergency readiness	Emergency readiness	1	kept
Emergency rescue	emergency rescue	emergency rescue	emergency rescue	Emergency rescue	1	kept
emergency rescue database	emergency rescue database	emergency rescue database	emergency rescue database	emergency rescue database	1	kept
emergency rescue room	emergency rescue room	emergency rescue room	emergency rescue room	emergency rescue room	1	kept
emergency responders	emergency responders	emergency responders	emergency responders	Emergency Responders	1	case_or_format
emergency response centre	emergency response centre	emergency response centre	emergency response centre	emergency response centre	1	kept
Emergency response plan	emergency response plan	emergency response plan	emergency response plan	Emergency response plan	1	kept
Emergency response team calling criteria	emergency response team calling criteria	emergency response team calling criteria	emergency response team calling criteria	Emergency response team calling criteria	1	kept
EMERGENCY RESPONSE TEAMS	emergency response teams	emergency response teams	emergency response teams	EMERGENCY RESPONSE TEAMS	1	kept
Emergency response teams	emergency response teams	emergency response teams	emergency response teams	EMERGENCY RESPONSE TEAMS	1	case_or_format
Emergency resternotomy	emergency resternotomy	emergency resternotomy	emergency resternotomy	Emergency resternotomy	1	kept
Emergency resuscitation	emergency resuscitation	emergency resuscitation	emergency resuscitation	Emergency resuscitation	1	kept

emergency room visits	emergency room visits	emergency room visits	emergency room visits	Emergency Room Visits	1	case_or_format
emergency rooms	emergency rooms	emergency rooms	emergency room	emergency room	1	singular
Emergency score	emergency score	emergency score	emergency score	Emergency score	1	kept
Emergency Service Hospital	emergency service hospital	emergency service hospital	emergency service hospital	Emergency Service, Hospital	1	case_or_format
Emergency service hospital	emergency service hospital	emergency service hospital	emergency service hospital	Emergency Service, Hospital	1	case_or_format
emergency service organizations	emergency service organizations	emergency service organizations	emergency service organizations	emergency service organizations	1	kept
Emergency service, Hospital	emergency service hospital	emergency service hospital	emergency service hospital	Emergency Service, Hospital	1	case_or_format
emergency service, hospital	emergency service hospital	emergency service hospital	emergency service hospital	Emergency Service, Hospital	1	case_or_format
emergency services	emergency services	emergency services	emergency service	Emergency service	1	singular
Emergency set up	emergency set up	emergency set up	emergency set up	Emergency set up	1	kept
Emergency setting	emergency setting	emergency setting	emergency setting	Emergency setting	1	kept
Emergency Shelter	emergency shelter	emergency shelter	emergency shelter	emergency shelter	1	case_or_format
Emergency Situation	emergency situation	emergency situation	emergency situation	Emergency situation	1	case_or_format
emergency situation	emergency situation	emergency situation	emergency situation	Emergency situation	1	case_or_format
emergency standards	emergency standards	emergency standards	emergency standards	emergency standards	1	kept
emergency status	emergency status	emergency status	emergency status	emergency status	1	kept

emergency stroke care	emergency stroke care	emergency stroke care	emergency stroke care	emergency stroke care	1	kept
Emergency surgeries	emergency surgeries	emergency surgeries	emergency surgery	emergency surgery	1	singular
Emergency surgery	emergency surgery	emergency surgery	emergency surgery	emergency surgery	1	case_or_format
emergency team	emergency team	emergency team	emergency team	emergency team	1	kept
Emergency Team Dynamics	emergency team dynamics	emergency team dynamics	emergency team dynamics	Emergency Team Dynamics	1	kept
EMERGENCY TEAMS	emergency teams	emergency teams	emergency team	emergency team	1	singular
Emergency Training	emergency training	emergency training	emergency training	Emergency Training	1	kept
emergency training	emergency training	emergency training	emergency training	Emergency Training	1	case_or_format
Emergency transport	emergency transport	emergency transport	emergency transport	emergency transport	1	case_or_format
emergency triage	emergency triage	emergency triage	emergency triage	emergency triage	1	kept
Emergency Unit	emergency unit	emergency unit	emergency unit	Emergency Unit	1	kept
Emergency unit	emergency unit	emergency unit	emergency unit	Emergency Unit	1	case_or_format
emergency vascular access	emergency vascular access	emergency vascular access	emergency vascular access	emergency vascular access	1	kept
Emergency visits	emergency visits	emergency visits	emergency visit	emergency visit	1	singular
Emergency Ward	emergency ward	emergency ward	emergency ward	emergency ward	1	case_or_format
Emergency wards	emergency wards	emergency wards	emergency ward	emergency ward	1	singular

Emergency water supply	emergency water supply	emergency water supply	emergency water supply	Emergency water supply	1	kept
emergency water supply	emergency water supply	emergency water supply	emergency water supply	Emergency water supply	1	case_or_format
EMERGENCY-DEPARTMENT CLINICIAN	emergency department clinician	emergency department clinician	emergency department clinician	EMERGENCY-DEPARTMENT CLINICIAN	1	kept
EMERGENCY-DEPARTMENT WORK	emergency department work	emergency department work	emergency department work	EMERGENCY-DEPARTMENT WORK	1	kept
EMERGENCY-MEDICINE	emergency medicine	emergency medicine	emergency medicine	emergency medicine	1	case_or_format
Emergent change	emergent change	emergent change	emergent change	Emergent change	1	kept
Emergent infectious diseases	emergent infectious diseases	emergent infectious diseases	emergent infectious diseases	Emergent infectious diseases	1	kept
Emergent narrative	emergent narrative	emergent narrative	emergent narrative	Emergent narrative	1	kept
emergent re-sternotomy	emergent re sternotomy	emergent re sternotomy	emergent re sternotomy	emergent re-sternotomy	1	kept
emergent tracheostomy	emergent tracheostomy	emergent tracheostomy	emergent tracheostomy	emergent tracheostomy	1	kept
Emergent tracheotomy care	emergent tracheotomy care	emergent tracheotomy care	emergent tracheotomy care	Emergent tracheotomy care	1	kept
emergent transfer	emergent transfer	emergent transfer	emergent transfer	emergent transfer	1	kept
Emerging	emerging	emerging	emerging	Emerging	1	kept
emerging bacteria	emerging bacteria	emerging bacteria	emerging bacteria	emerging bacteria	1	kept
EMERGING FRONTIER	emerging frontier	emerging frontier	emerging frontier	EMERGING FRONTIER	1	kept
Emerging fungal infections	emerging fungal infections	emerging fungal infections	emerging fungal infections	Emerging fungal infections	1	kept

emerging fungal infections	emerging fungal infections	emerging fungal infections	emerging fungal infections	Emerging fungal infections	1	case_or_format
Emerging Infectious Disease	emerging infectious disease	emerging infectious disease	emerging infectious disease	Emerging Infectious Disease	1	kept
Emerging pathogens	emerging pathogens	emerging pathogens	emerging pathogens	Emerging pathogens	1	kept
Emerging technologies	emerging technologies	emerging technologies	emerging technologies	Emerging technologies	1	kept
Eminectomy	eminectomy	eminectomy	eminectomy	Eminectomy	1	kept
emm89	emm89	emm89	emm89	emm89	1	kept
Emollient	emollient	emollient	emollient	Emollients	1	case_or_format
EMONC	emonc	emonc	emonc	EMONC	1	kept
emotion ethics education	emotion ethics education	emotion ethics education	emotion ethics education	emotion ethics education	1	kept
emotion management	emotion management	emotion management	emotion management	emotion management	1	kept
Emotion work	emotion work	emotion work	emotion work	Emotion work	1	kept
emotional abuse	emotional abuse	emotional abuse	emotional abuse	emotional abuse	1	kept
Emotional adjustment	emotional adjustment	emotional adjustment	emotional adjustment	Emotional Adjustment	1	case_or_format
emotional adjustment	emotional adjustment	emotional adjustment	emotional adjustment	Emotional Adjustment	1	case_or_format
emotional and spiritual support factor score	emotional and spiritual support factor score	emotional and spiritual support factor score	emotional and spiritual support factor score	emotional and spiritual support factor score	1	kept
emotional burden	emotional burden	emotional burden	emotional burden	emotional burden	1	kept

emotional burnout and coronavirus	emotional burnout and coronavirus	emotional burnout and coronavirus	emotional burnout and coronavirus	emotional burnout and coronavirus	1	kept
emotional burnout in doctors	emotional burnout in doctors	emotional burnout in doctors	emotional burnout in doctors	emotional burnout in doctors	1	kept
Emotional burnout syndrome	emotional burnout syndrome	emotional burnout syndrome	emotional burnout syndrome	Emotional burnout syndrome	1	kept
Emotional closeness	emotional closeness	emotional closeness	emotional closeness	Emotional closeness	1	kept
emotional competencies	emotional competencies	emotional competencies	emotional competencies	emotional competencies	1	kept
Emotional conflict	emotional conflict	emotional conflict	emotional conflict	Emotional conflict	1	kept
emotional contagion	emotional contagion	emotional contagion	emotional contagion	emotional contagion	1	kept
emotional coping	emotional coping	emotional coping	emotional coping	emotional coping	1	kept
emotional deprivation	emotional deprivation	emotional deprivation	emotional deprivation	emotional deprivation	1	kept
emotional development	emotional development	emotional development	emotional development	emotional development	1	kept
emotional dissonance	emotional dissonance	emotional dissonance	emotional dissonance	emotional dissonance	1	kept
emotional dysregulation	emotional dysregulation	emotional dysregulation	emotional dysregulation	emotional dysregulation	1	kept
Emotional Eating	emotional eating	emotional eating	emotional eating	emotional eating	1	case_or_format
emotional exhaustion score	emotional exhaustion score	emotional exhaustion score	emotional exhaustion score	emotional exhaustion score	1	kept
Emotional factors	emotional factors	emotional factors	emotional factors	Emotional factors	1	kept
emotional functioning	emotional functioning	emotional functioning	emotional functioning	emotional functioning	1	kept

Emotional impact	emotional impact	emotional impact	emotional impact	Emotional impact	1	kept
EMOTIONAL INTELLIGENCE	emotional intelligence	emotional intelligence	emotional intelligence	emotional intelligence	1	case_or_format
Emotional Labour	emotional labour	emotional labour	emotional labour	Emotional Labour	1	kept
Emotional labour	emotional labour	emotional labour	emotional labour	Emotional Labour	1	case_or_format
emotional labour	emotional labour	emotional labour	emotional labour	Emotional Labour	1	case_or_format
Emotional needs of family	emotional needs of family	emotional needs of family	emotional needs of family	Emotional needs of family	1	kept
emotional perception	emotional perception	emotional perception	emotional perception	emotional perception	1	kept
emotional presence	emotional presence	emotional presence	emotional presence	emotional presence	1	kept
emotional production	emotional production	emotional production	emotional production	emotional production	1	kept
Emotional quality	emotional quality	emotional quality	emotional quality	Emotional quality	1	kept
Emotional reactions to illness	emotional reactions to illness	emotional reactions to illness	emotional reactions to illness	Emotional reactions to illness	1	kept
Emotional recovery	emotional recovery	emotional recovery	emotional recovery	Emotional recovery	1	kept
Emotional responses	emotional responses	emotional responses	emotional responses	Emotional responses	1	kept
Emotional scenario	emotional scenario	emotional scenario	emotional scenario	Emotional scenario	1	kept
Emotional talk	emotional talk	emotional talk	emotional talk	Emotional talk	1	kept
emotional toll	emotional toll	emotional toll	emotional toll	emotional toll	1	kept

emotional well being	emotional well being	emotional well being	emotional well being	emotional well-being	1	case_or_format
Emotional wellbeing	emotional wellbeing	emotional wellbeing	emotional wellbeing	Emotional wellbeing	1	kept
emotional work	emotional work	emotional work	emotional work	emotional work	1	kept
empagliflozin	empagliflozin	empagliflozin	empagliflozin	empagliflozin	1	kept
empathetic care	empathetic care	empathetic care	empathetic care	empathetic care	1	kept
empathic ability	empathic ability	empathic ability	empathic ability	empathic ability	1	kept
Empathic care	empathic care	empathic care	empathic care	Empathic care	1	kept
empathic care	empathic care	empathic care	empathic care	Empathic care	1	case_or_format
Empathic communication	empathic communication	empathic communication	empathic communication	empathic communication	1	case_or_format
EMPATHIC-30	empathic 30	empathic 30	empathic 30	EMPATHIC-30	1	kept
empathy for pain	empathy for pain	empathy for pain	empathy for pain	empathy for pain	1	kept
Empathy for Pain Scale	empathy for pain scale	empathy for pain scale	empathy for pain scale	Empathy for Pain Scale	1	kept
empathy satisfaction	empathy satisfaction	empathy satisfaction	empathy satisfaction	empathy satisfaction	1	kept
Empathy training	empathy training	empathy training	empathy training	empathy training	1	case_or_format
Empatía	empat a	empat a	empat a	Empatía	1	kept
Empedobacter brevis	empedobacter brevis	empedobacter brevis	empedobacter brevis	Empedobacter brevis	1	kept

EMPIRIC ANTIBIOTIC-THERAPY	empiric antibiotic therapy	empiric antibiotic therapy	empiric antibiotic therapy	EMPIRIC ANTIBIOTIC-THERAPY	1	kept
Empiric therapy	empiric therapy	empiric therapy	empiric therapy	empiric therapy	1	case_or_format
Empirical analysis	empirical analysis	empirical analysis	empirical analysis	Empirical analysis	1	kept
empirical analysis	empirical analysis	empirical analysis	empirical analysis	Empirical analysis	1	case_or_format
Empirical approach	empirical approach	empirical approach	empirical approach	Empirical Approach	1	case_or_format
empirical contact precaution	empirical contact precaution	empirical contact precaution	empirical contact precaution	empirical contact precaution	1	kept
Empirical ethics	empirical ethics	empirical ethics	empirical ethics	Empirical ethics	1	kept
Empirical examination	empirical examination	empirical examination	empirical examination	Empirical examination	1	kept
Empirical investigation	empirical investigation	empirical investigation	empirical investigation	Empirical investigation	1	kept
empirical operations	empirical operations	empirical operations	empirical operations	empirical operations	1	kept
empirical operations management	empirical operations management	empirical operations management	empirical operations management	empirical operations management	1	kept
empirical research report	empirical research report	empirical research report	empirical research report	Empirical research report	1	case_or_format
Empirical studies	empirical studies	empirical studies	empirical study	empirical study	1	singular
empirical study	empirical study	empirical study	empirical study	empirical study	1	kept
EMPIRICAL THERAPY	empirical therapy	empirical therapy	empirical therapy	EMPIRICAL THERAPY	1	kept
Empiricism	empiricism	empiricism	empiricism	empiricism	1	case_or_format

EMPLOYEE	employee	employee	employee	employee	1	case_or_format
Employee	employee	employee	employee	employee	1	case_or_format
Employee assistance programs	employee assistance programs	employee assistance programs	employee assistance programs	Employee assistance programs	1	kept
employee attitude	employee attitude	employee attitude	employee attitude	employee attitude	1	kept
Employee effort	employee effort	employee effort	employee effort	Employee effort	1	kept
Employee loyalty	employee loyalty	employee loyalty	employee loyalty	Employee loyalty	1	kept
employee participation	employee participation	employee participation	employee participation	employee participation	1	kept
EMPLOYEE RETENTION	employee retention	employee retention	employee retention	Employee retention	1	case_or_format
EMPLOYEE SILENCE	employee silence	employee silence	employee silence	EMPLOYEE SILENCE	1	kept
Employee well-being	employee well being	employee well being	employee well being	Employee well-being	1	kept
Employee wellbeing	employee wellbeing	employee wellbeing	employee wellbeing	Employee wellbeing	1	kept
Employee workload	employee workload	employee workload	employee workload	Employee workload	1	kept
Employee's performance report	employee s performance report	employee s performance report	employee s performance report	Employee's performance report	1	kept
Employee-oriented leadership	employee oriented leadership	employee oriented leadership	employee oriented leadership	Employee-oriented leadership	1	kept
Employer-based health care	employer based health care	employer based health care	employer based health care	Employer-based health care	1	kept
Employer-directed care	employer directed care	employer directed care	employer directed care	Employer-directed care	1	kept

employment contract	employment contract	employment contract	employment contract	employment contract	1	kept
employment duration	employment duration	employment duration	employment duration	employment duration	1	kept
Employment planning	employment planning	employment planning	employment planning	Employment planning	1	kept
Employment status	employment status	employment status	employment status	employment status	1	case_or_format
Empow-erment	empow erment	empow erment	empow erment	Empow-erment	1	kept
Empower mothers	empower mothers	empower mothers	empower mothers	Empower mothers	1	kept
Empowered caring	empowered caring	empowered caring	empowered caring	Empowered caring	1	kept
Empowering environment	empowering environment	empowering environment	empowering environment	Empowering environment	1	kept
Empowerment management	empowerment management	empowerment management	empowerment management	Empowerment management	1	kept
empowerment status, instrument development	empowerment status instrument development	empowerment status instrument development	empowerment status instrument development	empowerment status, instrument development	1	kept
Empyema	empyema	empyema	empyema	empyema	1	case_or_format
emr compliance training	emr compliance training	emr compliance training	emr compliance training	emr compliance training	1	kept
EMS Emergency Medical Services	ems emergency medical services	ems emergency medical services	ems emergency medical services	EMS Emergency Medical Services	1	kept
EMS resident involvement	ems resident involvement	ems resident involvement	ems resident involvement	EMS resident involvement	1	kept
EMS, emergency medical services	ems emergency medical services	ems emergency medical services	ems emergency medical services	EMS Emergency Medical Services	1	case_or_format
EMSE	emse	emse	emse	EMSE	1	kept

Emtricitabine	emtricitabine	emtricitabine	emtricitabine	emtricitabine	1	case_or_format
emtricitabine plus tenofovir disoproxil	emtricitabine plus tenofovir disoproxil	emtricitabine plus tenofovir disoproxil	emtricitabine plus tenofovir disoproxil	emtricitabine plus tenofovir disoproxil	1	kept
EN complications	en complications	en complications	en complications	EN complications	1	kept
En route care	en route care	en route care	en route care	En route care	1	kept
en route care	en route care	en route care	en route care	En route care	1	case_or_format
enabling and constraining factors	enabling and constraining factors	enabling and constraining factors	enabling and constraining factors	enabling and constraining factors	1	kept
Enabling factors	enabling factors	enabling factors	enabling factors	Enabling factors	1	kept
Enabling parents	enabling parents	enabling parents	enabling parents	Enabling parents	1	kept
Enacting death	enacting death	enacting death	enacting death	enacting death	1	case_or_format
enactment	enactment	enactment	enactment	enactment	1	kept
encapsulation	encapsulation	encapsulation	encapsulation	encapsulation	1	kept
Encephalitis, Tick-Borne	encephalitis tick borne	encephalitis tick borne	encephalitis tick borne	Encephalitis, Tick-Borne	1	kept
encephalitozoonosis	encephalitozoonosis	encephalitozoonosis	encephalitozoonosis	encephalitozoonosis	1	kept
Encephalocele	encephalocele	encephalocele	encephalocele	Encephalocele	1	kept
Encephalofacial angiomatosis	encephalofacial angiomatosis	encephalofacial angiomatosis	encephalofacial angiomatosis	Encephalofacial angiomatosis	1	kept
Encephalomyelitis, Acute Disseminated	encephalomyelitis acute disseminated	encephalomyelitis acute disseminated	encephalomyelitis acute disseminated	Encephalomyelitis, Acute Disseminated	1	kept

Encounter	encounter	encounter	encounter	Encounter	1	kept
End of Life	end of life	end of life	end of life	End of life	1	case_or_format
End of life and palliative care	end of life and palliative care	end of life and palliative care	end of life and palliative care	End of life and palliative care	1	kept
End of Life Care	end of life care	end of life care	end of life care	end-of-life care	1	case_or_format
end of life care behaviors	end of life care behaviors	end of life care behaviors	end of life care behaviors	end of life care behaviors	1	kept
end of life care needs	end of life care needs	end of life care needs	end of life care needs	end of life care needs	1	kept
end of life hospitalization	end of life hospitalization	end of life hospitalization	end of life hospitalization	end of life hospitalization	1	kept
end of life in dementia scale	end of life in dementia scale	end of life in dementia scale	end of life in dementia scale	end of life in dementia scale	1	kept
end of life intensity	end of life intensity	end of life intensity	end of life intensity	end of life intensity	1	kept
End of lives	end of lives	end of lives	end of lives	End of lives	1	kept
end organ perfusion	end organ perfusion	end organ perfusion	end organ perfusion	end organ perfusion	1	kept
End stage liver disease	end stage liver disease	end stage liver disease	end stage liver disease	End Stage Liver Disease	1	case_or_format
End to end delay	end to end delay	end to end delay	end to end delay	End to end delay	1	kept
End user satisfaction	end user satisfaction	end user satisfaction	end user satisfaction	End user satisfaction	1	kept
end-diastolic velocity	end diastolic velocity	end diastolic velocity	end diastolic velocity	end-diastolic velocity	1	kept
END-EXPIRATORY PRESSURE	end expiratory pressure	end expiratory pressure	end expiratory pressure	END-EXPIRATORY PRESSURE	1	kept

End-life-care	end life care	end life care	end life care	End-life-care	1	kept
End-of life care	end of life care	end of life care	end of life care	end-of-life care	1	case_or_format
End-of life issues	end of life issues	end of life issues	end of life issues	end-of-life issues	1	case_or_format
End-of-Life	end of life	end of life	end of life	End of life	1	case_or_format
End-of-life Care	end of life care	end of life care	end of life care	end-of-life care	1	case_or_format
end-of-life care intensity	end of life care intensity	end of life care intensity	end of life care intensity	end-of-life care intensity	1	kept
End-of-life care planning	end of life care planning	end of life care planning	end of life care planning	End-of-life care planning	1	kept
End-of-life care protocol	end of life care protocol	end of life care protocol	end of life care protocol	End-of-life care protocol	1	kept
end-of-life communication	end of life communication	end of life communication	end of life communication	End-of-life communication	1	case_or_format
End-of-life conversations	end of life conversations	end of life conversations	end of life conversation	end-of-life conversation	1	singular
end-of-life core	end of life core	end of life core	end of life core	end-of-life core	1	kept
End-of-Life Decision Making and Associated Staff Stress	end of life decision making and associated staff stress	end of life decision making and associated staff stress	end of life decision making and associated staff stress	End-of-Life Decision Making and Associated Staff Stress	1	kept
end-of-life discussions and care	end of life discussions and care	end of life discussions and care	end of life discussions and care	end-of-life discussions and care	1	kept
end-of-life encounters	end of life encounters	end of life encounters	end of life encounters	end-of-life encounters	1	kept
End-of-life experience	end of life experience	end of life experience	end of life experience	End-of-life experience	1	kept
End-of-life practices	end of life practices	end of life practices	end of life practices	End-of-life practices	1	kept

end-of-life practices	end of life practices	end of life practices	end of life practices	End-of-life practices	1	case_or_format
end-of-life research	end of life research	end of life research	end of life research	end-of-life research	1	kept
End-of-life transitions	end of life transitions	end of life transitions	end of life transitions	End-of-life transitions	1	kept
end-of-life transitions	end of life transitions	end of life transitions	end of life transitions	End-of-life transitions	1	case_or_format
End-of-life-care	end of life care	end of life care	end of life care	end-of-life care	1	case_or_format
End-of-shift report	end of shift report	end of shift report	end of shift report	End-of-shift report	1	kept
end-oflife care	end oflife care	end oflife care	end oflife care	end-oflife care	1	kept
end-organ damage	end organ damage	end organ damage	end organ damage	end-organ damage	1	kept
End-organ failure	end organ failure	end organ failure	end organ failure	End-organ failure	1	kept
end-stage kidney disease	end stage kidney disease	end stage kidney disease	end stage kidney disease	end stage kidney disease	1	case_or_format
end-stage liver disease	end stage liver disease	end stage liver disease	end stage liver disease	End Stage Liver Disease	1	case_or_format
End-stage pulmonary diseases	end stage pulmonary diseases	end stage pulmonary diseases	end stage pulmonary diseases	End-stage pulmonary diseases	1	kept
End-stage renal disease	end stage renal disease	end stage renal disease	end stage renal disease	end stage renal disease	1	case_or_format
End-stage respiratory diseases	end stage respiratory diseases	end stage respiratory diseases	end stage respiratory diseases	End-stage respiratory diseases	1	kept
end-tidal CO<sub>2<	end tidal co sub 2	end tidal co sub 2	end tidal co sub 2	end-tidal CO<sub>2<	1	kept
end-user experience	end user experience	end user experience	end user experience	end-user experience	1	kept

End-users	end users	end users	end users	End-users	1	kept
endarterectomy	endarterectomy	endarterectomy	endarterectomy	Endarterectomy	1	case_or_format
Endemic COVID-19	endemic covid 19	endemic covid 19	endemic covid 19	Endemic COVID-19	1	kept
endemic species	endemic species	endemic species	endemic species	endemic species	1	kept
endemicity	endemicity	endemicity	endemicity	endemicity	1	kept
Endo-tracheal tube suctioning	endo tracheal tube suctioning	endo tracheal tube suctioning	endo tracheal tube suctioning	Endo-tracheal tube suctioning	1	kept
endobronchial intubation	endobronchial intubation	endobronchial intubation	endobronchial intubation	endobronchial intubation	1	kept
endobronchial ultrasonography	endobronchial ultrasonography	endobronchial ultrasonography	endobronchial ultrasonography	endobronchial ultrasonography	1	kept
Endocardial border definition	endocardial border definition	endocardial border definition	endocardial border definition	Endocardial border definition	1	kept
endocrine	endocrine	endocrine	endocrine	endocrine	1	kept
Endocrine disruptors	endocrine disruptors	endocrine disruptors	endocrine disruptors	Endocrine disruptors	1	kept
endocrine function	endocrine function	endocrine function	endocrine function	endocrine function	1	kept
Endocrine metabolic	endocrine metabolic	endocrine metabolic	endocrine metabolic	Endocrine metabolic	1	kept
endocrine surgery	endocrine surgery	endocrine surgery	endocrine surgery	endocrine surgery	1	kept
Endocrine System	endocrine system	endocrine system	endocrine system	Endocrine System	1	kept
endocrine tumor	endocrine tumor	endocrine tumor	endocrine tumor	endocrine tumor	1	kept

endoleak	endoleak	endoleak	endoleak	endoleak	1	kept
Endometrial Neoplasms	endometrial neoplasms	endometrial neoplasms	endometrial neoplasms	Endometrial Neoplasms	1	kept
Endometrial stromal sarcoma	endometrial stromal sarcoma	endometrial stromal sarcoma	endometrial stromal sarcoma	Endometrial stromal sarcoma	1	kept
Endometrium	endometrium	endometrium	endometrium	endometrium	1	case_or_format
endometrium cancer	endometrium cancer	endometrium cancer	endometrium cancer	endometrium cancer	1	kept
endometrium sarcoma	endometrium sarcoma	endometrium sarcoma	endometrium sarcoma	endometrium sarcoma	1	kept
endometrium tumor	endometrium tumor	endometrium tumor	endometrium tumor	endometrium tumor	1	kept
Endomyocardial biopsy	endomyocardial biopsy	endomyocardial biopsy	endomyocardial biopsy	Endomyocardial biopsy	1	kept
Endoport	endoport	endoport	endoport	Endoport	1	kept
Endoscope	endoscope	endoscope	endoscope	Endoscope	1	kept
endoscope	endoscope	endoscope	endoscope	Endoscope	1	case_or_format
Endoscopes	endoscopes	endoscopes	endoscope	Endoscope	1	singular
ENDOSCOPIC GASTROSTOMY TUBE	endoscopic gastrostomy tube	endoscopic gastrostomy tube	endoscopic gastrostomy tube	ENDOSCOPIC GASTROSTOMY TUBE	1	kept
Endoscopic retrograde cholangiopancreatography	endoscopic retrograde cholangiopancreatography	endoscopic retrograde cholangiopancreatography	endoscopic retrograde cholangiopancreatography	endoscopic retrograde cholangiopancreatography	1	case_or_format
endoscopic sinus surgery	endoscopic sinus surgery	endoscopic sinus surgery	endoscopic sinus surgery	endoscopic sinus surgery	1	kept
endoscopic sphincterotomy	endoscopic sphincterotomy	endoscopic sphincterotomy	endoscopic sphincterotomy	endoscopic sphincterotomy	1	kept

endoscopic ultrasound guided drainage	endoscopic ultrasound guided drainage	endoscopic ultrasound guided drainage	endoscopic ultrasound guided drainage	endoscopic ultrasound guided drainage	1	kept
Endoscopist	endoscopist	endoscopist	endoscopist	endoscopist	1	case_or_format
ENDOSCOPY	endoscopy	endoscopy	endoscopy	endoscopy	1	case_or_format
Endosonography	endosonography	endosonography	endosonography	Endosonography	1	kept
Endothelial glycocalyx	endothelial glycocalyx	endothelial glycocalyx	endothelial glycocalyx	Endothelial glycocalyx	1	kept
endothelial leukocyte adhesion molecule 1	endothelial leukocyte adhesion molecule 1	endothelial leukocyte adhesion molecule 1	endothelial leukocyte adhesion molecule 1	endothelial leukocyte adhesion molecule 1	1	kept
ENDOTHELIAL-CELLS	endothelial cells	endothelial cells	endothelial cells	ENDOTHELIAL-CELLS	1	kept
Endothelin A Receptor Antagonists	endothelin a receptor antagonists	endothelin a receptor antagonists	endothelin a receptor antagonists	Endothelin A Receptor Antagonists	1	kept
Endothelium	endothelium	endothelium	endothelium	endothelium	1	case_or_format
endothelium lesion	endothelium lesion	endothelium lesion	endothelium lesion	endothelium lesion	1	kept
endotoxin	endotoxin	endotoxin	endotoxin	endotoxin	1	kept
ENDOTRACHEAL	endotracheal	endotracheal	endotracheal	Endotracheal	1	case_or_format
endotracheal anesthesia	endotracheal anesthesia	endotracheal anesthesia	endotracheal anesthesia	endotracheal anesthesia	1	kept
Endotracheal aspiration	endotracheal aspiration	endotracheal aspiration	endotracheal aspiration	Endotracheal aspiration	1	kept
endotracheal aspiration	endotracheal aspiration	endotracheal aspiration	endotracheal aspiration	Endotracheal aspiration	1	case_or_format
endotracheal aspiration duration	endotracheal aspiration duration	endotracheal aspiration duration	endotracheal aspiration duration	endotracheal aspiration duration	1	kept

endotracheal cuff pressure measurement	endotracheal cuff pressure measurement	endotracheal cuff pressure measurement	endotracheal cuff pressure measurement	endotracheal cuff pressure measurement	1	kept
Endotracheal Intubation	endotracheal intubation	endotracheal intubation	endotracheal intubation	endotracheal intubation	1	case_or_format
Endotracheal intubations	endotracheal intubations	endotracheal intubations	endotracheal intubation	endotracheal intubation	1	singular
Endotracheal self-extubation	endotracheal self extubation	endotracheal self extubation	endotracheal self extubation	Endotracheal self-extubation	1	kept
ENDOTRACHEAL SUCTION	endotracheal suction	endotracheal suction	endotracheal suction	Endotracheal suction	1	case_or_format
Endotracheal Suction	endotracheal suction	endotracheal suction	endotracheal suction	Endotracheal suction	1	case_or_format
endotracheal tube carina distance	endotracheal tube carina distance	endotracheal tube carina distance	endotracheal tube carina distance	endotracheal tube carina distance	1	kept
Endotracheal tube cuff pressure	endotracheal tube cuff pressure	endotracheal tube cuff pressure	endotracheal tube cuff pressure	Endotracheal tube cuff pressure	1	kept
endotracheal tube cuff pressure	endotracheal tube cuff pressure	endotracheal tube cuff pressure	endotracheal tube cuff pressure	Endotracheal tube cuff pressure	1	case_or_format
endotracheal tube securing method	endotracheal tube securing method	endotracheal tube securing method	endotracheal tube securing method	endotracheal tube securing method	1	kept
endotracheal tube suction	endotracheal tube suction	endotracheal tube suction	endotracheal tube suction	endotracheal tube suction	1	kept
Endotracheal tube suctioning	endotracheal tube suctioning	endotracheal tube suctioning	endotracheal tube suctioning	endotracheal tube suctioning	1	case_or_format
Endovascular aortic repair	endovascular aortic repair	endovascular aortic repair	endovascular aortic repair	Endovascular aortic repair	1	kept
endovascular coiling	endovascular coiling	endovascular coiling	endovascular coiling	endovascular coiling	1	kept
endovascular stroke therapy	endovascular stroke therapy	endovascular stroke therapy	endovascular stroke therapy	endovascular stroke therapy	1	kept
Endovascular treatment	endovascular treatment	endovascular treatment	endovascular treatment	Endovascular treatment	1	kept

Endpoints of resuscitation	endpoints of resuscitation	endpoints of resuscitation	endpoints of resuscitation	Endpoints of resuscitation	1	kept
Endsley's model	endsley s model	endsley s model	endsley s model	Endsley's model	1	kept
end‐of‐life decision‐making	end of life decision making	end of life decision making	end of life decision making	end-of-life decision-making	1	case_or_format
ENEC	enec	enec	enec	ENEC	1	kept
energy conservation	energy conservation	energy conservation	energy conservation	energy conservation	1	kept
Energy deficit	energy deficit	energy deficit	energy deficit	energy deficit	1	case_or_format
Energy Drinks	energy drinks	energy drinks	energy drinks	Energy Drinks	1	kept
Energy requirement	energy requirement	energy requirement	energy requirement	Energy requirement	1	kept
energy supply	energy supply	energy supply	energy supply	energy supply	1	kept
Energy therapy	energy therapy	energy therapy	energy therapy	Energy therapy	1	kept
energy transfer	energy transfer	energy transfer	energy transfer	energy transfer	1	kept
eneterocutaneous fistula	eneterocutaneous fistula	eneterocutaneous fistula	eneterocutaneous fistula	eneterocutaneous fistula	1	kept
Enfermagem Cardiovascular	enfermagem cardiovascular	enfermagem cardiovascular	enfermagem cardiovascular	Enfermagem Cardiovascular	1	kept
Enfermagem de Cuidados Críticos	enfermagem de cuidados cr ticos	enfermagem de cuidados cr ticos	enfermagem de cuidados cr ticos	Enfermagem de Cuidados Críticos	1	kept
Enfermagem de cuidados críticos	enfermagem de cuidados cr ticos	enfermagem de cuidados cr ticos	enfermagem de cuidados cr ticos	Enfermagem de Cuidados Críticos	1	case_or_format
Enfermagem de Cuidados Paliativos	enfermagem de cuidados paliativos	enfermagem de cuidados paliativos	enfermagem de cuidados paliativos	Enfermagem de Cuidados Paliativos	1	kept

Enfermagem do Trabalho	enfermagem do trabalho	enfermagem do trabalho	enfermagem do trabalho	Enfermagem do Trabalho	1	kept
Enfermagem obstétrica	enfermagem obst trica	enfermagem obst trica	enfermagem obst trica	Enfermagem obstétrica	1	kept
Enfermagem Pediátrica	enfermagem pedi trica	enfermagem pedi trica	enfermagem pedi trica	Enfermagem Pediátrica	1	kept
Enfermagem pediátrica	enfermagem pedi trica	enfermagem pedi trica	enfermagem pedi trica	Enfermagem Pediátrica	1	case_or_format
Enfermedad crónica	enfermedad cr nica	enfermedad cr nica	enfermedad cr nica	Enfermedad crónica	1	kept
Enfermedad inflamatoria inmunomediada	enfermedad inflamatoria inmunomediada	enfermedad inflamatoria inmunomediada	enfermedad inflamatoria inmunomediada	Enfermedad inflamatoria inmunomediada	1	kept
Enfermeiras e Enfermeiros	enfermeiras e enfermeiros	enfermeiras e enfermeiros	enfermeiras e enfermeiros	Enfermeiras e Enfermeiros	1	kept
Enfermeiras e enfermeiros	enfermeiras e enfermeiros	enfermeiras e enfermeiros	enfermeiras e enfermeiros	Enfermeiras e Enfermeiros	1	case_or_format
Enfermeiro	enfermeiro	enfermeiro	enfermeiro	Enfermeiro	1	kept
Enfermera neonatal	enfermera neonatal	enfermera neonatal	enfermera neonatal	Enfermera neonatal	1	kept
Enfermera registrada	enfermera registrada	enfermera registrada	enfermera registrada	Enfermera registrada	1	kept
Enfermeras de cuidados críticos	enfermeras de cuidados cr ticos	enfermeras de cuidados cr ticos	enfermeras de cuidados cr ticos	Enfermeras de cuidados críticos	1	kept
Enfermeras de práctica avanzada	enfermeras de pr ctica avanzada	enfermeras de pr ctica avanzada	enfermeras de pr ctica avanzada	Enfermeras de práctica avanzada	1	kept
Enfermeras noveles	enfermeras noveles	enfermeras noveles	enfermeras noveles	Enfermeras noveles	1	kept
Enfermero	enfermero	enfermero	enfermero	Enfermeros	1	case_or_format
Enfermeros y enfermeras	enfermeros y enfermeras	enfermeros y enfermeras	enfermeros y enfermeras	Enfermeros y enfermeras	1	kept

Enfermería Cardiovascular	enfermer a cardiovascular	enfermer a cardiovascular	enfermer a cardiovascular	Enfermería Cardiovascular	1	kept
Enfermería cardiovascular	enfermer a cardiovascular	enfermer a cardiovascular	enfermer a cardiovascular	Enfermería Cardiovascular	1	case_or_format
Enfermería de Cuidados Paliativos	enfermer a de cuidados paliativos	enfermer a de cuidados paliativos	enfermer a de cuidados paliativos	Enfermería de Cuidados Paliativos	1	kept
Enfermería de familia	enfermer a de familia	enfermer a de familia	enfermer a de familia	Enfermería de familia	1	kept
Enfermería de rehabilitación	enfermer a de rehabilitaci n	enfermer a de rehabilitaci n	enfermer a de rehabilitaci n	Enfermería de rehabilitación	1	kept
Enfermería del Trabajo	enfermer a del trabajo	enfermer a del trabajo	enfermer a del trabajo	Enfermería del Trabajo	1	kept
Enfermería intensiva	enfermer a intensiva	enfermer a intensiva	enfermer a intensiva	Enfermería intensiva	1	kept
Enfermería militar	enfermer a militar	enfermer a militar	enfermer a militar	Enfermería militar	1	kept
Enfermería.	enfermer a	enfermer a	enfermer a	Enfermería	1	case_or_format
enflurane	enflurane	enflurane	enflurane	enflurane	1	kept
enfluvir suspension	enfluvir suspension	enfluvir suspension	enfluvir suspension	enfluvir suspension	1	kept
Enfoques de apoyo	enfoques de apoyo	enfoques de apoyo	enfoques de apoyo	Enfoques de apoyo	1	kept
Engage mothers	engage mothers	engage mothers	engage mothers	Engage mothers	1	kept
engagement in care	engagement in care	engagement in care	engagement in care	engagement in care	1	kept
Engaging the team	engaging the team	engaging the team	engaging the team	Engaging the team	1	kept
Engineered solutions	engineered solutions	engineered solutions	engineered solutions	Engineered solutions	1	kept

Engineering physics	engineering physics	engineering physics	engineering physics	Engineering physics	1	kept
Engineering research	engineering research	engineering research	engineering research	Engineering research	1	kept
Englishman	englishman	englishman	englishman	Englishman	1	kept
engraftment syndrome	engraftment syndrome	engraftment syndrome	engraftment syndrome	engraftment syndrome	1	kept
Enhanced	enhanced	enhanced	enhanced	Enhanced	1	kept
enhanced care	enhanced care	enhanced care	enhanced care	enhanced care	1	kept
Enhanced cleaning	enhanced cleaning	enhanced cleaning	enhanced cleaning	Enhanced cleaning	1	kept
enhanced cleaning	enhanced cleaning	enhanced cleaning	enhanced cleaning	Enhanced cleaning	1	case_or_format
Enhanced comfort theory	enhanced comfort theory	enhanced comfort theory	enhanced comfort theory	Enhanced comfort theory	1	kept
Enhanced environmental cleaning	enhanced environmental cleaning	enhanced environmental cleaning	enhanced environmental cleaning	Enhanced environmental cleaning	1	kept
Enhanced external counterpulsation	enhanced external counterpulsation	enhanced external counterpulsation	enhanced external counterpulsation	Enhanced external counterpulsation	1	kept
Enhanced Recovery After Cardiac Surgery	enhanced recovery after cardiac surgery	enhanced recovery after cardiac surgery	enhanced recovery after cardiac surgery	Enhanced Recovery After Cardiac Surgery	1	kept
Enhanced Recovery after Surgery	enhanced recovery after surgery	enhanced recovery after surgery	enhanced recovery after surgery	enhanced recovery after surgery	1	case_or_format
Enhanced recovery after surgery cardiac	enhanced recovery after surgery cardiac	enhanced recovery after surgery cardiac	enhanced recovery after surgery cardiac	Enhanced recovery after surgery cardiac	1	kept
enhanced recovery after surgery concept	enhanced recovery after surgery concept	enhanced recovery after surgery concept	enhanced recovery after surgery concept	enhanced recovery after surgery concept	1	kept
Enhanced Recovery After Surgery program	enhanced recovery after surgery program	enhanced recovery after surgery program	enhanced recovery after surgery program	Enhanced Recovery After Surgery program	1	kept

Enhanced recovery after surgery program	enhanced recovery after surgery program	enhanced recovery after surgery program	enhanced recovery after surgery program	Enhanced Recovery After Surgery program	1	case_or_format
enhanced recovery for cardiac surgery	enhanced recovery for cardiac surgery	enhanced recovery for cardiac surgery	enhanced recovery for cardiac surgery	enhanced recovery for cardiac surgery	1	kept
Enhanced recovery pathways	enhanced recovery pathways	enhanced recovery pathways	enhanced recovery pathways	Enhanced recovery pathways	1	kept
enhanced recovery protocol	enhanced recovery protocol	enhanced recovery protocol	enhanced recovery protocol	enhanced recovery protocol	1	kept
enhancement	enhancement	enhancement	enhancement	enhancement	1	kept
enhancing personal protection	enhancing personal protection	enhancing personal protection	enhancing personal protection	enhancing personal protection	1	kept
ENLS	enls	enls	enls	ENLS	1	kept
enmetazobactam	enmetazobactam	enmetazobactam	enmetazobactam	enmetazobactam	1	kept
enophthalmos	enophthalmos	enophthalmos	enophthalmos	enophthalmos	1	kept
Enoxaparina	enoxaparina	enoxaparina	enoxaparina	Enoxaparina	1	kept
Enr	enr	enr	enr	Enr	1	kept
ENROFLOXACIN	enrofloxacin	enrofloxacin	enrofloxacin	enrofloxacin	1	case_or_format
ENROLLEES	enrollees	enrollees	enrollees	ENROLLEES	1	kept
Ensayo clínico	ensayo cl nico	ensayo cl nico	ensayo cl nico	Ensayo clínico	1	kept
Enseñanza Mediante Simulación de Alta Fidelidad	ense anza mediante simulaci n de alta fidelidad	ense anza mediante simulaci n de alta fidelidad	ense anza mediante simulaci n de alta fidelidad	Enseñanza Mediante Simulación de Alta Fidelidad	1	kept
entanglement	entanglement	entanglement	entanglement	entanglement	1	kept

Enteral access	enteral access	enteral access	enteral access	Enteral access	1	kept
enteral access	enteral access	enteral access	enteral access	Enteral access	1	case_or_format
Enteral feeding algorithm	enteral feeding algorithm	enteral feeding algorithm	enteral feeding algorithm	Enteral feeding algorithm	1	kept
Enteral feeding catheter	enteral feeding catheter	enteral feeding catheter	enteral feeding catheter	Enteral feeding catheter	1	kept
enteral feeding interruption	enteral feeding interruption	enteral feeding interruption	enteral feeding interruption	enteral feeding interruption	1	kept
Enteral feeding tube	enteral feeding tube	enteral feeding tube	enteral feeding tube	enteral feeding tube	1	case_or_format
ENTERAL FEEDING TUBES	enteral feeding tubes	enteral feeding tubes	enteral feeding tube	enteral feeding tube	1	singular
enteral feeding tubes	enteral feeding tubes	enteral feeding tubes	enteral feeding tube	enteral feeding tube	1	singular
enteral feeds	enteral feeds	enteral feeds	enteral feeds	enteral feeds	1	kept
enteral immunomodulatory formula	enteral immunomodulatory formula	enteral immunomodulatory formula	enteral immunomodulatory formula	enteral immunomodulatory formula	1	kept
Enteral medication	enteral medication	enteral medication	enteral medication	Enteral medication	1	kept
enteral medication administration	enteral medication administration	enteral medication administration	enteral medication administration	enteral medication administration	1	kept
enteral nutrition catheterization	enteral nutrition catheterization	enteral nutrition catheterization	enteral nutrition catheterization	enteral nutrition catheterization	1	kept
enteral nutrition feeding intolerance	enteral nutrition feeding intolerance	enteral nutrition feeding intolerance	enteral nutrition feeding intolerance	enteral nutrition feeding intolerance	1	kept
enteral nutrition intolerance	enteral nutrition intolerance	enteral nutrition intolerance	enteral nutrition intolerance	enteral nutrition intolerance	1	kept
Enteral nutrition support	enteral nutrition support	enteral nutrition support	enteral nutrition support	enteral nutrition support	1	case_or_format

enteral nutrition suspension	enteral nutrition suspension	enteral nutrition suspension	enteral nutrition suspension	enteral nutrition suspension	1	kept
Enteral nutrition therapy	enteral nutrition therapy	enteral nutrition therapy	enteral nutrition therapy	Enteral nutrition therapy	1	kept
Enteral nutrition tolerance monitoring	enteral nutrition tolerance monitoring	enteral nutrition tolerance monitoring	enteral nutrition tolerance monitoring	Enteral nutrition tolerance monitoring	1	kept
Enteral nutritional support	enteral nutritional support	enteral nutritional support	enteral nutritional support	enteral nutritional support	1	case_or_format
Enteral Tube Feeding	enteral tube feeding	enteral tube feeding	enteral tube feeding	Enteral Tube Feeding	1	kept
enteral tube feeding	enteral tube feeding	enteral tube feeding	enteral tube feeding	Enteral Tube Feeding	1	case_or_format
Enteral Tubes	enteral tubes	enteral tubes	enteral tubes	Enteral Tubes	1	kept
Enteral tubes	enteral tubes	enteral tubes	enteral tubes	Enteral Tubes	1	case_or_format
ENTERIC COLONIZATION	enteric colonization	enteric colonization	enteric colonization	ENTERIC COLONIZATION	1	kept
enteric duplication cyst	enteric duplication cyst	enteric duplication cyst	enteric duplication cyst	enteric duplication cyst	1	kept
ENTERICA SEROTYPE TYPHI	enterica serotype typhi	enterica serotype typhi	enterica serotype typhi	ENTERICA SEROTYPE TYPHI	1	kept
Entero-atmospheric fistula	entero atmospheric fistula	entero atmospheric fistula	entero atmospheric fistula	Entero-atmospheric fistula	1	kept
Enterobacter asburiae	enterobacter asburiae	enterobacter asburiae	enterobacter asburiae	Enterobacter asburiae	1	kept
Enterobacter gergoviae	enterobacter gergoviae	enterobacter gergoviae	enterobacter gergoviae	Enterobacter gergoviae	1	kept
enterococcus	enterococcus	enterococcus	enterococcus	Enterococcus	1	case_or_format
Enterococcus faecalis infection	enterococcus faecalis infection	enterococcus faecalis infection	enterococcus faecalis infection	Enterococcus faecalis infection	1	kept

Enterococcus gallinarum	enterococcus gallinarum	enterococcus gallinarum	enterococcus gallinarum	Enterococcus gallinarum	1	kept
Enterocutaneous fistula	enterocutaneous fistula	enterocutaneous fistula	enterocutaneous fistula	enterocutaneous fistula	1	case_or_format
Enterohemorrhagic E coli	enterohemorrhagic e coli	enterohemorrhagic e coli	enterohemorrhagic e coli	Enterohemorrhagic E coli	1	kept
enterohepatic circulation	enterohepatic circulation	enterohepatic circulation	enterohepatic circulation	enterohepatic circulation	1	kept
Enterostomal therapy	enterostomal therapy	enterostomal therapy	enterostomal therapy	Enterostomal therapy	1	kept
enterotoxin	enterotoxin	enterotoxin	enterotoxin	enterotoxin	1	kept
Enterprise resource management	enterprise resource management	enterprise resource management	enterprise resource management	Enterprise resource management	1	kept
entorhinal cortex	entorhinal cortex	entorhinal cortex	entorhinal cortex	entorhinal cortex	1	kept
Entorno	entorno	entorno	entorno	Entorno	1	kept
Entornos con recursos limitados	entornos con recursos limitados	entornos con recursos limitados	entornos con recursos limitados	Entornos con recursos limitados	1	kept
ENTRAINMENT	entrainment	entrainment	entrainment	ENTRAINMENT	1	kept
Entrenamiento	entrenamiento	entrenamiento	entrenamiento	Entrenamiento	1	kept
Entrepreneurship	entrepreneurship	entrepreneurship	entrepreneurship	Entrepreneurship	1	kept
entrevues orales	entrevues orales	entrevues orales	entrevues orales	entrevues orales	1	kept
Entrophy-based Grey Relational Analysis	entrophy based grey relational analysis	entrophy based grey relational analysis	entrophy based grey relational analysis	Entrophy-based Grey Relational Analysis	1	kept
Entropy	entropy	entropy	entropy	entropy	1	case_or_format

entrustable professional activities	entrustable professional activities	entrustable professional activities	entrustable professional activity	Entrustable professional activity	1	singular
Entrustment decisions	entrustment decisions	entrustment decisions	entrustment decisions	Entrustment decisions	1	kept
entry into practice	entry into practice	entry into practice	entry into practice	entry into practice	1	kept
Enuresis	enuresis	enuresis	enuresis	Enuresis	1	kept
enuresis	enuresis	enuresis	enuresis	Enuresis	1	case_or_format
Enuresis alarm	enuresis alarm	enuresis alarm	enuresis alarm	Enuresis alarm	1	kept
Envejecimiento	envejecimiento	envejecimiento	envejecimiento	Envejecimiento	1	kept
Envelhecimento	envelhecimento	envelhecimento	envelhecimento	Envelhecimento	1	kept
Envenomation	envenomation	envenomation	envenomation	envenomation	1	case_or_format
Envenomations	envenomations	envenomations	envenomation	envenomation	1	singular
environment design	environment design	environment design	environment design	Environment Design	1	case_or_format
environment of care	environment of care	environment of care	environment of care	environment of care	1	kept
Environmental	environmental	environmental	environmental	Environmental	1	kept
environmental audit tool high care	environmental audit tool high care	environmental audit tool high care	environmental audit tool high care	environmental audit tool high care	1	kept
Environmental Audit Tool High Care Japanese version	environmental audit tool high care japanese version	environmental audit tool high care japanese version	environmental audit tool high care japanese version	Environmental Audit Tool High Care Japanese version	1	kept
Environmental burden	environmental burden	environmental burden	environmental burden	Environmental burden	1	kept

environmental cognition	environmental cognition	environmental cognition	environmental cognition	environmental cognition	1	kept
environmental control	environmental control	environmental control	environmental control	environmental control	1	kept
environmental culture	environmental culture	environmental culture	environmental culture	environmental culture	1	kept
Environmental exposure	environmental exposure	environmental exposure	environmental exposure	Environmental Exposure	1	case_or_format
environmental fate	environmental fate	environmental fate	environmental fate	environmental fate	1	kept
environmental hazard	environmental hazard	environmental hazard	environmental hazard	environmental hazard	1	kept
Environmental health	environmental health	environmental health	environmental health	environmental health	1	case_or_format
environmental health worker	environmental health worker	environmental health worker	environmental health worker	environmental health worker	1	kept
Environmental Impact	environmental impact	environmental impact	environmental impact	environmental impact	1	case_or_format
environmental intervention	environmental intervention	environmental intervention	environmental intervention	environmental intervention	1	kept
environmental medicine	environmental medicine	environmental medicine	environmental medicine	environmental medicine	1	kept
environmental music therapy	environmental music therapy	environmental music therapy	environmental music therapy	environmental music therapy	1	kept
environmental reservoir	environmental reservoir	environmental reservoir	environmental reservoir	environmental reservoir	1	kept
Environmental Restoration and Remediation	environmental restoration and remediation	environmental restoration and remediation	environmental restoration and remediation	Environmental Restoration and Remediation	1	kept
Environmental risk and developmental follow-up for infants	environmental risk and developmental follow up for infants	environmental risk and developmental follow up for infants	environmental risk and developmental follow up for infants	Environmental risk and developmental follow-up for infants	1	kept
environmental safety	environmental safety	environmental safety	environmental safety	environmental safety	1	kept

environmental sampling	environmental sampling	environmental sampling	environmental sampling	environmental sampling	1	kept
Environmental sensor	environmental sensor	environmental sensor	environmental sensor	Environmental sensor	1	kept
Environmental services	environmental services	environmental services	environmental services	Environmental services	1	kept
environmental services staff	environmental services staff	environmental services staff	environmental services staff	environmental services staff	1	kept
Environmental standards	environmental standards	environmental standards	environmental standards	Environmental standards	1	kept
environmental stewardship	environmental stewardship	environmental stewardship	environmental stewardship	environmental stewardship	1	kept
environmental stressor	environmental stressor	environmental stressor	environmental stressor	Environmental stressor	1	case_or_format
Environmental stressors	environmental stressors	environmental stressors	environmental stressor	Environmental stressor	1	singular
environmental stressors	environmental stressors	environmental stressors	environmental stressor	Environmental stressor	1	singular
ENVIRONMENTAL SURFACES	environmental surfaces	environmental surfaces	environmental surfaces	ENVIRONMENTAL SURFACES	1	kept
environmental surfaces	environmental surfaces	environmental surfaces	environmental surfaces	ENVIRONMENTAL SURFACES	1	case_or_format
environmental surveillance	environmental surveillance	environmental surveillance	environmental surveillance	environmental surveillance	1	kept
Environmental uncertainty	environmental uncertainty	environmental uncertainty	environmental uncertainty	Environmental uncertainty	1	kept
Environmental variables	environmental variables	environmental variables	environmental variables	Environmental variables	1	kept
Environmental visibility	environmental visibility	environmental visibility	environmental visibility	Environmental visibility	1	kept
ENVIRONMENTAL-QUALITY	environmental quality	environmental quality	environmental quality	ENVIRONMENTAL-QUALITY	1	kept

environments	environments	environments	environment	Environment	1	singular
enzymatic assay	enzymatic assay	enzymatic assay	enzymatic assay	enzymatic assay	1	kept
enzyme	enzyme	enzyme	enzyme	enzyme	1	kept
enzyme assay	enzyme assay	enzyme assay	enzyme assay	enzyme assay	1	kept
enzyme blood level	enzyme blood level	enzyme blood level	enzyme blood level	enzyme blood level	1	kept
Enzyme inhibition	enzyme inhibition	enzyme inhibition	enzyme inhibition	Enzyme inhibition	1	kept
Enzyme linked immunosorbent assay	enzyme linked immunosorbent assay	enzyme linked immunosorbent assay	enzyme linked immunosorbent assay	enzyme linked immunosorbent assay	1	case_or_format
enzyme release	enzyme release	enzyme release	enzyme release	enzyme release	1	kept
enzyme replacement	enzyme replacement	enzyme replacement	enzyme replacement	enzyme replacement	1	kept
enzyme replacement therapy	enzyme replacement therapy	enzyme replacement therapy	enzyme replacement therapy	enzyme replacement therapy	1	kept
EOL care	eol care	eol care	eol care	EOL care	1	kept
EOLC	eolc	eolc	eolc	EOLC	1	kept
eon	eon	eon	eon	eon	1	kept
Eosinophilia	eosinophilia	eosinophilia	eosinophilia	eosinophilia	1	case_or_format
Eosinophilic Esophagitis	eosinophilic esophagitis	eosinophilic esophagitis	eosinophilic esophagitis	eosinophilic esophagitis	1	case_or_format
EP lab, Sedation	ep lab sedation	ep lab sedation	ep lab sedation	EP lab, Sedation	1	kept

Ependymoma	ependymoma	ependymoma	ependymoma	Ependymoma	1	kept
ependymoma	ependymoma	ependymoma	ependymoma	Ependymoma	1	case_or_format
Ephedra extract	ephedra extract	ephedra extract	ephedra extract	Ephedra extract	1	kept
epherenone	epherenone	epherenone	epherenone	epherenone	1	kept
Epicardial	epicardial	epicardial	epicardial	Epicardial	1	kept
epicardial pacemaker	epicardial pacemaker	epicardial pacemaker	epicardial pacemaker	epicardial pacemaker	1	kept
Epicenter	epicenter	epicenter	epicenter	Epicenter	1	kept
Epidemic management	epidemic management	epidemic management	epidemic management	Epidemic management	1	kept
epidemic meningitis	epidemic meningitis	epidemic meningitis	epidemic meningitis	epidemic meningitis	1	kept
epidemics	epidemics	epidemics	epidemic	epidemic	1	singular
Epidemio-logical surveillance	epidemio logical surveillance	epidemio logical surveillance	epidemio logical surveillance	Epidemio-logical surveillance	1	kept
EPIDEMIOLOGIC ANALYSIS	epidemiologic analysis	epidemiologic analysis	epidemiologic analysis	EPIDEMIOLOGIC ANALYSIS	1	kept
epidemiologic factors	epidemiologic factors	epidemiologic factors	epidemiologic factors	epidemiologic factors	1	kept
Epidemiologic methods	epidemiologic methods	epidemiologic methods	epidemiologic methods	Epidemiologic Methods	1	case_or_format
Epidemiologic Research Design	epidemiologic research design	epidemiologic research design	epidemiologic research design	Epidemiologic Research Design	1	kept
EPIDEMIOLOGIC STUDIES	epidemiologic studies	epidemiologic studies	epidemiologic studies	Epidemiologic Studies	1	case_or_format

epidemiologic studies	epidemiologic studies	epidemiologic studies	epidemiologic studies	Epidemiologic Studies	1	case_or_format
Epidemiologic surveillance	epidemiologic surveillance	epidemiologic surveillance	epidemiologic surveillance	Epidemiologic surveillance	1	kept
Epidemiological factors	epidemiological factors	epidemiological factors	epidemiological factors	Epidemiological factors	1	kept
Epidemiological studies	epidemiological studies	epidemiological studies	epidemiological studies	Epidemiological studies	1	kept
Epidemiological surveillance	epidemiological surveillance	epidemiological surveillance	epidemiological surveillance	epidemiological surveillance	1	case_or_format
epidemiology, fungal infection	epidemiology fungal infection	epidemiology fungal infection	epidemiology fungal infection	epidemiology, fungal infection	1	kept
Epidemiology, Molecular	epidemiology molecular	epidemiology molecular	epidemiology molecular	Epidemiology, Molecular	1	kept
Epidemiology, pulmonary	epidemiology pulmonary	epidemiology pulmonary	epidemiology pulmonary	Epidemiology, pulmonary	1	kept
Epidemiología	epidemiolog a	epidemiolog a	epidemiolog a	Epidemiología	1	kept
epidermal growth factor	epidermal growth factor	epidermal growth factor	epidermal growth factor	epidermal growth factor	1	kept
epidermal growth factor receptor 2	epidermal growth factor receptor 2	epidermal growth factor receptor 2	epidermal growth factor receptor 2	epidermal growth factor receptor 2	1	kept
Epidermal necrolysis	epidermal necrolysis	epidermal necrolysis	epidermal necrolysis	Epidermal necrolysis	1	kept
epidermis cell	epidermis cell	epidermis cell	epidermis cell	epidermis cell	1	kept
Epidermolisys Bullosa	epidermolisys bullosa	epidermolisys bullosa	epidermolisys bullosa	Epidermolisys Bullosa	1	kept
Epidermolisys bullosa	epidermolisys bullosa	epidermolisys bullosa	epidermolisys bullosa	Epidermolisys Bullosa	1	case_or_format
Epidermolysis bullosa	epidermolysis bullosa	epidermolysis bullosa	epidermolysis bullosa	Epidermolysis Bullosa	1	case_or_format

epidermolytic hyperkeratosis	epidermolytic hyperkeratosis	epidermolytic hyperkeratosis	epidermolytic hyperkeratosis	epidermolytic hyperkeratosis	1	kept
Epidural anaesthesia	epidural anaesthesia	epidural anaesthesia	epidural anaesthesia	Epidural anaesthesia	1	kept
epidural anaesthesia	epidural anaesthesia	epidural anaesthesia	epidural anaesthesia	Epidural anaesthesia	1	case_or_format
Epidural analgesia	epidural analgesia	epidural analgesia	epidural analgesia	epidural analgesia	1	case_or_format
Epidural Infusion	epidural infusion	epidural infusion	epidural infusion	Epidural Infusion	1	kept
epidural space	epidural space	epidural space	epidural space	epidural space	1	kept
epidémie de polio en Alberta	epid mie de polio en alberta	epid mie de polio en alberta	epid mie de polio en alberta	epidémie de polio en Alberta	1	kept
Epigastric Arteries	epigastric arteries	epigastric arteries	epigastric artery	epigastric artery	1	singular
epigastric artery	epigastric artery	epigastric artery	epigastric artery	epigastric artery	1	kept
Epigenesis, Genetic	epigenesis genetic	epigenesis genetic	epigenesis genetic	Epigenesis, Genetic	1	kept
Epigenetics and developmental psychology	epigenetics and developmental psychology	epigenetics and developmental psychology	epigenetics and developmental psychology	Epigenetics and developmental psychology	1	kept
epiglottis cancer	epiglottis cancer	epiglottis cancer	epiglottis cancer	epiglottis cancer	1	kept
Epilepsia Partialis Continua	epilepsia partialis continua	epilepsia partialis continua	epilepsia partialis continua	Epilepsia Partialis Continua	1	kept
epilepsy monitoring	epilepsy monitoring	epilepsy monitoring	epilepsy monitoring	epilepsy monitoring	1	kept
Epilepsy monitoring unit	epilepsy monitoring unit	epilepsy monitoring unit	epilepsy monitoring unit	epilepsy monitoring unit	1	case_or_format
Epilepsy, Generalized	epilepsy generalized	epilepsy generalized	epilepsy generalized	Epilepsy, Generalized	1	kept

Epilepsy, Post-Traumatic	epilepsy post traumatic	epilepsy post traumatic	epilepsy post traumatic	Epilepsy, Post-Traumatic	1	kept
Epilepsy, Tonic-Clonic	epilepsy tonic clonic	epilepsy tonic clonic	epilepsy tonic clonic	Epilepsy, Tonic-Clonic	1	kept
Epileptic disorder	epileptic disorder	epileptic disorder	epileptic disorder	Epileptic disorder	1	kept
Epileptic seizure detection	epileptic seizure detection	epileptic seizure detection	epileptic seizure detection	Epileptic seizure detection	1	kept
Epileptiform discharges	epileptiform discharges	epileptiform discharges	epileptiform discharges	Epileptiform discharges	1	kept
epileptiform discharges	epileptiform discharges	epileptiform discharges	epileptiform discharges	Epileptiform discharges	1	case_or_format
EPINet	epinet	epinet	epinet	Epinet	1	case_or_format
epiphora	epiphora	epiphora	epiphora	epiphora	1	kept
Epiphysis	epiphysis	epiphysis	epiphysis	Epiphysis	1	kept
epiphysis	epiphysis	epiphysis	epiphysis	Epiphysis	1	case_or_format
episodes of care	episodes of care	episodes of care	episodes of care	episodes of care	1	kept
episodic vital sign collection	episodic vital sign collection	episodic vital sign collection	episodic vital sign collection	episodic vital sign collection	1	kept
epispadias	epispadias	epispadias	epispadias	epispadias	1	kept
Epistemic network analysis	epistemic network analysis	epistemic network analysis	epistemic network analysis	Epistemic network analysis	1	kept
Epistemics	epistemics	epistemics	epistemics	Epistemics	1	kept
EPISTEMOLOGY	epistemology	epistemology	epistemology	epistemology	1	case_or_format

Epistemology	epistemology	epistemology	epistemology	epistemology	1	case_or_format
epithelial mesenchymal transition	epithelial mesenchymal transition	epithelial mesenchymal transition	epithelial mesenchymal transition	epithelial mesenchymal transition	1	kept
Epithelium	epithelium	epithelium	epithelium	Epithelium	1	kept
epithelium cell	epithelium cell	epithelium cell	epithelium cell	epithelium cell	1	kept
epithelium lesion	epithelium lesion	epithelium lesion	epithelium lesion	epithelium lesion	1	kept
Epithelium, Corneal	epithelium corneal	epithelium corneal	epithelium corneal	Epithelium, Corneal	1	kept
epithelization	epithelization	epithelization	epithelization	epithelization	1	kept
eplerenone	eplerenone	eplerenone	eplerenone	eplerenone	1	kept
epoprostenol	epoprostenol	epoprostenol	epoprostenol	Epoprostenol	1	case_or_format
Epoxy Compounds	epoxy compounds	epoxy compounds	epoxy compounds	Epoxy Compounds	1	kept
eptifibatide	eptifibatide	eptifibatide	eptifibatide	eptifibatide	1	kept
EPUAP	epuap	epuap	epuap	EPUAP	1	kept
EQ-5D	eq 5d	eq 5d	eq 5d	EQ-5D	1	kept
equalizing noise level	equalizing noise level	equalizing noise level	equalizing noise level	equalizing noise level	1	kept
Equation Structural Modeling	equation structural modeling	equation structural modeling	equation structural modeling	Equation Structural Modeling	1	kept
Equid herpesvirus 1	equid herpesvirus 1	equid herpesvirus 1	equid herpesvirus 1	Equid herpesvirus 1	1	kept

equilibrium constant	equilibrium constant	equilibrium constant	equilibrium constant	equilibrium constant	1	kept
Equimosis	equimosis	equimosis	equimosis	Equimosis	1	kept
Equine	equine	equine	equine	Equine	1	kept
equine infectious anemia	equine infectious anemia	equine infectious anemia	equine infectious anemia	equine infectious anemia	1	kept
equine muscle spasm	equine muscle spasm	equine muscle spasm	equine muscle spasm	equine muscle spasm	1	kept
EQUIPMENT	equipment	equipment	equipment	equipment	1	case_or_format
Equipment and supplies	equipment and supplies	equipment and supplies	equipment and supplies	Equipment and Supplies	1	case_or_format
Equipment and Supplies Utilization	equipment and supplies utilization	equipment and supplies utilization	equipment and supplies utilization	equipment and supplies utilization	1	case_or_format
Equipment evaluation	equipment evaluation	equipment evaluation	equipment evaluation	Equipment evaluation	1	kept
Equipment failure	equipment failure	equipment failure	equipment failure	Equipment Failure	1	case_or_format
equipment failure analysis	equipment failure analysis	equipment failure analysis	equipment failure analysis	Equipment Failure Analysis	1	case_or_format
Equipment improvisation	equipment improvisation	equipment improvisation	equipment improvisation	Equipment improvisation	1	kept
equipment maintenance	equipment maintenance	equipment maintenance	equipment maintenance	equipment maintenance	1	kept
equipment safety	equipment safety	equipment safety	equipment safety	Equipment Safety	1	case_or_format
equipment selection	equipment selection	equipment selection	equipment selection	equipment selection	1	kept
Equipment-cuffs	equipment cuffs	equipment cuffs	equipment cuffs	Equipment-cuffs	1	kept

equipment: safety	equipment safety	equipment safety	equipment safety	Equipment Safety	1	case_or_format
Equipo de atención al paciente	equipo de atenci n al paciente	equipo de atenci n al paciente	equipo de atenci n al paciente	Equipo de atención al paciente	1	kept
Equipo e insumos hospitalarios	equipo e insumos hospitalarios	equipo e insumos hospitalarios	equipo e insumos hospitalarios	Equipo e insumos hospitalarios	1	kept
Equipos de respuesta rápida	equipos de respuesta r pida	equipos de respuesta r pida	equipos de respuesta r pida	Equipos de respuesta rápida	1	kept
equitable palliative care	equitable palliative care	equitable palliative care	equitable palliative care	equitable palliative care	1	kept
equivalent continuous sound level	equivalent continuous sound level	equivalent continuous sound level	equivalent continuous sound level	equivalent continuous sound level	1	kept
ER, Emergency room	er emergency room	er emergency room	er emergency room	ER, Emergency room	1	kept
ERA	era	era	era	ERAS	1	case_or_format
Eradication of nosocomial infections	eradication of nosocomial infections	eradication of nosocomial infections	eradication of nosocomial infections	Eradication of nosocomial infections	1	kept
ERAS = enhanced recovery after surgery	eras enhanced recovery after surgery	eras enhanced recovery after surgery	eras enhanced recovery after surgery	ERAS = enhanced recovery after surgery	1	kept
ERAS®	eras	eras	era	ERAS	1	singular
eravacycline	eravacycline	eravacycline	eravacycline	eravacycline	1	kept
erector spinae muscle	erector spinae muscle	erector spinae muscle	erector spinae muscle	erector spinae muscle	1	kept
Erector spinae-plane block	erector spinae plane block	erector spinae plane block	erector spinae plane block	Erector spinae-plane block	1	kept
Ergocalciferols	ergocalciferols	ergocalciferols	ergocalciferol	ergocalciferol	1	singular
ergology	ergology	ergology	ergology	ergology	1	kept

ergometry	ergometry	ergometry	ergometry	Ergometry	1	case_or_format
Ergonomic practice	ergonomic practice	ergonomic practice	ergonomic practice	Ergonomic practice	1	kept
Ergonomic risk management program	ergonomic risk management program	ergonomic risk management program	ergonomic risk management program	Ergonomic risk management program	1	kept
ERI	eri	eri	eri	ERI	1	kept
eritoran	eritoran	eritoran	eritoran	eritoran	1	kept
ermB gene	ermb gene	ermb gene	ermb gene	ermB gene	1	kept
Erosion	erosion	erosion	erosion	erosion	1	case_or_format
erreur médicamenteuse	erreur m dicamenteuse	erreur m dicamenteuse	erreur m dicamenteuse	erreur médicamenteuse	1	kept
Error and incident	error and incident	error and incident	error and incident	Error and incident	1	kept
error and risk analysis	error and risk analysis	error and risk analysis	error and risk analysis	error and risk analysis	1	kept
Error cause	error cause	error cause	error cause	Error cause	1	kept
Error communication	error communication	error communication	error communication	Error communication	1	kept
error cost	error cost	error cost	error cost	error cost	1	kept
error detectors	error detectors	error detectors	error detectors	error detectors	1	kept
Error in medicine	error in medicine	error in medicine	error in medicine	Error in medicine	1	kept
Error microphones	error microphones	error microphones	error microphones	Error microphones	1	kept

Error rates	error rates	error rates	error rates	Error rates	1	kept
error recovery	error recovery	error recovery	error recovery	error recovery	1	kept
Error type I	error type i	error type i	error type i	Error type I	1	kept
Error type II	error type ii	error type ii	error type ii	Error type II	1	kept
Errores de Medicación	errores de medicaci n	errores de medicaci n	errores de medicaci n	Errores de Medicación	1	kept
Errores de medicación	errores de medicaci n	errores de medicaci n	errores de medicaci n	Errores de Medicación	1	case_or_format
errors in medication	errors in medication	errors in medication	errors in medication	errors in medication	1	kept
errors of commission	errors of commission	errors of commission	errors of commission	errors of commission	1	kept
erythema elevatum diutinum	erythema elevatum diutinum	erythema elevatum diutinum	erythema elevatum diutinum	erythema elevatum diutinum	1	kept
Erythema multiforme	erythema multiforme	erythema multiforme	erythema multiforme	Erythema Multiforme	1	case_or_format
erythroblast	erythroblast	erythroblast	erythroblast	erythroblast	1	kept
Erythroblastosis fetalis	erythroblastosis fetalis	erythroblastosis fetalis	erythroblastosis fetalis	Erythroblastosis fetalis	1	kept
erythrocytapheresis	erythrocytapheresis	erythrocytapheresis	erythrocytapheresis	erythrocytapheresis	1	kept
Erythrocyte Count	erythrocyte count	erythrocyte count	erythrocyte count	erythrocyte count	1	case_or_format
erythrocyte level	erythrocyte level	erythrocyte level	erythrocyte level	erythrocyte level	1	kept
Erythrocyte Volume	erythrocyte volume	erythrocyte volume	erythrocyte volume	erythrocyte volume	1	case_or_format

erythrodermic psoriasis	erythrodermic psoriasis	erythrodermic psoriasis	erythrodermic psoriasis	erythrodermic psoriasis	1	kept
erythromelalgia	erythromelalgia	erythromelalgia	erythromelalgia	erythromelalgia	1	kept
erythropenia	erythropenia	erythropenia	erythropenia	erythropenia	1	kept
Erythropoietin-stimulating agents	erythropoietin stimulating agents	erythropoietin stimulating agents	erythropoietin stimulating agents	Erythropoietin-stimulating agents	1	kept
ESBL-producer	esbl producer	esbl producer	esbl producer	ESBL-producer	1	kept
ESBL-producing Enterobacteriaceae	esbl producing enterobacteriaceae	esbl producing enterobacteriaceae	esbl producing enterobacteriaceae	ESBL-producing Enterobacteriaceae	1	kept
Escalas conductuales	escalas conductuales	escalas conductuales	escalas conductuales	Escalas conductuales	1	kept
Escalas pronósticas	escalas pron sticas	escalas pron sticas	escalas pron sticas	Escalas pronósticas	1	kept
ESCALATING AGGRESSIVE-BEHAVIOR	escalating aggressive behavior	escalating aggressive behavior	escalating aggressive behavior	ESCALATING AGGRESSIVE-BEHAVIOR	1	kept
escalation protocols	escalation protocols	escalation protocols	escalation protocols	escalation protocols	1	kept
Escalations in care	escalations in care	escalations in care	escalations in care	Escalations in care	1	kept
escape room	escape room	escape room	escape room	Escape room	1	case_or_format
escharectomy	escharectomy	escharectomy	escharectomy	escharectomy	1	kept
escharotomy	escharotomy	escharotomy	escharotomy	Escharotomy	1	case_or_format
Escherichia	escherichia	escherichia	escherichia	Escherichia	1	kept
Escherichia coli protein	escherichia coli protein	escherichia coli protein	escherichia coli protein	Escherichia coli protein	1	kept

Escherichia coli Proteins	escherichia coli proteins	escherichia coli proteins	escherichia coli protein	Escherichia coli protein	1	singular
Escherichia coli ST131 C	escherichia coli st131 c	escherichia coli st131 c	escherichia coli st131 c	Escherichia coli ST131 C	1	kept
ESCHERICHIA-COLI BACTEREMIA	escherichia coli bacteremia	escherichia coli bacteremia	escherichia coli bacteremia	ESCHERICHIA-COLI BACTEREMIA	1	kept
Esgotamento Emocional	esgotamento emocional	esgotamento emocional	esgotamento emocional	Esgotamento Emocional	1	kept
ESI	esi	esi	esi	ESI	1	kept
ESKAPE pathogen	eskape pathogen	eskape pathogen	eskape pathogen	ESKAPE pathogen	1	kept
Eskimo	eskimo	eskimo	eskimo	Eskimo	1	kept
esophageal	esophageal	esophageal	esophageal	esophageal	1	kept
Esophageal Achalasia	esophageal achalasia	esophageal achalasia	esophageal achalasia	Esophageal Achalasia	1	kept
Esophageal cooling	esophageal cooling	esophageal cooling	esophageal cooling	Esophageal cooling	1	kept
ESOPHAGEAL DOPPLER ULTRASOUND	esophageal doppler ultrasound	esophageal doppler ultrasound	esophageal doppler ultrasound	ESOPHAGEAL DOPPLER ULTRASOUND	1	kept
Esophageal heat transfer	esophageal heat transfer	esophageal heat transfer	esophageal heat transfer	Esophageal heat transfer	1	kept
esophageal manometer	esophageal manometer	esophageal manometer	esophageal manometer	esophageal manometer	1	kept
Esophageal pH Monitoring	esophageal ph monitoring	esophageal ph monitoring	esophageal ph monitoring	Esophageal pH Monitoring	1	kept
Esophageal pressure measurements	esophageal pressure measurements	esophageal pressure measurements	esophageal pressure measurements	Esophageal pressure measurements	1	kept
Esophageal rupture	esophageal rupture	esophageal rupture	esophageal rupture	Esophageal rupture	1	kept

esophageal squamous cell carcinoma	esophageal squamous cell carcinoma	esophageal squamous cell carcinoma	esophageal squamous cell carcinoma	esophageal squamous cell carcinoma	1	kept
esophageal stent	esophageal stent	esophageal stent	esophageal stent	esophageal stent	1	kept
esophagography	esophagography	esophagography	esophagography	esophagography	1	kept
Esophagoscopy	esophagoscopy	esophagoscopy	esophagoscopy	Esophagoscopy	1	kept
esophagoscopy	esophagoscopy	esophagoscopy	esophagoscopy	Esophagoscopy	1	case_or_format
esophagostomy	esophagostomy	esophagostomy	esophagostomy	esophagostomy	1	kept
esophagus burn	esophagus burn	esophagus burn	esophagus burn	esophagus burn	1	kept
esophagus carcinoma	esophagus carcinoma	esophagus carcinoma	esophagus carcinoma	esophagus carcinoma	1	kept
esophagus dilatation	esophagus dilatation	esophagus dilatation	esophagus dilatation	esophagus dilatation	1	kept
esophagus dysplasia	esophagus dysplasia	esophagus dysplasia	esophagus dysplasia	esophagus dysplasia	1	kept
esophagus fistula	esophagus fistula	esophagus fistula	esophagus fistula	esophagus fistula	1	kept
esophagus function disorder	esophagus function disorder	esophagus function disorder	esophagus function disorder	esophagus function disorder	1	kept
esophagus motility	esophagus motility	esophagus motility	esophagus motility	esophagus motility	1	kept
esophagus surgery	esophagus surgery	esophagus surgery	esophagus surgery	esophagus surgery	1	kept
esophagus varices bleeding	esophagus varices bleeding	esophagus varices bleeding	esophagus varices bleeding	esophagus varices bleeding	1	kept
ESP8266	esp8266	esp8266	esp8266	ESP8266	1	kept

España	espa a	espa a	espa a	España	1	kept
Especialización	especializaci n	especializaci n	especializaci n	Especialización	1	kept
ESPEN	espen	espen	espen	ESPEN	1	kept
ESPEN Clinical Practice Guidelines	espen clinical practice guidelines	espen clinical practice guidelines	espen clinical practice guidelines	ESPEN Clinical Practice Guidelines	1	kept
ESPEN GUIDELINES	espen guidelines	espen guidelines	espen guidelines	ESPEN GUIDELINES	1	kept
Espiritualidad	espiritualidad	espiritualidad	espiritualidad	Espiritualidad	1	kept
Essence	essence	essence	essence	Essence	1	kept
Essential Care of Small Baby	essential care of small baby	essential care of small baby	essential care of small baby	Essential Care of Small Baby	1	kept
Essential component	essential component	essential component	essential component	Essential component	1	kept
essential drug	essential drug	essential drug	essential drug	essential drug	1	kept
essential fatty acid deficiency	essential fatty acid deficiency	essential fatty acid deficiency	essential fatty acid deficiency	essential fatty acid deficiency	1	kept
Essential health services	essential health services	essential health services	essential health services	Essential health services	1	kept
Essential medicines list	essential medicines list	essential medicines list	essential medicines list	Essential medicines list	1	kept
ESSENTIAL OIL	essential oil	essential oil	essential oil	essential oil	1	case_or_format
Essential oil lavender	essential oil lavender	essential oil lavender	essential oil lavender	Essential oil lavender	1	kept
Essential principles	essential principles	essential principles	essential principles	Essential principles	1	kept

essential worker	essential worker	essential worker	essential worker	essential worker	1	kept
ESSENTIALS	essentials	essentials	essentials	ESSENTIALS	1	kept
Essentials of Magnetism II	essentials of magnetism ii	essentials of magnetism ii	essentials of magnetism ii	Essentials of Magnetism II	1	kept
Estado de salud	estado de salud	estado de salud	estado de salud	Estado de salud	1	kept
esthetic surgery	esthetic surgery	esthetic surgery	esthetic surgery	esthetic surgery	1	kept
estimated cerebral perfusion pressure	estimated cerebral perfusion pressure	estimated cerebral perfusion pressure	estimated cerebral perfusion pressure	estimated cerebral perfusion pressure	1	kept
estimated fetal weight	estimated fetal weight	estimated fetal weight	estimated fetal weight	estimated fetal weight	1	kept
estimated fetus weight	estimated fetus weight	estimated fetus weight	estimated fetus weight	estimated fetus weight	1	kept
Estimation	estimation	estimation	estimation	Estimation	1	kept
estimation theory	estimation theory	estimation theory	estimation theory	estimation theory	1	kept
estimation weight	estimation weight	estimation weight	estimation weight	estimation weight	1	kept
Estimations	estimations	estimations	estimation	Estimation	1	singular
ESTIMATOR	estimator	estimator	estimator	ESTIMATOR	1	kept
Estrategias	estrategias	estrategias	estrategias	Estrategias	1	kept
Estrategias de ahorro de sangre	estrategias de ahorro de sangre	estrategias de ahorro de sangre	estrategias de ahorro de sangre	Estrategias de ahorro de sangre	1	kept
Estratégias	estrat gias	estrat gias	estrat gias	Estratégias	1	kept

Estresse Ocupacional	estresse ocupacional	estresse ocupacional	estresse ocupacional	Estresse Ocupacional	1	kept
Estresse Psicológico	estresse psicol gico	estresse psicol gico	estresse psicol gico	Estresse Psicológico	1	kept
Estriol	estriol	estriol	estriol	estriol	1	case_or_format
estrogen receptor alpha	estrogen receptor alpha	estrogen receptor alpha	estrogen receptor alpha	estrogen receptor alpha	1	kept
Estrogen Replacement Therapy	estrogen replacement therapy	estrogen replacement therapy	estrogen replacement therapy	Estrogen Replacement Therapy	1	kept
estrogen therapy	estrogen therapy	estrogen therapy	estrogen therapy	estrogen therapy	1	kept
Estrés Laboral	estr s laboral	estr s laboral	estr s laboral	Estrés Laboral	1	kept
Estudio cuasiexperimental	estudio cuasiexperimental	estudio cuasiexperimental	estudio cuasiexperimental	Estudio cuasiexperimental	1	kept
Estudio de Validación	estudio de validaci n	estudio de validaci n	estudio de validaci n	Estudio de validación	1	case_or_format
Estudio multicéntrico	estudio multic ntrico	estudio multic ntrico	estudio multic ntrico	Estudio multicéntrico	1	kept
Estudios de Validación	estudios de validaci n	estudios de validaci n	estudios de validaci n	Estudios de Validación	1	kept
Estudios de validación	estudios de validaci n	estudios de validaci n	estudios de validaci n	Estudios de Validación	1	case_or_format
Estudo de Validação	estudo de valida o	estudo de valida o	estudo de valida o	Estudo de Validação	1	kept
Estudos de Validação	estudos de valida o	estudos de valida o	estudos de valida o	Estudos de Validação	1	kept
Estudos de validação como assunto	estudos de valida o como assunto	estudos de valida o como assunto	estudos de valida o como assunto	Estudos de validação como assunto	1	kept
Estándar de calidad	est ndar de calidad	est ndar de calidad	est ndar de calidad	Estándar de calidad	1	kept

etacrynic acid	etacrynic acid	etacrynic acid	etacrynic acid	etacrynic acid	1	kept
etesevimab	etesevimab	etesevimab	etesevimab	etesevimab	1	kept
ethanol	ethanol	ethanol	ethanol	Ethanol	1	case_or_format
Ethanol-based hand rub	ethanol based hand rub	ethanol based hand rub	ethanol based hand rub	Ethanol-based hand rub	1	kept
Ethic	ethic	ethic	ethic	ethics	1	case_or_format
Ethic minority	ethic minority	ethic minority	ethic minority	Ethic minority	1	kept
Ethical action	ethical action	ethical action	ethical action	Ethical action	1	kept
Ethical analysis	ethical analysis	ethical analysis	ethical analysis	Ethical Analysis	1	case_or_format
Ethical aspects	ethical aspects	ethical aspects	ethical aspects	Ethical aspects	1	kept
ethical attitudes	ethical attitudes	ethical attitudes	ethical attitude	Ethical attitude	1	singular
Ethical awareness	ethical awareness	ethical awareness	ethical awareness	ethical awareness	1	case_or_format
ethical awareness scale	ethical awareness scale	ethical awareness scale	ethical awareness scale	ethical awareness scale	1	kept
Ethical behavior of a co-worker	ethical behavior of a co worker	ethical behavior of a co worker	ethical behavior of a co worker	Ethical behavior of a co-worker	1	kept
Ethical care	ethical care	ethical care	ethical care	ethical care	1	case_or_format
ethical caring	ethical caring	ethical caring	ethical caring	ethical caring	1	kept
ethical case intervention	ethical case intervention	ethical case intervention	ethical case intervention	ethical case intervention	1	kept

Ethical climate questionnaire	ethical climate questionnaire	ethical climate questionnaire	ethical climate questionnaire	Ethical climate questionnaire	1	kept
Ethical competences	ethical competences	ethical competences	ethical competences	Ethical competences	1	kept
ethical competency	ethical competency	ethical competency	ethical competency	ethical competency	1	kept
ethical conduct	ethical conduct	ethical conduct	ethical conduct	ethical conduct	1	kept
ethical confidence	ethical confidence	ethical confidence	ethical confidence	ethical confidence	1	kept
ethical conflict model	ethical conflict model	ethical conflict model	ethical conflict model	ethical conflict model	1	kept
Ethical conflicts	ethical conflicts	ethical conflicts	ethical conflict	Ethical conflict	1	singular
ETHICAL CONSIDERATIONS	ethical considerations	ethical considerations	ethical considerations	ETHICAL CONSIDERATIONS	1	kept
Ethical Considerations	ethical considerations	ethical considerations	ethical considerations	ETHICAL CONSIDERATIONS	1	case_or_format
Ethical considerations	ethical considerations	ethical considerations	ethical considerations	ETHICAL CONSIDERATIONS	1	case_or_format
Ethical decision-making	ethical decision making	ethical decision making	ethical decision making	ethical decision making	1	case_or_format
ethical decision-making ability	ethical decision making ability	ethical decision making ability	ethical decision making ability	ethical decision-making ability	1	kept
Ethical decision-making model	ethical decision making model	ethical decision making model	ethical decision making model	Ethical decision-making model	1	kept
ethical demands	ethical demands	ethical demands	ethical demands	ethical demands	1	kept
ethical dimensions	ethical dimensions	ethical dimensions	ethical dimensions	ethical dimensions	1	kept
Ethical discussions	ethical discussions	ethical discussions	ethical discussions	Ethical discussions	1	kept

Ethical environment	ethical environment	ethical environment	ethical environment	Ethical environment	1	kept
ethical experiences	ethical experiences	ethical experiences	ethical experiences	ethical experiences	1	kept
Ethical frameworks	ethical frameworks	ethical frameworks	ethical frameworks	Ethical frameworks	1	kept
ethical frameworks	ethical frameworks	ethical frameworks	ethical frameworks	Ethical frameworks	1	case_or_format
Ethical guidelines	ethical guidelines	ethical guidelines	ethical guidelines	Ethical guidelines	1	kept
Ethical humility	ethical humility	ethical humility	ethical humility	Ethical humility	1	kept
Ethical implications	ethical implications	ethical implications	ethical implications	Ethical implications	1	kept
ethical implications	ethical implications	ethical implications	ethical implications	Ethical implications	1	case_or_format
ethical implications of AI in health care	ethical implications of ai in health care	ethical implications of ai in health care	ethical implications of ai in health care	ethical implications of AI in health care	1	kept
Ethical Issues	ethical issues	ethical issues	ethical issues	ethical issues	1	case_or_format
Ethical Leadership	ethical leadership	ethical leadership	ethical leadership	ethical leadership	1	case_or_format
ethical making decision	ethical making decision	ethical making decision	ethical making decision	ethical making decision	1	kept
ETHICAL PRACTICE	ethical practice	ethical practice	ethical practice	ethical practice	1	case_or_format
Ethical practice	ethical practice	ethical practice	ethical practice	ethical practice	1	case_or_format
ethical problem	ethical problem	ethical problem	ethical problem	ethical problem	1	kept
Ethical problems	ethical problems	ethical problems	ethical problem	ethical problem	1	singular

ETHICAL QUESTIONNAIRE	ethical questionnaire	ethical questionnaire	ethical questionnaire	ETHICAL QUESTIONNAIRE	1	kept
Ethical Review	ethical review	ethical review	ethical review	Ethical Review	1	kept
Ethical work environment	ethical work environment	ethical work environment	ethical work environment	Ethical work environment	1	kept
ETHICAL-ISSUES	ethical issues	ethical issues	ethical issues	ethical issues	1	case_or_format
ethics and children in care	ethics and children in care	ethics and children in care	ethics and children in care	ethics and children in care	1	kept
Ethics and law	ethics and law	ethics and law	ethics and law	Ethics and law	1	kept
Ethics committee	ethics committee	ethics committee	ethics committee	Ethics Committees	1	case_or_format
Ethics committees	ethics committees	ethics committees	ethics committee	Ethics Committees	1	singular
ethics consultation services	ethics consultation services	ethics consultation services	ethics consultation services	ethics consultation services	1	kept
Ethics education	ethics education	ethics education	ethics education	ethics education	1	case_or_format
Ethics intervention	ethics intervention	ethics intervention	ethics intervention	Ethics intervention	1	kept
Ethics issues	ethics issues	ethics issues	ethics issues	Ethics issues	1	kept
Ethics principles	ethics principles	ethics principles	ethics principles	Ethics principles	1	kept
ethics responsibilities	ethics responsibilities	ethics responsibilities	ethics responsibilities	ethics responsibilities	1	kept
Ethics support services	ethics support services	ethics support services	ethics support services	Ethics support services	1	kept
ethics - professional	ethics professional	ethics professional	ethics professional	Ethics, Professional	1	case_or_format

ethicss	ethicss	ethicss	ethicss	ethicss	1	kept
Ethik	ethik	ethik	ethik	Ethik	1	kept
ethionamide	ethionamide	ethionamide	ethionamide	ethionamide	1	kept
ETHIOPIA	ethiopia	ethiopia	ethiopia	Ethiopia	1	case_or_format
Ethiopian	ethiopian	ethiopian	ethiopian	Ethiopian	1	kept
ethmoid sinus	ethmoid sinus	ethmoid sinus	ethmoid sinus	ethmoid sinus	1	kept
ethnic background	ethnic background	ethnic background	ethnic background	ethnic background	1	kept
ETHNIC DISPARITIES	ethnic disparities	ethnic disparities	ethnic disparity	ethnic disparity	1	singular
ethnic disparity	ethnic disparity	ethnic disparity	ethnic disparity	ethnic disparity	1	kept
ethnic diversity	ethnic diversity	ethnic diversity	ethnic diversity	ethnic diversity	1	kept
Ethnic groups	ethnic groups	ethnic groups	ethnic group	ethnic group	1	singular
Ethnic minorities	ethnic minorities	ethnic minorities	ethnic minority	Ethnic minority	1	singular
Ethnic minority	ethnic minority	ethnic minority	ethnic minority	Ethnic minority	1	kept
ETHNIC-DIFFERENCES	ethnic differences	ethnic differences	ethnic difference	ethnic difference	1	singular
Ethnogeriatrics	ethnogeriatrics	ethnogeriatrics	ethnogeriatrics	Ethnogeriatrics	1	kept
Ethnographic	ethnographic	ethnographic	ethnographic	Ethnographic	1	kept

ethnographic	ethnographic	ethnographic	ethnographic	Ethnographic	1	case_or_format
ethnographic methods	ethnographic methods	ethnographic methods	ethnographic methods	ethnographic methods	1	kept
Ethnographic research	ethnographic research	ethnographic research	ethnographic research	ethnographic research	1	case_or_format
Ethnogrpahy	ethnogrpahy	ethnogrpahy	ethnogrpahy	Ethnogrpahy	1	kept
Ethnology	ethnology	ethnology	ethnology	ethnology	1	case_or_format
Ethnomethodology	ethnomethodology	ethnomethodology	ethnomethodology	ethnomethodology	1	case_or_format
ethyl alcohol	ethyl alcohol	ethyl alcohol	ethyl alcohol	ethyl alcohol	1	kept
ethylene glycol intoxication	ethylene glycol intoxication	ethylene glycol intoxication	ethylene glycol intoxication	ethylene glycol intoxication	1	kept
Etiology study of bed sore	etiology study of bed sore	etiology study of bed sore	etiology study of bed sore	Etiology study of bed sore	1	kept
etonogestrel	etonogestrel	etonogestrel	etonogestrel	etonogestrel	1	kept
Etoposide	etoposide	etoposide	etoposide	etoposide	1	case_or_format
etoricoxib	etoricoxib	etoricoxib	etoricoxib	etoricoxib	1	kept
ETT	ett	ett	ett	ETT	1	kept
ETT, Endotracheal tube	ett endotracheal tube	ett endotracheal tube	ett endotracheal tube	ETT, Endotracheal tube	1	kept
Eucalyptus	eucalyptus	eucalyptus	eucalyptus	Eucalyptus	1	kept
Eucalyptus camaldulensis	eucalyptus camaldulensis	eucalyptus camaldulensis	eucalyptus camaldulensis	Eucalyptus camaldulensis	1	kept

eugenics	eugenics	eugenics	eugenics	eugenics	1	kept
Eukaryota	eukaryota	eukaryota	eukaryota	Eukaryota	1	kept
eukaryotic flagellum	eukaryotic flagellum	eukaryotic flagellum	eukaryotic flagellum	eukaryotic flagellum	1	kept
Eurasia	eurasia	eurasia	eurasia	Eurasia	1	kept
Euro SCORE II	euro score ii	euro score ii	euro score ii	Euro SCORE II	1	kept
Europe, Eastern	europe eastern	europe eastern	europe eastern	Europe, Eastern	1	kept
European Countries	european countries	european countries	european countries	European Countries	1	kept
European countries	european countries	european countries	european countries	European Countries	1	case_or_format
european economic area	european economic area	european economic area	european economic area	european economic area	1	kept
european heart failure self care behaviour questionnaire	european heart failure self care behaviour questionnaire	european heart failure self care behaviour questionnaire	european heart failure self care behaviour questionnaire	european heart failure self care behaviour questionnaire	1	kept
European Medicines Agency	european medicines agency	european medicines agency	european medicines agency	European Medicines Agency	1	kept
European Organization for Research and Treatment of Cancer	european organization for research and treatment of cancer	european organization for research and treatment of cancer	european organization for research and treatment of cancer	European Organization for Research and Treatment of Cancer	1	kept
European People	european people	european people	european people	European People	1	kept
European Pressure Ulcer Advisory Panel scale	european pressure ulcer advisory panel scale	european pressure ulcer advisory panel scale	european pressure ulcer advisory panel scale	European Pressure Ulcer Advisory Panel scale	1	kept
EUROPEAN RESUSCITATION	european resuscitation	european resuscitation	european resuscitation	EUROPEAN RESUSCITATION	1	kept
european society for clinical nutrition and metabolism criteria	european society for clinical nutrition and metabolism criteria	european society for clinical nutrition and metabolism criteria	european society for clinical nutrition and metabolism criteria	european society for clinical nutrition and metabolism criteria	1	kept

EUROPEAN-RESUSCITATION-COUNCIL	european resuscitation council	european resuscitation council	european resuscitation council	EUROPEAN-RESUSCITATION-COUNCIL	1	kept
EuroQOL Five Dimensions Questionnaire	euroqol five dimensions questionnaire	euroqol five dimensions questionnaire	euroqol five dimensions questionnaire	EuroQOL Five Dimensions Questionnaire	1	kept
Euthanasia, Active, Voluntary	euthanasia active voluntary	euthanasia active voluntary	euthanasia active voluntary	Euthanasia, Active, Voluntary	1	kept
Euthenasia	euthenasia	euthenasia	euthenasia	Euthenasia	1	kept
Euthyroid Sick Syndromes	euthyroid sick syndromes	euthyroid sick syndromes	euthyroid sick syndromes	Euthyroid Sick Syndromes	1	kept
eutrophia	eutrophia	eutrophia	eutrophia	eutrophia	1	kept
Evacuación aeromédica	evacuaci n aerom dica	evacuaci n aerom dica	evacuaci n aerom dica	Evacuación aeromédica	1	kept
Evacuation resources	evacuation resources	evacuation resources	evacuation resources	Evacuation resources	1	kept
EVALI	evali	evali	evali	EVALI	1	kept
Evaluación de resultado	evaluaci n de resultado	evaluaci n de resultado	evaluaci n de resultado	Evaluación de resultado	1	kept
Evaluación de resultados del paciente	evaluaci n de resultados del paciente	evaluaci n de resultados del paciente	evaluaci n de resultados del paciente	Evaluación de resultados del paciente	1	kept
Evaluación en enfermería	evaluaci n en enfermer a	evaluaci n en enfermer a	evaluaci n en enfermer a	Evaluación en enfermería	1	kept
Evaluación en salud	evaluaci n en salud	evaluaci n en salud	evaluaci n en salud	Evaluación en salud	1	kept
Evaluación resultados cuidados	evaluaci n resultados cuidados	evaluaci n resultados cuidados	evaluaci n resultados cuidados	Evaluación resultados cuidados	1	kept
EVALUATE	evaluate	evaluate	evaluate	EVALUATE	1	kept
EVALUATING COMPLEX INTERVENTIONS	evaluating complex interventions	evaluating complex interventions	evaluating complex interventions	EVALUATING COMPLEX INTERVENTIONS	1	kept

Evaluation feedback	evaluation feedback	evaluation feedback	evaluation feedback	Evaluation feedback	1	kept
Evaluation framework	evaluation framework	evaluation framework	evaluation framework	Evaluation framework	1	kept
evaluation instrument	evaluation instrument	evaluation instrument	evaluation instrument	evaluation instrument	1	kept
evaluation methodology	evaluation methodology	evaluation methodology	evaluation methodology	Evaluation methodology	1	case_or_format
Evaluation methods	evaluation methods	evaluation methods	evaluation methods	Evaluation methods	1	kept
evaluation of care	evaluation of care	evaluation of care	evaluation of care	evaluation of care	1	kept
Evaluation of care outcomes	evaluation of care outcomes	evaluation of care outcomes	evaluation of care outcomes	Evaluation of care outcomes	1	kept
Evaluation of issues	evaluation of issues	evaluation of issues	evaluation of issues	Evaluation of issues	1	kept
evaluation of issues	evaluation of issues	evaluation of issues	evaluation of issues	Evaluation of issues	1	case_or_format
Evaluation of neonatal death	evaluation of neonatal death	evaluation of neonatal death	evaluation of neonatal death	Evaluation of neonatal death	1	kept
evaluation of practice	evaluation of practice	evaluation of practice	evaluation of practice	evaluation of practice	1	kept
Evaluation of results of therapeutic interventions	evaluation of results of therapeutic interventions	evaluation of results of therapeutic interventions	evaluation of results of therapeutic interventions	Evaluation of results of therapeutic interventions	1	kept
Evaluation of the cerebral vessels	evaluation of the cerebral vessels	evaluation of the cerebral vessels	evaluation of the cerebral vessels	Evaluation of the cerebral vessels	1	kept
Evaluation of the processes and results in health care	evaluation of the processes and results in health care	evaluation of the processes and results in health care	evaluation of the processes and results in health care	Evaluation of the processes and results in health care	1	kept
EVALUATION PROTOCOL	evaluation protocol	evaluation protocol	evaluation protocol	EVALUATION PROTOCOL	1	kept
Evaluation studies	evaluation studies	evaluation studies	evaluation study	evaluation study	1	singular

Evaluation study	evaluation study	evaluation study	evaluation study	evaluation study	1	case_or_format
evaporation	evaporation	evaporation	evaporation	evaporation	1	kept
EVARUCI	evaruci	evaruci	evaruci	EVARUCI	1	kept
EVARUCI scale	evaruci scale	evaruci scale	evaruci scale	EVARUCI scale	1	kept
EVAT: Evacuating Scoring Tool	evat evacuating scoring tool	evat evacuating scoring tool	evat evacuating scoring tool	EVAT: Evacuating Scoring Tool	1	kept
EVD management	evd management	evd management	evd management	EVD management	1	kept
Eveningness	eveningness	eveningness	eveningness	Eveningness	1	kept
Event	event	event	event	EVENTS	1	case_or_format
event analysis	event analysis	event analysis	event analysis	event analysis	1	kept
Event based teaching programme	event based teaching programme	event based teaching programme	event based teaching programme	Event based teaching programme	1	kept
Event log	event log	event log	event log	Event log	1	kept
event related rumination inventory	event related rumination inventory	event related rumination inventory	event related rumination inventory	event related rumination inventory	1	kept
EVENT SCALE	event scale	event scale	event scale	EVENT SCALE	1	kept
Evento adverso	evento adverso	evento adverso	evento adverso	Evento adverso	1	kept
Eventos adversos	eventos adversos	eventos adversos	eventos adversos	Eventos adversos	1	kept
everolimus	everolimus	everolimus	everolimus	everolimus	1	kept

everyday ethics	everyday ethics	everyday ethics	everyday ethics	everyday ethics	1	kept
EVERYDAY LIFE	everyday life	everyday life	everyday life	EVERYDAY LIFE	1	kept
Evidence Base	evidence base	evidence base	evidence base	Evidence Base	1	kept
evidence based	evidence based	evidence based	evidence based	Evidence-based	1	case_or_format
Evidence Based Attitudes Attitude Questionnaire	evidence based attitudes attitude questionnaire	evidence based attitudes attitude questionnaire	evidence based attitudes attitude questionnaire	Evidence Based Attitudes Attitude Questionnaire	1	kept
evidence based emergency medicine	evidence based emergency medicine	evidence based emergency medicine	evidence based emergency medicine	evidence based emergency medicine	1	kept
evidence based healthcare	evidence based healthcare	evidence based healthcare	evidence based healthcare	evidence based healthcare	1	kept
evidence based interventions	evidence based interventions	evidence based interventions	evidence based intervention	evidence-based intervention	1	singular
Evidence Based Practice	evidence based practice	evidence based practice	evidence based practice	evidence based practice	1	case_or_format
Evidence Based Practice Attitudes Scale	evidence based practice attitudes scale	evidence based practice attitudes scale	evidence based practice attitudes scale	Evidence Based Practice Attitudes Scale	1	kept
Evidence map	evidence map	evidence map	evidence map	Evidence map	1	kept
Evidence mapping	evidence mapping	evidence mapping	evidence mapping	Evidence mapping	1	kept
Evidence synthesis	evidence synthesis	evidence synthesis	evidence synthesis	evidence synthesis	1	case_or_format
Evidence to decision	evidence to decision	evidence to decision	evidence to decision	Evidence to decision	1	kept
evidence-based continuous quality improvement	evidence based continuous quality improvement	evidence based continuous quality improvement	evidence based continuous quality improvement	evidence-based continuous quality improvement	1	kept
Evidence-Based Facility Design	evidence based facility design	evidence based facility design	evidence based facility design	Evidence-Based Facility Design	1	kept

evidence-based fever management	evidence based fever management	evidence based fever management	evidence based fever management	evidence-based fever management	1	kept
evidence-based framework	evidence based framework	evidence based framework	evidence based framework	evidence-based framework	1	kept
evidence-based health care	evidence based health care	evidence based health care	evidence based health care	evidence-based health care	1	kept
Evidence-based healthcare	evidence based healthcare	evidence based healthcare	evidence based healthcare	evidence based healthcare	1	case_or_format
evidence-based implementation	evidence based implementation	evidence based implementation	evidence based implementation	evidence-based implementation	1	kept
evidence-based intervention	evidence based intervention	evidence based intervention	evidence based intervention	evidence-based intervention	1	kept
Evidence-based interventions	evidence based interventions	evidence based interventions	evidence based intervention	evidence-based intervention	1	singular
Evidence-based management	evidence based management	evidence based management	evidence based management	evidence-based management	1	case_or_format
Evidence-based method	evidence based method	evidence based method	evidence based method	Evidence-based method	1	kept
Evidence-based perinatal care	evidence based perinatal care	evidence based perinatal care	evidence based perinatal care	Evidence-based perinatal care	1	kept
Evidence-based Practice	evidence based practice	evidence based practice	evidence based practice	evidence based practice	1	case_or_format
Evidence-based practice guidelines	evidence based practice guidelines	evidence based practice guidelines	evidence based practice guidelines	Evidence-based practice guidelines	1	kept
evidence-based practice strategies	evidence based practice strategies	evidence based practice strategies	evidence based practice strategies	evidence-based practice strategies	1	kept
Evidence-based practise	evidence based practise	evidence based practise	evidence based practise	Evidence-based practise	1	kept
Evidence-based prevention measures	evidence based prevention measures	evidence based prevention measures	evidence based prevention measures	Evidence-based prevention measures	1	kept
evidence-based professional practice	evidence based professional practice	evidence based professional practice	evidence based professional practice	evidence-based professional practice	1	kept

evidence-based quality improvements	evidence based quality improvements	evidence based quality improvements	evidence based quality improvement	evidence-based quality improvement	1	singular
Evidence-based recommendations	evidence based recommendations	evidence based recommendations	evidence based recommendations	Evidence-based recommendations	1	kept
evidence-generating practice	evidence generating practice	evidence generating practice	evidence generating practice	evidence-generating practice	1	kept
Evidenced-based practice	evidenced based practice	evidenced based practice	evidenced based practice	Evidenced-based practice	1	kept
evidenced-based practice	evidenced based practice	evidenced based practice	evidenced based practice	Evidenced-based practice	1	case_or_format
evidence - based	evidence based	evidence based	evidence based	Evidence-based	1	case_or_format
evidence - based practice implementation	evidence based practice implementation	evidence based practice implementation	evidence based practice implementation	evidence - based practice implementation	1	kept
evidence - based recommendations	evidence based recommendations	evidence based recommendations	evidence based recommendations	Evidence-based recommendations	1	case_or_format
evoked cortical response	evoked cortical response	evoked cortical response	evoked cortical response	evoked cortical response	1	kept
evoked muscle response	evoked muscle response	evoked muscle response	evoked muscle response	evoked muscle response	1	kept
evoked otoacoustic emission	evoked otoacoustic emission	evoked otoacoustic emission	evoked otoacoustic emission	evoked otoacoustic emission	1	kept
Evoked Potentials, Somatosensory	evoked potentials somatosensory	evoked potentials somatosensory	evoked potentials somatosensory	Evoked Potentials, Somatosensory	1	kept
Evoked Potentials, Visual	evoked potentials visual	evoked potentials visual	evoked potentials visual	Evoked Potentials, Visual	1	kept
evoked response	evoked response	evoked response	evoked response	evoked response	1	kept
Evolution path	evolution path	evolution path	evolution path	Evolution path	1	kept
evolutionary method	evolutionary method	evolutionary method	evolutionary method	evolutionary method	1	kept

EWS	ews	ews	ews	EWS	1	kept
EWS protocols	ews protocols	ews protocols	ews protocols	EWS protocols	1	kept
ex utero intrapartum treatment	ex utero intrapartum treatment	ex utero intrapartum treatment	ex utero intrapartum treatment	Ex Utero Intrapartum Treatment	1	case_or_format
exacerbation	exacerbation	exacerbation	exacerbation	exacerbation	1	kept
examination room	examination room	examination room	examination room	examination room	1	kept
Excessive fluid resuscitation	excessive fluid resuscitation	excessive fluid resuscitation	excessive fluid resuscitation	Excessive fluid resuscitation	1	kept
excessive hair growth	excessive hair growth	excessive hair growth	excessive hair growth	excessive hair growth	1	kept
excessive hours	excessive hours	excessive hours	excessive hours	excessive hours	1	kept
Excessive sleepiness	excessive sleepiness	excessive sleepiness	excessive sleepiness	Excessive sleepiness	1	kept
EXCHANGE	exchange	exchange	exchange	EXCHANGE	1	kept
excipial protect	excipial protect	excipial protect	excipial protect	excipial protect	1	kept
Excision	excision	excision	excision	excision	1	case_or_format
excitability	excitability	excitability	excitability	excitability	1	kept
excitation	excitation	excitation	excitation	excitation	1	kept
Excitatory Amino Acids	excitatory amino acids	excitatory amino acids	excitatory amino acids	Excitatory Amino Acids	1	kept
exclusion criteria	exclusion criteria	exclusion criteria	exclusion criteria	exclusion criteria	1	kept

exclusive activity	exclusive activity	exclusive activity	exclusive activity	exclusive activity	1	kept
EXERCISE	exercise	exercise	exercise	exercise	1	case_or_format
Exercise and Movement Techniques	exercise and movement techniques	exercise and movement techniques	exercise and movement techniques	Exercise and Movement Techniques	1	kept
exercise capacity	exercise capacity	exercise capacity	exercise capacity	Exercise capacity	1	case_or_format
exercise intervention	exercise intervention	exercise intervention	exercise intervention	exercise intervention	1	kept
exercise movement techniques	exercise movement techniques	exercise movement techniques	exercise movement techniques	exercise movement techniques	1	kept
exercise physiologist	exercise physiologist	exercise physiologist	exercise physiologist	exercise physiologist	1	kept
Exercise rehabilitation	exercise rehabilitation	exercise rehabilitation	exercise rehabilitation	Exercise rehabilitation	1	kept
Exercise Tolerance	exercise tolerance	exercise tolerance	exercise tolerance	exercise tolerance	1	case_or_format
Exercise training	exercise training	exercise training	exercise training	Exercise training	1	kept
exercise training	exercise training	exercise training	exercise training	Exercise training	1	case_or_format
Exertional heat stroke	exertional heat stroke	exertional heat stroke	exertional heat stroke	Exertional heat stroke	1	kept
exertional heat stroke	exertional heat stroke	exertional heat stroke	exertional heat stroke	Exertional heat stroke	1	case_or_format
exertional hypoxia	exertional hypoxia	exertional hypoxia	exertional hypoxia	exertional hypoxia	1	kept
exhalation	exhalation	exhalation	exhalation	Exhalation	1	case_or_format
Exhaled air ventilation	exhaled air ventilation	exhaled air ventilation	exhaled air ventilation	Exhaled air ventilation	1	kept

Exhaled breath condensate	exhaled breath condensate	exhaled breath condensate	exhaled breath condensate	Exhaled breath condensate	1	kept
Existence	existence	existence	existence	Existence	1	kept
Existential	existential	existential	existential	Existential	1	kept
Existential and meaning of life issues	existential and meaning of life issues	existential and meaning of life issues	existential and meaning of life issues	Existential and meaning of life issues	1	kept
existential anxiety	existential anxiety	existential anxiety	existential anxiety	existential anxiety	1	kept
EXISTENTIAL CARE	existential care	existential care	existential care	EXISTENTIAL CARE	1	kept
existential health	existential health	existential health	existential health	existential health	1	kept
existential orientation	existential orientation	existential orientation	existential orientation	existential orientation	1	kept
Existential suffering	existential suffering	existential suffering	existential suffering	Existential suffering	1	kept
existentialism	existentialism	existentialism	existentialism	Existentialism	1	case_or_format
existentialist	existentialist	existentialist	existentialist	existentialist	1	kept
Exit procedure	exit procedure	exit procedure	exit procedure	Exit procedure	1	kept
Exocrine Pancreatic Insufficiency	exocrine pancreatic insufficiency	exocrine pancreatic insufficiency	exocrine pancreatic insufficiency	Exocrine Pancreatic Insufficiency	1	kept
exogenous surfactant	exogenous surfactant	exogenous surfactant	exogenous surfactant	exogenous surfactant	1	kept
Exogenous VAP	exogenous vap	exogenous vap	exogenous vap	Exogenous VAP	1	kept
exome	exome	exome	exome	exome	1	kept

exon skipping	exon skipping	exon skipping	exon skipping	exon skipping	1	kept
Exophthalmos	exophthalmos	exophthalmos	exophthalmos	exophthalmos	1	case_or_format
Exosome	exosome	exosome	exosome	Exosome	1	kept
exosome	exosome	exosome	exosome	Exosome	1	case_or_format
exotic animal	exotic animal	exotic animal	exotic animal	exotic animal	1	kept
Exotic animal neurology	exotic animal neurology	exotic animal neurology	exotic animal neurology	Exotic animal neurology	1	kept
Expanded Disability Status Scale	expanded disability status scale	expanded disability status scale	expanded disability status scale	Expanded Disability Status Scale	1	kept
Expansive learning theory	expansive learning theory	expansive learning theory	expansive learning theory	Expansive learning theory	1	kept
expectant mother	expectant mother	expectant mother	expectant mother	expectant mother	1	kept
Expectation of families	expectation of families	expectation of families	expectation of families	Expectation of families	1	kept
expectation-maximization algorithm	expectation maximization algorithm	expectation maximization algorithm	expectation maximization algorithm	expectation-maximization algorithm	1	kept
Expected risk	expected risk	expected risk	expected risk	Expected risk	1	kept
expectorant agent	expectorant agent	expectorant agent	expectorant agent	expectorant agent	1	kept
Expenditure	expenditure	expenditure	expenditure	Expenditure	1	kept
expenditure	expenditure	expenditure	expenditure	Expenditure	1	case_or_format
Expenses	expenses	expenses	expenses	Expenses	1	kept

Experience Based Codesign	experience based codesign	experience based codesign	experience based codesign	Experience Based Codesign	1	kept
Experience design	experience design	experience design	experience design	Experience design	1	kept
experience design	experience design	experience design	experience design	Experience design	1	case_or_format
Experience of care	experience of care	experience of care	experience of care	Experience of care	1	kept
Experience of health care treatments	experience of health care treatments	experience of health care treatments	experience of health care treatments	Experience of health care treatments	1	kept
Experience of mothers	experience of mothers	experience of mothers	experience of mothers	Experience of mothers	1	kept
experience-based co-design	experience based co design	experience based co design	experience based co design	experience-based co-design	1	kept
Experience-complexity gap	experience complexity gap	experience complexity gap	experience complexity gap	Experience-complexity gap	1	kept
experience-expectant neural plasticity	experience expectant neural plasticity	experience expectant neural plasticity	experience expectant neural plasticity	experience-expectant neural plasticity	1	kept
experience-sampling method	experience sampling method	experience sampling method	experience sampling method	experience sampling method	1	case_or_format
experiences and perceptions	experiences and perceptions	experiences and perceptions	experiences and perceptions	experiences and perceptions	1	kept
experiences and support	experiences and support	experiences and support	experiences and support	experiences and support	1	kept
Experiences of care	experiences of care	experiences of care	experiences of care	Experiences of care	1	kept
Experiences of parents	experiences of parents	experiences of parents	experiences of parents	Experiences of parents	1	kept
Experiencias	experiencias	experiencias	experiencias	Experiencias	1	kept
Experiential design	experiential design	experiential design	experiential design	Experiential design	1	kept

Experiential learning preferences	experiential learning preferences	experiential learning preferences	experiential learning preferences	Experiential learning preferences	1	kept
EXPERIENTIAL QUALITY	experiential quality	experiential quality	experiential quality	EXPERIENTIAL QUALITY	1	kept
Experiment study	experiment study	experiment study	experiment study	Experiment study	1	kept
experimental animal	experimental animal	experimental animal	experimental animal	experimental animal	1	kept
Experimental groups	experimental groups	experimental groups	experimental groups	Experimental groups	1	kept
experimental hypothermia	experimental hypothermia	experimental hypothermia	experimental hypothermia	experimental hypothermia	1	kept
experimental pig	experimental pig	experimental pig	experimental pig	experimental pig	1	kept
Experimental study	experimental study	experimental study	experimental study	experimental study	1	case_or_format
Experiments	experiments	experiments	experiment	experiment	1	singular
Expert	expert	expert	expert	EXPERT	1	case_or_format
EXPERT CONSENSUS STATEMENT	expert consensus statement	expert consensus statement	expert consensus statement	EXPERT CONSENSUS STATEMENT	1	kept
Expert interviews	expert interviews	expert interviews	expert interviews	Expert interviews	1	kept
Expert involvement	expert involvement	expert involvement	expert involvement	Expert involvement	1	kept
expert involvement	expert involvement	expert involvement	expert involvement	Expert involvement	1	case_or_format
Expert opinion	expert opinion	expert opinion	expert opinion	Expert opinion	1	kept
Expert panel	expert panel	expert panel	expert panel	Expert panel	1	kept

Expert Performance Approach	expert performance approach	expert performance approach	expert performance approach	Expert Performance Approach	1	kept
Expert performance approach	expert performance approach	expert performance approach	expert performance approach	Expert Performance Approach	1	case_or_format
Expert testimony	expert testimony	expert testimony	expert testimony	Expert Testimony	1	case_or_format
Expertise development	expertise development	expertise development	expertise development	Expertise development	1	kept
Experts	experts	experts	expert	EXPERT	1	singular
expiratory chest wall vibration	expiratory chest wall vibration	expiratory chest wall vibration	expiratory chest wall vibration	expiratory chest wall vibration	1	kept
expiratory flow	expiratory flow	expiratory flow	expiratory flow	expiratory flow	1	kept
expiratory flow rate	expiratory flow rate	expiratory flow rate	expiratory flow rate	expiratory flow rate	1	kept
expiratory grunting	expiratory grunting	expiratory grunting	expiratory grunting	expiratory grunting	1	kept
Expiratory ribcage compression	expiratory ribcage compression	expiratory ribcage compression	expiratory ribcage compression	Expiratory ribcage compression	1	kept
expired minute ventilation monitoring	expired minute ventilation monitoring	expired minute ventilation monitoring	expired minute ventilation monitoring	expired minute ventilation monitoring	1	kept
explainable	explainable	explainable	explainable	explainable	1	kept
Explainable AI	explainable ai	explainable ai	explainable ai	Explainable AI	1	kept
explainable AI	explainable ai	explainable ai	explainable ai	Explainable AI	1	case_or_format
explanatory factor	explanatory factor	explanatory factor	explanatory factor	explanatory factor	1	kept
Explant	explant	explant	explant	Explant	1	kept

explant	explant	explant	explant	Explant	1	case_or_format
exploration mixed-methods	exploration mixed methods	exploration mixed methods	exploration mixed methods	exploration mixed-methods	1	kept
explorative study	explorative study	explorative study	explorative study	explorative study	1	kept
Exploratory	exploratory	exploratory	exploratory	Exploratory	1	kept
EXPLORATORY ANALYSIS	exploratory analysis	exploratory analysis	exploratory analysis	EXPLORATORY ANALYSIS	1	kept
exploratory and validatory factor analyses	exploratory and validatory factor analyses	exploratory and validatory factor analyses	exploratory and validatory factor analyses	exploratory and validatory factor analyses	1	kept
Exploratory Behavior	exploratory behavior	exploratory behavior	exploratory behavior	exploratory behavior	1	case_or_format
Exploratory descriptive study	exploratory descriptive study	exploratory descriptive study	exploratory descriptive study	Exploratory descriptive study	1	kept
EXPLORATORY FACTOR-ANALYSIS	exploratory factor analysis	exploratory factor analysis	exploratory factor analysis	exploratory factor analysis	1	case_or_format
exploratory laparotomy	exploratory laparotomy	exploratory laparotomy	exploratory laparotomy	exploratory laparotomy	1	kept
Exploratory Sequential Analysis	exploratory sequential analysis	exploratory sequential analysis	exploratory sequential analysis	Exploratory Sequential Analysis	1	kept
Explosive and blast incidents	explosive and blast incidents	explosive and blast incidents	explosive and blast incidents	Explosive and blast incidents	1	kept
Explosive devices	explosive devices	explosive devices	explosive devices	Explosive devices	1	kept
Exponential random graph	exponential random graph	exponential random graph	exponential random graph	Exponential random graph	1	kept
exponential random graph	exponential random graph	exponential random graph	exponential random graph	Exponential random graph	1	case_or_format
Exposición profesional	exposici n profesional	exposici n profesional	exposici n profesional	Exposición profesional	1	kept

Exposure	exposure	exposure	exposure	exposure	1	case_or_format
EXPOSURE KERATOPATHY	exposure keratopathy	exposure keratopathy	exposure keratopathy	Exposure keratopathy	1	case_or_format
Exposure level	exposure level	exposure level	exposure level	Exposure level	1	kept
exposure prevention information network	exposure prevention information network	exposure prevention information network	exposure prevention information network	exposure prevention information network	1	kept
exposure risk	exposure risk	exposure risk	exposure risk	exposure risk	1	kept
expressed emotion	expressed emotion	expressed emotion	expressed emotion	Expressed Emotion	1	case_or_format
Expression recognition	expression recognition	expression recognition	expression recognition	Expression recognition	1	kept
expressions of gratitude	expressions of gratitude	expressions of gratitude	expressions of gratitude	expressions of gratitude	1	kept
Expressive	expressive	expressive	expressive	Expressive	1	kept
Expressive touch	expressive touch	expressive touch	expressive touch	Expressive touch	1	kept
expressive writing	expressive writing	expressive writing	expressive writing	expressive writing	1	kept
exsanguination	exsanguination	exsanguination	exsanguination	exsanguination	1	kept
extended care	extended care	extended care	extended care	extended care	1	kept
Extended care benefit	extended care benefit	extended care benefit	extended care benefit	Extended care benefit	1	kept
Extended daily dialysis	extended daily dialysis	extended daily dialysis	extended daily dialysis	extended daily dialysis	1	case_or_format
Extended days	extended days	extended days	extended days	Extended days	1	kept

Extended entity relationship diagram	extended entity relationship diagram	extended entity relationship diagram	extended entity relationship diagram	Extended entity relationship diagram	1	kept
Extended prone positioning	extended prone positioning	extended prone positioning	extended prone positioning	Extended prone positioning	1	kept
Extended role	extended role	extended role	extended role	Extended role	1	kept
extended spectrum beta lactamase producing Escherichia coli	extended spectrum beta lactamase producing escherichia coli	extended spectrum beta lactamase producing escherichia coli	extended spectrum beta lactamase producing escherichia coli	extended spectrum beta lactamase producing Escherichia coli	1	kept
extended spectrum beta lactamase producing Gram negative	extended spectrum beta lactamase producing gram negative	extended spectrum beta lactamase producing gram negative	extended spectrum beta lactamase producing gram negative	extended spectrum beta lactamase producing Gram negative	1	kept
extended spectrum beta-lactamase	extended spectrum beta lactamase	extended spectrum beta lactamase	extended spectrum beta lactamase	extended spectrum beta lactamase	1	case_or_format
Extended spectrum β-lactamase	extended spectrum lactamase	extended spectrum lactamase	extended spectrum lactamase	Extended spectrum β-lactamase	1	kept
extended work hours	extended work hours	extended work hours	extended work hours	extended work hours	1	kept
EXTENDED-INFUSION	extended infusion	extended infusion	extended infusion	EXTENDED-INFUSION	1	kept
extended-infusion	extended infusion	extended infusion	extended infusion	EXTENDED-INFUSION	1	case_or_format
Extended-spectrum	extended spectrum	extended spectrum	extended spectrum	Extended-spectrum	1	kept
Extended-spectrum ß-lactamase producing enterobacteriaceae	extended spectrum lactamase producing enterobacteriaceae	extended spectrum lactamase producing enterobacteriaceae	extended spectrum lactamase producing enterobacteriaceae	Extended-spectrum ß-lactamase producing enterobacteriaceae	1	kept
extended-spectrum β-lactamase	extended spectrum lactamase	extended spectrum lactamase	extended spectrum lactamase	Extended spectrum β-lactamase	1	case_or_format
Extended-spectrum β-lactamases	extended spectrum lactamases	extended spectrum lactamases	extended spectrum lactamase	Extended spectrum β-lactamase	1	singular
EXTENDERS	extenders	extenders	extenders	EXTENDERS	1	kept
extending tubing	extending tubing	extending tubing	extending tubing	extending tubing	1	kept

Extension tube	extension tube	extension tube	extension tube	Extension tube	1	kept
Extensive drug resistance	extensive drug resistance	extensive drug resistance	extensive drug resistance	Extensive drug resistance	1	kept
extensively-drug resistant	extensively drug resistant	extensively drug resistant	extensively drug resistant	extensively-drug resistant	1	kept
external auditory canal	external auditory canal	external auditory canal	external auditory canal	external auditory canal	1	kept
external beam radiotherapy	external beam radiotherapy	external beam radiotherapy	external beam radiotherapy	external beam radiotherapy	1	kept
external capsule	external capsule	external capsule	external capsule	external capsule	1	kept
External cardiac compression	external cardiac compression	external cardiac compression	external cardiac compression	External cardiac compression	1	kept
External Causes	external causes	external causes	external causes	External Causes	1	kept
external cephalic version	external cephalic version	external cephalic version	external cephalic version	external cephalic version	1	kept
external defibrillation	external defibrillation	external defibrillation	external defibrillation	external defibrillation	1	kept
EXTERNAL DEFIBRILLATOR	external defibrillator	external defibrillator	external defibrillator	EXTERNAL DEFIBRILLATOR	1	kept
external fixation	external fixation	external fixation	external fixation	external fixation	1	kept
external limiting membrane	external limiting membrane	external limiting membrane	external limiting membrane	external limiting membrane	1	kept
external lumbar drain	external lumbar drain	external lumbar drain	external lumbar drain	external lumbar drain	1	kept
External memory aids	external memory aids	external memory aids	external memory aids	External memory aids	1	kept
external oblique intercostal block	external oblique intercostal block	external oblique intercostal block	external oblique intercostal block	external oblique intercostal block	1	kept

external otitis	external otitis	external otitis	external otitis	external otitis	1	kept
external quality assessment	external quality assessment	external quality assessment	external quality assessment	external quality assessment	1	kept
External sensors	external sensors	external sensors	external sensors	External sensors	1	kept
External stressors	external stressors	external stressors	external stressors	External stressors	1	kept
External validation	external validation	external validation	external validation	External validation	1	kept
external validation	external validation	external validation	external validation	External validation	1	case_or_format
external whistleblowing	external whistleblowing	external whistleblowing	external whistleblowing	external whistleblowing	1	kept
Extra corporeal membrane oxygenation	extra corporeal membrane oxygenation	extra corporeal membrane oxygenation	extra corporeal membrane oxygenation	extra corporeal membrane oxygenation	1	case_or_format
Extra uterine growth	extra uterine growth	extra uterine growth	extra uterine growth	Extra uterine growth	1	kept
extra-clinical ventilation	extra clinical ventilation	extra clinical ventilation	extra clinical ventilation	extra-clinical ventilation	1	kept
extra-corporeal life support	extra corporeal life support	extra corporeal life support	extra corporeal life support	extra-corporeal life support	1	kept
Extra-uterine environment	extra uterine environment	extra uterine environment	extra uterine environment	Extra-uterine environment	1	kept
extracellular	extracellular	extracellular	extracellular	extracellular	1	kept
Extracellular Fluid	extracellular fluid	extracellular fluid	extracellular fluid	extracellular fluid	1	case_or_format
Extracellular fluid	extracellular fluid	extracellular fluid	extracellular fluid	extracellular fluid	1	case_or_format
EXTRACELLULAR SLIME SUBSTANCE	extracellular slime substance	extracellular slime substance	extracellular slime substance	EXTRACELLULAR SLIME SUBSTANCE	1	kept

Extracellular vesicle	extracellular vesicle	extracellular vesicle	extracellular vesicle	extracellular vesicle	1	case_or_format
Extracellular Vesicles	extracellular vesicles	extracellular vesicles	extracellular vesicle	extracellular vesicle	1	singular
Extracorporal membrane oxygenation	extracorporal membrane oxygenation	extracorporal membrane oxygenation	extracorporal membrane oxygenation	Extracorporal membrane oxygenation	1	kept
Extracorporeal	extracorporeal	extracorporeal	extracorporeal	Extracorporeal	1	kept
extracorporeal blood purification	extracorporeal blood purification	extracorporeal blood purification	extracorporeal blood purification	extracorporeal blood purification	1	kept
Extracorporeal blood purification techniques	extracorporeal blood purification techniques	extracorporeal blood purification techniques	extracorporeal blood purification techniques	Extracorporeal blood purification techniques	1	kept
extracorporeal carbon dioxide removal	extracorporeal carbon dioxide removal	extracorporeal carbon dioxide removal	extracorporeal carbon dioxide removal	extracorporeal carbon dioxide removal	1	kept
Extracorporeal cardio-pulmonary resuscitation	extracorporeal cardio pulmonary resuscitation	extracorporeal cardio pulmonary resuscitation	extracorporeal cardio pulmonary resuscitation	Extracorporeal cardio-pulmonary resuscitation	1	kept
Extracorporeal CO<sub>2<	extracorporeal co sub 2	extracorporeal co sub 2	extracorporeal co sub 2	Extracorporeal CO<sub>2<	1	kept
extracorporeal devices	extracorporeal devices	extracorporeal devices	extracorporeal devices	extracorporeal devices	1	kept
extracorporeal hemoadsorption	extracorporeal hemoadsorption	extracorporeal hemoadsorption	extracorporeal hemoadsorption	extracorporeal hemoadsorption	1	kept
Extracorporeal Life Support	extracorporeal life support	extracorporeal life support	extracorporeal life support	Extracorporeal life support	1	case_or_format
extracorporeal life support implantation	extracorporeal life support implantation	extracorporeal life support implantation	extracorporeal life support implantation	extracorporeal life support implantation	1	kept
Extracorporeal Life Support Organization	extracorporeal life support organization	extracorporeal life support organization	extracorporeal life support organization	Extracorporeal Life Support Organization	1	kept
Extracorporeal liver support	extracorporeal liver support	extracorporeal liver support	extracorporeal liver support	Extracorporeal liver support	1	kept
Extracorporeal membrane oxygenation at noncardiac surgery	extracorporeal membrane oxygenation at noncardiac surgery	extracorporeal membrane oxygenation at noncardiac surgery	extracorporeal membrane oxygenation at noncardiac surgery	Extracorporeal membrane oxygenation at noncardiac surgery	1	kept

extracorporeal<br>membrane oxygenation<br>at noncardiac surgery	extracorporeal<br>membrane oxygenation<br>at noncardiac surgery	extracorporeal<br>membrane oxygenation<br>at noncardiac surgery	extracorporeal<br>membrane oxygenation<br>at noncardiac surgery	Extracorporeal<br>membrane oxygenation<br>at noncardiac surgery	1	case_or_format
extracorporeal<br>membrane oxygenation<br>cardiopulmonary	extracorporeal<br>membrane oxygenation<br>cardiopulmonary	extracorporeal<br>membrane oxygenation<br>cardiopulmonary	extracorporeal<br>membrane oxygenation<br>cardiopulmonary	extracorporeal<br>membrane oxygenation<br>cardiopulmonary	1	kept
Extracorporeal<br>membrane oxygenation<br>care	extracorporeal<br>membrane oxygenation<br>care	extracorporeal<br>membrane oxygenation<br>care	extracorporeal<br>membrane oxygenation<br>care	Extracorporeal<br>membrane oxygenation<br>care	1	kept
Extracorporeal<br>membrane oxygenation<br>complications	extracorporeal<br>membrane oxygenation<br>complications	extracorporeal<br>membrane oxygenation<br>complications	extracorporeal<br>membrane oxygenation<br>complications	Extracorporeal<br>membrane oxygenation<br>complications	1	kept
extracorporeal<br>membrane oxygenation<br>education	extracorporeal<br>membrane oxygenation<br>education	extracorporeal<br>membrane oxygenation<br>education	extracorporeal<br>membrane oxygenation<br>education	extracorporeal<br>membrane oxygenation<br>education	1	kept
Extracorporeal<br>membrane oxygenation<br>program	extracorporeal<br>membrane oxygenation<br>program	extracorporeal<br>membrane oxygenation<br>program	extracorporeal<br>membrane oxygenation<br>program	Extracorporeal<br>membrane oxygenation<br>program	1	kept
extracorporeal<br>membrane oxygenation<br>program	extracorporeal<br>membrane oxygenation<br>program	extracorporeal<br>membrane oxygenation<br>program	extracorporeal<br>membrane oxygenation<br>program	Extracorporeal<br>membrane oxygenation<br>program	1	case_or_format
extracorporeal<br>membrane oxygenation<br>simulation	extracorporeal<br>membrane oxygenation<br>simulation	extracorporeal<br>membrane oxygenation<br>simulation	extracorporeal<br>membrane oxygenation<br>simulation	extracorporeal<br>membrane oxygenation<br>simulation	1	kept
Extracorporeal<br>membrane oxygenation<br>specialist	extracorporeal<br>membrane oxygenation<br>specialist	extracorporeal<br>membrane oxygenation<br>specialist	extracorporeal<br>membrane oxygenation<br>specialist	Extracorporeal<br>membrane oxygenation<br>specialist	1	kept
extracorporeal<br>membrane oxygenation<br>specialist	extracorporeal<br>membrane oxygenation<br>specialist	extracorporeal<br>membrane oxygenation<br>specialist	extracorporeal<br>membrane oxygenation<br>specialist	Extracorporeal<br>membrane oxygenation<br>specialist	1	case_or_format
Extracorporeal<br>membrane oxygenation<br>transport	extracorporeal<br>membrane oxygenation<br>transport	extracorporeal<br>membrane oxygenation<br>transport	extracorporeal<br>membrane oxygenation<br>transport	Extracorporeal<br>membrane oxygenation<br>transport	1	kept
extracorporeal<br>membrane oxygenation<br>transport	extracorporeal<br>membrane oxygenation<br>transport	extracorporeal<br>membrane oxygenation<br>transport	extracorporeal<br>membrane oxygenation<br>transport	Extracorporeal<br>membrane oxygenation<br>transport	1	case_or_format
Extracorporeal<br>membrane oxygenation<br>without cardiothoracic	extracorporeal<br>membrane oxygenation<br>without cardiothoracic	extracorporeal<br>membrane oxygenation<br>without cardiothoracic	extracorporeal<br>membrane oxygenation<br>without cardiothoracic	Extracorporeal<br>membrane oxygenation<br>without cardiothoracic	1	kept
extracorporeal<br>membrane oxygenation<br>without cardiothoracic	extracorporeal<br>membrane oxygenation<br>without cardiothoracic	extracorporeal<br>membrane oxygenation<br>without cardiothoracic	extracorporeal<br>membrane oxygenation<br>without cardiothoracic	Extracorporeal<br>membrane oxygenation<br>without cardiothoracic	1	case_or_format
EXTRACORPOREAL<br>MEMBRANE-OXYGENATION	extracorporeal<br>membrane oxygenation	extracorporeal<br>membrane oxygenation	extracorporeal<br>membrane oxygenation	Extracorporeal<br>Membrane Oxygenation	1	case_or_format
Extracorporeal<br>membranous oxygenation	extracorporeal<br>membranous oxygenation	extracorporeal<br>membranous oxygenation	extracorporeal<br>membranous oxygenation	Extracorporeal<br>membranous oxygenation	1	kept

extracorporeal membranous oxygenation	extracorporeal membranous oxygenation	extracorporeal membranous oxygenation	extracorporeal membranous oxygenation	Extracorporeal membranous oxygenation	1	case_or_format
extracorporeal oxygenation device	extracorporeal oxygenation device	extracorporeal oxygenation device	extracorporeal oxygenation device	extracorporeal oxygenation device	1	kept
extracorporeal photopheresis	extracorporeal photopheresis	extracorporeal photopheresis	extracorporeal photopheresis	extracorporeal photopheresis	1	kept
Extracorporeal resuscitation	extracorporeal resuscitation	extracorporeal resuscitation	extracorporeal resuscitation	Extracorporeal resuscitation	1	kept
extracorporeal shock wave lithotripsy	extracorporeal shock wave lithotripsy	extracorporeal shock wave lithotripsy	extracorporeal shock wave lithotripsy	extracorporeal shock wave lithotripsy	1	kept
extracorporeal ultrafiltration	extracorporeal ultrafiltration	extracorporeal ultrafiltration	extracorporeal ultrafiltration	extracorporeal ultrafiltration	1	kept
extracranial event	extracranial event	extracranial event	extracranial event	extracranial event	1	kept
extracranial hemorrhage	extracranial hemorrhage	extracranial hemorrhage	extracranial hemorrhage	extracranial hemorrhage	1	kept
Extract informations	extract informations	extract informations	extract informations	Extract informations	1	kept
Extracting features	extracting features	extracting features	extracting features	Extracting features	1	kept
Extraction	extraction	extraction	extraction	extraction	1	case_or_format
extrahepatic bile duct obstruction	extrahepatic bile duct obstruction	extrahepatic bile duct obstruction	extrahepatic bile duct obstruction	extrahepatic bile duct obstruction	1	kept
extrarenal malignant rhabdoid tumor	extrarenal malignant rhabdoid tumor	extrarenal malignant rhabdoid tumor	extrarenal malignant rhabdoid tumor	extrarenal malignant rhabdoid tumor	1	kept
extrauterine life	extrauterine life	extrauterine life	extrauterine life	extrauterine life	1	kept
Extravasación de Materiales Terapé uticos y Diagnósticos	extravasaci n de materiales terap uticos y diagn sticos	extravasaci n de materiales terap uticos y diagn sticos	extravasaci n de materiales terap uticos y diagn sticos	Extravasación de Materiales Terapé uticos y Diagnósticos	1	kept
Extravasamento de Materiais Terapêuticos e Diagnósticos	extravasamento de materiais terap uticos e diagn sticos	extravasamento de materiais terap uticos e diagn sticos	extravasamento de materiais terap uticos e diagn sticos	Extravasamento de Materiais Terapêuticos e Diagnósticos	1	kept

EXTRAVASATION	extravasation	extravasation	extravasation	extravasation	1	case_or_format
extravasation injury	extravasation injury	extravasation injury	extravasation injury	extravasation injury	1	kept
Extravascular Lung Water	extravascular lung water	extravascular lung water	extravascular lung water	Extravascular Lung Water	1	kept
extravascular lung water	extravascular lung water	extravascular lung water	extravascular lung water	Extravascular Lung Water	1	case_or_format
extreme environment	extreme environment	extreme environment	extreme environment	extreme environment	1	kept
extreme event	extreme event	extreme event	extreme event	extreme event	1	kept
extreme heat	extreme heat	extreme heat	extreme heat	extreme heat	1	kept
Extreme heat events	extreme heat events	extreme heat events	extreme heat events	Extreme heat events	1	kept
extreme hot weather	extreme hot weather	extreme hot weather	extreme hot weather	extreme hot weather	1	kept
Extreme preterm	extreme preterm	extreme preterm	extreme preterm	Extreme preterm	1	kept
Extreme situations	extreme situations	extreme situations	extreme situations	Extreme situations	1	kept
extreme weight	extreme weight	extreme weight	extreme weight	extreme weight	1	kept
Extremely	extremely	extremely	extremely	Extremely	1	kept
Extremely low birth weight infants	extremely low birth weight infants	extremely low birth weight infants	extremely low birth weight infant	extremely low birth-weight infant	1	singular
Extremely low birth-weight infant	extremely low birth weight infant	extremely low birth weight infant	extremely low birth weight infant	extremely low birth-weight infant	1	case_or_format
extremely low birthweight infant	extremely low birthweight infant	extremely low birthweight infant	extremely low birthweight infant	extremely low birthweight infant	1	kept

Extremely low body weight	extremely low body weight	extremely low body weight	extremely low body weight	Extremely low body weight	1	kept
Extremely low frequencies	extremely low frequencies	extremely low frequencies	extremely low frequencies	Extremely low frequencies	1	kept
Extremely low frequency magnetic fields	extremely low frequency magnetic fields	extremely low frequency magnetic fields	extremely low frequency magnetic fields	Extremely low frequency magnetic fields	1	kept
extremely low gestational age neonate	extremely low gestational age neonate	extremely low gestational age neonate	extremely low gestational age neonate	extremely low gestational age neonate	1	kept
Extremely premature babies	extremely premature babies	extremely premature babies	extremely premature babies	Extremely premature babies	1	kept
Extremely premature infant	extremely premature infant	extremely premature infant	extremely premature infant	extremely premature infants	1	case_or_format
Extremely premature infants	extremely premature infants	extremely premature infants	extremely premature infant	extremely premature infants	1	singular
Extremely premature newborn	extremely premature newborn	extremely premature newborn	extremely premature newborn	Extremely premature newborn	1	kept
Extremely preterm	extremely preterm	extremely preterm	extremely preterm	Extremely preterm	1	kept
extremely preterm	extremely preterm	extremely preterm	extremely preterm	Extremely preterm	1	case_or_format
EXTREMELY PRETERM BIRTH	extremely preterm birth	extremely preterm birth	extremely preterm birth	EXTREMELY PRETERM BIRTH	1	kept
EXTREMELY PRETERM INFANTS	extremely preterm infants	extremely preterm infants	extremely preterm infant	Extremely preterm infants	1	singular
Extremely severe burn	extremely severe burn	extremely severe burn	extremely severe burn	Extremely severe burn	1	kept
Extremity Injury	extremity injury	extremity injury	extremity injury	Extremity Injury	1	kept
Extrinsic contamination	extrinsic contamination	extrinsic contamination	extrinsic contamination	Extrinsic contamination	1	kept
extrinsic motivation	extrinsic motivation	extrinsic motivation	extrinsic motivation	extrinsic motivation	1	kept

extrinsic thromboelastometry	extrinsic thromboelastometry	extrinsic thromboelastometry	extrinsic thromboelastometry	extrinsic thromboelastometry	1	kept
extrême prématurité	extr me pr maturit	extr me pr maturit	extr me pr maturit	extrême prématurité	1	kept
Extubación Traqueal	extubaci n traqueal	extubaci n traqueal	extubaci n traqueal	Extubación Traqueal	1	kept
extubating	extubating	extubating	extubating	extubating	1	kept
Extubation readiness	extubation readiness	extubation readiness	extubation readiness	Extubation readiness	1	kept
extubation readiness parameter	extubation readiness parameter	extubation readiness parameter	extubation readiness parameter	extubation readiness parameter	1	kept
extubation time	extubation time	extubation time	extubation time	extubation time	1	kept
Extubação	extuba o	extuba o	extuba o	Extubação	1	kept
Exudates and Transudates	exudates and transudates	exudates and transudates	exudates and transudates	Exudates and Transudates	1	kept
exudative phase	exudative phase	exudative phase	exudative phase	exudative phase	1	kept
Eye Abnormalities	eye abnormalities	eye abnormalities	eye abnormalities	Eye Abnormalities	1	kept
Eye banking	eye banking	eye banking	eye banking	Eye banking	1	kept
eye banking	eye banking	eye banking	eye banking	Eye banking	1	case_or_format
eye burn	eye burn	eye burn	eye burn	eye burn	1	kept
eye care bundle	eye care bundle	eye care bundle	eye care bundle	eye care bundle	1	kept
Eye care practice	eye care practice	eye care practice	eye care practice	Eye care practice	1	kept

eye care professional	eye care professional	eye care professional	eye care professional	eye care professional	1	kept
eye complication	eye complication	eye complication	eye complication	eye complication	1	kept
eye complications	eye complications	eye complications	eye complication	eye complication	1	singular
Eye donation	eye donation	eye donation	eye donation	Eye donation	1	kept
Eye dryness	eye dryness	eye dryness	eye dryness	Eye dryness	1	kept
eye fixation	eye fixation	eye fixation	eye fixation	eye fixation	1	kept
Eye Infections	eye infections	eye infections	eye infection	eye infection	1	singular
Eye injury	eye injury	eye injury	eye injury	Eye Injuries	1	case_or_format
eye irritation	eye irritation	eye irritation	eye irritation	eye irritation	1	kept
eye malformation	eye malformation	eye malformation	eye malformation	eye malformation	1	kept
Eye Mask	eye mask	eye mask	eye mask	EYE MASKS	1	case_or_format
eye movements	eye movements	eye movements	eye movement	Eye Movements	1	singular
Eye neoplasms	eye neoplasms	eye neoplasms	eye neoplasms	Eye neoplasms	1	kept
Eye patch	eye patch	eye patch	eye patch	Eye patch	1	kept
Eye protection	eye protection	eye protection	eye protection	eye protection	1	case_or_format
Eye tracking technology	eye tracking technology	eye tracking technology	eye tracking technology	Eye-Tracking Technology	1	case_or_format

eye tracking technology	eye tracking technology	eye tracking technology	eye tracking technology	Eye-Tracking Technology	1	case_or_format
eye tumor	eye tumor	eye tumor	eye tumor	eye tumor	1	kept
Eye, Artificial	eye artificial	eye artificial	eye artificial	Eye, Artificial	1	kept
Eye-masks	eye masks	eye masks	eye mask	EYE MASKS	1	singular
EYE-TRACKING	eye tracking	eye tracking	eye tracking	eye tracking	1	case_or_format
Eye-tracking	eye tracking	eye tracking	eye tracking	eye tracking	1	case_or_format
Eye-tracking technology	eye tracking technology	eye tracking technology	eye tracking technology	Eye-Tracking Technology	1	case_or_format
eyelid	eyelid	eyelid	eyelid	eyelid	1	kept
eyelid closure	eyelid closure	eyelid closure	eyelid closure	eyelid closure	1	kept
eyelid reconstruction	eyelid reconstruction	eyelid reconstruction	eyelid reconstruction	eyelid reconstruction	1	kept
eyewear	eyewear	eyewear	eyewear	eyewear	1	kept
Eysenck Personality Questionnaire	eysenck personality questionnaire	eysenck personality questionnaire	eysenck personality questionnaire	Eysenck Personality Questionnaire	1	kept
e－Delphi technique	e delphi technique	e delphi technique	e delphi technique	e-Delphi technique	1	case_or_format
E－learning	e learning	e learning	e learning	e-learning	1	case_or_format
e－learning	e learning	e learning	e learning	e-learning	1	case_or_format
F1 scores	f1 scores	f1 scores	f1 scores	F1 scores	1	kept

fabric glove	fabric glove	fabric glove	fabric glove	fabric glove	1	kept
Fabric materials	fabric materials	fabric materials	fabric materials	Fabric materials	1	kept
Fabry disease	fabry disease	fabry disease	fabry disease	Fabry disease	1	kept
FACE	face	face	face	face	1	case_or_format
face asymmetry	face asymmetry	face asymmetry	face asymmetry	face asymmetry	1	kept
face burn	face burn	face burn	face burn	face burn	1	kept
face detection	face detection	face detection	face detection	face detection	1	kept
face erythema	face erythema	face erythema	face erythema	face erythema	1	kept
face interview	face interview	face interview	face interview	face interview	1	kept
face mask ventilation	face mask ventilation	face mask ventilation	face mask ventilation	face mask ventilation	1	kept
Face monitoring	face monitoring	face monitoring	face monitoring	Face monitoring	1	kept
face monitoring	face monitoring	face monitoring	face monitoring	Face monitoring	1	case_or_format
face muscle	face muscle	face muscle	face muscle	face muscle	1	kept
face profile	face profile	face profile	face profile	face profile	1	kept
Face protective gear	face protective gear	face protective gear	face protective gear	Face protective gear	1	kept
Face scanning	face scanning	face scanning	face scanning	Face scanning	1	kept

Face shield	face shield	face shield	face shield	Face shield	1	kept
Face to face	face to face	face to face	face to face	Face to face	1	kept
face to face counseling	face to face counseling	face to face counseling	face to face counseling	face to face counseling	1	kept
face to face interview	face to face interview	face to face interview	face to face interview	face-to-face interview	1	case_or_format
face to face training	face to face training	face to face training	face to face training	face to face training	1	kept
face ulcer	face ulcer	face ulcer	face ulcer	face ulcer	1	kept
face validation	face validation	face validation	face validation	face validation	1	kept
face washing	face washing	face washing	face washing	face washing	1	kept
Face-to-face conversation	face to face conversation	face to face conversation	face to face conversation	Face-to-face conversation	1	kept
Face-to-face counselling	face to face counselling	face to face counselling	face to face counselling	Face-to-face counselling	1	kept
facebook	facebook	facebook	facebook	facebook	1	kept
Facemask ventilation	facemask ventilation	facemask ventilation	facemask ventilation	Facemask ventilation	1	kept
Faces Anxiety Scale	faces anxiety scale	faces anxiety scale	faces anxiety scale	Faces Anxiety Scale	1	kept
Faces detection	faces detection	faces detection	faces detection	Faces detection	1	kept
Faces pain scale	faces pain scale	faces pain scale	faces pain scale	Faces Pain Scale	1	case_or_format
Facial burns	facial burns	facial burns	facial burns	Facial burns	1	kept

Facial Dermatoses	facial dermatoses	facial dermatoses	facial dermatoses	Facial Dermatoses	1	kept
Facial expression	facial expression	facial expression	facial expression	facial expression	1	case_or_format
Facial expressions	facial expressions	facial expressions	facial expression	facial expression	1	singular
Facial feature	facial feature	facial feature	facial feature	Facial feature	1	kept
facial fractures	facial fractures	facial fractures	facial fractures	facial fractures	1	kept
Facial injuries	facial injuries	facial injuries	facial injuries	Facial Injuries	1	case_or_format
Facial Nerve	facial nerve	facial nerve	facial nerve	Facial Nerve	1	kept
facial occlusion	facial occlusion	facial occlusion	facial occlusion	facial occlusion	1	kept
Facial occlusions	facial occlusions	facial occlusions	facial occlusion	facial occlusion	1	singular
Facial Pain	facial pain	facial pain	facial pain	Facial Pain	1	kept
facial plastic & reconstructive surgery	facial plastic and reconstructive surgery	facial plastic and reconstructive surgery	facial plastic and reconstructive surgery	facial plastic & reconstructive surgery	1	kept
Facial port-wine stains	facial port wine stains	facial port wine stains	facial port wine stains	Facial port-wine stains	1	kept
facial response	facial response	facial response	facial response	facial response	1	kept
Facial scars	facial scars	facial scars	facial scars	Facial scars	1	kept
facial skin symptoms	facial skin symptoms	facial skin symptoms	facial skin symptoms	facial skin symptoms	1	kept
facial swelling	facial swelling	facial swelling	facial swelling	facial swelling	1	kept

Facial video analyse	facial video analyse	facial video analyse	facial video analyse	Facial video analyse	1	kept
Facial Video Analysis	facial video analysis	facial video analysis	facial video analysis	Facial Video Analysis	1	kept
facial video analysis	facial video analysis	facial video analysis	facial video analysis	Facial Video Analysis	1	case_or_format
Facies	facies	facies	facies	facies	1	case_or_format
Facilators	facilators	facilators	facilators	Facilators	1	kept
Facilitated goals of care discussions	facilitated goals of care discussions	facilitated goals of care discussions	facilitated goals of care discussions	Facilitated goals of care discussions	1	kept
facilitated tucking by parents	facilitated tucking by parents	facilitated tucking by parents	facilitated tucking by parents	Facilitated tucking by parents	1	case_or_format
FACILITATED TUCKING POSITION	facilitated tucking position	facilitated tucking position	facilitated tucking position	FACILITATED TUCKING POSITION	1	kept
Facilitated tucking position	facilitated tucking position	facilitated tucking position	facilitated tucking position	FACILITATED TUCKING POSITION	1	case_or_format
Facilitating behaviors	facilitating behaviors	facilitating behaviors	facilitating behaviors	Facilitating behaviors	1	kept
Facilitating conditions	facilitating conditions	facilitating conditions	facilitating conditions	Facilitating conditions	1	kept
facilitating connection	facilitating connection	facilitating connection	facilitating connection	facilitating connection	1	kept
Facilitating factors and barriers	facilitating factors and barriers	facilitating factors and barriers	facilitating factors and barriers	Facilitating factors and barriers	1	kept
FACILITATION	facilitation	facilitation	facilitation	facilitation	1	case_or_format
Facilitators and barriers	facilitators and barriers	facilitators and barriers	facilitators and barriers	facilitators and barriers	1	case_or_format
Facilitators of EBP	facilitators of ebp	facilitators of ebp	facilitators of ebp	Facilitators of EBP	1	kept

facilitators of EBP	facilitators of ebp	facilitators of ebp	facilitators of ebp	Facilitators of EBP	1	case_or_format
facilitators of palliative care	facilitators of palliative care	facilitators of palliative care	facilitators of palliative care	facilitators of palliative care	1	kept
Facilities	facilities	facilities	facility	FACILITIES	1	singular
Facilities and services utilization	facilities and services utilization	facilities and services utilization	facilities and services utilization	Facilities and Services Utilization	1	case_or_format
Facilities Management	facilities management	facilities management	facilities management	Facilities Management	1	kept
FACILITY	facility	facility	facility	FACILITIES	1	case_or_format
facility	facility	facility	facility	FACILITIES	1	case_or_format
facility based death review committee	facility based death review committee	facility based death review committee	facility based death review committee	facility based death review committee	1	kept
Facility design	facility design	facility design	facility design	Facility design	1	kept
facility type	facility type	facility type	facility type	facility type	1	kept
Facings	facings	facings	facings	Facings	1	kept
Facteurs humains	facteurs humains	facteurs humains	facteurs humains	Facteurs humains	1	kept
Factitious disease	factitious disease	factitious disease	factitious disease	factitious disease	1	case_or_format
Factitious Disorders	factitious disorders	factitious disorders	factitious disorders	Factitious Disorders	1	kept
Factitious hypoglycemia	factitious hypoglycemia	factitious hypoglycemia	factitious hypoglycemia	Factitious hypoglycemia	1	kept
Factor	factor	factor	factor	Factors	1	case_or_format

factor analyses	factor analyses	factor analyses	factor analysis	factor analysis	1	singular
Factor analysis, statistical	factor analysis statistical	factor analysis statistical	factor analysis statistical	Factor Analysis, Statistical	1	case_or_format
factor analysis, statistical	factor analysis statistical	factor analysis statistical	factor analysis statistical	Factor Analysis, Statistical	1	case_or_format
Factor measurement	factor measurement	factor measurement	factor measurement	Factor measurement	1	kept
Factor VII	factor vii	factor vii	factor vii	Factor VII	1	kept
Factor VIIa	factor viia	factor viia	factor viia	Factor VIIa	1	kept
factor Xa	factor xa	factor xa	factor xa	Factor Xa	1	case_or_format
Factor Xa inhibitor	factor xa inhibitor	factor xa inhibitor	factor xa inhibitor	Factor Xa Inhibitors	1	case_or_format
Factor Xa inhibitors	factor xa inhibitors	factor xa inhibitors	factor xa inhibitor	Factor Xa Inhibitors	1	singular
factor Xa inhibitors	factor xa inhibitors	factor xa inhibitors	factor xa inhibitor	Factor Xa Inhibitors	1	singular
Factorial analysis	factorial analysis	factorial analysis	factorial analysis	factorial analysis	1	case_or_format
Factorial survey	factorial survey	factorial survey	factorial survey	Factorial survey	1	kept
factors affecting intention	factors affecting intention	factors affecting intention	factors affecting intention	factors affecting intention	1	kept
factors influence	factors influence	factors influence	factors influence	factors influence	1	kept
factory analysis	factory analysis	factory analysis	factory analysis	factory analysis	1	kept
factual	factual	factual	factual	factual	1	kept

Factual database	factual database	factual database	factual database	factual database	1	case_or_format
factual memory	factual memory	factual memory	factual memory	factual memory	1	kept
FACULTY	faculty	faculty	faculty	Faculty	1	case_or_format
faculty	faculty	faculty	faculty	Faculty	1	case_or_format
faculty development	faculty development	faculty development	faculty development	faculty development	1	kept
Faculty staffing	faculty staffing	faculty staffing	faculty staffing	Faculty staffing	1	kept
Faecal collection devices	faecal collection devices	faecal collection devices	faecal collection devices	Faecal collection devices	1	kept
faecal diversion	faecal diversion	faecal diversion	faecal diversion	faecal diversion	1	kept
Faecal incontinence with diarrhoea	faecal incontinence with diarrhoea	faecal incontinence with diarrhoea	faecal incontinence with diarrhoea	Faecal incontinence with diarrhoea	1	kept
Faeces	faeces	faeces	faeces	Faeces	1	kept
Fagerstrom Test for Nicotine Dependence	fagerstrom test for nicotine dependence	fagerstrom test for nicotine dependence	fagerstrom test for nicotine dependence	Fagerstrom Test for Nicotine Dependence	1	kept
Failed extubation	failed extubation	failed extubation	failed extubation	Failed extubation	1	kept
failed TOLAC	failed tolac	failed tolac	failed tolac	failed TOLAC	1	kept
failure	failure	failure	failure	FAILURE	1	case_or_format
Failure Mode and Effects Analysis	failure mode and effects analysis	failure mode and effects analysis	failure mode and effects analysis	failure mode and effects analysis	1	case_or_format
Failure mode effects analysis	failure mode effects analysis	failure mode effects analysis	failure mode effects analysis	Failure mode effects analysis	1	kept

failure modes and effects analysis	failure modes and effects analysis	failure modes and effects analysis	failure modes and effects analysis	failure modes and effects analysis	1	kept
Failure of extubation	failure of extubation	failure of extubation	failure of extubation	Failure of extubation	1	kept
failure of extubation	failure of extubation	failure of extubation	failure of extubation	Failure of extubation	1	case_or_format
failure to monitor	failure to monitor	failure to monitor	failure to monitor	failure to monitor	1	kept
Failure to rescue quality	failure to rescue quality	failure to rescue quality	failure to rescue quality	Failure to rescue quality	1	kept
failure to rescue rate	failure to rescue rate	failure to rescue rate	failure to rescue rate	failure to rescue rate	1	kept
failure to wean	failure to wean	failure to wean	failure to wean	failure to wean	1	kept
fairness	fairness	fairness	fairness	fairness	1	kept
faith-based practices	faith based practices	faith based practices	faith based practices	faith-based practices	1	kept
falciform ligament	falciform ligament	falciform ligament	falciform ligament	falciform ligament	1	kept
falcine and tentorial subdural hematoma	falcine and tentorial subdural hematoma	falcine and tentorial subdural hematoma	falcine and tentorial subdural hematoma	falcine and tentorial subdural hematoma	1	kept
Fall accident	fall accident	fall accident	fall accident	Fall accident	1	kept
fall assessment tools	fall assessment tools	fall assessment tools	fall assessment tools	fall assessment tools	1	kept
FALL DETECTION SYSTEM	fall detection system	fall detection system	fall detection system	FALL DETECTION SYSTEM	1	kept
fall from bed	fall from bed	fall from bed	fall from bed	fall from bed	1	kept
fall prevention	fall prevention	fall prevention	fall prevention	fall prevention	1	kept

fall reduction	fall reduction	fall reduction	fall reduction	fall reduction	1	kept
Fall risk assessment	fall risk assessment	fall risk assessment	fall risk assessment	fall risk assessment	1	case_or_format
Fall-related major injury	fall related major injury	fall related major injury	fall related major injury	Fall-related major injury	1	kept
Falling coconuts	falling coconuts	falling coconuts	falling coconuts	Falling coconuts	1	kept
falls injury	falls injury	falls injury	falls injury	falls injury	1	kept
False alarm detection	false alarm detection	false alarm detection	false alarm detection	False alarm detection	1	kept
false alarm rate	false alarm rate	false alarm rate	false alarm rate	false alarm rate	1	kept
FALSE DISCOVERY RATE	false discovery rate	false discovery rate	false discovery rate	FALSE DISCOVERY RATE	1	kept
False news	false news	false news	false news	False news	1	kept
False positive blood cultures	false positive blood cultures	false positive blood cultures	false positive blood cultures	False positive blood cultures	1	kept
False positive cultures	false positive cultures	false positive cultures	false positive cultures	False positive cultures	1	kept
False-negative	false negative	false negative	false negative	False-negative	1	kept
Falta de pessoal	falta de pessoal	falta de pessoal	falta de pessoal	Falta de pessoal	1	kept
familial amyloid polyneuropathy	familial amyloid polyneuropathy	familial amyloid polyneuropathy	familial amyloid polyneuropathy	familial amyloid polyneuropathy	1	kept
Familial gastrointestinal stromal tumor	familial gastrointestinal stromal tumor	familial gastrointestinal stromal tumor	familial gastrointestinal stromal tumor	Familial gastrointestinal stromal tumor	1	kept
Familienzentrierte Pflege	familienzentrierte pflege	familienzentrierte pflege	familienzentrierte pflege	Familienzentrierte Pflege	1	kept

Families during illness	families during illness	families during illness	families during illness	Families during illness	1	kept
families with dependent children	families with dependent children	families with dependent children	families with dependent children	families with dependent children	1	kept
Family Accomodation Scale for Obsessive Compulsive Disorder	family accomodation scale for obsessive compulsive disorder	family accomodation scale for obsessive compulsive disorder	family accomodation scale for obsessive compulsive disorder	Family Accomodation Scale for Obsessive Compulsive Disorder	1	kept
family activated medical emergency team	family activated medical emergency team	family activated medical emergency team	family activated medical emergency team	family activated medical emergency team	1	kept
family advisory council	family advisory council	family advisory council	family advisory council	family advisory council	1	kept
Family agreement	family agreement	family agreement	family agreement	Family agreement	1	kept
Family and work balance	family and work balance	family and work balance	family and work balance	Family and work balance	1	kept
Family area	family area	family area	family area	Family area	1	kept
Family Assessment Device 12 Item Version	family assessment device 12 item version	family assessment device 12 item version	family assessment device 12 item version	Family Assessment Device 12 Item Version	1	kept
Family assistance	family assistance	family assistance	family assistance	Family assistance	1	kept
Family attitude of health personnel	family attitude of health personnel	family attitude of health personnel	family attitude of health personnel	Family attitude of health personnel	1	kept
FAMILY ATTITUDE SCALE	family attitude scale	family attitude scale	family attitude scale	FAMILY ATTITUDE SCALE	1	kept
FAMILY BOOKLET	family booklet	family booklet	family booklet	FAMILY BOOKLET	1	kept
family burden	family burden	family burden	family burden	family burden	1	kept
family care conference	family care conference	family care conference	family care conference	family care conference	1	kept
Family care giver	family care giver	family care giver	family care giver	Family care giver	1	kept

Family Caregiver	family caregiver	family caregiver	family caregiver	Family caregivers	1	case_or_format
Family carers	family carers	family carers	family carers	Family carers	1	kept
family centered	family centered	family centered	family centered	Family-centered	1	case_or_format
family centered developmental care	family centered developmental care	family centered developmental care	family centered developmental care	family centered developmental care	1	kept
Family centered rounds	family centered rounds	family centered rounds	family centered rounds	family-centered rounds	1	case_or_format
family centered-care	family centered care	family centered care	family centered care	family centered care	1	case_or_format
family centered-rounds	family centered rounds	family centered rounds	family centered rounds	family-centered rounds	1	case_or_format
Family Centred Care	family centred care	family centred care	family centred care	Family-centred care	1	case_or_format
family centred care	family centred care	family centred care	family centred care	Family-centred care	1	case_or_format
Family centred neonatal care	family centred neonatal care	family centred neonatal care	family centred neonatal care	Family centred neonatal care	1	kept
Family Centred-Care	family centred care	family centred care	family centred care	Family-centred care	1	case_or_format
Family characteristics	family characteristics	family characteristics	family characteristics	Family Characteristics	1	case_or_format
family clinic and municipality intervention	family clinic and municipality intervention	family clinic and municipality intervention	family clinic and municipality intervention	family clinic and municipality intervention	1	kept
Family communication	family communication	family communication	family communication	family communication	1	case_or_format
family concerns	family concerns	family concerns	family concerns	family concerns	1	kept
FAMILY CONFERENCES	family conferences	family conferences	family conference	family conference	1	singular

Family conferences	family conferences	family conferences	family conference	family conference	1	singular
Family conversations	family conversations	family conversations	family conversations	Family conversations	1	kept
Family decision	family decision	family decision	family decision	Family decision	1	kept
family dynamics	family dynamics	family dynamics	family dynamics	family dynamics	1	kept
Family empowerment care	family empowerment care	family empowerment care	family empowerment care	Family empowerment care	1	kept
Family Engagement	family engagement	family engagement	family engagement	family engagement	1	case_or_format
Family environment	family environment	family environment	family environment	Family environment	1	kept
Family experiences	family experiences	family experiences	family experience	Family experience	1	singular
Family facilitator during resuscitation	family facilitator during resuscitation	family facilitator during resuscitation	family facilitator during resuscitation	Family facilitator during resuscitation	1	kept
family factor	family factor	family factor	family factor	family factor	1	kept
family flyer	family flyer	family flyer	family flyer	family flyer	1	kept
family focused	family focused	family focused	family focused	family focused	1	kept
FAMILY FUNCTION	family function	family function	family function	Family function	1	case_or_format
Family functions	family functions	family functions	family function	Family function	1	singular
Family grieving	family grieving	family grieving	family grieving	Family grieving	1	kept
Family guidance	family guidance	family guidance	family guidance	Family guidance	1	kept

family guidance	family guidance	family guidance	family guidance	Family guidance	1	case_or_format
Family Health Conversation	family health conversation	family health conversation	family health conversation	Family Health Conversation	1	kept
family health conversations	family health conversations	family health conversations	family health conversation	Family Health Conversation	1	singular
Family HELP	family help	family help	family help	Family HELP	1	kept
family HELP	family help	family help	family help	Family HELP	1	case_or_format
Family impact	family impact	family impact	family impact	Family impact	1	kept
family Integrated Care	family integrated care	family integrated care	family integrated care	Family integrated care	1	case_or_format
Family integrity	family integrity	family integrity	family integrity	Family integrity	1	kept
Family management	family management	family management	family management	family management	1	case_or_format
Family management style	family management style	family management style	family management style	Family management style	1	kept
Family Medicine	family medicine	family medicine	family medicine	family medicine	1	case_or_format
Family membe	family membe	family membe	family membe	Family membe	1	kept
family member experience	family member experience	family member experience	family member experience	family member experience	1	kept
Family member involvement	family member involvement	family member involvement	family member involvement	Family member involvement	1	kept
family member needs	family member needs	family member needs	family member needs	family member needs	1	kept
Family member satisfaction	family member satisfaction	family member satisfaction	family member satisfaction	Family member satisfaction	1	kept

FAMILY MEMBERS	family members	family members	family member	Family members	1	singular
Family members´ satisfaction in the end-of-life care	family members satisfaction in the end of life care	family members satisfaction in the end of life care	family members satisfaction in the end of life care	Family members´ satisfaction in the end-of-life care	1	kept
Family members´ empowerment and satisfaction	family members empowerment and satisfaction	family members empowerment and satisfaction	family members empowerment and satisfaction	Family members´ empowerment and satisfaction	1	kept
family members' perception	family members perception	family members perception	family members perception	family members' perception	1	kept
Family Needs	family needs	family needs	family need	Family needs	1	singular
Family needs met	family needs met	family needs met	family needs met	Family needs met	1	kept
family network	family network	family network	family network	family network	1	kept
family nurture intervention	family nurture intervention	family nurture intervention	family nurture intervention	family nurture intervention	1	kept
Family outcomes	family outcomes	family outcomes	family outcomes	Family outcomes	1	kept
FAMILY PARTICIPATION	family participation	family participation	family participation	family participation	1	case_or_format
family participation in care	family participation in care	family participation in care	family participation in care	family participation in care	1	kept
Family participatory care	family participatory care	family participatory care	family participatory care	Family participatory care	1	kept
family participatory care	family participatory care	family participatory care	family participatory care	Family participatory care	1	case_or_format
Family participatory care, child rights, newborn-parent unity	family participatory care child rights newborn parent unity	family participatory care child rights newborn parent unity	family participatory care child rights newborn parent unity	Family participatory care, child rights, newborn-parent unity	1	kept
family perceptions	family perceptions	family perceptions	family perceptions	family perceptions	1	kept
Family Planning Policy	family planning policy	family planning policy	family planning policy	Family Planning Policy	1	kept

Family practice	family practice	family practice	family practice	Family Practice	1	case_or_format
Family Presence During Resuscitation	family presence during resuscitation	family presence during resuscitation	family presence during resuscitation	Family presence during resuscitation	1	case_or_format
Family Presence during Resuscitation	family presence during resuscitation	family presence during resuscitation	family presence during resuscitation	Family presence during resuscitation	1	case_or_format
family presence during resuscitation	family presence during resuscitation	family presence during resuscitation	family presence during resuscitation	Family presence during resuscitation	1	case_or_format
family questionnaire	family questionnaire	family questionnaire	family questionnaire	family questionnaire	1	kept
FAMILY REFUSAL	family refusal	family refusal	family refusal	FAMILY REFUSAL	1	kept
Family refusal	family refusal	family refusal	family refusal	FAMILY REFUSAL	1	case_or_format
family relations	family relations	family relations	family relation	Family Relations	1	singular
Family relationship	family relationship	family relationship	family relationship	Family relationship	1	kept
Family relationships	family relationships	family relationships	family relationship	Family relationship	1	singular
family relationships	family relationships	family relationships	family relationship	Family relationship	1	singular
family relocation stress	family relocation stress	family relocation stress	family relocation stress	family relocation stress	1	kept
Family Research	family research	family research	family research	Family Research	1	kept
family research	family research	family research	family research	Family Research	1	case_or_format
Family resiliency	family resiliency	family resiliency	family resiliency	Family resiliency	1	kept
Family roles	family roles	family roles	family roles	Family roles	1	kept

family satisfaction survey	family satisfaction survey	family satisfaction survey	family satisfaction survey	family satisfaction survey	1	kept
Family Separation	family separation	family separation	family separation	family separation	1	case_or_format
family stress thermometer	family stress thermometer	family stress thermometer	family stress thermometer	family stress thermometer	1	kept
Family support intervention	family support intervention	family support intervention	family support intervention	Family support intervention	1	kept
family support person	family support person	family support person	family support person	family support person	1	kept
family support programme	family support programme	family support programme	family support programme	family support programme	1	kept
family systems theory	family systems theory	family systems theory	family systems theory	family systems theory	1	kept
family theory	family theory	family theory	family theory	family theory	1	kept
Family traduction	family traduction	family traduction	family traduction	Family traduction	1	kept
Family understanding	family understanding	family understanding	family understanding	Family understanding	1	kept
Family unit	family unit	family unit	family unit	Family unit	1	kept
family veto	family veto	family veto	family veto	family veto	1	kept
Family voice	family voice	family voice	family voice	Family voice	1	kept
family witnessed resuscitation	family witnessed resuscitation	family witnessed resuscitation	family witnessed resuscitation	Family-witnessed resuscitation	1	case_or_format
family worry	family worry	family worry	family worry	family worry	1	kept
Family-centered approach	family centered approach	family centered approach	family centered approach	Family-centered approach	1	kept

family-centered approach	family centered approach	family centered approach	family centered approach	Family-centered approach	1	case_or_format
family-centered care practices	family centered care practices	family centered care practices	family centered care practices	family-centered care practices	1	kept
family-centered care theory	family centered care theory	family centered care theory	family centered care theory	family-centered care theory	1	kept
Family-centered-care	family centered care	family centered care	family centered care	family centered care	1	case_or_format
Family-centric	family centric	family centric	family centric	Family-centric	1	kept
FAMILY-INTEGRATED CARE	family integrated care	family integrated care	family integrated care	Family integrated care	1	case_or_format
Family-integrated-care	family integrated care	family integrated care	family integrated care	Family integrated care	1	case_or_format
Family-led interventions	family led interventions	family led interventions	family led interventions	Family-led interventions	1	kept
family-led interventions	family led interventions	family led interventions	family led interventions	Family-led interventions	1	case_or_format
FAMILY-MEMBERS EXPERIENCES	family members experiences	family members experiences	family members experiences	FAMILY-MEMBERS EXPERIENCES	1	kept
Family-physician relationship	family physician relationship	family physician relationship	family physician relationship	Family-physician relationship	1	kept
family-professional relationship	family professional relationship	family professional relationship	family professional relationship	family-professional relationship	1	kept
Family-relatives	family relatives	family relatives	family relatives	Family-relatives	1	kept
Familycentered	familycentered	familycentered	familycentered	Familycentered	1	kept
family‐led interventions	family led interventions	family led interventions	family led interventions	Family-led interventions	1	case_or_format
FAMIREA	famirea	famirea	famirea	FAMIREA	1	kept

Famous Persons	famous persons	famous persons	famous persons	Famous Persons	1	kept
Família	fam lia	fam lia	fam lia	Família	1	kept
Fantasies	fantasies	fantasies	fantasy	fantasy	1	singular
Fantasme	fantasme	fantasme	fantasme	Fantasme	1	kept
Fantasy	fantasy	fantasy	fantasy	fantasy	1	case_or_format
Fantoni translaryngeal method	fantoni translaryngeal method	fantoni translaryngeal method	fantoni translaryngeal method	Fantoni translaryngeal method	1	kept
Fantoni translaryngeal tracheostomy	fantoni translaryngeal tracheostomy	fantoni translaryngeal tracheostomy	fantoni translaryngeal tracheostomy	Fantoni translaryngeal tracheostomy	1	kept
FAQs = frequently asked questions	faqs frequently asked questions	faqs frequently asked questions	faqs frequently asked questions	FAQs = frequently asked questions	1	kept
Farmacia clínica	farmacia cl nica	farmacia cl nica	farmacia cl nica	Farmacia clínica	1	kept
Farmacia hospitalaria	farmacia hospitalaria	farmacia hospitalaria	farmacia hospitalaria	Farmacia hospitalaria	1	kept
Farmacéutico clínico	farmac utico cl nico	farmac utico cl nico	farmac utico cl nico	Farmacéutico clínico	1	kept
farmers attitude	farmers attitude	farmers attitude	farmers attitude	farmers attitude	1	kept
FAS ligand	fas ligand	fas ligand	fas ligand	FAS ligand	1	kept
Fas Ligand Protein	fas ligand protein	fas ligand protein	fas ligand protein	Fas Ligand Protein	1	kept
Fascia	fascia	fascia	fascia	Fascia	1	kept
fascia	fascia	fascia	fascia	Fascia	1	case_or_format

fascia iliaca block	fascia iliaca block	fascia iliaca block	fascia iliaca block	fascia iliaca block	1	kept
fasciitis	fasciitis	fasciitis	fasciitis	fasciitis	1	kept
Fasciocutaneous flap transplantation	fasciocutaneous flap transplantation	fasciocutaneous flap transplantation	fasciocutaneous flap transplantation	Fasciocutaneous flap transplantation	1	kept
Fasciotomy	fasciotomy	fasciotomy	fasciotomy	fasciotomy	1	case_or_format
fast track	fast track	fast track	fast track	Fast-track	1	case_or_format
fast track cardiac care	fast track cardiac care	fast track cardiac care	fast track cardiac care	fast track cardiac care	1	kept
Fast Track Extubation	fast track extubation	fast track extubation	fast track extubation	Fast Track Extubation	1	kept
fast tracking	fast tracking	fast tracking	fast tracking	fast-tracking	1	case_or_format
fast-track	fast track	fast track	fast track	Fast-track	1	case_or_format
Fast-track cardiac anesthesia	fast track cardiac anesthesia	fast track cardiac anesthesia	fast track cardiac anesthesia	Fast-track cardiac anesthesia	1	kept
Fast-track cardiac surgery	fast track cardiac surgery	fast track cardiac surgery	fast track cardiac surgery	Fast-track cardiac surgery	1	kept
fast-track surgery	fast track surgery	fast track surgery	fast track surgery	Fast track surgery	1	case_or_format
fast-track surgery and hepatopancreatobiliary surgery	fast track surgery and hepatopancreatobiliary surgery	fast track surgery and hepatopancreatobiliary surgery	fast track surgery and hepatopancreatobiliary surgery	fast-track surgery and hepatopancreatobiliary surgery	1	kept
FASTHUG	fasthug	fasthug	fasthug	FASTHUG	1	kept
fasting plasma glucose	fasting plasma glucose	fasting plasma glucose	fasting plasma glucose	fasting plasma glucose	1	kept
fasudil	fasudil	fasudil	fasudil	fasudil	1	kept

fat embolism	fat embolism	fat embolism	fat embolism	fat embolism	1	kept
fat emulsion	fat emulsion	fat emulsion	fat emulsion	fat emulsion	1	kept
fat emulsion, intravenous	fat emulsion intravenous	fat emulsion intravenous	fat emulsion intravenous	fat emulsion, intravenous	1	kept
fat-overload	fat overload	fat overload	fat overload	fat-overload	1	kept
fatal bleeding	fatal bleeding	fatal bleeding	fatal bleeding	fatal bleeding	1	kept
fatal grape aspiration	fatal grape aspiration	fatal grape aspiration	fatal grape aspiration	fatal grape aspiration	1	kept
Fatal intoxication	fatal intoxication	fatal intoxication	fatal intoxication	Fatal intoxication	1	kept
Fatal outcome	fatal outcome	fatal outcome	fatal outcome	Fatal Outcome	1	case_or_format
FATCOD	fatcod	fatcod	fatcod	FATCOD	1	kept
father and infant	father and infant	father and infant	father and infant	father and infant	1	kept
father involvement questionnaire	father involvement questionnaire	father involvement questionnaire	father involvement questionnaire	father involvement questionnaire	1	kept
Father's potential	father s potential	father s potential	father s potential	Father's potential	1	kept
Father-Child relations	father child relations	father child relations	father child relation	Father-Child Relations	1	singular
Father-child relationship	father child relationship	father child relationship	father child relationship	Father-child relationship	1	kept
Fatherhood	fatherhood	fatherhood	fatherhood	Fatherhood	1	kept
fatherhood	fatherhood	fatherhood	fatherhood	Fatherhood	1	case_or_format

fathers of premature infants	fathers of premature infants	fathers of premature infants	fathers of premature infants	fathers of premature infants	1	kept
Fathers' experiences	fathers experiences	fathers experiences	fathers experiences	Fathers' experiences	1	kept
fathers' involvement	fathers involvement	fathers involvement	fathers involvement	fathers' involvement	1	kept
Fathers' experiences	fathers experiences	fathers experiences	fathers experiences	Fathers' experiences	1	case_or_format
Fatiga	fatiga	fatiga	fatiga	Fatiga	1	kept
Fatiga de alarma	fatiga de alarma	fatiga de alarma	fatiga de alarma	Fatiga de alarma	1	kept
Fatiga por compasión	fatiga por compasi n	fatiga por compasi n	fatiga por compasi n	Fatiga por compasión	1	kept
fatigue alarm	fatigue alarm	fatigue alarm	fatigue alarm	fatigue alarm	1	kept
fatigue assessment	fatigue assessment	fatigue assessment	fatigue assessment	fatigue assessment	1	kept
fatigue impairment	fatigue impairment	fatigue impairment	fatigue impairment	fatigue impairment	1	kept
fatigue level	fatigue level	fatigue level	fatigue level	fatigue level	1	kept
Fatigue syndrome, chronic	fatigue syndrome chronic	fatigue syndrome chronic	fatigue syndrome chronic	Fatigue Syndrome, Chronic	1	case_or_format
fatty acid blood level	fatty acid blood level	fatty acid blood level	fatty acid blood level	fatty acid blood level	1	kept
Fatty Acids	fatty acids	fatty acids	fatty acid	fatty acid	1	singular
Fatty Acids, Omega-3	fatty acids omega 3	fatty acids omega 3	fatty acids omega 3	Fatty Acids, Omega-3	1	kept
Fatty liver disease	fatty liver disease	fatty liver disease	fatty liver disease	Fatty liver disease	1	kept

fatty liver of pregnancy	fatty liver of pregnancy	fatty liver of pregnancy	fatty liver of pregnancy	fatty liver of pregnancy	1	kept
faucet handle	faucet handle	faucet handle	faucet handle	faucet handle	1	kept
FCC guidelines	fcc guidelines	fcc guidelines	fcc guidelines	FCC guidelines	1	kept
FCCS course	fccs course	fccs course	fccs course	FCCS course	1	kept
FCCS, Family-Centered Care Scale	fccs family centered care scale	fccs family centered care scale	fccs family centered care scale	FCCS, Family-Centered Care Scale	1	kept
FDA	fda	fda	fda	FDA	1	kept
FE3+	fe3	fe3	fe3	FE3+	1	kept
Fear avoidance	fear avoidance	fear avoidance	fear avoidance	Fear avoidance	1	kept
fear conditioning	fear conditioning	fear conditioning	fear conditioning	fear conditioning	1	kept
fear of covid 19 scale	fear of covid 19 scale	fear of covid 19 scale	fear of covid 19 scale	fear of COVID-19 scale	1	case_or_format
fear of COVID-19	fear of covid 19	fear of covid 19	fear of covid 19	fear of COVID-19	1	kept
Fear of death	fear of death	fear of death	fear of death	fear of death	1	case_or_format
fear of dementia	fear of dementia	fear of dementia	fear of dementia	fear of dementia	1	kept
Fear of disease progression	fear of disease progression	fear of disease progression	fear of disease progression	Fear of disease progression	1	kept
fear of missing out	fear of missing out	fear of missing out	fear of missing out	fear of missing out	1	kept
fear of surgery	fear of surgery	fear of surgery	fear of surgery	fear of surgery	1	kept

fear of workplace	fear of workplace	fear of workplace	fear of workplace	fear of workplace	1	kept
Fear Scale for Healthcare Professionals	fear scale for healthcare professionals	fear scale for healthcare professionals	fear scale for healthcare professionals	Fear Scale for Healthcare Professionals	1	kept
Fears	fears	fears	fear	fear	1	singular
fears	fears	fears	fear	fear	1	singular
Feasibilities studies	feasibilities studies	feasibilities studies	feasibilities studies	Feasibilities studies	1	kept
feasibility assessment	feasibility assessment	feasibility assessment	feasibility assessment	feasibility assessment	1	kept
feasibility evaluation	feasibility evaluation	feasibility evaluation	feasibility evaluation	feasibility evaluation	1	kept
feasibility intervention measure	feasibility intervention measure	feasibility intervention measure	feasibility intervention measure	feasibility intervention measure	1	kept
FEASIBILITY STUDIES	feasibility studies	feasibility studies	feasibility study	Feasibility Studies	1	singular
Feasible schedule	feasible schedule	feasible schedule	feasible schedule	Feasible schedule	1	kept
feature	feature	feature	feature	feature	1	kept
Features extraction	features extraction	features extraction	features extraction	Features extraction	1	kept
Features recognition	features recognition	features recognition	features recognition	Features recognition	1	kept
febrile bone marrow aplasia	febrile bone marrow aplasia	febrile bone marrow aplasia	febrile bone marrow aplasia	febrile bone marrow aplasia	1	kept
FEBRILE NEUTROPENIA	febrile neutropenia	febrile neutropenia	febrile neutropenia	febrile neutropenia	1	case_or_format
febrile non-hemolytic transfusion reactions	febrile non hemolytic transfusion reactions	febrile non hemolytic transfusion reactions	febrile non hemolytic transfusion reactions	febrile non-hemolytic transfusion reactions	1	kept

febrile ulceronecrotic Mucha Habermann disease	febrile ulceronecrotic mucha habermann disease	febrile ulceronecrotic mucha habermann disease	febrile ulceronecrotic mucha habermann disease	febrile ulceronecrotic Mucha Habermann disease	1	kept
Febrile ulceronecrotic Mucha-Habermann disease	febrile ulceronecrotic mucha habermann disease	febrile ulceronecrotic mucha habermann disease	febrile ulceronecrotic mucha habermann disease	febrile ulceronecrotic Mucha Habermann disease	1	case_or_format
FECAL CARRIAGE	fecal carriage	fecal carriage	fecal carriage	FECAL CARRIAGE	1	kept
FECAL INCONTINENCE	fecal incontinence	fecal incontinence	fecal incontinence	Fecal Incontinence	1	case_or_format
Fecal management system	fecal management system	fecal management system	fecal management system	Fecal management system	1	kept
fecal management system	fecal management system	fecal management system	fecal management system	Fecal management system	1	case_or_format
Fecal Microbiota Transplantation	fecal microbiota transplantation	fecal microbiota transplantation	fecal microbiota transplantation	fecal microbiota transplantation	1	case_or_format
fecal transplantation	fecal transplantation	fecal transplantation	fecal transplantation	fecal transplantation	1	kept
feces composition	feces composition	feces composition	feces composition	feces composition	1	kept
feces microflora	feces microflora	feces microflora	feces microflora	feces microflora	1	kept
federal guidance	federal guidance	federal guidance	federal guidance	federal guidance	1	kept
Federal Joint Committee	federal joint committee	federal joint committee	federal joint committee	Federal Joint Committee	1	kept
Federated learning	federated learning	federated learning	federated learning	Federated learning	1	kept
federation	federation	federation	federation	federation	1	kept
fee	fee	fee	fee	fee	1	kept
Fee Schedules	fee schedules	fee schedules	fee schedules	Fee Schedules	1	kept

FEE-FOR-SERVICE	fee for service	fee for service	fee for service	FEE-FOR-SERVICE	1	kept
Feed	feed	feed	feed	Feed	1	kept
Feed and wrap	feed and wrap	feed and wrap	feed and wrap	Feed and wrap	1	kept
Feed back information	feed back information	feed back information	feed back information	Feed back information	1	kept
Feed intolerance	feed intolerance	feed intolerance	feed intolerance	feed intolerance	1	case_or_format
feedback and evaluation	feedback and evaluation	feedback and evaluation	feedback and evaluation	feedback and evaluation	1	kept
Feedback control	feedback control	feedback control	feedback control	Feedback control	1	kept
feedback device	feedback device	feedback device	feedback device	feedback device	1	kept
Feedback strategies	feedback strategies	feedback strategies	feedback strategies	Feedback strategies	1	kept
Feedback system	feedback system	feedback system	feedback system	feedback system	1	case_or_format
Feedback, Sensory	feedback sensory	feedback sensory	feedback sensory	Feedback, Sensory	1	kept
feedforward control theory	feedforward control theory	feedforward control theory	feedforward control theory	feedforward control theory	1	kept
Feeding behaviors	feeding behaviors	feeding behaviors	feeding behavior	Feeding Behavior	1	singular
feeding bundle	feeding bundle	feeding bundle	feeding bundle	feeding bundle	1	kept
Feeding experience	feeding experience	feeding experience	feeding experience	Feeding experience	1	kept
feeding experience	feeding experience	feeding experience	feeding experience	Feeding experience	1	case_or_format

feeding failure	feeding failure	feeding failure	feeding failure	feeding failure	1	kept
feeding interruption	feeding interruption	feeding interruption	feeding interruption	feeding interruption	1	kept
feeding intervals	feeding intervals	feeding intervals	feeding intervals	feeding intervals	1	kept
Feeding method	feeding method	feeding method	feeding method	Feeding Methods	1	case_or_format
FEEDING OPTIONS	feeding options	feeding options	feeding options	FEEDING OPTIONS	1	kept
feeding outcomes	feeding outcomes	feeding outcomes	feeding outcomes	feeding outcomes	1	kept
feeding practices	feeding practices	feeding practices	feeding practices	feeding practices	1	kept
Feeding process	feeding process	feeding process	feeding process	Feeding process	1	kept
Feeding protocols	feeding protocols	feeding protocols	feeding protocol	Feeding protocol	1	singular
feeding protocols	feeding protocols	feeding protocols	feeding protocol	Feeding protocol	1	singular
Feeding rate	feeding rate	feeding rate	feeding rate	Feeding rate	1	kept
Feeding refusal	feeding refusal	feeding refusal	feeding refusal	Feeding refusal	1	kept
Feeding relationship	feeding relationship	feeding relationship	feeding relationship	Feeding relationship	1	kept
Feeding risk factor scale	feeding risk factor scale	feeding risk factor scale	feeding risk factor scale	Feeding risk factor scale	1	kept
Feeding schedule	feeding schedule	feeding schedule	feeding schedule	Feeding schedule	1	kept
feeding supplementation	feeding supplementation	feeding supplementation	feeding supplementation	feeding supplementation	1	kept

Feeding tolerance	feeding tolerance	feeding tolerance	feeding tolerance	Feeding tolerance	1	kept
feeding tolerance	feeding tolerance	feeding tolerance	feeding tolerance	Feeding tolerance	1	case_or_format
Feeding tube placement	feeding tube placement	feeding tube placement	feeding tube placement	feeding tube placement	1	case_or_format
feeding tube stylet	feeding tube stylet	feeding tube stylet	feeding tube stylet	feeding tube stylet	1	kept
feeding tubes	feeding tubes	feeding tubes	feeding tube	Feeding tube	1	singular
FEEDING-TUBE	feeding tube	feeding tube	feeding tube	Feeding tube	1	case_or_format
Feeding-tube dependence	feeding tube dependence	feeding tube dependence	feeding tube dependence	Feeding-tube dependence	1	kept
FEEDING-TUBE INSERTION	feeding tube insertion	feeding tube insertion	feeding tube insertion	FEEDING-TUBE INSERTION	1	kept
FEEDING-TUBE PLACEMENT	feeding tube placement	feeding tube placement	feeding tube placement	feeding tube placement	1	case_or_format
FEEDING-TUBE USE	feeding tube use	feeding tube use	feeding tube use	FEEDING-TUBE USE	1	kept
Feeling safe	feeling safe	feeling safe	feeling safe	feeling safe	1	case_or_format
Feeling toward baby	feeling toward baby	feeling toward baby	feeling toward baby	Feeling toward baby	1	kept
Feelings	feelings	feelings	feelings	feelings	1	case_or_format
Fees and charges	fees and charges	fees and charges	fees and charges	Fees and Charges	1	case_or_format
FEIBA	feiba	feiba	feiba	FEIBA	1	kept
feline	feline	feline	feline	feline	1	kept

Felis catus	felis catus	felis catus	felis catus	Felis catus	1	kept
FELIS-SILVESTRIS-CATUS	felis silvestris catus	felis silvestris catus	felis silvestris catus	FELIS-SILVESTRIS-CATUS	1	kept
Fellow education	fellow education	fellow education	fellow education	Fellow education	1	kept
fellow training	fellow training	fellow training	fellow training	fellow training	1	kept
FELLOWS	fellows	fellows	fellow	Fellow	1	singular
FELT ACCOUNTABILITY	felt accountability	felt accountability	felt accountability	FELT ACCOUNTABILITY	1	kept
Female genital mutilation	female genital mutilation	female genital mutilation	female genital mutilation	Female genital mutilation	1	kept
female genital system	female genital system	female genital system	female genital system	female genital system	1	kept
female genital tract tumor	female genital tract tumor	female genital tract tumor	female genital tract tumor	female genital tract tumor	1	kept
Female healthcare workers	female healthcare workers	female healthcare workers	female healthcare workers	Female healthcare workers	1	kept
female pediatricians	female pediatricians	female pediatricians	female pediatricians	female pediatricians	1	kept
female sterilization	female sterilization	female sterilization	female sterilization	female sterilization	1	kept
female to male transgender	female to male transgender	female to male transgender	female to male transgender	female to male transgender	1	kept
FEMALE URINARY-INCONTINENCE	female urinary incontinence	female urinary incontinence	female urinary incontinence	FEMALE URINARY-INCONTINENCE	1	kept
Females	females	females	females	Females	1	kept
femininity	femininity	femininity	femininity	femininity	1	kept

feminism	feminism	feminism	feminism	Feminism	1	case_or_format
feminist standpoint theory	feminist standpoint theory	feminist standpoint theory	feminist standpoint theory	feminist standpoint theory	1	kept
feminization	feminization	feminization	feminization	feminization	1	kept
femme enceinte	femme enceinte	femme enceinte	femme enceinte	femme enceinte	1	kept
femoral access	femoral access	femoral access	femoral access	femoral access	1	kept
femoral block	femoral block	femoral block	femoral block	femoral block	1	kept
femoral catheterization	femoral catheterization	femoral catheterization	femoral catheterization	femoral catheterization	1	kept
Femoral fractures	femoral fractures	femoral fractures	femoral fractures	Femoral Fractures	1	case_or_format
Femoral Fractures, Distal	femoral fractures distal	femoral fractures distal	femoral fractures distal	Femoral Fractures, Distal	1	kept
Femoral neck fracture	femoral neck fracture	femoral neck fracture	femoral neck fracture	femoral neck fracture	1	case_or_format
Femoral neck fractures	femoral neck fractures	femoral neck fractures	femoral neck fracture	femoral neck fracture	1	singular
Femoral shaft treatment in children	femoral shaft treatment in children	femoral shaft treatment in children	femoral shaft treatment in children	Femoral shaft treatment in children	1	kept
femoral to abdomen	femoral to abdomen	femoral to abdomen	femoral to abdomen	femoral to abdomen	1	kept
femoral to abdomen tunneling	femoral to abdomen tunneling	femoral to abdomen tunneling	femoral to abdomen tunneling	femoral to abdomen tunneling	1	kept
FEMORAL-NECK	femoral neck	femoral neck	femoral neck	FEMORAL-NECK	1	kept
femoro jugular approach	femoro jugular approach	femoro jugular approach	femoro jugular approach	femoro jugular approach	1	kept

femoropopliteal bypass	femoropopliteal bypass	femoropopliteal bypass	femoropopliteal bypass	femoropopliteal bypass	1	kept
Femoropopliteal bypass graft	femoropopliteal bypass graft	femoropopliteal bypass graft	femoropopliteal bypass graft	Femoropopliteal bypass graft	1	kept
femur head necrosis	femur head necrosis	femur head necrosis	femur head necrosis	femur head necrosis	1	kept
femur neck	femur neck	femur neck	femur neck	femur neck	1	kept
femur shaft fracture	femur shaft fracture	femur shaft fracture	femur shaft fracture	femur shaft fracture	1	kept
fenfluramine	fenfluramine	fenfluramine	fenfluramine	fenfluramine	1	kept
feng shui	feng shui	feng shui	feng shui	feng shui	1	kept
fennel caraway oil	fennel caraway oil	fennel caraway oil	fennel caraway oil	fennel caraway oil	1	kept
fenofibrate	fenofibrate	fenofibrate	fenofibrate	fenofibrate	1	kept
Fenoldopam	fenoldopam	fenoldopam	fenoldopam	Fenoldopam	1	kept
fenoldopam	fenoldopam	fenoldopam	fenoldopam	Fenoldopam	1	case_or_format
Fenomenología	fenomenolog a	fenomenolog a	fenomenolog a	Fenomenología	1	kept
fenoterol	fenoterol	fenoterol	fenoterol	fenoterol	1	kept
fenoterol plus ipratropium bromide	fenoterol plus ipratropium bromide	fenoterol plus ipratropium bromide	fenoterol plus ipratropium bromide	fenoterol plus ipratropium bromide	1	kept
fentadon	fentadon	fentadon	fentadon	fentadon	1	kept
FENTANYL	fentanyl	fentanyl	fentanyl	fentanyl	1	case_or_format

fermentation technique	fermentation technique	fermentation technique	fermentation technique	fermentation technique	1	kept
ferro sanol drops	ferro sanol drops	ferro sanol drops	ferro sanol drops	ferro sanol drops	1	kept
Ferroptosis	ferroptosis	ferroptosis	ferroptosis	Ferroptosis	1	kept
ferrous gluconate	ferrous gluconate	ferrous gluconate	ferrous gluconate	ferrous gluconate	1	kept
fertilization	fertilization	fertilization	fertilization	fertilization	1	kept
FES recovery	fes recovery	fes recovery	fes recovery	FES recovery	1	kept
Fetal	fetal	fetal	fetal	FETAL	1	case_or_format
Fetal auditory development	fetal auditory development	fetal auditory development	fetal auditory development	Fetal auditory development	1	kept
fetal center	fetal center	fetal center	fetal center	fetal center	1	kept
fetal echocardiography	fetal echocardiography	fetal echocardiography	fetal echocardiography	fetal echocardiography	1	kept
fetal growth restriction	fetal growth restriction	fetal growth restriction	fetal growth restriction	fetal growth restriction	1	kept
Fetal heart	fetal heart	fetal heart	fetal heart	Fetal heart	1	kept
Fetal heart image	fetal heart image	fetal heart image	fetal heart image	Fetal heart image	1	kept
Fetal heart images	fetal heart images	fetal heart images	fetal heart image	Fetal heart image	1	singular
Fetal heart tone	fetal heart tone	fetal heart tone	fetal heart tone	Fetal heart tone	1	kept
Fetal hemoglobin	fetal hemoglobin	fetal hemoglobin	fetal hemoglobin	Fetal hemoglobin	1	kept

fetal hemoglobin	fetal hemoglobin	fetal hemoglobin	fetal hemoglobin	Fetal hemoglobin	1	case_or_format
Fetal hypoxemia	fetal hypoxemia	fetal hypoxemia	fetal hypoxemia	Fetal hypoxemia	1	kept
Fetal hypoxia	fetal hypoxia	fetal hypoxia	fetal hypoxia	Fetal Hypoxia	1	case_or_format
fetal intervention	fetal intervention	fetal intervention	fetal intervention	fetal intervention	1	kept
fetal magnetocardiography	fetal magnetocardiography	fetal magnetocardiography	fetal magnetocardiography	fetal magnetocardiography	1	kept
Fetal medicine	fetal medicine	fetal medicine	fetal medicine	Fetal medicine	1	kept
fetal medicine	fetal medicine	fetal medicine	fetal medicine	Fetal medicine	1	case_or_format
Fetal Mortality	fetal mortality	fetal mortality	fetal mortality	Fetal Mortality	1	kept
Fetal Organ Maturity	fetal organ maturity	fetal organ maturity	fetal organ maturity	Fetal Organ Maturity	1	kept
Fetal oxygenation	fetal oxygenation	fetal oxygenation	fetal oxygenation	Fetal oxygenation	1	kept
fetal pharmacology	fetal pharmacology	fetal pharmacology	fetal pharmacology	fetal pharmacology	1	kept
fetal physiology	fetal physiology	fetal physiology	fetal physiology	fetal physiology	1	kept
Fetal sonographies	fetal sonographies	fetal sonographies	fetal sonography	Fetal sonography	1	singular
Fetal sonography	fetal sonography	fetal sonography	fetal sonography	Fetal sonography	1	kept
Fetal surveillance	fetal surveillance	fetal surveillance	fetal surveillance	Fetal surveillance	1	kept
fetal tachycardia	fetal tachycardia	fetal tachycardia	fetal tachycardia	fetal tachycardia	1	kept

Fetal weight	fetal weight	fetal weight	fetal weight	Fetal Weight	1	case_or_format
feticide	feticide	feticide	feticide	feticide	1	kept
Fetofetal Transfusion	fetofetal transfusion	fetofetal transfusion	fetofetal transfusion	Fetofetal Transfusion	1	kept
fetoscopy	fetoscopy	fetoscopy	fetoscopy	fetoscopy	1	kept
FETUS	fetus	fetus	fetus	fetus	1	case_or_format
fetus blood sampling	fetus blood sampling	fetus blood sampling	fetus blood sampling	fetus blood sampling	1	kept
fetus circulation	fetus circulation	fetus circulation	fetus circulation	fetus circulation	1	kept
fetus development	fetus development	fetus development	fetus development	fetus development	1	kept
fetus lung maturation	fetus lung maturation	fetus lung maturation	fetus lung maturation	fetus lung maturation	1	kept
fetus membrane	fetus membrane	fetus membrane	fetus membrane	fetus membrane	1	kept
FEV1	fev1	fev1	fev1	FEV1	1	kept
Fever control	fever control	fever control	fever control	Fever control	1	kept
fever in labor	fever in labor	fever in labor	fever in labor	fever in labor	1	kept
Fever kowledge	fever kowledge	fever kowledge	fever kowledge	Fever kowledge	1	kept
Fever of Unknown Origin	fever of unknown origin	fever of unknown origin	fever of unknown origin	Fever of Unknown Origin	1	kept
Fever treatment	fever treatment	fever treatment	fever treatment	Fever treatment	1	kept

fexb bacterial gene	fexb bacterial gene	fexb bacterial gene	fexb bacterial gene	fexb bacterial gene	1	kept
fexofenadine	fexofenadine	fexofenadine	fexofenadine	fexofenadine	1	kept
FGDs	fgds	fgds	fgd	FGD	1	singular
Fiabilidad	fiabilidad	fiabilidad	fiabilidad	Fiabilidad	1	kept
fiber	fiber	fiber	fiber	fiber	1	kept
fiber con	fiber con	fiber con	fiber con	fiber con	1	kept
Fiber Optics	fiber optics	fiber optics	fiber optics	fiber optics	1	case_or_format
Fiberendoscopic evaluation of swallowing	fiberendoscopic evaluation of swallowing	fiberendoscopic evaluation of swallowing	fiberendoscopic evaluation of swallowing	Fiberendoscopic evaluation of swallowing	1	kept
fiberoptic assisted tracheoscopy	fiberoptic assisted tracheoscopy	fiberoptic assisted tracheoscopy	fiberoptic assisted tracheoscopy	fiberoptic assisted tracheoscopy	1	kept
fiberoptic intubation	fiberoptic intubation	fiberoptic intubation	fiberoptic intubation	fiberoptic intubation	1	kept
fiberoscope	fiberoscope	fiberoscope	fiberoscope	fiberoscope	1	kept
Fibre optic	fibre optic	fibre optic	fibre optic	Fibre optic	1	kept
fibric acid derivative	fibric acid derivative	fibric acid derivative	fibric acid derivative	fibric acid derivative	1	kept
Fibrinogen concentrate	fibrinogen concentrate	fibrinogen concentrate	fibrinogen concentrate	fibrinogen concentrate	1	case_or_format
fibrinogen receptor antagonist	fibrinogen receptor antagonist	fibrinogen receptor antagonist	fibrinogen receptor antagonist	fibrinogen receptor antagonist	1	kept
fibrinogen thromboelastometry	fibrinogen thromboelastometry	fibrinogen thromboelastometry	fibrinogen thromboelastometry	fibrinogen thromboelastometry	1	kept

Fibrinolytic agents	fibrinolytic agents	fibrinolytic agents	fibrinolytic agent	Fibrinolytic Agents	1	singular
Fibrinolytic therapy	fibrinolytic therapy	fibrinolytic therapy	fibrinolytic therapy	fibrinolytic therapy	1	case_or_format
fibroblast	fibroblast	fibroblast	fibroblast	fibroblast	1	kept
fibroblast growth factor 2	fibroblast growth factor 2	fibroblast growth factor 2	fibroblast growth factor 2	fibroblast growth factor 2	1	kept
Fibroblasts	fibroblasts	fibroblasts	fibroblast	fibroblast	1	singular
fibronectin	fibronectin	fibronectin	fibronectin	fibronectin	1	kept
Fidelity	fidelity	fidelity	fidelity	FIDELITY	1	case_or_format
fidelity assessment	fidelity assessment	fidelity assessment	fidelity assessment	fidelity assessment	1	kept
fidelity checklist	fidelity checklist	fidelity checklist	fidelity checklist	fidelity checklist	1	kept
Fidelity of delivery	fidelity of delivery	fidelity of delivery	fidelity of delivery	Fidelity of delivery	1	kept
FIDELITY SIMULATION	fidelity simulation	fidelity simulation	fidelity simulation	FIDELITY SIMULATION	1	kept
Field hospital	field hospital	field hospital	field hospital	field hospital	1	case_or_format
Field Hospitals	field hospitals	field hospitals	field hospital	field hospital	1	singular
Field hospitals	field hospitals	field hospitals	field hospital	field hospital	1	singular
Field study	field study	field study	field study	field study	1	case_or_format
Field triage	field triage	field triage	field triage	Field triage	1	kept

Field trips	field trips	field trips	field trips	Field trips	1	kept
Field work	field work	field work	field work	field work	1	case_or_format
FIENS	fiens	fiens	fiens	FIENS	1	kept
Filariasis	filariasis	filariasis	filariasis	Filariasis	1	kept
file transfer protocol	file transfer protocol	file transfer protocol	file transfer protocol	file transfer protocol	1	kept
Filgrastim	filgrastim	filgrastim	filgrastim	Filgrastim	1	kept
filipino	filipino	filipino	filipino	filipino	1	kept
filling pressures	filling pressures	filling pressures	filling pressures	filling pressures	1	kept
film coating	film coating	film coating	film coating	film coating	1	kept
Filobasidiella	filobasidiella	filobasidiella	filobasidiella	Filobasidiella	1	kept
Filobasidium	filobasidium	filobasidium	filobasidium	Filobasidium	1	kept
filovirus infection	filovirus infection	filovirus infection	filovirus infection	filovirus infection	1	kept
Filter	filter	filter	filter	filter	1	case_or_format
Filter changes	filter changes	filter changes	filter changes	Filter changes	1	kept
Filter exchange	filter exchange	filter exchange	filter exchange	Filter exchange	1	kept
filter life	filter life	filter life	filter life	Filter life	1	case_or_format

Filter needle	filter needle	filter needle	filter needle	Filter needle	1	kept
filter pressure	filter pressure	filter pressure	filter pressure	filter pressure	1	kept
Filtering face piece respirator	filtering face piece respirator	filtering face piece respirator	filtering face piece respirator	Filtering face piece respirator	1	kept
Filters	filters	filters	filter	filter	1	singular
fin de vie	fin de vie	fin de vie	fin de vie	Fin de vie	1	case_or_format
Final Rule	final rule	final rule	final rule	Final Rule	1	kept
Final stage psychological support	final stage psychological support	final stage psychological support	final stage psychological support	Final stage psychological support	1	kept
final verification	final verification	final verification	final verification	final verification	1	kept
Finance	finance	finance	finance	finance	1	case_or_format
finances	finances	finances	finance	finance	1	singular
financial	financial	financial	financial	financial	1	kept
Financial analysis	financial analysis	financial analysis	financial analysis	Financial analysis	1	kept
financial aspect	financial aspect	financial aspect	financial aspect	financial aspect	1	kept
Financial Audit	financial audit	financial audit	financial audit	Financial Audit	1	kept
financial burden	financial burden	financial burden	financial burden	financial burden	1	kept
Financial constraints	financial constraints	financial constraints	financial constraints	Financial constraints	1	kept

financial crisis	financial crisis	financial crisis	financial crisis	financial crisis	1	kept
financial deficit	financial deficit	financial deficit	financial deficit	financial deficit	1	kept
Financial impacts	financial impacts	financial impacts	financial impacts	Financial impacts	1	kept
FINANCIAL INCENTIVES	financial incentives	financial incentives	financial incentives	FINANCIAL INCENTIVES	1	kept
Financial loss	financial loss	financial loss	financial loss	Financial loss	1	kept
Financial Management	financial management	financial management	financial management	financial management	1	case_or_format
Financial management, hospital	financial management hospital	financial management hospital	financial management hospital	Financial Management, Hospital	1	case_or_format
Financial performance	financial performance	financial performance	financial performance	Financial performance	1	kept
Financial stress	financial stress	financial stress	financial stress	financial stress	1	case_or_format
Financial Support	financial support	financial support	financial support	Financial Support	1	kept
Fine grained	fine grained	fine grained	fine grained	Fine grained	1	kept
Finegoldia magna	finegoldia magna	finegoldia magna	finegoldia magna	Finegoldia magna	1	kept
finger dislocation	finger dislocation	finger dislocation	finger dislocation	finger dislocation	1	kept
Finger Injuries	finger injuries	finger injuries	finger injury	finger injury	1	singular
finger span test	finger span test	finger span test	finger span test	finger span test	1	kept
finger swab	finger swab	finger swab	finger swab	finger swab	1	kept

finger tapping test	finger tapping test	finger tapping test	finger tapping test	finger tapping test	1	kept
finger ulcer	finger ulcer	finger ulcer	finger ulcer	finger ulcer	1	kept
Fingerstick	fingerstick	fingerstick	fingerstick	Fingerstick	1	kept
fingertip blood glucose measurement frequency	fingertip blood glucose measurement frequency	fingertip blood glucose measurement frequency	fingertip blood glucose measurement frequency	fingertip blood glucose measurement frequency	1	kept
Finite element analyse	finite element analyse	finite element analyse	finite element analyse	Finite element analyse	1	kept
Finite mixture model	finite mixture model	finite mixture model	finite mixture model	Finite mixture model	1	kept
Finite state machine	finite state machine	finite state machine	finite state machine	Finite state machine	1	kept
Finitude	finitude	finitude	finitude	Finitude	1	kept
Finn	finn	finn	finn	Finn	1	kept
Finnegan	finnegan	finnegan	finnegan	Finnegan	1	kept
finnegan score	finnegan score	finnegan score	finnegan score	finnegan score	1	kept
Finnegan Scoring Tool	finnegan scoring tool	finnegan scoring tool	finnegan scoring tool	Finnegan Scoring Tool	1	kept
Finnish language	finnish language	finnish language	finnish language	Finnish language	1	kept
FIO2	fio2	fio2	fio2	FIO2	1	kept
Firefighters	firefighters	firefighters	firefighters	Firefighters	1	kept
fires	fires	fires	fire	Fires	1	singular

first bath	first bath	first bath	first bath	first bath	1	kept
first bathing	first bathing	first bathing	first bathing	first bathing	1	kept
first cervical vertebra	first cervical vertebra	first cervical vertebra	first cervical vertebra	first cervical vertebra	1	kept
First episode psychosis	first episode psychosis	first episode psychosis	first episode psychosis	First episode psychosis	1	kept
first intention high frequency oscillatory ventilation	first intention high frequency oscillatory ventilation	first intention high frequency oscillatory ventilation	first intention high frequency oscillatory ventilation	first intention high frequency oscillatory ventilation	1	kept
First Nation	first nation	first nation	first nation	First Nation	1	kept
first pass effect	first pass effect	first pass effect	first pass effect	first pass effect	1	kept
first stage of labor	first stage of labor	first stage of labor	first stage of labor	first stage of labor	1	kept
first-night effect	first night effect	first night effect	first night effect	first-night effect	1	kept
first-time insertion	first time insertion	first time insertion	first time insertion	first-time insertion	1	kept
Fishbone	fishbone	fishbone	fishbone	Fishbone	1	kept
Fishbone method	fishbone method	fishbone method	fishbone method	Fishbone method	1	kept
Fisher vectors	fisher vectors	fisher vectors	fisher vectors	Fisher vectors	1	kept
Fisher's exact test	fisher s exact test	fisher s exact test	fisher s exact test	Fisher's exact test	1	kept
Fistula prevention	fistula prevention	fistula prevention	fistula prevention	Fistula prevention	1	kept
Fistuloclysis	fistuloclysis	fistuloclysis	fistuloclysis	Fistuloclysis	1	kept

Fit	fit	fit	fit	Fit	1	kept
Fit for purpose	fit for purpose	fit for purpose	fit for purpose	Fit for purpose	1	kept
FIT INDEXES	fit indexes	fit indexes	fit indexes	FIT INDEXES	1	kept
Fit-testing	fit testing	fit testing	fit testing	Fit-testing	1	kept
fitness center	fitness center	fitness center	fitness center	fitness center	1	kept
Fitness to practice	fitness to practice	fitness to practice	fitness to practice	Fitness to practice	1	kept
fitness to practice	fitness to practice	fitness to practice	fitness to practice	Fitness to practice	1	case_or_format
fitness tracker	fitness tracker	fitness tracker	fitness tracker	fitness tracker	1	kept
Fitness trackers	fitness trackers	fitness trackers	fitness tracker	fitness tracker	1	singular
Fitting	fitting	fitting	fitting	Fitting	1	kept
five facets mindfulness questionnaire	five facets mindfulness questionnaire	five facets mindfulness questionnaire	five facets mindfulness questionnaire	five facets mindfulness questionnaire	1	kept
five moments of hand hygiene	five moments of hand hygiene	five moments of hand hygiene	five moments of hand hygiene	five moments of hand hygiene	1	kept
Fixation	fixation	fixation	fixation	Fixation	1	kept
fixation band	fixation band	fixation band	fixation band	fixation band	1	kept
Fixation stability	fixation stability	fixation stability	fixation stability	Fixation stability	1	kept
fixation strength	fixation strength	fixation strength	fixation strength	fixation strength	1	kept

fixed concentration	fixed concentration	fixed concentration	fixed concentration	fixed concentration	1	kept
fixed rate titration	fixed rate titration	fixed rate titration	fixed rate titration	fixed rate titration	1	kept
fixed-effect model	fixed effect model	fixed effect model	fixed effect model	fixed-effect model	1	kept
fixomull fixation	fixomull fixation	fixomull fixation	fixomull fixation	fixomull fixation	1	kept
FLACC	flacc	flacc	flacc	FLACC	1	kept
FLACC pain scale	flacc pain scale	flacc pain scale	flacc pain scale	FLACC pain scale	1	kept
FLACC score	flacc score	flacc score	flacc score	FLACC score	1	kept
flap debulking	flap debulking	flap debulking	flap debulking	flap debulking	1	kept
flatus	flatus	flatus	flatus	flatus	1	kept
flavectomy	flavectomy	flavectomy	flavectomy	flavectomy	1	kept
Flavivirus infection	flavivirus infection	flavivirus infection	flavivirus infection	Flavivirus infection	1	kept
Flavoring Agents	flavoring agents	flavoring agents	flavoring agents	Flavoring Agents	1	kept
flea	flea	flea	flea	flea	1	kept
Flecainide	flecainide	flecainide	flecainide	flecainide	1	case_or_format
Fleet operations	fleet operations	fleet operations	fleet operations	Fleet operations	1	kept
flexible	flexible	flexible	flexible	flexible	1	kept

flexible bronchoscope	flexible bronchoscope	flexible bronchoscope	flexible bronchoscope	flexible bronchoscope	1	kept
Flexible clinical teams	flexible clinical teams	flexible clinical teams	flexible clinical teams	Flexible clinical teams	1	kept
Flexible fibreoptic endoscopy	flexible fibreoptic endoscopy	flexible fibreoptic endoscopy	flexible fibreoptic endoscopy	Flexible fibreoptic endoscopy	1	kept
flexible nasal endoscopy	flexible nasal endoscopy	flexible nasal endoscopy	flexible nasal endoscopy	flexible nasal endoscopy	1	kept
Flexible units	flexible units	flexible units	flexible units	Flexible units	1	kept
Flexible visitation	flexible visitation	flexible visitation	flexible visitation	Flexible visitation	1	kept
flexible visitation	flexible visitation	flexible visitation	flexible visitation	Flexible visitation	1	case_or_format
Flexible visitation system	flexible visitation system	flexible visitation system	flexible visitation system	Flexible visitation system	1	kept
flexible visiting	flexible visiting	flexible visiting	flexible visiting	flexible visiting	1	kept
flexible visiting hours	flexible visiting hours	flexible visiting hours	flexible visiting hours	flexible visiting hours	1	kept
flexural dermatitis	flexural dermatitis	flexural dermatitis	flexural dermatitis	flexural dermatitis	1	kept
Flickering	flickering	flickering	flickering	Flickering	1	kept
Flight medicine	flight medicine	flight medicine	flight medicine	Flight medicine	1	kept
flight medicine	flight medicine	flight medicine	flight medicine	Flight medicine	1	case_or_format
Flipped approach	flipped approach	flipped approach	flipped approach	Flipped approach	1	kept
flipped classroom	flipped classroom	flipped classroom	flipped classroom	Flipped classroom	1	case_or_format

Flipped learning process	flipped learning process	flipped learning process	flipped learning process	Flipped learning process	1	kept
float pools	float pools	float pools	float pools	float pools	1	kept
floating	floating	floating	floating	floating	1	kept
Flodén ATODAI	flod n atodai	flod n atodai	flod n atodai	Flodén ATODAI	1	kept
flonidan	flonidan	flonidan	flonidan	flonidan	1	kept
flooding	flooding	flooding	flooding	flooding	1	kept
Floods	floods	floods	floods	Floods	1	kept
Floor	floor	floor	floor	Floors	1	case_or_format
Floor of mouth	floor of mouth	floor of mouth	floor of mouth	Floor of mouth	1	kept
Flooring	flooring	flooring	flooring	floorings	1	case_or_format
FLORA	flora	flora	flora	FLORA	1	kept
flora	flora	flora	flora	FLORA	1	case_or_format
Floral essences	floral essences	floral essences	floral essences	Floral essences	1	kept
Florence	florence	florence	florence	Florence	1	kept
flotation bed	flotation bed	flotation bed	flotation bed	flotation bed	1	kept
Flow	flow	flow	flow	FLOW	1	case_or_format

flow	flow	flow	flow	FLOW	1	case_or_format
flow disruptions	flow disruptions	flow disruptions	flow disruptions	flow disruptions	1	kept
flow dynamics	flow dynamics	flow dynamics	flow dynamics	flow dynamics	1	kept
flow experience	flow experience	flow experience	flow experience	flow experience	1	kept
flow kinetics	flow kinetics	flow kinetics	flow kinetics	flow kinetics	1	kept
flow measurement	flow measurement	flow measurement	flow measurement	flow measurement	1	kept
flow shop scheduling	flow shop scheduling	flow shop scheduling	flow shop scheduling	flow shop scheduling	1	kept
Flow velocity	flow velocity	flow velocity	flow velocity	Flow velocity	1	kept
Flowcharting	flowcharting	flowcharting	flowcharting	Flowcharting	1	kept
Flowers	flowers	flowers	flowers	Flowers	1	kept
Flowmeters	flowmeters	flowmeters	flowmeters	Flowmeters	1	kept
FLUCONAZOLE PROPHYLAXIS	fluconazole prophylaxis	fluconazole prophylaxis	fluconazole prophylaxis	FLUCONAZOLE PROPHYLAXIS	1	kept
fluctuating mental status evaluation	fluctuating mental status evaluation	fluctuating mental status evaluation	fluctuating mental status evaluation	fluctuating mental status evaluation	1	kept
fludarabine	fludarabine	fludarabine	fludarabine	fludarabine	1	kept
fluibron ped syrup	fluibron ped syrup	fluibron ped syrup	fluibron ped syrup	fluibron ped syrup	1	kept
fluid analysis	fluid analysis	fluid analysis	fluid analysis	fluid analysis	1	kept

Fluid balance charts	fluid balance charts	fluid balance charts	fluid balance charts	Fluid balance charts	1	kept
fluid balance monitoring	fluid balance monitoring	fluid balance monitoring	fluid balance monitoring	fluid balance monitoring	1	kept
fluid bolus	fluid bolus	fluid bolus	fluid bolus	fluid bolus	1	kept
fluid bolus therapy	fluid bolus therapy	fluid bolus therapy	fluid bolus therapy	fluid bolus therapy	1	kept
Fluid challenge	fluid challenge	fluid challenge	fluid challenge	Fluid challenge	1	kept
fluid distribution	fluid distribution	fluid distribution	fluid distribution	fluid distribution	1	kept
fluid electrolyte and acid-base disturbances	fluid electrolyte and acid base disturbances	fluid electrolyte and acid base disturbances	fluid electrolyte and acid base disturbances	fluid electrolyte and acid-base disturbances	1	kept
fluid intake minus output	fluid intake minus output	fluid intake minus output	fluid intake minus output	fluid intake minus output	1	kept
Fluid leakage	fluid leakage	fluid leakage	fluid leakage	Fluid leakage	1	kept
fluid management	fluid management	fluid management	fluid management	Fluid management	1	case_or_format
FLUID OVERLOAD	fluid overload	fluid overload	fluid overload	Fluid overload	1	case_or_format
Fluid removal	fluid removal	fluid removal	fluid removal	Fluid removal	1	kept
FLUID RESPONSIVENESS	fluid responsiveness	fluid responsiveness	fluid responsiveness	Fluid responsiveness	1	case_or_format
fluid responsiveness	fluid responsiveness	fluid responsiveness	fluid responsiveness	Fluid responsiveness	1	case_or_format
fluid stewardship	fluid stewardship	fluid stewardship	fluid stewardship	fluid stewardship	1	kept
Fluid teams	fluid teams	fluid teams	fluid teams	Fluid teams	1	kept

fluid therapies	fluid therapies	fluid therapies	fluid therapy	Fluid Therapy	1	singular
fluid transport	fluid transport	fluid transport	fluid transport	fluid transport	1	kept
FLUID-MANAGEMENT STRATEGIES	fluid management strategies	fluid management strategies	fluid management strategies	FLUID-MANAGEMENT STRATEGIES	1	kept
Fluidization	fluidization	fluidization	fluidization	Fluidization	1	kept
Fluidized positioner	fluidized positioner	fluidized positioner	fluidized positioner	Fluidized positioner	1	kept
Fluidoterapia	fluidoterapia	fluidoterapia	fluidoterapia	Fluidoterapia	1	kept
Fluids and electrolytes	fluids and electrolytes	fluids and electrolytes	fluids and electrolytes	Fluids and electrolytes	1	kept
Fluids and feeding	fluids and feeding	fluids and feeding	fluids and feeding	Fluids and feeding	1	kept
fluindostatin	fluindostatin	fluindostatin	fluindostatin	fluindostatin	1	kept
Flunitrazepam	flunitrazepam	flunitrazepam	flunitrazepam	flunitrazepam	1	case_or_format
flunixin meglumine	flunixin meglumine	flunixin meglumine	flunixin meglumine	flunixin meglumine	1	kept
fluoresceine	fluoresceine	fluoresceine	fluoresceine	fluoresceine	1	kept
FLUORESCENCE	fluorescence	fluorescence	fluorescence	fluorescence	1	case_or_format
fluorescence activated cell sorting	fluorescence activated cell sorting	fluorescence activated cell sorting	fluorescence activated cell sorting	fluorescence activated cell sorting	1	kept
fluorescence angiography	fluorescence angiography	fluorescence angiography	fluorescence angiography	fluorescence angiography	1	kept
fluorescent	fluorescent	fluorescent	fluorescent	fluorescent	1	kept

fluorescent antibody technique	fluorescent antibody technique	fluorescent antibody technique	fluorescent antibody technique	fluorescent antibody technique	1	kept
Fluorescent Antibody Technique, Direct	fluorescent antibody technique direct	fluorescent antibody technique direct	fluorescent antibody technique direct	Fluorescent Antibody Technique, Direct	1	kept
Fluorescent screens	fluorescent screens	fluorescent screens	fluorescent screens	Fluorescent screens	1	kept
fluorine 18	fluorine 18	fluorine 18	fluorine 18	fluorine 18	1	kept
Fluoroquinolone antibiotics	fluoroquinolone antibiotics	fluoroquinolone antibiotics	fluoroquinolone antibiotics	Fluoroquinolone antibiotics	1	kept
Fluorouracil	fluorouracil	fluorouracil	fluorouracil	fluorouracil	1	case_or_format
flupentixol	flupentixol	flupentixol	flupentixol	flupentixol	1	kept
flupentixol decanoate	flupentixol decanoate	flupentixol decanoate	flupentixol decanoate	flupentixol decanoate	1	kept
fluralaner	fluralaner	fluralaner	fluralaner	fluralaner	1	kept
flurbiprofen	flurbiprofen	flurbiprofen	flurbiprofen	flurbiprofen	1	kept
flush bag change	flush bag change	flush bag change	flush bag change	flush bag change	1	kept
flush test	flush test	flush test	flush test	flush test	1	kept
flushing and locking techniques	flushing and locking techniques	flushing and locking techniques	flushing and locking techniques	flushing and locking techniques	1	kept
flushing interval	flushing interval	flushing interval	flushing interval	flushing interval	1	kept
fluticasone furoate	fluticasone furoate	fluticasone furoate	fluticasone furoate	fluticasone furoate	1	kept
fluticasone propionate plus salmeterol xinafoate	fluticasone propionate plus salmeterol xinafoate	fluticasone propionate plus salmeterol xinafoate	fluticasone propionate plus salmeterol xinafoate	fluticasone propionate plus salmeterol xinafoate	1	kept

FMC2B first-medical-contact-to-balloon	fmc2b first medical contact to balloon	fmc2b first medical contact to balloon	fmc2b first medical contact to balloon	FMC2B first-medical-contact-to-balloon	1	kept
FMECA	fmeca	fmeca	fmeca	FMECA	1	kept
fMRI	fmri	fmri	fmri	fMRI	1	kept
foam dressings	foam dressings	foam dressings	foam dressings	foam dressings	1	kept
foaming	foaming	foaming	foaming	foaming	1	kept
Focal assessment	focal assessment	focal assessment	focal assessment	Focal assessment	1	kept
focal group	focal group	focal group	focal group	focal group	1	kept
Focalization	focalization	focalization	focalization	Focalization	1	kept
focus group analysis	focus group analysis	focus group analysis	focus group analysis	focus group analysis	1	kept
Focus group discussion	focus group discussion	focus group discussion	focus group discussion	focus group discussion	1	case_or_format
focus group interview	focus group interview	focus group interview	focus group interview	Focus group interviews	1	case_or_format
Focus groups interview	focus groups interview	focus groups interview	focus groups interview	Focus groups interview	1	kept
focus of the discipline	focus of the discipline	focus of the discipline	focus of the discipline	focus of the discipline	1	kept
focus-group interviews	focus group interviews	focus group interviews	focus group interview	Focus group interviews	1	singular
Focus-groups	focus groups	focus groups	focus group	Focus Groups	1	singular
Focused cardiac ultrasound	focused cardiac ultrasound	focused cardiac ultrasound	focused cardiac ultrasound	Focused cardiac ultrasound	1	kept

focused cardiac ultrasound	focused cardiac ultrasound	focused cardiac ultrasound	focused cardiac ultrasound	Focused cardiac ultrasound	1	case_or_format
Focused older adult assessment	focused older adult assessment	focused older adult assessment	focused older adult assessment	Focused older adult assessment	1	kept
focused transthoracic echocardiography	focused transthoracic echocardiography	focused transthoracic echocardiography	focused transthoracic echocardiography	focused transthoracic echocardiography	1	kept
FOCUSED TRIGGER TOOL	focused trigger tool	focused trigger tool	focused trigger tool	FOCUSED TRIGGER TOOL	1	kept
focused ultrasound	focused ultrasound	focused ultrasound	focused ultrasound	Focused ultrasound	1	case_or_format
Fogging	fogging	fogging	fogging	Fogging	1	kept
Fogging impairing vision	fogging impairing vision	fogging impairing vision	fogging impairing vision	Fogging impairing vision	1	kept
fogging impairing vision	fogging impairing vision	fogging impairing vision	fogging impairing vision	Fogging impairing vision	1	case_or_format
Foley catheter securement	foley catheter securement	foley catheter securement	foley catheter securement	Foley catheter securement	1	kept
Foley catheters	foley catheters	foley catheters	foley catheter	Foley catheter	1	singular
folic acid antagonist	folic acid antagonist	folic acid antagonist	folic acid antagonist	folic acid antagonist	1	kept
folic acid blood level	folic acid blood level	folic acid blood level	folic acid blood level	folic acid blood level	1	kept
folinate calcium	folinate calcium	folinate calcium	folinate calcium	folinate calcium	1	kept
follow-through care	follow through care	follow through care	follow through care	follow-through care	1	kept
Follow-up clinics	follow up clinics	follow up clinics	follow up clinic	Follow-up clinic	1	singular
Follow-up clinics for at-risk infants	follow up clinics for at risk infants	follow up clinics for at risk infants	follow up clinics for at risk infants	Follow-up clinics for at-risk infants	1	kept

follow-up conversations	follow up conversations	follow up conversations	follow up conversations	follow-up conversations	1	kept
Follow-up program	follow up program	follow up program	follow up program	Follow-up program	1	kept
follow-up strategies phenomenological hermeneutical method	follow up strategies phenomenological hermeneutical method	follow up strategies phenomenological hermeneutical method	follow up strategies phenomenological hermeneutical method	follow-up strategies phenomenological hermeneutical method	1	kept
follow-up studies	follow up studies	follow up studies	follow up studies	Follow-Up Studies	1	case_or_format
Follow-up visits	follow up visits	follow up visits	follow up visits	Follow-up visits	1	kept
follow-up, health services	follow up health services	follow up health services	follow up health services	follow-up, health services	1	kept
Follow-ups studies	follow ups studies	follow ups studies	follow ups studies	Follow-ups studies	1	kept
FOLLOWER	follower	follower	follower	FOLLOWER	1	kept
Followership	followership	followership	followership	Followership	1	kept
Fomites, Nosocomial infections, Acinetobacter	fomites nosocomial infections acinetobacter	fomites nosocomial infections acinetobacter	fomites nosocomial infections acinetobacter	Fomites, Nosocomial infections, Acinetobacter	1	kept
fonction d'altérance	fonction d alt rance	fonction d alt rance	fonction d alt rance	fonction d'altérance	1	kept
Fonctionnement social	fonctionnement social	fonctionnement social	fonctionnement social	Fonctionnement social	1	kept
Fondaparinux	fondaparinux	fondaparinux	fondaparinux	fondaparinux	1	case_or_format
fontan	fontan	fontan	fontan	Fontan	1	case_or_format
Fontan procedure	fontan procedure	fontan procedure	fontan procedure	Fontan Procedure	1	case_or_format
fontanel	fontanel	fontanel	fontanel	fontanel	1	kept

Food allergy	food allergy	food allergy	food allergy	food allergy	1	case_or_format
food borne	food borne	food borne	food borne	food borne	1	kept
Food challenge	food challenge	food challenge	food challenge	Food challenge	1	kept
Food chemistry	food chemistry	food chemistry	food chemistry	Food chemistry	1	kept
food control	food control	food control	food control	food control	1	kept
food fortification	food fortification	food fortification	food fortification	food fortification	1	kept
food freezing	food freezing	food freezing	food freezing	food freezing	1	kept
Food Habits	food habits	food habits	food habits	Food Habits	1	kept
Food hygiene	food hygiene	food hygiene	food hygiene	Food hygiene	1	kept
food immunotherapy	food immunotherapy	food immunotherapy	food immunotherapy	food immunotherapy	1	kept
Food industries	food industries	food industries	food industry	Food Industry	1	singular
food industry	food industry	food industry	food industry	Food Industry	1	case_or_format
Food Intake Level Scale	food intake level scale	food intake level scale	food intake level scale	Food Intake Level Scale	1	kept
food poisoning	food poisoning	food poisoning	food poisoning	food poisoning	1	kept
Food Preservation	food preservation	food preservation	food preservation	Food Preservation	1	kept
food processing	food processing	food processing	food processing	food processing	1	kept

Food proteins	food proteins	food proteins	food proteins	Food proteins	1	kept
Food Safety	food safety	food safety	food safety	food safety	1	case_or_format
Food safety	food safety	food safety	food safety	food safety	1	case_or_format
Food Services	food services	food services	food services	Food Services	1	kept
food services staff	food services staff	food services staff	food services staff	food services staff	1	kept
food storage	food storage	food storage	food storage	Food Storage	1	case_or_format
Food suffocation	food suffocation	food suffocation	food suffocation	Food suffocation	1	kept
food waste	food waste	food waste	food waste	food waste	1	kept
Food-away-from-home	food away from home	food away from home	food away from home	Food-away-from-home	1	kept
Food-drug interactions	food drug interactions	food drug interactions	food drug interaction	food drug interaction	1	singular
food-drug interactions	food drug interactions	food drug interactions	food drug interaction	food drug interaction	1	singular
Foodborne Diseases	foodborne diseases	foodborne diseases	foodborne diseases	Foodborne Diseases	1	kept
Foot burns	foot burns	foot burns	foot burns	Foot burns	1	kept
foot care	foot care	foot care	foot care	foot care	1	kept
foot disease	foot disease	foot disease	foot disease	Foot Diseases	1	case_or_format
foot functional index	foot functional index	foot functional index	foot functional index	foot functional index	1	kept

foot massage	foot massage	foot massage	foot massage	foot massage	1	kept
foot reflexology	foot reflexology	foot reflexology	foot reflexology	Foot reflexology	1	case_or_format
foot reflexology massage	foot reflexology massage	foot reflexology massage	foot reflexology massage	foot reflexology massage	1	kept
Footstep	footstep	footstep	footstep	Footsteps	1	case_or_format
Footwear	footwear	footwear	footwear	footwear	1	case_or_format
FOR-DISEASE-CONTROL	for disease control	for disease control	for disease control	FOR-DISEASE-CONTROL	1	kept
FOR-GESTATIONAL-AGE	for gestational age	for gestational age	for gestational age	FOR-GESTATIONAL-AGE	1	kept
for-profit hospice	for profit hospice	for profit hospice	for profit hospice	for-profit hospice	1	kept
foramen magnum	foramen magnum	foramen magnum	foramen magnum	foramen magnum	1	kept
Force sensing	force sensing	force sensing	force sensing	Force sensing	1	kept
Force sensing resistor	force sensing resistor	force sensing resistor	force sensing resistor	Force sensing resistor	1	kept
force sensing resistor array	force sensing resistor array	force sensing resistor array	force sensing resistor array	force sensing resistor array	1	kept
Force-sensing array	force sensing array	force sensing array	force sensing array	force-sensing array	1	case_or_format
forced expiration	forced expiration	forced expiration	forced expiration	forced expiration	1	kept
forced expiratory flow	forced expiratory flow	forced expiratory flow	forced expiratory flow	forced expiratory flow	1	kept
Forced Expiratory Flow Rates	forced expiratory flow rates	forced expiratory flow rates	forced expiratory flow rates	Forced Expiratory Flow Rates	1	kept

Forceps	forceps	forceps	forceps	forceps	1	case_or_format
Forearm	forearm	forearm	forearm	forearm	1	case_or_format
forearm blood pressure	forearm blood pressure	forearm blood pressure	forearm blood pressure	forearm blood pressure	1	kept
forearm fracture	forearm fracture	forearm fracture	forearm fracture	forearm fracture	1	kept
Foreign bodies	foreign bodies	foreign bodies	foreign body	foreign body	1	singular
foreign bodies	foreign bodies	foreign bodies	foreign body	foreign body	1	singular
Foreign body airway obstruction	foreign body airway obstruction	foreign body airway obstruction	foreign body airway obstruction	Foreign body airway obstruction	1	kept
foreign body reaction	foreign body reaction	foreign body reaction	foreign body reaction	foreign body reaction	1	kept
foreign worker	foreign worker	foreign worker	foreign worker	foreign worker	1	kept
forelimb	forelimb	forelimb	forelimb	forelimb	1	kept
forensic	forensic	forensic	forensic	forensic	1	kept
Forensic autopsy	forensic autopsy	forensic autopsy	forensic autopsy	Forensic autopsy	1	kept
Forensic Ballistics	forensic ballistics	forensic ballistics	forensic ballistics	Forensic Ballistics	1	kept
forensic evidence	forensic evidence	forensic evidence	forensic evidence	forensic evidence	1	kept
Forensic experts	forensic experts	forensic experts	forensic experts	Forensic experts	1	kept
Forensic Mental Health	forensic mental health	forensic mental health	forensic mental health	Forensic Mental Health	1	kept

Forensic Pathology	forensic pathology	forensic pathology	forensic pathology	Forensic Pathology	1	kept
Forensic pathology	forensic pathology	forensic pathology	forensic pathology	Forensic Pathology	1	case_or_format
forensic pathology	forensic pathology	forensic pathology	forensic pathology	Forensic Pathology	1	case_or_format
FORENSIC PSYCHIATRY	forensic psychiatry	forensic psychiatry	forensic psychiatry	Forensic Psychiatry	1	case_or_format
forensic psychiatry	forensic psychiatry	forensic psychiatry	forensic psychiatry	Forensic Psychiatry	1	case_or_format
FORENSIC PSYCHOLOGY	forensic psychology	forensic psychology	forensic psychology	FORENSIC PSYCHOLOGY	1	kept
forensic scientist	forensic scientist	forensic scientist	forensic scientist	forensic scientist	1	kept
forensic toxicology	forensic toxicology	forensic toxicology	forensic toxicology	forensic toxicology	1	kept
forensic wards	forensic wards	forensic wards	forensic wards	forensic wards	1	kept
foresight	foresight	foresight	foresight	foresight	1	kept
Forestry	forestry	forestry	forestry	Forestry	1	kept
Forests	forests	forests	forest	forest	1	singular
forgetfullness	forgetfullness	forgetfullness	forgetfullness	forgetfullness	1	kept
Forgoing cancer-specific treatment	forgoing cancer specific treatment	forgoing cancer specific treatment	forgoing cancer specific treatment	Forgoing cancer-specific treatment	1	kept
FORGOTTEN GRIEVERS	forgotten grievers	forgotten grievers	forgotten grievers	FORGOTTEN GRIEVERS	1	kept
FORM B	form b	form b	form b	FORM B	1	kept

Formación en enfermería	formaci n en enfermera	formaci n en enfermera	formaci n en enfermera	Formación en enfermería	1	kept
formal caregiver	formal caregiver	formal caregiver	formal caregiver	formal caregiver	1	kept
Formal model	formal model	formal model	formal model	Formal model	1	kept
formation locale de la communauté	formation locale de la communaut	formation locale de la communaut	formation locale de la communaut	formation locale de la communauté	1	kept
Formative feedback	formative feedback	formative feedback	formative feedback	Formative Feedback	1	case_or_format
formative feedback	formative feedback	formative feedback	formative feedback	Formative Feedback	1	case_or_format
formoterol	formoterol	formoterol	formoterol	formoterol	1	kept
Forms and records control	forms and records control	forms and records control	forms and records control	Forms and Records Control	1	case_or_format
Forms as Topic	forms as topic	forms as topic	forms as topic	Forms as Topic	1	kept
Forms of aggression	forms of aggression	forms of aggression	forms of aggression	Forms of aggression	1	kept
formula	formula	formula	formula	formula	1	kept
Formulary management	formulary management	formulary management	formulary management	Formulary management	1	kept
formulary management	formulary management	formulary management	formulary management	Formulary management	1	case_or_format
FORMULATIONS	formulations	formulations	formulations	FORMULATIONS	1	kept
Fortbildung	fortbildung	fortbildung	fortbildung	Fortbildung	1	kept
FORTIFICATION	fortification	fortification	fortification	FORTIFICATION	1	kept

Fortification	fortification	fortification	fortification	FORTIFICATION	1	case_or_format
fortification	fortification	fortification	fortification	FORTIFICATION	1	case_or_format
fortified	fortified	fortified	fortified	fortified	1	kept
Forward surgical team	forward surgical team	forward surgical team	forward surgical team	Forward surgical team	1	kept
forward translation	forward translation	forward translation	forward translation	forward translation	1	kept
FOSFOMYCIN	fosfomycin	fosfomycin	fosfomycin	fosfomycin	1	case_or_format
foster child	foster child	foster child	foster child	foster child	1	kept
Fostering anti-discriminatory practice	fostering anti discriminatory practice	fostering anti discriminatory practice	fostering anti discriminatory practice	Fostering anti-discriminatory practice	1	kept
Foucault	foucault	foucault	foucault	Foucault	1	kept
foucault	foucault	foucault	foucault	Foucault	1	case_or_format
FOUNDATION	foundation	foundation	foundation	Foundations	1	case_or_format
Foundation for International Education in [illegible]	foundation for international education in [illegible]	foundation for international education in [illegible]	foundation for international education in [illegible]	Foundation for International Education in [illegible]	1	kept
FOUR	four	four	four	FOUR	1	kept
four point measurement	four point measurement	four point measurement	four point measurement	four point measurement	1	kept
four topics approach	four topics approach	four topics approach	four topics approach	four topics approach	1	kept
Fourier transform infrared spectroscopy	fourier transform infrared spectroscopy	fourier transform infrared spectroscopy	fourier transform infrared spectroscopy	Fourier transform infrared spectroscopy	1	kept

Fourier transformation	fourier transformation	fourier transformation	fourier transformation	Fourier transformation	1	kept
Fournier Gangrene	fournier gangrene	fournier gangrene	fournier gangrene	Fournier gangrene	1	case_or_format
Fournier's gangrene	fournier s gangrene	fournier s gangrene	fournier s gangrene	Fournier's gangrene	1	kept
Fournier' s gangrene	fournier s gangrene	fournier s gangrene	fournier s gangrene	Fournier's gangrene	1	case_or_format
fraction absorbed	fraction absorbed	fraction absorbed	fraction absorbed	fraction absorbed	1	kept
fractional anisotropy	fractional anisotropy	fractional anisotropy	fractional anisotropy	fractional anisotropy	1	kept
fractional flow reserve	fractional flow reserve	fractional flow reserve	fractional flow reserve	fractional flow reserve	1	kept
fractional inhibitory concentration index	fractional inhibitory concentration index	fractional inhibitory concentration index	fractional inhibitory concentration index	fractional inhibitory concentration index	1	kept
Fracturas del Fémur	fracturas del f mur	fracturas del f mur	fracturas del f mur	Fracturas del Fémur	1	kept
FRACTURE	fracture	fracture	fracture	fracture	1	case_or_format
Fracture	fracture	fracture	fracture	fracture	1	case_or_format
Fracture Dislocation	fracture dislocation	fracture dislocation	fracture dislocation	fracture dislocation	1	case_or_format
Fracture Healing	fracture healing	fracture healing	fracture healing	fracture healing	1	case_or_format
fracture risk assessment	fracture risk assessment	fracture risk assessment	fracture risk assessment	fracture risk assessment	1	kept
FRACTURES	fractures	fractures	fracture	fracture	1	singular
Fractures	fractures	fractures	fracture	fracture	1	singular

fractures, bone	fractures bone	fractures bone	fractures bone	Fractures, Bone	1	case_or_format
Fractures, Stress	fractures stress	fractures stress	fractures stress	Fractures, Stress	1	kept
Fractures, Ununited	fractures ununited	fractures ununited	fractures ununited	Fractures, Ununited	1	kept
fragile hospital environments	fragile hospital environments	fragile hospital environments	fragile hospital environments	fragile hospital environments	1	kept
fragilité	fragilit	fragilit	fragilit	fragilité	1	kept
fragmented sleep	fragmented sleep	fragmented sleep	fragmented sleep	fragmented sleep	1	kept
Fraiberg	fraiberg	fraiberg	fraiberg	Fraiberg	1	kept
Frail	frail	frail	frail	Frail	1	kept
Frail elders	frail elders	frail elders	frail elders	Frail elders	1	kept
Frail Scale	frail scale	frail scale	frail scale	Frail Scale	1	kept
FRAIL Spain	frail spain	frail spain	frail spain	FRAIL Spain	1	kept
Frailty Index Acute Care	frailty index acute care	frailty index acute care	frailty index acute care	Frailty Index Acute Care	1	kept
frailty index from common laboratory values and vital signs	frailty index from common laboratory values and vital signs	frailty index from common laboratory values and vital signs	frailty index from common laboratory values and vital signs	frailty index from common laboratory values and vital signs	1	kept
Frailty score	frailty score	frailty score	frailty score	Frailty score	1	kept
Framework	framework	framework	framework	FRAMEWORK	1	case_or_format
framework	framework	framework	framework	FRAMEWORK	1	case_or_format

Framework structures	framework structures	framework structures	framework structures	Framework structures	1	kept
frameworks	frameworks	frameworks	framework	FRAMEWORK	1	singular
framing bias	framing bias	framing bias	framing bias	framing bias	1	kept
Framingham risk score	framingham risk score	framingham risk score	framingham risk score	Framingham risk score	1	kept
Fraturas do Fêmur	fraturas do f mur	fraturas do f mur	fraturas do f mur	Fraturas do Fêmur	1	kept
fraud	fraud	fraud	fraud	fraud	1	kept
Frazier free water protocol	frazier free water protocol	frazier free water protocol	frazier free water protocol	Frazier free water protocol	1	kept
Free fibula flap	free fibula flap	free fibula flap	free fibula flap	Free fibula flap	1	kept
Free Flap	free flap	free flap	free flap	Free flap	1	case_or_format
free flap monitoring	free flap monitoring	free flap monitoring	free flap monitoring	free flap monitoring	1	kept
free flap reconstruction	free flap reconstruction	free flap reconstruction	free flap reconstruction	Free flap reconstruction	1	case_or_format
free flaps	free flaps	free flaps	free flap	Free flap	1	singular
Free texts	free texts	free texts	free text	Free-text	1	singular
Free tissue flaps	free tissue flaps	free tissue flaps	free tissue flaps	Free Tissue Flaps	1	case_or_format
free-standing children’s hospital	free standing children s hospital	free standing children s hospital	free standing children s hospital	free-standing children’s hospital	1	kept
Free-text	free text	free text	free text	Free-text	1	kept

Free-text responses	free text responses	free text responses	free text responses	Free-text responses	1	kept
Freedom driver	freedom driver	freedom driver	freedom driver	Freedom driver	1	kept
FreeO2 system	freeo2 system	freeo2 system	freeo2 system	FreeO2 system	1	kept
Freestanding children's hospital	freestanding children s hospital	freestanding children s hospital	freestanding children s hospital	Freestanding children's hospital	1	kept
Freestanding midwifery unit	freestanding midwifery unit	freestanding midwifery unit	freestanding midwifery unit	Freestanding midwifery unit	1	kept
freezing	freezing	freezing	freezing	freezing	1	kept
Freire	freire	freire	freire	Freire	1	kept
French Allergology Society	french allergology society	french allergology society	french allergology society	French Allergology Society	1	kept
French Polynesia	french polynesia	french polynesia	french polynesia	French Polynesia	1	kept
French Society of Emergency Medicine	french society of emergency medicine	french society of emergency medicine	french society of emergency medicine	French Society of Emergency Medicine	1	kept
FRENCH-NATIONAL-SOCIETY	french national society	french national society	french national society	FRENCH-NATIONAL-SOCIETY	1	kept
frenotomy	frenotomy	frenotomy	frenotomy	frenotomy	1	kept
Frequency counts	frequency counts	frequency counts	frequency counts	Frequency counts	1	kept
Frequency domain analysis	frequency domain analysis	frequency domain analysis	frequency domain analysis	Frequency domain analysis	1	kept
Frequency domain approaches	frequency domain approaches	frequency domain approaches	frequency domain approaches	Frequency domain approaches	1	kept
frequently asked questions	frequently asked questions	frequently asked questions	frequently asked questions	frequently asked questions	1	kept

Fresh tracheostomy	fresh tracheostomy	fresh tracheostomy	fresh tracheostomy	Fresh tracheostomy	1	kept
Friedrich Theodor von Frerichs	friedrich theodor von frerichs	friedrich theodor von frerichs	friedrich theodor von frerichs	Friedrich Theodor von Frerichs	1	kept
Frommelt Attitudes Toward Care of the Dying Scale	frommelt attitudes toward care of the dying scale	frommelt attitudes toward care of the dying scale	frommelt attitudes toward care of the dying scale	Frommelt Attitudes Toward Care of the Dying Scale	1	kept
Frommelt FATCOD	frommelt fatcod	frommelt fatcod	frommelt fatcod	Frommelt FATCOD	1	kept
Frommelt fatcod	frommelt fatcod	frommelt fatcod	frommelt fatcod	Frommelt FATCOD	1	case_or_format
front-line provider	front line provider	front line provider	front line provider	front-line provider	1	kept
frontal cortex	frontal cortex	frontal cortex	frontal cortex	frontal cortex	1	kept
frontal variant frontotemporal dementia	frontal variant frontotemporal dementia	frontal variant frontotemporal dementia	frontal variant frontotemporal dementia	frontal variant frontotemporal dementia	1	kept
Frontline	frontline	frontline	frontline	frontline	1	case_or_format
Frontline clinicians	frontline clinicians	frontline clinicians	frontline clinicians	Frontline clinicians	1	kept
Frontline health workers	frontline health workers	frontline health workers	frontline health workers	Frontline health workers	1	kept
Frontline healthcare workers	frontline healthcare workers	frontline healthcare workers	frontline healthcare workers	Frontline healthcare workers	1	kept
frontline healthcare workers	frontline healthcare workers	frontline healthcare workers	frontline healthcare workers	Frontline healthcare workers	1	case_or_format
Frontline physicians	frontline physicians	frontline physicians	frontline physicians	Frontline physicians	1	kept
Frontline workers	frontline workers	frontline workers	frontline workers	Frontline workers	1	kept
frontline workers	frontline workers	frontline workers	frontline workers	Frontline workers	1	case_or_format

Frova percutaneous tracheostomy	frova percutaneous tracheostomy	frova percutaneous tracheostomy	frova percutaneous tracheostomy	Frova percutaneous tracheostomy	1	kept
Frozen abdomen	frozen abdomen	frozen abdomen	frozen abdomen	Frozen abdomen	1	kept
frozen food	frozen food	frozen food	frozen food	frozen food	1	kept
Frozen Foods	frozen foods	frozen foods	frozen food	frozen food	1	singular
frozen saline	frozen saline	frozen saline	frozen saline	frozen saline	1	kept
fructose	fructose	fructose	fructose	fructose	1	kept
Fruit	fruit	fruit	fruit	Fruit	1	kept
fruit	fruit	fruit	fruit	Fruit	1	case_or_format
Fruit allergy	fruit allergy	fruit allergy	fruit allergy	Fruit allergy	1	kept
Frusemide	frusemide	frusemide	frusemide	Frusemide	1	kept
Frühgeborene	fr hgeborene	fr hgeborene	fr hgeborene	Frühgeborene	1	kept
Frührehabilitation	fr hrehabilitation	fr hrehabilitation	fr hrehabilitation	Frührehabilitation	1	kept
FSGS	fsgs	fsgs	fsgs	FSGS	1	kept
fuel	fuel	fuel	fuel	fuel	1	kept
Fuerza laboral en salud	fuerza laboral en salud	fuerza laboral en salud	fuerza laboral en salud	Fuerza laboral en salud	1	kept
Fugl-Meyer assessment	fugl meyer assessment	fugl meyer assessment	fugl meyer assessment	Fugl-Meyer assessment	1	kept

Fujita Health University	fujita health university	fujita health university	fujita health university	Fujita Health University	1	kept
Fulfillment	fulfillment	fulfillment	fulfillment	Fulfillment	1	kept
full bed bath	full bed bath	full bed bath	full bed bath	full bed bath	1	kept
full breastfeeding	full breastfeeding	full breastfeeding	full breastfeeding	full breastfeeding	1	kept
Full diversity	full diversity	full diversity	full diversity	Full diversity	1	kept
Full feeds	full feeds	full feeds	full feeds	Full feeds	1	kept
Full Outline of Un-Responsiveness Scale	full outline of un responsiveness scale	full outline of un responsiveness scale	full outline of un responsiveness scale	Full Outline of Un-Responsiveness Scale	1	kept
Full Outline of Unresponsiveness	full outline of unresponsiveness	full outline of unresponsiveness	full outline of unresponsiveness	Full Outline of UnResponsiveness	1	case_or_format
Full outline of unresponsiveness	full outline of unresponsiveness	full outline of unresponsiveness	full outline of unresponsiveness	Full Outline of UnResponsiveness	1	case_or_format
Full Outline of UnResponsiveness scale	full outline of unresponsiveness scale	full outline of unresponsiveness scale	full outline of unresponsiveness scale	Full Outline of UnResponsiveness scale	1	kept
full outline of unresponsiveness score	full outline of unresponsiveness score	full outline of unresponsiveness score	full outline of unresponsiveness score	Full Outline of Unresponsiveness score	1	case_or_format
Full Outline of Unresponsiveness score coma scale	full outline of unresponsiveness score coma scale	full outline of unresponsiveness score coma scale	full outline of unresponsiveness score coma scale	Full Outline of Unresponsiveness score coma scale	1	kept
full outline of unresponsivness score	full outline of unresponsivness score	full outline of unresponsivness score	full outline of unresponsivness score	full outline of unresponsivness score	1	kept
Full Outline Un-Responsiveness	full outline un responsiveness	full outline un responsiveness	full outline un responsiveness	Full Outline Un-Responsiveness	1	kept
full population	full population	full population	full population	full population	1	kept
Full thickness	full thickness	full thickness	full thickness	Full thickness	1	kept

full weight bearing	full weight bearing	full weight bearing	full weight bearing	full weight bearing	1	kept
Full-born	full born	full born	full born	Full-born	1	kept
Full-empty system	full empty system	full empty system	full empty system	Full-empty system	1	kept
Full-term infant	full term infant	full term infant	full term infant	Full-term infant	1	kept
FULL-TERM INFANTS	full term infants	full term infants	full term infant	Full-term infant	1	singular
Fulminant hepatic failure	fulminant hepatic failure	fulminant hepatic failure	fulminant hepatic failure	fulminant hepatic failure	1	case_or_format
Fulminant myocarditis	fulminant myocarditis	fulminant myocarditis	fulminant myocarditis	Fulminant myocarditis	1	kept
fulminant myocarditis	fulminant myocarditis	fulminant myocarditis	fulminant myocarditis	Fulminant myocarditis	1	case_or_format
Fulminant type 1 diabetes mellitus	fulminant type 1 diabetes mellitus	fulminant type 1 diabetes mellitus	fulminant type 1 diabetes mellitus	Fulminant type 1 diabetes mellitus	1	kept
Fumigation	fumigation	fumigation	fumigation	Fumigation	1	kept
fumigation	fumigation	fumigation	fumigation	Fumigation	1	case_or_format
Function	function	function	function	Function	1	kept
Function of ritualized acts	function of ritualized acts	function of ritualized acts	function of ritualized acts	Function of ritualized acts	1	kept
Functional ability	functional ability	functional ability	functional ability	Functional ability	1	kept
functional ability	functional ability	functional ability	functional ability	Functional ability	1	case_or_format
functional capability	functional capability	functional capability	functional capability	functional capability	1	kept

Functional Capacity Index	functional capacity index	functional capacity index	functional capacity index	Functional Capacity Index	1	kept
Functional change	functional change	functional change	functional change	Functional change	1	kept
FUNCTIONAL CONSEQUENCES	functional consequences	functional consequences	functional consequences	FUNCTIONAL CONSEQUENCES	1	kept
FUNCTIONAL DECLINE	functional decline	functional decline	functional decline	Functional decline	1	case_or_format
functional fitness test	functional fitness test	functional fitness test	functional fitness test	functional fitness test	1	kept
functional gastrointestinal disorder	functional gastrointestinal disorder	functional gastrointestinal disorder	functional gastrointestinal disorder	functional gastrointestinal disorder	1	kept
FUNCTIONAL HEALTH	functional health	functional health	functional health	FUNCTIONAL HEALTH	1	kept
functional health status	functional health status	functional health status	functional health status	functional health status	1	kept
Functional hemodynamic indicators	functional hemodynamic indicators	functional hemodynamic indicators	functional hemodynamic indicators	Functional hemodynamic indicators	1	kept
Functional hemodynamic monitoring	functional hemodynamic monitoring	functional hemodynamic monitoring	functional hemodynamic monitoring	Functional hemodynamic monitoring	1	kept
Functional improvements	functional improvements	functional improvements	functional improvements	Functional improvements	1	kept
functional independence	functional independence	functional independence	functional independence	functional independence	1	kept
Functional independence measure	functional independence measure	functional independence measure	functional independence measure	Functional Independence Measure	1	case_or_format
functional independence measure	functional independence measure	functional independence measure	functional independence measure	Functional Independence Measure	1	case_or_format
Functional information	functional information	functional information	functional information	Functional information	1	kept
Functional limitation	functional limitation	functional limitation	functional limitation	Functional limitation	1	kept

Functional mobility	functional mobility	functional mobility	functional mobility	Functional mobility	1	kept
Functional Neuroimaging	functional neuroimaging	functional neuroimaging	functional neuroimaging	functional neuroimaging	1	case_or_format
functional neurological disorder	functional neurological disorder	functional neurological disorder	functional neurological disorder	functional neurological disorder	1	kept
Functional outcomes	functional outcomes	functional outcomes	functional outcome	Functional outcome	1	singular
Functional recommendations	functional recommendations	functional recommendations	functional recommendations	Functional recommendations	1	kept
functional recovery	functional recovery	functional recovery	functional recovery	Functional recovery	1	case_or_format
Functional requirement	functional requirement	functional requirement	functional requirement	Functional requirements	1	case_or_format
functional resonance analysis method	functional resonance analysis method	functional resonance analysis method	functional resonance analysis method	functional resonance analysis method	1	kept
Functional specification	functional specification	functional specification	functional specification	Functional specification	1	kept
functional staging	functional staging	functional staging	functional staging	functional staging	1	kept
FUNCTIONAL STATUS	functional status	functional status	functional status	functional status	1	case_or_format
Functional ultrasound	functional ultrasound	functional ultrasound	functional ultrasound	Functional ultrasound	1	kept
Functionality based hospital	functionality based hospital	functionality based hospital	functionality based hospital	Functionality based hospital	1	kept
Functioning	functioning	functioning	functioning	Functioning	1	kept
function‐focused care	function focused care	function focused care	function focused care	function‐focused care	1	kept
Fundamental care	fundamental care	fundamental care	fundamental care	fundamental care	1	case_or_format

Fundamental tools	fundamental tools	fundamental tools	fundamental tools	Fundamental tools	1	kept
fundamentals of care framework	fundamentals of care framework	fundamentals of care framework	fundamentals of care framework	fundamentals of care framework	1	kept
Funding sources	funding sources	funding sources	funding sources	Funding sources	1	kept
Funding streams	funding streams	funding streams	funding streams	Funding streams	1	kept
Fundoplication	fundoplication	fundoplication	fundoplication	Fundoplication	1	kept
fundus camera	fundus camera	fundus camera	fundus camera	fundus camera	1	kept
Fundus Oculi	fundus oculi	fundus oculi	fundus oculi	Fundus Oculi	1	kept
funeral rites	funeral rites	funeral rites	funeral rites	Funeral Rites	1	case_or_format
fungal	fungal	fungal	fungal	Fungal	1	case_or_format
fungal cell wall	fungal cell wall	fungal cell wall	fungal cell wall	fungal cell wall	1	kept
fungal DNA	fungal dna	fungal dna	fungal dna	fungal DNA	1	kept
fungal genetics	fungal genetics	fungal genetics	fungal genetics	fungal genetics	1	kept
Fungal infection	fungal infection	fungal infection	fungal infection	fungal infection	1	case_or_format
Fungal infections	fungal infections	fungal infections	fungal infection	fungal infection	1	singular
Fungal sepsis	fungal sepsis	fungal sepsis	fungal sepsis	Fungal sepsis	1	kept
FUNGI	fungi	fungi	fungi	Fungi	1	case_or_format

fungicide	fungicide	fungicide	fungicide	fungicide	1	kept
Fungus	fungus	fungus	fungus	fungus	1	case_or_format
fungus antibody	fungus antibody	fungus antibody	fungus antibody	fungus antibody	1	kept
fungus hyphae	fungus hyphae	fungus hyphae	fungus hyphae	fungus hyphae	1	kept
Funnel Chest	funnel chest	funnel chest	funnel chest	Funnel Chest	1	kept
funnel chest	funnel chest	funnel chest	funnel chest	Funnel Chest	1	case_or_format
Fusarium	fusarium	fusarium	fusarium	Fusarium	1	kept
fused fiber filament	fused fiber filament	fused fiber filament	fused fiber filament	fused fiber filament	1	kept
fusidate sodium	fusidate sodium	fusidate sodium	fusidate sodium	fusidate sodium	1	kept
fusion	fusion	fusion	fusion	fusion	1	kept
Fusión cognitiva	fusi n cognitiva	fusi n cognitiva	fusi n cognitiva	Fusión cognitiva	1	kept
Fusobacterium infection	fusobacterium infection	fusobacterium infection	fusobacterium infection	Fusobacterium Infections	1	case_or_format
Futile	futile	futile	futile	Futile	1	kept
FUTILE CARE	futile care	futile care	futile care	Futile care	1	case_or_format
Futile medical care	futile medical care	futile medical care	futile medical care	Futile medical care	1	kept
futile therapy	futile therapy	futile therapy	futile therapy	futile therapy	1	kept

Futile treatment	futile treatment	futile treatment	futile treatment	futile treatment	1	case_or_format
Futility of care	futility of care	futility of care	futility of care	Futility of care	1	kept
FUTURE	future	future	future	FUTURE	1	kept
Future designs	future designs	future designs	future designs	Future designs	1	kept
Future of health care	future of health care	future of health care	future of health care	Future of health care	1	kept
Future of work	future of work	future of work	future of work	Future of work	1	kept
future of work	future of work	future of work	future of work	Future of work	1	case_or_format
Fuzzy analysis network process	fuzzy analysis network process	fuzzy analysis network process	fuzzy analysis network process	Fuzzy analysis network process	1	kept
fuzzy c means clustering	fuzzy c means clustering	fuzzy c means clustering	fuzzy c means clustering	fuzzy c means clustering	1	kept
Fuzzy constraint satisfaction	fuzzy constraint satisfaction	fuzzy constraint satisfaction	fuzzy constraint satisfaction	Fuzzy constraint satisfaction	1	kept
Fuzzy Delphi Method	fuzzy delphi method	fuzzy delphi method	fuzzy delphi method	Fuzzy Delphi Method	1	kept
Fuzzy rules	fuzzy rules	fuzzy rules	fuzzy rules	Fuzzy rules	1	kept
Fuzzy sets	fuzzy sets	fuzzy sets	fuzzy sets	Fuzzy sets	1	kept
fuzzy system	fuzzy system	fuzzy system	fuzzy system	fuzzy system	1	kept
Fuzzy systems	fuzzy systems	fuzzy systems	fuzzy system	fuzzy system	1	singular
Fuzzy VIKOR	fuzzy vikor	fuzzy vikor	fuzzy vikor	Fuzzy VIKOR	1	kept

fuzzy VIKOR	fuzzy vikor	fuzzy vikor	fuzzy vikor	Fuzzy VIKOR	1	case_or_format
Fuzzy-constraint	fuzzy constraint	fuzzy constraint	fuzzy constraint	Fuzzy-constraint	1	kept
FVC	fvc	fvc	fvc	FVC	1	kept
FWR	fwr	fwr	fwr	FWR	1	kept
Fármacos analgésicos	f rmacos analg sicos	f rmacos analg sicos	f rmacos analg sicos	Fármacos analgésicos	1	kept
fédération	f d ration	f d ration	f d ration	fédération	1	kept
g 10	g 10	g 10	g 10	g 10	1	kept
GABA Agents	gaba agents	gaba agents	gaba agents	GABA Agents	1	kept
GABAergic system	gabaergic system	gabaergic system	gabaergic system	GABAergic system	1	kept
GABAergic transmission	gabaergic transmission	gabaergic transmission	gabaergic transmission	GABAergic transmission	1	kept
GABAPENTIN	gabapentin	gabapentin	gabapentin	gabapentin	1	case_or_format
gacyclidine	gacyclidine	gacyclidine	gacyclidine	gacyclidine	1	kept
Gadolinium	gadolinium	gadolinium	gadolinium	gadolinium	1	case_or_format
gag reflex	gag reflex	gag reflex	gag reflex	gag reflex	1	kept
galantamine	galantamine	galantamine	galantamine	galantamine	1	kept
Galenics	galenics	galenics	galenics	Galenics	1	kept

galidesivir	galidesivir	galidesivir	galidesivir	galidesivir	1	kept
Gallbladder	gallbladder	gallbladder	gallbladder	Gallbladder	1	kept
gallbladder	gallbladder	gallbladder	gallbladder	Gallbladder	1	case_or_format
gallbladder intussusception	gallbladder intussusception	gallbladder intussusception	gallbladder intussusception	gallbladder intussusception	1	kept
Gallstones	gallstones	gallstones	gallstone	gallstone	1	singular
Galvanic skin response	galvanic skin response	galvanic skin response	galvanic skin response	Galvanic Skin Response	1	case_or_format
Gambia	gambia	gambia	gambia	Gambia	1	kept
Gambro Prismaflex machine	gambro prismaflex machine	gambro prismaflex machine	gambro prismaflex machine	Gambro Prismaflex machine	1	kept
Game	game	game	game	GAME	1	case_or_format
game changers	game changers	game changers	game changers	game changers	1	kept
Game theory	game theory	game theory	game theory	Game Theory	1	case_or_format
Games	games	games	game	GAME	1	singular
Games, Experimental	games experimental	games experimental	games experimental	Games, Experimental	1	kept
Games, Recreational	games recreational	games recreational	games recreational	Games, Recreational	1	kept
Gamification-based training	gamification based training	gamification based training	gamification based training	Gamification-based training	1	kept
gamma butyrolactone	gamma butyrolactone	gamma butyrolactone	gamma butyrolactone	gamma butyrolactone	1	kept

gamma knife radiosurgery	gamma knife radiosurgery	gamma knife radiosurgery	gamma knife radiosurgery	gamma knife radiosurgery	1	kept
gamma radiation	gamma radiation	gamma radiation	gamma radiation	gamma radiation	1	kept
gamma-aminobutyric acid	gamma aminobutyric acid	gamma aminobutyric acid	gamma aminobutyric acid	gamma-Aminobutyric Acid	1	case_or_format
gang	gang	gang	gang	gang	1	kept
ganglioside GM1	ganglioside gm1	ganglioside gm1	ganglioside gm1	ganglioside GM1	1	kept
gangrenous cellulitis	gangrenous cellulitis	gangrenous cellulitis	gangrenous cellulitis	gangrenous cellulitis	1	kept
GAP	gap	gap	gap	GAP	1	kept
GAPN intervention	gapn intervention	gapn intervention	gapn intervention	GAPN intervention	1	kept
Gaps in drug knowledge	gaps in drug knowledge	gaps in drug knowledge	gaps in drug knowledge	Gaps in drug knowledge	1	kept
garamycine	garamycine	garamycine	garamycine	garamycine	1	kept
gardening	gardening	gardening	gardening	gardening	1	kept
garlic extract	garlic extract	garlic extract	garlic extract	garlic extract	1	kept
gas analysis	gas analysis	gas analysis	gas analysis	gas analysis	1	kept
gas embolism	gas embolism	gas embolism	gas embolism	gas embolism	1	kept
gas exchange disorder	gas exchange disorder	gas exchange disorder	gas exchange disorder	gas exchange disorder	1	kept
gas flaring	gas flaring	gas flaring	gas flaring	gas flaring	1	kept

GAS-EXCHANGE	gas exchange	gas exchange	gas exchange	gas exchange	1	case_or_format
gasping	gasping	gasping	gasping	gasping	1	kept
Gastric	gastric	gastric	gastric	Gastric	1	kept
gastric acid	gastric acid	gastric acid	gastric acid	gastric acid	1	kept
gastric aspirates	gastric aspirates	gastric aspirates	gastric aspirates	gastric aspirates	1	kept
Gastric Balloon	gastric balloon	gastric balloon	gastric balloon	Gastric Balloon	1	kept
gastric banding	gastric banding	gastric banding	gastric banding	gastric banding	1	kept
Gastric bypass	gastric bypass	gastric bypass	gastric bypass	Gastric Bypass	1	case_or_format
gastric bypass	gastric bypass	gastric bypass	gastric bypass	Gastric Bypass	1	case_or_format
gastric bypass surgery	gastric bypass surgery	gastric bypass surgery	gastric bypass surgery	gastric bypass surgery	1	kept
Gastric cancers	gastric cancers	gastric cancers	gastric cancer	Gastric cancer	1	singular
gastric catheter retention	gastric catheter retention	gastric catheter retention	gastric catheter retention	gastric catheter retention	1	kept
Gastric catheterization	gastric catheterization	gastric catheterization	gastric catheterization	Gastric catheterization	1	kept
gastric conduit necrosis	gastric conduit necrosis	gastric conduit necrosis	gastric conduit necrosis	gastric conduit necrosis	1	kept
GASTRIC CONTENTS	gastric contents	gastric contents	gastric contents	GASTRIC CONTENTS	1	kept
Gastric Dilatation	gastric dilatation	gastric dilatation	gastric dilatation	Gastric Dilatation	1	kept

gastric dysmotility	gastric dysmotility	gastric dysmotility	gastric dysmotility	gastric dysmotility	1	kept
gastric fluid leak	gastric fluid leak	gastric fluid leak	gastric fluid leak	gastric fluid leak	1	kept
Gastric fluid reinfusion	gastric fluid reinfusion	gastric fluid reinfusion	gastric fluid reinfusion	Gastric fluid reinfusion	1	kept
Gastric inflation	gastric inflation	gastric inflation	gastric inflation	Gastric inflation	1	kept
gastric insufflation	gastric insufflation	gastric insufflation	gastric insufflation	gastric insufflation	1	kept
gastric intussusception	gastric intussusception	gastric intussusception	gastric intussusception	gastric intussusception	1	kept
gastric residence time	gastric residence time	gastric residence time	gastric residence time	gastric residence time	1	kept
Gastric residual	gastric residual	gastric residual	gastric residual	Gastric residual	1	kept
gastric residual	gastric residual	gastric residual	gastric residual	Gastric residual	1	case_or_format
gastric residual volumes	gastric residual volumes	gastric residual volumes	gastric residual volume	gastric residual volume	1	singular
Gastric residuals	gastric residuals	gastric residuals	gastric residual	Gastric residual	1	singular
gastric stasis	gastric stasis	gastric stasis	gastric stasis	gastric stasis	1	kept
gastric tube	gastric tube	gastric tube	gastric tube	gastric tube	1	kept
gastric tube decompression	gastric tube decompression	gastric tube decompression	gastric tube decompression	gastric tube decompression	1	kept
Gastric ulcers	gastric ulcers	gastric ulcers	gastric ulcers	Gastric ulcers	1	kept
gastric volume	gastric volume	gastric volume	gastric volume	gastric volume	1	kept

gastroccult	gastroccult	gastroccult	gastroccult	gastroccult	1	kept
gastrochisis	gastrochisis	gastrochisis	gastrochisis	gastrochisis	1	kept
gastroduodenal pseudoaneurysm	gastroduodenal pseudoaneurysm	gastroduodenal pseudoaneurysm	gastroduodenal pseudoaneurysm	gastroduodenal pseudoaneurysm	1	kept
gastroduodenostomy	gastroduodenostomy	gastroduodenostomy	gastroduodenostomy	gastroduodenostomy	1	kept
gastroenterological procedure	gastroenterological procedure	gastroenterological procedure	gastroenterological procedure	gastroenterological procedure	1	kept
Gastroenterologist	gastroenterologist	gastroenterologist	gastroenterologist	gastroenterologist	1	case_or_format
gastroenterology department	gastroenterology department	gastroenterology department	gastroenterology department	gastroenterology department	1	kept
gastroesophageal junction	gastroesophageal junction	gastroesophageal junction	gastroesophageal junction	gastroesophageal junction	1	kept
Gastroesophageal reflux disease	gastroesophageal reflux disease	gastroesophageal reflux disease	gastroesophageal reflux disease	Gastroesophageal reflux disease	1	kept
GASTROESOPHAGEAL-REFLUX	gastroesophageal reflux	gastroesophageal reflux	gastroesophageal reflux	gastroesophageal reflux	1	case_or_format
gastrohemostasis	gastrohemostasis	gastrohemostasis	gastrohemostasis	gastrohemostasis	1	kept
gastrointestinal absorption	gastrointestinal absorption	gastrointestinal absorption	gastrointestinal absorption	gastrointestinal absorption	1	kept
gastrointestinal bleed	gastrointestinal bleed	gastrointestinal bleed	gastrointestinal bleed	gastrointestinal bleed	1	kept
Gastrointestinal cancer	gastrointestinal cancer	gastrointestinal cancer	gastrointestinal cancer	gastrointestinal cancer	1	case_or_format
Gastrointestinal complaint	gastrointestinal complaint	gastrointestinal complaint	gastrointestinal complaint	Gastrointestinal complaint	1	kept
Gastrointestinal Disease	gastrointestinal disease	gastrointestinal disease	gastrointestinal disease	gastrointestinal disease	1	case_or_format

Gastrointestinal diseases	gastrointestinal diseases	gastrointestinal diseases	gastrointestinal disease	gastrointestinal disease	1	singular
gastrointestinal disorders	gastrointestinal disorders	gastrointestinal disorders	gastrointestinal disorders	gastrointestinal disorders	1	kept
Gastrointestinal dysfunction	gastrointestinal dysfunction	gastrointestinal dysfunction	gastrointestinal dysfunction	Gastrointestinal dysfunction	1	kept
gastrointestinal endoscope	gastrointestinal endoscope	gastrointestinal endoscope	gastrointestinal endoscope	gastrointestinal endoscope	1	kept
Gastrointestinal function	gastrointestinal function	gastrointestinal function	gastrointestinal function	gastrointestinal function	1	case_or_format
gastrointestinal functioning	gastrointestinal functioning	gastrointestinal functioning	gastrointestinal functioning	gastrointestinal functioning	1	kept
Gastrointestinal haemorrhage	gastrointestinal haemorrhage	gastrointestinal haemorrhage	gastrointestinal haemorrhage	Gastrointestinal haemorrhage	1	kept
Gastrointestinal illness	gastrointestinal illness	gastrointestinal illness	gastrointestinal illness	Gastrointestinal illness	1	kept
Gastrointestinal Intubation	gastrointestinal intubation	gastrointestinal intubation	gastrointestinal intubation	Gastrointestinal intubation	1	case_or_format
GASTROINTESTINAL MICROBIOTA	gastrointestinal microbiota	gastrointestinal microbiota	gastrointestinal microbiota	GASTROINTESTINAL MICROBIOTA	1	kept
Gastrointestinal motility	gastrointestinal motility	gastrointestinal motility	gastrointestinal motility	Gastrointestinal Motility	1	case_or_format
gastrointestinal motility disorder	gastrointestinal motility disorder	gastrointestinal motility disorder	gastrointestinal motility disorder	gastrointestinal motility disorder	1	kept
gastrointestinal obstruction	gastrointestinal obstruction	gastrointestinal obstruction	gastrointestinal obstruction	gastrointestinal obstruction	1	kept
gastrointestinal pain	gastrointestinal pain	gastrointestinal pain	gastrointestinal pain	gastrointestinal pain	1	kept
gastrointestinal secretion	gastrointestinal secretion	gastrointestinal secretion	gastrointestinal secretion	gastrointestinal secretion	1	kept
gastrointestinal symptoms	gastrointestinal symptoms	gastrointestinal symptoms	gastrointestinal symptom	gastrointestinal symptom	1	singular

Gastrointestinal system	gastrointestinal system	gastrointestinal system	gastrointestinal system	Gastrointestinal system	1	kept
gastrointestinal system	gastrointestinal system	gastrointestinal system	gastrointestinal system	Gastrointestinal system	1	case_or_format
Gastrointestinal System Symptoms Scale	gastrointestinal system symptoms scale	gastrointestinal system symptoms scale	gastrointestinal system symptoms scale	Gastrointestinal System Symptoms Scale	1	kept
gastrointestinal tolerance	gastrointestinal tolerance	gastrointestinal tolerance	gastrointestinal tolerance	gastrointestinal tolerance	1	kept
gastrointestinal tube	gastrointestinal tube	gastrointestinal tube	gastrointestinal tube	gastrointestinal tube	1	kept
Gastrojejunostomy	gastrojejunostomy	gastrojejunostomy	gastrojejunostomy	gastrojejunostomy	1	case_or_format
gastrojejunostomy tubes	gastrojejunostomy tubes	gastrojejunostomy tubes	gastrojejunostomy tubes	gastrojejunostomy tubes	1	kept
gastroparesis	gastroparesis	gastroparesis	gastroparesis	Gastroparesis	1	case_or_format
Gastropexy	gastropexy	gastropexy	gastropexy	gastropexy	1	case_or_format
gastroscope	gastroscope	gastroscope	gastroscope	gastroscope	1	kept
Gastrostomy tube	gastrostomy tube	gastrostomy tube	gastrostomy tube	Gastrostomy tube	1	kept
Gastrostomy tube feeding	gastrostomy tube feeding	gastrostomy tube feeding	gastrostomy tube feeding	Gastrostomy tube feeding	1	kept
Gated Blood-Pool Imaging	gated blood pool imaging	gated blood pool imaging	gated blood pool imaging	Gated Blood-Pool Imaging	1	kept
Gatifloxacin	gatifloxacin	gatifloxacin	gatifloxacin	gatifloxacin	1	case_or_format
Gaucher disease	gaucher disease	gaucher disease	gaucher disease	Gaucher disease	1	kept
Gaultheria procumbens	gaultheria procumbens	gaultheria procumbens	gaultheria procumbens	Gaultheria procumbens	1	kept

Gaussian background	gaussian background	gaussian background	gaussian background	Gaussian background	1	kept
Gaussian noise	gaussian noise	gaussian noise	gaussian noise	Gaussian noise	1	kept
Gaussians	gaussians	gaussians	gaussians	Gaussians	1	kept
Gauteng province	gauteng province	gauteng province	gauteng province	Gauteng province	1	kept
gavage	gavage	gavage	gavage	gavage	1	kept
Gavage feeding	gavage feeding	gavage feeding	gavage feeding	Gavage feeding	1	kept
Gavage feeds	gavage feeds	gavage feeds	gavage feeds	Gavage feeds	1	kept
GAVeCeLT	gavecelt	gavecelt	gavecelt	GAVeCeLT	1	kept
Gaza strip	gaza strip	gaza strip	gaza strip	Gaza Strip	1	case_or_format
gaze paralysis	gaze paralysis	gaze paralysis	gaze paralysis	gaze paralysis	1	kept
GBM	gbm	gbm	gbm	GBM	1	kept
GBP	gbp	gbp	gbp	GBP	1	kept
GBS	gbs	gbs	gbs	GBS	1	kept
gcs	gcs	gcs	gcs	GCS	1	case_or_format
GCS = Glasgow Coma Scale	gcs glasgow coma scale	gcs glasgow coma scale	gcs glasgow coma scale	GCS = Glasgow Coma Scale	1	kept
GCS-M = Glasgow Coma Scale - Motor	gcs m glasgow coma scale motor	gcs m glasgow coma scale motor	gcs m glasgow coma scale motor	GCS-M = Glasgow Coma Scale - Motor	1	kept

Gdansk [Poland]	gdansk poland	gdansk poland	gdansk poland	Gdansk [Poland]	1	kept
gear motor	gear motor	gear motor	gear motor	gear motor	1	kept
Geisinger palliative and supportive medicine consult tool	geisinger palliative and supportive medicine consult tool	geisinger palliative and supportive medicine consult tool	geisinger palliative and supportive medicine consult tool	Geisinger palliative and supportive medicine consult tool	1	kept
GEL	gel	gel	gel	Gels	1	case_or_format
Gel pillow	gel pillow	gel pillow	gel pillow	Gel pillow	1	kept
gelatin	gelatin	gelatin	gelatin	gelatin	1	kept
gemtuzumab	gemtuzumab	gemtuzumab	gemtuzumab	gemtuzumab	1	kept
Gender bias	gender bias	gender bias	gender bias	gender bias	1	case_or_format
Gender disparities	gender disparities	gender disparities	gender disparity	Gender disparity	1	singular
Gender gap	gender gap	gender gap	gender gap	Gender gap	1	kept
Gender identity	gender identity	gender identity	gender identity	Gender Identity	1	case_or_format
gender inequities	gender inequities	gender inequities	gender inequities	gender inequities	1	kept
Gender perspective	gender perspective	gender perspective	gender perspective	Gender perspective	1	kept
Gender role	gender role	gender role	gender role	Gender role	1	kept
gender role	gender role	gender role	gender role	Gender role	1	case_or_format
Gender Role Orientation	gender role orientation	gender role orientation	gender role orientation	Gender Role Orientation	1	kept

Gender roles	gender roles	gender roles	gender role	Gender role	1	singular
gender roles	gender roles	gender roles	gender role	Gender role	1	singular
Gender Stereotypes	gender stereotypes	gender stereotypes	gender stereotypes	Gender Stereotypes	1	kept
GENDER-DIFFERENCES	gender differences	gender differences	gender differences	GENDER-DIFFERENCES	1	kept
Gender-specific	gender specific	gender specific	gender specific	Gender-specific	1	kept
Gene Amplification	gene amplification	gene amplification	gene amplification	gene amplification	1	case_or_format
gene conversion	gene conversion	gene conversion	gene conversion	gene conversion	1	kept
Gene Deletion	gene deletion	gene deletion	gene deletion	Gene Deletion	1	kept
gene deletion	gene deletion	gene deletion	gene deletion	Gene Deletion	1	case_or_format
gene editing	gene editing	gene editing	gene editing	gene editing	1	kept
Gene Expression	gene expression	gene expression	gene expression	gene expression	1	case_or_format
gene expression profiling	gene expression profiling	gene expression profiling	gene expression profiling	Gene Expression Profiling	1	case_or_format
gene frequency	gene frequency	gene frequency	gene frequency	gene frequency	1	kept
gene function	gene function	gene function	gene function	gene function	1	kept
gene induction	gene induction	gene induction	gene induction	gene induction	1	kept
Gene Library	gene library	gene library	gene library	Gene Library	1	kept

gene locus	gene locus	gene locus	gene locus	gene locus	1	kept
gene overexpression	gene overexpression	gene overexpression	gene overexpression	gene overexpression	1	kept
gene transfer	gene transfer	gene transfer	gene transfer	gene transfer	1	kept
Gene Transfer, Horizontal	gene transfer horizontal	gene transfer horizontal	gene transfer horizontal	Gene Transfer, Horizontal	1	kept
gene translocation	gene translocation	gene translocation	gene translocation	gene translocation	1	kept
GENE-EXPRESSION	gene expression	gene expression	gene expression	gene expression	1	case_or_format
General	general	general	general	general	1	case_or_format
General & acute	general and acute	general and acute	general and acute	General & acute	1	kept
general anaesthesia	general anaesthesia	general anaesthesia	general anaesthesia	general anaesthesia	1	kept
general apparatus, equipment and supplies	general apparatus equipment and supplies	general apparatus equipment and supplies	general apparatus equipment and supplies	general apparatus, equipment and supplies	1	kept
General guidance on prescribing	general guidance on prescribing	general guidance on prescribing	general guidance on prescribing	General guidance on prescribing	1	kept
General Health Questionnaire 28	general health questionnaire 28	general health questionnaire 28	general health questionnaire 28	General Health Questionnaire 28	1	kept
General Health Questionnaire-12	general health questionnaire 12	general health questionnaire 12	general health questionnaire 12	General Health Questionnaire-12	1	kept
General hospital psychiatry	general hospital psychiatry	general hospital psychiatry	general hospital psychiatry	General hospital psychiatry	1	kept
General Hospitals	general hospitals	general hospitals	general hospital	general hospital	1	singular
general medical surgical ward	general medical surgical ward	general medical surgical ward	general medical surgical ward	general medical surgical ward	1	kept

general medical wards	general medical wards	general medical wards	general medical wards	general medical wards	1	kept
General medicine	general medicine	general medicine	general medicine	general medicine	1	case_or_format
General movement assessment	general movement assessment	general movement assessment	general movement assessment	General movement assessment	1	kept
general movement assessment	general movement assessment	general movement assessment	general movement assessment	General movement assessment	1	case_or_format
GENERAL MOVEMENTS	general movements	general movements	general movements	GENERAL MOVEMENTS	1	kept
General Movements Assessment	general movements assessment	general movements assessment	general movements assessment	General Movements Assessment	1	kept
general or family practice physician	general or family practice physician	general or family practice physician	general or family practice physician	general or family practice physician	1	kept
General Paediatrics	general paediatrics	general paediatrics	general paediatrics	general paediatrics	1	case_or_format
general pathological parameters	general pathological parameters	general pathological parameters	general pathological parameters	general pathological parameters	1	kept
General practice and primary care	general practice and primary care	general practice and primary care	general practice and primary care	General practice and primary care	1	kept
General psychological distress	general psychological distress	general psychological distress	general psychological distress	General psychological distress	1	kept
General psychopathology level	general psychopathology level	general psychopathology level	general psychopathology level	General psychopathology level	1	kept
General radiology	general radiology	general radiology	general radiology	General radiology	1	kept
general self-efficacy	general self efficacy	general self efficacy	general self efficacy	general self-efficacy	1	kept
General surgery residency training	general surgery residency training	general surgery residency training	general surgery residency training	General surgery residency training	1	kept
general surgery services	general surgery services	general surgery services	general surgery services	general surgery services	1	kept

general surgery unit	general surgery unit	general surgery unit	general surgery unit	general surgery unit	1	kept
General System Theory	general system theory	general system theory	general system theory	General System Theory	1	kept
General unit	general unit	general unit	general unit	General unit	1	kept
General ward setting	general ward setting	general ward setting	general ward setting	General ward setting	1	kept
GENERAL WARDS	general wards	general wards	general ward	general ward	1	singular
GENERAL-POPULATION	general population	general population	general population	General population	1	case_or_format
GENERAL-PRACTICE	general practice	general practice	general practice	general practice	1	case_or_format
GENERAL-PRACTITIONER	general practitioner	general practitioner	general practitioner	general practitioner	1	case_or_format
generalisability	generalisability	generalisability	generalisability	generalisability	1	kept
Generalist	generalist	generalist	generalist	Generalist	1	kept
Generalist palliative care	generalist palliative care	generalist palliative care	generalist palliative care	Generalist palliative care	1	kept
generalizability	generalizability	generalizability	generalizability	generalizability	1	kept
Generalized	generalized	generalized	generalized	Generalized	1	kept
Generalized linear models	generalized linear models	generalized linear models	generalized linear models	Generalized linear models	1	kept
generalized linear models	generalized linear models	generalized linear models	generalized linear models	Generalized linear models	1	case_or_format
Generalized mixed-effects regression trees	generalized mixed effects regression trees	generalized mixed effects regression trees	generalized mixed effects regression trees	Generalized mixed-effects regression trees	1	kept

generalized rhythmic delta activity	generalized rhythmic delta activity	generalized rhythmic delta activity	generalized rhythmic delta activity	generalized rhythmic delta activity	1	kept
generation effects	generation effects	generation effects	generation effects	generation effects	1	kept
Generation process	generation process	generation process	generation process	Generation process	1	kept
Generational differences	generational differences	generational differences	generational differences	Generational differences	1	kept
Generations	generations	generations	generations	Generations	1	kept
Generative Artificial Intelligence	generative artificial intelligence	generative artificial intelligence	generative artificial intelligence	Generative artificial intelligence	1	case_or_format
generative language models	generative language models	generative language models	generative language models	generative language models	1	kept
generative pretrained transformer	generative pretrained transformer	generative pretrained transformer	generative pretrained transformer	generative pretrained transformer	1	kept
Generic approach	generic approach	generic approach	generic approach	Generic approach	1	kept
generic clinic model	generic clinic model	generic clinic model	generic clinic model	generic clinic model	1	kept
Generic patterns	generic patterns	generic patterns	generic patterns	Generic patterns	1	kept
Generic Quality of Life Inventory 74 scale	generic quality of life inventory 74 scale	generic quality of life inventory 74 scale	generic quality of life inventory 74 scale	Generic Quality of Life Inventory 74 scale	1	kept
Genes	genes	genes	gene	gene	1	singular
Genes, Dominant	genes dominant	genes dominant	genes dominant	Genes, Dominant	1	kept
Genetic algorithm	genetic algorithm	genetic algorithm	genetic algorithm	genetic algorithm	1	case_or_format
genetic association study	genetic association study	genetic association study	genetic association study	genetic association study	1	kept

genetic correlation	genetic correlation	genetic correlation	genetic correlation	genetic correlation	1	kept
genetic counselling	genetic counselling	genetic counselling	genetic counselling	genetic counselling	1	kept
genetic counselor	genetic counselor	genetic counselor	genetic counselor	genetic counselor	1	kept
genetic distance	genetic distance	genetic distance	genetic distance	genetic distance	1	kept
genetic epidemiology	genetic epidemiology	genetic epidemiology	genetic epidemiology	genetic epidemiology	1	kept
genetic fundamentals	genetic fundamentals	genetic fundamentals	genetic fundamentals	genetic fundamentals	1	kept
genetic heterogeneity	genetic heterogeneity	genetic heterogeneity	genetic heterogeneity	genetic heterogeneity	1	kept
genetic marker	genetic marker	genetic marker	genetic marker	genetic marker	1	kept
Genetic Markers	genetic markers	genetic markers	genetic marker	genetic marker	1	singular
Genetic Matching	genetic matching	genetic matching	genetic matching	Genetic Matching	1	kept
genetic procedures	genetic procedures	genetic procedures	genetic procedures	genetic procedures	1	kept
genetic profile	genetic profile	genetic profile	genetic profile	genetic profile	1	kept
genetic relatedness	genetic relatedness	genetic relatedness	genetic relatedness	genetic relatedness	1	kept
Genetic relations	genetic relations	genetic relations	genetic relations	Genetic relations	1	kept
Genetic Research	genetic research	genetic research	genetic research	Genetic Research	1	kept
genetic service providers	genetic service providers	genetic service providers	genetic service providers	genetic service providers	1	kept

genetic therapy	genetic therapy	genetic therapy	genetic therapy	genetic therapy	1	kept
genetic variant	genetic variant	genetic variant	genetic variant	genetic variant	1	kept
genital injury	genital injury	genital injury	genital injury	genital injury	1	kept
genital system	genital system	genital system	genital system	genital system	1	kept
genital tract infection	genital tract infection	genital tract infection	genital tract infection	genital tract infection	1	kept
genitourinary symptom	genitourinary symptom	genitourinary symptom	genitourinary symptom	genitourinary symptom	1	kept
Genitourinary trauma	genitourinary trauma	genitourinary trauma	genitourinary trauma	Genitourinary trauma	1	kept
genodermatosis	genodermatosis	genodermatosis	genodermatosis	genodermatosis	1	kept
genome analysis	genome analysis	genome analysis	genome analysis	genome analysis	1	kept
genome macrorestriction pulsed field gel	genome macrorestriction pulsed field gel	genome macrorestriction pulsed field gel	genome macrorestriction pulsed field gel	genome macrorestriction pulsed field gel	1	kept
Genome macrorestriction-pulsed-field gel	genome macrorestriction pulsed field gel	genome macrorestriction pulsed field gel	genome macrorestriction pulsed field gel	genome macrorestriction pulsed field gel	1	case_or_format
genome macrorestriction-pulsed-field gel	genome macrorestriction pulsed field gel	genome macrorestriction pulsed field gel	genome macrorestriction pulsed field gel	genome macrorestriction pulsed field gel	1	case_or_format
Genome, Viral	genome viral	genome viral	genome viral	Genome, Viral	1	kept
Genome-Wide Association Study	genome wide association study	genome wide association study	genome wide association study	genome-wide association study	1	case_or_format
genomic epidemiology	genomic epidemiology	genomic epidemiology	genomic epidemiology	genomic epidemiology	1	kept
GENOMIC MEDICINE	genomic medicine	genomic medicine	genomic medicine	GENOMIC MEDICINE	1	kept

genomic RNA	genomic rna	genomic rna	genomic rna	genomic RNA	1	kept
Genotyping Techniques	genotyping techniques	genotyping techniques	genotyping technique	genotyping technique	1	singular
genpril	genpril	genpril	genpril	genpril	1	kept
Gentamicin	gentamicin	gentamicin	gentamicin	gentamicin	1	case_or_format
gentamicin resistance	gentamicin resistance	gentamicin resistance	gentamicin resistance	gentamicin resistance	1	kept
geographic cohorting	geographic cohorting	geographic cohorting	geographic cohorting	geographic cohorting	1	kept
Geographic distribution	geographic distribution	geographic distribution	geographic distribution	geographic distribution	1	case_or_format
geographic information system	geographic information system	geographic information system	geographic information system	Geographic information systems	1	case_or_format
GEOGRAPHIC INFORMATION SYSTEMS	geographic information systems	geographic information systems	geographic information system	Geographic information systems	1	singular
Geographic Information Systems	geographic information systems	geographic information systems	geographic information system	Geographic information systems	1	singular
geographic variation	geographic variation	geographic variation	geographic variation	geographic variation	1	kept
GEOGRAPHIC-DISTRIBUTION	geographic distribution	geographic distribution	geographic distribution	geographic distribution	1	case_or_format
geographical thought	geographical thought	geographical thought	geographical thought	geographical thought	1	kept
Geography, Medical	geography medical	geography medical	geography medical	Geography, Medical	1	kept
Geometric conditions	geometric conditions	geometric conditions	geometric conditions	Geometric conditions	1	kept
geriatric admission	geriatric admission	geriatric admission	geriatric admission	geriatric admission	1	kept

Geriatric admissions	geriatric admissions	geriatric admissions	geriatric admission	geriatric admission	1	singular
Geriatric assessment tools	geriatric assessment tools	geriatric assessment tools	geriatric assessment tools	Geriatric assessment tools	1	kept
geriatric cardiology specialist	geriatric cardiology specialist	geriatric cardiology specialist	geriatric cardiology specialist	geriatric cardiology specialist	1	kept
Geriatric care	geriatric care	geriatric care	geriatric care	geriatric care	1	case_or_format
Geriatric convalescence	geriatric convalescence	geriatric convalescence	geriatric convalescence	Geriatric convalescence	1	kept
geriatric criteria	geriatric criteria	geriatric criteria	geriatric criteria	geriatric criteria	1	kept
geriatric dentistry	geriatric dentistry	geriatric dentistry	geriatric dentistry	geriatric dentistry	1	kept
Geriatric Depression Scale	geriatric depression scale	geriatric depression scale	geriatric depression scale	Geriatric Depression Scale	1	kept
Geriatric Emergency Medicine	geriatric emergency medicine	geriatric emergency medicine	geriatric emergency medicine	Geriatric Emergency Medicine	1	kept
Geriatric femur fractures	geriatric femur fractures	geriatric femur fractures	geriatric femur fractures	Geriatric femur fractures	1	kept
geriatric frailty	geriatric frailty	geriatric frailty	geriatric frailty	geriatric frailty	1	kept
Geriatric G-60	geriatric g 60	geriatric g 60	geriatric g 60	Geriatric G-60	1	kept
geriatric government	geriatric government	geriatric government	geriatric government	geriatric government	1	kept
Geriatric healthcare	geriatric healthcare	geriatric healthcare	geriatric healthcare	Geriatric healthcare	1	kept
Geriatric nutritional risk index	geriatric nutritional risk index	geriatric nutritional risk index	geriatric nutritional risk index	Geriatric Nutritional Risk Index	1	case_or_format
geriatric nutritional risk index	geriatric nutritional risk index	geriatric nutritional risk index	geriatric nutritional risk index	Geriatric Nutritional Risk Index	1	case_or_format

Geriatric palliative care	geriatric palliative care	geriatric palliative care	geriatric palliative care	Geriatric palliative care	1	kept
geriatric protocols	geriatric protocols	geriatric protocols	geriatric protocols	geriatric protocols	1	kept
geriatric psychiatry	geriatric psychiatry	geriatric psychiatry	geriatric psychiatry	Geriatric Psychiatry	1	case_or_format
Geriatric rehabilitation	geriatric rehabilitation	geriatric rehabilitation	geriatric rehabilitation	geriatric rehabilitation	1	case_or_format
Geriatric rib fractures	geriatric rib fractures	geriatric rib fractures	geriatric rib fractures	Geriatric rib fractures	1	kept
Geriatric surgery	geriatric surgery	geriatric surgery	geriatric surgery	geriatric surgery	1	case_or_format
geriatric syndrome	geriatric syndrome	geriatric syndrome	geriatric syndrome	geriatric syndrome	1	kept
geriatric training	geriatric training	geriatric training	geriatric training	geriatric training	1	kept
GERIATRIC TRAUMA	geriatric trauma	geriatric trauma	geriatric trauma	geriatric trauma	1	case_or_format
geriatric trauma clinical pathway	geriatric trauma clinical pathway	geriatric trauma clinical pathway	geriatric trauma clinical pathway	geriatric trauma clinical pathway	1	kept
Geriatric Trauma Screening Tool	geriatric trauma screening tool	geriatric trauma screening tool	geriatric trauma screening tool	Geriatric Trauma Screening Tool	1	kept
geriatric traumatology	geriatric traumatology	geriatric traumatology	geriatric traumatology	Geriatric traumatology	1	case_or_format
Geriatrician	geriatrician	geriatrician	geriatrician	geriatrician	1	case_or_format
GERIATRICS	geriatrics	geriatrics	geriatric	Geriatrics	1	singular
germ cell tumor	germ cell tumor	germ cell tumor	germ cell tumor	germ cell tumor	1	kept
germ line	germ line	germ line	germ line	germ line	1	kept

german cardiac arrest registry	german cardiac arrest registry	german cardiac arrest registry	german cardiac arrest registry	german cardiac arrest registry	1	kept
German diagnosis related groups	german diagnosis related groups	german diagnosis related groups	german diagnosis related groups	German diagnosis related groups	1	kept
German Environmental Audit Tool	german environmental audit tool	german environmental audit tool	german environmental audit tool	German Environmental Audit Tool	1	kept
German Federal Joint Commission	german federal joint commission	german federal joint commission	german federal joint commission	German Federal Joint Commission	1	kept
German federal joint commission	german federal joint commission	german federal joint commission	german federal joint commission	German Federal Joint Commission	1	case_or_format
German Interdisciplinary Society for Ambulatory	german interdisciplinary society for ambulatory	german interdisciplinary society for ambulatory	german interdisciplinary society for ambulatory	German Interdisciplinary Society for Ambulatory	1	kept
German Society of Internal Medicine	german society of internal medicine	german society of internal medicine	german society of internal medicine	German Society of Internal Medicine	1	kept
German Socio-Economic Panel	german socio economic panel	german socio economic panel	german socio economic panel	German Socio-Economic Panel	1	kept
Germline kit mutation	germline kit mutation	germline kit mutation	germline kit mutation	Germline kit mutation	1	kept
germline mutation	germline mutation	germline mutation	germline mutation	germline mutation	1	kept
GERONTOLOGY	gerontology	gerontology	gerontology	gerontology	1	case_or_format
Gerontology	gerontology	gerontology	gerontology	gerontology	1	case_or_format
gerontology hospitalisation	gerontology hospitalisation	gerontology hospitalisation	gerontology hospitalisation	gerontology hospitalisation	1	kept
Gestation	gestation	gestation	gestation	GESTATION	1	case_or_format
gestion de crise	gestion de crise	gestion de crise	gestion de crise	gestion de crise	1	kept
gestion des antimicrobiens	gestion des antimicrobiens	gestion des antimicrobiens	gestion des antimicrobiens	gestion des antimicrobiens	1	kept

gestion des risques	gestion des risques	gestion des risques	gestion des risques	gestion des risques	1	kept
Gestión de la información en salud	gesti n de la informaci n en salud	gesti n de la informaci n en salud	gesti n de la informaci n en salud	Gestión de la información en salud	1	kept
Gestión de personal	gesti n de personal	gesti n de personal	gesti n de personal	Gestión de personal	1	kept
Gesture recognition	gesture recognition	gesture recognition	gesture recognition	Gesture recognition	1	kept
Gestão do Conhecimento	gest o do conhecimento	gest o do conhecimento	gest o do conhecimento	Gestão do Conhecimento	1	kept
Get to Know Me board	get to know me board	get to know me board	get to know me board	Get to Know Me board	1	kept
Get With the Guidelines	get with the guidelines	get with the guidelines	get with the guidelines	Get With the Guidelines	1	kept
Ghpu	ghpu	ghpu	ghpu	Ghpu	1	kept
GHQ	ghq	ghq	ghq	GHQ	1	kept
GHQ 28	ghq 28	ghq 28	ghq 28	GHQ 28	1	kept
GI bleeding	gi bleeding	gi bleeding	gi bleeding	GI bleeding	1	kept
GI failure	gi failure	gi failure	gi failure	GI failure	1	kept
giant cell	giant cell	giant cell	giant cell	giant cell	1	kept
giant lumbar pseudomeningocele	giant lumbar pseudomeningocele	giant lumbar pseudomeningocele	giant lumbar pseudomeningocele	giant lumbar pseudomeningocele	1	kept
giant omphalocele	giant omphalocele	giant omphalocele	giant omphalocele	giant omphalocele	1	kept
Gift	gift	gift	gift	Gift	1	kept

gift	gift	gift	gift	Gift	1	case_or_format
Gift Giving	gift giving	gift giving	gift giving	gift giving	1	case_or_format
gimsilumab	gimsilumab	gimsilumab	gimsilumab	gimsilumab	1	kept
gingiva bleeding	gingiva bleeding	gingiva bleeding	gingiva bleeding	gingiva bleeding	1	kept
gingiva hyperplasia	gingiva hyperplasia	gingiva hyperplasia	gingiva hyperplasia	gingiva hyperplasia	1	kept
gingiva hypertrophy	gingiva hypertrophy	gingiva hypertrophy	gingiva hypertrophy	gingiva hypertrophy	1	kept
gingival index	gingival index	gingival index	gingival index	gingival index	1	kept
Ginkgo biloba extract	ginkgo biloba extract	ginkgo biloba extract	ginkgo biloba extract	Ginkgo biloba extract	1	kept
ginseng extract	ginseng extract	ginseng extract	ginseng extract	ginseng extract	1	kept
Giorgi phenomenological	giorgi phenomenological	giorgi phenomenological	giorgi phenomenological	Giorgi phenomenological	1	kept
GIS	gis	gis	gis	GIS	1	kept
GIS-based analysis	gis based analysis	gis based analysis	gis based analysis	GIS-based analysis	1	kept
giving compassionate care	giving compassionate care	giving compassionate care	giving compassionate care	giving compassionate care	1	kept
Giving information	giving information	giving information	giving information	Giving information	1	kept
giving information	giving information	giving information	giving information	Giving information	1	case_or_format
GKV-IPReG	gkv ipreg	gkv ipreg	gkv ipreg	GKV-IPReG	1	kept

GLASGOW COMA SCALE	glasgow coma scale	glasgow coma scale	glasgow coma scale	Glasgow coma scale	1	case_or_format
Glasgow coma score	glasgow coma score	glasgow coma score	glasgow coma score	Glasgow coma score	1	kept
Glasgow Liege scale	glasgow liege scale	glasgow liege scale	glasgow liege scale	Glasgow Liege scale	1	kept
Glasgow Outcome Score	glasgow outcome score	glasgow outcome score	glasgow outcome score	Glasgow Outcome Score	1	kept
Glass ampule	glass ampule	glass ampule	glass ampule	Glass ampule	1	kept
glass ionomer	glass ionomer	glass ionomer	glass ionomer	glass ionomer	1	kept
GLAUCOMA	glaucoma	glaucoma	glaucoma	glaucoma	1	case_or_format
Glial Fibrillary Acidic Protein	glial fibrillary acidic protein	glial fibrillary acidic protein	glial fibrillary acidic protein	glial fibrillary acidic protein	1	case_or_format
gliclazide	gliclazide	gliclazide	gliclazide	gliclazide	1	kept
glimepiride	glimepiride	glimepiride	glimepiride	glimepiride	1	kept
glisson capsulectomy	glisson capsulectomy	glisson capsulectomy	glisson capsulectomy	glisson capsulectomy	1	kept
glitazone derivative	glitazone derivative	glitazone derivative	glitazone derivative	glitazone derivative	1	kept
GLM	glm	glm	glm	GLM	1	kept
Global anesthesia	global anesthesia	global anesthesia	global anesthesia	Global anesthesia	1	kept
GLOBAL BURDEN	global burden	global burden	global burden	GLOBAL BURDEN	1	kept
Global burden of ROP	global burden of rop	global burden of rop	global burden of rop	Global burden of ROP	1	kept

global change	global change	global change	global change	global change	1	kept
global deterioration rating scale	global deterioration rating scale	global deterioration rating scale	global deterioration rating scale	global deterioration rating scale	1	kept
global disaster	global disaster	global disaster	global disaster	global disaster	1	kept
global disparities	global disparities	global disparities	global disparities	global disparities	1	kept
global education	global education	global education	global education	global education	1	kept
Global European Initiative	global european initiative	global european initiative	global european initiative	Global European Initiative	1	kept
Global functioning	global functioning	global functioning	global functioning	Global functioning	1	kept
Global Health Emergency	global health emergency	global health emergency	global health emergency	Global Health Emergency	1	kept
Global Health Policy	global health policy	global health policy	global health policy	Global Health Policy	1	kept
Global Health Security	global health security	global health security	global health security	Global Health Security	1	kept
Global perspective	global perspective	global perspective	global perspective	global perspective	1	case_or_format
global perspectives	global perspectives	global perspectives	global perspective	global perspective	1	singular
global policy	global policy	global policy	global policy	global policy	1	kept
global sleep quality	global sleep quality	global sleep quality	global sleep quality	global sleep quality	1	kept
global trigger tool	global trigger tool	global trigger tool	global trigger tool	global trigger tool	1	kept
Global Trigger Tool method	global trigger tool method	global trigger tool method	global trigger tool method	Global Trigger Tool method	1	kept

Global village	global village	global village	global village	Global village	1	kept
global warming	global warming	global warming	global warming	global warming	1	kept
global warming potential	global warming potential	global warming potential	global warming potential	global warming potential	1	kept
globe rupture	globe rupture	globe rupture	globe rupture	globe rupture	1	kept
globoid cell leukodystrophy	globoid cell leukodystrophy	globoid cell leukodystrophy	globoid cell leukodystrophy	globoid cell leukodystrophy	1	kept
glomerular filtration rate	glomerular filtration rate	glomerular filtration rate	glomerular filtration rate	Glomerular Filtration Rate	1	case_or_format
glomerulonephritis	glomerulonephritis	glomerulonephritis	glomerulonephritis	glomerulonephritis	1	kept
Glomerulosclerosis, Focal Segmental	glomerulosclerosis focal segmental	glomerulosclerosis focal segmental	glomerulosclerosis focal segmental	Glomerulosclerosis, Focal Segmental	1	kept
glomerulus basement membrane	glomerulus basement membrane	glomerulus basement membrane	glomerulus basement membrane	glomerulus basement membrane	1	kept
glomerulus capillary	glomerulus capillary	glomerulus capillary	glomerulus capillary	glomerulus capillary	1	kept
glossectomy	glossectomy	glossectomy	glossectomy	glossectomy	1	kept
glottal cleaning maneuver	glottal cleaning maneuver	glottal cleaning maneuver	glottal cleaning maneuver	glottal cleaning maneuver	1	kept
Gloucester comfort score	gloucester comfort score	gloucester comfort score	gloucester comfort score	Gloucester comfort score	1	kept
glove juice technique	glove juice technique	glove juice technique	glove juice technique	glove juice technique	1	kept
Glove use	glove use	glove use	glove use	Glove use	1	kept
glucagon blood level	glucagon blood level	glucagon blood level	glucagon blood level	glucagon blood level	1	kept

glucagon like peptide 1	glucagon like peptide 1	glucagon like peptide 1	glucagon like peptide 1	glucagon like peptide 1	1	kept
glucagon like peptide 1 derivative	glucagon like peptide 1 derivative	glucagon like peptide 1 derivative	glucagon like peptide 1 derivative	glucagon like peptide 1 derivative	1	kept
Glucagon-Like Peptide 1	glucagon like peptide 1	glucagon like peptide 1	glucagon like peptide 1	glucagon like peptide 1	1	case_or_format
Glucagon-like peptide-1 receptor agonist	glucagon like peptide 1 receptor agonist	glucagon like peptide 1 receptor agonist	glucagon like peptide 1 receptor agonist	glucagon like peptide 1 receptor agonist	1	case_or_format
Glucemia	glucemia	glucemia	glucemia	Glucemia	1	kept
Glucometer	glucometer	glucometer	glucometer	Glucometer	1	kept
Glucommander	glucommander	glucommander	glucommander	Glucommander	1	kept
Glucommander system	glucommander system	glucommander system	glucommander system	Glucommander system	1	kept
Gluconates	gluconates	gluconates	gluconates	Gluconates	1	kept
gluconeogenesis	gluconeogenesis	gluconeogenesis	gluconeogenesis	gluconeogenesis	1	kept
gluconic acid	gluconic acid	gluconic acid	gluconic acid	gluconic acid	1	kept
GLUCOSE	glucose	glucose	glucose	glucose	1	case_or_format
glucose cerebrospinal fluid level	glucose cerebrospinal fluid level	glucose cerebrospinal fluid level	glucose cerebrospinal fluid level	glucose cerebrospinal fluid level	1	kept
glucose clamp technique	glucose clamp technique	glucose clamp technique	glucose clamp technique	glucose clamp technique	1	kept
Glucose Dehydrogenases	glucose dehydrogenases	glucose dehydrogenases	glucose dehydrogenase	glucose dehydrogenase	1	singular
glucose gel	glucose gel	glucose gel	glucose gel	glucose gel	1	kept

GLUCOSE INFUSION	glucose infusion	glucose infusion	glucose infusion	glucose infusion	1	case_or_format
Glucose management	glucose management	glucose management	glucose management	Glucose management	1	kept
glucose management	glucose management	glucose management	glucose management	Glucose management	1	case_or_format
glucose meters	glucose meters	glucose meters	glucose meters	glucose meters	1	kept
Glucose monitoring	glucose monitoring	glucose monitoring	glucose monitoring	Glucose monitoring	1	kept
glucose monitoring	glucose monitoring	glucose monitoring	glucose monitoring	Glucose monitoring	1	case_or_format
GLUCOSE MONITORING-SYSTEM	glucose monitoring system	glucose monitoring system	glucose monitoring system	GLUCOSE MONITORING-SYSTEM	1	kept
Glucose Oxidase	glucose oxidase	glucose oxidase	glucose oxidase	glucose oxidase	1	case_or_format
glucose point of care testing	glucose point of care testing	glucose point of care testing	glucose point of care testing	glucose point of care testing	1	kept
Glucose regulation	glucose regulation	glucose regulation	glucose regulation	glucose regulation	1	case_or_format
glucose regulation system	glucose regulation system	glucose regulation system	glucose regulation system	glucose regulation system	1	kept
Glucose stabilization	glucose stabilization	glucose stabilization	glucose stabilization	Glucose stabilization	1	kept
glucose stabilization trial	glucose stabilization trial	glucose stabilization trial	glucose stabilization trial	glucose stabilization trial	1	kept
Glucose telemetry	glucose telemetry	glucose telemetry	glucose telemetry	Glucose telemetry	1	kept
glucose tolerance	glucose tolerance	glucose tolerance	glucose tolerance	glucose tolerance	1	kept
glucose transporter 4	glucose transporter 4	glucose transporter 4	glucose transporter 4	glucose transporter 4	1	kept

glucose utilization	glucose utilization	glucose utilization	glucose utilization	glucose utilization	1	kept
glucose-to-lymphocyte ratio	glucose to lymphocyte ratio	glucose to lymphocyte ratio	glucose to lymphocyte ratio	glucose-to-lymphocyte ratio	1	kept
glucosuria	glucosuria	glucosuria	glucosuria	glucosuria	1	kept
glutamate decarboxylase 65	glutamate decarboxylase 65	glutamate decarboxylase 65	glutamate decarboxylase 65	glutamate decarboxylase 65	1	kept
GLUTARALDEHYDE	glutaraldehyde	glutaraldehyde	glutaraldehyde	glutaraldehyde	1	case_or_format
glutaric acid	glutaric acid	glutaric acid	glutaric acid	glutaric acid	1	kept
glutathione	glutathione	glutathione	glutathione	glutathione	1	kept
GLUTATHIONE-PEROXIDASE	glutathione peroxidase	glutathione peroxidase	glutathione peroxidase	GLUTATHIONE-PEROXIDASE	1	kept
gluteal	gluteal	gluteal	gluteal	gluteal	1	kept
gluteus muscle	gluteus muscle	gluteus muscle	gluteus muscle	gluteus muscle	1	kept
Glycaemia	glycaemia	glycaemia	glycaemia	Glycaemia	1	kept
Glycaemia control	glycaemia control	glycaemia control	glycaemia control	Glycaemia control	1	kept
Glycaemic lability	glycaemic lability	glycaemic lability	glycaemic lability	Glycaemic lability	1	kept
Glycated haemoglobin A1c	glycated haemoglobin a1c	glycated haemoglobin a1c	glycated haemoglobin a1c	Glycated haemoglobin A1c	1	kept
Glycated Hemoglobin A	glycated hemoglobin a	glycated hemoglobin a	glycated hemoglobin a	Glycated Hemoglobin A	1	kept
Glycation	glycation	glycation	glycation	Glycation	1	kept

glycemia prediction	glycemia prediction	glycemia prediction	glycemia prediction	glycemia prediction	1	kept
glycemic control protocol	glycemic control protocol	glycemic control protocol	glycemic control protocol	glycemic control protocol	1	kept
glycemic management	glycemic management	glycemic management	glycemic management	glycemic management	1	kept
Glycemic penalty index	glycemic penalty index	glycemic penalty index	glycemic penalty index	Glycemic penalty index	1	kept
glycemic penalty index	glycemic penalty index	glycemic penalty index	glycemic penalty index	Glycemic penalty index	1	case_or_format
Glycemic variability	glycemic variability	glycemic variability	glycemic variability	glycemic variability	1	case_or_format
Glycerin	glycerin	glycerin	glycerin	Glycerin	1	kept
glycerol nitrate	glycerol nitrate	glycerol nitrate	glycerol nitrate	glycerol nitrate	1	kept
Glycerophosphates	glycerophosphates	glycerophosphates	glycerophosphates	Glycerophosphates	1	kept
Glycocalyx	glycocalyx	glycocalyx	glycocalyx	Glycocalyx	1	kept
glycocalyx	glycocalyx	glycocalyx	glycocalyx	Glycocalyx	1	case_or_format
glycogen	glycogen	glycogen	glycogen	glycogen	1	kept
glycogen storage disease type 2	glycogen storage disease type 2	glycogen storage disease type 2	glycogen storage disease type 2	glycogen storage disease type 2	1	kept
Glycol	glycol	glycol	glycol	Glycol	1	kept
glycolipid	glycolipid	glycolipid	glycolipid	glycolipid	1	kept
Glycolipids	glycolipids	glycolipids	glycolipid	glycolipid	1	singular

Glycopeptide	glycopeptide	glycopeptide	glycopeptide	glycopeptide	1	case_or_format
glycophorin A	glycophorin a	glycophorin a	glycophorin a	glycophorin A	1	kept
glycoprotein	glycoprotein	glycoprotein	glycoprotein	glycoprotein	1	kept
glycosidase inhibitor	glycosidase inhibitor	glycosidase inhibitor	glycosidase inhibitor	glycosidase inhibitor	1	kept
glycosylation	glycosylation	glycosylation	glycosylation	glycosylation	1	kept
glycosyltransferase	glycosyltransferase	glycosyltransferase	glycosyltransferase	glycosyltransferase	1	kept
GME	gme	gme	gme	GME	1	kept
GNA11 gene	gna11 gene	gna11 gene	gna11 gene	GNA11 gene	1	kept
GNAQ gene	gnaq gene	gnaq gene	gnaq gene	GNAQ gene	1	kept
goal concordant care	goal concordant care	goal concordant care	goal concordant care	Goal Concordant Care	1	case_or_format
goal directed lung physiotherapy	goal directed lung physiotherapy	goal directed lung physiotherapy	goal directed lung physiotherapy	goal-directed lung physiotherapy	1	case_or_format
Goal orientation	goal orientation	goal orientation	goal orientation	Goal orientation	1	kept
Goal Programming	goal programming	goal programming	goal programming	Goal programming	1	case_or_format
goal programming	goal programming	goal programming	goal programming	Goal programming	1	case_or_format
Goal setting	goal setting	goal setting	goal setting	Goal setting	1	kept
Goal-Concordant Care	goal concordant care	goal concordant care	goal concordant care	Goal Concordant Care	1	case_or_format

goal-directed	goal directed	goal directed	goal directed	goal-directed	1	kept
Goal-directed fluid therapy	goal directed fluid therapy	goal directed fluid therapy	goal directed fluid therapy	Goal-directed fluid therapy	1	kept
Goal-directed lung physiotherapy	goal directed lung physiotherapy	goal directed lung physiotherapy	goal directed lung physiotherapy	goal-directed lung physiotherapy	1	case_or_format
GOAL-DIRECTED RESUSCITATION	goal directed resuscitation	goal directed resuscitation	goal directed resuscitation	GOAL-DIRECTED RESUSCITATION	1	kept
GOAL-DIRECTED SEDATION	goal directed sedation	goal directed sedation	goal directed sedation	GOAL-DIRECTED SEDATION	1	kept
goal-directed therapy	goal directed therapy	goal directed therapy	goal directed therapy	Goal-directed therapy	1	case_or_format
Goal-oriented care	goal oriented care	goal oriented care	goal oriented care	Goal-oriented care	1	kept
goal-oriented therapy	goal oriented therapy	goal oriented therapy	goal oriented therapy	goal-oriented therapy	1	kept
Goal-programming	goal programming	goal programming	goal programming	Goal programming	1	case_or_format
goals of care conversations	goals of care conversations	goals of care conversations	goals of care conversations	goals of care conversations	1	kept
goals of care discussion	goals of care discussion	goals of care discussion	goals of care discussion	goals of care discussion	1	kept
goals of care discussions	goals of care discussions	goals of care discussions	goals of care discussion	goals of care discussion	1	singular
goggle-mask related skin disease	goggle mask related skin disease	goggle mask related skin disease	goggle mask related skin disease	goggle-mask related skin disease	1	kept
goggles	goggles	goggles	goggles	goggles	1	kept
goiter	goiter	goiter	goiter	Goiter	1	case_or_format
gold nanoparticle	gold nanoparticle	gold nanoparticle	gold nanoparticle	gold nanoparticle	1	kept

gold rose lavender oil	gold rose lavender oil	gold rose lavender oil	gold rose lavender oil	gold rose lavender oil	1	kept
Golden-Hour	golden hour	golden hour	golden hour	Golden Hour	1	case_or_format
Goldenhar syndrome	goldenhar syndrome	goldenhar syndrome	goldenhar syndrome	Goldenhar syndrome	1	kept
golimumab	golimumab	golimumab	golimumab	golimumab	1	kept
Good	good	good	good	Good	1	kept
Good clinical practice guidelines	good clinical practice guidelines	good clinical practice guidelines	good clinical practice guidelines	Good clinical practice guidelines	1	kept
Good Death	good death	good death	good death	Good death	1	case_or_format
Good dying	good dying	good dying	good dying	Good dying	1	kept
good general condition	good general condition	good general condition	good general condition	good general condition	1	kept
good manufacturing practice	good manufacturing practice	good manufacturing practice	good manufacturing practice	good manufacturing practice	1	kept
Good parent	good parent	good parent	good parent	Good parent	1	kept
Good practice	good practice	good practice	good practice	Good practice	1	kept
Goodpasture syndrome	goodpasture syndrome	goodpasture syndrome	goodpasture syndrome	Goodpasture syndrome	1	kept
GOOGLE GLASS	google glass	google glass	google glass	GOOGLE GLASS	1	kept
Gorham disease	gorham disease	gorham disease	gorham disease	Gorham disease	1	kept
Gorham-Stout Disease	gorham stout disease	gorham stout disease	gorham stout disease	Gorham-Stout Disease	1	kept

Gorontalo health system	gorontalo health system	gorontalo health system	gorontalo health system	Gorontalo health system	1	kept
Gossypiboma	gossypiboma	gossypiboma	gossypiboma	Gossypiboma	1	kept
Governing Board	governing board	governing board	governing board	Governing Board	1	kept
governing healthcare	governing healthcare	governing healthcare	governing healthcare	governing healthcare	1	kept
government employee	government employee	government employee	government employee	government employee	1	kept
GOVERNMENT HOSPITALS	government hospitals	government hospitals	government hospitals	GOVERNMENT HOSPITALS	1	kept
Government Programs	government programs	government programs	government programs	Government Programs	1	kept
gown	gown	gown	gown	gown	1	kept
gracilis flap	gracilis flap	gracilis flap	gracilis flap	gracilis flap	1	kept
Gracilis Muscle	gracilis muscle	gracilis muscle	gracilis muscle	Gracilis Muscle	1	kept
gracilis muscle	gracilis muscle	gracilis muscle	gracilis muscle	Gracilis Muscle	1	case_or_format
GRADE Approach	grade approach	grade approach	grade approach	GRADE approach	1	case_or_format
GRADE criteria	grade criteria	grade criteria	grade criteria	GRADE criteria	1	kept
Grade hospital	grade hospital	grade hospital	grade hospital	Grade hospital	1	kept
GRADE methodology	grade methodology	grade methodology	grade methodology	GRADE methodology	1	kept
GRADE system	grade system	grade system	grade system	GRADE system	1	kept

GRADE, Grading of Recommendations, Assessment	grade grading of recommendations assessment development	grade grading of recommendations assessment development	grade grading of recommendations assessment development	GRADE, Grading of Recommendations, Assessment	1	kept
GRADE, Grading of Recommendations, Assessment	grade grading of recommendations assessment development	grade grading of recommendations assessment development	grade grading of recommendations assessment development	GRADE, Grading of Recommendations, Assessment	1	singular
Grades of Recommendations, Assessment	grades of recommendations assessment development	grades of recommendations assessment development	grades of recommendations assessment development	Grades of Recommendations, Assessment	1	kept
gradient boosting machine	gradient boosting machine	gradient boosting machine	gradient boosting machine	gradient boosting machine	1	kept
Gradient feature	gradient feature	gradient feature	gradient feature	Gradient feature	1	kept
grading of recommendation assessment development	grading of recommendation assessment development	grading of recommendation assessment development	grading of recommendation assessment development	grading of recommendation assessment development	1	kept
grading of recommendations assessment	grading of recommendations assessment	grading of recommendations assessment	grading of recommendations assessment	grading of recommendations assessment	1	kept
Grading of Recommendations Assessment Development	grading of recommendations assessment development	grading of recommendations assessment development	grading of recommendations assessment development	Grading of Recommendations Assessment Development	1	kept
Grading of Recommendations Assessment	grading of recommendations assessment development	grading of recommendations assessment development	grading of recommendations assessment development	Grading of Recommendations Assessment Development	1	case_or_format
grading of recommendations assessment	grading of recommendations assessment development	grading of recommendations assessment development	grading of recommendations assessment development	Grading of Recommendations Assessment Development	1	case_or_format
Grading of Recommendations Assessment Development	grading of recommendations assessment development	grading of recommendations assessment development	grading of recommendations assessment development	Grading of Recommendations Assessment Development	1	kept
Grading of Recommendations, Assessment	grading of recommendations assessment development	grading of recommendations assessment development	grading of recommendations assessment development	Grading of Recommendations Assessment Development	1	singular
grading scale	grading scale	grading scale	grading scale	grading scale	1	kept
Graduate	graduate	graduate	graduate	graduate	1	case_or_format
Graduate Attributes	graduate attributes	graduate attributes	graduate attributes	Graduate Attributes	1	kept
Graduate education	graduate education	graduate education	graduate education	graduate education	1	case_or_format

Graduate Medical Education	graduate medical education	graduate medical education	graduate medical education	graduate medical education	1	case_or_format
graduate-level education	graduate level education	graduate level education	graduate level education	graduate-level education	1	kept
Graduates	graduates	graduates	graduate	graduate	1	singular
graft	graft	graft	graft	Graft	1	case_or_format
graft disruption	graft disruption	graft disruption	graft disruption	graft disruption	1	kept
graft perfusion	graft perfusion	graft perfusion	graft perfusion	graft perfusion	1	kept
Graft rejection	graft rejection	graft rejection	graft rejection	Graft Rejection	1	case_or_format
Graft Surgery	graft surgery	graft surgery	graft surgery	Graft Surgery	1	kept
Graft versus host disease	graft versus host disease	graft versus host disease	graft versus host disease	Graft versus host disease	1	kept
Graft-versus-host disease	graft versus host disease	graft versus host disease	graft versus host disease	Graft versus host disease	1	case_or_format
Gram negative anaerobic bacteria	gram negative anaerobic bacteria	gram negative anaerobic bacteria	gram negative anaerobic bacteria	Gram negative anaerobic bacteria	1	kept
Gram negative bacteria	gram negative bacteria	gram negative bacteria	gram negative bacteria	Gram-Negative Bacteria	1	case_or_format
gram negative bacteria carriage	gram negative bacteria carriage	gram negative bacteria carriage	gram negative bacteria carriage	gram negative bacteria carriage	1	kept
Gram positive asporogenous rod-shaped bacteria	gram positive asporogenous rod shaped bacteria	gram positive asporogenous rod shaped bacteria	gram positive asporogenous rod shaped bacteria	Gram positive asporogenous rod-shaped bacteria	1	kept
GRAM STAIN	gram stain	gram stain	gram stain	GRAM STAIN	1	kept
Gram-Negative Aerobic Bacteria	gram negative aerobic bacteria	gram negative aerobic bacteria	gram negative aerobic bacteria	Gram-Negative Aerobic Bacteria	1	kept

GRAM-NEGATIVE BACILLI	gram negative bacilli	gram negative bacilli	gram negative bacilli	Gram-negative bacilli	1	case_or_format
GRAM-NEGATIVE BACTEREMIA	gram negative bacteremia	gram negative bacteremia	gram negative bacteremia	GRAM-NEGATIVE BACTEREMIA	1	kept
Gram-negative non-fermenter	gram negative non fermenter	gram negative non fermenter	gram negative non fermenter	Gram-negative non-fermenter	1	kept
gram-negative organisms	gram negative organisms	gram negative organisms	gram negative organisms	gram-negative organisms	1	kept
GRAM-NEGATIVE PATHOGENS	gram negative pathogens	gram negative pathogens	gram negative pathogens	GRAM-NEGATIVE PATHOGENS	1	kept
Gram-positive bacterium	gram positive bacterium	gram positive bacterium	gram positive bacterium	Gram positive bacterium	1	case_or_format
grand mal epilepsy	grand mal epilepsy	grand mal epilepsy	grand mal epilepsy	grand mal epilepsy	1	kept
grand mal seizure	grand mal seizure	grand mal seizure	grand mal seizure	grand mal seizure	1	kept
GRANDMOTHERS	grandmothers	grandmothers	grandmother	grandmother	1	singular
grandmothers	grandmothers	grandmothers	grandmother	grandmother	1	singular
grandparents	grandparents	grandparents	grandparent	Grandparents	1	singular
Graneheim and Lundman ’s Method	graneheim and lundman s method	graneheim and lundman s method	graneheim and lundman s method	Graneheim and Lundman ’s Method	1	kept
Graneheim and Lundman ’s method	graneheim and lundman s method	graneheim and lundman s method	graneheim and lundman s method	Graneheim and Lundman ’s Method	1	case_or_format
granisetron	granisetron	granisetron	granisetron	granisetron	1	kept
Granular levels	granular levels	granular levels	granular levels	Granular levels	1	kept
Granulicatella	granulicatella	granulicatella	granulicatella	Granulicatella	1	kept

Granulocyte Colony-Stimulating Factor	granulocyte colony stimulating factor	granulocyte colony stimulating factor	granulocyte colony stimulating factor	granulocyte colony stimulating factor	1	case_or_format
Granulocytes	granulocytes	granulocytes	granulocyte	granulocyte	1	singular
granuloma annulare	granuloma annulare	granuloma annulare	granuloma annulare	granuloma annulare	1	kept
Granulomatosis with Polyangiitis	granulomatosis with polyangiitis	granulomatosis with polyangiitis	granulomatosis with polyangiitis	Granulomatosis with Polyangiitis	1	kept
Grape	grape	grape	grape	Grape	1	kept
grape	grape	grape	grape	Grape	1	case_or_format
Graph theory	graph theory	graph theory	graph theory	Graph theory	1	kept
Graphic data displays	graphic data displays	graphic data displays	graphic data displays	Graphic data displays	1	kept
Graphic design	graphic design	graphic design	graphic design	Graphic design	1	kept
Graphic methods	graphic methods	graphic methods	graphic methods	Graphic methods	1	kept
Graphic user interface	graphic user interface	graphic user interface	graphic user interface	Graphic user interface	1	kept
Graphical display	graphical display	graphical display	graphical display	Graphical display	1	kept
Graphical displays	graphical displays	graphical displays	graphical display	Graphical display	1	singular
Graphical format	graphical format	graphical format	graphical format	Graphical format	1	kept
Graphical information	graphical information	graphical information	graphical information	Graphical information	1	kept
grass pollen extract	grass pollen extract	grass pollen extract	grass pollen extract	grass pollen extract	1	kept

Gravedad del paciente	gravedad del paciente	gravedad del paciente	gravedad del paciente	Gravedad del paciente	1	kept
Graves disease	graves disease	graves disease	graves disease	Graves Disease	1	case_or_format
Graves’ disease	graves disease	graves disease	graves disease	Graves Disease	1	case_or_format
Gravidade do Paciente	gravidade do paciente	gravidade do paciente	gravidade do paciente	Gravidade do Paciente	1	kept
gravimetry	gravimetry	gravimetry	gravimetry	gravimetry	1	kept
Gravity	gravity	gravity	gravity	gravity	1	case_or_format
Gravity feeds	gravity feeds	gravity feeds	gravity feeds	Gravity feeds	1	kept
Greek	greek	greek	greek	Greek	1	kept
Greek hospital	greek hospital	greek hospital	greek hospital	Greek hospital	1	kept
Greek World	greek world	greek world	greek world	Greek World	1	kept
green behaviour	green behaviour	green behaviour	green behaviour	green behaviour	1	kept
green chemistry	green chemistry	green chemistry	green chemistry	green chemistry	1	kept
Green Cross	green cross	green cross	green cross	Green Cross	1	kept
greffon	greffon	greffon	greffon	greffon	1	kept
Grenham and Lundman	grenham and lundman	grenham and lundman	grenham and lundman	Grenham and Lundman	1	kept
Grey correlation model	grey correlation model	grey correlation model	grey correlation model	Grey correlation model	1	kept

GRIEF	grief	grief	grief	Grief	1	case_or_format
Grief symptoms	grief symptoms	grief symptoms	grief symptoms	Grief symptoms	1	kept
grief theory	grief theory	grief theory	grief theory	grief theory	1	kept
Grief trajectories	grief trajectories	grief trajectories	grief trajectories	Grief trajectories	1	kept
Grief treatment	grief treatment	grief treatment	grief treatment	Grief treatment	1	kept
GRIEVING FATHERS	grieving fathers	grieving fathers	grieving fathers	GRIEVING FATHERS	1	kept
Grieving process	grieving process	grieving process	grieving process	Grieving process	1	kept
Griggs percutaneous tracheostomy	griggs percutaneous tracheostomy	griggs percutaneous tracheostomy	griggs percutaneous tracheostomy	Griggs percutaneous tracheostomy	1	kept
Griggs technique	griggs technique	griggs technique	griggs technique	Griggs technique	1	kept
Grip on Challenging Behavior care program	grip on challenging behavior care program	grip on challenging behavior care program	grip on challenging behavior care program	Grip on Challenging Behavior care program	1	kept
grip on challenging behavior care program	grip on challenging behavior care program	grip on challenging behavior care program	grip on challenging behavior care program	Grip on Challenging Behavior care program	1	case_or_format
Grip strength	grip strength	grip strength	grip strength	grip strength	1	case_or_format
griseofulvin	griseofulvin	griseofulvin	griseofulvin	griseofulvin	1	kept
GRIT	grit	grit	grit	GRIT	1	kept
grit	grit	grit	grit	GRIT	1	case_or_format
groin	groin	groin	groin	Groin	1	case_or_format

grooved pegboard test	grooved pegboard test	grooved pegboard test	grooved pegboard test	grooved pegboard test	1	kept
Gross costing	gross costing	gross costing	gross costing	Gross costing	1	kept
Ground glass opacities	ground glass opacities	ground glass opacities	ground glass opacity	ground glass opacity	1	singular
Ground level falls	ground level falls	ground level falls	ground level fall	ground-level fall	1	singular
ground medical transport	ground medical transport	ground medical transport	ground medical transport	ground medical transport	1	kept
ground-level fall	ground level fall	ground level fall	ground level fall	ground-level fall	1	kept
ground-level falls	ground level falls	ground level falls	ground level fall	ground-level fall	1	singular
Grounded theory approach	grounded theory approach	grounded theory approach	grounded theory approach	grounded theory approach	1	case_or_format
Group A streptococcal disease	group a streptococcal disease	group a streptococcal disease	group a streptococcal disease	Group A streptococcal disease	1	kept
Group A Streptococcus	group a streptococcus	group a streptococcus	group a streptococcus	Group A Streptococcus	1	kept
Group A streptococcus	group a streptococcus	group a streptococcus	group a streptococcus	Group A Streptococcus	1	case_or_format
Group B Streptococcus	group b streptococcus	group b streptococcus	group b streptococcus	Group B Streptococcus	1	kept
Group cohesion	group cohesion	group cohesion	group cohesion	group cohesion	1	case_or_format
group decision making	group decision making	group decision making	group decision making	group decision making	1	kept
Group feedback	group feedback	group feedback	group feedback	Group feedback	1	kept
Group intervention	group intervention	group intervention	group intervention	Group intervention	1	kept

Group meetings	group meetings	group meetings	group meetings	Group meetings	1	kept
group processes	group processes	group processes	group processes	Group Processes	1	case_or_format
Group-based trajectory modeling	group based trajectory modeling	group based trajectory modeling	group based trajectory modeling	Group-based trajectory modeling	1	kept
GROUP-SIZE	group size	group size	group size	GROUP-SIZE	1	kept
grown up congenital heart disease	grown up congenital heart disease	grown up congenital heart disease	grown up congenital heart disease	grown up congenital heart disease	1	kept
Growth & development	growth and development	growth and development	growth and development	Growth and development	1	case_or_format
Growth and Development	growth and development	growth and development	growth and development	Growth and development	1	case_or_format
growth curve	growth curve	growth curve	growth curve	growth curve	1	kept
Growth disturbance	growth disturbance	growth disturbance	growth disturbance	Growth disturbance	1	kept
growth factor	growth factor	growth factor	growth factor	growth factor	1	kept
Growth faltering	growth faltering	growth faltering	growth faltering	Growth faltering	1	kept
growth hormone replacement therapy	growth hormone replacement therapy	growth hormone replacement therapy	growth hormone replacement therapy	growth hormone replacement therapy	1	kept
growth hormone therapy	growth hormone therapy	growth hormone therapy	growth hormone therapy	growth hormone therapy	1	kept
Growth measurement	growth measurement	growth measurement	growth measurement	Growth measurement	1	kept
growth monitoring	growth monitoring	growth monitoring	growth monitoring	growth monitoring	1	kept
Growth plate	growth plate	growth plate	growth plate	Growth plate	1	kept

growth plate	growth plate	growth plate	growth plate	Growth plate	1	case_or_format
growth restriction	growth restriction	growth restriction	growth restriction	growth restriction	1	kept
Growth Substances	growth substances	growth substances	growth substances	Growth Substances	1	kept
growth velocity (g	growth velocity g	growth velocity g	growth velocity g	growth velocity (g	1	kept
Grupo de Enfermería	grupo de enfermer a	grupo de enfermer a	grupo de enfermer a	Grupo de Enfermería	1	kept
Grupos focales	grupos focales	grupos focales	grupos focales	Grupos focales	1	kept
GSM mobile	gsm mobile	gsm mobile	gsm mobile	GSM mobile	1	kept
GSM module	gsm module	gsm module	gsm module	GSM module	1	kept
GSM SIM900A	gsm sim900a	gsm sim900a	gsm sim900a	GSM SIM900A	1	kept
GSM Technology	gsm technology	gsm technology	gsm technology	GSM Technology	1	kept
GSR	gsr	gsr	gsr	GSR	1	kept
GTT - Global Trigger Tool	gtt global trigger tool	gtt global trigger tool	gtt global trigger tool	GTT - Global Trigger Tool	1	kept
Guaiac	guaiac	guaiac	guaiac	Guaiac	1	kept
guaiac positive	guaiac positive	guaiac positive	guaiac positive	guaiac positive	1	kept
guanethidine	guanethidine	guanethidine	guanethidine	guanethidine	1	kept
Guangdong	guangdong	guangdong	guangdong	Guangdong	1	kept

Guangxi	guangxi	guangxi	guangxi	Guangxi	1	kept
guanidine derivative	guanidine derivative	guanidine derivative	guanidine derivative	guanidine derivative	1	kept
guanidine i 131	guanidine i 131	guanidine i 131	guanidine i 131	guanidine i 131	1	kept
Guanidines	guanidines	guanidines	guanidine	guanidine	1	singular
guanylate cyclase activator	guanylate cyclase activator	guanylate cyclase activator	guanylate cyclase activator	guanylate cyclase activator	1	kept
Guarantee of the quality of the health care actions	guarantee of the quality of the health care actions	guarantee of the quality of the health care actions	guarantee of the quality of the health care actions	Guarantee of the quality of the health care actions	1	kept
guardian's psychological mood	guardian s psychological mood	guardian s psychological mood	guardian s psychological mood	guardian's psychological mood	1	kept
guardianship	guardianship	guardianship	guardianship	guardianship	1	kept
gugging swallowing screen	gugging swallowing screen	gugging swallowing screen	gugging swallowing screen	gugging swallowing screen	1	kept
guide	guide	guide	guide	GUIDE	1	case_or_format
GUIDED CANNULATION	guided cannulation	guided cannulation	guided cannulation	GUIDED CANNULATION	1	kept
Guided imagery	guided imagery	guided imagery	guided imagery	guided imagery	1	case_or_format
guided participation theory	guided participation theory	guided participation theory	guided participation theory	guided participation theory	1	kept
guided self-determination	guided self determination	guided self determination	guided self determination	guided self-determination	1	kept
Guideline access	guideline access	guideline access	guideline access	Guideline access	1	kept
guideline adaptation	guideline adaptation	guideline adaptation	guideline adaptation	guideline adaptation	1	kept

Guideline compliance	guideline compliance	guideline compliance	guideline compliance	Guideline compliance	1	kept
guideline compliance	guideline compliance	guideline compliance	guideline compliance	Guideline compliance	1	case_or_format
Guideline quality	guideline quality	guideline quality	guideline quality	Guideline quality	1	kept
guideline related training duration	guideline related training duration	guideline related training duration	guideline related training duration	guideline related training duration	1	kept
guideline structure	guideline structure	guideline structure	guideline structure	guideline structure	1	kept
Guideline-concordant antibiotic therapy	guideline concordant antibiotic therapy	guideline concordant antibiotic therapy	guideline concordant antibiotic therapy	Guideline-concordant antibiotic therapy	1	kept
Guidelines adherence	guidelines adherence	guidelines adherence	guidelines adherence	Guidelines adherence	1	kept
Guidelines as topic	guidelines as topic	guidelines as topic	guidelines as topic	Guidelines as Topic	1	case_or_format
Guidelines for Essential Trauma Care	guidelines for essential trauma care	guidelines for essential trauma care	guidelines for essential trauma care	Guidelines for Essential Trauma Care	1	kept
Guidelines implementation	guidelines implementation	guidelines implementation	guidelines implementation	Guidelines implementation	1	kept
guidewire exchange	guidewire exchange	guidewire exchange	guidewire exchange	guidewire exchange	1	kept
Guidewire exchange versus new [illegible]	guidewire exchange versus new [illegible]	guidewire exchange versus new [illegible]	guidewire exchange versus new [illegible]	Guidewire exchange versus new [illegible]	1	kept
Guilan	guilan	guilan	guilan	Guilan	1	kept
Guillain-Barre syndrome	guillain barre syndrome	guillain barre syndrome	guillain barre syndrome	Guillain-Barre Syndrome	1	case_or_format
Guillain-barre syndrome	guillain barre syndrome	guillain barre syndrome	guillain barre syndrome	Guillain-Barre Syndrome	1	case_or_format
Guillain-Barré Syndrome	guillain barr syndrome	guillain barr syndrome	guillain barr syndrome	Guillain-Barré syndrome	1	case_or_format

guillotine amputation	guillotine amputation	guillotine amputation	guillotine amputation	guillotine amputation	1	kept
guilty	guilty	guilty	guilty	guilty	1	kept
guinea pig	guinea pig	guinea pig	guinea pig	guinea pig	1	kept
GUIs	guis	guis	guis	GUIs	1	kept
Gujarat	gujarat	gujarat	gujarat	Gujarat	1	kept
GUM ELASTIC BOUGIE	gum elastic bougie	gum elastic bougie	gum elastic bougie	GUM ELASTIC BOUGIE	1	kept
Gun Violence	gun violence	gun violence	gun violence	gun violence	1	case_or_format
gunpowder	gunpowder	gunpowder	gunpowder	gunpowder	1	kept
gunshot	gunshot	gunshot	gunshot	gunshot	1	kept
Gunshot wound	gunshot wound	gunshot wound	gunshot wound	Gunshot wound	1	kept
Gunshot Wounds	gunshot wounds	gunshot wounds	gunshot wound	Gunshot wound	1	singular
gunshot wounds	gunshot wounds	gunshot wounds	gunshot wound	Gunshot wound	1	singular
guselkumab	guselkumab	guselkumab	guselkumab	guselkumab	1	kept
Gustafson Kessel algorithm	gustafson kessel algorithm	gustafson kessel algorithm	gustafson kessel algorithm	Gustafson Kessel algorithm	1	kept
gustatory nervous system	gustatory nervous system	gustatory nervous system	gustatory nervous system	gustatory nervous system	1	kept
gustatory system	gustatory system	gustatory system	gustatory system	gustatory system	1	kept

Gut microbiome	gut microbiome	gut microbiome	gut microbiome	Gut microbiome	1	kept
gut microbiome	gut microbiome	gut microbiome	gut microbiome	Gut microbiome	1	case_or_format
Gut Microflora	gut microflora	gut microflora	gut microflora	Gut Microflora	1	kept
GUT MUCOSAL HYPOPERFUSION	gut mucosal hypoperfusion	gut mucosal hypoperfusion	gut mucosal hypoperfusion	GUT MUCOSAL HYPOPERFUSION	1	kept
GUT-BRAIN AXIS	gut brain axis	gut brain axis	gut brain axis	GUT-BRAIN AXIS	1	kept
GutCheckNEC	gutchecknec	gutchecknec	gutchecknec	GutCheckNEC	1	kept
gut‑brain axis	gut brain axis	gut brain axis	gut brain axis	GUT-BRAIN AXIS	1	case_or_format
Guía	gu a	gu a	gu a	Guía	1	kept
Guías	gu as	gu as	gu as	Guías	1	kept
gymnastics	gymnastics	gymnastics	gymnastics	gymnastics	1	kept
gynaecology	gynaecology	gynaecology	gynaecology	gynaecology	1	kept
gynaecology ward	gynaecology ward	gynaecology ward	gynaecology ward	gynaecology ward	1	kept
gynecologic care	gynecologic care	gynecologic care	gynecologic care	gynecologic care	1	kept
gynecological cancer	gynecological cancer	gynecological cancer	gynecological cancer	gynecological cancer	1	kept
gynecological examination	gynecological examination	gynecological examination	gynecological examination	gynecological examination	1	kept
GYNECOLOGY	gynecology	gynecology	gynecology	gynecology	1	case_or_format

Gyssen algorithm	gyssen algorithm	gyssen algorithm	gyssen algorithm	Gyssen algorithm	1	kept
H -K -Exchanging ATPase	h k exchanging atpase	h k exchanging atpase	h k exchanging atpase	H -K -Exchanging ATPase	1	kept
H-2 BLOCKERS	h 2 blockers	h 2 blockers	h 2 blockers	H-2 BLOCKERS	1	kept
H-2-RECEPTOR ANTAGONIST	h 2 receptor antagonist	h 2 receptor antagonist	h 2 receptor antagonist	H-2-RECEPTOR ANTAGONIST	1	kept
h-b FES-induced muscle recovery	h b fes induced muscle recovery	h b fes induced muscle recovery	h b fes induced muscle recovery	h-b FES-induced muscle recovery	1	kept
H-HOPE behavioral intervention	h hope behavioral intervention	h hope behavioral intervention	h hope behavioral intervention	H-HOPE behavioral intervention	1	kept
H-index	h index	h index	h index	H-index	1	kept
H. pylori infection	h pylori infection	h pylori infection	h pylori infection	H. pylori infection	1	kept
H1n1	h1n1	h1n1	h1n1	H1N1	1	case_or_format
H1N1 (swine flu	h1n1 swine flu	h1n1 swine flu	h1n1 swine flu	H1N1 (swine flu	1	kept
H1N1 subtype	h1n1 subtype	h1n1 subtype	h1n1 subtype	H1N1 subtype	1	kept
H2RA	h2ra	h2ra	h2ra	H2RA	1	kept
H7N9	h7n9	h7n9	h7n9	H7N9	1	kept
Habilidades	habilidades	habilidades	habilidades	Habilidades	1	kept
Habilidades sociales	habilidades sociales	habilidades sociales	habilidades sociales	Habilidades sociales	1	kept
Habilitation	habilitation	habilitation	habilitation	Habilitation	1	kept

habitual sleep efficiency	habitual sleep efficiency	habitual sleep efficiency	habitual sleep efficiency	habitual sleep efficiency	1	kept
HACCP	haccp	haccp	haccp	HACCP	1	kept
HACOR score	hacor score	hacor score	hacor score	HACOR score	1	kept
HADS	hads	hads	hads	HADS	1	kept
HADS Hospital Anxiety and Depression Scale	hads hospital anxiety and depression scale	hads hospital anxiety and depression scale	hads hospital anxiety and depression scale	HADS Hospital Anxiety and Depression Scale	1	kept
HAdV-55	hadv 55	hadv 55	hadv 55	HAdV-55	1	kept
Haematoma	haematoma	haematoma	haematoma	Haematoma	1	kept
haematotoxicity	haematotoxicity	haematotoxicity	haematotoxicity	haematotoxicity	1	kept
Haeme-oxygenase-1	haeme oxygenase 1	haeme oxygenase 1	haeme oxygenase 1	Haeme-oxygenase-1	1	kept
Haemodynamic	haemodynamic	haemodynamic	haemodynamic	Haemodynamics	1	case_or_format
Haemodynamic impairment	haemodynamic impairment	haemodynamic impairment	haemodynamic impairment	Haemodynamic impairment	1	kept
Haemodynamic management	haemodynamic management	haemodynamic management	haemodynamic management	Haemodynamic management	1	kept
Haemodynamic parameters	haemodynamic parameters	haemodynamic parameters	haemodynamic parameters	haemodynamic parameters	1	case_or_format
Haemodynamic phenomena	haemodynamic phenomena	haemodynamic phenomena	haemodynamic phenomena	Haemodynamic phenomena	1	kept
Haemodynamic stability	haemodynamic stability	haemodynamic stability	haemodynamic stability	Haemodynamic stability	1	kept
Haemodynamic status	haemodynamic status	haemodynamic status	haemodynamic status	Haemodynamic status	1	kept

haemofiltration	haemofiltration	haemofiltration	haemofiltration	Haemofiltration	1	case_or_format
Haemophilus	haemophilus	haemophilus	haemophilus	Haemophilus	1	kept
Haemophilus influenzae type b	haemophilus influenzae type b	haemophilus influenzae type b	haemophilus influenzae type b	Haemophilus influenzae type b	1	kept
Haemophilus influenzae type b vaccine	haemophilus influenzae type b vaccine	haemophilus influenzae type b vaccine	haemophilus influenzae type b vaccine	Haemophilus influenzae type b vaccine	1	kept
haemorrhage	haemorrhage	haemorrhage	haemorrhage	Haemorrhage	1	case_or_format
Haemorrhagic fever	haemorrhagic fever	haemorrhagic fever	haemorrhagic fever	Haemorrhagic fever	1	kept
haemorrhagic shock	haemorrhagic shock	haemorrhagic shock	haemorrhagic shock	Haemorrhagic shock	1	case_or_format
haemorrhagic transformation	haemorrhagic transformation	haemorrhagic transformation	haemorrhagic transformation	haemorrhagic transformation	1	kept
Haemotherapy	haemotherapy	haemotherapy	haemotherapy	Haemotherapy	1	kept
haemovigilance	haemovigilance	haemovigilance	haemovigilance	haemovigilance	1	kept
HAI = healthcare-associated infection	hai healthcare associated infection	hai healthcare associated infection	hai healthcare associated infection	HAI = healthcare-associated infection	1	kept
Hailey-Hailey	hailey hailey	hailey hailey	hailey hailey	Hailey-Hailey	1	kept
Hainan Province	hainan province	hainan province	hainan province	Hainan Province	1	kept
hair cap	hair cap	hair cap	hair cap	hair cap	1	kept
hair disease	hair disease	hair disease	hair disease	hair disease	1	kept
hair growth	hair growth	hair growth	hair growth	hair growth	1	kept

hair level	hair level	hair level	hair level	hair level	1	kept
Hair nicotine biomarkers	hair nicotine biomarkers	hair nicotine biomarkers	hair nicotine biomarkers	Hair nicotine biomarkers	1	kept
hair pull test	hair pull test	hair pull test	hair pull test	hair pull test	1	kept
hair removal	hair removal	hair removal	hair removal	hair removal	1	kept
HAITI	haiti	haiti	haiti	Haiti	1	case_or_format
Hajj	hajj	hajj	hajj	Hajj	1	kept
Hajj perspectives on Hajj rotations	hajj perspectives on hajj rotations	hajj perspectives on hajj rotations	hajj perspectives on hajj rotations	Hajj perspectives on Hajj rotations	1	kept
Half-open geriatric ward	half open geriatric ward	half open geriatric ward	half open geriatric ward	Half-open geriatric ward	1	kept
halfway house	halfway house	halfway house	halfway house	halfway house	1	kept
Halfway Houses	halfway houses	halfway houses	halfway house	halfway house	1	singular
hallucinations	hallucinations	hallucinations	hallucination	hallucination	1	singular
Hallucinogens	hallucinogens	hallucinogens	hallucinogens	Hallucinogens	1	kept
Halo vest	halo vest	halo vest	halo vest	Halo vest	1	kept
haloperidol decanoate	haloperidol decanoate	haloperidol decanoate	haloperidol decanoate	haloperidol decanoate	1	kept
Hamburg [Germany]	hamburg germany	hamburg germany	hamburg germany	Hamburg [Germany]	1	kept
hamburg parkinson day clinic	hamburg parkinson day clinic	hamburg parkinson day clinic	hamburg parkinson day clinic	hamburg parkinson day clinic	1	kept

Hammersmith Neonatal Neurological Examination And	hammersmith neonatal neurological examination and premie	hammersmith neonatal neurological examination and premie	hammersmith neonatal neurological examination and premie	Hammersmith Neonatal Neurological Examination And	1	kept
hamstring muscle	hamstring muscle	hamstring muscle	hamstring muscle	hamstring muscle	1	kept
Han Chinese	han chinese	han chinese	han chinese	Han Chinese	1	kept
Hand antisepsis adherence	hand antisepsis adherence	hand antisepsis adherence	hand antisepsis adherence	Hand antisepsis adherence	1	kept
Hand antisepsis compliance	hand antisepsis compliance	hand antisepsis compliance	hand antisepsis compliance	Hand antisepsis compliance	1	kept
Hand antisepsis training tool	hand antisepsis training tool	hand antisepsis training tool	hand antisepsis training tool	Hand antisepsis training tool	1	kept
hand burn	hand burn	hand burn	hand burn	hand burn	1	kept
Hand carriage	hand carriage	hand carriage	hand carriage	Hand carriage	1	kept
Hand contamination	hand contamination	hand contamination	hand contamination	Hand contamination	1	kept
hand contamination	hand contamination	hand contamination	hand contamination	Hand contamination	1	case_or_format
hand disinfectant dispenser	hand disinfectant dispenser	hand disinfectant dispenser	hand disinfectant dispenser	hand disinfectant dispenser	1	kept
Hand eczema	hand eczema	hand eczema	hand eczema	hand eczema	1	case_or_format
hand flora	hand flora	hand flora	hand flora	hand flora	1	kept
hand hygience	hand hygience	hand hygience	hand hygience	hand hygience	1	kept
Hand hygiene adherence	hand hygiene adherence	hand hygiene adherence	hand hygiene adherence	Hand hygiene adherence	1	kept
Hand hygiene attitude	hand hygiene attitude	hand hygiene attitude	hand hygiene attitude	Hand hygiene attitude	1	kept

Hand hygiene audit	hand hygiene audit	hand hygiene audit	hand hygiene audit	Hand hygiene audit	1	kept
hand hygiene audit in COVID 19 care	hand hygiene audit in covid 19 care	hand hygiene audit in covid 19 care	hand hygiene audit in covid 19 care	hand hygiene audit in COVID 19 care	1	kept
Hand hygiene complete adherence rate	hand hygiene complete adherence rate	hand hygiene complete adherence rate	hand hygiene complete adherence rate	Hand hygiene complete adherence rate	1	kept
hand hygiene complete adherence rate	hand hygiene complete adherence rate	hand hygiene complete adherence rate	hand hygiene complete adherence rate	Hand hygiene complete adherence rate	1	case_or_format
Hand Hygiene Compliance Score	hand hygiene compliance score	hand hygiene compliance score	hand hygiene compliance score	Hand Hygiene Compliance Score	1	kept
Hand hygiene guidelines	hand hygiene guidelines	hand hygiene guidelines	hand hygiene guidelines	Hand hygiene guidelines	1	kept
Hand Hygiene Knowledge Questionnaire	hand hygiene knowledge questionnaire	hand hygiene knowledge questionnaire	hand hygiene knowledge questionnaire	Hand Hygiene Knowledge Questionnaire	1	kept
Hand hygiene measurement	hand hygiene measurement	hand hygiene measurement	hand hygiene measurement	Hand hygiene measurement	1	kept
Hand hygiene observation	hand hygiene observation	hand hygiene observation	hand hygiene observation	Hand hygiene observation	1	kept
Hand hygiene opportunities	hand hygiene opportunities	hand hygiene opportunities	hand hygiene opportunity	Hand hygiene opportunity	1	singular
hand hygiene opportunities	hand hygiene opportunities	hand hygiene opportunities	hand hygiene opportunity	Hand hygiene opportunity	1	singular
Hand hygiene opportunity	hand hygiene opportunity	hand hygiene opportunity	hand hygiene opportunity	Hand hygiene opportunity	1	kept
Hand hygiene partial adherence rate	hand hygiene partial adherence rate	hand hygiene partial adherence rate	hand hygiene partial adherence rate	Hand hygiene partial adherence rate	1	kept
hand hygiene partial aherence rate	hand hygiene partial aherence rate	hand hygiene partial aherence rate	hand hygiene partial aherence rate	hand hygiene partial aherence rate	1	kept
Hand hygiene practice	hand hygiene practice	hand hygiene practice	hand hygiene practice	Hand hygiene practice	1	kept
Hand hygiene practices	hand hygiene practices	hand hygiene practices	hand hygiene practice	Hand hygiene practice	1	singular

Hand hygiene predictors	hand hygiene predictors	hand hygiene predictors	hand hygiene predictors	Hand hygiene predictors	1	kept
Hand Hygiene Self Assessment Questionnaire	hand hygiene self assessment questionnaire	hand hygiene self assessment questionnaire	hand hygiene self assessment questionnaire	Hand Hygiene Self Assessment Questionnaire	1	kept
hand hygiene total adherence rate	hand hygiene total adherence rate	hand hygiene total adherence rate	hand hygiene total adherence rate	hand hygiene total adherence rate	1	kept
Hand laceration repair	hand laceration repair	hand laceration repair	hand laceration repair	Hand laceration repair	1	kept
Hand microbiota	hand microbiota	hand microbiota	hand microbiota	Hand microbiota	1	kept
hand muscle strength	hand muscle strength	hand muscle strength	hand muscle strength	hand muscle strength	1	kept
Hand over	hand over	hand over	hand over	Hand-over	1	case_or_format
hand photographs	hand photographs	hand photographs	hand photographs	hand photographs	1	kept
hand rub	hand rub	hand rub	hand rub	Hand rub	1	case_or_format
hand rubbing	hand rubbing	hand rubbing	hand rubbing	hand rubbing	1	kept
Hand sanitizers	hand sanitizers	hand sanitizers	hand sanitizer	hand sanitizer	1	singular
hand smear	hand smear	hand smear	hand smear	hand smear	1	kept
Hand strength	hand strength	hand strength	hand strength	Hand Strength	1	case_or_format
hand strength	hand strength	hand strength	hand strength	Hand Strength	1	case_or_format
Hand Wash	hand wash	hand wash	hand wash	Hand Wash	1	kept
Hand wash	hand wash	hand wash	hand wash	Hand Wash	1	case_or_format

hand wash with soap and water	hand wash with soap and water	hand wash with soap and water	hand wash with soap and water	hand wash with soap and water	1	kept
Hand washing technique	hand washing technique	hand washing technique	hand washing technique	Hand washing technique	1	kept
hand-held echocardiography videos	hand held echocardiography videos	hand held echocardiography videos	hand held echocardiography videos	hand-held echocardiography videos	1	kept
HAND-HYGIENE	hand hygiene	hand hygiene	hand hygiene	Hand Hygiene	1	case_or_format
Hand-hygiene	hand hygiene	hand hygiene	hand hygiene	Hand Hygiene	1	case_or_format
Hand-hygiene compliance	hand hygiene compliance	hand hygiene compliance	hand hygiene compliance	Hand hygiene compliance	1	case_or_format
Hand-massage	hand massage	hand massage	hand massage	Hand-massage	1	kept
Hand-offs	hand offs	hand offs	hand off	hand-off	1	singular
Hand-washing permanent monitoring	hand washing permanent monitoring	hand washing permanent monitoring	hand washing permanent monitoring	Hand-washing permanent monitoring	1	kept
Handbooks	handbooks	handbooks	handbooks	Handbooks	1	kept
handheld decision support device	handheld decision support device	handheld decision support device	handheld decision support device	handheld decision support device	1	kept
handheld echocardiography	handheld echocardiography	handheld echocardiography	handheld echocardiography	handheld echocardiography	1	kept
Handheld scanner	handheld scanner	handheld scanner	handheld scanner	Handheld scanner	1	kept
Handheld ultrasound	handheld ultrasound	handheld ultrasound	handheld ultrasound	Handheld ultrasound	1	kept
Handheld ultrasound device	handheld ultrasound device	handheld ultrasound device	handheld ultrasound device	Handheld ultrasound device	1	kept
Handhelds	handhelds	handhelds	handheld	handheld	1	singular

Handling	handling	handling	handling	Handling	1	kept
handoff and rounding tool	handoff and rounding tool	handoff and rounding tool	handoff and rounding tool	handoff and rounding tool	1	kept
handoff approach	handoff approach	handoff approach	handoff approach	handoff approach	1	kept
handoff communication	handoff communication	handoff communication	handoff communication	handoff communication	1	kept
handoff metrics	handoff metrics	handoff metrics	handoff metrics	handoff metrics	1	kept
handoff performance	handoff performance	handoff performance	handoff performance	handoff performance	1	kept
handoff process	handoff process	handoff process	handoff process	handoff process	1	kept
HANDOFF TOOL	handoff tool	handoff tool	handoff tool	HANDOFF TOOL	1	kept
handoff tool	handoff tool	handoff tool	handoff tool	HANDOFF TOOL	1	case_or_format
handover evaluation scale	handover evaluation scale	handover evaluation scale	handover evaluation scale	handover evaluation scale	1	kept
handover of care	handover of care	handover of care	handover of care	handover of care	1	kept
handover process	handover process	handover process	handover process	handover process	1	kept
handover strategies	handover strategies	handover strategies	handover strategies	handover strategies	1	kept
handovers	handovers	handovers	handover	Handover	1	singular
Hands-free	hands free	hands free	hands free	Hands-free	1	kept
Hands-free communications	hands free communications	hands free communications	hands free communications	Hands-free communications	1	kept

Hands-free devices	hands free devices	hands free devices	hands free devices	Hands-free devices	1	kept
hands-on	hands on	hands on	hands on	hands-on	1	kept
handshake	handshake	handshake	handshake	handshake	1	kept
handshake free zone	handshake free zone	handshake free zone	handshake free zone	handshake free zone	1	kept
hang time	hang time	hang time	hang time	hang time	1	kept
hanging	hanging	hanging	hanging	Hanging	1	case_or_format
Haptic simulations	haptic simulations	haptic simulations	haptic simulations	Haptic simulations	1	kept
HAPU	hapu	hapu	hapu	HAPU	1	kept
Harassment	harassment	harassment	harassment	harassment	1	case_or_format
Harassment, Non-Sexual	harassment non sexual	harassment non sexual	harassment non sexual	Harassment, Non-Sexual	1	kept
Harborview Medical Center	harborview medical center	harborview medical center	harborview medical center	Harborview Medical Center	1	kept
hard palate	hard palate	hard palate	hard palate	hard palate	1	kept
hard work	hard work	hard work	hard work	hard work	1	kept
HARDINESS	hardiness	hardiness	hardiness	hardiness	1	case_or_format
hardness	hardness	hardness	hardness	hardness	1	kept
Hardware	hardware	hardware	hardware	Hardware	1	kept

Hardware and software	hardware and software	hardware and software	hardware and software	Hardware and software	1	kept
Hardware and software components	hardware and software components	hardware and software components	hardware and software components	Hardware and software components	1	kept
Hardware platform	hardware platform	hardware platform	hardware platform	Hardware platform	1	kept
hardware removal	hardware removal	hardware removal	hardware removal	hardware removal	1	kept
HARLEQUIN ICHTHYOSIS	harlequin ichthyosis	harlequin ichthyosis	harlequin ichthyosis	HARLEQUIN ICHTHYOSIS	1	kept
harlequin ichthyosis	harlequin ichthyosis	harlequin ichthyosis	harlequin ichthyosis	HARLEQUIN ICHTHYOSIS	1	case_or_format
Harlequin syndrome	harlequin syndrome	harlequin syndrome	harlequin syndrome	Harlequin syndrome	1	kept
harm prevention	harm prevention	harm prevention	harm prevention	harm prevention	1	kept
Harm Reduction	harm reduction	harm reduction	harm reduction	harm reduction	1	case_or_format
Harm reduction	harm reduction	harm reduction	harm reduction	harm reduction	1	case_or_format
harmonization	harmonization	harmonization	harmonization	harmonization	1	kept
Harmony	harmony	harmony	harmony	Harmony	1	kept
HARMS	harms	harms	harm	Harm	1	singular
harp music	harp music	harp music	harp music	harp music	1	kept
hartmann procedure	hartmann procedure	hartmann procedure	hartmann procedure	hartmann procedure	1	kept
Hartmann solution	hartmann solution	hartmann solution	hartmann solution	Hartmann solution	1	kept

Haryana	haryana	haryana	haryana	Haryana	1	kept
HAS BLED score	has bled score	has bled score	has bled score	HAS BLED score	1	kept
Hasegawa Dementia Scale Revised	hasegawa dementia scale revised	hasegawa dementia scale revised	hasegawa dementia scale revised	Hasegawa Dementia Scale Revised	1	kept
hasyayoga	hasyayoga	hasyayoga	hasyayoga	hasyayoga	1	kept
hat-trick	hat trick	hat trick	hat trick	hat-trick	1	kept
Hate	hate	hate	hate	Hate	1	kept
HATRICC	hatricc	hatricc	hatricc	HATRICC	1	kept
Havelock Theory of Change	havelock theory of change	havelock theory of change	havelock theory of change	Havelock Theory of Change	1	kept
having children	having children	having children	having children	having children	1	kept
Hawaiian	hawaiian	hawaiian	hawaiian	Hawaiian	1	kept
HAZARD	hazard	hazard	hazard	Hazard	1	case_or_format
hazardous substances	hazardous substances	hazardous substances	hazardous substances	Hazardous Substances	1	case_or_format
Hazards	hazards	hazards	hazard	Hazard	1	singular
hazards	hazards	hazards	hazard	Hazard	1	singular
hblA gene	hbla gene	hbla gene	hbla gene	hblA gene	1	kept
hblD gene	hbld gene	hbld gene	hbld gene	hblD gene	1	kept

HBV	hbv	hbv	hbv	HBV	1	kept
HCAI	hcai	hcai	hcai	HCAI	1	kept
HCI	hci	hci	hci	HCI	1	kept
HCW, health care worker	hcw health care worker	hcw health care worker	hcw health care worker	HCW, health care worker	1	kept
HCWs behavior	hcws behavior	hcws behavior	hcws behavior	HCWs behavior	1	kept
HCWs, Healthcare workers	hcws healthcare workers	hcws healthcare workers	hcws healthcare workers	HCWs, Healthcare workers	1	kept
HDU	hdu	hdu	hdu	HDU	1	kept
Head & Neck	head and neck	head and neck	head and neck	Head & Neck	1	kept
head & neck surgery	head and neck surgery	head and neck surgery	head and neck surgery	head and neck surgery	1	case_or_format
head and neck	head and neck	head and neck	head and neck	Head & Neck	1	case_or_format
Head and neck reconstruction	head and neck reconstruction	head and neck reconstruction	head and neck reconstruction	Head and neck reconstruction	1	kept
head and neck reconstruction	head and neck reconstruction	head and neck reconstruction	head and neck reconstruction	Head and neck reconstruction	1	case_or_format
head and neck unit	head and neck unit	head and neck unit	head and neck unit	head and neck unit	1	kept
Head circumference	head circumference	head circumference	head circumference	head circumference	1	case_or_format
head growth	head growth	head growth	head growth	head growth	1	kept
head height	head height	head height	head height	head height	1	kept

head impulse test	head impulse test	head impulse test	head impulse test	head impulse test	1	kept
head injuries	head injuries	head injuries	head injury	head injury	1	singular
Head molding	head molding	head molding	head molding	Head molding	1	kept
Head of bed elevation	head of bed elevation	head of bed elevation	head of bed elevation	head of bed elevation	1	case_or_format
head of bed position	head of bed position	head of bed position	head of bed position	head of bed position	1	kept
Head position	head position	head position	head position	head position	1	case_or_format
Head rotation	head rotation	head rotation	head rotation	Head rotation	1	kept
head tilting	head tilting	head tilting	head tilting	head tilting	1	kept
Head tracking system	head tracking system	head tracking system	head tracking system	Head tracking system	1	kept
head-end elevation	head end elevation	head end elevation	head end elevation	head-end elevation	1	kept
HEAD-INJURY	head injury	head injury	head injury	head injury	1	case_or_format
Head-molding helmet therapy	head molding helmet therapy	head molding helmet therapy	head molding helmet therapy	Head-molding helmet therapy	1	kept
head-of-bed angle	head of bed angle	head of bed angle	head of bed angle	head-of-bed angle	1	kept
Head-to-toe assessment	head to toe assessment	head to toe assessment	head to toe assessment	Head-to-toe assessment	1	kept
Headbox	headbox	headbox	headbox	Headbox	1	kept
Healing	healing	healing	healing	healing	1	case_or_format

healing modalities	healing modalities	healing modalities	healing modalities	healing modalities	1	kept
Healing practices	healing practices	healing practices	healing practices	Healing practices	1	kept
Healing Touch	healing touch	healing touch	healing touch	Healing Touch	1	kept
healing touch	healing touch	healing touch	healing touch	Healing Touch	1	case_or_format
Health administration	health administration	health administration	health administration	Health administration	1	kept
HEALTH ADVOCACY	health advocacy	health advocacy	health advocacy	HEALTH ADVOCACY	1	kept
Health and care workforce	health and care workforce	health and care workforce	health and care workforce	Health and care workforce	1	kept
Health and development	health and development	health and development	health and development	Health and development	1	kept
Health and economic burden	health and economic burden	health and economic burden	health and economic burden	Health and economic burden	1	kept
Health and wellness coaching	health and wellness coaching	health and wellness coaching	health and wellness coaching	Health and wellness coaching	1	kept
Health Assessment	health assessment	health assessment	health assessment	Health assessment	1	case_or_format
health assistant	health assistant	health assistant	health assistant	health assistant	1	kept
HEALTH BEHAVIOR-CHANGE	health behavior change	health behavior change	health behavior change	HEALTH BEHAVIOR-CHANGE	1	kept
Health behaviors	health behaviors	health behaviors	health behavior	health behavior	1	singular
Health behaviour	health behaviour	health behaviour	health behaviour	Health behaviour	1	kept
health belief model	health belief model	health belief model	health belief model	Health Belief Model	1	case_or_format

Health beliefs	health beliefs	health beliefs	health belief	health belief	1	singular
health beliefs	health beliefs	health beliefs	health belief	health belief	1	singular
Health care administration	health care administration	health care administration	health care administration	Health care administration	1	kept
health care administration	health care administration	health care administration	health care administration	Health care administration	1	case_or_format
health care and simulation	health care and simulation	health care and simulation	health care and simulation	health care and simulation	1	kept
Health care application	health care application	health care application	health care application	Health care application	1	kept
Health care apps	health care apps	health care apps	health care apps	Health care apps	1	kept
health care associated infective endocarditis	health care associated infective endocarditis	health care associated infective endocarditis	health care associated infective endocarditis	health care associated infective endocarditis	1	kept
Health care at the end of life	health care at the end of life	health care at the end of life	health care at the end of life	Health care at the end of life	1	kept
Health Care Barriers	health care barriers	health care barriers	health care barriers	Health Care Barriers	1	kept
health care capacity	health care capacity	health care capacity	health care capacity	health care capacity	1	kept
Health care cost control	health care cost control	health care cost control	health care cost control	Health care cost control	1	kept
Health Care Delivery	health care delivery	health care delivery	health care delivery	health care delivery	1	case_or_format
health care delivery methods	health care delivery methods	health care delivery methods	health care delivery methods	health care delivery methods	1	kept
health care delivery network fragmentation	health care delivery network fragmentation	health care delivery network fragmentation	health care delivery network fragmentation	health care delivery network fragmentation	1	kept
health care design	health care design	health care design	health care design	health care design	1	kept

health care disparities	health care disparities	health care disparities	health care disparity	health care disparity	1	singular
Health Care Economics and Organizations	health care economics and organizations	health care economics and organizations	health care economics and organizations	Health Care Economics and Organizations	1	kept
Health care economics and organizations	health care economics and organizations	health care economics and organizations	health care economics and organizations	Health Care Economics and Organizations	1	case_or_format
health care environment	health care environment	health care environment	health care environment	health care environment	1	kept
Health care errors	health care errors	health care errors	health care errors	Health care errors	1	kept
health care errors	health care errors	health care errors	health care errors	Health care errors	1	case_or_format
Health care ethics consultation	health care ethics consultation	health care ethics consultation	health care ethics consultation	Health care ethics consultation	1	kept
Health care evaluation mechanisms	health care evaluation mechanisms	health care evaluation mechanisms	health care evaluation mechanisms	Health care evaluation mechanisms	1	kept
Health care expenditures	health care expenditures	health care expenditures	health care expenditures	Health care expenditures	1	kept
health care facilities	health care facilities	health care facilities	health care facility	health care facility	1	singular
Health care facility	health care facility	health care facility	health care facility	health care facility	1	case_or_format
Health care failure mode and effect analysis	health care failure mode and effect analysis	health care failure mode and effect analysis	health care failure mode and effect analysis	Health care failure mode and effect analysis	1	kept
health care failure mode and effect analysis	health care failure mode and effect analysis	health care failure mode and effect analysis	health care failure mode and effect analysis	Health care failure mode and effect analysis	1	case_or_format
health care funding	health care funding	health care funding	health care funding	health care funding	1	kept
Health care impact	health care impact	health care impact	health care impact	Health care impact	1	kept
Health care informatics	health care informatics	health care informatics	health care informatics	Health care informatics	1	kept

Health care information system	health care information system	health care information system	health care information system	Health care information system	1	kept
Health Care Information Technology	health care information technology	health care information technology	health care information technology	Health Care Information Technology	1	kept
health care infrastructure	health care infrastructure	health care infrastructure	health care infrastructure	health care infrastructure	1	kept
Health care innovation	health care innovation	health care innovation	health care innovation	Health care innovation	1	kept
Health care intervention	health care intervention	health care intervention	health care intervention	Health care intervention	1	kept
Health care leader	health care leader	health care leader	health care leader	Health care leader	1	kept
health care model	health care model	health care model	health care model	health care model	1	kept
health care optimisation	health care optimisation	health care optimisation	health care optimisation	health care optimisation	1	kept
Health care organisation	health care organisation	health care organisation	health care organisation	Health care organisation	1	kept
Health Care Organization	health care organization	health care organization	health care organization	health care organization	1	case_or_format
Health care organization	health care organization	health care organization	health care organization	health care organization	1	case_or_format
Health care organization structures	health care organization structures	health care organization structures	health care organization structures	Health care organization structures	1	kept
health care organizations	health care organizations	health care organizations	health care organization	health care organization	1	singular
health care outcome and process assessment	health care outcome and process assessment	health care outcome and process assessment	health care outcome and process assessment	health care outcome and process assessment	1	kept
health care outcomes	health care outcomes	health care outcomes	health care outcomes	health care outcomes	1	kept
Health care policy	health care policy	health care policy	health care policy	health care policy	1	case_or_format

health care process assessment	health care process assessment	health care process assessment	health care process assessment	health care process assessment	1	kept
Health care products	health care products	health care products	health care products	Health care products	1	kept
health care provider actions around child death	health care provider actions around child death	health care provider actions around child death	health care provider actions around child death	health care provider actions around child death	1	kept
Health care providers' opinions	health care providers opinions	health care providers opinions	health care providers opinions	Health care providers' opinions	1	kept
health care providers' reflection	health care providers reflection	health care providers reflection	health care providers reflection	health care providers' reflection	1	kept
Health Care Quality	health care quality	health care quality	health care quality	health care quality	1	case_or_format
Health care quality improvement	health care quality improvement	health care quality improvement	health care quality improvement	Health care quality improvement	1	kept
health care quality indicators	health care quality indicators	health care quality indicators	health care quality indicators	health care quality indicators	1	kept
health care quality, access, and evaluation	health care quality access and evaluation	health care quality access and evaluation	health care quality access and evaluation	health care quality, access, and evaluation	1	kept
Health care rationing	health care rationing	health care rationing	health care rationing	Health Care Rationing	1	case_or_format
Health care reform	health care reform	health care reform	health care reform	Health Care Reform	1	case_or_format
Health care research	health care research	health care research	health care research	Health care research	1	kept
health care resource allocation	health care resource allocation	health care resource allocation	health care resource allocation	health care resource allocation	1	kept
Health care resource planning	health care resource planning	health care resource planning	health care resource planning	Health care resource planning	1	kept
health care resource use	health care resource use	health care resource use	health care resource use	health care resource use	1	kept
Health care satisfaction	health care satisfaction	health care satisfaction	health care satisfaction	Health care satisfaction	1	kept

Health care services digitalization	health care services digitalization	health care services digitalization	health care services digitalization	Health care services digitalization	1	kept
Health care settings	health care settings	health care settings	health care setting	Health-care setting	1	singular
health care settings	health care settings	health care settings	health care setting	Health-care setting	1	singular
health care simulation	health care simulation	health care simulation	health care simulation	health care simulation	1	kept
Health care staff	health care staff	health care staff	health care staff	health care staff	1	case_or_format
Health care staff vaccination	health care staff vaccination	health care staff vaccination	health care staff vaccination	Health care staff vaccination	1	kept
Health care team	health care team	health care team	health care team	health care team	1	case_or_format
health care team education	health care team education	health care team education	health care team education	health care team education	1	kept
Health care technology	health care technology	health care technology	health care technology	health care technology	1	case_or_format
Health care technology evaluation	health care technology evaluation	health care technology evaluation	health care technology evaluation	Health care technology evaluation	1	kept
health care transition	health care transition	health care transition	health care transition	health care transition	1	kept
health care transitions	health care transitions	health care transitions	health care transition	health care transition	1	singular
health care work	health care work	health care work	health care work	health care work	1	kept
health care worker exposure	health care worker exposure	health care worker exposure	health care worker exposure	health care worker exposure	1	kept
Health Care Workers	health care workers	health care workers	health care worker	Health care workers	1	singular
health care workstation	health care workstation	health care workstation	health care workstation	health care workstation	1	kept

health care, interprofessional	health care interprofessional	health care interprofessional	health care interprofessional	health care, interprofessional	1	kept
health care, teamwork	health care teamwork	health care teamwork	health care teamwork	health care, teamwork	1	kept
health care, work environment	health care work environment	health care work environment	health care work environment	health care, work environment	1	kept
Health care-acquired infection	health care acquired infection	health care acquired infection	health care acquired infection	Health care-acquired infections	1	case_or_format
health care-acquired infections	health care acquired infections	health care acquired infections	health care acquired infection	Health care-acquired infections	1	singular
Health care-associated	health care associated	health care associated	health care associated	Health care-associated	1	kept
health care-associated infection	health care associated infection	health care associated infection	health care associated infection	Health care-associated infections	1	case_or_format
health care-associated infections	health care associated infections	health care associated infections	health care associated infection	Health care-associated infections	1	singular
Health care-associated pneumonia	health care associated pneumonia	health care associated pneumonia	health care associated pneumonia	health care associated pneumonia	1	case_or_format
health care-associated pneumonia	health care associated pneumonia	health care associated pneumonia	health care associated pneumonia	health care associated pneumonia	1	case_or_format
Health care - associated infections	health care associated infections	health care associated infections	health care associated infection	Health care-associated infections	1	singular
Health centers	health centers	health centers	health center	health center	1	singular
health checkup	health checkup	health checkup	health checkup	health checkup	1	kept
health checkups	health checkups	health checkups	health checkup	health checkup	1	singular
Health condition	health condition	health condition	health condition	Health condition	1	kept
Health conditions	health conditions	health conditions	health condition	Health condition	1	singular

health crises	health crises	health crises	health crises	health crises	1	kept
health crisis	health crisis	health crisis	health crisis	health crisis	1	kept
Health data systems	health data systems	health data systems	health data systems	Health data systems	1	kept
Health diaries	health diaries	health diaries	health diaries	Health diaries	1	kept
Health disparity	health disparity	health disparity	health disparity	health disparity	1	case_or_format
Health Economics	health economics	health economics	health economics	health economics	1	case_or_format
Health economy	health economy	health economy	health economy	Health economy	1	kept
Health education competence	health education competence	health education competence	health education competence	Health education competence	1	kept
Health education models	health education models	health education models	health education models	Health education models	1	kept
health education, dental	health education dental	health education dental	health education dental	health education, dental	1	kept
Health educational program	health educational program	health educational program	health educational program	Health educational program	1	kept
Health effects	health effects	health effects	health effects	Health effects	1	kept
Health emergencies	health emergencies	health emergencies	health emergency	health emergency	1	singular
health emergency	health emergency	health emergency	health emergency	health emergency	1	kept
Health employee	health employee	health employee	health employee	Health employee	1	kept
Health etc	health etc	health etc	health etc	Health etc	1	kept

Health evaluation	health evaluation	health evaluation	health evaluation	Health Evaluation	1	case_or_format
Health expenditure	health expenditure	health expenditure	health expenditure	Health Expenditures	1	case_or_format
Health expenditures	health expenditures	health expenditures	health expenditure	Health Expenditures	1	singular
health facilities	health facilities	health facilities	health facilities	Health Facilities	1	case_or_format
Health Facilities, Proprietary	health facilities proprietary	health facilities proprietary	health facilities proprietary	Health Facilities, Proprietary	1	kept
Health Facility Administrators	health facility administrators	health facility administrators	health facility administrators	Health Facility Administrators	1	kept
health facility environment	health facility environment	health facility environment	health facility environment	Health Facility Environment	1	case_or_format
health geography	health geography	health geography	health geography	health geography	1	kept
Health hazards	health hazards	health hazards	health hazard	health hazard	1	singular
Health Impact Assessment	health impact assessment	health impact assessment	health impact assessment	health impact assessment	1	case_or_format
Health indicators	health indicators	health indicators	health indicators	Health indicators	1	kept
Health inequalities	health inequalities	health inequalities	health inequalities	Health inequalities	1	kept
health inequities	health inequities	health inequities	health inequity	Health inequity	1	singular
Health inequity	health inequity	health inequity	health inequity	Health inequity	1	kept
health inequity	health inequity	health inequity	health inequity	Health inequity	1	case_or_format
Health Information	health information	health information	health information	Health Information	1	kept

Health information	health information	health information	health information	Health Information	1	case_or_format
Health Information Exchange	health information exchange	health information exchange	health information exchange	Health Information Exchange	1	kept
Health information exchange	health information exchange	health information exchange	health information exchange	Health Information Exchange	1	case_or_format
Health information exchanges	health information exchanges	health information exchanges	health information exchange	Health Information Exchange	1	singular
health information management	health information management	health information management	health information management	Health Information Management	1	case_or_format
health information system	health information system	health information system	health information system	Health Information Systems	1	case_or_format
Health Information Technology Workaround	health information technology workaround	health information technology workaround	health information technology workaround	Health Information Technology Workaround	1	kept
HEALTH INFORMATION-TECHNOLOGY	health information technology	health information technology	health information technology	Health information technology	1	case_or_format
health insurance claims	health insurance claims	health insurance claims	health insurance claims	health insurance claims	1	kept
health insurance eligibility	health insurance eligibility	health insurance eligibility	health insurance eligibility	health insurance eligibility	1	kept
Health insurance reimbursement	health insurance reimbursement	health insurance reimbursement	health insurance reimbursement	Health insurance reimbursement	1	kept
Health Insurance Review and Assessment Service	health insurance review and assessment service	health insurance review and assessment service	health insurance review and assessment service	Health Insurance Review and Assessment Service	1	kept
Health intervention	health intervention	health intervention	health intervention	health intervention	1	case_or_format
Health Issues	health issues	health issues	health issues	Health Issues	1	kept
Health IT	health it	health it	health it	Health IT	1	kept
health knowledge	health knowledge	health knowledge	health knowledge	Health knowledge	1	case_or_format

Health knowledge, attitudes practice	health knowledge attitudes practice	health knowledge attitudes practice	health knowledge attitudes practice	Health Knowledge, Attitudes, Practice	1	case_or_format
Health knowledge, Attitudes, Practice	health knowledge attitudes practice	health knowledge attitudes practice	health knowledge attitudes practice	Health Knowledge, Attitudes, Practice	1	case_or_format
Health knowledge, attitudes, practice	health knowledge attitudes practice	health knowledge attitudes practice	health knowledge attitudes practice	Health Knowledge, Attitudes, Practice	1	case_or_format
health knowledge, attitudes, practice	health knowledge attitudes practice	health knowledge attitudes practice	health knowledge attitudes practice	Health Knowledge, Attitudes, Practice	1	case_or_format
Health Management Organization	health management organization	health management organization	health management organization	Health Management Organization	1	kept
Health manpower	health manpower	health manpower	health manpower	Health Manpower	1	case_or_format
health manpower	health manpower	health manpower	health manpower	Health Manpower	1	case_or_format
Health Monitoring	health monitoring	health monitoring	health monitoring	Health monitoring	1	case_or_format
health monitoring	health monitoring	health monitoring	health monitoring	Health monitoring	1	case_or_format
Health Monitoring System	health monitoring system	health monitoring system	health monitoring system	Health monitoring system	1	case_or_format
Health occupations	health occupations	health occupations	health occupations	Health Occupations	1	case_or_format
health occupations	health occupations	health occupations	health occupations	Health Occupations	1	case_or_format
Health of the elderly	health of the elderly	health of the elderly	health of the elderly	Health of the elderly	1	kept
health officer	health officer	health officer	health officer	health officer	1	kept
health organisations	health organisations	health organisations	health organisations	health organisations	1	kept
Health outcome	health outcome	health outcome	health outcome	health outcome	1	case_or_format

HEALTH OUTCOMES	health outcomes	health outcomes	health outcome	health outcome	1	singular
Health personnel attitudes	health personnel attitudes	health personnel attitudes	health personnel attitudes	Health personnel attitudes	1	kept
health personnel education	health personnel education	health personnel education	health personnel education	health personnel education	1	kept
Health personnel management	health personnel management	health personnel management	health personnel management	Health personnel management	1	kept
Health Planning Councils	health planning councils	health planning councils	health planning councils	Health Planning Councils	1	kept
Health Planning guidelines	health planning guidelines	health planning guidelines	health planning guidelines	Health Planning Guidelines	1	case_or_format
Health planning guidelines	health planning guidelines	health planning guidelines	health planning guidelines	Health Planning Guidelines	1	case_or_format
Health police	health police	health police	health police	Health police	1	kept
health Policy	health policy	health policy	health policy	Health Policy	1	case_or_format
health policy analysis	health policy analysis	health policy analysis	health policy analysis	health policy analysis	1	kept
health practices	health practices	health practices	health practices	health practices	1	kept
Health problems	health problems	health problems	health problems	Health problems	1	kept
health problems	health problems	health problems	health problems	Health problems	1	case_or_format
Health process	health process	health process	health process	Health process	1	kept
health process model	health process model	health process model	health process model	health process model	1	kept
health processionals	health processionals	health processionals	health processionals	health processionals	1	kept

health professional	health professional	health professional	health professional	Health professionals	1	case_or_format
Health professional loss and grieving	health professional loss and grieving	health professional loss and grieving	health professional loss and grieving	Health professional loss and grieving	1	kept
health professional well-being	health professional well being	health professional well being	health professional well being	health professional well-being	1	kept
health professionals follow up study	health professionals follow up study	health professionals follow up study	health professionals follow up study	health professionals follow up study	1	kept
Health professions	health professions	health professions	health profession	Health profession	1	singular
Health promoting hospital	health promoting hospital	health promoting hospital	health promoting hospital	Health promoting hospital	1	kept
Health promoting lifestyle	health promoting lifestyle	health promoting lifestyle	health promoting lifestyle	Health promoting lifestyle	1	kept
Health Promotion Administration	health promotion administration	health promotion administration	health promotion administration	Health Promotion Administration	1	kept
Health promotion behavior’s	health promotion behavior s	health promotion behavior s	health promotion behavior s	Health promotion behavior’s	1	kept
Health promotion positive actions	health promotion positive actions	health promotion positive actions	health promotion positive actions	Health promotion positive actions	1	kept
health promotion program	health promotion program	health promotion program	health promotion program	health promotion program	1	kept
Health provider	health provider	health provider	health provider	Health provider	1	kept
Health providers’ attitudes	health providers attitudes	health providers attitudes	health providers attitudes	Health providers’ attitudes	1	kept
health psychology	health psychology	health psychology	health psychology	health psychology	1	kept
health quality of care	health quality of care	health quality of care	health quality of care	health quality of care	1	kept
HEALTH RECORD	health record	health record	health record	Health records	1	case_or_format

Health record	health record	health record	health record	Health records	1	case_or_format
health record	health record	health record	health record	Health records	1	case_or_format
health records	health records	health records	health record	Health records	1	singular
Health Records, Personal	health records personal	health records personal	health records personal	Health Records, Personal	1	kept
Health Reform	health reform	health reform	health reform	Health Reform	1	kept
Health Related Quality of Life	health related quality of life	health related quality of life	health related quality of life	health-related quality of life	1	case_or_format
Health related quality of life	health related quality of life	health related quality of life	health related quality of life	health-related quality of life	1	case_or_format
health related quality of life	health related quality of life	health related quality of life	health related quality of life	health-related quality of life	1	case_or_format
health rights	health rights	health rights	health rights	health rights	1	kept
Health risk assessments	health risk assessments	health risk assessments	health risk assessment	health risk assessment	1	singular
health sciences	health sciences	health sciences	health science	health science	1	singular
health screening	health screening	health screening	health screening	health screening	1	kept
health sector in Turkey	health sector in turkey	health sector in turkey	health sector in turkey	health sector in Turkey	1	kept
Health service	health service	health service	health service	health service	1	case_or_format
Health Service Administration	health service administration	health service administration	health service administration	Health Service Administration	1	kept
Health service administration	health service administration	health service administration	health service administration	Health Service Administration	1	case_or_format

Health service costs	health service costs	health service costs	health service costs	Health service costs	1	kept
Health service delivery	health service delivery	health service delivery	health service delivery	Health service delivery	1	kept
health service delivery	health service delivery	health service delivery	health service delivery	Health service delivery	1	case_or_format
Health service evaluation	health service evaluation	health service evaluation	health service evaluation	Health service evaluation	1	kept
Health Service Management	health service management	health service management	health service management	Health Service Management	1	kept
health service use	health service use	health service use	health service use	health service use	1	kept
Health service utilization	health service utilization	health service utilization	health service utilization	Health service utilization	1	kept
Health services administration	health services administration	health services administration	health services administration	health services administration	1	case_or_format
health services administration and management	health services administration and management	health services administration and management	health services administration and management	health services administration & management	1	case_or_format
Health services efficiency	health services efficiency	health services efficiency	health services efficiency	Health services efficiency	1	kept
health services for the aged	health services for the aged	health services for the aged	health services for the aged	Health Services for the Aged	1	case_or_format
Health Services Integration	health services integration	health services integration	health services integration	Health Services Integration	1	kept
health services management	health services management	health services management	health services management	health services management	1	kept
health services misuse	health services misuse	health services misuse	health services misuse	Health Services Misuse	1	case_or_format
Health services needs and demand	health services needs and demand	health services needs and demand	health services needs and demand	Health Services Needs and Demand	1	case_or_format
health services needs and demand	health services needs and demand	health services needs and demand	health services needs and demand	Health Services Needs and Demand	1	case_or_format

Health services needs and demands	health services needs and demands	health services needs and demands	health services needs and demand	Health Services Needs and Demand	1	singular
Health Services, Indigenous	health services indigenous	health services indigenous	health services indigenous	Health Services, Indigenous	1	kept
Health simulation	health simulation	health simulation	health simulation	Health simulation	1	kept
health spending	health spending	health spending	health spending	Health Spending	1	case_or_format
health statistics	health statistics	health statistics	health statistics	health statistics	1	kept
Health status assessment	health status assessment	health status assessment	health status assessment	Health status assessment	1	kept
Health status indicators	health status indicators	health status indicators	health status indicator	Health Status Indicators	1	singular
health status indicators	health status indicators	health status indicators	health status indicator	Health Status Indicators	1	singular
Health students	health students	health students	health student	health student	1	singular
Health surveillance	health surveillance	health surveillance	health surveillance	Health surveillance	1	kept
health surveillance	health surveillance	health surveillance	health surveillance	Health surveillance	1	case_or_format
Health surveillances	health surveillances	health surveillances	health surveillance	Health surveillance	1	singular
HEALTH SURVEY	health survey	health survey	health survey	health survey	1	case_or_format
Health survey	health survey	health survey	health survey	health survey	1	case_or_format
Health system	health system	health system	health system	Health systems	1	case_or_format
health system	health system	health system	health system	Health systems	1	case_or_format

Health system change	health system change	health system change	health system change	Health system change	1	kept
Health system efficiency	health system efficiency	health system efficiency	health system efficiency	Health system efficiency	1	kept
Health system survey methodology	health system survey methodology	health system survey methodology	health system survey methodology	Health system survey methodology	1	kept
Health Systems	health systems	health systems	health system	Health systems	1	singular
Health systems evaluation	health systems evaluation	health systems evaluation	health systems evaluation	Health systems evaluation	1	kept
Health Systems Plans	health systems plans	health systems plans	health systems plans	Health Systems Plans	1	kept
Health Systems Strengthening	health systems strengthening	health systems strengthening	health systems strengthening	Health Systems Strengthening	1	kept
health team communication	health team communication	health team communication	health team communication	health team communication	1	kept
health technician	health technician	health technician	health technician	health technician	1	kept
Health Technology	health technology	health technology	health technology	health technology	1	case_or_format
Health Technology Assessment	health technology assessment	health technology assessment	health technology assessment	Health Technology Assessment	1	kept
health technology assessment	health technology assessment	health technology assessment	health technology assessment	Health Technology Assessment	1	case_or_format
Health Transition	health transition	health transition	health transition	Health Transition	1	kept
health transition	health transition	health transition	health transition	Health Transition	1	case_or_format
Health transitions	health transitions	health transitions	health transition	Health Transition	1	singular
health transitions	health transitions	health transitions	health transition	Health Transition	1	singular

Health worker	health worker	health worker	health worker	health worker	1	case_or_format
Health Worker Capacity-Building	health worker capacity building	health worker capacity building	health worker capacity building	Health Worker Capacity-Building	1	kept
Health workforce planning	health workforce planning	health workforce planning	health workforce planning	Health workforce planning	1	kept
Health Workforce Policy	health workforce policy	health workforce policy	health workforce policy	Health Workforce Policy	1	kept
Health working environment	health working environment	health working environment	health working environment	Health working environment	1	kept
Health-care associated infection	health care associated infection	health care associated infection	health care associated infection	Health care-associated infections	1	case_or_format
Health-care associated infections	health care associated infections	health care associated infections	health care associated infection	Health care-associated infections	1	singular
Health-care costs	health care costs	health care costs	health care cost	health care cost	1	singular
Health-care decisions	health care decisions	health care decisions	health care decisions	Health-care decisions	1	kept
Health-care delivery	health care delivery	health care delivery	health care delivery	health care delivery	1	case_or_format
health-care economics	health care economics	health care economics	health care economics	health care economics	1	case_or_format
HEALTH-CARE EPIDEMIOLOGY	health care epidemiology	health care epidemiology	health care epidemiology	HEALTH-CARE EPIDEMIOLOGY	1	kept
Health-care expenditures	health care expenditures	health care expenditures	health care expenditures	Health care expenditures	1	case_or_format
health-care management	health care management	health care management	health care management	health care management	1	case_or_format
Health-care managements	health care managements	health care managements	health care management	health care management	1	singular
health-care organization	health care organization	health care organization	health care organization	health care organization	1	case_or_format

Health-care personnel	health care personnel	health care personnel	health care personnel	health care personnel	1	case_or_format
health-care personnel	health care personnel	health care personnel	health care personnel	health care personnel	1	case_or_format
Health-care provider	health care provider	health care provider	health care provider	health care providers	1	case_or_format
HEALTH-CARE PROVIDERS	health care providers	health care providers	health care provider	health care providers	1	singular
health-care providers' attitude	health care providers attitude	health care providers attitude	health care providers attitude	health-care providers' attitude	1	kept
HEALTH-CARE QUALITY	health care quality	health care quality	health care quality	health care quality	1	case_or_format
Health-care quality	health care quality	health care quality	health care quality	health care quality	1	case_or_format
Health-care quality improvement	health care quality improvement	health care quality improvement	health care quality improvement	Health care quality improvement	1	case_or_format
Health-care safety	health care safety	health care safety	health care safety	Health-care safety	1	kept
Health-care setting	health care setting	health care setting	health care setting	Health-care setting	1	kept
Health-care staff	health care staff	health care staff	health care staff	health care staff	1	case_or_format
HEALTH-CARE SYSTEM	health care system	health care system	health care system	health care system	1	case_or_format
health-care utilization	health care utilization	health care utilization	health care utilization	health care utilization	1	case_or_format
health-care-associated infection	health care associated infection	health care associated infection	health care associated infection	Health care-associated infections	1	case_or_format
HEALTH-CARE-SYSTEM	health care system	health care system	health care system	health care system	1	case_or_format
Health-disease process	health disease process	health disease process	health disease process	Health-disease process	1	kept

Health-ITUES	health itues	health itues	health itues	Health-ITUES	1	kept
HEALTH-PROBLEMS	health problems	health problems	health problems	Health problems	1	case_or_format
HEALTH-PROFESSIONALS	health professionals	health professionals	health professional	Health professionals	1	singular
HEALTH-SERVICES RESEARCH	health services research	health services research	health services research	Health Services Research	1	case_or_format
HealthCare	healthcare	healthcare	healthcare	Healthcare	1	case_or_format
healthcare access	healthcare access	healthcare access	healthcare access	healthcare access	1	kept
Healthcare acquired infection	healthcare acquired infection	healthcare acquired infection	healthcare acquired infection	Healthcare acquired infection	1	kept
Healthcare and medicine	healthcare and medicine	healthcare and medicine	healthcare and medicine	Healthcare and medicine	1	kept
Healthcare and Technology Synergy Framework	healthcare and technology synergy framework	healthcare and technology synergy framework	healthcare and technology synergy framework	Healthcare and Technology Synergy Framework	1	kept
healthcare architecture	healthcare architecture	healthcare architecture	healthcare architecture	Healthcare architecture	1	case_or_format
healthcare assistant	healthcare assistant	healthcare assistant	healthcare assistant	healthcare assistant	1	kept
healthcare associated pneumonia	healthcare associated pneumonia	healthcare associated pneumonia	healthcare associated pneumonia	Healthcare-Associated Pneumonia	1	case_or_format
healthcare burnout	healthcare burnout	healthcare burnout	healthcare burnout	healthcare burnout	1	kept
healthcare collaboration	healthcare collaboration	healthcare collaboration	healthcare collaboration	healthcare collaboration	1	kept
Healthcare complaints	healthcare complaints	healthcare complaints	healthcare complaints	Healthcare complaints	1	kept
Healthcare cost	healthcare cost	healthcare cost	healthcare cost	Healthcare costs	1	case_or_format

Healthcare Costs	healthcare costs	healthcare costs	healthcare cost	Healthcare costs	1	singular
Healthcare deliv-ery system	healthcare deliv ery system	healthcare deliv ery system	healthcare deliv ery system	Healthcare deliv-ery system	1	kept
Healthcare delivery challenges	healthcare delivery challenges	healthcare delivery challenges	healthcare delivery challenges	Healthcare delivery challenges	1	kept
healthcare delivery strain	healthcare delivery strain	healthcare delivery strain	healthcare delivery strain	healthcare delivery strain	1	kept
Healthcare design	healthcare design	healthcare design	healthcare design	healthcare design	1	case_or_format
healthcare disparity	healthcare disparity	healthcare disparity	healthcare disparity	Healthcare Disparities	1	case_or_format
Healthcare domains	healthcare domains	healthcare domains	healthcare domains	Healthcare domains	1	kept
Healthcare economics	healthcare economics	healthcare economics	healthcare economics	Healthcare economics	1	kept
Healthcare effectiveness	healthcare effectiveness	healthcare effectiveness	healthcare effectiveness	Healthcare effectiveness	1	kept
healthcare employees	healthcare employees	healthcare employees	healthcare employees	healthcare employees	1	kept
healthcare environment design	healthcare environment design	healthcare environment design	healthcare environment design	healthcare environment design	1	kept
healthcare expenditures	healthcare expenditures	healthcare expenditures	healthcare expenditures	healthcare expenditures	1	kept
healthcare experience	healthcare experience	healthcare experience	healthcare experience	healthcare experience	1	kept
Healthcare facilities	healthcare facilities	healthcare facilities	healthcare facility	Healthcare facility	1	singular
healthcare facilities	healthcare facilities	healthcare facilities	healthcare facility	Healthcare facility	1	singular
Healthcare facility	healthcare facility	healthcare facility	healthcare facility	Healthcare facility	1	kept

Healthcare facility design	healthcare facility design	healthcare facility design	healthcare facility design	Healthcare facility design	1	kept
healthcare facility planning	healthcare facility planning	healthcare facility planning	healthcare facility planning	healthcare facility planning	1	kept
healthcare failure mode and effects analysis	healthcare failure mode and effects analysis	healthcare failure mode and effects analysis	healthcare failure mode and effects analysis	healthcare failure mode and effects analysis	1	kept
healthcare hierarchy	healthcare hierarchy	healthcare hierarchy	healthcare hierarchy	healthcare hierarchy	1	kept
healthcare industry	healthcare industry	healthcare industry	healthcare industry	Healthcare industry	1	case_or_format
healthcare inequalities	healthcare inequalities	healthcare inequalities	healthcare inequality	healthcare inequality	1	singular
healthcare inequality	healthcare inequality	healthcare inequality	healthcare inequality	healthcare inequality	1	kept
Healthcare Informatics	healthcare informatics	healthcare informatics	healthcare informatics	Healthcare Informatics	1	kept
Healthcare information technology	healthcare information technology	healthcare information technology	healthcare information technology	Healthcare information technology	1	kept
healthcare infrastructure	healthcare infrastructure	healthcare infrastructure	healthcare infrastructure	healthcare infrastructure	1	kept
Healthcare innovation	healthcare innovation	healthcare innovation	healthcare innovation	Healthcare innovation	1	kept
Healthcare institutions	healthcare institutions	healthcare institutions	healthcare institutions	Healthcare institutions	1	kept
healthcare institutions	healthcare institutions	healthcare institutions	healthcare institutions	Healthcare institutions	1	case_or_format
Healthcare interventions	healthcare interventions	healthcare interventions	healthcare interventions	Healthcare interventions	1	kept
Healthcare IT	healthcare it	healthcare it	healthcare it	Healthcare IT	1	kept
healthcare leader	healthcare leader	healthcare leader	healthcare leader	healthcare leader	1	kept

healthcare leadership	healthcare leadership	healthcare leadership	healthcare leadership	healthcare leadership	1	kept
Healthcare logistical management	healthcare logistical management	healthcare logistical management	healthcare logistical management	Healthcare logistical management	1	kept
healthcare near misses	healthcare near misses	healthcare near misses	healthcare near misses	healthcare near misses	1	kept
Healthcare needs	healthcare needs	healthcare needs	healthcare needs	Healthcare needs	1	kept
Healthcare occupations	healthcare occupations	healthcare occupations	healthcare occupations	Healthcare occupations	1	kept
Healthcare organisation	healthcare organisation	healthcare organisation	healthcare organisation	Healthcare organisation	1	kept
healthcare organisation	healthcare organisation	healthcare organisation	healthcare organisation	Healthcare organisation	1	case_or_format
Healthcare organizations	healthcare organizations	healthcare organizations	healthcare organization	Healthcare organization	1	singular
healthcare organizations	healthcare organizations	healthcare organizations	healthcare organization	Healthcare organization	1	singular
Healthcare Organizations, critical skills, Case [illegible]	healthcare organizations critical skills case [illegible]	healthcare organizations critical skills case [illegible]	healthcare organizations critical skills case [illegible]	Healthcare Organizations, critical skills, Case [illegible]	1	kept
healthcare outcomes	healthcare outcomes	healthcare outcomes	healthcare outcomes	Healthcare outcomes	1	case_or_format
Healthcare personal	healthcare personal	healthcare personal	healthcare personal	Healthcare personal	1	kept
Healthcare personnel satisfaction	healthcare personnel satisfaction	healthcare personnel satisfaction	healthcare personnel satisfaction	Healthcare personnel satisfaction	1	kept
healthcare planning	healthcare planning	healthcare planning	healthcare planning	healthcare planning	1	kept
Healthcare policy	healthcare policy	healthcare policy	healthcare policy	healthcare policy	1	case_or_format
Healthcare practices	healthcare practices	healthcare practices	healthcare practices	Healthcare practices	1	kept

healthcare privatization	healthcare privatization	healthcare privatization	healthcare privatization	healthcare privatization	1	kept
healthcare professional experiences	healthcare professional experiences	healthcare professional experiences	healthcare professional experiences	healthcare professional experiences	1	kept
healthcare professional perceptions	healthcare professional perceptions	healthcare professional perceptions	healthcare professional perceptions	healthcare professional perceptions	1	kept
Healthcare professional shortage	healthcare professional shortage	healthcare professional shortage	healthcare professional shortage	Healthcare professional shortage	1	kept
healthcare professional students	healthcare professional students	healthcare professional students	healthcare professional students	healthcare professional students	1	kept
Healthcare professionals stress	healthcare professionals stress	healthcare professionals stress	healthcare professionals stress	Healthcare professionals stress	1	kept
healthcare professionals’ attitudes	healthcare professionals attitudes	healthcare professionals attitudes	healthcare professionals attitudes	healthcare professionals’ attitudes	1	kept
healthcare professionals’ awareness	healthcare professionals awareness	healthcare professionals awareness	healthcare professionals awareness	healthcare professionals’ awareness	1	kept
Healthcare Provider	healthcare provider	healthcare provider	healthcare provider	Healthcare providers	1	case_or_format
Healthcare Provider and Family Bidirectional Digital	healthcare provider and family bidirectional digital	healthcare provider and family bidirectional digital	healthcare provider and family bidirectional digital	Healthcare Provider and Family Bidirectional Digital	1	kept
Healthcare Providers	healthcare providers	healthcare providers	healthcare provider	Healthcare providers	1	singular
Healthcare quality assessment	healthcare quality assessment	healthcare quality assessment	healthcare quality assessment	Healthcare quality assessment	1	kept
Healthcare Quality Improvement	healthcare quality improvement	healthcare quality improvement	healthcare quality improvement	healthcare quality improvement	1	case_or_format
healthcare quality improvement circle	healthcare quality improvement circle	healthcare quality improvement circle	healthcare quality improvement circle	healthcare quality improvement circle	1	kept
healthcare rationing	healthcare rationing	healthcare rationing	healthcare rationing	Healthcare rationing	1	case_or_format
healthcare reform	healthcare reform	healthcare reform	healthcare reform	Healthcare reform	1	case_or_format

healthcare regulation	healthcare regulation	healthcare regulation	healthcare regulation	healthcare regulation	1	kept
healthcare resource	healthcare resource	healthcare resource	healthcare resource	Healthcare resources	1	case_or_format
healthcare resource allocation	healthcare resource allocation	healthcare resource allocation	healthcare resource allocation	healthcare resource allocation	1	kept
healthcare resource utilisation	healthcare resource utilisation	healthcare resource utilisation	healthcare resource utilisation	healthcare resource utilisation	1	kept
healthcare resources utilization	healthcare resources utilization	healthcare resources utilization	healthcare resources utilization	healthcare resources utilization	1	kept
healthcare resourcing	healthcare resourcing	healthcare resourcing	healthcare resourcing	healthcare resourcing	1	kept
Healthcare safety culture	healthcare safety culture	healthcare safety culture	healthcare safety culture	Healthcare safety culture	1	kept
healthcare satisfaction	healthcare satisfaction	healthcare satisfaction	healthcare satisfaction	healthcare satisfaction	1	kept
Healthcare sectors	healthcare sectors	healthcare sectors	healthcare sectors	Healthcare sectors	1	kept
Healthcare service robots	healthcare service robots	healthcare service robots	healthcare service robots	Healthcare service robots	1	kept
healthcare setting	healthcare setting	healthcare setting	healthcare setting	healthcare setting	1	kept
Healthcare simulation	healthcare simulation	healthcare simulation	healthcare simulation	Healthcare simulation	1	kept
healthcare simulation	healthcare simulation	healthcare simulation	healthcare simulation	Healthcare simulation	1	case_or_format
Healthcare spending	healthcare spending	healthcare spending	healthcare spending	Healthcare spending	1	kept
Healthcare students	healthcare students	healthcare students	healthcare students	Healthcare students	1	kept
Healthcare survey	healthcare survey	healthcare survey	healthcare survey	healthcare survey	1	case_or_format

healthcare surveys	healthcare surveys	healthcare surveys	healthcare survey	healthcare survey	1	singular
Healthcare sustainability	healthcare sustainability	healthcare sustainability	healthcare sustainability	Healthcare sustainability	1	kept
Healthcare system improvement	healthcare system improvement	healthcare system improvement	healthcare system improvement	Healthcare system improvement	1	kept
healthcare targets	healthcare targets	healthcare targets	healthcare targets	healthcare targets	1	kept
healthcare teams	healthcare teams	healthcare teams	healthcare team	Healthcare team	1	singular
Healthcare training	healthcare training	healthcare training	healthcare training	Healthcare training	1	kept
Healthcare use	healthcare use	healthcare use	healthcare use	Healthcare use	1	kept
healthcare use	healthcare use	healthcare use	healthcare use	Healthcare use	1	case_or_format
Healthcare Utilisation databases	healthcare utilisation databases	healthcare utilisation databases	healthcare utilisation databases	Healthcare Utilisation databases	1	kept
Healthcare worker behaviour	healthcare worker behaviour	healthcare worker behaviour	healthcare worker behaviour	Healthcare worker behaviour	1	kept
healthcare worker training	healthcare worker training	healthcare worker training	healthcare worker training	healthcare worker training	1	kept
Healthcare workers behaviour	healthcare workers behaviour	healthcare workers behaviour	healthcare workers behaviour	Healthcare workers behaviour	1	kept
healthcare workers' experiences	healthcare workers experiences	healthcare workers experiences	healthcare workers experiences	healthcare workers' experiences	1	kept
Healthcare worker's habits	healthcare worker s habits	healthcare worker s habits	healthcare worker s habits	Healthcare worker's habits	1	kept
Healthcare-acquired infection	healthcare acquired infection	healthcare acquired infection	healthcare acquired infection	Healthcare acquired infection	1	case_or_format
healthcare-acquired infections	healthcare acquired infections	healthcare acquired infections	healthcare acquired infection	Healthcare acquired infection	1	singular

Healthcare-associated	healthcare associated	healthcare associated	healthcare associated	Healthcare-associated	1	kept
Healthcare-Associated Infection	healthcare associated infection	healthcare associated infection	healthcare associated infection	healthcare associated infection	1	case_or_format
Healthcare-associated Infections	healthcare associated infections	healthcare associated infections	healthcare associated infection	healthcare associated infection	1	singular
Healthcare-professionals	healthcare professionals	healthcare professionals	healthcare professional	healthcare professionals	1	singular
Healthcare-simulation	healthcare simulation	healthcare simulation	healthcare simulation	Healthcare simulation	1	case_or_format
healthcare-workers	healthcare workers	healthcare workers	healthcare worker	Healthcare workers	1	singular
healthcare‐associated infection	healthcare associated infection	healthcare associated infection	healthcare associated infection	healthcare associated infection	1	case_or_format
Healthcare-associated infections	healthcare associated infections	healthcare associated infections	healthcare associated infection	healthcare associated infection	1	singular
HEALTHIEST	healthiest	healthiest	healthiest	HEALTHIEST	1	kept
Healthy	healthy	healthy	healthy	Healthy	1	kept
Healthy Aging	healthy aging	healthy aging	healthy aging	Healthy Aging	1	kept
healthy aging brain care monitor	healthy aging brain care monitor	healthy aging brain care monitor	healthy aging brain care monitor	healthy aging brain care monitor	1	kept
healthy diet	healthy diet	healthy diet	healthy diet	healthy diet	1	kept
Healthy full-term infants	healthy full term infants	healthy full term infants	healthy full term infants	Healthy full-term infants	1	kept
HEALTHY HORSES	healthy horses	healthy horses	healthy horses	HEALTHY HORSES	1	kept
HEALTHY LIFE EXPECTANCY	healthy life expectancy	healthy life expectancy	healthy life expectancy	HEALTHY LIFE EXPECTANCY	1	kept

healthy life expectancy	healthy life expectancy	healthy life expectancy	healthy life expectancy	HEALTHY LIFE EXPECTANCY	1	case_or_format
Healthy newborn	healthy newborn	healthy newborn	healthy newborn	Healthy newborn	1	kept
Healthy practice environment	healthy practice environment	healthy practice environment	healthy practice environment	Healthy practice environment	1	kept
Healthy Teams model	healthy teams model	healthy teams model	healthy teams model	Healthy Teams model	1	kept
Healthy work hours	healthy work hours	healthy work hours	healthy work hours	Healthy work hours	1	kept
healthy working environment	healthy working environment	healthy working environment	healthy working environment	healthy working environment	1	kept
Healthy workplace	healthy workplace	healthy workplace	healthy workplace	Healthy workplace	1	kept
Health‐care worker	health care worker	health care worker	health care worker	Health care workers	1	case_or_format
Health‐related quality of life	health related quality of life	health related quality of life	health related quality of life	health-related quality of life	1	case_or_format
hearing impaired person	hearing impaired person	hearing impaired person	hearing impaired person	hearing impaired person	1	kept
hearing screening	hearing screening	hearing screening	hearing screening	hearing screening	1	kept
hearing voices	hearing voices	hearing voices	hearing voices	hearing voices	1	kept
heart anatomy	heart anatomy	heart anatomy	heart anatomy	heart anatomy	1	kept
heart aneurysm	heart aneurysm	heart aneurysm	heart aneurysm	heart aneurysm	1	kept
Heart arrhythmias	heart arrhythmias	heart arrhythmias	heart arrhythmia	heart arrhythmia	1	singular
Heart assist devices	heart assist devices	heart assist devices	heart assist device	Heart-Assist Devices	1	singular

heart atrium appendage	heart atrium appendage	heart atrium appendage	heart atrium appendage	heart atrium appendage	1	kept
Heart beat	heart beat	heart beat	heart beat	heart beat	1	case_or_format
Heart Beat Measurement	heart beat measurement	heart beat measurement	heart beat measurement	Heart Beat Measurement	1	kept
Heart beat rates	heart beat rates	heart beat rates	heart beat rates	Heart beat rates	1	kept
Heart beat sensor	heart beat sensor	heart beat sensor	heart beat sensor	Heart beat sensor	1	kept
Heart beat sensors	heart beat sensors	heart beat sensors	heart beat sensor	Heart beat sensor	1	singular
Heart beats	heart beats	heart beats	heart beat	heart beat	1	singular
Heart Block	heart block	heart block	heart block	heart block	1	case_or_format
Heart catheterisation	heart catheterisation	heart catheterisation	heart catheterisation	Heart catheterisation	1	kept
heart catheterisation	heart catheterisation	heart catheterisation	heart catheterisation	Heart catheterisation	1	case_or_format
heart contusion	heart contusion	heart contusion	heart contusion	heart contusion	1	kept
Heart dataset	heart dataset	heart dataset	heart dataset	Heart dataset	1	kept
Heart defects, congenital	heart defects congenital	heart defects congenital	heart defects congenital	Heart Defects, Congenital	1	case_or_format
heart development	heart development	heart development	heart development	heart development	1	kept
heart dilatation	heart dilatation	heart dilatation	heart dilatation	heart dilatation	1	kept
heart donor	heart donor	heart donor	heart donor	heart donor	1	kept

heart edema	heart edema	heart edema	heart edema	heart edema	1	kept
Heart failure care	heart failure care	heart failure care	heart failure care	Heart failure care	1	kept
Heart failure clinic	heart failure clinic	heart failure clinic	heart failure clinic	Heart failure clinic	1	kept
Heart failure management programs	heart failure management programs	heart failure management programs	heart failure management programs	Heart failure management programs	1	kept
Heart failure monitoring	heart failure monitoring	heart failure monitoring	heart failure monitoring	Heart failure monitoring	1	kept
Heart failure treatment	heart failure treatment	heart failure treatment	heart failure treatment	Heart failure treatment	1	kept
Heart failure with preserved ejection fraction	heart failure with preserved ejection fraction	heart failure with preserved ejection fraction	heart failure with preserved ejection fraction	heart failure with preserved ejection fraction	1	case_or_format
Heart failure with preserved systolic function	heart failure with preserved systolic function	heart failure with preserved systolic function	heart failure with preserved systolic function	Heart failure with preserved systolic function	1	kept
Heart failure with reduced ejection fraction	heart failure with reduced ejection fraction	heart failure with reduced ejection fraction	heart failure with reduced ejection fraction	heart failure with reduced ejection fraction	1	case_or_format
heart fibrillation	heart fibrillation	heart fibrillation	heart fibrillation	heart fibrillation	1	kept
heart infarction localization	heart infarction localization	heart infarction localization	heart infarction localization	heart infarction localization	1	kept
heart innervation	heart innervation	heart innervation	heart innervation	heart innervation	1	kept
heart left atrium pressure	heart left atrium pressure	heart left atrium pressure	heart left atrium pressure	heart left atrium pressure	1	kept
heart left ventricle aneurysm	heart left ventricle aneurysm	heart left ventricle aneurysm	heart left ventricle aneurysm	heart left ventricle aneurysm	1	kept
heart left ventricle contraction	heart left ventricle contraction	heart left ventricle contraction	heart left ventricle contraction	heart left ventricle contraction	1	kept
heart left ventricle ejection time	heart left ventricle ejection time	heart left ventricle ejection time	heart left ventricle ejection time	heart left ventricle ejection time	1	kept

heart left ventricle enddiastolic pressure	heart left ventricle enddiastolic pressure	heart left ventricle enddiastolic pressure	heart left ventricle enddiastolic pressure	heart left ventricle enddiastolic pressure	1	kept
heart left ventricle enddiastolic volume	heart left ventricle enddiastolic volume	heart left ventricle enddiastolic volume	heart left ventricle enddiastolic volume	heart left ventricle enddiastolic volume	1	kept
heart left ventricle outflow tract	heart left ventricle outflow tract	heart left ventricle outflow tract	heart left ventricle outflow tract	heart left ventricle outflow tract	1	kept
heart left ventricle outflow tract obstruction	heart left ventricle outflow tract obstruction	heart left ventricle outflow tract obstruction	heart left ventricle outflow tract obstruction	heart left ventricle outflow tract obstruction	1	kept
heart left ventricle performance	heart left ventricle performance	heart left ventricle performance	heart left ventricle performance	heart left ventricle performance	1	kept
heart left ventricle volume	heart left ventricle volume	heart left ventricle volume	heart left ventricle volume	heart left ventricle volume	1	kept
heart lung transplantation	heart lung transplantation	heart lung transplantation	heart lung transplantation	Heart-Lung Transplantation	1	case_or_format
Heart massage	heart massage	heart massage	heart massage	Heart Massage	1	case_or_format
Heart Massage [E02.365.647.375]	heart massage e02 365 647 375	heart massage e02 365 647 375	heart massage e02 365 647 375	Heart Massage [E02.365.647.375]	1	kept
heart mitochondrion	heart mitochondrion	heart mitochondrion	heart mitochondrion	heart mitochondrion	1	kept
Heart Murmurs	heart murmurs	heart murmurs	heart murmur	heart murmur	1	singular
heart muscle capillary	heart muscle capillary	heart muscle capillary	heart muscle capillary	heart muscle capillary	1	kept
heart muscle metabolism	heart muscle metabolism	heart muscle metabolism	heart muscle metabolism	heart muscle metabolism	1	kept
heart muscle necrosis	heart muscle necrosis	heart muscle necrosis	heart muscle necrosis	heart muscle necrosis	1	kept
heart muscle oxygen consumption	heart muscle oxygen consumption	heart muscle oxygen consumption	heart muscle oxygen consumption	heart muscle oxygen consumption	1	kept
heart muscle perfusion	heart muscle perfusion	heart muscle perfusion	heart muscle perfusion	heart muscle perfusion	1	kept

heart outflow tract obstruction	heart outflow tract obstruction	heart outflow tract obstruction	heart outflow tract obstruction	heart outflow tract obstruction	1	kept
heart output measurement	heart output measurement	heart output measurement	heart output measurement	heart output measurement	1	kept
Heart pain	heart pain	heart pain	heart pain	Heart pain	1	kept
Heart period	heart period	heart period	heart period	Heart period	1	kept
heart preejection period	heart preejection period	heart preejection period	heart preejection period	heart preejection period	1	kept
heart preservation	heart preservation	heart preservation	heart preservation	heart preservation	1	kept
Heart problems	heart problems	heart problems	heart problems	Heart problems	1	kept
heart rate characteristic monitoring	heart rate characteristic monitoring	heart rate characteristic monitoring	heart rate characteristic monitoring	heart rate characteristic monitoring	1	kept
Heart rate variability signals	heart rate variability signals	heart rate variability signals	heart rate variability signals	Heart rate variability signals	1	kept
heart reinfarction	heart reinfarction	heart reinfarction	heart reinfarction	heart reinfarction	1	kept
heart repolarization	heart repolarization	heart repolarization	heart repolarization	heart repolarization	1	kept
heart rupture	heart rupture	heart rupture	heart rupture	heart rupture	1	kept
Heart Rupture, Post-Infarction	heart rupture post infarction	heart rupture post infarction	heart rupture post infarction	Heart Rupture, Post-Infarction	1	kept
HEART score	heart score	heart score	heart score	HEART score	1	kept
heart surgery department	heart surgery department	heart surgery department	heart surgery department	heart surgery department	1	kept
heart tissue	heart tissue	heart tissue	heart tissue	heart tissue	1	kept

Heart transplants	heart transplants	heart transplants	heart transplant	Heart transplant	1	singular
Heart valve surgery	heart valve surgery	heart valve surgery	heart valve surgery	heart valve surgery	1	case_or_format
heart ventricle aneurysm	heart ventricle aneurysm	heart ventricle aneurysm	heart ventricle aneurysm	heart ventricle aneurysm	1	kept
heart ventricle bigeminy	heart ventricle bigeminy	heart ventricle bigeminy	heart ventricle bigeminy	heart ventricle bigeminy	1	kept
heart ventricle conduction	heart ventricle conduction	heart ventricle conduction	heart ventricle conduction	heart ventricle conduction	1	kept
heart ventricle filling	heart ventricle filling	heart ventricle filling	heart ventricle filling	heart ventricle filling	1	kept
heart ventricle overload	heart ventricle overload	heart ventricle overload	heart ventricle overload	heart ventricle overload	1	kept
heart ventricle pacing	heart ventricle pacing	heart ventricle pacing	heart ventricle pacing	heart ventricle pacing	1	kept
heart ventricle perforation	heart ventricle perforation	heart ventricle perforation	heart ventricle perforation	heart ventricle perforation	1	kept
heart-assist	heart assist	heart assist	heart assist	heart-assist	1	kept
Heart-assist device	heart assist device	heart assist device	heart assist device	Heart-Assist Devices	1	case_or_format
HEART-RATE	heart rate	heart rate	heart rate	heart rate	1	case_or_format
Heart-rate monitoring	heart rate monitoring	heart rate monitoring	heart rate monitoring	Heart-rate monitoring	1	kept
HEART-RATE-VARIABILITY	heart rate variability	heart rate variability	heart rate variability	heart rate variability	1	case_or_format
HEART-SURGERY	heart surgery	heart surgery	heart surgery	heart surgery	1	case_or_format
heartbeat-music intervention	heartbeat music intervention	heartbeat music intervention	heartbeat music intervention	heartbeat-music intervention	1	kept

heart－lung interactions	heart lung interactions	heart lung interactions	heart lung interactions	heart－lung interactions	1	kept
heat and moisture exchange	heat and moisture exchange	heat and moisture exchange	heat and moisture exchange	heat and moisture exchange	1	kept
Heat and moisture exchanger	heat and moisture exchanger	heat and moisture exchanger	heat and moisture exchanger	Heat and moisture exchanger	1	kept
heat application	heat application	heat application	heat application	heat application	1	kept
Heat emergencies	heat emergencies	heat emergencies	heat emergencies	Heat emergencies	1	kept
heat exchanger	heat exchanger	heat exchanger	heat exchanger	heat exchanger	1	kept
Heat exposure	heat exposure	heat exposure	heat exposure	Heat exposure	1	kept
Heat loss	heat loss	heat loss	heat loss	heat loss	1	case_or_format
heat pack	heat pack	heat pack	heat pack	heat pack	1	kept
heat sensation	heat sensation	heat sensation	heat sensation	heat sensation	1	kept
heat shock protein	heat shock protein	heat shock protein	heat shock protein	heat shock protein	1	kept
heat stress	heat stress	heat stress	heat stress	heat stress	1	kept
Heat-moisture exchange	heat moisture exchange	heat moisture exchange	heat moisture exchange	Heat-moisture exchange	1	kept
heated humidified high flow nasal cannula	heated humidified high flow nasal cannula	heated humidified high flow nasal cannula	heated humidified high flow nasal cannula	heated humidified high flow nasal cannula	1	kept
heated humidified oxygen	heated humidified oxygen	heated humidified oxygen	heated humidified oxygen	heated humidified oxygen	1	kept
Heated humidifier	heated humidifier	heated humidifier	heated humidifier	Heated humidifier	1	kept

Heath care economics	heath care economics	heath care economics	heath care economics	Heath care economics	1	kept
Heath care worker	heath care worker	heath care worker	heath care worker	Heath care worker	1	kept
Heavy Case Conferences	heavy case conferences	heavy case conferences	heavy case conferences	Heavy Case Conferences	1	kept
heavy drinker	heavy drinker	heavy drinker	heavy drinker	heavy drinker	1	kept
heavy metal poisoning	heavy metal poisoning	heavy metal poisoning	heavy metal poisoning	heavy metal poisoning	1	kept
hedge language	hedge language	hedge language	hedge language	hedge language	1	kept
hedonism	hedonism	hedonism	hedonism	hedonism	1	kept
heel blood sampling	heel blood sampling	heel blood sampling	heel blood sampling	heel blood sampling	1	kept
HEEL LANCE	heel lance	heel lance	heel lance	Heel lance	1	case_or_format
Heel lancing	heel lancing	heel lancing	heel lancing	heel lancing	1	case_or_format
heel pain	heel pain	heel pain	heel pain	heel pain	1	kept
Heel pressure injuries	heel pressure injuries	heel pressure injuries	heel pressure injury	heel pressure injuries	1	singular
heel pressure injury	heel pressure injury	heel pressure injury	heel pressure injury	heel pressure injuries	1	case_or_format
Heel Prick	heel prick	heel prick	heel prick	heel prick	1	case_or_format
heel protector	heel protector	heel protector	heel protector	heel protector	1	kept
heel puncture	heel puncture	heel puncture	heel puncture	heel puncture	1	kept

Heel stick	heel stick	heel stick	heel stick	heel stick	1	case_or_format
Heel warming	heel warming	heel warming	heel warming	Heel warming	1	kept
HEEL-LANCE	heel lance	heel lance	heel lance	Heel lance	1	case_or_format
Height determination	height determination	height determination	height determination	Height determination	1	kept
height measurement	height measurement	height measurement	height measurement	height measurement	1	kept
Heindrich scale	heindrich scale	heindrich scale	heindrich scale	Heindrich scale	1	kept
Helicolenus hilgendorfi	helicolenus hilgendorfi	helicolenus hilgendorfi	helicolenus hilgendorfi	Helicolenus hilgendorfi	1	kept
Helicopter emergency medical service	helicopter emergency medical service	helicopter emergency medical service	helicopter emergency medical service	helicopter emergency medical service	1	case_or_format
helicopter emergency medical service physician	helicopter emergency medical service physician	helicopter emergency medical service physician	helicopter emergency medical service physician	helicopter emergency medical service physician	1	kept
helicopter emergency services	helicopter emergency services	helicopter emergency services	helicopter emergency services	helicopter emergency services	1	kept
Helicopter rescue transfer	helicopter rescue transfer	helicopter rescue transfer	helicopter rescue transfer	Helicopter rescue transfer	1	kept
heliotherapy	heliotherapy	heliotherapy	heliotherapy	heliotherapy	1	kept
heliox	heliox	heliox	heliox	heliox	1	kept
Helipad	helipad	helipad	helipad	Helipad	1	kept
Heller Myotomy	heller myotomy	heller myotomy	heller myotomy	Heller Myotomy	1	kept
HELLP	hellp	hellp	hellp	HELLP	1	kept

helmet continuous positive airway pressure	helmet continuous positive airway pressure	helmet continuous positive airway pressure	helmet continuous positive airway pressure	helmet continuous positive airway pressure	1	kept
Helmet CPAP	helmet cpap	helmet cpap	helmet cpap	Helmet CPAP	1	kept
help	help	help	help	help	1	kept
Help-seeking	help seeking	help seeking	help seeking	Help-seeking	1	kept
help-seeking behavior	help seeking behavior	help seeking behavior	help seeking behavior	help seeking behavior	1	case_or_format
Helping Babies Breath Program	helping babies breath program	helping babies breath program	helping babies breath program	Helping Babies Breath Program	1	kept
helping babies breathe	helping babies breathe	helping babies breathe	helping babies breathe	helping babies breathe	1	kept
Helping behaviors	helping behaviors	helping behaviors	helping behavior	Helping Behavior	1	singular
helping behaviors	helping behaviors	helping behaviors	helping behavior	Helping Behavior	1	singular
Helping method	helping method	helping method	helping method	Helping method	1	kept
Helping professions	helping professions	helping professions	helping professions	Helping professions	1	kept
Hem	hem	hem	hem	HEMS	1	case_or_format
hemagglutinin	hemagglutinin	hemagglutinin	hemagglutinin	hemagglutinin	1	kept
Hemangioblastoma	hemangioblastoma	hemangioblastoma	hemangioblastoma	Hemangioblastoma	1	kept
hemangioblastoma	hemangioblastoma	hemangioblastoma	hemangioblastoma	Hemangioblastoma	1	case_or_format
Hemangioendothelioma	hemangioendothelioma	hemangioendothelioma	hemangioendothelioma	Hemangioendothelioma	1	kept

hemangioendothelioma	hemangioendothelioma	hemangioendothelioma	hemangioendothelioma	Hemangioendothelioma	1	case_or_format
Hematochezia	hematochezia	hematochezia	hematochezia	hematochezia	1	case_or_format
hematocolpos	hematocolpos	hematocolpos	hematocolpos	hematocolpos	1	kept
Hematologic cancer	hematologic cancer	hematologic cancer	hematologic cancer	Hematologic cancer	1	kept
Hematologic malignancies	hematologic malignancies	hematologic malignancies	hematologic malignancy	hematologic malignancy	1	singular
Hematologic malignancy	hematologic malignancy	hematologic malignancy	hematologic malignancy	hematologic malignancy	1	case_or_format
hematologic oncologists	hematologic oncologists	hematologic oncologists	hematologic oncologists	hematologic oncologists	1	kept
hematologist-oncologist	hematologist oncologist	hematologist oncologist	hematologist oncologist	hematologist-oncologist	1	kept
hematology analyzer	hematology analyzer	hematology analyzer	hematology analyzer	hematology analyzer	1	kept
hematology oncology	hematology oncology	hematology oncology	hematology oncology	hematology oncology	1	kept
hematology parameters	hematology parameters	hematology parameters	hematology parameters	hematology parameters	1	kept
Hematoma Evacuation	hematoma evacuation	hematoma evacuation	hematoma evacuation	hematoma evacuation	1	case_or_format
Hematoma, Subdural, Chronic	hematoma subdural chronic	hematoma subdural chronic	hematoma subdural chronic	Hematoma, Subdural, Chronic	1	kept
Hematoma, Subdural, Spinal	hematoma subdural spinal	hematoma subdural spinal	hematoma subdural spinal	Hematoma, Subdural, Spinal	1	kept
Hematopoiesis	hematopoiesis	hematopoiesis	hematopoiesis	Hematopoiesis	1	kept
Hematopoietic Cell Growth Factors	hematopoietic cell growth factors	hematopoietic cell growth factors	hematopoietic cell growth factors	Hematopoietic Cell Growth Factors	1	kept

Hematopoietic cell transplant	hematopoietic cell transplant	hematopoietic cell transplant	hematopoietic cell transplant	Hematopoietic cell transplant	1	kept
hematopoietic cell transplantation	hematopoietic cell transplantation	hematopoietic cell transplantation	hematopoietic cell transplantation	Hematopoietic cell transplantation	1	case_or_format
Hematopoietic Stem Cells Transplantation	hematopoietic stem cells transplantation	hematopoietic stem cells transplantation	hematopoietic stem cells transplantation	Hematopoietic Stem Cells Transplantation	1	kept
Hematopoietic stem cells transplantation	hematopoietic stem cells transplantation	hematopoietic stem cells transplantation	hematopoietic stem cells transplantation	Hematopoietic Stem Cells Transplantation	1	case_or_format
hematopoietic system	hematopoietic system	hematopoietic system	hematopoietic system	hematopoietic system	1	kept
hematuria color scale	hematuria color scale	hematuria color scale	hematuria color scale	hematuria color scale	1	kept
Hemiarthroplasty	hemiarthroplasty	hemiarthroplasty	hemiarthroplasty	hemiarthroplasty	1	case_or_format
hemiconvulsion hemiatrophy and epilepsy	hemiconvulsion hemiatrophy and epilepsy	hemiconvulsion hemiatrophy and epilepsy	hemiconvulsion hemiatrophy and epilepsy	hemiconvulsion hemiatrophy and epilepsy	1	kept
hemiconvulsions	hemiconvulsions	hemiconvulsions	hemiconvulsions	hemiconvulsions	1	kept
hemiconvulsive hemiatrophy syndrome	hemiconvulsive hemiatrophy syndrome	hemiconvulsive hemiatrophy syndrome	hemiconvulsive hemiatrophy syndrome	hemiconvulsive hemiatrophy syndrome	1	kept
Hemicraniectomy	hemicraniectomy	hemicraniectomy	hemicraniectomy	hemicraniectomy	1	case_or_format
hemifacial microsomia	hemifacial microsomia	hemifacial microsomia	hemifacial microsomia	hemifacial microsomia	1	kept
hemihepatectomy	hemihepatectomy	hemihepatectomy	hemihepatectomy	hemihepatectomy	1	kept
hemipelvectomy	hemipelvectomy	hemipelvectomy	hemipelvectomy	hemipelvectomy	1	kept
hemisection	hemisection	hemisection	hemisection	hemisection	1	kept
Hemispherectomy	hemispherectomy	hemispherectomy	hemispherectomy	Hemispherectomy	1	kept

hemisternotomy	hemisternotomy	hemisternotomy	hemisternotomy	hemisternotomy	1	kept
Hemoadsorption	hemoadsorption	hemoadsorption	hemoadsorption	Hemoadsorption	1	kept
hemoadsorption technology	hemoadsorption technology	hemoadsorption technology	hemoadsorption technology	hemoadsorption technology	1	kept
hemochromatosis	hemochromatosis	hemochromatosis	hemochromatosis	Hemochromatosis	1	case_or_format
hemoconcentration	hemoconcentration	hemoconcentration	hemoconcentration	hemoconcentration	1	kept
Hemocultivo	hemocultivo	hemocultivo	hemocultivo	Hemocultivo	1	kept
HEMODIALYSIS	hemodialysis	hemodialysis	hemodialysis	hemodialysis	1	case_or_format
hemodialysis associated pneumonia	hemodialysis associated pneumonia	hemodialysis associated pneumonia	hemodialysis associated pneumonia	hemodialysis associated pneumonia	1	kept
hemodialysis fluid	hemodialysis fluid	hemodialysis fluid	hemodialysis fluid	hemodialysis fluid	1	kept
Hemodialysis hospital units	hemodialysis hospital units	hemodialysis hospital units	hemodialysis hospital units	Hemodialysis hospital units	1	kept
Hemodialysis, Home	hemodialysis home	hemodialysis home	hemodialysis home	Hemodialysis, Home	1	kept
Hemodialyzers	hemodialyzers	hemodialyzers	hemodialyzers	Hemodialyzers	1	kept
hemodynamic	hemodynamic	hemodynamic	hemodynamic	hemodynamics	1	case_or_format
Hemodynamic compromise	hemodynamic compromise	hemodynamic compromise	hemodynamic compromise	Hemodynamic compromise	1	kept
hemodynamic instability	hemodynamic instability	hemodynamic instability	hemodynamic instability	Hemodynamic instability	1	case_or_format
Hemodynamic Parameters	hemodynamic parameters	hemodynamic parameters	hemodynamic parameters	hemodynamic parameters	1	case_or_format

Hemodynamic parameters	hemodynamic parameters	hemodynamic parameters	hemodynamic parameters	hemodynamic parameters	1	case_or_format
Hemodynamic profile	hemodynamic profile	hemodynamic profile	hemodynamic profile	Hemodynamic profile	1	kept
Hemodynamic stability	hemodynamic stability	hemodynamic stability	hemodynamic stability	hemodynamic stability	1	case_or_format
Hemodynamic status	hemodynamic status	hemodynamic status	hemodynamic status	Hemodynamic status	1	kept
hemodynamic status	hemodynamic status	hemodynamic status	hemodynamic status	Hemodynamic status	1	case_or_format
hemodynamic stress	hemodynamic stress	hemodynamic stress	hemodynamic stress	hemodynamic stress	1	kept
Hemodynamic variables	hemodynamic variables	hemodynamic variables	hemodynamic variables	Hemodynamic variables	1	kept
Hemodynamic waveform analysis	hemodynamic waveform analysis	hemodynamic waveform analysis	hemodynamic waveform analysis	Hemodynamic waveform analysis	1	kept
Hemofilter	hemofilter	hemofilter	hemofilter	Hemofilter	1	kept
HEMOFILTRATION	hemofiltration	hemofiltration	hemofiltration	Hemofiltration	1	case_or_format
Hemoglobin	hemoglobin	hemoglobin	hemoglobin	hemoglobin	1	case_or_format
Hemoglobin A	hemoglobin a	hemoglobin a	hemoglobin a	hemoglobin A	1	case_or_format
hemoglobin analysis	hemoglobin analysis	hemoglobin analysis	hemoglobin analysis	hemoglobin analysis	1	kept
Hemoglobin oxygen saturation	hemoglobin oxygen saturation	hemoglobin oxygen saturation	hemoglobin oxygen saturation	Hemoglobin oxygen saturation	1	kept
hemoglobin SC disease	hemoglobin sc disease	hemoglobin sc disease	hemoglobin sc disease	hemoglobin SC disease	1	kept
Hemoglobinometry	hemoglobinometry	hemoglobinometry	hemoglobinometry	Hemoglobinometry	1	kept

hemoglobinopathies	hemoglobinopathies	hemoglobinopathies	hemoglobinopathies	hemoglobinopathies	1	kept
hemolysin	hemolysin	hemolysin	hemolysin	hemolysin	1	kept
hemolysis rate	hemolysis rate	hemolysis rate	hemolysis rate	hemolysis rate	1	kept
Hemolysis with IVIG	hemolysis with ivig	hemolysis with ivig	hemolysis with ivig	Hemolysis with IVIG	1	kept
Hemolytic disease of newborn	hemolytic disease of newborn	hemolytic disease of newborn	hemolytic disease of newborn	Hemolytic disease of newborn	1	kept
Hemolyzed	hemolyzed	hemolyzed	hemolyzed	Hemolyzed	1	kept
hemoperfusion	hemoperfusion	hemoperfusion	hemoperfusion	hemoperfusion	1	kept
hemopericardium	hemopericardium	hemopericardium	hemopericardium	hemopericardium	1	kept
hemophagocytic lymphohistiocytosis	hemophagocytic lymphohistiocytosis	hemophagocytic lymphohistiocytosis	hemophagocytic lymphohistiocytosis	Hemophagocytic lymphohistiocytosis	1	case_or_format
Hemophagocytosis	hemophagocytosis	hemophagocytosis	hemophagocytosis	Hemophagocytosis	1	kept
hemopoietic growth factor	hemopoietic growth factor	hemopoietic growth factor	hemopoietic growth factor	hemopoietic growth factor	1	kept
Hemorheology	hemorheology	hemorheology	hemorheology	Hemorheology	1	kept
Hemorrhage Measurement scoring system	hemorrhage measurement scoring system	hemorrhage measurement scoring system	hemorrhage measurement scoring system	Hemorrhage Measurement scoring system	1	kept
hemorrhage risk assessment	hemorrhage risk assessment	hemorrhage risk assessment	hemorrhage risk assessment	hemorrhage risk assessment	1	kept
Hemorrhagic	hemorrhagic	hemorrhagic	hemorrhagic	Hemorrhagic	1	kept
hemorrhagic cystitis	hemorrhagic cystitis	hemorrhagic cystitis	hemorrhagic cystitis	hemorrhagic cystitis	1	kept

Hemorrhagic stroke	hemorrhagic stroke	hemorrhagic stroke	hemorrhagic stroke	hemorrhagic stroke	1	case_or_format
hemostatic efficacy	hemostatic efficacy	hemostatic efficacy	hemostatic efficacy	hemostatic efficacy	1	kept
hemostatic technique	hemostatic technique	hemostatic technique	hemostatic technique	Hemostatic Techniques	1	case_or_format
Hemotransfusion	hemotransfusion	hemotransfusion	hemotransfusion	Hemotransfusion	1	kept
hemovigilance	hemovigilance	hemovigilance	hemovigilance	Hemovigilance	1	case_or_format
HEN	hen	hen	hen	HEN	1	kept
hen	hen	hen	hen	HEN	1	case_or_format
Henan province	henan province	henan province	henan province	Henan province	1	kept
Hendrich II Fall Risk Model	hendrich ii fall risk model	hendrich ii fall risk model	hendrich ii fall risk model	Hendrich II Fall Risk Model	1	kept
henna	henna	henna	henna	henna	1	kept
Henri lefebvre	henri lefebvre	henri lefebvre	henri lefebvre	Henri lefebvre	1	kept
heparin abuse	heparin abuse	heparin abuse	heparin abuse	heparin abuse	1	kept
heparin calcium	heparin calcium	heparin calcium	heparin calcium	heparin calcium	1	kept
Heparin lock	heparin lock	heparin lock	heparin lock	Heparin lock	1	kept
heparin plus vancomycin	heparin plus vancomycin	heparin plus vancomycin	heparin plus vancomycin	heparin plus vancomycin	1	kept
Heparin resistance	heparin resistance	heparin resistance	heparin resistance	Heparin resistance	1	kept

Heparin safety	heparin safety	heparin safety	heparin safety	Heparin safety	1	kept
Heparin saline	heparin saline	heparin saline	heparin saline	Heparin saline	1	kept
Heparin-induced thrombocytopenia	heparin induced thrombocytopenia	heparin induced thrombocytopenia	heparin induced thrombocytopenia	heparin induced thrombocytopenia	1	case_or_format
Heparinoid	heparinoid	heparinoid	heparinoid	heparinoid	1	case_or_format
Heparinoids	heparinoids	heparinoids	heparinoid	heparinoid	1	singular
heparins	heparins	heparins	heparin	heparin	1	singular
Hepatic	hepatic	hepatic	hepatic	Hepatic	1	kept
Hepatic Artery	hepatic artery	hepatic artery	hepatic artery	Hepatic Artery	1	kept
Hepatic artery thrombosis	hepatic artery thrombosis	hepatic artery thrombosis	hepatic artery thrombosis	hepatic artery thrombosis	1	case_or_format
hepatic availability	hepatic availability	hepatic availability	hepatic availability	hepatic availability	1	kept
hepatic coma	hepatic coma	hepatic coma	hepatic coma	hepatic coma	1	kept
Hepatic Failure	hepatic failure	hepatic failure	hepatic failure	Hepatic Failure	1	kept
Hepatic failure	hepatic failure	hepatic failure	hepatic failure	Hepatic Failure	1	case_or_format
Hepatic necrosis	hepatic necrosis	hepatic necrosis	hepatic necrosis	Hepatic necrosis	1	kept
hepatic necrosis	hepatic necrosis	hepatic necrosis	hepatic necrosis	Hepatic necrosis	1	case_or_format
hepatic portal vein	hepatic portal vein	hepatic portal vein	hepatic portal vein	hepatic portal vein	1	kept

hepatic portal venous gas	hepatic portal venous gas	hepatic portal venous gas	hepatic portal venous gas	hepatic portal venous gas	1	kept
hepatic reperfusion	hepatic reperfusion	hepatic reperfusion	hepatic reperfusion	hepatic reperfusion	1	kept
hepatic subcapsular hematoma	hepatic subcapsular hematoma	hepatic subcapsular hematoma	hepatic subcapsular hematoma	hepatic subcapsular hematoma	1	kept
Hepatitis A Antibodies	hepatitis a antibodies	hepatitis a antibodies	hepatitis a antibody	hepatitis A antibody	1	singular
Hepatitis B Antibodies	hepatitis b antibodies	hepatitis b antibodies	hepatitis b antibody	hepatitis B antibody	1	singular
hepatitis C antigen	hepatitis c antigen	hepatitis c antigen	hepatitis c antigen	hepatitis C antigen	1	kept
hepatitis vaccine	hepatitis vaccine	hepatitis vaccine	hepatitis vaccine	hepatitis vaccine	1	kept
hepatobiliary system	hepatobiliary system	hepatobiliary system	hepatobiliary system	hepatobiliary system	1	kept
hepatobiliary system cancer	hepatobiliary system cancer	hepatobiliary system cancer	hepatobiliary system cancer	hepatobiliary system cancer	1	kept
hepatobiliary system infection	hepatobiliary system infection	hepatobiliary system infection	hepatobiliary system infection	hepatobiliary system infection	1	kept
hepatojejunostomy	hepatojejunostomy	hepatojejunostomy	hepatojejunostomy	hepatojejunostomy	1	kept
Hepatorenal	hepatorenal	hepatorenal	hepatorenal	Hepatorenal	1	kept
Hepatorenal syndrome	hepatorenal syndrome	hepatorenal syndrome	hepatorenal syndrome	Hepatorenal Syndrome	1	case_or_format
hepatosplenomegaly	hepatosplenomegaly	hepatosplenomegaly	hepatosplenomegaly	hepatosplenomegaly	1	kept
hepcidin	hepcidin	hepcidin	hepcidin	hepcidin	1	kept
herb	herb	herb	herb	herb	1	kept

Herbal And Dietary Supplements	herbal and dietary supplements	herbal and dietary supplements	herbal and dietary supplements	Herbal And Dietary Supplements	1	kept
Herd immunity	herd immunity	herd immunity	herd immunity	herd immunity	1	case_or_format
Hereditary angioedema	hereditary angioedema	hereditary angioedema	hereditary angioedema	Hereditary angioedema	1	kept
hereditary spinal muscular atrophy	hereditary spinal muscular atrophy	hereditary spinal muscular atrophy	hereditary spinal muscular atrophy	hereditary spinal muscular atrophy	1	kept
hereditary transthyretin amyloidosis	hereditary transthyretin amyloidosis	hereditary transthyretin amyloidosis	hereditary transthyretin amyloidosis	hereditary transthyretin amyloidosis	1	kept
Hereditary transthyretin amyloidosis with [illegible]	hereditary transthyretin amyloidosis with [illegible]	hereditary transthyretin amyloidosis with [illegible]	hereditary transthyretin amyloidosis with [illegible]	Hereditary transthyretin amyloidosis with [illegible]	1	kept
Heredodegenerative Disorders, Nervous System	heredodegenerative disorders nervous system	heredodegenerative disorders nervous system	heredodegenerative disorders nervous system	Heredodegenerative Disorders, Nervous System	1	kept
Heridas y Lesiones	heridas y lesiones	heridas y lesiones	heridas y lesiones	Heridas y Lesiones	1	kept
Heridas y Traumatismos	heridas y traumatismos	heridas y traumatismos	heridas y traumatismos	Heridas y Traumatismos	1	kept
Hermeneutic	hermeneutic	hermeneutic	hermeneutic	Hermeneutics	1	case_or_format
Hermeneutic phenomenological study	hermeneutic phenomenological study	hermeneutic phenomenological study	hermeneutic phenomenological study	Hermeneutic phenomenological study	1	kept
Hermeneutical phenomenology	hermeneutical phenomenology	hermeneutical phenomenology	hermeneutical phenomenology	Hermeneutical phenomenology	1	kept
hermeneutical phenomenology	hermeneutical phenomenology	hermeneutical phenomenology	hermeneutical phenomenology	Hermeneutical phenomenology	1	case_or_format
Hermeneutics - phenomenological	hermeneutics phenomenological	hermeneutics phenomenological	hermeneutics phenomenological	Hermeneutics - phenomenological	1	kept
Hermenéutica	hermen utica	hermen utica	hermen utica	Hermenéutica	1	kept
HERNIA REPAIR	hernia repair	hernia repair	hernia repair	HERNIA REPAIR	1	kept

hernia strangulation	hernia strangulation	hernia strangulation	hernia strangulation	hernia strangulation	1	kept
herniorrhaphy	herniorrhaphy	herniorrhaphy	herniorrhaphy	Herniorrhaphy	1	case_or_format
herniotomy	herniotomy	herniotomy	herniotomy	herniotomy	1	kept
Hero	hero	hero	hero	Hero	1	kept
Hero narrative	hero narrative	hero narrative	hero narrative	Hero narrative	1	kept
herpangina	herpangina	herpangina	herpangina	herpangina	1	kept
herpes	herpes	herpes	herpes	herpes	1	kept
Herpes simplex virus 1	herpes simplex virus 1	herpes simplex virus 1	herpes simplex virus 1	Herpes simplex virus 1	1	kept
Herpes simplex virus 2	herpes simplex virus 2	herpes simplex virus 2	herpes simplex virus 2	Herpes simplex virus 2	1	kept
herpes simplex virus hepatitis	herpes simplex virus hepatitis	herpes simplex virus hepatitis	herpes simplex virus hepatitis	herpes simplex virus hepatitis	1	kept
Herpesviridae	herpesviridae	herpesviridae	herpesviridae	Herpesviridae	1	kept
hertz	hertz	hertz	hertz	hertz	1	kept
Herzbergs job satisfaction questionnaire	herzbergs job satisfaction questionnaire	herzbergs job satisfaction questionnaire	herzbergs job satisfaction questionnaire	Herzbergs job satisfaction questionnaire	1	kept
HerzMobil Tirol programme	herzmobil tirol programme	herzmobil tirol programme	herzmobil tirol programme	HerzMobil Tirol programme	1	kept
HESI Exit Exam	hesi exit exam	hesi exit exam	hesi exit exam	HESI Exit Exam	1	kept
HESI Specialty Exams	hesi specialty exams	hesi specialty exams	hesi specialty exams	HESI Specialty Exams	1	kept

Hesitancy	hesitancy	hesitancy	hesitancy	Hesitancy	1	kept
HETEROGENEITY	heterogeneity	heterogeneity	heterogeneity	HETEROGENEITY	1	kept
Heterogeneity	heterogeneity	heterogeneity	heterogeneity	HETEROGENEITY	1	case_or_format
heterogeneity	heterogeneity	heterogeneity	heterogeneity	HETEROGENEITY	1	case_or_format
Heterogeneous data sources	heterogeneous data sources	heterogeneous data sources	heterogeneous data sources	Heterogeneous data sources	1	kept
Heterogeneous networks	heterogeneous networks	heterogeneous networks	heterogeneous networks	Heterogeneous networks	1	kept
Heterometallic complexes	heterometallic complexes	heterometallic complexes	heterometallic complexes	Heterometallic complexes	1	kept
Heterometals	heterometals	heterometals	heterometals	Heterometals	1	kept
Heterotaxy Syndrome	heterotaxy syndrome	heterotaxy syndrome	heterotaxy syndrome	Heterotaxy Syndrome	1	kept
Heterotaxy syndrome	heterotaxy syndrome	heterotaxy syndrome	heterotaxy syndrome	Heterotaxy Syndrome	1	case_or_format
heterozygosity	heterozygosity	heterozygosity	heterozygosity	heterozygosity	1	kept
Heterozygote	heterozygote	heterozygote	heterozygote	heterozygote	1	case_or_format
heterozygote detection	heterozygote detection	heterozygote detection	heterozygote detection	heterozygote detection	1	kept
Heuristic	heuristic	heuristic	heuristic	Heuristics	1	case_or_format
Heuristic approach	heuristic approach	heuristic approach	heuristic approach	Heuristic approach	1	kept
HEURISTIC EVALUATION	heuristic evaluation	heuristic evaluation	heuristic evaluation	HEURISTIC EVALUATION	1	kept

Heuristic evaluation	heuristic evaluation	heuristic evaluation	heuristic evaluation	HEURISTIC EVALUATION	1	case_or_format
Heuristic methods	heuristic methods	heuristic methods	heuristic method	heuristic method	1	singular
Heuristic programming	heuristic programming	heuristic programming	heuristic programming	Heuristic programming	1	kept
HEURISTICS	heuristics	heuristics	heuristic	Heuristics	1	singular
hexacycline	hexacycline	hexacycline	hexacycline	hexacycline	1	kept
Hexosyltransferases	hexosyltransferases	hexosyltransferases	hexosyltransferases	Hexosyltransferases	1	kept
HH compliance	hh compliance	hh compliance	hh compliance	HH compliance	1	kept
HHHFNC	hhhfnc	hhhfnc	hhhfnc	HHHFNC	1	kept
Hiatal hernia	hiatal hernia	hiatal hernia	hiatal hernia	hiatal hernia	1	case_or_format
Hiatus	hiatus	hiatus	hiatus	Hiatus	1	kept
Hiatus Hernia	hiatus hernia	hiatus hernia	hiatus hernia	hiatus hernia	1	case_or_format
HICARE-network	hicare network	hicare network	hicare network	HICARE-network	1	kept
Hickman catheter	hickman catheter	hickman catheter	hickman catheter	Hickman catheter	1	kept
hidradenitis	hidradenitis	hidradenitis	hidradenitis	hidradenitis	1	kept
HIE and developmental follow-up for at-risk infants	hie and developmental follow up for at risk infants	hie and developmental follow up for at risk infants	hie and developmental follow up for at risk infants	HIE and developmental follow-up for at-risk infants	1	kept
Hierarchical clustering	hierarchical clustering	hierarchical clustering	hierarchical clustering	hierarchical clustering	1	case_or_format

Hierarchical dirichlet process	hierarchical dirichlet process	hierarchical dirichlet process	hierarchical dirichlet process	Hierarchical dirichlet process	1	kept
hierarchical modelling	hierarchical modelling	hierarchical modelling	hierarchical modelling	hierarchical modelling	1	kept
hierarchical models	hierarchical models	hierarchical models	hierarchical models	hierarchical models	1	kept
Hierarchy	hierarchy	hierarchy	hierarchy	Hierarchy	1	kept
hierarchy	hierarchy	hierarchy	hierarchy	Hierarchy	1	case_or_format
Hierarchy of Controls	hierarchy of controls	hierarchy of controls	hierarchy of controls	Hierarchy of Controls	1	kept
Hierarchy, Social	hierarchy social	hierarchy social	hierarchy social	Hierarchy, Social	1	kept
High acuity setting	high acuity setting	high acuity setting	high acuity setting	High acuity settings	1	case_or_format
high alert medications	high alert medications	high alert medications	high alert medication	high alert medication	1	singular
HIGH ANORECTAL MALFORMATION	high anorectal malformation	high anorectal malformation	high anorectal malformation	HIGH ANORECTAL MALFORMATION	1	kept
High attitude	high attitude	high attitude	high attitude	High attitude	1	kept
high birth weight	high birth weight	high birth weight	high birth weight	high birth weight	1	kept
High contexts	high contexts	high contexts	high contexts	High contexts	1	kept
high density lipoprotein	high density lipoprotein	high density lipoprotein	high density lipoprotein	high density lipoprotein	1	kept
High dependency	high dependency	high dependency	high dependency	High dependency	1	kept
high dependency care unit	high dependency care unit	high dependency care unit	high dependency care unit	high dependency care unit	1	kept

High dependency unit	high dependency unit	high dependency unit	high dependency unit	high dependency unit	1	case_or_format
High dependency units	high dependency units	high dependency units	high dependency unit	high dependency unit	1	singular
High dimensional data	high dimensional data	high dimensional data	high dimensional data	High dimensional data	1	kept
High dose	high dose	high dose	high dose	High dose	1	kept
high exposure risk at work	high exposure risk at work	high exposure risk at work	high exposure risk at work	high exposure risk at work	1	kept
high exposure risk outside work	high exposure risk outside work	high exposure risk outside work	high exposure risk outside work	high exposure risk outside work	1	kept
high fidelity	high fidelity	high fidelity	high fidelity	high-fidelity	1	case_or_format
High Fidelity Simulation	high fidelity simulation	high fidelity simulation	high fidelity simulation	high-fidelity simulation	1	case_or_format
High fidelity simulation based-learning	high fidelity simulation based learning	high fidelity simulation based learning	high fidelity simulation based learning	High fidelity simulation based-learning	1	kept
High flow	high flow	high flow	high flow	High flow	1	kept
High flow cannula	high flow cannula	high flow cannula	high flow cannula	High flow cannula	1	kept
High Flow Nasal Cannula	high flow nasal cannula	high flow nasal cannula	high flow nasal cannula	High-flow nasal cannula	1	case_or_format
high Flow Nasal Cannula	high flow nasal cannula	high flow nasal cannula	high flow nasal cannula	High-flow nasal cannula	1	case_or_format
High flow nasal humidification	high flow nasal humidification	high flow nasal humidification	high flow nasal humidification	High flow nasal humidification	1	kept
High flow nasal therapy	high flow nasal therapy	high flow nasal therapy	high flow nasal therapy	High flow nasal therapy	1	kept
high flow nsal cannula therapy	high flow nsal cannula therapy	high flow nsal cannula therapy	high flow nsal cannula therapy	high flow nsal cannula therapy	1	kept

High flow oxygen	high flow oxygen	high flow oxygen	high flow oxygen	High flow oxygen	1	kept
High frequency oscillatory ventilation	high frequency oscillatory ventilation	high frequency oscillatory ventilation	high frequency oscillatory ventilation	high frequency oscillatory ventilation	1	case_or_format
high frequency percussive ventilation	high frequency percussive ventilation	high frequency percussive ventilation	high frequency percussive ventilation	high frequency percussive ventilation	1	kept
High frequency ultrasound	high frequency ultrasound	high frequency ultrasound	high frequency ultrasound	high frequency ultrasound	1	case_or_format
high grade dysplasia	high grade dysplasia	high grade dysplasia	high grade dysplasia	high grade dysplasia	1	kept
High income countries	high income countries	high income countries	high income country	high income country	1	singular
high income countries	high income countries	high income countries	high income country	high income country	1	singular
high intensity situation exposure	high intensity situation exposure	high intensity situation exposure	high intensity situation exposure	high intensity situation exposure	1	kept
high level mupirocin resistance	high level mupirocin resistance	high level mupirocin resistance	high level mupirocin resistance	high level mupirocin resistance	1	kept
High mortality rate	high mortality rate	high mortality rate	high mortality rate	High mortality rate	1	kept
High noise levels	high noise levels	high noise levels	high noise levels	High noise levels	1	kept
high oxygen pressure	high oxygen pressure	high oxygen pressure	high oxygen pressure	high oxygen pressure	1	kept
High peak airway pressures	high peak airway pressures	high peak airway pressures	high peak airway pressures	High peak airway pressures	1	kept
High pressure	high pressure	high pressure	high pressure	High pressure	1	kept
High quality images	high quality images	high quality images	high quality images	High quality images	1	kept
High rate	high rate	high rate	high rate	High rate	1	kept

high reliability organization	high reliability organization	high reliability organization	high reliability organization	High reliability organizations	1	case_or_format
high reliability organizations	high reliability organizations	high reliability organizations	high reliability organization	High reliability organizations	1	singular
high resolution computed tomography scan	high resolution computed tomography scan	high resolution computed tomography scan	high resolution computed tomography scan	high resolution computed tomography scan	1	kept
high resolution computer tomography	high resolution computer tomography	high resolution computer tomography	high resolution computer tomography	high resolution computer tomography	1	kept
high resolution transmission electron microscopy	high resolution transmission electron microscopy	high resolution transmission electron microscopy	high resolution transmission electron microscopy	high resolution transmission electron microscopy	1	kept
High risk infant	high risk infant	high risk infant	high risk infant	high risk infant	1	case_or_format
high risk obstetric service	high risk obstetric service	high risk obstetric service	high risk obstetric service	high risk obstetric service	1	kept
High risk pregnancy	high risk pregnancy	high risk pregnancy	high risk pregnancy	high risk pregnancy	1	case_or_format
high school graduate	high school graduate	high school graduate	high school graduate	high school graduate	1	kept
High Speed	high speed	high speed	high speed	High Speed	1	kept
High speed train	high speed train	high speed train	high speed train	High speed train	1	kept
High stakes evaluation	high stakes evaluation	high stakes evaluation	high stakes evaluation	High stakes evaluation	1	kept
High stress	high stress	high stress	high stress	High stress	1	kept
High technology environment	high technology environment	high technology environment	high technology environment	High technology environment	1	kept
high tidal volume ventilation	high tidal volume ventilation	high tidal volume ventilation	high tidal volume ventilation	high tidal volume ventilation	1	kept
high volume low pressure	high volume low pressure	high volume low pressure	high volume low pressure	high volume low pressure	1	kept

high volume surgeon	high volume surgeon	high volume surgeon	high volume surgeon	high volume surgeon	1	kept
High volumes	high volumes	high volumes	high volumes	High volumes	1	kept
high-alert medication	high alert medication	high alert medication	high alert medication	high alert medication	1	case_or_format
HIGH-ALERT MEDICATIONS	high alert medications	high alert medications	high alert medication	high alert medication	1	singular
high-altitude pulmonary edema	high altitude pulmonary edema	high altitude pulmonary edema	high altitude pulmonary edema	high-altitude pulmonary edema	1	kept
High-dependency care	high dependency care	high dependency care	high dependency care	High dependency care	1	case_or_format
high-dependency care area	high dependency care area	high dependency care area	high dependency care area	high-dependency care area	1	kept
High-dependency care unit	high dependency care unit	high dependency care unit	high dependency care unit	high dependency care unit	1	case_or_format
High-dependency unit	high dependency unit	high dependency unit	high dependency unit	high dependency unit	1	case_or_format
high-fidelity manikin	high fidelity manikin	high fidelity manikin	high fidelity manikin	high-fidelity manikin	1	kept
High-Fidelity simulation	high fidelity simulation	high fidelity simulation	high fidelity simulation	high-fidelity simulation	1	case_or_format
high-fidelity simulator	high fidelity simulator	high fidelity simulator	high fidelity simulator	high-fidelity simulator	1	kept
high-fidelity situation simulation	high fidelity situation simulation	high fidelity situation simulation	high fidelity situation simulation	high-fidelity situation simulation	1	kept
HIGH-FLOW NASAL CANNULA THERAPY	high flow nasal cannula therapy	high flow nasal cannula therapy	high flow nasal cannula therapy	high flow nasal cannula therapy	1	case_or_format
High-flow nasal cannula therapy	high flow nasal cannula therapy	high flow nasal cannula therapy	high flow nasal cannula therapy	high flow nasal cannula therapy	1	case_or_format
high-flow nasal cannulae	high flow nasal cannulae	high flow nasal cannulae	high flow nasal cannulae	high-flow nasal cannulae	1	kept

high-flow nasal oxygen	high flow nasal oxygen	high flow nasal oxygen	high flow nasal oxygen	High-flow nasal oxygen	1	case_or_format
High-flow oxygen via nasal cannula	high flow oxygen via nasal cannula	high flow oxygen via nasal cannula	high flow oxygen via nasal cannula	High-flow oxygen via nasal cannula	1	kept
high-frequency chest wall oscillation	high frequency chest wall oscillation	high frequency chest wall oscillation	high frequency chest wall oscillation	high-frequency chest wall oscillation	1	kept
high-frequency oscillatory ventilation	high frequency oscillatory ventilation	high frequency oscillatory ventilation	high frequency oscillatory ventilation	high frequency oscillatory ventilation	1	case_or_format
High-frequency ventilation	high frequency ventilation	high frequency ventilation	high frequency ventilation	High-Frequency Ventilation	1	case_or_format
high-grade endometrial stromal sarcoma	high grade endometrial stromal sarcoma	high grade endometrial stromal sarcoma	high grade endometrial stromal sarcoma	high-grade endometrial stromal sarcoma	1	kept
high-grade glioma	high grade glioma	high grade glioma	high grade glioma	high-grade glioma	1	kept
High-impact trauma	high impact trauma	high impact trauma	high impact trauma	High-impact trauma	1	kept
High-level Mupirocin Resistance	high level mupirocin resistance	high level mupirocin resistance	high level mupirocin resistance	high level mupirocin resistance	1	case_or_format
High-Line	high line	high line	high line	High-Line	1	kept
HIGH-OSMOLALITY	high osmolality	high osmolality	high osmolality	HIGH-OSMOLALITY	1	kept
High-precision	high precision	high precision	high precision	High-precision	1	kept
HIGH-PROTEIN-INTAKE	high protein intake	high protein intake	high protein intake	HIGH-PROTEIN-INTAKE	1	kept
high-quality care	high quality care	high quality care	high quality care	high-quality care	1	kept
HIGH-RELIABILITY	high reliability	high reliability	high reliability	High reliability	1	case_or_format
high-reliability unit	high reliability unit	high reliability unit	high reliability unit	high-reliability unit	1	kept

High-resolution scanning electron microscopies	high resolution scanning electron microscopies	high resolution scanning electron microscopies	high resolution scanning electron microscopies	High-resolution scanning electron microscopies	1	kept
High-risk babies	high risk babies	high risk babies	high risk babies	High-risk babies	1	kept
high-risk factors	high risk factors	high risk factors	high risk factors	high-risk factors	1	kept
High-risk infant follow-up	high risk infant follow up	high risk infant follow up	high risk infant follow up	High-risk infant follow-up	1	kept
HIGH-RISK INFANTS	high risk infants	high risk infants	high risk infant	high risk infant	1	singular
High-risk medications	high risk medications	high risk medications	high risk medications	High-risk medications	1	kept
High-risk neonatal	high risk neonatal	high risk neonatal	high risk neonatal	High-risk neonatal	1	kept
High-risk neonate	high risk neonate	high risk neonate	high risk neonate	high-risk neonate	1	case_or_format
High-risk neonates	high risk neonates	high risk neonates	high risk neonate	high-risk neonate	1	singular
high-risk newborn	high risk newborn	high risk newborn	high risk newborn	high-risk newborn	1	kept
HIGH-RISK NEWBORNS	high risk newborns	high risk newborns	high risk newborn	high-risk newborn	1	singular
High-risk population	high risk population	high risk population	high risk population	high risk population	1	case_or_format
High-risk pre-eclampsia infant	high risk pre eclampsia infant	high risk pre eclampsia infant	high risk pre eclampsia infant	High-risk pre-eclampsia infant	1	kept
HIGH-RISK PREGNANCY	high risk pregnancy	high risk pregnancy	high risk pregnancy	high risk pregnancy	1	case_or_format
high-risk professionals	high risk professionals	high risk professionals	high risk professionals	high-risk professionals	1	kept
High-risk surgery	high risk surgery	high risk surgery	high risk surgery	High-risk surgery	1	kept

HIGH-SELECTIVITY	high selectivity	high selectivity	high selectivity	HIGH-SELECTIVITY	1	kept
High-tech	high tech	high tech	high tech	High-tech	1	kept
High-touch	high touch	high touch	high touch	High-touch	1	kept
high-touch surfaces	high touch surfaces	high touch surfaces	high touch surfaces	High-touch surfaces	1	case_or_format
High-value care	high value care	high value care	high value care	High-value care	1	kept
high-value care	high value care	high value care	high value care	High-value care	1	case_or_format
Higher education institution	higher education institution	higher education institution	higher education institution	Higher education institution	1	kept
Higher efficiency	higher efficiency	higher efficiency	higher efficiency	Higher efficiency	1	kept
Higher level of care	higher level of care	higher level of care	higher level of care	Higher level of care	1	kept
higher parental mental burden	higher parental mental burden	higher parental mental burden	higher parental mental burden	higher parental mental burden	1	kept
Higher-order thinking	higher order thinking	higher order thinking	higher order thinking	Higher-order thinking	1	kept
highly infectious diseases	highly infectious diseases	highly infectious diseases	highly infectious diseases	highly infectious diseases	1	kept
Highly specialized hospitals	highly specialized hospitals	highly specialized hospitals	highly specialized hospitals	Highly specialized hospitals	1	kept
high‑fidelity simulation training	high fidelity simulation training	high fidelity simulation training	high fidelity simulation training	High Fidelity Simulation Training	1	case_or_format
Higiene das mãos	higiene das m os	higiene das m os	higiene das m os	Higiene das mãos	1	kept
Higiene de las manos	higiene de las manos	higiene de las manos	higiene de las manos	Higiene de las manos	1	kept

hikikomori	hikikomori	hikikomori	hikikomori	hikikomori	1	kept
Himachal Pradesh	himachal pradesh	himachal pradesh	himachal pradesh	Himachal Pradesh	1	kept
Hindi	hindi	hindi	hindi	Hindi	1	kept
Hindlimb	hindlimb	hindlimb	hindlimb	Hindlimb	1	kept
Hindu	hindu	hindu	hindu	Hindu	1	kept
Hip	hip	hip	hip	hip	1	case_or_format
hip dysplasia	hip dysplasia	hip dysplasia	hip dysplasia	hip dysplasia	1	kept
hip flexion	hip flexion	hip flexion	hip flexion	hip flexion	1	kept
hip fracture database	hip fracture database	hip fracture database	hip fracture database	hip fracture database	1	kept
Hip fracture patterns	hip fracture patterns	hip fracture patterns	hip fracture patterns	Hip fracture patterns	1	kept
hip fractures	hip fractures	hip fractures	hip fracture	hip fracture	1	singular
hip injury	hip injury	hip injury	hip injury	hip injury	1	kept
Hip Prosthesis	hip prosthesis	hip prosthesis	hip prosthesis	Hip Prosthesis	1	kept
hip prosthesis	hip prosthesis	hip prosthesis	hip prosthesis	Hip Prosthesis	1	case_or_format
HIPAA Privacy Rule	hipaa privacy rule	hipaa privacy rule	hipaa privacy rule	HIPAA Privacy Rule	1	kept
Hiperglucemia por estrés	hiperglucemia por estrs	hiperglucemia por estrs	hiperglucemia por estrs	Hiperglucemia por estrés	1	kept

Hipertensão	hipertens o	hipertens o	hipertens o	Hipertensão	1	kept
Hipnóticos y sedantes	hipn ticos y sedantes	hipn ticos y sedantes	hipn ticos y sedantes	Hipnóticos y sedantes	1	kept
Hipotensão Ortostática	hipotens o ortost tica	hipotens o ortost tica	hipotens o ortost tica	Hipotensão Ortostática	1	kept
hippocampal CA1 region	hippocampal ca1 region	hippocampal ca1 region	hippocampal ca1 region	hippocampal CA1 region	1	kept
hippocampal sclerosis	hippocampal sclerosis	hippocampal sclerosis	hippocampal sclerosis	hippocampal sclerosis	1	kept
HIPPOCAMPUS	hippocampus	hippocampus	hippocampus	hippocampus	1	case_or_format
HIRA Plan	hira plan	hira plan	hira plan	HIRA Plan	1	kept
Hiring	hiring	hiring	hiring	Hiring	1	kept
hirudin	hirudin	hirudin	hirudin	Hirudins	1	case_or_format
hirudin derivative	hirudin derivative	hirudin derivative	hirudin derivative	hirudin derivative	1	kept
hirulog	hirulog	hirulog	hirulog	hirulog	1	kept
His bundle	his bundle	his bundle	his bundle	His bundle	1	kept
histamine 2 receptor antagonist	histamine 2 receptor antagonist	histamine 2 receptor antagonist	histamine 2 receptor antagonist	histamine 2 receptor antagonist	1	kept
histamine agonist	histamine agonist	histamine agonist	histamine agonist	histamine agonist	1	kept
Histamine Agonists	histamine agonists	histamine agonists	histamine agonist	histamine agonist	1	singular
Histamine Antagonists	histamine antagonists	histamine antagonists	histamine antagonists	Histamine Antagonists	1	kept

Histamine antagonists	histamine antagonists	histamine antagonists	histamine antagonists	Histamine Antagonists	1	case_or_format
histamine H1 receptor antagonist	histamine h1 receptor antagonist	histamine h1 receptor antagonist	histamine h1 receptor antagonist	histamine H1 receptor antagonist	1	kept
Histamine-2	histamine 2	histamine 2	histamine 2	Histamine-2	1	kept
histocompatibility	histocompatibility	histocompatibility	histocompatibility	histocompatibility	1	kept
Histocompatibility Testing	histocompatibility testing	histocompatibility testing	histocompatibility testing	Histocompatibility Testing	1	kept
Histoplasma	histoplasma	histoplasma	histoplasma	Histoplasma	1	kept
histoplasmosis	histoplasmosis	histoplasmosis	histoplasmosis	Histoplasmosis	1	case_or_format
Historical data	historical data	historical data	historical data	Historical data	1	kept
historical research	historical research	historical research	historical research	historical research	1	kept
historical trauma	historical trauma	historical trauma	historical trauma	historical trauma	1	kept
HISTORY	history	history	history	history	1	case_or_format
History of Medicine	history of medicine	history of medicine	history of medicine	history of medicine	1	case_or_format
History of medicine	history of medicine	history of medicine	history of medicine	history of medicine	1	case_or_format
History of neonatal resuscitation	history of neonatal resuscitation	history of neonatal resuscitation	history of neonatal resuscitation	History of neonatal resuscitation	1	kept
History of Neurosurgery	history of neurosurgery	history of neurosurgery	history of neurosurgery	History of Neurosurgery	1	kept
history taking	history taking	history taking	history taking	history taking	1	kept

History, 15th Century	history 15th century	history 15th century	history 15th century	History, 15th Century	1	kept
Hiv	hiv	hiv	hiv	HIV	1	case_or_format
hiv	hiv	hiv	hiv	HIV	1	case_or_format
HIV and AIDS information	hiv and aids information	hiv and aids information	hiv and aids information	HIV and AIDS information	1	kept
HIV and AIDS management	hiv and aids management	hiv and aids management	hiv and aids management	HIV and AIDS management	1	kept
HIV comorbidities	hiv comorbidities	hiv comorbidities	hiv comorbidities	HIV comorbidities	1	kept
HIV physiology	hiv physiology	hiv physiology	hiv physiology	HIV physiology	1	kept
HIV test	hiv test	hiv test	hiv test	HIV test	1	kept
HIV-1	hiv 1	hiv 1	hiv 1	HIV-1	1	kept
Hiv-1	hiv 1	hiv 1	hiv 1	HIV-1	1	case_or_format
HLA antibody	hla antibody	hla antibody	hla antibody	HLA antibody	1	kept
HLA antibody positivity	hla antibody positivity	hla antibody positivity	hla antibody positivity	HLA antibody positivity	1	kept
HLA antigen class 1	hla antigen class 1	hla antigen class 1	hla antigen class 1	HLA antigen class 1	1	kept
hla gene	hla gene	hla gene	hla gene	hla gene	1	kept
HLA matching	hla matching	hla matching	hla matching	HLA matching	1	kept
HLA system	hla system	hla system	hla system	HLA system	1	kept

hlb gene	hlb gene	hlb gene	hlb gene	hlb gene	1	kept
hld gene	hld gene	hld gene	hld gene	hld gene	1	kept
hlg2 gene	hlg2 gene	hlg2 gene	hlg2 gene	hlg2 gene	1	kept
HMBANA	hmbana	hmbana	hmbana	HMBANA	1	kept
Hockey	hockey	hockey	hockey	Hockey	1	kept
Hodges’ model	hodges model	hodges model	hodges model	Hodges’ model	1	kept
Hoehn and Yahr scale	hoehn and yahr scale	hoehn and yahr scale	hoehn and yahr scale	Hoehn and Yahr scale	1	kept
holding	holding	holding	holding	Holding	1	case_or_format
holistic and cultural care	holistic and cultural care	holistic and cultural care	holistic and cultural care	holistic and cultural care	1	kept
holistic approach	holistic approach	holistic approach	holistic approach	holistic approach	1	kept
Holistic approaches	holistic approaches	holistic approaches	holistic approach	holistic approach	1	singular
Holistic balance	holistic balance	holistic balance	holistic balance	Holistic balance	1	kept
Holistic Care	holistic care	holistic care	holistic care	holistic care	1	case_or_format
Holistic healing	holistic healing	holistic healing	holistic healing	Holistic healing	1	kept
holistic health	holistic health	holistic health	holistic health	Holistic Health	1	case_or_format
hollow fiber membrane	hollow fiber membrane	hollow fiber membrane	hollow fiber membrane	hollow fiber membrane	1	kept

hollow viscus perforation	hollow viscus perforation	hollow viscus perforation	hollow viscus perforation	hollow viscus perforation	1	kept
hollyhock oil	hollyhock oil	hollyhock oil	hollyhock oil	hollyhock oil	1	kept
Holocaust	holocaust	holocaust	holocaust	Holocaust	1	kept
holocaust	holocaust	holocaust	holocaust	Holocaust	1	case_or_format
Holocaust studies	holocaust studies	holocaust studies	holocaust studies	Holocaust studies	1	kept
Holocaust survivors	holocaust survivors	holocaust survivors	holocaust survivors	Holocaust survivors	1	kept
Holograms	holograms	holograms	holograms	Holograms	1	kept
Holt-Winter	holt winter	holt winter	holt winter	Holt-Winter	1	kept
Holt-Winters	holt winters	holt winters	holt winter	Holt-Winter	1	singular
Holter	holter	holter	holter	Holter	1	kept
home accident	home accident	home accident	home accident	home accident	1	kept
home and community-based care and services	home and community based care and services	home and community based care and services	home and community based care and services	home and community-based care and services	1	kept
Home based cardiac rehabilitation	home based cardiac rehabilitation	home based cardiac rehabilitation	home based cardiac rehabilitation	Home based cardiac rehabilitation	1	kept
home based care model	home based care model	home based care model	home based care model	home based care model	1	kept
Home birth	home birth	home birth	home birth	home birth	1	case_or_format
home call	home call	home call	home call	home call	1	kept

Home Care	home care	home care	home care	home care	1	case_or_format
home care agencies	home care agencies	home care agencies	home care agencies	Home Care Agencies	1	case_or_format
home childbirth	home childbirth	home childbirth	home childbirth	Home Childbirth	1	case_or_format
home death	home death	home death	home death	home death	1	kept
home discharge	home discharge	home discharge	home discharge	home discharge	1	kept
HOME EARLIER	home earlier	home earlier	home earlier	HOME EARLIER	1	kept
Home enteral feeding	home enteral feeding	home enteral feeding	home enteral feeding	Home enteral feeding	1	kept
Home environment	home environment	home environment	home environment	home environment	1	case_or_format
HOME FOLLOW-UP	home follow up	home follow up	home follow up	HOME FOLLOW-UP	1	kept
home gavage	home gavage	home gavage	home gavage	home gavage	1	kept
Home health	home health	home health	home health	home health	1	case_or_format
Home health rehabilitation	home health rehabilitation	home health rehabilitation	home health rehabilitation	Home health rehabilitation	1	kept
home healthcare	home healthcare	home healthcare	home healthcare	Home healthcare	1	case_or_format
home healthcare service use	home healthcare service use	home healthcare service use	home healthcare service use	home healthcare service use	1	kept
Home hemodialysis	home hemodialysis	home hemodialysis	home hemodialysis	Home hemodialysis	1	kept
Home hospitalization	home hospitalization	home hospitalization	home hospitalization	Home hospitalization	1	kept

Home hospitalization system	home hospitalization system	home hospitalization system	home hospitalization system	Home hospitalization system	1	kept
home hospitalization unit	home hospitalization unit	home hospitalization unit	home hospitalization unit	home hospitalization unit	1	kept
home infusion	home infusion	home infusion	home infusion	home infusion	1	kept
Home Infusion Therapy	home infusion therapy	home infusion therapy	home infusion therapy	Home Infusion Therapy	1	kept
Home infusion therapy	home infusion therapy	home infusion therapy	home infusion therapy	Home Infusion Therapy	1	case_or_format
home infusion therapy	home infusion therapy	home infusion therapy	home infusion therapy	Home Infusion Therapy	1	case_or_format
home intravenous therapy	home intravenous therapy	home intravenous therapy	home intravenous therapy	home intravenous therapy	1	kept
Home Mechanical Ventilation	home mechanical ventilation	home mechanical ventilation	home mechanical ventilation	Home mechanical ventilation	1	case_or_format
Home parenteral	home parenteral	home parenteral	home parenteral	Home parenteral	1	kept
Home parenteral nutrition	home parenteral nutrition	home parenteral nutrition	home parenteral nutrition	Home parenteral nutrition	1	kept
home parenteral nutrition	home parenteral nutrition	home parenteral nutrition	home parenteral nutrition	Home parenteral nutrition	1	case_or_format
home rehabilitation	home rehabilitation	home rehabilitation	home rehabilitation	home rehabilitation	1	kept
Home remedies	home remedies	home remedies	home remedies	Home remedies	1	kept
HOME RESIDENTS	home residents	home residents	home residents	HOME RESIDENTS	1	kept
Home respiration	home respiration	home respiration	home respiration	Home respiration	1	kept
home return	home return	home return	home return	Home return	1	case_or_format

home sleep apnea testing	home sleep apnea testing	home sleep apnea testing	home sleep apnea testing	home sleep apnea testing	1	kept
HOME STAFF	home staff	home staff	home staff	HOME STAFF	1	kept
HOME SUPPORT	home support	home support	home support	HOME SUPPORT	1	kept
home telecare	home telecare	home telecare	home telecare	home telecare	1	kept
Home treatment	home treatment	home treatment	home treatment	Home treatment	1	kept
Home visit	home visit	home visit	home visit	home visit	1	case_or_format
Home visit program	home visit program	home visit program	home visit program	Home visit program	1	kept
home visiting	home visiting	home visiting	home visiting	home visiting	1	kept
HOME VISITING PROGRAM	home visiting program	home visiting program	home visiting program	HOME VISITING PROGRAM	1	kept
home visitors	home visitors	home visitors	home visitors	home visitors	1	kept
home visits	home visits	home visits	home visit	home visit	1	singular
home ward	home ward	home ward	home ward	home ward	1	kept
Home-based	home based	home based	home based	Home-based	1	kept
Home-based care	home based care	home based care	home based care	home-based care	1	case_or_format
home-based community care	home based community care	home based community care	home based community care	home-based community care	1	kept
home-based palliative care	home based palliative care	home based palliative care	home based palliative care	home-based palliative care	1	kept

Home-based treatment	home based treatment	home based treatment	home based treatment	Home-based treatment	1	kept
Homebound HCT	homebound hct	homebound hct	homebound hct	Homebound HCT	1	kept
Homecare	homecare	homecare	homecare	Homecare	1	kept
Homecare program	homecare program	homecare program	homecare program	Homecare program	1	kept
homecare services	homecare services	homecare services	homecare services	homecare services	1	kept
homeless	homeless	homeless	homeless	homeless	1	kept
homeless man	homeless man	homeless man	homeless man	homeless man	1	kept
Homeless Persons	homeless persons	homeless persons	homeless person	homeless person	1	singular
homeobox	homeobox	homeobox	homeobox	homeobox	1	kept
homeodomain protein	homeodomain protein	homeodomain protein	homeodomain protein	homeodomain protein	1	kept
Homeodomain Proteins	homeodomain proteins	homeodomain proteins	homeodomain protein	homeodomain protein	1	singular
homeopathic agent	homeopathic agent	homeopathic agent	homeopathic agent	homeopathic agent	1	kept
Homeopathy	homeopathy	homeopathy	homeopathy	Homeopathy	1	kept
homeopathy	homeopathy	homeopathy	homeopathy	Homeopathy	1	case_or_format
HOMES	homes	homes	home	home	1	singular
Homocysteine	homocysteine	homocysteine	homocysteine	homocysteine	1	case_or_format

homonymous hemianopia	homonymous hemianopia	homonymous hemianopia	homonymous hemianopia	homonymous hemianopia	1	kept
homophobia	homophobia	homophobia	homophobia	homophobia	1	kept
Homosexuality, Female	homosexuality female	homosexuality female	homosexuality female	Homosexuality, Female	1	kept
Homozygote	homozygote	homozygote	homozygote	Homozygote	1	kept
homozygote	homozygote	homozygote	homozygote	Homozygote	1	case_or_format
HONEY	honey	honey	honey	Honey	1	case_or_format
HONG-KONG	hong kong	hong kong	hong kong	Hong Kong	1	case_or_format
Honor Walk	honor walk	honor walk	honor walk	Honor Walk	1	kept
Honshu	honshu	honshu	honshu	Honshu	1	kept
Hood	hood	hood	hood	Hood	1	kept
Hopefulness	hopefulness	hopefulness	hopefulness	Hopefulness	1	kept
HOPELESSLY ILL	hopelessly ill	hopelessly ill	hopelessly ill	HOPELESSLY ILL	1	kept
hoping	hoping	hoping	hoping	hoping	1	kept
HOPKINS PRECURSORS	hopkins precursors	hopkins precursors	hopkins precursors	HOPKINS PRECURSORS	1	kept
Hopkins Symptom Checklist 10	hopkins symptom checklist 10	hopkins symptom checklist 10	hopkins symptom checklist 10	Hopkins Symptom Checklist 10	1	kept
Hoplobrotula armata	hoplobrotula armata	hoplobrotula armata	hoplobrotula armata	Hoplobrotula armata	1	kept

Hopsital Anxiety and Depression scale	hopsital anxiety and depression scale	hopsital anxiety and depression scale	hopsital anxiety and depression scale	Hopsital Anxiety and Depression scale	1	kept
horizontal disease transmission	horizontal disease transmission	horizontal disease transmission	horizontal disease transmission	horizontal disease transmission	1	kept
horizontal infection	horizontal infection	horizontal infection	horizontal infection	horizontal infection	1	kept
horizontal transmission	horizontal transmission	horizontal transmission	horizontal transmission	horizontal transmission	1	kept
HORIZONTAL VIOLENCE	horizontal violence	horizontal violence	horizontal violence	horizontal violence	1	case_or_format
hormonal regulation	hormonal regulation	hormonal regulation	hormonal regulation	hormonal regulation	1	kept
Hormone Replacement Therapy	hormone replacement therapy	hormone replacement therapy	hormone replacement therapy	Hormone Replacement Therapy	1	kept
horrible disease	horrible disease	horrible disease	horrible disease	horrible disease	1	kept
horse disease	horse disease	horse disease	horse disease	horse disease	1	kept
horseback riding	horseback riding	horseback riding	horseback riding	horseback riding	1	kept
hospice at home	hospice at home	hospice at home	hospice at home	hospice at home	1	kept
hospice at home service	hospice at home service	hospice at home service	hospice at home service	hospice at home service	1	kept
HOSPICE CARE	hospice care	hospice care	hospice care	Hospice Care	1	case_or_format
hospice enrollment	hospice enrollment	hospice enrollment	hospice enrollment	hospice enrollment	1	kept
hospice length of stay	hospice length of stay	hospice length of stay	hospice length of stay	hospice length of stay	1	kept
Hospice medicine	hospice medicine	hospice medicine	hospice medicine	Hospice medicine	1	kept

hospice preferred practices	hospice preferred practices	hospice preferred practices	hospice preferred practices	hospice preferred practices	1	kept
hospital acquired anemia	hospital acquired anemia	hospital acquired anemia	hospital acquired anemia	hospital acquired anemia	1	kept
hospital acquired bloodstream infection	hospital acquired bloodstream infection	hospital acquired bloodstream infection	hospital acquired bloodstream infection	hospital acquired bloodstream infection	1	kept
hospital acquired decubitus	hospital acquired decubitus	hospital acquired decubitus	hospital acquired decubitus	hospital acquired decubitus	1	kept
Hospital Acquired Infection	hospital acquired infection	hospital acquired infection	hospital acquired infection	Hospital-acquired infections	1	case_or_format
Hospital Acquired Infections	hospital acquired infections	hospital acquired infections	hospital acquired infection	Hospital-acquired infections	1	singular
hospital acquired pressure injury	hospital acquired pressure injury	hospital acquired pressure injury	hospital acquired pressure injury	hospital-acquired pressure injury	1	case_or_format
hospital acquired pressure ulcer	hospital acquired pressure ulcer	hospital acquired pressure ulcer	hospital acquired pressure ulcer	Hospital-acquired pressure ulcers	1	case_or_format
Hospital administration	hospital administration	hospital administration	hospital administration	Hospital Administration	1	case_or_format
Hospital administrator	hospital administrator	hospital administrator	hospital administrator	hospital administrator	1	case_or_format
Hospital admission time	hospital admission time	hospital admission time	hospital admission time	Hospital admission time	1	kept
Hospital Admissions	hospital admissions	hospital admissions	hospital admission	hospital admission	1	singular
Hospital admissions	hospital admissions	hospital admissions	hospital admission	hospital admission	1	singular
hospital alternative	hospital alternative	hospital alternative	hospital alternative	hospital alternative	1	kept
Hospital anxiety	hospital anxiety	hospital anxiety	hospital anxiety	HOSPITAL ANXIETY	1	case_or_format
Hospital associated	hospital associated	hospital associated	hospital associated	Hospital associated	1	kept

hospital associated complications of older people	hospital associated complications of older people	hospital associated complications of older people	hospital associated complications of older people	hospital associated complications of older people	1	kept
Hospital associated infections	hospital associated infections	hospital associated infections	hospital associated infection	hospital-associated infection	1	singular
Hospital at Home	hospital at home	hospital at home	hospital at home	hospital at home	1	case_or_format
Hospital at home	hospital at home	hospital at home	hospital at home	hospital at home	1	case_or_format
hospital auxiliary	hospital auxiliary	hospital auxiliary	hospital auxiliary	hospital auxiliary	1	kept
hospital based cohort study	hospital based cohort study	hospital based cohort study	hospital based cohort study	hospital based cohort study	1	kept
hospital based shooting	hospital based shooting	hospital based shooting	hospital based shooting	hospital based shooting	1	kept
Hospital bed design	hospital bed design	hospital bed design	hospital bed design	Hospital bed design	1	kept
hospital bed modelling	hospital bed modelling	hospital bed modelling	hospital bed modelling	hospital bed modelling	1	kept
hospital bed occupancy	hospital bed occupancy	hospital bed occupancy	hospital bed occupancy	hospital bed occupancy	1	kept
Hospital bed-blocking	hospital bed blocking	hospital bed blocking	hospital bed blocking	Hospital bed-blocking	1	kept
hospital birth	hospital birth	hospital birth	hospital birth	hospital birth	1	kept
Hospital capacities	hospital capacities	hospital capacities	hospital capacity	hospital capacity	1	singular
Hospital capacity	hospital capacity	hospital capacity	hospital capacity	hospital capacity	1	case_or_format
Hospital capacity planning	hospital capacity planning	hospital capacity planning	hospital capacity planning	Hospital capacity planning	1	kept
hospital capacity planning	hospital capacity planning	hospital capacity planning	hospital capacity planning	Hospital capacity planning	1	case_or_format

HOSPITAL CARDIOPULMONARY-RESUSCITATION	hospital cardiopulmonary resuscitation	hospital cardiopulmonary resuscitation	hospital cardiopulmonary resuscitation	HOSPITAL CARDIOPULMONARY-RESUSCITATION	1	kept
Hospital Care	hospital care	hospital care	hospital care	hospital care	1	case_or_format
hospital care for children	hospital care for children	hospital care for children	hospital care for children	hospital care for children	1	kept
Hospital care levels	hospital care levels	hospital care levels	hospital care levels	Hospital care levels	1	kept
hospital CEO	hospital ceo	hospital ceo	hospital ceo	hospital CEO	1	kept
Hospital characteristic	hospital characteristic	hospital characteristic	hospital characteristic	Hospital characteristic	1	kept
HOSPITAL CHARACTERISTICS	hospital characteristics	hospital characteristics	hospital characteristic	Hospital characteristic	1	singular
hospital charges	hospital charges	hospital charges	hospital charge	Hospital Charges	1	singular
Hospital cleaning	hospital cleaning	hospital cleaning	hospital cleaning	Hospital cleaning	1	kept
Hospital cleanliness	hospital cleanliness	hospital cleanliness	hospital cleanliness	Hospital cleanliness	1	kept
Hospital communication	hospital communication	hospital communication	hospital communication	Hospital communication	1	kept
HOSPITAL CONSULTANTS	hospital consultants	hospital consultants	hospital consultants	HOSPITAL CONSULTANTS	1	kept
Hospital Consumer Assessment of Healthcare Providers	hospital consumer assessment of healthcare providers	hospital consumer assessment of healthcare providers	hospital consumer assessment of healthcare providers	Hospital Consumer Assessment of Healthcare Providers	1	kept
Hospital cost	hospital cost	hospital cost	hospital cost	Hospital Costs	1	case_or_format
Hospital course	hospital course	hospital course	hospital course	Hospital course	1	kept
hospital course	hospital course	hospital course	hospital course	Hospital course	1	case_or_format

Hospital death	hospital death	hospital death	hospital death	Hospital death	1	kept
hospital death	hospital death	hospital death	hospital death	Hospital death	1	case_or_format
Hospital deaths	hospital deaths	hospital deaths	hospital death	Hospital death	1	singular
Hospital dentistry	hospital dentistry	hospital dentistry	hospital dentistry	Hospital dentistry	1	kept
hospital dentistry	hospital dentistry	hospital dentistry	hospital dentistry	Hospital dentistry	1	case_or_format
hospital dermatology	hospital dermatology	hospital dermatology	hospital dermatology	hospital dermatology	1	kept
hospital diabetes	hospital diabetes	hospital diabetes	hospital diabetes	hospital diabetes	1	kept
hospital disaster simulation	hospital disaster simulation	hospital disaster simulation	hospital disaster simulation	hospital disaster simulation	1	kept
hospital disposition	hospital disposition	hospital disposition	hospital disposition	hospital disposition	1	kept
Hospital Distribution Systems	hospital distribution systems	hospital distribution systems	hospital distribution systems	Hospital Distribution Systems	1	kept
hospital distribution systems	hospital distribution systems	hospital distribution systems	hospital distribution systems	Hospital Distribution Systems	1	case_or_format
hospital drug distribution systems	hospital drug distribution systems	hospital drug distribution systems	hospital drug distribution systems	hospital drug distribution systems	1	kept
Hospital drug supply chain	hospital drug supply chain	hospital drug supply chain	hospital drug supply chain	Hospital drug supply chain	1	kept
Hospital Elder Life Program	hospital elder life program	hospital elder life program	hospital elder life program	Hospital Elder Life Program	1	kept
Hospital elder life program	hospital elder life program	hospital elder life program	hospital elder life program	Hospital Elder Life Program	1	case_or_format
hospital elder life program	hospital elder life program	hospital elder life program	hospital elder life program	Hospital Elder Life Program	1	case_or_format

Hospital emergency	hospital emergency	hospital emergency	hospital emergency	Hospital emergency	1	kept
Hospital emergency health services	hospital emergency health services	hospital emergency health services	hospital emergency health services	Hospital emergency health services	1	kept
Hospital Emergency Services	hospital emergency services	hospital emergency services	hospital emergency service	hospital emergency service	1	singular
Hospital Emergency Ward	hospital emergency ward	hospital emergency ward	hospital emergency ward	Hospital Emergency Ward	1	kept
hospital environments	hospital environments	hospital environments	hospital environment	Hospital environment	1	singular
Hospital equipment and supplies	hospital equipment and supplies	hospital equipment and supplies	hospital equipment and supplies	Hospital equipment and supplies	1	kept
hospital ethics climate	hospital ethics climate	hospital ethics climate	hospital ethics climate	hospital ethics climate	1	kept
Hospital ethics committee	hospital ethics committee	hospital ethics committee	hospital ethics committee	Hospital ethics committee	1	kept
hospital ethics committee	hospital ethics committee	hospital ethics committee	hospital ethics committee	Hospital ethics committee	1	case_or_format
hospital ethnography	hospital ethnography	hospital ethnography	hospital ethnography	hospital ethnography	1	kept
hospital evacuation	hospital evacuation	hospital evacuation	hospital evacuation	hospital evacuation	1	kept
hospital exchange	hospital exchange	hospital exchange	hospital exchange	hospital exchange	1	kept
Hospital facilities and services	hospital facilities and services	hospital facilities and services	hospital facilities and services	Hospital facilities and services	1	kept
Hospital for Special Surgery knee rating score	hospital for special surgery knee rating score	hospital for special surgery knee rating score	hospital for special surgery knee rating score	Hospital for Special Surgery knee rating score	1	kept
Hospital frailty risk measure	hospital frailty risk measure	hospital frailty risk measure	hospital frailty risk measure	Hospital frailty risk measure	1	kept
hospital frailty risk score	hospital frailty risk score	hospital frailty risk score	hospital frailty risk score	hospital frailty risk score	1	kept

hospital funding	hospital funding	hospital funding	hospital funding	hospital funding	1	kept
Hospital hygienes	hospital hygienes	hospital hygienes	hospital hygiene	hospital hygiene	1	singular
hospital IBCLC staffing	hospital ibclc staffing	hospital ibclc staffing	hospital ibclc staffing	hospital IBCLC staffing	1	kept
Hospital in the Home	hospital in the home	hospital in the home	hospital in the home	Hospital in the Home	1	kept
Hospital in the home	hospital in the home	hospital in the home	hospital in the home	Hospital in the Home	1	case_or_format
hospital in the home model	hospital in the home model	hospital in the home model	hospital in the home model	hospital in the home model	1	kept
Hospital incident	hospital incident	hospital incident	hospital incident	Hospital incident	1	kept
hospital incident reporting	hospital incident reporting	hospital incident reporting	hospital incident reporting	Hospital incident reporting	1	case_or_format
Hospital Infection	hospital infection	hospital infection	hospital infection	hospital infection	1	case_or_format
Hospital infections	hospital infections	hospital infections	hospital infection	hospital infection	1	singular
Hospital information	hospital information	hospital information	hospital information	Hospital information	1	kept
Hospital information system	hospital information system	hospital information system	hospital information system	hospital information system	1	case_or_format
hospital information systems	hospital information systems	hospital information systems	hospital information system	hospital information system	1	singular
hospital levels	hospital levels	hospital levels	hospital levels	Hospital levels	1	case_or_format
Hospital libraries	hospital libraries	hospital libraries	hospital libraries	Hospital libraries	1	kept
Hospital logistics	hospital logistics	hospital logistics	hospital logistics	Hospital logistics	1	kept

Hospital malnutrition	hospital malnutrition	hospital malnutrition	hospital malnutrition	Hospital malnutrition	1	kept
hospital medical emergency team	hospital medical emergency team	hospital medical emergency team	hospital medical emergency team	hospital medical emergency team	1	kept
HOSPITAL NOISE	hospital noise	hospital noise	hospital noise	Hospital noise	1	case_or_format
hospital noise	hospital noise	hospital noise	hospital noise	Hospital noise	1	case_or_format
hospital occupancy	hospital occupancy	hospital occupancy	hospital occupancy	hospital occupancy	1	kept
hospital occupational health	hospital occupational health	hospital occupational health	hospital occupational health	hospital occupational health	1	kept
Hospital operations	hospital operations	hospital operations	hospital operations	hospital operations	1	case_or_format
hospital organisation	hospital organisation	hospital organisation	hospital organisation	hospital organisation	1	kept
Hospital organization	hospital organization	hospital organization	hospital organization	hospital organization	1	case_or_format
hospital organization and administration	hospital organization and administration	hospital organization and administration	hospital organization and administration	hospital organization and administration	1	kept
Hospital outcome	hospital outcome	hospital outcome	hospital outcome	Hospital outcome	1	kept
hospital outcomes	hospital outcomes	hospital outcomes	hospital outcome	Hospital outcome	1	singular
hospital palliative care	hospital palliative care	hospital palliative care	hospital palliative care	hospital palliative care	1	kept
Hospital Performance	hospital performance	hospital performance	hospital performance	Hospital Performance	1	kept
Hospital performance	hospital performance	hospital performance	hospital performance	Hospital Performance	1	case_or_format
Hospital perspective	hospital perspective	hospital perspective	hospital perspective	Hospital perspective	1	kept

Hospital pharmacist	hospital pharmacist	hospital pharmacist	hospital pharmacist	hospital pharmacist	1	case_or_format
HOSPITAL PHARMACISTS	hospital pharmacists	hospital pharmacists	hospital pharmacist	hospital pharmacist	1	singular
Hospital pharmacy service	hospital pharmacy service	hospital pharmacy service	hospital pharmacy service	Hospital pharmacy service	1	kept
Hospital pharmacy services	hospital pharmacy services	hospital pharmacy services	hospital pharmacy service	Hospital pharmacy service	1	singular
Hospital pharmacy technician	hospital pharmacy technician	hospital pharmacy technician	hospital pharmacy technician	Hospital pharmacy technician	1	kept
Hospital physical therapy service	hospital physical therapy service	hospital physical therapy service	hospital physical therapy service	Hospital physical therapy service	1	kept
Hospital physicians	hospital physicians	hospital physicians	hospital physician	hospital physician	1	singular
Hospital policies	hospital policies	hospital policies	hospital policy	hospital policy	1	singular
HOSPITAL PRACTICES	hospital practices	hospital practices	hospital practices	HOSPITAL PRACTICES	1	kept
Hospital preparedness	hospital preparedness	hospital preparedness	hospital preparedness	Hospital preparedness	1	kept
hospital preparedness	hospital preparedness	hospital preparedness	hospital preparedness	Hospital preparedness	1	case_or_format
Hospital procurement	hospital procurement	hospital procurement	hospital procurement	Hospital procurement	1	kept
hospital profiling	hospital profiling	hospital profiling	hospital profiling	hospital profiling	1	kept
Hospital protection program	hospital protection program	hospital protection program	hospital protection program	Hospital protection program	1	kept
hospital rapid response system	hospital rapid response system	hospital rapid response system	hospital rapid response system	hospital rapid response system	1	kept
hospital readmissions	hospital readmissions	hospital readmissions	hospital readmission	hospital readmission	1	singular

Hospital record	hospital record	hospital record	hospital record	Hospital Records	1	case_or_format
Hospital records	hospital records	hospital records	hospital record	Hospital Records	1	singular
Hospital resources	hospital resources	hospital resources	hospital resources	Hospital resources	1	kept
Hospital restriction policy	hospital restriction policy	hospital restriction policy	hospital restriction policy	Hospital restriction policy	1	kept
hospital safety	hospital safety	hospital safety	hospital safety	hospital safety	1	kept
Hospital segundo y tercer nivel	hospital segundo y tercer nivel	hospital segundo y tercer nivel	hospital segundo y tercer nivel	Hospital segundo y tercer nivel	1	kept
Hospital Setting	hospital setting	hospital setting	hospital setting	Hospital settings	1	case_or_format
hospital setting	hospital setting	hospital setting	hospital setting	Hospital settings	1	case_or_format
Hospital Shared Services	hospital shared services	hospital shared services	hospital shared services	Hospital Shared Services	1	kept
hospital software design features	hospital software design features	hospital software design features	hospital software design features	hospital software design features	1	kept
Hospital soundscape	hospital soundscape	hospital soundscape	hospital soundscape	Hospital soundscape	1	kept
Hospital Staff	hospital staff	hospital staff	hospital staff	Hospital staff	1	case_or_format
hospital staff support	hospital staff support	hospital staff support	hospital staff support	hospital staff support	1	kept
hospital staffing model	hospital staffing model	hospital staffing model	hospital staffing model	hospital staffing model	1	kept
hospital strain	hospital strain	hospital strain	hospital strain	hospital strain	1	kept
hospital survivor	hospital survivor	hospital survivor	hospital survivor	hospital survivor	1	kept

Hospital system	hospital system	hospital system	hospital system	Hospital system	1	kept
Hospital systems	hospital systems	hospital systems	hospital system	Hospital system	1	singular
hospital table	hospital table	hospital table	hospital table	hospital table	1	kept
Hospital tiers	hospital tiers	hospital tiers	hospital tiers	Hospital tiers	1	kept
Hospital to Home Transition	hospital to home transition	hospital to home transition	hospital to home transition	hospital to home transition	1	case_or_format
Hospital traineeship	hospital traineeship	hospital traineeship	hospital traineeship	Hospital traineeship	1	kept
hospital transfers	hospital transfers	hospital transfers	hospital transfers	hospital transfers	1	kept
hospital type	hospital type	hospital type	hospital type	hospital type	1	kept
HOSPITAL UTILIZATION	hospital utilization	hospital utilization	hospital utilization	hospital utilization	1	case_or_format
hospital visitors	hospital visitors	hospital visitors	hospital visitors	hospital visitors	1	kept
Hospital volume	hospital volume	hospital volume	hospital volume	hospital volume	1	case_or_format
hospital volunteers	hospital volunteers	hospital volunteers	hospital volunteer	Hospital Volunteers	1	singular
Hospital ward	hospital ward	hospital ward	hospital ward	Hospital wards	1	case_or_format
HOSPITAL WARDS	hospital wards	hospital wards	hospital ward	Hospital wards	1	singular
hospital wards	hospital wards	hospital wards	hospital ward	Hospital wards	1	singular
hospital wide prevention bundle	hospital wide prevention bundle	hospital wide prevention bundle	hospital wide prevention bundle	hospital wide prevention bundle	1	kept

Hospital, private	hospital private	hospital private	hospital private	Hospital, private	1	kept
Hospital, Public sector	hospital public sector	hospital public sector	hospital public sector	Hospital, Public sector	1	kept
hospital-acquired	hospital acquired	hospital acquired	hospital acquired	hospital acquired	1	case_or_format
Hospital-acquired complications	hospital acquired complications	hospital acquired complications	hospital acquired complications	Hospital-acquired complications	1	kept
Hospital-Acquired Condition	hospital acquired condition	hospital acquired condition	hospital acquired condition	hospital-acquired conditions	1	case_or_format
Hospital-acquired condition	hospital acquired condition	hospital acquired condition	hospital acquired condition	hospital-acquired conditions	1	case_or_format
Hospital-Acquired Condition Reduction Program	hospital acquired condition reduction program	hospital acquired condition reduction program	hospital acquired condition reduction program	Hospital-Acquired Condition Reduction Program	1	kept
Hospital-acquired conditions	hospital acquired conditions	hospital acquired conditions	hospital acquired condition	hospital-acquired conditions	1	singular
hospital-acquired delirium	hospital acquired delirium	hospital acquired delirium	hospital acquired delirium	hospital-acquired delirium	1	kept
hospital-acquired disability	hospital acquired disability	hospital acquired disability	hospital acquired disability	hospital-acquired disability	1	kept
hospital-acquired hypoglycemia	hospital acquired hypoglycemia	hospital acquired hypoglycemia	hospital acquired hypoglycemia	hospital-acquired hypoglycemia	1	kept
hospital-acquired infection surveillance	hospital acquired infection surveillance	hospital acquired infection surveillance	hospital acquired infection surveillance	hospital-acquired infection surveillance	1	kept
Hospital-Acquired Infections	hospital acquired infections	hospital acquired infections	hospital acquired infection	Hospital-acquired infections	1	singular
hospital-acquired pressure ulcers	hospital acquired pressure ulcers	hospital acquired pressure ulcers	hospital acquired pressure ulcer	Hospital-acquired pressure ulcers	1	singular
Hospital-acquired venous thromboembolism	hospital acquired venous thromboembolism	hospital acquired venous thromboembolism	hospital acquired venous thromboembolism	hospital-acquired venous thromboembolism	1	case_or_format
Hospital-acquired-infection	hospital acquired infection	hospital acquired infection	hospital acquired infection	Hospital-acquired infections	1	case_or_format

Hospital-admitted	hospital admitted	hospital admitted	hospital admitted	Hospital-admitted	1	kept
hospital-associated disability	hospital associated disability	hospital associated disability	hospital associated disability	hospital-associated disability	1	kept
hospital-associated infection	hospital associated infection	hospital associated infection	hospital associated infection	hospital-associated infection	1	kept
Hospital-associated infection mitigation	hospital associated infection mitigation	hospital associated infection mitigation	hospital associated infection mitigation	Hospital-associated infection mitigation	1	kept
hospital-associated infections	hospital associated infections	hospital associated infections	hospital associated infection	hospital-associated infection	1	singular
Hospital-associated pneumonia	hospital associated pneumonia	hospital associated pneumonia	hospital associated pneumonia	Hospital-associated pneumonia	1	kept
Hospital-at-home	hospital at home	hospital at home	hospital at home	hospital at home	1	case_or_format
Hospital-based ambulatory team	hospital based ambulatory team	hospital based ambulatory team	hospital based ambulatory team	Hospital-based ambulatory team	1	kept
hospital-based education	hospital based education	hospital based education	hospital based education	hospital-based education	1	kept
Hospital-based emergency department	hospital based emergency department	hospital based emergency department	hospital based emergency department	Hospital-based emergency department	1	kept
hospital-based home care	hospital based home care	hospital based home care	hospital based home care	hospital-based home care	1	kept
Hospital-based medicine	hospital based medicine	hospital based medicine	hospital based medicine	Hospital-based medicine	1	kept
hospital-based palliative care	hospital based palliative care	hospital based palliative care	hospital based palliative care	hospital-based palliative care	1	kept
HOSPITAL-CARE	hospital care	hospital care	hospital care	hospital care	1	case_or_format
Hospital-managed home-care	hospital managed home care	hospital managed home care	hospital managed home care	Hospital-managed home-care	1	kept
Hospital-onset	hospital onset	hospital onset	hospital onset	Hospital-onset	1	kept

Hospital-specific palliative care issues	hospital specific palliative care issues	hospital specific palliative care issues	hospital specific palliative care issues	Hospital-specific palliative care issues	1	kept
hospital-specific palliative care issues	hospital specific palliative care issues	hospital specific palliative care issues	hospital specific palliative care issues	Hospital-specific palliative care issues	1	case_or_format
HOSPITAL-SURVEY	hospital survey	hospital survey	hospital survey	HOSPITAL-SURVEY	1	kept
hospital-to-home transition	hospital to home transition	hospital to home transition	hospital to home transition	hospital to home transition	1	case_or_format
hospital-wide alert system	hospital wide alert system	hospital wide alert system	hospital wide alert system	hospital-wide alert system	1	kept
Hospitale	hospitale	hospitale	hospitale	Hospitale	1	kept
Hospitales	hospitales	hospitales	hospitale	Hospitale	1	singular
Hospitalidad	hospitalidad	hospitalidad	hospitalidad	Hospitalidad	1	kept
Hospitalisation cost and readmission rate	hospitalisation cost and readmission rate	hospitalisation cost and readmission rate	hospitalisation cost and readmission rate	Hospitalisation cost and readmission rate	1	kept
hospitalisation costs	hospitalisation costs	hospitalisation costs	hospitalisation costs	hospitalisation costs	1	kept
hospitalised child	hospitalised child	hospitalised child	hospitalised child	hospitalised child	1	kept
hospitalised neonates	hospitalised neonates	hospitalised neonates	hospitalised neonates	hospitalised neonates	1	kept
HOSPITALISTS	hospitalists	hospitalists	hospitalist	Hospitalists	1	singular
hospitality industry	hospitality industry	hospitality industry	hospitality industry	hospitality industry	1	kept
Hospitalization associated infection	hospitalization associated infection	hospitalization associated infection	hospitalization associated infection	Hospitalization associated infection	1	kept
Hospitalization cost	hospitalization cost	hospitalization cost	hospitalization cost	hospitalization cost	1	case_or_format

Hospitalization cost and length of stay	hospitalization cost and length of stay	hospitalization cost and length of stay	hospitalization cost and length of stay	Hospitalization cost and length of stay	1	kept
hospitalization units	hospitalization units	hospitalization units	hospitalization units	hospitalization units	1	kept
HOSPITALIZATIONS	hospitalizations	hospitalizations	hospitalization	hospitalization	1	singular
Hospitalizations	hospitalizations	hospitalizations	hospitalization	hospitalization	1	singular
hospitalized adolescent	hospitalized adolescent	hospitalized adolescent	hospitalized adolescent	hospitalized adolescent	1	kept
HOSPITALIZED ADULTS	hospitalized adults	hospitalized adults	hospitalized adults	HOSPITALIZED ADULTS	1	kept
Hospitalized Child	hospitalized child	hospitalized child	hospitalized child	hospitalized child	1	case_or_format
Hospitalized Children	hospitalized children	hospitalized children	hospitalized children	HOSPITALIZED CHILDREN	1	case_or_format
Hospitalized children	hospitalized children	hospitalized children	hospitalized children	HOSPITALIZED CHILDREN	1	case_or_format
hospitalized COVID-19	hospitalized covid 19	hospitalized covid 19	hospitalized covid 19	hospitalized COVID-19	1	kept
HOSPITALIZED ELDERS	hospitalized elders	hospitalized elders	hospitalized elders	HOSPITALIZED ELDERS	1	kept
hospitalized older adults	hospitalized older adults	hospitalized older adults	hospitalized older adults	hospitalized older adults	1	kept
HOSPITALIZED OLDER-ADULTS	hospitalized older adults	hospitalized older adults	hospitalized older adults	hospitalized older adults	1	case_or_format
Hospitalized smokers	hospitalized smokers	hospitalized smokers	hospitalized smokers	Hospitalized smokers	1	kept
hospitalized subjects	hospitalized subjects	hospitalized subjects	hospitalized subjects	hospitalized subjects	1	kept
Hospitals isolation	hospitals isolation	hospitals isolation	hospitals isolation	Hospitals isolation	1	kept

hospitals isolation	hospitals isolation	hospitals isolation	hospitals isolation	Hospitals isolation	1	case_or_format
hospitals psychiatric	hospitals psychiatric	hospitals psychiatric	hospitals psychiatric	Hospitals, Psychiatric	1	case_or_format
Hospitals teaching	hospitals teaching	hospitals teaching	hospitals teaching	Hospitals, Teaching	1	case_or_format
Hospitals, Isolation	hospitals isolation	hospitals isolation	hospitals isolation	Hospitals isolation	1	case_or_format
hospitals, paediatric	hospitals paediatric	hospitals paediatric	hospitals paediatric	hospitals, paediatric	1	kept
Hospitals, Proprietary	hospitals proprietary	hospitals proprietary	hospitals proprietary	Hospitals, Proprietary	1	kept
Hospitals, public	hospitals public	hospitals public	hospitals public	Hospitals, Public	1	case_or_format
hospitals, public	hospitals public	hospitals public	hospitals public	Hospitals, Public	1	case_or_format
Hospitals, Satellite	hospitals satellite	hospitals satellite	hospitals satellite	Hospitals, Satellite	1	kept
Hospitals, special	hospitals special	hospitals special	hospitals special	Hospitals, Special	1	case_or_format
hospitalspediatric	hospitalspediatric	hospitalspediatric	hospitalspediatric	hospitalspediatric	1	kept
hospital‑acquired pressure injury	hospital acquired pressure injury	hospital acquired pressure injury	hospital acquired pressure injury	hospital-acquired pressure injury	1	case_or_format
hospital‑associated pneumonia	hospital associated pneumonia	hospital associated pneumonia	hospital associated pneumonia	Hospital-associated pneumonia	1	case_or_format
Host Microbial Interactions	host microbial interactions	host microbial interactions	host microbial interactions	Host Microbial Interactions	1	kept
HOST-CELL INVASION	host cell invasion	host cell invasion	host cell invasion	HOST-CELL INVASION	1	kept
Host-Parasite Interactions	host parasite interactions	host parasite interactions	host parasite interactions	Host-Parasite Interactions	1	kept

Hostile	hostile	hostile	hostile	Hostile	1	kept
Hot debrief	hot debrief	hot debrief	hot debrief	Hot debrief	1	kept
Hot debriefing	hot debriefing	hot debriefing	hot debriefing	Hot debriefing	1	kept
Hot research topics	hot research topics	hot research topics	hot research topics	Hot research topics	1	kept
hot tub lung	hot tub lung	hot tub lung	hot tub lung	hot tub lung	1	kept
Hotspots	hotspots	hotspots	hotspots	hotspots	1	case_or_format
house calls	house calls	house calls	house calls	House Calls	1	case_or_format
house officer	house officer	house officer	house officer	House officers	1	case_or_format
HOUSE OFFICER YEAR	house officer year	house officer year	house officer year	HOUSE OFFICER YEAR	1	kept
Household Expenditure and Income Survey	household expenditure and income survey	household expenditure and income survey	household expenditure and income survey	Household Expenditure and Income Survey	1	kept
Household Products	household products	household products	household products	Household Products	1	kept
Household Work	household work	household work	household work	Household Work	1	kept
Houses	houses	houses	houses	Houses	1	kept
HOUSES index	houses index	houses index	houses index	HOUSES index	1	kept
Housestaff	housestaff	housestaff	housestaff	housestaff	1	case_or_format
housing instability	housing instability	housing instability	housing instability	housing instability	1	kept

housing quality	housing quality	housing quality	housing quality	housing quality	1	kept
how to prepare	how to prepare	how to prepare	how to prepare	how to prepare	1	kept
Hox protein	hox protein	hox protein	hox protein	Hox protein	1	kept
hox11 protein	hox11 protein	hox11 protein	hox11 protein	hox11 protein	1	kept
HPLC	hplc	hplc	hplc	HPLC	1	kept
HPN	hpn	hpn	hpn	HPN	1	kept
HPNA	hpna	hpna	hpna	HPNA	1	kept
HQIC	hqic	hqic	hqic	HQIC	1	kept
HRQOL	hrqol	hrqol	hrqol	HRQoL	1	case_or_format
hrqol	hrqol	hrqol	hrqol	HRQoL	1	case_or_format
HRV, heart rate variability	hrv heart rate variability	hrv heart rate variability	hrv heart rate variability	HRV, heart rate variability	1	kept
hsPDA	hspda	hspda	hspda	hsPDA	1	kept
HSROC model	hsroc model	hsroc model	hsroc model	HSROC model	1	kept
HT, healthcare technology	ht healthcare technology	ht healthcare technology	ht healthcare technology	HT, healthcare technology	1	kept
hta method	hta method	hta method	hta method	hta method	1	kept
HTLV-1 infection	htlv 1 infection	htlv 1 infection	htlv 1 infection	HTLV-1 infection	1	kept

HTTP	http	http	http	HTTP	1	kept
Hub & Spoke healthcare	hub and spoke healthcare	hub and spoke healthcare	hub and spoke healthcare	Hub & Spoke healthcare	1	kept
Hub and spoke networking Primary PTCA	hub and spoke networking primary ptca	hub and spoke networking primary ptca	hub and spoke networking primary ptca	Hub and spoke networking Primary PTCA	1	kept
hub and spoke pancreatitis network	hub and spoke pancreatitis network	hub and spoke pancreatitis network	hub and spoke pancreatitis network	hub and spoke pancreatitis network	1	kept
Hub colonization	hub colonization	hub colonization	hub colonization	Hub colonization	1	kept
Hub culture	hub culture	hub culture	hub culture	Hub culture	1	kept
Hub disinfection	hub disinfection	hub disinfection	hub disinfection	Hub disinfection	1	kept
huddle	huddle	huddle	huddle	huddle	1	kept
huddle spaces	huddle spaces	huddle spaces	huddle spaces	huddle spaces	1	kept
Hue City	hue city	hue city	hue city	Hue City	1	kept
hugging	hugging	hugging	hugging	hugging	1	kept
humalog mix 50	humalog mix 50	humalog mix 50	humalog mix 50	humalog mix 50	1	kept
Humerus	humerus	humerus	humerus	Humerus	1	kept
humerus	humerus	humerus	humerus	Humerus	1	case_or_format
HUMIDIFICATION	humidification	humidification	humidification	humidification	1	case_or_format
humidified	humidified	humidified	humidified	humidified	1	kept

Humidified high-flow nasal cannula	humidified high flow nasal cannula	humidified high flow nasal cannula	humidified high flow nasal cannula	Humidified high-flow nasal cannula	1	kept
humidified low flow oxygen therapy	humidified low flow oxygen therapy	humidified low flow oxygen therapy	humidified low flow oxygen therapy	humidified low flow oxygen therapy	1	kept
Humidifier System	humidifier system	humidifier system	humidifier system	Humidifier System	1	kept
humidifiers	humidifiers	humidifiers	humidifier	humidifier	1	singular
HUMIDITY	humidity	humidity	humidity	Humidity	1	case_or_format
Humidity control	humidity control	humidity control	humidity control	Humidity control	1	kept
humidity in a rural incubator	humidity in a rural incubator	humidity in a rural incubator	humidity in a rural incubator	humidity in a rural incubator	1	kept
humification	humification	humification	humification	humification	1	kept
Humor	humor	humor	humor	humor	1	case_or_format
humoral immunity	humoral immunity	humoral immunity	humoral immunity	humoral immunity	1	kept
humulin m 3	humulin m 3	humulin m 3	humulin m 3	humulin m 3	1	kept
Hunan	hunan	hunan	hunan	Hunan	1	kept
Hunan province	hunan province	hunan province	hunan province	Hunan province	1	kept
Hunt and Hess SAH Severity Scale	hunt and hess sah severity scale	hunt and hess sah severity scale	hunt and hess sah severity scale	Hunt and Hess SAH Severity Scale	1	kept
Huntingtin	huntingtin	huntingtin	huntingtin	Huntingtin	1	kept
huntingtin	huntingtin	huntingtin	huntingtin	Huntingtin	1	case_or_format

Huntington disease	huntington disease	huntington disease	huntington disease	Huntington disease	1	kept
Hurdle models	hurdle models	hurdle models	hurdle models	Hurdle models	1	kept
Hurricane	hurricane	hurricane	hurricane	hurricane	1	case_or_format
hurricanes	hurricanes	hurricanes	hurricane	hurricane	1	singular
hurting	hurting	hurting	hurting	hurting	1	kept
HUS	hus	hus	hus	HUS	1	kept
HUSSERL	husserl	husserl	husserl	HUSSERL	1	kept
HVAC-SYSTEMS	hvac systems	hvac systems	hvac systems	HVAC-SYSTEMS	1	kept
Hyaline membrane disease	hyaline membrane disease	hyaline membrane disease	hyaline membrane disease	hyaline membrane disease	1	case_or_format
hyaluronic acid filler	hyaluronic acid filler	hyaluronic acid filler	hyaluronic acid filler	hyaluronic acid filler	1	kept
hyaluronoglucosaminidase	hyaluronoglucosaminidase	hyaluronoglucosaminidase	hyaluronoglucosaminidase	Hyaluronoglucosaminidase	1	case_or_format
Hybrid closed-loop	hybrid closed loop	hybrid closed loop	hybrid closed loop	Hybrid closed-loop	1	kept
Hybrid cloud server	hybrid cloud server	hybrid cloud server	hybrid cloud server	Hybrid cloud server	1	kept
Hybrid effectiveness-implementation trials	hybrid effectiveness implementation trials	hybrid effectiveness implementation trials	hybrid effectiveness implementation trials	Hybrid effectiveness-implementation trials	1	kept
hybrid incubator	hybrid incubator	hybrid incubator	hybrid incubator	hybrid incubator	1	kept
hybrid intervention	hybrid intervention	hybrid intervention	hybrid intervention	hybrid intervention	1	kept

Hybrid model	hybrid model	hybrid model	hybrid model	hybrid model	1	case_or_format
Hybrid model of concept development	hybrid model of concept development	hybrid model of concept development	hybrid model of concept development	Hybrid model of concept development	1	kept
hybrid model simulation	hybrid model simulation	hybrid model simulation	hybrid model simulation	hybrid model simulation	1	kept
hybrid of online and offline learning model	hybrid of online and offline learning model	hybrid of online and offline learning model	hybrid of online and offline learning model	hybrid of online and offline learning model	1	kept
Hybrid simulation	hybrid simulation	hybrid simulation	hybrid simulation	Hybrid simulation	1	kept
hybrid single ventricle palliation surgery	hybrid single ventricle palliation surgery	hybrid single ventricle palliation surgery	hybrid single ventricle palliation surgery	hybrid single ventricle palliation surgery	1	kept
hybrid style clinical round	hybrid style clinical round	hybrid style clinical round	hybrid style clinical round	hybrid style clinical round	1	kept
hybrid system	hybrid system	hybrid system	hybrid system	hybrid system	1	kept
hybridization	hybridization	hybridization	hybridization	hybridization	1	kept
Hybridization, Genetic	hybridization genetic	hybridization genetic	hybridization genetic	Hybridization, Genetic	1	kept
hydralazine plus isosorbide dinitrate	hydralazine plus isosorbide dinitrate	hydralazine plus isosorbide dinitrate	hydralazine plus isosorbide dinitrate	hydralazine plus isosorbide dinitrate	1	kept
Hydraulic Fracking	hydraulic fracking	hydraulic fracking	hydraulic fracking	Hydraulic Fracking	1	kept
Hydric balance	hydric balance	hydric balance	hydric balance	Hydric balance	1	kept
Hydrides	hydrides	hydrides	hydrides	Hydrides	1	kept
hydroalcohol	hydroalcohol	hydroalcohol	hydroalcohol	hydroalcohol	1	kept
Hydrocarbons	hydrocarbons	hydrocarbons	hydrocarbon	hydrocarbon	1	singular

Hydrocarbons, Brominated	hydrocarbons brominated	hydrocarbons brominated	hydrocarbons brominated	Hydrocarbons, Brominated	1	kept
hydrocele	hydrocele	hydrocele	hydrocele	hydrocele	1	kept
hydrochlorothiazide plus spironolactone	hydrochlorothiazide plus spironolactone	hydrochlorothiazide plus spironolactone	hydrochlorothiazide plus spironolactone	hydrochlorothiazide plus spironolactone	1	kept
hydrocodone bitartrate plus ibuprofen	hydrocodone bitartrate plus ibuprofen	hydrocodone bitartrate plus ibuprofen	hydrocodone bitartrate plus ibuprofen	hydrocodone bitartrate plus ibuprofen	1	kept
hydrodistillation	hydrodistillation	hydrodistillation	hydrodistillation	hydrodistillation	1	kept
hydrodynamics	hydrodynamics	hydrodynamics	hydrodynamics	Hydrodynamics	1	case_or_format
hydrofluoric acid	hydrofluoric acid	hydrofluoric acid	hydrofluoric acid	hydrofluoric acid	1	kept
HYDROGEL	hydrogel	hydrogel	hydrogel	hydrogel	1	case_or_format
Hydrogel, Polyethylene Glycol Dimethacrylate	hydrogel polyethylene glycol dimethacrylate	hydrogel polyethylene glycol dimethacrylate	hydrogel polyethylene glycol dimethacrylate	Hydrogel, Polyethylene Glycol Dimethacrylate	1	kept
hydrogen	hydrogen	hydrogen	hydrogen	hydrogen	1	kept
hydrogen bond	hydrogen bond	hydrogen bond	hydrogen bond	hydrogen bond	1	kept
Hydrogen peroxide	hydrogen peroxide	hydrogen peroxide	hydrogen peroxide	hydrogen peroxide	1	case_or_format
hydrogen potassium adenosine triphosphatase	hydrogen potassium adenosine triphosphatase	hydrogen potassium adenosine triphosphatase	hydrogen potassium adenosine triphosphatase	hydrogen potassium adenosine triphosphatase	1	kept
Hydrogen-ion concentration	hydrogen ion concentration	hydrogen ion concentration	hydrogen ion concentration	Hydrogen-Ion Concentration	1	case_or_format
hydrolysis	hydrolysis	hydrolysis	hydrolysis	hydrolysis	1	kept
hydrops fetalis	hydrops fetalis	hydrops fetalis	hydrops fetalis	Hydrops Fetalis	1	case_or_format

hydrostat	hydrostat	hydrostat	hydrostat	hydrostat	1	kept
hydroxybutyric acid	hydroxybutyric acid	hydroxybutyric acid	hydroxybutyric acid	hydroxybutyric acid	1	kept
hydroxyethyl starch	hydroxyethyl starch	hydroxyethyl starch	hydroxyethyl starch	hydroxyethyl starch	1	kept
HYDROXYETHYL STARCH 130	hydroxyethyl starch 130	hydroxyethyl starch 130	hydroxyethyl starch 130	HYDROXYETHYL STARCH 130	1	kept
Hydroxyethyl Starch Derivatives	hydroxyethyl starch derivatives	hydroxyethyl starch derivatives	hydroxyethyl starch derivatives	Hydroxyethyl Starch Derivatives	1	kept
hydroxyl radical	hydroxyl radical	hydroxyl radical	hydroxyl radical	hydroxyl radical	1	kept
hydroxylamine	hydroxylamine	hydroxylamine	hydroxylamine	hydroxylamine	1	kept
Hydroxylamines	hydroxylamines	hydroxylamines	hydroxylamine	hydroxylamine	1	singular
hydroxyprogesterone	hydroxyprogesterone	hydroxyprogesterone	hydroxyprogesterone	hydroxyprogesterone	1	kept
hydroxyprogesterone caproate	hydroxyprogesterone caproate	hydroxyprogesterone caproate	hydroxyprogesterone caproate	hydroxyprogesterone caproate	1	kept
hydroxyzine embonate	hydroxyzine embonate	hydroxyzine embonate	hydroxyzine embonate	hydroxyzine embonate	1	kept
hygiene and comfort care	hygiene and comfort care	hygiene and comfort care	hygiene and comfort care	hygiene and comfort care	1	kept
hygiene hypothesis	hygiene hypothesis	hygiene hypothesis	hygiene hypothesis	hygiene hypothesis	1	kept
hygiene management	hygiene management	hygiene management	hygiene management	hygiene management	1	kept
Hygiene Practices	hygiene practices	hygiene practices	hygiene practices	Hygiene Practices	1	kept
hygiene, infection control	hygiene infection control	hygiene infection control	hygiene infection control	hygiene, infection control	1	kept

Hygienic care	hygienic care	hygienic care	hygienic care	Hygienic care	1	kept
hyoid bone	hyoid bone	hyoid bone	hyoid bone	hyoid bone	1	kept
hyoscine n oxide	hyoscine n oxide	hyoscine n oxide	hyoscine n oxide	hyoscine n oxide	1	kept
hyoscine N-oxide	hyoscine n oxide	hyoscine n oxide	hyoscine n oxide	hyoscine n oxide	1	case_or_format
hyoscyamine	hyoscyamine	hyoscyamine	hyoscyamine	hyoscyamine	1	kept
Hyper-acute stroke care	hyper acute stroke care	hyper acute stroke care	hyper acute stroke care	Hyper-acute stroke care	1	kept
Hyperaldosteronism	hyperaldosteronism	hyperaldosteronism	hyperaldosteronism	Hyperaldosteronism	1	kept
hyperaldosteronism	hyperaldosteronism	hyperaldosteronism	hyperaldosteronism	Hyperaldosteronism	1	case_or_format
Hyperalgesia	hyperalgesia	hyperalgesia	hyperalgesia	hyperalgesia	1	case_or_format
Hyperbaric	hyperbaric	hyperbaric	hyperbaric	Hyperbaric	1	kept
hyperbaric	hyperbaric	hyperbaric	hyperbaric	Hyperbaric	1	case_or_format
hyperbaric chamber	hyperbaric chamber	hyperbaric chamber	hyperbaric chamber	hyperbaric chamber	1	kept
Hyperbaric facilities	hyperbaric facilities	hyperbaric facilities	hyperbaric facilities	Hyperbaric facilities	1	kept
Hyperbaric oxygenation therapy	hyperbaric oxygenation therapy	hyperbaric oxygenation therapy	hyperbaric oxygenation therapy	Hyperbaric oxygenation therapy	1	kept
Hyperbaric research	hyperbaric research	hyperbaric research	hyperbaric research	Hyperbaric research	1	kept
hyperbaric therapy	hyperbaric therapy	hyperbaric therapy	hyperbaric therapy	hyperbaric therapy	1	kept

hyperbarism	hyperbarism	hyperbarism	hyperbarism	hyperbarism	1	kept
hyperbilirubinemia in pregnancy	hyperbilirubinemia in pregnancy	hyperbilirubinemia in pregnancy	hyperbilirubinemia in pregnancy	hyperbilirubinemia in pregnancy	1	kept
hypercalciuria	hypercalciuria	hypercalciuria	hypercalciuria	hypercalciuria	1	kept
Hypercapnic coma	hypercapnic coma	hypercapnic coma	hypercapnic coma	Hypercapnic coma	1	kept
hypercapnic coma	hypercapnic coma	hypercapnic coma	hypercapnic coma	Hypercapnic coma	1	case_or_format
Hypercoagulability	hypercoagulability	hypercoagulability	hypercoagulability	hypercoagulability	1	case_or_format
hypercreatininemia	hypercreatininemia	hypercreatininemia	hypercreatininemia	hypercreatininemia	1	kept
hypereosinophilia	hypereosinophilia	hypereosinophilia	hypereosinophilia	hypereosinophilia	1	kept
hyperferritinemia	hyperferritinemia	hyperferritinemia	hyperferritinemia	hyperferritinemia	1	kept
hypergastrinemia	hypergastrinemia	hypergastrinemia	hypergastrinemia	hypergastrinemia	1	kept
hyperglycaemia hypoglycaemia	hyperglycaemia hypoglycaemia	hyperglycaemia hypoglycaemia	hyperglycaemia hypoglycaemia	hyperglycaemia hypoglycaemia	1	kept
Hyperglycaemic hyperosmolar syndrome	hyperglycaemic hyperosmolar syndrome	hyperglycaemic hyperosmolar syndrome	hyperglycaemic hyperosmolar syndrome	Hyperglycaemic hyperosmolar syndrome	1	kept
Hyperglycaemic state	hyperglycaemic state	hyperglycaemic state	hyperglycaemic state	Hyperglycaemic state	1	kept
HYPERGLYCEMIA	hyperglycemia	hyperglycemia	hyperglycemia	Hyperglycemia	1	case_or_format
hyperglycemia management	hyperglycemia management	hyperglycemia management	hyperglycemia management	hyperglycemia management	1	kept
hyperglycemic index	hyperglycemic index	hyperglycemic index	hyperglycemic index	hyperglycemic index	1	kept

Hyperhomocysteinemia	hyperhomocysteinemia	hyperhomocysteinemia	hyperhomocysteinemia	Hyperhomocysteinemia	1	kept
hyperhomocysteinemia	hyperhomocysteinemia	hyperhomocysteinemia	hyperhomocysteinemia	Hyperhomocysteinemia	1	case_or_format
hyperinflammatory syndrome	hyperinflammatory syndrome	hyperinflammatory syndrome	hyperinflammatory syndrome	hyperinflammatory syndrome	1	kept
Hyperinsulinemia	hyperinsulinemia	hyperinsulinemia	hyperinsulinemia	hyperinsulinemia	1	case_or_format
hyperinsulinemia-euglycemia therapy	hyperinsulinemia euglycemia therapy	hyperinsulinemia euglycemia therapy	hyperinsulinemia euglycemia therapy	hyperinsulinemia-euglycemia therapy	1	kept
Hyperkalaemia	hyperkalaemia	hyperkalaemia	hyperkalaemia	Hyperkalaemia	1	kept
hyperkeratosis	hyperkeratosis	hyperkeratosis	hyperkeratosis	hyperkeratosis	1	kept
hyperkinesia	hyperkinesia	hyperkinesia	hyperkinesia	hyperkinesia	1	kept
hyperleukocytosis	hyperleukocytosis	hyperleukocytosis	hyperleukocytosis	hyperleukocytosis	1	kept
Hyperoglyphe japonica	hyperoglyphe japonica	hyperoglyphe japonica	hyperoglyphe japonica	Hyperoglyphe japonica	1	kept
Hyperoxaemia	hyperoxaemia	hyperoxaemia	hyperoxaemia	Hyperoxaemia	1	kept
hyperoxaemia	hyperoxaemia	hyperoxaemia	hyperoxaemia	Hyperoxaemia	1	case_or_format
Hyperoxaluria	hyperoxaluria	hyperoxaluria	hyperoxaluria	Hyperoxaluria	1	kept
hyperoxaluria	hyperoxaluria	hyperoxaluria	hyperoxaluria	Hyperoxaluria	1	case_or_format
Hyperoxygenation	hyperoxygenation	hyperoxygenation	hyperoxygenation	Hyperoxygenation	1	kept
Hyperphagia	hyperphagia	hyperphagia	hyperphagia	Hyperphagia	1	kept

hyperpyrexia	hyperpyrexia	hyperpyrexia	hyperpyrexia	hyperpyrexia	1	kept
Hypersensibility: latex	hypersensibility latex	hypersensibility latex	hypersensibility latex	Hypersensibility: latex	1	kept
hypersensitivity pneumonitis	hypersensitivity pneumonitis	hypersensitivity pneumonitis	hypersensitivity pneumonitis	hypersensitivity pneumonitis	1	kept
Hypersensitivity reactions	hypersensitivity reactions	hypersensitivity reactions	hypersensitivity reaction	hypersensitivity reaction	1	singular
Hypersensitivity, Delayed	hypersensitivity delayed	hypersensitivity delayed	hypersensitivity delayed	Hypersensitivity, Delayed	1	kept
Hypersensitivity, Immediate	hypersensitivity immediate	hypersensitivity immediate	hypersensitivity immediate	Hypersensitivity, Immediate	1	kept
hypersexuality	hypersexuality	hypersexuality	hypersexuality	hypersexuality	1	kept
hypersplenism	hypersplenism	hypersplenism	hypersplenism	hypersplenism	1	kept
hypertelorism	hypertelorism	hypertelorism	hypertelorism	hypertelorism	1	kept
hypertension encephalopathy	hypertension encephalopathy	hypertension encephalopathy	hypertension encephalopathy	hypertension encephalopathy	1	kept
hypertension portale	hypertension portale	hypertension portale	hypertension portale	hypertension portale	1	kept
Hypertension, Malignant	hypertension malignant	hypertension malignant	hypertension malignant	Hypertension, Malignant	1	kept
Hypertension, Renal	hypertension renal	hypertension renal	hypertension renal	Hypertension, Renal	1	kept
Hypertensive	hypertensive	hypertensive	hypertensive	Hypertensive	1	kept
Hypertensive crisis	hypertensive crisis	hypertensive crisis	hypertensive crisis	hypertensive crisis	1	case_or_format
Hypertensive disorders	hypertensive disorders	hypertensive disorders	hypertensive disorders	Hypertensive disorders	1	kept

Hypertensive disorders of pregnancy	hypertensive disorders of pregnancy	hypertensive disorders of pregnancy	hypertensive disorders of pregnancy	Hypertensive disorders of pregnancy	1	kept
Hypertensive emergencies	hypertensive emergencies	hypertensive emergencies	hypertensive emergency	hypertensive emergency	1	singular
Hypertensive urgency	hypertensive urgency	hypertensive urgency	hypertensive urgency	Hypertensive urgency	1	kept
hypertensive urgency	hypertensive urgency	hypertensive urgency	hypertensive urgency	Hypertensive urgency	1	case_or_format
Hyperthermia device	hyperthermia device	hyperthermia device	hyperthermia device	Hyperthermia device	1	kept
HYPERTHERMIC INTRAPERITONEAL CHEMOTHERAPY	hyperthermic intraperitoneal chemotherapy	hyperthermic intraperitoneal chemotherapy	hyperthermic intraperitoneal chemotherapy	hyperthermic intraperitoneal chemotherapy	1	case_or_format
Hyperthermic intraperitoneal chemotherapy	hyperthermic intraperitoneal chemotherapy	hyperthermic intraperitoneal chemotherapy	hyperthermic intraperitoneal chemotherapy	hyperthermic intraperitoneal chemotherapy	1	case_or_format
Hyperthermic rebound	hyperthermic rebound	hyperthermic rebound	hyperthermic rebound	Hyperthermic rebound	1	kept
Hypertonic	hypertonic	hypertonic	hypertonic	Hypertonic	1	kept
hypertranslucent lung	hypertranslucent lung	hypertranslucent lung	hypertranslucent lung	hypertranslucent lung	1	kept
hypertrophic obstructive cardiomyopathy	hypertrophic obstructive cardiomyopathy	hypertrophic obstructive cardiomyopathy	hypertrophic obstructive cardiomyopathy	hypertrophic obstructive cardiomyopathy	1	kept
hypertrophic pyloric stenosis	hypertrophic pyloric stenosis	hypertrophic pyloric stenosis	hypertrophic pyloric stenosis	hypertrophic pyloric stenosis	1	kept
Hypertrophic scar	hypertrophic scar	hypertrophic scar	hypertrophic scar	hypertrophic scar	1	case_or_format
Hypertrophies	hypertrophies	hypertrophies	hypertrophy	Hypertrophy	1	singular
hypertrophy	hypertrophy	hypertrophy	hypertrophy	Hypertrophy	1	case_or_format
hyperviscosity syndrome	hyperviscosity syndrome	hyperviscosity syndrome	hyperviscosity syndrome	hyperviscosity syndrome	1	kept

Hypervolemia	hypervolemia	hypervolemia	hypervolemia	hypervolemia	1	case_or_format
Hypesthesia	hypesthesia	hypesthesia	hypesthesia	hypesthesia	1	case_or_format
Hypnosis, Anesthetic	hypnosis anesthetic	hypnosis anesthetic	hypnosis anesthetic	Hypnosis, Anesthetic	1	kept
hypnotics	hypnotics	hypnotics	hypnotics	hypnotics	1	kept
Hypnotics, sedatives	hypnotics sedatives	hypnotics sedatives	hypnotics sedatives	Hypnotics, sedatives	1	kept
hypoactive POD	hypoactive pod	hypoactive pod	hypoactive pod	hypoactive POD	1	kept
hypoactivity	hypoactivity	hypoactivity	hypoactivity	hypoactivity	1	kept
hypoacusis	hypoacusis	hypoacusis	hypoacusis	hypoacusis	1	kept
hypoadrenocorticism	hypoadrenocorticism	hypoadrenocorticism	hypoadrenocorticism	hypoadrenocorticism	1	kept
hypobaric hypoxic hypoxia	hypobaric hypoxic hypoxia	hypobaric hypoxic hypoxia	hypobaric hypoxic hypoxia	hypobaric hypoxic hypoxia	1	kept
hypobulimia	hypobulimia	hypobulimia	hypobulimia	hypobulimia	1	kept
Hypocalcaemia	hypocalcaemia	hypocalcaemia	hypocalcaemia	Hypocalcaemia	1	kept
hypochlorous acid	hypochlorous acid	hypochlorous acid	hypochlorous acid	hypochlorous acid	1	kept
Hypocupremia	hypocupremia	hypocupremia	hypocupremia	Hypocupremia	1	kept
hypocupremia	hypocupremia	hypocupremia	hypocupremia	Hypocupremia	1	case_or_format
hypodermoclysis	hypodermoclysis	hypodermoclysis	hypodermoclysis	hypodermoclysis	1	kept

hypofibrinogenemia	hypofibrinogenemia	hypofibrinogenemia	hypofibrinogenemia	hypofibrinogenemia	1	kept
Hypofractionation	hypofractionation	hypofractionation	hypofractionation	Hypofractionation	1	kept
Hypogastric artery ligation	hypogastric artery ligation	hypogastric artery ligation	hypogastric artery ligation	Hypogastric artery ligation	1	kept
Hypoglicemia	hypoglicemia	hypoglicemia	hypoglicemia	Hypoglicemia	1	kept
HYPOGLYCEMIA	hypoglycemia	hypoglycemia	hypoglycemia	Hypoglycemia	1	case_or_format
Hypoglycemic	hypoglycemic	hypoglycemic	hypoglycemic	Hypoglycemic	1	kept
hypoglycemic episodes	hypoglycemic episodes	hypoglycemic episodes	hypoglycemic episodes	hypoglycemic episodes	1	kept
Hypokalaemia	hypokalaemia	hypokalaemia	hypokalaemia	Hypokalaemia	1	kept
hypokalaemia	hypokalaemia	hypokalaemia	hypokalaemia	Hypokalaemia	1	case_or_format
Hypokataemia	hypokataemia	hypokataemia	hypokataemia	Hypokataemia	1	kept
hypomania	hypomania	hypomania	hypomania	hypomania	1	kept
Hyponatraemia	hyponatraemia	hyponatraemia	hyponatraemia	Hyponatraemia	1	kept
hyponatraemia	hyponatraemia	hyponatraemia	hyponatraemia	Hyponatraemia	1	case_or_format
Hyponatremia incidence	hyponatremia incidence	hyponatremia incidence	hyponatremia incidence	Hyponatremia incidence	1	kept
Hyponatremia prevalence	hyponatremia prevalence	hyponatremia prevalence	hyponatremia prevalence	Hyponatremia prevalence	1	kept
hypoosmolarity	hypoosmolarity	hypoosmolarity	hypoosmolarity	hypoosmolarity	1	kept

hypoparathyroidism	hypoparathyroidism	hypoparathyroidism	hypoparathyroidism	hypoparathyroidism	1	kept
Hypophosphataemia	hypophosphataemia	hypophosphataemia	hypophosphataemia	Hypophosphataemia	1	kept
hypophosphatasia	hypophosphatasia	hypophosphatasia	hypophosphatasia	hypophosphatasia	1	kept
hypophysis disease	hypophysis disease	hypophysis disease	hypophysis disease	hypophysis disease	1	kept
hypophysis thyroid system	hypophysis thyroid system	hypophysis thyroid system	hypophysis thyroid system	hypophysis thyroid system	1	kept
hypopituitarism	hypopituitarism	hypopituitarism	hypopituitarism	Hypopituitarism	1	case_or_format
hypoplastic right heart	hypoplastic right heart	hypoplastic right heart	hypoplastic right heart	hypoplastic right heart	1	kept
Hypopneas	hypopneas	hypopneas	hypopneas	Hypopneas	1	kept
hypoprothrombinemia	hypoprothrombinemia	hypoprothrombinemia	hypoprothrombinemia	hypoprothrombinemia	1	kept
hyposmia	hyposmia	hyposmia	hyposmia	hyposmia	1	kept
Hypostasis	hypostasis	hypostasis	hypostasis	Hypostasis	1	kept
Hypotension, other drugs	hypotension other drugs	hypotension other drugs	hypotension other drugs	Hypotension, other drugs	1	kept
Hypotensive shock	hypotensive shock	hypotensive shock	hypotensive shock	Hypotensive shock	1	kept
Hypothalamus	hypothalamus	hypothalamus	hypothalamus	Hypothalamus	1	kept
hypothalamus	hypothalamus	hypothalamus	hypothalamus	Hypothalamus	1	case_or_format
Hypothermia in newborn infants	hypothermia in newborn infants	hypothermia in newborn infants	hypothermia in newborn infants	Hypothermia in newborn infants	1	kept

Hypothermia Induced	hypothermia induced	hypothermia induced	hypothermia induced	Hypothermia, Induced	1	case_or_format
Hypothermia management	hypothermia management	hypothermia management	hypothermia management	Hypothermia management	1	kept
Hypothermia on admission	hypothermia on admission	hypothermia on admission	hypothermia on admission	Hypothermia on admission	1	kept
hypothermia treatment	hypothermia treatment	hypothermia treatment	hypothermia treatment	Hypothermia treatment	1	case_or_format
Hypothesis	hypothesis	hypothesis	hypothesis	hypothesis	1	case_or_format
Hypothesis formation	hypothesis formation	hypothesis formation	hypothesis formation	Hypothesis formation	1	kept
Hypothesis testing	hypothesis testing	hypothesis testing	hypothesis testing	Hypothesis testing	1	kept
hypothetical	hypothetical	hypothetical	hypothetical	hypothetical	1	kept
Hypotonia	hypotonia	hypotonia	hypotonia	Hypotonia	1	kept
hypovigilance	hypovigilance	hypovigilance	hypovigilance	hypovigilance	1	kept
Hypovolaemia	hypovolaemia	hypovolaemia	hypovolaemia	Hypovolaemia	1	kept
hypovolemic hyponatremia	hypovolemic hyponatremia	hypovolemic hyponatremia	hypovolemic hyponatremia	hypovolemic hyponatremia	1	kept
hypoxemia severity	hypoxemia severity	hypoxemia severity	hypoxemia severity	hypoxemia severity	1	kept
hypoxemic cardiac arrest	hypoxemic cardiac arrest	hypoxemic cardiac arrest	hypoxemic cardiac arrest	hypoxemic cardiac arrest	1	kept
Hypoxemic episodes	hypoxemic episodes	hypoxemic episodes	hypoxemic episodes	Hypoxemic episodes	1	kept
HYPOXEMIC RESPIRATORY-FAILURE	hypoxemic respiratory failure	hypoxemic respiratory failure	hypoxemic respiratory failure	hypoxemic respiratory failure	1	case_or_format

hypoxia challenge test	hypoxia challenge test	hypoxia challenge test	hypoxia challenge test	hypoxia challenge test	1	kept
hypoxia inducible factor 1	hypoxia inducible factor 1	hypoxia inducible factor 1	hypoxia inducible factor 1	hypoxia inducible factor 1	1	kept
hypoxia-ischemia, brain	hypoxia ischemia brain	hypoxia ischemia brain	hypoxia ischemia brain	Hypoxia-Ischemia, Brain	1	case_or_format
Hypoxic brain injury	hypoxic brain injury	hypoxic brain injury	hypoxic brain injury	hypoxic brain injury	1	case_or_format
hypoxic ischemic injury	hypoxic ischemic injury	hypoxic ischemic injury	hypoxic ischemic injury	hypoxic ischemic injury	1	kept
Hypoxic-ischaemic encephalopathy	hypoxic ischaemic encephalopathy	hypoxic ischaemic encephalopathy	hypoxic ischaemic encephalopathy	Hypoxic-ischaemic encephalopathy	1	kept
HYPOXIC-ISCHEMIC ENCEPHALOPATHY	hypoxic ischemic encephalopathy	hypoxic ischemic encephalopathy	hypoxic ischemic encephalopathy	hypoxic ischemic encephalopathy	1	case_or_format
hysterotomy	hysterotomy	hysterotomy	hysterotomy	hysterotomy	1	kept
Hématurie	h maturie	h maturie	h maturie	Hématurie	1	kept
hémorragie	h morragie	h morragie	h morragie	hémorragie	1	kept
hépatectomie	h patectomie	h patectomie	h patectomie	hépatectomie	1	kept
Höpital de l´ Université de l’ Alberta	h pital de l universit de l alberta	h pital de l universit de l alberta	h pital de l universit de l alberta	Höpital de l´ Université de l’ Alberta	1	kept
hôpital modulaire	h pital modulaire	h pital modulaire	h pital modulaire	hôpital modulaire	1	kept
I TOUCH	i touch	i touch	i touch	I TOUCH	1	kept
I-PASS	i pass	i pass	i pass	I-PASS	1	kept
i-Stat	i stat	i stat	i stat	i-Stat	1	kept

i.v. piggyback	i v piggyback	i v piggyback	i v piggyback	i.v. piggyback	1	kept
i.v. push	i v push	i v push	i v push	i.v. push	1	kept
I00	i00	i00	i00	I00	1	kept
I10	i10	i10	i10	I10	1	kept
I11	i11	i11	i11	I11	1	kept
I12	i12	i12	i12	I12	1	kept
IAD	iad	iad	iad	IAD	1	kept
IAP	iap	iap	iap	IAP	1	kept
Iatrogenic anaemia	iatrogenic anaemia	iatrogenic anaemia	iatrogenic anaemia	Iatrogenic anaemia	1	kept
Iatrogenic bleeding	iatrogenic bleeding	iatrogenic bleeding	iatrogenic bleeding	Iatrogenic bleeding	1	kept
iatrogenic blood loss	iatrogenic blood loss	iatrogenic blood loss	iatrogenic blood loss	iatrogenic blood loss	1	kept
iatrogenic harm	iatrogenic harm	iatrogenic harm	iatrogenic harm	iatrogenic harm	1	kept
Iatrogenic injury	iatrogenic injury	iatrogenic injury	iatrogenic injury	Iatrogenic injury	1	kept
iatrogenic severe hypoglycemia	iatrogenic severe hypoglycemia	iatrogenic severe hypoglycemia	iatrogenic severe hypoglycemia	iatrogenic severe hypoglycemia	1	kept
Iatrogenic withdrawal	iatrogenic withdrawal	iatrogenic withdrawal	iatrogenic withdrawal	Iatrogenic withdrawal	1	kept
iatrogenic withdrawal	iatrogenic withdrawal	iatrogenic withdrawal	iatrogenic withdrawal	Iatrogenic withdrawal	1	case_or_format

IBI	ibi	ibi	ibi	IBI	1	kept
IBUPROFEN	ibuprofen	ibuprofen	ibuprofen	ibuprofen	1	case_or_format
Ibuprofen	ibuprofen	ibuprofen	ibuprofen	ibuprofen	1	case_or_format
iburamin cold syrup	iburamin cold syrup	iburamin cold syrup	iburamin cold syrup	iburamin cold syrup	1	kept
Ibutilide	ibutilide	ibutilide	ibutilide	ibutilide	1	case_or_format
ICC: intraclass correlation coefficient	icc intraclass correlation coefficient	icc intraclass correlation coefficient	icc intraclass correlation coefficient	ICC: intraclass correlation coefficient	1	kept
ICCU	iccu	iccu	iccu	ICCU	1	kept
ICD deactivation	icd deactivation	icd deactivation	icd deactivation	ICD deactivation	1	kept
ICD therapy	icd therapy	icd therapy	icd therapy	ICD therapy	1	kept
ICD-10-PCS	icd 10 pcs	icd 10 pcs	icd 10 pcs	ICD-10-PCS	1	kept
ICD-9 code group prediction	icd 9 code group prediction	icd 9 code group prediction	icd 9 code group prediction	ICD-9 code group prediction	1	kept
ICD-classification	icd classification	icd classification	icd classification	ICD-classification	1	kept
ICD-O-3	icd o 3	icd o 3	icd o 3	ICD-O-3	1	kept
Ice pack	ice pack	ice pack	ice pack	Ice pack	1	kept
Ice-cold water	ice cold water	ice cold water	ice cold water	Ice-cold water	1	kept
ICF code	icf code	icf code	icf code	ICF code	1	kept

ICH	ich	ich	ich	ICH	1	kept
Ichthyosis	ichthyosis	ichthyosis	ichthyosis	ichthyosis	1	case_or_format
ICNA	icna	icna	icna	ICNA	1	kept
ICNARC	icnarc	icnarc	icnarc	ICNARC	1	kept
icon	icon	icon	icon	Icon	1	case_or_format
ICP	icp	icp	icp	ICP	1	kept
icp monitoring	icp monitoring	icp monitoring	icp monitoring	ICP monitoring	1	case_or_format
ICP, interactive care plan	icp interactive care plan	icp interactive care plan	icp interactive care plan	ICP, interactive care plan	1	kept
ICT	ict	ict	ict	ICT	1	kept
ID consultation	id consultation	id consultation	id consultation	ID consultation	1	kept
idarubicin	idarubicin	idarubicin	idarubicin	idarubicin	1	kept
Idarucizumab	idarucizumab	idarucizumab	idarucizumab	idarucizumab	1	case_or_format
IDEATION	ideation	ideation	ideation	IDEATION	1	kept
Identifiability	identifiability	identifiability	identifiability	Identifiability	1	kept
Identification accuracy	identification accuracy	identification accuracy	identification accuracy	Identification accuracy	1	kept
identification key	identification key	identification key	identification key	identification key	1	kept

identification method	identification method	identification method	identification method	identification method	1	kept
Identification, Psychological	identification psychological	identification psychological	identification psychological	Identification, Psychological	1	kept
IDENTIFY	identify	identify	identify	IDENTIFY	1	kept
IDENTIFYING CHILDREN	identifying children	identifying children	identifying children	IDENTIFYING CHILDREN	1	kept
IDENTITIES	identities	identities	identity	identity	1	singular
IDENTITY	identity	identity	identity	identity	1	case_or_format
Identity information	identity information	identity information	identity information	Identity information	1	kept
ideology	ideology	ideology	ideology	ideology	1	kept
idiopathic pulmonary fibrosis cell line	idiopathic pulmonary fibrosis cell line	idiopathic pulmonary fibrosis cell line	idiopathic pulmonary fibrosis cell line	idiopathic pulmonary fibrosis cell line	1	kept
idiopathic pulmonary thromboembolism	idiopathic pulmonary thromboembolism	idiopathic pulmonary thromboembolism	idiopathic pulmonary thromboembolism	idiopathic pulmonary thromboembolism	1	kept
idiopathic scoliosis	idiopathic scoliosis	idiopathic scoliosis	idiopathic scoliosis	idiopathic scoliosis	1	kept
Idiopathic tracheal stenosis	idiopathic tracheal stenosis	idiopathic tracheal stenosis	idiopathic tracheal stenosis	Idiopathic tracheal stenosis	1	kept
IDM	idm	idm	idm	IDM	1	kept
idoxuridine	idoxuridine	idoxuridine	idoxuridine	idoxuridine	1	kept
IFIC	ific	ific	ific	IFIC	1	kept
IgE	ige	ige	ige	IgE	1	kept

IgE antibodies	ige antibodies	ige antibodies	ige antibodies	IgE antibodies	1	kept
IgG	igg	igg	igg	IgG	1	kept
Igh-performance work systems	igh performance work systems	igh performance work systems	igh performance work systems	Igh-performance work systems	1	kept
IgM	igm	igm	igm	IgM	1	kept
Ignatzschineria larvae	ignatzschineria larvae	ignatzschineria larvae	ignatzschineria larvae	Ignatzschineria larvae	1	kept
Ignatzschineria larvae sepsis	ignatzschineria larvae sepsis	ignatzschineria larvae sepsis	ignatzschineria larvae sepsis	Ignatzschineria larvae sepsis	1	kept
Ignatzschineria ureiclastica	ignatzschineria ureiclastica	ignatzschineria ureiclastica	ignatzschineria ureiclastica	Ignatzschineria ureiclastica	1	kept
IGRA	igra	igra	igra	IGRA	1	kept
IHD	ihd	ihd	ihd	IHD	1	kept
IHI - Institute Healthcare for Improvement	ihi institute healthcare for improvement	ihi institute healthcare for improvement	ihi institute healthcare for improvement	IHI - Institute Healthcare for Improvement	1	kept
IIQ-7	iiq 7	iiq 7	iiq 7	IIQ-7	1	kept
IL-6	il 6	il 6	il 6	IL-6	1	kept
IL-8	il 8	il 8	il 8	IL-8	1	kept
ILCOR guidelines	ilcor guidelines	ilcor guidelines	ilcor guidelines	ILCOR guidelines	1	kept
ileal perforation	ileal perforation	ileal perforation	ileal perforation	ileal perforation	1	kept
ileal pouch-anal anastomosis	ileal pouch anal anastomosis	ileal pouch anal anastomosis	ileal pouch anal anastomosis	ileal pouch-anal anastomosis	1	kept

ileocecectomy	ileocecectomy	ileocecectomy	ileocecectomy	ileocecectomy	1	kept
ileorectal anastomosis	ileorectal anastomosis	ileorectal anastomosis	ileorectal anastomosis	ileorectal anastomosis	1	kept
Ileum	ileum	ileum	ileum	Ileum	1	kept
ILI: influenza-like illness	ili influenza like illness	ili influenza like illness	ili influenza like illness	ILI: influenza-like illness	1	kept
Iliac Artery	iliac artery	iliac artery	iliac artery	iliac artery	1	case_or_format
iliac vein	iliac vein	iliac vein	iliac vein	iliac vein	1	kept
iliopsoas muscle	iliopsoas muscle	iliopsoas muscle	iliopsoas muscle	iliopsoas muscle	1	kept
ILL HOSPITALIZED ADULTS	ill hospitalized adults	ill hospitalized adults	ill hospitalized adults	ILL HOSPITALIZED ADULTS	1	kept
Ill infants	ill infants	ill infants	ill infants	Ill infants	1	kept
ill lighting syndrome	ill lighting syndrome	ill lighting syndrome	ill lighting syndrome	ill lighting syndrome	1	kept
Illegible prescription	illegible prescription	illegible prescription	illegible prescription	Illegible prescription	1	kept
Illegitimate tasks	illegitimate tasks	illegitimate tasks	illegitimate tasks	Illegitimate tasks	1	kept
illicit drug use	illicit drug use	illicit drug use	illicit drug use	illicit drug use	1	kept
illicit drugs	illicit drugs	illicit drugs	illicit drug	illicit drug	1	singular
Illinois Perinatal Quality Collaborative	illinois perinatal quality collaborative	illinois perinatal quality collaborative	illinois perinatal quality collaborative	Illinois Perinatal Quality Collaborative	1	kept
Illness beliefs	illness beliefs	illness beliefs	illness beliefs	Illness beliefs	1	kept

Illness blogs	illness blogs	illness blogs	illness blogs	Illness blogs	1	kept
illness burden	illness burden	illness burden	illness burden	illness burden	1	kept
illness communication	illness communication	illness communication	illness communication	illness communication	1	kept
Illness experience	illness experience	illness experience	illness experience	Illness experience	1	kept
ILLNESS PERCEPTION	illness perception	illness perception	illness perception	illness perception	1	case_or_format
ILLNESS RATING-SCALE	illness rating scale	illness rating scale	illness rating scale	ILLNESS RATING-SCALE	1	kept
Illness representations	illness representations	illness representations	illness representations	Illness representations	1	kept
Illness severity	illness severity	illness severity	illness severity	Illness severity	1	kept
Illness-severity	illness severity	illness severity	illness severity	Illness severity	1	case_or_format
ILLNESSES	illnesses	illnesses	illnesses	ILLNESSES	1	kept
illumina sequencing	illumina sequencing	illumina sequencing	illumina sequencing	illumina sequencing	1	kept
illustration	illustration	illustration	illustration	illustration	1	kept
image acquisition	image acquisition	image acquisition	image acquisition	image acquisition	1	kept
Image analysis	image analysis	image analysis	image analysis	image analysis	1	case_or_format
Image enhancement	image enhancement	image enhancement	image enhancement	Image Enhancement	1	case_or_format
image enhancement	image enhancement	image enhancement	image enhancement	Image Enhancement	1	case_or_format

image guided radiotherapy	image guided radiotherapy	image guided radiotherapy	image guided radiotherapy	image guided radiotherapy	1	kept
image intensifier	image intensifier	image intensifier	image intensifier	image intensifier	1	kept
Image interpretation	image interpretation	image interpretation	image interpretation	Image interpretation	1	kept
Image interpretation checklist	image interpretation checklist	image interpretation checklist	image interpretation checklist	Image interpretation checklist	1	kept
Image processing	image processing	image processing	image processing	image processing	1	case_or_format
image reconstruction	image reconstruction	image reconstruction	image reconstruction	Image reconstruction	1	case_or_format
Image resolution	image resolution	image resolution	image resolution	Image resolution	1	kept
Image segmentation	image segmentation	image segmentation	image segmentation	image segmentation	1	case_or_format
Image super-resolution reconstruction	image super resolution reconstruction	image super resolution reconstruction	image super resolution reconstruction	Image super-resolution reconstruction	1	kept
Imagination	imagination	imagination	imagination	imagination	1	case_or_format
imaging and display	imaging and display	imaging and display	imaging and display	imaging and display	1	kept
imaging device	imaging device	imaging device	imaging device	imaging device	1	kept
Imaging of stroke complications of stroke	imaging of stroke complications of stroke	imaging of stroke complications of stroke	imaging of stroke complications of stroke	Imaging of stroke complications of stroke	1	kept
imaging software	imaging software	imaging software	imaging software	imaging software	1	kept
Imaging Test	imaging test	imaging test	imaging test	Imaging Test	1	kept
imaging to needle time	imaging to needle time	imaging to needle time	imaging to needle time	imaging to needle time	1	kept

Imaging, three-dimensional	imaging three dimensional	imaging three dimensional	imaging three dimensional	Imaging, Three-Dimensional	1	case_or_format
imatinib	imatinib	imatinib	imatinib	imatinib	1	kept
Imatinib therapy	imatinib therapy	imatinib therapy	imatinib therapy	Imatinib therapy	1	kept
IMG = international medical graduate	img international medical graduate	img international medical graduate	img international medical graduate	IMG = international medical graduate	1	kept
Imidazoles	imidazoles	imidazoles	imidazoles	Imidazoles	1	kept
Immaturity	immaturity	immaturity	immaturity	immaturity	1	case_or_format
IMMEDIATE	immediate	immediate	immediate	IMMEDIATE	1	kept
Immediate environment	immediate environment	immediate environment	immediate environment	Immediate environment	1	kept
Immediate extubation	immediate extubation	immediate extubation	immediate extubation	Immediate extubation	1	kept
Immediate hypersensitivity reactions	immediate hypersensitivity reactions	immediate hypersensitivity reactions	immediate hypersensitivity reactions	Immediate hypersensitivity reactions	1	kept
Immediate life support	immediate life support	immediate life support	immediate life support	Immediate life support	1	kept
Immediate Life Support course	immediate life support course	immediate life support course	immediate life support course	Immediate Life Support course	1	kept
Immediate outcome	immediate outcome	immediate outcome	immediate outcome	Immediate outcome	1	kept
Immediate post-operative period	immediate post operative period	immediate post operative period	immediate post operative period	Immediate post-operative period	1	kept
immediate reactions	immediate reactions	immediate reactions	immediate reactions	immediate reactions	1	kept
Immediate-Early Proteins	immediate early proteins	immediate early proteins	immediate early proteins	Immediate-Early Proteins	1	kept

Immersive education	immersive education	immersive education	immersive education	Immersive education	1	kept
Immersive virtual environments	immersive virtual environments	immersive virtual environments	immersive virtual environments	Immersive virtual environments	1	kept
Immigrant	immigrant	immigrant	immigrant	immigrant	1	case_or_format
immigrants	immigrants	immigrants	immigrant	immigrant	1	singular
Immigration	immigration	immigration	immigration	immigration	1	case_or_format
immigration detention	immigration detention	immigration detention	immigration detention	immigration detention	1	kept
IMMINENT DEATH	imminent death	imminent death	imminent death	IMMINENT DEATH	1	kept
imminent death	imminent death	imminent death	imminent death	IMMINENT DEATH	1	case_or_format
IMMINENT VIOLENCE	imminent violence	imminent violence	imminent violence	IMMINENT VIOLENCE	1	kept
Immobility	immobility	immobility	immobility	immobility	1	case_or_format
Immobility complications	immobility complications	immobility complications	immobility complications	Immobility complications	1	kept
immobilization stress	immobilization stress	immobilization stress	immobilization stress	immobilization stress	1	kept
Immune	immune	immune	immune	Immune	1	kept
Immune dysfunction	immune dysfunction	immune dysfunction	immune dysfunction	Immune dysfunction	1	kept
immune dysregulation	immune dysregulation	immune dysregulation	immune dysregulation	immune dysregulation	1	kept
immune effector cell associated neurotoxicity syndrome	immune effector cell associated neurotoxicity syndrome	immune effector cell associated neurotoxicity syndrome	immune effector cell associated neurotoxicity syndrome	immune effector cell associated neurotoxicity syndrome	1	kept

Immune function	immune function	immune function	immune function	Immune function	1	kept
immune function	immune function	immune function	immune function	Immune function	1	case_or_format
Immune globulin	immune globulin	immune globulin	immune globulin	Immune globulin	1	kept
immune responses	immune responses	immune responses	immune response	immune response	1	singular
immune signatures	immune signatures	immune signatures	immune signatures	immune signatures	1	kept
IMMUNE SUPPRESSION	immune suppression	immune suppression	immune suppression	IMMUNE SUPPRESSION	1	kept
IMMUNE-RESPONSE	immune response	immune response	immune response	immune response	1	case_or_format
Immunisation	immunisation	immunisation	immunisation	Immunisation	1	kept
immunisation	immunisation	immunisation	immunisation	Immunisation	1	case_or_format
Immunisation programmes	immunisation programmes	immunisation programmes	immunisation programmes	Immunisation programmes	1	kept
Immunity, Humoral	immunity humoral	immunity humoral	immunity humoral	Immunity, Humoral	1	kept
immunization error	immunization error	immunization error	immunization error	immunization error	1	kept
Immunization rates	immunization rates	immunization rates	immunization rates	Immunization rates	1	kept
Immunization, Passive	immunization passive	immunization passive	immunization passive	Immunization, Passive	1	kept
immunoadsorption	immunoadsorption	immunoadsorption	immunoadsorption	immunoadsorption	1	kept
immunoaffinity chromatography	immunoaffinity chromatography	immunoaffinity chromatography	immunoaffinity chromatography	immunoaffinity chromatography	1	kept

Immunoassay	immunoassay	immunoassay	immunoassay	immunoassay	1	case_or_format
Immunochemistry	immunochemistry	immunochemistry	immunochemistry	Immunochemistry	1	kept
immunochemotherapy	immunochemotherapy	immunochemotherapy	immunochemotherapy	immunochemotherapy	1	kept
immunocompetent cell	immunocompetent cell	immunocompetent cell	immunocompetent cell	immunocompetent cell	1	kept
immunocompromised	immunocompromised	immunocompromised	immunocompromised	Immunocompromised	1	case_or_format
immunodiffusion	immunodiffusion	immunodiffusion	immunodiffusion	immunodiffusion	1	kept
Immunoenzyme Techniques	immunoenzyme techniques	immunoenzyme techniques	immunoenzyme techniques	Immunoenzyme Techniques	1	kept
immunofluorescence	immunofluorescence	immunofluorescence	immunofluorescence	immunofluorescence	1	kept
immunofluorescence assay	immunofluorescence assay	immunofluorescence assay	immunofluorescence assay	immunofluorescence assay	1	kept
immunofluorescence test	immunofluorescence test	immunofluorescence test	immunofluorescence test	immunofluorescence test	1	kept
Immunoglobulin	immunoglobulin	immunoglobulin	immunoglobulin	immunoglobulin	1	case_or_format
immunoglobulin A antibody	immunoglobulin a antibody	immunoglobulin a antibody	immunoglobulin a antibody	immunoglobulin A antibody	1	kept
immunoglobulin A deficiency	immunoglobulin a deficiency	immunoglobulin a deficiency	immunoglobulin a deficiency	immunoglobulin A deficiency	1	kept
immunoglobulin A nephropathy	immunoglobulin a nephropathy	immunoglobulin a nephropathy	immunoglobulin a nephropathy	immunoglobulin A nephropathy	1	kept
immunoglobulin A paraproteinemia	immunoglobulin a paraproteinemia	immunoglobulin a paraproteinemia	immunoglobulin a paraproteinemia	immunoglobulin A paraproteinemia	1	kept
immunoglobulin E receptor	immunoglobulin e receptor	immunoglobulin e receptor	immunoglobulin e receptor	immunoglobulin E receptor	1	kept

immunoglobulin F fragment	immunoglobulin f fragment	immunoglobulin f fragment	immunoglobulin f fragment	immunoglobulin F fragment	1	kept
Immunoglobulin Fab Fragments	immunoglobulin fab fragments	immunoglobulin fab fragments	immunoglobulin fab fragments	Immunoglobulin Fab Fragments	1	kept
immunoglobulin G1	immunoglobulin g1	immunoglobulin g1	immunoglobulin g1	immunoglobulin G1	1	kept
immunoglobulin G4 related disease	immunoglobulin g4 related disease	immunoglobulin g4 related disease	immunoglobulin g4 related disease	immunoglobulin G4 related disease	1	kept
Immunoglobulin Light-chain Amyloidosis	immunoglobulin light chain amyloidosis	immunoglobulin light chain amyloidosis	immunoglobulin light chain amyloidosis	Immunoglobulin Light-chain Amyloidosis	1	kept
Immunologic	immunologic	immunologic	immunologic	Immunologic	1	kept
Immunologic Tests	immunologic tests	immunologic tests	immunologic tests	Immunologic Tests	1	kept
immunological consequences	immunological consequences	immunological consequences	immunological consequences	immunological consequences	1	kept
immunological parameters	immunological parameters	immunological parameters	immunological parameters	immunological parameters	1	kept
immunological procedures	immunological procedures	immunological procedures	immunological procedures	immunological procedures	1	kept
immunological products and vaccines	immunological products and vaccines	immunological products and vaccines	immunological products and vaccines	immunological products and vaccines	1	kept
Immunomodulatory drugs	immunomodulatory drugs	immunomodulatory drugs	immunomodulatory drugs	Immunomodulatory drugs	1	kept
immunonutrition	immunonutrition	immunonutrition	immunonutrition	immunonutrition	1	kept
Immunoparalysis	immunoparalysis	immunoparalysis	immunoparalysis	Immunoparalysis	1	kept
Immunophenotyping	immunophenotyping	immunophenotyping	immunophenotyping	immunophenotyping	1	case_or_format
immunoreactivity	immunoreactivity	immunoreactivity	immunoreactivity	immunoreactivity	1	kept

Immunosenescence	immunosenescence	immunosenescence	immunosenescence	immunosenescence	1	case_or_format
immunostimulating agent	immunostimulating agent	immunostimulating agent	immunostimulating agent	immunostimulating agent	1	kept
immunosuppressed	immunosuppressed	immunosuppressed	immunosuppressed	immunosuppressed	1	kept
Immunosuppressive therapy	immunosuppressive therapy	immunosuppressive therapy	immunosuppressive therapy	Immunosuppressive therapy	1	kept
Immunotherapy, adoptive	immunotherapy adoptive	immunotherapy adoptive	immunotherapy adoptive	Immunotherapy, Adoptive	1	case_or_format
immunoturbidimetry	immunoturbidimetry	immunoturbidimetry	immunoturbidimetry	immunoturbidimetry	1	kept
immunsuppression catabolism syndrome	immunsuppression catabolism syndrome	immunsuppression catabolism syndrome	immunsuppression catabolism syndrome	immunsuppression catabolism syndrome	1	kept
IMP-8	imp 8	imp 8	imp 8	IMP-8	1	kept
IMP13 protein	imp13 protein	imp13 protein	imp13 protein	IMP13 protein	1	kept
Impact Event Scale Revisited	impact event scale revisited	impact event scale revisited	impact event scale revisited	Impact Event Scale Revisited	1	kept
impact of compassion	impact of compassion	impact of compassion	impact of compassion	impact of compassion	1	kept
Impact of life event scale- Revised questionnaire	impact of life event scale revised questionnaire	impact of life event scale revised questionnaire	impact of life event scale revised questionnaire	Impact of life event scale- Revised questionnaire	1	kept
impact on public welfare	impact on public welfare	impact on public welfare	impact on public welfare	impact on public welfare	1	kept
impaired bladder emptying	impaired bladder emptying	impaired bladder emptying	impaired bladder emptying	impaired bladder emptying	1	kept
Impaired Practice	impaired practice	impaired practice	impaired practice	Impaired Practice	1	kept
impaired practice	impaired practice	impaired practice	impaired practice	Impaired Practice	1	case_or_format

impaired professionals	impaired professionals	impaired professionals	impaired professionals	impaired professionals	1	kept
Impaired subjects	impaired subjects	impaired subjects	impaired subjects	Impaired subjects	1	kept
Impaired uteroplacental perfusion	impaired uteroplacental perfusion	impaired uteroplacental perfusion	impaired uteroplacental perfusion	Impaired uteroplacental perfusion	1	kept
Impedance cardiography	impedance cardiography	impedance cardiography	impedance cardiography	impedance cardiography	1	case_or_format
impedance plethysmography	impedance plethysmography	impedance plethysmography	impedance plethysmography	impedance plethysmography	1	kept
Impella CP Smart Assist	impella cp smart assist	impella cp smart assist	impella cp smart assist	Impella CP Smart Assist	1	kept
Impetigo	impetigo	impetigo	impetigo	impetigo	1	case_or_format
impetigo bullosa	impetigo bullosa	impetigo bullosa	impetigo bullosa	impetigo bullosa	1	kept
Implant infection	implant infection	implant infection	implant infection	Implant infection	1	kept
implant stenosis	implant stenosis	implant stenosis	implant stenosis	implant stenosis	1	kept
implantable clip	implantable clip	implantable clip	implantable clip	implantable clip	1	kept
Implantable hemodynamic monitoring	implantable hemodynamic monitoring	implantable hemodynamic monitoring	implantable hemodynamic monitoring	Implantable hemodynamic monitoring	1	kept
implantable port system	implantable port system	implantable port system	implantable port system	implantable port system	1	kept
Implantación de prótesis de válvulas cardíacas	implantaci n de pr tesis de v lvulas card acas	implantaci n de pr tesis de v lvulas card acas	implantaci n de pr tesis de v lvulas card acas	Implantación de prótesis de válvulas cardíacas	1	kept
IMPLANTATION	implantation	implantation	implantation	implantation	1	case_or_format
Implantation sequence	implantation sequence	implantation sequence	implantation sequence	Implantation sequence	1	kept

Implanted cardiac device	implanted cardiac device	implanted cardiac device	implanted cardiac device	Implanted cardiac device	1	kept
Implementation and deployment	implementation and deployment	implementation and deployment	implementation and deployment	implementation and deployment	1	case_or_format
implementation barriers	implementation barriers	implementation barriers	implementation barriers	implementation barriers	1	kept
Implementation Climate Scale	implementation climate scale	implementation climate scale	implementation climate scale	Implementation Climate Scale	1	kept
IMPLEMENTATION FIDELITY	implementation fidelity	implementation fidelity	implementation fidelity	IMPLEMENTATION FIDELITY	1	kept
implementation frameworks	implementation frameworks	implementation frameworks	implementation frameworks	implementation frameworks	1	kept
Implementation guideline	implementation guideline	implementation guideline	implementation guideline	Implementation guideline	1	kept
implementation guidelines	implementation guidelines	implementation guidelines	implementation guideline	Implementation guideline	1	singular
Implementation of pediatric anesthesia in Africa	implementation of pediatric anesthesia in africa	implementation of pediatric anesthesia in africa	implementation of pediatric anesthesia in africa	Implementation of pediatric anesthesia in Africa	1	kept
implementation of research to practice	implementation of research to practice	implementation of research to practice	implementation of research to practice	implementation of research to practice	1	kept
implementation practice	implementation practice	implementation practice	implementation practice	implementation practice	1	kept
Implementation research	implementation research	implementation research	implementation research	implementation research	1	case_or_format
implementation status	implementation status	implementation status	implementation status	implementation status	1	kept
Implementation strategy	implementation strategy	implementation strategy	implementation strategy	Implementation strategies	1	case_or_format
implementation strategy	implementation strategy	implementation strategy	implementation strategy	Implementation strategies	1	case_or_format
Implementation, recovery of function	implementation recovery of function	implementation recovery of function	implementation recovery of function	Implementation, recovery of function	1	kept

IMPLEMENTING EVIDENCE	implementing evidence	implementing evidence	implementing evidence	IMPLEMENTING EVIDENCE	1	kept
Implications	implications	implications	implications	implications	1	case_or_format
implicit	implicit	implicit	implicit	implicit	1	kept
Implicit absenteeism	implicit absenteeism	implicit absenteeism	implicit absenteeism	Implicit absenteeism	1	kept
Implicit bias	implicit bias	implicit bias	implicit bias	implicit bias	1	case_or_format
implicit bias mitigation strategies	implicit bias mitigation strategies	implicit bias mitigation strategies	implicit bias mitigation strategies	implicit bias mitigation strategies	1	kept
implicit bias recognition and management	implicit bias recognition and management	implicit bias recognition and management	implicit bias recognition and management	implicit bias recognition and management	1	kept
Implicit rationing	implicit rationing	implicit rationing	implicit rationing	Implicit rationing	1	kept
Implosive Therapy	implosive therapy	implosive therapy	implosive therapy	Implosive Therapy	1	kept
importance of clear communication with medical provider	importance of clear communication with medical provider	importance of clear communication with medical provider	importance of clear communication with medical provider	importance of clear communication with medical provider	1	kept
IMPORTANT DIFFERENCE	important difference	important difference	important difference	IMPORTANT DIFFERENCE	1	kept
impostor phenomenon	impostor phenomenon	impostor phenomenon	impostor phenomenon	impostor phenomenon	1	kept
impotence	impotence	impotence	impotence	impotence	1	kept
impregnated sponge	impregnated sponge	impregnated sponge	impregnated sponge	impregnated sponge	1	kept
improper dose	improper dose	improper dose	improper dose	improper dose	1	kept
improper dose error	improper dose error	improper dose error	improper dose error	improper dose error	1	kept

IMPROVE DOCUMENTATION	improve documentation	improve documentation	improve documentation	IMPROVE DOCUMENTATION	1	kept
IMPROVE OUTCOMES	improve outcomes	improve outcomes	improve outcomes	IMPROVE OUTCOMES	1	kept
IMPROVE QUALITY	improve quality	improve quality	improve quality	IMPROVE QUALITY	1	kept
Improved clinical outcomes	improved clinical outcomes	improved clinical outcomes	improved clinical outcomes	Improved clinical outcomes	1	kept
improved communication	improved communication	improved communication	improved communication	improved communication	1	kept
Improved designs	improved designs	improved designs	improved designs	Improved designs	1	kept
Improved early warning score	improved early warning score	improved early warning score	improved early warning score	Improved early warning score	1	kept
Improved ETT cuff pressure measurement procedure	improved ett cuff pressure measurement procedure	improved ett cuff pressure measurement procedure	improved ett cuff pressure measurement procedure	Improved ETT cuff pressure measurement procedure	1	kept
improved outcomes	improved outcomes	improved outcomes	improved outcomes	Improved outcomes	1	case_or_format
IMPROVEMENT SCIENCE	improvement science	improvement science	improvement science	IMPROVEMENT SCIENCE	1	kept
Improvement Science	improvement science	improvement science	improvement science	IMPROVEMENT SCIENCE	1	case_or_format
improvement science	improvement science	improvement science	improvement science	IMPROVEMENT SCIENCE	1	case_or_format
Improvement, quality	improvement quality	improvement quality	improvement quality	Improvement, quality	1	kept
Improvements	improvements	improvements	improvement	IMPROVEMENT	1	singular
Improving care	improving care	improving care	improving care	Improving care	1	kept
IMPROVING COMMUNICATION	improving communication	improving communication	improving communication	IMPROVING COMMUNICATION	1	kept

IMPROVING HEAD GROWTH	improving head growth	improving head growth	improving head growth	IMPROVING HEAD GROWTH	1	kept
Improving outcomes	improving outcomes	improving outcomes	improving outcomes	Improving outcomes	1	kept
improving safety culture	improving safety culture	improving safety culture	improving safety culture	improving safety culture	1	kept
improvisation	improvisation	improvisation	improvisation	improvisation	1	kept
Impulse Response	impulse response	impulse response	impulse response	Impulse Response	1	kept
Impulsive sounds	impulsive sounds	impulsive sounds	impulsive sounds	Impulsive sounds	1	kept
in depth interview	in depth interview	in depth interview	in depth interview	In-depth interview	1	case_or_format
In hospital cardiac arrest	in hospital cardiac arrest	in hospital cardiac arrest	in hospital cardiac arrest	In-hospital cardiac arrest	1	case_or_format
in hospital fellow coverage	in hospital fellow coverage	in hospital fellow coverage	in hospital fellow coverage	in hospital fellow coverage	1	kept
In hospital mortality	in hospital mortality	in hospital mortality	in hospital mortality	in-hospital mortality	1	case_or_format
In Hospital Setting	in hospital setting	in hospital setting	in hospital setting	In Hospital Setting	1	kept
in hospital stroke	in hospital stroke	in hospital stroke	in hospital stroke	in hospital stroke	1	kept
In service training	in service training	in service training	in service training	in service training	1	case_or_format
In silico intervention	in silico intervention	in silico intervention	in silico intervention	In silico intervention	1	kept
in situ	in situ	in situ	in situ	In situ	1	case_or_format
In situ processing	in situ processing	in situ processing	in situ processing	In situ processing	1	kept

In Situ Simulation	in situ simulation	in situ simulation	in situ simulation	in situ simulation	1	case_or_format
in situ simulation training	in situ simulation training	in situ simulation training	in situ simulation training	in situ simulation training	1	kept
In situ training	in situ training	in situ training	in situ training	In situ training	1	kept
in transport mortality	in transport mortality	in transport mortality	in transport mortality	in transport mortality	1	kept
in vitro fertilisation	in vitro fertilisation	in vitro fertilisation	in vitro fertilisation	in vitro fertilisation	1	kept
In vitro fertilization-embryo transfer	in vitro fertilization embryo transfer	in vitro fertilization embryo transfer	in vitro fertilization embryo transfer	In vitro fertilization-embryo transfer	1	kept
In Vitro Techniques	in vitro techniques	in vitro techniques	in vitro techniques	In Vitro Techniques	1	kept
In vivo microscopy	in vivo microscopy	in vivo microscopy	in vivo microscopy	In vivo microscopy	1	kept
In-arms-holding	in arms holding	in arms holding	in arms holding	In-arms-holding	1	kept
In-Degree	in degree	in degree	in degree	In-Degree	1	kept
In-depth interviews	in depth interviews	in depth interviews	in depth interview	In-depth interview	1	singular
IN-HOME CARE	in home care	in home care	in home care	IN-HOME CARE	1	kept
in-hospital	in hospital	in hospital	in hospital	In-hospital	1	case_or_format
In-hospital arrest	in hospital arrest	in hospital arrest	in hospital arrest	In-hospital arrest	1	kept
In-Hospital Cardiac Arrests	in hospital cardiac arrests	in hospital cardiac arrests	in hospital cardiac arrest	In-hospital cardiac arrest	1	singular
in-hospital cost	in hospital cost	in hospital cost	in hospital cost	in-hospital cost	1	kept

In-hospital CPR	in hospital cpr	in hospital cpr	in hospital cpr	In-hospital CPR	1	kept
in-hospital diabetes	in hospital diabetes	in hospital diabetes	in hospital diabetes	in-hospital diabetes	1	kept
in-hospital falls	in hospital falls	in hospital falls	in hospital falls	in-hospital falls	1	kept
In-hospital Mortality	in hospital mortality	in hospital mortality	in hospital mortality	in-hospital mortality	1	case_or_format
In-hospital outcome	in hospital outcome	in hospital outcome	in hospital outcome	In-hospital outcome	1	kept
In-hospital Stroke	in hospital stroke	in hospital stroke	in hospital stroke	in hospital stroke	1	case_or_format
in-hospital stroke	in hospital stroke	in hospital stroke	in hospital stroke	in hospital stroke	1	case_or_format
In-hospital survivals	in hospital survivals	in hospital survivals	in hospital survivals	In-hospital survivals	1	kept
in-hospital transport	in hospital transport	in hospital transport	in hospital transport	in-hospital transport	1	kept
In-person interpreting	in person interpreting	in person interpreting	in person interpreting	In-person interpreting	1	kept
In-service	in service	in service	in service	in-service	1	case_or_format
In-service courses	in service courses	in service courses	in service courses	In-service courses	1	kept
IN-SITU	in situ	in situ	in situ	In situ	1	case_or_format
In-Situ	in situ	in situ	in situ	In situ	1	case_or_format
In-situ simulation	in situ simulation	in situ simulation	in situ simulation	in situ simulation	1	case_or_format
In-situ simulation training	in situ simulation training	in situ simulation training	in situ simulation training	in situ simulation training	1	case_or_format

in-stent restenosis	in stent restenosis	in stent restenosis	in stent restenosis	in-stent restenosis	1	kept
IN-UTERO EXPOSURE	in utero exposure	in utero exposure	in utero exposure	IN-UTERO EXPOSURE	1	kept
IN-VITRO	in vitro	in vitro	in vitro	IN-VITRO	1	kept
Inadequate resources	inadequate resources	inadequate resources	inadequate resources	Inadequate resources	1	kept
inadequate volume	inadequate volume	inadequate volume	inadequate volume	inadequate volume	1	kept
Inappropriate	inappropriate	inappropriate	inappropriate	Inappropriate	1	kept
Inappropriate antibiotics	inappropriate antibiotics	inappropriate antibiotics	inappropriate antibiotics	Inappropriate antibiotics	1	kept
INAPPROPRIATE ANTIDIURETIC-HORMONE	inappropriate antidiuretic hormone	inappropriate antidiuretic hormone	inappropriate antidiuretic hormone	INAPPROPRIATE ANTIDIURETIC-HORMONE	1	kept
Inappropriate care	inappropriate care	inappropriate care	inappropriate care	inappropriate care	1	case_or_format
inappropriate levels of care	inappropriate levels of care	inappropriate levels of care	inappropriate levels of care	inappropriate levels of care	1	kept
Inappropriate treatment	inappropriate treatment	inappropriate treatment	inappropriate treatment	Inappropriate treatment	1	kept
INAPPROPRIATE USE	inappropriate use	inappropriate use	inappropriate use	INAPPROPRIATE USE	1	kept
inattention	inattention	inattention	inattention	Inattention	1	case_or_format
Inattentional blindness	inattentional blindness	inattentional blindness	inattentional blindness	Inattentional blindness	1	kept
inborn errors of metabolism	inborn errors of metabolism	inborn errors of metabolism	inborn errors of metabolism	inborn errors of metabolism	1	kept
Inbreeding	inbreeding	inbreeding	inbreeding	Inbreeding	1	kept

inbreeding	inbreeding	inbreeding	inbreeding	Inbreeding	1	case_or_format
incarceration	incarceration	incarceration	incarceration	incarceration	1	kept
Incentive	incentive	incentive	incentive	Incentive	1	kept
incentive	incentive	incentive	incentive	Incentive	1	case_or_format
Incentive Reimbursement	incentive reimbursement	incentive reimbursement	incentive reimbursement	Incentive Reimbursement	1	kept
Incentives	incentives	incentives	incentive	Incentive	1	singular
incentives	incentives	incentives	incentive	Incentive	1	singular
Incidence density	incidence density	incidence density	incidence density	Incidence density	1	kept
incidence density rate	incidence density rate	incidence density rate	incidence density rate	incidence density rate	1	kept
incidence density reduction	incidence density reduction	incidence density reduction	incidence density reduction	incidence density reduction	1	kept
incidence of delirium	incidence of delirium	incidence of delirium	incidence of delirium	incidence of delirium	1	kept
Incidence Rate	incidence rate	incidence rate	incidence rate	Incidence rates	1	case_or_format
incidence rate	incidence rate	incidence rate	incidence rate	Incidence rates	1	case_or_format
Incidence rate and mortality	incidence rate and mortality	incidence rate and mortality	incidence rate and mortality	Incidence rate and mortality	1	kept
incidence rate ratio	incidence rate ratio	incidence rate ratio	incidence rate ratio	incidence rate ratio	1	kept
Incidence study	incidence study	incidence study	incidence study	Incidence study	1	kept

Incident command system	incident command system	incident command system	incident command system	Incident command system	1	kept
Incident command systems	incident command systems	incident command systems	incident command system	Incident command system	1	singular
Incident management	incident management	incident management	incident management	Incident management	1	kept
Incident report	incident report	incident report	incident report	incident report	1	case_or_format
INCIDENT REPORTING SYSTEM	incident reporting system	incident reporting system	incident reporting system	Incident reporting system	1	case_or_format
INCIDENT REPORTS	incident reports	incident reports	incident report	incident report	1	singular
Incidents	incidents	incidents	incident	INCIDENTS	1	singular
Incidents errors	incidents errors	incidents errors	incidents errors	Incidents errors	1	kept
incision site	incision site	incision site	incision site	incision site	1	kept
incisional hernia	incisional hernia	incisional hernia	incisional hernia	incisional hernia	1	kept
incisor	incisor	incisor	incisor	incisor	1	kept
INCIVILITY	incivility	incivility	incivility	Incivility	1	case_or_format
INCLUSION	inclusion	inclusion	inclusion	inclusion	1	case_or_format
inclusivity	inclusivity	inclusivity	inclusivity	inclusivity	1	kept
income group	income group	income group	income group	income group	1	kept
Incompatibility	incompatibility	incompatibility	incompatibility	Incompatibility	1	kept

Incompatibility of Drugs	incompatibility of drugs	incompatibility of drugs	incompatibility of drugs	Incompatibility of Drugs	1	kept
incomplete spinal cord injury	incomplete spinal cord injury	incomplete spinal cord injury	incomplete spinal cord injury	incomplete spinal cord injury	1	kept
inconsistencies	inconsistencies	inconsistencies	inconsistency	INCONSISTENCY	1	singular
INCONSISTENCY	inconsistency	inconsistency	inconsistency	INCONSISTENCY	1	kept
Incontinence Associated Dermatitis and its Severity	incontinence associated dermatitis and its severity	incontinence associated dermatitis and its severity	incontinence associated dermatitis and its severity	Incontinence Associated Dermatitis and its Severity	1	kept
Incontinence Associated Dermatitis and its Severity score	incontinence associated dermatitis and its severity score	incontinence associated dermatitis and its severity score	incontinence associated dermatitis and its severity score	Incontinence Associated Dermatitis and its Severity score	1	kept
incontinence associated dermatitis invention tool	incontinence associated dermatitis invention tool	incontinence associated dermatitis invention tool	incontinence associated dermatitis invention tool	incontinence associated dermatitis invention tool	1	kept
INCONTINENCE CARE	incontinence care	incontinence care	incontinence care	INCONTINENCE CARE	1	kept
incontinence care	incontinence care	incontinence care	incontinence care	INCONTINENCE CARE	1	case_or_format
Incontinence Pads	incontinence pads	incontinence pads	incontinence pads	Incontinence Pads	1	kept
Incontinence Severity Index	incontinence severity index	incontinence severity index	incontinence severity index	Incontinence Severity Index	1	kept
Incontinenceassociated dermatitis	incontinenceassociated dermatitis	incontinenceassociated dermatitis	incontinenceassociated dermatitis	Incontinenceassociated dermatitis	1	kept
incontinence-associated dermatitis	incontinence associated dermatitis	incontinence associated dermatitis	incontinence associated dermatitis	Incontinence-associated dermatitis	1	case_or_format
incorrect dose	incorrect dose	incorrect dose	incorrect dose	incorrect dose	1	kept
incorrect duration	incorrect duration	incorrect duration	incorrect duration	incorrect duration	1	kept
incorrect frequency	incorrect frequency	incorrect frequency	incorrect frequency	incorrect frequency	1	kept

incorrect medication	incorrect medication	incorrect medication	incorrect medication	incorrect medication	1	kept
incorrect time	incorrect time	incorrect time	incorrect time	incorrect time	1	kept
INCREASE HOPE	increase hope	increase hope	increase hope	INCREASE HOPE	1	kept
increased appetite	increased appetite	increased appetite	increased appetite	increased appetite	1	kept
INCREASES	increases	increases	increase	INCREASE	1	singular
incremental cost	incremental cost	incremental cost	incremental cost	incremental cost	1	kept
Incremental cost-effectiveness ratio	incremental cost effectiveness ratio	incremental cost effectiveness ratio	incremental cost effectiveness ratio	Incremental cost-effectiveness ratio	1	kept
Incretins	incretins	incretins	incretins	Incretins	1	kept
incubation temperature	incubation temperature	incubation temperature	incubation temperature	incubation temperature	1	kept
INCUBATOR	incubator	incubator	incubator	incubator	1	case_or_format
incubator-baby shows	incubator baby shows	incubator baby shows	incubator baby shows	incubator-baby shows	1	kept
incubators, infant	incubators infant	incubators infant	incubators infant	Incubators, Infant	1	case_or_format
Incurable cancer	incurable cancer	incurable cancer	incurable cancer	Incurable cancer	1	kept
indefinite lockdown	indefinite lockdown	indefinite lockdown	indefinite lockdown	indefinite lockdown	1	kept
INDEPENDENCE	independence	independence	independence	independence	1	case_or_format
INDEPENDENCE MEASURE SCORE	independence measure score	independence measure score	independence measure score	INDEPENDENCE MEASURE SCORE	1	kept

independent component analysis	independent component analysis	independent component analysis	independent component analysis	independent component analysis	1	kept
Independent double check	independent double check	independent double check	independent double check	Independent double check	1	kept
Independent living	independent living	independent living	independent living	independent living	1	case_or_format
independent motherhood	independent motherhood	independent motherhood	independent motherhood	independent motherhood	1	kept
INDEPENDENT PREDICTOR	independent predictor	independent predictor	independent predictor	INDEPENDENT PREDICTOR	1	kept
Independent variables	independent variables	independent variables	independent variable	independent variable	1	singular
indepth interview	indepth interview	indepth interview	indepth interview	indepth interview	1	kept
index	index	index	index	INDEX	1	case_or_format
Index development index	index development index	index development index	index development index	Index development index	1	kept
index method	index method	index method	index method	index method	1	kept
Index system	index system	index system	index system	Index system	1	kept
Index values	index values	index values	index values	Index values	1	kept
india	india	india	india	India	1	case_or_format
Indicadores de calidad	indicadores de calidad	indicadores de calidad	indicadores de calidad	Indicadores de calidad	1	kept
Indicadores de calidad asistencial	indicadores de calidad asistencial	indicadores de calidad asistencial	indicadores de calidad asistencial	Indicadores de calidad asistencial	1	kept
Indicadores de calidad de la atención de salud	indicadores de calidad de la atenci n de salud	indicadores de calidad de la atenci n de salud	indicadores de calidad de la atenci n de salud	Indicadores de calidad de la atención de salud	1	kept

Indicated	indicated	indicated	indicated	Indicated	1	kept
Indication	indication	indication	indication	indication	1	case_or_format
Indications	indications	indications	indication	indication	1	singular
indications	indications	indications	indication	indication	1	singular
Indications for hand hygiene	indications for hand hygiene	indications for hand hygiene	indications for hand hygiene	Indications for hand hygiene	1	kept
Indications for transport	indications for transport	indications for transport	indications for transport	Indications for transport	1	kept
Indicator dilution	indicator dilution	indicator dilution	indicator dilution	Indicator dilution	1	kept
indicator dilution curve	indicator dilution curve	indicator dilution curve	indicator dilution curve	indicator dilution curve	1	kept
Indicators and Reagents	indicators and reagents	indicators and reagents	indicators and reagents	Indicators and Reagents	1	kept
Indices	indices	indices	indices	Indices	1	kept
Indigenous	indigenous	indigenous	indigenous	Indigenous	1	kept
indigenous	indigenous	indigenous	indigenous	Indigenous	1	case_or_format
Indigenous Australian	indigenous australian	indigenous australian	indigenous australian	Indigenous Australian	1	kept
indigenous peoples	indigenous peoples	indigenous peoples	indigenous people	indigenous people	1	singular
indigenous voices	indigenous voices	indigenous voices	indigenous voices	indigenous voices	1	kept
indigestion	indigestion	indigestion	indigestion	indigestion	1	kept

Indirect Calorimetry	indirect calorimetry	indirect calorimetry	indirect calorimetry	indirect calorimetry	1	case_or_format
Indirect cost	indirect cost	indirect cost	indirect cost	indirect costs	1	case_or_format
indirect cost	indirect cost	indirect cost	indirect cost	indirect costs	1	case_or_format
indirect ophthalmoscopy	indirect ophthalmoscopy	indirect ophthalmoscopy	indirect ophthalmoscopy	indirect ophthalmoscopy	1	kept
INDIRECT PROTECTION	indirect protection	indirect protection	indirect protection	INDIRECT PROTECTION	1	kept
individual behavior assessment	individual behavior assessment	individual behavior assessment	individual behavior assessment	individual behavior assessment	1	kept
Individual characteristics	individual characteristics	individual characteristics	individual characteristics	Individual characteristics	1	kept
Individual conflict	individual conflict	individual conflict	individual conflict	Individual conflict	1	kept
individual factors	individual factors	individual factors	individual factors	individual factors	1	kept
Individual Family Service Plan for at-risk infants	individual family service plan for at risk infants	individual family service plan for at risk infants	individual family service plan for at risk infants	Individual Family Service Plan for at-risk infants	1	kept
Individual feedback	individual feedback	individual feedback	individual feedback	Individual feedback	1	kept
individual feedback	individual feedback	individual feedback	individual feedback	Individual feedback	1	case_or_format
individual interviews	individual interviews	individual interviews	individual interviews	individual interviews	1	kept
individualised care	individualised care	individualised care	individualised care	individualised care	1	kept
individualised care plan	individualised care plan	individualised care plan	individualised care plan	individualised care plan	1	kept
Individualised Care Scale	individualised care scale	individualised care scale	individualised care scale	Individualised Care Scale	1	kept

individualised developmental care	individualised developmental care	individualised developmental care	individualised developmental care	Individualised developmental care	1	case_or_format
Individualised glucose control	individualised glucose control	individualised glucose control	individualised glucose control	Individualised glucose control	1	kept
Individualized aging	individualized aging	individualized aging	individualized aging	Individualized aging	1	kept
Individualized Care	individualized care	individualized care	individualized care	Individualized care	1	case_or_format
individualized care plan	individualized care plan	individualized care plan	individualized care plan	individualized care plan	1	kept
Individualized Education Plan for at-risk infants	individualized education plan for at risk infants	individualized education plan for at risk infants	individualized education plan for at risk infants	Individualized Education Plan for at-risk infants	1	kept
Individualized repositioning frequency	individualized repositioning frequency	individualized repositioning frequency	individualized repositioning frequency	Individualized repositioning frequency	1	kept
Individualized Supportive Developmental Care	individualized supportive developmental care	individualized supportive developmental care	individualized supportive developmental care	Individualized Supportive Developmental Care	1	kept
individualized teaching	individualized teaching	individualized teaching	individualized teaching	individualized teaching	1	kept
Individualized thirst management	individualized thirst management	individualized thirst management	individualized thirst management	Individualized thirst management	1	kept
Individually compounded nutrition via multi-chamber bags	individually compounded nutrition via multi chamber bags	individually compounded nutrition via multi chamber bags	individually compounded nutrition via multi chamber bags	Individually compounded nutrition via multi-chamber bags	1	kept
indocyanine green	indocyanine green	indocyanine green	indocyanine green	indocyanine green	1	kept
indocyanine green angiography	indocyanine green angiography	indocyanine green angiography	indocyanine green angiography	indocyanine green angiography	1	kept
indole derivative	indole derivative	indole derivative	indole derivative	indole derivative	1	kept
Indoles	indoles	indoles	indoles	Indoles	1	kept
indomethacin	indomethacin	indomethacin	indomethacin	Indomethacin	1	case_or_format

Indonesia health system	indonesia health system	indonesia health system	indonesia health system	Indonesia health system	1	kept
Indonesian version	indonesian version	indonesian version	indonesian version	Indonesian version	1	kept
Indoor	indoor	indoor	indoor	Indoor	1	kept
indoor air	indoor air	indoor air	indoor air	Indoor air	1	case_or_format
Indoor air monitoring	indoor air monitoring	indoor air monitoring	indoor air monitoring	Indoor air monitoring	1	kept
INDOOR AIR-QUALITY	indoor air quality	indoor air quality	indoor air quality	Indoor air quality	1	case_or_format
Indoor environment	indoor environment	indoor environment	indoor environment	indoor environment	1	case_or_format
Indoor Localization	indoor localization	indoor localization	indoor localization	Indoor localization	1	case_or_format
indoor localization	indoor localization	indoor localization	indoor localization	Indoor localization	1	case_or_format
Indoor localization systems	indoor localization systems	indoor localization systems	indoor localization systems	Indoor localization systems	1	kept
indoor sound quality	indoor sound quality	indoor sound quality	indoor sound quality	indoor sound quality	1	kept
indoor soundscape assessment	indoor soundscape assessment	indoor soundscape assessment	indoor soundscape assessment	indoor soundscape assessment	1	kept
indoor soundscape modelling	indoor soundscape modelling	indoor soundscape modelling	indoor soundscape modelling	indoor soundscape modelling	1	kept
indoprofen	indoprofen	indoprofen	indoprofen	indoprofen	1	kept
induced coma	induced coma	induced coma	induced coma	induced coma	1	kept
Induced Hypothermia	induced hypothermia	induced hypothermia	induced hypothermia	induced hypothermia	1	case_or_format

INDUCED INTESTINAL INJURY	induced intestinal injury	induced intestinal injury	induced intestinal injury	INDUCED INTESTINAL INJURY	1	kept
INDUCED NORMOTHERMIA	induced normothermia	induced normothermia	induced normothermia	INDUCED NORMOTHERMIA	1	kept
INDUCED RESPIRATORY DEPRESSION	induced respiratory depression	induced respiratory depression	induced respiratory depression	INDUCED RESPIRATORY DEPRESSION	1	kept
INDUCTION	induction	induction	induction	induction	1	case_or_format
Induction	induction	induction	induction	induction	1	case_or_format
induction methods	induction methods	induction methods	induction methods	induction methods	1	kept
Induction of labor	induction of labor	induction of labor	induction of labor	induction of labor	1	case_or_format
Induction of labour	induction of labour	induction of labour	induction of labour	Induction of labour	1	kept
Induction rates	induction rates	induction rates	induction rates	Induction rates	1	kept
Inductive	inductive	inductive	inductive	Inductive	1	kept
Inductive Content Analysis	inductive content analysis	inductive content analysis	inductive content analysis	Inductive Content Analysis	1	kept
Inductive content analysis	inductive content analysis	inductive content analysis	inductive content analysis	Inductive Content Analysis	1	case_or_format
inductive content analysis	inductive content analysis	inductive content analysis	inductive content analysis	Inductive Content Analysis	1	case_or_format
inductive reasoning	inductive reasoning	inductive reasoning	inductive reasoning	inductive reasoning	1	kept
Industrial design	industrial design	industrial design	industrial design	Industrial design	1	kept
industrial design	industrial design	industrial design	industrial design	Industrial design	1	case_or_format

Industrial electronics	industrial electronics	industrial electronics	industrial electronics	Industrial electronics	1	kept
industrial psychology	industrial psychology	industrial psychology	industrial psychology	industrial psychology	1	kept
INDUSTRIAL-RELATIONS	industrial relations	industrial relations	industrial relations	INDUSTRIAL-RELATIONS	1	kept
industrialization	industrialization	industrialization	industrialization	industrialization	1	kept
Industry	industry	industry	industry	Industry	1	kept
industry	industry	industry	industry	Industry	1	case_or_format
INDWELLING FOLEY CATHETERS	indwelling foley catheters	indwelling foley catheters	indwelling foley catheters	INDWELLING FOLEY CATHETERS	1	kept
indwelling urinary catheter securement	indwelling urinary catheter securement	indwelling urinary catheter securement	indwelling urinary catheter securement	indwelling urinary catheter securement	1	kept
indwelling urinary catheters	indwelling urinary catheters	indwelling urinary catheters	indwelling urinary catheter	indwelling urinary catheter	1	singular
Ineffective Breathing Pattern	ineffective breathing pattern	ineffective breathing pattern	ineffective breathing pattern	Ineffective Breathing Pattern	1	kept
Ineffective breathing pattern	ineffective breathing pattern	ineffective breathing pattern	ineffective breathing pattern	Ineffective Breathing Pattern	1	case_or_format
INEFFICIENCIES	inefficiencies	inefficiencies	inefficiencies	INEFFICIENCIES	1	kept
InEK matrix	inek matrix	inek matrix	inek matrix	InEK matrix	1	kept
inequalities	inequalities	inequalities	inequality	inequality	1	singular
Inequalities in care	inequalities in care	inequalities in care	inequalities in care	Inequalities in care	1	kept
inequalities in health	inequalities in health	inequalities in health	inequalities in health	inequalities in health	1	kept

inequity	inequity	inequity	inequity	inequity	1	kept
Infant [M01.060.703]	infant m01 060 703	infant m01 060 703	infant m01 060 703	Infant [M01.060.703]	1	kept
Infant and family-centered care	infant and family centered care	infant and family centered care	infant and family centered care	Infant and family-centered care	1	kept
infant behavior	infant behavior	infant behavior	infant behavior	Infant Behavior	1	case_or_format
Infant Behavior Questionnaire-Revised	infant behavior questionnaire revised	infant behavior questionnaire revised	infant behavior questionnaire revised	Infant Behavior Questionnaire-Revised	1	kept
infant bonding	infant bonding	infant bonding	infant bonding	infant bonding	1	kept
Infant born prematurely	infant born prematurely	infant born prematurely	infant born prematurely	Infant born prematurely	1	kept
Infant bpremature	infant bpremature	infant bpremature	infant bpremature	Infant bpremature	1	kept
infant closeness	infant closeness	infant closeness	infant closeness	infant closeness	1	kept
infant cries	infant cries	infant cries	infant cry	Infant cry	1	singular
Infant cry	infant cry	infant cry	infant cry	Infant cry	1	kept
infant crying plan	infant crying plan	infant crying plan	infant crying plan	infant crying plan	1	kept
infant death	infant death	infant death	infant death	Infant Death	1	case_or_format
Infant Development	infant development	infant development	infant development	Infant development	1	case_or_format
infant development	infant development	infant development	infant development	Infant development	1	case_or_format
Infant developmental	infant developmental	infant developmental	infant developmental	Infant developmental	1	kept

infant end-of-life	infant end of life	infant end of life	infant end of life	infant end-of-life	1	kept
Infant feeding advancement	infant feeding advancement	infant feeding advancement	infant feeding advancement	Infant feeding advancement	1	kept
Infant feeding practices	infant feeding practices	infant feeding practices	infant feeding practices	Infant feeding practices	1	kept
Infant feeding protocol	infant feeding protocol	infant feeding protocol	infant feeding protocol	Infant feeding protocol	1	kept
infant food	infant food	infant food	infant food	Infant Food	1	case_or_format
Infant formula	infant formula	infant formula	infant formula	Infant Formula	1	case_or_format
Infant health	infant health	infant health	infant health	Infant Health	1	case_or_format
infant incubators	infant incubators	infant incubators	infant incubators	infant incubators	1	kept
Infant loss	infant loss	infant loss	infant loss	Infant loss	1	kept
infant massage	infant massage	infant massage	infant massage	infant massage	1	kept
infant medical trauma	infant medical trauma	infant medical trauma	infant medical trauma	infant medical trauma	1	kept
INFANT MORTALITY	infant mortality	infant mortality	infant mortality	Infant Mortality	1	case_or_format
Infant Neurological International Battery scale	infant neurological international battery scale	infant neurological international battery scale	infant neurological international battery scale	Infant Neurological International Battery scale	1	kept
infant nutrition disorders	infant nutrition disorders	infant nutrition disorders	infant nutrition disorders	Infant Nutrition Disorders	1	case_or_format
Infant nutritional physiologic	infant nutritional physiologic	infant nutritional physiologic	infant nutritional physiologic	Infant nutritional physiologic	1	kept
infant nutritional physiological phenomena	infant nutritional physiological phenomena	infant nutritional physiological phenomena	infant nutritional physiological phenomena	Infant Nutritional Physiological Phenomena	1	case_or_format

infant pain	infant pain	infant pain	infant pain	Infant pain	1	case_or_format
infant physiology	infant physiology	infant physiology	infant physiology	infant physiology	1	kept
Infant Positioning Assessment Tool	infant positioning assessment tool	infant positioning assessment tool	infant positioning assessment tool	Infant Positioning Assessment Tool	1	kept
Infant positioning*	infant positioning	infant positioning	infant positioning	Infant positioning*	1	kept
Infant Premature	infant premature	infant premature	infant premature	Infant, Premature	1	case_or_format
Infant preterm	infant preterm	infant preterm	infant preterm	Infant preterm	1	kept
infant resuscitation mannequin	infant resuscitation mannequin	infant resuscitation mannequin	infant resuscitation mannequin	infant resuscitation mannequin	1	kept
Infant safe sleep	infant safe sleep	infant safe sleep	infant safe sleep	Infant safe sleep	1	kept
Infant safe sleep practices	infant safe sleep practices	infant safe sleep practices	infant safe sleep practices	Infant safe sleep practices	1	kept
Infant safe sleep*	infant safe sleep	infant safe sleep	infant safe sleep	Infant safe sleep	1	case_or_format
infant skin damage	infant skin damage	infant skin damage	infant skin damage	infant skin damage	1	kept
Infant sleep	infant sleep	infant sleep	infant sleep	Infant sleep	1	kept
Infant Stress Scale	infant stress scale	infant stress scale	infant stress scale	Infant Stress Scale	1	kept
infant transfer	infant transfer	infant transfer	infant transfer	infant transfer	1	kept
infant, high risk	infant high risk	infant high risk	infant high risk	infant, high risk	1	kept
Infant, newborn, diseases	infant newborn diseases	infant newborn diseases	infant newborn diseases	Infant, Newborn, Diseases	1	case_or_format

Infant, Premature, Diseases [prevention & control]	infant premature diseases prevention and control	infant premature diseases prevention and control	infant premature diseases prevention and control	Infant, Premature, Diseases [prevention & control]	1	kept
Infant, preterm	infant preterm	infant preterm	infant preterm	Infant preterm	1	case_or_format
Infant,premature	infant premature	infant premature	infant premature	Infant, Premature	1	case_or_format
Infant-directed vocalisation	infant directed vocalisation	infant directed vocalisation	infant directed vocalisation	Infant-directed vocalisation	1	kept
Infant-Driven Feeding	infant driven feeding	infant driven feeding	infant driven feeding	Infant-Driven Feeding	1	kept
infant-driven feeding	infant driven feeding	infant driven feeding	infant driven feeding	Infant-Driven Feeding	1	case_or_format
Infant-parent-interaction	infant parent interaction	infant parent interaction	infant parent interaction	Infant-parent-interaction	1	kept
infant: newborn	infant newborn	infant newborn	infant newborn	Infant, Newborn	1	case_or_format
infant: premature	infant premature	infant premature	infant premature	Infant, Premature	1	case_or_format
Infante	infante	infante	infante	Infante	1	kept
infantile hypertrophic pyloric stenosis	infantile hypertrophic pyloric stenosis	infantile hypertrophic pyloric stenosis	infantile hypertrophic pyloric stenosis	infantile hypertrophic pyloric stenosis	1	kept
infants and families	infants and families	infants and families	infants and families	infants and families	1	kept
Infants and toddlers	infants and toddlers	infants and toddlers	infants and toddlers	Infants and toddlers	1	kept
Infants and young children	infants and young children	infants and young children	infants and young children	Infants and young children	1	kept
infants at end-of-life	infants at end of life	infants at end of life	infants at end of life	infants at end-of-life	1	kept
INFANTS BORN	infants born	infants born	infants born	INFANTS BORN	1	kept

infants born to mothers with drugs abuse	infants born to mothers with drugs abuse	infants born to mothers with drugs abuse	infants born to mothers with drugs abuse	infants born to mothers with drugs abuse	1	kept
Infants exposed to substances	infants exposed to substances	infants exposed to substances	infants exposed to substances	Infants exposed to substances	1	kept
Infants pain	infants pain	infants pain	infants pain	Infants pain	1	kept
Infants seropositive for HIV	infants seropositive for hiv	infants seropositive for hiv	infants seropositive for hiv	Infants seropositive for HIV	1	kept
Infants with Congenital Anomalies	infants with congenital anomalies	infants with congenital anomalies	infants with congenital anomalies	Infants with Congenital Anomalies	1	kept
infants' procedural pain	infants procedural pain	infants procedural pain	infants procedural pain	infants' procedural pain	1	kept
infants, high-risk	infants high risk	infants high risk	infants high risk	infants, high-risk	1	kept
Infants, newborns	infants newborns	infants newborns	infants newborns	Infants, newborns	1	kept
infarctus du myocarde	infarctus du myocarde	infarctus du myocarde	infarctus du myocarde	infarctus du myocarde	1	kept
Infecciones	infecciones	infecciones	infecciones	Infecciones	1	kept
Infecciones por Coronavirus	infecciones por coronavirus	infecciones por coronavirus	infecciones por coronavirus	Infecciones por coronavirus	1	case_or_format
Infección	infecci n	infecci n	infecci n	Infección	1	kept
infected solution	infected solution	infected solution	infected solution	infected solution	1	kept
Infection algorithms	infection algorithms	infection algorithms	infection algorithms	Infection algorithms	1	kept
infection and prevention control measures	infection and prevention control measures	infection and prevention control measures	infection and prevention control measures	infection and prevention control measures	1	kept
infection care	infection care	infection care	infection care	infection care	1	kept

Infection Control and Prevention	infection control and prevention	infection control and prevention	infection control and prevention	infection control and prevention	1	case_or_format
Infection control committee	infection control committee	infection control committee	infection control committee	Infection control committee	1	kept
infection control guidelines	infection control guidelines	infection control guidelines	infection control guidelines	Infection control guidelines	1	case_or_format
Infection control infringement	infection control infringement	infection control infringement	infection control infringement	Infection control infringement	1	kept
Infection control liaison	infection control liaison	infection control liaison	infection control liaison	Infection control liaison	1	kept
infection control professional	infection control professional	infection control professional	infection control professional	infection control professional	1	kept
Infection control programme	infection control programme	infection control programme	infection control programme	Infection control programme	1	kept
infection control staff	infection control staff	infection control staff	infection control staff	infection control staff	1	kept
Infection control strategies	infection control strategies	infection control strategies	infection control strategies	Infection control strategies	1	kept
Infection Control Team	infection control team	infection control team	infection control team	Infection Control Team	1	kept
infection control team	infection control team	infection control team	infection control team	Infection Control Team	1	case_or_format
Infection control training	infection control training	infection control training	infection control training	Infection control training	1	kept
Infection controls	infection controls	infection controls	infection control	Infection Control	1	singular
infection fatality rate	infection fatality rate	infection fatality rate	infection fatality rate	infection fatality rate	1	kept
infection fatality ratio	infection fatality ratio	infection fatality ratio	infection fatality ratio	infection fatality ratio	1	kept
INFECTION HAZARD	infection hazard	infection hazard	infection hazard	INFECTION HAZARD	1	kept

Infection nosocomiale	infection nosocomiale	infection nosocomiale	infection nosocomiale	Infection nosocomiale	1	kept
infection prevention and control measures	infection prevention and control measures	infection prevention and control measures	infection prevention and control measures	infection prevention and control measures	1	kept
infection prevention climate	infection prevention climate	infection prevention climate	infection prevention climate	infection prevention climate	1	kept
Infection Probability Score	infection probability score	infection probability score	infection probability score	Infection Probability Score	1	kept
Infection surveillance	infection surveillance	infection surveillance	infection surveillance	Infection surveillance	1	kept
infection surveillance	infection surveillance	infection surveillance	infection surveillance	Infection surveillance	1	case_or_format
Infection transmission	infection transmission	infection transmission	infection transmission	Infection transmission	1	kept
infection wave	infection wave	infection wave	infection wave	infection wave	1	kept
Infection, nosocomial	infection nosocomial	infection nosocomial	infection nosocomial	Infection, nosocomial	1	kept
INFECTION-CONTROL MEASURES	infection control measures	infection control measures	infection control measures	infection control measures	1	case_or_format
INFECTION-CONTROL PRACTICES	infection control practices	infection control practices	infection control practices	Infection control practices	1	case_or_format
Infection-control-and-prevention	infection control and prevention	infection control and prevention	infection control and prevention	infection control and prevention	1	case_or_format
infection-related ventilator-associated complication	infection related ventilator associated complication	infection related ventilator associated complication	infection related ventilator associated complication	infection-related ventilator-associated complication	1	kept
infections acquired in hospital	infections acquired in hospital	infections acquired in hospital	infections acquired in hospital	infections acquired in hospital	1	kept
Infections in healthcare workers	infections in healthcare workers	infections in healthcare workers	infections in healthcare workers	Infections in healthcare workers	1	kept
Infections related to the catheter	infections related to the catheter	infections related to the catheter	infections related to the catheter	Infections related to the catheter	1	kept

INFECTIONS SURVEILLANCE SYSTEM	infections surveillance system	infections surveillance system	infections surveillance system	INFECTIONS SURVEILLANCE SYSTEM	1	kept
infectious	infectious	infectious	infectious	infectious	1	kept
Infectious Disease Outbreak	infectious disease outbreak	infectious disease outbreak	infectious disease outbreak	Infectious Disease Outbreak	1	kept
infectious disease transmission	infectious disease transmission	infectious disease transmission	infectious disease transmission	Infectious disease transmission	1	case_or_format
INFECTIOUS DISEASES	infectious diseases	infectious diseases	infectious disease	infectious disease	1	singular
infectious diseases & infestations	infectious diseases and infestations	infectious diseases and infestations	infectious diseases and infestations	infectious diseases & infestations	1	kept
Infectious Diseases Society of America AmericanThoracic	infectious diseases society of america americanthoracic	infectious diseases society of america americanthoracic	infectious diseases society of america americanthoracic	Infectious Diseases Society of America AmericanThoracic	1	kept
infectious diseases ward	infectious diseases ward	infectious diseases ward	infectious diseases ward	infectious diseases ward	1	kept
Infectious etiology	infectious etiology	infectious etiology	infectious etiology	Infectious etiology	1	kept
infectious keratitis	infectious keratitis	infectious keratitis	infectious keratitis	infectious keratitis	1	kept
infectious medical waste	infectious medical waste	infectious medical waste	infectious medical waste	infectious medical waste	1	kept
infectious pancreatic necrosis	infectious pancreatic necrosis	infectious pancreatic necrosis	infectious pancreatic necrosis	infectious pancreatic necrosis	1	kept
infectious pregnancy complication	infectious pregnancy complication	infectious pregnancy complication	infectious pregnancy complication	infectious pregnancy complication	1	kept
Infectious risk	infectious risk	infectious risk	infectious risk	Infectious risk	1	kept
Infectious shock	infectious shock	infectious shock	infectious shock	Infectious shock	1	kept
Infectious shock induced	infectious shock induced	infectious shock induced	infectious shock induced	Infectious shock induced	1	kept

INFECTIOUS-DISEASES	infectious diseases	infectious diseases	infectious disease	infectious disease	1	singular
infectivity	infectivity	infectivity	infectivity	infectivity	1	kept
Infecton control	infecton control	infecton control	infecton control	Infecton control	1	kept
Infecção	infec o	infec o	infec o	Infecção	1	kept
Inference engines	inference engines	inference engines	inference engines	Inference engines	1	kept
INFERENCES	inferences	inferences	inferences	INFERENCES	1	kept
Inferential models	inferential models	inferential models	inferential models	Inferential models	1	kept
Inferential statistics	inferential statistics	inferential statistics	inferential statistics	inferential statistics	1	case_or_format
inferior cava vein obstruction	inferior cava vein obstruction	inferior cava vein obstruction	inferior cava vein obstruction	inferior cava vein obstruction	1	kept
inferior medial migration	inferior medial migration	inferior medial migration	inferior medial migration	inferior medial migration	1	kept
inferior vena cava	inferior vena cava	inferior vena cava	inferior vena cava	inferior vena cava	1	kept
inferior vena cava filters	inferior vena cava filters	inferior vena cava filters	inferior vena cava filters	inferior vena cava filters	1	kept
infirmier anesthésiste	infirmier anesth siste	infirmier anesth siste	infirmier anesth siste	infirmier anesthésiste	1	kept
infirmier de réanimation	infirmier de r animation	infirmier de r animation	infirmier de r animation	infirmier de réanimation	1	kept
infirmier perfusionniste	infirmier perfusionniste	infirmier perfusionniste	infirmier perfusionniste	infirmier perfusionniste	1	kept
infirmière	infirmi re	infirmi re	infirmi re	infirmière	1	kept

infirmière coordinatrice	infirmi re coordinatrice	infirmi re coordinatrice	infirmi re coordinatrice	infirmière coordinatrice	1	kept
Infl ammation	infl ammation	infl ammation	infl ammation	Infl ammation	1	kept
Inflamación	inflamaci n	inflamaci n	inflamaci n	Inflamación	1	kept
inflammasome	inflammasome	inflammasome	inflammasome	inflammasome	1	kept
Inflammation marker	inflammation marker	inflammation marker	inflammation marker	Inflammation marker	1	kept
inflammatory	inflammatory	inflammatory	inflammatory	inflammatory	1	kept
Inflammatory biomarker	inflammatory biomarker	inflammatory biomarker	inflammatory biomarker	Inflammatory biomarker	1	kept
inflammatory biomarkers	inflammatory biomarkers	inflammatory biomarkers	inflammatory biomarker	Inflammatory biomarker	1	singular
inflammatory cytokines	inflammatory cytokines	inflammatory cytokines	inflammatory cytokines	inflammatory cytokines	1	kept
Inflammatory disease	inflammatory disease	inflammatory disease	inflammatory disease	Inflammatory disease	1	kept
inflammatory markers	inflammatory markers	inflammatory markers	inflammatory markers	inflammatory markers	1	kept
Inflammatory response	inflammatory response	inflammatory response	inflammatory response	inflammatory response	1	case_or_format
INFLAMMATORY RESPONSE SYNDROME	inflammatory response syndrome	inflammatory response syndrome	inflammatory response syndrome	INFLAMMATORY RESPONSE SYNDROME	1	kept
Inflammatory skin condition	inflammatory skin condition	inflammatory skin condition	inflammatory skin condition	Inflammatory skin condition	1	kept
Inflation	inflation	inflation	inflation	Inflation	1	kept
Infliximab	infliximab	infliximab	infliximab	infliximab	1	case_or_format

Influence factor	influence factor	influence factor	influence factor	influence factors	1	case_or_format
Influence patterns	influence patterns	influence patterns	influence patterns	Influence patterns	1	kept
influencer	influencer	influencer	influencer	influencer	1	kept
Influences of technology	influences of technology	influences of technology	influences of technology	Influences of technology	1	kept
influencing factors analysis	influencing factors analysis	influencing factors analysis	influencing factors analysis	influencing factors analysis	1	kept
influential factor	influential factor	influential factor	influential factor	influential factor	1	kept
Influential Factors	influential factors	influential factors	influential factor	influential factor	1	singular
influential factors	influential factors	influential factors	influential factor	influential factor	1	singular
Influenza A Virus, H3N2 Subtype	influenza a virus h3n2 subtype	influenza a virus h3n2 subtype	influenza a virus h3n2 subtype	Influenza A Virus, H3N2 Subtype	1	kept
Influenza A Virus, H5N1 Subtype	influenza a virus h5n1 subtype	influenza a virus h5n1 subtype	influenza a virus h5n1 subtype	Influenza A Virus, H5N1 Subtype	1	kept
Influenza A Virus, H7N9 Subtype	influenza a virus h7n9 subtype	influenza a virus h7n9 subtype	influenza a virus h7n9 subtype	Influenza A Virus, H7N9 Subtype	1	kept
Influenza AH1N1	influenza ah1n1	influenza ah1n1	influenza ah1n1	Influenza AH1N1	1	kept
influenza antiviral treatment	influenza antiviral treatment	influenza antiviral treatment	influenza antiviral treatment	influenza antiviral treatment	1	kept
influenza hospitalizations	influenza hospitalizations	influenza hospitalizations	influenza hospitalizations	influenza hospitalizations	1	kept
Influenza immunization	influenza immunization	influenza immunization	influenza immunization	Influenza immunization	1	kept
Influenza Pandemic, 1918-1919	influenza pandemic 1918 1919	influenza pandemic 1918 1919	influenza pandemic 1918 1919	Influenza Pandemic, 1918-1919	1	kept

INFLUENZA SEASON	influenza season	influenza season	influenza season	INFLUENZA SEASON	1	kept
Influenza surveillance	influenza surveillance	influenza surveillance	influenza surveillance	Influenza surveillance	1	kept
influenza vaccines	influenza vaccines	influenza vaccines	influenza vaccine	influenza vaccine	1	singular
Influenza virus A H5N1	influenza virus a h5n1	influenza virus a h5n1	influenza virus a h5n1	Influenza virus A H5N1	1	kept
influx of victims	influx of victims	influx of victims	influx of victims	influx of victims	1	kept
infodemic	infodemic	infodemic	infodemic	infodemic	1	kept
Información	informaci n	informaci n	informaci n	Información	1	kept
Informal care	informal care	informal care	informal care	informal care	1	case_or_format
Informal caregiver	informal caregiver	informal caregiver	informal caregiver	INFORMAL CAREGIVERS	1	case_or_format
informal caregiver	informal caregiver	informal caregiver	informal caregiver	INFORMAL CAREGIVERS	1	case_or_format
INFORMAL CAREGIVER BURDEN	informal caregiver burden	informal caregiver burden	informal caregiver burden	INFORMAL CAREGIVER BURDEN	1	kept
Informal caregiving	informal caregiving	informal caregiving	informal caregiving	Informal caregiving	1	kept
informal caregiving	informal caregiving	informal caregiving	informal caregiving	Informal caregiving	1	case_or_format
INFORMAL COERCION	informal coercion	informal coercion	informal coercion	INFORMAL COERCION	1	kept
Informal Governance Mechanisms	informal governance mechanisms	informal governance mechanisms	informal governance mechanisms	Informal Governance Mechanisms	1	kept
Informal learning	informal learning	informal learning	informal learning	Informal learning	1	kept

Informal support	informal support	informal support	informal support	Informal support	1	kept
Informant Questionnaire of Cognitive Decline in	informant questionnaire of cognitive decline in	informant questionnaire of cognitive decline in	informant questionnaire of cognitive decline in	Informant Questionnaire of Cognitive Decline in	1	kept
Informatics framework	informatics framework	informatics framework	informatics framework	Informatics framework	1	kept
Information and communication	information and communication	information and communication	information and communication	Information and communication	1	kept
information and communication technology program	information and communication technology program	information and communication technology program	information and communication technology program	information and communication technology program	1	kept
information behaviour	information behaviour	information behaviour	information behaviour	information behaviour	1	kept
Information broker	information broker	information broker	information broker	Information broker	1	kept
Information Centers	information centers	information centers	information center	information center	1	singular
Information communication technology	information communication technology	information communication technology	information communication technology	Information communication technology	1	kept
Information concerning	information concerning	information concerning	information concerning	Information concerning	1	kept
Information development and evaluation	information development and evaluation	information development and evaluation	information development and evaluation	Information development and evaluation	1	kept
Information extraction	information extraction	information extraction	information extraction	Information extraction	1	kept
Information factor	information factor	information factor	information factor	Information factor	1	kept
Information filtering	information filtering	information filtering	information filtering	Information filtering	1	kept
Information gathering	information gathering	information gathering	information gathering	Information gathering	1	kept
Information giving	information giving	information giving	information giving	Information giving	1	kept

Information Literacy	information literacy	information literacy	information literacy	Information literacy	1	case_or_format
Information loss	information loss	information loss	information loss	Information loss	1	kept
information management	information management	information management	information management	Information management	1	case_or_format
information need	information need	information need	information need	Information needs	1	case_or_format
information needs	information needs	information needs	information need	Information needs	1	singular
Information organisation	information organisation	information organisation	information organisation	Information organisation	1	kept
information overload	information overload	information overload	information overload	information overload	1	kept
Information overloads	information overloads	information overloads	information overload	information overload	1	singular
information processing bias	information processing bias	information processing bias	information processing bias	information processing bias	1	kept
Information provision	information provision	information provision	information provision	Information provision	1	kept
information provision	information provision	information provision	information provision	Information provision	1	case_or_format
Information reporting	information reporting	information reporting	information reporting	Information reporting	1	kept
Information retrieval systems	information retrieval systems	information retrieval systems	information retrieval systems	Information retrieval systems	1	kept
Information Science	information science	information science	information science	information science	1	case_or_format
Information seeking behavior	information seeking behavior	information seeking behavior	information seeking behavior	Information Seeking Behavior	1	case_or_format
Information seeking behaviors	information seeking behaviors	information seeking behaviors	information seeking behavior	Information Seeking Behavior	1	singular

Information sharing	information sharing	information sharing	information sharing	information sharing	1	case_or_format
Information Sources	information sources	information sources	information source	information source	1	singular
Information sources	information sources	information sources	information source	information source	1	singular
Information storage and retrieval	information storage and retrieval	information storage and retrieval	information storage and retrieval	Information Storage and Retrieval	1	case_or_format
Information System	information system	information system	information system	information system	1	case_or_format
Information systems research framework	information systems research framework	information systems research framework	information systems research framework	Information systems research framework	1	kept
information systems research framework	information systems research framework	information systems research framework	information systems research framework	Information systems research framework	1	case_or_format
information technology in health	information technology in health	information technology in health	information technology in health	information technology in health	1	kept
Information technology professionals	information technology professionals	information technology professionals	information technology professionals	Information technology professionals	1	kept
Information Theory	information theory	information theory	information theory	Information Theory	1	kept
Information to parents	information to parents	information to parents	information to parents	Information to parents	1	kept
Information tools	information tools	information tools	information tools	Information tools	1	kept
Information transfers	information transfers	information transfers	information transfer	Information transfer	1	singular
Information visualization	information visualization	information visualization	information visualization	Information visualization	1	kept
INFORMATION-SEEKING BEHAVIOR	information seeking behavior	information seeking behavior	information seeking behavior	Information Seeking Behavior	1	case_or_format
information-seeking behavior	information seeking behavior	information seeking behavior	information seeking behavior	Information Seeking Behavior	1	case_or_format

Information-seeking behaviors	information seeking behaviors	information seeking behaviors	information seeking behavior	Information Seeking Behavior	1	singular
INFORMATION-SYSTEM	information system	information system	information system	information system	1	case_or_format
INFORMATION-SYSTEMS	information systems	information systems	information system	information system	1	singular
INFORMATION-TRANSFER	information transfer	information transfer	information transfer	Information transfer	1	case_or_format
informational consistency	informational consistency	informational consistency	informational consistency	informational consistency	1	kept
Informational continuity	informational continuity	informational continuity	informational continuity	Informational continuity	1	kept
Informational need	informational need	informational need	informational need	Informational need	1	kept
informational needs	informational needs	informational needs	informational need	Informational need	1	singular
Informational quality	informational quality	informational quality	informational quality	Informational quality	1	kept
Informationists	informationists	informationists	informationists	Informationists	1	kept
informative systems	informative systems	informative systems	informative systems	informative systems	1	kept
Informed choice	informed choice	informed choice	informed choice	Informed choice	1	kept
informed consent form	informed consent form	informed consent form	informed consent form	informed consent form	1	kept
informed decision-making	informed decision making	informed decision making	informed decision making	informed decision-making	1	kept
informed practice	informed practice	informed practice	informed practice	informed practice	1	kept
Informing	informing	informing	informing	Informing	1	kept

Infra-red sensor	infra red sensor	infra red sensor	infra red sensor	Infra-red sensor	1	kept
Infrared	infrared	infrared	infrared	Infrared	1	kept
Infrared detectors	infrared detectors	infrared detectors	infrared detectors	Infrared detectors	1	kept
INFRARED EAR THERMOMETRY	infrared ear thermometry	infrared ear thermometry	infrared ear thermometry	INFRARED EAR THERMOMETRY	1	kept
Infrared imaging	infrared imaging	infrared imaging	infrared imaging	Infrared imaging	1	kept
infrared thermography	infrared thermography	infrared thermography	infrared thermography	infrared thermography	1	kept
infrastructure analysis	infrastructure analysis	infrastructure analysis	infrastructure analysis	infrastructure analysis	1	kept
INFUSION	infusion	infusion	infusion	infusion	1	case_or_format
Infusion alarm	infusion alarm	infusion alarm	infusion alarm	Infusion alarm	1	kept
Infusion center	infusion center	infusion center	infusion center	Infusion center	1	kept
infusion device	infusion device	infusion device	infusion device	infusion device	1	kept
Infusion device safety	infusion device safety	infusion device safety	infusion device safety	Infusion device safety	1	kept
infusion error	infusion error	infusion error	infusion error	infusion error	1	kept
infusion maintenance	infusion maintenance	infusion maintenance	infusion maintenance	infusion maintenance	1	kept
infusion protocols	infusion protocols	infusion protocols	infusion protocols	infusion protocols	1	kept
Infusion Pumps, Implantable	infusion pumps implantable	infusion pumps implantable	infusion pumps implantable	Infusion Pumps, Implantable	1	kept

infusion regimen	infusion regimen	infusion regimen	infusion regimen	infusion regimen	1	kept
Infusion set	infusion set	infusion set	infusion set	Infusion set	1	kept
Infusion systems	infusion systems	infusion systems	infusion system	infusion system	1	singular
Infusion team	infusion team	infusion team	infusion team	Infusion team	1	kept
Infusion tubing	infusion tubing	infusion tubing	infusion tubing	Infusion tubing	1	kept
Infusion with a syringe	infusion with a syringe	infusion with a syringe	infusion with a syringe	Infusion with a syringe	1	kept
Infusions, Intra-Arterial	infusions intra arterial	infusions intra arterial	infusions intra arterial	Infusions, Intra-Arterial	1	kept
Infusions, intravenous	infusions intravenous	infusions intravenous	infusions intravenous	Infusions, Intravenous	1	case_or_format
infusions, intravenous	infusions intravenous	infusions intravenous	infusions intravenous	Infusions, Intravenous	1	case_or_format
inguinal lymph node	inguinal lymph node	inguinal lymph node	inguinal lymph node	inguinal lymph node	1	kept
inguinal pain	inguinal pain	inguinal pain	inguinal pain	inguinal pain	1	kept
INHALATION	inhalation	inhalation	inhalation	inhalation	1	case_or_format
Inhalation burns	inhalation burns	inhalation burns	inhalation burns	Inhalation burns	1	kept
inhalational anaesthesia agent: desflurane	inhalational anaesthesia agent desflurane	inhalational anaesthesia agent desflurane	inhalational anaesthesia agent desflurane	inhalational anaesthesia agent: desflurane	1	kept
inhalational anaesthesia agent: sevoflurane	inhalational anaesthesia agent sevoflurane	inhalational anaesthesia agent sevoflurane	inhalational anaesthesia agent sevoflurane	inhalational anaesthesia agent: sevoflurane	1	kept
Inhalational therapy	inhalational therapy	inhalational therapy	inhalational therapy	Inhalational therapy	1	kept

inhaled nitric oxide	inhaled nitric oxide	inhaled nitric oxide	inhaled nitric oxide	inhaled nitric oxide	1	kept
INHALED NITRIC-OXIDE	inhaled nitric oxide	inhaled nitric oxide	inhaled nitric oxide	inhaled nitric oxide	1	case_or_format
inhaler	inhaler	inhaler	inhaler	inhaler	1	kept
inhaler adapters	inhaler adapters	inhaler adapters	inhaler adapters	inhaler adapters	1	kept
inhaler connectors	inhaler connectors	inhaler connectors	inhaler connectors	inhaler connectors	1	kept
Inhaler technique	inhaler technique	inhaler technique	inhaler technique	Inhaler technique	1	kept
Inherited metabolic disease	inherited metabolic disease	inherited metabolic disease	inherited metabolic disease	Inherited metabolic disease	1	kept
INHERITED METABOLIC-DISORDERS	inherited metabolic disorders	inherited metabolic disorders	inherited metabolic disorders	INHERITED METABOLIC-DISORDERS	1	kept
Inhibition	inhibition	inhibition	inhibition	inhibition	1	case_or_format
inhibition zone	inhibition zone	inhibition zone	inhibition zone	inhibition zone	1	kept
inhibitory control	inhibitory control	inhibitory control	inhibitory control	inhibitory control	1	kept
Inhospital mortality	inhospital mortality	inhospital mortality	inhospital mortality	Inhospital mortality	1	kept
INITIAL EVALUATION	initial evaluation	initial evaluation	initial evaluation	INITIAL EVALUATION	1	kept
Initial infusion	initial infusion	initial infusion	initial infusion	Initial infusion	1	kept
initial results	initial results	initial results	initial results	initial results	1	kept
INITIAL VALIDATION	initial validation	initial validation	initial validation	INITIAL VALIDATION	1	kept

Initiation	initiation	initiation	initiation	Initiation	1	kept
initiative behavior	initiative behavior	initiative behavior	initiative behavior	initiative behavior	1	kept
Injectable glucose-lowering therapies	injectable glucose lowering therapies	injectable glucose lowering therapies	injectable glucose lowering therapies	Injectable glucose-lowering therapies	1	kept
Injectable lipid emulsions	injectable lipid emulsions	injectable lipid emulsions	injectable lipid emulsions	Injectable lipid emulsions	1	kept
Injection laryngoplasty	injection laryngoplasty	injection laryngoplasty	injection laryngoplasty	Injection laryngoplasty	1	kept
Injection Preparation	injection preparation	injection preparation	injection preparation	Injection Preparation	1	kept
Injection safety	injection safety	injection safety	injection safety	Injection safety	1	kept
injection safety	injection safety	injection safety	injection safety	Injection safety	1	case_or_format
injection site contusion	injection site contusion	injection site contusion	injection site contusion	injection site contusion	1	kept
injection site edema	injection site edema	injection site edema	injection site edema	injection site edema	1	kept
injection site erythema	injection site erythema	injection site erythema	injection site erythema	injection site erythema	1	kept
injection site irritation	injection site irritation	injection site irritation	injection site irritation	injection site irritation	1	kept
injection site pruritus	injection site pruritus	injection site pruritus	injection site pruritus	injection site pruritus	1	kept
injection site rash	injection site rash	injection site rash	injection site rash	injection site rash	1	kept
Injection site reaction	injection site reaction	injection site reaction	injection site reaction	injection site reaction	1	case_or_format
injection site scar	injection site scar	injection site scar	injection site scar	injection site scar	1	kept

INJECTION-DRUG USERS	injection drug users	injection drug users	injection drug user	injection drug user	1	singular
Injury deaths	injury deaths	injury deaths	injury deaths	Injury deaths	1	kept
Injury mortality	injury mortality	injury mortality	injury mortality	Injury mortality	1	kept
Injury pattern	injury pattern	injury pattern	injury pattern	Injury pattern	1	kept
Injury patterns	injury patterns	injury patterns	injury pattern	Injury pattern	1	singular
INJURY PREVALENCE	injury prevalence	injury prevalence	injury prevalence	INJURY PREVALENCE	1	kept
injury prevention	injury prevention	injury prevention	injury prevention	Injury prevention	1	case_or_format
INJURY SEVERITY	injury severity	injury severity	injury severity	injury severity	1	case_or_format
Injury Severity	injury severity	injury severity	injury severity	injury severity	1	case_or_format
Injury Severity Scale	injury severity scale	injury severity scale	injury severity scale	Injury Severity Scale	1	kept
injury severity score	injury severity score	injury severity score	injury severity score	Injury Severity Score	1	case_or_format
injury severity scores	injury severity scores	injury severity scores	injury severity score	Injury Severity Score	1	singular
Inmates	inmates	inmates	inmates	Inmates	1	kept
Inmersión	inmersi n	inmersi n	inmersi n	Inmersión	1	kept
innate immune response	innate immune response	innate immune response	innate immune response	innate immune response	1	kept
Inner cannula	inner cannula	inner cannula	inner cannula	Inner cannula	1	kept

inner conflict	inner conflict	inner conflict	inner conflict	inner conflict	1	kept
inner-city	inner city	inner city	inner city	inner-city	1	kept
innervation	innervation	innervation	innervation	innervation	1	kept
INNOVATION	innovation	innovation	innovation	innovation	1	case_or_format
innovation attributes	innovation attributes	innovation attributes	innovation attributes	innovation attributes	1	kept
innovation diffusion	innovation diffusion	innovation diffusion	innovation diffusion	innovation diffusion	1	kept
innovations	innovations	innovations	innovation	innovation	1	singular
Innovative approaches	innovative approaches	innovative approaches	innovative approaches	Innovative approaches	1	kept
innovative rural healthcare models	innovative rural healthcare models	innovative rural healthcare models	innovative rural healthcare models	innovative rural healthcare models	1	kept
Innovative technology	innovative technology	innovative technology	innovative technology	Innovative technology	1	kept
innovative technology	innovative technology	innovative technology	innovative technology	Innovative technology	1	case_or_format
Innovative work behavior	innovative work behavior	innovative work behavior	innovative work behavior	Innovative work behavior	1	kept
Inosine Pranobex	inosine pranobex	inosine pranobex	inosine pranobex	Inosine Pranobex	1	kept
Inositol	inositol	inositol	inositol	Inositol	1	kept
inositol	inositol	inositol	inositol	Inositol	1	case_or_format
Inotrope	inotrope	inotrope	inotrope	Inotropes	1	case_or_format

inotropic	inotropic	inotropic	inotropic	inotropic	1	kept
inotropic support	inotropic support	inotropic support	inotropic support	inotropic support	1	kept
INQUIRY	inquiry	inquiry	inquiry	INQUIRY	1	kept
Inquiry	inquiry	inquiry	inquiry	INQUIRY	1	case_or_format
inquiry-based learning	inquiry based learning	inquiry based learning	inquiry based learning	inquiry-based learning	1	kept
INR	inr	inr	inr	INR	1	kept
INR, international normalized ratio	inr international normalized ratio	inr international normalized ratio	inr international normalized ratio	INR, international normalized ratio	1	kept
Insect Control	insect control	insect control	insect control	Insect Control	1	kept
insect sting	insect sting	insect sting	insect sting	insect sting	1	kept
insecticide	insecticide	insecticide	insecticide	Insecticides	1	case_or_format
insecticides	insecticides	insecticides	insecticide	Insecticides	1	singular
Insensible water loss	insensible water loss	insensible water loss	insensible water loss	Insensible water loss	1	kept
insertion	insertion	insertion	insertion	INSERTION	1	case_or_format
INSERTION BUNDLE	insertion bundle	insertion bundle	insertion bundle	INSERTION BUNDLE	1	kept
Insertion checklist	insertion checklist	insertion checklist	insertion checklist	Insertion checklist	1	kept
Insertion site	insertion site	insertion site	insertion site	Insertion site	1	kept

insertion skill	insertion skill	insertion skill	insertion skill	insertion skill	1	kept
Inservice	inservice	inservice	inservice	Inservice	1	kept
inservice education	inservice education	inservice education	inservice education	inservice education	1	kept
INSOMNIA	insomnia	insomnia	insomnia	insomnia	1	case_or_format
insomnia disorder	insomnia disorder	insomnia disorder	insomnia disorder	insomnia disorder	1	kept
insomnia symptoms	insomnia symptoms	insomnia symptoms	insomnia symptoms	insomnia symptoms	1	kept
Inspiratory effort	inspiratory effort	inspiratory effort	inspiratory effort	Inspiratory effort	1	kept
Inspiratory flow rate	inspiratory flow rate	inspiratory flow rate	inspiratory flow rate	Inspiratory flow rate	1	kept
inspiratory muscle strength training	inspiratory muscle strength training	inspiratory muscle strength training	inspiratory muscle strength training	inspiratory muscle strength training	1	kept
Inspiratory muscle training	inspiratory muscle training	inspiratory muscle training	inspiratory muscle training	Inspiratory muscle training	1	kept
inspiratory muscle training	inspiratory muscle training	inspiratory muscle training	inspiratory muscle training	Inspiratory muscle training	1	case_or_format
Inspiratory positive pressure ventilation	inspiratory positive pressure ventilation	inspiratory positive pressure ventilation	inspiratory positive pressure ventilation	Inspiratory positive pressure ventilation	1	kept
inspiratory reserve volume	inspiratory reserve volume	inspiratory reserve volume	inspiratory reserve volume	inspiratory reserve volume	1	kept
Instability	instability	instability	instability	instability	1	case_or_format
INSTITUTE	institute	institute	institute	INSTITUTE	1	kept
institution	institution	institution	institution	INSTITUTIONS	1	case_or_format

institutional culture	institutional culture	institutional culture	institutional culture	institutional culture	1	kept
Institutional decision making	institutional decision making	institutional decision making	institutional decision making	Institutional decision making	1	kept
institutional implementation	institutional implementation	institutional implementation	institutional implementation	institutional implementation	1	kept
Institutional isomorphism	institutional isomorphism	institutional isomorphism	institutional isomorphism	Institutional isomorphism	1	kept
institutional ordering	institutional ordering	institutional ordering	institutional ordering	institutional ordering	1	kept
institutional outcome	institutional outcome	institutional outcome	institutional outcome	institutional outcome	1	kept
institutional participation	institutional participation	institutional participation	institutional participation	institutional participation	1	kept
institutional review board	institutional review board	institutional review board	institutional review board	institutional review board	1	kept
Institutionalisation	institutionalisation	institutionalisation	institutionalisation	Institutionalisation	1	kept
institutionalized elderly	institutionalized elderly	institutionalized elderly	institutionalized elderly	institutionalized elderly	1	kept
Instrucciones previas	instrucciones previas	instrucciones previas	instrucciones previas	Instrucciones previas	1	kept
Instruction	instruction	instruction	instruction	Instruction	1	kept
Instructional films and video	instructional films and video	instructional films and video	instructional films and video	Instructional films and video	1	kept
instructional module	instructional module	instructional module	instructional module	instructional module	1	kept
Instructional Program	instructional program	instructional program	instructional program	Instructional Program	1	kept
Instructor-led training	instructor led training	instructor led training	instructor led training	Instructor-led training	1	kept

Instructors Team	instructors team	instructors team	instructors team	Instructors Team	1	kept
Instrument development and validation	instrument development and validation	instrument development and validation	instrument development and validation	Instrument development and validation	1	kept
instrument for measuring different types of cognitive	instrument for measuring different types of cognitive	instrument for measuring different types of cognitive	instrument for measuring different types of cognitive	instrument for measuring different types of cognitive	1	kept
INSTRUMENT TRANSLATION PROCESS	instrument translation process	instrument translation process	instrument translation process	INSTRUMENT TRANSLATION PROCESS	1	kept
INSTRUMENTAL ACTIVITIES	instrumental activities	instrumental activities	instrumental activities	INSTRUMENTAL ACTIVITIES	1	kept
Instrumental Activities of Daily Living Questionnaire	instrumental activities of daily living questionnaire	instrumental activities of daily living questionnaire	instrumental activities of daily living questionnaire	Instrumental Activities of Daily Living Questionnaire	1	kept
Instrumental birth	instrumental birth	instrumental birth	instrumental birth	Instrumental birth	1	kept
Instrumental pressure injury	instrumental pressure injury	instrumental pressure injury	instrumental pressure injury	Instrumental pressure injury	1	kept
Instrumental study	instrumental study	instrumental study	instrumental study	Instrumental study	1	kept
Instrumental touch	instrumental touch	instrumental touch	instrumental touch	Instrumental touch	1	kept
instrumental touching	instrumental touching	instrumental touching	instrumental touching	instrumental touching	1	kept
instrumental variable analysis	instrumental variable analysis	instrumental variable analysis	instrumental variable analysis	instrumental variable analysis	1	kept
Instrumental variables	instrumental variables	instrumental variables	instrumental variables	Instrumental variables	1	kept
instrumental variables	instrumental variables	instrumental variables	instrumental variables	Instrumental variables	1	case_or_format
Instrumentation	instrumentation	instrumentation	instrumentation	instrumentation	1	case_or_format
Instrumentation amplifier	instrumentation amplifier	instrumentation amplifier	instrumentation amplifier	Instrumentation amplifier	1	kept

instrumentation]	instrumentation	instrumentation	instrumentation	instrumentation	1	case_or_format
Insufficiently sampled	insufficiently sampled	insufficiently sampled	insufficiently sampled	Insufficiently sampled	1	kept
insufflation	insufflation	insufflation	insufflation	Insufflation	1	case_or_format
INSUFFLATION VENTILATORY EXCHANGE	insufflation ventilatory exchange	insufflation ventilatory exchange	insufflation ventilatory exchange	INSUFFLATION VENTILATORY EXCHANGE	1	kept
Insuficiencia de la vá lvula mitral	insuficiencia de la v lvula mitral	insuficiencia de la v lvula mitral	insuficiencia de la v lvula mitral	Insuficiencia de la vá lvula mitral	1	kept
Insuficiencia respiratoria	insuficiencia respiratoria	insuficiencia respiratoria	insuficiencia respiratoria	Insuficiencia respiratoria	1	kept
insula	insula	insula	insula	insula	1	kept
insulin administration	insulin administration	insulin administration	insulin administration	insulin administration	1	kept
insulin calculator	insulin calculator	insulin calculator	insulin calculator	insulin calculator	1	kept
insulin degludec	insulin degludec	insulin degludec	insulin degludec	insulin degludec	1	kept
Insulin drip	insulin drip	insulin drip	insulin drip	Insulin drip	1	kept
insulin drip	insulin drip	insulin drip	insulin drip	Insulin drip	1	case_or_format
insulin infusion protocol	insulin infusion protocol	insulin infusion protocol	insulin infusion protocol	insulin infusion protocol	1	kept
Insulin infusion system	insulin infusion system	insulin infusion system	insulin infusion system	Insulin Infusion Systems	1	case_or_format
insulin infusion systems	insulin infusion systems	insulin infusion systems	insulin infusion system	Insulin Infusion Systems	1	singular
insulin initiation	insulin initiation	insulin initiation	insulin initiation	insulin initiation	1	kept

insulin level	insulin level	insulin level	insulin level	insulin level	1	kept
insulin like growth factor binding protein 7	insulin like growth factor binding protein 7	insulin like growth factor binding protein 7	insulin like growth factor binding protein 7	insulin like growth factor binding protein 7	1	kept
insulin management	insulin management	insulin management	insulin management	insulin management	1	kept
insulin metabolism	insulin metabolism	insulin metabolism	insulin metabolism	insulin metabolism	1	kept
insulin pens	insulin pens	insulin pens	insulin pens	insulin pens	1	kept
Insulin pump	insulin pump	insulin pump	insulin pump	insulin pump	1	case_or_format
Insulin Pump Therapy	insulin pump therapy	insulin pump therapy	insulin pump therapy	Insulin Pump Therapy	1	kept
Insulin regimen	insulin regimen	insulin regimen	insulin regimen	Insulin regimen	1	kept
insulin release	insulin release	insulin release	insulin release	insulin release	1	kept
Insulin resistance	insulin resistance	insulin resistance	insulin resistance	insulin resistance	1	case_or_format
Insulin sensitivity	insulin sensitivity	insulin sensitivity	insulin sensitivity	insulin sensitivity	1	case_or_format
insulin sensitivity coefficient	insulin sensitivity coefficient	insulin sensitivity coefficient	insulin sensitivity coefficient	insulin sensitivity coefficient	1	kept
insulin shock	insulin shock	insulin shock	insulin shock	insulin shock	1	kept
Insulin Therapy	insulin therapy	insulin therapy	insulin therapy	Insulin therapy	1	case_or_format
Insulin utilisation	insulin utilisation	insulin utilisation	insulin utilisation	Insulin utilisation	1	kept
insulin vial and syringes	insulin vial and syringes	insulin vial and syringes	insulin vial and syringes	insulin vial and syringes	1	kept

Insulin-Like Growth Factor Binding Proteins	insulin like growth factor binding proteins	insulin like growth factor binding proteins	insulin like growth factor binding proteins	Insulin-Like Growth Factor Binding Proteins	1	kept
Insulin-Like Growth Factor I	insulin like growth factor i	insulin like growth factor i	insulin like growth factor i	Insulin-Like Growth Factor I	1	kept
insulin-like growth factor-1	insulin like growth factor 1	insulin like growth factor 1	insulin like growth factor 1	insulin-like growth factor-1	1	kept
insulin-like growth factor-binding protein 7	insulin like growth factor binding protein 7	insulin like growth factor binding protein 7	insulin like growth factor binding protein 7	insulin like growth factor binding protein 7	1	case_or_format
Insulina	insulina	insulina	insulina	Insulina	1	kept
insulinothérapie	insulinoth rapie	insulinoth rapie	insulinoth rapie	insulinothérapie	1	kept
insulin‐dependent	insulin dependent	insulin dependent	insulin dependent	insulin‐dependent	1	kept
insuman comb 50	insuman comb 50	insuman comb 50	insuman comb 50	insuman comb 50	1	kept
insurance coverage	insurance coverage	insurance coverage	insurance coverage	Insurance Coverage	1	case_or_format
insurance disparities	insurance disparities	insurance disparities	insurance disparities	insurance disparities	1	kept
insurance status	insurance status	insurance status	insurance status	insurance status	1	kept
Insurance, Accident	insurance accident	insurance accident	insurance accident	Insurance, Accident	1	kept
Insurance, Liability	insurance liability	insurance liability	insurance liability	Insurance, Liability	1	kept
Intake and Output	intake and output	intake and output	intake and output	Intake and Output	1	kept
INTcare system	intcare system	intcare system	intcare system	INTCare system	1	case_or_format
INTCare tracking systems	intcare tracking systems	intcare tracking systems	intcare tracking systems	INTCare tracking systems	1	kept

Integer linear programming	integer linear programming	integer linear programming	integer linear programming	Integer Linear Programming	1	case_or_format
integer linear programming	integer linear programming	integer linear programming	integer linear programming	Integer Linear Programming	1	case_or_format
Integer nonlinear programming	integer nonlinear programming	integer nonlinear programming	integer nonlinear programming	Integer nonlinear programming	1	kept
Integer programming models	integer programming models	integer programming models	integer programming models	Integer programming models	1	kept
Integra	integra	integra	integra	Integra	1	kept
Integración	integraci n	integraci n	integraci n	Integración	1	kept
Integrated Advanced Information Management Systems	integrated advanced information management systems	integrated advanced information management systems	integrated advanced information management systems	Integrated Advanced Information Management Systems	1	kept
integrated approach	integrated approach	integrated approach	integrated approach	integrated approach	1	kept
Integrated approach to e-learning	integrated approach to e learning	integrated approach to e learning	integrated approach to e learning	Integrated approach to e-learning	1	kept
Integrated care	integrated care	integrated care	integrated care	integrated care	1	case_or_format
integrated care management program	integrated care management program	integrated care management program	integrated care management program	integrated care management program	1	kept
Integrated circuit design	integrated circuit design	integrated circuit design	integrated circuit design	Integrated circuit design	1	kept
Integrated circuits	integrated circuits	integrated circuits	integrated circuits	Integrated circuits	1	kept
Integrated control	integrated control	integrated control	integrated control	Integrated control	1	kept
integrated delivery of health care	integrated delivery of health care	integrated delivery of health care	integrated delivery of health care	integrated delivery of health care	1	kept
Integrated Discrimination Index	integrated discrimination index	integrated discrimination index	integrated discrimination index	Integrated Discrimination Index	1	kept

integrated graphical information display	integrated graphical information display	integrated graphical information display	integrated graphical information display	integrated graphical information display	1	kept
Integrated health care	integrated health care	integrated health care	integrated health care	Integrated health care	1	kept
Integrated health information systems	integrated health information systems	integrated health information systems	integrated health information systems	Integrated health information systems	1	kept
Integrated Health Monitoring	integrated health monitoring	integrated health monitoring	integrated health monitoring	Integrated Health Monitoring	1	kept
integrated home care	integrated home care	integrated home care	integrated home care	integrated home care	1	kept
INTEGRATED KNOWLEDGE TRANSLATION	integrated knowledge translation	integrated knowledge translation	integrated knowledge translation	INTEGRATED KNOWLEDGE TRANSLATION	1	kept
Integrated learning experience	integrated learning experience	integrated learning experience	integrated learning experience	Integrated learning experience	1	kept
integrated neonatal care unit	integrated neonatal care unit	integrated neonatal care unit	integrated neonatal care unit	integrated neonatal care unit	1	kept
Integrated Operations	integrated operations	integrated operations	integrated operations	Integrated Operations	1	kept
integrated palliative care team	integrated palliative care team	integrated palliative care team	integrated palliative care team	integrated palliative care team	1	kept
Integrated pest management	integrated pest management	integrated pest management	integrated pest management	Integrated pest management	1	kept
Integrated planning	integrated planning	integrated planning	integrated planning	Integrated planning	1	kept
Integrated practice unit	integrated practice unit	integrated practice unit	integrated practice unit	Integrated practice unit	1	kept
integrated pulmonary index	integrated pulmonary index	integrated pulmonary index	integrated pulmonary index	integrated pulmonary index	1	kept
Integrated review	integrated review	integrated review	integrated review	Integrated review	1	kept
Integrated simulators	integrated simulators	integrated simulators	integrated simulators	Integrated simulators	1	kept

Integrated treatment	integrated treatment	integrated treatment	integrated treatment	Integrated treatment	1	kept
integrated unit	integrated unit	integrated unit	integrated unit	integrated unit	1	kept
Integrating systems	integrating systems	integrating systems	integrating systems	Integrating systems	1	kept
Integration time	integration time	integration time	integration time	Integration time	1	kept
Integrative	integrative	integrative	integrative	Integrative	1	kept
integrative	integrative	integrative	integrative	Integrative	1	case_or_format
Integrative assessment	integrative assessment	integrative assessment	integrative assessment	Integrative assessment	1	kept
integrative care program	integrative care program	integrative care program	integrative care program	integrative care program	1	kept
Integrative model	integrative model	integrative model	integrative model	Integrative model	1	kept
integrative pain management	integrative pain management	integrative pain management	integrative pain management	integrative pain management	1	kept
Integrative psychological support	integrative psychological support	integrative psychological support	integrative psychological support	Integrative psychological support	1	kept
Integrative Review	integrative review	integrative review	integrative review	integrative review	1	case_or_format
INTEGRATIVE REVIEWS	integrative reviews	integrative reviews	integrative review	integrative review	1	singular
integrative therapies	integrative therapies	integrative therapies	integrative therapies	Integrative therapies	1	case_or_format
Integração	integra o	integra o	integra o	Integração	1	kept
integron	integron	integron	integron	integron	1	kept

Integrons	integrons	integrons	integron	integron	1	singular
Inteligencia artificial	inteligencia artificial	inteligencia artificial	inteligencia artificial	Inteligencia artificial	1	kept
intellect	intellect	intellect	intellect	intellect	1	kept
intellectual delays	intellectual delays	intellectual delays	intellectual delays	intellectual delays	1	kept
intellectual developmental disabilities	intellectual developmental disabilities	intellectual developmental disabilities	intellectual developmental disabilities	intellectual developmental disabilities	1	kept
Intellectual disabilities	intellectual disabilities	intellectual disabilities	intellectual disability	Intellectual Disability	1	singular
intellectual disabilities	intellectual disabilities	intellectual disabilities	intellectual disability	Intellectual Disability	1	singular
Intellectual disability	intellectual disability	intellectual disability	intellectual disability	Intellectual Disability	1	case_or_format
intelligence artificielle	intelligence artificielle	intelligence artificielle	intelligence artificielle	intelligence artificielle	1	kept
Intelligence Care	intelligence care	intelligence care	intelligence care	Intelligence Care	1	kept
Intelligence care	intelligence care	intelligence care	intelligence care	Intelligence Care	1	case_or_format
Intelligence models	intelligence models	intelligence models	intelligence models	Intelligence models	1	kept
intelligent control	intelligent control	intelligent control	intelligent control	Intelligent control	1	case_or_format
intelligent management	intelligent management	intelligent management	intelligent management	intelligent management	1	kept
Intelligent monitoring	intelligent monitoring	intelligent monitoring	intelligent monitoring	Intelligent monitoring	1	kept
Intelligent robots	intelligent robots	intelligent robots	intelligent robot	Intelligent robot	1	singular

intelligent threshold	intelligent threshold	intelligent threshold	intelligent threshold	intelligent threshold	1	kept
Intellivent	intellivent	intellivent	intellivent	Intellivent	1	kept
INTELLiVENT-ASV	intellivent asv	intellivent asv	intellivent asv	INTELLiVENT-ASV	1	kept
Intención de abandono de la profesión	intenci n de abandono de la profesi n	intenci n de abandono de la profesi n	intenci n de abandono de la profesi n	Intención de abandono de la profesión	1	kept
Intención de rotar	intenci n de rotar	intenci n de rotar	intenci n de rotar	Intención de rotar	1	kept
Intensidad de cuidados	intensidad de cuidados	intensidad de cuidados	intensidad de cuidados	Intensidad de cuidados	1	kept
INTENSITY	intensity	intensity	intensity	INTENSITY	1	kept
Intensity detection	intensity detection	intensity detection	intensity detection	Intensity detection	1	kept
Intensity levels	intensity levels	intensity levels	intensity levels	Intensity levels	1	kept
Intensity of care	intensity of care	intensity of care	intensity of care	Intensity of care	1	kept
intensity of treatment	intensity of treatment	intensity of treatment	intensity of treatment	Intensity of treatment	1	case_or_format
intensive are unit	intensive are unit	intensive are unit	intensive are unit	intensive are unit	1	kept
Intensive cardiac care	intensive cardiac care	intensive cardiac care	intensive cardiac care	Intensive cardiac care	1	kept
Intensive cardiac care unit	intensive cardiac care unit	intensive cardiac care unit	intensive cardiac care unit	Intensive Cardiac Care Unit	1	case_or_format
intensive cardiovascular care units	intensive cardiovascular care units	intensive cardiovascular care units	intensive cardiovascular care units	intensive cardiovascular care units	1	kept
intensive home care	intensive home care	intensive home care	intensive home care	intensive home care	1	kept

intensive insulin	intensive insulin	intensive insulin	intensive insulin	intensive insulin	1	kept
Intensive Insulin Protocols	intensive insulin protocols	intensive insulin protocols	intensive insulin protocols	Intensive Insulin Protocols	1	kept
Intensive Insulin therapy	intensive insulin therapy	intensive insulin therapy	intensive insulin therapy	Intensive insulin therapy	1	case_or_format
Intensive management	intensive management	intensive management	intensive management	Intensive management	1	kept
Intensive Medicine	intensive medicine	intensive medicine	intensive medicine	Intensive Medicine	1	kept
intensive palliative care unit	intensive palliative care unit	intensive palliative care unit	intensive palliative care unit	intensive palliative care unit	1	kept
intensive psychiatry	intensive psychiatry	intensive psychiatry	intensive psychiatry	intensive psychiatry	1	kept
intensive rehabilitation	intensive rehabilitation	intensive rehabilitation	intensive rehabilitation	intensive rehabilitation	1	kept
intensive rehabilitation ward	intensive rehabilitation ward	intensive rehabilitation ward	intensive rehabilitation ward	intensive rehabilitation ward	1	kept
intensive respiratory care	intensive respiratory care	intensive respiratory care	intensive respiratory care	intensive respiratory care	1	kept
Intensive room	intensive room	intensive room	intensive room	Intensive room	1	kept
Intensive Short-Term Dynamic Psychotherapy	intensive short term dynamic psychotherapy	intensive short term dynamic psychotherapy	intensive short term dynamic psychotherapy	Intensive Short-Term Dynamic Psychotherapy	1	kept
Intensive Therapy	intensive therapy	intensive therapy	intensive therapy	Intensive therapy	1	case_or_format
Intensive therapy unit	intensive therapy unit	intensive therapy unit	intensive therapy unit	Intensive therapy units	1	case_or_format
intensive treatment	intensive treatment	intensive treatment	intensive treatment	intensive treatment	1	kept
Intensive unit acquired weakness	intensive unit acquired weakness	intensive unit acquired weakness	intensive unit acquired weakness	Intensive unit acquired weakness	1	kept

Intensive unit caregivers	intensive unit caregivers	intensive unit caregivers	intensive unit caregivers	Intensive unit caregivers	1	kept
intensive vascular care	intensive vascular care	intensive vascular care	intensive vascular care	intensive vascular care	1	kept
intensive-insulin therapy	intensive insulin therapy	intensive insulin therapy	intensive insulin therapy	Intensive insulin therapy	1	case_or_format
INTENSIVIST	intensivist	intensivist	intensivist	intensivist	1	case_or_format
Intensivist model	intensivist model	intensivist model	intensivist model	Intensivist model	1	kept
Intensivist presence	intensivist presence	intensivist presence	intensivist presence	Intensivist presence	1	kept
INTENSIVIST SHORTAGE	intensivist shortage	intensivist shortage	intensivist shortage	INTENSIVIST SHORTAGE	1	kept
intensivists	intensivists	intensivists	intensivist	intensivist	1	singular
Intensivstationen	intensivstationen	intensivstationen	intensivstationen	Intensivstationen	1	kept
intensvie care unit	intensvie care unit	intensvie care unit	intensvie care unit	intensvie care unit	1	kept
INTENT	intent	intent	intent	INTENT	1	kept
intent to report	intent to report	intent to report	intent to report	intent to report	1	kept
intent to stay	intent to stay	intent to stay	intent to stay	Intent to stay	1	case_or_format
intent to stay scale	intent to stay scale	intent to stay scale	intent to stay scale	intent to stay scale	1	kept
Intent-to-leave	intent to leave	intent to leave	intent to leave	Intent to leave	1	case_or_format
intention of voluntary report	intention of voluntary report	intention of voluntary report	intention of voluntary report	intention of voluntary report	1	kept

Intention to care	intention to care	intention to care	intention to care	Intention to care	1	kept
Intention to leave profession	intention to leave profession	intention to leave profession	intention to leave profession	Intention to leave profession	1	kept
intention to practice	intention to practice	intention to practice	intention to practice	intention to practice	1	kept
intention to remain employed	intention to remain employed	intention to remain employed	intention to remain employed	intention to remain employed	1	kept
Intention-to-leave	intention to leave	intention to leave	intention to leave	Intention to leave	1	case_or_format
Intention-to-treat	intention to treat	intention to treat	intention to treat	Intention-to-treat	1	kept
Intentional injury	intentional injury	intentional injury	intentional injury	Intentional injury	1	kept
intentional life-ending acts	intentional life ending acts	intentional life ending acts	intentional life ending acts	intentional life-ending acts	1	kept
intentional rounding	intentional rounding	intentional rounding	intentional rounding	intentional rounding	1	kept
intentional silence	intentional silence	intentional silence	intentional silence	intentional silence	1	kept
Intentions to leave	intentions to leave	intentions to leave	intentions to leave	Intentions to leave	1	kept
Intepretive	intepretive	intepretive	intepretive	Intepretive	1	kept
Inter changeable operator model	inter changeable operator model	inter changeable operator model	inter changeable operator model	Inter changeable operator model	1	kept
inter facility transfer	inter facility transfer	inter facility transfer	inter facility transfer	inter facility transfer	1	kept
Inter hospital	inter hospital	inter hospital	inter hospital	Inter hospital	1	kept
Inter-disciplinary team	inter disciplinary team	inter disciplinary team	inter disciplinary team	Inter-disciplinary team	1	kept

Inter-facility transfer	inter facility transfer	inter facility transfer	inter facility transfer	inter facility transfer	1	case_or_format
Inter-facility transport	inter facility transport	inter facility transport	inter facility transport	Inter-facility transport	1	kept
Inter-hospital transport	inter hospital transport	inter hospital transport	inter hospital transport	inter-hospital transport	1	case_or_format
Inter-observer agreement	inter observer agreement	inter observer agreement	inter observer agreement	Inter-observer agreement	1	kept
inter-observer reliability	inter observer reliability	inter observer reliability	inter observer reliability	inter-observer reliability	1	kept
Inter-professioal collaboration	inter professioal collaboration	inter professioal collaboration	inter professioal collaboration	Inter-professioal collaboration	1	kept
Inter-professional	inter professional	inter professional	inter professional	Inter professionals	1	case_or_format
Inter-professional care	inter professional care	inter professional care	inter professional care	Inter-professional care	1	kept
Inter-professional Collaboration	inter professional collaboration	inter professional collaboration	inter professional collaboration	Inter-professional collaboration	1	case_or_format
Inter-professional communication	inter professional communication	inter professional communication	inter professional communication	Inter-professional communication	1	kept
Inter-professional cooperation	inter professional cooperation	inter professional cooperation	inter professional cooperation	Inter-professional cooperation	1	kept
inter-professional cooperation	inter professional cooperation	inter professional cooperation	inter professional cooperation	Inter-professional cooperation	1	case_or_format
inter-professional ethics	inter professional ethics	inter professional ethics	inter professional ethics	inter-professional ethics	1	kept
Inter-professional interactions	inter professional interactions	inter professional interactions	inter professional interactions	Inter-professional interactions	1	kept
inter-professional interactions	inter professional interactions	inter professional interactions	inter professional interactions	Inter-professional interactions	1	case_or_format
Inter-professional relationship	inter professional relationship	inter professional relationship	inter professional relationship	Inter-professional relationship	1	kept

Inter-professional relationships	inter professional relationships	inter professional relationships	inter professional relationship	Inter-professional relationship	1	singular
Inter-professional teams	inter professional teams	inter professional teams	inter professional teams	Inter-professional teams	1	kept
Inter-rater Reliability	inter rater reliability	inter rater reliability	inter rater reliability	inter-rater reliability	1	case_or_format
Inter-rater reliability	inter rater reliability	inter rater reliability	inter rater reliability	inter-rater reliability	1	case_or_format
Inter-rater variability	inter rater variability	inter rater variability	inter rater variability	inter-rater variability	1	case_or_format
inter-shift handoff	inter shift handoff	inter shift handoff	inter shift handoff	inter-shift handoff	1	kept
interaction analysis	interaction analysis	interaction analysis	interaction analysis	interaction analysis	1	kept
Interaction device	interaction device	interaction device	interaction device	Interaction device	1	kept
Interaction devices	interaction devices	interaction devices	interaction device	Interaction device	1	singular
interaction with family	interaction with family	interaction with family	interaction with family	interaction with family	1	kept
Interaction-related behaviors	interaction related behaviors	interaction related behaviors	interaction related behaviors	Interaction-related behaviors	1	kept
Interactional factor	interactional factor	interactional factor	interactional factor	Interactional factor	1	kept
interactional justice	interactional justice	interactional justice	interactional justice	Interactional justice	1	case_or_format
interactions	interactions	interactions	interaction	Interaction	1	singular
Interactive discussion group	interactive discussion group	interactive discussion group	interactive discussion group	Interactive discussion group	1	kept
Interactive e-book	interactive e book	interactive e book	interactive e book	Interactive e-book	1	kept

Interactive handgrip game	interactive handgrip game	interactive handgrip game	interactive handgrip game	Interactive handgrip game	1	kept
Interactive infiltration	interactive infiltration	interactive infiltration	interactive infiltration	Interactive infiltration	1	kept
Interactive learning environment	interactive learning environment	interactive learning environment	interactive learning environment	Interactive learning environment	1	kept
Interactive platform	interactive platform	interactive platform	interactive platform	Interactive platform	1	kept
Interactive practice	interactive practice	interactive practice	interactive practice	Interactive practice	1	kept
Interactive scenarios	interactive scenarios	interactive scenarios	interactive scenarios	Interactive scenarios	1	kept
interactive technology	interactive technology	interactive technology	interactive technology	interactive technology	1	kept
Interactive Training	interactive training	interactive training	interactive training	Interactive Training	1	kept
interactive workshops	interactive workshops	interactive workshops	interactive workshops	interactive workshops	1	kept
interatrial block	interatrial block	interatrial block	interatrial block	interatrial block	1	kept
Intercellular adhesion molecule 1	intercellular adhesion molecule 1	intercellular adhesion molecule 1	intercellular adhesion molecule 1	intercellular adhesion molecule 1	1	case_or_format
Intercellular Adhesion Molecule-1	intercellular adhesion molecule 1	intercellular adhesion molecule 1	intercellular adhesion molecule 1	intercellular adhesion molecule 1	1	case_or_format
interclass correlation coefficient	interclass correlation coefficient	interclass correlation coefficient	interclass correlation coefficient	interclass correlation coefficient	1	kept
intercostal nerve blocking	intercostal nerve blocking	intercostal nerve blocking	intercostal nerve blocking	intercostal nerve blocking	1	kept
Intercultural care	intercultural care	intercultural care	intercultural care	Intercultural care	1	kept
Intercultural translation	intercultural translation	intercultural translation	intercultural translation	Intercultural translation	1	kept

interdepartmental care	interdepartmental care	interdepartmental care	interdepartmental care	interdepartmental care	1	kept
interdepartmental communication	interdepartmental communication	interdepartmental communication	interdepartmental communication	interdepartmental communication	1	kept
interdigitation zone	interdigitation zone	interdigitation zone	interdigitation zone	interdigitation zone	1	kept
Interdisciplinary applied science	interdisciplinary applied science	interdisciplinary applied science	interdisciplinary applied science	Interdisciplinary applied science	1	kept
interdisciplinary bedside rounds	interdisciplinary bedside rounds	interdisciplinary bedside rounds	interdisciplinary bedside rounds	interdisciplinary bedside rounds	1	kept
INTERDISCIPLINARY COLLABORATION	interdisciplinary collaboration	interdisciplinary collaboration	interdisciplinary collaboration	Interdisciplinary collaboration	1	case_or_format
INTERDISCIPLINARY COMMUNICATION	interdisciplinary communication	interdisciplinary communication	interdisciplinary communication	Interdisciplinary Communication	1	case_or_format
Interdisciplinary communications	interdisciplinary communications	interdisciplinary communications	interdisciplinary communication	Interdisciplinary Communication	1	singular
interdisciplinary cooperation	interdisciplinary cooperation	interdisciplinary cooperation	interdisciplinary cooperation	Interdisciplinary cooperation	1	case_or_format
interdisciplinary debriefing	interdisciplinary debriefing	interdisciplinary debriefing	interdisciplinary debriefing	interdisciplinary debriefing	1	kept
Interdisciplinary education	interdisciplinary education	interdisciplinary education	interdisciplinary education	interdisciplinary education	1	case_or_format
interdisciplinary palliative care education	interdisciplinary palliative care education	interdisciplinary palliative care education	interdisciplinary palliative care education	interdisciplinary palliative care education	1	kept
Interdisciplinary reflection	interdisciplinary reflection	interdisciplinary reflection	interdisciplinary reflection	Interdisciplinary reflection	1	kept
interdisciplinary relationships	interdisciplinary relationships	interdisciplinary relationships	interdisciplinary relationships	interdisciplinary relationships	1	kept
INTERDISCIPLINARY ROUNDS	interdisciplinary rounds	interdisciplinary rounds	interdisciplinary rounds	interdisciplinary rounds	1	case_or_format
Interdisciplinary rounds	interdisciplinary rounds	interdisciplinary rounds	interdisciplinary rounds	interdisciplinary rounds	1	case_or_format

Interdisciplinary team collaboration	interdisciplinary team collaboration	interdisciplinary team collaboration	interdisciplinary team collaboration	Interdisciplinary team collaboration	1	kept
Interdisciplinary team supervision	interdisciplinary team supervision	interdisciplinary team supervision	interdisciplinary team supervision	Interdisciplinary team supervision	1	kept
Interdisciplinary Teamwork	interdisciplinary teamwork	interdisciplinary teamwork	interdisciplinary teamwork	interdisciplinary teamwork	1	case_or_format
Interdisciplinary teamwork in health care	interdisciplinary teamwork in health care	interdisciplinary teamwork in health care	interdisciplinary teamwork in health care	Interdisciplinary teamwork in health care	1	kept
Interdisciplinary training	interdisciplinary training	interdisciplinary training	interdisciplinary training	Interdisciplinary training	1	kept
interest	interest	interest	interest	interest	1	kept
Interface	interface	interface	interface	Interface	1	kept
INTERFACE DESIGN	interface design	interface design	interface design	INTERFACE DESIGN	1	kept
Interface design	interface design	interface design	interface design	INTERFACE DESIGN	1	case_or_format
Interface functions	interface functions	interface functions	interface functions	Interface functions	1	kept
interface terminology	interface terminology	interface terminology	interface terminology	interface terminology	1	kept
Interface usability	interface usability	interface usability	interface usability	Interface usability	1	kept
interface usability	interface usability	interface usability	interface usability	Interface usability	1	case_or_format
Interfaces	interfaces	interfaces	interface	Interface	1	singular
Interfaces and usability	interfaces and usability	interfaces and usability	interfaces and usability	Interfaces and usability	1	kept
Interfacility transfer	interfacility transfer	interfacility transfer	interfacility transfer	interfacility transfer	1	case_or_format

interfacility transfers	interfacility transfers	interfacility transfers	interfacility transfer	interfacility transfer	1	singular
Interfacility transfers, handovers	interfacility transfers handovers	interfacility transfers handovers	interfacility transfers handovers	Interfacility transfers, handovers	1	kept
INTERFACILITY TRANSPORT	interfacility transport	interfacility transport	interfacility transport	Interfacility transport	1	case_or_format
interfaith chaplaincy	interfaith chaplaincy	interfaith chaplaincy	interfaith chaplaincy	interfaith chaplaincy	1	kept
Interfase	interfase	interfase	interfase	Interfase	1	kept
interferometry	interferometry	interferometry	interferometry	interferometry	1	kept
Interferon gamma	interferon gamma	interferon gamma	interferon gamma	Interferon-gamma	1	case_or_format
interferon range	interferon range	interferon range	interferon range	interferon range	1	kept
interferon regulatory factor	interferon regulatory factor	interferon regulatory factor	interferon regulatory factor	interferon regulatory factor	1	kept
Interferon-gamma Release Tests	interferon gamma release tests	interferon gamma release tests	interferon gamma release tests	Interferon-gamma Release Tests	1	kept
Interferon-gamma release tests	interferon gamma release tests	interferon gamma release tests	interferon gamma release tests	Interferon-gamma Release Tests	1	case_or_format
interferon-gamma release tests	interferon gamma release tests	interferon gamma release tests	interferon gamma release tests	Interferon-gamma Release Tests	1	case_or_format
Interferons	interferons	interferons	interferon	interferon	1	singular
intergenerational relations	intergenerational relations	intergenerational relations	intergenerational relations	Intergenerational Relations	1	case_or_format
intergovernmental organization	intergovernmental organization	intergovernmental organization	intergovernmental organization	intergovernmental organization	1	kept
Interhospital	interhospital	interhospital	interhospital	Interhospital	1	kept

interhospital	interhospital	interhospital	interhospital	Interhospital	1	case_or_format
INTERHOSPITAL TRANSFER	interhospital transfer	interhospital transfer	interhospital transfer	Interhospital transfer	1	case_or_format
INTERHOSPITAL TRANSFERS	interhospital transfers	interhospital transfers	interhospital transfer	Interhospital transfer	1	singular
interhospital transport	interhospital transport	interhospital transport	interhospital transport	Interhospital transport	1	case_or_format
Interhospital transport of critically ill infants	interhospital transport of critically ill infants	interhospital transport of critically ill infants	interhospital transport of critically ill infants	Interhospital transport of critically ill infants	1	kept
Interindividual variation	interindividual variation	interindividual variation	interindividual variation	Interindividual variation	1	kept
Interior design and furnishings	interior design and furnishings	interior design and furnishings	interior design and furnishings	Interior Design and Furnishings	1	case_or_format
interior design and furnishings	interior design and furnishings	interior design and furnishings	interior design and furnishings	Interior Design and Furnishings	1	case_or_format
interleukin 1 receptor type II	interleukin 1 receptor type ii	interleukin 1 receptor type ii	interleukin 1 receptor type ii	interleukin 1 receptor type II	1	kept
interleukin 12	interleukin 12	interleukin 12	interleukin 12	interleukin 12	1	kept
interleukin 16	interleukin 16	interleukin 16	interleukin 16	interleukin 16	1	kept
interleukin 18	interleukin 18	interleukin 18	interleukin 18	interleukin 18	1	kept
interleukin 2 receptor antibody	interleukin 2 receptor antibody	interleukin 2 receptor antibody	interleukin 2 receptor antibody	interleukin 2 receptor antibody	1	kept
interleukin 6 inhibitor	interleukin 6 inhibitor	interleukin 6 inhibitor	interleukin 6 inhibitor	interleukin 6 inhibitor	1	kept
Interleukin-1	interleukin 1	interleukin 1	interleukin 1	interleukin 1	1	case_or_format
interleukin-1 beta	interleukin 1 beta	interleukin 1 beta	interleukin 1 beta	interleukin-1 beta	1	kept

Interleukin-18	interleukin 18	interleukin 18	interleukin 18	interleukin 18	1	case_or_format
interleukin-2	interleukin 2	interleukin 2	interleukin 2	interleukin 2	1	case_or_format
interleukin-6	interleukin 6	interleukin 6	interleukin 6	interleukin 6	1	case_or_format
Interleukins	interleukins	interleukins	interleukins	Interleukins	1	kept
Interlink	interlink	interlink	interlink	Interlink	1	kept
intermediate	intermediate	intermediate	intermediate	intermediate	1	kept
intermediate care facilities	intermediate care facilities	intermediate care facilities	intermediate care facility	Intermediate Care Facilities	1	singular
Intermediate care facilities [economics]	intermediate care facilities economics	intermediate care facilities economics	intermediate care facilities economics	Intermediate care facilities [economics]	1	kept
intermediate care facility	intermediate care facility	intermediate care facility	intermediate care facility	Intermediate Care Facilities	1	case_or_format
Intermediate Care Units	intermediate care units	intermediate care units	intermediate care unit	intermediate care unit	1	singular
Intermediate critical wards	intermediate critical wards	intermediate critical wards	intermediate critical wards	Intermediate critical wards	1	kept
intermediate dependency care unit	intermediate dependency care unit	intermediate dependency care unit	intermediate dependency care unit	intermediate dependency care unit	1	kept
Intermediate effect.	intermediate effect	intermediate effect	intermediate effect	Intermediate effect.	1	kept
intermediate medical care unit	intermediate medical care unit	intermediate medical care unit	intermediate medical care unit	intermediate medical care unit	1	kept
Intermediate respiratory care units	intermediate respiratory care units	intermediate respiratory care units	intermediate respiratory care unit	intermediate respiratory care unit	1	singular
Intermediate-care facilities	intermediate care facilities	intermediate care facilities	intermediate care facility	Intermediate Care Facilities	1	singular

intermittent feeding	intermittent feeding	intermittent feeding	intermittent feeding	intermittent feeding	1	kept
Intermittent flushing	intermittent flushing	intermittent flushing	intermittent flushing	Intermittent flushing	1	kept
INTERMITTENT HEMODIALYSIS	intermittent hemodialysis	intermittent hemodialysis	intermittent hemodialysis	Intermittent hemodialysis	1	case_or_format
INTERMITTENT INFUSION	intermittent infusion	intermittent infusion	intermittent infusion	INTERMITTENT INFUSION	1	kept
Intermittent phototherapy	intermittent phototherapy	intermittent phototherapy	intermittent phototherapy	Intermittent phototherapy	1	kept
intermittent phototherapy	intermittent phototherapy	intermittent phototherapy	intermittent phototherapy	Intermittent phototherapy	1	case_or_format
Intermittent pneumatic compression devices	intermittent pneumatic compression devices	intermittent pneumatic compression devices	intermittent pneumatic compression device	Intermittent Pneumatic Compression Devices	1	singular
intermittent pneumatic compression devices	intermittent pneumatic compression devices	intermittent pneumatic compression devices	intermittent pneumatic compression device	Intermittent Pneumatic Compression Devices	1	singular
Intermittent Positive-Pressure Breathing	intermittent positive pressure breathing	intermittent positive pressure breathing	intermittent positive pressure breathing	Intermittent Positive-Pressure Breathing	1	kept
intermittent positive-pressure ventilation	intermittent positive pressure ventilation	intermittent positive pressure ventilation	intermittent positive pressure ventilation	Intermittent Positive-Pressure Ventilation	1	case_or_format
intermittent sedation	intermittent sedation	intermittent sedation	intermittent sedation	intermittent sedation	1	kept
intermittent sedation interruption	intermittent sedation interruption	intermittent sedation interruption	intermittent sedation interruption	intermittent sedation interruption	1	kept
Intermodernism	intermodernism	intermodernism	intermodernism	Intermodernism	1	kept
internal carotid artery	internal carotid artery	internal carotid artery	internal carotid artery	internal carotid artery	1	kept
internal carotid artery stenosis	internal carotid artery stenosis	internal carotid artery stenosis	internal carotid artery stenosis	internal carotid artery stenosis	1	kept
Internal consistency	internal consistency	internal consistency	internal consistency	internal consistency	1	case_or_format

Internal consistency reliability	internal consistency reliability	internal consistency reliability	internal consistency reliability	Internal consistency reliability	1	kept
internal consistency reliability	internal consistency reliability	internal consistency reliability	internal consistency reliability	Internal consistency reliability	1	case_or_format
internal crisis communication	internal crisis communication	internal crisis communication	internal crisis communication	internal crisis communication	1	kept
internal disease	internal disease	internal disease	internal disease	internal disease	1	kept
internal fixation	internal fixation	internal fixation	internal fixation	internal fixation	1	kept
Internal fixation treatment	internal fixation treatment	internal fixation treatment	internal fixation treatment	Internal fixation treatment	1	kept
internal fixator	internal fixator	internal fixator	internal fixator	Internal Fixators	1	case_or_format
internal jugular venous access	internal jugular venous access	internal jugular venous access	internal jugular venous access	internal jugular venous access	1	kept
INTERNAL JUGULAR-VEIN	internal jugular vein	internal jugular vein	internal jugular vein	internal jugular vein	1	case_or_format
internal limiting membrane	internal limiting membrane	internal limiting membrane	internal limiting membrane	internal limiting membrane	1	kept
internal medical ward	internal medical ward	internal medical ward	internal medical ward	internal medical ward	1	kept
Internal medicine department	internal medicine department	internal medicine department	internal medicine department	Internal medicine department	1	kept
internal medicine ward	internal medicine ward	internal medicine ward	internal medicine ward	internal medicine ward	1	kept
internal medicine-pediatrics	internal medicine pediatrics	internal medicine pediatrics	internal medicine pediatrics	internal medicine-pediatrics	1	kept
Internal organs	internal organs	internal organs	internal organs	Internal organs	1	kept
internal quality control	internal quality control	internal quality control	internal quality control	internal quality control	1	kept

internal transfer	internal transfer	internal transfer	internal transfer	internal transfer	1	kept
internal whistleblowing	internal whistleblowing	internal whistleblowing	internal whistleblowing	internal whistleblowing	1	kept
Internalizing	internalizing	internalizing	internalizing	Internalizing	1	kept
Internalizing and externalizing behavior	internalizing and externalizing behavior	internalizing and externalizing behavior	internalizing and externalizing behavior	Internalizing and externalizing behavior	1	kept
internalizing disorder	internalizing disorder	internalizing disorder	internalizing disorder	internalizing disorder	1	kept
international aspects	international aspects	international aspects	international aspects	international aspects	1	kept
International benchmark	international benchmark	international benchmark	international benchmark	International benchmark	1	kept
international benchmarking	international benchmarking	international benchmarking	international benchmarking	international benchmarking	1	kept
International Board Certified Lactation Consultant	international board certified lactation consultant	international board certified lactation consultant	international board certified lactation consultant	International Board Certified Lactation Consultant	1	kept
International Building Code	international building code	international building code	international building code	International Building Code	1	kept
INTERNATIONAL CLASSIFICATION	international classification	international classification	international classification	INTERNATIONAL CLASSIFICATION	1	kept
International Classification of Disease	international classification of disease	international classification of disease	international classification of disease	International Classification of Diseases	1	case_or_format
International classification of diseases	international classification of diseases	international classification of diseases	international classification of disease	International Classification of Diseases	1	singular
international classification of diseases-10	international classification of diseases 10	international classification of diseases 10	international classification of diseases 10	international classification of diseases-10	1	kept
international classification of functioning	international classification of functioning	international classification of functioning	international classification of functioning	international classification of functioning	1	kept
International Code Council	international code council	international code council	international code council	International Code Council	1	kept

International Collaboration	international collaboration	international collaboration	international collaboration	International Collaboration	1	kept
international collaboration	international collaboration	international collaboration	international collaboration	International Collaboration	1	case_or_format
international comparison	international comparison	international comparison	international comparison	international comparison	1	kept
international development	international development	international development	international development	international development	1	kept
international dollar	international dollar	international dollar	international dollar	international dollar	1	kept
international educational experiences	international educational experiences	international educational experiences	international educational experiences	international educational experiences	1	kept
International Federation of Gynecology and	international federation of gynecology and	international federation of gynecology and	international federation of gynecology and	International Federation of Gynecology and	1	kept
INTERNATIONAL GUIDELINES	international guidelines	international guidelines	international guidelines	INTERNATIONAL GUIDELINES	1	kept
International guidelines	international guidelines	international guidelines	international guidelines	INTERNATIONAL GUIDELINES	1	case_or_format
International Guidelines for Management of Sepsis	international guidelines for management of sepsis	international guidelines for management of sepsis	international guidelines for management of sepsis	International Guidelines for Management of Sepsis	1	kept
International Health	international health	international health	international health	International Health	1	kept
international health	international health	international health	international health	International Health	1	case_or_format
International Health Services	international health services	international health services	international health services	international health services	1	case_or_format
International health services	international health services	international health services	international health services	international health services	1	case_or_format
International Liaison Committee on Resuscitation	international liaison committee on resuscitation	international liaison committee on resuscitation	international liaison committee on resuscitation	International Liaison Committee on Resuscitation	1	kept
international medical graduates	international medical graduates	international medical graduates	international medical graduates	international medical graduates	1	kept

International pediatric emergency medicine	international pediatric emergency medicine	international pediatric emergency medicine	international pediatric emergency medicine	International pediatric emergency medicine	1	kept
international practice	international practice	international practice	international practice	international practice	1	kept
International Pressure Ulcer Prevalence Survey	international pressure ulcer prevalence survey	international pressure ulcer prevalence survey	international pressure ulcer prevalence survey	International Pressure Ulcer Prevalence Survey	1	kept
international prospective study	international prospective study	international prospective study	international prospective study	international prospective study	1	kept
International Quality of Life Assessment scale	international quality of life assessment scale	international quality of life assessment scale	international quality of life assessment scale	International Quality of Life Assessment scale	1	kept
international research	international research	international research	international research	international research	1	kept
International Staging System	international staging system	international staging system	international staging system	International Staging System	1	kept
International standard	international standard	international standard	international standard	International standards	1	case_or_format
international symposium	international symposium	international symposium	international symposium	international symposium	1	kept
International transfers	international transfers	international transfers	international transfers	International transfers	1	kept
international transport	international transport	international transport	international transport	international transport	1	kept
Internes	internes	internes	internes	Internes	1	kept
Internet connectivity	internet connectivity	internet connectivity	internet connectivity	Internet connectivity	1	kept
Internet education	internet education	internet education	internet education	Internet education	1	kept
Internet of healthcare things	internet of healthcare things	internet of healthcare things	internet of healthcare things	Internet of healthcare things	1	kept
Internet of thing	internet of thing	internet of thing	internet of thing	Internet of things	1	case_or_format

internet of things	internet of things	internet of things	internet of thing	Internet of things	1	singular
internet-based cognitive behavioral therapy	internet based cognitive behavioral therapy	internet based cognitive behavioral therapy	internet based cognitive behavioral therapy	internet-based cognitive behavioral therapy	1	kept
Internet-of-things	internet of things	internet of things	internet of thing	Internet of things	1	singular
Internetbased learning	internetbased learning	internetbased learning	internetbased learning	Internetbased learning	1	kept
INTERNS	interns	interns	interns	INTERNS	1	kept
internship and residency	internship and residency	internship and residency	internship and residency	Internship and Residency	1	case_or_format
Internship experience	internship experience	internship experience	internship experience	Internship experience	1	kept
internship nonmedical	internship nonmedical	internship nonmedical	internship nonmedical	Internship, Nonmedical	1	case_or_format
internships	internships	internships	internship	Internship	1	singular
Interobserver	interobserver	interobserver	interobserver	Interobserver	1	kept
interpersonal and communication skills	interpersonal and communication skills	interpersonal and communication skills	interpersonal and communication skills	Interpersonal and Communication Skills	1	case_or_format
Interpersonal communication competence	interpersonal communication competence	interpersonal communication competence	interpersonal communication competence	Interpersonal communication competence	1	kept
interpersonal conflict	interpersonal conflict	interpersonal conflict	interpersonal conflict	interpersonal conflict	1	kept
INTERPERSONAL CONFLICTS	interpersonal conflicts	interpersonal conflicts	interpersonal conflict	interpersonal conflict	1	singular
interpersonal conflicts	interpersonal conflicts	interpersonal conflicts	interpersonal conflict	interpersonal conflict	1	singular
Interpersonal help process	interpersonal help process	interpersonal help process	interpersonal help process	Interpersonal help process	1	kept

Interpersonal problems	interpersonal problems	interpersonal problems	interpersonal problems	Interpersonal problems	1	kept
interpersonal psychotherapy	interpersonal psychotherapy	interpersonal psychotherapy	interpersonal psychotherapy	interpersonal psychotherapy	1	kept
interpersonal reactivity	interpersonal reactivity	interpersonal reactivity	interpersonal reactivity	interpersonal reactivity	1	kept
interpersonal skill	interpersonal skill	interpersonal skill	interpersonal skill	interpersonal skill	1	kept
Interpersonal Skills and Communication	interpersonal skills and communication	interpersonal skills and communication	interpersonal skills and communication	Interpersonal Skills and Communication	1	kept
Interpersonal support	interpersonal support	interpersonal support	interpersonal support	Interpersonal support	1	kept
INTERPERSONAL TOUCH	interpersonal touch	interpersonal touch	interpersonal touch	INTERPERSONAL TOUCH	1	kept
interpersonal trust	interpersonal trust	interpersonal trust	interpersonal trust	interpersonal trust	1	kept
Interpersonal violence	interpersonal violence	interpersonal violence	interpersonal violence	Interpersonal violence	1	kept
interphalangeal joint	interphalangeal joint	interphalangeal joint	interphalangeal joint	interphalangeal joint	1	kept
interpleural blocking	interpleural blocking	interpleural blocking	interpleural blocking	interpleural blocking	1	kept
interpretative	interpretative	interpretative	interpretative	interpretative	1	kept
interpretative biography	interpretative biography	interpretative biography	interpretative biography	interpretative biography	1	kept
Interpreter modality	interpreter modality	interpreter modality	interpreter modality	Interpreter modality	1	kept
Interpreter mode	interpreter mode	interpreter mode	interpreter mode	Interpreter mode	1	kept
Interpretive	interpretive	interpretive	interpretive	Interpretive	1	kept

interpretive biography	interpretive biography	interpretive biography	interpretive biography	interpretive biography	1	kept
interpretive descriptive	interpretive descriptive	interpretive descriptive	interpretive descriptive	interpretive descriptive	1	kept
Interpretive methods	interpretive methods	interpretive methods	interpretive methods	Interpretive methods	1	kept
Interprofessional assessment	interprofessional assessment	interprofessional assessment	interprofessional assessment	Interprofessional assessment	1	kept
Interprofessional collaborative care	interprofessional collaborative care	interprofessional collaborative care	interprofessional collaborative care	Interprofessional collaborative care	1	kept
interprofessional collaborative care	interprofessional collaborative care	interprofessional collaborative care	interprofessional collaborative care	Interprofessional collaborative care	1	case_or_format
Interprofessional Collaborative Practice	interprofessional collaborative practice	interprofessional collaborative practice	interprofessional collaborative practice	Interprofessional Collaborative Practice	1	kept
interprofessional collaborative practices	interprofessional collaborative practices	interprofessional collaborative practices	interprofessional collaborative practice	Interprofessional Collaborative Practice	1	singular
INTERPROFESSIONAL COMMUNICATION	interprofessional communication	interprofessional communication	interprofessional communication	interprofessional communication	1	case_or_format
Interprofessional communication and collaboration	interprofessional communication and collaboration	interprofessional communication and collaboration	interprofessional communication and collaboration	Interprofessional communication and collaboration	1	kept
interprofessional competencies	interprofessional competencies	interprofessional competencies	interprofessional competencies	interprofessional competencies	1	kept
Interprofessional cooperation	interprofessional cooperation	interprofessional cooperation	interprofessional cooperation	Interprofessional cooperation	1	kept
interprofessional decision-making	interprofessional decision making	interprofessional decision making	interprofessional decision making	interprofessional decision-making	1	kept
interprofessional educational collaborative	interprofessional educational collaborative	interprofessional educational collaborative	interprofessional educational collaborative	interprofessional educational collaborative	1	kept
interprofessional health care	interprofessional health care	interprofessional health care	interprofessional health care	interprofessional health care	1	kept
interprofessional high acuity response team	interprofessional high acuity response team	interprofessional high acuity response team	interprofessional high acuity response team	interprofessional high acuity response team	1	kept

interprofessional interactions	interprofessional interactions	interprofessional interactions	interprofessional interactions	interprofessional interactions	1	kept
Interprofessional learning	interprofessional learning	interprofessional learning	interprofessional learning	Interprofessional learning	1	kept
interprofessional learning	interprofessional learning	interprofessional learning	interprofessional learning	Interprofessional learning	1	case_or_format
interprofessional outcomes	interprofessional outcomes	interprofessional outcomes	interprofessional outcomes	interprofessional outcomes	1	kept
Interprofessional Practice	interprofessional practice	interprofessional practice	interprofessional practice	interprofessional practice	1	case_or_format
interprofessional Practice	interprofessional practice	interprofessional practice	interprofessional practice	interprofessional practice	1	case_or_format
Interprofessional relationship	interprofessional relationship	interprofessional relationship	interprofessional relationship	Interprofessional relationships	1	case_or_format
interprofessional relationships	interprofessional relationships	interprofessional relationships	interprofessional relationship	Interprofessional relationships	1	singular
INTERPROFESSIONAL ROUNDS	interprofessional rounds	interprofessional rounds	interprofessional rounds	INTERPROFESSIONAL ROUNDS	1	kept
Interprofessional rounds	interprofessional rounds	interprofessional rounds	interprofessional rounds	INTERPROFESSIONAL ROUNDS	1	case_or_format
interprofessional rounds	interprofessional rounds	interprofessional rounds	interprofessional rounds	INTERPROFESSIONAL ROUNDS	1	case_or_format
interprofessional self-perceived collaboration	interprofessional self perceived collaboration	interprofessional self perceived collaboration	interprofessional self perceived collaboration	interprofessional self-perceived collaboration	1	kept
INTERPROFESSIONAL SIMULATION	interprofessional simulation	interprofessional simulation	interprofessional simulation	interprofessional simulation	1	case_or_format
Interprofessional simulation	interprofessional simulation	interprofessional simulation	interprofessional simulation	interprofessional simulation	1	case_or_format
Interprofessional staff engagement	interprofessional staff engagement	interprofessional staff engagement	interprofessional staff engagement	Interprofessional staff engagement	1	kept
Interprofessional team approach	interprofessional team approach	interprofessional team approach	interprofessional team approach	Interprofessional team approach	1	kept

Interprofessional team management	interprofessional team management	interprofessional team management	interprofessional team management	Interprofessional team management	1	kept
Interprofessional team training	interprofessional team training	interprofessional team training	interprofessional team training	Interprofessional team training	1	kept
Interprofessional Teamwork	interprofessional teamwork	interprofessional teamwork	interprofessional teamwork	Interprofessional teamwork	1	case_or_format
Interprofessional training	interprofessional training	interprofessional training	interprofessional training	Interprofessional training	1	kept
interprofessional training	interprofessional training	interprofessional training	interprofessional training	Interprofessional training	1	case_or_format
interprofessionality	interprofessionality	interprofessionality	interprofessionality	interprofessionality	1	kept
interprofessionelle Zusammenarbeit	interprofessionelle zusammenarbeit	interprofessionelle zusammenarbeit	interprofessionelle zusammenarbeit	interprofessionelle Zusammenarbeit	1	kept
interprofessionnalité	interprofessionnalit	interprofessionnalit	interprofessionnalit	interprofessionnalité	1	kept
interquartile range	interquartile range	interquartile range	interquartile range	interquartile range	1	kept
interRAI Acute Care	interrai acute care	interrai acute care	interrai acute care	interRAI Acute Care	1	kept
Interrater agreement	interrater agreement	interrater agreement	interrater agreement	Interrater agreement	1	kept
INTERRELATIONSHIP	interrelationship	interrelationship	interrelationship	INTERRELATIONSHIP	1	kept
INTERRUPTED TIME SERIES	interrupted time series	interrupted time series	interrupted time series	Interrupted time series	1	case_or_format
interrupted time series	interrupted time series	interrupted time series	interrupted time series	Interrupted time series	1	case_or_format
INTERRUPTED TIME-SERIES	interrupted time series	interrupted time series	interrupted time series	Interrupted time series	1	case_or_format
Interrupted time-series	interrupted time series	interrupted time series	interrupted time series	Interrupted time series	1	case_or_format

INTERRUPTION	interruption	interruption	interruption	Interruptions	1	case_or_format
interruptive communication	interruptive communication	interruptive communication	interruptive communication	interruptive communication	1	kept
intersectionality	intersectionality	intersectionality	intersectionality	intersectionality	1	kept
Interspecies difference	interspecies difference	interspecies difference	interspecies difference	Interspecies difference	1	kept
interstage	interstage	interstage	interstage	interstage	1	kept
interstitial	interstitial	interstitial	interstitial	Interstitial	1	case_or_format
interstitial idiopathic pulmonary fibrosis	interstitial idiopathic pulmonary fibrosis	interstitial idiopathic pulmonary fibrosis	interstitial idiopathic pulmonary fibrosis	interstitial idiopathic pulmonary fibrosis	1	kept
Interstitial lung disease	interstitial lung disease	interstitial lung disease	interstitial lung disease	interstitial lung disease	1	case_or_format
interstitial nephritis	interstitial nephritis	interstitial nephritis	interstitial nephritis	interstitial nephritis	1	kept
Interstitial pneumonia	interstitial pneumonia	interstitial pneumonia	interstitial pneumonia	interstitial pneumonia	1	case_or_format
intersubjectivity	intersubjectivity	intersubjectivity	intersubjectivity	intersubjectivity	1	kept
Intertriginous dermatitis	intertriginous dermatitis	intertriginous dermatitis	intertriginous dermatitis	Intertriginous dermatitis	1	kept
intertriginous dermatitis	intertriginous dermatitis	intertriginous dermatitis	intertriginous dermatitis	Intertriginous dermatitis	1	case_or_format
intertrochanteric femur fracture	intertrochanteric femur fracture	intertrochanteric femur fracture	intertrochanteric femur fracture	intertrochanteric femur fracture	1	kept
interval exercise	interval exercise	interval exercise	interval exercise	interval exercise	1	kept
intervening variable	intervening variable	intervening variable	intervening variable	intervening variable	1	kept

Intervention activities	intervention activities	intervention activities	intervention activities	Intervention activities	1	kept
Intervention adaptations	intervention adaptations	intervention adaptations	intervention adaptations	Intervention adaptations	1	kept
intervention bundle	intervention bundle	intervention bundle	intervention bundle	intervention bundle	1	kept
Intervention fidelity	intervention fidelity	intervention fidelity	intervention fidelity	Intervention fidelity	1	kept
intervention implementation	intervention implementation	intervention implementation	intervention implementation	Intervention implementation	1	case_or_format
Intervention Mapping	intervention mapping	intervention mapping	intervention mapping	Intervention Mapping	1	kept
Intervention mapping	intervention mapping	intervention mapping	intervention mapping	Intervention Mapping	1	case_or_format
intervention order	intervention order	intervention order	intervention order	intervention order	1	kept
Intervention period	intervention period	intervention period	intervention period	Intervention period	1	kept
INTERVENTION PROGRAM	intervention program	intervention program	intervention program	intervention program	1	case_or_format
Intervention Program	intervention program	intervention program	intervention program	intervention program	1	case_or_format
Intervention program	intervention program	intervention program	intervention program	intervention program	1	case_or_format
Intervention programs	intervention programs	intervention programs	intervention program	intervention program	1	singular
Intervention score	intervention score	intervention score	intervention score	Intervention score	1	kept
Intervention strategy	intervention strategy	intervention strategy	intervention strategy	Intervention strategy	1	kept
intervention studies	intervention studies	intervention studies	intervention study	intervention study	1	singular

intervention testing	intervention testing	intervention testing	intervention testing	intervention testing	1	kept
Interventional cardiology	interventional cardiology	interventional cardiology	interventional cardiology	Interventional cardiology	1	kept
interventional cardiology	interventional cardiology	interventional cardiology	interventional cardiology	Interventional cardiology	1	case_or_format
interventional magnetic resonance imaging	interventional magnetic resonance imaging	interventional magnetic resonance imaging	interventional magnetic resonance imaging	interventional magnetic resonance imaging	1	kept
interventional neuroradiology	interventional neuroradiology	interventional neuroradiology	interventional neuroradiology	interventional neuroradiology	1	kept
Interventional radiology procedure sedation	interventional radiology procedure sedation	interventional radiology procedure sedation	interventional radiology procedure sedation	Interventional radiology procedure sedation	1	kept
Intervertebral Disc Degeneration	intervertebral disc degeneration	intervertebral disc degeneration	intervertebral disc degeneration	Intervertebral Disc Degeneration	1	kept
intervertebral disk degeneration	intervertebral disk degeneration	intervertebral disk degeneration	intervertebral disk degeneration	intervertebral disk degeneration	1	kept
intervertebral disk disease	intervertebral disk disease	intervertebral disk disease	intervertebral disk disease	intervertebral disk disease	1	kept
intervertebral disk hernia	intervertebral disk hernia	intervertebral disk hernia	intervertebral disk hernia	intervertebral disk hernia	1	kept
intervertebral diskectomy	intervertebral diskectomy	intervertebral diskectomy	intervertebral diskectomy	intervertebral diskectomy	1	kept
Interview survey	interview survey	interview survey	interview survey	Interview survey	1	kept
INTERVIEWS	interviews	interviews	interview	interview	1	singular
interviews, semi-structured	interviews semi structured	interviews semi structured	interviews semi structured	interviews, semi-structured	1	kept
Intesive Care Units	intesive care units	intesive care units	intesive care units	Intesive Care Units	1	kept
Intestinal Atresia	intestinal atresia	intestinal atresia	intestinal atresia	Intestinal Atresia	1	kept

intestinal dysmotility	intestinal dysmotility	intestinal dysmotility	intestinal dysmotility	intestinal dysmotility	1	kept
intestinal failure-associated liver disease	intestinal failure associated liver disease	intestinal failure associated liver disease	intestinal failure associated liver disease	intestinal failure-associated liver disease	1	kept
intestinal fibrosis	intestinal fibrosis	intestinal fibrosis	intestinal fibrosis	intestinal fibrosis	1	kept
Intestinal insufficiency	intestinal insufficiency	intestinal insufficiency	intestinal insufficiency	Intestinal insufficiency	1	kept
intestinal loop	intestinal loop	intestinal loop	intestinal loop	intestinal loop	1	kept
Intestinal Neoplasms	intestinal neoplasms	intestinal neoplasms	intestinal neoplasms	Intestinal Neoplasms	1	kept
Intestinal Pseudo-Obstruction	intestinal pseudo obstruction	intestinal pseudo obstruction	intestinal pseudo obstruction	Intestinal Pseudo-Obstruction	1	kept
intestinal rehabilitation	intestinal rehabilitation	intestinal rehabilitation	intestinal rehabilitation	intestinal rehabilitation	1	kept
intestinal secretions	intestinal secretions	intestinal secretions	intestinal secretions	intestinal secretions	1	kept
INTESTINAL-OBSTRUCTION	intestinal obstruction	intestinal obstruction	intestinal obstruction	Intestinal Obstruction	1	case_or_format
Intestine	intestine	intestine	intestine	Intestines	1	case_or_format
intestine anastomosis	intestine anastomosis	intestine anastomosis	intestine anastomosis	intestine anastomosis	1	kept
intestine biopsy	intestine biopsy	intestine biopsy	intestine biopsy	intestine biopsy	1	kept
intestine blood flow	intestine blood flow	intestine blood flow	intestine blood flow	intestine blood flow	1	kept
intestine distension	intestine distension	intestine distension	intestine distension	intestine distension	1	kept
intestine endoscopy	intestine endoscopy	intestine endoscopy	intestine endoscopy	intestine endoscopy	1	kept

intestine graft	intestine graft	intestine graft	intestine graft	intestine graft	1	kept
intestine lavage	intestine lavage	intestine lavage	intestine lavage	intestine lavage	1	kept
intestine malformation	intestine malformation	intestine malformation	intestine malformation	intestine malformation	1	kept
intestine pseudoobstruction	intestine pseudoobstruction	intestine pseudoobstruction	intestine pseudoobstruction	intestine pseudoobstruction	1	kept
intestine stenosis	intestine stenosis	intestine stenosis	intestine stenosis	intestine stenosis	1	kept
intestine transit time	intestine transit time	intestine transit time	intestine transit time	intestine transit time	1	kept
intestine transplantation	intestine transplantation	intestine transplantation	intestine transplantation	intestine transplantation	1	kept
INTIMACY	intimacy	intimacy	intimacy	intimacy	1	case_or_format
Intimate care	intimate care	intimate care	intimate care	Intimate care	1	kept
Intimate Partner Violence	intimate partner violence	intimate partner violence	intimate partner violence	Intimate Partner Violence	1	kept
Intimate partner violence	intimate partner violence	intimate partner violence	intimate partner violence	Intimate Partner Violence	1	case_or_format
INTOLERANCE	intolerance	intolerance	intolerance	INTOLERANCE	1	kept
Intoxications	intoxications	intoxications	intoxication	intoxication	1	singular
intra	intra	intra	intra	intra	1	kept
intra aortic balloon counterpulsation	intra aortic balloon counterpulsation	intra aortic balloon counterpulsation	intra aortic balloon counterpulsation	intra aortic balloon counterpulsation	1	kept
intra cavitary electrocardiogram	intra cavitary electrocardiogram	intra cavitary electrocardiogram	intra cavitary electrocardiogram	intra cavitary electrocardiogram	1	kept

intra cuff pressure	intra cuff pressure	intra cuff pressure	intra cuff pressure	intra cuff pressure	1	kept
Intra hospital transport practices	intra hospital transport practices	intra hospital transport practices	intra hospital transport practices	Intra hospital transport practices	1	kept
Intra-abdominal	intra abdominal	intra abdominal	intra abdominal	Intra-abdominal	1	kept
Intra-abdominal candidiasis	intra abdominal candidiasis	intra abdominal candidiasis	intra abdominal candidiasis	Intra-abdominal candidiasis	1	kept
intra-abdominal hypertension	intra abdominal hypertension	intra abdominal hypertension	intra abdominal hypertension	Intra-Abdominal Hypertension	1	case_or_format
intra-abdominal surgery	intra abdominal surgery	intra abdominal surgery	intra abdominal surgery	intra-abdominal surgery	1	kept
Intra-aortic balloon counterpulsation	intra aortic balloon counterpulsation	intra aortic balloon counterpulsation	intra aortic balloon counterpulsation	intra aortic balloon counterpulsation	1	case_or_format
Intra-aortic balloon pump	intra aortic balloon pump	intra aortic balloon pump	intra aortic balloon pump	Intra-Aortic Balloon Pump	1	case_or_format
intra-aortic balloon pump complication	intra aortic balloon pump complication	intra aortic balloon pump complication	intra aortic balloon pump complication	intra-aortic balloon pump complication	1	kept
Intra-arterial	intra arterial	intra arterial	intra arterial	Intra-arterial	1	kept
Intra-arterial blood pressure	intra arterial blood pressure	intra arterial blood pressure	intra arterial blood pressure	Intra-arterial blood pressure	1	kept
Intra-arterial line	intra arterial line	intra arterial line	intra arterial line	Intra-arterial line	1	kept
Intra-arterial thrombolysis	intra arterial thrombolysis	intra arterial thrombolysis	intra arterial thrombolysis	Intra-arterial thrombolysis	1	kept
Intra-cuff pressure of endotracheal tube	intra cuff pressure of endotracheal tube	intra cuff pressure of endotracheal tube	intra cuff pressure of endotracheal tube	Intra-cuff pressure of endotracheal tube	1	kept
Intra-hospital transitions	intra hospital transitions	intra hospital transitions	intra hospital transitions	Intra-hospital transitions	1	kept
Intra-operative	intra operative	intra operative	intra operative	Intra-operative	1	kept

Intra-professional	intra professional	intra professional	intra professional	Intra-professional	1	kept
INTRAABDOMINAL HYPERTENSION	intraabdominal hypertension	intraabdominal hypertension	intraabdominal hypertension	intraabdominal hypertension	1	case_or_format
Intraabdominal hypertension	intraabdominal hypertension	intraabdominal hypertension	intraabdominal hypertension	intraabdominal hypertension	1	case_or_format
intraabdominal surgery	intraabdominal surgery	intraabdominal surgery	intraabdominal surgery	intraabdominal surgery	1	kept
Intraaortic balloon pump	intraaortic balloon pump	intraaortic balloon pump	intraaortic balloon pump	intraaortic balloon pump	1	case_or_format
Intracanial bleeding	intracanial bleeding	intracanial bleeding	intracanial bleeding	Intracanial bleeding	1	kept
intracavitary- ECG	intracavitary ecg	intracavitary ecg	intracavitary ecg	intracavitary- ECG	1	kept
intracellular	intracellular	intracellular	intracellular	intracellular	1	kept
Intracellular fluid	intracellular fluid	intracellular fluid	intracellular fluid	Intracellular Fluid	1	case_or_format
intracellular signaling	intracellular signaling	intracellular signaling	intracellular signaling	intracellular signaling	1	kept
Intracellular Signaling Peptides and Proteins	intracellular signaling peptides and proteins	intracellular signaling peptides and proteins	intracellular signaling peptides and proteins	Intracellular Signaling Peptides and Proteins	1	kept
Intracerebral abscess	intracerebral abscess	intracerebral abscess	intracerebral abscess	Intracerebral abscess	1	kept
intracerebral hypertension	intracerebral hypertension	intracerebral hypertension	intracerebral hypertension	intracerebral hypertension	1	kept
intracerebral mass lesion	intracerebral mass lesion	intracerebral mass lesion	intracerebral mass lesion	intracerebral mass lesion	1	kept
Intracortical electroencephalography	intracortical electroencephalography	intracortical electroencephalography	intracortical electroencephalography	Intracortical electroencephalography	1	kept
Intracranial adaptive capacity	intracranial adaptive capacity	intracranial adaptive capacity	intracranial adaptive capacity	Intracranial adaptive capacity	1	kept

INTRACRANIAL COMPLICATION	intracranial complication	intracranial complication	intracranial complication	INTRACRANIAL COMPLICATION	1	kept
intracranial event	intracranial event	intracranial event	intracranial event	intracranial event	1	kept
Intracranial hemorrhages	intracranial hemorrhages	intracranial hemorrhages	intracranial hemorrhage	Intracranial Hemorrhages	1	singular
intracranial hypotension	intracranial hypotension	intracranial hypotension	intracranial hypotension	intracranial hypotension	1	kept
Intracranial pressure monitor	intracranial pressure monitor	intracranial pressure monitor	intracranial pressure monitor	Intracranial pressure monitor	1	kept
Intracranial pressure monitor placement	intracranial pressure monitor placement	intracranial pressure monitor placement	intracranial pressure monitor placement	Intracranial pressure monitor placement	1	kept
Intracranial space-occupying lesions	intracranial space occupying lesions	intracranial space occupying lesions	intracranial space occupying lesions	Intracranial space-occupying lesions	1	kept
intracranial teratoma	intracranial teratoma	intracranial teratoma	intracranial teratoma	intracranial teratoma	1	kept
Intracranial tumor	intracranial tumor	intracranial tumor	intracranial tumor	Intracranial tumor	1	kept
intracranial tumor	intracranial tumor	intracranial tumor	intracranial tumor	Intracranial tumor	1	case_or_format
INTRACRANIAL-PRESSURE	intracranial pressure	intracranial pressure	intracranial pressure	intracranial pressure	1	case_or_format
Intractable diarrhea infants	intractable diarrhea infants	intractable diarrhea infants	intractable diarrhea infants	Intractable diarrhea infants	1	kept
intractable retching	intractable retching	intractable retching	intractable retching	intractable retching	1	kept
Intracuff pressure	intracuff pressure	intracuff pressure	intracuff pressure	Intracuff pressure	1	kept
intracutaneous test	intracutaneous test	intracutaneous test	intracutaneous test	intracutaneous test	1	kept
intracytoplasmic sperm injection	intracytoplasmic sperm injection	intracytoplasmic sperm injection	intracytoplasmic sperm injection	intracytoplasmic sperm injection	1	kept

Intradisciplinary collaboration	intradisciplinary collaboration	intradisciplinary collaboration	intradisciplinary collaboration	Intradisciplinary collaboration	1	kept
intraductal carcinoma	intraductal carcinoma	intraductal carcinoma	intraductal carcinoma	intraductal carcinoma	1	kept
Intrahepatic Cholestasis of Pregnancy	intrahepatic cholestasis of pregnancy	intrahepatic cholestasis of pregnancy	intrahepatic cholestasis of pregnancy	intrahepatic cholestasis of pregnancy	1	case_or_format
intrahospital	intrahospital	intrahospital	intrahospital	intrahospital	1	kept
intrahospital handover	intrahospital handover	intrahospital handover	intrahospital handover	intrahospital handover	1	kept
intrahospital traffic	intrahospital traffic	intrahospital traffic	intrahospital traffic	intrahospital traffic	1	kept
Intrahospital transfer	intrahospital transfer	intrahospital transfer	intrahospital transfer	intrahospital transfer	1	case_or_format
intrahospital transfer protocol	intrahospital transfer protocol	intrahospital transfer protocol	intrahospital transfer protocol	intrahospital transfer protocol	1	kept
Intrahospital transports	intrahospital transports	intrahospital transports	intrahospital transport	Intrahospital transport	1	singular
Intralipid	intralipid	intralipid	intralipid	Intralipid	1	kept
Intramedullary nail	intramedullary nail	intramedullary nail	intramedullary nail	intramedullary nail	1	case_or_format
Intramedullary nailing	intramedullary nailing	intramedullary nailing	intramedullary nailing	intramedullary nailing	1	case_or_format
Intramolecular Oxidoreductases	intramolecular oxidoreductases	intramolecular oxidoreductases	intramolecular oxidoreductases	Intramolecular Oxidoreductases	1	kept
Intramural	intramural	intramural	intramural	Intramural	1	kept
intranasal drug administration	intranasal drug administration	intranasal drug administration	intranasal drug administration	intranasal drug administration	1	kept
intraocular hemorrhage	intraocular hemorrhage	intraocular hemorrhage	intraocular hemorrhage	intraocular hemorrhage	1	kept

intraocular hypertension	intraocular hypertension	intraocular hypertension	intraocular hypertension	intraocular hypertension	1	kept
Intraoperative	intraoperative	intraoperative	intraoperative	Intraoperative	1	kept
intraoperative awareness	intraoperative awareness	intraoperative awareness	intraoperative awareness	intraoperative awareness	1	kept
Intraoperative bleeding	intraoperative bleeding	intraoperative bleeding	intraoperative bleeding	Intraoperative bleeding	1	kept
Intraoperative blood transfusion	intraoperative blood transfusion	intraoperative blood transfusion	intraoperative blood transfusion	Intraoperative blood transfusion	1	kept
intraoperative blood transfusion	intraoperative blood transfusion	intraoperative blood transfusion	intraoperative blood transfusion	Intraoperative blood transfusion	1	case_or_format
Intraoperative cardiac arrest	intraoperative cardiac arrest	intraoperative cardiac arrest	intraoperative cardiac arrest	Intraoperative cardiac arrest	1	kept
Intraoperative care	intraoperative care	intraoperative care	intraoperative care	Intraoperative Care	1	case_or_format
intraoperative checklist	intraoperative checklist	intraoperative checklist	intraoperative checklist	intraoperative checklist	1	kept
intraoperative complications	intraoperative complications	intraoperative complications	intraoperative complications	Intraoperative Complications	1	case_or_format
INTRAOPERATIVE HYPOTHERMIA	intraoperative hypothermia	intraoperative hypothermia	intraoperative hypothermia	INTRAOPERATIVE HYPOTHERMIA	1	kept
intraoperative monitoring and management	intraoperative monitoring and management	intraoperative monitoring and management	intraoperative monitoring and management	intraoperative monitoring and management	1	kept
Intraosseous access	intraosseous access	intraosseous access	intraosseous access	Intraosseous access	1	kept
intraosseous device	intraosseous device	intraosseous device	intraosseous device	intraosseous device	1	kept
INTRAOSSEOUS VASCULAR ACCESS	intraosseous vascular access	intraosseous vascular access	intraosseous vascular access	INTRAOSSEOUS VASCULAR ACCESS	1	kept
Intraparenchymal hemorrhage	intraparenchymal hemorrhage	intraparenchymal hemorrhage	intraparenchymal hemorrhage	Intraparenchymal hemorrhage	1	kept

intrapartum antibiotics	intrapartum antibiotics	intrapartum antibiotics	intrapartum antibiotics	intrapartum antibiotics	1	kept
intrapartum hemorrhage	intrapartum hemorrhage	intrapartum hemorrhage	intrapartum hemorrhage	intrapartum hemorrhage	1	kept
intrapartum monitoring	intrapartum monitoring	intrapartum monitoring	intrapartum monitoring	intrapartum monitoring	1	kept
Intrapartum mortality	intrapartum mortality	intrapartum mortality	intrapartum mortality	Intrapartum mortality	1	kept
intrapartum mortality	intrapartum mortality	intrapartum mortality	intrapartum mortality	Intrapartum mortality	1	case_or_format
intraperitoneal hematoma	intraperitoneal hematoma	intraperitoneal hematoma	intraperitoneal hematoma	intraperitoneal hematoma	1	kept
Intraprofessional collaboration	intraprofessional collaboration	intraprofessional collaboration	intraprofessional collaboration	Intraprofessional collaboration	1	kept
intraprofessional collaboration	intraprofessional collaboration	intraprofessional collaboration	intraprofessional collaboration	Intraprofessional collaboration	1	case_or_format
intraprofessional communication	intraprofessional communication	intraprofessional communication	intraprofessional communication	intraprofessional communication	1	kept
Intrapulmonary factor VIIa	intrapulmonary factor viia	intrapulmonary factor viia	intrapulmonary factor viia	Intrapulmonary factor VIIa	1	kept
intrapulmonary percussion	intrapulmonary percussion	intrapulmonary percussion	intrapulmonary percussion	intrapulmonary percussion	1	kept
intrapulmonary percussive ventilation	intrapulmonary percussive ventilation	intrapulmonary percussive ventilation	intrapulmonary percussive ventilation	intrapulmonary percussive ventilation	1	kept
intraspinal	intraspinal	intraspinal	intraspinal	intraspinal	1	kept
intraspinal pressure	intraspinal pressure	intraspinal pressure	intraspinal pressure	intraspinal pressure	1	kept
intrathecal analgesia	intrathecal analgesia	intrathecal analgesia	intrathecal analgesia	intrathecal analgesia	1	kept
intrathecal baclofen	intrathecal baclofen	intrathecal baclofen	intrathecal baclofen	intrathecal baclofen	1	kept

Intrathecal morphine	intrathecal morphine	intrathecal morphine	intrathecal morphine	Intrathecal morphine	1	kept
intrathecal opioids	intrathecal opioids	intrathecal opioids	intrathecal opioids	intrathecal opioids	1	kept
Intrathoracic impedance	intrathoracic impedance	intrathoracic impedance	intrathoracic impedance	Intrathoracic impedance	1	kept
Intrauterine existence	intrauterine existence	intrauterine existence	intrauterine existence	Intrauterine existence	1	kept
Intrauterine growth retardation	intrauterine growth retardation	intrauterine growth retardation	intrauterine growth retardation	intrauterine growth retardation	1	case_or_format
intrauterine infections	intrauterine infections	intrauterine infections	intrauterine infection	intrauterine infection	1	singular
intravascular	intravascular	intravascular	intravascular	intravascular	1	kept
Intravascular catheter	intravascular catheter	intravascular catheter	intravascular catheter	intravascular catheter	1	case_or_format
Intravascular catheter-related infections	intravascular catheter related infections	intravascular catheter related infections	intravascular catheter related infections	Intravascular catheter-related infections	1	kept
Intravascular catheters	intravascular catheters	intravascular catheters	intravascular catheter	intravascular catheter	1	singular
Intravascular line related infections	intravascular line related infections	intravascular line related infections	intravascular line related infections	Intravascular line related infections	1	kept
intravascular space	intravascular space	intravascular space	intravascular space	intravascular space	1	kept
intravenous access	intravenous access	intravenous access	intravenous access	Intravenous access	1	case_or_format
intravenous application	intravenous application	intravenous application	intravenous application	intravenous application	1	kept
Intravenous Blood Glucose system	intravenous blood glucose system	intravenous blood glucose system	intravenous blood glucose system	Intravenous Blood Glucose system	1	kept
intravenous cannulation	intravenous cannulation	intravenous cannulation	intravenous cannulation	Intravenous cannulation	1	case_or_format

intravenous catheter related bloodstream infection	intravenous catheter related bloodstream infection	intravenous catheter related bloodstream infection	intravenous catheter related bloodstream infection	intravenous catheter related bloodstream infection	1	kept
Intravenous catheterization	intravenous catheterization	intravenous catheterization	intravenous catheterization	Intravenous catheterization	1	kept
INTRAVENOUS CATHETERS	intravenous catheters	intravenous catheters	intravenous catheter	intravenous catheter	1	singular
INTRAVENOUS CIPROFLOXACIN	intravenous ciprofloxacin	intravenous ciprofloxacin	intravenous ciprofloxacin	INTRAVENOUS CIPROFLOXACIN	1	kept
INTRAVENOUS COLISTIN	intravenous colistin	intravenous colistin	intravenous colistin	INTRAVENOUS COLISTIN	1	kept
INTRAVENOUS DEXMEDETOMIDINE	intravenous dexmedetomidine	intravenous dexmedetomidine	intravenous dexmedetomidine	INTRAVENOUS DEXMEDETOMIDINE	1	kept
intravenous drug abuse	intravenous drug abuse	intravenous drug abuse	intravenous drug abuse	intravenous drug abuse	1	kept
intravenous drug use	intravenous drug use	intravenous drug use	intravenous drug use	intravenous drug use	1	kept
Intravenous drugs	intravenous drugs	intravenous drugs	intravenous drugs	Intravenous drugs	1	kept
intravenous fluid	intravenous fluid	intravenous fluid	intravenous fluid	Intravenous fluid	1	case_or_format
Intravenous fluid administration	intravenous fluid administration	intravenous fluid administration	intravenous fluid administration	Intravenous fluid administration	1	kept
intravenous fluid administration	intravenous fluid administration	intravenous fluid administration	intravenous fluid administration	Intravenous fluid administration	1	case_or_format
Intravenous fluid electrolyte	intravenous fluid electrolyte	intravenous fluid electrolyte	intravenous fluid electrolyte	Intravenous fluid electrolyte	1	kept
Intravenous fluid warmer	intravenous fluid warmer	intravenous fluid warmer	intravenous fluid warmer	Intravenous fluid warmer	1	kept
intravenous immunoglobulin	intravenous immunoglobulin	intravenous immunoglobulin	intravenous immunoglobulin	Intravenous immunoglobulin	1	case_or_format
Intravenous immunoglobulins	intravenous immunoglobulins	intravenous immunoglobulins	intravenous immunoglobulin	Intravenous immunoglobulin	1	singular

Intravenous insulin infusion	intravenous insulin infusion	intravenous insulin infusion	intravenous insulin infusion	Intravenous insulin infusion	1	kept
Intravenous insulin protocol	intravenous insulin protocol	intravenous insulin protocol	intravenous insulin protocol	Intravenous insulin protocol	1	kept
INTRAVENOUS KETAMINE	intravenous ketamine	intravenous ketamine	intravenous ketamine	INTRAVENOUS KETAMINE	1	kept
intravenous lipid emulsion	intravenous lipid emulsion	intravenous lipid emulsion	intravenous lipid emulsion	intravenous lipid emulsion	1	kept
Intravenous medication	intravenous medication	intravenous medication	intravenous medication	INTRAVENOUS MEDICATION	1	case_or_format
intravenous medication administration	intravenous medication administration	intravenous medication administration	intravenous medication administration	intravenous medication administration	1	kept
Intravenous Medication Administration Errors	intravenous medication administration errors	intravenous medication administration errors	intravenous medication administration errors	Intravenous Medication Administration Errors	1	kept
Intravenous medication protocol	intravenous medication protocol	intravenous medication protocol	intravenous medication protocol	Intravenous medication protocol	1	kept
Intravenous medicine	intravenous medicine	intravenous medicine	intravenous medicine	Intravenous medicine	1	kept
intravenous mixtures and parenteral nutrition	intravenous mixtures and parenteral nutrition	intravenous mixtures and parenteral nutrition	intravenous mixtures and parenteral nutrition	intravenous mixtures and parenteral nutrition	1	kept
intravenous needleless connector hub	intravenous needleless connector hub	intravenous needleless connector hub	intravenous needleless connector hub	intravenous needleless connector hub	1	kept
INTRAVENOUS NUTRITION	intravenous nutrition	intravenous nutrition	intravenous nutrition	INTRAVENOUS NUTRITION	1	kept
intravenous pole	intravenous pole	intravenous pole	intravenous pole	intravenous pole	1	kept
Intravenous remimazolam	intravenous remimazolam	intravenous remimazolam	intravenous remimazolam	Intravenous remimazolam	1	kept
intravenous smart pump	intravenous smart pump	intravenous smart pump	intravenous smart pump	intravenous smart pump	1	kept
Intravenous smart pumps	intravenous smart pumps	intravenous smart pumps	intravenous smart pump	intravenous smart pump	1	singular

Intravenous therapies	intravenous therapies	intravenous therapies	intravenous therapy	Intravenous therapy	1	singular
INTRAVENOUS THROMBOLYSIS	intravenous thrombolysis	intravenous thrombolysis	intravenous thrombolysis	Intravenous thrombolysis	1	case_or_format
intravenous thrombolysis	intravenous thrombolysis	intravenous thrombolysis	intravenous thrombolysis	Intravenous thrombolysis	1	case_or_format
Intravital microscopy	intravital microscopy	intravital microscopy	intravital microscopy	Intravital microscopy	1	kept
intra - abdominal pressure	intra abdominal pressure	intra abdominal pressure	intra abdominal pressure	Intra-abdominal pressure	1	case_or_format
intra - abdominal pressure management	intra abdominal pressure management	intra abdominal pressure management	intra abdominal pressure management	intra - abdominal pressure management	1	kept
intra - aortic balloon pumping	intra aortic balloon pumping	intra aortic balloon pumping	intra aortic balloon pumping	Intra-Aortic Balloon Pumping	1	case_or_format
intra - professional	intra professional	intra professional	intra professional	Intra-professional	1	case_or_format
Intrinsic	intrinsic	intrinsic	intrinsic	Intrinsic	1	kept
intrinsic	intrinsic	intrinsic	intrinsic	Intrinsic	1	case_or_format
intrinsic factor	intrinsic factor	intrinsic factor	intrinsic factor	intrinsic factor	1	kept
intrinsic thromboelastometry	intrinsic thromboelastometry	intrinsic thromboelastometry	intrinsic thromboelastometry	intrinsic thromboelastometry	1	kept
Intrinsic work motivation	intrinsic work motivation	intrinsic work motivation	intrinsic work motivation	Intrinsic work motivation	1	kept
introducer technique	introducer technique	introducer technique	introducer technique	introducer technique	1	kept
Introductory programs	introductory programs	introductory programs	introductory programs	Introductory programs	1	kept
Introns	introns	introns	intron	intron	1	singular

Intrusive memories	intrusive memories	intrusive memories	intrusive memories	Intrusive memories	1	kept
intrusive memories	intrusive memories	intrusive memories	intrusive memories	Intrusive memories	1	case_or_format
INTUBATED ADULTS	intubated adults	intubated adults	intubated adults	INTUBATED ADULTS	1	kept
Intubation gastrointestinal	intubation gastrointestinal	intubation gastrointestinal	intubation gastrointestinal	Intubation, Gastrointestinal	1	case_or_format
intubation injury	intubation injury	intubation injury	intubation injury	intubation injury	1	kept
Intubation intratracheal	intubation intratracheal	intubation intratracheal	intubation intratracheal	Intubation, Intratracheal	1	case_or_format
intubation length of stay	intubation length of stay	intubation length of stay	intubation length of stay	intubation length of stay	1	kept
Intubation readiness	intubation readiness	intubation readiness	intubation readiness	Intubation readiness	1	kept
Intubation, endotracheal	intubation endotracheal	intubation endotracheal	intubation endotracheal	Intubation, endotracheal	1	kept
INTUITION	intuition	intuition	intuition	Intuition	1	case_or_format
Intuitive user interface	intuitive user interface	intuitive user interface	intuitive user interface	Intuitive user interface	1	kept
Intussusception	intussusception	intussusception	intussusception	intussusception	1	case_or_format
intégration	int gration	int gration	int gration	intégration	1	kept
Inuits	inuits	inuits	inuits	Inuits	1	kept
Invasive airway management	invasive airway management	invasive airway management	invasive airway management	Invasive airway management	1	kept
Invasive aspergillosis	invasive aspergillosis	invasive aspergillosis	invasive aspergillosis	invasive aspergillosis	1	case_or_format

INVASIVE CANDIDIASIS	invasive candidiasis	invasive candidiasis	invasive candidiasis	invasive candidiasis	1	case_or_format
Invasive candidiasis	invasive candidiasis	invasive candidiasis	invasive candidiasis	invasive candidiasis	1	case_or_format
Invasive device associated infections	invasive device associated infections	invasive device associated infections	invasive device associated infections	Invasive device associated infections	1	kept
invasive device placement	invasive device placement	invasive device placement	invasive device placement	invasive device placement	1	kept
Invasive Escherichia coli Disease	invasive escherichia coli disease	invasive escherichia coli disease	invasive escherichia coli disease	Invasive Escherichia coli Disease	1	kept
Invasive Escherichia coli disease	invasive escherichia coli disease	invasive escherichia coli disease	invasive escherichia coli disease	Invasive Escherichia coli Disease	1	case_or_format
Invasive fungal diseases	invasive fungal diseases	invasive fungal diseases	invasive fungal diseases	Invasive fungal diseases	1	kept
Invasive fungal infections	invasive fungal infections	invasive fungal infections	invasive fungal infection	Invasive Fungal Infections	1	singular
invasive fungal infections	invasive fungal infections	invasive fungal infections	invasive fungal infection	Invasive Fungal Infections	1	singular
Invasive home mechanical ventilation	invasive home mechanical ventilation	invasive home mechanical ventilation	invasive home mechanical ventilation	Invasive home mechanical ventilation	1	kept
invasive infections	invasive infections	invasive infections	invasive infections	invasive infections	1	kept
Invasive monitoring	invasive monitoring	invasive monitoring	invasive monitoring	invasive monitoring	1	case_or_format
Invasive neuromonitoring	invasive neuromonitoring	invasive neuromonitoring	invasive neuromonitoring	Invasive neuromonitoring	1	kept
INVASIVE PNEUMOCOCCAL DISEASE	invasive pneumococcal disease	invasive pneumococcal disease	invasive pneumococcal disease	INVASIVE PNEUMOCOCCAL DISEASE	1	kept
invasive positive pressure ventilation	invasive positive pressure ventilation	invasive positive pressure ventilation	invasive positive pressure ventilation	invasive positive pressure ventilation	1	kept
INVASIVE PROCEDURES	invasive procedures	invasive procedures	invasive procedure	invasive procedure	1	singular

Invasive Pulmonary Aspergillosis	invasive pulmonary aspergillosis	invasive pulmonary aspergillosis	invasive pulmonary aspergillosis	Invasive Pulmonary Aspergillosis	1	kept
invasive technique	invasive technique	invasive technique	invasive technique	invasive technique	1	kept
inventions	inventions	inventions	invention	Inventions	1	singular
Inventory control	inventory control	inventory control	inventory control	Inventory control	1	kept
inventory control	inventory control	inventory control	inventory control	Inventory control	1	case_or_format
Inventory Depression and Anxiety Symptoms General Depression	inventory depression and anxiety symptoms general depression	inventory depression and anxiety symptoms general depression	inventory depression and anxiety symptoms general depression	Inventory Depression and Anxiety Symptoms General Depression	1	kept
Inventory management	inventory management	inventory management	inventory management	Inventory management	1	kept
inventory of aggressivity	inventory of aggressivity	inventory of aggressivity	inventory of aggressivity	inventory of aggressivity	1	kept
Inventory policies	inventory policies	inventory policies	inventory policies	Inventory policies	1	kept
Inverse correlation	inverse correlation	inverse correlation	inverse correlation	Inverse correlation	1	kept
inverse probability of treatment weight	inverse probability of treatment weight	inverse probability of treatment weight	inverse probability of treatment weight	inverse probability of treatment weight	1	kept
inverse probability weighting	inverse probability weighting	inverse probability weighting	inverse probability weighting	inverse probability weighting	1	kept
inverse ratio ventilation	inverse ratio ventilation	inverse ratio ventilation	inverse ratio ventilation	inverse ratio ventilation	1	kept
inverse relationship	inverse relationship	inverse relationship	inverse relationship	inverse relationship	1	kept
inverse variance weighted method	inverse variance weighted method	inverse variance weighted method	inverse variance weighted method	inverse variance weighted method	1	kept
invert sugar	invert sugar	invert sugar	invert sugar	invert sugar	1	kept

Invertebrata	invertebrata	invertebrata	invertebrata	Invertebrata	1	kept
invertebrate	invertebrate	invertebrate	invertebrate	invertebrate	1	kept
Investigación en Enfermería	investigaci n en enfermer a	investigaci n en enfermer a	investigaci n en enfermer a	Investigación en Enfermería	1	kept
Investigación en evaluación de enfermer ía	investigaci n en evaluaci n de enfermer a	investigaci n en evaluaci n de enfermer a	investigaci n en evaluaci n de enfermer a	Investigación en evaluación de enfermer ía	1	kept
Investigation	investigation	investigation	investigation	Investigation	1	kept
investigation	investigation	investigation	investigation	Investigation	1	case_or_format
investigative procedures	investigative procedures	investigative procedures	investigative procedures	investigative procedures	1	kept
involuntary commitment	involuntary commitment	involuntary commitment	involuntary commitment	involuntary commitment	1	kept
INVOLUNTARY MEDICATION	involuntary medication	involuntary medication	involuntary medication	INVOLUNTARY MEDICATION	1	kept
Involvement in care	involvement in care	involvement in care	involvement in care	Involvement in care	1	kept
Involving mothers	involving mothers	involving mothers	involving mothers	Involving mothers	1	kept
Inégalités de parcours de vie	in galit s de parcours de vie	in galit s de parcours de vie	in galit s de parcours de vie	Inégalités de parcours de vie	1	kept
in‐hospital stroke	in hospital stroke	in hospital stroke	in hospital stroke	in hospital stroke	1	case_or_format
IOCA	ioca	ioca	ioca	IOCA	1	kept
Iodinated contrast material	iodinated contrast material	iodinated contrast material	iodinated contrast material	Iodinated contrast material	1	kept
iodinated contrast medium	iodinated contrast medium	iodinated contrast medium	iodinated contrast medium	iodinated contrast medium	1	kept

iodine	iodine	iodine	iodine	iodine	1	kept
Iodine Radioisotopes	iodine radioisotopes	iodine radioisotopes	iodine radioisotopes	Iodine Radioisotopes	1	kept
Iodine-131	iodine 131	iodine 131	iodine 131	Iodine-131	1	kept
Iodophor	iodophor	iodophor	iodophor	Iodophor	1	kept
iodophor	iodophor	iodophor	iodophor	Iodophor	1	case_or_format
Iodophors	iodophors	iodophors	iodophor	Iodophor	1	singular
iodothyronine	iodothyronine	iodothyronine	iodothyronine	iodothyronine	1	kept
iohexol	iohexol	iohexol	iohexol	iohexol	1	kept
Ionic solutions	ionic solutions	ionic solutions	ionic solutions	Ionic solutions	1	kept
IONS	ions	ions	ions	IONS	1	kept
iopamidol	iopamidol	iopamidol	iopamidol	iopamidol	1	kept
IOT	iot	iot	iot	IoT	1	case_or_format
IOT applications	iot applications	iot applications	iot applications	IOT applications	1	kept
IoT cloud	iot cloud	iot cloud	iot cloud	IoT cloud	1	kept
Iowa model of EBP	iowa model of ebp	iowa model of ebp	iowa model of ebp	Iowa model of EBP	1	kept
IPASS	ipass	ipass	ipass	IPASS	1	kept

IPAT	ipat	ipat	ipat	IPAT	1	kept
IPCU	ipcu	ipcu	ipcu	IPCU	1	kept
Ipilimumab	ipilimumab	ipilimumab	ipilimumab	ipilimumab	1	case_or_format
Ipsilateral	ipsilateral	ipsilateral	ipsilateral	Ipsilateral	1	kept
IPTW = inverse probability of treatment assignment	iptw inverse probability of treatment assignment	iptw inverse probability of treatment assignment	iptw inverse probability of treatment assignment	IPTW = inverse probability of treatment assignment	1	kept
IQR - interquartile range	iqr interquartile range	iqr interquartile range	iqr interquartile range	IQR, interquartile range	1	case_or_format
IQR, interquartile range	iqr interquartile range	iqr interquartile range	iqr interquartile range	IQR, interquartile range	1	kept
Ira Gunn	ira gunn	ira gunn	ira gunn	Ira Gunn	1	kept
Iran.	iran	iran	iran	Iran	1	case_or_format
Iranian culture	iranian culture	iranian culture	iranian culture	Iranian culture	1	kept
IRAQ	iraq	iraq	iraq	Iraq	1	case_or_format
Iraqi	iraqi	iraqi	iraqi	Iraqi	1	kept
Iraqi Kurdistan	iraqi kurdistan	iraqi kurdistan	iraqi kurdistan	Iraqi Kurdistan	1	kept
IRB, Institutional review board	irb institutional review board	irb institutional review board	irb institutional review board	IRB, Institutional review board	1	kept
irbesartan	irbesartan	irbesartan	irbesartan	irbesartan	1	kept
IRELAND	ireland	ireland	ireland	Ireland	1	case_or_format

Iris	iris	iris	iris	Iris	1	kept
iro gene	iro gene	iro gene	iro gene	iro gene	1	kept
iro protein	iro protein	iro protein	iro protein	iro protein	1	kept
Iron Chelating Agents	iron chelating agents	iron chelating agents	iron chelating agents	Iron Chelating Agents	1	kept
iron deficiency	iron deficiency	iron deficiency	iron deficiency	iron deficiency	1	kept
iron dextran	iron dextran	iron dextran	iron dextran	iron dextran	1	kept
Iron lung	iron lung	iron lung	iron lung	Iron lung	1	kept
iron metabolism	iron metabolism	iron metabolism	iron metabolism	iron metabolism	1	kept
Iron Metabolism Disorders	iron metabolism disorders	iron metabolism disorders	iron metabolism disorders	Iron Metabolism Disorders	1	kept
Iron poisoning	iron poisoning	iron poisoning	iron poisoning	Iron poisoning	1	kept
iron saccharate	iron saccharate	iron saccharate	iron saccharate	iron saccharate	1	kept
Iron supplement	iron supplement	iron supplement	iron supplement	Iron supplement	1	kept
iron supplementation	iron supplementation	iron supplementation	iron supplementation	iron supplementation	1	kept
Iron therapy	iron therapy	iron therapy	iron therapy	iron therapy	1	case_or_format
Iron, Dietary	iron dietary	iron dietary	iron dietary	Iron, Dietary	1	kept
IRRAflow	irraflow	irraflow	irraflow	IRRAflow	1	kept

Irregular sampling	irregular sampling	irregular sampling	irregular sampling	Irregular sampling	1	kept
IRRIGATION	irrigation	irrigation	irrigation	Irrigation	1	case_or_format
irrigation solution	irrigation solution	irrigation solution	irrigation solution	irrigation solution	1	kept
irritable colon	irritable colon	irritable colon	irritable colon	irritable colon	1	kept
IS technologies	is technologies	is technologies	is technologies	IS technologies	1	kept
is the cure worse than the disease	is the cure worse than the disease	is the cure worse than the disease	is the cure worse than the disease	is the cure worse than the disease	1	kept
isavuconazole	isavuconazole	isavuconazole	isavuconazole	isavuconazole	1	kept
Ischaemia	ischaemia	ischaemia	ischaemia	Ischaemia	1	kept
ischaemia	ischaemia	ischaemia	ischaemia	Ischaemia	1	case_or_format
ISCHEMIA-REPERFUSION INJURY	ischemia reperfusion injury	ischemia reperfusion injury	ischemia reperfusion injury	ISCHEMIA-REPERFUSION INJURY	1	kept
Ischemic	ischemic	ischemic	ischemic	Ischemic	1	kept
Ischemic changes	ischemic changes	ischemic changes	ischemic changes	Ischemic changes	1	kept
Ischemic colitis	ischemic colitis	ischemic colitis	ischemic colitis	ischemic colitis	1	case_or_format
ischemic hepatitis	ischemic hepatitis	ischemic hepatitis	ischemic hepatitis	ischemic hepatitis	1	kept
ischemic mitral valve regurgitation	ischemic mitral valve regurgitation	ischemic mitral valve regurgitation	ischemic mitral valve regurgitation	ischemic mitral valve regurgitation	1	kept
Ischemic necrosis	ischemic necrosis	ischemic necrosis	ischemic necrosis	Ischemic necrosis	1	kept

ischemic optic neuropathy	ischemic optic neuropathy	ischemic optic neuropathy	ischemic optic neuropathy	ischemic optic neuropathy	1	kept
Ischemic Preconditioning	ischemic preconditioning	ischemic preconditioning	ischemic preconditioning	Ischemic Preconditioning	1	kept
ischemic preconditioning	ischemic preconditioning	ischemic preconditioning	ischemic preconditioning	Ischemic Preconditioning	1	case_or_format
Ischemic strokes	ischemic strokes	ischemic strokes	ischemic stroke	ischemic stroke	1	singular
ISCHEMIC-STROKE	ischemic stroke	ischemic stroke	ischemic stroke	ischemic stroke	1	case_or_format
ischium	ischium	ischium	ischium	ischium	1	kept
iseganan	iseganan	iseganan	iseganan	iseganan	1	kept
Islam-based caring	islam based caring	islam based caring	islam based caring	Islam-based caring	1	kept
Islamic beliefs	islamic beliefs	islamic beliefs	islamic beliefs	Islamic beliefs	1	kept
Islamic Caring	islamic caring	islamic caring	islamic caring	Islamic Caring	1	kept
Islamic ethics	islamic ethics	islamic ethics	islamic ethics	Islamic ethics	1	kept
Islamic Republic of Iran	islamic republic of iran	islamic republic of iran	islamic republic of iran	Islamic Republic of Iran	1	kept
Islamic rituals	islamic rituals	islamic rituals	islamic rituals	Islamic rituals	1	kept
Islamic spiritual care	islamic spiritual care	islamic spiritual care	islamic spiritual care	Islamic spiritual care	1	kept
Islamic values	islamic values	islamic values	islamic values	Islamic values	1	kept
Islamic-based	islamic based	islamic based	islamic based	Islamic-based	1	kept

island	island	island	island	island	1	kept
island flap	island flap	island flap	island flap	island flap	1	kept
Isle of Man	isle of man	isle of man	isle of man	Isle of Man	1	kept
islet antigen 2	islet antigen 2	islet antigen 2	islet antigen 2	islet antigen 2	1	kept
Isoantibodies	isoantibodies	isoantibodies	isoantibodies	Isoantibodies	1	kept
ISOBAR	isobar	isobar	isobar	ISOBAR	1	kept
isocitrate dehydrogenase	isocitrate dehydrogenase	isocitrate dehydrogenase	isocitrate dehydrogenase	isocitrate dehydrogenase	1	kept
Isoelectric Focusing	isoelectric focusing	isoelectric focusing	isoelectric focusing	isoelectric focusing	1	case_or_format
isoelectric point	isoelectric point	isoelectric point	isoelectric point	isoelectric point	1	kept
isolated rooms	isolated rooms	isolated rooms	isolated rooms	isolated rooms	1	kept
isolated traumatic occipital condyle fracture	isolated traumatic occipital condyle fracture	isolated traumatic occipital condyle fracture	isolated traumatic occipital condyle fracture	isolated traumatic occipital condyle fracture	1	kept
Isolation facility	isolation facility	isolation facility	isolation facility	isolation facility	1	case_or_format
Isolation gown	isolation gown	isolation gown	isolation gown	Isolation gown	1	kept
isolation gown	isolation gown	isolation gown	isolation gown	Isolation gown	1	case_or_format
Isolation hospital	isolation hospital	isolation hospital	isolation hospital	isolation hospital	1	case_or_format
Isolation hospitals	isolation hospitals	isolation hospitals	isolation hospital	isolation hospital	1	singular

Isolation precaution	isolation precaution	isolation precaution	isolation precaution	ISOLATION PRECAUTIONS	1	case_or_format
isolation precaution	isolation precaution	isolation precaution	isolation precaution	ISOLATION PRECAUTIONS	1	case_or_format
Isolation rooms	isolation rooms	isolation rooms	isolation room	isolation room	1	singular
isolation ward	isolation ward	isolation ward	isolation ward	Isolation wards	1	case_or_format
isolette	isolette	isolette	isolette	isolette	1	kept
Isomers	isomers	isomers	isomers	Isomers	1	kept
isopropionyltoluidonium bromide	isopropionyltoluidonium bromide	isopropionyltoluidonium bromide	isopropionyltoluidonium bromide	isopropionyltoluidonium bromide	1	kept
Isopropyl alcohol	isopropyl alcohol	isopropyl alcohol	isopropyl alcohol	Isopropyl alcohol	1	kept
isopropyl alcohol	isopropyl alcohol	isopropyl alcohol	isopropyl alcohol	Isopropyl alcohol	1	case_or_format
Isoquinolines	isoquinolines	isoquinolines	isoquinolines	Isoquinolines	1	kept
isosorbide	isosorbide	isosorbide	isosorbide	isosorbide	1	kept
isosorbide dinitrate	isosorbide dinitrate	isosorbide dinitrate	isosorbide dinitrate	isosorbide dinitrate	1	kept
Isotonic saline	isotonic saline	isotonic saline	isotonic saline	Isotonic saline	1	kept
ISPOR translation	ispor translation	ispor translation	ispor translation	ISPOR translation	1	kept
isradipine	isradipine	isradipine	isradipine	isradipine	1	kept
ISS	iss	iss	iss	ISS	1	kept

ISS = Injury Severity Score	iss injury severity score	iss injury severity score	iss injury severity score	ISS = Injury Severity Score	1	kept
Issue resolutions	issue resolutions	issue resolutions	issue resolutions	Issue resolutions	1	kept
Issues	issues	issues	issues	ISSUES	1	case_or_format
issues	issues	issues	issues	ISSUES	1	case_or_format
IT adoption	it adoption	it adoption	it adoption	IT adoption	1	kept
IT benchmarking	it benchmarking	it benchmarking	it benchmarking	IT benchmarking	1	kept
IT infrastructures	it infrastructures	it infrastructures	it infrastructures	IT infrastructures	1	kept
iTAKi fall risk scale	itaki fall risk scale	itaki fall risk scale	itaki fall risk scale	iTAKi fall risk scale	1	kept
ITALIAN GROUP	italian group	italian group	italian group	ITALIAN GROUP	1	kept
Italian healthcare	italian healthcare	italian healthcare	italian healthcare	Italian healthcare	1	kept
Italian healthcare system	italian healthcare system	italian healthcare system	italian healthcare system	Italian healthcare system	1	kept
Italian Moral Distress Scale Revised	italian moral distress scale revised	italian moral distress scale revised	italian moral distress scale revised	Italian Moral Distress Scale Revised	1	kept
Italian National Law 38	italian national law 38	italian national law 38	italian national law 38	Italian National Law 38	1	kept
Italian people	italian people	italian people	italian people	Italian people	1	kept
Italian regions	italian regions	italian regions	italian regions	Italian regions	1	kept
Italian sanitary cooperation	italian sanitary cooperation	italian sanitary cooperation	italian sanitary cooperation	Italian sanitary cooperation	1	kept

ITB	itb	itb	itb	ITB	1	kept
Item analysis	item analysis	item analysis	item analysis	Item analysis	1	kept
item analysis	item analysis	item analysis	item analysis	Item analysis	1	case_or_format
item discrimination	item discrimination	item discrimination	item discrimination	item discrimination	1	kept
iterative analysis	iterative analysis	iterative analysis	iterative analysis	iterative analysis	1	kept
iterative fruit fly optimization algorithm	iterative fruit fly optimization algorithm	iterative fruit fly optimization algorithm	iterative fruit fly optimization algorithm	iterative fruit fly optimization algorithm	1	kept
Itolizumab	itolizumab	itolizumab	itolizumab	Itolizumab	1	kept
ITS	its	its	its	ITS	1	kept
ITS applications	its applications	its applications	its applications	ITS applications	1	kept
iuc gene	iuc gene	iuc gene	iuc gene	iuc gene	1	kept
iuc protein	iuc protein	iuc protein	iuc protein	iuc protein	1	kept
IUGR	iugr	iugr	iugr	IUGR	1	kept
IV = intravenous	iv intravenous	iv intravenous	iv intravenous	IV, intravenous	1	case_or_format
IV administration sets	iv administration sets	iv administration sets	iv administration sets	IV administration sets	1	kept
IV care bundle	iv care bundle	iv care bundle	iv care bundle	IV care bundle	1	kept
IV connector	iv connector	iv connector	iv connector	IV connector	1	kept

IV connectors	iv connectors	iv connectors	iv connector	IV connector	1	singular
IV infiltration	iv infiltration	iv infiltration	iv infiltration	IV infiltration	1	kept
IV infusions	iv infusions	iv infusions	iv infusion	IV infusion	1	singular
IV insertion	iv insertion	iv insertion	iv insertion	IV insertion	1	kept
IV medication	iv medication	iv medication	iv medication	IV medication	1	kept
IV smart pumps	iv smart pumps	iv smart pumps	iv smart pump	IV smart pump	1	singular
IV team	iv team	iv team	iv team	IV team	1	kept
IV therapy	iv therapy	iv therapy	iv therapy	IV therapy	1	kept
IV vitamin C	iv vitamin c	iv vitamin c	iv vitamin c	IV vitamin C	1	kept
IVC	ivc	ivc	ivc	IVC	1	kept
IVH	ivh	ivh	ivh	IVH	1	kept
IVIG	ivig	ivig	ivig	IVIG	1	kept
ixekizumab	ixekizumab	ixekizumab	ixekizumab	ixekizumab	1	kept
J-point	j point	j point	j point	J-point	1	kept
J11	j11	j11	j11	J11	1	kept
Jackson	jackson	jackson	jackson	Jackson	1	kept

jackson heart study	jackson heart study	jackson heart study	jackson heart study	jackson heart study	1	kept
Jackson networks	jackson networks	jackson networks	jackson networks	Jackson networks	1	kept
Jackson-Cubbin scale	jackson cubbin scale	jackson cubbin scale	jackson cubbin scale	Jackson-Cubbin scale	1	kept
Jackson－Cubbin scale	jackson cubbin scale	jackson cubbin scale	jackson cubbin scale	Jackson-Cubbin scale	1	case_or_format
JAK-STAT signaling	jak stat signaling	jak stat signaling	jak stat signaling	JAK-STAT signaling	1	kept
James Lind Alliance	james lind alliance	james lind alliance	james lind alliance	James Lind Alliance	1	kept
Japan Coma Scale score	japan coma scale score	japan coma scale score	japan coma scale score	Japan Coma Scale score	1	kept
Japanese Encephalitis	japanese encephalitis	japanese encephalitis	japanese encephalitis	Japanese Encephalitis	1	kept
Japanese encephalitis	japanese encephalitis	japanese encephalitis	japanese encephalitis	Japanese Encephalitis	1	case_or_format
Japanese encephalitis virus	japanese encephalitis virus	japanese encephalitis virus	japanese encephalitis virus	Japanese encephalitis virus	1	kept
Japanese healthcare	japanese healthcare	japanese healthcare	japanese healthcare	Japanese healthcare	1	kept
Japanese Orthopedic Association score	japanese orthopedic association score	japanese orthopedic association score	japanese orthopedic association score	Japanese Orthopedic Association score	1	kept
Jardiance	jardiance	jardiance	jardiance	Jardiance	1	kept
Jaundice, neonatal	jaundice neonatal	jaundice neonatal	jaundice neonatal	Jaundice, Neonatal	1	case_or_format
jaw fracture	jaw fracture	jaw fracture	jaw fracture	jaw fracture	1	kept
Jaw Fractures	jaw fractures	jaw fractures	jaw fracture	jaw fracture	1	singular

jaw pain	jaw pain	jaw pain	jaw pain	jaw pain	1	kept
Jazan	jazan	jazan	jazan	Jazan	1	kept
JD-R MODEL	jd r model	jd r model	jd r model	JD-R model	1	case_or_format
Jealousy	jealousy	jealousy	jealousy	Jealousy	1	kept
Jeddah	jeddah	jeddah	jeddah	Jeddah	1	kept
jeddah saudi arbia	jeddah saudi arbia	jeddah saudi arbia	jeddah saudi arbia	jeddah saudi arbia	1	kept
Jefferson Scale of Empathy-Health Professionals	jefferson scale of empathy health professionals	jefferson scale of empathy health professionals	jefferson scale of empathy health professionals	Jefferson Scale of Empathy-Health Professionals	1	kept
Jehovah's Witness	jehovah s witness	jehovah s witness	jehovah s witness	Jehovah's witness	1	case_or_format
Jehovah' s Witnesses	jehovah s witnesses	jehovah s witnesses	jehovah s witnesses	Jehovah's Witnesses	1	case_or_format
jejunoileal bypass	jejunoileal bypass	jejunoileal bypass	jejunoileal bypass	jejunoileal bypass	1	kept
jejunostomy catheter	jejunostomy catheter	jejunostomy catheter	jejunostomy catheter	jejunostomy catheter	1	kept
Jejunostomy tube	jejunostomy tube	jejunostomy tube	jejunostomy tube	Jejunostomy tube	1	kept
jejunostomy tube	jejunostomy tube	jejunostomy tube	jejunostomy tube	Jejunostomy tube	1	case_or_format
Jew	jew	jew	jew	jew	1	case_or_format
jewelry	jewelry	jewelry	jewelry	Jewelry	1	case_or_format
JH-HLM, Johns Hopkins-Highest Level of Mobility	jh hlm johns hopkins highest level of mobility	jh hlm johns hopkins highest level of mobility	jh hlm johns hopkins highest level of mobility	JH-HLM, Johns Hopkins-Highest Level of Mobility	1	kept

Jharkhand	jharkhand	jharkhand	jharkhand	Jharkhand	1	kept
jigsaw cooperative learning method	jigsaw cooperative learning method	jigsaw cooperative learning method	jigsaw cooperative learning method	jigsaw cooperative learning method	1	kept
Jimma Medical Center	jimma medical center	jimma medical center	jimma medical center	Jimma Medical Center	1	kept
JOB	job	job	job	Job	1	case_or_format
job	job	job	job	Job	1	case_or_format
job accommodation	job accommodation	job accommodation	job accommodation	job accommodation	1	kept
Job autonomy	job autonomy	job autonomy	job autonomy	Job autonomy	1	kept
job autonomy	job autonomy	job autonomy	job autonomy	Job autonomy	1	case_or_format
JOB BURNOUT	job burnout	job burnout	job burnout	job burnout	1	case_or_format
Job Burnout	job burnout	job burnout	job burnout	job burnout	1	case_or_format
job change	job change	job change	job change	job change	1	kept
Job characteristics	job characteristics	job characteristics	job characteristics	job characteristics	1	case_or_format
job cognitive failures	job cognitive failures	job cognitive failures	job cognitive failures	job cognitive failures	1	kept
job content	job content	job content	job content	job content	1	kept
Job Content Questionnaire	job content questionnaire	job content questionnaire	job content questionnaire	Job Content Questionnaire	1	kept
job control	job control	job control	job control	job control	1	kept

Job demand-control model	job demand control model	job demand control model	job demand control model	Job demand-control model	1	kept
JOB DEMANDS	job demands	job demands	job demands	JOB DEMANDS	1	kept
job demands	job demands	job demands	job demands	JOB DEMANDS	1	case_or_format
Job demands-resources	job demands resources	job demands resources	job demands resources	job demands-resources	1	case_or_format
Job demands-resources model	job demands resources model	job demands resources model	job demands resources model	Job demands‐resources model	1	case_or_format
Job Descriptive Index	job descriptive index	job descriptive index	job descriptive index	Job Descriptive Index	1	kept
job designation	job designation	job designation	job designation	job designation	1	kept
Job dissatisfaction	job dissatisfaction	job dissatisfaction	job dissatisfaction	job dissatisfaction	1	case_or_format
job engagement	job engagement	job engagement	job engagement	job engagement	1	kept
job enjoyment	job enjoyment	job enjoyment	job enjoyment	job enjoyment	1	kept
job enrichment	job enrichment	job enrichment	job enrichment	job enrichment	1	kept
job finding	job finding	job finding	job finding	job finding	1	kept
Job incorporation	job incorporation	job incorporation	job incorporation	Job incorporation	1	kept
Job market	job market	job market	job market	job market	1	case_or_format
Job motivation	job motivation	job motivation	job motivation	Job motivation	1	kept
job motivation	job motivation	job motivation	job motivation	Job motivation	1	case_or_format

Job outcomes	job outcomes	job outcomes	job outcomes	JOB OUTCOMES	1	case_or_format
Job Performance	job performance	job performance	job performance	job performance	1	case_or_format
job position	job position	job position	job position	job position	1	kept
job satisfaction assessment	job satisfaction assessment	job satisfaction assessment	job satisfaction assessment	job satisfaction assessment	1	kept
Job Satisfaction Questionnaire	job satisfaction questionnaire	job satisfaction questionnaire	job satisfaction questionnaire	Job Satisfaction Questionnaire	1	kept
Job security	job security	job security	job security	job security	1	case_or_format
JOB STRAIN	job strain	job strain	job strain	Job strain	1	case_or_format
job stress scale	job stress scale	job stress scale	job stress scale	job stress scale	1	kept
job stressors	job stressors	job stressors	job stressors	Job stressors	1	case_or_format
job transfer	job transfer	job transfer	job transfer	job transfer	1	kept
job turnover	job turnover	job turnover	job turnover	job turnover	1	kept
job type	job type	job type	job type	job type	1	kept
Job-related Affective Well-being Scale	job related affective well being scale	job related affective well being scale	job related affective well being scale	Job-related Affective Well-being Scale	1	kept
Job-related stress	job related stress	job related stress	job related stress	Job-related stress	1	kept
job-related stress	job related stress	job related stress	job related stress	Job-related stress	1	case_or_format
JOB-STRESS	job stress	job stress	job stress	job stress	1	case_or_format

jobless	jobless	jobless	jobless	jobless	1	kept
jobs and business suffer	jobs and business suffer	jobs and business suffer	jobs and business suffer	jobs and business suffer	1	kept
job - demand resource model	job demand resource model	job demand resource model	job demand resource model	job - demand resource model	1	kept
John Doe	john doe	john doe	john doe	John Doe	1	kept
JOINT	joint	joint	joint	joint	1	case_or_format
joint capsulotomy	joint capsulotomy	joint capsulotomy	joint capsulotomy	joint capsulotomy	1	kept
Joint Commission	joint commission	joint commission	joint commission	Joint Commission	1	kept
Joint distributions	joint distributions	joint distributions	joint distributions	Joint distributions	1	kept
Joint infections	joint infections	joint infections	joint infections	Joint infections	1	kept
joint limitation	joint limitation	joint limitation	joint limitation	joint limitation	1	kept
Joint medical service	joint medical service	joint medical service	joint medical service	Joint medical service	1	kept
Joint pair leadership	joint pair leadership	joint pair leadership	joint pair leadership	Joint pair leadership	1	kept
Joint participation	joint participation	joint participation	joint participation	Joint participation	1	kept
Joint participation Model	joint participation model	joint participation model	joint participation model	Joint participation Model	1	kept
Joint replacement	joint replacement	joint replacement	joint replacement	Joint replacement	1	kept
joint replacement registry	joint replacement registry	joint replacement registry	joint replacement registry	joint replacement registry	1	kept

joint surgery	joint surgery	joint surgery	joint surgery	joint surgery	1	kept
joint swelling	joint swelling	joint swelling	joint swelling	joint swelling	1	kept
Joint trauma	joint trauma	joint trauma	joint trauma	Joint trauma	1	kept
Joint working	joint working	joint working	joint working	Joint working	1	kept
Jordania	jordania	jordania	jordania	Jordania	1	kept
JOT	jot	jot	jot	JOT	1	kept
Journal articles	journal articles	journal articles	journal articles	Journal articles	1	kept
Journal clubs	journal clubs	journal clubs	journal clubs	Journal clubs	1	kept
Journal of Trauma and Acute Care Surgery	journal of trauma and acute care surgery	journal of trauma and acute care surgery	journal of trauma and acute care surgery	Journal of Trauma and Acute Care Surgery	1	kept
journaling	journaling	journaling	journaling	journaling	1	kept
Journey box	journey box	journey box	journey box	Journey box	1	kept
joy	joy	joy	joy	joy	1	kept
joy in work	joy in work	joy in work	joy in work	joy in work	1	kept
Judgement	judgement	judgement	judgement	Judgement	1	kept
judgement analysis	judgement analysis	judgement analysis	judgement analysis	judgement analysis	1	kept
Judgement performance	judgement performance	judgement performance	judgement performance	Judgement performance	1	kept

judgment	judgment	judgment	judgment	Judgment	1	case_or_format
Judgment analysis	judgment analysis	judgment analysis	judgment analysis	Judgment analysis	1	kept
Judicialization of health	judicialization of health	judicialization of health	judicialization of health	Judicialization of health	1	kept
judiciarization	judiciarization	judiciarization	judiciarization	judiciarization	1	kept
Jugular	jugular	jugular	jugular	Jugular	1	kept
Juice	juice	juice	juice	Juice	1	kept
jumping	jumping	jumping	jumping	jumping	1	kept
junctional adhesion molecule C	junctional adhesion molecule c	junctional adhesion molecule c	junctional adhesion molecule c	junctional adhesion molecule C	1	kept
Junctional ectopic tachycardia	junctional ectopic tachycardia	junctional ectopic tachycardia	junctional ectopic tachycardia	Junctional ectopic tachycardia	1	kept
Jungian Theory	jungian theory	jungian theory	jungian theory	Jungian Theory	1	kept
Junior doctor	junior doctor	junior doctor	junior doctor	JUNIOR DOCTORS	1	case_or_format
Junior residents	junior residents	junior residents	junior residents	Junior residents	1	kept
junior residents	junior residents	junior residents	junior residents	Junior residents	1	case_or_format
junior staff	junior staff	junior staff	junior staff	junior staff	1	kept
Jurisdiction	jurisdiction	jurisdiction	jurisdiction	Jurisdiction	1	kept
Jurisprudencia	jurisprudencia	jurisprudencia	jurisprudencia	Jurisprudencia	1	kept

Just Culture	just culture	just culture	just culture	Just Culture	1	kept
Just culture	just culture	just culture	just culture	Just Culture	1	case_or_format
just culture	just culture	just culture	just culture	Just Culture	1	case_or_format
Just in time	just in time	just in time	just in time	JUST-IN-TIME	1	case_or_format
Just in time learning	just in time learning	just in time learning	just in time learning	Just in time learning	1	kept
Just in time production	just in time production	just in time production	just in time production	Just in time production	1	kept
Just-in-time learning	just in time learning	just in time learning	just in time learning	Just in time learning	1	case_or_format
justice system involvement	justice system involvement	justice system involvement	justice system involvement	justice system involvement	1	kept
Juvenile Delinquency	juvenile delinquency	juvenile delinquency	juvenile delinquency	Juvenile Delinquency	1	kept
juvenile delinquency	juvenile delinquency	juvenile delinquency	juvenile delinquency	Juvenile Delinquency	1	case_or_format
juvenile rheumatoid arthritis	juvenile rheumatoid arthritis	juvenile rheumatoid arthritis	juvenile rheumatoid arthritis	juvenile rheumatoid arthritis	1	kept
k fold cross validation	k fold cross validation	k fold cross validation	k fold cross validation	k fold cross validation	1	kept
K ras protein	k ras protein	k ras protein	k ras protein	K ras protein	1	kept
K-means	k means	k means	k means	K-means	1	kept
K-SEIR model	k seir model	k seir model	k seir model	K-SEIR model	1	kept
Kahoot	kahoot	kahoot	kahoot	Kahoot	1	kept

kaizen	kaizen	kaizen	kaizen	kaizen	1	kept
kallikrein kinin system	kallikrein kinin system	kallikrein kinin system	kallikrein kinin system	kallikrein kinin system	1	kept
Kamishibai Card	kamishibai card	kamishibai card	kamishibai card	Kamishibai Card	1	kept
Kampot	kampot	kampot	kampot	Kampot	1	kept
KANGAROO	kangaroo	kangaroo	kangaroo	kangaroo	1	case_or_format
Kangaroo Method	kangaroo method	kangaroo method	kangaroo method	Kangaroo Method	1	kept
Kangaroo method	kangaroo method	kangaroo method	kangaroo method	Kangaroo Method	1	case_or_format
kangaroo mother care method	kangaroo mother care method	kangaroo mother care method	kangaroo mother care method	Kangaroo-Mother Care Method	1	case_or_format
kangaroo mother care methods	kangaroo mother care methods	kangaroo mother care methods	kangaroo mother care method	Kangaroo-Mother Care Method	1	singular
Kangaroo-care	kangaroo care	kangaroo care	kangaroo care	kangaroo care	1	case_or_format
Kangaroo-mother care	kangaroo mother care	kangaroo mother care	kangaroo mother care	Kangaroo mother care	1	case_or_format
Kangaroo-Mother care method	kangaroo mother care method	kangaroo mother care method	kangaroo mother care method	Kangaroo-Mother Care Method	1	case_or_format
Kangaroo-mother care method	kangaroo mother care method	kangaroo mother care method	kangaroo mother care method	Kangaroo-Mother Care Method	1	case_or_format
Kangroo care	kangroo care	kangroo care	kangroo care	Kangroo care	1	kept
Kano model	kano model	kano model	kano model	Kano model	1	kept
Kansas City Cardiomyopathy Questionnaire	kansas city cardiomyopathy questionnaire	kansas city cardiomyopathy questionnaire	kansas city cardiomyopathy questionnaire	Kansas City Cardiomyopathy Questionnaire	1	kept

Kanter	kanter	kanter	kanter	Kanter	1	kept
kaolin pectin	kaolin pectin	kaolin pectin	kaolin pectin	kaolin pectin	1	kept
KAP (Knowledge	kap knowledge	kap knowledge	kap knowledge	KAP (Knowledge	1	kept
KAP study	kap study	kap study	kap study	KAP study	1	kept
KAP survey	kap survey	kap survey	kap survey	KAP survey	1	kept
Kaplan-Meiers Estimate	kaplan meiers estimate	kaplan meiers estimate	kaplan meiers estimate	Kaplan-Meiers Estimate	1	kept
Kaposi sarcoma	kaposi sarcoma	kaposi sarcoma	kaposi sarcoma	Kaposi sarcoma	1	kept
Kaposi’s sarcoma	kaposi s sarcoma	kaposi s sarcoma	kaposi s sarcoma	Kaposi’s sarcoma	1	kept
Kappa coefficients	kappa coefficients	kappa coefficients	kappa coefficients	Kappa coefficients	1	kept
Karachi	karachi	karachi	karachi	Karachi	1	kept
Karasek	karasek	karasek	karasek	Karasek	1	kept
KardiaMobile	kardiamobile	kardiamobile	kardiamobile	KardiaMobile	1	kept
KardiaStation	kardiastation	kardiastation	kardiastation	KardiaStation	1	kept
Karnataka-India	karnataka india	karnataka india	karnataka india	Karnataka-India	1	kept
KARNOFSKY	karnofsky	karnofsky	karnofsky	KARNOFSKY	1	kept
KARNOFSKY PERFORMANCE STATUS	karnofsky performance status	karnofsky performance status	karnofsky performance status	Karnofsky Performance Status	1	case_or_format

Karolinska Psychodynamic Profile	karolinska psychodynamic profile	karolinska psychodynamic profile	karolinska psychodynamic profile	Karolinska Psychodynamic Profile	1	kept
karyotyping	karyotyping	karyotyping	karyotyping	Karyotyping	1	case_or_format
Kasabach-Merritt Syndrome	kasabach merritt syndrome	kasabach merritt syndrome	kasabach merritt syndrome	Kasabach Merritt syndrome	1	case_or_format
Katz Index of Activity of Daily Living	katz index of activity of daily living	katz index of activity of daily living	katz index of activity of daily living	Katz Index of Activity of Daily Living	1	kept
Kawasaki Disease	kawasaki disease	kawasaki disease	kawasaki disease	Kawasaki Disease	1	kept
KAWASAKI-DISEASE	kawasaki disease	kawasaki disease	kawasaki disease	Kawasaki Disease	1	case_or_format
KDIGO	kdigo	kdigo	kdigo	KDIGO	1	kept
keepsakes	keepsakes	keepsakes	keepsake	Keepsake	1	singular
Keloid	keloid	keloid	keloid	Keloid	1	kept
keloid	keloid	keloid	keloid	Keloid	1	case_or_format
Kennedy Terminal Ulcer	kennedy terminal ulcer	kennedy terminal ulcer	kennedy terminal ulcer	Kennedy terminal ulcer	1	case_or_format
Kep	kep	kep	kep	Kep	1	kept
Keratin-19	keratin 19	keratin 19	keratin 19	Keratin-19	1	kept
KERATOPATHY	keratopathy	keratopathy	keratopathy	keratopathy	1	case_or_format
Keratopathy	keratopathy	keratopathy	keratopathy	keratopathy	1	case_or_format
Kerman [Iran]	kerman iran	kerman iran	kerman iran	Kerman [Iran]	1	kept

Kessler psychological distress scale	kessler psychological distress scale	kessler psychological distress scale	kessler psychological distress scale	Kessler Psychological Distress Scale	1	case_or_format
Ketamine infusion	ketamine infusion	ketamine infusion	ketamine infusion	Ketamine infusion	1	kept
KETAMINE SEDATION	ketamine sedation	ketamine sedation	ketamine sedation	KETAMINE SEDATION	1	kept
ketanserin	ketanserin	ketanserin	ketanserin	ketanserin	1	kept
Ketoacidosis	ketoacidosis	ketoacidosis	ketoacidosis	ketoacidosis	1	case_or_format
Ketonaemia	ketonaemia	ketonaemia	ketonaemia	Ketonaemia	1	kept
Ketone Bodies	ketone bodies	ketone bodies	ketone body	ketone body	1	singular
ketone body	ketone body	ketone body	ketone body	ketone body	1	kept
ketoprofen	ketoprofen	ketoprofen	ketoprofen	ketoprofen	1	kept
Ketorolac	ketorolac	ketorolac	ketorolac	ketorolac	1	case_or_format
Ketosis	ketosis	ketosis	ketosis	Ketosis	1	kept
KEY	key	key	key	KEY	1	kept
Key driver diagram	key driver diagram	key driver diagram	key driver diagram	Key driver diagram	1	kept
Key indicators	key indicators	key indicators	key indicators	Key indicators	1	kept
Key performance indicator	key performance indicator	key performance indicator	key performance indicator	key performance indicator	1	case_or_format
Key performance indicators	key performance indicators	key performance indicators	key performance indicator	key performance indicator	1	singular

key performance indicators	key performance indicators	key performance indicators	key performance indicator	key performance indicator	1	singular
Key wordsDevelopmental care	key wordsdevelopmental care	key wordsdevelopmental care	key wordsdevelopmental care	Key wordsDevelopmental care	1	kept
keyboard	keyboard	keyboard	keyboard	keyboard	1	kept
Keyboards	keyboards	keyboards	keyboard	keyboard	1	singular
KEYWORDS	keywords	keywords	keywords	KEYWORDS	1	kept
Keywords: cardiopulmonary resuscitation	keywords cardiopulmonary resuscitation	keywords cardiopulmonary resuscitation	keywords cardiopulmonary resuscitation	Keywords: cardiopulmonary resuscitation	1	kept
Khartoum	khartoum	khartoum	khartoum	Khartoum	1	kept
khat chewing	khat chewing	khat chewing	khat chewing	khat chewing	1	kept
Khorasan	khorasan	khorasan	khorasan	Khorasan	1	kept
Ki 67 antigen	ki 67 antigen	ki 67 antigen	ki 67 antigen	Ki 67 antigen	1	kept
kidney agenesis	kidney agenesis	kidney agenesis	kidney agenesis	kidney agenesis	1	kept
kidney allograft	kidney allograft	kidney allograft	kidney allograft	kidney allograft	1	kept
kidney autotransplantation	kidney autotransplantation	kidney autotransplantation	kidney autotransplantation	kidney autotransplantation	1	kept
kidney blood vessel	kidney blood vessel	kidney blood vessel	kidney blood vessel	kidney blood vessel	1	kept
kidney calcification	kidney calcification	kidney calcification	kidney calcification	kidney calcification	1	kept
kidney carcinoma	kidney carcinoma	kidney carcinoma	kidney carcinoma	kidney carcinoma	1	kept

kidney circulation	kidney circulation	kidney circulation	kidney circulation	kidney circulation	1	kept
kidney clearance	kidney clearance	kidney clearance	kidney clearance	kidney clearance	1	kept
kidney colic	kidney colic	kidney colic	kidney colic	kidney colic	1	kept
kidney collecting tubule	kidney collecting tubule	kidney collecting tubule	kidney collecting tubule	kidney collecting tubule	1	kept
kidney concentrating capacity	kidney concentrating capacity	kidney concentrating capacity	kidney concentrating capacity	kidney concentrating capacity	1	kept
kidney cortex	kidney cortex	kidney cortex	kidney cortex	kidney cortex	1	kept
Kidney dialysis	kidney dialysis	kidney dialysis	kidney dialysis	Kidney dialysis	1	kept
Kidney disease	kidney disease	kidney disease	kidney disease	kidney disease	1	case_or_format
kidney education	kidney education	kidney education	kidney education	kidney education	1	kept
kidney examination	kidney examination	kidney examination	kidney examination	kidney examination	1	kept
kidney failure, chronic	kidney failure chronic	kidney failure chronic	kidney failure chronic	Kidney Failure, Chronic	1	case_or_format
kidney graft rejection	kidney graft rejection	kidney graft rejection	kidney graft rejection	kidney graft rejection	1	kept
kidney hemangiomyolipoma	kidney hemangiomyolipoma	kidney hemangiomyolipoma	kidney hemangiomyolipoma	kidney hemangiomyolipoma	1	kept
kidney infection	kidney infection	kidney infection	kidney infection	kidney infection	1	kept
kidney ischemia	kidney ischemia	kidney ischemia	kidney ischemia	kidney ischemia	1	kept
kidney medulla	kidney medulla	kidney medulla	kidney medulla	kidney medulla	1	kept

kidney parenchyma	kidney parenchyma	kidney parenchyma	kidney parenchyma	kidney parenchyma	1	kept
Kidney recovery	kidney recovery	kidney recovery	kidney recovery	Kidney recovery	1	kept
kidney sarcoma	kidney sarcoma	kidney sarcoma	kidney sarcoma	kidney sarcoma	1	kept
Kidney stones	kidney stones	kidney stones	kidney stones	Kidney stones	1	kept
kidney transplant unit	kidney transplant unit	kidney transplant unit	kidney transplant unit	kidney transplant unit	1	kept
kidney tubule	kidney tubule	kidney tubule	kidney tubule	kidney tubule	1	kept
kidney tubule acidosis	kidney tubule acidosis	kidney tubule acidosis	kidney tubule acidosis	kidney tubule acidosis	1	kept
Kidney Tubules	kidney tubules	kidney tubules	kidney tubule	kidney tubule	1	singular
kidney tumor	kidney tumor	kidney tumor	kidney tumor	kidney tumor	1	kept
kidney vein	kidney vein	kidney vein	kidney vein	kidney vein	1	kept
kidney vein thrombosis	kidney vein thrombosis	kidney vein thrombosis	kidney vein thrombosis	kidney vein thrombosis	1	kept
Kidney, Artificial	kidney artificial	kidney artificial	kidney artificial	Kidney, Artificial	1	kept
kidney, transplantation	kidney transplantation	kidney transplantation	kidney transplantation	kidney transplantation	1	case_or_format
kidneys	kidneys	kidneys	kidney	Kidney	1	singular
Kielland's	kielland s	kielland s	kielland s	Kielland's	1	kept
kindergarten	kindergarten	kindergarten	kindergarten	kindergarten	1	kept

kindness	kindness	kindness	kindness	kindness	1	kept
kinesiology	kinesiology	kinesiology	kinesiology	kinesiology	1	kept
Kinesiophobia	kinesiophobia	kinesiophobia	kinesiophobia	Kinesiophobia	1	kept
kinesiophobia	kinesiophobia	kinesiophobia	kinesiophobia	Kinesiophobia	1	case_or_format
Kinesthesis	kinesthesis	kinesthesis	kinesthesis	Kinesthesis	1	kept
Kinesthetic	kinesthetic	kinesthetic	kinesthetic	kinesthetic	1	case_or_format
kinesthetic stimulation	kinesthetic stimulation	kinesthetic stimulation	kinesthetic stimulation	kinesthetic stimulation	1	kept
kinetic time	kinetic time	kinetic time	kinetic time	kinetic time	1	kept
kinetics	kinetics	kinetics	kinetics	Kinetics	1	case_or_format
kinetosome	kinetosome	kinetosome	kinetosome	kinetosome	1	kept
king parkinson disease pain scale	king parkinson disease pain scale	king parkinson disease pain scale	king parkinson disease pain scale	king parkinson disease pain scale	1	kept
Kings Outcome Scale for Childhood Head Injury	kings outcome scale for childhood head injury	kings outcome scale for childhood head injury	kings outcome scale for childhood head injury	Kings Outcome Scale for Childhood Head Injury	1	kept
King’s theory of goal attainment	king s theory of goal attainment	king s theory of goal attainment	king s theory of goal attainment	King’s theory of goal attainment	1	kept
kinin	kinin	kinin	kinin	kinin	1	kept
Kinins	kinins	kinins	kinin	kinin	1	singular
Kinki	kinki	kinki	kinki	Kinki	1	kept

kinlessness	kinlessness	kinlessness	kinlessness	kinlessness	1	kept
KINSHIP	kinship	kinship	kinship	KINSHIP	1	kept
kinésithérapeute	kin sith rapeute	kin sith rapeute	kin sith rapeute	kinésithérapeute	1	kept
kirby bauer method	kirby bauer method	kirby bauer method	kirby bauer method	kirby bauer method	1	kept
Kirkpatrick's elvaluation model	kirkpatrick s elvaluation model	kirkpatrick s elvaluation model	kirkpatrick s elvaluation model	Kirkpatrick's elvaluation model	1	kept
Kirkpatrick's Evaluation	kirkpatrick s evaluation	kirkpatrick s evaluation	kirkpatrick s evaluation	Kirkpatrick's Evaluation	1	kept
Kirkpatrick's model	kirkpatrick s model	kirkpatrick s model	kirkpatrick s model	Kirkpatrick's model	1	kept
kitchen	kitchen	kitchen	kitchen	kitchen	1	kept
Kitten	kitten	kitten	kitten	Kitten	1	kept
kitten	kitten	kitten	kitten	Kitten	1	case_or_format
kiwifruit	kiwifruit	kiwifruit	kiwifruit	kiwifruit	1	kept
kişisel koruyucu ekipman	ki isel koruyucu ekipman	ki isel koruyucu ekipman	ki isel koruyucu ekipman	kişisel koruyucu ekipman	1	kept
KJ method	kj method	kj method	kj method	KJ method	1	kept
Kjelland	kjelland	kjelland	kjelland	Kjelland	1	kept
KLEBSIELLA	klebsiella	klebsiella	klebsiella	Klebsiella	1	case_or_format
Klebsiella pneumonia	klebsiella pneumonia	klebsiella pneumonia	klebsiella pneumonia	Klebsiella pneumonia	1	kept

Klebsiella pneumoniae 002b	klebsiella pneumoniae 002b	klebsiella pneumoniae 002b	klebsiella pneumoniae 002b	Klebsiella pneumoniae 002b	1	kept
Klebsiella pneumoniae ST412	klebsiella pneumoniae st412	klebsiella pneumoniae st412	klebsiella pneumoniae st412	Klebsiella pneumoniae ST412	1	kept
Klebsiella pneumoniae ST86	klebsiella pneumoniae st86	klebsiella pneumoniae st86	klebsiella pneumoniae st86	Klebsiella pneumoniae ST86	1	kept
KLEBSIELLA-PNEUMONIAE BACTEREMIA	klebsiella pneumoniae bacteremia	klebsiella pneumoniae bacteremia	klebsiella pneumoniae bacteremia	KLEBSIELLA-PNEUMONIAE BACTEREMIA	1	kept
KLEBSIELLA-PNEUMONIAE INFECTION	klebsiella pneumoniae infection	klebsiella pneumoniae infection	klebsiella pneumoniae infection	Klebsiella pneumoniae infection	1	case_or_format
klinische Entscheidungsfindung	klinische entscheidungsfindung	klinische entscheidungsfindung	klinische entscheidungsfindung	klinische Entscheidungsfindung	1	kept
klonipin	klonipin	klonipin	klonipin	klonipin	1	kept
KMC chair	kmc chair	kmc chair	kmc chair	KMC chair	1	kept
KMC, Kangaroo mother care	kmc kangaroo mother care	kmc kangaroo mother care	kmc kangaroo mother care	KMC, Kangaroo mother care	1	kept
knee amputation	knee amputation	knee amputation	knee amputation	knee amputation	1	kept
Knee arthroplasty	knee arthroplasty	knee arthroplasty	knee arthroplasty	knee arthroplasty	1	case_or_format
knee extension	knee extension	knee extension	knee extension	knee extension	1	kept
knee height	knee height	knee height	knee height	knee height	1	kept
Knee Joint	knee joint	knee joint	knee joint	Knee Joint	1	kept
knee meniscus rupture	knee meniscus rupture	knee meniscus rupture	knee meniscus rupture	knee meniscus rupture	1	kept
knee osteoarthritis	knee osteoarthritis	knee osteoarthritis	knee osteoarthritis	knee osteoarthritis	1	kept

KNEE PAIN	knee pain	knee pain	knee pain	knee pain	1	case_or_format
knee surgery	knee surgery	knee surgery	knee surgery	knee surgery	1	kept
Knowledge acquisition and representation	knowledge acquisition and representation	knowledge acquisition and representation	knowledge acquisition and representation	Knowledge acquisition and representation	1	kept
Knowledge acquisition tools	knowledge acquisition tools	knowledge acquisition tools	knowledge acquisition tools	Knowledge acquisition tools	1	kept
Knowledge and attitude	knowledge and attitude	knowledge and attitude	knowledge and attitude	knowledge and attitude	1	case_or_format
knowledge and attitude survey regarding pain	knowledge and attitude survey regarding pain	knowledge and attitude survey regarding pain	knowledge and attitude survey regarding pain	knowledge and attitude survey regarding pain	1	kept
knowledge and attitudes summary statement	knowledge and attitudes summary statement	knowledge and attitudes summary statement	knowledge and attitudes summary statement	knowledge and attitudes summary statement	1	kept
knowledge and attitudes survey regarding pain revised	knowledge and attitudes survey regarding pain revised	knowledge and attitudes survey regarding pain revised	knowledge and attitudes survey regarding pain revised	knowledge and attitudes survey regarding pain revised	1	kept
Knowledge and experience	knowledge and experience	knowledge and experience	knowledge and experience	Knowledge and experience	1	kept
Knowledge and performance	knowledge and performance	knowledge and performance	knowledge and performance	Knowledge and performance	1	kept
Knowledge and practices	knowledge and practices	knowledge and practices	knowledge and practices	Knowledge and practices	1	kept
Knowledge and skills	knowledge and skills	knowledge and skills	knowledge and skills	Knowledge and skills	1	kept
Knowledge attitude and practice	knowledge attitude and practice	knowledge attitude and practice	knowledge attitude and practice	knowledge, attitude, and practice	1	case_or_format
knowledge attitude behavior questionnaire	knowledge attitude behavior questionnaire	knowledge attitude behavior questionnaire	knowledge attitude behavior questionnaire	knowledge attitude behavior questionnaire	1	kept
knowledge deficit	knowledge deficit	knowledge deficit	knowledge deficit	knowledge deficit	1	kept
Knowledge degradation	knowledge degradation	knowledge degradation	knowledge degradation	Knowledge degradation	1	kept

Knowledge Discovery	knowledge discovery	knowledge discovery	knowledge discovery	Knowledge Discovery	1	kept
knowledge discovery rules	knowledge discovery rules	knowledge discovery rules	knowledge discovery rules	knowledge discovery rules	1	kept
knowledge dissemination	knowledge dissemination	knowledge dissemination	knowledge dissemination	knowledge dissemination	1	kept
Knowledge enhancement	knowledge enhancement	knowledge enhancement	knowledge enhancement	Knowledge enhancement	1	kept
KNOWLEDGE GAPS	knowledge gaps	knowledge gaps	knowledge gap	knowledge gap	1	singular
Knowledge gaps	knowledge gaps	knowledge gaps	knowledge gap	knowledge gap	1	singular
knowledge gaps	knowledge gaps	knowledge gaps	knowledge gap	knowledge gap	1	singular
knowledge graph guided double attention long short term memory	knowledge graph guided double attention long short term memory	knowledge graph guided double attention long short term memory	knowledge graph guided double attention long short term memory	knowledge graph guided double attention long short term memory	1	kept
knowledge guided time aware long short term memory network	knowledge guided time aware long short term memory network	knowledge guided time aware long short term memory network	knowledge guided time aware long short term memory network	knowledge guided time aware long short term memory network	1	kept
Knowledge management	knowledge management	knowledge management	knowledge management	knowledge management	1	case_or_format
Knowledge management.	knowledge management	knowledge management	knowledge management	knowledge management	1	case_or_format
Knowledge of pain	knowledge of pain	knowledge of pain	knowledge of pain	Knowledge of pain	1	kept
Knowledge of Results, Psychological	knowledge of results psychological	knowledge of results psychological	knowledge of results psychological	Knowledge of Results, Psychological	1	kept
Knowledge on HIV	knowledge on hiv	knowledge on hiv	knowledge on hiv	Knowledge on HIV	1	kept
Knowledge on Practice	knowledge on practice	knowledge on practice	knowledge on practice	Knowledge on Practice	1	kept
Knowledge reasoning	knowledge reasoning	knowledge reasoning	knowledge reasoning	Knowledge reasoning	1	kept

Knowledge representation	knowledge representation	knowledge representation	knowledge representation	Knowledge representation	1	kept
knowledge retention	knowledge retention	knowledge retention	knowledge retention	knowledge retention	1	kept
knowledge score	knowledge score	knowledge score	knowledge score	knowledge score	1	kept
Knowledge sharing	knowledge sharing	knowledge sharing	knowledge sharing	Knowledge sharing	1	kept
knowledge sharing	knowledge sharing	knowledge sharing	knowledge sharing	Knowledge sharing	1	case_or_format
knowledge sharing behaviour	knowledge sharing behaviour	knowledge sharing behaviour	knowledge sharing behaviour	knowledge sharing behaviour	1	kept
Knowledge structures	knowledge structures	knowledge structures	knowledge structures	Knowledge structures	1	kept
Knowledge synthesis	knowledge synthesis	knowledge synthesis	knowledge synthesis	Knowledge synthesis	1	kept
Knowledge test	knowledge test	knowledge test	knowledge test	knowledge test	1	case_or_format
Knowledge that informs practice	knowledge that informs practice	knowledge that informs practice	knowledge that informs practice	Knowledge that informs practice	1	kept
Knowledge to Action Framework	knowledge to action framework	knowledge to action framework	knowledge to action framework	Knowledge to Action Framework	1	kept
Knowledge tranfer	knowledge tranfer	knowledge tranfer	knowledge tranfer	Knowledge tranfer	1	kept
knowledge, attitude, and behavior	knowledge attitude and behavior	knowledge attitude and behavior	knowledge attitude and behavior	knowledge, attitude, and behavior	1	kept
Knowledge, attitude, and practice	knowledge attitude and practice	knowledge attitude and practice	knowledge attitude and practice	knowledge, attitude, and practice	1	case_or_format
Knowledge, attitudes, Practic	knowledge attitudes practic	knowledge attitudes practic	knowledge attitudes practic	Knowledge, attitudes, Practic	1	kept
Knowledge, attitudes, practice	knowledge attitudes practice	knowledge attitudes practice	knowledge attitudes practice	Knowledge, attitudes, practice	1	kept

Knowledge-attitude-belief-practice	knowledge attitude belief practice	knowledge attitude belief practice	knowledge attitude belief practice	Knowledge-attitude-belief-practice	1	kept
Knowledge-Belief-Beha-vior	knowledge belief beha vior	knowledge belief beha vior	knowledge belief beha vior	Knowledge-Belief-Beha-vior	1	kept
Knowledge-discovery-rules	knowledge discovery rules	knowledge discovery rules	knowledge discovery rules	knowledge discovery rules	1	case_or_format
Knowledge-to-action	knowledge to action	knowledge to action	knowledge to action	Knowledge-to-action	1	kept
knowledge-to-action	knowledge to action	knowledge to action	knowledge to action	Knowledge-to-action	1	case_or_format
Knowledge-to-action framework	knowledge to action framework	knowledge to action framework	knowledge to action framework	Knowledge to Action Framework	1	case_or_format
Knowledge-to-care gap	knowledge to care gap	knowledge to care gap	knowledge to care gap	Knowledge-to-care gap	1	kept
knowledge‐attitude‐practice	knowledge attitude practice	knowledge attitude practice	knowledge attitude practice	Knowledge-attitude-practice	1	case_or_format
Knowles' Adult Learning Theory	knowles adult learning theory	knowles adult learning theory	knowles adult learning theory	Knowles' Adult Learning Theory	1	kept
Kocuria	kocuria	kocuria	kocuria	Kocuria	1	kept
Korean families	korean families	korean families	korean families	Korean families	1	kept
Korean Occupational Stress Scale	korean occupational stress scale	korean occupational stress scale	korean occupational stress scale	Korean Occupational Stress Scale	1	kept
Korean triage	korean triage	korean triage	korean triage	Korean triage	1	kept
Korean Triage and Acuity Scale	korean triage and acuity scale	korean triage and acuity scale	korean triage and acuity scale	Korean Triage and Acuity Scale	1	kept
Korean version	korean version	korean version	korean version	Korean version	1	kept
Korean War	korean war	korean war	korean war	Korean War	1	kept

Korsakoff psychosis	korsakoff psychosis	korsakoff psychosis	korsakoff psychosis	Korsakoff psychosis	1	kept
Kosovo	kosovo	kosovo	kosovo	Kosovo	1	kept
kotter’ s eight-step change model	kotter s eight step change model	kotter s eight step change model	kotter s eight step change model	kotter’ s eight-step change model	1	kept
Kounis syndrome	kounis syndrome	kounis syndrome	kounis syndrome	Kounis Syndrome	1	case_or_format
KPC	kpc	kpc	kpc	KPC	1	kept
KPC-3	kpc 3	kpc 3	kpc 3	KPC-3	1	kept
KPIs	kpis	kpis	kpis	KPIs	1	kept
Kratom	kratom	kratom	kratom	Kratom	1	kept
Krippendorff's alpha coefficient	krippendorff s alpha coefficient	krippendorff s alpha coefficient	krippendorff s alpha coefficient	Krippendorff's alpha coefficient	1	kept
Krohwinkel	krohwinkel	krohwinkel	krohwinkel	Krohwinkel	1	kept
KSA	ksa	ksa	ksa	KSA	1	kept
Kunshan explosion	kunshan explosion	kunshan explosion	kunshan explosion	Kunshan explosion	1	kept
KVO	kvo	kvo	kvo	KVO	1	kept
kwashiorkor	kwashiorkor	kwashiorkor	kwashiorkor	kwashiorkor	1	kept
Kyphoplasty	kyphoplasty	kyphoplasty	kyphoplasty	Kyphoplasty	1	kept
kyphoplasty	kyphoplasty	kyphoplasty	kyphoplasty	Kyphoplasty	1	case_or_format

kyphoscoliosis	kyphoscoliosis	kyphoscoliosis	kyphoscoliosis	kyphoscoliosis	1	kept
kyphosis	kyphosis	kyphosis	kyphosis	Kyphosis	1	case_or_format
L-Carnitine	l carnitine	l carnitine	l carnitine	L-Carnitine	1	kept
La Crosse virus	la crosse virus	la crosse virus	la crosse virus	La Crosse virus	1	kept
LA Los Angeles	la los angeles	la los angeles	la los angeles	LA Los Angeles	1	kept
Lab-view softwares	lab view softwares	lab view softwares	lab view softwares	Lab-view softwares	1	kept
Label design	label design	label design	label design	Label design	1	kept
Labeling	labeling	labeling	labeling	Labeling	1	kept
labelling	labelling	labelling	labelling	labelling	1	kept
labelling error	labelling error	labelling error	labelling error	labelling error	1	kept
Labial Frenum	labial frenum	labial frenum	labial frenum	Labial Frenum	1	kept
Labor and birth	labor and birth	labor and birth	labor and birth	Labor and birth	1	kept
labor coaching	labor coaching	labor coaching	labor coaching	labor coaching	1	kept
Labor conflicts	labor conflicts	labor conflicts	labor conflicts	Labor conflicts	1	kept
labor dystocia	labor dystocia	labor dystocia	labor dystocia	labor dystocia	1	kept
labor education	labor education	labor education	labor education	labor education	1	kept

Labor market	labor market	labor market	labor market	Labor market	1	kept
labor market	labor market	labor market	labor market	Labor market	1	case_or_format
Labor market policy	labor market policy	labor market policy	labor market policy	Labor market policy	1	kept
labor policy	labor policy	labor policy	labor policy	labor policy	1	kept
labor stage	labor stage	labor stage	labor stage	labor stage	1	kept
Labor Stage, Third	labor stage third	labor stage third	labor stage third	Labor Stage, Third	1	kept
Labor Stress	labor stress	labor stress	labor stress	Labor Stress	1	kept
Labor Unions	labor unions	labor unions	labor unions	Labor Unions	1	kept
Laboratory	laboratory	laboratory	laboratory	laboratory	1	case_or_format
laboratory automation	laboratory automation	laboratory automation	laboratory automation	laboratory automation	1	kept
Laboratory based Acute Physiology Score version 2	laboratory based acute physiology score version 2	laboratory based acute physiology score version 2	laboratory based acute physiology score version 2	Laboratory based Acute Physiology Score version 2	1	kept
Laboratory Critical Values	laboratory critical values	laboratory critical values	laboratory critical values	Laboratory Critical Values	1	kept
Laboratory Error Classification System	laboratory error classification system	laboratory error classification system	laboratory error classification system	Laboratory Error Classification System	1	kept
Laboratory examinations and diagnoses	laboratory examinations and diagnoses	laboratory examinations and diagnoses	laboratory examinations and diagnoses	Laboratory examinations and diagnoses	1	kept
Laboratory features	laboratory features	laboratory features	laboratory features	Laboratory features	1	kept
Laboratory Infection	laboratory infection	laboratory infection	laboratory infection	Laboratory Infection	1	kept

laboratory investigations	laboratory investigations	laboratory investigations	laboratory investigations	laboratory investigations	1	kept
laboratory method	laboratory method	laboratory method	laboratory method	laboratory method	1	kept
laboratory monitoring	laboratory monitoring	laboratory monitoring	laboratory monitoring	laboratory monitoring	1	kept
Laboratory organization and management	laboratory organization and management	laboratory organization and management	laboratory organization and management	Laboratory organization and management	1	kept
Laboratory parameters	laboratory parameters	laboratory parameters	laboratory parameters	Laboratory parameters	1	kept
Laboratory Teams	laboratory teams	laboratory teams	laboratory teams	Laboratory Teams	1	kept
Laboratory Test	laboratory test	laboratory test	laboratory test	laboratory test	1	case_or_format
Laboratory test	laboratory test	laboratory test	laboratory test	laboratory test	1	case_or_format
Laboratory testing	laboratory testing	laboratory testing	laboratory testing	Laboratory testing	1	kept
Laboratory tests	laboratory tests	laboratory tests	laboratory test	laboratory test	1	singular
laboratory tests	laboratory tests	laboratory tests	laboratory test	laboratory test	1	singular
Laboratory virtual instrument engineering work-bench	laboratory virtual instrument engineering work bench	laboratory virtual instrument engineering work bench	laboratory virtual instrument engineering work bench	Laboratory virtual instrument engineering work-bench	1	kept
laboratory worker	laboratory worker	laboratory worker	laboratory worker	laboratory worker	1	kept
Labour	labour	labour	labour	labour	1	case_or_format
Labour care	labour care	labour care	labour care	Labour care	1	kept
labour induction	labour induction	labour induction	labour induction	labour induction	1	kept

labour pain	labour pain	labour pain	labour pain	labour pain	1	kept
labyrinthitis	labyrinthitis	labyrinthitis	labyrinthitis	labyrinthitis	1	kept
LACE	lace	lace	lace	LACE	1	kept
lack of energy and motivation	lack of energy and motivation	lack of energy and motivation	lack of energy and motivation	lack of energy and motivation	1	kept
lack of hospice and palliative care	lack of hospice and palliative care	lack of hospice and palliative care	lack of hospice and palliative care	lack of hospice and palliative care	1	kept
Lack of occupational coping self-efficacy	lack of occupational coping self efficacy	lack of occupational coping self efficacy	lack of occupational coping self efficacy	Lack of occupational coping self-efficacy	1	kept
Lack of personal accomplishment	lack of personal accomplishment	lack of personal accomplishment	lack of personal accomplishment	Lack of personal accomplishment	1	kept
Lack of physicians	lack of physicians	lack of physicians	lack of physicians	Lack of physicians	1	kept
LACTAMASE-PRODUCING ENTEROBACTERIACEAE	lactamase producing enterobacteriaceae	lactamase producing enterobacteriaceae	lactamase producing enterobacteriaceae	LACTAMASE-PRODUCING ENTEROBACTERIACEAE	1	kept
LACTAMASES	lactamases	lactamases	lactamase	LACTAMASES	1	singular
LACTATE	lactate	lactate	lactate	Lactates	1	case_or_format
lactate dehydrogenase to albumin ratio	lactate dehydrogenase to albumin ratio	lactate dehydrogenase to albumin ratio	lactate dehydrogenase to albumin ratio	lactate dehydrogenase to albumin ratio	1	kept
LACTATE LEVELS	lactate levels	lactate levels	lactate levels	LACTATE LEVELS	1	kept
lactate shock	lactate shock	lactate shock	lactate shock	lactate shock	1	kept
lactate to albumin ratio	lactate to albumin ratio	lactate to albumin ratio	lactate to albumin ratio	lactate to albumin ratio	1	kept
lactated Ringer’ s	lactated ringer s	lactated ringer s	lactated ringer s	lactated Ringer’ s	1	kept

LACTATING WOMEN	lactating women	lactating women	lactating women	LACTATING WOMEN	1	kept
lactation acuity	lactation acuity	lactation acuity	lactation acuity	lactation acuity	1	kept
lactation aid	lactation aid	lactation aid	lactation aid	lactation aid	1	kept
Lactation consultant	lactation consultant	lactation consultant	lactation consultant	lactation consultant	1	case_or_format
lactation inhibition	lactation inhibition	lactation inhibition	lactation inhibition	lactation inhibition	1	kept
lactation support	lactation support	lactation support	lactation support	Lactation support	1	case_or_format
lactation suppression	lactation suppression	lactation suppression	lactation suppression	lactation suppression	1	kept
lactation-associated morbidity	lactation associated morbidity	lactation associated morbidity	lactation associated morbidity	lactation-associated morbidity	1	kept
lactational exposure	lactational exposure	lactational exposure	lactational exposure	lactational exposure	1	kept
lactic acidemia	lactic acidemia	lactic acidemia	lactic acidemia	lactic acidemia	1	kept
Lactobacillus minutus	lactobacillus minutus	lactobacillus minutus	lactobacillus minutus	Lactobacillus minutus	1	kept
Lactobacillus paracasei	lactobacillus paracasei	lactobacillus paracasei	lactobacillus paracasei	Lactobacillus paracasei	1	kept
LACTOBACILLUS-CASEI	lactobacillus casei	lactobacillus casei	lactobacillus casei	Lactobacillus casei	1	case_or_format
LACTOBACILLUS-REUTERI	lactobacillus reuteri	lactobacillus reuteri	lactobacillus reuteri	LACTOBACILLUS-REUTERI	1	kept
LACTOGENESIS STAGE-II	lactogenesis stage ii	lactogenesis stage ii	lactogenesis stage ii	LACTOGENESIS STAGE-II	1	kept
lactose intolerance	lactose intolerance	lactose intolerance	lactose intolerance	lactose intolerance	1	kept

LADDER	ladder	ladder	ladder	LADDER	1	kept
ladder	ladder	ladder	ladder	LADDER	1	case_or_format
Lagenaria siceraria	lagenaria siceraria	lagenaria siceraria	lagenaria siceraria	Lagenaria siceraria	1	kept
Lagenaria siceraria juice	lagenaria siceraria juice	lagenaria siceraria juice	lagenaria siceraria juice	Lagenaria siceraria juice	1	kept
Lagenaria sicraria	lagenaria sicraria	lagenaria sicraria	lagenaria sicraria	Lagenaria sicraria	1	kept
Lagophtalmos	lagophtalmos	lagophtalmos	lagophtalmos	Lagophtalmos	1	kept
Lahore	lahore	lahore	lahore	Lahore	1	kept
LAHSHAL	lahshal	lahshal	lahshal	LAHSHAL	1	kept
LAHSHAL classification system	lahshal classification system	lahshal classification system	lahshal classification system	LAHSHAL classification system	1	kept
Lakshadweep	lakshadweep	lakshadweep	lakshadweep	Lakshadweep	1	kept
Lamellar	lamellar	lamellar	lamellar	Lamellar	1	kept
Lamin Type A	lamin type a	lamin type a	lamin type a	Lamin Type A	1	kept
laminar airflow	laminar airflow	laminar airflow	laminar airflow	laminar airflow	1	kept
Laminectomy	laminectomy	laminectomy	laminectomy	laminectomy	1	case_or_format
lamivudine plus nevirapine plus zidovudine	lamivudine plus nevirapine plus zidovudine	lamivudine plus nevirapine plus zidovudine	lamivudine plus nevirapine plus zidovudine	lamivudine plus nevirapine plus zidovudine	1	kept
lanadelumab	lanadelumab	lanadelumab	lanadelumab	lanadelumab	1	kept

Lancashire	lancashire	lancashire	lancashire	Lancashire	1	kept
Lancet	lancet	lancet	lancet	Lancet	1	kept
Lancet Puncture	lancet puncture	lancet puncture	lancet puncture	Lancet Puncture	1	kept
land use	land use	land use	land use	land use	1	kept
landiolol	landiolol	landiolol	landiolol	landiolol	1	kept
landslide	landslide	landslide	landslide	landslide	1	kept
Landslides	landslides	landslides	landslide	landslide	1	singular
LANGUAGE	language	language	language	language	1	case_or_format
language and communications	language and communications	language and communications	language and communications	language and communications	1	kept
Language and hearing	language and hearing	language and hearing	language and hearing	Language and hearing	1	kept
Language barrier	language barrier	language barrier	language barrier	language barriers	1	case_or_format
Language constructs	language constructs	language constructs	language constructs	Language constructs	1	kept
Language Development Disorders	language development disorders	language development disorders	language development disorders	Language Development Disorders	1	kept
Language development of preterm infants	language development of preterm infants	language development of preterm infants	language development of preterm infants	Language development of preterm infants	1	kept
language environment	language environment	language environment	language environment	language environment	1	kept
language model	language model	language model	language model	language model	1	kept

LANGUAGE PROFICIENCY	language proficiency	language proficiency	language proficiency	language proficiency	1	case_or_format
Language technology	language technology	language technology	language technology	Language technology	1	kept
language test	language test	language test	language test	language test	1	kept
Language Therapy	language therapy	language therapy	language therapy	language therapy	1	case_or_format
language-discordant care	language discordant care	language discordant care	language discordant care	language-discordant care	1	kept
language‐discordant care	language discordant care	language discordant care	language discordant care	language-discordant care	1	case_or_format
lanolin alcohol	lanolin alcohol	lanolin alcohol	lanolin alcohol	lanolin alcohol	1	kept
Lansoprazole	lansoprazole	lansoprazole	lansoprazole	lansoprazole	1	case_or_format
Lao PDR	lao pdr	lao pdr	lao pdr	Lao PDR	1	kept
laparoscope	laparoscope	laparoscope	laparoscope	laparoscope	1	kept
laparoscopic appendectomy	laparoscopic appendectomy	laparoscopic appendectomy	laparoscopic appendectomy	laparoscopic appendectomy	1	kept
laparoscopic distal gastrectomy	laparoscopic distal gastrectomy	laparoscopic distal gastrectomy	laparoscopic distal gastrectomy	laparoscopic distal gastrectomy	1	kept
laparoscopic gastrointestinal surgery	laparoscopic gastrointestinal surgery	laparoscopic gastrointestinal surgery	laparoscopic gastrointestinal surgery	laparoscopic gastrointestinal surgery	1	kept
laparoscopic hysterectomy	laparoscopic hysterectomy	laparoscopic hysterectomy	laparoscopic hysterectomy	laparoscopic hysterectomy	1	kept
laparoscopic inversion esophagectomy	laparoscopic inversion esophagectomy	laparoscopic inversion esophagectomy	laparoscopic inversion esophagectomy	laparoscopic inversion esophagectomy	1	kept
Laparoscopic radical prostatectomy	laparoscopic radical prostatectomy	laparoscopic radical prostatectomy	laparoscopic radical prostatectomy	Laparoscopic radical prostatectomy	1	kept

laparoscopic sleeve gastrectomy	laparoscopic sleeve gastrectomy	laparoscopic sleeve gastrectomy	laparoscopic sleeve gastrectomy	laparoscopic sleeve gastrectomy	1	kept
LAPAROSCOPIC SURGERY	laparoscopic surgery	laparoscopic surgery	laparoscopic surgery	laparoscopic surgery	1	case_or_format
laparoscopic total gastrectomy	laparoscopic total gastrectomy	laparoscopic total gastrectomy	laparoscopic total gastrectomy	laparoscopic total gastrectomy	1	kept
Laparostomy	laparostomy	laparostomy	laparostomy	Laparostomy	1	kept
Laptop computers	laptop computers	laptop computers	laptop computers	Laptop computers	1	kept
LAR	lar	lar	lar	LAR	1	kept
Large amounts	large amounts	large amounts	large amounts	Large amounts	1	kept
large blood pressure cuffs	large blood pressure cuffs	large blood pressure cuffs	large blood pressure cuffs	large blood pressure cuffs	1	kept
Large clinical data	large clinical data	large clinical data	large clinical data	Large clinical data	1	kept
large clinical data	large clinical data	large clinical data	large clinical data	Large clinical data	1	case_or_format
Large datasets	large datasets	large datasets	large datasets	Large datasets	1	kept
large intestine	large intestine	large intestine	large intestine	large intestine	1	kept
Large Language Model	large language model	large language model	large language model	large language models	1	case_or_format
large language model	large language model	large language model	large language model	large language models	1	case_or_format
Large language models	large language models	large language models	large language model	large language models	1	singular
large size arms	large size arms	large size arms	large size arms	large size arms	1	kept

Large vessel occlusion	large vessel occlusion	large vessel occlusion	large vessel occlusion	Large vessel occlusion	1	kept
Larva	larva	larva	larva	Larva	1	kept
larva	larva	larva	larva	Larva	1	case_or_format
Larvae	larvae	larvae	larvae	Larvae	1	kept
Laryngeal Cartilages	laryngeal cartilages	laryngeal cartilages	laryngeal cartilages	Laryngeal Cartilages	1	kept
LARYNGEAL MASK AIRWAY	laryngeal mask airway	laryngeal mask airway	laryngeal mask airway	LARYNGEAL MASK AIRWAY	1	kept
Laryngeal mask airway	laryngeal mask airway	laryngeal mask airway	laryngeal mask airway	LARYNGEAL MASK AIRWAY	1	case_or_format
laryngeal mask airway	laryngeal mask airway	laryngeal mask airway	laryngeal mask airway	LARYNGEAL MASK AIRWAY	1	case_or_format
Laryngeal Mucosa	laryngeal mucosa	laryngeal mucosa	laryngeal mucosa	Laryngeal Mucosa	1	kept
Laryngeal Neoplasms	laryngeal neoplasms	laryngeal neoplasms	laryngeal neoplasms	Laryngeal Neoplasms	1	kept
laryngeal nerve neuropathy	laryngeal nerve neuropathy	laryngeal nerve neuropathy	laryngeal nerve neuropathy	laryngeal nerve neuropathy	1	kept
laryngeal tube	laryngeal tube	laryngeal tube	laryngeal tube	laryngeal tube	1	kept
laryngomalacia	laryngomalacia	laryngomalacia	laryngomalacia	Laryngomalacia	1	case_or_format
laryngoscope blade	laryngoscope blade	laryngoscope blade	laryngoscope blade	laryngoscope blade	1	kept
laryngospasm	laryngospasm	laryngospasm	laryngospasm	laryngospasm	1	kept
Laryngotracheal reconstruction	laryngotracheal reconstruction	laryngotracheal reconstruction	laryngotracheal reconstruction	laryngotracheal reconstruction	1	case_or_format

larynx atresia	larynx atresia	larynx atresia	larynx atresia	larynx atresia	1	kept
larynx cartilage	larynx cartilage	larynx cartilage	larynx cartilage	larynx cartilage	1	kept
larynx mucosa	larynx mucosa	larynx mucosa	larynx mucosa	larynx mucosa	1	kept
larynx squamous cell carcinoma	larynx squamous cell carcinoma	larynx squamous cell carcinoma	larynx squamous cell carcinoma	larynx squamous cell carcinoma	1	kept
Lasater	lasater	lasater	lasater	Lasater	1	kept
Laser	laser	laser	laser	Laser	1	kept
laser	laser	laser	laser	Laser	1	case_or_format
Laser beams	laser beams	laser beams	laser beams	Laser beams	1	kept
Laser Doppler flowmetry	laser doppler flowmetry	laser doppler flowmetry	laser doppler flowmetry	Laser-Doppler Flowmetry	1	case_or_format
laser Doppler flowmetry	laser doppler flowmetry	laser doppler flowmetry	laser doppler flowmetry	Laser-Doppler Flowmetry	1	case_or_format
Laser lead extraction	laser lead extraction	laser lead extraction	laser lead extraction	Laser lead extraction	1	kept
Laser photocoagulation	laser photocoagulation	laser photocoagulation	laser photocoagulation	Laser photocoagulation	1	kept
Laser surgery	laser surgery	laser surgery	laser surgery	laser surgery	1	case_or_format
Laser therapy	laser therapy	laser therapy	laser therapy	Laser Therapy	1	case_or_format
Laser tissue interaction	laser tissue interaction	laser tissue interaction	laser tissue interaction	Laser tissue interaction	1	kept
Lassa virus	lassa virus	lassa virus	lassa virus	Lassa virus	1	kept

LAST 3 MONTHS	last 3 months	last 3 months	last 3 months	LAST 3 MONTHS	1	kept
LAST HOURS	last hours	last hours	last hours	LAST HOURS	1	kept
Last offices	last offices	last offices	last offices	Last offices	1	kept
Last rites	last rites	last rites	last rites	Last rites	1	kept
Last utterances	last utterances	last utterances	last utterances	Last utterances	1	kept
LAST YEAR	last year	last year	last year	LAST YEAR	1	kept
latanoprost	latanoprost	latanoprost	latanoprost	latanoprost	1	kept
LATCH	latch	latch	latch	LATCH	1	kept
latch audible swallowing type of nipple comfort hold	latch audible swallowing type of nipple comfort hold	latch audible swallowing type of nipple comfort hold	latch audible swallowing type of nipple comfort hold	latch audible swallowing type of nipple comfort hold	1	kept
Late	late	late	late	Late	1	kept
late complications	late complications	late complications	late complications	late complications	1	kept
late effects	late effects	late effects	late effects	late effects	1	kept
Late effects of polio	late effects of polio	late effects of polio	late effects of polio	Late effects of polio	1	kept
Late enrollment in hospice care	late enrollment in hospice care	late enrollment in hospice care	late enrollment in hospice care	Late enrollment in hospice care	1	kept
late life depression	late life depression	late life depression	late life depression	late life depression	1	kept
late mortality	late mortality	late mortality	late mortality	late mortality	1	kept

late onset	late onset	late onset	late onset	late onset	1	kept
Late onset sepsis	late onset sepsis	late onset sepsis	late onset sepsis	LATE-ONSET SEPSIS	1	case_or_format
LATE PARENTERAL-NUTRITION	late parenteral nutrition	late parenteral nutrition	late parenteral nutrition	LATE PARENTERAL-NUTRITION	1	kept
late premature infants	late premature infants	late premature infants	late premature infants	late premature infants	1	kept
Late preterm babies	late preterm babies	late preterm babies	late preterm babies	Late preterm babies	1	kept
Late preterm infant	late preterm infant	late preterm infant	late preterm infant	late preterm infant	1	case_or_format
late preterm infants	late preterm infants	late preterm infants	late preterm infant	late preterm infant	1	singular
late referral	late referral	late referral	late referral	late referral	1	kept
Late tracheostomy	late tracheostomy	late tracheostomy	late tracheostomy	Late tracheostomy	1	kept
late tracheostomy	late tracheostomy	late tracheostomy	late tracheostomy	Late tracheostomy	1	case_or_format
LATE-LIFE DEPRESSION	late life depression	late life depression	late life depression	late life depression	1	case_or_format
late-life populations	late life populations	late life populations	late life populations	late-life populations	1	kept
LATE-ONSET INFECTION	late onset infection	late onset infection	late onset infection	LATE-ONSET INFECTION	1	kept
Latent class analysis	latent class analysis	latent class analysis	latent class analysis	Latent Class Analysis	1	case_or_format
Latent class growth model	latent class growth model	latent class growth model	latent class growth model	Latent class growth model	1	kept
latent class growth model	latent class growth model	latent class growth model	latent class growth model	Latent class growth model	1	case_or_format

latent period	latent period	latent period	latent period	latent period	1	kept
Latent profiles	latent profiles	latent profiles	latent profile	latent profile	1	singular
latent profiles	latent profiles	latent profiles	latent profile	latent profile	1	singular
latent safety threat	latent safety threat	latent safety threat	latent safety threat	latent safety threat	1	kept
Latent safety threats	latent safety threats	latent safety threats	latent safety threat	latent safety threat	1	singular
latent syphilis	latent syphilis	latent syphilis	latent syphilis	latent syphilis	1	kept
Latent tuberculosis infection	latent tuberculosis infection	latent tuberculosis infection	latent tuberculosis infection	Latent tuberculosis infection	1	kept
latent tuberculosis infection	latent tuberculosis infection	latent tuberculosis infection	latent tuberculosis infection	Latent tuberculosis infection	1	case_or_format
lateral femoral cutaneous block	lateral femoral cutaneous block	lateral femoral cutaneous block	lateral femoral cutaneous block	lateral femoral cutaneous block	1	kept
Lateral femur plate	lateral femur plate	lateral femur plate	lateral femur plate	Lateral femur plate	1	kept
Lateral hostilities	lateral hostilities	lateral hostilities	lateral hostilities	Lateral hostilities	1	kept
lateral migration	lateral migration	lateral migration	lateral migration	lateral migration	1	kept
LATERAL POSITION	lateral position	lateral position	lateral position	LATERAL POSITION	1	kept
lateral position	lateral position	lateral position	lateral position	LATERAL POSITION	1	case_or_format
LATERAL ROTATION	lateral rotation	lateral rotation	lateral rotation	LATERAL ROTATION	1	kept
lateral sacral artery	lateral sacral artery	lateral sacral artery	lateral sacral artery	lateral sacral artery	1	kept

Lateral tilt position	lateral tilt position	lateral tilt position	lateral tilt position	Lateral tilt position	1	kept
Lateral turning	lateral turning	lateral turning	lateral turning	Lateral turning	1	kept
Lateral violence	lateral violence	lateral violence	lateral violence	Lateral violence	1	kept
lateral violence	lateral violence	lateral violence	lateral violence	Lateral violence	1	case_or_format
Lateralized rhythmic delta activity	lateralized rhythmic delta activity	lateralized rhythmic delta activity	lateralized rhythmic delta activity	Lateralized rhythmic delta activity	1	kept
lateralized rhythmic delta activity	lateralized rhythmic delta activity	lateralized rhythmic delta activity	lateralized rhythmic delta activity	Lateralized rhythmic delta activity	1	case_or_format
latex agglutination test	latex agglutination test	latex agglutination test	latex agglutination test	latex agglutination test	1	kept
Latex hypersensitivity	latex hypersensitivity	latex hypersensitivity	latex hypersensitivity	Latex Hypersensitivity	1	case_or_format
Latin America and the Caribbean	latin america and the caribbean	latin america and the caribbean	latin america and the caribbean	Latin America and the Caribbean	1	kept
Latin Americans	latin americans	latin americans	latin americans	Latin Americans	1	kept
latissimus dorsi flap	latissimus dorsi flap	latissimus dorsi flap	latissimus dorsi flap	latissimus dorsi flap	1	kept
laughter yoga	laughter yoga	laughter yoga	laughter yoga	laughter yoga	1	kept
laundry	laundry	laundry	laundry	laundry	1	kept
Lavender aromatherapy	lavender aromatherapy	lavender aromatherapy	lavender aromatherapy	Lavender aromatherapy	1	kept
Lavender oil	lavender oil	lavender oil	lavender oil	lavender oil	1	case_or_format
Law decree	law decree	law decree	law decree	Law decree	1	kept

Lawsonia inermis	lawsonia inermis	lawsonia inermis	lawsonia inermis	Lawsonia inermis	1	kept
Laxatives	laxatives	laxatives	laxative	laxative	1	singular
lay caregivers	lay caregivers	lay caregivers	lay caregivers	lay caregivers	1	kept
LAY RESPONDERS	lay responders	lay responders	lay responders	LAY RESPONDERS	1	kept
Layout visualization	layout visualization	layout visualization	layout visualization	Layout visualization	1	kept
Lazio	lazio	lazio	lazio	Lazio	1	kept
LBP Top Algorithm	lbp top algorithm	lbp top algorithm	lbp top algorithm	LBP Top Algorithm	1	kept
LBW	lbw	lbw	lbw	LBW	1	kept
LCA	lca	lca	lca	LCA	1	kept
LCD	lcd	lcd	lcd	LCD	1	kept
LCP	lcp	lcp	lcp	LCP	1	kept
LD50	ld50	ld50	ld50	LD50	1	kept
LDH, lactate dehydrogenase	ldh lactate dehydrogenase	ldh lactate dehydrogenase	ldh lactate dehydrogenase	LDH, lactate dehydrogenase	1	kept
Lead	lead	lead	lead	Lead	1	kept
leader	leader	leader	leader	leader	1	kept
Leader behavior	leader behavior	leader behavior	leader behavior	Leader behavior	1	kept

Leaderboard	leaderboard	leaderboard	leaderboard	Leaderboard	1	kept
LEADERS	leaders	leaders	leader	leader	1	singular
Leaders	leaders	leaders	leader	leader	1	singular
leadership for change programme launching ceremony	leadership for change programme launching ceremony	leadership for change programme launching ceremony	leadership for change programme launching ceremony	leadership for change programme launching ceremony	1	kept
leadership models	leadership models	leadership models	leadership models	leadership models	1	kept
leadership support	leadership support	leadership support	leadership support	leadership support	1	kept
LEADING CHANGE	leading change	leading change	leading change	LEADING CHANGE	1	kept
Leading with care	leading with care	leading with care	leading with care	Leading with care	1	kept
leadless CRT	leadless crt	leadless crt	leadless crt	leadless CRT	1	kept
leadless pacer	leadless pacer	leadless pacer	leadless pacer	leadless pacer	1	kept
League tables	league tables	league tables	league tables	League tables	1	kept
Leakage	leakage	leakage	leakage	Leakage	1	kept
LEAN	lean	lean	lean	Lean	1	case_or_format
Lean approach	lean approach	lean approach	lean approach	Lean approach	1	kept
Lean batch IV	lean batch iv	lean batch iv	lean batch iv	Lean batch IV	1	kept
lean body weight	lean body weight	lean body weight	lean body weight	lean body weight	1	kept

lean design	lean design	lean design	lean design	lean design	1	kept
Lean Healthcare	lean healthcare	lean healthcare	lean healthcare	Lean Healthcare	1	kept
Lean Six Sigma	lean six sigma	lean six sigma	lean six sigma	Lean Six Sigma	1	kept
Leapfrog Group	leapfrog group	leapfrog group	leapfrog group	Leapfrog Group	1	kept
Leapfrog Hospital Safety Score	leapfrog hospital safety score	leapfrog hospital safety score	leapfrog hospital safety score	Leapfrog Hospital Safety Score	1	kept
learned helplessness	learned helplessness	learned helplessness	learned helplessness	learned helplessness	1	kept
learner	learner	learner	learner	learner	1	kept
learner engagement	learner engagement	learner engagement	learner engagement	learner engagement	1	kept
learners	learners	learners	learner	learner	1	singular
Learning achievement	learning achievement	learning achievement	learning achievement	Learning achievement	1	kept
learning aids	learning aids	learning aids	learning aids	learning aids	1	kept
learning and emotion	learning and emotion	learning and emotion	learning and emotion	learning and emotion	1	kept
LEARNING APPROACH	learning approach	learning approach	learning approach	LEARNING APPROACH	1	kept
learning collaborative	learning collaborative	learning collaborative	learning collaborative	learning collaborative	1	kept
Learning collaboratives	learning collaboratives	learning collaboratives	learning collaborative	learning collaborative	1	singular
learning community	learning community	learning community	learning community	learning community	1	kept

learning compassionate care	learning compassionate care	learning compassionate care	learning compassionate care	learning compassionate care	1	kept
Learning curve	learning curve	learning curve	learning curve	learning curve	1	case_or_format
learning disabilities	learning disabilities	learning disabilities	learning disability	Learning Disabilities	1	singular
Learning effect	learning effect	learning effect	learning effect	Learning effect	1	kept
learning effectiveness	learning effectiveness	learning effectiveness	learning effectiveness	learning effectiveness	1	kept
Learning environments	learning environments	learning environments	learning environment	learning environment	1	singular
Learning experiences	learning experiences	learning experiences	learning experiences	Learning experiences	1	kept
Learning frameworks	learning frameworks	learning frameworks	learning frameworks	Learning frameworks	1	kept
learning health	learning health	learning health	learning health	learning health	1	kept
Learning health system.	learning health system	learning health system	learning health system	learning health system	1	case_or_format
Learning motivation	learning motivation	learning motivation	learning motivation	learning motivation	1	case_or_format
Learning needs	learning needs	learning needs	learning needs	Learning needs	1	kept
Learning needs assessment	learning needs assessment	learning needs assessment	learning needs assessment	Learning needs assessment	1	kept
Learning outcome	learning outcome	learning outcome	learning outcome	learning outcomes	1	case_or_format
Learning paradigms	learning paradigms	learning paradigms	learning paradigms	Learning paradigms	1	kept
Learning performance	learning performance	learning performance	learning performance	Learning performance	1	kept

Learning results	learning results	learning results	learning results	Learning results	1	kept
Learning simulation	learning simulation	learning simulation	learning simulation	Learning simulation	1	kept
Learning strategies	learning strategies	learning strategies	learning strategy	Learning strategy	1	singular
Learning strategy	learning strategy	learning strategy	learning strategy	Learning strategy	1	kept
Learning style	learning style	learning style	learning style	learning style	1	case_or_format
learning styles	learning styles	learning styles	learning style	learning style	1	singular
Learning text	learning text	learning text	learning text	Learning text	1	kept
learning theory	learning theory	learning theory	learning theory	learning theory	1	kept
Learning tool	learning tool	learning tool	learning tool	Learning tool	1	kept
Learning-by-concordance tool	learning by concordance tool	learning by concordance tool	learning by concordance tool	Learning-by-concordance tool	1	kept
LEARNING-ENVIRONMENT	learning environment	learning environment	learning environment	learning environment	1	case_or_format
Learning－training	learning training	learning training	learning training	Learning－training	1	kept
Least developed countries	least developed countries	least developed countries	least developed countries	Least developed countries	1	kept
least developed countries	least developed countries	least developed countries	least developed countries	Least developed countries	1	case_or_format
Least Square	least square	least square	least square	Least Square	1	kept
least square analysis	least square analysis	least square analysis	least square analysis	least square analysis	1	kept

LEAST-SQUARES	least squares	least squares	least square	Least Square	1	singular
leave one out cross validation	leave one out cross validation	leave one out cross validation	leave one out cross validation	leave one out cross validation	1	kept
Leavers	leavers	leavers	leavers	Leavers	1	kept
leaving intentions	leaving intentions	leaving intentions	leaving intentions	leaving intentions	1	kept
Lebanese	lebanese	lebanese	lebanese	Lebanese	1	kept
lecturer perceptions	lecturer perceptions	lecturer perceptions	lecturer perceptions	lecturer perceptions	1	kept
Lectures	lectures	lectures	lecture	Lecture	1	singular
LED MEDICINES MANAGEMENT	led medicines management	led medicines management	led medicines management	LED MEDICINES MANAGEMENT	1	kept
leech	leech	leech	leech	leech	1	kept
Leeches	leeches	leeches	leech	leech	1	singular
left lung	left lung	left lung	left lung	left lung	1	kept
left subclavian artery	left subclavian artery	left subclavian artery	left subclavian artery	left subclavian artery	1	kept
LEFT UNDONE	left undone	left undone	left undone	LEFT UNDONE	1	kept
left ventricvular assist devices	left ventricvular assist devices	left ventricvular assist devices	left ventricvular assist devices	left ventricvular assist devices	1	kept
leg angiography	leg angiography	leg angiography	leg angiography	leg angiography	1	kept
leg blood flow	leg blood flow	leg blood flow	leg blood flow	leg blood flow	1	kept

leg cramp	leg cramp	leg cramp	leg cramp	leg cramp	1	kept
leg fracture	leg fracture	leg fracture	leg fracture	leg fracture	1	kept
Leg Ulcer	leg ulcer	leg ulcer	leg ulcer	leg ulcer	1	case_or_format
leg varicosis	leg varicosis	leg varicosis	leg varicosis	leg varicosis	1	kept
Legal and ethical	legal and ethical	legal and ethical	legal and ethical	Legal and ethical	1	kept
Legal aspects	legal aspects	legal aspects	legal aspect	legal aspect	1	singular
legal competence	legal competence	legal competence	legal competence	legal competence	1	kept
Legal considerations	legal considerations	legal considerations	legal considerations	Legal considerations	1	kept
legal duty	legal duty	legal duty	legal duty	legal duty	1	kept
legal frame	legal frame	legal frame	legal frame	legal frame	1	kept
Legal Guardian	legal guardian	legal guardian	legal guardian	Legal Guardians	1	case_or_format
Legal guardians	legal guardians	legal guardians	legal guardian	Legal Guardians	1	singular
Legal issues	legal issues	legal issues	legal issues	Legal issues	1	kept
LEGAL LANDSCAPE	legal landscape	legal landscape	legal landscape	LEGAL LANDSCAPE	1	kept
Legal medicine	legal medicine	legal medicine	legal medicine	Legal medicine	1	kept
legal risk	legal risk	legal risk	legal risk	legal risk	1	kept

Legibility	legibility	legibility	legibility	Legibility	1	kept
legionella anisa	legionella anisa	legionella anisa	legionella anisa	legionella anisa	1	kept
Legionellosis	legionellosis	legionellosis	legionellosis	Legionellosis	1	kept
Legisla- tion as Topic	legisla tion as topic	legisla tion as topic	legisla tion as topic	Legisla- tion as Topic	1	kept
Legislación de Enfermería	legislaci n de enfermer a	legislaci n de enfermer a	legislaci n de enfermer a	Legislación de Enfermería	1	kept
Legislação de Enfermagem	legisla o de enfermagem	legisla o de enfermagem	legisla o de enfermagem	Legislação de Enfermagem	1	kept
LEGS	legs	legs	leg	leg	1	singular
legs and head weight	legs and head weight	legs and head weight	legs and head weight	legs and head weight	1	kept
leiomyoblastoma	leiomyoblastoma	leiomyoblastoma	leiomyoblastoma	leiomyoblastoma	1	kept
Leisure	leisure	leisure	leisure	leisure	1	case_or_format
leisure crafting	leisure crafting	leisure crafting	leisure crafting	leisure crafting	1	kept
Leisure time physical activity	leisure time physical activity	leisure time physical activity	leisure time physical activity	Leisure time physical activity	1	kept
lemon oil	lemon oil	lemon oil	lemon oil	lemon oil	1	kept
length	length	length	length	LENGTH	1	case_or_format
length board	length board	length board	length board	length board	1	kept
Length of CCU stay	length of ccu stay	length of ccu stay	length of ccu stay	Length of CCU stay	1	kept

Length of Hospital Stay	length of hospital stay	length of hospital stay	length of hospital stay	Length of hospital stay	1	case_or_format
Length of hospitalization	length of hospitalization	length of hospitalization	length of hospitalization	Length of hospitalization	1	kept
length of postoperative stay	length of postoperative stay	length of postoperative stay	length of postoperative stay	length of postoperative stay	1	kept
Length of slay	length of slay	length of slay	length of slay	Length of slay	1	kept
length of stay in the hospital	length of stay in the hospital	length of stay in the hospital	length of stay in the hospital	length of stay in the hospital	1	kept
length-of-stay	length of stay	length of stay	length of stay	Length of Stay	1	case_or_format
Lenguaje enfermero estandarizado	lenguaje enfermero estandarizado	lenguaje enfermero estandarizado	lenguaje enfermero estandarizado	Lenguaje enfermero estandarizado	1	kept
Lennox Gastaut syndrome	lennox gastaut syndrome	lennox gastaut syndrome	lennox gastaut syndrome	Lennox Gastaut syndrome	1	kept
lentiform nucleus	lentiform nucleus	lentiform nucleus	lentiform nucleus	lentiform nucleus	1	kept
LEP	lep	lep	lep	LEP	1	kept
leptomeningeal disease	leptomeningeal disease	leptomeningeal disease	leptomeningeal disease	leptomeningeal disease	1	kept
leptomeningeal malignancy	leptomeningeal malignancy	leptomeningeal malignancy	leptomeningeal malignancy	leptomeningeal malignancy	1	kept
leptomeningeal metastasis	leptomeningeal metastasis	leptomeningeal metastasis	leptomeningeal metastasis	leptomeningeal metastasis	1	kept
leptomeningitis	leptomeningitis	leptomeningitis	leptomeningitis	leptomeningitis	1	kept
Leptospirosis	leptospirosis	leptospirosis	leptospirosis	leptospirosis	1	case_or_format
leronlimab	leronlimab	leronlimab	leronlimab	leronlimab	1	kept

Lesiones cutáneas	lesiones cut neas	lesiones cut neas	lesiones cut neas	Lesiones cutáneas	1	kept
Lesiones cutáneas asociadas a la humedad	lesiones cut neas asociadas a la humedad	lesiones cut neas asociadas a la humedad	lesiones cut neas asociadas a la humedad	Lesiones cutáneas asociadas a la humedad	1	kept
lesions and defects	lesions and defects	lesions and defects	lesions and defects	lesions and defects	1	kept
Less developed countries	less developed countries	less developed countries	less developed countries	Less developed countries	1	kept
lesser trochanter	lesser trochanter	lesser trochanter	lesser trochanter	lesser trochanter	1	kept
lessons	lessons	lessons	lessons	LESSONS	1	case_or_format
less‑invasive surfactant administration	less invasive surfactant administration	less invasive surfactant administration	less invasive surfactant administration	less invasive surfactant administration	1	case_or_format
lethal poisoning	lethal poisoning	lethal poisoning	lethal poisoning	lethal poisoning	1	kept
lethal skeletal dysplasia	lethal skeletal dysplasia	lethal skeletal dysplasia	lethal skeletal dysplasia	lethal skeletal dysplasia	1	kept
Lethal triad	lethal triad	lethal triad	lethal triad	Lethal triad	1	kept
Letter of condolence	letter of condolence	letter of condolence	letter of condolence	Letter of condolence	1	kept
Leucine	leucine	leucine	leucine	Leucine	1	kept
leucine	leucine	leucine	leucine	Leucine	1	case_or_format
Leucovorin	leucovorin	leucovorin	leucovorin	Leucovorin	1	kept
leukemia relapse	leukemia relapse	leukemia relapse	leukemia relapse	leukemia relapse	1	kept
Leukemia, Lymphocytic, Acute, L1	leukemia lymphocytic acute l1	leukemia lymphocytic acute l1	leukemia lymphocytic acute l1	Leukemia, Lymphocytic, Acute, L1	1	kept

Leukemia, Myelocytic, Acute	leukemia myelocytic acute	leukemia myelocytic acute	leukemia myelocytic acute	Leukemia, Myelocytic, Acute	1	kept
Leukocyte counts	leukocyte counts	leukocyte counts	leukocyte count	leukocyte count	1	singular
Leukocyte Elastase	leukocyte elastase	leukocyte elastase	leukocyte elastase	Leukocyte Elastase	1	kept
leukocyte elastase	leukocyte elastase	leukocyte elastase	leukocyte elastase	Leukocyte Elastase	1	case_or_format
leukocyte function	leukocyte function	leukocyte function	leukocyte function	leukocyte function	1	kept
Leukocyte reduction	leukocyte reduction	leukocyte reduction	leukocyte reduction	Leukocyte reduction	1	kept
Leukocytes	leukocytes	leukocytes	leukocyte	leukocyte	1	singular
leukocytes	leukocytes	leukocytes	leukocyte	leukocyte	1	singular
Leukocytes, Mononuclear	leukocytes mononuclear	leukocytes mononuclear	leukocytes mononuclear	Leukocytes, Mononuclear	1	kept
leukodystrophy	leukodystrophy	leukodystrophy	leukodystrophy	leukodystrophy	1	kept
Leukodystrophy, Globoid Cell	leukodystrophy globoid cell	leukodystrophy globoid cell	leukodystrophy globoid cell	Leukodystrophy, Globoid Cell	1	kept
Leukoencephalopathies	leukoencephalopathies	leukoencephalopathies	leukoencephalopathy	leukoencephalopathy	1	singular
Leukotriene B4	leukotriene b4	leukotriene b4	leukotriene b4	leukotriene B4	1	case_or_format
leukotriene D4	leukotriene d4	leukotriene d4	leukotriene d4	leukotriene D4	1	kept
leuprorelin	leuprorelin	leuprorelin	leuprorelin	leuprorelin	1	kept
Leuven study	leuven study	leuven study	leuven study	Leuven study	1	kept

Level	level	level	level	LEVEL	1	case_or_format
level	level	level	level	LEVEL	1	case_or_format
Level 2 beds	level 2 beds	level 2 beds	level 2 beds	Level 2 beds	1	kept
Level difference	level difference	level difference	level difference	Level difference	1	kept
Level III care	level iii care	level iii care	level iii care	Level III care	1	kept
level of consciousness	level of consciousness	level of consciousness	level of consciousness	Level of consciousness	1	case_or_format
level of evidence	level of evidence	level of evidence	level of evidence	level of evidence	1	kept
level of rehabilitation	level of rehabilitation	level of rehabilitation	level of rehabilitation	level of rehabilitation	1	kept
Level of relaxation	level of relaxation	level of relaxation	level of relaxation	Level of relaxation	1	kept
level of stress	level of stress	level of stress	level of stress	level of stress	1	kept
level-of-care	level of care	level of care	level of care	level of care	1	case_or_format
Levels	levels	levels	level	LEVEL	1	singular
levels of adoption	levels of adoption	levels of adoption	levels of adoption	levels of adoption	1	kept
Levels of care	levels of care	levels of care	levels of care	Levels of care	1	kept
levels of care	levels of care	levels of care	levels of care	Levels of care	1	case_or_format
levera	levera	levera	levera	levera	1	kept

Levinas	levinas	levinas	levinas	Levinas	1	kept
levmentin	levmentin	levmentin	levmentin	levmentin	1	kept
Levobupivacaine	levobupivacaine	levobupivacaine	levobupivacaine	Levobupivacaine	1	kept
levobupivacaine	levobupivacaine	levobupivacaine	levobupivacaine	Levobupivacaine	1	case_or_format
levocetirizine	levocetirizine	levocetirizine	levocetirizine	levocetirizine	1	kept
levodopa methyl ester	levodopa methyl ester	levodopa methyl ester	levodopa methyl ester	levodopa methyl ester	1	kept
Levofloxacin	levofloxacin	levofloxacin	levofloxacin	levofloxacin	1	case_or_format
levoleucovorin	levoleucovorin	levoleucovorin	levoleucovorin	levoleucovorin	1	kept
Lewy body	lewy body	lewy body	lewy body	Lewy body	1	kept
Lewy body disease	lewy body disease	lewy body disease	lewy body disease	Lewy body disease	1	kept
Lexicographic goal programming	lexicographic goal programming	lexicographic goal programming	lexicographic goal programming	Lexicographic goal programming	1	kept
Lexicometric analysis	lexicometric analysis	lexicometric analysis	lexicometric analysis	Lexicometric analysis	1	kept
LGBT	lgbt	lgbt	lgbt	LGBT	1	kept
LGBT people	lgbt people	lgbt people	lgbt people	LGBT people	1	kept
LGBTQ	lgbtq	lgbtq	lgbtq	LGBTQ	1	kept
LGBTQ people	lgbtq people	lgbtq people	lgbtq people	LGBTQ people	1	kept

li fi	li fi	li fi	li fi	li fi	1	kept
Li-Fi	li fi	li fi	li fi	li fi	1	case_or_format
liability	liability	liability	liability	liability	1	kept
Liberal oxygenation	liberal oxygenation	liberal oxygenation	liberal oxygenation	Liberal oxygenation	1	kept
Liberation from mechanical ventilator	liberation from mechanical ventilator	liberation from mechanical ventilator	liberation from mechanical ventilator	Liberation from mechanical ventilator	1	kept
librarian	librarian	librarian	librarian	Librarians	1	case_or_format
librarians, clinical	librarians clinical	librarians clinical	librarians clinical	librarians, clinical	1	kept
librarians, embedded	librarians embedded	librarians embedded	librarians embedded	librarians, embedded	1	kept
librarians, medical	librarians medical	librarians medical	librarians medical	librarians, medical	1	kept
Libraries, Medical	libraries medical	libraries medical	libraries medical	Libraries, Medical	1	kept
Library Science	library science	library science	library science	Library Science	1	kept
library science	library science	library science	library science	Library Science	1	case_or_format
Library Services	library services	library services	library services	Library Services	1	kept
lichenoid	lichenoid	lichenoid	lichenoid	lichenoid	1	kept
lichenoid eruption	lichenoid eruption	lichenoid eruption	lichenoid eruption	lichenoid eruption	1	kept
Lichtheimia	lichtheimia	lichtheimia	lichtheimia	Lichtheimia	1	kept

Licox	licox	licox	licox	Licox	1	kept
Liderança	lideran a	lideran a	lideran a	Liderança	1	kept
LIDOCAINE	lidocaine	lidocaine	lidocaine	lidocaine	1	case_or_format
lidocaine plus prilocaine	lidocaine plus prilocaine	lidocaine plus prilocaine	lidocaine plus prilocaine	lidocaine plus prilocaine	1	kept
Lidocaine, Prilocaine Drug Combination	lidocaine prilocaine drug combination	lidocaine prilocaine drug combination	lidocaine prilocaine drug combination	Lidocaine, Prilocaine Drug Combination	1	kept
Lieu de décès	lieu de d c s	lieu de d c s	lieu de d c s	Lieu de décès	1	kept
Lieux de décès	lieux de d c s	lieux de d c s	lieux de d c s	Lieux de décès	1	kept
Life -sustaining treatment	life sustaining treatment	life sustaining treatment	life sustaining treatment	life sustaining treatment	1	case_or_format
Life attitude	life attitude	life attitude	life attitude	Life attitude	1	kept
Life course inequalities	life course inequalities	life course inequalities	life course inequalities	Life course inequalities	1	kept
life course perspective	life course perspective	life course perspective	life course perspective	life course perspective	1	kept
Life cycle	life cycle	life cycle	life cycle	life cycle	1	case_or_format
Life cycle analysis	life cycle analysis	life cycle analysis	life cycle analysis	Life cycle analysis	1	kept
Life Cycle Assessment	life cycle assessment	life cycle assessment	life cycle assessment	life cycle assessment	1	case_or_format
LIFE EXPECTANCY	life expectancy	life expectancy	life expectancy	life expectancy	1	case_or_format
Life expectancyLife Expectancy	life expectancylife expectancy	life expectancylife expectancy	life expectancylife expectancy	Life expectancyLife Expectancy	1	kept

Life Extension Medical Decision law	life extension medical decision law	life extension medical decision law	life extension medical decision law	Life Extension Medical Decision law	1	kept
life impact	life impact	life impact	life impact	life impact	1	kept
Life Opportunity	life opportunity	life opportunity	life opportunity	Life Opportunity	1	kept
life quality	life quality	life quality	life quality	Life quality	1	case_or_format
life shortening illness	life shortening illness	life shortening illness	life shortening illness	life shortening illness	1	kept
life stress	life stress	life stress	life stress	life stress	1	kept
life style	life style	life style	life style	Life Style	1	case_or_format
Life support course	life support course	life support course	life support course	Life support course	1	kept
Life support instructors	life support instructors	life support instructors	life support instructors	Life support instructors	1	kept
life support plan	life support plan	life support plan	life support plan	life support plan	1	kept
Life support plans	life support plans	life support plans	life support plan	life support plan	1	singular
life support systems	life support systems	life support systems	life support systems	Life Support Systems	1	case_or_format
Life support ventillator	life support ventillator	life support ventillator	life support ventillator	Life support ventillator	1	kept
life sustaining intervention	life sustaining intervention	life sustaining intervention	life sustaining intervention	life sustaining intervention	1	kept
Life sustaining treatment	life sustaining treatment	life sustaining treatment	life sustaining treatment	life sustaining treatment	1	case_or_format
life table	life table	life table	life table	life table	1	kept

Life-Changing Events	life changing events	life changing events	life changing events	Life-changing events	1	case_or_format
life-extending procedures	life extending procedures	life extending procedures	life extending procedures	life-extending procedures	1	kept
life-limiting	life limiting	life limiting	life limiting	life-limiting	1	kept
Life-limiting condition	life limiting condition	life limiting condition	life limiting condition	Life-limiting condition	1	kept
life-limiting condition	life limiting condition	life limiting condition	life limiting condition	Life-limiting condition	1	case_or_format
life-limiting diseases	life limiting diseases	life limiting diseases	life limiting diseases	life-limiting diseases	1	kept
life-limiting dysplasia	life limiting dysplasia	life limiting dysplasia	life limiting dysplasia	life-limiting dysplasia	1	kept
life-prolonging	life prolonging	life prolonging	life prolonging	life-prolonging	1	kept
life-support care	life support care	life support care	life support care	Life Support Care	1	case_or_format
life-sustaining therapies	life sustaining therapies	life sustaining therapies	life sustaining therapy	life-sustaining therapy	1	singular
life-sustaining therapy	life sustaining therapy	life sustaining therapy	life sustaining therapy	life-sustaining therapy	1	kept
Life-sustaining treatment decisions	life sustaining treatment decisions	life sustaining treatment decisions	life sustaining treatment decisions	Life-sustaining treatment decisions	1	kept
Life-sustaining treatment utilization	life sustaining treatment utilization	life sustaining treatment utilization	life sustaining treatment utilization	Life-sustaining treatment utilization	1	kept
Life-threatening condition	life threatening condition	life threatening condition	life threatening condition	Life threatening conditions	1	case_or_format
Life-threatening diseases	life threatening diseases	life threatening diseases	life threatening diseases	life-threatening diseases	1	case_or_format
life-threatening hemoptysis	life threatening hemoptysis	life threatening hemoptysis	life threatening hemoptysis	life-threatening hemoptysis	1	kept

Life-threatening hemorrhage	life threatening hemorrhage	life threatening hemorrhage	life threatening hemorrhage	Life-threatening hemorrhage	1	kept
LIFE-THREATENING ILLNESS	life threatening illness	life threatening illness	life threatening illness	Life-threatening illness	1	case_or_format
Lifelong learning	lifelong learning	lifelong learning	lifelong learning	Lifelong learning	1	kept
lifelong learning	lifelong learning	lifelong learning	lifelong learning	Lifelong learning	1	case_or_format
lifesaving intervention	lifesaving intervention	lifesaving intervention	lifesaving intervention	lifesaving intervention	1	kept
Lifestyle	lifestyle	lifestyle	lifestyle	lifestyle	1	case_or_format
lifetime risk	lifetime risk	lifetime risk	lifetime risk	lifetime risk	1	kept
life - changing events	life changing events	life changing events	life changing events	Life-changing events	1	case_or_format
LiFi	lifi	lifi	lifi	LiFi	1	kept
Lift equipment use	lift equipment use	lift equipment use	lift equipment use	Lift equipment use	1	kept
Lift use	lift use	lift use	lift use	Lift use	1	kept
ligament injury	ligament injury	ligament injury	ligament injury	ligament injury	1	kept
LIGANDS	ligands	ligands	ligands	LIGANDS	1	kept
Ligation	ligation	ligation	ligation	ligation	1	case_or_format
LIGHT	light	light	light	Light	1	case_or_format
light dark cycle	light dark cycle	light dark cycle	light dark cycle	light-dark cycles	1	case_or_format

Light distribution	light distribution	light distribution	light distribution	Light distribution	1	kept
Light entrainment	light entrainment	light entrainment	light entrainment	Light entrainment	1	kept
light environment	light environment	light environment	light environment	Light environment	1	case_or_format
LIGHT EXPOSURE	light exposure	light exposure	light exposure	light exposure	1	case_or_format
Light exposure	light exposure	light exposure	light exposure	light exposure	1	case_or_format
light gradient boosting machine	light gradient boosting machine	light gradient boosting machine	light gradient boosting machine	light gradient boosting machine	1	kept
Light management	light management	light management	light management	Light management	1	kept
light pollution	light pollution	light pollution	light pollution	light pollution	1	kept
light reduction	light reduction	light reduction	light reduction	light reduction	1	kept
light related phenomena	light related phenomena	light related phenomena	light related phenomena	light related phenomena	1	kept
light-dark cycle	light dark cycle	light dark cycle	light dark cycle	light-dark cycles	1	case_or_format
Light-emitting diode	light emitting diode	light emitting diode	light emitting diode	light emitting diode	1	case_or_format
lighter	lighter	lighter	lighter	lighter	1	kept
LightGBM, Light Gradient Boosting Machine	lightgbm light gradient boosting machine	lightgbm light gradient boosting machine	lightgbm light gradient boosting machine	LightGBM, Light Gradient Boosting Machine	1	kept
Lighting designs	lighting designs	lighting designs	lighting designs	Lighting designs	1	kept
Lighting environment	lighting environment	lighting environment	lighting environment	Lighting environment	1	kept

Lighting fixtures	lighting fixtures	lighting fixtures	lighting fixtures	Lighting fixtures	1	kept
Lighting systems	lighting systems	lighting systems	lighting systems	Lighting systems	1	kept
LightRegularity Index	lightregularity index	lightregularity index	lightregularity index	LightRegularity Index	1	kept
light－dark cycle	light dark cycle	light dark cycle	light dark cycle	light-dark cycles	1	case_or_format
LIGNOCAINE	lignocaine	lignocaine	lignocaine	LIGNOCAINE	1	kept
likelihood ratio test	likelihood ratio test	likelihood ratio test	likelihood ratio test	likelihood ratio test	1	kept
LIKERT	likert	likert	likert	LIKERT	1	kept
limb	limb	limb	limb	limb	1	kept
limb disease	limb disease	limb disease	limb disease	limb disease	1	kept
Limb ischaemia	limb ischaemia	limb ischaemia	limb ischaemia	Limb ischaemia	1	kept
Limb ischemia	limb ischemia	limb ischemia	limb ischemia	limb ischemia	1	case_or_format
limb paralysis	limb paralysis	limb paralysis	limb paralysis	limb paralysis	1	kept
limb perfusion	limb perfusion	limb perfusion	limb perfusion	limb perfusion	1	kept
limb replantation	limb replantation	limb replantation	limb replantation	limb replantation	1	kept
limb specific outcome	limb specific outcome	limb specific outcome	limb specific outcome	limb specific outcome	1	kept
limb strength training	limb strength training	limb strength training	limb strength training	limb strength training	1	kept

limb surgery	limb surgery	limb surgery	limb surgery	limb surgery	1	kept
limb swelling	limb swelling	limb swelling	limb swelling	limb swelling	1	kept
LIME	lime	lime	lime	LIME	1	kept
liminality	liminality	liminality	liminality	Liminality	1	case_or_format
limit of agreement	limit of agreement	limit of agreement	limit of agreement	limit of agreement	1	kept
Limit of viability	limit of viability	limit of viability	limit of viability	Limit of viability	1	kept
Limitación de tratamiento de soporte vital	limitaci n de tratamiento de soporte vital	limitaci n de tratamiento de soporte vital	limitaci n de tratamiento de soporte vital	Limitación de tratamiento de soporte vital	1	kept
LIMITATION	limitation	limitation	limitation	LIMITATION	1	kept
Limitation	limitation	limitation	limitation	LIMITATION	1	case_or_format
Limitation des thé rapeutiques	limitation des th rapeutiques	limitation des th rapeutiques	limitation des th rapeutiques	Limitation des thé rapeutiques	1	kept
limitation of futile therapy	limitation of futile therapy	limitation of futile therapy	limitation of futile therapy	limitation of futile therapy	1	kept
Limitation of life support treatment	limitation of life support treatment	limitation of life support treatment	limitation of life support treatment	Limitation of life support treatment	1	kept
Limitation of life-sustaining treatment	limitation of life sustaining treatment	limitation of life sustaining treatment	limitation of life sustaining treatment	Limitation of life-sustaining treatment	1	kept
Limitation of medical therapy	limitation of medical therapy	limitation of medical therapy	limitation of medical therapy	Limitation of medical therapy	1	kept
Limitation of medical treatment	limitation of medical treatment	limitation of medical treatment	limitation of medical treatment	Limitation of medical treatment	1	kept
limitation of medical treatment	limitation of medical treatment	limitation of medical treatment	limitation of medical treatment	Limitation of medical treatment	1	case_or_format

limitation of therapy	limitation of therapy	limitation of therapy	limitation of therapy	limitation of therapy	1	kept
limitations	limitations	limitations	limitation	LIMITATION	1	singular
LIMITED ABILITY	limited ability	limited ability	limited ability	LIMITED ABILITY	1	kept
Limited attentions	limited attentions	limited attentions	limited attentions	Limited attentions	1	kept
Limited channel EEG	limited channel eeg	limited channel eeg	limited channel eeg	Limited channel EEG	1	kept
Limited communication range	limited communication range	limited communication range	limited communication range	Limited communication range	1	kept
Limited english proficiency	limited english proficiency	limited english proficiency	limited english proficiency	Limited English Proficiency	1	case_or_format
limited experiences	limited experiences	limited experiences	limited experiences	limited experiences	1	kept
limited mutual language proficiency	limited mutual language proficiency	limited mutual language proficiency	limited mutual language proficiency	limited mutual language proficiency	1	kept
Limited neonatal autopsy	limited neonatal autopsy	limited neonatal autopsy	limited neonatal autopsy	Limited neonatal autopsy	1	kept
Limited resource	limited resource	limited resource	limited resource	limited resources	1	case_or_format
limited resource	limited resource	limited resource	limited resource	limited resources	1	case_or_format
limited resuscitation	limited resuscitation	limited resuscitation	limited resuscitation	limited resuscitation	1	kept
limited training exposure	limited training exposure	limited training exposure	limited training exposure	limited training exposure	1	kept
Limited visiting hours	limited visiting hours	limited visiting hours	limited visiting hours	Limited visiting hours	1	kept
Limiting life sustaining treatment	limiting life sustaining treatment	limiting life sustaining treatment	limiting life sustaining treatment	Limiting life sustaining treatment	1	kept

LINE	line	line	line	LINE	1	kept
Line access	line access	line access	line access	Line access	1	kept
line care bundle	line care bundle	line care bundle	line care bundle	line care bundle	1	kept
Line change	line change	line change	line change	Line change	1	kept
line maintenance	line maintenance	line maintenance	line maintenance	line maintenance	1	kept
LINE PLACEMENT	line placement	line placement	line placement	LINE PLACEMENT	1	kept
Linear integer programming	linear integer programming	linear integer programming	linear integer programming	Linear integer programming	1	kept
LINEAR JUDGMENT	linear judgment	linear judgment	linear judgment	LINEAR JUDGMENT	1	kept
linear mixed models	linear mixed models	linear mixed models	linear mixed models	linear mixed models	1	kept
Linear programming models	linear programming models	linear programming models	linear programming models	Linear programming models	1	kept
Linear regression modelling	linear regression modelling	linear regression modelling	linear regression modelling	Linear regression modelling	1	kept
LINES	lines	lines	line	LINE	1	singular
linezolid resistance	linezolid resistance	linezolid resistance	linezolid resistance	linezolid resistance	1	kept
linezolid synergy	linezolid synergy	linezolid synergy	linezolid synergy	linezolid synergy	1	kept
lingual angioedema	lingual angioedema	lingual angioedema	lingual angioedema	lingual angioedema	1	kept
Lingual Frenum	lingual frenum	lingual frenum	lingual frenum	Lingual Frenum	1	kept

Linguistic Inquiry	linguistic inquiry	linguistic inquiry	linguistic inquiry	Linguistic Inquiry	1	kept
Linguistic inquiry	linguistic inquiry	linguistic inquiry	linguistic inquiry	Linguistic Inquiry	1	case_or_format
Linguistic validation	linguistic validation	linguistic validation	linguistic validation	Linguistic validation	1	kept
Linkage	linkage	linkage	linkage	Linkage	1	kept
LINKED SURVEY	linked survey	linked survey	linked survey	LINKED SURVEY	1	kept
LINKING OUTCOMES	linking outcomes	linking outcomes	linking outcomes	LINKING OUTCOMES	1	kept
linola oil	linola oil	linola oil	linola oil	linola oil	1	kept
Lip	lip	lip	lip	lip	1	case_or_format
lip carcinoma	lip carcinoma	lip carcinoma	lip carcinoma	lip carcinoma	1	kept
lip injury	lip injury	lip injury	lip injury	lip injury	1	kept
lip reconstruction	lip reconstruction	lip reconstruction	lip reconstruction	lip reconstruction	1	kept
lip swelling	lip swelling	lip swelling	lip swelling	lip swelling	1	kept
Liparis tanakae	liparis tanakae	liparis tanakae	liparis tanakae	Liparis tanakae	1	kept
Lipase	lipase	lipase	lipase	Lipase	1	kept
lipase	lipase	lipase	lipase	Lipase	1	case_or_format
lipemia	lipemia	lipemia	lipemia	lipemia	1	kept

Lipid	lipid	lipid	lipid	lipid	1	case_or_format
lipid absorption	lipid absorption	lipid absorption	lipid absorption	lipid absorption	1	kept
lipid diet	lipid diet	lipid diet	lipid diet	lipid diet	1	kept
Lipid emulsion	lipid emulsion	lipid emulsion	lipid emulsion	lipid emulsion	1	case_or_format
lipid infusion	lipid infusion	lipid infusion	lipid infusion	lipid infusion	1	kept
Lipid Metabolism, Inborn Errors	lipid metabolism inborn errors	lipid metabolism inborn errors	lipid metabolism inborn errors	Lipid Metabolism, Inborn Errors	1	kept
Lipid Peroxidation	lipid peroxidation	lipid peroxidation	lipid peroxidation	Lipid Peroxidation	1	kept
Lipid peroxidation	lipid peroxidation	lipid peroxidation	lipid peroxidation	Lipid Peroxidation	1	case_or_format
lipid peroxidation	lipid peroxidation	lipid peroxidation	lipid peroxidation	Lipid Peroxidation	1	case_or_format
LIPID-LADEN MACROPHAGES	lipid laden macrophages	lipid laden macrophages	lipid laden macrophages	LIPID-LADEN MACROPHAGES	1	kept
Lipolysis	lipolysis	lipolysis	lipolysis	Lipolysis	1	kept
lipoma	lipoma	lipoma	lipoma	lipoma	1	kept
lipopolysaccharide	lipopolysaccharide	lipopolysaccharide	lipopolysaccharide	Lipopolysaccharide	1	case_or_format
Lipopolysaccharides	lipopolysaccharides	lipopolysaccharides	lipopolysaccharide	Lipopolysaccharide	1	singular
Liposomes	liposomes	liposomes	liposome	liposome	1	singular
liquefied natural gas	liquefied natural gas	liquefied natural gas	liquefied natural gas	liquefied natural gas	1	kept

liquefied petroleum gas	liquefied petroleum gas	liquefied petroleum gas	liquefied petroleum gas	liquefied petroleum gas	1	kept
liquid chromatography	liquid chromatography	liquid chromatography	liquid chromatography	liquid chromatography	1	kept
Liquid chromatography tandem mass spectrometry	liquid chromatography tandem mass spectrometry	liquid chromatography tandem mass spectrometry	liquid chromatography tandem mass spectrometry	Liquid chromatography tandem mass spectrometry	1	kept
liquid crystal	liquid crystal	liquid crystal	liquid crystal	liquid crystal	1	kept
Liquid crystal displays	liquid crystal displays	liquid crystal displays	liquid crystal displays	Liquid crystal displays	1	kept
LIQUID NUTRITION SUPPLEMENT	liquid nutrition supplement	liquid nutrition supplement	liquid nutrition supplement	LIQUID NUTRITION SUPPLEMENT	1	kept
liquid paraffin	liquid paraffin	liquid paraffin	liquid paraffin	liquid paraffin	1	kept
Liquid polymer acrylate	liquid polymer acrylate	liquid polymer acrylate	liquid polymer acrylate	Liquid polymer acrylate	1	kept
liquid soap	liquid soap	liquid soap	liquid soap	liquid soap	1	kept
Liquid stools	liquid stools	liquid stools	liquid stools	Liquid stools	1	kept
liquid ventilation	liquid ventilation	liquid ventilation	liquid ventilation	Liquid Ventilation	1	case_or_format
liquid waste	liquid waste	liquid waste	liquid waste	liquid waste	1	kept
liraglutide	liraglutide	liraglutide	liraglutide	liraglutide	1	kept
Lisinopril	lisinopril	lisinopril	lisinopril	lisinopril	1	case_or_format
lisofylline	lisofylline	lisofylline	lisofylline	lisofylline	1	kept
listening to music	listening to music	listening to music	listening to music	listening to music	1	kept

listening visit	listening visit	listening visit	listening visit	Listening Visits	1	case_or_format
listening visits	listening visits	listening visits	listening visit	Listening Visits	1	singular
listerine	listerine	listerine	listerine	listerine	1	kept
Listeriosis	listeriosis	listeriosis	listeriosis	Listeriosis	1	kept
listeriosis	listeriosis	listeriosis	listeriosis	Listeriosis	1	case_or_format
Literacia em Saúde	literacia em sa de	literacia em sa de	literacia em sa de	Literacia em Saúde	1	kept
Literature	literature	literature	literature	literature	1	case_or_format
Literature researches	literature researches	literature researches	literature researches	Literature researches	1	kept
Literature search	literature search	literature search	literature search	Literature search	1	kept
Lithiasis	lithiasis	lithiasis	lithiasis	Lithiasis	1	kept
Lithium	lithium	lithium	lithium	lithium	1	case_or_format
Lithium alloys	lithium alloys	lithium alloys	lithium alloys	Lithium alloys	1	kept
lithium carbonate	lithium carbonate	lithium carbonate	lithium carbonate	lithium carbonate	1	kept
Lithium Compounds	lithium compounds	lithium compounds	lithium compounds	Lithium Compounds	1	kept
Lithium-ion batteries	lithium ion batteries	lithium ion batteries	lithium ion batteries	Lithium-ion batteries	1	kept
lithotomy	lithotomy	lithotomy	lithotomy	lithotomy	1	kept

lithotomy position	lithotomy position	lithotomy position	lithotomy position	lithotomy position	1	kept
little finger	little finger	little finger	little finger	little finger	1	kept
live case	live case	live case	live case	live case	1	kept
LIVE MUSIC	live music	live music	live music	LIVE MUSIC	1	kept
live performed music therapy	live performed music therapy	live performed music therapy	live performed music therapy	live performed music therapy	1	kept
live vaccine	live vaccine	live vaccine	live vaccine	live vaccine	1	kept
Live-stream	live stream	live stream	live stream	Live-stream	1	kept
LIVED EXPERIENCES	lived experiences	lived experiences	lived experience	lived experience	1	singular
lived experiences	lived experiences	lived experiences	lived experience	lived experience	1	singular
liver and kidney transplantation	liver and kidney transplantation	liver and kidney transplantation	liver and kidney transplantation	liver and kidney transplantation	1	kept
liver biopsy	liver biopsy	liver biopsy	liver biopsy	liver biopsy	1	kept
liver blood flow	liver blood flow	liver blood flow	liver blood flow	liver blood flow	1	kept
Liver cirrhosis	liver cirrhosis	liver cirrhosis	liver cirrhosis	liver cirrhosis	1	case_or_format
Liver dysfunction	liver dysfunction	liver dysfunction	liver dysfunction	liver dysfunction	1	case_or_format
Liver enzymes	liver enzymes	liver enzymes	liver enzyme	liver enzyme	1	singular
liver fibrosis	liver fibrosis	liver fibrosis	liver fibrosis	liver fibrosis	1	kept

liver hemangiomatosis	liver hemangiomatosis	liver hemangiomatosis	liver hemangiomatosis	liver hemangiomatosis	1	kept
liver hematoma	liver hematoma	liver hematoma	liver hematoma	liver hematoma	1	kept
liver hemorrhage	liver hemorrhage	liver hemorrhage	liver hemorrhage	liver hemorrhage	1	kept
Liver impairment	liver impairment	liver impairment	liver impairment	Liver impairment	1	kept
Liver injury	liver injury	liver injury	liver injury	liver injury	1	case_or_format
liver lobectomy	liver lobectomy	liver lobectomy	liver lobectomy	liver lobectomy	1	kept
liver metabolism	liver metabolism	liver metabolism	liver metabolism	liver metabolism	1	kept
liver nodule	liver nodule	liver nodule	liver nodule	liver nodule	1	kept
liver perfusion	liver perfusion	liver perfusion	liver perfusion	liver perfusion	1	kept
Liver resection	liver resection	liver resection	liver resection	liver resection	1	case_or_format
liver sinusoid	liver sinusoid	liver sinusoid	liver sinusoid	liver sinusoid	1	kept
Liver support	liver support	liver support	liver support	liver support	1	case_or_format
liver surgery	liver surgery	liver surgery	liver surgery	liver surgery	1	kept
liver tissue	liver tissue	liver tissue	liver tissue	liver tissue	1	kept
liver transplant	liver transplant	liver transplant	liver transplant	Liver Transplant	1	case_or_format
liver vein	liver vein	liver vein	liver vein	liver vein	1	kept

liver vein obstruction	liver vein obstruction	liver vein obstruction	liver vein obstruction	liver vein obstruction	1	kept
liver weight	liver weight	liver weight	liver weight	liver weight	1	kept
Liver, Artificial	liver artificial	liver artificial	liver artificial	Liver, Artificial	1	kept
LIVER-TRANSPLANTATION	liver transplantation	liver transplantation	liver transplantation	Liver Transplantation	1	case_or_format
liverpool care pathway	liverpool care pathway	liverpool care pathway	liverpool care pathway	liverpool care pathway	1	kept
livestock	livestock	livestock	livestock	livestock	1	kept
Living donors	living donors	living donors	living donor	Living Donors	1	singular
living lab	living lab	living lab	living lab	living lab	1	kept
living wage	living wage	living wage	living wage	living wage	1	kept
Ljubljana	ljubljana	ljubljana	ljubljana	Ljubljana	1	kept
LMA	lma	lma	lma	LMA	1	kept
LMS tables	lms tables	lms tables	lms tables	LMS tables	1	kept
load cell	load cell	load cell	load cell	load cell	1	kept
Load index	load index	load index	load index	Load index	1	kept
Loading dose	loading dose	loading dose	loading dose	Loading dose	1	kept
LOADS	loads	loads	load	LOAD	1	singular

Lobectomy	lobectomy	lobectomy	lobectomy	lobectomy	1	case_or_format
local adaptation	local adaptation	local adaptation	local adaptation	local adaptation	1	kept
Local anesthetics	local anesthetics	local anesthetics	local anesthetics	Local anesthetics	1	kept
local anesthetics	local anesthetics	local anesthetics	local anesthetics	Local anesthetics	1	case_or_format
local cold application	local cold application	local cold application	local cold application	local cold application	1	kept
Local concentration antibiotics	local concentration antibiotics	local concentration antibiotics	local concentration antibiotics	Local concentration antibiotics	1	kept
local currency	local currency	local currency	local currency	local currency	1	kept
Local influence	local influence	local influence	local influence	Local influence	1	kept
local knowledge	local knowledge	local knowledge	local knowledge	local knowledge	1	kept
Local language	local language	local language	local language	Local language	1	kept
local participation	local participation	local participation	local participation	local participation	1	kept
Local Safety Standards for Invasive Procedures	local safety standards for invasive procedures	local safety standards for invasive procedures	local safety standards for invasive procedures	Local Safety Standards for Invasive Procedures	1	kept
Locally advanced pelvic malignancy	locally advanced pelvic malignancy	locally advanced pelvic malignancy	locally advanced pelvic malignancy	Locally advanced pelvic malignancy	1	kept
Locally advanced rectal cancer	locally advanced rectal cancer	locally advanced rectal cancer	locally advanced rectal cancer	Locally advanced rectal cancer	1	kept
Location	location	location	location	Location	1	kept
Location based	location based	location based	location based	Location based	1	kept

Location Directories and Signs	location directories and signs	location directories and signs	location directories and signs	Location Directories and Signs	1	kept
Location of death	location of death	location of death	location of death	Location of death	1	kept
location of death	location of death	location of death	location of death	Location of death	1	case_or_format
Location system	location system	location system	location system	Location system	1	kept
Location systems	location systems	location systems	location system	Location system	1	singular
lockdowns	lockdowns	lockdowns	lockdown	lockdown	1	singular
locked unit	locked unit	locked unit	locked unit	locked unit	1	kept
LOCKED-IN SYNDROME	locked in syndrome	locked in syndrome	locked in syndrome	locked in syndrome	1	case_or_format
LOCKING	locking	locking	locking	LOCKING	1	kept
Locking solution	locking solution	locking solution	locking solution	Locking solution	1	kept
locking solution	locking solution	locking solution	locking solution	Locking solution	1	case_or_format
Loculated pleural effusion	loculated pleural effusion	loculated pleural effusion	loculated pleural effusion	Loculated pleural effusion	1	kept
Loeffler pneumonia	loeffler pneumonia	loeffler pneumonia	loeffler pneumonia	Loeffler pneumonia	1	kept
Loewenstein Occupational Therapy Cognitive Assessment	loewenstein occupational therapy cognitive assessment	loewenstein occupational therapy cognitive assessment	loewenstein occupational therapy cognitive assessment	Loewenstein Occupational Therapy Cognitive Assessment	1	kept
Log data	log data	log data	log data	Log data	1	kept
log rank test	log rank test	log rank test	log rank test	log rank test	1	kept

Log-roll	log roll	log roll	log roll	Log-roll	1	kept
logEuroSCORE, logistic European System for Cardiac Operative Risk	logeuroscore logistic european system for cardiac operative risk	logeuroscore logistic european system for cardiac operative risk	logeuroscore logistic european system for cardiac operative risk	logEuroSCORE, logistic European System for Cardiac Operative Risk	1	kept
logging	logging	logging	logging	logging	1	kept
LOGIC	logic	logic	logic	Logic	1	case_or_format
LOGIC ARTIFICIAL PANCREAS	logic artificial pancreas	logic artificial pancreas	logic artificial pancreas	LOGIC ARTIFICIAL PANCREAS	1	kept
Logic devices	logic devices	logic devices	logic devices	Logic devices	1	kept
LOGIC MODEL METHODS	logic model methods	logic model methods	logic model methods	LOGIC MODEL METHODS	1	kept
Logic module	logic module	logic module	logic module	Logic module	1	kept
Logic programming	logic programming	logic programming	logic programming	Logic programming	1	kept
logical decision tree regression	logical decision tree regression	logical decision tree regression	logical decision tree regression	logical decision tree regression	1	kept
logical reasoning	logical reasoning	logical reasoning	logical reasoning	logical reasoning	1	kept
Logistic capability	logistic capability	logistic capability	logistic capability	Logistic capability	1	kept
Logistic models	logistic models	logistic models	logistic models	Logistic Models	1	case_or_format
logistic models	logistic models	logistic models	logistic models	Logistic Models	1	case_or_format
logistic regression modelling	logistic regression modelling	logistic regression modelling	logistic regression modelling	logistic regression modelling	1	kept
LOGISTIC-REGRESSION	logistic regression	logistic regression	logistic regression	Logistic regression	1	case_or_format

logistics	logistics	logistics	logistics	Logistics	1	case_or_format
Logit regression	logit regression	logit regression	logit regression	Logit regression	1	kept
logo test	logo test	logo test	logo test	logo test	1	kept
logotherapy	logotherapy	logotherapy	logotherapy	logotherapy	1	kept
Lombardy	lombardy	lombardy	lombardy	Lombardy	1	kept
Lombardy region	lombardy region	lombardy region	lombardy region	Lombardy region	1	kept
lomefloxacin	lomefloxacin	lomefloxacin	lomefloxacin	lomefloxacin	1	kept
lomustine	lomustine	lomustine	lomustine	lomustine	1	kept
London trauma school	london trauma school	london trauma school	london trauma school	London trauma school	1	kept
LONELINESS	loneliness	loneliness	loneliness	Loneliness	1	case_or_format
Long -term care	long term care	long term care	long term care	long term care	1	case_or_format
long care term facility	long care term facility	long care term facility	long care term facility	long care term facility	1	kept
long cephalomedullary nail	long cephalomedullary nail	long cephalomedullary nail	long cephalomedullary nail	long cephalomedullary nail	1	kept
long COVID-19	long covid 19	long covid 19	long covid 19	long COVID-19	1	kept
long distance neonatal interhospital transport	long distance neonatal interhospital transport	long distance neonatal interhospital transport	long distance neonatal interhospital transport	long distance neonatal interhospital transport	1	kept
Long peripherical catheter	long peripherical catheter	long peripherical catheter	long peripherical catheter	Long peripherical catheter	1	kept

long shift tolerance	long shift tolerance	long shift tolerance	long shift tolerance	long shift tolerance	1	kept
Long shifts	long shifts	long shifts	long shifts	Long shifts	1	kept
long term	long term	long term	long term	LONG-TERM	1	case_or_format
long term acute care	long term acute care	long term acute care	long term acute care	long term acute care	1	kept
long term antibiotic	long term antibiotic	long term antibiotic	long term antibiotic	long term antibiotic	1	kept
Long term care facilities	long term care facilities	long term care facilities	long term care facility	long-term care facilities	1	singular
long term care facility	long term care facility	long term care facility	long term care facility	long-term care facilities	1	case_or_format
long term chronic ventilation	long term chronic ventilation	long term chronic ventilation	long term chronic ventilation	long term chronic ventilation	1	kept
long term EEG	long term eeg	long term eeg	long term eeg	long term EEG	1	kept
Long term effects	long term effects	long term effects	long term effects	Long term effects	1	kept
long term mechanical ventilation	long term mechanical ventilation	long term mechanical ventilation	long term mechanical ventilation	Long-term mechanical ventilation	1	case_or_format
Long term monitoring	long term monitoring	long term monitoring	long term monitoring	Long term monitoring	1	kept
Long Term Oxygen Therapy	long term oxygen therapy	long term oxygen therapy	long term oxygen therapy	Long Term Oxygen Therapy	1	kept
long term potentiation	long term potentiation	long term potentiation	long term potentiation	long term potentiation	1	kept
long term stress	long term stress	long term stress	long term stress	long term stress	1	kept
long-acting antiretroviral	long acting antiretroviral	long acting antiretroviral	long acting antiretroviral	long-acting antiretroviral	1	kept

Long-chain 3-ketoacyl-CoA thiolase	long chain 3 ketoacyl coa thiolase	long chain 3 ketoacyl coa thiolase	long chain 3 ketoacyl coa thiolase	Long-chain 3-ketoacyl-CoA thiolase	1	kept
long-gap esophageal atresia	long gap esophageal atresia	long gap esophageal atresia	long gap esophageal atresia	long-gap esophageal atresia	1	kept
long-stay	long stay	long stay	long stay	long-stay	1	kept
Long-term	long term	long term	long term	LONG-TERM	1	case_or_format
long-term	long term	long term	long term	LONG-TERM	1	case_or_format
Long-term acute care hospital	long term acute care hospital	long term acute care hospital	long term acute care hospital	long term acute care hospital	1	case_or_format
long-term acute care hospital	long term acute care hospital	long term acute care hospital	long term acute care hospital	long term acute care hospital	1	case_or_format
Long-term care beds	long term care beds	long term care beds	long term care beds	Long-term care beds	1	kept
Long-Term Care facility	long term care facility	long term care facility	long term care facility	long-term care facilities	1	case_or_format
long-term care facility employees	long term care facility employees	long term care facility employees	long term care facility employees	long-term care facility employees	1	kept
Long-term care hospital	long term care hospital	long term care hospital	long term care hospital	Long-term care hospital	1	kept
Long-term care outcomes	long term care outcomes	long term care outcomes	long term care outcomes	Long-term care outcomes	1	kept
long-term care reimbursement	long term care reimbursement	long term care reimbursement	long term care reimbursement	long-term care reimbursement	1	kept
long-term care.	long term care	long term care	long term care	long term care	1	case_or_format
long-term cognitive impairment	long term cognitive impairment	long term cognitive impairment	long term cognitive impairment	long-term cognitive impairment	1	kept
Long-term conditions	long term conditions	long term conditions	long term conditions	Long-term conditions	1	kept

Long-term continuous EEG monitoring	long term continuous eeg monitoring	long term continuous eeg monitoring	long term continuous eeg monitoring	Long-term continuous EEG monitoring	1	kept
long-term denervated muscles	long term denervated muscles	long term denervated muscles	long term denervated muscles	long-term denervated muscles	1	kept
Long-term effects	long term effects	long term effects	long term effects	Long term effects	1	case_or_format
Long-term follow-up	long term follow up	long term follow up	long term follow up	Long-term follow-up	1	kept
long-term follow-up	long term follow up	long term follow up	long term follow up	Long-term follow-up	1	case_or_format
long-term home ventilation	long term home ventilation	long term home ventilation	long term home ventilation	long-term home ventilation	1	kept
long-term hospital	long term hospital	long term hospital	long term hospital	long-term hospital	1	kept
Long-term impacts	long term impacts	long term impacts	long term impacts	Long-term impacts	1	kept
long-term intravenous device	long term intravenous device	long term intravenous device	long term intravenous device	long-term intravenous device	1	kept
Long-term invasive mechanical ventilation	long term invasive mechanical ventilation	long term invasive mechanical ventilation	long term invasive mechanical ventilation	Long-term invasive mechanical ventilation	1	kept
Long-term monitoring	long term monitoring	long term monitoring	long term monitoring	Long term monitoring	1	case_or_format
LONG-TERM MORTALITY	long term mortality	long term mortality	long term mortality	LONG-TERM MORTALITY	1	kept
Long-term outcomes of sepsis	long term outcomes of sepsis	long term outcomes of sepsis	long term outcomes of sepsis	Long-term outcomes of sepsis	1	kept
long-term oxygen therapy	long term oxygen therapy	long term oxygen therapy	long term oxygen therapy	Long Term Oxygen Therapy	1	case_or_format
Long-term prophylaxis	long term prophylaxis	long term prophylaxis	long term prophylaxis	Long-term prophylaxis	1	kept
Long-term results	long term results	long term results	long term results	Long-term results	1	kept

Long-term survivors	long term survivors	long term survivors	long term survivors	Long-term survivors	1	kept
long-term survivors	long term survivors	long term survivors	long term survivors	Long-term survivors	1	case_or_format
LONG-TERM USE	long term use	long term use	long term use	LONG-TERM USE	1	kept
long-term-care hospitals	long term care hospitals	long term care hospitals	long term care hospital	Long-term care hospital	1	singular
longitudinal follow-up	longitudinal follow up	longitudinal follow up	longitudinal follow up	longitudinal follow-up	1	kept
longterm and postacute care	longterm and postacute care	longterm and postacute care	longterm and postacute care	longterm and postacute care	1	kept
long‐term home ventilation	long term home ventilation	long term home ventilation	long term home ventilation	long-term home ventilation	1	case_or_format
Looking	looking	looking	looking	Looking	1	kept
LOOP DIURETICS	loop diuretics	loop diuretics	loop diuretics	LOOP DIURETICS	1	kept
loop electrosurgical excision	loop electrosurgical excision	loop electrosurgical excision	loop electrosurgical excision	loop electrosurgical excision	1	kept
LOOP INSULIN DELIVERY	loop insulin delivery	loop insulin delivery	loop insulin delivery	LOOP INSULIN DELIVERY	1	kept
Loose anagen hair	loose anagen hair	loose anagen hair	loose anagen hair	Loose anagen hair	1	kept
loose anagen hair syndrome	loose anagen hair syndrome	loose anagen hair syndrome	loose anagen hair syndrome	loose anagen hair syndrome	1	kept
Loperamide	loperamide	loperamide	loperamide	loperamide	1	case_or_format
Lophius litulon	lophius litulon	lophius litulon	lophius litulon	Lophius litulon	1	kept
lormetazepam	lormetazepam	lormetazepam	lormetazepam	lormetazepam	1	kept

LoS	los	los	los	LOS	1	case_or_format
LOS, length of stay	los length of stay	los length of stay	los length of stay	LOS = length of stay	1	case_or_format
Losartan	losartan	losartan	losartan	losartan	1	case_or_format
Loss functions	loss functions	loss functions	loss functions	Loss functions	1	kept
Loss of consciousness	loss of consciousness	loss of consciousness	loss of consciousness	Loss of consciousness	1	kept
loss of control	loss of control	loss of control	loss of control	loss of control	1	kept
loss of function mutation	loss of function mutation	loss of function mutation	loss of function mutation	loss of function mutation	1	kept
Loss of independence	loss of independence	loss of independence	loss of independence	Loss of independence	1	kept
Loss of independency	loss of independency	loss of independency	loss of independency	Loss of independency	1	kept
Loss of motive	loss of motive	loss of motive	loss of motive	Loss of motive	1	kept
LOSS PREVENTION	loss prevention	loss prevention	loss prevention	LOSS PREVENTION	1	kept
lotilaner	lotilaner	lotilaner	lotilaner	lotilaner	1	kept
lotion	lotion	lotion	lotion	lotion	1	kept
Lotus Room	lotus room	lotus room	lotus room	Lotus Room	1	kept
Loudness perception	loudness perception	loudness perception	loudness perception	Loudness Perception	1	case_or_format
loved ones	loved ones	loved ones	loved ones	loved ones	1	kept

Lovingkindness	lovingkindness	lovingkindness	lovingkindness	Lovingkindness	1	kept
low acuity triage score	low acuity triage score	low acuity triage score	low acuity triage score	low acuity triage score	1	kept
Low and Middle Income Countries	low and middle income countries	low and middle income countries	low and middle income country	low- and middle-income countries	1	singular
Low and middle income country	low and middle income country	low and middle income country	low and middle income country	low- and middle-income countries	1	case_or_format
low and middle income country	low and middle income country	low and middle income country	low and middle income country	low- and middle-income countries	1	case_or_format
Low anterior resection	low anterior resection	low anterior resection	low anterior resection	Low anterior resection	1	kept
Low back injuries	low back injuries	low back injuries	low back injuries	Low back injuries	1	kept
Low birth weighs	low birth weighs	low birth weighs	low birth weighs	Low birth weighs	1	kept
Low birth weight and developmental follow-up for infants	low birth weight and developmental follow up for infants	low birth weight and developmental follow up for infants	low birth weight and developmental follow up for infants	Low birth weight and developmental follow-up for infants	1	kept
Low Birth Weight Babies	low birth weight babies	low birth weight babies	low birth weight babies	Low Birth Weight Babies	1	kept
low birth weight infants	low birth weight infants	low birth weight infants	low birth weight infant	Low birth weight infant	1	singular
low birth-weight infant	low birth weight infant	low birth weight infant	low birth weight infant	Low birth weight infant	1	case_or_format
Low birthweight	low birthweight	low birthweight	low birthweight	Low birthweight	1	kept
Low birthweight neonates	low birthweight neonates	low birthweight neonates	low birthweight neonates	Low birthweight neonates	1	kept
low calorie diet	low calorie diet	low calorie diet	low calorie diet	low calorie diet	1	kept
Low cardiac output	low cardiac output	low cardiac output	low cardiac output	Low cardiac output	1	kept

low cardiac output syndrome	low cardiac output syndrome	low cardiac output syndrome	low cardiac output syndrome	Low cardiac output syndrome	1	case_or_format
Low color temperatures	low color temperatures	low color temperatures	low color temperatures	Low color temperatures	1	kept
Low cost	low cost	low cost	low cost	Low cost	1	kept
Low dose	low dose	low dose	low dose	Low dose	1	kept
low dose high frequency training	low dose high frequency training	low dose high frequency training	low dose high frequency training	low-dose high-frequency training	1	case_or_format
low energy trauma	low energy trauma	low energy trauma	low energy trauma	low energy trauma	1	kept
low frequency noise	low frequency noise	low frequency noise	low frequency noise	low frequency noise	1	kept
Low functional reserve	low functional reserve	low functional reserve	low functional reserve	Low functional reserve	1	kept
Low health literacy	low health literacy	low health literacy	low health literacy	Low health literacy	1	kept
low income population	low income population	low income population	low income population	low income population	1	kept
low intensity exercise	low intensity exercise	low intensity exercise	low intensity exercise	low intensity exercise	1	kept
low internal jugular vein	low internal jugular vein	low internal jugular vein	low internal jugular vein	low internal jugular vein	1	kept
Low level laser therapy	low level laser therapy	low level laser therapy	low level laser therapy	low level laser therapy	1	case_or_format
low level mupirocin resistance	low level mupirocin resistance	low level mupirocin resistance	low level mupirocin resistance	low level mupirocin resistance	1	kept
Low literacy	low literacy	low literacy	low literacy	Low literacy	1	kept
Low middle-income country	low middle income country	low middle income country	low middle income country	Low middle-income country	1	kept

low middle-income country	low middle income country	low middle income country	low middle income country	Low middle-income country	1	case_or_format
LOW MOBILITY	low mobility	low mobility	low mobility	LOW MOBILITY	1	kept
Low molecular weight heparin	low molecular weight heparin	low molecular weight heparin	low molecular weight heparin	low molecular weight heparin	1	case_or_format
low professional achievement task	low professional achievement task	low professional achievement task	low professional achievement task	low professional achievement task	1	kept
Low QRS voltage	low qrs voltage	low qrs voltage	low qrs voltage	Low QRS voltage	1	kept
Low resource	low resource	low resource	low resource	low resource	1	case_or_format
low resource setting	low resource setting	low resource setting	low resource setting	Low-resource settings	1	case_or_format
Low resource settings	low resource settings	low resource settings	low resource setting	Low-resource settings	1	singular
low risk	low risk	low risk	low risk	low risk	1	kept
low risk maternity care	low risk maternity care	low risk maternity care	low risk maternity care	low risk maternity care	1	kept
low secure services	low secure services	low secure services	low secure services	low secure services	1	kept
low set ear	low set ear	low set ear	low set ear	low set ear	1	kept
low socioeconomic status	low socioeconomic status	low socioeconomic status	low socioeconomic status	low socioeconomic status	1	kept
Low speed	low speed	low speed	low speed	Low speed	1	kept
Low threshold	low threshold	low threshold	low threshold	Low threshold	1	kept
low vision	low vision	low vision	low vision	low vision	1	kept

Low, high, and super gelers	low high and super gelers	low high and super gelers	low high and super gelers	Low, high, and super gelers	1	kept
Low- and Middle-income countries	low and middle income countries	low and middle income countries	low and middle income country	low- and middle-income countries	1	singular
low- and middle-income country	low and middle income country	low and middle income country	low and middle income country	low- and middle-income countries	1	case_or_format
Low-air-loss	low air loss	low air loss	low air loss	Low-air-loss	1	kept
Low-amplitude	low amplitude	low amplitude	low amplitude	Low-amplitude	1	kept
Low-and-middle-income countries	low and middle income countries	low and middle income countries	low and middle income country	low- and middle-income countries	1	singular
Low-back pain	low back pain	low back pain	low back pain	low back pain	1	case_or_format
low-back pain	low back pain	low back pain	low back pain	low back pain	1	case_or_format
Low-birth weight	low birth weight	low birth weight	low birth weight	low birth weight	1	case_or_format
Low-birth-weight infant	low birth weight infant	low birth weight infant	low birth weight infant	Low birth weight infant	1	case_or_format
Low-birth-weight infants	low birth weight infants	low birth weight infants	low birth weight infant	Low birth weight infant	1	singular
Low-birthweight	low birthweight	low birthweight	low birthweight	Low birthweight	1	case_or_format
Low-birthweight infants	low birthweight infants	low birthweight infants	low birthweight infants	Low-birthweight infants	1	kept
Low-birthweight preterm infants	low birthweight preterm infants	low birthweight preterm infants	low birthweight preterm infants	Low-birthweight preterm infants	1	kept
Low-cost	low cost	low cost	low cost	Low cost	1	case_or_format
low-cost	low cost	low cost	low cost	Low cost	1	case_or_format

low-cost simulation model	low cost simulation model	low cost simulation model	low cost simulation model	low-cost simulation model	1	kept
Low-costs	low costs	low costs	low cost	Low cost	1	singular
low-dose radiotherapy	low dose radiotherapy	low dose radiotherapy	low dose radiotherapy	low-dose radiotherapy	1	kept
LOW-EFFICIENCY DIALYSIS	low efficiency dialysis	low efficiency dialysis	low efficiency dialysis	LOW-EFFICIENCY DIALYSIS	1	kept
low-energy trauma	low energy trauma	low energy trauma	low energy trauma	low energy trauma	1	case_or_format
Low-fidelity simulation	low fidelity simulation	low fidelity simulation	low fidelity simulation	low fidelity simulation	1	case_or_format
low-fidelity simulation	low fidelity simulation	low fidelity simulation	low fidelity simulation	low fidelity simulation	1	case_or_format
low-flow oxygen	low flow oxygen	low flow oxygen	low flow oxygen	low-flow oxygen	1	kept
low-income	low income	low income	low income	low-income	1	kept
low-income and middle-income countries	low income and middle income countries	low income and middle income countries	low income and middle income countries	low-income and middle-income countries	1	kept
low-income and middleincome countries	low income and middleincome countries	low income and middleincome countries	low income and middleincome countries	low-income and middleincome countries	1	kept
low-income countries	low income countries	low income countries	low income country	low income country	1	singular
low-income country	low income country	low income country	low income country	low income country	1	case_or_format
low-level laser therapy	low level laser therapy	low level laser therapy	low level laser therapy	low level laser therapy	1	case_or_format
Low-Level Light Therapy	low level light therapy	low level light therapy	low level light therapy	Low-Level Light Therapy	1	kept
Low-level Mupirocin Resistance	low level mupirocin resistance	low level mupirocin resistance	low level mupirocin resistance	low level mupirocin resistance	1	case_or_format

Low-middle income country	low middle income country	low middle income country	low middle income country	Low middle-income country	1	case_or_format
low-middle income country	low middle income country	low middle income country	low middle income country	Low middle-income country	1	case_or_format
low-middle-income countries	low middle income countries	low middle income countries	low middle income country	Low middle-income country	1	singular
Low-molecular weight heparin	low molecular weight heparin	low molecular weight heparin	low molecular weight heparin	low molecular weight heparin	1	case_or_format
Low-molecular-weight heparin	low molecular weight heparin	low molecular weight heparin	low molecular weight heparin	low molecular weight heparin	1	case_or_format
LOW-PREVALENCE	low prevalence	low prevalence	low prevalence	LOW-PREVALENCE	1	kept
Low-risk birth	low risk birth	low risk birth	low risk birth	Low-risk birth	1	kept
low-risk birth	low risk birth	low risk birth	low risk birth	Low-risk birth	1	case_or_format
Low-risk pregnancy	low risk pregnancy	low risk pregnancy	low risk pregnancy	Low-risk pregnancy	1	kept
low-tech	low tech	low tech	low tech	low-tech	1	kept
low-technology support surfaces	low technology support surfaces	low technology support surfaces	low technology support surfaces	low-technology support surfaces	1	kept
lower body negative pressure	lower body negative pressure	lower body negative pressure	lower body negative pressure	lower body negative pressure	1	kept
lower calyceal stones	lower calyceal stones	lower calyceal stones	lower calyceal stones	lower calyceal stones	1	kept
lower esophagus sphincter pressure	lower esophagus sphincter pressure	lower esophagus sphincter pressure	lower esophagus sphincter pressure	lower esophagus sphincter pressure	1	kept
Lower extremity	lower extremity	lower extremity	lower extremity	Lower Extremity	1	case_or_format
lower extremity	lower extremity	lower extremity	lower extremity	Lower Extremity	1	case_or_format

Lower extremity fracture	lower extremity fracture	lower extremity fracture	lower extremity fracture	Lower extremity fracture	1	kept
lower extremity infection	lower extremity infection	lower extremity infection	lower extremity infection	lower extremity infection	1	kept
lower extremity injury	lower extremity injury	lower extremity injury	lower extremity injury	lower extremity injury	1	kept
Lower extremity muscle strength	lower extremity muscle strength	lower extremity muscle strength	lower extremity muscle strength	Lower extremity muscle strength	1	kept
lower eyelid	lower eyelid	lower eyelid	lower eyelid	lower eyelid	1	kept
Lower gastrointestinal bleeding	lower gastrointestinal bleeding	lower gastrointestinal bleeding	lower gastrointestinal bleeding	Lower gastrointestinal bleeding	1	kept
Lower Gastrointestinal Tract	lower gastrointestinal tract	lower gastrointestinal tract	lower gastrointestinal tract	Lower Gastrointestinal Tract	1	kept
Lower Limb crSaO2 Detection1	lower limb crsao2 detection1	lower limb crsao2 detection1	lower limb crsao2 detection1	Lower Limb crSaO2 Detection1	1	kept
Lower Limb crSaO<sub>2<	lower limb crsao sub 2	lower limb crsao sub 2	lower limb crsao sub 2	Lower Limb crSaO<sub>2<	1	kept
lower limb muscle strength	lower limb muscle strength	lower limb muscle strength	lower limb muscle strength	lower limb muscle strength	1	kept
lower lip	lower lip	lower lip	lower lip	lower lip	1	kept
lower motor neurons	lower motor neurons	lower motor neurons	lower motor neurons	lower motor neurons	1	kept
Lower resolution	lower resolution	lower resolution	lower resolution	Lower resolution	1	kept
Lower respiratory illness	lower respiratory illness	lower respiratory illness	lower respiratory illness	Lower respiratory illness	1	kept
Lower respiratory infections	lower respiratory infections	lower respiratory infections	lower respiratory infections	Lower respiratory infections	1	kept
lower respiratory infections	lower respiratory infections	lower respiratory infections	lower respiratory infections	Lower respiratory infections	1	case_or_format

lower respiratory tract	lower respiratory tract	lower respiratory tract	lower respiratory tract	lower respiratory tract	1	kept
lower respiratory tract infections	lower respiratory tract infections	lower respiratory tract infections	lower respiratory tract infection	lower respiratory tract infection	1	singular
Lower- and middle-income countries	lower and middle income countries	lower and middle income countries	lower and middle income countries	Lower- and middle-income countries	1	kept
low‐resource settings	low resource settings	low resource settings	low resource setting	Low-resource settings	1	singular
low‐risk women	low risk women	low risk women	low risk women	low‐risk women	1	kept
LOYALTY	loyalty	loyalty	loyalty	LOYALTY	1	kept
LPI	lpi	lpi	lpi	LPI	1	kept
LRE	lre	lre	lre	LRE	1	kept
LTE network	lte network	lte network	lte network	LTE network	1	kept
LTE Networks	lte networks	lte networks	lte network	LTE network	1	singular
Lubricant Eye Drops	lubricant eye drops	lubricant eye drops	lubricant eye drops	Lubricant Eye Drops	1	kept
Lubricants	lubricants	lubricants	lubricants	Lubricants	1	kept
Lucid interval	lucid interval	lucid interval	lucid interval	Lucid interval	1	kept
Ludwig’s angina	ludwig s angina	ludwig s angina	ludwig s angina	Ludwig's Angina	1	case_or_format
luer connector	luer connector	luer connector	luer connector	luer connector	1	kept
lumbar disc arthroplasty	lumbar disc arthroplasty	lumbar disc arthroplasty	lumbar disc arthroplasty	lumbar disc arthroplasty	1	kept

lumbar disk hernia	lumbar disk hernia	lumbar disk hernia	lumbar disk hernia	lumbar disk hernia	1	kept
lumbar drain	lumbar drain	lumbar drain	lumbar drain	lumbar drain	1	kept
lumbar microdiscectomy	lumbar microdiscectomy	lumbar microdiscectomy	lumbar microdiscectomy	lumbar microdiscectomy	1	kept
lumbar spinal cord injury	lumbar spinal cord injury	lumbar spinal cord injury	lumbar spinal cord injury	lumbar spinal cord injury	1	kept
Lumbar spinal stenosis	lumbar spinal stenosis	lumbar spinal stenosis	lumbar spinal stenosis	lumbar spinal stenosis	1	case_or_format
Lumber	lumber	lumber	lumber	Lumber	1	kept
lumbosacral spine	lumbosacral spine	lumbosacral spine	lumbosacral spine	lumbosacral spine	1	kept
Luminance	luminance	luminance	luminance	Luminance	1	kept
Luminance distributions	luminance distributions	luminance distributions	luminance distributions	Luminance distributions	1	kept
Luminescent Measurements	luminescent measurements	luminescent measurements	luminescent measurements	Luminescent Measurements	1	kept
Luminous flux	luminous flux	luminous flux	luminous flux	Luminous flux	1	kept
LUNG	lung	lung	lung	Lung	1	case_or_format
Lung Abscess	lung abscess	lung abscess	lung abscess	lung abscess	1	case_or_format
lung adenocarcinoma	lung adenocarcinoma	lung adenocarcinoma	lung adenocarcinoma	lung adenocarcinoma	1	kept
lung allocation score	lung allocation score	lung allocation score	lung allocation score	lung allocation score	1	kept
lung alveolus carbon dioxide tension	lung alveolus carbon dioxide tension	lung alveolus carbon dioxide tension	lung alveolus carbon dioxide tension	lung alveolus carbon dioxide tension	1	kept

lung alveolus cell carcinoma	lung alveolus cell carcinoma	lung alveolus cell carcinoma	lung alveolus cell carcinoma	lung alveolus cell carcinoma	1	kept
lung alveolus epithelium	lung alveolus epithelium	lung alveolus epithelium	lung alveolus epithelium	lung alveolus epithelium	1	kept
lung alveolus epithelium cell	lung alveolus epithelium cell	lung alveolus epithelium cell	lung alveolus epithelium cell	lung alveolus epithelium cell	1	kept
lung alveolus hypoventilation	lung alveolus hypoventilation	lung alveolus hypoventilation	lung alveolus hypoventilation	lung alveolus hypoventilation	1	kept
lung alveolus oxygen tension	lung alveolus oxygen tension	lung alveolus oxygen tension	lung alveolus oxygen tension	lung alveolus oxygen tension	1	kept
lung and airways physiology	lung and airways physiology	lung and airways physiology	lung and airways physiology	lung and airways physiology	1	kept
lung artery banding	lung artery banding	lung artery banding	lung artery banding	lung artery banding	1	kept
lung barotrauma	lung barotrauma	lung barotrauma	lung barotrauma	lung barotrauma	1	kept
lung cancer surgery	lung cancer surgery	lung cancer surgery	lung cancer surgery	lung cancer surgery	1	kept
lung capacity	lung capacity	lung capacity	lung capacity	lung capacity	1	kept
lung capillary	lung capillary	lung capillary	lung capillary	lung capillary	1	kept
lung capillary pressure	lung capillary pressure	lung capillary pressure	lung capillary pressure	lung capillary pressure	1	kept
lung collapse	lung collapse	lung collapse	lung collapse	lung collapse	1	kept
lung donor	lung donor	lung donor	lung donor	lung donor	1	kept
lung epithelium	lung epithelium	lung epithelium	lung epithelium	lung epithelium	1	kept
lung examination	lung examination	lung examination	lung examination	lung examination	1	kept

lung flow volume curve	lung flow volume curve	lung flow volume curve	lung flow volume curve	lung flow volume curve	1	kept
lung fluid	lung fluid	lung fluid	lung fluid	lung fluid	1	kept
lung fluid clearance	lung fluid clearance	lung fluid clearance	lung fluid clearance	lung fluid clearance	1	kept
Lung function	lung function	lung function	lung function	lung function	1	case_or_format
Lung graft	lung graft	lung graft	lung graft	Lung graft	1	kept
lung graft rejection	lung graft rejection	lung graft rejection	lung graft rejection	lung graft rejection	1	kept
Lung herniation	lung herniation	lung herniation	lung herniation	Lung herniation	1	kept
Lung imaging	lung imaging	lung imaging	lung imaging	Lung imaging	1	kept
lung imaging	lung imaging	lung imaging	lung imaging	Lung imaging	1	case_or_format
lung inflation	lung inflation	lung inflation	lung inflation	lung inflation	1	kept
LUNG INJURY	lung injury	lung injury	lung injury	lung injury	1	case_or_format
Lung injury	lung injury	lung injury	lung injury	lung injury	1	case_or_format
LUNG MICROBIOME	lung microbiome	lung microbiome	lung microbiome	LUNG MICROBIOME	1	kept
Lung protective strategies	lung protective strategies	lung protective strategies	lung protective strategy	Lung protective strategy	1	singular
Lung protective strategy	lung protective strategy	lung protective strategy	lung protective strategy	Lung protective strategy	1	kept
lung protective ventilation	lung protective ventilation	lung protective ventilation	lung protective ventilation	Lung protective ventilation	1	case_or_format

lung recruitment	lung recruitment	lung recruitment	lung recruitment	Lung recruitment	1	case_or_format
lung recruitment therapy	lung recruitment therapy	lung recruitment therapy	lung recruitment therapy	lung recruitment therapy	1	kept
lung small cell cancer	lung small cell cancer	lung small cell cancer	lung small cell cancer	lung small cell cancer	1	kept
lung symptoms	lung symptoms	lung symptoms	lung symptoms	lung symptoms	1	kept
lung transplant	lung transplant	lung transplant	lung transplant	Lung transplant	1	case_or_format
Lung Ultrasound Score	lung ultrasound score	lung ultrasound score	lung ultrasound score	lung ultrasound score	1	case_or_format
Lung ventilation	lung ventilation	lung ventilation	lung ventilation	lung ventilation	1	case_or_format
lung ventilation distribution	lung ventilation distribution	lung ventilation distribution	lung ventilation distribution	lung ventilation distribution	1	kept
lung ventilation perfusion ratio	lung ventilation perfusion ratio	lung ventilation perfusion ratio	lung ventilation perfusion ratio	lung ventilation perfusion ratio	1	kept
lung weight	lung weight	lung weight	lung weight	lung weight	1	kept
LUNG-CANCER	lung cancer	lung cancer	lung cancer	lung cancer	1	case_or_format
LUNG-FUNCTION	lung function	lung function	lung function	lung function	1	case_or_format
Lung-protective ventilation	lung protective ventilation	lung protective ventilation	lung protective ventilation	Lung protective ventilation	1	case_or_format
lungfish	lungfish	lungfish	lungfish	lungfish	1	kept
lupus erythematosus	lupus erythematosus	lupus erythematosus	lupus erythematosus	lupus erythematosus	1	kept
lupus podocytopathy	lupus podocytopathy	lupus podocytopathy	lupus podocytopathy	lupus podocytopathy	1	kept

lupus vulgaris	lupus vulgaris	lupus vulgaris	lupus vulgaris	lupus vulgaris	1	kept
Luria-Nebraska Neuropsychological Battery	luria nebraska neuropsychological battery	luria nebraska neuropsychological battery	luria nebraska neuropsychological battery	Luria-Nebraska Neuropsychological Battery	1	kept
LUS	lus	lus	lus	LUS	1	kept
Lusaka [Zambia]	lusaka zambia	lusaka zambia	lusaka zambia	Lusaka [Zambia]	1	kept
Lutte des âges	lutte des ges	lutte des ges	lutte des ges	Lutte des âges	1	kept
Luxembourg	luxembourg	luxembourg	luxembourg	Luxembourg	1	kept
LVADs	lvads	lvads	lvad	LVAD	1	singular
LVHG	lvhg	lvhg	lvhg	LVHG	1	kept
LVO	lvo	lvo	lvo	LVO	1	kept
Lycopersicon esculentum	lycopersicon esculentum	lycopersicon esculentum	lycopersicon esculentum	Lycopersicon esculentum	1	kept
Lyme disease	lyme disease	lyme disease	lyme disease	Lyme Disease	1	case_or_format
lymph vessel metastasis	lymph vessel metastasis	lymph vessel metastasis	lymph vessel metastasis	lymph vessel metastasis	1	kept
Lymphadenitis	lymphadenitis	lymphadenitis	lymphadenitis	Lymphadenitis	1	kept
Lymphangiectasis	lymphangiectasis	lymphangiectasis	lymphangiectasis	Lymphangiectasis	1	kept
lymphangioleiomyomatosis	lymphangioleiomyomatosis	lymphangioleiomyomatosis	lymphangioleiomyomatosis	lymphangioleiomyomatosis	1	kept
Lymphangioma	lymphangioma	lymphangioma	lymphangioma	lymphangioma	1	case_or_format

Lymphatic Abnormalities	lymphatic abnormalities	lymphatic abnormalities	lymphatic abnormalities	Lymphatic Abnormalities	1	kept
lymphatic drainage	lymphatic drainage	lymphatic drainage	lymphatic drainage	lymphatic drainage	1	kept
Lymphatic Metastasis	lymphatic metastasis	lymphatic metastasis	lymphatic metastasis	Lymphatic Metastasis	1	kept
lymphatic system disease	lymphatic system disease	lymphatic system disease	lymphatic system disease	lymphatic system disease	1	kept
Lymphatic Vessels	lymphatic vessels	lymphatic vessels	lymphatic vessels	Lymphatic Vessels	1	kept
Lymphedema	lymphedema	lymphedema	lymphedema	lymphedema	1	case_or_format
Lymphocyte Activation	lymphocyte activation	lymphocyte activation	lymphocyte activation	Lymphocyte Activation	1	kept
Lymphocyte Count	lymphocyte count	lymphocyte count	lymphocyte count	lymphocyte count	1	case_or_format
Lymphocyte neutrophil ratio	lymphocyte neutrophil ratio	lymphocyte neutrophil ratio	lymphocyte neutrophil ratio	Lymphocyte neutrophil ratio	1	kept
Lymphocyte subsets	lymphocyte subsets	lymphocyte subsets	lymphocyte subsets	Lymphocyte subsets	1	kept
Lymphoedema	lymphoedema	lymphoedema	lymphoedema	Lymphoedema	1	kept
Lymphohistiocytosis	lymphohistiocytosis	lymphohistiocytosis	lymphohistiocytosis	Lymphohistiocytosis	1	kept
lymphokine activated killer cell	lymphokine activated killer cell	lymphokine activated killer cell	lymphokine activated killer cell	lymphokine activated killer cell	1	kept
Lymphokine-activated killer cell	lymphokine activated killer cell	lymphokine activated killer cell	lymphokine activated killer cell	lymphokine activated killer cell	1	case_or_format
Lymphokine-activated killer cells	lymphokine activated killer cells	lymphokine activated killer cells	lymphokine activated killer cell	lymphokine activated killer cell	1	singular
lysosomal storage disorder	lysosomal storage disorder	lysosomal storage disorder	lysosomal storage disorder	lysosomal storage disorder	1	kept

lysosome	lysosome	lysosome	lysosome	lysosome	1	kept
Lysosomes	lysosomes	lysosomes	lysosome	lysosome	1	singular
lysozyme	lysozyme	lysozyme	lysozyme	lysozyme	1	kept
M score	m score	m score	m score	M score	1	kept
M Technique	m technique	m technique	m technique	M Technique	1	kept
M-health	m health	m health	m health	M-health	1	kept
Maastricht 3	maastricht 3	maastricht 3	maastricht 3	Maastricht 3	1	kept
MAC	mac	mac	mac	MAC	1	kept
MAC-SIM cards	mac sim cards	mac sim cards	mac sim cards	MAC-SIM cards	1	kept
machine learning algorithms	machine learning algorithms	machine learning algorithms	machine learning algorithm	machine learning algorithm	1	singular
machine learning.	machine learning	machine learning	machine learning	machine learning	1	case_or_format
Machine Listening	machine listening	machine listening	machine listening	Machine Listening	1	kept
Machines	machines	machines	machine	machine	1	singular
macrocephaly	macrocephaly	macrocephaly	macrocephaly	macrocephaly	1	kept
macrocognition	macrocognition	macrocognition	macrocognition	macrocognition	1	kept
Macrocyclic lactones	macrocyclic lactones	macrocyclic lactones	macrocyclic lactones	Macrocyclic lactones	1	kept

Macroeconomic conditions	macroeconomic conditions	macroeconomic conditions	macroeconomic conditions	Macroeconomic conditions	1	kept
macroergonomic analysis	macroergonomic analysis	macroergonomic analysis	macroergonomic analysis	macroergonomic analysis	1	kept
Macroergonomic and the environment	macroergonomic and the environment	macroergonomic and the environment	macroergonomic and the environment	Macroergonomic and the environment	1	kept
macroergonomics	macroergonomics	macroergonomics	macroergonomics	Macroergonomics	1	case_or_format
macroergonomics and the environment	macroergonomics and the environment	macroergonomics and the environment	macroergonomics and the environment	macroergonomics and the environment	1	kept
macroglossia	macroglossia	macroglossia	macroglossia	Macroglossia	1	case_or_format
macronutrients	macronutrients	macronutrients	macronutrient	macronutrient	1	singular
Macrophage	macrophage	macrophage	macrophage	macrophage	1	case_or_format
Macrophage Activation Syndrome	macrophage activation syndrome	macrophage activation syndrome	macrophage activation syndrome	Macrophage Activation Syndrome	1	kept
Macrophage activation syndrome	macrophage activation syndrome	macrophage activation syndrome	macrophage activation syndrome	Macrophage Activation Syndrome	1	case_or_format
macrophage activation syndrome	macrophage activation syndrome	macrophage activation syndrome	macrophage activation syndrome	Macrophage Activation Syndrome	1	case_or_format
macrophages	macrophages	macrophages	macrophage	macrophage	1	singular
Macropodidae	macropodidae	macropodidae	macropodidae	Macropodidae	1	kept
Macular function	macular function	macular function	macular function	Macular function	1	kept
Macular sensitivity	macular sensitivity	macular sensitivity	macular sensitivity	Macular sensitivity	1	kept
Madhya Pradesh	madhya pradesh	madhya pradesh	madhya pradesh	Madhya Pradesh	1	kept

mafenide	mafenide	mafenide	mafenide	mafenide	1	kept
Maggot	maggot	maggot	maggot	Maggot	1	kept
magnesium aspartate	magnesium aspartate	magnesium aspartate	magnesium aspartate	magnesium aspartate	1	kept
magnesium oxide	magnesium oxide	magnesium oxide	magnesium oxide	magnesium oxide	1	kept
magnesium sulfate for fetal neuroprotection	magnesium sulfate for fetal neuroprotection	magnesium sulfate for fetal neuroprotection	magnesium sulfate for fetal neuroprotection	magnesium sulfate for fetal neuroprotection	1	kept
Magnesium sulphate	magnesium sulphate	magnesium sulphate	magnesium sulphate	Magnesium sulphate	1	kept
magnesium sulphate	magnesium sulphate	magnesium sulphate	magnesium sulphate	Magnesium sulphate	1	case_or_format
MAGNESIUM-SULFATE	magnesium sulfate	magnesium sulfate	magnesium sulfate	magnesium sulfate	1	case_or_format
Magnet	magnet	magnet	magnet	magnet	1	case_or_format
Magnet hospital	magnet hospital	magnet hospital	magnet hospital	MAGNET HOSPITALS	1	case_or_format
magnet hospital	magnet hospital	magnet hospital	magnet hospital	MAGNET HOSPITALS	1	case_or_format
Magnet Hospitals	magnet hospitals	magnet hospitals	magnet hospital	MAGNET HOSPITALS	1	singular
Magnet hospitals	magnet hospitals	magnet hospitals	magnet hospital	MAGNET HOSPITALS	1	singular
magnetic field therapy	magnetic field therapy	magnetic field therapy	magnetic field therapy	magnetic field therapy	1	kept
Magnetic Fields	magnetic fields	magnetic fields	magnetic field	magnetic field	1	singular
magnetic resonance cholangiopancreatograp[illegible]	magnetic resonance cholangiopancreatograp[illegible]	magnetic resonance cholangiopancreatograp[illegible]	magnetic resonance cholangiopancreatograp[illegible]	magnetic resonance cholangiopancreatograp[illegible]	1	kept

Magnetic Resonance Spectroscopy	magnetic resonance spectroscopy	magnetic resonance spectroscopy	magnetic resonance spectroscopy	Magnetic Resonance Spectroscopy	1	kept
magnetic resonance venography	magnetic resonance venography	magnetic resonance venography	magnetic resonance venography	magnetic resonance venography	1	kept
magnetic stimulation	magnetic stimulation	magnetic stimulation	magnetic stimulation	magnetic stimulation	1	kept
Magnetic-field	magnetic field	magnetic field	magnetic field	magnetic field	1	case_or_format
magnetism	magnetism	magnetism	magnetism	magnetism	1	kept
magnetoencephalography	magnetoencephalography	magnetoencephalography	magnetoencephalography	magnetoencephalography	1	kept
Magnet® hospital	magnet hospital	magnet hospital	magnet hospital	MAGNET HOSPITALS	1	case_or_format
Magnifier	magnifier	magnifier	magnifier	Magnifier	1	kept
magnitude	magnitude	magnitude	magnitude	magnitude	1	kept
MAIL SURVEY	mail survey	mail survey	mail survey	MAIL SURVEY	1	kept
MAIL SURVEYS	mail surveys	mail surveys	mail survey	MAIL SURVEY	1	singular
main caregiver ability evaluation index system	main caregiver ability evaluation index system	main caregiver ability evaluation index system	main caregiver ability evaluation index system	main caregiver ability evaluation index system	1	kept
Main effect	main effect	main effect	main effect	Main effect	1	kept
Mainland China	mainland china	mainland china	mainland china	Mainland China	1	kept
maintenance	maintenance	maintenance	maintenance	Maintenance	1	case_or_format
Maintenance checklist	maintenance checklist	maintenance checklist	maintenance checklist	Maintenance checklist	1	kept

maintenance chemotherapy	maintenance chemotherapy	maintenance chemotherapy	maintenance chemotherapy	maintenance chemotherapy	1	kept
maintenance of certification	maintenance of certification	maintenance of certification	maintenance of certification	maintenance of certification	1	kept
maintenance of respiration and circulation	maintenance of respiration and circulation	maintenance of respiration and circulation	maintenance of respiration and circulation	maintenance of respiration and circulation	1	kept
Major abdominal surgery	major abdominal surgery	major abdominal surgery	major abdominal surgery	Major abdominal surgery	1	kept
major abdominal surgery	major abdominal surgery	major abdominal surgery	major abdominal surgery	Major abdominal surgery	1	case_or_format
major adverse cardiac event	major adverse cardiac event	major adverse cardiac event	major adverse cardiac event	major adverse cardiac event	1	kept
major aortopulmonary collaterals	major aortopulmonary collaterals	major aortopulmonary collaterals	major aortopulmonary collaterals	major aortopulmonary collaterals	1	kept
Major body systems assessment	major body systems assessment	major body systems assessment	major body systems assessment	Major body systems assessment	1	kept
MAJOR COMPLICATIONS	major complications	major complications	major complications	MAJOR COMPLICATIONS	1	kept
Major depression disorder	major depression disorder	major depression disorder	major depression disorder	Major depression disorder	1	kept
major desaturation	major desaturation	major desaturation	major desaturation	major desaturation	1	kept
Major factors	major factors	major factors	major factors	Major factors	1	kept
Major heart surgery	major heart surgery	major heart surgery	major heart surgery	Major heart surgery	1	kept
major histocompatibility complex	major histocompatibility complex	major histocompatibility complex	major histocompatibility complex	major histocompatibility complex	1	kept
Major Hospital	major hospital	major hospital	major hospital	Major Hospital	1	kept
major incident planning	major incident planning	major incident planning	major incident planning	major incident planning	1	kept

major incidents	major incidents	major incidents	major incidents	major incidents	1	kept
Major respiratory eventP	major respiratory eventp	major respiratory eventp	major respiratory eventp	Major respiratory eventP	1	kept
major trauma	major trauma	major trauma	major trauma	Major trauma	1	case_or_format
major trauma management	major trauma management	major trauma management	major trauma management	major trauma management	1	kept
MAKERS	makers	makers	makers	MAKERS	1	kept
making end of life	making end of life	making end of life	making end of life	making end of life	1	kept
MAKING MODELS	making models	making models	making models	MAKING MODELS	1	kept
Makkah [Saudi Arabia]	makkah saudi arabia	makkah saudi arabia	makkah saudi arabia	Makkah [Saudi Arabia]	1	kept
maladjustment	maladjustment	maladjustment	maladjustment	maladjustment	1	kept
Malakichthys wakiyae	malakichthys wakiyae	malakichthys wakiyae	malakichthys wakiyae	Malakichthys wakiyae	1	kept
malar rash	malar rash	malar rash	malar rash	malar rash	1	kept
Malaria	malaria	malaria	malaria	malaria	1	case_or_format
Malaria-leptospira co-infection	malaria leptospira co infection	malaria leptospira co infection	malaria leptospira co infection	Malaria-leptospira co-infection	1	kept
Malassezia species	malassezia species	malassezia species	malassezia species	Malassezia species	1	kept
Malawian	malawian	malawian	malawian	Malawian	1	kept
Malaysian	malaysian	malaysian	malaysian	Malaysians	1	case_or_format

Malaysian hospital	malaysian hospital	malaysian hospital	malaysian hospital	Malaysian hospital	1	kept
Maldives	maldives	maldives	maldives	Maldives	1	kept
male infertility	male infertility	male infertility	male infertility	male infertility	1	kept
male students	male students	male students	male students	male students	1	kept
male type alopecia	male type alopecia	male type alopecia	male type alopecia	male type alopecia	1	kept
Malformation	malformation	malformation	malformation	Malformation	1	kept
malformation syndrome	malformation syndrome	malformation syndrome	malformation syndrome	malformation syndrome	1	kept
Malformations of Cortical Development	malformations of cortical development	malformations of cortical development	malformations of cortical development	Malformations of Cortical Development	1	kept
Mali	mali	mali	mali	Mali	1	kept
malic acid	malic acid	malic acid	malic acid	malic acid	1	kept
MALIGNANCIES	malignancies	malignancies	malignancy	Malignancy	1	singular
Malignancy	malignancy	malignancy	malignancy	Malignancy	1	kept
malignant arrhythmia	malignant arrhythmia	malignant arrhythmia	malignant arrhythmia	malignant arrhythmia	1	kept
MALIGNANT BOWEL OBSTRUCTION	malignant bowel obstruction	malignant bowel obstruction	malignant bowel obstruction	MALIGNANT BOWEL OBSTRUCTION	1	kept
Malignant cell metabolism	malignant cell metabolism	malignant cell metabolism	malignant cell metabolism	Malignant cell metabolism	1	kept
Malignant cerebral edema	malignant cerebral edema	malignant cerebral edema	malignant cerebral edema	Malignant cerebral edema	1	kept

Malignant hyperthermia	malignant hyperthermia	malignant hyperthermia	malignant hyperthermia	Malignant Hyperthermia	1	case_or_format
malignant lymphoma	malignant lymphoma	malignant lymphoma	malignant lymphoma	malignant lymphoma	1	kept
malignant meningioma	malignant meningioma	malignant meningioma	malignant meningioma	malignant meningioma	1	kept
malignant syndrome	malignant syndrome	malignant syndrome	malignant syndrome	malignant syndrome	1	kept
Malingering	malingering	malingering	malingering	Malingering	1	kept
Malnourished	malnourished	malnourished	malnourished	Malnourished	1	kept
Malnourishment and fasting in anesthesia	malnourishment and fasting in anesthesia	malnourishment and fasting in anesthesia	malnourishment and fasting in anesthesia	Malnourishment and fasting in anesthesia	1	kept
Malnutrition in the elderly	malnutrition in the elderly	malnutrition in the elderly	malnutrition in the elderly	Malnutrition in the elderly	1	kept
Malnutrition Screening Tool	malnutrition screening tool	malnutrition screening tool	malnutrition screening tool	Malnutrition Screening Tool	1	kept
malnutrition screening tool	malnutrition screening tool	malnutrition screening tool	malnutrition screening tool	Malnutrition Screening Tool	1	case_or_format
Malnutrition Universal Screening Tool	malnutrition universal screening tool	malnutrition universal screening tool	malnutrition universal screening tool	Malnutrition Universal Screening Tool	1	kept
malocclusion	malocclusion	malocclusion	malocclusion	malocclusion	1	kept
Malondialdehyde	malondialdehyde	malondialdehyde	malondialdehyde	Malondialdehyde	1	kept
Mammalia	mammalia	mammalia	mammalia	Mammalia	1	kept
mammalian target of rapamycin inhibitor	mammalian target of rapamycin inhibitor	mammalian target of rapamycin inhibitor	mammalian target of rapamycin inhibitor	mammalian target of rapamycin inhibitor	1	kept
Mammals	mammals	mammals	mammals	Mammals	1	kept

mammary gland growth	mammary gland growth	mammary gland growth	mammary gland growth	mammary gland growth	1	kept
mammography	mammography	mammography	mammography	mammography	1	kept
Man-machine systems	man machine systems	man machine systems	man machine systems	Man-Machine Systems	1	case_or_format
MANAGED CARE	managed care	managed care	managed care	managed care	1	case_or_format
Managed care	managed care	managed care	managed care	managed care	1	case_or_format
management alarm	management alarm	management alarm	management alarm	management alarm	1	kept
MANAGEMENT ALGORITHM	management algorithm	management algorithm	management algorithm	MANAGEMENT ALGORITHM	1	kept
Management and staffing	management and staffing	management and staffing	management and staffing	Management and staffing	1	kept
Management aspects	management aspects	management aspects	management aspects	Management aspects	1	kept
management commitment	management commitment	management commitment	management commitment	management commitment	1	kept
Management commitment to safeties	management commitment to safeties	management commitment to safeties	management commitment to safeties	Management commitment to safeties	1	kept
Management consulting	management consulting	management consulting	management consulting	Management consulting	1	kept
MANAGEMENT CONTROL-SYSTEMS	management control systems	management control systems	management control systems	MANAGEMENT CONTROL-SYSTEMS	1	kept
Management costs	management costs	management costs	management costs	Management costs	1	kept
Management Flight Simulator	management flight simulator	management flight simulator	management flight simulator	Management Flight Simulator	1	kept
Management interventions	management interventions	management interventions	management interventions	Management interventions	1	kept

Management IS	management is	management is	management is	Management IS	1	kept
management mode	management mode	management mode	management mode	management mode	1	kept
management of bleeding event	management of bleeding event	management of bleeding event	management of bleeding event	management of bleeding event	1	kept
management of knowledge	management of knowledge	management of knowledge	management of knowledge	management of knowledge	1	kept
Management of logistics	management of logistics	management of logistics	management of logistics	Management of logistics	1	kept
Management of neuroemergency	management of neuroemergency	management of neuroemergency	management of neuroemergency	Management of neuroemergency	1	kept
management of stress	management of stress	management of stress	management of stress	management of stress	1	kept
management of tracheostomy	management of tracheostomy	management of tracheostomy	management of tracheostomy	management of tracheostomy	1	kept
management platform	management platform	management platform	management platform	management platform	1	kept
Management practices	management practices	management practices	management practices	Management practices	1	kept
management protocol	management protocol	management protocol	management protocol	management protocol	1	kept
management quality	management quality	management quality	management quality	management quality	1	kept
MANAGEMENT STRATEGIES	management strategies	management strategies	management strategies	Management strategies	1	case_or_format
management style	management style	management style	management style	management style	1	kept
Management support	management support	management support	management support	Management support	1	kept
MANAGEMENT SYSTEMS	management systems	management systems	management system	Management system	1	singular

Management tool	management tool	management tool	management tool	Management tool	1	kept
MANAGEMENT-SYSTEM	management system	management system	management system	Management system	1	case_or_format
Manager	manager	manager	manager	manager	1	case_or_format
Manager education	manager education	manager education	manager education	Manager education	1	kept
Managerial activity	managerial activity	managerial activity	managerial activity	Managerial activity	1	kept
Managerial model	managerial model	managerial model	managerial model	Managerial model	1	kept
MANAGERS	managers	managers	manager	manager	1	singular
managers	managers	managers	manager	manager	1	singular
MANAGING CLIENTS	managing clients	managing clients	managing clients	MANAGING CLIENTS	1	kept
managing disruptive behavior	managing disruptive behavior	managing disruptive behavior	managing disruptive behavior	managing disruptive behavior	1	kept
Managing fatigue and shift work	managing fatigue and shift work	managing fatigue and shift work	managing fatigue and shift work	Managing fatigue and shift work	1	kept
MANAGING JOB STRESS	managing job stress	managing job stress	managing job stress	MANAGING JOB STRESS	1	kept
mandala coloring	mandala coloring	mandala coloring	mandala coloring	mandala coloring	1	kept
Mandated Use	mandated use	mandated use	mandated use	Mandated Use	1	kept
Mandates	mandates	mandates	mandates	Mandates	1	kept
Mandatory regulations	mandatory regulations	mandatory regulations	mandatory regulations	Mandatory regulations	1	kept

mandatory testing	mandatory testing	mandatory testing	mandatory testing	Mandatory testing	1	case_or_format
Mandatory training	mandatory training	mandatory training	mandatory training	Mandatory training	1	kept
Mandible	mandible	mandible	mandible	mandible	1	case_or_format
mandible condyle	mandible condyle	mandible condyle	mandible condyle	mandible condyle	1	kept
mandible condyle disclocation	mandible condyle disclocation	mandible condyle disclocation	mandible condyle disclocation	mandible condyle disclocation	1	kept
mandible reconstruction	mandible reconstruction	mandible reconstruction	mandible reconstruction	mandible reconstruction	1	kept
mandible resection	mandible resection	mandible resection	mandible resection	mandible resection	1	kept
mandibular combat trauma	mandibular combat trauma	mandibular combat trauma	mandibular combat trauma	mandibular combat trauma	1	kept
Mandibular Condyle	mandibular condyle	mandibular condyle	mandibular condyle	Mandibular Condyle	1	kept
Mandibular condyle	mandibular condyle	mandibular condyle	mandibular condyle	Mandibular Condyle	1	case_or_format
Mandibular Injuries	mandibular injuries	mandibular injuries	mandibular injuries	Mandibular Injuries	1	kept
mandibular traction	mandibular traction	mandibular traction	mandibular traction	mandibular traction	1	kept
Manejo del dolor	manejo del dolor	manejo del dolor	manejo del dolor	Manejo del dolor	1	kept
Manganese	manganese	manganese	manganese	Manganese	1	kept
manganese	manganese	manganese	manganese	Manganese	1	case_or_format
manganese superoxide dismutase	manganese superoxide dismutase	manganese superoxide dismutase	manganese superoxide dismutase	manganese superoxide dismutase	1	kept

Mania	mania	mania	mania	mania	1	case_or_format
manifestation	manifestation	manifestation	manifestation	manifestation	1	kept
manifestations of stress	manifestations of stress	manifestations of stress	manifestations of stress	manifestations of stress	1	kept
Manikin study	manikin study	manikin study	manikin study	Manikin study	1	kept
manikin-based simulation	manikin based simulation	manikin based simulation	manikin based simulation	manikin-based simulation	1	kept
MANIPULATION	manipulation	manipulation	manipulation	MANIPULATION	1	kept
manipulation	manipulation	manipulation	manipulation	MANIPULATION	1	case_or_format
Manipulation, Chiropractic	manipulation chiropractic	manipulation chiropractic	manipulation chiropractic	Manipulation, Chiropractic	1	kept
Manipulation, Osteopathic	manipulation osteopathic	manipulation osteopathic	manipulation osteopathic	Manipulation, Osteopathic	1	kept
Mann-Whitney U test	mann whitney u test	mann whitney u test	mann whitney u test	Mann-Whitney U test	1	kept
mannan	mannan	mannan	mannan	mannan	1	kept
mannan antibody	mannan antibody	mannan antibody	mannan antibody	mannan antibody	1	kept
Mannan antigen	mannan antigen	mannan antigen	mannan antigen	Mannan antigen	1	kept
mannan antigen	mannan antigen	mannan antigen	mannan antigen	Mannan antigen	1	case_or_format
Mannans	mannans	mannans	mannan	mannan	1	singular
Mannequin	mannequin	mannequin	mannequin	mannequin	1	case_or_format

Mannequin study	mannequin study	mannequin study	mannequin study	Mannequin study	1	kept
Mannequin-Based Simulation	mannequin based simulation	mannequin based simulation	mannequin based simulation	Mannequin-Based Simulation	1	kept
mannosidosis	mannosidosis	mannosidosis	mannosidosis	mannosidosis	1	kept
Mann–Whitney-test	mann whitney test	mann whitney test	mann whitney test	Mann–Whitney-test	1	kept
manometer	manometer	manometer	manometer	manometer	1	kept
manual	manual	manual	manual	manual	1	kept
Manual accidents	manual accidents	manual accidents	manual accidents	Manual accidents	1	kept
manual bedside placement	manual bedside placement	manual bedside placement	manual bedside placement	manual bedside placement	1	kept
manual cardiopulmonary resuscitation	manual cardiopulmonary resuscitation	manual cardiopulmonary resuscitation	manual cardiopulmonary resuscitation	manual cardiopulmonary resuscitation	1	kept
Manual coding	manual coding	manual coding	manual coding	Manual coding	1	kept
Manual Communication	manual communication	manual communication	manual communication	Manual Communication	1	kept
manual communication	manual communication	manual communication	manual communication	Manual Communication	1	case_or_format
manual cuff pressure control	manual cuff pressure control	manual cuff pressure control	manual cuff pressure control	manual cuff pressure control	1	kept
Manual handling	manual handling	manual handling	manual handling	Manual handling	1	kept
manual hyperinflation	manual hyperinflation	manual hyperinflation	manual hyperinflation	manual hyperinflation	1	kept
manual inflation	manual inflation	manual inflation	manual inflation	manual inflation	1	kept

manual labor	manual labor	manual labor	manual labor	manual labor	1	kept
manual lung hyperinflation	manual lung hyperinflation	manual lung hyperinflation	manual lung hyperinflation	manual lung hyperinflation	1	kept
Manual lymphatic drainage	manual lymphatic drainage	manual lymphatic drainage	manual lymphatic drainage	Manual lymphatic drainage	1	kept
Manual method	manual method	manual method	manual method	Manual method	1	kept
Manual Muscle Testing Scale	manual muscle testing scale	manual muscle testing scale	manual muscle testing scale	Manual Muscle Testing Scale	1	kept
manual restraint	manual restraint	manual restraint	manual restraint	manual restraint	1	kept
manual rib cage compression	manual rib cage compression	manual rib cage compression	manual rib cage compression	manual rib cage compression	1	kept
Manual ventilation	manual ventilation	manual ventilation	manual ventilation	manual ventilation	1	case_or_format
Manufacture	manufacture	manufacture	manufacture	Manufacture	1	kept
manuscript	manuscript	manuscript	manuscript	manuscript	1	kept
Manuscripts	manuscripts	manuscripts	manuscript	manuscript	1	singular
Manuscripts, Medical as Topic	manuscripts medical as topic	manuscripts medical as topic	manuscripts medical as topic	Manuscripts, Medical as Topic	1	kept
Maori People	maori people	maori people	maori people	Maori People	1	kept
Maori people	maori people	maori people	maori people	Maori People	1	case_or_format
Maorse Fall Scale	maorse fall scale	maorse fall scale	maorse fall scale	Maorse Fall Scale	1	kept
MAP = mean arterial pressure	map mean arterial pressure	map mean arterial pressure	map mean arterial pressure	MAP = mean arterial pressure	1	kept

MAP Kinase Kinase 2	map kinase kinase 2	map kinase kinase 2	map kinase kinase 2	MAP Kinase Kinase 2	1	kept
MAP kinase signaling	map kinase signaling	map kinase signaling	map kinase signaling	MAP kinase signaling	1	kept
MAP2K1	map2k1	map2k1	map2k1	MAP2K1	1	kept
MAP2K2	map2k2	map2k2	map2k2	MAP2K2	1	kept
Mapleson C circuit	mapleson c circuit	mapleson c circuit	mapleson c circuit	Mapleson C circuit	1	kept
Mapping	mapping	mapping	mapping	Mapping	1	kept
mapping	mapping	mapping	mapping	Mapping	1	case_or_format
MAPS	maps	maps	maps	MAPS	1	kept
Marathon Running	marathon running	marathon running	marathon running	Marathon Running	1	kept
Marburg	marburg	marburg	marburg	Marburg	1	kept
Marburg Heart Score	marburg heart score	marburg heart score	marburg heart score	Marburg Heart Score	1	kept
marburg heart score	marburg heart score	marburg heart score	marburg heart score	Marburg Heart Score	1	case_or_format
Marburg hemorrhagic fever	marburg hemorrhagic fever	marburg hemorrhagic fever	marburg hemorrhagic fever	Marburg hemorrhagic fever	1	kept
Marburg vaccine	marburg vaccine	marburg vaccine	marburg vaccine	Marburg vaccine	1	kept
Marc de Leval	marc de leval	marc de leval	marc de leval	Marc de Leval	1	kept
margin of viability	margin of viability	margin of viability	margin of viability	margin of viability	1	kept

marginalization	marginalization	marginalization	marginalization	marginalization	1	kept
Marijuana Use	marijuana use	marijuana use	marijuana use	Marijuana Use	1	kept
marine environment	marine environment	marine environment	marine environment	marine environment	1	kept
Marital intimacy	marital intimacy	marital intimacy	marital intimacy	Marital intimacy	1	kept
marital status	marital status	marital status	marital status	Marital Status	1	case_or_format
Maritime	maritime	maritime	maritime	Maritime	1	kept
MARKER	marker	marker	marker	MARKER	1	kept
marker	marker	marker	marker	MARKER	1	case_or_format
Markov chain	markov chain	markov chain	markov chain	Markov Chains	1	case_or_format
Markov chain Monte Carlo method	markov chain monte carlo method	markov chain monte carlo method	markov chain monte carlo method	Markov chain Monte Carlo method	1	kept
Markov decision model	markov decision model	markov decision model	markov decision model	Markov decision model	1	kept
Markov decision process	markov decision process	markov decision process	markov decision process	Markov decision process	1	kept
MARS®	mars	mars	mars	MARS®	1	kept
Martinsen	martinsen	martinsen	martinsen	Martinsen	1	kept
MASCULINITY	masculinity	masculinity	masculinity	Masculinity	1	case_or_format
MASK	mask	mask	mask	Masks	1	case_or_format

Mask compliance	mask compliance	mask compliance	mask compliance	Mask compliance	1	kept
mask leakage	mask leakage	mask leakage	mask leakage	mask leakage	1	kept
mask treatment	mask treatment	mask treatment	mask treatment	mask treatment	1	kept
masked hypertension	masked hypertension	masked hypertension	masked hypertension	masked hypertension	1	kept
masks	masks	masks	mask	Masks	1	singular
Maslach	maslach	maslach	maslach	Maslach	1	kept
maslach burnout inventory questionnaire	maslach burnout inventory questionnaire	maslach burnout inventory questionnaire	maslach burnout inventory questionnaire	maslach burnout inventory questionnaire	1	kept
Maslach Burnout Scale	maslach burnout scale	maslach burnout scale	maslach burnout scale	Maslach Burnout Scale	1	kept
maslach jackson burnout inventory	maslach jackson burnout inventory	maslach jackson burnout inventory	maslach jackson burnout inventory	maslach jackson burnout inventory	1	kept
Maslach's burnout scale	maslach s burnout scale	maslach s burnout scale	maslach s burnout scale	Maslach's burnout scale	1	kept
mass casualties	mass casualties	mass casualties	mass casualty	Mass casualty	1	singular
mass casualty incident	mass casualty incident	mass casualty incident	mass casualty incident	Mass Casualty Incidents	1	case_or_format
Mass casualty incidents	mass casualty incidents	mass casualty incidents	mass casualty incident	Mass Casualty Incidents	1	singular
Mass emergency	mass emergency	mass emergency	mass emergency	Mass emergency	1	kept
Mass gathering	mass gathering	mass gathering	mass gathering	mass gathering	1	case_or_format
Mass gathering event	mass gathering event	mass gathering event	mass gathering event	Mass gathering event	1	kept

Mass gathering medicine	mass gathering medicine	mass gathering medicine	mass gathering medicine	Mass gathering medicine	1	kept
Mass Gatherings	mass gatherings	mass gatherings	mass gathering	mass gathering	1	singular
Mass Immunization	mass immunization	mass immunization	mass immunization	mass immunization	1	case_or_format
Mass index	mass index	mass index	mass index	Mass index	1	kept
mass media	mass media	mass media	mass media	Mass Media	1	case_or_format
Mass Shooting Events	mass shooting events	mass shooting events	mass shooting events	Mass Shooting Events	1	kept
Mass Spectrometry	mass spectrometry	mass spectrometry	mass spectrometry	Mass spectrometry	1	case_or_format
Mass sports	mass sports	mass sports	mass sports	Mass sports	1	kept
Mass transfusion	mass transfusion	mass transfusion	mass transfusion	Mass transfusion	1	kept
Mass-casualty incident	mass casualty incident	mass casualty incident	mass casualty incident	Mass Casualty Incidents	1	case_or_format
Massachusetts Institute of Technology	massachusetts institute of technology	massachusetts institute of technology	massachusetts institute of technology	Massachusetts Institute of Technology	1	kept
massage therapist	massage therapist	massage therapist	massage therapist	massage therapist	1	kept
Massage Therapy	massage therapy	massage therapy	massage therapy	Massage therapy	1	case_or_format
Massage therapy by the mother	massage therapy by the mother	massage therapy by the mother	massage therapy by the mother	Massage therapy by the mother	1	kept
Masseter Muscle	masseter muscle	masseter muscle	masseter muscle	Masseter Muscle	1	kept
masseter muscle	masseter muscle	masseter muscle	masseter muscle	Masseter Muscle	1	case_or_format

Massive burn casualty	massive burn casualty	massive burn casualty	massive burn casualty	Massive burn casualty	1	kept
massive hemoptysis	massive hemoptysis	massive hemoptysis	massive hemoptysis	massive hemoptysis	1	kept
massive pulmonary embolism	massive pulmonary embolism	massive pulmonary embolism	massive pulmonary embolism	massive pulmonary embolism	1	kept
massive transfusion protocol	massive transfusion protocol	massive transfusion protocol	massive transfusion protocol	Massive transfusion protocol	1	case_or_format
mast cell	mast cell	mast cell	mast cell	mast cell	1	kept
Mastadenovirus	mastadenovirus	mastadenovirus	mastadenovirus	Mastadenovirus	1	kept
mastalgia	mastalgia	mastalgia	mastalgia	mastalgia	1	kept
Master Athletes	master athletes	master athletes	master athletes	Master Athletes	1	kept
master athletes	master athletes	master athletes	master athletes	Master Athletes	1	case_or_format
Master surgery scheduling	master surgery scheduling	master surgery scheduling	master surgery scheduling	Master surgery scheduling	1	kept
Master’ s program	master s program	master s program	master s program	Master’ s program	1	kept
master’ s program	master s program	master s program	master s program	Master’ s program	1	case_or_format
mastication	mastication	mastication	mastication	mastication	1	kept
Mastoiditis	mastoiditis	mastoiditis	mastoiditis	Mastoiditis	1	kept
mastoiditis	mastoiditis	mastoiditis	mastoiditis	Mastoiditis	1	case_or_format
mat	mat	mat	mat	mat	1	kept

Matching	matching	matching	matching	Matching	1	kept
Matching mechanisms	matching mechanisms	matching mechanisms	matching mechanisms	Matching mechanisms	1	kept
Matching Michigan	matching michigan	matching michigan	matching michigan	Matching Michigan	1	kept
material cost	material cost	material cost	material cost	material cost	1	kept
material jetting	material jetting	material jetting	material jetting	material jetting	1	kept
Material vigilance	material vigilance	material vigilance	material vigilance	Material vigilance	1	kept
materials	materials	materials	materials	materials	1	kept
Materials Management	materials management	materials management	materials management	materials management	1	case_or_format
materials testing	materials testing	materials testing	materials testing	Materials Testing	1	case_or_format
maternal ability	maternal ability	maternal ability	maternal ability	maternal ability	1	kept
maternal affect	maternal affect	maternal affect	maternal affect	maternal affect	1	kept
maternal analgesia	maternal analgesia	maternal analgesia	maternal analgesia	maternal analgesia	1	kept
Maternal and child care	maternal and child care	maternal and child care	maternal and child care	Maternal and child care	1	kept
Maternal and Child Health	maternal and child health	maternal and child health	maternal and child health	maternal and child health	1	case_or_format
Maternal and child health	maternal and child health	maternal and child health	maternal and child health	maternal and child health	1	case_or_format
Maternal and child health centers	maternal and child health centers	maternal and child health centers	maternal and child health centers	Maternal and child health centers	1	kept

maternal and child health services	maternal and child health services	maternal and child health services	maternal and child health services	maternal and child health services	1	kept
maternal and infant health outcome	maternal and infant health outcome	maternal and infant health outcome	maternal and infant health outcome	maternal and infant health outcome	1	kept
maternal and neonatal outcomes	maternal and neonatal outcomes	maternal and neonatal outcomes	maternal and neonatal outcome	Maternal and neonatal outcome	1	singular
Maternal and newborn health services	maternal and newborn health services	maternal and newborn health services	maternal and newborn health services	Maternal and newborn health services	1	kept
Maternal and perinatal deaths	maternal and perinatal deaths	maternal and perinatal deaths	maternal and perinatal deaths	Maternal and perinatal deaths	1	kept
maternal antibody	maternal antibody	maternal antibody	maternal antibody	maternal antibody	1	kept
maternal arrest in pregnancy	maternal arrest in pregnancy	maternal arrest in pregnancy	maternal arrest in pregnancy	maternal arrest in pregnancy	1	kept
MATERNAL ATTACHMENT	maternal attachment	maternal attachment	maternal attachment	MATERNAL ATTACHMENT	1	kept
Maternal attachment	maternal attachment	maternal attachment	maternal attachment	MATERNAL ATTACHMENT	1	case_or_format
maternal attachment	maternal attachment	maternal attachment	maternal attachment	MATERNAL ATTACHMENT	1	case_or_format
Maternal attitude	maternal attitude	maternal attitude	maternal attitude	maternal attitude	1	case_or_format
maternal bereavement	maternal bereavement	maternal bereavement	maternal bereavement	maternal bereavement	1	kept
Maternal birth outcome	maternal birth outcome	maternal birth outcome	maternal birth outcome	Maternal birth outcome	1	kept
maternal bonding	maternal bonding	maternal bonding	maternal bonding	maternal bonding	1	kept
Maternal care	maternal care	maternal care	maternal care	maternal care	1	case_or_format
Maternal confidence questionnaire	maternal confidence questionnaire	maternal confidence questionnaire	maternal confidence questionnaire	Maternal confidence questionnaire	1	kept

Maternal COVID-19	maternal covid 19	maternal covid 19	maternal covid 19	Maternal COVID-19	1	kept
maternal decompensation	maternal decompensation	maternal decompensation	maternal decompensation	maternal decompensation	1	kept
MATERNAL DEPRESSION	maternal depression	maternal depression	maternal depression	MATERNAL DEPRESSION	1	kept
maternal depression	maternal depression	maternal depression	maternal depression	MATERNAL DEPRESSION	1	case_or_format
maternal deprivation	maternal deprivation	maternal deprivation	maternal deprivation	maternal deprivation	1	kept
Maternal diabetes	maternal diabetes	maternal diabetes	maternal diabetes	Maternal diabetes	1	kept
maternal diaphragmatic hernia	maternal diaphragmatic hernia	maternal diaphragmatic hernia	maternal diaphragmatic hernia	maternal diaphragmatic hernia	1	kept
Maternal distress	maternal distress	maternal distress	maternal distress	Maternal distress	1	kept
maternal distress	maternal distress	maternal distress	maternal distress	Maternal distress	1	case_or_format
maternal diversity	maternal diversity	maternal diversity	maternal diversity	maternal diversity	1	kept
maternal early warning tool	maternal early warning tool	maternal early warning tool	maternal early warning tool	maternal early warning tool	1	kept
Maternal education	maternal education	maternal education	maternal education	Maternal education	1	kept
Maternal experience	maternal experience	maternal experience	maternal experience	Maternal experience	1	kept
maternal experience	maternal experience	maternal experience	maternal experience	Maternal experience	1	case_or_format
maternal experiences	maternal experiences	maternal experiences	maternal experience	Maternal experience	1	singular
maternal factors	maternal factors	maternal factors	maternal factors	maternal factors	1	kept

maternal fetal specialist	maternal fetal specialist	maternal fetal specialist	maternal fetal specialist	maternal fetal specialist	1	kept
maternal health	maternal health	maternal health	maternal health	Maternal health	1	case_or_format
Maternal health research	maternal health research	maternal health research	maternal health research	Maternal health research	1	kept
Maternal health services	maternal health services	maternal health services	maternal health service	Maternal Health Services	1	singular
maternal hemorrhage	maternal hemorrhage	maternal hemorrhage	maternal hemorrhage	maternal hemorrhage	1	kept
maternal holding	maternal holding	maternal holding	maternal holding	maternal holding	1	kept
maternal hypoglycemia	maternal hypoglycemia	maternal hypoglycemia	maternal hypoglycemia	maternal hypoglycemia	1	kept
Maternal identity	maternal identity	maternal identity	maternal identity	Maternal identity	1	kept
maternal infant health	maternal infant health	maternal infant health	maternal infant health	maternal infant health	1	kept
maternal infant hospitalization unit	maternal infant hospitalization unit	maternal infant hospitalization unit	maternal infant hospitalization unit	maternal infant hospitalization unit	1	kept
maternal infection	maternal infection	maternal infection	maternal infection	maternal infection	1	kept
Maternal medicine	maternal medicine	maternal medicine	maternal medicine	maternal medicine	1	case_or_format
Maternal mental health	maternal mental health	maternal mental health	maternal mental health	maternal mental health	1	case_or_format
maternal metabolic syndrome	maternal metabolic syndrome	maternal metabolic syndrome	maternal metabolic syndrome	maternal metabolic syndrome	1	kept
maternal morbidity and mortality	maternal morbidity and mortality	maternal morbidity and mortality	maternal morbidity and mortality	maternal morbidity and mortality	1	kept
maternal narcotic pain medication	maternal narcotic pain medication	maternal narcotic pain medication	maternal narcotic pain medication	maternal narcotic pain medication	1	kept

maternal near miss	maternal near miss	maternal near miss	maternal near miss	Maternal near miss	1	case_or_format
Maternal near missmortality ratio	maternal near missmortality ratio	maternal near missmortality ratio	maternal near missmortality ratio	Maternal near missmortality ratio	1	kept
Maternal near-miss	maternal near miss	maternal near miss	maternal near miss	Maternal near miss	1	case_or_format
Maternal needs	maternal needs	maternal needs	maternal needs	Maternal needs	1	kept
Maternal Nutritional Physiological Phenomena	maternal nutritional physiological phenomena	maternal nutritional physiological phenomena	maternal nutritional physiological phenomena	Maternal Nutritional Physiological Phenomena	1	kept
maternal nutritional physiological phenomena	maternal nutritional physiological phenomena	maternal nutritional physiological phenomena	maternal nutritional physiological phenomena	Maternal Nutritional Physiological Phenomena	1	case_or_format
maternal obesity	maternal obesity	maternal obesity	maternal obesity	maternal obesity	1	kept
Maternal oncology treatment	maternal oncology treatment	maternal oncology treatment	maternal oncology treatment	Maternal oncology treatment	1	kept
Maternal outcome	maternal outcome	maternal outcome	maternal outcome	Maternal outcomes	1	case_or_format
maternal outcome	maternal outcome	maternal outcome	maternal outcome	Maternal outcomes	1	case_or_format
maternal outcomes	maternal outcomes	maternal outcomes	maternal outcome	Maternal outcomes	1	singular
Maternal Participation Program	maternal participation program	maternal participation program	maternal participation program	Maternal Participation Program	1	kept
maternal place of care	maternal place of care	maternal place of care	maternal place of care	maternal place of care	1	kept
Maternal Postnatal Attachment	maternal postnatal attachment	maternal postnatal attachment	maternal postnatal attachment	Maternal Postnatal Attachment	1	kept
MATERNAL POSTTRAUMATIC STRESS	maternal posttraumatic stress	maternal posttraumatic stress	maternal posttraumatic stress	MATERNAL POSTTRAUMATIC STRESS	1	kept
Maternal presence	maternal presence	maternal presence	maternal presence	Maternal presence	1	kept

MATERNAL PSYCHOLOGICAL DISTRESS	maternal psychological distress	maternal psychological distress	maternal psychological distress	MATERNAL PSYCHOLOGICAL DISTRESS	1	kept
Maternal readiness to hospital discharge	maternal readiness to hospital discharge	maternal readiness to hospital discharge	maternal readiness to hospital discharge	Maternal readiness to hospital discharge	1	kept
maternal reflective functioning	maternal reflective functioning	maternal reflective functioning	maternal reflective functioning	maternal reflective functioning	1	kept
Maternal representations	maternal representations	maternal representations	maternal representations	Maternal representations	1	kept
maternal request	maternal request	maternal request	maternal request	maternal request	1	kept
Maternal role	maternal role	maternal role	maternal role	Maternal role	1	kept
Maternal role adaptation	maternal role adaptation	maternal role adaptation	maternal role adaptation	Maternal role adaptation	1	kept
Maternal role attainment	maternal role attainment	maternal role attainment	maternal role attainment	maternal role attainment	1	case_or_format
Maternal role development	maternal role development	maternal role development	maternal role development	Maternal role development	1	kept
Maternal satisfaction	maternal satisfaction	maternal satisfaction	maternal satisfaction	maternal satisfaction	1	case_or_format
Maternal self-esteem	maternal self esteem	maternal self esteem	maternal self esteem	Maternal self-esteem	1	kept
Maternal sense of competence	maternal sense of competence	maternal sense of competence	maternal sense of competence	Maternal sense of competence	1	kept
MATERNAL SEPARATION	maternal separation	maternal separation	maternal separation	MATERNAL SEPARATION	1	kept
Maternal Sepsis	maternal sepsis	maternal sepsis	maternal sepsis	Maternal Sepsis	1	kept
maternal sepsis	maternal sepsis	maternal sepsis	maternal sepsis	Maternal Sepsis	1	case_or_format
maternal singing	maternal singing	maternal singing	maternal singing	maternal singing	1	kept

maternal sound	maternal sound	maternal sound	maternal sound	maternal sound	1	kept
MATERNAL SPEECH	maternal speech	maternal speech	maternal speech	MATERNAL SPEECH	1	kept
MATERNAL STRESS	maternal stress	maternal stress	maternal stress	maternal stress	1	case_or_format
maternal transfer	maternal transfer	maternal transfer	maternal transfer	maternal transfer	1	kept
Maternal vascular malperfusion	maternal vascular malperfusion	maternal vascular malperfusion	maternal vascular malperfusion	Maternal vascular malperfusion	1	kept
MATERNAL VOICE	maternal voice	maternal voice	maternal voice	Maternal voice	1	case_or_format
maternal voice	maternal voice	maternal voice	maternal voice	Maternal voice	1	case_or_format
Maternal voice and music therapy	maternal voice and music therapy	maternal voice and music therapy	maternal voice and music therapy	Maternal voice and music therapy	1	kept
Maternal voice intervention	maternal voice intervention	maternal voice intervention	maternal voice intervention	Maternal voice intervention	1	kept
Maternal voice intervention for preterm infants	maternal voice intervention for preterm infants	maternal voice intervention for preterm infants	maternal voice intervention for preterm infants	Maternal voice intervention for preterm infants	1	kept
Maternal voice recognition	maternal voice recognition	maternal voice recognition	maternal voice recognition	Maternal voice recognition	1	kept
maternal well being	maternal well being	maternal well being	maternal well being	maternal well being	1	kept
Maternal-Acute Illness Management	maternal acute illness management	maternal acute illness management	maternal acute illness management	Maternal-Acute Illness Management	1	kept
MATERNAL-CARE	maternal care	maternal care	maternal care	maternal care	1	case_or_format
Maternal-child	maternal child	maternal child	maternal child	Maternal-child	1	kept
maternal-child health	maternal child health	maternal child health	maternal child health	maternal-child health	1	kept

Maternal-child relations	maternal child relations	maternal child relations	maternal child relations	Maternal-child relations	1	kept
Maternal-Fetal Exchange	maternal fetal exchange	maternal fetal exchange	maternal fetal exchange	Maternal-Fetal Exchange	1	kept
Maternal-Fetal Relations	maternal fetal relations	maternal fetal relations	maternal fetal relations	Maternal-Fetal Relations	1	kept
Maternal-fetal relations	maternal fetal relations	maternal fetal relations	maternal fetal relations	Maternal-Fetal Relations	1	case_or_format
Maternal-infant attachment	maternal infant attachment	maternal infant attachment	maternal infant attachment	Maternal-infant attachment	1	kept
maternal-infant separation	maternal infant separation	maternal infant separation	maternal infant separation	maternal-infant separation	1	kept
maternal‐infant health units	maternal infant health units	maternal infant health units	maternal infant health units	maternal‐infant health units	1	kept
Maternal‐newborn bonding	maternal newborn bonding	maternal newborn bonding	maternal newborn bonding	Maternal‐newborn bonding	1	kept
maternity	maternity	maternity	maternity	Maternity	1	case_or_format
maternity and neonatal hospital wards	maternity and neonatal hospital wards	maternity and neonatal hospital wards	maternity and neonatal hospital wards	maternity and neonatal hospital wards	1	kept
MATERNITY CARE	maternity care	maternity care	maternity care	Maternity care	1	case_or_format
Maternity Care	maternity care	maternity care	maternity care	Maternity care	1	case_or_format
maternity care models	maternity care models	maternity care models	maternity care models	maternity care models	1	kept
Maternity hospital	maternity hospital	maternity hospital	maternity hospital	Maternity hospital	1	kept
Maternity Hospitals	maternity hospitals	maternity hospitals	maternity hospital	Maternity hospital	1	singular
Maternity Model of Care	maternity model of care	maternity model of care	maternity model of care	Maternity Model of Care	1	kept

Maternity protection	maternity protection	maternity protection	maternity protection	Maternity protection	1	kept
maternity units	maternity units	maternity units	maternity units	maternity units	1	kept
maternity workload	maternity workload	maternity workload	maternity workload	maternity workload	1	kept
Mathematical	mathematical	mathematical	mathematical	Mathematical	1	kept
Mathematical Computing	mathematical computing	mathematical computing	mathematical computing	Mathematical Computing	1	kept
mathematical computing	mathematical computing	mathematical computing	mathematical computing	Mathematical Computing	1	case_or_format
mathematical modeling	mathematical modeling	mathematical modeling	mathematical modeling	mathematical modeling	1	kept
Mathematical modelling	mathematical modelling	mathematical modelling	mathematical modelling	Mathematical modelling	1	kept
Mathematical program	mathematical program	mathematical program	mathematical program	Mathematical program	1	kept
Matrix completion problems	matrix completion problems	matrix completion problems	matrix completion problems	Matrix completion problems	1	kept
Matrix Metalloproteinase 8	matrix metalloproteinase 8	matrix metalloproteinase 8	matrix metalloproteinase 8	Matrix Metalloproteinase 8	1	kept
Matrix Metalloproteinase 9	matrix metalloproteinase 9	matrix metalloproteinase 9	matrix metalloproteinase 9	Matrix Metalloproteinase 9	1	kept
MATTER	matter	matter	matter	MATTER	1	kept
Mattress	mattress	mattress	mattress	Mattress	1	kept
mattress	mattress	mattress	mattress	Mattress	1	case_or_format
mattresses	mattresses	mattresses	mattresses	mattresses	1	kept

maturation	maturation	maturation	maturation	maturation	1	kept
mature care	mature care	mature care	mature care	mature care	1	kept
Maugeri Foundation Respiratory Failure questionnaire	maugeri foundation respiratory failure questionnaire	maugeri foundation respiratory failure questionnaire	maugeri foundation respiratory failure questionnaire	Maugeri Foundation Respiratory Failure questionnaire	1	kept
maxilla resection	maxilla resection	maxilla resection	maxilla resection	maxilla resection	1	kept
maxillary sinus	maxillary sinus	maxillary sinus	maxillary sinus	maxillary sinus	1	kept
maxillofacial disorder	maxillofacial disorder	maxillofacial disorder	maxillofacial disorder	maxillofacial disorder	1	kept
maxillofacial injury	maxillofacial injury	maxillofacial injury	maxillofacial injury	maxillofacial injury	1	kept
Maximal barrier precaution	maximal barrier precaution	maximal barrier precaution	maximal barrier precaution	Maximal barrier precaution	1	kept
Maximization	maximization	maximization	maximization	Maximization	1	kept
maximum inspiratory diaphragm activity	maximum inspiratory diaphragm activity	maximum inspiratory diaphragm activity	maximum inspiratory diaphragm activity	maximum inspiratory diaphragm activity	1	kept
Maximum inspiratory pressure	maximum inspiratory pressure	maximum inspiratory pressure	maximum inspiratory pressure	Maximum inspiratory pressure	1	kept
maximum noise level	maximum noise level	maximum noise level	maximum noise level	maximum noise level	1	kept
Maximum noise levels	maximum noise levels	maximum noise levels	maximum noise level	maximum noise level	1	singular
Mayer-Rokitansky-Kuster-Hauser	mayer rokitansky kuster hauser	mayer rokitansky kuster hauser	mayer rokitansky kuster hauser	Mayer-Rokitansky-Kuster-Hauser	1	kept
Mayo delirium prediction	mayo delirium prediction	mayo delirium prediction	mayo delirium prediction	Mayo delirium prediction	1	kept
Mayo High Performance Teamwork Scale	mayo high performance teamwork scale	mayo high performance teamwork scale	mayo high performance teamwork scale	Mayo High Performance Teamwork Scale	1	kept

Mazandaran	mazandaran	mazandaran	mazandaran	Mazandaran	1	kept
mbarara surgical services quality assurance database	mbarara surgical services quality assurance database	mbarara surgical services quality assurance database	mbarara surgical services quality assurance database	mbarara surgical services quality assurance database	1	kept
MBI	mbi	mbi	mbi	MBI	1	kept
MBI-HSS	mbi hss	mbi hss	mbi hss	MBI-HSS	1	kept
McCabe score	mccabe score	mccabe score	mccabe score	McCabe score	1	kept
McFadden	mcfadden	mcfadden	mcfadden	McFadden	1	kept
McGill pain questionnaire	mcgill pain questionnaire	mcgill pain questionnaire	mcgill pain questionnaire	McGill Pain Questionnaire	1	case_or_format
McGrath MAC video laryngoscope	mcgrath mac video laryngoscope	mcgrath mac video laryngoscope	mcgrath mac video laryngoscope	McGrath MAC video laryngoscope	1	kept
MCI due to Alzheimer's disease	mci due to alzheimer s disease	mci due to alzheimer s disease	mci due to alzheimer s disease	MCI due to Alzheimer's disease	1	kept
McMaster FAD	mcmaster fad	mcmaster fad	mcmaster fad	McMaster FAD	1	kept
McNemar's test	mcnemar s test	mcnemar s test	mcnemar s test	McNemar's test	1	kept
MCS	mcs	mcs	mcs	MCS	1	kept
MCSDs	mcsds	mcsds	mcsds	MCSDs	1	kept
MD, mean difference	md mean difference	md mean difference	md mean difference	MD, mean difference	1	kept
MDR pathogen	mdr pathogen	mdr pathogen	mdr pathogen	MDR pathogen	1	kept
MDR pathogens	mdr pathogens	mdr pathogens	mdr pathogen	MDR pathogen	1	singular

MDRO	mdro	mdro	mdro	MDROs	1	case_or_format
mdro	mdro	mdro	mdro	MDROs	1	case_or_format
MDS-AC	mds ac	mds ac	mds ac	MDS-AC	1	kept
meal service	meal service	meal service	meal service	meal service	1	kept
MEALS	meals	meals	meal	meal	1	singular
mealtime	mealtime	mealtime	mealtime	mealtime	1	kept
Mean absolute error	mean absolute error	mean absolute error	mean absolute error	Mean absolute error	1	kept
mean absolute error	mean absolute error	mean absolute error	mean absolute error	Mean absolute error	1	case_or_format
mean airway pressure	mean airway pressure	mean airway pressure	mean airway pressure	mean airway pressure	1	kept
mean corpuscular hemoglobin	mean corpuscular hemoglobin	mean corpuscular hemoglobin	mean corpuscular hemoglobin	mean corpuscular hemoglobin	1	kept
mean injury severity score	mean injury severity score	mean injury severity score	mean injury severity score	mean injury severity score	1	kept
Mean percentage	mean percentage	mean percentage	mean percentage	Mean percentage	1	kept
Mean square error	mean square error	mean square error	mean square error	Mean square error	1	kept
mean transit time	mean transit time	mean transit time	mean transit time	mean transit time	1	kept
Meaning and joy	meaning and joy	meaning and joy	meaning and joy	Meaning and joy	1	kept
Meaning in life	meaning in life	meaning in life	meaning in life	meaning in life	1	case_or_format

Meaning of life	meaning of life	meaning of life	meaning of life	Meaning of life	1	kept
MEANINGFUL USE	meaningful use	meaningful use	meaningful use	Meaningful Use	1	case_or_format
Measles Vaccine	measles vaccine	measles vaccine	measles vaccine	measles vaccine	1	case_or_format
MEASURABLE OUTCOMES	measurable outcomes	measurable outcomes	measurable outcomes	MEASURABLE OUTCOMES	1	kept
Measure of collaboration	measure of collaboration	measure of collaboration	measure of collaboration	Measure of collaboration	1	kept
measure of processes of care for service provider tool	measure of processes of care for service provider tool	measure of processes of care for service provider tool	measure of processes of care for service provider tool	measure of processes of care for service provider tool	1	kept
Measured parameters	measured parameters	measured parameters	measured parameters	Measured parameters	1	kept
Measurement equivalence	measurement equivalence	measurement equivalence	measurement equivalence	Measurement equivalence	1	kept
Measurement frequency	measurement frequency	measurement frequency	measurement frequency	Measurement frequency	1	kept
Measurement instruments	measurement instruments	measurement instruments	measurement instrument	Measurement instrument	1	singular
Measurement of compliance	measurement of compliance	measurement of compliance	measurement of compliance	Measurement of compliance	1	kept
Measurement points	measurement points	measurement points	measurement points	Measurement points	1	kept
Measurement properties	measurement properties	measurement properties	measurement properties	Measurement properties	1	kept
Measurement scale	measurement scale	measurement scale	measurement scale	Measurement scale	1	kept
measurement scale	measurement scale	measurement scale	measurement scale	Measurement scale	1	case_or_format
measurement tool	measurement tool	measurement tool	measurement tool	measurement tool	1	kept

measurement tools	measurement tools	measurement tools	measurement tool	measurement tool	1	singular
measures	measures	measures	measures	measures	1	kept
Measures of Association, Exposure, Risk or Outcome	measures of association exposure risk or outcome	measures of association exposure risk or outcome	measures of association exposure risk or outcome	Measures of Association, Exposure, Risk or Outcome	1	kept
Measures of improvement	measures of improvement	measures of improvement	measures of improvement	Measures of improvement	1	kept
Measuring health care costs	measuring health care costs	measuring health care costs	measuring health care costs	Measuring health care costs	1	kept
Measuring instruments	measuring instruments	measuring instruments	measuring instruments	Measuring instruments	1	kept
Measuring points	measuring points	measuring points	measuring points	Measuring points	1	kept
Measuring sedation	measuring sedation	measuring sedation	measuring sedation	Measuring sedation	1	kept
Measuring systems	measuring systems	measuring systems	measuring systems	Measuring systems	1	kept
Meat	meat	meat	meat	Meat	1	kept
meat	meat	meat	meat	Meat	1	case_or_format
meatal pressure injury	meatal pressure injury	meatal pressure injury	meatal pressure injury	meatal pressure injury	1	kept
mecA gene	meca gene	meca gene	meca gene	MecA gene	1	case_or_format
Mechanic ventilation	mechanic ventilation	mechanic ventilation	mechanic ventilation	Mechanic ventilation	1	kept
mechanic ventilation	mechanic ventilation	mechanic ventilation	mechanic ventilation	Mechanic ventilation	1	case_or_format
Mechanical barrier	mechanical barrier	mechanical barrier	mechanical barrier	Mechanical barrier	1	kept

mechanical bladder	mechanical bladder	mechanical bladder	mechanical bladder	mechanical bladder	1	kept
mechanical cardiopulmonary resuscitation	mechanical cardiopulmonary resuscitation	mechanical cardiopulmonary resuscitation	mechanical cardiopulmonary resuscitation	mechanical cardiopulmonary resuscitation	1	kept
mechanical chest compressor	mechanical chest compressor	mechanical chest compressor	mechanical chest compressor	mechanical chest compressor	1	kept
Mechanical circulatory assist devices	mechanical circulatory assist devices	mechanical circulatory assist devices	mechanical circulatory assist devices	Mechanical circulatory assist devices	1	kept
Mechanical Circulatory Support Cardiopulmonary	mechanical circulatory support cardiopulmonary	mechanical circulatory support cardiopulmonary	mechanical circulatory support cardiopulmonary	Mechanical Circulatory Support Cardiopulmonary	1	kept
mechanical circulatory support devices	mechanical circulatory support devices	mechanical circulatory support devices	mechanical circulatory support devices	mechanical circulatory support devices	1	kept
mechanical compliance	mechanical compliance	mechanical compliance	mechanical compliance	mechanical compliance	1	kept
Mechanical complications	mechanical complications	mechanical complications	mechanical complications	Mechanical complications	1	kept
Mechanical containment	mechanical containment	mechanical containment	mechanical containment	Mechanical containment	1	kept
mechanical debridement	mechanical debridement	mechanical debridement	mechanical debridement	mechanical debridement	1	kept
Mechanical device	mechanical device	mechanical device	mechanical device	Mechanical device	1	kept
Mechanical expression	mechanical expression	mechanical expression	mechanical expression	Mechanical expression	1	kept
mechanical heart valve prosthesis	mechanical heart valve prosthesis	mechanical heart valve prosthesis	mechanical heart valve prosthesis	mechanical heart valve prosthesis	1	kept
Mechanical in-exhaler	mechanical in exhaler	mechanical in exhaler	mechanical in exhaler	Mechanical in-exhaler	1	kept
mechanical insufflation exsufflation	mechanical insufflation exsufflation	mechanical insufflation exsufflation	mechanical insufflation exsufflation	mechanical insufflation exsufflation	1	kept
Mechanical insufflation-exsufflation	mechanical insufflation exsufflation	mechanical insufflation exsufflation	mechanical insufflation exsufflation	mechanical insufflation exsufflation	1	case_or_format

mechanical methods	mechanical methods	mechanical methods	mechanical methods	mechanical methods	1	kept
mechanical prevention	mechanical prevention	mechanical prevention	mechanical prevention	mechanical prevention	1	kept
mechanical prophylaxis	mechanical prophylaxis	mechanical prophylaxis	mechanical prophylaxis	mechanical prophylaxis	1	kept
mechanical respiratory failure	mechanical respiratory failure	mechanical respiratory failure	mechanical respiratory failure	mechanical respiratory failure	1	kept
mechanical stiffness	mechanical stiffness	mechanical stiffness	mechanical stiffness	mechanical stiffness	1	kept
mechanical stimulation	mechanical stimulation	mechanical stimulation	mechanical stimulation	mechanical stimulation	1	kept
MECHANICAL THROMBECTOMY	mechanical thrombectomy	mechanical thrombectomy	mechanical thrombectomy	mechanical thrombectomy	1	case_or_format
Mechanical Thrombolysis	mechanical thrombolysis	mechanical thrombolysis	mechanical thrombolysis	Mechanical Thrombolysis	1	kept
mechanical thrombolysis	mechanical thrombolysis	mechanical thrombolysis	mechanical thrombolysis	Mechanical Thrombolysis	1	case_or_format
mechanical ventilatioin	mechanical ventilatioin	mechanical ventilatioin	mechanical ventilatioin	mechanical ventilatioin	1	kept
Mechanical ventilation circuit	mechanical ventilation circuit	mechanical ventilation circuit	mechanical ventilation circuit	Mechanical ventilation circuit	1	kept
Mechanical ventilation liberation	mechanical ventilation liberation	mechanical ventilation liberation	mechanical ventilation liberation	Mechanical ventilation liberation	1	kept
mechanical ventilation outcomes	mechanical ventilation outcomes	mechanical ventilation outcomes	mechanical ventilation outcomes	mechanical ventilation outcomes	1	kept
mechanical ventilation position	mechanical ventilation position	mechanical ventilation position	mechanical ventilation position	mechanical ventilation position	1	kept
Mechanical ventilation time	mechanical ventilation time	mechanical ventilation time	mechanical ventilation time	Mechanical ventilation time	1	kept
Mechanical ventilation weaning	mechanical ventilation weaning	mechanical ventilation weaning	mechanical ventilation weaning	Mechanical ventilation weaning	1	kept

mechanical ventilation weaning	mechanical ventilation weaning	mechanical ventilation weaning	mechanical ventilation weaning	Mechanical ventilation weaning	1	case_or_format
Mechanical Ventilation Weaning Pedagogy	mechanical ventilation weaning pedagogy	mechanical ventilation weaning pedagogy	mechanical ventilation weaning pedagogy	Mechanical Ventilation Weaning Pedagogy	1	kept
mechanical ventilation withdrawal	mechanical ventilation withdrawal	mechanical ventilation withdrawal	mechanical ventilation withdrawal	mechanical ventilation withdrawal	1	kept
Mechanical Ventilatlator	mechanical ventilatlator	mechanical ventilatlator	mechanical ventilatlator	Mechanical Ventilatlator	1	kept
Mechanical ventilator weaning	mechanical ventilator weaning	mechanical ventilator weaning	mechanical ventilator weaning	Mechanical ventilator weaning	1	kept
Mechanical ventilatory support	mechanical ventilatory support	mechanical ventilatory support	mechanical ventilatory support	Mechanical ventilatory support	1	kept
mechanical ventilatory therapy	mechanical ventilatory therapy	mechanical ventilatory therapy	mechanical ventilatory therapy	mechanical ventilatory therapy	1	kept
Mechanically ventilated children	mechanically ventilated children	mechanically ventilated children	mechanically ventilated children	Mechanically ventilated children	1	kept
Mechanically ventilation	mechanically ventilation	mechanically ventilation	mechanically ventilation	Mechanically ventilation	1	kept
MECHANICS	mechanics	mechanics	mechanics	mechanics	1	case_or_format
Meconium-stained amniotic fluid	meconium stained amniotic fluid	meconium stained amniotic fluid	meconium stained amniotic fluid	Meconium-stained amniotic fluid	1	kept
Med	med	med	med	Med	1	kept
mederantil	mederantil	mederantil	mederantil	mederantil	1	kept
media	media	media	media	Media	1	case_or_format
Media Influence	media influence	media influence	media influence	Media Influence	1	kept
medial prefrontal cortex	medial prefrontal cortex	medial prefrontal cortex	medial prefrontal cortex	medial prefrontal cortex	1	kept

medial septal nucleus	medial septal nucleus	medial septal nucleus	medial septal nucleus	medial septal nucleus	1	kept
medial temporal lobe	medial temporal lobe	medial temporal lobe	medial temporal lobe	medial temporal lobe	1	kept
median	median	median	median	median	1	kept
median nerve	median nerve	median nerve	median nerve	median nerve	1	kept
median test	median test	median test	median test	median test	1	kept
Mediastinal Diseases	mediastinal diseases	mediastinal diseases	mediastinal diseases	Mediastinal Diseases	1	kept
Mediastinal Emphysema	mediastinal emphysema	mediastinal emphysema	mediastinal emphysema	Mediastinal Emphysema	1	kept
mediastinal hematoma	mediastinal hematoma	mediastinal hematoma	mediastinal hematoma	mediastinal hematoma	1	kept
mediastinal mass	mediastinal mass	mediastinal mass	mediastinal mass	mediastinal mass	1	kept
MEDIASTINITIS	mediastinitis	mediastinitis	mediastinitis	mediastinitis	1	case_or_format
mediastinoscopy	mediastinoscopy	mediastinoscopy	mediastinoscopy	mediastinoscopy	1	kept
mediastinum lymph node	mediastinum lymph node	mediastinum lymph node	mediastinum lymph node	mediastinum lymph node	1	kept
mediastinum mass	mediastinum mass	mediastinum mass	mediastinum mass	mediastinum mass	1	kept
Mediating	mediating	mediating	mediating	Mediating	1	kept
Mediating effect	mediating effect	mediating effect	mediating effect	mediating effect	1	case_or_format
MEDIATING ROLE	mediating role	mediating role	mediating role	Mediating role	1	case_or_format

Mediating roles	mediating roles	mediating roles	mediating role	Mediating role	1	singular
mediation effect	mediation effect	mediation effect	mediation effect	Mediation effect	1	case_or_format
medic	medic	medic	medic	medic	1	kept
Medicaid eligibility	medicaid eligibility	medicaid eligibility	medicaid eligibility	Medicaid eligibility	1	kept
Medicaid reimbursement	medicaid reimbursement	medicaid reimbursement	medicaid reimbursement	Medicaid reimbursement	1	kept
medical accident	medical accident	medical accident	medical accident	medical accident	1	kept
medical adhesive related injuries	medical adhesive related injuries	medical adhesive related injuries	medical adhesive related injuries	medical adhesive related injuries	1	kept
Medical adhesive skin injury	medical adhesive skin injury	medical adhesive skin injury	medical adhesive skin injury	Medical adhesive skin injury	1	kept
Medical Adhesive-Related Skin Injury	medical adhesive related skin injury	medical adhesive related skin injury	medical adhesive related skin injury	medical adhesive-related skin injury	1	case_or_format
Medical adhesives	medical adhesives	medical adhesives	medical adhesive	medical adhesive	1	singular
medical adhesive - related skin injuries	medical adhesive related skin injuries	medical adhesive related skin injuries	medical adhesive related skin injury	medical adhesive-related skin injury	1	singular
medical adhesive - related skin injury	medical adhesive related skin injury	medical adhesive related skin injury	medical adhesive related skin injury	medical adhesive-related skin injury	1	case_or_format
Medical advances	medical advances	medical advances	medical advances	Medical advances	1	kept
Medical alarm system	medical alarm system	medical alarm system	medical alarm system	Medical alarm system	1	kept
Medical alarm systems	medical alarm systems	medical alarm systems	medical alarm system	Medical alarm system	1	singular
medical alarms	medical alarms	medical alarms	medical alarms	medical alarms	1	kept

medical and legal practices	medical and legal practices	medical and legal practices	medical and legal practices	medical and legal practices	1	kept
Medical and surgical units	medical and surgical units	medical and surgical units	medical and surgical units	Medical and surgical units	1	kept
medical apparatus, equipment and supplies	medical apparatus equipment and supplies	medical apparatus equipment and supplies	medical apparatus equipment and supplies	medical apparatus, equipment and supplies	1	kept
Medical Assistance in Dying	medical assistance in dying	medical assistance in dying	medical assistance in dying	Medical Assistance in Dying	1	kept
Medical assistance in dying	medical assistance in dying	medical assistance in dying	medical assistance in dying	Medical Assistance in Dying	1	case_or_format
medical assistance in dying	medical assistance in dying	medical assistance in dying	medical assistance in dying	Medical Assistance in Dying	1	case_or_format
Medical associate profession	medical associate profession	medical associate profession	medical associate profession	Medical associate profession	1	kept
Medical big data	medical big data	medical big data	medical big data	Medical big data	1	kept
Medical care capacity	medical care capacity	medical care capacity	medical care capacity	Medical care capacity	1	kept
medical care costs	medical care costs	medical care costs	medical care costs	medical care costs	1	kept
Medical care providers	medical care providers	medical care providers	medical care providers	Medical care providers	1	kept
Medical Care Team	medical care team	medical care team	medical care team	Medical Care Team	1	kept
Medical care team	medical care team	medical care team	medical care team	Medical Care Team	1	case_or_format
medical case construction	medical case construction	medical case construction	medical case construction	medical case construction	1	kept
medical chart	medical chart	medical chart	medical chart	medical chart	1	kept
Medical City	medical city	medical city	medical city	Medical City	1	kept

Medical command	medical command	medical command	medical command	Medical command	1	kept
Medical command training	medical command training	medical command training	medical command training	Medical command training	1	kept
medical complexities	medical complexities	medical complexities	medical complexity	MEDICAL COMPLEXITY	1	singular
MEDICAL COMPLICATIONS	medical complications	medical complications	medical complications	MEDICAL COMPLICATIONS	1	kept
Medical continuing education	medical continuing education	medical continuing education	medical continuing education	Medical continuing education	1	kept
Medical Coordination	medical coordination	medical coordination	medical coordination	Medical Coordination	1	kept
medical cost	medical cost	medical cost	medical cost	Medical Costs	1	case_or_format
medical crisis	medical crisis	medical crisis	medical crisis	medical crisis	1	kept
Medical data mining	medical data mining	medical data mining	medical data mining	Medical data mining	1	kept
medical data mining	medical data mining	medical data mining	medical data mining	Medical data mining	1	case_or_format
Medical database	medical database	medical database	medical database	Medical database	1	kept
medical debt	medical debt	medical debt	medical debt	medical debt	1	kept
medical department	medical department	medical department	medical department	medical department	1	kept
Medical departments	medical departments	medical departments	medical department	medical department	1	singular
Medical Device	medical device	medical device	medical device	medical device	1	case_or_format
Medical device design	medical device design	medical device design	medical device design	Medical device design	1	kept

medical device design	medical device design	medical device design	medical device design	Medical device design	1	case_or_format
Medical device development	medical device development	medical device development	medical device development	Medical device development	1	kept
medical device development	medical device development	medical device development	medical device development	Medical device development	1	case_or_format
Medical device evaluation	medical device evaluation	medical device evaluation	medical device evaluation	Medical device evaluation	1	kept
medical device regulation	medical device regulation	medical device regulation	medical device regulation	medical device regulation	1	kept
medical device related decubitus	medical device related decubitus	medical device related decubitus	medical device related decubitus	medical device related decubitus	1	kept
Medical device related pressure injuries	medical device related pressure injuries	medical device related pressure injuries	medical device related pressure injury	medical device-related pressure injury	1	singular
medical device size	medical device size	medical device size	medical device size	medical device size	1	kept
Medical device usability	medical device usability	medical device usability	medical device usability	Medical device usability	1	kept
medical device weight	medical device weight	medical device weight	medical device weight	medical device weight	1	kept
medical device-related pressure injuries	medical device related pressure injuries	medical device related pressure injuries	medical device related pressure injury	medical device-related pressure injury	1	singular
Medical device-related pressure ulcers	medical device related pressure ulcers	medical device related pressure ulcers	medical device related pressure ulcer	medical device related pressure ulcer	1	singular
Medical devices related injuries	medical devices related injuries	medical devices related injuries	medical devices related injuries	Medical devices related injuries	1	kept
medical device - related pressure injuries	medical device related pressure injuries	medical device related pressure injuries	medical device related pressure injury	medical device-related pressure injury	1	singular
medical device - related pressure injury	medical device related pressure injury	medical device related pressure injury	medical device related pressure injury	medical device-related pressure injury	1	case_or_format
MEDICAL DIAGNOSTIC KNOWLEDGE	medical diagnostic knowledge	medical diagnostic knowledge	medical diagnostic knowledge	MEDICAL DIAGNOSTIC KNOWLEDGE	1	kept

Medical direction	medical direction	medical direction	medical direction	Medical direction	1	kept
MEDICAL DIRECTOR	medical director	medical director	medical director	medical director	1	case_or_format
Medical directors	medical directors	medical directors	medical director	medical director	1	singular
Medical disaster	medical disaster	medical disaster	medical disaster	Medical disaster	1	kept
medical dispute	medical dispute	medical dispute	medical dispute	medical dispute	1	kept
Medical documentation	medical documentation	medical documentation	medical documentation	medical documentation	1	case_or_format
Medical documents	medical documents	medical documents	medical documents	Medical documents	1	kept
medical documents analysis	medical documents analysis	medical documents analysis	medical documents analysis	medical documents analysis	1	kept
Medical domains	medical domains	medical domains	medical domains	Medical domains	1	kept
Medical education & training	medical education and training	medical education and training	medical education and training	medical education & training	1	case_or_format
medical education and training	medical education and training	medical education and training	medical education and training	medical education & training	1	case_or_format
Medical Education Research Study Quality Instrument	medical education research study quality instrument	medical education research study quality instrument	medical education research study quality instrument	Medical Education Research Study Quality Instrument	1	kept
Medical education-graduate	medical education graduate	medical education graduate	medical education graduate	Medical education-graduate	1	kept
Medical electrical equipment	medical electrical equipment	medical electrical equipment	medical electrical equipment	Medical electrical equipment	1	kept
Medical embedded system	medical embedded system	medical embedded system	medical embedded system	Medical embedded system	1	kept
Medical emer-gency team	medical emer gency team	medical emer gency team	medical emer gency team	Medical emer-gency team	1	kept

Medical Emergencies	medical emergencies	medical emergencies	medical emergency	Medical emergency	1	singular
medical emergencies	medical emergencies	medical emergencies	medical emergency	Medical emergency	1	singular
Medical emergency response team	medical emergency response team	medical emergency response team	medical emergency response team	Medical emergency response team	1	kept
medical emergency treatment	medical emergency treatment	medical emergency treatment	medical emergency treatment	medical emergency treatment	1	kept
MEDICAL ENCOUNTERS	medical encounters	medical encounters	medical encounters	MEDICAL ENCOUNTERS	1	kept
Medical engineering	medical engineering	medical engineering	medical engineering	Medical engineering	1	kept
medical equipment	medical equipment	medical equipment	medical equipment	Medical equipment	1	case_or_format
Medical equipment alarms	medical equipment alarms	medical equipment alarms	medical equipment alarms	Medical equipment alarms	1	kept
medical error, measurement	medical error measurement	medical error measurement	medical error measurement	medical error, measurement	1	kept
MEDICAL ETHICS	medical ethics	medical ethics	medical ethics	medical ethics	1	case_or_format
Medical Ethics	medical ethics	medical ethics	medical ethics	medical ethics	1	case_or_format
Medical ethics education	medical ethics education	medical ethics education	medical ethics education	Medical ethics education	1	kept
Medical expenses	medical expenses	medical expenses	medical expenses	Medical expenses	1	kept
medical expenses	medical expenses	medical expenses	medical expenses	Medical expenses	1	case_or_format
Medical fields	medical fields	medical fields	medical fields	Medical fields	1	kept
medical gas	medical gas	medical gas	medical gas	medical gas	1	kept

medical geneticist	medical geneticist	medical geneticist	medical geneticist	medical geneticist	1	kept
MEDICAL GENETICS	medical genetics	medical genetics	medical genetics	MEDICAL GENETICS	1	kept
Medical High Dependency	medical high dependency	medical high dependency	medical high dependency	Medical High Dependency	1	kept
MEDICAL HOME	medical home	medical home	medical home	MEDICAL HOME	1	kept
Medical home	medical home	medical home	medical home	MEDICAL HOME	1	case_or_format
medical home	medical home	medical home	medical home	MEDICAL HOME	1	case_or_format
Medical illness	medical illness	medical illness	medical illness	Medical illness	1	kept
Medical Illustration	medical illustration	medical illustration	medical illustration	Medical Illustration	1	kept
medical illustration	medical illustration	medical illustration	medical illustration	Medical Illustration	1	case_or_format
medical image processing	medical image processing	medical image processing	medical image processing	medical image processing	1	kept
medical imaging technology	medical imaging technology	medical imaging technology	medical imaging technology	medical imaging technology	1	kept
Medical incidents	medical incidents	medical incidents	medical incidents	Medical incidents	1	kept
MEDICAL INFORMATION	medical information	medical information	medical information	medical information	1	case_or_format
Medical Information Network Distribution Service	medical information network distribution service	medical information network distribution service	medical information network distribution service	Medical Information Network Distribution Service	1	kept
Medical infrastructure and technology	medical infrastructure and technology	medical infrastructure and technology	medical infrastructure and technology	Medical infrastructure and technology	1	kept
medical innovations	medical innovations	medical innovations	medical innovations	medical innovations	1	kept

medical interpreter	medical interpreter	medical interpreter	medical interpreter	medical interpreter	1	kept
medical interpreter services	medical interpreter services	medical interpreter services	medical interpreter services	medical interpreter services	1	kept
Medical intervention	medical intervention	medical intervention	medical intervention	Medical intervention	1	kept
Medical knowledge	medical knowledge	medical knowledge	medical knowledge	Medical Knowledge	1	case_or_format
medical knowledge	medical knowledge	medical knowledge	medical knowledge	Medical Knowledge	1	case_or_format
Medical leaders	medical leaders	medical leaders	medical leaders	Medical leaders	1	kept
medical leadership	medical leadership	medical leadership	medical leadership	medical leadership	1	kept
medical line	medical line	medical line	medical line	medical line	1	kept
medical line entanglement	medical line entanglement	medical line entanglement	medical line entanglement	medical line entanglement	1	kept
Medical literatures	medical literatures	medical literatures	medical literature	medical literature	1	singular
Medical logic module	medical logic module	medical logic module	medical logic module	Medical logic module	1	kept
Medical Malpractice	medical malpractice	medical malpractice	medical malpractice	Medical malpractice	1	case_or_format
Medical management of blast injuries	medical management of blast injuries	medical management of blast injuries	medical management of blast injuries	Medical management of blast injuries	1	kept
Medical management of traumatic injuries	medical management of traumatic injuries	medical management of traumatic injuries	medical management of traumatic injuries	Medical management of traumatic injuries	1	kept
medical managers	medical managers	medical managers	medical managers	medical managers	1	kept
Medical Marijuana	medical marijuana	medical marijuana	medical marijuana	Medical Marijuana	1	kept

Medical mistakes	medical mistakes	medical mistakes	medical mistakes	Medical mistakes	1	kept
medical mobile device	medical mobile device	medical mobile device	medical mobile device	medical mobile device	1	kept
medical mobile devices	medical mobile devices	medical mobile devices	medical mobile device	medical mobile device	1	singular
medical nebulizer	medical nebulizer	medical nebulizer	medical nebulizer	medical nebulizer	1	kept
Medical necessity	medical necessity	medical necessity	medical necessity	Medical necessity	1	kept
Medical neglect	medical neglect	medical neglect	medical neglect	Medical neglect	1	kept
medical neglect	medical neglect	medical neglect	medical neglect	Medical neglect	1	case_or_format
Medical Negligence	medical negligence	medical negligence	medical negligence	Medical Negligence	1	kept
Medical negligence	medical negligence	medical negligence	medical negligence	Medical Negligence	1	case_or_format
medical office	medical office	medical office	medical office	medical office	1	kept
Medical oncology	medical oncology	medical oncology	medical oncology	Medical Oncology	1	case_or_format
Medical order entry system	medical order entry system	medical order entry system	medical order entry system	Medical Order Entry Systems	1	case_or_format
Medical outcome	medical outcome	medical outcome	medical outcome	Medical outcome	1	kept
Medical oversight	medical oversight	medical oversight	medical oversight	Medical oversight	1	kept
Medical overuse	medical overuse	medical overuse	medical overuse	Medical Overuse	1	case_or_format
Medical overuse quality improvement	medical overuse quality improvement	medical overuse quality improvement	medical overuse quality improvement	Medical overuse quality improvement	1	kept

medical physicist	medical physicist	medical physicist	medical physicist	medical physicist	1	kept
medical power	medical power	medical power	medical power	medical power	1	kept
Medical practice satisfaction	medical practice satisfaction	medical practice satisfaction	medical practice satisfaction	Medical practice satisfaction	1	kept
Medical practices	medical practices	medical practices	medical practice	medical practice	1	singular
Medical practises	medical practises	medical practises	medical practises	Medical practises	1	kept
Medical practitioner	medical practitioner	medical practitioner	medical practitioner	Medical practitioner	1	kept
medical problems in pregnancy	medical problems in pregnancy	medical problems in pregnancy	medical problems in pregnancy	medical problems in pregnancy	1	kept
MEDICAL PROGRESS	medical progress	medical progress	medical progress	MEDICAL PROGRESS	1	kept
medical psychology	medical psychology	medical psychology	medical psychology	medical psychology	1	kept
Medical quality	medical quality	medical quality	medical quality	Medical quality	1	kept
medical rapid response team	medical rapid response team	medical rapid response team	medical rapid response team	medical rapid response team	1	kept
Medical Receptionists	medical receptionists	medical receptionists	medical receptionist	medical receptionist	1	singular
MEDICAL RECORD	medical record	medical record	medical record	medical record	1	case_or_format
Medical record administration	medical record administration	medical record administration	medical record administration	Medical record administration	1	kept
medical record analysis	medical record analysis	medical record analysis	medical record analysis	medical record analysis	1	kept
medical record audit	medical record audit	medical record audit	medical record audit	medical record audit	1	kept

Medical record linkage	medical record linkage	medical record linkage	medical record linkage	Medical Record Linkage	1	case_or_format
Medical records software	medical records software	medical records software	medical records software	Medical records software	1	kept
Medical Records, Problem-Oriented	medical records problem oriented	medical records problem oriented	medical records problem oriented	Medical Records, Problem-Oriented	1	kept
medical rescue team	medical rescue team	medical rescue team	medical rescue team	medical rescue team	1	kept
Medical Research Council Dyspnea Scale	medical research council dyspnea scale	medical research council dyspnea scale	medical research council dyspnea scale	Medical Research Council Dyspnea Scale	1	kept
Medical Research Council Scale	medical research council scale	medical research council scale	medical research council scale	Medical Research Council Scale	1	kept
medical research council score	medical research council score	medical research council score	medical research council score	medical research council score	1	kept
Medical Research Council Sum Score	medical research council sum score	medical research council sum score	medical research council sum score	Medical Research Council Sum Score	1	kept
Medical residency	medical residency	medical residency	medical residency	medical residency	1	case_or_format
medical resident	medical resident	medical resident	medical resident	medical residents	1	case_or_format
medical resource utilization	medical resource utilization	medical resource utilization	medical resource utilization	Medical resource utilization	1	case_or_format
medical resources	medical resources	medical resources	medical resources	medical resources	1	kept
MEDICAL RESPONSE	medical response	medical response	medical response	MEDICAL RESPONSE	1	kept
medical resuscitation	medical resuscitation	medical resuscitation	medical resuscitation	medical resuscitation	1	kept
medical retina	medical retina	medical retina	medical retina	medical retina	1	kept
medical review	medical review	medical review	medical review	medical review	1	kept

Medical robotics	medical robotics	medical robotics	medical robotics	Medical robotics	1	kept
medical round	medical round	medical round	medical round	medical round	1	kept
medical rounds	medical rounds	medical rounds	medical round	medical round	1	singular
Medical safety	medical safety	medical safety	medical safety	Medical safety	1	kept
Medical Secretaries	medical secretaries	medical secretaries	medical secretary	medical secretary	1	singular
medical sensors	medical sensors	medical sensors	medical sensors	medical sensors	1	kept
Medical specialist	medical specialist	medical specialist	medical specialist	medical specialist	1	case_or_format
medical speciality	medical speciality	medical speciality	medical speciality	medical speciality	1	kept
Medical specialization	medical specialization	medical specialization	medical specialization	Medical specialization	1	kept
Medical Staff Privileges	medical staff privileges	medical staff privileges	medical staff privileges	Medical Staff Privileges	1	kept
medical staff questionnaire	medical staff questionnaire	medical staff questionnaire	medical staff questionnaire	medical staff questionnaire	1	kept
Medical staff, Hospital	medical staff hospital	medical staff hospital	medical staff hospital	Medical Staff, Hospital	1	case_or_format
Medical Staffers	medical staffers	medical staffers	medical staffers	Medical Staffers	1	kept
Medical staffing	medical staffing	medical staffing	medical staffing	medical staffing	1	case_or_format
Medical Student	medical student	medical student	medical student	medical student	1	case_or_format
Medical student and resident education	medical student and resident education	medical student and resident education	medical student and resident education	Medical student and resident education	1	kept

medical students	medical students	medical students	medical student	medical student	1	singular
medical team	medical team	medical team	medical team	medical team	1	kept
MEDICAL THERAPY	medical therapy	medical therapy	medical therapy	MEDICAL THERAPY	1	kept
medical transcription	medical transcription	medical transcription	medical transcription	medical transcription	1	kept
medical transport	medical transport	medical transport	medical transport	medical transport	1	kept
Medical treatment	medical treatment	medical treatment	medical treatment	Medical treatment	1	kept
medical uncertainty	medical uncertainty	medical uncertainty	medical uncertainty	medical uncertainty	1	kept
Medical units	medical units	medical units	medical units	medical units	1	case_or_format
Medical volunteerism	medical volunteerism	medical volunteerism	medical volunteerism	Medical volunteerism	1	kept
Medical ward	medical ward	medical ward	medical ward	medical ward	1	case_or_format
Medical wards	medical wards	medical wards	medical ward	medical ward	1	singular
Medical waste	medical waste	medical waste	medical waste	Medical Waste	1	case_or_format
medical waste	medical waste	medical waste	medical waste	Medical Waste	1	case_or_format
Medical worker	medical worker	medical worker	medical worker	Medical workers	1	case_or_format
Medical workforce	medical workforce	medical workforce	medical workforce	Medical workforce	1	kept
Medical Writing	medical writing	medical writing	medical writing	Medical Writing	1	kept

Medical-dental collaboration	medical dental collaboration	medical dental collaboration	medical dental collaboration	Medical-dental collaboration	1	kept
Medical-device related pressure ulcers	medical device related pressure ulcers	medical device related pressure ulcers	medical device related pressure ulcer	medical device related pressure ulcer	1	singular
Medical-device related skin damage	medical device related skin damage	medical device related skin damage	medical device related skin damage	Medical-device related skin damage	1	kept
medical-psychiatry units	medical psychiatry units	medical psychiatry units	medical psychiatry units	medical-psychiatry units	1	kept
MEDICAL-RECORD	medical record	medical record	medical record	medical record	1	case_or_format
MEDICAL-RECORDS	medical records	medical records	medical record	medical record	1	singular
MEDICAL-SERVICES	medical services	medical services	medical service	medical service	1	singular
medical-staff	medical staff	medical staff	medical staff	medical staff	1	case_or_format
medical-surgical	medical surgical	medical surgical	medical surgical	medical-surgical	1	kept
medical-surgical oncology	medical surgical oncology	medical surgical oncology	medical surgical oncology	medical-surgical oncology	1	kept
Medical-surgical unit	medical surgical unit	medical surgical unit	medical surgical unit	medical-surgical unit	1	case_or_format
Medical-surgical units	medical surgical units	medical surgical units	medical surgical unit	medical-surgical unit	1	singular
Medical-surgical ward	medical surgical ward	medical surgical ward	medical surgical ward	Medical-surgical ward	1	kept
Medical-surgical wards	medical surgical wards	medical surgical wards	medical surgical ward	Medical-surgical ward	1	singular
Medically complex	medically complex	medically complex	medically complex	Medically complex	1	kept
Medically complex child	medically complex child	medically complex child	medically complex child	Medically complex child	1	kept

medically complex infant	medically complex infant	medically complex infant	medically complex infant	medically complex infant	1	kept
Medically complex infants	medically complex infants	medically complex infants	medically complex infant	medically complex infant	1	singular
medically fragile children	medically fragile children	medically fragile children	medically fragile children	medically fragile children	1	kept
Medically fragile infants	medically fragile infants	medically fragile infants	medically fragile infant	medically fragile infant	1	singular
medically underserved	medically underserved	medically underserved	medically underserved	medically underserved	1	kept
Medically Unexplained Symptoms	medically unexplained symptoms	medically unexplained symptoms	medically unexplained symptoms	Medically Unexplained Symptoms	1	kept
MEDICARE	medicare	medicare	medicare	Medicare	1	case_or_format
Medicare Advantage	medicare advantage	medicare advantage	medicare advantage	Medicare Advantage	1	kept
Medicare advantage	medicare advantage	medicare advantage	medicare advantage	Medicare Advantage	1	case_or_format
Medicare Advantage plans	medicare advantage plans	medicare advantage plans	medicare advantage plans	Medicare Advantage plans	1	kept
medicare beneficiary	medicare beneficiary	medicare beneficiary	medicare beneficiary	medicare beneficiary	1	kept
Medicare Coordinated Care Demonstration	medicare coordinated care demonstration	medicare coordinated care demonstration	medicare coordinated care demonstration	Medicare Coordinated Care Demonstration	1	kept
Medicare expenditures	medicare expenditures	medicare expenditures	medicare expenditures	Medicare expenditures	1	kept
Medicare fee-for-service	medicare fee for service	medicare fee for service	medicare fee for service	Medicare fee-for-service	1	kept
MEDICARE MANAGED CARE	medicare managed care	medicare managed care	medicare managed care	MEDICARE MANAGED CARE	1	kept
Medicare occupancy	medicare occupancy	medicare occupancy	medicare occupancy	Medicare occupancy	1	kept

MEDICARE PAYMENT	medicare payment	medicare payment	medicare payment	MEDICARE PAYMENT	1	kept
Medicare Payment Advisory Commission	medicare payment advisory commission	medicare payment advisory commission	medicare payment advisory commission	Medicare Payment Advisory Commission	1	kept
Medicare policy	medicare policy	medicare policy	medicare policy	Medicare policy	1	kept
medication adherence assessment	medication adherence assessment	medication adherence assessment	medication adherence assessment	medication adherence assessment	1	kept
medication administration error	medication administration error	medication administration error	medication administration error	medication administration errors	1	case_or_format
Medication administration practices	medication administration practices	medication administration practices	medication administration practices	Medication administration practices	1	kept
Medication administration tasks	medication administration tasks	medication administration tasks	medication administration tasks	Medication administration tasks	1	kept
Medication administrations	medication administrations	medication administrations	medication administration	medication administration	1	singular
medication alert system	medication alert system	medication alert system	medication alert system	medication alert system	1	kept
Medication care	medication care	medication care	medication care	Medication care	1	kept
Medication class	medication class	medication class	medication class	Medication class	1	kept
MEDICATION COMMUNICATION	medication communication	medication communication	medication communication	MEDICATION COMMUNICATION	1	kept
Medication communication	medication communication	medication communication	medication communication	MEDICATION COMMUNICATION	1	case_or_format
medication communication	medication communication	medication communication	medication communication	MEDICATION COMMUNICATION	1	case_or_format
medication consolidation	medication consolidation	medication consolidation	medication consolidation	medication consolidation	1	kept
medication dead volume	medication dead volume	medication dead volume	medication dead volume	medication dead volume	1	kept

Medication delay	medication delay	medication delay	medication delay	Medication delay	1	kept
medication delay	medication delay	medication delay	medication delay	Medication delay	1	case_or_format
Medication delivery	medication delivery	medication delivery	medication delivery	Medication delivery	1	kept
medication dispensing process	medication dispensing process	medication dispensing process	medication dispensing process	medication dispensing process	1	kept
Medication distribution technology	medication distribution technology	medication distribution technology	medication distribution technology	Medication distribution technology	1	kept
MEDICATION ERROR	medication error	medication error	medication error	Medication Errors	1	case_or_format
Medication Error	medication error	medication error	medication error	Medication Errors	1	case_or_format
medication error checklist	medication error checklist	medication error checklist	medication error checklist	medication error checklist	1	kept
medication error prevalence rate	medication error prevalence rate	medication error prevalence rate	medication error prevalence rate	medication error prevalence rate	1	kept
medication error reporting	medication error reporting	medication error reporting	medication error reporting	medication error reporting	1	kept
medication errors, prevention and control	medication errors prevention and control	medication errors prevention and control	medication errors prevention and control	medication errors, prevention and control	1	kept
medication interaction	medication interaction	medication interaction	medication interaction	medication interaction	1	kept
medication knowledge	medication knowledge	medication knowledge	medication knowledge	medication knowledge	1	kept
Medication omission	medication omission	medication omission	medication omission	Medication omission	1	kept
Medication order set	medication order set	medication order set	medication order set	Medication order set	1	kept
medication preparation	medication preparation	medication preparation	medication preparation	medication preparation	1	kept

MEDICATION PRESCRIPTION ERRORS	medication prescription errors	medication prescription errors	medication prescription errors	MEDICATION PRESCRIPTION ERRORS	1	kept
Medication process	medication process	medication process	medication process	Medication process	1	kept
MEDICATION RECONCILIATION	medication reconciliation	medication reconciliation	medication reconciliation	Medication Reconciliation	1	case_or_format
Medication Review	medication review	medication review	medication review	Medication Review	1	kept
Medication review	medication review	medication review	medication review	Medication Review	1	case_or_format
medication review	medication review	medication review	medication review	Medication Review	1	case_or_format
medication room	medication room	medication room	medication room	medication room	1	kept
Medication Safety	medication safety	medication safety	medication safety	medication safety	1	case_or_format
medication shortage	medication shortage	medication shortage	medication shortage	medication shortage	1	kept
Medication supply	medication supply	medication supply	medication supply	Medication supply	1	kept
Medication systems in the hospital	medication systems in the hospital	medication systems in the hospital	medication systems in the hospital	Medication systems in the hospital	1	kept
MEDICATION SYSTEMS, HOSPITAL	medication systems hospital	medication systems hospital	medication systems hospital	Medication Systems, Hospital	1	case_or_format
MEDICATION THERAPY MANAGEMENT	medication therapy management	medication therapy management	medication therapy management	medication therapy management	1	case_or_format
medication training program	medication training program	medication training program	medication training program	medication training program	1	kept
medication usage	medication usage	medication usage	medication usage	medication usage	1	kept
medication use	medication use	medication use	medication use	medication use	1	kept

medication use process safety	medication use process safety	medication use process safety	medication use process safety	medication use process safety	1	kept
medication utilization	medication utilization	medication utilization	medication utilization	medication utilization	1	kept
Medication-errors	medication errors	medication errors	medication error	Medication Errors	1	singular
medication-related errors	medication related errors	medication related errors	medication related errors	medication-related errors	1	kept
medication-use system	medication use system	medication use system	medication use system	medication-use system	1	kept
MEDICATIONS	medications	medications	medication	Medication	1	singular
Medications to treat opioid use disorder	medications to treat opioid use disorder	medications to treat opioid use disorder	medications to treat opioid use disorder	Medications to treat opioid use disorder	1	kept
medicinal chemistry	medicinal chemistry	medicinal chemistry	medicinal chemistry	medicinal chemistry	1	kept
medicine administration	medicine administration	medicine administration	medicine administration	medicine administration	1	kept
Medicine and the Law	medicine and the law	medicine and the law	medicine and the law	Medicine and the Law	1	kept
MEDICINE CAM	medicine cam	medicine cam	medicine cam	MEDICINE CAM	1	kept
Medicine in literature	medicine in literature	medicine in literature	medicine in literature	Medicine in Literature	1	case_or_format
Medicine in the Arts	medicine in the arts	medicine in the arts	medicine in the arts	medicine in the arts	1	case_or_format
medicine modification	medicine modification	medicine modification	medicine modification	medicine modification	1	kept
Medicine preparation	medicine preparation	medicine preparation	medicine preparation	Medicine preparation	1	kept
MEDICINE SCCM	medicine sccm	medicine sccm	medicine sccm	MEDICINE SCCM	1	kept

Medicines	medicines	medicines	medicines	medicines	1	case_or_format
medico-legal	medico legal	medico legal	medico legal	medico-legal	1	kept
Medicolegal	medicolegal	medicolegal	medicolegal	medicolegal	1	case_or_format
Medidas de dolor	medidas de dolor	medidas de dolor	medidas de dolor	Medidas de dolor	1	kept
Medigoxin	medigoxin	medigoxin	medigoxin	Medigoxin	1	kept
MEDITATION	meditation	meditation	meditation	meditation	1	case_or_format
Mediterranean diet	mediterranean diet	mediterranean diet	mediterranean diet	Mediterranean diet	1	kept
Mediterranean Islands	mediterranean islands	mediterranean islands	mediterranean islands	Mediterranean Islands	1	kept
medium long term venous access	medium long term venous access	medium long term venous access	medium long term venous access	medium long term venous access	1	kept
medroxyprogesterone	medroxyprogesterone	medroxyprogesterone	medroxyprogesterone	medroxyprogesterone	1	kept
Medtronic CoreValve	medtronic corevalve	medtronic corevalve	medtronic corevalve	Medtronic CoreValve	1	kept
Medtronic Sentrino<sup>®<	medtronic sentrino sup	medtronic sentrino sup	medtronic sentrino sup	Medtronic Sentrino<sup>®<	1	kept
Medtronic Sentrino®	medtronic sentrino	medtronic sentrino	medtronic sentrino	Medtronic Sentrino®	1	kept
medulloblastoma	medulloblastoma	medulloblastoma	medulloblastoma	medulloblastoma	1	kept
Meeting	meeting	meeting	meeting	Meeting	1	kept
Meeting family needs	meeting family needs	meeting family needs	meeting family needs	Meeting family needs	1	kept

MEETINGS	meetings	meetings	meeting	Meeting	1	singular
Megacode simulation	megacode simulation	megacode simulation	megacode simulation	Megacode simulation	1	kept
megacolon	megacolon	megacolon	megacolon	megacolon	1	kept
megaesophagus	megaesophagus	megaesophagus	megaesophagus	megaesophagus	1	kept
megakaryocyte	megakaryocyte	megakaryocyte	megakaryocyte	megakaryocyte	1	kept
megestrol	megestrol	megestrol	megestrol	megestrol	1	kept
Megestrol Acetate	megestrol acetate	megestrol acetate	megestrol acetate	Megestrol Acetate	1	kept
meglumine diatrizoate plus sodium diatrizoate	meglumine diatrizoate plus sodium diatrizoate	meglumine diatrizoate plus sodium diatrizoate	meglumine diatrizoate plus sodium diatrizoate	meglumine diatrizoate plus sodium diatrizoate	1	kept
Meister's questionnaire	meister s questionnaire	meister s questionnaire	meister s questionnaire	Meister's questionnaire	1	kept
Melaleuca	melaleuca	melaleuca	melaleuca	Melaleuca	1	kept
Melaleuca alternifolia	melaleuca alternifolia	melaleuca alternifolia	melaleuca alternifolia	Melaleuca alternifolia	1	kept
melanocytosis	melanocytosis	melanocytosis	melanocytosis	melanocytosis	1	kept
MELANOPSIN	melanopsin	melanopsin	melanopsin	MELANOPSIN	1	kept
MELAS	melas	melas	melas	MELAS	1	kept
MELAS syndrome	melas syndrome	melas syndrome	melas syndrome	MELAS syndrome	1	kept
MELATONIN	melatonin	melatonin	melatonin	melatonin	1	case_or_format

melatonin receptor agonist	melatonin receptor agonist	melatonin receptor agonist	melatonin receptor agonist	melatonin receptor agonist	1	kept
MELATONIN SECRETION	melatonin secretion	melatonin secretion	melatonin secretion	MELATONIN SECRETION	1	kept
MELD SCORE	meld score	meld score	meld score	MELD SCORE	1	kept
MELD score	meld score	meld score	meld score	MELD SCORE	1	case_or_format
Meleis	meleis	meleis	meleis	Meleis	1	kept
Meleis Transformation Theory	meleis transformation theory	meleis transformation theory	meleis transformation theory	Meleis Transformation Theory	1	kept
Meleis transition theory	meleis transition theory	meleis transition theory	meleis transition theory	Meleis transition theory	1	kept
Melhoria de Qualidade	melhoria de qualidade	melhoria de qualidade	melhoria de qualidade	Melhoria de Qualidade	1	kept
Mellitus	mellitus	mellitus	mellitus	Mellitus	1	kept
Melodic alarms	melodic alarms	melodic alarms	melodic alarms	Melodic alarms	1	kept
melon	melon	melon	melon	melon	1	kept
melperone	melperone	melperone	melperone	melperone	1	kept
member	member	member	member	MEMBERS	1	case_or_format
MEMBER EXCHANGE	member exchange	member exchange	member exchange	MEMBER EXCHANGE	1	kept
Member involvement	member involvement	member involvement	member involvement	Member involvement	1	kept
membrane filter	membrane filter	membrane filter	membrane filter	membrane filter	1	kept

membrane microparticle	membrane microparticle	membrane microparticle	membrane microparticle	membrane microparticle	1	kept
membrane protein	membrane protein	membrane protein	membrane protein	membrane protein	1	kept
membrane rupture	membrane rupture	membrane rupture	membrane rupture	membrane rupture	1	kept
Memories	memories	memories	memory	memory	1	singular
MEMORY	memory	memory	memory	memory	1	case_or_format
Memory and Executive Screening	memory and executive screening	memory and executive screening	memory and executive screening	Memory and Executive Screening	1	kept
memory assessment	memory assessment	memory assessment	memory assessment	memory assessment	1	kept
memory box	memory box	memory box	memory box	memory box	1	kept
memory care	memory care	memory care	memory care	memory care	1	kept
memory care unit	memory care unit	memory care unit	memory care unit	memory care unit	1	kept
memory gaps	memory gaps	memory gaps	memory gaps	memory gaps	1	kept
memory T lymphocyte	memory t lymphocyte	memory t lymphocyte	memory t lymphocyte	memory T lymphocyte	1	kept
memory test	memory test	memory test	memory test	memory test	1	kept
Memory, Episodic	memory episodic	memory episodic	memory episodic	Memory, Episodic	1	kept
Memory, Short-Term	memory short term	memory short term	memory short term	Memory, Short-Term	1	kept
MEMS microphone	mems microphone	mems microphone	mems microphone	MEMS microphones	1	case_or_format

men	men	men	men	Men	1	case_or_format
Men's Health	men s health	men s health	men s health	Men's Health	1	kept
men's health	men s health	men s health	men s health	Men's Health	1	case_or_format
MEN2B syndrome	men2b syndrome	men2b syndrome	men2b syndrome	MEN2B syndrome	1	kept
menadione	menadione	menadione	menadione	menadione	1	kept
menadol	menadol	menadol	menadol	menadol	1	kept
Mendelian randomization	mendelian randomization	mendelian randomization	mendelian randomization	Mendelian randomization	1	kept
Mendelian Randomization Analysis	mendelian randomization analysis	mendelian randomization analysis	mendelian randomization analysis	Mendelian randomization analysis	1	case_or_format
Mendelsohn maneuver	mendelsohn maneuver	mendelsohn maneuver	mendelsohn maneuver	Mendelsohn maneuver	1	kept
meningeal melanocytoma	meningeal melanocytoma	meningeal melanocytoma	meningeal melanocytoma	meningeal melanocytoma	1	kept
Meningeal Neoplasms	meningeal neoplasms	meningeal neoplasms	meningeal neoplasms	Meningeal Neoplasms	1	kept
meningitis vaccine	meningitis vaccine	meningitis vaccine	meningitis vaccine	meningitis vaccine	1	kept
Meningitis, Pneumococcal	meningitis pneumococcal	meningitis pneumococcal	meningitis pneumococcal	Meningitis, Pneumococcal	1	kept
Meningitis, Viral	meningitis viral	meningitis viral	meningitis viral	Meningitis, Viral	1	kept
Meningococcal meningitis	meningococcal meningitis	meningococcal meningitis	meningococcal meningitis	Meningococcal meningitis	1	kept
Meningococcus vaccine	meningococcus vaccine	meningococcus vaccine	meningococcus vaccine	Meningococcus vaccine	1	kept

meningomyelocele	meningomyelocele	meningomyelocele	meningomyelocele	Meningomyelocele	1	case_or_format
Menores	menores	menores	menores	Menores	1	kept
Menstrual Cycle	menstrual cycle	menstrual cycle	menstrual cycle	menstrual cycle	1	case_or_format
mental	mental	mental	mental	mental	1	kept
mental compliance	mental compliance	mental compliance	mental compliance	mental compliance	1	kept
Mental Component Score	mental component score	mental component score	mental component score	Mental Component Score	1	kept
mental component summary	mental component summary	mental component summary	mental component summary	mental component summary	1	kept
mental concentration	mental concentration	mental concentration	mental concentration	mental concentration	1	kept
MENTAL DISORDER	mental disorder	mental disorder	mental disorder	Mental Disorders	1	case_or_format
Mental Disorder	mental disorder	mental disorder	mental disorder	Mental Disorders	1	case_or_format
Mental disorder	mental disorder	mental disorder	mental disorder	Mental Disorders	1	case_or_format
Mental disorders [therapy]	mental disorders therapy	mental disorders therapy	mental disorders therapy	Mental disorders [therapy]	1	kept
mental distress	mental distress	mental distress	mental distress	mental distress	1	kept
Mental Healing	mental healing	mental healing	mental healing	Mental Healing	1	kept
mental health and psychiatry	mental health and psychiatry	mental health and psychiatry	mental health and psychiatry	mental health and psychiatry	1	kept
Mental Health Care	mental health care	mental health care	mental health care	mental health care	1	case_or_format

mental health impact	mental health impact	mental health impact	mental health impact	mental health impact	1	kept
mental health inventory	mental health inventory	mental health inventory	mental health inventory	mental health inventory	1	kept
mental health issues	mental health issues	mental health issues	mental health issues	mental health issues	1	kept
Mental health outcome	mental health outcome	mental health outcome	mental health outcome	Mental health outcomes	1	case_or_format
mental health problems	mental health problems	mental health problems	mental health problem	mental health problem	1	singular
mental health services	mental health services	mental health services	mental health service	mental health service	1	singular
Mental health sleep quality	mental health sleep quality	mental health sleep quality	mental health sleep quality	Mental health sleep quality	1	kept
Mental health survey	mental health survey	mental health survey	mental health survey	Mental health survey	1	kept
mental healthcare workers	mental healthcare workers	mental healthcare workers	mental healthcare workers	mental healthcare workers	1	kept
mental illness	mental illness	mental illness	mental illness	Mental illness	1	case_or_format
mental impairment	mental impairment	mental impairment	mental impairment	mental impairment	1	kept
mental irritation	mental irritation	mental irritation	mental irritation	mental irritation	1	kept
Mental loads	mental loads	mental loads	mental load	mental load	1	singular
Mental model	mental model	mental model	mental model	Mental models	1	case_or_format
mental overstimulation	mental overstimulation	mental overstimulation	mental overstimulation	mental overstimulation	1	kept
mental reaction	mental reaction	mental reaction	mental reaction	mental reaction	1	kept

Mental recall	mental recall	mental recall	mental recall	Mental Recall	1	case_or_format
Mental state deterioration	mental state deterioration	mental state deterioration	mental state deterioration	Mental state deterioration	1	kept
mental states	mental states	mental states	mental states	mental states	1	kept
MENTAL STATUS	mental status	mental status	mental status	MENTAL STATUS	1	kept
Mental Suffering	mental suffering	mental suffering	mental suffering	Mental Suffering	1	kept
mental task	mental task	mental task	mental task	mental task	1	kept
mental well-being	mental well being	mental well being	mental well being	mental well-being	1	kept
MENTAL WORKLOAD	mental workload	mental workload	mental workload	mental workload	1	case_or_format
MENTAL-DISORDERS	mental disorders	mental disorders	mental disorder	Mental Disorders	1	singular
mental-health resources	mental health resources	mental health resources	mental health resources	mental-health resources	1	kept
mentally disabled person	mentally disabled person	mentally disabled person	mentally disabled person	mentally disabled person	1	kept
Mentation	mentation	mentation	mentation	Mentation	1	kept
Mentha	mentha	mentha	mentha	Mentha	1	kept
Mentha x piperita	mentha x piperita	mentha x piperita	mentha x piperita	Mentha x piperita	1	kept
menthol	menthol	menthol	menthol	Menthol	1	case_or_format
menthol gum	menthol gum	menthol gum	menthol gum	menthol gum	1	kept

Menu navigation	menu navigation	menu navigation	menu navigation	Menu navigation	1	kept
Menu Planning	menu planning	menu planning	menu planning	Menu Planning	1	kept
Mepilex Border heel dressing	mepilex border heel dressing	mepilex border heel dressing	mepilex border heel dressing	Mepilex Border heel dressing	1	kept
mepolizumab	mepolizumab	mepolizumab	mepolizumab	mepolizumab	1	kept
meprednisone	meprednisone	meprednisone	meprednisone	meprednisone	1	kept
MEQ	meq	meq	meq	MEQ	1	kept
mercaptopurine	mercaptopurine	mercaptopurine	mercaptopurine	mercaptopurine	1	kept
mercury	mercury	mercury	mercury	Mercury	1	case_or_format
Mercy killing	mercy killing	mercy killing	mercy killing	Mercy killing	1	kept
Mergers and acquisitions	mergers and acquisitions	mergers and acquisitions	mergers and acquisitions	Mergers and acquisitions	1	kept
mergers and acquisitions	mergers and acquisitions	mergers and acquisitions	mergers and acquisitions	Mergers and acquisitions	1	case_or_format
Merle Mishel	merle mishel	merle mishel	merle mishel	Merle Mishel	1	kept
meropenem hikma	meropenem hikma	meropenem hikma	meropenem hikma	meropenem hikma	1	kept
meropenem–vaborbactam	meropenem vaborbactam	meropenem vaborbactam	meropenem vaborbactam	meropenem–vaborbactam	1	kept
Merve Kızılırmak	merve k z l rmak	merve k z l rmak	merve k z l rmak	Merve Kızılırmak	1	kept
mesalazine	mesalazine	mesalazine	mesalazine	mesalazine	1	kept

mesenchymal stem cell transplantation	mesenchymal stem cell transplantation	mesenchymal stem cell transplantation	mesenchymal stem cell transplantation	mesenchymal stem cell transplantation	1	kept
mesenchymal stem cells	mesenchymal stem cells	mesenchymal stem cells	mesenchymal stem cell	mesenchymal stem cell	1	singular
Mesenteric Artery, Superior	mesenteric artery superior	mesenteric artery superior	mesenteric artery superior	Mesenteric Artery, Superior	1	kept
mesenteric blood vessel occlusion	mesenteric blood vessel occlusion	mesenteric blood vessel occlusion	mesenteric blood vessel occlusion	mesenteric blood vessel occlusion	1	kept
Mesenteric Vascular Occlusion	mesenteric vascular occlusion	mesenteric vascular occlusion	mesenteric vascular occlusion	Mesenteric Vascular Occlusion	1	kept
Mesentery	mesentery	mesentery	mesentery	Mesentery	1	kept
mesentery	mesentery	mesentery	mesentery	Mesentery	1	case_or_format
Mesh terms: Weights and Measures	mesh terms weights and measures	mesh terms weights and measures	mesh terms weights and measures	Mesh terms: Weights and Measures	1	kept
MESH-terms	mesh terms	mesh terms	mesh terms	MeSH Terms	1	case_or_format
MeSH: stroke	mesh stroke	mesh stroke	mesh stroke	MeSH: stroke	1	kept
Mesial temporal atrophy	mesial temporal atrophy	mesial temporal atrophy	mesial temporal atrophy	Mesial temporal atrophy	1	kept
mesoglycan	mesoglycan	mesoglycan	mesoglycan	mesoglycan	1	kept
Mesothelioma	mesothelioma	mesothelioma	mesothelioma	mesothelioma	1	case_or_format
Mesothelioma, Malignant	mesothelioma malignant	mesothelioma malignant	mesothelioma malignant	Mesothelioma, Malignant	1	kept
Message	message	message	message	Message	1	kept
Message board	message board	message board	message board	Message board	1	kept

MESSENGER-RNA	messenger rna	messenger rna	messenger rna	messenger RNA	1	case_or_format
mestil	mestil	mestil	mestil	mestil	1	kept
MET criteria	met criteria	met criteria	met criteria	MET criteria	1	kept
Met needs	met needs	met needs	met needs	Met needs	1	kept
Meta-analysis prevalence	meta analysis prevalence	meta analysis prevalence	meta analysis prevalence	Meta-analysis prevalence	1	kept
meta-ethnography	meta ethnography	meta ethnography	meta ethnography	meta-ethnography	1	kept
Meta-summary	meta summary	meta summary	meta summary	Meta-summary	1	kept
meta-summary	meta summary	meta summary	meta summary	Meta-summary	1	case_or_format
META-SYNTHESIS	meta synthesis	meta synthesis	meta synthesis	meta-synthesis	1	case_or_format
METAANALYTIC PATH-ANALYSIS	metaanalytic path analysis	metaanalytic path analysis	metaanalytic path analysis	METAANALYTIC PATH-ANALYSIS	1	kept
Metabolic	metabolic	metabolic	metabolic	Metabolic	1	kept
metabolic	metabolic	metabolic	metabolic	Metabolic	1	case_or_format
Metabolic acidemia	metabolic acidemia	metabolic acidemia	metabolic acidemia	Metabolic acidemia	1	kept
Metabolic acidosis	metabolic acidosis	metabolic acidosis	metabolic acidosis	metabolic acidosis	1	case_or_format
metabolic activation	metabolic activation	metabolic activation	metabolic activation	metabolic activation	1	kept
Metabolic causes of neonatal death	metabolic causes of neonatal death	metabolic causes of neonatal death	metabolic causes of neonatal death	Metabolic causes of neonatal death	1	kept

metabolic complications	metabolic complications	metabolic complications	metabolic complications	metabolic complications	1	kept
Metabolic control	metabolic control	metabolic control	metabolic control	Metabolic control	1	kept
METABOLIC CRISIS	metabolic crisis	metabolic crisis	metabolic crisis	METABOLIC CRISIS	1	kept
metabolic emergencies	metabolic emergencies	metabolic emergencies	metabolic emergencies	metabolic emergencies	1	kept
metabolic fatty liver	metabolic fatty liver	metabolic fatty liver	metabolic fatty liver	metabolic fatty liver	1	kept
Metabolic liver disease	metabolic liver disease	metabolic liver disease	metabolic liver disease	Metabolic liver disease	1	kept
Metabolic status	metabolic status	metabolic status	metabolic status	Metabolic status	1	kept
metabolic stress	metabolic stress	metabolic stress	metabolic stress	metabolic stress	1	kept
Metabolic suppression	metabolic suppression	metabolic suppression	metabolic suppression	Metabolic suppression	1	kept
metabolic suppression therapy	metabolic suppression therapy	metabolic suppression therapy	metabolic suppression therapy	metabolic suppression therapy	1	kept
metabolic surgery	metabolic surgery	metabolic surgery	metabolic surgery	metabolic surgery	1	kept
Metabolic syndrome	metabolic syndrome	metabolic syndrome	metabolic syndrome	Metabolic Syndrome	1	case_or_format
metabolic syndrome	metabolic syndrome	metabolic syndrome	metabolic syndrome	Metabolic Syndrome	1	case_or_format
Metabolic Syndrome X	metabolic syndrome x	metabolic syndrome x	metabolic syndrome x	metabolic syndrome X	1	case_or_format
METABOLISM	metabolism	metabolism	metabolism	metabolism	1	case_or_format
Metabolism and digestive system drugs	metabolism and digestive system drugs	metabolism and digestive system drugs	metabolism and digestive system drugs	Metabolism and digestive system drugs	1	kept

metabolites	metabolites	metabolites	metabolites	metabolites	1	kept
Metabolome	metabolome	metabolome	metabolome	Metabolome	1	kept
Metachromatic leukodystrophy	metachromatic leukodystrophy	metachromatic leukodystrophy	metachromatic leukodystrophy	metachromatic leukodystrophy	1	case_or_format
Metaclopramide	metaclopramide	metaclopramide	metaclopramide	Metaclopramide	1	kept
metacognition	metacognition	metacognition	metacognition	Metacognition	1	case_or_format
Metacognitive awareness	metacognitive awareness	metacognitive awareness	metacognitive awareness	Metacognitive awareness	1	kept
metadata	metadata	metadata	metadata	metadata	1	kept
metadrenalin	metadrenalin	metadrenalin	metadrenalin	metadrenalin	1	kept
METAGENOMICS	metagenomics	metagenomics	metagenomics	metagenomics	1	case_or_format
metal	metal	metal	metal	metal	1	kept
metal ion	metal ion	metal ion	metal ion	metal ion	1	kept
metal nanoparticle	metal nanoparticle	metal nanoparticle	metal nanoparticle	metal nanoparticle	1	kept
Metal Nanoparticles	metal nanoparticles	metal nanoparticles	metal nanoparticle	metal nanoparticle	1	singular
METAL-ORGANIC FRAMEWORKS	metal organic frameworks	metal organic frameworks	metal organic frameworks	METAL-ORGANIC FRAMEWORKS	1	kept
metallic implant corrosion	metallic implant corrosion	metallic implant corrosion	metallic implant corrosion	metallic implant corrosion	1	kept
metallo phosphide	metallo phosphide	metallo phosphide	metallo phosphide	metallo phosphide	1	kept

METALLO-BETA-LACTAMASE	metallo beta lactamase	metallo beta lactamase	metallo beta lactamase	metallo beta lactamase	1	case_or_format
metallo-beta-lactamase	metallo beta lactamase	metallo beta lactamase	metallo beta lactamase	metallo beta lactamase	1	case_or_format
metalloproteinase	metalloproteinase	metalloproteinase	metalloproteinase	metalloproteinase	1	kept
Metals	metals	metals	metal	metal	1	singular
METAMIZOL	metamizol	metamizol	metamizol	METAMIZOL	1	kept
metanalysis	metanalysis	metanalysis	metanalysis	metanalysis	1	kept
Metaparadigm	metaparadigm	metaparadigm	metaparadigm	Metaparadigm	1	kept
metaphor	metaphor	metaphor	metaphor	Metaphor	1	case_or_format
metaphysics	metaphysics	metaphysics	metaphysics	metaphysics	1	kept
Metaphysis	metaphysis	metaphysis	metaphysis	Metaphysis	1	kept
Metapneumovirus	metapneumovirus	metapneumovirus	metapneumovirus	Metapneumovirus	1	kept
metaraminol	metaraminol	metaraminol	metaraminol	metaraminol	1	kept
metastasis potential	metastasis potential	metastasis potential	metastasis potential	metastasis potential	1	kept
Metastatic brain tumor	metastatic brain tumor	metastatic brain tumor	metastatic brain tumor	Metastatic brain tumor	1	kept
metastatic breast cancer	metastatic breast cancer	metastatic breast cancer	metastatic breast cancer	metastatic breast cancer	1	kept
Metastatic disease	metastatic disease	metastatic disease	metastatic disease	Metastatic disease	1	kept

metastatic lung cancer	metastatic lung cancer	metastatic lung cancer	metastatic lung cancer	metastatic lung cancer	1	kept
Metastatic medullary thyroid carcinoma	metastatic medullary thyroid carcinoma	metastatic medullary thyroid carcinoma	metastatic medullary thyroid carcinoma	Metastatic medullary thyroid carcinoma	1	kept
metatarsal bone	metatarsal bone	metatarsal bone	metatarsal bone	metatarsal bone	1	kept
Metazoa	metazoa	metazoa	metazoa	Metazoa	1	kept
meta－ analysis	meta analysis	meta analysis	meta analysis	meta analysis	1	case_or_format
meta－ethnography	meta ethnography	meta ethnography	meta ethnography	meta-ethnography	1	case_or_format
Meteorological	meteorological	meteorological	meteorological	Meteorological	1	kept
meteorology	meteorology	meteorology	meteorology	meteorology	1	kept
Meter	meter	meter	meter	Meter	1	kept
Metered dose inhalers [MeSH]	metered dose inhalers mesh	metered dose inhalers mesh	metered dose inhalers mesh	Metered dose inhalers [MeSH]	1	kept
methacholine	methacholine	methacholine	methacholine	methacholine	1	kept
METHADONE	methadone	methadone	methadone	methadone	1	case_or_format
methadone maintenance	methadone maintenance	methadone maintenance	methadone maintenance	methadone maintenance	1	kept
Methadone maintenance therapy	methadone maintenance therapy	methadone maintenance therapy	methadone maintenance therapy	Methadone maintenance therapy	1	kept
methadone treatment	methadone treatment	methadone treatment	methadone treatment	methadone treatment	1	kept
methicillin resistance	methicillin resistance	methicillin resistance	methicillin resistance	Methicillin Resistance	1	case_or_format

Methicillin resistant staph aureus	methicillin resistant staph aureus	methicillin resistant staph aureus	methicillin resistant staph aureus	Methicillin resistant staph aureus	1	kept
Methicillin Resistant Staphylococcus aureus	methicillin resistant staphylococcus aureus	methicillin resistant staphylococcus aureus	methicillin resistant staphylococcus aureus	methicillin resistant Staphylococcus aureus	1	case_or_format
Methicillin resistant Staphylococcus aureus	methicillin resistant staphylococcus aureus	methicillin resistant staphylococcus aureus	methicillin resistant staphylococcus aureus	methicillin resistant Staphylococcus aureus	1	case_or_format
methicillin resistant Staphylococcus aureus pneumonia	methicillin resistant staphylococcus aureus pneumonia	methicillin resistant staphylococcus aureus pneumonia	methicillin resistant staphylococcus aureus pneumonia	methicillin resistant Staphylococcus aureus pneumonia	1	kept
Methicillin resistant Staphylococcus epidermis	methicillin resistant staphylococcus epidermis	methicillin resistant staphylococcus epidermis	methicillin resistant staphylococcus epidermis	Methicillin resistant Staphylococcus epidermis	1	kept
methicillin resistant Staphylococcus pseudintermedius	methicillin resistant staphylococcus pseudintermedius	methicillin resistant staphylococcus pseudintermedius	methicillin resistant staphylococcus pseudintermedius	methicillin resistant Staphylococcus pseudintermedius	1	kept
Methicillin sensitive Staphylococcus aureus	methicillin sensitive staphylococcus aureus	methicillin sensitive staphylococcus aureus	methicillin sensitive staphylococcus aureus	Methicillin sensitive Staphylococcus aureus	1	kept
methicillin sensitive Staphylococcus aureus	methicillin sensitive staphylococcus aureus	methicillin sensitive staphylococcus aureus	methicillin sensitive staphylococcus aureus	Methicillin sensitive Staphylococcus aureus	1	case_or_format
Methicillin-resistant	methicillin resistant	methicillin resistant	methicillin resistant	METHICILLIN-RESISTANT	1	case_or_format
methicillin-resistant	methicillin resistant	methicillin resistant	methicillin resistant	METHICILLIN-RESISTANT	1	case_or_format
methicillin-resistant coagulase-negative Staphylococcus	methicillin resistant coagulase negative staphylococcus	methicillin resistant coagulase negative staphylococcus	methicillin resistant coagulase negative staphylococcus	methicillin-resistant coagulase-negative Staphylococcus	1	kept
methicillin-resistant coagulase-negative staphylococcus	methicillin resistant coagulase negative staphylococcus	methicillin resistant coagulase negative staphylococcus	methicillin resistant coagulase negative staphylococcus	methicillin-resistant coagulase-negative Staphylococcus	1	case_or_format
Methicillin-resistant S. Aureus MRSA	methicillin resistant s aureus mrsa	methicillin resistant s aureus mrsa	methicillin resistant s aureus mrsa	Methicillin-resistant S. Aureus MRSA	1	kept
methicillin-resistant staphylococcus aureus	methicillin resistant staphylococcus aureus	methicillin resistant staphylococcus aureus	methicillin resistant staphylococcus aureus	methicillin resistant Staphylococcus aureus	1	case_or_format
Methicillin-resistant Staphylococcus aureus carriage	methicillin resistant staphylococcus aureus carriage	methicillin resistant staphylococcus aureus carriage	methicillin resistant staphylococcus aureus carriage	Methicillin-resistant Staphylococcus aureus carriage	1	kept
methicillin-resistant Staphylococcus epidermidis	methicillin resistant staphylococcus epidermidis	methicillin resistant staphylococcus epidermidis	methicillin resistant staphylococcus epidermidis	methicillin-resistant Staphylococcus epidermidis	1	kept

METHOD BIAS	method bias	method bias	method bias	METHOD BIAS	1	kept
method of counselling	method of counselling	method of counselling	method of counselling	method of counselling	1	kept
Methodical approach	methodical approach	methodical approach	methodical approach	Methodical approach	1	kept
Methodologic studies	methodologic studies	methodologic studies	methodologic studies	Methodologic studies	1	kept
Methodological	methodological	methodological	methodological	Methodological	1	kept
METHODOLOGICAL ANALYSIS	methodological analysis	methodological analysis	methodological analysis	METHODOLOGICAL ANALYSIS	1	kept
Methodological resources	methodological resources	methodological resources	methodological resources	Methodological resources	1	kept
Methodological tools	methodological tools	methodological tools	methodological tools	Methodological tools	1	kept
methods in qualitative inquiry	methods in qualitative inquiry	methods in qualitative inquiry	methods in qualitative inquiry	methods in qualitative inquiry	1	kept
Methods physiology	methods physiology	methods physiology	methods physiology	Methods physiology	1	kept
Methohexital	methohexital	methohexital	methohexital	Methohexital	1	kept
methohexital	methohexital	methohexital	methohexital	Methohexital	1	case_or_format
Methoxyhydroxyphenylglycol	methoxyhydroxyphenylglycol	methoxyhydroxyphenylglycol	methoxyhydroxyphenylglycol	Methoxyhydroxyphenylglycol	1	kept
methyl paraben	methyl paraben	methyl paraben	methyl paraben	methyl paraben	1	kept
methyl salicylate	methyl salicylate	methyl salicylate	methyl salicylate	methyl salicylate	1	kept
Methylcellulose	methylcellulose	methylcellulose	methylcellulose	Methylcellulose	1	kept

methylcellulose	methylcellulose	methylcellulose	methylcellulose	Methylcellulose	1	case_or_format
methylmalonic acid	methylmalonic acid	methylmalonic acid	methylmalonic acid	Methylmalonic Acid	1	case_or_format
Methylmalonic acidemia	methylmalonic acidemia	methylmalonic acidemia	methylmalonic acidemia	Methylmalonic acidemia	1	kept
methylmalonic acidemia	methylmalonic acidemia	methylmalonic acidemia	methylmalonic acidemia	Methylmalonic acidemia	1	case_or_format
Methylobacterium	methylobacterium	methylobacterium	methylobacterium	Methylobacterium	1	kept
Methylprednisolone Hemisuccinate	methylprednisolone hemisuccinate	methylprednisolone hemisuccinate	methylprednisolone hemisuccinate	Methylprednisolone Hemisuccinate	1	kept
metildigoxin	metildigoxin	metildigoxin	metildigoxin	metildigoxin	1	kept
metolazone	metolazone	metolazone	metolazone	metolazone	1	kept
Metoprolol	metoprolol	metoprolol	metoprolol	metoprolol	1	case_or_format
metronome	metronome	metronome	metronome	metronome	1	kept
metropolitan	metropolitan	metropolitan	metropolitan	metropolitan	1	kept
Mexican	mexican	mexican	mexican	Mexican	1	kept
Mexican-Americans	mexican americans	mexican americans	mexican americans	Mexican Americans	1	case_or_format
mexiletine	mexiletine	mexiletine	mexiletine	mexiletine	1	kept
mFI-5	mfi 5	mfi 5	mfi 5	mFI-5	1	kept
MFIS	mfis	mfis	mfis	MFIS	1	kept

mFLACC	mflacc	mflacc	mflacc	mFLACC	1	kept
MHDU	mhdu	mhdu	mhdu	MHDU	1	kept
MHealth	mhealth	mhealth	mhealth	mHealth	1	case_or_format
mHealth App Usability Questionnaire	mhealth app usability questionnaire	mhealth app usability questionnaire	mhealth app usability questionnaire	mHealth App Usability Questionnaire	1	kept
MI = minimally invasive	mi minimally invasive	mi minimally invasive	mi minimally invasive	MI = minimally invasive	1	kept
Mianserin	mianserin	mianserin	mianserin	Mianserin	1	kept
MIC	mic	mic	mic	MIC	1	kept
MIC = minimum inhibitory concentration	mic minimum inhibitory concentration	mic minimum inhibitory concentration	mic minimum inhibitory concentration	MIC = minimum inhibitory concentration	1	kept
Mice, Inbred BALB C	mice inbred balb c	mice inbred balb c	mice inbred balb c	Mice, Inbred BALB C	1	kept
micro blogging	micro blogging	micro blogging	micro blogging	micro blogging	1	kept
Micro-lectures	micro lectures	micro lectures	micro lectures	Micro-lectures	1	kept
Micro-perforated panels	micro perforated panels	micro perforated panels	micro perforated panels	Micro-perforated panels	1	kept
micro-RNA	micro rna	micro rna	micro rna	micro-RNA	1	kept
Micro-simulation	micro simulation	micro simulation	micro simulation	Micro-simulation	1	kept
Micro-structure absorption	micro structure absorption	micro structure absorption	micro structure absorption	Micro-structure absorption	1	kept
Microalbuminuria	microalbuminuria	microalbuminuria	microalbuminuria	Microalbuminuria	1	kept

microalbuminuria	microalbuminuria	microalbuminuria	microalbuminuria	Microalbuminuria	1	case_or_format
Microanalysis	microanalysis	microanalysis	microanalysis	Microanalysis	1	kept
Microbes	microbes	microbes	microbes	Microbes	1	kept
Microbial	microbial	microbial	microbial	Microbial	1	kept
microbial	microbial	microbial	microbial	Microbial	1	case_or_format
microbial activity	microbial activity	microbial activity	microbial activity	microbial activity	1	kept
Microbial communities	microbial communities	microbial communities	microbial community	microbial community	1	singular
Microbial composition	microbial composition	microbial composition	microbial composition	Microbial composition	1	kept
Microbial contamination of keyboards and	microbial contamination of keyboards and	microbial contamination of keyboards and	microbial contamination of keyboards and	Microbial contamination of keyboards and	1	kept
Microbial Drug Resistance	microbial drug resistance	microbial drug resistance	microbial drug resistance	Microbial Drug Resistance	1	kept
Microbial drug resistance	microbial drug resistance	microbial drug resistance	microbial drug resistance	Microbial Drug Resistance	1	case_or_format
Microbial dysbiosis	microbial dysbiosis	microbial dysbiosis	microbial dysbiosis	Microbial dysbiosis	1	kept
Microbial etiology	microbial etiology	microbial etiology	microbial etiology	Microbial etiology	1	kept
microbial immunity	microbial immunity	microbial immunity	microbial immunity	microbial immunity	1	kept
MICROBIAL-CONTAMINATION	microbial contamination	microbial contamination	microbial contamination	microbial contamination	1	case_or_format
Microbiological colonization	microbiological colonization	microbiological colonization	microbiological colonization	Microbiological colonization	1	kept

microbiological examinations	microbiological examinations	microbiological examinations	microbiological examination	microbiological examination	1	singular
Microbiological surveillance	microbiological surveillance	microbiological surveillance	microbiological surveillance	Microbiological surveillance	1	kept
MICROBIOLOGY	microbiology	microbiology	microbiology	microbiology	1	case_or_format
Microbiology laboratory	microbiology laboratory	microbiology laboratory	microbiology laboratory	Microbiology laboratory	1	kept
microbiota	microbiota	microbiota	microbiota	Microbiota	1	case_or_format
microcapsule	microcapsule	microcapsule	microcapsule	microcapsule	1	kept
Microchannels	microchannels	microchannels	microchannels	Microchannels	1	kept
microcirculation video	microcirculation video	microcirculation video	microcirculation video	microcirculation video	1	kept
MICROCIRCULATORY ALTERATIONS	microcirculatory alterations	microcirculatory alterations	microcirculatory alterations	MICROCIRCULATORY ALTERATIONS	1	kept
Microcirculatory changes	microcirculatory changes	microcirculatory changes	microcirculatory changes	Microcirculatory changes	1	kept
MICROCLIMATE	microclimate	microclimate	microclimate	microclimate	1	case_or_format
MICROCOMPUTER	microcomputer	microcomputer	microcomputer	microcomputer	1	case_or_format
Microcomputers	microcomputers	microcomputers	microcomputer	microcomputer	1	singular
Microcontrollers	microcontrollers	microcontrollers	microcontroller	Microcontroller	1	singular
microdeletion	microdeletion	microdeletion	microdeletion	microdeletion	1	kept
microdiscectomy	microdiscectomy	microdiscectomy	microdiscectomy	microdiscectomy	1	kept

Microelectrodes	microelectrodes	microelectrodes	microelectrodes	Microelectrodes	1	kept
Microelectronics	microelectronics	microelectronics	microelectronics	Microelectronics	1	kept
Microenvironment theory	microenvironment theory	microenvironment theory	microenvironment theory	Microenvironment theory	1	kept
Microenvironments	microenvironments	microenvironments	microenvironment	microenvironment	1	singular
microfiber	microfiber	microfiber	microfiber	microfiber	1	kept
microfilaria	microfilaria	microfilaria	microfilaria	Microfilaria	1	case_or_format
Microflora	microflora	microflora	microflora	microflora	1	case_or_format
Micrognathism	micrognathism	micrognathism	micrognathism	Micrognathism	1	kept
micromelia	micromelia	micromelia	micromelia	micromelia	1	kept
micronodue	micronodue	micronodue	micronodue	micronodue	1	kept
Microorganisms	microorganisms	microorganisms	microorganism	microorganism	1	singular
micropenis	micropenis	micropenis	micropenis	micropenis	1	kept
Microperimeter	microperimeter	microperimeter	microperimeter	Microperimeter	1	kept
microplastic	microplastic	microplastic	microplastic	microplastic	1	kept
micropreemies	micropreemies	micropreemies	micropreemies	micropreemies	1	kept
micropuncture	micropuncture	micropuncture	micropuncture	micropuncture	1	kept

Microscopy, Electron, Transmission	microscopy electron transmission	microscopy electron transmission	microscopy electron transmission	Microscopy, Electron, Transmission	1	kept
Microscopy, Video	microscopy video	microscopy video	microscopy video	Microscopy, Video	1	kept
Microsimulation	microsimulation	microsimulation	microsimulation	Microsimulation	1	kept
microsimulation	microsimulation	microsimulation	microsimulation	Microsimulation	1	case_or_format
Microsnare	microsnare	microsnare	microsnare	Microsnare	1	kept
Microsoft windows	microsoft windows	microsoft windows	microsoft windows	Microsoft windows	1	kept
Microstructure	microstructure	microstructure	microstructure	Microstructure	1	kept
microsystems	microsystems	microsystems	microsystems	microsystems	1	kept
Microvascular clamp	microvascular clamp	microvascular clamp	microvascular clamp	Microvascular clamp	1	kept
microvascular decompression	microvascular decompression	microvascular decompression	microvascular decompression	microvascular decompression	1	kept
MICROVASCULAR DYSFUNCTION	microvascular dysfunction	microvascular dysfunction	microvascular dysfunction	MICROVASCULAR DYSFUNCTION	1	kept
Microvascular free flap	microvascular free flap	microvascular free flap	microvascular free flap	Microvascular free flap	1	kept
microvascular free flap	microvascular free flap	microvascular free flap	microvascular free flap	Microvascular free flap	1	case_or_format
microvascular thrombosis	microvascular thrombosis	microvascular thrombosis	microvascular thrombosis	microvascular thrombosis	1	kept
Microvilli	microvilli	microvilli	microvilli	Microvilli	1	kept
Microvillous inclusion disease	microvillous inclusion disease	microvillous inclusion disease	microvillous inclusion disease	Microvillous inclusion disease	1	kept

microvillus	microvillus	microvillus	microvillus	microvillus	1	kept
microvillus inclusion disease	microvillus inclusion disease	microvillus inclusion disease	microvillus inclusion disease	microvillus inclusion disease	1	kept
Microwave integrated circuits	microwave integrated circuits	microwave integrated circuits	microwave integrated circuits	Microwave integrated circuits	1	kept
microwave thermotherapy	microwave thermotherapy	microwave thermotherapy	microwave thermotherapy	microwave thermotherapy	1	kept
Mid-frequencies	mid frequencies	mid frequencies	mid frequencies	Mid-frequencies	1	kept
Mid-level provider	mid level provider	mid level provider	mid level provider	Mid-level provider	1	kept
Midazolam Hydrochloride	midazolam hydrochloride	midazolam hydrochloride	midazolam hydrochloride	Midazolam Hydrochloride	1	kept
Middle Cerebral Artery	middle cerebral artery	middle cerebral artery	middle cerebral artery	middle cerebral artery	1	case_or_format
Middle cerebral artery	middle cerebral artery	middle cerebral artery	middle cerebral artery	middle cerebral artery	1	case_or_format
Middle cerebral artery infarction, malignant	middle cerebral artery infarction malignant	middle cerebral artery infarction malignant	middle cerebral artery infarction malignant	Middle cerebral artery infarction, malignant	1	kept
middle ear	middle ear	middle ear	middle ear	middle ear	1	kept
middle ear pressure	middle ear pressure	middle ear pressure	middle ear pressure	middle ear pressure	1	kept
middle ear surgery	middle ear surgery	middle ear surgery	middle ear surgery	middle ear surgery	1	kept
Middle Ear Ventilation	middle ear ventilation	middle ear ventilation	middle ear ventilation	Middle Ear Ventilation	1	kept
middle ear ventilation	middle ear ventilation	middle ear ventilation	middle ear ventilation	Middle Ear Ventilation	1	case_or_format
Middle East Respiratory Syndrome	middle east respiratory syndrome	middle east respiratory syndrome	middle east respiratory syndrome	Middle East respiratory syndrome	1	case_or_format

Middle fossa floor	middle fossa floor	middle fossa floor	middle fossa floor	Middle fossa floor	1	kept
middle meningeal artery	middle meningeal artery	middle meningeal artery	middle meningeal artery	middle meningeal artery	1	kept
middle school	middle school	middle school	middle school	middle school	1	kept
Middle-income countries	middle income countries	middle income countries	middle income country	middle income country	1	singular
middle-income countries	middle income countries	middle income countries	middle income country	middle income country	1	singular
middle-income country	middle income country	middle income country	middle income country	middle income country	1	case_or_format
midface hypoplasia	midface hypoplasia	midface hypoplasia	midface hypoplasia	midface hypoplasia	1	kept
midgut	midgut	midgut	midgut	midgut	1	kept
Midline diffuse glioma	midline diffuse glioma	midline diffuse glioma	midline diffuse glioma	Midline diffuse glioma	1	kept
midline head position	midline head position	midline head position	midline head position	midline head position	1	kept
Midline venous catheter	midline venous catheter	midline venous catheter	midline venous catheter	Midline venous catheter	1	kept
Midodrine	midodrine	midodrine	midodrine	Midodrine	1	kept
midregional proadrenomedullin	midregional proadrenomedullin	midregional proadrenomedullin	midregional proadrenomedullin	midregional proadrenomedullin	1	kept
MidU study	midu study	midu study	midu study	MidU study	1	kept
Midwife-led birth care	midwife led birth care	midwife led birth care	midwife led birth care	Midwife-led birth care	1	kept
midwife-led unit	midwife led unit	midwife led unit	midwife led unit	midwife-led unit	1	kept

midwife-to-infant ratio	midwife to infant ratio	midwife to infant ratio	midwife to infant ratio	midwife-to-infant ratio	1	kept
Midwifery care	midwifery care	midwifery care	midwifery care	Midwifery care	1	kept
midwifery care	midwifery care	midwifery care	midwifery care	Midwifery care	1	case_or_format
Midwifery students	midwifery students	midwifery students	midwifery student	midwifery student	1	singular
Midwifery training	midwifery training	midwifery training	midwifery training	Midwifery training	1	kept
midwifery unit	midwifery unit	midwifery unit	midwifery unit	midwifery unit	1	kept
midwifery-led birth center	midwifery led birth center	midwifery led birth center	midwifery led birth center	midwifery-led birth center	1	kept
Midwifery-led care	midwifery led care	midwifery led care	midwifery led care	Midwifery-led care	1	kept
midwifery‐led birth center	midwifery led birth center	midwifery led birth center	midwifery led birth center	midwifery-led birth center	1	case_or_format
MIDWIVES	midwives	midwives	midwives	Midwives	1	case_or_format
Midwives training	midwives training	midwives training	midwives training	Midwives training	1	kept
MIF	mif	mif	mif	MIF	1	kept
migalastat	migalastat	migalastat	migalastat	migalastat	1	kept
miglitol	miglitol	miglitol	miglitol	miglitol	1	kept
migraine with aura	migraine with aura	migraine with aura	migraine with aura	migraine with aura	1	kept
migraine without aura	migraine without aura	migraine without aura	migraine without aura	migraine without aura	1	kept

Migrant health	migrant health	migrant health	migrant health	Migrant health	1	kept
migrants	migrants	migrants	migrant	migrant	1	singular
Migration and health	migration and health	migration and health	migration and health	Migration and health	1	kept
Migration background	migration background	migration background	migration background	Migration background	1	kept
Milano [Lombardy]	milano lombardy	milano lombardy	milano lombardy	Milano [Lombardy]	1	kept
milbemycin	milbemycin	milbemycin	milbemycin	milbemycin	1	kept
mild	mild	mild	mild	mild	1	kept
MILD COGNITIVE IMPAIRMENT	mild cognitive impairment	mild cognitive impairment	mild cognitive impairment	mild cognitive impairment	1	case_or_format
Mild cognitive impairment	mild cognitive impairment	mild cognitive impairment	mild cognitive impairment	mild cognitive impairment	1	case_or_format
mild hepatic impairment	mild hepatic impairment	mild hepatic impairment	mild hepatic impairment	mild hepatic impairment	1	kept
Mild hypothermia	mild hypothermia	mild hypothermia	mild hypothermia	MILD HYPOTHERMIA	1	case_or_format
mild hypothermia	mild hypothermia	mild hypothermia	mild hypothermia	MILD HYPOTHERMIA	1	case_or_format
mild stroke	mild stroke	mild stroke	mild stroke	mild stroke	1	kept
mild traumatic brain injury	mild traumatic brain injury	mild traumatic brain injury	mild traumatic brain injury	mild traumatic brain injury	1	kept
Milestones	milestones	milestones	milestones	Milestones	1	kept
MILIEU	milieu	milieu	milieu	MILIEU	1	kept

milieu therapy	milieu therapy	milieu therapy	milieu therapy	milieu therapy	1	kept
military field hospital	military field hospital	military field hospital	military field hospital	military field hospital	1	kept
military health	military health	military health	military health	military health	1	kept
military health service	military health service	military health service	military health service	military health service	1	kept
Military Hospitals	military hospitals	military hospitals	military hospital	military hospital	1	singular
Military hospitals	military hospitals	military hospitals	military hospital	military hospital	1	singular
military hospitals	military hospitals	military hospitals	military hospital	military hospital	1	singular
military medical ethics	military medical ethics	military medical ethics	military medical ethics	military medical ethics	1	kept
Military personnel	military personnel	military personnel	military personnel	Military Personnel	1	case_or_format
military phenomena	military phenomena	military phenomena	military phenomena	military phenomena	1	kept
Military photography	military photography	military photography	military photography	Military photography	1	kept
military physician	military physician	military physician	military physician	military physician	1	kept
military service	military service	military service	military service	military service	1	kept
military student	military student	military student	military student	military student	1	kept
Military training	military training	military training	military training	Military training	1	kept
milk ejection	milk ejection	milk ejection	milk ejection	Milk Ejection	1	case_or_format

milk fat	milk fat	milk fat	milk fat	milk fat	1	kept
milk lab	milk lab	milk lab	milk lab	milk lab	1	kept
milk protein	milk protein	milk protein	milk protein	milk protein	1	kept
milk technician	milk technician	milk technician	milk technician	milk technician	1	kept
Milk yield	milk yield	milk yield	milk yield	Milk yield	1	kept
milk yield	milk yield	milk yield	milk yield	Milk yield	1	case_or_format
Milrinone	milrinone	milrinone	milrinone	milrinone	1	case_or_format
milronone	milronone	milronone	milronone	milronone	1	kept
MIMIC II	mimic ii	mimic ii	mimic ii	MIMIC II	1	kept
MIMIC IV	mimic iv	mimic iv	mimic iv	MIMIC-IV	1	case_or_format
Mimic-ii	mimic ii	mimic ii	mimic ii	MIMIC II	1	case_or_format
Mimicking arrthymias	mimicking arrthymias	mimicking arrthymias	mimicking arrthymias	Mimicking arrthymias	1	kept
MIMIC－IV	mimic iv	mimic iv	mimic iv	MIMIC-IV	1	case_or_format
MIMIC－IV	mimic iv	mimic iv	mimic iv	MIMIC-IV	1	case_or_format
minamol plus suspension	minamol plus suspension	minamol plus suspension	minamol plus suspension	minamol plus suspension	1	kept
MIND	mind	mind	mind	MIND	1	kept

MIND questionnaire	mind questionnaire	mind questionnaire	mind questionnaire	MIND questionnaire	1	kept
Mind-Body Relations	mind body relations	mind body relations	mind body relations	Mind-Body Relations	1	kept
Mindful Attention Awareness Scale	mindful attention awareness scale	mindful attention awareness scale	mindful attention awareness scale	Mindful Attention Awareness Scale	1	kept
mindful coloring	mindful coloring	mindful coloring	mindful coloring	mindful coloring	1	kept
Mindful organizing	mindful organizing	mindful organizing	mindful organizing	Mindful organizing	1	kept
Mindfulness intervention	mindfulness intervention	mindfulness intervention	mindfulness intervention	Mindfulness intervention	1	kept
mindfulness meditation	mindfulness meditation	mindfulness meditation	mindfulness meditation	mindfulness meditation	1	kept
Mindfulness Training	mindfulness training	mindfulness training	mindfulness training	Mindfulness Training	1	kept
mindfulness-based cognitive therapy	mindfulness based cognitive therapy	mindfulness based cognitive therapy	mindfulness based cognitive therapy	mindfulness-based cognitive therapy	1	kept
Mindfulness-Based Cognitive Therapy , Perceived Stress Scale	mindfulness based cognitive therapy perceived stress scale	mindfulness based cognitive therapy perceived stress scale	mindfulness based cognitive therapy perceived stress scale	Mindfulness-Based Cognitive Therapy , Perceived Stress Scale	1	kept
mindfulness-based intervention	mindfulness based intervention	mindfulness based intervention	mindfulness based intervention	Mindfulness-based interventions	1	case_or_format
MINDFULNESS-BASED INTERVENTIONS	mindfulness based interventions	mindfulness based interventions	mindfulness based intervention	Mindfulness-based interventions	1	singular
mindfulness‐based intervention	mindfulness based intervention	mindfulness based intervention	mindfulness based intervention	Mindfulness-based interventions	1	case_or_format
MINDS	minds	minds	mind	MIND	1	singular
Mineral Oil	mineral oil	mineral oil	mineral oil	Mineral Oil	1	kept
mineralization	mineralization	mineralization	mineralization	mineralization	1	kept

mineralocorticoid	mineralocorticoid	mineralocorticoid	mineralocorticoid	mineralocorticoid	1	kept
mineralocorticoid receptor	mineralocorticoid receptor	mineralocorticoid receptor	mineralocorticoid receptor	mineralocorticoid receptor	1	kept
Mineralocorticoid Receptor Antagonists	mineralocorticoid receptor antagonists	mineralocorticoid receptor antagonists	mineralocorticoid receptor antagonists	Mineralocorticoid Receptor Antagonists	1	kept
Mineralocorticoids	mineralocorticoids	mineralocorticoids	mineralocorticoid	mineralocorticoid	1	singular
mineralocorticoids	mineralocorticoids	mineralocorticoids	mineralocorticoid	mineralocorticoid	1	singular
mini clinical examination	mini clinical examination	mini clinical examination	mini clinical examination	mini clinical examination	1	kept
mini international neuropsychiatric interview	mini international neuropsychiatric interview	mini international neuropsychiatric interview	mini international neuropsychiatric interview	mini international neuropsychiatric interview	1	kept
mini mental state examination	mini mental state examination	mini mental state examination	mini mental state examination	Mini Mental State Examination	1	case_or_format
Mini-thoracotomy	mini thoracotomy	mini thoracotomy	mini thoracotomy	Mini-thoracotomy	1	kept
Miniaturization	miniaturization	miniaturization	miniaturization	Miniaturization	1	kept
miniaturization	miniaturization	miniaturization	miniaturization	Miniaturization	1	case_or_format
MINIBAG Plus delivery system	minibag plus delivery system	minibag plus delivery system	minibag plus delivery system	MINIBAG Plus delivery system	1	kept
MiniCog	minicog	minicog	minicog	MiniCog	1	kept
minimal infant motion	minimal infant motion	minimal infant motion	minimal infant motion	minimal infant motion	1	kept
Minimal invasive cardiac surgery	minimal invasive cardiac surgery	minimal invasive cardiac surgery	minimal invasive cardiac surgery	Minimal invasive cardiac surgery	1	kept
minimal model	minimal model	minimal model	minimal model	minimal model	1	kept

minimal occlusion volume	minimal occlusion volume	minimal occlusion volume	minimal occlusion volume	minimal occlusion volume	1	kept
minimal residual disease	minimal residual disease	minimal residual disease	minimal residual disease	minimal residual disease	1	kept
Minimal resources	minimal resources	minimal resources	minimal resources	Minimal resources	1	kept
minimal ventilatory pressure	minimal ventilatory pressure	minimal ventilatory pressure	minimal ventilatory pressure	minimal ventilatory pressure	1	kept
Minimalist approach	minimalist approach	minimalist approach	minimalist approach	Minimalist approach	1	kept
minimalist approach	minimalist approach	minimalist approach	minimalist approach	Minimalist approach	1	case_or_format
Minimally conscious state	minimally conscious state	minimally conscious state	minimally conscious state	minimally conscious state	1	case_or_format
minimally invasive aortic surgery	minimally invasive aortic surgery	minimally invasive aortic surgery	minimally invasive aortic surgery	minimally invasive aortic surgery	1	kept
Minimally invasive approach	minimally invasive approach	minimally invasive approach	minimally invasive approach	Minimally invasive approach	1	kept
Minimally invasive cardiac surgery	minimally invasive cardiac surgery	minimally invasive cardiac surgery	minimally invasive cardiac surgery	minimally invasive cardiac surgery	1	case_or_format
Minimally invasive clot evacuation	minimally invasive clot evacuation	minimally invasive clot evacuation	minimally invasive clot evacuation	Minimally invasive clot evacuation	1	kept
minimally invasive clot evacuation	minimally invasive clot evacuation	minimally invasive clot evacuation	minimally invasive clot evacuation	Minimally invasive clot evacuation	1	case_or_format
Minimally invasive esophagectomy	minimally invasive esophagectomy	minimally invasive esophagectomy	minimally invasive esophagectomy	Minimally invasive esophagectomy	1	kept
minimally invasive hematoma	minimally invasive hematoma	minimally invasive hematoma	minimally invasive hematoma	minimally invasive hematoma	1	kept
minimally invasive mitral surgery	minimally invasive mitral surgery	minimally invasive mitral surgery	minimally invasive mitral surgery	minimally invasive mitral surgery	1	kept
minimally invasive mitral valve surgery	minimally invasive mitral valve surgery	minimally invasive mitral valve surgery	minimally invasive mitral valve surgery	minimally invasive mitral valve surgery	1	kept

minimally invasive surfactant therapy	minimally invasive surfactant therapy	minimally invasive surfactant therapy	minimally invasive surfactant therapy	minimally invasive surfactant therapy	1	kept
minimally invasive surgical procedures	minimally invasive surgical procedures	minimally invasive surgical procedures	minimally invasive surgical procedures	Minimally Invasive Surgical Procedures	1	case_or_format
Minimally invasive techniques	minimally invasive techniques	minimally invasive techniques	minimally invasive techniques	Minimally invasive techniques	1	kept
Minimally invasive tissue sampling	minimally invasive tissue sampling	minimally invasive tissue sampling	minimally invasive tissue sampling	Minimally invasive tissue sampling	1	kept
minimally invasive tissue sampling	minimally invasive tissue sampling	minimally invasive tissue sampling	minimally invasive tissue sampling	Minimally invasive tissue sampling	1	case_or_format
minimise harm	minimise harm	minimise harm	minimise harm	minimise harm	1	kept
minimise restraints	minimise restraints	minimise restraints	minimise restraints	minimise restraints	1	kept
MINIMIZATION	minimization	minimization	minimization	MINIMIZATION	1	kept
Minimum Data Set	minimum data set	minimum data set	minimum data set	MINIMUM DATA SET	1	case_or_format
minimum data set	minimum data set	minimum data set	minimum data set	MINIMUM DATA SET	1	case_or_format
Minimum Data Set Cognition Scale	minimum data set cognition scale	minimum data set cognition scale	minimum data set cognition scale	Minimum Data Set Cognition Scale	1	kept
minimum detectable change	minimum detectable change	minimum detectable change	minimum detectable change	minimum detectable change	1	kept
minimum lung alveolus concentration	minimum lung alveolus concentration	minimum lung alveolus concentration	minimum lung alveolus concentration	minimum lung alveolus concentration	1	kept
minimum plasma concentration	minimum plasma concentration	minimum plasma concentration	minimum plasma concentration	minimum plasma concentration	1	kept
Minimum requirements	minimum requirements	minimum requirements	minimum requirements	Minimum requirements	1	kept
Mining techniques	mining techniques	mining techniques	mining techniques	Mining techniques	1	kept

minithoracotomy	minithoracotomy	minithoracotomy	minithoracotomy	minithoracotomy	1	kept
mink	mink	mink	mink	Mink	1	case_or_format
MINNESOTA	minnesota	minnesota	minnesota	Minnesota	1	case_or_format
Minnesota Living with Heart Failure Questionnaire	minnesota living with heart failure questionnaire	minnesota living with heart failure questionnaire	minnesota living with heart failure questionnaire	Minnesota Living with Heart Failure Questionnaire	1	kept
Minnesota Satisfaction Questionnaire	minnesota satisfaction questionnaire	minnesota satisfaction questionnaire	minnesota satisfaction questionnaire	minnesota satisfaction questionnaire	1	case_or_format
Minnesota Satisfaction Questionnaire short form	minnesota satisfaction questionnaire short form	minnesota satisfaction questionnaire short form	minnesota satisfaction questionnaire short form	Minnesota Satisfaction Questionnaire short form	1	kept
Minnesota Sedation Assessment Tool	minnesota sedation assessment tool	minnesota sedation assessment tool	minnesota sedation assessment tool	Minnesota Sedation Assessment Tool	1	kept
minor adverse effects	minor adverse effects	minor adverse effects	minor adverse effects	minor adverse effects	1	kept
Minor procedures	minor procedures	minor procedures	minor procedures	Minor procedures	1	kept
Minor Surgical Procedures	minor surgical procedures	minor surgical procedures	minor surgical procedures	Minor Surgical Procedures	1	kept
Minority health	minority health	minority health	minority health	Minority health	1	kept
Minors	minors	minors	minors	Minors	1	kept
MINUTE VENTILATION	minute ventilation	minute ventilation	minute ventilation	MINUTE VENTILATION	1	kept
MIPS	mips	mips	mips	MIPS	1	kept
mirabegron	mirabegron	mirabegron	mirabegron	mirabegron	1	kept
Mirabilite	mirabilite	mirabilite	mirabilite	Mirabilite	1	kept

miracle baby	miracle baby	miracle baby	miracle baby	miracle baby	1	kept
Mirror	mirror	mirror	mirror	Mirror	1	kept
Mirror syndrome	mirror syndrome	mirror syndrome	mirror syndrome	Mirror syndrome	1	kept
Miscarriage	miscarriage	miscarriage	miscarriage	Miscarriage	1	kept
miscommunication	miscommunication	miscommunication	miscommunication	miscommunication	1	kept
Misconceptions About Opioids	misconceptions about opioids	misconceptions about opioids	misconceptions about opioids	Misconceptions About Opioids	1	kept
misconduct	misconduct	misconduct	misconduct	misconduct	1	kept
Mishandling	mishandling	mishandling	mishandling	Mishandling	1	kept
Mishaps	mishaps	mishaps	mishaps	Mishaps	1	kept
Misinformation	misinformation	misinformation	misinformation	misinformation	1	case_or_format
mislabelling	mislabelling	mislabelling	mislabelling	mislabelling	1	kept
mismatch repair	mismatch repair	mismatch repair	mismatch repair	mismatch repair	1	kept
MISPLACEMENT	misplacement	misplacement	misplacement	MISPLACEMENT	1	kept
Misscare	misscare	misscare	misscare	Misscare	1	kept
MISSCARE Survey	misscare survey	misscare survey	misscare survey	MISSCARE Survey	1	kept
MISSCARE survey	misscare survey	misscare survey	misscare survey	MISSCARE Survey	1	case_or_format

Missed Care	missed care	missed care	missed care	Missed care	1	case_or_format
missed doses	missed doses	missed doses	missed doses	missed doses	1	kept
Missed injuries	missed injuries	missed injuries	missed injuries	Missed injuries	1	kept
missed placental accreta	missed placental accreta	missed placental accreta	missed placental accreta	missed placental accreta	1	kept
Missing	missing	missing	missing	Missing	1	kept
missing data	missing data	missing data	missing data	missing data	1	kept
Missing doses	missing doses	missing doses	missing doses	Missing doses	1	kept
missing doses	missing doses	missing doses	missing doses	Missing doses	1	case_or_format
mission	mission	mission	mission	mission	1	kept
Mission critical	mission critical	mission critical	mission critical	Mission critical	1	kept
Mission trips	mission trips	mission trips	mission trips	Mission trips	1	kept
MIST	mist	mist	mist	MIST	1	kept
MISTAKES	mistakes	mistakes	mistake	mistake	1	singular
misunderstanding	misunderstanding	misunderstanding	misunderstanding	misunderstanding	1	kept
MISUSERS	misusers	misusers	misusers	MISUSERS	1	kept
mitigation phase	mitigation phase	mitigation phase	mitigation phase	mitigation phase	1	kept

Mitigators	mitigators	mitigators	mitigators	Mitigators	1	kept
Mitochondrial	mitochondrial	mitochondrial	mitochondrial	Mitochondrial	1	kept
Mitochondrial Diseases	mitochondrial diseases	mitochondrial diseases	mitochondrial diseases	Mitochondrial Diseases	1	kept
mitochondrial encephalomyopathy	mitochondrial encephalomyopathy	mitochondrial encephalomyopathy	mitochondrial encephalomyopathy	mitochondrial encephalomyopathy	1	kept
mitochondrial trifunctional protein deficiency	mitochondrial trifunctional protein deficiency	mitochondrial trifunctional protein deficiency	mitochondrial trifunctional protein deficiency	mitochondrial trifunctional protein deficiency	1	kept
mitofen syrup	mitofen syrup	mitofen syrup	mitofen syrup	mitofen syrup	1	kept
mitogen activated protein kinase kinase 1	mitogen activated protein kinase kinase 1	mitogen activated protein kinase kinase 1	mitogen activated protein kinase kinase 1	mitogen activated protein kinase kinase 1	1	kept
mitogenesis	mitogenesis	mitogenesis	mitogenesis	mitogenesis	1	kept
mitomycin	mitomycin	mitomycin	mitomycin	mitomycin	1	kept
Mitosporic Fungi	mitosporic fungi	mitosporic fungi	mitosporic fungi	Mitosporic Fungi	1	kept
mitoxantrone	mitoxantrone	mitoxantrone	mitoxantrone	mitoxantrone	1	kept
MitraClip	mitraclip	mitraclip	mitraclip	MitraClip	1	kept
Mitragyna	mitragyna	mitragyna	mitragyna	Mitragyna	1	kept
Mitral regurgitation	mitral regurgitation	mitral regurgitation	mitral regurgitation	Mitral regurgitation	1	kept
mitral regurgitation	mitral regurgitation	mitral regurgitation	mitral regurgitation	Mitral regurgitation	1	case_or_format
Mitral stenosis	mitral stenosis	mitral stenosis	mitral stenosis	Mitral stenosis	1	kept

mitral valve annulus repair	mitral valve annulus repair	mitral valve annulus repair	mitral valve annulus repair	mitral valve annulus repair	1	kept
mitral valve prolapse	mitral valve prolapse	mitral valve prolapse	mitral valve prolapse	Mitral Valve Prolapse	1	case_or_format
Mivacurium	mivacurium	mivacurium	mivacurium	mivacurium	1	case_or_format
mixed anxiety and depression	mixed anxiety and depression	mixed anxiety and depression	mixed anxiety and depression	mixed anxiety and depression	1	kept
mixed cell culture	mixed cell culture	mixed cell culture	mixed cell culture	mixed cell culture	1	kept
mixed connective tissue disease	mixed connective tissue disease	mixed connective tissue disease	mixed connective tissue disease	mixed connective tissue disease	1	kept
mixed depression and dementia	mixed depression and dementia	mixed depression and dementia	mixed depression and dementia	mixed depression and dementia	1	kept
mixed effects models	mixed effects models	mixed effects models	mixed effects models	mixed effects models	1	kept
mixed hearing loss	mixed hearing loss	mixed hearing loss	mixed hearing loss	mixed hearing loss	1	kept
Mixed integer	mixed integer	mixed integer	mixed integer	Mixed integer	1	kept
Mixed integer linear programming	mixed integer linear programming	mixed integer linear programming	mixed integer linear programming	Mixed integer linear programming	1	kept
Mixed integer programming model	mixed integer programming model	mixed integer programming model	mixed integer programming model	Mixed integer programming model	1	kept
Mixed Method	mixed method	mixed method	mixed method	Mixed methods	1	case_or_format
Mixed method design	mixed method design	mixed method design	mixed method design	mixed-method design	1	case_or_format
mixed method design	mixed method design	mixed method design	mixed method design	mixed-method design	1	case_or_format
Mixed method evaluation	mixed method evaluation	mixed method evaluation	mixed method evaluation	Mixed method evaluation	1	kept

mixed methods approach	mixed methods approach	mixed methods approach	mixed methods approach	mixed methods approach	1	kept
mixed methods case study	mixed methods case study	mixed methods case study	mixed methods case study	mixed methods case study	1	kept
Mixed methods design	mixed methods design	mixed methods design	mixed methods design	Mixed methods design	1	kept
Mixed methods study	mixed methods study	mixed methods study	mixed methods study	Mixed methods study	1	kept
Mixed Reality	mixed reality	mixed reality	mixed reality	Mixed reality	1	case_or_format
Mixed shock	mixed shock	mixed shock	mixed shock	Mixed shock	1	kept
mixed-method	mixed method	mixed method	mixed method	Mixed methods	1	case_or_format
Mixed-method design	mixed method design	mixed method design	mixed method design	mixed-method design	1	case_or_format
mixed-method study	mixed method study	mixed method study	mixed method study	Mixed-method study	1	case_or_format
MIXED-METHODS	mixed methods	mixed methods	mixed method	Mixed methods	1	singular
mixed-methods approach	mixed methods approach	mixed methods approach	mixed methods approach	mixed methods approach	1	case_or_format
mixed-methods research	mixed methods research	mixed methods research	mixed methods research	Mixed-methods research	1	case_or_format
MIXED-METHODS STUDY	mixed methods study	mixed methods study	mixed methods study	Mixed methods study	1	case_or_format
Mixed-methods study	mixed methods study	mixed methods study	mixed methods study	Mixed methods study	1	case_or_format
mixed-methods study	mixed methods study	mixed methods study	mixed methods study	Mixed methods study	1	case_or_format
mixed－method	mixed method	mixed method	mixed method	Mixed methods	1	case_or_format

mixed‑method design	mixed method design	mixed method design	mixed method design	mixed-method design	1	case_or_format
Mixing	mixing	mixing	mixing	Mixing	1	kept
mixing of injection	mixing of injection	mixing of injection	mixing of injection	mixing of injection	1	kept
MLP	mlp	mlp	mlp	MLP	1	kept
MLST	mlst	mlst	mlst	MLST	1	kept
MMD-HP	mmd hp	mmd hp	mmd hp	MMD-HP	1	kept
MMD-HP-SPA	mmd hp spa	mmd hp spa	mmd hp spa	MMD-HP-SPA	1	kept
MMD‑HP	mmd hp	mmd hp	mmd hp	MMD-HP	1	case_or_format
Mnemonic	mnemonic	mnemonic	mnemonic	Mnemonic	1	kept
mnemonic	mnemonic	mnemonic	mnemonic	Mnemonic	1	case_or_format
mNUTRIC score	mnutric score	mnutric score	mnutric score	mNUTRIC score	1	kept
mobbing	mobbing	mobbing	mobbing	mobbing	1	kept
Mobile	mobile	mobile	mobile	Mobile	1	kept
mobile	mobile	mobile	mobile	Mobile	1	case_or_format
Mobile communications	mobile communications	mobile communications	mobile communications	Mobile communications	1	kept
Mobile computing	mobile computing	mobile computing	mobile computing	Mobile computing	1	kept

mobile computing	mobile computing	mobile computing	mobile computing	Mobile computing	1	case_or_format
Mobile Device	mobile device	mobile device	mobile device	Mobile device	1	case_or_format
mobile device deployment	mobile device deployment	mobile device deployment	mobile device deployment	mobile device deployment	1	kept
Mobile electronic medical record	mobile electronic medical record	mobile electronic medical record	mobile electronic medical record	Mobile electronic medical record	1	kept
mobile enhanced family integrated care	mobile enhanced family integrated care	mobile enhanced family integrated care	mobile enhanced family integrated care	mobile enhanced family integrated care	1	kept
mobile eye tracker	mobile eye tracker	mobile eye tracker	mobile eye tracker	mobile eye tracker	1	kept
Mobile health	mobile health	mobile health	mobile health	mobile health	1	case_or_format
mobile health application	mobile health application	mobile health application	mobile health application	Mobile health application	1	case_or_format
mobile learning	mobile learning	mobile learning	mobile learning	Mobile learning	1	case_or_format
Mobile libraries	mobile libraries	mobile libraries	mobile libraries	Mobile libraries	1	kept
mobile medical teams	mobile medical teams	mobile medical teams	mobile medical teams	mobile medical teams	1	kept
mobile MRI	mobile mri	mobile mri	mobile mri	mobile MRI	1	kept
Mobile neuroendoscopy	mobile neuroendoscopy	mobile neuroendoscopy	mobile neuroendoscopy	Mobile neuroendoscopy	1	kept
mobile palliative care	mobile palliative care	mobile palliative care	mobile palliative care	mobile palliative care	1	kept
Mobile phone APP	mobile phone app	mobile phone app	mobile phone app	Mobile phone APP	1	kept
MOBILE PHONE APPLICATIONS	mobile phone applications	mobile phone applications	mobile phone applications	MOBILE PHONE APPLICATIONS	1	kept

mobile phone applications	mobile phone applications	mobile phone applications	mobile phone applications	MOBILE PHONE APPLICATIONS	1	case_or_format
Mobile phone text messaging	mobile phone text messaging	mobile phone text messaging	mobile phone text messaging	Mobile phone text messaging	1	kept
mobile phones	mobile phones	mobile phones	mobile phone	mobile phone	1	singular
Mobile robots	mobile robots	mobile robots	mobile robots	Mobile robots	1	kept
Mobile stroke unit	mobile stroke unit	mobile stroke unit	mobile stroke unit	Mobile stroke unit	1	kept
Mobile team	mobile team	mobile team	mobile team	Mobile team	1	kept
Mobile technology	mobile technology	mobile technology	mobile technology	Mobile technology	1	kept
Mobile telecommunication systems	mobile telecommunication systems	mobile telecommunication systems	mobile telecommunication systems	Mobile telecommunication systems	1	kept
Mobile X-ray	mobile x ray	mobile x ray	mobile x ray	Mobile X-ray	1	kept
Mobile x-ray	mobile x ray	mobile x ray	mobile x ray	Mobile X-ray	1	case_or_format
MOBILISATION	mobilisation	mobilisation	mobilisation	Mobilisation	1	case_or_format
Mobility champion	mobility champion	mobility champion	mobility champion	Mobility champion	1	kept
Mobility control	mobility control	mobility control	mobility control	Mobility control	1	kept
mobility limitation	mobility limitation	mobility limitation	mobility limitation	Mobility Limitation	1	case_or_format
Mobility physical therapy	mobility physical therapy	mobility physical therapy	mobility physical therapy	Mobility physical therapy	1	kept
Mobility programs	mobility programs	mobility programs	mobility programs	Mobility programs	1	kept

Mobility protocol	mobility protocol	mobility protocol	mobility protocol	Mobility protocol	1	kept
mobility work	mobility work	mobility work	mobility work	mobility work	1	kept
MOBILIZATION PRACTICES	mobilization practices	mobilization practices	mobilization practices	MOBILIZATION PRACTICES	1	kept
mobilization time	mobilization time	mobilization time	mobilization time	mobilization time	1	kept
Mobilization with turning Bispectral index	mobilization with turning bispectral index	mobilization with turning bispectral index	mobilization with turning bispectral index	Mobilization with turning Bispectral index	1	kept
Mochi	mochi	mochi	mochi	Mochi	1	kept
mochi	mochi	mochi	mochi	Mochi	1	case_or_format
Mock code	mock code	mock code	mock code	Mock code	1	kept
mock code	mock code	mock code	mock code	Mock code	1	case_or_format
modafinil	modafinil	modafinil	modafinil	modafinil	1	kept
Modality	modality	modality	modality	Modality	1	kept
modality	modality	modality	modality	Modality	1	case_or_format
mode of delivery	mode of delivery	mode of delivery	mode of delivery	mode of delivery	1	kept
Mode of transport	mode of transport	mode of transport	mode of transport	Mode of transport	1	kept
Model and analysis	model and analysis	model and analysis	model and analysis	Model and analysis	1	kept
Model based controls	model based controls	model based controls	model based control	Model-based control	1	singular

Model for improvement	model for improvement	model for improvement	model for improvement	Model for improvement	1	kept
model for improvement	model for improvement	model for improvement	model for improvement	Model for improvement	1	case_or_format
Model of delivery	model of delivery	model of delivery	model of delivery	Model of delivery	1	kept
Model validation	model validation	model validation	model validation	Model validation	1	kept
model, statistical	model statistical	model statistical	model statistical	model, statistical	1	kept
Model-based control	model based control	model based control	model based control	Model-based control	1	kept
Model-based Protocol	model based protocol	model based protocol	model based protocol	Model-based protocol	1	case_or_format
model-based protocol	model based protocol	model based protocol	model based protocol	Model-based protocol	1	case_or_format
Model-based virtual trial	model based virtual trial	model based virtual trial	model based virtual trial	Model-based virtual trial	1	kept
Modeling approach	modeling approach	modeling approach	modeling approach	Modeling approach	1	kept
MODELING DEVELOPMENT	modeling development	modeling development	modeling development	MODELING DEVELOPMENT	1	kept
modelling and scenario building	modelling and scenario building	modelling and scenario building	modelling and scenario building	modelling and scenario building	1	kept
Models, Biopsychosocial	models biopsychosocial	models biopsychosocial	models biopsychosocial	Models, Biopsychosocial	1	kept
models, statistical	models statistical	models statistical	models statistical	Models, Statistical	1	case_or_format
Models: organizational	models organizational	models organizational	models organizational	Models, Organizational	1	case_or_format
Modems	modems	modems	modems	Modems	1	kept

MODERATE	moderate	moderate	moderate	MODERATE	1	kept
moderate	moderate	moderate	moderate	MODERATE	1	case_or_format
Moderate and severe craniocerebral injury	moderate and severe craniocerebral injury	moderate and severe craniocerebral injury	moderate and severe craniocerebral injury	Moderate and severe craniocerebral injury	1	kept
Moderate Fidelity In Situ Simulation	moderate fidelity in situ simulation	moderate fidelity in situ simulation	moderate fidelity in situ simulation	Moderate Fidelity In Situ Simulation	1	kept
moderate preterm	moderate preterm	moderate preterm	moderate preterm	moderate preterm	1	kept
moderate sedation	moderate sedation	moderate sedation	moderate sedation	Moderate sedation	1	case_or_format
Moderate to severe traumatic brain injury	moderate to severe traumatic brain injury	moderate to severe traumatic brain injury	moderate to severe traumatic brain injury	Moderate to severe traumatic brain injury	1	kept
moderate to severe traumatic brain injury	moderate to severe traumatic brain injury	moderate to severe traumatic brain injury	moderate to severe traumatic brain injury	Moderate to severe traumatic brain injury	1	case_or_format
moderated mediation	moderated mediation	moderated mediation	moderated mediation	moderated mediation	1	kept
moderated mediation model	moderated mediation model	moderated mediation model	moderated mediation model	moderated mediation model	1	kept
moderately preterm infant	moderately preterm infant	moderately preterm infant	moderately preterm infant	moderately preterm infant	1	kept
Moderating	moderating	moderating	moderating	Moderating	1	kept
Moderating effects	moderating effects	moderating effects	moderating effect	Moderating effect	1	singular
MODERATING ROLE	moderating role	moderating role	moderating role	MODERATING ROLE	1	kept
moderating variables	moderating variables	moderating variables	moderating variables	moderating variables	1	kept
moderation	moderation	moderation	moderation	moderation	1	kept

moderation effect	moderation effect	moderation effect	moderation effect	moderation effect	1	kept
MODERATOR	moderator	moderator	moderator	MODERATOR	1	kept
moderator variable	moderator variable	moderator variable	moderator variable	moderator variable	1	kept
Moderators	moderators	moderators	moderator	MODERATOR	1	singular
Modern health system	modern health system	modern health system	modern health system	Modern health system	1	kept
Modern medicine	modern medicine	modern medicine	modern medicine	Modern medicine	1	kept
modern times	modern times	modern times	modern times	modern times	1	kept
Moderna	moderna	moderna	moderna	Moderna	1	kept
Modernisation agency	modernisation agency	modernisation agency	modernisation agency	Modernisation agency	1	kept
Modification	modification	modification	modification	Modification	1	kept
Modifications of vital signs	modifications of vital signs	modifications of vital signs	modifications of vital signs	Modifications of vital signs	1	kept
modified aldrete recovery score	modified aldrete recovery score	modified aldrete recovery score	modified aldrete recovery score	modified aldrete recovery score	1	kept
modified and adapted oral sensorimotor intervention	modified and adapted oral sensorimotor intervention	modified and adapted oral sensorimotor intervention	modified and adapted oral sensorimotor intervention	modified and adapted oral sensorimotor intervention	1	kept
Modified Ashworth Scale	modified ashworth scale	modified ashworth scale	modified ashworth scale	Modified Ashworth Scale	1	kept
Modified Ashworth Scale 2	modified ashworth scale 2	modified ashworth scale 2	modified ashworth scale 2	Modified Ashworth Scale 2	1	kept
Modified Beck Oral Assessment Scale	modified beck oral assessment scale	modified beck oral assessment scale	modified beck oral assessment scale	Modified Beck Oral Assessment Scale	1	kept

modified Blalock-Thomas-Taussig shunt	modified blalock thomas taussig shunt	modified blalock thomas taussig shunt	modified blalock thomas taussig shunt	modified Blalock-Thomas-Taussig shunt	1	kept
modified Borg dyspnea scale	modified borg dyspnea scale	modified borg dyspnea scale	modified borg dyspnea scale	modified Borg dyspnea scale	1	kept
modified Clinical Pulmonary Infection Score	modified clinical pulmonary infection score	modified clinical pulmonary infection score	modified clinical pulmonary infection score	modified Clinical Pulmonary Infection Score	1	kept
Modified Delphi	modified delphi	modified delphi	modified delphi	modified Delphi	1	case_or_format
modified delphi consensus study	modified delphi consensus study	modified delphi consensus study	modified delphi consensus study	modified delphi consensus study	1	kept
Modified Delphi method	modified delphi method	modified delphi method	modified delphi method	Modified Delphi method	1	kept
Modified early warning score	modified early warning score	modified early warning score	modified early warning score	Modified Early Warning Score	1	case_or_format
modified early warning scoring	modified early warning scoring	modified early warning scoring	modified early warning scoring	modified early warning scoring	1	kept
Modified early warning system	modified early warning system	modified early warning system	modified early warning system	Modified early warning system	1	kept
modified exchange blood transfusion therapy	modified exchange blood transfusion therapy	modified exchange blood transfusion therapy	modified exchange blood transfusion therapy	modified exchange blood transfusion therapy	1	kept
Modified frailty index	modified frailty index	modified frailty index	modified frailty index	modified frailty index	1	case_or_format
modified freedman questionnaire	modified freedman questionnaire	modified freedman questionnaire	modified freedman questionnaire	modified freedman questionnaire	1	kept
Modified Glasgow coma scale motor response	modified glasgow coma scale motor response	modified glasgow coma scale motor response	modified glasgow coma scale motor response	Modified Glasgow coma scale motor response	1	kept
modified high flow nasal cannula therapy	modified high flow nasal cannula therapy	modified high flow nasal cannula therapy	modified high flow nasal cannula therapy	modified high flow nasal cannula therapy	1	kept
modified knowledge and attitude survey regarding pain	modified knowledge and attitude survey regarding pain	modified knowledge and attitude survey regarding pain	modified knowledge and attitude survey regarding pain	modified knowledge and attitude survey regarding pain	1	kept
Modified NUTRIC score	modified nutric score	modified nutric score	modified nutric score	Modified NUTRIC score	1	kept

modified nutritional risk in the critical ill score	modified nutritional risk in the critical ill score	modified nutritional risk in the critical ill score	modified nutritional risk in the critical ill score	modified nutritional risk in the critical ill score	1	kept
Modified Obstetric Early Warning System	modified obstetric early warning system	modified obstetric early warning system	modified obstetric early warning system	Modified Obstetric Early Warning System	1	kept
modified Perceived Devaluation Discrimination scale	modified perceived devaluation discrimination scale	modified perceived devaluation discrimination scale	modified perceived devaluation discrimination scale	modified Perceived Devaluation Discrimination scale	1	kept
Modified PIC score	modified pic score	modified pic score	modified pic score	Modified PIC score	1	kept
modified postoperative pain questionnaire	modified postoperative pain questionnaire	modified postoperative pain questionnaire	modified postoperative pain questionnaire	modified postoperative pain questionnaire	1	kept
modified pressure ulcer staging system checklist	modified pressure ulcer staging system checklist	modified pressure ulcer staging system checklist	modified pressure ulcer staging system checklist	modified pressure ulcer staging system checklist	1	kept
Modified Rankin Scale	modified rankin scale	modified rankin scale	modified rankin scale	Modified Rankin Scale	1	kept
modified Rankin Scale	modified rankin scale	modified rankin scale	modified rankin scale	Modified Rankin Scale	1	case_or_format
modified rankin scale	modified rankin scale	modified rankin scale	modified rankin scale	Modified Rankin Scale	1	case_or_format
modified respiratory clinical score	modified respiratory clinical score	modified respiratory clinical score	modified respiratory clinical score	modified respiratory clinical score	1	kept
modified Schofield guideline	modified schofield guideline	modified schofield guideline	modified schofield guideline	modified Schofield guideline	1	kept
Modified Seldinger technique	modified seldinger technique	modified seldinger technique	modified seldinger technique	Modified Seldinger technique	1	kept
modified Seldinger technique	modified seldinger technique	modified seldinger technique	modified seldinger technique	Modified Seldinger technique	1	case_or_format
Modified Self Assessment	modified self assessment	modified self assessment	modified self assessment	Modified Self Assessment	1	kept
modified Sequential Organ Failure Assessment score	modified sequential organ failure assessment score	modified sequential organ failure assessment score	modified sequential organ failure assessment score	modified Sequential Organ Failure Assessment score	1	kept
Modified Shock Index	modified shock index	modified shock index	modified shock index	Modified Shock Index	1	kept

modified the Pain, Inspiratory Effort, Cough score	modified the pain inspiratory effort cough score	modified the pain inspiratory effort cough score	modified the pain inspiratory effort cough score	modified the Pain, Inspiratory Effort, Cough score	1	kept
modified trunk rotation test	modified trunk rotation test	modified trunk rotation test	modified trunk rotation test	modified trunk rotation test	1	kept
modified water swallowing test score	modified water swallowing test score	modified water swallowing test score	modified water swallowing test score	modified water swallowing test score	1	kept
MODS score	mods score	mods score	mods score	MODS score	1	kept
Modular ethical treatment allocation process model	modular ethical treatment allocation process model	modular ethical treatment allocation process model	modular ethical treatment allocation process model	Modular ethical treatment allocation process model	1	kept
Modularized teaching	modularized teaching	modularized teaching	modularized teaching	Modularized teaching	1	kept
Modulation	modulation	modulation	modulation	Modulation	1	kept
Module	module	module	module	Module	1	kept
mogamulizumab	mogamulizumab	mogamulizumab	mogamulizumab	mogamulizumab	1	kept
MOI = mechanism of injury	moi mechanism of injury	moi mechanism of injury	moi mechanism of injury	MOI = mechanism of injury	1	kept
Moisture lesion	moisture lesion	moisture lesion	moisture lesion	moisture lesion	1	case_or_format
Moisture lesion prescription sticker	moisture lesion prescription sticker	moisture lesion prescription sticker	moisture lesion prescription sticker	Moisture lesion prescription sticker	1	kept
Moisture-associated damage	moisture associated damage	moisture associated damage	moisture associated damage	Moisture-associated damage	1	kept
Moisture-associated skin damage	moisture associated skin damage	moisture associated skin damage	moisture associated skin damage	moisture-associated skin damage	1	case_or_format
Moisturiser	moisturiser	moisturiser	moisturiser	Moisturiser	1	kept
moisturising	moisturising	moisturising	moisturising	moisturising	1	kept

moisturizer	moisturizer	moisturizer	moisturizer	moisturizer	1	kept
Molar	molar	molar	molar	Molar	1	kept
molecular beacon	molecular beacon	molecular beacon	molecular beacon	molecular beacon	1	kept
Molecular Biology	molecular biology	molecular biology	molecular biology	molecular biology	1	case_or_format
molecular characterization	molecular characterization	molecular characterization	molecular characterization	molecular characterization	1	kept
molecular cloning	molecular cloning	molecular cloning	molecular cloning	molecular cloning	1	kept
molecular diagnosis for infectious diseases	molecular diagnosis for infectious diseases	molecular diagnosis for infectious diseases	molecular diagnosis for infectious diseases	molecular diagnosis for infectious diseases	1	kept
Molecular Diagnostic Techniques	molecular diagnostic techniques	molecular diagnostic techniques	molecular diagnostic techniques	Molecular Diagnostic Techniques	1	kept
molecular diagnostics	molecular diagnostics	molecular diagnostics	molecular diagnostics	molecular diagnostics	1	kept
molecular docking	molecular docking	molecular docking	molecular docking	molecular docking	1	kept
MOLECULAR EPIDEMIOLOGY	molecular epidemiology	molecular epidemiology	molecular epidemiology	molecular epidemiology	1	case_or_format
molecular genetics	molecular genetics	molecular genetics	molecular genetics	molecular genetics	1	kept
molecular interaction	molecular interaction	molecular interaction	molecular interaction	molecular interaction	1	kept
molecular model	molecular model	molecular model	molecular model	molecular model	1	kept
molecular phylogeny	molecular phylogeny	molecular phylogeny	molecular phylogeny	molecular phylogeny	1	kept
Molecular Sequence Data	molecular sequence data	molecular sequence data	molecular sequence data	Molecular Sequence Data	1	kept

molecularly targeted therapy	molecularly targeted therapy	molecularly targeted therapy	molecularly targeted therapy	molecularly targeted therapy	1	kept
molnupiravir	molnupiravir	molnupiravir	molnupiravir	molnupiravir	1	kept
molybdenum	molybdenum	molybdenum	molybdenum	molybdenum	1	kept
Moments of hand hygiene	moments of hand hygiene	moments of hand hygiene	moments of hand hygiene	Moments of hand hygiene	1	kept
mometasone furoate	mometasone furoate	mometasone furoate	mometasone furoate	mometasone furoate	1	kept
MONASH Bioethic Review	monash bioethic review	monash bioethic review	monash bioethic review	MONASH Bioethic Review	1	kept
moni-toring, physiologic	moni toring physiologic	moni toring physiologic	moni toring physiologic	moni-toring, physiologic	1	kept
monitor alarm	monitor alarm	monitor alarm	monitor alarm	monitor alarm	1	kept
MONITOR ALARMS	monitor alarms	monitor alarms	monitor alarm	monitor alarm	1	singular
Monitor alarms	monitor alarms	monitor alarms	monitor alarm	monitor alarm	1	singular
monitor alarms	monitor alarms	monitor alarms	monitor alarm	monitor alarm	1	singular
monitor surveillance	monitor surveillance	monitor surveillance	monitor surveillance	monitor surveillance	1	kept
monitorage	monitorage	monitorage	monitorage	monitorage	1	kept
Monitoring and surveillance	monitoring and surveillance	monitoring and surveillance	monitoring and surveillance	Monitoring and surveillance	1	kept
Monitoring and tracking	monitoring and tracking	monitoring and tracking	monitoring and tracking	Monitoring and tracking	1	kept
Monitoring applications	monitoring applications	monitoring applications	monitoring applications	Monitoring applications	1	kept

Monitoring centers	monitoring centers	monitoring centers	monitoring centers	Monitoring centers	1	kept
monitoring devices	monitoring devices	monitoring devices	monitoring device	Monitoring device	1	singular
Monitoring displays	monitoring displays	monitoring displays	monitoring displays	Monitoring displays	1	kept
monitoring error	monitoring error	monitoring error	monitoring error	monitoring error	1	kept
Monitoring mechanisms	monitoring mechanisms	monitoring mechanisms	monitoring mechanisms	Monitoring mechanisms	1	kept
Monitoring parameters	monitoring parameters	monitoring parameters	monitoring parameters	Monitoring parameters	1	kept
Monitoring signals	monitoring signals	monitoring signals	monitoring signals	Monitoring signals	1	kept
MONITORING SYSTEMS	monitoring systems	monitoring systems	monitoring system	Monitoring system	1	singular
Monitoring systems	monitoring systems	monitoring systems	monitoring system	Monitoring system	1	singular
monitoring systems	monitoring systems	monitoring systems	monitoring system	Monitoring system	1	singular
Monitoring technologies	monitoring technologies	monitoring technologies	monitoring technologies	Monitoring technologies	1	kept
monitoring technologies	monitoring technologies	monitoring technologies	monitoring technologies	Monitoring technologies	1	case_or_format
Monitoring, bispectral index	monitoring bispectral index	monitoring bispectral index	monitoring bispectral index	Monitoring, bispectral index	1	kept
Monitoring-cuff pressure	monitoring cuff pressure	monitoring cuff pressure	monitoring cuff pressure	Monitoring-cuff pressure	1	kept
Monitorización	monitorizaci n	monitorizaci n	monitorizaci n	Monitorización	1	kept
Monitorización continua de glucosa	monitorizaci n continua de glucosa	monitorizaci n continua de glucosa	monitorizaci n continua de glucosa	Monitorización continua de glucosa	1	kept

Monitorización dolor-agitación-delirio	monitorizaci n dolor agitaci n delirio	monitorizaci n dolor agitaci n delirio	monitorizaci n dolor agitaci n delirio	Monitorización dolor-agitación-delirio	1	kept
Monitorización electrofisiológica	monitorizaci n electrofisiol gica	monitorizaci n electrofisiol gica	monitorizaci n electrofisiol gica	Monitorización electrofisiológica	1	kept
Monitorization	monitorization	monitorization	monitorization	Monitorization	1	kept
monitors	monitors	monitors	monitor	monitor	1	singular
Monkeypox virus	monkeypox virus	monkeypox virus	monkeypox virus	Monkeypox virus	1	kept
monoamine	monoamine	monoamine	monoamine	monoamine	1	kept
Monoamine oxidase inhibitors	monoamine oxidase inhibitors	monoamine oxidase inhibitors	monoamine oxidase inhibitors	Monoamine oxidase inhibitors	1	kept
monochorionic twins	monochorionic twins	monochorionic twins	monochorionic twins	monochorionic twins	1	kept
monoclonal antibodies	monoclonal antibodies	monoclonal antibodies	monoclonal antibody	monoclonal antibody	1	singular
Monoclonal antibody	monoclonal antibody	monoclonal antibody	monoclonal antibody	monoclonal antibody	1	case_or_format
monoclonal antibody therapies	monoclonal antibody therapies	monoclonal antibody therapies	monoclonal antibody therapy	monoclonal antibody therapy	1	singular
monocomponent insulin	monocomponent insulin	monocomponent insulin	monocomponent insulin	monocomponent insulin	1	kept
Monocyte	monocyte	monocyte	monocyte	monocyte	1	case_or_format
MONOCYTE CHEMOATTRACTANT PROTEIN-1	monocyte chemoattractant protein 1	monocyte chemoattractant protein 1	monocyte chemoattractant protein 1	MONOCYTE CHEMOATTRACTANT PROTEIN-1	1	kept
monocyte chemotactic protein	monocyte chemotactic protein	monocyte chemotactic protein	monocyte chemotactic protein	monocyte chemotactic protein	1	kept
monocyte count	monocyte count	monocyte count	monocyte count	monocyte count	1	kept

monocytosis	monocytosis	monocytosis	monocytosis	monocytosis	1	kept
monomodal analgesia	monomodal analgesia	monomodal analgesia	monomodal analgesia	monomodal analgesia	1	kept
mononuclear cell	mononuclear cell	mononuclear cell	mononuclear cell	mononuclear cell	1	kept
Mononucleotide polymorphism	mononucleotide polymorphism	mononucleotide polymorphism	mononucleotide polymorphism	Mononucleotide polymorphism	1	kept
Monotonic properties	monotonic properties	monotonic properties	monotonic properties	Monotonic properties	1	kept
monounsaturated fatty acid	monounsaturated fatty acid	monounsaturated fatty acid	monounsaturated fatty acid	monounsaturated fatty acid	1	kept
Montana	montana	montana	montana	Montana	1	kept
Monte carlo	monte carlo	monte carlo	monte carlo	Monte carlo	1	kept
montebello rehabilitation factor score revised	montebello rehabilitation factor score revised	montebello rehabilitation factor score revised	montebello rehabilitation factor score revised	montebello rehabilitation factor score revised	1	kept
montefiore medical center	montefiore medical center	montefiore medical center	montefiore medical center	montefiore medical center	1	kept
MOOC	mooc	mooc	mooc	MOOC	1	kept
Mood disorder	mood disorder	mood disorder	mood disorder	mood disorder	1	case_or_format
mood stabilizer	mood stabilizer	mood stabilizer	mood stabilizer	mood stabilizer	1	kept
mood state	mood state	mood state	mood state	Mood states	1	case_or_format
mood swing	mood swing	mood swing	mood swing	mood swing	1	kept
Mor-bidity	mor bidity	mor bidity	mor bidity	Mor-bidity	1	kept

moral case deliberation	moral case deliberation	moral case deliberation	moral case deliberation	moral case deliberation	1	kept
moral certainty	moral certainty	moral certainty	moral certainty	moral certainty	1	kept
Moral challenges	moral challenges	moral challenges	moral challenges	Moral challenges	1	kept
moral climate	moral climate	moral climate	moral climate	moral climate	1	kept
moral conflict assessment	moral conflict assessment	moral conflict assessment	moral conflict assessment	moral conflict assessment	1	kept
Moral deliberative method	moral deliberative method	moral deliberative method	moral deliberative method	Moral deliberative method	1	kept
moral development	moral development	moral development	moral development	Moral Development	1	case_or_format
Moral dilemma	moral dilemma	moral dilemma	moral dilemma	moral dilemma	1	case_or_format
Moral dilemmas	moral dilemmas	moral dilemmas	moral dilemma	moral dilemma	1	singular
moral distress level	moral distress level	moral distress level	moral distress level	moral distress level	1	kept
Moral Distress Scale Revised	moral distress scale revised	moral distress scale revised	moral distress scale revised	Moral Distress Scale Revised	1	kept
moral distress scale revised	moral distress scale revised	moral distress scale revised	moral distress scale revised	Moral Distress Scale Revised	1	case_or_format
Moral Distress Scale-Revised	moral distress scale revised	moral distress scale revised	moral distress scale revised	Moral Distress Scale Revised	1	case_or_format
Moral distress scale-revised	moral distress scale revised	moral distress scale revised	moral distress scale revised	Moral Distress Scale Revised	1	case_or_format
Moral Distress Score	moral distress score	moral distress score	moral distress score	Moral Distress Score	1	kept
moral distress theory	moral distress theory	moral distress theory	moral distress theory	moral distress theory	1	kept

Moral distress thermometer	moral distress thermometer	moral distress thermometer	moral distress thermometer	Moral distress thermometer	1	kept
Moral duty	moral duty	moral duty	moral duty	Moral duty	1	kept
moral emotions	moral emotions	moral emotions	moral emotions	Moral emotions	1	case_or_format
moral foundations	moral foundations	moral foundations	moral foundations	moral foundations	1	kept
moral integrity	moral integrity	moral integrity	moral integrity	moral integrity	1	kept
Moral intelligence	moral intelligence	moral intelligence	moral intelligence	Moral intelligence	1	kept
moral intelligence	moral intelligence	moral intelligence	moral intelligence	Moral intelligence	1	case_or_format
moral judgement	moral judgement	moral judgement	moral judgement	moral judgement	1	kept
MORAL JUDGMENT	moral judgment	moral judgment	moral judgment	MORAL JUDGMENT	1	kept
Moral judgment	moral judgment	moral judgment	moral judgment	MORAL JUDGMENT	1	case_or_format
Moral judgments	moral judgments	moral judgments	moral judgment	MORAL JUDGMENT	1	singular
Moral laboratory	moral laboratory	moral laboratory	moral laboratory	Moral laboratory	1	kept
moral leader	moral leader	moral leader	moral leader	moral leader	1	kept
moral motivation	moral motivation	moral motivation	moral motivation	moral motivation	1	kept
moral obligation	moral obligation	moral obligation	moral obligation	Moral Obligations	1	case_or_format
Moral obligations	moral obligations	moral obligations	moral obligation	Moral Obligations	1	singular

moral practice	moral practice	moral practice	moral practice	moral practice	1	kept
moral reasoning	moral reasoning	moral reasoning	moral reasoning	moral reasoning	1	kept
MORAL REASONING SKILLS	moral reasoning skills	moral reasoning skills	moral reasoning skills	MORAL REASONING SKILLS	1	kept
Moral residue	moral residue	moral residue	moral residue	Moral residue	1	kept
moral residue	moral residue	moral residue	moral residue	Moral residue	1	case_or_format
Moral resiliency	moral resiliency	moral resiliency	moral resiliency	Moral resiliency	1	kept
moral resiliency	moral resiliency	moral resiliency	moral resiliency	Moral resiliency	1	case_or_format
MORAL SENSITIVITY	moral sensitivity	moral sensitivity	moral sensitivity	moral sensitivity	1	case_or_format
Moral stress	moral stress	moral stress	moral stress	moral stress	1	case_or_format
moral struggle	moral struggle	moral struggle	moral struggle	moral struggle	1	kept
moral suffering	moral suffering	moral suffering	moral suffering	moral suffering	1	kept
morale	morale	morale	morale	Morale	1	case_or_format
Moraxella catahallis	moraxella catahallis	moraxella catahallis	moraxella catahallis	Moraxella catahallis	1	kept
Moraxella infection	moraxella infection	moraxella infection	moraxella infection	Moraxella infection	1	kept
morbid	morbid	morbid	morbid	morbid	1	kept
MORBID-OBESITY	morbid obesity	morbid obesity	morbid obesity	morbid obesity	1	case_or_format

Morbidade	morbidade	morbidade	morbidade	Morbidade	1	kept
morbidity and mortality	morbidity and mortality	morbidity and mortality	morbidity and mortality	Morbidity and mortality	1	case_or_format
Morbidity and Mortality Acute Predictor	morbidity and mortality acute predictor	morbidity and mortality acute predictor	morbidity and mortality acute predictor	Morbidity and Mortality Acute Predictor	1	kept
Morbidly obese	morbidly obese	morbidly obese	morbidly obese	Morbidly obese	1	kept
Morbilidad	morbilidad	morbilidad	morbilidad	Morbilidad	1	kept
Morgue	morgue	morgue	morgue	morgue	1	case_or_format
Morisky Medication Adherence Scale	morisky medication adherence scale	morisky medication adherence scale	morisky medication adherence scale	Morisky Medication Adherence Scale	1	kept
Morningness	morningness	morningness	morningness	Morningness	1	kept
Morningness Eveningness Questionnaire	morningness eveningness questionnaire	morningness eveningness questionnaire	morningness eveningness questionnaire	Morningness Eveningness Questionnaire	1	kept
morningness-eveningness	morningness eveningness	morningness eveningness	morningness eveningness	morningness-eveningness	1	kept
Morningness-Eveningness Questionnaire	morningness eveningness questionnaire	morningness eveningness questionnaire	morningness eveningness questionnaire	Morningness Eveningness Questionnaire	1	case_or_format
Morocc0	morocc0	morocc0	morocc0	Morocc0	1	kept
Moroccan	moroccan	moroccan	moroccan	Moroccan	1	kept
morocco	morocco	morocco	morocco	Morocco	1	case_or_format
morphine 3 glucuronide	morphine 3 glucuronide	morphine 3 glucuronide	morphine 3 glucuronide	morphine 3 glucuronide	1	kept
morphine 6 glucuronide	morphine 6 glucuronide	morphine 6 glucuronide	morphine 6 glucuronide	morphine 6 glucuronide	1	kept

morphine derivative	morphine derivative	morphine derivative	morphine derivative	Morphine Derivatives	1	case_or_format
morphine glucuronide	morphine glucuronide	morphine glucuronide	morphine glucuronide	morphine glucuronide	1	kept
Morphology	morphology	morphology	morphology	morphology	1	case_or_format
Morris Activities of Daily Living Scale	morris activities of daily living scale	morris activities of daily living scale	morris activities of daily living scale	Morris Activities of Daily Living Scale	1	kept
Morris scale	morris scale	morris scale	morris scale	Morris scale	1	kept
morse fall scale	morse fall scale	morse fall scale	morse fall scale	Morse Fall Scale	1	case_or_format
Morse scale	morse scale	morse scale	morse scale	Morse scale	1	kept
Mort	mort	mort	mort	Mort	1	kept
mort	mort	mort	mort	Mort	1	case_or_format
Mortalidad hospitalaria	mortalidad hospitalaria	mortalidad hospitalaria	mortalidad hospitalaria	Mortalidad hospitalaria	1	kept
Mortalidad materna	mortalidad materna	mortalidad materna	mortalidad materna	Mortalidad materna	1	kept
Mortalidade	mortalidade	mortalidade	mortalidade	Mortalidade	1	kept
Mortalidade materna	mortalidade materna	mortalidade materna	mortalidade materna	Mortalidade materna	1	kept
mortality and major disability	mortality and major disability	mortality and major disability	mortality and major disability	mortality and major disability	1	kept
mortality and morbidity	mortality and morbidity	mortality and morbidity	mortality and morbidity	mortality and morbidity	1	kept
mortality benefit	mortality benefit	mortality benefit	mortality benefit	mortality benefit	1	kept

Mortality determinants	mortality determinants	mortality determinants	mortality determinants	Mortality determinants	1	kept
mortality in surgery	mortality in surgery	mortality in surgery	mortality in surgery	mortality in surgery	1	kept
Mortality index	mortality index	mortality index	mortality index	Mortality index	1	kept
Mortality Prediction	mortality prediction	mortality prediction	mortality prediction	Mortality prediction	1	case_or_format
mortality predictors	mortality predictors	mortality predictors	mortality predictors	mortality predictors	1	kept
mortality probability model	mortality probability model	mortality probability model	mortality probability model	mortality probability model	1	kept
MORTALITY RATES	mortality rates	mortality rates	mortality rate	mortality rate	1	singular
Mortality statistics	mortality statistics	mortality statistics	mortality statistics	Mortality statistics	1	kept
mortality) < measurement of quality	mortality measurement of quality	mortality measurement of quality	mortality measurement of quality	mortality) < measurement of quality	1	kept
Mortality, Premature	mortality premature	mortality premature	mortality premature	Mortality, Premature	1	kept
MORTALITY-RATE	mortality rate	mortality rate	mortality rate	mortality rate	1	case_or_format
MORTALITY-RATES	mortality rates	mortality rates	mortality rate	mortality rate	1	singular
Morte	morte	morte	morte	Morte	1	kept
Morte Súbita	morte s bita	morte s bita	morte s bita	Morte Súbita	1	kept
Mosaicism	mosaicism	mosaicism	mosaicism	Mosaicism	1	kept
mosapride citrate	mosapride citrate	mosapride citrate	mosapride citrate	mosapride citrate	1	kept

mosquito	mosquito	mosquito	mosquito	mosquito	1	kept
mosquito bite	mosquito bite	mosquito bite	mosquito bite	mosquito bite	1	kept
Most likely	most likely	most likely	most likely	Most likely	1	kept
motavizumab	motavizumab	motavizumab	motavizumab	motavizumab	1	kept
mothballs	mothballs	mothballs	mothballs	mothballs	1	kept
Mother and baby closeness	mother and baby closeness	mother and baby closeness	mother and baby closeness	Mother and baby closeness	1	kept
MOTHER CARE	mother care	mother care	mother care	MOTHER CARE	1	kept
mother fetus relationship	mother fetus relationship	mother fetus relationship	mother fetus relationship	mother fetus relationship	1	kept
Mother information needs	mother information needs	mother information needs	mother information needs	Mother information needs	1	kept
Mother interactions	mother interactions	mother interactions	mother interactions	Mother interactions	1	kept
mother participation	mother participation	mother participation	mother participation	mother participation	1	kept
Mother relationship	mother relationship	mother relationship	mother relationship	Mother relationship	1	kept
mother to infant bonding scale	mother to infant bonding scale	mother to infant bonding scale	mother to infant bonding scale	mother to infant bonding scale	1	kept
mother who cannot breastfeed	mother who cannot breastfeed	mother who cannot breastfeed	mother who cannot breastfeed	mother who cannot breastfeed	1	kept
Mother's	mother s	mother s	mother s	Mother's	1	kept
Mother's anxiety	mother s anxiety	mother s anxiety	mother s anxiety	Mother's anxiety	1	kept

Mother's heart beat	mother s heart beat	mother s heart beat	mother s heart beat	Mother's heart beat	1	kept
mother's own milk	mother s own milk	mother s own milk	mother s own milk	mother's own milk	1	kept
mother's own milk feeding	mother s own milk feeding	mother s own milk feeding	mother s own milk feeding	mother's own milk feeding	1	kept
mother's own milk provision	mother s own milk provision	mother s own milk provision	mother s own milk provision	mother's own milk provision	1	kept
mother's voice	mother s voice	mother s voice	mother s voice	Mother's voice	1	case_or_format
Mother-baby separation	mother baby separation	mother baby separation	mother baby separation	Mother-baby separation	1	kept
mother-baby separation	mother baby separation	mother baby separation	mother baby separation	Mother-baby separation	1	case_or_format
Mother-child interaction	mother child interaction	mother child interaction	mother child interaction	Mother-child interaction	1	kept
Mother-Child Relationships	mother child relationships	mother child relationships	mother child relationship	Mother－child relationship	1	singular
Mother-child relationships	mother child relationships	mother child relationships	mother child relationship	Mother－child relationship	1	singular
mother-infant attachment	mother infant attachment	mother infant attachment	mother infant attachment	Mother-infant attachment	1	case_or_format
Mother-infant relationship	mother infant relationship	mother infant relationship	mother infant relationship	Mother-infant relationship	1	kept
Mother-infant synchrony	mother infant synchrony	mother infant synchrony	mother infant synchrony	Mother-infant synchrony	1	kept
motherhood	motherhood	motherhood	motherhood	Motherhood	1	case_or_format
Motherhood awareness	motherhood awareness	motherhood awareness	motherhood awareness	Motherhood awareness	1	kept
Motherhood development	motherhood development	motherhood development	motherhood development	Motherhood development	1	kept

Motherhood satisfaction	motherhood satisfaction	motherhood satisfaction	motherhood satisfaction	Motherhood satisfaction	1	kept
mothers continued view of their toddlers as vulnerable and always	mothers continued view of their toddlers as vulnerable and always	mothers continued view of their toddlers as vulnerable and always	mothers continued view of their toddlers as vulnerable and always	mothers continued view of their toddlers as vulnerable and always	1	kept
MOTHERS EXPERIENCES	mothers experiences	mothers experiences	mothers experience	mothers’ experiences	1	singular
mothers of premature infant	mothers of premature infant	mothers of premature infant	mothers of premature infant	mothers of premature infants	1	case_or_format
mothers with premature infant	mothers with premature infant	mothers with premature infant	mothers with premature infant	mothers with premature infant	1	kept
Mothers’ experience	mothers experience	mothers experience	mothers experience	mothers’ experiences	1	case_or_format
Mothers’ experiences	mothers experiences	mothers experiences	mothers experience	mothers’ experiences	1	singular
mothers’ experiences	mothers experiences	mothers experiences	mothers experience	mothers’ experiences	1	singular
mothers, mothering	mothers mothering	mothers mothering	mothers mothering	mothers, mothering	1	kept
Motherâ€″infant interaction	mother infant interaction	mother infant interaction	mother infant interaction	Mother-infant interaction	1	case_or_format
Mother－child relationship	mother child relationship	mother child relationship	mother child relationship	Mother－child relationship	1	kept
mother－infant attachment	mother infant attachment	mother infant attachment	mother infant attachment	Mother-infant attachment	1	case_or_format
mother－infant bonding	mother infant bonding	mother infant bonding	mother infant bonding	mother－infant bonding	1	kept
mother－infant interaction	mother infant interaction	mother infant interaction	mother infant interaction	Mother-infant interaction	1	case_or_format
mother’s feeling	mother s feeling	mother s feeling	mother s feeling	mother’s feeling	1	kept
mother’s heartbeat	mother s heartbeat	mother s heartbeat	mother s heartbeat	mother’s heartbeat	1	kept

Mother’ s lullabies	mother s lullabies	mother s lullabies	mother s lullabies	Mother’ s lullabies	1	kept
mother’ s own milk	mother s own milk	mother s own milk	mother s own milk	mother’s own milk	1	case_or_format
Mother’ s voice	mother s voice	mother s voice	mother s voice	Mother’s voice	1	case_or_format
MOTION	motion	motion	motion	motion	1	case_or_format
Motion analysis	motion analysis	motion analysis	motion analysis	Motion analysis	1	kept
Motion control	motion control	motion control	motion control	Motion control	1	kept
Motion detection	motion detection	motion detection	motion detection	Motion detection	1	kept
Motion Pictures	motion pictures	motion pictures	motion pictures	Motion Pictures	1	kept
motion sickness	motion sickness	motion sickness	motion sickness	motion sickness	1	kept
Motion tracking	motion tracking	motion tracking	motion tracking	Motion tracking	1	kept
Motion-Time Studies	motion time studies	motion time studies	motion time studies	Motion-Time Studies	1	kept
Motivation Factors	motivation factors	motivation factors	motivation factors	Motivation Factors	1	kept
Motivation to learn	motivation to learn	motivation to learn	motivation to learn	Motivation to learn	1	kept
Motivational Interview	motivational interview	motivational interview	motivational interview	Motivational Interview	1	kept
Motivational psychological intervention	motivational psychological intervention	motivational psychological intervention	motivational psychological intervention	Motivational psychological intervention	1	kept
motoneuron	motoneuron	motoneuron	motoneuron	motoneuron	1	kept

motor abnormalities	motor abnormalities	motor abnormalities	motor abnormalities	motor abnormalities	1	kept
Motor activity assessment scale	motor activity assessment scale	motor activity assessment scale	motor activity assessment scale	Motor activity assessment scale	1	kept
motor coordination	motor coordination	motor coordination	motor coordination	motor coordination	1	kept
motor cortex	motor cortex	motor cortex	motor cortex	motor cortex	1	kept
motor dysfunction assessment	motor dysfunction assessment	motor dysfunction assessment	motor dysfunction assessment	motor dysfunction assessment	1	kept
motor evoked potential	motor evoked potential	motor evoked potential	motor evoked potential	motor evoked potential	1	kept
Motor evoked potentials	motor evoked potentials	motor evoked potentials	motor evoked potential	motor evoked potential	1	singular
motor fluctuations	motor fluctuations	motor fluctuations	motor fluctuations	motor fluctuations	1	kept
Motor function	motor function	motor function	motor function	Motor function	1	kept
motor function	motor function	motor function	motor function	Motor function	1	case_or_format
motor function test	motor function test	motor function test	motor function test	motor function test	1	kept
motor learning	motor learning	motor learning	motor learning	motor learning	1	kept
motor nerve conduction	motor nerve conduction	motor nerve conduction	motor nerve conduction	motor nerve conduction	1	kept
Motor Neurons	motor neurons	motor neurons	motor neurons	Motor Neurons	1	kept
motor neuropathy	motor neuropathy	motor neuropathy	motor neuropathy	motor neuropathy	1	kept
motor reaction time	motor reaction time	motor reaction time	motor reaction time	motor reaction time	1	kept

motor retardation	motor retardation	motor retardation	motor retardation	motor retardation	1	kept
Motor Skills Disorders [prevention & control]	motor skills disorders prevention and control	motor skills disorders prevention and control	motor skills disorders prevention and control	Motor Skills Disorders [prevention & control]	1	kept
motor subtype	motor subtype	motor subtype	motor subtype	motor subtype	1	kept
motor system	motor system	motor system	motor system	motor system	1	kept
Motor vehicle accident	motor vehicle accident	motor vehicle accident	motor vehicle accident	Motor vehicle accident	1	kept
Motor vehicle accidents	motor vehicle accidents	motor vehicle accidents	motor vehicle accident	Motor vehicle accident	1	singular
motor vehicle accidents	motor vehicle accidents	motor vehicle accidents	motor vehicle accident	Motor vehicle accident	1	singular
motor vehicle collision	motor vehicle collision	motor vehicle collision	motor vehicle collision	motor vehicle collision	1	kept
Motor vehicle crashes	motor vehicle crashes	motor vehicle crashes	motor vehicle crashes	Motor vehicle crashes	1	kept
motor vehicle drivers	motor vehicle drivers	motor vehicle drivers	motor vehicle drivers	motor vehicle drivers	1	kept
motor vehicle passengers	motor vehicle passengers	motor vehicle passengers	motor vehicle passengers	motor vehicle passengers	1	kept
motor weakness	motor weakness	motor weakness	motor weakness	motor weakness	1	kept
Motorcycle	motorcycle	motorcycle	motorcycle	Motorcycles	1	case_or_format
motorcycle	motorcycle	motorcycle	motorcycle	Motorcycles	1	case_or_format
motorcyclist	motorcyclist	motorcyclist	motorcyclist	motorcyclist	1	kept
motorized transport	motorized transport	motorized transport	motorized transport	motorized transport	1	kept

motrin ib	motrin ib	motrin ib	motrin ib	motrin ib	1	kept
Mots clés : vieillissement	mots cl s vieillissement	mots cl s vieillissement	mots cl s vieillissement	Mots clés : vieillissement	1	kept
Mounted sensors	mounted sensors	mounted sensors	mounted sensors	Mounted sensors	1	kept
MOUSE MODEL	mouse model	mouse model	mouse model	MOUSE MODEL	1	kept
mouth assessment	mouth assessment	mouth assessment	mouth assessment	mouth assessment	1	kept
MOUTH CARE	mouth care	mouth care	mouth care	Mouth care	1	case_or_format
mouth diagnostic	mouth diagnostic	mouth diagnostic	mouth diagnostic	mouth diagnostic	1	kept
mouth floor	mouth floor	mouth floor	mouth floor	Mouth Floor	1	case_or_format
mouth inflammation	mouth inflammation	mouth inflammation	mouth inflammation	mouth inflammation	1	kept
Mouth neoplasms	mouth neoplasms	mouth neoplasms	mouth neoplasms	Mouth Neoplasms	1	case_or_format
Mouth rinse	mouth rinse	mouth rinse	mouth rinse	Mouth rinse	1	kept
mouth squamous cell carcinoma	mouth squamous cell carcinoma	mouth squamous cell carcinoma	mouth squamous cell carcinoma	mouth squamous cell carcinoma	1	kept
mouth to bag resuscitator	mouth to bag resuscitator	mouth to bag resuscitator	mouth to bag resuscitator	mouth to bag resuscitator	1	kept
Move	move	move	move	Move	1	kept
Movement Disorders [prevention & control]	movement disorders prevention and control	movement disorders prevention and control	movement disorders prevention and control	Movement Disorders [prevention & control]	1	kept
movement time	movement time	movement time	movement time	movement time	1	kept

MOVEMENTS	movements	movements	movement	Movement	1	singular
moving	moving	moving	moving	moving	1	kept
Moving a hospital	moving a hospital	moving a hospital	moving a hospital	Moving a hospital	1	kept
Moving window	moving window	moving window	moving window	Moving window	1	kept
Moxifloxacin	moxifloxacin	moxifloxacin	moxifloxacin	moxifloxacin	1	case_or_format
moyamoya	moyamoya	moyamoya	moyamoya	moyamoya	1	kept
Mozambican	mozambican	mozambican	mozambican	Mozambican	1	kept
Mozart music	mozart music	mozart music	mozart music	Mozart music	1	kept
mPIC score	mpic score	mpic score	mpic score	mPIC score	1	kept
MPOC-SP tool	mpoc sp tool	mpoc sp tool	mpoc sp tool	MPOC-SP tool	1	kept
Mpox	mpox	mpox	mpox	Mpox	1	kept
MR-imaging	mr imaging	mr imaging	mr imaging	MR-imaging	1	kept
MRCP	mrcp	mrcp	mrcp	MRCP	1	kept
MRDOs	mrdos	mrdos	mrdos	MRDOs	1	kept
mrna 1273	mrna 1273	mrna 1273	mrna 1273	mrna 1273	1	kept
mRNA vaccine	mrna vaccine	mrna vaccine	mrna vaccine	mRNA vaccine	1	kept

mrsa	mrsa	mrsa	mrsa	MRSA	1	case_or_format
MRSA active surveillance	mrsa active surveillance	mrsa active surveillance	mrsa active surveillance	MRSA active surveillance	1	kept
MRSA carrier	mrsa carrier	mrsa carrier	mrsa carrier	MRSA carrier	1	kept
MRSA INFECTIONS	mrsa infections	mrsa infections	mrsa infections	MRSA INFECTIONS	1	kept
MRSA nasal swabs	mrsa nasal swabs	mrsa nasal swabs	mrsa nasal swabs	MRSA nasal swabs	1	kept
MRSA pneumonia	mrsa pneumonia	mrsa pneumonia	mrsa pneumonia	MRSA pneumonia	1	kept
MRSA reduction	mrsa reduction	mrsa reduction	mrsa reduction	MRSA reduction	1	kept
MRSA transmission rate	mrsa transmission rate	mrsa transmission rate	mrsa transmission rate	MRSA transmission rate	1	kept
MSAF	msaf	msaf	msaf	MSAF	1	kept
MSCs transplantation	mscs transplantation	mscs transplantation	mscs transplantation	MSCs transplantation	1	kept
MSLT	mslt	mslt	mslt	MSLT	1	kept
MSTU	mstu	mstu	mstu	MSTU	1	kept
MTI Peshawar	mti peshawar	mti peshawar	mti peshawar	MTI Peshawar	1	kept
mucin binding assay	mucin binding assay	mucin binding assay	mucin binding assay	mucin binding assay	1	kept
Mucoactive agents	mucoactive agents	mucoactive agents	mucoactive agents	Mucoactive agents	1	kept
mucociliary transport	mucociliary transport	mucociliary transport	mucociliary transport	mucociliary transport	1	kept

Mucocutaneous exposure	mucocutaneous exposure	mucocutaneous exposure	mucocutaneous exposure	Mucocutaneous exposure	1	kept
Mucolipidoses	mucolipidoses	mucolipidoses	mucolipidoses	Mucolipidoses	1	kept
mucolipidosis	mucolipidosis	mucolipidosis	mucolipidosis	mucolipidosis	1	kept
mucopolysaccharidosis	mucopolysaccharidosis	mucopolysaccharidosis	mucopolysaccharidosis	mucopolysaccharidosis	1	kept
mucosal barrier injury laboratory-confirmed bloodstream infection	mucosal barrier injury laboratory confirmed bloodstream infection	mucosal barrier injury laboratory confirmed bloodstream infection	mucosal barrier injury laboratory confirmed bloodstream infection	mucosal barrier injury laboratory-confirmed bloodstream infection	1	kept
mucosal cancer	mucosal cancer	mucosal cancer	mucosal cancer	mucosal cancer	1	kept
mucosal dryness	mucosal dryness	mucosal dryness	mucosal dryness	mucosal dryness	1	kept
Mucosal exposure	mucosal exposure	mucosal exposure	mucosal exposure	Mucosal exposure	1	kept
MUCOSAL LESIONS	mucosal lesions	mucosal lesions	mucosal lesions	MUCOSAL LESIONS	1	kept
Mucosal membrane pressure injury	mucosal membrane pressure injury	mucosal membrane pressure injury	mucosal membrane pressure injury	mucosal membrane pressure injury	1	case_or_format
Mucosal Plaque Score	mucosal plaque score	mucosal plaque score	mucosal plaque score	Mucosal Plaque Score	1	kept
MUCOSITIS	mucositis	mucositis	mucositis	MUCOSITIS	1	kept
Mucositis	mucositis	mucositis	mucositis	MUCOSITIS	1	case_or_format
mucositis	mucositis	mucositis	mucositis	MUCOSITIS	1	case_or_format
Mucositis Assessment Scale	mucositis assessment scale	mucositis assessment scale	mucositis assessment scale	Mucositis Assessment Scale	1	kept
mucous fistula refeeding	mucous fistula refeeding	mucous fistula refeeding	mucous fistula refeeding	mucous fistula refeeding	1	kept

Mucous membrane	mucous membrane	mucous membrane	mucous membrane	Mucous Membrane	1	case_or_format
mucous membrane	mucous membrane	mucous membrane	mucous membrane	Mucous Membrane	1	case_or_format
mucous membrane pemphigoid	mucous membrane pemphigoid	mucous membrane pemphigoid	mucous membrane pemphigoid	mucous membrane pemphigoid	1	kept
Mucus fistula refeeding	mucus fistula refeeding	mucus fistula refeeding	mucus fistula refeeding	Mucus fistula refeeding	1	kept
mucus plugging	mucus plugging	mucus plugging	mucus plugging	mucus plugging	1	kept
mucus secretion	mucus secretion	mucus secretion	mucus secretion	mucus secretion	1	kept
Muellerian duct	muellerian duct	muellerian duct	muellerian duct	Muellerian duct	1	kept
Muerte neonatal	muerte neonatal	muerte neonatal	muerte neonatal	Muerte neonatal	1	kept
Muerte súbita	muerte s bita	muerte s bita	muerte s bita	Muerte súbita	1	kept
Mulago	mulago	mulago	mulago	Mulago	1	kept
mule	mule	mule	mule	mule	1	kept
Mule foals	mule foals	mule foals	mule foals	Mule foals	1	kept
Mullerian Ducts	mullerian ducts	mullerian ducts	mullerian ducts	Mullerian Ducts	1	kept
Multi agent systems	multi agent systems	multi agent systems	multi agent system	Multi-Agent system	1	singular
Multi criteria decision-making	multi criteria decision making	multi criteria decision making	multi criteria decision making	Multi-criteria decision making	1	case_or_format
Multi drug resistance	multi drug resistance	multi drug resistance	multi drug resistance	Multi-drug resistance	1	case_or_format

Multi Independence Dimensions questionnaire	multi independence dimensions questionnaire	multi independence dimensions questionnaire	multi independence dimensions questionnaire	Multi Independence Dimensions questionnaire	1	kept
multi sensory stimulation	multi sensory stimulation	multi sensory stimulation	multi sensory stimulation	Multi-sensory stimulation	1	case_or_format
Multi variables	multi variables	multi variables	multi variables	Multi variables	1	kept
Multi- drug resistance bacteria	multi drug resistance bacteria	multi drug resistance bacteria	multi drug resistance bacteria	Multi- drug resistance bacteria	1	kept
Multi-Agent Model	multi agent model	multi agent model	multi agent model	Multi-Agent Model	1	kept
Multi-Agent system	multi agent system	multi agent system	multi agent system	Multi-Agent system	1	kept
Multi-bed occupancy rooms	multi bed occupancy rooms	multi bed occupancy rooms	multi bed occupancy rooms	Multi-bed occupancy rooms	1	kept
Multi-camera environments	multi camera environments	multi camera environments	multi camera environments	Multi-camera environments	1	kept
Multi-cameras	multi cameras	multi cameras	multi cameras	Multi-cameras	1	kept
multi-center study	multi center study	multi center study	multi center study	multi-center study	1	kept
Multi-centre	multi centre	multi centre	multi centre	Multi-centre	1	kept
Multi-centre study	multi centre study	multi centre study	multi centre study	Multi-centre study	1	kept
Multi-component interventions	multi component interventions	multi component interventions	multi component interventions	Multi-component interventions	1	kept
multi-country survey	multi country survey	multi country survey	multi country survey	multi-country survey	1	kept
Multi-criteria decision analysis	multi criteria decision analysis	multi criteria decision analysis	multi criteria decision analysis	multi-criteria decision analysis	1	case_or_format
Multi-Criteria Decision-Making	multi criteria decision making	multi criteria decision making	multi criteria decision making	Multi-criteria decision making	1	case_or_format

Multi-disciplinary research	multi disciplinary research	multi disciplinary research	multi disciplinary research	Multi-disciplinary research	1	kept
Multi-disciplinary team	multi disciplinary team	multi disciplinary team	multi disciplinary team	Multi-disciplinary teams	1	case_or_format
multi-disciplinary teams	multi disciplinary teams	multi disciplinary teams	multi disciplinary team	Multi-disciplinary teams	1	singular
Multi-drug resistant	multi drug resistant	multi drug resistant	multi drug resistant	Multi-drug resistant	1	kept
multi-drug resistant	multi drug resistant	multi drug resistant	multi drug resistant	Multi-drug resistant	1	case_or_format
Multi-drug resistant Enterobacteriaceae	multi drug resistant enterobacteriaceae	multi drug resistant enterobacteriaceae	multi drug resistant enterobacteriaceae	Multi-drug resistant Enterobacteriaceae	1	kept
Multi-drug resistant organisms	multi drug resistant organisms	multi drug resistant organisms	multi drug resistant organisms	Multi-drug resistant organisms	1	kept
multi-drug resistant organisms	multi drug resistant organisms	multi drug resistant organisms	multi drug resistant organisms	Multi-drug resistant organisms	1	case_or_format
Multi-drug resistant pathogen	multi drug resistant pathogen	multi drug resistant pathogen	multi drug resistant pathogen	Multi-drug resistant pathogen	1	kept
Multi-drug-resistant bacteria infection	multi drug resistant bacteria infection	multi drug resistant bacteria infection	multi drug resistant bacteria infection	Multi-drug-resistant bacteria infection	1	kept
multi-drug-resistant bacteria infection	multi drug resistant bacteria infection	multi drug resistant bacteria infection	multi drug resistant bacteria infection	Multi-drug-resistant bacteria infection	1	case_or_format
Multi-drug-resistant organisms	multi drug resistant organisms	multi drug resistant organisms	multi drug resistant organisms	Multi-drug resistant organisms	1	case_or_format
Multi-infusion	multi infusion	multi infusion	multi infusion	Multi-infusion	1	kept
Multi-layer network	multi layer network	multi layer network	multi layer network	Multi-layer network	1	kept
multi-level	multi level	multi level	multi level	multi-level	1	kept
Multi-level case study	multi level case study	multi level case study	multi level case study	Multi-level case study	1	kept

Multi-media	multi media	multi media	multi media	Multi-media	1	kept
multi-method approach	multi method approach	multi method approach	multi method approach	multi-method approach	1	kept
Multi-methods	multi methods	multi methods	multi methods	Multi-methods	1	kept
Multi-mode intervention	multi mode intervention	multi mode intervention	multi mode intervention	Multi-mode intervention	1	kept
multi-objective optimization	multi objective optimization	multi objective optimization	multi objective optimization	multi-objective optimization	1	kept
multi-parameter fusion	multi parameter fusion	multi parameter fusion	multi parameter fusion	multi-parameter fusion	1	kept
multi-professional team	multi professional team	multi professional team	multi professional team	multi-professional team	1	kept
Multi-professional training	multi professional training	multi professional training	multi professional training	Multi-professional training	1	kept
Multi-sensory stimulation intervention	multi sensory stimulation intervention	multi sensory stimulation intervention	multi sensory stimulation intervention	Multi-sensory stimulation intervention	1	kept
Multi-Sources	multi sources	multi sources	multi sources	Multi-Sources	1	kept
Multi-species	multi species	multi species	multi species	Multi-species	1	kept
Multi-task model	multi task model	multi task model	multi task model	Multi-task model	1	kept
Multi-team systems	multi team systems	multi team systems	multi team systems	Multi-team systems	1	kept
multibed	multibed	multibed	multibed	multibed	1	kept
Multibottle system	multibottle system	multibottle system	multibottle system	Multibottle system	1	kept
multicenter prospective cohort study	multicenter prospective cohort study	multicenter prospective cohort study	multicenter prospective cohort study	multicenter prospective cohort study	1	kept

Multicenter randomized clinical controlled trial	multicenter randomized clinical controlled trial	multicenter randomized clinical controlled trial	multicenter randomized clinical controlled trial	Multicenter randomized clinical controlled trial	1	kept
Multicenter Studies	multicenter studies	multicenter studies	multicenter study	multicenter study	1	singular
Multicenter studies	multicenter studies	multicenter studies	multicenter study	multicenter study	1	singular
multicenter survey	multicenter survey	multicenter survey	multicenter survey	multicenter survey	1	kept
multicenter trials	multicenter trials	multicenter trials	multicenter trials	multicenter trials	1	kept
Multicentre	multicentre	multicentre	multicentre	Multicentre	1	kept
multicentre study	multicentre study	multicentre study	multicentre study	Multicentre study	1	case_or_format
MULTICOMPONENT	multicomponent	multicomponent	multicomponent	MULTICOMPONENT	1	kept
multicomponent approach	multicomponent approach	multicomponent approach	multicomponent approach	multicomponent approach	1	kept
MULTICOMPONENT EXERCISE PROGRAM	multicomponent exercise program	multicomponent exercise program	multicomponent exercise program	MULTICOMPONENT EXERCISE PROGRAM	1	kept
Multicriteria decision-making	multicriteria decision making	multicriteria decision making	multicriteria decision making	Multicriteria decision-making	1	kept
Multicriterion decision makings	multicriterion decision makings	multicriterion decision makings	multicriterion decision makings	Multicriterion decision makings	1	kept
Multidepartment	multidepartment	multidepartment	multidepartment	Multidepartment	1	kept
Multidimensional evaluation	multidimensional evaluation	multidimensional evaluation	multidimensional evaluation	Multidimensional evaluation	1	kept
multidimensional post discharge disease management program	multidimensional post discharge disease management program	multidimensional post discharge disease management program	multidimensional post discharge disease management program	multidimensional post discharge disease management program	1	kept
multidimensional post discharge disease management programme	multidimensional post discharge disease management programme	multidimensional post discharge disease management programme	multidimensional post discharge disease management programme	multidimensional post discharge disease management programme	1	kept

MULTIDIMENSIONAL PROGNOSTIC INDEX	multidimensional prognostic index	multidimensional prognostic index	multidimensional prognostic index	MULTIDIMENSIONAL PROGNOSTIC INDEX	1	kept
Multidimensional Prognostic Index	multidimensional prognostic index	multidimensional prognostic index	multidimensional prognostic index	MULTIDIMENSIONAL PROGNOSTIC INDEX	1	case_or_format
Multidimensional space	multidimensional space	multidimensional space	multidimensional space	Multidimensional space	1	kept
MULTIDISCIPLINARY	multidisciplinary	multidisciplinary	multidisciplinary	multidisciplinary	1	case_or_format
multidisciplinary approaches	multidisciplinary approaches	multidisciplinary approaches	multidisciplinary approach	Multidisciplinary approach	1	singular
MULTIDISCIPLINARY CANCER TEAMS	multidisciplinary cancer teams	multidisciplinary cancer teams	multidisciplinary cancer teams	MULTIDISCIPLINARY CANCER TEAMS	1	kept
Multidisciplinary Care Team	multidisciplinary care team	multidisciplinary care team	multidisciplinary care team	Multidisciplinary Care Team	1	kept
Multidisciplinary care team	multidisciplinary care team	multidisciplinary care team	multidisciplinary care team	Multidisciplinary Care Team	1	case_or_format
Multidisciplinary care teams	multidisciplinary care teams	multidisciplinary care teams	multidisciplinary care team	Multidisciplinary Care Team	1	singular
multidisciplinary clinic	multidisciplinary clinic	multidisciplinary clinic	multidisciplinary clinic	multidisciplinary clinic	1	kept
Multidisciplinary communication	multidisciplinary communication	multidisciplinary communication	multidisciplinary communication	multidisciplinary communication	1	case_or_format
multidisciplinary decision making	multidisciplinary decision making	multidisciplinary decision making	multidisciplinary decision making	multidisciplinary decision making	1	kept
Multidisciplinary effort	multidisciplinary effort	multidisciplinary effort	multidisciplinary effort	Multidisciplinary effort	1	kept
Multidisciplinary evaluation	multidisciplinary evaluation	multidisciplinary evaluation	multidisciplinary evaluation	Multidisciplinary evaluation	1	kept
Multidisciplinary health team	multidisciplinary health team	multidisciplinary health team	multidisciplinary health team	Multidisciplinary health team	1	kept
multidisciplinary healthcare team	multidisciplinary healthcare team	multidisciplinary healthcare team	multidisciplinary healthcare team	multidisciplinary healthcare team	1	kept

multidisciplinary in hospital team	multidisciplinary in hospital team	multidisciplinary in hospital team	multidisciplinary in hospital team	multidisciplinary in hospital team	1	kept
Multidisciplinary intervention	multidisciplinary intervention	multidisciplinary intervention	multidisciplinary intervention	Multidisciplinary intervention	1	kept
Multidisciplinary management	multidisciplinary management	multidisciplinary management	multidisciplinary management	multidisciplinary management	1	case_or_format
Multidisciplinary mobility team	multidisciplinary mobility team	multidisciplinary mobility team	multidisciplinary mobility team	Multidisciplinary mobility team	1	kept
Multidisciplinary nutritional support	multidisciplinary nutritional support	multidisciplinary nutritional support	multidisciplinary nutritional support	Multidisciplinary nutritional support	1	kept
multidisciplinary perceptions	multidisciplinary perceptions	multidisciplinary perceptions	multidisciplinary perceptions	multidisciplinary perceptions	1	kept
multidisciplinary rehabilitation team	multidisciplinary rehabilitation team	multidisciplinary rehabilitation team	multidisciplinary rehabilitation team	multidisciplinary rehabilitation team	1	kept
Multidisciplinary research	multidisciplinary research	multidisciplinary research	multidisciplinary research	Multidisciplinary research	1	kept
multidisciplinary round	multidisciplinary round	multidisciplinary round	multidisciplinary round	Multidisciplinary rounds	1	case_or_format
multidisciplinary rounds	multidisciplinary rounds	multidisciplinary rounds	multidisciplinary round	Multidisciplinary rounds	1	singular
multidisciplinary safety rounds	multidisciplinary safety rounds	multidisciplinary safety rounds	multidisciplinary safety rounds	multidisciplinary safety rounds	1	kept
multidisciplinary simulation	multidisciplinary simulation	multidisciplinary simulation	multidisciplinary simulation	multidisciplinary simulation	1	kept
multidisciplinary simulation training	multidisciplinary simulation training	multidisciplinary simulation training	multidisciplinary simulation training	multidisciplinary simulation training	1	kept
multidisciplinary staff	multidisciplinary staff	multidisciplinary staff	multidisciplinary staff	multidisciplinary staff	1	kept
Multidisciplinary support team	multidisciplinary support team	multidisciplinary support team	multidisciplinary support team	Multidisciplinary support team	1	kept
Multidisciplinary Team	multidisciplinary team	multidisciplinary team	multidisciplinary team	multidisciplinary team	1	case_or_format

multidisciplinary team care	multidisciplinary team care	multidisciplinary team care	multidisciplinary team care	multidisciplinary team care	1	kept
multidisciplinary team involvement	multidisciplinary team involvement	multidisciplinary team involvement	multidisciplinary team involvement	multidisciplinary team involvement	1	kept
multidisciplinary team management	multidisciplinary team management	multidisciplinary team management	multidisciplinary team management	multidisciplinary team management	1	kept
multidisciplinary team model	multidisciplinary team model	multidisciplinary team model	multidisciplinary team model	multidisciplinary team model	1	kept
Multidisciplinary Team observation assessment tool	multidisciplinary team observation assessment tool	multidisciplinary team observation assessment tool	multidisciplinary team observation assessment tool	Multidisciplinary Team observation assessment tool	1	kept
Multidisciplinary team-care	multidisciplinary team care	multidisciplinary team care	multidisciplinary team care	multidisciplinary team care	1	case_or_format
multidose injection vials	multidose injection vials	multidose injection vials	multidose injection vials	multidose injection vials	1	kept
Multidose vials	multidose vials	multidose vials	multidose vials	Multidose vials	1	kept
Multidrug Resistance	multidrug resistance	multidrug resistance	multidrug resistance	multidrug resistance	1	case_or_format
Multidrug resistant	multidrug resistant	multidrug resistant	multidrug resistant	Multidrug-resistant	1	case_or_format
multidrug resistant	multidrug resistant	multidrug resistant	multidrug resistant	Multidrug-resistant	1	case_or_format
multidrug resistant Acinetobacter baumannii	multidrug resistant acinetobacter baumannii	multidrug resistant acinetobacter baumannii	multidrug resistant acinetobacter baumannii	multidrug resistant Acinetobacter baumannii	1	kept
multidrug resistant acinetobacter baumannii	multidrug resistant acinetobacter baumannii	multidrug resistant acinetobacter baumannii	multidrug resistant acinetobacter baumannii	multidrug resistant Acinetobacter baumannii	1	case_or_format
Multidrug resistant bacteria	multidrug resistant bacteria	multidrug resistant bacteria	multidrug resistant bacteria	Multidrug-resistant bacteria	1	case_or_format
multidrug resistant Escherichia coli	multidrug resistant escherichia coli	multidrug resistant escherichia coli	multidrug resistant escherichia coli	multidrug resistant Escherichia coli	1	kept
multidrug resistant gram negative bacteria	multidrug resistant gram negative bacteria	multidrug resistant gram negative bacteria	multidrug resistant gram negative bacteria	multidrug resistant gram negative bacteria	1	kept

multidrug resistant gram negative bacterial infection	multidrug resistant gram negative bacterial infection	multidrug resistant gram negative bacterial infection	multidrug resistant gram negative bacterial infection	multidrug resistant gram negative bacterial infection	1	kept
multidrug resistant Gram positive bacterium	multidrug resistant gram positive bacterium	multidrug resistant gram positive bacterium	multidrug resistant gram positive bacterium	multidrug resistant Gram positive bacterium	1	kept
multidrug resistant organisms	multidrug resistant organisms	multidrug resistant organisms	multidrug resistant organism	Multidrug-resistant organisms	1	singular
MULTIDRUG-RESISTANCE	multidrug resistance	multidrug resistance	multidrug resistance	multidrug resistance	1	case_or_format
multidrug-resistant	multidrug resistant	multidrug resistant	multidrug resistant	Multidrug-resistant	1	case_or_format
multidrug-resistant Acinetobacter baumannii	multidrug resistant acinetobacter baumannii	multidrug resistant acinetobacter baumannii	multidrug resistant acinetobacter baumannii	multidrug resistant Acinetobacter baumannii	1	case_or_format
multidrug-resistant bacteria	multidrug resistant bacteria	multidrug resistant bacteria	multidrug resistant bacteria	Multidrug-resistant bacteria	1	case_or_format
multidrug-resistant endocarditis	multidrug resistant endocarditis	multidrug resistant endocarditis	multidrug resistant endocarditis	multidrug-resistant endocarditis	1	kept
Multidrug-resistant gram-negative	multidrug resistant gram negative	multidrug resistant gram negative	multidrug resistant gram negative	Multidrug-resistant gram-negative	1	kept
Multidrug-resistant gram-negative bacilli	multidrug resistant gram negative bacilli	multidrug resistant gram negative bacilli	multidrug resistant gram negative bacilli	Multidrug-resistant gram-negative bacilli	1	kept
Multidrug-resistant gram-negative bacteria	multidrug resistant gram negative bacteria	multidrug resistant gram negative bacteria	multidrug resistant gram negative bacteria	multidrug resistant gram negative bacteria	1	case_or_format
multidrug-resistant Gram-negative bacteria	multidrug resistant gram negative bacteria	multidrug resistant gram negative bacteria	multidrug resistant gram negative bacteria	multidrug resistant gram negative bacteria	1	case_or_format
multidrug-resistant gram-negative bacteria	multidrug resistant gram negative bacteria	multidrug resistant gram negative bacteria	multidrug resistant gram negative bacteria	multidrug resistant gram negative bacteria	1	case_or_format
multidrug-resistant microorganisms	multidrug resistant microorganisms	multidrug resistant microorganisms	multidrug resistant microorganisms	multidrug-resistant microorganisms	1	kept
Multidrug-resistant organism	multidrug resistant organism	multidrug resistant organism	multidrug resistant organism	Multidrug-resistant organisms	1	case_or_format
Multidrugresistant	multidrugresistant	multidrugresistant	multidrugresistant	Multidrugresistant	1	kept

multidrug‐resistant organism infections	multidrug resistant organism infections	multidrug resistant organism infections	multidrug resistant organism infections	multidrug‐resistant organism infections	1	kept
multidrug‐resistant organisms	multidrug resistant organisms	multidrug resistant organisms	multidrug resistant organism	Multidrug-resistant organisms	1	singular
Multienzyme Complexes	multienzyme complexes	multienzyme complexes	multienzyme complexes	Multienzyme Complexes	1	kept
Multifaceted approach	multifaceted approach	multifaceted approach	multifaceted approach	Multifaceted approach	1	kept
Multifaceted education	multifaceted education	multifaceted education	multifaceted education	Multifaceted education	1	kept
multifaceted intervention	multifaceted intervention	multifaceted intervention	multifaceted intervention	multifaceted intervention	1	kept
Multifaceted interventions	multifaceted interventions	multifaceted interventions	multifaceted intervention	multifaceted intervention	1	singular
multifacetted intervention	multifacetted intervention	multifacetted intervention	multifacetted intervention	multifacetted intervention	1	kept
multifacility collaboration	multifacility collaboration	multifacility collaboration	multifacility collaboration	multifacility collaboration	1	kept
multifactor dimensionality reduction	multifactor dimensionality reduction	multifactor dimensionality reduction	multifactor dimensionality reduction	multifactor dimensionality reduction	1	kept
multifactorial genetic disorder	multifactorial genetic disorder	multifactorial genetic disorder	multifactorial genetic disorder	multifactorial genetic disorder	1	kept
multifactorial inheritance	multifactorial inheritance	multifactorial inheritance	multifactorial inheritance	multifactorial inheritance	1	kept
MULTIFACTORIAL INTERVENTION	multifactorial intervention	multifactorial intervention	multifactorial intervention	MULTIFACTORIAL INTERVENTION	1	kept
multifetal pregnancy reduction	multifetal pregnancy reduction	multifetal pregnancy reduction	multifetal pregnancy reduction	multifetal pregnancy reduction	1	kept
Multifunctional rehabilitation	multifunctional rehabilitation	multifunctional rehabilitation	multifunctional rehabilitation	Multifunctional rehabilitation	1	kept
multiinstitutional systems	multiinstitutional systems	multiinstitutional systems	multiinstitutional systems	multiinstitutional systems	1	kept

Multilaterally shared leadership	multilaterally shared leadership	multilaterally shared leadership	multilaterally shared leadership	Multilaterally shared leadership	1	kept
Multilayer neural network	multilayer neural network	multilayer neural network	multilayer neural network	Multilayer neural network	1	kept
Multilayer Perceptrons	multilayer perceptrons	multilayer perceptrons	multilayer perceptron	multilayer perceptron	1	singular
Multilayers	multilayers	multilayers	multilayers	Multilayers	1	kept
multilevel impact	multilevel impact	multilevel impact	multilevel impact	multilevel impact	1	kept
multilevel modeling	multilevel modeling	multilevel modeling	multilevel modeling	multilevel modeling	1	kept
multilevel modelling	multilevel modelling	multilevel modelling	multilevel modelling	Multilevel modelling	1	case_or_format
Multilevel models	multilevel models	multilevel models	multilevel models	Multilevel models	1	kept
Multilevels	multilevels	multilevels	multilevels	Multilevels	1	kept
multilocus enzyme electrophoresis	multilocus enzyme electrophoresis	multilocus enzyme electrophoresis	multilocus enzyme electrophoresis	multilocus enzyme electrophoresis	1	kept
MULTIMEDIA	multimedia	multimedia	multimedia	Multimedia	1	case_or_format
multimedia electronic training	multimedia electronic training	multimedia electronic training	multimedia electronic training	multimedia electronic training	1	kept
multimedia training	multimedia training	multimedia training	multimedia training	multimedia training	1	kept
multimethod research	multimethod research	multimethod research	multimethod research	multimethod research	1	kept
Multimethod study	multimethod study	multimethod study	multimethod study	Multimethod study	1	kept
Multimodal alarms	multimodal alarms	multimodal alarms	multimodal alarms	Multimodal alarms	1	kept

Multimodal approach	multimodal approach	multimodal approach	multimodal approach	Multimodal approach	1	kept
Multimodal approaches	multimodal approaches	multimodal approaches	multimodal approach	Multimodal approach	1	singular
Multimodal biomedical sensor network	multimodal biomedical sensor network	multimodal biomedical sensor network	multimodal biomedical sensor network	Multimodal biomedical sensor network	1	kept
multimodal brain function monitoring	multimodal brain function monitoring	multimodal brain function monitoring	multimodal brain function monitoring	multimodal brain function monitoring	1	kept
Multimodal complex treatment	multimodal complex treatment	multimodal complex treatment	multimodal complex treatment	Multimodal complex treatment	1	kept
multimodal data collection and distribution	multimodal data collection and distribution	multimodal data collection and distribution	multimodal data collection and distribution	multimodal data collection and distribution	1	kept
multimodal imaging	multimodal imaging	multimodal imaging	multimodal imaging	multimodal imaging	1	kept
Multimodal Interaction	multimodal interaction	multimodal interaction	multimodal interaction	Multimodal Interaction	1	kept
Multimodal interaction user-centered design	multimodal interaction user centered design	multimodal interaction user centered design	multimodal interaction user centered design	Multimodal interaction user-centered design	1	kept
Multimodal interaction user-centred design	multimodal interaction user centred design	multimodal interaction user centred design	multimodal interaction user centred design	Multimodal interaction user-centred design	1	kept
Multimodal intervention	multimodal intervention	multimodal intervention	multimodal intervention	multimodal intervention	1	case_or_format
multimodal management	multimodal management	multimodal management	multimodal management	multimodal management	1	kept
Multimodal neuromonitoring	multimodal neuromonitoring	multimodal neuromonitoring	multimodal neuromonitoring	Multimodal neuromonitoring	1	kept
multimodal research and education	multimodal research and education	multimodal research and education	multimodal research and education	multimodal research and education	1	kept
multimodal strategies	multimodal strategies	multimodal strategies	multimodal strategies	multimodal strategies	1	kept
multimodal teaching	multimodal teaching	multimodal teaching	multimodal teaching	multimodal teaching	1	kept

Multimodality monitoring	multimodality monitoring	multimodality monitoring	multimodality monitoring	Multimodality monitoring	1	kept
multimodality monitoring	multimodality monitoring	multimodality monitoring	multimodality monitoring	Multimodality monitoring	1	case_or_format
Multimodality neuromonitoring	multimodality neuromonitoring	multimodality neuromonitoring	multimodality neuromonitoring	Multimodality neuromonitoring	1	kept
Multimorbid	multimorbid	multimorbid	multimorbid	Multimorbid	1	kept
Multinomial distributions	multinomial distributions	multinomial distributions	multinomial distributions	Multinomial distributions	1	kept
multiomics	multiomics	multiomics	multiomics	multiomics	1	kept
Multiorgan dysfunction	multiorgan dysfunction	multiorgan dysfunction	multiorgan dysfunction	Multiorgan dysfunction	1	kept
multiorgan dysfunction	multiorgan dysfunction	multiorgan dysfunction	multiorgan dysfunction	Multiorgan dysfunction	1	case_or_format
Multiorgan dysfunction syndrome	multiorgan dysfunction syndrome	multiorgan dysfunction syndrome	multiorgan dysfunction syndrome	Multiorgan dysfunction syndrome	1	kept
Multiorgan failure	multiorgan failure	multiorgan failure	multiorgan failure	multiorgan failure	1	case_or_format
multiparous women	multiparous women	multiparous women	multiparous women	multiparous women	1	kept
Multipath	multipath	multipath	multipath	Multipath	1	kept
Multiple birth	multiple birth	multiple birth	multiple birth	Multiple births	1	case_or_format
multiple birth	multiple birth	multiple birth	multiple birth	Multiple births	1	case_or_format
Multiple channels	multiple channels	multiple channels	multiple channels	Multiple channels	1	kept
Multiple correlation coefficients	multiple correlation coefficients	multiple correlation coefficients	multiple correlation coefficients	Multiple correlation coefficients	1	kept

Multiple Daily Injections	multiple daily injections	multiple daily injections	multiple daily injections	Multiple Daily Injections	1	kept
Multiple deliveries	multiple deliveries	multiple deliveries	multiple deliveries	Multiple deliveries	1	kept
Multiple disciplines treatment	multiple disciplines treatment	multiple disciplines treatment	multiple disciplines treatment	Multiple disciplines treatment	1	kept
Multiple drug resistance	multiple drug resistance	multiple drug resistance	multiple drug resistance	Multiple drug resistance	1	kept
Multiple drug-resistant organism	multiple drug resistant organism	multiple drug resistant organism	multiple drug resistant organism	Multiple drug-resistant organism	1	kept
multiple drug-resistant organism	multiple drug resistant organism	multiple drug resistant organism	multiple drug resistant organism	Multiple drug-resistant organism	1	case_or_format
Multiple Endocrine Neoplasia Type 2b	multiple endocrine neoplasia type 2b	multiple endocrine neoplasia type 2b	multiple endocrine neoplasia type 2b	Multiple Endocrine Neoplasia Type 2b	1	kept
Multiple features	multiple features	multiple features	multiple features	Multiple features	1	kept
Multiple grief	multiple grief	multiple grief	multiple grief	Multiple grief	1	kept
multiple infections	multiple infections	multiple infections	multiple infections	multiple infections	1	kept
Multiple Infrared Sensors	multiple infrared sensors	multiple infrared sensors	multiple infrared sensors	Multiple Infrared Sensors	1	kept
Multiple injuries	multiple injuries	multiple injuries	multiple injuries	Multiple injuries	1	kept
multiple interventions	multiple interventions	multiple interventions	multiple interventions	multiple interventions	1	kept
multiple intravenous infusion	multiple intravenous infusion	multiple intravenous infusion	multiple intravenous infusion	multiple intravenous infusion	1	kept
multiple job holding	multiple job holding	multiple job holding	multiple job holding	multiple job holding	1	kept
Multiple logistic regression	multiple logistic regression	multiple logistic regression	multiple logistic regression	Multiple logistic regression	1	kept

multiple logistic regression analysis	multiple logistic regression analysis	multiple logistic regression analysis	multiple logistic regression analysis	multiple logistic regression analysis	1	kept
Multiple loss	multiple loss	multiple loss	multiple loss	Multiple loss	1	kept
multiple lumen tube	multiple lumen tube	multiple lumen tube	multiple lumen tube	multiple lumen tube	1	kept
multiple mediation analysis	multiple mediation analysis	multiple mediation analysis	multiple mediation analysis	multiple mediation analysis	1	kept
Multiple myeloma	multiple myeloma	multiple myeloma	multiple myeloma	multiple myeloma	1	case_or_format
Multiple ontologies	multiple ontologies	multiple ontologies	multiple ontologies	multiple ontologies	1	case_or_format
Multiple organ dysfunction	multiple organ dysfunction	multiple organ dysfunction	multiple organ dysfunction	Multiple organ dysfunction	1	kept
Multiple organ dysfunction score	multiple organ dysfunction score	multiple organ dysfunction score	multiple organ dysfunction score	Multiple organ dysfunction score	1	kept
Multiple organ dysfunction syndrome	multiple organ dysfunction syndrome	multiple organ dysfunction syndrome	multiple organ dysfunction syndrome	multiple organ dysfunction syndrome	1	case_or_format
multiple organ infarction	multiple organ infarction	multiple organ infarction	multiple organ infarction	multiple organ infarction	1	kept
Multiple organ injuries	multiple organ injuries	multiple organ injuries	multiple organ injuries	Multiple organ injuries	1	kept
Multiple Organs Dysfunctional Score	multiple organs dysfunctional score	multiple organs dysfunctional score	multiple organs dysfunctional score	Multiple Organs Dysfunctional Score	1	kept
multiple plane imaging	multiple plane imaging	multiple plane imaging	multiple plane imaging	multiple plane imaging	1	kept
Multiple pregnancy	multiple pregnancy	multiple pregnancy	multiple pregnancy	multiple pregnancy	1	case_or_format
multiple pulmonary nodules	multiple pulmonary nodules	multiple pulmonary nodules	multiple pulmonary nodules	multiple pulmonary nodules	1	kept
multiple regression analysis	multiple regression analysis	multiple regression analysis	multiple regression analysis	multiple regression analysis	1	kept

Multiple resources	multiple resources	multiple resources	multiple resources	Multiple resources	1	kept
Multiple sclerosis	multiple sclerosis	multiple sclerosis	multiple sclerosis	multiple sclerosis	1	case_or_format
multiple social-environmental risks	multiple social environmental risks	multiple social environmental risks	multiple social environmental risks	multiple social-environmental risks	1	kept
multiple system atrophy	multiple system atrophy	multiple system atrophy	multiple system atrophy	multiple system atrophy	1	kept
Multiple translocations	multiple translocations	multiple translocations	multiple translocations	Multiple translocations	1	kept
Multiple types of healthcare workers	multiple types of healthcare workers	multiple types of healthcare workers	multiple types of healthcare workers	Multiple types of healthcare workers	1	kept
multiple-choice	multiple choice	multiple choice	multiple choice	multiple-choice	1	kept
Multiple-choice test	multiple choice test	multiple choice test	multiple choice test	multiple choice test	1	case_or_format
Multiple-criteria decision analysis	multiple criteria decision analysis	multiple criteria decision analysis	multiple criteria decision analysis	Multiple-criteria decision analysis	1	kept
multiple-group measurement	multiple group measurement	multiple group measurement	multiple group measurement	multiple-group measurement	1	kept
MULTIPLE-ITEM	multiple item	multiple item	multiple item	MULTIPLE-ITEM	1	kept
Multiples roles	multiples roles	multiples roles	multiples roles	Multiples roles	1	kept
Multiplex PCR	multiplex pcr	multiplex pcr	multiplex pcr	Multiplex PCR	1	kept
multiplex PCR	multiplex pcr	multiplex pcr	multiplex pcr	Multiplex PCR	1	case_or_format
Multiplicative scatter correction	multiplicative scatter correction	multiplicative scatter correction	multiplicative scatter correction	Multiplicative scatter correction	1	kept
multiplier titration	multiplier titration	multiplier titration	multiplier titration	multiplier titration	1	kept

multipotent stem cell	multipotent stem cell	multipotent stem cell	multipotent stem cell	multipotent stem cell	1	kept
Multipotent Stem Cells	multipotent stem cells	multipotent stem cells	multipotent stem cell	multipotent stem cell	1	singular
Multiprofessional	multiprofessional	multiprofessional	multiprofessional	multiprofessional	1	case_or_format
multiprofessional course	multiprofessional course	multiprofessional course	multiprofessional course	multiprofessional course	1	kept
multiprofessional education	multiprofessional education	multiprofessional education	multiprofessional education	Multiprofessional education	1	case_or_format
multiprofessional survey	multiprofessional survey	multiprofessional survey	multiprofessional survey	multiprofessional survey	1	kept
multiprofessional teams	multiprofessional teams	multiprofessional teams	multiprofessional teams	multiprofessional teams	1	kept
multiprofessional training	multiprofessional training	multiprofessional training	multiprofessional training	multiprofessional training	1	kept
MULTIRESISTANT ACINETOBACTER-BAUMANNII	multiresistant acinetobacter baumannii	multiresistant acinetobacter baumannii	multiresistant acinetobacter baumannii	MULTIRESISTANT ACINETOBACTER-BAUMANNII	1	kept
Multiresistant microorganisms	multiresistant microorganisms	multiresistant microorganisms	multiresistant microorganisms	Multiresistant microorganisms	1	kept
Multirresistance	multirresistance	multirresistance	multirresistance	Multirresistance	1	kept
multirésistance	multir sistance	multir sistance	multir sistance	multirésistance	1	kept
Multiscale vision transformer	multiscale vision transformer	multiscale vision transformer	multiscale vision transformer	Multiscale vision transformer	1	kept
multiscale vision transformer	multiscale vision transformer	multiscale vision transformer	multiscale vision transformer	Multiscale vision transformer	1	case_or_format
multisensory stimulation	multisensory stimulation	multisensory stimulation	multisensory stimulation	multisensory stimulation	1	kept
Multisensory stimulus	multisensory stimulus	multisensory stimulus	multisensory stimulus	Multisensory stimulus	1	kept

MULTISITE	multisite	multisite	multisite	MULTISITE	1	kept
Multisources	multisources	multisources	multisources	Multisources	1	kept
Multispecialty Care	multispecialty care	multispecialty care	multispecialty care	Multispecialty Care	1	kept
multispecialty mortality and morbidity conference	multispecialty mortality and morbidity conference	multispecialty mortality and morbidity conference	multispecialty mortality and morbidity conference	multispecialty mortality and morbidity conference	1	kept
multisystem chronic illnesses	multisystem chronic illnesses	multisystem chronic illnesses	multisystem chronic illnesses	multisystem chronic illnesses	1	kept
Multisystem Inflammatory Syndrome in Children	multisystem inflammatory syndrome in children	multisystem inflammatory syndrome in children	multisystem inflammatory syndrome in children	Multisystem Inflammatory Syndrome in Children	1	kept
Multisystem inflammatory syndrome in children	multisystem inflammatory syndrome in children	multisystem inflammatory syndrome in children	multisystem inflammatory syndrome in children	Multisystem Inflammatory Syndrome in Children	1	case_or_format
multisystem organ failure	multisystem organ failure	multisystem organ failure	multisystem organ failure	multisystem organ failure	1	kept
MULTITASKING	multitasking	multitasking	multitasking	MULTITASKING	1	kept
Multitrauma	multitrauma	multitrauma	multitrauma	Multitrauma	1	kept
multitrauma care unit	multitrauma care unit	multitrauma care unit	multitrauma care unit	multitrauma care unit	1	kept
Multivariate classification	multivariate classification	multivariate classification	multivariate classification	Multivariate classification	1	kept
multivariate Cox model	multivariate cox model	multivariate cox model	multivariate cox model	multivariate Cox model	1	kept
Multivariate cox regression	multivariate cox regression	multivariate cox regression	multivariate cox regression	Multivariate cox regression	1	kept
Multivariate regression	multivariate regression	multivariate regression	multivariate regression	Multivariate regression	1	kept
multivariate statistics	multivariate statistics	multivariate statistics	multivariate statistics	multivariate statistics	1	kept

multivisceral failure syndrome	multivisceral failure syndrome	multivisceral failure syndrome	multivisceral failure syndrome	multivisceral failure syndrome	1	kept
mumps vaccine	mumps vaccine	mumps vaccine	mumps vaccine	mumps vaccine	1	kept
municipality	municipality	municipality	municipality	municipality	1	kept
MUPIROCIN RESISTANCE	mupirocin resistance	mupirocin resistance	mupirocin resistance	MUPIROCIN RESISTANCE	1	kept
mupirocin-resistant coagulase-negative staphylococcus	mupirocin resistant coagulase negative staphylococcus	mupirocin resistant coagulase negative staphylococcus	mupirocin resistant coagulase negative staphylococcus	mupirocin-resistant coagulase-negative staphylococcus	1	kept
mupirocin-resistant staphylococcus aureus	mupirocin resistant staphylococcus aureus	mupirocin resistant staphylococcus aureus	mupirocin resistant staphylococcus aureus	mupirocin-resistant staphylococcus aureus	1	kept
Muramoylpentapeptide Carboxypeptidase	muramoylpentapeptide carboxypeptidase	muramoylpentapeptide carboxypeptidase	muramoylpentapeptide carboxypeptidase	Muramoylpentapeptide Carboxypeptidase	1	kept
Murmur	murmur	murmur	murmur	Murmur	1	kept
muscle action potential	muscle action potential	muscle action potential	muscle action potential	muscle action potential	1	kept
Muscle and bone maturation	muscle and bone maturation	muscle and bone maturation	muscle and bone maturation	Muscle and bone maturation	1	kept
muscle cell	muscle cell	muscle cell	muscle cell	muscle cell	1	kept
Muscle Color Computed Tomography	muscle color computed tomography	muscle color computed tomography	muscle color computed tomography	Muscle Color Computed Tomography	1	kept
Muscle Contraction	muscle contraction	muscle contraction	muscle contraction	muscle contraction	1	case_or_format
muscle contracture	muscle contracture	muscle contracture	muscle contracture	muscle contracture	1	kept
muscle cramps	muscle cramps	muscle cramps	muscle cramp	muscle cramp	1	singular
muscle denervation	muscle denervation	muscle denervation	muscle denervation	muscle denervation	1	kept

muscle enzyme	muscle enzyme	muscle enzyme	muscle enzyme	muscle enzyme	1	kept
Muscle Fibers, Skeletal	muscle fibers skeletal	muscle fibers skeletal	muscle fibers skeletal	Muscle Fibers, Skeletal	1	kept
muscle fibril	muscle fibril	muscle fibril	muscle fibril	muscle fibril	1	kept
Muscle growth	muscle growth	muscle growth	muscle growth	Muscle growth	1	kept
muscle growth	muscle growth	muscle growth	muscle growth	Muscle growth	1	case_or_format
muscle infection	muscle infection	muscle infection	muscle infection	muscle infection	1	kept
muscle invasive bladder cancer	muscle invasive bladder cancer	muscle invasive bladder cancer	muscle invasive bladder cancer	muscle invasive bladder cancer	1	kept
muscle layer thickness	muscle layer thickness	muscle layer thickness	muscle layer thickness	muscle layer thickness	1	kept
MUSCLE MASS	muscle mass	muscle mass	muscle mass	muscle mass	1	case_or_format
muscle metabolism	muscle metabolism	muscle metabolism	muscle metabolism	muscle metabolism	1	kept
Muscle power	muscle power	muscle power	muscle power	Muscle power	1	kept
muscle quadriceps muscle layer thickness	muscle quadriceps muscle layer thickness	muscle quadriceps muscle layer thickness	muscle quadriceps muscle layer thickness	muscle quadriceps muscle layer thickness	1	kept
muscle relaxant	muscle relaxant	muscle relaxant	muscle relaxant	muscle relaxant	1	kept
muscle resection	muscle resection	muscle resection	muscle resection	muscle resection	1	kept
muscle rest time	muscle rest time	muscle rest time	muscle rest time	muscle rest time	1	kept
muscle stiffness	muscle stiffness	muscle stiffness	muscle stiffness	muscle stiffness	1	kept

Muscle Strength Dynamometer	muscle strength dynamometer	muscle strength dynamometer	muscle strength dynamometer	Muscle Strength Dynamometer	1	kept
Muscle tension	muscle tension	muscle tension	muscle tension	Muscle tension	1	kept
muscle tension	muscle tension	muscle tension	muscle tension	Muscle tension	1	case_or_format
muscle tissue	muscle tissue	muscle tissue	muscle tissue	muscle tissue	1	kept
muscle twitch	muscle twitch	muscle twitch	muscle twitch	muscle twitch	1	kept
muscle wasting	muscle wasting	muscle wasting	muscle wasting	muscle wasting	1	kept
muscle weakness Resumo	muscle weakness resumo	muscle weakness resumo	muscle weakness resumo	muscle weakness Resumo	1	kept
Muscle, skeletal	muscle skeletal	muscle skeletal	muscle skeletal	Muscle, Skeletal	1	case_or_format
Muscular Atrophy, Spinal	muscular atrophy spinal	muscular atrophy spinal	muscular atrophy spinal	Muscular Atrophy, Spinal	1	kept
muscular dystrophies	muscular dystrophies	muscular dystrophies	muscular dystrophy	muscular dystrophy	1	singular
Muscular dystrophy	muscular dystrophy	muscular dystrophy	muscular dystrophy	muscular dystrophy	1	case_or_format
Muscular failure	muscular failure	muscular failure	muscular failure	Muscular failure	1	kept
MUSCULAR-DYSTROPHY	muscular dystrophy	muscular dystrophy	muscular dystrophy	muscular dystrophy	1	case_or_format
Musculoskeletal	musculoskeletal	musculoskeletal	musculoskeletal	musculoskeletal	1	case_or_format
musculoskeletal abnormalities	musculoskeletal abnormalities	musculoskeletal abnormalities	musculoskeletal abnormalities	musculoskeletal abnormalities	1	kept
Musculoskeletal Development	musculoskeletal development	musculoskeletal development	musculoskeletal development	Musculoskeletal Development	1	kept

Musculoskeletal discomfort	musculoskeletal discomfort	musculoskeletal discomfort	musculoskeletal discomfort	Musculoskeletal discomfort	1	kept
musculoskeletal discomfort	musculoskeletal discomfort	musculoskeletal discomfort	musculoskeletal discomfort	Musculoskeletal discomfort	1	case_or_format
Musculoskeletal diseases	musculoskeletal diseases	musculoskeletal diseases	musculoskeletal disease	Musculoskeletal Diseases	1	singular
Musculoskeletal disorders prevalence	musculoskeletal disorders prevalence	musculoskeletal disorders prevalence	musculoskeletal disorders prevalence	Musculoskeletal disorders prevalence	1	kept
musculoskeletal infection	musculoskeletal infection	musculoskeletal infection	musculoskeletal infection	musculoskeletal infection	1	kept
Musculoskeletal injuries	musculoskeletal injuries	musculoskeletal injuries	musculoskeletal injury	musculoskeletal injury	1	singular
MUSCULOSKELETAL INJURY	musculoskeletal injury	musculoskeletal injury	musculoskeletal injury	musculoskeletal injury	1	case_or_format
Musculoskeletal injury	musculoskeletal injury	musculoskeletal injury	musculoskeletal injury	musculoskeletal injury	1	case_or_format
Musculoskeletal oncology	musculoskeletal oncology	musculoskeletal oncology	musculoskeletal oncology	Musculoskeletal oncology	1	kept
Musculoskeletal Physiological Phenomena	musculoskeletal physiological phenomena	musculoskeletal physiological phenomena	musculoskeletal physiological phenomena	Musculoskeletal Physiological Phenomena	1	kept
Musculoskeletal problems	musculoskeletal problems	musculoskeletal problems	musculoskeletal problems	Musculoskeletal problems	1	kept
musculoskeletal symptom	musculoskeletal symptom	musculoskeletal symptom	musculoskeletal symptom	musculoskeletal symptom	1	kept
MUSCULOSKELETAL SYMPTOMS	musculoskeletal symptoms	musculoskeletal symptoms	musculoskeletal symptom	musculoskeletal symptom	1	singular
musculoskeletal system parameters	musculoskeletal system parameters	musculoskeletal system parameters	musculoskeletal system parameters	musculoskeletal system parameters	1	kept
mushroom poisoning	mushroom poisoning	mushroom poisoning	mushroom poisoning	Mushroom Poisoning	1	case_or_format
music voice	music voice	music voice	music voice	music voice	1	kept

Musical instruments	musical instruments	musical instruments	musical instruments	Musical instruments	1	kept
Musical intervention	musical intervention	musical intervention	musical intervention	Musical intervention	1	kept
musical intervention	musical intervention	musical intervention	musical intervention	Musical intervention	1	case_or_format
musical prompt	musical prompt	musical prompt	musical prompt	musical prompt	1	kept
musical stimulation	musical stimulation	musical stimulation	musical stimulation	musical stimulation	1	kept
Muslims	muslims	muslims	muslim	Muslim	1	singular
Mustard Gas	mustard gas	mustard gas	mustard gas	Mustard Gas	1	kept
Mustela vison	mustela vison	mustela vison	mustela vison	Mustela vison	1	kept
Mutagenesis, Insertional	mutagenesis insertional	mutagenesis insertional	mutagenesis insertional	Mutagenesis, Insertional	1	kept
Mutant strain	mutant strain	mutant strain	mutant strain	Mutant strain	1	kept
Mutation, Missense	mutation missense	mutation missense	mutation missense	Mutation, Missense	1	kept
mutual experience	mutual experience	mutual experience	mutual experience	mutual experience	1	kept
Mutual feedback	mutual feedback	mutual feedback	mutual feedback	Mutual feedback	1	kept
Mutual storytelling	mutual storytelling	mutual storytelling	mutual storytelling	Mutual storytelling	1	kept
mutual trust	mutual trust	mutual trust	mutual trust	mutual trust	1	kept
Mutual-touch	mutual touch	mutual touch	mutual touch	Mutual-touch	1	kept

Myalgia	myalgia	myalgia	myalgia	myalgia	1	case_or_format
Myalgias	myalgias	myalgias	myalgia	myalgia	1	singular
myasthenia	myasthenia	myasthenia	myasthenia	myasthenia	1	kept
myasthenia crisis	myasthenia crisis	myasthenia crisis	myasthenia crisis	myasthenia crisis	1	kept
Myasthenia gravis	myasthenia gravis	myasthenia gravis	myasthenia gravis	myasthenia gravis	1	case_or_format
mycetoma	mycetoma	mycetoma	mycetoma	mycetoma	1	kept
mycobacteriosis	mycobacteriosis	mycobacteriosis	mycobacteriosis	mycobacteriosis	1	kept
Mycobacterium avium complex	mycobacterium avium complex	mycobacterium avium complex	mycobacterium avium complex	Mycobacterium avium complex	1	kept
Mycobacterium avium complex infection	mycobacterium avium complex infection	mycobacterium avium complex infection	mycobacterium avium complex infection	Mycobacterium avium complex infection	1	kept
Mycobacterium intracellulare avium	mycobacterium intracellulare avium	mycobacterium intracellulare avium	mycobacterium intracellulare avium	Mycobacterium intracellulare avium	1	kept
Mycobacterium marinum	mycobacterium marinum	mycobacterium marinum	mycobacterium marinum	Mycobacterium marinum	1	kept
MYCOBACTERIUM-TUBERCULOSIS	mycobacterium tuberculosis	mycobacterium tuberculosis	mycobacterium tuberculosis	Mycobacterium tuberculosis	1	case_or_format
mycophenolic acid 2 morpholinoethyl ester	mycophenolic acid 2 morpholinoethyl ester	mycophenolic acid 2 morpholinoethyl ester	mycophenolic acid 2 morpholinoethyl ester	mycophenolic acid 2 morpholinoethyl ester	1	kept
mycoplasma pneumonia	mycoplasma pneumonia	mycoplasma pneumonia	mycoplasma pneumonia	Mycoplasma pneumonia	1	case_or_format
Mycosis Fungoides	mycosis fungoides	mycosis fungoides	mycosis fungoides	mycosis fungoides	1	case_or_format
mycotic aneurysm	mycotic aneurysm	mycotic aneurysm	mycotic aneurysm	mycotic aneurysm	1	kept

mycotoxin	mycotoxin	mycotoxin	mycotoxin	mycotoxin	1	kept
mydriatic agent	mydriatic agent	mydriatic agent	mydriatic agent	mydriatic agent	1	kept
Mydriatics	mydriatics	mydriatics	mydriatics	Mydriatics	1	kept
myelin basic protein	myelin basic protein	myelin basic protein	myelin basic protein	myelin basic protein	1	kept
Myelinolysis	myelinolysis	myelinolysis	myelinolysis	Myelinolysis	1	kept
Myelinolysis, Central Pontine	myelinolysis central pontine	myelinolysis central pontine	myelinolysis central pontine	Myelinolysis, Central Pontine	1	kept
myelitis	myelitis	myelitis	myelitis	myelitis	1	kept
myeloablative agent	myeloablative agent	myeloablative agent	myeloablative agent	myeloablative agent	1	kept
myeloblastin	myeloblastin	myeloblastin	myeloblastin	myeloblastin	1	kept
myelodysplasia	myelodysplasia	myelodysplasia	myelodysplasia	myelodysplasia	1	kept
Myelodysplastic Syndromes	myelodysplastic syndromes	myelodysplastic syndromes	myelodysplastic syndrome	myelodysplastic syndrome	1	singular
myeloid leukemia	myeloid leukemia	myeloid leukemia	myeloid leukemia	myeloid leukemia	1	kept
myeloid metaplasia	myeloid metaplasia	myeloid metaplasia	myeloid metaplasia	myeloid metaplasia	1	kept
myeloma cast nephropathy	myeloma cast nephropathy	myeloma cast nephropathy	myeloma cast nephropathy	myeloma cast nephropathy	1	kept
Myelophthisis	myelophthisis	myelophthisis	myelophthisis	Myelophthisis	1	kept
myeloproliferative disorder	myeloproliferative disorder	myeloproliferative disorder	myeloproliferative disorder	Myeloproliferative Disorders	1	case_or_format

myh11 gene	myh11 gene	myh11 gene	myh11 gene	myh11 gene	1	kept
myiasis	myiasis	myiasis	myiasis	myiasis	1	kept
Myocardial contrast echocardiography	myocardial contrast echocardiography	myocardial contrast echocardiography	myocardial contrast echocardiography	Myocardial contrast echocardiography	1	kept
MYOCARDIAL DYSFUNCTION	myocardial dysfunction	myocardial dysfunction	myocardial dysfunction	MYOCARDIAL DYSFUNCTION	1	kept
Myocardial dysfunction	myocardial dysfunction	myocardial dysfunction	myocardial dysfunction	MYOCARDIAL DYSFUNCTION	1	case_or_format
Myocardial injury	myocardial injury	myocardial injury	myocardial injury	Myocardial injury	1	kept
Myocardial ischemia	myocardial ischemia	myocardial ischemia	myocardial ischemia	Myocardial Ischemia	1	case_or_format
myocardial ischemia	myocardial ischemia	myocardial ischemia	myocardial ischemia	Myocardial Ischemia	1	case_or_format
Myocardial ischemia and infarction	myocardial ischemia and infarction	myocardial ischemia and infarction	myocardial ischemia and infarction	Myocardial ischemia and infarction	1	kept
myocardial ischemia reperfusion injury	myocardial ischemia reperfusion injury	myocardial ischemia reperfusion injury	myocardial ischemia reperfusion injury	myocardial ischemia reperfusion injury	1	kept
Myocardial perfusion imaging	myocardial perfusion imaging	myocardial perfusion imaging	myocardial perfusion imaging	Myocardial perfusion imaging	1	kept
myocardial perfusion imaging	myocardial perfusion imaging	myocardial perfusion imaging	myocardial perfusion imaging	Myocardial perfusion imaging	1	case_or_format
Myocardial Reperfusion Injury	myocardial reperfusion injury	myocardial reperfusion injury	myocardial reperfusion injury	Myocardial Reperfusion Injury	1	kept
Myocardial tissue	myocardial tissue	myocardial tissue	myocardial tissue	Myocardial tissue	1	kept
Myocardium	myocardium	myocardium	myocardium	Myocardium	1	kept
Myocutaneous Flap	myocutaneous flap	myocutaneous flap	myocutaneous flap	Myocutaneous Flap	1	kept

myocutaneous flap	myocutaneous flap	myocutaneous flap	myocutaneous flap	Myocutaneous Flap	1	case_or_format
myocyte disarray	myocyte disarray	myocyte disarray	myocyte disarray	myocyte disarray	1	kept
myofasciitis	myofasciitis	myofasciitis	myofasciitis	myofasciitis	1	kept
myometrium	myometrium	myometrium	myometrium	myometrium	1	kept
myosin	myosin	myosin	myosin	myosin	1	kept
myosin heavy chain	myosin heavy chain	myosin heavy chain	myosin heavy chain	myosin heavy chain	1	kept
Myosin Heavy Chains	myosin heavy chains	myosin heavy chains	myosin heavy chain	myosin heavy chain	1	singular
Myosins	myosins	myosins	myosin	myosin	1	singular
myositis	myositis	myositis	myositis	myositis	1	kept
Myosteatosis	myosteatosis	myosteatosis	myosteatosis	Myosteatosis	1	kept
myotome	myotome	myotome	myotome	myotome	1	kept
Myotomy	myotomy	myotomy	myotomy	Myotomy	1	kept
myotomy	myotomy	myotomy	myotomy	Myotomy	1	case_or_format
myotonia	myotonia	myotonia	myotonia	myotonia	1	kept
myotonic dystrophy	myotonic dystrophy	myotonic dystrophy	myotonic dystrophy	myotonic dystrophy	1	kept
Myotrauma	myotrauma	myotrauma	myotrauma	Myotrauma	1	kept

MYSTIC PROGRAM	mystic program	mystic program	mystic program	MYSTIC PROGRAM	1	kept
myth and culture	myth and culture	myth and culture	myth and culture	myth and culture	1	kept
Myxedema coma	myxedema coma	myxedema coma	myxedema coma	Myxedema coma	1	kept
myxoma	myxoma	myxoma	myxoma	myxoma	1	kept
Máscaras	m scaras	m scaras	m scaras	Máscaras	1	kept
mécanisme de défense	m canisme de d fense	m canisme de d fense	m canisme de d fense	mécanisme de défense	1	kept
Médecin	m decin	m decin	m decin	Médecin	1	kept
médecin anesthésiste r éanimateur	m decin anesth siste r animateur	m decin anesth siste r animateur	m decin anesth siste r animateur	médecin anesthésiste r éanimateur	1	kept
médecin anesthésiste-r éanimateur	m decin anesth siste r animateur	m decin anesth siste r animateur	m decin anesth siste r animateur	médecin anesthésiste r éanimateur	1	case_or_format
médecine périopé ratoire	m decine p riop ratoire	m decine p riop ratoire	m decine p riop ratoire	médecine périopé ratoire	1	kept
Médico	m dico	m dico	m dico	Médicos	1	case_or_format
Médico quirúrgica	m dico quir rgica	m dico quir rgica	m dico quir rgica	Médico quirúrgica	1	kept
N gene	n gene	n gene	n gene	N gene	1	kept
n methyl dextro aspartic acid	n methyl dextro aspartic acid	n methyl dextro aspartic acid	n methyl dextro aspartic acid	n methyl dextro aspartic acid	1	kept
n methyl dextro aspartic acid receptor antibody	n methyl dextro aspartic acid receptor antibody	n methyl dextro aspartic acid receptor antibody	n methyl dextro aspartic acid receptor antibody	n methyl dextro aspartic acid receptor antibody	1	kept
n methyl dextro aspartic acid receptor blocking agent	n methyl dextro aspartic acid receptor blocking agent	n methyl dextro aspartic acid receptor blocking agent	n methyl dextro aspartic acid receptor blocking agent	n methyl dextro aspartic acid receptor blocking agent	1	kept

N- spiperone	n spiperone	n spiperone	n spiperone	N- spiperone	1	kept
N-ACETYLCYSTEINE	n acetylcysteine	n acetylcysteine	n acetylcysteine	N-ACETYLCYSTEINE	1	kept
N-acetylcysteine	n acetylcysteine	n acetylcysteine	n acetylcysteine	N-ACETYLCYSTEINE	1	case_or_format
N-Acetylneuraminic Acid	n acetylneuraminic acid	n acetylneuraminic acid	n acetylneuraminic acid	N-Acetylneuraminic Acid	1	kept
N-BUTYL PHTHALATE	n butyl phthalate	n butyl phthalate	n butyl phthalate	N-BUTYL PHTHALATE	1	kept
N-methyl-3	n methyl 3	n methyl 3	n methyl 3	N-methyl-3	1	kept
N.I.N.E.	n i n e	n i n e	n i n e	N.I.N.E.	1	kept
N95	n95	n95	n95	N95	1	kept
N95 MASK	n95 mask	n95 mask	n95 mask	N95 masks	1	case_or_format
N95 respirator	n95 respirator	n95 respirator	n95 respirator	N95 Respirators	1	case_or_format
N95-mask	n95 mask	n95 mask	n95 mask	N95 masks	1	case_or_format
naed	naed	naed	naed	naed	1	kept
nafamstat mesilate	nafamstat mesilate	nafamstat mesilate	nafamstat mesilate	nafamstat mesilate	1	kept
Nager acrofacial dysostosis	nager acrofacial dysostosis	nager acrofacial dysostosis	nager acrofacial dysostosis	Nager acrofacial dysostosis	1	kept
Nail Diseases	nail diseases	nail diseases	nail diseases	Nail Diseases	1	kept
nail infection	nail infection	nail infection	nail infection	nail infection	1	kept

Nail plate construct	nail plate construct	nail plate construct	nail plate construct	Nail plate construct	1	kept
Nail plate fixation	nail plate fixation	nail plate fixation	nail plate fixation	Nail plate fixation	1	kept
Nakaseomyces	nakaseomyces	nakaseomyces	nakaseomyces	Nakaseomyces	1	kept
naked eye assessment	naked eye assessment	naked eye assessment	naked eye assessment	naked eye assessment	1	kept
NALOXONE USE	naloxone use	naloxone use	naloxone use	NALOXONE USE	1	kept
Naltrexone	naltrexone	naltrexone	naltrexone	naltrexone	1	case_or_format
named groups by marital status	named groups by marital status	named groups by marital status	named groups by marital status	named groups by marital status	1	kept
named groups by pregnancy	named groups by pregnancy	named groups by pregnancy	named groups by pregnancy	named groups by pregnancy	1	kept
named groups of persons	named groups of persons	named groups of persons	named groups of persons	named groups of persons	1	kept
Nan, missing value	nan missing value	nan missing value	nan missing value	Nan, missing value	1	kept
NANDA-I terminology	nanda i terminology	nanda i terminology	nanda i terminology	NANDA-I terminology	1	kept
nandrolone	nandrolone	nandrolone	nandrolone	nandrolone	1	kept
NANOCLUSTERS	nanoclusters	nanoclusters	nanoclusters	NANOCLUSTERS	1	kept
Nanocrystalline	nanocrystalline	nanocrystalline	nanocrystalline	Nanocrystalline	1	kept
Nanoparticle sizes	nanoparticle sizes	nanoparticle sizes	nanoparticle sizes	Nanoparticle sizes	1	kept
nanoparticle tracking analysis	nanoparticle tracking analysis	nanoparticle tracking analysis	nanoparticle tracking analysis	nanoparticle tracking analysis	1	kept

Nanoparticles formations	nanoparticles formations	nanoparticles formations	nanoparticles formations	Nanoparticles formations	1	kept
Nanosilver	nanosilver	nanosilver	nanosilver	Nanosilver	1	kept
Nanotechnology	nanotechnology	nanotechnology	nanotechnology	Nanotechnology	1	kept
NAP1	nap1	nap1	nap1	NAP1	1	kept
Naples Heart Project	naples heart project	naples heart project	naples heart project	Naples Heart Project	1	kept
Napping	napping	napping	napping	napping	1	case_or_format
Napping policy	napping policy	napping policy	napping policy	Napping policy	1	kept
napping policy	napping policy	napping policy	napping policy	Napping policy	1	case_or_format
Naps at work	naps at work	naps at work	naps at work	Naps at work	1	kept
Naps during nightshifts	naps during nightshifts	naps during nightshifts	naps during nightshifts	Naps during nightshifts	1	kept
narcissism	narcissism	narcissism	narcissism	Narcissism	1	case_or_format
narcotic dependence	narcotic dependence	narcotic dependence	narcotic dependence	narcotic dependence	1	kept
narcotic drugs	narcotic drugs	narcotic drugs	narcotic drugs	narcotic drugs	1	kept
narcotics anonymous	narcotics anonymous	narcotics anonymous	narcotics anonymous	narcotics anonymous	1	kept
narrative analysis	narrative analysis	narrative analysis	narrative analysis	narrative analysis	1	kept
Narrative care	narrative care	narrative care	narrative care	Narrative care	1	kept

Narrative Inquiry	narrative inquiry	narrative inquiry	narrative inquiry	narrative inquiry	1	case_or_format
Narrative intervention	narrative intervention	narrative intervention	narrative intervention	Narrative intervention	1	kept
Narrative Medicine	narrative medicine	narrative medicine	narrative medicine	narrative medicine	1	case_or_format
Narrative medicine	narrative medicine	narrative medicine	narrative medicine	narrative medicine	1	case_or_format
Narrative overview	narrative overview	narrative overview	narrative overview	Narrative overview	1	kept
NARRATIVE PEDAGOGY	narrative pedagogy	narrative pedagogy	narrative pedagogy	NARRATIVE PEDAGOGY	1	kept
Narrative pedagogy	narrative pedagogy	narrative pedagogy	narrative pedagogy	NARRATIVE PEDAGOGY	1	case_or_format
narrative study	narrative study	narrative study	narrative study	narrative study	1	kept
Narrative Therapy	narrative therapy	narrative therapy	narrative therapy	narrative therapy	1	case_or_format
Narrative writing	narrative writing	narrative writing	narrative writing	Narrative writing	1	kept
narrative writing	narrative writing	narrative writing	narrative writing	Narrative writing	1	case_or_format
narrative‑medicine	narrative medicine	narrative medicine	narrative medicine	narrative medicine	1	case_or_format
narrativism	narrativism	narrativism	narrativism	narrativism	1	kept
Narritive	narritive	narritive	narritive	Narritive	1	kept
narrow band imaging	narrow band imaging	narrow band imaging	narrow band imaging	narrow band imaging	1	kept
Narrow QRS tachycardia	narrow qrs tachycardia	narrow qrs tachycardia	narrow qrs tachycardia	Narrow QRS tachycardia	1	kept

NASA-TLX score	nasa tlx score	nasa tlx score	nasa tlx score	NASA-TLX score	1	kept
nasal biphasic positive airway pressure	nasal biphasic positive airway pressure	nasal biphasic positive airway pressure	nasal biphasic positive airway pressure	nasal biphasic positive airway pressure	1	kept
Nasal cannula	nasal cannula	nasal cannula	nasal cannula	Nasal cannula	1	kept
Nasal cannulae	nasal cannulae	nasal cannulae	nasal cannulae	Nasal cannulae	1	kept
NASAL CARRIAGE	nasal carriage	nasal carriage	nasal carriage	NASAL CARRIAGE	1	kept
Nasal Carriage	nasal carriage	nasal carriage	nasal carriage	NASAL CARRIAGE	1	case_or_format
nasal carriage	nasal carriage	nasal carriage	nasal carriage	NASAL CARRIAGE	1	case_or_format
nasal carrier	nasal carrier	nasal carrier	nasal carrier	nasal carrier	1	kept
NASAL COLONIZATION	nasal colonization	nasal colonization	nasal colonization	NASAL COLONIZATION	1	kept
nasal colonization	nasal colonization	nasal colonization	nasal colonization	NASAL COLONIZATION	1	case_or_format
nasal endoscopy	nasal endoscopy	nasal endoscopy	nasal endoscopy	nasal endoscopy	1	kept
nasal feeding	nasal feeding	nasal feeding	nasal feeding	nasal feeding	1	kept
Nasal high flow	nasal high flow	nasal high flow	nasal high flow	Nasal high flow	1	kept
nasal high‑flow oxygen	nasal high flow oxygen	nasal high flow oxygen	nasal high flow oxygen	nasal high‑flow oxygen	1	kept
Nasal hygiene	nasal hygiene	nasal hygiene	nasal hygiene	Nasal hygiene	1	kept
Nasal injuries	nasal injuries	nasal injuries	nasal injury	Nasal injury	1	singular

nasal lavage	nasal lavage	nasal lavage	nasal lavage	nasal lavage	1	kept
nasal mucosal bleeding	nasal mucosal bleeding	nasal mucosal bleeding	nasal mucosal bleeding	nasal mucosal bleeding	1	kept
Nasal mupirocin	nasal mupirocin	nasal mupirocin	nasal mupirocin	Nasal mupirocin	1	kept
Nasal obstruction	nasal obstruction	nasal obstruction	nasal obstruction	Nasal obstruction	1	kept
nasal packing	nasal packing	nasal packing	nasal packing	nasal packing	1	kept
nasal positive end expiratory pressure	nasal positive end expiratory pressure	nasal positive end expiratory pressure	nasal positive end expiratory pressure	nasal positive end expiratory pressure	1	kept
Nasal prongs	nasal prongs	nasal prongs	nasal prongs	Nasal prongs	1	kept
nasal redness	nasal redness	nasal redness	nasal redness	nasal redness	1	kept
Nasal Septum	nasal septum	nasal septum	nasal septum	Nasal Septum	1	kept
Nasal septum	nasal septum	nasal septum	nasal septum	Nasal Septum	1	case_or_format
Nasal skin breakdown	nasal skin breakdown	nasal skin breakdown	nasal skin breakdown	Nasal skin breakdown	1	kept
Nasal Sprays	nasal sprays	nasal sprays	nasal sprays	Nasal Sprays	1	kept
Nasal swabs	nasal swabs	nasal swabs	nasal swabs	Nasal swabs	1	kept
nasal tissue	nasal tissue	nasal tissue	nasal tissue	nasal tissue	1	kept
nasal trauma	nasal trauma	nasal trauma	nasal trauma	nasal trauma	1	kept
nasally placed feeding tube	nasally placed feeding tube	nasally placed feeding tube	nasally placed feeding tube	nasally placed feeding tube	1	kept

Nasoenteral tubes	nasoenteral tubes	nasoenteral tubes	nasoenteral tubes	Nasoenteral tubes	1	kept
nasoenteric tube	nasoenteric tube	nasoenteric tube	nasoenteric tube	nasoenteric tube	1	kept
Nasoenteric tubes	nasoenteric tubes	nasoenteric tubes	nasoenteric tube	nasoenteric tube	1	singular
Nasogastric	nasogastric	nasogastric	nasogastric	Nasogastric	1	kept
nasogastric feeding	nasogastric feeding	nasogastric feeding	nasogastric feeding	Nasogastric feeding	1	case_or_format
Nasogastric intubation	nasogastric intubation	nasogastric intubation	nasogastric intubation	Nasogastric intubation	1	kept
nasogastric probe	nasogastric probe	nasogastric probe	nasogastric probe	nasogastric probe	1	kept
NASOGASTRIC TUBE	nasogastric tube	nasogastric tube	nasogastric tube	nasogastric tube	1	case_or_format
Nasogastric Tube	nasogastric tube	nasogastric tube	nasogastric tube	nasogastric tube	1	case_or_format
nasogastric tube blockage	nasogastric tube blockage	nasogastric tube blockage	nasogastric tube blockage	nasogastric tube blockage	1	kept
nasogastric tube dislodgment	nasogastric tube dislodgment	nasogastric tube dislodgment	nasogastric tube dislodgment	nasogastric tube dislodgment	1	kept
Nasogastric tube feeding at home	nasogastric tube feeding at home	nasogastric tube feeding at home	nasogastric tube feeding at home	Nasogastric tube feeding at home	1	kept
nasogastric tube placement	nasogastric tube placement	nasogastric tube placement	nasogastric tube placement	nasogastric tube placement	1	kept
Nasogastric tube placement technique	nasogastric tube placement technique	nasogastric tube placement technique	nasogastric tube placement technique	Nasogastric tube placement technique	1	kept
Nasogastric tubes	nasogastric tubes	nasogastric tubes	nasogastric tube	nasogastric tube	1	singular
nasointestinal tube obstruction	nasointestinal tube obstruction	nasointestinal tube obstruction	nasointestinal tube obstruction	nasointestinal tube obstruction	1	kept

nasojejunal tube	nasojejunal tube	nasojejunal tube	nasojejunal tube	nasojejunal tube	1	kept
Nasopharyngeal Neoplasms	nasopharyngeal neoplasms	nasopharyngeal neoplasms	nasopharyngeal neoplasms	Nasopharyngeal Neoplasms	1	kept
nasopharyngeal suctioning	nasopharyngeal suctioning	nasopharyngeal suctioning	nasopharyngeal suctioning	nasopharyngeal suctioning	1	kept
nasopharyngeal swabs	nasopharyngeal swabs	nasopharyngeal swabs	nasopharyngeal swab	nasopharyngeal swab	1	singular
nasopharyngoscopy	nasopharyngoscopy	nasopharyngoscopy	nasopharyngoscopy	nasopharyngoscopy	1	kept
nasopharynx airway	nasopharynx airway	nasopharynx airway	nasopharynx airway	nasopharynx airway	1	kept
nasotracheal	nasotracheal	nasotracheal	nasotracheal	nasotracheal	1	kept
nateglinide	nateglinide	nateglinide	nateglinide	nateglinide	1	kept
National Aeronautics and Space Administration Task	national aeronautics and space administration task	national aeronautics and space administration task	national aeronautics and space administration task	National Aeronautics and Space Administration Task	1	kept
National Aeronautics and Space Administration–Task	national aeronautics and space administration task	national aeronautics and space administration task	national aeronautics and space administration task	National Aeronautics and Space Administration Task	1	case_or_format
NATIONAL AUDIT	national audit	national audit	national audit	NATIONAL AUDIT	1	kept
NATIONAL AUDIT PROJECT	national audit project	national audit project	national audit project	NATIONAL AUDIT PROJECT	1	kept
national benchmarking	national benchmarking	national benchmarking	national benchmarking	national benchmarking	1	kept
National Bereaved Family Survey	national bereaved family survey	national bereaved family survey	national bereaved family survey	National Bereaved Family Survey	1	kept
National campaign	national campaign	national campaign	national campaign	National campaign	1	kept
National Capital Territory of Delhi	national capital territory of delhi	national capital territory of delhi	national capital territory of delhi	National Capital Territory of Delhi	1	kept

national cohort	national cohort	national cohort	national cohort	national cohort	1	kept
National data	national data	national data	national data	National data	1	kept
National database	national database	national database	national database	National database	1	kept
NATIONAL EARLY WARNING SCORE	national early warning score	national early warning score	national early warning score	National Early Warning Score	1	case_or_format
National Emergency Access Target	national emergency access target	national emergency access target	national emergency access target	National Emergency Access Target	1	kept
National Emergency Access Target,	national emergency access target	national emergency access target	national emergency access target	National Emergency Access Target	1	case_or_format
national emergency department overcrowding scale	national emergency department overcrowding scale	national emergency department overcrowding scale	national emergency department overcrowding scale	national emergency department overcrowding scale	1	kept
National guidelines	national guidelines	national guidelines	national guidelines	National guidelines	1	kept
national health alert	national health alert	national health alert	national health alert	national health alert	1	kept
national health and aging trends survey end of life planning	national health and aging trends survey end of life planning	national health and aging trends survey end of life planning	national health and aging trends survey end of life planning	national health and aging trends survey end of life planning	1	kept
national health coverage	national health coverage	national health coverage	national health coverage	national health coverage	1	kept
National Health Insurance Service	national health insurance service	national health insurance service	national health insurance service	National Health Insurance Service	1	kept
national health programmes	national health programmes	national health programmes	national health programmes	national health programmes	1	kept
national health system	national health system	national health system	national health system	national health system	1	kept
national healthcare decisions month	national healthcare decisions month	national healthcare decisions month	national healthcare decisions month	national healthcare decisions month	1	kept
National Healthcare Safety Network	national healthcare safety network	national healthcare safety network	national healthcare safety network	National Healthcare Safety Network	1	kept

National Healthcare Safety Network Surveillance system	national healthcare safety network surveillance system	national healthcare safety network surveillance system	national healthcare safety network surveillance system	National Healthcare Safety Network Surveillance system	1	kept
National Hospital Kandy	national hospital kandy	national hospital kandy	national hospital kandy	National Hospital Kandy	1	kept
National Institute for Health Research	national institute for health research	national institute for health research	national institute for health research	National Institute for Health Research	1	kept
National Instruments	national instruments	national instruments	national instruments	National Instruments	1	kept
National Organ Donation Collaborative	national organ donation collaborative	national organ donation collaborative	national organ donation collaborative	National Organ Donation Collaborative	1	kept
National Provider Identifier	national provider identifier	national provider identifier	national provider identifier	National Provider Identifier	1	kept
National Quality Forum	national quality forum	national quality forum	national quality forum	National Quality Forum	1	kept
National recommendations	national recommendations	national recommendations	national recommendations	National recommendations	1	kept
National referral hospital	national referral hospital	national referral hospital	national referral hospital	National referral hospital	1	kept
National service framework	national service framework	national service framework	national service framework	National service framework	1	kept
National standard	national standard	national standard	national standard	National standard	1	kept
National surgical planning	national surgical planning	national surgical planning	national surgical planning	National surgical planning	1	kept
NATIONAL SURVEILLANCE	national surveillance	national surveillance	national surveillance	NATIONAL SURVEILLANCE	1	kept
National Trauma Data Bank	national trauma data bank	national trauma data bank	national trauma data bank	National Trauma Data Bank	1	kept
national trends	national trends	national trends	national trends	national trends	1	kept
National weighted activity unit	national weighted activity unit	national weighted activity unit	national weighted activity unit	National weighted activity unit	1	kept

national-level	national level	national level	national level	national-level	1	kept
Nations	nations	nations	nations	Nations	1	kept
NATIONWIDE	nationwide	nationwide	nationwide	NATIONWIDE	1	kept
nationwide	nationwide	nationwide	nationwide	NATIONWIDE	1	case_or_format
nationwide questionnaire in Japan	nationwide questionnaire in japan	nationwide questionnaire in japan	nationwide questionnaire in japan	Nationwide questionnaire in Japan	1	case_or_format
Nationwide survey	nationwide survey	nationwide survey	nationwide survey	nationwide survey	1	case_or_format
Native American	native american	native american	native american	Native American	1	kept
Native Hawaiian or Pacific Islander	native hawaiian or pacific islander	native hawaiian or pacific islander	native hawaiian or pacific islander	Native Hawaiian or Pacific Islander	1	kept
Natriuresis	natriuresis	natriuresis	natriuresis	natriuresis	1	case_or_format
Natriuretic peptide	natriuretic peptide	natriuretic peptide	natriuretic peptide	Natriuretic peptides	1	case_or_format
NATRIURETIC PEPTIDE LEVELS	natriuretic peptide levels	natriuretic peptide levels	natriuretic peptide levels	NATRIURETIC PEPTIDE LEVELS	1	kept
natriuretic peptide type C	natriuretic peptide type c	natriuretic peptide type c	natriuretic peptide type c	natriuretic peptide type C	1	kept
Natural Disasters	natural disasters	natural disasters	natural disaster	natural disaster	1	singular
Natural light	natural light	natural light	natural light	Natural light	1	kept
natural light	natural light	natural light	natural light	Natural light	1	case_or_format
Natural Orifice Endoscopic Surgery	natural orifice endoscopic surgery	natural orifice endoscopic surgery	natural orifice endoscopic surgery	Natural Orifice Endoscopic Surgery	1	kept

Natural orifice transluminal endoscopic surgery	natural orifice transluminal endoscopic surgery	natural orifice transluminal endoscopic surgery	natural orifice transluminal endoscopic surgery	natural orifice transluminal endoscopic surgery	1	case_or_format
natural product	natural product	natural product	natural product	natural product	1	kept
natural resistance associated macrophage protein 2	natural resistance associated macrophage protein 2	natural resistance associated macrophage protein 2	natural resistance associated macrophage protein 2	natural resistance associated macrophage protein 2	1	kept
natural resource	natural resource	natural resource	natural resource	natural resource	1	kept
Natural rubber allergy	natural rubber allergy	natural rubber allergy	natural rubber allergy	Natural rubber allergy	1	kept
Natural rubber latex	natural rubber latex	natural rubber latex	natural rubber latex	Natural rubber latex	1	kept
Naturalistic decision making	naturalistic decision making	naturalistic decision making	naturalistic decision making	Naturalistic decision making	1	kept
naturalistic decision making	naturalistic decision making	naturalistic decision making	naturalistic decision making	Naturalistic decision making	1	case_or_format
naturalistic inquiry	naturalistic inquiry	naturalistic inquiry	naturalistic inquiry	naturalistic inquiry	1	kept
Nature	nature	nature	nature	Nature	1	kept
nature	nature	nature	nature	Nature	1	case_or_format
Nature music	nature music	nature music	nature music	Nature music	1	kept
nature music	nature music	nature music	nature music	Nature music	1	case_or_format
nature of the work	nature of the work	nature of the work	nature of the work	nature of the work	1	kept
Nature sound	nature sound	nature sound	nature sound	Nature sound	1	kept
nature sound	nature sound	nature sound	nature sound	Nature sound	1	case_or_format

nature‐based sounds	nature based sounds	nature based sounds	nature based sounds	nature‐based sounds	1	kept
naturopathy	naturopathy	naturopathy	naturopathy	naturopathy	1	kept
Nausea-vomiting	nausea vomiting	nausea vomiting	nausea vomiting	Nausea-vomiting	1	kept
navigation-assisted surgery	navigation assisted surgery	navigation assisted surgery	navigation assisted surgery	navigation-assisted surgery	1	kept
Navigator Program	navigator program	navigator program	navigator program	Navigator Program	1	kept
navy	navy	navy	navy	navy	1	kept
NC, nasal cannula	nc nasal cannula	nc nasal cannula	nc nasal cannula	NC, nasal cannula	1	kept
NCCU	nccu	nccu	nccu	NCCU	1	kept
NCLEX-RN success	nclex rn success	nclex rn success	nclex rn success	NCLEX-RN success	1	kept
ncov	ncov	ncov	ncov	ncov	1	kept
NCP	ncp	ncp	ncp	NCP	1	kept
nCPAP ventilation	ncpap ventilation	ncpap ventilation	ncpap ventilation	nCPAP ventilation	1	kept
NCT06201962	nct06201962	nct06201962	nct06201962	NCT06201962	1	kept
NDM	ndm	ndm	ndm	NDM	1	kept
NDNQI pressure ulcer education	ndnqi pressure ulcer education	ndnqi pressure ulcer education	ndnqi pressure ulcer education	NDNQI pressure ulcer education	1	kept
NDNQI unit-level data	ndnqi unit level data	ndnqi unit level data	ndnqi unit level data	NDNQI unit-level data	1	kept

Near Drowning	near drowning	near drowning	near drowning	near drowning	1	case_or_format
near fatal grape aspiration	near fatal grape aspiration	near fatal grape aspiration	near fatal grape aspiration	near fatal grape aspiration	1	kept
near infrared light	near infrared light	near infrared light	near infrared light	near infrared light	1	kept
Near infrared spectroscopy	near infrared spectroscopy	near infrared spectroscopy	near infrared spectroscopy	near infrared spectroscopy	1	case_or_format
near infrared therapy	near infrared therapy	near infrared therapy	near infrared therapy	near infrared therapy	1	kept
Near Miss Infants	near miss infants	near miss infants	near miss infants	Near Miss Infants	1	kept
Near miss ratio	near miss ratio	near miss ratio	near miss ratio	Near miss ratio	1	kept
Near miss Salud	near miss salud	near miss salud	near miss salud	Near miss Salud	1	kept
near real time hospital occupany data	near real time hospital occupany data	near real time hospital occupany data	near real time hospital occupany data	near real time hospital occupany data	1	kept
near-continuous glucose monitoring system	near continuous glucose monitoring system	near continuous glucose monitoring system	near continuous glucose monitoring system	near-continuous glucose monitoring system	1	kept
NEAR-DEATH	near death	near death	near death	NEAR-DEATH	1	kept
Near-death experience	near death experience	near death experience	near death experience	near-death experiences	1	case_or_format
Near-field electromagnetic ranging system	near field electromagnetic ranging system	near field electromagnetic ranging system	near field electromagnetic ranging system	Near-field electromagnetic ranging system	1	kept
Near-infrared electromagnetic radiation	near infrared electromagnetic radiation	near infrared electromagnetic radiation	near infrared electromagnetic radiation	Near-infrared electromagnetic radiation	1	kept
Near-infrared light	near infrared light	near infrared light	near infrared light	near infrared light	1	case_or_format
Near-infrared spectroscopy physiological	near infrared spectroscopy physiological	near infrared spectroscopy physiological	near infrared spectroscopy physiological	Near-infrared spectroscopy physiological	1	kept

Near-miss errors	near miss errors	near miss errors	near miss errors	Near-miss errors	1	kept
near-miss events	near miss events	near miss events	near miss events	near-miss events	1	kept
Near-misses	near misses	near misses	near misses	Near-misses	1	kept
NEAR-TERM INFANTS	near term infants	near term infants	near term infants	NEAR-TERM INFANTS	1	kept
nearest neighbor matching	nearest neighbor matching	nearest neighbor matching	nearest neighbor matching	nearest neighbor matching	1	kept
NearMiss	nearmiss	nearmiss	nearmiss	NearMiss	1	kept
nebulized antibiotics	nebulized antibiotics	nebulized antibiotics	nebulized antibiotics	nebulized antibiotics	1	kept
Nebulizers	nebulizers	nebulizers	nebulizer	nebulizer	1	singular
Nebulizers and vaporizers [MeSH]	nebulizers and vaporizers mesh	nebulizers and vaporizers mesh	nebulizers and vaporizers mesh	Nebulizers and vaporizers [MeSH]	1	kept
NEC score	nec score	nec score	nec score	NEC score	1	kept
NEC-zero	nec zero	nec zero	nec zero	NEC-zero	1	kept
neck control	neck control	neck control	neck control	neck control	1	kept
neck disease	neck disease	neck disease	neck disease	neck disease	1	kept
Neck Dissection	neck dissection	neck dissection	neck dissection	neck dissection	1	case_or_format
neck mass	neck mass	neck mass	neck mass	neck mass	1	kept
Neck Muscles	neck muscles	neck muscles	neck muscles	Neck Muscles	1	kept

Neck muscles	neck muscles	neck muscles	neck muscles	Neck Muscles	1	case_or_format
Neck pain	neck pain	neck pain	neck pain	neck pain	1	case_or_format
neck rash	neck rash	neck rash	neck rash	neck rash	1	kept
neck tumor	neck tumor	neck tumor	neck tumor	neck tumor	1	kept
NECK-CANCER	neck cancer	neck cancer	neck cancer	NECK-CANCER	1	kept
Necropolitics	necropolitics	necropolitics	necropolitics	Necropolitics	1	kept
Necrotic bowel	necrotic bowel	necrotic bowel	necrotic bowel	Necrotic bowel	1	kept
Necrotising	necrotising	necrotising	necrotising	Necrotising	1	kept
necrotising fasciitis	necrotising fasciitis	necrotising fasciitis	necrotising fasciitis	Necrotising fasciitis	1	case_or_format
Necrotising infections	necrotising infections	necrotising infections	necrotising infections	Necrotising infections	1	kept
necrotising soft tissue infection	necrotising soft tissue infection	necrotising soft tissue infection	necrotising soft tissue infection	necrotising soft tissue infection	1	kept
necrotizing cellulitis	necrotizing cellulitis	necrotizing cellulitis	necrotizing cellulitis	necrotizing cellulitis	1	kept
necrotizing colitis	necrotizing colitis	necrotizing colitis	necrotizing colitis	necrotizing colitis	1	kept
necrotizing enteritis	necrotizing enteritis	necrotizing enteritis	necrotizing enteritis	necrotizing enteritis	1	kept
Necrotizing skin and soft tissue infections	necrotizing skin and soft tissue infections	necrotizing skin and soft tissue infections	necrotizing skin and soft tissue infections	Necrotizing skin and soft tissue infections	1	kept
Necrotizing soft tissue infection	necrotizing soft tissue infection	necrotizing soft tissue infection	necrotizing soft tissue infection	Necrotizing soft tissue infection	1	kept

Nectrotising enterocolitis	nectrotising enterocolitis	nectrotising enterocolitis	nectrotising enterocolitis	Nectrotising enterocolitis	1	kept
NED	ned	ned	ned	NED	1	kept
NEDS	neds	neds	ned	NED	1	singular
neecham confusion scale	neecham confusion scale	neecham confusion scale	neecham confusion scale	NEECHAM Confusion Scale	1	case_or_format
NEECHAM tool	neecham tool	neecham tool	neecham tool	NEECHAM tool	1	kept
need assessment	need assessment	need assessment	need assessment	need assessment	1	kept
Need Based Oral Care	need based oral care	need based oral care	need based oral care	Need Based Oral Care	1	kept
Need findings	need findings	need findings	need findings	Need findings	1	kept
need for comfort	need for comfort	need for comfort	need for comfort	need for comfort	1	kept
need for information	need for information	need for information	need for information	need for information	1	kept
need for proximity	need for proximity	need for proximity	need for proximity	need for proximity	1	kept
need for support	need for support	need for support	need for support	need for support	1	kept
Need for trauma intervention	need for trauma intervention	need for trauma intervention	need for trauma intervention	Need for trauma intervention	1	kept
need of assurance	need of assurance	need of assurance	need of assurance	need of assurance	1	kept
need satisfaction	need satisfaction	need satisfaction	need satisfaction	need satisfaction	1	kept
needle biopsy	needle biopsy	needle biopsy	needle biopsy	needle biopsy	1	kept

needle decompression	needle decompression	needle decompression	needle decompression	needle decompression	1	kept
Needle positioning system	needle positioning system	needle positioning system	needle positioning system	Needle positioning system	1	kept
needle prick infection	needle prick infection	needle prick infection	needle prick infection	needle prick infection	1	kept
needle stick injury	needle stick injury	needle stick injury	needle stick injury	Needle stick injuries	1	case_or_format
Needle sticks injuries	needle sticks injuries	needle sticks injuries	needle sticks injuries	Needle sticks injuries	1	kept
Needle-free connectors	needle free connectors	needle free connectors	needle free connectors	Needle-free connectors	1	kept
Needleless catheter	needleless catheter	needleless catheter	needleless catheter	Needleless catheter	1	kept
needles	needles	needles	needle	needle	1	singular
needlestick and sharps injury	needlestick and sharps injury	needlestick and sharps injury	needlestick and sharps injury	needlestick and sharps injury	1	kept
Needlestick injures and sharp injury	needlestick injures and sharp injury	needlestick injures and sharp injury	needlestick injures and sharp injury	Needlestick injures and sharp injury	1	kept
Needlestick injuries	needlestick injuries	needlestick injuries	needlestick injury	needlestick injury	1	singular
needlesticks	needlesticks	needlesticks	needlestick	Needlestick	1	singular
needs analysis	needs analysis	needs analysis	needs analysis	needs analysis	1	kept
Needs at the End of Life Screening Tool	needs at the end of life screening tool	needs at the end of life screening tool	needs at the end of life screening tool	Needs at the End of Life Screening Tool	1	kept
Needs estimate	needs estimate	needs estimate	needs estimate	Needs estimate	1	kept
Needs of Children Questionnaire	needs of children questionnaire	needs of children questionnaire	needs of children questionnaire	Needs of Children Questionnaire	1	kept

Needs of family members	needs of family members	needs of family members	needs of family members	Needs of family members	1	kept
Needs of parents	needs of parents	needs of parents	needs of parents	Needs of parents	1	kept
Neelon and Champagne Confusion Scale	neelon and champagne confusion scale	neelon and champagne confusion scale	neelon and champagne confusion scale	Neelon and Champagne Confusion Scale	1	kept
Neelon and Champagne confusion scale	neelon and champagne confusion scale	neelon and champagne confusion scale	neelon and champagne confusion scale	Neelon and Champagne Confusion Scale	1	case_or_format
neelon and champagne confusion scale	neelon and champagne confusion scale	neelon and champagne confusion scale	neelon and champagne confusion scale	Neelon and Champagne Confusion Scale	1	case_or_format
Neelon and Champagne Scale	neelon and champagne scale	neelon and champagne scale	neelon and champagne scale	Neelon and Champagne Scale	1	kept
nefazodone	nefazodone	nefazodone	nefazodone	nefazodone	1	kept
Nefopam	nefopam	nefopam	nefopam	nefopam	1	case_or_format
Negative Act Questionnaire‐Revised	negative act questionnaire revised	negative act questionnaire revised	negative act questionnaire revised	Negative Act Questionnaire‐Revised	1	kept
NEGATIVE ACTS QUESTIONNAIRE	negative acts questionnaire	negative acts questionnaire	negative acts questionnaire	NEGATIVE ACTS QUESTIONNAIRE	1	kept
Negative affect	negative affect	negative affect	negative affect	Negative affect	1	kept
Negative affectivity	negative affectivity	negative affectivity	negative affectivity	Negative affectivity	1	kept
negative blood culture	negative blood culture	negative blood culture	negative blood culture	negative blood culture	1	kept
negative cases and controls	negative cases and controls	negative cases and controls	negative cases and controls	negative cases and controls	1	kept
Negative correlation	negative correlation	negative correlation	negative correlation	Negative correlation	1	kept
Negative emotion	negative emotion	negative emotion	negative emotion	negative emotion	1	case_or_format

negative emotional symptoms	negative emotional symptoms	negative emotional symptoms	negative emotional symptoms	negative emotional symptoms	1	kept
Negative emotions	negative emotions	negative emotions	negative emotion	negative emotion	1	singular
negative emotions	negative emotions	negative emotions	negative emotion	negative emotion	1	singular
negative feedback	negative feedback	negative feedback	negative feedback	negative feedback	1	kept
negative pressure isolation ward	negative pressure isolation ward	negative pressure isolation ward	negative pressure isolation ward	negative pressure isolation ward	1	kept
Negative pressures	negative pressures	negative pressures	negative pressures	Negative pressures	1	kept
Negativist	negativist	negativist	negativist	Negativist	1	kept
Negibacteria	negibacteria	negibacteria	negibacteria	Negibacteria	1	kept
Neglected diseases	neglected diseases	neglected diseases	neglected diseases	Neglected diseases	1	kept
NEGOTIATED ORDER	negotiated order	negotiated order	negotiated order	NEGOTIATED ORDER	1	kept
Negotiated partnership	negotiated partnership	negotiated partnership	negotiated partnership	Negotiated partnership	1	kept
Negro	negro	negro	negro	Negro	1	kept
Neighborhood Characteristics	neighborhood characteristics	neighborhood characteristics	neighborhood characteristic	neighborhood characteristic	1	singular
neighborhood socioeconomic deprivation	neighborhood socioeconomic deprivation	neighborhood socioeconomic deprivation	neighborhood socioeconomic deprivation	neighborhood socioeconomic deprivation	1	kept
neighborhoods	neighborhoods	neighborhoods	neighborhood	neighborhood	1	singular
Neighbourhood	neighbourhood	neighbourhood	neighbourhood	Neighbourhood	1	kept

Nekrotizan enterokolit	nekrotizan enterokolit	nekrotizan enterokolit	nekrotizan enterokolit	Nekrotizan enterokolit	1	kept
Nellcor OxiMax N-600x	nellcor oximax n 600x	nellcor oximax n 600x	nellcor oximax n 600x	Nellcor OxiMax N-600x	1	kept
nemaline myopathy	nemaline myopathy	nemaline myopathy	nemaline myopathy	nemaline myopathy	1	kept
NEMU	nemu	nemu	nemu	NEMU	1	kept
Nennatal intencive care u initc	nennatal intencive care u initc	nennatal intencive care u initc	nennatal intencive care u initc	Nennatal intencive care u initc	1	kept
NeO stress score	neo stress score	neo stress score	neo stress score	NeO stress score	1	kept
Neoadjuvant chemotherapy	neoadjuvant chemotherapy	neoadjuvant chemotherapy	neoadjuvant chemotherapy	neoadjuvant chemotherapy	1	case_or_format
neolyte	neolyte	neolyte	neolyte	neolyte	1	kept
Neonat<sup> <	neonat sup	neonat sup	neonat sup	Neonat<sup> <	1	kept
Neonatal [methods]	neonatal methods	neonatal methods	neonatal methods	Neonatal [methods]	1	kept
neonatal ABO hemolysis	neonatal abo hemolysis	neonatal abo hemolysis	neonatal abo hemolysis	neonatal ABO hemolysis	1	kept
Neonatal abstinence scoring system	neonatal abstinence scoring system	neonatal abstinence scoring system	neonatal abstinence scoring system	Neonatal abstinence scoring system	1	kept
NEONATAL ABSTINENCE SYNDROME	neonatal abstinence syndrome	neonatal abstinence syndrome	neonatal abstinence syndrome	Neonatal Abstinence Syndrome	1	case_or_format
neonatal abstinence syndrome - prevention and control	neonatal abstinence syndrome prevention and control	neonatal abstinence syndrome prevention and control	neonatal abstinence syndrome prevention and control	neonatal abstinence syndrome - prevention and control	1	kept
neonatal abstinence syndrome rating score	neonatal abstinence syndrome rating score	neonatal abstinence syndrome rating score	neonatal abstinence syndrome rating score	neonatal abstinence syndrome rating score	1	kept
Neonatal adaptation	neonatal adaptation	neonatal adaptation	neonatal adaptation	Neonatal adaptation	1	kept

neonatal adaptation disorders	neonatal adaptation disorders	neonatal adaptation disorders	neonatal adaptation disorders	neonatal adaptation disorders	1	kept
neonatal addiction	neonatal addiction	neonatal addiction	neonatal addiction	neonatal addiction	1	kept
neonatal adverse event severity scale	neonatal adverse event severity scale	neonatal adverse event severity scale	neonatal adverse event severity scale	neonatal adverse event severity scale	1	kept
Neonatal alertness state	neonatal alertness state	neonatal alertness state	neonatal alertness state	Neonatal alertness state	1	kept
neonatal alertness state	neonatal alertness state	neonatal alertness state	neonatal alertness state	Neonatal alertness state	1	case_or_format
neonatal and infant	neonatal and infant	neonatal and infant	neonatal and infant	neonatal and infant	1	kept
neonatal apnea	neonatal apnea	neonatal apnea	neonatal apnea	neonatal apnea	1	kept
Neonatal asphyxia	neonatal asphyxia	neonatal asphyxia	neonatal asphyxia	Neonatal asphyxia	1	kept
neonatal asphyxia	neonatal asphyxia	neonatal asphyxia	neonatal asphyxia	Neonatal asphyxia	1	case_or_format
Neonatal autopsy	neonatal autopsy	neonatal autopsy	neonatal autopsy	Neonatal autopsy	1	kept
Neonatal Behavioral Assessment Scale	neonatal behavioral assessment scale	neonatal behavioral assessment scale	neonatal behavioral assessment scale	Neonatal Behavioral Assessment Scale	1	kept
Neonatal behavioral assessment scale	neonatal behavioral assessment scale	neonatal behavioral assessment scale	neonatal behavioral assessment scale	Neonatal Behavioral Assessment Scale	1	case_or_format
neonatal behavioral assessment scale	neonatal behavioral assessment scale	neonatal behavioral assessment scale	neonatal behavioral assessment scale	Neonatal Behavioral Assessment Scale	1	case_or_format
neonatal behavioral neurological score	neonatal behavioral neurological score	neonatal behavioral neurological score	neonatal behavioral neurological score	neonatal behavioral neurological score	1	kept
Neonatal birth outcome	neonatal birth outcome	neonatal birth outcome	neonatal birth outcome	Neonatal birth outcome	1	kept
Neonatal brain	neonatal brain	neonatal brain	neonatal brain	Neonatal brain	1	kept

Neonatal brain function	neonatal brain function	neonatal brain function	neonatal brain function	Neonatal brain function	1	kept
neonatal brain injury	neonatal brain injury	neonatal brain injury	neonatal brain injury	neonatal brain injury	1	kept
Neonatal burn injury	neonatal burn injury	neonatal burn injury	neonatal burn injury	Neonatal burn injury	1	kept
neonatal calculator	neonatal calculator	neonatal calculator	neonatal calculator	neonatal calculator	1	kept
NEONATAL CARE	neonatal care	neonatal care	neonatal care	neonatal care	1	case_or_format
Neonatal Care	neonatal care	neonatal care	neonatal care	neonatal care	1	case_or_format
Neonatal care bundle	neonatal care bundle	neonatal care bundle	neonatal care bundle	neonatal care bundle	1	case_or_format
neonatal care standards	neonatal care standards	neonatal care standards	neonatal care standards	neonatal care standards	1	kept
Neonatal care unit	neonatal care unit	neonatal care unit	neonatal care unit	neonatal care unit	1	case_or_format
neonatal care units	neonatal care units	neonatal care units	neonatal care unit	neonatal care unit	1	singular
neonatal chronic lung disease	neonatal chronic lung disease	neonatal chronic lung disease	neonatal chronic lung disease	neonatal chronic lung disease	1	kept
neonatal CKRT	neonatal ckrt	neonatal ckrt	neonatal ckrt	neonatal CKRT	1	kept
Neonatal clinicians	neonatal clinicians	neonatal clinicians	neonatal clinicians	Neonatal clinicians	1	kept
neonatal codes	neonatal codes	neonatal codes	neonatal codes	neonatal codes	1	kept
Neonatal competencies	neonatal competencies	neonatal competencies	neonatal competencies	Neonatal competencies	1	kept
neonatal couplet care	neonatal couplet care	neonatal couplet care	neonatal couplet care	neonatal couplet care	1	kept

neonatal delivery room management	neonatal delivery room management	neonatal delivery room management	neonatal delivery room management	neonatal delivery room management	1	kept
neonatal depression	neonatal depression	neonatal depression	neonatal depression	neonatal depression	1	kept
Neonatal development	neonatal development	neonatal development	neonatal development	Neonatal development	1	kept
neonatal early-onset sepsis	neonatal early onset sepsis	neonatal early onset sepsis	neonatal early onset sepsis	neonatal early-onset sepsis	1	kept
Neonatal ECMO education	neonatal ecmo education	neonatal ecmo education	neonatal ecmo education	Neonatal ECMO education	1	kept
Neonatal end-of-life care	neonatal end of life care	neonatal end of life care	neonatal end of life care	Neonatal end-of-life care	1	kept
neonatal end－of－life care	neonatal end of life care	neonatal end of life care	neonatal end of life care	Neonatal end-of-life care	1	case_or_format
neonatal extracorporeal membrane oxygenation	neonatal extracorporeal membrane oxygenation	neonatal extracorporeal membrane oxygenation	neonatal extracorporeal membrane oxygenation	neonatal extracorporeal membrane oxygenation	1	kept
neonatal feeding	neonatal feeding	neonatal feeding	neonatal feeding	neonatal feeding	1	kept
neonatal focal ischemic stroke	neonatal focal ischemic stroke	neonatal focal ischemic stroke	neonatal focal ischemic stroke	neonatal focal ischemic stroke	1	kept
Neonatal follow up	neonatal follow up	neonatal follow up	neonatal follow up	Neonatal follow up	1	kept
neonatal follow up clinic	neonatal follow up clinic	neonatal follow up clinic	neonatal follow up clinic	neonatal follow up clinic	1	kept
Neonatal follow-up	neonatal follow up	neonatal follow up	neonatal follow up	Neonatal follow up	1	case_or_format
neonatal follow-up	neonatal follow up	neonatal follow up	neonatal follow up	Neonatal follow up	1	case_or_format
neonatal growth	neonatal growth	neonatal growth	neonatal growth	neonatal growth	1	kept
neonatal health care	neonatal health care	neonatal health care	neonatal health care	neonatal health care	1	kept

neonatal health care professionals	neonatal health care professionals	neonatal health care professionals	neonatal health care professionals	neonatal health care professionals	1	kept
Neonatal health outcomes	neonatal health outcomes	neonatal health outcomes	neonatal health outcomes	Neonatal health outcomes	1	kept
neonatal healthcare professionals	neonatal healthcare professionals	neonatal healthcare professionals	neonatal healthcare professionals	neonatal healthcare professionals	1	kept
Neonatal healthcare providers	neonatal healthcare providers	neonatal healthcare providers	neonatal healthcare providers	Neonatal healthcare providers	1	kept
Neonatal hemochromatosis	neonatal hemochromatosis	neonatal hemochromatosis	neonatal hemochromatosis	Neonatal hemochromatosis	1	kept
neonatal hypertension	neonatal hypertension	neonatal hypertension	neonatal hypertension	neonatal hypertension	1	kept
neonatal hypoglycaemia	neonatal hypoglycaemia	neonatal hypoglycaemia	neonatal hypoglycaemia	neonatal hypoglycaemia	1	kept
Neonatal hypoxic ischemic encephalopathies	neonatal hypoxic ischemic encephalopathies	neonatal hypoxic ischemic encephalopathies	neonatal hypoxic ischemic encephalopathy	Neonatal hypoxic ischemic encephalopathy	1	singular
Neonatal hypoxic ischemic encephalopathy	neonatal hypoxic ischemic encephalopathy	neonatal hypoxic ischemic encephalopathy	neonatal hypoxic ischemic encephalopathy	Neonatal hypoxic ischemic encephalopathy	1	kept
Neonatal immunity	neonatal immunity	neonatal immunity	neonatal immunity	Neonatal immunity	1	kept
Neonatal incubators	neonatal incubators	neonatal incubators	neonatal incubator	neonatal incubator	1	singular
Neonatal individualized developmental care	neonatal individualized developmental care	neonatal individualized developmental care	neonatal individualized developmental care	Neonatal individualized developmental care	1	kept
Neonatal Infant Braden Q scale	neonatal infant braden q scale	neonatal infant braden q scale	neonatal infant braden q scale	Neonatal Infant Braden Q scale	1	kept
Neonatal infant pain scale	neonatal infant pain scale	neonatal infant pain scale	neonatal infant pain scale	Neonatal Infant Pain Scale	1	case_or_format
neonatal infant pain scale	neonatal infant pain scale	neonatal infant pain scale	neonatal infant pain scale	Neonatal Infant Pain Scale	1	case_or_format
Neonatal Infant, Newborn	neonatal infant newborn	neonatal infant newborn	neonatal infant newborn	Neonatal Infant, Newborn	1	kept

Neonatal infections	neonatal infections	neonatal infections	neonatal infection	Neonatal infection	1	singular
Neonatal informatics	neonatal informatics	neonatal informatics	neonatal informatics	Neonatal informatics	1	kept
neonatal innovation	neonatal innovation	neonatal innovation	neonatal innovation	neonatal innovation	1	kept
Neonatal intensive and special care unit	neonatal intensive and special care unit	neonatal intensive and special care unit	neonatal intensive and special care unit	Neonatal intensive and special care unit	1	kept
neonatal intensive therapy	neonatal intensive therapy	neonatal intensive therapy	neonatal intensive therapy	neonatal intensive therapy	1	kept
neonatal intensive unit	neonatal intensive unit	neonatal intensive unit	neonatal intensive unit	neonatal intensive unit	1	kept
Neonatal intermediate care unit	neonatal intermediate care unit	neonatal intermediate care unit	neonatal intermediate care unit	Neonatal intermediate care unit	1	kept
Neonatal intestinal	neonatal intestinal	neonatal intestinal	neonatal intestinal	Neonatal intestinal	1	kept
Neonatal intubation	neonatal intubation	neonatal intubation	neonatal intubation	Neonatal intubation	1	kept
Neonatal Jaundice	neonatal jaundice	neonatal jaundice	neonatal jaundice	Neonatal jaundice	1	case_or_format
neonatal late-onset sepsis	neonatal late onset sepsis	neonatal late onset sepsis	neonatal late onset sepsis	neonatal late-onset sepsis	1	kept
Neonatal length of stay	neonatal length of stay	neonatal length of stay	neonatal length of stay	Neonatal length of stay	1	kept
neonatal levels of care	neonatal levels of care	neonatal levels of care	neonatal levels of care	neonatal levels of care	1	kept
Neonatal Life Support Task Force	neonatal life support task force	neonatal life support task force	neonatal life support task force	Neonatal Life Support Task Force	1	kept
Neonatal Monitoring	neonatal monitoring	neonatal monitoring	neonatal monitoring	Neonatal monitoring	1	case_or_format
neonatal monitoring	neonatal monitoring	neonatal monitoring	neonatal monitoring	Neonatal monitoring	1	case_or_format

neonatal morbidity	neonatal morbidity	neonatal morbidity	neonatal morbidity	Neonatal morbidity	1	case_or_format
neonatal MRI	neonatal mri	neonatal mri	neonatal mri	neonatal MRI	1	kept
Neonatal near miss	neonatal near miss	neonatal near miss	neonatal near miss	Neonatal near miss	1	kept
Neonatal necrotizing enterocolitis	neonatal necrotizing enterocolitis	neonatal necrotizing enterocolitis	neonatal necrotizing enterocolitis	Neonatal necrotizing enterocolitis	1	kept
neonatal neurological examination	neonatal neurological examination	neonatal neurological examination	neonatal neurological examination	neonatal neurological examination	1	kept
neonatal nutrition	neonatal nutrition	neonatal nutrition	neonatal nutrition	Neonatal nutrition	1	case_or_format
neonatal occupational therapy	neonatal occupational therapy	neonatal occupational therapy	neonatal occupational therapy	neonatal occupational therapy	1	kept
Neonatal Opioid Withdrawal Syndrome	neonatal opioid withdrawal syndrome	neonatal opioid withdrawal syndrome	neonatal opioid withdrawal syndrome	neonatal opioid withdrawal syndrome	1	case_or_format
Neonatal opioid withdrawal syndrome	neonatal opioid withdrawal syndrome	neonatal opioid withdrawal syndrome	neonatal opioid withdrawal syndrome	neonatal opioid withdrawal syndrome	1	case_or_format
Neonatal organ donation	neonatal organ donation	neonatal organ donation	neonatal organ donation	Neonatal organ donation	1	kept
Neonatal outcome	neonatal outcome	neonatal outcome	neonatal outcome	neonatal outcome	1	case_or_format
NEONATAL OUTCOMES	neonatal outcomes	neonatal outcomes	neonatal outcome	neonatal outcome	1	singular
Neonatal Pain	neonatal pain	neonatal pain	neonatal pain	Neonatal pain	1	case_or_format
Neonatal pain assessment	neonatal pain assessment	neonatal pain assessment	neonatal pain assessment	neonatal pain assessment	1	case_or_format
neonatal pain care	neonatal pain care	neonatal pain care	neonatal pain care	neonatal pain care	1	kept
neonatal pain management	neonatal pain management	neonatal pain management	neonatal pain management	Neonatal pain management	1	case_or_format

Neonatal pain, Pgitation and Sedation Scale	neonatal pain pgitation and sedation scale	neonatal pain pgitation and sedation scale	neonatal pain pgitation and sedation scale	Neonatal pain, Pgitation and Sedation Scale	1	kept
Neonatal palliation	neonatal palliation	neonatal palliation	neonatal palliation	Neonatal palliation	1	kept
neonatal palliative care attitude scale	neonatal palliative care attitude scale	neonatal palliative care attitude scale	neonatal palliative care attitude scale	neonatal palliative care attitude scale	1	kept
Neonatal palliative care protocol	neonatal palliative care protocol	neonatal palliative care protocol	neonatal palliative care protocol	Neonatal palliative care protocol	1	kept
neonatal palliative care team	neonatal palliative care team	neonatal palliative care team	neonatal palliative care team	neonatal palliative care team	1	kept
Neonatal parent support	neonatal parent support	neonatal parent support	neonatal parent support	Neonatal parent support	1	kept
neonatal parents	neonatal parents	neonatal parents	neonatal parents	neonatal parents	1	kept
neonatal pediatric health	neonatal pediatric health	neonatal pediatric health	neonatal pediatric health	neonatal pediatric health	1	kept
Neonatal period	neonatal period	neonatal period	neonatal period	Neonatal period	1	kept
neonatal period	neonatal period	neonatal period	neonatal period	Neonatal period	1	case_or_format
Neonatal phantom	neonatal phantom	neonatal phantom	neonatal phantom	Neonatal phantom	1	kept
neonatal phantom	neonatal phantom	neonatal phantom	neonatal phantom	Neonatal phantom	1	case_or_format
Neonatal physician	neonatal physician	neonatal physician	neonatal physician	Neonatal physician	1	kept
neonatal physiotherapy	neonatal physiotherapy	neonatal physiotherapy	neonatal physiotherapy	neonatal physiotherapy	1	kept
Neonatal population	neonatal population	neonatal population	neonatal population	Neonatal population	1	kept
neonatal postresuscitation care	neonatal postresuscitation care	neonatal postresuscitation care	neonatal postresuscitation care	neonatal postresuscitation care	1	kept

neonatal pressure injury	neonatal pressure injury	neonatal pressure injury	neonatal pressure injury	neonatal pressure injury	1	kept
Neonatal pressure ulcer	neonatal pressure ulcer	neonatal pressure ulcer	neonatal pressure ulcer	Neonatal pressure ulcer	1	kept
Neonatal procedural pain	neonatal procedural pain	neonatal procedural pain	neonatal procedural pain	Neonatal procedural pain	1	kept
neonatal provider workforce	neonatal provider workforce	neonatal provider workforce	neonatal provider workforce	neonatal provider workforce	1	kept
Neonatal psychiatry	neonatal psychiatry	neonatal psychiatry	neonatal psychiatry	Neonatal psychiatry	1	kept
neonatal readmissions	neonatal readmissions	neonatal readmissions	neonatal readmissions	neonatal readmissions	1	kept
Neonatal recurrent hypoglycemia	neonatal recurrent hypoglycemia	neonatal recurrent hypoglycemia	neonatal recurrent hypoglycemia	Neonatal recurrent hypoglycemia	1	kept
Neonatal research trials	neonatal research trials	neonatal research trials	neonatal research trials	Neonatal research trials	1	kept
neonatal respiratory distress	neonatal respiratory distress	neonatal respiratory distress	neonatal respiratory distress	neonatal respiratory distress	1	kept
Neonatal Respiratory Distress Syndrome	neonatal respiratory distress syndrome	neonatal respiratory distress syndrome	neonatal respiratory distress syndrome	neonatal respiratory distress syndrome	1	case_or_format
Neonatal respiratory distress syndrome	neonatal respiratory distress syndrome	neonatal respiratory distress syndrome	neonatal respiratory distress syndrome	neonatal respiratory distress syndrome	1	case_or_format
Neonatal respiratory support	neonatal respiratory support	neonatal respiratory support	neonatal respiratory support	neonatal respiratory support	1	case_or_format
Neonatal Resuscitation	neonatal resuscitation	neonatal resuscitation	neonatal resuscitation	neonatal resuscitation	1	case_or_format
neonatal resuscitation programme	neonatal resuscitation programme	neonatal resuscitation programme	neonatal resuscitation programme	neonatal resuscitation programme	1	kept
neonatal resuscitation team	neonatal resuscitation team	neonatal resuscitation team	neonatal resuscitation team	neonatal resuscitation team	1	kept
Neonatal safety	neonatal safety	neonatal safety	neonatal safety	Neonatal safety	1	kept

neonatal score	neonatal score	neonatal score	neonatal score	neonatal score	1	kept
NEONATAL SERVICES	neonatal services	neonatal services	neonatal services	NEONATAL SERVICES	1	kept
neonatal simulation	neonatal simulation	neonatal simulation	neonatal simulation	neonatal simulation	1	kept
neonatal skin care	neonatal skin care	neonatal skin care	neonatal skin care	neonatal skin care	1	kept
neonatal skin risk assessment scale	neonatal skin risk assessment scale	neonatal skin risk assessment scale	neonatal skin risk assessment scale	neonatal skin risk assessment scale	1	kept
Neonatal sleep	neonatal sleep	neonatal sleep	neonatal sleep	neonatal sleep	1	case_or_format
neonatal specialist	neonatal specialist	neonatal specialist	neonatal specialist	neonatal specialist	1	kept
neonatal stabilization	neonatal stabilization	neonatal stabilization	neonatal stabilization	neonatal stabilization	1	kept
neonatal standard	neonatal standard	neonatal standard	neonatal standard	neonatal standards	1	case_or_format
Neonatal stress	neonatal stress	neonatal stress	neonatal stress	neonatal stress	1	case_or_format
neonatal stress reduction	neonatal stress reduction	neonatal stress reduction	neonatal stress reduction	neonatal stress reduction	1	kept
neonatal substance abuse	neonatal substance abuse	neonatal substance abuse	neonatal substance abuse	neonatal substance abuse	1	kept
Neonatal surgery,	neonatal surgery	neonatal surgery	neonatal surgery	Neonatal surgery	1	case_or_format
Neonatal tissue donation	neonatal tissue donation	neonatal tissue donation	neonatal tissue donation	Neonatal tissue donation	1	kept
Neonatal transitional care	neonatal transitional care	neonatal transitional care	neonatal transitional care	Neonatal transitional care	1	kept
Neonatal transport challenges	neonatal transport challenges	neonatal transport challenges	neonatal transport challenges	Neonatal transport challenges	1	kept

neonatal transport network	neonatal transport network	neonatal transport network	neonatal transport network	neonatal transport network	1	kept
Neonatal transport system KPIs	neonatal transport system kpis	neonatal transport system kpis	neonatal transport system kpis	Neonatal transport system KPIs	1	kept
Neonatal transport team	neonatal transport team	neonatal transport team	neonatal transport team	Neonatal transport team	1	kept
neonatal transport team	neonatal transport team	neonatal transport team	neonatal transport team	Neonatal transport team	1	case_or_format
Neonatal transport team workforce	neonatal transport team workforce	neonatal transport team workforce	neonatal transport team workforce	Neonatal transport team workforce	1	kept
Neonatal tuberculosis	neonatal tuberculosis	neonatal tuberculosis	neonatal tuberculosis	Neonatal tuberculosis	1	kept
Neonatal unit admission	neonatal unit admission	neonatal unit admission	neonatal unit admission	Neonatal unit admission	1	kept
neonatal units, United Kingdom	neonatal units united kingdom	neonatal units united kingdom	neonatal units united kingdom	neonatal units, United Kingdom	1	kept
neonatal ventilator	neonatal ventilator	neonatal ventilator	neonatal ventilator	neonatal ventilator	1	kept
Neonatal vital sign measurement	neonatal vital sign measurement	neonatal vital sign measurement	neonatal vital sign measurement	Neonatal vital sign measurement	1	kept
neonatal withdrawal	neonatal withdrawal	neonatal withdrawal	neonatal withdrawal	neonatal withdrawal	1	kept
neonatal withdrawal syndrome	neonatal withdrawal syndrome	neonatal withdrawal syndrome	neonatal withdrawal syndrome	neonatal withdrawal syndrome	1	kept
NEONATAL-MORTALITY	neonatal mortality	neonatal mortality	neonatal mortality	Neonatal mortality	1	case_or_format
Neonatal-Perinatal medicine	neonatal perinatal medicine	neonatal perinatal medicine	neonatal perinatal medicine	Neonatal-Perinatal medicine	1	kept
Neonatal. Catheterization	neonatal catheterization	neonatal catheterization	neonatal catheterization	Neonatal. Catheterization	1	kept
neonatalogist	neonatalogist	neonatalogist	neonatalogist	neonatalogist	1	kept

Neonate health	neonate health	neonate health	neonate health	Neonate health	1	kept
Neonate Practice	neonate practice	neonate practice	neonate practice	Neonate Practice	1	kept
Neonate resuscitation	neonate resuscitation	neonate resuscitation	neonate resuscitation	Neonate resuscitation	1	kept
neonate, family	neonate family	neonate family	neonate family	neonate, family	1	kept
Neonates and children	neonates and children	neonates and children	neonates and children	Neonates and children	1	kept
Neonato	neonato	neonato	neonato	Neonato	1	kept
Neonatologia	neonatologia	neonatologia	neonatologia	Neonatologia	1	kept
neonatologists	neonatologists	neonatologists	neonatologist	neonatologist	1	singular
Neonatology and infant development	neonatology and infant development	neonatology and infant development	neonatology and infant development	Neonatology and infant development	1	kept
Neonatology for at-risk infants	neonatology for at risk infants	neonatology for at risk infants	neonatology for at risk infants	Neonatology for at-risk infants	1	kept
Neonatology Survey of Interdisciplinary Groups in Healthcare	neonatology survey of interdisciplinary groups in healthcare	neonatology survey of interdisciplinary groups in healthcare	neonatology survey of interdisciplinary groups in healthcare	Neonatology Survey of Interdisciplinary Groups in Healthcare	1	kept
neonatology training	neonatology training	neonatology training	neonatology training	neonatology training	1	kept
NeonatologyPrimary Health Care	neonatologyprimary health care	neonatologyprimary health care	neonatologyprimary health care	NeonatologyPrimary Health Care	1	kept
Neonatología	neonatolog a	neonatolog a	neonatolog a	Neonatología	1	kept
Neoplasia	neoplasia	neoplasia	neoplasia	Neoplasia	1	kept
Neoplasm	neoplasm	neoplasm	neoplasm	Neoplasms	1	case_or_format

Neoplasm Grading	neoplasm grading	neoplasm grading	neoplasm grading	Neoplasm Grading	1	kept
Neoplasm Proteins	neoplasm proteins	neoplasm proteins	neoplasm proteins	Neoplasm Proteins	1	kept
Neoplasms, Second Primary	neoplasms second primary	neoplasms second primary	neoplasms second primary	Neoplasms, Second Primary	1	kept
NEOPLASTIC FEVER	neoplastic fever	neoplastic fever	neoplastic fever	NEOPLASTIC FEVER	1	kept
Neopuff	neopuff	neopuff	neopuff	Neopuff	1	kept
neovagina	neovagina	neovagina	neovagina	neovagina	1	kept
Nephelometry and Turbidimetry	nephelometry and turbidimetry	nephelometry and turbidimetry	nephelometry and turbidimetry	Nephelometry and Turbidimetry	1	kept
nephew	nephew	nephew	nephew	nephew	1	kept
Nephrectomy	nephrectomy	nephrectomy	nephrectomy	nephrectomy	1	case_or_format
nephro hospitalist	nephro hospitalist	nephro hospitalist	nephro hospitalist	nephro hospitalist	1	kept
nephrogenic diabetes insipidus	nephrogenic diabetes insipidus	nephrogenic diabetes insipidus	nephrogenic diabetes insipidus	nephrogenic diabetes insipidus	1	kept
Nephrolithotomy, Percutaneous	nephrolithotomy percutaneous	nephrolithotomy percutaneous	nephrolithotomy percutaneous	Nephrolithotomy, Percutaneous	1	kept
Nephrology consultation	nephrology consultation	nephrology consultation	nephrology consultation	Nephrology consultation	1	kept
nephrology unit	nephrology unit	nephrology unit	nephrology unit	nephrology unit	1	kept
nephron	nephron	nephron	nephron	nephron	1	kept
Nephrons	nephrons	nephrons	nephron	nephron	1	singular

Nephropathy	nephropathy	nephropathy	nephropathy	Nephropathy	1	kept
Nephrosis	nephrosis	nephrosis	nephrosis	Nephrosis	1	kept
nephrosis	nephrosis	nephrosis	nephrosis	Nephrosis	1	case_or_format
nephrostomy	nephrostomy	nephrostomy	nephrostomy	nephrostomy	1	kept
nephrostomy tube	nephrostomy tube	nephrostomy tube	nephrostomy tube	nephrostomy tube	1	kept
nephrotoxic drugs	nephrotoxic drugs	nephrotoxic drugs	nephrotoxic drugs	nephrotoxic drugs	1	kept
nerolidol	nerolidol	nerolidol	nerolidol	nerolidol	1	kept
Nerve block catheter	nerve block catheter	nerve block catheter	nerve block catheter	Nerve block catheter	1	kept
nerve cell	nerve cell	nerve cell	nerve cell	nerve cell	1	kept
nerve cell excitability	nerve cell excitability	nerve cell excitability	nerve cell excitability	nerve cell excitability	1	kept
nerve cell membrane	nerve cell membrane	nerve cell membrane	nerve cell membrane	nerve cell membrane	1	kept
nerve compression	nerve compression	nerve compression	nerve compression	nerve compression	1	kept
nerve conduction study	nerve conduction study	nerve conduction study	nerve conduction study	Nerve conduction studies	1	case_or_format
nerve conduction velocity test	nerve conduction velocity test	nerve conduction velocity test	nerve conduction velocity test	nerve conduction velocity test	1	kept
nerve entrapment	nerve entrapment	nerve entrapment	nerve entrapment	nerve entrapment	1	kept
nerve excitability	nerve excitability	nerve excitability	nerve excitability	nerve excitability	1	kept

nerve growth factor	nerve growth factor	nerve growth factor	nerve growth factor	nerve growth factor	1	kept
nerve paralysis	nerve paralysis	nerve paralysis	nerve paralysis	nerve paralysis	1	kept
nerve regeneration	nerve regeneration	nerve regeneration	nerve regeneration	nerve regeneration	1	kept
nerve root	nerve root	nerve root	nerve root	nerve root	1	kept
Nerve sensitization	nerve sensitization	nerve sensitization	nerve sensitization	Nerve sensitization	1	kept
nerve sheath	nerve sheath	nerve sheath	nerve sheath	nerve sheath	1	kept
nerve stimulation	nerve stimulation	nerve stimulation	nerve stimulation	nerve stimulation	1	kept
nerve tract	nerve tract	nerve tract	nerve tract	nerve tract	1	kept
Nerves	nerves	nerves	nerves	Nerves	1	kept
Nervous system	nervous system	nervous system	nervous system	nervous system	1	case_or_format
Nervous system diseases	nervous system diseases	nervous system diseases	nervous system diseases	Nervous System Diseases	1	case_or_format
nervous system diseases	nervous system diseases	nervous system diseases	nervous system diseases	Nervous System Diseases	1	case_or_format
Nesiritide	nesiritide	nesiritide	nesiritide	nesiritide	1	case_or_format
nested case-control study	nested case control study	nested case control study	nested case control study	nested case-control study	1	kept
Nested polymerase chain reaction	nested polymerase chain reaction	nested polymerase chain reaction	nested polymerase chain reaction	Nested polymerase chain reaction	1	kept
nested polymerase chain reaction	nested polymerase chain reaction	nested polymerase chain reaction	nested polymerase chain reaction	Nested polymerase chain reaction	1	case_or_format

Nesting	nesting	nesting	nesting	nesting	1	case_or_format
net promotor score	net promotor score	net promotor score	net promotor score	net promotor score	1	kept
Net Reclassification Improvement	net reclassification improvement	net reclassification improvement	net reclassification improvement	Net Reclassification Improvement	1	kept
netilmicin	netilmicin	netilmicin	netilmicin	netilmicin	1	kept
netupitant	netupitant	netupitant	netupitant	netupitant	1	kept
Network analyses	network analyses	network analyses	network analysis	network analysis	1	singular
Network architecture	network architecture	network architecture	network architecture	Network architecture	1	kept
Network centralities	network centralities	network centralities	network centralities	Network centralities	1	kept
Network design	network design	network design	network design	Network design	1	kept
Network layers	network layers	network layers	network layers	Network layers	1	kept
network learning	network learning	network learning	network learning	network learning	1	kept
network meta analysis	network meta analysis	network meta analysis	network meta analysis	Network meta-analysis	1	case_or_format
Network Meta-Analysis as Topic	network meta analysis as topic	network meta analysis as topic	network meta analysis as topic	Network Meta-Analysis as Topic	1	kept
Network Neurobehavioral Scale	network neurobehavioral scale	network neurobehavioral scale	network neurobehavioral scale	Network Neurobehavioral Scale	1	kept
Network of networks	network of networks	network of networks	network of networks	Network of networks	1	kept
Network of spaces	network of spaces	network of spaces	network of spaces	Network of spaces	1	kept

Network operator	network operator	network operator	network operator	Network operator	1	kept
Network routing	network routing	network routing	network routing	Network routing	1	kept
Network security	network security	network security	network security	Network security	1	kept
Network strategy	network strategy	network strategy	network strategy	Network strategy	1	kept
Network structures	network structures	network structures	network structures	Network structures	1	kept
Networking	networking	networking	networking	Networking	1	kept
Neugeborene	neugeborene	neugeborene	neugeborene	Neugeborene	1	kept
Neuman Systems Model	neuman systems model	neuman systems model	neuman systems model	Neuman Systems Model	1	kept
Neuman’s Systems Model	neuman s systems model	neuman s systems model	neuman s systems model	Neuman’s Systems Model	1	kept
Neumonía	neumon a	neumon a	neumon a	Neumonía	1	kept
Neumonía asociada a ventilación mecánica	neumon a asociada a ventilaci n mec nica	neumon a asociada a ventilaci n mec nica	neumon a asociada a ventilaci n mec nica	Neumonía asociada a ventilación mecánica	1	kept
Neural Crest	neural crest	neural crest	neural crest	Neural Crest	1	kept
Neural crest	neural crest	neural crest	neural crest	Neural Crest	1	case_or_format
neural crest	neural crest	neural crest	neural crest	Neural Crest	1	case_or_format
Neural network	neural network	neural network	neural network	Neural networks	1	case_or_format
Neural Networks	neural networks	neural networks	neural network	Neural networks	1	singular

neural processes	neural processes	neural processes	neural processes	neural processes	1	kept
neural tube defect	neural tube defect	neural tube defect	neural tube defect	neural tube defect	1	kept
NEURAL-NETWORKS	neural networks	neural networks	neural network	Neural networks	1	singular
Neural-networks	neural networks	neural networks	neural network	Neural networks	1	singular
neurally adjusted ventilator assist	neurally adjusted ventilator assist	neurally adjusted ventilator assist	neurally adjusted ventilator assist	neurally adjusted ventilator assist	1	kept
neurally adjusted ventilator assistance	neurally adjusted ventilator assistance	neurally adjusted ventilator assistance	neurally adjusted ventilator assistance	neurally adjusted ventilator assistance	1	kept
Neurally adjusted ventilatory assist	neurally adjusted ventilatory assist	neurally adjusted ventilatory assist	neurally adjusted ventilatory assist	Neurally adjusted ventilatory assist	1	kept
Neuraxial analgesia	neuraxial analgesia	neuraxial analgesia	neuraxial analgesia	Neuraxial analgesia	1	kept
neuraxial block	neuraxial block	neuraxial block	neuraxial block	neuraxial block	1	kept
Neuro acute units	neuro acute units	neuro acute units	neuro acute units	Neuro acute units	1	kept
neuro check	neuro check	neuro check	neuro check	neuro check	1	kept
neuro development	neuro development	neuro development	neuro development	neuro development	1	kept
neuro oncologic treatment	neuro oncologic treatment	neuro oncologic treatment	neuro oncologic treatment	neuro oncologic treatment	1	kept
Neuro Psychiatric Inventoryusing Home edition	neuro psychiatric inventoryusing home edition	neuro psychiatric inventoryusing home edition	neuro psychiatric inventoryusing home edition	Neuro Psychiatric Inventoryusing Home edition	1	kept
neuro pulmonology unit	neuro pulmonology unit	neuro pulmonology unit	neuro pulmonology unit	neuro pulmonology unit	1	kept
Neuro-anesthesia	neuro anesthesia	neuro anesthesia	neuro anesthesia	Neuro-anesthesia	1	kept

neuro-intervention	neuro intervention	neuro intervention	neuro intervention	neuro-intervention	1	kept
neuro-muscular rehabilitation	neuro muscular rehabilitation	neuro muscular rehabilitation	neuro muscular rehabilitation	neuro-muscular rehabilitation	1	kept
Neuro-ophthalmology	neuro ophthalmology	neuro ophthalmology	neuro ophthalmology	Neuro-ophthalmology	1	kept
Neuroanatomy	neuroanatomy	neuroanatomy	neuroanatomy	neuroanatomy	1	case_or_format
Neuroanesthesia	neuroanesthesia	neuroanesthesia	neuroanesthesia	Neuroanesthesia	1	kept
neurobehavior	neurobehavior	neurobehavior	neurobehavior	Neurobehavior	1	case_or_format
Neurobehavioral	neurobehavioral	neurobehavioral	neurobehavioral	Neurobehavioral	1	kept
NEUROBEHAVIORAL DEVELOPMENT	neurobehavioral development	neurobehavioral development	neurobehavioral development	Neurobehavioral development	1	case_or_format
neurobehavioral development	neurobehavioral development	neurobehavioral development	neurobehavioral development	Neurobehavioral development	1	case_or_format
Neurobehavioral outcome	neurobehavioral outcome	neurobehavioral outcome	neurobehavioral outcome	Neurobehavioral outcome	1	kept
NEUROBEHAVIORAL OUTCOMES	neurobehavioral outcomes	neurobehavioral outcomes	neurobehavioral outcome	Neurobehavioral outcome	1	singular
neuroception	neuroception	neuroception	neuroception	neuroception	1	kept
neurocheck	neurocheck	neurocheck	neurocheck	neurocheck	1	kept
neurocheck frequency	neurocheck frequency	neurocheck frequency	neurocheck frequency	neurocheck frequency	1	kept
neurocognitive function	neurocognitive function	neurocognitive function	neurocognitive function	neurocognitive function	1	kept
Neurocritical	neurocritical	neurocritical	neurocritical	neurocritical	1	case_or_format

neurocritical illness	neurocritical illness	neurocritical illness	neurocritical illness	neurocritical illness	1	kept
Neurocutaneous syndrome	neurocutaneous syndrome	neurocutaneous syndrome	neurocutaneous syndrome	Neurocutaneous syndrome	1	kept
neurodegenerative	neurodegenerative	neurodegenerative	neurodegenerative	neurodegenerative	1	kept
neurodegenerative disease	neurodegenerative disease	neurodegenerative disease	neurodegenerative disease	Neurodegenerative diseases	1	case_or_format
Neurodegenerative Diseases	neurodegenerative diseases	neurodegenerative diseases	neurodegenerative disease	Neurodegenerative diseases	1	singular
Neurodevelopment of at-risk infants	neurodevelopment of at risk infants	neurodevelopment of at risk infants	neurodevelopment of at risk infants	Neurodevelopment of at-risk infants	1	kept
Neurodevelopment of preterm infants	neurodevelopment of preterm infants	neurodevelopment of preterm infants	neurodevelopment of preterm infants	Neurodevelopment of preterm infants	1	kept
Neurodevelopmental	neurodevelopmental	neurodevelopmental	neurodevelopmental	Neurodevelopmental	1	kept
Neurodevelopmental care	neurodevelopmental care	neurodevelopmental care	neurodevelopmental care	Neurodevelopmental care	1	kept
neurodevelopmental disability	neurodevelopmental disability	neurodevelopmental disability	neurodevelopmental disability	neurodevelopmental disability	1	kept
neurodevelopmental disorder	neurodevelopmental disorder	neurodevelopmental disorder	neurodevelopmental disorder	Neurodevelopmental Disorders	1	case_or_format
neurodevelopmental disorders	neurodevelopmental disorders	neurodevelopmental disorders	neurodevelopmental disorder	Neurodevelopmental Disorders	1	singular
neurodevelopmental follow-up	neurodevelopmental follow up	neurodevelopmental follow up	neurodevelopmental follow up	neurodevelopmental follow-up	1	kept
neurodevelopmental outcomes	neurodevelopmental outcomes	neurodevelopmental outcomes	neurodevelopmental outcome	neurodevelopmental outcome	1	singular
Neurodevelopmental supportive care	neurodevelopmental supportive care	neurodevelopmental supportive care	neurodevelopmental supportive care	Neurodevelopmental supportive care	1	kept
neurodiagnostic technician	neurodiagnostic technician	neurodiagnostic technician	neurodiagnostic technician	neurodiagnostic technician	1	kept

neurodiagnostic technologist	neurodiagnostic technologist	neurodiagnostic technologist	neurodiagnostic technologist	neurodiagnostic technologist	1	kept
Neurodiagnostics	neurodiagnostics	neurodiagnostics	neurodiagnostics	Neurodiagnostics	1	kept
neuroectoderm tumor	neuroectoderm tumor	neuroectoderm tumor	neuroectoderm tumor	neuroectoderm tumor	1	kept
Neuroectodermal Tumors	neuroectodermal tumors	neuroectodermal tumors	neuroectodermal tumors	Neuroectodermal Tumors	1	kept
neuroendocrinology	neuroendocrinology	neuroendocrinology	neuroendocrinology	neuroendocrinology	1	kept
Neuroendoscopic lavage	neuroendoscopic lavage	neuroendoscopic lavage	neuroendoscopic lavage	Neuroendoscopic lavage	1	kept
neurofeedback	neurofeedback	neurofeedback	neurofeedback	neurofeedback	1	kept
neurofibromatosis type 1	neurofibromatosis type 1	neurofibromatosis type 1	neurofibromatosis type 1	neurofibromatosis type 1	1	kept
neurofilament light polypeptide	neurofilament light polypeptide	neurofilament light polypeptide	neurofilament light polypeptide	neurofilament light polypeptide	1	kept
neurofilament protein	neurofilament protein	neurofilament protein	neurofilament protein	neurofilament protein	1	kept
Neurogenesis	neurogenesis	neurogenesis	neurogenesis	Neurogenesis	1	kept
Neurogenic bladder	neurogenic bladder	neurogenic bladder	neurogenic bladder	neurogenic bladder	1	case_or_format
neurogenic bowel	neurogenic bowel	neurogenic bowel	neurogenic bowel	neurogenic bowel	1	kept
neurogenic pulmonary edema	neurogenic pulmonary edema	neurogenic pulmonary edema	neurogenic pulmonary edema	neurogenic pulmonary edema	1	kept
neurogenic shock	neurogenic shock	neurogenic shock	neurogenic shock	neurogenic shock	1	kept
Neurohospitalist	neurohospitalist	neurohospitalist	neurohospitalist	Neurohospitalist	1	kept

neuroimmunology	neuroimmunology	neuroimmunology	neuroimmunology	neuroimmunology	1	kept
Neuroinfection	neuroinfection	neuroinfection	neuroinfection	Neuroinfection	1	kept
Neuroinflammation	neuroinflammation	neuroinflammation	neuroinflammation	Neuroinflammation	1	kept
Neuroinflammatory Diseases	neuroinflammatory diseases	neuroinflammatory diseases	neuroinflammatory diseases	Neuroinflammatory Diseases	1	kept
Neurointensivist	neurointensivist	neurointensivist	neurointensivist	Neurointensivist	1	kept
Neurointensivists	neurointensivists	neurointensivists	neurointensivist	Neurointensivist	1	singular
NEUROLEPTIC MALIGNANT SYNDROME	neuroleptic malignant syndrome	neuroleptic malignant syndrome	neuroleptic malignant syndrome	neuroleptic malignant syndrome	1	case_or_format
neurolinguistic programming	neurolinguistic programming	neurolinguistic programming	neurolinguistic programming	neurolinguistic programming	1	kept
Neurologic assessments	neurologic assessments	neurologic assessments	neurologic assessment	Neurologic assessment	1	singular
neurologic assessments	neurologic assessments	neurologic assessments	neurologic assessment	Neurologic assessment	1	singular
neurologic death	neurologic death	neurologic death	neurologic death	neurologic death	1	kept
Neurologic deterioration	neurologic deterioration	neurologic deterioration	neurologic deterioration	Neurologic deterioration	1	kept
neurologic emergencies	neurologic emergencies	neurologic emergencies	neurologic emergency	Neurologic emergency	1	singular
Neurologic emergency	neurologic emergency	neurologic emergency	neurologic emergency	Neurologic emergency	1	kept
Neurologic injury	neurologic injury	neurologic injury	neurologic injury	Neurologic injury	1	kept
neurologic injury	neurologic injury	neurologic injury	neurologic injury	Neurologic injury	1	case_or_format

neurologic toxicities	neurologic toxicities	neurologic toxicities	neurologic toxicities	neurologic toxicities	1	kept
neurological assessment	neurological assessment	neurological assessment	neurological assessment	Neurological assessment	1	case_or_format
Neurological assessments,	neurological assessments	neurological assessments	neurological assessment	Neurological assessment	1	singular
neurological determination of death	neurological determination of death	neurological determination of death	neurological determination of death	neurological determination of death	1	kept
Neurological disease	neurological disease	neurological disease	neurological disease	Neurological disease	1	kept
neurological disease	neurological disease	neurological disease	neurological disease	Neurological disease	1	case_or_format
Neurological diseases	neurological diseases	neurological diseases	neurological disease	Neurological disease	1	singular
neurological diseases	neurological diseases	neurological diseases	neurological disease	Neurological disease	1	singular
neurological disorders	neurological disorders	neurological disorders	neurological disorders	Neurological disorders	1	case_or_format
neurological early rehabilitation	neurological early rehabilitation	neurological early rehabilitation	neurological early rehabilitation	neurological early rehabilitation	1	kept
neurological endoscope	neurological endoscope	neurological endoscope	neurological endoscope	neurological endoscope	1	kept
neurological exam	neurological exam	neurological exam	neurological exam	neurological exam	1	kept
NEUROLOGICAL FUNCTION	neurological function	neurological function	neurological function	neurological function	1	case_or_format
Neurological illness	neurological illness	neurological illness	neurological illness	Neurological illness	1	kept
Neurological injury	neurological injury	neurological injury	neurological injury	neurological injury	1	case_or_format
neurological intensive	neurological intensive	neurological intensive	neurological intensive	neurological intensive	1	kept

Neurological monitoring	neurological monitoring	neurological monitoring	neurological monitoring	Neurological monitoring	1	kept
Neurological post-acute rehabilitation	neurological post acute rehabilitation	neurological post acute rehabilitation	neurological post acute rehabilitation	Neurological post-acute rehabilitation	1	kept
neurological problems	neurological problems	neurological problems	neurological problems	Neurological problems	1	case_or_format
Neurological Pupil index	neurological pupil index	neurological pupil index	neurological pupil index	Neurological Pupil index	1	kept
neurological pupil index	neurological pupil index	neurological pupil index	neurological pupil index	Neurological Pupil index	1	case_or_format
neurological recovery	neurological recovery	neurological recovery	neurological recovery	Neurological recovery	1	case_or_format
Neurological specialty	neurological specialty	neurological specialty	neurological specialty	Neurological specialty	1	kept
neurological status	neurological status	neurological status	neurological status	neurological status	1	kept
Neurology administration	neurology administration	neurology administration	neurology administration	Neurology administration	1	kept
Neurology residency	neurology residency	neurology residency	neurology residency	Neurology residency	1	kept
Neurology ward	neurology ward	neurology ward	neurology ward	Neurology ward	1	kept
neurology ward	neurology ward	neurology ward	neurology ward	Neurology ward	1	case_or_format
neurology-specific areas of palliative care	neurology specific areas of palliative care	neurology specific areas of palliative care	neurology specific areas of palliative care	neurology-specific areas of palliative care	1	kept
neuromarker	neuromarker	neuromarker	neuromarker	neuromarker	1	kept
neurometabolic disease	neurometabolic disease	neurometabolic disease	neurometabolic disease	neurometabolic disease	1	kept
NEUROMUSCULAR BLOCKADE	neuromuscular blockade	neuromuscular blockade	neuromuscular blockade	Neuromuscular Blockade	1	case_or_format

Neuromuscular blocking agent	neuromuscular blocking agent	neuromuscular blocking agent	neuromuscular blocking agent	neuromuscular blocking agent	1	case_or_format
neuromuscular blocking medications	neuromuscular blocking medications	neuromuscular blocking medications	neuromuscular blocking medications	neuromuscular blocking medications	1	kept
Neuromuscular Complex Care Centre	neuromuscular complex care centre	neuromuscular complex care centre	neuromuscular complex care centre	Neuromuscular Complex Care Centre	1	kept
neuromuscular depolarizing agent	neuromuscular depolarizing agent	neuromuscular depolarizing agent	neuromuscular depolarizing agent	neuromuscular depolarizing agent	1	kept
Neuromuscular Depolarizing Agents	neuromuscular depolarizing agents	neuromuscular depolarizing agents	neuromuscular depolarizing agent	neuromuscular depolarizing agent	1	singular
Neuromuscular Electrical Stimulation	neuromuscular electrical stimulation	neuromuscular electrical stimulation	neuromuscular electrical stimulation	neuromuscular electrical stimulation	1	case_or_format
Neuromuscular electrical stimulation	neuromuscular electrical stimulation	neuromuscular electrical stimulation	neuromuscular electrical stimulation	neuromuscular electrical stimulation	1	case_or_format
neuromuscular function	neuromuscular function	neuromuscular function	neuromuscular function	neuromuscular function	1	kept
Neuromuscular models of care	neuromuscular models of care	neuromuscular models of care	neuromuscular models of care	Neuromuscular models of care	1	kept
Neuromuscular Monitoring	neuromuscular monitoring	neuromuscular monitoring	neuromuscular monitoring	Neuromuscular Monitoring	1	kept
neuromuscular monitoring	neuromuscular monitoring	neuromuscular monitoring	neuromuscular monitoring	Neuromuscular Monitoring	1	case_or_format
neuromuscular scoliosis	neuromuscular scoliosis	neuromuscular scoliosis	neuromuscular scoliosis	neuromuscular scoliosis	1	kept
neuromuscular spinal deformity	neuromuscular spinal deformity	neuromuscular spinal deformity	neuromuscular spinal deformity	neuromuscular spinal deformity	1	kept
neuromuscular synapse	neuromuscular synapse	neuromuscular synapse	neuromuscular synapse	neuromuscular synapse	1	kept
neuromuscular weakness	neuromuscular weakness	neuromuscular weakness	neuromuscular weakness	neuromuscular weakness	1	kept
Neurons	neurons	neurons	neurons	Neurons	1	kept

Neuropathy	neuropathy	neuropathy	neuropathy	neuropathy	1	case_or_format
Neuropeptides	neuropeptides	neuropeptides	neuropeptides	Neuropeptides	1	kept
neuropharmacology	neuropharmacology	neuropharmacology	neuropharmacology	neuropharmacology	1	kept
Neurophychological tests	neurophychological tests	neurophychological tests	neurophychological tests	Neurophychological tests	1	kept
neurophysiologist	neurophysiologist	neurophysiologist	neurophysiologist	neurophysiologist	1	kept
NEUROPHYSIOLOGY	neurophysiology	neurophysiology	neurophysiology	neurophysiology	1	case_or_format
Neuroprognostication	neuroprognostication	neuroprognostication	neuroprognostication	Neuroprognostication	1	kept
Neuroprotection bundle	neuroprotection bundle	neuroprotection bundle	neuroprotection bundle	Neuroprotection bundle	1	kept
neuroprotective	neuroprotective	neuroprotective	neuroprotective	Neuroprotective	1	case_or_format
neuroprotective strategies	neuroprotective strategies	neuroprotective strategies	neuroprotective strategies	neuroprotective strategies	1	kept
Neuropsychiatric symptoms	neuropsychiatric symptoms	neuropsychiatric symptoms	neuropsychiatric symptoms	neuropsychiatric symptoms	1	case_or_format
Neuropsychological stress	neuropsychological stress	neuropsychological stress	neuropsychological stress	Neuropsychological stress	1	kept
neuropsychological tests	neuropsychological tests	neuropsychological tests	neuropsychological test	Neuropsychological Tests	1	singular
neuropsychologist	neuropsychologist	neuropsychologist	neuropsychologist	neuropsychologist	1	kept
neuroreanimation	neuroreanimation	neuroreanimation	neuroreanimation	neuroreanimation	1	kept
neuroréanimation	neuror animation	neuror animation	neuror animation	neuroréanimation	1	kept

neurosarcoidosis	neurosarcoidosis	neurosarcoidosis	neurosarcoidosis	neurosarcoidosis	1	kept
NEUROSCIENCE	neuroscience	neuroscience	neuroscience	neuroscience	1	case_or_format
Neuroscience and Neuroanaesthesia	neuroscience and neuroanaesthesia	neuroscience and neuroanaesthesia	neuroscience and neuroanaesthesia	Neuroscience and Neuroanaesthesia	1	kept
Neuroscience unit	neuroscience unit	neuroscience unit	neuroscience unit	Neuroscience unit	1	kept
neuroscience unit	neuroscience unit	neuroscience unit	neuroscience unit	Neuroscience unit	1	case_or_format
Neuroscience ward	neuroscience ward	neuroscience ward	neuroscience ward	Neuroscience ward	1	kept
neurosecretion	neurosecretion	neurosecretion	neurosecretion	neurosecretion	1	kept
neurosensitive development	neurosensitive development	neurosensitive development	neurosensitive development	neurosensitive development	1	kept
Neurosurgeons’ role	neurosurgeons role	neurosurgeons role	neurosurgeons role	Neurosurgeons’ role	1	kept
Neurosurgery education	neurosurgery education	neurosurgery education	neurosurgery education	Neurosurgery education	1	kept
Neurosurgery Education and Development Foundation	neurosurgery education and development foundation	neurosurgery education and development foundation	neurosurgery education and development foundation	Neurosurgery Education and Development Foundation	1	kept
neurosurgery emergency observation wards	neurosurgery emergency observation wards	neurosurgery emergency observation wards	neurosurgery emergency observation wards	neurosurgery emergency observation wards	1	kept
Neurosurgery residencies	neurosurgery residencies	neurosurgery residencies	neurosurgery residencies	Neurosurgery residencies	1	kept
neurosurgery unit	neurosurgery unit	neurosurgery unit	neurosurgery unit	neurosurgery unit	1	kept
Neurosurgical	neurosurgical	neurosurgical	neurosurgical	Neurosurgical	1	kept
neurosurgical	neurosurgical	neurosurgical	neurosurgical	Neurosurgical	1	case_or_format

Neurosurgical anesthesia	neurosurgical anesthesia	neurosurgical anesthesia	neurosurgical anesthesia	Neurosurgical anesthesia	1	kept
neurosurgical consultation	neurosurgical consultation	neurosurgical consultation	neurosurgical consultation	neurosurgical consultation	1	kept
neurosurgical disease	neurosurgical disease	neurosurgical disease	neurosurgical disease	neurosurgical disease	1	kept
Neurosurgical emergency	neurosurgical emergency	neurosurgical emergency	neurosurgical emergency	Neurosurgical emergency	1	kept
Neurosurgical training	neurosurgical training	neurosurgical training	neurosurgical training	Neurosurgical training	1	kept
Neurosurgical unit	neurosurgical unit	neurosurgical unit	neurosurgical unit	Neurosurgical unit	1	kept
neurosurgical unit	neurosurgical unit	neurosurgical unit	neurosurgical unit	Neurosurgical unit	1	case_or_format
neurosurgical ward	neurosurgical ward	neurosurgical ward	neurosurgical ward	neurosurgical ward	1	kept
neurosurgy	neurosurgy	neurosurgy	neurosurgy	neurosurgy	1	kept
neurotherapy	neurotherapy	neurotherapy	neurotherapy	neurotherapy	1	kept
neurotrophin	neurotrophin	neurotrophin	neurotrophin	neurotrophin	1	kept
neurotropism	neurotropism	neurotropism	neurotropism	neurotropism	1	kept
neurovascular function	neurovascular function	neurovascular function	neurovascular function	neurovascular function	1	kept
neurovascular unit	neurovascular unit	neurovascular unit	neurovascular unit	Neurovascular unit	1	case_or_format
neutral pressure connector	neutral pressure connector	neutral pressure connector	neutral pressure connector	neutral pressure connector	1	kept
neutropenic enterocolitis	neutropenic enterocolitis	neutropenic enterocolitis	neutropenic enterocolitis	neutropenic enterocolitis	1	kept

neutropenic fever	neutropenic fever	neutropenic fever	neutropenic fever	neutropenic fever	1	kept
Neutrophil	neutrophil	neutrophil	neutrophil	neutrophil	1	case_or_format
neutrophil chemotaxis	neutrophil chemotaxis	neutrophil chemotaxis	neutrophil chemotaxis	neutrophil chemotaxis	1	kept
neutrophil extracellular trap	neutrophil extracellular trap	neutrophil extracellular trap	neutrophil extracellular trap	neutrophil extracellular trap	1	kept
Neutrophil-to-lymphocyte ratio	neutrophil to lymphocyte ratio	neutrophil to lymphocyte ratio	neutrophil to lymphocyte ratio	Neutrophil-to-lymphocyte ratio	1	kept
NEVER	never	never	never	NEVER	1	kept
Never Events	never events	never events	never events	Never events	1	case_or_format
never events	never events	never events	never events	Never events	1	case_or_format
new antibiotic	new antibiotic	new antibiotic	new antibiotic	new antibiotic	1	kept
New born babies	new born babies	new born babies	new born babies	New born babies	1	kept
New clinical training system	new clinical training system	new clinical training system	new clinical training system	New clinical training system	1	kept
New Delhi metallo beta lactamase	new delhi metallo beta lactamase	new delhi metallo beta lactamase	new delhi metallo beta lactamase	New Delhi metallo beta lactamase	1	kept
new drug	new drug	new drug	new drug	new drug	1	kept
new facility	new facility	new facility	new facility	new facility	1	kept
New graduate	new graduate	new graduate	new graduate	New graduate	1	kept
new graduate RNSs	new graduate rnss	new graduate rnss	new graduate rnss	new graduate RNSs	1	kept

new methods	new methods	new methods	new methods	new methods	1	kept
New non-sutured biological heart valve	new non sutured biological heart valve	new non sutured biological heart valve	new non sutured biological heart valve	New non-sutured biological heart valve	1	kept
New normal	new normal	new normal	new normal	New normal	1	kept
New Pandemic	new pandemic	new pandemic	new pandemic	New Pandemic	1	kept
New public management	new public management	new public management	new public management	New public management	1	kept
New RNs	new rns	new rns	new rns	New RNs	1	kept
New roles in practice	new roles in practice	new roles in practice	new roles in practice	New roles in practice	1	kept
New services	new services	new services	new services	New services	1	kept
new species	new species	new species	new species	new species	1	kept
New ways of working	new ways of working	new ways of working	new ways of working	New ways of working	1	kept
new work	new work	new work	new work	new work	1	kept
new york city	new york city	new york city	new york city	New York City	1	case_or_format
New zealand	new zealand	new zealand	new zealand	New Zealand	1	case_or_format
new-born	new born	new born	new born	New-born	1	case_or_format
New-onset diabetes mellitus after transplantation	new onset diabetes mellitus after transplantation	new onset diabetes mellitus after transplantation	new onset diabetes mellitus after transplantation	New-onset diabetes mellitus after transplantation	1	kept
NEW-SOUTH-WALES	new south wales	new south wales	new south wales	New South Wales	1	case_or_format

Newborn ADD ACUTE RENAL FAILURE	newborn add acute renal failure	newborn add acute renal failure	newborn add acute renal failure	Newborn ADD ACUTE RENAL FAILURE	1	kept
Newborn and preterm	newborn and preterm	newborn and preterm	newborn and preterm	Newborn and preterm	1	kept
Newborn Babies	newborn babies	newborn babies	newborn baby	NEWBORN BABY	1	singular
NEWBORN BABY	newborn baby	newborn baby	newborn baby	NEWBORN BABY	1	kept
Newborn care, family-centered care	newborn care family centered care	newborn care family centered care	newborn care family centered care	Newborn care, family-centered care	1	kept
newborn cochlear implant	newborn cochlear implant	newborn cochlear implant	newborn cochlear implant	newborn cochlear implant	1	kept
newborn diabetes mellitus	newborn diabetes mellitus	newborn diabetes mellitus	newborn diabetes mellitus	newborn diabetes mellitus	1	kept
newborn exfoliative dermatitis	newborn exfoliative dermatitis	newborn exfoliative dermatitis	newborn exfoliative dermatitis	newborn exfoliative dermatitis	1	kept
Newborn Handover Verification	newborn handover verification	newborn handover verification	newborn handover verification	Newborn Handover Verification	1	kept
Newborn health	newborn health	newborn health	newborn health	Newborn health	1	kept
Newborn health services	newborn health services	newborn health services	newborn health services	Newborn health services	1	kept
newborn hospitalization	newborn hospitalization	newborn hospitalization	newborn hospitalization	newborn hospitalization	1	kept
Newborn hypoglycemia	newborn hypoglycemia	newborn hypoglycemia	newborn hypoglycemia	Newborn hypoglycemia	1	kept
Newborn individualized developmental care and assessment program	newborn individualized developmental care and assessment program	newborn individualized developmental care and assessment program	newborn individualized developmental care and assessment program	Newborn individualized developmental care and assessment program	1	kept
Newborn Infant	newborn infant	newborn infant	newborn infant	Newborn infant	1	case_or_format
newborn infants	newborn infants	newborn infants	newborn infant	Newborn infant	1	singular

newborn injury	newborn injury	newborn injury	newborn injury	newborn injury	1	kept
newborn life support techniques	newborn life support techniques	newborn life support techniques	newborn life support techniques	newborn life support techniques	1	kept
newborn management	newborn management	newborn management	newborn management	newborn management	1	kept
newborn massage	newborn massage	newborn massage	newborn massage	newborn massage	1	kept
newborn opioid withdrawal syndrome	newborn opioid withdrawal syndrome	newborn opioid withdrawal syndrome	newborn opioid withdrawal syndrome	newborn opioid withdrawal syndrome	1	kept
Newborn Outcome	newborn outcome	newborn outcome	newborn outcome	Newborn Outcome	1	kept
Newborn outcomes	newborn outcomes	newborn outcomes	newborn outcome	Newborn Outcome	1	singular
newborn respiratory distress	newborn respiratory distress	newborn respiratory distress	newborn respiratory distress	newborn respiratory distress	1	kept
Newborn Resuscitation	newborn resuscitation	newborn resuscitation	newborn resuscitation	newborn resuscitation	1	case_or_format
newborn resuscitation at birth	newborn resuscitation at birth	newborn resuscitation at birth	newborn resuscitation at birth	newborn resuscitation at birth	1	kept
Newborn risk factors	newborn risk factors	newborn risk factors	newborn risk factors	Newborn risk factors	1	kept
Newborn screening	newborn screening	newborn screening	newborn screening	newborn screening	1	case_or_format
Newborn screening pulse oximetry	newborn screening pulse oximetry	newborn screening pulse oximetry	newborn screening pulse oximetry	Newborn screening pulse oximetry	1	kept
Newborn sleep	newborn sleep	newborn sleep	newborn sleep	Newborn sleep	1	kept
Newborn stabilization	newborn stabilization	newborn stabilization	newborn stabilization	Newborn stabilization	1	kept
newborn tetanus	newborn tetanus	newborn tetanus	newborn tetanus	newborn tetanus	1	kept

newborn transition	newborn transition	newborn transition	newborn transition	newborn transition	1	kept
newborn transitional care	newborn transitional care	newborn transitional care	newborn transitional care	newborn transitional care	1	kept
Newborn Transportation	newborn transportation	newborn transportation	newborn transportation	Newborn Transportation	1	kept
newborn vital parameter	newborn vital parameter	newborn vital parameter	newborn vital parameter	newborn vital parameter	1	kept
Newborn, Infant	newborn infant	newborn infant	newborn infant	Newborn infant	1	case_or_format
NEWBORN-CARE	newborn care	newborn care	newborn care	newborn care	1	case_or_format
NEWBORN-INFANTS	newborn infants	newborn infants	newborn infant	Newborn infant	1	singular
Newborne	newborne	newborne	newborne	Newborne	1	kept
Newcastle disease	newcastle disease	newcastle disease	newcastle disease	Newcastle disease	1	kept
Newcastle Ottawa Score	newcastle ottawa score	newcastle ottawa score	newcastle ottawa score	Newcastle Ottawa Score	1	kept
newly graduated	newly graduated	newly graduated	newly graduated	newly graduated	1	kept
news	news	news	news	NEWS	1	case_or_format
NEWSPAPER EMPLOYEES	newspaper employees	newspaper employees	newspaper employees	NEWSPAPER EMPLOYEES	1	kept
Newspapers	newspapers	newspapers	newspapers	Newspapers	1	kept
Newsvendor models	newsvendor models	newsvendor models	newsvendor model	newsvendor model	1	singular
Newsvendors	newsvendors	newsvendors	newsvendors	Newsvendors	1	kept

Next generation sequencing	next generation sequencing	next generation sequencing	next generation sequencing	Next generation sequencing	1	kept
Next of kin	next of kin	next of kin	next of kin	next of kin	1	case_or_format
NF-KAPPA-B	nf kappa b	nf kappa b	nf kappa b	NF-KAPPA-B	1	kept
Ngo	ngo	ngo	ngo	Ngo	1	kept
NGS	ngs	ngs	ngs	NGS	1	kept
NHAP	nhap	nhap	nhap	NHAP	1	kept
NHS	nhs	nhs	nhs	NHS	1	kept
NHS cancels routine surgery and therapies	nhs cancels routine surgery and therapies	nhs cancels routine surgery and therapies	nhs cancels routine surgery and therapies	NHS cancels routine surgery and therapies	1	kept
NHSN	nhsn	nhsn	nhsn	NHSN	1	kept
NHW = non-Hispanic white	nhw non hispanic white	nhw non hispanic white	nhw non hispanic white	NHW = non-Hispanic white	1	kept
NI MyRIO	ni myrio	ni myrio	ni myrio	NI MyRIO	1	kept
NIAPAS	niapas	niapas	niapas	NIAPAS	1	kept
NIC classification	nic classification	nic classification	nic classification	NIC classification	1	kept
NICE	nice	nice	nice	NICE	1	kept
NICE guidance	nice guidance	nice guidance	nice guidance	NICE guidance	1	kept
NICE guidelines	nice guidelines	nice guidelines	nice guidelines	NICE guidelines	1	kept

NICE-SUGAR	nice sugar	nice sugar	nice sugar	NICE-SUGAR	1	kept
NICHD	nichd	nichd	nichd	NICHD	1	kept
Nickel alloys	nickel alloys	nickel alloys	nickel alloys	Nickel alloys	1	kept
nicotinamide adenine dinucleotide	nicotinamide adenine dinucleotide	nicotinamide adenine dinucleotide	nicotinamide adenine dinucleotide	nicotinamide adenine dinucleotide	1	kept
nicotinamide adenine dinucleotide adenosine diphosphate	nicotinamide adenine dinucleotide adenosine diphosphate	nicotinamide adenine dinucleotide adenosine diphosphate	nicotinamide adenine dinucleotide adenosine diphosphate	nicotinamide adenine dinucleotide adenosine diphosphate	1	kept
Nicotine replacement therapy	nicotine replacement therapy	nicotine replacement therapy	nicotine replacement therapy	nicotine replacement therapy	1	case_or_format
nicotinic agent	nicotinic agent	nicotinic agent	nicotinic agent	nicotinic agent	1	kept
Niemann Pick disease	niemann pick disease	niemann pick disease	niemann pick disease	Niemann Pick disease	1	kept
Nigerian newborns	nigerian newborns	nigerian newborns	nigerian newborns	Nigerian newborns	1	kept
NIGHT	night	night	night	night	1	case_or_format
Night care	night care	night care	night care	Night Care	1	case_or_format
night care facility	night care facility	night care facility	night care facility	night care facility	1	kept
Night Eating Syndrome	night eating syndrome	night eating syndrome	night eating syndrome	Night Eating Syndrome	1	kept
night eating syndrome	night eating syndrome	night eating syndrome	night eating syndrome	Night Eating Syndrome	1	case_or_format
night hospitalist	night hospitalist	night hospitalist	night hospitalist	night hospitalist	1	kept
Night Shift	night shift	night shift	night shift	night shift	1	case_or_format

Night shift countermeasures	night shift countermeasures	night shift countermeasures	night shift countermeasures	Night shift countermeasures	1	kept
night shift work	night shift work	night shift work	night shift work	night shift work	1	kept
night shifts	night shifts	night shifts	night shift	night shift	1	singular
night vision	night vision	night vision	night vision	night vision	1	kept
Night work	night work	night work	night work	night work	1	case_or_format
NIGHT-SHIFT	night shift	night shift	night shift	night shift	1	case_or_format
Night-Shift	night shift	night shift	night shift	night shift	1	case_or_format
Night-time dialysis	night time dialysis	night time dialysis	night time dialysis	Night-time dialysis	1	kept
nights	nights	nights	night	night	1	singular
nighttime	nighttime	nighttime	nighttime	nighttime	1	kept
NIGHTTIME INCONTINENCE	nighttime incontinence	nighttime incontinence	nighttime incontinence	NIGHTTIME INCONTINENCE	1	kept
NIGHTTIME NOISE	nighttime noise	nighttime noise	nighttime noise	NIGHTTIME NOISE	1	kept
nighttime sleep duration	nighttime sleep duration	nighttime sleep duration	nighttime sleep duration	nighttime sleep duration	1	kept
nighttime telemedicine	nighttime telemedicine	nighttime telemedicine	nighttime telemedicine	nighttime telemedicine	1	kept
Nightwork	nightwork	nightwork	nightwork	Nightwork	1	kept
NIHR	nihr	nihr	nihr	NIHR	1	kept

Nil by mouth	nil by mouth	nil by mouth	nil by mouth	Nil by mouth	1	kept
nil per os	nil per os	nil per os	nil per os	nil per os	1	kept
nilbex	nilbex	nilbex	nilbex	nilbex	1	kept
NIMART	nimart	nimart	nimart	NIMART	1	kept
nine-item list	nine item list	nine item list	nine item list	nine-item list	1	kept
Niphon spinosus	niphon spinosus	niphon spinosus	niphon spinosus	Niphon spinosus	1	kept
Nipple shield	nipple shield	nipple shield	nipple shield	Nipple shield	1	kept
nipple shields	nipple shields	nipple shields	nipple shield	Nipple shield	1	singular
NIPPV	nippv	nippv	nippv	NIPPV	1	kept
NIPS pain scale	nips pain scale	nips pain scale	nips pain scale	NIPS pain scale	1	kept
NIR SPECTROSCOPY	nir spectroscopy	nir spectroscopy	nir spectroscopy	NIR SPECTROSCOPY	1	kept
NIR spectrum	nir spectrum	nir spectrum	nir spectrum	NIR spectrum	1	kept
Nirsevimab	nirsevimab	nirsevimab	nirsevimab	nirsevimab	1	case_or_format
nitazoxanide	nitazoxanide	nitazoxanide	nitazoxanide	nitazoxanide	1	kept
nitinol stent	nitinol stent	nitinol stent	nitinol stent	nitinol stent	1	kept
Nitrates	nitrates	nitrates	nitrate	nitrate	1	singular

nitric oxide donor	nitric oxide donor	nitric oxide donor	nitric oxide donor	nitric oxide donor	1	kept
nitric oxide synthase	nitric oxide synthase	nitric oxide synthase	nitric oxide synthase	nitric oxide synthase	1	kept
Nitriles	nitriles	nitriles	nitriles	Nitriles	1	kept
Nitrites	nitrites	nitrites	nitrite	nitrite	1	singular
Nitrogen excretion	nitrogen excretion	nitrogen excretion	nitrogen excretion	Nitrogen excretion	1	kept
nitrogen excretion	nitrogen excretion	nitrogen excretion	nitrogen excretion	Nitrogen excretion	1	case_or_format
nitrogen urine level	nitrogen urine level	nitrogen urine level	nitrogen urine level	nitrogen urine level	1	kept
NITROGEN-BALANCE	nitrogen balance	nitrogen balance	nitrogen balance	nitrogen balance	1	case_or_format
nitrosourea derivative	nitrosourea derivative	nitrosourea derivative	nitrosourea derivative	nitrosourea derivative	1	kept
Nitrous oxide	nitrous oxide	nitrous oxide	nitrous oxide	nitrous oxide	1	case_or_format
Nivel de atención	nivel de atenci n	nivel de atenci n	nivel de atenci n	Nivel de atención	1	kept
Nivolumab	nivolumab	nivolumab	nivolumab	nivolumab	1	case_or_format
nizatidine	nizatidine	nizatidine	nizatidine	nizatidine	1	kept
Niño Hospitalizado	ni o hospitalizado	ni o hospitalizado	ni o hospitalizado	Niño Hospitalizado	1	kept
NLM)	nlm	nlm	nlm	NLM)	1	kept
NLP	nlp	nlp	nlp	NLP	1	kept

NLS TF, Neonatal Life Support Task Force	nls tf neonatal life support task force	nls tf neonatal life support task force	nls tf neonatal life support task force	NLS TF, Neonatal Life Support Task Force	1	kept
nm2cab	nm2cab	nm2cab	nm2cab	nm2cab	1	kept
NMC standards	nmc standards	nmc standards	nmc standards	NMC standards	1	kept
NMDA receptor antibody encephalitis	nmda receptor antibody encephalitis	nmda receptor antibody encephalitis	nmda receptor antibody encephalitis	NMDA receptor antibody encephalitis	1	kept
NNP	nnp	nnp	nnp	NNP	1	kept
NNP staffing issues	nnp staffing issues	nnp staffing issues	nnp staffing issues	NNP staffing issues	1	kept
NNT, number needed to treat	nnt number needed to treat	nnt number needed to treat	nnt number needed to treat	NNT, number needed to treat	1	kept
No comunicativo	no comunicativo	no comunicativo	no comunicativo	No comunicativo	1	kept
No indication	no indication	no indication	no indication	No indication	1	kept
no visitor	no visitor	no visitor	no visitor	no visitor	1	kept
No-sedation	no sedation	no sedation	no sedation	No sedation	1	case_or_format
No-Shift-Lag	no shift lag	no shift lag	no shift lag	No-Shift-Lag	1	kept
no-show	no show	no show	no show	no-show	1	kept
NOAC	noac	noac	noac	NOAC	1	kept
NOC terminology	noc terminology	noc terminology	noc terminology	NOC terminology	1	kept
Nocardia	nocardia	nocardia	nocardia	Nocardia	1	kept

Nociception Coma Scale	nociception coma scale	nociception coma scale	nociception coma scale	Nociception Coma Scale	1	kept
Nociception Coma Scale-revised	nociception coma scale revised	nociception coma scale revised	nociception coma scale revised	Nociception Coma Scale-revised	1	kept
NOCICEPTION LEVEL	nociception level	nociception level	nociception level	NOCICEPTION LEVEL	1	kept
Nociceptive procedure	nociceptive procedure	nociceptive procedure	nociceptive procedure	Nociceptive procedure	1	kept
Nociceptors	nociceptors	nociceptors	nociceptors	Nociceptors	1	kept
nocturnal awakening	nocturnal awakening	nocturnal awakening	nocturnal awakening	nocturnal awakening	1	kept
nocturnal epilepsy	nocturnal epilepsy	nocturnal epilepsy	nocturnal epilepsy	nocturnal epilepsy	1	kept
nocturnal hypoglycemia	nocturnal hypoglycemia	nocturnal hypoglycemia	nocturnal hypoglycemia	nocturnal hypoglycemia	1	kept
Nocturnal intensivist	nocturnal intensivist	nocturnal intensivist	nocturnal intensivist	Nocturnal intensivist	1	kept
NOCTURNAL SLEEP	nocturnal sleep	nocturnal sleep	nocturnal sleep	NOCTURNAL SLEEP	1	kept
Nocturnist	nocturnist	nocturnist	nocturnist	Nocturnist	1	kept
Noddings’ philosophy of education	noddings philosophy of education	noddings philosophy of education	noddings philosophy of education	Noddings’ philosophy of education	1	kept
NOISE ANNOYANCE	noise annoyance	noise annoyance	noise annoyance	NOISE ANNOYANCE	1	kept
Noise cancellation	noise cancellation	noise cancellation	noise cancellation	Noise cancellation	1	kept
noise detection algorithm	noise detection algorithm	noise detection algorithm	noise detection algorithm	noise detection algorithm	1	kept
Noise distribution	noise distribution	noise distribution	noise distribution	Noise distribution	1	kept

Noise environment	noise environment	noise environment	noise environment	Noise environment	1	kept
Noise environments	noise environments	noise environments	noise environment	Noise environment	1	singular
Noise events	noise events	noise events	noise events	Noise events	1	kept
Noise exposure	noise exposure	noise exposure	noise exposure	Noise exposure	1	kept
noise exposure	noise exposure	noise exposure	noise exposure	Noise exposure	1	case_or_format
Noise index	noise index	noise index	noise index	Noise index	1	kept
noise injury	noise injury	noise injury	noise injury	noise injury	1	kept
Noise level data loggers	noise level data loggers	noise level data loggers	noise level data loggers	Noise level data loggers	1	kept
Noise level determinants	noise level determinants	noise level determinants	noise level determinants	Noise level determinants	1	kept
noise management	noise management	noise management	noise management	Noise management	1	case_or_format
Noise measurement	noise measurement	noise measurement	noise measurement	noise measurement	1	case_or_format
Noise monitoring	noise monitoring	noise monitoring	noise monitoring	Noise monitoring	1	kept
noise perception	noise perception	noise perception	noise perception	noise perception	1	kept
Noise-cancelling headphones	noise cancelling headphones	noise cancelling headphones	noise cancelling headphones	Noise-cancelling headphones	1	kept
Noise-free environments	noise free environments	noise free environments	noise free environments	Noise-free environments	1	kept
Noises	noises	noises	noise	Noise	1	singular

Nomenclature	nomenclature	nomenclature	nomenclature	nomenclature	1	case_or_format
nominal group	nominal group	nominal group	nominal group	nominal group	1	kept
NOMINAL GROUP TECHNIQUE	nominal group technique	nominal group technique	nominal group technique	Nominal group technique	1	case_or_format
Nominal Group Technique	nominal group technique	nominal group technique	nominal group technique	Nominal group technique	1	case_or_format
Nomogra	nomogra	nomogra	nomogra	Nomogra	1	kept
non accidental trauma	non accidental trauma	non accidental trauma	non accidental trauma	non accidental trauma	1	kept
non acute disease	non acute disease	non acute disease	non acute disease	non acute disease	1	kept
non Albicans Candida yeast	non albicans candida yeast	non albicans candida yeast	non albicans candida yeast	non Albicans Candida yeast	1	kept
non catheter urinary tract infection	non catheter urinary tract infection	non catheter urinary tract infection	non catheter urinary tract infection	non catheter urinary tract infection	1	kept
non clinical interruption	non clinical interruption	non clinical interruption	non clinical interruption	non clinical interruption	1	kept
non contact monitoring	non contact monitoring	non contact monitoring	non contact monitoring	non contact monitoring	1	kept
non invasive mechanical ventilation	non invasive mechanical ventilation	non invasive mechanical ventilation	non invasive mechanical ventilation	Non-invasive mechanical ventilation	1	case_or_format
Non invasive Ventilation	non invasive ventilation	non invasive ventilation	non invasive ventilation	Non-invasive ventilation	1	case_or_format
non invasive ventilation	non invasive ventilation	non invasive ventilation	non invasive ventilation	Non-invasive ventilation	1	case_or_format
non maleficence	non maleficence	non maleficence	non maleficence	non maleficence	1	kept
non motor symptoms questionnaire	non motor symptoms questionnaire	non motor symptoms questionnaire	non motor symptoms questionnaire	non motor symptoms questionnaire	1	kept

non neonatal specialist	non neonatal specialist	non neonatal specialist	non neonatal specialist	non neonatal specialist	1	kept
non nutritive sucking	non nutritive sucking	non nutritive sucking	non nutritive sucking	Non-nutritive sucking	1	case_or_format
non palliative care	non palliative care	non palliative care	non palliative care	non palliative care	1	kept
non pharmacological analgesia therapy	non pharmacological analgesia therapy	non pharmacological analgesia therapy	non pharmacological analgesia therapy	non pharmacological analgesia therapy	1	kept
non pharmacological intervention	non pharmacological intervention	non pharmacological intervention	non pharmacological intervention	Non-pharmacological interventions	1	case_or_format
non pharmacological interventions	non pharmacological interventions	non pharmacological interventions	non pharmacological intervention	Non-pharmacological interventions	1	singular
non probability purposive sample	non probability purposive sample	non probability purposive sample	non probability purposive sample	non probability purposive sample	1	kept
non probability sampling method	non probability sampling method	non probability sampling method	non probability sampling method	non probability sampling method	1	kept
non rebreathing valve	non rebreathing valve	non rebreathing valve	non rebreathing valve	non rebreathing valve	1	kept
non resource intensive intervention	non resource intensive intervention	non resource intensive intervention	non resource intensive intervention	non resource intensive intervention	1	kept
non st segment elevation acute coronary syndrome	non st segment elevation acute coronary syndrome	non st segment elevation acute coronary syndrome	non st segment elevation acute coronary syndrome	non st segment elevation acute coronary syndrome	1	kept
non sterile glove	non sterile glove	non sterile glove	non sterile glove	non sterile glove	1	kept
non tunneled venous access catheter	non tunneled venous access catheter	non tunneled venous access catheter	non tunneled venous access catheter	non tunneled venous access catheter	1	kept
non ventilator associated hospital acquired pneumonia	non ventilator associated hospital acquired pneumonia	non ventilator associated hospital acquired pneumonia	non ventilator associated hospital acquired pneumonia	non ventilator associated hospital acquired pneumonia	1	kept
non ventilator associated pneumonia	non ventilator associated pneumonia	non ventilator associated pneumonia	non ventilator associated pneumonia	non ventilator associated pneumonia	1	kept
non-academia	non academia	non academia	non academia	non-academia	1	kept

Non-accidental trauma	non accidental trauma	non accidental trauma	non accidental trauma	non accidental trauma	1	case_or_format
Non-alcoholic Fatty Liver Disease	non alcoholic fatty liver disease	non alcoholic fatty liver disease	non alcoholic fatty liver disease	Non-alcoholic Fatty Liver Disease	1	kept
non-alcoholic fatty liver disease	non alcoholic fatty liver disease	non alcoholic fatty liver disease	non alcoholic fatty liver disease	Non-alcoholic Fatty Liver Disease	1	case_or_format
non-American	non american	non american	non american	non-American	1	kept
Non-anesthesiologist	non anesthesiologist	non anesthesiologist	non anesthesiologist	Non-anesthesiologist	1	kept
NON-ANESTHESIOLOGISTS	non anesthesiologists	non anesthesiologists	non anesthesiologist	Non-anesthesiologist	1	singular
Non-anion-gap metabolic acidemia	non anion gap metabolic acidemia	non anion gap metabolic acidemia	non anion gap metabolic acidemia	non anion gap metabolic acidemia	1	case_or_format
Non-antibiotic	non antibiotic	non antibiotic	non antibiotic	Non-antibiotic	1	kept
non-beneficial	non beneficial	non beneficial	non beneficial	non-beneficial	1	kept
Non-beneficial care	non beneficial care	non beneficial care	non beneficial care	Non-beneficial care	1	kept
non-cardiac	non cardiac	non cardiac	non cardiac	non-cardiac	1	kept
non-cardiac cause	non cardiac cause	non cardiac cause	non cardiac cause	non-cardiac cause	1	kept
non-comorbid respiratory	non comorbid respiratory	non comorbid respiratory	non comorbid respiratory	non-comorbid respiratory	1	kept
Non-contact	non contact	non contact	non contact	Non-contact	1	kept
non-contact technologies	non contact technologies	non contact technologies	non contact technologies	non-contact technologies	1	kept
Non-COVID HCFs	non covid hcfs	non covid hcfs	non covid hcfs	Non-COVID HCFs	1	kept

non-COVID-19 admissions	non covid 19 admissions	non covid 19 admissions	non covid 19 admissions	non-COVID-19 admissions	1	kept
non-depolarizing muscle relaxants	non depolarizing muscle relaxants	non depolarizing muscle relaxants	non depolarizing muscle relaxants	non-depolarizing muscle relaxants	1	kept
non-dialytic measures	non dialytic measures	non dialytic measures	non dialytic measures	non-dialytic measures	1	kept
Non-divulgence	non divulgence	non divulgence	non divulgence	Non-divulgence	1	kept
Non-emergency groups	non emergency groups	non emergency groups	non emergency groups	Non-emergency groups	1	kept
Non-Genetic Healthcare Provider	non genetic healthcare provider	non genetic healthcare provider	non genetic healthcare provider	Non-Genetic Healthcare Provider	1	kept
Non-home discharge	non home discharge	non home discharge	non home discharge	non-home discharge	1	case_or_format
Non-intended death	non intended death	non intended death	non intended death	Non-intended death	1	kept
non-intubated	non intubated	non intubated	non intubated	non-intubated	1	kept
Non-invasive diagnostics	non invasive diagnostics	non invasive diagnostics	non invasive diagnostics	Non-invasive diagnostics	1	kept
Non-invasive hemodynamic monitoring	non invasive hemodynamic monitoring	non invasive hemodynamic monitoring	non invasive hemodynamic monitoring	Non-invasive hemodynamic monitoring	1	kept
Non-Invasive Positive Pressure Ventilation	non invasive positive pressure ventilation	non invasive positive pressure ventilation	non invasive positive pressure ventilation	non-invasive positive pressure ventilation	1	case_or_format
Non-invasive positive pressure ventilation	non invasive positive pressure ventilation	non invasive positive pressure ventilation	non invasive positive pressure ventilation	non-invasive positive pressure ventilation	1	case_or_format
NON-INVASIVE RESPIRATORY SUPPORT	non invasive respiratory support	non invasive respiratory support	non invasive respiratory support	Non-invasive respiratory support	1	case_or_format
non-invasive respiratory support	non invasive respiratory support	non invasive respiratory support	non invasive respiratory support	Non-invasive respiratory support	1	case_or_format
Non-invasive respiratory support care	non invasive respiratory support care	non invasive respiratory support care	non invasive respiratory support care	Non-invasive respiratory support care	1	kept

Non-invasive ventilation sequential therapy	non invasive ventilation sequential therapy	non invasive ventilation sequential therapy	non invasive ventilation sequential therapy	Non-invasive ventilation sequential therapy	1	kept
Non-linear relationships	non linear relationships	non linear relationships	non linear relationships	Non-linear relationships	1	kept
Non-Medical Prescribing	non medical prescribing	non medical prescribing	non medical prescribing	Non-medical prescribing	1	case_or_format
non-medical student	non medical student	non medical student	non medical student	non-medical student	1	kept
non-mortality	non mortality	non mortality	non mortality	non-mortality	1	kept
Non-nociceptive procedure	non nociceptive procedure	non nociceptive procedure	non nociceptive procedure	Non-nociceptive procedure	1	kept
Non-nutritious sucking	non nutritious sucking	non nutritious sucking	non nutritious sucking	Non-nutritious sucking	1	kept
non-nutritive sucking	non nutritive sucking	non nutritive sucking	non nutritive sucking	Non-nutritive sucking	1	case_or_format
non-obstructive coronary artery disease	non obstructive coronary artery disease	non obstructive coronary artery disease	non obstructive coronary artery disease	non-obstructive coronary artery disease	1	kept
non-operating room anesthesia	non operating room anesthesia	non operating room anesthesia	non operating room anesthesia	non-operating room anesthesia	1	kept
Non-opioid approaches	non opioid approaches	non opioid approaches	non opioid approaches	Non-opioid approaches	1	kept
Non-palliative care professionals	non palliative care professionals	non palliative care professionals	non palliative care professionals	Non-palliative care professionals	1	kept
Non-parametric statistics	non parametric statistics	non parametric statistics	non parametric statistics	Non-parametric statistics	1	kept
Non-pharmaceutical interventions	non pharmaceutical interventions	non pharmaceutical interventions	non pharmaceutical interventions	non-pharmaceutical interventions	1	case_or_format
non-pharmacologic	non pharmacologic	non pharmacologic	non pharmacologic	non-pharmacologic	1	kept
Non-pharmacologic pain management	non pharmacologic pain management	non pharmacologic pain management	non pharmacologic pain management	Non-pharmacologic pain management	1	kept

Non-pharmacological and pharmacological interventions	non pharmacological and pharmacological interventions	non pharmacological and pharmacological interventions	non pharmacological and pharmacological interventions	Non-pharmacological and pharmacological interventions	1	kept
Non-pharmacological delirium prevention practice	non pharmacological delirium prevention practice	non pharmacological delirium prevention practice	non pharmacological delirium prevention practice	Non-pharmacological delirium prevention practice	1	kept
non-pharmacological intervention	non pharmacological intervention	non pharmacological intervention	non pharmacological intervention	Non-pharmacological interventions	1	case_or_format
Non-pharmacological management	non pharmacological management	non pharmacological management	non pharmacological management	Non-pharmacological management	1	kept
Non-pharmacological measures	non pharmacological measures	non pharmacological measures	non pharmacological measures	Non-pharmacological measures	1	kept
Non-pharmacological methods	non pharmacological methods	non pharmacological methods	non pharmacological methods	Non-pharmacological methods	1	kept
Non-pharmacological pain management methods	non pharmacological pain management methods	non pharmacological pain management methods	non pharmacological pain management methods	Non-pharmacological pain management methods	1	kept
Non-pharmacological protocol	non pharmacological protocol	non pharmacological protocol	non pharmacological protocol	Non-pharmacological protocol	1	kept
Non-pharmacological sleep intervention	non pharmacological sleep intervention	non pharmacological sleep intervention	non pharmacological sleep intervention	Non-pharmacological sleep intervention	1	kept
Non-pharmacological therapy	non pharmacological therapy	non pharmacological therapy	non pharmacological therapy	Non-pharmacological therapy	1	kept
Non-physician provider	non physician provider	non physician provider	non physician provider	Non-physician provider	1	kept
non-physician team members	non physician team members	non physician team members	non physician team members	non-physician team members	1	kept
Non-plaque-induced gingivitis	non plaque induced gingivitis	non plaque induced gingivitis	non plaque induced gingivitis	Non-plaque-induced gingivitis	1	kept
Non-programmatic	non programmatic	non programmatic	non programmatic	Non-programmatic	1	kept
Non-punitive	non punitive	non punitive	non punitive	Non-punitive	1	kept
Non-randomised controlled trial	non randomised controlled trial	non randomised controlled trial	non randomised controlled trial	Non-randomised controlled trial	1	kept

non-randomised controlled trial	non randomised controlled trial	non randomised controlled trial	non randomised controlled trial	Non-randomised controlled trial	1	case_or_format
NON-RANDOMISED INTERVENTIONAL STUDY	non randomised interventional study	non randomised interventional study	non randomised interventional study	NON-RANDOMISED INTERVENTIONAL STUDY	1	kept
Non-randomized controlled trials as topic	non randomized controlled trials as topic	non randomized controlled trials as topic	non randomized controlled trials as topic	Non-Randomized Controlled Trials as Topic	1	case_or_format
non-reproductive endocrine glands	non reproductive endocrine glands	non reproductive endocrine glands	non reproductive endocrine glands	non-reproductive endocrine glands	1	kept
Non-routine events	non routine events	non routine events	non routine events	Non-routine events	1	kept
non-sedation	non sedation	non sedation	non sedation	non-sedation	1	kept
non-small cell lung cancer	non small cell lung cancer	non small cell lung cancer	non small cell lung cancer	non small cell lung cancer	1	case_or_format
non-ST-segment elevation MI	non st segment elevation mi	non st segment elevation mi	non st segment elevation mi	non-ST-segment elevation MI	1	kept
Non-steroidal anti-inflammatory analgesics	non steroidal anti inflammatory analgesics	non steroidal anti inflammatory analgesics	non steroidal anti inflammatory analgesics	Non-steroidal anti-inflammatory analgesics	1	kept
Non-stress test	non stress test	non stress test	non stress test	Non-stress test	1	kept
non-survivors	non survivors	non survivors	non survivors	non-survivors	1	kept
non-technical skill	non technical skill	non technical skill	non technical skill	Non-technical skills	1	case_or_format
non-therapeutic care	non therapeutic care	non therapeutic care	non therapeutic care	non-therapeutic care	1	kept
Non-thyroidal illness syndrome	non thyroidal illness syndrome	non thyroidal illness syndrome	non thyroidal illness syndrome	Non-thyroidal illness syndrome	1	kept
non-time-critical	non time critical	non time critical	non time critical	non-time-critical	1	kept
Non-treatment decisions	non treatment decisions	non treatment decisions	non treatment decisions	Non-treatment decisions	1	kept

Non-ventilator pneumonia	non ventilator pneumonia	non ventilator pneumonia	non ventilator pneumonia	Non-ventilator pneumonia	1	kept
Non-verbal communications	non verbal communications	non verbal communications	non verbal communication	non-verbal communication	1	singular
Non-Verbal Pain Scale	non verbal pain scale	non verbal pain scale	non verbal pain scale	Non-Verbal Pain Scale	1	kept
Non-water birth	non water birth	non water birth	non water birth	Non-water birth	1	kept
non-Western	non western	non western	non western	non-Western	1	kept
Nonaccidental trauma	nonaccidental trauma	nonaccidental trauma	nonaccidental trauma	Nonaccidental trauma	1	kept
nonaccidental trauma	nonaccidental trauma	nonaccidental trauma	nonaccidental trauma	Nonaccidental trauma	1	case_or_format
nonactionable alarm	nonactionable alarm	nonactionable alarm	nonactionable alarm	nonactionable alarm	1	kept
nonactionable alarms	nonactionable alarms	nonactionable alarms	nonactionable alarm	nonactionable alarm	1	singular
nonadherence practice	nonadherence practice	nonadherence practice	nonadherence practice	nonadherence practice	1	kept
Nonagenarians	nonagenarians	nonagenarians	nonagenarians	Nonagenarians	1	kept
Nonalcoholic fatty liver disease	nonalcoholic fatty liver disease	nonalcoholic fatty liver disease	nonalcoholic fatty liver disease	Nonalcoholic fatty liver disease	1	kept
nonalcoholic fatty liver disease	nonalcoholic fatty liver disease	nonalcoholic fatty liver disease	nonalcoholic fatty liver disease	Nonalcoholic fatty liver disease	1	case_or_format
nonalcoholic steatohepatitis	nonalcoholic steatohepatitis	nonalcoholic steatohepatitis	nonalcoholic steatohepatitis	nonalcoholic steatohepatitis	1	kept
Nonbacteremia	nonbacteremia	nonbacteremia	nonbacteremia	Nonbacteremia	1	kept
Nonbeneficial care	nonbeneficial care	nonbeneficial care	nonbeneficial care	nonbeneficial care	1	case_or_format

nonbeneficial treatment	nonbeneficial treatment	nonbeneficial treatment	nonbeneficial treatment	nonbeneficial treatment	1	kept
noncancer diagnosis	noncancer diagnosis	noncancer diagnosis	noncancer diagnosis	noncancer diagnosis	1	kept
noncancer palliative care	noncancer palliative care	noncancer palliative care	noncancer palliative care	noncancer palliative care	1	kept
NONCARDIAC SURGERY	noncardiac surgery	noncardiac surgery	noncardiac surgery	NONCARDIAC SURGERY	1	kept
noncardiogenic lung edema	noncardiogenic lung edema	noncardiogenic lung edema	noncardiogenic lung edema	noncardiogenic lung edema	1	kept
noncardiogenic pulmonary edema	noncardiogenic pulmonary edema	noncardiogenic pulmonary edema	noncardiogenic pulmonary edema	noncardiogenic pulmonary edema	1	kept
NONCARDIOTHORACIC SURGERY	noncardiothoracic surgery	noncardiothoracic surgery	noncardiothoracic surgery	NONCARDIOTHORACIC SURGERY	1	kept
Noncommunicable diseases	noncommunicable diseases	noncommunicable diseases	noncommunicable disease	noncommunicable disease	1	singular
noncompliance	noncompliance	noncompliance	noncompliance	Noncompliance	1	case_or_format
noncompressible torso hemorrhage	noncompressible torso hemorrhage	noncompressible torso hemorrhage	noncompressible torso hemorrhage	noncompressible torso hemorrhage	1	kept
NONCONVEX	nonconvex	nonconvex	nonconvex	NONCONVEX	1	kept
Noncritically ill	noncritically ill	noncritically ill	noncritically ill	Noncritically ill	1	kept
Noncuffed	noncuffed	noncuffed	noncuffed	Noncuffed	1	kept
Nondelivery hospital	nondelivery hospital	nondelivery hospital	nondelivery hospital	Nondelivery hospital	1	kept
Nondisjunction, Genetic	nondisjunction genetic	nondisjunction genetic	nondisjunction genetic	Nondisjunction, Genetic	1	kept
nonemergent intubation	nonemergent intubation	nonemergent intubation	nonemergent intubation	nonemergent intubation	1	kept

nonepileptic seizure	nonepileptic seizure	nonepileptic seizure	nonepileptic seizure	nonepileptic seizure	1	kept
nonfemoral access	nonfemoral access	nonfemoral access	nonfemoral access	nonfemoral access	1	kept
Nonfermenters	nonfermenters	nonfermenters	nonfermenters	Nonfermenters	1	kept
nongovernmental organization	nongovernmental organization	nongovernmental organization	nongovernmental organization	nongovernmental organization	1	kept
nonhumidified low flow oxygen therapy	nonhumidified low flow oxygen therapy	nonhumidified low flow oxygen therapy	nonhumidified low flow oxygen therapy	nonhumidified low flow oxygen therapy	1	kept
Nonimmune hydrops fetalis	nonimmune hydrops fetalis	nonimmune hydrops fetalis	nonimmune hydrops fetalis	Nonimmune hydrops fetalis	1	kept
nonindicated use	nonindicated use	nonindicated use	nonindicated use	nonindicated use	1	kept
noninterruptive technology	noninterruptive technology	noninterruptive technology	noninterruptive technology	noninterruptive technology	1	kept
nonintubated and intubated	nonintubated and intubated	nonintubated and intubated	nonintubated and intubated	nonintubated and intubated	1	kept
Nonintubated thoracic surgery	nonintubated thoracic surgery	nonintubated thoracic surgery	nonintubated thoracic surgery	Nonintubated thoracic surgery	1	kept
Noninvasive	noninvasive	noninvasive	noninvasive	noninvasive	1	case_or_format
Noninvasive blood pressure	noninvasive blood pressure	noninvasive blood pressure	noninvasive blood pressure	noninvasive blood pressure	1	case_or_format
Noninvasive hemodynamics	noninvasive hemodynamics	noninvasive hemodynamics	noninvasive hemodynamics	Noninvasive hemodynamics	1	kept
Noninvasive mechanical ventilation respiratory	noninvasive mechanical ventilation respiratory	noninvasive mechanical ventilation respiratory	noninvasive mechanical ventilation respiratory	Noninvasive mechanical ventilation respiratory	1	kept
Noninvasive methods	noninvasive methods	noninvasive methods	noninvasive methods	Noninvasive methods	1	kept
noninvasive neurally adjusted ventilatory assist	noninvasive neurally adjusted ventilatory assist	noninvasive neurally adjusted ventilatory assist	noninvasive neurally adjusted ventilatory assist	noninvasive neurally adjusted ventilatory assist	1	kept

noninvasive neuromodulation	noninvasive neuromodulation	noninvasive neuromodulation	noninvasive neuromodulation	noninvasive neuromodulation	1	kept
Noninvasive positive pressure ventilation	noninvasive positive pressure ventilation	noninvasive positive pressure ventilation	noninvasive positive pressure ventilation	noninvasive positive pressure ventilation	1	case_or_format
Noninvasive positive-pressure ventilation	noninvasive positive pressure ventilation	noninvasive positive pressure ventilation	noninvasive positive pressure ventilation	noninvasive positive pressure ventilation	1	case_or_format
noninvasive prenatal testing	noninvasive prenatal testing	noninvasive prenatal testing	noninvasive prenatal testing	noninvasive prenatal testing	1	kept
NONINVASIVE RESPIRATORY SUPPORT	noninvasive respiratory support	noninvasive respiratory support	noninvasive respiratory support	Noninvasive respiratory support	1	case_or_format
Noninvasive respiratory volume monitor	noninvasive respiratory volume monitor	noninvasive respiratory volume monitor	noninvasive respiratory volume monitor	Noninvasive respiratory volume monitor	1	kept
noninvasive support therapies	noninvasive support therapies	noninvasive support therapies	noninvasive support therapies	noninvasive support therapies	1	kept
noninvasive technique	noninvasive technique	noninvasive technique	noninvasive technique	noninvasive technique	1	kept
noninvasive ventilation device related pressure skin	noninvasive ventilation device related pressure skin	noninvasive ventilation device related pressure skin	noninvasive ventilation device related pressure skin	noninvasive ventilation device related pressure skin	1	kept
noninvasive ventilation treatment	noninvasive ventilation treatment	noninvasive ventilation treatment	noninvasive ventilation treatment	noninvasive ventilation treatment	1	kept
nonionic surfactant	nonionic surfactant	nonionic surfactant	nonionic surfactant	nonionic surfactant	1	kept
nonmechanically ventilated	nonmechanically ventilated	nonmechanically ventilated	nonmechanically ventilated	nonmechanically ventilated	1	kept
nonnucleoside reverse transcriptase inhibitor	nonnucleoside reverse transcriptase inhibitor	nonnucleoside reverse transcriptase inhibitor	nonnucleoside reverse transcriptase inhibitor	nonnucleoside reverse transcriptase inhibitor	1	kept
Nonocclusive	nonocclusive	nonocclusive	nonocclusive	Nonocclusive	1	kept
nonoxinol	nonoxinol	nonoxinol	nonoxinol	nonoxinol	1	kept
Nonoxynol	nonoxynol	nonoxynol	nonoxynol	Nonoxynol	1	kept

nonpharmacologic bundles	nonpharmacologic bundles	nonpharmacologic bundles	nonpharmacologic bundles	nonpharmacologic bundles	1	kept
Nonpharmacologic care	nonpharmacologic care	nonpharmacologic care	nonpharmacologic care	Nonpharmacologic care	1	kept
Nonpharmacologic intervention	nonpharmacologic intervention	nonpharmacologic intervention	nonpharmacologic intervention	nonpharmacologic intervention	1	case_or_format
nonpharmacologic pain intervention	nonpharmacologic pain intervention	nonpharmacologic pain intervention	nonpharmacologic pain intervention	nonpharmacologic pain intervention	1	kept
Nonpharmacologic pain management	nonpharmacologic pain management	nonpharmacologic pain management	nonpharmacologic pain management	Nonpharmacologic pain management	1	kept
Nonpharmacologic pain relief	nonpharmacologic pain relief	nonpharmacologic pain relief	nonpharmacologic pain relief	Nonpharmacologic pain relief	1	kept
nonpharmacologic pain relief	nonpharmacologic pain relief	nonpharmacologic pain relief	nonpharmacologic pain relief	Nonpharmacologic pain relief	1	case_or_format
nonpharmacological	nonpharmacological	nonpharmacological	nonpharmacological	Nonpharmacological	1	case_or_format
nonpharmacological treatment	nonpharmacological treatment	nonpharmacological treatment	nonpharmacological treatment	nonpharmacological treatment	1	kept
Nonphysician anesthesia provider	nonphysician anesthesia provider	nonphysician anesthesia provider	nonphysician anesthesia provider	Nonphysician anesthesia provider	1	kept
nonphysician anesthesia provider	nonphysician anesthesia provider	nonphysician anesthesia provider	nonphysician anesthesia provider	Nonphysician anesthesia provider	1	case_or_format
NONPHYSICIAN CLINICIAN	nonphysician clinician	nonphysician clinician	nonphysician clinician	NONPHYSICIAN CLINICIAN	1	kept
nonphysician clinicians	nonphysician clinicians	nonphysician clinicians	nonphysician clinician	NONPHYSICIAN CLINICIAN	1	singular
NONPHYSICIAN PRACTITIONERS	nonphysician practitioners	nonphysician practitioners	nonphysician practitioners	NONPHYSICIAN PRACTITIONERS	1	kept
Nonprescription Drugs	nonprescription drugs	nonprescription drugs	nonprescription drugs	Nonprescription Drugs	1	kept
NONRESPONSE	nonresponse	nonresponse	nonresponse	NONRESPONSE	1	kept

nonseparation	nonseparation	nonseparation	nonseparation	nonseparation	1	kept
nonsevere medication errors	nonsevere medication errors	nonsevere medication errors	nonsevere medication errors	nonsevere medication errors	1	kept
Nonstationary	nonstationary	nonstationary	nonstationary	Nonstationary	1	kept
nonstructural protein 1	nonstructural protein 1	nonstructural protein 1	nonstructural protein 1	nonstructural protein 1	1	kept
nonstructural protein 2	nonstructural protein 2	nonstructural protein 2	nonstructural protein 2	nonstructural protein 2	1	kept
nonsurgical invasive therapy	nonsurgical invasive therapy	nonsurgical invasive therapy	nonsurgical invasive therapy	nonsurgical invasive therapy	1	kept
nonsurvival	nonsurvival	nonsurvival	nonsurvival	nonsurvival	1	kept
NONTECHNICAL SKILLS ASSESSMENT	nontechnical skills assessment	nontechnical skills assessment	nontechnical skills assessment	NONTECHNICAL SKILLS ASSESSMENT	1	kept
Nontuberculous Mycobacteria	nontuberculous mycobacteria	nontuberculous mycobacteria	nontuberculous mycobacteria	Nontuberculous Mycobacteria	1	kept
Nontuberculous mycobacteria	nontuberculous mycobacteria	nontuberculous mycobacteria	nontuberculous mycobacteria	Nontuberculous Mycobacteria	1	case_or_format
nontuberculous mycobacteria	nontuberculous mycobacteria	nontuberculous mycobacteria	nontuberculous mycobacteria	Nontuberculous Mycobacteria	1	case_or_format
nontunneled catheter	nontunneled catheter	nontunneled catheter	nontunneled catheter	nontunneled catheter	1	kept
nonvariceal upper gastrointestinal bleeding	nonvariceal upper gastrointestinal bleeding	nonvariceal upper gastrointestinal bleeding	nonvariceal upper gastrointestinal bleeding	nonvariceal upper gastrointestinal bleeding	1	kept
nonventilator hospital acquired pneumonia	nonventilator hospital acquired pneumonia	nonventilator hospital acquired pneumonia	nonventilator hospital acquired pneumonia	nonventilator hospital acquired pneumonia	1	kept
Nonventilator hospital-acquired pneumonia	nonventilator hospital acquired pneumonia	nonventilator hospital acquired pneumonia	nonventilator hospital acquired pneumonia	nonventilator hospital acquired pneumonia	1	case_or_format
nonventilator hospital-acquired pneumonia	nonventilator hospital acquired pneumonia	nonventilator hospital acquired pneumonia	nonventilator hospital acquired pneumonia	nonventilator hospital acquired pneumonia	1	case_or_format

nonverbal indicator	nonverbal indicator	nonverbal indicator	nonverbal indicator	nonverbal indicator	1	kept
Nonverbal Pain Assessment Tool	nonverbal pain assessment tool	nonverbal pain assessment tool	nonverbal pain assessment tool	Nonverbal Pain Assessment Tool	1	kept
Nonverbal Pain Scale	nonverbal pain scale	nonverbal pain scale	nonverbal pain scale	Nonverbal pain scale	1	case_or_format
nonverbal pain scale	nonverbal pain scale	nonverbal pain scale	nonverbal pain scale	Nonverbal pain scale	1	case_or_format
nonvertebral fracture	nonvertebral fracture	nonvertebral fracture	nonvertebral fracture	nonvertebral fracture	1	kept
nonvitamin K antagonist oral anticoagulant	nonvitamin k antagonist oral anticoagulant	nonvitamin k antagonist oral anticoagulant	nonvitamin k antagonist oral anticoagulant	nonvitamin K antagonist oral anticoagulant	1	kept
nonvocal	nonvocal	nonvocal	nonvocal	nonvocal	1	kept
non - invasive positive pressure ventilation	non invasive positive pressure ventilation	non invasive positive pressure ventilation	non invasive positive pressure ventilation	non-invasive positive pressure ventilation	1	case_or_format
non - invasive respiratory support	non invasive respiratory support	non invasive respiratory support	non invasive respiratory support	Non-invasive respiratory support	1	case_or_format
non - invasive ventilation	non invasive ventilation	non invasive ventilation	non invasive ventilation	Non-invasive ventilation	1	case_or_format
non - pharmacological	non pharmacological	non pharmacological	non pharmacological	Non-pharmacological	1	case_or_format
non - pharmacological management	non pharmacological management	non pharmacological management	non pharmacological management	Non-pharmacological management	1	case_or_format
non - pharmacological methods	non pharmacological methods	non pharmacological methods	non pharmacological methods	Non-pharmacological methods	1	case_or_format
non - technical language	non technical language	non technical language	non technical language	non - technical language	1	kept
Non - device-related pneumonia	non device related pneumonia	non device related pneumonia	non device related pneumonia	Non - device-related pneumonia	1	kept
nootropil syrup	nootropil syrup	nootropil syrup	nootropil syrup	nootropil syrup	1	kept

noradrenalin bitartrate	noradrenalin bitartrate	noradrenalin bitartrate	noradrenalin bitartrate	noradrenalin bitartrate	1	kept
noradrenalin blood level	noradrenalin blood level	noradrenalin blood level	noradrenalin blood level	noradrenalin blood level	1	kept
noradrenalin release	noradrenalin release	noradrenalin release	noradrenalin release	noradrenalin release	1	kept
noradrenalin uptake inhibitor	noradrenalin uptake inhibitor	noradrenalin uptake inhibitor	noradrenalin uptake inhibitor	noradrenalin uptake inhibitor	1	kept
noradrenaline	noradrenaline	noradrenaline	noradrenaline	Noradrenaline	1	case_or_format
Nordic Diagnosis Related Groups	nordic diagnosis related groups	nordic diagnosis related groups	nordic diagnosis related groups	Nordic Diagnosis Related Groups	1	kept
Nordic questionnaire	nordic questionnaire	nordic questionnaire	nordic questionnaire	Nordic questionnaire	1	kept
Norepinefrina	norepinefrina	norepinefrina	norepinefrina	Norepinefrina	1	kept
NORepinephrine	norepinephrine	norepinephrine	norepinephrine	Norepinephrine	1	case_or_format
norepinephrine base	norepinephrine base	norepinephrine base	norepinephrine base	norepinephrine base	1	kept
norepinephrine tartrate	norepinephrine tartrate	norepinephrine tartrate	norepinephrine tartrate	norepinephrine tartrate	1	kept
normal body temperature	normal body temperature	normal body temperature	normal body temperature	normal body temperature	1	kept
normal care unit	normal care unit	normal care unit	normal care unit	normal care unit	1	kept
Normal flora	normal flora	normal flora	normal flora	Normal flora	1	kept
normal range	normal range	normal range	normal range	normal range	1	kept
Normal Saline	normal saline	normal saline	normal saline	Normal saline	1	case_or_format

normalising research	normalising research	normalising research	normalising research	normalising research	1	kept
Normalization MeAsure Development survey	normalization measure development survey	normalization measure development survey	normalization measure development survey	Normalization MeAsure Development survey	1	kept
Normalization of deviance pneumothorax	normalization of deviance pneumothorax	normalization of deviance pneumothorax	normalization of deviance pneumothorax	Normalization of deviance pneumothorax	1	kept
NORMALIZATION PROCESS THEORY	normalization process theory	normalization process theory	normalization process theory	normalization process theory	1	case_or_format
Normalization process theory	normalization process theory	normalization process theory	normalization process theory	normalization process theory	1	case_or_format
NORMATIVE DATA	normative data	normative data	normative data	NORMATIVE DATA	1	kept
Normativity	normativity	normativity	normativity	Normativity	1	kept
normativity	normativity	normativity	normativity	Normativity	1	case_or_format
normetadrenalin	normetadrenalin	normetadrenalin	normetadrenalin	normetadrenalin	1	kept
Normocapnia	normocapnia	normocapnia	normocapnia	Normocapnia	1	kept
Normoglycaemia	normoglycaemia	normoglycaemia	normoglycaemia	Normoglycaemia	1	kept
normotensive hydrocephalus	normotensive hydrocephalus	normotensive hydrocephalus	normotensive hydrocephalus	normotensive hydrocephalus	1	kept
normothermia	normothermia	normothermia	normothermia	Normothermia	1	case_or_format
norms	norms	norms	norms	norms	1	kept
norovirus	norovirus	norovirus	norovirus	Norovirus	1	case_or_format
norovirus infection	norovirus infection	norovirus infection	norovirus infection	norovirus infection	1	kept

North African	north african	north african	north african	North African	1	kept
north american neonatal health care professional	north american neonatal health care professional	north american neonatal health care professional	north american neonatal health care professional	north american neonatal health care professional	1	kept
North American People	north american people	north american people	north american people	North American People	1	kept
North China	north china	north china	north china	North China	1	kept
NORTH-AMERICA	north america	north america	north america	North America	1	case_or_format
north-south partnership	north south partnership	north south partnership	north south partnership	north-south partnership	1	kept
Northeast Ethiopia	northeast ethiopia	northeast ethiopia	northeast ethiopia	Northeast Ethiopia	1	kept
northeast united states	northeast united states	northeast united states	northeast united states	northeast united states	1	kept
Northern Europe	northern europe	northern europe	northern europe	Northern Europe	1	kept
Northern Hemisphere	northern hemisphere	northern hemisphere	northern hemisphere	Northern Hemisphere	1	kept
NORTHERN MANHATTAN STROKE	northern manhattan stroke	northern manhattan stroke	northern manhattan stroke	NORTHERN MANHATTAN STROKE	1	kept
Northwestern Nigeria	northwestern nigeria	northwestern nigeria	northwestern nigeria	Northwestern Nigeria	1	kept
norton index	norton index	norton index	norton index	norton index	1	kept
NORTON SCALE	norton scale	norton scale	norton scale	Norton Scale	1	case_or_format
Norton scale	norton scale	norton scale	norton scale	Norton Scale	1	case_or_format
Nortriptyline	nortriptyline	nortriptyline	nortriptyline	nortriptyline	1	case_or_format

NORWALK VIRUS	norwalk virus	norwalk virus	norwalk virus	NORWALK VIRUS	1	kept
nose carcinoma	nose carcinoma	nose carcinoma	nose carcinoma	nose carcinoma	1	kept
nose congestion	nose congestion	nose congestion	nose congestion	nose congestion	1	kept
nose fracture	nose fracture	nose fracture	nose fracture	nose fracture	1	kept
nose secretion	nose secretion	nose secretion	nose secretion	nose secretion	1	kept
nose septum reconstruction	nose septum reconstruction	nose septum reconstruction	nose septum reconstruction	nose septum reconstruction	1	kept
nose surgery	nose surgery	nose surgery	nose surgery	nose surgery	1	kept
nosebleed	nosebleed	nosebleed	nosebleed	nosebleed	1	kept
nosocomial acquisition	nosocomial acquisition	nosocomial acquisition	nosocomial acquisition	nosocomial acquisition	1	kept
NOSOCOMIAL BACTEREMIA	nosocomial bacteremia	nosocomial bacteremia	nosocomial bacteremia	NOSOCOMIAL BACTEREMIA	1	kept
Nosocomial diarrhea	nosocomial diarrhea	nosocomial diarrhea	nosocomial diarrhea	Nosocomial diarrhea	1	kept
NOSOCOMIAL FUNGAL-INFECTIONS	nosocomial fungal infections	nosocomial fungal infections	nosocomial fungal infections	NOSOCOMIAL FUNGAL-INFECTIONS	1	kept
Nosocomial infection control	nosocomial infection control	nosocomial infection control	nosocomial infection control	Nosocomial infection control	1	kept
nosocomial Influenza	nosocomial influenza	nosocomial influenza	nosocomial influenza	nosocomial Influenza	1	kept
NOSOCOMIAL KLEBSIELLA	nosocomial klebsiella	nosocomial klebsiella	nosocomial klebsiella	NOSOCOMIAL KLEBSIELLA	1	kept
NOSOCOMIAL OUTBREAK	nosocomial outbreak	nosocomial outbreak	nosocomial outbreak	NOSOCOMIAL OUTBREAK	1	kept

NOSOCOMIAL PATHOGENS	nosocomial pathogens	nosocomial pathogens	nosocomial pathogens	NOSOCOMIAL PATHOGENS	1	kept
NOSOCOMIAL PNEUMONIA	nosocomial pneumonia	nosocomial pneumonia	nosocomial pneumonia	nosocomial pneumonia	1	case_or_format
Nosocomial pressure ulcers	nosocomial pressure ulcers	nosocomial pressure ulcers	nosocomial pressure ulcers	Nosocomial pressure ulcers	1	kept
Nosocomial sepsis	nosocomial sepsis	nosocomial sepsis	nosocomial sepsis	Nosocomial sepsis	1	kept
Nosocomial sinusitis	nosocomial sinusitis	nosocomial sinusitis	nosocomial sinusitis	Nosocomial sinusitis	1	kept
NOSOCOMIAL TRANSMISSION	nosocomial transmission	nosocomial transmission	nosocomial transmission	nosocomial transmission	1	case_or_format
Nosocomial-MRSA	nosocomial mrsa	nosocomial mrsa	nosocomial mrsa	Nosocomial-MRSA	1	kept
not applicable	not applicable	not applicable	not applicable	not applicable	1	kept
Not For Resuscitation	not for resuscitation	not for resuscitation	not for resuscitation	Not For Resuscitation	1	kept
Not for resuscitation	not for resuscitation	not for resuscitation	not for resuscitation	Not For Resuscitation	1	case_or_format
Not monitoring	not monitoring	not monitoring	not monitoring	Not monitoring	1	kept
not the Plague	not the plague	not the plague	not the plague	not the Plague	1	kept
Notation	notation	notation	notation	Notation	1	kept
Notch	notch	notch	notch	Notch	1	kept
NOTECHS rating scale	notechs rating scale	notechs rating scale	notechs rating scale	NOTECHS rating scale	1	kept
Notificación	notificaci n	notificaci n	notificaci n	Notificación	1	kept

NotSanG	notsang	notsang	notsang	NotSanG	1	kept
notuss syrup	notuss syrup	notuss syrup	notuss syrup	notuss syrup	1	kept
nouveau antibiotique	nouveau antibiotique	nouveau antibiotique	nouveau antibiotique	nouveau antibiotique	1	kept
Novel algorithm	novel algorithm	novel algorithm	novel algorithm	Novel algorithm	1	kept
novel approaches	novel approaches	novel approaches	novel approaches	novel approaches	1	kept
Novel clustering	novel clustering	novel clustering	novel clustering	Novel clustering	1	kept
novel coronavirus disease	novel coronavirus disease	novel coronavirus disease	novel coronavirus disease	novel coronavirus disease	1	kept
novel coronavirus pneumonia	novel coronavirus pneumonia	novel coronavirus pneumonia	novel coronavirus pneumonia	novel coronavirus pneumonia	1	kept
Novel design	novel design	novel design	novel design	Novel design	1	kept
Novel devices	novel devices	novel devices	novel devices	Novel devices	1	kept
novel H1N1	novel h1n1	novel h1n1	novel h1n1	novel H1N1	1	kept
Novel infectious diseases	novel infectious diseases	novel infectious diseases	novel infectious diseases	Novel infectious diseases	1	kept
Novel influenza A	novel influenza a	novel influenza a	novel influenza a	Novel influenza A	1	kept
Novel psychoactive substances	novel psychoactive substances	novel psychoactive substances	novel psychoactive substances	Novel psychoactive substances	1	kept
Novel technologies	novel technologies	novel technologies	novel technologies	Novel technologies	1	kept
novelty effect	novelty effect	novelty effect	novelty effect	novelty effect	1	kept

NOVICE	novice	novice	novice	novice	1	case_or_format
Novice	novice	novice	novice	novice	1	case_or_format
Novice Computer Decision Support	novice computer decision support	novice computer decision support	novice computer decision support	Novice Computer Decision Support	1	kept
Novice user	novice user	novice user	novice user	Novice user	1	kept
novices	novices	novices	novice	novice	1	singular
NovoSeven	novoseven	novoseven	novoseven	NovoSeven	1	kept
novospirin	novospirin	novospirin	novospirin	novospirin	1	kept
NOWS	nows	nows	nows	NOWS	1	kept
NP-led clinic	np led clinic	np led clinic	np led clinic	NP-led clinic	1	kept
NPs	nps	nps	nps	NPs	1	kept
NPs testing	nps testing	nps testing	nps testing	NPs testing	1	kept
NPUAP grading system	npuap grading system	npuap grading system	npuap grading system	NPUAP grading system	1	kept
NREM	nrem	nrem	nrem	NREM	1	kept
NREM sleep	nrem sleep	nrem sleep	nrem sleep	NREM sleep	1	kept
NRF2-ARE PATHWAY	nrf2 are pathway	nrf2 are pathway	nrf2 are pathway	NRF2-ARE PATHWAY	1	kept
NRP®	nrp	nrp	nrp	NRP	1	case_or_format

NRP® algorithm	nrp algorithm	nrp algorithm	nrp algorithm	NRP® algorithm	1	kept
NRS	nrs	nrs	nrs	NRS	1	kept
NRS score	nrs score	nrs score	nrs score	NRS score	1	kept
NRS2002	nrs2002	nrs2002	nrs2002	NRS2002	1	kept
NRULA	nrula	nrula	nrula	NRULA	1	kept
NSAID = nonsteroidal anti-inflammatory drug	nsaid nonsteroidal anti inflammatory drug	nsaid nonsteroidal anti inflammatory drug	nsaid nonsteroidal anti inflammatory drug	NSAID = nonsteroidal anti-inflammatory drug	1	kept
NSCIR-IR	nscir ir	nscir ir	nscir ir	NSCIR-IR	1	kept
NSCLC	nsclc	nsclc	nsclc	NSCLC	1	kept
NSQIP	nsqip	nsqip	nsqip	NSQIP	1	kept
NSQIP occurrences	nsqip occurrences	nsqip occurrences	nsqip occurrences	NSQIP occurrences	1	kept
NST implementation	nst implementation	nst implementation	nst implementation	NST implementation	1	kept
NSTEMI	nstemi	nstemi	nstemi	NSTEMI	1	kept
NTCU = neuro transitional care unit	ntcu neuro transitional care unit	ntcu neuro transitional care unit	ntcu neuro transitional care unit	NTCU = neuro transitional care unit	1	kept
NTDB RDS = National Trauma Data Bank Research Data Set	ntdb rds national trauma data bank research data set	ntdb rds national trauma data bank research data set	ntdb rds national trauma data bank research data set	NTDB RDS = National Trauma Data Bank Research Data Set	1	kept
NTS, non-technical skills	nts non technical skills	nts non technical skills	nts non technical skills	NTS, non-technical skills	1	kept
NTSV	ntsv	ntsv	ntsv	NTSV	1	kept

Nu DESC	nu desc	nu desc	nu desc	Nu-DESC	1	case_or_format
Nubian	nubian	nubian	nubian	Nubian	1	kept
nuchal rigidity	nuchal rigidity	nuchal rigidity	nuchal rigidity	nuchal rigidity	1	kept
Nuclear	nuclear	nuclear	nuclear	Nuclear	1	kept
nuclear magnetic resonance scanner	nuclear magnetic resonance scanner	nuclear magnetic resonance scanner	nuclear magnetic resonance scanner	nuclear magnetic resonance scanner	1	kept
Nuclear Weapons	nuclear weapons	nuclear weapons	nuclear weapons	Nuclear Weapons	1	kept
nucleic acid	nucleic acid	nucleic acid	nucleic acid	nucleic acid	1	kept
Nucleic Acid Hybridization	nucleic acid hybridization	nucleic acid hybridization	nucleic acid hybridization	Nucleic Acid Hybridization	1	kept
Nucleocapsid	nucleocapsid	nucleocapsid	nucleocapsid	Nucleocapsid	1	kept
nucleoside diphosphate kinase	nucleoside diphosphate kinase	nucleoside diphosphate kinase	nucleoside diphosphate kinase	nucleoside diphosphate kinase	1	kept
nucleus accumbens	nucleus accumbens	nucleus accumbens	nucleus accumbens	nucleus accumbens	1	kept
nucleus tracti solitarii	nucleus tracti solitarii	nucleus tracti solitarii	nucleus tracti solitarii	nucleus tracti solitarii	1	kept
Nudging	nudging	nudging	nudging	Nudging	1	kept
Nuisance alarms	nuisance alarms	nuisance alarms	nuisance alarms	nuisance alarms	1	case_or_format
Number of beds	number of beds	number of beds	number of beds	Number of beds	1	kept
Number of false alarms	number of false alarms	number of false alarms	number of false alarms	Number of false alarms	1	kept

number of infection	number of infection	number of infection	number of infection	number of infection	1	kept
Number of peoples	number of peoples	number of peoples	number of peoples	Number of peoples	1	kept
Number Trauma Scale	number trauma scale	number trauma scale	number trauma scale	Number Trauma Scale	1	kept
NUMBERS	numbers	numbers	numbers	NUMBERS	1	kept
numbers needed to treat	numbers needed to treat	numbers needed to treat	numbers needed to treat	numbers needed to treat	1	kept
NUMERACY	numeracy	numeracy	numeracy	NUMERACY	1	kept
numeric pain scale	numeric pain scale	numeric pain scale	numeric pain scale	numeric pain scale	1	kept
Numeric Rating Scale	numeric rating scale	numeric rating scale	numeric rating scale	numeric rating scale	1	case_or_format
Numerical experiments	numerical experiments	numerical experiments	numerical experiments	Numerical experiments	1	kept
Numerical methods	numerical methods	numerical methods	numerical methods	Numerical methods	1	kept
numerical model	numerical model	numerical model	numerical model	numerical model	1	kept
Numerical Rating Scale	numerical rating scale	numerical rating scale	numerical rating scale	Numerical Rating Scale	1	kept
numerical rating scale	numerical rating scale	numerical rating scale	numerical rating scale	Numerical Rating Scale	1	case_or_format
Nurturing anti-racist behaviours	nurturing anti racist behaviours	nurturing anti racist behaviours	nurturing anti racist behaviours	Nurturing anti-racist behaviours	1	kept
Nurturing care	nurturing care	nurturing care	nurturing care	Nurturing care	1	kept
nut	nut	nut	nut	nut	1	kept

NUTRIC score	nutric score	nutric score	nutric score	NUTRIC score	1	kept
Nutrición	nutrici n	nutrici n	nutrici n	Nutrición	1	kept
NUTRIENT	nutrient	nutrient	nutrient	nutrient	1	case_or_format
Nutrient oxidation	nutrient oxidation	nutrient oxidation	nutrient oxidation	Nutrient oxidation	1	kept
nutrient oxidation	nutrient oxidation	nutrient oxidation	nutrient oxidation	Nutrient oxidation	1	case_or_format
nutrient solution	nutrient solution	nutrient solution	nutrient solution	nutrient solution	1	kept
nutrient supply	nutrient supply	nutrient supply	nutrient supply	nutrient supply	1	kept
Nutrigenomics	nutrigenomics	nutrigenomics	nutrigenomics	Nutrigenomics	1	kept
nutrition alert form	nutrition alert form	nutrition alert form	nutrition alert form	nutrition alert form	1	kept
Nutrition care	nutrition care	nutrition care	nutrition care	Nutrition care	1	kept
nutrition counseling	nutrition counseling	nutrition counseling	nutrition counseling	nutrition counseling	1	kept
Nutrition COVID-19	nutrition covid 19	nutrition covid 19	nutrition covid 19	Nutrition COVID-19	1	kept
nutrition guidelines	nutrition guidelines	nutrition guidelines	nutrition guidelines	nutrition guidelines	1	kept
Nutrition intervention	nutrition intervention	nutrition intervention	nutrition intervention	Nutrition intervention	1	kept
Nutrition management	nutrition management	nutrition management	nutrition management	Nutrition management	1	kept
nutrition management	nutrition management	nutrition management	nutrition management	Nutrition management	1	case_or_format

Nutrition Physiology	nutrition physiology	nutrition physiology	nutrition physiology	Nutrition Physiology	1	kept
nutrition policy	nutrition policy	nutrition policy	nutrition policy	Nutrition Policy	1	case_or_format
Nutrition practice	nutrition practice	nutrition practice	nutrition practice	Nutrition practice	1	kept
nutrition prescription	nutrition prescription	nutrition prescription	nutrition prescription	nutrition prescription	1	kept
Nutrition protocol	nutrition protocol	nutrition protocol	nutrition protocol	Nutrition protocol	1	kept
nutrition protocol	nutrition protocol	nutrition protocol	nutrition protocol	Nutrition protocol	1	case_or_format
nutrition protocols	nutrition protocols	nutrition protocols	nutrition protocol	Nutrition protocol	1	singular
Nutrition risk factors	nutrition risk factors	nutrition risk factors	nutrition risk factors	Nutrition risk factors	1	kept
nutrition risk in critically ill	nutrition risk in critically ill	nutrition risk in critically ill	nutrition risk in critically ill	nutrition risk in critically ill	1	kept
Nutrition Risk Screening	nutrition risk screening	nutrition risk screening	nutrition risk screening	Nutrition Risk Screening	1	kept
Nutrition screening	nutrition screening	nutrition screening	nutrition screening	nutrition screening	1	case_or_format
Nutrition screening tool	nutrition screening tool	nutrition screening tool	nutrition screening tool	Nutrition screening tool	1	kept
NUTRITION SUPPORT	nutrition support	nutrition support	nutrition support	nutrition support	1	case_or_format
NUTRITION SUPPORT TEAM	nutrition support team	nutrition support team	nutrition support team	nutrition support teams	1	case_or_format
Nutrition support team	nutrition support team	nutrition support team	nutrition support team	nutrition support teams	1	case_or_format
nutrition support team	nutrition support team	nutrition support team	nutrition support team	nutrition support teams	1	case_or_format

nutrition support team therapy	nutrition support team therapy	nutrition support team therapy	nutrition support team therapy	nutrition support team therapy	1	kept
NUTRITION SUPPORT THERAPY	nutrition support therapy	nutrition support therapy	nutrition support therapy	NUTRITION SUPPORT THERAPY	1	kept
NUTRITION THERAPY	nutrition therapy	nutrition therapy	nutrition therapy	Nutrition Therapy	1	case_or_format
Nutrition, emotion, and epigenetic effects on brain development	nutrition emotion and epigenetic effects on brain development	nutrition emotion and epigenetic effects on brain development	nutrition emotion and epigenetic effects on brain development	Nutrition, emotion, and epigenetic effects on brain development	1	kept
nutritional	nutritional	nutritional	nutritional	nutritional	1	kept
Nutritional assessment	nutritional assessment	nutritional assessment	nutritional assessment	nutritional assessment	1	case_or_format
Nutritional guideline	nutritional guideline	nutritional guideline	nutritional guideline	Nutritional guideline	1	kept
Nutritional habits	nutritional habits	nutritional habits	nutritional habits	Nutritional habits	1	kept
nutritional indicators	nutritional indicators	nutritional indicators	nutritional indicators	Nutritional indicators	1	case_or_format
Nutritional management	nutritional management	nutritional management	nutritional management	Nutritional management	1	kept
nutritional parameters	nutritional parameters	nutritional parameters	nutritional parameters	nutritional parameters	1	kept
Nutritional requirements	nutritional requirements	nutritional requirements	nutritional requirement	Nutritional Requirements	1	singular
nutritional requirements	nutritional requirements	nutritional requirements	nutritional requirement	Nutritional Requirements	1	singular
nutritional risk factor	nutritional risk factor	nutritional risk factor	nutritional risk factor	nutritional risk factor	1	kept
Nutritional Risk Screening 2002	nutritional risk screening 2002	nutritional risk screening 2002	nutritional risk screening 2002	Nutritional Risk Screening 2002	1	kept
nutritional science	nutritional science	nutritional science	nutritional science	nutritional science	1	kept

Nutritional Sciences	nutritional sciences	nutritional sciences	nutritional science	nutritional science	1	singular
nutritional screening	nutritional screening	nutritional screening	nutritional screening	nutritional screening	1	kept
Nutritional screening tools	nutritional screening tools	nutritional screening tools	nutritional screening tools	Nutritional screening tools	1	kept
nutritional state	nutritional state	nutritional state	nutritional state	Nutritional state	1	case_or_format
NUTRITIONAL SUPPLEMENTATION	nutritional supplementation	nutritional supplementation	nutritional supplementation	NUTRITIONAL SUPPLEMENTATION	1	kept
NUTRITIONAL SUPPLEMENTS	nutritional supplements	nutritional supplements	nutritional supplements	NUTRITIONAL SUPPLEMENTS	1	kept
nutritional support impact	nutritional support impact	nutritional support impact	nutritional support impact	nutritional support impact	1	kept
Nutritional support team	nutritional support team	nutritional support team	nutritional support team	Nutritional support team	1	kept
nutritional support team	nutritional support team	nutritional support team	nutritional support team	Nutritional support team	1	case_or_format
nutritional therapist	nutritional therapist	nutritional therapist	nutritional therapist	nutritional therapist	1	kept
Nutritive Value	nutritive value	nutritive value	nutritive value	Nutritive Value	1	kept
Nutrição	nutri o	nutri o	nutri o	Nutrição	1	kept
Nutrição enteral	nutri o enteral	nutri o enteral	nutri o enteral	Nutrição enteral	1	kept
NVPS	nvps	nvps	nvps	NVPS	1	kept
NWI	nwi	nwi	nwi	NWI	1	kept
NWSQ	nwsq	nwsq	nwsq	NWSQ	1	kept

nxiety	nxiety	nxiety	nxiety	nxiety	1	kept
NxStage System One machine	nxstage system one machine	nxstage system one machine	nxstage system one machine	NxStage System One machine	1	kept
Nyqvist KH	nyqvist kh	nyqvist kh	nyqvist kh	Nyqvist KH	1	kept
nystagmus	nystagmus	nystagmus	nystagmus	nystagmus	1	kept
négligence	n gligence	n gligence	n gligence	négligence	1	kept
OASIS score	oasis score	oasis score	oasis score	OASIS score	1	kept
OB teleflex	ob teleflex	ob teleflex	ob teleflex	OB teleflex	1	kept
Obamacare	obamacare	obamacare	obamacare	Obamacare	1	kept
Obese	obese	obese	obese	Obese	1	kept
Obesity in burn care unit	obesity in burn care unit	obesity in burn care unit	obesity in burn care unit	Obesity in burn care unit	1	kept
Obesity, Abdominal	obesity abdominal	obesity abdominal	obesity abdominal	Obesity, Abdominal	1	kept
obinutuzumab	obinutuzumab	obinutuzumab	obinutuzumab	obinutuzumab	1	kept
Object surface	object surface	object surface	object surface	Object surface	1	kept
Objective and subjective evaluations	objective and subjective evaluations	objective and subjective evaluations	objective and subjective evaluations	Objective and subjective evaluations	1	kept
Objective measurement	objective measurement	objective measurement	objective measurement	Objective measurement	1	kept
objective sleep	objective sleep	objective sleep	objective sleep	objective sleep	1	kept

Objective sleep parameters	objective sleep parameters	objective sleep parameters	objective sleep parameters	Objective sleep parameters	1	kept
Objective Structured Clinical Assessment	objective structured clinical assessment	objective structured clinical assessment	objective structured clinical assessment	Objective Structured Clinical Assessment	1	kept
Objective Structured Clinical Examination	objective structured clinical examination	objective structured clinical examination	objective structured clinical examination	Objective Structured Clinical Examination	1	kept
Objective structured clinical examination	objective structured clinical examination	objective structured clinical examination	objective structured clinical examination	Objective Structured Clinical Examination	1	case_or_format
objective structured clinical examination	objective structured clinical examination	objective structured clinical examination	objective structured clinical examination	Objective Structured Clinical Examination	1	case_or_format
oblimin rotation	oblimin rotation	oblimin rotation	oblimin rotation	oblimin rotation	1	kept
OBSERVATION FREQUENCY	observation frequency	observation frequency	observation frequency	OBSERVATION FREQUENCY	1	kept
observational	observational	observational	observational	Observational	1	case_or_format
OBSERVATIONAL COHORT	observational cohort	observational cohort	observational cohort	OBSERVATIONAL COHORT	1	kept
Observational design	observational design	observational design	observational design	Observational design	1	kept
observational registry study	observational registry study	observational registry study	observational registry study	observational registry study	1	kept
observational scales	observational scales	observational scales	observational scales	observational scales	1	kept
observational studies	observational studies	observational studies	observational study	observational study	1	singular
OBSERVATIONAL STUDY	observational study	observational study	observational study	observational study	1	case_or_format
Observational Study as Topic	observational study as topic	observational study as topic	observational study as topic	Observational Study as Topic	1	kept
observer	observer	observer	observer	observer	1	kept

observer bias	observer bias	observer bias	observer bias	observer bias	1	kept
Observer variation	observer variation	observer variation	observer variation	Observer Variation	1	case_or_format
observer-reported outcome measures	observer reported outcome measures	observer reported outcome measures	observer reported outcome measures	observer-reported outcome measures	1	kept
Obsessive and compulsive behaviors	obsessive and compulsive behaviors	obsessive and compulsive behaviors	obsessive and compulsive behaviors	Obsessive and compulsive behaviors	1	kept
Obsessive compulsive disorder	obsessive compulsive disorder	obsessive compulsive disorder	obsessive compulsive disorder	obsessive compulsive disorder	1	case_or_format
obsessive-compulsive	obsessive compulsive	obsessive compulsive	obsessive compulsive	obsessive-compulsive	1	kept
Obsessive-compulsive disorder	obsessive compulsive disorder	obsessive compulsive disorder	obsessive compulsive disorder	obsessive compulsive disorder	1	case_or_format
Obstacle factors	obstacle factors	obstacle factors	obstacle factors	Obstacle factors	1	kept
obstacles	obstacles	obstacles	obstacles	Obstacles	1	case_or_format
Obstacles to end-of-life care	obstacles to end of life care	obstacles to end of life care	obstacles to end of life care	Obstacles to end-of-life care	1	kept
obstacles to oral care	obstacles to oral care	obstacles to oral care	obstacles to oral care	obstacles to oral care	1	kept
Obstetric	obstetric	obstetric	obstetric	Obstetrics	1	case_or_format
obstetric	obstetric	obstetric	obstetric	Obstetrics	1	case_or_format
obstetric anaesthesia	obstetric anaesthesia	obstetric anaesthesia	obstetric anaesthesia	obstetric anaesthesia	1	kept
Obstetric anesthesia	obstetric anesthesia	obstetric anesthesia	obstetric anesthesia	obstetric anesthesia	1	case_or_format
Obstetric cardiac arrest	obstetric cardiac arrest	obstetric cardiac arrest	obstetric cardiac arrest	Obstetric cardiac arrest	1	kept

Obstetric care	obstetric care	obstetric care	obstetric care	obstetric care	1	case_or_format
Obstetric critical illness	obstetric critical illness	obstetric critical illness	obstetric critical illness	Obstetric critical illness	1	kept
obstetric education	obstetric education	obstetric education	obstetric education	obstetric education	1	kept
obstetric emergency team	obstetric emergency team	obstetric emergency team	obstetric emergency team	obstetric emergency team	1	kept
obstetric forceps	obstetric forceps	obstetric forceps	obstetric forceps	obstetric forceps	1	kept
Obstetric hemorrhage	obstetric hemorrhage	obstetric hemorrhage	obstetric hemorrhage	obstetric hemorrhage	1	case_or_format
obstetric indication	obstetric indication	obstetric indication	obstetric indication	obstetric indication	1	kept
obstetric labor complications	obstetric labor complications	obstetric labor complications	obstetric labor complications	Obstetric Labor Complications	1	case_or_format
Obstetric labour complication	obstetric labour complication	obstetric labour complication	obstetric labour complication	Obstetric labour complication	1	kept
obstetric operation	obstetric operation	obstetric operation	obstetric operation	obstetric operation	1	kept
obstetric outcomes	obstetric outcomes	obstetric outcomes	obstetric outcomes	obstetric outcomes	1	kept
Obstetric Racism	obstetric racism	obstetric racism	obstetric racism	Obstetric Racism	1	kept
obstetric rapid response team	obstetric rapid response team	obstetric rapid response team	obstetric rapid response team	obstetric rapid response team	1	kept
obstetric simulation	obstetric simulation	obstetric simulation	obstetric simulation	obstetric simulation	1	kept
Obstetric Surgical Procedures	obstetric surgical procedures	obstetric surgical procedures	obstetric surgical procedures	Obstetric Surgical Procedures	1	kept
obstetric traumas	obstetric traumas	obstetric traumas	obstetric traumas	obstetric traumas	1	kept

Obstetrical costs	obstetrical costs	obstetrical costs	obstetrical costs	Obstetrical costs	1	kept
Obstetrical Forceps	obstetrical forceps	obstetrical forceps	obstetrical forceps	Obstetrical Forceps	1	kept
Obstetrician-led care	obstetrician led care	obstetrician led care	obstetrician led care	Obstetrician-led care	1	kept
obstetrics and gynaecology	obstetrics and gynaecology	obstetrics and gynaecology	obstetrics and gynaecology	Obstetrics and gynaecology	1	case_or_format
Obstetrics and gynecology department, hospital	obstetrics and gynecology department hospital	obstetrics and gynecology department hospital	obstetrics and gynecology department hospital	Obstetrics and Gynecology Department, Hospital	1	case_or_format
obstetrics hospitalization unit	obstetrics hospitalization unit	obstetrics hospitalization unit	obstetrics hospitalization unit	obstetrics hospitalization unit	1	kept
obstetrics-neonatal care	obstetrics neonatal care	obstetrics neonatal care	obstetrics neonatal care	obstetrics-neonatal care	1	kept
obstructive	obstructive	obstructive	obstructive	obstructive	1	kept
obstructive defecation	obstructive defecation	obstructive defecation	obstructive defecation	obstructive defecation	1	kept
obstructive jaundice	obstructive jaundice	obstructive jaundice	obstructive jaundice	obstructive jaundice	1	kept
obstructive pulmonary disease assessment	obstructive pulmonary disease assessment	obstructive pulmonary disease assessment	obstructive pulmonary disease assessment	obstructive pulmonary disease assessment	1	kept
obstructive sleep apnoea	obstructive sleep apnoea	obstructive sleep apnoea	obstructive sleep apnoea	obstructive sleep apnoea	1	kept
OBSTRUCTIVE SLEEP-APNEA	obstructive sleep apnea	obstructive sleep apnea	obstructive sleep apnea	Obstructive sleep apnea	1	case_or_format
obstructive uropathy	obstructive uropathy	obstructive uropathy	obstructive uropathy	obstructive uropathy	1	kept
obturator artery injury	obturator artery injury	obturator artery injury	obturator artery injury	obturator artery injury	1	kept
occipital bone	occipital bone	occipital bone	occipital bone	occipital bone	1	kept

Occipital condyle	occipital condyle	occipital condyle	occipital condyle	Occipital condyle	1	kept
Occipital Lobe	occipital lobe	occipital lobe	occipital lobe	occipital lobe	1	case_or_format
occipitofrontal circumference	occipitofrontal circumference	occipitofrontal circumference	occipitofrontal circumference	occipitofrontal circumference	1	kept
occiput posterior position	occiput posterior position	occiput posterior position	occiput posterior position	occiput posterior position	1	kept
occlusion management	occlusion management	occlusion management	occlusion management	occlusion management	1	kept
Occlusive	occlusive	occlusive	occlusive	Occlusive	1	kept
Occlusive wrap	occlusive wrap	occlusive wrap	occlusive wrap	Occlusive wrap	1	kept
occult	occult	occult	occult	occult	1	kept
occult invasive bacterial infection	occult invasive bacterial infection	occult invasive bacterial infection	occult invasive bacterial infection	occult invasive bacterial infection	1	kept
occupancy fluctuations	occupancy fluctuations	occupancy fluctuations	occupancy fluctuations	occupancy fluctuations	1	kept
Occupants behaviours	occupants behaviours	occupants behaviours	occupants behaviours	Occupants behaviours	1	kept
occupation-related infections	occupation related infections	occupation related infections	occupation related infections	occupation-related infections	1	kept
occupational	occupational	occupational	occupational	Occupational	1	case_or_format
OCCUPATIONAL & INDUSTRIAL MEDICINE	occupational and industrial medicine	occupational and industrial medicine	occupational and industrial medicine	occupational & industrial medicine	1	case_or_format
Occupational accident	occupational accident	occupational accident	occupational accident	occupational accident	1	case_or_format
Occupational accidents	occupational accidents	occupational accidents	occupational accident	occupational accident	1	singular

occupational allergic contact dermatitis	occupational allergic contact dermatitis	occupational allergic contact dermatitis	occupational allergic contact dermatitis	occupational allergic contact dermatitis	1	kept
OCCUPATIONAL ASTHMA	occupational asthma	occupational asthma	occupational asthma	OCCUPATIONAL ASTHMA	1	kept
Occupational blood exposures	occupational blood exposures	occupational blood exposures	occupational blood exposures	Occupational blood exposures	1	kept
Occupational Burnout	occupational burnout	occupational burnout	occupational burnout	occupational burnout	1	case_or_format
OCCUPATIONAL DERMATITIS	occupational dermatitis	occupational dermatitis	occupational dermatitis	OCCUPATIONAL DERMATITIS	1	kept
occupational dermatitis	occupational dermatitis	occupational dermatitis	occupational dermatitis	OCCUPATIONAL DERMATITIS	1	case_or_format
occupational factors	occupational factors	occupational factors	occupational factors	occupational factors	1	kept
occupational grief	occupational grief	occupational grief	occupational grief	occupational grief	1	kept
Occupational group	occupational group	occupational group	occupational group	Occupational group	1	kept
Occupational groups	occupational groups	occupational groups	occupational group	Occupational group	1	singular
Occupational hazards	occupational hazards	occupational hazards	occupational hazard	occupational hazard	1	singular
occupational health and safety	occupational health and safety	occupational health and safety	occupational health and safety	occupational health and safety	1	kept
occupational health and safety team	occupational health and safety team	occupational health and safety team	occupational health and safety team	occupational health and safety team	1	kept
Occupational health risk	occupational health risk	occupational health risk	occupational health risk	Occupational health risk	1	kept
occupational health: waste anaesthesia gas	occupational health waste anaesthesia gas	occupational health waste anaesthesia gas	occupational health waste anaesthesia gas	occupational health: waste anaesthesia gas	1	kept
Occupational heatlh	occupational heatlh	occupational heatlh	occupational heatlh	Occupational heatlh	1	kept

Occupational injuries	occupational injuries	occupational injuries	occupational injury	Occupational Injuries	1	singular
Occupational injury	occupational injury	occupational injury	occupational injury	Occupational Injuries	1	case_or_format
occupational injury	occupational injury	occupational injury	occupational injury	Occupational Injuries	1	case_or_format
occupational mental health	occupational mental health	occupational mental health	occupational mental health	occupational mental health	1	kept
occupational noise	occupational noise	occupational noise	occupational noise	Occupational noise	1	case_or_format
Occupational outbreak	occupational outbreak	occupational outbreak	occupational outbreak	Occupational outbreak	1	kept
Occupational physical activity	occupational physical activity	occupational physical activity	occupational physical activity	Occupational physical activity	1	kept
occupational physician	occupational physician	occupational physician	occupational physician	occupational physician	1	kept
occupational risk analysis and management	occupational risk analysis and management	occupational risk analysis and management	occupational risk analysis and management	occupational risk analysis and management	1	kept
Occupational risk factor	occupational risk factor	occupational risk factor	occupational risk factor	occupational risk factor	1	case_or_format
Occupational Risks	occupational risks	occupational risks	occupational risk	Occupational risks	1	singular
occupational safety & health	occupational safety and health	occupational safety and health	occupational safety and health	occupational safety & health	1	kept
Occupational Safety and Health	occupational safety and health	occupational safety and health	occupational safety and health	occupational safety & health	1	case_or_format
occupational safety and health	occupational safety and health	occupational safety and health	occupational safety and health	occupational safety & health	1	case_or_format
Occupational Safety and Health Administration	occupational safety and health administration	occupational safety and health administration	occupational safety and health administration	Occupational Safety and Health Administration	1	kept
Occupational satisfaction	occupational satisfaction	occupational satisfaction	occupational satisfaction	Occupational satisfaction	1	kept

Occupational stressors	occupational stressors	occupational stressors	occupational stressors	Occupational stressors	1	kept
Occupational therapist	occupational therapist	occupational therapist	occupational therapist	occupational therapist	1	case_or_format
Occupational therapists	occupational therapists	occupational therapists	occupational therapist	occupational therapist	1	singular
Occupational tumors	occupational tumors	occupational tumors	occupational tumors	Occupational tumors	1	kept
OCCUPATIONAL VIOLENCE	occupational violence	occupational violence	occupational violence	OCCUPATIONAL VIOLENCE	1	kept
Occupational violence	occupational violence	occupational violence	occupational violence	OCCUPATIONAL VIOLENCE	1	case_or_format
occupational violence	occupational violence	occupational violence	occupational violence	OCCUPATIONAL VIOLENCE	1	case_or_format
Occupational wellbeing	occupational wellbeing	occupational wellbeing	occupational wellbeing	Occupational wellbeing	1	kept
OCCUPATIONAL-EXPOSURE	occupational exposure	occupational exposure	occupational exposure	occupational exposure	1	case_or_format
Occupied Philippines	occupied philippines	occupied philippines	occupied philippines	Occupied Philippines	1	kept
occurrence	occurrence	occurrence	occurrence	occurrence	1	kept
Occurrence of medication errors	occurrence of medication errors	occurrence of medication errors	occurrence of medication errors	Occurrence of medication errors	1	kept
Occurrence of Pneumonia	occurrence of pneumonia	occurrence of pneumonia	occurrence of pneumonia	Occurrence of Pneumonia	1	kept
occurrence reporting	occurrence reporting	occurrence reporting	occurrence reporting	occurrence reporting	1	kept
OCT	oct	oct	oct	OCT	1	kept
Octenidine	octenidine	octenidine	octenidine	Octenidine	1	kept

octenidine plus phenoxyethanol	octenidine plus phenoxyethanol	octenidine plus phenoxyethanol	octenidine plus phenoxyethanol	octenidine plus phenoxyethanol	1	kept
Octogenarian	octogenarian	octogenarian	octogenarian	Octogenarians	1	case_or_format
octopus	octopus	octopus	octopus	octopus	1	kept
Octopus vulgaris	octopus vulgaris	octopus vulgaris	octopus vulgaris	Octopus vulgaris	1	kept
Octreotide	octreotide	octreotide	octreotide	octreotide	1	case_or_format
ocular involvement	ocular involvement	ocular involvement	ocular involvement	ocular involvement	1	kept
ocular lesion	ocular lesion	ocular lesion	ocular lesion	ocular lesion	1	kept
Ocular Physiological Phenomena	ocular physiological phenomena	ocular physiological phenomena	ocular physiological phenomena	Ocular Physiological Phenomena	1	kept
ocular surface care	ocular surface care	ocular surface care	ocular surface care	ocular surface care	1	kept
Ocular surface disease	ocular surface disease	ocular surface disease	ocular surface disease	Ocular surface disease	1	kept
ocular surface disease	ocular surface disease	ocular surface disease	ocular surface disease	Ocular surface disease	1	case_or_format
ocular surface disease index	ocular surface disease index	ocular surface disease index	ocular surface disease index	ocular surface disease index	1	kept
Ocular surface disorder	ocular surface disorder	ocular surface disorder	ocular surface disorder	ocular surface disorder	1	case_or_format
ODI = Oswestry Disability Index	odi oswestry disability index	odi oswestry disability index	odi oswestry disability index	ODI = Oswestry Disability Index	1	kept
Odorants	odorants	odorants	odorants	Odorants	1	kept
OECD	oecd	oecd	oecd	OECD	1	kept

oesophageal pressure	oesophageal pressure	oesophageal pressure	oesophageal pressure	oesophageal pressure	1	kept
oesophagus	oesophagus	oesophagus	oesophagus	oesophagus	1	kept
of health care	of health care	of health care	of health care	of health care	1	kept
OF-CARE GLUCOMETER	of care glucometer	of care glucometer	of care glucometer	OF-CARE GLUCOMETER	1	kept
OF-LIFE-CARE	of life care	of life care	of life care	OF-LIFE CARE	1	case_or_format
ofatumumab	ofatumumab	ofatumumab	ofatumumab	ofatumumab	1	kept
off-label	off label	off label	off label	off-label	1	kept
Off-Label Use	off label use	off label use	off label use	Off-Label Use	1	kept
Off-pump bypass	off pump bypass	off pump bypass	off pump bypass	Off-pump bypass	1	kept
off-pump cabg	off pump cabg	off pump cabg	off pump cabg	off-pump cabg	1	kept
office	office	office	office	office	1	kept
Office Automation	office automation	office automation	office automation	Office Automation	1	kept
Office environments	office environments	office environments	office environments	Office environments	1	kept
Office preparedness	office preparedness	office preparedness	office preparedness	Office preparedness	1	kept
office worker	office worker	office worker	office worker	office worker	1	kept
OIG	oig	oig	oig	OIG	1	kept

oil	oil	oil	oil	oil	1	kept
oil-compresses	oil compresses	oil compresses	oil compresses	oil-compresses	1	kept
Olanzapine	olanzapine	olanzapine	olanzapine	olanzapine	1	case_or_format
olaratumab	olaratumab	olaratumab	olaratumab	olaratumab	1	kept
Old age	old age	old age	old age	Old age	1	kept
Old man	old man	old man	old man	Old man	1	kept
OLD PEOPLES HOMES	old peoples homes	old peoples homes	old peoples homes	OLD PEOPLES HOMES	1	kept
OLDER ADULTS	older adults	older adults	older adult	Older adults	1	singular
Older assessment	older assessment	older assessment	older assessment	Older assessment	1	kept
OLDER CHILDREN	older children	older children	older children	OLDER CHILDREN	1	kept
Older person	older person	older person	older person	older person	1	case_or_format
Older persons	older persons	older persons	older person	older person	1	singular
oldest	oldest	oldest	oldest	oldest	1	kept
oldest old	oldest old	oldest old	oldest old	oldest old	1	kept
oldest old and thrombolysis	oldest old and thrombolysis	oldest old and thrombolysis	oldest old and thrombolysis	oldest old and thrombolysis	1	kept
olecranon	olecranon	olecranon	olecranon	olecranon	1	kept

Olfactory environment	olfactory environment	olfactory environment	olfactory environment	Olfactory environment	1	kept
olfactory nerve	olfactory nerve	olfactory nerve	olfactory nerve	olfactory nerve	1	kept
Olfactory perception	olfactory perception	olfactory perception	olfactory perception	Olfactory perception	1	kept
olfactory system	olfactory system	olfactory system	olfactory system	olfactory system	1	kept
olfactory tract	olfactory tract	olfactory tract	olfactory tract	olfactory tract	1	kept
Oligodendroglia	oligodendroglia	oligodendroglia	oligodendroglia	Oligodendroglia	1	kept
oligodendroglia	oligodendroglia	oligodendroglia	oligodendroglia	Oligodendroglia	1	case_or_format
oligodendroglioma	oligodendroglioma	oligodendroglioma	oligodendroglioma	oligodendroglioma	1	kept
oligonucleotide	oligonucleotide	oligonucleotide	oligonucleotide	oligonucleotide	1	kept
Oligonucleotide Array Sequence Analysis	oligonucleotide array sequence analysis	oligonucleotide array sequence analysis	oligonucleotide array sequence analysis	Oligonucleotide Array Sequence Analysis	1	kept
oligophrenia	oligophrenia	oligophrenia	oligophrenia	oligophrenia	1	kept
oligosaccharide	oligosaccharide	oligosaccharide	oligosaccharide	oligosaccharide	1	kept
Olive Oil	olive oil	olive oil	olive oil	olive oil	1	case_or_format
Olive oil	olive oil	olive oil	olive oil	olive oil	1	case_or_format
olmesartan	olmesartan	olmesartan	olmesartan	olmesartan	1	kept
Olmstead decision	olmstead decision	olmstead decision	olmstead decision	Olmstead decision	1	kept

Omadacycline	omadacycline	omadacycline	omadacycline	omadacycline	1	case_or_format
Omaha System	omaha system	omaha system	omaha system	Omaha System	1	kept
omaha system	omaha system	omaha system	omaha system	Omaha System	1	case_or_format
omega 6 fatty acid	omega 6 fatty acid	omega 6 fatty acid	omega 6 fatty acid	omega 6 fatty acid	1	kept
omega-3 fatty acid	omega 3 fatty acid	omega 3 fatty acid	omega 3 fatty acid	omega 3 fatty acid	1	case_or_format
omega-3 fatty acids	omega 3 fatty acids	omega 3 fatty acids	omega 3 fatty acid	omega 3 fatty acid	1	singular
OMEPRAZOLE	omeprazole	omeprazole	omeprazole	omeprazole	1	case_or_format
Omeprazole	omeprazole	omeprazole	omeprazole	omeprazole	1	case_or_format
omission error	omission error	omission error	omission error	omission errors	1	case_or_format
omission of medication	omission of medication	omission of medication	omission of medication	omission of medication	1	kept
Omitted care	omitted care	omitted care	omitted care	Omitted care	1	kept
Ommastrephidae	ommastrephidae	ommastrephidae	ommastrephidae	Ommastrephidae	1	kept
Omphalocele	omphalocele	omphalocele	omphalocele	omphalocele	1	case_or_format
On demands	on demands	on demands	on demand	on-demand	1	singular
On-body	on body	on body	on body	On-body	1	kept
On-call	on call	on call	on call	On-call	1	kept

on-demand	on demand	on demand	on demand	on-demand	1	kept
On-demand CXR	on demand cxr	on demand cxr	on demand cxr	On-demand CXR	1	kept
On-line access	on line access	on line access	on line access	On-line access	1	kept
On-line survey	on line survey	on line survey	on line survey	On-line survey	1	kept
On-pump	on pump	on pump	on pump	On-pump	1	kept
on-site caregivers	on site caregivers	on site caregivers	on site caregivers	on-site caregivers	1	kept
on-site strategy	on site strategy	on site strategy	on site strategy	on-site strategy	1	kept
Onco-nephrology	onco nephrology	onco nephrology	onco nephrology	Onco-nephrology	1	kept
oncogene N ras	oncogene n ras	oncogene n ras	oncogene n ras	oncogene N ras	1	kept
Oncologic emergencies	oncologic emergencies	oncologic emergencies	oncologic emergency	Oncologic emergency	1	singular
oncologic emergencies	oncologic emergencies	oncologic emergencies	oncologic emergency	Oncologic emergency	1	singular
Oncologic emergency	oncologic emergency	oncologic emergency	oncologic emergency	Oncologic emergency	1	kept
Oncological emergencies	oncological emergencies	oncological emergencies	oncological emergencies	Oncological emergencies	1	kept
Oncology centre	oncology centre	oncology centre	oncology centre	Oncology centre	1	kept
oncology hospital	oncology hospital	oncology hospital	oncology hospital	oncology hospital	1	kept
ONCOLOGY RESEARCH-CENTER	oncology research center	oncology research center	oncology research center	ONCOLOGY RESEARCH-CENTER	1	kept

Ondine’s curse	ondine s curse	ondine s curse	ondine s curse	Ondine’s curse	1	kept
One-day workshops	one day workshops	one day workshops	one day workshops	One-day workshops	1	kept
One-hour sepsis bundle	one hour sepsis bundle	one hour sepsis bundle	one hour sepsis bundle	One-hour sepsis bundle	1	kept
ONE-Sim	one sim	one sim	one sim	ONE-Sim	1	kept
One-tailed test	one tailed test	one tailed test	one tailed test	One-tailed test	1	kept
one-year follow-up	one year follow up	one year follow up	one year follow up	one-year follow-up	1	kept
ONE-YEAR OUTCOMES	one year outcomes	one year outcomes	one year outcomes	ONE-YEAR OUTCOMES	1	kept
one-year prognosis	one year prognosis	one year prognosis	one year prognosis	one-year prognosis	1	kept
oneself	oneself	oneself	oneself	oneself	1	kept
Ongoing education	ongoing education	ongoing education	ongoing education	Ongoing education	1	kept
Ongoing employment	ongoing employment	ongoing employment	ongoing employment	Ongoing employment	1	kept
online debriefing	online debriefing	online debriefing	online debriefing	online debriefing	1	kept
Online education	online education	online education	online education	online education	1	case_or_format
Online intervention	online intervention	online intervention	online intervention	Online intervention	1	kept
online intervention	online intervention	online intervention	online intervention	Online intervention	1	case_or_format
online learning	online learning	online learning	online learning	Online learning	1	case_or_format

Online Learning Package	online learning package	online learning package	online learning package	Online Learning Package	1	kept
online monitoring	online monitoring	online monitoring	online monitoring	online monitoring	1	kept
online network	online network	online network	online network	online network	1	kept
online research	online research	online research	online research	online research	1	kept
online screening	online screening	online screening	online screening	online screening	1	kept
online social network	online social network	online social network	online social network	online social network	1	kept
Online support	online support	online support	online support	Online support	1	kept
Online survey software	online survey software	online survey software	online survey software	Online survey software	1	kept
Online systems	online systems	online systems	online system	online system	1	singular
Online training	online training	online training	online training	Online training	1	kept
online training module	online training module	online training module	online training module	online training module	1	kept
online working	online working	online working	online working	online working	1	kept
onsolidated criteria for reporting qualitative research	onsolidated criteria for reporting qualitative research	onsolidated criteria for reporting qualitative research	onsolidated criteria for reporting qualitative research	onsolidated criteria for reporting qualitative research	1	kept
onychomycosis	onychomycosis	onychomycosis	onychomycosis	onychomycosis	1	kept
oocyte development	oocyte development	oocyte development	oocyte development	oocyte development	1	kept
Oocyte Donation	oocyte donation	oocyte donation	oocyte donation	oocyte donation	1	case_or_format

Oorganizational citizenship behavior	oorganizational citizenship behavior	oorganizational citizenship behavior	oorganizational citizenship behavior	Oorganizational citizenship behavior	1	kept
op 1118	op 1118	op 1118	op 1118	op 1118	1	kept
OPAT	opat	opat	opat	OPAT	1	kept
open abdomen technique	open abdomen technique	open abdomen technique	open abdomen technique	open abdomen technique	1	kept
Open Abdomen Techniques	open abdomen techniques	open abdomen techniques	open abdomen technique	open abdomen technique	1	singular
Open access	open access	open access	open access	open access	1	case_or_format
OPEN APPENDECTOMY	open appendectomy	open appendectomy	open appendectomy	OPEN APPENDECTOMY	1	kept
Open bay	open bay	open bay	open bay	Open bay	1	kept
open bay	open bay	open bay	open bay	Open bay	1	case_or_format
Open Bite	open bite	open bite	open bite	Open Bite	1	kept
open bite	open bite	open bite	open bite	Open Bite	1	case_or_format
open chest extracorporeal oxygenation	open chest extracorporeal oxygenation	open chest extracorporeal oxygenation	open chest extracorporeal oxygenation	open chest extracorporeal oxygenation	1	kept
open colectomy	open colectomy	open colectomy	open colectomy	open colectomy	1	kept
Open distance learning	open distance learning	open distance learning	open distance learning	Open distance learning	1	kept
open endotracheal suction	open endotracheal suction	open endotracheal suction	open endotracheal suction	open endotracheal suction	1	kept
Open femoral fractures in children	open femoral fractures in children	open femoral fractures in children	open femoral fractures in children	Open femoral fractures in children	1	kept

open femoral shaft fracture	open femoral shaft fracture	open femoral shaft fracture	open femoral shaft fracture	open femoral shaft fracture	1	kept
Open fracture	open fracture	open fracture	open fracture	open fracture	1	case_or_format
Open heart	open heart	open heart	open heart	Open heart	1	kept
Open Heart Surgery	open heart surgery	open heart surgery	open heart surgery	open heart surgery	1	case_or_format
Open heart surgery	open heart surgery	open heart surgery	open heart surgery	open heart surgery	1	case_or_format
Open loop control	open loop control	open loop control	open loop control	Open loop control	1	kept
Open lung	open lung	open lung	open lung	Open lung	1	kept
open notes	open notes	open notes	open notes	open notes	1	kept
open pelvic fractures	open pelvic fractures	open pelvic fractures	open pelvic fractures	open pelvic fractures	1	kept
open plan	open plan	open plan	open plan	open plan	1	kept
open premature care	open premature care	open premature care	open premature care	open premature care	1	kept
open reduction and internal fixation	open reduction and internal fixation	open reduction and internal fixation	open reduction and internal fixation	open reduction and internal fixation	1	kept
Open reduction-internal fixation	open reduction internal fixation	open reduction internal fixation	open reduction internal fixation	Open reduction-internal fixation	1	kept
open suction	open suction	open suction	open suction	open suction	1	kept
Open suction system	open suction system	open suction system	open suction system	Open suction system	1	kept
Open suctioning	open suctioning	open suctioning	open suctioning	Open suctioning	1	kept

Open system suction method	open system suction method	open system suction method	open system suction method	Open system suction method	1	kept
Open system suctioning	open system suctioning	open system suctioning	open system suctioning	Open system suctioning	1	kept
Open tracheal suction system	open tracheal suction system	open tracheal suction system	open tracheal suction system	Open tracheal suction system	1	kept
Open visit	open visit	open visit	open visit	Open visit	1	kept
Open visiting hours	open visiting hours	open visiting hours	open visiting hours	Open visiting hours	1	kept
open visiting policy	open visiting policy	open visiting policy	open visiting policy	open visiting policy	1	kept
Open-bay	open bay	open bay	open bay	Open bay	1	case_or_format
Open-bay design	open bay design	open bay design	open bay design	Open-bay design	1	kept
open-ended questionnaire	open ended questionnaire	open ended questionnaire	open ended questionnaire	open ended questionnaire	1	case_or_format
OPEN-HEART-SURGERY	open heart surgery	open heart surgery	open heart surgery	open heart surgery	1	case_or_format
open-plan	open plan	open plan	open plan	open plan	1	case_or_format
open-plan layout	open plan layout	open plan layout	open plan layout	open-plan layout	1	kept
OpenNotes	opennotes	opennotes	opennotes	OpenNotes	1	kept
open‐heart surgery	open heart surgery	open heart surgery	open heart surgery	open heart surgery	1	case_or_format
operant conditioning	operant conditioning	operant conditioning	operant conditioning	operant conditioning	1	kept
Operating angle	operating angle	operating angle	operating angle	Operating angle	1	kept

Operating capacity	operating capacity	operating capacity	operating capacity	Operating capacity	1	kept
operating cost	operating cost	operating cost	operating cost	operating cost	1	kept
Operating efficiency	operating efficiency	operating efficiency	operating efficiency	Operating efficiency	1	kept
Operating room economics	operating room economics	operating room economics	operating room economics	Operating room economics	1	kept
operating room efficiency	operating room efficiency	operating room efficiency	operating room efficiency	operating room efficiency	1	kept
operating room information systems	operating room information systems	operating room information systems	operating room information systems	Operating Room Information Systems	1	case_or_format
operating room planning	operating room planning	operating room planning	operating room planning	operating room planning	1	kept
operating room resource management	operating room resource management	operating room resource management	operating room resource management	operating room resource management	1	kept
Operating theatre scheduling	operating theatre scheduling	operating theatre scheduling	operating theatre scheduling	Operating theatre scheduling	1	kept
OPERATING-ROOM EFFICIENCY	operating room efficiency	operating room efficiency	operating room efficiency	operating room efficiency	1	case_or_format
OPERATING-ROOM TEMPERATURE	operating room temperature	operating room temperature	operating room temperature	OPERATING-ROOM TEMPERATURE	1	kept
Operation	operation	operation	operation	OPERATIONS	1	case_or_format
operation efficiency	operation efficiency	operation efficiency	operation efficiency	operation efficiency	1	kept
Operation management	operation management	operation management	operation management	Operation management	1	kept
operation Resilience	operation resilience	operation resilience	operation resilience	operation Resilience	1	kept
Operation room	operation room	operation room	operation room	Operation room	1	kept

operation room	operation room	operation room	operation room	Operation room	1	case_or_format
operation stick	operation stick	operation stick	operation stick	operation stick	1	kept
operation theatre	operation theatre	operation theatre	operation theatre	operation theatre	1	kept
Operational amplifiers	operational amplifiers	operational amplifiers	operational amplifiers	Operational amplifiers	1	kept
Operational and medical management of disasters	operational and medical management of disasters	operational and medical management of disasters	operational and medical management of disasters	Operational and medical management of disasters	1	kept
Operational characteristics	operational characteristics	operational characteristics	operational characteristics	Operational characteristics	1	kept
Operational decisions	operational decisions	operational decisions	operational decisions	Operational decisions	1	kept
operational efficiency	operational efficiency	operational efficiency	operational efficiency	operational efficiency	1	kept
operational failures	operational failures	operational failures	operational failures	operational failures	1	kept
Operational interruptions	operational interruptions	operational interruptions	operational interruptions	Operational interruptions	1	kept
operational management	operational management	operational management	operational management	operational management	1	kept
operational medicine	operational medicine	operational medicine	operational medicine	operational medicine	1	kept
Operational Research	operational research	operational research	operational research	Operational Research	1	kept
operational research	operational research	operational research	operational research	Operational Research	1	case_or_format
Operational standards	operational standards	operational standards	operational standards	Operational standards	1	kept
Operational Workflow	operational workflow	operational workflow	operational workflow	Operational Workflow	1	kept

Operationalization and measurement	operationalization and measurement	operationalization and measurement	operationalization and measurement	Operationalization and measurement	1	kept
Operations	operations	operations	operation	OPERATIONS	1	singular
operations	operations	operations	operation	OPERATIONS	1	singular
operations engineering	operations engineering	operations engineering	operations engineering	operations engineering	1	kept
Operative Blood Salvage	operative blood salvage	operative blood salvage	operative blood salvage	Operative Blood Salvage	1	kept
OPERATIVE EXPERIENCE	operative experience	operative experience	operative experience	OPERATIVE EXPERIENCE	1	kept
Operative management	operative management	operative management	operative management	Operative management	1	kept
OPERATIVE MORTALITY	operative mortality	operative mortality	operative mortality	OPERATIVE MORTALITY	1	kept
operative treatment	operative treatment	operative treatment	operative treatment	operative treatment	1	kept
operative vaginal delivery	operative vaginal delivery	operative vaginal delivery	operative vaginal delivery	operative vaginal delivery	1	kept
OPERATOR	operator	operator	operator	OPERATOR	1	kept
operator	operator	operator	operator	OPERATOR	1	case_or_format
Operator’s extension	operator s extension	operator s extension	operator s extension	Operator’s extension	1	kept
Ophthalmic Services guidance	ophthalmic services guidance	ophthalmic services guidance	ophthalmic services guidance	Ophthalmic Services guidance	1	kept
ophthalmology reorganization	ophthalmology reorganization	ophthalmology reorganization	ophthalmology reorganization	ophthalmology reorganization	1	kept
Opiate	opiate	opiate	opiate	opiate	1	case_or_format

opiate agonist	opiate agonist	opiate agonist	opiate agonist	opiate agonist	1	kept
opiate antagonist	opiate antagonist	opiate antagonist	opiate antagonist	opiate antagonist	1	kept
Opiate crisis	opiate crisis	opiate crisis	opiate crisis	Opiate crisis	1	kept
opiate receptor	opiate receptor	opiate receptor	opiate receptor	opiate receptor	1	kept
opiate substitution treatment	opiate substitution treatment	opiate substitution treatment	opiate substitution treatment	Opiate Substitution Treatment	1	case_or_format
opiate substitutions treatment	opiate substitutions treatment	opiate substitutions treatment	opiate substitutions treatment	opiate substitutions treatment	1	kept
Opiate treatment program	opiate treatment program	opiate treatment program	opiate treatment program	Opiate treatment program	1	kept
opicapone	opicapone	opicapone	opicapone	opicapone	1	kept
OPINION	opinion	opinion	opinion	Opinion	1	case_or_format
opinion	opinion	opinion	opinion	Opinion	1	case_or_format
Opinion leaders	opinion leaders	opinion leaders	opinion leaders	Opinion leaders	1	kept
opinion survey	opinion survey	opinion survey	opinion survey	opinion survey	1	kept
OPINIONS	opinions	opinions	opinion	Opinion	1	singular
opinions	opinions	opinions	opinion	Opinion	1	singular
Opinión	opini n	opini n	opini n	Opinión	1	kept
Opioid abuse	opioid abuse	opioid abuse	opioid abuse	Opioid abuse	1	kept

opioid addiction	opioid addiction	opioid addiction	opioid addiction	opioid addiction	1	kept
Opioid analgesic	opioid analgesic	opioid analgesic	opioid analgesic	opioid analgesics	1	case_or_format
Opioid analgesics	opioid analgesics	opioid analgesics	opioid analgesic	opioid analgesics	1	singular
opioid antagonists	opioid antagonists	opioid antagonists	opioid antagonists	opioid antagonists	1	kept
opioid consumption	opioid consumption	opioid consumption	opioid consumption	opioid consumption	1	kept
opioid dependence	opioid dependence	opioid dependence	opioid dependence	opioid dependence	1	kept
Opioid dependency	opioid dependency	opioid dependency	opioid dependency	Opioid dependency	1	kept
Opioid Epidemic	opioid epidemic	opioid epidemic	opioid epidemic	Opioid Epidemic	1	kept
opioid epidemic	opioid epidemic	opioid epidemic	opioid epidemic	Opioid Epidemic	1	case_or_format
Opioid induced respiratory depression	opioid induced respiratory depression	opioid induced respiratory depression	opioid induced respiratory depression	opioid-induced respiratory depression	1	case_or_format
opioid induced respiratory depression	opioid induced respiratory depression	opioid induced respiratory depression	opioid induced respiratory depression	opioid-induced respiratory depression	1	case_or_format
Opioid infusion	opioid infusion	opioid infusion	opioid infusion	opioid infusion	1	case_or_format
opioid maintenance therapy	opioid maintenance therapy	opioid maintenance therapy	opioid maintenance therapy	opioid maintenance therapy	1	kept
Opioid misuse	opioid misuse	opioid misuse	opioid misuse	Opioid misuse	1	kept
opioid overdose	opioid overdose	opioid overdose	opioid overdose	opioid overdose	1	kept
Opioid poisoning	opioid poisoning	opioid poisoning	opioid poisoning	Opioid poisoning	1	kept

opioid prescription	opioid prescription	opioid prescription	opioid prescription	opioid prescription	1	kept
Opioid risk assessment	opioid risk assessment	opioid risk assessment	opioid risk assessment	Opioid risk assessment	1	kept
Opioid sparing	opioid sparing	opioid sparing	opioid sparing	opioid sparing	1	case_or_format
Opioid taper	opioid taper	opioid taper	opioid taper	Opioid taper	1	kept
Opioid titration	opioid titration	opioid titration	opioid titration	Opioid titration	1	kept
opioid tolerance	opioid tolerance	opioid tolerance	opioid tolerance	opioid tolerance	1	kept
Opioid Use Disorder	opioid use disorder	opioid use disorder	opioid use disorder	Opioid use disorder	1	case_or_format
Opioid utilization	opioid utilization	opioid utilization	opioid utilization	Opioid utilization	1	kept
OPIOID WITHDRAWAL	opioid withdrawal	opioid withdrawal	opioid withdrawal	opioid withdrawal	1	case_or_format
Opioid withdrawal	opioid withdrawal	opioid withdrawal	opioid withdrawal	opioid withdrawal	1	case_or_format
opioid withdrawal management	opioid withdrawal management	opioid withdrawal management	opioid withdrawal management	opioid withdrawal management	1	kept
Opioid withdrawal symptoms	opioid withdrawal symptoms	opioid withdrawal symptoms	opioid withdrawal symptoms	Opioid withdrawal symptoms	1	kept
opioid withdrawal syndrome	opioid withdrawal syndrome	opioid withdrawal syndrome	opioid withdrawal syndrome	opioid withdrawal syndrome	1	kept
opioid-free anesthesia	opioid free anesthesia	opioid free anesthesia	opioid free anesthesia	opioid-free anesthesia	1	kept
OPIOID-INDUCED CONSTIPATION	opioid induced constipation	opioid induced constipation	opioid induced constipation	opioid induced constipation	1	case_or_format
opioid-induced sedation	opioid induced sedation	opioid induced sedation	opioid induced sedation	opioid-induced sedation	1	kept

Opioid-related disorders	opioid related disorders	opioid related disorders	opioid related disorders	Opioid-Related Disorders	1	case_or_format
Opioid-remifentanil	opioid remifentanil	opioid remifentanil	opioid remifentanil	Opioid-remifentanil	1	kept
opisthotonus	opisthotonus	opisthotonus	opisthotonus	opisthotonus	1	kept
Opium	opium	opium	opium	Opium	1	kept
Opoids	opoids	opoids	opoids	Opoids	1	kept
Opoptosis	opoptosis	opoptosis	opoptosis	Opoptosis	1	kept
opportunistic mycosis	opportunistic mycosis	opportunistic mycosis	opportunistic mycosis	opportunistic mycosis	1	kept
opportunities	opportunities	opportunities	opportunity	Opportunities	1	singular
Opportunity	opportunity	opportunity	opportunity	Opportunities	1	case_or_format
Opportunity Costs	opportunity costs	opportunity costs	opportunity cost	opportunity cost	1	singular
Oppression	oppression	oppression	oppression	Oppression	1	kept
Optic Disk	optic disk	optic disk	optic disk	Optic Disk	1	kept
optic disk	optic disk	optic disk	optic disk	Optic Disk	1	case_or_format
optic nerve atrophy	optic nerve atrophy	optic nerve atrophy	optic nerve atrophy	optic nerve atrophy	1	kept
optic nerve diameter	optic nerve diameter	optic nerve diameter	optic nerve diameter	optic nerve diameter	1	kept
Optic Nerve Diseases	optic nerve diseases	optic nerve diseases	optic nerve disease	optic nerve disease	1	singular

Optic nerve diseases	optic nerve diseases	optic nerve diseases	optic nerve disease	optic nerve disease	1	singular
optic nerve fiber	optic nerve fiber	optic nerve fiber	optic nerve fiber	optic nerve fiber	1	kept
optic nerve glioma	optic nerve glioma	optic nerve glioma	optic nerve glioma	optic nerve glioma	1	kept
optic nerve hypoplasia	optic nerve hypoplasia	optic nerve hypoplasia	optic nerve hypoplasia	optic nerve hypoplasia	1	kept
Optic nerve involvement	optic nerve involvement	optic nerve involvement	optic nerve involvement	Optic nerve involvement	1	kept
Optic nerve sheath	optic nerve sheath	optic nerve sheath	optic nerve sheath	Optic nerve sheath	1	kept
optic nerve sheath	optic nerve sheath	optic nerve sheath	optic nerve sheath	Optic nerve sheath	1	case_or_format
Optic neuropathy	optic neuropathy	optic neuropathy	optic neuropathy	Optic neuropathy	1	kept
optical density	optical density	optical density	optical density	optical density	1	kept
optical detection	optical detection	optical detection	optical detection	optical detection	1	kept
Optical Fibers	optical fibers	optical fibers	optical fibers	Optical Fibers	1	kept
Optical flow techniques	optical flow techniques	optical flow techniques	optical flow techniques	Optical flow techniques	1	kept
Optical flows	optical flows	optical flows	optical flows	Optical flows	1	kept
optical instrumentation	optical instrumentation	optical instrumentation	optical instrumentation	optical instrumentation	1	kept
Optical sensors	optical sensors	optical sensors	optical sensors	Optical sensors	1	kept
optical spectroscopy	optical spectroscopy	optical spectroscopy	optical spectroscopy	optical spectroscopy	1	kept

optics	optics	optics	optics	optics	1	kept
Optimal allocation	optimal allocation	optimal allocation	optimal allocation	Optimal allocation	1	kept
Optimal conditions	optimal conditions	optimal conditions	optimal conditions	Optimal conditions	1	kept
OPTIMAL DOSING REGIMEN	optimal dosing regimen	optimal dosing regimen	optimal dosing regimen	OPTIMAL DOSING REGIMEN	1	kept
Optimal environment	optimal environment	optimal environment	optimal environment	Optimal environment	1	kept
Optimal evaluations	optimal evaluations	optimal evaluations	optimal evaluations	Optimal evaluations	1	kept
Optimal initial care	optimal initial care	optimal initial care	optimal initial care	Optimal initial care	1	kept
Optimal modes	optimal modes	optimal modes	optimal modes	Optimal modes	1	kept
Optimal operating point	optimal operating point	optimal operating point	optimal operating point	Optimal operating point	1	kept
Optimal planning	optimal planning	optimal planning	optimal planning	Optimal planning	1	kept
Optimal schedule	optimal schedule	optimal schedule	optimal schedule	Optimal schedule	1	kept
optimal schedule	optimal schedule	optimal schedule	optimal schedule	Optimal schedule	1	case_or_format
Optimal staffing	optimal staffing	optimal staffing	optimal staffing	Optimal staffing	1	kept
Optimal target	optimal target	optimal target	optimal target	Optimal target	1	kept
optimal treatment	optimal treatment	optimal treatment	optimal treatment	optimal treatment	1	kept
optimisation	optimisation	optimisation	optimisation	Optimisations	1	case_or_format

Optimisation models	optimisation models	optimisation models	optimisation models	Optimisation models	1	kept
OPTIMIZATION	optimization	optimization	optimization	Optimization	1	case_or_format
Optimization method	optimization method	optimization method	optimization method	Optimization method	1	kept
Optimization problems	optimization problems	optimization problems	optimization problems	Optimization problems	1	kept
Optimization research	optimization research	optimization research	optimization research	Optimization research	1	kept
Optimize efficiency	optimize efficiency	optimize efficiency	optimize efficiency	Optimize efficiency	1	kept
optimize pattern	optimize pattern	optimize pattern	optimize pattern	optimize pattern	1	kept
optimum occupancy	optimum occupancy	optimum occupancy	optimum occupancy	optimum occupancy	1	kept
opération Résilience	op ration r silience	op ration r silience	op ration r silience	opération Résilience	1	kept
OR = operating room	or operating room	or operating room	or operating room	OR, operating room	1	case_or_format
OR in health services	or in health services	or in health services	or in health services	OR in health services	1	kept
OR time	or time	or time	or time	OR time	1	kept
OR utilization	or utilization	or utilization	or utilization	OR utilization	1	kept
OR, operating room	or operating room	or operating room	or operating room	OR, operating room	1	kept
ORAL ACETYLCYSTEINE	oral acetylcysteine	oral acetylcysteine	oral acetylcysteine	ORAL ACETYLCYSTEINE	1	kept
Oral and maxillofacial malignancies	oral and maxillofacial malignancies	oral and maxillofacial malignancies	oral and maxillofacial malignancies	Oral and maxillofacial malignancies	1	kept

Oral Assessment	oral assessment	oral assessment	oral assessment	Oral Assessment	1	kept
oral assessment	oral assessment	oral assessment	oral assessment	Oral Assessment	1	case_or_format
Oral assessment scale	oral assessment scale	oral assessment scale	oral assessment scale	Oral assessment scale	1	kept
oral baclofen	oral baclofen	oral baclofen	oral baclofen	oral baclofen	1	kept
Oral bacteria	oral bacteria	oral bacteria	oral bacteria	Oral bacteria	1	kept
oral bacteria	oral bacteria	oral bacteria	oral bacteria	Oral bacteria	1	case_or_format
oral blister	oral blister	oral blister	oral blister	oral blister	1	kept
Oral Care	oral care	oral care	oral care	Oral care	1	case_or_format
oral care frequency	oral care frequency	oral care frequency	oral care frequency	oral care frequency	1	kept
Oral care intervention	oral care intervention	oral care intervention	oral care intervention	Oral care intervention	1	kept
Oral care practice	oral care practice	oral care practice	oral care practice	Oral care practice	1	kept
oral care protocol	oral care protocol	oral care protocol	oral care protocol	oral care protocol	1	kept
oral care quality	oral care quality	oral care quality	oral care quality	oral care quality	1	kept
Oral care solutions	oral care solutions	oral care solutions	oral care solutions	Oral care solutions	1	kept
oral care tools	oral care tools	oral care tools	oral care tools	oral care tools	1	kept
Oral cavity	oral cavity	oral cavity	oral cavity	Oral cavity	1	kept

oral colostrum	oral colostrum	oral colostrum	oral colostrum	oral colostrum	1	kept
oral contraceptive use	oral contraceptive use	oral contraceptive use	oral contraceptive use	oral contraceptive use	1	kept
oral cryotherapy	oral cryotherapy	oral cryotherapy	oral cryotherapy	oral cryotherapy	1	kept
Oral decontamination	oral decontamination	oral decontamination	oral decontamination	Oral decontamination	1	kept
oral dextrose gel	oral dextrose gel	oral dextrose gel	oral dextrose gel	oral dextrose gel	1	kept
ORAL DIETARY-SUPPLEMENTS	oral dietary supplements	oral dietary supplements	oral dietary supplements	ORAL DIETARY-SUPPLEMENTS	1	kept
oral diseases	oral diseases	oral diseases	oral diseases	oral diseases	1	kept
oral dosage forms	oral dosage forms	oral dosage forms	oral dosage forms	oral dosage forms	1	kept
Oral Feeding	oral feeding	oral feeding	oral feeding	Oral feeding	1	case_or_format
Oral feeding readiness	oral feeding readiness	oral feeding readiness	oral feeding readiness	oral feeding readiness	1	case_or_format
Oral feeding skills	oral feeding skills	oral feeding skills	oral feeding skills	Oral feeding skills	1	kept
Oral feedings	oral feedings	oral feedings	oral feeding	Oral feeding	1	singular
Oral Frailty Index 8	oral frailty index 8	oral frailty index 8	oral frailty index 8	Oral Frailty Index 8	1	kept
Oral glucose	oral glucose	oral glucose	oral glucose	ORAL GLUCOSE	1	case_or_format
Oral glucose solution	oral glucose solution	oral glucose solution	oral glucose solution	Oral glucose solution	1	kept
Oral Health Assessment Tool	oral health assessment tool	oral health assessment tool	oral health assessment tool	Oral Health Assessment Tool	1	kept

Oral health care	oral health care	oral health care	oral health care	Oral health care	1	kept
oral Health Care	oral health care	oral health care	oral health care	Oral health care	1	case_or_format
oral health care	oral health care	oral health care	oral health care	Oral health care	1	case_or_format
Oral health care program	oral health care program	oral health care program	oral health care program	Oral health care program	1	kept
Oral Health Status	oral health status	oral health status	oral health status	Oral Health Status	1	kept
Oral healthcare	oral healthcare	oral healthcare	oral healthcare	Oral healthcare	1	kept
oral hygiene care	oral hygiene care	oral hygiene care	oral hygiene care	Oral hygiene care	1	case_or_format
Oral Hygiene Index	oral hygiene index	oral hygiene index	oral hygiene index	Oral Hygiene Index	1	kept
Oral Hygiene Scoring System	oral hygiene scoring system	oral hygiene scoring system	oral hygiene scoring system	Oral Hygiene Scoring System	1	kept
Oral immune therapy	oral immune therapy	oral immune therapy	oral immune therapy	Oral immune therapy	1	kept
oral intake	oral intake	oral intake	oral intake	Oral intake	1	case_or_format
oral intervention	oral intervention	oral intervention	oral intervention	oral intervention	1	kept
oral interviews	oral interviews	oral interviews	oral interviews	oral interviews	1	kept
Oral intubation	oral intubation	oral intubation	oral intubation	Oral intubation	1	kept
Oral malodour	oral malodour	oral malodour	oral malodour	Oral malodour	1	kept
Oral manifestations	oral manifestations	oral manifestations	oral manifestations	Oral manifestations	1	kept

Oral massage	oral massage	oral massage	oral massage	Oral massage	1	kept
oral medication	oral medication	oral medication	oral medication	oral medication	1	kept
Oral medication administration	oral medication administration	oral medication administration	oral medication administration	Oral medication administration	1	kept
ORAL MEDICATIONS	oral medications	oral medications	oral medication	oral medication	1	singular
oral medicine	oral medicine	oral medicine	oral medicine	oral medicine	1	kept
oral medicines	oral medicines	oral medicines	oral medicine	oral medicine	1	singular
ORAL MELATONIN	oral melatonin	oral melatonin	oral melatonin	ORAL MELATONIN	1	kept
Oral motor	oral motor	oral motor	oral motor	Oral motor	1	kept
Oral motor intervention	oral motor intervention	oral motor intervention	oral motor intervention	Oral motor intervention	1	kept
oral motor intervention	oral motor intervention	oral motor intervention	oral motor intervention	Oral motor intervention	1	case_or_format
Oral Motor Stimulation	oral motor stimulation	oral motor stimulation	oral motor stimulation	oral motor stimulation	1	case_or_format
oral mucosa	oral mucosa	oral mucosa	oral mucosa	Oral mucosa	1	case_or_format
oral mucosal pressure injury	oral mucosal pressure injury	oral mucosal pressure injury	oral mucosal pressure injury	oral mucosal pressure injury	1	kept
Oral Mucositis Assessment Scale	oral mucositis assessment scale	oral mucositis assessment scale	oral mucositis assessment scale	Oral Mucositis Assessment Scale	1	kept
oral nutrition	oral nutrition	oral nutrition	oral nutrition	Oral nutrition	1	case_or_format
Oral nutritional supplement	oral nutritional supplement	oral nutritional supplement	oral nutritional supplement	Oral nutritional supplement	1	kept

oral nutritional supplements	oral nutritional supplements	oral nutritional supplements	oral nutritional supplement	Oral nutritional supplement	1	singular
oral pH	oral ph	oral ph	oral ph	oral pH	1	kept
Oral prophylaxis	oral prophylaxis	oral prophylaxis	oral prophylaxis	Oral prophylaxis	1	kept
ORAL RINSE	oral rinse	oral rinse	oral rinse	ORAL RINSE	1	kept
Oral spray	oral spray	oral spray	oral spray	Oral spray	1	kept
Oral squamous cell carcinoma	oral squamous cell carcinoma	oral squamous cell carcinoma	oral squamous cell carcinoma	Oral squamous cell carcinoma	1	kept
Oral sucrose	oral sucrose	oral sucrose	oral sucrose	Oral sucrose	1	kept
Oral suction	oral suction	oral suction	oral suction	Oral suction	1	kept
Oral supportive nutrition	oral supportive nutrition	oral supportive nutrition	oral supportive nutrition	Oral supportive nutrition	1	kept
Oral suspensions	oral suspensions	oral suspensions	oral suspensions	Oral suspensions	1	kept
oral swab	oral swab	oral swab	oral swab	oral swab	1	kept
Oral therapeutic delivery	oral therapeutic delivery	oral therapeutic delivery	oral therapeutic delivery	Oral therapeutic delivery	1	kept
Oral therapy	oral therapy	oral therapy	oral therapy	Oral therapy	1	kept
oral therapy	oral therapy	oral therapy	oral therapy	Oral therapy	1	case_or_format
oral therapy unit	oral therapy unit	oral therapy unit	oral therapy unit	oral therapy unit	1	kept
oral thermometer	oral thermometer	oral thermometer	oral thermometer	oral thermometer	1	kept

Oral tracheal intubation	oral tracheal intubation	oral tracheal intubation	oral tracheal intubation	Oral tracheal intubation	1	kept
Oral-feeding	oral feeding	oral feeding	oral feeding	Oral feeding	1	case_or_format
Orality	orality	orality	orality	Orality	1	kept
Orbit	orbit	orbit	orbit	Orbit	1	kept
orbit disease	orbit disease	orbit disease	orbit disease	orbit disease	1	kept
orbit injury	orbit injury	orbit injury	orbit injury	orbit injury	1	kept
orbit pseudotumor	orbit pseudotumor	orbit pseudotumor	orbit pseudotumor	orbit pseudotumor	1	kept
orbital congestion	orbital congestion	orbital congestion	orbital congestion	orbital congestion	1	kept
orchidopexy	orchidopexy	orchidopexy	orchidopexy	orchidopexy	1	kept
orchiectomy	orchiectomy	orchiectomy	orchiectomy	orchiectomy	1	kept
orchitis	orchitis	orchitis	orchitis	orchitis	1	kept
orciprenaline	orciprenaline	orciprenaline	orciprenaline	orciprenaline	1	kept
Order entry	order entry	order entry	order entry	ORDER ENTRY	1	case_or_format
order review	order review	order review	order review	order review	1	kept
ORDER SET	order set	order set	order set	ORDER SET	1	kept
Order set	order set	order set	order set	ORDER SET	1	case_or_format

order sets	order sets	order sets	order set	ORDER SET	1	singular
order verification	order verification	order verification	order verification	order verification	1	kept
ORDER-ENTRY	order entry	order entry	order entry	ORDER ENTRY	1	case_or_format
Ordering medica-tion	ordering medica tion	ordering medica tion	ordering medica tion	Ordering medica-tion	1	kept
Ordering process	ordering process	ordering process	ordering process	Ordering process	1	kept
ORDERS	orders	orders	orders	ORDERS	1	kept
Orem Self Care Model	orem self care model	orem self care model	orem self care model	Orem self-care model	1	case_or_format
orexin receptor antagonist	orexin receptor antagonist	orexin receptor antagonist	orexin receptor antagonist	orexin receptor antagonist	1	kept
organ blood flow	organ blood flow	organ blood flow	organ blood flow	organ blood flow	1	kept
organ care	organ care	organ care	organ care	organ care	1	kept
organ donation after cardiocirculatory determination of death	organ donation after cardiocirculatory determination of death	organ donation after cardiocirculatory determination of death	organ donation after cardiocirculatory determination of death	organ donation after cardiocirculatory determination of death	1	kept
organ donation after circulatory determination of death	organ donation after circulatory determination of death	organ donation after circulatory determination of death	organ donation after circulatory determination of death	organ donation after circulatory determination of death	1	kept
organ donation resistance	organ donation resistance	organ donation resistance	organ donation resistance	organ donation resistance	1	kept
Organ donor rate	organ donor rate	organ donor rate	organ donor rate	Organ donor rate	1	kept
Organ dysfunction	organ dysfunction	organ dysfunction	organ dysfunction	organ dysfunction	1	case_or_format
ORGAN DYSFUNCTION SYSTEM	organ dysfunction system	organ dysfunction system	organ dysfunction system	ORGAN DYSFUNCTION SYSTEM	1	kept

ORGAN FAILURE	organ failure	organ failure	organ failure	Organ failure	1	case_or_format
organ failure	organ failure	organ failure	organ failure	Organ failure	1	case_or_format
organ harvesting	organ harvesting	organ harvesting	organ harvesting	organ harvesting	1	kept
organ procurement and allocation	organ procurement and allocation	organ procurement and allocation	organ procurement and allocation	organ procurement and allocation	1	kept
organ procurement center	organ procurement center	organ procurement center	organ procurement center	organ procurement center	1	kept
Organ procurement system	organ procurement system	organ procurement system	organ procurement system	Organ procurement system	1	kept
Organ protective management	organ protective management	organ protective management	organ protective management	Organ protective management	1	kept
organ retrieval	organ retrieval	organ retrieval	organ retrieval	Organ retrieval	1	case_or_format
organ size	organ size	organ size	organ size	Organ Size	1	case_or_format
organ space infection	organ space infection	organ space infection	organ space infection	organ space infection	1	kept
Organ support	organ support	organ support	organ support	Organ support	1	kept
organ support	organ support	organ support	organ support	Organ support	1	case_or_format
Organ transplant	organ transplant	organ transplant	organ transplant	Organ transplant	1	kept
organ transplant	organ transplant	organ transplant	organ transplant	Organ transplant	1	case_or_format
Organ transplant coordinators	organ transplant coordinators	organ transplant coordinators	organ transplant coordinators	Organ transplant coordinators	1	kept
organ transplant coordinators	organ transplant coordinators	organ transplant coordinators	organ transplant coordinators	Organ transplant coordinators	1	case_or_format

Organ transplantation barriers	organ transplantation barriers	organ transplantation barriers	organ transplantation barriers	Organ transplantation barriers	1	kept
organ transplantation in general	organ transplantation in general	organ transplantation in general	organ transplantation in general	organ transplantation in general	1	kept
organic brain syndome scale	organic brain syndome scale	organic brain syndome scale	organic brain syndome scale	organic brain syndome scale	1	kept
Organic pollutants	organic pollutants	organic pollutants	organic pollutants	Organic pollutants	1	kept
Organic polymers	organic polymers	organic polymers	organic polymers	Organic polymers	1	kept
organisation des soins	organisation des soins	organisation des soins	organisation des soins	organisation des soins	1	kept
Organisation Of Health Services	organisation of health services	organisation of health services	organisation of health services	organisation of health services	1	case_or_format
Organisation of health services	organisation of health services	organisation of health services	organisation of health services	organisation of health services	1	case_or_format
Organisational	organisational	organisational	organisational	Organisational	1	kept
Organisational behaviour	organisational behaviour	organisational behaviour	organisational behaviour	Organisational behaviour	1	kept
organisational behaviour	organisational behaviour	organisational behaviour	organisational behaviour	Organisational behaviour	1	case_or_format
organisational challenges	organisational challenges	organisational challenges	organisational challenges	organisational challenges	1	kept
Organisational change	organisational change	organisational change	organisational change	Organisational change	1	kept
Organisational characteristics	organisational characteristics	organisational characteristics	organisational characteristics	Organisational characteristics	1	kept
Organisational commitment	organisational commitment	organisational commitment	organisational commitment	Organisational commitment	1	kept
Organisational context	organisational context	organisational context	organisational context	Organisational context	1	kept

organisational ethics	organisational ethics	organisational ethics	organisational ethics	organisational ethics	1	kept
Organisational factors	organisational factors	organisational factors	organisational factors	organisational factors	1	case_or_format
Organisational innovation	organisational innovation	organisational innovation	organisational innovation	Organisational innovation	1	kept
organisational innovation	organisational innovation	organisational innovation	organisational innovation	Organisational innovation	1	case_or_format
Organisational models	organisational models	organisational models	organisational models	Organisational models	1	kept
Organisational policy	organisational policy	organisational policy	organisational policy	Organisational policy	1	kept
Organisational readiness	organisational readiness	organisational readiness	organisational readiness	Organisational readiness	1	kept
Organisational supports	organisational supports	organisational supports	organisational support	organisational support	1	singular
Organisational survey	organisational survey	organisational survey	organisational survey	Organisational survey	1	kept
organisations	organisations	organisations	organisation	Organisation	1	singular
Organism Hydration Status	organism hydration status	organism hydration status	organism hydration status	Organism Hydration Status	1	kept
Organism hydration status	organism hydration status	organism hydration status	organism hydration status	Organism Hydration Status	1	case_or_format
ORGANISMS	organisms	organisms	organisms	organisms	1	case_or_format
Organization And Administration	organization and administration	organization and administration	organization and administration	Organization and Administration	1	case_or_format
Organization and administration	organization and administration	organization and administration	organization and administration	Organization and Administration	1	case_or_format
Organization and management	organization and management	organization and management	organization and management	organization and management	1	case_or_format

organization level	organization level	organization level	organization level	organization level	1	kept
organization of care	organization of care	organization of care	organization of care	Organization of care	1	case_or_format
organizational	organizational	organizational	organizational	Organizational	1	case_or_format
organizational accident model	organizational accident model	organizational accident model	organizational accident model	organizational accident model	1	kept
Organizational assessment	organizational assessment	organizational assessment	organizational assessment	Organizational assessment	1	kept
organizational attributes	organizational attributes	organizational attributes	organizational attributes	organizational attributes	1	kept
Organizational behavior	organizational behavior	organizational behavior	organizational behavior	organizational behavior	1	case_or_format
Organizational burnout	organizational burnout	organizational burnout	organizational burnout	Organizational burnout	1	kept
Organizational change	organizational change	organizational change	organizational change	organizational change	1	case_or_format
organizational changes	organizational changes	organizational changes	organizational change	organizational change	1	singular
organizational citizenship	organizational citizenship	organizational citizenship	organizational citizenship	organizational citizenship	1	kept
Organizational citizenship behavior	organizational citizenship behavior	organizational citizenship behavior	organizational citizenship behavior	ORGANIZATIONAL CITIZENSHIP BEHAVIOR	1	case_or_format
organizational citizenship behavior	organizational citizenship behavior	organizational citizenship behavior	organizational citizenship behavior	ORGANIZATIONAL CITIZENSHIP BEHAVIOR	1	case_or_format
Organizational constraints	organizational constraints	organizational constraints	organizational constraints	Organizational constraints	1	kept
organizational context	organizational context	organizational context	organizational context	organizational context	1	kept
Organizational context factors	organizational context factors	organizational context factors	organizational context factors	Organizational context factors	1	kept

ORGANIZATIONAL CONTROL	organizational control	organizational control	organizational control	ORGANIZATIONAL CONTROL	1	kept
Organizational creativity	organizational creativity	organizational creativity	organizational creativity	Organizational creativity	1	kept
Organizational Cynicism Scale	organizational cynicism scale	organizational cynicism scale	organizational cynicism scale	Organizational Cynicism Scale	1	kept
organizational development	organizational development	organizational development	organizational development	organizational development	1	kept
organizational dynamics	organizational dynamics	organizational dynamics	organizational dynamics	organizational dynamics	1	kept
Organizational efficiency	organizational efficiency	organizational efficiency	organizational efficiency	organizational efficiency	1	case_or_format
organizational ethical climate	organizational ethical climate	organizational ethical climate	organizational ethical climate	organizational ethical climate	1	kept
ORGANIZATIONAL ETHICS	organizational ethics	organizational ethics	organizational ethics	organizational ethics	1	case_or_format
Organizational ethics	organizational ethics	organizational ethics	organizational ethics	organizational ethics	1	case_or_format
organizational framework	organizational framework	organizational framework	organizational framework	organizational framework	1	kept
organizational innovation	organizational innovation	organizational innovation	organizational innovation	Organizational Innovation	1	case_or_format
organizational issue	organizational issue	organizational issue	organizational issue	organizational issue	1	kept
Organizational Justice	organizational justice	organizational justice	organizational justice	Organizational justice	1	case_or_format
Organizational learning	organizational learning	organizational learning	organizational learning	Organizational learning	1	kept
organizational learning	organizational learning	organizational learning	organizational learning	Organizational learning	1	case_or_format
Organizational Life	organizational life	organizational life	organizational life	Organizational Life	1	kept

Organizational model	organizational model	organizational model	organizational model	Organizational model	1	kept
organizational model	organizational model	organizational model	organizational model	Organizational model	1	case_or_format
organizational organizational constraints scale	organizational organizational constraints scale	organizational organizational constraints scale	organizational organizational constraints scale	organizational organizational constraints scale	1	kept
Organizational performance	organizational performance	organizational performance	organizational performance	Organizational performance	1	kept
organizational psychology	organizational psychology	organizational psychology	organizational psychology	Organizational psychology	1	case_or_format
Organizational readiness	organizational readiness	organizational readiness	organizational readiness	Organizational readiness	1	kept
Organizational resilience	organizational resilience	organizational resilience	organizational resilience	Organizational resilience	1	kept
organizational resilience	organizational resilience	organizational resilience	organizational resilience	Organizational resilience	1	case_or_format
organizational retention	organizational retention	organizational retention	organizational retention	organizational retention	1	kept
ORGANIZATIONAL SOCIALIZATION	organizational socialization	organizational socialization	organizational socialization	ORGANIZATIONAL SOCIALIZATION	1	kept
Organizational strategy	organizational strategy	organizational strategy	organizational strategy	Organizational strategy	1	kept
Organizational structure	organizational structure	organizational structure	organizational structure	organizational structure	1	case_or_format
Organizational structures	organizational structures	organizational structures	organizational structure	organizational structure	1	singular
Organizational studies	organizational studies	organizational studies	organizational studies	Organizational studies	1	kept
Organizational Support Questionnaire	organizational support questionnaire	organizational support questionnaire	organizational support questionnaire	Organizational Support Questionnaire	1	kept
organizational trust	organizational trust	organizational trust	organizational trust	organizational trust	1	kept

Organizational Well-being	organizational well being	organizational well being	organizational well being	Organizational Well-being	1	kept
organizations	organizations	organizations	organization	organization	1	singular
ORGANIZED CARE	organized care	organized care	organized care	ORGANIZED CARE	1	kept
Organized stroke care	organized stroke care	organized stroke care	organized stroke care	Organized stroke care	1	kept
organizing tubing	organizing tubing	organizing tubing	organizing tubing	organizing tubing	1	kept
ORGANO-PHOSPHATE	organo phosphate	organo phosphate	organo phosphate	ORGANO-PHOSPHATE	1	kept
Organophosphate mortality	organophosphate mortality	organophosphate mortality	organophosphate mortality	Organophosphate mortality	1	kept
Organophosphate poisoning	organophosphate poisoning	organophosphate poisoning	organophosphate poisoning	Organophosphate Poisoning	1	case_or_format
Organophosphorus compound	organophosphorus compound	organophosphorus compound	organophosphorus compound	Organophosphorus compound	1	kept
Organophosphorus Compounds	organophosphorus compounds	organophosphorus compounds	organophosphorus compound	Organophosphorus compound	1	singular
Organoplatinum Compounds	organoplatinum compounds	organoplatinum compounds	organoplatinum compounds	Organoplatinum Compounds	1	kept
Organosilicon Compounds	organosilicon compounds	organosilicon compounds	organosilicon compounds	Organosilicon Compounds	1	kept
Orientation message	orientation message	orientation message	orientation message	Orientation message	1	kept
Orientee	orientee	orientee	orientee	Orientee	1	kept
Origanum majorana extract	origanum majorana extract	origanum majorana extract	origanum majorana extract	Origanum majorana extract	1	kept
original nonverbal pain scale	original nonverbal pain scale	original nonverbal pain scale	original nonverbal pain scale	original nonverbal pain scale	1	kept

oritavancin	oritavancin	oritavancin	oritavancin	oritavancin	1	kept
ornidazole	ornidazole	ornidazole	ornidazole	ornidazole	1	kept
Oro-motor stimulation	oro motor stimulation	oro motor stimulation	oro motor stimulation	Oro-motor stimulation	1	kept
orofacial dyskinesia	orofacial dyskinesia	orofacial dyskinesia	orofacial dyskinesia	orofacial dyskinesia	1	kept
orofacial epiparoxysm	orofacial epiparoxysm	orofacial epiparoxysm	orofacial epiparoxysm	orofacial epiparoxysm	1	kept
Orogastric	orogastric	orogastric	orogastric	Orogastric	1	kept
Oromotor	oromotor	oromotor	oromotor	Oromotor	1	kept
Oromotor restriction	oromotor restriction	oromotor restriction	oromotor restriction	Oromotor restriction	1	kept
Oromucosal adhesive paste	oromucosal adhesive paste	oromucosal adhesive paste	oromucosal adhesive paste	Oromucosal adhesive paste	1	kept
oropharyngeal cancer	oropharyngeal cancer	oropharyngeal cancer	oropharyngeal cancer	oropharyngeal cancer	1	kept
OROPHARYNGEAL DYSPHAGIA	oropharyngeal dysphagia	oropharyngeal dysphagia	oropharyngeal dysphagia	oropharyngeal dysphagia	1	case_or_format
oropharyngeal immune therapy	oropharyngeal immune therapy	oropharyngeal immune therapy	oropharyngeal immune therapy	oropharyngeal immune therapy	1	kept
Oropharyngeal Neoplasms	oropharyngeal neoplasms	oropharyngeal neoplasms	oropharyngeal neoplasms	Oropharyngeal Neoplasms	1	kept
Oropharyngeal sampling	oropharyngeal sampling	oropharyngeal sampling	oropharyngeal sampling	Oropharyngeal sampling	1	kept
oropharyngeal therapy	oropharyngeal therapy	oropharyngeal therapy	oropharyngeal therapy	oropharyngeal therapy	1	kept
oropharynx airway	oropharynx airway	oropharynx airway	oropharynx airway	oropharynx airway	1	kept

oropharynx cancer	oropharynx cancer	oropharynx cancer	oropharynx cancer	oropharynx cancer	1	kept
oropharynx tumor	oropharynx tumor	oropharynx tumor	oropharynx tumor	oropharynx tumor	1	kept
Orotracheal Intubation	orotracheal intubation	orotracheal intubation	orotracheal intubation	Orotracheal intubation	1	case_or_format
orotracheal intubation	orotracheal intubation	orotracheal intubation	orotracheal intubation	Orotracheal intubation	1	case_or_format
orthoclone okt	orthoclone okt	orthoclone okt	orthoclone okt	orthoclone okt	1	kept
orthogeriatric pathway	orthogeriatric pathway	orthogeriatric pathway	orthogeriatric pathway	orthogeriatric pathway	1	kept
orthogeriatrics	orthogeriatrics	orthogeriatrics	orthogeriatrics	orthogeriatrics	1	kept
orthopaedic	orthopaedic	orthopaedic	orthopaedic	Orthopaedic	1	case_or_format
Orthopaedic and trauma hospital care	orthopaedic and trauma hospital care	orthopaedic and trauma hospital care	orthopaedic and trauma hospital care	Orthopaedic and trauma hospital care	1	kept
Orthopaedic and trauma surgery	orthopaedic and trauma surgery	orthopaedic and trauma surgery	orthopaedic and trauma surgery	Orthopaedic and trauma surgery	1	kept
Orthopaedic doctor	orthopaedic doctor	orthopaedic doctor	orthopaedic doctor	Orthopaedic doctor	1	kept
Orthopaedic oncology	orthopaedic oncology	orthopaedic oncology	orthopaedic oncology	Orthopaedic oncology	1	kept
orthopaedic redeployment	orthopaedic redeployment	orthopaedic redeployment	orthopaedic redeployment	orthopaedic redeployment	1	kept
orthopaedic specialty hospital	orthopaedic specialty hospital	orthopaedic specialty hospital	orthopaedic specialty hospital	orthopaedic specialty hospital	1	kept
orthopaedic surgery	orthopaedic surgery	orthopaedic surgery	orthopaedic surgery	orthopaedic surgery	1	kept
Orthopaedics	orthopaedics	orthopaedics	orthopaedic	Orthopaedic	1	singular

orthopaedics	orthopaedics	orthopaedics	orthopaedic	Orthopaedic	1	singular
Orthopedic Equipment	orthopedic equipment	orthopedic equipment	orthopedic equipment	Orthopedic Equipment	1	kept
orthopedic equipment	orthopedic equipment	orthopedic equipment	orthopedic equipment	Orthopedic Equipment	1	case_or_format
orthopedic implant	orthopedic implant	orthopedic implant	orthopedic implant	orthopedic implant	1	kept
orthopedic specialist	orthopedic specialist	orthopedic specialist	orthopedic specialist	orthopedic specialist	1	kept
Orthopedic Surgeons	orthopedic surgeons	orthopedic surgeons	orthopedic surgeon	orthopedic surgeon	1	singular
Orthopedics procedures	orthopedics procedures	orthopedics procedures	orthopedics procedures	Orthopedics procedures	1	kept
Orthosis	orthosis	orthosis	orthosis	orthosis	1	case_or_format
Orthotic brace	orthotic brace	orthotic brace	orthotic brace	Orthotic brace	1	kept
Orthotopic liver transplantation	orthotopic liver transplantation	orthotopic liver transplantation	orthotopic liver transplantation	orthotopic liver transplantation	1	case_or_format
orudis kt	orudis kt	orudis kt	orudis kt	orudis kt	1	kept
Oryza	oryza	oryza	oryza	Oryza	1	kept
OSA	osa	osa	osa	OSA	1	kept
OSAHS	osahs	osahs	osahs	OSAHS	1	kept
Osaka [Kinki]	osaka kinki	osaka kinki	osaka kinki	Osaka [Kinki]	1	kept
OSCILLATION	oscillation	oscillation	oscillation	OSCILLATION	1	kept

oscillation	oscillation	oscillation	oscillation	OSCILLATION	1	case_or_format
Oscillibacter ruminantium	oscillibacter ruminantium	oscillibacter ruminantium	oscillibacter ruminantium	Oscillibacter ruminantium	1	kept
Oscillometry	oscillometry	oscillometry	oscillometry	oscillometry	1	case_or_format
Oseltamivir phosphate capsule	oseltamivir phosphate capsule	oseltamivir phosphate capsule	oseltamivir phosphate capsule	Oseltamivir phosphate capsule	1	kept
Oslo social support scale	oslo social support scale	oslo social support scale	oslo social support scale	Oslo social support scale	1	kept
Osmoregulation	osmoregulation	osmoregulation	osmoregulation	Osmoregulation	1	kept
osmoregulation	osmoregulation	osmoregulation	osmoregulation	Osmoregulation	1	case_or_format
Osmosis	osmosis	osmosis	osmosis	osmosis	1	case_or_format
osmotic agent	osmotic agent	osmotic agent	osmotic agent	osmotic agent	1	kept
Osmotic demyelination syndrome	osmotic demyelination syndrome	osmotic demyelination syndrome	osmotic demyelination syndrome	Osmotic demyelination syndrome	1	kept
osmotic demyelination syndrome	osmotic demyelination syndrome	osmotic demyelination syndrome	osmotic demyelination syndrome	Osmotic demyelination syndrome	1	case_or_format
osmotic diuresis	osmotic diuresis	osmotic diuresis	osmotic diuresis	osmotic diuresis	1	kept
osmotic therapy	osmotic therapy	osmotic therapy	osmotic therapy	osmotic therapy	1	kept
Ospitalized	ospitalized	ospitalized	ospitalized	Ospitalized	1	kept
osteitis	osteitis	osteitis	osteitis	osteitis	1	kept
osteocalcin	osteocalcin	osteocalcin	osteocalcin	osteocalcin	1	kept

Osteochondrodysplasias	osteochondrodysplasias	osteochondrodysplasias	osteochondrodysplasias	Osteochondrodysplasias	1	kept
osteocutaneous	osteocutaneous	osteocutaneous	osteocutaneous	osteocutaneous	1	kept
Osteogenesis Imperfecta	osteogenesis imperfecta	osteogenesis imperfecta	osteogenesis imperfecta	osteogenesis imperfecta	1	case_or_format
Osteogenesis, Distraction	osteogenesis distraction	osteogenesis distraction	osteogenesis distraction	Osteogenesis, Distraction	1	kept
Osteonecrosis	osteonecrosis	osteonecrosis	osteonecrosis	Osteonecrosis	1	kept
osteopathic physician	osteopathic physician	osteopathic physician	osteopathic physician	osteopathic physician	1	kept
Osteoporosis	osteoporosis	osteoporosis	osteoporosis	osteoporosis	1	case_or_format
Osteoporotic fractures	osteoporotic fractures	osteoporotic fractures	osteoporotic fractures	Osteoporotic Fractures	1	case_or_format
Osteotomy	osteotomy	osteotomy	osteotomy	Osteotomy	1	kept
Osteotomy, Sagittal Split Ramus	osteotomy sagittal split ramus	osteotomy sagittal split ramus	osteotomy sagittal split ramus	Osteotomy, Sagittal Split Ramus	1	kept
Ostracism	ostracism	ostracism	ostracism	Ostracism	1	kept
Oswestry Low Back Pain and Disability Questionnaire	oswestry low back pain and disability questionnaire	oswestry low back pain and disability questionnaire	oswestry low back pain and disability questionnaire	Oswestry Low Back Pain and Disability Questionnaire	1	kept
OTAS rating scale	otas rating scale	otas rating scale	otas rating scale	OTAS rating scale	1	kept
othering	othering	othering	othering	othering	1	kept
Otherness	otherness	otherness	otherness	Otherness	1	kept
otherness function	otherness function	otherness function	otherness function	otherness function	1	kept

other—zero level	other zero level	other zero level	other zero level	other—zero level	1	kept
Otitis Media	otitis media	otitis media	otitis media	otitis media	1	case_or_format
otoacoustic emission	otoacoustic emission	otoacoustic emission	otoacoustic emission	otoacoustic emission	1	kept
OTOACOUSTIC EMISSIONS	otoacoustic emissions	otoacoustic emissions	otoacoustic emission	otoacoustic emission	1	singular
otoacoustic emissions	otoacoustic emissions	otoacoustic emissions	otoacoustic emission	otoacoustic emission	1	singular
Otoacoustic Emissions, Spontaneous	otoacoustic emissions spontaneous	otoacoustic emissions spontaneous	otoacoustic emissions spontaneous	Otoacoustic Emissions, Spontaneous	1	kept
otolaryngo urology unit	otolaryngo urology unit	otolaryngo urology unit	otolaryngo urology unit	otolaryngo urology unit	1	kept
Otoloryngology	otoloryngology	otoloryngology	otoloryngology	Otoloryngology	1	kept
Otorrinolaringología	otorrinolaringolog a	otorrinolaringolog a	otorrinolaringolog a	Otorrinolaringología	1	kept
Ototoxicity	ototoxicity	ototoxicity	ototoxicity	ototoxicity	1	case_or_format
ottawa crisis resource management global rating scale	ottawa crisis resource management global rating scale	ottawa crisis resource management global rating scale	ottawa crisis resource management global rating scale	ottawa crisis resource management global rating scale	1	kept
Otus	otus	otus	otus	Otus	1	kept
Ouarzazate province	ouarzazate province	ouarzazate province	ouarzazate province	Ouarzazate province	1	kept
Out -of -hospital cardiac arrest	out of hospital cardiac arrest	out of hospital cardiac arrest	out of hospital cardiac arrest	out of hospital cardiac arrest	1	case_or_format
out of hospital care	out of hospital care	out of hospital care	out of hospital care	out of hospital care	1	kept
out of hospital emergency care	out of hospital emergency care	out of hospital emergency care	out of hospital emergency care	out of hospital emergency care	1	kept

Out of scope event	out of scope event	out of scope event	out of scope event	Out of scope event	1	kept
Out-of-hospital arrest	out of hospital arrest	out of hospital arrest	out of hospital arrest	Out-of-hospital arrest	1	kept
out-of-hospital birth	out of hospital birth	out of hospital birth	out of hospital birth	out-of-hospital birth	1	kept
Out-of-hospital ventilation	out of hospital ventilation	out of hospital ventilation	out of hospital ventilation	Out-of-hospital ventilation	1	kept
Out-of-hours	out of hours	out of hours	out of hours	Out-of-hours	1	kept
out-of-hours	out of hours	out of hours	out of hours	Out-of-hours	1	case_or_format
Out-ofhospital cardiac arrest	out ofhospital cardiac arrest	out ofhospital cardiac arrest	out ofhospital cardiac arrest	Out-ofhospital cardiac arrest	1	kept
outbreak detection	outbreak detection	outbreak detection	outbreak detection	outbreak detection	1	kept
Outbreak investigation	outbreak investigation	outbreak investigation	outbreak investigation	Outbreak investigation	1	kept
Outbreak management	outbreak management	outbreak management	outbreak management	Outbreak management	1	kept
Outbreak multidrug-resistant bacteria	outbreak multidrug resistant bacteria	outbreak multidrug resistant bacteria	outbreak multidrug resistant bacteria	Outbreak multidrug-resistant bacteria	1	kept
Outbreak simulation	outbreak simulation	outbreak simulation	outbreak simulation	Outbreak simulation	1	kept
OUTBREAKS	outbreaks	outbreaks	outbreak	Outbreak	1	singular
Outcome assessment in Healthcare	outcome assessment in healthcare	outcome assessment in healthcare	outcome assessment in healthcare	Outcome assessment in Healthcare	1	kept
outcome at 28 days	outcome at 28 days	outcome at 28 days	outcome at 28 days	outcome at 28 days	1	kept
outcome evaluation	outcome evaluation	outcome evaluation	outcome evaluation	outcome evaluation	1	kept

outcome measure	outcome measure	outcome measure	outcome measure	outcome measures	1	case_or_format
Outcome measurement	outcome measurement	outcome measurement	outcome measurement	Outcome measurement	1	kept
outcome of early versus late tracheostomy	outcome of early versus late tracheostomy	outcome of early versus late tracheostomy	outcome of early versus late tracheostomy	outcome of early versus late tracheostomy	1	kept
Outcome predictor	outcome predictor	outcome predictor	outcome predictor	Outcome predictor	1	kept
Outcome present test model	outcome present test model	outcome present test model	outcome present test model	Outcome present test model	1	kept
outcome reporting	outcome reporting	outcome reporting	outcome reporting	outcome reporting	1	kept
Outcome research	outcome research	outcome research	outcome research	Outcome research	1	kept
OUTCOME SCORE	outcome score	outcome score	outcome score	OUTCOME SCORE	1	kept
outcome studies	outcome studies	outcome studies	outcome studies	outcome studies	1	kept
Outcome variables	outcome variables	outcome variables	outcome variable	outcome variable	1	singular
Outcomes analysis	outcomes analysis	outcomes analysis	outcomes analysis	Outcomes analysis	1	kept
outcomes assessment	outcomes assessment	outcomes assessment	outcomes assessment	Outcomes assessment	1	case_or_format
outcomes database	outcomes database	outcomes database	outcomes database	outcomes database	1	kept
Outcomes management	outcomes management	outcomes management	outcomes management	Outcomes management	1	kept
outcomes measurements	outcomes measurements	outcomes measurements	outcomes measurements	outcomes measurements	1	kept
Outcomes of care	outcomes of care	outcomes of care	outcomes of care	Outcomes of care	1	kept

outcomes of care	outcomes of care	outcomes of care	outcomes of care	Outcomes of care	1	case_or_format
Outcomes of critical illness	outcomes of critical illness	outcomes of critical illness	outcomes of critical illness	Outcomes of critical illness	1	kept
outcomes of high-risk infants	outcomes of high risk infants	outcomes of high risk infants	outcomes of high risk infants	outcomes of high-risk infants	1	kept
Outcomes of neonates	outcomes of neonates	outcomes of neonates	outcomes of neonates	Outcomes of neonates	1	kept
outer membrane protein	outer membrane protein	outer membrane protein	outer membrane protein	Outer membrane protein	1	case_or_format
Outer membrane protein A	outer membrane protein a	outer membrane protein a	outer membrane protein a	Outer membrane protein A	1	kept
OUTPUT	output	output	output	OUTPUT	1	kept
Output feedback sliding mode	output feedback sliding mode	output feedback sliding mode	output feedback sliding mode	Output feedback sliding mode	1	kept
OUTREACH	outreach	outreach	outreach	outreach	1	case_or_format
Outreach team	outreach team	outreach team	outreach team	Outreach team	1	kept
Outsole material	outsole material	outsole material	outsole material	Outsole material	1	kept
Outsourced cleaning services	outsourced cleaning services	outsourced cleaning services	outsourced cleaning services	Outsourced cleaning services	1	kept
Outsourced Services	outsourced services	outsourced services	outsourced services	Outsourced Services	1	kept
ovarian neoplasms	ovarian neoplasms	ovarian neoplasms	ovarian neoplasms	Ovarian Neoplasms	1	case_or_format
Ovarian Reserve	ovarian reserve	ovarian reserve	ovarian reserve	Ovarian Reserve	1	kept
ovarian torsion	ovarian torsion	ovarian torsion	ovarian torsion	ovarian torsion	1	kept

ovariectomy	ovariectomy	ovariectomy	ovariectomy	ovariectomy	1	kept
ovary cyst	ovary cyst	ovary cyst	ovary cyst	ovary cyst	1	kept
ovary torsion	ovary torsion	ovary torsion	ovary torsion	ovary torsion	1	kept
ovary tumor	ovary tumor	ovary tumor	ovary tumor	ovary tumor	1	kept
Over	over	over	over	Over	1	kept
Over infusion	over infusion	over infusion	over infusion	Over infusion	1	kept
over the head position	over the head position	over the head position	over the head position	over the head position	1	kept
Overactive bladder	overactive bladder	overactive bladder	overactive bladder	Overactive bladder	1	kept
overactive bladder	overactive bladder	overactive bladder	overactive bladder	Overactive bladder	1	case_or_format
Overall survival	overall survival	overall survival	overall survival	overall survival	1	case_or_format
overburdened	overburdened	overburdened	overburdened	overburdened	1	kept
Overcoming exhaustion	overcoming exhaustion	overcoming exhaustion	overcoming exhaustion	Overcoming exhaustion	1	kept
overcoming exhaustion	overcoming exhaustion	overcoming exhaustion	overcoming exhaustion	Overcoming exhaustion	1	case_or_format
Overcommitment	overcommitment	overcommitment	overcommitment	Overcommitment	1	kept
overdiagnosis	overdiagnosis	overdiagnosis	overdiagnosis	overdiagnosis	1	kept
Overfeeding	overfeeding	overfeeding	overfeeding	Overfeeding	1	kept

overfeeding	overfeeding	overfeeding	overfeeding	Overfeeding	1	case_or_format
overhead lift system	overhead lift system	overhead lift system	overhead lift system	overhead lift system	1	kept
overnight staffing	overnight staffing	overnight staffing	overnight staffing	overnight staffing	1	kept
overprescribing	overprescribing	overprescribing	overprescribing	overprescribing	1	kept
overseas recruitment	overseas recruitment	overseas recruitment	overseas recruitment	overseas recruitment	1	kept
OVERSEDATION	oversedation	oversedation	oversedation	OVERSEDATION	1	kept
oversedation	oversedation	oversedation	oversedation	OVERSEDATION	1	case_or_format
Overt observation	overt observation	overt observation	overt observation	Overt observation	1	kept
overt observation	overt observation	overt observation	overt observation	Overt observation	1	case_or_format
OVERTIME	overtime	overtime	overtime	overtime	1	case_or_format
overtime work	overtime work	overtime work	overtime work	overtime work	1	kept
overtreatment	overtreatment	overtreatment	overtreatment	overtreatment	1	kept
Overuse	overuse	overuse	overuse	Overuse	1	kept
overview	overview	overview	overview	overview	1	kept
Own experience	own experience	own experience	own experience	Own experience	1	kept
OWN NAME	own name	own name	own name	OWN NAME	1	kept

OXA 1 gene	oxa 1 gene	oxa 1 gene	oxa 1 gene	OXA 1 gene	1	kept
OXA-181 Escherichia coli	oxa 181 escherichia coli	oxa 181 escherichia coli	oxa 181 escherichia coli	OXA-181 Escherichia coli	1	kept
oxacillinase	oxacillinase	oxacillinase	oxacillinase	oxacillinase	1	kept
oxalate	oxalate	oxalate	oxalate	oxalate	1	kept
Oxalates	oxalates	oxalates	oxalate	oxalate	1	singular
oxalic acid	oxalic acid	oxalic acid	oxalic acid	oxalic acid	1	kept
oxalic acid derivative	oxalic acid derivative	oxalic acid derivative	oxalic acid derivative	oxalic acid derivative	1	kept
oxazine derivative	oxazine derivative	oxazine derivative	oxazine derivative	oxazine derivative	1	kept
Oxazines	oxazines	oxazines	oxazines	Oxazines	1	kept
oxcarbazepine	oxcarbazepine	oxcarbazepine	oxcarbazepine	oxcarbazepine	1	kept
Oxford Knee Score	oxford knee score	oxford knee score	oxford knee score	Oxford Knee Score	1	kept
Oxford scale	oxford scale	oxford scale	oxford scale	Oxford scale	1	kept
oxicam derivative	oxicam derivative	oxicam derivative	oxicam derivative	oxicam derivative	1	kept
Oxidants	oxidants	oxidants	oxidants	Oxidants	1	kept
Oxidants, Photochemical	oxidants photochemical	oxidants photochemical	oxidants photochemical	Oxidants, Photochemical	1	kept
OXIDATION	oxidation	oxidation	oxidation	OXIDATION	1	kept

oxidation	oxidation	oxidation	oxidation	OXIDATION	1	case_or_format
oxidative damage	oxidative damage	oxidative damage	oxidative damage	oxidative damage	1	kept
oxidative phosphorylation	oxidative phosphorylation	oxidative phosphorylation	oxidative phosphorylation	oxidative phosphorylation	1	kept
oxide	oxide	oxide	oxide	Oxides	1	case_or_format
oxidizing agent	oxidizing agent	oxidizing agent	oxidizing agent	oxidizing agent	1	kept
oxidoreductase	oxidoreductase	oxidoreductase	oxidoreductase	oxidoreductase	1	kept
Oxigenação por Membrana Extracorpórea	oxigena o por membrana extracorp rea	oxigena o por membrana extracorp rea	oxigena o por membrana extracorp rea	Oxigenação por Membrana Extracorpórea	1	kept
oxprenolol	oxprenolol	oxprenolol	oxprenolol	oxprenolol	1	kept
oxxa syrup	oxxa syrup	oxxa syrup	oxxa syrup	oxxa syrup	1	kept
oxy ir	oxy ir	oxy ir	oxy ir	oxy ir	1	kept
oxybate sodium	oxybate sodium	oxybate sodium	oxybate sodium	oxybate sodium	1	kept
oxybuprocaine	oxybuprocaine	oxybuprocaine	oxybuprocaine	oxybuprocaine	1	kept
oxybutynin	oxybutynin	oxybutynin	oxybutynin	oxybutynin	1	kept
oxycodone plus paracetamol	oxycodone plus paracetamol	oxycodone plus paracetamol	oxycodone plus paracetamol	oxycodone plus paracetamol	1	kept
oxygen administration kit	oxygen administration kit	oxygen administration kit	oxygen administration kit	oxygen administration kit	1	kept
Oxygen administration safety	oxygen administration safety	oxygen administration safety	oxygen administration safety	Oxygen administration safety	1	kept

oxygen analyzer	oxygen analyzer	oxygen analyzer	oxygen analyzer	oxygen analyzer	1	kept
oxygen and carbon dioxide partial pressure	oxygen and carbon dioxide partial pressure	oxygen and carbon dioxide partial pressure	oxygen and carbon dioxide partial pressure	oxygen and carbon dioxide partial pressure	1	kept
Oxygen consumption	oxygen consumption	oxygen consumption	oxygen consumption	oxygen consumption	1	case_or_format
Oxygen control	oxygen control	oxygen control	oxygen control	Oxygen control	1	kept
oxygen cylinder	oxygen cylinder	oxygen cylinder	oxygen cylinder	oxygen cylinder	1	kept
Oxygen delivery devices	oxygen delivery devices	oxygen delivery devices	oxygen delivery device	oxygen delivery device	1	singular
oxygen desaturation (A	oxygen desaturation a	oxygen desaturation a	oxygen desaturation a	oxygen desaturation (A	1	kept
oxygen desaturation index	oxygen desaturation index	oxygen desaturation index	oxygen desaturation index	oxygen desaturation index	1	kept
oxygen dissociation curve	oxygen dissociation curve	oxygen dissociation curve	oxygen dissociation curve	oxygen dissociation curve	1	kept
Oxygen management	oxygen management	oxygen management	oxygen management	Oxygen management	1	kept
Oxygen masks	oxygen masks	oxygen masks	oxygen mask	oxygen mask	1	singular
oxygen partial pressure	oxygen partial pressure	oxygen partial pressure	oxygen partial pressure	oxygen partial pressure	1	kept
Oxygen practices	oxygen practices	oxygen practices	oxygen practices	Oxygen practices	1	kept
oxygen saturation level	oxygen saturation level	oxygen saturation level	oxygen saturation level	oxygen saturation level	1	kept
Oxygen saturation monitoring	oxygen saturation monitoring	oxygen saturation monitoring	oxygen saturation monitoring	oxygen saturation monitoring	1	case_or_format
oxygen saturation: PaO <sub>2<	oxygen saturation pao sub 2	oxygen saturation pao sub 2	oxygen saturation pao sub 2	oxygen saturation: PaO <sub>2<	1	kept

Oxygen support	oxygen support	oxygen support	oxygen support	Oxygen support	1	kept
Oxygen therapy, unemployment, uncertainty	oxygen therapy unemployment uncertainty	oxygen therapy unemployment uncertainty	oxygen therapy unemployment uncertainty	Oxygen therapy, unemployment, uncertainty	1	kept
oxygen tissue level	oxygen tissue level	oxygen tissue level	oxygen tissue level	oxygen tissue level	1	kept
oxygen tissue saturation	oxygen tissue saturation	oxygen tissue saturation	oxygen tissue saturation	oxygen tissue saturation	1	kept
oxygen treatment	oxygen treatment	oxygen treatment	oxygen treatment	oxygen treatment	1	kept
oxygen use	oxygen use	oxygen use	oxygen use	oxygen use	1	kept
OXYGEN-THERAPY	oxygen therapy	oxygen therapy	oxygen therapy	oxygen therapy	1	case_or_format
Oxygenated blood	oxygenated blood	oxygenated blood	oxygenated blood	Oxygenated blood	1	kept
OXYGENATION	oxygenation	oxygenation	oxygenation	oxygenation	1	case_or_format
Oxygenator failure	oxygenator failure	oxygenator failure	oxygenator failure	Oxygenator failure	1	kept
Oxygenators	oxygenators	oxygenators	oxygenator	oxygenator	1	singular
oxygen‐related therapy	oxygen related therapy	oxygen related therapy	oxygen related therapy	oxygen‐related therapy	1	kept
Oxyhemoglobins	oxyhemoglobins	oxyhemoglobins	oxyhemoglobins	Oxyhemoglobins	1	kept
oxymetazoline	oxymetazoline	oxymetazoline	oxymetazoline	oxymetazoline	1	kept
Oxymetry	oxymetry	oxymetry	oxymetry	Oxymetry	1	kept
oxymorphone	oxymorphone	oxymorphone	oxymorphone	oxymorphone	1	kept

oxytetracycline	oxytetracycline	oxytetracycline	oxytetracycline	oxytetracycline	1	kept
oxytocin augmentation	oxytocin augmentation	oxytocin augmentation	oxytocin augmentation	oxytocin augmentation	1	kept
oxytocin checklist	oxytocin checklist	oxytocin checklist	oxytocin checklist	oxytocin checklist	1	kept
oxytocin receptor	oxytocin receptor	oxytocin receptor	oxytocin receptor	oxytocin receptor	1	kept
ozonated water mouthwash	ozonated water mouthwash	ozonated water mouthwash	ozonated water mouthwash	ozonated water mouthwash	1	kept
p-dichlorobenzene	p dichlorobenzene	p dichlorobenzene	p dichlorobenzene	p-dichlorobenzene	1	kept
P-values	p values	p values	p values	P-values	1	kept
p16 levels	p16 levels	p16 levels	p16 levels	p16 levels	1	kept
P2-mask	p2 mask	p2 mask	p2 mask	P2-mask	1	kept
Pabon Lasso Mode	pabon lasso mode	pabon lasso mode	pabon lasso mode	Pabon Lasso Mode	1	kept
pabrinex	pabrinex	pabrinex	pabrinex	pabrinex	1	kept
Pacemaker failure	pacemaker failure	pacemaker failure	pacemaker failure	Pacemaker failure	1	kept
pacemaker failure	pacemaker failure	pacemaker failure	pacemaker failure	Pacemaker failure	1	case_or_format
pacemaker implantation	pacemaker implantation	pacemaker implantation	pacemaker implantation	pacemaker implantation	1	kept
Pacemakers	pacemakers	pacemakers	pacemaker	pacemaker	1	singular
Paciente	paciente	paciente	paciente	Paciente	1	kept

Paciente crítico	paciente cr tico	paciente cr tico	paciente cr tico	Paciente crítico	1	kept
Pacientes	pacientes	pacientes	paciente	Paciente	1	singular
Pacientes Internados	pacientes internados	pacientes internados	pacientes internados	Pacientes Internados	1	kept
Pacientes no comunicativos	pacientes no comunicativos	pacientes no comunicativos	pacientes no comunicativos	Pacientes no comunicativos	1	kept
Pacientes traqueostomizados	pacientes traqueostomizados	pacientes traqueostomizados	pacientes traqueostomizados	Pacientes traqueostomizados	1	kept
Pacific Islands	pacific islands	pacific islands	pacific islands	Pacific islands	1	case_or_format
Pacifier	pacifier	pacifier	pacifier	Pacifiers	1	case_or_format
pacifiers	pacifiers	pacifiers	pacifier	Pacifiers	1	singular
pacing & electrophysiology	pacing and electrophysiology	pacing and electrophysiology	pacing and electrophysiology	pacing & electrophysiology	1	kept
package insert	package insert	package insert	package insert	package insert	1	kept
Packaging	packaging	packaging	packaging	packaging	1	case_or_format
packing	packing	packing	packing	packing	1	kept
PACS	pacs	pacs	pacs	PACS	1	kept
PACT	pact	pact	pact	PACT	1	kept
Pacu	pacu	pacu	pacu	PACU	1	case_or_format
PACU = post-anesthesia care unit	pacu post anesthesia care unit	pacu post anesthesia care unit	pacu post anesthesia care unit	PACU = post-anesthesia care unit	1	kept

PACU length of stay	pacu length of stay	pacu length of stay	pacu length of stay	PACU length of stay	1	kept
PACU resource optimization	pacu resource optimization	pacu resource optimization	pacu resource optimization	PACU resource optimization	1	kept
PAD assessment	pad assessment	pad assessment	pad assessment	PAD assessment	1	kept
PAD Bundle	pad bundle	pad bundle	pad bundle	PAD Bundle	1	kept
PAD management	pad management	pad management	pad management	PAD management	1	kept
PADIS guidelines	padis guidelines	padis guidelines	padis guidelines	PADIS guidelines	1	kept
Padres	padres	padres	padres	Padres	1	kept
Padrão de cuidado	padr o de cuidado	padr o de cuidado	padr o de cuidado	Padrão de cuidado	1	kept
PAED scale	paed scale	paed scale	paed scale	PAED scale	1	kept
paediatric A&E and ambulatory care	paediatric a and e and ambulatory care	paediatric a and e and ambulatory care	paediatric a and e and ambulatory care	paediatric A&E and ambulatory care	1	kept
Paediatric advanced warning score	paediatric advanced warning score	paediatric advanced warning score	paediatric advanced warning score	Paediatric advanced warning score	1	kept
paediatric cardiac arrest	paediatric cardiac arrest	paediatric cardiac arrest	paediatric cardiac arrest	paediatric cardiac arrest	1	kept
Paediatric cardiac surgery	paediatric cardiac surgery	paediatric cardiac surgery	paediatric cardiac surgery	Paediatric cardiac surgery	1	kept
paediatric cardiac surgery	paediatric cardiac surgery	paediatric cardiac surgery	paediatric cardiac surgery	Paediatric cardiac surgery	1	case_or_format
Paediatric cardiac surgical wound care practices	paediatric cardiac surgical wound care practices	paediatric cardiac surgical wound care practices	paediatric cardiac surgical wound care practices	Paediatric cardiac surgical wound care practices	1	kept
Paediatric care	paediatric care	paediatric care	paediatric care	paediatric care	1	case_or_format

Paediatric cuff pressure monitoring	paediatric cuff pressure monitoring	paediatric cuff pressure monitoring	paediatric cuff pressure monitoring	Paediatric cuff pressure monitoring	1	kept
paediatric cuff pressure monitoring	paediatric cuff pressure monitoring	paediatric cuff pressure monitoring	paediatric cuff pressure monitoring	Paediatric cuff pressure monitoring	1	case_or_format
Paediatric Delirium	paediatric delirium	paediatric delirium	paediatric delirium	paediatric delirium	1	case_or_format
Paediatric Early Warning	paediatric early warning	paediatric early warning	paediatric early warning	Paediatric Early Warning	1	kept
Paediatric early warning assessment tools	paediatric early warning assessment tools	paediatric early warning assessment tools	paediatric early warning assessment tools	Paediatric early warning assessment tools	1	kept
Paediatric early warning score	paediatric early warning score	paediatric early warning score	paediatric early warning score	Paediatric early warning score	1	kept
paediatric early warning score	paediatric early warning score	paediatric early warning score	paediatric early warning score	Paediatric early warning score	1	case_or_format
paediatric early warning system	paediatric early warning system	paediatric early warning system	paediatric early warning system	paediatric early warning system	1	kept
PAEDIATRIC EARLY WARNING SYSTEMS	paediatric early warning systems	paediatric early warning systems	paediatric early warning system	paediatric early warning system	1	singular
Paediatric Early Warning Tool	paediatric early warning tool	paediatric early warning tool	paediatric early warning tool	Paediatric Early Warning Tool	1	kept
Paediatric emergencies	paediatric emergencies	paediatric emergencies	paediatric emergencies	Paediatric emergencies	1	kept
paediatric emergency medicine	paediatric emergency medicine	paediatric emergency medicine	paediatric emergency medicine	paediatric emergency medicine	1	kept
Paediatric Emergency Warning Score	paediatric emergency warning score	paediatric emergency warning score	paediatric emergency warning score	Paediatric Emergency Warning Score	1	kept
Paediatric ENT services	paediatric ent services	paediatric ent services	paediatric ent services	Paediatric ENT services	1	kept
Paediatric extracorporeal life support	paediatric extracorporeal life support	paediatric extracorporeal life support	paediatric extracorporeal life support	Paediatric extracorporeal life support	1	kept
paediatric home care	paediatric home care	paediatric home care	paediatric home care	paediatric home care	1	kept

paediatric hospital	paediatric hospital	paediatric hospital	paediatric hospital	paediatric hospitals	1	case_or_format
Paediatric Hospitals	paediatric hospitals	paediatric hospitals	paediatric hospital	paediatric hospitals	1	singular
paediatric infectious disease & immunisation	paediatric infectious disease and immunisation	paediatric infectious disease and immunisation	paediatric infectious disease and immunisation	paediatric infectious disease & immunisation	1	kept
Paediatric Intensive Critical Unit	paediatric intensive critical unit	paediatric intensive critical unit	paediatric intensive critical unit	Paediatric Intensive Critical Unit	1	kept
paediatric life support	paediatric life support	paediatric life support	paediatric life support	paediatric life support	1	kept
paediatric neurology	paediatric neurology	paediatric neurology	paediatric neurology	paediatric neurology	1	kept
paediatric oncology	paediatric oncology	paediatric oncology	paediatric oncology	Paediatric oncology	1	case_or_format
Paediatric ophthalmology	paediatric ophthalmology	paediatric ophthalmology	paediatric ophthalmology	Paediatric ophthalmology	1	kept
paediatric organ donation	paediatric organ donation	paediatric organ donation	paediatric organ donation	paediatric organ donation	1	kept
Paediatric palliative care	paediatric palliative care	paediatric palliative care	paediatric palliative care	paediatric palliative care	1	case_or_format
Paediatric practice	paediatric practice	paediatric practice	paediatric practice	Paediatric Practice	1	case_or_format
Paediatric prescribing	paediatric prescribing	paediatric prescribing	paediatric prescribing	Paediatric prescribing	1	kept
PAEDIATRIC REHABILITATION	paediatric rehabilitation	paediatric rehabilitation	paediatric rehabilitation	PAEDIATRIC REHABILITATION	1	kept
paediatric resuscitation	paediatric resuscitation	paediatric resuscitation	paediatric resuscitation	paediatric resuscitation	1	kept
Paediatric retrieval	paediatric retrieval	paediatric retrieval	paediatric retrieval	Paediatric retrieval	1	kept
paediatric retrieval	paediatric retrieval	paediatric retrieval	paediatric retrieval	Paediatric retrieval	1	case_or_format

paediatric retrieval service	paediatric retrieval service	paediatric retrieval service	paediatric retrieval service	paediatric retrieval service	1	kept
Paediatric rheumatology	paediatric rheumatology	paediatric rheumatology	paediatric rheumatology	Paediatric rheumatology	1	kept
Paediatric Surgery	paediatric surgery	paediatric surgery	paediatric surgery	Paediatric surgery	1	case_or_format
paediatric thoracic medicine	paediatric thoracic medicine	paediatric thoracic medicine	paediatric thoracic medicine	paediatric thoracic medicine	1	kept
paediatric trauma	paediatric trauma	paediatric trauma	paediatric trauma	paediatric trauma	1	kept
Paenibacillus	paenibacillus	paenibacillus	paenibacillus	Paenibacillus	1	kept
paffenbarger physical activity questionnaire	paffenbarger physical activity questionnaire	paffenbarger physical activity questionnaire	paffenbarger physical activity questionnaire	paffenbarger physical activity questionnaire	1	kept
pager explosion	pager explosion	pager explosion	pager explosion	pager explosion	1	kept
Pagers	pagers	pagers	pager	pager	1	singular
Paget-Schroetter	paget schroetter	paget schroetter	paget schroetter	Paget-Schroetter	1	kept
Paging	paging	paging	paging	Paging	1	kept
Pagrus	pagrus	pagrus	pagrus	Pagrus	1	kept
Pagrus major	pagrus major	pagrus major	pagrus major	Pagrus major	1	kept
pain agitation and delirium guideline	pain agitation and delirium guideline	pain agitation and delirium guideline	pain agitation and delirium guideline	pain agitation and delirium guideline	1	kept
Pain alleviation	pain alleviation	pain alleviation	pain alleviation	Pain alleviation	1	kept
Pain and Impaired Cognition 15	pain and impaired cognition 15	pain and impaired cognition 15	pain and impaired cognition 15	Pain and Impaired Cognition 15	1	kept

pain and pain management	pain and pain management	pain and pain management	pain and pain management	pain and pain management	1	kept
Pain assessing methods	pain assessing methods	pain assessing methods	pain assessing methods	Pain assessing methods	1	kept
Pain Assessment and Intervention Notation Algorithm	pain assessment and intervention notation algorithm	pain assessment and intervention notation algorithm	pain assessment and intervention notation algorithm	Pain Assessment and Intervention Notation Algorithm	1	kept
Pain Assessment in Advanced Dementia	pain assessment in advanced dementia	pain assessment in advanced dementia	pain assessment in advanced dementia	Pain Assessment in Advanced Dementia	1	kept
pain assessment scale	pain assessment scale	pain assessment scale	pain assessment scale	pain assessment scale	1	kept
Pain assessment scales	pain assessment scales	pain assessment scales	pain assessment scale	pain assessment scale	1	singular
pain assessment tool	pain assessment tool	pain assessment tool	pain assessment tool	Pain assessment tool	1	case_or_format
Pain Behavior Assessment Tool	pain behavior assessment tool	pain behavior assessment tool	pain behavior assessment tool	Pain Behavior Assessment Tool	1	kept
pain behaviour	pain behaviour	pain behaviour	pain behaviour	pain behaviour	1	kept
Pain by body region	pain by body region	pain by body region	pain by body region	Pain by body region	1	kept
pain care	pain care	pain care	pain care	pain care	1	kept
Pain Care Quality Interdisciplinary	pain care quality interdisciplinary	pain care quality interdisciplinary	pain care quality interdisciplinary	Pain Care Quality Interdisciplinary	1	kept
Pain catheterization, central venous	pain catheterization central venous	pain catheterization central venous	pain catheterization central venous	Pain catheterization, central venous	1	kept
Pain classification	pain classification	pain classification	pain classification	Pain classification	1	kept
pain classification	pain classification	pain classification	pain classification	Pain classification	1	case_or_format
Pain clinics	pain clinics	pain clinics	pain clinics	Pain Clinics	1	case_or_format

pain complication	pain complication	pain complication	pain complication	pain complication	1	kept
Pain demographics	pain demographics	pain demographics	pain demographics	Pain demographics	1	kept
Pain detection	pain detection	pain detection	pain detection	Pain detection	1	kept
pain detection	pain detection	pain detection	pain detection	Pain detection	1	case_or_format
Pain dimension	pain dimension	pain dimension	pain dimension	Pain dimension	1	kept
pain disability index	pain disability index	pain disability index	pain disability index	pain disability index	1	kept
Pain evaluation	pain evaluation	pain evaluation	pain evaluation	Pain evaluation	1	kept
pain examination	pain examination	pain examination	pain examination	pain examination	1	kept
pain experience	pain experience	pain experience	pain experience	pain experience	1	kept
pain experiences	pain experiences	pain experiences	pain experience	pain experience	1	singular
Pain in Advanced Dementia	pain in advanced dementia	pain in advanced dementia	pain in advanced dementia	Pain in Advanced Dementia	1	kept
Pain intensity	pain intensity	pain intensity	pain intensity	pain intensity	1	case_or_format
Pain intensity detection	pain intensity detection	pain intensity detection	pain intensity detection	Pain intensity detection	1	kept
Pain intensity measurement	pain intensity measurement	pain intensity measurement	pain intensity measurement	Pain intensity measurement	1	kept
Pain management certification	pain management certification	pain management certification	pain management certification	Pain management certification	1	kept
pain management guideline	pain management guideline	pain management guideline	pain management guideline	pain management guideline	1	kept

Pain management in elders	pain management in elders	pain management in elders	pain management in elders	Pain management in elders	1	kept
Pain management program	pain management program	pain management program	pain management program	Pain management program	1	kept
pain management.	pain management	pain management	pain management	Pain Management	1	case_or_format
pain managements	pain managements	pain managements	pain management	Pain Management	1	singular
Pain measurement tool	pain measurement tool	pain measurement tool	pain measurement tool	Pain measurement tool	1	kept
Pain measurements	pain measurements	pain measurements	pain measurement	Pain Measurement	1	singular
pain measures	pain measures	pain measures	pain measures	pain measures	1	kept
Pain medicine	pain medicine	pain medicine	pain medicine	Pain medicine	1	kept
Pain perception	pain perception	pain perception	pain perception	Pain Perception	1	case_or_format
pain presence	pain presence	pain presence	pain presence	pain presence	1	kept
Pain protocol	pain protocol	pain protocol	pain protocol	Pain protocol	1	kept
Pain questionnaire	pain questionnaire	pain questionnaire	pain questionnaire	Pain questionnaire	1	kept
Pain relief	pain relief	pain relief	pain relief	Pain relief	1	kept
Pain relief care	pain relief care	pain relief care	pain relief care	Pain relief care	1	kept
Pain responses	pain responses	pain responses	pain responses	Pain responses	1	kept
Pain scale	pain scale	pain scale	pain scale	Pain scales	1	case_or_format

pain scale	pain scale	pain scale	pain scale	Pain scales	1	case_or_format
Pain score	pain score	pain score	pain score	Pain score	1	kept
Pain scores	pain scores	pain scores	pain score	Pain score	1	singular
Pain the elderly	pain the elderly	pain the elderly	pain the elderly	Pain the elderly	1	kept
pain tool	pain tool	pain tool	pain tool	pain tool	1	kept
Pain treatment	pain treatment	pain treatment	pain treatment	pain treatment	1	case_or_format
Pain treatment, non-pharmcological	pain treatment non pharmcological	pain treatment non pharmcological	pain treatment non pharmcological	Pain treatment, non-pharmcological	1	kept
Pain vignette	pain vignette	pain vignette	pain vignette	Pain vignette	1	kept
Pain, postoperative	pain postoperative	pain postoperative	pain postoperative	Pain, Postoperative	1	case_or_format
painful bladder syndrome	painful bladder syndrome	painful bladder syndrome	painful bladder syndrome	painful bladder syndrome	1	kept
painful procedures	painful procedures	painful procedures	painful procedure	Painful procedures	1	singular
painting	painting	painting	painting	painting	1	kept
Pain - postoperative	pain postoperative	pain postoperative	pain postoperative	Pain, Postoperative	1	case_or_format
paired associate learning	paired associate learning	paired associate learning	paired associate learning	paired associate learning	1	kept
paired like homeobox 2B gene	paired like homeobox 2b gene	paired like homeobox 2b gene	paired like homeobox 2b gene	paired like homeobox 2B gene	1	kept
Pakistani	pakistani	pakistani	pakistani	Pakistani	1	kept

PAL	pal	pal	pal	PAL	1	kept
paladai	paladai	paladai	paladai	paladai	1	kept
Palate	palate	palate	palate	palate	1	case_or_format
palate malformation	palate malformation	palate malformation	palate malformation	palate malformation	1	kept
palestine	palestine	palestine	palestine	Palestine	1	case_or_format
palliation	palliation	palliation	palliation	palliation	1	kept
Palliative attitude	palliative attitude	palliative attitude	palliative attitude	Palliative attitude	1	kept
PALLIATIVE CANCER CARE	palliative cancer care	palliative cancer care	palliative cancer care	PALLIATIVE CANCER CARE	1	kept
palliative care access	palliative care access	palliative care access	palliative care access	palliative care access	1	kept
Palliative care and neonatal palliative care	palliative care and neonatal palliative care	palliative care and neonatal palliative care	palliative care and neonatal palliative care	Palliative care and neonatal palliative care	1	kept
palliative care approach	palliative care approach	palliative care approach	palliative care approach	palliative care approach	1	kept
Palliative care concepts	palliative care concepts	palliative care concepts	palliative care concepts	Palliative care concepts	1	kept
palliative care consultation service	palliative care consultation service	palliative care consultation service	palliative care consultation service	palliative care consultation service	1	kept
palliative care consultation timing	palliative care consultation timing	palliative care consultation timing	palliative care consultation timing	palliative care consultation timing	1	kept
Palliative care core competencies	palliative care core competencies	palliative care core competencies	palliative care core competencies	Palliative care core competencies	1	kept
Palliative care education	palliative care education	palliative care education	palliative care education	Palliative care education	1	kept

palliative care expert	palliative care expert	palliative care expert	palliative care expert	palliative care expert	1	kept
palliative care facility	palliative care facility	palliative care facility	palliative care facility	palliative care facility	1	kept
palliative care in oncology	palliative care in oncology	palliative care in oncology	palliative care in oncology	palliative care in oncology	1	kept
palliative care medicine	palliative care medicine	palliative care medicine	palliative care medicine	palliative care medicine	1	kept
palliative care needs	palliative care needs	palliative care needs	palliative care needs	palliative care needs	1	kept
Palliative care pathway	palliative care pathway	palliative care pathway	palliative care pathway	Palliative care pathway	1	kept
palliative care practices	palliative care practices	palliative care practices	palliative care practices	palliative care practices	1	kept
Palliative care protocol	palliative care protocol	palliative care protocol	palliative care protocol	Palliative care protocol	1	kept
palliative care referral criteria	palliative care referral criteria	palliative care referral criteria	palliative care referral criteria	palliative care referral criteria	1	kept
Palliative care self-efficacy	palliative care self efficacy	palliative care self efficacy	palliative care self efficacy	Palliative care self-efficacy	1	kept
palliative care social work	palliative care social work	palliative care social work	palliative care social work	palliative care social work	1	kept
palliative care specialist	palliative care specialist	palliative care specialist	palliative care specialist	palliative care specialist	1	kept
Palliative care team	palliative care team	palliative care team	palliative care team	Palliative care team	1	kept
palliative care teams	palliative care teams	palliative care teams	palliative care team	Palliative care team	1	singular
palliative care toolkit	palliative care toolkit	palliative care toolkit	palliative care toolkit	palliative care toolkit	1	kept
Palliative care trends	palliative care trends	palliative care trends	palliative care trends	Palliative care trends	1	kept

Palliative care triggers	palliative care triggers	palliative care triggers	palliative care triggers	Palliative care triggers	1	kept
palliative care understanding	palliative care understanding	palliative care understanding	palliative care understanding	palliative care understanding	1	kept
palliative comfort care	palliative comfort care	palliative comfort care	palliative comfort care	palliative comfort care	1	kept
PALLIATIVE HOME-CARE	palliative home care	palliative home care	palliative home care	PALLIATIVE HOME-CARE	1	kept
palliative homecare support team	palliative homecare support team	palliative homecare support team	palliative homecare support team	palliative homecare support team	1	kept
palliative outcomes	palliative outcomes	palliative outcomes	palliative outcomes	palliative outcomes	1	kept
Palliative pain management	palliative pain management	palliative pain management	palliative pain management	Palliative pain management	1	kept
PALLIATIVE SEDATION	palliative sedation	palliative sedation	palliative sedation	Palliative sedation	1	case_or_format
Palliative social worker	palliative social worker	palliative social worker	palliative social worker	Palliative social worker	1	kept
Palliative supportive care	palliative supportive care	palliative supportive care	palliative supportive care	Palliative supportive care	1	kept
palliative surgery	palliative surgery	palliative surgery	palliative surgery	palliative surgery	1	kept
Palliative therapy	palliative therapy	palliative therapy	palliative therapy	palliative therapy	1	case_or_format
palliative transport	palliative transport	palliative transport	palliative transport	palliative transport	1	kept
palliative ventilator withdrawal	palliative ventilator withdrawal	palliative ventilator withdrawal	palliative ventilator withdrawal	palliative ventilator withdrawal	1	kept
Pallitative care	pallitative care	pallitative care	pallitative care	Pallitative care	1	kept
palpation method	palpation method	palpation method	palpation method	palpation method	1	kept

PALS	pals	pals	pal	PAL	1	singular
pamate	pamate	pamate	pamate	pamate	1	kept
Pamphlet	pamphlet	pamphlet	pamphlet	Pamphlets	1	case_or_format
Pan troglodytes	pan troglodytes	pan troglodytes	pan troglodytes	Pan troglodytes	1	kept
pan-drug resistance	pan drug resistance	pan drug resistance	pan drug resistance	pan-drug resistance	1	kept
PANAS	panas	panas	panas	PANAS	1	kept
PANAS Positive and Negative Affect Scale	panas positive and negative affect scale	panas positive and negative affect scale	panas positive and negative affect scale	PANAS Positive and Negative Affect Scale	1	kept
pancreas calcification	pancreas calcification	pancreas calcification	pancreas calcification	pancreas calcification	1	kept
pancreas carcinoma	pancreas carcinoma	pancreas carcinoma	pancreas carcinoma	pancreas carcinoma	1	kept
pancreas disease	pancreas disease	pancreas disease	pancreas disease	pancreas disease	1	kept
pancreas enzyme	pancreas enzyme	pancreas enzyme	pancreas enzyme	pancreas enzyme	1	kept
pancreas exocrine insufficiency	pancreas exocrine insufficiency	pancreas exocrine insufficiency	pancreas exocrine insufficiency	pancreas exocrine insufficiency	1	kept
pancreas hemorrhage	pancreas hemorrhage	pancreas hemorrhage	pancreas hemorrhage	pancreas hemorrhage	1	kept
pancreas islet cell antibody	pancreas islet cell antibody	pancreas islet cell antibody	pancreas islet cell antibody	pancreas islet cell antibody	1	kept
pancreas juice	pancreas juice	pancreas juice	pancreas juice	pancreas juice	1	kept
pancreas pseudocyst	pancreas pseudocyst	pancreas pseudocyst	pancreas pseudocyst	pancreas pseudocyst	1	kept

pancreas surgery	pancreas surgery	pancreas surgery	pancreas surgery	pancreas surgery	1	kept
Pancreatic Diseases	pancreatic diseases	pancreatic diseases	pancreatic disease	pancreatic disease	1	singular
Pancreatic ductal adenocarcinoma	pancreatic ductal adenocarcinoma	pancreatic ductal adenocarcinoma	pancreatic ductal adenocarcinoma	Pancreatic ductal adenocarcinoma	1	kept
Pancreatic Elastase	pancreatic elastase	pancreatic elastase	pancreatic elastase	Pancreatic Elastase	1	kept
Pancreatic Fistula	pancreatic fistula	pancreatic fistula	pancreatic fistula	Pancreatic Fistula	1	kept
pancreatic sepsis	pancreatic sepsis	pancreatic sepsis	pancreatic sepsis	pancreatic sepsis	1	kept
pancreatic surgery	pancreatic surgery	pancreatic surgery	pancreatic surgery	Pancreatic surgery	1	case_or_format
Pancreatic tumors	pancreatic tumors	pancreatic tumors	pancreatic tumors	Pancreatic tumors	1	kept
Pancuronium	pancuronium	pancuronium	pancuronium	pancuronium	1	case_or_format
Pancytopenia	pancytopenia	pancytopenia	pancytopenia	pancytopenia	1	case_or_format
pandemi	pandemi	pandemi	pandemi	pandemi	1	kept
Pandemia	pandemia	pandemia	pandemia	Pandemia	1	kept
Pandemia de COVID-19	pandemia de covid 19	pandemia de covid 19	pandemia de covid 19	Pandemia de COVID-19	1	kept
pandemic awareness	pandemic awareness	pandemic awareness	pandemic awareness	pandemic awareness	1	kept
pandemic awareness scale	pandemic awareness scale	pandemic awareness scale	pandemic awareness scale	pandemic awareness scale	1	kept
pandemic best practices	pandemic best practices	pandemic best practices	pandemic best practices	pandemic best practices	1	kept

Pandemic influenza vaccine	pandemic influenza vaccine	pandemic influenza vaccine	pandemic influenza vaccine	Pandemic influenza vaccine	1	kept
Pandemic influenza virus	pandemic influenza virus	pandemic influenza virus	pandemic influenza virus	Pandemic influenza virus	1	kept
Pandemic perceptions	pandemic perceptions	pandemic perceptions	pandemic perceptions	Pandemic perceptions	1	kept
Pandemic preparedness	pandemic preparedness	pandemic preparedness	pandemic preparedness	pandemic preparedness	1	case_or_format
pandemic preparedness plan for Radiation Oncology-PPP-RO	pandemic preparedness plan for radiation oncology ppp ro	pandemic preparedness plan for radiation oncology ppp ro	pandemic preparedness plan for radiation oncology ppp ro	pandemic preparedness plan for Radiation Oncology-PPP-RO	1	kept
Pandemic preparedness, psychological distress	pandemic preparedness psychological distress	pandemic preparedness psychological distress	pandemic preparedness psychological distress	Pandemic preparedness, psychological distress	1	kept
Pandemic response	pandemic response	pandemic response	pandemic response	pandemic response	1	case_or_format
Pandemic.	pandemic	pandemic	pandemic	Pandemics	1	case_or_format
pandemics policy	pandemics policy	pandemics policy	pandemics policy	pandemics policy	1	kept
Pandemie	pandemie	pandemie	pandemie	Pandemie	1	kept
panel data	panel data	panel data	panel data	panel data	1	kept
panel data econometrics	panel data econometrics	panel data econometrics	panel data econometrics	panel data econometrics	1	kept
Panel discussion	panel discussion	panel discussion	panel discussion	Panel discussion	1	kept
panel study	panel study	panel study	panel study	panel study	1	kept
Panic	panic	panic	panic	panic	1	case_or_format
panipenem	panipenem	panipenem	panipenem	panipenem	1	kept

panopticon	panopticon	panopticon	panopticon	panopticon	1	kept
panoramic radiography	panoramic radiography	panoramic radiography	panoramic radiography	panoramic radiography	1	kept
Pantoea	pantoea	pantoea	pantoea	Pantoea	1	kept
Pantoea agglomerans	pantoea agglomerans	pantoea agglomerans	pantoea agglomerans	Pantoea agglomerans	1	kept
Panton-Valentine leucocidin	panton valentine leucocidin	panton valentine leucocidin	panton valentine leucocidin	Panton-Valentine leucocidin	1	kept
Panton-Valentine leukocidin-Staphylococcus aureus	panton valentine leukocidin staphylococcus aureus	panton valentine leukocidin staphylococcus aureus	panton valentine leukocidin staphylococcus aureus	Panton-Valentine leukocidin-Staphylococcus aureus	1	kept
papaverine	papaverine	papaverine	papaverine	Papaverine	1	case_or_format
PAPER	paper	paper	paper	Paper	1	case_or_format
paper based checklist	paper based checklist	paper based checklist	paper based checklist	paper based checklist	1	kept
paper based medication administration	paper based medication administration	paper based medication administration	paper based medication administration	paper based medication administration	1	kept
paper diffusion method	paper diffusion method	paper diffusion method	paper diffusion method	paper diffusion method	1	kept
Paper documents	paper documents	paper documents	paper documents	Paper documents	1	kept
paper resuscitation record	paper resuscitation record	paper resuscitation record	paper resuscitation record	paper resuscitation record	1	kept
Paper simulation	paper simulation	paper simulation	paper simulation	Paper simulation	1	kept
Paper-based checklist	paper based checklist	paper based checklist	paper based checklist	paper based checklist	1	case_or_format
papilledema	papilledema	papilledema	papilledema	papilledema	1	kept

papillomavirus infection	papillomavirus infection	papillomavirus infection	papillomavirus infection	papillomavirus infection	1	kept
Paquetes de medidas	paquetes de medidas	paquetes de medidas	paquetes de medidas	Paquetes de medidas	1	kept
Par-ents	par ents	par ents	par ents	Par-ents	1	kept
paracetamol derivative	paracetamol derivative	paracetamol derivative	paracetamol derivative	paracetamol derivative	1	kept
paractamol overdose	paractamol overdose	paractamol overdose	paractamol overdose	paractamol overdose	1	kept
Parada cardiorrespiratoria	parada cardiorrespiratoria	parada cardiorrespiratoria	parada cardiorrespiratoria	Parada cardiorrespiratoria	1	kept
Parada Cardíaca	parada card aca	parada card aca	parada card aca	Parada Cardíaca	1	kept
paradichlorobenzene	paradichlorobenzene	paradichlorobenzene	paradichlorobenzene	paradichlorobenzene	1	kept
PARADIGM	paradigm	paradigm	paradigm	PARADIGM	1	kept
Paradox theory	paradox theory	paradox theory	paradox theory	Paradox theory	1	kept
paradoxical pulse	paradoxical pulse	paradoxical pulse	paradoxical pulse	paradoxical pulse	1	kept
paraffin	paraffin	paraffin	paraffin	paraffin	1	kept
paraffin embedding	paraffin embedding	paraffin embedding	paraffin embedding	paraffin embedding	1	kept
Paraganglioma extra-adrenal pheochro-mocytoma vasopressin	paraganglioma extra adrenal pheochro mocytoma vasopressin	paraganglioma extra adrenal pheochro mocytoma vasopressin	paraganglioma extra adrenal pheochro mocytoma vasopressin	Paraganglioma extra-adrenal pheochro-mocytoma vasopressin	1	kept
Parainfluenza virus 1	parainfluenza virus 1	parainfluenza virus 1	parainfluenza virus 1	Parainfluenza virus 1	1	kept
Parainfluenza virus 2	parainfluenza virus 2	parainfluenza virus 2	parainfluenza virus 2	Parainfluenza virus 2	1	kept

Parainfluenza virus 3	parainfluenza virus 3	parainfluenza virus 3	parainfluenza virus 3	Parainfluenza virus 3	1	kept
Parainfluenza virus 4	parainfluenza virus 4	parainfluenza virus 4	parainfluenza virus 4	Parainfluenza virus 4	1	kept
paraldehyde	paraldehyde	paraldehyde	paraldehyde	paraldehyde	1	kept
Parallel processing	parallel processing	parallel processing	parallel processing	Parallel processing	1	kept
paramedic education	paramedic education	paramedic education	paramedic education	paramedic education	1	kept
Paramedic Training	paramedic training	paramedic training	paramedic training	Paramedic Training	1	kept
Paramedic-only staffed ambulances	paramedic only staffed ambulances	paramedic only staffed ambulances	paramedic only staffed ambulances	Paramedic-only staffed ambulances	1	kept
Paramedical	paramedical	paramedical	paramedical	Paramedical	1	kept
paramedical	paramedical	paramedical	paramedical	Paramedical	1	case_or_format
Paramedical education	paramedical education	paramedical education	paramedical education	paramedical education	1	case_or_format
Paramedicine	paramedicine	paramedicine	paramedicine	Paramedicine	1	kept
paramedics	paramedics	paramedics	paramedic	Paramedic	1	singular
parameter estimation	parameter estimation	parameter estimation	parameter estimation	parameter estimation	1	kept
Parameter range	parameter range	parameter range	parameter range	Parameter range	1	kept
Parameters	parameters	parameters	parameter	parameters	1	singular
Parametric and semiparametric mixed-effects logit models	parametric and semiparametric mixed effects logit models	parametric and semiparametric mixed effects logit models	parametric and semiparametric mixed effects logit models	Parametric and semiparametric mixed-effects logit models	1	kept

Parametric statistics	parametric statistics	parametric statistics	parametric statistics	Parametric statistics	1	kept
Paramyxovirinae	paramyxovirinae	paramyxovirinae	paramyxovirinae	Paramyxovirinae	1	kept
paramédical	param dical	param dical	param dical	paramédical	1	kept
Paranasal sinus	paranasal sinus	paranasal sinus	paranasal sinus	Paranasal sinus	1	kept
paranasal sinus	paranasal sinus	paranasal sinus	paranasal sinus	Paranasal sinus	1	case_or_format
paraneoplastic encephalitis	paraneoplastic encephalitis	paraneoplastic encephalitis	paraneoplastic encephalitis	paraneoplastic encephalitis	1	kept
Paraproteinemias	paraproteinemias	paraproteinemias	paraproteinemia	paraproteinemia	1	singular
PARAPSILOSIS	parapsilosis	parapsilosis	parapsilosis	PARAPSILOSIS	1	kept
Paraquat	paraquat	paraquat	paraquat	Paraquat	1	kept
paraquat	paraquat	paraquat	paraquat	Paraquat	1	case_or_format
Paraquat poisoning	paraquat poisoning	paraquat poisoning	paraquat poisoning	Paraquat poisoning	1	kept
parasitic	parasitic	parasitic	parasitic	parasitic	1	kept
paraspinal muscle	paraspinal muscle	paraspinal muscle	paraspinal muscle	paraspinal muscle	1	kept
Paraspinal Muscles	paraspinal muscles	paraspinal muscles	paraspinal muscle	paraspinal muscle	1	singular
parastomal hernia	parastomal hernia	parastomal hernia	parastomal hernia	parastomal hernia	1	kept
Parasuicide	parasuicide	parasuicide	parasuicide	Parasuicide	1	kept

Parasympathetic activity	parasympathetic activity	parasympathetic activity	parasympathetic activity	Parasympathetic activity	1	kept
parasympathetic tone	parasympathetic tone	parasympathetic tone	parasympathetic tone	parasympathetic tone	1	kept
parathyroid adenoma	parathyroid adenoma	parathyroid adenoma	parathyroid adenoma	parathyroid adenoma	1	kept
parathyroid hormone blood level	parathyroid hormone blood level	parathyroid hormone blood level	parathyroid hormone blood level	parathyroid hormone blood level	1	kept
parathyroid hormone related protein	parathyroid hormone related protein	parathyroid hormone related protein	parathyroid hormone related protein	parathyroid hormone related protein	1	kept
parathyroid hormone[1-34]	parathyroid hormone 1 34	parathyroid hormone 1 34	parathyroid hormone 1 34	parathyroid hormone[1-34]	1	kept
paravertebral analgesia	paravertebral analgesia	paravertebral analgesia	paravertebral analgesia	paravertebral analgesia	1	kept
paravertebral block	paravertebral block	paravertebral block	paravertebral block	paravertebral block	1	kept
parecoxib	parecoxib	parecoxib	parecoxib	parecoxib	1	kept
parenchyma	parenchyma	parenchyma	parenchyma	parenchyma	1	kept
Parenchymal laceration	parenchymal laceration	parenchymal laceration	parenchymal laceration	Parenchymal laceration	1	kept
parenchymal lesion	parenchymal lesion	parenchymal lesion	parenchymal lesion	parenchymal lesion	1	kept
parent administered sensorimotor intervention	parent administered sensorimotor intervention	parent administered sensorimotor intervention	parent administered sensorimotor intervention	parent administered sensorimotor intervention	1	kept
Parent coping	parent coping	parent coping	parent coping	Parent coping	1	kept
parent coping	parent coping	parent coping	parent coping	Parent coping	1	case_or_format
Parent counseling	parent counseling	parent counseling	parent counseling	parent counseling	1	case_or_format

Parent decision making	parent decision making	parent decision making	parent decision making	Parent decision making	1	kept
parent delivered intervention	parent delivered intervention	parent delivered intervention	parent delivered intervention	parent delivered intervention	1	kept
Parent Distress	parent distress	parent distress	parent distress	Parent Distress	1	kept
parent empowerment	parent empowerment	parent empowerment	parent empowerment	parent empowerment	1	kept
Parent experiences	parent experiences	parent experiences	parent experience	Parent experience	1	singular
Parent gender	parent gender	parent gender	parent gender	Parent gender	1	kept
Parent Health Literacy Activities Test	parent health literacy activities test	parent health literacy activities test	parent health literacy activities test	Parent Health Literacy Activities Test	1	kept
parent infant relationships	parent infant relationships	parent infant relationships	parent infant relationship	parent-infant relationship	1	singular
parent involvement	parent involvement	parent involvement	parent involvement	parent involvement	1	kept
parent mental health	parent mental health	parent mental health	parent mental health	parent mental health	1	kept
PARENT PARTICIPATION	parent participation	parent participation	parent participation	PARENT PARTICIPATION	1	kept
Parent participation	parent participation	parent participation	parent participation	PARENT PARTICIPATION	1	case_or_format
parent partnered care	parent partnered care	parent partnered care	parent partnered care	parent partnered care	1	kept
Parent perceptions	parent perceptions	parent perceptions	parent perceptions	Parent perceptions	1	kept
Parent perspective	parent perspective	parent perspective	parent perspective	parent perspective	1	case_or_format
parent preference	parent preference	parent preference	parent preference	parent preference	1	kept

parent psychosocial functioning	parent psychosocial functioning	parent psychosocial functioning	parent psychosocial functioning	parent psychosocial functioning	1	kept
Parent role	parent role	parent role	parent role	Parent role	1	kept
PARENT SATISFACTION	parent satisfaction	parent satisfaction	parent satisfaction	Parent satisfaction	1	case_or_format
PARENT STRESS	parent stress	parent stress	parent stress	PARENT STRESS	1	kept
Parent stress	parent stress	parent stress	parent stress	PARENT STRESS	1	case_or_format
Parent Stressor Scale	parent stressor scale	parent stressor scale	parent stressor scale	Parent Stressor Scale	1	kept
parent teaching	parent teaching	parent teaching	parent teaching	parent teaching	1	kept
parent training	parent training	parent training	parent training	parent training	1	kept
parent transfer	parent transfer	parent transfer	parent transfer	parent transfer	1	kept
parent's perception of provider actions	parent s perception of provider actions	parent s perception of provider actions	parent s perception of provider actions	parent's perception of provider actions	1	kept
Parent's program	parent s program	parent s program	parent s program	Parent's program	1	kept
Parent-child	parent child	parent child	parent child	Parent-child	1	kept
parent-child bonding	parent child bonding	parent child bonding	parent child bonding	parent-child bonding	1	kept
Parent-child interaction	parent child interaction	parent child interaction	parent child interaction	Parent-child interaction	1	kept
parent-child interaction	parent child interaction	parent child interaction	parent child interaction	Parent-child interaction	1	case_or_format
Parent-child relation	parent child relation	parent child relation	parent child relation	Parent-Child Relations	1	case_or_format

parent-child relation	parent child relation	parent child relation	parent child relation	Parent-Child Relations	1	case_or_format
Parent-child relations	parent child relations	parent child relations	parent child relation	Parent-Child Relations	1	singular
parent-child relationships	parent child relationships	parent child relationships	parent child relationship	parent-child relationship	1	singular
Parent-child-relation	parent child relation	parent child relation	parent child relation	Parent-Child Relations	1	case_or_format
parent-delivered intervention	parent delivered intervention	parent delivered intervention	parent delivered intervention	parent delivered intervention	1	case_or_format
Parent-healthcare provider relationships	parent healthcare provider relationships	parent healthcare provider relationships	parent healthcare provider relationships	Parent-healthcare provider relationships	1	kept
parent-infant interactions	parent infant interactions	parent infant interactions	parent infant interaction	parent－infant interaction	1	singular
parent-infant relation	parent infant relation	parent infant relation	parent infant relation	parent-infant relation	1	kept
parent-infant relationship	parent infant relationship	parent infant relationship	parent infant relationship	parent-infant relationship	1	kept
parent-infant separation	parent infant separation	parent infant separation	parent infant separation	parent-infant separation	1	kept
parent-to-parent	parent to parent	parent to parent	parent to parent	parent-to-parent	1	kept
Parent<sup> <	parent sup	parent sup	parent sup	Parent<sup> <	1	kept
Parental accompaniment	parental accompaniment	parental accompaniment	parental accompaniment	Parental accompaniment	1	kept
parental attachment	parental attachment	parental attachment	parental attachment	parental attachment	1	kept
parental authority	parental authority	parental authority	parental authority	parental authority	1	kept
parental autonomy	parental autonomy	parental autonomy	parental autonomy	parental autonomy	1	kept

parental caregiver	parental caregiver	parental caregiver	parental caregiver	parental caregiver	1	kept
Parental conflicts and effects on embryo	parental conflicts and effects on embryo	parental conflicts and effects on embryo	parental conflicts and effects on embryo	Parental conflicts and effects on embryo	1	kept
Parental consent	parental consent	parental consent	parental consent	Parental Consent	1	case_or_format
parental coping	parental coping	parental coping	parental coping	parental coping	1	kept
parental decision making	parental decision making	parental decision making	parental decision making	parental decision making	1	kept
parental decision-making	parental decision making	parental decision making	parental decision making	parental decision making	1	case_or_format
Parental depression	parental depression	parental depression	parental depression	Parental depression	1	kept
parental deprivation	parental deprivation	parental deprivation	parental deprivation	parental deprivation	1	kept
parental dimension	parental dimension	parental dimension	parental dimension	parental dimension	1	kept
Parental experience	parental experience	parental experience	parental experience	parental experiences	1	case_or_format
parental experience of support	parental experience of support	parental experience of support	parental experience of support	parental experience of support	1	kept
parental factors	parental factors	parental factors	parental factors	parental factors	1	kept
PARENTAL GRIEF	parental grief	parental grief	parental grief	PARENTAL GRIEF	1	kept
parental guidance	parental guidance	parental guidance	parental guidance	parental guidance	1	kept
parental interaction with infants	parental interaction with infants	parental interaction with infants	parental interaction with infants	parental interaction with infants	1	kept
Parental interventions and developmental care programs	parental interventions and developmental care programs	parental interventions and developmental care programs	parental interventions and developmental care programs	Parental interventions and developmental care programs	1	kept

Parental Leave	parental leave	parental leave	parental leave	Parental Leave	1	kept
parental leave	parental leave	parental leave	parental leave	Parental Leave	1	case_or_format
parental need	parental need	parental need	parental need	parental need	1	kept
Parental needs	parental needs	parental needs	parental need	parental need	1	singular
parental needs	parental needs	parental needs	parental need	parental need	1	singular
Parental notification	parental notification	parental notification	parental notification	Parental notification	1	kept
parental notification	parental notification	parental notification	parental notification	Parental notification	1	case_or_format
parental nutrition	parental nutrition	parental nutrition	parental nutrition	parental nutrition	1	kept
parental paid leave	parental paid leave	parental paid leave	parental paid leave	parental paid leave	1	kept
parental participation	parental participation	parental participation	parental participation	Parental participation	1	case_or_format
Parental participation in care	parental participation in care	parental participation in care	parental participation in care	Parental participation in care	1	kept
parental perception	parental perception	parental perception	parental perception	Parental perceptions	1	case_or_format
Parental perception of infant illness	parental perception of infant illness	parental perception of infant illness	parental perception of infant illness	Parental perception of infant illness	1	kept
parental perceptions	parental perceptions	parental perceptions	parental perception	Parental perceptions	1	singular
Parental performances	parental performances	parental performances	parental performances	Parental performances	1	kept
parental permission	parental permission	parental permission	parental permission	parental permission	1	kept

PARENTAL PREFERENCES	parental preferences	parental preferences	parental preferences	PARENTAL PREFERENCES	1	kept
parental Presence	parental presence	parental presence	parental presence	Parental presence	1	case_or_format
Parental psychosocial support	parental psychosocial support	parental psychosocial support	parental psychosocial support	Parental psychosocial support	1	kept
Parental readiness	parental readiness	parental readiness	parental readiness	Parental readiness	1	kept
Parental reflective functioning	parental reflective functioning	parental reflective functioning	parental reflective functioning	Parental reflective functioning	1	kept
parental relational engagement	parental relational engagement	parental relational engagement	parental relational engagement	parental relational engagement	1	kept
parental responsibilities	parental responsibilities	parental responsibilities	parental responsibilities	parental responsibilities	1	kept
Parental role	parental role	parental role	parental role	Parental role	1	kept
parental self efficacy	parental self efficacy	parental self efficacy	parental self efficacy	Parental self-efficacy	1	case_or_format
Parental sleep	parental sleep	parental sleep	parental sleep	Parental sleep	1	kept
Parental stressor	parental stressor	parental stressor	parental stressor	Parental stressor	1	kept
Parental Stressor Scale	parental stressor scale	parental stressor scale	parental stressor scale	Parental Stressor Scale	1	kept
Parental stressors	parental stressors	parental stressors	parental stressor	Parental stressor	1	singular
parental support	parental support	parental support	parental support	Parental support	1	case_or_format
parental support programme	parental support programme	parental support programme	parental support programme	parental support programme	1	kept
Parental training	parental training	parental training	parental training	Parental training	1	kept

parental training program	parental training program	parental training program	parental training program	parental training program	1	kept
parental visitation program	parental visitation program	parental visitation program	parental visitation program	parental visitation program	1	kept
parental well being	parental well being	parental well being	parental well being	parental well being	1	kept
PARENTERAL ANTIBIOTIC-THERAPY	parenteral antibiotic therapy	parenteral antibiotic therapy	parenteral antibiotic therapy	PARENTERAL ANTIBIOTIC-THERAPY	1	kept
Parenteral infusion	parenteral infusion	parenteral infusion	parenteral infusion	Parenteral infusion	1	kept
Parenteral medication	parenteral medication	parenteral medication	parenteral medication	Parenteral medication	1	kept
parenteral nutrition support	parenteral nutrition support	parenteral nutrition support	parenteral nutrition support	parenteral nutrition support	1	kept
parenteral nutrition-associated cholestasis	parenteral nutrition associated cholestasis	parenteral nutrition associated cholestasis	parenteral nutrition associated cholestasis	parenteral nutrition-associated cholestasis	1	kept
Parenting education	parenting education	parenting education	parenting education	parenting education	1	case_or_format
parenting resilience	parenting resilience	parenting resilience	parenting resilience	parenting resilience	1	kept
Parenting role	parenting role	parenting role	parenting role	Parenting role	1	kept
Parenting self-efficacy	parenting self efficacy	parenting self efficacy	parenting self efficacy	Parenting self-efficacy	1	kept
parenting support	parenting support	parenting support	parenting support	parenting support	1	kept
parenting, families	parenting families	parenting families	parenting families	parenting, families	1	kept
PARENTS EXPERIENCES	parents experiences	parents experiences	parents experience	PARENTS EXPERIENCES	1	singular
Parents of preterm infant	parents of preterm infant	parents of preterm infant	parents of preterm infant	Parents of preterm infant	1	kept

Parents of preterm infants	parents of preterm infants	parents of preterm infants	parents of preterm infant	Parents of preterm infant	1	singular
Parents satisfaction	parents satisfaction	parents satisfaction	parents satisfaction	Parents' satisfaction	1	case_or_format
parents satisfaction	parents satisfaction	parents satisfaction	parents satisfaction	Parents' satisfaction	1	case_or_format
parents' experience	parents experience	parents experience	parents experience	PARENTS EXPERIENCES	1	case_or_format
Parents' experiences	parents experiences	parents experiences	parents experience	PARENTS EXPERIENCES	1	singular
parents' participation	parents participation	parents participation	parents participation	parents' participation	1	kept
Parents' satisfaction	parents satisfaction	parents satisfaction	parents satisfaction	Parents' satisfaction	1	case_or_format
Parents' views	parents views	parents views	parents views	Parents' views	1	kept
parents' voice	parents voice	parents voice	parents voice	parents' voice	1	kept
Parents' experience	parents experience	parents experience	parents experience	PARENTS EXPERIENCES	1	case_or_format
parents' experience	parents experience	parents experience	parents experience	PARENTS EXPERIENCES	1	case_or_format
parents' mental health	parents mental health	parents mental health	parents mental health	parents' mental health	1	kept
Parents' perspective	parents perspective	parents perspective	parents perspective	Parents' perspective	1	kept
parent–child relations	parent child relations	parent child relations	parent child relation	Parent-Child Relations	1	singular
parent–health care provider relationship	parent health care provider relationship	parent health care provider relationship	parent health care provider relationship	parent–health care provider relationship	1	kept
parent–infant	parent infant	parent infant	parent infant	parent–infant	1	kept

parent－infant dyad	parent infant dyad	parent infant dyad	parent infant dyad	parent－infant dyad	1	kept
parent－infant interaction	parent infant interaction	parent infant interaction	parent infant interaction	parent－infant interaction	1	kept
Parent′s need	parent s need	parent s need	parent s need	Parent′s need	1	kept
Parent*	parent	parent	parent	Parents	1	case_or_format
Pareto chart	pareto chart	pareto chart	pareto chart	Pareto chart	1	kept
Parientes	parientes	parientes	parientes	Parientes	1	kept
PARKINSON DISEASE	parkinson disease	parkinson disease	parkinson disease	Parkinson disease	1	case_or_format
parkinson disease questionnaire	parkinson disease questionnaire	parkinson disease questionnaire	parkinson disease questionnaire	parkinson disease questionnaire	1	kept
parkinson disease sleep scale	parkinson disease sleep scale	parkinson disease sleep scale	parkinson disease sleep scale	parkinson disease sleep scale	1	kept
Parkinson´s disease medication safety acute care setting	parkinson s disease medication safety acute care setting	parkinson s disease medication safety acute care setting	parkinson s disease medication safety acute care setting	Parkinson´s disease medication safety acute care setting	1	kept
PARKINSONS-DISEASE	parkinsons disease	parkinsons disease	parkinsons disease	PARKINSONS-DISEASE	1	kept
Paro cardíaco	paro card aco	paro card aco	paro card aco	Paro cardíaco	1	kept
Paro Cardíoaco	paro card oaco	paro card oaco	paro card oaco	Paro Cardíoaco	1	kept
parotitis	parotitis	parotitis	parotitis	parotitis	1	kept
paroxysmal sympathetic activity	paroxysmal sympathetic activity	paroxysmal sympathetic activity	paroxysmal sympathetic activity	paroxysmal sympathetic activity	1	kept
Paroxysmal sympathetic hyperactivity	paroxysmal sympathetic hyperactivity	paroxysmal sympathetic hyperactivity	paroxysmal sympathetic hyperactivity	Paroxysmal sympathetic hyperactivity	1	kept

parrhêsia	parrh sia	parrh sia	parrh sia	parrhêsia	1	kept
Parse	parse	parse	parse	Parse	1	kept
parsimony analysis	parsimony analysis	parsimony analysis	parsimony analysis	parsimony analysis	1	kept
PART	part	part	part	PART	1	kept
Partería	parter a	parter a	parter a	Partería	1	kept
parthenogenesis	parthenogenesis	parthenogenesis	parthenogenesis	parthenogenesis	1	kept
partial body weight	partial body weight	partial body weight	partial body weight	partial body weight	1	kept
Partial disability	partial disability	partial disability	partial disability	Partial disability	1	kept
partial gastrectomy	partial gastrectomy	partial gastrectomy	partial gastrectomy	partial gastrectomy	1	kept
partial hepatectomy	partial hepatectomy	partial hepatectomy	partial hepatectomy	partial hepatectomy	1	kept
partial least squares regression	partial least squares regression	partial least squares regression	partial least squares regression	partial least squares regression	1	kept
partial mastectomy	partial mastectomy	partial mastectomy	partial mastectomy	partial mastectomy	1	kept
Partial pancreatectomy	partial pancreatectomy	partial pancreatectomy	partial pancreatectomy	Partial pancreatectomy	1	kept
partial peritonectomy	partial peritonectomy	partial peritonectomy	partial peritonectomy	partial peritonectomy	1	kept
partial pressure of carbon dioxide	partial pressure of carbon dioxide	partial pressure of carbon dioxide	partial pressure of carbon dioxide	partial pressure of carbon dioxide	1	kept
partial thickness burn	partial thickness burn	partial thickness burn	partial thickness burn	partial thickness burn	1	kept

Partially paralytic	partially paralytic	partially paralytic	partially paralytic	Partially paralytic	1	kept
Participación del paciente	participaci n del paciente	participaci n del paciente	participaci n del paciente	Participación del paciente	1	kept
Participant	participant	participant	participant	participant	1	case_or_format
participant enrollment	participant enrollment	participant enrollment	participant enrollment	participant enrollment	1	kept
participant information sheet	participant information sheet	participant information sheet	participant information sheet	participant information sheet	1	kept
PARTICIPANT OBSERVATION	participant observation	participant observation	participant observation	participant observation	1	case_or_format
Participant retention	participant retention	participant retention	participant retention	Participant retention	1	kept
participation in care	participation in care	participation in care	participation in care	participation in care	1	kept
Participation in caregiving	participation in caregiving	participation in caregiving	participation in caregiving	Participation in caregiving	1	kept
participatory photographic research	participatory photographic research	participatory photographic research	participatory photographic research	participatory photographic research	1	kept
participatory research	participatory research	participatory research	participatory research	participatory research	1	kept
Participatory training programme	participatory training programme	participatory training programme	participatory training programme	Participatory training programme	1	kept
Particle contamination	particle contamination	particle contamination	particle contamination	Particle contamination	1	kept
Particle swarm optimization	particle swarm optimization	particle swarm optimization	particle swarm optimization	Particle swarm optimization	1	kept
Partitional calorimetry	partitional calorimetry	partitional calorimetry	partitional calorimetry	Partitional calorimetry	1	kept
Partnered-research	partnered research	partnered research	partnered research	Partnered-research	1	kept

partnering	partnering	partnering	partnering	partnering	1	kept
PARTNERS	partners	partners	partners	Partners	1	case_or_format
partners' experiences	partners experiences	partners experiences	partners experiences	partners' experiences	1	kept
PARTNERSHIP MODEL	partnership model	partnership model	partnership model	PARTNERSHIP MODEL	1	kept
partnership working	partnership working	partnership working	partnership working	partnership working	1	kept
PartnershipPractice	partnershippractice	partnershippractice	partnershippractice	PartnershipPractice	1	kept
Partnerships	partnerships	partnerships	partnership	partnership	1	singular
partnerships	partnerships	partnerships	partnership	partnership	1	singular
Parto normal	parto normal	parto normal	parto normal	Parto normal	1	kept
Parturient	parturient	parturient	parturient	Parturient	1	kept
parturient	parturient	parturient	parturient	Parturient	1	case_or_format
Parvimonas micra	parvimonas micra	parvimonas micra	parvimonas micra	Parvimonas micra	1	kept
Parvoviridae	parvoviridae	parvoviridae	parvoviridae	Parvoviridae	1	kept
parvovirus infection	parvovirus infection	parvovirus infection	parvovirus infection	parvovirus infection	1	kept
Pase de guardia	pase de guardia	pase de guardia	pase de guardia	Pase de guardia	1	kept
Pasero Opioid Sedation Scale	pasero opioid sedation scale	pasero opioid sedation scale	pasero opioid sedation scale	Pasero Opioid Sedation Scale	1	kept

Pasireotide	pasireotide	pasireotide	pasireotide	Pasireotide	1	kept
PASSAGE	passage	passage	passage	PASSAGE	1	kept
Passiflora	passiflora	passiflora	passiflora	Passiflora	1	kept
passion for work	passion for work	passion for work	passion for work	passion for work	1	kept
passive hypothermia	passive hypothermia	passive hypothermia	passive hypothermia	passive hypothermia	1	kept
Passive leg raise	passive leg raise	passive leg raise	passive leg raise	passive leg raise	1	case_or_format
Passive leg raise test	passive leg raise test	passive leg raise test	passive leg raise test	Passive leg raise test	1	kept
passive leg raise test	passive leg raise test	passive leg raise test	passive leg raise test	Passive leg raise test	1	case_or_format
passive leg raising	passive leg raising	passive leg raising	passive leg raising	passive leg raising	1	kept
Passive mobilization	passive mobilization	passive mobilization	passive mobilization	Passive mobilization	1	kept
passive mobilization	passive mobilization	passive mobilization	passive mobilization	Passive mobilization	1	case_or_format
PASSIVE PHARYNX	passive pharynx	passive pharynx	passive pharynx	PASSIVE PHARYNX	1	kept
passive resistance training	passive resistance training	passive resistance training	passive resistance training	passive resistance training	1	kept
passive surveillance	passive surveillance	passive surveillance	passive surveillance	passive surveillance	1	kept
passive transport	passive transport	passive transport	passive transport	passive transport	1	kept
Passy Muir speaking valve	passy muir speaking valve	passy muir speaking valve	passy muir speaking valve	Passy Muir speaking valve	1	kept

pasteurized milk	pasteurized milk	pasteurized milk	pasteurized milk	pasteurized milk	1	kept
pastry	pastry	pastry	pastry	pastry	1	kept
Patan Hospital	patan hospital	patan hospital	patan hospital	Patan Hospital	1	kept
patch monitoring	patch monitoring	patch monitoring	patch monitoring	patch monitoring	1	kept
patch test	patch test	patch test	patch test	patch test	1	kept
patella fracture	patella fracture	patella fracture	patella fracture	patella fracture	1	kept
patellofemoral osteoarthritis	patellofemoral osteoarthritis	patellofemoral osteoarthritis	patellofemoral osteoarthritis	patellofemoral osteoarthritis	1	kept
Patency	patency	patency	patency	Patency	1	kept
patent	patent	patent	patent	patent	1	kept
Patent ductus arteriosus	patent ductus arteriosus	patent ductus arteriosus	patent ductus arteriosus	patent ductus arteriosus	1	case_or_format
patent ductus arteriosus ligation	patent ductus arteriosus ligation	patent ductus arteriosus ligation	patent ductus arteriosus ligation	patent ductus arteriosus ligation	1	kept
Paternal	paternal	paternal	paternal	Paternal	1	kept
Paternal bonding	paternal bonding	paternal bonding	paternal bonding	Paternal bonding	1	kept
paternal bonding	paternal bonding	paternal bonding	paternal bonding	Paternal bonding	1	case_or_format
paternal care	paternal care	paternal care	paternal care	paternal care	1	kept
paternal involvement	paternal involvement	paternal involvement	paternal involvement	paternal involvement	1	kept

paternal needs	paternal needs	paternal needs	paternal needs	paternal needs	1	kept
paternal pregnancy	paternal pregnancy	paternal pregnancy	paternal pregnancy	paternal pregnancy	1	kept
Paternal role	paternal role	paternal role	paternal role	Paternal role	1	kept
Paternal Skin Care	paternal skin care	paternal skin care	paternal skin care	Paternal Skin Care	1	kept
Paternal skin care	paternal skin care	paternal skin care	paternal skin care	Paternal Skin Care	1	case_or_format
Paternal-infant attachment	paternal infant attachment	paternal infant attachment	paternal infant attachment	Paternal-infant attachment	1	kept
paternity	paternity	paternity	paternity	Paternity	1	case_or_format
Path -based oral care	path based oral care	path based oral care	path based oral care	Path-based oral care	1	case_or_format
Path-based oral care	path based oral care	path based oral care	path based oral care	Path-based oral care	1	kept
Pathogen dissemination	pathogen dissemination	pathogen dissemination	pathogen dissemination	Pathogen dissemination	1	kept
pathogen genomics	pathogen genomics	pathogen genomics	pathogen genomics	pathogen genomics	1	kept
Pathogen surveillance	pathogen surveillance	pathogen surveillance	pathogen surveillance	Pathogen surveillance	1	kept
pathogen transmission	pathogen transmission	pathogen transmission	pathogen transmission	pathogen transmission	1	kept
Pathogenic	pathogenic	pathogenic	pathogenic	Pathogenic	1	kept
pathogenic bacteria	pathogenic bacteria	pathogenic bacteria	pathogenic bacteria	Pathogenic bacteria	1	case_or_format
PATHOGENS	pathogens	pathogens	pathogen	pathogen	1	singular

pathogens	pathogens	pathogens	pathogen	pathogen	1	singular
pathological anatomy	pathological anatomy	pathological anatomy	pathological anatomy	pathological anatomy	1	kept
Pathological jaundice	pathological jaundice	pathological jaundice	pathological jaundice	Pathological jaundice	1	kept
Pathophysiologic effects of immobility	pathophysiologic effects of immobility	pathophysiologic effects of immobility	pathophysiologic effects of immobility	Pathophysiologic effects of immobility	1	kept
Pathophysiological mechanisms	pathophysiological mechanisms	pathophysiological mechanisms	pathophysiological mechanisms	Pathophysiological mechanisms	1	kept
Pathway analysis	pathway analysis	pathway analysis	pathway analysis	pathway analysis	1	case_or_format
patronage systems	patronage systems	patronage systems	patronage systems	patronage systems	1	kept
pattern	pattern	pattern	pattern	PATTERNS	1	case_or_format
Pattern recognition	pattern recognition	pattern recognition	pattern recognition	pattern recognition	1	case_or_format
Pattern Recognition, Physiological	pattern recognition physiological	pattern recognition physiological	pattern recognition physiological	Pattern Recognition, Physiological	1	kept
patterns of errors contributing to trauma mortality	patterns of errors contributing to trauma mortality	patterns of errors contributing to trauma mortality	patterns of errors contributing to trauma mortality	patterns of errors contributing to trauma mortality	1	kept
Patterns of injury	patterns of injury	patterns of injury	patterns of injury	Patterns of injury	1	kept
Pauses	pauses	pauses	pauses	Pauses	1	kept
PAY	pay	pay	pay	PAY	1	kept
pay	pay	pay	pay	PAY	1	case_or_format
Pay gap	pay gap	pay gap	pay gap	Pay gap	1	kept

pay-for-performance	pay for performance	pay for performance	pay for performance	pay-for-performance	1	kept
Payer status	payer status	payer status	payer status	Payer status	1	kept
Payment error rate measurement	payment error rate measurement	payment error rate measurement	payment error rate measurement	Payment error rate measurement	1	kept
Payment reform	payment reform	payment reform	payment reform	payment reform	1	case_or_format
payor disposition	payor disposition	payor disposition	payor disposition	payor disposition	1	kept
Payroll-Based Journaling	payroll based journaling	payroll based journaling	payroll based journaling	Payroll-Based Journaling	1	kept
PCAP	pcap	pcap	pcap	PCAP	1	kept
PCC	pcc	pcc	pcc	PCC	1	kept
PCC3	pcc3	pcc3	pcc3	PCC3	1	kept
PCI percutaneous coronary intervention	pci percutaneous coronary intervention	pci percutaneous coronary intervention	pci percutaneous coronary intervention	PCI percutaneous coronary intervention	1	kept
PCICS	pcics	pcics	pcics	PCICS	1	kept
PCL-C	pcl c	pcl c	pcl c	PCL-C	1	kept
PCPC, Pediatric Cerebral Performance Category	pcpc pediatric cerebral performance category	pcpc pediatric cerebral performance category	pcpc pediatric cerebral performance category	PCPC, Pediatric Cerebral Performance Category	1	kept
PCQN	pcqn	pcqn	pcqn	PCQN	1	kept
PDCA cycle	pdca cycle	pdca cycle	pdca cycle	PDCA cycle	1	kept
PDCA cycle model management	pdca cycle model management	pdca cycle model management	pdca cycle model management	PDCA cycle model management	1	kept

PDCA cycle of care	pdca cycle of care	pdca cycle of care	pdca cycle of care	PDCA cycle of care	1	kept
pdsa cycle	pdsa cycle	pdsa cycle	pdsa cycle	PDSA cycle	1	case_or_format
PDT	pdt	pdt	pdt	PDT	1	kept
PE = pulmonary embolus	pe pulmonary embolus	pe pulmonary embolus	pe pulmonary embolus	PE = pulmonary embolus	1	kept
peace and power	peace and power	peace and power	peace and power	peace and power	1	kept
Peaceful death	peaceful death	peaceful death	peaceful death	Peaceful death	1	kept
Peadiatrics	peadiatrics	peadiatrics	peadiatrics	Peadiatrics	1	kept
Peak airway pressure	peak airway pressure	peak airway pressure	peak airway pressure	Peak airway pressure	1	kept
peak airway pressure	peak airway pressure	peak airway pressure	peak airway pressure	Peak airway pressure	1	case_or_format
PEAK EXPIRATORY FLOW	peak expiratory flow	peak expiratory flow	peak expiratory flow	peak expiratory flow	1	case_or_format
Peak Expiratory Flow Rate	peak expiratory flow rate	peak expiratory flow rate	peak expiratory flow rate	Peak Expiratory Flow Rate	1	kept
Peak inspiratory pressure	peak inspiratory pressure	peak inspiratory pressure	peak inspiratory pressure	peak inspiratory pressure	1	case_or_format
peak oxygen uptake	peak oxygen uptake	peak oxygen uptake	peak oxygen uptake	peak oxygen uptake	1	kept
peak systolic velocity	peak systolic velocity	peak systolic velocity	peak systolic velocity	peak systolic velocity	1	kept
Pearson's Chi-square	pearson s chi square	pearson s chi square	pearson s chi square	Pearson's Chi-square	1	kept
Pearson's r	pearson s r	pearson s r	pearson s r	Pearson's r	1	kept

pectoralis major muscle	pectoralis major muscle	pectoralis major muscle	pectoralis major muscle	pectoralis major muscle	1	kept
PEDAGOGY	pedagogy	pedagogy	pedagogy	PEDAGOGY	1	kept
pedagogy	pedagogy	pedagogy	pedagogy	PEDAGOGY	1	case_or_format
Pedestrian	pedestrian	pedestrian	pedestrian	pedestrian	1	case_or_format
pedestrian safety	pedestrian safety	pedestrian safety	pedestrian safety	pedestrian safety	1	kept
Pedestrian street crossings	pedestrian street crossings	pedestrian street crossings	pedestrian street crossings	Pedestrian street crossings	1	kept
Pedestrians	pedestrians	pedestrians	pedestrian	pedestrian	1	singular
pediatric ABCDEF bundle	pediatric abcdef bundle	pediatric abcdef bundle	pediatric abcdef bundle	pediatric ABCDEF bundle	1	kept
Pediatric acute care unit	pediatric acute care unit	pediatric acute care unit	pediatric acute care unit	Pediatric acute care unit	1	kept
Pediatric acute chest syndrome	pediatric acute chest syndrome	pediatric acute chest syndrome	pediatric acute chest syndrome	Pediatric acute chest syndrome	1	kept
Pediatric acute liver failure	pediatric acute liver failure	pediatric acute liver failure	pediatric acute liver failure	Pediatric acute liver failure	1	kept
pediatric acute lung injury and sepsis investigator	pediatric acute lung injury and sepsis investigator	pediatric acute lung injury and sepsis investigator	pediatric acute lung injury and sepsis investigator	pediatric acute lung injury and sepsis investigator	1	kept
Pediatric Acute Respiratory Distress Syndrome	pediatric acute respiratory distress syndrome	pediatric acute respiratory distress syndrome	pediatric acute respiratory distress syndrome	Pediatric Acute Respiratory Distress Syndrome	1	kept
pediatric acute respiratory distress syndrome	pediatric acute respiratory distress syndrome	pediatric acute respiratory distress syndrome	pediatric acute respiratory distress syndrome	Pediatric Acute Respiratory Distress Syndrome	1	case_or_format
pediatric advance care planning	pediatric advance care planning	pediatric advance care planning	pediatric advance care planning	pediatric advance care planning	1	kept
pediatric aerosol	pediatric aerosol	pediatric aerosol	pediatric aerosol	pediatric aerosol	1	kept

pediatric airway	pediatric airway	pediatric airway	pediatric airway	pediatric airway	1	kept
Pediatric airway management	pediatric airway management	pediatric airway management	pediatric airway management	Pediatric airway management	1	kept
PEDIATRIC ANESTHESIA	pediatric anesthesia	pediatric anesthesia	pediatric anesthesia	pediatric anesthesia	1	case_or_format
Pediatric Anesthesia	pediatric anesthesia	pediatric anesthesia	pediatric anesthesia	pediatric anesthesia	1	case_or_format
Pediatric anesthesia	pediatric anesthesia	pediatric anesthesia	pediatric anesthesia	pediatric anesthesia	1	case_or_format
Pediatric Anesthesia Emergence Delirium	pediatric anesthesia emergence delirium	pediatric anesthesia emergence delirium	pediatric anesthesia emergence delirium	Pediatric Anesthesia Emergence Delirium	1	kept
pediatric anesthesiologist	pediatric anesthesiologist	pediatric anesthesiologist	pediatric anesthesiologist	pediatric anesthesiologist	1	kept
Pediatric anesthesiology	pediatric anesthesiology	pediatric anesthesiology	pediatric anesthesiology	Pediatric anesthesiology	1	kept
pediatric anesthesiology	pediatric anesthesiology	pediatric anesthesiology	pediatric anesthesiology	Pediatric anesthesiology	1	case_or_format
pediatric aortic trauma	pediatric aortic trauma	pediatric aortic trauma	pediatric aortic trauma	pediatric aortic trauma	1	kept
Pediatric arrhythmia	pediatric arrhythmia	pediatric arrhythmia	pediatric arrhythmia	Pediatric arrhythmia	1	kept
Pediatric Assistants	pediatric assistants	pediatric assistants	pediatric assistants	Pediatric Assistants	1	kept
Pediatric brain tumors	pediatric brain tumors	pediatric brain tumors	pediatric brain tumors	Pediatric brain tumors	1	kept
PEDIATRIC BURNS	pediatric burns	pediatric burns	pediatric burns	PEDIATRIC BURNS	1	kept
pediatric canadian triage acuity scale	pediatric canadian triage acuity scale	pediatric canadian triage acuity scale	pediatric canadian triage acuity scale	pediatric canadian triage acuity scale	1	kept
Pediatric Cardiac Acute Care Unit	pediatric cardiac acute care unit	pediatric cardiac acute care unit	pediatric cardiac acute care unit	Pediatric Cardiac Acute Care Unit	1	kept

Pediatric cardiac arrest	pediatric cardiac arrest	pediatric cardiac arrest	pediatric cardiac arrest	Pediatric cardiac arrest	1	kept
pediatric cardiac critical	pediatric cardiac critical	pediatric cardiac critical	pediatric cardiac critical	pediatric cardiac critical	1	kept
Pediatric Cardiac Program	pediatric cardiac program	pediatric cardiac program	pediatric cardiac program	Pediatric Cardiac Program	1	kept
Pediatric Cardiac Quality of Life Inventory	pediatric cardiac quality of life inventory	pediatric cardiac quality of life inventory	pediatric cardiac quality of life inventory	Pediatric Cardiac Quality of Life Inventory	1	kept
pediatric cardiac service	pediatric cardiac service	pediatric cardiac service	pediatric cardiac service	pediatric cardiac service	1	kept
Pediatric cardiology	pediatric cardiology	pediatric cardiology	pediatric cardiology	pediatric cardiology	1	case_or_format
pediatric care provider	pediatric care provider	pediatric care provider	pediatric care provider	pediatric care provider	1	kept
pediatric care providers	pediatric care providers	pediatric care providers	pediatric care provider	pediatric care provider	1	singular
Pediatric Cerebral Performance Category Scale	pediatric cerebral performance category scale	pediatric cerebral performance category scale	pediatric cerebral performance category scale	Pediatric Cerebral Performance Category Scale	1	kept
Pediatric child hospitalized	pediatric child hospitalized	pediatric child hospitalized	pediatric child hospitalized	Pediatric child hospitalized	1	kept
pediatric chronic conditions	pediatric chronic conditions	pediatric chronic conditions	pediatric chronic conditions	pediatric chronic conditions	1	kept
pediatric chronic kidney disease	pediatric chronic kidney disease	pediatric chronic kidney disease	pediatric chronic kidney disease	pediatric chronic kidney disease	1	kept
pediatric CKRT	pediatric ckrt	pediatric ckrt	pediatric ckrt	pediatric CKRT	1	kept
pediatric communication issues	pediatric communication issues	pediatric communication issues	pediatric communication issues	pediatric communication issues	1	kept
pediatric craniopharyngioma	pediatric craniopharyngioma	pediatric craniopharyngioma	pediatric craniopharyngioma	pediatric craniopharyngioma	1	kept
PEDIATRIC CRITICAL ILLNESS	pediatric critical illness	pediatric critical illness	pediatric critical illness	PEDIATRIC CRITICAL ILLNESS	1	kept

PEDIATRIC DELIRIUM	pediatric delirium	pediatric delirium	pediatric delirium	pediatric delirium	1	case_or_format
pediatric dentist	pediatric dentist	pediatric dentist	pediatric dentist	pediatric dentist	1	kept
pediatric dialysis	pediatric dialysis	pediatric dialysis	pediatric dialysis	pediatric dialysis	1	kept
pediatric disaster service	pediatric disaster service	pediatric disaster service	pediatric disaster service	pediatric disaster service	1	kept
Pediatric DKA	pediatric dka	pediatric dka	pediatric dka	Pediatric DKA	1	kept
Pediatric Early Warning Scores	pediatric early warning scores	pediatric early warning scores	pediatric early warning score	Pediatric Early Warning Score	1	singular
pediatric early warning system score	pediatric early warning system score	pediatric early warning system score	pediatric early warning system score	pediatric early warning system score	1	kept
Pediatric Early Warning Systems	pediatric early warning systems	pediatric early warning systems	pediatric early warning system	Pediatric Early Warning System	1	singular
Pediatric early warning systems	pediatric early warning systems	pediatric early warning systems	pediatric early warning system	Pediatric Early Warning System	1	singular
Pediatric emergencies	pediatric emergencies	pediatric emergencies	pediatric emergency	pediatric emergency	1	singular
pediatric emergencies	pediatric emergencies	pediatric emergencies	pediatric emergency	pediatric emergency	1	singular
Pediatric emergency care	pediatric emergency care	pediatric emergency care	pediatric emergency care	Pediatric emergency care	1	kept
Pediatric emergency care education	pediatric emergency care education	pediatric emergency care education	pediatric emergency care education	Pediatric emergency care education	1	kept
Pediatric Emergency Department	pediatric emergency department	pediatric emergency department	pediatric emergency department	Pediatric emergency department	1	case_or_format
pediatric emergency department	pediatric emergency department	pediatric emergency department	pediatric emergency department	Pediatric emergency department	1	case_or_format
Pediatric Emergency Departments	pediatric emergency departments	pediatric emergency departments	pediatric emergency department	Pediatric emergency department	1	singular

Pediatric Emergency Medicine [H02 403 670 450]	pediatric emergency medicine h02 403 670 450	pediatric emergency medicine h02 403 670 450	pediatric emergency medicine h02 403 670 450	Pediatric Emergency Medicine [H02 403 670 450]	1	kept
pediatric emergency physician	pediatric emergency physician	pediatric emergency physician	pediatric emergency physician	pediatric emergency physician	1	kept
Pediatric end-of-life care	pediatric end of life care	pediatric end of life care	pediatric end of life care	Pediatric end-of-life care	1	kept
pediatric end-of-life care	pediatric end of life care	pediatric end of life care	pediatric end of life care	Pediatric end-of-life care	1	case_or_format
Pediatric Endocrinology	pediatric endocrinology	pediatric endocrinology	pediatric endocrinology	Pediatric Endocrinology	1	kept
pediatric equipment	pediatric equipment	pediatric equipment	pediatric equipment	pediatric equipment	1	kept
Pediatric feeding disorders	pediatric feeding disorders	pediatric feeding disorders	pediatric feeding disorders	Pediatric feeding disorders	1	kept
Pediatric field hospital	pediatric field hospital	pediatric field hospital	pediatric field hospital	Pediatric field hospital	1	kept
pediatric fluid management	pediatric fluid management	pediatric fluid management	pediatric fluid management	pediatric fluid management	1	kept
Pediatric formulation	pediatric formulation	pediatric formulation	pediatric formulation	Pediatric formulation	1	kept
Pediatric FOUR score scale	pediatric four score scale	pediatric four score scale	pediatric four score scale	Pediatric FOUR score scale	1	kept
Pediatric gastroenteritis	pediatric gastroenteritis	pediatric gastroenteritis	pediatric gastroenteritis	Pediatric gastroenteritis	1	kept
Pediatric gastroenterology	pediatric gastroenterology	pediatric gastroenterology	pediatric gastroenterology	Pediatric gastroenterology	1	kept
Pediatric guidelines	pediatric guidelines	pediatric guidelines	pediatric guidelines	Pediatric guidelines	1	kept
Pediatric head injury	pediatric head injury	pediatric head injury	pediatric head injury	Pediatric head injury	1	kept
pediatric heart failure	pediatric heart failure	pediatric heart failure	pediatric heart failure	pediatric heart failure	1	kept

pediatric hematology	pediatric hematology	pediatric hematology	pediatric hematology	pediatric hematology	1	kept
pediatric hospice care	pediatric hospice care	pediatric hospice care	pediatric hospice care	pediatric hospice care	1	kept
pediatric hospital medicine	pediatric hospital medicine	pediatric hospital medicine	pediatric hospital medicine	Pediatric hospital medicine	1	case_or_format
pediatric hospital palliative care	pediatric hospital palliative care	pediatric hospital palliative care	pediatric hospital palliative care	pediatric hospital palliative care	1	kept
Pediatric hospitalization	pediatric hospitalization	pediatric hospitalization	pediatric hospitalization	Pediatric hospitalization	1	kept
pediatric illness care	pediatric illness care	pediatric illness care	pediatric illness care	pediatric illness care	1	kept
pediatric index of mortality	pediatric index of mortality	pediatric index of mortality	pediatric index of mortality	Pediatric Index of Mortality	1	case_or_format
pediatric index of mortality 2 score	pediatric index of mortality 2 score	pediatric index of mortality 2 score	pediatric index of mortality 2 score	pediatric index of mortality 2 score	1	kept
Pediatric infectious diseases	pediatric infectious diseases	pediatric infectious diseases	pediatric infectious diseases	Pediatric infectious diseases	1	kept
pediatric injury	pediatric injury	pediatric injury	pediatric injury	pediatric injury	1	kept
pediatric intensive unit	pediatric intensive unit	pediatric intensive unit	pediatric intensive unit	pediatric intensive unit	1	kept
pediatric intermediate care unit	pediatric intermediate care unit	pediatric intermediate care unit	pediatric intermediate care unit	pediatric intermediate care unit	1	kept
Pediatric Korean Triage and Acuity Scale	pediatric korean triage and acuity scale	pediatric korean triage and acuity scale	pediatric korean triage and acuity scale	Pediatric Korean Triage and Acuity Scale	1	kept
Pediatric level I trauma center	pediatric level i trauma center	pediatric level i trauma center	pediatric level i trauma center	Pediatric level I trauma center	1	kept
pediatric logistic organ dysfunction	pediatric logistic organ dysfunction	pediatric logistic organ dysfunction	pediatric logistic organ dysfunction	pediatric logistic organ dysfunction	1	kept
pediatric logistic organ dysfunction score	pediatric logistic organ dysfunction score	pediatric logistic organ dysfunction score	pediatric logistic organ dysfunction score	pediatric logistic organ dysfunction score	1	kept

pediatric long term extended care facilities	pediatric long term extended care facilities	pediatric long term extended care facilities	pediatric long term extended care facilities	pediatric long term extended care facilities	1	kept
Pediatric long-term care	pediatric long term care	pediatric long term care	pediatric long term care	Pediatric long-term care	1	kept
Pediatric loss	pediatric loss	pediatric loss	pediatric loss	Pediatric loss	1	kept
pediatric medical subspecialists	pediatric medical subspecialists	pediatric medical subspecialists	pediatric medical subspecialists	pediatric medical subspecialists	1	kept
pediatric medical surgical unit	pediatric medical surgical unit	pediatric medical surgical unit	pediatric medical surgical unit	pediatric medical surgical unit	1	kept
PEDIATRIC MEDICATION ERRORS	pediatric medication errors	pediatric medication errors	pediatric medication errors	PEDIATRIC MEDICATION ERRORS	1	kept
Pediatric minimally invasive surgery	pediatric minimally invasive surgery	pediatric minimally invasive surgery	pediatric minimally invasive surgery	Pediatric minimally invasive surgery	1	kept
pediatric mobilization	pediatric mobilization	pediatric mobilization	pediatric mobilization	pediatric mobilization	1	kept
pediatric mock code	pediatric mock code	pediatric mock code	pediatric mock code	pediatric mock code	1	kept
pediatric neonatal transport team	pediatric neonatal transport team	pediatric neonatal transport team	pediatric neonatal transport team	pediatric neonatal transport team	1	kept
Pediatric Neurohospitalist	pediatric neurohospitalist	pediatric neurohospitalist	pediatric neurohospitalist	Pediatric Neurohospitalist	1	kept
Pediatric Neurology	pediatric neurology	pediatric neurology	pediatric neurology	Pediatric Neurology	1	kept
pediatric nutrition	pediatric nutrition	pediatric nutrition	pediatric nutrition	pediatric nutrition	1	kept
Pediatric Nutrition Risk Score	pediatric nutrition risk score	pediatric nutrition risk score	pediatric nutrition risk score	Pediatric Nutrition Risk Score	1	kept
pediatric nutrition risk score	pediatric nutrition risk score	pediatric nutrition risk score	pediatric nutrition risk score	Pediatric Nutrition Risk Score	1	case_or_format
Pediatric Nutrition Screen Tool	pediatric nutrition screen tool	pediatric nutrition screen tool	pediatric nutrition screen tool	Pediatric Nutrition Screen Tool	1	kept

Pediatric oncologic emergency	pediatric oncologic emergency	pediatric oncologic emergency	pediatric oncologic emergency	Pediatric oncologic emergency	1	kept
PEDIATRIC ONCOLOGY	pediatric oncology	pediatric oncology	pediatric oncology	pediatric oncology	1	case_or_format
pediatric otolaryngology	pediatric otolaryngology	pediatric otolaryngology	pediatric otolaryngology	pediatric otolaryngology	1	kept
pediatric overall performance category	pediatric overall performance category	pediatric overall performance category	pediatric overall performance category	pediatric overall performance category	1	kept
pediatric pain	pediatric pain	pediatric pain	pediatric pain	Pediatric pain	1	case_or_format
pediatric pain control	pediatric pain control	pediatric pain control	pediatric pain control	pediatric pain control	1	kept
Pediatric pain management	pediatric pain management	pediatric pain management	pediatric pain management	Pediatric pain management	1	kept
pediatric pain management	pediatric pain management	pediatric pain management	pediatric pain management	Pediatric pain management	1	case_or_format
PEDIATRIC PALLIATIVE CARE	pediatric palliative care	pediatric palliative care	pediatric palliative care	pediatric palliative care	1	case_or_format
pediatric palliative care consult	pediatric palliative care consult	pediatric palliative care consult	pediatric palliative care consult	pediatric palliative care consult	1	kept
pediatric palliative care services	pediatric palliative care services	pediatric palliative care services	pediatric palliative care services	pediatric palliative care services	1	kept
Pediatric post-acute care	pediatric post acute care	pediatric post acute care	pediatric post acute care	Pediatric post-acute care	1	kept
pediatric practice	pediatric practice	pediatric practice	pediatric practice	pediatric practice	1	kept
Pediatric pressure injury	pediatric pressure injury	pediatric pressure injury	pediatric pressure injury	Pediatric pressure injury	1	kept
Pediatric pressure reduction surfaces	pediatric pressure reduction surfaces	pediatric pressure reduction surfaces	pediatric pressure reduction surfaces	Pediatric pressure reduction surfaces	1	kept
Pediatric pressure ulcers	pediatric pressure ulcers	pediatric pressure ulcers	pediatric pressure ulcers	Pediatric pressure ulcers	1	kept

Pediatric providers	pediatric providers	pediatric providers	pediatric providers	Pediatric providers	1	kept
pediatric pulmonary hypertension network	pediatric pulmonary hypertension network	pediatric pulmonary hypertension network	pediatric pulmonary hypertension network	pediatric pulmonary hypertension network	1	kept
Pediatric Pulmonology	pediatric pulmonology	pediatric pulmonology	pediatric pulmonology	Pediatric Pulmonology	1	kept
Pediatric radiology	pediatric radiology	pediatric radiology	pediatric radiology	pediatric radiology	1	case_or_format
pediatric rape	pediatric rape	pediatric rape	pediatric rape	pediatric rape	1	kept
Pediatric readiness	pediatric readiness	pediatric readiness	pediatric readiness	Pediatric readiness	1	kept
pediatric readiness	pediatric readiness	pediatric readiness	pediatric readiness	Pediatric readiness	1	case_or_format
Pediatric reanimation	pediatric reanimation	pediatric reanimation	pediatric reanimation	Pediatric reanimation	1	kept
pediatric research ward	pediatric research ward	pediatric research ward	pediatric research ward	pediatric research ward	1	kept
pediatric resident	pediatric resident	pediatric resident	pediatric resident	pediatric resident	1	kept
Pediatric respiratory distress	pediatric respiratory distress	pediatric respiratory distress	pediatric respiratory distress	Pediatric respiratory distress	1	kept
pediatric restraint orders	pediatric restraint orders	pediatric restraint orders	pediatric restraint orders	pediatric restraint orders	1	kept
PEDIATRIC RESUSCITATION	pediatric resuscitation	pediatric resuscitation	pediatric resuscitation	Pediatric resuscitation	1	case_or_format
pediatric rheumatology	pediatric rheumatology	pediatric rheumatology	pediatric rheumatology	pediatric rheumatology	1	kept
pediatric risk of mortality	pediatric risk of mortality	pediatric risk of mortality	pediatric risk of mortality	Pediatric Risk of Mortality	1	case_or_format
Pediatric Risk of Mortality I111 score	pediatric risk of mortality i111 score	pediatric risk of mortality i111 score	pediatric risk of mortality i111 score	Pediatric Risk of Mortality I111 score	1	kept

Pediatric Risk of Mortality III score	pediatric risk of mortality iii score	pediatric risk of mortality iii score	pediatric risk of mortality iii score	Pediatric Risk of Mortality III score	1	kept
Pediatric Risk of Mortality score	pediatric risk of mortality score	pediatric risk of mortality score	pediatric risk of mortality score	Pediatric Risk of Mortality score	1	kept
pediatric risk of mortality score iii	pediatric risk of mortality score iii	pediatric risk of mortality score iii	pediatric risk of mortality score iii	pediatric risk of mortality score iii	1	kept
Pediatric robotically assisted surgery	pediatric robotically assisted surgery	pediatric robotically assisted surgery	pediatric robotically assisted surgery	Pediatric robotically assisted surgery	1	kept
Pediatric sedation program	pediatric sedation program	pediatric sedation program	pediatric sedation program	Pediatric sedation program	1	kept
Pediatric sedation protocol	pediatric sedation protocol	pediatric sedation protocol	pediatric sedation protocol	Pediatric sedation protocol	1	kept
Pediatric septic shock	pediatric septic shock	pediatric septic shock	pediatric septic shock	Pediatric septic shock	1	kept
Pediatric Stroke	pediatric stroke	pediatric stroke	pediatric stroke	Pediatric Stroke	1	kept
Pediatric stroke	pediatric stroke	pediatric stroke	pediatric stroke	Pediatric Stroke	1	case_or_format
Pediatric support surfaces	pediatric support surfaces	pediatric support surfaces	pediatric support surfaces	Pediatric support surfaces	1	kept
Pediatric surgical emergencies	pediatric surgical emergencies	pediatric surgical emergencies	pediatric surgical emergencies	Pediatric surgical emergencies	1	kept
pediatric surgical specialists	pediatric surgical specialists	pediatric surgical specialists	pediatric surgical specialists	pediatric surgical specialists	1	kept
pediatric telehealth	pediatric telehealth	pediatric telehealth	pediatric telehealth	pediatric telehealth	1	kept
pediatric TEVAR	pediatric tevar	pediatric tevar	pediatric tevar	pediatric TEVAR	1	kept
Pediatric transport	pediatric transport	pediatric transport	pediatric transport	Pediatric transport	1	kept
Pediatric transport team	pediatric transport team	pediatric transport team	pediatric transport team	Pediatric transport team	1	kept

Pediatric Trauma	pediatric trauma	pediatric trauma	pediatric trauma	Pediatric trauma	1	case_or_format
Pediatric trauma cases	pediatric trauma cases	pediatric trauma cases	pediatric trauma cases	Pediatric trauma cases	1	kept
pediatric trauma resuscitation module	pediatric trauma resuscitation module	pediatric trauma resuscitation module	pediatric trauma resuscitation module	pediatric trauma resuscitation module	1	kept
Pediatric traumatic brain injury	pediatric traumatic brain injury	pediatric traumatic brain injury	pediatric traumatic brain injury	Pediatric traumatic brain injury	1	kept
pediatric traumatic brain injury	pediatric traumatic brain injury	pediatric traumatic brain injury	pediatric traumatic brain injury	Pediatric traumatic brain injury	1	case_or_format
Pediatric vascular access	pediatric vascular access	pediatric vascular access	pediatric vascular access	Pediatric vascular access	1	kept
Pediatric Yorkhill Malnutrition Score	pediatric yorkhill malnutrition score	pediatric yorkhill malnutrition score	pediatric yorkhill malnutrition score	Pediatric Yorkhill Malnutrition Score	1	kept
Pediatrician	pediatrician	pediatrician	pediatrician	pediatrician	1	case_or_format
pediatricians	pediatricians	pediatricians	pediatrician	pediatrician	1	singular
Pediatrics care	pediatrics care	pediatrics care	pediatrics care	Pediatrics care	1	kept
Pediatrics Translating	pediatrics translating	pediatrics translating	pediatrics translating	Pediatrics Translating	1	kept
Pediatrix	pediatrix	pediatrix	pediatrix	Pediatrix	1	kept
Pedicle Flap	pedicle flap	pedicle flap	pedicle flap	Pedicle Flap	1	kept
pedicle screw	pedicle screw	pedicle screw	pedicle screw	pedicle screw	1	kept
Pedicle Screws	pedicle screws	pedicle screws	pedicle screw	pedicle screw	1	singular
Pedometer	pedometer	pedometer	pedometer	pedometer	1	case_or_format

PEDRO SCALE	pedro scale	pedro scale	pedro scale	PEDRO SCALE	1	kept
PEEP, positive end expiratory pressure	peep positive end expiratory pressure	peep positive end expiratory pressure	peep positive end expiratory pressure	PEEP, positive end expiratory pressure	1	kept
Peer assistance	peer assistance	peer assistance	peer assistance	Peer assistance	1	kept
peer counselling	peer counselling	peer counselling	peer counselling	peer counselling	1	kept
Peer counselor	peer counselor	peer counselor	peer counselor	Peer counselor	1	kept
peer education	peer education	peer education	peer education	peer education	1	kept
Peer group	peer group	peer group	peer group	Peer Group	1	case_or_format
Peer groups	peer groups	peer groups	peer group	Peer Group	1	singular
peer mentors	peer mentors	peer mentors	peer mentors	peer mentors	1	kept
peer reviewer feedback	peer reviewer feedback	peer reviewer feedback	peer reviewer feedback	peer reviewer feedback	1	kept
PEER SUPPORT	peer support	peer support	peer support	peer support	1	case_or_format
peer support workers	peer support workers	peer support workers	peer support workers	peer support workers	1	kept
Peer-leader	peer leader	peer leader	peer leader	Peer-leader	1	kept
peer-support	peer support	peer support	peer support	peer support	1	case_or_format
pefloxacin	pefloxacin	pefloxacin	pefloxacin	pefloxacin	1	kept
PEI	pei	pei	pei	PEI	1	kept

Peircardial effusion	peircardial effusion	peircardial effusion	peircardial effusion	Peircardial effusion	1	kept
Pelargonium	pelargonium	pelargonium	pelargonium	Pelargonium	1	kept
Pelargonium graveolens	pelargonium graveolens	pelargonium graveolens	pelargonium graveolens	Pelargonium graveolens	1	kept
Pele	pele	pele	pele	Pele	1	kept
Pelizaeus Merzbacher disease	pelizaeus merzbacher disease	pelizaeus merzbacher disease	pelizaeus merzbacher disease	Pelizaeus Merzbacher disease	1	kept
Pelvic Exenteration	pelvic exenteration	pelvic exenteration	pelvic exenteration	Pelvic Exenteration	1	kept
Pelvic exenteration	pelvic exenteration	pelvic exenteration	pelvic exenteration	Pelvic Exenteration	1	case_or_format
pelvic floor muscle training	pelvic floor muscle training	pelvic floor muscle training	pelvic floor muscle training	pelvic floor muscle training	1	kept
Pelvic fractures	pelvic fractures	pelvic fractures	pelvic fracture	pelvic fracture	1	singular
Pelvic Neoplasms	pelvic neoplasms	pelvic neoplasms	pelvic neoplasms	Pelvic Neoplasms	1	kept
pelvic organ prolapse	pelvic organ prolapse	pelvic organ prolapse	pelvic organ prolapse	pelvic organ prolapse	1	kept
Pelvic-perineal pathway	pelvic perineal pathway	pelvic perineal pathway	pelvic perineal pathway	Pelvic-perineal pathway	1	kept
pelvis cancer	pelvis cancer	pelvis cancer	pelvis cancer	pelvis cancer	1	kept
pelvis exenteration	pelvis exenteration	pelvis exenteration	pelvis exenteration	pelvis exenteration	1	kept
pelvis floor	pelvis floor	pelvis floor	pelvis floor	pelvis floor	1	kept
pelvis surgery	pelvis surgery	pelvis surgery	pelvis surgery	pelvis surgery	1	kept

pelvis tumor	pelvis tumor	pelvis tumor	pelvis tumor	pelvis tumor	1	kept
pembrolizumab	pembrolizumab	pembrolizumab	pembrolizumab	pembrolizumab	1	kept
pemetrexed	pemetrexed	pemetrexed	pemetrexed	pemetrexed	1	kept
pemphigoid	pemphigoid	pemphigoid	pemphigoid	pemphigoid	1	kept
pemphigoid gestationis	pemphigoid gestationis	pemphigoid gestationis	pemphigoid gestationis	pemphigoid gestationis	1	kept
pemphigus foliaceus	pemphigus foliaceus	pemphigus foliaceus	pemphigus foliaceus	pemphigus foliaceus	1	kept
Pemphigus, Benign Familial	pemphigus benign familial	pemphigus benign familial	pemphigus benign familial	Pemphigus, Benign Familial	1	kept
penciclovir	penciclovir	penciclovir	penciclovir	penciclovir	1	kept
Penetrating spinal cord injury	penetrating spinal cord injury	penetrating spinal cord injury	penetrating spinal cord injury	Penetrating spinal cord injury	1	kept
penetration aspiration scale	penetration aspiration scale	penetration aspiration scale	penetration aspiration scale	penetration aspiration scale	1	kept
Penicillamine	penicillamine	penicillamine	penicillamine	Penicillamine	1	kept
penicillin binding protein	penicillin binding protein	penicillin binding protein	penicillin binding protein	Penicillin-Binding Proteins	1	case_or_format
Penicillin G	penicillin g	penicillin g	penicillin g	penicillin G	1	case_or_format
penicillin G potassium	penicillin g potassium	penicillin g potassium	penicillin g potassium	penicillin G potassium	1	kept
penicillin V	penicillin v	penicillin v	penicillin v	penicillin V	1	kept
penicillin V potassium	penicillin v potassium	penicillin v potassium	penicillin v potassium	penicillin V potassium	1	kept

penicilliosis	penicilliosis	penicilliosis	penicilliosis	penicilliosis	1	kept
PENICILLIUM	penicillium	penicillium	penicillium	PENICILLIUM	1	kept
Penis	penis	penis	penis	Penis	1	kept
penis carcinoma	penis carcinoma	penis carcinoma	penis carcinoma	penis carcinoma	1	kept
Penn Arthroplasty Risk Score	penn arthroplasty risk score	penn arthroplasty risk score	penn arthroplasty risk score	Penn Arthroplasty Risk Score	1	kept
Pentamidine	pentamidine	pentamidine	pentamidine	pentamidine	1	case_or_format
Pentasaccharide	pentasaccharide	pentasaccharide	pentasaccharide	Pentasaccharide	1	kept
pentetate calcium trisodium	pentetate calcium trisodium	pentetate calcium trisodium	pentetate calcium trisodium	pentetate calcium trisodium	1	kept
PENTOBARBITAL SODIUM	pentobarbital sodium	pentobarbital sodium	pentobarbital sodium	PENTOBARBITAL SODIUM	1	kept
pentoxifylline	pentoxifylline	pentoxifylline	pentoxifylline	pentoxifylline	1	kept
People	people	people	people	PEOPLE	1	case_or_format
people by drinking status	people by drinking status	people by drinking status	people by drinking status	people by drinking status	1	kept
PEOPLE DIE	people die	people die	people die	PEOPLE DIE	1	kept
people with dementia	people with dementia	people with dementia	people with dementia	people with dementia	1	kept
people with disabilities	people with disabilities	people with disabilities	people with disabilities	people with disabilities	1	kept
PEPPA framework	peppa framework	peppa framework	peppa framework	PEPPA framework	1	kept

peppermint	peppermint	peppermint	peppermint	peppermint	1	kept
Peppermint aroma therapy	peppermint aroma therapy	peppermint aroma therapy	peppermint aroma therapy	Peppermint aroma therapy	1	kept
peppermint oil	peppermint oil	peppermint oil	peppermint oil	peppermint oil	1	kept
Pepsin	pepsin	pepsin	pepsin	Pepsin	1	kept
Pepsin A	pepsin a	pepsin a	pepsin a	pepsin A	1	case_or_format
Peptic	peptic	peptic	peptic	Peptic	1	kept
peptic ulcer bleeding	peptic ulcer bleeding	peptic ulcer bleeding	peptic ulcer bleeding	peptic ulcer bleeding	1	kept
peptide hydrolase	peptide hydrolase	peptide hydrolase	peptide hydrolase	peptide hydrolase	1	kept
Peptide Hydrolases	peptide hydrolases	peptide hydrolases	peptide hydrolase	peptide hydrolase	1	singular
Peptide Nucleic Acids	peptide nucleic acids	peptide nucleic acids	peptide nucleic acids	Peptide Nucleic Acids	1	kept
Peptidyl Transferases	peptidyl transferases	peptidyl transferases	peptidyl transferases	Peptidyl Transferases	1	kept
peptidyltransferase	peptidyltransferase	peptidyltransferase	peptidyltransferase	peptidyltransferase	1	kept
peracetic acid	peracetic acid	peracetic acid	peracetic acid	peracetic acid	1	kept
Perceived barrier	perceived barrier	perceived barrier	perceived barrier	Perceived barriers	1	case_or_format
Perceived behavioral control	perceived behavioral control	perceived behavioral control	perceived behavioral control	Perceived behavioral control	1	kept
perceived benefits	perceived benefits	perceived benefits	perceived benefits	perceived benefits	1	kept

Perceived excessive care	perceived excessive care	perceived excessive care	perceived excessive care	Perceived excessive care	1	kept
Perceived health	perceived health	perceived health	perceived health	Perceived health	1	kept
perceived health	perceived health	perceived health	perceived health	Perceived health	1	case_or_format
Perceived inappropriate care	perceived inappropriate care	perceived inappropriate care	perceived inappropriate care	Perceived inappropriate care	1	kept
Perceived Inventory	perceived inventory	perceived inventory	perceived inventory	Perceived Inventory	1	kept
Perceived level of stress	perceived level of stress	perceived level of stress	perceived level of stress	Perceived level of stress	1	kept
Perceived Maternal Parenting Self Efficacy	perceived maternal parenting self efficacy	perceived maternal parenting self efficacy	perceived maternal parenting self efficacy	Perceived Maternal Parenting Self Efficacy	1	kept
Perceived need	perceived need	perceived need	perceived need	Perceived needs	1	case_or_format
PERCEIVED NEEDS	perceived needs	perceived needs	perceived need	Perceived needs	1	singular
perceived needs	perceived needs	perceived needs	perceived need	Perceived needs	1	singular
perceived organizational innovativeness	perceived organizational innovativeness	perceived organizational innovativeness	perceived organizational innovativeness	perceived organizational innovativeness	1	kept
perceived organizational justice	perceived organizational justice	perceived organizational justice	perceived organizational justice	perceived organizational justice	1	kept
PERCEIVED ORGANIZATIONAL SUPPORT	perceived organizational support	perceived organizational support	perceived organizational support	Perceived organizational support	1	case_or_format
perceived overqualification	perceived overqualification	perceived overqualification	perceived overqualification	perceived overqualification	1	kept
PERCEIVED PRODUCTIVITY	perceived productivity	perceived productivity	perceived productivity	PERCEIVED PRODUCTIVITY	1	kept
perceived professional benefits	perceived professional benefits	perceived professional benefits	perceived professional benefits	perceived professional benefits	1	kept

Perceived respect	perceived respect	perceived respect	perceived respect	Perceived respect	1	kept
Perceived self-efficacy	perceived self efficacy	perceived self efficacy	perceived self efficacy	Perceived self-efficacy	1	kept
perceived support	perceived support	perceived support	perceived support	perceived support	1	kept
perceived value	perceived value	perceived value	perceived value	perceived value	1	kept
Perceived work stressors	perceived work stressors	perceived work stressors	perceived work stressors	Perceived work stressors	1	kept
percentage of glottis opening scale score	percentage of glottis opening scale score	percentage of glottis opening scale score	percentage of glottis opening scale score	percentage of glottis opening scale score	1	kept
percentile distribution	percentile distribution	percentile distribution	percentile distribution	percentile distribution	1	kept
percentile sound level	percentile sound level	percentile sound level	percentile sound level	percentile sound level	1	kept
perception of illness	perception of illness	perception of illness	perception of illness	perception of illness	1	kept
perception of management	perception of management	perception of management	perception of management	perception of management	1	kept
Perception of pregnancy risk	perception of pregnancy risk	perception of pregnancy risk	perception of pregnancy risk	Perception of pregnancy risk	1	kept
Perception of risk	perception of risk	perception of risk	perception of risk	Perception of risk	1	kept
Perception of stressors	perception of stressors	perception of stressors	perception of stressors	Perception of stressors	1	kept
Perceptions of engineering	perceptions of engineering	perceptions of engineering	perceptions of engineering	Perceptions of engineering	1	kept
Perceptions of needs	perceptions of needs	perceptions of needs	perceptions of needs	Perceptions of needs	1	kept
Perceptions of stressors	perceptions of stressors	perceptions of stressors	perceptions of stressors	Perceptions of stressors	1	kept

perceptual annoyance	perceptual annoyance	perceptual annoyance	perceptual annoyance	perceptual annoyance	1	kept
Perceptual bias	perceptual bias	perceptual bias	perceptual bias	Perceptual bias	1	kept
Perceptual Disorders	perceptual disorders	perceptual disorders	perceptual disorders	Perceptual Disorders	1	kept
Perceptual disturbance	perceptual disturbance	perceptual disturbance	perceptual disturbance	Perceptual disturbance	1	kept
Perceptual pressure	perceptual pressure	perceptual pressure	perceptual pressure	Perceptual pressure	1	kept
perceptual pressure	perceptual pressure	perceptual pressure	perceptual pressure	Perceptual pressure	1	case_or_format
Percieved Social Support	percieved social support	percieved social support	percieved social support	Percieved Social Support	1	kept
PERCUSSION	percussion	percussion	percussion	percussion	1	case_or_format
Percussion	percussion	percussion	percussion	percussion	1	case_or_format
Percutaneous	percutaneous	percutaneous	percutaneous	Percutaneous	1	kept
percutaneous	percutaneous	percutaneous	percutaneous	Percutaneous	1	case_or_format
percutaneous access	percutaneous access	percutaneous access	percutaneous access	percutaneous access	1	kept
percutaneous biopsy	percutaneous biopsy	percutaneous biopsy	percutaneous biopsy	percutaneous biopsy	1	kept
percutaneous cardiopulmonary assistance	percutaneous cardiopulmonary assistance	percutaneous cardiopulmonary assistance	percutaneous cardiopulmonary assistance	percutaneous cardiopulmonary assistance	1	kept
PERCUTANEOUS CATHETERIZATION	percutaneous catheterization	percutaneous catheterization	percutaneous catheterization	PERCUTANEOUS CATHETERIZATION	1	kept
Percutaneous coronary interventions	percutaneous coronary interventions	percutaneous coronary interventions	percutaneous coronary intervention	percutaneous coronary intervention	1	singular

percutaneous dilatation tracheostomy	percutaneous dilatation tracheostomy	percutaneous dilatation tracheostomy	percutaneous dilatation tracheostomy	percutaneous dilatation tracheostomy	1	kept
percutaneous dilatational methods	percutaneous dilatational methods	percutaneous dilatational methods	percutaneous dilatational methods	percutaneous dilatational methods	1	kept
Percutaneous dilatational tracheostomy#	percutaneous dilatational tracheostomy	percutaneous dilatational tracheostomy	percutaneous dilatational tracheostomy	percutaneous dilatational tracheostomy	1	case_or_format
Percutaneous dilational tracheostomy	percutaneous dilational tracheostomy	percutaneous dilational tracheostomy	percutaneous dilational tracheostomy	percutaneous dilational tracheostomy	1	case_or_format
Percutaneous endoscopic gastrostomy	percutaneous endoscopic gastrostomy	percutaneous endoscopic gastrostomy	percutaneous endoscopic gastrostomy	percutaneous endoscopic gastrostomy	1	case_or_format
Percutaneous endoscopic gastrostomy placement	percutaneous endoscopic gastrostomy placement	percutaneous endoscopic gastrostomy placement	percutaneous endoscopic gastrostomy placement	Percutaneous endoscopic gastrostomy placement	1	kept
Percutaneous exposure	percutaneous exposure	percutaneous exposure	percutaneous exposure	Percutaneous exposure	1	kept
percutaneous jejunostomy	percutaneous jejunostomy	percutaneous jejunostomy	percutaneous jejunostomy	percutaneous jejunostomy	1	kept
percutaneous mitral valve repair	percutaneous mitral valve repair	percutaneous mitral valve repair	percutaneous mitral valve repair	percutaneous mitral valve repair	1	kept
Percutaneous nephrolithotomy	percutaneous nephrolithotomy	percutaneous nephrolithotomy	percutaneous nephrolithotomy	percutaneous nephrolithotomy	1	case_or_format
Percutaneous tracheostomy	percutaneous tracheostomy	percutaneous tracheostomy	percutaneous tracheostomy	percutaneous tracheostomy	1	case_or_format
percutaneous transhepatic drainage	percutaneous transhepatic drainage	percutaneous transhepatic drainage	percutaneous transhepatic drainage	percutaneous transhepatic drainage	1	kept
percutaneous vertebroplasty	percutaneous vertebroplasty	percutaneous vertebroplasty	percutaneous vertebroplasty	percutaneous vertebroplasty	1	kept
Percutaneously inserted	percutaneously inserted	percutaneously inserted	percutaneously inserted	Percutaneously inserted	1	kept
percutwist technique	percutwist technique	percutwist technique	percutwist technique	percutwist technique	1	kept
Perfil laboral	perfil laboral	perfil laboral	perfil laboral	Perfil laboral	1	kept

perforated viscus	perforated viscus	perforated viscus	perforated viscus	perforated viscus	1	kept
perforation injury	perforation injury	perforation injury	perforation injury	perforation injury	1	kept
Perforator Flap	perforator flap	perforator flap	perforator flap	Perforator Flap	1	kept
perforator flap	perforator flap	perforator flap	perforator flap	Perforator Flap	1	case_or_format
Performance and Spinal cord injury	performance and spinal cord injury	performance and spinal cord injury	performance and spinal cord injury	Performance and Spinal cord injury	1	kept
Performance appraisal	performance appraisal	performance appraisal	performance appraisal	performance appraisal	1	case_or_format
Performance assessment	performance assessment	performance assessment	performance assessment	performance assessment	1	case_or_format
Performance Based Development System	performance based development system	performance based development system	performance based development system	Performance Based Development System	1	kept
performance bias	performance bias	performance bias	performance bias	performance bias	1	kept
performance checklist	performance checklist	performance checklist	performance checklist	performance checklist	1	kept
PERFORMANCE EVALUATION	performance evaluation	performance evaluation	performance evaluation	PERFORMANCE EVALUATION	1	kept
Performance evaluation	performance evaluation	performance evaluation	performance evaluation	PERFORMANCE EVALUATION	1	case_or_format
Performance improvements	performance improvements	performance improvements	performance improvement	Performance improvement	1	singular
Performance measure	performance measure	performance measure	performance measure	performance measures	1	case_or_format
Performance measurement	performance measurement	performance measurement	performance measurement	Performance measurement	1	kept
performance measurement	performance measurement	performance measurement	performance measurement	Performance measurement	1	case_or_format

Performance metrices	performance metrices	performance metrices	performance metrices	Performance metrices	1	kept
performance obstacle	performance obstacle	performance obstacle	performance obstacle	Performance obstacles	1	case_or_format
performance obstacles	performance obstacles	performance obstacles	performance obstacle	Performance obstacles	1	singular
Performance Oriented Mobility Assessment	performance oriented mobility assessment	performance oriented mobility assessment	performance oriented mobility assessment	Performance Oriented Mobility Assessment	1	kept
performance reporting	performance reporting	performance reporting	performance reporting	performance reporting	1	kept
PERFORMANCE-MEASURES	performance measures	performance measures	performance measure	performance measures	1	singular
performing artist	performing artist	performing artist	performing artist	performing artist	1	kept
Perfused boundary region	perfused boundary region	perfused boundary region	perfused boundary region	Perfused boundary region	1	kept
perfusion education.	perfusion education	perfusion education	perfusion education	perfusion education.	1	kept
perfusion index	perfusion index	perfusion index	perfusion index	perfusion index	1	kept
perfusion practice	perfusion practice	perfusion practice	perfusion practice	perfusion practice	1	kept
peri-implant fracture	peri implant fracture	peri implant fracture	peri implant fracture	peri-implant fracture	1	kept
peri-operative	peri operative	peri operative	peri operative	peri-operative	1	kept
Peri-operative Physicians	peri operative physicians	peri operative physicians	peri operative physicians	Peri-operative Physicians	1	kept
perianal	perianal	perianal	perianal	perianal	1	kept
perianal assessment tool	perianal assessment tool	perianal assessment tool	perianal assessment tool	perianal assessment tool	1	kept

pericardial anatomy	pericardial anatomy	pericardial anatomy	pericardial anatomy	pericardial anatomy	1	kept
pericardial disease	pericardial disease	pericardial disease	pericardial disease	pericardial disease	1	kept
pericardial drain	pericardial drain	pericardial drain	pericardial drain	pericardial drain	1	kept
Pericardial effusion	pericardial effusion	pericardial effusion	pericardial effusion	pericardial effusion	1	case_or_format
Pericardial window	pericardial window	pericardial window	pericardial window	Pericardial window	1	kept
Pericardium	pericardium	pericardium	pericardium	Pericardium	1	kept
pericardium	pericardium	pericardium	pericardium	Pericardium	1	case_or_format
Perinatal Asphyxia	perinatal asphyxia	perinatal asphyxia	perinatal asphyxia	perinatal asphyxia	1	case_or_format
Perinatal asphyxia	perinatal asphyxia	perinatal asphyxia	perinatal asphyxia	perinatal asphyxia	1	case_or_format
perinatal complications	perinatal complications	perinatal complications	perinatal complications	perinatal complications	1	kept
Perinatal continuum	perinatal continuum	perinatal continuum	perinatal continuum	Perinatal continuum	1	kept
Perinatal depression	perinatal depression	perinatal depression	perinatal depression	Perinatal depression	1	kept
perinatal depression	perinatal depression	perinatal depression	perinatal depression	Perinatal depression	1	case_or_format
perinatal emergency	perinatal emergency	perinatal emergency	perinatal emergency	perinatal emergency	1	kept
Perinatal experiences and transgenerational consequences	perinatal experiences and transgenerational consequences	perinatal experiences and transgenerational consequences	perinatal experiences and transgenerational consequences	Perinatal experiences and transgenerational consequences	1	kept
perinatal exposure	perinatal exposure	perinatal exposure	perinatal exposure	perinatal exposure	1	kept

perinatal health equity	perinatal health equity	perinatal health equity	perinatal health equity	perinatal health equity	1	kept
Perinatal health research	perinatal health research	perinatal health research	perinatal health research	Perinatal health research	1	kept
Perinatal healthcare	perinatal healthcare	perinatal healthcare	perinatal healthcare	Perinatal healthcare	1	kept
Perinatal inflammation	perinatal inflammation	perinatal inflammation	perinatal inflammation	Perinatal inflammation	1	kept
perinatal loss	perinatal loss	perinatal loss	perinatal loss	Perinatal loss	1	case_or_format
perinatal mental health	perinatal mental health	perinatal mental health	perinatal mental health	perinatal mental health	1	kept
perinatal mood disorders	perinatal mood disorders	perinatal mood disorders	perinatal mood disorders	perinatal mood disorders	1	kept
Perinatal outcome	perinatal outcome	perinatal outcome	perinatal outcome	perinatal outcome	1	case_or_format
Perinatal outcomes	perinatal outcomes	perinatal outcomes	perinatal outcome	perinatal outcome	1	singular
perinatal outcomes	perinatal outcomes	perinatal outcomes	perinatal outcome	perinatal outcome	1	singular
Perinatal Palliative Care	perinatal palliative care	perinatal palliative care	perinatal palliative care	Perinatal palliative care	1	case_or_format
perinatal palliative care	perinatal palliative care	perinatal palliative care	perinatal palliative care	Perinatal palliative care	1	case_or_format
Perinatal Post Traumatic Stress Disorder Questionnaire	perinatal post traumatic stress disorder questionnaire	perinatal post traumatic stress disorder questionnaire	perinatal post traumatic stress disorder questionnaire	Perinatal Post Traumatic Stress Disorder Questionnaire	1	kept
Perinatal Problem Identification Programme	perinatal problem identification programme	perinatal problem identification programme	perinatal problem identification programme	Perinatal Problem Identification Programme	1	kept
perinatal psychology	perinatal psychology	perinatal psychology	perinatal psychology	perinatal psychology	1	kept
perinatal quality	perinatal quality	perinatal quality	perinatal quality	perinatal quality	1	kept

Perinatal regionalization	perinatal regionalization	perinatal regionalization	perinatal regionalization	Perinatal regionalization	1	kept
perinatal systemic health	perinatal systemic health	perinatal systemic health	perinatal systemic health	perinatal systemic health	1	kept
PERINATAL-MORTALITY	perinatal mortality	perinatal mortality	perinatal mortality	Perinatal Mortality	1	case_or_format
perineal assessment tool	perineal assessment tool	perineal assessment tool	perineal assessment tool	Perineal Assessment Tool	1	case_or_format
perineal care	perineal care	perineal care	perineal care	perineal care	1	kept
Perineal dermatitis	perineal dermatitis	perineal dermatitis	perineal dermatitis	Perineal dermatitis	1	kept
perineal laceration	perineal laceration	perineal laceration	perineal laceration	perineal laceration	1	kept
perineal trauma	perineal trauma	perineal trauma	perineal trauma	perineal trauma	1	kept
perineum laceration	perineum laceration	perineum laceration	perineum laceration	perineum laceration	1	kept
Periodic discharge	periodic discharge	periodic discharge	periodic discharge	Periodic discharge	1	kept
periodic hypersomnia	periodic hypersomnia	periodic hypersomnia	periodic hypersomnia	periodic hypersomnia	1	kept
periodic paralysis	periodic paralysis	periodic paralysis	periodic paralysis	periodic paralysis	1	kept
Periodicals	periodicals	periodicals	periodicals	Periodicals	1	kept
periodontal treatment	periodontal treatment	periodontal treatment	periodontal treatment	periodontal treatment	1	kept
PERIODONTAL-DISEASE	periodontal disease	periodontal disease	periodontal disease	Periodontal Diseases	1	case_or_format
PERIOPERATIVE ANXIETY	perioperative anxiety	perioperative anxiety	perioperative anxiety	PERIOPERATIVE ANXIETY	1	kept

Perioperative assessment	perioperative assessment	perioperative assessment	perioperative assessment	Perioperative assessment	1	kept
PERIOPERATIVE BETA-BLOCKADE	perioperative beta blockade	perioperative beta blockade	perioperative beta blockade	PERIOPERATIVE BETA-BLOCKADE	1	kept
Perioperative cardiac assessment	perioperative cardiac assessment	perioperative cardiac assessment	perioperative cardiac assessment	Perioperative cardiac assessment	1	kept
Perioperative cardiac screening	perioperative cardiac screening	perioperative cardiac screening	perioperative cardiac screening	Perioperative cardiac screening	1	kept
PERIOPERATIVE CARDIAC-ARREST	perioperative cardiac arrest	perioperative cardiac arrest	perioperative cardiac arrest	PERIOPERATIVE CARDIAC-ARREST	1	kept
Perioperative care bundle	perioperative care bundle	perioperative care bundle	perioperative care bundle	Perioperative care bundle	1	kept
PERIOPERATIVE COMPLICATIONS	perioperative complications	perioperative complications	perioperative complication	perioperative complication	1	singular
Perioperative education	perioperative education	perioperative education	perioperative education	Perioperative education	1	kept
perioperative epidemiology	perioperative epidemiology	perioperative epidemiology	perioperative epidemiology	perioperative epidemiology	1	kept
perioperative glycemic management	perioperative glycemic management	perioperative glycemic management	perioperative glycemic management	perioperative glycemic management	1	kept
Perioperative infection control	perioperative infection control	perioperative infection control	perioperative infection control	Perioperative infection control	1	kept
Perioperative intervention	perioperative intervention	perioperative intervention	perioperative intervention	Perioperative intervention	1	kept
Perioperative Medicine	perioperative medicine	perioperative medicine	perioperative medicine	perioperative medicine	1	case_or_format
Perioperative morbidity and mortality	perioperative morbidity and mortality	perioperative morbidity and mortality	perioperative morbidity and mortality	Perioperative morbidity and mortality	1	kept
perioperative mortality	perioperative mortality	perioperative mortality	perioperative mortality	perioperative mortality	1	kept
perioperative neurocognitive disorders	perioperative neurocognitive disorders	perioperative neurocognitive disorders	perioperative neurocognitive disorders	perioperative neurocognitive disorders	1	kept

Perioperative optimisation	perioperative optimisation	perioperative optimisation	perioperative optimisation	Perioperative optimisation	1	kept
perioperative process	perioperative process	perioperative process	perioperative process	perioperative process	1	kept
perioperative risk assessment	perioperative risk assessment	perioperative risk assessment	perioperative risk assessment	perioperative risk assessment	1	kept
perioperative surgical home	perioperative surgical home	perioperative surgical home	perioperative surgical home	perioperative surgical home	1	kept
perioperative urinary management	perioperative urinary management	perioperative urinary management	perioperative urinary management	perioperative urinary management	1	kept
periorbital hematoma	periorbital hematoma	periorbital hematoma	periorbital hematoma	periorbital hematoma	1	kept
peripartum	peripartum	peripartum	peripartum	Peripartum	1	case_or_format
peripartum hemorrhage	peripartum hemorrhage	peripartum hemorrhage	peripartum hemorrhage	peripartum hemorrhage	1	kept
Peripartum Period	peripartum period	peripartum period	peripartum period	Peripartum Period	1	kept
Peripheral arterial catheter	peripheral arterial catheter	peripheral arterial catheter	peripheral arterial catheter	Peripheral arterial catheter	1	kept
Peripheral arterial catheterisation	peripheral arterial catheterisation	peripheral arterial catheterisation	peripheral arterial catheterisation	Peripheral arterial catheterisation	1	kept
Peripheral arterial catheterization	peripheral arterial catheterization	peripheral arterial catheterization	peripheral arterial catheterization	Peripheral arterial catheterization	1	kept
peripheral arterial catheterization	peripheral arterial catheterization	peripheral arterial catheterization	peripheral arterial catheterization	Peripheral arterial catheterization	1	case_or_format
Peripheral arterial catheters	peripheral arterial catheters	peripheral arterial catheters	peripheral arterial catheter	Peripheral arterial catheter	1	singular
Peripheral arterial disease	peripheral arterial disease	peripheral arterial disease	peripheral arterial disease	Peripheral Arterial Disease	1	case_or_format
peripheral blood mononuclear cell	peripheral blood mononuclear cell	peripheral blood mononuclear cell	peripheral blood mononuclear cell	peripheral blood mononuclear cell	1	kept

Peripheral blood T-lymphocytes	peripheral blood t lymphocytes	peripheral blood t lymphocytes	peripheral blood t lymphocytes	Peripheral blood T-lymphocytes	1	kept
peripheral catheterisation	peripheral catheterisation	peripheral catheterisation	peripheral catheterisation	Peripheral catheterisation	1	case_or_format
Peripheral devices	peripheral devices	peripheral devices	peripheral devices	Peripheral devices	1	kept
peripheral infiltration injury	peripheral infiltration injury	peripheral infiltration injury	peripheral infiltration injury	peripheral infiltration injury	1	kept
Peripheral Interaction	peripheral interaction	peripheral interaction	peripheral interaction	Peripheral interaction	1	case_or_format
Peripheral interactions	peripheral interactions	peripheral interactions	peripheral interaction	Peripheral interaction	1	singular
Peripheral intravascular catheters	peripheral intravascular catheters	peripheral intravascular catheters	peripheral intravascular catheters	Peripheral intravascular catheters	1	kept
peripheral intravenous	peripheral intravenous	peripheral intravenous	peripheral intravenous	peripheral intravenous	1	kept
Peripheral intravenous access	peripheral intravenous access	peripheral intravenous access	peripheral intravenous access	Peripheral intravenous access	1	kept
peripheral intravenous cannula	peripheral intravenous cannula	peripheral intravenous cannula	peripheral intravenous cannula	peripheral intravenous cannula	1	kept
peripheral intravenous catheter	peripheral intravenous catheter	peripheral intravenous catheter	peripheral intravenous catheter	Peripheral intravenous catheter	1	case_or_format
peripheral intravenous catheterisation	peripheral intravenous catheterisation	peripheral intravenous catheterisation	peripheral intravenous catheterisation	peripheral intravenous catheterisation	1	kept
Peripheral intravenous catheterization	peripheral intravenous catheterization	peripheral intravenous catheterization	peripheral intravenous catheterization	Peripheral intravenous catheterization	1	kept
peripheral intravenous catheterization	peripheral intravenous catheterization	peripheral intravenous catheterization	peripheral intravenous catheterization	Peripheral intravenous catheterization	1	case_or_format
PERIPHERAL INTRAVENOUS CATHETERS	peripheral intravenous catheters	peripheral intravenous catheters	peripheral intravenous catheter	Peripheral intravenous catheter	1	singular
peripheral intravenous catheters	peripheral intravenous catheters	peripheral intravenous catheters	peripheral intravenous catheter	Peripheral intravenous catheter	1	singular

Peripheral intravenous therapy	peripheral intravenous therapy	peripheral intravenous therapy	peripheral intravenous therapy	Peripheral intravenous therapy	1	kept
peripheral ischemia	peripheral ischemia	peripheral ischemia	peripheral ischemia	peripheral ischemia	1	kept
peripheral line	peripheral line	peripheral line	peripheral line	peripheral line	1	kept
peripheral nerve	peripheral nerve	peripheral nerve	peripheral nerve	Peripheral Nerves	1	case_or_format
Peripheral nerve block	peripheral nerve block	peripheral nerve block	peripheral nerve block	Peripheral nerve block	1	kept
peripheral nerve block	peripheral nerve block	peripheral nerve block	peripheral nerve block	Peripheral nerve block	1	case_or_format
Peripheral Nerve Injuries	peripheral nerve injuries	peripheral nerve injuries	peripheral nerve injury	peripheral nerve injury	1	singular
Peripheral nerve injuries	peripheral nerve injuries	peripheral nerve injuries	peripheral nerve injury	peripheral nerve injury	1	singular
peripheral nerve injury	peripheral nerve injury	peripheral nerve injury	peripheral nerve injury	peripheral nerve injury	1	kept
Peripheral Nervous System	peripheral nervous system	peripheral nervous system	peripheral nervous system	Peripheral Nervous System	1	kept
Peripheral perfusion	peripheral perfusion	peripheral perfusion	peripheral perfusion	Peripheral perfusion	1	kept
Peripheral perfusion indicators	peripheral perfusion indicators	peripheral perfusion indicators	peripheral perfusion indicators	Peripheral perfusion indicators	1	kept
Peripheral vascular access	peripheral vascular access	peripheral vascular access	peripheral vascular access	Peripheral vascular access	1	kept
peripheral vascular access	peripheral vascular access	peripheral vascular access	peripheral vascular access	Peripheral vascular access	1	case_or_format
Peripheral vascular catheter	peripheral vascular catheter	peripheral vascular catheter	peripheral vascular catheter	Peripheral vascular catheter	1	kept
peripheral vascular catheter	peripheral vascular catheter	peripheral vascular catheter	peripheral vascular catheter	Peripheral vascular catheter	1	case_or_format

peripheral vascular line	peripheral vascular line	peripheral vascular line	peripheral vascular line	peripheral vascular line	1	kept
Peripheral veins	peripheral veins	peripheral veins	peripheral vein	peripheral vein	1	singular
peripheral venous access	peripheral venous access	peripheral venous access	peripheral venous access	peripheral venous access	1	kept
Peripheral venous catheter	peripheral venous catheter	peripheral venous catheter	peripheral venous catheter	peripheral venous catheter	1	case_or_format
Peripheral venous catheterization	peripheral venous catheterization	peripheral venous catheterization	peripheral venous catheterization	peripheral venous catheterization	1	case_or_format
Peripheral venous catheters	peripheral venous catheters	peripheral venous catheters	peripheral venous catheter	peripheral venous catheter	1	singular
Peripheral venues catheter	peripheral venues catheter	peripheral venues catheter	peripheral venues catheter	Peripheral venues catheter	1	kept
peripheral volume of distribution	peripheral volume of distribution	peripheral volume of distribution	peripheral volume of distribution	peripheral volume of distribution	1	kept
Peripheral-inserted central venous line	peripheral inserted central venous line	peripheral inserted central venous line	peripheral inserted central venous line	Peripheral-inserted central venous line	1	kept
Peripheral-line-associated bloodstream infection	peripheral line associated bloodstream infection	peripheral line associated bloodstream infection	peripheral line associated bloodstream infection	Peripheral-line-associated bloodstream infection	1	kept
peripheral-line-associated bloodstream infection	peripheral line associated bloodstream infection	peripheral line associated bloodstream infection	peripheral line associated bloodstream infection	Peripheral-line-associated bloodstream infection	1	case_or_format
peripherally inserted central catheter complication	peripherally inserted central catheter complication	peripherally inserted central catheter complication	peripherally inserted central catheter complication	peripherally inserted central catheter complication	1	kept
peripherally inserted central catheter tip malposition	peripherally inserted central catheter tip malposition	peripherally inserted central catheter tip malposition	peripherally inserted central catheter tip malposition	peripherally inserted central catheter tip malposition	1	kept
Peripherally inserted central catheters Phlebitis	peripherally inserted central catheters phlebitis	peripherally inserted central catheters phlebitis	peripherally inserted central catheters phlebitis	Peripherally inserted central catheters Phlebitis	1	kept
Peripherally inserted central venous catheter	peripherally inserted central venous catheter	peripherally inserted central venous catheter	peripherally inserted central venous catheter	peripherally inserted central venous catheter	1	case_or_format
periprocedural	periprocedural	periprocedural	periprocedural	periprocedural	1	kept

Periprosthetic knee replacement fracture	periprosthetic knee replacement fracture	periprosthetic knee replacement fracture	periprosthetic knee replacement fracture	Periprosthetic knee replacement fracture	1	kept
Peristalsis	peristalsis	peristalsis	peristalsis	Peristalsis	1	kept
peristalsis	peristalsis	peristalsis	peristalsis	Peristalsis	1	case_or_format
Peristaltic motion	peristaltic motion	peristaltic motion	peristaltic motion	Peristaltic motion	1	kept
Peristaltic pump	peristaltic pump	peristaltic pump	peristaltic pump	Peristaltic pump	1	kept
peristaltic pump	peristaltic pump	peristaltic pump	peristaltic pump	Peristaltic pump	1	case_or_format
Peristaltic pumps	peristaltic pumps	peristaltic pumps	peristaltic pump	Peristaltic pump	1	singular
Peristomal care	peristomal care	peristomal care	peristomal care	Peristomal care	1	kept
peritoneal ascites	peritoneal ascites	peritoneal ascites	peritoneal ascites	peritoneal ascites	1	kept
peritoneal biopsy	peritoneal biopsy	peritoneal biopsy	peritoneal biopsy	peritoneal biopsy	1	kept
Peritoneal cancer	peritoneal cancer	peritoneal cancer	peritoneal cancer	Peritoneal cancer	1	kept
Peritoneal carcinomatosis	peritoneal carcinomatosis	peritoneal carcinomatosis	peritoneal carcinomatosis	Peritoneal carcinomatosis	1	kept
Peritoneal Cavity	peritoneal cavity	peritoneal cavity	peritoneal cavity	Peritoneal Cavity	1	kept
peritoneal cavity	peritoneal cavity	peritoneal cavity	peritoneal cavity	Peritoneal Cavity	1	case_or_format
peritoneal disease	peritoneal disease	peritoneal disease	peritoneal disease	peritoneal disease	1	kept
Peritoneal Lavage	peritoneal lavage	peritoneal lavage	peritoneal lavage	Peritoneal Lavage	1	kept

Peritoneal lavage	peritoneal lavage	peritoneal lavage	peritoneal lavage	Peritoneal Lavage	1	case_or_format
Peritoneal lavages	peritoneal lavages	peritoneal lavages	peritoneal lavage	Peritoneal Lavage	1	singular
peritoneal metastases	peritoneal metastases	peritoneal metastases	peritoneal metastases	peritoneal metastases	1	kept
peritoneal sac rupture	peritoneal sac rupture	peritoneal sac rupture	peritoneal sac rupture	peritoneal sac rupture	1	kept
Peritoneum	peritoneum	peritoneum	peritoneum	Peritoneum	1	kept
peritoneum	peritoneum	peritoneum	peritoneum	Peritoneum	1	case_or_format
peritoneum cancer	peritoneum cancer	peritoneum cancer	peritoneum cancer	peritoneum cancer	1	kept
peritoneum lavage	peritoneum lavage	peritoneum lavage	peritoneum lavage	peritoneum lavage	1	kept
peritoneum metastasis	peritoneum metastasis	peritoneum metastasis	peritoneum metastasis	peritoneum metastasis	1	kept
peritoneum pseudomyxoma	peritoneum pseudomyxoma	peritoneum pseudomyxoma	peritoneum pseudomyxoma	peritoneum pseudomyxoma	1	kept
Peritraumatic Dissociative Experiences	peritraumatic dissociative experiences	peritraumatic dissociative experiences	peritraumatic dissociative experiences	Peritraumatic Dissociative Experiences	1	kept
Periurethral cleansing	periurethral cleansing	periurethral cleansing	periurethral cleansing	Periurethral cleansing	1	kept
Periviable	periviable	periviable	periviable	Periviable	1	kept
periviable	periviable	periviable	periviable	Periviable	1	case_or_format
periviable delivery	periviable delivery	periviable delivery	periviable delivery	periviable delivery	1	kept
Permanent Education	permanent education	permanent education	permanent education	Permanent Education	1	kept

Permanent employees	permanent employees	permanent employees	permanent employees	Permanent employees	1	kept
Permanent Medical Complexity	permanent medical complexity	permanent medical complexity	permanent medical complexity	Permanent Medical Complexity	1	kept
Perme Score	perme score	perme score	perme score	Perme Score	1	kept
permethrin	permethrin	permethrin	permethrin	permethrin	1	kept
PERMISSION	permission	permission	permission	PERMISSION	1	kept
Permission Limited Information Specific Suggestions Intensive	permission limited information specific suggestions intensive	permission limited information specific suggestions intensive	permission limited information specific suggestions intensive	Permission Limited Information Specific Suggestions Intensive	1	kept
Pernicious anemia	pernicious anemia	pernicious anemia	pernicious anemia	Pernicious anemia	1	kept
peroneal nerve	peroneal nerve	peroneal nerve	peroneal nerve	peroneal nerve	1	kept
peroneal nerve paralysis	peroneal nerve paralysis	peroneal nerve paralysis	peroneal nerve paralysis	peroneal nerve paralysis	1	kept
peroneal neuropathies	peroneal neuropathies	peroneal neuropathies	peroneal neuropathies	peroneal neuropathies	1	kept
peroneus nerve	peroneus nerve	peroneus nerve	peroneus nerve	peroneus nerve	1	kept
peroral endoscopic myotomy	peroral endoscopic myotomy	peroral endoscopic myotomy	peroral endoscopic myotomy	peroral endoscopic myotomy	1	kept
perospirone	perospirone	perospirone	perospirone	perospirone	1	kept
peroxidase	peroxidase	peroxidase	peroxidase	peroxidase	1	kept
peroxisome proliferator activated receptor gamma agonist	peroxisome proliferator activated receptor gamma agonist	peroxisome proliferator activated receptor gamma agonist	peroxisome proliferator activated receptor gamma agonist	peroxisome proliferator activated receptor gamma agonist	1	kept
perpetrator	perpetrator	perpetrator	perpetrator	perpetrator	1	kept

persecutory delusion	persecutory delusion	persecutory delusion	persecutory delusion	persecutory delusion	1	kept
Persian version	persian version	persian version	persian version	Persian version	1	kept
persistant inflammation	persistant inflammation	persistant inflammation	persistant inflammation	persistant inflammation	1	kept
persistent acute kidney injury	persistent acute kidney injury	persistent acute kidney injury	persistent acute kidney injury	persistent acute kidney injury	1	kept
persistent atrial fibrillation	persistent atrial fibrillation	persistent atrial fibrillation	persistent atrial fibrillation	persistent atrial fibrillation	1	kept
persistent fetus circulation	persistent fetus circulation	persistent fetus circulation	persistent fetus circulation	persistent fetus circulation	1	kept
Persistent hoarseness	persistent hoarseness	persistent hoarseness	persistent hoarseness	Persistent hoarseness	1	kept
Persistent Hyperinsulinemia Hypoglycemia of	persistent hyperinsulinemia hypoglycemia of	persistent hyperinsulinemia hypoglycemia of	persistent hyperinsulinemia hypoglycemia of	Persistent Hyperinsulinemia Hypoglycemia of	1	kept
persistent hyperinsulinemic hypoglycemia of	persistent hyperinsulinemic hypoglycemia of	persistent hyperinsulinemic hypoglycemia of	persistent hyperinsulinemic hypoglycemia of	persistent hyperinsulinemic hypoglycemia of	1	kept
persistent inflammation immunosuppression	persistent inflammation immunosuppression	persistent inflammation immunosuppression	persistent inflammation immunosuppression	persistent inflammation immunosuppression	1	kept
persistent inflammatory-immunosuppressive and	persistent inflammatory immunosuppressive and	persistent inflammatory immunosuppressive and	persistent inflammatory immunosuppressive and	persistent inflammatory-immunosuppressive and	1	kept
persistent oligohydramnios	persistent oligohydramnios	persistent oligohydramnios	persistent oligohydramnios	persistent oligohydramnios	1	kept
Persistent pulmonary hypertension of the newborn	persistent pulmonary hypertension of the newborn	persistent pulmonary hypertension of the newborn	persistent pulmonary hypertension of the newborn	Persistent pulmonary hypertension of the newborn	1	kept
PERSISTENT VEGETATIVE STATE	persistent vegetative state	persistent vegetative state	persistent vegetative state	persistent vegetative state	1	case_or_format
Persistent vegetative state	persistent vegetative state	persistent vegetative state	persistent vegetative state	persistent vegetative state	1	case_or_format
Persisting immunity	persisting immunity	persisting immunity	persisting immunity	Persisting immunity	1	kept

person	person	person	person	person	1	kept
person centred	person centred	person centred	person centred	person-centred	1	case_or_format
Person centred care	person centred care	person centred care	person centred care	person-centred care	1	case_or_format
person separation index	person separation index	person separation index	person separation index	person separation index	1	kept
Person-centered medicine	person centered medicine	person centered medicine	person centered medicine	Person-centered medicine	1	kept
Person-Centered Psychotherapy	person centered psychotherapy	person centered psychotherapy	person centered psychotherapy	Person-Centered Psychotherapy	1	kept
Person-Centred Care	person centred care	person centred care	person centred care	person-centred care	1	case_or_format
Person-centred practice framework	person centred practice framework	person centred practice framework	person centred practice framework	Person-centred practice framework	1	kept
Person-centredness	person centredness	person centredness	person centredness	person-centredness	1	case_or_format
Persona en Situación Crítica	persona en situaci n cr tica	persona en situaci n cr tica	persona en situaci n cr tica	Persona en Situación Crítica	1	kept
Personal	personal	personal	personal	Personal	1	kept
personal	personal	personal	personal	Personal	1	case_or_format
Personal Accomplishment	personal accomplishment	personal accomplishment	personal accomplishment	Personal accomplishment	1	case_or_format
Personal achievement	personal achievement	personal achievement	personal achievement	Personal achievement	1	kept
Personal anxiety	personal anxiety	personal anxiety	personal anxiety	Personal anxiety	1	kept
Personal attitudes	personal attitudes	personal attitudes	personal attitudes	Personal attitudes	1	kept

Personal autonomy	personal autonomy	personal autonomy	personal autonomy	Personal Autonomy	1	case_or_format
Personal burden	personal burden	personal burden	personal burden	Personal burden	1	kept
personal characteristics	personal characteristics	personal characteristics	personal characteristics	Personal characteristics	1	case_or_format
personal communication,	personal communication	personal communication	personal communication	personal communication,	1	kept
Personal de salud	personal de salud	personal de salud	personal de salud	Personal de salud	1	kept
personal development	personal development	personal development	personal development	personal development	1	kept
Personal digital assistants	personal digital assistants	personal digital assistants	personal digital assistant	personal digital assistant	1	singular
Personal exchange	personal exchange	personal exchange	personal exchange	Personal exchange	1	kept
personal failure	personal failure	personal failure	personal failure	personal failure	1	kept
personal finance	personal finance	personal finance	personal finance	personal finance	1	kept
Personal glucose meter	personal glucose meter	personal glucose meter	personal glucose meter	Personal glucose meter	1	kept
Personal glucose meters	personal glucose meters	personal glucose meters	personal glucose meter	Personal glucose meter	1	singular
personal growth	personal growth	personal growth	personal growth	personal growth	1	kept
Personal hygiene	personal hygiene	personal hygiene	personal hygiene	personal hygiene	1	case_or_format
personal innovativeness	personal innovativeness	personal innovativeness	personal innovativeness	personal innovativeness	1	kept
Personal integrity	personal integrity	personal integrity	personal integrity	Personal integrity	1	kept

Personal interaction	personal interaction	personal interaction	personal interaction	Personal interaction	1	kept
personal narratives	personal narratives	personal narratives	personal narratives	Personal narratives	1	case_or_format
personal pet visitation	personal pet visitation	personal pet visitation	personal pet visitation	personal pet visitation	1	kept
Personal preferences	personal preferences	personal preferences	personal preferences	Personal preferences	1	kept
Personal protection equipment	personal protection equipment	personal protection equipment	personal protection equipment	Personal protection equipment	1	kept
personal protection equipment	personal protection equipment	personal protection equipment	personal protection equipment	Personal protection equipment	1	case_or_format
Personal protective measures	personal protective measures	personal protective measures	personal protective measures	Personal protective measures	1	kept
Personal resources	personal resources	personal resources	personal resources	Personal resources	1	kept
personal risk	personal risk	personal risk	personal risk	personal risk	1	kept
Personal Strengths	personal strengths	personal strengths	personal strengths	Personal Strengths	1	kept
personal trainer	personal trainer	personal trainer	personal trainer	personal trainer	1	kept
Personal Turnover	personal turnover	personal turnover	personal turnover	Personal Turnover	1	kept
Personal turnover	personal turnover	personal turnover	personal turnover	Personal Turnover	1	case_or_format
Personal ventilation	personal ventilation	personal ventilation	personal ventilation	Personal ventilation	1	kept
personal well-being	personal well being	personal well being	personal well being	personal well-being	1	kept
Personalidad	personalidad	personalidad	personalidad	Personalidad	1	kept

Personalisation	personalisation	personalisation	personalisation	Personalisation	1	kept
Personalised care	personalised care	personalised care	personalised care	Personalised care	1	kept
Personalised medicine	personalised medicine	personalised medicine	personalised medicine	Personalised medicine	1	kept
personalised ventilation	personalised ventilation	personalised ventilation	personalised ventilation	personalised ventilation	1	kept
Personality Assessment	personality assessment	personality assessment	personality assessment	personality assessment	1	case_or_format
Personality characteristics	personality characteristics	personality characteristics	personality characteristics	Personality characteristics	1	kept
Personality Disorders	personality disorders	personality disorders	personality disorder	personality disorder	1	singular
Personality needs	personality needs	personality needs	personality needs	Personality needs	1	kept
Personality trait	personality trait	personality trait	personality trait	Personality traits	1	case_or_format
Personality Traits	personality traits	personality traits	personality trait	Personality traits	1	singular
personality traits	personality traits	personality traits	personality trait	Personality traits	1	singular
Personality traits-job satisfaction – reference values	personality traits job satisfaction reference values system	personality traits job satisfaction reference values system	personality traits job satisfaction reference values system	Personality traits-job satisfaction – reference values	1	kept
personalized cancer therapy	personalized cancer therapy	personalized cancer therapy	personalized cancer therapy	personalized cancer therapy	1	kept
Personalized care	personalized care	personalized care	personalized care	personalized care	1	case_or_format
Personalized diagnosis	personalized diagnosis	personalized diagnosis	personalized diagnosis	Personalized diagnosis	1	kept
Personalized glycemic control	personalized glycemic control	personalized glycemic control	personalized glycemic control	Personalized glycemic control	1	kept

Personalized model	personalized model	personalized model	personalized model	Personalized model	1	kept
personalized therapy	personalized therapy	personalized therapy	personalized therapy	personalized therapy	1	kept
Personel downsizing	personel downsizing	personel downsizing	personel downsizing	Personel downsizing	1	kept
Personel staffing	personel staffing	personel staffing	personel staffing	Personel staffing	1	kept
personnel cost	personnel cost	personnel cost	personnel cost	personnel cost	1	kept
Personnel delegation	personnel delegation	personnel delegation	personnel delegation	Personnel Delegation	1	case_or_format
Personnel Downsizing	personnel downsizing	personnel downsizing	personnel downsizing	Personnel downsizing	1	case_or_format
personnel recruitment	personnel recruitment	personnel recruitment	personnel recruitment	personnel recruitment	1	kept
personnel relationship	personnel relationship	personnel relationship	personnel relationship	personnel relationship	1	kept
personnel selection	personnel selection	personnel selection	personnel selection	Personnel Selection	1	case_or_format
Personnel Staffing	personnel staffing	personnel staffing	personnel staffing	personnel staffing	1	case_or_format
PERSONNEL STAFFING AND SCHEDULING	personnel staffing and scheduling	personnel staffing and scheduling	personnel staffing and scheduling	Personnel Staffing and Scheduling	1	case_or_format
Personnes âgées	personnes g es	personnes g es	personnes g es	Personnes âgées	1	kept
persons with disabilities	persons with disabilities	persons with disabilities	persons with disabilities	Persons with Disabilities	1	case_or_format
Persons with Hearing Disabilities	persons with hearing disabilities	persons with hearing disabilities	persons with hearing disabilities	Persons with Hearing Disabilities	1	kept
perspective-taking	perspective taking	perspective taking	perspective taking	perspective-taking	1	kept

perspectives on death	perspectives on death	perspectives on death	perspectives on death	perspectives on death	1	kept
Pertrochanteric fracture	pertrochanteric fracture	pertrochanteric fracture	pertrochanteric fracture	Pertrochanteric fracture	1	kept
pertussis toxin	pertussis toxin	pertussis toxin	pertussis toxin	pertussis toxin	1	kept
Pertussis vaccine	pertussis vaccine	pertussis vaccine	pertussis vaccine	pertussis vaccine	1	case_or_format
Pervasive devices	pervasive devices	pervasive devices	pervasive devices	Pervasive devices	1	kept
Pesquisa em Enfermagem	pesquisa em enfermagem	pesquisa em enfermagem	pesquisa em enfermagem	Pesquisa em Enfermagem	1	kept
Pessimism	pessimism	pessimism	pessimism	pessimism	1	case_or_format
Pessoa em Situação Crítica	pessoa em situa o cr tica	pessoa em situa o cr tica	pessoa em situa o cr tica	Pessoa em Situação Crítica	1	kept
pest control	pest control	pest control	pest control	pest control	1	kept
Pesticide	pesticide	pesticide	pesticide	Pesticides	1	case_or_format
pesticide	pesticide	pesticide	pesticide	Pesticides	1	case_or_format
pet assisted intervention	pet assisted intervention	pet assisted intervention	pet assisted intervention	pet assisted intervention	1	kept
pet therapy	pet therapy	pet therapy	pet therapy	pet therapy	1	kept
petal	petal	petal	petal	petal	1	kept
petroleum	petroleum	petroleum	petroleum	petroleum	1	kept
Pets	pets	pets	pets	Pets	1	kept

PETS protocol	pets protocol	pets protocol	pets protocol	PETS protocol	1	kept
PEW	pew	pew	pew	PEWS	1	case_or_format
Peyton’ s four-step approach	peyton s four step approach	peyton s four step approach	peyton s four step approach	Peyton’ s four-step approach	1	kept
PFGE typing	pfge typing	pfge typing	pfge typing	PFGE typing	1	kept
Pfizer-BioNTech	pfizer biontech	pfizer biontech	pfizer biontech	Pfizer-BioNTech	1	kept
Pflege	pflege	pflege	pflege	Pflege	1	kept
Pflegende	pflegende	pflegende	pflegende	Pflegende	1	kept
Pflegeplanung	pflegeplanung	pflegeplanung	pflegeplanung	Pflegeplanung	1	kept
PGC-1α	pgc 1	pgc 1	pgc 1	PGC-1α	1	kept
pH testing	ph testing	ph testing	ph testing	pH testing	1	kept
phagocyte	phagocyte	phagocyte	phagocyte	phagocyte	1	kept
phagocytosis	phagocytosis	phagocytosis	phagocytosis	phagocytosis	1	kept
phantom	phantom	phantom	phantom	Phantoms	1	case_or_format
Phantom study	phantom study	phantom study	phantom study	Phantom study	1	kept
PHARMACEUTICAL CARE	pharmaceutical care	pharmaceutical care	pharmaceutical care	pharmaceutical care	1	case_or_format
pharmaceutical care management in corona virus crisis model	pharmaceutical care management in corona virus crisis model	pharmaceutical care management in corona virus crisis model	pharmaceutical care management in corona virus crisis model	pharmaceutical care management in corona virus crisis model	1	kept

Pharmaceutical cost	pharmaceutical cost	pharmaceutical cost	pharmaceutical cost	Pharmaceutical cost	1	kept
Pharmaceutical knowledge	pharmaceutical knowledge	pharmaceutical knowledge	pharmaceutical knowledge	Pharmaceutical knowledge	1	kept
Pharmaceutical preparations	pharmaceutical preparations	pharmaceutical preparations	pharmaceutical preparations	Pharmaceutical Preparations	1	case_or_format
pharmaceutical service	pharmaceutical service	pharmaceutical service	pharmaceutical service	Pharmaceutical services	1	case_or_format
PHARMACEUTICAL SERVICES	pharmaceutical services	pharmaceutical services	pharmaceutical service	Pharmaceutical services	1	singular
pharmaceutical services	pharmaceutical services	pharmaceutical services	pharmaceutical service	Pharmaceutical services	1	singular
Pharmaceutical services, Pharmacists, hospital, Telepharmacy	pharmaceutical services pharmacists hospital telepharmacy	pharmaceutical services pharmacists hospital telepharmacy	pharmaceutical services pharmacists hospital telepharmacy	Pharmaceutical services, Pharmacists, hospital, Telepharmacy	1	kept
Pharmaceutical Vehicles	pharmaceutical vehicles	pharmaceutical vehicles	pharmaceutical vehicles	Pharmaceutical Vehicles	1	kept
Pharmaceutical waste	pharmaceutical waste	pharmaceutical waste	pharmaceutical waste	Pharmaceutical waste	1	kept
Pharmaceuticalisation	pharmaceuticalisation	pharmaceuticalisation	pharmaceuticalisation	Pharmaceuticalisation	1	kept
PHARMACIST	pharmacist	pharmacist	pharmacist	pharmacist	1	case_or_format
pharmacist driven termination protocol	pharmacist driven termination protocol	pharmacist driven termination protocol	pharmacist driven termination protocol	pharmacist driven termination protocol	1	kept
pharmacist interventions	pharmacist interventions	pharmacist interventions	pharmacist intervention	Pharmacist intervention	1	singular
pharmacist outcomes	pharmacist outcomes	pharmacist outcomes	pharmacist outcomes	pharmacist outcomes	1	kept
pharmacist value	pharmacist value	pharmacist value	pharmacist value	pharmacist value	1	kept
PHARMACISTS	pharmacists	pharmacists	pharmacist	pharmacist	1	singular

pharmaco-economics	pharmaco economics	pharmaco economics	pharmaco economics	pharmaco-economics	1	kept
pharmacodynamic parameters	pharmacodynamic parameters	pharmacodynamic parameters	pharmacodynamic parameters	pharmacodynamic parameters	1	kept
PHARMACOEPIDEMIOLOGY	pharmacoepidemiology	pharmacoepidemiology	pharmacoepidemiology	pharmacoepidemiology	1	case_or_format
Pharmacoepidemiology	pharmacoepidemiology	pharmacoepidemiology	pharmacoepidemiology	pharmacoepidemiology	1	case_or_format
pharmacokinetic	pharmacokinetic	pharmacokinetic	pharmacokinetic	pharmacokinetics	1	case_or_format
Pharmacokinetics covariates	pharmacokinetics covariates	pharmacokinetics covariates	pharmacokinetics covariates	Pharmacokinetics covariates	1	kept
Pharmacologic intervention	pharmacologic intervention	pharmacologic intervention	pharmacologic intervention	Pharmacologic intervention	1	kept
Pharmacologic pain management	pharmacologic pain management	pharmacologic pain management	pharmacologic pain management	Pharmacologic pain management	1	kept
Pharmacologic strategies to prevent PTSD	pharmacologic strategies to prevent ptsd	pharmacologic strategies to prevent ptsd	pharmacologic strategies to prevent ptsd	Pharmacologic strategies to prevent PTSD	1	kept
pharmacologic therapy	pharmacologic therapy	pharmacologic therapy	pharmacologic therapy	Pharmacologic therapy	1	case_or_format
Pharmacological action	pharmacological action	pharmacological action	pharmacological action	Pharmacological action	1	kept
Pharmacological intervention	pharmacological intervention	pharmacological intervention	pharmacological intervention	Pharmacological intervention	1	kept
Pharmacological interventions	pharmacological interventions	pharmacological interventions	pharmacological intervention	Pharmacological intervention	1	singular
pharmacological knowledge	pharmacological knowledge	pharmacological knowledge	pharmacological knowledge	pharmacological knowledge	1	kept
pharmacological parameters	pharmacological parameters	pharmacological parameters	pharmacological parameters	pharmacological parameters	1	kept
pharmacological procedures	pharmacological procedures	pharmacological procedures	pharmacological procedures	pharmacological procedures	1	kept

pharmacological treatment	pharmacological treatment	pharmacological treatment	pharmacological treatment	pharmacological treatment	1	kept
PHARMACOLOGY	pharmacology	pharmacology	pharmacology	pharmacology	1	case_or_format
Pharmacology department	pharmacology department	pharmacology department	pharmacology department	Pharmacology department	1	kept
Pharmacology of paediatric brain injury	pharmacology of paediatric brain injury	pharmacology of paediatric brain injury	pharmacology of paediatric brain injury	Pharmacology of paediatric brain injury	1	kept
pharmacology of paediatric brain injury	pharmacology of paediatric brain injury	pharmacology of paediatric brain injury	pharmacology of paediatric brain injury	Pharmacology of paediatric brain injury	1	case_or_format
Pharmacology safety	pharmacology safety	pharmacology safety	pharmacology safety	Pharmacology safety	1	kept
Pharmacomarker	pharmacomarker	pharmacomarker	pharmacomarker	Pharmacomarker	1	kept
Pharmaconutrient	pharmaconutrient	pharmaconutrient	pharmaconutrient	Pharmaconutrient	1	kept
PHARMACOTHERAPY	pharmacotherapy	pharmacotherapy	pharmacotherapy	Pharmacotherapy	1	case_or_format
PHARMACY ADMINISTRATION	pharmacy administration	pharmacy administration	pharmacy administration	PHARMACY ADMINISTRATION	1	kept
pharmacy administration	pharmacy administration	pharmacy administration	pharmacy administration	PHARMACY ADMINISTRATION	1	case_or_format
pharmacy and therapeutics committee	pharmacy and therapeutics committee	pharmacy and therapeutics committee	pharmacy and therapeutics committee	Pharmacy and Therapeutics Committee	1	case_or_format
pharmacy cost	pharmacy cost	pharmacy cost	pharmacy cost	pharmacy cost	1	kept
pharmacy delivery robot	pharmacy delivery robot	pharmacy delivery robot	pharmacy delivery robot	pharmacy delivery robot	1	kept
Pharmacy Department	pharmacy department	pharmacy department	pharmacy department	Pharmacy Department	1	kept
Pharmacy education	pharmacy education	pharmacy education	pharmacy education	pharmacy education	1	case_or_format

pharmacy operations	pharmacy operations	pharmacy operations	pharmacy operations	pharmacy operations	1	kept
pharmacy practice advancement	pharmacy practice advancement	pharmacy practice advancement	pharmacy practice advancement	pharmacy practice advancement	1	kept
pharmacy research	pharmacy research	pharmacy research	pharmacy research	pharmacy research	1	kept
Pharmacy Residencies	pharmacy residencies	pharmacy residencies	pharmacy residency	pharmacy residency	1	singular
pharmacy residency	pharmacy residency	pharmacy residency	pharmacy residency	pharmacy residency	1	kept
PHARMACY SERVICE, HOSPITAL	pharmacy service hospital	pharmacy service hospital	pharmacy service hospital	Pharmacy Service, Hospital	1	case_or_format
Pharmacy technician	pharmacy technician	pharmacy technician	pharmacy technician	pharmacy technician	1	case_or_format
Pharyngeal Diseases	pharyngeal diseases	pharyngeal diseases	pharyngeal diseases	Pharyngeal Diseases	1	kept
pharyngectomy	pharyngectomy	pharyngectomy	pharyngectomy	pharyngectomy	1	kept
pharyngolaryngectomy	pharyngolaryngectomy	pharyngolaryngectomy	pharyngolaryngectomy	pharyngolaryngectomy	1	kept
pharynx hemorrhage	pharynx hemorrhage	pharynx hemorrhage	pharynx hemorrhage	pharynx hemorrhage	1	kept
phase shift	phase shift	phase shift	phase shift	phase shift	1	kept
phases of emotional burnout	phases of emotional burnout	phases of emotional burnout	phases of emotional burnout	phases of emotional burnout	1	kept
phenazopyridine	phenazopyridine	phenazopyridine	phenazopyridine	phenazopyridine	1	kept
phenelzine	phenelzine	phenelzine	phenelzine	phenelzine	1	kept
phenethylamine derivative	phenethylamine derivative	phenethylamine derivative	phenethylamine derivative	phenethylamine derivative	1	kept

Phenethylamines	phenethylamines	phenethylamines	phenethylamines	Phenethylamines	1	kept
Phenols	phenols	phenols	phenols	Phenols	1	kept
phenomenological	phenomenological	phenomenological	phenomenological	phenomenological	1	kept
Phenomenological approach	phenomenological approach	phenomenological approach	phenomenological approach	Phenomenological approach	1	kept
phenomenological approach	phenomenological approach	phenomenological approach	phenomenological approach	Phenomenological approach	1	case_or_format
phenomenological design	phenomenological design	phenomenological design	phenomenological design	phenomenological design	1	kept
phenomenological hermeneutic	phenomenological hermeneutic	phenomenological hermeneutic	phenomenological hermeneutic	Phenomenological hermeneutic	1	case_or_format
phenomenological hermeneutic method	phenomenological hermeneutic method	phenomenological hermeneutic method	phenomenological hermeneutic method	phenomenological hermeneutic method	1	kept
phenomenological hermeneutic research	phenomenological hermeneutic research	phenomenological hermeneutic research	phenomenological hermeneutic research	phenomenological hermeneutic research	1	kept
phenomenological hermeneutics	phenomenological hermeneutics	phenomenological hermeneutics	phenomenological hermeneutic	Phenomenological hermeneutic	1	singular
phenomenological inquiry	phenomenological inquiry	phenomenological inquiry	phenomenological inquiry	phenomenological inquiry	1	kept
Phenomenological-Hermeneutic	phenomenological hermeneutic	phenomenological hermeneutic	phenomenological hermeneutic	Phenomenological hermeneutic	1	case_or_format
Phenomenological-hermeneutic	phenomenological hermeneutic	phenomenological hermeneutic	phenomenological hermeneutic	Phenomenological hermeneutic	1	case_or_format
phenomenological-hermeneutic	phenomenological hermeneutic	phenomenological hermeneutic	phenomenological hermeneutic	Phenomenological hermeneutic	1	case_or_format
Phenomenological-hermeneutic research	phenomenological hermeneutic research	phenomenological hermeneutic research	phenomenological hermeneutic research	phenomenological hermeneutic research	1	case_or_format
phenomenological-hermeneutic research	phenomenological hermeneutic research	phenomenological hermeneutic research	phenomenological hermeneutic research	phenomenological hermeneutic research	1	case_or_format

phenomenological-hermeneutic	phenomenological hermeneutic	phenomenological hermeneutic	phenomenological hermeneutic	Phenomenological hermeneutic	1	case_or_format
phenomenology and hermeneutics	phenomenology and hermeneutics	phenomenology and hermeneutics	phenomenology and hermeneutics	phenomenology and hermeneutics	1	kept
Phenomenology study	phenomenology study	phenomenology study	phenomenology study	Phenomenology study	1	kept
phenomenology study	phenomenology study	phenomenology study	phenomenology study	Phenomenology study	1	case_or_format
Phenomenon of heroism	phenomenon of heroism	phenomenon of heroism	phenomenon of heroism	Phenomenon of heroism	1	kept
phenomonological, ontology, Løgstrup	phenomonological ontology l gstrup	phenomonological ontology l gstrup	phenomonological ontology l gstrup	phenomonological, ontology, Løgstrup	1	kept
phenothiazine derivative	phenothiazine derivative	phenothiazine derivative	phenothiazine derivative	phenothiazine derivative	1	kept
phenothiazines	phenothiazines	phenothiazines	phenothiazines	phenothiazines	1	kept
Phenotyping	phenotyping	phenotyping	phenotyping	Phenotyping	1	kept
phenotyping	phenotyping	phenotyping	phenotyping	Phenotyping	1	case_or_format
phenprocoumon	phenprocoumon	phenprocoumon	phenprocoumon	phenprocoumon	1	kept
phenserine	phenserine	phenserine	phenserine	phenserine	1	kept
phentermine	phentermine	phentermine	phentermine	phentermine	1	kept
phenylbutazone	phenylbutazone	phenylbutazone	phenylbutazone	phenylbutazone	1	kept
Phi Correlation	phi correlation	phi correlation	phi correlation	Phi Correlation	1	kept
Philosophical Approach	philosophical approach	philosophical approach	philosophical approach	Philosophical Approach	1	kept

philosophical hermeneutic interpretation	philosophical hermeneutic interpretation	philosophical hermeneutic interpretation	philosophical hermeneutic interpretation	philosophical hermeneutic interpretation	1	kept
PHILOSOPHICAL-PERSPECTIVES	philosophical perspectives	philosophical perspectives	philosophical perspectives	PHILOSOPHICAL-PERSPECTIVES	1	kept
Phlebostatic axis	phlebostatic axis	phlebostatic axis	phlebostatic axis	Phlebostatic axis	1	kept
Phones	phones	phones	phones	Phones	1	kept
Phonetics	phonetics	phonetics	phonetics	Phonetics	1	kept
phonetics	phonetics	phonetics	phonetics	Phonetics	1	case_or_format
phosphate supplementation	phosphate supplementation	phosphate supplementation	phosphate supplementation	phosphate supplementation	1	kept
Phosphates	phosphates	phosphates	phosphate	phosphate	1	singular
phosphine derivative	phosphine derivative	phosphine derivative	phosphine derivative	phosphine derivative	1	kept
phosphine gas intoxication	phosphine gas intoxication	phosphine gas intoxication	phosphine gas intoxication	phosphine gas intoxication	1	kept
Phosphines	phosphines	phosphines	phosphine	phosphine	1	singular
Phosphocreatine	phosphocreatine	phosphocreatine	phosphocreatine	Phosphocreatine	1	kept
phosphodiesterase III inhibitor	phosphodiesterase iii inhibitor	phosphodiesterase iii inhibitor	phosphodiesterase iii inhibitor	phosphodiesterase III inhibitor	1	kept
phosphodiesterase V inhibitor	phosphodiesterase v inhibitor	phosphodiesterase v inhibitor	phosphodiesterase v inhibitor	phosphodiesterase V inhibitor	1	kept
phospholipase	phospholipase	phospholipase	phospholipase	phospholipase	1	kept
Phospholipids	phospholipids	phospholipids	phospholipid	phospholipid	1	singular

phosphonic acid derivative	phosphonic acid derivative	phosphonic acid derivative	phosphonic acid derivative	phosphonic acid derivative	1	kept
Phosphoric Acid Esters	phosphoric acid esters	phosphoric acid esters	phosphoric acid esters	Phosphoric Acid Esters	1	kept
photochemical smog	photochemical smog	photochemical smog	photochemical smog	photochemical smog	1	kept
photon correlation spectroscopy	photon correlation spectroscopy	photon correlation spectroscopy	photon correlation spectroscopy	photon correlation spectroscopy	1	kept
photopheresis	photopheresis	photopheresis	photopheresis	photopheresis	1	kept
photoreceptor	photoreceptor	photoreceptor	photoreceptor	photoreceptor	1	kept
photosynthesis	photosynthesis	photosynthesis	photosynthesis	photosynthesis	1	kept
phototherapy treatment	phototherapy treatment	phototherapy treatment	phototherapy treatment	phototherapy treatment	1	kept
phototoxicity	phototoxicity	phototoxicity	phototoxicity	phototoxicity	1	kept
photovoice	photovoice	photovoice	photovoice	photovoice	1	kept
PHOX2B	phox2b	phox2b	phox2b	PHOX2B	1	kept
PHQ-9	phq 9	phq 9	phq 9	PHQ-9	1	kept
Phrenic Nerve	phrenic nerve	phrenic nerve	phrenic nerve	Phrenic Nerve	1	kept
phrenic nerve paralysis	phrenic nerve paralysis	phrenic nerve paralysis	phrenic nerve paralysis	phrenic nerve paralysis	1	kept
phthalates	phthalates	phthalates	phthalates	phthalates	1	kept
phthalic acid bis ester	phthalic acid bis ester	phthalic acid bis ester	phthalic acid bis ester	phthalic acid bis ester	1	kept

Phylogenetic relationship	phylogenetic relationship	phylogenetic relationship	phylogenetic relationship	Phylogenetic relationship	1	kept
Phylogeography	phylogeography	phylogeography	phylogeography	Phylogeography	1	kept
Physiatrists	physiatrists	physiatrists	physiatrist	physiatrist	1	singular
Physiatry	physiatry	physiatry	physiatry	Physiatry	1	kept
physiatry led stroke consult service	physiatry led stroke consult service	physiatry led stroke consult service	physiatry led stroke consult service	physiatry led stroke consult service	1	kept
Physical Abuse	physical abuse	physical abuse	physical abuse	physical abuse	1	case_or_format
Physical abuse	physical abuse	physical abuse	physical abuse	physical abuse	1	case_or_format
Physical activity behaviours	physical activity behaviours	physical activity behaviours	physical activity behaviours	Physical activity behaviours	1	kept
physical activity determinants	physical activity determinants	physical activity determinants	physical activity determinants	physical activity determinants	1	kept
Physical activity health paradox	physical activity health paradox	physical activity health paradox	physical activity health paradox	Physical activity health paradox	1	kept
physical aggression	physical aggression	physical aggression	physical aggression	physical aggression	1	kept
physical and visual access	physical and visual access	physical and visual access	physical and visual access	physical and visual access	1	kept
physical and visual accessibilities	physical and visual accessibilities	physical and visual accessibilities	physical and visual accessibilities	physical and visual accessibilities	1	kept
Physical assessment	physical assessment	physical assessment	physical assessment	PHYSICAL ASSESSMENT	1	case_or_format
Physical Burden	physical burden	physical burden	physical burden	Physical Burden	1	kept
Physical changes	physical changes	physical changes	physical changes	Physical changes	1	kept

physical chemistry	physical chemistry	physical chemistry	physical chemistry	physical chemistry	1	kept
Physical Component Score	physical component score	physical component score	physical component score	Physical Component Score	1	kept
physical component summary	physical component summary	physical component summary	physical component summary	physical component summary	1	kept
physical constitution and health	physical constitution and health	physical constitution and health	physical constitution and health	physical constitution and health	1	kept
Physical demands	physical demands	physical demands	physical demands	Physical demands	1	kept
Physical design	physical design	physical design	physical design	Physical design	1	kept
PHYSICAL DESIGN-FEATURES	physical design features	physical design features	physical design features	PHYSICAL DESIGN-FEATURES	1	kept
physical development	physical development	physical development	physical development	physical development	1	kept
physical distancing	physical distancing	physical distancing	physical distancing	Physical Distancing	1	case_or_format
Physical domain	physical domain	physical domain	physical domain	Physical domain	1	kept
Physical Education and Training	physical education and training	physical education and training	physical education and training	Physical Education and Training	1	kept
Physical Environmental Stress Scale	physical environmental stress scale	physical environmental stress scale	physical environmental stress scale	Physical Environmental Stress Scale	1	kept
Physical examination	physical examination	physical examination	physical examination	physical examination	1	case_or_format
Physical Fatigue	physical fatigue	physical fatigue	physical fatigue	Physical Fatigue	1	kept
Physical fatigue	physical fatigue	physical fatigue	physical fatigue	Physical Fatigue	1	case_or_format
Physical Fitness	physical fitness	physical fitness	physical fitness	Physical Fitness	1	kept

Physical fitness	physical fitness	physical fitness	physical fitness	Physical Fitness	1	case_or_format
PHYSICAL FUNCTION	physical function	physical function	physical function	physical function	1	case_or_format
physical function score	physical function score	physical function score	physical function score	physical function score	1	kept
Physical functional performance	physical functional performance	physical functional performance	physical functional performance	Physical Functional Performance	1	case_or_format
Physical functioning	physical functioning	physical functioning	physical functioning	physical functioning	1	case_or_format
physical goals	physical goals	physical goals	physical goals	physical goals	1	kept
physical impairment	physical impairment	physical impairment	physical impairment	Physical impairments	1	case_or_format
physical impairments	physical impairments	physical impairments	physical impairment	Physical impairments	1	singular
Physical medicine and rehabilitation	physical medicine and rehabilitation	physical medicine and rehabilitation	physical medicine and rehabilitation	Physical medicine and rehabilitation	1	kept
physical mobilisation	physical mobilisation	physical mobilisation	physical mobilisation	physical mobilisation	1	kept
Physical mobility	physical mobility	physical mobility	physical mobility	physical mobility	1	case_or_format
physical models	physical models	physical models	physical models	physical models	1	kept
Physical morbidity	physical morbidity	physical morbidity	physical morbidity	Physical morbidity	1	kept
Physical parameters	physical parameters	physical parameters	physical parameters	physical parameters	1	case_or_format
physical phenomena	physical phenomena	physical phenomena	physical phenomena	physical phenomena	1	kept
physical recovery	physical recovery	physical recovery	physical recovery	Physical recovery	1	case_or_format

PHYSICAL REHABILITATION	physical rehabilitation	physical rehabilitation	physical rehabilitation	physical rehabilitation	1	case_or_format
PHYSICAL RESTRAINT	physical restraint	physical restraint	physical restraint	physical restraint	1	case_or_format
Physical Restraint Practices	physical restraint practices	physical restraint practices	physical restraint practices	Physical Restraint Practices	1	kept
physical restraint reduction	physical restraint reduction	physical restraint reduction	physical restraint reduction	physical restraint reduction	1	kept
Physical restrictors	physical restrictors	physical restrictors	physical restrictors	Physical restrictors	1	kept
Physical Self Maintenance Scale	physical self maintenance scale	physical self maintenance scale	physical self maintenance scale	Physical Self Maintenance Scale	1	kept
Physical sensors	physical sensors	physical sensors	physical sensors	Physical sensors	1	kept
Physical setting	physical setting	physical setting	physical setting	Physical setting	1	kept
Physical simulation	physical simulation	physical simulation	physical simulation	Physical simulation	1	kept
PHYSICAL SPACE	physical space	physical space	physical space	PHYSICAL SPACE	1	kept
physical status classification system	physical status classification system	physical status classification system	physical status classification system	physical status classification system	1	kept
Physical stress	physical stress	physical stress	physical stress	physical stress	1	case_or_format
Physical suffering	physical suffering	physical suffering	physical suffering	Physical suffering	1	kept
Physical therapist	physical therapist	physical therapist	physical therapist	Physical Therapists	1	case_or_format
physical therapist	physical therapist	physical therapist	physical therapist	Physical Therapists	1	case_or_format
Physical Therapy Department	physical therapy department	physical therapy department	physical therapy department	Physical Therapy Department	1	kept

physical therapy education	physical therapy education	physical therapy education	physical therapy education	physical therapy education	1	kept
Physical therapy teaching	physical therapy teaching	physical therapy teaching	physical therapy teaching	Physical therapy teaching	1	kept
Physical therapy techniques	physical therapy techniques	physical therapy techniques	physical therapy techniques	Physical therapy techniques	1	kept
physical therapy techniques	physical therapy techniques	physical therapy techniques	physical therapy techniques	Physical therapy techniques	1	case_or_format
Physical variables	physical variables	physical variables	physical variables	Physical variables	1	kept
Physical Violence	physical violence	physical violence	physical violence	physical violence	1	case_or_format
physical wellbeing	physical wellbeing	physical wellbeing	physical wellbeing	physical wellbeing	1	kept
PHYSICAL WORK	physical work	physical work	physical work	PHYSICAL WORK	1	kept
physical workload	physical workload	physical workload	physical workload	physical workload	1	kept
Physical workloads	physical workloads	physical workloads	physical workload	physical workload	1	singular
physical workloads	physical workloads	physical workloads	physical workload	physical workload	1	singular
PHYSICAL-ENVIRONMENT	physical environment	physical environment	physical environment	physical environment	1	case_or_format
PHYSICAL-THERAPY	physical therapy	physical therapy	physical therapy	Physical therapy	1	case_or_format
Physician Active Shift Signout in the Emergency Department	physician active shift signout in the emergency department	physician active shift signout in the emergency department	physician active shift signout in the emergency department	Physician Active Shift Signout in the Emergency Department	1	kept
physician age	physician age	physician age	physician age	physician age	1	kept
Physician associate	physician associate	physician associate	physician associate	physician associate	1	case_or_format

Physician associates	physician associates	physician associates	physician associate	physician associate	1	singular
physician burden	physician burden	physician burden	physician burden	physician burden	1	kept
PHYSICIAN BURNOUT	physician burnout	physician burnout	physician burnout	PHYSICIAN BURNOUT	1	kept
Physician burnout	physician burnout	physician burnout	physician burnout	PHYSICIAN BURNOUT	1	case_or_format
physician burnout	physician burnout	physician burnout	physician burnout	PHYSICIAN BURNOUT	1	case_or_format
Physician collaboration network	physician collaboration network	physician collaboration network	physician collaboration network	Physician collaboration network	1	kept
physician collaboration network	physician collaboration network	physician collaboration network	physician collaboration network	Physician collaboration network	1	case_or_format
physician documentation	physician documentation	physician documentation	physician documentation	Physician documentation	1	case_or_format
Physician extender	physician extender	physician extender	physician extender	Physician extender	1	kept
Physician Health	physician health	physician health	physician health	Physician Health	1	kept
physician health	physician health	physician health	physician health	Physician Health	1	case_or_format
Physician intensity	physician intensity	physician intensity	physician intensity	Physician intensity	1	kept
physician level	physician level	physician level	physician level	physician level	1	kept
physician moral distress	physician moral distress	physician moral distress	physician moral distress	physician moral distress	1	kept
PHYSICIAN NETWORKS	physician networks	physician networks	physician networks	PHYSICIAN NETWORKS	1	kept
PHYSICIAN ORDERS	physician orders	physician orders	physician orders	PHYSICIAN ORDERS	1	kept

physician orders for life sustaining treatment	physician orders for life sustaining treatment	physician orders for life sustaining treatment	physician orders for life sustaining treatment	physician orders for life sustaining treatment	1	kept
Physician payment	physician payment	physician payment	physician payment	Physician payment	1	kept
Physician practice patterns	physician practice patterns	physician practice patterns	physician practice patterns	physician practice patterns	1	case_or_format
PHYSICIAN PROFILES	physician profiles	physician profiles	physician profiles	PHYSICIAN PROFILES	1	kept
Physician reports	physician reports	physician reports	physician reports	Physician reports	1	kept
Physician roles	physician roles	physician roles	physician roles	Physician roles	1	kept
Physician rounds	physician rounds	physician rounds	physician rounds	Physician rounds	1	kept
Physician shortage	physician shortage	physician shortage	physician shortage	physician shortage	1	case_or_format
Physician Staffing Pattern	physician staffing pattern	physician staffing pattern	physician staffing pattern	Physician Staffing Pattern	1	kept
Physician training	physician training	physician training	physician training	physician training	1	case_or_format
Physician versatility	physician versatility	physician versatility	physician versatility	Physician versatility	1	kept
Physician wellbeing	physician wellbeing	physician wellbeing	physician wellbeing	Physician wellbeing	1	kept
physician workforce	physician workforce	physician workforce	physician workforce	physician workforce	1	kept
physician’s	physician s	physician s	physician s	physician’s	1	kept
Physician’s acceptance	physician s acceptance	physician s acceptance	physician s acceptance	Physician’s acceptance	1	kept
Physician’s orders	physician s orders	physician s orders	physician s orders	Physician’s orders	1	kept

PHYSICIAN-ASSISTANTS	physician assistants	physician assistants	physician assistant	physician assistant	1	singular
physician-assisted death	physician assisted death	physician assisted death	physician assisted death	physician-assisted death	1	kept
Physician-assisted suicide	physician assisted suicide	physician assisted suicide	physician assisted suicide	PHYSICIAN-ASSISTED SUICIDE	1	case_or_format
Physician-staffed ambulances	physician staffed ambulances	physician staffed ambulances	physician staffed ambulances	Physician-staffed ambulances	1	kept
PHYSICIANS REACTIONS	physicians reactions	physicians reactions	physicians reactions	PHYSICIANS REACTIONS	1	kept
Physicians scheduling	physicians scheduling	physicians scheduling	physicians scheduling	Physicians scheduling	1	kept
physicians scheduling	physicians scheduling	physicians scheduling	physicians scheduling	Physicians scheduling	1	case_or_format
Physicians' Offices	physicians offices	physicians offices	physicians offices	Physicians' Offices	1	kept
physicians' traits	physicians traits	physicians traits	physicians traits	physicians' traits	1	kept
physician's	physician s	physician s	physician s	physician's	1	case_or_format
physicist	physicist	physicist	physicist	physicist	1	kept
physiologic birth	physiologic birth	physiologic birth	physiologic birth	physiologic birth	1	kept
Physiologic changes with aging	physiologic changes with aging	physiologic changes with aging	physiologic changes with aging	Physiologic changes with aging	1	kept
physiologic criteria	physiologic criteria	physiologic criteria	physiologic criteria	physiologic criteria	1	kept
Physiologic deterioration	physiologic deterioration	physiologic deterioration	physiologic deterioration	Physiologic deterioration	1	kept
physiologic labor	physiologic labor	physiologic labor	physiologic labor	physiologic labor	1	kept

Physiologic Monitor Alarms	physiologic monitor alarms	physiologic monitor alarms	physiologic monitor alarms	Physiologic Monitor Alarms	1	kept
Physiologic monitor alarms	physiologic monitor alarms	physiologic monitor alarms	physiologic monitor alarms	Physiologic Monitor Alarms	1	case_or_format
Physiologic monitoring alarms	physiologic monitoring alarms	physiologic monitoring alarms	physiologic monitoring alarms	Physiologic monitoring alarms	1	kept
physiologic parameter	physiologic parameter	physiologic parameter	physiologic parameter	physiologic parameter	1	kept
Physiologic parameters	physiologic parameters	physiologic parameters	physiologic parameter	physiologic parameter	1	singular
physiologic parameters	physiologic parameters	physiologic parameters	physiologic parameter	physiologic parameter	1	singular
physiologic responses	physiologic responses	physiologic responses	physiologic responses	physiologic responses	1	kept
Physiological	physiological	physiological	physiological	physiological	1	case_or_format
Physiological adaptation	physiological adaptation	physiological adaptation	physiological adaptation	physiological adaptation	1	case_or_format
physiological birth practices	physiological birth practices	physiological birth practices	physiological birth practices	physiological birth practices	1	kept
Physiological changes	physiological changes	physiological changes	physiological changes	Physiological changes	1	kept
Physiological criteria	physiological criteria	physiological criteria	physiological criteria	Physiological criteria	1	kept
Physiological data	physiological data	physiological data	physiological data	Physiological data	1	kept
Physiological deterioration	physiological deterioration	physiological deterioration	physiological deterioration	Physiological deterioration	1	kept
physiological development	physiological development	physiological development	physiological development	physiological development	1	kept
Physiological effects	physiological effects	physiological effects	physiological effects	Physiological effects	1	kept

physiological effects	physiological effects	physiological effects	physiological effects	Physiological effects	1	case_or_format
Physiological feedback	physiological feedback	physiological feedback	physiological feedback	Physiological feedback	1	kept
physiological health and psychological health	physiological health and psychological health	physiological health and psychological health	physiological health and psychological health	physiological health and psychological health	1	kept
Physiological indicators	physiological indicators	physiological indicators	physiological indicators	PHYSIOLOGICAL INDICATORS	1	case_or_format
Physiological indices	physiological indices	physiological indices	physiological indices	Physiological indices	1	kept
Physiological informations	physiological informations	physiological informations	physiological informations	Physiological informations	1	kept
physiological losses	physiological losses	physiological losses	physiological losses	physiological losses	1	kept
Physiological measurement	physiological measurement	physiological measurement	physiological measurement	Physiological measurement	1	kept
physiological measurement	physiological measurement	physiological measurement	physiological measurement	Physiological measurement	1	case_or_format
Physiological measurements	physiological measurements	physiological measurements	physiological measurement	Physiological measurement	1	singular
physiological measurements	physiological measurements	physiological measurements	physiological measurement	Physiological measurement	1	singular
Physiological measures	physiological measures	physiological measures	physiological measures	Physiological measures	1	kept
physiological monitor	physiological monitor	physiological monitor	physiological monitor	physiological monitor	1	kept
physiological monitor alarms	physiological monitor alarms	physiological monitor alarms	physiological monitor alarms	physiological monitor alarms	1	kept
Physiological Monitors	physiological monitors	physiological monitors	physiological monitor	physiological monitor	1	singular
Physiological pattern recognition	physiological pattern recognition	physiological pattern recognition	physiological pattern recognition	Physiological pattern recognition	1	kept

physiological pattern recognition	physiological pattern recognition	physiological pattern recognition	physiological pattern recognition	Physiological pattern recognition	1	case_or_format
physiological perception	physiological perception	physiological perception	physiological perception	physiological perception	1	kept
Physiological reactions	physiological reactions	physiological reactions	physiological reactions	Physiological reactions	1	kept
physiological recovery	physiological recovery	physiological recovery	physiological recovery	physiological recovery	1	kept
physiological response	physiological response	physiological response	physiological response	Physiological response	1	case_or_format
Physiological responses of pain	physiological responses of pain	physiological responses of pain	physiological responses of pain	Physiological responses of pain	1	kept
Physiological sensors	physiological sensors	physiological sensors	physiological sensors	Physiological sensors	1	kept
Physiological Signals	physiological signals	physiological signals	physiological signals	Physiological signals	1	case_or_format
physiological status detection	physiological status detection	physiological status detection	physiological status detection	physiological status detection	1	kept
Physiological surveillance system	physiological surveillance system	physiological surveillance system	physiological surveillance system	Physiological surveillance system	1	kept
PHYSIOLOGICAL TRACK	physiological track	physiological track	physiological track	PHYSIOLOGICAL TRACK	1	kept
Physiological variable	physiological variable	physiological variable	physiological variable	physiological variable	1	case_or_format
physiotherapist assistant	physiotherapist assistant	physiotherapist assistant	physiotherapist assistant	physiotherapist assistant	1	kept
PHYSIOTHERAPY	physiotherapy	physiotherapy	physiotherapy	physiotherapy	1	case_or_format
Physiotherapy students	physiotherapy students	physiotherapy students	physiotherapy students	Physiotherapy students	1	kept
physiotherapy students	physiotherapy students	physiotherapy students	physiotherapy students	Physiotherapy students	1	case_or_format

Physis	physis	physis	physis	Physis	1	kept
phytohemagglutinin	phytohemagglutinin	phytohemagglutinin	phytohemagglutinin	phytohemagglutinin	1	kept
phytotherapy	phytotherapy	phytotherapy	phytotherapy	Phytotherapy	1	case_or_format
phytotoxicity	phytotoxicity	phytotoxicity	phytotoxicity	phytotoxicity	1	kept
PIC score	pic score	pic score	pic score	PIC score	1	kept
PICaNET	picanet	picanet	picanet	PICaNET	1	kept
PICC dressing	picc dressing	picc dressing	picc dressing	PICC dressing	1	kept
PICC team	picc team	picc team	picc team	PICC team	1	kept
Pichia anomala	pichia anomala	pichia anomala	pichia anomala	Pichia anomala	1	kept
Pichia kudriavzevii	pichia kudriavzevii	pichia kudriavzevii	pichia kudriavzevii	Pichia kudriavzevii	1	kept
picibanil	picibanil	picibanil	picibanil	picibanil	1	kept
PICNET	picnet	picnet	picnet	PICNET	1	kept
PICO, population, intervention, comparison, outcome	pico population intervention comparison outcome	pico population intervention comparison outcome	pico population intervention comparison outcome	PICO, population, intervention, comparison, outcome	1	kept
Piconet	piconet	piconet	piconet	Piconet	1	kept
Piconets	piconets	piconets	piconet	Piconet	1	singular
pics	pics	pics	pics	PICS	1	case_or_format

PICS clinic	pics clinic	pics clinic	pics clinic	PICS clinic	1	kept
PICS clinics	pics clinics	pics clinics	pics clinic	PICS clinic	1	singular
PICS-p	pics p	pics p	pics p	PICS-p	1	kept
PICS‑p	pics p	pics p	pics p	PICS-p	1	case_or_format
PICS–F	pics f	pics f	pics f	PICS-F	1	case_or_format
Pictogram	pictogram	pictogram	pictogram	Pictogram	1	kept
Pictograma	pictograma	pictograma	pictograma	Pictograma	1	kept
Piedmont [Italy]	piedmont italy	piedmont italy	piedmont italy	Piedmont [Italy]	1	kept
Piel	piel	piel	piel	Piel	1	kept
Pieper Pressure Ulcer Knowledge test	pieper pressure ulcer knowledge test	pieper pressure ulcer knowledge test	pieper pressure ulcer knowledge test	Pieper Pressure Ulcer Knowledge test	1	kept
pieper zulkowski pressure injury knowledge	pieper zulkowski pressure injury knowledge	pieper zulkowski pressure injury knowledge	pieper zulkowski pressure injury knowledge	pieper zulkowski pressure injury knowledge	1	kept
Pierre-Robin syndrome	pierre robin syndrome	pierre robin syndrome	pierre robin syndrome	Pierre Robin syndrome	1	case_or_format
Piezoelectric	piezoelectric	piezoelectric	piezoelectric	Piezoelectric	1	kept
pigeon	pigeon	pigeon	pigeon	pigeon	1	kept
pigeon breeder lung	pigeon breeder lung	pigeon breeder lung	pigeon breeder lung	pigeon breeder lung	1	kept
pigeons and doves	pigeons and doves	pigeons and doves	pigeons and doves	pigeons and doves	1	kept

Piggyback technique	piggyback technique	piggyback technique	piggyback technique	Piggyback technique	1	kept
pigment disorder	pigment disorder	pigment disorder	pigment disorder	pigment disorder	1	kept
pigmentation	pigmentation	pigmentation	pigmentation	pigmentation	1	kept
PIGS	pigs	pigs	pig	pig	1	singular
pilates	pilates	pilates	pilates	pilates	1	kept
pill	pill	pill	pill	pill	1	kept
Pill count	pill count	pill count	pill count	Pill count	1	kept
pillow	pillow	pillow	pillow	pillow	1	kept
Pilot hospital-based	pilot hospital based	pilot hospital based	pilot hospital based	Pilot hospital-based	1	kept
pilot project	pilot project	pilot project	pilot project	Pilot Projects	1	case_or_format
pilot projects	pilot projects	pilot projects	pilot project	Pilot Projects	1	singular
Pilot RCT	pilot rct	pilot rct	pilot rct	Pilot RCT	1	kept
Pilot reports	pilot reports	pilot reports	pilot reports	Pilot reports	1	kept
pilot studies	pilot studies	pilot studies	pilot study	pilot study	1	singular
pilot study design	pilot study design	pilot study design	pilot study design	pilot study design	1	kept
Pilot study.	pilot study	pilot study	pilot study	pilot study	1	case_or_format

pilot trial	pilot trial	pilot trial	pilot trial	Pilot trial	1	case_or_format
Pilots	pilots	pilots	pilot	pilot	1	singular
pimobendan	pimobendan	pimobendan	pimobendan	pimobendan	1	kept
pinch strength	pinch strength	pinch strength	pinch strength	pinch strength	1	kept
pineal body	pineal body	pineal body	pineal body	pineal body	1	kept
pineal body tumor	pineal body tumor	pineal body tumor	pineal body tumor	pineal body tumor	1	kept
PIOMI	piomi	piomi	piomi	PIOMI	1	kept
pioneers	pioneers	pioneers	pioneers	pioneers	1	kept
pipecolic acid derivative	pipecolic acid derivative	pipecolic acid derivative	pipecolic acid derivative	pipecolic acid derivative	1	kept
Pipecolic Acids	pipecolic acids	pipecolic acids	pipecolic acids	Pipecolic Acids	1	kept
pipeline	pipeline	pipeline	pipeline	pipeline	1	kept
Pipelines	pipelines	pipelines	pipeline	pipeline	1	singular
piperacillin, tazobactam drug combination	piperacillin tazobactam drug combination	piperacillin tazobactam drug combination	piperacillin tazobactam drug combination	Piperacillin, Tazobactam Drug Combination	1	case_or_format
piperacillin-tazobactam	piperacillin tazobactam	piperacillin tazobactam	piperacillin tazobactam	PIPERACILLIN-TAZOBACTAM	1	case_or_format
piperazinedione	piperazinedione	piperazinedione	piperazinedione	piperazinedione	1	kept
piperidine derivative	piperidine derivative	piperidine derivative	piperidine derivative	piperidine derivative	1	kept

pirfenidone	pirfenidone	pirfenidone	pirfenidone	pirfenidone	1	kept
Pirinitramide	pirinitramide	pirinitramide	pirinitramide	Pirinitramide	1	kept
piroplasmosis	piroplasmosis	piroplasmosis	piroplasmosis	piroplasmosis	1	kept
Pitch Discrimination	pitch discrimination	pitch discrimination	pitch discrimination	Pitch Discrimination	1	kept
pitfalls	pitfalls	pitfalls	pitfalls	Pitfalls	1	case_or_format
pitfalls of protocols	pitfalls of protocols	pitfalls of protocols	pitfalls of protocols	pitfalls of protocols	1	kept
Pitocin	pitocin	pitocin	pitocin	Pitocin	1	kept
pitt bacteremia score	pitt bacteremia score	pitt bacteremia score	pitt bacteremia score	pitt bacteremia score	1	kept
Pittsburgh sleep quality index	pittsburgh sleep quality index	pittsburgh sleep quality index	pittsburgh sleep quality index	Pittsburgh Sleep Quality Index	1	case_or_format
pituitary disorders	pituitary disorders	pituitary disorders	pituitary disorders	pituitary disorders	1	kept
pityriasis lichenoides	pityriasis lichenoides	pityriasis lichenoides	pityriasis lichenoides	pityriasis lichenoides	1	kept
pityriasis rubra pilaris	pityriasis rubra pilaris	pityriasis rubra pilaris	pityriasis rubra pilaris	pityriasis rubra pilaris	1	kept
PIV catheter dwell time	piv catheter dwell time	piv catheter dwell time	piv catheter dwell time	PIV catheter dwell time	1	kept
PIVCs	pivcs	pivcs	pivcs	PIVCs	1	kept
PIVs	pivs	pivs	piv	PIV	1	singular
PKAT	pkat	pkat	pkat	PKAT	1	kept

Place	place	place	place	PLACE	1	case_or_format
Place of birth	place of birth	place of birth	place of birth	place of birth	1	case_or_format
place of care	place of care	place of care	place of care	place of care	1	kept
place type	place type	place type	place type	place type	1	kept
place-making	place making	place making	place making	place-making	1	kept
Placebo-controlled trial	placebo controlled trial	placebo controlled trial	placebo controlled trial	Placebo-controlled trial	1	kept
PLACEMAKING	placemaking	placemaking	placemaking	PLACEMAKING	1	kept
placenta accreta placenta increta	placenta accreta placenta increta	placenta accreta placenta increta	placenta accreta placenta increta	placenta accreta placenta increta	1	kept
placenta circulation	placenta circulation	placenta circulation	placenta circulation	placenta circulation	1	kept
placenta development	placenta development	placenta development	placenta development	placenta development	1	kept
Placenta Diseases	placenta diseases	placenta diseases	placenta diseases	Placenta Diseases	1	kept
placenta membranacea	placenta membranacea	placenta membranacea	placenta membranacea	placenta membranacea	1	kept
placenta percreta	placenta percreta	placenta percreta	placenta percreta	placenta percreta	1	kept
placenta perfusion	placenta perfusion	placenta perfusion	placenta perfusion	placenta perfusion	1	kept
Placenta Previa	placenta previa	placenta previa	placenta previa	placenta previa	1	case_or_format
placenta tissue	placenta tissue	placenta tissue	placenta tissue	placenta tissue	1	kept

placental abnormality	placental abnormality	placental abnormality	placental abnormality	placental abnormality	1	kept
Placental barrier	placental barrier	placental barrier	placental barrier	Placental barrier	1	kept
Placental blood	placental blood	placental blood	placental blood	Placental blood	1	kept
placental delivery	placental delivery	placental delivery	placental delivery	placental delivery	1	kept
placental thickness	placental thickness	placental thickness	placental thickness	placental thickness	1	kept
Placentation	placentation	placentation	placentation	Placentation	1	kept
placentomegaly	placentomegaly	placentomegaly	placentomegaly	placentomegaly	1	kept
Places of death	places of death	places of death	places of death	Places of death	1	kept
Plagiarism	plagiarism	plagiarism	plagiarism	Plagiarism	1	kept
Plague	plague	plague	plague	plague	1	case_or_format
Plan de cuidados de enfermería	plan de cuidados de enfermer a	plan de cuidados de enfermer a	plan de cuidados de enfermer a	Plan de cuidados de enfermería	1	kept
Plan de estudios	plan de estudios	plan de estudios	plan de estudios	Plan de estudios	1	kept
Plan geometry	plan geometry	plan geometry	plan geometry	Plan geometry	1	kept
Plan-Do-Check-Act cycle	plan do check act cycle	plan do check act cycle	plan do check act cycle	Plan-Do-Check-Act cycle	1	kept
Plan-Do-Study-Act	plan do study act	plan do study act	plan do study act	plan do study act	1	case_or_format
plan-do-study-act	plan do study act	plan do study act	plan do study act	plan do study act	1	case_or_format

Plan-Do-Study-Act cycle	plan do study act cycle	plan do study act cycle	plan do study act cycle	Plan-Do-Study-Act cycle	1	kept
Plan-Do-Study-Act cycles	plan do study act cycles	plan do study act cycles	plan do study act cycle	Plan-Do-Study-Act cycle	1	singular
Planejamento de assistência ao paciente	planejamento de assist ncia ao paciente	planejamento de assist ncia ao paciente	planejamento de assist ncia ao paciente	Planejamento de assistência ao paciente	1	kept
planetary health	planetary health	planetary health	planetary health	planetary health	1	kept
Planificación de la atención al paciente	planificaci n de la atenci n al paciente	planificaci n de la atenci n al paciente	planificaci n de la atenci n al paciente	Planificación de la atención al paciente	1	kept
Planned behaviors	planned behaviors	planned behaviors	planned behavior	PLANNED BEHAVIOR	1	singular
Planned change	planned change	planned change	planned change	Planned change	1	kept
planned event	planned event	planned event	planned event	planned event	1	kept
Planning admission	planning admission	planning admission	planning admission	Planning admission	1	kept
Planning horizons	planning horizons	planning horizons	planning horizons	Planning horizons	1	kept
Planning meetings	planning meetings	planning meetings	planning meetings	Planning meetings	1	kept
planning method	planning method	planning method	planning method	planning method	1	kept
Plant Leaves	plant leaves	plant leaves	plant leaves	Plant Leaves	1	kept
plant-based milk	plant based milk	plant based milk	plant based milk	plant-based milk	1	kept
plantar fasciitis	plantar fasciitis	plantar fasciitis	plantar fasciitis	plantar fasciitis	1	kept
plantar flexion contracture	plantar flexion contracture	plantar flexion contracture	plantar flexion contracture	plantar flexion contracture	1	kept

plantar heel pain	plantar heel pain	plantar heel pain	plantar heel pain	plantar heel pain	1	kept
Plantar Plate	plantar plate	plantar plate	plantar plate	Plantar Plate	1	kept
Plants, Medicinal	plants medicinal	plants medicinal	plants medicinal	Plants, Medicinal	1	kept
plasma cell	plasma cell	plasma cell	plasma cell	plasma cell	1	kept
plasma cell dyscrasia	plasma cell dyscrasia	plasma cell dyscrasia	plasma cell dyscrasia	plasma cell dyscrasia	1	kept
plasma clearance	plasma clearance	plasma clearance	plasma clearance	plasma clearance	1	kept
plasma half life	plasma half life	plasma half life	plasma half life	plasma half life	1	kept
plasma protein	plasma protein	plasma protein	plasma protein	plasma protein	1	kept
Plasma pseudocolinesterase	plasma pseudocolinesterase	plasma pseudocolinesterase	plasma pseudocolinesterase	Plasma pseudocolinesterase	1	kept
plasma renin activity	plasma renin activity	plasma renin activity	plasma renin activity	plasma renin activity	1	kept
plasma wastage	plasma wastage	plasma wastage	plasma wastage	plasma wastage	1	kept
PLASMA-CORTISOL	plasma cortisol	plasma cortisol	plasma cortisol	PLASMA-CORTISOL	1	kept
PLASMA-LEVELS	plasma levels	plasma levels	plasma levels	PLASMA-LEVELS	1	kept
plasma-lyte	plasma lyte	plasma lyte	plasma lyte	plasma-lyte	1	kept
Plasmacytoma	plasmacytoma	plasmacytoma	plasmacytoma	plasmacytoma	1	case_or_format
Plasmaféresis terapé utica	plasmaf resis terap utica	plasmaf resis terap utica	plasmaf resis terap utica	Plasmaféresis terapé utica	1	kept

Plasmid	plasmid	plasmid	plasmid	plasmid	1	case_or_format
plasmid DNA	plasmid dna	plasmid dna	plasmid dna	plasmid DNA	1	kept
Plasminogen activator	plasminogen activator	plasminogen activator	plasminogen activator	plasminogen activator	1	case_or_format
plasminogen activator inhibitor	plasminogen activator inhibitor	plasminogen activator inhibitor	plasminogen activator inhibitor	plasminogen activator inhibitor	1	kept
plasminogen activator inhibitor 1	plasminogen activator inhibitor 1	plasminogen activator inhibitor 1	plasminogen activator inhibitor 1	plasminogen activator inhibitor 1	1	kept
Plasminogen Activators	plasminogen activators	plasminogen activators	plasminogen activator	plasminogen activator	1	singular
plaster cast	plaster cast	plaster cast	plaster cast	plaster cast	1	kept
Plastic	plastic	plastic	plastic	plastic	1	case_or_format
Plastic bronchitis	plastic bronchitis	plastic bronchitis	plastic bronchitis	Plastic bronchitis	1	kept
plasticizer	plasticizer	plasticizer	plasticizer	Plasticizers	1	case_or_format
plateau fracture	plateau fracture	plateau fracture	plateau fracture	plateau fracture	1	kept
Platelet Activation	platelet activation	platelet activation	platelet activation	Platelet Activation	1	kept
Platelet distribution width	platelet distribution width	platelet distribution width	platelet distribution width	Platelet distribution width	1	kept
platelet distribution width	platelet distribution width	platelet distribution width	platelet distribution width	Platelet distribution width	1	case_or_format
platelet endothelial cell adhesion molecule 1	platelet endothelial cell adhesion molecule 1	platelet endothelial cell adhesion molecule 1	platelet endothelial cell adhesion molecule 1	platelet endothelial cell adhesion molecule 1	1	kept
Platelet Glycoprotein GPIIb-IIIa Complex	platelet glycoprotein gpiib iiia complex	platelet glycoprotein gpiib iiia complex	platelet glycoprotein gpiib iiia complex	Platelet Glycoprotein GPIIb-IIIa Complex	1	kept

platelet microparticle	platelet microparticle	platelet microparticle	platelet microparticle	platelet microparticle	1	kept
Platelet reactivity	platelet reactivity	platelet reactivity	platelet reactivity	Platelet reactivity	1	kept
platelet reactivity	platelet reactivity	platelet reactivity	platelet reactivity	Platelet reactivity	1	case_or_format
platelet transfusion refractoriness	platelet transfusion refractoriness	platelet transfusion refractoriness	platelet transfusion refractoriness	platelet transfusion refractoriness	1	kept
platelet volume	platelet volume	platelet volume	platelet volume	platelet volume	1	kept
Platelet-Rich Plasma	platelet rich plasma	platelet rich plasma	platelet rich plasma	Platelet-Rich Plasma	1	kept
Platelet-to-lymphocyte ratio	platelet to lymphocyte ratio	platelet to lymphocyte ratio	platelet to lymphocyte ratio	Platelet-to-lymphocyte ratio	1	kept
plateletcrit	plateletcrit	plateletcrit	plateletcrit	plateletcrit	1	kept
Platelets count	platelets count	platelets count	platelets count	Platelets count	1	kept
platin hypersensitivity	platin hypersensitivity	platin hypersensitivity	platin hypersensitivity	platin hypersensitivity	1	kept
Platinum	platinum	platinum	platinum	platinum	1	case_or_format
platinum coil	platinum coil	platinum coil	platinum coil	platinum coil	1	kept
platinum complex	platinum complex	platinum complex	platinum complex	platinum complex	1	kept
platinum compounds	platinum compounds	platinum compounds	platinum compounds	platinum compounds	1	kept
Play and playthings	play and playthings	play and playthings	play and playthings	Play and Playthings	1	case_or_format
Play Therapy	play therapy	play therapy	play therapy	Play Therapy	1	kept

play therapy	play therapy	play therapy	play therapy	Play Therapy	1	case_or_format
Playthings	playthings	playthings	playthings	Playthings	1	kept
plazomicin	plazomicin	plazomicin	plazomicin	plazomicin	1	kept
pleconaril	pleconaril	pleconaril	pleconaril	pleconaril	1	kept
pleiotropy	pleiotropy	pleiotropy	pleiotropy	pleiotropy	1	kept
Plethysmography	plethysmography	plethysmography	plethysmography	Plethysmography	1	kept
plethysmography	plethysmography	plethysmography	plethysmography	Plethysmography	1	case_or_format
Plethysmography, Impedance	plethysmography impedance	plethysmography impedance	plethysmography impedance	Plethysmography, Impedance	1	kept
pleura	pleura	pleura	pleura	Pleura	1	case_or_format
pleura biopsy	pleura biopsy	pleura biopsy	pleura biopsy	pleura biopsy	1	kept
pleura mesothelioma	pleura mesothelioma	pleura mesothelioma	pleura mesothelioma	pleura mesothelioma	1	kept
pleura tumor	pleura tumor	pleura tumor	pleura tumor	pleura tumor	1	kept
Pleural Diseases	pleural diseases	pleural diseases	pleural diseases	Pleural Diseases	1	kept
Pleural Neoplasms	pleural neoplasms	pleural neoplasms	pleural neoplasms	Pleural Neoplasms	1	kept
pleural pressure	pleural pressure	pleural pressure	pleural pressure	pleural pressure	1	kept
Pleural space	pleural space	pleural space	pleural space	Pleural space	1	kept

pleurisy	pleurisy	pleurisy	pleurisy	pleurisy	1	kept
pleuromutilin	pleuromutilin	pleuromutilin	pleuromutilin	pleuromutilin	1	kept
PLIF = posterior lumbar interbody fusion	plif posterior lumbar interbody fusion	plif posterior lumbar interbody fusion	plif posterior lumbar interbody fusion	PLIF = posterior lumbar interbody fusion	1	kept
plots and curves	plots and curves	plots and curves	plots and curves	plots and curves	1	kept
pluripotent stem cell	pluripotent stem cell	pluripotent stem cell	pluripotent stem cell	pluripotent stem cell	1	kept
Pluripotent Stem Cells	pluripotent stem cells	pluripotent stem cells	pluripotent stem cell	pluripotent stem cell	1	singular
pneumatic dilation	pneumatic dilation	pneumatic dilation	pneumatic dilation	pneumatic dilation	1	kept
pneumatic tool	pneumatic tool	pneumatic tool	pneumatic tool	pneumatic tool	1	kept
Pneumocephalus	pneumocephalus	pneumocephalus	pneumocephalus	Pneumocephalus	1	kept
pneumocephalus	pneumocephalus	pneumocephalus	pneumocephalus	Pneumocephalus	1	case_or_format
Pneumococcal	pneumococcal	pneumococcal	pneumococcal	Pneumococcal	1	kept
Pneumococcal HUS	pneumococcal hus	pneumococcal hus	pneumococcal hus	Pneumococcal HUS	1	kept
Pneumococcal urine antigen test	pneumococcal urine antigen test	pneumococcal urine antigen test	pneumococcal urine antigen test	Pneumococcal urine antigen test	1	kept
Pneumococcal vaccination	pneumococcal vaccination	pneumococcal vaccination	pneumococcal vaccination	Pneumococcal vaccination	1	kept
pneumococcal vaccination	pneumococcal vaccination	pneumococcal vaccination	pneumococcal vaccination	Pneumococcal vaccination	1	case_or_format
Pneumocystis carinii	pneumocystis carinii	pneumocystis carinii	pneumocystis carinii	Pneumocystis carinii	1	kept

Pneumocystis carinii pneumonia	pneumocystis carinii pneumonia	pneumocystis carinii pneumonia	pneumocystis carinii pneumonia	Pneumocystis carinii pneumonia	1	kept
Pneumocystis jirovecii pneumonia	pneumocystis jirovecii pneumonia	pneumocystis jirovecii pneumonia	pneumocystis jirovecii pneumonia	Pneumocystis jirovecii pneumonia	1	kept
pneumodynamics	pneumodynamics	pneumodynamics	pneumodynamics	pneumodynamics	1	kept
pneumography	pneumography	pneumography	pneumography	pneumography	1	kept
Pneumonia Associated with Mechanical Ventilation	pneumonia associated with mechanical ventilation	pneumonia associated with mechanical ventilation	pneumonia associated with mechanical ventilation	Pneumonia Associated with Mechanical Ventilation	1	kept
pneumonia care	pneumonia care	pneumonia care	pneumonia care	pneumonia care	1	kept
Pneumonia diagnosis	pneumonia diagnosis	pneumonia diagnosis	pneumonia diagnosis	Pneumonia diagnosis	1	kept
pneumonia outcome research trial severity index	pneumonia outcome research trial severity index	pneumonia outcome research trial severity index	pneumonia outcome research trial severity index	pneumonia outcome research trial severity index	1	kept
Pneumonia prevention	pneumonia prevention	pneumonia prevention	pneumonia prevention	Pneumonia prevention	1	kept
pneumonia prevention	pneumonia prevention	pneumonia prevention	pneumonia prevention	Pneumonia prevention	1	case_or_format
pneumonia severity index	pneumonia severity index	pneumonia severity index	pneumonia severity index	Pneumonia Severity Index	1	case_or_format
Pneumonia Ventilator-Associated	pneumonia ventilator associated	pneumonia ventilator associated	pneumonia ventilator associated	Pneumonia, Ventilator-Associated	1	case_or_format
pneumonia ventilator-Associated	pneumonia ventilator associated	pneumonia ventilator associated	pneumonia ventilator associated	Pneumonia, Ventilator-Associated	1	case_or_format
Pneumonia zero	pneumonia zero	pneumonia zero	pneumonia zero	Pneumonia zero	1	kept
Pneumonia, bacterial	pneumonia bacterial	pneumonia bacterial	pneumonia bacterial	Pneumonia, Bacterial	1	case_or_format
Pneumonia, Necrotizing	pneumonia necrotizing	pneumonia necrotizing	pneumonia necrotizing	Pneumonia, Necrotizing	1	kept

pneumonia, ventilator-associated	pneumonia ventilator associated	pneumonia ventilator associated	pneumonia ventilator associated	Pneumonia, Ventilator-Associated	1	case_or_format
pneumopathie	pneumopathie	pneumopathie	pneumopathie	pneumopathie	1	kept
pneumopathy	pneumopathy	pneumopathy	pneumopathy	pneumopathy	1	kept
pneumopericardium	pneumopericardium	pneumopericardium	pneumopericardium	Pneumopericardium	1	case_or_format
pneumosepsis	pneumosepsis	pneumosepsis	pneumosepsis	pneumosepsis	1	kept
pneumotachygraphy	pneumotachygraphy	pneumotachygraphy	pneumotachygraphy	pneumotachygraphy	1	kept
Pneumovirus	pneumovirus	pneumovirus	pneumovirus	Pneumovirus	1	kept
PoCT	poct	poct	poct	POCT	1	case_or_format
POCUS education	pocus education	pocus education	pocus education	POCUS education	1	kept
POCUS program	pocus program	pocus program	pocus program	POCUS program	1	kept
Podcast	podcast	podcast	podcast	podcast	1	case_or_format
Podiatry	podiatry	podiatry	podiatry	Podiatry	1	kept
Podocytes	podocytes	podocytes	podocyte	podocyte	1	singular
Point of care	point of care	point of care	point of care	point of care	1	case_or_format
point of care devices	point of care devices	point of care devices	point of care devices	point of care devices	1	kept
point of care glucose measurement	point of care glucose measurement	point of care glucose measurement	point of care glucose measurement	point of care glucose measurement	1	kept

point of care quality improvement approach	point of care quality improvement approach	point of care quality improvement approach	point of care quality improvement approach	point of care quality improvement approach	1	kept
Point of Care Ultrasound	point of care ultrasound	point of care ultrasound	point of care ultrasound	point of care ultrasound	1	case_or_format
Point of care ultrasound	point of care ultrasound	point of care ultrasound	point of care ultrasound	point of care ultrasound	1	case_or_format
point of use	point of use	point of use	point of use	point of use	1	kept
Point Prevalence Survey	point prevalence survey	point prevalence survey	point prevalence survey	Point Prevalence Survey	1	kept
Point prevalence survey	point prevalence survey	point prevalence survey	point prevalence survey	Point Prevalence Survey	1	case_or_format
Point-of-care	point of care	point of care	point of care	point of care	1	case_or_format
point-of-care documentation	point of care documentation	point of care documentation	point of care documentation	point-of-care documentation	1	kept
Point-of-Care systems	point of care systems	point of care systems	point of care system	Point-of-Care Systems	1	singular
Point-of-care troponin	point of care troponin	point of care troponin	point of care troponin	Point-of-care troponin	1	kept
Point-of-care ultrasonography	point of care ultrasonography	point of care ultrasonography	point of care ultrasonography	Point-of-care ultrasonography	1	kept
Point-of-care-testing	point of care testing	point of care testing	point of care testing	point of care testing	1	case_or_format
poison	poison	poison	poison	poison	1	kept
Poison center	poison center	poison center	poison center	poison center	1	case_or_format
Poison information center	poison information center	poison information center	poison information center	Poison information center	1	kept
POISONING ADMISSIONS	poisoning admissions	poisoning admissions	poisoning admissions	POISONING ADMISSIONS	1	kept

polarography	polarography	polarography	polarography	polarography	1	kept
Police data	police data	police data	police data	Police data	1	kept
Police officers	police officers	police officers	police officers	Police officers	1	kept
Policies	policies	policies	policy	policy	1	singular
policies	policies	policies	policy	policy	1	singular
Policies and procedures	policies and procedures	policies and procedures	policies and procedures	Policies and procedures	1	kept
Policy analysis	policy analysis	policy analysis	policy analysis	Policy analysis	1	kept
policy and procedures	policy and procedures	policy and procedures	policy and procedures	policy and procedures	1	kept
policy approach	policy approach	policy approach	policy approach	policy approach	1	kept
Policy compliance	policy compliance	policy compliance	policy compliance	Policy compliance	1	kept
policy development	policy development	policy development	policy development	policy development	1	kept
Policy Implementation	policy implementation	policy implementation	policy implementation	policy implementation	1	case_or_format
policy maker	policy maker	policy maker	policy maker	policy maker	1	kept
policy makers	policy makers	policy makers	policy maker	policy maker	1	singular
Policy making	policy making	policy making	policy making	Policy Making	1	case_or_format
Policy options	policy options	policy options	policy options	Policy options	1	kept

Policy solutions	policy solutions	policy solutions	policy solutions	Policy solutions	1	kept
policy-based evidence	policy based evidence	policy based evidence	policy based evidence	policy-based evidence	1	kept
poliglecaprone	poliglecaprone	poliglecaprone	poliglecaprone	poliglecaprone	1	kept
poliomyélite	poliomy lite	poliomy lite	poliomy lite	poliomyélite	1	kept
Polish observational study	polish observational study	polish observational study	polish observational study	Polish observational study	1	kept
Polish validation study	polish validation study	polish validation study	polish validation study	Polish validation study	1	kept
politef	politef	politef	politef	politef	1	kept
political decision-making	political decision making	political decision making	political decision making	political decision-making	1	kept
Political implications	political implications	political implications	political implications	Political implications	1	kept
political participation	political participation	political participation	political participation	political participation	1	kept
political process	political process	political process	political process	political process	1	kept
POLITICS	politics	politics	politics	politics	1	case_or_format
POLLUTION	pollution	pollution	pollution	pollution	1	case_or_format
poloxamer	poloxamer	poloxamer	poloxamer	poloxamer	1	kept
POLST form	polst form	polst form	polst form	POLST form	1	kept
Poly Polymerases	poly polymerases	poly polymerases	poly polymerases	Poly Polymerases	1	kept

Polyclonal	polyclonal	polyclonal	polyclonal	Polyclonal	1	kept
polyclonal antibody	polyclonal antibody	polyclonal antibody	polyclonal antibody	polyclonal antibody	1	kept
polydeoxyribonucleotide	polydeoxyribonucleotide	polydeoxyribonucleotide	polydeoxyribonucleotide	polydeoxyribonucleotide	1	kept
Polydeoxyribonucleotides	polydeoxyribonucleotides	polydeoxyribonucleotides	polydeoxyribonucleotide	polydeoxyribonucleotide	1	singular
Polydipsia	polydipsia	polydipsia	polydipsia	polydipsia	1	case_or_format
polyene	polyene	polyene	polyene	polyene	1	kept
Polyesters	polyesters	polyesters	polyester	polyester	1	singular
polyetherurethan	polyetherurethan	polyetherurethan	polyetherurethan	polyetherurethan	1	kept
POLYETHYLENE COVERS	polyethylene covers	polyethylene covers	polyethylene covers	POLYETHYLENE COVERS	1	kept
polyethylene wrap	polyethylene wrap	polyethylene wrap	polyethylene wrap	polyethylene wrap	1	kept
Polymer	polymer	polymer	polymer	polymer	1	case_or_format
POLYMERASE-CHAIN-REACTION	polymerase chain reaction	polymerase chain reaction	polymerase chain reaction	polymerase chain reaction	1	case_or_format
polymerization	polymerization	polymerization	polymerization	polymerization	1	kept
polymicrobial	polymicrobial	polymicrobial	polymicrobial	polymicrobial	1	kept
polymonography	polymonography	polymonography	polymonography	polymonography	1	kept
Polymorphism, Genetic	polymorphism genetic	polymorphism genetic	polymorphism genetic	Polymorphism, Genetic	1	kept

polymorphisms	polymorphisms	polymorphisms	polymorphisms	polymorphisms	1	kept
polymorphonuclear cell	polymorphonuclear cell	polymorphonuclear cell	polymorphonuclear cell	polymorphonuclear cell	1	kept
polymyxin derivative	polymyxin derivative	polymyxin derivative	polymyxin derivative	polymyxin derivative	1	kept
polypeptide	polypeptide	polypeptide	polypeptide	polypeptide	1	kept
Polypeptides	polypeptides	polypeptides	polypeptide	polypeptide	1	singular
POLYPHARMACY	polypharmacy	polypharmacy	polypharmacy	polypharmacy	1	case_or_format
polyphenol	polyphenol	polyphenol	polyphenol	polyphenol	1	kept
Polyploidy	polyploidy	polyploidy	polyploidy	Polyploidy	1	kept
Polypropylene Oxygenators	polypropylene oxygenators	polypropylene oxygenators	polypropylene oxygenators	Polypropylene Oxygenators	1	kept
polysaccharide vaccine	polysaccharide vaccine	polysaccharide vaccine	polysaccharide vaccine	polysaccharide vaccine	1	kept
Polysensory neuropathy	polysensory neuropathy	polysensory neuropathy	polysensory neuropathy	Polysensory neuropathy	1	kept
POLYSOMNOGRAPHY	polysomnography	polysomnography	polysomnography	Polysomnography	1	case_or_format
polysporin	polysporin	polysporin	polysporin	polysporin	1	kept
polystyrenesulfonate calcium	polystyrenesulfonate calcium	polystyrenesulfonate calcium	polystyrenesulfonate calcium	polystyrenesulfonate calcium	1	kept
polysulfone	polysulfone	polysulfone	polysulfone	polysulfone	1	kept
polysulfone membrane	polysulfone membrane	polysulfone membrane	polysulfone membrane	polysulfone membrane	1	kept

Polytetrafluoroethylene	polytetrafluoroethylene	polytetrafluoroethylene	polytetrafluoroethylene	Polytetrafluoroethylene	1	kept
POLYTRAUMA	polytrauma	polytrauma	polytrauma	polytrauma	1	case_or_format
Polytrauma	polytrauma	polytrauma	polytrauma	polytrauma	1	case_or_format
polytraumatisme	polytraumatisme	polytraumatisme	polytraumatisme	polytraumatisme	1	kept
polyunsaturated fatty acid	polyunsaturated fatty acid	polyunsaturated fatty acid	polyunsaturated fatty acid	polyunsaturated fatty acid	1	kept
Polyurethane	polyurethane	polyurethane	polyurethane	Polyurethanes	1	case_or_format
polyurethane	polyurethane	polyurethane	polyurethane	Polyurethanes	1	case_or_format
Polyurethane endotracheal tube	polyurethane endotracheal tube	polyurethane endotracheal tube	polyurethane endotracheal tube	Polyurethane endotracheal tube	1	kept
polyuria	polyuria	polyuria	polyuria	polyuria	1	kept
polyvalent	polyvalent	polyvalent	polyvalent	polyvalent	1	kept
Polyvinyl Chloride	polyvinyl chloride	polyvinyl chloride	polyvinyl chloride	Polyvinyl Chloride	1	kept
polyvinyl chloride	polyvinyl chloride	polyvinyl chloride	polyvinyl chloride	Polyvinyl Chloride	1	case_or_format
Política sanitaria	pol tica sanitaria	pol tica sanitaria	pol tica sanitaria	Política sanitaria	1	kept
pons	pons	pons	pons	pons	1	kept
pons angle	pons angle	pons angle	pons angle	pons angle	1	kept
pons angle tumor	pons angle tumor	pons angle tumor	pons angle tumor	pons angle tumor	1	kept

PONV Intensity Scale	ponv intensity scale	ponv intensity scale	ponv intensity scale	PONV Intensity Scale	1	kept
poor communication	poor communication	poor communication	poor communication	poor communication	1	kept
poor documentation	poor documentation	poor documentation	poor documentation	poor documentation	1	kept
poor general condition	poor general condition	poor general condition	poor general condition	poor general condition	1	kept
poor glycemic control	poor glycemic control	poor glycemic control	poor glycemic control	poor glycemic control	1	kept
Poor hygiene	poor hygiene	poor hygiene	poor hygiene	Poor hygiene	1	kept
poor prognosis	poor prognosis	poor prognosis	poor prognosis	poor prognosis	1	kept
Poor sleep	poor sleep	poor sleep	poor sleep	Poor sleep	1	kept
Poor sucking	poor sucking	poor sucking	poor sucking	Poor sucking	1	kept
poor team experience	poor team experience	poor team experience	poor team experience	poor team experience	1	kept
poor work ethics	poor work ethics	poor work ethics	poor work ethics	poor work ethics	1	kept
Poorly resourced hospitals	poorly resourced hospitals	poorly resourced hospitals	poorly resourced hospitals	Poorly resourced hospitals	1	kept
POPC, Pediatric Overall Performance Category	popc pediatric overall performance category	popc pediatric overall performance category	popc pediatric overall performance category	POPC, Pediatric Overall Performance Category	1	kept
Population	population	population	population	population	1	case_or_format
population abundance	population abundance	population abundance	population abundance	population abundance	1	kept
population and population related phenomena	population and population related phenomena	population and population related phenomena	population and population related phenomena	population and population related phenomena	1	kept

population distribution	population distribution	population distribution	population distribution	population distribution	1	kept
Population dynamics	population dynamics	population dynamics	population dynamics	Population Dynamics	1	case_or_format
population dynamics	population dynamics	population dynamics	population dynamics	Population Dynamics	1	case_or_format
population genetics	population genetics	population genetics	population genetics	population genetics	1	kept
population group	population group	population group	population group	population group	1	kept
Population Groups	population groups	population groups	population group	population group	1	singular
Population Growth	population growth	population growth	population growth	population growth	1	case_or_format
Population Health	population health	population health	population health	population health	1	case_or_format
Population health	population health	population health	population health	population health	1	case_or_format
population level	population level	population level	population level	population level	1	kept
population model	population model	population model	population model	population model	1	kept
POPULATION PHARMACOKINETIC ANALYSIS	population pharmacokinetic analysis	population pharmacokinetic analysis	population pharmacokinetic analysis	POPULATION PHARMACOKINETIC ANALYSIS	1	kept
population risk	population risk	population risk	population risk	population risk	1	kept
population-based	population based	population based	population based	Population-based	1	case_or_format
POPULATION-BASED COHORT	population based cohort	population based cohort	population based cohort	POPULATION-BASED COHORT	1	kept
population-based study	population based study	population based study	population based study	population-based study	1	kept

poractant alfa	poractant alfa	poractant alfa	poractant alfa	poractant alfa	1	kept
Porcine respiratory coronavirus	porcine respiratory coronavirus	porcine respiratory coronavirus	porcine respiratory coronavirus	Porcine respiratory coronavirus	1	kept
pork	pork	pork	pork	pork	1	kept
porphyria	porphyria	porphyria	porphyria	porphyria	1	kept
Porphyria, Acute Intermittent	porphyria acute intermittent	porphyria acute intermittent	porphyria acute intermittent	Porphyria, Acute Intermittent	1	kept
Porphyromonas	porphyromonas	porphyromonas	porphyromonas	Porphyromonas	1	kept
port catheter	port catheter	port catheter	port catheter	port catheter	1	kept
Port-of-call	port of call	port of call	port of call	Port-of-call	1	kept
Portable bladder ultrasound	portable bladder ultrasound	portable bladder ultrasound	portable bladder ultrasound	Portable bladder ultrasound	1	kept
Portable chest radiograph	portable chest radiograph	portable chest radiograph	portable chest radiograph	Portable chest radiograph	1	kept
portable computed tomography scanner	portable computed tomography scanner	portable computed tomography scanner	portable computed tomography scanner	portable computed tomography scanner	1	kept
Portable dental unit	portable dental unit	portable dental unit	portable dental unit	Portable dental unit	1	kept
Portable Device Technology	portable device technology	portable device technology	portable device technology	Portable Device Technology	1	kept
Portable medical device	portable medical device	portable medical device	portable medical device	Portable medical device	1	kept
Portable radiograph	portable radiograph	portable radiograph	portable radiograph	Portable radiograph	1	kept
Portable radiographs	portable radiographs	portable radiographs	portable radiograph	Portable radiograph	1	singular

Portable radiography	portable radiography	portable radiography	portable radiography	Portable radiography	1	kept
portable radiography	portable radiography	portable radiography	portable radiography	Portable radiography	1	case_or_format
Portable toilets	portable toilets	portable toilets	portable toilets	Portable toilets	1	kept
Portable ultrasound	portable ultrasound	portable ultrasound	portable ultrasound	Portable ultrasound	1	kept
Portal Pressure	portal pressure	portal pressure	portal pressure	Portal Pressure	1	kept
portal vein	portal vein	portal vein	portal vein	Portal Vein	1	case_or_format
portal vein obstruction	portal vein obstruction	portal vein obstruction	portal vein obstruction	portal vein obstruction	1	kept
Portasystemic Shunt, Transjugular Intrahepatic	portasystemic shunt transjugular intrahepatic	portasystemic shunt transjugular intrahepatic	portasystemic shunt transjugular intrahepatic	Portasystemic Shunt, Transjugular Intrahepatic	1	kept
porter	porter	porter	porter	porter	1	kept
Portfolio research	portfolio research	portfolio research	portfolio research	Portfolio research	1	kept
portoenterostomy	portoenterostomy	portoenterostomy	portoenterostomy	portoenterostomy	1	kept
portopulmonary hypertension	portopulmonary hypertension	portopulmonary hypertension	portopulmonary hypertension	portopulmonary hypertension	1	kept
Portrait Value Questionnaire	portrait value questionnaire	portrait value questionnaire	portrait value questionnaire	Portrait Value Questionnaire	1	kept
Portraits	portraits	portraits	portraits	Portraits	1	kept
Portraits as Topic	portraits as topic	portraits as topic	portraits as topic	Portraits as Topic	1	kept
Portuguese language	portuguese language	portuguese language	portuguese language	Portuguese language	1	kept

Posibacteria	posibacteria	posibacteria	posibacteria	Posibacteria	1	kept
Posición prona	posici n prona	posici n prona	posici n prona	Posición prona	1	kept
Position change	position change	position change	position change	Position change	1	kept
POSITION STATEMENT	position statement	position statement	position statement	Position statement	1	case_or_format
position tracking	position tracking	position tracking	position tracking	position tracking	1	kept
positional head deformity	positional head deformity	positional head deformity	positional head deformity	positional head deformity	1	kept
positional head shape deformity	positional head shape deformity	positional head shape deformity	positional head shape deformity	positional head shape deformity	1	kept
Positional plagiocephaly	positional plagiocephaly	positional plagiocephaly	positional plagiocephaly	Positional plagiocephaly	1	kept
positioner	positioner	positioner	positioner	positioner	1	kept
Positioning devices	positioning devices	positioning devices	positioning devices	Positioning devices	1	kept
Positioning therapy	positioning therapy	positioning therapy	positioning therapy	Positioning therapy	1	kept
Positions	positions	positions	position	position	1	singular
Positive airway pressure	positive airway pressure	positive airway pressure	positive airway pressure	POSITIVE AIRWAY PRESSURE	1	case_or_format
positive airway pressure	positive airway pressure	positive airway pressure	positive airway pressure	POSITIVE AIRWAY PRESSURE	1	case_or_format
positive and negative affect	positive and negative affect	positive and negative affect	positive and negative affect	positive and negative affect	1	kept
Positive Aspects of Caregiving	positive aspects of caregiving	positive aspects of caregiving	positive aspects of caregiving	Positive Aspects of Caregiving	1	kept

Positive attitude	positive attitude	positive attitude	positive attitude	Positive attitude	1	kept
positive case rate	positive case rate	positive case rate	positive case rate	positive case rate	1	kept
positive catheter tip culture	positive catheter tip culture	positive catheter tip culture	positive catheter tip culture	positive catheter tip culture	1	kept
positive communication	positive communication	positive communication	positive communication	positive communication	1	kept
positive deviance	positive deviance	positive deviance	positive deviance	positive deviance	1	kept
positive end expiratory pressure ventilator	positive end expiratory pressure ventilator	positive end expiratory pressure ventilator	positive end expiratory pressure ventilator	positive end expiratory pressure ventilator	1	kept
Positive end-expiratory pressure	positive end expiratory pressure	positive end expiratory pressure	positive end expiratory pressure	positive end expiratory pressure	1	case_or_format
Positive experiences	positive experiences	positive experiences	positive experiences	Positive experiences	1	kept
Positive impacts	positive impacts	positive impacts	positive impacts	Positive impacts	1	kept
Positive maternal attitudes and infant and child development	positive maternal attitudes and infant and child development	positive maternal attitudes and infant and child development	positive maternal attitudes and infant and child development	Positive maternal attitudes and infant and child development	1	kept
positive outcome expectation	positive outcome expectation	positive outcome expectation	positive outcome expectation	positive outcome expectation	1	kept
positive parent touch	positive parent touch	positive parent touch	positive parent touch	positive parent touch	1	kept
POSITIVE PRACTICE ENVIRONMENTS	positive practice environments	positive practice environments	positive practice environments	POSITIVE PRACTICE ENVIRONMENTS	1	kept
Positive Practice Environments	positive practice environments	positive practice environments	positive practice environments	POSITIVE PRACTICE ENVIRONMENTS	1	case_or_format
Positive practice environments	positive practice environments	positive practice environments	positive practice environments	POSITIVE PRACTICE ENVIRONMENTS	1	case_or_format
Positive pressure	positive pressure	positive pressure	positive pressure	Positive pressure	1	kept

Positive pressure isolation rooms	positive pressure isolation rooms	positive pressure isolation rooms	positive pressure isolation rooms	Positive pressure isolation rooms	1	kept
Positive psychology intervention	positive psychology intervention	positive psychology intervention	positive psychology intervention	Positive psychology intervention	1	kept
positive syndrome	positive syndrome	positive syndrome	positive syndrome	positive syndrome	1	kept
Positive-end-expiratory respiration	positive end expiratory respiration	positive end expiratory respiration	positive end expiratory respiration	Positive-end-expiratory respiration	1	kept
POSITIVE-PRESSURE VENTILATION	positive pressure ventilation	positive pressure ventilation	positive pressure ventilation	positive pressure ventilation	1	case_or_format
Positive-pressure ventilation	positive pressure ventilation	positive pressure ventilation	positive pressure ventilation	positive pressure ventilation	1	case_or_format
Positron Emission Tomography Computed Tomography	positron emission tomography computed tomography	positron emission tomography computed tomography	positron emission tomography computed tomography	positron emission tomography-computed tomography	1	case_or_format
POSS	poss	poss	poss	POSS	1	kept
post acute care	post acute care	post acute care	post acute care	Post-acute care	1	case_or_format
Post anaesthesia care	post anaesthesia care	post anaesthesia care	post anaesthesia care	Post anaesthesia care	1	kept
post anaesthetic care	post anaesthetic care	post anaesthetic care	post anaesthetic care	post anaesthetic care	1	kept
post anaesthetic care unit	post anaesthetic care unit	post anaesthetic care unit	post anaesthetic care unit	post anaesthetic care unit	1	kept
Post anesthesia care unit	post anesthesia care unit	post anesthesia care unit	post anesthesia care unit	Post anesthesia care unit	1	kept
post cardiac arrest syndrome	post cardiac arrest syndrome	post cardiac arrest syndrome	post cardiac arrest syndrome	post cardiac arrest syndrome	1	kept
post cardiotomy delirium	post cardiotomy delirium	post cardiotomy delirium	post cardiotomy delirium	post cardiotomy delirium	1	kept
Post CCU discharge mortality	post ccu discharge mortality	post ccu discharge mortality	post ccu discharge mortality	Post CCU discharge mortality	1	kept

Post cholecystectomy management	post cholecystectomy management	post cholecystectomy management	post cholecystectomy management	Post cholecystectomy management	1	kept
post infection complication	post infection complication	post infection complication	post infection complication	post infection complication	1	kept
post intervention survey	post intervention survey	post intervention survey	post intervention survey	post intervention survey	1	kept
Post interventions	post interventions	post interventions	post interventions	Post interventions	1	kept
Post liver transplantation management	post liver transplantation management	post liver transplantation management	post liver transplantation management	Post liver transplantation management	1	kept
Post mortem care	post mortem care	post mortem care	post mortem care	Post-mortem care	1	case_or_format
Post natal depression	post natal depression	post natal depression	post natal depression	Post natal depression	1	kept
Post natal growth	post natal growth	post natal growth	post natal growth	Post natal growth	1	kept
post operative care	post operative care	post operative care	post operative care	post-operative care	1	case_or_format
Post operative endotracheal tube tolerance	post operative endotracheal tube tolerance	post operative endotracheal tube tolerance	post operative endotracheal tube tolerance	Post operative endotracheal tube tolerance	1	kept
post operative surveillance team	post operative surveillance team	post operative surveillance team	post operative surveillance team	post operative surveillance team	1	kept
Post pyloric feeding	post pyloric feeding	post pyloric feeding	post pyloric feeding	Post pyloric feeding	1	kept
Post resuscitation care	post resuscitation care	post resuscitation care	post resuscitation care	Post-resuscitation care	1	case_or_format
Post stroke complications	post stroke complications	post stroke complications	post stroke complications	Post stroke complications	1	kept
post traumatic amnesia	post traumatic amnesia	post traumatic amnesia	post traumatic amnesia	post traumatic amnesia	1	kept
post traumatic brain injury	post traumatic brain injury	post traumatic brain injury	post traumatic brain injury	post traumatic brain injury	1	kept

post traumatic hydrocephalus	post traumatic hydrocephalus	post traumatic hydrocephalus	post traumatic hydrocephalus	post traumatic hydrocephalus	1	kept
post traumatic stress	post traumatic stress	post traumatic stress	post traumatic stress	Post-traumatic stress	1	case_or_format
Post traumatic stress Disorder	post traumatic stress disorder	post traumatic stress disorder	post traumatic stress disorder	Post-traumatic stress disorder	1	case_or_format
Post traumatic stress disorder	post traumatic stress disorder	post traumatic stress disorder	post traumatic stress disorder	Post-traumatic stress disorder	1	case_or_format
post traumatic stress disorder	post traumatic stress disorder	post traumatic stress disorder	post traumatic stress disorder	Post-traumatic stress disorder	1	case_or_format
Post Traumatic Stress Disorder Screening Scale	post traumatic stress disorder screening scale	post traumatic stress disorder screening scale	post traumatic stress disorder screening scale	Post Traumatic Stress Disorder Screening Scale	1	kept
post traumatic stress syndrome 10 questionnaire	post traumatic stress syndrome 10 questionnaire	post traumatic stress syndrome 10 questionnaire	post traumatic stress syndrome 10 questionnaire	post traumatic stress syndrome 10 questionnaire	1	kept
Post- Operative Care	post operative care	post operative care	post operative care	post-operative care	1	case_or_format
Post-acute COVID-19 syndrome	post acute covid 19 syndrome	post acute covid 19 syndrome	post acute covid 19 syndrome	Post-Acute COVID-19 Syndrome	1	case_or_format
Post-acute sequelae of COVID-19	post acute sequelae of covid 19	post acute sequelae of covid 19	post acute sequelae of covid 19	Post-acute sequelae of COVID-19	1	kept
post-acute SNF	post acute snf	post acute snf	post acute snf	post-acute SNF	1	kept
post-anaesthesia recovery room	post anaesthesia recovery room	post anaesthesia recovery room	post anaesthesia recovery room	post-anaesthesia recovery room	1	kept
Post-anaesthetic care	post anaesthetic care	post anaesthetic care	post anaesthetic care	post anaesthetic care	1	case_or_format
post-analytical phase	post analytical phase	post analytical phase	post analytical phase	post-analytical phase	1	kept
post-anesthetic care unit	post anesthetic care unit	post anesthetic care unit	post anesthetic care unit	post-anesthetic care unit	1	kept
Post-basic	post basic	post basic	post basic	Post-basic	1	kept

post-CABG	post cabg	post cabg	post cabg	post-CABG	1	kept
Post-cardiac arrest syndrome	post cardiac arrest syndrome	post cardiac arrest syndrome	post cardiac arrest syndrome	post cardiac arrest syndrome	1	case_or_format
post-cardiac arrest syndrome	post cardiac arrest syndrome	post cardiac arrest syndrome	post cardiac arrest syndrome	post cardiac arrest syndrome	1	case_or_format
post-cardiac surgery	post cardiac surgery	post cardiac surgery	post cardiac surgery	post-cardiac surgery	1	kept
Post-cardiac surgery delirium	post cardiac surgery delirium	post cardiac surgery delirium	post cardiac surgery delirium	Post-cardiac surgery delirium	1	kept
Post-cardiotomy	post cardiotomy	post cardiotomy	post cardiotomy	Post-cardiotomy	1	kept
post-COVID syndrome 3 rehabilitation pain	post covid syndrome 3 rehabilitation pain	post covid syndrome 3 rehabilitation pain	post covid syndrome 3 rehabilitation pain	post-COVID syndrome 3 rehabilitation pain	1	kept
Post-COVID-19	post covid 19	post covid 19	post covid 19	Post-COVID-19	1	kept
post-COVID-19	post covid 19	post covid 19	post covid 19	Post-COVID-19	1	case_or_format
post-craniotomy fever	post craniotomy fever	post craniotomy fever	post craniotomy fever	post-craniotomy fever	1	kept
post-decolonization	post decolonization	post decolonization	post decolonization	post-decolonization	1	kept
Post-Dural Puncture Headache	post dural puncture headache	post dural puncture headache	post dural puncture headache	Post-Dural Puncture Headache	1	kept
Post-exposure prophylaxis	post exposure prophylaxis	post exposure prophylaxis	post exposure prophylaxis	post exposure prophylaxis	1	case_or_format
Post-extubation dysphagia	post extubation dysphagia	post extubation dysphagia	post extubation dysphagia	Post-extubation dysphagia	1	kept
post-extubation dysphagia	post extubation dysphagia	post extubation dysphagia	post extubation dysphagia	Post-extubation dysphagia	1	case_or_format
Post-extubation swallowing function	post extubation swallowing function	post extubation swallowing function	post extubation swallowing function	Post-extubation swallowing function	1	kept

post-graduate training	post graduate training	post graduate training	post graduate training	post-graduate training	1	kept
Post-hemorrhagic hydrocephalus	post hemorrhagic hydrocephalus	post hemorrhagic hydrocephalus	post hemorrhagic hydrocephalus	Post-hemorrhagic hydrocephalus	1	kept
post-hemorrhagic hydrocephalus	post hemorrhagic hydrocephalus	post hemorrhagic hydrocephalus	post hemorrhagic hydrocephalus	Post-hemorrhagic hydrocephalus	1	case_or_format
Post-hospitalization	post hospitalization	post hospitalization	post hospitalization	Post-hospitalization	1	kept
Post-infarction management	post infarction management	post infarction management	post infarction management	Post-infarction management	1	kept
post-infarction management	post infarction management	post infarction management	post infarction management	Post-infarction management	1	case_or_format
Post-kidney transplantation	post kidney transplantation	post kidney transplantation	post kidney transplantation	Post-kidney transplantation	1	kept
post-mortem care	post mortem care	post mortem care	post mortem care	Post-mortem care	1	case_or_format
Post-neonatal tetanus	post neonatal tetanus	post neonatal tetanus	post neonatal tetanus	post neonatal tetanus	1	case_or_format
Post-occupancy evaluation	post occupancy evaluation	post occupancy evaluation	post occupancy evaluation	Post occupancy evaluation	1	case_or_format
post-occupancy evaluation	post occupancy evaluation	post occupancy evaluation	post occupancy evaluation	Post occupancy evaluation	1	case_or_format
post-operative communication	post operative communication	post operative communication	post operative communication	post-operative communication	1	kept
Post-operative complications	post operative complications	post operative complications	post operative complications	Post-operative complications	1	kept
post-operative delirium	post operative delirium	post operative delirium	post operative delirium	post-operative delirium	1	kept
post-operative outcome in cardiac surgery	post operative outcome in cardiac surgery	post operative outcome in cardiac surgery	post operative outcome in cardiac surgery	post-operative outcome in cardiac surgery	1	kept
post-operative pain	post operative pain	post operative pain	post operative pain	post-operative pain	1	kept

Post-operative pain score	post operative pain score	post operative pain score	post operative pain score	Post-operative pain score	1	kept
Post-operative period	post operative period	post operative period	post operative period	Post-operative period	1	kept
post-operative unit	post operative unit	post operative unit	post operative unit	post-operative unit	1	kept
Post-pandemic challenges	post pandemic challenges	post pandemic challenges	post pandemic challenges	Post-pandemic challenges	1	kept
Post-Partum Hemorrhage	post partum hemorrhage	post partum hemorrhage	post partum hemorrhage	Post-Partum Hemorrhage	1	kept
Post-registration education	post registration education	post registration education	post registration education	post-registration education	1	case_or_format
Post-sepsis syndrome	post sepsis syndrome	post sepsis syndrome	post sepsis syndrome	Post-Sepsis-Syndrome	1	case_or_format
post-stroke depression	post stroke depression	post stroke depression	post stroke depression	post-stroke depression	1	kept
post-stroke rehabilitation	post stroke rehabilitation	post stroke rehabilitation	post stroke rehabilitation	post-stroke rehabilitation	1	kept
post-surgical	post surgical	post surgical	post surgical	post-surgical	1	kept
Post-surgical pain	post surgical pain	post surgical pain	post surgical pain	Post-surgical pain	1	kept
post-sympathectomy care	post sympathectomy care	post sympathectomy care	post sympathectomy care	post-sympathectomy care	1	kept
Post-test	post test	post test	post test	Post-test	1	kept
Post-trauma	post trauma	post trauma	post trauma	Post-trauma	1	kept
post-traumatic amnesia	post traumatic amnesia	post traumatic amnesia	post traumatic amnesia	post traumatic amnesia	1	case_or_format
Post-traumatic Check-List Scale	post traumatic check list scale	post traumatic check list scale	post traumatic check list scale	Post-traumatic Check-List Scale	1	kept

Post-traumatic disorder	post traumatic disorder	post traumatic disorder	post traumatic disorder	Post-traumatic disorder	1	kept
Post-traumatic seizure	post traumatic seizure	post traumatic seizure	post traumatic seizure	Post-traumatic seizure	1	kept
POST-TRAUMATIC STRESS DISORDER	post traumatic stress disorder	post traumatic stress disorder	post traumatic stress disorder	Post-traumatic stress disorder	1	case_or_format
Post-traumatic Stress Disorders	post traumatic stress disorders	post traumatic stress disorders	post traumatic stress disorder	Post-traumatic stress disorder	1	singular
Post-traumatic stress disorders	post traumatic stress disorders	post traumatic stress disorders	post traumatic stress disorder	Post-traumatic stress disorder	1	singular
post-traumatic stress symptoms	post traumatic stress symptoms	post traumatic stress symptoms	post traumatic stress symptoms	Post-traumatic stress symptoms	1	case_or_format
post-vaccine events	post vaccine events	post vaccine events	post vaccine events	post-vaccine events	1	kept
POSTACUTE CARE	postacute care	postacute care	postacute care	postacute care	1	case_or_format
Postacute Care	postacute care	postacute care	postacute care	postacute care	1	case_or_format
Postacute care	postacute care	postacute care	postacute care	postacute care	1	case_or_format
Postacute care coordination	postacute care coordination	postacute care coordination	postacute care coordination	Postacute care coordination	1	kept
postacute facility care	postacute facility care	postacute facility care	postacute facility care	postacute facility care	1	kept
Postal survey	postal survey	postal survey	postal survey	Postal survey	1	kept
POSTAL-SURVEYS	postal surveys	postal surveys	postal survey	Postal survey	1	singular
postanaesthetic care unit	postanaesthetic care unit	postanaesthetic care unit	postanaesthetic care unit	postanaesthetic care unit	1	kept
Postanesthesia care	postanesthesia care	postanesthesia care	postanesthesia care	postanesthesia care	1	case_or_format

POSTANESTHESIA CARE-UNIT	postanesthesia care unit	postanesthesia care unit	postanesthesia care unit	postanesthesia care unit	1	case_or_format
Postanesthesia recovery	postanesthesia recovery	postanesthesia recovery	postanesthesia recovery	postanesthesia recovery	1	case_or_format
Postanoxic coma	postanoxic coma	postanoxic coma	postanoxic coma	Postanoxic coma	1	kept
Postanoxic encephalopathy	postanoxic encephalopathy	postanoxic encephalopathy	postanoxic encephalopathy	Postanoxic encephalopathy	1	kept
Postcardiac arrest	postcardiac arrest	postcardiac arrest	postcardiac arrest	Postcardiac arrest	1	kept
postcardiotomy syndrome	postcardiotomy syndrome	postcardiotomy syndrome	postcardiotomy syndrome	postcardiotomy syndrome	1	kept
Postcardiotomy-extracorporeal membrane oxygenation	postcardiotomy extracorporeal membrane oxygenation	postcardiotomy extracorporeal membrane oxygenation	postcardiotomy extracorporeal membrane oxygenation	Postcardiotomy-extracorporeal membrane oxygenation	1	kept
postcentral gyrus	postcentral gyrus	postcentral gyrus	postcentral gyrus	postcentral gyrus	1	kept
postdischarge	postdischarge	postdischarge	postdischarge	postdischarge	1	kept
postdural puncture headache	postdural puncture headache	postdural puncture headache	postdural puncture headache	postdural puncture headache	1	kept
posterior	posterior	posterior	posterior	posterior	1	kept
posterior approach thoracolumbar fusion	posterior approach thoracolumbar fusion	posterior approach thoracolumbar fusion	posterior approach thoracolumbar fusion	posterior approach thoracolumbar fusion	1	kept
posterior cervical decompression and fusion	posterior cervical decompression and fusion	posterior cervical decompression and fusion	posterior cervical decompression and fusion	posterior cervical decompression and fusion	1	kept
Posterior component release	posterior component release	posterior component release	posterior component release	Posterior component release	1	kept
posterior cranial fossa tumor	posterior cranial fossa tumor	posterior cranial fossa tumor	posterior cranial fossa tumor	posterior cranial fossa tumor	1	kept
Posterior reversible leukoencephalopathy syndrome	posterior reversible leukoencephalopathy syndrome	posterior reversible leukoencephalopathy syndrome	posterior reversible leukoencephalopathy syndrome	Posterior reversible leukoencephalopathy syndrome	1	kept

posterior thoracolumbar decompression	posterior thoracolumbar decompression	posterior thoracolumbar decompression	posterior thoracolumbar decompression	posterior thoracolumbar decompression	1	kept
posterior tibial nerve	posterior tibial nerve	posterior tibial nerve	posterior tibial nerve	posterior tibial nerve	1	kept
posterolateral thoracotomy	posterolateral thoracotomy	posterolateral thoracotomy	posterolateral thoracotomy	posterolateral thoracotomy	1	kept
Postevent debriefing	postevent debriefing	postevent debriefing	postevent debriefing	Postevent debriefing	1	kept
Postexposure	postexposure	postexposure	postexposure	Postexposure	1	kept
Postexposure prophylaxis	postexposure prophylaxis	postexposure prophylaxis	postexposure prophylaxis	Postexposure prophylaxis	1	kept
postextubation	postextubation	postextubation	postextubation	postextubation	1	kept
Postextubation Dysphagia	postextubation dysphagia	postextubation dysphagia	postextubation dysphagia	postextubation dysphagia	1	case_or_format
Postextubation failure	postextubation failure	postextubation failure	postextubation failure	Postextubation failure	1	kept
Postgraduate fellowships	postgraduate fellowships	postgraduate fellowships	postgraduate fellowships	Postgraduate fellowships	1	kept
Postgraduate medical education	postgraduate medical education	postgraduate medical education	postgraduate medical education	postgraduate medical education	1	case_or_format
Postgraduate Program in Tropical Medicine	postgraduate program in tropical medicine	postgraduate program in tropical medicine	postgraduate program in tropical medicine	Postgraduate Program in Tropical Medicine	1	kept
Postgraduate Student	postgraduate student	postgraduate student	postgraduate student	postgraduate student	1	case_or_format
postinfectious encephalopathy	postinfectious encephalopathy	postinfectious encephalopathy	postinfectious encephalopathy	postinfectious encephalopathy	1	kept
postinfectious glomerulonephritis	postinfectious glomerulonephritis	postinfectious glomerulonephritis	postinfectious glomerulonephritis	postinfectious glomerulonephritis	1	kept
postinjury mental health condition	postinjury mental health condition	postinjury mental health condition	postinjury mental health condition	postinjury mental health condition	1	kept

postintubation sedation	postintubation sedation	postintubation sedation	postintubation sedation	postintubation sedation	1	kept
postintubation tracheal stenosis	postintubation tracheal stenosis	postintubation tracheal stenosis	postintubation tracheal stenosis	postintubation tracheal stenosis	1	kept
postmenstrual age	postmenstrual age	postmenstrual age	postmenstrual age	postmenstrual age	1	kept
postmodernism	postmodernism	postmodernism	postmodernism	Postmodernism	1	case_or_format
Postmortem care	postmortem care	postmortem care	postmortem care	Postmortem care	1	kept
postmortem care	postmortem care	postmortem care	postmortem care	Postmortem care	1	case_or_format
postmortem change	postmortem change	postmortem change	postmortem change	postmortem change	1	kept
Postmortem Changes	postmortem changes	postmortem changes	postmortem change	postmortem change	1	singular
POSTNATAL AGE	postnatal age	postnatal age	postnatal age	POSTNATAL AGE	1	kept
Postnatal care	postnatal care	postnatal care	postnatal care	Postnatal Care	1	case_or_format
Postnatal depression	postnatal depression	postnatal depression	postnatal depression	postnatal depression	1	case_or_format
Postnatal development	postnatal development	postnatal development	postnatal development	postnatal development	1	case_or_format
Postnatal exposure	postnatal exposure	postnatal exposure	postnatal exposure	Postnatal exposure	1	kept
postnatal infection	postnatal infection	postnatal infection	postnatal infection	postnatal infection	1	kept
postnatal pyrexia	postnatal pyrexia	postnatal pyrexia	postnatal pyrexia	postnatal pyrexia	1	kept
Postnatal stabilization	postnatal stabilization	postnatal stabilization	postnatal stabilization	Postnatal stabilization	1	kept

postnatal testing	postnatal testing	postnatal testing	postnatal testing	postnatal testing	1	kept
Postnatal weight gain	postnatal weight gain	postnatal weight gain	postnatal weight gain	Postnatal weight gain	1	kept
Postoccupancy evaluation	postoccupancy evaluation	postoccupancy evaluation	postoccupancy evaluation	Postoccupancy evaluation	1	kept
postoperative adverse events	postoperative adverse events	postoperative adverse events	postoperative adverse events	postoperative adverse events	1	kept
postoperative atelectasis	postoperative atelectasis	postoperative atelectasis	postoperative atelectasis	postoperative atelectasis	1	kept
Postoperative atrial fibrillation	postoperative atrial fibrillation	postoperative atrial fibrillation	postoperative atrial fibrillation	Postoperative atrial fibrillation	1	kept
Postoperative cardiac surgery	postoperative cardiac surgery	postoperative cardiac surgery	postoperative cardiac surgery	Postoperative cardiac surgery	1	kept
postoperative care and recovery	postoperative care and recovery	postoperative care and recovery	postoperative care and recovery	postoperative care and recovery	1	kept
postoperative care unit	postoperative care unit	postoperative care unit	postoperative care unit	postoperative care unit	1	kept
postoperative cognitive decline	postoperative cognitive decline	postoperative cognitive decline	postoperative cognitive decline	postoperative cognitive decline	1	kept
Postoperative complication	postoperative complication	postoperative complication	postoperative complication	Postoperative Complications	1	case_or_format
Postoperative dehydration	postoperative dehydration	postoperative dehydration	postoperative dehydration	Postoperative dehydration	1	kept
postoperative deterioration	postoperative deterioration	postoperative deterioration	postoperative deterioration	postoperative deterioration	1	kept
POSTOPERATIVE ENTERAL NUTRITION	postoperative enteral nutrition	postoperative enteral nutrition	postoperative enteral nutrition	POSTOPERATIVE ENTERAL NUTRITION	1	kept
Postoperative feeding care	postoperative feeding care	postoperative feeding care	postoperative feeding care	Postoperative feeding care	1	kept
postoperative feeding care	postoperative feeding care	postoperative feeding care	postoperative feeding care	Postoperative feeding care	1	case_or_format

postoperative gastrointestinal mobility	postoperative gastrointestinal mobility	postoperative gastrointestinal mobility	postoperative gastrointestinal mobility	postoperative gastrointestinal mobility	1	kept
Postoperative haemorrhage	postoperative haemorrhage	postoperative haemorrhage	postoperative haemorrhage	Postoperative haemorrhage	1	kept
postoperative handoff	postoperative handoff	postoperative handoff	postoperative handoff	postoperative handoff	1	kept
POSTOPERATIVE HANDOVER	postoperative handover	postoperative handover	postoperative handover	Postoperative handover	1	case_or_format
postoperative handover quality	postoperative handover quality	postoperative handover quality	postoperative handover quality	postoperative handover quality	1	kept
postoperative Hip fracture	postoperative hip fracture	postoperative hip fracture	postoperative hip fracture	postoperative Hip fracture	1	kept
postoperative hypotension	postoperative hypotension	postoperative hypotension	postoperative hypotension	postoperative hypotension	1	kept
POSTOPERATIVE HYPOTHERMIA	postoperative hypothermia	postoperative hypothermia	postoperative hypothermia	POSTOPERATIVE HYPOTHERMIA	1	kept
postoperative hypothermia	postoperative hypothermia	postoperative hypothermia	postoperative hypothermia	POSTOPERATIVE HYPOTHERMIA	1	case_or_format
postoperative inflammation	postoperative inflammation	postoperative inflammation	postoperative inflammation	postoperative inflammation	1	kept
postoperative intermediate care	postoperative intermediate care	postoperative intermediate care	postoperative intermediate care	postoperative intermediate care	1	kept
Postoperative management	postoperative management	postoperative management	postoperative management	postoperative management	1	case_or_format
postoperative neurocognitive disorder	postoperative neurocognitive disorder	postoperative neurocognitive disorder	postoperative neurocognitive disorder	postoperative neurocognitive disorder	1	kept
Postoperative outcome	postoperative outcome	postoperative outcome	postoperative outcome	postoperative outcomes	1	case_or_format
postoperative outcome	postoperative outcome	postoperative outcome	postoperative outcome	postoperative outcomes	1	case_or_format
Postoperative outcomes	postoperative outcomes	postoperative outcomes	postoperative outcome	postoperative outcomes	1	singular

postoperative pain assessment	postoperative pain assessment	postoperative pain assessment	postoperative pain assessment	postoperative pain assessment	1	kept
Postoperative prophylaxis	postoperative prophylaxis	postoperative prophylaxis	postoperative prophylaxis	Postoperative prophylaxis	1	kept
Postoperative psychological support	postoperative psychological support	postoperative psychological support	postoperative psychological support	Postoperative psychological support	1	kept
Postoperative psychopathology	postoperative psychopathology	postoperative psychopathology	postoperative psychopathology	Postoperative psychopathology	1	kept
Postoperative pulmonary complication	postoperative pulmonary complication	postoperative pulmonary complication	postoperative pulmonary complication	Postoperative pulmonary complications	1	case_or_format
POSTOPERATIVE PULMONARY COMPLICATIONS	postoperative pulmonary complications	postoperative pulmonary complications	postoperative pulmonary complication	Postoperative pulmonary complications	1	singular
postoperative rehabilitation	postoperative rehabilitation	postoperative rehabilitation	postoperative rehabilitation	postoperative rehabilitation	1	kept
postoperative residual neuromuscular blockade	postoperative residual neuromuscular blockade	postoperative residual neuromuscular blockade	postoperative residual neuromuscular blockade	postoperative residual neuromuscular blockade	1	kept
Postoperative sedation	postoperative sedation	postoperative sedation	postoperative sedation	Postoperative sedation	1	kept
postoperative surgery	postoperative surgery	postoperative surgery	postoperative surgery	postoperative surgery	1	kept
postoperative surgical ward care	postoperative surgical ward care	postoperative surgical ward care	postoperative surgical ward care	postoperative surgical ward care	1	kept
Postoperative tracheostomy	postoperative tracheostomy	postoperative tracheostomy	postoperative tracheostomy	Postoperative tracheostomy	1	kept
postoperative vasoplegia	postoperative vasoplegia	postoperative vasoplegia	postoperative vasoplegia	postoperative vasoplegia	1	kept
Postoperative wellbeing	postoperative wellbeing	postoperative wellbeing	postoperative wellbeing	Postoperative wellbeing	1	kept
Postoperatorio	postoperatorio	postoperatorio	postoperatorio	Postoperatorio	1	kept
Postpancreatic resection	postpancreatic resection	postpancreatic resection	postpancreatic resection	Postpancreatic resection	1	kept

Postpartum adaptation	postpartum adaptation	postpartum adaptation	postpartum adaptation	Postpartum adaptation	1	kept
Postpartum blues	postpartum blues	postpartum blues	postpartum blues	Postpartum blues	1	kept
postpartum care center	postpartum care center	postpartum care center	postpartum care center	postpartum care center	1	kept
Postpartum Depression Screening Scale	postpartum depression screening scale	postpartum depression screening scale	postpartum depression screening scale	Postpartum Depression Screening Scale	1	kept
postpartum depression symptoms	postpartum depression symptoms	postpartum depression symptoms	postpartum depression symptoms	postpartum depression symptoms	1	kept
Postpartum depression, anxiety, mother-child interactions	postpartum depression anxiety mother child interactions	postpartum depression anxiety mother child interactions	postpartum depression anxiety mother child interactions	Postpartum depression, anxiety, mother-child interactions	1	kept
Postpartum haemorrhage	postpartum haemorrhage	postpartum haemorrhage	postpartum haemorrhage	Postpartum haemorrhage	1	kept
postpartum haemorrhage	postpartum haemorrhage	postpartum haemorrhage	postpartum haemorrhage	Postpartum haemorrhage	1	case_or_format
Postpartum health care services	postpartum health care services	postpartum health care services	postpartum health care services	Postpartum health care services	1	kept
Postpartum hemorrhage	postpartum hemorrhage	postpartum hemorrhage	postpartum hemorrhage	postpartum hemorrhage	1	case_or_format
postpartum mental health screening	postpartum mental health screening	postpartum mental health screening	postpartum mental health screening	postpartum mental health screening	1	kept
postpartum mood and anxiety	postpartum mood and anxiety	postpartum mood and anxiety	postpartum mood and anxiety	postpartum mood and anxiety	1	kept
postpartum mood and anxiety disorders	postpartum mood and anxiety disorders	postpartum mood and anxiety disorders	postpartum mood and anxiety disorders	postpartum mood and anxiety disorders	1	kept
postpartum mood disorders	postpartum mood disorders	postpartum mood disorders	postpartum mood disorders	postpartum mood disorders	1	kept
Postpartum mothers	postpartum mothers	postpartum mothers	postpartum mothers	Postpartum mothers	1	kept
postpartum posttraumatic stress	postpartum posttraumatic stress	postpartum posttraumatic stress	postpartum posttraumatic stress	postpartum posttraumatic stress	1	kept

postpartum screening	postpartum screening	postpartum screening	postpartum screening	postpartum screening	1	kept
POSTPARTUM STRESS	postpartum stress	postpartum stress	postpartum stress	POSTPARTUM STRESS	1	kept
postpartum stress	postpartum stress	postpartum stress	postpartum stress	POSTPARTUM STRESS	1	case_or_format
Postpartum trauma	postpartum trauma	postpartum trauma	postpartum trauma	Postpartum trauma	1	kept
Postpartum visit	postpartum visit	postpartum visit	postpartum visit	Postpartum visit	1	kept
postpericardiotomy syndrome	postpericardiotomy syndrome	postpericardiotomy syndrome	postpericardiotomy syndrome	postpericardiotomy syndrome	1	kept
postpolio syndrome	postpolio syndrome	postpolio syndrome	postpolio syndrome	postpolio syndrome	1	kept
Postpoliomyelitis Syndrome	postpoliomyelitis syndrome	postpoliomyelitis syndrome	postpoliomyelitis syndrome	Postpoliomyelitis Syndrome	1	kept
Postpoliomyelitis syndrome	postpoliomyelitis syndrome	postpoliomyelitis syndrome	postpoliomyelitis syndrome	Postpoliomyelitis Syndrome	1	case_or_format
postpoliomyelitis syndrome	postpoliomyelitis syndrome	postpoliomyelitis syndrome	postpoliomyelitis syndrome	Postpoliomyelitis Syndrome	1	case_or_format
Postprandial Period	postprandial period	postprandial period	postprandial period	Postprandial Period	1	kept
postprandial state	postprandial state	postprandial state	postprandial state	postprandial state	1	kept
Postprocedural care	postprocedural care	postprocedural care	postprocedural care	Postprocedural care	1	kept
postprocedural complications	postprocedural complications	postprocedural complications	postprocedural complications	postprocedural complications	1	kept
Postpyloric	postpyloric	postpyloric	postpyloric	Postpyloric	1	kept
postpyloric placement	postpyloric placement	postpyloric placement	postpyloric placement	postpyloric placement	1	kept

Postregistration qualification	postregistration qualification	postregistration qualification	postregistration qualification	Postregistration qualification	1	kept
postregistration qualifications	postregistration qualifications	postregistration qualifications	postregistration qualification	Postregistration qualification	1	singular
Postrenal	postrenal	postrenal	postrenal	Postrenal	1	kept
Poststroke complications	poststroke complications	poststroke complications	poststroke complications	Poststroke complications	1	kept
poststroke delirium	poststroke delirium	poststroke delirium	poststroke delirium	poststroke delirium	1	kept
Postsurgical infection	postsurgical infection	postsurgical infection	postsurgical infection	Postsurgical infection	1	kept
POSTSURGICAL PAIN	postsurgical pain	postsurgical pain	postsurgical pain	POSTSURGICAL PAIN	1	kept
POSTTHORACOTOMY PAIN	postthoracotomy pain	postthoracotomy pain	postthoracotomy pain	POSTTHORACOTOMY PAIN	1	kept
postthrombosis syndrome	postthrombosis syndrome	postthrombosis syndrome	postthrombosis syndrome	postthrombosis syndrome	1	kept
Posttransplant infections	posttransplant infections	posttransplant infections	posttransplant infections	Posttransplant infections	1	kept
posttransplant lymphoproliferative disease	posttransplant lymphoproliferative disease	posttransplant lymphoproliferative disease	posttransplant lymphoproliferative disease	posttransplant lymphoproliferative disease	1	kept
Posttransplant lymphoproliferative disorder	posttransplant lymphoproliferative disorder	posttransplant lymphoproliferative disorder	posttransplant lymphoproliferative disorder	Posttransplant lymphoproliferative disorder	1	kept
Posttraumatic amnesia	posttraumatic amnesia	posttraumatic amnesia	posttraumatic amnesia	Posttraumatic amnesia	1	kept
posttraumatic complication	posttraumatic complication	posttraumatic complication	posttraumatic complication	posttraumatic complication	1	kept
Posttraumatic Stress	posttraumatic stress	posttraumatic stress	posttraumatic stress	POSTTRAUMATIC STRESS	1	case_or_format
Posttraumatic Stress Disorder	posttraumatic stress disorder	posttraumatic stress disorder	posttraumatic stress disorder	posttraumatic stress disorder	1	case_or_format

posttraumatic stress disorder checklist 5	posttraumatic stress disorder checklist 5	posttraumatic stress disorder checklist 5	posttraumatic stress disorder checklist 5	posttraumatic stress disorder checklist 5	1	kept
posttraumatic stress disorders	posttraumatic stress disorders	posttraumatic stress disorders	posttraumatic stress disorder	posttraumatic stress disorder	1	singular
Posttraumatic stress symptoms	posttraumatic stress symptoms	posttraumatic stress symptoms	posttraumatic stress symptoms	POSTTRAUMATIC STRESS SYMPTOMS	1	case_or_format
Posttraumatic stress syndrome	posttraumatic stress syndrome	posttraumatic stress syndrome	posttraumatic stress syndrome	Posttraumatic stress syndrome	1	kept
Posttraumatic Symptom Scale	posttraumatic symptom scale	posttraumatic symptom scale	posttraumatic symptom scale	Posttraumatic Symptom Scale	1	kept
Postural hypotension	postural hypotension	postural hypotension	postural hypotension	Postural hypotension	1	kept
Posture Comfort	posture comfort	posture comfort	posture comfort	Posture Comfort	1	kept
Postvoidal residual volume	postvoidal residual volume	postvoidal residual volume	postvoidal residual volume	Postvoidal residual volume	1	kept
post‑graduate programme	post graduate programme	post graduate programme	post graduate programme	post‑graduate programme	1	kept
post‑operative	post operative	post operative	post operative	post-operative	1	case_or_format
post‑partum	post partum	post partum	post partum	post‑partum	1	kept
post‑traumatic growth	post traumatic growth	post traumatic growth	post traumatic growth	post-traumatic growth	1	case_or_format
post‑acute care	post acute care	post acute care	post acute care	Post-acute care	1	case_or_format
Post‑COVID-19	post covid 19	post covid 19	post covid 19	Post-COVID-19	1	case_or_format
Post‑COVID-19 Syndrome	post covid 19 syndrome	post covid 19 syndrome	post covid 19 syndrome	Post‑COVID-19 Syndrome	1	kept
Potable water	potable water	potable water	potable water	Potable water	1	kept

Potassium Acetate	potassium acetate	potassium acetate	potassium acetate	Potassium Acetate	1	kept
Potassium Channel Blockers	potassium channel blockers	potassium channel blockers	potassium channel blockers	Potassium Channel Blockers	1	kept
potassium channel blocking agent	potassium channel blocking agent	potassium channel blocking agent	potassium channel blocking agent	potassium channel blocking agent	1	kept
potassium chloride administration	potassium chloride administration	potassium chloride administration	potassium chloride administration	potassium chloride administration	1	kept
potassium concentrations	potassium concentrations	potassium concentrations	potassium concentrations	potassium concentrations	1	kept
potassium derivative	potassium derivative	potassium derivative	potassium derivative	potassium derivative	1	kept
potassium intake	potassium intake	potassium intake	potassium intake	potassium intake	1	kept
Potassium replacement	potassium replacement	potassium replacement	potassium replacement	Potassium replacement	1	kept
potassium transport	potassium transport	potassium transport	potassium transport	potassium transport	1	kept
potassium urine level	potassium urine level	potassium urine level	potassium urine level	potassium urine level	1	kept
Potential benefits	potential benefits	potential benefits	potential benefits	Potential benefits	1	kept
potential crisis events	potential crisis events	potential crisis events	potential crisis events	potential crisis events	1	kept
potential drugs	potential drugs	potential drugs	potential drugs	potential drugs	1	kept
Potential errors	potential errors	potential errors	potential errors	Potential errors	1	kept
Potential failure mode	potential failure mode	potential failure mode	potential failure mode	Potential failure mode	1	kept
Potential pathogen transmission	potential pathogen transmission	potential pathogen transmission	potential pathogen transmission	Potential pathogen transmission	1	kept

Potential values	potential values	potential values	potential values	Potential values	1	kept
POTENTIAL VECTOR	potential vector	potential vector	potential vector	POTENTIAL VECTOR	1	kept
Potentially avoidable hospitalizations	potentially avoidable hospitalizations	potentially avoidable hospitalizations	potentially avoidable hospitalizations	Potentially avoidable hospitalizations	1	kept
potentially better practice	potentially better practice	potentially better practice	potentially better practice	potentially better practice	1	kept
potentially inappropriate end of life care	potentially inappropriate end of life care	potentially inappropriate end of life care	potentially inappropriate end of life care	potentially inappropriate end of life care	1	kept
POTENTIALLY INAPPROPRIATE MEDICATION	potentially inappropriate medication	potentially inappropriate medication	potentially inappropriate medication	potentially inappropriate medication	1	case_or_format
potentially inappropriate medication list	potentially inappropriate medication list	potentially inappropriate medication list	potentially inappropriate medication list	Potentially Inappropriate Medication List	1	case_or_format
POTENTIALLY INAPPROPRIATE MEDICATIONS	potentially inappropriate medications	potentially inappropriate medications	potentially inappropriate medication	potentially inappropriate medication	1	singular
potentially inappropriate transfer	potentially inappropriate transfer	potentially inappropriate transfer	potentially inappropriate transfer	potentially inappropriate transfer	1	kept
Potentially inappropriate treatment	potentially inappropriate treatment	potentially inappropriate treatment	potentially inappropriate treatment	potentially inappropriate treatment	1	case_or_format
POTENTIALLY INEFFECTIVE CARE	potentially ineffective care	potentially ineffective care	potentially ineffective care	POTENTIALLY INEFFECTIVE CARE	1	kept
potentially psychologically traumatic events	potentially psychologically traumatic events	potentially psychologically traumatic events	potentially psychologically traumatic events	potentially psychologically traumatic events	1	kept
potentially unsafe medication practice	potentially unsafe medication practice	potentially unsafe medication practice	potentially unsafe medication practice	potentially unsafe medication practice	1	kept
Poultry	poultry	poultry	poultry	Poultry	1	kept
poultry	poultry	poultry	poultry	Poultry	1	case_or_format
Povidone iodine	povidone iodine	povidone iodine	povidone iodine	povidone iodine	1	case_or_format

powder bed fusion	powder bed fusion	powder bed fusion	powder bed fusion	powder bed fusion	1	kept
POWER	power	power	power	Power	1	case_or_format
power calculations	power calculations	power calculations	power calculations	power calculations	1	kept
Power disparities	power disparities	power disparities	power disparities	Power disparities	1	kept
power distance	power distance	power distance	power distance	power distance	1	kept
Power Doppler imaging	power doppler imaging	power doppler imaging	power doppler imaging	Power Doppler imaging	1	kept
power imbalance	power imbalance	power imbalance	power imbalance	power imbalance	1	kept
power quality	power quality	power quality	power quality	power quality	1	kept
Power relations	power relations	power relations	power relations	power relations	1	case_or_format
Power spectrum	power spectrum	power spectrum	power spectrum	Power spectrum	1	kept
powered air-purifying respirator	powered air purifying respirator	powered air purifying respirator	powered air purifying respirator	powered air-purifying respirator	1	kept
powered emergency ventilator	powered emergency ventilator	powered emergency ventilator	powered emergency ventilator	powered emergency ventilator	1	kept
powered wheelchair	powered wheelchair	powered wheelchair	powered wheelchair	powered wheelchair	1	kept
Powerful	powerful	powerful	powerful	Powerful	1	kept
powerless	powerless	powerless	powerless	powerless	1	kept
Powerlessness	powerlessness	powerlessness	powerlessness	powerlessness	1	case_or_format

POWs	pows	pows	pows	POWs	1	kept
poxta bacterial gene	poxta bacterial gene	poxta bacterial gene	poxta bacterial gene	poxta bacterial gene	1	kept
ppe	ppe	ppe	ppe	PPE	1	case_or_format
PPE adherence	ppe adherence	ppe adherence	ppe adherence	PPE adherence	1	kept
PPE conservation	ppe conservation	ppe conservation	ppe conservation	PPE conservation	1	kept
PPE supply chain	ppe supply chain	ppe supply chain	ppe supply chain	PPE supply chain	1	kept
PPE-cognitive	ppe cognitive	ppe cognitive	ppe cognitive	PPE-cognitive	1	kept
PPE-related dermatitis	ppe related dermatitis	ppe related dermatitis	ppe related dermatitis	PPE-related dermatitis	1	kept
PPE-SAFE	ppe safe	ppe safe	ppe safe	PPE-SAFE	1	kept
PPHN	pphn	pphn	pphn	PPHN	1	kept
PR interval	pr interval	pr interval	pr interval	PR interval	1	kept
Practical care	practical care	practical care	practical care	Practical care	1	kept
Practical guide	practical guide	practical guide	practical guide	Practical guide	1	kept
Practical Intelligence	practical intelligence	practical intelligence	practical intelligence	Practical Intelligence	1	kept
Practical issues	practical issues	practical issues	practical issues	Practical issues	1	kept
practical knowledge	practical knowledge	practical knowledge	practical knowledge	practical knowledge	1	kept

practical model	practical model	practical model	practical model	practical model	1	kept
practical procedure	practical procedure	practical procedure	practical procedure	practical procedure	1	kept
Practical skills	practical skills	practical skills	practical skills	Practical skills	1	kept
practical skills	practical skills	practical skills	practical skills	Practical skills	1	case_or_format
practice & procedure	practice and procedure	practice and procedure	practice and procedure	practice & procedure	1	kept
practice analysis	practice analysis	practice analysis	practice analysis	practice analysis	1	kept
Practice and risk stratification	practice and risk stratification	practice and risk stratification	practice and risk stratification	Practice and risk stratification	1	kept
Practice Based Learning and Improvement	practice based learning and improvement	practice based learning and improvement	practice based learning and improvement	Practice-Based Learning and Improvement	1	case_or_format
practice changes	practice changes	practice changes	practice change	Practice change	1	singular
Practice Development	practice development	practice development	practice development	Practice development	1	case_or_format
Practice development resuscitation	practice development resuscitation	practice development resuscitation	practice development resuscitation	Practice development resuscitation	1	kept
practice discrepancies	practice discrepancies	practice discrepancies	practice discrepancies	practice discrepancies	1	kept
practice doctorate	practice doctorate	practice doctorate	practice doctorate	practice doctorate	1	kept
Practice domains	practice domains	practice domains	practice domains	Practice domains	1	kept
Practice Environment Scale	practice environment scale	practice environment scale	practice environment scale	PRACTICE ENVIRONMENT SCALE	1	case_or_format
practice environment scale	practice environment scale	practice environment scale	practice environment scale	PRACTICE ENVIRONMENT SCALE	1	case_or_format

practice environment score	practice environment score	practice environment score	practice environment score	practice environment score	1	kept
PRACTICE ENVIRONMENTS	practice environments	practice environments	practice environment	practice environment	1	singular
Practice evaluation tool	practice evaluation tool	practice evaluation tool	practice evaluation tool	Practice evaluation tool	1	kept
Practice evidence	practice evidence	practice evidence	practice evidence	Practice evidence	1	kept
Practice guidebook	practice guidebook	practice guidebook	practice guidebook	Practice guidebook	1	kept
Practice Guideline	practice guideline	practice guideline	practice guideline	practice guideline	1	case_or_format
Practice guideline as topic	practice guideline as topic	practice guideline as topic	practice guideline as topic	Practice guideline as topic	1	kept
Practice implications	practice implications	practice implications	practice implications	practice implications	1	case_or_format
practice innovation	practice innovation	practice innovation	practice innovation	practice innovation	1	kept
practice learning	practice learning	practice learning	practice learning	practice learning	1	kept
practice managment	practice managment	practice managment	practice managment	practice managment	1	kept
Practice misalignment	practice misalignment	practice misalignment	practice misalignment	Practice misalignment	1	kept
practice misalignment	practice misalignment	practice misalignment	practice misalignment	Practice misalignment	1	case_or_format
Practice normalization	practice normalization	practice normalization	practice normalization	Practice normalization	1	kept
Practice parameters	practice parameters	practice parameters	practice parameters	Practice parameters	1	kept
Practice pattern	practice pattern	practice pattern	practice pattern	practice patterns	1	case_or_format

PRACTICE PATTERNS	practice patterns	practice patterns	practice pattern	practice patterns	1	singular
Practice Patterns, Dentists'	practice patterns dentists	practice patterns dentists	practice patterns dentists	Practice Patterns, Dentists'	1	kept
Practice patterns, Physicians'	practice patterns physicians	practice patterns physicians	practice patterns physicians	Practice Patterns, Physicians'	1	case_or_format
Practice projects	practice projects	practice projects	practice projects	Practice projects	1	kept
Practice Readiness	practice readiness	practice readiness	practice readiness	practice readiness	1	case_or_format
Practice skills	practice skills	practice skills	practice skills	Practice skills	1	kept
Practice sustainment	practice sustainment	practice sustainment	practice sustainment	Practice sustainment	1	kept
Practice teachings	practice teachings	practice teachings	practice teachings	Practice teachings	1	kept
practice variability	practice variability	practice variability	practice variability	practice variability	1	kept
practice variation	practice variation	practice variation	practice variation	Practice variation	1	case_or_format
practice)	practice	practice	practice	Practice	1	case_or_format
Practice, Psychological	practice psychological	practice psychological	practice psychological	Practice, Psychological	1	kept
practice-based learning	practice based learning	practice based learning	practice based learning	practice-based learning	1	kept
practice-based learning and [illegible]	practice based learning and [illegible]	practice based learning and [illegible]	practice based learning and [illegible]	Practice-Based Learning and [illegible]	1	case_or_format
Practice-intervention research	practice intervention research	practice intervention research	practice intervention research	Practice-intervention research	1	kept
Practice-primed decision model	practice primed decision model	practice primed decision model	practice primed decision model	Practice-primed decision model	1	kept

Practice-readiness	practice readiness	practice readiness	practice readiness	practice readiness	1	case_or_format
practices urinary catheterization	practices urinary catheterization	practices urinary catheterization	practices urinary catheterization	practices urinary catheterization	1	kept
practise defensive medicine	practise defensive medicine	practise defensive medicine	practise defensive medicine	practise defensive medicine	1	kept
Practises	practises	practises	practises	Practises	1	kept
PRACTITIONER	practitioner	practitioner	practitioner	PRACTITIONERS	1	case_or_format
Practitioner	practitioner	practitioner	practitioner	PRACTITIONERS	1	case_or_format
practitioner	practitioner	practitioner	practitioner	PRACTITIONERS	1	case_or_format
practitioner behavior	practitioner behavior	practitioner behavior	practitioner behavior	practitioner behavior	1	kept
practitioner focused intervention	practitioner focused intervention	practitioner focused intervention	practitioner focused intervention	practitioner focused intervention	1	kept
PRACTITIONER PROGRAM	practitioner program	practitioner program	practitioner program	PRACTITIONER PROGRAM	1	kept
Practitioners' attitudes	practitioners attitudes	practitioners attitudes	practitioners attitudes	Practitioners' attitudes	1	kept
Prader-Willi syndrome	prader willi syndrome	prader willi syndrome	prader willi syndrome	Prader-Willi Syndrome	1	case_or_format
pradofloxacin	pradofloxacin	pradofloxacin	pradofloxacin	pradofloxacin	1	kept
Pragmatic Clinical Trial	pragmatic clinical trial	pragmatic clinical trial	pragmatic clinical trial	Pragmatic clinical trial	1	case_or_format
Pragmatic trial	pragmatic trial	pragmatic trial	pragmatic trial	pragmatic trial	1	case_or_format
Pragmatism	pragmatism	pragmatism	pragmatism	Pragmatism	1	kept

pramipexole	pramipexole	pramipexole	pramipexole	pramipexole	1	kept
prasterone sulfate	prasterone sulfate	prasterone sulfate	prasterone sulfate	prasterone sulfate	1	kept
Prasugrel Hydrochloride	prasugrel hydrochloride	prasugrel hydrochloride	prasugrel hydrochloride	Prasugrel Hydrochloride	1	kept
pratique professionnelle	pratique professionnelle	pratique professionnelle	pratique professionnelle	pratique professionnelle	1	kept
prayer	prayer	prayer	prayer	prayer	1	kept
pre B cell leukemia transcription factor 1	pre b cell leukemia transcription factor 1	pre b cell leukemia transcription factor 1	pre b cell leukemia transcription factor 1	pre B cell leukemia transcription factor 1	1	kept
pre hospital emergency medicine	pre hospital emergency medicine	pre hospital emergency medicine	pre hospital emergency medicine	pre hospital emergency medicine	1	kept
Pre hospital setting	pre hospital setting	pre hospital setting	pre hospital setting	Pre hospital setting	1	kept
pre post design	pre post design	pre post design	pre post design	pre post design	1	kept
Pre Sleep Arousal Scale	pre sleep arousal scale	pre sleep arousal scale	pre sleep arousal scale	Pre Sleep Arousal Scale	1	kept
Pre term infants	pre term infants	pre term infants	pre term infant	Pre-term infant	1	singular
pre term infants	pre term infants	pre term infants	pre term infant	Pre-term infant	1	singular
Pre-	pre	pre	pre	Pre-	1	kept
pre-	pre	pre	pre	Pre-	1	case_or_format
Pre- and post-study design	pre and post study design	pre and post study design	pre and post study design	Pre- and post-study design	1	kept
Pre-Analytical Phase	pre analytical phase	pre analytical phase	pre analytical phase	pre-analytical phase	1	case_or_format

pre-cardiopulmonary arrest	pre cardiopulmonary arrest	pre cardiopulmonary arrest	pre cardiopulmonary arrest	pre-cardiopulmonary arrest	1	kept
Pre-Deliric model	pre deliric model	pre deliric model	pre deliric model	Pre-Deliric model	1	kept
Pre-Deployment	pre deployment	pre deployment	pre deployment	Pre-Deployment	1	kept
Pre-emptive isolation	pre emptive isolation	pre emptive isolation	pre emptive isolation	Pre-emptive isolation	1	kept
Pre-existing peripheral intravenous cannula	pre existing peripheral intravenous cannula	pre existing peripheral intravenous cannula	pre existing peripheral intravenous cannula	Pre-existing peripheral intravenous cannula	1	kept
Pre-filled syringe	pre filled syringe	pre filled syringe	pre filled syringe	Pre-filled syringe	1	kept
pre-hospital	pre hospital	pre hospital	pre hospital	Pre-hospital	1	case_or_format
Pre-hospital care	pre hospital care	pre hospital care	pre hospital care	Pre-hospital care	1	kept
pre-hospital care	pre hospital care	pre hospital care	pre hospital care	Pre-hospital care	1	case_or_format
Pre-hospital environment	pre hospital environment	pre hospital environment	pre hospital environment	Pre-hospital environment	1	kept
Pre-hospital triage	pre hospital triage	pre hospital triage	pre hospital triage	Pre-hospital triage	1	kept
pre-implementation	pre implementation	pre implementation	pre implementation	pre-implementation	1	kept
Pre-impregnated wipes	pre impregnated wipes	pre impregnated wipes	pre impregnated wipes	Pre-impregnated wipes	1	kept
pre-operative assessment	pre operative assessment	pre operative assessment	pre operative assessment	pre-operative assessment	1	kept
Pre-operative care	pre operative care	pre operative care	pre operative care	pre-operative care	1	case_or_format
pre-registration education	pre registration education	pre registration education	pre registration education	pre-registration education	1	kept

Pre-registration interprofessional education	pre registration interprofessional education	pre registration interprofessional education	pre registration interprofessional education	Pre-registration interprofessional education	1	kept
Pre-symptomatic carriers	pre symptomatic carriers	pre symptomatic carriers	pre symptomatic carriers	Pre-symptomatic carriers	1	kept
pre-symptomatic carriers	pre symptomatic carriers	pre symptomatic carriers	pre symptomatic carriers	Pre-symptomatic carriers	1	case_or_format
Pre-term birth	pre term birth	pre term birth	pre term birth	Pre-term birth	1	kept
Pre-term infant	pre term infant	pre term infant	pre term infant	Pre-term infant	1	kept
pre-term infant	pre term infant	pre term infant	pre term infant	Pre-term infant	1	case_or_format
Pre-term infants	pre term infants	pre term infants	pre term infant	Pre-term infant	1	singular
Pre-term new borns	pre term new borns	pre term new borns	pre term new borns	Pre-term new borns	1	kept
Pre-weaning kit growth	pre weaning kit growth	pre weaning kit growth	pre weaning kit growth	Pre-weaning kit growth	1	kept
pre-weaning kit growth	pre weaning kit growth	pre weaning kit growth	pre weaning kit growth	Pre-weaning kit growth	1	case_or_format
PREALBUMIN	prealbumin	prealbumin	prealbumin	prealbumin	1	case_or_format
preanalytical	preanalytical	preanalytical	preanalytical	preanalytical	1	kept
Preanalytical phase	preanalytical phase	preanalytical phase	preanalytical phase	Preanalytical phase	1	kept
Preanalytics	preanalytics	preanalytics	preanalytics	Preanalytics	1	kept
preauthorization	preauthorization	preauthorization	preauthorization	preauthorization	1	kept
prebiotic and probiotic therapy	prebiotic and probiotic therapy	prebiotic and probiotic therapy	prebiotic and probiotic therapy	prebiotic and probiotic therapy	1	kept

precariousness	precariousness	precariousness	precariousness	precariousness	1	kept
precautions	precautions	precautions	precautions	precautions	1	kept
PRECEDE	precede	precede	precede	PRECEDE	1	kept
Precepting	precepting	precepting	precepting	Precepting	1	kept
precepting	precepting	precepting	precepting	Precepting	1	case_or_format
Preceptor program	preceptor program	preceptor program	preceptor program	Preceptor program	1	kept
Preceptors	preceptors	preceptors	preceptor	Preceptor	1	singular
Preceptorship handbook	preceptorship handbook	preceptorship handbook	preceptorship handbook	Preceptorship handbook	1	kept
Preceptors' experiences	preceptors experiences	preceptors experiences	preceptors experiences	Preceptors' experiences	1	kept
Prechtl General Movements Assessment	prechtl general movements assessment	prechtl general movements assessment	prechtl general movements assessment	Prechtl General Movements Assessment	1	kept
PRECIPITANTS	precipitants	precipitants	precipitants	PRECIPITANTS	1	kept
PRECIPITATING FACTORS	precipitating factors	precipitating factors	precipitating factors	Precipitating Factors	1	case_or_format
PRECISION	precision	precision	precision	PRECISION	1	kept
Preclinical work	preclinical work	preclinical work	preclinical work	Preclinical work	1	kept
PRECONCEPTION	preconception	preconception	preconception	PRECONCEPTION	1	kept
Preconception and periconception in prenatal psychology	preconception and periconception in prenatal psychology	preconception and periconception in prenatal psychology	preconception and periconception in prenatal psychology	Preconception and periconception in prenatal psychology	1	kept

Preconception care	preconception care	preconception care	preconception care	Preconception Care	1	case_or_format
Precursor Cell Lymphoblastic Leukemia-Lymphoma	precursor cell lymphoblastic leukemia lymphoma	precursor cell lymphoblastic leukemia lymphoma	precursor cell lymphoblastic leukemia lymphoma	Precursor Cell Lymphoblastic Leukemia-Lymphoma	1	kept
predation	predation	predation	predation	predation	1	kept
Predict	predict	predict	predict	PREDICT	1	case_or_format
predict serious clinical deterioration	predict serious clinical deterioration	predict serious clinical deterioration	predict serious clinical deterioration	predict serious clinical deterioration	1	kept
predicted body weight	predicted body weight	predicted body weight	predicted body weight	predicted body weight	1	kept
predicted VBAC success	predicted vbac success	predicted vbac success	predicted vbac success	predicted VBAC success	1	kept
PREDICTING HOSPITAL MORTALITY	predicting hospital mortality	predicting hospital mortality	predicting hospital mortality	PREDICTING HOSPITAL MORTALITY	1	kept
PREDICTING LENGTH	predicting length	predicting length	predicting length	PREDICTING LENGTH	1	kept
Predicting Location after Arthroplasty Nomogram	predicting location after arthroplasty nomogram	predicting location after arthroplasty nomogram	predicting location after arthroplasty nomogram	Predicting Location after Arthroplasty Nomogram	1	kept
Predicting mortality	predicting mortality	predicting mortality	predicting mortality	Predicting mortality	1	kept
Predicting outcomes	predicting outcomes	predicting outcomes	predicting outcomes	Predicting outcomes	1	kept
prediction algorithm	prediction algorithm	prediction algorithm	prediction algorithm	prediction algorithm	1	kept
Prediction model development	prediction model development	prediction model development	prediction model development	Prediction model development	1	kept
prediction modeling	prediction modeling	prediction modeling	prediction modeling	prediction modeling	1	kept
prediction of prognosis	prediction of prognosis	prediction of prognosis	prediction of prognosis	prediction of prognosis	1	kept

prediction of team performance	prediction of team performance	prediction of team performance	prediction of team performance	prediction of team performance	1	kept
Prediction problem	prediction problem	prediction problem	prediction problem	Prediction problem	1	kept
prediction score	prediction score	prediction score	prediction score	prediction score	1	kept
Prediction tasks	prediction tasks	prediction tasks	prediction tasks	Prediction tasks	1	kept
prediction tool	prediction tool	prediction tool	prediction tool	prediction tool	1	kept
Prediction tools	prediction tools	prediction tools	prediction tool	prediction tool	1	singular
predictive	predictive	predictive	predictive	predictive	1	kept
Predictive capabilities	predictive capabilities	predictive capabilities	predictive capabilities	Predictive capabilities	1	kept
PREDICTIVE CONTROL	predictive control	predictive control	predictive control	PREDICTIVE CONTROL	1	kept
PREDICTIVE CONTROL ALGORITHM	predictive control algorithm	predictive control algorithm	predictive control algorithm	PREDICTIVE CONTROL ALGORITHM	1	kept
Predictive control systems	predictive control systems	predictive control systems	predictive control systems	Predictive control systems	1	kept
Predictive equations	predictive equations	predictive equations	predictive equations	Predictive equations	1	kept
Predictive factors	predictive factors	predictive factors	predictive factors	predictive factors	1	case_or_format
Predictive modelling	predictive modelling	predictive modelling	predictive modelling	Predictive modelling	1	kept
predictive modelling	predictive modelling	predictive modelling	predictive modelling	Predictive modelling	1	case_or_format
Predictive models	predictive models	predictive models	predictive model	predictive model	1	singular

predictive models	predictive models	predictive models	predictive model	predictive model	1	singular
Predictive monitoring	predictive monitoring	predictive monitoring	predictive monitoring	Predictive monitoring	1	kept
predictive monitoring	predictive monitoring	predictive monitoring	predictive monitoring	Predictive monitoring	1	case_or_format
predictive nomogram	predictive nomogram	predictive nomogram	predictive nomogram	predictive nomogram	1	kept
Predictive performance	predictive performance	predictive performance	predictive performance	Predictive performance	1	kept
predictive research	predictive research	predictive research	predictive research	predictive research	1	kept
predictive scale	predictive scale	predictive scale	predictive scale	predictive scale	1	kept
Predictive value	predictive value	predictive value	predictive value	predictive value	1	case_or_format
predictive value of tests	predictive value of tests	predictive value of tests	predictive value of tests	Predictive Value of Tests	1	case_or_format
PREDICTIVE-VALUE	predictive value	predictive value	predictive value	predictive value	1	case_or_format
predictors of readmission	predictors of readmission	predictors of readmission	predictors of readmission	predictors of readmission	1	kept
Predisposing and precipitating risk factors	predisposing and precipitating risk factors	predisposing and precipitating risk factors	predisposing and precipitating risk factors	Predisposing and precipitating risk factors	1	kept
Predisposing factors	predisposing factors	predisposing factors	predisposing factors	Predisposing factors	1	kept
predisposing factors	predisposing factors	predisposing factors	predisposing factors	Predisposing factors	1	case_or_format
predisposition infection response organ dysfunction	predisposition infection response organ dysfunction	predisposition infection response organ dysfunction	predisposition infection response organ dysfunction	predisposition infection response organ dysfunction	1	kept
predisposition insult response organ dysfunction community	predisposition insult response organ dysfunction community	predisposition insult response organ dysfunction community	predisposition insult response organ dysfunction community	predisposition insult response organ dysfunction community	1	kept

Prednisolone	prednisolone	prednisolone	prednisolone	prednisolone	1	case_or_format
preeclampsia education	preeclampsia education	preeclampsia education	preeclampsia education	preeclampsia education	1	kept
Preeclampsia with severe features	preeclampsia with severe features	preeclampsia with severe features	preeclampsia with severe features	Preeclampsia with severe features	1	kept
preeclamptic mother	preeclamptic mother	preeclamptic mother	preeclamptic mother	preeclamptic mother	1	kept
PREEMPTIVE CONTACT PRECAUTIONS	preemptive contact precautions	preemptive contact precautions	preemptive contact precautions	PREEMPTIVE CONTACT PRECAUTIONS	1	kept
preexisting condition	preexisting condition	preexisting condition	preexisting condition	preexisting condition	1	kept
PREFERENCE	preference	preference	preference	PREFERENCES	1	case_or_format
Preference	preference	preference	preference	PREFERENCES	1	case_or_format
Preferences regarding life-sustaining [illegible]	preferences regarding life sustaining [illegible]	preferences regarding life sustaining [illegible]	preferences regarding life sustaining [illegible]	Preferences regarding life-sustaining [illegible]	1	kept
Preferred place of death	preferred place of death	preferred place of death	preferred place of death	Preferred place of death	1	kept
prefilled medication syringes	prefilled medication syringes	prefilled medication syringes	prefilled medication syringes	prefilled medication syringes	1	kept
prefilled syringe	prefilled syringe	prefilled syringe	prefilled syringe	prefilled syringe	1	kept
Preformed silo	preformed silo	preformed silo	preformed silo	Preformed silo	1	kept
preformed silo	preformed silo	preformed silo	preformed silo	Preformed silo	1	case_or_format
Pregabalin	pregabalin	pregabalin	pregabalin	pregabalin	1	case_or_format
Pregnancy complication	pregnancy complication	pregnancy complication	pregnancy complication	Pregnancy Complications	1	case_or_format

Pregnancy Complications, Hematologic	pregnancy complications hematologic	pregnancy complications hematologic	pregnancy complications hematologic	Pregnancy Complications, Hematologic	1	kept
pregnancy education	pregnancy education	pregnancy education	pregnancy education	pregnancy education	1	kept
Pregnancy in adolescence	pregnancy in adolescence	pregnancy in adolescence	pregnancy in adolescence	Pregnancy in Adolescence	1	case_or_format
pregnancy interval	pregnancy interval	pregnancy interval	pregnancy interval	pregnancy interval	1	kept
Pregnancy outcome	pregnancy outcome	pregnancy outcome	pregnancy outcome	Pregnancy Outcome	1	case_or_format
Pregnancy outcomes	pregnancy outcomes	pregnancy outcomes	pregnancy outcome	Pregnancy Outcome	1	singular
Pregnancy periods	pregnancy periods	pregnancy periods	pregnancy periods	Pregnancy periods	1	kept
Pregnancy recognition	pregnancy recognition	pregnancy recognition	pregnancy recognition	Pregnancy recognition	1	kept
pregnancy screening	pregnancy screening	pregnancy screening	pregnancy screening	pregnancy screening	1	kept
Pregnancy stress	pregnancy stress	pregnancy stress	pregnancy stress	Pregnancy stress	1	kept
PREGNANCY TERMINATION	pregnancy termination	pregnancy termination	pregnancy termination	pregnancy termination	1	case_or_format
Pregnancy termination	pregnancy termination	pregnancy termination	pregnancy termination	pregnancy termination	1	case_or_format
pregnancy toxemia	pregnancy toxemia	pregnancy toxemia	pregnancy toxemia	pregnancy toxemia	1	kept
Pregnancy Trimester, First	pregnancy trimester first	pregnancy trimester first	pregnancy trimester first	Pregnancy Trimester, First	1	kept
Pregnancy Trimesters	pregnancy trimesters	pregnancy trimesters	pregnancy trimesters	Pregnancy Trimesters	1	kept
Pregnancy, Prolonged	pregnancy prolonged	pregnancy prolonged	pregnancy prolonged	Pregnancy, Prolonged	1	kept

Pregnancy-induced hypertension	pregnancy induced hypertension	pregnancy induced hypertension	pregnancy induced hypertension	pregnancy induced hypertension	1	case_or_format
pregnancy-related complications	pregnancy related complications	pregnancy related complications	pregnancy related complications	pregnancy-related complications	1	kept
pregnant	pregnant	pregnant	pregnant	Pregnant	1	case_or_format
Pregnant people	pregnant people	pregnant people	pregnant people	Pregnant People	1	case_or_format
pregnant person	pregnant person	pregnant person	pregnant person	pregnant person	1	kept
Pregnant women	pregnant women	pregnant women	pregnant women	Pregnant Women	1	case_or_format
Prehabilitation	prehabilitation	prehabilitation	prehabilitation	prehabilitation	1	case_or_format
Prehospital care of bleeding	prehospital care of bleeding	prehospital care of bleeding	prehospital care of bleeding	Prehospital care of bleeding	1	kept
Prehospital care of traumatic bleeding	prehospital care of traumatic bleeding	prehospital care of traumatic bleeding	prehospital care of traumatic bleeding	Prehospital care of traumatic bleeding	1	kept
Prehospital care system	prehospital care system	prehospital care system	prehospital care system	Prehospital care system	1	kept
prehospital emergency care	prehospital emergency care	prehospital emergency care	prehospital emergency care	Prehospital emergency care	1	case_or_format
Prehospital emergency medicine	prehospital emergency medicine	prehospital emergency medicine	prehospital emergency medicine	Prehospital emergency medicine	1	kept
prehospital medicine	prehospital medicine	prehospital medicine	prehospital medicine	prehospital medicine	1	kept
Prehospital monitoring	prehospital monitoring	prehospital monitoring	prehospital monitoring	Prehospital monitoring	1	kept
Prehospital physicians	prehospital physicians	prehospital physicians	prehospital physicians	Prehospital physicians	1	kept
Prehospital resuscitation	prehospital resuscitation	prehospital resuscitation	prehospital resuscitation	Prehospital resuscitation	1	kept

prehospital screen	prehospital screen	prehospital screen	prehospital screen	prehospital screen	1	kept
prehospital thrombolysis	prehospital thrombolysis	prehospital thrombolysis	prehospital thrombolysis	prehospital thrombolysis	1	kept
Prehospital transfusion	prehospital transfusion	prehospital transfusion	prehospital transfusion	Prehospital transfusion	1	kept
Prehospital treatment of coagulopathy	prehospital treatment of coagulopathy	prehospital treatment of coagulopathy	prehospital treatment of coagulopathy	Prehospital treatment of coagulopathy	1	kept
Prehospitalcare	prehospitalcare	prehospitalcare	prehospitalcare	Prehospitalcare	1	kept
prehospitalisation care	prehospitalisation care	prehospitalisation care	prehospitalisation care	prehospitalisation care	1	kept
prehypertension	prehypertension	prehypertension	prehypertension	prehypertension	1	kept
preinjury mental health condition	preinjury mental health condition	preinjury mental health condition	preinjury mental health condition	preinjury mental health condition	1	kept
Prelabor rupture of membranes	prelabor rupture of membranes	prelabor rupture of membranes	prelabor rupture of membranes	Prelabor rupture of membranes	1	kept
preliminary communication	preliminary communication	preliminary communication	preliminary communication	preliminary communication	1	kept
Preliminary Data	preliminary data	preliminary data	preliminary data	preliminary data	1	case_or_format
Preload	preload	preload	preload	Preload	1	kept
Premature [growth and development]	premature growth and development	premature growth and development	premature growth and development	Premature [growth and development]	1	kept
premature aging	premature aging	premature aging	premature aging	premature aging	1	kept
Premature delivery and child development	premature delivery and child development	premature delivery and child development	premature delivery and child development	Premature delivery and child development	1	kept
premature diaper	premature diaper	premature diaper	premature diaper	premature diaper	1	kept

premature infant development	premature infant development	premature infant development	premature infant development	premature infant development	1	kept
Premature infant oral motor intervention	premature infant oral motor intervention	premature infant oral motor intervention	premature infant oral motor intervention	Premature infant oral motor intervention	1	kept
premature infant pain profile scale	premature infant pain profile scale	premature infant pain profile scale	premature infant pain profile scale	premature infant pain profile scale	1	kept
Premature Infant Pain Profile Scale Revised	premature infant pain profile scale revised	premature infant pain profile scale revised	premature infant pain profile scale revised	Premature Infant Pain Profile Scale Revised	1	kept
Premature Infant Pain Profile-Revised	premature infant pain profile revised	premature infant pain profile revised	premature infant pain profile revised	Premature Infant Pain Profile Revised	1	case_or_format
Premature infant pain profile-revised	premature infant pain profile revised	premature infant pain profile revised	premature infant pain profile revised	Premature Infant Pain Profile Revised	1	case_or_format
premature infant skin care	premature infant skin care	premature infant skin care	premature infant skin care	premature infant skin care	1	kept
premature mortality	premature mortality	premature mortality	premature mortality	premature mortality	1	kept
Premature Neonate	premature neonate	premature neonate	premature neonate	Premature neonate	1	case_or_format
Premature Newborn	premature newborn	premature newborn	premature newborn	Premature newborn	1	case_or_format
premature newborn	premature newborn	premature newborn	premature newborn	Premature newborn	1	case_or_format
Premature newborn development	premature newborn development	premature newborn development	premature newborn development	Premature newborn development	1	kept
Premature newborns	premature newborns	premature newborns	premature newborn	Premature newborn	1	singular
premature oral feeding readiness assessment scale	premature oral feeding readiness assessment scale	premature oral feeding readiness assessment scale	premature oral feeding readiness assessment scale	premature oral feeding readiness assessment scale	1	kept
premature step-down	premature step down	premature step down	premature step down	premature step-down	1	kept
premature twins	premature twins	premature twins	premature twins	premature twins	1	kept

PREMATURE-INFANT	premature infant	premature infant	premature infant	Premature infant	1	case_or_format
Prematurebirth and developmental follow-up for at-risk infants	prematurebirth and developmental follow up for at risk infants	prematurebirth and developmental follow up for at risk infants	prematurebirth and developmental follow up for at risk infants	Prematurebirth and developmental follow-up for at-risk infants	1	kept
Prematurely born baby	prematurely born baby	prematurely born baby	prematurely born baby	Prematurely born baby	1	kept
PREMATURITY	prematurity	prematurity	prematurity	prematurity	1	case_or_format
prematüre	premat re	premat re	premat re	prematüre	1	kept
prematüre bebek	premat re bebek	premat re bebek	premat re bebek	prematüre bebek	1	kept
Premedication effects and con-5 of 14	premedication effects and con 5 of 14	premedication effects and con 5 of 14	premedication effects and con 5 of 14	Premedication effects and con-5 of 14	1	kept
Premenopause	premenopause	premenopause	premenopause	Premenopause	1	kept
premenopause	premenopause	premenopause	premenopause	Premenopause	1	case_or_format
Premenstrual syndrome	premenstrual syndrome	premenstrual syndrome	premenstrual syndrome	Premenstrual syndrome	1	kept
premix drug	premix drug	premix drug	premix drug	premix drug	1	kept
premix drug preparation	premix drug preparation	premix drug preparation	premix drug preparation	premix drug preparation	1	kept
Prenatal and perinatal origins of childhood diseases and disorders	prenatal and perinatal origins of childhood diseases and disorders	prenatal and perinatal origins of childhood diseases and disorders	prenatal and perinatal origins of childhood diseases and disorders	Prenatal and perinatal origins of childhood diseases and disorders	1	kept
Prenatal and perinatal shock	prenatal and perinatal shock	prenatal and perinatal shock	prenatal and perinatal shock	Prenatal and perinatal shock	1	kept
Prenatal and perinatal traumas and birth culture	prenatal and perinatal traumas and birth culture	prenatal and perinatal traumas and birth culture	prenatal and perinatal traumas and birth culture	Prenatal and perinatal traumas and birth culture	1	kept
Prenatal attachment	prenatal attachment	prenatal attachment	prenatal attachment	Prenatal attachment	1	kept

Prenatal care coordination	prenatal care coordination	prenatal care coordination	prenatal care coordination	Prenatal care coordination	1	kept
Prenatal care initiation	prenatal care initiation	prenatal care initiation	prenatal care initiation	Prenatal care initiation	1	kept
Prenatal care, birthing center	prenatal care birthing center	prenatal care birthing center	prenatal care birthing center	Prenatal care, birthing center	1	kept
Prenatal counseling, prevention, and pregnancy	prenatal counseling prevention and pregnancy	prenatal counseling prevention and pregnancy	prenatal counseling prevention and pregnancy	Prenatal counseling, prevention, and pregnancy	1	kept
Prenatal counselling	prenatal counselling	prenatal counselling	prenatal counselling	Prenatal counselling	1	kept
prenatal education	prenatal education	prenatal education	prenatal education	Prenatal Education	1	case_or_format
Prenatal entry	prenatal entry	prenatal entry	prenatal entry	Prenatal entry	1	kept
Prenatal experiences and development across the lifespan	prenatal experiences and development across the lifespan	prenatal experiences and development across the lifespan	prenatal experiences and development across the lifespan	Prenatal experiences and development across the lifespan	1	kept
prenatal genetic test	prenatal genetic test	prenatal genetic test	prenatal genetic test	prenatal genetic test	1	kept
Prenatal music exposure	prenatal music exposure	prenatal music exposure	prenatal music exposure	Prenatal music exposure	1	kept
Prenatal opioid exposure	prenatal opioid exposure	prenatal opioid exposure	prenatal opioid exposure	Prenatal opioid exposure	1	kept
Prenatal psychology, stress, and brain development	prenatal psychology stress and brain development	prenatal psychology stress and brain development	prenatal psychology stress and brain development	Prenatal psychology, stress, and brain development	1	kept
Prenatal Ultrasonography	prenatal ultrasonography	prenatal ultrasonography	prenatal ultrasonography	Prenatal Ultrasonography	1	kept
PRENATAL-DIAGNOSIS	prenatal diagnosis	prenatal diagnosis	prenatal diagnosis	Prenatal Diagnosis	1	case_or_format
Preoperative	preoperative	preoperative	preoperative	preoperative	1	case_or_format
preoperative anemia	preoperative anemia	preoperative anemia	preoperative anemia	preoperative anemia	1	kept

PREOPERATIVE ANXIETY	preoperative anxiety	preoperative anxiety	preoperative anxiety	PREOPERATIVE ANXIETY	1	kept
Preoperative anxiety	preoperative anxiety	preoperative anxiety	preoperative anxiety	PREOPERATIVE ANXIETY	1	case_or_format
preoperative anxiety	preoperative anxiety	preoperative anxiety	preoperative anxiety	PREOPERATIVE ANXIETY	1	case_or_format
Preoperative assessment	preoperative assessment	preoperative assessment	preoperative assessment	preoperative assessment	1	case_or_format
Preoperative care	preoperative care	preoperative care	preoperative care	Preoperative Care	1	case_or_format
preoperative complication	preoperative complication	preoperative complication	preoperative complication	preoperative complication	1	kept
PREOPERATIVE EDUCATION	preoperative education	preoperative education	preoperative education	preoperative education	1	case_or_format
Preoperative education	preoperative education	preoperative education	preoperative education	preoperative education	1	case_or_format
Preoperative fasting	preoperative fasting	preoperative fasting	preoperative fasting	Preoperative fasting	1	kept
preoperative HF screening	preoperative hf screening	preoperative hf screening	preoperative hf screening	preoperative HF screening	1	kept
preoperative monitoring	preoperative monitoring	preoperative monitoring	preoperative monitoring	preoperative monitoring	1	kept
Preoperative optimisation	preoperative optimisation	preoperative optimisation	preoperative optimisation	Preoperative optimisation	1	kept
PREOPERATIVE OPTIMIZATION	preoperative optimization	preoperative optimization	preoperative optimization	preoperative optimization	1	case_or_format
Preoperative period	preoperative period	preoperative period	preoperative period	preoperative period	1	case_or_format
Preoperative preparation	preoperative preparation	preoperative preparation	preoperative preparation	Preoperative preparation	1	kept
Preoperative psychological assessment and	preoperative psychological assessment and	preoperative psychological assessment and	preoperative psychological assessment and	Preoperative psychological assessment and	1	kept

preoperative visit	preoperative visit	preoperative visit	preoperative visit	preoperative visit	1	kept
Preoxygenation	preoxygenation	preoxygenation	preoxygenation	Preoxygenation	1	kept
Preparation error	preparation error	preparation error	preparation error	Preparation errors	1	case_or_format
preparation errors	preparation errors	preparation errors	preparation error	Preparation errors	1	singular
Preparation for discharge	preparation for discharge	preparation for discharge	preparation for discharge	Preparation for discharge	1	kept
Preparation for transport	preparation for transport	preparation for transport	preparation for transport	Preparation for transport	1	kept
Prepared	prepared	prepared	prepared	Prepared	1	kept
Preparedness and response	preparedness and response	preparedness and response	preparedness and response	Preparedness and response	1	kept
Preprocessing techniques	preprocessing techniques	preprocessing techniques	preprocessing techniques	Preprocessing techniques	1	kept
preproendothelin 1	preproendothelin 1	preproendothelin 1	preproendothelin 1	preproendothelin 1	1	kept
Prepyloric	prepyloric	prepyloric	prepyloric	Prepyloric	1	kept
Preregistration clinical placement	preregistration clinical placement	preregistration clinical placement	preregistration clinical placement	Preregistration clinical placement	1	kept
Preregistration education	preregistration education	preregistration education	preregistration education	Preregistration education	1	kept
Prerenal	prerenal	prerenal	prerenal	Prerenal	1	kept
Presbyterian	presbyterian	presbyterian	presbyterian	Presbyterian	1	kept
Preschool	preschool	preschool	preschool	preschool	1	case_or_format

Preschool children	preschool children	preschool children	preschool children	Preschool children	1	kept
Preschool-aged children	preschool aged children	preschool aged children	preschool aged children	Preschool-aged children	1	kept
Prescriber	prescriber	prescriber	prescriber	Prescriber	1	kept
Prescribing and administration documentation errors	prescribing and administration documentation errors	prescribing and administration documentation errors	prescribing and administration documentation errors	Prescribing and administration documentation errors	1	kept
prescribing and administration documentation errors	prescribing and administration documentation errors	prescribing and administration documentation errors	prescribing and administration documentation errors	Prescribing and administration documentation errors	1	case_or_format
prescribing behaviors	prescribing behaviors	prescribing behaviors	prescribing behaviors	prescribing behaviors	1	kept
prescribing guideline	prescribing guideline	prescribing guideline	prescribing guideline	prescribing guideline	1	kept
Prescribing patterns	prescribing patterns	prescribing patterns	prescribing patterns	Prescribing patterns	1	kept
PRESCRIPTION	prescription	prescription	prescription	prescription	1	case_or_format
prescription drug diversion	prescription drug diversion	prescription drug diversion	prescription drug diversion	prescription drug diversion	1	kept
prescription drug monitoring program	prescription drug monitoring program	prescription drug monitoring program	prescription drug monitoring program	prescription drug monitoring program	1	kept
prescription error	prescription error	prescription error	prescription error	PRESCRIPTION ERRORS	1	case_or_format
Prescriptions, Non-Drug	prescriptions non drug	prescriptions non drug	prescriptions non drug	Prescriptions, Non-Drug	1	kept
prescriptive authority	prescriptive authority	prescriptive authority	prescriptive authority	prescriptive authority	1	kept
Presence	presence	presence	presence	presence	1	case_or_format
Presence at the bedside	presence at the bedside	presence at the bedside	presence at the bedside	Presence at the bedside	1	kept

Presence technologies	presence technologies	presence technologies	presence technologies	Presence technologies	1	kept
Present Self Assessment	present self assessment	present self assessment	present self assessment	Present Self Assessment	1	kept
presentation	presentation	presentation	presentation	presentation	1	kept
PRESENTEEISM	presenteeism	presenteeism	presenteeism	Presenteeism	1	case_or_format
presenting symptoms	presenting symptoms	presenting symptoms	presenting symptoms	presenting symptoms	1	kept
preservation	preservation	preservation	preservation	preservation	1	kept
preservation solution	preservation solution	preservation solution	preservation solution	preservation solution	1	kept
Presión arterial	presi n arterial	presi n arterial	presi n arterial	Presión arterial	1	kept
Presión positiva continua en la vía aé rea nasal	presi n positiva continua en la v a a rea nasal	presi n positiva continua en la v a a rea nasal	presi n positiva continua en la v a a rea nasal	Presión positiva continua en la vía aé rea nasal	1	kept
Pressor	pressor	pressor	pressor	Pressor	1	kept
pressor response	pressor response	pressor response	pressor response	pressor response	1	kept
Pressure [adverse effects]	pressure adverse effects	pressure adverse effects	pressure adverse effects	Pressure [adverse effects]	1	kept
Pressure amplitude	pressure amplitude	pressure amplitude	pressure amplitude	Pressure amplitude	1	kept
Pressure and temperature	pressure and temperature	pressure and temperature	pressure and temperature	Pressure and temperature	1	kept
pressure and tension	pressure and tension	pressure and tension	pressure and tension	pressure and tension	1	kept
pressure chambers	pressure chambers	pressure chambers	pressure chambers	pressure chambers	1	kept

Pressure control	pressure control	pressure control	pressure control	Pressure control	1	kept
pressure control	pressure control	pressure control	pressure control	Pressure control	1	case_or_format
pressure control ventilation	pressure control ventilation	pressure control ventilation	pressure control ventilation	pressure control ventilation	1	kept
pressure dressing	pressure dressing	pressure dressing	pressure dressing	pressure dressing	1	kept
Pressure effects	pressure effects	pressure effects	pressure effects	Pressure effects	1	kept
pressure induced angioedema	pressure induced angioedema	pressure induced angioedema	pressure induced angioedema	pressure induced angioedema	1	kept
PRESSURE INJURY	pressure injury	pressure injury	pressure injury	pressure injury	1	case_or_format
Pressure Injury	pressure injury	pressure injury	pressure injury	pressure injury	1	case_or_format
pressure injury care bundle	pressure injury care bundle	pressure injury care bundle	pressure injury care bundle	pressure injury care bundle	1	kept
Pressure injury development	pressure injury development	pressure injury development	pressure injury development	Pressure injury development	1	kept
Pressure injury risk	pressure injury risk	pressure injury risk	pressure injury risk	Pressure injury risk	1	kept
Pressure injury risk assessment	pressure injury risk assessment	pressure injury risk assessment	pressure injury risk assessment	Pressure injury risk assessment	1	kept
pressure injury risk assessment	pressure injury risk assessment	pressure injury risk assessment	pressure injury risk assessment	Pressure injury risk assessment	1	case_or_format
Pressure injury staging	pressure injury staging	pressure injury staging	pressure injury staging	Pressure injury staging	1	kept
pressure leakage	pressure leakage	pressure leakage	pressure leakage	pressure leakage	1	kept
Pressure limitation	pressure limitation	pressure limitation	pressure limitation	Pressure limitation	1	kept

Pressure measuring	pressure measuring	pressure measuring	pressure measuring	Pressure measuring	1	kept
Pressure monitoring	pressure monitoring	pressure monitoring	pressure monitoring	Pressure monitoring	1	kept
Pressure monitors	pressure monitors	pressure monitors	pressure monitors	Pressure monitors	1	kept
Pressure redistribution	pressure redistribution	pressure redistribution	pressure redistribution	Pressure redistribution	1	kept
Pressure reducing crib mattress	pressure reducing crib mattress	pressure reducing crib mattress	pressure reducing crib mattress	Pressure reducing crib mattress	1	kept
Pressure reduction	pressure reduction	pressure reduction	pressure reduction	Pressure reduction	1	kept
pressure relief	pressure relief	pressure relief	pressure relief	pressure relief	1	kept
Pressure relief specialty beds	pressure relief specialty beds	pressure relief specialty beds	pressure relief specialty beds	Pressure relief specialty beds	1	kept
pressure risk assessment scales	pressure risk assessment scales	pressure risk assessment scales	pressure risk assessment scales	Pressure risk assessment scales	1	case_or_format
Pressure sensor	pressure sensor	pressure sensor	pressure sensor	Pressure sensor	1	kept
pressure sensor	pressure sensor	pressure sensor	pressure sensor	Pressure sensor	1	case_or_format
Pressure Sore	pressure sore	pressure sore	pressure sore	pressure sore	1	case_or_format
Pressure sore	pressure sore	pressure sore	pressure sore	pressure sore	1	case_or_format
pressure sores grade	pressure sores grade	pressure sores grade	pressure sores grade	pressure sores grade	1	kept
PRESSURE SUPPORT	pressure support	pressure support	pressure support	PRESSURE SUPPORT	1	kept
Pressure system	pressure system	pressure system	pressure system	Pressure system	1	kept

Pressure Ulcer Care	pressure ulcer care	pressure ulcer care	pressure ulcer care	pressure ulcer care	1	case_or_format
Pressure ulcer classification	pressure ulcer classification	pressure ulcer classification	pressure ulcer classification	Pressure ulcer classification	1	kept
Pressure Ulcer Knowledge Assessment Tool 2.0	pressure ulcer knowledge assessment tool 2.0	pressure ulcer knowledge assessment tool 2.0	pressure ulcer knowledge assessment tool 2.0	Pressure Ulcer Knowledge Assessment Tool 2.0	1	kept
Pressure Ulcer Knowledge Assessment Tool version 2.0	pressure ulcer knowledge assessment tool version 2.0	pressure ulcer knowledge assessment tool version 2.0	pressure ulcer knowledge assessment tool version 2.0	Pressure Ulcer Knowledge Assessment Tool version 2.0	1	kept
pressure ulcer management	pressure ulcer management	pressure ulcer management	pressure ulcer management	pressure ulcer management	1	kept
Pressure ulcer prevalence	pressure ulcer prevalence	pressure ulcer prevalence	pressure ulcer prevalence	Pressure ulcer prevalence	1	kept
PRESSURE ULCER PREVENTION	pressure ulcer prevention	pressure ulcer prevention	pressure ulcer prevention	pressure ulcer prevention	1	case_or_format
Pressure ulcer prevention and control	pressure ulcer prevention and control	pressure ulcer prevention and control	pressure ulcer prevention and control	Pressure ulcer prevention and control	1	kept
Pressure ulcer prevention bundle	pressure ulcer prevention bundle	pressure ulcer prevention bundle	pressure ulcer prevention bundle	Pressure ulcer prevention bundle	1	kept
Pressure Ulcer Prevention Knowledge Assessment Instrument	pressure ulcer prevention knowledge assessment instrument	pressure ulcer prevention knowledge assessment instrument	pressure ulcer prevention knowledge assessment instrument	Pressure Ulcer Prevention Knowledge Assessment Instrument	1	kept
Pressure ulcer prevention pediatrics	pressure ulcer prevention pediatrics	pressure ulcer prevention pediatrics	pressure ulcer prevention pediatrics	Pressure ulcer prevention pediatrics	1	kept
pressure ulcer prophylaxis	pressure ulcer prophylaxis	pressure ulcer prophylaxis	pressure ulcer prophylaxis	pressure ulcer prophylaxis	1	kept
PRESSURE ULCER RISK	pressure ulcer risk	pressure ulcer risk	pressure ulcer risk	PRESSURE ULCER RISK	1	kept
Pressure ulcer risk assessment	pressure ulcer risk assessment	pressure ulcer risk assessment	pressure ulcer risk assessment	Pressure ulcer risk assessment	1	kept
pressure ulcer risk assessment	pressure ulcer risk assessment	pressure ulcer risk assessment	pressure ulcer risk assessment	Pressure ulcer risk assessment	1	case_or_format
Pressure ulcer risk factors	pressure ulcer risk factors	pressure ulcer risk factors	pressure ulcer risk factors	Pressure ulcer risk factors	1	kept

pressure ulcer risk factors	pressure ulcer risk factors	pressure ulcer risk factors	pressure ulcer risk factors	Pressure ulcer risk factors	1	case_or_format
Pressure Ulcer Risk Scale	pressure ulcer risk scale	pressure ulcer risk scale	pressure ulcer risk scale	Pressure Ulcer Risk Scale	1	kept
Pressure ulcer staging	pressure ulcer staging	pressure ulcer staging	pressure ulcer staging	Pressure ulcer staging	1	kept
pressure ulcer staging form	pressure ulcer staging form	pressure ulcer staging form	pressure ulcer staging form	pressure ulcer staging form	1	kept
Pressure ulceration	pressure ulceration	pressure ulceration	pressure ulceration	pressure ulceration	1	case_or_format
Pressure ulcers prevention	pressure ulcers prevention	pressure ulcers prevention	pressure ulcers prevention	Pressure ulcers prevention	1	kept
Pressure ventilation	pressure ventilation	pressure ventilation	pressure ventilation	Pressure ventilation	1	kept
Pressure wound	pressure wound	pressure wound	pressure wound	Pressure wound	1	kept
PRESSURE-CONTROLLED VENTILATION	pressure controlled ventilation	pressure controlled ventilation	pressure controlled ventilation	pressure controlled ventilation	1	case_or_format
Pressure-induced urticaria	pressure induced urticaria	pressure induced urticaria	pressure induced urticaria	Pressure-induced urticaria	1	kept
Pressure-resistant cap cotton cover	pressure resistant cap cotton cover	pressure resistant cap cotton cover	pressure resistant cap cotton cover	Pressure-resistant cap cotton cover	1	kept
Pressure-ulcer prevention	pressure ulcer prevention	pressure ulcer prevention	pressure ulcer prevention	pressure ulcer prevention	1	case_or_format
pressured induced angioedema	pressured induced angioedema	pressured induced angioedema	pressured induced angioedema	pressured induced angioedema	1	kept
Prestige	prestige	prestige	prestige	Prestige	1	kept
Presurgical optimisation programme	presurgical optimisation programme	presurgical optimisation programme	presurgical optimisation programme	Presurgical optimisation programme	1	kept
Preterm babies	preterm babies	preterm babies	preterm baby	Preterm baby	1	singular

Preterm birth and developmental follow-up for at-risk infants	preterm birth and developmental follow up for at risk infants	preterm birth and developmental follow up for at risk infants	preterm birth and developmental follow up for at risk infants	Preterm birth and developmental follow-up for at-risk infants	1	kept
preterm birth knowledge	preterm birth knowledge	preterm birth knowledge	preterm birth knowledge	preterm birth knowledge	1	kept
preterm birth prevention	preterm birth prevention	preterm birth prevention	preterm birth prevention	preterm birth prevention	1	kept
preterm birth rate	preterm birth rate	preterm birth rate	preterm birth rate	preterm birth rate	1	kept
preterm care	preterm care	preterm care	preterm care	preterm care	1	kept
Preterm children	preterm children	preterm children	preterm children	Preterm children	1	kept
Preterm delivery	preterm delivery	preterm delivery	preterm delivery	Preterm delivery	1	kept
preterm feeding intolerance	preterm feeding intolerance	preterm feeding intolerance	preterm feeding intolerance	preterm feeding intolerance	1	kept
preterm home care	preterm home care	preterm home care	preterm home care	preterm home care	1	kept
preterm infant and neonate	preterm infant and neonate	preterm infant and neonate	preterm infant and neonate	preterm infant and neonate	1	kept
Preterm infant behavior and development	preterm infant behavior and development	preterm infant behavior and development	preterm infant behavior and development	Preterm infant behavior and development	1	kept
preterm infant care	preterm infant care	preterm infant care	preterm infant care	preterm infant care	1	kept
preterm infant retina	preterm infant retina	preterm infant retina	preterm infant retina	preterm infant retina	1	kept
preterm infants care	preterm infants care	preterm infants care	preterm infants care	preterm infants care	1	kept
Preterm neonatal care	preterm neonatal care	preterm neonatal care	preterm neonatal care	Preterm neonatal care	1	kept
Preterm neonate Protocolized nasal skin care	preterm neonate protocolized nasal skin care	preterm neonate protocolized nasal skin care	preterm neonate protocolized nasal skin care	Preterm neonate Protocolized nasal skin care	1	kept

preterm neonates	preterm neonates	preterm neonates	preterm neonate	Preterm neonates	1	singular
preterm newborns	preterm newborns	preterm newborns	preterm newborn	Preterm newborn	1	singular
preterm prelabor rupture of fetal membranes	preterm prelabor rupture of fetal membranes	preterm prelabor rupture of fetal membranes	preterm prelabor rupture of fetal membranes	preterm prelabor rupture of fetal membranes	1	kept
PRETERM PREMATURE RUPTURE	preterm premature rupture	preterm premature rupture	preterm premature rupture	PRETERM PREMATURE RUPTURE	1	kept
PRETERM-BIRTH	preterm birth	preterm birth	preterm birth	Preterm birth	1	case_or_format
preterm.	preterm	preterm	preterm	Preterm	1	case_or_format
PretermInfantOral Feeding Readiness Assessment scale	preterminfantoral feeding readiness assessment scale	preterminfantoral feeding readiness assessment scale	preterminfantoral feeding readiness assessment scale	PretermInfantOral Feeding Readiness Assessment scale	1	kept
Pretest	pretest	pretest	pretest	Pretest	1	kept
pretest posttest control group design	pretest posttest control group design	pretest posttest control group design	pretest posttest control group design	pretest posttest control group design	1	kept
Pretoria	pretoria	pretoria	pretoria	Pretoria	1	kept
Pretransfusion verification	pretransfusion verification	pretransfusion verification	pretransfusion verification	Pretransfusion verification	1	kept
Prevalence of delirium	prevalence of delirium	prevalence of delirium	prevalence of delirium	Prevalence of delirium	1	kept
Prevalence Risk assessment	prevalence risk assessment	prevalence risk assessment	prevalence risk assessment	Prevalence Risk assessment	1	kept
prevalence study	prevalence study	prevalence study	prevalence study	prevalence study	1	kept
PREVALENCE SURVEY	prevalence survey	prevalence survey	prevalence survey	PREVALENCE SURVEY	1	kept
Prevalence survey	prevalence survey	prevalence survey	prevalence survey	PREVALENCE SURVEY	1	case_or_format

Prevalence surveys	prevalence surveys	prevalence surveys	prevalence survey	PREVALENCE SURVEY	1	singular
Prevalencia	prevalencia	prevalencia	prevalencia	Prevalencia	1	kept
Prevalência	preval ncia	preval ncia	preval ncia	Prevalência	1	kept
Prevención	prevenci n	prevenci n	prevenci n	Prevención	1	kept
Prevención primaria	prevenci n primaria	prevenci n primaria	prevenci n primaria	Prevención primaria	1	kept
prevent	prevent	prevent	prevent	prevent	1	kept
PREVENT NECROTIZING ENTEROCOLITIS	prevent necrotizing enterocolitis	prevent necrotizing enterocolitis	prevent necrotizing enterocolitis	PREVENT NECROTIZING ENTEROCOLITIS	1	kept
PREVENTABILITY	preventability	preventability	preventability	PREVENTABILITY	1	kept
preventability	preventability	preventability	preventability	PREVENTABILITY	1	case_or_format
Preventable	preventable	preventable	preventable	Preventable	1	kept
Preventable adverse drug event	preventable adverse drug event	preventable adverse drug event	preventable adverse drug event	Preventable adverse drug event	1	kept
Preventative measures	preventative measures	preventative measures	preventative measures	Preventative measures	1	kept
Preventing admissions with sepsis	preventing admissions with sepsis	preventing admissions with sepsis	preventing admissions with sepsis	Preventing admissions with sepsis	1	kept
PREVENTING DELIRIUM	preventing delirium	preventing delirium	preventing delirium	PREVENTING DELIRIUM	1	kept
PREVENTING NECROTIZING ENTEROCOLITIS	preventing necrotizing enterocolitis	preventing necrotizing enterocolitis	preventing necrotizing enterocolitis	PREVENTING NECROTIZING ENTEROCOLITIS	1	kept
PREVENTING NOSOCOMIAL TRANSMISSION	preventing nosocomial transmission	preventing nosocomial transmission	preventing nosocomial transmission	PREVENTING NOSOCOMIAL TRANSMISSION	1	kept

Preventing pressure sores	preventing pressure sores	preventing pressure sores	preventing pressure sores	Preventing pressure sores	1	kept
Prevention & Control	prevention and control	prevention and control	prevention and control	prevention and control	1	case_or_format
prevention & control	prevention and control	prevention and control	prevention and control	prevention and control	1	case_or_format
prevention algorithm	prevention algorithm	prevention algorithm	prevention algorithm	prevention algorithm	1	kept
Prevention and	prevention and	prevention and	prevention and	Prevention and	1	kept
Prevention and Control	prevention and control	prevention and control	prevention and control	prevention and control	1	case_or_format
Prevention bundle	prevention bundle	prevention bundle	prevention bundle	Prevention bundle	1	kept
Prevention healthcare-associated infections	prevention healthcare associated infections	prevention healthcare associated infections	prevention healthcare associated infections	Prevention healthcare-associated infections	1	kept
Prevention interventions for pressure injury	prevention interventions for pressure injury	prevention interventions for pressure injury	prevention interventions for pressure injury	Prevention interventions for pressure injury	1	kept
prevention measure	prevention measure	prevention measure	prevention measure	Prevention measures	1	case_or_format
prevention of adverse events and errors	prevention of adverse events and errors	prevention of adverse events and errors	prevention of adverse events and errors	prevention of adverse events and errors	1	kept
Prevention of candidiasis	prevention of candidiasis	prevention of candidiasis	prevention of candidiasis	Prevention of candidiasis	1	kept
prevention of catheter-associated	prevention of catheter associated	prevention of catheter associated	prevention of catheter associated	prevention of catheter-associated	1	kept
prevention of central-line-associated bloodstream infections	prevention of central line associated bloodstream infections	prevention of central line associated bloodstream infections	prevention of central line associated bloodstream infections	prevention of central-line-associated bloodstream infections	1	kept
Prevention of delirium	prevention of delirium	prevention of delirium	prevention of delirium	Prevention of delirium	1	kept
Prevention of errors	prevention of errors	prevention of errors	prevention of errors	Prevention of errors	1	kept

Prevention of post stroke sequelae	prevention of post stroke sequelae	prevention of post stroke sequelae	prevention of post stroke sequelae	Prevention of post stroke sequelae	1	kept
Prevention of prematurity	prevention of prematurity	prevention of prematurity	prevention of prematurity	Prevention of prematurity	1	kept
Prevention of pressure wounds in pediatrics	prevention of pressure wounds in pediatrics	prevention of pressure wounds in pediatrics	prevention of pressure wounds in pediatrics	Prevention of pressure wounds in pediatrics	1	kept
Prevention of ROP	prevention of rop	prevention of rop	prevention of rop	Prevention of ROP	1	kept
prevention of Ventilator-Associated Pneumonia	prevention of ventilator associated pneumonia	prevention of ventilator associated pneumonia	prevention of ventilator associated pneumonia	prevention of Ventilator-Associated Pneumonia	1	kept
PREVENTION PRACTICES	prevention practices	prevention practices	prevention practice	Prevention practice	1	singular
prevention practices	prevention practices	prevention practices	prevention practice	Prevention practice	1	singular
Prevention program	prevention program	prevention program	prevention program	Prevention program	1	kept
prevention programs	prevention programs	prevention programs	prevention program	Prevention program	1	singular
Prevention respiratory therapy	prevention respiratory therapy	prevention respiratory therapy	prevention respiratory therapy	Prevention respiratory therapy	1	kept
Prevention strategy	prevention strategy	prevention strategy	prevention strategy	prevention strategies	1	case_or_format
prevention strategy	prevention strategy	prevention strategy	prevention strategy	prevention strategies	1	case_or_format
prevention study	prevention study	prevention study	prevention study	prevention study	1	kept
prevention y control	prevention y control	prevention y control	prevention y control	prevention y control	1	kept
Preventive	preventive	preventive	preventive	Preventive	1	kept
preventive	preventive	preventive	preventive	Preventive	1	case_or_format

preventive adverse event	preventive adverse event	preventive adverse event	preventive adverse event	preventive adverse event	1	kept
PREVENTIVE CARE	preventive care	preventive care	preventive care	PREVENTIVE CARE	1	kept
Preventive care	preventive care	preventive care	preventive care	PREVENTIVE CARE	1	case_or_format
Preventive dressings	preventive dressings	preventive dressings	preventive dressings	Preventive dressings	1	kept
preventive dressings	preventive dressings	preventive dressings	preventive dressings	Preventive dressings	1	case_or_format
Preventive Guidelines	preventive guidelines	preventive guidelines	preventive guidelines	Preventive Guidelines	1	kept
Preventive Health services	preventive health services	preventive health services	preventive health service	preventive health service	1	singular
Preventive health services	preventive health services	preventive health services	preventive health service	preventive health service	1	singular
Preventive mattresses	preventive mattresses	preventive mattresses	preventive mattresses	Preventive mattresses	1	kept
preventive measures and practice	preventive measures and practice	preventive measures and practice	preventive measures and practice	preventive measures and practice	1	kept
Preventive Medicine and Hospital Hygiene	preventive medicine and hospital hygiene	preventive medicine and hospital hygiene	preventive medicine and hospital hygiene	Preventive Medicine and Hospital Hygiene	1	kept
Preventive practices	preventive practices	preventive practices	preventive practices	Preventive practices	1	kept
preventive practices	preventive practices	preventive practices	preventive practices	Preventive practices	1	case_or_format
preventive procedures	preventive procedures	preventive procedures	preventive procedures	preventive procedures	1	kept
preventive strategy	preventive strategy	preventive strategy	preventive strategy	preventive strategy	1	kept
Previable period	previable period	previable period	previable period	Previable period	1	kept

Previous experience	previous experience	previous experience	previous experience	Previous experience	1	kept
Prevotella	prevotella	prevotella	prevotella	Prevotella	1	kept
Prevotella melaninogenica	prevotella melaninogenica	prevotella melaninogenica	prevotella melaninogenica	Prevotella melaninogenica	1	kept
pre - hospital	pre hospital	pre hospital	pre hospital	Pre-hospital	1	case_or_format
price	price	price	price	price	1	kept
PRICE APPROACH	price approach	price approach	price approach	PRICE APPROACH	1	kept
pricing policy	pricing policy	pricing policy	pricing policy	pricing policy	1	kept
pride	pride	pride	pride	pride	1	kept
Primary adrenal insufficiency	primary adrenal insufficiency	primary adrenal insufficiency	primary adrenal insufficiency	Primary adrenal insufficiency	1	kept
Primary amputation revascularization	primary amputation revascularization	primary amputation revascularization	primary amputation revascularization	Primary amputation revascularization	1	kept
Primary and secondary injury	primary and secondary injury	primary and secondary injury	primary and secondary injury	Primary and secondary injury	1	kept
PRIMARY ANGIOPLASTY	primary angioplasty	primary angioplasty	primary angioplasty	PRIMARY ANGIOPLASTY	1	kept
primary assessment	primary assessment	primary assessment	primary assessment	primary assessment	1	kept
Primary Biliary Cholangitis	primary biliary cholangitis	primary biliary cholangitis	primary biliary cholangitis	Primary Biliary Cholangitis	1	kept
primary biliary cirrhosis	primary biliary cirrhosis	primary biliary cirrhosis	primary biliary cirrhosis	primary biliary cirrhosis	1	kept
Primary blast lung	primary blast lung	primary blast lung	primary blast lung	Primary blast lung	1	kept

Primary Care	primary care	primary care	primary care	primary care	1	case_or_format
primary care and social interface	primary care and social interface	primary care and social interface	primary care and social interface	primary care and social interface	1	kept
Primary Care Evaluation of Mental Disorders	primary care evaluation of mental disorders	primary care evaluation of mental disorders	primary care evaluation of mental disorders	Primary Care Evaluation of Mental Disorders	1	kept
Primary Care Hospital	primary care hospital	primary care hospital	primary care hospital	Primary Care Hospital	1	kept
Primary care innovation	primary care innovation	primary care innovation	primary care innovation	Primary care innovation	1	kept
Primary care physician	primary care physician	primary care physician	primary care physician	Primary care physician	1	kept
Primary care physicians	primary care physicians	primary care physicians	primary care physician	Primary care physician	1	singular
primary care provider	primary care provider	primary care provider	primary care provider	primary care provider	1	kept
primary care providers	primary care providers	primary care providers	primary care provider	primary care provider	1	singular
Primary caregiver	primary caregiver	primary caregiver	primary caregiver	Primary caregiver	1	kept
Primary caregivers	primary caregivers	primary caregivers	primary caregiver	Primary caregiver	1	singular
primary caregivers	primary caregivers	primary caregivers	primary caregiver	Primary caregiver	1	singular
Primary data	primary data	primary data	primary data	Primary data	1	kept
Primary family caregivers	primary family caregivers	primary family caregivers	primary family caregivers	Primary family caregivers	1	kept
primary headache	primary headache	primary headache	primary headache	primary headache	1	kept
Primary health care setting	primary health care setting	primary health care setting	primary health care setting	Primary health care setting	1	kept

Primary health centers	primary health centers	primary health centers	primary health centers	Primary health centers	1	kept
Primary healthcare	primary healthcare	primary healthcare	primary healthcare	Primary healthcare	1	kept
primary healthcare	primary healthcare	primary healthcare	primary healthcare	Primary healthcare	1	case_or_format
Primary hospital	primary hospital	primary hospital	primary hospital	Primary hospital	1	kept
primary insomnia	primary insomnia	primary insomnia	primary insomnia	primary insomnia	1	kept
Primary knee arthroplasty	primary knee arthroplasty	primary knee arthroplasty	primary knee arthroplasty	Primary knee arthroplasty	1	kept
primary knee arthroplasty	primary knee arthroplasty	primary knee arthroplasty	primary knee arthroplasty	Primary knee arthroplasty	1	case_or_format
Primary level hospital	primary level hospital	primary level hospital	primary level hospital	Primary level hospital	1	kept
primary medicine	primary medicine	primary medicine	primary medicine	primary medicine	1	kept
primary mitochondrial disease	primary mitochondrial disease	primary mitochondrial disease	primary mitochondrial disease	primary mitochondrial disease	1	kept
primary motor cortex	primary motor cortex	primary motor cortex	primary motor cortex	primary motor cortex	1	kept
Primary objective	primary objective	primary objective	primary objective	Primary objective	1	kept
Primary outcome	primary outcome	primary outcome	primary outcome	primary outcome	1	case_or_format
primary outcomes	primary outcomes	primary outcomes	primary outcome	primary outcome	1	singular
Primary percutaneous coronary intervention	primary percutaneous coronary intervention	primary percutaneous coronary intervention	primary percutaneous coronary intervention	Primary percutaneous coronary intervention	1	kept
primary respiratory care	primary respiratory care	primary respiratory care	primary respiratory care	primary respiratory care	1	kept

primary school	primary school	primary school	primary school	primary school	1	kept
Primary Sclerosing Cholangitis	primary sclerosing cholangitis	primary sclerosing cholangitis	primary sclerosing cholangitis	primary sclerosing cholangitis	1	case_or_format
Primary stroke center	primary stroke center	primary stroke center	primary stroke center	Primary stroke center	1	kept
primary total hip arthroplasty	primary total hip arthroplasty	primary total hip arthroplasty	primary total hip arthroplasty	primary total hip arthroplasty	1	kept
primary total knee arthroplasty	primary total knee arthroplasty	primary total knee arthroplasty	primary total knee arthroplasty	primary total knee arthroplasty	1	kept
primary tumor	primary tumor	primary tumor	primary tumor	primary tumor	1	kept
PRIMARY-CARE	primary care	primary care	primary care	primary care	1	case_or_format
PRIMARY-HEALTH-CARE	primary health care	primary health care	primary health care	Primary Health Care	1	case_or_format
PRIMATE INFANTS	primate infants	primate infants	primate infants	PRIMATE INFANTS	1	kept
primigravidae	primigravidae	primigravidae	primigravidae	primigravidae	1	kept
primitive reflex	primitive reflex	primitive reflex	primitive reflex	primitive reflex	1	kept
PRINCA	princa	princa	princa	PRINCA	1	kept
Prince Edward Island	prince edward island	prince edward island	prince edward island	Prince Edward Island	1	kept
principal-agent theory	principal agent theory	principal agent theory	principal agent theory	principal-agent theory	1	kept
principle of least interest	principle of least interest	principle of least interest	principle of least interest	principle of least interest	1	kept
Principle of seamless management	principle of seamless management	principle of seamless management	principle of seamless management	Principle of seamless management	1	kept

principle of seamless management	principle of seamless management	principle of seamless management	principle of seamless management	Principle of seamless management	1	case_or_format
Principles	principles	principles	principles	Principles	1	kept
Principles and practices	principles and practices	principles and practices	principles and practices	Principles and practices	1	kept
principles of controlled airway management	principles of controlled airway management	principles of controlled airway management	principles of controlled airway management	principles of controlled airway management	1	kept
principles of emergent airway management	principles of emergent airway management	principles of emergent airway management	principles of emergent airway management	principles of emergent airway management	1	kept
Printed circuit boards	printed circuit boards	printed circuit boards	printed circuit boards	Printed circuit boards	1	kept
Printed papers	printed papers	printed papers	printed papers	Printed papers	1	kept
Printing, three-dimensional	printing three dimensional	printing three dimensional	printing three dimensional	Printing, Three-Dimensional	1	case_or_format
prion	prion	prion	prion	prion	1	kept
prion disease	prion disease	prion disease	prion disease	prion disease	1	kept
PRIOR-AUTHORIZATION	prior authorization	prior authorization	prior authorization	PRIOR-AUTHORIZATION	1	kept
Priorities	priorities	priorities	priorities	Priorities	1	kept
priorities	priorities	priorities	priorities	Priorities	1	case_or_format
Prioritisation	prioritisation	prioritisation	prioritisation	Prioritisation	1	kept
Prioritising	prioritising	prioritising	prioritising	Prioritising	1	kept
prioritization	prioritization	prioritization	prioritization	Prioritization	1	case_or_format

Priority setting	priority setting	priority setting	priority setting	Priority setting	1	kept
PRIS	pris	pris	pris	PRIS	1	kept
prise en charge du diabète en milieu hospitalier	prise en charge du diab te en milieu hospitalier	prise en charge du diab te en milieu hospitalier	prise en charge du diab te en milieu hospitalier	prise en charge du diabète en milieu hospitalier	1	kept
prism	prism	prism	prism	prism	1	kept
PRISM III severity score	prism iii severity score	prism iii severity score	prism iii severity score	PRISM III severity score	1	kept
PRISM score	prism score	prism score	prism score	PRISM score	1	kept
Prisma statement	prisma statement	prisma statement	prisma statement	Prisma statement	1	kept
prisomocitrate	prisomocitrate	prisomocitrate	prisomocitrate	prisomocitrate	1	kept
Prison Policy	prison policy	prison policy	prison policy	Prison Policy	1	kept
Prison System in Ceará	prison system in cear	prison system in cear	prison system in cear	Prison System in Ceará	1	kept
PRIVACY	privacy	privacy	privacy	privacy	1	case_or_format
Privacy-preserving techniques	privacy preserving techniques	privacy preserving techniques	privacy preserving techniques	Privacy-preserving techniques	1	kept
Private care	private care	private care	private care	Private care	1	kept
Private clouds	private clouds	private clouds	private clouds	Private clouds	1	kept
Private Hospital(s	private hospital s	private hospital s	private hospital s	Private Hospital(s	1	kept
private room	private room	private room	private room	Private room	1	case_or_format

private rooms	private rooms	private rooms	private room	Private room	1	singular
private-pay	private pay	private pay	private pay	private-pay	1	kept
Private-pay occupancy	private pay occupancy	private pay occupancy	private pay occupancy	Private-pay occupancy	1	kept
Privatization	privatization	privatization	privatization	Privatization	1	kept
proactive C-L	proactive c l	proactive c l	proactive c l	proactive C-L	1	kept
proactive consultation liaison psychiatry	proactive consultation liaison psychiatry	proactive consultation liaison psychiatry	proactive consultation liaison psychiatry	proactive consultation liaison psychiatry	1	kept
Proactive monitoring	proactive monitoring	proactive monitoring	proactive monitoring	Proactive monitoring	1	kept
Proactive rounding	proactive rounding	proactive rounding	proactive rounding	Proactive rounding	1	kept
proadipocyte	proadipocyte	proadipocyte	proadipocyte	proadipocyte	1	kept
Probabilistic topic models	probabilistic topic models	probabilistic topic models	probabilistic topic models	Probabilistic topic models	1	kept
Probability distributions	probability distributions	probability distributions	probability distributions	Probability distributions	1	kept
Probability learning	probability learning	probability learning	probability learning	Probability learning	1	kept
probability learning	probability learning	probability learning	probability learning	Probability learning	1	case_or_format
probability sample	probability sample	probability sample	probability sample	probability sample	1	kept
Probability sampling	probability sampling	probability sampling	probability sampling	Probability sampling	1	kept
Probability theory	probability theory	probability theory	probability theory	Probability theory	1	kept

probiotic supplementation	probiotic supplementation	probiotic supplementation	probiotic supplementation	probiotic supplementation	1	kept
Problem size	problem size	problem size	problem size	Problem size	1	kept
problem-based learning	problem based learning	problem based learning	problem based learning	Problem-Based Learning	1	case_or_format
problem-focused coping	problem focused coping	problem focused coping	problem focused coping	problem-focused coping	1	kept
Problem-Solving	problem solving	problem solving	problem solving	Problem Solving	1	case_or_format
problem-solving	problem solving	problem solving	problem solving	Problem Solving	1	case_or_format
Problem-solving abilities	problem solving abilities	problem solving abilities	problem solving abilities	Problem-solving abilities	1	kept
problem-solving skills	problem solving skills	problem solving skills	problem solving skills	problem-solving skills	1	kept
Problemas gastrointestinales	problemas gastrointestinales	problemas gastrointestinales	problemas gastrointestinales	Problemas gastrointestinales	1	kept
problem‐solving	problem solving	problem solving	problem solving	Problem Solving	1	case_or_format
probucol	probucol	probucol	probucol	probucol	1	kept
Procedimiento de Glenn	procedimiento de glenn	procedimiento de glenn	procedimiento de glenn	Procedimiento de Glenn	1	kept
procedural	procedural	procedural	procedural	Procedural	1	case_or_format
Procedural exposure	procedural exposure	procedural exposure	procedural exposure	Procedural exposure	1	kept
Procedural intervention	procedural intervention	procedural intervention	procedural intervention	Procedural intervention	1	kept
procedural justice	procedural justice	procedural justice	procedural justice	procedural justice	1	kept

procedural memory	procedural memory	procedural memory	procedural memory	procedural memory	1	kept
Procedural pain management	procedural pain management	procedural pain management	procedural pain management	procedural pain management	1	case_or_format
Procedural pain, Neonates, Pain management	procedural pain neonates pain management	procedural pain neonates pain management	procedural pain neonates pain management	Procedural pain, Neonates, Pain management	1	kept
Procedural sedation	procedural sedation	procedural sedation	procedural sedation	Procedural Sedation	1	case_or_format
PROCEDURAL SKILLS	procedural skills	procedural skills	procedural skills	PROCEDURAL SKILLS	1	kept
procedural skills	procedural skills	procedural skills	procedural skills	PROCEDURAL SKILLS	1	case_or_format
procedural skills training	procedural skills training	procedural skills training	procedural skills training	procedural skills training	1	kept
procedural terminology codes	procedural terminology codes	procedural terminology codes	procedural terminology codes	procedural terminology codes	1	kept
procedural ultrasound	procedural ultrasound	procedural ultrasound	procedural ultrasound	procedural ultrasound	1	kept
procedure based assessments	procedure based assessments	procedure based assessments	procedure based assessments	procedure based assessments	1	kept
Procedure failure	procedure failure	procedure failure	procedure failure	Procedure failure	1	kept
procedure pain	procedure pain	procedure pain	procedure pain	procedure pain	1	kept
procedures.	procedures	procedures	procedure	procedures	1	singular
Proceso	proceso	proceso	proceso	Proceso	1	kept
Proceso de atención de enfermería	proceso de atenci n de enfermer a	proceso de atenci n de enfermer a	proceso de atenci n de enfermer a	Proceso de atención de enfermería	1	kept
Proceso de enfermería	proceso de enfermer a	proceso de enfermer a	proceso de enfermer a	Proceso de enfermería	1	kept

Process change	process change	process change	process change	Process change	1	kept
Process control	process control	process control	process control	process control	1	case_or_format
Process Cost Analysis	process cost analysis	process cost analysis	process cost analysis	Process Cost Analysis	1	kept
Process design	process design	process design	process design	process design	1	case_or_format
Process engineering	process engineering	process engineering	process engineering	Process engineering	1	kept
Process Improvement	process improvement	process improvement	process improvement	Process improvement	1	case_or_format
process indicators	process indicators	process indicators	process indicators	process indicators	1	kept
Process management	process management	process management	process management	Process management	1	kept
Process measures	process measures	process measures	process measures	Process measures	1	kept
Process of care	process of care	process of care	process of care	Process of care	1	kept
process of delivery	process of delivery	process of delivery	process of delivery	process of delivery	1	kept
process reengineering	process reengineering	process reengineering	process reengineering	process reengineering	1	kept
process standardization	process standardization	process standardization	process standardization	process standardization	1	kept
Process time	process time	process time	process time	Process time	1	kept
Process-models	process models	process models	process model	process model	1	singular
Processes	processes	processes	processes	Processes	1	kept

processes of care	processes of care	processes of care	processes of care	processes of care	1	kept
prochlorperazine maleate	prochlorperazine maleate	prochlorperazine maleate	prochlorperazine maleate	prochlorperazine maleate	1	kept
Procoagulants	procoagulants	procoagulants	procoagulants	Procoagulants	1	kept
procord	procord	procord	procord	procord	1	kept
proctalgia	proctalgia	proctalgia	proctalgia	proctalgia	1	kept
Proctoclysis	proctoclysis	proctoclysis	proctoclysis	Proctoclysis	1	kept
Proctoscopy	proctoscopy	proctoscopy	proctoscopy	Proctoscopy	1	kept
Procurement decisions	procurement decisions	procurement decisions	procurement decisions	Procurement decisions	1	kept
Prodromal Symptoms	prodromal symptoms	prodromal symptoms	prodromal symptom	prodromal symptom	1	singular
Product interaction	product interaction	product interaction	product interaction	Product interaction	1	kept
Product Packaging	product packaging	product packaging	product packaging	Product Packaging	1	kept
product parameters	product parameters	product parameters	product parameters	product parameters	1	kept
Product Surveillance, Postmarketing	product surveillance postmarketing	product surveillance postmarketing	product surveillance postmarketing	Product Surveillance, Postmarketing	1	kept
product vigilance	product vigilance	product vigilance	product vigilance	product vigilance	1	kept
production function	production function	production function	production function	production function	1	kept
productivity cost	productivity cost	productivity cost	productivity cost	productivity cost	1	kept

Productivity costs	productivity costs	productivity costs	productivity cost	productivity cost	1	singular
Profesional de la salud	profesional de la salud	profesional de la salud	profesional de la salud	Profesional de la salud	1	kept
Profesionales de enfermería	profesionales de enfermer a	profesionales de enfermer a	profesionales de enfermer a	Profesionales de enfermería	1	kept
Profesionales en enfermería	profesionales en enfermer a	profesionales en enfermer a	profesionales en enfermer a	Profesionales en enfermería	1	kept
Profesionales sanitarios	profesionales sanitarios	profesionales sanitarios	profesionales sanitarios	Profesionales sanitarios	1	kept
Profession	profession	profession	profession	PROFESSION	1	case_or_format
profession rater reliability	profession rater reliability	profession rater reliability	profession rater reliability	profession rater reliability	1	kept
professional - professional relationship	professional professional relationship	professional professional relationship	professional professional relationship	professional - professional relationship	1	kept
professional adaptation	professional adaptation	professional adaptation	professional adaptation	professional adaptation	1	kept
PROFESSIONAL AUTONOMY	professional autonomy	professional autonomy	professional autonomy	Professional Autonomy	1	case_or_format
Professional autonomy.	professional autonomy	professional autonomy	professional autonomy	Professional Autonomy	1	case_or_format
professional behavior	professional behavior	professional behavior	professional behavior	Professional behavior	1	case_or_format
Professional bereavement	professional bereavement	professional bereavement	professional bereavement	Professional bereavement	1	kept
Professional bodies	professional bodies	professional bodies	professional bodies	Professional bodies	1	kept
professional boundaries	professional boundaries	professional boundaries	professional boundaries	Professional boundaries	1	case_or_format
Professional Burnout	professional burnout	professional burnout	professional burnout	professional burnout	1	case_or_format

professional burnout syndrome	professional burnout syndrome	professional burnout syndrome	professional burnout syndrome	professional burnout syndrome	1	kept
Professional caregiver	professional caregiver	professional caregiver	professional caregiver	Professional caregiver	1	kept
professional category	professional category	professional category	professional category	professional category	1	kept
Professional category, Saudi Arabia	professional category saudi arabia	professional category saudi arabia	professional category saudi arabia	Professional category, Saudi Arabia	1	kept
professional codes	professional codes	professional codes	professional codes	professional codes	1	kept
professional collaboration	professional collaboration	professional collaboration	professional collaboration	professional collaboration	1	kept
Professional com- petence	professional com petence	professional com petence	professional com petence	Professional com- petence	1	kept
Professional Commitment	professional commitment	professional commitment	professional commitment	professional commitment	1	case_or_format
Professional communication	professional communication	professional communication	professional communication	Professional communication	1	kept
professional communication	professional communication	professional communication	professional communication	Professional communication	1	case_or_format
professional comportment	professional comportment	professional comportment	professional comportment	professional comportment	1	kept
Professional Conduct	professional conduct	professional conduct	professional conduct	Professional Conduct	1	kept
Professional conduct	professional conduct	professional conduct	professional conduct	Professional Conduct	1	case_or_format
Professional considerations	professional considerations	professional considerations	professional considerations	Professional considerations	1	kept
PROFESSIONAL COURSE ALERT	professional course alert	professional course alert	professional course alert	PROFESSIONAL COURSE ALERT	1	kept
professional culture	professional culture	professional culture	professional culture	professional culture	1	kept

Professional Education	professional education	professional education	professional education	Professional education	1	case_or_format
Professional efficacy	professional efficacy	professional efficacy	professional efficacy	Professional efficacy	1	kept
Professional engagement	professional engagement	professional engagement	professional engagement	Professional engagement	1	kept
Professional environment	professional environment	professional environment	professional environment	Professional environment	1	kept
professional environment	professional environment	professional environment	professional environment	Professional environment	1	case_or_format
professional environmental assessment procedure	professional environmental assessment procedure	professional environmental assessment procedure	professional environmental assessment procedure	professional environmental assessment procedure	1	kept
Professional ethics	professional ethics	professional ethics	professional ethics	professional ethics	1	case_or_format
Professional ethics in medicine	professional ethics in medicine	professional ethics in medicine	professional ethics in medicine	Professional ethics in medicine	1	kept
Professional Exhaustion	professional exhaustion	professional exhaustion	professional exhaustion	Professional exhaustion	1	case_or_format
professional experience	professional experience	professional experience	professional experience	Professional experience	1	case_or_format
Professional expertise	professional expertise	professional expertise	professional expertise	Professional expertise	1	kept
Professional family relations	professional family relations	professional family relations	professional family relations	Professional-Family Relations	1	case_or_format
professional functioning	professional functioning	professional functioning	professional functioning	professional functioning	1	kept
professional governance	professional governance	professional governance	professional governance	professional governance	1	kept
professional identification	professional identification	professional identification	professional identification	professional identification	1	kept
professional image	professional image	professional image	professional image	professional image	1	kept

professional interpreter	professional interpreter	professional interpreter	professional interpreter	professional interpreter	1	kept
Professional issues in radiology	professional issues in radiology	professional issues in radiology	professional issues in radiology	Professional issues in radiology	1	kept
Professional judgement	professional judgement	professional judgement	professional judgement	Professional judgement	1	kept
Professional life	professional life	professional life	professional life	Professional life	1	kept
Professional life quality	professional life quality	professional life quality	professional life quality	Professional life quality	1	kept
professional midwives	professional midwives	professional midwives	professional midwives	professional midwives	1	kept
professional misconduct	professional misconduct	professional misconduct	professional misconduct	Professional Misconduct	1	case_or_format
Professional needs	professional needs	professional needs	professional needs	Professional needs	1	kept
Professional obligations	professional obligations	professional obligations	professional obligations	Professional obligations	1	kept
Professional organization	professional organization	professional organization	professional organization	Professional organization	1	kept
professional players	professional players	professional players	professional players	professional players	1	kept
Professional Practice Environment	professional practice environment	professional practice environment	professional practice environment	Professional practice environment	1	case_or_format
Professional Practice Environment scale	professional practice environment scale	professional practice environment scale	professional practice environment scale	Professional Practice Environment scale	1	kept
PROFESSIONAL PRACTICE ENVIRONMENTS	professional practice environments	professional practice environments	professional practice environment	Professional practice environment	1	singular
Professional practice evaluation	professional practice evaluation	professional practice evaluation	professional practice evaluation	Professional practice evaluation	1	kept
professional practice evaluation	professional practice evaluation	professional practice evaluation	professional practice evaluation	Professional practice evaluation	1	case_or_format

Professional Practice Management	professional practice management	professional practice management	professional practice management	Professional Practice Management	1	kept
Professional practice model	professional practice model	professional practice model	professional practice model	Professional practice model	1	kept
Professional practice models	professional practice models	professional practice models	professional practice model	Professional practice model	1	singular
Professional practices	professional practices	professional practices	professional practice	professional practice	1	singular
Professional Quality of Life	professional quality of life	professional quality of life	professional quality of life	professional quality of life	1	case_or_format
Professional Quality of Life 5	professional quality of life 5	professional quality of life 5	professional quality of life 5	Professional Quality of Life 5	1	kept
professional quality of life 5	professional quality of life 5	professional quality of life 5	professional quality of life 5	Professional Quality of Life 5	1	case_or_format
professional quality of life questionnaire	professional quality of life questionnaire	professional quality of life questionnaire	professional quality of life questionnaire	professional quality of life questionnaire	1	kept
Professional Quality of Life Scale	professional quality of life scale	professional quality of life scale	professional quality of life scale	professional quality of life scale	1	case_or_format
Professional quality of life scale	professional quality of life scale	professional quality of life scale	professional quality of life scale	professional quality of life scale	1	case_or_format
Professional Quality of Life Scale Version 5	professional quality of life scale version 5	professional quality of life scale version 5	professional quality of life scale version 5	Professional Quality of Life Scale Version 5	1	kept
Professional regulation	professional regulation	professional regulation	professional regulation	Professional regulation	1	kept
Professional relations	professional relations	professional relations	professional relations	Professional relations	1	kept
Professional Review Organizations	professional review organizations	professional review organizations	professional review organizations	Professional Review Organizations	1	kept
professional risk factor	professional risk factor	professional risk factor	professional risk factor	professional risk factor	1	kept
Professional satisfaction	professional satisfaction	professional satisfaction	professional satisfaction	Professional satisfaction	1	kept

professional satisfiers	professional satisfiers	professional satisfiers	professional satisfiers	professional satisfiers	1	kept
Professional Self-concept	professional self concept	professional self concept	professional self concept	Professional Self-concept	1	kept
professional self - concept	professional self concept	professional self concept	professional self concept	Professional Self-concept	1	case_or_format
professional skills	professional skills	professional skills	professional skills	professional skills	1	kept
Professional staff	professional staff	professional staff	professional staff	Professional staff	1	kept
Professional standards	professional standards	professional standards	professional standard	professional standard	1	singular
professional standards review organization	professional standards review organization	professional standards review organization	professional standards review organization	professional standards review organization	1	kept
Professional status	professional status	professional status	professional status	Professional status	1	kept
professional support	professional support	professional support	professional support	professional support	1	kept
Professional team	professional team	professional team	professional team	Professional team	1	kept
Professional upgrading	professional upgrading	professional upgrading	professional upgrading	Professional upgrading	1	kept
professional values	professional values	professional values	professional value	Professional value	1	singular
Professional variables	professional variables	professional variables	professional variables	Professional variables	1	kept
professional variation	professional variation	professional variation	professional variation	professional variation	1	kept
PROFESSIONAL WORK	professional work	professional work	professional work	PROFESSIONAL WORK	1	kept
professional worries and concerns	professional worries and concerns	professional worries and concerns	professional worries and concerns	professional worries and concerns	1	kept

Professional-Family relations	professional family relations	professional family relations	professional family relations	Professional-Family Relations	1	case_or_format
professional-family relationship	professional family relationship	professional family relationship	professional family relationship	professional-family relationship	1	kept
Professional-family relationships	professional family relationships	professional family relationships	professional family relationship	professional-family relationship	1	singular
professionalfamily relationships	professionalfamily relationships	professionalfamily relationships	professionalfamily relationships	professionalfamily relationships	1	kept
Professionalization	professionalization	professionalization	professionalization	Professionalization	1	kept
Professionals	professionals	professionals	professional	professional	1	singular
professionals	professionals	professionals	professional	professional	1	singular
PROFESSIONALS PERCEPTIONS	professionals perceptions	professionals perceptions	professionals perceptions	PROFESSIONALS PERCEPTIONS	1	kept
Professionals’ perspectives	professionals perspectives	professionals perspectives	professionals perspectives	Professionals’ perspectives	1	kept
professional‑family relationship	professional family relationship	professional family relationship	professional family relationship	professional-family relationship	1	case_or_format
professions	professions	professions	profession	PROFESSION	1	singular
Proffesional	proffesional	proffesional	proffesional	Proffesional	1	kept
proficiency	proficiency	proficiency	proficiency	proficiency	1	kept
Proficiency testing	proficiency testing	proficiency testing	proficiency testing	Proficiency testing	1	kept
PROFILE	profile	profile	profile	PROFILE	1	kept
progaf	progaf	progaf	progaf	progaf	1	kept

Progeria	progeria	progeria	progeria	Progeria	1	kept
progesterone blood level	progesterone blood level	progesterone blood level	progesterone blood level	progesterone blood level	1	kept
prognosis calculator	prognosis calculator	prognosis calculator	prognosis calculator	prognosis calculator	1	kept
Prognostic	prognostic	prognostic	prognostic	Prognostic	1	kept
PROGNOSTIC ACCURACY	prognostic accuracy	prognostic accuracy	prognostic accuracy	PROGNOSTIC ACCURACY	1	kept
Prognostic awareness	prognostic awareness	prognostic awareness	prognostic awareness	Prognostic awareness	1	kept
prognostic biomarker	prognostic biomarker	prognostic biomarker	prognostic biomarker	prognostic biomarker	1	kept
Prognostic effect	prognostic effect	prognostic effect	prognostic effect	Prognostic effect	1	kept
Prognostic factor	prognostic factor	prognostic factor	prognostic factor	prognostic factors	1	case_or_format
prognostic indices	prognostic indices	prognostic indices	prognostic indices	prognostic indices	1	kept
Prognostic marker	prognostic marker	prognostic marker	prognostic marker	Prognostic marker	1	kept
Prognostic markers	prognostic markers	prognostic markers	prognostic marker	Prognostic marker	1	singular
prognostic model	prognostic model	prognostic model	prognostic model	prognostic model	1	kept
Prognostic models	prognostic models	prognostic models	prognostic model	prognostic model	1	singular
Prognostic review	prognostic review	prognostic review	prognostic review	Prognostic review	1	kept
Prognostic uncertainty	prognostic uncertainty	prognostic uncertainty	prognostic uncertainty	Prognostic uncertainty	1	kept

PROGNOSTIC VALUE	prognostic value	prognostic value	prognostic value	Prognostic value	1	case_or_format
prognoz	prognoz	prognoz	prognoz	prognoz	1	kept
program cost effectiveness	program cost effectiveness	program cost effectiveness	program cost effectiveness	program cost effectiveness	1	kept
Program documentation	program documentation	program documentation	program documentation	Program documentation	1	kept
program impact	program impact	program impact	program impact	program impact	1	kept
Program initiation	program initiation	program initiation	program initiation	Program initiation	1	kept
Program logic model	program logic model	program logic model	program logic model	Program logic model	1	kept
Program organization	program organization	program organization	program organization	Program organization	1	kept
Program structure	program structure	program structure	program structure	Program structure	1	kept
Program sustainability	program sustainability	program sustainability	program sustainability	program sustainability	1	case_or_format
program theory	program theory	program theory	program theory	program theory	1	kept
PROGRAM-DIRECTORS	program directors	program directors	program directors	PROGRAM-DIRECTORS	1	kept
Programmable logic controllers	programmable logic controllers	programmable logic controllers	programmable logic controllers	Programmable logic controllers	1	kept
Programmable System on Chip	programmable system on chip	programmable system on chip	programmable system on chip	Programmable System on Chip	1	kept
Programmable system on chips	programmable system on chips	programmable system on chips	programmable system on chip	Programmable System on Chip	1	singular
programme theory	programme theory	programme theory	programme theory	programme theory	1	kept

Programmed Instruction as Topic	programmed instruction as topic	programmed instruction as topic	programmed instruction as topic	Programmed Instruction as Topic	1	kept
programming errors	programming errors	programming errors	programming errors	programming errors	1	kept
Programming languages	programming languages	programming languages	programming languages	Programming Languages	1	case_or_format
Programming tools	programming tools	programming tools	programming tools	Programming tools	1	kept
PROGRAMMING-MODEL	programming model	programming model	programming model	PROGRAMMING-MODEL	1	kept
Programs	programs	programs	program	PROGRAM	1	singular
PROGRESS-Plus	progress plus	progress plus	progress plus	PROGRESS-Plus	1	kept
PROGRESSION	progression	progression	progression	PROGRESSION	1	kept
Progression of Pneumonia	progression of pneumonia	progression of pneumonia	progression of pneumonia	Progression of Pneumonia	1	kept
Progressive care	progressive care	progressive care	progressive care	progressive care	1	case_or_format
Progressive care unit	progressive care unit	progressive care unit	progressive care unit	progressive care unit	1	case_or_format
Progressive early rehabilitation training	progressive early rehabilitation training	progressive early rehabilitation training	progressive early rehabilitation training	Progressive early rehabilitation training	1	kept
progressive early rehabilitation training	progressive early rehabilitation training	progressive early rehabilitation training	progressive early rehabilitation training	Progressive early rehabilitation training	1	case_or_format
progressive exercise	progressive exercise	progressive exercise	progressive exercise	progressive exercise	1	kept
progressive mobility	progressive mobility	progressive mobility	progressive mobility	Progressive mobility	1	case_or_format
Progressive muscle relaxation exercise	progressive muscle relaxation exercise	progressive muscle relaxation exercise	progressive muscle relaxation exercise	Progressive muscle relaxation exercise	1	kept

Progressive supranuclear palsy	progressive supranuclear palsy	progressive supranuclear palsy	progressive supranuclear palsy	progressive supranuclear palsy	1	case_or_format
progressive tonic monoparesis of the right hindlimb	progressive tonic monoparesis of the right hindlimb	progressive tonic monoparesis of the right hindlimb	progressive tonic monoparesis of the right hindlimb	progressive tonic monoparesis of the right hindlimb	1	kept
Progressive web app	progressive web app	progressive web app	progressive web app	Progressive web app	1	kept
Prohibitins	prohibitins	prohibitins	prohibitins	Prohibitins	1	kept
Prohibition of employment	prohibition of employment	prohibition of employment	prohibition of employment	Prohibition of employment	1	kept
Project ECHO	project echo	project echo	project echo	Project ECHO	1	kept
PROJECTED WORKFORCE	projected workforce	projected workforce	projected workforce	PROJECTED WORKFORCE	1	kept
Projectiles	projectiles	projectiles	projectiles	Projectiles	1	kept
projections	projections	projections	projections	projections	1	kept
Projecto no Coração de Nápoles	projecto no cora o de n poles	projecto no cora o de n poles	projecto no cora o de n poles	Projecto no Coração de Nápoles	1	kept
Prokaryota	prokaryota	prokaryota	prokaryota	Prokaryota	1	kept
Prolactin	prolactin	prolactin	prolactin	prolactin	1	case_or_format
Prolaryn Gel	prolaryn gel	prolaryn gel	prolaryn gel	Prolaryn Gel	1	kept
Prolidase Deficiency	prolidase deficiency	prolidase deficiency	prolidase deficiency	Prolidase Deficiency	1	kept
prolidase deficiency	prolidase deficiency	prolidase deficiency	prolidase deficiency	Prolidase Deficiency	1	case_or_format
proliferative phase	proliferative phase	proliferative phase	proliferative phase	proliferative phase	1	kept

Proline	proline	proline	proline	Proline	1	kept
prolonged critical illness	prolonged critical illness	prolonged critical illness	prolonged critical illness	prolonged critical illness	1	kept
Prolonged ECMO support	prolonged ecmo support	prolonged ecmo support	prolonged ecmo support	Prolonged ECMO support	1	kept
Prolonged hospitalization	prolonged hospitalization	prolonged hospitalization	prolonged hospitalization	Prolonged hospitalization	1	kept
Prolonged intermittent renal replacement therapy	prolonged intermittent renal replacement therapy	prolonged intermittent renal replacement therapy	prolonged intermittent renal replacement therapy	Prolonged intermittent renal replacement therapy	1	kept
prolonged intermittent renal replacement therapy	prolonged intermittent renal replacement therapy	prolonged intermittent renal replacement therapy	prolonged intermittent renal replacement therapy	Prolonged intermittent renal replacement therapy	1	case_or_format
Prolonged mechanical	prolonged mechanical	prolonged mechanical	prolonged mechanical	Prolonged mechanical	1	kept
Prolonged prone positioning	prolonged prone positioning	prolonged prone positioning	prolonged prone positioning	Prolonged prone positioning	1	kept
Prolonged respiratory failure	prolonged respiratory failure	prolonged respiratory failure	prolonged respiratory failure	Prolonged respiratory failure	1	kept
PROLONGED STAY	prolonged stay	prolonged stay	prolonged stay	PROLONGED STAY	1	kept
Prolonged therapy	prolonged therapy	prolonged therapy	prolonged therapy	Prolonged therapy	1	kept
Prolonged ventilation	prolonged ventilation	prolonged ventilation	prolonged ventilation	Prolonged ventilation	1	kept
prolonged ventilation	prolonged ventilation	prolonged ventilation	prolonged ventilation	Prolonged ventilation	1	case_or_format
prolonged weaning	prolonged weaning	prolonged weaning	prolonged weaning	Prolonged weaning	1	case_or_format
PROLONGED-INFUSION	prolonged infusion	prolonged infusion	prolonged infusion	PROLONGED-INFUSION	1	kept
prolonged-infusion	prolonged infusion	prolonged infusion	prolonged infusion	PROLONGED-INFUSION	1	case_or_format

PROLONGING TREATMENTS	prolonging treatments	prolonging treatments	prolonging treatments	PROLONGING TREATMENTS	1	kept
prolymphocytic leukemia	prolymphocytic leukemia	prolymphocytic leukemia	prolymphocytic leukemia	prolymphocytic leukemia	1	kept
promax rotation	promax rotation	promax rotation	promax rotation	promax rotation	1	kept
Promoting Action on Research Implementation in	promoting action on research implementation in	promoting action on research implementation in	promoting action on research implementation in	Promoting Action on Research Implementation in	1	kept
Promoting factors	promoting factors	promoting factors	promoting factors	Promoting factors	1	kept
PROMOTING SLEEP	promoting sleep	promoting sleep	promoting sleep	PROMOTING SLEEP	1	kept
PROMOTION	promotion	promotion	promotion	PROMOTION	1	kept
Promotion	promotion	promotion	promotion	PROMOTION	1	case_or_format
Pronación	pronaci n	pronaci n	pronaci n	Pronación	1	kept
Pronação	prona o	prona o	prona o	Pronação	1	kept
PRONE POSITION	prone position	prone position	prone position	prone position	1	case_or_format
Prone position ventilation	prone position ventilation	prone position ventilation	prone position ventilation	prone position ventilation	1	case_or_format
prone possition	prone possition	prone possition	prone possition	prone possition	1	kept
prone team	prone team	prone team	prone team	prone team	1	kept
Prone Ventilation	prone ventilation	prone ventilation	prone ventilation	prone ventilation	1	case_or_format
Proning	proning	proning	proning	proning	1	case_or_format

Pronostic neurologique	pronostic neurologique	pronostic neurologique	pronostic neurologique	Pronostic neurologique	1	kept
Proof of concept	proof of concept	proof of concept	proof of concept	proof of concept	1	case_or_format
PROPACETAMOL	propacetamol	propacetamol	propacetamol	PROPACETAMOL	1	kept
propafenone	propafenone	propafenone	propafenone	propafenone	1	kept
Proparacaine	proparacaine	proparacaine	proparacaine	Proparacaine	1	kept
Propensity matching	propensity matching	propensity matching	propensity matching	Propensity matching	1	kept
propensity model	propensity model	propensity model	propensity model	propensity model	1	kept
Propensity score matching	propensity score matching	propensity score matching	propensity score matching	propensity score matching	1	case_or_format
propensity score weighting	propensity score weighting	propensity score weighting	propensity score weighting	propensity score weighting	1	kept
proper use	proper use	proper use	proper use	proper use	1	kept
Propess®	propess	propess	propess	Propess®	1	kept
PROPHYLACTIC ANTIBIOTIC-THERAPY	prophylactic antibiotic therapy	prophylactic antibiotic therapy	prophylactic antibiotic therapy	PROPHYLACTIC ANTIBIOTIC-THERAPY	1	kept
prophylactic antibiotics	prophylactic antibiotics	prophylactic antibiotics	prophylactic antibiotics	prophylactic antibiotics	1	kept
prophylactic dressing	prophylactic dressing	prophylactic dressing	prophylactic dressing	prophylactic dressing	1	kept
prophylactic lumbar drainage	prophylactic lumbar drainage	prophylactic lumbar drainage	prophylactic lumbar drainage	prophylactic lumbar drainage	1	kept
PROPHYLACTIC PROBIOTICS	prophylactic probiotics	prophylactic probiotics	prophylactic probiotics	PROPHYLACTIC PROBIOTICS	1	kept

Propionates	propionates	propionates	propionates	Propionates	1	kept
Propionic Acidemia	propionic acidemia	propionic acidemia	propionic acidemia	propionic acidemia	1	case_or_format
propionic aciduria	propionic aciduria	propionic aciduria	propionic aciduria	propionic aciduria	1	kept
PROPOFOL	propofol	propofol	propofol	propofol	1	case_or_format
PROPOFOL ANESTHESIA	propofol anesthesia	propofol anesthesia	propofol anesthesia	PROPOFOL ANESTHESIA	1	kept
proportion	proportion	proportion	proportion	proportion	1	kept
proportional assist ventilation	proportional assist ventilation	proportional assist ventilation	proportional assist ventilation	proportional assist ventilation	1	kept
proportional distribution	proportional distribution	proportional distribution	proportional distribution	proportional distribution	1	kept
proportional Meta-analysis	proportional meta analysis	proportional meta analysis	proportional meta analysis	proportional Meta-analysis	1	kept
PROPORTIONAL-ASSIST VENTILATION	proportional assist ventilation	proportional assist ventilation	proportional assist ventilation	proportional assist ventilation	1	case_or_format
propranolol derivative	propranolol derivative	propranolol derivative	propranolol derivative	propranolol derivative	1	kept
Prospect	prospect	prospect	prospect	Prospect	1	kept
prospect	prospect	prospect	prospect	Prospect	1	case_or_format
PROSPECTIVE AUDIT	prospective audit	prospective audit	prospective audit	PROSPECTIVE AUDIT	1	kept
PROSPECTIVE COHORT	prospective cohort	prospective cohort	prospective cohort	PROSPECTIVE COHORT	1	kept
prospective cohort	prospective cohort	prospective cohort	prospective cohort	PROSPECTIVE COHORT	1	case_or_format

prospective cohort study	prospective cohort study	prospective cohort study	prospective cohort study	prospective cohort study	1	kept
prospective cost	prospective cost	prospective cost	prospective cost	prospective cost	1	kept
Prospective epidemiological study	prospective epidemiological study	prospective epidemiological study	prospective epidemiological study	Prospective epidemiological study	1	kept
Prospective hospital-acquired pressure ulcer	prospective hospital acquired pressure ulcer	prospective hospital acquired pressure ulcer	prospective hospital acquired pressure ulcer	Prospective hospital-acquired pressure ulcer	1	kept
Prospective interventional study	prospective interventional study	prospective interventional study	prospective interventional study	Prospective interventional study	1	kept
Prospective payment system	prospective payment system	prospective payment system	prospective payment system	Prospective Payment System	1	case_or_format
PROSPECTIVE RANDOMIZED-TRIAL	prospective randomized trial	prospective randomized trial	prospective randomized trial	PROSPECTIVE RANDOMIZED-TRIAL	1	kept
Prospective repeated measures	prospective repeated measures	prospective repeated measures	prospective repeated measures	Prospective repeated measures	1	kept
prospective risk analysis	prospective risk analysis	prospective risk analysis	prospective risk analysis	prospective risk analysis	1	kept
Prospectives	prospectives	prospectives	prospective	Prospective	1	singular
PROSPERO registration #CRD42016049285	prospero registration crd42016049285	prospero registration crd42016049285	prospero registration crd42016049285	PROSPERO registration #CRD42016049285	1	kept
Prostacyclin	prostacyclin	prostacyclin	prostacyclin	prostacyclin	1	case_or_format
prostaglandin blood level	prostaglandin blood level	prostaglandin blood level	prostaglandin blood level	prostaglandin blood level	1	kept
prostaglandin derivative	prostaglandin derivative	prostaglandin derivative	prostaglandin derivative	prostaglandin derivative	1	kept
prostaglandin F2 alpha	prostaglandin f2 alpha	prostaglandin f2 alpha	prostaglandin f2 alpha	prostaglandin F2 alpha	1	kept
Prostaglandins	prostaglandins	prostaglandins	prostaglandin	prostaglandin	1	singular

prostaglandins	prostaglandins	prostaglandins	prostaglandin	prostaglandin	1	singular
Prostaglandins I	prostaglandins i	prostaglandins i	prostaglandins i	Prostaglandins I	1	kept
prostate adenocarcinoma	prostate adenocarcinoma	prostate adenocarcinoma	prostate adenocarcinoma	prostate adenocarcinoma	1	kept
Prostate cancer	prostate cancer	prostate cancer	prostate cancer	prostate cancer	1	case_or_format
prostate carcinoma	prostate carcinoma	prostate carcinoma	prostate carcinoma	prostate carcinoma	1	kept
Prostatic Hyperplasia	prostatic hyperplasia	prostatic hyperplasia	prostatic hyperplasia	Prostatic Hyperplasia	1	kept
Prostatic neoplasm	prostatic neoplasm	prostatic neoplasm	prostatic neoplasm	Prostatic Neoplasms	1	case_or_format
Prostheses and Implants	prostheses and implants	prostheses and implants	prostheses and implants	Prostheses and Implants	1	kept
prosthesis design	prosthesis design	prosthesis design	prosthesis design	Prosthesis Design	1	case_or_format
prosthesis mispositioning	prosthesis mispositioning	prosthesis mispositioning	prosthesis mispositioning	prosthesis mispositioning	1	kept
prosthesis thrombosis	prosthesis thrombosis	prosthesis thrombosis	prosthesis thrombosis	prosthesis thrombosis	1	kept
prosthetic device stenosis	prosthetic device stenosis	prosthetic device stenosis	prosthetic device stenosis	prosthetic device stenosis	1	kept
Protamine	protamine	protamine	protamine	protamine	1	case_or_format
Protease Inhibitors	protease inhibitors	protease inhibitors	protease inhibitors	Protease Inhibitors	1	kept
Protected PCI	protected pci	protected pci	protected pci	Protected PCI	1	kept
protected sampling	protected sampling	protected sampling	protected sampling	protected sampling	1	kept

PROTECTION	protection	protection	protection	protection	1	case_or_format
Protection mechanisms	protection mechanisms	protection mechanisms	protection mechanisms	Protection mechanisms	1	kept
Protections	protections	protections	protection	protection	1	singular
Protective agents	protective agents	protective agents	protective agent	protective agent	1	singular
Protective devices	protective devices	protective devices	protective devices	Protective Devices	1	case_or_format
Protective eyewear	protective eyewear	protective eyewear	protective eyewear	Protective eyewear	1	kept
protective eyewear fogging	protective eyewear fogging	protective eyewear fogging	protective eyewear fogging	protective eyewear fogging	1	kept
Protective factors	protective factors	protective factors	protective factors	Protective Factors	1	case_or_format
protective factors indent: first line: 1.27 cm formatted: [illegible]	protective factors indent first line 1 27 cm formatted normal [illegible]	protective factors indent first line 1 27 cm formatted normal [illegible]	protective factors indent first line 1 27 cm formatted normal [illegible]	protective factors indent: first line: 1.27 cm formatted: [illegible]	1	kept
Protective gloves	protective gloves	protective gloves	protective glove	protective glove	1	singular
Protective health behaviors	protective health behaviors	protective health behaviors	protective health behaviors	Protective health behaviors	1	kept
PROTECTIVE ISOLATION	protective isolation	protective isolation	protective isolation	PROTECTIVE ISOLATION	1	kept
Protective lead garments	protective lead garments	protective lead garments	protective lead garments	Protective lead garments	1	kept
Protective loop ileostomy	protective loop ileostomy	protective loop ileostomy	protective loop ileostomy	Protective loop ileostomy	1	kept
protective loop ileostomy	protective loop ileostomy	protective loop ileostomy	protective loop ileostomy	Protective loop ileostomy	1	case_or_format
Protective lung strategy	protective lung strategy	protective lung strategy	protective lung strategy	Protective lung strategies	1	case_or_format

Protective masks	protective masks	protective masks	protective masks	Protective masks	1	kept
Protective ventilation	protective ventilation	protective ventilation	protective ventilation	protective ventilation	1	case_or_format
PROTECTS	protects	protects	protects	PROTECTS	1	kept
protein analysis	protein analysis	protein analysis	protein analysis	protein analysis	1	kept
protein binding	protein binding	protein binding	protein binding	protein binding	1	kept
Protein Biosynthesis	protein biosynthesis	protein biosynthesis	protein biosynthesis	Protein Biosynthesis	1	kept
Protein C Deficiency	protein c deficiency	protein c deficiency	protein c deficiency	protein C deficiency	1	case_or_format
protein c jun	protein c jun	protein c jun	protein c jun	protein c jun	1	kept
protein concentrate plus carbohydrates plus lipids plus [illegible]	protein concentrate plus carbohydrates plus lipids plus [illegible]	protein concentrate plus carbohydrates plus lipids plus [illegible]	protein concentrate plus carbohydrates plus lipids plus [illegible]	protein concentrate plus carbohydrates plus lipids plus [illegible]	1	kept
protein content	protein content	protein content	protein content	protein content	1	kept
Protein Deficiency	protein deficiency	protein deficiency	protein deficiency	Protein Deficiency	1	kept
Protein deficiency	protein deficiency	protein deficiency	protein deficiency	Protein Deficiency	1	case_or_format
Protein delivery	protein delivery	protein delivery	protein delivery	Protein delivery	1	kept
protein depletion	protein depletion	protein depletion	protein depletion	protein depletion	1	kept
protein determination	protein determination	protein determination	protein determination	protein determination	1	kept
protein electrophoresis	protein electrophoresis	protein electrophoresis	protein electrophoresis	protein electrophoresis	1	kept

protein energy provision	protein energy provision	protein energy provision	protein energy provision	protein energy provision	1	kept
protein histidine kinase	protein histidine kinase	protein histidine kinase	protein histidine kinase	protein histidine kinase	1	kept
Protein intake	protein intake	protein intake	protein intake	protein intake	1	case_or_format
protein ParC	protein parc	protein parc	protein parc	protein ParC	1	kept
protein ParE	protein pare	protein pare	protein pare	protein ParE	1	kept
protein phosphorylation	protein phosphorylation	protein phosphorylation	protein phosphorylation	protein phosphorylation	1	kept
Protein precursors	protein precursors	protein precursors	protein precursor	Protein Precursors	1	singular
protein purification	protein purification	protein purification	protein purification	protein purification	1	kept
Protein requirement	protein requirement	protein requirement	protein requirement	Protein requirement	1	kept
protein requirements	protein requirements	protein requirements	protein requirement	Protein requirement	1	singular
protein S deficiency	protein s deficiency	protein s deficiency	protein s deficiency	protein S deficiency	1	kept
protein supply	protein supply	protein supply	protein supply	protein supply	1	kept
Protein synthesis	protein synthesis	protein synthesis	protein synthesis	protein synthesis	1	case_or_format
protein targeting	protein targeting	protein targeting	protein targeting	protein targeting	1	kept
protein targets	protein targets	protein targets	protein targets	protein targets	1	kept
protein Tbx1	protein tbx1	protein tbx1	protein tbx1	protein Tbx1	1	kept

protein to creatinine ratio	protein to creatinine ratio	protein to creatinine ratio	protein to creatinine ratio	protein to creatinine ratio	1	kept
protein tyrosine phosphatase	protein tyrosine phosphatase	protein tyrosine phosphatase	protein tyrosine phosphatase	protein tyrosine phosphatase	1	kept
Proteolysis	proteolysis	proteolysis	proteolysis	Proteolysis	1	kept
proteolysis	proteolysis	proteolysis	proteolysis	Proteolysis	1	case_or_format
Proteome	proteome	proteome	proteome	Proteome	1	kept
Proteomic	proteomic	proteomic	proteomic	Proteomics	1	case_or_format
protest	protest	protest	protest	protest	1	kept
Proteus infection	proteus infection	proteus infection	proteus infection	Proteus infection	1	kept
Proteus mirabilis infection	proteus mirabilis infection	proteus mirabilis infection	proteus mirabilis infection	Proteus mirabilis infection	1	kept
Proteus vulgaris	proteus vulgaris	proteus vulgaris	proteus vulgaris	Proteus vulgaris	1	kept
Prothrombin	prothrombin	prothrombin	prothrombin	prothrombin	1	case_or_format
Prothrombin complex concentrate	prothrombin complex concentrate	prothrombin complex concentrate	prothrombin complex concentrate	prothrombin complex concentrates	1	case_or_format
prothrombin complex concentrate	prothrombin complex concentrate	prothrombin complex concentrate	prothrombin complex concentrate	prothrombin complex concentrates	1	case_or_format
Prothrombin complex concentrates	prothrombin complex concentrates	prothrombin complex concentrates	prothrombin complex concentrate	prothrombin complex concentrates	1	singular
protionamide	protionamide	protionamide	protionamide	protionamide	1	kept
protirelin	protirelin	protirelin	protirelin	protirelin	1	kept

protocol adherence	protocol adherence	protocol adherence	protocol adherence	protocol adherence	1	kept
Protocol analysis	protocol analysis	protocol analysis	protocol analysis	Protocol analysis	1	kept
Protocol compliance	protocol compliance	protocol compliance	protocol compliance	protocol compliance	1	case_or_format
Protocol development	protocol development	protocol development	protocol development	Protocol development	1	kept
protocol driven	protocol driven	protocol driven	protocol driven	protocol driven	1	kept
protocol implementation	protocol implementation	protocol implementation	protocol implementation	protocol implementation	1	kept
protocol monitoring	protocol monitoring	protocol monitoring	protocol monitoring	protocol monitoring	1	kept
protocol registration	protocol registration	protocol registration	protocol registration	protocol registration	1	kept
Protocol requirements	protocol requirements	protocol requirements	protocol requirements	Protocol requirements	1	kept
protocol-based care	protocol based care	protocol based care	protocol based care	Protocol-based care	1	case_or_format
Protocol-directed weaning	protocol directed weaning	protocol directed weaning	protocol directed weaning	Protocol-directed weaning	1	kept
protocole de coopé ration	protocole de coop ration	protocole de coop ration	protocole de coop ration	protocole de coopé ration	1	kept
protocole de gestion	protocole de gestion	protocole de gestion	protocole de gestion	protocole de gestion	1	kept
Protocolised management	protocolised management	protocolised management	protocolised management	Protocolised management	1	kept
Protocolized vs. non-protocolized weaning	protocolized vs non protocolized weaning	protocolized vs non protocolized weaning	protocolized vs non protocolized weaning	Protocolized vs. non-protocolized weaning	1	kept
Protocolos	protocolos	protocolos	protocolos	Protocolos	1	kept

protocols and guidelines	protocols and guidelines	protocols and guidelines	protocols and guidelines	protocols & guidelines	1	case_or_format
proton	proton	proton	proton	proton	1	kept
proton pump	proton pump	proton pump	proton pump	proton pump	1	kept
PROTON PUMP INHIBITORS	proton pump inhibitors	proton pump inhibitors	proton pump inhibitor	proton pump inhibitor	1	singular
Proton Pumps	proton pumps	proton pumps	proton pump	proton pump	1	singular
proton therapy	proton therapy	proton therapy	proton therapy	proton therapy	1	kept
prototype analysis	prototype analysis	prototype analysis	prototype analysis	prototype analysis	1	kept
prototype BloodNav system	prototype bloodnav system	prototype bloodnav system	prototype bloodnav system	prototype BloodNav system	1	kept
Prototype designs	prototype designs	prototype designs	prototype designs	Prototype designs	1	kept
prototype electronic stethoscope	prototype electronic stethoscope	prototype electronic stethoscope	prototype electronic stethoscope	prototype electronic stethoscope	1	kept
Provide guidances	provide guidances	provide guidances	provide guidances	Provide guidances	1	kept
PROVIDE RELIABLE COUNTS	provide reliable counts	provide reliable counts	provide reliable counts	PROVIDE RELIABLE COUNTS	1	kept
Providencia stuartii	providencia stuartii	providencia stuartii	providencia stuartii	Providencia stuartii	1	kept
PROVIDER	provider	provider	provider	PROVIDERS	1	case_or_format
Provider bias	provider bias	provider bias	provider bias	Provider bias	1	kept
Provider communication	provider communication	provider communication	provider communication	Provider communication	1	kept

Provider in triage	provider in triage	provider in triage	provider in triage	Provider in triage	1	kept
Provider practice	provider practice	provider practice	provider practice	Provider practice	1	kept
provider response time	provider response time	provider response time	provider response time	provider response time	1	kept
provider satisfaction	provider satisfaction	provider satisfaction	provider satisfaction	provider satisfaction	1	kept
Provider stigma	provider stigma	provider stigma	provider stigma	Provider stigma	1	kept
Provider training	provider training	provider training	provider training	Provider training	1	kept
Provider-SDM communication, Qualitative data	provider sdm communication qualitative data	provider sdm communication qualitative data	provider sdm communication qualitative data	Provider-SDM communication, Qualitative data	1	kept
Providers awareness	providers awareness	providers awareness	providers awareness	Providers awareness	1	kept
PROVIDING CARE	providing care	providing care	providing care	PROVIDING CARE	1	kept
provision of care	provision of care	provision of care	provision of care	provision of care	1	kept
Proxies	proxies	proxies	proxy	Proxy	1	singular
Proximal Femoral Fractures	proximal femoral fractures	proximal femoral fractures	proximal femoral fractures	Proximal Femoral Fractures	1	kept
Proximal femoral fractures	proximal femoral fractures	proximal femoral fractures	proximal femoral fractures	Proximal Femoral Fractures	1	case_or_format
proximal femoral replacement	proximal femoral replacement	proximal femoral replacement	proximal femoral replacement	proximal femoral replacement	1	kept
Proximity	proximity	proximity	proximity	proximity	1	case_or_format
proximity in dying	proximity in dying	proximity in dying	proximity in dying	proximity in dying	1	kept

proxy reporters	proxy reporters	proxy reporters	proxy reporters	proxy reporters	1	kept
Proxy score	proxy score	proxy score	proxy score	Proxy score	1	kept
Proxy test for delirium	proxy test for delirium	proxy test for delirium	proxy test for delirium	Proxy test for delirium	1	kept
Proyecto del Corazón de Nápoles	proyecto del coraz n de n poles	proyecto del coraz n de n poles	proyecto del coraz n de n poles	Proyecto del Corazón de Nápoles	1	kept
Proyectos zero	proyectos zero	proyectos zero	proyectos zero	Proyectos zero	1	kept
Prozessverantwortliche Pflege	prozessverantwortliche pflege	prozessverantwortliche pflege	prozessverantwortliche pflege	Prozessverantwortliche Pflege	1	kept
Pruebas de laboratorio	pruebas de laboratorio	pruebas de laboratorio	pruebas de laboratorio	Pruebas de laboratorio	1	kept
Prunus dulcis	prunus dulcis	prunus dulcis	prunus dulcis	Prunus dulcis	1	kept
pruritis	pruritis	pruritis	pruritis	pruritis	1	kept
Práctica avanzada	pr ctica avanzada	pr ctica avanzada	pr ctica avanzada	Práctica avanzada	1	kept
Práctica clínica basada en la evidencia	pr ctica cl nica basada en la evidencia	pr ctica cl nica basada en la evidencia	pr ctica cl nica basada en la evidencia	Práctica clínica basada en la evidencia	1	kept
Práctica colaborativa	pr ctica colaborativa	pr ctica colaborativa	pr ctica colaborativa	Práctica colaborativa	1	kept
Práctica Profesional	pr ctica profesional	pr ctica profesional	pr ctica profesional	Práctica Profesional	1	kept
Prática Avançada de Enfermagem	pr tica avan ada de enfermagem	pr tica avan ada de enfermagem	pr tica avan ada de enfermagem	Prática Avançada de Enfermagem	1	kept
Prática Profissional	pr tica profissional	pr tica profissional	pr tica profissional	Prática Profissional	1	kept
prévention	pr vention	pr vention	pr vention	prévention	1	kept

Psenopsis anomala	psenopsis anomala	psenopsis anomala	psenopsis anomala	Psenopsis anomala	1	kept
Pseudo resistant seizure	pseudo resistant seizure	pseudo resistant seizure	pseudo resistant seizure	Pseudo resistant seizure	1	kept
Pseudo-fusion	pseudo fusion	pseudo fusion	pseudo fusion	Pseudo-fusion	1	kept
Pseudo-notch	pseudo notch	pseudo notch	pseudo notch	Pseudo-notch	1	kept
Pseudo-unplanned extubation	pseudo unplanned extubation	pseudo unplanned extubation	pseudo unplanned extubation	Pseudo-unplanned extubation	1	kept
pseudoallergy	pseudoallergy	pseudoallergy	pseudoallergy	pseudoallergy	1	kept
pseudobulbar palsy	pseudobulbar palsy	pseudobulbar palsy	pseudobulbar palsy	pseudobulbar palsy	1	kept
pseudocyst	pseudocyst	pseudocyst	pseudocyst	pseudocyst	1	kept
Pseudoephedrine	pseudoephedrine	pseudoephedrine	pseudoephedrine	pseudoephedrine	1	case_or_format
pseudohyperglycemia	pseudohyperglycemia	pseudohyperglycemia	pseudohyperglycemia	pseudohyperglycemia	1	kept
pseudohyperkalemia	pseudohyperkalemia	pseudohyperkalemia	pseudohyperkalemia	pseudohyperkalemia	1	kept
Pseudomembranous colitis	pseudomembranous colitis	pseudomembranous colitis	pseudomembranous colitis	pseudomembranous colitis	1	case_or_format
pseudomonas	pseudomonas	pseudomonas	pseudomonas	Pseudomonas	1	case_or_format
Pseudomonas fluorescens	pseudomonas fluorescens	pseudomonas fluorescens	pseudomonas fluorescens	Pseudomonas fluorescens	1	kept
Pseudomonas spp	pseudomonas spp	pseudomonas spp	pseudomonas spp	Pseudomonas spp	1	kept
PSEUDOMONAS-AERUGINOSA BACTEREMIA	pseudomonas aeruginosa bacteremia	pseudomonas aeruginosa bacteremia	pseudomonas aeruginosa bacteremia	PSEUDOMONAS-AERUGINOSA BACTEREMIA	1	kept

PSEUDOMONAS-AERUGINOSA INFECTION	pseudomonas aeruginosa infection	pseudomonas aeruginosa infection	pseudomonas aeruginosa infection	PSEUDOMONAS-AERUGINOSA INFECTION	1	kept
PSEUDOMONAS-CEPACIA	pseudomonas cepacia	pseudomonas cepacia	pseudomonas cepacia	PSEUDOMONAS-CEPACIA	1	kept
pseudopodium	pseudopodium	pseudopodium	pseudopodium	pseudopodium	1	kept
pseudosepsis	pseudosepsis	pseudosepsis	pseudosepsis	pseudosepsis	1	kept
Psicologist counseling	psicologist counseling	psicologist counseling	psicologist counseling	Psicologist counseling	1	kept
psikososyal tepki	psikososyal tepki	psikososyal tepki	psikososyal tepki	psikososyal tepki	1	kept
Psilocybin	psilocybin	psilocybin	psilocybin	Psilocybin	1	kept
PSM	psm	psm	psm	PSM	1	kept
PSS-14	pss 14	pss 14	pss 14	PSS-14	1	kept
PSS:IH stress	pss ih stress	pss ih stress	pss ih stress	PSS:IH stress	1	kept
psychiatric care	psychiatric care	psychiatric care	psychiatric care	psychiatric care	1	kept
Psychiatric care beds	psychiatric care beds	psychiatric care beds	psychiatric care beds	Psychiatric care beds	1	kept
psychiatric department, hospital	psychiatric department hospital	psychiatric department hospital	psychiatric department hospital	Psychiatric Department, Hospital	1	case_or_format
Psychiatric disorder	psychiatric disorder	psychiatric disorder	psychiatric disorder	Psychiatric disorder	1	kept
Psychiatric disorders	psychiatric disorders	psychiatric disorders	psychiatric disorder	Psychiatric disorder	1	singular
PSYCHIATRIC EMERGENCIES	psychiatric emergencies	psychiatric emergencies	psychiatric emergency	psychiatric emergency	1	singular

Psychiatric emergencies	psychiatric emergencies	psychiatric emergencies	psychiatric emergency	psychiatric emergency	1	singular
psychiatric emergency	psychiatric emergency	psychiatric emergency	psychiatric emergency	psychiatric emergency	1	kept
psychiatric emergency department	psychiatric emergency department	psychiatric emergency department	psychiatric emergency department	psychiatric emergency department	1	kept
Psychiatric facility design	psychiatric facility design	psychiatric facility design	psychiatric facility design	Psychiatric facility design	1	kept
psychiatric hospital	psychiatric hospital	psychiatric hospital	psychiatric hospital	psychiatric hospitals	1	case_or_format
Psychiatric illness	psychiatric illness	psychiatric illness	psychiatric illness	Psychiatric illness	1	kept
psychiatric illness	psychiatric illness	psychiatric illness	psychiatric illness	Psychiatric illness	1	case_or_format
PSYCHIATRIC SCREENING QUESTIONNAIRE	psychiatric screening questionnaire	psychiatric screening questionnaire	psychiatric screening questionnaire	PSYCHIATRIC SCREENING QUESTIONNAIRE	1	kept
Psychiatric status rating scales	psychiatric status rating scales	psychiatric status rating scales	psychiatric status rating scales	Psychiatric Status Rating Scales	1	case_or_format
PSYCHIATRIC-DISORDERS	psychiatric disorders	psychiatric disorders	psychiatric disorder	Psychiatric disorder	1	singular
PSYCHIATRIC-HOSPITAL DESIGN	psychiatric hospital design	psychiatric hospital design	psychiatric hospital design	PSYCHIATRIC-HOSPITAL DESIGN	1	kept
PSYCHIATRIC-HOSPITALS	psychiatric hospitals	psychiatric hospitals	psychiatric hospital	psychiatric hospitals	1	singular
Psycho-education	psycho education	psycho education	psycho education	Psycho-education	1	kept
psycho-educational training	psycho educational training	psycho educational training	psycho educational training	psycho-educational training	1	kept
Psycho-emotional states	psycho emotional states	psycho emotional states	psycho emotional states	Psycho-emotional states	1	kept
psycho-neurological development	psycho neurological development	psycho neurological development	psycho neurological development	psycho-neurological development	1	kept

Psycho-Oncology	psycho oncology	psycho oncology	psycho oncology	psycho-oncology	1	case_or_format
Psycho-oncology care	psycho oncology care	psycho oncology care	psycho oncology care	Psycho-oncology care	1	kept
Psycho-social aspects of care	psycho social aspects of care	psycho social aspects of care	psycho social aspects of care	Psycho-social aspects of care	1	kept
Psycho-social support systems	psycho social support systems	psycho social support systems	psycho social support systems	Psycho-social support systems	1	kept
psycho-socio-emotional needs	psycho socio emotional needs	psycho socio emotional needs	psycho socio emotional needs	psycho-socio-emotional needs	1	kept
Psychoacoustics	psychoacoustics	psychoacoustics	psychoacoustics	Psychoacoustics	1	kept
Psychoanalysis	psychoanalysis	psychoanalysis	psychoanalysis	psychoanalysis	1	case_or_format
Psychodrama	psychodrama	psychodrama	psychodrama	Psychodrama	1	kept
psychodynamics	psychodynamics	psychodynamics	psychodynamics	psychodynamics	1	kept
psychodynamics of work	psychodynamics of work	psychodynamics of work	psychodynamics of work	psychodynamics of work	1	kept
psychoemotional stress	psychoemotional stress	psychoemotional stress	psychoemotional stress	psychoemotional stress	1	kept
psychogenic non epileptiform seizures	psychogenic non epileptiform seizures	psychogenic non epileptiform seizures	psychogenic non epileptiform seizures	psychogenic non epileptiform seizures	1	kept
psychogenic nonepileptic seizure	psychogenic nonepileptic seizure	psychogenic nonepileptic seizure	psychogenic nonepileptic seizure	psychogenic nonepileptic seizure	1	kept
psychologic distress	psychologic distress	psychologic distress	psychologic distress	psychologic distress	1	kept
psychologic pressure	psychologic pressure	psychologic pressure	psychologic pressure	psychologic pressure	1	kept
psychologic safety	psychologic safety	psychologic safety	psychologic safety	psychologic safety	1	kept

Psychologic stress	psychologic stress	psychologic stress	psychologic stress	Psychologic stress	1	kept
Psychological Adaptation	psychological adaptation	psychological adaptation	psychological adaptation	Psychological adaptation	1	case_or_format
psychological aid	psychological aid	psychological aid	psychological aid	psychological aid	1	kept
psychological assessment	psychological assessment	psychological assessment	psychological assessment	psychological assessment	1	kept
psychological attributes	psychological attributes	psychological attributes	psychological attributes	psychological attributes	1	kept
psychological balance	psychological balance	psychological balance	psychological balance	psychological balance	1	kept
Psychological burnout	psychological burnout	psychological burnout	psychological burnout	psychological burnout	1	case_or_format
psychological capital appreciation	psychological capital appreciation	psychological capital appreciation	psychological capital appreciation	psychological capital appreciation	1	kept
Psychological care	psychological care	psychological care	psychological care	psychological care	1	case_or_format
psychological development	psychological development	psychological development	psychological development	psychological development	1	kept
Psychological disorder	psychological disorder	psychological disorder	psychological disorder	Psychological disorder	1	kept
Psychological disorders	psychological disorders	psychological disorders	psychological disorder	Psychological disorder	1	singular
psychological disorders	psychological disorders	psychological disorders	psychological disorder	Psychological disorder	1	singular
Psychological Distance	psychological distance	psychological distance	psychological distance	Psychological Distance	1	kept
psychological distance	psychological distance	psychological distance	psychological distance	Psychological Distance	1	case_or_format
Psychological emotion	psychological emotion	psychological emotion	psychological emotion	Psychological emotion	1	kept

Psychological empowerments	psychological empowerments	psychological empowerments	psychological empowerment	Psychological empowerment	1	singular
psychological exhaustion	psychological exhaustion	psychological exhaustion	psychological exhaustion	psychological exhaustion	1	kept
Psychological Factors	psychological factors	psychological factors	psychological factors	Psychological Factors	1	kept
psychological factors	psychological factors	psychological factors	psychological factors	Psychological Factors	1	case_or_format
psychological feedback	psychological feedback	psychological feedback	psychological feedback	psychological feedback	1	kept
Psychological First Aid	psychological first aid	psychological first aid	psychological first aid	psychological first aid	1	case_or_format
psychological goals	psychological goals	psychological goals	psychological goals	psychological goals	1	kept
Psychological hardiness	psychological hardiness	psychological hardiness	psychological hardiness	Psychological hardiness	1	kept
psychological hardiness	psychological hardiness	psychological hardiness	psychological hardiness	Psychological hardiness	1	case_or_format
Psychological Hardiness Questionnaire	psychological hardiness questionnaire	psychological hardiness questionnaire	psychological hardiness questionnaire	Psychological Hardiness Questionnaire	1	kept
Psychological immune competency	psychological immune competency	psychological immune competency	psychological immune competency	Psychological immune competency	1	kept
Psychological inflexibility	psychological inflexibility	psychological inflexibility	psychological inflexibility	Psychological inflexibility	1	kept
psychological inflexibility	psychological inflexibility	psychological inflexibility	psychological inflexibility	Psychological inflexibility	1	case_or_format
PSYCHOLOGICAL INTERVENTION	psychological intervention	psychological intervention	psychological intervention	psychological intervention	1	case_or_format
psychological intervention for emergencies and crises	psychological intervention for emergencies and crises	psychological intervention for emergencies and crises	psychological intervention for emergencies and crises	psychological intervention for emergencies and crises	1	kept
psychological interventions	psychological interventions	psychological interventions	psychological intervention	psychological intervention	1	singular

Psychological issues	psychological issues	psychological issues	psychological issues	Psychological issues	1	kept
Psychological morbidity	psychological morbidity	psychological morbidity	psychological morbidity	Psychological morbidity	1	kept
psychological need satisfaction	psychological need satisfaction	psychological need satisfaction	psychological need satisfaction	psychological need satisfaction	1	kept
psychological needs	psychological needs	psychological needs	psychological needs	Psychological needs	1	case_or_format
Psychological outcomes	psychological outcomes	psychological outcomes	psychological outcome	psychological outcomes	1	singular
psychological pain	psychological pain	psychological pain	psychological pain	psychological pain	1	kept
Psychological preparation	psychological preparation	psychological preparation	psychological preparation	Psychological preparation	1	kept
Psychological Problems	psychological problems	psychological problems	psychological problems	Psychological Problems	1	kept
psychological problems	psychological problems	psychological problems	psychological problems	Psychological Problems	1	case_or_format
Psychological processes	psychological processes	psychological processes	psychological processes	Psychological processes	1	kept
Psychological reactions	psychological reactions	psychological reactions	psychological reactions	Psychological reactions	1	kept
psychological rehabilitation	psychological rehabilitation	psychological rehabilitation	psychological rehabilitation	psychological rehabilitation	1	kept
Psychological Resilience	psychological resilience	psychological resilience	psychological resilience	psychological resilience	1	case_or_format
Psychological resiliency	psychological resiliency	psychological resiliency	psychological resiliency	Psychological resiliency	1	kept
Psychological response	psychological response	psychological response	psychological response	Psychological response	1	kept
psychological responses	psychological responses	psychological responses	psychological response	Psychological response	1	singular

PSYCHOLOGICAL SAFETY	psychological safety	psychological safety	psychological safety	psychological safety	1	case_or_format
Psychological screening	psychological screening	psychological screening	psychological screening	Psychological screening	1	kept
psychological sequelae	psychological sequelae	psychological sequelae	psychological sequelae	psychological sequelae	1	kept
psychological state	psychological state	psychological state	psychological state	psychological state	1	kept
psychological stress reduction model	psychological stress reduction model	psychological stress reduction model	psychological stress reduction model	psychological stress reduction model	1	kept
Psychological Stress Scale	psychological stress scale	psychological stress scale	psychological stress scale	Psychological Stress Scale	1	kept
psychological suffering	psychological suffering	psychological suffering	psychological suffering	psychological suffering	1	kept
psychological support system	psychological support system	psychological support system	psychological support system	psychological support system	1	kept
Psychological tailoring	psychological tailoring	psychological tailoring	psychological tailoring	Psychological tailoring	1	kept
Psychological tests	psychological tests	psychological tests	psychological tests	Psychological Tests	1	case_or_format
Psychological trauma	psychological trauma	psychological trauma	psychological trauma	Psychological Trauma	1	case_or_format
Psychological Trauma and trauma-informed care	psychological trauma and trauma informed care	psychological trauma and trauma informed care	psychological trauma and trauma informed care	Psychological Trauma and trauma-informed care	1	kept
psychological upset	psychological upset	psychological upset	psychological upset	psychological upset	1	kept
Psychological wellbeing	psychological wellbeing	psychological wellbeing	psychological wellbeing	psychological wellbeing	1	case_or_format
psychological wellbeing assessment	psychological wellbeing assessment	psychological wellbeing assessment	psychological wellbeing assessment	psychological wellbeing assessment	1	kept
psychologist dedicated to caregivers	psychologist dedicated to caregivers	psychologist dedicated to caregivers	psychologist dedicated to caregivers	psychologist dedicated to caregivers	1	kept

Psychologists	psychologists	psychologists	psychologist	psychologist	1	singular
psychologue dédié aux soignants	psychologue d di aux soignants	psychologue d di aux soignants	psychologue d di aux soignants	psychologue dédié aux soignants	1	kept
Psychology, Applied	psychology applied	psychology applied	psychology applied	Psychology, Applied	1	kept
Psychology, Clinical	psychology clinical	psychology clinical	psychology clinical	Psychology, Clinical	1	kept
Psychology, Developmental	psychology developmental	psychology developmental	psychology developmental	Psychology, Developmental	1	kept
Psychology, social	psychology social	psychology social	psychology social	Psychology, social	1	kept
Psychometric analysis	psychometric analysis	psychometric analysis	psychometric analysis	Psychometric analysis	1	kept
Psychometric characteristic	psychometric characteristic	psychometric characteristic	psychometric characteristic	Psychometric characteristic	1	kept
PSYCHOMETRIC DEVELOPMENT	psychometric development	psychometric development	psychometric development	PSYCHOMETRIC DEVELOPMENT	1	kept
psychometric evaluation.	psychometric evaluation	psychometric evaluation	psychometric evaluation	PSYCHOMETRIC EVALUATION	1	case_or_format
Psychometric validation	psychometric validation	psychometric validation	psychometric validation	PSYCHOMETRIC VALIDATION	1	case_or_format
psychomotor	psychomotor	psychomotor	psychomotor	psychomotor	1	kept
Psychomotor agitation [therapy]	psychomotor agitation therapy	psychomotor agitation therapy	psychomotor agitation therapy	Psychomotor agitation [therapy]	1	kept
Psychomotor Disorders [prevention & control]	psychomotor disorders prevention and control	psychomotor disorders prevention and control	psychomotor disorders prevention and control	Psychomotor Disorders [prevention & control]	1	kept
psychomotor therapist	psychomotor therapist	psychomotor therapist	psychomotor therapist	psychomotor therapist	1	kept
PSYCHOMOTOR VIGILANCE	psychomotor vigilance	psychomotor vigilance	psychomotor vigilance	PSYCHOMOTOR VIGILANCE	1	kept

psychomotor vigilance task	psychomotor vigilance task	psychomotor vigilance task	psychomotor vigilance task	psychomotor vigilance task	1	kept
Psychopathology	psychopathology	psychopathology	psychopathology	psychopathology	1	case_or_format
PSYCHOPATHY CHECKLIST	psychopathy checklist	psychopathy checklist	psychopathy checklist	PSYCHOPATHY CHECKLIST	1	kept
psychopharmacology	psychopharmacology	psychopharmacology	psychopharmacology	psychopharmacology	1	kept
Psychophysical performance	psychophysical performance	psychophysical performance	psychophysical performance	Psychophysical performance	1	kept
Psychophysics	psychophysics	psychophysics	psychophysics	Psychophysics	1	kept
Psychosocial adjustment	psychosocial adjustment	psychosocial adjustment	psychosocial adjustment	psychosocial adjustment	1	case_or_format
Psychosocial aspects of coping with loss	psychosocial aspects of coping with loss	psychosocial aspects of coping with loss	psychosocial aspects of coping with loss	Psychosocial aspects of coping with loss	1	kept
psychosocial assistance	psychosocial assistance	psychosocial assistance	psychosocial assistance	psychosocial assistance	1	kept
Psychosocial crisis	psychosocial crisis	psychosocial crisis	psychosocial crisis	Psychosocial crisis	1	kept
psychosocial effects	psychosocial effects	psychosocial effects	psychosocial effects	psychosocial effects	1	kept
PSYCHOSOCIAL FACTORS	psychosocial factors	psychosocial factors	psychosocial factors	psychosocial factors	1	case_or_format
psychosocial goals	psychosocial goals	psychosocial goals	psychosocial goals	psychosocial goals	1	kept
Psychosocial health	psychosocial health	psychosocial health	psychosocial health	Psychosocial health	1	kept
psychosocial health	psychosocial health	psychosocial health	psychosocial health	Psychosocial health	1	case_or_format
Psychosocial Health Risks and Resources	psychosocial health risks and resources	psychosocial health risks and resources	psychosocial health risks and resources	Psychosocial Health Risks and Resources	1	kept

Psychosocial health risks and resources	psychosocial health risks and resources	psychosocial health risks and resources	psychosocial health risks and resources	Psychosocial Health Risks and Resources	1	case_or_format
psychosocial impact.	psychosocial impact	psychosocial impact	psychosocial impact	psychosocial impact	1	case_or_format
psychosocial interventions	psychosocial interventions	psychosocial interventions	psychosocial intervention	psychosocial intervention	1	singular
Psychosocial Issues	psychosocial issues	psychosocial issues	psychosocial issues	psychosocial issues	1	case_or_format
psychosocial job stressors	psychosocial job stressors	psychosocial job stressors	psychosocial job stressors	psychosocial job stressors	1	kept
psychosocial life	psychosocial life	psychosocial life	psychosocial life	psychosocial life	1	kept
Psychosocial needs	psychosocial needs	psychosocial needs	psychosocial needs	psychosocial needs	1	case_or_format
Psychosocial recovery	psychosocial recovery	psychosocial recovery	psychosocial recovery	Psychosocial recovery	1	kept
psychosocial rehabilitation	psychosocial rehabilitation	psychosocial rehabilitation	psychosocial rehabilitation	psychosocial rehabilitation	1	kept
psychosocial response	psychosocial response	psychosocial response	psychosocial response	psychosocial response	1	kept
psychosocial stress	psychosocial stress	psychosocial stress	psychosocial stress	Psychosocial stress	1	case_or_format
Psychosocial Support	psychosocial support	psychosocial support	psychosocial support	psychosocial support	1	case_or_format
psychosocial support systems	psychosocial support systems	psychosocial support systems	psychosocial support systems	Psychosocial Support Systems	1	case_or_format
Psychosocial variables	psychosocial variables	psychosocial variables	psychosocial variables	Psychosocial variables	1	kept
psychosocial well‐being	psychosocial well being	psychosocial well being	psychosocial well being	psychosocial well-being	1	case_or_format
PSYCHOSOCIAL WORK FACTORS	psychosocial work factors	psychosocial work factors	psychosocial work factors	PSYCHOSOCIAL WORK FACTORS	1	kept

psychosocial work factors	psychosocial work factors	psychosocial work factors	psychosocial work factors	PSYCHOSOCIAL WORK FACTORS	1	case_or_format
PSYCHOSOCIAL WORK-ENVIRONMENT	psychosocial work environment	psychosocial work environment	psychosocial work environment	Psychosocial work environment	1	case_or_format
psychosomatic complications	psychosomatic complications	psychosomatic complications	psychosomatic complications	psychosomatic complications	1	kept
psychosomatics	psychosomatics	psychosomatics	psychosomatics	psychosomatics	1	kept
PSYCHOTHERAPY	psychotherapy	psychotherapy	psychotherapy	psychotherapy	1	case_or_format
Psychotherapy training	psychotherapy training	psychotherapy training	psychotherapy training	Psychotherapy training	1	kept
Psychotherapy, Brief	psychotherapy brief	psychotherapy brief	psychotherapy brief	Psychotherapy, Brief	1	kept
psychotic disorders	psychotic disorders	psychotic disorders	psychotic disorder	Psychotic Disorders	1	singular
Psychotropic chemicals	psychotropic chemicals	psychotropic chemicals	psychotropic chemicals	Psychotropic chemicals	1	kept
psychotropic drug use	psychotropic drug use	psychotropic drug use	psychotropic drug use	psychotropic drug use	1	kept
Psycosocial support systems	psycosocial support systems	psycosocial support systems	psycosocial support systems	Psycosocial support systems	1	kept
PTA	pta	pta	pta	PTA	1	kept
PTAI	ptai	ptai	ptai	PTAI	1	kept
PTDR	ptdr	ptdr	ptdr	PTDR	1	kept
ptosis	ptosis	ptosis	ptosis	ptosis	1	kept
ptsd	ptsd	ptsd	ptsd	PTSD	1	case_or_format

PTSD COACH	ptsd coach	ptsd coach	ptsd coach	PTSD COACH	1	kept
PTSD symptoms	ptsd symptoms	ptsd symptoms	ptsd symptoms	PTSD symptoms	1	kept
PTT, partial thromboplastin time	ptt partial thromboplastin time	ptt partial thromboplastin time	ptt partial thromboplastin time	PTT, partial thromboplastin time	1	kept
Pubic Bone	pubic bone	pubic bone	pubic bone	Pubic Bone	1	kept
pubic bone	pubic bone	pubic bone	pubic bone	Pubic Bone	1	case_or_format
pubic ramus fracture	pubic ramus fracture	pubic ramus fracture	pubic ramus fracture	pubic ramus fracture	1	kept
pubic symphysis diastasis	pubic symphysis diastasis	pubic symphysis diastasis	pubic symphysis diastasis	pubic symphysis diastasis	1	kept
pubis symphysis	pubis symphysis	pubis symphysis	pubis symphysis	pubis symphysis	1	kept
public and global mental health	public and global mental health	public and global mental health	public and global mental health	public and global mental health	1	kept
Public competition	public competition	public competition	public competition	Public competition	1	kept
public discourse	public discourse	public discourse	public discourse	public discourse	1	kept
Public Facilities	public facilities	public facilities	public facilities	Public Facilities	1	kept
Public Health Administration	public health administration	public health administration	public health administration	Public Health Administration	1	kept
public health and welfare	public health and welfare	public health and welfare	public health and welfare	public health and welfare	1	kept
public health emergency	public health emergency	public health emergency	public health emergency	public health emergencies	1	case_or_format
public health legislation	public health legislation	public health legislation	public health legislation	public health legislation	1	kept

public health message	public health message	public health message	public health message	public health message	1	kept
Public health practice	public health practice	public health practice	public health practice	Public Health Practice	1	case_or_format
public health preparedness	public health preparedness	public health preparedness	public health preparedness	Public health preparedness	1	case_or_format
Public Health Preparedness and Response	public health preparedness and response	public health preparedness and response	public health preparedness and response	Public Health Preparedness and Response	1	kept
Public health problem	public health problem	public health problem	public health problem	public health problem	1	case_or_format
Public health professional	public health professional	public health professional	public health professional	Public health professional	1	kept
public health professional	public health professional	public health professional	public health professional	Public health professional	1	case_or_format
public health shielding policy	public health shielding policy	public health shielding policy	public health shielding policy	public health shielding policy	1	kept
public healthcare	public healthcare	public healthcare	public healthcare	public healthcare	1	kept
Public hospitals in mogadishu-Somalia	public hospitals in mogadishu somalia	public hospitals in mogadishu somalia	public hospitals in mogadishu somalia	Public hospitals in mogadishu-Somalia	1	kept
PUBLIC IMAGE	public image	public image	public image	PUBLIC IMAGE	1	kept
Public mass shooting	public mass shooting	public mass shooting	public mass shooting	Public mass shooting	1	kept
Public policy	public policy	public policy	public policy	public policy	1	case_or_format
Public Relations	public relations	public relations	public relations	public relations	1	case_or_format
PUBLIC SAFETY PERSONNEL	public safety personnel	public safety personnel	public safety personnel	PUBLIC SAFETY PERSONNEL	1	kept
public sector accounting	public sector accounting	public sector accounting	public sector accounting	public sector accounting	1	kept

Public service	public service	public service	public service	Public service	1	kept
public service announcement	public service announcement	public service announcement	public service announcement	public service announcement	1	kept
Public space	public space	public space	public space	Public space	1	kept
public sphere	public sphere	public sphere	public sphere	public sphere	1	kept
Publicaciones científicas	publicaciones cient ficas	publicaciones cient ficas	publicaciones cient ficas	Publicaciones científicas	1	kept
Publication	publication	publication	publication	publication	1	case_or_format
PUBLICATION BIAS	publication bias	publication bias	publication bias	publication bias	1	case_or_format
publications	publications	publications	publication	publication	1	singular
Puducherry	puducherry	puducherry	puducherry	Puducherry	1	kept
puerperal sepsis	puerperal sepsis	puerperal sepsis	puerperal sepsis	puerperal sepsis	1	kept
Puerperal sepsis. Sepsis	puerperal sepsis sepsis	puerperal sepsis sepsis	puerperal sepsis sepsis	Puerperal sepsis. Sepsis	1	kept
Puerperium	puerperium	puerperium	puerperium	puerperium	1	case_or_format
puffy cheek	puffy cheek	puffy cheek	puffy cheek	puffy cheek	1	kept
PUKAT 2.0	pukat 2 0	pukat 2 0	pukat 2 0	PUKAT 2.0	1	kept
pull technique	pull technique	pull technique	pull technique	pull technique	1	kept
pulmistat syrup	pulmistat syrup	pulmistat syrup	pulmistat syrup	pulmistat syrup	1	kept

PULMONARY	pulmonary	pulmonary	pulmonary	Pulmonary	1	case_or_format
pulmonary alveolar proteinosis	pulmonary alveolar proteinosis	pulmonary alveolar proteinosis	pulmonary alveolar proteinosis	Pulmonary Alveolar Proteinosis	1	case_or_format
Pulmonary arterial hypertension	pulmonary arterial hypertension	pulmonary arterial hypertension	pulmonary arterial hypertension	Pulmonary Arterial Hypertension	1	case_or_format
pulmonary artery catheter education	pulmonary artery catheter education	pulmonary artery catheter education	pulmonary artery catheter education	pulmonary artery catheter education	1	kept
pulmonary artery embolectomy	pulmonary artery embolectomy	pulmonary artery embolectomy	pulmonary artery embolectomy	pulmonary artery embolectomy	1	kept
Pulmonary artery pressure	pulmonary artery pressure	pulmonary artery pressure	pulmonary artery pressure	Pulmonary artery pressure	1	kept
pulmonary artery pressure	pulmonary artery pressure	pulmonary artery pressure	pulmonary artery pressure	Pulmonary artery pressure	1	case_or_format
pulmonary artery stenosis	pulmonary artery stenosis	pulmonary artery stenosis	pulmonary artery stenosis	pulmonary artery stenosis	1	kept
Pulmonary Aspergillosis	pulmonary aspergillosis	pulmonary aspergillosis	pulmonary aspergillosis	Pulmonary Aspergillosis	1	kept
Pulmonary atelectasis	pulmonary atelectasis	pulmonary atelectasis	pulmonary atelectasis	Pulmonary Atelectasis	1	case_or_format
Pulmonary benign metastasizing leiomyoma	pulmonary benign metastasizing leiomyoma	pulmonary benign metastasizing leiomyoma	pulmonary benign metastasizing leiomyoma	Pulmonary benign metastasizing leiomyoma	1	kept
Pulmonary capillary wedge pressure	pulmonary capillary wedge pressure	pulmonary capillary wedge pressure	pulmonary capillary wedge pressure	Pulmonary capillary wedge pressure	1	kept
Pulmonary care	pulmonary care	pulmonary care	pulmonary care	Pulmonary care	1	kept
pulmonary care	pulmonary care	pulmonary care	pulmonary care	Pulmonary care	1	case_or_format
pulmonary comorbidities	pulmonary comorbidities	pulmonary comorbidities	pulmonary comorbidities	pulmonary comorbidities	1	kept
pulmonary crackles	pulmonary crackles	pulmonary crackles	pulmonary crackles	pulmonary crackles	1	kept

pulmonary disease	pulmonary disease	pulmonary disease	pulmonary disease	Pulmonary diseases	1	case_or_format
pulmonary disease educator	pulmonary disease educator	pulmonary disease educator	pulmonary disease educator	pulmonary disease educator	1	kept
Pulmonary disease, chronic obstructive	pulmonary disease chronic obstructive	pulmonary disease chronic obstructive	pulmonary disease chronic obstructive	Pulmonary Disease, Chronic Obstructive	1	case_or_format
Pulmonary emboli	pulmonary emboli	pulmonary emboli	pulmonary emboli	Pulmonary emboli	1	kept
Pulmonary embolism response team	pulmonary embolism response team	pulmonary embolism response team	pulmonary embolism response team	Pulmonary embolism response team	1	kept
Pulmonary Embolism Severity Index	pulmonary embolism severity index	pulmonary embolism severity index	pulmonary embolism severity index	Pulmonary Embolism Severity Index	1	kept
Pulmonary function	pulmonary function	pulmonary function	pulmonary function	pulmonary function	1	case_or_format
Pulmonary function test	pulmonary function test	pulmonary function test	pulmonary function test	Pulmonary function test	1	kept
pulmonary function testing	pulmonary function testing	pulmonary function testing	pulmonary function testing	pulmonary function testing	1	kept
Pulmonary function tests	pulmonary function tests	pulmonary function tests	pulmonary function test	Pulmonary function test	1	singular
PULMONARY GRANULOMA-FORMATION	pulmonary granuloma formation	pulmonary granuloma formation	pulmonary granuloma formation	PULMONARY GRANULOMA-FORMATION	1	kept
Pulmonary graphical display	pulmonary graphical display	pulmonary graphical display	pulmonary graphical display	Pulmonary graphical display	1	kept
pulmonary hemorrhages	pulmonary hemorrhages	pulmonary hemorrhages	pulmonary hemorrhages	pulmonary hemorrhages	1	kept
pulmonary hypoplasia	pulmonary hypoplasia	pulmonary hypoplasia	pulmonary hypoplasia	pulmonary hypoplasia	1	kept
pulmonary illness	pulmonary illness	pulmonary illness	pulmonary illness	pulmonary illness	1	kept
PULMONARY INFECTION SCORE	pulmonary infection score	pulmonary infection score	pulmonary infection score	PULMONARY INFECTION SCORE	1	kept

PULMONARY INFECTIONS	pulmonary infections	pulmonary infections	pulmonary infection	Pulmonary infection	1	singular
Pulmonary infections	pulmonary infections	pulmonary infections	pulmonary infection	Pulmonary infection	1	singular
pulmonary intravascular coagulopathy	pulmonary intravascular coagulopathy	pulmonary intravascular coagulopathy	pulmonary intravascular coagulopathy	pulmonary intravascular coagulopathy	1	kept
Pulmonary issues	pulmonary issues	pulmonary issues	pulmonary issues	Pulmonary issues	1	kept
Pulmonary manifestation	pulmonary manifestation	pulmonary manifestation	pulmonary manifestation	Pulmonary manifestation	1	kept
pulmonary metastasis	pulmonary metastasis	pulmonary metastasis	pulmonary metastasis	pulmonary metastasis	1	kept
Pulmonary oedema	pulmonary oedema	pulmonary oedema	pulmonary oedema	Pulmonary oedema	1	kept
pulmonary rehabilitation care	pulmonary rehabilitation care	pulmonary rehabilitation care	pulmonary rehabilitation care	pulmonary rehabilitation care	1	kept
pulmonary sepsis	pulmonary sepsis	pulmonary sepsis	pulmonary sepsis	pulmonary sepsis	1	kept
Pulmonary simulator	pulmonary simulator	pulmonary simulator	pulmonary simulator	Pulmonary simulator	1	kept
pulmonary simulator	pulmonary simulator	pulmonary simulator	pulmonary simulator	Pulmonary simulator	1	case_or_format
Pulmonary Surgical Procedures	pulmonary surgical procedures	pulmonary surgical procedures	pulmonary surgical procedures	Pulmonary Surgical Procedures	1	kept
Pulmonary thromboembolism	pulmonary thromboembolism	pulmonary thromboembolism	pulmonary thromboembolism	Pulmonary thromboembolism	1	kept
Pulmonary thromboendarterectomy	pulmonary thromboendarterectomy	pulmonary thromboendarterectomy	pulmonary thromboendarterectomy	Pulmonary thromboendarterectomy	1	kept
Pulmonary Tuberculosis	pulmonary tuberculosis	pulmonary tuberculosis	pulmonary tuberculosis	Pulmonary Tuberculosis	1	kept
Pulmonary tuberculosis	pulmonary tuberculosis	pulmonary tuberculosis	pulmonary tuberculosis	Pulmonary Tuberculosis	1	case_or_format

pulmonary valve replacement	pulmonary valve replacement	pulmonary valve replacement	pulmonary valve replacement	pulmonary valve replacement	1	kept
Pulmonary Valve Stenosis	pulmonary valve stenosis	pulmonary valve stenosis	pulmonary valve stenosis	pulmonary valve stenosis	1	case_or_format
pulmonary vasodilator medications	pulmonary vasodilator medications	pulmonary vasodilator medications	pulmonary vasodilator medications	pulmonary vasodilator medications	1	kept
pulmonary vein isolation	pulmonary vein isolation	pulmonary vein isolation	pulmonary vein isolation	pulmonary vein isolation	1	kept
pulmonary vein thrombosis	pulmonary vein thrombosis	pulmonary vein thrombosis	pulmonary vein thrombosis	pulmonary vein thrombosis	1	kept
pulmonary ventilation	pulmonary ventilation	pulmonary ventilation	pulmonary ventilation	Pulmonary Ventilation	1	case_or_format
Pulmonary ventilators	pulmonary ventilators	pulmonary ventilators	pulmonary ventilators	Pulmonary ventilators	1	kept
pulmonary ventilatory function	pulmonary ventilatory function	pulmonary ventilatory function	pulmonary ventilatory function	pulmonary ventilatory function	1	kept
Pulmonary wedge pressure	pulmonary wedge pressure	pulmonary wedge pressure	pulmonary wedge pressure	Pulmonary Wedge Pressure	1	case_or_format
pulmonary wheezes	pulmonary wheezes	pulmonary wheezes	pulmonary wheezes	pulmonary wheezes	1	kept
PULMONARY-ARTERY CATHETERIZATION	pulmonary artery catheterization	pulmonary artery catheterization	pulmonary artery catheterization	pulmonary artery catheterization	1	case_or_format
PULMONARY-ARTERY TEMPERATURES	pulmonary artery temperatures	pulmonary artery temperatures	pulmonary artery temperatures	PULMONARY-ARTERY TEMPERATURES	1	kept
Pulmonaryembolism	pulmonaryembolism	pulmonaryembolism	pulmonaryembolism	Pulmonaryembolism	1	kept
Pulmonologist	pulmonologist	pulmonologist	pulmonologist	pulmonologist	1	case_or_format
Pulmonologist care	pulmonologist care	pulmonologist care	pulmonologist care	Pulmonologist care	1	kept
pulsatile flow	pulsatile flow	pulsatile flow	pulsatile flow	pulsatile flow	1	kept

Pulsatile oral stimulation	pulsatile oral stimulation	pulsatile oral stimulation	pulsatile oral stimulation	Pulsatile oral stimulation	1	kept
Pulsatility	pulsatility	pulsatility	pulsatility	Pulsatility	1	kept
Pulsation therapy	pulsation therapy	pulsation therapy	pulsation therapy	Pulsation therapy	1	kept
pulsation therapy	pulsation therapy	pulsation therapy	pulsation therapy	Pulsation therapy	1	case_or_format
Pulse decomposition analysis	pulse decomposition analysis	pulse decomposition analysis	pulse decomposition analysis	Pulse decomposition analysis	1	kept
pulse flush method	pulse flush method	pulse flush method	pulse flush method	pulse flush method	1	kept
PULSE OXIMETRY	pulse oximetry	pulse oximetry	pulse oximetry	pulse oximetry	1	case_or_format
Pulse oximetry monitoring	pulse oximetry monitoring	pulse oximetry monitoring	pulse oximetry monitoring	Pulse oximetry monitoring	1	kept
Pulse oximetry screening	pulse oximetry screening	pulse oximetry screening	pulse oximetry screening	Pulse oximetry screening	1	kept
pulse oximetry screening	pulse oximetry screening	pulse oximetry screening	pulse oximetry screening	Pulse oximetry screening	1	case_or_format
Pulse pressure	pulse pressure	pulse pressure	pulse pressure	pulse pressure	1	case_or_format
Pulse pressure-based monitoring	pulse pressure based monitoring	pulse pressure based monitoring	pulse pressure based monitoring	Pulse pressure-based monitoring	1	kept
Pulse rate	pulse rate	pulse rate	pulse rate	pulse rate	1	case_or_format
Pulse Therapy, Drug	pulse therapy drug	pulse therapy drug	pulse therapy drug	Pulse Therapy, Drug	1	kept
pulse transit time	pulse transit time	pulse transit time	pulse transit time	Pulse transit time	1	case_or_format
Pulse-oximetry	pulse oximetry	pulse oximetry	pulse oximetry	pulse oximetry	1	case_or_format

Pulsed field gel electrophoresis	pulsed field gel electrophoresis	pulsed field gel electrophoresis	pulsed field gel electrophoresis	pulsed field gel electrophoresis	1	case_or_format
pulsed technique	pulsed technique	pulsed technique	pulsed technique	pulsed technique	1	kept
Pulseless electrical activity	pulseless electrical activity	pulseless electrical activity	pulseless electrical activity	pulseless electrical activity	1	case_or_format
PUMP	pump	pump	pump	pump	1	case_or_format
Pump and dump	pump and dump	pump and dump	pump and dump	Pump and dump	1	kept
pump design	pump design	pump design	pump design	pump design	1	kept
Pump parameters	pump parameters	pump parameters	pump parameters	Pump parameters	1	kept
pump power	pump power	pump power	pump power	pump power	1	kept
Pump program	pump program	pump program	pump program	Pump program	1	kept
PUMP TECHNOLOGY	pump technology	pump technology	pump technology	PUMP TECHNOLOGY	1	kept
Pump thrombosis	pump thrombosis	pump thrombosis	pump thrombosis	Pump thrombosis	1	kept
pump thrombosis	pump thrombosis	pump thrombosis	pump thrombosis	Pump thrombosis	1	case_or_format
Pumping	pumping	pumping	pumping	Pumping	1	kept
pumps	pumps	pumps	pump	pump	1	singular
PUNCTURE	puncture	puncture	puncture	Punctures	1	case_or_format
Puntuación de alerta temprana	puntuaci n de alerta temprana	puntuaci n de alerta temprana	puntuaci n de alerta temprana	Puntuación de alerta temprana	1	kept

pupillary assessment	pupillary assessment	pupillary assessment	pupillary assessment	pupillary assessment	1	kept
pupillary function	pupillary function	pupillary function	pupillary function	pupillary function	1	kept
Pupillary light reflex	pupillary light reflex	pupillary light reflex	pupillary light reflex	Pupillary light reflex	1	kept
pupillary light reflex	pupillary light reflex	pupillary light reflex	pupillary light reflex	Pupillary light reflex	1	case_or_format
Pupillary reactivity	pupillary reactivity	pupillary reactivity	pupillary reactivity	Pupillary reactivity	1	kept
Pupillary reflex	pupillary reflex	pupillary reflex	pupillary reflex	Pupillary reflex	1	kept
Pupillary size	pupillary size	pupillary size	pupillary size	Pupillary size	1	kept
Pupillometry	pupillometry	pupillometry	pupillometry	pupillometry	1	case_or_format
PUPKAI-T	pupkai t	pupkai t	pupkai t	PUPKAI-T	1	kept
Puppy	puppy	puppy	puppy	puppy	1	case_or_format
Pure NOTES	pure notes	pure notes	pure notes	Pure NOTES	1	kept
pure tone audiometry	pure tone audiometry	pure tone audiometry	pure tone audiometry	pure tone audiometry	1	kept
purge solution	purge solution	purge solution	purge solution	purge solution	1	kept
Purification	purification	purification	purification	purification	1	case_or_format
Purinergic P2Y Receptor Antagonists	purinergic p2y receptor antagonists	purinergic p2y receptor antagonists	purinergic p2y receptor antagonist	purinergic P2Y receptor antagonist	1	singular
puromycin	puromycin	puromycin	puromycin	puromycin	1	kept

purple urine bag syndrome	purple urine bag syndrome	purple urine bag syndrome	purple urine bag syndrome	purple urine bag syndrome	1	kept
Purpura, Thrombotic Thrombocytopenic	purpura thrombotic thrombocytopenic	purpura thrombotic thrombocytopenic	purpura thrombotic thrombocytopenic	Purpura, Thrombotic Thrombocytopenic	1	kept
purpurin	purpurin	purpurin	purpurin	purpurin	1	kept
pus	pus	pus	pus	pus	1	kept
push pull method	push pull method	push pull method	push pull method	push pull method	1	kept
push-out effect	push out effect	push out effect	push out effect	push-out effect	1	kept
Push-pull	push pull	push pull	push pull	Push-pull	1	kept
push-pull technique	push pull technique	push pull technique	push pull technique	push-pull technique	1	kept
pushing	pushing	pushing	pushing	pushing	1	kept
pustule	pustule	pustule	pustule	pustule	1	kept
pustulosis	pustulosis	pustulosis	pustulosis	pustulosis	1	kept
pustulosis palmoplantaris	pustulosis palmoplantaris	pustulosis palmoplantaris	pustulosis palmoplantaris	pustulosis palmoplantaris	1	kept
pyelectasis	pyelectasis	pyelectasis	pyelectasis	pyelectasis	1	kept
pyeloplasty	pyeloplasty	pyeloplasty	pyeloplasty	pyeloplasty	1	kept
pygopagus	pygopagus	pygopagus	pygopagus	pygopagus	1	kept
Pyloric Antrum	pyloric antrum	pyloric antrum	pyloric antrum	Pyloric Antrum	1	kept

Pyloric atresia	pyloric atresia	pyloric atresia	pyloric atresia	Pyloric atresia	1	kept
Pyloric obstruction	pyloric obstruction	pyloric obstruction	pyloric obstruction	Pyloric obstruction	1	kept
pyloromyotomy	pyloromyotomy	pyloromyotomy	pyloromyotomy	pyloromyotomy	1	kept
pyoderma	pyoderma	pyoderma	pyoderma	pyoderma	1	kept
pyoderma gangrenosum	pyoderma gangrenosum	pyoderma gangrenosum	pyoderma gangrenosum	pyoderma gangrenosum	1	kept
pyopneumothorax	pyopneumothorax	pyopneumothorax	pyopneumothorax	pyopneumothorax	1	kept
Pyramid process	pyramid process	pyramid process	pyramid process	Pyramid process	1	kept
pyramidal nerve cell	pyramidal nerve cell	pyramidal nerve cell	pyramidal nerve cell	pyramidal nerve cell	1	kept
pyramidal tract	pyramidal tract	pyramidal tract	pyramidal tract	pyramidal tract	1	kept
pyran derivative	pyran derivative	pyran derivative	pyran derivative	pyran derivative	1	kept
Pyrans	pyrans	pyrans	pyrans	Pyrans	1	kept
pyrexia	pyrexia	pyrexia	pyrexia	pyrexia	1	kept
pyrexia idiopathica	pyrexia idiopathica	pyrexia idiopathica	pyrexia idiopathica	pyrexia idiopathica	1	kept
pyridoxine deficiency	pyridoxine deficiency	pyridoxine deficiency	pyridoxine deficiency	pyridoxine deficiency	1	kept
pyriform sinus	pyriform sinus	pyriform sinus	pyriform sinus	pyriform sinus	1	kept
pyrosequencing	pyrosequencing	pyrosequencing	pyrosequencing	pyrosequencing	1	kept

Q method	q method	q method	q method	Q method	1	kept
Q wave	q wave	q wave	q wave	Q wave	1	kept
Q, quartile	q quartile	q quartile	q quartile	Q, quartile	1	kept
Q-methodology	q methodology	q methodology	q methodology	Q methodology	1	case_or_format
Q-rounds	q rounds	q rounds	q rounds	Q-rounds	1	kept
QacA	qaca	qaca	qaca	QacA	1	kept
qacA	qaca	qaca	qaca	QacA	1	case_or_format
QASC TRIAL	qasc trial	qasc trial	qasc trial	QASC TRIAL	1	kept
Qassim Region	qassim region	qassim region	qassim region	Qassim Region	1	kept
qatar	qatar	qatar	qatar	Qatar	1	case_or_format
Qazvin	qazvin	qazvin	qazvin	Qazvin	1	kept
qCSI	qcsi	qcsi	qcsi	qCSI	1	kept
qEEG	qeeg	qeeg	qeeg	QEEG	1	case_or_format
QEEW	qeew	qeew	qeew	QEEW	1	kept
QFIFE	qfife	qfife	qfife	QFIFE	1	kept
QI intervention	qi intervention	qi intervention	qi intervention	QI intervention	1	kept

QI project	qi project	qi project	qi project	QI project	1	kept
QNWL	qnwl	qnwl	qnwl	QNWL	1	kept
QoL, Quality of Life	qol quality of life	qol quality of life	qol quality of life	QoL, Quality of Life	1	kept
QR-code	qr code	qr code	qr code	QR-code	1	kept
QRS amplitude	qrs amplitude	qrs amplitude	qrs amplitude	QRS amplitude	1	kept
QRS complexes	qrs complexes	qrs complexes	qrs complex	QRS complex	1	singular
QTc	qtc	qtc	qtc	QTc	1	kept
QTc interval prolongation	qtc interval prolongation	qtc interval prolongation	qtc interval prolongation	QTc interval prolongation	1	kept
quadratus lumborum block	quadratus lumborum block	quadratus lumborum block	quadratus lumborum block	quadratus lumborum block	1	kept
Quadruple aim	quadruple aim	quadruple aim	quadruple aim	Quadruple aim	1	kept
Quail	quail	quail	quail	Quail	1	kept
quail	quail	quail	quail	Quail	1	case_or_format
quailitative study	quailitative study	quailitative study	quailitative study	quailitative study	1	kept
Qualification	qualification	qualification	qualification	Qualification	1	kept
qualification	qualification	qualification	qualification	Qualification	1	case_or_format
Qualification process	qualification process	qualification process	qualification process	Qualification process	1	kept

QUALITATIVE	qualitative	qualitative	qualitative	qualitative	1	case_or_format
qualitative case study	qualitative case study	qualitative case study	qualitative case study	qualitative case study	1	kept
qualitative data	qualitative data	qualitative data	qualitative data	qualitative data	1	kept
Qualitative data analysis	qualitative data analysis	qualitative data analysis	qualitative data analysis	Qualitative data analysis	1	kept
qualitative data analysis	qualitative data analysis	qualitative data analysis	qualitative data analysis	Qualitative data analysis	1	case_or_format
qualitative description	qualitative description	qualitative description	qualitative description	Qualitative description	1	case_or_format
Qualitative descriptive	qualitative descriptive	qualitative descriptive	qualitative descriptive	qualitative descriptive	1	case_or_format
qualitative descriptive enquiry	qualitative descriptive enquiry	qualitative descriptive enquiry	qualitative descriptive enquiry	qualitative descriptive enquiry	1	kept
Qualitative descriptive study	qualitative descriptive study	qualitative descriptive study	qualitative descriptive study	Qualitative descriptive study	1	kept
Qualitative enquiry	qualitative enquiry	qualitative enquiry	qualitative enquiry	Qualitative enquiry	1	kept
qualitative evaluation	qualitative evaluation	qualitative evaluation	qualitative evaluation	Qualitative evaluation	1	case_or_format
Qualitative exploration	qualitative exploration	qualitative exploration	qualitative exploration	Qualitative exploration	1	kept
Qualitative exploratory research	qualitative exploratory research	qualitative exploratory research	qualitative exploratory research	Qualitative exploratory research	1	kept
Qualitative Inhaltsanalyse	qualitative inhaltsanalyse	qualitative inhaltsanalyse	qualitative inhaltsanalyse	Qualitative Inhaltsanalyse	1	kept
Qualitative interpretive methods	qualitative interpretive methods	qualitative interpretive methods	qualitative interpretive methods	Qualitative interpretive methods	1	kept
Qualitative interview	qualitative interview	qualitative interview	qualitative interview	Qualitative interviews	1	case_or_format

qualitative interview study	qualitative interview study	qualitative interview study	qualitative interview study	qualitative interview study	1	kept
Qualitative investigation	qualitative investigation	qualitative investigation	qualitative investigation	Qualitative investigation	1	kept
qualitative metasummary	qualitative metasummary	qualitative metasummary	qualitative metasummary	qualitative metasummary	1	kept
qualitative metasynthesis	qualitative metasynthesis	qualitative metasynthesis	qualitative metasynthesis	qualitative metasynthesis	1	kept
qualitative methodology	qualitative methodology	qualitative methodology	qualitative methodology	Qualitative methodology	1	case_or_format
qualitative multiple case study	qualitative multiple case study	qualitative multiple case study	qualitative multiple case study	qualitative multiple case study	1	kept
Qualitative research design	qualitative research design	qualitative research design	qualitative research design	Qualitative research design	1	kept
qualitative research interviewing	qualitative research interviewing	qualitative research interviewing	qualitative research interviewing	qualitative research interviewing	1	kept
Qualitative research methods	qualitative research methods	qualitative research methods	qualitative research methods	Qualitative research methods	1	kept
Qualitative research, Qualitative studies	qualitative research qualitative studies	qualitative research qualitative studies	qualitative research qualitative studies	Qualitative research, Qualitative studies	1	kept
Qualitative review	qualitative review	qualitative review	qualitative review	qualitative review	1	case_or_format
Qualitative surveys	qualitative surveys	qualitative surveys	qualitative surveys	Qualitative surveys	1	kept
Qualitative Synthesis	qualitative synthesis	qualitative synthesis	qualitative synthesis	Qualitative synthesis	1	case_or_format
qualitative thematic content analysis	qualitative thematic content analysis	qualitative thematic content analysis	qualitative thematic content analysis	Qualitative thematic content analysis	1	case_or_format
Qualitative thematic content analyze	qualitative thematic content analyze	qualitative thematic content analyze	qualitative thematic content analyze	Qualitative thematic content analyze	1	kept
qualitative validity	qualitative validity	qualitative validity	qualitative validity	qualitative validity	1	kept

qualitative, hermeneutics	qualitative hermeneutics	qualitative hermeneutics	qualitative hermeneutics	qualitative, hermeneutics	1	kept
QUALITATIVE-ANALYSIS	qualitative analysis	qualitative analysis	qualitative analysis	qualitative analysis	1	case_or_format
quality & safety	quality and safety	quality and safety	quality and safety	quality and safety	1	case_or_format
quality and safe use of medicines	quality and safe use of medicines	quality and safe use of medicines	quality and safe use of medicines	quality and safe use of medicines	1	kept
Quality and safeties	quality and safeties	quality and safeties	quality and safety	quality and safety	1	singular
Quality and value of life	quality and value of life	quality and value of life	quality and value of life	Quality and value of life	1	kept
quality assessment in healthcare	quality assessment in healthcare	quality assessment in healthcare	quality assessment in healthcare	quality assessment in healthcare	1	kept
Quality Assessment of Diagnostic Accuracy Studies	quality assessment of diagnostic accuracy studies	quality assessment of diagnostic accuracy studies	quality assessment of diagnostic accuracy studies	Quality Assessment of Diagnostic Accuracy Studies	1	kept
QUALITY ASSURANCE	quality assurance	quality assurance	quality assurance	Quality assurance	1	case_or_format
Quality Assurance	quality assurance	quality assurance	quality assurance	Quality assurance	1	case_or_format
quality bundle	quality bundle	quality bundle	quality bundle	quality bundle	1	kept
QUALITY CARE	quality care	quality care	quality care	Quality care	1	case_or_format
Quality care outcomes	quality care outcomes	quality care outcomes	quality care outcomes	Quality care outcomes	1	kept
quality challenges	quality challenges	quality challenges	quality challenges	quality challenges	1	kept
quality champion	quality champion	quality champion	quality champion	quality champion	1	kept
Quality conceptualization	quality conceptualization	quality conceptualization	quality conceptualization	Quality conceptualization	1	kept

Quality end-of-life care	quality end of life care	quality end of life care	quality end of life care	Quality end-of-life care	1	kept
Quality engineering	quality engineering	quality engineering	quality engineering	Quality engineering	1	kept
Quality evaluation index	quality evaluation index	quality evaluation index	quality evaluation index	Quality evaluation index	1	kept
quality guideline of care delivery of preterm and mature	quality guideline of care delivery of preterm and mature	quality guideline of care delivery of preterm and mature	quality guideline of care delivery of preterm and mature	quality guideline of care delivery of preterm and mature	1	kept
quality improvement collaborative	quality improvement collaborative	quality improvement collaborative	quality improvement collaborative	quality improvement collaborative	1	kept
quality improvement effectiveness	quality improvement effectiveness	quality improvement effectiveness	quality improvement effectiveness	quality improvement effectiveness	1	kept
Quality improvement in health care	quality improvement in health care	quality improvement in health care	quality improvement in health care	Quality improvement in health care	1	kept
quality improvement initiative	quality improvement initiative	quality improvement initiative	quality improvement initiative	quality improvement initiative	1	kept
quality improvement interventions	quality improvement interventions	quality improvement interventions	quality improvement interventions	quality improvement interventions	1	kept
quality improvement methods	quality improvement methods	quality improvement methods	quality improvement methods	quality improvement methods	1	kept
quality improvement pediatrics	quality improvement pediatrics	quality improvement pediatrics	quality improvement pediatrics	quality improvement pediatrics	1	kept
QUALITY IMPROVEMENT PROGRAM	quality improvement program	quality improvement program	quality improvement program	Quality improvement program	1	case_or_format
QUALITY IMPROVEMENT PROJECT	quality improvement project	quality improvement project	quality improvement project	quality improvement project	1	case_or_format
Quality Improvements	quality improvements	quality improvements	quality improvement	Quality Improvement	1	singular
Quality improvements	quality improvements	quality improvements	quality improvement	Quality Improvement	1	singular
quality improvements	quality improvements	quality improvements	quality improvement	Quality Improvement	1	singular

Quality imrovement	quality imrovement	quality imrovement	quality imrovement	Quality imrovement	1	kept
Quality In Health Care	quality in health care	quality in health care	quality in health care	quality in health care	1	case_or_format
quality In healthcare	quality in healthcare	quality in healthcare	quality in healthcare	quality in healthcare	1	case_or_format
Quality Indicator	quality indicator	quality indicator	quality indicator	Quality indicators	1	case_or_format
Quality indicators in health care	quality indicators in health care	quality indicators in health care	quality indicators in health care	Quality indicators in health care	1	kept
Quality indicators, Health care	quality indicators health care	quality indicators health care	quality indicators health care	Quality Indicators, Health Care	1	case_or_format
Quality indicators - health care	quality indicators health care	quality indicators health care	quality indicators health care	Quality Indicators, Health Care	1	case_or_format
Quality initiative	quality initiative	quality initiative	quality initiative	quality initiative	1	case_or_format
Quality initiatives	quality initiatives	quality initiatives	quality initiative	quality initiative	1	singular
Quality inspection of health care	quality inspection of health care	quality inspection of health care	quality inspection of health care	Quality inspection of health care	1	kept
QUALITY MANAGEMENT	quality management	quality management	quality management	Quality management	1	case_or_format
Quality Management	quality management	quality management	quality management	Quality management	1	case_or_format
Quality management system	quality management system	quality management system	quality management system	Quality management system	1	kept
Quality measure	quality measure	quality measure	quality measure	quality measures	1	case_or_format
Quality measurement	quality measurement	quality measurement	quality measurement	quality measurement	1	case_or_format
Quality metrics	quality metrics	quality metrics	quality metrics	Quality metrics	1	kept

Quality monitoring	quality monitoring	quality monitoring	quality monitoring	Quality monitoring	1	kept
Quality newborn care	quality newborn care	quality newborn care	quality newborn care	Quality newborn care	1	kept
Quality of blood glucose control	quality of blood glucose control	quality of blood glucose control	quality of blood glucose control	Quality of blood glucose control	1	kept
Quality of care indicators	quality of care indicators	quality of care indicators	quality of care indicators	Quality of care indicators	1	kept
quality of care provision	quality of care provision	quality of care provision	quality of care provision	quality of care provision	1	kept
quality of communication	quality of communication	quality of communication	quality of communication	quality of communication	1	kept
Quality of context	quality of context	quality of context	quality of context	Quality of context	1	kept
Quality of contexts	quality of contexts	quality of contexts	quality of context	Quality of context	1	singular
Quality of death and dying	quality of death and dying	quality of death and dying	quality of death and dying	Quality of death and dying	1	kept
Quality of documentation	quality of documentation	quality of documentation	quality of documentation	Quality of documentation	1	kept
Quality of dying	quality of dying	quality of dying	quality of dying	quality of dying	1	case_or_format
Quality of Dying and Death Score	quality of dying and death score	quality of dying and death score	quality of dying and death score	Quality of Dying and Death Score	1	kept
quality of end-of-life care	quality of end of life care	quality of end of life care	quality of end of life care	quality of end-of-life care	1	kept
QUALITY OF HEALTH CARE	quality of health care	quality of health care	quality of health care	Quality of Health Care	1	case_or_format
Quality of life at the end of life	quality of life at the end of life	quality of life at the end of life	quality of life at the end of life	Quality of life at the end of life	1	kept
quality of life in dementia questionnaire	quality of life in dementia questionnaire	quality of life in dementia questionnaire	quality of life in dementia questionnaire	quality of life in dementia questionnaire	1	kept

Quality of Life Index	quality of life index	quality of life index	quality of life index	Quality of Life Index	1	kept
quality of life index	quality of life index	quality of life index	quality of life index	Quality of Life Index	1	case_or_format
Quality of Life Questionnaire	quality of life questionnaire	quality of life questionnaire	quality of life questionnaire	Quality of Life Questionnaire	1	kept
quality of professional life	quality of professional life	quality of professional life	quality of professional life	quality of professional life	1	kept
quality of service	quality of service	quality of service	quality of service	Quality of service	1	case_or_format
Quality of Service constraints	quality of service constraints	quality of service constraints	quality of service constraints	Quality of Service constraints	1	kept
quality of services	quality of services	quality of services	quality of service	Quality of service	1	singular
Quality of the guidelines	quality of the guidelines	quality of the guidelines	quality of the guidelines	Quality of the guidelines	1	kept
quality of work environment	quality of work environment	quality of work environment	quality of work environment	quality of work environment	1	kept
quality performance	quality performance	quality performance	quality performance	quality performance	1	kept
Quality performance measures	quality performance measures	quality performance measures	quality performance measures	Quality performance measures	1	kept
Quality protocol	quality protocol	quality protocol	quality protocol	Quality protocol	1	kept
Quality requirement framework	quality requirement framework	quality requirement framework	quality requirement framework	Quality requirement framework	1	kept
quality review	quality review	quality review	quality review	quality review	1	kept
Quality safety	quality safety	quality safety	quality safety	Quality safety	1	kept
Quality standards	quality standards	quality standards	quality standards	quality standards	1	case_or_format

quality standards in health	quality standards in health	quality standards in health	quality standards in health	quality standards in health	1	kept
quality work environments	quality work environments	quality work environments	quality work environments	quality work environments	1	kept
Quality - hospital staff	quality hospital staff	quality hospital staff	quality hospital staff	Quality - hospital staff	1	kept
Quality-adjusted life years	quality adjusted life years	quality adjusted life years	quality adjusted life year	quality adjusted life year	1	singular
quality-of-care	quality of care	quality of care	quality of care	Quality of care	1	case_or_format
Quality-of-service	quality of service	quality of service	quality of service	Quality of service	1	case_or_format
QualitySteps	qualitysteps	qualitysteps	qualitysteps	QualitySteps	1	kept
Qualitä tsentwicklungsprojekt	qualit tsentwicklungsprojekt	qualit tsentwicklungsprojekt	qualit tsentwicklungsprojekt	Qualitä tsentwicklungsprojekt	1	kept
qualité	qualit	qualit	qualit	qualité	1	kept
qualité de vie	qualit de vie	qualit de vie	qualit de vie	qualité de vie	1	kept
quantifying care provision	quantifying care provision	quantifying care provision	quantifying care provision	quantifying care provision	1	kept
Quantitative approaches	quantitative approaches	quantitative approaches	quantitative approaches	quantitative approaches	1	case_or_format
Quantitative assessments	quantitative assessments	quantitative assessments	quantitative assessments	Quantitative assessments	1	kept
Quantitative description	quantitative description	quantitative description	quantitative description	Quantitative description	1	kept
Quantitative design	quantitative design	quantitative design	quantitative design	Quantitative design	1	kept
quantitative design	quantitative design	quantitative design	quantitative design	Quantitative design	1	case_or_format

quantitative electroencephalogram	quantitative electroencephalogram	quantitative electroencephalogram	quantitative electroencephalogram	quantitative electroencephalogram	1	kept
quantitative fit testing	quantitative fit testing	quantitative fit testing	quantitative fit testing	quantitative fit testing	1	kept
quantitative imaging biomarkers	quantitative imaging biomarkers	quantitative imaging biomarkers	quantitative imaging biomarkers	quantitative imaging biomarkers	1	kept
quantitative method	quantitative method	quantitative method	quantitative method	Quantitative method	1	case_or_format
Quantitative methodology	quantitative methodology	quantitative methodology	quantitative methodology	Quantitative methodology	1	kept
quantitative modeling	quantitative modeling	quantitative modeling	quantitative modeling	quantitative modeling	1	kept
quantitative monitoring	quantitative monitoring	quantitative monitoring	quantitative monitoring	quantitative monitoring	1	kept
QUANTITATIVE PUPILLOMETRY	quantitative pupillometry	quantitative pupillometry	quantitative pupillometry	QUANTITATIVE PUPILLOMETRY	1	kept
Quantitative Research	quantitative research	quantitative research	quantitative research	quantitative research	1	case_or_format
quantitative text analysis	quantitative text analysis	quantitative text analysis	quantitative text analysis	quantitative text analysis	1	kept
QUANTITATIVE ULTRASOUND	quantitative ultrasound	quantitative ultrasound	quantitative ultrasound	QUANTITATIVE ULTRASOUND	1	kept
Quantum-learning methodology	quantum learning methodology	quantum learning methodology	quantum learning methodology	Quantum-learning methodology	1	kept
quarantine policies	quarantine policies	quarantine policies	quarantine policies	quarantine policies	1	kept
quarter prone	quarter prone	quarter prone	quarter prone	quarter prone	1	kept
Quasi experimental	quasi experimental	quasi experimental	quasi experimental	quasi-experimental	1	case_or_format
quasi experimental	quasi experimental	quasi experimental	quasi experimental	quasi-experimental	1	case_or_format

quasi experimental evaluation	quasi experimental evaluation	quasi experimental evaluation	quasi experimental evaluation	quasi experimental evaluation	1	kept
Quasi-experimental	quasi experimental	quasi experimental	quasi experimental	quasi-experimental	1	case_or_format
quasiexperimental	quasiexperimental	quasiexperimental	quasiexperimental	quasiexperimental	1	kept
quaternary care medical facility	quaternary care medical facility	quaternary care medical facility	quaternary care medical facility	quaternary care medical facility	1	kept
Quebec [Canada]	quebec canada	quebec canada	quebec canada	Quebec [Canada]	1	kept
Quemadura	quemadura	quemadura	quemadura	Quemadura	1	kept
Questioning	questioning	questioning	questioning	Questioning	1	kept
questioning attitude	questioning attitude	questioning attitude	questioning attitude	questioning attitude	1	kept
QUESTIONNAIRE CHQ	questionnaire chq	questionnaire chq	questionnaire chq	QUESTIONNAIRE CHQ	1	kept
questionnaire for impulsive compulsive disorders in parkinson	questionnaire for impulsive compulsive disorders in parkinson	questionnaire for impulsive compulsive disorders in parkinson	questionnaire for impulsive compulsive disorders in parkinson	questionnaire for impulsive compulsive disorders in parkinson	1	kept
questionnaire study	questionnaire study	questionnaire study	questionnaire study	questionnaire study	1	kept
Questionnaire survey design	questionnaire survey design	questionnaire survey design	questionnaire survey design	Questionnaire survey design	1	kept
Questionnaire validation	questionnaire validation	questionnaire validation	questionnaire validation	Questionnaire validation	1	kept
questionnaire validation	questionnaire validation	questionnaire validation	questionnaire validation	Questionnaire validation	1	case_or_format
Questionnaires and surveys	questionnaires and surveys	questionnaires and surveys	questionnaires and surveys	Questionnaires and surveys	1	kept
Questionnaires of Impact of Event Scale	questionnaires of impact of event scale	questionnaires of impact of event scale	questionnaires of impact of event scale	Questionnaires of Impact of Event Scale	1	kept

Quetiapine	quetiapine	quetiapine	quetiapine	quetiapine	1	case_or_format
QUEUING THEORY	queuing theory	queuing theory	queuing theory	QUEUING THEORY	1	kept
queuing theory	queuing theory	queuing theory	queuing theory	QUEUING THEORY	1	case_or_format
Quick-dischange wards	quick dischange wards	quick dischange wards	quick dischange wards	Quick-dischange wards	1	kept
quick-Sequential Organ Failure Assessment criteria	quick sequential organ failure assessment criteria	quick sequential organ failure assessment criteria	quick sequential organ failure assessment criteria	quick-Sequential Organ Failure Assessment criteria	1	kept
quiet-time	quiet time	quiet time	quiet time	Quiet time	1	case_or_format
quilt	quilt	quilt	quilt	quilt	1	kept
quinidine	quinidine	quinidine	quinidine	quinidine	1	kept
quinidine gluconate	quinidine gluconate	quinidine gluconate	quinidine gluconate	quinidine gluconate	1	kept
quinidine polygalacturonate	quinidine polygalacturonate	quinidine polygalacturonate	quinidine polygalacturonate	quinidine polygalacturonate	1	kept
Quinolines	quinolines	quinolines	quinolines	Quinolines	1	kept
quinoxaline derivative	quinoxaline derivative	quinoxaline derivative	quinoxaline derivative	quinoxaline derivative	1	kept
Quinoxalines	quinoxalines	quinoxalines	quinoxalines	Quinoxalines	1	kept
QUIZ	quiz	quiz	quiz	QUIZ	1	kept
QUIZ KOP-Q	quiz kop q	quiz kop q	quiz kop q	QUIZ KOP-Q	1	kept
quota sample	quota sample	quota sample	quota sample	quota sample	1	kept

Qur'an reader	qur an reader	qur an reader	qur an reader	Qur'an reader	1	kept
Qur'an recitation	qur an recitation	qur an recitation	qur an recitation	Qur'an recitation	1	kept
Quran recitation	quran recitation	quran recitation	quran recitation	Quran recitation	1	kept
Quranic therapy	quranic therapy	quranic therapy	quranic therapy	Quranic therapy	1	kept
R F M, Respiratory Function Monitoring	r f m respiratory function monitoring	r f m respiratory function monitoring	r f m respiratory function monitoring	R F M, Respiratory Function Monitoring	1	kept
R software	r software	r software	r software	R software	1	kept
R&B = room and board	r and b room and board	r and b room and board	r and b room and board	R&B = room and board	1	kept
R-KMC, Reverse kangaroo mother care	r kmc reverse kangaroo mother care	r kmc reverse kangaroo mother care	r kmc reverse kangaroo mother care	R-KMC, Reverse kangaroo mother care	1	kept
RAAA Timeout	raaa timeout	raaa timeout	raaa timeout	RAAA Timeout	1	kept
Rabbits	rabbits	rabbits	rabbits	Rabbits	1	kept
rabies immunoglobulin	rabies immunoglobulin	rabies immunoglobulin	rabies immunoglobulin	rabies immunoglobulin	1	kept
Rabies Vaccines	rabies vaccines	rabies vaccines	rabies vaccine	rabies vaccine	1	singular
RAC	rac	rac	rac	RAC	1	kept
Race Disparities	race disparities	race disparities	race disparities	Race Disparities	1	kept
Race factors	race factors	race factors	race factors	Race factors	1	kept
race factors	race factors	race factors	race factors	Race factors	1	case_or_format

Racemic adrenaline	racemic adrenaline	racemic adrenaline	racemic adrenaline	Racemic adrenaline	1	kept
racepinefrine	racepinefrine	racepinefrine	racepinefrine	racepinefrine	1	kept
Racepinephrine	racepinephrine	racepinephrine	racepinephrine	Racepinephrine	1	kept
racial and ethnic diversity	racial and ethnic diversity	racial and ethnic diversity	racial and ethnic diversity	racial and ethnic diversity	1	kept
racial discrimination	racial discrimination	racial discrimination	racial discrimination	racial discrimination	1	kept
Racial disparity	racial disparity	racial disparity	racial disparity	racial disparity	1	case_or_format
Racial groups	racial groups	racial groups	racial groups	Racial Groups	1	case_or_format
racial identity	racial identity	racial identity	racial identity	racial identity	1	kept
RACIAL-DIFFERENCES	racial differences	racial differences	racial differences	racial differences	1	case_or_format
radar	radar	radar	radar	radar	1	kept
Radial angiography	radial angiography	radial angiography	radial angiography	Radial angiography	1	kept
Radial artery cannulation	radial artery cannulation	radial artery cannulation	radial artery cannulation	Radial artery cannulation	1	kept
radial artery occlusion	radial artery occlusion	radial artery occlusion	radial artery occlusion	radial artery occlusion	1	kept
Radial artery puncture	radial artery puncture	radial artery puncture	radial artery puncture	Radial artery puncture	1	kept
radial forearm flap	radial forearm flap	radial forearm flap	radial forearm flap	radial forearm flap	1	kept
Radial Forearm Free Flap	radial forearm free flap	radial forearm free flap	radial forearm free flap	Radial Forearm Free Flap	1	kept

RADIANT	radiant	radiant	radiant	RADIANT	1	kept
Radiant heat exchange	radiant heat exchange	radiant heat exchange	radiant heat exchange	Radiant heat exchange	1	kept
radiant warmer	radiant warmer	radiant warmer	radiant warmer	Radiant warmer	1	case_or_format
radiation beam	radiation beam	radiation beam	radiation beam	radiation beam	1	kept
radiation burn	radiation burn	radiation burn	radiation burn	radiation burn	1	kept
radiation dose reduction	radiation dose reduction	radiation dose reduction	radiation dose reduction	radiation dose reduction	1	kept
Radiation Dosimeters	radiation dosimeters	radiation dosimeters	radiation dosimeters	Radiation Dosimeters	1	kept
radiation field	radiation field	radiation field	radiation field	radiation field	1	kept
radiation induced neoplasm	radiation induced neoplasm	radiation induced neoplasm	radiation induced neoplasm	radiation induced neoplasm	1	kept
radiation physics	radiation physics	radiation physics	radiation physics	radiation physics	1	kept
radiation pneumonia	radiation pneumonia	radiation pneumonia	radiation pneumonia	radiation pneumonia	1	kept
Radiation Therapy	radiation therapy	radiation therapy	radiation therapy	radiation therapy	1	case_or_format
RADIATION-DOSE BENCHMARKS	radiation dose benchmarks	radiation dose benchmarks	radiation dose benchmarks	RADIATION-DOSE BENCHMARKS	1	kept
Radiation-Protective Agents	radiation protective agents	radiation protective agents	radiation protective agents	Radiation-Protective Agents	1	kept
Radical cystectomy	radical cystectomy	radical cystectomy	radical cystectomy	radical cystectomy	1	case_or_format
radical nephroureterectomy	radical nephroureterectomy	radical nephroureterectomy	radical nephroureterectomy	radical nephroureterectomy	1	kept

radical prostatectomy	radical prostatectomy	radical prostatectomy	radical prostatectomy	radical prostatectomy	1	kept
radio frequency identification device	radio frequency identification device	radio frequency identification device	radio frequency identification device	Radio Frequency Identification Device	1	case_or_format
Radio frequency identification technology	radio frequency identification technology	radio frequency identification technology	radio frequency identification technology	Radio frequency identification technology	1	kept
Radio Waves	radio waves	radio waves	radio waves	Radio Waves	1	kept
Radio-frequency identification device technologies	radio frequency identification device technologies	radio frequency identification device technologies	radio frequency identification device technologies	Radio-frequency identification device technologies	1	kept
Radio-opaque marker	radio opaque marker	radio opaque marker	radio opaque marker	Radio-opaque marker	1	kept
Radioactive	radioactive	radioactive	radioactive	Radioactive	1	kept
radioallergosorbent test	radioallergosorbent test	radioallergosorbent test	radioallergosorbent test	radioallergosorbent test	1	kept
RADIOCONTRAST-INDUCED NEPHROPATHY	radiocontrast induced nephropathy	radiocontrast induced nephropathy	radiocontrast induced nephropathy	RADIOCONTRAST-INDUCED NEPHROPATHY	1	kept
radioembolization	radioembolization	radioembolization	radioembolization	radioembolization	1	kept
Radiofrequency Ablation	radiofrequency ablation	radiofrequency ablation	radiofrequency ablation	radiofrequency ablation	1	case_or_format
radiographers	radiographers	radiographers	radiographer	radiographer	1	singular
Radiographic assessment	radiographic assessment	radiographic assessment	radiographic assessment	Radiographic assessment	1	kept
RADIOGRAPHIC DETECTION	radiographic detection	radiographic detection	radiographic detection	RADIOGRAPHIC DETECTION	1	kept
Radiographic Image Interpretation, Computer-Assisted	radiographic image interpretation computer assisted	radiographic image interpretation computer assisted	radiographic image interpretation computer assisted	Radiographic Image Interpretation, Computer-Assisted	1	kept
Radiographic imaging	radiographic imaging	radiographic imaging	radiographic imaging	Radiographic imaging	1	kept

Radiographs	radiographs	radiographs	radiographs	Radiographs	1	kept
radiographs	radiographs	radiographs	radiographs	Radiographs	1	case_or_format
Radiograpy	radiograpy	radiograpy	radiograpy	Radiograpy	1	kept
Radioimmunoassay	radioimmunoassay	radioimmunoassay	radioimmunoassay	Radioimmunoassay	1	kept
radioimmunoassay	radioimmunoassay	radioimmunoassay	radioimmunoassay	Radioimmunoassay	1	case_or_format
Radiological staff	radiological staff	radiological staff	radiological staff	Radiological staff	1	kept
radiologist assistant	radiologist assistant	radiologist assistant	radiologist assistant	radiologist assistant	1	kept
Radiology department	radiology department	radiology department	radiology department	radiology department	1	case_or_format
Radiology procedure	radiology procedure	radiology procedure	radiology procedure	Radiology procedure	1	kept
Radiology staff	radiology staff	radiology staff	radiology staff	Radiology staff	1	kept
radiometric dating	radiometric dating	radiometric dating	radiometric dating	radiometric dating	1	kept
radiomics	radiomics	radiomics	radiomics	radiomics	1	kept
Radionuclide Imaging	radionuclide imaging	radionuclide imaging	radionuclide imaging	Radionuclide Imaging	1	kept
Radiopharmaceutical	radiopharmaceutical	radiopharmaceutical	radiopharmaceutical	Radiopharmaceuticals	1	case_or_format
radiotherapist	radiotherapist	radiotherapist	radiotherapist	radiotherapist	1	kept
Radiotherapy Dosage	radiotherapy dosage	radiotherapy dosage	radiotherapy dosage	Radiotherapy Dosage	1	kept

radiotherapy dosage	radiotherapy dosage	radiotherapy dosage	radiotherapy dosage	Radiotherapy Dosage	1	case_or_format
Radiotherapy Planning, Computer-Assisted	radiotherapy planning computer assisted	radiotherapy planning computer assisted	radiotherapy planning computer assisted	Radiotherapy Planning, Computer-Assisted	1	kept
radius fracture	radius fracture	radius fracture	radius fracture	radius fracture	1	kept
RAFAELA®	rafaela	rafaela	rafaela	RAFAELA®	1	kept
Rahim Organizational Conflict Inventory II	rahim organizational conflict inventory ii	rahim organizational conflict inventory ii	rahim organizational conflict inventory ii	Rahim Organizational Conflict Inventory II	1	kept
rail roading technique	rail roading technique	rail roading technique	rail roading technique	rail roading technique	1	kept
rain forest	rain forest	rain forest	rain forest	rain forest	1	kept
RAIN questionnaire	rain questionnaire	rain questionnaire	rain questionnaire	RAIN questionnaire	1	kept
raised intracranial pressure	raised intracranial pressure	raised intracranial pressure	raised intracranial pressure	raised intracranial pressure	1	kept
RAISING GRANDCHILDREN	raising grandchildren	raising grandchildren	raising grandchildren	RAISING GRANDCHILDREN	1	kept
raisonnement clinique	raisonnement clinique	raisonnement clinique	raisonnement clinique	raisonnement clinique	1	kept
Ralstonia	ralstonia	ralstonia	ralstonia	Ralstonia	1	kept
Ramadan fasting	ramadan fasting	ramadan fasting	ramadan fasting	Ramadan fasting	1	kept
Ramp up rapid response system	ramp up rapid response system	ramp up rapid response system	ramp up rapid response system	Ramp up rapid response system	1	kept
ramsay	ramsay	ramsay	ramsay	ramsay	1	kept
Ramsay Scale	ramsay scale	ramsay scale	ramsay scale	Ramsay scale	1	case_or_format

ramsay scale	ramsay scale	ramsay scale	ramsay scale	Ramsay scale	1	case_or_format
Ramsay sedation score	ramsay sedation score	ramsay sedation score	ramsay sedation score	Ramsay sedation score	1	kept
Ramsey Mobilization Protocol	ramsey mobilization protocol	ramsey mobilization protocol	ramsey mobilization protocol	Ramsey Mobilization Protocol	1	kept
Ramsey mobilization protocol	ramsey mobilization protocol	ramsey mobilization protocol	ramsey mobilization protocol	Ramsey Mobilization Protocol	1	case_or_format
ramucirumab	ramucirumab	ramucirumab	ramucirumab	ramucirumab	1	kept
RAND	rand	rand	rand	RAND	1	kept
RAND 36 questionnaire	rand 36 questionnaire	rand 36 questionnaire	rand 36 questionnaire	RAND 36 questionnaire	1	kept
RAND-36	rand 36	rand 36	rand 36	RAND-36	1	kept
random audit	random audit	random audit	random audit	random audit	1	kept
Random effects,benchmarking	random effects benchmarking	random effects benchmarking	random effects benchmarking	Random effects,benchmarking	1	kept
Random processes	random processes	random processes	random processes	Random processes	1	kept
random walk	random walk	random walk	random walk	random walk	1	kept
Randomised control trial	randomised control trial	randomised control trial	randomised control trial	Randomised control trial	1	kept
randomised control trial	randomised control trial	randomised control trial	randomised control trial	Randomised control trial	1	case_or_format
Randomised controlled trail	randomised controlled trail	randomised controlled trail	randomised controlled trail	Randomised controlled trail	1	kept
Randomised Controlled Trial	randomised controlled trial	randomised controlled trial	randomised controlled trial	Randomised controlled trial	1	case_or_format

Randomised Controlled Trials	randomised controlled trials	randomised controlled trials	randomised controlled trial	Randomised controlled trial	1	singular
Randomised trial	randomised trial	randomised trial	randomised trial	Randomised trial	1	kept
Randomized control trial	randomized control trial	randomized control trial	randomized control trial	Randomized control trial	1	kept
randomized controlled pragmatic trial	randomized controlled pragmatic trial	randomized controlled pragmatic trial	randomized controlled pragmatic trial	randomized controlled pragmatic trial	1	kept
RANDOMIZED CONTROLLED TRIAL	randomized controlled trial	randomized controlled trial	randomized controlled trial	randomized controlled trial	1	case_or_format
randomized controlled trial as topic	randomized controlled trial as topic	randomized controlled trial as topic	randomized controlled trial as topic	randomized controlled trial as topic	1	kept
randomized controlled trial study	randomized controlled trial study	randomized controlled trial study	randomized controlled trial study	randomized controlled trial study	1	kept
RANDOMIZED CONTROLLED-TRIALS	randomized controlled trials	randomized controlled trials	randomized controlled trial	randomized controlled trial	1	singular
RANDOMIZED PROSPECTIVE TRIAL	randomized prospective trial	randomized prospective trial	randomized prospective trial	RANDOMIZED PROSPECTIVE TRIAL	1	kept
RANDOMIZED TRIAL	randomized trial	randomized trial	randomized trial	RANDOMIZED-TRIAL	1	case_or_format
Randomized trials	randomized trials	randomized trials	randomized trial	RANDOMIZED-TRIAL	1	singular
Range finding	range finding	range finding	range finding	Range finding	1	kept
Range of motions	range of motions	range of motions	range of motion	range of motion	1	singular
Ranging systems	ranging systems	ranging systems	ranging systems	Ranging systems	1	kept
RANITIDINE	ranitidine	ranitidine	ranitidine	ranitidine	1	case_or_format
Ranking	ranking	ranking	ranking	Ranking	1	kept

ranolazine	ranolazine	ranolazine	ranolazine	ranolazine	1	kept
rape	rape	rape	rape	Rape	1	case_or_format
rapid assessment of physical activity	rapid assessment of physical activity	rapid assessment of physical activity	rapid assessment of physical activity	rapid assessment of physical activity	1	kept
rapid atrial swirl sign	rapid atrial swirl sign	rapid atrial swirl sign	rapid atrial swirl sign	rapid atrial swirl sign	1	kept
rapid blood transfusion protocol	rapid blood transfusion protocol	rapid blood transfusion protocol	rapid blood transfusion protocol	rapid blood transfusion protocol	1	kept
rapid burnout screening tool	rapid burnout screening tool	rapid burnout screening tool	rapid burnout screening tool	rapid burnout screening tool	1	kept
Rapid Cycle Deliberate Practice	rapid cycle deliberate practice	rapid cycle deliberate practice	rapid cycle deliberate practice	rapid cycle deliberate practice	1	case_or_format
Rapid cycle improvement method	rapid cycle improvement method	rapid cycle improvement method	rapid cycle improvement method	Rapid cycle improvement method	1	kept
Rapid deceleration injuries	rapid deceleration injuries	rapid deceleration injuries	rapid deceleration injuries	Rapid deceleration injuries	1	kept
RAPID DEPLOYMENT	rapid deployment	rapid deployment	rapid deployment	RAPID DEPLOYMENT	1	kept
Rapid desensitization	rapid desensitization	rapid desensitization	rapid desensitization	Rapid desensitization	1	kept
rapid drug desensitization	rapid drug desensitization	rapid drug desensitization	rapid drug desensitization	rapid drug desensitization	1	kept
rapid genome sequencing	rapid genome sequencing	rapid genome sequencing	rapid genome sequencing	rapid genome sequencing	1	kept
Rapid Genome-Wide Sequencing	rapid genome wide sequencing	rapid genome wide sequencing	rapid genome wide sequencing	Rapid Genome-Wide Sequencing	1	kept
rapid genome-wide sequencing	rapid genome wide sequencing	rapid genome wide sequencing	rapid genome wide sequencing	Rapid Genome-Wide Sequencing	1	case_or_format
Rapid improvement event	rapid improvement event	rapid improvement event	rapid improvement event	Rapid improvement event	1	kept

Rapid infusion system	rapid infusion system	rapid infusion system	rapid infusion system	Rapid infusion system	1	kept
Rapid multiplex PCR diagnostic	rapid multiplex pcr diagnostic	rapid multiplex pcr diagnostic	rapid multiplex pcr diagnostic	Rapid multiplex PCR diagnostic	1	kept
rapid prototyping	rapid prototyping	rapid prototyping	rapid prototyping	rapid prototyping	1	kept
Rapid response EEG	rapid response eeg	rapid response eeg	rapid response eeg	Rapid response EEG	1	kept
RAPID RESPONSE TEAM	rapid response team	rapid response team	rapid response team	rapid response team	1	case_or_format
Rapid response team barriers	rapid response team barriers	rapid response team barriers	rapid response team barriers	Rapid response team barriers	1	kept
Rapid response team of hospitals	rapid response team of hospitals	rapid response team of hospitals	rapid response team of hospitals	Rapid response team of hospitals	1	kept
RAPID RESPONSE TEAMS	rapid response teams	rapid response teams	rapid response team	rapid response team	1	singular
Rapid Response Teams	rapid response teams	rapid response teams	rapid response team	rapid response team	1	singular
Rapid response time	rapid response time	rapid response time	rapid response time	Rapid response time	1	kept
rapid sequence endotracheal intubatio	rapid sequence endotracheal intubatio	rapid sequence endotracheal intubatio	rapid sequence endotracheal intubatio	rapid sequence endotracheal intubatio	1	kept
rapid sequence intubation	rapid sequence intubation	rapid sequence intubation	rapid sequence intubation	rapid sequence intubation	1	kept
Rapid Sequence Intubation and Induction	rapid sequence intubation and induction	rapid sequence intubation and induction	rapid sequence intubation and induction	Rapid Sequence Intubation and Induction	1	kept
Rapid shallow breathing index	rapid shallow breathing index	rapid shallow breathing index	rapid shallow breathing index	rapid shallow breathing index	1	case_or_format
rapid spread	rapid spread	rapid spread	rapid spread	rapid spread	1	kept
rapid tests	rapid tests	rapid tests	rapid tests	rapid tests	1	kept

Rapid Triage and Prediction Assistance Model	rapid triage and prediction assistance model	rapid triage and prediction assistance model	rapid triage and prediction assistance model	Rapid Triage and Prediction Assistance Model	1	kept
rapid upper limb assessment	rapid upper limb assessment	rapid upper limb assessment	rapid upper limb assessment	Rapid Upper Limb Assessment	1	case_or_format
rapid whole genome sequencing	rapid whole genome sequencing	rapid whole genome sequencing	rapid whole genome sequencing	rapid whole genome sequencing	1	kept
Rapid-acting insulin analogues	rapid acting insulin analogues	rapid acting insulin analogues	rapid acting insulin analogues	Rapid-acting insulin analogues	1	kept
rapid-cycle deliberate practice	rapid cycle deliberate practice	rapid cycle deliberate practice	rapid cycle deliberate practice	rapid cycle deliberate practice	1	case_or_format
RAPID-SEQUENCE INTUBATION	rapid sequence intubation	rapid sequence intubation	rapid sequence intubation	rapid sequence intubation	1	case_or_format
rare extrarenal malignant rhabdoid tumor	rare extrarenal malignant rhabdoid tumor	rare extrarenal malignant rhabdoid tumor	rare extrarenal malignant rhabdoid tumor	rare extrarenal malignant rhabdoid tumor	1	kept
Ras-MAP kinase	ras map kinase	ras map kinase	ras map kinase	Ras-MAP kinase	1	kept
rasagiline	rasagiline	rasagiline	rasagiline	rasagiline	1	kept
rasburicase	rasburicase	rasburicase	rasburicase	rasburicase	1	kept
Rasopathies	rasopathies	rasopathies	rasopathies	Rasopathies	1	kept
Raspberry pi	raspberry pi	raspberry pi	raspberry pi	Raspberry pi	1	kept
RASS score	rass score	rass score	rass score	RASS score	1	kept
rate	rate	rate	rate	RATES	1	case_or_format
Rate of compressions	rate of compressions	rate of compressions	rate of compressions	Rate of compressions	1	kept
Rate Setting and Review	rate setting and review	rate setting and review	rate setting and review	Rate Setting and Review	1	kept

RATIO	ratio	ratio	ratio	Ratio	1	case_or_format
Ratio enfermera-paciente	ratio enfermera paciente	ratio enfermera paciente	ratio enfermera paciente	Ratio enfermera-paciente	1	kept
ratio of oxygen saturation index	ratio of oxygen saturation index	ratio of oxygen saturation index	ratio of oxygen saturation index	ratio of oxygen saturation index	1	kept
Ratio pitfalls of units to measures	ratio pitfalls of units to measures	ratio pitfalls of units to measures	ratio pitfalls of units to measures	Ratio pitfalls of units to measures	1	kept
Rational Experiential Inventory 40	rational experiential inventory 40	rational experiential inventory 40	rational experiential inventory 40	Rational Experiential Inventory 40	1	kept
rational quality	rational quality	rational quality	rational quality	rational quality	1	kept
Rational use of blood	rational use of blood	rational use of blood	rational use of blood	Rational use of blood	1	kept
rationalization	rationalization	rationalization	rationalization	rationalization	1	kept
rationed care	rationed care	rationed care	rationed care	Rationed care	1	case_or_format
rationing of care	rationing of care	rationing of care	rationing of care	rationing of care	1	kept
RATIOS	ratios	ratios	ratio	Ratio	1	singular
Rats, Sprague-Dawley	rats sprague dawley	rats sprague dawley	rats sprague dawley	Rats, Sprague-Dawley	1	kept
Rattlesnake	rattlesnake	rattlesnake	rattlesnake	Rattlesnake	1	kept
RAW 264.7 Cells	raw 264 7 cells	raw 264 7 cells	raw 264 7 cells	RAW 264.7 Cells	1	kept
Raytec gauze	raytec gauze	raytec gauze	raytec gauze	Raytec gauze	1	kept
RBaux score	rbaux score	rbaux score	rbaux score	RBaux score	1	kept

RBC transfusion	rbc transfusion	rbc transfusion	rbc transfusion	RBC transfusion	1	kept
RCP = respiratory care practitioner	rcp respiratory care practitioner	rcp respiratory care practitioner	rcp respiratory care practitioner	RCP = respiratory care practitioner	1	kept
RCTs, randomized controlled trials	rcts randomized controlled trials	rcts randomized controlled trials	rcts randomized controlled trials	RCTs, randomized controlled trials	1	kept
rdpene	rdpene	rdpene	rdpene	rdpene	1	kept
RDS	rds	rds	rds	RDS	1	kept
Re B	re b	re b	re b	Re B	1	kept
re-accessed	re accessed	re accessed	re accessed	re-accessed	1	kept
re-dosing	re dosing	re dosing	re dosing	re-dosing	1	kept
Re-establishing	re establishing	re establishing	re establishing	Re-establishing	1	kept
Re-hospitalization	re hospitalization	re hospitalization	re hospitalization	Re-hospitalization	1	kept
Re-integration	re integration	re integration	re integration	Re-integration	1	kept
re-intubation	re intubation	re intubation	re intubation	re-intubation	1	kept
re-site	re site	re site	re site	re-site	1	kept
Reaction	reaction	reaction	reaction	reaction	1	case_or_format
Reaction level scale	reaction level scale	reaction level scale	reaction level scale	Reaction level scale	1	kept
Reactivating - rehabilitative interventions	reactivating rehabilitative interventions	reactivating rehabilitative interventions	reactivating rehabilitative interventions	Reactivating - rehabilitative interventions	1	kept

Reactive Attachment Disorder	reactive attachment disorder	reactive attachment disorder	reactive attachment disorder	Reactive Attachment Disorder	1	kept
Reactive oxygen species	reactive oxygen species	reactive oxygen species	reactive oxygen species	Reactive Oxygen Species	1	case_or_format
reactive oxygen species	reactive oxygen species	reactive oxygen species	reactive oxygen species	Reactive Oxygen Species	1	case_or_format
REACTIVITY	reactivity	reactivity	reactivity	REACTIVITY	1	kept
Readability	readability	readability	readability	Readability	1	kept
READINESS	readiness	readiness	readiness	Readiness	1	case_or_format
readiness for interprofessional learning scale	readiness for interprofessional learning scale	readiness for interprofessional learning scale	readiness for interprofessional learning scale	readiness for interprofessional learning scale	1	kept
readiness to care	readiness to care	readiness to care	readiness to care	readiness to care	1	kept
Reading programs	reading programs	reading programs	reading programs	Reading programs	1	kept
Readmission intensity	readmission intensity	readmission intensity	readmission intensity	Readmission intensity	1	kept
Readmission rate	readmission rate	readmission rate	readmission rate	Readmission rate	1	kept
readmission rate	readmission rate	readmission rate	readmission rate	Readmission rate	1	case_or_format
READMISSION RATES	readmission rates	readmission rates	readmission rate	Readmission rate	1	singular
readmission risk	readmission risk	readmission risk	readmission risk	readmission risk	1	kept
readmissions < complications	readmissions complications	readmissions complications	readmissions complications	readmissions < complications	1	kept
Readmissions, Length of Stay	readmissions length of stay	readmissions length of stay	readmissions length of stay	Readmissions, Length of Stay	1	kept

READMISSIONS-REDUCTION PROGRAM	readmissions reduction program	readmissions reduction program	readmissions reduction program	READMISSIONS-REDUCTION PROGRAM	1	kept
Ready-to-administer	ready to administer	ready to administer	ready to administer	Ready-to-administer	1	kept
Ready-to-administer infusions	ready to administer infusions	ready to administer infusions	ready to administer infusions	Ready-to-administer infusions	1	kept
Ready-to-use three-chamber-bags	ready to use three chamber bags	ready to use three chamber bags	ready to use three chamber bags	Ready-to-use three-chamber-bags	1	kept
reagent	reagent	reagent	reagent	reagent	1	kept
Reagent Kits, Diagnostic	reagent kits diagnostic	reagent kits diagnostic	reagent kits diagnostic	Reagent Kits, Diagnostic	1	kept
Reagent Strips	reagent strips	reagent strips	reagent strips	Reagent Strips	1	kept
Reagins	reagins	reagins	reagins	Reagins	1	kept
real food solution	real food solution	real food solution	real food solution	real food solution	1	kept
Real time data display	real time data display	real time data display	real time data display	Real time data display	1	kept
real time echography	real time echography	real time echography	real time echography	real time echography	1	kept
real time glucose tracking	real time glucose tracking	real time glucose tracking	real time glucose tracking	real time glucose tracking	1	kept
real time PCR	real time pcr	real time pcr	real time pcr	real time PCR	1	kept
Real time remote monitoring	real time remote monitoring	real time remote monitoring	real time remote monitoring	Real time remote monitoring	1	kept
real time tracking system	real time tracking system	real time tracking system	real time tracking system	real time tracking system	1	kept
real time video and audio communication	real time video and audio communication	real time video and audio communication	real time video and audio communication	real time video and audio communication	1	kept

real-time continuous glucose monitoring	real time continuous glucose monitoring	real time continuous glucose monitoring	real time continuous glucose monitoring	real-time continuous glucose monitoring	1	kept
Real-time contrast echocardiography	real time contrast echocardiography	real time contrast echocardiography	real time contrast echocardiography	Real-time contrast echocardiography	1	kept
Real-time data	real time data	real time data	real time data	real-time data	1	case_or_format
real-time e-alert	real time e alert	real time e alert	real time e alert	real-time e-alert	1	kept
Real-time feedback	real time feedback	real time feedback	real time feedback	Real-time feedback	1	kept
real-time feedback	real time feedback	real time feedback	real time feedback	Real-time feedback	1	case_or_format
Real-time location tracking systems	real time location tracking systems	real time location tracking systems	real time location tracking systems	Real-time location tracking systems	1	kept
Real-time monitoring	real time monitoring	real time monitoring	real time monitoring	Real time monitoring	1	case_or_format
real-time monitoring	real time monitoring	real time monitoring	real time monitoring	Real time monitoring	1	case_or_format
Real-time observation	real time observation	real time observation	real time observation	Real-time observation	1	kept
Real-time recording	real time recording	real time recording	real time recording	Real-time recording	1	kept
Real-time remote monitoring	real time remote monitoring	real time remote monitoring	real time remote monitoring	Real time remote monitoring	1	case_or_format
Real-time, automated brainstem and cerebral monitoring	real time automated brainstem and cerebral monitoring	real time automated brainstem and cerebral monitoring	real time automated brainstem and cerebral monitoring	Real-time, automated brainstem and cerebral monitoring	1	kept
real-world	real world	real world	real world	Real-world	1	case_or_format
Real-world data	real world data	real world data	real world data	Real-world data	1	kept
Real-world evidence	real world evidence	real world evidence	real world evidence	Real World Evidence	1	case_or_format

real-world study	real world study	real world study	real world study	real-world study	1	kept
realist evaluation	realist evaluation	realist evaluation	realist evaluation	Realist evaluation	1	case_or_format
realist methodology	realist methodology	realist methodology	realist methodology	realist methodology	1	kept
Realistic Evaluation	realistic evaluation	realistic evaluation	realistic evaluation	Realistic Evaluation	1	kept
realistic evaluation	realistic evaluation	realistic evaluation	realistic evaluation	Realistic Evaluation	1	case_or_format
Realistic scenario	realistic scenario	realistic scenario	realistic scenario	Realistic scenario	1	kept
reality awareness training	reality awareness training	reality awareness training	reality awareness training	reality awareness training	1	kept
Reality orientation	reality orientation	reality orientation	reality orientation	Reality orientation	1	kept
Reality shock	reality shock	reality shock	reality shock	Reality shock	1	kept
Reality Testing	reality testing	reality testing	reality testing	Reality Testing	1	kept
Reanimación	reanimaci n	reanimaci n	reanimaci n	Reanimación	1	kept
Reanimación Cardiopulmonar	reanimaci n cardiopulmonar	reanimaci n cardiopulmonar	reanimaci n cardiopulmonar	Reanimación Cardiopulmonar	1	kept
Reanimation	reanimation	reanimation	reanimation	Reanimation	1	kept
reanimation	reanimation	reanimation	reanimation	Reanimation	1	case_or_format
Reanimação Cardiopulmonar	reanima o cardiopulmonar	reanima o cardiopulmonar	reanima o cardiopulmonar	Reanimação Cardiopulmonar	1	kept
Reanimiation	reanimiation	reanimiation	reanimiation	Reanimiation	1	kept

REASONS	reasons	reasons	reasons	REASONS	1	kept
Reasons	reasons	reasons	reasons	REASONS	1	case_or_format
reasons for missed care	reasons for missed care	reasons for missed care	reasons for missed care	reasons for missed care	1	kept
Reassessment	reassessment	reassessment	reassessment	Reassessment	1	kept
reassessment	reassessment	reassessment	reassessment	Reassessment	1	case_or_format
reavement	reavement	reavement	reavement	reavement	1	kept
Rebound	rebound	rebound	rebound	rebound	1	case_or_format
rebreathing	rebreathing	rebreathing	rebreathing	rebreathing	1	kept
RecA gene	reca gene	reca gene	reca gene	RecA gene	1	kept
RECage project	recage project	recage project	recage project	RECage project	1	kept
recalled experience of death	recalled experience of death	recalled experience of death	recalled experience of death	recalled experience of death	1	kept
Recatheterization	recatheterization	recatheterization	recatheterization	Recatheterization	1	kept
RECEIPT	receipt	receipt	receipt	RECEIPT	1	kept
Receiver operating characteristic	receiver operating characteristic	receiver operating characteristic	receiver operating characteristic	receiver operating characteristic	1	case_or_format
Receiver operating curves	receiver operating curves	receiver operating curves	receiver operating curves	Receiver operating curves	1	kept
Receiver positions	receiver positions	receiver positions	receiver positions	Receiver positions	1	kept

RECEIVING HOSPICE	receiving hospice	receiving hospice	receiving hospice	RECEIVING HOSPICE	1	kept
RECEIVING HOSPICE CARE	receiving hospice care	receiving hospice care	receiving hospice care	RECEIVING HOSPICE CARE	1	kept
RECEIVING MECHANICAL VENTILATION	receiving mechanical ventilation	receiving mechanical ventilation	receiving mechanical ventilation	RECEIVING MECHANICAL VENTILATION	1	kept
receiving performa	receiving performa	receiving performa	receiving performa	receiving performa	1	kept
Recent researches	recent researches	recent researches	recent researches	Recent researches	1	kept
Reception	reception	reception	reception	Reception	1	kept
Reception area for life-threatening emergencies	reception area for life threatening emergencies	reception area for life threatening emergencies	reception area for life threatening emergencies	Reception area for life-threatening emergencies	1	kept
receptive field	receptive field	receptive field	receptive field	receptive field	1	kept
receptor affinity	receptor affinity	receptor affinity	receptor affinity	receptor affinity	1	kept
Receptors, Adrenergic	receptors adrenergic	receptors adrenergic	receptors adrenergic	Receptors, Adrenergic	1	kept
Receptors, Adrenergic, alpha	receptors adrenergic alpha	receptors adrenergic alpha	receptors adrenergic alpha	Receptors, Adrenergic, alpha	1	kept
Receptors, Adrenergic, beta-1	receptors adrenergic beta 1	receptors adrenergic beta 1	receptors adrenergic beta 1	Receptors, Adrenergic, beta-1	1	kept
Receptors, Adrenergic, beta-2	receptors adrenergic beta 2	receptors adrenergic beta 2	receptors adrenergic beta 2	Receptors, Adrenergic, beta-2	1	kept
Receptors, Chimeric Antigen	receptors chimeric antigen	receptors chimeric antigen	receptors chimeric antigen	Receptors, Chimeric Antigen	1	kept
Receptors, chimeric antigen	receptors chimeric antigen	receptors chimeric antigen	receptors chimeric antigen	Receptors, Chimeric Antigen	1	case_or_format
Receptors, Glutamate	receptors glutamate	receptors glutamate	receptors glutamate	Receptors, Glutamate	1	kept

Receptors, N-Methyl-D-Aspartate	receptors n methyl d aspartate	receptors n methyl d aspartate	receptors n methyl d aspartate	Receptors, N-Methyl-D-Aspartate	1	kept
Receptors, Tumor Necrosis Factor, Type I	receptors tumor necrosis factor type i	receptors tumor necrosis factor type i	receptors tumor necrosis factor type i	Receptors, Tumor Necrosis Factor, Type I	1	kept
Receptors, Virus	receptors virus	receptors virus	receptors virus	Receptors, Virus	1	kept
recessive gene	recessive gene	recessive gene	recessive gene	recessive gene	1	kept
recherche	recherche	recherche	recherche	recherche	1	kept
recherche clinique	recherche clinique	recherche clinique	recherche clinique	recherche clinique	1	kept
recipe4health	recipe4health	recipe4health	recipe4health	recipe4health	1	kept
Recipient	recipient	recipient	recipient	recipient	1	case_or_format
RECIPIENTS	recipients	recipients	recipient	recipient	1	singular
Recirculations	recirculations	recirculations	recirculations	Recirculations	1	kept
Recién Nacido de Bajo Peso	reci n nacido de bajo peso	reci n nacido de bajo peso	reci n nacido de bajo peso	Recién Nacido de Bajo Peso	1	kept
Recién nacido prematuro	reci n nacido prematuro	reci n nacido prematuro	reci n nacido prematuro	Recién Nacido Prematuro	1	case_or_format
Recién-Nacido	reci n nacido	reci n nacido	reci n nacido	Recién Nacido	1	case_or_format
recognise	recognise	recognise	recognise	recognise	1	kept
Recognising and responding to clinical deterioration	recognising and responding to clinical deterioration	recognising and responding to clinical deterioration	recognising and responding to clinical deterioration	Recognising and responding to clinical deterioration	1	kept
Recognition and response	recognition and response	recognition and response	recognition and response	Recognition and response	1	kept

recognition and response	recognition and response	recognition and response	recognition and response	Recognition and response	1	case_or_format
Recognition Primed Decision Model	recognition primed decision model	recognition primed decision model	recognition primed decision model	Recognition Primed Decision Model	1	kept
Recognition, Psychology	recognition psychology	recognition psychology	recognition psychology	Recognition, Psychology	1	kept
recognizer	recognizer	recognizer	recognizer	recognizer	1	kept
Recolección de muestras de sangre	recolecci n de muestras de sangre	recolecci n de muestras de sangre	recolecci n de muestras de sangre	Recolección de muestras de sangre	1	kept
RECOLLECTION	recollection	recollection	recollection	recollections	1	case_or_format
Recollection	recollection	recollection	recollection	recollections	1	case_or_format
recombinant activated factor vii	recombinant activated factor vii	recombinant activated factor vii	recombinant activated factor vii	recombinant activated factor vii	1	kept
recombinant alpha2b interferon	recombinant alpha2b interferon	recombinant alpha2b interferon	recombinant alpha2b interferon	recombinant alpha2b interferon	1	kept
recombinant blood clotting factor 9	recombinant blood clotting factor 9	recombinant blood clotting factor 9	recombinant blood clotting factor 9	recombinant blood clotting factor 9	1	kept
recombinant granulocyte colony stimulating factor	recombinant granulocyte colony stimulating factor	recombinant granulocyte colony stimulating factor	recombinant granulocyte colony stimulating factor	recombinant granulocyte colony stimulating factor	1	kept
recombinant growth hormone	recombinant growth hormone	recombinant growth hormone	recombinant growth hormone	recombinant growth hormone	1	kept
recombinant interferon	recombinant interferon	recombinant interferon	recombinant interferon	recombinant interferon	1	kept
recombinant interleukin 2	recombinant interleukin 2	recombinant interleukin 2	recombinant interleukin 2	recombinant interleukin 2	1	kept
recombinant proteins	recombinant proteins	recombinant proteins	recombinant protein	Recombinant Proteins	1	singular
recombinant thrombopoietin	recombinant thrombopoietin	recombinant thrombopoietin	recombinant thrombopoietin	recombinant thrombopoietin	1	kept

Recombinant tissue plasminogen activator	recombinant tissue plasminogen activator	recombinant tissue plasminogen activator	recombinant tissue plasminogen activator	Recombinant tissue plasminogen activator	1	kept
Recomendaciones	recomendaciones	recomendaciones	recomendaciones	Recomendaciones	1	kept
recommendation practice	recommendation practice	recommendation practice	recommendation practice	recommendation practice	1	kept
Recommendations for hand hygiene	recommendations for hand hygiene	recommendations for hand hygiene	recommendations for hand hygiene	Recommendations for hand hygiene	1	kept
Recommendations for practice	recommendations for practice	recommendations for practice	recommendations for practice	Recommendations for practice	1	kept
Recommendations for research	recommendations for research	recommendations for research	recommendations for research	Recommendations for research	1	kept
Recommended values	recommended values	recommended values	recommended values	Recommended values	1	kept
Reconceptualized Uncertainty in Illness Theory	reconceptualized uncertainty in illness theory	reconceptualized uncertainty in illness theory	reconceptualized uncertainty in illness theory	Reconceptualized Uncertainty in Illness Theory	1	kept
reconsolidation intervention	reconsolidation intervention	reconsolidation intervention	reconsolidation intervention	reconsolidation intervention	1	kept
Reconstructing beliefs	reconstructing beliefs	reconstructing beliefs	reconstructing beliefs	Reconstructing beliefs	1	kept
RECONSTRUCTION	reconstruction	reconstruction	reconstruction	RECONSTRUCTION	1	kept
Reconstruction	reconstruction	reconstruction	reconstruction	RECONSTRUCTION	1	case_or_format
reconstruction	reconstruction	reconstruction	reconstruction	RECONSTRUCTION	1	case_or_format
Reconstructive surgery	reconstructive surgery	reconstructive surgery	reconstructive surgery	reconstructive surgery	1	case_or_format
reconstructive surgical procedures	reconstructive surgical procedures	reconstructive surgical procedures	reconstructive surgical procedures	Reconstructive Surgical Procedures	1	case_or_format
record	record	record	record	Records	1	case_or_format

Record keeping	record keeping	record keeping	record keeping	Record keeping	1	kept
Record linkage	record linkage	record linkage	record linkage	Record linkage	1	kept
Record review	record review	record review	record review	Record review	1	kept
Record sheet	record sheet	record sheet	record sheet	Record sheet	1	kept
RECORD SYSTEM	record system	record system	record system	RECORD SYSTEM	1	kept
record-keeping	record keeping	record keeping	record keeping	Record keeping	1	case_or_format
Recorded music	recorded music	recorded music	recorded music	Recorded music	1	kept
Recorded signals	recorded signals	recorded signals	recorded signals	Recorded signals	1	kept
Recording systems	recording systems	recording systems	recording systems	Recording systems	1	kept
records	records	records	record	Records	1	singular
Recours aux soins	recours aux soins	recours aux soins	recours aux soins	Recours aux soins	1	kept
recovery assessment	recovery assessment	recovery assessment	recovery assessment	recovery assessment	1	kept
recovery experience	recovery experience	recovery experience	recovery experience	recovery experience	1	kept
recovery experiences	recovery experiences	recovery experiences	recovery experience	recovery experience	1	singular
Recovery factors	recovery factors	recovery factors	recovery factors	Recovery factors	1	kept
Recovery framework	recovery framework	recovery framework	recovery framework	Recovery framework	1	kept

Recovery from Addiction	recovery from addiction	recovery from addiction	recovery from addiction	Recovery from Addiction	1	kept
recovery from addiction	recovery from addiction	recovery from addiction	recovery from addiction	Recovery from Addiction	1	case_or_format
Recovery mechanisms	recovery mechanisms	recovery mechanisms	recovery mechanisms	Recovery mechanisms	1	kept
recovery of function	recovery of function	recovery of function	recovery of function	Recovery of Function	1	case_or_format
Recovery oriented	recovery oriented	recovery oriented	recovery oriented	Recovery oriented	1	kept
Recovery position	recovery position	recovery position	recovery position	Recovery position	1	kept
recovery rate	recovery rate	recovery rate	recovery rate	recovery rate	1	kept
RECOVERY ROOM	recovery room	recovery room	recovery room	Recovery Room	1	case_or_format
Recovery self-efficacy	recovery self efficacy	recovery self efficacy	recovery self efficacy	Recovery self-efficacy	1	kept
Recovery time	recovery time	recovery time	recovery time	Recovery time	1	kept
Recovery Unit	recovery unit	recovery unit	recovery unit	Recovery Unit	1	kept
Recovery unit	recovery unit	recovery unit	recovery unit	Recovery Unit	1	case_or_format
recreational drug use	recreational drug use	recreational drug use	recreational drug use	recreational drug use	1	kept
recreational game	recreational game	recreational game	recreational game	recreational game	1	kept
recreational therapist	recreational therapist	recreational therapist	recreational therapist	recreational therapist	1	kept
recreational therapy	recreational therapy	recreational therapy	recreational therapy	recreational therapy	1	kept

recruitment	recruitment	recruitment	recruitment	Recruitment	1	case_or_format
Recruitment and retention	recruitment and retention	recruitment and retention	recruitment and retention	Recruitment and retention	1	kept
recruitment and retention	recruitment and retention	recruitment and retention	recruitment and retention	Recruitment and retention	1	case_or_format
Rectal cancer	rectal cancer	rectal cancer	rectal cancer	Rectal cancer	1	kept
Rectal neoplasm	rectal neoplasm	rectal neoplasm	rectal neoplasm	Rectal Neoplasms	1	case_or_format
Rectal rehydration	rectal rehydration	rectal rehydration	rectal rehydration	Rectal rehydration	1	kept
Rectal screening	rectal screening	rectal screening	rectal screening	Rectal screening	1	kept
Rectal temperature	rectal temperature	rectal temperature	rectal temperature	rectal temperature	1	case_or_format
Rectal trumpet	rectal trumpet	rectal trumpet	rectal trumpet	Rectal trumpet	1	kept
rectogluteal fistula	rectogluteal fistula	rectogluteal fistula	rectogluteal fistula	rectogluteal fistula	1	kept
rectum abdominoperineal resection	rectum abdominoperineal resection	rectum abdominoperineal resection	rectum abdominoperineal resection	rectum abdominoperineal resection	1	kept
rectum anastomosis	rectum anastomosis	rectum anastomosis	rectum anastomosis	rectum anastomosis	1	kept
rectum biopsy	rectum biopsy	rectum biopsy	rectum biopsy	rectum biopsy	1	kept
rectum carcinoma	rectum carcinoma	rectum carcinoma	rectum carcinoma	rectum carcinoma	1	kept
rectum disease	rectum disease	rectum disease	rectum disease	rectum disease	1	kept
rectum fistula	rectum fistula	rectum fistula	rectum fistula	rectum fistula	1	kept

rectum injury	rectum injury	rectum injury	rectum injury	rectum injury	1	kept
rectum resection	rectum resection	rectum resection	rectum resection	rectum resection	1	kept
rectus femoris muscle	rectus femoris muscle	rectus femoris muscle	rectus femoris muscle	rectus femoris muscle	1	kept
Recumbent length	recumbent length	recumbent length	recumbent length	Recumbent length	1	kept
Recuperación funcional	recuperaci n funcional	recuperaci n funcional	recuperaci n funcional	Recuperación funcional	1	kept
RECURRENCE	recurrence	recurrence	recurrence	Recurrence	1	case_or_format
recurrent airway infection	recurrent airway infection	recurrent airway infection	recurrent airway infection	recurrent airway infection	1	kept
Recurrent CDI	recurrent cdi	recurrent cdi	recurrent cdi	Recurrent CDI	1	kept
recurrent clostridium difficile infection	recurrent clostridium difficile infection	recurrent clostridium difficile infection	recurrent clostridium difficile infection	recurrent clostridium difficile infection	1	kept
recurrent dislocation	recurrent dislocation	recurrent dislocation	recurrent dislocation	recurrent dislocation	1	kept
Recurrent events	recurrent events	recurrent events	recurrent events	Recurrent events	1	kept
recurrent laryngospasm	recurrent laryngospasm	recurrent laryngospasm	recurrent laryngospasm	recurrent laryngospasm	1	kept
Recursive feature elimination	recursive feature elimination	recursive feature elimination	recursive feature elimination	Recursive feature elimination	1	kept
recursive feature elimination	recursive feature elimination	recursive feature elimination	recursive feature elimination	Recursive feature elimination	1	case_or_format
Recursos	recursos	recursos	recursos	Recursos	1	kept
Recém-nascido	rec m nascido	rec m nascido	rec m nascido	Recém-Nascido	1	case_or_format

Recém-Nascido de Baixo Peso	rec m nascido de baixo peso	rec m nascido de baixo peso	rec m nascido de baixo peso	Recém-Nascido de Baixo Peso	1	kept
Recém-Nascido Prematuro	rec m nascido prematuro	rec m nascido prematuro	rec m nascido prematuro	Recém-Nascido Prematuro	1	kept
Recém-nascido Pré-termo	rec m nascido pr termo	rec m nascido pr termo	rec m nascido pr termo	Recém-nascido Pré-termo	1	kept
Red blood cells	red blood cells	red blood cells	red blood cells	Red blood cells	1	kept
Red cell component	red cell component	red cell component	red cell component	Red cell component	1	kept
red cell component	red cell component	red cell component	red cell component	Red cell component	1	case_or_format
red cross	red cross	red cross	red cross	red cross	1	kept
Red distributions width	red distributions width	red distributions width	red distributions width	Red distributions width	1	kept
red flag	red flag	red flag	red flag	red flag	1	kept
REDCap	redcap	redcap	redcap	REDCap	1	kept
Redeployed	redeployed	redeployed	redeployed	Redeployed	1	kept
Redeployment pathway	redeployment pathway	redeployment pathway	redeployment pathway	Redeployment pathway	1	kept
REDISTRIBUTING SUPPORT SURFACES	redistributing support surfaces	redistributing support surfaces	redistributing support surfaces	REDISTRIBUTING SUPPORT SURFACES	1	kept
REDUCE HOSPITALIZATIONS	reduce hospitalizations	reduce hospitalizations	reduce hospitalizations	REDUCE HOSPITALIZATIONS	1	kept
REDUCED MORTALITY	reduced mortality	reduced mortality	reduced mortality	REDUCED MORTALITY	1	kept
reduced personal accomplishment	reduced personal accomplishment	reduced personal accomplishment	reduced personal accomplishment	reduced personal accomplishment	1	kept

Reduced rule base	reduced rule base	reduced rule base	reduced rule base	Reduced rule base	1	kept
Reducing noise	reducing noise	reducing noise	reducing noise	Reducing noise	1	kept
REDUCING PAIN	reducing pain	reducing pain	reducing pain	REDUCING PAIN	1	kept
REDUCING SECLUSION	reducing seclusion	reducing seclusion	reducing seclusion	REDUCING SECLUSION	1	kept
reducing time	reducing time	reducing time	reducing time	reducing time	1	kept
Reduction	reduction	reduction	reduction	REDUCTION	1	case_or_format
reduction model	reduction model	reduction model	reduction model	reduction model	1	kept
Reduction process	reduction process	reduction process	reduction process	Reduction process	1	kept
Reduction strategy	reduction strategy	reduction strategy	reduction strategy	Reduction strategy	1	kept
Redundant sensory intervention	redundant sensory intervention	redundant sensory intervention	redundant sensory intervention	Redundant sensory intervention	1	kept
reference checklist manual	reference checklist manual	reference checklist manual	reference checklist manual	reference checklist manual	1	kept
reference heart rate value	reference heart rate value	reference heart rate value	reference heart rate value	reference heart rate value	1	kept
Reference network	reference network	reference network	reference network	Reference network	1	kept
Reference services	reference services	reference services	reference services	Reference services	1	kept
Reference source	reference source	reference source	reference source	Reference source	1	kept
reference standard	reference standard	reference standard	reference standard	Reference Standards	1	case_or_format

Reference values	reference values	reference values	reference value	Reference Values	1	singular
Reference:	reference	reference	reference	Reference:	1	kept
Referral chain	referral chain	referral chain	referral chain	Referral chain	1	kept
Referral criteria	referral criteria	referral criteria	referral criteria	Referral criteria	1	kept
referral warranting retinopathy of prematurity	referral warranting retinopathy of prematurity	referral warranting retinopathy of prematurity	referral warranting retinopathy of prematurity	referral warranting retinopathy of prematurity	1	kept
refill prescription	refill prescription	refill prescription	refill prescription	refill prescription	1	kept
Refined care	refined care	refined care	refined care	Refined care	1	kept
REFINEMENT	refinement	refinement	refinement	REFINEMENT	1	kept
REFLECTION	reflection	reflection	reflection	Reflection	1	case_or_format
Reflection of ELSO registry	reflection of elso registry	reflection of elso registry	reflection of elso registry	Reflection of ELSO registry	1	kept
Reflective	reflective	reflective	reflective	Reflective	1	kept
Reflective journaling technique	reflective journaling technique	reflective journaling technique	reflective journaling technique	Reflective journaling technique	1	kept
Reflective journals	reflective journals	reflective journals	reflective journals	Reflective journals	1	kept
reflective learning	reflective learning	reflective learning	reflective learning	reflective learning	1	kept
Reflective learning conversations	reflective learning conversations	reflective learning conversations	reflective learning conversations	Reflective learning conversations	1	kept
reflective lifeworld research	reflective lifeworld research	reflective lifeworld research	reflective lifeworld research	Reflective lifeworld research	1	case_or_format

Reflective model	reflective model	reflective model	reflective model	Reflective model	1	kept
REFLECTIVE PRACTICE	reflective practice	reflective practice	reflective practice	Reflective practice	1	case_or_format
Reflex, Abnormal	reflex abnormal	reflex abnormal	reflex abnormal	Reflex, Abnormal	1	kept
reflux esophagitis	reflux esophagitis	reflux esophagitis	reflux esophagitis	reflux esophagitis	1	kept
REFORM	reform	reform	reform	REFORM	1	kept
refraction error	refraction error	refraction error	refraction error	refraction error	1	kept
Refractory agitation	refractory agitation	refractory agitation	refractory agitation	Refractory agitation	1	kept
REFRACTORY BREATHLESSNESS	refractory breathlessness	refractory breathlessness	refractory breathlessness	REFRACTORY BREATHLESSNESS	1	kept
Refractory CDI	refractory cdi	refractory cdi	refractory cdi	Refractory CDI	1	kept
refractory cough	refractory cough	refractory cough	refractory cough	refractory cough	1	kept
refractory hyperkalemia	refractory hyperkalemia	refractory hyperkalemia	refractory hyperkalemia	refractory hyperkalemia	1	kept
Refractory insomnia	refractory insomnia	refractory insomnia	refractory insomnia	Refractory insomnia	1	kept
refractory lung failure	refractory lung failure	refractory lung failure	refractory lung failure	refractory lung failure	1	kept
Refractory materials	refractory materials	refractory materials	refractory materials	Refractory materials	1	kept
refractory pain	refractory pain	refractory pain	refractory pain	refractory pain	1	kept
Refresher	refresher	refresher	refresher	Refresher	1	kept

Refresher program	refresher program	refresher program	refresher program	Refresher program	1	kept
REFUGEES	refugees	refugees	refugee	Refugees	1	singular
refugees	refugees	refugees	refugee	Refugees	1	singular
refund	refund	refund	refund	refund	1	kept
refusal	refusal	refusal	refusal	refusal	1	kept
Refusal of treatment	refusal of treatment	refusal of treatment	refusal of treatment	Refusal of treatment	1	kept
refusal to feed	refusal to feed	refusal to feed	refusal to feed	refusal to feed	1	kept
refusing treatments	refusing treatments	refusing treatments	refusing treatments	refusing treatments	1	kept
Regards	regards	regards	regards	Regards	1	kept
Regen-COV	regen cov	regen cov	regen cov	Regen-COV	1	kept
REGIMENS	regimens	regimens	regimens	REGIMENS	1	kept
region	region	region	region	region	1	kept
Region size	region size	region size	region size	Region size	1	kept
Regional	regional	regional	regional	regional	1	case_or_format
Regional anaesthesia	regional anaesthesia	regional anaesthesia	regional anaesthesia	regional anaesthesia	1	case_or_format
Regional analgesia	regional analgesia	regional analgesia	regional analgesia	Regional analgesia	1	kept

regional analgesia	regional analgesia	regional analgesia	regional analgesia	Regional analgesia	1	case_or_format
REGIONAL CANCER CENTER	regional cancer center	regional cancer center	regional cancer center	REGIONAL CANCER CENTER	1	kept
regional care	regional care	regional care	regional care	regional care	1	kept
regional care network	regional care network	regional care network	regional care network	regional care network	1	kept
Regional differences	regional differences	regional differences	regional differences	Regional differences	1	kept
regional homogeneity	regional homogeneity	regional homogeneity	regional homogeneity	regional homogeneity	1	kept
Regional Hospital	regional hospital	regional hospital	regional hospital	Regional Hospital	1	kept
Regional location	regional location	regional location	regional location	Regional location	1	kept
Regional lung ventilation	regional lung ventilation	regional lung ventilation	regional lung ventilation	Regional lung ventilation	1	kept
regional oxygenation	regional oxygenation	regional oxygenation	regional oxygenation	regional oxygenation	1	kept
regional perfusion discrepancy	regional perfusion discrepancy	regional perfusion discrepancy	regional perfusion discrepancy	regional perfusion discrepancy	1	kept
Regional public hospital	regional public hospital	regional public hospital	regional public hospital	Regional public hospital	1	kept
regional selection	regional selection	regional selection	regional selection	regional selection	1	kept
regional variation	regional variation	regional variation	regional variation	Regional variation	1	case_or_format
REGIONAL WEANING CENTER	regional weaning center	regional weaning center	regional weaning center	REGIONAL WEANING CENTER	1	kept
REGIONAL-VARIATIONS	regional variations	regional variations	regional variation	Regional variation	1	singular

regionalised transfer	regionalised transfer	regionalised transfer	regionalised transfer	regionalised transfer	1	kept
regionalization of health	regionalization of health	regionalization of health	regionalization of health	regionalization of health	1	kept
Regionalization of health care	regionalization of health care	regionalization of health care	regionalization of health care	Regionalization of health care	1	kept
Regionalized health service	regionalized health service	regionalized health service	regionalized health service	Regionalized health service	1	kept
REGIONALIZED SYSTEM	regionalized system	regionalized system	regionalized system	REGIONALIZED SYSTEM	1	kept
registered dietitian	registered dietitian	registered dietitian	registered dietitian	registered dietitian	1	kept
Registered respiratory therapist	registered respiratory therapist	registered respiratory therapist	registered respiratory therapist	Registered respiratory therapists	1	case_or_format
registered respiratory therapist	registered respiratory therapist	registered respiratory therapist	registered respiratory therapist	Registered respiratory therapists	1	case_or_format
Registrar	registrar	registrar	registrar	Registrar	1	kept
Registration Burden	registration burden	registration burden	registration burden	Registration Burden	1	kept
registries	registries	registries	registry	Registries	1	singular
Registro de enfermería	registro de enfermer a	registro de enfermer a	registro de enfermer a	Registro de enfermería	1	kept
Registros	registros	registros	registros	Registros	1	kept
REGISTRY	registry	registry	registry	Registries	1	case_or_format
REGRESSION	regression	regression	regression	REGRESSION	1	kept
Regression tests	regression tests	regression tests	regression tests	Regression tests	1	kept

REGRESSION TREE	regression tree	regression tree	regression tree	REGRESSION TREE	1	kept
regression-discontinuity design	regression discontinuity design	regression discontinuity design	regression discontinuity design	regression-discontinuity design	1	kept
regression - discontinuity design	regression discontinuity design	regression discontinuity design	regression discontinuity design	regression-discontinuity design	1	case_or_format
Regret	regret	regret	regret	Regret	1	kept
regular discharge	regular discharge	regular discharge	regular discharge	regular discharge	1	kept
Regulation	regulation	regulation	regulation	Regulations	1	case_or_format
REGULATIONS	regulations	regulations	regulation	Regulations	1	singular
Regulator	regulator	regulator	regulator	Regulator	1	kept
Regulatory agencies	regulatory agencies	regulatory agencies	regulatory agencies	Regulatory agencies	1	kept
Regulatory bodies	regulatory bodies	regulatory bodies	regulatory bodies	Regulatory bodies	1	kept
regulatory emotional self efficacy	regulatory emotional self efficacy	regulatory emotional self efficacy	regulatory emotional self efficacy	regulatory emotional self efficacy	1	kept
regulatory guidance	regulatory guidance	regulatory guidance	regulatory guidance	regulatory guidance	1	kept
regulatory toxicology	regulatory toxicology	regulatory toxicology	regulatory toxicology	regulatory toxicology	1	kept
Regurgitation	regurgitation	regurgitation	regurgitation	Regurgitation	1	kept
Regurgitation, Gastric	regurgitation gastric	regurgitation gastric	regurgitation gastric	Regurgitation, Gastric	1	kept
Rehabilitation assignment	rehabilitation assignment	rehabilitation assignment	rehabilitation assignment	Rehabilitation assignment	1	kept

Rehabilitation Coronavirus	rehabilitation coronavirus	rehabilitation coronavirus	rehabilitation coronavirus	Rehabilitation Coronavirus	1	kept
rehabilitation engineer	rehabilitation engineer	rehabilitation engineer	rehabilitation engineer	rehabilitation engineer	1	kept
Rehabilitation epidemiology	rehabilitation epidemiology	rehabilitation epidemiology	rehabilitation epidemiology	Rehabilitation epidemiology	1	kept
Rehabilitation exercise	rehabilitation exercise	rehabilitation exercise	rehabilitation exercise	Rehabilitation exercise	1	kept
rehabilitation exercise	rehabilitation exercise	rehabilitation exercise	rehabilitation exercise	Rehabilitation exercise	1	case_or_format
Rehabilitation methods	rehabilitation methods	rehabilitation methods	rehabilitation methods	Rehabilitation methods	1	kept
Rehabilitation of blast injuries	rehabilitation of blast injuries	rehabilitation of blast injuries	rehabilitation of blast injuries	Rehabilitation of blast injuries	1	kept
Rehabilitation of paediatric brain injury	rehabilitation of paediatric brain injury	rehabilitation of paediatric brain injury	rehabilitation of paediatric brain injury	Rehabilitation of paediatric brain injury	1	kept
rehabilitation of paediatric brain injury	rehabilitation of paediatric brain injury	rehabilitation of paediatric brain injury	rehabilitation of paediatric brain injury	Rehabilitation of paediatric brain injury	1	case_or_format
REHABILITATION PROGRAM	rehabilitation program	rehabilitation program	rehabilitation program	Rehabilitation programs	1	case_or_format
rehabilitation research	rehabilitation research	rehabilitation research	rehabilitation research	rehabilitation research	1	kept
rehabilitation team	rehabilitation team	rehabilitation team	rehabilitation team	rehabilitation team	1	kept
rehabilitation therapist	rehabilitation therapist	rehabilitation therapist	rehabilitation therapist	rehabilitation therapist	1	kept
rehabilitation training	rehabilitation training	rehabilitation training	rehabilitation training	rehabilitation training	1	kept
rehabilitation unit	rehabilitation unit	rehabilitation unit	rehabilitation unit	rehabilitation unit	1	kept
Rehabilitation, Vocational	rehabilitation vocational	rehabilitation vocational	rehabilitation vocational	Rehabilitation, Vocational	1	kept

Rehospitalisation	rehospitalisation	rehospitalisation	rehospitalisation	Rehospitalisation	1	kept
Rehospitalization	rehospitalization	rehospitalization	rehospitalization	rehospitalization	1	case_or_format
REHOSPITALIZATIONS	rehospitalizations	rehospitalizations	rehospitalization	rehospitalization	1	singular
Rehydration	rehydration	rehydration	rehydration	rehydration	1	case_or_format
REIKI THERAPY	reiki therapy	reiki therapy	reiki therapy	REIKI THERAPY	1	kept
Reimbursement, Disproportionate Share	reimbursement disproportionate share	reimbursement disproportionate share	reimbursement disproportionate share	Reimbursement, Disproportionate Share	1	kept
REINFECTION	reinfection	reinfection	reinfection	reinfection	1	case_or_format
Reinforcement workshop	reinforcement workshop	reinforcement workshop	reinforcement workshop	Reinforcement workshop	1	kept
reinforcing	reinforcing	reinforcing	reinforcing	reinforcing	1	kept
Relaciones Familiares	relaciones familiares	relaciones familiares	relaciones familiares	Relaciones familiares	1	case_or_format
Relaciones interpersonales	relaciones interpersonales	relaciones interpersonales	relaciones interpersonales	Relaciones interpersonales	1	kept
related pressure injury	related pressure injury	related pressure injury	related pressure injury	related pressure injury	1	kept
relation building	relation building	relation building	relation building	relation building	1	kept
relation to the self	relation to the self	relation to the self	relation to the self	relation to the self	1	kept
Relational care	relational care	relational care	relational care	Relational care	1	kept
Relational sociology	relational sociology	relational sociology	relational sociology	Relational sociology	1	kept

relational tool	relational tool	relational tool	relational tool	relational tool	1	kept
Relations	relations	relations	relations	Relations	1	kept
Relationship centered	relationship centered	relationship centered	relationship centered	Relationship centered	1	kept
Relationship dynamics	relationship dynamics	relationship dynamics	relationship dynamics	Relationship dynamics	1	kept
Relationship Scales Questionnaire	relationship scales questionnaire	relationship scales questionnaire	relationship scales questionnaire	Relationship Scales Questionnaire	1	kept
Relationship-based	relationship based	relationship based	relationship based	Relationship-based	1	kept
Relationship-based care	relationship based care	relationship based care	relationship based care	Relationship-based care	1	kept
relationship-based care	relationship based care	relationship based care	relationship based care	Relationship-based care	1	case_or_format
relative afferent pupillary defect	relative afferent pupillary defect	relative afferent pupillary defect	relative afferent pupillary defect	relative afferent pupillary defect	1	kept
relative arm acceleration	relative arm acceleration	relative arm acceleration	relative arm acceleration	relative arm acceleration	1	kept
Relative contribution	relative contribution	relative contribution	relative contribution	Relative contribution	1	kept
RELATIVE CONTRIBUTIONS	relative contributions	relative contributions	relative contribution	Relative contribution	1	singular
relative environmental assessment lens indicator	relative environmental assessment lens indicator	relative environmental assessment lens indicator	relative environmental assessment lens indicator	relative environmental assessment lens indicator	1	kept
Relative experiences	relative experiences	relative experiences	relative experiences	Relative experiences	1	kept
relative involvement	relative involvement	relative involvement	relative involvement	relative involvement	1	kept
relative light unit	relative light unit	relative light unit	relative light unit	relative light unit	1	kept

Relative need	relative need	relative need	relative need	Relative need	1	kept
RELATIVE PERFORMANCE INFORMATION	relative performance information	relative performance information	relative performance information	RELATIVE PERFORMANCE INFORMATION	1	kept
Relative value scales	relative value scales	relative value scales	relative value scales	Relative Value Scales	1	case_or_format
Relative value unit	relative value unit	relative value unit	relative value unit	Relative value unit	1	kept
Relatives' experience	relatives experience	relatives experience	relatives experience	Relatives' experience	1	kept
Relatives' experiences	relatives experiences	relatives experiences	relatives experience	Relatives' experience	1	singular
relatives-presence during resuscitation	relatives presence during resuscitation	relatives presence during resuscitation	relatives presence during resuscitation	relatives-presence during resuscitation	1	kept
Relatos de Casos	relatos de casos	relatos de casos	relatos de casos	Relatos de Casos	1	kept
relaxation of research rules	relaxation of research rules	relaxation of research rules	relaxation of research rules	relaxation of research rules	1	kept
Relaxation Techniques	relaxation techniques	relaxation techniques	relaxation techniques	Relaxation Techniques	1	kept
Relaxation-inducing therapies	relaxation inducing therapies	relaxation inducing therapies	relaxation inducing therapies	Relaxation-inducing therapies	1	kept
relaxin	relaxin	relaxin	relaxin	relaxin	1	kept
Relações Familiares	rela es familiares	rela es familiares	rela es familiares	Relações Familiares	1	kept
Relevant factors	relevant factors	relevant factors	relevant factors	Relevant factors	1	kept
Reliability analysis	reliability analysis	reliability analysis	reliability analysis	Reliability analysis	1	kept
reliability analysis	reliability analysis	reliability analysis	reliability analysis	Reliability analysis	1	case_or_format

Reliability of results	reliability of results	reliability of results	reliability of results	Reliability of results	1	kept
Reliable methods	reliable methods	reliable methods	reliable methods	Reliable methods	1	kept
reliance agreement	reliance agreement	reliance agreement	reliance agreement	reliance agreement	1	kept
Religion and spirituality	religion and spirituality	religion and spirituality	religion and spirituality	Religion and spirituality	1	kept
religious	religious	religious	religious	Religious	1	case_or_format
Religious Adaptation	religious adaptation	religious adaptation	religious adaptation	Religious Adaptation	1	kept
Religious attitude and behavior	religious attitude and behavior	religious attitude and behavior	religious attitude and behavior	Religious attitude and behavior	1	kept
religious background	religious background	religious background	religious background	religious background	1	kept
Religious Beliefs	religious beliefs	religious beliefs	religious beliefs	Religious Beliefs	1	kept
Religious beliefs	religious beliefs	religious beliefs	religious beliefs	Religious Beliefs	1	case_or_format
religious beliefs	religious beliefs	religious beliefs	religious beliefs	Religious Beliefs	1	case_or_format
Religious commitment	religious commitment	religious commitment	religious commitment	Religious commitment	1	kept
religious coping	religious coping	religious coping	religious coping	religious coping	1	kept
religious health	religious health	religious health	religious health	religious health	1	kept
religious personnel	religious personnel	religious personnel	religious personnel	religious personnel	1	kept
RELIGIOUSNESS	religiousness	religiousness	religiousness	religiousness	1	case_or_format

relocation stress syndrome	relocation stress syndrome	relocation stress syndrome	relocation stress syndrome	relocation stress syndrome	1	kept
REM	rem	rem	rem	REM	1	kept
REM sleep deprivation	rem sleep deprivation	rem sleep deprivation	rem sleep deprivation	REM sleep deprivation	1	kept
Remdesivir	remdesivir	remdesivir	remdesivir	remdesivir	1	case_or_format
REMIFENTANIL	remifentanil	remifentanil	remifentanil	remifentanil	1	case_or_format
Remimazolam	remimazolam	remimazolam	remimazolam	remimazolam	1	case_or_format
Remimazolam tosylate	remimazolam tosylate	remimazolam tosylate	remimazolam tosylate	Remimazolam tosylate	1	kept
reminiscence group therapy	reminiscence group therapy	reminiscence group therapy	reminiscence group therapy	reminiscence group therapy	1	kept
Remission Induction	remission induction	remission induction	remission induction	Remission Induction	1	kept
Remission, Spontaneous	remission spontaneous	remission spontaneous	remission spontaneous	Remission, Spontaneous	1	kept
Remodeling	remodeling	remodeling	remodeling	Remodeling	1	kept
remote anesthesia	remote anesthesia	remote anesthesia	remote anesthesia	remote anesthesia	1	kept
Remote care	remote care	remote care	remote care	Remote care	1	kept
remote care	remote care	remote care	remote care	Remote care	1	case_or_format
remote caregivers	remote caregivers	remote caregivers	remote caregivers	remote caregivers	1	kept
Remote consultation	remote consultation	remote consultation	remote consultation	Remote Consultation	1	case_or_format

Remote control	remote control	remote control	remote control	Remote control	1	kept
Remote damage control resuscitation	remote damage control resuscitation	remote damage control resuscitation	remote damage control resuscitation	Remote damage control resuscitation	1	kept
remote didactic education	remote didactic education	remote didactic education	remote didactic education	remote didactic education	1	kept
remote health care	remote health care	remote health care	remote health care	remote health care	1	kept
Remote home care	remote home care	remote home care	remote home care	Remote home care	1	kept
remote intensive management	remote intensive management	remote intensive management	remote intensive management	remote intensive management	1	kept
remote ischemic preconditioning	remote ischemic preconditioning	remote ischemic preconditioning	remote ischemic preconditioning	remote ischemic preconditioning	1	kept
Remote learning	remote learning	remote learning	remote learning	Remote learning	1	kept
remote medicine	remote medicine	remote medicine	remote medicine	remote medicine	1	kept
Remote monitoring programme	remote monitoring programme	remote monitoring programme	remote monitoring programme	Remote monitoring programme	1	kept
Remote network system	remote network system	remote network system	remote network system	Remote network system	1	kept
Remote networks	remote networks	remote networks	remote networks	Remote networks	1	kept
remote nutrition care	remote nutrition care	remote nutrition care	remote nutrition care	remote nutrition care	1	kept
remote respiration sensing	remote respiration sensing	remote respiration sensing	remote respiration sensing	remote respiration sensing	1	kept
Remote sensing technology	remote sensing technology	remote sensing technology	remote sensing technology	Remote Sensing Technology	1	case_or_format
remote sensing technology	remote sensing technology	remote sensing technology	remote sensing technology	Remote Sensing Technology	1	case_or_format

Remote support	remote support	remote support	remote support	Remote support	1	kept
remote tele-mentored ultrasound	remote tele mentored ultrasound	remote tele mentored ultrasound	remote tele mentored ultrasound	remote tele-mentored ultrasound	1	kept
Removal	removal	removal	removal	REMOVAL	1	case_or_format
Removal of the Endotracheal Tube	removal of the endotracheal tube	removal of the endotracheal tube	removal of the endotracheal tube	Removal of the Endotracheal Tube	1	kept
removal of trans radial band	removal of trans radial band	removal of trans radial band	removal of trans radial band	removal of trans radial band	1	kept
Remuneration	remuneration	remuneration	remuneration	remuneration	1	case_or_format
Renal	renal	renal	renal	renal	1	case_or_format
Renal Agents	renal agents	renal agents	renal agents	Renal Agents	1	kept
Renal angina	renal angina	renal angina	renal angina	Renal angina	1	kept
Renal Artery Obstruction	renal artery obstruction	renal artery obstruction	renal artery obstruction	Renal Artery Obstruction	1	kept
Renal autotransplant	renal autotransplant	renal autotransplant	renal autotransplant	Renal autotransplant	1	kept
renal autotransplant	renal autotransplant	renal autotransplant	renal autotransplant	Renal autotransplant	1	case_or_format
renal blood flow	renal blood flow	renal blood flow	renal blood flow	renal blood flow	1	kept
Renal Circulation	renal circulation	renal circulation	renal circulation	Renal Circulation	1	kept
Renal complications	renal complications	renal complications	renal complications	Renal complications	1	kept
Renal disease	renal disease	renal disease	renal disease	Renal disease	1	kept

Renal Failure	renal failure	renal failure	renal failure	Renal failure	1	case_or_format
renal fluid responsiveness	renal fluid responsiveness	renal fluid responsiveness	renal fluid responsiveness	renal fluid responsiveness	1	kept
Renal function	renal function	renal function	renal function	Renal function	1	kept
renal leiomyosarcoma	renal leiomyosarcoma	renal leiomyosarcoma	renal leiomyosarcoma	Renal leiomyosarcoma	1	case_or_format
Renal medicine	renal medicine	renal medicine	renal medicine	Renal medicine	1	kept
renal oxygenation	renal oxygenation	renal oxygenation	renal oxygenation	renal oxygenation	1	kept
Renal physiology	renal physiology	renal physiology	renal physiology	Renal physiology	1	kept
renal system	renal system	renal system	renal system	renal system	1	kept
Renal therapy	renal therapy	renal therapy	renal therapy	Renal therapy	1	kept
Renal transplant	renal transplant	renal transplant	renal transplant	Renal transplant	1	kept
Renal transplantation	renal transplantation	renal transplantation	renal transplantation	Renal transplantation	1	kept
Renal tubular acidosis	renal tubular acidosis	renal tubular acidosis	renal tubular acidosis	Renal tubular acidosis	1	kept
Rendering	rendering	rendering	rendering	Rendering	1	kept
renewable energy	renewable energy	renewable energy	renewable energy	renewable energy	1	kept
Renin-angiotensin-aldosterone system	renin angiotensin aldosterone system	renin angiotensin aldosterone system	renin angiotensin aldosterone system	renin angiotensin aldosterone system	1	case_or_format
renin-angiotensin-aldosterone system	renin angiotensin aldosterone system	renin angiotensin aldosterone system	renin angiotensin aldosterone system	renin angiotensin aldosterone system	1	case_or_format

renin-angiotensin-aldosterone system blockers	renin angiotensin aldosterone system blockers	renin angiotensin aldosterone system blockers	renin angiotensin aldosterone system blockers	renin-angiotensin-aldosterone system blockers	1	kept
renography	renography	renography	renography	renography	1	kept
Renoprotection	renoprotection	renoprotection	renoprotection	Renoprotection	1	kept
Renovascular	renovascular	renovascular	renovascular	Renovascular	1	kept
renovascular disease	renovascular disease	renovascular disease	renovascular disease	renovascular disease	1	kept
renovascular hypertension	renovascular hypertension	renovascular hypertension	renovascular hypertension	renovascular hypertension	1	kept
Renu Gel	renu gel	renu gel	renu gel	Renu Gel	1	kept
Reocardiography	reocardiography	reocardiography	reocardiography	Reocardiography	1	kept
reorganisation	reorganisation	reorganisation	reorganisation	reorganisation	1	kept
Reorganization	reorganization	reorganization	reorganization	Reorganization	1	kept
reoxygenation	reoxygenation	reoxygenation	reoxygenation	reoxygenation	1	kept
repaglinide	repaglinide	repaglinide	repaglinide	repaglinide	1	kept
Repeat caesarean section	repeat caesarean section	repeat caesarean section	repeat caesarean section	Repeat caesarean section	1	kept
repeat cesarean section	repeat cesarean section	repeat cesarean section	repeat cesarean section	repeat cesarean section	1	kept
Repeat MET calls	repeat met calls	repeat met calls	repeat met calls	Repeat MET calls	1	kept
Repeatable	repeatable	repeatable	repeatable	Repeatable	1	kept

Repeatable Battery for the Assessment of [illegible]	repeatable battery for the assessment of [illegible]	repeatable battery for the assessment of [illegible]	repeatable battery for the assessment of [illegible]	Repeatable Battery for the Assessment of [illegible]	1	kept
repeated education	repeated education	repeated education	repeated education	repeated education	1	kept
Repeated measurement	repeated measurement	repeated measurement	repeated measurement	Repeated measurement	1	kept
Repeated measures	repeated measures	repeated measures	repeated measures	Repeated measures	1	kept
repeated measures	repeated measures	repeated measures	repeated measures	Repeated measures	1	case_or_format
Repeated simulation	repeated simulation	repeated simulation	repeated simulation	Repeated simulation	1	kept
repeated simulations	repeated simulations	repeated simulations	repeated simulation	Repeated simulation	1	singular
Repeating simulations	repeating simulations	repeating simulations	repeating simulations	Repeating simulations	1	kept
reperfusion hépatique	reperfusion h patique	reperfusion h patique	reperfusion h patique	reperfusion hépatique	1	kept
repetitive immersive simulation	repetitive immersive simulation	repetitive immersive simulation	repetitive immersive simulation	repetitive immersive simulation	1	kept
Repetitive Saliva Swallowing Test Score	repetitive saliva swallowing test score	repetitive saliva swallowing test score	repetitive saliva swallowing test score	Repetitive Saliva Swallowing Test Score	1	kept
Repetitive Sequences, Nucleic Acid	repetitive sequences nucleic acid	repetitive sequences nucleic acid	repetitive sequences nucleic acid	Repetitive Sequences, Nucleic Acid	1	kept
repetitive transcranial magnetic stimulation	repetitive transcranial magnetic stimulation	repetitive transcranial magnetic stimulation	repetitive transcranial magnetic stimulation	repetitive transcranial magnetic stimulation	1	kept
replacement	replacement	replacement	replacement	replacement	1	kept
REPLACEMENT THERAPY	replacement therapy	replacement therapy	replacement therapy	REPLACEMENT THERAPY	1	kept
replacement therapy	replacement therapy	replacement therapy	replacement therapy	REPLACEMENT THERAPY	1	case_or_format

Replicability	replicability	replicability	replicability	replicability	1	case_or_format
Replicable	replicable	replicable	replicable	Replicable	1	kept
Repolarization	repolarization	repolarization	repolarization	Repolarization	1	kept
report	report	report	report	report	1	kept
report control	report control	report control	report control	report control	1	kept
report standardization	report standardization	report standardization	report standardization	report standardization	1	kept
report-writing	report writing	report writing	report writing	report-writing	1	kept
Reported incidence	reported incidence	reported incidence	reported incidence	Reported incidence	1	kept
Reported medication errors	reported medication errors	reported medication errors	reported medication errors	Reported medication errors	1	kept
Reportes del caso	reportes del caso	reportes del caso	reportes del caso	Reportes del caso	1	kept
reporting bias	reporting bias	reporting bias	reporting bias	reporting bias	1	kept
REPORTING PROGRAM	reporting program	reporting program	reporting program	REPORTING PROGRAM	1	kept
REPORTING QUALITATIVE RESEARCH	reporting qualitative research	reporting qualitative research	reporting qualitative research	REPORTING QUALITATIVE RESEARCH	1	kept
Reporting radiographer	reporting radiographer	reporting radiographer	reporting radiographer	Reporting radiographer	1	kept
reporting rate	reporting rate	reporting rate	reporting rate	reporting rate	1	kept
Reports	reports	reports	report	report	1	singular

reposition	reposition	reposition	reposition	reposition	1	kept
repositioning mattress	repositioning mattress	repositioning mattress	repositioning mattress	repositioning mattress	1	kept
Represent	represent	represent	represent	Represent	1	kept
representation	representation	representation	representation	Representation	1	case_or_format
representative claim	representative claim	representative claim	representative claim	representative claim	1	kept
Reprocessed single-use suction catheter	reprocessed single use suction catheter	reprocessed single use suction catheter	reprocessed single use suction catheter	Reprocessed single-use suction catheter	1	kept
Reproducibilities	reproducibilities	reproducibilities	reproducibility	reproducibility	1	singular
REPRODUCIBILITY	reproducibility	reproducibility	reproducibility	reproducibility	1	case_or_format
Reproducibility	reproducibility	reproducibility	reproducibility	reproducibility	1	case_or_format
Reproduction	reproduction	reproduction	reproduction	reproduction	1	case_or_format
reptile	reptile	reptile	reptile	reptile	1	kept
Republic of Moldova	republic of moldova	republic of moldova	republic of moldova	Republic of Moldova	1	kept
Republic of Srpska	republic of srpska	republic of srpska	republic of srpska	Republic of Srpska	1	kept
Requester	requester	requester	requester	Requester	1	kept
requirements analysis and design	requirements analysis and design	requirements analysis and design	requirements analysis and design	requirements analysis and design	1	kept
REQUIRING MECHANICAL VENTILATION	requiring mechanical ventilation	requiring mechanical ventilation	requiring mechanical ventilation	REQUIRING MECHANICAL VENTILATION	1	kept

rescue simulation	rescue simulation	rescue simulation	rescue simulation	rescue simulation	1	kept
Rescue therapy	rescue therapy	rescue therapy	rescue therapy	Rescue therapy	1	kept
Rescue work	rescue work	rescue work	rescue work	Rescue Work	1	case_or_format
Rescuer fatigue	rescuer fatigue	rescuer fatigue	rescuer fatigue	Rescuer fatigue	1	kept
Research activities	research activities	research activities	research activities	Research activities	1	kept
Research agenda	research agenda	research agenda	research agenda	Research agenda	1	kept
research agenda	research agenda	research agenda	research agenda	Research agenda	1	case_or_format
research capacity	research capacity	research capacity	research capacity	research capacity	1	kept
Research consortium	research consortium	research consortium	research consortium	Research consortium	1	kept
Research critique	research critique	research critique	research critique	Research critique	1	kept
research culture	research culture	research culture	research culture	research culture	1	kept
research delivery	research delivery	research delivery	research delivery	research delivery	1	kept
Research design stage	research design stage	research design stage	research design stage	Research design stage	1	kept
Research Diagnostic Criteria	research diagnostic criteria	research diagnostic criteria	research diagnostic criteria	Research Diagnostic Criteria	1	kept
research evaluation	research evaluation	research evaluation	research evaluation	research evaluation	1	kept
Research frontier	research frontier	research frontier	research frontier	Research frontier	1	kept

research gap	research gap	research gap	research gap	research gap	1	kept
research institute	research institute	research institute	research institute	research institute	1	kept
Research instrument	research instrument	research instrument	research instrument	Research instrument	1	kept
research intensity	research intensity	research intensity	research intensity	research intensity	1	kept
research method	research method	research method	research method	research methods	1	case_or_format
Research methods	research methods	research methods	research method	research methods	1	singular
research metrics	research metrics	research metrics	research metrics	research metrics	1	kept
Research models	research models	research models	research models	Research models	1	kept
Research papers	research papers	research papers	research papers	Research papers	1	kept
research practice	research practice	research practice	research practice	research practice	1	kept
RESEARCH PRIORITIES	research priorities	research priorities	research priority	Research priorities	1	singular
Research Priorities	research priorities	research priorities	research priority	Research priorities	1	singular
Research prioritisation	research prioritisation	research prioritisation	research prioritisation	Research prioritisation	1	kept
research prioritization exercise	research prioritization exercise	research prioritization exercise	research prioritization exercise	research prioritization exercise	1	kept
Research progress	research progress	research progress	research progress	Research progress	1	kept
Research protocol	research protocol	research protocol	research protocol	research protocol	1	case_or_format

Research regulatory	research regulatory	research regulatory	research regulatory	Research regulatory	1	kept
Research results	research results	research results	research results	Research results	1	kept
research setting	research setting	research setting	research setting	research setting	1	kept
research staff physician	research staff physician	research staff physician	research staff physician	research staff physician	1	kept
research strategy	research strategy	research strategy	research strategy	research strategy	1	kept
Research studies	research studies	research studies	research studies	Research studies	1	kept
research studies	research studies	research studies	research studies	Research studies	1	case_or_format
Research subjects	research subjects	research subjects	research subject	Research Subjects	1	singular
Research Support	research support	research support	research support	Research Support	1	kept
Research teams	research teams	research teams	research teams	Research teams	1	kept
research techniques	research techniques	research techniques	research techniques	research techniques	1	kept
Research tools	research tools	research tools	research tools	Research tools	1	kept
research tools	research tools	research tools	research tools	Research tools	1	case_or_format
Research topic	research topic	research topic	research topic	Research topic	1	kept
Research translation	research translation	research translation	research translation	Research translation	1	kept
research use	research use	research use	research use	research use	1	kept

research utilization	research utilization	research utilization	research utilization	Research utilization	1	case_or_format
research, qualitative	research qualitative	research qualitative	research qualitative	research, qualitative	1	kept
research-informed design	research informed design	research informed design	research informed design	research-informed design	1	kept
research-quantitative	research quantitative	research quantitative	research quantitative	research-quantitative	1	kept
Research: quantitative	research quantitative	research quantitative	research quantitative	research-quantitative	1	case_or_format
researcher - participant relationship	researcher participant relationship	researcher participant relationship	researcher participant relationship	researcher - participant relationship	1	kept
Resection	resection	resection	resection	Resection	1	kept
reserpine	reserpine	reserpine	reserpine	reserpine	1	kept
Residence time	residence time	residence time	residence time	Residence time	1	kept
RESIDENCY PROGRAMS	residency programs	residency programs	residency programs	RESIDENCY PROGRAMS	1	kept
Residency programs	residency programs	residency programs	residency programs	RESIDENCY PROGRAMS	1	case_or_format
RESIDENT	resident	resident	resident	resident	1	case_or_format
Resident Assessment Instrument Home Care	resident assessment instrument home care	resident assessment instrument home care	resident assessment instrument home care	Resident Assessment Instrument Home Care	1	kept
resident champion	resident champion	resident champion	resident champion	resident champion	1	kept
resident competency	resident competency	resident competency	resident competency	resident competency	1	kept
resident duty hours	resident duty hours	resident duty hours	resident duty hours	resident duty hours	1	kept

resident evaluation	resident evaluation	resident evaluation	resident evaluation	resident evaluation	1	kept
RESIDENT OUTCOMES	resident outcomes	resident outcomes	resident outcomes	RESIDENT OUTCOMES	1	kept
resident performance	resident performance	resident performance	resident performance	resident performance	1	kept
resident research	resident research	resident research	resident research	resident research	1	kept
RESIDENT SATISFACTION	resident satisfaction	resident satisfaction	resident satisfaction	RESIDENT SATISFACTION	1	kept
Resident scheduling	resident scheduling	resident scheduling	resident scheduling	Resident scheduling	1	kept
Resident training	resident training	resident training	resident training	resident training	1	case_or_format
Residential aged care	residential aged care	residential aged care	residential aged care	Residential aged care	1	kept
Residential aged care facilities	residential aged care facilities	residential aged care facilities	residential aged care facilities	Residential aged care facilities	1	kept
Residential care centre	residential care centre	residential care centre	residential care centre	Residential care centre	1	kept
Residential care facilities	residential care facilities	residential care facilities	residential care facilities	Residential care facilities	1	kept
residential facilities	residential facilities	residential facilities	residential facilities	Residential Facilities	1	case_or_format
residential mobility	residential mobility	residential mobility	residential mobility	residential mobility	1	kept
RESIDENTS FAMILIES	residents families	residents families	residents families	RESIDENTS FAMILIES	1	kept
RESIDUAL GASTRIC VOLUME	residual gastric volume	residual gastric volume	residual gastric volume	RESIDUAL GASTRIC VOLUME	1	kept
Residual lesion score	residual lesion score	residual lesion score	residual lesion score	Residual lesion score	1	kept

residual paralysis	residual paralysis	residual paralysis	residual paralysis	residual paralysis	1	kept
Residual risk	residual risk	residual risk	residual risk	Residual risk	1	kept
residual risk	residual risk	residual risk	residual risk	Residual risk	1	case_or_format
residual urine	residual urine	residual urine	residual urine	residual urine	1	kept
Residual volume	residual volume	residual volume	residual volume	residual volume	1	case_or_format
residue analysis	residue analysis	residue analysis	residue analysis	residue analysis	1	kept
resign	resign	resign	resign	resign	1	kept
resign intention	resign intention	resign intention	resign intention	resign intention	1	kept
resilience and death anxiety	resilience and death anxiety	resilience and death anxiety	resilience and death anxiety	resilience and death anxiety	1	kept
Resilience engineerings	resilience engineerings	resilience engineerings	resilience engineering	Resilience engineering	1	singular
Resilience in healthcare	resilience in healthcare	resilience in healthcare	resilience in healthcare	Resilience in healthcare	1	kept
Resilient systems	resilient systems	resilient systems	resilient systems	Resilient systems	1	kept
resillience	resillience	resillience	resillience	resillience	1	kept
resin	resin	resin	resin	resin	1	kept
resistance and hierarchy	resistance and hierarchy	resistance and hierarchy	resistance and hierarchy	resistance and hierarchy	1	kept
resistance exercise	resistance exercise	resistance exercise	resistance exercise	resistance exercise	1	kept

Resistance patterns	resistance patterns	resistance patterns	resistance patterns	Resistance patterns	1	kept
Resistance surveillance	resistance surveillance	resistance surveillance	resistance surveillance	Resistance surveillance	1	kept
Resistance to change	resistance to change	resistance to change	resistance to change	Resistance to change	1	kept
Resistant	resistant	resistant	resistant	RESISTANT	1	case_or_format
resistant bacteremia	resistant bacteremia	resistant bacteremia	resistant bacteremia	resistant bacteremia	1	kept
RESISTANT ESCHERICHIA-COLI	resistant escherichia coli	resistant escherichia coli	resistant escherichia coli	RESISTANT ESCHERICHIA-COLI	1	kept
RESISTANT KLEBSIELLA-PNEUMONIAE	resistant klebsiella pneumoniae	resistant klebsiella pneumoniae	resistant klebsiella pneumoniae	RESISTANT KLEBSIELLA-PNEUMONIAE	1	kept
RESISTANT PSEUDOMONAS-AERUGINOSA	resistant pseudomonas aeruginosa	resistant pseudomonas aeruginosa	resistant pseudomonas aeruginosa	RESISTANT PSEUDOMONAS-AERUGINOSA	1	kept
resistin	resistin	resistin	resistin	resistin	1	kept
resistive exercise	resistive exercise	resistive exercise	resistive exercise	resistive exercise	1	kept
Resistor arrays	resistor arrays	resistor arrays	resistor arrays	Resistor arrays	1	kept
RESOLUTION	resolution	resolution	resolution	RESOLUTION	1	kept
Resource	resource	resource	resource	Resources	1	case_or_format
Resource and care	resource and care	resource and care	resource and care	Resource and care	1	kept
Resource capacity	resource capacity	resource capacity	resource capacity	Resource capacity	1	kept
Resource constraints	resource constraints	resource constraints	resource constraints	Resource constraints	1	kept

resource cost	resource cost	resource cost	resource cost	resource cost	1	kept
Resource depletion	resource depletion	resource depletion	resource depletion	Resource depletion	1	kept
resource deployment	resource deployment	resource deployment	resource deployment	resource deployment	1	kept
Resource distribution	resource distribution	resource distribution	resource distribution	Resource distribution	1	kept
resource limitation	resource limitation	resource limitation	resource limitation	resource limitation	1	kept
Resource limitations	resource limitations	resource limitations	resource limitation	resource limitation	1	singular
resource limited	resource limited	resource limited	resource limited	resource limited	1	kept
Resource management	resource management	resource management	resource management	resource management	1	case_or_format
resource restricted setting	resource restricted setting	resource restricted setting	resource restricted setting	resource restricted setting	1	kept
Resource sharing	resource sharing	resource sharing	resource sharing	Resource sharing	1	kept
resource teams	resource teams	resource teams	resource teams	resource teams	1	kept
RESOURCE USE	resource use	resource use	resource use	resource use	1	case_or_format
resource variable	resource variable	resource variable	resource variable	resource variable	1	kept
Resource-limited	resource limited	resource limited	resource limited	resource limited	1	case_or_format
resource-limited	resource limited	resource limited	resource limited	resource limited	1	case_or_format
Resource-limited setting	resource limited setting	resource limited setting	resource limited setting	resource limited setting	1	case_or_format

Resource-saving	resource saving	resource saving	resource saving	Resource-saving	1	kept
resourcefulness	resourcefulness	resourcefulness	resourcefulness	resourcefulness	1	kept
resources adequacy	resources adequacy	resources adequacy	resources adequacy	resources adequacy	1	kept
Resources on pain management	resources on pain management	resources on pain management	resources on pain management	Resources on pain management	1	kept
RESPECT	respect	respect	respect	respect	1	case_or_format
Respectful communication	respectful communication	respectful communication	respectful communication	Respectful communication	1	kept
Respectful maternity care	respectful maternity care	respectful maternity care	respectful maternity care	Respectful maternity care	1	kept
Respiración artificial	respiraci n artificial	respiraci n artificial	respiraci n artificial	Respiración artificial	1	kept
Respiration Artificial	respiration artificial	artificial ventilation	artificial ventilation	Artificial Ventilation	1	alias
respiration artificial	respiration artificial	artificial ventilation	artificial ventilation	Artificial Ventilation	1	alias
Respiration disorders	respiration disorders	respiration disorders	respiration disorders	Respiration Disorders	1	case_or_format
Respiration rate	respiration rate	respiration rate	respiration rate	Respiration rate	1	kept
respiration support	respiration support	respiration support	respiration support	respiration support	1	kept
respiration, artificial	respiration artificial	artificial ventilation	artificial ventilation	Artificial Ventilation	1	alias
Respiration, artificial [MeSH]	respiration artificial mesh	respiration artificial mesh	respiration artificial mesh	Respiration, artificial [MeSH]	1	kept
Respiration, Artificial [methods]	respiration artificial methods	respiration artificial methods	respiration artificial methods	Respiration, Artificial [methods]	1	kept

Respiration-artificial	respiration artificial	artificial ventilation	artificial ventilation	Artificial Ventilation	1	alias
respirations	respirations	respirations	respiration	Respiration	1	singular
respirator fit	respirator fit	respirator fit	respirator fit	respirator fit	1	kept
Respiratory Aerosols and Droplets	respiratory aerosols and droplets	respiratory aerosols and droplets	respiratory aerosols and droplets	Respiratory Aerosols and Droplets	1	kept
Respiratory airways	respiratory airways	respiratory airways	respiratory airways	Respiratory airways	1	kept
Respiratory and motor rehabilitation	respiratory and motor rehabilitation	respiratory and motor rehabilitation	respiratory and motor rehabilitation	Respiratory and motor rehabilitation	1	kept
Respiratory aspiration	respiratory aspiration	respiratory aspiration	respiratory aspiration	Respiratory Aspiration	1	case_or_format
respiratory aspiration	respiratory aspiration	respiratory aspiration	respiratory aspiration	Respiratory Aspiration	1	case_or_format
Respiratory assessment	respiratory assessment	respiratory assessment	respiratory assessment	Respiratory assessment	1	kept
Respiratory asynchronies	respiratory asynchronies	respiratory asynchronies	respiratory asynchronies	Respiratory asynchronies	1	kept
respiratory care center	respiratory care center	respiratory care center	respiratory care center	respiratory care center	1	kept
Respiratory care practice	respiratory care practice	respiratory care practice	respiratory care practice	respiratory care practice	1	case_or_format
Respiratory care units	respiratory care units	respiratory care units	respiratory care units	Respiratory Care Units	1	case_or_format
respiratory care units	respiratory care units	respiratory care units	respiratory care units	Respiratory Care Units	1	case_or_format
Respiratory complication	respiratory complication	respiratory complication	respiratory complication	Respiratory complication	1	kept
respiratory complication	respiratory complication	respiratory complication	respiratory complication	Respiratory complication	1	case_or_format

respiratory culture	respiratory culture	respiratory culture	respiratory culture	respiratory culture	1	kept
Respiratory department	respiratory department	respiratory department	respiratory department	Respiratory department	1	kept
RESPIRATORY DEPRESSION	respiratory depression	respiratory depression	respiratory depression	respiratory depression	1	case_or_format
Respiratory depression	respiratory depression	respiratory depression	respiratory depression	respiratory depression	1	case_or_format
Respiratory device-related pressure injury	respiratory device related pressure injury	respiratory device related pressure injury	respiratory device related pressure injury	Respiratory device-related pressure injury	1	kept
Respiratory disease	respiratory disease	respiratory disease	respiratory disease	Respiratory diseases	1	case_or_format
respiratory disorder	respiratory disorder	respiratory disorder	respiratory disorder	respiratory disorder	1	kept
Respiratory disorders	respiratory disorders	respiratory disorders	respiratory disorder	respiratory disorder	1	singular
Respiratory Distress	respiratory distress	respiratory distress	respiratory distress	respiratory distress	1	case_or_format
respiratory distress observation scale	respiratory distress observation scale	respiratory distress observation scale	respiratory distress observation scale	respiratory distress observation scale	1	kept
Respiratory distress syndrom	respiratory distress syndrom	respiratory distress syndrom	respiratory distress syndrom	Respiratory distress syndrom	1	kept
respiratory distress syndrome of preterm infant	respiratory distress syndrome of preterm infant	respiratory distress syndrome of preterm infant	respiratory distress syndrome of preterm infant	respiratory distress syndrome of preterm infant	1	kept
Respiratory distress syndrome RDS)	respiratory distress syndrome rds	respiratory distress syndrome rds	respiratory distress syndrome rds	Respiratory distress syndrome RDS)	1	kept
respiratory distress syndrome, acute	respiratory distress syndrome acute	respiratory distress syndrome acute	respiratory distress syndrome acute	respiratory distress syndrome, acute	1	kept
Respiratory Distress Syndrome, Adult [mortality	respiratory distress syndrome adult mortality	respiratory distress syndrome adult mortality	respiratory distress syndrome adult mortality	Respiratory Distress Syndrome, Adult [mortality	1	kept
respiratory disturbance index	respiratory disturbance index	respiratory disturbance index	respiratory disturbance index	respiratory disturbance index	1	kept

Respiratory dysfunction	respiratory dysfunction	respiratory dysfunction	respiratory dysfunction	Respiratory dysfunction	1	kept
respiratory dystrophy	respiratory dystrophy	respiratory dystrophy	respiratory dystrophy	respiratory dystrophy	1	kept
respiratory effort	respiratory effort	respiratory effort	respiratory effort	respiratory effort	1	kept
Respiratory exacerbation	respiratory exacerbation	respiratory exacerbation	respiratory exacerbation	Respiratory exacerbation	1	kept
Respiratory Failure	respiratory failure	respiratory failure	respiratory failure	respiratory failure	1	case_or_format
respiratory features	respiratory features	respiratory features	respiratory features	respiratory features	1	kept
Respiratory function monitor	respiratory function monitor	respiratory function monitor	respiratory function monitor	Respiratory function monitor	1	kept
respiratory function monitor	respiratory function monitor	respiratory function monitor	respiratory function monitor	Respiratory function monitor	1	case_or_format
Respiratory function monitoring	respiratory function monitoring	respiratory function monitoring	respiratory function monitoring	Respiratory function monitoring	1	kept
Respiratory function tests	respiratory function tests	respiratory function tests	respiratory function tests	Respiratory Function Tests	1	case_or_format
respiratory functions	respiratory functions	respiratory functions	respiratory function	respiratory function	1	singular
RESPIRATORY HEALTH	respiratory health	respiratory health	respiratory health	RESPIRATORY HEALTH	1	kept
Respiratory high dependency units	respiratory high dependency units	respiratory high dependency units	respiratory high dependency units	Respiratory high dependency units	1	kept
respiratory hypersensitivity	respiratory hypersensitivity	respiratory hypersensitivity	respiratory hypersensitivity	respiratory hypersensitivity	1	kept
RESPIRATORY ILLNESS	respiratory illness	respiratory illness	respiratory illness	RESPIRATORY ILLNESS	1	kept
respiratory illness screening tool	respiratory illness screening tool	respiratory illness screening tool	respiratory illness screening tool	respiratory illness screening tool	1	kept

respiratory incident	respiratory incident	respiratory incident	respiratory incident	respiratory incident	1	kept
Respiratory Indices	respiratory indices	respiratory indices	respiratory indices	Respiratory Indices	1	kept
respiratory inductance plethysmography	respiratory inductance plethysmography	respiratory inductance plethysmography	respiratory inductance plethysmography	respiratory inductance plethysmography	1	kept
Respiratory infection	respiratory infection	respiratory infection	respiratory infection	respiratory infections	1	case_or_format
respiratory Infection	respiratory infection	respiratory infection	respiratory infection	respiratory infections	1	case_or_format
Respiratory infectious disease	respiratory infectious disease	respiratory infectious disease	respiratory infectious disease	Respiratory infectious disease	1	kept
respiratory instability	respiratory instability	respiratory instability	respiratory instability	respiratory instability	1	kept
respiratory insufficience	respiratory insufficience	respiratory insufficience	respiratory insufficience	respiratory insufficience	1	kept
RESPIRATORY INSUFFICIENCY	respiratory insufficiency	respiratory insufficiency	respiratory insufficiency	Respiratory Insufficiency	1	case_or_format
Respiratory insufficiency or failure	respiratory insufficiency or failure	respiratory insufficiency or failure	respiratory insufficiency or failure	Respiratory insufficiency or failure	1	kept
Respiratory insuficiency	respiratory insuficiency	respiratory insuficiency	respiratory insuficiency	Respiratory insuficiency	1	kept
Respiratory intermediate care unit	respiratory intermediate care unit	respiratory intermediate care unit	respiratory intermediate care unit	Respiratory intermediate care unit	1	kept
respiratory intermediate care unit	respiratory intermediate care unit	respiratory intermediate care unit	respiratory intermediate care unit	Respiratory intermediate care unit	1	case_or_format
Respiratory intermediate unit	respiratory intermediate unit	respiratory intermediate unit	respiratory intermediate unit	Respiratory intermediate unit	1	kept
respiratory intermediate unit	respiratory intermediate unit	respiratory intermediate unit	respiratory intermediate unit	Respiratory intermediate unit	1	case_or_format
Respiratory Kinesitherapy	respiratory kinesitherapy	respiratory kinesitherapy	respiratory kinesitherapy	Respiratory Kinesitherapy	1	kept

RESPIRATORY MANAGEMENT	respiratory management	respiratory management	respiratory management	Respiratory management	1	case_or_format
Respiratory mechanics	respiratory mechanics	respiratory mechanics	respiratory mechanics	Respiratory Mechanics	1	case_or_format
Respiratory mechanics indicators	respiratory mechanics indicators	respiratory mechanics indicators	respiratory mechanics indicators	Respiratory mechanics indicators	1	kept
Respiratory Medicine	respiratory medicine	respiratory medicine	respiratory medicine	RESPIRATORY MEDICINE	1	case_or_format
Respiratory medicine	respiratory medicine	respiratory medicine	respiratory medicine	RESPIRATORY MEDICINE	1	case_or_format
Respiratory Monitoring	respiratory monitoring	respiratory monitoring	respiratory monitoring	Respiratory Monitoring	1	kept
Respiratory monitoring	respiratory monitoring	respiratory monitoring	respiratory monitoring	Respiratory Monitoring	1	case_or_format
Respiratory muscle	respiratory muscle	respiratory muscle	respiratory muscle	Respiratory Muscles	1	case_or_format
respiratory muscle	respiratory muscle	respiratory muscle	respiratory muscle	Respiratory Muscles	1	case_or_format
Respiratory muscle function	respiratory muscle function	respiratory muscle function	respiratory muscle function	Respiratory muscle function	1	kept
Respiratory muscle weakness	respiratory muscle weakness	respiratory muscle weakness	respiratory muscle weakness	Respiratory muscle weakness	1	kept
respiratory muscle weakness	respiratory muscle weakness	respiratory muscle weakness	respiratory muscle weakness	Respiratory muscle weakness	1	case_or_format
respiratory outcome	respiratory outcome	respiratory outcome	respiratory outcome	respiratory outcome	1	kept
respiratory parameters	respiratory parameters	respiratory parameters	respiratory parameters	respiratory parameters	1	kept
respiratory physiology	respiratory physiology	respiratory physiology	respiratory physiology	Respiratory Physiology	1	case_or_format
Respiratory physiotherapist	respiratory physiotherapist	respiratory physiotherapist	respiratory physiotherapist	Respiratory physiotherapist	1	kept

Respiratory physiotherapy	respiratory physiotherapy	respiratory physiotherapy	respiratory physiotherapy	Respiratory physiotherapy	1	kept
Respiratory problems	respiratory problems	respiratory problems	respiratory problems	Respiratory problems	1	kept
respiratory prosthesis	respiratory prosthesis	respiratory prosthesis	respiratory prosthesis	respiratory prosthesis	1	kept
respiratory protection	respiratory protection	respiratory protection	respiratory protection	respiratory protection	1	kept
respiratory quotient	respiratory quotient	respiratory quotient	respiratory quotient	respiratory quotient	1	kept
respiratory rate oxygenation	respiratory rate oxygenation	respiratory rate oxygenation	respiratory rate oxygenation	respiratory rate oxygenation	1	kept
Respiratory rates	respiratory rates	respiratory rates	respiratory rate	Respiratory Rate	1	singular
respiratory rehabilitation	respiratory rehabilitation	respiratory rehabilitation	respiratory rehabilitation	Respiratory rehabilitation	1	case_or_format
respiratory score	respiratory score	respiratory score	respiratory score	respiratory score	1	kept
Respiratory sensor systems	respiratory sensor systems	respiratory sensor systems	respiratory sensor systems	Respiratory sensor systems	1	kept
respiratory Sequential Organ Failure Assessment score	respiratory sequential organ failure assessment score	respiratory sequential organ failure assessment score	respiratory sequential organ failure assessment score	respiratory Sequential Organ Failure Assessment score	1	kept
respiratory sinus arrhythmia	respiratory sinus arrhythmia	respiratory sinus arrhythmia	respiratory sinus arrhythmia	respiratory sinus arrhythmia	1	kept
respiratory status	respiratory status	respiratory status	respiratory status	respiratory status	1	kept
Respiratory symptoms	respiratory symptoms	respiratory symptoms	respiratory symptoms	Respiratory symptoms	1	kept
respiratory symptoms	respiratory symptoms	respiratory symptoms	respiratory symptoms	Respiratory symptoms	1	case_or_format
respiratory syncytial virus infections	respiratory syncytial virus infections	respiratory syncytial virus infections	respiratory syncytial virus infection	Respiratory Syncytial Virus Infections	1	singular

respiratory syncytial virus maternal vaccine	respiratory syncytial virus maternal vaccine	respiratory syncytial virus maternal vaccine	respiratory syncytial virus maternal vaccine	respiratory syncytial virus maternal vaccine	1	kept
Respiratory Syncytial Virus Vaccines	respiratory syncytial virus vaccines	respiratory syncytial virus vaccines	respiratory syncytial virus vaccine	respiratory syncytial virus vaccine	1	singular
Respiratory syncytial viruses	respiratory syncytial viruses	respiratory syncytial viruses	respiratory syncytial viruses	Respiratory Syncytial Viruses	1	case_or_format
respiratory system and disorders	respiratory system and disorders	respiratory system and disorders	respiratory system and disorders	respiratory system and disorders	1	kept
Respiratory system leakage	respiratory system leakage	respiratory system leakage	respiratory system leakage	Respiratory system leakage	1	kept
Respiratory Therapist	respiratory therapist	respiratory therapist	respiratory therapist	respiratory therapist	1	case_or_format
respiratory therapist attitude	respiratory therapist attitude	respiratory therapist attitude	respiratory therapist attitude	respiratory therapist attitude	1	kept
Respiratory Therapists	respiratory therapists	respiratory therapists	respiratory therapist	respiratory therapist	1	singular
Respiratory therapy students	respiratory therapy students	respiratory therapy students	respiratory therapy students	Respiratory therapy students	1	kept
respiratory therapy students	respiratory therapy students	respiratory therapy students	respiratory therapy students	Respiratory therapy students	1	case_or_format
Respiratory tract	respiratory tract	respiratory tract	respiratory tract	Respiratory tract	1	kept
respiratory tract allergy	respiratory tract allergy	respiratory tract allergy	respiratory tract allergy	respiratory tract allergy	1	kept
respiratory tract diseases	respiratory tract diseases	respiratory tract diseases	respiratory tract disease	respiratory tract disease	1	singular
Respiratory tract disorders	respiratory tract disorders	respiratory tract disorders	respiratory tract disorders	Respiratory tract disorders	1	kept
respiratory tract inflammation	respiratory tract inflammation	respiratory tract inflammation	respiratory tract inflammation	respiratory tract inflammation	1	kept
Respiratory training	respiratory training	respiratory training	respiratory training	Respiratory training	1	kept

Respiratory Transport	respiratory transport	respiratory transport	respiratory transport	Respiratory Transport	1	kept
Respiratory variables	respiratory variables	respiratory variables	respiratory variables	Respiratory variables	1	kept
respiratory viral pathogens	respiratory viral pathogens	respiratory viral pathogens	respiratory viral pathogens	respiratory viral pathogens	1	kept
Respiratory virus	respiratory virus	respiratory virus	respiratory virus	respiratory virus	1	case_or_format
Respiratory weaning	respiratory weaning	respiratory weaning	respiratory weaning	Respiratory weaning	1	kept
RESPIRATORY-DISTRESS	respiratory distress	respiratory distress	respiratory distress	respiratory distress	1	case_or_format
RESPIRATORY-INFECTION	respiratory infection	respiratory infection	respiratory infection	respiratory infections	1	case_or_format
RESPIRATORY-TRACT	respiratory tract	respiratory tract	respiratory tract	Respiratory tract	1	case_or_format
respirometry	respirometry	respirometry	respirometry	respirometry	1	kept
respite	respite	respite	respite	respite	1	kept
respite rooms	respite rooms	respite rooms	respite rooms	respite rooms	1	kept
respond	respond	respond	respond	respond	1	kept
responder	responder	responder	responder	responder	1	kept
Responding capabilities	responding capabilities	responding capabilities	responding capabilities	Responding capabilities	1	kept
responsabilité	responsabilit	responsabilit	responsabilit	responsabilité	1	kept
response bias	response bias	response bias	response bias	response bias	1	kept

Response proportions	response proportions	response proportions	response proportions	Response proportions	1	kept
response rate	response rate	response rate	response rate	Response rate	1	case_or_format
RESPONSE RATES	response rates	response rates	response rate	Response rate	1	singular
response rates	response rates	response rates	response rate	Response rate	1	singular
Response systems	response systems	response systems	response systems	Response systems	1	kept
RESPONSE TEAM	response team	response team	response team	RESPONSE TEAM	1	kept
Response time	response time	response time	response time	response time	1	case_or_format
response variable	response variable	response variable	response variable	response variable	1	kept
responses rates	responses rates	responses rates	responses rates	responses rates	1	kept
RESPONSIBILITY	responsibility	responsibility	responsibility	responsibility	1	case_or_format
RESPONSIBLE CLINICIAN	responsible clinician	responsible clinician	responsible clinician	RESPONSIBLE CLINICIAN	1	kept
Responsive bassinet	responsive bassinet	responsive bassinet	responsive bassinet	responsive bassinet	1	case_or_format
Responsiveness	responsiveness	responsiveness	responsiveness	Responsiveness	1	kept
responsiveness	responsiveness	responsiveness	responsiveness	Responsiveness	1	case_or_format
Ressource management	ressource management	ressource management	ressource management	Ressource management	1	kept
Ressources	ressources	ressources	ressources	Ressources	1	kept

Rest time	rest time	rest time	rest time	Rest time	1	kept
Restaurants	restaurants	restaurants	restaurant	restaurant	1	singular
Restaurants' opening hours	restaurants opening hours	restaurants opening hours	restaurants opening hours	Restaurants' opening hours	1	kept
restenosis	restenosis	restenosis	restenosis	restenosis	1	kept
resternotomy	resternotomy	resternotomy	resternotomy	resternotomy	1	kept
Resting heart rate	resting heart rate	resting heart rate	resting heart rate	Resting heart rate	1	kept
resting heart rate	resting heart rate	resting heart rate	resting heart rate	Resting heart rate	1	case_or_format
resting motor threshold	resting motor threshold	resting motor threshold	resting motor threshold	resting motor threshold	1	kept
Restlessness	restlessness	restlessness	restlessness	restlessness	1	case_or_format
Restoring	restoring	restoring	restoring	Restoring	1	kept
restorying	restorying	restorying	restorying	restorying	1	kept
Restraint application	restraint application	restraint application	restraint application	Restraint application	1	kept
restraint assessment	restraint assessment	restraint assessment	restraint assessment	restraint assessment	1	kept
restraint management bundle	restraint management bundle	restraint management bundle	restraint management bundle	restraint management bundle	1	kept
restraint minimisation	restraint minimisation	restraint minimisation	restraint minimisation	restraint minimisation	1	kept
Restraint minimization	restraint minimization	restraint minimization	restraint minimization	Restraint minimization	1	kept

restraint minimization	restraint minimization	restraint minimization	restraint minimization	Restraint minimization	1	case_or_format
restraint monitoring	restraint monitoring	restraint monitoring	restraint monitoring	restraint monitoring	1	kept
Restraint reduction	restraint reduction	restraint reduction	restraint reduction	Restraint reduction	1	kept
Restricción Física	restricci n f sica	restricci n f sica	restricci n f sica	Restricción física	1	case_or_format
restricted family presence	restricted family presence	restricted family presence	restricted family presence	restricted family presence	1	kept
restricted visitation policy	restricted visitation policy	restricted visitation policy	restricted visitation policy	restricted visitation policy	1	kept
restriction	restriction	restriction	restriction	RESTRICTION	1	case_or_format
restriction fragment	restriction fragment	restriction fragment	restriction fragment	restriction fragment	1	kept
restriction fragment length polymorphism	restriction fragment length polymorphism	restriction fragment length polymorphism	restriction fragment length polymorphism	restriction fragment length polymorphism	1	kept
restriction mapping	restriction mapping	restriction mapping	restriction mapping	restriction mapping	1	kept
RESTRICTIONS	restrictions	restrictions	restriction	RESTRICTION	1	singular
restrictive cardiomyopathy	restrictive cardiomyopathy	restrictive cardiomyopathy	restrictive cardiomyopathy	restrictive cardiomyopathy	1	kept
Restrictive practices	restrictive practices	restrictive practices	restrictive practice	restrictive practice	1	singular
Restrictive transfusion strategy	restrictive transfusion strategy	restrictive transfusion strategy	restrictive transfusion strategy	Restrictive transfusion strategy	1	kept
restrictive visiting	restrictive visiting	restrictive visiting	restrictive visiting	restrictive visiting	1	kept
Restructure	restructure	restructure	restructure	Restructure	1	kept

Rest’ s model	rest s model	rest s model	rest s model	Rest’ s model	1	kept
Resucitación cardiopulmonar	resucitaci n cardiopulmonar	resucitaci n cardiopulmonar	resucitaci n cardiopulmonar	Resucitación cardiopulmonar	1	kept
RESUSCITATE	resuscitate	resuscitate	resuscitate	RESUSCITATE	1	kept
Resuscitation cardiopulmonary	resuscitation cardiopulmonary	resuscitation cardiopulmonary	resuscitation cardiopulmonary	Resuscitation cardiopulmonary	1	kept
resuscitation data point scoring	resuscitation data point scoring	resuscitation data point scoring	resuscitation data point scoring	resuscitation data point scoring	1	kept
resuscitation decision	resuscitation decision	resuscitation decision	resuscitation decision	Resuscitation decision	1	case_or_format
Resuscitation decisions	resuscitation decisions	resuscitation decisions	resuscitation decision	Resuscitation decision	1	singular
resuscitation education	resuscitation education	resuscitation education	resuscitation education	resuscitation education	1	kept
Resuscitation knowledge level	resuscitation knowledge level	resuscitation knowledge level	resuscitation knowledge level	Resuscitation knowledge level	1	kept
resuscitation knowledge level	resuscitation knowledge level	resuscitation knowledge level	resuscitation knowledge level	Resuscitation knowledge level	1	case_or_format
Resuscitation officers	resuscitation officers	resuscitation officers	resuscitation officers	Resuscitation officers	1	kept
Resuscitation practice	resuscitation practice	resuscitation practice	resuscitation practice	Resuscitation practice	1	kept
resuscitation preference	resuscitation preference	resuscitation preference	resuscitation preference	resuscitation preference	1	kept
RESUSCITATION-COUNCIL GUIDELINES	resuscitation council guidelines	resuscitation council guidelines	resuscitation council guidelines	RESUSCITATION-COUNCIL GUIDELINES	1	kept
Resuscitation.	resuscitation	resuscitation	resuscitation	resuscitation	1	case_or_format
Resuscitative	resuscitative	resuscitative	resuscitative	Resuscitative	1	kept

Resuscitative Endovascular Balloon of the Aorta	resuscitative endovascular balloon of the aorta	resuscitative endovascular balloon of the aorta	resuscitative endovascular balloon of the aorta	Resuscitative Endovascular Balloon of the Aorta	1	kept
resuscitative thoracotomy	resuscitative thoracotomy	resuscitative thoracotomy	resuscitative thoracotomy	Resuscitative thoracotomy	1	case_or_format
Resuscitators	resuscitators	resuscitators	resuscitators	Resuscitators	1	kept
resuscitators	resuscitators	resuscitators	resuscitators	Resuscitators	1	case_or_format
resveratrol	resveratrol	resveratrol	resveratrol	resveratrol	1	kept
Retained Airway Secretions	retained airway secretions	retained airway secretions	retained airway secretions	Retained Airway Secretions	1	kept
Retained catheter	retained catheter	retained catheter	retained catheter	Retained catheter	1	kept
retained fragment	retained fragment	retained fragment	retained fragment	retained fragment	1	kept
retained inappropriate	retained inappropriate	retained inappropriate	retained inappropriate	retained inappropriate	1	kept
Retained surgical items	retained surgical items	retained surgical items	retained surgical item	retained surgical item	1	singular
Retained wire	retained wire	retained wire	retained wire	Retained wire	1	kept
retapamulin	retapamulin	retapamulin	retapamulin	retapamulin	1	kept
RETARDATION	retardation	retardation	retardation	RETARDATION	1	kept
retching	retching	retching	retching	retching	1	kept
Retention enema	retention enema	retention enema	retention enema	Retention enema	1	kept
Retention enema device	retention enema device	retention enema device	retention enema device	Retention enema device	1	kept

retention in care	retention in care	retention in care	retention in care	retention in care	1	kept
Retention of subjects	retention of subjects	retention of subjects	retention of subjects	Retention of subjects	1	kept
retina blood vessel occlusion	retina blood vessel occlusion	retina blood vessel occlusion	retina blood vessel occlusion	retina blood vessel occlusion	1	kept
retina detachment	retina detachment	retina detachment	retina detachment	retina detachment	1	kept
retina examination	retina examination	retina examination	retina examination	retina examination	1	kept
retina fovea	retina fovea	retina fovea	retina fovea	retina fovea	1	kept
retina hemorrhage	retina hemorrhage	retina hemorrhage	retina hemorrhage	retina hemorrhage	1	kept
retina injury	retina injury	retina injury	retina injury	retina injury	1	kept
retina macula cystoid edema	retina macula cystoid edema	retina macula cystoid edema	retina macula cystoid edema	retina macula cystoid edema	1	kept
retina neovascularization	retina neovascularization	retina neovascularization	retina neovascularization	retina neovascularization	1	kept
retina tissue	retina tissue	retina tissue	retina tissue	retina tissue	1	kept
retina vasculitis	retina vasculitis	retina vasculitis	retina vasculitis	retina vasculitis	1	kept
retina vein occlusion	retina vein occlusion	retina vein occlusion	retina vein occlusion	retina vein occlusion	1	kept
Retinal Detachment	retinal detachment	retinal detachment	retinal detachment	Retinal Detachment	1	kept
Retinal fundus image	retinal fundus image	retinal fundus image	retinal fundus image	Retinal fundus image	1	kept
Retinal fundus images	retinal fundus images	retinal fundus images	retinal fundus image	Retinal fundus image	1	singular

retinal ganglion cell layer	retinal ganglion cell layer	retinal ganglion cell layer	retinal ganglion cell layer	retinal ganglion cell layer	1	kept
Retinal Hemorrhage	retinal hemorrhage	retinal hemorrhage	retinal hemorrhage	Retinal Hemorrhage	1	kept
retinal inner nuclear layer	retinal inner nuclear layer	retinal inner nuclear layer	retinal inner nuclear layer	retinal inner nuclear layer	1	kept
retinal inner plexiform layer	retinal inner plexiform layer	retinal inner plexiform layer	retinal inner plexiform layer	retinal inner plexiform layer	1	kept
retinal nerve fiber layer thickness	retinal nerve fiber layer thickness	retinal nerve fiber layer thickness	retinal nerve fiber layer thickness	retinal nerve fiber layer thickness	1	kept
retinal outer nuclear layer	retinal outer nuclear layer	retinal outer nuclear layer	retinal outer nuclear layer	retinal outer nuclear layer	1	kept
retinal outer plexiform layer	retinal outer plexiform layer	retinal outer plexiform layer	retinal outer plexiform layer	retinal outer plexiform layer	1	kept
retinal pigment epithelium	retinal pigment epithelium	retinal pigment epithelium	retinal pigment epithelium	retinal pigment epithelium	1	kept
retinal thickness	retinal thickness	retinal thickness	retinal thickness	retinal thickness	1	kept
Retinal vasculitis	retinal vasculitis	retinal vasculitis	retinal vasculitis	Retinal vasculitis	1	kept
Retinal Vessels	retinal vessels	retinal vessels	retinal vessels	Retinal Vessels	1	kept
retinoid	retinoid	retinoid	retinoid	retinoid	1	kept
retinol binding protein	retinol binding protein	retinol binding protein	retinol binding protein	retinol binding protein	1	kept
Retinopathy screening	retinopathy screening	retinopathy screening	retinopathy screening	Retinopathy screening	1	kept
retinoscope	retinoscope	retinoscope	retinoscope	retinoscope	1	kept
Retinoscopes	retinoscopes	retinoscopes	retinoscope	retinoscope	1	singular

retract reorder error	retract reorder error	retract reorder error	retract reorder error	retract reorder error	1	kept
Retractable safety needle	retractable safety needle	retractable safety needle	retractable safety needle	Retractable safety needle	1	kept
retractor	retractor	retractor	retractor	retractor	1	kept
Retransplantation	retransplantation	retransplantation	retransplantation	Retransplantation	1	kept
retransplantation	retransplantation	retransplantation	retransplantation	Retransplantation	1	case_or_format
RETRIEVAL	retrieval	retrieval	retrieval	retrieval	1	case_or_format
Retrieval co-ordinator	retrieval co ordinator	retrieval co ordinator	retrieval co ordinator	Retrieval co-ordinator	1	kept
retrieval medicine	retrieval medicine	retrieval medicine	retrieval medicine	Retrieval medicine	1	case_or_format
Retrieval teams	retrieval teams	retrieval teams	retrieval teams	Retrieval teams	1	kept
retrieval-augmented generation process	retrieval augmented generation process	retrieval augmented generation process	retrieval augmented generation process	retrieval-augmented generation process	1	kept
retrobulbar haemorrhage	retrobulbar haemorrhage	retrobulbar haemorrhage	retrobulbar haemorrhage	retrobulbar haemorrhage	1	kept
Retrognathia	retrognathia	retrognathia	retrognathia	retrognathia	1	case_or_format
retrograde amnesia	retrograde amnesia	retrograde amnesia	retrograde amnesia	retrograde amnesia	1	kept
retrograde intrarenal surgery	retrograde intrarenal surgery	retrograde intrarenal surgery	retrograde intrarenal surgery	retrograde intrarenal surgery	1	kept
retroperitoneal cancer	retroperitoneal cancer	retroperitoneal cancer	retroperitoneal cancer	retroperitoneal cancer	1	kept
retroperitoneal fibrosis	retroperitoneal fibrosis	retroperitoneal fibrosis	retroperitoneal fibrosis	retroperitoneal fibrosis	1	kept

retroperitoneal space hemorrhage	retroperitoneal space hemorrhage	retroperitoneal space hemorrhage	retroperitoneal space hemorrhage	retroperitoneal space hemorrhage	1	kept
retrospective analyse	retrospective analyse	retrospective analyse	retrospective analyse	retrospective analyse	1	kept
RETROSPECTIVE ANALYSIS	retrospective analysis	retrospective analysis	retrospective analysis	RETROSPECTIVE ANALYSIS	1	kept
Retrospective analysis	retrospective analysis	retrospective analysis	retrospective analysis	RETROSPECTIVE ANALYSIS	1	case_or_format
retrospective analysis	retrospective analysis	retrospective analysis	retrospective analysis	RETROSPECTIVE ANALYSIS	1	case_or_format
Retrospective audit	retrospective audit	retrospective audit	retrospective audit	Retrospective audit	1	kept
Retrospective case-control study	retrospective case control study	retrospective case control study	retrospective case control study	Retrospective case-control study	1	kept
retrospective cohort study	retrospective cohort study	retrospective cohort study	retrospective cohort study	retrospective cohort study	1	kept
retrospective comparative study	retrospective comparative study	retrospective comparative study	retrospective comparative study	retrospective comparative study	1	kept
Retrospective document analysis	retrospective document analysis	retrospective document analysis	retrospective document analysis	Retrospective document analysis	1	kept
retrospective observational study	retrospective observational study	retrospective observational study	retrospective observational study	retrospective observational study	1	kept
retrospective reporting	retrospective reporting	retrospective reporting	retrospective reporting	retrospective reporting	1	kept
retrospective research	retrospective research	retrospective research	retrospective research	retrospective research	1	kept
Retrotransfer	retrotransfer	retrotransfer	retrotransfer	Retrotransfer	1	kept
Return medicine	return medicine	return medicine	return medicine	Return medicine	1	kept
Return of spontaneous circulation	return of spontaneous circulation	return of spontaneous circulation	return of spontaneous circulation	return of spontaneous circulation	1	case_or_format

return of spontaneous circulation after cardiac arrest score	return of spontaneous circulation after cardiac arrest score	return of spontaneous circulation after cardiac arrest score	return of spontaneous circulation after cardiac arrest score	return of spontaneous circulation after cardiac arrest score	1	kept
Return on Investment	return on investment	return on investment	return on investment	Return on Investment	1	kept
Return on investment	return on investment	return on investment	return on investment	Return on Investment	1	case_or_format
return-to-work	return to work	return to work	return to work	return to work	1	case_or_format
Reusable linens	reusable linens	reusable linens	reusable linens	Reusable linens	1	kept
Reusable personal protective equipment	reusable personal protective equipment	reusable personal protective equipment	reusable personal protective equipment	Reusable personal protective equipment	1	kept
reusable PPE	reusable ppe	reusable ppe	reusable ppe	reusable PPE	1	kept
reusable respirator	reusable respirator	reusable respirator	reusable respirator	reusable respirator	1	kept
revaccination	revaccination	revaccination	revaccination	revaccination	1	kept
Reverberation	reverberation	reverberation	reverberation	Reverberation	1	kept
reversal	reversal	reversal	reversal	Reversal	1	case_or_format
Reversal agents	reversal agents	reversal agents	reversal agents	Reversal agents	1	kept
reverse shoulder arthroplasty	reverse shoulder arthroplasty	reverse shoulder arthroplasty	reverse shoulder arthroplasty	reverse shoulder arthroplasty	1	kept
reverse Trendelenburg	reverse trendelenburg	reverse trendelenburg	reverse trendelenburg	reverse Trendelenburg	1	kept
Reversible respiratory failure	reversible respiratory failure	reversible respiratory failure	reversible respiratory failure	Reversible respiratory failure	1	kept
reversible spinal cord ischemia	reversible spinal cord ischemia	reversible spinal cord ischemia	reversible spinal cord ischemia	reversible spinal cord ischemia	1	kept

revertant	revertant	revertant	revertant	revertant	1	kept
revetase	revetase	revetase	revetase	revetase	1	kept
Review article	review article	review article	review article	Review article	1	kept
review article	review article	review article	review article	Review article	1	case_or_format
review literature	review literature	review literature	review literature	Review literature	1	case_or_format
review literature as topic	review literature as topic	review literature as topic	review literature as topic	Review Literature as Topic	1	case_or_format
Revised clinical respiratory score	revised clinical respiratory score	revised clinical respiratory score	revised clinical respiratory score	Revised clinical respiratory score	1	kept
Revised Delirium Rating Scale	revised delirium rating scale	revised delirium rating scale	revised delirium rating scale	Revised Delirium Rating Scale	1	kept
revised nonverbal pain scale	revised nonverbal pain scale	revised nonverbal pain scale	revised nonverbal pain scale	revised nonverbal pain scale	1	kept
Revised Organ Transplant Act	revised organ transplant act	revised organ transplant act	revised organ transplant act	Revised Organ Transplant Act	1	kept
revision	revision	revision	revision	revision	1	kept
Revision surgery	revision surgery	revision surgery	revision surgery	Revision surgery	1	kept
REVISIT	revisit	revisit	revisit	Revisit	1	case_or_format
Revisión	revisi n	revisi n	revisi n	Revisión	1	kept
Revisión de alcance	revisi n de alcance	revisi n de alcance	revisi n de alcance	Revisión de alcance	1	kept
Revisión integrativa	revisi n integrativa	revisi n integrativa	revisi n integrativa	Revisión integrativa	1	kept

rewards	rewards	rewards	reward	Reward	1	singular
rewarming	rewarming	rewarming	rewarming	Rewarming	1	case_or_format
rewriting guidelines and protocol	rewriting guidelines and protocol	rewriting guidelines and protocol	rewriting guidelines and protocol	rewriting guidelines and protocol	1	kept
re - deployment	re deployment	re deployment	re deployment	re - deployment	1	kept
RF-ID tags	rf id tags	rf id tags	rf id tags	RF-ID tags	1	kept
RFDS	rfds	rfds	rfds	RFDS	1	kept
RFID Technology	rfid technology	rfid technology	rfid technology	RFID Technology	1	kept
rFLACC score	rflacc score	rflacc score	rflacc score	rFLACC score	1	kept
rFVIIa	rfviia	rfviia	rfviia	rFVIIa	1	kept
rhabdoid tumor	rhabdoid tumor	rhabdoid tumor	rhabdoid tumor	rhabdoid tumor	1	kept
rhabdomyomas	rhabdomyomas	rhabdomyomas	rhabdomyomas	rhabdomyomas	1	kept
Rhenium alloys	rhenium alloys	rhenium alloys	rhenium alloys	Rhenium alloys	1	kept
Rhenium compounds	rhenium compounds	rhenium compounds	rhenium compounds	Rhenium compounds	1	kept
Rheology	rheology	rheology	rheology	Rheology	1	kept
rhesus incompatibility	rhesus incompatibility	rhesus incompatibility	rhesus incompatibility	rhesus incompatibility	1	kept
Rheumatic Diseases	rheumatic diseases	rheumatic diseases	rheumatic disease	rheumatic disease	1	singular

Rheumatoid arthritis	rheumatoid arthritis	rheumatoid arthritis	rheumatoid arthritis	rheumatoid arthritis	1	case_or_format
rheumatoid factor	rheumatoid factor	rheumatoid factor	rheumatoid factor	rheumatoid factor	1	kept
rheumatologically diseases	rheumatologically diseases	rheumatologically diseases	rheumatologically diseases	rheumatologically diseases	1	kept
Rheumatology	rheumatology	rheumatology	rheumatology	rheumatology	1	case_or_format
rhinovirus	rhinovirus	rhinovirus	rhinovirus	Rhinovirus	1	case_or_format
Rhinovirus infection	rhinovirus infection	rhinovirus infection	rhinovirus infection	Rhinovirus infection	1	kept
Rhizopus oryzae	rhizopus oryzae	rhizopus oryzae	rhizopus oryzae	Rhizopus oryzae	1	kept
Rhodococcus hoagie	rhodococcus hoagie	rhodococcus hoagie	rhodococcus hoagie	Rhodococcus hoagie	1	kept
Rhodococcus hoagii	rhodococcus hoagii	rhodococcus hoagii	rhodococcus hoagii	Rhodococcus hoagii	1	kept
Rhodotorula	rhodotorula	rhodotorula	rhodotorula	Rhodotorula	1	kept
rhonchus	rhonchus	rhonchus	rhonchus	rhonchus	1	kept
rhPTH<sup>1-34<	rhpth sup 1 34	rhpth sup 1 34	rhpth sup 1 34	rhPTH<sup>1-34<	1	kept
Rhythmic and periodic patterns	rhythmic and periodic patterns	rhythmic and periodic patterns	rhythmic and periodic patterns	Rhythmic and periodic patterns	1	kept
rhythmic embrocation	rhythmic embrocation	rhythmic embrocation	rhythmic embrocation	rhythmic embrocation	1	kept
Rib fracture	rib fracture	rib fracture	rib fracture	rib fracture	1	case_or_format
Rib fracture scoring systems	rib fracture scoring systems	rib fracture scoring systems	rib fracture scoring systems	Rib fracture scoring systems	1	kept

Rib plating	rib plating	rib plating	rib plating	Rib plating	1	kept
riboflavin deficiency	riboflavin deficiency	riboflavin deficiency	riboflavin deficiency	riboflavin deficiency	1	kept
ribosome	ribosome	ribosome	ribosome	ribosome	1	kept
ribosome protein	ribosome protein	ribosome protein	ribosome protein	ribosome protein	1	kept
ribotyping	ribotyping	ribotyping	ribotyping	Ribotyping	1	case_or_format
rice	rice	rice	rice	rice	1	kept
Richards	richards	richards	richards	Richards	1	kept
richards campbell sleep questionnaire	richards campbell sleep questionnaire	richards campbell sleep questionnaire	richards campbell sleep questionnaire	Richards Campbell Sleep Questionnaire	1	case_or_format
Richards-Campbell sleep questionnaire	richards campbell sleep questionnaire	richards campbell sleep questionnaire	richards campbell sleep questionnaire	Richards Campbell Sleep Questionnaire	1	case_or_format
Richards－Campbell Sleep Questionnaire	richards campbell sleep questionnaire	richards campbell sleep questionnaire	richards campbell sleep questionnaire	Richards Campbell Sleep Questionnaire	1	case_or_format
Richmond agitation and sedation scale	richmond agitation and sedation scale	richmond agitation and sedation scale	richmond agitation and sedation scale	Richmond Agitation and Sedation Scale	1	case_or_format
Richmond agitation sedation scale	richmond agitation sedation scale	richmond agitation sedation scale	richmond agitation sedation scale	Richmond Agitation Sedation Scale	1	case_or_format
richmond agitation sedation scale	richmond agitation sedation scale	richmond agitation sedation scale	richmond agitation sedation scale	Richmond Agitation Sedation Scale	1	case_or_format
Richmond agitation-sedation scale	richmond agitation sedation scale	richmond agitation sedation scale	richmond agitation sedation scale	Richmond Agitation Sedation Scale	1	case_or_format
Richmond Agitation-Sedation Scale - RASS-PAL	richmond agitation sedation scale rass pal	richmond agitation sedation scale rass pal	richmond agitation sedation scale rass pal	Richmond Agitation-Sedation Scale - RASS-PAL	1	kept
Richmond Sedation Agitation Scale	richmond sedation agitation scale	richmond sedation agitation scale	richmond sedation agitation scale	Richmond Sedation Agitation Scale	1	kept

Richmond Sedation Scale	richmond sedation scale	richmond sedation scale	richmond sedation scale	Richmond Sedation Scale	1	kept
Rickettsia conorii	rickettsia conorii	rickettsia conorii	rickettsia conorii	Rickettsia conorii	1	kept
Rickettsia Infections	rickettsia infections	rickettsia infections	rickettsia infections	Rickettsia Infections	1	kept
Rickettsia prowazekii	rickettsia prowazekii	rickettsia prowazekii	rickettsia prowazekii	Rickettsia prowazekii	1	kept
Rickettsia typhi	rickettsia typhi	rickettsia typhi	rickettsia typhi	Rickettsia typhi	1	kept
Rickettsioses	rickettsioses	rickettsioses	rickettsioses	Rickettsioses	1	kept
rickettsiosis	rickettsiosis	rickettsiosis	rickettsiosis	rickettsiosis	1	kept
Ricoeur	ricoeur	ricoeur	ricoeur	Ricoeur	1	kept
rifampicin resistance	rifampicin resistance	rifampicin resistance	rifampicin resistance	rifampicin resistance	1	kept
rifamycin	rifamycin	rifamycin	rifamycin	rifamycin	1	kept
RIFLE	rifle	rifle	rifle	RIFLE	1	kept
rifle	rifle	rifle	rifle	RIFLE	1	case_or_format
RIFLE CRITERIA	rifle criteria	rifle criteria	rifle criteria	RIFLE CRITERIA	1	kept
Rift Valley Fever	rift valley fever	rift valley fever	rift valley fever	Rift Valley fever	1	case_or_format
Rift valley fever	rift valley fever	rift valley fever	rift valley fever	Rift Valley fever	1	case_or_format
Right angle connector	right angle connector	right angle connector	right angle connector	Right angle connector	1	kept

right atrial enlargement	right atrial enlargement	right atrial enlargement	right atrial enlargement	right atrial enlargement	1	kept
right gastroepiploic artery	right gastroepiploic artery	right gastroepiploic artery	right gastroepiploic artery	right gastroepiploic artery	1	kept
right groin	right groin	right groin	right groin	right groin	1	kept
right handedness	right handedness	right handedness	right handedness	right handedness	1	kept
Right heart failure	right heart failure	right heart failure	right heart failure	Right heart failure	1	kept
right lung	right lung	right lung	right lung	right lung	1	kept
right pulmonary artery	right pulmonary artery	right pulmonary artery	right pulmonary artery	right pulmonary artery	1	kept
Right to die	right to die	right to die	right to die	Right to Die	1	case_or_format
right to freedom of movement	right to freedom of movement	right to freedom of movement	right to freedom of movement	right to freedom of movement	1	kept
right to health	right to health	right to health	right to health	right to health	1	kept
right to life	right to life	right to life	right to life	Right to life	1	case_or_format
Right to pain relief	right to pain relief	right to pain relief	right to pain relief	Right to pain relief	1	kept
Right ventricle assessment	right ventricle assessment	right ventricle assessment	right ventricle assessment	Right ventricle assessment	1	kept
right ventricle to pulmonary artery conduit	right ventricle to pulmonary artery conduit	right ventricle to pulmonary artery conduit	right ventricle to pulmonary artery conduit	right ventricle to pulmonary artery conduit	1	kept
Right-sided infective endocarditis	right sided infective endocarditis	right sided infective endocarditis	right sided infective endocarditis	Right-sided infective endocarditis	1	kept
RIGHT-TO-DIE	right to die	right to die	right to die	Right to Die	1	case_or_format

Riker scale	riker scale	riker scale	riker scale	Riker scale	1	kept
Rikerscale	rikerscale	rikerscale	rikerscale	Rikerscale	1	kept
Rikersedation-agitation	rikersedation agitation	rikersedation agitation	rikersedation agitation	Rikersedation-agitation	1	kept
Rilpivirine	rilpivirine	rilpivirine	rilpivirine	Rilpivirine	1	kept
rilpivirine	rilpivirine	rilpivirine	rilpivirine	Rilpivirine	1	case_or_format
rimactance	rimactance	rimactance	rimactance	rimactance	1	kept
rimonabant	rimonabant	rimonabant	rimonabant	rimonabant	1	kept
ring finger	ring finger	ring finger	ring finger	ring finger	1	kept
Ring theory of personhood	ring theory of personhood	ring theory of personhood	ring theory of personhood	Ring theory of personhood	1	kept
ring wearing	ring wearing	ring wearing	ring wearing	ring wearing	1	kept
Ringer's lactate	ringer s lactate	ringer s lactate	ringer s lactate	Ringer's Lactate	1	case_or_format
RINSE	rinse	rinse	rinse	RINSE	1	kept
risankizumab	risankizumab	risankizumab	risankizumab	risankizumab	1	kept
risedronic acid	risedronic acid	risedronic acid	risedronic acid	risedronic acid	1	kept
Risk adjustment	risk adjustment	risk adjustment	risk adjustment	Risk Adjustment	1	case_or_format
risk adjustment	risk adjustment	risk adjustment	risk adjustment	Risk Adjustment	1	case_or_format

Risk adjustments	risk adjustments	risk adjustments	risk adjustment	Risk Adjustment	1	singular
RISK ANALYSIS	risk analysis	risk analysis	risk analysis	RISK ANALYSIS	1	kept
Risk analysis	risk analysis	risk analysis	risk analysis	RISK ANALYSIS	1	case_or_format
Risk Assessment and Prediction Tool	risk assessment and prediction tool	risk assessment and prediction tool	risk assessment and prediction tool	Risk Assessment and Prediction Tool	1	kept
risk assessment and screening	risk assessment and screening	risk assessment and screening	risk assessment and screening	risk assessment and screening	1	kept
RISK ASSESSMENT SCALES	risk assessment scales	risk assessment scales	risk assessment scale	Risk assessment scale	1	singular
Risk assessment systems	risk assessment systems	risk assessment systems	risk assessment systems	Risk assessment systems	1	kept
Risk assessment tool	risk assessment tool	risk assessment tool	risk assessment tool	Risk assessment tool	1	kept
risk assessment tool	risk assessment tool	risk assessment tool	risk assessment tool	Risk assessment tool	1	case_or_format
Risk assessment tools	risk assessment tools	risk assessment tools	risk assessment tool	Risk assessment tool	1	singular
risk behavior	risk behavior	risk behavior	risk behavior	risk behavior	1	kept
risk communication	risk communication	risk communication	risk communication	risk communication	1	kept
Risk control	risk control	risk control	risk control	Risk control	1	kept
risk control	risk control	risk control	risk control	Risk control	1	case_or_format
risk değerlendirmesi	risk de erlendirmesi	risk de erlendirmesi	risk de erlendirmesi	risk değerlendirmesi	1	kept
Risk difference	risk difference	risk difference	risk difference	Risk difference	1	kept

risk estimation	risk estimation	risk estimation	risk estimation	risk estimation	1	kept
risk exposure assessment	risk exposure assessment	risk exposure assessment	risk exposure assessment	risk exposure assessment	1	kept
Risk Factor	risk factor	risk factor	risk factor	Risk Factors	1	case_or_format
Risk factor analysis	risk factor analysis	risk factor analysis	risk factor analysis	Risk factor analysis	1	kept
Risk factors for postoperative delirium	risk factors for postoperative delirium	risk factors for postoperative delirium	risk factors for postoperative delirium	Risk factors for postoperative delirium	1	kept
Risk Factors for Substance Abuse	risk factors for substance abuse	risk factors for substance abuse	risk factors for substance abuse	Risk Factors for Substance Abuse	1	kept
risk factors for substance abuse	risk factors for substance abuse	risk factors for substance abuse	risk factors for substance abuse	Risk Factors for Substance Abuse	1	case_or_format
Risk factors of severity for rib fractures	risk factors of severity for rib fractures	risk factors of severity for rib fractures	risk factors of severity for rib fractures	Risk factors of severity for rib fractures	1	kept
Risk group	risk group	risk group	risk group	Risk group	1	kept
Risk groups	risk groups	risk groups	risk group	Risk group	1	singular
risk index	risk index	risk index	risk index	Risk index	1	case_or_format
Risk management strategies	risk management strategies	risk management strategies	risk management strategies	Risk management strategies	1	kept
Risk measure	risk measure	risk measure	risk measure	Risk measure	1	kept
risk minimization action	risk minimization action	risk minimization action	risk minimization action	risk minimization action	1	kept
Risk mitigation	risk mitigation	risk mitigation	risk mitigation	Risk mitigation	1	kept
risk mitigation	risk mitigation	risk mitigation	risk mitigation	Risk mitigation	1	case_or_format

risk mitigation measurement	risk mitigation measurement	risk mitigation measurement	risk mitigation measurement	risk mitigation measurement	1	kept
Risk monitors	risk monitors	risk monitors	risk monitors	Risk monitors	1	kept
Risk of Dysphagia in Cardiac Surgery	risk of dysphagia in cardiac surgery	risk of dysphagia in cardiac surgery	risk of dysphagia in cardiac surgery	Risk of Dysphagia in Cardiac Surgery	1	kept
risk of falling	risk of falling	risk of falling	risk of falling	risk of falling	1	kept
Risk of infection	risk of infection	risk of infection	risk of infection	Risk of infection	1	kept
Risk of mortality	risk of mortality	risk of mortality	risk of mortality	Risk of mortality	1	kept
risk of pressure ulcers	risk of pressure ulcers	risk of pressure ulcers	risk of pressure ulcers	risk of pressure ulcers	1	kept
Risk of VAP	risk of vap	risk of vap	risk of vap	Risk of VAP	1	kept
risk of VAP	risk of vap	risk of vap	risk of vap	Risk of VAP	1	case_or_format
RISK PERCEPTION	risk perception	risk perception	risk perception	Risk perception	1	case_or_format
risk predicting	risk predicting	risk predicting	risk predicting	risk predicting	1	kept
risk prediction mode	risk prediction mode	risk prediction mode	risk prediction mode	risk prediction mode	1	kept
Risk predictions	risk predictions	risk predictions	risk prediction	risk prediction	1	singular
Risk profile	risk profile	risk profile	risk profile	Risk profile	1	kept
Risk quantification	risk quantification	risk quantification	risk quantification	Risk quantification	1	kept
Risk Reduction	risk reduction	risk reduction	risk reduction	risk reduction	1	case_or_format

Risk reduction	risk reduction	risk reduction	risk reduction	risk reduction	1	case_or_format
Risk scale	risk scale	risk scale	risk scale	risk scale	1	case_or_format
Risk score: violence risk assessment	risk score violence risk assessment	risk score violence risk assessment	risk score violence risk assessment	Risk score: violence risk assessment	1	kept
Risk scores	risk scores	risk scores	risk score	Risk score	1	singular
risk scores	risk scores	risk scores	risk score	Risk score	1	singular
Risk screening	risk screening	risk screening	risk screening	Risk screening	1	kept
risk standardized mortality rate	risk standardized mortality rate	risk standardized mortality rate	risk standardized mortality rate	risk standardized mortality rate	1	kept
Risk Stratification	risk stratification	risk stratification	risk stratification	Risk stratification	1	case_or_format
risk stratification tool	risk stratification tool	risk stratification tool	risk stratification tool	risk stratification tool	1	kept
Risk translation	risk translation	risk translation	risk translation	Risk translation	1	kept
risk work	risk work	risk work	risk work	risk work	1	kept
RISK-ASSESSMENT-SCALES	risk assessment scales	risk assessment scales	risk assessment scale	Risk assessment scale	1	singular
Risk-based	risk based	risk based	risk based	Risk-based	1	kept
risk-prediction tool	risk prediction tool	risk prediction tool	risk prediction tool	risk-prediction tool	1	kept
Risk-stratifying tools	risk stratifying tools	risk stratifying tools	risk stratifying tools	Risk-stratifying tools	1	kept
RISKS	risks	risks	risk	risk	1	singular

Risks of transport	risks of transport	risks of transport	risks of transport	Risks of transport	1	kept
RISPERIDONE	risperidone	risperidone	risperidone	risperidone	1	case_or_format
Risperidone	risperidone	risperidone	risperidone	risperidone	1	case_or_format
ritodrine	ritodrine	ritodrine	ritodrine	ritodrine	1	kept
Ritonavir	ritonavir	ritonavir	ritonavir	ritonavir	1	case_or_format
Ritualization	ritualization	ritualization	ritualization	Ritualization	1	kept
rituals	rituals	rituals	ritual	ritual	1	singular
river basin	river basin	river basin	river basin	river basin	1	kept
river water	river water	river water	river water	river water	1	kept
rmp gene	rmp gene	rmp gene	rmp gene	rmp gene	1	kept
rmp protein	rmp protein	rmp protein	rmp protein	rmp protein	1	kept
RNA analysis	rna analysis	rna analysis	rna analysis	RNA analysis	1	kept
RNA directed RNA polymerase inhibitor	rna directed rna polymerase inhibitor	rna directed rna polymerase inhibitor	rna directed rna polymerase inhibitor	RNA directed RNA polymerase inhibitor	1	kept
RNA EXPRESSION	rna expression	rna expression	rna expression	RNA EXPRESSION	1	kept
RNA polymerase	rna polymerase	rna polymerase	rna polymerase	RNA polymerase	1	kept
RNA sequencing	rna sequencing	rna sequencing	rna sequencing	RNA sequencing	1	kept

RNA splice site	rna splice site	rna splice site	rna splice site	RNA Splice Sites	1	case_or_format
RNA Splicing	rna splicing	rna splicing	rna splicing	RNA splicing	1	case_or_format
RNA virus	rna virus	rna virus	rna virus	RNA virus	1	kept
RNA virus infection	rna virus infection	rna virus infection	rna virus infection	RNA virus infection	1	kept
RNA, Small Untranslated	rna small untranslated	rna small untranslated	rna small untranslated	RNA, Small Untranslated	1	kept
Road traffic	road traffic	road traffic	road traffic	Road traffic	1	kept
Road traffic accidents	road traffic accidents	road traffic accidents	road traffic accidents	Road traffic accidents	1	kept
road traffic accidents	road traffic accidents	road traffic accidents	road traffic accidents	Road traffic accidents	1	case_or_format
Road Traffic Injury	road traffic injury	road traffic injury	road traffic injury	road traffic injuries	1	case_or_format
ROAD TRAFFIC NOISE	road traffic noise	road traffic noise	road traffic noise	ROAD TRAFFIC NOISE	1	kept
road traffic safety	road traffic safety	road traffic safety	road traffic safety	road traffic safety	1	kept
RoB, risk of bias	rob risk of bias	rob risk of bias	rob risk of bias	RoB, risk of bias	1	kept
ROBIN-SEQUENCE	robin sequence	robin sequence	robin sequence	ROBIN-SEQUENCE	1	kept
robins 1 tool	robins 1 tool	robins 1 tool	robins 1 tool	robins 1 tool	1	kept
robot assisted radical gastrectomy	robot assisted radical gastrectomy	robot assisted radical gastrectomy	robot assisted radical gastrectomy	robot assisted radical gastrectomy	1	kept
Robot Operating System	robot operating system	robot operating system	robot operating system	Robot Operating System	1	kept

Robot programming	robot programming	robot programming	robot programming	Robot programming	1	kept
Robotic arms	robotic arms	robotic arms	robotic arms	Robotic arms	1	kept
Robotic simulator	robotic simulator	robotic simulator	robotic simulator	Robotic simulator	1	kept
Robotic systems	robotic systems	robotic systems	robotic systems	Robotic systems	1	kept
Robotic telepresence	robotic telepresence	robotic telepresence	robotic telepresence	Robotic telepresence	1	kept
robotic telepresence	robotic telepresence	robotic telepresence	robotic telepresence	Robotic telepresence	1	case_or_format
Robotic units	robotic units	robotic units	robotic units	Robotic units	1	kept
Robotic-assisted laparoscopic surgery	robotic assisted laparoscopic surgery	robotic assisted laparoscopic surgery	robotic assisted laparoscopic surgery	Robotic-assisted laparoscopic surgery	1	kept
robotically assisted surgery	robotically assisted surgery	robotically assisted surgery	robotically assisted surgery	robotically assisted surgery	1	kept
robotization	robotization	robotization	robotization	robotization	1	kept
Robots	robots	robots	robot	Robot	1	singular
Robson group 1	robson group 1	robson group 1	robson group 1	Robson group 1	1	kept
Robust algorithm	robust algorithm	robust algorithm	robust algorithm	robust algorithm	1	case_or_format
Robustness	robustness	robustness	robustness	Robustness	1	kept
robustness	robustness	robustness	robustness	Robustness	1	case_or_format
ROC	roc	roc	roc	ROC	1	kept

ROC AUC, area under the receiver operating characteristics curve	roc auc area under the receiver operating characteristics curve	roc auc area under the receiver operating characteristics curve	roc auc area under the receiver operating characteristics curve	ROC AUC, area under the receiver operating characteristics curve	1	kept
ROC, receiver operating curve	roc receiver operating curve	roc receiver operating curve	roc receiver operating curve	ROC, receiver operating curve	1	kept
rodent	rodent	rodent	rodent	rodent	1	kept
Rodgers’ s Concept Analysis	rodgers s concept analysis	rodgers s concept analysis	rodgers s concept analysis	Rodgers’ s Concept Analysis	1	kept
rofecoxib	rofecoxib	rofecoxib	rofecoxib	rofecoxib	1	kept
Rogers’ Diffusion of Innovation Theory	rogers diffusion of innovation theory	rogers diffusion of innovation theory	rogers diffusion of innovation theory	Rogers’ Diffusion of Innovation Theory	1	kept
Rogers’ Diffusion of Innovations framework	rogers diffusion of innovations framework	rogers diffusion of innovations framework	rogers diffusion of innovations framework	Rogers’ Diffusion of Innovations framework	1	kept
Rokitansky	rokitansky	rokitansky	rokitansky	Rokitansky	1	kept
Rokitansky syndrome	rokitansky syndrome	rokitansky syndrome	rokitansky syndrome	Rokitansky syndrome	1	kept
Rol de la enfermera	rol de la enfermera	rol de la enfermera	rol de la enfermera	Rol de la enfermera	1	kept
role ambiguity	role ambiguity	role ambiguity	role ambiguity	Role ambiguity	1	case_or_format
role and responsibility	role and responsibility	role and responsibility	role and responsibility	role and responsibility	1	kept
role assignment	role assignment	role assignment	role assignment	role assignment	1	kept
Role change	role change	role change	role change	Role change	1	kept
role clarity	role clarity	role clarity	role clarity	role clarity	1	kept
role conflict and ambiguity	role conflict and ambiguity	role conflict and ambiguity	role conflict and ambiguity	role conflict and ambiguity	1	kept

role conflicts	role conflicts	role conflicts	role conflict	Role conflict	1	singular
role confusion	role confusion	role confusion	role confusion	role confusion	1	kept
role expectations	role expectations	role expectations	role expectations	role expectations	1	kept
role identity	role identity	role identity	role identity	role identity	1	kept
Role modeling	role modeling	role modeling	role modeling	Role modeling	1	kept
Role morality	role morality	role morality	role morality	Role morality	1	kept
Role of appellate courts	role of appellate courts	role of appellate courts	role of appellate courts	Role of appellate courts	1	kept
role of health care professionals	role of health care professionals	role of health care professionals	role of health care professionals	Role of health care professionals	1	case_or_format
role of multidisciplinary team	role of multidisciplinary team	role of multidisciplinary team	role of multidisciplinary team	role of multidisciplinary team	1	kept
role overload	role overload	role overload	role overload	role overload	1	kept
Role performance	role performance	role performance	role performance	Role performance	1	kept
role performance	role performance	role performance	role performance	Role performance	1	case_or_format
Role strain	role strain	role strain	role strain	Role strain	1	kept
Role stress	role stress	role stress	role stress	Role stress	1	kept
role stress	role stress	role stress	role stress	Role stress	1	case_or_format
ROLE-CLARITY	role clarity	role clarity	role clarity	role clarity	1	case_or_format

roller pumps	roller pumps	roller pumps	roller pump	roller pump	1	singular
rolling circle amplification	rolling circle amplification	rolling circle amplification	rolling circle amplification	rolling circle amplification	1	kept
Rolling horizon	rolling horizon	rolling horizon	rolling horizon	Rolling horizon	1	kept
Roma [Lazio]	roma lazio	roma lazio	roma lazio	Roma [Lazio]	1	kept
Romani	romani	romani	romani	Romani	1	kept
rome iv scale	rome iv scale	rome iv scale	rome iv scale	rome iv scale	1	kept
romlusevimab	romlusevimab	romlusevimab	romlusevimab	romlusevimab	1	kept
Ronchi brief evaluation battery	ronchi brief evaluation battery	ronchi brief evaluation battery	ronchi brief evaluation battery	Ronchi brief evaluation battery	1	kept
Room air	room air	room air	room air	Room air	1	kept
ROOM THORACOTOMY	room thoracotomy	room thoracotomy	room thoracotomy	ROOM THORACOTOMY	1	kept
room topography	room topography	room topography	room topography	room topography	1	kept
Room ventilations	room ventilations	room ventilations	room ventilation	room ventilation	1	singular
rooming-in	rooming in	rooming in	rooming in	rooming in	1	case_or_format
rooming-in care	rooming in care	rooming in care	rooming in care	Rooming-in Care	1	case_or_format
ROOMS	rooms	rooms	room	ROOM	1	singular
Root-cause analysis	root cause analysis	root cause analysis	root cause analysis	root cause analysis	1	case_or_format

ROP screening	rop screening	rop screening	rop screening	ROP screening	1	kept
ropimol	ropimol	ropimol	ropimol	ropimol	1	kept
ropinirole	ropinirole	ropinirole	ropinirole	ropinirole	1	kept
Rosa	rosa	rosa	rosa	Rosa	1	kept
rosacea	rosacea	rosacea	rosacea	rosacea	1	kept
rose	rose	rose	rose	rose	1	kept
Rosenberg Self-Esteem Scale	rosenberg self esteem scale	rosenberg self esteem scale	rosenberg self esteem scale	Rosenberg Self-Esteem Scale	1	kept
rosiglitazone	rosiglitazone	rosiglitazone	rosiglitazone	rosiglitazone	1	kept
rosin	rosin	rosin	rosin	rosin	1	kept
Rossetti fundoplication	rossetti fundoplication	rossetti fundoplication	rossetti fundoplication	Rossetti fundoplication	1	kept
Rotación de personal	rotaci n de personal	rotaci n de personal	rotaci n de personal	Rotación de personal	1	kept
rotary blood pump	rotary blood pump	rotary blood pump	rotary blood pump	rotary blood pump	1	kept
ROTATING SHIFT WORK	rotating shift work	rotating shift work	rotating shift work	ROTATING SHIFT WORK	1	kept
rotating shift worker	rotating shift worker	rotating shift worker	rotating shift worker	rotating shift worker	1	kept
rotating shifts	rotating shifts	rotating shifts	rotating shift	rotating shift	1	singular
rotating shiftwork	rotating shiftwork	rotating shiftwork	rotating shiftwork	rotating shiftwork	1	kept

rotational bed	rotational bed	rotational bed	rotational bed	rotational bed	1	kept
Rotational vaginal delivery	rotational vaginal delivery	rotational vaginal delivery	rotational vaginal delivery	Rotational vaginal delivery	1	kept
Rotations	rotations	rotations	rotation	Rotation	1	singular
ROTAVIRUS VACCINATION	rotavirus vaccination	rotavirus vaccination	rotavirus vaccination	ROTAVIRUS VACCINATION	1	kept
Rotavirus Vaccines	rotavirus vaccines	rotavirus vaccines	rotavirus vaccine	Rotavirus vaccine	1	singular
Rothia mucilaginosa	rothia mucilaginosa	rothia mucilaginosa	rothia mucilaginosa	Rothia mucilaginosa	1	kept
Rotokinetic beds	rotokinetic beds	rotokinetic beds	rotokinetic beds	Rotokinetic beds	1	kept
rounding	rounding	rounding	rounding	Rounding	1	case_or_format
rounding checklist	rounding checklist	rounding checklist	rounding checklist	rounding checklist	1	kept
ROUTE	route	route	route	ROUTE	1	kept
Route of feeding	route of feeding	route of feeding	route of feeding	Route of feeding	1	kept
Route transmission	route transmission	route transmission	route transmission	Route transmission	1	kept
routes of feeding	routes of feeding	routes of feeding	routes of feeding	routes of feeding	1	kept
Routine practice	routine practice	routine practice	routine practice	Routine practice	1	kept
Routines	routines	routines	routines	Routines	1	kept
routinisation of RCTs	routinisation of rcts	routinisation of rcts	routinisation of rcts	routinisation of RCTs	1	kept

Roy's Adaptation Model	roy s adaptation model	roy s adaptation model	roy s adaptation model	Roy’ s adaptation model	1	case_or_format
Royal College of Anaesthetists CPD Matrix: 2A07, 3G00	royal college of anaesthetists cpd matrix 2a07 3g00	royal college of anaesthetists cpd matrix 2a07 3g00	royal college of anaesthetists cpd matrix 2a07 3g00	Royal College of Anaesthetists CPD Matrix: 2A07, 3G00	1	kept
Royal Flying Doctor Service	royal flying doctor service	royal flying doctor service	royal flying doctor service	Royal Flying Doctor Service	1	kept
ROYAL-COLLEGE	royal college	royal college	royal college	ROYAL-COLLEGE	1	kept
Roy’ s Adaptation Model	roy s adaptation model	roy s adaptation model	roy s adaptation model	Roy’ s adaptation model	1	case_or_format
Roy’ s adaptation theory	roy s adaptation theory	roy s adaptation theory	roy s adaptation theory	Roy’ s adaptation theory	1	kept
RP, risk plot	rp risk plot	rp risk plot	rp risk plot	RP, risk plot	1	kept
RPL	rpl	rpl	rpl	RPL	1	kept
RPL classification system	rpl classification system	rpl classification system	rpl classification system	RPL classification system	1	kept
RR, risk ratio	rr risk ratio	rr risk ratio	rr risk ratio	RR, risk ratio	1	kept
RRC = Residency Review Committee	rrc residency review committee	rrc residency review committee	rrc residency review committee	RRC = Residency Review Committee	1	kept
RRF	rrf	rrf	rrf	RRF	1	kept
RRI, the interval between R waves in the ECG	rri the interval between r waves in the ecg	rri the interval between r waves in the ecg	rri the interval between r waves in the ecg	RRI, the interval between R waves in the ECG	1	kept
Rs10757278	rs10757278	rs10757278	rs10757278	Rs10757278	1	kept
Rs1333049	rs1333049	rs1333049	rs1333049	Rs1333049	1	kept
Rs1376251	rs1376251	rs1376251	rs1376251	Rs1376251	1	kept

Rs17465637	rs17465637	rs17465637	rs17465637	Rs17465637	1	kept
Rs2549513	rs2549513	rs2549513	rs2549513	Rs2549513	1	kept
Rs4804611	rs4804611	rs4804611	rs4804611	Rs4804611	1	kept
Rs499818	rs499818	rs499818	rs499818	Rs499818	1	kept
Rs619203	rs619203	rs619203	rs619203	Rs619203	1	kept
RSBI	rsbi	rsbi	rsbi	RSBI	1	kept
rsi	rsi	rsi	rsi	rsi	1	kept
RSV bronchiolitis	rsv bronchiolitis	rsv bronchiolitis	rsv bronchiolitis	RSV bronchiolitis	1	kept
RSV hospitalization	rsv hospitalization	rsv hospitalization	rsv hospitalization	RSV hospitalization	1	kept
RSV population-based incidence	rsv population based incidence	rsv population based incidence	rsv population based incidence	RSV population-based incidence	1	kept
RSV prophylaxis	rsv prophylaxis	rsv prophylaxis	rsv prophylaxis	RSV prophylaxis	1	kept
RSV therapy	rsv therapy	rsv therapy	rsv therapy	RSV therapy	1	kept
RSV vaccines	rsv vaccines	rsv vaccines	rsv vaccine	RSV vaccine	1	singular
rtCGM	rtcgm	rtcgm	rtcgm	rtCGM	1	kept
RTMUS	rtmus	rtmus	rtmus	RTMUS	1	kept
rtPA	rtpa	rtpa	rtpa	rtPA	1	kept

RUB CONSUMPTION	rub consumption	rub consumption	rub consumption	RUB CONSUMPTION	1	kept
rubella vaccine	rubella vaccine	rubella vaccine	rubella vaccine	rubella vaccine	1	kept
Rubric	rubric	rubric	rubric	Rubric	1	kept
rugby	rugby	rugby	rugby	rugby	1	kept
Ruido	ruido	ruido	ruido	Ruido	1	kept
Rule-based expert system	rule based expert system	rule based expert system	rule based expert system	rule-based expert system	1	case_or_format
RULES	rules	rules	rules	RULES	1	kept
Rules	rules	rules	rules	RULES	1	case_or_format
Rumination, Cognitive	rumination cognitive	rumination cognitive	rumination cognitive	Rumination, Cognitive	1	kept
ruptured abdominal aortic aneurysm	ruptured abdominal aortic aneurysm	ruptured abdominal aortic aneurysm	ruptured abdominal aortic aneurysm	ruptured abdominal aortic aneurysm	1	kept
ruptured uterus	ruptured uterus	ruptured uterus	ruptured uterus	ruptured uterus	1	kept
Rural acute care	rural acute care	rural acute care	rural acute care	Rural acute care	1	kept
Rural and remote healthcare	rural and remote healthcare	rural and remote healthcare	rural and remote healthcare	Rural and remote healthcare	1	kept
rural behavioral health telehealth	rural behavioral health telehealth	rural behavioral health telehealth	rural behavioral health telehealth	rural behavioral health telehealth	1	kept
Rural emergency medicine	rural emergency medicine	rural emergency medicine	rural emergency medicine	Rural emergency medicine	1	kept
rural emergency medicine	rural emergency medicine	rural emergency medicine	rural emergency medicine	Rural emergency medicine	1	case_or_format

rural health services	rural health services	rural health services	rural health services	Rural Health Services	1	case_or_format
Rural healthcare	rural healthcare	rural healthcare	rural healthcare	Rural healthcare	1	kept
rural healthcare	rural healthcare	rural healthcare	rural healthcare	Rural healthcare	1	case_or_format
Rural hospital	rural hospital	rural hospital	rural hospital	rural hospital	1	case_or_format
rural incubator	rural incubator	rural incubator	rural incubator	rural incubator	1	kept
rural medicine	rural medicine	rural medicine	rural medicine	rural medicine	1	kept
Rural population	rural population	rural population	rural population	Rural Population	1	case_or_format
Rural practice	rural practice	rural practice	rural practice	Rural practice	1	kept
rural radiologist	rural radiologist	rural radiologist	rural radiologist	rural radiologist	1	kept
rural radiology	rural radiology	rural radiology	rural radiology	rural radiology	1	kept
rural regions	rural regions	rural regions	rural regions	rural regions	1	kept
rural surgery	rural surgery	rural surgery	rural surgery	rural surgery	1	kept
Rural trauma	rural trauma	rural trauma	rural trauma	Rural trauma	1	kept
rural Veterans	rural veterans	rural veterans	rural veterans	rural Veterans	1	kept
rural women	rural women	rural women	rural women	rural women	1	kept
Russian	russian	russian	russian	Russian	1	kept

Ruído	ru do	ru do	ru do	Ruído	1	kept
réadaptation	r adaptation	r adaptation	r adaptation	réadaptation	1	kept
Réglette	r glette	r glette	r glette	Réglette	1	kept
régulation de la glycémie	r gulation de la glyc mie	r gulation de la glyc mie	r gulation de la glyc mie	régulation de la glycémie	1	kept
réhabilitation	r habilitation	r habilitation	r habilitation	réhabilitation	1	kept
réintégration communautaire	r int gration communautaire	r int gration communautaire	r int gration communautaire	réintégration communautaire	1	kept
réorganisation	r organisation	r organisation	r organisation	réorganisation	1	kept
Résilience	r silience	r silience	r silience	Résilience	1	kept
résultats	r sultats	r sultats	r sultats	résultats	1	kept
S. Aureus	s aureus	s aureus	s aureus	S. aureus	1	case_or_format
S.aureus	s aureus	s aureus	s aureus	S. aureus	1	case_or_format
S.S. scale	s s scale	s s scale	s s scale	S.S. scale	1	kept
SAAC = Society of Academic Anesthesiology Chairs	saac society of academic anesthesiology chairs	saac society of academic anesthesiology chairs	saac society of academic anesthesiology chairs	SAAC = Society of Academic Anesthesiology Chairs	1	kept
sabbatical	sabbatical	sabbatical	sabbatical	sabbatical	1	kept
Saccharomyces boulardii	saccharomyces boulardii	saccharomyces boulardii	saccharomyces boulardii	Saccharomyces boulardii	1	kept
Saccharomyces cerevisiae	saccharomyces cerevisiae	saccharomyces cerevisiae	saccharomyces cerevisiae	Saccharomyces cerevisiae	1	kept

SACCHAROMYCES-BOULARDII	saccharomyces boulardii	saccharomyces boulardii	saccharomyces boulardii	Saccharomyces boulardii	1	case_or_format
sacral bone exposure	sacral bone exposure	sacral bone exposure	sacral bone exposure	sacral bone exposure	1	kept
Sacral dressing	sacral dressing	sacral dressing	sacral dressing	Sacral dressing	1	kept
sacrectomy	sacrectomy	sacrectomy	sacrectomy	sacrectomy	1	kept
sacred pause	sacred pause	sacred pause	sacred pause	sacred pause	1	kept
SACRIFICE	sacrifice	sacrifice	sacrifice	SACRIFICE	1	kept
Sacrifice	sacrifice	sacrifice	sacrifice	SACRIFICE	1	case_or_format
Sacrococcygeal teratoma	sacrococcygeal teratoma	sacrococcygeal teratoma	sacrococcygeal teratoma	sacrococcygeal teratoma	1	case_or_format
sacrococcyx tumor	sacrococcyx tumor	sacrococcyx tumor	sacrococcyx tumor	sacrococcyx tumor	1	kept
sacroiliac joint	sacroiliac joint	sacroiliac joint	sacroiliac joint	sacroiliac joint	1	kept
sacroiliac joint fusion	sacroiliac joint fusion	sacroiliac joint fusion	sacroiliac joint fusion	sacroiliac joint fusion	1	kept
sacubitril plus valsartan	sacubitril plus valsartan	sacubitril plus valsartan	sacubitril plus valsartan	sacubitril plus valsartan	1	kept
Sadness	sadness	sadness	sadness	sadness	1	case_or_format
SAE	sae	sae	sae	SAE	1	kept
Safe Care	safe care	safe care	safe care	Safe Care	1	kept
Safe handling	safe handling	safe handling	safe handling	Safe handling	1	kept

Safe injection practice	safe injection practice	safe injection practice	safe injection practice	Safe injection practice	1	kept
Safe injection practices	safe injection practices	safe injection practices	safe injection practice	Safe injection practice	1	singular
Safe management	safe management	safe management	safe management	Safe managements	1	case_or_format
Safe motherhood	safe motherhood	safe motherhood	safe motherhood	Safe motherhood	1	kept
SAFE PRACTICE	safe practice	safe practice	safe practice	SAFE PRACTICE	1	kept
Safe practices	safe practices	safe practices	safe practice	SAFE PRACTICE	1	singular
Safe sleep	safe sleep	safe sleep	safe sleep	safe sleep	1	case_or_format
safe sleep knowledge	safe sleep knowledge	safe sleep knowledge	safe sleep knowledge	safe sleep knowledge	1	kept
Safe sleep practice	safe sleep practice	safe sleep practice	safe sleep practice	safe sleep practices	1	case_or_format
safe sleep program	safe sleep program	safe sleep program	safe sleep program	safe sleep program	1	kept
Safe surgery	safe surgery	safe surgery	safe surgery	Safe surgery	1	kept
Safe transport	safe transport	safe transport	safe transport	Safe transport	1	kept
safe transportation	safe transportation	safe transportation	safe transportation	safe transportation	1	kept
Safe work behavior	safe work behavior	safe work behavior	safe work behavior	Safe work behavior	1	kept
Safe work practice	safe work practice	safe work practice	safe work practice	Safe work practice	1	kept
SafeBoosC	safeboosc	safeboosc	safeboosc	SafeBoosC	1	kept

safeguarding	safeguarding	safeguarding	safeguarding	safeguarding	1	kept
safekeeping	safekeeping	safekeeping	safekeeping	safekeeping	1	kept
SAFETY ALERTS	safety alerts	safety alerts	safety alerts	SAFETY ALERTS	1	kept
Safety and efficiencies	safety and efficiencies	safety and efficiencies	safety and efficiencies	Safety and efficiencies	1	kept
Safety and healths	safety and healths	safety and healths	safety and healths	Safety and healths	1	kept
Safety and quality	safety and quality	safety and quality	safety and quality	Safety and quality	1	kept
safety and quality climate	safety and quality climate	safety and quality climate	safety and quality climate	safety and quality climate	1	kept
Safety assurance	safety assurance	safety assurance	safety assurance	Safety assurance	1	kept
Safety attendants	safety attendants	safety attendants	safety attendants	Safety attendants	1	kept
Safety attitude questionnaire	safety attitude questionnaire	safety attitude questionnaire	safety attitude questionnaire	Safety Attitude Questionnaire	1	case_or_format
Safety attitudes questionnaire	safety attitudes questionnaire	safety attitudes questionnaire	safety attitudes questionnaire	safety attitudes questionnaire	1	case_or_format
Safety briefing	safety briefing	safety briefing	safety briefing	Safety briefing	1	kept
safety briefing	safety briefing	safety briefing	safety briefing	Safety briefing	1	case_or_format
safety bundle	safety bundle	safety bundle	safety bundle	safety bundle	1	kept
safety bundles	safety bundles	safety bundles	safety bundle	safety bundle	1	singular
SAFETY CLIMATE	safety climate	safety climate	safety climate	safety climate	1	case_or_format

Safety compliances	safety compliances	safety compliances	safety compliance	Safety compliance	1	singular
safety concepts	safety concepts	safety concepts	safety concepts	safety concepts	1	kept
Safety criteria	safety criteria	safety criteria	safety criteria	Safety criteria	1	kept
Safety criterion	safety criterion	safety criterion	safety criterion	Safety criterion	1	kept
Safety culture survey	safety culture survey	safety culture survey	safety culture survey	Safety culture survey	1	kept
Safety equipment	safety equipment	safety equipment	safety equipment	Safety equipment	1	kept
safety evaluation	safety evaluation	safety evaluation	safety evaluation	safety evaluation	1	kept
Safety experts	safety experts	safety experts	safety experts	Safety experts	1	kept
Safety factor	safety factor	safety factor	safety factor	Safety factor	1	kept
safety hazards	safety hazards	safety hazards	safety hazards	safety hazards	1	kept
Safety huddle	safety huddle	safety huddle	safety huddle	Safety huddle	1	kept
Safety in the imaging areas	safety in the imaging areas	safety in the imaging areas	safety in the imaging areas	Safety in the imaging areas	1	kept
Safety incidents	safety incidents	safety incidents	safety incidents	Safety incidents	1	kept
Safety instructions	safety instructions	safety instructions	safety instructions	Safety instructions	1	kept
Safety issue	safety issue	safety issue	safety issue	Safety issues	1	case_or_format
Safety measures	safety measures	safety measures	safety measures	Safety measures	1	kept

safety measures	safety measures	safety measures	safety measures	Safety measures	1	case_or_format
Safety of care	safety of care	safety of care	safety of care	Safety of care	1	kept
Safety organizing	safety organizing	safety organizing	safety organizing	Safety organizing	1	kept
SAFETY OUTCOMES	safety outcomes	safety outcomes	safety outcomes	SAFETY OUTCOMES	1	kept
Safety participations	safety participations	safety participations	safety participation	Safety participation	1	singular
safety pause	safety pause	safety pause	safety pause	safety pause	1	kept
Safety programs	safety programs	safety programs	safety programs	Safety programs	1	kept
safety related evidence based protocol	safety related evidence based protocol	safety related evidence based protocol	safety related evidence based protocol	safety related evidence based protocol	1	kept
Safety software	safety software	safety software	safety software	Safety software	1	kept
SAFETY SUPPORT-SYSTEM	safety support system	safety support system	safety support system	SAFETY SUPPORT-SYSTEM	1	kept
SAFETY THREATS	safety threats	safety threats	safety threats	SAFETY THREATS	1	kept
Safety training package	safety training package	safety training package	safety training package	Safety training package	1	kept
Safety transition	safety transition	safety transition	safety transition	Safety transition	1	kept
Safety,	safety	safety	safety	safety	1	case_or_format
Safety-critical domain	safety critical domain	safety critical domain	safety critical domain	Safety-critical domain	1	kept
Safety-net hospital	safety net hospital	safety net hospital	safety net hospital	safety net hospital	1	case_or_format

safinamide	safinamide	safinamide	safinamide	safinamide	1	kept
SAGAT	sagat	sagat	sagat	SAGAT	1	kept
sagittal plane	sagittal plane	sagittal plane	sagittal plane	sagittal plane	1	kept
SAH	sah	sah	sah	SAH	1	kept
SAHS	sahs	sahs	sah	SAH	1	singular
Salbutamol	salbutamol	salbutamol	salbutamol	salbutamol	1	case_or_format
salcatonin	salcatonin	salcatonin	salcatonin	salcatonin	1	kept
Salicylic Acid	salicylic acid	salicylic acid	salicylic acid	Salicylic Acid	1	kept
salicylic acid	salicylic acid	salicylic acid	salicylic acid	Salicylic Acid	1	case_or_format
salicylic acid derivative	salicylic acid derivative	salicylic acid derivative	salicylic acid derivative	salicylic acid derivative	1	kept
salicylism	salicylism	salicylism	salicylism	salicylism	1	kept
Salient belief	salient belief	salient belief	salient belief	Salient belief	1	kept
Salient beliefs	salient beliefs	salient beliefs	salient belief	Salient belief	1	singular
saline	saline	saline	saline	saline	1	kept
SALIVA	saliva	saliva	saliva	Saliva	1	case_or_format
saliva substitute	saliva substitute	saliva substitute	saliva substitute	saliva substitute	1	kept

Salivary alpha amylase	salivary alpha amylase	salivary alpha amylase	salivary alpha amylase	Salivary alpha amylase	1	kept
salivary gland	salivary gland	salivary gland	salivary gland	salivary gland	1	kept
Salivary secretory IgA	salivary secretory iga	salivary secretory iga	salivary secretory iga	Salivary secretory IgA	1	kept
Salivation	salivation	salivation	salivation	salivation	1	case_or_format
salle de cathétérisme	salle de cath t risme	salle de cath t risme	salle de cath t risme	salle de cathétérisme	1	kept
Salmonella enterica serovar typhimurium	salmonella enterica serovar typhimurium	salmonella enterica serovar typhimurium	salmonella enterica serovar typhimurium	Salmonella enterica serovar typhimurium	1	kept
Salmonella typhi	salmonella typhi	salmonella typhi	salmonella typhi	Salmonella typhi	1	kept
Salmonella typhimurium	salmonella typhimurium	salmonella typhimurium	salmonella typhimurium	Salmonella typhimurium	1	kept
salsalate	salsalate	salsalate	salsalate	salsalate	1	kept
Salud laboral	salud laboral	salud laboral	salud laboral	Salud laboral	1	kept
Salud mental	salud mental	salud mental	salud mental	Salud mental	1	kept
salutogenesis	salutogenesis	salutogenesis	salutogenesis	salutogenesis	1	kept
Salutogenic	salutogenic	salutogenic	salutogenic	Salutogenic	1	kept
salvage	salvage	salvage	salvage	salvage	1	kept
Salvia sclaria	salvia sclaria	salvia sclaria	salvia sclaria	Salvia sclaria	1	kept
SAME-DAY DISCHARGE	same day discharge	same day discharge	same day discharge	SAME-DAY DISCHARGE	1	kept

Same-day discharge	same day discharge	same day discharge	same day discharge	SAME-DAY DISCHARGE	1	case_or_format
Samoa	samoa	samoa	samoa	Samoa	1	kept
Sample size	sample size	sample size	sample size	sample size	1	case_or_format
SAMPLE-SIZE CALCULATION	sample size calculation	sample size calculation	sample size calculation	SAMPLE-SIZE CALCULATION	1	kept
sampling error	sampling error	sampling error	sampling error	sampling error	1	kept
Sampling frequencies	sampling frequencies	sampling frequencies	sampling frequencies	Sampling frequencies	1	kept
SAMPLING METHOD	sampling method	sampling method	sampling method	SAMPLING METHOD	1	kept
Sampling method	sampling method	sampling method	sampling method	SAMPLING METHOD	1	case_or_format
Sampling technique	sampling technique	sampling technique	sampling technique	Sampling technique	1	kept
Sampling units	sampling units	sampling units	sampling units	Sampling units	1	kept
SAMU	samu	samu	samu	SAMU	1	kept
SANDELOWSKI	sandelowski	sandelowski	sandelowski	SANDELOWSKI	1	kept
sandwich ELISA	sandwich elisa	sandwich elisa	sandwich elisa	sandwich ELISA	1	kept
Sangre	sangre	sangre	sangre	Sangre	1	kept
Sanitary containment	sanitary containment	sanitary containment	sanitary containment	Sanitary containment	1	kept
sanitary equipment	sanitary equipment	sanitary equipment	sanitary equipment	sanitary equipment	1	kept

Sanitary personnel	sanitary personnel	sanitary personnel	sanitary personnel	Sanitary personnel	1	kept
Sanitization	sanitization	sanitization	sanitization	Sanitization	1	kept
Sanitizing products	sanitizing products	sanitizing products	sanitizing products	Sanitizing products	1	kept
Sankey diagrams	sankey diagrams	sankey diagrams	sankey diagrams	Sankey diagrams	1	kept
Santa Catarina	santa catarina	santa catarina	santa catarina	Santa Catarina	1	kept
santa clara strength of religious faith questionnaire	santa clara strength of religious faith questionnaire	santa clara strength of religious faith questionnaire	santa clara strength of religious faith questionnaire	santa clara strength of religious faith questionnaire	1	kept
Sao Paulo , Brazil	sao paulo brazil	sao paulo brazil	sao paulo brazil	Sao Paulo , Brazil	1	kept
SaO2	sao2	sao2	sao2	SaO2	1	kept
SaO<sub>2<	sao sub 2	sao sub 2	sao sub 2	SaO<sub>2<	1	kept
Saphenous Vein	saphenous vein	saphenous vein	saphenous vein	saphenous vein	1	case_or_format
saphenous vein graft	saphenous vein graft	saphenous vein graft	saphenous vein graft	saphenous vein graft	1	kept
Saponins	saponins	saponins	saponins	Saponins	1	kept
saprotroph	saprotroph	saprotroph	saprotroph	saprotroph	1	kept
SAPS 3	saps 3	saps 3	saps 3	SAPS 3	1	kept
SAPS I score	saps i score	saps i score	saps i score	SAPS I score	1	kept
SAPS II	saps ii	saps ii	saps ii	SAPS II	1	kept

SAPS-II	saps ii	saps ii	saps ii	SAPS II	1	case_or_format
SAPS2	saps2	saps2	saps2	SAPS2	1	kept
SAQ = Safety Attitudes Questionnaire	saq safety attitudes questionnaire	saq safety attitudes questionnaire	saq safety attitudes questionnaire	SAQ = Safety Attitudes Questionnaire	1	kept
SAR-CoV-2	sar cov 2	sar cov 2	sar cov 2	SAR-CoV-2	1	kept
SARC-F	sarc f	sarc f	sarc f	SARC-F	1	kept
Sarcoidosis	sarcoidosis	sarcoidosis	sarcoidosis	sarcoidosis	1	case_or_format
sarcoma	sarcoma	sarcoma	sarcoma	sarcoma	1	kept
Sarcoma, Ewing	sarcoma ewing	sarcoma ewing	sarcoma ewing	Sarcoma, Ewing	1	kept
Sarcoma, Kaposi	sarcoma kaposi	sarcoma kaposi	sarcoma kaposi	Sarcoma, Kaposi	1	kept
SARCOPENIA	sarcopenia	sarcopenia	sarcopenia	Sarcopenia	1	case_or_format
Sarcopenia in hip fracture	sarcopenia in hip fracture	sarcopenia in hip fracture	sarcopenia in hip fracture	Sarcopenia in hip fracture	1	kept
sarcopenic obesity	sarcopenic obesity	sarcopenic obesity	sarcopenic obesity	sarcopenic obesity	1	kept
SARI	sari	sari	sari	SARI	1	kept
SARI: severe acute respiratory infection	sari severe acute respiratory infection	sari severe acute respiratory infection	sari severe acute respiratory infection	SARI: severe acute respiratory infection	1	kept
sarolaner	sarolaner	sarolaner	sarolaner	sarolaner	1	kept
SARS CoV-2 variants	sars cov 2 variants	sars cov 2 variants	sars cov 2 variants	SARS-CoV-2 variants	1	case_or_format

SARS CoV2	sars cov2	sars cov2	sars cov2	SARS-CoV2	1	case_or_format
Sars CoV2	sars cov2	sars cov2	sars cov2	SARS-CoV2	1	case_or_format
SARS TRANSMISSION	sars transmission	sars transmission	sars transmission	SARS TRANSMISSION	1	kept
SARS virus	sars virus	sars virus	sars virus	SARS Virus	1	case_or_format
SARS-COV 2	sars cov 2	sars cov 2	sars cov 2	SARS-CoV-2	1	case_or_format
SARS-Cov-2	sars cov 2	sars cov 2	sars cov 2	SARS-CoV-2	1	case_or_format
Sars-cov-2	sars cov 2	sars cov 2	sars cov 2	SARS-CoV-2	1	case_or_format
sars-cov-2	sars cov 2	sars cov 2	sars cov 2	SARS-CoV-2	1	case_or_format
SARS-Cov-2 coronavirus	sars cov 2 coronavirus	sars cov 2 coronavirus	sars cov 2 coronavirus	SARS-Cov-2 coronavirus	1	kept
SARS-CoV-2 Gamma	sars cov 2 gamma	sars cov 2 gamma	sars cov 2 gamma	SARS-CoV-2 Gamma	1	kept
SARS-CoV-2 specific antibodies	sars cov 2 specific antibodies	sars cov 2 specific antibodies	sars cov 2 specific antibodies	SARS-CoV-2 specific antibodies	1	kept
SARS-CoV-2 transmission	sars cov 2 transmission	sars cov 2 transmission	sars cov 2 transmission	SARS-CoV-2 transmission	1	kept
SARS-CoV-2, Severe acute respiratory syndrome coronavirus-2	sars cov 2 severe acute respiratory syndrome coronavirus 2	sars cov 2 severe acute respiratory syndrome coronavirus 2	sars cov 2 severe acute respiratory syndrome coronavirus 2	SARS-CoV-2, Severe acute respiratory syndrome coronavirus-2	1	kept
SARS-CoV-2-IgG	sars cov 2 igg	sars cov 2 igg	sars cov 2 igg	SARS-CoV-2-IgG	1	kept
SARS-CoV-2: severe acute	sars cov 2 severe acute	sars cov 2 severe acute	sars cov 2 severe acute	SARS-CoV-2: severe acute	1	kept
SARS-CoV2 IgG	sars cov2 igg	sars cov2 igg	sars cov2 igg	SARS-CoV2 IgG	1	kept

SARSCoV-2	sarscov 2	sarscov 2	sarscov 2	SARSCoV-2	1	kept
sartorius muscle	sartorius muscle	sartorius muscle	sartorius muscle	sartorius muscle	1	kept
SAS	sas	sas	sas	SAS	1	kept
satellite	satellite	satellite	satellite	satellite	1	kept
satellite imagery	satellite imagery	satellite imagery	satellite imagery	satellite imagery	1	kept
satellite pharmacist	satellite pharmacist	satellite pharmacist	satellite pharmacist	satellite pharmacist	1	kept
Satiety	satiety	satiety	satiety	Satiety	1	kept
satiety	satiety	satiety	satiety	Satiety	1	case_or_format
Satisfacción	satisfacci n	satisfacci n	satisfacci n	Satisfacción	1	kept
Satisfacción del paciente	satisfacci n del paciente	satisfacci n del paciente	satisfacci n del paciente	Satisfacción del paciente	1	kept
Satisfacción laboral	satisfacci n laboral	satisfacci n laboral	satisfacci n laboral	Satisfacción laboral	1	kept
Satisfacción usuaria	satisfacci n usuaria	satisfacci n usuaria	satisfacci n usuaria	Satisfacción usuaria	1	kept
Satisfaction and trust	satisfaction and trust	satisfaction and trust	satisfaction and trust	Satisfaction and trust	1	kept
Satisfaction Assessment Questionnaire	satisfaction assessment questionnaire	satisfaction assessment questionnaire	satisfaction assessment questionnaire	Satisfaction Assessment Questionnaire	1	kept
satisfaction of compassion	satisfaction of compassion	satisfaction of compassion	satisfaction of compassion	satisfaction of compassion	1	kept
Satisfaction rates	satisfaction rates	satisfaction rates	satisfaction rate	satisfaction rate	1	singular

Satisfaction with care	satisfaction with care	satisfaction with care	satisfaction with care	satisfaction with care	1	case_or_format
Satisfaction with life	satisfaction with life	satisfaction with life	satisfaction with life	Satisfaction with life	1	kept
Satisfactory degree	satisfactory degree	satisfactory degree	satisfactory degree	Satisfactory degree	1	kept
satisficing	satisficing	satisficing	satisficing	satisficing	1	kept
Satisfied	satisfied	satisfied	satisfied	Satisfied	1	kept
SatSeconds alarm settings	satseconds alarm settings	satseconds alarm settings	satseconds alarm settings	SatSeconds alarm settings	1	kept
Saturated oxygen	saturated oxygen	saturated oxygen	saturated oxygen	Saturated oxygen	1	kept
saturation	saturation	saturation	saturation	SATURATION	1	case_or_format
saturation monitoring	saturation monitoring	saturation monitoring	saturation monitoring	saturation monitoring	1	kept
saturation probe injury	saturation probe injury	saturation probe injury	saturation probe injury	saturation probe injury	1	kept
Satı Demir	sat demir	sat demir	sat demir	Satı Demir	1	kept
Saudi Arabi	saudi arabi	saudi arabi	saudi arabi	Saudi Arabi	1	kept
Saudi arabia	saudi arabia	saudi arabia	saudi arabia	Saudi Arabia	1	case_or_format
Saudi Career Development Program	saudi career development program	saudi career development program	saudi career development program	Saudi Career Development Program	1	kept
Saudi family needs	saudi family needs	saudi family needs	saudi family needs	Saudi family needs	1	kept
SaudiArabia	saudiarabia	saudiarabia	saudiarabia	SaudiArabia	1	kept

SAVE	save	save	save	SAVE	1	kept
Saves time	saves time	saves time	saves time	Saves time	1	kept
Saving Protocol	saving protocol	saving protocol	saving protocol	Saving Protocol	1	kept
savoir	savoir	savoir	savoir	savoir	1	kept
SBAR checklist	sbar checklist	sbar checklist	sbar checklist	SBAR checklist	1	kept
SBAR Communication	sbar communication	sbar communication	sbar communication	SBAR Communication	1	kept
SBAR communication	sbar communication	sbar communication	sbar communication	SBAR Communication	1	case_or_format
SBAR communication system	sbar communication system	sbar communication system	sbar communication system	SBAR communication system	1	kept
SBAR COMMUNICATION TECHNIQUE	sbar communication technique	sbar communication technique	sbar communication technique	SBAR COMMUNICATION TECHNIQUE	1	kept
SBAR concept	sbar concept	sbar concept	sbar concept	SBAR concept	1	kept
SBAR form	sbar form	sbar form	sbar form	SBAR form	1	kept
SBAR model	sbar model	sbar model	sbar model	SBAR model	1	kept
SBI	sbi	sbi	sbi	SBI	1	kept
SBRT	sbrt	sbrt	sbrt	SBRT	1	kept
SCA-Alp protocol	sca alp protocol	sca alp protocol	sca alp protocol	SCA-Alp protocol	1	kept
scalability	scalability	scalability	scalability	scalability	1	kept

Scalability test	scalability test	scalability test	scalability test	Scalability test	1	kept
Scalds	scalds	scalds	scald	scald	1	singular
SCALE AITCS	scale aitcs	scale aitcs	scale aitcs	SCALE AITCS	1	kept
Scale levels	scale levels	scale levels	scale levels	Scale levels	1	kept
scale reliability	scale reliability	scale reliability	scale reliability	scale reliability	1	kept
Scale Validation	scale validation	scale validation	scale validation	Scale Validation	1	kept
scale validity	scale validity	scale validity	scale validity	scale validity	1	kept
scale-driven	scale driven	scale driven	scale driven	scale-driven	1	kept
scales of control	scales of control	scales of control	scales of control	scales of control	1	kept
scalp hair	scalp hair	scalp hair	scalp hair	scalp hair	1	kept
scalpel	scalpel	scalpel	scalpel	scalpel	1	kept
SCANDINAVIA	scandinavia	scandinavia	scandinavia	Scandinavia	1	case_or_format
Scanning electron microscopy	scanning electron microscopy	scanning electron microscopy	scanning electron microscopy	scanning electron microscopy	1	case_or_format
Scans	scans	scans	scans	Scans	1	kept
SCAP	scap	scap	scap	SCAP	1	kept
Scapegoat	scapegoat	scapegoat	scapegoat	Scapegoat	1	kept

scaphocephaly	scaphocephaly	scaphocephaly	scaphocephaly	scaphocephaly	1	kept
scar	scar	scar	scar	scar	1	kept
scar tissue	scar tissue	scar tissue	scar tissue	scar tissue	1	kept
Scar-free surgery	scar free surgery	scar free surgery	scar free surgery	Scar-free surgery	1	kept
Scarce resources	scarce resources	scarce resources	scarce resources	Scarce resources	1	kept
Scattering effects	scattering effects	scattering effects	scattering effects	Scattering effects	1	kept
Scatternet Network	scatternet network	scatternet network	scatternet network	Scatternet Network	1	kept
Scatternets	scatternets	scatternets	scatternets	Scatternets	1	kept
scavenging	scavenging	scavenging	scavenging	scavenging	1	kept
SCCM clinical practice guidelines	sccm clinical practice guidelines	sccm clinical practice guidelines	sccm clinical practice guidelines	SCCM clinical practice guidelines	1	kept
SCCM guidelines	sccm guidelines	sccm guidelines	sccm guidelines	SCCM guidelines	1	kept
SCD, sudden cardiac death	scd sudden cardiac death	scd sudden cardiac death	scd sudden cardiac death	SCD, sudden cardiac death	1	kept
scenario analysis	scenario analysis	scenario analysis	scenario analysis	scenario analysis	1	kept
Scenario planning	scenario planning	scenario planning	scenario planning	Scenario planning	1	kept
Scenario-based	scenario based	scenario based	scenario based	Scenario-based	1	kept
Scenario-based learning	scenario based learning	scenario based learning	scenario based learning	Scenario-based learning	1	kept

Scenario-based simulations	scenario based simulations	scenario based simulations	scenario based simulations	Scenario-based simulations	1	kept
Scenarios	scenarios	scenarios	scenario	Scenario	1	singular
SCHEDULE	schedule	schedule	schedule	SCHEDULE	1	kept
schedule	schedule	schedule	schedule	SCHEDULE	1	case_or_format
Schedule development	schedule development	schedule development	schedule development	Schedule development	1	kept
schedule modes	schedule modes	schedule modes	schedule modes	schedule modes	1	kept
scheduled naps	scheduled naps	scheduled naps	scheduled naps	scheduled naps	1	kept
Scheduling application	scheduling application	scheduling application	scheduling application	Scheduling application	1	kept
SCHEDULING FLEXIBLE SERVERS	scheduling flexible servers	scheduling flexible servers	scheduling flexible servers	SCHEDULING FLEXIBLE SERVERS	1	kept
Scheduling periods	scheduling periods	scheduling periods	scheduling periods	Scheduling periods	1	kept
Scheduling policies	scheduling policies	scheduling policies	scheduling policies	Scheduling policies	1	kept
SCHEDULING PROBLEM	scheduling problem	scheduling problem	scheduling problem	SCHEDULING PROBLEM	1	kept
Scheduling problem	scheduling problem	scheduling problem	scheduling problem	SCHEDULING PROBLEM	1	case_or_format
schizencephaly	schizencephaly	schizencephaly	schizencephaly	schizencephaly	1	kept
Schizophrenic Psychology	schizophrenic psychology	schizophrenic psychology	schizophrenic psychology	Schizophrenic Psychology	1	kept
Schmerz	schmerz	schmerz	schmerz	Schmerz	1	kept

scholarly communication	scholarly communication	scholarly communication	scholarly communication	scholarly communication	1	kept
scholarship	scholarship	scholarship	scholarship	scholarship	1	kept
school closure	school closure	school closure	school closure	school closure	1	kept
school health education	school health education	school health education	school health education	school health education	1	kept
School performance	school performance	school performance	school performance	School performance	1	kept
School refusal	school refusal	school refusal	school refusal	School refusal	1	kept
SCHOOL-AGE	school age	school age	school age	SCHOOL-AGE	1	kept
Schools, Dental	schools dental	schools dental	schools dental	Schools, Dental	1	kept
Schwartz Bartter syndrome	schwartz bartter syndrome	schwartz bartter syndrome	schwartz bartter syndrome	Schwartz Bartter syndrome	1	kept
schéma insulinique de type basal-bolus	sch ma insulinique de type basal bolus	sch ma insulinique de type basal bolus	sch ma insulinique de type basal bolus	schéma insulinique de type basal-bolus	1	kept
SCI = spinal cord injury	sci spinal cord injury	sci spinal cord injury	sci spinal cord injury	SCI = spinal cord injury	1	kept
sciatica	sciatica	sciatica	sciatica	sciatica	1	kept
Science and Technology	science and technology	science and technology	science and technology	Science and Technology	1	kept
science infirmière	science infirmi re	science infirmi re	science infirmi re	science infirmière	1	kept
science of health care delivery	science of health care delivery	science of health care delivery	science of health care delivery	science of health care delivery	1	kept
Sciences review	sciences review	sciences review	sciences review	Sciences review	1	kept

scientific principle	scientific principle	scientific principle	scientific principle	scientific principle	1	kept
Scientific Products	scientific products	scientific products	scientific products	Scientific Products	1	kept
scintigraphy	scintigraphy	scintigraphy	scintigraphy	scintigraphy	1	kept
SCIP measure	scip measure	scip measure	scip measure	SCIP measure	1	kept
Scleral Buckling	scleral buckling	scleral buckling	scleral buckling	Scleral Buckling	1	kept
scleredema	scleredema	scleredema	scleredema	scleredema	1	kept
Scleroderma	scleroderma	scleroderma	scleroderma	Scleroderma	1	kept
scleroderma	scleroderma	scleroderma	scleroderma	Scleroderma	1	case_or_format
Scleroderma, Systemic	scleroderma systemic	scleroderma systemic	scleroderma systemic	Scleroderma, Systemic	1	kept
Sclerodermum	sclerodermum	sclerodermum	sclerodermum	Sclerodermum	1	kept
Sclinical judgment	sclinical judgment	sclinical judgment	sclinical judgment	Sclinical judgment	1	kept
Scope	scope	scope	scope	Scope	1	kept
scope	scope	scope	scope	Scope	1	case_or_format
scope-of-practice	scope of practice	scope of practice	scope of practice	scope of practice	1	case_or_format
Scoping literature review	scoping literature review	scoping literature review	scoping literature review	Scoping literature review	1	kept
scopolamine derivative	scopolamine derivative	scopolamine derivative	scopolamine derivative	scopolamine derivative	1	kept

Scopolamine Derivatives	scopolamine derivatives	scopolamine derivatives	scopolamine derivative	scopolamine derivative	1	singular
SCOPUS	scopus	scopus	scopus	Scopus	1	case_or_format
Score for neonatal acute physiology ii	score for neonatal acute physiology ii	score for neonatal acute physiology ii	score for neonatal acute physiology ii	Score for neonatal acute physiology ii	1	kept
score for neonatal acute physiology version II	score for neonatal acute physiology version ii	score for neonatal acute physiology version ii	score for neonatal acute physiology version ii	score for neonatal acute physiology version II	1	kept
score for toxic epidermal necrosis	score for toxic epidermal necrosis	score for toxic epidermal necrosis	score for toxic epidermal necrosis	score for toxic epidermal necrosis	1	kept
SCORE SAPS-II	score saps ii	score saps ii	score saps ii	SCORE SAPS-II	1	kept
SCORES	scores	scores	score	SCORE	1	singular
Scores	scores	scores	score	SCORE	1	singular
scores	scores	scores	score	SCORE	1	singular
Scoring methods	scoring methods	scoring methods	scoring methods	Scoring methods	1	kept
SCORING SYSTEMS	scoring systems	scoring systems	scoring system	scoring system	1	singular
scoring tool	scoring tool	scoring tool	scoring tool	scoring tool	1	kept
Scorpion	scorpion	scorpion	scorpion	Scorpion	1	kept
scorpion	scorpion	scorpion	scorpion	Scorpion	1	case_or_format
Scorpion envenomation	scorpion envenomation	scorpion envenomation	scorpion envenomation	Scorpion envenomation	1	kept
scorpion envenomation	scorpion envenomation	scorpion envenomation	scorpion envenomation	Scorpion envenomation	1	case_or_format

scorpion sting	scorpion sting	scorpion sting	scorpion sting	scorpion sting	1	kept
Scorpion sting knowledge	scorpion sting knowledge	scorpion sting knowledge	scorpion sting knowledge	Scorpion sting knowledge	1	kept
Scorpion Stings	scorpion stings	scorpion stings	scorpion sting	scorpion sting	1	singular
scorpion venom	scorpion venom	scorpion venom	scorpion venom	scorpion venom	1	kept
Scorpions	scorpions	scorpions	scorpion	Scorpion	1	singular
SCRAs	scras	scras	scras	SCRAs	1	kept
SCREEN	screen	screen	screen	SCREEN	1	kept
screen	screen	screen	screen	SCREEN	1	case_or_format
Screen based simulator	screen based simulator	screen based simulator	screen based simulator	Screen based simulator	1	kept
screen culture	screen culture	screen culture	screen culture	screen culture	1	kept
Screen Time	screen time	screen time	screen time	Screen Time	1	kept
Screen time	screen time	screen time	screen time	Screen Time	1	case_or_format
screen time	screen time	screen time	screen time	Screen Time	1	case_or_format
Screen-based simulation	screen based simulation	screen based simulation	screen based simulation	Screen-based simulation	1	kept
screen-based simulation	screen based simulation	screen based simulation	screen based simulation	Screen-based simulation	1	case_or_format
screen-detected diabetes	screen detected diabetes	screen detected diabetes	screen detected diabetes	screen-detected diabetes	1	kept

SCREENING CHECKLIST	screening checklist	screening checklist	screening checklist	SCREENING CHECKLIST	1	kept
Screening criteria	screening criteria	screening criteria	screening criteria	Screening criteria	1	kept
Screening device	screening device	screening device	screening device	Screening device	1	kept
Screening method	screening method	screening method	screening method	Screening method	1	kept
SCREENING TOOL	screening tool	screening tool	screening tool	Screening tool	1	case_or_format
Screening Tool for Risk On Nutritional Status and Growth	screening tool for risk on nutritional status and growth	screening tool for risk on nutritional status and growth	screening tool for risk on nutritional status and growth	Screening Tool for Risk On Nutritional Status and Growth	1	kept
Screening Tool for the Assessment of Malnutrition in	screening tool for the assessment of malnutrition in	screening tool for the assessment of malnutrition in	screening tool for the assessment of malnutrition in	Screening Tool for the Assessment of Malnutrition in	1	kept
screening tools	screening tools	screening tools	screening tool	Screening tool	1	singular
Script concordance test	script concordance test	script concordance test	script concordance test	Script concordance test	1	kept
Script strategy	script strategy	script strategy	script strategy	Script strategy	1	kept
scrotal swelling	scrotal swelling	scrotal swelling	scrotal swelling	scrotal swelling	1	kept
Scrotum	scrotum	scrotum	scrotum	Scrotum	1	kept
scrotum	scrotum	scrotum	scrotum	Scrotum	1	case_or_format
Scrub Typhus	scrub typhus	scrub typhus	scrub typhus	scrub typhus	1	case_or_format
Scrum methodologies	scrum methodologies	scrum methodologies	scrum methodologies	Scrum methodologies	1	kept
SCT management	sct management	sct management	sct management	SCT management	1	kept

ScvO2	scvo2	scvo2	scvo2	ScvO2	1	kept
ScvO<sub>2<	scvo sub 2	scvo sub 2	scvo sub 2	ScvO<sub>2<	1	kept
SD, Standard deviation	sd standard deviation	sd standard deviation	sd standard deviation	SD, Standard deviation	1	kept
SDD	sdd	sdd	sdd	SDD	1	kept
SDF, Supported Diagonal Flexion	sdf supported diagonal flexion	sdf supported diagonal flexion	sdf supported diagonal flexion	SDF, Supported Diagonal Flexion	1	kept
SDH	sdh	sdh	sdh	SDH	1	kept
SDRA	sdra	sdra	sdra	SDRA	1	kept
sea	sea	sea	sea	sea	1	kept
sea surface temperature	sea surface temperature	sea surface temperature	sea surface temperature	sea surface temperature	1	kept
SEARCH	search	search	search	SEARCH	1	kept
Search and destroy policy	search and destroy policy	search and destroy policy	search and destroy policy	Search and destroy policy	1	kept
Search out Severity Score	search out severity score	search out severity score	search out severity score	Search out Severity Score	1	kept
seasonalinfluenza	seasonalinfluenza	seasonalinfluenza	seasonalinfluenza	seasonalinfluenza	1	kept
seat	seat	seat	seat	seat	1	kept
seatbelt injury	seatbelt injury	seatbelt injury	seatbelt injury	seatbelt injury	1	kept
Seattle-King County	seattle king county	seattle king county	seattle king county	Seattle-King County	1	kept

seborrheic dermatitis	seborrheic dermatitis	seborrheic dermatitis	seborrheic dermatitis	seborrheic dermatitis	1	kept
seclusion and restraint	seclusion and restraint	seclusion and restraint	seclusion and restraint	seclusion and restraint	1	kept
second cancer	second cancer	second cancer	second cancer	second cancer	1	kept
second cervical vertebra	second cervical vertebra	second cervical vertebra	second cervical vertebra	second cervical vertebra	1	kept
second look surgery	second look surgery	second look surgery	second look surgery	second look surgery	1	kept
Second phase	second phase	second phase	second phase	Second phase	1	kept
second stage	second stage	second stage	second stage	second stage	1	kept
second stage of labor	second stage of labor	second stage of labor	second stage of labor	second stage of labor	1	kept
second victim experience and support survey	second victim experience and support survey	second victim experience and support survey	second victim experience and support survey	second victim experience and support survey	1	kept
Second victims	second victims	second victims	second victim	Second victim	1	singular
second victims	second victims	second victims	second victim	Second victim	1	singular
Second Victims in German speaking countries	second victims in german speaking countries	second victims in german speaking countries	second victims in german speaking countries	Second Victims in German speaking countries	1	kept
second wave	second wave	second wave	second wave	second wave	1	kept
second-line treatment	second line treatment	second line treatment	second line treatment	second-line treatment	1	kept
SECONDARY	secondary	secondary	secondary	secondary	1	case_or_format
Secondary adrenal insufficiency	secondary adrenal insufficiency	secondary adrenal insufficiency	secondary adrenal insufficiency	Secondary adrenal insufficiency	1	kept

secondary aeromedical services	secondary aeromedical services	secondary aeromedical services	secondary aeromedical services	secondary aeromedical services	1	kept
secondary amenorrhea	secondary amenorrhea	secondary amenorrhea	secondary amenorrhea	secondary amenorrhea	1	kept
Secondary analysis	secondary analysis	secondary analysis	secondary analysis	secondary analysis	1	case_or_format
Secondary and tertiary care hospital	secondary and tertiary care hospital	secondary and tertiary care hospital	secondary and tertiary care hospital	Secondary and tertiary care hospital	1	kept
secondary assessment	secondary assessment	secondary assessment	secondary assessment	secondary assessment	1	kept
Secondary brain damage	secondary brain damage	secondary brain damage	secondary brain damage	Secondary brain damage	1	kept
secondary brain injury	secondary brain injury	secondary brain injury	secondary brain injury	secondary brain injury	1	kept
Secondary brain injury of systemic origin	secondary brain injury of systemic origin	secondary brain injury of systemic origin	secondary brain injury of systemic origin	Secondary brain injury of systemic origin	1	kept
Secondary Care	secondary care	secondary care	secondary care	Secondary Care	1	kept
Secondary Care Centers	secondary care centers	secondary care centers	secondary care center	secondary care center	1	singular
Secondary Data Analysis	secondary data analysis	secondary data analysis	secondary data analysis	Secondary data analysis	1	case_or_format
Secondary Health Care	secondary health care	secondary health care	secondary health care	secondary health care	1	case_or_format
Secondary injuries	secondary injuries	secondary injuries	secondary injuries	Secondary injuries	1	kept
secondary injury prevention	secondary injury prevention	secondary injury prevention	secondary injury prevention	secondary injury prevention	1	kept
secondary insult	secondary insult	secondary insult	secondary insult	secondary insult	1	kept
secondary medication administration	secondary medication administration	secondary medication administration	secondary medication administration	secondary medication administration	1	kept

Secondary obstetrician involvement	secondary obstetrician involvement	secondary obstetrician involvement	secondary obstetrician involvement	Secondary obstetrician involvement	1	kept
secondary post-traumatic stress	secondary post traumatic stress	secondary post traumatic stress	secondary post traumatic stress	secondary post-traumatic stress	1	kept
Secondary prevention adherence stroke	secondary prevention adherence stroke	secondary prevention adherence stroke	secondary prevention adherence stroke	Secondary prevention adherence stroke	1	kept
Secondary prevention.	secondary prevention	secondary prevention	secondary prevention	secondary prevention	1	case_or_format
secondary qualitative analysis	secondary qualitative analysis	secondary qualitative analysis	secondary qualitative analysis	secondary qualitative analysis	1	kept
secondary sclerosing cholangitis	secondary sclerosing cholangitis	secondary sclerosing cholangitis	secondary sclerosing cholangitis	secondary sclerosing cholangitis	1	kept
secondary spine injury	secondary spine injury	secondary spine injury	secondary spine injury	secondary spine injury	1	kept
secondary surfactant deficiency	secondary surfactant deficiency	secondary surfactant deficiency	secondary surfactant deficiency	secondary surfactant deficiency	1	kept
secondary syphilis	secondary syphilis	secondary syphilis	secondary syphilis	secondary syphilis	1	kept
Secondary transportation	secondary transportation	secondary transportation	secondary transportation	Secondary transportation	1	kept
secondary trauma stress	secondary trauma stress	secondary trauma stress	secondary trauma stress	secondary trauma stress	1	kept
Secondary traumatic stress scale	secondary traumatic stress scale	secondary traumatic stress scale	secondary traumatic stress scale	Secondary Traumatic Stress Scale	1	case_or_format
secondary traumatization	secondary traumatization	secondary traumatization	secondary traumatization	secondary traumatization	1	kept
secondary use	secondary use	secondary use	secondary use	secondary use	1	kept
Secondhand smoke exposure	secondhand smoke exposure	secondhand smoke exposure	secondhand smoke exposure	Secondhand smoke exposure	1	kept
secretagogue	secretagogue	secretagogue	secretagogue	secretagogue	1	kept

Secretion management	secretion management	secretion management	secretion management	Secretion management	1	kept
secretion management	secretion management	secretion management	secretion management	Secretion management	1	case_or_format
SECRETIONS	secretions	secretions	secretion	secretion	1	singular
Secretions	secretions	secretions	secretion	secretion	1	singular
secretory activation	secretory activation	secretory activation	secretory activation	secretory activation	1	kept
secretory immunoglobulin	secretory immunoglobulin	secretory immunoglobulin	secretory immunoglobulin	secretory immunoglobulin	1	kept
sectra	sectra	sectra	sectra	sectra	1	kept
secukinumab	secukinumab	secukinumab	secukinumab	secukinumab	1	kept
SECULAR TRENDS	secular trends	secular trends	secular trends	SECULAR TRENDS	1	kept
secular trends	secular trends	secular trends	secular trends	SECULAR TRENDS	1	case_or_format
Secularisation	secularisation	secularisation	secularisation	Secularisation	1	kept
Secure data storage	secure data storage	secure data storage	secure data storage	Secure data storage	1	kept
Secure messaging	secure messaging	secure messaging	secure messaging	Secure messaging	1	kept
Secured dementia unit	secured dementia unit	secured dementia unit	secured dementia unit	Secured dementia unit	1	kept
securement	securement	securement	securement	Securement	1	case_or_format
security and training	security and training	security and training	security and training	security and training	1	kept

Security of data	security of data	security of data	security of data	Security of data	1	kept
Sedación inhalada	sedaci n inhalada	sedaci n inhalada	sedaci n inhalada	Sedación inhalada	1	kept
Sedación inhalatoria	sedaci n inhalatoria	sedaci n inhalatoria	sedaci n inhalatoria	Sedación inhalatoria	1	kept
Sedantes	sedantes	sedantes	sedantes	Sedantes	1	kept
Sedated	sedated	sedated	sedated	sedated	1	case_or_format
sedation agitation scale	sedation agitation scale	sedation agitation scale	sedation agitation scale	Sedation Agitation Scale	1	case_or_format
Sedation alternative	sedation alternative	sedation alternative	sedation alternative	Sedation alternative	1	kept
sedation analgesia management	sedation analgesia management	sedation analgesia management	sedation analgesia management	sedation analgesia management	1	kept
Sedation assessment and management	sedation assessment and management	sedation assessment and management	sedation assessment and management	Sedation assessment and management	1	kept
sedation dose	sedation dose	sedation dose	sedation dose	sedation dose	1	kept
sedation guidelines	sedation guidelines	sedation guidelines	sedation guidelines	sedation guidelines	1	kept
Sedation hold	sedation hold	sedation hold	sedation hold	Sedation hold	1	kept
Sedation holds	sedation holds	sedation holds	sedation hold	Sedation hold	1	singular
Sedation infusions	sedation infusions	sedation infusions	sedation infusions	Sedation infusions	1	kept
Sedation Intensity Score	sedation intensity score	sedation intensity score	sedation intensity score	Sedation Intensity Score	1	kept
Sedation interruption	sedation interruption	sedation interruption	sedation interruption	Sedation interruption	1	kept

sedation level	sedation level	sedation level	sedation level	sedation level	1	kept
SEDATION PRACTICE RECOMMENDATIONS	sedation practice recommendations	sedation practice recommendations	sedation practice recommendations	SEDATION PRACTICE RECOMMENDATIONS	1	kept
Sedation practices	sedation practices	sedation practices	sedation practices	Sedation practices	1	kept
Sedation protocol	sedation protocol	sedation protocol	sedation protocol	sedation protocol	1	case_or_format
Sedation score	sedation score	sedation score	sedation score	Sedation score	1	kept
Sedation tools	sedation tools	sedation tools	sedation tools	Sedation tools	1	kept
Sedation weaning	sedation weaning	sedation weaning	sedation weaning	Sedation weaning	1	kept
Sedation-analgesia	sedation analgesia	sedation analgesia	sedation analgesia	Sedation-analgesia	1	kept
sedative	sedative	sedative	sedative	Sedatives	1	case_or_format
Sedative effects	sedative effects	sedative effects	sedative effects	Sedative effects	1	kept
Sedative interruption	sedative interruption	sedative interruption	sedative interruption	Sedative interruption	1	kept
Sedative withdrawal	sedative withdrawal	sedative withdrawal	sedative withdrawal	Sedative withdrawal	1	kept
SEDATIVES	sedatives	sedatives	sedative	Sedatives	1	singular
sedatives and hypnotics	sedatives and hypnotics	sedatives and hypnotics	sedatives and hypnotics	sedatives and hypnotics	1	kept
SedLine	sedline	sedline	sedline	SedLine	1	kept
Sedline	sedline	sedline	sedline	SedLine	1	case_or_format

sedo analgesia management	sedo analgesia management	sedo analgesia management	sedo analgesia management	sedo analgesia management	1	kept
Sedo-analgesia	sedo analgesia	sedo analgesia	sedo analgesia	Sedo-analgesia	1	kept
seech therapist	seech therapist	seech therapist	seech therapist	seech therapist	1	kept
SEEKING	seeking	seeking	seeking	SEEKING	1	kept
seeking help	seeking help	seeking help	seeking help	seeking help	1	kept
seeking safety	seeking safety	seeking safety	seeking safety	seeking safety	1	kept
SEER	seer	seer	seer	SEER	1	kept
segmental colectomy	segmental colectomy	segmental colectomy	segmental colectomy	segmental colectomy	1	kept
SEGMENTATION	segmentation	segmentation	segmentation	SEGMENTATION	1	kept
Segregation	segregation	segregation	segregation	Segregation	1	kept
segregation analysis	segregation analysis	segregation analysis	segregation analysis	segregation analysis	1	kept
Segurança do paciente	seguran a do paciente	seguran a do paciente	seguran a do paciente	Segurança do Paciente	1	case_or_format
Seguridad	seguridad	seguridad	seguridad	Seguridad	1	kept
Seguridad clínica	seguridad cl nica	seguridad cl nica	seguridad cl nica	Seguridad clínica	1	kept
Seguridad de los pacientes	seguridad de los pacientes	seguridad de los pacientes	seguridad de los pacientes	Seguridad de los pacientes	1	kept
SEIPS model	seips model	seips model	seips model	SEIPS model	1	kept

SEIR model	seir model	seir model	seir model	SEIR model	1	kept
Seismic waves	seismic waves	seismic waves	seismic waves	Seismic waves	1	kept
Seizure detection	seizure detection	seizure detection	seizure detection	Seizure detection	1	kept
seizure identification	seizure identification	seizure identification	seizure identification	seizure identification	1	kept
seizure protocol	seizure protocol	seizure protocol	seizure protocol	seizure protocol	1	kept
seizure susceptibility	seizure susceptibility	seizure susceptibility	seizure susceptibility	seizure susceptibility	1	kept
seizures rhythmic and periodic patterns	seizures rhythmic and periodic patterns	seizures rhythmic and periodic patterns	seizures rhythmic and periodic patterns	seizures rhythmic and periodic patterns	1	kept
sekrol syrup	sekrol syrup	sekrol syrup	sekrol syrup	sekrol syrup	1	kept
Seldinger catheterization	seldinger catheterization	seldinger catheterization	seldinger catheterization	Seldinger catheterization	1	kept
seldinger technique	seldinger technique	seldinger technique	seldinger technique	seldinger technique	1	kept
Selection Committee	selection committee	selection committee	selection committee	Selection Committee	1	kept
selection mechanisms	selection mechanisms	selection mechanisms	selection mechanisms	selection mechanisms	1	kept
Selective	selective	selective	selective	Selective	1	kept
Selective decontamination	selective decontamination	selective decontamination	selective decontamination	Selective decontamination	1	kept
Selective digestive decontamination	selective digestive decontamination	selective digestive decontamination	selective digestive decontamination	Selective digestive decontamination	1	kept
Selective order management	selective order management	selective order management	selective order management	Selective order management	1	kept

Selective oropharyngeal decontamination	selective oropharyngeal decontamination	selective oropharyngeal decontamination	selective oropharyngeal decontamination	Selective oropharyngeal decontamination	1	kept
selegiline	selegiline	selegiline	selegiline	selegiline	1	kept
SELENIUM	selenium	selenium	selenium	selenium	1	case_or_format
SELF	self	self	self	self	1	case_or_format
Self	self	self	self	self	1	case_or_format
self actualization	self actualization	self actualization	self actualization	self actualization	1	kept
self administered online questionnaire	self administered online questionnaire	self administered online questionnaire	self administered online questionnaire	self administered online questionnaire	1	kept
self alienation	self alienation	self alienation	self alienation	self alienation	1	kept
self assessed educational need	self assessed educational need	self assessed educational need	self assessed educational need	self assessed educational need	1	kept
self assessment	self assessment	self assessment	self assessment	Self-Assessment	1	case_or_format
Self awareness	self awareness	self awareness	self awareness	self-awareness	1	case_or_format
self care heart failure index	self care heart failure index	self care heart failure index	self care heart failure index	self care heart failure index	1	kept
self compassion	self compassion	self compassion	self compassion	Self-compassion	1	case_or_format
self confidence	self confidence	self confidence	self confidence	Self-confidence	1	case_or_format
Self efficacy	self efficacy	self efficacy	self efficacy	Self Efficacy	1	case_or_format
self efficacy scale	self efficacy scale	self efficacy scale	self efficacy scale	self efficacy scale	1	kept

self expanding stent	self expanding stent	self expanding stent	self expanding stent	self expanding stent	1	kept
self extubation prevention	self extubation prevention	self extubation prevention	self extubation prevention	self extubation prevention	1	kept
self instructed video education	self instructed video education	self instructed video education	self instructed video education	self instructed video education	1	kept
Self instructional module	self instructional module	self instructional module	self instructional module	Self instructional module	1	kept
self knowledge	self knowledge	self knowledge	self knowledge	self knowledge	1	kept
Self made guiding device	self made guiding device	self made guiding device	self made guiding device	Self made guiding device	1	kept
self made questionnaire	self made questionnaire	self made questionnaire	self made questionnaire	self made questionnaire	1	kept
Self Medication	self medication	self medication	self medication	Self Medication	1	kept
self medication	self medication	self medication	self medication	Self Medication	1	case_or_format
Self Organizing Map algorithm	self organizing map algorithm	self organizing map algorithm	self organizing map algorithm	Self Organizing Map algorithm	1	kept
self protection	self protection	self protection	self protection	self protection	1	kept
Self Psychology	self psychology	self psychology	self psychology	Self Psychology	1	kept
Self Rating Depression Scale	self rating depression scale	self rating depression scale	self rating depression scale	Self-rating Depression Scale	1	case_or_format
self rating scale	self rating scale	self rating scale	self rating scale	self rating scale	1	kept
self rating sleeping situation scale	self rating sleeping situation scale	self rating sleeping situation scale	self rating sleeping situation scale	self rating sleeping situation scale	1	kept
self referrals	self referrals	self referrals	self referrals	self referrals	1	kept

Self reporting surveys	self reporting surveys	self reporting surveys	self reporting surveys	Self reporting surveys	1	kept
self toughness	self toughness	self toughness	self toughness	self toughness	1	kept
Self- made arterial indwelling needle	self made arterial indwelling needle	self made arterial indwelling needle	self made arterial indwelling needle	Self- made arterial indwelling needle	1	kept
Self-actualization	self actualization	self actualization	self actualization	self actualization	1	case_or_format
self-adjusting insulin administration	self adjusting insulin administration	self adjusting insulin administration	self adjusting insulin administration	self-adjusting insulin administration	1	kept
Self-administered questionnaire	self administered questionnaire	self administered questionnaire	self administered questionnaire	self administered questionnaire	1	case_or_format
Self-administration of medication	self administration of medication	self administration of medication	self administration of medication	Self-administration of medication	1	kept
self-admission	self admission	self admission	self admission	self-admission	1	kept
Self-assessment competence tool	self assessment competence tool	self assessment competence tool	self assessment competence tool	Self-assessment competence tool	1	kept
self-assessment competence tool	self assessment competence tool	self assessment competence tool	self assessment competence tool	Self-assessment competence tool	1	case_or_format
self-blame	self blame	self blame	self blame	self blame	1	case_or_format
SELF-CARE	self care	self care	self care	self care	1	case_or_format
Self-Care	self care	self care	self care	self care	1	case_or_format
Self-care readiness	self care readiness	self care readiness	self care readiness	Self-care readiness	1	kept
Self-Care Units	self care units	self care units	self care units	Self-Care Units	1	kept
Self-catheterization	self catheterization	self catheterization	self catheterization	Self-catheterization	1	kept

Self-checking table	self checking table	self checking table	self checking table	Self-checking table	1	kept
Self-Compassion	self compassion	self compassion	self compassion	Self-compassion	1	case_or_format
Self-Compassion Scale	self compassion scale	self compassion scale	self compassion scale	Self-Compassion Scale	1	kept
self-competence	self competence	self competence	self competence	self-competence	1	kept
self-concept assessment	self concept assessment	self concept assessment	self concept assessment	self-concept assessment	1	kept
self-contact	self contact	self contact	self contact	self-contact	1	kept
Self-contained systems	self contained systems	self contained systems	self contained systems	Self-contained systems	1	kept
Self-Control	self control	self control	self control	self control	1	case_or_format
Self-destructive behavior	self destructive behavior	self destructive behavior	self destructive behavior	Self-destructive behavior	1	kept
SELF-DETERMINATION THEORY	self determination theory	self determination theory	self determination theory	SELF-DETERMINATION THEORY	1	kept
Self-Determination Theory	self determination theory	self determination theory	self determination theory	SELF-DETERMINATION THEORY	1	case_or_format
self-determination theory	self determination theory	self determination theory	self determination theory	SELF-DETERMINATION THEORY	1	case_or_format
self-directed	self directed	self directed	self directed	self-directed	1	kept
Self-directed learning	self directed learning	self directed learning	self directed learning	self-directed learning	1	case_or_format
Self-Directed Learning as Topic	self directed learning as topic	self directed learning as topic	self directed learning as topic	Self-Directed Learning as Topic	1	kept
Self-driven empowerment model	self driven empowerment model	self driven empowerment model	self driven empowerment model	Self-driven empowerment model	1	kept

Self-Efficacy	self efficacy	self efficacy	self efficacy	Self Efficacy	1	case_or_format
self-ethical behavior	self ethical behavior	self ethical behavior	self ethical behavior	self-ethical behavior	1	kept
Self-evaluation	self evaluation	self evaluation	self evaluation	self evaluation	1	case_or_format
Self-evaluation programs	self evaluation programs	self evaluation programs	self evaluation programs	Self-Evaluation Programs	1	case_or_format
self-extubation	self extubation	self extubation	self extubation	Self-extubation	1	case_or_format
self-guided	self guided	self guided	self guided	self-guided	1	kept
self-harm	self harm	self harm	self harm	Self-harm	1	case_or_format
Self-help groups	self help groups	self help groups	self help groups	Self-Help Groups	1	case_or_format
self-identity	self identity	self identity	self identity	self-identity	1	kept
Self-inflating bag	self inflating bag	self inflating bag	self inflating bag	Self-inflating bag	1	kept
Self-inflating bag valve resuscitator	self inflating bag valve resuscitator	self inflating bag valve resuscitator	self inflating bag valve resuscitator	Self-inflating bag valve resuscitator	1	kept
Self-injurious behavior	self injurious behavior	self injurious behavior	self injurious behavior	Self-Injurious Behavior	1	case_or_format
SELF-INJURIOUS BEHAVIOUR	self injurious behaviour	self injurious behaviour	self injurious behaviour	SELF-INJURIOUS BEHAVIOUR	1	kept
self-instructional module	self instructional module	self instructional module	self instructional module	Self instructional module	1	case_or_format
Self-integration	self integration	self integration	self integration	Self-integration	1	kept
self-knowledge	self knowledge	self knowledge	self knowledge	self knowledge	1	case_or_format

Self-leadership	self leadership	self leadership	self leadership	self-leadership	1	case_or_format
Self-learning module	self learning module	self learning module	self learning module	Self-learning module	1	kept
Self-learning package	self learning package	self learning package	self learning package	Self-learning package	1	kept
SELF-MANAGEMENT	self management	self management	self management	Self-Management	1	case_or_format
Self-monitoring	self monitoring	self monitoring	self monitoring	self monitoring	1	case_or_format
Self-perception	self perception	self perception	self perception	Self-perception	1	kept
self-perception	self perception	self perception	self perception	Self-perception	1	case_or_format
Self-poisoning	self poisoning	self poisoning	self poisoning	self poisoning	1	case_or_format
self-protection	self protection	self protection	self protection	self protection	1	case_or_format
Self-reflection	self reflection	self reflection	self reflection	self-reflection	1	case_or_format
Self-reflection and insight scale	self reflection and insight scale	self reflection and insight scale	self reflection and insight scale	Self-reflection and insight scale	1	kept
self-registration	self registration	self registration	self registration	self-registration	1	kept
self-regulation	self regulation	self regulation	self regulation	Self-regulation	1	case_or_format
self-regulation ability	self regulation ability	self regulation ability	self regulation ability	self-regulation ability	1	kept
self-regulatory feeding	self regulatory feeding	self regulatory feeding	self regulatory feeding	self-regulatory feeding	1	kept
Self-reliance	self reliance	self reliance	self reliance	Self-reliance	1	kept

SELF-REPORT	self report	self report	self report	self report	1	case_or_format
self-report measure	self report measure	self report measure	self report measure	self-report measure	1	kept
self-reported adverse events	self reported adverse events	self reported adverse events	self reported adverse events	self-reported adverse events	1	kept
self-reported competencies	self reported competencies	self reported competencies	self reported competencies	self-reported competencies	1	kept
self-reported hours	self reported hours	self reported hours	self reported hours	self-reported hours	1	kept
self-reported medical errors	self reported medical errors	self reported medical errors	self reported medical errors	self-reported medical errors	1	kept
self-reported outcomes	self reported outcomes	self reported outcomes	self reported outcomes	self-reported outcomes	1	kept
SELF-REPORTED PRACTICES	self reported practices	self reported practices	self reported practices	SELF-REPORTED PRACTICES	1	kept
Self-reported practices	self reported practices	self reported practices	self reported practices	SELF-REPORTED PRACTICES	1	case_or_format
self-reported questionnaire	self reported questionnaire	self reported questionnaire	self reported questionnaire	self-reported questionnaire	1	kept
Self-reported symptoms	self reported symptoms	self reported symptoms	self reported symptoms	Self-reported symptoms	1	kept
Self-selected goals	self selected goals	self selected goals	self selected goals	Self-selected goals	1	kept
self-study and simulation	self study and simulation	self study and simulation	self study and simulation	self-study and simulation	1	kept
Self-sufficiency	self sufficiency	self sufficiency	self sufficiency	Self-sufficiency	1	kept
self-transcendence	self transcendence	self transcendence	self transcendence	self transcendence	1	case_or_format
Selfcare	selfcare	selfcare	selfcare	Selfcare	1	kept

Selfconfidence	selfconfidence	selfconfidence	selfconfidence	Selfconfidence	1	kept
Selfefficacy	selfefficacy	selfefficacy	selfefficacy	Selfefficacy	1	kept
self‐compassion	self compassion	self compassion	self compassion	Self-compassion	1	case_or_format
self‐esteem	self esteem	self esteem	self esteem	self esteem	1	case_or_format
Sem	sem	sem	sem	Sem	1	kept
Semantic descriptions	semantic descriptions	semantic descriptions	semantic descriptions	Semantic descriptions	1	kept
semantic differential method	semantic differential method	semantic differential method	semantic differential method	semantic differential method	1	kept
Semantic differential scale	semantic differential scale	semantic differential scale	semantic differential scale	Semantic differential scale	1	kept
semantic differential scale	semantic differential scale	semantic differential scale	semantic differential scale	Semantic differential scale	1	case_or_format
semi recumbent position	semi recumbent position	semi recumbent position	semi recumbent position	semi recumbent position	1	kept
semi structured questionnaire	semi structured questionnaire	semi structured questionnaire	semi structured questionnaire	semi structured questionnaire	1	kept
Semi-elemental	semi elemental	semi elemental	semi elemental	Semi-elemental	1	kept
Semi-Fowler position	semi fowler position	semi fowler position	semi fowler position	Semi-Fowler position	1	kept
Semi-Markov decision process model	semi markov decision process model	semi markov decision process model	semi markov decision process model	Semi-Markov decision process model	1	kept
semi-recumbent	semi recumbent	semi recumbent	semi recumbent	semi-recumbent	1	kept
Semi-Structured interviews	semi structured interviews	semi structured interviews	semi structured interview	semi structured interview	1	singular

Semi-structured interviews	semi structured interviews	semi structured interviews	semi structured interview	semi structured interview	1	singular
semi-structured interviews	semi structured interviews	semi structured interviews	semi structured interview	semi structured interview	1	singular
Semiautomatic methods	semiautomatic methods	semiautomatic methods	semiautomatic methods	Semiautomatic methods	1	kept
semidemand feeding	semidemand feeding	semidemand feeding	semidemand feeding	semidemand feeding	1	kept
Semirecumbent	semirecumbent	semirecumbent	semirecumbent	Semirecumbent	1	kept
semirecumbent position	semirecumbent position	semirecumbent position	semirecumbent position	semirecumbent position	1	kept
semisitting position	semisitting position	semisitting position	semisitting position	Semisitting position	1	case_or_format
Semistructured	semistructured	semistructured	semistructured	Semistructured	1	kept
Semistructured interviews	semistructured interviews	semistructured interviews	semistructured interviews	Semistructured interviews	1	kept
Semistructured questionnaires	semistructured questionnaires	semistructured questionnaires	semistructured questionnaires	Semistructured questionnaires	1	kept
semi - structured interview	semi structured interview	semi structured interview	semi structured interview	semi structured interview	1	case_or_format
senekot	senekot	senekot	senekot	senekot	1	kept
Senescence	senescence	senescence	senescence	senescence	1	case_or_format
senile dementia	senile dementia	senile dementia	senile dementia	senile dementia	1	kept
senility	senility	senility	senility	senility	1	kept
Senior	senior	senior	senior	Senior	1	kept

Senior health care workers	senior health care workers	senior health care workers	senior health care workers	Senior health care workers	1	kept
senior residents	senior residents	senior residents	senior residents	senior residents	1	kept
seniority	seniority	seniority	seniority	seniority	1	kept
Sense making	sense making	sense making	sense making	Sense making	1	kept
Sense of Coherence Scale	sense of coherence scale	sense of coherence scale	sense of coherence scale	Sense of Coherence Scale	1	kept
sense organ disorder	sense organ disorder	sense organ disorder	sense organ disorder	sense organ disorder	1	kept
Sense Organs	sense organs	sense organs	sense organs	Sense Organs	1	kept
sensemaking	sensemaking	sensemaking	sensemaking	sensemaking	1	kept
Sensibilidad	sensibilidad	sensibilidad	sensibilidad	Sensibilidad	1	kept
Sensing devices	sensing devices	sensing devices	sensing devices	Sensing devices	1	kept
sensitive period	sensitive period	sensitive period	sensitive period	sensitive period	1	kept
sensitivity of screening	sensitivity of screening	sensitivity of screening	sensitivity of screening	sensitivity of screening	1	kept
sensitivity to change	sensitivity to change	sensitivity to change	sensitivity to change	sensitivity to change	1	kept
sensitivity training	sensitivity training	sensitivity training	sensitivity training	sensitivity training	1	kept
SENSITIVITY-ANALYSIS	sensitivity analysis	sensitivity analysis	sensitivity analysis	sensitivity analysis	1	case_or_format
Sensor	sensor	sensor	sensor	sensor	1	case_or_format

Sensor applications	sensor applications	sensor applications	sensor applications	Sensor applications	1	kept
Sensor behavior	sensor behavior	sensor behavior	sensor behavior	Sensor behavior	1	kept
Sensor data analysis	sensor data analysis	sensor data analysis	sensor data analysis	Sensor data analysis	1	kept
sensor data analysis	sensor data analysis	sensor data analysis	sensor data analysis	Sensor data analysis	1	case_or_format
sensor devices and platforms	sensor devices and platforms	sensor devices and platforms	sensor devices and platforms	sensor devices and platforms	1	kept
Sensor installation	sensor installation	sensor installation	sensor installation	Sensor installation	1	kept
sensor mats	sensor mats	sensor mats	sensor mats	sensor mats	1	kept
Sensor network design	sensor network design	sensor network design	sensor network design	Sensor network design	1	kept
Sensor networks	sensor networks	sensor networks	sensor networks	Sensor networks	1	kept
Sensor readings	sensor readings	sensor readings	sensor readings	Sensor readings	1	kept
SENSORS	sensors	sensors	sensor	sensor	1	singular
sensors	sensors	sensors	sensor	sensor	1	singular
Sensors network	sensors network	sensors network	sensors network	Sensors network	1	kept
sensors networks	sensors networks	sensors networks	sensors network	Sensors network	1	singular
sensory abnormalities	sensory abnormalities	sensory abnormalities	sensory abnormalities	sensory abnormalities	1	kept
Sensory Aids	sensory aids	sensory aids	sensory aid	sensory aid	1	singular

Sensory Art Therapies	sensory art therapies	sensory art therapies	sensory art therapies	Sensory Art Therapies	1	kept
sensory defensiveness	sensory defensiveness	sensory defensiveness	sensory defensiveness	sensory defensiveness	1	kept
sensory deficit	sensory deficit	sensory deficit	sensory deficit	sensory deficit	1	kept
sensory deprivation and sensory overload	sensory deprivation and sensory overload	sensory deprivation and sensory overload	sensory deprivation and sensory overload	sensory deprivation and sensory overload	1	kept
Sensory feedback	sensory feedback	sensory feedback	sensory feedback	sensory feedback	1	case_or_format
Sensory interventions	sensory interventions	sensory interventions	sensory interventions	Sensory interventions	1	kept
sensory nerve	sensory nerve	sensory nerve	sensory nerve	sensory nerve	1	kept
sensory nerve conduction	sensory nerve conduction	sensory nerve conduction	sensory nerve conduction	sensory nerve conduction	1	kept
Sensory perception	sensory perception	sensory perception	sensory perception	Sensory perception	1	kept
Sensory processing sensitivity	sensory processing sensitivity	sensory processing sensitivity	sensory processing sensitivity	Sensory processing sensitivity	1	kept
sensory rehabilitation	sensory rehabilitation	sensory rehabilitation	sensory rehabilitation	sensory rehabilitation	1	kept
Sensory Stimulation	sensory stimulation	sensory stimulation	sensory stimulation	sensory stimulation	1	case_or_format
sensory stimulations	sensory stimulations	sensory stimulations	sensory stimulation	sensory stimulation	1	singular
sensory stimuli	sensory stimuli	sensory stimuli	sensory stimuli	sensory stimuli	1	kept
Sensory stimulus	sensory stimulus	sensory stimulus	sensory stimulus	Sensory stimulus	1	kept
sensory system examination	sensory system examination	sensory system examination	sensory system examination	sensory system examination	1	kept

SENSORY-ROOM	sensory room	sensory room	sensory room	SENSORY-ROOM	1	kept
sensory‑based interventions	sensory based interventions	sensory based interventions	sensory based interventions	sensory-based interventions	1	case_or_format
sensory‑supported intervention	sensory supported intervention	sensory supported intervention	sensory supported intervention	sensory‑supported intervention	1	kept
sentiment analysis	sentiment analysis	sentiment analysis	sentiment analysis	Sentiment Analysis	1	case_or_format
sentiment scores	sentiment scores	sentiment scores	sentiment scores	sentiment scores	1	kept
sentinel	sentinel	sentinel	sentinel	sentinel	1	kept
Sentinel events	sentinel events	sentinel events	sentinel events	Sentinel events	1	kept
Separate algorithm	separate algorithm	separate algorithm	separate algorithm	Separate algorithm	1	kept
SEPARATION	separation	separation	separation	Separation	1	case_or_format
Sepsis 6	sepsis 6	sepsis 6	sepsis 6	Sepsis 6	1	kept
sepsis alert	sepsis alert	sepsis alert	sepsis alert	sepsis alert	1	kept
sepsis alert system	sepsis alert system	sepsis alert system	sepsis alert system	sepsis alert system	1	kept
Sepsis and biomarkers of sepsis	sepsis and biomarkers of sepsis	sepsis and biomarkers of sepsis	sepsis and biomarkers of sepsis	Sepsis and biomarkers of sepsis	1	kept
Sepsis and heart failure	sepsis and heart failure	sepsis and heart failure	sepsis and heart failure	Sepsis and heart failure	1	kept
Sepsis and shock	sepsis and shock	sepsis and shock	sepsis and shock	Sepsis and shock	1	kept
sepsis biomarkers	sepsis biomarkers	sepsis biomarkers	sepsis biomarkers	sepsis biomarkers	1	kept

sepsis bundle	sepsis bundle	sepsis bundle	sepsis bundle	Sepsis bundle	1	case_or_format
Sepsis campaign	sepsis campaign	sepsis campaign	sepsis campaign	Sepsis campaign	1	kept
sepsis care bundle	sepsis care bundle	sepsis care bundle	sepsis care bundle	sepsis care bundle	1	kept
Sepsis care bundles	sepsis care bundles	sepsis care bundles	sepsis care bundle	sepsis care bundle	1	singular
sepsis care bundles	sepsis care bundles	sepsis care bundles	sepsis care bundle	sepsis care bundle	1	singular
Sepsis costs	sepsis costs	sepsis costs	sepsis costs	Sepsis costs	1	kept
sepsis education	sepsis education	sepsis education	sepsis education	Sepsis education	1	case_or_format
Sepsis guidelines	sepsis guidelines	sepsis guidelines	sepsis guidelines	Sepsis guidelines	1	kept
sepsis guidelines	sepsis guidelines	sepsis guidelines	sepsis guidelines	Sepsis guidelines	1	case_or_format
Sepsis mortality	sepsis mortality	sepsis mortality	sepsis mortality	Sepsis mortality	1	kept
Sepsis outcomes	sepsis outcomes	sepsis outcomes	sepsis outcomes	Sepsis outcomes	1	kept
Sepsis protocol	sepsis protocol	sepsis protocol	sepsis protocol	Sepsis protocol	1	kept
Sepsis recognition	sepsis recognition	sepsis recognition	sepsis recognition	Sepsis recognition	1	kept
sepsis related organ dysfunction	sepsis related organ dysfunction	sepsis related organ dysfunction	sepsis related organ dysfunction	sepsis related organ dysfunction	1	kept
Sepsis resuscitation	sepsis resuscitation	sepsis resuscitation	sepsis resuscitation	Sepsis resuscitation	1	kept
Sepsis Screening	sepsis screening	sepsis screening	sepsis screening	Sepsis Screening	1	kept

Sepsis screening	sepsis screening	sepsis screening	sepsis screening	Sepsis Screening	1	case_or_format
sepsis screening tool	sepsis screening tool	sepsis screening tool	sepsis screening tool	sepsis screening tool	1	kept
Sepsis sequelae	sepsis sequelae	sepsis sequelae	sepsis sequelae	Sepsis sequelae	1	kept
Sepsis shock	sepsis shock	sepsis shock	sepsis shock	Sepsis shock	1	kept
Sepsis Six	sepsis six	sepsis six	sepsis six	Sepsis Six	1	kept
sepsis six	sepsis six	sepsis six	sepsis six	Sepsis Six	1	case_or_format
sepsis six guidelines	sepsis six guidelines	sepsis six guidelines	sepsis six guidelines	sepsis six guidelines	1	kept
Sepsis surveillance	sepsis surveillance	sepsis surveillance	sepsis surveillance	Sepsis surveillance	1	kept
sepsis survivors	sepsis survivors	sepsis survivors	sepsis survivors	sepsis survivors	1	kept
Sepsis syndrome	sepsis syndrome	sepsis syndrome	sepsis syndrome	Sepsis syndrome	1	kept
sepsis trajectories	sepsis trajectories	sepsis trajectories	sepsis trajectory	Sepsis trajectory	1	singular
Sepsis trajectory	sepsis trajectory	sepsis trajectory	sepsis trajectory	Sepsis trajectory	1	kept
sepsis transition and recovery program	sepsis transition and recovery program	sepsis transition and recovery program	sepsis transition and recovery program	sepsis transition and recovery program	1	kept
sepsis treatment guidelines	sepsis treatment guidelines	sepsis treatment guidelines	sepsis treatment guidelines	sepsis treatment guidelines	1	kept
sepsis-associated coagulopathy	sepsis associated coagulopathy	sepsis associated coagulopathy	sepsis associated coagulopathy	sepsis-associated coagulopathy	1	kept
Sepsis-associated delirium	sepsis associated delirium	sepsis associated delirium	sepsis associated delirium	Sepsis-associated delirium	1	kept

sepsis-associated delirium	sepsis associated delirium	sepsis associated delirium	sepsis associated delirium	Sepsis-associated delirium	1	case_or_format
Sepsis-associated encephalopathy	sepsis associated encephalopathy	sepsis associated encephalopathy	sepsis associated encephalopathy	Sepsis-associated encephalopathy	1	kept
Sepsis-induced cardiomyopathy	sepsis induced cardiomyopathy	sepsis induced cardiomyopathy	sepsis induced cardiomyopathy	Sepsis-induced cardiomyopathy	1	kept
sepsis－associated delirium	sepsis associated delirium	sepsis associated delirium	sepsis associated delirium	Sepsis-associated delirium	1	case_or_format
septal myectomy	septal myectomy	septal myectomy	septal myectomy	septal myectomy	1	kept
septal occluder	septal occluder	septal occluder	septal occluder	septal occluder	1	kept
Septal Occluder Device	septal occluder device	septal occluder device	septal occluder device	Septal Occluder Device	1	kept
September 11 Terrorist Attacks	september 11 terrorist attacks	september 11 terrorist attacks	september 11 terrorist attacks	September 11 Terrorist Attacks	1	kept
Septic diseases	septic diseases	septic diseases	septic diseases	Septic diseases	1	kept
septic embolism	septic embolism	septic embolism	septic embolism	septic embolism	1	kept
septic encephalopathy	septic encephalopathy	septic encephalopathy	septic encephalopathy	septic encephalopathy	1	kept
Septic paradoxical embolism	septic paradoxical embolism	septic paradoxical embolism	septic paradoxical embolism	Septic paradoxical embolism	1	kept
Septic pulmonary embolism	septic pulmonary embolism	septic pulmonary embolism	septic pulmonary embolism	Septic pulmonary embolism	1	kept
septic pulmonary embolism	septic pulmonary embolism	septic pulmonary embolism	septic pulmonary embolism	Septic pulmonary embolism	1	case_or_format
septic shock associated acute kidney injury	septic shock associated acute kidney injury	septic shock associated acute kidney injury	septic shock associated acute kidney injury	septic shock associated acute kidney injury	1	kept
Septic shock resuscitation	septic shock resuscitation	septic shock resuscitation	septic shock resuscitation	Septic shock resuscitation	1	kept

septic thrombophlebitis	septic thrombophlebitis	septic thrombophlebitis	septic thrombophlebitis	septic thrombophlebitis	1	kept
Septoplasty	septoplasty	septoplasty	septoplasty	Septoplasty	1	kept
sequence	sequence	sequence	sequence	sequence	1	kept
sequence alignment	sequence alignment	sequence alignment	sequence alignment	sequence alignment	1	kept
Sequence Analysis	sequence analysis	sequence analysis	sequence analysis	sequence analysis	1	case_or_format
sequence homology	sequence homology	sequence homology	sequence homology	sequence homology	1	kept
Sequential activities	sequential activities	sequential activities	sequential activities	Sequential activities	1	kept
Sequential analysis	sequential analysis	sequential analysis	sequential analysis	Sequential analysis	1	kept
Sequential organ failure assessment	sequential organ failure assessment	sequential organ failure assessment	sequential organ failure assessment	Sequential Organ Failure Assessment	1	case_or_format
sequential organ failure assessment	sequential organ failure assessment	sequential organ failure assessment	sequential organ failure assessment	Sequential Organ Failure Assessment	1	case_or_format
sequential segmental analysis	sequential segmental analysis	sequential segmental analysis	sequential segmental analysis	sequential segmental analysis	1	kept
serine proteinase	serine proteinase	serine proteinase	serine proteinase	serine proteinase	1	kept
serious	serious	serious	serious	serious	1	kept
Serious adverse event	serious adverse event	serious adverse event	serious adverse event	Serious adverse event	1	kept
SERIOUS ADVERSE EVENTS	serious adverse events	serious adverse events	serious adverse event	Serious adverse event	1	singular
Serious disease	serious disease	serious disease	serious disease	Serious disease	1	kept

SERIOUS GAME	serious game	serious game	serious game	Serious game	1	case_or_format
Serious games	serious games	serious games	serious game	Serious game	1	singular
serious gaming	serious gaming	serious gaming	serious gaming	serious gaming	1	kept
serious illness care program	serious illness care program	serious illness care program	serious illness care program	serious illness care program	1	kept
serious illness support tool	serious illness support tool	serious illness support tool	serious illness support tool	serious illness support tool	1	kept
serious mental illness	serious mental illness	serious mental illness	serious mental illness	serious mental illness	1	kept
seriously ill	seriously ill	seriously ill	seriously ill	SERIOUSLY ILL	1	case_or_format
seroepidemiological studies	seroepidemiological studies	seroepidemiological studies	seroepidemiological studies	seroepidemiological studies	1	kept
Serologic Tests	serologic tests	serologic tests	serologic tests	Serologic Tests	1	kept
serological tests	serological tests	serological tests	serological tests	serological tests	1	kept
seropositivity	seropositivity	seropositivity	seropositivity	seropositivity	1	kept
Seroprevalencia	seroprevalencia	seroprevalencia	seroprevalencia	Seroprevalencia	1	kept
serotonin agonist	serotonin agonist	serotonin agonist	serotonin agonist	serotonin agonist	1	kept
serotonin antagonist	serotonin antagonist	serotonin antagonist	serotonin antagonist	serotonin antagonist	1	kept
Serotonin Antagonists	serotonin antagonists	serotonin antagonists	serotonin antagonist	serotonin antagonist	1	singular
serotonin syndrome	serotonin syndrome	serotonin syndrome	serotonin syndrome	Serotonin Syndrome	1	case_or_format

Serotonin toxicity	serotonin toxicity	serotonin toxicity	serotonin toxicity	Serotonin toxicity	1	kept
serotonin transporter	serotonin transporter	serotonin transporter	serotonin transporter	serotonin transporter	1	kept
Serotonin Uptake Inhibitors	serotonin uptake inhibitors	serotonin uptake inhibitors	serotonin uptake inhibitor	serotonin uptake inhibitor	1	singular
Serratia liquefaciens	serratia liquefaciens	serratia liquefaciens	serratia liquefaciens	Serratia liquefaciens	1	kept
SERRATIA-MARCESCENS	serratia marcescens	serratia marcescens	serratia marcescens	Serratia marcescens	1	case_or_format
SERRATIA-MARCESCENS BACTEREMIA	serratia marcescens bacteremia	serratia marcescens bacteremia	serratia marcescens bacteremia	SERRATIA-MARCESCENS BACTEREMIA	1	kept
serratus anterior muscle	serratus anterior muscle	serratus anterior muscle	serratus anterior muscle	serratus anterior muscle	1	kept
serratus anterior plane block	serratus anterior plane block	serratus anterior plane block	serratus anterior plane block	serratus anterior plane block	1	kept
SERUM	serum	serum	serum	serum	1	case_or_format
Serum albumin	serum albumin	serum albumin	serum albumin	serum albumin	1	case_or_format
serum amyloid A	serum amyloid a	serum amyloid a	serum amyloid a	serum amyloid A	1	kept
SERUM ANTICHOLINERGIC ACTIVITY	serum anticholinergic activity	serum anticholinergic activity	serum anticholinergic activity	SERUM ANTICHOLINERGIC ACTIVITY	1	kept
Serum anticholinergic activity	serum anticholinergic activity	serum anticholinergic activity	serum anticholinergic activity	SERUM ANTICHOLINERGIC ACTIVITY	1	case_or_format
Serum biomarkers	serum biomarkers	serum biomarkers	serum biomarkers	Serum biomarkers	1	kept
serum coagulation assay	serum coagulation assay	serum coagulation assay	serum coagulation assay	serum coagulation assay	1	kept
Serum cortisol level	serum cortisol level	serum cortisol level	serum cortisol level	Serum cortisol level	1	kept

serum IgA	serum iga	serum iga	serum iga	serum IgA	1	kept
Serum inflammatory factors	serum inflammatory factors	serum inflammatory factors	serum inflammatory factors	Serum inflammatory factors	1	kept
SERUM LACTATE LEVEL	serum lactate level	serum lactate level	serum lactate level	SERUM LACTATE LEVEL	1	kept
Serum lipid levels	serum lipid levels	serum lipid levels	serum lipid levels	Serum lipid levels	1	kept
Serum MMP-9 levels	serum mmp 9 levels	serum mmp 9 levels	serum mmp 9 levels	Serum MMP-9 levels	1	kept
serum osmolarity	serum osmolarity	serum osmolarity	serum osmolarity	serum osmolarity	1	kept
SERUM SODIUM CONCENTRATION	serum sodium concentration	serum sodium concentration	serum sodium concentration	SERUM SODIUM CONCENTRATION	1	kept
servant leadership	servant leadership	servant leadership	servant leadership	servant leadership	1	kept
service de santé des armées	service de sant des arm es	service de sant des arm es	service de sant des arm es	service de santé des armées	1	kept
service delivery	service delivery	service delivery	service delivery	Service delivery	1	case_or_format
service development	service development	service development	service development	Service development	1	case_or_format
service dog	service dog	service dog	service dog	service dog	1	kept
service education partnerships	service education partnerships	service education partnerships	service education partnerships	service education partnerships	1	kept
Service gaps	service gaps	service gaps	service gaps	Service gaps	1	kept
Service indicators	service indicators	service indicators	service indicators	Service indicators	1	kept
service intensity	service intensity	service intensity	service intensity	service intensity	1	kept

Service line	service line	service line	service line	Service line	1	kept
service management and workforce planning	service management and workforce planning	service management and workforce planning	service management and workforce planning	service management and workforce planning	1	kept
service organisation	service organisation	service organisation	service organisation	service organisation	1	kept
Service organization	service organization	service organization	service organization	Service organization	1	kept
Service oriented architecture	service oriented architecture	service oriented architecture	service oriented architecture	Service oriented architecture	1	kept
service planning	service planning	service planning	service planning	service planning	1	kept
Service platforms	service platforms	service platforms	service platforms	Service platforms	1	kept
service provision	service provision	service provision	service provision	Service provision	1	case_or_format
Service quality	service quality	service quality	service quality	Service Quality	1	case_or_format
service satisfaction	service satisfaction	service satisfaction	service satisfaction	service satisfaction	1	kept
service specific information	service specific information	service specific information	service specific information	service specific information	1	kept
SERVICE SYSTEMS	service systems	service systems	service systems	SERVICE SYSTEMS	1	kept
service time	service time	service time	service time	service time	1	kept
Service utilization rates	service utilization rates	service utilization rates	service utilization rates	Service utilization rates	1	kept
Servicio de urgencias hospitalarias	servicio de urgencias hospitalarias	servicio de urgencias hospitalarias	servicio de urgencias hospitalarias	Servicio de urgencias hospitalarias	1	kept
Servicios médicos de Urgencias	servicios m dicos de urgencias	servicios m dicos de urgencias	servicios m dicos de urgencias	Servicios médicos de Urgencias	1	kept

Serving	serving	serving	serving	Serving	1	kept
servo control	servo control	servo control	servo control	servo control	1	kept
Servo-control	servo control	servo control	servo control	servo control	1	case_or_format
Servqual evaluation method	servqual evaluation method	servqual evaluation method	servqual evaluation method	Servqual evaluation method	1	kept
sesame oil	sesame oil	sesame oil	sesame oil	Sesame Oil	1	case_or_format
Setting outs	setting outs	setting outs	setting outs	Setting outs	1	kept
seven-day working	seven day working	seven day working	seven day working	seven-day working	1	kept
seventh day adventist	seventh day adventist	seventh day adventist	seventh day adventist	seventh day adventist	1	kept
Sever acute respiratory syndrome	sever acute respiratory syndrome	sever acute respiratory syndrome	sever acute respiratory syndrome	Sever acute respiratory syndrome	1	kept
severe acute brain injury	severe acute brain injury	severe acute brain injury	severe acute brain injury	severe acute brain injury	1	kept
Severe Acute Respiratory Infection	severe acute respiratory infection	severe acute respiratory infection	severe acute respiratory infection	Severe acute respiratory infection	1	case_or_format
Severe acute respiratory syndrome corona virus-2	severe acute respiratory syndrome corona virus 2	severe acute respiratory syndrome corona virus 2	severe acute respiratory syndrome corona virus 2	Severe acute respiratory syndrome corona virus-2	1	kept
Severe acute respiratory syndrome corona virus-2	severe acute respiratory syndrome corona virus 2	severe acute respiratory syndrome corona virus 2	severe acute respiratory syndrome corona virus 2	Severe acute respiratory syndrome corona virus-2	1	kept
Severe acute respiratory syndrome coronavirus 2 pandemic	severe acute respiratory syndrome coronavirus 2 pandemic	severe acute respiratory syndrome coronavirus 2 pandemic	severe acute respiratory syndrome coronavirus 2 pandemic	Severe acute respiratory syndrome coronavirus 2 pandemic	1	kept
Severe acute respiratory syndrome coronavirus 2 vaccine	severe acute respiratory syndrome coronavirus 2 vaccine	severe acute respiratory syndrome coronavirus 2 vaccine	severe acute respiratory syndrome coronavirus 2 vaccine	Severe acute respiratory syndrome coronavirus 2 vaccine	1	kept
Severe acute respiratory syndrome coronavirus-2	severe acute respiratory syndrome coronavirus 2	severe acute respiratory syndrome coronavirus 2	severe acute respiratory syndrome coronavirus 2	Severe acute respiratory syndrome coronavirus 2	1	case_or_format

severe acute respiratory syndrome vaccine	severe acute respiratory syndrome vaccine	severe acute respiratory syndrome vaccine	severe acute respiratory syndrome vaccine	severe acute respiratory syndrome vaccine	1	kept
Severe acute respiratory syndrome-related coronavirus	severe acute respiratory syndrome related coronavirus	severe acute respiratory syndrome related coronavirus	severe acute respiratory syndrome related coronavirus	Severe acute respiratory syndrome-related coronavirus	1	kept
Severe adult respiratory failure	severe adult respiratory failure	severe adult respiratory failure	severe adult respiratory failure	Severe adult respiratory failure	1	kept
Severe adverse medical event	severe adverse medical event	severe adverse medical event	severe adverse medical event	Severe adverse medical event	1	kept
severe aneurysmal subarachnoid hemorrhage	severe aneurysmal subarachnoid hemorrhage	severe aneurysmal subarachnoid hemorrhage	severe aneurysmal subarachnoid hemorrhage	severe aneurysmal subarachnoid hemorrhage	1	kept
Severe asthma	severe asthma	severe asthma	severe asthma	severe asthma	1	case_or_format
Severe brain injury	severe brain injury	severe brain injury	severe brain injury	Severe brain injury	1	kept
Severe brain trauma	severe brain trauma	severe brain trauma	severe brain trauma	Severe brain trauma	1	kept
severe burn injuries	severe burn injuries	severe burn injuries	severe burn injuries	severe burn injuries	1	kept
severe CAP	severe cap	severe cap	severe cap	severe CAP	1	kept
severe childhood asthma	severe childhood asthma	severe childhood asthma	severe childhood asthma	severe childhood asthma	1	kept
Severe Clostridium difficile infection	severe clostridium difficile infection	severe clostridium difficile infection	severe clostridium difficile infection	Severe Clostridium difficile infection	1	kept
SEVERE COGNITIVE IMPAIRMENT	severe cognitive impairment	severe cognitive impairment	severe cognitive impairment	SEVERE COGNITIVE IMPAIRMENT	1	kept
severe combined immunodeficiency	severe combined immunodeficiency	severe combined immunodeficiency	severe combined immunodeficiency	Severe Combined Immunodeficiency	1	case_or_format
Severe COVID-19 respiratory failure	severe covid 19 respiratory failure	severe covid 19 respiratory failure	severe covid 19 respiratory failure	Severe COVID-19 respiratory failure	1	kept
Severe craniocerebral injury	severe craniocerebral injury	severe craniocerebral injury	severe craniocerebral injury	Severe craniocerebral injury	1	kept

severe cutaneous adverse reaction	severe cutaneous adverse reaction	severe cutaneous adverse reaction	severe cutaneous adverse reaction	Severe cutaneous adverse reactions	1	case_or_format
Severe dehydration	severe dehydration	severe dehydration	severe dehydration	Severe dehydration	1	kept
Severe encephalopathy	severe encephalopathy	severe encephalopathy	severe encephalopathy	Severe encephalopathy	1	kept
severe encephalopathy	severe encephalopathy	severe encephalopathy	severe encephalopathy	Severe encephalopathy	1	case_or_format
Severe Head Injury	severe head injury	severe head injury	severe head injury	SEVERE HEAD-INJURY	1	case_or_format
severe head injury	severe head injury	severe head injury	severe head injury	SEVERE HEAD-INJURY	1	case_or_format
severe head trauma	severe head trauma	severe head trauma	severe head trauma	severe head trauma	1	kept
severe heart failure	severe heart failure	severe heart failure	severe heart failure	severe heart failure	1	kept
severe hepatic impairment	severe hepatic impairment	severe hepatic impairment	severe hepatic impairment	severe hepatic impairment	1	kept
Severe hypoglycemia	severe hypoglycemia	severe hypoglycemia	severe hypoglycemia	Severe hypoglycemia	1	kept
severe illness	severe illness	severe illness	severe illness	severe illness	1	kept
Severe infection	severe infection	severe infection	severe infection	Severe infection	1	kept
severe infectious diseases	severe infectious diseases	severe infectious diseases	severe infectious diseases	severe infectious diseases	1	kept
SEVERE INFECTIOUS PURPURA	severe infectious purpura	severe infectious purpura	severe infectious purpura	SEVERE INFECTIOUS PURPURA	1	kept
Severe injury	severe injury	severe injury	severe injury	Severe injury	1	kept
severe lymphopenia	severe lymphopenia	severe lymphopenia	severe lymphopenia	severe lymphopenia	1	kept

Severe malaria	severe malaria	severe malaria	severe malaria	Severe malaria	1	kept
Severe maternal outcome	severe maternal outcome	severe maternal outcome	severe maternal outcome	Severe maternal outcome	1	kept
Severe medication errors	severe medication errors	severe medication errors	severe medication errors	Severe medication errors	1	kept
SEVERE MENINGOCOCCAL SEPSIS	severe meningococcal sepsis	severe meningococcal sepsis	severe meningococcal sepsis	SEVERE MENINGOCOCCAL SEPSIS	1	kept
Severe mental health problems	severe mental health problems	severe mental health problems	severe mental health problems	Severe mental health problems	1	kept
SEVERE MENTAL-ILLNESS	severe mental illness	severe mental illness	severe mental illness	SEVERE MENTAL-ILLNESS	1	kept
severe motor and intellectual disabilities	severe motor and intellectual disabilities	severe motor and intellectual disabilities	severe motor and intellectual disabilities	severe motor and intellectual disabilities	1	kept
severe myoclonic epilepsy in infancy	severe myoclonic epilepsy in infancy	severe myoclonic epilepsy in infancy	severe myoclonic epilepsy in infancy	severe myoclonic epilepsy in infancy	1	kept
Severe neurological disease	severe neurological disease	severe neurological disease	severe neurological disease	Severe neurological disease	1	kept
severe neurological disease	severe neurological disease	severe neurological disease	severe neurological disease	Severe neurological disease	1	case_or_format
Severe neurosurgery	severe neurosurgery	severe neurosurgery	severe neurosurgery	Severe neurosurgery	1	kept
SEVERE NOSOCOMIAL PNEUMONIA	severe nosocomial pneumonia	severe nosocomial pneumonia	severe nosocomial pneumonia	SEVERE NOSOCOMIAL PNEUMONIA	1	kept
severe obstructive sleep apnea	severe obstructive sleep apnea	severe obstructive sleep apnea	severe obstructive sleep apnea	severe obstructive sleep apnea	1	kept
severe osteogenesis imperfecta	severe osteogenesis imperfecta	severe osteogenesis imperfecta	severe osteogenesis imperfecta	severe osteogenesis imperfecta	1	kept
Severe paediatric OSAS	severe paediatric osas	severe paediatric osas	severe paediatric osas	Severe paediatric OSAS	1	kept
Severe Pneumonia	severe pneumonia	severe pneumonia	severe pneumonia	severe pneumonia	1	case_or_format

severe respiratory disease	severe respiratory disease	severe respiratory disease	severe respiratory disease	severe respiratory disease	1	kept
severe TBI	severe tbi	severe tbi	severe tbi	severe TBI	1	kept
Severe trauma	severe trauma	severe trauma	severe trauma	Severe trauma	1	kept
severe traumatic spinal cord injury	severe traumatic spinal cord injury	severe traumatic spinal cord injury	severe traumatic spinal cord injury	severe traumatic spinal cord injury	1	kept
Severity assessment	severity assessment	severity assessment	severity assessment	Severity assessment	1	kept
severity assessment score	severity assessment score	severity assessment score	severity assessment score	severity assessment score	1	kept
Severity classification	severity classification	severity classification	severity classification	Severity classification	1	kept
severity classification	severity classification	severity classification	severity classification	Severity classification	1	case_or_format
SEVERITY INDEX	severity index	severity index	severity index	SEVERITY INDEX	1	kept
severity index	severity index	severity index	severity index	SEVERITY INDEX	1	case_or_format
severity of disease scoring system	severity of disease scoring system	severity of disease scoring system	severity of disease scoring system	severity of disease scoring system	1	kept
Severity of Ethanol Withdrawal Scale	severity of ethanol withdrawal scale	severity of ethanol withdrawal scale	severity of ethanol withdrawal scale	Severity of Ethanol Withdrawal Scale	1	kept
severity of illness score	severity of illness score	severity of illness score	severity of illness score	severity of illness score	1	kept
Severity of illness, Back-translation	severity of illness back translation	severity of illness back translation	severity of illness back translation	Severity of illness, Back-translation	1	kept
Severity of infant illness	severity of infant illness	severity of infant illness	severity of infant illness	Severity of infant illness	1	kept
Severity score	severity score	severity score	severity score	Severity scores	1	case_or_format

Severity scoring	severity scoring	severity scoring	severity scoring	Severity scoring	1	kept
severity scoring systems	severity scoring systems	severity scoring systems	severity scoring systems	severity scoring systems	1	kept
Severity-of-illness scoring system	severity of illness scoring system	severity of illness scoring system	severity of illness scoring system	Severity-of-illness scoring system	1	kept
Sevoflurane anesthesia	sevoflurane anesthesia	sevoflurane anesthesia	sevoflurane anesthesia	Sevoflurane anesthesia	1	kept
sevrage	sevrage	sevrage	sevrage	sevrage	1	kept
sewage disposal	sewage disposal	sewage disposal	sewage disposal	sewage disposal	1	kept
SEWS	sews	sews	sews	SEWS	1	kept
SEX	sex	sex	sex	sex	1	case_or_format
sex allocation	sex allocation	sex allocation	sex allocation	sex allocation	1	kept
Sex Counseling	sex counseling	sex counseling	sex counseling	Sex Counseling	1	kept
Sex Differentiation	sex differentiation	sex differentiation	sex differentiation	Sex Differentiation	1	kept
Sex Education	sex education	sex education	sex education	Sex Education	1	kept
Sex factors	sex factors	sex factors	sex factor	Sex Factors	1	singular
sex hormone binding globulin	sex hormone binding globulin	sex hormone binding globulin	sex hormone binding globulin	sex hormone binding globulin	1	kept
Sex hormones	sex hormones	sex hormones	sex hormone	sex hormone	1	singular
sex hormones	sex hormones	sex hormones	sex hormone	sex hormone	1	singular

sex-related differences	sex related differences	sex related differences	sex related differences	sex-related differences	1	kept
Sexual and Gender Disorders	sexual and gender disorders	sexual and gender disorders	sexual and gender disorders	Sexual and Gender Disorders	1	kept
sexual and gender minority	sexual and gender minority	sexual and gender minority	sexual and gender minority	Sexual and Gender Minorities	1	case_or_format
sexual arousal disorder	sexual arousal disorder	sexual arousal disorder	sexual arousal disorder	sexual arousal disorder	1	kept
Sexual Behavior	sexual behavior	sexual behavior	sexual behavior	sexual behavior	1	case_or_format
sexual counseling	sexual counseling	sexual counseling	sexual counseling	sexual counseling	1	kept
sexual crime	sexual crime	sexual crime	sexual crime	sexual crime	1	kept
Sexual disparity	sexual disparity	sexual disparity	sexual disparity	Sexual disparity	1	kept
sexual disparity	sexual disparity	sexual disparity	sexual disparity	Sexual disparity	1	case_or_format
Sexual dysfunction	sexual dysfunction	sexual dysfunction	sexual dysfunction	sexual dysfunction	1	case_or_format
Sexual Dysfunction, Physiological	sexual dysfunction physiological	sexual dysfunction physiological	sexual dysfunction physiological	Sexual Dysfunction, Physiological	1	kept
Sexual health	sexual health	sexual health	sexual health	sexual health	1	case_or_format
Sexual Health Care	sexual health care	sexual health care	sexual health care	Sexual Health Care	1	kept
sexual minorities	sexual minorities	sexual minorities	sexual minorities	sexual minorities	1	kept
Sexual Partners	sexual partners	sexual partners	sexual partners	Sexual Partners	1	kept
Sexual problems	sexual problems	sexual problems	sexual problems	Sexual problems	1	kept

sexual risk behavior	sexual risk behavior	sexual risk behavior	sexual risk behavior	sexual risk behavior	1	kept
sexual transmission	sexual transmission	sexual transmission	sexual transmission	sexual transmission	1	kept
Sezary Syndrome	sezary syndrome	sezary syndrome	sezary syndrome	Sezary Syndrome	1	kept
Sezary syndrome	sezary syndrome	sezary syndrome	sezary syndrome	Sezary Syndrome	1	case_or_format
SF 8 Health Survey	sf 8 health survey	sf 8 health survey	sf 8 health survey	SF 8 Health Survey	1	kept
SF-12	sf 12	sf 12	sf 12	SF-12	1	kept
SGLT-2 inhibitor	sglt 2 inhibitor	sglt 2 inhibitor	sglt 2 inhibitor	SGLT-2 inhibitor	1	kept
shadowing	shadowing	shadowing	shadowing	shadowing	1	kept
Shahadatain	shahadatain	shahadatain	shahadatain	Shahadatain	1	kept
shaker exercise	shaker exercise	shaker exercise	shaker exercise	shaker exercise	1	kept
Shakti Divas	shakti divas	shakti divas	shakti divas	Shakti Divas	1	kept
shallow tracheal suctioning	shallow tracheal suctioning	shallow tracheal suctioning	shallow tracheal suctioning	shallow tracheal suctioning	1	kept
shampoo	shampoo	shampoo	shampoo	shampoo	1	kept
Shandong	shandong	shandong	shandong	Shandong	1	kept
Shanna Ratnesar-Shumate	shanna ratnesar shumate	shanna ratnesar shumate	shanna ratnesar shumate	Shanna Ratnesar-Shumate	1	kept
Shanna ratnesar-shumate	shanna ratnesar shumate	shanna ratnesar shumate	shanna ratnesar shumate	Shanna Ratnesar-Shumate	1	case_or_format

Shannon index	shannon index	shannon index	shannon index	Shannon index	1	kept
SHAP	shap	shap	shap	SHAP	1	kept
SHAP, SHapley Additive exPlanations	shap shapley additive explanations	shap shapley additive explanations	shap shapley additive explanations	SHAP, SHapley Additive exPlanations	1	kept
Shape	shape	shape	shape	Shape	1	kept
shape	shape	shape	shape	Shape	1	case_or_format
Shapley additive explanation	shapley additive explanation	shapley additive explanation	shapley additive explanation	Shapley additive explanation	1	kept
Shapley value	shapley value	shapley value	shapley value	Shapley Value	1	case_or_format
shared decision	shared decision	shared decision	shared decision	shared decision	1	kept
Shared decision makings	shared decision makings	shared decision makings	shared decision making	shared decision making	1	singular
SHARED DECISION-MAKING	shared decision making	shared decision making	shared decision making	shared decision making	1	case_or_format
Shared decision-making	shared decision making	shared decision making	shared decision making	shared decision making	1	case_or_format
Shared learning	shared learning	shared learning	shared learning	Shared learning	1	kept
shared mental model	shared mental model	shared mental model	shared mental model	shared mental model	1	kept
Shared Mental Models	shared mental models	shared mental models	shared mental model	shared mental model	1	singular
shared mental models	shared mental models	shared mental models	shared mental model	shared mental model	1	singular
shared notes	shared notes	shared notes	shared notes	shared notes	1	kept

Shared Process	shared process	shared process	shared process	Shared Process	1	kept
shared psychotic disorder	shared psychotic disorder	shared psychotic disorder	shared psychotic disorder	shared psychotic disorder	1	kept
shared reading program	shared reading program	shared reading program	shared reading program	shared reading program	1	kept
Shared resources	shared resources	shared resources	shared resources	Shared resources	1	kept
Shared situational awareness	shared situational awareness	shared situational awareness	shared situational awareness	Shared situational awareness	1	kept
shared understanding	shared understanding	shared understanding	shared understanding	shared understanding	1	kept
Shared-decision making	shared decision making	shared decision making	shared decision making	shared decision making	1	case_or_format
shared-decision making	shared decision making	shared decision making	shared decision making	shared decision making	1	case_or_format
Sharing	sharing	sharing	sharing	Sharing	1	kept
Sharjah	sharjah	sharjah	sharjah	Sharjah	1	kept
SHARPS INJURIES	sharps injuries	sharps injuries	sharps injury	Sharps injuries	1	singular
Sharps object injuries	sharps object injuries	sharps object injuries	sharps object injuries	Sharps object injuries	1	kept
SHEA	shea	shea	shea	SHEA	1	kept
Shear	shear	shear	shear	Shear	1	kept
shear rate	shear rate	shear rate	shear rate	shear rate	1	kept
Shear Strength	shear strength	shear strength	shear strength	Shear Strength	1	kept

sheath removal	sheath removal	sheath removal	sheath removal	sheath removal	1	kept
sheep	sheep	sheep	sheep	Sheep	1	case_or_format
Sheffield Care Environment Assessment Matrix Swedish version	sheffield care environment assessment matrix swedish version	sheffield care environment assessment matrix swedish version	sheffield care environment assessment matrix swedish version	Sheffield Care Environment Assessment Matrix Swedish version	1	kept
sheltered employment	sheltered employment	sheltered employment	sheltered employment	sheltered employment	1	kept
sheltered workshop	sheltered workshop	sheltered workshop	sheltered workshop	sheltered workshop	1	kept
shenmai injection	shenmai injection	shenmai injection	shenmai injection	shenmai injection	1	kept
Sherlock 3CG Tip Confirmation System	sherlock 3cg tip confirmation system	sherlock 3cg tip confirmation system	sherlock 3cg tip confirmation system	Sherlock 3CG Tip Confirmation System	1	kept
SHERPA	sherpa	sherpa	sherpa	SHERPA	1	kept
sherpa method	sherpa method	sherpa method	sherpa method	sherpa method	1	kept
Shetland sheepdog	shetland sheepdog	shetland sheepdog	shetland sheepdog	Shetland sheepdog	1	kept
SHG	shg	shg	shg	SHG	1	kept
SHIFT	shift	shift	shift	Shift	1	case_or_format
SHIFT CHANGEOVERS	shift changeovers	shift changeovers	shift changeovers	SHIFT CHANGEOVERS	1	kept
shift handover	shift handover	shift handover	shift handover	Shift handover	1	case_or_format
shift leader	shift leader	shift leader	shift leader	shift leader	1	kept
SHIFT LENGTH	shift length	shift length	shift length	SHIFT LENGTH	1	kept

Shift length	shift length	shift length	shift length	SHIFT LENGTH	1	case_or_format
shift safety check	shift safety check	shift safety check	shift safety check	shift safety check	1	kept
SHIFT SYSTEMS	shift systems	shift systems	shift systems	SHIFT SYSTEMS	1	kept
Shift team	shift team	shift team	shift team	Shift team	1	kept
shift team	shift team	shift team	shift team	Shift team	1	case_or_format
shift to the left	shift to the left	shift to the left	shift to the left	shift to the left	1	kept
shift type	shift type	shift type	shift type	shift type	1	kept
shift work sleep disorder	shift work sleep disorder	shift work sleep disorder	shift work sleep disorder	Shift work sleep disorder	1	case_or_format
Shift work sleep disorders	shift work sleep disorders	shift work sleep disorders	shift work sleep disorder	Shift work sleep disorder	1	singular
Shift work - sleep	shift work sleep	shift work sleep	shift work sleep	Shift work - sleep	1	kept
Shift worker	shift worker	shift worker	shift worker	shift worker	1	case_or_format
shift worker sleep disorder	shift worker sleep disorder	shift worker sleep disorder	shift worker sleep disorder	shift worker sleep disorder	1	kept
Shift workers	shift workers	shift workers	shift worker	shift worker	1	singular
Shift workIN	shift workin	shift workin	shift workin	Shift workIN	1	kept
Shift-handover	shift handover	shift handover	shift handover	Shift handover	1	case_or_format
Shift-report	shift report	shift report	shift report	SHIFT REPORT	1	case_or_format

shift-to-shift	shift to shift	shift to shift	shift to shift	shift-to-shift	1	kept
shift-work satisfaction	shift work satisfaction	shift work satisfaction	shift work satisfaction	shift-work satisfaction	1	kept
shifting performa	shifting performa	shifting performa	shifting performa	shifting performa	1	kept
SHIFTS	shifts	shifts	shift	Shift	1	singular
shifts	shifts	shifts	shift	Shift	1	singular
ShiftWorkSchedule	shiftworkschedule	shiftworkschedule	shiftworkschedule	ShiftWorkSchedule	1	kept
Shiga toxin‑producing E coli	shiga toxin producing e coli	shiga toxin producing e coli	shiga toxin producing e coli	Shiga toxin‑producing E coli	1	kept
Ship	ship	ship	ship	Ships	1	case_or_format
shiver prevention	shiver prevention	shiver prevention	shiver prevention	shiver prevention	1	kept
Shock cardiogénico	shock cardiog nico	shock cardiog nico	shock cardiog nico	Shock cardiogénico	1	kept
Shock teams	shock teams	shock teams	shock teams	Shock teams	1	kept
Shock trauma center	shock trauma center	shock trauma center	shock trauma center	Shock trauma center	1	kept
shock wave	shock wave	shock wave	shock wave	shock wave	1	kept
shock, cardiogenic	shock cardiogenic	shock cardiogenic	shock cardiogenic	Shock, Cardiogenic	1	case_or_format
Shock, epidemiology	shock epidemiology	shock epidemiology	shock epidemiology	Shock, epidemiology	1	kept
Shock, mortality	shock mortality	shock mortality	shock mortality	Shock, mortality	1	kept

shockable rhythm	shockable rhythm	shockable rhythm	shockable rhythm	shockable rhythm	1	kept
shoe	shoe	shoe	shoe	shoe	1	kept
shoe cover	shoe cover	shoe cover	shoe cover	shoe cover	1	kept
shoes	shoes	shoes	shoes	Shoes	1	case_or_format
Shone syndrome	shone syndrome	shone syndrome	shone syndrome	Shone syndrome	1	kept
short acting insulin	short acting insulin	short acting insulin	short acting insulin	short acting insulin	1	kept
Short form	short form	short form	short form	Short form	1	kept
Short Form 36 Physical Function Domain Score	short form 36 physical function domain score	short form 36 physical function domain score	short form 36 physical function domain score	Short Form 36 Physical Function Domain Score	1	kept
Short Form 36 Physical Health Component Score	short form 36 physical health component score	short form 36 physical health component score	short form 36 physical health component score	Short Form 36 Physical Health Component Score	1	kept
Short Form Health Survey	short form health survey	short form health survey	short form health survey	Short Form Health Survey	1	kept
Short form health survey-36 questionnaires	short form health survey 36 questionnaires	short form health survey 36 questionnaires	short form health survey 36 questionnaires	Short form health survey-36 questionnaires	1	kept
Short intervention	short intervention	short intervention	short intervention	Short intervention	1	kept
Short Orientation Memory Concentration Test	short orientation memory concentration test	short orientation memory concentration test	short orientation memory concentration test	Short Orientation Memory Concentration Test	1	kept
Short peripheral cannula	short peripheral cannula	short peripheral cannula	short peripheral cannula	Short peripheral cannula	1	kept
short peripheral venous catheter	short peripheral venous catheter	short peripheral venous catheter	short peripheral venous catheter	short peripheral venous catheter	1	kept
short peripherical catheter	short peripherical catheter	short peripherical catheter	short peripherical catheter	short peripherical catheter	1	kept

short physical performance battery	short physical performance battery	short physical performance battery	short physical performance battery	short physical performance battery	1	kept
Short standardized advance directive	short standardized advance directive	short standardized advance directive	short standardized advance directive	Short standardized advance directive	1	kept
short stay	short stay	short stay	short stay	short stay	1	kept
short term exposure	short term exposure	short term exposure	short term exposure	short term exposure	1	kept
Short term memory	short term memory	short term memory	short term memory	short term memory	1	case_or_format
Short Warwick-Edinburgh Mental Well-Being Scale	short warwick edinburgh mental well being scale	short warwick edinburgh mental well being scale	short warwick edinburgh mental well being scale	Short Warwick-Edinburgh Mental Well-Being Scale	1	kept
Short-stay	short stay	short stay	short stay	short stay	1	case_or_format
Short-term mortality	short term mortality	short term mortality	short term mortality	Short-term mortality	1	kept
Short-term outcomes	short term outcomes	short term outcomes	short term outcomes	Short-term outcomes	1	kept
SHORT-TERM PREDICTION	short term prediction	short term prediction	short term prediction	SHORT-TERM PREDICTION	1	kept
short-term prognosis	short term prognosis	short term prognosis	short term prognosis	short-term prognosis	1	kept
Short-term prophylaxis	short term prophylaxis	short term prophylaxis	short term prophylaxis	Short-term prophylaxis	1	kept
short-term variation	short term variation	short term variation	short term variation	short-term variation	1	kept
SHORTAGE	shortage	shortage	shortage	Shortage	1	case_or_format
shortage	shortage	shortage	shortage	Shortage	1	case_or_format
Shortage of intensivists	shortage of intensivists	shortage of intensivists	shortage of intensivists	Shortage of intensivists	1	kept

SHORTAGES	shortages	shortages	shortage	Shortage	1	singular
Shortened application	shortened application	shortened application	shortened application	Shortened application	1	kept
shortening time	shortening time	shortening time	shortening time	shortening time	1	kept
Should occur	should occur	should occur	should occur	Should occur	1	kept
Shoulder Dystocia	shoulder dystocia	shoulder dystocia	shoulder dystocia	shoulder dystocia	1	case_or_format
shoulder dystocia checklist	shoulder dystocia checklist	shoulder dystocia checklist	shoulder dystocia checklist	shoulder dystocia checklist	1	kept
shoulder fracture	shoulder fracture	shoulder fracture	shoulder fracture	shoulder fracture	1	kept
Shoulder Fractures	shoulder fractures	shoulder fractures	shoulder fracture	shoulder fracture	1	singular
Shoulder Joint	shoulder joint	shoulder joint	shoulder joint	Shoulder Joint	1	kept
Shoulder Pain	shoulder pain	shoulder pain	shoulder pain	shoulder pain	1	case_or_format
Shower bath	shower bath	shower bath	shower bath	Shower bath	1	kept
shunt	shunt	shunt	shunt	Shunt	1	case_or_format
shunting	shunting	shunting	shunting	shunting	1	kept
shyness	shyness	shyness	shyness	shyness	1	kept
SIAARTI	siaarti	siaarti	siaarti	SIAARTI	1	kept
SIADH	siadh	siadh	siadh	SIADH	1	kept

sialic acid derivative	sialic acid derivative	sialic acid derivative	sialic acid derivative	sialic acid derivative	1	kept
Sialic Acids	sialic acids	sialic acids	sialic acids	Sialic Acids	1	kept
sialolithiasis	sialolithiasis	sialolithiasis	sialolithiasis	sialolithiasis	1	kept
Sichuan province	sichuan province	sichuan province	sichuan province	Sichuan province	1	kept
sick	sick	sick	sick	sick	1	kept
Sick building syndrome	sick building syndrome	sick building syndrome	sick building syndrome	sick building syndrome	1	case_or_format
Sick newborn	sick newborn	sick newborn	sick newborn	Sick newborn	1	kept
Sick newborns	sick newborns	sick newborns	sick newborn	Sick newborn	1	singular
Sick preterm infant	sick preterm infant	sick preterm infant	sick preterm infant	Sick preterm infant	1	kept
sickle cell	sickle cell	sickle cell	sickle cell	Sickle cell	1	case_or_format
sickle cell crisis	sickle cell crisis	sickle cell crisis	sickle cell crisis	sickle cell crisis	1	kept
Sickle cell disease	sickle cell disease	sickle cell disease	sickle cell disease	sickle cell disease	1	case_or_format
Sickness absenteeism	sickness absenteeism	sickness absenteeism	sickness absenteeism	Sickness absenteeism	1	kept
Side-effects	side effects	side effects	side effect	side effect	1	singular
side-effects	side effects	side effects	side effect	side effect	1	singular
Sidestream dark field imaging	sidestream dark field imaging	sidestream dark field imaging	sidestream dark field imaging	Sidestream dark field imaging	1	kept

Sidestream darkfield microscopy	sidestream darkfield microscopy	sidestream darkfield microscopy	sidestream darkfield microscopy	Sidestream darkfield microscopy	1	kept
SIDS prevention	sids prevention	sids prevention	sids prevention	SIDS prevention	1	kept
sigmoid colostomy	sigmoid colostomy	sigmoid colostomy	sigmoid colostomy	sigmoid colostomy	1	kept
Sigmoid volvulus	sigmoid volvulus	sigmoid volvulus	sigmoid volvulus	sigmoid volvulus	1	case_or_format
sigmoidectomy	sigmoidectomy	sigmoidectomy	sigmoidectomy	sigmoidectomy	1	kept
Sigmoidoscopy	sigmoidoscopy	sigmoidoscopy	sigmoidoscopy	sigmoidoscopy	1	case_or_format
Sign	sign	sign	sign	SIGNS	1	case_or_format
sign language interpreter	sign language interpreter	sign language interpreter	sign language interpreter	sign language interpreter	1	kept
Sign management	sign management	sign management	sign management	Sign management	1	kept
sign test	sign test	sign test	sign test	sign test	1	kept
Signal Detection, Psychological	signal detection psychological	signal detection psychological	signal detection psychological	Signal Detection, Psychological	1	kept
Signal measurement	signal measurement	signal measurement	signal measurement	Signal measurement	1	kept
signal peptide	signal peptide	signal peptide	signal peptide	signal peptide	1	kept
Signal quality assessment	signal quality assessment	signal quality assessment	signal quality assessment	Signal quality assessment	1	kept
Signal selection	signal selection	signal selection	signal selection	Signal selection	1	kept
Signal to noise ratio	signal to noise ratio	signal to noise ratio	signal to noise ratio	Signal to noise ratio	1	kept

Signal Transduction	signal transduction	signal transduction	signal transduction	signal transduction	1	case_or_format
Signal variability	signal variability	signal variability	signal variability	Signal variability	1	kept
Signal-To-Noise Ratio	signal to noise ratio	signal to noise ratio	signal to noise ratio	Signal to noise ratio	1	case_or_format
signal-to-noise ratio	signal to noise ratio	signal to noise ratio	signal to noise ratio	Signal to noise ratio	1	case_or_format
signet ring carcinoma	signet ring carcinoma	signet ring carcinoma	signet ring carcinoma	signet ring carcinoma	1	kept
Significance	significance	significance	significance	Significance	1	kept
significant event	significant event	significant event	significant event	significant event	1	kept
Significant harm	significant harm	significant harm	significant harm	Significant harm	1	kept
Signos Vitales	signos vitales	signos vitales	signos vitales	Signos Vitales	1	kept
Signout and Sign-out	signout and sign out	signout and sign out	signout and sign out	Signout and Sign-out	1	kept
Signs	signs	signs	sign	SIGNS	1	singular
Signs and Symptoms	signs and symptoms	signs and symptoms	signs and symptoms	signs and symptoms	1	case_or_format
silane derivative	silane derivative	silane derivative	silane derivative	silane derivative	1	kept
Silanes	silanes	silanes	silanes	Silanes	1	kept
silastic	silastic	silastic	silastic	silastic	1	kept
SILENCE	silence	silence	silence	SILENCE	1	kept

Silence Kills	silence kills	silence kills	silence kills	Silence Kills	1	kept
Silent aspiration	silent aspiration	silent aspiration	silent aspiration	Silent aspiration	1	kept
Silent transmission	silent transmission	silent transmission	silent transmission	Silent transmission	1	kept
silent transmission	silent transmission	silent transmission	silent transmission	Silent transmission	1	case_or_format
Silent type	silent type	silent type	silent type	Silent type	1	kept
silica nanoparticle	silica nanoparticle	silica nanoparticle	silica nanoparticle	silica nanoparticle	1	kept
Silicon	silicon	silicon	silicon	silicon	1	case_or_format
Silicon Dioxide	silicon dioxide	silicon dioxide	silicon dioxide	Silicon Dioxide	1	kept
silicon dioxide	silicon dioxide	silicon dioxide	silicon dioxide	Silicon Dioxide	1	case_or_format
Silicone adhesive	silicone adhesive	silicone adhesive	silicone adhesive	Silicone adhesive	1	kept
Silicone adhesives	silicone adhesives	silicone adhesives	silicone adhesive	Silicone adhesive	1	singular
silicone foam	silicone foam	silicone foam	silicone foam	silicone foam	1	kept
silicone foam border dressing	silicone foam border dressing	silicone foam border dressing	silicone foam border dressing	silicone foam border dressing	1	kept
silicone polymer	silicone polymer	silicone polymer	silicone polymer	silicone polymer	1	kept
Silicone polymers	silicone polymers	silicone polymers	silicone polymer	silicone polymer	1	singular
Silicone-based adhesive remover spray	silicone based adhesive remover spray	silicone based adhesive remover spray	silicone based adhesive remover spray	Silicone-based adhesive remover spray	1	kept

silicosis	silicosis	silicosis	silicosis	silicosis	1	kept
silicristin	silicristin	silicristin	silicristin	silicristin	1	kept
silidianin	silidianin	silidianin	silidianin	silidianin	1	kept
Silk	silk	silk	silk	Silk	1	kept
silk	silk	silk	silk	Silk	1	case_or_format
siloplasty	siloplasty	siloplasty	siloplasty	siloplasty	1	kept
silver impregnation	silver impregnation	silver impregnation	silver impregnation	silver impregnation	1	kept
silver nanoparticle	silver nanoparticle	silver nanoparticle	silver nanoparticle	silver nanoparticle	1	kept
Silver Sulfadiazine	silver sulfadiazine	silver sulfadiazine	silver sulfadiazine	Silver Sulfadiazine	1	kept
silver trauma	silver trauma	silver trauma	silver trauma	silver trauma	1	kept
SILVER-NANOPARTICLES	silver nanoparticles	silver nanoparticles	silver nanoparticle	silver nanoparticle	1	singular
silverdin	silverdin	silverdin	silverdin	silverdin	1	kept
Silverman and Andersen index	silverman and andersen index	silverman and andersen index	silverman and andersen index	Silverman and Andersen index	1	kept
Silverman Andersen Score	silverman andersen score	silverman andersen score	silverman andersen score	Silverman Andersen Score	1	kept
Silverman Score	silverman score	silverman score	silverman score	Silverman Score	1	kept
Silymarin	silymarin	silymarin	silymarin	silymarin	1	case_or_format

simethicone	simethicone	simethicone	simethicone	simethicone	1	kept
similac	similac	similac	similac	similac	1	kept
Similarity	similarity	similarity	similarity	Similarity	1	kept
Similarity analysis	similarity analysis	similarity analysis	similarity analysis	Similarity analysis	1	kept
simman 3G	simman 3g	simman 3g	simman 3g	simman 3G	1	kept
Simple lymphatic drainage	simple lymphatic drainage	simple lymphatic drainage	simple lymphatic drainage	Simple lymphatic drainage	1	kept
Simple triage and rapid treatment	simple triage and rapid treatment	simple triage and rapid treatment	simple triage and rapid treatment	Simple triage and rapid treatment	1	kept
Simplified Acute Physiological Score	simplified acute physiological score	simplified acute physiological score	simplified acute physiological score	Simplified Acute Physiological Score	1	kept
simplified acute physiology score	simplified acute physiology score	simplified acute physiology score	simplified acute physiology score	Simplified Acute Physiology Score	1	case_or_format
Simplified Acute Physiology Score II	simplified acute physiology score ii	simplified acute physiology score ii	simplified acute physiology score ii	Simplified Acute Physiology Score II	1	kept
simplified Chinese script	simplified chinese script	simplified chinese script	simplified chinese script	simplified Chinese script	1	kept
simplified coping style questionnaire	simplified coping style questionnaire	simplified coping style questionnaire	simplified coping style questionnaire	simplified coping style questionnaire	1	kept
Simplified motor scale	simplified motor scale	simplified motor scale	simplified motor scale	Simplified motor scale	1	kept
Simplified Motor score	simplified motor score	simplified motor score	simplified motor score	Simplified Motor score	1	kept
Simulación	simulaci n	simulaci n	simulaci n	Simulación	1	kept
SIMULATED ENVIRONMENT	simulated environment	simulated environment	simulated environment	SIMULATED ENVIRONMENT	1	kept

Simulated environment	simulated environment	simulated environment	simulated environment	SIMULATED ENVIRONMENT	1	case_or_format
simulated participant	simulated participant	simulated participant	simulated participant	simulated participant	1	kept
Simulated scenario teaching	simulated scenario teaching	simulated scenario teaching	simulated scenario teaching	Simulated scenario teaching	1	kept
simulatin centre operations	simulatin centre operations	simulatin centre operations	simulatin centre operations	simulatin centre operations	1	kept
simulation and games	simulation and games	simulation and games	simulation and games	simulation and games	1	kept
Simulation and modeling	simulation and modeling	simulation and modeling	simulation and modeling	Simulation and modeling	1	kept
Simulation approach	simulation approach	simulation approach	simulation approach	Simulation approach	1	kept
simulation as teaching methodology	simulation as teaching methodology	simulation as teaching methodology	simulation as teaching methodology	simulation as teaching methodology	1	kept
simulation based education	simulation based education	simulation based education	simulation based education	Simulation-based education	1	case_or_format
simulation based implementation method	simulation based implementation method	simulation based implementation method	simulation based implementation method	simulation based implementation method	1	kept
simulation based learning	simulation based learning	simulation based learning	simulation based learning	simulation-based learning	1	case_or_format
simulation based learning activity	simulation based learning activity	simulation based learning activity	simulation based learning activity	simulation based learning activity	1	kept
simulation center	simulation center	simulation center	simulation center	simulation center	1	kept
Simulation context	simulation context	simulation context	simulation context	Simulation context	1	kept
Simulation design	simulation design	simulation design	simulation design	Simulation design	1	kept
SIMULATION EDUCATION	simulation education	simulation education	simulation education	SIMULATION EDUCATION	1	kept

Simulation Education	simulation education	simulation education	simulation education	SIMULATION EDUCATION	1	case_or_format
Simulation education	simulation education	simulation education	simulation education	SIMULATION EDUCATION	1	case_or_format
simulation education	simulation education	simulation education	simulation education	SIMULATION EDUCATION	1	case_or_format
Simulation exercise	simulation exercise	simulation exercise	simulation exercise	Simulation exercise	1	kept
simulation in health care	simulation in health care	simulation in health care	simulation in health care	simulation in health care	1	kept
Simulation in healthcare	simulation in healthcare	simulation in healthcare	simulation in healthcare	simulation in healthcare	1	case_or_format
simulation knowledge retention	simulation knowledge retention	simulation knowledge retention	simulation knowledge retention	simulation knowledge retention	1	kept
Simulation laboratory	simulation laboratory	simulation laboratory	simulation laboratory	Simulation laboratory	1	kept
Simulation learning effectiveness scale	simulation learning effectiveness scale	simulation learning effectiveness scale	simulation learning effectiveness scale	Simulation learning effectiveness scale	1	kept
simulation learning system	simulation learning system	simulation learning system	simulation learning system	simulation learning system	1	kept
Simulation medicine	simulation medicine	simulation medicine	simulation medicine	Simulation medicine	1	kept
Simulation meta-modeling	simulation meta modeling	simulation meta modeling	simulation meta modeling	Simulation meta-modeling	1	kept
Simulation method	simulation method	simulation method	simulation method	Simulation method	1	kept
simulation model	simulation model	simulation model	simulation model	simulation model	1	kept
Simulation study	simulation study	simulation study	simulation study	Simulation studies	1	case_or_format
Simulation survey	simulation survey	simulation survey	simulation survey	Simulation survey	1	kept

simulation teaching	simulation teaching	simulation teaching	simulation teaching	simulation teaching	1	kept
Simulation teaching method	simulation teaching method	simulation teaching method	simulation teaching method	Simulation teaching method	1	kept
Simulation technique	simulation technique	simulation technique	simulation technique	Simulation technique	1	kept
Simulation theory	simulation theory	simulation theory	simulation theory	Simulation theory	1	kept
simulation tool	simulation tool	simulation tool	simulation tool	simulation tool	1	kept
Simulation-based assessment	simulation based assessment	simulation based assessment	simulation based assessment	Simulation-based assessment	1	kept
Simulation-based clinical systems testing	simulation based clinical systems testing	simulation based clinical systems testing	simulation based clinical systems testing	Simulation-based clinical systems testing	1	kept
SIMULATION-BASED EDUCATION	simulation based education	simulation based education	simulation based education	Simulation-based education	1	case_or_format
Simulation-based learning experience	simulation based learning experience	simulation based learning experience	simulation based learning experience	Simulation based learning experiences	1	case_or_format
Simulation-based medical education	simulation based medical education	simulation based medical education	simulation based medical education	Simulation-based medical education	1	kept
Simulation-optimization	simulation optimization	simulation optimization	simulation optimization	Simulation-optimization	1	kept
Simulations	simulations	simulations	simulation	simulation	1	singular
simulation‐based	simulation based	simulation based	simulation based	simulation‐based	1	kept
Simultaneous monitoring	simultaneous monitoring	simultaneous monitoring	simultaneous monitoring	Simultaneous monitoring	1	kept
Sinais Vitais	sinais vitais	sinais vitais	sinais vitais	Sinais Vitais	1	kept
Sind	sind	sind	sind	Sind	1	kept

SINEH cardiopulmonary triage scale	sineh cardiopulmonary triage scale	sineh cardiopulmonary triage scale	sineh cardiopulmonary triage scale	SINEH cardiopulmonary triage scale	1	kept
Singaporean environmental assessment tool	singaporean environmental assessment tool	singaporean environmental assessment tool	singaporean environmental assessment tool	Singaporean environmental assessment tool	1	kept
Singing	singing	singing	singing	singing	1	case_or_format
SINGLE	single	single	single	single	1	case_or_format
single anastomosis duodeno-ileal bypass with sleeve	single anastomosis duodeno ileal bypass with sleeve	single anastomosis duodeno ileal bypass with sleeve	single anastomosis duodeno ileal bypass with sleeve	single anastomosis duodeno-ileal bypass with sleeve	1	kept
single center study	single center study	single center study	single center study	single center study	1	kept
single centre study	single centre study	single centre study	single centre study	single centre study	1	kept
Single family room	single family room	single family room	single family room	single family room	1	case_or_format
single Gaussian background	single gaussian background	single gaussian background	single gaussian background	single Gaussian background	1	kept
single institutional review board	single institutional review board	single institutional review board	single institutional review board	single institutional review board	1	kept
Single item burnout measure	single item burnout measure	single item burnout measure	single item burnout measure	Single item burnout measure	1	kept
Single Item Burnout Question	single item burnout question	single item burnout question	single item burnout question	Single Item Burnout Question	1	kept
Single Item Measure of Burnout	single item measure of burnout	single item measure of burnout	single item measure of burnout	Single Item Measure of Burnout	1	kept
single nucleotide polymorphisms	single nucleotide polymorphisms	single nucleotide polymorphisms	single nucleotide polymorphism	single nucleotide polymorphism	1	singular
Single occupancy rooms	single occupancy rooms	single occupancy rooms	single occupancy rooms	Single occupancy rooms	1	kept
single parent	single parent	single parent	single parent	single parent	1	kept

SINGLE PROLONGED STRESS	single prolonged stress	single prolonged stress	single prolonged stress	SINGLE PROLONGED STRESS	1	kept
single room care unit	single room care unit	single room care unit	single room care unit	single room care unit	1	kept
single stranded DNA	single stranded dna	single stranded dna	single stranded dna	single stranded DNA	1	kept
single ventricle	single ventricle	single ventricle	single ventricle	single ventricle	1	kept
single woman	single woman	single woman	single woman	single woman	1	kept
SINGLE-BED	single bed	single bed	single bed	SINGLE-BED	1	kept
single-case study	single case study	single case study	single case study	single-case study	1	kept
Single-family room	single family room	single family room	single family room	single family room	1	case_or_format
Single-family room, developmental, core measure	single family room developmental core measure	single family room developmental core measure	single family room developmental core measure	Single-family room, developmental, core measure	1	kept
SINGLE-ITEM	single item	single item	single item	SINGLE-ITEM	1	kept
Single-room	single room	single room	single room	Single room	1	case_or_format
single-shot interscalene block	single shot interscalene block	single shot interscalene block	single shot interscalene block	single-shot interscalene block	1	kept
SINGLE-STAGE LARYNGOTRACHEOPLASTY	single stage laryngotracheoplasty	single stage laryngotracheoplasty	single stage laryngotracheoplasty	SINGLE-STAGE LARYNGOTRACHEOPLASTY	1	kept
Sink	sink	sink	sink	Sink	1	kept
Sinks	sinks	sinks	sink	Sink	1	singular
Sinoatrial Node	sinoatrial node	sinoatrial node	sinoatrial node	Sinoatrial Node	1	kept

Sinonasal cancer	sinonasal cancer	sinonasal cancer	sinonasal cancer	Sinonasal cancer	1	kept
sinovac	sinovac	sinovac	sinovac	sinovac	1	kept
sinus arrest	sinus arrest	sinus arrest	sinus arrest	sinus arrest	1	kept
sinus congestion	sinus congestion	sinus congestion	sinus congestion	sinus congestion	1	kept
Sinus of Valsalva	sinus of valsalva	sinus of valsalva	sinus of valsalva	Sinus of Valsalva	1	kept
sinus of valsalva aneurysm	sinus of valsalva aneurysm	sinus of valsalva aneurysm	sinus of valsalva aneurysm	sinus of valsalva aneurysm	1	kept
Sinus of Valsalva fistula	sinus of valsalva fistula	sinus of valsalva fistula	sinus of valsalva fistula	Sinus of Valsalva fistula	1	kept
sinus of valsalva fistula	sinus of valsalva fistula	sinus of valsalva fistula	sinus of valsalva fistula	Sinus of Valsalva fistula	1	case_or_format
sinus surgery	sinus surgery	sinus surgery	sinus surgery	sinus surgery	1	kept
Sinusitis	sinusitis	sinusitis	sinusitis	sinusitis	1	case_or_format
Sinusoidal noise	sinusoidal noise	sinusoidal noise	sinusoidal noise	Sinusoidal noise	1	kept
Sinusoidal obstruction syndrome	sinusoidal obstruction syndrome	sinusoidal obstruction syndrome	sinusoidal obstruction syndrome	Sinusoidal obstruction syndrome	1	kept
SipTilSend	siptilsend	siptilsend	siptilsend	SipTilSend	1	kept
SIR model	sir model	sir model	sir model	SIR model	1	kept
Siriraj hospital	siriraj hospital	siriraj hospital	siriraj hospital	Siriraj hospital	1	kept
sirolimus	sirolimus	sirolimus	sirolimus	Sirolimus	1	case_or_format

SIRPIDs	sirpids	sirpids	sirpids	SIRPIDs	1	kept
sisomicin	sisomicin	sisomicin	sisomicin	sisomicin	1	kept
Sistema de gestión	sistema de gesti n	sistema de gesti n	sistema de gesti n	Sistema de gestión	1	kept
Sistema de respuesta rápida	sistema de respuesta r pida	sistema de respuesta r pida	sistema de respuesta r pida	Sistema de respuesta rápida	1	kept
Sistema de valoración	sistema de valoraci n	sistema de valoraci n	sistema de valoraci n	Sistema de valoración	1	kept
Sistemas de retorno de sangre	sistemas de retorno de sangre	sistemas de retorno de sangre	sistemas de retorno de sangre	Sistemas de retorno de sangre	1	kept
sister-in-law	sister in law	sister in law	sister in law	sister-in-law	1	kept
sit-to-stand test	sit to stand test	sit to stand test	sit to stand test	sit-to-stand test	1	kept
sitagliptin	sitagliptin	sitagliptin	sitagliptin	sitagliptin	1	kept
site of death	site of death	site of death	site of death	site of death	1	kept
Sitting Position	sitting position	sitting position	sitting position	Sitting Position	1	kept
situated practice	situated practice	situated practice	situated practice	situated practice	1	kept
Situation Awareness Global Assessment Technique	situation awareness global assessment technique	situation awareness global assessment technique	situation awareness global assessment technique	Situation Awareness Global Assessment Technique	1	kept
situation background assessment recommendation	situation background assessment recommendation	situation background assessment recommendation	situation background assessment recommendation	Situation-Background-Assessment-Recommendation	1	case_or_format
Situational	situational	situational	situational	Situational	1	kept
situational analysis	situational analysis	situational analysis	situational analysis	situational analysis	1	kept

situational anxiety	situational anxiety	situational anxiety	situational anxiety	situational anxiety	1	kept
Situational ethics	situational ethics	situational ethics	situational ethics	Situational ethics	1	kept
six sigma methodology	six sigma methodology	six sigma methodology	six sigma methodology	six sigma methodology	1	kept
Six Sigma quality management	six sigma quality management	six sigma quality management	six sigma quality management	Six Sigma quality management	1	kept
Six Sigma, VAP Bundle	six sigma vap bundle	six sigma vap bundle	six sigma vap bundle	Six Sigma, VAP Bundle	1	kept
sixth cervical vertebra	sixth cervical vertebra	sixth cervical vertebra	sixth cervical vertebra	sixth cervical vertebra	1	kept
SIZE	size	size	size	size	1	case_or_format
Skating	skating	skating	skating	Skating	1	kept
skeleton	skeleton	skeleton	skeleton	skeleton	1	kept
skeleton traction	skeleton traction	skeleton traction	skeleton traction	skeleton traction	1	kept
Skill competency	skill competency	skill competency	skill competency	Skill competency	1	kept
skill enhancement	skill enhancement	skill enhancement	skill enhancement	skill enhancement	1	kept
Skill levels	skill levels	skill levels	skill levels	Skill levels	1	kept
skill mismatch	skill mismatch	skill mismatch	skill mismatch	skill mismatch	1	kept
SKILL MIX	skill mix	skill mix	skill mix	skill mix	1	case_or_format
Skill training	skill training	skill training	skill training	Skill training	1	kept

Skilled	skilled	skilled	skilled	Skilled	1	kept
Skilled attendant	skilled attendant	skilled attendant	skilled attendant	Skilled attendant	1	kept
skilled communication	skilled communication	skilled communication	skilled communication	skilled communication	1	kept
Skills assessment	skills assessment	skills assessment	skills assessment	Skills assessment	1	kept
Skills competency programme	skills competency programme	skills competency programme	skills competency programme	Skills competency programme	1	kept
skills shortage	skills shortage	skills shortage	skills shortage	skills shortage	1	kept
Skills testing	skills testing	skills testing	skills testing	Skills testing	1	kept
Skills-based workshops	skills based workshops	skills based workshops	skills based workshops	Skills-based workshops	1	kept
Skin absorption	skin absorption	skin absorption	skin absorption	Skin Absorption	1	case_or_format
skin absorption	skin absorption	skin absorption	skin absorption	Skin Absorption	1	case_or_format
Skin aging	skin aging	skin aging	skin aging	Skin aging	1	kept
skin aging	skin aging	skin aging	skin aging	Skin aging	1	case_or_format
Skin and wound assessment teams	skin and wound assessment teams	skin and wound assessment teams	skin and wound assessment teams	Skin and wound assessment teams	1	kept
skin antisepsis	skin antisepsis	skin antisepsis	skin antisepsis	skin antisepsis	1	kept
skin antiseptics	skin antiseptics	skin antiseptics	skin antiseptics	skin antiseptics	1	kept
Skin assessment	skin assessment	skin assessment	skin assessment	skin assessment	1	case_or_format

Skin Assessment Tool	skin assessment tool	skin assessment tool	skin assessment tool	Skin Assessment Tool	1	kept
Skin assessments	skin assessments	skin assessments	skin assessment	skin assessment	1	singular
skin autograft	skin autograft	skin autograft	skin autograft	skin autograft	1	kept
Skin barrier	skin barrier	skin barrier	skin barrier	skin barrier	1	case_or_format
SKIN BARRIER FUNCTION	skin barrier function	skin barrier function	skin barrier function	SKIN BARRIER FUNCTION	1	kept
skin bleeding	skin bleeding	skin bleeding	skin bleeding	skin bleeding	1	kept
skin blood flow	skin blood flow	skin blood flow	skin blood flow	skin blood flow	1	kept
skin burning sensation	skin burning sensation	skin burning sensation	skin burning sensation	skin burning sensation	1	kept
skin carcinoma	skin carcinoma	skin carcinoma	skin carcinoma	skin carcinoma	1	kept
Skin care protocol	skin care protocol	skin care protocol	skin care protocol	Skin care protocol	1	kept
Skin Changes at Life's End	skin changes at life s end	skin changes at life s end	skin changes at life s end	Skin Changes at Life's End	1	kept
skin chapping	skin chapping	skin chapping	skin chapping	skin chapping	1	kept
skin cleanser	skin cleanser	skin cleanser	skin cleanser	skin cleanser	1	kept
Skin commensals	skin commensals	skin commensals	skin commensals	Skin commensals	1	kept
skin conductance responses	skin conductance responses	skin conductance responses	skin conductance responses	skin conductance responses	1	kept
skin contusion	skin contusion	skin contusion	skin contusion	skin contusion	1	kept

SKIN DAMAGE	skin damage	skin damage	skin damage	SKIN DAMAGE	1	kept
Skin damage	skin damage	skin damage	skin damage	SKIN DAMAGE	1	case_or_format
skin discomfort	skin discomfort	skin discomfort	skin discomfort	skin discomfort	1	kept
skin diseases	skin diseases	skin diseases	skin disease	Skin Diseases	1	singular
Skin disorders	skin disorders	skin disorders	skin disorders	Skin disorders	1	kept
skin dyspigmentation	skin dyspigmentation	skin dyspigmentation	skin dyspigmentation	skin dyspigmentation	1	kept
skin epithelium	skin epithelium	skin epithelium	skin epithelium	skin epithelium	1	kept
skin fissure	skin fissure	skin fissure	skin fissure	skin fissure	1	kept
skin fistula	skin fistula	skin fistula	skin fistula	skin fistula	1	kept
skin flap	skin flap	skin flap	skin flap	skin flap	1	kept
skin friction erosion	skin friction erosion	skin friction erosion	skin friction erosion	skin friction erosion	1	kept
skin infiltrate	skin infiltrate	skin infiltrate	skin infiltrate	skin infiltrate	1	kept
Skin Injuries	skin injuries	skin injuries	skin injury	skin injury	1	singular
skin injuries	skin injuries	skin injuries	skin injury	skin injury	1	singular
Skin Integrity	skin integrity	skin integrity	skin integrity	Skin integrity	1	case_or_format
skin integrity	skin integrity	skin integrity	skin integrity	Skin integrity	1	case_or_format

skin interface	skin interface	skin interface	skin interface	skin interface	1	kept
Skin Irritancy Tests	skin irritancy tests	skin irritancy tests	skin irritancy tests	Skin Irritancy Tests	1	kept
skin lupus erythematosus	skin lupus erythematosus	skin lupus erythematosus	skin lupus erythematosus	skin lupus erythematosus	1	kept
Skin microbiome	skin microbiome	skin microbiome	skin microbiome	Skin microbiome	1	kept
SKIN mode	skin mode	skin mode	skin mode	SKIN mode	1	kept
Skin mottling score	skin mottling score	skin mottling score	skin mottling score	Skin mottling score	1	kept
skin nodule	skin nodule	skin nodule	skin nodule	skin nodule	1	kept
skin perfusion	skin perfusion	skin perfusion	skin perfusion	skin perfusion	1	kept
Skin pH	skin ph	skin ph	skin ph	Skin pH	1	kept
skin physiology	skin physiology	skin physiology	skin physiology	Skin physiology	1	case_or_format
skin picking disorder	skin picking disorder	skin picking disorder	skin picking disorder	skin picking disorder	1	kept
Skin popping	skin popping	skin popping	skin popping	Skin popping	1	kept
skin problem	skin problem	skin problem	skin problem	skin problem	1	kept
Skin protectant	skin protectant	skin protectant	skin protectant	Skin protectant	1	kept
skin protectant	skin protectant	skin protectant	skin protectant	Skin protectant	1	case_or_format
Skin protection	skin protection	skin protection	skin protection	skin protection	1	case_or_format

skin protective agent	skin protective agent	skin protective agent	skin protective agent	skin protective agent	1	kept
skin sensation	skin sensation	skin sensation	skin sensation	skin sensation	1	kept
skin sensitivity	skin sensitivity	skin sensitivity	skin sensitivity	skin sensitivity	1	kept
Skin sensors	skin sensors	skin sensors	skin sensors	Skin sensors	1	kept
Skin stripping	skin stripping	skin stripping	skin stripping	Skin stripping	1	kept
skin structure	skin structure	skin structure	skin structure	skin structure	1	kept
Skin surface temperature	skin surface temperature	skin surface temperature	skin surface temperature	Skin surface temperature	1	kept
skin surgery	skin surgery	skin surgery	skin surgery	skin surgery	1	kept
skin team	skin team	skin team	skin team	skin team	1	kept
Skin Tear Knowledge Assessment Instrument	skin tear knowledge assessment instrument	skin tear knowledge assessment instrument	skin tear knowledge assessment instrument	Skin Tear Knowledge Assessment Instrument	1	kept
Skin temperatures	skin temperatures	skin temperatures	skin temperature	Skin Temperature	1	singular
Skin to skin care	skin to skin care	skin to skin care	skin to skin care	skin-to-skin care	1	case_or_format
skin to skin care	skin to skin care	skin to skin care	skin to skin care	skin-to-skin care	1	case_or_format
Skin tolerability	skin tolerability	skin tolerability	skin tolerability	Skin tolerability	1	kept
Skin tone	skin tone	skin tone	skin tone	Skin tone	1	kept
Skin ulcer	skin ulcer	skin ulcer	skin ulcer	skin ulcer	1	case_or_format

skin water loss	skin water loss	skin water loss	skin water loss	skin water loss	1	kept
Skin wounds and injuries	skin wounds and injuries	skin wounds and injuries	skin wounds and injuries	Skin wounds and injuries	1	kept
Skin-prick test	skin prick test	skin prick test	skin prick test	Skin-prick test	1	kept
Skin-to-skin method	skin to skin method	skin to skin method	skin to skin method	Skin-to-skin method	1	kept
skin-to-skin-care	skin to skin care	skin to skin care	skin to skin care	skin-to-skin care	1	case_or_format
Skincare bundle	skincare bundle	skincare bundle	skincare bundle	Skincare bundle	1	kept
skindex-16	skindex 16	skindex 16	skindex 16	skindex-16	1	kept
skinfold	skinfold	skinfold	skinfold	skinfold	1	kept
Skull base	skull base	skull base	skull base	Skull base	1	kept
skull base	skull base	skull base	skull base	Skull base	1	case_or_format
skull development	skull development	skull development	skull development	skull development	1	kept
skull injury	skull injury	skull injury	skull injury	skull injury	1	kept
Skull traction	skull traction	skull traction	skull traction	Skull traction	1	kept
SLACK RESOURCES	slack resources	slack resources	slack resources	SLACK RESOURCES	1	kept
slaughterhouse	slaughterhouse	slaughterhouse	slaughterhouse	slaughterhouse	1	kept
SLE	sle	sle	sle	SLE	1	kept

SLEDD	sledd	sledd	sledd	SLEDD	1	kept
Sleep alterations	sleep alterations	sleep alterations	sleep alterations	Sleep alterations	1	kept
sleep and arousal	sleep and arousal	sleep and arousal	sleep and arousal	sleep and arousal	1	kept
sleep and safety	sleep and safety	sleep and safety	sleep and safety	sleep and safety	1	kept
Sleep and wake states	sleep and wake states	sleep and wake states	sleep and wake states	Sleep and wake states	1	kept
Sleep Apnea	sleep apnea	sleep apnea	sleep apnea	Sleep apnea	1	case_or_format
sleep assessment	sleep assessment	sleep assessment	sleep assessment	Sleep assessment	1	case_or_format
sleep bout length	sleep bout length	sleep bout length	sleep bout length	sleep bout length	1	kept
sleep care	sleep care	sleep care	sleep care	sleep care	1	kept
sleep care bundle	sleep care bundle	sleep care bundle	sleep care bundle	sleep care bundle	1	kept
Sleep care guidelines	sleep care guidelines	sleep care guidelines	sleep care guidelines	Sleep care guidelines	1	kept
Sleep deprivations	sleep deprivations	sleep deprivations	sleep deprivation	Sleep Deprivation	1	singular
Sleep disordered breathing	sleep disordered breathing	sleep disordered breathing	sleep disordered breathing	sleep disordered breathing	1	case_or_format
Sleep disorders, circadian rhythm	sleep disorders circadian rhythm	sleep disorders circadian rhythm	sleep disorders circadian rhythm	Sleep Disorders, Circadian Rhythm	1	case_or_format
sleep duration	sleep duration	sleep duration	sleep duration	Sleep Duration	1	case_or_format
sleep education	sleep education	sleep education	sleep education	sleep education	1	kept

sleep effort	sleep effort	sleep effort	sleep effort	sleep effort	1	kept
Sleep enhancement	sleep enhancement	sleep enhancement	sleep enhancement	Sleep enhancement	1	kept
sleep evaluation	sleep evaluation	sleep evaluation	sleep evaluation	sleep evaluation	1	kept
sleep fragmentation	sleep fragmentation	sleep fragmentation	sleep fragmentation	sleep fragmentation	1	kept
sleep frequency	sleep frequency	sleep frequency	sleep frequency	sleep frequency	1	kept
sleep health	sleep health	sleep health	sleep health	sleep health	1	kept
Sleep interventions	sleep interventions	sleep interventions	sleep intervention	Sleep intervention	1	singular
Sleep Latency	sleep latency	sleep latency	sleep latency	sleep latency	1	case_or_format
Sleep measurement	sleep measurement	sleep measurement	sleep measurement	Sleep measurement	1	kept
sleep measurement	sleep measurement	sleep measurement	sleep measurement	Sleep measurement	1	case_or_format
SLEEP MEDICINE	sleep medicine	sleep medicine	sleep medicine	sleep medicine	1	case_or_format
Sleep pattern	sleep pattern	sleep pattern	sleep pattern	sleep pattern	1	case_or_format
sleep protection	sleep protection	sleep protection	sleep protection	Sleep protection	1	case_or_format
sleep protocol	sleep protocol	sleep protocol	sleep protocol	sleep protocol	1	kept
sleep quality & quantity	sleep quality and quantity	sleep quality and quantity	sleep quality and quantity	sleep quality & quantity	1	kept
sleep quality and quality of life	sleep quality and quality of life	sleep quality and quality of life	sleep quality and quality of life	sleep quality and quality of life	1	kept

sleep related deaths	sleep related deaths	sleep related deaths	sleep related deaths	sleep related deaths	1	kept
Sleep scales	sleep scales	sleep scales	sleep scales	Sleep scales	1	kept
Sleep staging	sleep staging	sleep staging	sleep staging	Sleep staging	1	kept
Sleep state	sleep state	sleep state	sleep state	Sleep state	1	kept
sleep therapy	sleep therapy	sleep therapy	sleep therapy	sleep therapy	1	kept
sleep variables	sleep variables	sleep variables	sleep variables	sleep variables	1	kept
Sleep,	sleep	sleep	sleep	Sleep	1	case_or_format
Sleep-disturbing factors	sleep disturbing factors	sleep disturbing factors	sleep disturbing factors	Sleep-disturbing factors	1	kept
SLEEP-WAKE BEHAVIORS	sleep wake behaviors	sleep wake behaviors	sleep wake behaviors	SLEEP-WAKE BEHAVIORS	1	kept
sleep-wake cycle	sleep wake cycle	sleep wake cycle	sleep wake cycle	Sleep-wake cycle	1	case_or_format
Sleep-wake cycling	sleep wake cycling	sleep wake cycling	sleep wake cycling	Sleep-wake cycling	1	kept
Sleep-Wake States	sleep wake states	sleep wake states	sleep wake states	Sleep-Wake States	1	kept
sleep-wakefulness	sleep wakefulness	sleep wakefulness	sleep wakefulness	sleep-wakefulness	1	kept
Sleephygieneinterventions	sleephygieneinterventions	sleephygieneinterventions	sleephygieneinterventions	Sleephygieneinterventions	1	kept
Sleeplessness	sleeplessness	sleeplessness	sleeplessness	Sleeplessness	1	kept
SleepQuality	sleepquality	sleepquality	sleepquality	SleepQuality	1	kept

Sleepy driving	sleepy driving	sleepy driving	sleepy driving	Sleepy driving	1	kept
sleepy therapist	sleepy therapist	sleepy therapist	sleepy therapist	sleepy therapist	1	kept
sleep–wake states	sleep wake states	sleep wake states	sleep wake states	Sleep-Wake States	1	case_or_format
Slide rule	slide rule	slide rule	slide rule	Slide rule	1	kept
slide rule	slide rule	slide rule	slide rule	Slide rule	1	case_or_format
sliding lung sign	sliding lung sign	sliding lung sign	sliding lung sign	sliding lung sign	1	kept
Sliding mode control	sliding mode control	sliding mode control	sliding mode control	sliding mode control	1	case_or_format
SLIP	slip	slip	slip	slips	1	case_or_format
Slip	slip	slip	slip	slips	1	case_or_format
slipping	slipping	slipping	slipping	slipping	1	kept
slow brain wave	slow brain wave	slow brain wave	slow brain wave	slow brain wave	1	kept
Slow code	slow code	slow code	slow code	slow code	1	case_or_format
slow codes	slow codes	slow codes	slow code	slow code	1	singular
slow vital capacity	slow vital capacity	slow vital capacity	slow vital capacity	slow vital capacity	1	kept
Slow wave sleep	slow wave sleep	slow wave sleep	slow wave sleep	Slow wave sleep	1	kept
slowing infection spread	slowing infection spread	slowing infection spread	slowing infection spread	slowing infection spread	1	kept

slowness	slowness	slowness	slowness	slowness	1	kept
SLP	slp	slp	slp	SLP	1	kept
SLT	slt	slt	slt	SLT	1	kept
SLURP	slurp	slurp	slurp	SLURP	1	kept
SMA	sma	sma	sma	SMA	1	kept
small animals	small animals	small animals	small animals	small animals	1	kept
Small baby programs	small baby programs	small baby programs	small baby programs	Small baby programs	1	kept
small bowel feeding tube	small bowel feeding tube	small bowel feeding tube	small bowel feeding tube	small bowel feeding tube	1	kept
small bowel intussusception	small bowel intussusception	small bowel intussusception	small bowel intussusception	small bowel intussusception	1	kept
small bowel necrosis	small bowel necrosis	small bowel necrosis	small bowel necrosis	small bowel necrosis	1	kept
Small bowel obstruction	small bowel obstruction	small bowel obstruction	small bowel obstruction	small bowel obstruction	1	case_or_format
Small intestine	small intestine	small intestine	small intestine	small intestine	1	case_or_format
small intestine content	small intestine content	small intestine content	small intestine content	small intestine content	1	kept
small intestine disease	small intestine disease	small intestine disease	small intestine disease	small intestine disease	1	kept
small mammal	small mammal	small mammal	small mammal	small mammal	1	kept
small untranslated RNA	small untranslated rna	small untranslated rna	small untranslated rna	small untranslated RNA	1	kept

small volume tubes	small volume tubes	small volume tubes	small volume tubes	Small volume tubes	1	case_or_format
small-bore connector	small bore connector	small bore connector	small bore connector	small-bore connector	1	kept
Small-bowel fistula	small bowel fistula	small bowel fistula	small bowel fistula	Small-bowel fistula	1	kept
SMALL-SAMPLE ADJUSTMENTS	small sample adjustments	small sample adjustments	small sample adjustments	SMALL-SAMPLE ADJUSTMENTS	1	kept
Small-vessel occlusion	small vessel occlusion	small vessel occlusion	small vessel occlusion	Small-vessel occlusion	1	kept
small-world networks	small world networks	small world networks	small world networks	small-world networks	1	kept
Smart	smart	smart	smart	Smart	1	kept
Smart band	smart band	smart band	smart band	Smart band	1	kept
Smart Bracelet	smart bracelet	smart bracelet	smart bracelet	Smart Bracelet	1	kept
smart card	smart card	smart card	smart card	smart card	1	kept
smart care	smart care	smart care	smart care	smart care	1	kept
SMART COP	smart cop	smart cop	smart cop	SMART COP	1	kept
Smart devices	smart devices	smart devices	smart devices	Smart devices	1	kept
Smart glasses	smart glasses	smart glasses	smart glasses	Smart Glasses	1	case_or_format
smart glasses	smart glasses	smart glasses	smart glasses	Smart Glasses	1	case_or_format
Smart health	smart health	smart health	smart health	Smart health	1	kept

smart healthcare	smart healthcare	smart healthcare	smart healthcare	smart healthcare	1	kept
Smart hospital	smart hospital	smart hospital	smart hospital	Smart hospital	1	kept
smart infusion technology	smart infusion technology	smart infusion technology	smart infusion technology	smart infusion technology	1	kept
SMART IRB	smart irb	smart irb	smart irb	SMART IRB	1	kept
smart medicine	smart medicine	smart medicine	smart medicine	smart medicine	1	kept
smart phone	smart phone	smart phone	smart phone	smart phone	1	kept
smart pump	smart pump	smart pump	smart pump	Smart pump	1	case_or_format
Smart pumps	smart pumps	smart pumps	smart pump	Smart pump	1	singular
Smart syringe pump	smart syringe pump	smart syringe pump	smart syringe pump	Smart syringe pump	1	kept
smart ventilator	smart ventilator	smart ventilator	smart ventilator	smart ventilator	1	kept
Smart watch	smart watch	smart watch	smart watch	Smart watch	1	kept
smart watch	smart watch	smart watch	smart watch	Smart watch	1	case_or_format
Smart wristband	smart wristband	smart wristband	smart wristband	Smart wristband	1	kept
Smartcare	smartcare	smartcare	smartcare	Smartcare	1	kept
Smartphone app	smartphone app	smartphone app	smartphone app	Smartphone app	1	kept
smartphone-based application	smartphone based application	smartphone based application	smartphone based application	smartphone-based application	1	kept

Smartwatch	smartwatch	smartwatch	smartwatch	Smartwatch	1	kept
Smell	smell	smell	smell	Smell	1	kept
smell	smell	smell	smell	Smell	1	case_or_format
smell and taste parameters	smell and taste parameters	smell and taste parameters	smell and taste parameters	smell and taste parameters	1	kept
smelling disorder	smelling disorder	smelling disorder	smelling disorder	smelling disorder	1	kept
Smiling	smiling	smiling	smiling	Smiling	1	kept
SMN	smn	smn	smn	SMN	1	kept
SMOFlipid	smoflipid	smoflipid	smoflipid	SMOFlipid	1	kept
Smoke-Free Policy	smoke free policy	smoke free policy	smoke free policy	Smoke-Free Policy	1	kept
Smoker	smoker	smoker	smoker	Smoker	1	kept
Smokers	smokers	smokers	smoker	Smoker	1	singular
Smoking and pregnancy	smoking and pregnancy	smoking and pregnancy	smoking and pregnancy	Smoking and pregnancy	1	kept
Smoking ban in hospital	smoking ban in hospital	smoking ban in hospital	smoking ban in hospital	Smoking ban in hospital	1	kept
Smoking cessations	smoking cessations	smoking cessations	smoking cessation	smoking cessation	1	singular
smoldering multiple myeloma	smoldering multiple myeloma	smoldering multiple myeloma	smoldering multiple myeloma	smoldering multiple myeloma	1	kept
smooth muscle relaxation	smooth muscle relaxation	smooth muscle relaxation	smooth muscle relaxation	smooth muscle relaxation	1	kept

SMOTE	smote	smote	smote	SMOTE	1	kept
SMS message	sms message	sms message	sms message	SMS message	1	kept
sMVAP index	smvap index	smvap index	smvap index	sMVAP index	1	kept
snake	snake	snake	snake	snake	1	kept
snake venom antiserum	snake venom antiserum	snake venom antiserum	snake venom antiserum	snake venom antiserum	1	kept
snake venom detection test	snake venom detection test	snake venom detection test	snake venom detection test	snake venom detection test	1	kept
Snakebite	snakebite	snakebite	snakebite	snakebite	1	case_or_format
Snakebite envenomations	snakebite envenomations	snakebite envenomations	snakebite envenomations	Snakebite envenomations	1	kept
Snakes	snakes	snakes	snake	snake	1	singular
SNAP	snap	snap	snap	SNAP	1	kept
SNF	snf	snf	snf	SNF	1	kept
Sniffin Sticks test	sniffin sticks test	sniffin sticks test	sniffin sticks test	Sniffin Sticks test	1	kept
snoring	snoring	snoring	snoring	snoring	1	kept
snow	snow	snow	snow	Snow	1	case_or_format
snowmobile	snowmobile	snowmobile	snowmobile	snowmobile	1	kept
Soap	soap	soap	soap	soap	1	case_or_format

Soap and water	soap and water	soap and water	soap and water	Soap and water	1	kept
Soap-coating	soap coating	soap coating	soap coating	Soap-coating	1	kept
SOAP-M	soap m	soap m	soap m	SOAP-M	1	kept
Sobriety	sobriety	sobriety	sobriety	Sobriety	1	kept
Social	social	social	social	Social	1	kept
Social adaptation	social adaptation	social adaptation	social adaptation	social adaptation	1	case_or_format
Social analyzer	social analyzer	social analyzer	social analyzer	Social analyzer	1	kept
Social and Occupational Functioning Assessment	social and occupational functioning assessment	social and occupational functioning assessment	social and occupational functioning assessment	Social and Occupational Functioning Assessment	1	kept
social approach	social approach	social approach	social approach	social approach	1	kept
social attitude	social attitude	social attitude	social attitude	social attitude	1	kept
Social awkwardness	social awkwardness	social awkwardness	social awkwardness	Social awkwardness	1	kept
social background	social background	social background	social background	social background	1	kept
Social Behavior Disorders	social behavior disorders	social behavior disorders	social behavior disorders	Social Behavior Disorders	1	kept
social bond	social bond	social bond	social bond	social bond	1	kept
social change	social change	social change	social change	social change	1	kept
social cognitive theory	social cognitive theory	social cognitive theory	social cognitive theory	Social Cognitive Theory	1	case_or_format

Social competence	social competence	social competence	social competence	social competence	1	case_or_format
Social Conditions	social conditions	social conditions	social conditions	Social Conditions	1	kept
social construction	social construction	social construction	social construction	social construction	1	kept
social constructionism	social constructionism	social constructionism	social constructionism	social constructionism	1	kept
Social Control Policies	social control policies	social control policies	social control policies	Social Control Policies	1	kept
Social Control, Informal	social control informal	social control informal	social control informal	Social Control, Informal	1	kept
social Darwinism	social darwinism	social darwinism	social darwinism	social Darwinism	1	kept
social dependency	social dependency	social dependency	social dependency	social dependency	1	kept
Social determinants	social determinants	social determinants	social determinants	Social determinants	1	kept
social dimensions of pulmonary medicine	social dimensions of pulmonary medicine	social dimensions of pulmonary medicine	social dimensions of pulmonary medicine	social dimensions of pulmonary medicine	1	kept
Social Discrimination	social discrimination	social discrimination	social discrimination	social discrimination	1	case_or_format
Social distancing	social distancing	social distancing	social distancing	social distancing	1	case_or_format
social drivers of health	social drivers of health	social drivers of health	social drivers of health	social drivers of health	1	kept
social ecology	social ecology	social ecology	social ecology	social ecology	1	kept
Social Emergency	social emergency	social emergency	social emergency	Social Emergency	1	kept
social experience	social experience	social experience	social experience	social experience	1	kept

Social functioning	social functioning	social functioning	social functioning	Social functioning	1	kept
social group	social group	social group	social group	social group	1	kept
Social Health	social health	social health	social health	Social Health	1	kept
social identification	social identification	social identification	social identification	Social Identification	1	case_or_format
SOCIAL IDENTITY	social identity	social identity	social identity	social identity	1	case_or_format
Social identity	social identity	social identity	social identity	social identity	1	case_or_format
social inclusion	social inclusion	social inclusion	social inclusion	social inclusion	1	kept
Social influence	social influence	social influence	social influence	Social influence	1	kept
social innovation	social innovation	social innovation	social innovation	social innovation	1	kept
social insurance	social insurance	social insurance	social insurance	social insurance	1	kept
Social Integration	social integration	social integration	social integration	Social Integration	1	kept
Social Interaction	social interaction	social interaction	social interaction	social interaction	1	case_or_format
social interactions	social interactions	social interactions	social interaction	social interaction	1	singular
social isolation or quarantine	social isolation or quarantine	social isolation or quarantine	social isolation or quarantine	social isolation or quarantine	1	kept
Social issues	social issues	social issues	social issues	Social issues	1	kept
SOCIAL JUDGMENT	social judgment	social judgment	social judgment	SOCIAL JUDGMENT	1	kept

Social learning	social learning	social learning	social learning	social learning	1	case_or_format
Social Marketing	social marketing	social marketing	social marketing	Social Marketing	1	kept
social marketing	social marketing	social marketing	social marketing	Social Marketing	1	case_or_format
social media violations	social media violations	social media violations	social media violations	social media violations	1	kept
social mobilization	social mobilization	social mobilization	social mobilization	social mobilization	1	kept
Social networking app	social networking app	social networking app	social networking app	Social networking app	1	kept
social networking sites	social networking sites	social networking sites	social networking sites	social networking sites	1	kept
Social networks	social networks	social networks	social network	social network	1	singular
Social Norms	social norms	social norms	social norm	social norm	1	singular
Social organizations	social organizations	social organizations	social organizations	Social organizations	1	kept
social outcomes	social outcomes	social outcomes	social outcomes	social outcomes	1	kept
social phenomenological	social phenomenological	social phenomenological	social phenomenological	social phenomenological	1	kept
Social protection	social protection	social protection	social protection	Social protection	1	kept
social reinforcement	social reinforcement	social reinforcement	social reinforcement	social reinforcement	1	kept
social rejection	social rejection	social rejection	social rejection	social rejection	1	kept
Social relations	social relations	social relations	social relations	Social relations	1	kept

social relationships	social relationships	social relationships	social relationships	social relationships	1	kept
Social Responsiveness Scale	social responsiveness scale	social responsiveness scale	social responsiveness scale	Social Responsiveness Scale	1	kept
Social return on investment	social return on investment	social return on investment	social return on investment	Social return on investment	1	kept
SOCIAL ROBOTS	social robots	social robots	social robots	SOCIAL ROBOTS	1	kept
social roles	social roles	social roles	social roles	social roles	1	kept
social science	social science	social science	social science	social science	1	kept
Social science research	social science research	social science research	social science research	Social science research	1	kept
Social Sciences	social sciences	social sciences	social science	social science	1	singular
social sciences	social sciences	social sciences	social science	social science	1	singular
social service staff	social service staff	social service staff	social service staff	social service staff	1	kept
Social services	social services	social services	social services	Social services	1	kept
Social stories	social stories	social stories	social stories	Social stories	1	kept
social stratification	social stratification	social stratification	social stratification	social stratification	1	kept
Social stress	social stress	social stress	social stress	social stress	1	case_or_format
Social structure	social structure	social structure	social structure	social structure	1	case_or_format
Social Support Rating Scale	social support rating scale	social support rating scale	social support rating scale	Social Support Rating Scale	1	kept

Social supports	social supports	social supports	social support	Social Support	1	singular
social value	social value	social value	social value	Social Values	1	case_or_format
social vulnerability	social vulnerability	social vulnerability	social vulnerability	social vulnerability	1	kept
Social Work Department, Hospital	social work department hospital	social work department hospital	social work department hospital	Social Work Department, Hospital	1	kept
social work education	social work education	social work education	social work education	social work education	1	kept
Social work environment	social work environment	social work environment	social work environment	Social work environment	1	kept
social work referral	social work referral	social work referral	social work referral	social work referral	1	kept
Social work referrals	social work referrals	social work referrals	social work referral	social work referral	1	singular
Social worker	social worker	social worker	social worker	social worker	1	case_or_format
social workers	social workers	social workers	social worker	social worker	1	singular
social-ecological model	social ecological model	social ecological model	social ecological model	social-ecological model	1	kept
SOCIAL-WORK	social work	social work	social work	social work	1	case_or_format
socially at risk	socially at risk	socially at risk	socially at risk	socially at risk	1	kept
socially at-risk	socially at risk	socially at risk	socially at risk	socially at risk	1	case_or_format
Societal issues	societal issues	societal issues	societal issues	Societal issues	1	kept
Societal Turmoil	societal turmoil	societal turmoil	societal turmoil	Societal Turmoil	1	kept

Societies and institutions	societies and institutions	societies and institutions	societies and institutions	Societies and institutions	1	kept
Societies, Pharmaceutical	societies pharmaceutical	societies pharmaceutical	societies pharmaceutical	Societies, Pharmaceutical	1	kept
society and environment	society and environment	society and environment	society and environment	society and environment	1	kept
Society of Thoracic Surgeons Predicted Risk of Operative	society of thoracic surgeons predicted risk of operative	society of thoracic surgeons predicted risk of operative	society of thoracic surgeons predicted risk of operative	Society of Thoracic Surgeons Predicted Risk of Operative	1	kept
Socio-Demographic	socio demographic	socio demographic	socio demographic	Socio-demographic	1	case_or_format
Socio-ecological model	socio ecological model	socio ecological model	socio ecological model	Socio-ecological model	1	kept
Socio-economic status	socio economic status	socio economic status	socio economic status	Socio-economic status	1	kept
Socio-technical factors	socio technical factors	socio technical factors	socio technical factors	Socio-technical factors	1	kept
Socio-technical systems	socio technical systems	socio technical systems	socio technical systems	Socio-technical systems	1	kept
Socioadaptive interventions	socioadaptive interventions	socioadaptive interventions	socioadaptive interventions	Socioadaptive interventions	1	kept
sociodemographic characteristics	sociodemographic characteristics	sociodemographic characteristics	sociodemographic characteristics	sociodemographic characteristics	1	kept
Sociodemographic factors	sociodemographic factors	sociodemographic factors	sociodemographic factors	Sociodemographic Factors	1	case_or_format
socioecological model	socioecological model	socioecological model	socioecological model	socioecological model	1	kept
Socioeconomic	socioeconomic	socioeconomic	socioeconomic	socioeconomics	1	case_or_format
Socioeconomic Disparities in Health	socioeconomic disparities in health	socioeconomic disparities in health	socioeconomic disparities in health	Socioeconomic Disparities in Health	1	kept
socioeconomic parameters	socioeconomic parameters	socioeconomic parameters	socioeconomic parameters	socioeconomic parameters	1	kept

Sociogram	sociogram	sociogram	sociogram	Sociogram	1	kept
sociogram	sociogram	sociogram	sociogram	Sociogram	1	case_or_format
Sociological Factors	sociological factors	sociological factors	sociological factors	Sociological Factors	1	kept
sociological theory	sociological theory	sociological theory	sociological theory	sociological theory	1	kept
Sociometric Techniques	sociometric techniques	sociometric techniques	sociometric techniques	Sociometric Techniques	1	kept
sociotechnical aspects of information technology	sociotechnical aspects of information technology	sociotechnical aspects of information technology	sociotechnical aspects of information technology	sociotechnical aspects of information technology	1	kept
sociotechnical system	sociotechnical system	sociotechnical system	sociotechnical system	Sociotechnical systems	1	case_or_format
sociotechnical systems	sociotechnical systems	sociotechnical systems	sociotechnical system	Sociotechnical systems	1	singular
socio - demographic factors	socio demographic factors	socio demographic factors	socio demographic factors	socio-demographic factors	1	case_or_format
sodium absorption	sodium absorption	sodium absorption	sodium absorption	sodium absorption	1	kept
sodium bicarbonate	sodium bicarbonate	sodium bicarbonate	sodium bicarbonate	Sodium Bicarbonate	1	case_or_format
sodium channel	sodium channel	sodium channel	sodium channel	sodium channel	1	kept
sodium channel blocking agent	sodium channel blocking agent	sodium channel blocking agent	sodium channel blocking agent	sodium channel blocking agent	1	kept
Sodium citrate	sodium citrate	sodium citrate	sodium citrate	Sodium citrate	1	kept
Sodium hypochlorite	sodium hypochlorite	sodium hypochlorite	sodium hypochlorite	Sodium Hypochlorite	1	case_or_format
sodium ion	sodium ion	sodium ion	sodium ion	sodium ion	1	kept

Sodium Lactate	sodium lactate	sodium lactate	sodium lactate	Sodium Lactate	1	kept
sodium nitrite	sodium nitrite	sodium nitrite	sodium nitrite	sodium nitrite	1	kept
Sodium Oxybate	sodium oxybate	sodium oxybate	sodium oxybate	Sodium Oxybate	1	kept
Sodium pentothal	sodium pentothal	sodium pentothal	sodium pentothal	Sodium pentothal	1	kept
Sodium Potassium Chloride Symporter Inhibitors	sodium potassium chloride symporter inhibitors	sodium potassium chloride symporter inhibitors	sodium potassium chloride symporter inhibitors	Sodium Potassium Chloride Symporter Inhibitors	1	kept
sodium restriction	sodium restriction	sodium restriction	sodium restriction	sodium restriction	1	kept
sodium transport	sodium transport	sodium transport	sodium transport	sodium transport	1	kept
Sodium valproate	sodium valproate	sodium valproate	sodium valproate	Sodium valproate	1	kept
SODIUM-BICARBONATE	sodium bicarbonate	sodium bicarbonate	sodium bicarbonate	Sodium Bicarbonate	1	case_or_format
SOFA Score	sofa score	sofa score	sofa score	SOFA score	1	case_or_format
Soft computing	soft computing	soft computing	soft computing	Soft computing	1	kept
Soft constraint	soft constraint	soft constraint	soft constraint	Soft constraint	1	kept
Soft constraints	soft constraints	soft constraints	soft constraint	Soft constraint	1	singular
soft constraints	soft constraints	soft constraints	soft constraint	Soft constraint	1	singular
soft diet	soft diet	soft diet	soft diet	soft diet	1	kept
Soft tissue calcification	soft tissue calcification	soft tissue calcification	soft tissue calcification	Soft tissue calcification	1	kept

soft tissue infections	soft tissue infections	soft tissue infections	soft tissue infection	soft tissue infection	1	singular
soft tissue injuries	soft tissue injuries	soft tissue injuries	soft tissue injury	soft tissue injury	1	singular
Soft-tissue injury	soft tissue injury	soft tissue injury	soft tissue injury	soft tissue injury	1	case_or_format
SOFT-TISSUE STIFFNESS	soft tissue stiffness	soft tissue stiffness	soft tissue stiffness	SOFT-TISSUE STIFFNESS	1	kept
Software algorithms	software algorithms	software algorithms	software algorithms	Software algorithms	1	kept
software assisted screening	software assisted screening	software assisted screening	software assisted screening	software assisted screening	1	kept
Software design	software design	software design	software design	Software Design	1	case_or_format
Software engineering	software engineering	software engineering	software engineering	software engineering	1	case_or_format
Software evaluation	software evaluation	software evaluation	software evaluation	Software evaluation	1	kept
Software platforms	software platforms	software platforms	software platforms	Software platforms	1	kept
Software prototyping	software prototyping	software prototyping	software prototyping	Software prototyping	1	kept
Software reliability	software reliability	software reliability	software reliability	Software reliability	1	kept
soignant	soignant	soignant	soignant	soignant	1	kept
soignants	soignants	soignants	soignant	soignant	1	singular
soin critique	soin critique	soin critique	soin critique	soin critique	1	kept
soin de bouche	soin de bouche	soin de bouche	soin de bouche	soin de bouche	1	kept

soin intensif	soin intensif	soin intensif	soin intensif	soin intensif	1	kept
soins	soins	soins	soins	soins	1	kept
soins aigus	soins aigus	soins aigus	soins aigus	soins aigus	1	kept
soins critiques	soins critiques	soins critiques	soins critiques	soins critiques	1	kept
soins d'hygiène et de confort	soins d hygi ne et de confort	soins d hygi ne et de confort	soins d hygi ne et de confort	soins d'hygiène et de confort	1	kept
soins infirmiers	soins infirmiers	soins infirmiers	soins infirmiers	soins infirmiers	1	kept
Soins intensifs	soins intensifs	soins intensifs	soins intensifs	Soins intensifs	1	kept
soins intensifs	soins intensifs	soins intensifs	soins intensifs	Soins intensifs	1	case_or_format
soins interdisciplinaires	soins interdisciplinaires	soins interdisciplinaires	soins interdisciplinaires	soins interdisciplinaires	1	kept
Soins palliatifs	soins palliatifs	soins palliatifs	soins palliatifs	Soins palliatifs	1	kept
Sojourn time	sojourn time	sojourn time	sojourn time	Sojourn time	1	kept
Solar energy	solar energy	solar energy	solar energy	Solar energy	1	kept
solar energy	solar energy	solar energy	solar energy	Solar energy	1	case_or_format
Solenoids	solenoids	solenoids	solenoids	Solenoids	1	kept
Solid modelling	solid modelling	solid modelling	solid modelling	Solid modelling	1	kept
solid oral dosage forms	solid oral dosage forms	solid oral dosage forms	solid oral dosage forms	solid oral dosage forms	1	kept

Solid organ donation	solid organ donation	solid organ donation	solid organ donation	Solid organ donation	1	kept
Solid organ transplantation	solid organ transplantation	solid organ transplantation	solid organ transplantation	Solid organ transplantation	1	kept
solid organ transplantation	solid organ transplantation	solid organ transplantation	solid organ transplantation	Solid organ transplantation	1	case_or_format
Solid tumor	solid tumor	solid tumor	solid tumor	solid tumor	1	case_or_format
solid waste management	solid waste management	solid waste management	solid waste management	solid waste management	1	kept
SOLID-ORGAN TRANSPLANTATION	solid organ transplantation	solid organ transplantation	solid organ transplantation	Solid organ transplantation	1	case_or_format
SOLID-PHASE EXTRACTION	solid phase extraction	solid phase extraction	solid phase extraction	SOLID-PHASE EXTRACTION	1	kept
Solitary kidney	solitary kidney	solitary kidney	solitary kidney	Solitary kidney	1	kept
Solubility	solubility	solubility	solubility	Solubility	1	kept
solutions	solutions	solutions	solutions	Solutions	1	case_or_format
SOLVENT	solvent	solvent	solvent	solvent	1	case_or_format
Solvents	solvents	solvents	solvent	solvent	1	singular
Solvothermal reactions	solvothermal reactions	solvothermal reactions	solvothermal reactions	Solvothermal reactions	1	kept
Somalia Hospital	somalia hospital	somalia hospital	somalia hospital	Somalia Hospital	1	kept
somatic delusion	somatic delusion	somatic delusion	somatic delusion	somatic delusion	1	kept
somatic disease	somatic disease	somatic disease	somatic disease	somatic disease	1	kept

somatic symptoms	somatic symptoms	somatic symptoms	somatic symptoms	somatic symptoms	1	kept
somatoform disorder	somatoform disorder	somatoform disorder	somatoform disorder	somatoform disorder	1	kept
Somatosensory evoked potentials	somatosensory evoked potentials	somatosensory evoked potentials	somatosensory evoked potential	somatosensory evoked potential	1	singular
son	son	son	son	son	1	kept
son-in-law	son in law	son in law	son in law	son-in-law	1	kept
Songklanagarind Pediatric Triage Model	songklanagarind pediatric triage model	songklanagarind pediatric triage model	songklanagarind pediatric triage model	Songklanagarind Pediatric Triage Model	1	kept
Sonography	sonography	sonography	sonography	Sonography	1	kept
sonography	sonography	sonography	sonography	Sonography	1	case_or_format
soothing touch	soothing touch	soothing touch	soothing touch	soothing touch	1	kept
Sophia observation withdrawal symptoms-paediatric Delirium	sophia observation withdrawal symptoms paediatric delirium	sophia observation withdrawal symptoms paediatric delirium	sophia observation withdrawal symptoms paediatric delirium	Sophia observation withdrawal symptoms-paediatric Delirium	1	kept
Sophia withdrawal scale	sophia withdrawal scale	sophia withdrawal scale	sophia withdrawal scale	Sophia withdrawal scale	1	kept
sophrologie	sophrologie	sophrologie	sophrologie	sophrologie	1	kept
sophrology	sophrology	sophrology	sophrology	sophrology	1	kept
sopor	sopor	sopor	sopor	sopor	1	kept
SOPS	sops	sops	sops	SOPS	1	kept
Sorafenib	sorafenib	sorafenib	sorafenib	sorafenib	1	case_or_format

SORE RISK	sore risk	sore risk	sore risk	SORE RISK	1	kept
Sorrorigenic	sorrorigenic	sorrorigenic	sorrorigenic	Sorrorigenic	1	kept
sorrorigenic wound	sorrorigenic wound	sorrorigenic wound	sorrorigenic wound	sorrorigenic wound	1	kept
sorrow	sorrow	sorrow	sorrow	sorrow	1	kept
SORT IT	sort it	sort it	sort it	SORT IT	1	kept
Sotalol	sotalol	sotalol	sotalol	sotalol	1	case_or_format
Sotos Syndrome	sotos syndrome	sotos syndrome	sotos syndrome	Sotos syndrome	1	case_or_format
Sound absorption	sound absorption	sound absorption	sound absorption	Sound absorption	1	kept
sound desensitization	sound desensitization	sound desensitization	sound desensitization	sound desensitization	1	kept
SOUND ENVIRONMENT	sound environment	sound environment	sound environment	Sound environment	1	case_or_format
Sound insulating materials	sound insulating materials	sound insulating materials	sound insulating materials	Sound insulating materials	1	kept
Sound isolation	sound isolation	sound isolation	sound isolation	Sound isolation	1	kept
sound level	sound level	sound level	sound level	Sound level	1	case_or_format
sound level meter	sound level meter	sound level meter	sound level meter	sound level meter	1	kept
sound level meters	sound level meters	sound level meters	sound level meter	sound level meter	1	singular
sound level pressure	sound level pressure	sound level pressure	sound level pressure	sound level pressure	1	kept

sound levels	sound levels	sound levels	sound level	Sound level	1	singular
Sound pressure	sound pressure	sound pressure	sound pressure	sound pressure	1	case_or_format
sound pressure levels	sound pressure levels	sound pressure levels	sound pressure level	Sound pressure level	1	singular
Sound Recordings	sound recordings	sound recordings	sound recordings	Sound Recordings	1	kept
sound sources	sound sources	sound sources	sound source	Sound source	1	singular
SOUNDSCAPE	soundscape	soundscape	soundscape	Soundscapes	1	case_or_format
soundscape	soundscape	soundscape	soundscape	Soundscapes	1	case_or_format
Soundscape Dimensions I-INCE Classification of Subjects Number :	soundscape dimensions i ince classification of subjects number 61	soundscape dimensions i ince classification of subjects number 61	soundscape dimensions i ince classification of subjects number 61	Soundscape Dimensions I-INCE Classification of Subjects Number :	1	kept
Soundscape intervention	soundscape intervention	soundscape intervention	soundscape intervention	Soundscape intervention	1	kept
soundscape perception	soundscape perception	soundscape perception	soundscape perception	soundscape perception	1	kept
sour seven questionnaire	sour seven questionnaire	sour seven questionnaire	sour seven questionnaire	Sour Seven Questionnaire	1	case_or_format
Sources of hope	sources of hope	sources of hope	sources of hope	Sources of hope	1	kept
Sources of informations	sources of informations	sources of informations	sources of informations	Sources of informations	1	kept
sources of moral distress	sources of moral distress	sources of moral distress	sources of moral distress	sources of moral distress	1	kept
Sources of stress	sources of stress	sources of stress	sources of stress	Sources of stress	1	kept
South asian mothers	south asian mothers	south asian mothers	south asian mothers	South asian mothers	1	kept

South Cumbria	south cumbria	south cumbria	south cumbria	South Cumbria	1	kept
South East Asia	south east asia	south east asia	south east asia	South East Asia	1	kept
South East Iran	south east iran	south east iran	south east iran	South East Iran	1	kept
South India	south india	south india	south india	South India	1	kept
South West Pacific clone	south west pacific clone	south west pacific clone	south west pacific clone	South West Pacific clone	1	kept
Southern Hemisphere	southern hemisphere	southern hemisphere	southern hemisphere	Southern Hemisphere	1	kept
southern United States	southern united states	southern united states	southern united states	southern United States	1	kept
Southern-region	southern region	southern region	southern region	Southern-region	1	kept
Southwest China	southwest china	southwest china	southwest china	Southwest China	1	kept
southwestern Nigeria	southwestern nigeria	southwestern nigeria	southwestern nigeria	southwestern Nigeria	1	kept
SOWATS	sowats	sowats	sowats	SOWATS	1	kept
Soybean Oil	soybean oil	soybean oil	soybean oil	soybean oil	1	case_or_format
Soybean oil	soybean oil	soybean oil	soybean oil	soybean oil	1	case_or_format
Space	space	space	space	space	1	case_or_format
space and place	space and place	space and place	space and place	space and place	1	kept
space glucoseControl system	space glucosecontrol system	space glucosecontrol system	space glucosecontrol system	space glucoseControl system	1	kept

Space Simulation	space simulation	space simulation	space simulation	Space Simulation	1	kept
Space simulation	space simulation	space simulation	space simulation	Space Simulation	1	case_or_format
Space Syntax	space syntax	space syntax	space syntax	Space syntax	1	case_or_format
space syntax	space syntax	space syntax	space syntax	Space syntax	1	case_or_format
SpaceGlucose control	spaceglucose control	spaceglucose control	spaceglucose control	SpaceGlucose control	1	kept
spacers	spacers	spacers	spacers	spacers	1	kept
SPAIN	spain	spain	spain	Spain	1	case_or_format
SPANISH	spanish	spanish	spanish	Spanish	1	case_or_format
Spanish flu	spanish flu	spanish flu	spanish flu	Spanish flu	1	kept
Spanish National Health System	spanish national health system	spanish national health system	spanish national health system	Spanish National Health System	1	kept
Spanish-speaking	spanish speaking	spanish speaking	spanish speaking	Spanish-speaking	1	kept
spar h method	spar h method	spar h method	spar h method	spar h method	1	kept
SPARCO	sparco	sparco	sparco	SPARCO	1	kept
sparfloxacin	sparfloxacin	sparfloxacin	sparfloxacin	sparfloxacin	1	kept
Sparse recovery	sparse recovery	sparse recovery	sparse recovery	sparse recovery	1	case_or_format
Spasm	spasm	spasm	spasm	Spasm	1	kept

spasmolysis	spasmolysis	spasmolysis	spasmolysis	spasmolysis	1	kept
spasmolytic agent	spasmolytic agent	spasmolytic agent	spasmolytic agent	spasmolytic agent	1	kept
Spasms, Infantile	spasms infantile	spasms infantile	spasms infantile	Spasms, Infantile	1	kept
Spastic paralysis	spastic paralysis	spastic paralysis	spastic paralysis	Spastic paralysis	1	kept
Spatial	spatial	spatial	spatial	Spatial	1	kept
spatial disorientation	spatial disorientation	spatial disorientation	spatial disorientation	spatial disorientation	1	kept
Spatial filterings	spatial filterings	spatial filterings	spatial filterings	Spatial filterings	1	kept
SPATIAL LAYOUT	spatial layout	spatial layout	spatial layout	SPATIAL LAYOUT	1	kept
spatial learning	spatial learning	spatial learning	spatial learning	spatial learning	1	kept
spatial ordering	spatial ordering	spatial ordering	spatial ordering	spatial ordering	1	kept
spatial orientation	spatial orientation	spatial orientation	spatial orientation	spatial orientation	1	kept
Spatial planning	spatial planning	spatial planning	spatial planning	Spatial planning	1	kept
Spatial practices	spatial practices	spatial practices	spatial practices	Spatial practices	1	kept
Spatial span test	spatial span test	spatial span test	spatial span test	Spatial span test	1	kept
Spatiotemporal behavior	spatiotemporal behavior	spatiotemporal behavior	spatiotemporal behavior	Spatiotemporal behavior	1	kept
Spatiotemporal behaviors	spatiotemporal behaviors	spatiotemporal behaviors	spatiotemporal behavior	Spatiotemporal behavior	1	singular

Speak for Myself	speak for myself	speak for myself	speak for myself	Speak for Myself	1	kept
Speak for Myself Voice	speak for myself voice	speak for myself voice	speak for myself voice	Speak for Myself Voice	1	kept
Speakers’ policy	speakers policy	speakers policy	speakers policy	Speakers’ policy	1	kept
speaking about goals and expectations program	speaking about goals and expectations program	speaking about goals and expectations program	speaking about goals and expectations program	speaking about goals and expectations program	1	kept
Speaking valves	speaking valves	speaking valves	speaking valves	Speaking valves	1	kept
Spearman's r	spearman s r	spearman s r	spearman s r	Spearman's r	1	kept
Spearman's rho	spearman s rho	spearman s rho	spearman s rho	Spearman's rho	1	kept
special care transport	special care transport	special care transport	special care transport	special care transport	1	kept
special delivery unit	special delivery unit	special delivery unit	special delivery unit	special delivery unit	1	kept
special education	special education	special education	special education	special education	1	kept
special neonatal care units	special neonatal care units	special neonatal care units	special neonatal care units	special neonatal care units	1	kept
Special newborn care unit	special newborn care unit	special newborn care unit	special newborn care unit	Special newborn care unit	1	kept
special newborn care unit	special newborn care unit	special newborn care unit	special newborn care unit	Special newborn care unit	1	case_or_format
special risk groups	special risk groups	special risk groups	special risk groups	special risk groups	1	kept
Special units	special units	special units	special units	Special units	1	kept
Specialisation	specialisation	specialisation	specialisation	Specialisation	1	kept

Specialism	specialism	specialism	specialism	Specialism	1	kept
Specialist centres	specialist centres	specialist centres	specialist centres	Specialist centres	1	kept
Specialist departments	specialist departments	specialist departments	specialist departments	Specialist departments	1	kept
Specialist palliative care	specialist palliative care	specialist palliative care	specialist palliative care	specialist palliative care	1	case_or_format
specialist pleural service	specialist pleural service	specialist pleural service	specialist pleural service	specialist pleural service	1	kept
SPECIALIST RETRIEVAL TEAM	specialist retrieval team	specialist retrieval team	specialist retrieval team	SPECIALIST RETRIEVAL TEAM	1	kept
Speciality practice	speciality practice	speciality practice	speciality practice	Speciality practice	1	kept
speciality status	speciality status	speciality status	speciality status	speciality status	1	kept
Specialized care unit	specialized care unit	specialized care unit	specialized care unit	Specialized care unit	1	kept
specialized continuous renal replacement therapy team	specialized continuous renal replacement therapy team	specialized continuous renal replacement therapy team	specialized continuous renal replacement therapy team	specialized continuous renal replacement therapy team	1	kept
specialized courses	specialized courses	specialized courses	specialized courses	specialized courses	1	kept
Specialized CRRT team	specialized crrt team	specialized crrt team	specialized crrt team	Specialized CRRT team	1	kept
Specialized knowledge	specialized knowledge	specialized knowledge	specialized knowledge	Specialized knowledge	1	kept
Specialized nutrition	specialized nutrition	specialized nutrition	specialized nutrition	Specialized nutrition	1	kept
Specialized palliative homecare	specialized palliative homecare	specialized palliative homecare	specialized palliative homecare	Specialized palliative homecare	1	kept
Specialized unit	specialized unit	specialized unit	specialized unit	Specialized unit	1	kept

Specialties, Dental	specialties dental	specialties dental	specialties dental	Specialties, Dental	1	kept
SPECIALTY	specialty	specialty	specialty	specialty	1	case_or_format
Specialty	specialty	specialty	specialty	specialty	1	case_or_format
Specialty care	specialty care	specialty care	specialty care	Specialty care	1	kept
specialty choice	specialty choice	specialty choice	specialty choice	specialty choice	1	kept
specialty dementia care units	specialty dementia care units	specialty dementia care units	specialty dementia care units	specialty dementia care units	1	kept
specialty learning outcomes	specialty learning outcomes	specialty learning outcomes	specialty learning outcomes	specialty learning outcomes	1	kept
specialty pharmacy	specialty pharmacy	specialty pharmacy	specialty pharmacy	specialty pharmacy	1	kept
Specialty team	specialty team	specialty team	specialty team	Specialty team	1	kept
species	species	species	species	species	1	kept
species composition	species composition	species composition	species composition	species composition	1	kept
species diversity	species diversity	species diversity	species diversity	species diversity	1	kept
Specific competences	specific competences	specific competences	specific competences	Specific competences	1	kept
specific conditions	specific conditions	specific conditions	specific conditions	specific conditions	1	kept
Specific loads	specific loads	specific loads	specific loads	Specific loads	1	kept
Specific location	specific location	specific location	specific location	Specific location	1	kept

Specific professional training	specific professional training	specific professional training	specific professional training	Specific professional training	1	kept
Specific protection	specific protection	specific protection	specific protection	Specific protection	1	kept
Specific sensors	specific sensors	specific sensors	specific sensors	Specific sensors	1	kept
Specific training	specific training	specific training	specific training	Specific training	1	kept
Specifications	specifications	specifications	specifications	Specifications	1	kept
SPECIFICITY	specificity	specificity	specificity	specificity	1	case_or_format
Specimen rejection rate	specimen rejection rate	specimen rejection rate	specimen rejection rate	Specimen rejection rate	1	kept
spectrin	spectrin	spectrin	spectrin	spectrin	1	kept
spectrometer	spectrometer	spectrometer	spectrometer	spectrometer	1	kept
Spectroscopy, Fourier Transform Infrared	spectroscopy fourier transform infrared	spectroscopy fourier transform infrared	spectroscopy fourier transform infrared	Spectroscopy, Fourier Transform Infrared	1	kept
Spectrum Analysis	spectrum analysis	spectrum analysis	spectrum analysis	Spectrum Analysis	1	kept
SPECTRUM BETA-LACTAMASES	spectrum beta lactamases	spectrum beta lactamases	spectrum beta lactamase	SPECTRUM BETA-LACTAMASE	1	singular
SPEECH	speech	speech	speech	speech	1	case_or_format
Speech Acoustics	speech acoustics	speech acoustics	speech acoustics	Speech Acoustics	1	kept
Speech and language pathologist	speech and language pathologist	speech and language pathologist	speech and language pathologist	Speech and language pathologist	1	kept
Speech and language pathologists	speech and language pathologists	speech and language pathologists	speech and language pathologist	Speech and language pathologist	1	singular

Speech and nutritional rehabilitation	speech and nutritional rehabilitation	speech and nutritional rehabilitation	speech and nutritional rehabilitation	Speech and nutritional rehabilitation	1	kept
speech delay	speech delay	speech delay	speech delay	speech delay	1	kept
Speech disorders	speech disorders	speech disorders	speech disorder	speech disorder	1	singular
Speech enhancement	speech enhancement	speech enhancement	speech enhancement	Speech enhancement	1	kept
speech impairment	speech impairment	speech impairment	speech impairment	speech impairment	1	kept
Speech intelligibility	speech intelligibility	speech intelligibility	speech intelligibility	Speech Intelligibility	1	case_or_format
Speech language pathologists	speech language pathologists	speech language pathologists	speech language pathologist	speech language pathologist	1	singular
Speech pathology	speech pathology	speech pathology	speech pathology	Speech pathology	1	kept
speech pathology	speech pathology	speech pathology	speech pathology	Speech pathology	1	case_or_format
speech perception	speech perception	speech perception	speech perception	Speech Perception	1	case_or_format
Speech privacy	speech privacy	speech privacy	speech privacy	Speech privacy	1	kept
Speech privacy distance	speech privacy distance	speech privacy distance	speech privacy distance	Speech privacy distance	1	kept
SPEECH RECOGNITION	speech recognition	speech recognition	speech recognition	SPEECH RECOGNITION	1	kept
Speech Recognition Software	speech recognition software	speech recognition software	speech recognition software	Speech Recognition Software	1	kept
speech rehabilitation	speech rehabilitation	speech rehabilitation	speech rehabilitation	speech rehabilitation	1	kept
speech sound disorder	speech sound disorder	speech sound disorder	speech sound disorder	speech sound disorder	1	kept

speech therapist	speech therapist	speech therapist	speech therapist	speech therapist	1	kept
Speech transmission	speech transmission	speech transmission	speech transmission	Speech transmission	1	kept
Speech, Language and Hearing Sciences	speech language and hearing sciences	speech language and hearing sciences	speech language and hearing sciences	Speech, Language and Hearing Sciences	1	kept
speech-language pathologist	speech language pathologist	speech language pathologist	speech language pathologist	speech language pathologist	1	case_or_format
Speech-language pathologists	speech language pathologists	speech language pathologists	speech language pathologist	speech language pathologist	1	singular
speech-recognition	speech recognition	speech recognition	speech recognition	SPEECH RECOGNITION	1	case_or_format
Speech-to-text	speech to text	speech to text	speech to text	Speech-to-text	1	kept
SPEED	speed	speed	speed	SPEED	1	kept
sphenoid	sphenoid	sphenoid	sphenoid	sphenoid	1	kept
Sphenoid Sinus	sphenoid sinus	sphenoid sinus	sphenoid sinus	Sphenoid Sinus	1	kept
sphincteroplasty	sphincteroplasty	sphincteroplasty	sphincteroplasty	sphincteroplasty	1	kept
Sphingobacterium multivorum	sphingobacterium multivorum	sphingobacterium multivorum	sphingobacterium multivorum	Sphingobacterium multivorum	1	kept
Sphingomonas	sphingomonas	sphingomonas	sphingomonas	Sphingomonas	1	kept
Sphygmomanometers	sphygmomanometers	sphygmomanometers	sphygmomanometer	sphygmomanometer	1	singular
spice	spice	spice	spice	spice	1	kept
SPICE-R tool, simulation	spice r tool simulation	spice r tool simulation	spice r tool simulation	SPICE-R tool, simulation	1	kept

spider	spider	spider	spider	spider	1	kept
spider bite	spider bite	spider bite	spider bite	spider bite	1	kept
spider venom	spider venom	spider venom	spider venom	spider venom	1	kept
Spider Venoms	spider venoms	spider venoms	spider venom	spider venom	1	singular
Spike Glycoprotein, Coronavirus	spike glycoprotein coronavirus	spike glycoprotein coronavirus	spike glycoprotein coronavirus	Spike Glycoprotein, Coronavirus	1	kept
spina bifida	spina bifida	spina bifida	spina bifida	spina bifida	1	kept
Spinal abscess	spinal abscess	spinal abscess	spinal abscess	Spinal abscess	1	kept
spinal abscess	spinal abscess	spinal abscess	spinal abscess	Spinal abscess	1	case_or_format
spinal anaesthesia	spinal anaesthesia	spinal anaesthesia	spinal anaesthesia	spinal anaesthesia	1	kept
Spinal anesthesia	spinal anesthesia	spinal anesthesia	spinal anesthesia	spinal anesthesia	1	case_or_format
spinal cord atrophy	spinal cord atrophy	spinal cord atrophy	spinal cord atrophy	spinal cord atrophy	1	kept
spinal cord biopsy	spinal cord biopsy	spinal cord biopsy	spinal cord biopsy	spinal cord biopsy	1	kept
spinal cord blood flow	spinal cord blood flow	spinal cord blood flow	spinal cord blood flow	spinal cord blood flow	1	kept
Spinal cord compression	spinal cord compression	spinal cord compression	spinal cord compression	spinal cord compression	1	case_or_format
spinal cord dorsal horn	spinal cord dorsal horn	spinal cord dorsal horn	spinal cord dorsal horn	spinal cord dorsal horn	1	kept
Spinal cord impairment	spinal cord impairment	spinal cord impairment	spinal cord impairment	Spinal cord impairment	1	kept

spinal cord injury unit	spinal cord injury unit	spinal cord injury unit	spinal cord injury unit	spinal cord injury unit	1	kept
Spinal Cord Ischemia	spinal cord ischemia	spinal cord ischemia	spinal cord ischemia	spinal cord ischemia	1	case_or_format
Spinal cord ischemia	spinal cord ischemia	spinal cord ischemia	spinal cord ischemia	spinal cord ischemia	1	case_or_format
spinal cord nerve cell	spinal cord nerve cell	spinal cord nerve cell	spinal cord nerve cell	spinal cord nerve cell	1	kept
spinal cord perfusion pressure	spinal cord perfusion pressure	spinal cord perfusion pressure	spinal cord perfusion pressure	spinal cord perfusion pressure	1	kept
spinal cord services	spinal cord services	spinal cord services	spinal cord services	spinal cord services	1	kept
spinal cord tumor	spinal cord tumor	spinal cord tumor	spinal cord tumor	spinal cord tumor	1	kept
Spinal epidural abscess	spinal epidural abscess	spinal epidural abscess	spinal epidural abscess	Spinal epidural abscess	1	kept
Spinal fixation	spinal fixation	spinal fixation	spinal fixation	Spinal fixation	1	kept
Spinal fracture	spinal fracture	spinal fracture	spinal fracture	Spinal Fractures	1	case_or_format
spinal fracture	spinal fracture	spinal fracture	spinal fracture	Spinal Fractures	1	case_or_format
Spinal fractures	spinal fractures	spinal fractures	spinal fracture	Spinal Fractures	1	singular
spinal fusion surgery	spinal fusion surgery	spinal fusion surgery	spinal fusion surgery	spinal fusion surgery	1	kept
spinal ganglion	spinal ganglion	spinal ganglion	spinal ganglion	spinal ganglion	1	kept
Spinal immobilisation	spinal immobilisation	spinal immobilisation	spinal immobilisation	Spinal immobilisation	1	kept
Spinal infection	spinal infection	spinal infection	spinal infection	Spinal infection	1	kept

spinal innervation	spinal innervation	spinal innervation	spinal innervation	spinal innervation	1	kept
spinal instrumentation	spinal instrumentation	spinal instrumentation	spinal instrumentation	spinal instrumentation	1	kept
spinal nerve	spinal nerve	spinal nerve	spinal nerve	spinal nerve	1	kept
Spinal Nerves	spinal nerves	spinal nerves	spinal nerve	spinal nerve	1	singular
spinal shock	spinal shock	spinal shock	spinal shock	spinal shock	1	kept
spinal shock trauma	spinal shock trauma	spinal shock trauma	spinal shock trauma	spinal shock trauma	1	kept
spinal surgery complications	spinal surgery complications	spinal surgery complications	spinal surgery complications	spinal surgery complications	1	kept
spine disease	spine disease	spine disease	spine disease	spine disease	1	kept
spine fractures	spine fractures	spine fractures	spine fracture	spine fracture	1	singular
spine immobilisation	spine immobilisation	spine immobilisation	spine immobilisation	spine immobilisation	1	kept
spine instability	spine instability	spine instability	spine instability	spine instability	1	kept
spine metastasis	spine metastasis	spine metastasis	spine metastasis	spine metastasis	1	kept
Spine surgery	spine surgery	spine surgery	spine surgery	spine surgery	1	case_or_format
Spine trauma	spine trauma	spine trauma	spine trauma	Spine trauma	1	kept
spine trauma	spine trauma	spine trauma	spine trauma	Spine trauma	1	case_or_format
Spiperone	spiperone	spiperone	spiperone	Spiperone	1	kept

spiperone	spiperone	spiperone	spiperone	Spiperone	1	case_or_format
spiral computer assisted tomography	spiral computer assisted tomography	spiral computer assisted tomography	spiral computer assisted tomography	spiral computer assisted tomography	1	kept
Spiral nasal catheter blind insertion technique	spiral nasal catheter blind insertion technique	spiral nasal catheter blind insertion technique	spiral nasal catheter blind insertion technique	Spiral nasal catheter blind insertion technique	1	kept
spiritual	spiritual	spiritual	spiritual	Spiritual	1	case_or_format
Spiritual assessment	spiritual assessment	spiritual assessment	spiritual assessment	Spiritual assessment	1	kept
Spiritual attention	spiritual attention	spiritual attention	spiritual attention	Spiritual attention	1	kept
Spiritual Care	spiritual care	spiritual care	spiritual care	spiritual care	1	case_or_format
Spiritual care assessment	spiritual care assessment	spiritual care assessment	spiritual care assessment	Spiritual care assessment	1	kept
spiritual care assessment	spiritual care assessment	spiritual care assessment	spiritual care assessment	Spiritual care assessment	1	case_or_format
Spiritual care competence	spiritual care competence	spiritual care competence	spiritual care competence	Spiritual care competence	1	kept
Spiritual Care Competency	spiritual care competency	spiritual care competency	spiritual care competency	spiritual care competencies	1	case_or_format
Spiritual Care Competency Scale	spiritual care competency scale	spiritual care competency scale	spiritual care competency scale	Spiritual Care Competency Scale	1	kept
spiritual caregiving	spiritual caregiving	spiritual caregiving	spiritual caregiving	spiritual caregiving	1	kept
Spiritual caring behaviors	spiritual caring behaviors	spiritual caring behaviors	spiritual caring behaviors	Spiritual caring behaviors	1	kept
Spiritual Commitment	spiritual commitment	spiritual commitment	spiritual commitment	Spiritual Commitment	1	kept
spiritual coping	spiritual coping	spiritual coping	spiritual coping	spiritual coping	1	kept

spiritual distress	spiritual distress	spiritual distress	spiritual distress	spiritual distress	1	kept
Spiritual Health	spiritual health	spiritual health	spiritual health	Spiritual health	1	case_or_format
Spiritual Health Scale Short Form	spiritual health scale short form	spiritual health scale short form	spiritual health scale short form	Spiritual Health Scale Short Form	1	kept
spiritual intelligence	spiritual intelligence	spiritual intelligence	spiritual intelligence	Spiritual Intelligence	1	case_or_format
spiritual intelligence self report inventory	spiritual intelligence self report inventory	spiritual intelligence self report inventory	spiritual intelligence self report inventory	spiritual intelligence self report inventory	1	kept
Spiritual suffering	spiritual suffering	spiritual suffering	spiritual suffering	Spiritual suffering	1	kept
Spiritual therapies	spiritual therapies	spiritual therapies	spiritual therapies	Spiritual Therapies	1	case_or_format
spiritual well being questionnaire	spiritual well being questionnaire	spiritual well being questionnaire	spiritual well being questionnaire	spiritual well being questionnaire	1	kept
Spiritual Well-Being	spiritual well being	spiritual well being	spiritual well being	spiritual well-being	1	case_or_format
spiritualism	spiritualism	spiritualism	spiritualism	Spiritualism	1	case_or_format
SPIRITUALITY	spirituality	spirituality	spirituality	Spirituality	1	case_or_format
spirituality validation studies	spirituality validation studies	spirituality validation studies	spirituality validation studies	spirituality validation studies	1	kept
spirometer	spirometer	spirometer	spirometer	spirometer	1	kept
Spironolactone	spironolactone	spironolactone	spironolactone	spironolactone	1	case_or_format
spleen	spleen	spleen	spleen	Spleen	1	case_or_format
spleen disease	spleen disease	spleen disease	spleen disease	spleen disease	1	kept

Splenic	splenic	splenic	splenic	Splenic	1	kept
Splenic artery embolisation	splenic artery embolisation	splenic artery embolisation	splenic artery embolisation	Splenic artery embolisation	1	kept
Splenic artery rupture	splenic artery rupture	splenic artery rupture	splenic artery rupture	Splenic artery rupture	1	kept
Splenic Infarction	splenic infarction	splenic infarction	splenic infarction	Splenic Infarction	1	kept
splenic involvement	splenic involvement	splenic involvement	splenic involvement	splenic involvement	1	kept
Splenic Rupture	splenic rupture	splenic rupture	splenic rupture	Splenic Rupture	1	kept
splenium	splenium	splenium	splenium	splenium	1	kept
Splenomegaly	splenomegaly	splenomegaly	splenomegaly	splenomegaly	1	case_or_format
splicing defect	splicing defect	splicing defect	splicing defect	splicing defect	1	kept
Split flow	split flow	split flow	split flow	Split flow	1	kept
Split-flow triage process	split flow triage process	split flow triage process	split flow triage process	Split-flow triage process	1	kept
splitting	splitting	splitting	splitting	splitting	1	kept
SpO2 monitoring	spo2 monitoring	spo2 monitoring	spo2 monitoring	SpO2 monitoring	1	kept
SpO2 rate	spo2 rate	spo2 rate	spo2 rate	SpO2 rate	1	kept
SPO<sub>2<	spo sub 2	spo sub 2	spo sub 2	SPO<sub>2<	1	kept
spondylarthritis	spondylarthritis	spondylarthritis	spondylarthritis	spondylarthritis	1	kept

spondylitis	spondylitis	spondylitis	spondylitis	spondylitis	1	kept
spondylogenic pain syndrome	spondylogenic pain syndrome	spondylogenic pain syndrome	spondylogenic pain syndrome	spondylogenic pain syndrome	1	kept
spondylolisthesis	spondylolisthesis	spondylolisthesis	spondylolisthesis	Spondylolisthesis	1	case_or_format
spondyloptosis	spondyloptosis	spondyloptosis	spondyloptosis	spondyloptosis	1	kept
Sponge bath	sponge bath	sponge bath	sponge bath	Sponge bath	1	kept
Sponge bathing	sponge bathing	sponge bathing	sponge bathing	Sponge bathing	1	kept
SPONGES	sponges	sponges	sponges	SPONGES	1	kept
Spontaneous abortion	spontaneous abortion	spontaneous abortion	spontaneous abortion	spontaneous abortion	1	case_or_format
Spontaneous awakening	spontaneous awakening	spontaneous awakening	spontaneous awakening	Spontaneous awakening	1	kept
spontaneous awakening	spontaneous awakening	spontaneous awakening	spontaneous awakening	Spontaneous awakening	1	case_or_format
Spontaneous awakening trial	spontaneous awakening trial	spontaneous awakening trial	spontaneous awakening trial	spontaneous awakening trial	1	case_or_format
Spontaneous awakening trials	spontaneous awakening trials	spontaneous awakening trials	spontaneous awakening trial	spontaneous awakening trial	1	singular
spontaneous awakening trials	spontaneous awakening trials	spontaneous awakening trials	spontaneous awakening trial	spontaneous awakening trial	1	singular
spontaneous bacterial peritonitis	spontaneous bacterial peritonitis	spontaneous bacterial peritonitis	spontaneous bacterial peritonitis	spontaneous bacterial peritonitis	1	kept
spontaneous birth	spontaneous birth	spontaneous birth	spontaneous birth	spontaneous birth	1	kept
spontaneous breathing	spontaneous breathing	spontaneous breathing	spontaneous breathing	Spontaneous breathing	1	case_or_format

Spontaneous breathing trials	spontaneous breathing trials	spontaneous breathing trials	spontaneous breathing trial	Spontaneous breathing trial	1	singular
Spontaneous circulation	spontaneous circulation	spontaneous circulation	spontaneous circulation	Spontaneous circulation	1	kept
Spontaneous coronary artery dissection	spontaneous coronary artery dissection	spontaneous coronary artery dissection	spontaneous coronary artery dissection	Spontaneous coronary artery dissection	1	kept
Spontaneous hemothorax	spontaneous hemothorax	spontaneous hemothorax	spontaneous hemothorax	Spontaneous hemothorax	1	kept
spontaneous hemothorax	spontaneous hemothorax	spontaneous hemothorax	spontaneous hemothorax	Spontaneous hemothorax	1	case_or_format
Spontaneous intracerebral haemorrhage	spontaneous intracerebral haemorrhage	spontaneous intracerebral haemorrhage	spontaneous intracerebral haemorrhage	Spontaneous intracerebral haemorrhage	1	kept
spontaneous newborn skull fracture	spontaneous newborn skull fracture	spontaneous newborn skull fracture	spontaneous newborn skull fracture	spontaneous newborn skull fracture	1	kept
spontaneous preterm birth guideline	spontaneous preterm birth guideline	spontaneous preterm birth guideline	spontaneous preterm birth guideline	spontaneous preterm birth guideline	1	kept
spontaneously	spontaneously	spontaneously	spontaneously	spontaneously	1	kept
Spores	spores	spores	spores	Spores	1	kept
sporotrichosis	sporotrichosis	sporotrichosis	sporotrichosis	sporotrichosis	1	kept
sport facility	sport facility	sport facility	sport facility	sport facility	1	kept
sports drink	sports drink	sports drink	sports drink	sports drink	1	kept
Spotted Fever Group Rickettsiosis	spotted fever group rickettsiosis	spotted fever group rickettsiosis	spotted fever group rickettsiosis	Spotted Fever Group Rickettsiosis	1	kept
Spouse	spouse	spouse	spouse	Spouses	1	case_or_format
Spouse caregiver	spouse caregiver	spouse caregiver	spouse caregiver	Spouse caregiver	1	kept

SPREAD	spread	spread	spread	SPREAD	1	kept
Spreading Cortical Depression	spreading cortical depression	spreading cortical depression	spreading cortical depression	Spreading Cortical Depression	1	kept
spreading cortical depression	spreading cortical depression	spreading cortical depression	spreading cortical depression	Spreading Cortical Depression	1	case_or_format
Spreitzer	spreitzer	spreitzer	spreitzer	Spreitzer	1	kept
SpringerLink	springerlink	springerlink	springerlink	SpringerLink	1	kept
SPSS, Statistical Package for Social Sciences	spss statistical package for social sciences	spss statistical package for social sciences	spss statistical package for social sciences	SPSS, Statistical Package for Social Sciences	1	kept
sputum discoloration	sputum discoloration	sputum discoloration	sputum discoloration	sputum discoloration	1	kept
sputum induction	sputum induction	sputum induction	sputum induction	sputum induction	1	kept
Sputum suction	sputum suction	sputum suction	sputum suction	Sputum suction	1	kept
sputum suctioning care	sputum suctioning care	sputum suctioning care	sputum suctioning care	sputum suctioning care	1	kept
squamous cell	squamous cell	squamous cell	squamous cell	squamous cell	1	kept
Squamous Cell Carcinoma of Head and Neck	squamous cell carcinoma of head and neck	squamous cell carcinoma of head and neck	squamous cell carcinoma of head and neck	Squamous Cell Carcinoma of Head and Neck	1	kept
squamous cell lung carcinoma	squamous cell lung carcinoma	squamous cell lung carcinoma	squamous cell lung carcinoma	squamous cell lung carcinoma	1	kept
squid	squid	squid	squid	squid	1	kept
SRC STEMI Receiving Center	src stemi receiving center	src stemi receiving center	src stemi receiving center	SRC STEMI Receiving Center	1	kept
SRI-LANKA	sri lanka	sri lanka	sri lanka	Sri Lanka	1	case_or_format

Srinagarind hospital	srinagarind hospital	srinagarind hospital	srinagarind hospital	Srinagarind hospital	1	kept
SRNA	srna	srna	srna	SRNA	1	kept
SSE	sse	sse	sse	SSE	1	kept
ST analysis	st analysis	st analysis	st analysis	ST analysis	1	kept
ST elevation	st elevation	st elevation	st elevation	ST elevation	1	kept
St Josephs Healthcare Hamilton	st josephs healthcare hamilton	st josephs healthcare hamilton	st josephs healthcare hamilton	St Josephs Healthcare Hamilton	1	kept
ST segments	st segments	st segments	st segment	ST segment	1	singular
ST-segment monitoring	st segment monitoring	st segment monitoring	st segment monitoring	ST-segment monitoring	1	kept
St. George Respiratory Questionnaire	st george respiratory questionnaire	st george respiratory questionnaire	st george respiratory questionnaire	St. George Respiratory Questionnaire	1	kept
ST147	st147	st147	st147	ST147	1	kept
ST307 high-risk clone	st307 high risk clone	st307 high risk clone	st307 high risk clone	ST307 high-risk clone	1	kept
Stability	stability	stability	stability	STABILITY	1	case_or_format
stability	stability	stability	stability	STABILITY	1	case_or_format
Stability Test	stability test	stability test	stability test	Stability Test	1	kept
stabilization	stabilization	stabilization	stabilization	Stabilization	1	case_or_format
stabilization care	stabilization care	stabilization care	stabilization care	stabilization care	1	kept

stable isotope	stable isotope	stable isotope	stable isotope	stable isotope	1	kept
STABLE Program	stable program	stable program	stable program	STABLE Program	1	kept
stable sections	stable sections	stable sections	stable sections	stable sections	1	kept
Stacked ensemble learning	stacked ensemble learning	stacked ensemble learning	stacked ensemble learning	Stacked ensemble learning	1	kept
Staff and family education	staff and family education	staff and family education	staff and family education	Staff and family education	1	kept
staff attitude	staff attitude	staff attitude	staff attitude	staff attitudes	1	case_or_format
Staff attitudes	staff attitudes	staff attitudes	staff attitude	staff attitudes	1	singular
Staff availability and attitude in geriatric facilities	staff availability and attitude in geriatric facilities	staff availability and attitude in geriatric facilities	staff availability and attitude in geriatric facilities	Staff availability and attitude in geriatric facilities	1	kept
Staff awareness	staff awareness	staff awareness	staff awareness	staff awareness	1	case_or_format
Staff behavioural change	staff behavioural change	staff behavioural change	staff behavioural change	Staff behavioural change	1	kept
STAFF BURNOUT	staff burnout	staff burnout	staff burnout	STAFF BURNOUT	1	kept
Staff burnout	staff burnout	staff burnout	staff burnout	STAFF BURNOUT	1	case_or_format
staff burnout	staff burnout	staff burnout	staff burnout	STAFF BURNOUT	1	case_or_format
Staff cost	staff cost	staff cost	staff cost	Staff cost	1	kept
Staff empowerment	staff empowerment	staff empowerment	staff empowerment	staff empowerment	1	case_or_format
Staff engagement	staff engagement	staff engagement	staff engagement	Staff engagement	1	kept

STAFF EXPERIENCE	staff experience	staff experience	staff experience	STAFF EXPERIENCE	1	kept
staff experience	staff experience	staff experience	staff experience	STAFF EXPERIENCE	1	case_or_format
Staff experiences	staff experiences	staff experiences	staff experience	STAFF EXPERIENCE	1	singular
Staff health	staff health	staff health	staff health	staff health	1	case_or_format
staff interaction and teamwork	staff interaction and teamwork	staff interaction and teamwork	staff interaction and teamwork	staff interaction and teamwork	1	kept
STAFF KNOWLEDGE	staff knowledge	staff knowledge	staff knowledge	STAFF KNOWLEDGE	1	kept
Staff movement	staff movement	staff movement	staff movement	Staff movement	1	kept
Staff Observation Aggression Scale Revised	staff observation aggression scale revised	staff observation aggression scale revised	staff observation aggression scale revised	Staff Observation Aggression Scale Revised	1	kept
staff observation aggression scale revised	staff observation aggression scale revised	staff observation aggression scale revised	staff observation aggression scale revised	Staff Observation Aggression Scale Revised	1	case_or_format
staff perception	staff perception	staff perception	staff perception	Staff perception	1	case_or_format
Staff perceptions and attitudes	staff perceptions and attitudes	staff perceptions and attitudes	staff perceptions and attitudes	Staff perceptions and attitudes	1	kept
staff performance	staff performance	staff performance	staff performance	staff performance	1	kept
Staff perspectives	staff perspectives	staff perspectives	staff perspectives	staff perspectives	1	case_or_format
Staff reallocation	staff reallocation	staff reallocation	staff reallocation	Staff reallocation	1	kept
Staff recruitment	staff recruitment	staff recruitment	staff recruitment	Staff recruitment	1	kept
staff reflection	staff reflection	staff reflection	staff reflection	staff reflection	1	kept

staff restroom	staff restroom	staff restroom	staff restroom	staff restroom	1	kept
Staff retention	staff retention	staff retention	staff retention	staff retention	1	case_or_format
staff satisfaction questionnaire	staff satisfaction questionnaire	staff satisfaction questionnaire	staff satisfaction questionnaire	staff satisfaction questionnaire	1	kept
staff shortage	staff shortage	staff shortage	staff shortage	staff shortage	1	kept
Staff shortages	staff shortages	staff shortages	staff shortage	staff shortage	1	singular
staff shortages	staff shortages	staff shortages	staff shortage	staff shortage	1	singular
Staff sizing	staff sizing	staff sizing	staff sizing	Staff sizing	1	kept
staff stress	staff stress	staff stress	staff stress	staff stress	1	kept
STAFF TIME	staff time	staff time	staff time	STAFF TIME	1	kept
Staff trajectory	staff trajectory	staff trajectory	staff trajectory	Staff trajectory	1	kept
STAFF TURNOVER	staff turnover	staff turnover	staff turnover	Staff turnover	1	case_or_format
staff turnover	staff turnover	staff turnover	staff turnover	Staff turnover	1	case_or_format
Staff views	staff views	staff views	staff views	Staff views	1	kept
staff well being	staff well being	staff well being	staff well being	Staff well-being	1	case_or_format
staff well‐being	staff well being	staff well being	staff well being	Staff well-being	1	case_or_format
Staff work area	staff work area	staff work area	staff work area	Staff work area	1	kept

Staff workload	staff workload	staff workload	staff workload	STAFF WORKLOAD	1	case_or_format
staff-support	staff support	staff support	staff support	Staff support	1	case_or_format
Staffing adequacy	staffing adequacy	staffing adequacy	staffing adequacy	staffing adequacy	1	case_or_format
staffing and scheduling	staffing and scheduling	staffing and scheduling	staffing and scheduling	staffing and scheduling	1	kept
Staffing features	staffing features	staffing features	staffing features	Staffing features	1	kept
Staffing level	staffing level	staffing level	staffing level	STAFFING LEVELS	1	case_or_format
Staffing model	staffing model	staffing model	staffing model	staffing models	1	case_or_format
Staffing needs	staffing needs	staffing needs	staffing needs	Staffing needs	1	kept
STAFFING RATIOS	staffing ratios	staffing ratios	staffing ratio	staffing ratio	1	singular
staffing ratios	staffing ratios	staffing ratios	staffing ratio	staffing ratio	1	singular
staffing skill mix	staffing skill mix	staffing skill mix	staffing skill mix	staffing skill mix	1	kept
Staffing strategies	staffing strategies	staffing strategies	staffing strategies	Staffing strategies	1	kept
STAFFS KNOWLEDGE	staffs knowledge	staffs knowledge	staffs knowledge	STAFFS KNOWLEDGE	1	kept
stage	stage	stage	stage	STAGE	1	case_or_format
stage 1 kidney disease	stage 1 kidney disease	stage 1 kidney disease	stage 1 kidney disease	stage 1 kidney disease	1	kept
stage clinique	stage clinique	stage clinique	stage clinique	stage clinique	1	kept

staged surgical approach	staged surgical approach	staged surgical approach	staged surgical approach	staged surgical approach	1	kept
Staining and Labeling	staining and labeling	staining and labeling	staining and labeling	Staining and Labeling	1	kept
Stainless Steel	stainless steel	stainless steel	stainless steel	Stainless Steel	1	kept
stainless steel	stainless steel	stainless steel	stainless steel	Stainless Steel	1	case_or_format
Stakeholder	stakeholder	stakeholder	stakeholder	Stakeholders	1	case_or_format
Stakeholder analysis	stakeholder analysis	stakeholder analysis	stakeholder analysis	Stakeholder analysis	1	kept
Stakeholder engagement	stakeholder engagement	stakeholder engagement	stakeholder engagement	stakeholder engagement	1	case_or_format
stakeholder interview	stakeholder interview	stakeholder interview	stakeholder interview	stakeholder interview	1	kept
Stakeholder involvement	stakeholder involvement	stakeholder involvement	stakeholder involvement	Stakeholder involvement	1	kept
Stakeholders' views	stakeholders views	stakeholders views	stakeholders views	Stakeholders' views	1	kept
stance with eyes and functional reach test	stance with eyes and functional reach test	stance with eyes and functional reach test	stance with eyes and functional reach test	stance with eyes and functional reach test	1	kept
Standard algorithms	standard algorithms	standard algorithms	standard algorithms	Standard algorithms	1	kept
standard breastfeeding performance questionnaire	standard breastfeeding performance questionnaire	standard breastfeeding performance questionnaire	standard breastfeeding performance questionnaire	standard breastfeeding performance questionnaire	1	kept
standard concentration	standard concentration	standard concentration	standard concentration	standard concentration	1	kept
Standard concentrations	standard concentrations	standard concentrations	standard concentration	standard concentration	1	singular
standard concentrations	standard concentrations	standard concentrations	standard concentration	standard concentration	1	singular

standard deviation	standard deviation	standard deviation	standard deviation	Standard deviation	1	case_or_format
standard hygiene precaution	standard hygiene precaution	standard hygiene precaution	standard hygiene precaution	standard hygiene precaution	1	kept
Standard hygiene precautions	standard hygiene precautions	standard hygiene precautions	standard hygiene precaution	standard hygiene precaution	1	singular
Standard infusion	standard infusion	standard infusion	standard infusion	Standard infusion	1	kept
Standard mattress	standard mattress	standard mattress	standard mattress	Standard mattress	1	kept
standard of practice	standard of practice	standard of practice	standard of practice	standard of practice	1	kept
Standard of quality	standard of quality	standard of quality	standard of quality	Standard of quality	1	kept
Standard Operating Procedure	standard operating procedure	standard operating procedure	standard operating procedure	standard operating procedure	1	case_or_format
Standard operating procedures	standard operating procedures	standard operating procedures	standard operating procedure	standard operating procedure	1	singular
standard operational protocol	standard operational protocol	standard operational protocol	standard operational protocol	standard operational protocol	1	kept
Standard operative procedures	standard operative procedures	standard operative procedures	standard operative procedures	Standard operative procedures	1	kept
standard operative protocol	standard operative protocol	standard operative protocol	standard operative protocol	standard operative protocol	1	kept
Standard practice	standard practice	standard practice	standard practice	Standard practice	1	kept
Standard practices	standard practices	standard practices	standard practice	Standard practice	1	singular
Standard protocols	standard protocols	standard protocols	standard protocols	Standard protocols	1	kept
Standard Shiftwork Index	standard shiftwork index	standard shiftwork index	standard shiftwork index	Standard Shiftwork Index	1	kept

Standard system	standard system	standard system	standard system	Standard system	1	kept
standard time utilization	standard time utilization	standard time utilization	standard time utilization	standard time utilization	1	kept
Standardisation	standardisation	standardisation	standardisation	Standardisation	1	kept
standardisation	standardisation	standardisation	standardisation	Standardisation	1	case_or_format
standardised care	standardised care	standardised care	standardised care	standardised care	1	kept
standardised care plan	standardised care plan	standardised care plan	standardised care plan	standardised care plan	1	kept
standardised data collection tool	standardised data collection tool	standardised data collection tool	standardised data collection tool	standardised data collection tool	1	kept
Standardised framework	standardised framework	standardised framework	standardised framework	Standardised framework	1	kept
standardize	standardize	standardize	standardize	standardize	1	kept
standardized	standardized	standardized	standardized	standardized	1	kept
Standardized care	standardized care	standardized care	standardized care	Standardized care	1	kept
Standardized care plan	standardized care plan	standardized care plan	standardized care plan	Standardized care plan	1	kept
standardized care plan	standardized care plan	standardized care plan	standardized care plan	Standardized care plan	1	case_or_format
standardized handoff tool	standardized handoff tool	standardized handoff tool	standardized handoff tool	standardized handoff tool	1	kept
Standardized infection ratio	standardized infection ratio	standardized infection ratio	standardized infection ratio	Standardized infection ratio	1	kept
standardized infection ratio	standardized infection ratio	standardized infection ratio	standardized infection ratio	Standardized infection ratio	1	case_or_format

standardized label	standardized label	standardized label	standardized label	standardized label	1	kept
Standardized mortality ratio	standardized mortality ratio	standardized mortality ratio	standardized mortality ratio	standardized mortality ratio	1	case_or_format
standardized operational procedure	standardized operational procedure	standardized operational procedure	standardized operational procedure	standardized operational procedure	1	kept
Standardized protocol	standardized protocol	standardized protocol	standardized protocol	Standardized protocol	1	kept
standardized protocol	standardized protocol	standardized protocol	standardized protocol	Standardized protocol	1	case_or_format
standardized protocols	standardized protocols	standardized protocols	standardized protocol	Standardized protocol	1	singular
standardized questionnaire	standardized questionnaire	standardized questionnaire	standardized questionnaire	standardized questionnaire	1	kept
Standardized testing	standardized testing	standardized testing	standardized testing	Standardized testing	1	kept
standardized testing	standardized testing	standardized testing	standardized testing	Standardized testing	1	case_or_format
standardized titration protocol	standardized titration protocol	standardized titration protocol	standardized titration protocol	standardized titration protocol	1	kept
standardized tool	standardized tool	standardized tool	standardized tool	standardized tool	1	kept
standardized training	standardized training	standardized training	standardized training	Standardized training	1	case_or_format
Standardized utilization ratio	standardized utilization ratio	standardized utilization ratio	standardized utilization ratio	Standardized utilization ratio	1	kept
standards for reporting qualitative research 21 item	standards for reporting qualitative research 21 item	standards for reporting qualitative research 21 item	standards for reporting qualitative research 21 item	standards for reporting qualitative research 21 item	1	kept
standards of practice	standards of practice	standards of practice	standards of practice	standards of practice	1	kept
Standing Position	standing position	standing position	standing position	Standing Position	1	kept

standing posture	standing posture	standing posture	standing posture	standing posture	1	kept
Stanford presenteeism scale-6	stanford presenteeism scale 6	stanford presenteeism scale 6	stanford presenteeism scale 6	Stanford presenteeism scale-6	1	kept
Stanford proxy test for delirium	stanford proxy test for delirium	stanford proxy test for delirium	stanford proxy test for delirium	Stanford proxy test for delirium	1	kept
Stanford type A aortic dissection	stanford type a aortic dissection	stanford type a aortic dissection	stanford type a aortic dissection	Stanford type A aortic dissection	1	kept
Staphylococcal infections	staphylococcal infections	staphylococcal infections	staphylococcal infections	Staphylococcal Infections	1	case_or_format
staphylococcal scald skin syndrome	staphylococcal scald skin syndrome	staphylococcal scald skin syndrome	staphylococcal scald skin syndrome	staphylococcal scald skin syndrome	1	kept
staphylococcal scalded skin syndrome	staphylococcal scalded skin syndrome	staphylococcal scalded skin syndrome	staphylococcal scalded skin syndrome	staphylococcal scalded skin syndrome	1	kept
Staphylococcal Skin Infections	staphylococcal skin infections	staphylococcal skin infections	staphylococcal skin infections	Staphylococcal Skin Infections	1	kept
staphylococcal toxic shock syndrome	staphylococcal toxic shock syndrome	staphylococcal toxic shock syndrome	staphylococcal toxic shock syndrome	staphylococcal toxic shock syndrome	1	kept
Staphylococci	staphylococci	staphylococci	staphylococci	Staphylococci	1	kept
Staphylococcus acidominimus	staphylococcus acidominimus	staphylococcus acidominimus	staphylococcus acidominimus	Staphylococcus acidominimus	1	kept
Staphylococcus Aureus	staphylococcus aureus	staphylococcus aureus	staphylococcus aureus	Staphylococcus aureus	1	case_or_format
Staphylococcus aureus colonization	staphylococcus aureus colonization	staphylococcus aureus colonization	staphylococcus aureus colonization	Staphylococcus aureus colonization	1	kept
Staphylococcus caprae	staphylococcus caprae	staphylococcus caprae	staphylococcus caprae	Staphylococcus caprae	1	kept
Staphylococcus cohini	staphylococcus cohini	staphylococcus cohini	staphylococcus cohini	Staphylococcus cohini	1	kept
Staphylococcus epidermidis infection	staphylococcus epidermidis infection	staphylococcus epidermidis infection	staphylococcus epidermidis infection	Staphylococcus epidermidis infection	1	kept

Staphylococcus pseudintermedius	staphylococcus pseudintermedius	staphylococcus pseudintermedius	staphylococcus pseudintermedius	Staphylococcus pseudintermedius	1	kept
Staphylococcus schleiferi	staphylococcus schleiferi	staphylococcus schleiferi	staphylococcus schleiferi	Staphylococcus schleiferi	1	kept
Staphylococcus spp	staphylococcus spp	staphylococcus spp	staphylococcus spp	Staphylococcus spp	1	kept
Staphylococcus warneri	staphylococcus warneri	staphylococcus warneri	staphylococcus warneri	Staphylococcus warneri	1	kept
Starch	starch	starch	starch	Starch	1	kept
START	start	start	start	START	1	kept
startle epilepsy	startle epilepsy	startle epilepsy	startle epilepsy	startle epilepsy	1	kept
Starvation	starvation	starvation	starvation	starvation	1	case_or_format
STATA, Statistical software for data science	stata statistical software for data science	stata statistical software for data science	stata statistical software for data science	STATA, Statistical software for data science	1	kept
State anxiety	state anxiety	state anxiety	state anxiety	State anxiety	1	kept
state anxiety	state anxiety	state anxiety	state anxiety	State anxiety	1	case_or_format
state bans	state bans	state bans	state bans	state bans	1	kept
State behavioral scale	state behavioral scale	state behavioral scale	state behavioral scale	State Behavioral Scale	1	case_or_format
State classification	state classification	state classification	state classification	State classification	1	kept
State Health Plans	state health plans	state health plans	state health plans	State Health Plans	1	kept
State of body function	state of body function	state of body function	state of body function	State of body function	1	kept

state of consciousness	state of consciousness	state of consciousness	state of consciousness	state of consciousness	1	kept
State Police Aviation Division	state police aviation division	state police aviation division	state police aviation division	State Police Aviation Division	1	kept
state-space collapse	state space collapse	state space collapse	state space collapse	state-space collapse	1	kept
State-Trait Anxiety Inventory	state trait anxiety inventory	state trait anxiety inventory	state trait anxiety inventory	State Trait Anxiety Inventory	1	case_or_format
STATEMENT	statement	statement	statement	STATEMENT	1	kept
States	states	states	state	STATES	1	singular
static air support surfaces	static air support surfaces	static air support surfaces	static air support surfaces	static air support surfaces	1	kept
Static factors	static factors	static factors	static factors	Static factors	1	kept
statine	statine	statine	statine	statine	1	kept
STATINS	statins	statins	statins	Statins	1	case_or_format
Statistical analysis plan	statistical analysis plan	statistical analysis plan	statistical analysis plan	Statistical analysis plan	1	kept
statistical concepts	statistical concepts	statistical concepts	statistical concepts	statistical concepts	1	kept
statistical data analysis	statistical data analysis	statistical data analysis	statistical data analysis	statistical data analysis	1	kept
STATISTICAL DISTRIBUTIONS	statistical distributions	statistical distributions	statistical distributions	STATISTICAL DISTRIBUTIONS	1	kept
Statistical Factor Analysis	statistical factor analysis	statistical factor analysis	statistical factor analysis	Statistical Factor Analysis	1	kept
Statistical methods	statistical methods	statistical methods	statistical methods	Statistical methods	1	kept

Statistical models	statistical models	statistical models	statistical model	statistical model	1	singular
statistical process control	statistical process control	statistical process control	statistical process control	statistical process control	1	kept
Statistically significant difference	statistically significant difference	statistically significant difference	statistically significant difference	Statistically significant difference	1	kept
Statistics & numerical data	statistics and numerical data	statistics and numerical data	statistics and numerical data	statistics and numerical data	1	case_or_format
statistics & research methods	statistics and research methods	statistics and research methods	statistics and research methods	STATISTICS & RESEARCH METHODS	1	case_or_format
statistics and research methods	statistics and research methods	statistics and research methods	statistics and research methods	STATISTICS & RESEARCH METHODS	1	case_or_format
Status of waters	status of waters	status of waters	status of waters	Status of waters	1	kept
status survey	status survey	status survey	status survey	status survey	1	kept
Status update	status update	status update	status update	Status update	1	kept
stay-at-home order	stay at home order	stay at home order	stay at home order	stay-at-home order	1	kept
staying home	staying home	staying home	staying home	staying home	1	kept
Steady state	steady state	steady state	steady state	steady state	1	case_or_format
Steam	steam	steam	steam	Steam	1	kept
STEC-HUS	stec hus	stec hus	stec hus	STEC-HUS	1	kept
Steelmaking	steelmaking	steelmaking	steelmaking	Steelmaking	1	kept
Stem cell	stem cell	stem cell	stem cell	Stem cell	1	kept

stem cell	stem cell	stem cell	stem cell	Stem cell	1	case_or_format
Stem Cells	stem cells	stem cells	stem cell	Stem cell	1	singular
STEM-CELL TRANSPLANTATION	stem cell transplantation	stem cell transplantation	stem cell transplantation	stem cell transplantation	1	case_or_format
STEMI ST-elevation myocardial infarction	stemi st elevation myocardial infarction	stemi st elevation myocardial infarction	stemi st elevation myocardial infarction	STEMI ST-elevation myocardial infarction	1	kept
Stenosis	stenosis	stenosis	stenosis	STENOSIS	1	case_or_format
Stenosis, Pulmonary Vein	stenosis pulmonary vein	stenosis pulmonary vein	stenosis pulmonary vein	Stenosis, Pulmonary Vein	1	kept
Stenotrophomonas maltophila	stenotrophomonas maltophila	stenotrophomonas maltophila	stenotrophomonas maltophila	Stenotrophomonas maltophila	1	kept
stent implantation	stent implantation	stent implantation	stent implantation	stent implantation	1	kept
stent migration	stent migration	stent migration	stent migration	stent migration	1	kept
stent thrombosis	stent thrombosis	stent thrombosis	stent thrombosis	stent thrombosis	1	kept
Stent-retrieval devices	stent retrieval devices	stent retrieval devices	stent retrieval devices	Stent-retrieval devices	1	kept
Stent-retriever	stent retriever	stent retriever	stent retriever	Stent-retriever	1	kept
Step down unit	step down unit	step down unit	step down unit	step down unit	1	case_or_format
Step wedge randomised control trial	step wedge randomised control trial	step wedge randomised control trial	step wedge randomised control trial	Step wedge randomised control trial	1	kept
step wise multiple regression	step wise multiple regression	step wise multiple regression	step wise multiple regression	step wise multiple regression	1	kept
step-by-step care delegation	step by step care delegation	step by step care delegation	step by step care delegation	step-by-step care delegation	1	kept

Step-down	step down	step down	step down	Step-down	1	kept
step-down	step down	step down	step down	Step-down	1	case_or_format
Step-down beds	step down beds	step down beds	step down beds	Step-down beds	1	kept
step-down unit	step down unit	step down unit	step down unit	step down unit	1	case_or_format
Step-down units	step down units	step down units	step down unit	step down unit	1	singular
stepdown care	stepdown care	stepdown care	stepdown care	stepdown care	1	kept
Stepdown unit	stepdown unit	stepdown unit	stepdown unit	Stepdown unit	1	kept
Stepped wedge randomized controlled trial	stepped wedge randomized controlled trial	stepped wedge randomized controlled trial	stepped wedge randomized controlled trial	Stepped wedge randomized controlled trial	1	kept
Stepped wedge trials	stepped wedge trials	stepped wedge trials	stepped wedge trials	Stepped wedge trials	1	kept
Stepped-wedge cluster randomised trials	stepped wedge cluster randomised trials	stepped wedge cluster randomised trials	stepped wedge cluster randomised trial	Stepped-wedge cluster-randomised trial	1	singular
Steps	steps	steps	steps	Steps	1	kept
stereolithography	stereolithography	stereolithography	stereolithography	stereolithography	1	kept
stereotactic procedure	stereotactic procedure	stereotactic procedure	stereotactic procedure	stereotactic procedure	1	kept
Sterile barrier	sterile barrier	sterile barrier	sterile barrier	Sterile barrier	1	kept
STERILE BARRIER PRECAUTIONS	sterile barrier precautions	sterile barrier precautions	sterile barrier precautions	STERILE BARRIER PRECAUTIONS	1	kept
Sterile compounding	sterile compounding	sterile compounding	sterile compounding	Sterile compounding	1	kept

sterile cotton swab	sterile cotton swab	sterile cotton swab	sterile cotton swab	sterile cotton swab	1	kept
sterile glove	sterile glove	sterile glove	sterile glove	sterile glove	1	kept
Sterile practices	sterile practices	sterile practices	sterile practices	Sterile practices	1	kept
Sterile reprocessing	sterile reprocessing	sterile reprocessing	sterile reprocessing	Sterile reprocessing	1	kept
Sterile technique	sterile technique	sterile technique	sterile technique	Sterile technique	1	kept
Sterile tubing change	sterile tubing change	sterile tubing change	sterile tubing change	Sterile tubing change	1	kept
Sternal non-union	sternal non union	sternal non union	sternal non union	Sternal non-union	1	kept
sternal region	sternal region	sternal region	sternal region	sternal region	1	kept
Sternal wound infections	sternal wound infections	sternal wound infections	sternal wound infections	Sternal wound infections	1	kept
sternotomy pain	sternotomy pain	sternotomy pain	sternotomy pain	sternotomy pain	1	kept
steroid hormone	steroid hormone	steroid hormone	steroid hormone	steroid hormone	1	kept
STESS	stess	stess	stess	STESS	1	kept
stethoscopes	stethoscopes	stethoscopes	stethoscope	Stethoscopes	1	singular
Stethoscopes decontamination	stethoscopes decontamination	stethoscopes decontamination	stethoscopes decontamination	Stethoscopes decontamination	1	kept
Stevens - Johnson syndrome	stevens johnson syndrome	stevens johnson syndrome	stevens johnson syndrome	Stevens-Johnson Syndrome	1	case_or_format
stewarding	stewarding	stewarding	stewarding	stewarding	1	kept

STEWARDSHIP	stewardship	stewardship	stewardship	stewardship	1	case_or_format
stewardship intervention program	stewardship intervention program	stewardship intervention program	stewardship intervention program	stewardship intervention program	1	kept
STEWARDSHIP PROGRAM	stewardship program	stewardship program	stewardship program	STEWARDSHIP PROGRAM	1	kept
STG	stg	stg	stg	STG	1	kept
Sti	sti	sti	sti	Sti	1	kept
stiff neck	stiff neck	stiff neck	stiff neck	stiff neck	1	kept
stigmatization	stigmatization	stigmatization	stigmatization	stigmatization	1	kept
STIMULATION	stimulation	stimulation	stimulation	stimulation	1	case_or_format
Stimulation	stimulation	stimulation	stimulation	stimulation	1	case_or_format
stimulation frequency	stimulation frequency	stimulation frequency	stimulation frequency	stimulation frequency	1	kept
stimuli reduction	stimuli reduction	stimuli reduction	stimuli reduction	stimuli reduction	1	kept
stimulus induced rhythmic periodic or ictal discharge	stimulus induced rhythmic periodic or ictal discharge	stimulus induced rhythmic periodic or ictal discharge	stimulus induced rhythmic periodic or ictal discharge	stimulus induced rhythmic periodic or ictal discharge	1	kept
Stimulus-induced seizures	stimulus induced seizures	stimulus induced seizures	stimulus induced seizures	Stimulus-induced seizures	1	kept
stinging sensation	stinging sensation	stinging sensation	stinging sensation	stinging sensation	1	kept
stochastic gradient descent	stochastic gradient descent	stochastic gradient descent	stochastic gradient descent	stochastic gradient descent	1	kept
Stochastic settings	stochastic settings	stochastic settings	stochastic settings	Stochastic settings	1	kept

Stochastic targeted protocol	stochastic targeted protocol	stochastic targeted protocol	stochastic targeted protocol	Stochastic targeted protocol	1	kept
stochasticity	stochasticity	stochasticity	stochasticity	stochasticity	1	kept
Stochastics	stochastics	stochastics	stochastics	Stochastics	1	kept
Stock management	stock management	stock management	stock management	Stock management	1	kept
Stocks in hospitals	stocks in hospitals	stocks in hospitals	stocks in hospitals	Stocks in hospitals	1	kept
stoicism	stoicism	stoicism	stoicism	stoicism	1	kept
stoma cleaning	stoma cleaning	stoma cleaning	stoma cleaning	stoma cleaning	1	kept
Stoma management	stoma management	stoma management	stoma management	Stoma management	1	kept
stomach acid secretion	stomach acid secretion	stomach acid secretion	stomach acid secretion	stomach acid secretion	1	kept
stomach adenocarcinoma	stomach adenocarcinoma	stomach adenocarcinoma	stomach adenocarcinoma	stomach adenocarcinoma	1	kept
stomach biopsy	stomach biopsy	stomach biopsy	stomach biopsy	stomach biopsy	1	kept
stomach discomfort	stomach discomfort	stomach discomfort	stomach discomfort	stomach discomfort	1	kept
stomach function	stomach function	stomach function	stomach function	stomach function	1	kept
Stomach inflation	stomach inflation	stomach inflation	stomach inflation	Stomach inflation	1	kept
stomach mucosa lesion	stomach mucosa lesion	stomach mucosa lesion	stomach mucosa lesion	stomach mucosa lesion	1	kept
stomach muscle	stomach muscle	stomach muscle	stomach muscle	stomach muscle	1	kept

Stomach neoplasms	stomach neoplasms	stomach neoplasms	stomach neoplasms	Stomach Neoplasms	1	case_or_format
stomach polyp	stomach polyp	stomach polyp	stomach polyp	stomach polyp	1	kept
stomach protection	stomach protection	stomach protection	stomach protection	stomach protection	1	kept
stomach residual volume	stomach residual volume	stomach residual volume	stomach residual volume	stomach residual volume	1	kept
stomach secretion	stomach secretion	stomach secretion	stomach secretion	stomach secretion	1	kept
stomach transposition	stomach transposition	stomach transposition	stomach transposition	stomach transposition	1	kept
Stomach Volvulus	stomach volvulus	stomach volvulus	stomach volvulus	Stomach Volvulus	1	kept
stomach volvulus	stomach volvulus	stomach volvulus	stomach volvulus	Stomach Volvulus	1	case_or_format
stone-free rate	stone free rate	stone free rate	stone free rate	stone-free rate	1	kept
stool	stool	stool	stool	stool	1	kept
Stool studies	stool studies	stool studies	stool studies	Stool studies	1	kept
stop codon	stop codon	stop codon	stop codon	stop codon	1	kept
STOP-Bang questionnaire	stop bang questionnaire	stop bang questionnaire	stop bang questionnaire	STOP-Bang questionnaire	1	kept
Stop-Bang Questionnaire	stop bang questionnaire	stop bang questionnaire	stop bang questionnaire	STOP-Bang questionnaire	1	case_or_format
stopcock	stopcock	stopcock	stopcock	stopcock	1	kept
stopcock test	stopcock test	stopcock test	stopcock test	stopcock test	1	kept

STOPP criteria	stopp criteria	stopp criteria	stopp criteria	STOPP criteria	1	kept
Storage	storage	storage	storage	storage	1	case_or_format
storage temperature	storage temperature	storage temperature	storage temperature	storage temperature	1	kept
Stories	stories	stories	stories	Stories	1	kept
STORY COMPREHENSION	story comprehension	story comprehension	story comprehension	STORY COMPREHENSION	1	kept
storyboarding	storyboarding	storyboarding	storyboarding	storyboarding	1	kept
Storytelling	storytelling	storytelling	storytelling	storytelling	1	case_or_format
STP = Safe Transitions Pathway	stp safe transitions pathway	stp safe transitions pathway	stp safe transitions pathway	STP = Safe Transitions Pathway	1	kept
Strabismus	strabismus	strabismus	strabismus	Strabismus	1	kept
straight sinus	straight sinus	straight sinus	straight sinus	straight sinus	1	kept
STRAIN	strain	strain	strain	Strain	1	case_or_format
strain difference	strain difference	strain difference	strain difference	strain difference	1	kept
Strain of Care for Delirium Index	strain of care for delirium index	strain of care for delirium index	strain of care for delirium index	Strain of Care for Delirium Index	1	kept
Strait jacket	strait jacket	strait jacket	strait jacket	Strait jacket	1	kept
Strategic Behavior	strategic behavior	strategic behavior	strategic behavior	Strategic Behavior	1	kept
Strategic development	strategic development	strategic development	strategic development	Strategic development	1	kept

Strategic diagram	strategic diagram	strategic diagram	strategic diagram	Strategic diagram	1	kept
strategic plan	strategic plan	strategic plan	strategic plan	strategic plan	1	kept
Strategic Planning	strategic planning	strategic planning	strategic planning	strategic planning	1	case_or_format
strategic resource allocation	strategic resource allocation	strategic resource allocation	strategic resource allocation	strategic resource allocation	1	kept
Strategies Used by People to Promote Health Scale	strategies used by people to promote health scale	strategies used by people to promote health scale	strategies used by people to promote health scale	Strategies Used by People to Promote Health Scale	1	kept
Strategy	strategy	strategy	strategy	STRATEGIES	1	case_or_format
STRATIFICATION	stratification	stratification	stratification	stratification	1	case_or_format
stratum corneum	stratum corneum	stratum corneum	stratum corneum	stratum corneum	1	kept
stray dog	stray dog	stray dog	stray dog	stray dog	1	kept
Street crimes	street crimes	street crimes	street crimes	Street crimes	1	kept
Street traffic control	street traffic control	street traffic control	street traffic control	Street traffic control	1	kept
streptococcal bacteremia	streptococcal bacteremia	streptococcal bacteremia	streptococcal bacteremia	streptococcal bacteremia	1	kept
streptococcal toxic shock syndrome	streptococcal toxic shock syndrome	streptococcal toxic shock syndrome	streptococcal toxic shock syndrome	streptococcal toxic shock syndrome	1	kept
Streptococcus dysgalactiae	streptococcus dysgalactiae	streptococcus dysgalactiae	streptococcus dysgalactiae	Streptococcus dysgalactiae	1	kept
Streptococcus group G	streptococcus group g	streptococcus group g	streptococcus group g	Streptococcus group G	1	kept
Streptococcus intermedius	streptococcus intermedius	streptococcus intermedius	streptococcus intermedius	Streptococcus intermedius	1	kept

Streptococcus parasanguinis	streptococcus parasanguinis	streptococcus parasanguinis	streptococcus parasanguinis	Streptococcus parasanguinis	1	kept
Streptococcus pyogenes venomous bites	streptococcus pyogenes venomous bites	streptococcus pyogenes venomous bites	streptococcus pyogenes venomous bites	Streptococcus pyogenes venomous bites	1	kept
Streptococcus salivarius	streptococcus salivarius	streptococcus salivarius	streptococcus salivarius	Streptococcus salivarius	1	kept
Streptococcus vaccine	streptococcus vaccine	streptococcus vaccine	streptococcus vaccine	Streptococcus vaccine	1	kept
streptogramin derivative	streptogramin derivative	streptogramin derivative	streptogramin derivative	streptogramin derivative	1	kept
stress and well-being	stress and well being	stress and well being	stress and well being	stress and well-being	1	kept
Stress anxiety	stress anxiety	stress anxiety	stress anxiety	Stress anxiety	1	kept
stress biomarkers	stress biomarkers	stress biomarkers	stress biomarkers	stress biomarkers	1	kept
stress cardiomyopathy	stress cardiomyopathy	stress cardiomyopathy	stress cardiomyopathy	Stress cardiomyopathy	1	case_or_format
Stress coping	stress coping	stress coping	stress coping	Stress coping	1	kept
stress coping	stress coping	stress coping	stress coping	Stress coping	1	case_or_format
stress coping strategies	stress coping strategies	stress coping strategies	stress coping strategy	Stress Coping Strategy	1	singular
Stress Coping Strategy	stress coping strategy	stress coping strategy	stress coping strategy	Stress Coping Strategy	1	kept
Stress coping styles	stress coping styles	stress coping styles	stress coping styles	Stress coping styles	1	kept
stress disorder	stress disorder	stress disorder	stress disorder	Stress disorders	1	case_or_format
stress disorders post - traumatic	stress disorders post traumatic	stress disorders post traumatic	stress disorders post traumatic	Stress Disorders, Post-Traumatic	1	case_or_format

Stress echocardiography	stress echocardiography	stress echocardiography	stress echocardiography	stress echocardiography	1	case_or_format
stress experiences	stress experiences	stress experiences	stress experiences	stress experiences	1	kept
stress factors	stress factors	stress factors	stress factors	stress factors	1	kept
stress fracture	stress fracture	stress fracture	stress fracture	stress fracture	1	kept
Stress hyperglycaemia	stress hyperglycaemia	stress hyperglycaemia	stress hyperglycaemia	Stress hyperglycaemia	1	kept
STRESS HYPERGLYCEMIA	stress hyperglycemia	stress hyperglycemia	stress hyperglycemia	stress hyperglycemia	1	case_or_format
Stress incontinence	stress incontinence	stress incontinence	stress incontinence	Stress incontinence	1	kept
stress incontinence	stress incontinence	stress incontinence	stress incontinence	Stress incontinence	1	case_or_format
Stress induction	stress induction	stress induction	stress induction	Stress induction	1	kept
Stress level	stress level	stress level	stress level	Stress level	1	kept
Stress Levels	stress levels	stress levels	stress level	Stress level	1	singular
stress levels	stress levels	stress levels	stress level	Stress level	1	singular
stress levels and surgery	stress levels and surgery	stress levels and surgery	stress levels and surgery	stress levels and surgery	1	kept
Stress levels associated with delivering and [illegible]	stress levels associated with delivering and [illegible]	stress levels associated with delivering and [illegible]	stress levels associated with delivering and [illegible]	Stress levels associated with delivering and [illegible]	1	kept
stress management intervention	stress management intervention	stress management intervention	stress management intervention	stress management intervention	1	kept
Stress measure	stress measure	stress measure	stress measure	Stress measure	1	kept

Stress monitoring	stress monitoring	stress monitoring	stress monitoring	Stress monitoring	1	kept
stress of conscience	stress of conscience	stress of conscience	stress of conscience	Stress of conscience	1	case_or_format
Stress of higher status	stress of higher status	stress of higher status	stress of higher status	Stress of higher status	1	kept
Stress physiological	stress physiological	stress physiological	stress physiological	Stress, Physiological	1	case_or_format
STRESS PROCESS	stress process	stress process	stress process	STRESS PROCESS	1	kept
stress psychologic	stress psychologic	stress psychologic	stress psychologic	stress psychologic	1	kept
Stress Psychological	stress psychological	stress psychological	stress psychological	Stress, Psychological	1	case_or_format
stress recognition	stress recognition	stress recognition	stress recognition	stress recognition	1	kept
Stress related mucosal disease	stress related mucosal disease	stress related mucosal disease	stress related mucosal disease	Stress related mucosal disease	1	kept
stress related mucosal disease	stress related mucosal disease	stress related mucosal disease	stress related mucosal disease	Stress related mucosal disease	1	case_or_format
Stress symptoms	stress symptoms	stress symptoms	stress symptoms	Stress symptoms	1	kept
stress ulcer prophylaxis	stress ulcer prophylaxis	stress ulcer prophylaxis	stress ulcer prophylaxis	Stress ulcer prophylaxis	1	case_or_format
stress, psychological	stress psychological	stress psychological	stress psychological	Stress, Psychological	1	case_or_format
Stress-induced cardiomyopathy	stress induced cardiomyopathy	stress induced cardiomyopathy	stress induced cardiomyopathy	Stress-induced cardiomyopathy	1	kept
Stress-induced hyperglycaemia	stress induced hyperglycaemia	stress induced hyperglycaemia	stress induced hyperglycaemia	Stress-induced hyperglycaemia	1	kept
stress-induced hyperglycemia.	stress induced hyperglycemia	stress induced hyperglycemia	stress induced hyperglycemia	stress-induced hyperglycemia.	1	kept

stress-monitor	stress monitor	stress monitor	stress monitor	stress-monitor	1	kept
Stress-related mucosal bleeding	stress related mucosal bleeding	stress related mucosal bleeding	stress related mucosal bleeding	Stress-related mucosal bleeding	1	kept
Stress-related mucosal disease	stress related mucosal disease	stress related mucosal disease	stress related mucosal disease	Stress related mucosal disease	1	case_or_format
StressAnxiety	stressanxiety	stressanxiety	stressanxiety	StressAnxiety	1	kept
Stresses	stresses	stresses	stresses	Stresses	1	kept
Stresses response	stresses response	stresses response	stresses response	Stresses response	1	kept
stretcher CPR	stretcher cpr	stretcher cpr	stretcher cpr	stretcher CPR	1	kept
Stricture	stricture	stricture	stricture	Stricture	1	kept
strobe checklist	strobe checklist	strobe checklist	strobe checklist	strobe checklist	1	kept
Stroke and cerebrovascular disease	stroke and cerebrovascular disease	stroke and cerebrovascular disease	stroke and cerebrovascular disease	Stroke and cerebrovascular disease	1	kept
stroke and cerebrovascular disease	stroke and cerebrovascular disease	stroke and cerebrovascular disease	stroke and cerebrovascular disease	Stroke and cerebrovascular disease	1	case_or_format
stroke and covid-19	stroke and covid 19	stroke and covid 19	stroke and covid 19	stroke and covid-19	1	kept
Stroke associated pneumonia	stroke associated pneumonia	stroke associated pneumonia	stroke associated pneumonia	Stroke associated pneumonia	1	kept
stroke awareness	stroke awareness	stroke awareness	stroke awareness	stroke awareness	1	kept
Stroke care	stroke care	stroke care	stroke care	Stroke care	1	kept
stroke care	stroke care	stroke care	stroke care	Stroke care	1	case_or_format

Stroke care unit	stroke care unit	stroke care unit	stroke care unit	Stroke care unit	1	kept
Stroke centers	stroke centers	stroke centers	stroke centers	Stroke centers	1	kept
Stroke certification	stroke certification	stroke certification	stroke certification	Stroke certification	1	kept
Stroke code	stroke code	stroke code	stroke code	Stroke code	1	kept
stroke complications	stroke complications	stroke complications	stroke complications	stroke complications	1	kept
Stroke epidemiology	stroke epidemiology	stroke epidemiology	stroke epidemiology	Stroke epidemiology	1	kept
STROKE FAMILY CAREGIVER	stroke family caregiver	stroke family caregiver	stroke family caregiver	STROKE FAMILY CAREGIVER	1	kept
stroke in elderly	stroke in elderly	stroke in elderly	stroke in elderly	stroke in elderly	1	kept
stroke knowledge	stroke knowledge	stroke knowledge	stroke knowledge	stroke knowledge	1	kept
STROKE MEDICINE	stroke medicine	stroke medicine	stroke medicine	STROKE MEDICINE	1	kept
Stroke outcome	stroke outcome	stroke outcome	stroke outcome	Stroke outcomes	1	case_or_format
stroke outcome	stroke outcome	stroke outcome	stroke outcome	Stroke outcomes	1	case_or_format
STROKE PREVENTION	stroke prevention	stroke prevention	stroke prevention	STROKE PREVENTION	1	kept
Stroke prevention	stroke prevention	stroke prevention	stroke prevention	STROKE PREVENTION	1	case_or_format
stroke recovery	stroke recovery	stroke recovery	stroke recovery	stroke recovery	1	kept
Stroke registries	stroke registries	stroke registries	stroke registries	Stroke registries	1	kept

Stroke rehabilitation	stroke rehabilitation	stroke rehabilitation	stroke rehabilitation	Stroke Rehabilitation	1	case_or_format
Stroke syndromes	stroke syndromes	stroke syndromes	stroke syndromes	Stroke syndromes	1	kept
Stroke systems of care	stroke systems of care	stroke systems of care	stroke systems of care	Stroke systems of care	1	kept
Stroke teams	stroke teams	stroke teams	stroke teams	Stroke teams	1	kept
stroke teams	stroke teams	stroke teams	stroke teams	Stroke teams	1	case_or_format
Stroke therapy	stroke therapy	stroke therapy	stroke therapy	Stroke therapy	1	kept
Stroke unit care	stroke unit care	stroke unit care	stroke unit care	Stroke unit care	1	kept
stroke units	stroke units	stroke units	stroke unit	stroke unit	1	singular
stroke volume index	stroke volume index	stroke volume index	stroke volume index	stroke volume index	1	kept
Stroke volume optimization	stroke volume optimization	stroke volume optimization	stroke volume optimization	Stroke volume optimization	1	kept
Stroke volume variation	stroke volume variation	stroke volume variation	stroke volume variation	Stroke volume variation	1	kept
Stroke, acute	stroke acute	stroke acute	stroke acute	Stroke, acute	1	kept
stroma	stroma	stroma	stroma	stroma	1	kept
Strong model	strong model	strong model	strong model	STRONG MODEL	1	case_or_format
strontium ranelate	strontium ranelate	strontium ranelate	strontium ranelate	strontium ranelate	1	kept
Stroop test	stroop test	stroop test	stroop test	Stroop test	1	kept

Structural characterization	structural characterization	structural characterization	structural characterization	Structural characterization	1	kept
STRUCTURAL EMPOWERMENT	structural empowerment	structural empowerment	structural empowerment	Structural empowerment	1	case_or_format
Structural equation model	structural equation model	structural equation model	structural equation model	structural equation model	1	case_or_format
structural logistics	structural logistics	structural logistics	structural logistics	structural logistics	1	kept
STRUCTURAL POWER	structural power	structural power	structural power	STRUCTURAL POWER	1	kept
Structural rearrangement	structural rearrangement	structural rearrangement	structural rearrangement	Structural rearrangement	1	kept
Structural requirements	structural requirements	structural requirements	structural requirements	Structural requirements	1	kept
structural requirements	structural requirements	structural requirements	structural requirements	Structural requirements	1	case_or_format
structure collapse	structure collapse	structure collapse	structure collapse	structure collapse	1	kept
structure equation model	structure equation model	structure equation model	structure equation model	structure equation model	1	kept
Structure factors	structure factors	structure factors	structure factors	Structure factors	1	kept
structure of morbidity	structure of morbidity	structure of morbidity	structure of morbidity	structure of morbidity	1	kept
Structure-based	structure based	structure based	structure based	Structure-based	1	kept
Structure-learning	structure learning	structure learning	structure learning	Structure-learning	1	kept
Structured	structured	structured	structured	Structured	1	kept
Structured care	structured care	structured care	structured care	Structured care	1	kept

structured documentation	structured documentation	structured documentation	structured documentation	structured documentation	1	kept
structured interviews	structured interviews	structured interviews	structured interview	structured interview	1	singular
structured knowledge questionnaire	structured knowledge questionnaire	structured knowledge questionnaire	structured knowledge questionnaire	structured knowledge questionnaire	1	kept
structured review	structured review	structured review	structured review	structured review	1	kept
structured vs. unstructured data	structured vs unstructured data	structured vs unstructured data	structured vs unstructured data	structured vs. unstructured data	1	kept
Struggle of the ages	struggle of the ages	struggle of the ages	struggle of the ages	Struggle of the ages	1	kept
STSS	stss	stss	stss	STSS	1	kept
Student assessment	student assessment	student assessment	student assessment	Student assessment	1	kept
Student engagement	student engagement	student engagement	student engagement	Student engagement	1	kept
Student experience	student experience	student experience	student experience	Student experience	1	kept
Student Health Services	student health services	student health services	student health services	Student Health Services	1	kept
Student levels	student levels	student levels	student levels	Student levels	1	kept
Student midwives	student midwives	student midwives	student midwives	Student midwives	1	kept
student perceptions	student perceptions	student perceptions	student perceptions	student perceptions	1	kept
Student Perceptions of Interprofessional Clinical Education–	student perceptions of interprofessional clinical education	student perceptions of interprofessional clinical education	student perceptions of interprofessional clinical education	Student Perceptions of Interprofessional Clinical Education–	1	kept
Student Performance Appraisal	student performance appraisal	student performance appraisal	student performance appraisal	Student Performance Appraisal	1	kept

student recruitment	student recruitment	student recruitment	student recruitment	student recruitment	1	kept
STUDENTS KNOWLEDGE	students knowledge	students knowledge	students knowledge	STUDENTS KNOWLEDGE	1	kept
Students' feedback	students feedback	students feedback	students feedback	Students' feedback	1	kept
Students' knowledge	students knowledge	students knowledge	students knowledge	STUDENTS KNOWLEDGE	1	case_or_format
Students' Perception	students perception	students perception	students perception	Students' Perception	1	kept
Students, Dental	students dental	students dental	students dental	Students, Dental	1	kept
Students, pharmacy	students pharmacy	students pharmacy	students pharmacy	Students, Pharmacy	1	case_or_format
Study Guide as Topic	study guide as topic	study guide as topic	study guide as topic	Study Guide as Topic	1	kept
study protocol	study protocol	study protocol	study protocol	Study protocol	1	case_or_format
study start-up	study start up	study start up	study start up	study start-up	1	kept
Study to Understand Prognoses and References for [illegible]	study to understand prognoses and references for [illegible]	study to understand prognoses and references for [illegible]	study to understand prognoses and references for [illegible]	Study to Understand Prognoses and References for [illegible]	1	kept
Stunning	stunning	stunning	stunning	Stunning	1	kept
stunting	stunting	stunting	stunting	stunting	1	kept
Sturge Weber syndrome	sturge weber syndrome	sturge weber syndrome	sturge weber syndrome	Sturge-Weber Syndrome	1	case_or_format
Sturge-Weber syndrome	sturge weber syndrome	sturge weber syndrome	sturge weber syndrome	Sturge-Weber Syndrome	1	case_or_format
STYLES	styles	styles	style	STYLE	1	singular

Stylet	stylet	stylet	stylet	Stylet	1	kept
styling	styling	styling	styling	styling	1	kept
sub optimal oxygen blending	sub optimal oxygen blending	sub optimal oxygen blending	sub optimal oxygen blending	sub optimal oxygen blending	1	kept
Sub-acute care	sub acute care	sub acute care	sub acute care	Sub-acute care	1	kept
sub-acute care	sub acute care	sub acute care	sub acute care	Sub-acute care	1	case_or_format
Sub-epidermal moisture	sub epidermal moisture	sub epidermal moisture	sub epidermal moisture	Sub-epidermal moisture	1	kept
Sub-plateau area	sub plateau area	sub plateau area	sub plateau area	Sub-plateau area	1	kept
Sub-Saharan Africa,	sub saharan africa	sub saharan africa	sub saharan africa	Sub-Saharan Africa	1	case_or_format
sub-Saharan African	sub saharan african	sub saharan african	sub saharan african	sub-Saharan African	1	kept
subacute model of geriatric care	subacute model of geriatric care	subacute model of geriatric care	subacute model of geriatric care	subacute model of geriatric care	1	kept
subacute rehabilitation	subacute rehabilitation	subacute rehabilitation	subacute rehabilitation	subacute rehabilitation	1	kept
Subarachnoid anaesthesia	subarachnoid anaesthesia	subarachnoid anaesthesia	subarachnoid anaesthesia	Subarachnoid anaesthesia	1	kept
Subarachnoid Space	subarachnoid space	subarachnoid space	subarachnoid space	Subarachnoid Space	1	kept
Subarachnoid space	subarachnoid space	subarachnoid space	subarachnoid space	Subarachnoid Space	1	case_or_format
subarachnoid space	subarachnoid space	subarachnoid space	subarachnoid space	Subarachnoid Space	1	case_or_format
subclavian	subclavian	subclavian	subclavian	subclavian	1	kept

subclavian access	subclavian access	subclavian access	subclavian access	subclavian access	1	kept
Subclavian Artery	subclavian artery	subclavian artery	subclavian artery	subclavian artery	1	case_or_format
Subclavian vein	subclavian vein	subclavian vein	subclavian vein	Subclavian Vein	1	case_or_format
Subcutaneous continuous glucose monitoring	subcutaneous continuous glucose monitoring	subcutaneous continuous glucose monitoring	subcutaneous continuous glucose monitoring	Subcutaneous continuous glucose monitoring	1	kept
Subcutaneous Emphysema	subcutaneous emphysema	subcutaneous emphysema	subcutaneous emphysema	subcutaneous emphysema	1	case_or_format
Subcutaneous emphysema	subcutaneous emphysema	subcutaneous emphysema	subcutaneous emphysema	subcutaneous emphysema	1	case_or_format
Subcutaneous Fat	subcutaneous fat	subcutaneous fat	subcutaneous fat	Subcutaneous Fat	1	kept
Subcutaneous injection	subcutaneous injection	subcutaneous injection	subcutaneous injection	Subcutaneous injection	1	kept
subcutaneous injection	subcutaneous injection	subcutaneous injection	subcutaneous injection	Subcutaneous injection	1	case_or_format
subcutaneous monoclonal antibodies	subcutaneous monoclonal antibodies	subcutaneous monoclonal antibodies	subcutaneous monoclonal antibodies	subcutaneous monoclonal antibodies	1	kept
subcutaneous trauma	subcutaneous trauma	subcutaneous trauma	subcutaneous trauma	subcutaneous trauma	1	kept
Subdermal EEG	subdermal eeg	subdermal eeg	subdermal eeg	Subdermal EEG	1	kept
Subdural hemorrhage	subdural hemorrhage	subdural hemorrhage	subdural hemorrhage	Subdural hemorrhage	1	kept
suberythroderma	suberythroderma	suberythroderma	suberythroderma	suberythroderma	1	kept
SUBGLOTTIC SECRETION DRAINAGE	subglottic secretion drainage	subglottic secretion drainage	subglottic secretion drainage	Subglottic secretion drainage	1	case_or_format
Subglottic stenosis	subglottic stenosis	subglottic stenosis	subglottic stenosis	subglottic stenosis	1	case_or_format

Subglottic suction	subglottic suction	subglottic suction	subglottic suction	Subglottic suction	1	kept
subject matter expert	subject matter expert	subject matter expert	subject matter expert	subject matter expert	1	kept
subject-centered outcomes	subject centered outcomes	subject centered outcomes	subject centered outcomes	subject-centered outcomes	1	kept
Subjective aspects	subjective aspects	subjective aspects	subjective aspects	Subjective aspects	1	kept
subjective cues	subjective cues	subjective cues	subjective cues	subjective cues	1	kept
Subjective evaluations	subjective evaluations	subjective evaluations	subjective evaluations	Subjective evaluations	1	kept
Subjective Global Nutrition Assessment	subjective global nutrition assessment	subjective global nutrition assessment	subjective global nutrition assessment	Subjective Global Nutrition Assessment	1	kept
Subjective Happiness Scale	subjective happiness scale	subjective happiness scale	subjective happiness scale	Subjective Happiness Scale	1	kept
Subjective information	subjective information	subjective information	subjective information	Subjective information	1	kept
Subjective sleep	subjective sleep	subjective sleep	subjective sleep	Subjective sleep	1	kept
subjective sleep	subjective sleep	subjective sleep	subjective sleep	Subjective sleep	1	case_or_format
subjective sleep quality	subjective sleep quality	subjective sleep quality	subjective sleep quality	subjective sleep quality	1	kept
Subjective strain	subjective strain	subjective strain	subjective strain	Subjective strain	1	kept
Subjective stress	subjective stress	subjective stress	subjective stress	Subjective stress	1	kept
subjective well-being	subjective well being	subjective well being	subjective well being	Subjective well-being	1	case_or_format
subjective workload	subjective workload	subjective workload	subjective workload	Subjective workload	1	case_or_format

Subjectivity	subjectivity	subjectivity	subjectivity	Subjectivity	1	kept
Subjects	subjects	subjects	subjects	Subjects	1	kept
Sublimation	sublimation	sublimation	sublimation	Sublimation	1	kept
sublingual gland	sublingual gland	sublingual gland	sublingual gland	sublingual gland	1	kept
Sublingual Immunotherapy	sublingual immunotherapy	sublingual immunotherapy	sublingual immunotherapy	Sublingual Immunotherapy	1	kept
sublingual immunotherapy	sublingual immunotherapy	sublingual immunotherapy	sublingual immunotherapy	Sublingual Immunotherapy	1	case_or_format
sublingual microcirculation	sublingual microcirculation	sublingual microcirculation	sublingual microcirculation	sublingual microcirculation	1	kept
sublingual sufentanil tablet system	sublingual sufentanil tablet system	sublingual sufentanil tablet system	sublingual sufentanil tablet system	sublingual sufentanil tablet system	1	kept
Submental Island Flap	submental island flap	submental island flap	submental island flap	Submental Island Flap	1	kept
submental island flap reconstruction	submental island flap reconstruction	submental island flap reconstruction	submental island flap reconstruction	submental island flap reconstruction	1	kept
submersion	submersion	submersion	submersion	submersion	1	kept
submucosa	submucosa	submucosa	submucosa	submucosa	1	kept
suboccipital craniectomy	suboccipital craniectomy	suboccipital craniectomy	suboccipital craniectomy	suboccipital craniectomy	1	kept
Suboptimal care	suboptimal care	suboptimal care	suboptimal care	Suboptimal care	1	kept
suboptimal communication	suboptimal communication	suboptimal communication	suboptimal communication	suboptimal communication	1	kept
Suboptimal ward care	suboptimal ward care	suboptimal ward care	suboptimal ward care	Suboptimal ward care	1	kept

subretinal neovascularization	subretinal neovascularization	subretinal neovascularization	subretinal neovascularization	subretinal neovascularization	1	kept
subsarcolemmal mitochondria	subsarcolemmal mitochondria	subsarcolemmal mitochondria	subsarcolemmal mitochondria	subsarcolemmal mitochondria	1	kept
subsartorial artery	subsartorial artery	subsartorial artery	subsartorial artery	subsartorial artery	1	kept
Subscale	subscale	subscale	subscale	Subscale	1	kept
Subsequent contralateral hip fracture	subsequent contralateral hip fracture	subsequent contralateral hip fracture	subsequent contralateral hip fracture	Subsequent contralateral hip fracture	1	kept
subspeciality	subspeciality	subspeciality	subspeciality	subspeciality	1	kept
subspecialty palliative care	subspecialty palliative care	subspecialty palliative care	subspecialty palliative care	subspecialty palliative care	1	kept
Substance Abuse	substance abuse	substance abuse	substance abuse	substance abuse	1	case_or_format
substance abuse treatment	substance abuse treatment	substance abuse treatment	substance abuse treatment	substance abuse treatment	1	kept
substance addiction	substance addiction	substance addiction	substance addiction	substance addiction	1	kept
substance misuse	substance misuse	substance misuse	substance misuse	substance misuse	1	kept
substance P	substance p	substance p	substance p	substance P	1	kept
Substance Use & Misuse	substance use and misuse	substance use and misuse	substance use and misuse	Substance Use & Misuse	1	kept
substance use & misuse	substance use and misuse	substance use and misuse	substance use and misuse	Substance Use & Misuse	1	case_or_format
Substance Use Disorder	substance use disorder	substance use disorder	substance use disorder	substance use disorder	1	case_or_format
Substance use during pregnancy	substance use during pregnancy	substance use during pregnancy	substance use during pregnancy	Substance use during pregnancy	1	kept

substance use in pregnancy	substance use in pregnancy	substance use in pregnancy	substance use in pregnancy	substance use in pregnancy	1	kept
Substance withdrawal	substance withdrawal	substance withdrawal	substance withdrawal	Substance withdrawal	1	kept
substance withdrawal syndrome	substance withdrawal syndrome	substance withdrawal syndrome	substance withdrawal syndrome	Substance Withdrawal Syndrome	1	case_or_format
Substance-Related disorders	substance related disorders	substance related disorders	substance related disorders	Substance-Related Disorders	1	case_or_format
Substance-related disorders	substance related disorders	substance related disorders	substance related disorders	Substance-Related Disorders	1	case_or_format
Substances Abstinence Syndrome	substances abstinence syndrome	substances abstinence syndrome	substances abstinence syndrome	Substances Abstinence Syndrome	1	kept
substance‐exposed pregnancy	substance exposed pregnancy	substance exposed pregnancy	substance exposed pregnancy	substance-exposed pregnancy	1	case_or_format
Substandard	substandard	substandard	substandard	Substandard	1	kept
substantiated complaint	substantiated complaint	substantiated complaint	substantiated complaint	substantiated complaint	1	kept
substernal chest pain	substernal chest pain	substernal chest pain	substernal chest pain	substernal chest pain	1	kept
substernal pain	substernal pain	substernal pain	substernal pain	substernal pain	1	kept
substitute decision-maker	substitute decision maker	substitute decision maker	substitute decision maker	substitute decision-maker	1	kept
Substitute decision-makers	substitute decision makers	substitute decision makers	substitute decision maker	substitute decision-maker	1	singular
substituted judgment	substituted judgment	substituted judgment	substituted judgment	substituted judgment	1	kept
substitution	substitution	substitution	substitution	substitution	1	kept
subthalamic nucleus	subthalamic nucleus	subthalamic nucleus	subthalamic nucleus	Subthalamic Nucleus	1	case_or_format

Subtle	subtle	subtle	subtle	Subtle	1	kept
Subtle control	subtle control	subtle control	subtle control	Subtle control	1	kept
subtype classification	subtype classification	subtype classification	subtype classification	subtype classification	1	kept
Suburban	suburban	suburban	suburban	Suburban	1	kept
Suburban Population	suburban population	suburban population	suburban population	Suburban Population	1	kept
suburban population	suburban population	suburban population	suburban population	Suburban Population	1	case_or_format
Suburethral Slings	suburethral slings	suburethral slings	suburethral slings	Suburethral Slings	1	kept
Success	success	success	success	Success	1	kept
succimer	succimer	succimer	succimer	succimer	1	kept
Suck central pattern generator	suck central pattern generator	suck central pattern generator	suck central pattern generator	Suck central pattern generator	1	kept
Suck cycle period	suck cycle period	suck cycle period	suck cycle period	Suck cycle period	1	kept
SUCKING	sucking	sucking	sucking	sucking	1	case_or_format
Sucking behavior	sucking behavior	sucking behavior	sucking behavior	Sucking Behavior	1	case_or_format
sucking organization	sucking organization	sucking organization	sucking organization	sucking organization	1	kept
sucking skills	sucking skills	sucking skills	sucking skills	sucking skills	1	kept
sucking success	sucking success	sucking success	sucking success	Sucking success	1	case_or_format

sucrase	sucrase	sucrase	sucrase	sucrase	1	kept
Suction assessment	suction assessment	suction assessment	suction assessment	Suction assessment	1	kept
Suction catheter	suction catheter	suction catheter	suction catheter	Suction catheter	1	kept
Suction device	suction device	suction device	suction device	Suction device	1	kept
Suction devices	suction devices	suction devices	suction device	Suction device	1	singular
Suction education	suction education	suction education	suction education	Suction education	1	kept
suction event	suction event	suction event	suction event	suction event	1	kept
Suction needs	suction needs	suction needs	suction needs	Suction needs	1	kept
Suction pressure	suction pressure	suction pressure	suction pressure	Suction pressure	1	kept
suction pump	suction pump	suction pump	suction pump	suction pump	1	kept
Suction systems	suction systems	suction systems	suction systems	Suction systems	1	kept
Suction tool	suction tool	suction tool	suction tool	Suction tool	1	kept
suction toothbrush	suction toothbrush	suction toothbrush	suction toothbrush	suction toothbrush	1	kept
suction-related complications	suction related complications	suction related complications	suction related complications	suction-related complications	1	kept
SUCTIONING TECHNIQUES	suctioning techniques	suctioning techniques	suctioning techniques	SUCTIONING TECHNIQUES	1	kept
Sudden bereavement	sudden bereavement	sudden bereavement	sudden bereavement	Sudden bereavement	1	kept

sudden birth	sudden birth	sudden birth	sudden birth	sudden birth	1	kept
Sudden infant death	sudden infant death	sudden infant death	sudden infant death	Sudden Infant Death	1	case_or_format
Sudden Infant Death Syndrome	sudden infant death syndrome	sudden infant death syndrome	sudden infant death syndrome	sudden infant death syndrome	1	case_or_format
Sudden infant death*	sudden infant death	sudden infant death	sudden infant death	Sudden Infant Death	1	case_or_format
sudden speechlessness	sudden speechlessness	sudden speechlessness	sudden speechlessness	sudden speechlessness	1	kept
Sudden Unexpected Death in Epilepsy	sudden unexpected death in epilepsy	sudden unexpected death in epilepsy	sudden unexpected death in epilepsy	Sudden Unexpected Death in Epilepsy	1	kept
sudden unexpected death in epilepsy	sudden unexpected death in epilepsy	sudden unexpected death in epilepsy	sudden unexpected death in epilepsy	Sudden Unexpected Death in Epilepsy	1	case_or_format
Sudden unexpected infant death	sudden unexpected infant death	sudden unexpected infant death	sudden unexpected infant death	Sudden unexpected infant death	1	kept
Sudden unexplained death	sudden unexplained death	sudden unexplained death	sudden unexplained death	Sudden unexplained death	1	kept
sudden unexplained death	sudden unexplained death	sudden unexplained death	sudden unexplained death	Sudden unexplained death	1	case_or_format
Sufentanyl	sufentanyl	sufentanyl	sufentanyl	Sufentanyl	1	kept
Suffering of parents	suffering of parents	suffering of parents	suffering of parents	Suffering of parents	1	kept
sugar	sugar	sugar	sugar	sugar	1	kept
sugar phosphate	sugar phosphate	sugar phosphate	sugar phosphate	sugar phosphate	1	kept
Sugar Phosphates	sugar phosphates	sugar phosphates	sugar phosphate	sugar phosphate	1	singular
sugar-sweetened beverage	sugar sweetened beverage	sugar sweetened beverage	sugar sweetened beverage	sugar-sweetened beverage	1	kept

Suicidal behavior	suicidal behavior	suicidal behavior	suicidal behavior	suicidal behavior	1	case_or_format
suicidal ideation scale	suicidal ideation scale	suicidal ideation scale	suicidal ideation scale	suicidal ideation scale	1	kept
Suicidal thoughts	suicidal thoughts	suicidal thoughts	suicidal thoughts	Suicidal thoughts	1	kept
suicidal thoughts	suicidal thoughts	suicidal thoughts	suicidal thoughts	Suicidal thoughts	1	case_or_format
Suicide & self-harm	suicide and self harm	suicide and self harm	suicide and self harm	Suicide & self-harm	1	kept
suicide & self-harm	suicide and self harm	suicide and self harm	suicide and self harm	Suicide & self-harm	1	case_or_format
Suicide prevention training	suicide prevention training	suicide prevention training	suicide prevention training	Suicide prevention training	1	kept
SUIDS	suids	suids	suids	SUIDS	1	kept
Suita	suita	suita	suita	Suita	1	kept
Sulbactam	sulbactam	sulbactam	sulbactam	sulbactam	1	case_or_format
sulcus bleeding	sulcus bleeding	sulcus bleeding	sulcus bleeding	sulcus bleeding	1	kept
SULFADIAZINE	sulfadiazine	sulfadiazine	sulfadiazine	SULFADIAZINE	1	kept
Sulfadiazine	sulfadiazine	sulfadiazine	sulfadiazine	SULFADIAZINE	1	case_or_format
sulfadiazine	sulfadiazine	sulfadiazine	sulfadiazine	SULFADIAZINE	1	case_or_format
Sulfasalazine	sulfasalazine	sulfasalazine	sulfasalazine	Sulfasalazine	1	kept
sulfate transporter	sulfate transporter	sulfate transporter	sulfate transporter	sulfate transporter	1	kept

Sulfonylurea	sulfonylurea	sulfonylurea	sulfonylurea	sulfonylurea	1	case_or_format
sulfur derivative	sulfur derivative	sulfur derivative	sulfur derivative	sulfur derivative	1	kept
Sulfur mustard	sulfur mustard	sulfur mustard	sulfur mustard	Sulfur mustard	1	kept
sulphate	sulphate	sulphate	sulphate	sulphate	1	kept
sulphatoxymelatonin	sulphatoxymelatonin	sulphatoxymelatonin	sulphatoxymelatonin	sulphatoxymelatonin	1	kept
sulpiride	sulpiride	sulpiride	sulpiride	sulpiride	1	kept
sumatriptan	sumatriptan	sumatriptan	sumatriptan	sumatriptan	1	kept
Summary diabetes self care activity	summary diabetes self care activity	summary diabetes self care activity	summary diabetes self care activity	Summary diabetes self care activity	1	kept
Summary of diagnostic tests chart	summary of diagnostic tests chart	summary of diagnostic tests chart	summary of diagnostic tests chart	Summary of diagnostic tests chart	1	kept
summary reporting	summary reporting	summary reporting	summary reporting	summary reporting	1	kept
Summative evaluation	summative evaluation	summative evaluation	summative evaluation	Summative evaluation	1	kept
summative evaluation	summative evaluation	summative evaluation	summative evaluation	Summative evaluation	1	case_or_format
sun	sun	sun	sun	sun	1	kept
sun exposure	sun exposure	sun exposure	sun exposure	sun exposure	1	kept
SUN, serum urea nitrogen	sun serum urea nitrogen	sun serum urea nitrogen	sun serum urea nitrogen	SUN, serum urea nitrogen	1	kept
sunburn	sunburn	sunburn	sunburn	sunburn	1	kept

Sunflower oil	sunflower oil	sunflower oil	sunflower oil	Sunflower Oil	1	case_or_format
sunflower oil	sunflower oil	sunflower oil	sunflower oil	Sunflower Oil	1	case_or_format
Sungai Buloh Hospital	sungai buloh hospital	sungai buloh hospital	sungai buloh hospital	Sungai Buloh Hospital	1	kept
sunlight	sunlight	sunlight	sunlight	Sunlight	1	case_or_format
Sunni	sunni	sunni	sunni	Sunni	1	kept
sunscreen	sunscreen	sunscreen	sunscreen	sunscreen	1	kept
SUP	sup	sup	sup	SUP	1	kept
Super resolution	super resolution	super resolution	super resolution	Super resolution	1	kept
super users	super users	super users	super users	super users	1	kept
Super-specialty	super specialty	super specialty	super specialty	Super-specialty	1	kept
superbugs	superbugs	superbugs	superbugs	superbugs	1	kept
Superficial Back Muscles	superficial back muscles	superficial back muscles	superficial back muscles	Superficial Back Muscles	1	kept
superficial blood vessel colour change measurement	superficial blood vessel colour change measurement	superficial blood vessel colour change measurement	superficial blood vessel colour change measurement	superficial blood vessel colour change measurement	1	kept
superficial blood vessel volume change measurement	superficial blood vessel volume change measurement	superficial blood vessel volume change measurement	superficial blood vessel volume change measurement	superficial blood vessel volume change measurement	1	kept
Superficial culture	superficial culture	superficial culture	superficial culture	Superficial culture	1	kept
superficial surgical site infection	superficial surgical site infection	superficial surgical site infection	superficial surgical site infection	superficial surgical site infection	1	kept

Superficial temporal artery	superficial temporal artery	superficial temporal artery	superficial temporal artery	Superficial temporal artery	1	kept
superficial temporal artery	superficial temporal artery	superficial temporal artery	superficial temporal artery	Superficial temporal artery	1	case_or_format
Superficial veins	superficial veins	superficial veins	superficial veins	Superficial veins	1	kept
superficial wound infection	superficial wound infection	superficial wound infection	superficial wound infection	superficial wound infection	1	kept
superimposed preeclampsia	superimposed preeclampsia	superimposed preeclampsia	superimposed preeclampsia	superimposed preeclampsia	1	kept
superior cerebellar peduncle	superior cerebellar peduncle	superior cerebellar peduncle	superior cerebellar peduncle	superior cerebellar peduncle	1	kept
superior epigastric artery	superior epigastric artery	superior epigastric artery	superior epigastric artery	superior epigastric artery	1	kept
superior laryngeal nerve	superior laryngeal nerve	superior laryngeal nerve	superior laryngeal nerve	superior laryngeal nerve	1	kept
superior laryngeal nerve block	superior laryngeal nerve block	superior laryngeal nerve block	superior laryngeal nerve block	superior laryngeal nerve block	1	kept
Superior mesenteric artery	superior mesenteric artery	superior mesenteric artery	superior mesenteric artery	Superior mesenteric artery	1	kept
superior mesenteric artery	superior mesenteric artery	superior mesenteric artery	superior mesenteric artery	Superior mesenteric artery	1	case_or_format
superior sagittal sinus	superior sagittal sinus	superior sagittal sinus	superior sagittal sinus	superior sagittal sinus	1	kept
superior vena cava syndrome	superior vena cava syndrome	superior vena cava syndrome	superior vena cava syndrome	Superior Vena Cava Syndrome	1	case_or_format
superiority trial	superiority trial	superiority trial	superiority trial	superiority trial	1	kept
supernatant	supernatant	supernatant	supernatant	supernatant	1	kept
Supernumerary status	supernumerary status	supernumerary status	supernumerary status	Supernumerary status	1	kept

superoxide dismutase 2	superoxide dismutase 2	superoxide dismutase 2	superoxide dismutase 2	superoxide dismutase 2	1	kept
Superresolution	superresolution	superresolution	superresolution	Superresolution	1	kept
superspreading event	superspreading event	superspreading event	superspreading event	superspreading event	1	kept
Superstitions	superstitions	superstitions	superstitions	Superstitions	1	kept
supervised flat-sharing communities	supervised flat sharing communities	supervised flat sharing communities	supervised flat sharing communities	supervised flat-sharing communities	1	kept
Supervised learning	supervised learning	supervised learning	supervised learning	Supervised learning	1	kept
Supervised Machine Learning	supervised machine learning	supervised machine learning	supervised machine learning	Supervised machine learning	1	case_or_format
Supervision	supervision	supervision	supervision	supervision	1	case_or_format
supervisory	supervisory	supervisory	supervisory	Supervisory	1	case_or_format
Supervisory control	supervisory control	supervisory control	supervisory control	Supervisory control	1	kept
supervisory control	supervisory control	supervisory control	supervisory control	Supervisory control	1	case_or_format
supine measurement	supine measurement	supine measurement	supine measurement	supine measurement	1	kept
SUPPLEMENT	supplement	supplement	supplement	SUPPLEMENT	1	kept
supplemental feeding	supplemental feeding	supplemental feeding	supplemental feeding	supplemental feeding	1	kept
supplemental feeding device	supplemental feeding device	supplemental feeding device	supplemental feeding device	supplemental feeding device	1	kept
Supplemental oxygen	supplemental oxygen	supplemental oxygen	supplemental oxygen	Supplemental oxygen	1	kept

supplemental oxygen	supplemental oxygen	supplemental oxygen	supplemental oxygen	Supplemental oxygen	1	case_or_format
supplements	supplements	supplements	supplement	SUPPLEMENT	1	singular
Supplies	supplies	supplies	supply	supply	1	singular
SupplMeshName: Acute fatty liver of pregnancy, UI:	supplmeshname acute fatty liver of pregnancy ui c537957	supplmeshname acute fatty liver of pregnancy ui c537957	supplmeshname acute fatty liver of pregnancy ui c537957	SupplMeshName: Acute fatty liver of pregnancy, UI:	1	kept
SupplMeshName: Canadian people, UI: C000726214, Type:	supplmeshname canadian people ui c000726214 type population	supplmeshname canadian people ui c000726214 type population	supplmeshname canadian people ui c000726214 type population	SupplMeshName: Canadian people, UI: C000726214, Type:	1	kept
SupplMeshName: Chromosome 1q43-Q44 Deletion Syndrome, UI:	supplmeshname chromosome 1q43 q44 deletion syndrome ui	supplmeshname chromosome 1q43 q44 deletion syndrome ui	supplmeshname chromosome 1q43 q44 deletion syndrome ui	SupplMeshName: Chromosome 1q43-Q44 Deletion Syndrome, UI:	1	kept
SupplMeshName: Connor-Davidson Resilience Scale, UI: C000728153	supplmeshname connor davidson resilience scale ui c000728153	supplmeshname connor davidson resilience scale ui c000728153	supplmeshname connor davidson resilience scale ui c000728153	SupplMeshName: Connor-Davidson Resilience Scale, UI: C000728153	1	kept
SupplMeshName: COVID-19 reinfection, UI: C000722979, Type:	supplmeshname covid 19 reinfection ui c000722979 type	supplmeshname covid 19 reinfection ui c000722979 type	supplmeshname covid 19 reinfection ui c000722979 type	SupplMeshName: COVID-19 reinfection, UI: C000722979, Type:	1	kept
SupplMeshName: Esophageal atresia with or without	supplmeshname esophageal atresia with or without	supplmeshname esophageal atresia with or without	supplmeshname esophageal atresia with or without	SupplMeshName: Esophageal atresia with or without	1	kept
SupplMeshName: Iranian people, UI: C000724774, Type:	supplmeshname iranian people ui c000724774 type population	supplmeshname iranian people ui c000724774 type population	supplmeshname iranian people ui c000724774 type population	SupplMeshName: Iranian people, UI: C000724774, Type:	1	kept
SupplMeshName: Laryngeal cleft, UI: C537875, Type: Disease	supplmeshname laryngeal cleft ui c537875 type disease	supplmeshname laryngeal cleft ui c537875 type disease	supplmeshname laryngeal cleft ui c537875 type disease	SupplMeshName: Laryngeal cleft, UI: C537875, Type: Disease	1	kept
SupplMeshName: Naganishia albida, UI: C000685160, Type:	supplmeshname naganishia albida ui c000685160 type	supplmeshname naganishia albida ui c000685160 type	supplmeshname naganishia albida ui c000685160 type	SupplMeshName: Naganishia albida, UI: C000685160, Type:	1	kept
supply	supply	supply	supply	supply	1	kept
Supply and distribution	supply and distribution	supply and distribution	supply and distribution	supply and distribution	1	case_or_format
Supply chain limitations	supply chain limitations	supply chain limitations	supply chain limitations	Supply chain limitations	1	kept
supply chain management	supply chain management	supply chain management	supply chain management	Supply chain management	1	case_or_format

supply management	supply management	supply management	supply management	supply management	1	kept
supply use	supply use	supply use	supply use	supply use	1	kept
supply-side barriers	supply side barriers	supply side barriers	supply side barriers	supply-side barriers	1	kept
Support and care	support and care	support and care	support and care	Support and care	1	kept
Support devices	support devices	support devices	support devices	Support devices	1	kept
Support group	support group	support group	support group	support group	1	case_or_format
SUPPORT GROUPS	support groups	support groups	support group	support group	1	singular
Support groups	support groups	support groups	support group	support group	1	singular
support groups	support groups	support groups	support group	support group	1	singular
SUPPORT NEEDS	support needs	support needs	support needs	Support needs	1	case_or_format
support of the French pediatric pneumology and allergology	support of the french pediatric pneumology and allergology	support of the french pediatric pneumology and allergology	support of the french pediatric pneumology and allergology	support of the French pediatric pneumology and allergology	1	kept
Support programs	support programs	support programs	support programs	Support programs	1	kept
support service	support service	support service	support service	Support services	1	case_or_format
Support staff	support staff	support staff	support staff	Support staff	1	kept
support staff	support staff	support staff	support staff	Support staff	1	case_or_format
Support surface	support surface	support surface	support surface	Support surfaces	1	case_or_format

SUPPORT THERAPY	support therapy	support therapy	support therapy	SUPPORT THERAPY	1	kept
Support vector classifiers	support vector classifiers	support vector classifiers	support vector classifiers	Support vector classifiers	1	kept
Support vectors machine	support vectors machine	support vectors machine	support vectors machine	Support vectors machine	1	kept
SUPPORT VENTILATION	support ventilation	support ventilation	support ventilation	SUPPORT VENTILATION	1	kept
Support work	support work	support work	support work	Support work	1	kept
Support worker	support worker	support worker	support worker	Support worker	1	kept
support worker	support worker	support worker	support worker	Support worker	1	case_or_format
support workers in ITU	support workers in itu	support workers in itu	support workers in itu	support workers in ITU	1	kept
Support, Non-U.S. Gov't	support non u s gov t	support non u s gov t	support non u s gov t	Support, Non-U.S. Gov't	1	kept
support-seeking	support seeking	support seeking	support seeking	support-seeking	1	kept
Supported	supported	supported	supported	Supported	1	kept
supported independence	supported independence	supported independence	supported independence	supported independence	1	kept
supported weight bearing	supported weight bearing	supported weight bearing	supported weight bearing	supported weight bearing	1	kept
Supporter	supporter	supporter	supporter	Supporter	1	kept
Supportive	supportive	supportive	supportive	Supportive	1	kept
Supportive and palliative care indicators tool	supportive and palliative care indicators tool	supportive and palliative care indicators tool	supportive and palliative care indicators tool	Supportive and palliative care indicators tool	1	kept

Supportive behaviours	supportive behaviours	supportive behaviours	supportive behaviours	Supportive behaviours	1	kept
supportive behaviours	supportive behaviours	supportive behaviours	supportive behaviours	Supportive behaviours	1	case_or_format
SupporTive Care At Home Research	supportive care at home research	supportive care at home research	supportive care at home research	SupporTive Care At Home Research	1	kept
supportive care bundle	supportive care bundle	supportive care bundle	supportive care bundle	supportive care bundle	1	kept
supportive clinical instructor	supportive clinical instructor	supportive clinical instructor	supportive clinical instructor	supportive clinical instructor	1	kept
Supportive communication	supportive communication	supportive communication	supportive communication	Supportive communication	1	kept
Supportive developmental position	supportive developmental position	supportive developmental position	supportive developmental position	Supportive developmental position	1	kept
Supportive Educational Program	supportive educational program	supportive educational program	supportive educational program	Supportive Educational Program	1	kept
supportive environment	supportive environment	supportive environment	supportive environment	supportive environment	1	kept
supportive intervention	supportive intervention	supportive intervention	supportive intervention	supportive intervention	1	kept
SUPPORTIVE MEDICATION USE	supportive medication use	supportive medication use	supportive medication use	SUPPORTIVE MEDICATION USE	1	kept
Supportive position	supportive position	supportive position	supportive position	Supportive position	1	kept
supportive positioning	supportive positioning	supportive positioning	supportive positioning	supportive positioning	1	kept
Supportive Program	supportive program	supportive program	supportive program	Supportive Program	1	kept
supportive services	supportive services	supportive services	supportive services	supportive services	1	kept
Supportive Therapeutic Excursion Program	supportive therapeutic excursion program	supportive therapeutic excursion program	supportive therapeutic excursion program	Supportive Therapeutic Excursion Program	1	kept

supportive training intervention	supportive training intervention	supportive training intervention	supportive training intervention	supportive training intervention	1	kept
Supports	supports	supports	support	SUPPORT	1	singular
Suppositories	suppositories	suppositories	suppository	suppository	1	singular
SUPPRESSANTS	suppressants	suppressants	suppressants	SUPPRESSANTS	1	kept
suppressing effect	suppressing effect	suppressing effect	suppressing effect	suppressing effect	1	kept
SUPPRESSION	suppression	suppression	suppression	SUPPRESSION	1	kept
SUPPRESSOR-CELLS	suppressor cells	suppressor cells	suppressor cells	SUPPRESSOR-CELLS	1	kept
SUPRACHIASMATIC NUCLEUS	suprachiasmatic nucleus	suprachiasmatic nucleus	suprachiasmatic nucleus	SUPRACHIASMATIC NUCLEUS	1	kept
Supraglottic airway	supraglottic airway	supraglottic airway	supraglottic airway	Supraglottic airway	1	kept
Supraglottoplasty	supraglottoplasty	supraglottoplasty	supraglottoplasty	Supraglottoplasty	1	kept
Supranuclear Palsy, Progressive	supranuclear palsy progressive	supranuclear palsy progressive	supranuclear palsy progressive	Supranuclear Palsy, Progressive	1	kept
suprapubic aspiration	suprapubic aspiration	suprapubic aspiration	suprapubic aspiration	suprapubic aspiration	1	kept
suprapubic tube placement	suprapubic tube placement	suprapubic tube placement	suprapubic tube placement	suprapubic tube placement	1	kept
suprarenal vein	suprarenal vein	suprarenal vein	suprarenal vein	suprarenal vein	1	kept
Supratentorial Neoplasms	supratentorial neoplasms	supratentorial neoplasms	supratentorial neoplasms	Supratentorial Neoplasms	1	kept
Supreme Court Decisions	supreme court decisions	supreme court decisions	supreme court decisions	Supreme Court Decisions	1	kept

Surah Ar-Rahman	surah ar rahman	surah ar rahman	surah ar rahman	Surah Ar-Rahman	1	kept
surface	surface	surface	surface	surface	1	kept
Surface acting	surface acting	surface acting	surface acting	Surface acting	1	kept
Surface contact	surface contact	surface contact	surface contact	Surface contact	1	kept
Surface contacts	surface contacts	surface contacts	surface contact	Surface contact	1	singular
Surface contamination	surface contamination	surface contamination	surface contamination	Surface contamination	1	kept
surface electromyography	surface electromyography	surface electromyography	surface electromyography	surface electromyography	1	kept
Surface hygiene	surface hygiene	surface hygiene	surface hygiene	Surface hygiene	1	kept
Surface sample	surface sample	surface sample	surface sample	Surface sample	1	kept
surface transmission	surface transmission	surface transmission	surface transmission	surface transmission	1	kept
SURFACE-TENSION	surface tension	surface tension	surface tension	SURFACE-TENSION	1	kept
surfaces	surfaces	surfaces	surface	surface	1	singular
surfactant administration	surfactant administration	surfactant administration	surfactant administration	surfactant administration	1	kept
surfactant distribution	surfactant distribution	surfactant distribution	surfactant distribution	surfactant distribution	1	kept
surfactant echocardiogram	surfactant echocardiogram	surfactant echocardiogram	surfactant echocardiogram	surfactant echocardiogram	1	kept
surfactant inhalation	surfactant inhalation	surfactant inhalation	surfactant inhalation	surfactant inhalation	1	kept

surfactant viscosity	surfactant viscosity	surfactant viscosity	surfactant viscosity	surfactant viscosity	1	kept
Surfactants	surfactants	surfactants	surfactant	surfactant	1	singular
surge plan	surge plan	surge plan	surge plan	surge plan	1	kept
Surge planning	surge planning	surge planning	surge planning	surge planning	1	case_or_format
surge staffing	surge staffing	surge staffing	surge staffing	surge staffing	1	kept
surge-level indicators	surge level indicators	surge level indicators	surge level indicators	surge-level indicators	1	kept
surgeon attitude	surgeon attitude	surgeon attitude	surgeon attitude	surgeon attitude	1	kept
Surgeon collaboration	surgeon collaboration	surgeon collaboration	surgeon collaboration	Surgeon collaboration	1	kept
Surgeon reallocation	surgeon reallocation	surgeon reallocation	surgeon reallocation	Surgeon reallocation	1	kept
Surgeon-in-charge	surgeon in charge	surgeon in charge	surgeon in charge	Surgeon-in-charge	1	kept
surgeons	surgeons	surgeons	surgeon	surgeon	1	singular
Surgeries	surgeries	surgeries	surgery	surgery	1	singular
Surgery - Cardiothoracic	surgery cardiothoracic	surgery cardiothoracic	surgery cardiothoracic	Surgery - Cardiothoracic	1	kept
surgery and gynecology unit	surgery and gynecology unit	surgery and gynecology unit	surgery and gynecology unit	surgery and gynecology unit	1	kept
surgery boot camp	surgery boot camp	surgery boot camp	surgery boot camp	surgery boot camp	1	kept
surgery frailty	surgery frailty	surgery frailty	surgery frailty	surgery frailty	1	kept

surgery registry	surgery registry	surgery registry	surgery registry	surgery registry	1	kept
surgery trauma	surgery trauma	surgery trauma	surgery trauma	surgery trauma	1	kept
Surgery Unit	surgery unit	surgery unit	surgery unit	surgery unit	1	case_or_format
Surgery, abdominal: bariatric	surgery abdominal bariatric	surgery abdominal bariatric	surgery abdominal bariatric	Surgery, abdominal: bariatric	1	kept
surgery, transplantation	surgery transplantation	surgery transplantation	surgery transplantation	surgery, transplantation	1	kept
Surgical abdomen	surgical abdomen	surgical abdomen	surgical abdomen	Surgical abdomen	1	kept
Surgical Apgar Score	surgical apgar score	surgical apgar score	surgical apgar score	Surgical Apgar Score	1	kept
Surgical approach	surgical approach	surgical approach	surgical approach	surgical approach	1	case_or_format
Surgical Attire	surgical attire	surgical attire	surgical attire	Surgical Attire	1	kept
surgical attire	surgical attire	surgical attire	surgical attire	Surgical Attire	1	case_or_format
surgical backlogs	surgical backlogs	surgical backlogs	surgical backlogs	surgical backlogs	1	kept
Surgical Capacity	surgical capacity	surgical capacity	surgical capacity	Surgical Capacity	1	kept
SURGICAL CARE	surgical care	surgical care	surgical care	surgical care	1	case_or_format
Surgical Care Improvement Project	surgical care improvement project	surgical care improvement project	surgical care improvement project	Surgical Care Improvement Project	1	kept
Surgical Care Practitioner	surgical care practitioner	surgical care practitioner	surgical care practitioner	Surgical Care Practitioner	1	kept
surgical clip ligation	surgical clip ligation	surgical clip ligation	surgical clip ligation	surgical clip ligation	1	kept

surgical complaints procedures	surgical complaints procedures	surgical complaints procedures	surgical complaints procedures	surgical complaints procedures	1	kept
surgical complexity	surgical complexity	surgical complexity	surgical complexity	surgical complexity	1	kept
Surgical delivery	surgical delivery	surgical delivery	surgical delivery	Surgical delivery	1	kept
surgical department	surgical department	surgical department	surgical department	surgical department	1	kept
surgical deterioration	surgical deterioration	surgical deterioration	surgical deterioration	surgical deterioration	1	kept
Surgical Drapes	surgical drapes	surgical drapes	surgical drapes	Surgical Drapes	1	kept
Surgical Equipment	surgical equipment	surgical equipment	surgical equipment	surgical equipment	1	case_or_format
surgical fascial closure	surgical fascial closure	surgical fascial closure	surgical fascial closure	surgical fascial closure	1	kept
surgical fixation devices	surgical fixation devices	surgical fixation devices	surgical fixation devices	surgical fixation devices	1	kept
surgical glue	surgical glue	surgical glue	surgical glue	surgical glue	1	kept
Surgical health outcomes research	surgical health outcomes research	surgical health outcomes research	surgical health outcomes research	Surgical health outcomes research	1	kept
surgical high dependency	surgical high dependency	surgical high dependency	surgical high dependency	surgical high dependency	1	kept
surgical information	surgical information	surgical information	surgical information	surgical information	1	kept
surgical instrument	surgical instrument	surgical instrument	surgical instrument	Surgical Instruments	1	case_or_format
Surgical instruments	surgical instruments	surgical instruments	surgical instrument	Surgical Instruments	1	singular
Surgical intervention	surgical intervention	surgical intervention	surgical intervention	Surgical intervention	1	kept

surgical mesh	surgical mesh	surgical mesh	surgical mesh	Surgical Mesh	1	case_or_format
surgical microscope	surgical microscope	surgical microscope	surgical microscope	surgical microscope	1	kept
surgical mission	surgical mission	surgical mission	surgical mission	surgical mission	1	kept
Surgical neonate	surgical neonate	surgical neonate	surgical neonate	Surgical neonate	1	kept
Surgical nutrition	surgical nutrition	surgical nutrition	surgical nutrition	Surgical nutrition	1	kept
Surgical Optimal Mobility Score	surgical optimal mobility score	surgical optimal mobility score	surgical optimal mobility score	Surgical Optimal Mobility Score	1	kept
Surgical optimisation	surgical optimisation	surgical optimisation	surgical optimisation	Surgical optimisation	1	kept
surgical outcome	surgical outcome	surgical outcome	surgical outcome	surgical outcomes	1	case_or_format
Surgical outcomes	surgical outcomes	surgical outcomes	surgical outcome	surgical outcomes	1	singular
Surgical Procedures, Minor	surgical procedures minor	surgical procedures minor	surgical procedures minor	Surgical Procedures, Minor	1	kept
surgical prophylaxis	surgical prophylaxis	surgical prophylaxis	surgical prophylaxis	surgical prophylaxis	1	kept
surgical quality improvement	surgical quality improvement	surgical quality improvement	surgical quality improvement	surgical quality improvement	1	kept
SURGICAL RECOVERY	surgical recovery	surgical recovery	surgical recovery	SURGICAL RECOVERY	1	kept
Surgical repair	surgical repair	surgical repair	surgical repair	Surgical repair	1	kept
Surgical resection	surgical resection	surgical resection	surgical resection	Surgical resection	1	kept
surgical resection	surgical resection	surgical resection	surgical resection	Surgical resection	1	case_or_format

Surgical reservoir	surgical reservoir	surgical reservoir	surgical reservoir	Surgical reservoir	1	kept
surgical residency	surgical residency	surgical residency	surgical residency	surgical residency	1	kept
Surgical residents	surgical residents	surgical residents	surgical residents	Surgical residents	1	kept
surgical residents	surgical residents	surgical residents	surgical residents	Surgical residents	1	case_or_format
Surgical revascularization	surgical revascularization	surgical revascularization	surgical revascularization	Surgical revascularization	1	kept
Surgical safety	surgical safety	surgical safety	surgical safety	Surgical safety	1	kept
surgical scheduling	surgical scheduling	surgical scheduling	surgical scheduling	surgical scheduling	1	kept
Surgical separation	surgical separation	surgical separation	surgical separation	Surgical separation	1	kept
surgical separation	surgical separation	surgical separation	surgical separation	Surgical separation	1	case_or_format
surgical sepsis	surgical sepsis	surgical sepsis	surgical sepsis	surgical sepsis	1	kept
SURGICAL SITE INFECTIONS	surgical site infections	surgical site infections	surgical site infection	SURGICAL SITE INFECTION	1	singular
surgical specialty hospital	surgical specialty hospital	surgical specialty hospital	surgical specialty hospital	surgical specialty hospital	1	kept
surgical sponge	surgical sponge	surgical sponge	surgical sponge	surgical sponge	1	kept
Surgical Sponges	surgical sponges	surgical sponges	surgical sponge	surgical sponge	1	singular
Surgical Stapling	surgical stapling	surgical stapling	surgical stapling	Surgical Stapling	1	kept
surgical stapling	surgical stapling	surgical stapling	surgical stapling	Surgical Stapling	1	case_or_format

Surgical subspecialisation	surgical subspecialisation	surgical subspecialisation	surgical subspecialisation	Surgical subspecialisation	1	kept
surgical swab	surgical swab	surgical swab	surgical swab	surgical swab	1	kept
Surgical system strengthening	surgical system strengthening	surgical system strengthening	surgical system strengthening	Surgical system strengthening	1	kept
Surgical tapes	surgical tapes	surgical tapes	surgical tape	Surgical Tape	1	singular
surgical tapes	surgical tapes	surgical tapes	surgical tape	Surgical Tape	1	singular
surgical teaching	surgical teaching	surgical teaching	surgical teaching	surgical teaching	1	kept
surgical technology	surgical technology	surgical technology	surgical technology	surgical technology	1	kept
surgical thrombectomy	surgical thrombectomy	surgical thrombectomy	surgical thrombectomy	surgical thrombectomy	1	kept
surgical timing	surgical timing	surgical timing	surgical timing	surgical timing	1	kept
Surgical tracheotomy	surgical tracheotomy	surgical tracheotomy	surgical tracheotomy	Surgical tracheotomy	1	kept
Surgical training	surgical training	surgical training	surgical training	surgical training	1	case_or_format
Surgical treatment of blast injuries	surgical treatment of blast injuries	surgical treatment of blast injuries	surgical treatment of blast injuries	Surgical treatment of blast injuries	1	kept
Surgical triage	surgical triage	surgical triage	surgical triage	Surgical triage	1	kept
surgical units	surgical units	surgical units	surgical units	surgical units	1	kept
Surgical volume	surgical volume	surgical volume	surgical volume	surgical volume	1	case_or_format
Surgical wards	surgical wards	surgical wards	surgical ward	surgical ward	1	singular

Surgical-site infection	surgical site infection	surgical site infection	surgical site infection	SURGICAL SITE INFECTION	1	case_or_format
SURGICAL-SITE INFECTIONS	surgical site infections	surgical site infections	surgical site infection	SURGICAL SITE INFECTION	1	singular
Surprise Question	surprise question	surprise question	surprise question	Surprise Question	1	kept
surprise question	surprise question	surprise question	surprise question	Surprise Question	1	case_or_format
surrogate consent	surrogate consent	surrogate consent	surrogate consent	surrogate consent	1	kept
surrogate decision	surrogate decision	surrogate decision	surrogate decision	surrogate decision	1	kept
surrogate decision makers	surrogate decision makers	surrogate decision makers	surrogate decision maker	Surrogate decision maker	1	singular
surrogate decision-maker	surrogate decision maker	surrogate decision maker	surrogate decision maker	Surrogate decision maker	1	case_or_format
surrogate mother	surrogate mother	surrogate mother	surrogate mother	surrogate mother	1	kept
Surrogates	surrogates	surrogates	surrogate	Surrogate	1	singular
Surveillance data	surveillance data	surveillance data	surveillance data	Surveillance data	1	kept
surveillance error grid	surveillance error grid	surveillance error grid	surveillance error grid	surveillance error grid	1	kept
Surveillance indicators	surveillance indicators	surveillance indicators	surveillance indicators	Surveillance indicators	1	kept
surveillance neurologique infirmière	surveillance neurologique infirmire	surveillance neurologique infirmire	surveillance neurologique infirmire	surveillance neurologique infirmière	1	kept
Surveillance programs	surveillance programs	surveillance programs	surveillance programs	Surveillance programs	1	kept
Surveillance video	surveillance video	surveillance video	surveillance video	Surveillance video	1	kept

Survery	survery	survery	survery	Survery	1	kept
survey and questionnaire	survey and questionnaire	survey and questionnaire	survey and questionnaire	Survey and questionnaires	1	case_or_format
survey design	survey design	survey design	survey design	survey design	1	kept
Survey instrument	survey instrument	survey instrument	survey instrument	Survey instrument	1	kept
Survey instruments	survey instruments	survey instruments	survey instrument	Survey instrument	1	singular
survey method	survey method	survey method	survey method	survey methods	1	case_or_format
Survey methodology	survey methodology	survey methodology	survey methodology	survey methodology	1	case_or_format
Survey of practice	survey of practice	survey of practice	survey of practice	Survey of practice	1	kept
Survey policy	survey policy	survey policy	survey policy	Survey policy	1	kept
Survey Research	survey research	survey research	survey research	Survey research	1	case_or_format
SURVEY RESPONSE RATES	survey response rates	survey response rates	survey response rates	SURVEY RESPONSE RATES	1	kept
SURVEY RETURN RATES	survey return rates	survey return rates	survey return rates	SURVEY RETURN RATES	1	kept
Survey safety	survey safety	survey safety	survey safety	Survey safety	1	kept
Survey Study	survey study	survey study	survey study	Survey study	1	case_or_format
survey study	survey study	survey study	survey study	Survey study	1	case_or_format
SurveyMonkey	surveymonkey	surveymonkey	surveymonkey	SurveyMonkey	1	kept

Survival analysis, nosocomial infection	survival analysis nosocomial infection	survival analysis nosocomial infection	survival analysis nosocomial infection	Survival analysis, nosocomial infection	1	kept
Survival convective clearance	survival convective clearance	survival convective clearance	survival convective clearance	Survival convective clearance	1	kept
Survival curve	survival curve	survival curve	survival curve	Survival curve	1	kept
survival curve	survival curve	survival curve	survival curve	Survival curve	1	case_or_format
survival index	survival index	survival index	survival index	survival index	1	kept
Survival journey	survival journey	survival journey	survival journey	Survival journey	1	kept
survival motor neuron protein	survival motor neuron protein	survival motor neuron protein	survival motor neuron protein	survival motor neuron protein	1	kept
SURVIVAL OUTCOMES	survival outcomes	survival outcomes	survival outcomes	SURVIVAL OUTCOMES	1	kept
Survival to discharge	survival to discharge	survival to discharge	survival to discharge	Survival to discharge	1	kept
survival trajectory analysis	survival trajectory analysis	survival trajectory analysis	survival trajectory analysis	survival trajectory analysis	1	kept
SURVIVING SEPSIS	surviving sepsis	surviving sepsis	surviving sepsis	SURVIVING SEPSIS	1	kept
Surviving Sepsis guideline	surviving sepsis guideline	surviving sepsis guideline	surviving sepsis guideline	Surviving Sepsis guideline	1	kept
survivorship care	survivorship care	survivorship care	survivorship care	survivorship care	1	kept
Survivorship prog	survivorship prog	survivorship prog	survivorship prog	Survivorship prog	1	kept
SUS	sus	sus	sus	SUS	1	kept
SUSCEPTIBILITY PATTERNS	susceptibility patterns	susceptibility patterns	susceptibility patterns	SUSCEPTIBILITY PATTERNS	1	kept

Sushi	sushi	sushi	sushi	Sushi	1	kept
SUSPECTED INFECTION	suspected infection	suspected infection	suspected infection	SUSPECTED INFECTION	1	kept
suspension laryngoscopy	suspension laryngoscopy	suspension laryngoscopy	suspension laryngoscopy	suspension laryngoscopy	1	kept
Suspensions	suspensions	suspensions	suspension	suspension	1	singular
SUSTAINABILITY	sustainability	sustainability	sustainability	sustainability	1	case_or_format
Sustainability Transitions	sustainability transitions	sustainability transitions	sustainability transitions	Sustainability Transitions	1	kept
Sustainable Development Goals	sustainable development goals	sustainable development goals	sustainable development goal	sustainable development goal	1	singular
Sustainable growth rate	sustainable growth rate	sustainable growth rate	sustainable growth rate	Sustainable growth rate	1	kept
sustainable growth rate	sustainable growth rate	sustainable growth rate	sustainable growth rate	Sustainable growth rate	1	case_or_format
Sustainable performance improvements	sustainable performance improvements	sustainable performance improvements	sustainable performance improvements	Sustainable performance improvements	1	kept
sustainable rural health services	sustainable rural health services	sustainable rural health services	sustainable rural health services	sustainable rural health services	1	kept
sustained care	sustained care	sustained care	sustained care	sustained care	1	kept
Sustained inflation	sustained inflation	sustained inflation	sustained inflation	Sustained inflation	1	kept
Sustained low efficiency dialysis	sustained low efficiency dialysis	sustained low efficiency dialysis	sustained low efficiency dialysis	Sustained low-efficiency dialysis	1	case_or_format
sustained release formulation	sustained release formulation	sustained release formulation	sustained release formulation	sustained release formulation	1	kept
Sustained vigilance	sustained vigilance	sustained vigilance	sustained vigilance	Sustained vigilance	1	kept

sustained vigilance	sustained vigilance	sustained vigilance	sustained vigilance	Sustained vigilance	1	case_or_format
sustained vigilance model	sustained vigilance model	sustained vigilance model	sustained vigilance model	sustained vigilance model	1	kept
sustained virologic response	sustained virologic response	sustained virologic response	sustained virologic response	sustained virologic response	1	kept
sustaining	sustaining	sustaining	sustaining	sustaining	1	kept
SUSTAINING THERAPY	sustaining therapy	sustaining therapy	sustaining therapy	SUSTAINING THERAPY	1	kept
sustenance	sustenance	sustenance	sustenance	sustenance	1	kept
suture	suture	suture	suture	suture	1	kept
sutureless	sutureless	sutureless	sutureless	sutureless	1	kept
SVB	svb	svb	svb	SVB	1	kept
SVEST	svest	svest	svest	SVEST	1	kept
SVT	svt	svt	svt	SVT	1	kept
Swab	swab	swab	swab	Swab	1	kept
swaddling	swaddling	swaddling	swaddling	Swaddling	1	case_or_format
swallowing exercise	swallowing exercise	swallowing exercise	swallowing exercise	swallowing exercise	1	kept
swallowing stimulation	swallowing stimulation	swallowing stimulation	swallowing stimulation	swallowing stimulation	1	kept
Swallowing Therapy	swallowing therapy	swallowing therapy	swallowing therapy	Swallowing Therapy	1	kept

swamp	swamp	swamp	swamp	swamp	1	kept
Swan-Ganz	swan ganz	swan ganz	swan ganz	Swan-Ganz	1	kept
swan-Ganz catheter	swan ganz catheter	swan ganz catheter	swan ganz catheter	Swan Ganz catheter	1	case_or_format
SWAN-GANZ CATHETERS	swan ganz catheters	swan ganz catheters	swan ganz catheter	Swan Ganz catheter	1	singular
Sweat	sweat	sweat	sweat	Sweat	1	kept
sweat	sweat	sweat	sweat	Sweat	1	case_or_format
Swedish citizen	swedish citizen	swedish citizen	swedish citizen	Swedish citizen	1	kept
Sweet almonds oil	sweet almonds oil	sweet almonds oil	sweet almonds oil	Sweet almonds oil	1	kept
sweet orange	sweet orange	sweet orange	sweet orange	sweet orange	1	kept
Swimming	swimming	swimming	swimming	Swimming	1	kept
swimming	swimming	swimming	swimming	Swimming	1	case_or_format
Swimming Pools	swimming pools	swimming pools	swimming pool	swimming pool	1	singular
swimming position	swimming position	swimming position	swimming position	swimming position	1	kept
swine	swine	swine	swine	Swine	1	case_or_format
Swine, Miniature	swine miniature	swine miniature	swine miniature	Swine, Miniature	1	kept
Swiss cheese theory	swiss cheese theory	swiss cheese theory	swiss cheese theory	Swiss cheese theory	1	kept

Switch operation	switch operation	switch operation	switch operation	Switch operation	1	kept
Switches	switches	switches	switches	Switches	1	kept
SYBR green	sybr green	sybr green	sybr green	SYBR green	1	kept
Syllabus	syllabus	syllabus	syllabus	Syllabus	1	kept
symbolic immortality	symbolic immortality	symbolic immortality	symbolic immortality	symbolic immortality	1	kept
Symbolic interactionism	symbolic interactionism	symbolic interactionism	symbolic interactionism	symbolic interactionism	1	case_or_format
sympathetic blocking	sympathetic blocking	sympathetic blocking	sympathetic blocking	sympathetic blocking	1	kept
sympathetic dysfunction	sympathetic dysfunction	sympathetic dysfunction	sympathetic dysfunction	sympathetic dysfunction	1	kept
sympathetic nerve	sympathetic nerve	sympathetic nerve	sympathetic nerve	sympathetic nerve	1	kept
Sympatholysis	sympatholysis	sympatholysis	sympatholysis	Sympatholysis	1	kept
sympathy	sympathy	sympathy	sympathy	sympathy	1	kept
Symptom	symptom	symptom	symptom	symptom	1	case_or_format
Symptom Burden	symptom burden	symptom burden	symptom burden	symptom burden	1	case_or_format
Symptom burden	symptom burden	symptom burden	symptom burden	symptom burden	1	case_or_format
symptom cluster	symptom cluster	symptom cluster	symptom cluster	symptom cluster	1	kept
symptom clusters	symptom clusters	symptom clusters	symptom cluster	symptom cluster	1	singular

symptom experiences and	symptom experiences and	symptom experiences and	symptom experiences and	symptom experiences and	1	kept
Symptom Flare Up	symptom flare up	symptom flare up	symptom flare up	Symptom Flare Up	1	kept
Symptom recognition	symptom recognition	symptom recognition	symptom recognition	Symptom recognition	1	kept
Symptom science	symptom science	symptom science	symptom science	Symptom science	1	kept
Symptomatic aneurysms	symptomatic aneurysms	symptomatic aneurysms	symptomatic aneurysms	Symptomatic aneurysms	1	kept
symptomatic epilepsy	symptomatic epilepsy	symptomatic epilepsy	symptomatic epilepsy	symptomatic epilepsy	1	kept
Symptoms of burnout	symptoms of burnout	symptoms of burnout	symptoms of burnout	Symptoms of burnout	1	kept
symptoms of depression	symptoms of depression	symptoms of depression	symptoms of depression	symptoms of depression	1	kept
symptoms of posttraumatic stress	symptoms of posttraumatic stress	symptoms of posttraumatic stress	symptoms of posttraumatic stress	symptoms of posttraumatic stress	1	kept
symptoms related criteria	symptoms related criteria	symptoms related criteria	symptoms related criteria	symptoms related criteria	1	kept
synactive theory	synactive theory	synactive theory	synactive theory	synactive theory	1	kept
Synagis	synagis	synagis	synagis	Synagis	1	kept
synapse	synapse	synapse	synapse	synapse	1	kept
Synapses	synapses	synapses	synapse	synapse	1	singular
synbiotic agent	synbiotic agent	synbiotic agent	synbiotic agent	synbiotic agent	1	kept
synchronous	synchronous	synchronous	synchronous	synchronous	1	kept

synchrony	synchrony	synchrony	synchrony	synchrony	1	kept
syndactyly	syndactyly	syndactyly	syndactyly	syndactyly	1	kept
syndaver®	syndaver	syndaver	syndaver	syndaver®	1	kept
syndrome coronaire aigu	syndrome coronaire aigu	syndrome coronaire aigu	syndrome coronaire aigu	syndrome coronaire aigu	1	kept
syndrome de dé faillance multiviscé rale	syndrome de d faillance multivisc rale	syndrome de d faillance multivisc rale	syndrome de d faillance multivisc rale	syndrome de dé faillance multiviscé rale	1	kept
syndrome delineation	syndrome delineation	syndrome delineation	syndrome delineation	syndrome delineation	1	kept
syndrome du bébé secou é	syndrome du b b secou	syndrome du b b secou	syndrome du b b secou	syndrome du bébé secou é	1	kept
synergistic effect	synergistic effect	synergistic effect	synergistic effect	synergistic effect	1	kept
SYNERGISTIC SEDATION	synergistic sedation	synergistic sedation	synergistic sedation	SYNERGISTIC SEDATION	1	kept
synergy model	synergy model	synergy model	synergy model	Synergy Model	1	case_or_format
synovectomy	synovectomy	synovectomy	synovectomy	synovectomy	1	kept
Syntactic properties	syntactic properties	syntactic properties	syntactic properties	Syntactic properties	1	kept
SYNTAX score	syntax score	syntax score	syntax score	SYNTAX score	1	kept
Synthetic cannabinoids	synthetic cannabinoids	synthetic cannabinoids	synthetic cannabinoids	Synthetic cannabinoids	1	kept
Syphilis	syphilis	syphilis	syphilis	syphilis	1	case_or_format
Syrian	syrian	syrian	syrian	Syrian	1	kept

Syrian crisis	syrian crisis	syrian crisis	syrian crisis	Syrian crisis	1	kept
Syrian refugees	syrian refugees	syrian refugees	syrian refugees	Syrian refugees	1	kept
Syringe	syringe	syringe	syringe	Syringes	1	case_or_format
syringe feeding	syringe feeding	syringe feeding	syringe feeding	syringe feeding	1	kept
syringe needle	syringe needle	syringe needle	syringe needle	syringe needle	1	kept
Syringe pump	syringe pump	syringe pump	syringe pump	syringe pump	1	case_or_format
Syringe pumps	syringe pumps	syringe pumps	syringe pump	syringe pump	1	singular
SYRINGES	syringes	syringes	syringe	Syringes	1	singular
syringes	syringes	syringes	syringe	Syringes	1	singular
syringomyelia	syringomyelia	syringomyelia	syringomyelia	syringomyelia	1	kept
system	system	system	system	SYSTEM	1	case_or_format
system approaches	system approaches	system approaches	system approaches	system approaches	1	kept
System architecture design	system architecture design	system architecture design	system architecture design	System architecture design	1	kept
System Based Practice	system based practice	system based practice	system based practice	System Based Practice	1	kept
System configurations	system configurations	system configurations	system configurations	System configurations	1	kept
system design	system design	system design	system design	System design	1	case_or_format

system design and analysis	system design and analysis	system design and analysis	system design and analysis	system design and analysis	1	kept
System elements	system elements	system elements	system elements	System elements	1	kept
System functions	system functions	system functions	system functions	System functions	1	kept
System interfaces	system interfaces	system interfaces	system interfaces	System interfaces	1	kept
System of prevention	system of prevention	system of prevention	system of prevention	System of prevention	1	kept
System on chips	system on chips	system on chips	system on chip	System-on-chip	1	singular
system outcomes	system outcomes	system outcomes	system outcomes	system outcomes	1	kept
System planning	system planning	system planning	system planning	System planning	1	kept
system probing	system probing	system probing	system probing	system probing	1	kept
System requirements	system requirements	system requirements	system requirements	System requirements	1	kept
System resiliences	system resiliences	system resiliences	system resiliences	System resiliences	1	kept
System robustness	system robustness	system robustness	system robustness	System robustness	1	kept
SYSTEM SCORE	system score	system score	system score	SYSTEM SCORE	1	kept
system simulation	system simulation	system simulation	system simulation	system simulation	1	kept
System Stagewise Health Education	system stagewise health education	system stagewise health education	system stagewise health education	System Stagewise Health Education	1	kept
System strain	system strain	system strain	system strain	System strain	1	kept

System-level approach	system level approach	system level approach	system level approach	System-level approach	1	kept
SYSTEMATIC ANALYSIS	systematic analysis	systematic analysis	systematic analysis	SYSTEMATIC ANALYSIS	1	kept
Systematic management	systematic management	systematic management	systematic management	Systematic management	1	kept
systematic management	systematic management	systematic management	systematic management	Systematic management	1	case_or_format
systematic mapping	systematic mapping	systematic mapping	systematic mapping	systematic mapping	1	kept
Systematic nutrition assessment	systematic nutrition assessment	systematic nutrition assessment	systematic nutrition assessment	Systematic nutrition assessment	1	kept
Systematic process	systematic process	systematic process	systematic process	Systematic process	1	kept
systematic restraints	systematic restraints	systematic restraints	systematic restraints	systematic restraints	1	kept
systemic approach	systemic approach	systemic approach	systemic approach	systemic approach	1	kept
Systemic health	systemic health	systemic health	systemic health	Systemic health	1	kept
Systemic immune-inflammatory index	systemic immune inflammatory index	systemic immune inflammatory index	systemic immune inflammatory index	Systemic immune-inflammatory index	1	kept
systemic inflammation response index	systemic inflammation response index	systemic inflammation response index	systemic inflammation response index	systemic inflammation response index	1	kept
SYSTEMIC INFLAMMATORY INTERLEUKIN-1-BETA	systemic inflammatory interleukin 1 beta	systemic inflammatory interleukin 1 beta	systemic inflammatory interleukin 1 beta	SYSTEMIC INFLAMMATORY INTERLEUKIN-1-BETA	1	kept
systemic mycosis	systemic mycosis	systemic mycosis	systemic mycosis	systemic mycosis	1	kept
systemic pulmonary shunt	systemic pulmonary shunt	systemic pulmonary shunt	systemic pulmonary shunt	systemic pulmonary shunt	1	kept
Systemic Racism	systemic racism	systemic racism	systemic racism	Systemic Racism	1	kept

systemic racism	systemic racism	systemic racism	systemic racism	Systemic Racism	1	case_or_format
Systemic sclerosis	systemic sclerosis	systemic sclerosis	systemic sclerosis	systemic sclerosis	1	case_or_format
systemic thrombolysis	systemic thrombolysis	systemic thrombolysis	systemic thrombolysis	systemic thrombolysis	1	kept
systemic vasculitis	systemic vasculitis	systemic vasculitis	systemic vasculitis	systemic vasculitis	1	kept
Systems approach	systems approach	systems approach	systems approach	Systems approach	1	kept
Systems Based Practice	systems based practice	systems based practice	systems based practice	Systems-Based Practice	1	case_or_format
systems change	systems change	systems change	systems change	systems change	1	kept
systems development	systems development	systems development	systems development	Systems development	1	case_or_format
Systems development life cycle	systems development life cycle	systems development life cycle	systems development life cycle	Systems development life cycle	1	kept
systems integration	systems integration	systems integration	systems integration	Systems Integration	1	case_or_format
Systems Management	systems management	systems management	systems management	Systems Management	1	kept
systems of care	systems of care	systems of care	systems of care	systems of care	1	kept
systems psychodynamics	systems psychodynamics	systems psychodynamics	systems psychodynamics	systems psychodynamics	1	kept
systems theory	systems theory	systems theory	systems theory	Systems Theory	1	case_or_format
Systems thinking	systems thinking	systems thinking	systems thinking	Systems thinking	1	kept
systems-based practice	systems based practice	systems based practice	systems based practice	Systems-Based Practice	1	case_or_format

systems-level error	systems level error	systems level error	systems level error	systems-level error	1	kept
système de santé	syst me de sant	syst me de sant	syst me de sant	système de santé	1	kept
Syzygium	syzygium	syzygium	syzygium	Syzygium	1	kept
Sécurité	s curit	s curit	s curit	sécurité	1	case_or_format
sédation	s dation	s dation	s dation	sédation	1	kept
Síndrome de Abstinência a Substâncias	s ndrome de abstin ncia a subst ncias	s ndrome de abstin ncia a subst ncias	s ndrome de abstin ncia a subst ncias	Síndrome de Abstinência a Substâncias	1	kept
Síndrome de Burnout	s ndrome de burnout	s ndrome de burnout	s ndrome de burnout	Síndrome de Burnout	1	kept
Síndrome post agudo de COVID-19	s ndrome post agudo de covid 19	s ndrome post agudo de covid 19	s ndrome post agudo de covid 19	Síndrome post agudo de COVID-19	1	kept
Síndrome post cuidados intensivos	s ndrome post cuidados intensivos	s ndrome post cuidados intensivos	s ndrome post cuidados intensivos	Síndrome post cuidados intensivos	1	kept
Síndrome post-cuidados intensivos	s ndrome post cuidados intensivos	s ndrome post cuidados intensivos	s ndrome post cuidados intensivos	Síndrome post cuidados intensivos	1	case_or_format
T cell	t cell	t cell	t cell	T cell	1	kept
T-Lymphocytes, Cytotoxic	t lymphocytes cytotoxic	t lymphocytes cytotoxic	t lymphocytes cytotoxic	T-Lymphocytes, Cytotoxic	1	kept
T-NOTECHS	t notechs	t notechs	t notechs	T-NOTECHS	1	kept
t-PA	t pa	t pa	t pa	t-PA	1	kept
T-piece resuscitator	t piece resuscitator	t piece resuscitator	t piece resuscitator	T-piece resuscitator	1	kept
T-TAQ	t taq	t taq	t taq	T-TAQ	1	kept

T-Test	t test	t test	t test	T-Test	1	kept
T6 health systems	t6 health systems	t6 health systems	t6 health systems	T6 health systems	1	kept
tabagism	tabagism	tabagism	tabagism	tabagism	1	kept
Table of organization	table of organization	table of organization	table of organization	Table of organization	1	kept
table of organization	table of organization	table of organization	table of organization	Table of organization	1	case_or_format
table-top simulator	table top simulator	table top simulator	table top simulator	table-top simulator	1	kept
Tablet	tablet	tablet	tablet	tablet	1	case_or_format
Tablet computer	tablet computer	tablet computer	tablet computer	Tablet computer	1	kept
tablet computer	tablet computer	tablet computer	tablet computer	Tablet computer	1	case_or_format
TABLET COMPUTERS	tablet computers	tablet computers	tablet computer	Tablet computer	1	singular
tablet manufacture	tablet manufacture	tablet manufacture	tablet manufacture	tablet manufacture	1	kept
tabletop simulator	tabletop simulator	tabletop simulator	tabletop simulator	tabletop simulator	1	kept
tabloid	tabloid	tabloid	tabloid	tabloid	1	kept
taboo	taboo	taboo	taboo	taboo	1	kept
Tabriz	tabriz	tabriz	tabriz	Tabriz	1	kept
Tachyarrhythmia	tachyarrhythmia	tachyarrhythmia	tachyarrhythmia	Tachyarrhythmia	1	kept

Tacit knowledge	tacit knowledge	tacit knowledge	tacit knowledge	Tacit knowledge	1	kept
tacit knowledge	tacit knowledge	tacit knowledge	tacit knowledge	Tacit knowledge	1	case_or_format
TACO	taco	taco	taco	TACO	1	kept
Tactical planning	tactical planning	tactical planning	tactical planning	Tactical planning	1	kept
Tactile cues	tactile cues	tactile cues	tactile cues	Tactile cues	1	kept
tactile kinesthetic stimulation	tactile kinesthetic stimulation	tactile kinesthetic stimulation	tactile kinesthetic stimulation	tactile kinesthetic stimulation	1	kept
Tactile stimuli	tactile stimuli	tactile stimuli	tactile stimuli	Tactile stimuli	1	kept
Tactile touch	tactile touch	tactile touch	tactile touch	Tactile touch	1	kept
TACTILE-KINESTHETIC STIMULATION	tactile kinesthetic stimulation	tactile kinesthetic stimulation	tactile kinesthetic stimulation	tactile kinesthetic stimulation	1	case_or_format
Taian	taian	taian	taian	Taian	1	kept
tailored	tailored	tailored	tailored	tailored	1	kept
Tailored implementation	tailored implementation	tailored implementation	tailored implementation	Tailored implementation	1	kept
Tailored training	tailored training	tailored training	tailored training	Tailored training	1	kept
Taiwan Society of Emergency Medicine	taiwan society of emergency medicine	taiwan society of emergency medicine	taiwan society of emergency medicine	Taiwan Society of Emergency Medicine	1	kept
taking care	taking care	taking care	taking care	taking care	1	kept
TAKOTSUBO CARDIOMYOPATHY	takotsubo cardiomyopathy	takotsubo cardiomyopathy	takotsubo cardiomyopathy	takotsubo cardiomyopathy	1	case_or_format

Talent attraction	talent attraction	talent attraction	talent attraction	Talent attraction	1	kept
Talent management	talent management	talent management	talent management	Talent management	1	kept
Talk	talk	talk	talk	Talk	1	kept
talk	talk	talk	talk	Talk	1	case_or_format
talking points	talking points	talking points	talking points	talking points	1	kept
TAM	tam	tam	tam	TAM	1	kept
TAMIS	tamis	tamis	tamis	TAMIS	1	kept
Tandem	tandem	tandem	tandem	Tandem	1	kept
tandem mass spectrometry	tandem mass spectrometry	tandem mass spectrometry	tandem mass spectrometry	Tandem Mass Spectrometry	1	case_or_format
TANDEM MASS-SPECTROMETRY	tandem mass spectrometry	tandem mass spectrometry	tandem mass spectrometry	Tandem Mass Spectrometry	1	case_or_format
taniborbactam	taniborbactam	taniborbactam	taniborbactam	taniborbactam	1	kept
tanzania	tanzania	tanzania	tanzania	Tanzania	1	case_or_format
Tap contamination	tap contamination	tap contamination	tap contamination	Tap contamination	1	kept
TAP-WATER	tap water	tap water	tap water	tap water	1	case_or_format
Tape blister	tape blister	tape blister	tape blister	Tape blister	1	kept
tape loosening	tape loosening	tape loosening	tape loosening	tape loosening	1	kept

tape measure	tape measure	tape measure	tape measure	tape measure	1	kept
tape recorder	tape recorder	tape recorder	tape recorder	tape recorder	1	kept
tapentadol	tapentadol	tapentadol	tapentadol	tapentadol	1	kept
taper	taper	taper	taper	taper	1	kept
TAR	tar	tar	tar	TAR	1	kept
tar	tar	tar	tar	TAR	1	case_or_format
tardive dyskinesia	tardive dyskinesia	tardive dyskinesia	tardive dyskinesia	tardive dyskinesia	1	kept
TARGET	target	target	target	TARGET	1	kept
target organ	target organ	target organ	target organ	target organ	1	kept
Target temperature management	target temperature management	target temperature management	target temperature management	Target temperature management	1	kept
target temperature management	target temperature management	target temperature management	target temperature management	Target temperature management	1	case_or_format
target-controlled infusion	target controlled infusion	target controlled infusion	target controlled infusion	target-controlled infusion	1	kept
targeted interventions	targeted interventions	targeted interventions	targeted interventions	targeted interventions	1	kept
targeted neonatal echocardiography	targeted neonatal echocardiography	targeted neonatal echocardiography	targeted neonatal echocardiography	targeted neonatal echocardiography	1	kept
targeted surveillance	targeted surveillance	targeted surveillance	targeted surveillance	targeted surveillance	1	kept
Targeted therapeutics	targeted therapeutics	targeted therapeutics	targeted therapeutics	Targeted therapeutics	1	kept

targeted therapy	targeted therapy	targeted therapy	targeted therapy	targeted therapy	1	kept
targeted training	targeted training	targeted training	targeted training	targeted training	1	kept
Targeting oxygen	targeting oxygen	targeting oxygen	targeting oxygen	Targeting oxygen	1	kept
Targeting policies	targeting policies	targeting policies	targeting policies	Targeting policies	1	kept
TARGETS	targets	targets	target	TARGET	1	singular
Tarification	tarification	tarification	tarification	Tarification	1	kept
tarlatamab	tarlatamab	tarlatamab	tarlatamab	tarlatamab	1	kept
task	task	task	task	tasks	1	case_or_format
Task advice networks	task advice networks	task advice networks	task advice networks	Task advice networks	1	kept
Task completion time	task completion time	task completion time	task completion time	Task Completion Time	1	case_or_format
Task delegation	task delegation	task delegation	task delegation	Task delegation	1	kept
task documentation	task documentation	task documentation	task documentation	task documentation	1	kept
task interruption	task interruption	task interruption	task interruption	task interruption	1	kept
task performance and analysis	task performance and analysis	task performance and analysis	task performance and analysis	Task Performance and Analysis	1	case_or_format
Task performance ond analysis	task performance ond analysis	task performance ond analysis	task performance ond analysis	Task performance ond analysis	1	kept
Task sharing	task sharing	task sharing	task sharing	Task sharing	1	kept

Task shift	task shift	task shift	task shift	Task shift	1	kept
task switching	task switching	task switching	task switching	task switching	1	kept
task trainers	task trainers	task trainers	task trainers	task trainers	1	kept
Task workloads	task workloads	task workloads	task workloads	Task workloads	1	kept
Task-based	task based	task based	task based	Task-based	1	kept
Task-oriented leadership	task oriented leadership	task oriented leadership	task oriented leadership	Task-oriented leadership	1	kept
Task-shifting	task shifting	task shifting	task shifting	task shifting	1	case_or_format
Task-specific models	task specific models	task specific models	task specific models	Task-specific models	1	kept
task-specific training	task specific training	task specific training	task specific training	task-specific training	1	kept
Tasks	tasks	tasks	task	tasks	1	singular
Tasks undone	tasks undone	tasks undone	tasks undone	Tasks undone	1	kept
tattoo	tattoo	tattoo	tattoo	tattoo	1	kept
Tattooing	tattooing	tattooing	tattooing	Tattooing	1	kept
tau protein	tau protein	tau protein	tau protein	tau protein	1	kept
taurine cattle	taurine cattle	taurine cattle	taurine cattle	taurine cattle	1	kept
taxane-induced pain	taxane induced pain	taxane induced pain	taxane induced pain	taxane-induced pain	1	kept

taxing job demands	taxing job demands	taxing job demands	taxing job demands	taxing job demands	1	kept
taxoid	taxoid	taxoid	taxoid	taxoid	1	kept
Taxoids	taxoids	taxoids	taxoid	taxoid	1	singular
taxonomic rank	taxonomic rank	taxonomic rank	taxonomic rank	taxonomic rank	1	kept
Taxonomies	taxonomies	taxonomies	taxonomy	taxonomy	1	singular
Taxonomy	taxonomy	taxonomy	taxonomy	taxonomy	1	case_or_format
Taxonomy of error	taxonomy of error	taxonomy of error	taxonomy of error	Taxonomy of error	1	kept
Taylor	taylor	taylor	taylor	Taylor	1	kept
TB stigma scale	tb stigma scale	tb stigma scale	tb stigma scale	TB stigma scale	1	kept
TBI, traumatic brain injury	tbi traumatic brain injury	tbi traumatic brain injury	tbi traumatic brain injury	TBI = traumatic brain injury	1	case_or_format
TC-POFRAS	tc pofras	tc pofras	tc pofras	TC-POFRAS	1	kept
TCAR	tcar	tcar	tcar	TCAR	1	kept
TCPC	tcpc	tcpc	tcpc	TCPC	1	kept
TDF	tdf	tdf	tdf	TDF	1	kept
TDvax	tdvax	tdvax	tdvax	TDvax	1	kept
tea consumption	tea consumption	tea consumption	tea consumption	tea consumption	1	kept

Tea Tree Oil	tea tree oil	tea tree oil	tea tree oil	Tea Tree Oil	1	kept
tea tree oil	tea tree oil	tea tree oil	tea tree oil	Tea Tree Oil	1	case_or_format
Teach back	teach back	teach back	teach back	Teach back	1	kept
teacher training	teacher training	teacher training	teacher training	teacher training	1	kept
Teacher Training [I02.358.984]	teacher training i02 358 984	teacher training i02 358 984	teacher training i02 358 984	Teacher Training [I02.358.984]	1	kept
TEACHERS	teachers	teachers	teacher	teacher	1	singular
Teaching and Learning	teaching and learning	teaching and learning	teaching and learning	Teaching and learning	1	case_or_format
Teaching and research	teaching and research	teaching and research	teaching and research	Teaching and research	1	kept
teaching assistant	teaching assistant	teaching assistant	teaching assistant	teaching assistant	1	kept
Teaching communication	teaching communication	teaching communication	teaching communication	Teaching communication	1	kept
teaching effectiveness	teaching effectiveness	teaching effectiveness	teaching effectiveness	teaching effectiveness	1	kept
Teaching exercise	teaching exercise	teaching exercise	teaching exercise	Teaching exercise	1	kept
Teaching Hospital	teaching hospital	teaching hospital	teaching hospital	teaching hospital	1	case_or_format
teaching hospitals	teaching hospitals	teaching hospitals	teaching hospital	teaching hospital	1	singular
Teaching interventions	teaching interventions	teaching interventions	teaching interventions	Teaching interventions	1	kept
teaching methodology simulation	teaching methodology simulation	teaching methodology simulation	teaching methodology simulation	teaching methodology simulation	1	kept

Teaching Methods	teaching methods	teaching methods	teaching methods	teaching methods	1	case_or_format
Teaching module	teaching module	teaching module	teaching module	Teaching module	1	kept
Teaching Programme	teaching programme	teaching programme	teaching programme	Teaching Programme	1	kept
Teaching reform	teaching reform	teaching reform	teaching reform	Teaching reform	1	kept
TEACHING SURGICAL SKILLS	teaching surgical skills	teaching surgical skills	teaching surgical skills	TEACHING SURGICAL SKILLS	1	kept
TEACHING THINKING	teaching thinking	teaching thinking	teaching thinking	TEACHING THINKING	1	kept
Teaching-learning process	teaching learning process	teaching learning process	teaching learning process	Teaching-learning process	1	kept
Teaching-learning strategies	teaching learning strategies	teaching learning strategies	teaching learning strategies	Teaching-learning strategies	1	kept
Team approach	team approach	team approach	team approach	Team approach	1	kept
team approach	team approach	team approach	team approach	Team approach	1	case_or_format
Team assessment	team assessment	team assessment	team assessment	Team assessment	1	kept
team based intervention	team based intervention	team based intervention	team based intervention	team based intervention	1	kept
Team briefing	team briefing	team briefing	team briefing	Team briefing	1	kept
team briefing	team briefing	team briefing	team briefing	Team briefing	1	case_or_format
team cardiopulmonary resuscitation	team cardiopulmonary resuscitation	team cardiopulmonary resuscitation	team cardiopulmonary resuscitation	team cardiopulmonary resuscitation	1	kept
team cohesion	team cohesion	team cohesion	team cohesion	team cohesion	1	kept

Team collaboration	team collaboration	team collaboration	team collaboration	Team collaboration	1	kept
Team composition	team composition	team composition	team composition	Team composition	1	kept
team composition	team composition	team composition	team composition	Team composition	1	case_or_format
team conference	team conference	team conference	team conference	team conference	1	kept
team debrief	team debrief	team debrief	team debrief	team debrief	1	kept
Team discordance	team discordance	team discordance	team discordance	Team discordance	1	kept
team emergency assessment measure	team emergency assessment measure	team emergency assessment measure	team emergency assessment measure	Team Emergency Assessment Measure	1	case_or_format
Team factors	team factors	team factors	team factors	Team factors	1	kept
TEAM FAMILIARITY	team familiarity	team familiarity	team familiarity	TEAM FAMILIARITY	1	kept
Team function	team function	team function	team function	Team function	1	kept
Team functioning	team functioning	team functioning	team functioning	Team functioning	1	kept
team intelligence	team intelligence	team intelligence	team intelligence	team intelligence	1	kept
Team leaders	team leaders	team leaders	team leaders	Team leaders	1	kept
Team leadership	team leadership	team leadership	team leadership	Team leadership	1	kept
Team management	team management	team management	team management	Team management	1	kept
team meeting	team meeting	team meeting	team meeting	team meeting	1	kept

Team meetings	team meetings	team meetings	team meeting	team meeting	1	singular
team meetings	team meetings	team meetings	team meeting	team meeting	1	singular
team member effectiveness	team member effectiveness	team member effectiveness	team member effectiveness	team member effectiveness	1	kept
TEAM MEMBERS	team members	team members	team members	TEAM MEMBERS	1	kept
Team members	team members	team members	team members	TEAM MEMBERS	1	case_or_format
team outcomes	team outcomes	team outcomes	team outcomes	team outcomes	1	kept
team performance	team performance	team performance	team performance	Team performance	1	case_or_format
team performance observation tool	team performance observation tool	team performance observation tool	team performance observation tool	team performance observation tool	1	kept
Team processes	team processes	team processes	team processes	Team processes	1	kept
Team reflexivity	team reflexivity	team reflexivity	team reflexivity	Team reflexivity	1	kept
team reflexivity	team reflexivity	team reflexivity	team reflexivity	Team reflexivity	1	case_or_format
Team resource management	team resource management	team resource management	team resource management	Team resource management	1	kept
team resource management	team resource management	team resource management	team resource management	Team resource management	1	case_or_format
Team resuscitation	team resuscitation	team resuscitation	team resuscitation	Team resuscitation	1	kept
team science	team science	team science	team science	team science	1	kept
Team situation awareness	team situation awareness	team situation awareness	team situation awareness	Team situation awareness	1	kept

Team size and composition	team size and composition	team size and composition	team size and composition	Team size and composition	1	kept
Team skills	team skills	team skills	team skills	Team skills	1	kept
team STEPPS	team stepps	team stepps	team stepps	team STEPPS	1	kept
team strategy	team strategy	team strategy	team strategy	team strategy	1	kept
team structure	team structure	team structure	team structure	team structure	1	kept
Team Survey Objective	team survey objective	team survey objective	team survey objective	Team Survey Objective	1	kept
Team work climate	team work climate	team work climate	team work climate	Team work climate	1	kept
Team Working	team working	team working	team working	Team working	1	case_or_format
Team working and integration	team working and integration	team working and integration	team working and integration	Team working and integration	1	kept
Team-based care	team based care	team based care	team based care	team-based care	1	case_or_format
Team-based learning	team based learning	team based learning	team based learning	Team-Based Learning	1	case_or_format
team-based models of care	team based models of care	team based models of care	team based models of care	team-based models of care	1	kept
team-Based Practice	team based practice	team based practice	team based practice	team-Based Practice	1	kept
team-based staffing	team based staffing	team based staffing	team based staffing	team-based staffing	1	kept
team-based training	team based training	team based training	team based training	team-based training	1	kept
TeamSTEPPS<sup>®<	teamstepps sup	teamstepps sup	teamstepps sup	TeamSTEPPS<sup>®<	1	kept

teamwork and communication	teamwork and communication	teamwork and communication	teamwork and communication	teamwork and communication	1	kept
teamwork attitude questionnaire	teamwork attitude questionnaire	teamwork attitude questionnaire	teamwork attitude questionnaire	teamwork attitude questionnaire	1	kept
teamwork climate	teamwork climate	teamwork climate	teamwork climate	teamwork climate	1	kept
teamwork in medicine	teamwork in medicine	teamwork in medicine	teamwork in medicine	teamwork in medicine	1	kept
Teamwork measuring	teamwork measuring	teamwork measuring	teamwork measuring	Teamwork measuring	1	kept
teamwork measuring	teamwork measuring	teamwork measuring	teamwork measuring	Teamwork measuring	1	case_or_format
teamwork perceptions	teamwork perceptions	teamwork perceptions	teamwork perceptions	teamwork perceptions	1	kept
Teamwork skills	teamwork skills	teamwork skills	teamwork skills	Teamwork skills	1	kept
teamwork skills	teamwork skills	teamwork skills	teamwork skills	Teamwork skills	1	case_or_format
teamwork training	teamwork training	teamwork training	teamwork training	teamwork training	1	kept
TEARS	tears	tears	tears	Tears	1	case_or_format
technical aid	technical aid	technical aid	technical aid	technical aid	1	kept
technical and domestic health care assistance	technical and domestic health care assistance	technical and domestic health care assistance	technical and domestic health care assistance	technical and domestic health care assistance	1	kept
Technical and non-technical skills	technical and non technical skills	technical and non technical skills	technical and non technical skills	Technical and non-technical skills	1	kept
Technical aspects of research	technical aspects of research	technical aspects of research	technical aspects of research	Technical aspects of research	1	kept
Technical development	technical development	technical development	technical development	Technical development	1	kept

TECHNICAL QUALITY	technical quality	technical quality	technical quality	TECHNICAL QUALITY	1	kept
technical service	technical service	technical service	technical service	technical service	1	kept
Technical skill	technical skill	technical skill	technical skill	Technical skill	1	kept
Technical skills	technical skills	technical skills	technical skill	Technical skill	1	singular
Technical systems	technical systems	technical systems	technical systems	Technical systems	1	kept
technician	technician	technician	technician	technician	1	kept
Technicians	technicians	technicians	technician	technician	1	singular
technique for order preference by similarity to ideal	technique for order preference by similarity to ideal	technique for order preference by similarity to ideal	technique for order preference by similarity to ideal	technique for order preference by similarity to ideal	1	kept
Techniques	techniques	techniques	technique	technique	1	singular
techniques	techniques	techniques	technique	technique	1	singular
Techniques & procedures	techniques and procedures	techniques and procedures	techniques and procedure	techniques and procedures	1	singular
techniques and procedure	techniques and procedure	techniques and procedure	techniques and procedure	techniques and procedures	1	case_or_format
technological advancement	technological advancement	technological advancement	technological advancement	technological advancement	1	kept
Technological caring	technological caring	technological caring	technological caring	Technological caring	1	kept
Technological competence	technological competence	technological competence	technological competence	Technological competence	1	kept
Technological competency	technological competency	technological competency	technological competency	Technological competency	1	kept

Technological Development	technological development	technological development	technological development	Technological Development	1	kept
Technological innovation	technological innovation	technological innovation	technological innovation	technological innovation	1	case_or_format
Technological innovations	technological innovations	technological innovations	technological innovation	technological innovation	1	singular
technological innovations	technological innovations	technological innovations	technological innovation	technological innovation	1	singular
Technological instruments	technological instruments	technological instruments	technological instruments	Technological instruments	1	kept
technological instruments	technological instruments	technological instruments	technological instruments	Technological instruments	1	case_or_format
Technological knowing	technological knowing	technological knowing	technological knowing	Technological knowing	1	kept
Technological resources	technological resources	technological resources	technological resources	Technological resources	1	kept
TECHNOLOGIES	technologies	technologies	technology	Technology	1	singular
Technologies	technologies	technologies	technology	Technology	1	singular
Technologies and applications	technologies and applications	technologies and applications	technologies and applications	Technologies and applications	1	kept
technologist	technologist	technologist	technologist	technologist	1	kept
technology adoption	technology adoption	technology adoption	technology adoption	Technology adoption	1	case_or_format
Technology adoption, information technology	technology adoption information technology	technology adoption information technology	technology adoption information technology	Technology adoption, information technology	1	kept
Technology Burden	technology burden	technology burden	technology burden	Technology Burden	1	kept
technology dependence	technology dependence	technology dependence	technology dependence	technology dependence	1	kept

Technology implementation	technology implementation	technology implementation	technology implementation	Technology implementation	1	kept
Technology in operating room	technology in operating room	technology in operating room	technology in operating room	Technology in operating room	1	kept
Technology interactions	technology interactions	technology interactions	technology interactions	Technology interactions	1	kept
Technology readiness index	technology readiness index	technology readiness index	technology readiness index	Technology readiness index	1	kept
technology selection	technology selection	technology selection	technology selection	technology selection	1	kept
Technology-based	technology based	technology based	technology based	Technology-based	1	kept
Technology-based care	technology based care	technology based care	technology based care	Technology-based care	1	kept
Technology-based education	technology based education	technology based education	technology based education	Technology-based education	1	kept
technology-based education	technology based education	technology based education	technology based education	Technology-based education	1	case_or_format
Technology-based interventions	technology based interventions	technology based interventions	technology based interventions	Technology-based interventions	1	kept
technology-dependent child	technology dependent child	technology dependent child	technology dependent child	technology-dependent child	1	kept
Technology-dependent infant	technology dependent infant	technology dependent infant	technology dependent infant	Technology-dependent infant	1	kept
technology-enhanced education	technology enhanced education	technology enhanced education	technology enhanced education	technology-enhanced education	1	kept
Tecnologia	tecnologia	tecnologia	tecnologia	Tecnologia	1	kept
Tecnologia Educacional	tecnologia educacional	tecnologia educacional	tecnologia educacional	Tecnologia Educacional	1	kept
Tecnología	tecnolog a	tecnolog a	tecnolog a	Tecnología	1	kept

Tecnología Educacional	tecnolog a educacional	tecnolog a educacional	tecnolog a educacional	Tecnología Educacional	1	kept
teeth brushing	teeth brushing	teeth brushing	teeth brushing	teeth brushing	1	kept
TEG	teg	teg	teg	TEG	1	kept
Tegaderm	tegaderm	tegaderm	tegaderm	Tegaderm	1	kept
Teicoplanin	teicoplanin	teicoplanin	teicoplanin	teicoplanin	1	case_or_format
telavancin	telavancin	telavancin	telavancin	telavancin	1	kept
tele education	tele education	tele education	tele education	tele education	1	kept
tele-CCM	tele ccm	tele ccm	tele ccm	tele-CCM	1	kept
Tele-education	tele education	tele education	tele education	tele education	1	case_or_format
Tele-Health	tele health	tele health	tele health	Tele-Health	1	kept
tele-intensivist	tele intensivist	tele intensivist	tele intensivist	Tele-intensivist	1	case_or_format
Tele-medicine	tele medicine	tele medicine	tele medicine	Tele-medicine	1	kept
tele-medicine	tele medicine	tele medicine	tele medicine	Tele-medicine	1	case_or_format
tele-operated android robot	tele operated android robot	tele operated android robot	tele operated android robot	tele-operated android robot	1	kept
tele-pharmacy	tele pharmacy	tele pharmacy	tele pharmacy	tele-pharmacy	1	kept
Tele-rounding	tele rounding	tele rounding	tele rounding	Tele-rounding	1	kept

Tele-support	tele support	tele support	tele support	Tele-support	1	kept
Tele-ultrasound	tele ultrasound	tele ultrasound	tele ultrasound	Tele-ultrasound	1	kept
tele-ultrasound	tele ultrasound	tele ultrasound	tele ultrasound	Tele-ultrasound	1	case_or_format
Telecare	telecare	telecare	telecare	telecare	1	case_or_format
telecoaching	telecoaching	telecoaching	telecoaching	telecoaching	1	kept
Telecommunication networks	telecommunication networks	telecommunication networks	telecommunication networks	Telecommunication networks	1	kept
Telecommunication services	telecommunication services	telecommunication services	telecommunication services	Telecommunication services	1	kept
Teledebriefing	teledebriefing	teledebriefing	teledebriefing	Teledebriefing	1	kept
teledebriefing	teledebriefing	teledebriefing	teledebriefing	Teledebriefing	1	case_or_format
TELEDERMATOLOGY	teledermatology	teledermatology	teledermatology	TELEDERMATOLOGY	1	kept
Telematics	telematics	telematics	telematics	Telematics	1	kept
TELEMEDICINE	telemedicine	telemedicine	telemedicine	Telemedicine	1	case_or_format
telemedicine adoption	telemedicine adoption	telemedicine adoption	telemedicine adoption	telemedicine adoption	1	kept
Telemedicine application	telemedicine application	telemedicine application	telemedicine application	Telemedicine application	1	kept
telemedicine innovations	telemedicine innovations	telemedicine innovations	telemedicine innovations	telemedicine innovations	1	kept
Telemedicine systems	telemedicine systems	telemedicine systems	telemedicine systems	Telemedicine systems	1	kept

TELEMETRY	telemetry	telemetry	telemetry	Telemetry	1	case_or_format
TELEMETRY BEDS	telemetry beds	telemetry beds	telemetry beds	TELEMETRY BEDS	1	kept
Telemetry monitoring	telemetry monitoring	telemetry monitoring	telemetry monitoring	Telemetry monitoring	1	kept
Telemetry unit	telemetry unit	telemetry unit	telemetry unit	Telemetry unit	1	kept
Telemonitoring	telemonitoring	telemonitoring	telemonitoring	telemonitoring	1	case_or_format
teleophthalmology	teleophthalmology	teleophthalmology	teleophthalmology	teleophthalmology	1	kept
teleost	teleost	teleost	teleost	teleost	1	kept
Teleostei	teleostei	teleostei	teleostei	Teleostei	1	kept
telephone calls	telephone calls	telephone calls	telephone calls	telephone calls	1	kept
Telephone circuits	telephone circuits	telephone circuits	telephone circuits	Telephone circuits	1	kept
telephone counseling	telephone counseling	telephone counseling	telephone counseling	telephone counseling	1	kept
Telephone counselling	telephone counselling	telephone counselling	telephone counselling	Telephone counselling	1	kept
telephone follow up	telephone follow up	telephone follow up	telephone follow up	telephone follow up	1	kept
Telephone follow-up	telephone follow up	telephone follow up	telephone follow up	telephone follow up	1	case_or_format
Telephone helpline	telephone helpline	telephone helpline	telephone helpline	Telephone helpline	1	kept
Telephone interview	telephone interview	telephone interview	telephone interview	telephone interview	1	case_or_format

telephone recorded interview	telephone recorded interview	telephone recorded interview	telephone recorded interview	telephone recorded interview	1	kept
Telephone sets	telephone sets	telephone sets	telephone sets	Telephone sets	1	kept
telepresence	telepresence	telepresence	telepresence	Telepresence	1	case_or_format
telepsychotherapy	telepsychotherapy	telepsychotherapy	telepsychotherapy	telepsychotherapy	1	kept
Teleradiology	teleradiology	teleradiology	teleradiology	teleradiology	1	case_or_format
Telerounding	telerounding	telerounding	telerounding	Telerounding	1	kept
telescope	telescope	telescope	telescope	telescope	1	kept
telescreening	telescreening	telescreening	telescreening	telescreening	1	kept
Telestroke	telestroke	telestroke	telestroke	Telestroke	1	kept
telestroke	telestroke	telestroke	telestroke	Telestroke	1	case_or_format
Teleworking	teleworking	teleworking	teleworking	Teleworking	1	kept
telithromycin	telithromycin	telithromycin	telithromycin	telithromycin	1	kept
TELL US	tell us	tell us	tell us	TELL US	1	kept
Tellurium compounds	tellurium compounds	tellurium compounds	tellurium compounds	Tellurium compounds	1	kept
Teléfonos inteligentes	tel fonos inteligentes	tel fonos inteligentes	tel fonos inteligentes	Teléfonos inteligentes	1	kept
TEM	tem	tem	tem	TEM	1	kept

TEM-206	tem 206	tem 206	tem 206	TEM-206	1	kept
temperature management device	temperature management device	temperature management device	temperature management device	temperature management device	1	kept
Temperature parameters	temperature parameters	temperature parameters	temperature parameters	Temperature parameters	1	kept
temperature regulation	temperature regulation	temperature regulation	temperature regulation	temperature regulation	1	kept
Temperature sensor	temperature sensor	temperature sensor	temperature sensor	Temperature sensor	1	kept
Temperature sensors	temperature sensors	temperature sensors	temperature sensor	Temperature sensor	1	singular
temperature stress	temperature stress	temperature stress	temperature stress	temperature stress	1	kept
template analysis	template analysis	template analysis	template analysis	template analysis	1	kept
templates	templates	templates	templates	templates	1	kept
temporal analysis	temporal analysis	temporal analysis	temporal analysis	temporal analysis	1	kept
Temporal artery temperature	temporal artery temperature	temporal artery temperature	temporal artery temperature	Temporal artery temperature	1	kept
temporal artery temperature	temporal artery temperature	temporal artery temperature	temporal artery temperature	Temporal artery temperature	1	case_or_format
temporal artery thermometer	temporal artery thermometer	temporal artery thermometer	temporal artery thermometer	temporal artery thermometer	1	kept
temporal cortex	temporal cortex	temporal cortex	temporal cortex	temporal cortex	1	kept
Temporal evolution	temporal evolution	temporal evolution	temporal evolution	Temporal evolution	1	kept
temporal lobe epilepsy	temporal lobe epilepsy	temporal lobe epilepsy	temporal lobe epilepsy	temporal lobe epilepsy	1	kept

Temporal network analysis	temporal network analysis	temporal network analysis	temporal network analysis	Temporal network analysis	1	kept
Temporal profile	temporal profile	temporal profile	temporal profile	Temporal profile	1	kept
temporal stability	temporal stability	temporal stability	temporal stability	temporal stability	1	kept
temporal variation	temporal variation	temporal variation	temporal variation	temporal variation	1	kept
temporalis muscle	temporalis muscle	temporalis muscle	temporalis muscle	temporalis muscle	1	kept
temporality	temporality	temporality	temporality	temporality	1	kept
Temporary agency work	temporary agency work	temporary agency work	temporary agency work	Temporary agency work	1	kept
Temporary agency workers	temporary agency workers	temporary agency workers	temporary agency workers	Temporary agency workers	1	kept
temporary employment	temporary employment	temporary employment	temporary employment	temporary employment	1	kept
temporary hospital	temporary hospital	temporary hospital	temporary hospital	temporary hospital	1	kept
temporary mechanical circulatory support	temporary mechanical circulatory support	temporary mechanical circulatory support	temporary mechanical circulatory support	temporary mechanical circulatory support	1	kept
Temporary work	temporary work	temporary work	temporary work	Temporary work	1	kept
TEN	ten	ten	ten	TEN	1	kept
Ten true rights	ten true rights	ten true rights	ten true rights	Ten true rights	1	kept
tena bed plus pad	tena bed plus pad	tena bed plus pad	tena bed plus pad	tena bed plus pad	1	kept
tena bed super pad	tena bed super pad	tena bed super pad	tena bed super pad	tena bed super pad	1	kept

tendency to make medical errors	tendency to make medical errors	tendency to make medical errors	tendency to make medical errors	tendency to make medical errors	1	kept
tendon injury	tendon injury	tendon injury	tendon injury	tendon injury	1	kept
tenesmus	tenesmus	tenesmus	tenesmus	tenesmus	1	kept
Tenivac	tenivac	tenivac	tenivac	Tenivac	1	kept
tennis elbow	tennis elbow	tennis elbow	tennis elbow	tennis elbow	1	kept
Tenofovir	tenofovir	tenofovir	tenofovir	tenofovir	1	case_or_format
tenofovir alafenamide	tenofovir alafenamide	tenofovir alafenamide	tenofovir alafenamide	tenofovir alafenamide	1	kept
tenofovir disoproxil fumarate	tenofovir disoproxil fumarate	tenofovir disoproxil fumarate	tenofovir disoproxil fumarate	tenofovir disoproxil fumarate	1	kept
TENSION	tension	tension	tension	tension	1	case_or_format
Tension	tension	tension	tension	tension	1	case_or_format
Tension pneumothorax	tension pneumothorax	tension pneumothorax	tension pneumothorax	tension pneumothorax	1	case_or_format
tensor fascia lata muscle	tensor fascia lata muscle	tensor fascia lata muscle	tensor fascia lata muscle	tensor fascia lata muscle	1	kept
tenure	tenure	tenure	tenure	tenure	1	kept
Teoría de las transiciones	teor a de las transiciones	teor a de las transiciones	teor a de las transiciones	Teoría de las transiciones	1	kept
Teoría de rango medio de enfermería	teor a de rango medio de enfermer a	teor a de rango medio de enfermer a	teor a de rango medio de enfermer a	Teoría de rango medio de enfermería	1	kept
Teoría de Watson	teor a de watson	teor a de watson	teor a de watson	Teoría de Watson	1	kept

Terapia física	terapia f sica	terapia f sica	terapia f sica	Terapia física	1	kept
Terapia Intensiva Neonatal	terapia intensiva neonatal	terapia intensiva neonatal	terapia intensiva neonatal	Terapia Intensiva Neonatal	1	kept
teratogenicity	teratogenicity	teratogenicity	teratogenicity	teratogenicity	1	kept
Teratology	teratology	teratology	teratology	Teratology	1	kept
terazosin	terazosin	terazosin	terazosin	terazosin	1	kept
terbutaline sulfate	terbutaline sulfate	terbutaline sulfate	terbutaline sulfate	terbutaline sulfate	1	kept
TERCAP	tercap	tercap	tercap	TERCAP	1	kept
terfenadine	terfenadine	terfenadine	terfenadine	terfenadine	1	kept
Terlipressin	terlipressin	terlipressin	terlipressin	terlipressin	1	case_or_format
TERM FOLLOW-UP	term follow up	term follow up	term follow up	TERM FOLLOW-UP	1	kept
TERM INDWELLING CATHETERS	term indwelling catheters	term indwelling catheters	term indwelling catheters	TERM INDWELLING CATHETERS	1	kept
term infant	term infant	term infant	term infant	Term infant	1	case_or_format
Term infants	term infants	term infants	term infant	Term infant	1	singular
term newborn	term newborn	term newborn	term newborn	term newborn	1	kept
TERM OUTCOMES	term outcomes	term outcomes	term outcomes	TERM OUTCOMES	1	kept
termaset plus suspension	termaset plus suspension	termaset plus suspension	termaset plus suspension	termaset plus suspension	1	kept

TERMINAL EXTUBATION	terminal extubation	terminal extubation	terminal extubation	terminal extubation	1	case_or_format
Terminal Extubation	terminal extubation	terminal extubation	terminal extubation	terminal extubation	1	case_or_format
TERMINAL ILLNESS	terminal illness	terminal illness	terminal illness	Terminal illness	1	case_or_format
terminal illnesses	terminal illnesses	terminal illnesses	terminal illnesses	terminal illnesses	1	kept
Terminally ill children	terminally ill children	terminally ill children	terminally ill children	Terminally ill children	1	kept
terminally ill children	terminally ill children	terminally ill children	terminally ill children	Terminally ill children	1	case_or_format
TERMINALLY-ILL	terminally ill	terminally ill	terminally ill	Terminally Ill	1	case_or_format
termination of cardiopulmonary resuscitation	termination of cardiopulmonary resuscitation	termination of cardiopulmonary resuscitation	termination of cardiopulmonary resuscitation	termination of cardiopulmonary resuscitation	1	kept
termination of caring relationship	termination of caring relationship	termination of caring relationship	termination of caring relationship	termination of caring relationship	1	kept
termination of life without explicit request	termination of life without explicit request	termination of life without explicit request	termination of life without explicit request	termination of life without explicit request	1	kept
Termination of pregnancy	termination of pregnancy	termination of pregnancy	termination of pregnancy	Termination of pregnancy	1	kept
termination of pregnancy	termination of pregnancy	termination of pregnancy	termination of pregnancy	Termination of pregnancy	1	case_or_format
Terminologia Padronizada em Enfermagem	terminologia padronizada em enfermagem	terminologia padronizada em enfermagem	terminologia padronizada em enfermagem	Terminologia Padronizada em Enfermagem	1	kept
terminologies	terminologies	terminologies	terminology	Terminology	1	singular
terminology	terminology	terminology	terminology	Terminology	1	case_or_format
Terminología de enfermería estandarizada	terminolog a de enfermer a estandarizada	terminolog a de enfermer a estandarizada	terminolog a de enfermer a estandarizada	Terminología de enfermería estandarizada	1	kept

Terminología Normalizada de Enfermería	terminolog a normalizada de enfermer a	terminolog a normalizada de enfermer a	terminolog a normalizada de enfermer a	Terminología Normalizada de Enfermería	1	kept
Terminología normalizada de enfermería	terminolog a normalizada de enfermer a	terminolog a normalizada de enfermer a	terminolog a normalizada de enfermer a	Terminología Normalizada de Enfermería	1	case_or_format
terms	terms	terms	term	TERM	1	singular
Terror attack	terror attack	terror attack	terror attack	Terror attack	1	kept
Terrorism and medical emergencies	terrorism and medical emergencies	terrorism and medical emergencies	terrorism and medical emergencies	Terrorism and medical emergencies	1	kept
Tertiary Care Centres	tertiary care centres	tertiary care centres	tertiary care centres	Tertiary Care Centres	1	kept
Tertiary care facility	tertiary care facility	tertiary care facility	tertiary care facility	Tertiary care facility	1	kept
Tertiary care management process	tertiary care management process	tertiary care management process	tertiary care management process	Tertiary care management process	1	kept
Tertiary Hospital	tertiary hospital	tertiary hospital	tertiary hospital	Tertiary hospital	1	case_or_format
Tertiary Hospitals	tertiary hospitals	tertiary hospitals	tertiary hospital	Tertiary hospital	1	singular
tertiary hospitals	tertiary hospitals	tertiary hospitals	tertiary hospital	Tertiary hospital	1	singular
tertiary hospitals disaster simulation	tertiary hospitals disaster simulation	tertiary hospitals disaster simulation	tertiary hospitals disaster simulation	tertiary hospitals disaster simulation	1	kept
Tertiary level care	tertiary level care	tertiary level care	tertiary level care	Tertiary level care	1	kept
Tertiary Public Hospital	tertiary public hospital	tertiary public hospital	tertiary public hospital	Tertiary Public Hospital	1	kept
Test Anxiety Scale	test anxiety scale	test anxiety scale	test anxiety scale	Test Anxiety Scale	1	kept
Test construction	test construction	test construction	test construction	Test construction	1	kept

Test development	test development	test development	test development	Test development	1	kept
Test for goodness of fit	test for goodness of fit	test for goodness of fit	test for goodness of fit	Test for goodness of fit	1	kept
Test for independence	test for independence	test for independence	test for independence	Test for independence	1	kept
Test of Infant Motor Performance	test of infant motor performance	test of infant motor performance	test of infant motor performance	Test of Infant Motor Performance	1	kept
Test reliability	test reliability	test reliability	test reliability	Test reliability	1	kept
test strip	test strip	test strip	test strip	test strip	1	kept
Test validation	test validation	test validation	test validation	Test validation	1	kept
test validity	test validity	test validity	test validity	test validity	1	kept
test-retest	test retest	test retest	test retest	test-retest	1	kept
test-weighing	test weighing	test weighing	test weighing	test-weighing	1	kept
Testamento vital	testamento vital	testamento vital	testamento vital	Testamento vital	1	kept
TESTING KANTER THEORY	testing kanter theory	testing kanter theory	testing kanter theory	TESTING KANTER THEORY	1	kept
Testing pediatric emergency department	testing pediatric emergency department	testing pediatric emergency department	testing pediatric emergency department	Testing pediatric emergency department	1	kept
testis injury	testis injury	testis injury	testis injury	testis injury	1	kept
testis torsion	testis torsion	testis torsion	testis torsion	testis torsion	1	kept
TESTS	tests	tests	tests	TESTS	1	kept

Test‐retest reliability	test retest reliability	test retest reliability	test retest reliability	test retest reliability	1	case_or_format
tet bacterial gene	tet bacterial gene	tet bacterial gene	tet bacterial gene	tet bacterial gene	1	kept
Tetanus Antitoxin	tetanus antitoxin	tetanus antitoxin	tetanus antitoxin	Tetanus Antitoxin	1	kept
Tetanus Toxin	tetanus toxin	tetanus toxin	tetanus toxin	Tetanus Toxin	1	kept
tetanus toxin	tetanus toxin	tetanus toxin	tetanus toxin	Tetanus Toxin	1	case_or_format
tetM gene	tetm gene	tetm gene	tetm gene	tetM gene	1	kept
tetrahydrolipstatin	tetrahydrolipstatin	tetrahydrolipstatin	tetrahydrolipstatin	tetrahydrolipstatin	1	kept
tetraplegia	tetraplegia	tetraplegia	tetraplegia	tetraplegia	1	kept
Texas Instruments	texas instruments	texas instruments	texas instruments	Texas Instruments	1	kept
text analysis	text analysis	text analysis	text analysis	text analysis	1	kept
Text based classification	text based classification	text based classification	text based classification	Text based classification	1	kept
text mining	text mining	text mining	text mining	Text mining	1	case_or_format
Text network analysis	text network analysis	text network analysis	text network analysis	Text network analysis	1	kept
Text processing	text processing	text processing	text processing	Text processing	1	kept
text-mining study	text mining study	text mining study	text mining study	text-mining study	1	kept
Texture feature extraction	texture feature extraction	texture feature extraction	texture feature extraction	Texture feature extraction	1	kept

Textures	textures	textures	textures	Textures	1	kept
TFA	tfa	tfa	tfa	TFA	1	kept
Th17 cell	th17 cell	th17 cell	th17 cell	Th17 cell	1	kept
Th2 cell	th2 cell	th2 cell	th2 cell	Th2 cell	1	kept
Thai Buddhist	thai buddhist	thai buddhist	thai buddhist	Thai Buddhist	1	kept
Thai version	thai version	thai version	thai version	Thai version	1	kept
ThailandEnd-of-life care	thailandend of life care	thailandend of life care	thailandend of life care	ThailandEnd-of-life care	1	kept
Thalamic function	thalamic function	thalamic function	thalamic function	Thalamic function	1	kept
Thalamic Nuclei	thalamic nuclei	thalamic nuclei	thalamic nuclei	Thalamic Nuclei	1	kept
thalamic pain	thalamic pain	thalamic pain	thalamic pain	thalamic pain	1	kept
thalassemia	thalassemia	thalassemia	thalassemia	thalassemia	1	kept
Thallium Radioisotopes	thallium radioisotopes	thallium radioisotopes	thallium radioisotopes	Thallium Radioisotopes	1	kept
Thamnaconus modestus	thamnaconus modestus	thamnaconus modestus	thamnaconus modestus	Thamnaconus modestus	1	kept
Thanatophoric Dysplasia	thanatophoric dysplasia	thanatophoric dysplasia	thanatophoric dysplasia	Thanatophoric Dysplasia	1	kept
THC	thc	thc	thc	THC	1	kept
The 3-milliliter water swallowing test	the 3 milliliter water swallowing test	the 3 milliliter water swallowing test	the 3 milliliter water swallowing test	The 3-milliliter water swallowing test	1	kept

The chronically critically ill	the chronically critically ill	the chronically critically ill	the chronically critically ill	The chronically critically ill	1	kept
The CO-PARTNER tool	the co partner tool	the co partner tool	the co partner tool	The CO-PARTNER tool	1	kept
the early years	the early years	the early years	the early years	the early years	1	kept
The elderly	the elderly	the elderly	the elderly	The elderly	1	kept
the elderly	the elderly	the elderly	the elderly	The elderly	1	case_or_format
the elderly population	the elderly population	the elderly population	the elderly population	the elderly population	1	kept
The ethics of care	the ethics of care	the ethics of care	the ethics of care	The ethics of care	1	kept
The factors affecting sleep pattern	the factors affecting sleep pattern	the factors affecting sleep pattern	the factors affecting sleep pattern	The factors affecting sleep pattern	1	kept
the FOUR scale	the four scale	the four scale	the four scale	the FOUR scale	1	kept
The Gambia hospitals	the gambia hospitals	the gambia hospitals	the gambia hospitals	The Gambia hospitals	1	kept
the Glasgow Coma Scale	the glasgow coma scale	the glasgow coma scale	the glasgow coma scale	the Glasgow Coma Scale	1	kept
The Hawthorne effect	the hawthorne effect	the hawthorne effect	the hawthorne effect	The Hawthorne effect	1	kept
The heart rate variability	the heart rate variability	the heart rate variability	the heart rate variability	The heart rate variability	1	kept
The Hospital Anxiety and Depression Scale	the hospital anxiety and depression scale	the hospital anxiety and depression scale	the hospital anxiety and depression scale	The Hospital Anxiety and Depression Scale	1	kept
The International Organization for Standardization	the international organization for standardization	the international organization for standardization	the international organization for standardization	The International Organization for Standardization	1	kept
The Johns Hopkins Adapted Cognitive Exam	the johns hopkins adapted cognitive exam	the johns hopkins adapted cognitive exam	the johns hopkins adapted cognitive exam	The Johns Hopkins Adapted Cognitive Exam	1	kept

The KAP model	the kap model	the kap model	the kap model	The KAP model	1	kept
The model for symptom management	the model for symptom management	the model for symptom management	the model for symptom management	The model for symptom management	1	kept
The Moral Distress Survey Revised	the moral distress survey revised	the moral distress survey revised	the moral distress survey revised	The Moral Distress Survey Revised	1	kept
The quality of life	the quality of life	the quality of life	the quality of life	The quality of life	1	kept
the quality of life	the quality of life	the quality of life	the quality of life	The quality of life	1	case_or_format
the RAPIT study	the rapit study	the rapit study	the rapit study	the RAPIT study	1	kept
the spiritual attitude and involvement list	the spiritual attitude and involvement list	the spiritual attitude and involvement list	the spiritual attitude and involvement list	the spiritual attitude and involvement list	1	kept
The study was performed in a ten-week period, twice a	the study was performed in a ten week period twice a	the study was performed in a ten week period twice a	the study was performed in a ten week period twice a	The study was performed in a ten-week period, twice a	1	kept
THEATER	theater	theater	theater	THEATER	1	kept
Theatres	theatres	theatres	theatres	Theatres	1	kept
theft	theft	theft	theft	theft	1	kept
Thematic analyses	thematic analyses	thematic analyses	thematic analysis	thematic analysis	1	singular
Thematic Apperception Test	thematic apperception test	thematic apperception test	thematic apperception test	Thematic Apperception Test	1	kept
Thematic synthesis	thematic synthesis	thematic synthesis	thematic synthesis	Thematic synthesis	1	kept
thematic synthesis	thematic synthesis	thematic synthesis	thematic synthesis	Thematic synthesis	1	case_or_format
Themes	themes	themes	themes	Themes	1	kept

themes	themes	themes	themes	Themes	1	case_or_format
Theodore Striker	theodore striker	theodore striker	theodore striker	Theodore Striker	1	kept
THEOPHYLLINE PROPHYLAXIS	theophylline prophylaxis	theophylline prophylaxis	theophylline prophylaxis	THEOPHYLLINE PROPHYLAXIS	1	kept
Theorem proving	theorem proving	theorem proving	theorem proving	Theorem proving	1	kept
Theoretical	theoretical	theoretical	theoretical	Theoretical	1	kept
Theoretical approach	theoretical approach	theoretical approach	theoretical approach	Theoretical approach	1	kept
Theoretical domain framework	theoretical domain framework	theoretical domain framework	theoretical domain framework	Theoretical domain framework	1	kept
theoretical domain framework	theoretical domain framework	theoretical domain framework	theoretical domain framework	Theoretical domain framework	1	case_or_format
theoretical framework	theoretical framework	theoretical framework	theoretical framework	Theoretical framework	1	case_or_format
theoretical knowledge and pattern recognition	theoretical knowledge and pattern recognition	theoretical knowledge and pattern recognition	theoretical knowledge and pattern recognition	theoretical knowledge and pattern recognition	1	kept
Theoretical sampling	theoretical sampling	theoretical sampling	theoretical sampling	Theoretical sampling	1	kept
theories	theories	theories	theory	theory	1	singular
Theory	theory	theory	theory	theory	1	case_or_format
theory analysis	theory analysis	theory analysis	theory analysis	theory analysis	1	kept
Theory and practice	theory and practice	theory and practice	theory and practice	Theory and practice	1	kept
Theory critique	theory critique	theory critique	theory critique	Theory critique	1	kept

Theory of change	theory of change	theory of change	theory of change	Theory of change	1	kept
Theory of Mind	theory of mind	theory of mind	theory of mind	Theory of Mind	1	kept
theory of planned behavior .	theory of planned behavior	theory of planned behavior	theory of planned behavior	Theory of Planned Behavior	1	case_or_format
Theory of Planned Behaviour	theory of planned behaviour	theory of planned behaviour	theory of planned behaviour	Theory of planned behaviour	1	case_or_format
Theory of Reasoned Action	theory of reasoned action	theory of reasoned action	theory of reasoned action	Theory of Reasoned Action	1	kept
Theory of self-regulation	theory of self regulation	theory of self regulation	theory of self regulation	Theory of self-regulation	1	kept
theory of unpleasant symptoms	theory of unpleasant symptoms	theory of unpleasant symptoms	theory of unpleasant symptoms	theory of unpleasant symptoms	1	kept
theory synthesis	theory synthesis	theory synthesis	theory synthesis	theory synthesis	1	kept
theory-guided practice	theory guided practice	theory guided practice	theory guided practice	theory-guided practice	1	kept
Theory-practice-caring gap	theory practice caring gap	theory practice caring gap	theory practice caring gap	Theory-practice-caring gap	1	kept
Therapeutic Alliance	therapeutic alliance	therapeutic alliance	therapeutic alliance	Therapeutic Alliance	1	kept
Therapeutic alliance	therapeutic alliance	therapeutic alliance	therapeutic alliance	Therapeutic Alliance	1	case_or_format
therapeutic alliance	therapeutic alliance	therapeutic alliance	therapeutic alliance	Therapeutic Alliance	1	case_or_format
therapeutic anticoagulation	therapeutic anticoagulation	therapeutic anticoagulation	therapeutic anticoagulation	therapeutic anticoagulation	1	kept
therapeutic apheresis	therapeutic apheresis	therapeutic apheresis	therapeutic apheresis	therapeutic apheresis	1	kept
Therapeutic artifacts	therapeutic artifacts	therapeutic artifacts	therapeutic artifacts	Therapeutic artifacts	1	kept

Therapeutic barium enema	therapeutic barium enema	therapeutic barium enema	therapeutic barium enema	Therapeutic barium enema	1	kept
Therapeutic bronchoscopies	therapeutic bronchoscopies	therapeutic bronchoscopies	therapeutic bronchoscopy	Therapeutic bronchoscopy	1	singular
Therapeutic bronchoscopy	therapeutic bronchoscopy	therapeutic bronchoscopy	therapeutic bronchoscopy	Therapeutic bronchoscopy	1	kept
Therapeutic Community	therapeutic community	therapeutic community	therapeutic community	Therapeutic Community	1	kept
Therapeutic cuddling	therapeutic cuddling	therapeutic cuddling	therapeutic cuddling	Therapeutic cuddling	1	kept
therapeutic dose	therapeutic dose	therapeutic dose	therapeutic dose	therapeutic dose	1	kept
Therapeutic drug monitoring	therapeutic drug monitoring	therapeutic drug monitoring	therapeutic drug monitoring	therapeutic drug monitoring	1	case_or_format
therapeutic education	therapeutic education	therapeutic education	therapeutic education	therapeutic education	1	kept
therapeutic efficacy	therapeutic efficacy	therapeutic efficacy	therapeutic efficacy	therapeutic efficacy	1	kept
therapeutic engagement	therapeutic engagement	therapeutic engagement	therapeutic engagement	therapeutic engagement	1	kept
Therapeutic Equivalency	therapeutic equivalency	therapeutic equivalency	therapeutic equivalency	Therapeutic Equivalency	1	kept
Therapeutic failure	therapeutic failure	therapeutic failure	therapeutic failure	Therapeutic failure	1	kept
therapeutic hyperventilation	therapeutic hyperventilation	therapeutic hyperventilation	therapeutic hyperventilation	therapeutic hyperventilation	1	kept
THERAPEUTIC HYPOTHERMIA	therapeutic hypothermia	therapeutic hypothermia	therapeutic hypothermia	therapeutic hypothermia	1	case_or_format
Therapeutic hypothermias	therapeutic hypothermias	therapeutic hypothermias	therapeutic hypothermia	therapeutic hypothermia	1	singular
Therapeutic immobilization	therapeutic immobilization	therapeutic immobilization	therapeutic immobilization	Therapeutic immobilization	1	kept

therapeutic index	therapeutic index	therapeutic index	therapeutic index	therapeutic index	1	kept
Therapeutic intervention	therapeutic intervention	therapeutic intervention	therapeutic intervention	Therapeutic intervention	1	kept
Therapeutic intervention scores	therapeutic intervention scores	therapeutic intervention scores	therapeutic intervention scores	Therapeutic intervention scores	1	kept
Therapeutic Intervention Scoring System 10	therapeutic intervention scoring system 10	therapeutic intervention scoring system 10	therapeutic intervention scoring system 10	Therapeutic Intervention Scoring System 10	1	kept
Therapeutic Intervention Scoring System 28	therapeutic intervention scoring system 28	therapeutic intervention scoring system 28	therapeutic intervention scoring system 28	Therapeutic Intervention Scoring System 28	1	kept
Therapeutic Intervention Scoring System for Critically	therapeutic intervention scoring system for critically	therapeutic intervention scoring system for critically	therapeutic intervention scoring system for critically	Therapeutic Intervention Scoring System for Critically	1	kept
Therapeutic interventional scoring system in stratifying	therapeutic interventional scoring system in stratifying	therapeutic interventional scoring system in stratifying	therapeutic interventional scoring system in stratifying	Therapeutic interventional scoring system in stratifying	1	kept
Therapeutic interventions	therapeutic interventions	therapeutic interventions	therapeutic intervention	Therapeutic intervention	1	singular
Therapeutic Interventions Scoring System	therapeutic interventions scoring system	therapeutic interventions scoring system	therapeutic interventions scoring system	Therapeutic Interventions Scoring System	1	kept
therapeutic listening	therapeutic listening	therapeutic listening	therapeutic listening	therapeutic listening	1	kept
Therapeutic Misconception	therapeutic misconception	therapeutic misconception	therapeutic misconception	therapeutic misconception	1	case_or_format
Therapeutic music listening	therapeutic music listening	therapeutic music listening	therapeutic music listening	therapeutic music listening	1	case_or_format
Therapeutic nihilism	therapeutic nihilism	therapeutic nihilism	therapeutic nihilism	Therapeutic nihilism	1	kept
Therapeutic play	therapeutic play	therapeutic play	therapeutic play	Therapeutic play	1	kept
THERAPEUTIC PROCEDURES	therapeutic procedures	therapeutic procedures	therapeutic procedures	THERAPEUTIC PROCEDURES	1	kept
therapeutic process	therapeutic process	therapeutic process	therapeutic process	therapeutic process	1	kept

therapeutic professions	therapeutic professions	therapeutic professions	therapeutic professions	therapeutic professions	1	kept
Therapeutic relationship	therapeutic relationship	therapeutic relationship	therapeutic relationship	therapeutic relationship	1	case_or_format
therapeutic relationships	therapeutic relationships	therapeutic relationships	therapeutic relationship	therapeutic relationship	1	singular
therapeutic support limitation	therapeutic support limitation	therapeutic support limitation	therapeutic support limitation	therapeutic support limitation	1	kept
THERAPEUTIC TARGET	therapeutic target	therapeutic target	therapeutic target	THERAPEUTIC TARGET	1	kept
therapeutic team	therapeutic team	therapeutic team	therapeutic team	therapeutic team	1	kept
THERAPEUTIC TOUCH	therapeutic touch	therapeutic touch	therapeutic touch	Therapeutic Touch	1	case_or_format
Therapeutic toys	therapeutic toys	therapeutic toys	therapeutic toys	Therapeutic toys	1	kept
THERAPEUTICS	therapeutics	therapeutics	therapeutic	Therapeutics	1	singular
THERAPIES	therapies	therapies	therapy	therapy	1	singular
Therapy devices	therapy devices	therapy devices	therapy devices	Therapy devices	1	kept
Therapy disruption	therapy disruption	therapy disruption	therapy disruption	Therapy disruption	1	kept
Therapy dogs	therapy dogs	therapy dogs	therapy dogs	Therapy dogs	1	kept
therapy dogs	therapy dogs	therapy dogs	therapy dogs	Therapy dogs	1	case_or_format
therapy intensity	therapy intensity	therapy intensity	therapy intensity	therapy intensity	1	kept
therapy resistance	therapy resistance	therapy resistance	therapy resistance	therapy resistance	1	kept

Therapy standards	therapy standards	therapy standards	therapy standards	Therapy standards	1	kept
Therapy: Withdrawal	therapy withdrawal	therapy withdrawal	therapy withdrawal	Therapy: Withdrawal	1	kept
Thermal burns	thermal burns	thermal burns	thermal burns	Thermal burns	1	kept
Thermal care	thermal care	thermal care	thermal care	Thermal care	1	kept
Thermal comfort	thermal comfort	thermal comfort	thermal comfort	Thermal comfort	1	kept
thermal comfort	thermal comfort	thermal comfort	thermal comfort	Thermal comfort	1	case_or_format
Thermal Conductivity	thermal conductivity	thermal conductivity	thermal conductivity	Thermal Conductivity	1	kept
thermal diffusion	thermal diffusion	thermal diffusion	thermal diffusion	thermal diffusion	1	kept
Thermal diffusion flowmetry	thermal diffusion flowmetry	thermal diffusion flowmetry	thermal diffusion flowmetry	Thermal diffusion flowmetry	1	kept
thermal image	thermal image	thermal image	thermal image	thermal image	1	kept
thermal imaging	thermal imaging	thermal imaging	thermal imaging	thermal imaging	1	kept
Thermal management	thermal management	thermal management	thermal management	Thermal management	1	kept
thermal management	thermal management	thermal management	thermal management	Thermal management	1	case_or_format
thermal regulation	thermal regulation	thermal regulation	thermal regulation	thermal regulation	1	kept
thermal stress	thermal stress	thermal stress	thermal stress	thermal stress	1	kept
thermal time	thermal time	thermal time	thermal time	thermal time	1	kept

Thermal-imaging	thermal imaging	thermal imaging	thermal imaging	thermal imaging	1	case_or_format
thermoluminescence dosimeter	thermoluminescence dosimeter	thermoluminescence dosimeter	thermoluminescence dosimeter	thermoluminescence dosimeter	1	kept
Thermometer	thermometer	thermometer	thermometer	Thermometers	1	case_or_format
Thermoneutral environment	thermoneutral environment	thermoneutral environment	thermoneutral environment	Thermoneutral environment	1	kept
THERMOREGULATION	thermoregulation	thermoregulation	thermoregulation	thermoregulation	1	case_or_format
thermostability	thermostability	thermostability	thermostability	thermostability	1	kept
thiazole derivative	thiazole derivative	thiazole derivative	thiazole derivative	thiazole derivative	1	kept
Thiazoles	thiazoles	thiazoles	thiazoles	Thiazoles	1	kept
thiazolidine derivative	thiazolidine derivative	thiazolidine derivative	thiazolidine derivative	thiazolidine derivative	1	kept
Thiazolidines	thiazolidines	thiazolidines	thiazolidines	Thiazolidines	1	kept
Thick	thick	thick	thick	Thick	1	kept
thickened placenta	thickened placenta	thickened placenta	thickened placenta	thickened placenta	1	kept
thickness	thickness	thickness	thickness	thickness	1	kept
thienamycin derivative	thienamycin derivative	thienamycin derivative	thienamycin derivative	thienamycin derivative	1	kept
Thienamycins	thienamycins	thienamycins	thienamycins	Thienamycins	1	kept
thiepin derivative	thiepin derivative	thiepin derivative	thiepin derivative	thiepin derivative	1	kept

Thiepins	thiepins	thiepins	thiepins	Thiepins	1	kept
Thigh	thigh	thigh	thigh	Thigh	1	kept
thigh	thigh	thigh	thigh	Thigh	1	case_or_format
thigh pain	thigh pain	thigh pain	thigh pain	thigh pain	1	kept
Thin endometrium	thin endometrium	thin endometrium	thin endometrium	Thin endometrium	1	kept
thin layer chromatography	thin layer chromatography	thin layer chromatography	thin layer chromatography	thin layer chromatography	1	kept
Think aloud	think aloud	think aloud	think aloud	Think aloud	1	kept
think aloud	think aloud	think aloud	think aloud	Think aloud	1	case_or_format
think aloud technique	think aloud technique	think aloud technique	think aloud technique	think aloud technique	1	kept
Think-aloud interviews	think aloud interviews	think aloud interviews	think aloud interviews	Think-aloud interviews	1	kept
thinking impairment	thinking impairment	thinking impairment	thinking impairment	thinking impairment	1	kept
Thinness	thinness	thinness	thinness	Thinness	1	kept
thioacetazone	thioacetazone	thioacetazone	thioacetazone	thioacetazone	1	kept
Thiopental	thiopental	thiopental	thiopental	thiopental	1	case_or_format
thiosulfate	thiosulfate	thiosulfate	thiosulfate	thiosulfate	1	kept
Thiosulfates	thiosulfates	thiosulfates	thiosulfate	thiosulfate	1	singular

thiotepa	thiotepa	thiotepa	thiotepa	thiotepa	1	kept
third generation cephalosporin	third generation cephalosporin	third generation cephalosporin	third generation cephalosporin	third generation cephalosporin	1	kept
third generation cephalosporin resistant	third generation cephalosporin resistant	third generation cephalosporin resistant	third generation cephalosporin resistant	third generation cephalosporin resistant	1	kept
Third party consent	third party consent	third party consent	third party consent	Third-Party Consent	1	case_or_format
third party consent	third party consent	third party consent	third party consent	Third-Party Consent	1	case_or_format
Third Ventricle	third ventricle	third ventricle	third ventricle	Third Ventricle	1	kept
Third world	third world	third world	third world	Third world	1	kept
Third-generation cephalosporin resistance	third generation cephalosporin resistance	third generation cephalosporin resistance	third generation cephalosporin resistance	Third-generation cephalosporin resistance	1	kept
Third-party consent	third party consent	third party consent	third party consent	Third-Party Consent	1	case_or_format
THIRST	thirst	thirst	thirst	Thirst	1	case_or_format
thirst intensity	thirst intensity	thirst intensity	thirst intensity	thirst intensity	1	kept
This article has been subject to double-blind peer review	this article has been subject to double blind peer review	this article has been subject to double blind peer review	this article has been subject to double blind peer review	This article has been subject to double-blind peer review	1	kept
Thoracic	thoracic	thoracic	thoracic	Thoracic	1	kept
thoracic	thoracic	thoracic	thoracic	Thoracic	1	case_or_format
Thoracic aorta injuries	thoracic aorta injuries	thoracic aorta injuries	thoracic aorta injuries	Thoracic aorta injuries	1	kept
thoracic aortic trauma	thoracic aortic trauma	thoracic aortic trauma	thoracic aortic trauma	thoracic aortic trauma	1	kept

thoracic cancer	thoracic cancer	thoracic cancer	thoracic cancer	thoracic cancer	1	kept
Thoracic Cavity	thoracic cavity	thoracic cavity	thoracic cavity	thoracic cavity	1	case_or_format
Thoracic endovascular aortic repair	thoracic endovascular aortic repair	thoracic endovascular aortic repair	thoracic endovascular aortic repair	Thoracic endovascular aortic repair	1	kept
thoracic spinal cord injury	thoracic spinal cord injury	thoracic spinal cord injury	thoracic spinal cord injury	thoracic spinal cord injury	1	kept
Thoracic spine	thoracic spine	thoracic spine	thoracic spine	Thoracic spine	1	kept
thoracic spine	thoracic spine	thoracic spine	thoracic spine	Thoracic spine	1	case_or_format
Thoracic surgical procedures.	thoracic surgical procedures	thoracic surgical procedures	thoracic surgical procedures	Thoracic Surgical Procedures	1	case_or_format
thoracic sympathectomy	thoracic sympathectomy	thoracic sympathectomy	thoracic sympathectomy	thoracic sympathectomy	1	kept
thoracic sympathetic nerve ablation	thoracic sympathetic nerve ablation	thoracic sympathetic nerve ablation	thoracic sympathetic nerve ablation	thoracic sympathetic nerve ablation	1	kept
thoracic trauma	thoracic trauma	thoracic trauma	thoracic trauma	Thoracic trauma	1	case_or_format
thoracoabdominal aorta aneurysm	thoracoabdominal aorta aneurysm	thoracoabdominal aorta aneurysm	thoracoabdominal aorta aneurysm	thoracoabdominal aorta aneurysm	1	kept
Thoracoabdominal aortic aneurysm	thoracoabdominal aortic aneurysm	thoracoabdominal aortic aneurysm	thoracoabdominal aortic aneurysm	Thoracoabdominal aortic aneurysm	1	kept
thoracoabdominal aortic surgery	thoracoabdominal aortic surgery	thoracoabdominal aortic surgery	thoracoabdominal aortic surgery	thoracoabdominal aortic surgery	1	kept
thoracoabdominal surgery	thoracoabdominal surgery	thoracoabdominal surgery	thoracoabdominal surgery	thoracoabdominal surgery	1	kept
thoracolumbar scoliosis	thoracolumbar scoliosis	thoracolumbar scoliosis	thoracolumbar scoliosis	thoracolumbar scoliosis	1	kept
thoracolumbar spine	thoracolumbar spine	thoracolumbar spine	thoracolumbar spine	thoracolumbar spine	1	kept

thoracolumbar spine fractures	thoracolumbar spine fractures	thoracolumbar spine fractures	thoracolumbar spine fractures	thoracolumbar spine fractures	1	kept
Thoracolumbar spine injury	thoracolumbar spine injury	thoracolumbar spine injury	thoracolumbar spine injury	Thoracolumbar spine injury	1	kept
thoracoomphalopagus	thoracoomphalopagus	thoracoomphalopagus	thoracoomphalopagus	thoracoomphalopagus	1	kept
thoracoscopic surgery	thoracoscopic surgery	thoracoscopic surgery	thoracoscopic surgery	thoracoscopic surgery	1	kept
Thoracoscopy	thoracoscopy	thoracoscopy	thoracoscopy	thoracoscopy	1	case_or_format
Thoracostomy tube	thoracostomy tube	thoracostomy tube	thoracostomy tube	Thoracostomy tube	1	kept
THORACOTOMY	thoracotomy	thoracotomy	thoracotomy	thoracotomy	1	case_or_format
thorax cancer	thorax cancer	thorax cancer	thorax cancer	thorax cancer	1	kept
thorax deformity	thorax deformity	thorax deformity	thorax deformity	thorax deformity	1	kept
thorax examination	thorax examination	thorax examination	thorax examination	thorax examination	1	kept
thorax outlet syndrome	thorax outlet syndrome	thorax outlet syndrome	thorax outlet syndrome	thorax outlet syndrome	1	kept
thorax penetrating trauma	thorax penetrating trauma	thorax penetrating trauma	thorax penetrating trauma	thorax penetrating trauma	1	kept
thought control	thought control	thought control	thought control	thought control	1	kept
Thought stopping	thought stopping	thought stopping	thought stopping	Thought stopping	1	kept
thought. Dec's Bireme	thought dec s bireme	thought dec s bireme	thought dec s bireme	thought. Dec's Bireme	1	kept
thoughts	thoughts	thoughts	thoughts	thoughts	1	kept

three bottle suction	three bottle suction	three bottle suction	three bottle suction	three bottle suction	1	kept
three dimensional echocardiography	three dimensional echocardiography	three dimensional echocardiography	three dimensional echocardiography	three dimensional echocardiography	1	kept
three dimensional imaging	three dimensional imaging	three dimensional imaging	three dimensional imaging	three-dimensional imaging	1	case_or_format
Three networks	three networks	three networks	three networks	Three networks	1	kept
Three-bottle suction	three bottle suction	three bottle suction	three bottle suction	three bottle suction	1	case_or_format
Three-dimensional quality structure	three dimensional quality structure	three dimensional quality structure	three dimensional quality structure	Three-dimensional quality structure	1	kept
Three-level triage algorithm	three level triage algorithm	three level triage algorithm	three level triage algorithm	Three-level triage algorithm	1	kept
Three-way stopcock	three way stopcock	three way stopcock	three way stopcock	Three-way stopcock	1	kept
threshold concepts	threshold concepts	threshold concepts	threshold concepts	threshold concepts	1	kept
threshold of viability	threshold of viability	threshold of viability	threshold of viability	threshold of viability	1	kept
thriving	thriving	thriving	thriving	Thriving	1	case_or_format
throat edema	throat edema	throat edema	throat edema	throat edema	1	kept
throat surgery	throat surgery	throat surgery	throat surgery	throat surgery	1	kept
throat tightness	throat tightness	throat tightness	throat tightness	throat tightness	1	kept
Thrombin	thrombin	thrombin	thrombin	thrombin	1	case_or_format
thrombin antithrombin complex	thrombin antithrombin complex	thrombin antithrombin complex	thrombin antithrombin complex	thrombin antithrombin complex	1	kept

THROMBINS2	thrombins2	thrombins2	thrombins2	THROMBINS2	1	kept
thrombocyte activating factor	thrombocyte activating factor	thrombocyte activating factor	thrombocyte activating factor	thrombocyte activating factor	1	kept
thrombocyte aggregation	thrombocyte aggregation	thrombocyte aggregation	thrombocyte aggregation	thrombocyte aggregation	1	kept
thrombocyte preservation	thrombocyte preservation	thrombocyte preservation	thrombocyte preservation	thrombocyte preservation	1	kept
thrombocyte rich plasma	thrombocyte rich plasma	thrombocyte rich plasma	thrombocyte rich plasma	thrombocyte rich plasma	1	kept
thrombocythemia	thrombocythemia	thrombocythemia	thrombocythemia	thrombocythemia	1	kept
thrombocytopheresis	thrombocytopheresis	thrombocytopheresis	thrombocytopheresis	thrombocytopheresis	1	kept
thrombocytopoiesis	thrombocytopoiesis	thrombocytopoiesis	thrombocytopoiesis	thrombocytopoiesis	1	kept
thromboelastogram assay	thromboelastogram assay	thromboelastogram assay	thromboelastogram assay	thromboelastogram assay	1	kept
Thromboembolic	thromboembolic	thromboembolic	thromboembolic	Thromboembolic	1	kept
thrombogenesis	thrombogenesis	thrombogenesis	thrombogenesis	thrombogenesis	1	kept
thrombolytic	thrombolytic	thrombolytic	thrombolytic	Thrombolytic	1	case_or_format
Thrombolytic therapy	thrombolytic therapy	thrombolytic therapy	thrombolytic therapy	Thrombolytic Therapy	1	case_or_format
thrombolytic therapy	thrombolytic therapy	thrombolytic therapy	thrombolytic therapy	Thrombolytic Therapy	1	case_or_format
thromboplastin	thromboplastin	thromboplastin	thromboplastin	Thromboplastin	1	case_or_format
thromboprophylaxis	thromboprophylaxis	thromboprophylaxis	thromboprophylaxis	Thromboprophylaxis	1	case_or_format

Thrombose	thrombose	thrombose	thrombose	Thrombose	1	kept
thrombospondin 1	thrombospondin 1	thrombospondin 1	thrombospondin 1	thrombospondin 1	1	kept
Thrombotic	thrombotic	thrombotic	thrombotic	Thrombotic	1	kept
Thrombotic Microangiopathy	thrombotic microangiopathy	thrombotic microangiopathy	thrombotic microangiopathy	Thrombotic Microangiopathies	1	case_or_format
thrombotic microangiopathy	thrombotic microangiopathy	thrombotic microangiopathy	thrombotic microangiopathy	Thrombotic Microangiopathies	1	case_or_format
throughput	throughput	throughput	throughput	Throughput	1	case_or_format
thumb	thumb	thumb	thumb	thumb	1	kept
thumb injury	thumb injury	thumb injury	thumb injury	thumb injury	1	kept
Thymic Neoplasm	thymic neoplasm	thymic neoplasm	thymic neoplasm	Thymic Neoplasm	1	kept
thymol	thymol	thymol	thymol	thymol	1	kept
Thymoma	thymoma	thymoma	thymoma	Thymoma	1	kept
thymosin alpha1	thymosin alpha1	thymosin alpha1	thymosin alpha1	thymosin alpha1	1	kept
ThymusCarcinoma	thymuscarcinoma	thymuscarcinoma	thymuscarcinoma	ThymusCarcinoma	1	kept
thyrohyoid muscle	thyrohyoid muscle	thyrohyoid muscle	thyrohyoid muscle	thyrohyoid muscle	1	kept
Thyroid	thyroid	thyroid	thyroid	Thyroid	1	kept
thyroid	thyroid	thyroid	thyroid	Thyroid	1	case_or_format

Thyroid Cancer, Papillary	thyroid cancer papillary	thyroid cancer papillary	thyroid cancer papillary	Thyroid Cancer, Papillary	1	kept
thyroid cartilage	thyroid cartilage	thyroid cartilage	thyroid cartilage	thyroid cartilage	1	kept
thyroid dysfunction	thyroid dysfunction	thyroid dysfunction	thyroid dysfunction	thyroid dysfunction	1	kept
thyroid function test	thyroid function test	thyroid function test	thyroid function test	thyroid function test	1	kept
Thyroid Function Tests	thyroid function tests	thyroid function tests	thyroid function test	thyroid function test	1	singular
Thyroid hormone	thyroid hormone	thyroid hormone	thyroid hormone	thyroid hormone	1	case_or_format
thyroid hormone blood level	thyroid hormone blood level	thyroid hormone blood level	thyroid hormone blood level	thyroid hormone blood level	1	kept
thyroid mass	thyroid mass	thyroid mass	thyroid mass	thyroid mass	1	kept
thyroid medullary carcinoma	thyroid medullary carcinoma	thyroid medullary carcinoma	thyroid medullary carcinoma	thyroid medullary carcinoma	1	kept
Thyroid Nodule	thyroid nodule	thyroid nodule	thyroid nodule	Thyroid Nodule	1	kept
Thyroid storm	thyroid storm	thyroid storm	thyroid storm	Thyroid storm	1	kept
thyroid transcription factor 1	thyroid transcription factor 1	thyroid transcription factor 1	thyroid transcription factor 1	thyroid transcription factor 1	1	kept
thyroid tumor	thyroid tumor	thyroid tumor	thyroid tumor	thyroid tumor	1	kept
thyroidectomy	thyroidectomy	thyroidectomy	thyroidectomy	Thyroidectomy	1	case_or_format
thyrotoxicosis	thyrotoxicosis	thyrotoxicosis	thyrotoxicosis	Thyrotoxicosis	1	case_or_format
thyrotropin blood level	thyrotropin blood level	thyrotropin blood level	thyrotropin blood level	thyrotropin blood level	1	kept

thyroxine binding globulin	thyroxine binding globulin	thyroxine binding globulin	thyroxine binding globulin	thyroxine binding globulin	1	kept
thyroxine binding protein	thyroxine binding protein	thyroxine binding protein	thyroxine binding protein	thyroxine binding protein	1	kept
Thyroxine-Binding Proteins	thyroxine binding proteins	thyroxine binding proteins	thyroxine binding protein	thyroxine binding protein	1	singular
théorie ancrée	th orie ancr e	th orie ancr e	th orie ancr e	théorie ancrée	1	kept
Tibet	tibet	tibet	tibet	Tibet	1	kept
TIBIA	tibia	tibia	tibia	Tibia	1	case_or_format
tibia shaft fracture	tibia shaft fracture	tibia shaft fracture	tibia shaft fracture	tibia shaft fracture	1	kept
Tibial Fractures	tibial fractures	tibial fractures	tibial fractures	Tibial Fractures	1	kept
tibialis anterior muscle	tibialis anterior muscle	tibialis anterior muscle	tibialis anterior muscle	tibialis anterior muscle	1	kept
tic	tic	tic	tic	tic	1	kept
Tick	tick	tick	tick	Tick	1	kept
Tick borne encephalitis flavivirus	tick borne encephalitis flavivirus	tick borne encephalitis flavivirus	tick borne encephalitis flavivirus	Tick borne encephalitis flavivirus	1	kept
tick infestation	tick infestation	tick infestation	tick infestation	tick infestation	1	kept
Tick Paralysis	tick paralysis	tick paralysis	tick paralysis	tick paralysis	1	case_or_format
tick-borne encephalitis	tick borne encephalitis	tick borne encephalitis	tick borne encephalitis	tick borne encephalitis	1	case_or_format
Ticks	ticks	ticks	tick	Tick	1	singular

tidal volume ventilation	tidal volume ventilation	tidal volume ventilation	tidal volume ventilation	tidal volume ventilation	1	kept
TIDAL VOLUMES	tidal volumes	tidal volumes	tidal volume	tidal volume	1	singular
TIDieR	tidier	tidier	tidier	TIDieR	1	kept
Tiered levels	tiered levels	tiered levels	tiered levels	Tiered levels	1	kept
TIGER	tiger	tiger	tiger	TIGER	1	kept
tilidine	tilidine	tilidine	tilidine	tilidine	1	kept
tilt table	tilt table	tilt table	tilt table	tilt table	1	kept
Tilting	tilting	tilting	tilting	tilting	1	case_or_format
Timber	timber	timber	timber	Timber	1	kept
time 4 day	time 4 day	time 4 day	time 4 day	time 4 day	1	kept
time analysis	time analysis	time analysis	time analysis	time analysis	1	kept
Time and motion	time and motion	time and motion	time and motion	Time and motion	1	kept
time bound nature of task	time bound nature of task	time bound nature of task	time bound nature of task	time bound nature of task	1	kept
time constraint	time constraint	time constraint	time constraint	time constraint	1	kept
time constraints	time constraints	time constraints	time constraint	time constraint	1	singular
Time delays	time delays	time delays	time delays	Time delays	1	kept

time driven activity based costing	time driven activity based costing	time driven activity based costing	time driven activity based costing	time driven activity based costing	1	kept
Time efficiencies	time efficiencies	time efficiencies	time efficiency	time-efficiency	1	singular
Time for physiologic respiratory adaption	time for physiologic respiratory adaption	time for physiologic respiratory adaption	time for physiologic respiratory adaption	Time for physiologic respiratory adaption	1	kept
Time frequency analysis	time frequency analysis	time frequency analysis	time frequency analysis	Time frequency analysis	1	kept
Time horizons	time horizons	time horizons	time horizons	Time horizons	1	kept
time in range	time in range	time in range	time in range	time in range	1	kept
Time lag	time lag	time lag	time lag	Time lag	1	kept
Time level	time level	time level	time level	Time level	1	kept
Time measurements	time measurements	time measurements	time measurements	Time measurements	1	kept
Time of birth	time of birth	time of birth	time of birth	time of birth	1	case_or_format
Time Optimization	time optimization	time optimization	time optimization	Time Optimization	1	kept
time perspective	time perspective	time perspective	time perspective	time perspective	1	kept
Time pressure	time pressure	time pressure	time pressure	time pressure	1	case_or_format
Time pressures	time pressures	time pressures	time pressure	time pressure	1	singular
Time savings	time savings	time savings	time savings	Time savings	1	kept
time series modelling	time series modelling	time series modelling	time series modelling	time series modelling	1	kept

Time series prediction	time series prediction	time series prediction	time series prediction	Time series prediction	1	kept
Time shifts	time shifts	time shifts	time shifts	Time shifts	1	kept
Time study	time study	time study	time study	Time study	1	kept
time study	time study	time study	time study	Time study	1	case_or_format
time temperature curve	time temperature curve	time temperature curve	time temperature curve	time temperature curve	1	kept
Time to antibiotic administration	time to antibiotic administration	time to antibiotic administration	time to antibiotic administration	Time to antibiotic administration	1	kept
time to onset	time to onset	time to onset	time to onset	time to onset	1	kept
Time to task	time to task	time to task	time to task	Time to task	1	kept
time trade-off method	time trade off method	time trade off method	time trade off method	time trade-off method	1	kept
time trend	time trend	time trend	time trend	TIME TRENDS	1	case_or_format
Time with electronic medical records	time with electronic medical records	time with electronic medical records	time with electronic medical records	Time with electronic medical records	1	kept
Time-and-motion	time and motion	time and motion	time and motion	Time and motion	1	case_or_format
Time-and-motion techniques	time and motion techniques	time and motion techniques	time and motion techniques	Time-and-motion techniques	1	kept
Time-consuming tasks	time consuming tasks	time consuming tasks	time consuming tasks	Time-consuming tasks	1	kept
time-critical	time critical	time critical	time critical	time-critical	1	kept
Time-driven activity-based costing	time driven activity based costing	time driven activity based costing	time driven activity based costing	time driven activity based costing	1	case_or_format

Time-in-target	time in target	time in target	time in target	Time-in-target	1	kept
Time-pressure	time pressure	time pressure	time pressure	time pressure	1	case_or_format
Time-scales	time scales	time scales	time scales	Time-scales	1	kept
Time-series analyses	time series analyses	time series analyses	time series analysis	time series analysis	1	singular
Time-series modelling	time series modelling	time series modelling	time series modelling	time series modelling	1	case_or_format
time-to-antibiotics	time to antibiotics	time to antibiotics	time to antibiotics	time-to-antibiotics	1	kept
time-varying predictors	time varying predictors	time varying predictors	time varying predictors	time-varying predictors	1	kept
Timed Get up and Go score	timed get up and go score	timed get up and go score	timed get up and go score	Timed Get up and Go score	1	kept
Timed Up and Go test	timed up and go test	timed up and go test	timed up and go test	Timed Up and Go test	1	kept
timed up and go test	timed up and go test	timed up and go test	timed up and go test	Timed Up and Go test	1	case_or_format
Timeliness of care	timeliness of care	timeliness of care	timeliness of care	Timeliness of care	1	kept
timely discharge	timely discharge	timely discharge	timely discharge	timely discharge	1	kept
Timely management	timely management	timely management	timely management	Timely management	1	kept
time－weighted average	time weighted average	time weighted average	time weighted average	time－weighted average	1	kept
time－ motion studies	time motion studies	time motion studies	time motion study	Time-motion study	1	singular
time－motion analysis	time motion analysis	time motion analysis	time motion analysis	time－motion analysis	1	kept

Timing of dialysis	timing of dialysis	timing of dialysis	timing of dialysis	Timing of dialysis	1	kept
Tinning	tinning	tinning	tinning	Tinning	1	kept
Tinnitus	tinnitus	tinnitus	tinnitus	tinnitus	1	case_or_format
tinzaparin	tinzaparin	tinzaparin	tinzaparin	tinzaparin	1	kept
tioguanine	tioguanine	tioguanine	tioguanine	tioguanine	1	kept
Tip colonization	tip colonization	tip colonization	tip colonization	Tip colonization	1	kept
tip conformation system	tip conformation system	tip conformation system	tip conformation system	tip conformation system	1	kept
Tip location	tip location	tip location	tip location	Tip location	1	kept
tip location	tip location	tip location	tip location	Tip location	1	case_or_format
TIP POSITION	tip position	tip position	tip position	TIP POSITION	1	kept
Tip positioning	tip positioning	tip positioning	tip positioning	Tip positioning	1	kept
TISS	tiss	tiss	tiss	TISS	1	kept
TISSUE	tissue	tissue	tissue	Tissue	1	case_or_format
tissue adhesive	tissue adhesive	tissue adhesive	tissue adhesive	Tissue Adhesives	1	case_or_format
tissue blood flow	tissue blood flow	tissue blood flow	tissue blood flow	tissue blood flow	1	kept
tissue characterization	tissue characterization	tissue characterization	tissue characterization	tissue characterization	1	kept

Tissue damage	tissue damage	tissue damage	tissue damage	Tissue damage	1	kept
TISSUE DONATION	tissue donation	tissue donation	tissue donation	Tissue donation	1	case_or_format
Tissue Donation	tissue donation	tissue donation	tissue donation	Tissue donation	1	case_or_format
tissue donation	tissue donation	tissue donation	tissue donation	Tissue donation	1	case_or_format
Tissue factor	tissue factor	tissue factor	tissue factor	Tissue factor	1	kept
Tissue Inhibitor of Metalloproteinase-2	tissue inhibitor of metalloproteinase 2	tissue inhibitor of metalloproteinase 2	tissue inhibitor of metalloproteinase 2	tissue inhibitor of metalloproteinase 2	1	case_or_format
tissue inhibitor of metalloproteinases 2	tissue inhibitor of metalloproteinases 2	tissue inhibitor of metalloproteinases 2	tissue inhibitor of metalloproteinases 2	tissue inhibitor of metalloproteinases 2	1	kept
TISSUE OXYGEN-SATURATION	tissue oxygen saturation	tissue oxygen saturation	tissue oxygen saturation	TISSUE OXYGEN-SATURATION	1	kept
TISSUE OXYGENATION	tissue oxygenation	tissue oxygenation	tissue oxygenation	tissue oxygenation	1	case_or_format
Tissue perfusion	tissue perfusion	tissue perfusion	tissue perfusion	tissue perfusion	1	case_or_format
Tissue regeneration	tissue regeneration	tissue regeneration	tissue regeneration	tissue regeneration	1	case_or_format
Tissue repair	tissue repair	tissue repair	tissue repair	Tissue repair	1	kept
tissue repair	tissue repair	tissue repair	tissue repair	Tissue repair	1	case_or_format
tissue saturation	tissue saturation	tissue saturation	tissue saturation	tissue saturation	1	kept
Tissue Survival	tissue survival	tissue survival	tissue survival	tissue survival	1	case_or_format
tissue tolerance	tissue tolerance	tissue tolerance	tissue tolerance	tissue tolerance	1	kept

TISSUE-PLASMINOGEN ACTIVATOR	tissue plasminogen activator	tissue plasminogen activator	tissue plasminogen activator	tissue plasminogen activator	1	case_or_format
Tissues and organ procurement	tissues and organ procurement	tissues and organ procurement	tissues and organ procurement	Tissues and organ procurement	1	kept
Titanium	titanium	titanium	titanium	Titanium	1	kept
titanium	titanium	titanium	titanium	Titanium	1	case_or_format
titration	titration	titration	titration	Titration	1	case_or_format
TIVAD	tivad	tivad	tivad	TIVAD	1	kept
tizanidine	tizanidine	tizanidine	tizanidine	tizanidine	1	kept
TKM Ebola	tkm ebola	tkm ebola	tkm ebola	TKM Ebola	1	kept
TLIF = transforaminal lumbar interbody fusion	tlif transforaminal lumbar interbody fusion	tlif transforaminal lumbar interbody fusion	tlif transforaminal lumbar interbody fusion	TLIF = transforaminal lumbar interbody fusion	1	kept
TMS	tms	tms	tms	TMS	1	kept
TNF inhibitors	tnf inhibitors	tnf inhibitors	tnf inhibitors	TNF inhibitors	1	kept
TO effect	to effect	to effect	to effect	TO effect	1	kept
TO-RESCUE	to rescue	to rescue	to rescue	TO-RESCUE	1	kept
Tobacco	tobacco	tobacco	tobacco	tobacco	1	case_or_format
Tobacco cessation	tobacco cessation	tobacco cessation	tobacco cessation	Tobacco cessation	1	kept
tobacco industry	tobacco industry	tobacco industry	tobacco industry	tobacco industry	1	kept

tobacco smoke	tobacco smoke	tobacco smoke	tobacco smoke	tobacco smoke	1	kept
Tobacco Smoke Pollution	tobacco smoke pollution	tobacco smoke pollution	tobacco smoke pollution	Tobacco Smoke Pollution	1	kept
Tobacco Smoking	tobacco smoking	tobacco smoking	tobacco smoking	Tobacco Smoking	1	kept
tobacco treatment	tobacco treatment	tobacco treatment	tobacco treatment	tobacco treatment	1	kept
tocainide	tocainide	tocainide	tocainide	tocainide	1	kept
Tocantins	tocantins	tocantins	tocantins	Tocantins	1	kept
Tocolysis	tocolysis	tocolysis	tocolysis	tocolysis	1	case_or_format
toddlerhood	toddlerhood	toddlerhood	toddlerhood	toddlerhood	1	kept
toe	toe	toe	toe	toe	1	kept
toe injury	toe injury	toe injury	toe injury	toe injury	1	kept
Toes	toes	toes	toe	toe	1	singular
Togetherness	togetherness	togetherness	togetherness	Togetherness	1	kept
tolbutamide	tolbutamide	tolbutamide	tolbutamide	tolbutamide	1	kept
tolcapone	tolcapone	tolcapone	tolcapone	tolcapone	1	kept
TOLERANCE	tolerance	tolerance	tolerance	Tolerance	1	case_or_format
tolerance	tolerance	tolerance	tolerance	Tolerance	1	case_or_format

tolevamer	tolevamer	tolevamer	tolevamer	tolevamer	1	kept
toll like receptor	toll like receptor	toll like receptor	toll like receptor	toll like receptor	1	kept
toll like receptor 2	toll like receptor 2	toll like receptor 2	toll like receptor 2	toll like receptor 2	1	kept
Toll-Like Receptors	toll like receptors	toll like receptors	toll like receptor	toll like receptor	1	singular
tolterodine	tolterodine	tolterodine	tolterodine	tolterodine	1	kept
TOLVAPTAN	tolvaptan	tolvaptan	tolvaptan	TOLVAPTAN	1	kept
tomato	tomato	tomato	tomato	tomato	1	kept
tomography	tomography	tomography	tomography	Tomography	1	case_or_format
Tomography imaging	tomography imaging	tomography imaging	tomography imaging	Tomography imaging	1	kept
Tomography, X-ray computed	tomography x ray computed	tomography x ray computed	tomography x ray computed	Tomography, X-Ray Computed	1	case_or_format
tongue carcinoma	tongue carcinoma	tongue carcinoma	tongue carcinoma	tongue carcinoma	1	kept
tongue disease	tongue disease	tongue disease	tongue disease	tongue disease	1	kept
tongue edema	tongue edema	tongue edema	tongue edema	tongue edema	1	kept
tongue frenulum	tongue frenulum	tongue frenulum	tongue frenulum	tongue frenulum	1	kept
tongue mucosa	tongue mucosa	tongue mucosa	tongue mucosa	tongue mucosa	1	kept
tongue swelling	tongue swelling	tongue swelling	tongue swelling	tongue swelling	1	kept

tongue ulcer	tongue ulcer	tongue ulcer	tongue ulcer	tongue ulcer	1	kept
tongue-tie	tongue tie	tongue tie	tongue tie	tongue-tie	1	kept
tonsil cancer	tonsil cancer	tonsil cancer	tonsil cancer	tonsil cancer	1	kept
Tonsillar Neoplasms	tonsillar neoplasms	tonsillar neoplasms	tonsillar neoplasms	Tonsillar Neoplasms	1	kept
tonsillitis	tonsillitis	tonsillitis	tonsillitis	tonsillitis	1	kept
tool	tool	tool	tool	TOOL	1	case_or_format
tool measurement	tool measurement	tool measurement	tool measurement	tool measurement	1	kept
tool of palliative complexity	tool of palliative complexity	tool of palliative complexity	tool of palliative complexity	tool of palliative complexity	1	kept
tool validation	tool validation	tool validation	tool validation	tool validation	1	kept
toolkit	toolkit	toolkit	toolkit	toolkit	1	kept
tooth abscess	tooth abscess	tooth abscess	tooth abscess	tooth abscess	1	kept
Tooth Crown	tooth crown	tooth crown	tooth crown	tooth crown	1	case_or_format
Tooth Extraction	tooth extraction	tooth extraction	tooth extraction	tooth extraction	1	case_or_format
tooth filling	tooth filling	tooth filling	tooth filling	tooth filling	1	kept
tooth fracture	tooth fracture	tooth fracture	tooth fracture	tooth fracture	1	kept
tooth pain	tooth pain	tooth pain	tooth pain	tooth pain	1	kept

tooth periapical disease	tooth periapical disease	tooth periapical disease	tooth periapical disease	tooth periapical disease	1	kept
tooth radiography	tooth radiography	tooth radiography	tooth radiography	tooth radiography	1	kept
tooth root canal	tooth root canal	tooth root canal	tooth root canal	tooth root canal	1	kept
toothbrush	toothbrush	toothbrush	toothbrush	toothbrush	1	kept
top 100	top 100	top 100	top 100	top 100	1	kept
Top management	top management	top management	top management	Top management	1	kept
Top performer	top performer	top performer	top performer	Top performer	1	kept
top-down management	top down management	top down management	top down management	top-down management	1	kept
Topic model	topic model	topic model	topic model	Topic model	1	kept
Topic modeling	topic modeling	topic modeling	topic modeling	Topic Modeling	1	case_or_format
TOPICAL APPLICATION	topical application	topical application	topical application	TOPICAL APPLICATION	1	kept
topical skin care	topical skin care	topical skin care	topical skin care	topical skin care	1	kept
topography	topography	topography	topography	topography	1	kept
torasemide	torasemide	torasemide	torasemide	torasemide	1	kept
Torino [Piedmont]	torino piedmont	torino piedmont	torino piedmont	Torino [Piedmont]	1	kept
tornado	tornado	tornado	tornado	tornado	1	kept

Tornadoes	tornadoes	tornadoes	tornado	tornado	1	singular
TORONTO	toronto	toronto	toronto	TORONTO	1	kept
Toronto	toronto	toronto	toronto	TORONTO	1	case_or_format
Torsade de pointes	torsade de pointes	torsade de pointes	torsade de pointes	Torsade de pointes	1	kept
torsade de pointes	torsade de pointes	torsade de pointes	torsade de pointes	Torsade de pointes	1	case_or_format
torsades de pointes	torsades de pointes	torsades de pointes	torsades de pointes	Torsades de Pointes	1	case_or_format
torticollis	torticollis	torticollis	torticollis	Torticollis	1	case_or_format
Total body surface area	total body surface area	total body surface area	total body surface area	total body surface area	1	case_or_format
Total Certainty Score	total certainty score	total certainty score	total certainty score	Total Certainty Score	1	kept
total cholesterol	total cholesterol	total cholesterol	total cholesterol	total cholesterol	1	kept
total colon resection	total colon resection	total colon resection	total colon resection	total colon resection	1	kept
Total Deviation Score	total deviation score	total deviation score	total deviation score	Total Deviation Score	1	kept
Total Distress Score	total distress score	total distress score	total distress score	Total Distress Score	1	kept
Total institutions	total institutions	total institutions	total institutions	Total institutions	1	kept
total joint arthroplasty	total joint arthroplasty	total joint arthroplasty	total joint arthroplasty	total joint arthroplasty	1	kept
Total knee	total knee	total knee	total knee	Total knee	1	kept

Total knee arthroplasty	total knee arthroplasty	total knee arthroplasty	total knee arthroplasty	total knee arthroplasty	1	case_or_format
total laryngectomy	total laryngectomy	total laryngectomy	total laryngectomy	total laryngectomy	1	kept
Total lymphocyte count	total lymphocyte count	total lymphocyte count	total lymphocyte count	Total lymphocyte count	1	kept
total mesorectal excision	total mesorectal excision	total mesorectal excision	total mesorectal excision	total mesorectal excision	1	kept
total number of new staff	total number of new staff	total number of new staff	total number of new staff	total number of new staff	1	kept
total pain	total pain	total pain	total pain	total pain	1	kept
Total parental nutrition	total parental nutrition	total parental nutrition	total parental nutrition	Total parental nutrition	1	kept
Total Parenteral Nutrition	total parenteral nutrition	total parenteral nutrition	total parenteral nutrition	total parenteral nutrition	1	case_or_format
total protein	total protein	total protein	total protein	total protein	1	kept
Total psoas volume	total psoas volume	total psoas volume	total psoas volume	Total psoas volume	1	kept
Total quality management	total quality management	total quality management	total quality management	total quality management	1	case_or_format
total shoulder arthroplasty	total shoulder arthroplasty	total shoulder arthroplasty	total shoulder arthroplasty	total shoulder arthroplasty	1	kept
total skin electron therapy	total skin electron therapy	total skin electron therapy	total skin electron therapy	total skin electron therapy	1	kept
Total sleep time	total sleep time	total sleep time	total sleep time	Total sleep time	1	kept
total sleep time	total sleep time	total sleep time	total sleep time	Total sleep time	1	case_or_format
total spiritual health score	total spiritual health score	total spiritual health score	total spiritual health score	total spiritual health score	1	kept

total transport time	total transport time	total transport time	total transport time	total transport time	1	kept
TOTAL-COST	total cost	total cost	total cost	TOTAL-COST	1	kept
totally endoscopic mitral valve surgery	totally endoscopic mitral valve surgery	totally endoscopic mitral valve surgery	totally endoscopic mitral valve surgery	totally endoscopic mitral valve surgery	1	kept
totally implanted venous access catheter	totally implanted venous access catheter	totally implanted venous access catheter	totally implanted venous access catheter	totally implanted venous access catheter	1	kept
totipotent stem cell	totipotent stem cell	totipotent stem cell	totipotent stem cell	totipotent stem cell	1	kept
Totipotent Stem Cells	totipotent stem cells	totipotent stem cells	totipotent stem cell	totipotent stem cell	1	singular
TOUCH	touch	touch	touch	Touch	1	case_or_format
Touch Perception	touch perception	touch perception	touch perception	Touch Perception	1	kept
touch therapy	touch therapy	touch therapy	touch therapy	touch therapy	1	kept
tourism	tourism	tourism	tourism	tourism	1	kept
Tourniquet	tourniquet	tourniquet	tourniquet	tourniquet	1	case_or_format
Tourniquets	tourniquets	tourniquets	tourniquet	tourniquet	1	singular
Townsend deprivation index	townsend deprivation index	townsend deprivation index	townsend deprivation index	Townsend deprivation index	1	kept
toxic alcohol ingestion	toxic alcohol ingestion	toxic alcohol ingestion	toxic alcohol ingestion	toxic alcohol ingestion	1	kept
toxic alcohols	toxic alcohols	toxic alcohols	toxic alcohols	toxic alcohols	1	kept
toxic death	toxic death	toxic death	toxic death	toxic death	1	kept

toxic encephalopathies	toxic encephalopathies	toxic encephalopathies	toxic encephalopathies	toxic encephalopathies	1	kept
toxic inhalation	toxic inhalation	toxic inhalation	toxic inhalation	toxic inhalation	1	kept
toxic leadership	toxic leadership	toxic leadership	toxic leadership	toxic leadership	1	kept
toxic leukoencephalopathy	toxic leukoencephalopathy	toxic leukoencephalopathy	toxic leukoencephalopathy	toxic leukoencephalopathy	1	kept
toxic shock syndrome	toxic shock syndrome	toxic shock syndrome	toxic shock syndrome	toxic shock syndrome	1	kept
Toxic stress	toxic stress	toxic stress	toxic stress	toxic stress	1	case_or_format
toxic workplace	toxic workplace	toxic workplace	toxic workplace	toxic workplace	1	kept
toxicity and intoxication	toxicity and intoxication	toxicity and intoxication	toxicity and intoxication	toxicity and intoxication	1	kept
TOXICOLOGY	toxicology	toxicology	toxicology	toxicology	1	case_or_format
toxicosis	toxicosis	toxicosis	toxicosis	toxicosis	1	kept
Toxicovigilance	toxicovigilance	toxicovigilance	toxicovigilance	Toxicovigilance	1	kept
Toxigenic	toxigenic	toxigenic	toxigenic	Toxigenic	1	kept
toxoplasmosis	toxoplasmosis	toxoplasmosis	toxoplasmosis	toxoplasmosis	1	kept
TPA	tpa	tpa	tpa	tPA	1	case_or_format
tpa in elderly	tpa in elderly	tpa in elderly	tpa in elderly	tpa in elderly	1	kept
TPE	tpe	tpe	tpe	TPE	1	kept

TPE management	tpe management	tpe management	tpe management	TPE management	1	kept
Trabajo	trabajo	trabajo	trabajo	Trabajo	1	kept
Traceability	traceability	traceability	traceability	Traceability	1	kept
trachea disease	trachea disease	trachea disease	trachea disease	trachea disease	1	kept
trachea mucus	trachea mucus	trachea mucus	trachea mucus	trachea mucus	1	kept
trachea pressure	trachea pressure	trachea pressure	trachea pressure	trachea pressure	1	kept
trachea resection	trachea resection	trachea resection	trachea resection	trachea resection	1	kept
trachea surgery	trachea surgery	trachea surgery	trachea surgery	trachea surgery	1	kept
Tracheal aspiration	tracheal aspiration	tracheal aspiration	tracheal aspiration	Tracheal aspiration	1	kept
Tracheal bleeding	tracheal bleeding	tracheal bleeding	tracheal bleeding	Tracheal bleeding	1	kept
Tracheal cuff manometer	tracheal cuff manometer	tracheal cuff manometer	tracheal cuff manometer	Tracheal cuff manometer	1	kept
tracheal cuff pressure	tracheal cuff pressure	tracheal cuff pressure	tracheal cuff pressure	tracheal cuff pressure	1	kept
Tracheal damage	tracheal damage	tracheal damage	tracheal damage	Tracheal damage	1	kept
Tracheal dilatation	tracheal dilatation	tracheal dilatation	tracheal dilatation	Tracheal dilatation	1	kept
tracheal dilatation	tracheal dilatation	tracheal dilatation	tracheal dilatation	Tracheal dilatation	1	case_or_format
Tracheal Diseases	tracheal diseases	tracheal diseases	tracheal diseases	Tracheal Diseases	1	kept

Tracheal extubation	tracheal extubation	tracheal extubation	tracheal extubation	tracheal extubation	1	case_or_format
tracheal extubation criteria	tracheal extubation criteria	tracheal extubation criteria	tracheal extubation criteria	tracheal extubation criteria	1	kept
Tracheal Intubation	tracheal intubation	tracheal intubation	tracheal intubation	Tracheal intubation	1	case_or_format
Tracheal intubation fixation method	tracheal intubation fixation method	tracheal intubation fixation method	tracheal intubation fixation method	Tracheal intubation fixation method	1	kept
Tracheal perfusion	tracheal perfusion	tracheal perfusion	tracheal perfusion	Tracheal perfusion	1	kept
tracheal reintubation	tracheal reintubation	tracheal reintubation	tracheal reintubation	tracheal reintubation	1	kept
tracheal silicon	tracheal silicon	tracheal silicon	tracheal silicon	tracheal silicon	1	kept
Tracheal stenosis	tracheal stenosis	tracheal stenosis	tracheal stenosis	Tracheal Stenosis	1	case_or_format
tracheal suctioning	tracheal suctioning	tracheal suctioning	tracheal suctioning	Tracheal suctioning	1	case_or_format
Tracheal tube cuff pressures	tracheal tube cuff pressures	tracheal tube cuff pressures	tracheal tube cuff pressures	Tracheal tube cuff pressures	1	kept
tracheal tube cuff pressures	tracheal tube cuff pressures	tracheal tube cuff pressures	tracheal tube cuff pressures	Tracheal tube cuff pressures	1	case_or_format
Tracheo-team	tracheo team	tracheo team	tracheo team	Tracheo-team	1	kept
Tracheobronchial malacia	tracheobronchial malacia	tracheobronchial malacia	tracheobronchial malacia	Tracheobronchial malacia	1	kept
tracheobronchitis	tracheobronchitis	tracheobronchitis	tracheobronchitis	tracheobronchitis	1	kept
Tracheoinnnominate artery fistula	tracheoinnnominate artery fistula	tracheoinnnominate artery fistula	tracheoinnnominate artery fistula	Tracheoinnnominate artery fistula	1	kept
tracheoinnominate artery fistula	tracheoinnominate artery fistula	tracheoinnominate artery fistula	tracheoinnominate artery fistula	tracheoinnominate artery fistula	1	kept

Tracheomalacia	tracheomalacia	tracheomalacia	tracheomalacia	tracheomalacia	1	case_or_format
tracheostomy care ciliary beat frequency	tracheostomy care ciliary beat frequency	tracheostomy care ciliary beat frequency	tracheostomy care ciliary beat frequency	tracheostomy care ciliary beat frequency	1	kept
tracheostomy care knowledge	tracheostomy care knowledge	tracheostomy care knowledge	tracheostomy care knowledge	tracheostomy care knowledge	1	kept
Tracheostomy Complications	tracheostomy complications	tracheostomy complications	tracheostomy complications	Tracheostomy complications	1	case_or_format
Tracheostomy emergency	tracheostomy emergency	tracheostomy emergency	tracheostomy emergency	Tracheostomy emergency	1	kept
Tracheostomy guidelines	tracheostomy guidelines	tracheostomy guidelines	tracheostomy guidelines	Tracheostomy guidelines	1	kept
tracheostomy kit	tracheostomy kit	tracheostomy kit	tracheostomy kit	tracheostomy kit	1	kept
Tracheostomy management	tracheostomy management	tracheostomy management	tracheostomy management	Tracheostomy management	1	kept
tracheostomy review and management service	tracheostomy review and management service	tracheostomy review and management service	tracheostomy review and management service	tracheostomy review and management service	1	kept
TRACHEOSTOMY SERVICE	tracheostomy service	tracheostomy service	tracheostomy service	TRACHEOSTOMY SERVICE	1	kept
tracheostomy training	tracheostomy training	tracheostomy training	tracheostomy training	tracheostomy training	1	kept
Tracheostomy tube	tracheostomy tube	tracheostomy tube	tracheostomy tube	tracheostomy tube	1	case_or_format
Tracheostomy well-being score	tracheostomy well being score	tracheostomy well being score	tracheostomy well being score	Tracheostomy well-being score	1	kept
tracheotomy tube	tracheotomy tube	tracheotomy tube	tracheotomy tube	tracheotomy tube	1	kept
track and trigger	track and trigger	track and trigger	track and trigger	Track and trigger	1	case_or_format
track and trigger charts	track and trigger charts	track and trigger charts	track and trigger charts	track and trigger charts	1	kept

track and trigger scores	track and trigger scores	track and trigger scores	track and trigger scores	track and trigger scores	1	kept
Track and trigger system	track and trigger system	track and trigger system	track and trigger system	track and trigger system	1	case_or_format
Track and trigger tool	track and trigger tool	track and trigger tool	track and trigger tool	Track and trigger tool	1	kept
Track trigger systems	track trigger systems	track trigger systems	track trigger systems	Track trigger systems	1	kept
Track-and-trigger systems	track and trigger systems	track and trigger systems	track and trigger system	track and trigger system	1	singular
TRACK-AND-TRIGGER TOOLS	track and trigger tools	track and trigger tools	track and trigger tool	Track and trigger tool	1	singular
TRACKING	tracking	tracking	tracking	Tracking	1	case_or_format
Tracking system	tracking system	tracking system	tracking system	Tracking system	1	kept
TRACT PATHOGENS	tract pathogens	tract pathogens	tract pathogens	TRACT PATHOGENS	1	kept
TRACT-INFECTION	tract infection	tract infection	tract infection	TRACT-INFECTION	1	kept
Traction	traction	traction	traction	Traction	1	kept
Traction performance	traction performance	traction performance	traction performance	Traction performance	1	kept
TRADE-OFF	trade off	trade off	trade off	TRADE-OFF	1	kept
tradition-based practice	tradition based practice	tradition based practice	tradition based practice	tradition-based practice	1	kept
traditional birth attendant	traditional birth attendant	traditional birth attendant	traditional birth attendant	traditional birth attendant	1	kept
Traditional Chinese	traditional chinese	traditional chinese	traditional chinese	Traditional Chinese	1	kept

traditional Chinese medicine	traditional chinese medicine	traditional chinese medicine	traditional chinese medicine	Traditional Chinese Medicine	1	case_or_format
traditional classroom instruction	traditional classroom instruction	traditional classroom instruction	traditional classroom instruction	traditional classroom instruction	1	kept
Traditional education	traditional education	traditional education	traditional education	Traditional education	1	kept
Traditional educations	traditional educations	traditional educations	traditional education	Traditional education	1	singular
traditional feeding	traditional feeding	traditional feeding	traditional feeding	traditional feeding	1	kept
traditional healer	traditional healer	traditional healer	traditional healer	traditional healer	1	kept
traditional insertion	traditional insertion	traditional insertion	traditional insertion	traditional insertion	1	kept
Traditional medicine	traditional medicine	traditional medicine	traditional medicine	traditional medicine	1	case_or_format
traditional obesity parameters	traditional obesity parameters	traditional obesity parameters	traditional obesity parameters	traditional obesity parameters	1	kept
traditional open surgery	traditional open surgery	traditional open surgery	traditional open surgery	traditional open surgery	1	kept
traditional paper record	traditional paper record	traditional paper record	traditional paper record	traditional paper record	1	kept
traditional sample	traditional sample	traditional sample	traditional sample	traditional sample	1	kept
traditional therapy	traditional therapy	traditional therapy	traditional therapy	traditional therapy	1	kept
Traffic accidents	traffic accidents	traffic accidents	traffic accident	traffic accident	1	singular
Traffic noise	traffic noise	traffic noise	traffic noise	Traffic noise	1	kept
traffic safety	traffic safety	traffic safety	traffic safety	traffic safety	1	kept

train-of-four	train of four	train of four	train of four	train of four	1	case_or_format
train-the-trainer	train the trainer	train the trainer	train the trainer	train-the-trainer	1	kept
trainee allocation	trainee allocation	trainee allocation	trainee allocation	trainee allocation	1	kept
trainee doctors	trainee doctors	trainee doctors	trainee doctors	trainee doctors	1	kept
Trainee physicians	trainee physicians	trainee physicians	trainee physicians	Trainee physicians	1	kept
Training (see medical education	training see medical education	training see medical education	training see medical education	Training (see medical education	1	kept
Training and awareness	training and awareness	training and awareness	training and awareness	Training and awareness	1	kept
training and awareness	training and awareness	training and awareness	training and awareness	Training and awareness	1	case_or_format
Training applications	training applications	training applications	training applications	Training applications	1	kept
Training corpus	training corpus	training corpus	training corpus	Training corpus	1	kept
Training costs	training costs	training costs	training costs	Training costs	1	kept
TRAINING COURSE	training course	training course	training course	training course	1	case_or_format
Training Courses	training courses	training courses	training course	training course	1	singular
training demand	training demand	training demand	training demand	Training demands	1	case_or_format
Training devices	training devices	training devices	training devices	Training devices	1	kept
Training evaluation	training evaluation	training evaluation	training evaluation	Training evaluation	1	kept

Training experiences	training experiences	training experiences	training experiences	Training experiences	1	kept
training experiences	training experiences	training experiences	training experiences	Training experiences	1	case_or_format
Training guidelines	training guidelines	training guidelines	training guidelines	Training guidelines	1	kept
Training intervention	training intervention	training intervention	training intervention	Training intervention	1	kept
training management status	training management status	training management status	training management status	training management status	1	kept
TRAINING NEEDS	training needs	training needs	training needs	Training needs	1	case_or_format
Training platform	training platform	training platform	training platform	Training platform	1	kept
Training program for parents	training program for parents	training program for parents	training program for parents	Training program for parents	1	kept
training programme	training programme	training programme	training programme	training programme	1	kept
Training programmes	training programmes	training programmes	training programme	training programme	1	singular
Training Programs	training programs	training programs	training program	Training program	1	singular
training programs	training programs	training programs	training program	Training program	1	singular
Training purpose	training purpose	training purpose	training purpose	Training purpose	1	kept
Training seminar	training seminar	training seminar	training seminar	Training seminar	1	kept
Training Sessions	training sessions	training sessions	training sessions	Training Sessions	1	kept
Training sessions	training sessions	training sessions	training sessions	Training Sessions	1	case_or_format

Training staff	training staff	training staff	training staff	Training staff	1	kept
Training support	training support	training support	training support	Training Support	1	case_or_format
Training tools	training tools	training tools	training tools	Training tools	1	kept
Training)	training	training	training	training	1	case_or_format
Training-supportive program	training supportive program	training supportive program	training supportive program	Training-supportive program	1	kept
Trainings	trainings	trainings	training	training	1	singular
TRAIT ANXIETY	trait anxiety	trait anxiety	trait anxiety	TRAIT ANXIETY	1	kept
Trait anxiety	trait anxiety	trait anxiety	trait anxiety	TRAIT ANXIETY	1	case_or_format
traitement intraveineux continu	traitement intraveineux continu	traitement intraveineux continu	traitement intraveineux continu	traitement intraveineux continu	1	kept
Traits	traits	traits	traits	Traits	1	kept
TRAJECTORIES	trajectories	trajectories	trajectory	Trajectory	1	singular
Trajectories	trajectories	trajectories	trajectory	Trajectory	1	singular
Trajectory of illness	trajectory of illness	trajectory of illness	trajectory of illness	Trajectory of illness	1	kept
Trajectory test	trajectory test	trajectory test	trajectory test	Trajectory test	1	kept
TRALI	trali	trali	trali	TRALI	1	kept
tramadolor	tramadolor	tramadolor	tramadolor	tramadolor	1	kept

trandolapril	trandolapril	trandolapril	trandolapril	trandolapril	1	kept
Traning	traning	traning	traning	Traning	1	kept
Tranquilizing Agents	tranquilizing agents	tranquilizing agents	tranquilizing agents	Tranquilizing Agents	1	kept
trans sectoral cooperation	trans sectoral cooperation	trans sectoral cooperation	trans sectoral cooperation	trans-sectoral cooperation	1	case_or_format
Trans-Activators	trans activators	trans activators	trans activators	Trans-Activators	1	kept
Transactional Analysis	transactional analysis	transactional analysis	transactional analysis	Transactional Analysis	1	kept
transactive memory	transactive memory	transactive memory	transactive memory	transactive memory	1	kept
transactive memory systems	transactive memory systems	transactive memory systems	transactive memory systems	transactive memory systems	1	kept
Transaminase	transaminase	transaminase	transaminase	Transaminase	1	kept
Transaminases	transaminases	transaminases	transaminase	Transaminase	1	singular
Transanal Endoscopic Microsurgery	transanal endoscopic microsurgery	transanal endoscopic microsurgery	transanal endoscopic microsurgery	Transanal Endoscopic Microsurgery	1	kept
Transanal endoscopic microsurgery	transanal endoscopic microsurgery	transanal endoscopic microsurgery	transanal endoscopic microsurgery	Transanal Endoscopic Microsurgery	1	case_or_format
transanal endoscopic microsurgery	transanal endoscopic microsurgery	transanal endoscopic microsurgery	transanal endoscopic microsurgery	Transanal Endoscopic Microsurgery	1	case_or_format
transaxillary access	transaxillary access	transaxillary access	transaxillary access	transaxillary access	1	kept
transaxillary artery access	transaxillary artery access	transaxillary artery access	transaxillary artery access	transaxillary artery access	1	kept
transcatheter aortic valve replacement	transcatheter aortic valve replacement	transcatheter aortic valve replacement	transcatheter aortic valve replacement	Transcatheter Aortic Valve Replacement	1	case_or_format

Transcatheter Aortic Valve Replacemnet	transcatheter aortic valve replacemnet	transcatheter aortic valve replacemnet	transcatheter aortic valve replacemnet	Transcatheter Aortic Valve Replacemnet	1	kept
transcatheter mitral valve repair	transcatheter mitral valve repair	transcatheter mitral valve repair	transcatheter mitral valve repair	transcatheter mitral valve repair	1	kept
transcatheter patent ductus arteriosus closure	transcatheter patent ductus arteriosus closure	transcatheter patent ductus arteriosus closure	transcatheter patent ductus arteriosus closure	transcatheter patent ductus arteriosus closure	1	kept
transcaval access	transcaval access	transcaval access	transcaval access	transcaval access	1	kept
transcaval artery access	transcaval artery access	transcaval artery access	transcaval artery access	transcaval artery access	1	kept
transcellular fluid	transcellular fluid	transcellular fluid	transcellular fluid	transcellular fluid	1	kept
Transcendence	transcendence	transcendence	transcendence	Transcendence	1	kept
transcranial color doppler	transcranial color doppler	transcranial color doppler	transcranial color doppler	transcranial color doppler	1	kept
TRANSCRANIAL DOPPLER	transcranial doppler	transcranial doppler	transcranial doppler	TRANSCRANIAL DOPPLER	1	kept
Transcranial Doppler	transcranial doppler	transcranial doppler	transcranial doppler	TRANSCRANIAL DOPPLER	1	case_or_format
transcranial Doppler	transcranial doppler	transcranial doppler	transcranial doppler	TRANSCRANIAL DOPPLER	1	case_or_format
transcranial doppler	transcranial doppler	transcranial doppler	transcranial doppler	TRANSCRANIAL DOPPLER	1	case_or_format
transcranial magnetic stimulation	transcranial magnetic stimulation	transcranial magnetic stimulation	transcranial magnetic stimulation	Transcranial Magnetic Stimulation	1	case_or_format
transcript	transcript	transcript	transcript	transcript	1	kept
Transcription	transcription	transcription	transcription	Transcription	1	kept
Transcription errors	transcription errors	transcription errors	transcription errors	Transcription errors	1	kept

transcription factor	transcription factor	transcription factor	transcription factor	transcription factor	1	kept
transcription factor E2A	transcription factor e2a	transcription factor e2a	transcription factor e2a	transcription factor E2A	1	kept
transcription factor GATA	transcription factor gata	transcription factor gata	transcription factor gata	transcription factor GATA	1	kept
transcription factor RUNX1	transcription factor runx1	transcription factor runx1	transcription factor runx1	transcription factor RUNX1	1	kept
transcription regulation	transcription regulation	transcription regulation	transcription regulation	transcription regulation	1	kept
transcriptome	transcriptome	transcriptome	transcriptome	transcriptome	1	kept
Transcultural care	transcultural care	transcultural care	transcultural care	transcultural care	1	case_or_format
transcultural health	transcultural health	transcultural health	transcultural health	transcultural health	1	kept
transcutaneous	transcutaneous	transcutaneous	transcutaneous	transcutaneous	1	kept
Transcutaneous jaundice device	transcutaneous jaundice device	transcutaneous jaundice device	transcutaneous jaundice device	Transcutaneous jaundice device	1	kept
Transdermal Patch	transdermal patch	transdermal patch	transdermal patch	Transdermal Patch	1	kept
Transdiaphragmatic pressure	transdiaphragmatic pressure	transdiaphragmatic pressure	transdiaphragmatic pressure	Transdiaphragmatic pressure	1	kept
transdisciplinary	transdisciplinary	transdisciplinary	transdisciplinary	transdisciplinary	1	kept
Transdisciplinary care	transdisciplinary care	transdisciplinary care	transdisciplinary care	Transdisciplinary care	1	kept
transdisciplinary team	transdisciplinary team	transdisciplinary team	transdisciplinary team	transdisciplinary team	1	kept
Transducer	transducer	transducer	transducer	Transducers	1	case_or_format

Transepidermal water loss	transepidermal water loss	transepidermal water loss	transepidermal water loss	transepidermal water loss	1	case_or_format
TRANSEPIDERMAL WATER-LOSS	transepidermal water loss	transepidermal water loss	transepidermal water loss	transepidermal water loss	1	case_or_format
Transesophageal echocardiographies	transesophageal echocardiographies	transesophageal echocardiographies	transesophageal echocardiography	transesophageal echocardiography	1	singular
transfeminism	transfeminism	transfeminism	transfeminism	transfeminism	1	kept
transfemoral artery access	transfemoral artery access	transfemoral artery access	transfemoral artery access	transfemoral artery access	1	kept
transfemoral transcatheter aortic valve implantation	transfemoral transcatheter aortic valve implantation	transfemoral transcatheter aortic valve implantation	transfemoral transcatheter aortic valve implantation	transfemoral transcatheter aortic valve implantation	1	kept
transfer center	transfer center	transfer center	transfer center	transfer center	1	kept
Transfer functions	transfer functions	transfer functions	transfer functions	Transfer functions	1	kept
Transfer home to die	transfer home to die	transfer home to die	transfer home to die	Transfer home to die	1	kept
transfer of accountability	transfer of accountability	transfer of accountability	transfer of accountability	transfer of accountability	1	kept
Transfer of learning	transfer of learning	transfer of learning	transfer of learning	transfer of learning	1	case_or_format
transfer of skills	transfer of skills	transfer of skills	transfer of skills	transfer of skills	1	kept
Transfer process	transfer process	transfer process	transfer process	Transfer process	1	kept
transfer process	transfer process	transfer process	transfer process	Transfer process	1	case_or_format
Transfer protocol	transfer protocol	transfer protocol	transfer protocol	Transfer protocol	1	kept
transfer RNA	transfer rna	transfer rna	transfer rna	transfer RNA	1	kept

Transfer safety	transfer safety	transfer safety	transfer safety	Transfer safety	1	kept
TRANSFER SERVICE	transfer service	transfer service	transfer service	TRANSFER SERVICE	1	kept
transfer status	transfer status	transfer status	transfer status	transfer status	1	kept
transfer summary	transfer summary	transfer summary	transfer summary	transfer summary	1	kept
Transfer team	transfer team	transfer team	transfer team	Transfer team	1	kept
transference	transference	transference	transference	transference	1	kept
Transferencia de pacientes	transferencia de pacientes	transferencia de pacientes	transferencia de pacientes	Transferencia de pacientes	1	kept
transferred children	transferred children	transferred children	transferred children	transferred children	1	kept
Transferrin	transferrin	transferrin	transferrin	transferrin	1	case_or_format
transferrin saturation	transferrin saturation	transferrin saturation	transferrin saturation	transferrin saturation	1	kept
transferring medical providers	transferring medical providers	transferring medical providers	transferring medical providers	transferring medical providers	1	kept
Transferrins	transferrins	transferrins	transferrin	transferrin	1	singular
transfert de compé tences	transfert de comp tences	transfert de comp tences	transfert de comp tences	transfert de compé tences	1	kept
transfontanellar ear to ear distance	transfontanellar ear to ear distance	transfontanellar ear to ear distance	transfontanellar ear to ear distance	transfontanellar ear to ear distance	1	kept
transformation	transformation	transformation	transformation	Transformation	1	case_or_format
Transformationalism	transformationalism	transformationalism	transformationalism	Transformationalism	1	kept

Transformative learning	transformative learning	transformative learning	transformative learning	Transformative learning	1	kept
Transformer	transformer	transformer	transformer	Transformer	1	kept
Transforming care at the bedside	transforming care at the bedside	transforming care at the bedside	transforming care at the bedside	Transforming care at the bedside	1	kept
transforming growth factor beta	transforming growth factor beta	transforming growth factor beta	transforming growth factor beta	transforming growth factor beta	1	kept
Transfusion Knowledge Level	transfusion knowledge level	transfusion knowledge level	transfusion knowledge level	Transfusion Knowledge Level	1	kept
transfusion knowledge level	transfusion knowledge level	transfusion knowledge level	transfusion knowledge level	Transfusion Knowledge Level	1	case_or_format
Transfusion Medicine	transfusion medicine	transfusion medicine	transfusion medicine	transfusion medicine	1	case_or_format
Transfusion medicine	transfusion medicine	transfusion medicine	transfusion medicine	transfusion medicine	1	case_or_format
Transfusion of blood components	transfusion of blood components	transfusion of blood components	transfusion of blood components	Transfusion of blood components	1	kept
transfusion practice	transfusion practice	transfusion practice	transfusion practice	Transfusion practices	1	case_or_format
Transfusion process	transfusion process	transfusion process	transfusion process	Transfusion process	1	kept
transfusion trigger	transfusion trigger	transfusion trigger	transfusion trigger	transfusion trigger	1	kept
transfusion triggers	transfusion triggers	transfusion triggers	transfusion trigger	transfusion trigger	1	singular
transfusion-related acute lung injury	transfusion related acute lung injury	transfusion related acute lung injury	transfusion related acute lung injury	transfusion related acute lung injury	1	case_or_format
Transfusion: blood	transfusion blood	transfusion blood	transfusion blood	Transfusion: blood	1	kept
Transgastric NOTES	transgastric notes	transgastric notes	transgastric notes	Transgastric NOTES	1	kept

transgender	transgender	transgender	transgender	transgender	1	kept
Transgender health	transgender health	transgender health	transgender health	Transgender health	1	kept
transgenderism	transgenderism	transgenderism	transgenderism	transgenderism	1	kept
Transgression in recording methods	transgression in recording methods	transgression in recording methods	transgression in recording methods	Transgression in recording methods	1	kept
Transición de UCI	transici n de uci	transici n de uci	transici n de uci	Transición de UCI	1	kept
transient evoked otoacoustic emission	transient evoked otoacoustic emission	transient evoked otoacoustic emission	transient evoked otoacoustic emission	transient evoked otoacoustic emission	1	kept
transient ischaemic attack	transient ischaemic attack	transient ischaemic attack	transient ischaemic attack	transient ischaemic attack	1	kept
Transient Tachypnea of the Newborn	transient tachypnea of the newborn	transient tachypnea of the newborn	transient tachypnea of the newborn	transient tachypnea of the newborn	1	case_or_format
Transition Assessment Post Transfer program	transition assessment post transfer program	transition assessment post transfer program	transition assessment post transfer program	Transition Assessment Post Transfer program	1	kept
Transition care	transition care	transition care	transition care	Transition care	1	kept
transition care	transition care	transition care	transition care	Transition care	1	case_or_format
transition element	transition element	transition element	transition element	transition element	1	kept
Transition experience	transition experience	transition experience	transition experience	Transition experience	1	kept
transition from hospital to home	transition from hospital to home	transition from hospital to home	transition from hospital to home	transition from hospital to home	1	kept
transition guide	transition guide	transition guide	transition guide	transition guide	1	kept
Transition model	transition model	transition model	transition model	Transition model	1	kept

transition model	transition model	transition model	transition model	Transition model	1	case_or_format
Transition of care	transition of care	transition of care	transition of care	transition of care	1	case_or_format
transition period	transition period	transition period	transition period	transition period	1	kept
Transition phase	transition phase	transition phase	transition phase	Transition phase	1	kept
Transition procedure	transition procedure	transition procedure	transition procedure	Transition procedure	1	kept
Transition Process	transition process	transition process	transition process	transition process	1	case_or_format
Transition program	transition program	transition program	transition program	Transition program	1	kept
transition program	transition program	transition program	transition program	Transition program	1	case_or_format
transition programme	transition programme	transition programme	transition programme	transition programme	1	kept
Transition questionnaire	transition questionnaire	transition questionnaire	transition questionnaire	Transition questionnaire	1	kept
transition role	transition role	transition role	transition role	transition role	1	kept
transition shock	transition shock	transition shock	transition shock	Transition shock	1	case_or_format
Transition support	transition support	transition support	transition support	Transition support	1	kept
Transition Theory	transition theory	transition theory	transition theory	Transition Theory	1	kept
transition time	transition time	transition time	transition time	transition time	1	kept
Transition to home	transition to home	transition to home	transition to home	transition to home	1	case_or_format

Transition to motherhood	transition to motherhood	transition to motherhood	transition to motherhood	Transition to motherhood	1	kept
transition to motherhood	transition to motherhood	transition to motherhood	transition to motherhood	Transition to motherhood	1	case_or_format
Transition to Practice	transition to practice	transition to practice	transition to practice	Transition to Practice	1	kept
transition to practice	transition to practice	transition to practice	transition to practice	Transition to Practice	1	case_or_format
Transition to practice programs	transition to practice programs	transition to practice programs	transition to practice programs	Transition to practice programs	1	kept
Transition to specialty practice	transition to specialty practice	transition to specialty practice	transition to specialty practice	Transition to specialty practice	1	kept
Transition-to-practice programs	transition to practice programs	transition to practice programs	transition to practice programs	Transition to practice programs	1	case_or_format
Transitional	transitional	transitional	transitional	Transitional	1	kept
transitional care program	transitional care program	transitional care program	transitional care program	transitional care program	1	kept
transitional care unit	transitional care unit	transitional care unit	transitional care unit	transitional care unit	1	kept
transitional cell carcinoma	transitional cell carcinoma	transitional cell carcinoma	transitional cell carcinoma	transitional cell carcinoma	1	kept
transitional space	transitional space	transitional space	transitional space	transitional space	1	kept
transitions theory	transitions theory	transitions theory	transitions theory	Transitions theory	1	case_or_format
transitions to palliative care	transitions to palliative care	transitions to palliative care	transitions to palliative care	transitions to palliative care	1	kept
translaryngeal intubation	translaryngeal intubation	translaryngeal intubation	translaryngeal intubation	translaryngeal intubation	1	kept
Translating evidence into practice	translating evidence into practice	translating evidence into practice	translating evidence into practice	Translating evidence into practice	1	kept

translating political risk	translating political risk	translating political risk	translating political risk	translating political risk	1	kept
translating professional risk	translating professional risk	translating professional risk	translating professional risk	translating professional risk	1	kept
Translating Research into Practice Framework	translating research into practice framework	translating research into practice framework	translating research into practice framework	Translating Research into Practice Framework	1	kept
translating systemic risk	translating systemic risk	translating systemic risk	translating systemic risk	translating systemic risk	1	kept
translation research	translation research	translation research	translation research	translation research	1	kept
Translation, written materials	translation written materials	translation written materials	translation written materials	Translation, written materials	1	kept
Translational Research	translational research	translational research	translational research	translational research	1	case_or_format
translations	translations	translations	translation	Translations	1	singular
Translator	translator	translator	translator	Translator	1	kept
transmembrane pressure gradient	transmembrane pressure gradient	transmembrane pressure gradient	transmembrane pressure gradient	transmembrane pressure gradient	1	kept
transmembrane protease serine 2	transmembrane protease serine 2	transmembrane protease serine 2	transmembrane protease serine 2	transmembrane protease serine 2	1	kept
Transmission control	transmission control	transmission control	transmission control	Transmission control	1	kept
transmission dynamics	transmission dynamics	transmission dynamics	transmission dynamics	TRANSMISSION DYNAMICS	1	case_or_format
Transmission electron microscopy	transmission electron microscopy	transmission electron microscopy	transmission electron microscopy	transmission electron microscopy	1	case_or_format
Transmission mode	transmission mode	transmission mode	transmission mode	Transmission mode	1	kept
Transmission risk	transmission risk	transmission risk	transmission risk	transmission risk	1	case_or_format

transmission time	transmission time	transmission time	transmission time	transmission time	1	kept
Transmission-based precautions	transmission based precautions	transmission based precautions	transmission based precautions	Transmission-based precautions	1	kept
transmural pressure	transmural pressure	transmural pressure	transmural pressure	transmural pressure	1	kept
transnasal endoscopic repair	transnasal endoscopic repair	transnasal endoscopic repair	transnasal endoscopic repair	transnasal endoscopic repair	1	kept
Transnasal repair	transnasal repair	transnasal repair	transnasal repair	Transnasal repair	1	kept
transnational organizations	transnational organizations	transnational organizations	transnational organizations	transnational organizations	1	kept
transoral endotracheal intubation	transoral endotracheal intubation	transoral endotracheal intubation	transoral endotracheal intubation	transoral endotracheal intubation	1	kept
transorbital sonography	transorbital sonography	transorbital sonography	transorbital sonography	transorbital sonography	1	kept
Transparency	transparency	transparency	transparency	Transparency	1	kept
transparency	transparency	transparency	transparency	Transparency	1	case_or_format
transparent cover	transparent cover	transparent cover	transparent cover	transparent cover	1	kept
transplacental infection	transplacental infection	transplacental infection	transplacental infection	transplacental infection	1	kept
TRANSPLANT	transplant	transplant	transplant	Transplants	1	case_or_format
transplant candidacy	transplant candidacy	transplant candidacy	transplant candidacy	transplant candidacy	1	kept
Transplant candidates	transplant candidates	transplant candidates	transplant candidates	Transplant candidates	1	kept
transplant center	transplant center	transplant center	transplant center	transplant center	1	kept

transplant complication	transplant complication	transplant complication	transplant complication	transplant complication	1	kept
Transplant complications	transplant complications	transplant complications	transplant complication	transplant complication	1	singular
Transplant coordinator	transplant coordinator	transplant coordinator	transplant coordinator	transplant coordinator	1	case_or_format
transplant donor	transplant donor	transplant donor	transplant donor	transplant donor	1	kept
Transplant evaluation	transplant evaluation	transplant evaluation	transplant evaluation	Transplant evaluation	1	kept
transplant program organization	transplant program organization	transplant program organization	transplant program organization	transplant program organization	1	kept
Transplant recipients	transplant recipients	transplant recipients	transplant recipients	Transplant Recipients	1	case_or_format
transplantation cardio-pulmonaire	transplantation cardio pulmonaire	transplantation cardio pulmonaire	transplantation cardio pulmonaire	transplantation cardio-pulmonaire	1	kept
Transplantation law	transplantation law	transplantation law	transplantation law	Transplantation law	1	kept
transplantation, kidney	transplantation kidney	transplantation kidney	transplantation kidney	transplantation, kidney	1	kept
transplants	transplants	transplants	transplant	Transplants	1	singular
transport carts	transport carts	transport carts	transport carts	transport carts	1	kept
Transport efficiency	transport efficiency	transport efficiency	transport efficiency	Transport efficiency	1	kept
Transport guidelines	transport guidelines	transport guidelines	transport guidelines	Transport guidelines	1	kept
transport policy	transport policy	transport policy	transport policy	transport policy	1	kept
Transport safety	transport safety	transport safety	transport safety	Transport safety	1	kept

transport stress	transport stress	transport stress	transport stress	transport stress	1	kept
Transport team	transport team	transport team	transport team	Transport team	1	kept
transport teams	transport teams	transport teams	transport team	Transport team	1	singular
transport vesicle	transport vesicle	transport vesicle	transport vesicle	transport vesicle	1	kept
Transportation network	transportation network	transportation network	transportation network	Transportation network	1	kept
transportation staff	transportation staff	transportation staff	transportation staff	transportation staff	1	kept
Transportation vehicles	transportation vehicles	transportation vehicles	transportation vehicles	Transportation vehicles	1	kept
Transporte aeromédico	transporte aerom dico	transporte aerom dico	transporte aerom dico	Transporte aeromédico	1	kept
Transporte aéreo	transporte a reo	transporte a reo	transporte a reo	Transporte aéreo	1	kept
Transporte Inter-hospitalar	transporte inter hospitalar	transporte inter hospitalar	transporte inter hospitalar	Transporte Inter-hospitalar	1	kept
Transporte interhospitalario	transporte interhospitalario	transporte interhospitalario	transporte interhospitalario	Transporte interhospitalario	1	kept
Transporte intrahospitalario	transporte intrahospitalario	transporte intrahospitalario	transporte intrahospitalario	Transporte intrahospitalario	1	kept
Transposition of great arteries	transposition of great arteries	transposition of great arteries	transposition of great arteries	Transposition of great arteries	1	kept
transposition of the great arteries	transposition of the great arteries	transposition of the great arteries	transposition of the great arteries	transposition of the great arteries	1	kept
Transradial band	transradial band	transradial band	transradial band	Transradial band	1	kept
Transshipment safety	transshipment safety	transshipment safety	transshipment safety	Transshipment safety	1	kept

transsphenoidal surgery	transsphenoidal surgery	transsphenoidal surgery	transsphenoidal surgery	transsphenoidal surgery	1	kept
Transtentorial herniation	transtentorial herniation	transtentorial herniation	transtentorial herniation	Transtentorial herniation	1	kept
Transtheoretical model	transtheoretical model	transtheoretical model	transtheoretical model	Transtheoretical model	1	kept
transthoracic cardioversion	transthoracic cardioversion	transthoracic cardioversion	transthoracic cardioversion	transthoracic cardioversion	1	kept
Transthoracic echocardiography	transthoracic echocardiography	transthoracic echocardiography	transthoracic echocardiography	transthoracic echocardiography	1	case_or_format
transtracheal catheter	transtracheal catheter	transtracheal catheter	transtracheal catheter	transtracheal catheter	1	kept
TRANSURETHRAL CATHETERIZATION	transurethral catheterization	transurethral catheterization	transurethral catheterization	TRANSURETHRAL CATHETERIZATION	1	kept
Transverse abdominis plane block	transverse abdominis plane block	transverse abdominis plane block	transverse abdominis plane block	Transverse abdominis plane block	1	kept
transverse process	transverse process	transverse process	transverse process	transverse process	1	kept
transverse sinus	transverse sinus	transverse sinus	transverse sinus	transverse sinus	1	kept
Transversus abdominis plane block	transversus abdominis plane block	transversus abdominis plane block	transversus abdominis plane block	Transversus abdominis plane block	1	kept
transvertical ear to ear distance	transvertical ear to ear distance	transvertical ear to ear distance	transvertical ear to ear distance	transvertical ear to ear distance	1	kept
trapezius muscle	trapezius muscle	trapezius muscle	trapezius muscle	trapezius muscle	1	kept
Trapezius muscle activity	trapezius muscle activity	trapezius muscle activity	trapezius muscle activity	Trapezius muscle activity	1	kept
Traslado a la UCI	traslado a la uci	traslado a la uci	traslado a la uci	Traslado a la UCI	1	kept
Traspaso de información	traspaso de informacin	traspaso de informacin	traspaso de informacin	Traspaso de información	1	kept

Trasplante	trasplante	trasplante	trasplante	Trasplante	1	kept
Trasplante de pulmón	trasplante de pulm n	trasplante de pulm n	trasplante de pulm n	Trasplante de pulmón	1	kept
Trastornos de la deglución	trastornos de la degluci n	trastornos de la degluci n	trastornos de la degluci n	Trastornos de la deglución	1	kept
trauma activation	trauma activation	trauma activation	trauma activation	trauma activation	1	kept
Trauma and coagulopathy	trauma and coagulopathy	trauma and coagulopathy	trauma and coagulopathy	Trauma and coagulopathy	1	kept
Trauma and injury severity score	trauma and injury severity score	trauma and injury severity score	trauma and injury severity score	Trauma and injury severity score	1	kept
Trauma and Stressor Related Disorders	trauma and stressor related disorders	trauma and stressor related disorders	trauma and stressor related disorders	Trauma and Stressor Related Disorders	1	kept
Trauma call	trauma call	trauma call	trauma call	Trauma call	1	kept
Trauma care knowledge	trauma care knowledge	trauma care knowledge	trauma care knowledge	Trauma care knowledge	1	kept
Trauma care systems	trauma care systems	trauma care systems	trauma care systems	Trauma care systems	1	kept
Trauma caregiver	trauma caregiver	trauma caregiver	trauma caregiver	Trauma caregiver	1	kept
Trauma case management	trauma case management	trauma case management	trauma case management	Trauma case management	1	kept
TRAUMA CENTER	trauma center	trauma center	trauma center	Trauma Centers	1	case_or_format
Trauma center development	trauma center development	trauma center development	trauma center development	Trauma center development	1	kept
Trauma center verification	trauma center verification	trauma center verification	trauma center verification	Trauma center verification	1	kept
Trauma coordinator	trauma coordinator	trauma coordinator	trauma coordinator	Trauma coordinator	1	kept

Trauma database	trauma database	trauma database	trauma database	Trauma database	1	kept
trauma focused occupational therapy	trauma focused occupational therapy	trauma focused occupational therapy	trauma focused occupational therapy	trauma focused occupational therapy	1	kept
Trauma hemostasis and oxygenation	trauma hemostasis and oxygenation	trauma hemostasis and oxygenation	trauma hemostasis and oxygenation	Trauma hemostasis and oxygenation	1	kept
trauma hospitalist	trauma hospitalist	trauma hospitalist	trauma hospitalist	trauma hospitalist	1	kept
trauma informed care	trauma informed care	trauma informed care	trauma informed care	trauma-informed care	1	case_or_format
Trauma mechanism	trauma mechanism	trauma mechanism	trauma mechanism	Trauma mechanism	1	kept
Trauma medi	trauma medi	trauma medi	trauma medi	Trauma medi	1	kept
Trauma model	trauma model	trauma model	trauma model	Trauma model	1	kept
Trauma morbidity	trauma morbidity	trauma morbidity	trauma morbidity	Trauma morbidity	1	kept
Trauma Orchestration of Continuing Healthcare	trauma orchestration of continuing healthcare	trauma orchestration of continuing healthcare	trauma orchestration of continuing healthcare	Trauma Orchestration of Continuing Healthcare	1	kept
Trauma outcomes	trauma outcomes	trauma outcomes	trauma outcomes	Trauma outcomes	1	kept
Trauma providers' practice	trauma providers practice	trauma providers practice	trauma providers practice	Trauma providers' practice	1	kept
trauma quality improvement	trauma quality improvement	trauma quality improvement	trauma quality improvement	trauma quality improvement	1	kept
trauma rehabilitation	trauma rehabilitation	trauma rehabilitation	trauma rehabilitation	trauma rehabilitation	1	kept
Trauma resuscitation	trauma resuscitation	trauma resuscitation	trauma resuscitation	Trauma resuscitation	1	kept
Trauma room	trauma room	trauma room	trauma room	Trauma room	1	kept

trauma room	trauma room	trauma room	trauma room	Trauma room	1	case_or_format
Trauma rounds	trauma rounds	trauma rounds	trauma rounds	Trauma rounds	1	kept
Trauma screening	trauma screening	trauma screening	trauma screening	Trauma screening	1	kept
Trauma Screening Questionnaire	trauma screening questionnaire	trauma screening questionnaire	trauma screening questionnaire	Trauma Screening Questionnaire	1	kept
Trauma Surgery	trauma surgery	trauma surgery	trauma surgery	trauma surgery	1	case_or_format
Trauma system	trauma system	trauma system	trauma system	Trauma systems	1	case_or_format
trauma system	trauma system	trauma system	trauma system	Trauma systems	1	case_or_format
Trauma team activation	trauma team activation	trauma team activation	trauma team activation	Trauma team activation	1	kept
trauma team performance	trauma team performance	trauma team performance	trauma team performance	trauma team performance	1	kept
Trauma triage	trauma triage	trauma triage	trauma triage	Trauma triage	1	kept
Trauma units	trauma units	trauma units	trauma units	Trauma units	1	kept
trauma units	trauma units	trauma units	trauma units	Trauma units	1	case_or_format
trauma volume	trauma volume	trauma volume	trauma volume	trauma volume	1	kept
trauma workload	trauma workload	trauma workload	trauma workload	trauma workload	1	kept
Trauma, Triage	trauma triage	trauma triage	trauma triage	Trauma triage	1	case_or_format
Trauma-informed communication	trauma informed communication	trauma informed communication	trauma informed communication	Trauma-informed communication	1	kept

trauma-specific treatment	trauma specific treatment	trauma specific treatment	trauma specific treatment	trauma-specific treatment	1	kept
traumatic aortic rupture	traumatic aortic rupture	traumatic aortic rupture	traumatic aortic rupture	traumatic aortic rupture	1	kept
Traumatic birth	traumatic birth	traumatic birth	traumatic birth	Traumatic birth	1	kept
Traumatic Brain Iinjury	traumatic brain iinjury	traumatic brain iinjury	traumatic brain iinjury	Traumatic Brain Iinjury	1	kept
Traumatic chest injury	traumatic chest injury	traumatic chest injury	traumatic chest injury	Traumatic chest injury	1	kept
traumatic coma	traumatic coma	traumatic coma	traumatic coma	traumatic coma	1	kept
traumatic deaths	traumatic deaths	traumatic deaths	traumatic deaths	traumatic deaths	1	kept
traumatic delivery	traumatic delivery	traumatic delivery	traumatic delivery	traumatic delivery	1	kept
traumatic epilepsy	traumatic epilepsy	traumatic epilepsy	traumatic epilepsy	traumatic epilepsy	1	kept
traumatic experiences	traumatic experiences	traumatic experiences	traumatic experiences	traumatic experiences	1	kept
Traumatic fracture	traumatic fracture	traumatic fracture	traumatic fracture	Traumatic fracture	1	kept
Traumatic Head Injury	traumatic head injury	traumatic head injury	traumatic head injury	Traumatic Head Injury	1	kept
Traumatic head injury	traumatic head injury	traumatic head injury	traumatic head injury	Traumatic Head Injury	1	case_or_format
traumatic hematoma	traumatic hematoma	traumatic hematoma	traumatic hematoma	traumatic hematoma	1	kept
traumatic injuries	traumatic injuries	traumatic injuries	traumatic injury	Traumatic injury	1	singular
Traumatic intracranial hemorrhage	traumatic intracranial hemorrhage	traumatic intracranial hemorrhage	traumatic intracranial hemorrhage	Traumatic intracranial hemorrhage	1	kept

Traumatic rib fractures	traumatic rib fractures	traumatic rib fractures	traumatic rib fractures	Traumatic rib fractures	1	kept
Traumatic rupture	traumatic rupture	traumatic rupture	traumatic rupture	Traumatic rupture	1	kept
Traumatic stress	traumatic stress	traumatic stress	traumatic stress	TRAUMATIC STRESS	1	case_or_format
traumatic stressor	traumatic stressor	traumatic stressor	traumatic stressor	traumatic stressor	1	kept
Traumatic vertical atlantoaxial dislocation	traumatic vertical atlantoaxial dislocation	traumatic vertical atlantoaxial dislocation	traumatic vertical atlantoaxial dislocation	Traumatic vertical atlantoaxial dislocation	1	kept
traumatic vertical atlantoaxial dislocation	traumatic vertical atlantoaxial dislocation	traumatic vertical atlantoaxial dislocation	traumatic vertical atlantoaxial dislocation	Traumatic vertical atlantoaxial dislocation	1	case_or_format
traumatisme crânien grave	traumatisme cr nien grave	traumatisme cr nien grave	traumatisme cr nien grave	traumatisme crânien grave	1	kept
traumatization	traumatization	traumatization	traumatization	traumatization	1	kept
traumatology unit	traumatology unit	traumatology unit	traumatology unit	traumatology unit	1	kept
travel restriction	travel restriction	travel restriction	travel restriction	travel restriction	1	kept
Tre atment	tre atment	tre atment	tre atment	Tre atment	1	kept
Treatment Adherence and Compliance	treatment adherence and compliance	treatment adherence and compliance	treatment adherence and compliance	Treatment Adherence and Compliance	1	kept
treatment algorithm	treatment algorithm	treatment algorithm	treatment algorithm	treatment algorithm	1	kept
treatment beliefs	treatment beliefs	treatment beliefs	treatment beliefs	treatment beliefs	1	kept
treatment communication	treatment communication	treatment communication	treatment communication	treatment communication	1	kept
treatment compliance	treatment compliance	treatment compliance	treatment compliance	treatment compliance	1	kept

treatment decision	treatment decision	treatment decision	treatment decision	treatment decision	1	kept
treatment decision making	treatment decision making	treatment decision making	treatment decision making	treatment decision making	1	kept
Treatment decisionmaking	treatment decisionmaking	treatment decisionmaking	treatment decisionmaking	Treatment decisionmaking	1	kept
treatment effect heterogeneity	treatment effect heterogeneity	treatment effect heterogeneity	treatment effect heterogeneity	treatment effect heterogeneity	1	kept
treatment efficacy	treatment efficacy	treatment efficacy	treatment efficacy	treatment efficacy	1	kept
Treatment errors	treatment errors	treatment errors	treatment errors	Treatment errors	1	kept
treatment escalation	treatment escalation	treatment escalation	treatment escalation	treatment escalation	1	kept
Treatment escalation restriction	treatment escalation restriction	treatment escalation restriction	treatment escalation restriction	Treatment escalation restriction	1	kept
treatment expectation	treatment expectation	treatment expectation	treatment expectation	treatment expectation	1	kept
Treatment failure	treatment failure	treatment failure	treatment failure	treatment failure	1	case_or_format
Treatment goal compliance	treatment goal compliance	treatment goal compliance	treatment goal compliance	Treatment goal compliance	1	kept
Treatment guidelines	treatment guidelines	treatment guidelines	treatment guideline	treatment guideline	1	singular
treatment guidelines	treatment guidelines	treatment guidelines	treatment guideline	treatment guideline	1	singular
Treatment Guidelines for Sepsis and Septic Shock	treatment guidelines for sepsis and septic shock	treatment guidelines for sepsis and septic shock	treatment guidelines for sepsis and septic shock	Treatment Guidelines for Sepsis and Septic Shock	1	kept
treatment inappropriateness	treatment inappropriateness	treatment inappropriateness	treatment inappropriateness	treatment inappropriateness	1	kept
Treatment Interruption	treatment interruption	treatment interruption	treatment interruption	treatment interruption	1	case_or_format

treatment lasers	treatment lasers	treatment lasers	treatment lasers	treatment lasers	1	kept
treatment limitation	treatment limitation	treatment limitation	treatment limitation	Treatment limitation	1	case_or_format
treatment medical	treatment medical	treatment medical	treatment medical	treatment medical	1	kept
Treatment mistakes	treatment mistakes	treatment mistakes	treatment mistakes	Treatment mistakes	1	kept
Treatment of Disorder of Consciousness	treatment of disorder of consciousness	treatment of disorder of consciousness	treatment of disorder of consciousness	Treatment of Disorder of Consciousness	1	kept
treatment of Disorder of Consciousness	treatment of disorder of consciousness	treatment of disorder of consciousness	treatment of disorder of consciousness	Treatment of Disorder of Consciousness	1	case_or_format
TREATMENT ONSET	treatment onset	treatment onset	treatment onset	TREATMENT ONSET	1	kept
treatment orders	treatment orders	treatment orders	treatment orders	treatment orders	1	kept
Treatment outcomes	treatment outcomes	treatment outcomes	treatment outcome	Treatment Outcome	1	singular
Treatment patterns	treatment patterns	treatment patterns	treatment patterns	Treatment patterns	1	kept
TREATMENT PREFERENCES	treatment preferences	treatment preferences	treatment preferences	TREATMENT PREFERENCES	1	kept
Treatment refusal	treatment refusal	treatment refusal	treatment refusal	treatment refusal	1	case_or_format
Treatment strategies	treatment strategies	treatment strategies	treatment strategy	treatment strategy	1	singular
treatment strategy	treatment strategy	treatment strategy	treatment strategy	treatment strategy	1	kept
treatment success rate	treatment success rate	treatment success rate	treatment success rate	treatment success rate	1	kept
treatment switching	treatment switching	treatment switching	treatment switching	treatment switching	1	kept

treatment toxicities	treatment toxicities	treatment toxicities	treatment toxicities	treatment toxicities	1	kept
Treatment withholding	treatment withholding	treatment withholding	treatment withholding	Treatment withholding	1	kept
Treatment-gap	treatment gap	treatment gap	treatment gap	Treatment-gap	1	kept
Treatment-limitation decisions	treatment limitation decisions	treatment limitation decisions	treatment limitation decision	treatment limitation decision	1	singular
Treatment-limitation-decisions	treatment limitation decisions	treatment limitation decisions	treatment limitation decision	treatment limitation decision	1	singular
Treatments	treatments	treatments	treatment	Treatment	1	singular
tree	tree	tree	tree	Trees	1	case_or_format
Treinamento com Simulação de Alta Fidelidade	treinamento com simula o de alta fidelidade	treinamento com simula o de alta fidelidade	treinamento com simula o de alta fidelidade	Treinamento com Simulação de Alta Fidelidade	1	kept
Treino	treino	treino	treino	Treino	1	kept
tremors	tremors	tremors	tremor	tremor	1	singular
trend analysis	trend analysis	trend analysis	trend analysis	trend analysis	1	kept
TrendCare	trendcare	trendcare	trendcare	TrendCare	1	kept
trendelenburg position	trendelenburg position	trendelenburg position	trendelenburg position	trendelenburg position	1	kept
trends analysis	trends analysis	trends analysis	trends analysis	trends analysis	1	kept
trengths, Weaknesses, Opportunities, and Threat framework	trengths weaknesses opportunities and threat framework	trengths weaknesses opportunities and threat framework	trengths weaknesses opportunities and threat framework	trengths, Weaknesses, Opportunities, and Threat framework	1	kept
triacylglycerol level	triacylglycerol level	triacylglycerol level	triacylglycerol level	triacylglycerol level	1	kept

Triage and blast injuries	triage and blast injuries	triage and blast injuries	triage and blast injuries	Triage and blast injuries	1	kept
Triage at admission	triage at admission	triage at admission	triage at admission	Triage at admission	1	kept
triage category	triage category	triage category	triage category	triage category	1	kept
triage quality improvement	triage quality improvement	triage quality improvement	triage quality improvement	triage quality improvement	1	kept
triage tool	triage tool	triage tool	triage tool	triage tool	1	kept
triage validation	triage validation	triage validation	triage validation	triage validation	1	kept
Trial designs	trial designs	trial designs	trial designs	Trial designs	1	kept
Trial execution	trial execution	trial execution	trial execution	Trial execution	1	kept
Trial of Labor	trial of labor	trial of labor	trial of labor	trial of labor	1	case_or_format
Trial of labor after cesarean section	trial of labor after cesarean section	trial of labor after cesarean section	trial of labor after cesarean section	Trial of labor after cesarean section	1	kept
Trial of labour after caesarean	trial of labour after caesarean	trial of labour after caesarean	trial of labour after caesarean	Trial of labour after caesarean	1	kept
trial of therapy	trial of therapy	trial of therapy	trial of therapy	trial of therapy	1	kept
Trial registration: ChiCTR	trial registration chictr	trial registration chictr	trial registration chictr	Trial registration: ChiCTR	1	kept
trialling new therapies	trialling new therapies	trialling new therapies	trialling new therapies	trialling new therapies	1	kept
trials	trials	trials	trial	TRIAL	1	singular
trials.	trials	trials	trial	TRIAL	1	singular

triamterene	triamterene	triamterene	triamterene	triamterene	1	kept
Triandis	triandis	triandis	triandis	Triandis	1	kept
Triangulation	triangulation	triangulation	triangulation	triangulation	1	case_or_format
Triangulation methodology	triangulation methodology	triangulation methodology	triangulation methodology	Triangulation methodology	1	kept
triazolam	triazolam	triazolam	triazolam	triazolam	1	kept
tribal areas	tribal areas	tribal areas	tribal areas	tribal areas	1	kept
Tribulations	tribulations	tribulations	tribulations	Tribulations	1	kept
triceps surae muscle	triceps surae muscle	triceps surae muscle	triceps surae muscle	triceps surae muscle	1	kept
trichloroethylene	trichloroethylene	trichloroethylene	trichloroethylene	trichloroethylene	1	kept
Trichomonas vaginalis	trichomonas vaginalis	trichomonas vaginalis	trichomonas vaginalis	Trichomonas vaginalis	1	kept
trichomoniasis	trichomoniasis	trichomoniasis	trichomoniasis	trichomoniasis	1	kept
Trichosporon asahii	trichosporon asahii	trichosporon asahii	trichosporon asahii	Trichosporon asahii	1	kept
Triclosan	triclosan	triclosan	triclosan	triclosan	1	case_or_format
tricosal	tricosal	tricosal	tricosal	tricosal	1	kept
Tricyclic antidepressants	tricyclic antidepressants	tricyclic antidepressants	tricyclic antidepressants	Tricyclic antidepressants	1	kept
trier personality questionnaire	trier personality questionnaire	trier personality questionnaire	trier personality questionnaire	trier personality questionnaire	1	kept

trifluridine	trifluridine	trifluridine	trifluridine	trifluridine	1	kept
Trifunctional protein deficiency	trifunctional protein deficiency	trifunctional protein deficiency	trifunctional protein deficiency	Trifunctional protein deficiency	1	kept
Trigeminal nerve	trigeminal nerve	trigeminal nerve	trigeminal nerve	Trigeminal nerve	1	kept
trigeminus neuralgia	trigeminus neuralgia	trigeminus neuralgia	trigeminus neuralgia	trigeminus neuralgia	1	kept
Trigger factors	trigger factors	trigger factors	trigger factors	Trigger factors	1	kept
Trigger questions	trigger questions	trigger questions	trigger questions	Trigger questions	1	kept
trigger team activation	trigger team activation	trigger team activation	trigger team activation	trigger team activation	1	kept
TRIGGER TOOL	trigger tool	trigger tool	trigger tool	Trigger tools	1	case_or_format
trigger tool	trigger tool	trigger tool	trigger tool	Trigger tools	1	case_or_format
triggering receptor expressed on myeloid cells 1	triggering receptor expressed on myeloid cells 1	triggering receptor expressed on myeloid cells 1	triggering receptor expressed on myeloid cells 1	triggering receptor expressed on myeloid cells 1	1	kept
Triggers	triggers	triggers	trigger	trigger	1	singular
triglyceride glucose index	triglyceride glucose index	triglyceride glucose index	triglyceride glucose index	triglyceride glucose index	1	kept
triglycerides	triglycerides	triglycerides	triglycerides	Triglycerides	1	case_or_format
trihexyphenidyl	trihexyphenidyl	trihexyphenidyl	trihexyphenidyl	trihexyphenidyl	1	kept
trihibit	trihibit	trihibit	trihibit	trihibit	1	kept
TRIIODOTHYRONINE	triiodothyronine	triiodothyronine	triiodothyronine	Triiodothyronine	1	case_or_format

Trimethoprim-Sulfamethoxazole Combination	trimethoprim sulfamethoxazole combination	trimethoprim sulfamethoxazole combination	trimethoprim sulfamethoxazole combination	Trimethoprim-Sulfamethoxazole Combination	1	kept
trimethoprim - sulfamethoxazole	trimethoprim sulfamethoxazole	trimethoprim sulfamethoxazole	trimethoprim sulfamethoxazole	trimethoprim - sulfamethoxazole	1	kept
Trimodal therapy	trimodal therapy	trimodal therapy	trimodal therapy	Trimodal therapy	1	kept
Trinuclear	trinuclear	trinuclear	trinuclear	Trinuclear	1	kept
Tripeptidyl-Peptidase 1	tripeptidyl peptidase 1	tripeptidyl peptidase 1	tripeptidyl peptidase 1	Tripeptidyl-Peptidase 1	1	kept
Triple Aim	triple aim	triple aim	triple aim	Triple Aim	1	kept
triple aim	triple aim	triple aim	triple aim	Triple Aim	1	case_or_format
triple lumen catheter	triple lumen catheter	triple lumen catheter	triple lumen catheter	triple lumen catheter	1	kept
Triple-H	triple h	triple h	triple h	Triple-H	1	kept
triplet embeddings	triplet embeddings	triplet embeddings	triplet embeddings	triplet embeddings	1	kept
Triplet gestation	triplet gestation	triplet gestation	triplet gestation	Triplet gestation	1	kept
Triplet pregnancy	triplet pregnancy	triplet pregnancy	triplet pregnancy	triplet pregnancy	1	case_or_format
Triplet<sup> <	triplet sup	triplet sup	triplet sup	Triplet<sup> <	1	kept
triptan derivative	triptan derivative	triptan derivative	triptan derivative	triptan derivative	1	kept
Tripura	tripura	tripura	tripura	Tripura	1	kept
Trisomy 18	trisomy 18	trisomy 18	trisomy 18	trisomy 18	1	case_or_format

TRISS	triss	triss	triss	TRISS	1	kept
trocar	trocar	trocar	trocar	trocar	1	kept
troclosene	troclosene	troclosene	troclosene	troclosene	1	kept
Trombley-Brennan Terminal Tissue Injury	trombley brennan terminal tissue injury	trombley brennan terminal tissue injury	trombley brennan terminal tissue injury	Trombley-Brennan Terminal Tissue Injury	1	kept
Trombley-Brennan terminal tissue injury	trombley brennan terminal tissue injury	trombley brennan terminal tissue injury	trombley brennan terminal tissue injury	Trombley-Brennan Terminal Tissue Injury	1	case_or_format
trophoblast	trophoblast	trophoblast	trophoblast	trophoblast	1	kept
Tropical medicine	tropical medicine	tropical medicine	tropical medicine	Tropical Medicine	1	case_or_format
tropical medicine	tropical medicine	tropical medicine	tropical medicine	Tropical Medicine	1	case_or_format
Tropical surgery	tropical surgery	tropical surgery	tropical surgery	Tropical surgery	1	kept
tropics	tropics	tropics	tropics	tropics	1	kept
Troponin C	troponin c	troponin c	troponin c	Troponin C	1	kept
Troponin T	troponin t	troponin t	troponin t	troponin T	1	case_or_format
trospium chloride	trospium chloride	trospium chloride	trospium chloride	trospium chloride	1	kept
troubles psychologiques	troubles psychologiques	troubles psychologiques	troubles psychologiques	troubles psychologiques	1	kept
trovafloxacin	trovafloxacin	trovafloxacin	trovafloxacin	trovafloxacin	1	kept
True alarms	true alarms	true alarms	true alarms	True alarms	1	kept

truncus arteriosus	truncus arteriosus	truncus arteriosus	truncus arteriosus	truncus arteriosus	1	kept
trunk stability	trunk stability	trunk stability	trunk stability	trunk stability	1	kept
trust in AI	trust in ai	trust in ai	trust in ai	trust in AI	1	kept
Trust models	trust models	trust models	trust models	Trust models	1	kept
Truth disclosure	truth disclosure	truth disclosure	truth disclosure	Truth Disclosure	1	case_or_format
truth-telling	truth telling	truth telling	truth telling	truth-telling	1	kept
Trypanosoma cruzi	trypanosoma cruzi	trypanosoma cruzi	trypanosoma cruzi	Trypanosoma cruzi	1	kept
trypsin	trypsin	trypsin	trypsin	trypsin	1	kept
trypsin inhibitor	trypsin inhibitor	trypsin inhibitor	trypsin inhibitor	trypsin inhibitor	1	kept
Trypsin inhibitors	trypsin inhibitors	trypsin inhibitors	trypsin inhibitor	trypsin inhibitor	1	singular
tryptophan	tryptophan	tryptophan	tryptophan	tryptophan	1	kept
TSAGAT	tsagat	tsagat	tsagat	TSAGAT	1	kept
TSCI	tsci	tsci	tsci	TSCI	1	kept
Tshwane District	tshwane district	tshwane district	tshwane district	Tshwane District	1	kept
tst gene	tst gene	tst gene	tst gene	tst gene	1	kept
Tsunami	tsunami	tsunami	tsunami	Tsunami	1	kept

tsunami	tsunami	tsunami	tsunami	Tsunami	1	case_or_format
Tsutsugamushi disease	tsutsugamushi disease	tsutsugamushi disease	tsutsugamushi disease	Tsutsugamushi disease	1	kept
TTM	ttm	ttm	ttm	TTM	1	kept
tube block	tube block	tube block	tube block	tube block	1	kept
Tube Change	tube change	tube change	tube change	Tube Change	1	kept
tube cuff	tube cuff	tube cuff	tube cuff	tube cuff	1	kept
Tube Dislodgment	tube dislodgment	tube dislodgment	tube dislodgment	Tube Dislodgment	1	kept
Tube dislodgment	tube dislodgment	tube dislodgment	tube dislodgment	Tube Dislodgment	1	case_or_format
Tube Feeding	tube feeding	tube feeding	tube feeding	Tube feeding	1	case_or_format
tube feeds	tube feeds	tube feeds	tube feeds	tube feeds	1	kept
tube flow	tube flow	tube flow	tube flow	tube flow	1	kept
TUBE INSERTION	tube insertion	tube insertion	tube insertion	TUBE INSERTION	1	kept
TUBE PLACEMENT	tube placement	tube placement	tube placement	TUBE PLACEMENT	1	kept
tube placement	tube placement	tube placement	tube placement	TUBE PLACEMENT	1	case_or_format
Tube-feeding	tube feeding	tube feeding	tube feeding	Tube feeding	1	case_or_format
Tuberculin	tuberculin	tuberculin	tuberculin	tuberculin	1	case_or_format

Tuberculin Skin Test	tuberculin skin test	tuberculin skin test	tuberculin skin test	Tuberculin skin test	1	case_or_format
tuberculin skin test	tuberculin skin test	tuberculin skin test	tuberculin skin test	Tuberculin skin test	1	case_or_format
TUBERCULOSIS	tuberculosis	tuberculosis	tuberculosis	tuberculosis	1	case_or_format
TUBERCULOSIS CASE DETECTION	tuberculosis case detection	tuberculosis case detection	tuberculosis case detection	TUBERCULOSIS CASE DETECTION	1	kept
tuberculosis control	tuberculosis control	tuberculosis control	tuberculosis control	tuberculosis control	1	kept
Tuberculosis, pulmonary	tuberculosis pulmonary	tuberculosis pulmonary	tuberculosis pulmonary	Tuberculosis, Pulmonary	1	case_or_format
Tuberculosis-destroyed lung	tuberculosis destroyed lung	tuberculosis destroyed lung	tuberculosis destroyed lung	Tuberculosis-destroyed lung	1	kept
Tuberculous meningitis	tuberculous meningitis	tuberculous meningitis	tuberculous meningitis	tuberculous meningitis	1	case_or_format
tuberous sclerosis	tuberous sclerosis	tuberous sclerosis	tuberous sclerosis	tuberous sclerosis	1	kept
tuberous sclerosis complex	tuberous sclerosis complex	tuberous sclerosis complex	tuberous sclerosis complex	tuberous sclerosis complex	1	kept
Tubos de menor volumen	tubos de menor volumen	tubos de menor volumen	tubos de menor volumen	Tubos de menor volumen	1	kept
Tumeur cérébrale	tumeur c r brale	tumeur c r brale	tumeur c r brale	Tumeur cérébrale	1	kept
Tumor	tumor	tumor	tumor	tumor	1	case_or_format
tumor associated leukocyte	tumor associated leukocyte	tumor associated leukocyte	tumor associated leukocyte	tumor associated leukocyte	1	kept
tumor biopsy	tumor biopsy	tumor biopsy	tumor biopsy	tumor biopsy	1	kept
tumor cell	tumor cell	tumor cell	tumor cell	tumor cell	1	kept

tumor diagnosis	tumor diagnosis	tumor diagnosis	tumor diagnosis	tumor diagnosis	1	kept
tumor growth	tumor growth	tumor growth	tumor growth	tumor growth	1	kept
tumor invasion	tumor invasion	tumor invasion	tumor invasion	tumor invasion	1	kept
tumor localization	tumor localization	tumor localization	tumor localization	tumor localization	1	kept
tumor margin	tumor margin	tumor margin	tumor margin	tumor margin	1	kept
tumor marker	tumor marker	tumor marker	tumor marker	tumor marker	1	kept
Tumor necrosis factor	tumor necrosis factor	tumor necrosis factor	tumor necrosis factor	tumor necrosis factor	1	case_or_format
tumor necrosis factor antibody	tumor necrosis factor antibody	tumor necrosis factor antibody	tumor necrosis factor antibody	tumor necrosis factor antibody	1	kept
Tumor necrosis factor inhibitors	tumor necrosis factor inhibitors	tumor necrosis factor inhibitors	tumor necrosis factor inhibitor	tumor necrosis factor inhibitor	1	singular
tumor necrosis factor receptor	tumor necrosis factor receptor	tumor necrosis factor receptor	tumor necrosis factor receptor	tumor necrosis factor receptor	1	kept
tumor necrosis factor receptor associated factor 1	tumor necrosis factor receptor associated factor 1	tumor necrosis factor receptor associated factor 1	tumor necrosis factor receptor associated factor 1	tumor necrosis factor receptor associated factor 1	1	kept
tumor necrosis factor receptor associated factor 2	tumor necrosis factor receptor associated factor 2	tumor necrosis factor receptor associated factor 2	tumor necrosis factor receptor associated factor 2	tumor necrosis factor receptor associated factor 2	1	kept
tums	tums	tums	tums	tums	1	kept
tunellization	tunellization	tunellization	tunellization	tunellization	1	kept
Tunneled catheters	tunneled catheters	tunneled catheters	tunneled catheters	Tunneled catheters	1	kept
tunneled pleural catheter	tunneled pleural catheter	tunneled pleural catheter	tunneled pleural catheter	tunneled pleural catheter	1	kept

tunneled venous access catheter	tunneled venous access catheter	tunneled venous access catheter	tunneled venous access catheter	tunneled venous access catheter	1	kept
tunneling	tunneling	tunneling	tunneling	tunneling	1	kept
tunnelled catheters	tunnelled catheters	tunnelled catheters	tunnelled catheters	tunnelled catheters	1	kept
turbidity	turbidity	turbidity	turbidity	turbidity	1	kept
Turbinates	turbinates	turbinates	turbinates	Turbinates	1	kept
turbinoplasty	turbinoplasty	turbinoplasty	turbinoplasty	turbinoplasty	1	kept
Turbulence	turbulence	turbulence	turbulence	Turbulence	1	kept
Turin	turin	turin	turin	Turin	1	kept
Turkish healthcare settings	turkish healthcare settings	turkish healthcare settings	turkish healthcare settings	Turkish healthcare settings	1	kept
TURKISH VERSION	turkish version	turkish version	turkish version	TURKISH VERSION	1	kept
Turkish version of the developmental care scale	turkish version of the developmental care scale	turkish version of the developmental care scale	turkish version of the developmental care scale	Turkish version of the developmental care scale	1	kept
turn angle	turn angle	turn angle	turn angle	turn angle	1	kept
Turn team	turn team	turn team	turn team	Turn team	1	kept
Turn-around-time	turn around time	turn around time	turn around time	Turn-around-time	1	kept
turning frequency	turning frequency	turning frequency	turning frequency	turning frequency	1	kept
TURNOVER INTENT	turnover intent	turnover intent	turnover intent	TURNOVER INTENT	1	kept

turnover intention scale	turnover intention scale	turnover intention scale	turnover intention scale	turnover intention scale	1	kept
Turnover rate	turnover rate	turnover rate	turnover rate	turnover rate	1	case_or_format
tus q	tus q	tus q	tus q	tus q	1	kept
Tuscany	tuscany	tuscany	tuscany	Tuscany	1	kept
tuscod syrup	tuscod syrup	tuscod syrup	tuscod syrup	tuscod syrup	1	kept
TWA exposure	twa exposure	twa exposure	twa exposure	TWA exposure	1	kept
Twin gestation	twin gestation	twin gestation	twin gestation	Twin gestation	1	kept
twin twin transfusion syndrome	twin twin transfusion syndrome	twin twin transfusion syndrome	twin twin transfusion syndrome	twin twin transfusion syndrome	1	kept
Twin<sup> <	twin sup	twin sup	twin sup	Twin<sup> <	1	kept
Twisting algorithms	twisting algorithms	twisting algorithms	twisting algorithms	Twisting algorithms	1	kept
Twitter	twitter	twitter	twitter	Twitter	1	kept
two bag method	two bag method	two bag method	two bag method	two bag method	1	kept
two days after the operation	two days after the operation	two days after the operation	two days after the operation	two days after the operation	1	kept
Two samples	two samples	two samples	two samples	Two samples	1	kept
two-group design	two group design	two group design	two group design	two-group design	1	kept
Two-level model	two level model	two level model	two level model	Two-level model	1	kept

two-level model	two level model	two level model	two level model	Two-level model	1	case_or_format
Two-tailed test	two tailed test	two tailed test	two tailed test	Two-tailed test	1	kept
two-wave	two wave	two wave	two wave	two-wave	1	kept
TXA	txa	txa	txa	TXA	1	kept
TYMPANIC MEMBRANE TEMPERATURE	tympanic membrane temperature	tympanic membrane temperature	tympanic membrane temperature	TYMPANIC MEMBRANE TEMPERATURE	1	kept
type 1	type 1	type 1	type 1	type 1	1	kept
Type 1 diabetes	type 1 diabetes	type 1 diabetes	type 1 diabetes	Type 1 diabetes	1	kept
type 1 diabetes	type 1 diabetes	type 1 diabetes	type 1 diabetes	Type 1 diabetes	1	case_or_format
Type 1 Diabetes Mellitus	type 1 diabetes mellitus	type 1 diabetes mellitus	type 1 diabetes mellitus	Type 1 Diabetes Mellitus	1	kept
Type 1 diabetes mellitus	type 1 diabetes mellitus	type 1 diabetes mellitus	type 1 diabetes mellitus	Type 1 Diabetes Mellitus	1	case_or_format
type 1 hypersensitivity reaction	type 1 hypersensitivity reaction	type 1 hypersensitivity reaction	type 1 hypersensitivity reaction	type 1 hypersensitivity reaction	1	kept
type 2	type 2	type 2	type 2	type 2	1	kept
Type 2 respiratory failure	type 2 respiratory failure	type 2 respiratory failure	type 2 respiratory failure	Type 2 respiratory failure	1	kept
Type D Personality	type d personality	type d personality	type d personality	Type D Personality	1	kept
type groupings	type groupings	type groupings	type groupings	type groupings	1	kept
type of protective equipment required	type of protective equipment required	type of protective equipment required	type of protective equipment required	type of protective equipment required	1	kept

type of screening	type of screening	type of screening	type of screening	type of screening	1	kept
Type “C” esophageal atresia	type c esophageal atresia	type c esophageal atresia	type c esophageal atresia	Type “C” esophageal atresia	1	kept
Type-2 diabetes	type 2 diabetes	type 2 diabetes	type 2 diabetes	type 2 diabetes	1	case_or_format
Types	types	types	types	Types	1	kept
types of article	types of article	types of article	types of article	types of article	1	kept
Types of Error	types of error	types of error	types of error	Types of Error	1	kept
types of ethical conflict	types of ethical conflict	types of ethical conflict	types of ethical conflict	types of ethical conflict	1	kept
types of study	types of study	types of study	types of study	types of study	1	kept
typhoid vaccine	typhoid vaccine	typhoid vaccine	typhoid vaccine	typhoid vaccine	1	kept
Typology	typology	typology	typology	Typology	1	kept
Técnica Delphi	t cnica delphi	t cnica delphi	t cnica delphi	Técnica Delphi	1	kept
ubiquinone derivative	ubiquinone derivative	ubiquinone derivative	ubiquinone derivative	ubiquinone derivative	1	kept
ubiquitin thiolesterase	ubiquitin thiolesterase	ubiquitin thiolesterase	ubiquitin thiolesterase	ubiquitin thiolesterase	1	kept
Ubiquitous Computing	ubiquitous computing	ubiquitous computing	ubiquitous computing	Ubiquitous Computing	1	kept
Ubiquitous computing	ubiquitous computing	ubiquitous computing	ubiquitous computing	Ubiquitous Computing	1	case_or_format
UC = University of Cincinnati	uc university of cincinnati	uc university of cincinnati	uc university of cincinnati	UC = University of Cincinnati	1	kept

Uci quirúrgica	uci quir rgica	uci quir rgica	uci quir rgica	Uci quirúrgica	1	kept
UCI-A	uci a	uci a	uci a	UCI-A	1	kept
UCLA University of California	ucla university of california	ucla university of california	ucla university of california	UCLA University of California	1	kept
UCLA-GAS	ucla gas	ucla gas	ucla gas	UCLA-GAS	1	kept
UCSF = University of California, San Francisco	ucsf university of california san francisco	ucsf university of california san francisco	ucsf university of california san francisco	UCSF = University of California, San Francisco	1	kept
UEE	uee	uee	uee	UEE	1	kept
Ugandan	ugandan	ugandan	ugandan	Ugandan	1	kept
UK MEDICAL PRACTITIONERS	uk medical practitioners	uk medical practitioners	uk medical practitioners	UK MEDICAL PRACTITIONERS	1	kept
ULCER RISK	ulcer risk	ulcer risk	ulcer risk	ULCER RISK	1	kept
Ulcerative erosions	ulcerative erosions	ulcerative erosions	ulcerative erosions	Ulcerative erosions	1	kept
ulcerative esophagitis	ulcerative esophagitis	ulcerative esophagitis	ulcerative esophagitis	ulcerative esophagitis	1	kept
ulcerogenesis	ulcerogenesis	ulcerogenesis	ulcerogenesis	ulcerogenesis	1	kept
ulinastatin	ulinastatin	ulinastatin	ulinastatin	ulinastatin	1	kept
ulna fracture	ulna fracture	ulna fracture	ulna fracture	ulna fracture	1	kept
Ulnar Artery	ulnar artery	ulnar artery	ulnar artery	Ulnar Artery	1	kept
Ulnar length	ulnar length	ulnar length	ulnar length	Ulnar length	1	kept

ulnar length	ulnar length	ulnar length	ulnar length	Ulnar length	1	case_or_format
Ulnar Nerve	ulnar nerve	ulnar nerve	ulnar nerve	ulnar nerve	1	case_or_format
ultipot k	ultipot k	ultipot k	ultipot k	ultipot k	1	kept
Ultra-narrow bandwidth	ultra narrow bandwidth	ultra narrow bandwidth	ultra narrow bandwidth	Ultra-narrow bandwidth	1	kept
ultra-pure soft water	ultra pure soft water	ultra pure soft water	ultra pure soft water	ultra-pure soft water	1	kept
ultralow-field	ultralow field	ultralow field	ultralow field	ultralow-field	1	kept
Ultraprotective ventilation	ultraprotective ventilation	ultraprotective ventilation	ultraprotective ventilation	Ultraprotective ventilation	1	kept
Ultrasonic applications	ultrasonic applications	ultrasonic applications	ultrasonic applications	Ultrasonic applications	1	kept
ultrasonic drug delivery	ultrasonic drug delivery	ultrasonic drug delivery	ultrasonic drug delivery	ultrasonic drug delivery	1	kept
Ultrasonic sensor HC-SR04	ultrasonic sensor hc sr04	ultrasonic sensor hc sr04	ultrasonic sensor hc sr04	Ultrasonic sensor HC-SR04	1	kept
Ultrasonic sensors	ultrasonic sensors	ultrasonic sensors	ultrasonic sensors	Ultrasonic sensors	1	kept
ULTRASONOGRAPHY	ultrasonography	ultrasonography	ultrasonography	Ultrasonography	1	case_or_format
ultrasonography—Doppler evaluation	ultrasonography doppler evaluation	ultrasonography doppler evaluation	ultrasonography doppler evaluation	ultrasonography—Doppler evaluation	1	kept
ultrasound bladder scanner	ultrasound bladder scanner	ultrasound bladder scanner	ultrasound bladder scanner	ultrasound bladder scanner	1	kept
Ultrasound compression test	ultrasound compression test	ultrasound compression test	ultrasound compression test	Ultrasound compression test	1	kept
Ultrasound contrast agents	ultrasound contrast agents	ultrasound contrast agents	ultrasound contrast agents	Ultrasound contrast agents	1	kept

Ultrasound diagnosis	ultrasound diagnosis	ultrasound diagnosis	ultrasound diagnosis	Ultrasound diagnosis	1	kept
ULTRASOUND GUIDANCE	ultrasound guidance	ultrasound guidance	ultrasound guidance	ultrasound guidance	1	case_or_format
ultrasound guided	ultrasound guided	ultrasound guided	ultrasound guided	Ultrasound-guided	1	case_or_format
ultrasound guided PIV	ultrasound guided piv	ultrasound guided piv	ultrasound guided piv	ultrasound guided PIV	1	kept
ultrasound guided vascular access	ultrasound guided vascular access	ultrasound guided vascular access	ultrasound guided vascular access	ultrasound guided vascular access	1	kept
Ultrasound imaging	ultrasound imaging	ultrasound imaging	ultrasound imaging	Ultrasound imaging	1	kept
Ultrasound in neurological emergencies	ultrasound in neurological emergencies	ultrasound in neurological emergencies	ultrasound in neurological emergencies	Ultrasound in neurological emergencies	1	kept
Ultrasound probes	ultrasound probes	ultrasound probes	ultrasound probes	Ultrasound probes	1	kept
ultrasound scanner	ultrasound scanner	ultrasound scanner	ultrasound scanner	ultrasound scanner	1	kept
Ultrasound screening	ultrasound screening	ultrasound screening	ultrasound screening	Ultrasound screening	1	kept
Ultrasound training	ultrasound training	ultrasound training	ultrasound training	Ultrasound training	1	kept
ultrasound transducer	ultrasound transducer	ultrasound transducer	ultrasound transducer	ultrasound transducer	1	kept
Ultrasound-guided peripheral IV	ultrasound guided peripheral iv	ultrasound guided peripheral iv	ultrasound guided peripheral iv	Ultrasound-guided peripheral IV	1	kept
ultrasound-guided regional anestheia	ultrasound guided regional anestheia	ultrasound guided regional anestheia	ultrasound guided regional anestheia	Ultrasound-guided regional anestheia	1	case_or_format
Ultrasound-guided vascular access	ultrasound guided vascular access	ultrasound guided vascular access	ultrasound guided vascular access	ultrasound guided vascular access	1	case_or_format
ultrasound-guided vascular cannulation	ultrasound guided vascular cannulation	ultrasound guided vascular cannulation	ultrasound guided vascular cannulation	ultrasound-guided vascular cannulation	1	kept

ultraviolet spectrophotometry	ultraviolet spectrophotometry	ultraviolet spectrophotometry	ultraviolet spectrophotometry	ultraviolet spectrophotometry	1	kept
ultraviolet spectroscopy	ultraviolet spectroscopy	ultraviolet spectroscopy	ultraviolet spectroscopy	ultraviolet spectroscopy	1	kept
ultraviolet visible spectroscopy	ultraviolet visible spectroscopy	ultraviolet visible spectroscopy	ultraviolet visible spectroscopy	ultraviolet visible spectroscopy	1	kept
Umbilical arterial catheter	umbilical arterial catheter	umbilical arterial catheter	umbilical arterial catheter	Umbilical arterial catheter	1	kept
Umbilical artery catheter	umbilical artery catheter	umbilical artery catheter	umbilical artery catheter	umbilical artery catheter	1	case_or_format
umbilical catheter	umbilical catheter	umbilical catheter	umbilical catheter	umbilical catheter	1	kept
umbilical cord blood flow	umbilical cord blood flow	umbilical cord blood flow	umbilical cord blood flow	umbilical cord blood flow	1	kept
Umbilical cord care	umbilical cord care	umbilical cord care	umbilical cord care	Umbilical cord care	1	kept
umbilical cord catheterization	umbilical cord catheterization	umbilical cord catheterization	umbilical cord catheterization	umbilical cord catheterization	1	kept
umbilical cord milking	umbilical cord milking	umbilical cord milking	umbilical cord milking	umbilical cord milking	1	kept
umbilical malformation	umbilical malformation	umbilical malformation	umbilical malformation	umbilical malformation	1	kept
Umbilical vein catheter	umbilical vein catheter	umbilical vein catheter	umbilical vein catheter	Umbilical vein catheter	1	kept
umbilical vein catheter	umbilical vein catheter	umbilical vein catheter	umbilical vein catheter	Umbilical vein catheter	1	case_or_format
Umbilical venous catheter	umbilical venous catheter	umbilical venous catheter	umbilical venous catheter	Umbilical venous catheter	1	kept
umbilical venous catheter	umbilical venous catheter	umbilical venous catheter	umbilical venous catheter	Umbilical venous catheter	1	case_or_format
umifenovir	umifenovir	umifenovir	umifenovir	umifenovir	1	kept

Un-expected death	un expected death	un expected death	un expected death	Un-expected death	1	kept
Unable-to-assess	unable to assess	unable to assess	unable to assess	Unable-to-assess	1	kept
unauthorized drug error	unauthorized drug error	unauthorized drug error	unauthorized drug error	unauthorized drug error	1	kept
unbalanced	unbalanced	unbalanced	unbalanced	unbalanced	1	kept
Unbalanced panel data	unbalanced panel data	unbalanced panel data	unbalanced panel data	Unbalanced panel data	1	kept
unbefriended advance	unbefriended advance	unbefriended advance	unbefriended advance	unbefriended advance	1	kept
uncal herniation	uncal herniation	uncal herniation	uncal herniation	uncal herniation	1	kept
Uncertainty analysis	uncertainty analysis	uncertainty analysis	uncertainty analysis	Uncertainty analysis	1	kept
Uncertainty in illness	uncertainty in illness	uncertainty in illness	uncertainty in illness	Uncertainty in illness	1	kept
Uncertainty in illness for family member	uncertainty in illness for family member	uncertainty in illness for family member	uncertainty in illness for family member	Uncertainty in illness for family member	1	kept
Unconscious	unconscious	unconscious	unconscious	Unconscious	1	kept
Unconscious bias	unconscious bias	unconscious bias	unconscious bias	Unconscious bias	1	kept
Unconscious, Psychology	unconscious psychology	unconscious psychology	unconscious psychology	Unconscious, Psychology	1	kept
Unconstrained bio-sensing	unconstrained bio sensing	unconstrained bio sensing	unconstrained bio sensing	Unconstrained bio-sensing	1	kept
unconstrained biosensing	unconstrained biosensing	unconstrained biosensing	unconstrained biosensing	unconstrained biosensing	1	kept
Uncontrolled pain	uncontrolled pain	uncontrolled pain	uncontrolled pain	Uncontrolled pain	1	kept

unconventional language	unconventional language	unconventional language	unconventional language	unconventional language	1	kept
uncuffed tracheostomy tube	uncuffed tracheostomy tube	uncuffed tracheostomy tube	uncuffed tracheostomy tube	uncuffed tracheostomy tube	1	kept
under resourced settings	under resourced settings	under resourced settings	under resourced settings	under resourced settings	1	kept
Under staffing	under staffing	under staffing	under staffing	Under staffing	1	kept
Under-5 deaths	under 5 deaths	under 5 deaths	under 5 deaths	Under-5 deaths	1	kept
UNDER-5 MORTALITY	under 5 mortality	under 5 mortality	under 5 mortality	UNDER-5 MORTALITY	1	kept
Under-triage	under triage	under triage	under triage	Under-triage	1	kept
under-use and over-use	under use and over use	under use and over use	under use and over use	under-use and over-use	1	kept
Underachievement	underachievement	underachievement	underachievement	Underachievement	1	kept
Underage drinking	underage drinking	underage drinking	underage drinking	Underage drinking	1	kept
Underdeveloped countries	underdeveloped countries	underdeveloped countries	underdeveloped countries	Underdeveloped countries	1	kept
underfeeding	underfeeding	underfeeding	underfeeding	Underfeeding	1	case_or_format
Underfill	underfill	underfill	underfill	Underfill	1	kept
UNDERGRADUATE	undergraduate	undergraduate	undergraduate	Undergraduate	1	case_or_format
undergraduate	undergraduate	undergraduate	undergraduate	Undergraduate	1	case_or_format
undergraduate and postgraduate training	undergraduate and postgraduate training	undergraduate and postgraduate training	undergraduate and postgraduate training	undergraduate and postgraduate training	1	kept

Undergraduate medical education	undergraduate medical education	undergraduate medical education	undergraduate medical education	Undergraduate medical education	1	kept
UNDERNUTRITION	undernutrition	undernutrition	undernutrition	undernutrition	1	case_or_format
UNDERPAD	underpad	underpad	underpad	UNDERPAD	1	kept
underprescribing	underprescribing	underprescribing	underprescribing	underprescribing	1	kept
Underreporting	underreporting	underreporting	underreporting	underreporting	1	case_or_format
underrepresented populations	underrepresented populations	underrepresented populations	underrepresented populations	underrepresented populations	1	kept
underserved populations	underserved populations	underserved populations	underserved populations	underserved populations	1	kept
Understanding	understanding	understanding	understanding	understanding	1	case_or_format
Understanding spirituality	understanding spirituality	understanding spirituality	understanding spirituality	Understanding spirituality	1	kept
UNDERSTANDING SUCCESS	understanding success	understanding success	understanding success	UNDERSTANDING SUCCESS	1	kept
Underwater acoustics	underwater acoustics	underwater acoustics	underwater acoustics	Underwater acoustics	1	kept
UNDIFFERENTIATED HYPOTENSION	undifferentiated hypotension	undifferentiated hypotension	undifferentiated hypotension	UNDIFFERENTIATED HYPOTENSION	1	kept
undignified death	undignified death	undignified death	undignified death	undignified death	1	kept
UNDP ART-GOLD Indonesia	undp art gold indonesia	undp art gold indonesia	undp art gold indonesia	UNDP ART-GOLD Indonesia	1	kept
Unemployment rate	unemployment rate	unemployment rate	unemployment rate	Unemployment rate	1	kept
Unexpected cardiac arrest	unexpected cardiac arrest	unexpected cardiac arrest	unexpected cardiac arrest	Unexpected cardiac arrest	1	kept

unexpected cardiac arrest	unexpected cardiac arrest	unexpected cardiac arrest	unexpected cardiac arrest	Unexpected cardiac arrest	1	case_or_format
Unexpected death	unexpected death	unexpected death	unexpected death	Unexpected death	1	kept
unexpected event	unexpected event	unexpected event	unexpected event	unexpected event	1	kept
UNEXPECTED INCREASED MORTALITY	unexpected increased mortality	unexpected increased mortality	unexpected increased mortality	UNEXPECTED INCREASED MORTALITY	1	kept
Unfavorable cervix	unfavorable cervix	unfavorable cervix	unfavorable cervix	Unfavorable cervix	1	kept
Unfavourable outcomes	unfavourable outcomes	unfavourable outcomes	unfavourable outcomes	Unfavourable outcomes	1	kept
Unfolding case-study	unfolding case study	unfolding case study	unfolding case study	Unfolding case-study	1	kept
unfractionated heparin	unfractionated heparin	unfractionated heparin	unfractionated heparin	unfractionated heparin	1	kept
unhealthy diet	unhealthy diet	unhealthy diet	unhealthy diet	unhealthy diet	1	kept
Unidad de Cuidado Intensivo Pediátrico	unidad de cuidado intensivo pedi trico	unidad de cuidado intensivo pedi trico	unidad de cuidado intensivo pedi trico	Unidad de Cuidado Intensivo Pediátrico	1	kept
Unidad de cuidados intensivos neonatal	unidad de cuidados intensivos neonatal	unidad de cuidados intensivos neonatal	unidad de cuidados intensivos neonatal	Unidad de cuidados intensivos neonatal	1	kept
Unidad de recién nacidos	unidad de reci n nacidos	unidad de reci n nacidos	unidad de reci n nacidos	Unidad de recién nacidos	1	kept
Unidad de Terapia Intensiva Neonatal	unidad de terapia intensiva neonatal	unidad de terapia intensiva neonatal	unidad de terapia intensiva neonatal	Unidad de Terapia Intensiva Neonatal	1	kept
Unidad Transversal de Atención	unidad transversal de atenci n	unidad transversal de atenci n	unidad transversal de atenci n	Unidad Transversal de Atención	1	kept
Unidade de Cuidados Intensivos Neonatais	unidade de cuidados intensivos neonatais	unidade de cuidados intensivos neonatais	unidade de cuidados intensivos neonatais	Unidade de Cuidados Intensivos Neonatais	1	kept
Unidade de Terapia Intensiva Neonatal	unidade de terapia intensiva neonatal	unidade de terapia intensiva neonatal	unidade de terapia intensiva neonatal	Unidade de Terapia Intensiva Neonatal	1	kept

Unidades Cuidados Intensivos	unidades cuidados intensivos	unidades cuidados intensivos	unidades cuidados intensivos	Unidades Cuidados Intensivos	1	kept
Unidades de cuidados intensiva	unidades de cuidados intensiva	unidades de cuidados intensiva	unidades de cuidados intensiva	Unidades de cuidados intensiva	1	kept
Unidades de cuidados Intensivos	unidades de cuidados intensivos	unidades de cuidados intensivos	unidades de cuidados intensivos	Unidades de cuidados intensivos	1	case_or_format
unidades de cuidados intensivos	unidades de cuidados intensivos	unidades de cuidados intensivos	unidades de cuidados intensivos	Unidades de cuidados intensivos	1	case_or_format
Unidades de cuidados intensivos neonatales	unidades de cuidados intensivos neonatales	unidades de cuidados intensivos neonatales	unidades de cuidados intensivos neonatales	Unidades de cuidados intensivos neonatales	1	kept
Unidades de cuidados intensivos pediátricos	unidades de cuidados intensivos pedi tricos	unidades de cuidados intensivos pedi tricos	unidades de cuidados intensivos pedi tricos	Unidades de cuidados intensivos pediátricos	1	kept
Unidades de Terapia In-tensiva	unidades de terapia in tensiva	unidades de terapia in tensiva	unidades de terapia in tensiva	Unidades de Terapia In-tensiva	1	kept
Unidades de Terapia Intensiva Pediátrica	unidades de terapia intensiva pedi trica	unidades de terapia intensiva pedi trica	unidades de terapia intensiva pedi trica	Unidades de Terapia Intensiva Pediátrica	1	kept
Unidentified	unidentified	unidentified	unidentified	Unidentified	1	kept
Unified Medical Language System	unified medical language system	unified medical language system	unified medical language system	Unified Medical Language System	1	kept
unifocalization	unifocalization	unifocalization	unifocalization	unifocalization	1	kept
uniforms	uniforms	uniforms	uniforms	uniforms	1	kept
unilateral blindness	unilateral blindness	unilateral blindness	unilateral blindness	unilateral blindness	1	kept
Unilateral hearing loss	unilateral hearing loss	unilateral hearing loss	unilateral hearing loss	Unilateral hearing loss	1	kept
unilateral hearing loss	unilateral hearing loss	unilateral hearing loss	unilateral hearing loss	Unilateral hearing loss	1	case_or_format
Unilateral renal	unilateral renal	unilateral renal	unilateral renal	Unilateral renal	1	kept

uninsured	uninsured	uninsured	uninsured	uninsured	1	kept
Unintended consequences	unintended consequences	unintended consequences	unintended consequences	UNINTENDED CONSEQUENCES	1	case_or_format
unintended extubation	unintended extubation	unintended extubation	unintended extubation	unintended extubation	1	kept
unique situation	unique situation	unique situation	unique situation	unique situation	1	kept
Unit care	unit care	unit care	unit care	Unit care	1	kept
unit champions	unit champions	unit champions	unit champions	unit champions	1	kept
unit characteristics	unit characteristics	unit characteristics	unit characteristics	unit characteristics	1	kept
unit climate	unit climate	unit climate	unit climate	unit climate	1	kept
Unit Coordinating Council	unit coordinating council	unit coordinating council	unit coordinating council	Unit Coordinating Council	1	kept
Unit culture	unit culture	unit culture	unit culture	Unit culture	1	kept
unit culture	unit culture	unit culture	unit culture	Unit culture	1	case_or_format
Unit design	unit design	unit design	unit design	Unit design	1	kept
unit layout	unit layout	unit layout	unit layout	unit layout	1	kept
unit leadership	unit leadership	unit leadership	unit leadership	unit leadership	1	kept
Unit managers	unit managers	unit managers	unit managers	Unit managers	1	kept
Unit of mass	unit of mass	unit of mass	unit of mass	Unit of mass	1	kept

UNIT READMISSIONS	unit readmissions	unit readmissions	unit readmissions	UNIT READMISSIONS	1	kept
unit size	unit size	unit size	unit size	unit size	1	kept
Unit staff development	unit staff development	unit staff development	unit staff development	Unit staff development	1	kept
Unit stay	unit stay	unit stay	unit stay	Unit stay	1	kept
Unit structure	unit structure	unit structure	unit structure	Unit structure	1	kept
UNIT TELEMEDICINE PROGRAM	unit telemedicine program	unit telemedicine program	unit telemedicine program	UNIT TELEMEDICINE PROGRAM	1	kept
UNIT TRANSFER DELAY	unit transfer delay	unit transfer delay	unit transfer delay	UNIT TRANSFER DELAY	1	kept
Unit water consumption	unit water consumption	unit water consumption	unit water consumption	Unit water consumption	1	kept
unit water consumption	unit water consumption	unit water consumption	unit water consumption	Unit water consumption	1	case_or_format
Unit-acquired Infections	unit acquired infections	unit acquired infections	unit acquired infections	Unit-acquired Infections	1	kept
Unit-acquired pressure ulcers	unit acquired pressure ulcers	unit acquired pressure ulcers	unit acquired pressure ulcers	Unit-acquired pressure ulcers	1	kept
Unit-based	unit based	unit based	unit based	Unit-based	1	kept
unit-based council	unit based council	unit based council	unit based council	unit-based council	1	kept
unit-based quality improvement	unit based quality improvement	unit based quality improvement	unit based quality improvement	unit-based quality improvement	1	kept
Unit-based transport team	unit based transport team	unit based transport team	unit based transport team	Unit-based transport team	1	kept
unit-level workload	unit level workload	unit level workload	unit level workload	unit-level workload	1	kept

United arab emirates	united arab emirates	united arab emirates	united arab emirates	United Arab Emirates	1	case_or_format
United kingdom health policy	united kingdom health policy	united kingdom health policy	united kingdom health policy	United kingdom health policy	1	kept
United Nations Convention on the Rights of the Child	united nations convention on the rights of the child	united nations convention on the rights of the child	united nations convention on the rights of the child	United Nations Convention on the Rights of the Child	1	kept
United States National Aeronautics and Space Administration	united states national aeronautics and space administration	united states national aeronautics and space administration	united states national aeronautics and space administration	United States National Aeronautics and Space Administration	1	kept
United States Occupational Safety and Health	united states occupational safety and health	united states occupational safety and health	united states occupational safety and health	United States Occupational Safety and Health	1	kept
United states public health service	united states public health service	united states public health service	united states public health service	United states public health service	1	kept
unité des soins intensifs néonatals	unit des soins intensifs n onatals	unit des soins intensifs n onatals	unit des soins intensifs n onatals	unité des soins intensifs néonatals	1	kept
univariate Cox model	univariate cox model	univariate cox model	univariate cox model	univariate Cox model	1	kept
Universal	universal	universal	universal	Universal	1	kept
Universal bed	universal bed	universal bed	universal bed	Universal bed	1	kept
universal bed unit	universal bed unit	universal bed unit	universal bed unit	universal bed unit	1	kept
UNIVERSAL DECOLONIZATION	universal decolonization	universal decolonization	universal decolonization	UNIVERSAL DECOLONIZATION	1	kept
UNIVERSAL HEALTH COVERAGE	universal health coverage	universal health coverage	universal health coverage	Universal health coverage	1	case_or_format
Universal Health Coverage	universal health coverage	universal health coverage	universal health coverage	Universal health coverage	1	case_or_format
Universal Health Insurance	universal health insurance	universal health insurance	universal health insurance	universal health insurance	1	case_or_format
universal healthcare	universal healthcare	universal healthcare	universal healthcare	universal healthcare	1	kept

Universal Healthcare Precautions	universal healthcare precautions	universal healthcare precautions	universal healthcare precautions	Universal Healthcare Precautions	1	kept
universal masking	universal masking	universal masking	universal masking	universal masking	1	kept
Universal orecautions	universal orecautions	universal orecautions	universal orecautions	Universal orecautions	1	kept
universal precautions	universal precautions	universal precautions	universal precaution	Universal Precautions	1	singular
universal room	universal room	universal room	universal room	universal room	1	kept
Universal screening	universal screening	universal screening	universal screening	Universal screening	1	kept
universal screening	universal screening	universal screening	universal screening	Universal screening	1	case_or_format
universalism	universalism	universalism	universalism	universalism	1	kept
Universe of Developmental Care	universe of developmental care	universe of developmental care	universe of developmental care	Universe of Developmental Care	1	kept
University	university	university	university	university	1	case_or_format
University hospital	university hospital	university hospital	university hospital	university hospital	1	case_or_format
University Hospitals	university hospitals	university hospitals	university hospital	university hospital	1	singular
University of Alberta Hospital	university of alberta hospital	university of alberta hospital	university of alberta hospital	University of Alberta Hospital	1	kept
university of california at los angeles geriatrics [illegible]	university of california at los angeles geriatrics [illegible]	university of california at los angeles geriatrics [illegible]	university of california at los angeles geriatrics [illegible]	university of california at los angeles geriatrics [illegible]	1	kept
University of Florida	university of florida	university of florida	university of florida	University of Florida	1	kept
University of Maryland Medical Center	university of maryland medical center	university of maryland medical center	university of maryland medical center	University of Maryland Medical Center	1	kept

University of Michigan Sedation Scale	university of michigan sedation scale	university of michigan sedation scale	university of michigan sedation scale	University of Michigan Sedation Scale	1	kept
University of Toronto Centre for Bioethics	university of toronto centre for bioethics	university of toronto centre for bioethics	university of toronto centre for bioethics	University of Toronto Centre for Bioethics	1	kept
University of Virginia	university of virginia	university of virginia	university of virginia	University of Virginia	1	kept
university sector	university sector	university sector	university sector	university sector	1	kept
university student	university student	university student	university student	university student	1	kept
University teaching	university teaching	university teaching	university teaching	University teaching	1	kept
UNKNOWN ORIGIN	unknown origin	unknown origin	unknown origin	UNKNOWN ORIGIN	1	kept
unlicenced worker	unlicenced worker	unlicenced worker	unlicenced worker	unlicenced worker	1	kept
unlicensed drug use	unlicensed drug use	unlicensed drug use	unlicensed drug use	unlicensed drug use	1	kept
Unmet health care needs	unmet health care needs	unmet health care needs	unmet health care needs	Unmet health care needs	1	kept
unmet need	unmet need	unmet need	unmet need	unmet need	1	kept
unmet needs	unmet needs	unmet needs	unmet need	unmet need	1	singular
Unnecessary injections	unnecessary injections	unnecessary injections	unnecessary injections	Unnecessary injections	1	kept
unnoticed clinical deterioration	unnoticed clinical deterioration	unnoticed clinical deterioration	unnoticed clinical deterioration	unnoticed clinical deterioration	1	kept
Unplanned	unplanned	unplanned	unplanned	Unplanned	1	kept
unplanned	unplanned	unplanned	unplanned	Unplanned	1	case_or_format

Unplanned admissions	unplanned admissions	unplanned admissions	unplanned admission	UNPLANNED ADMISSION	1	singular
unplanned complications	unplanned complications	unplanned complications	unplanned complications	unplanned complications	1	kept
Unplanned device removal	unplanned device removal	unplanned device removal	unplanned device removal	Unplanned device removal	1	kept
unplanned endotracheal extubation	unplanned endotracheal extubation	unplanned endotracheal extubation	unplanned endotracheal extubation	unplanned endotracheal extubation	1	kept
Unplanned endotracheal tube extubation	unplanned endotracheal tube extubation	unplanned endotracheal tube extubation	unplanned endotracheal tube extubation	Unplanned endotracheal tube extubation	1	kept
Unplanned extu bation	unplanned extu bation	unplanned extu bation	unplanned extu bation	Unplanned extu bation	1	kept
unplanned extubations	unplanned extubations	unplanned extubations	unplanned extubation	unplanned extubation	1	singular
Unplanned hospital admissions	unplanned hospital admissions	unplanned hospital admissions	unplanned hospital admissions	Unplanned hospital admissions	1	kept
unplanned hospital admissions	unplanned hospital admissions	unplanned hospital admissions	unplanned hospital admissions	Unplanned hospital admissions	1	case_or_format
unplanned hospitalization	unplanned hospitalization	unplanned hospitalization	unplanned hospitalization	unplanned hospitalization	1	kept
Unplanned readmission	unplanned readmission	unplanned readmission	unplanned readmission	Unplanned readmission	1	kept
UNPLANNED READMISSIONS	unplanned readmissions	unplanned readmissions	unplanned readmission	Unplanned readmission	1	singular
Unplanned transfer	unplanned transfer	unplanned transfer	unplanned transfer	Unplanned transfer	1	kept
unplanned transfer	unplanned transfer	unplanned transfer	unplanned transfer	Unplanned transfer	1	case_or_format
unplanned treatment interruption	unplanned treatment interruption	unplanned treatment interruption	unplanned treatment interruption	unplanned treatment interruption	1	kept
Unprotected airway	unprotected airway	unprotected airway	unprotected airway	Unprotected airway	1	kept

unprovoked seizure	unprovoked seizure	unprovoked seizure	unprovoked seizure	unprovoked seizure	1	kept
Unreasonable use	unreasonable use	unreasonable use	unreasonable use	Unreasonable use	1	kept
unrecognized deterioration	unrecognized deterioration	unrecognized deterioration	unrecognized deterioration	unrecognized deterioration	1	kept
unrepresented	unrepresented	unrepresented	unrepresented	unrepresented	1	kept
Unresponsive wakefulness syndrome	unresponsive wakefulness syndrome	unresponsive wakefulness syndrome	unresponsive wakefulness syndrome	Unresponsive wakefulness syndrome	1	kept
unresponsive wakefulness syndrome	unresponsive wakefulness syndrome	unresponsive wakefulness syndrome	unresponsive wakefulness syndrome	Unresponsive wakefulness syndrome	1	case_or_format
Unrestricted access	unrestricted access	unrestricted access	unrestricted access	Unrestricted access	1	kept
Unrestricted visitation	unrestricted visitation	unrestricted visitation	unrestricted visitation	Unrestricted visitation	1	kept
unrestricted visitation	unrestricted visitation	unrestricted visitation	unrestricted visitation	Unrestricted visitation	1	case_or_format
unruptured intracranial aneurysm	unruptured intracranial aneurysm	unruptured intracranial aneurysm	unruptured intracranial aneurysm	unruptured intracranial aneurysm	1	kept
Unsafe practices	unsafe practices	unsafe practices	unsafe practices	Unsafe practices	1	kept
UNSCHEDULED RETURN VISITS	unscheduled return visits	unscheduled return visits	unscheduled return visits	UNSCHEDULED RETURN VISITS	1	kept
unstable	unstable	unstable	unstable	unstable	1	kept
unstable angina	unstable angina	unstable angina	unstable angina	Unstable angina	1	case_or_format
unsteady gait	unsteady gait	unsteady gait	unsteady gait	unsteady gait	1	kept
unstructured interview	unstructured interview	unstructured interview	unstructured interview	unstructured interview	1	kept

Unstructured text	unstructured text	unstructured text	unstructured text	Unstructured text	1	kept
unstructured text	unstructured text	unstructured text	unstructured text	Unstructured text	1	case_or_format
Unsupervised learning	unsupervised learning	unsupervised learning	unsupervised learning	Unsupervised learning	1	kept
unsupervised machine learning	unsupervised machine learning	unsupervised machine learning	unsupervised machine learning	Unsupervised Machine Learning	1	case_or_format
Untoward clinical events	untoward clinical events	untoward clinical events	untoward clinical events	Untoward clinical events	1	kept
Unused PVC	unused pvc	unused pvc	unused pvc	Unused PVC	1	kept
Unvaccinated individuals	unvaccinated individuals	unvaccinated individuals	unvaccinated individuals	Unvaccinated individuals	1	kept
unvaccinated person	unvaccinated person	unvaccinated person	unvaccinated person	unvaccinated person	1	kept
unveiling parent	unveiling parent	unveiling parent	unveiling parent	unveiling parent	1	kept
unwanted pregnancy	unwanted pregnancy	unwanted pregnancy	unwanted pregnancy	unwanted pregnancy	1	kept
UP INTERVENTION	up intervention	up intervention	up intervention	UP INTERVENTION	1	kept
Up-Regulation	up regulation	up regulation	up regulation	Up-Regulation	1	kept
up-skilling clinicians	up skilling clinicians	up skilling clinicians	up skilling clinicians	up-skilling clinicians	1	kept
UPDATE	update	update	update	UPDATE	1	kept
UPDATED BEERS CRITERIA	updated beers criteria	updated beers criteria	updated beers criteria	UPDATED BEERS CRITERIA	1	kept
Upfront surgery	upfront surgery	upfront surgery	upfront surgery	Upfront surgery	1	kept

UPPER AIRWAY	upper airway	upper airway	upper airway	UPPER AIRWAY	1	kept
upper extremity	upper extremity	upper extremity	upper extremity	Upper Extremity	1	case_or_format
Upper gastrointestinal cancer	upper gastrointestinal cancer	upper gastrointestinal cancer	upper gastrointestinal cancer	Upper gastrointestinal cancer	1	kept
upper limb muscle strength	upper limb muscle strength	upper limb muscle strength	upper limb muscle strength	upper limb muscle strength	1	kept
upper motor neurons	upper motor neurons	upper motor neurons	upper motor neurons	upper motor neurons	1	kept
upright position	upright position	upright position	upright position	upright position	1	kept
Upskill training	upskill training	upskill training	upskill training	Upskill training	1	kept
upstream outcome	upstream outcome	upstream outcome	upstream outcome	upstream outcome	1	kept
Uptake	uptake	uptake	uptake	Uptake	1	kept
Uranium alloys	uranium alloys	uranium alloys	uranium alloys	Uranium alloys	1	kept
Urban	urban	urban	urban	URBAN	1	case_or_format
urban	urban	urban	urban	URBAN	1	case_or_format
Urban growth	urban growth	urban growth	urban growth	Urban growth	1	kept
Urban soundscape	urban soundscape	urban soundscape	urban soundscape	Urban soundscape	1	kept
Urea creatinine ratio	urea creatinine ratio	urea creatinine ratio	urea creatinine ratio	Urea creatinine ratio	1	kept
urea creatinine ratio	urea creatinine ratio	urea creatinine ratio	urea creatinine ratio	Urea creatinine ratio	1	case_or_format

ureidopenicillin derivative	ureidopenicillin derivative	ureidopenicillin derivative	ureidopenicillin derivative	ureidopenicillin derivative	1	kept
uremic toxin	uremic toxin	uremic toxin	uremic toxin	uremic toxin	1	kept
ureter catheter	ureter catheter	ureter catheter	ureter catheter	ureter catheter	1	kept
ureter catheterization	ureter catheterization	ureter catheterization	ureter catheterization	ureter catheterization	1	kept
ureter obstruction	ureter obstruction	ureter obstruction	ureter obstruction	ureter obstruction	1	kept
ureter stone	ureter stone	ureter stone	ureter stone	ureter stone	1	kept
ureteric injury	ureteric injury	ureteric injury	ureteric injury	ureteric injury	1	kept
ureteropelvic junction obstruction	ureteropelvic junction obstruction	ureteropelvic junction obstruction	ureteropelvic junction obstruction	ureteropelvic junction obstruction	1	kept
Urethra	urethra	urethra	urethra	urethra	1	case_or_format
urethra disease	urethra disease	urethra disease	urethra disease	urethra disease	1	kept
urethra fistula	urethra fistula	urethra fistula	urethra fistula	urethra fistula	1	kept
urethra stricture	urethra stricture	urethra stricture	urethra stricture	urethra stricture	1	kept
urethra valve	urethra valve	urethra valve	urethra valve	urethra valve	1	kept
urethral bleeding	urethral bleeding	urethral bleeding	urethral bleeding	urethral bleeding	1	kept
urethral catheter	urethral catheter	urethral catheter	urethral catheter	urethral catheter	1	kept
Urgencias médicas	urgencias m dicas	urgencias m dicas	urgencias m dicas	Urgencias médicas	1	kept

urgency classification	urgency classification	urgency classification	urgency classification	urgency classification	1	kept
urgency status	urgency status	urgency status	urgency status	urgency status	1	kept
Urgent admission	urgent admission	urgent admission	urgent admission	Urgent admission	1	kept
urgent care center	urgent care center	urgent care center	urgent care center	urgent care center	1	kept
urgent start peritoneal dialysis	urgent start peritoneal dialysis	urgent start peritoneal dialysis	urgent start peritoneal dialysis	urgent start peritoneal dialysis	1	kept
Urgent surgery	urgent surgery	urgent surgery	urgent surgery	Urgent surgery	1	kept
Urgent-start	urgent start	urgent start	urgent start	Urgent-start	1	kept
urgent-start PD	urgent start pd	urgent start pd	urgent start pd	urgent-start PD	1	kept
uric acid nephropathy	uric acid nephropathy	uric acid nephropathy	uric acid nephropathy	uric acid nephropathy	1	kept
Urinary	urinary	urinary	urinary	Urinary	1	kept
Urinary Bladder Diseases	urinary bladder diseases	urinary bladder diseases	urinary bladder diseases	Urinary Bladder Diseases	1	kept
urinary bladder neoplasms	urinary bladder neoplasms	urinary bladder neoplasms	urinary bladder neoplasms	Urinary Bladder Neoplasms	1	case_or_format
Urinary Bladder, Neurogenic	urinary bladder neurogenic	urinary bladder neurogenic	urinary bladder neurogenic	Urinary Bladder, Neurogenic	1	kept
Urinary Bladder, Overactive	urinary bladder overactive	urinary bladder overactive	urinary bladder overactive	Urinary Bladder, Overactive	1	kept
urinary catherization	urinary catherization	urinary catherization	urinary catherization	urinary catherization	1	kept
URINARY CATHETER	urinary catheter	urinary catheter	urinary catheter	urinary catheter	1	case_or_format

Urinary catheter care	urinary catheter care	urinary catheter care	urinary catheter care	Urinary catheter care	1	kept
Urinary catheter DUR	urinary catheter dur	urinary catheter dur	urinary catheter dur	Urinary catheter DUR	1	kept
Urinary catheter management	urinary catheter management	urinary catheter management	urinary catheter management	Urinary catheter management	1	kept
urinary catheter removal process	urinary catheter removal process	urinary catheter removal process	urinary catheter removal process	urinary catheter removal process	1	kept
Urinary catheter securement	urinary catheter securement	urinary catheter securement	urinary catheter securement	Urinary catheter securement	1	kept
Urinary Catheterization [adverse effects	urinary catheterization adverse effects	urinary catheterization adverse effects	urinary catheterization adverse effects	Urinary Catheterization [adverse effects	1	kept
urinary catheters	urinary catheters	urinary catheters	urinary catheter	urinary catheter	1	singular
urinary culture contamination	urinary culture contamination	urinary culture contamination	urinary culture contamination	urinary culture contamination	1	kept
urinary diversion	urinary diversion	urinary diversion	urinary diversion	urinary diversion	1	kept
urinary excretion	urinary excretion	urinary excretion	urinary excretion	urinary excretion	1	kept
urinary fractional excretion	urinary fractional excretion	urinary fractional excretion	urinary fractional excretion	urinary fractional excretion	1	kept
Urinary incontinence	urinary incontinence	urinary incontinence	urinary incontinence	Urinary Incontinence	1	case_or_format
urinary stoma	urinary stoma	urinary stoma	urinary stoma	urinary stoma	1	kept
urinary tract disease assessment	urinary tract disease assessment	urinary tract disease assessment	urinary tract disease assessment	urinary tract disease assessment	1	kept
urinary tract fistula	urinary tract fistula	urinary tract fistula	urinary tract fistula	urinary tract fistula	1	kept
Urinary Tract Infections [etiology]	urinary tract infections etiology	urinary tract infections etiology	urinary tract infections etiology	Urinary Tract Infections [etiology]	1	kept

urinary tract obstruction	urinary tract obstruction	urinary tract obstruction	urinary tract obstruction	urinary tract obstruction	1	kept
urinary tract surgery	urinary tract surgery	urinary tract surgery	urinary tract surgery	urinary tract surgery	1	kept
urinary tract tumor	urinary tract tumor	urinary tract tumor	urinary tract tumor	urinary tract tumor	1	kept
URINARY-TRACT	urinary tract	urinary tract	urinary tract	urinary tract	1	case_or_format
Urination Disorders [therapy]	urination disorders therapy	urination disorders therapy	urination disorders therapy	Urination Disorders [therapy]	1	kept
urine acidification	urine acidification	urine acidification	urine acidification	urine acidification	1	kept
Urine culture	urine culture	urine culture	urine culture	urine culture	1	case_or_format
Urine cultures	urine cultures	urine cultures	urine culture	urine culture	1	singular
urine output	urine output	urine output	urine output	Urine output	1	case_or_format
urine output monitoring	urine output monitoring	urine output monitoring	urine output monitoring	urine output monitoring	1	kept
urine pH	urine ph	urine ph	urine ph	urine pH	1	kept
urine production	urine production	urine production	urine production	urine production	1	kept
Urine proteomics	urine proteomics	urine proteomics	urine proteomics	Urine proteomics	1	kept
Urine volume	urine volume	urine volume	urine volume	urine volume	1	case_or_format
URODYNAMIC EQUIPMENT	urodynamic equipment	urodynamic equipment	urodynamic equipment	URODYNAMIC EQUIPMENT	1	kept
urogenital emergency	urogenital emergency	urogenital emergency	urogenital emergency	urogenital emergency	1	kept

urogenital scintiscanning	urogenital scintiscanning	urogenital scintiscanning	urogenital scintiscanning	urogenital scintiscanning	1	kept
Urogenital System	urogenital system	urogenital system	urogenital system	Urogenital System	1	kept
urogenital system	urogenital system	urogenital system	urogenital system	Urogenital System	1	case_or_format
urogenital system examination	urogenital system examination	urogenital system examination	urogenital system examination	urogenital system examination	1	kept
urogenital tract injury	urogenital tract injury	urogenital tract injury	urogenital tract injury	urogenital tract injury	1	kept
Urokinase-Type Plasminogen Activator	urokinase type plasminogen activator	urokinase type plasminogen activator	urokinase type plasminogen activator	Urokinase-Type Plasminogen Activator	1	kept
urolithiasis	urolithiasis	urolithiasis	urolithiasis	urolithiasis	1	kept
Urologic Diseases	urologic diseases	urologic diseases	urologic diseases	Urologic Diseases	1	kept
Urologic Neoplasms	urologic neoplasms	urologic neoplasms	urologic neoplasms	Urologic Neoplasms	1	kept
urological equipment	urological equipment	urological equipment	urological equipment	urological equipment	1	kept
urological surgery	urological surgery	urological surgery	urological surgery	urological surgery	1	kept
urological surgical procedures	urological surgical procedures	urological surgical procedures	urological surgical procedures	urological surgical procedures	1	kept
Urologists	urologists	urologists	urologist	urologist	1	singular
Urology Department, Hospital	urology department hospital	urology department hospital	urology department hospital	Urology Department, Hospital	1	kept
urology unit	urology unit	urology unit	urology unit	urology unit	1	kept
Uroteuthis edulis	uroteuthis edulis	uroteuthis edulis	uroteuthis edulis	Uroteuthis edulis	1	kept

URPA	urpa	urpa	urpa	URPA	1	kept
Urse	urse	urse	urse	Urse	1	kept
Uruguayan	uruguayan	uruguayan	uruguayan	Uruguayan	1	kept
US Food and Drug Administration	us food and drug administration	us food and drug administration	us food and drug administration	US Food and Drug Administration	1	kept
US health care costs	us health care costs	us health care costs	us health care costs	US health care costs	1	kept
us navy	us navy	us navy	us navy	us navy	1	kept
US News and World Report	us news and world report	us news and world report	us news and world report	US News and World Report	1	kept
US News and World Report hospital ranking	us news and world report hospital ranking	us news and world report hospital ranking	us news and world report hospital ranking	US News and World Report hospital ranking	1	kept
US PHYSICIAN WORKFORCE	us physician workforce	us physician workforce	us physician workforce	US PHYSICIAN WORKFORCE	1	kept
USA	usa	usa	usa	USA	1	kept
USA, United States of America	usa united states of america	usa united states of america	usa united states of america	USA, United States of America	1	kept
usability engineering	usability engineering	usability engineering	usability engineering	Usability engineering	1	case_or_format
Usability evaluation	usability evaluation	usability evaluation	usability evaluation	Usability evaluation	1	kept
usability evaluation	usability evaluation	usability evaluation	usability evaluation	Usability evaluation	1	case_or_format
Usability problems	usability problems	usability problems	usability problems	Usability problems	1	kept
Usability score	usability score	usability score	usability score	Usability score	1	kept

Usability studies	usability studies	usability studies	usability studies	Usability studies	1	kept
USAGE	usage	usage	usage	USAGE	1	kept
Usage experience	usage experience	usage experience	usage experience	Usage experience	1	kept
Use of antibiotics	use of antibiotics	use of antibiotics	use of antibiotics	Use of antibiotics	1	kept
use of sleeping medication	use of sleeping medication	use of sleeping medication	use of sleeping medication	use of sleeping medication	1	kept
Use-related risk mitigation	use related risk mitigation	use related risk mitigation	use related risk mitigation	Use-related risk mitigation	1	kept
Use-related risks	use related risks	use related risks	use related risks	Use-related risks	1	kept
use-related risks mitigation	use related risks mitigation	use related risks mitigation	use related risks mitigation	use-related risks mitigation	1	kept
uselessness	uselessness	uselessness	uselessness	uselessness	1	kept
user acceptance	user acceptance	user acceptance	user acceptance	User acceptance	1	case_or_format
User acceptance and resistance	user acceptance and resistance	user acceptance and resistance	user acceptance and resistance	User acceptance and resistance	1	kept
user centered design	user centered design	user centered design	user centered design	user-centered design	1	case_or_format
User centered designs	user centered designs	user centered designs	user centered design	user-centered design	1	singular
User communication	user communication	user communication	user communication	User communication	1	kept
User evaluations	user evaluations	user evaluations	user evaluations	User evaluations	1	kept
User Experience	user experience	user experience	user experience	User experience	1	case_or_format

User experience design	user experience design	user experience design	user experience design	User experience design	1	kept
user experience design	user experience design	user experience design	user experience design	User experience design	1	case_or_format
user experience questionnaire	user experience questionnaire	user experience questionnaire	user experience questionnaire	user experience questionnaire	1	kept
User experience study	user experience study	user experience study	user experience study	User experience study	1	kept
User experiences	user experiences	user experiences	user experience	User experience	1	singular
User friendliness	user friendliness	user friendliness	user friendliness	User-friendliness	1	case_or_format
User interaction	user interaction	user interaction	user interaction	User interaction	1	kept
User need	user need	user need	user need	User need	1	kept
User needs	user needs	user needs	user need	User need	1	singular
User profile	user profile	user profile	user profile	User profile	1	kept
User requirements	user requirements	user requirements	user requirements	User requirements	1	kept
User Research	user research	user research	user research	User Research	1	kept
User satisfaction	user satisfaction	user satisfaction	user satisfaction	User satisfaction	1	kept
user satisfaction	user satisfaction	user satisfaction	user satisfaction	User satisfaction	1	case_or_format
User testing	user testing	user testing	user testing	User testing	1	kept
User tests	user tests	user tests	user test	User test	1	singular

user-centered	user centered	user centered	user centered	user-centered	1	kept
User-centered approach	user centered approach	user centered approach	user centered approach	User-centered approach	1	kept
User-centered design approaches	user centered design approaches	user centered design approaches	user centered design approaches	User-centered design approaches	1	kept
User-centered design techniques	user centered design techniques	user centered design techniques	user centered design techniques	User-centered design techniques	1	kept
User-centred design	user centred design	user centred design	user centred design	User-centred design	1	kept
user-centred design	user centred design	user centred design	user centred design	User-centred design	1	case_or_format
User-computer interface	user computer interface	user computer interface	user computer interface	User-Computer Interface	1	case_or_format
user-computer interface	user computer interface	user computer interface	user computer interface	User-Computer Interface	1	case_or_format
Users' acceptance	users acceptance	users acceptance	users acceptance	Users' acceptance	1	kept
Users' experiences	users experiences	users experiences	users experiences	Users' experiences	1	kept
users' experiences of health care	users experiences of health care	users experiences of health care	users experiences of health care	users' experiences of health care	1	kept
uses	uses	uses	uses	uses	1	kept
using artificial intelligence	using artificial intelligence	using artificial intelligence	using artificial intelligence	using artificial intelligence	1	kept
USMLE Step 2	usmle step 2	usmle step 2	usmle step 2	USMLE Step 2	1	kept
USMLE step 2	usmle step 2	usmle step 2	usmle step 2	USMLE Step 2	1	case_or_format
USMLE Step 3	usmle step 3	usmle step 3	usmle step 3	USMLE Step 3	1	kept

USMLE step 3	usmle step 3	usmle step 3	usmle step 3	USMLE Step 3	1	case_or_format
ustekinumab	ustekinumab	ustekinumab	ustekinumab	ustekinumab	1	kept
usual care	usual care	usual care	usual care	usual care	1	kept
Usual workplace	usual workplace	usual workplace	usual workplace	Usual workplace	1	kept
UT BMAF rating scale	ut bmaf rating scale	ut bmaf rating scale	ut bmaf rating scale	UT BMAF rating scale	1	kept
uterine arteriovenous malformation	uterine arteriovenous malformation	uterine arteriovenous malformation	uterine arteriovenous malformation	uterine arteriovenous malformation	1	kept
uterine artery	uterine artery	uterine artery	uterine artery	uterine artery	1	kept
Uterine atony	uterine atony	uterine atony	uterine atony	uterine atony	1	case_or_format
Uterine Balloon Tamponade	uterine balloon tamponade	uterine balloon tamponade	uterine balloon tamponade	Uterine Balloon Tamponade	1	kept
uterine balloon tamponade	uterine balloon tamponade	uterine balloon tamponade	uterine balloon tamponade	Uterine Balloon Tamponade	1	case_or_format
Uterine Cervical Neoplasms	uterine cervical neoplasms	uterine cervical neoplasms	uterine cervical neoplasms	Uterine Cervical Neoplasms	1	kept
uterine cervical neoplasms	uterine cervical neoplasms	uterine cervical neoplasms	uterine cervical neoplasms	Uterine Cervical Neoplasms	1	case_or_format
uterine cervix cerclage	uterine cervix cerclage	uterine cervix cerclage	uterine cervix cerclage	uterine cervix cerclage	1	kept
uterine cervix cytology	uterine cervix cytology	uterine cervix cytology	uterine cervix cytology	uterine cervix cytology	1	kept
uterine cervix incompetence	uterine cervix incompetence	uterine cervix incompetence	uterine cervix incompetence	uterine cervix incompetence	1	kept
uterine complication	uterine complication	uterine complication	uterine complication	uterine complication	1	kept

Uterine Contraction	uterine contraction	uterine contraction	uterine contraction	Uterine Contraction	1	kept
uterine hemorrhage	uterine hemorrhage	uterine hemorrhage	uterine hemorrhage	Uterine Hemorrhage	1	case_or_format
Uterine Monitoring	uterine monitoring	uterine monitoring	uterine monitoring	Uterine Monitoring	1	kept
uterine neoplasms	uterine neoplasms	uterine neoplasms	uterine neoplasms	Uterine Neoplasms	1	case_or_format
uterine rupture	uterine rupture	uterine rupture	uterine rupture	Uterine Rupture	1	case_or_format
uterine tachysystole	uterine tachysystole	uterine tachysystole	uterine tachysystole	uterine tachysystole	1	kept
uterine tamponade	uterine tamponade	uterine tamponade	uterine tamponade	uterine tamponade	1	kept
uterine tube ligation	uterine tube ligation	uterine tube ligation	uterine tube ligation	uterine tube ligation	1	kept
Uteroplacental apoplexy	uteroplacental apoplexy	uteroplacental apoplexy	uteroplacental apoplexy	Uteroplacental apoplexy	1	kept
uterotonics	uterotonics	uterotonics	uterotonics	uterotonics	1	kept
uterus bleeding	uterus bleeding	uterus bleeding	uterus bleeding	uterus bleeding	1	kept
uterus contractility	uterus contractility	uterus contractility	uterus contractility	uterus contractility	1	kept
uterus sarcoma	uterus sarcoma	uterus sarcoma	uterus sarcoma	uterus sarcoma	1	kept
uterus surgery	uterus surgery	uterus surgery	uterus surgery	uterus surgery	1	kept
UTI = urinary tract infection	uti urinary tract infection	uti urinary tract infection	uti urinary tract infection	UTI = urinary tract infection	1	kept
Utilisation	utilisation	utilisation	utilisation	Utilisation	1	kept

utility	utility	utility	utility	Utility	1	case_or_format
utilization drift	utilization drift	utilization drift	utilization drift	utilization drift	1	kept
utilization of services	utilization of services	utilization of services	utilization of services	utilization of services	1	kept
Utilization rates	utilization rates	utilization rates	utilization rates	Utilization rates	1	kept
Utopias	utopias	utopias	utopias	Utopias	1	kept
Utrecht work engagement scale	utrecht work engagement scale	utrecht work engagement scale	utrecht work engagement scale	Utrecht Work Engagement Scale	1	case_or_format
Utrecht Work Engagement Scale-9	utrecht work engagement scale 9	utrecht work engagement scale 9	utrecht work engagement scale 9	Utrecht Work Engagement Scale-9	1	kept
UV-C disinfection	uv c disinfection	uv c disinfection	uv c disinfection	UV-C disinfection	1	kept
UV-C LED	uv c led	uv c led	uv c led	UV-C LED	1	kept
UVC placement	uvc placement	uvc placement	uvc placement	UVC placement	1	kept
uveitis	uveitis	uveitis	uveitis	Uveitis	1	case_or_format
Uvuloplatopharyngoplasty	uvuloplatopharyngoplasty	uvuloplatopharyngoplasty	uvuloplatopharyngoplasty	Uvuloplatopharyngoplasty	1	kept
V receptors	v receptors	v receptors	v receptors	V receptors	1	kept
vaborbactam	vaborbactam	vaborbactam	vaborbactam	vaborbactam	1	kept
vacancy	vacancy	vacancy	vacancy	vacancy	1	kept
VACCINATION	vaccination	vaccination	vaccination	vaccination	1	case_or_format

Vaccination campaign	vaccination campaign	vaccination campaign	vaccination campaign	Vaccination campaign	1	kept
Vaccination Compliance Index	vaccination compliance index	vaccination compliance index	vaccination compliance index	Vaccination Compliance Index	1	kept
Vaccination Hesitancy	vaccination hesitancy	vaccination hesitancy	vaccination hesitancy	Vaccination Hesitancy	1	kept
Vaccination hesitancy	vaccination hesitancy	vaccination hesitancy	vaccination hesitancy	Vaccination Hesitancy	1	case_or_format
Vaccination rates	vaccination rates	vaccination rates	vaccination rates	Vaccination rates	1	kept
Vaccination status	vaccination status	vaccination status	vaccination status	Vaccination status	1	kept
vaccination uptake	vaccination uptake	vaccination uptake	vaccination uptake	vaccination uptake	1	kept
Vaccinations coverage	vaccinations coverage	vaccinations coverage	vaccinations coverage	Vaccinations coverage	1	kept
VACCINE	vaccine	vaccine	vaccine	vaccine	1	case_or_format
Vaccine accep-tance	vaccine accep tance	vaccine accep tance	vaccine accep tance	Vaccine accep-tance	1	kept
Vaccine acceptance	vaccine acceptance	vaccine acceptance	vaccine acceptance	Vaccine acceptance	1	kept
vaccine attribute	vaccine attribute	vaccine attribute	vaccine attribute	vaccine attribute	1	kept
vaccine beliefs	vaccine beliefs	vaccine beliefs	vaccine beliefs	vaccine beliefs	1	kept
Vaccine Confidence Index	vaccine confidence index	vaccine confidence index	vaccine confidence index	Vaccine Confidence Index	1	kept
Vaccine effectiveness	vaccine effectiveness	vaccine effectiveness	vaccine effectiveness	vaccine effectiveness	1	case_or_format
vaccine hesitant	vaccine hesitant	vaccine hesitant	vaccine hesitant	vaccine hesitant	1	kept

Vaccine impact	vaccine impact	vaccine impact	vaccine impact	Vaccine impact	1	kept
Vaccine procurement	vaccine procurement	vaccine procurement	vaccine procurement	Vaccine procurement	1	kept
Vaccine refusal	vaccine refusal	vaccine refusal	vaccine refusal	Vaccine refusal	1	kept
vaccine safety	vaccine safety	vaccine safety	vaccine safety	vaccine safety	1	kept
Vaccine safety concerns	vaccine safety concerns	vaccine safety concerns	vaccine safety concerns	Vaccine safety concerns	1	kept
Vaccine timeliness	vaccine timeliness	vaccine timeliness	vaccine timeliness	Vaccine timeliness	1	kept
vaccines	vaccines	vaccines	vaccine	vaccine	1	singular
vaccines protection	vaccines protection	vaccines protection	vaccines protection	vaccines protection	1	kept
vaccinia	vaccinia	vaccinia	vaccinia	vaccinia	1	kept
Vaccinia virus	vaccinia virus	vaccinia virus	vaccinia virus	Vaccinia virus	1	kept
Vaccinology	vaccinology	vaccinology	vaccinology	Vaccinology	1	kept
vaccinology	vaccinology	vaccinology	vaccinology	Vaccinology	1	case_or_format
Vacunas contra la COVID-19	vacunas contra la covid 19	vacunas contra la covid 19	vacunas contra la covid 19	Vacunas contra la COVID-19	1	kept
Vacuum	vacuum	vacuum	vacuum	vacuum	1	case_or_format
vacuum aspiration	vacuum aspiration	vacuum aspiration	vacuum aspiration	vacuum aspiration	1	kept
vacuum assisted closure device	vacuum assisted closure device	vacuum assisted closure device	vacuum assisted closure device	vacuum assisted closure device	1	kept

vacuum extractor	vacuum extractor	vacuum extractor	vacuum extractor	vacuum extractor	1	kept
Vacuum-assisted delivery	vacuum assisted delivery	vacuum assisted delivery	vacuum assisted delivery	Vacuum-assisted delivery	1	kept
VADs	vads	vads	vads	VADs	1	kept
vagal efficiency	vagal efficiency	vagal efficiency	vagal efficiency	vagal efficiency	1	kept
Vagal tone	vagal tone	vagal tone	vagal tone	Vagal tone	1	kept
vagina aplasia	vagina aplasia	vagina aplasia	vagina aplasia	vagina aplasia	1	kept
vagina cancer	vagina cancer	vagina cancer	vagina cancer	vagina cancer	1	kept
vagina disease	vagina disease	vagina disease	vagina disease	vagina disease	1	kept
vagina mycosis	vagina mycosis	vagina mycosis	vagina mycosis	vagina mycosis	1	kept
vagina pain	vagina pain	vagina pain	vagina pain	vagina pain	1	kept
vaginal agenesis	vaginal agenesis	vaginal agenesis	vaginal agenesis	vaginal agenesis	1	kept
Vaginal birth	vaginal birth	vaginal birth	vaginal birth	Vaginal birth	1	kept
Vaginal birth after caesarean	vaginal birth after caesarean	vaginal birth after caesarean	vaginal birth after caesarean	Vaginal birth after caesarean	1	kept
Vaginal births	vaginal births	vaginal births	vaginal birth	Vaginal birth	1	singular
vaginal dilation	vaginal dilation	vaginal dilation	vaginal dilation	vaginal dilation	1	kept
vaginal lavage	vaginal lavage	vaginal lavage	vaginal lavage	vaginal lavage	1	kept

vaginal secretion	vaginal secretion	vaginal secretion	vaginal secretion	vaginal secretion	1	kept
vaginitis	vaginitis	vaginitis	vaginitis	vaginitis	1	kept
vagolytic	vagolytic	vagolytic	vagolytic	vagolytic	1	kept
vagus nerve nucleus	vagus nerve nucleus	vagus nerve nucleus	vagus nerve nucleus	vagus nerve nucleus	1	kept
vagus tone	vagus tone	vagus tone	vagus tone	vagus tone	1	kept
VAI	vai	vai	vai	VAI	1	kept
valenf instrument	valenf instrument	valenf instrument	valenf instrument	valenf instrument	1	kept
Valerates	valerates	valerates	valerates	Valerates	1	kept
valerian	valerian	valerian	valerian	valerian	1	kept
valid and reliable	valid and reliable	valid and reliable	valid and reliable	valid and reliable	1	kept
VALID INDICATORS	valid indicators	valid indicators	valid indicators	VALID INDICATORS	1	kept
Validated	validated	validated	validated	Validated	1	kept
Validated tools	validated tools	validated tools	validated tools	Validated tools	1	kept
validation & verification 40	validation and verification 40	validation and verification 40	validation and verification 40	validation & verification 40	1	kept
Validation Questionnaire	validation questionnaire	validation questionnaire	validation questionnaire	Validation Questionnaire	1	kept
validation therapy	validation therapy	validation therapy	validation therapy	validation therapy	1	kept

validity and reliability	validity and reliability	validity and reliability	validity and reliability	Validity and reliability	1	case_or_format
Validity assessment	validity assessment	validity assessment	validity assessment	Validity assessment	1	kept
Validity of the tests	validity of the tests	validity of the tests	validity of the tests	Validity of the tests	1	kept
validity-reliability	validity reliability	validity reliability	validity reliability	validity-reliability	1	kept
valley fever	valley fever	valley fever	valley fever	valley fever	1	kept
Valoración enfermera	valoraci n enfermera	valoraci n enfermera	valoraci n enfermera	Valoración enfermera	1	kept
valorisation	valorisation	valorisation	valorisation	valorisation	1	kept
Valsalva maneuver	valsalva maneuver	valsalva maneuver	valsalva maneuver	Valsalva maneuver	1	kept
value based care	value based care	value based care	value based care	value based care	1	kept
value of application	value of application	value of application	value of application	value of application	1	kept
value proposition	value proposition	value proposition	value proposition	value proposition	1	kept
value-based care	value based care	value based care	value based care	value based care	1	case_or_format
value-based payment system	value based payment system	value based payment system	value based payment system	value-based payment system	1	kept
Value-Based Purchasing	value based purchasing	value based purchasing	value based purchasing	Value-Based Purchasing	1	kept
Value-based purchasing	value based purchasing	value based purchasing	value based purchasing	Value-Based Purchasing	1	case_or_format
value-based purchasing	value based purchasing	value based purchasing	value based purchasing	Value-Based Purchasing	1	case_or_format

Value-discernment	value discernment	value discernment	value discernment	Value-discernment	1	kept
values	values	values	value	value	1	singular
Values-based care	values based care	values based care	values based care	Values-based care	1	kept
Valve	valve	valve	valve	Valve	1	kept
valve	valve	valve	valve	Valve	1	case_or_format
valve deterioration	valve deterioration	valve deterioration	valve deterioration	valve deterioration	1	kept
valve surgery	valve surgery	valve surgery	valve surgery	valve surgery	1	kept
valve-in-valve transcatheter aortic valve implantation	valve in valve transcatheter aortic valve implantation	valve in valve transcatheter aortic valve implantation	valve in valve transcatheter aortic valve implantation	valve-in-valve transcatheter aortic valve implantation	1	kept
Valvular disease	valvular disease	valvular disease	valvular disease	Valvular disease	1	kept
Valvular diseases	valvular diseases	valvular diseases	valvular disease	Valvular disease	1	singular
Van der Woude syndrome	van der woude syndrome	van der woude syndrome	van der woude syndrome	Van der Woude syndrome	1	kept
Van Manen	van manen	van manen	van manen	Van Manen	1	kept
vana bacterial gene	vana bacterial gene	vana bacterial gene	vana bacterial gene	vana bacterial gene	1	kept
vanA protein	vana protein	vana protein	vana protein	vanA protein	1	kept
vanb bacterial gene	vanb bacterial gene	vanb bacterial gene	vanb bacterial gene	vanb bacterial gene	1	kept
vanB protein	vanb protein	vanb protein	vanb protein	vanB protein	1	kept

VanB protein, Enterococcus	vanb protein enterococcus	vanb protein enterococcus	vanb protein enterococcus	VanB protein, Enterococcus	1	kept
VANCOMYCIN RESISTANCE	vancomycin resistance	vancomycin resistance	vancomycin resistance	Vancomycin Resistance	1	case_or_format
vancomycin resistant Staphylococcus aureus	vancomycin resistant staphylococcus aureus	vancomycin resistant staphylococcus aureus	vancomycin resistant staphylococcus aureus	vancomycin resistant Staphylococcus aureus	1	kept
Vancomycin-resistant	vancomycin resistant	vancomycin resistant	vancomycin resistant	Vancomycin-resistant	1	kept
VANCOMYCIN-RESISTANT ENTEROCOCCUS	vancomycin resistant enterococcus	vancomycin resistant enterococcus	vancomycin resistant enterococcus	vancomycin resistant Enterococcus	1	case_or_format
vancomycin-resistant Enterococcus faecium	vancomycin resistant enterococcus faecium	vancomycin resistant enterococcus faecium	vancomycin resistant enterococcus faecium	vancomycin-resistant Enterococcus faecium	1	kept
vancomycin-resistant Enterococcus infection	vancomycin resistant enterococcus infection	vancomycin resistant enterococcus infection	vancomycin resistant enterococcus infection	vancomycin-resistant Enterococcus infection	1	kept
vancomycin-resistant Staphylococcus aureus	vancomycin resistant staphylococcus aureus	vancomycin resistant staphylococcus aureus	vancomycin resistant staphylococcus aureus	vancomycin resistant Staphylococcus aureus	1	case_or_format
Vancomycin-susceptible	vancomycin susceptible	vancomycin susceptible	vancomycin susceptible	Vancomycin-susceptible	1	kept
Vandalism	vandalism	vandalism	vandalism	Vandalism	1	kept
Vanuatu	vanuatu	vanuatu	vanuatu	Vanuatu	1	kept
Vap	vap	vap	vap	VAP	1	case_or_format
VAP = ventilator-associated pneumonia	vap ventilator associated pneumonia	vap ventilator associated pneumonia	vap ventilator associated pneumonia	VAP, Ventilator-associated pneumonia	1	case_or_format
VAP bundle prevention team	vap bundle prevention team	vap bundle prevention team	vap bundle prevention team	VAP bundle prevention team	1	kept
VAP Bundles	vap bundles	vap bundles	vap bundle	VAP bundle	1	singular
VAP bundles	vap bundles	vap bundles	vap bundle	VAP bundle	1	singular

VAP Prevention.	vap prevention	vap prevention	vap prevention	VAP prevention	1	case_or_format
VAP risk	vap risk	vap risk	vap risk	VAP risk	1	kept
VAP, Ventilator-associated pneumonia	vap ventilator associated pneumonia	vap ventilator associated pneumonia	vap ventilator associated pneumonia	VAP, Ventilator-associated pneumonia	1	kept
vaping associated lung injury	vaping associated lung injury	vaping associated lung injury	vaping associated lung injury	vaping associated lung injury	1	kept
vaporization	vaporization	vaporization	vaporization	vaporization	1	kept
vaporizer	vaporizer	vaporizer	vaporizer	vaporizer	1	kept
VAPS scale	vaps scale	vaps scale	vaps scale	VAPS scale	1	kept
Varanidae	varanidae	varanidae	varanidae	Varanidae	1	kept
varenicline	varenicline	varenicline	varenicline	varenicline	1	kept
Variability in practice	variability in practice	variability in practice	variability in practice	Variability in practice	1	kept
Variable lightings	variable lightings	variable lightings	variable lightings	Variable lightings	1	kept
Variable selection	variable selection	variable selection	variable selection	Variable selection	1	kept
variance	variance	variance	variance	variance	1	kept
variance analysis	variance analysis	variance analysis	variance analysis	variance analysis	1	kept
Variance explained	variance explained	variance explained	variance explained	Variance explained	1	kept
Variant	variant	variant	variant	Variant	1	kept

variant angina pectoris	variant angina pectoris	variant angina pectoris	variant angina pectoris	variant angina pectoris	1	kept
variant Creutzfeldt Jakob disease	variant creutzfeldt jakob disease	variant creutzfeldt jakob disease	variant creutzfeldt jakob disease	variant Creutzfeldt Jakob disease	1	kept
Variation	variation	variation	variation	Variations	1	case_or_format
variation	variation	variation	variation	Variations	1	case_or_format
variation in care	variation in care	variation in care	variation in care	variation in care	1	kept
variation in cost	variation in cost	variation in cost	variation in cost	variation in cost	1	kept
Variational inference	variational inference	variational inference	variational inference	Variational inference	1	kept
variceal bleeding	variceal bleeding	variceal bleeding	variceal bleeding	variceal bleeding	1	kept
Varicella zoster	varicella zoster	varicella zoster	varicella zoster	Varicella zoster	1	kept
varicella zoster vaccine	varicella zoster vaccine	varicella zoster vaccine	varicella zoster vaccine	varicella zoster vaccine	1	kept
varicella zoster virus	varicella zoster virus	varicella zoster virus	varicella zoster virus	Varicella zoster virus	1	case_or_format
Varicella Zoster Virus Infection	varicella zoster virus infection	varicella zoster virus infection	varicella zoster virus infection	Varicella Zoster Virus Infection	1	kept
Varicella-zoster	varicella zoster	varicella zoster	varicella zoster	Varicella zoster	1	case_or_format
Varicella-zoster outbreak	varicella zoster outbreak	varicella zoster outbreak	varicella zoster outbreak	Varicella-zoster outbreak	1	kept
varicella-zoster virus	varicella zoster virus	varicella zoster virus	varicella zoster virus	Varicella zoster virus	1	case_or_format
Varicose Ulcer	varicose ulcer	varicose ulcer	varicose ulcer	Varicose Ulcer	1	kept

varicose veins	varicose veins	varicose veins	varicose veins	varicose veins	1	kept
Variola virus	variola virus	variola virus	variola virus	Variola virus	1	kept
varus deformity	varus deformity	varus deformity	varus deformity	varus deformity	1	kept
varus derotation osteotomy	varus derotation osteotomy	varus derotation osteotomy	varus derotation osteotomy	varus derotation osteotomy	1	kept
VAS = visual analog scale	vas visual analog scale	vas visual analog scale	vas visual analog scale	VAS = visual analog scale	1	kept
Vasa Previa	vasa previa	vasa previa	vasa previa	vasa previa	1	case_or_format
vascular access complications	vascular access complications	vascular access complications	vascular access complications	vascular access complications	1	kept
Vascular access device	vascular access device	vascular access device	vascular access device	Vascular Access Devices	1	case_or_format
vascular access management	vascular access management	vascular access management	vascular access management	vascular access management	1	kept
Vascular access planning	vascular access planning	vascular access planning	vascular access planning	Vascular access planning	1	kept
vascular access score	vascular access score	vascular access score	vascular access score	vascular access score	1	kept
Vascular Access Service	vascular access service	vascular access service	vascular access service	Vascular Access Service	1	kept
vascular access specialist team	vascular access specialist team	vascular access specialist team	vascular access specialist team	vascular access specialist team	1	kept
Vascular access team	vascular access team	vascular access team	vascular access team	Vascular Access Team	1	case_or_format
Vascular access therapy	vascular access therapy	vascular access therapy	vascular access therapy	Vascular access therapy	1	kept
Vascular Access Unit	vascular access unit	vascular access unit	vascular access unit	Vascular Access Unit	1	kept

vascular amyloidosis	vascular amyloidosis	vascular amyloidosis	vascular amyloidosis	vascular amyloidosis	1	kept
Vascular attributes	vascular attributes	vascular attributes	vascular attributes	Vascular attributes	1	kept
Vascular calcification	vascular calcification	vascular calcification	vascular calcification	Vascular calcification	1	kept
Vascular catheters	vascular catheters	vascular catheters	vascular catheter	Vascular catheter	1	singular
Vascular cell adhesion molecule 1	vascular cell adhesion molecule 1	vascular cell adhesion molecule 1	vascular cell adhesion molecule 1	Vascular cell adhesion molecule 1	1	kept
vascular cell adhesion molecule 1	vascular cell adhesion molecule 1	vascular cell adhesion molecule 1	vascular cell adhesion molecule 1	Vascular cell adhesion molecule 1	1	case_or_format
Vascular Cell Adhesion Molecule-1	vascular cell adhesion molecule 1	vascular cell adhesion molecule 1	vascular cell adhesion molecule 1	Vascular cell adhesion molecule 1	1	case_or_format
Vascular crisis	vascular crisis	vascular crisis	vascular crisis	Vascular crisis	1	kept
vascular device	vascular device	vascular device	vascular device	Vascular Devices	1	case_or_format
Vascular disease	vascular disease	vascular disease	vascular disease	Vascular Diseases	1	case_or_format
Vascular diseases	vascular diseases	vascular diseases	vascular disease	Vascular Diseases	1	singular
vascular equipment	vascular equipment	vascular equipment	vascular equipment	vascular equipment	1	kept
vascular fragility	vascular fragility	vascular fragility	vascular fragility	vascular fragility	1	kept
vascular guide wire	vascular guide wire	vascular guide wire	vascular guide wire	vascular guide wire	1	kept
vascular infusion team	vascular infusion team	vascular infusion team	vascular infusion team	vascular infusion team	1	kept
Vascular Interventional Radiology	vascular interventional radiology	vascular interventional radiology	vascular interventional radiology	Vascular Interventional Radiology	1	kept

vascular interventional radiology	vascular interventional radiology	vascular interventional radiology	vascular interventional radiology	Vascular Interventional Radiology	1	case_or_format
Vascular lesions	vascular lesions	vascular lesions	vascular lesions	Vascular lesions	1	kept
Vascular pressure	vascular pressure	vascular pressure	vascular pressure	Vascular pressure	1	kept
vascular smooth muscle	vascular smooth muscle	vascular smooth muscle	vascular smooth muscle	vascular smooth muscle	1	kept
vascular stent	vascular stent	vascular stent	vascular stent	vascular stent	1	kept
Vascular surgery	vascular surgery	vascular surgery	vascular surgery	vascular surgery	1	case_or_format
vasculotropin antibody	vasculotropin antibody	vasculotropin antibody	vasculotropin antibody	vasculotropin antibody	1	kept
vasculotropin receptor	vasculotropin receptor	vasculotropin receptor	vasculotropin receptor	vasculotropin receptor	1	kept
Vaseline	vaseline	vaseline	vaseline	Vaseline	1	kept
Vasoactive	vasoactive	vasoactive	vasoactive	Vasoactive	1	kept
vasoactive agents	vasoactive agents	vasoactive agents	vasoactive agent	vasoactive agent	1	singular
vasoactive drugs	vasoactive drugs	vasoactive drugs	vasoactive drug	Vasoactive drugs	1	singular
vasoactive infusion	vasoactive infusion	vasoactive infusion	vasoactive infusion	vasoactive infusion	1	kept
vasoactive inotrope score	vasoactive inotrope score	vasoactive inotrope score	vasoactive inotrope score	vasoactive inotrope score	1	kept
Vasoactive medication	vasoactive medication	vasoactive medication	vasoactive medication	Vasoactive medication	1	kept
vasoactive medication	vasoactive medication	vasoactive medication	vasoactive medication	Vasoactive medication	1	case_or_format

Vasoactive medications	vasoactive medications	vasoactive medications	vasoactive medication	Vasoactive medication	1	singular
Vasodilation	vasodilation	vasodilation	vasodilation	Vasodilation	1	kept
Vasodilators	vasodilators	vasodilators	vasodilators	Vasodilators	1	kept
vasodilators	vasodilators	vasodilators	vasodilators	Vasodilators	1	case_or_format
Vasodilatory shock	vasodilatory shock	vasodilatory shock	vasodilatory shock	Vasodilatory shock	1	kept
vasodilatory shock	vasodilatory shock	vasodilatory shock	vasodilatory shock	Vasodilatory shock	1	case_or_format
vasolastine	vasolastine	vasolastine	vasolastine	vasolastine	1	kept
vasomotor reflex	vasomotor reflex	vasomotor reflex	vasomotor reflex	vasomotor reflex	1	kept
Vasopressin	vasopressin	vasopressin	vasopressin	vasopressin	1	case_or_format
vasopressin blood level	vasopressin blood level	vasopressin blood level	vasopressin blood level	vasopressin blood level	1	kept
VASOPRESSIN RECEPTOR ANTAGONIST	vasopressin receptor antagonist	vasopressin receptor antagonist	vasopressin receptor antagonist	VASOPRESSIN RECEPTOR ANTAGONIST	1	kept
Vasopressor agent	vasopressor agent	vasopressor agent	vasopressor agent	Vasopressor agent	1	kept
Vasopressor agents	vasopressor agents	vasopressor agents	vasopressor agent	Vasopressor agent	1	singular
Vasopressor dose	vasopressor dose	vasopressor dose	vasopressor dose	Vasopressor dose	1	kept
vasopressor, compounding	vasopressor compounding	vasopressor compounding	vasopressor compounding	vasopressor, compounding	1	kept
vastus lateralis muscle	vastus lateralis muscle	vastus lateralis muscle	vastus lateralis muscle	vastus lateralis muscle	1	kept

VAT	vat	vat	vat	VATS	1	case_or_format
vat polymerization	vat polymerization	vat polymerization	vat polymerization	vat polymerization	1	kept
VCR = vertebral column resection	vcr vertebral column resection	vcr vertebral column resection	vcr vertebral column resection	VCR = vertebral column resection	1	kept
Vector	vector	vector	vector	Vector	1	kept
vector	vector	vector	vector	Vector	1	case_or_format
Vector spaces	vector spaces	vector spaces	vector spaces	Vector spaces	1	kept
vector vaccine	vector vaccine	vector vaccine	vector vaccine	vector vaccine	1	kept
vegan diet	vegan diet	vegan diet	vegan diet	vegan diet	1	kept
Vegetables	vegetables	vegetables	vegetable	vegetable	1	singular
vegetarian diet	vegetarian diet	vegetarian diet	vegetarian diet	vegetarian diet	1	kept
vegetative stage	vegetative stage	vegetative stage	vegetative stage	vegetative stage	1	kept
VEHICLE	vehicle	vehicle	vehicle	VEHICLE	1	kept
Veillonella	veillonella	veillonella	veillonella	Veillonella	1	kept
vein compliance	vein compliance	vein compliance	vein compliance	vein compliance	1	kept
vein injury	vein injury	vein injury	vein injury	vein injury	1	kept
vein insufficiency	vein insufficiency	vein insufficiency	vein insufficiency	vein insufficiency	1	kept

Vein pattern	vein pattern	vein pattern	vein pattern	Vein pattern	1	kept
vein visualization device	vein visualization device	vein visualization device	vein visualization device	vein visualization device	1	kept
veins	veins	veins	vein	Veins	1	singular
venarterial	venarterial	venarterial	venarterial	venarterial	1	kept
VENIPUNCTURE	venipuncture	venipuncture	venipuncture	Venipuncture	1	case_or_format
venipuncture insertion	venipuncture insertion	venipuncture insertion	venipuncture insertion	venipuncture insertion	1	kept
venlafaxine	venlafaxine	venlafaxine	venlafaxine	venlafaxine	1	kept
veno-Arterial ECMO	veno arterial ecmo	veno arterial ecmo	veno arterial ecmo	veno-arterial ECMO	1	case_or_format
veno-arterial ecmo	veno arterial ecmo	veno arterial ecmo	veno arterial ecmo	veno-arterial ECMO	1	case_or_format
Veno-occlusive disease	veno occlusive disease	veno occlusive disease	veno occlusive disease	Veno-occlusive disease	1	kept
veno-occlusive disease	veno occlusive disease	veno occlusive disease	veno occlusive disease	Veno-occlusive disease	1	case_or_format
Veno-venoi extracorporeal membrane oxygenation	veno venoi extracorporeal membrane oxygenation	veno venoi extracorporeal membrane oxygenation	veno venoi extracorporeal membrane oxygenation	Veno-venoi extracorporeal membrane oxygenation	1	kept
veno-venous ecmo	veno venous ecmo	veno venous ecmo	veno venous ecmo	veno-venous ECMO	1	case_or_format
veno-venous extracorporeal membrane oxygenation	veno venous extracorporeal membrane oxygenation	veno venous extracorporeal membrane oxygenation	veno venous extracorporeal membrane oxygenation	veno-venous extracorporeal membrane oxygenation	1	kept
venoarterial extracorporeal corporeal oxygenation	venoarterial extracorporeal corporeal oxygenation	venoarterial extracorporeal corporeal oxygenation	venoarterial extracorporeal corporeal oxygenation	venoarterial extracorporeal corporeal oxygenation	1	kept
venoarterial extracorporeal membrane oxygenation	venoarterial extracorporeal membrane oxygenation	venoarterial extracorporeal membrane oxygenation	venoarterial extracorporeal membrane oxygenation	venoarterial extracorporeal membrane oxygenation	1	kept

VENOUS ACCESS	venous access	venous access	venous access	venous access	1	case_or_format
venous access occlusion	venous access occlusion	venous access occlusion	venous access occlusion	venous access occlusion	1	kept
venous cannulation	venous cannulation	venous cannulation	venous cannulation	venous cannulation	1	kept
Venous Cutdown	venous cutdown	venous cutdown	venous cutdown	Venous Cutdown	1	kept
venous leg ulcers	venous leg ulcers	venous leg ulcers	venous leg ulcers	venous leg ulcers	1	kept
Venous lines	venous lines	venous lines	venous lines	Venous lines	1	kept
venous occlusion	venous occlusion	venous occlusion	venous occlusion	venous occlusion	1	kept
Venous pool technique	venous pool technique	venous pool technique	venous pool technique	Venous pool technique	1	kept
Venous Pressure	venous pressure	venous pressure	venous pressure	venous pressure	1	case_or_format
Venous pressure	venous pressure	venous pressure	venous pressure	venous pressure	1	case_or_format
Venous puncture	venous puncture	venous puncture	venous puncture	Venous puncture	1	kept
venous sinus injury	venous sinus injury	venous sinus injury	venous sinus injury	venous sinus injury	1	kept
venous stent	venous stent	venous stent	venous stent	venous stent	1	kept
VENOUS THROMBOEMBOLISM	venous thromboembolism	venous thromboembolism	venous thromboembolism	venous thromboembolism	1	case_or_format
venovenous	venovenous	venovenous	venovenous	venovenous	1	kept
venovenous ECMO	venovenous ecmo	venovenous ecmo	venovenous ecmo	venovenous ECMO	1	kept

venovenous extracorporeal corporeal oxygenation	venovenous extracorporeal corporeal oxygenation	venovenous extracorporeal corporeal oxygenation	venovenous extracorporeal corporeal oxygenation	venovenous extracorporeal corporeal oxygenation	1	kept
Venovenous Extracorporeal Membrane Oxygenation	venovenous extracorporeal membrane oxygenation	venovenous extracorporeal membrane oxygenation	venovenous extracorporeal membrane oxygenation	venovenous extracorporeal membrane oxygenation	1	case_or_format
Ventilación mecánica no invasiva	ventilaci n mec nica no invasiva	ventilaci n mec nica no invasiva	ventilaci n mec nica no invasiva	Ventilación mecánica no invasiva	1	kept
Ventilación no invasiva	ventilaci n no invasiva	ventilaci n no invasiva	ventilaci n no invasiva	Ventilación no invasiva	1	kept
VENTILATED ADULT	ventilated adult	ventilated adult	ventilated adult	VENTILATED ADULT	1	kept
Ventilation bundle	ventilation bundle	ventilation bundle	ventilation bundle	Ventilation bundle	1	kept
ventilation distribution	ventilation distribution	ventilation distribution	ventilation distribution	ventilation distribution	1	kept
Ventilation distributions	ventilation distributions	ventilation distributions	ventilation distribution	ventilation distribution	1	singular
ventilation duration	ventilation duration	ventilation duration	ventilation duration	ventilation duration	1	kept
Ventilation mechanical	ventilation mechanical	ventilation mechanical	ventilation mechanical	Ventilation mechanical	1	kept
Ventilation mode	ventilation mode	ventilation mode	ventilation mode	Ventilation mode	1	kept
ventilation modes	ventilation modes	ventilation modes	ventilation mode	Ventilation mode	1	singular
Ventilation mécanique	ventilation m canique	ventilation m canique	ventilation m canique	Ventilation mécanique	1	kept
ventilation mécanique	ventilation m canique	ventilation m canique	ventilation m canique	Ventilation mécanique	1	case_or_format
ventilation safety	ventilation safety	ventilation safety	ventilation safety	ventilation safety	1	kept
ventilation sedation	ventilation sedation	ventilation sedation	ventilation sedation	ventilation sedation	1	kept

Ventilation strategy	ventilation strategy	ventilation strategy	ventilation strategy	Ventilation strategy	1	kept
Ventilation Techniques	ventilation techniques	ventilation techniques	ventilation techniques	Ventilation Techniques	1	kept
ventilation time	ventilation time	ventilation time	ventilation time	ventilation time	1	kept
ventilation tube occlusion	ventilation tube occlusion	ventilation tube occlusion	ventilation tube occlusion	ventilation tube occlusion	1	kept
ventilation-associated pneumonia	ventilation associated pneumonia	ventilation associated pneumonia	ventilation associated pneumonia	ventilation-associated pneumonia	1	kept
Ventilator acquired pneumonia	ventilator acquired pneumonia	ventilator acquired pneumonia	ventilator acquired pneumonia	Ventilator acquired pneumonia	1	kept
ventilator assisted individuals	ventilator assisted individuals	ventilator assisted individuals	ventilator assisted individuals	ventilator assisted individuals	1	kept
ventilator associated	ventilator associated	ventilator associated	ventilator associated	Ventilator-associated	1	case_or_format
ventilator associated complication	ventilator associated complication	ventilator associated complication	ventilator associated complication	ventilator associated complication	1	kept
ventilator associated complications	ventilator associated complications	ventilator associated complications	ventilator associated complication	ventilator associated complication	1	singular
Ventilator associated event	ventilator associated event	ventilator associated event	ventilator associated event	Ventilator-associated events	1	case_or_format
ventilator associated event	ventilator associated event	ventilator associated event	ventilator associated event	Ventilator-associated events	1	case_or_format
ventilator associated events	ventilator associated events	ventilator associated events	ventilator associated event	Ventilator-associated events	1	singular
ventilator associated oral complication	ventilator associated oral complication	ventilator associated oral complication	ventilator associated oral complication	ventilator associated oral complication	1	kept
Ventilator Associated Pneumonia	ventilator associated pneumonia	ventilator associated pneumonia	ventilator associated pneumonia	ventilator associated pneumonia	1	case_or_format
ventilator associated pneumonia barriers to oral care	ventilator associated pneumonia barriers to oral care	ventilator associated pneumonia barriers to oral care	ventilator associated pneumonia barriers to oral care	ventilator associated pneumonia barriers to oral care	1	kept

Ventilator associated pneumonia prevention	ventilator associated pneumonia prevention	ventilator associated pneumonia prevention	ventilator associated pneumonia prevention	Ventilator associated pneumonia prevention	1	kept
ventilator associatedpneumonia	ventilator associatedpneumonia	ventilator associatedpneumonia	ventilator associatedpneumonia	ventilator associatedpneumonia	1	kept
ventilator bundle checklist	ventilator bundle checklist	ventilator bundle checklist	ventilator bundle checklist	ventilator bundle checklist	1	kept
ventilator bundles	ventilator bundles	ventilator bundles	ventilator bundle	Ventilator bundle	1	singular
ventilator care	ventilator care	ventilator care	ventilator care	ventilator care	1	kept
Ventilator Care Bundle	ventilator care bundle	ventilator care bundle	ventilator care bundle	Ventilator care bundle	1	case_or_format
Ventilator care Bundle	ventilator care bundle	ventilator care bundle	ventilator care bundle	Ventilator care bundle	1	case_or_format
ventilator care bundles	ventilator care bundles	ventilator care bundles	ventilator care bundle	Ventilator care bundle	1	singular
Ventilator Dependence	ventilator dependence	ventilator dependence	ventilator dependence	Ventilator Dependence	1	kept
Ventilator dependent	ventilator dependent	ventilator dependent	ventilator dependent	Ventilator dependent	1	kept
ventilator dependent	ventilator dependent	ventilator dependent	ventilator dependent	Ventilator dependent	1	case_or_format
Ventilator duration	ventilator duration	ventilator duration	ventilator duration	Ventilator duration	1	kept
ventilator dyssynchrony	ventilator dyssynchrony	ventilator dyssynchrony	ventilator dyssynchrony	ventilator dyssynchrony	1	kept
Ventilator eye	ventilator eye	ventilator eye	ventilator eye	Ventilator eye	1	kept
ventilator graphics	ventilator graphics	ventilator graphics	ventilator graphics	ventilator graphics	1	kept
Ventilator induced diaphragm dysfunction	ventilator induced diaphragm dysfunction	ventilator induced diaphragm dysfunction	ventilator induced diaphragm dysfunction	Ventilator induced diaphragm dysfunction	1	kept

ventilator liberation	ventilator liberation	ventilator liberation	ventilator liberation	Ventilator liberation	1	case_or_format
ventilator management	ventilator management	ventilator management	ventilator management	Ventilator management	1	case_or_format
Ventilator mode	ventilator mode	ventilator mode	ventilator mode	Ventilator mode	1	kept
ventilator modes	ventilator modes	ventilator modes	ventilator mode	Ventilator mode	1	singular
Ventilator pneumonia prevention	ventilator pneumonia prevention	ventilator pneumonia prevention	ventilator pneumonia prevention	Ventilator pneumonia prevention	1	kept
Ventilator settings	ventilator settings	ventilator settings	ventilator settings	Ventilator settings	1	kept
ventilator shortage	ventilator shortage	ventilator shortage	ventilator shortage	ventilator shortage	1	kept
Ventilator time	ventilator time	ventilator time	ventilator time	Ventilator time	1	kept
ventilator tubing	ventilator tubing	ventilator tubing	ventilator tubing	ventilator tubing	1	kept
ventilator use	ventilator use	ventilator use	ventilator use	ventilator use	1	kept
Ventilator waveform	ventilator waveform	ventilator waveform	ventilator waveform	ventilator waveform	1	case_or_format
Ventilator withdrawal	ventilator withdrawal	ventilator withdrawal	ventilator withdrawal	Ventilator withdrawal	1	kept
Ventilator- associated pneumonia	ventilator associated pneumonia	ventilator associated pneumonia	ventilator associated pneumonia	ventilator associated pneumonia	1	case_or_format
Ventilator-associated condition	ventilator associated condition	ventilator associated condition	ventilator associated condition	Ventilator-associated condition	1	kept
ventilator-associated events bundle	ventilator associated events bundle	ventilator associated events bundle	ventilator associated events bundle	ventilator-associated events bundle	1	kept
Ventilator-associated gingivitis	ventilator associated gingivitis	ventilator associated gingivitis	ventilator associated gingivitis	Ventilator-associated gingivitis	1	kept

Ventilator-associated infection	ventilator associated infection	ventilator associated infection	ventilator associated infection	Ventilator-associated infection	1	kept
ventilator-associated infection	ventilator associated infection	ventilator associated infection	ventilator associated infection	Ventilator-associated infection	1	case_or_format
ventilator-Associated infections	ventilator associated infections	ventilator associated infections	ventilator associated infection	Ventilator-associated infection	1	singular
Ventilator-associated infectious complications	ventilator associated infectious complications	ventilator associated infectious complications	ventilator associated infectious complications	Ventilator-associated infectious complications	1	kept
ventilator-associated infectious complications	ventilator associated infectious complications	ventilator associated infectious complications	ventilator associated infectious complications	Ventilator-associated infectious complications	1	case_or_format
Ventilator-associated infectious pneumonia	ventilator associated infectious pneumonia	ventilator associated infectious pneumonia	ventilator associated infectious pneumonia	Ventilator-associated infectious pneumonia	1	kept
ventilator-associated infectious pneumonia	ventilator associated infectious pneumonia	ventilator associated infectious pneumonia	ventilator associated infectious pneumonia	Ventilator-associated infectious pneumonia	1	case_or_format
ventilator-associated lung injury	ventilator associated lung injury	ventilator associated lung injury	ventilator associated lung injury	ventilator-associated lung injury	1	kept
Ventilator-associated Pneumonia	ventilator associated pneumonia	ventilator associated pneumonia	ventilator associated pneumonia	ventilator associated pneumonia	1	case_or_format
Ventilator-Associated Pneumonia, VAP, Prevention	ventilator associated pneumonia vap prevention	ventilator associated pneumonia vap prevention	ventilator associated pneumonia vap prevention	Ventilator-Associated Pneumonia, VAP, Prevention	1	kept
Ventilator-associated problems	ventilator associated problems	ventilator associated problems	ventilator associated problems	Ventilator-associated problems	1	kept
Ventilator-associated tracheobronchitis	ventilator associated tracheobronchitis	ventilator associated tracheobronchitis	ventilator associated tracheobronchitis	Ventilator-associated tracheobronchitis	1	kept
Ventilator-dependent	ventilator dependent	ventilator dependent	ventilator dependent	Ventilator dependent	1	case_or_format
ventilator-dependent children	ventilator dependent children	ventilator dependent children	ventilator dependent children	ventilator-dependent children	1	kept
Ventilator-related error	ventilator related error	ventilator related error	ventilator related error	Ventilator-related error	1	kept
ventilatorassociated pneumonia	ventilatorassociated pneumonia	ventilatorassociated pneumonia	ventilatorassociated pneumonia	ventilatorassociated pneumonia	1	kept

Ventilators, Negative-Pressure	ventilators negative pressure	ventilators negative pressure	ventilators negative pressure	Ventilators, Negative-Pressure	1	kept
VENTILATORY EXCHANGE THRIVE	ventilatory exchange thrive	ventilatory exchange thrive	ventilatory exchange thrive	VENTILATORY EXCHANGE THRIVE	1	kept
ventilatory management	ventilatory management	ventilatory management	ventilatory management	ventilatory management	1	kept
Ventilatory mode	ventilatory mode	ventilatory mode	ventilatory mode	Ventilatory mode	1	kept
Ventilatory strategy	ventilatory strategy	ventilatory strategy	ventilatory strategy	Ventilatory strategy	1	kept
Ventilatory weaning protocol	ventilatory weaning protocol	ventilatory weaning protocol	ventilatory weaning protocol	Ventilatory weaning protocol	1	kept
Ventilação artificial	ventila o artificial	ventila o artificial	ventila o artificial	Ventilação artificial	1	kept
Ventilação com insuflador manual	ventila o com insuflador manual	ventila o com insuflador manual	ventila o com insuflador manual	Ventilação com insuflador manual	1	kept
Ventillator-associated pneumonia	ventillator associated pneumonia	ventillator associated pneumonia	ventillator associated pneumonia	Ventillator-associated pneumonia	1	kept
ventillatory management	ventillatory management	ventillatory management	ventillatory management	ventillatory management	1	kept
venting behavior	venting behavior	venting behavior	venting behavior	venting behavior	1	kept
Ventral decubitus	ventral decubitus	ventral decubitus	ventral decubitus	Ventral decubitus	1	kept
Ventral hernia	ventral hernia	ventral hernia	ventral hernia	Ventral hernia	1	kept
ventral vagal complex	ventral vagal complex	ventral vagal complex	ventral vagal complex	ventral vagal complex	1	kept
VERBAL ABUSE	verbal abuse	verbal abuse	verbal abuse	verbal abuse	1	case_or_format
Verbal abuse	verbal abuse	verbal abuse	verbal abuse	verbal abuse	1	case_or_format

verbal analog scale	verbal analog scale	verbal analog scale	verbal analog scale	verbal analog scale	1	kept
VERBAL COMMUNICATION	verbal communication	verbal communication	verbal communication	verbal communication	1	case_or_format
Verbal information	verbal information	verbal information	verbal information	Verbal information	1	kept
verbal memory	verbal memory	verbal memory	verbal memory	verbal memory	1	kept
Verbatim transcription	verbatim transcription	verbatim transcription	verbatim transcription	Verbatim transcription	1	kept
VERIFICATION	verification	verification	verification	VERIFICATION	1	kept
Verification method	verification method	verification method	verification method	Verification method	1	kept
vernix caseosa	vernix caseosa	vernix caseosa	vernix caseosa	vernix caseosa	1	kept
Vero cell line	vero cell line	vero cell line	vero cell line	Vero cell line	1	kept
Vero Cells	vero cells	vero cells	vero cells	Vero Cells	1	kept
Veronica	veronica	veronica	veronica	Veronica	1	kept
vertebra body	vertebra body	vertebra body	vertebra body	vertebra body	1	kept
vertebra dislocation	vertebra dislocation	vertebra dislocation	vertebra dislocation	vertebra dislocation	1	kept
vertebra fracture	vertebra fracture	vertebra fracture	vertebra fracture	vertebra fracture	1	kept
vertebral canal stenosis	vertebral canal stenosis	vertebral canal stenosis	vertebral canal stenosis	vertebral canal stenosis	1	kept
vertebral venous plexus	vertebral venous plexus	vertebral venous plexus	vertebral venous plexus	vertebral venous plexus	1	kept

Vertebroplasty	vertebroplasty	vertebroplasty	vertebroplasty	Vertebroplasty	1	kept
vertical band gastroplasty	vertical band gastroplasty	vertical band gastroplasty	vertical band gastroplasty	vertical band gastroplasty	1	kept
Vertical transformational leadership	vertical transformational leadership	vertical transformational leadership	vertical transformational leadership	Vertical transformational leadership	1	kept
Very early rehabilitation or intensive telemetry	very early rehabilitation or intensive telemetry	very early rehabilitation or intensive telemetry	very early rehabilitation or intensive telemetry	Very early rehabilitation or intensive telemetry	1	kept
very early-onset VAP	very early onset vap	very early onset vap	very early onset vap	very early-onset VAP	1	kept
very frequent	very frequent	very frequent	very frequent	very frequent	1	kept
Very Low Birth Weight	very low birth weight	very low birth weight	very low birth weight	very low birth weight	1	case_or_format
Very low birth weight neonates	very low birth weight neonates	very low birth weight neonates	very low birth weight neonates	Very low birth weight neonates	1	kept
very low birth weight neonates	very low birth weight neonates	very low birth weight neonates	very low birth weight neonates	Very low birth weight neonates	1	case_or_format
very low birth weight premature infants	very low birth weight premature infants	very low birth weight premature infants	very low birth weight premature infants	very low birth weight premature infants	1	kept
Very low birth-weight	very low birth weight	very low birth weight	very low birth weight	very low birth weight	1	case_or_format
Very low birth-weight infant	very low birth weight infant	very low birth weight infant	very low birth weight infant	Very low birth weight infant	1	case_or_format
Very low birthweight infants	very low birthweight infants	very low birthweight infants	very low birthweight infant	very low birthweight infant	1	singular
very low birthweight infants	very low birthweight infants	very low birthweight infants	very low birthweight infant	very low birthweight infant	1	singular
Very low weight newborn	very low weight newborn	very low weight newborn	very low weight newborn	Very low weight newborn	1	kept
very low-birth weight	very low birth weight	very low birth weight	very low birth weight	very low birth weight	1	case_or_format

Very low-birth-weight	very low birth weight	very low birth weight	very low birth weight	very low birth weight	1	case_or_format
Very low-birthweight infant	very low birthweight infant	very low birthweight infant	very low birthweight infant	very low birthweight infant	1	case_or_format
Very low-birthweight infants	very low birthweight infants	very low birthweight infants	very low birthweight infant	very low birthweight infant	1	singular
Very old	very old	very old	very old	very old	1	case_or_format
very premature newborn	very premature newborn	very premature newborn	very premature newborn	very premature newborn	1	kept
very preterm	very preterm	very preterm	very preterm	very preterm	1	kept
Very preterm infant	very preterm infant	very preterm infant	very preterm infant	Very preterm infants	1	case_or_format
Very-low birth weight infants	very low birth weight infants	very low birth weight infants	very low birth weight infant	Very low birth weight infant	1	singular
very-low birthweight infant	very low birthweight infant	very low birthweight infant	very low birthweight infant	very low birthweight infant	1	case_or_format
very-low-birth-weight	very low birth weight	very low birth weight	very low birth weight	very low birth weight	1	case_or_format
Very-low-birthweight	very low birthweight	very low birthweight	very low birthweight	Very low birthweight	1	case_or_format
vesicant	vesicant	vesicant	vesicant	vesicant	1	kept
vesicoureteral reflux	vesicoureteral reflux	vesicoureteral reflux	vesicoureteral reflux	vesicoureteral reflux	1	kept
vessel dilator	vessel dilator	vessel dilator	vessel dilator	vessel dilator	1	kept
VESTIBULAR INTERVENTION	vestibular intervention	vestibular intervention	vestibular intervention	VESTIBULAR INTERVENTION	1	kept
Vestibule, Labyrinth	vestibule labyrinth	vestibule labyrinth	vestibule labyrinth	Vestibule, Labyrinth	1	kept

veterans affairs	veterans affairs	veterans affairs	veterans affairs	veterans affairs	1	kept
Veterans Healthcare Administration	veterans healthcare administration	veterans healthcare administration	veterans healthcare administration	Veterans Healthcare Administration	1	kept
veterinary clinical trial	veterinary clinical trial	veterinary clinical trial	veterinary clinical trial	veterinary clinical trial	1	kept
Veterinary emergency room	veterinary emergency room	veterinary emergency room	veterinary emergency room	Veterinary emergency room	1	kept
veterinary emergency room	veterinary emergency room	veterinary emergency room	veterinary emergency room	Veterinary emergency room	1	case_or_format
veterinary hospitals	veterinary hospitals	veterinary hospitals	veterinary hospitals	veterinary hospitals	1	kept
veterinary student	veterinary student	veterinary student	veterinary student	veterinary student	1	kept
Veterinary technician	veterinary technician	veterinary technician	veterinary technician	veterinary technicians	1	case_or_format
veterinary technician	veterinary technician	veterinary technician	veterinary technician	veterinary technicians	1	case_or_format
vetivex	vetivex	vetivex	vetivex	vetivex	1	kept
VIABILITY	viability	viability	viability	VIABILITY	1	kept
Viability	viability	viability	viability	VIABILITY	1	case_or_format
Viable solutions	viable solutions	viable solutions	viable solutions	Viable solutions	1	kept
vial	vial	vial	vial	vial	1	kept
Vial Exchange Rate and Bag analysis	vial exchange rate and bag analysis	vial exchange rate and bag analysis	vial exchange rate and bag analysis	Vial Exchange Rate and Bag analysis	1	kept
VIBRATION	vibration	vibration	vibration	vibration	1	case_or_format

Vibration assisted	vibration assisted	vibration assisted	vibration assisted	Vibration assisted	1	kept
Vibration motor	vibration motor	vibration motor	vibration motor	Vibration motor	1	kept
Vibration pattern	vibration pattern	vibration pattern	vibration pattern	Vibration pattern	1	kept
vibration sense	vibration sense	vibration sense	vibration sense	vibration sense	1	kept
vibration therapy	vibration therapy	vibration therapy	vibration therapy	vibration therapy	1	kept
Vibrations	vibrations	vibrations	vibration	vibration	1	singular
Vibrio cholerae	vibrio cholerae	vibrio cholerae	vibrio cholerae	Vibrio cholerae	1	kept
VIBRO-TACTILE DISPLAY	vibro tactile display	vibro tactile display	vibro tactile display	VIBRO-TACTILE DISPLAY	1	kept
Vibrotactile	vibrotactile	vibrotactile	vibrotactile	Vibrotactile	1	kept
vicarious posttraumatic growth	vicarious posttraumatic growth	vicarious posttraumatic growth	vicarious posttraumatic growth	vicarious posttraumatic growth	1	kept
victims of child abuse	victims of child abuse	victims of child abuse	victims of child abuse	victims of child abuse	1	kept
Video analysis	video analysis	video analysis	video analysis	Video analysis	1	kept
Video assisted teaching module	video assisted teaching module	video assisted teaching module	video assisted teaching module	Video assisted teaching module	1	kept
video call system	video call system	video call system	video call system	video call system	1	kept
video calls	video calls	video calls	video call	video call	1	singular
Video camera footage	video camera footage	video camera footage	video camera footage	Video camera footage	1	kept

video camera footage	video camera footage	video camera footage	video camera footage	Video camera footage	1	case_or_format
Video cameras	video cameras	video cameras	video cameras	Video cameras	1	kept
video cameras	video cameras	video cameras	video cameras	Video cameras	1	case_or_format
video communication	video communication	video communication	video communication	video communication	1	kept
Video conferencing	video conferencing	video conferencing	video conferencing	Video conferencing	1	kept
Video education	video education	video education	video education	Video education	1	kept
Video game	video game	video game	video game	Video Games	1	case_or_format
Video information	video information	video information	video information	Video information	1	kept
video interview	video interview	video interview	video interview	video interview	1	kept
video laryngoscope	video laryngoscope	video laryngoscope	video laryngoscope	video laryngoscope	1	kept
video laryngoscopy	video laryngoscopy	video laryngoscopy	video laryngoscopy	video laryngoscopy	1	kept
video messaging service	video messaging service	video messaging service	video messaging service	video-messaging service	1	case_or_format
video microscopy	video microscopy	video microscopy	video microscopy	video microscopy	1	kept
Video monitoring	video monitoring	video monitoring	video monitoring	Video monitoring	1	kept
video reflexive ethnography	video reflexive ethnography	video reflexive ethnography	video reflexive ethnography	video reflexive ethnography	1	kept
Video remote interpreting	video remote interpreting	video remote interpreting	video remote interpreting	Video remote interpreting	1	kept

VIDEO SELF-INSTRUCTION	video self instruction	video self instruction	video self instruction	VIDEO SELF-INSTRUCTION	1	kept
video signal processing algorithms	video signal processing algorithms	video signal processing algorithms	video signal processing algorithms	video signal processing algorithms	1	kept
Video telemetry	video telemetry	video telemetry	video telemetry	Video telemetry	1	kept
video-assisted thoracoscopic surgery	video assisted thoracoscopic surgery	video assisted thoracoscopic surgery	video assisted thoracoscopic surgery	video assisted thoracoscopic surgery	1	case_or_format
video-assisted training	video assisted training	video assisted training	video assisted training	video-assisted training	1	kept
video-audio demonstration	video audio demonstration	video audio demonstration	video audio demonstration	video-audio demonstration	1	kept
Video-audio media	video audio media	video audio media	video audio media	Video-audio media	1	kept
video-based learning	video based learning	video based learning	video based learning	video-based learning	1	kept
video-based noncontact monitoring methods	video based noncontact monitoring methods	video based noncontact monitoring methods	video based noncontact monitoring methods	video-based noncontact monitoring methods	1	kept
Video-conferencing Tools	video conferencing tools	video conferencing tools	video conferencing tools	Video-conferencing Tools	1	kept
Video-EEG	video eeg	video eeg	video eeg	Video-EEG	1	kept
Video-electroencephalography	video electroencephalography	video electroencephalography	video electroencephalography	Video-electroencephalography	1	kept
VIDEO-LARYNGOSCOPY	video laryngoscopy	video laryngoscopy	video laryngoscopy	video laryngoscopy	1	case_or_format
Video-reflexive ethnography	video reflexive ethnography	video reflexive ethnography	video reflexive ethnography	video reflexive ethnography	1	case_or_format
video-reflexive ethnography	video reflexive ethnography	video reflexive ethnography	video reflexive ethnography	video reflexive ethnography	1	case_or_format
videoendoscopy	videoendoscopy	videoendoscopy	videoendoscopy	videoendoscopy	1	kept

videolaparoscopy	videolaparoscopy	videolaparoscopy	videolaparoscopy	videolaparoscopy	1	kept
Videos	videos	videos	video	Video	1	singular
videotape	videotape	videotape	videotape	videotape	1	kept
video－assisted education	video assisted education	video assisted education	video assisted education	video－assisted education	1	kept
video－audio demonstration	video audio demonstration	video audio demonstration	video audio demonstration	video−audio demonstration	1	case_or_format
vieillissment	vieillissment	vieillissment	vieillissment	vieillissment	1	kept
Vienna Protocol Neonatal Pain Agitation and Sedation	vienna protocol neonatal pain agitation and sedation	vienna protocol neonatal pain agitation and sedation	vienna protocol neonatal pain agitation and sedation	Vienna Protocol Neonatal Pain Agitation and Sedation	1	kept
Vietnam Conflict	vietnam conflict	vietnam conflict	vietnam conflict	Vietnam Conflict	1	kept
view to the outside	view to the outside	view to the outside	view to the outside	view to the outside	1	kept
Viewpoint	viewpoint	viewpoint	viewpoint	Viewpoint	1	kept
viewpoint	viewpoint	viewpoint	viewpoint	Viewpoint	1	case_or_format
views of parents	views of parents	views of parents	views of parents	views of parents	1	kept
vigilance	vigilance	vigilance	vigilance	VIGILANCE	1	case_or_format
vigilance task	vigilance task	vigilance task	vigilance task	vigilance task	1	kept
Vigilant attendance	vigilant attendance	vigilant attendance	vigilant attendance	Vigilant attendance	1	kept
Vignette	vignette	vignette	vignette	vignette	1	case_or_format

vignettes	vignettes	vignettes	vignette	vignette	1	singular
vigor	vigor	vigor	vigor	vigor	1	kept
Vinblastine	vinblastine	vinblastine	vinblastine	vinblastine	1	case_or_format
Vincristine	vincristine	vincristine	vincristine	vincristine	1	case_or_format
vincristine sulfate	vincristine sulfate	vincristine sulfate	vincristine sulfate	vincristine sulfate	1	kept
vinyl isolation bag	vinyl isolation bag	vinyl isolation bag	vinyl isolation bag	vinyl isolation bag	1	kept
vinyltrimethoxysilane	vinyltrimethoxysilane	vinyltrimethoxysilane	vinyltrimethoxysilane	vinyltrimethoxysilane	1	kept
violation	violation	violation	violation	violation	1	kept
violations	violations	violations	violation	violation	1	singular
Violence at Work	violence at work	violence at work	violence at work	Violence at Work	1	kept
violence at work	violence at work	violence at work	violence at work	Violence at Work	1	case_or_format
violence in healthcare	violence in healthcare	violence in healthcare	violence in healthcare	violence in healthcare	1	kept
Violence prevention	violence prevention	violence prevention	violence prevention	Violence prevention	1	kept
violence, abusive behaviour	violence abusive behaviour	violence abusive behaviour	violence abusive behaviour	violence, abusive behaviour	1	kept
Violencia laboral	violencia laboral	violencia laboral	violencia laboral	Violencia laboral	1	kept
Violent behaviour	violent behaviour	violent behaviour	violent behaviour	Violent behaviour	1	kept

Viral	viral	viral	viral	Viral	1	kept
viral	viral	viral	viral	Viral	1	case_or_format
viral clearance	viral clearance	viral clearance	viral clearance	viral clearance	1	kept
viral conjunctivitis	viral conjunctivitis	viral conjunctivitis	viral conjunctivitis	viral conjunctivitis	1	kept
viral croup	viral croup	viral croup	viral croup	viral croup	1	kept
viral disease	viral disease	viral disease	viral disease	viral disease	1	kept
viral encephalitis	viral encephalitis	viral encephalitis	viral encephalitis	viral encephalitis	1	kept
viral exanthema	viral exanthema	viral exanthema	viral exanthema	viral exanthema	1	kept
viral exposure	viral exposure	viral exposure	viral exposure	viral exposure	1	kept
Viral Fusion Proteins	viral fusion proteins	viral fusion proteins	viral fusion proteins	Viral Fusion Proteins	1	kept
viral gastroenteritis	viral gastroenteritis	viral gastroenteritis	viral gastroenteritis	viral gastroenteritis	1	kept
Viral haemorrhagic fever	viral haemorrhagic fever	viral haemorrhagic fever	viral haemorrhagic fever	Viral haemorrhagic fever	1	kept
Viral hemorrhagic fevers	viral hemorrhagic fevers	viral hemorrhagic fevers	viral hemorrhagic fevers	Viral hemorrhagic fevers	1	kept
Viral Hepatitis	viral hepatitis	viral hepatitis	viral hepatitis	Viral Hepatitis	1	kept
Viral hepatitis	viral hepatitis	viral hepatitis	viral hepatitis	Viral Hepatitis	1	case_or_format
viral hepatitis	viral hepatitis	viral hepatitis	viral hepatitis	Viral Hepatitis	1	case_or_format

Viral herpes	viral herpes	viral herpes	viral herpes	Viral herpes	1	kept
viral load	viral load	viral load	viral load	Viral Load	1	case_or_format
viral pneumonia	viral pneumonia	viral pneumonia	viral pneumonia	viral pneumonia	1	kept
viral protein	viral protein	viral protein	viral protein	viral protein	1	kept
Viral Proteins	viral proteins	viral proteins	viral protein	viral protein	1	singular
viral sepsis	viral sepsis	viral sepsis	viral sepsis	viral sepsis	1	kept
Viral Structural Proteins	viral structural proteins	viral structural proteins	viral structural proteins	Viral Structural Proteins	1	kept
Viral Tropism	viral tropism	viral tropism	viral tropism	Viral Tropism	1	kept
viral tropism	viral tropism	viral tropism	viral tropism	Viral Tropism	1	case_or_format
viral upper respiratory tract infection	viral upper respiratory tract infection	viral upper respiratory tract infection	viral upper respiratory tract infection	viral upper respiratory tract infection	1	kept
Viremia	viremia	viremia	viremia	viremia	1	case_or_format
Virological cure	virological cure	virological cure	virological cure	Virological cure	1	kept
Virtual car	virtual car	virtual car	virtual car	Virtual car	1	kept
Virtual care	virtual care	virtual care	virtual care	virtual care	1	case_or_format
Virtual contact options	virtual contact options	virtual contact options	virtual contact options	Virtual contact options	1	kept
Virtual debriefing	virtual debriefing	virtual debriefing	virtual debriefing	Virtual debriefing	1	kept

virtual debriefing	virtual debriefing	virtual debriefing	virtual debriefing	Virtual debriefing	1	case_or_format
virtual dissection table	virtual dissection table	virtual dissection table	virtual dissection table	virtual dissection table	1	kept
virtual environment	virtual environment	virtual environment	virtual environment	virtual environments	1	case_or_format
Virtual Environments	virtual environments	virtual environments	virtual environment	virtual environments	1	singular
virtual health	virtual health	virtual health	virtual health	virtual health	1	kept
Virtual Health Care	virtual health care	virtual health care	virtual health care	Virtual Health Care	1	kept
Virtual interactive practice	virtual interactive practice	virtual interactive practice	virtual interactive practice	Virtual interactive practice	1	kept
virtual interactive practice	virtual interactive practice	virtual interactive practice	virtual interactive practice	Virtual interactive practice	1	case_or_format
Virtual journal club	virtual journal club	virtual journal club	virtual journal club	Virtual journal club	1	kept
Virtual learning	virtual learning	virtual learning	virtual learning	Virtual learning	1	kept
virtual learning	virtual learning	virtual learning	virtual learning	Virtual learning	1	case_or_format
Virtual medicine	virtual medicine	virtual medicine	virtual medicine	virtual medicine	1	case_or_format
virtual mock-ups	virtual mock ups	virtual mock ups	virtual mock ups	virtual mock-ups	1	kept
Virtual radiology rounds	virtual radiology rounds	virtual radiology rounds	virtual radiology rounds	Virtual radiology rounds	1	kept
virtual reality exposure therapy	virtual reality exposure therapy	virtual reality exposure therapy	virtual reality exposure therapy	Virtual Reality Exposure Therapy	1	case_or_format
Virtual reality system	virtual reality system	virtual reality system	virtual reality system	Virtual reality system	1	kept

virtual rounds	virtual rounds	virtual rounds	virtual rounds	virtual rounds	1	kept
virtual rural generalist service versus usual care	virtual rural generalist service versus usual care	virtual rural generalist service versus usual care	virtual rural generalist service versus usual care	virtual rural generalist service versus usual care	1	kept
Virtual sensing	virtual sensing	virtual sensing	virtual sensing	Virtual sensing	1	kept
virtual sensing	virtual sensing	virtual sensing	virtual sensing	Virtual sensing	1	case_or_format
Virtual sensor	virtual sensor	virtual sensor	virtual sensor	Virtual sensor	1	kept
Virtual Systems	virtual systems	virtual systems	virtual systems	Virtual Systems	1	kept
Virtual teams	virtual teams	virtual teams	virtual teams	Virtual teams	1	kept
Virtual visitation	virtual visitation	virtual visitation	virtual visitation	Virtual visitation	1	kept
Virtual visiting	virtual visiting	virtual visiting	virtual visiting	Virtual visiting	1	kept
Virtue ethics	virtue ethics	virtue ethics	virtue ethics	Virtue ethics	1	kept
virtue ethics	virtue ethics	virtue ethics	virtue ethics	Virtue ethics	1	case_or_format
Virulence genes	virulence genes	virulence genes	virulence genes	Virulence genes	1	kept
Virulence patterns	virulence patterns	virulence patterns	virulence patterns	Virulence patterns	1	kept
VIRUS	virus	virus	virus	Virus	1	case_or_format
virus classification	virus classification	virus classification	virus classification	virus classification	1	kept
virus concentration	virus concentration	virus concentration	virus concentration	virus concentration	1	kept

virus etiology	virus etiology	virus etiology	virus etiology	virus etiology	1	kept
virus gene	virus gene	virus gene	virus gene	virus gene	1	kept
virus hemorrhagic fever	virus hemorrhagic fever	virus hemorrhagic fever	virus hemorrhagic fever	virus hemorrhagic fever	1	kept
virus identification	virus identification	virus identification	virus identification	virus identification	1	kept
virus immunity	virus immunity	virus immunity	virus immunity	virus immunity	1	kept
virus inactivation	virus inactivation	virus inactivation	virus inactivation	virus inactivation	1	kept
virus neutralization	virus neutralization	virus neutralization	virus neutralization	virus neutralization	1	kept
virus receptor	virus receptor	virus receptor	virus receptor	virus receptor	1	kept
Virus sampling	virus sampling	virus sampling	virus sampling	Virus sampling	1	kept
virus sampling	virus sampling	virus sampling	virus sampling	Virus sampling	1	case_or_format
Virus Shedding	virus shedding	virus shedding	virus shedding	virus shedding	1	case_or_format
virus typing	virus typing	virus typing	virus typing	virus typing	1	kept
VIRUS-INFECTION	virus infection	virus infection	virus infection	virus infection	1	case_or_format
VIRUS-INFECTIONS	virus infections	virus infections	virus infection	virus infection	1	singular
viscera	viscera	viscera	viscera	viscera	1	kept
Visceral obesity	visceral obesity	visceral obesity	visceral obesity	Visceral obesity	1	kept

VISCERAL PROTEINS	visceral proteins	visceral proteins	visceral proteins	VISCERAL PROTEINS	1	kept
VISCOELASTIC FOAM	viscoelastic foam	viscoelastic foam	viscoelastic foam	VISCOELASTIC FOAM	1	kept
Viscoelastic Substances	viscoelastic substances	viscoelastic substances	viscoelastic substances	Viscoelastic Substances	1	kept
viscoelasticity	viscoelasticity	viscoelasticity	viscoelasticity	viscoelasticity	1	kept
viscosity grade	viscosity grade	viscosity grade	viscosity grade	viscosity grade	1	kept
Visibility Design	visibility design	visibility design	visibility design	Visibility Design	1	kept
Vision Disorders	vision disorders	vision disorders	vision disorders	Vision Disorders	1	kept
Vision Tests	vision tests	vision tests	vision tests	Vision Tests	1	kept
Vision-based methods	vision based methods	vision based methods	vision based methods	Vision-based methods	1	kept
visit	visit	visit	visit	VISITS	1	case_or_format
visit limitation	visit limitation	visit limitation	visit limitation	visit limitation	1	kept
visit restriction	visit restriction	visit restriction	visit restriction	visit restriction	1	kept
visit-related infections	visit related infections	visit related infections	visit related infections	visit-related infections	1	kept
Visita familiar	visita familiar	visita familiar	visita familiar	Visita familiar	1	kept
visitation guidelines	visitation guidelines	visitation guidelines	visitation guidelines	visitation guidelines	1	kept
Visitation hours	visitation hours	visitation hours	visitation hours	Visitation hours	1	kept

visitation restriction	visitation restriction	visitation restriction	visitation restriction	visitation restriction	1	kept
Visiting family members	visiting family members	visiting family members	visiting family members	Visiting family members	1	kept
visiting hour	visiting hour	visiting hour	visiting hour	Visiting hours	1	case_or_format
visiting hours	visiting hours	visiting hours	visiting hour	Visiting hours	1	singular
visitor management	visitor management	visitor management	visitor management	visitor management	1	kept
Visitor policies	visitor policies	visitor policies	visitor policies	Visitor policies	1	kept
VISITOR RESTRICTIONS	visitor restrictions	visitor restrictions	visitor restrictions	VISITOR RESTRICTIONS	1	kept
Visitor Restrictions	visitor restrictions	visitor restrictions	visitor restrictions	VISITOR RESTRICTIONS	1	case_or_format
visitor restrictions	visitor restrictions	visitor restrictions	visitor restrictions	VISITOR RESTRICTIONS	1	case_or_format
visteril	visteril	visteril	visteril	visteril	1	kept
Visual Acuity	visual acuity	visual acuity	visual acuity	visual acuity	1	case_or_format
visual aid	visual aid	visual aid	visual aid	visual aid	1	kept
Visual Analog Scale for Confusion	visual analog scale for confusion	visual analog scale for confusion	visual analog scale for confusion	Visual Analog Scale for Confusion	1	kept
VISUAL ANALOG SCALES	visual analog scales	visual analog scales	visual analog scale	visual analog scale	1	singular
visual analogue fatigue scale	visual analogue fatigue scale	visual analogue fatigue scale	visual analogue fatigue scale	visual analogue fatigue scale	1	kept
visual analogue scale	visual analogue scale	visual analogue scale	visual analogue scale	Visual analogue scale	1	case_or_format

visual analysis	visual analysis	visual analysis	visual analysis	visual analysis	1	kept
visual approach	visual approach	visual approach	visual approach	visual approach	1	kept
Visual assessments	visual assessments	visual assessments	visual assessments	Visual assessments	1	kept
Visual Attention	visual attention	visual attention	visual attention	visual attention	1	case_or_format
visual basic	visual basic	visual basic	visual basic	visual basic	1	kept
Visual basic for applications	visual basic for applications	visual basic for applications	visual basic for applications	Visual basic for applications	1	kept
visual behavior	visual behavior	visual behavior	visual behavior	visual behavior	1	kept
Visual colors	visual colors	visual colors	visual colors	Visual colors	1	kept
Visual communication	visual communication	visual communication	visual communication	Visual communication	1	kept
Visual differential diagnosis	visual differential diagnosis	visual differential diagnosis	visual differential diagnosis	Visual differential diagnosis	1	kept
visual discrimination	visual discrimination	visual discrimination	visual discrimination	visual discrimination	1	kept
visual display unit	visual display unit	visual display unit	visual display unit	visual display unit	1	kept
Visual effects	visual effects	visual effects	visual effects	Visual effects	1	kept
Visual estimation	visual estimation	visual estimation	visual estimation	Visual estimation	1	kept
Visual evoked potential	visual evoked potential	visual evoked potential	visual evoked potential	visual evoked potential	1	case_or_format
visual field defect	visual field defect	visual field defect	visual field defect	visual field defect	1	kept

Visual impairment	visual impairment	visual impairment	visual impairment	visual impairment	1	case_or_format
Visual Infusion Phlebitis Score	visual infusion phlebitis score	visual infusion phlebitis score	visual infusion phlebitis score	Visual Infusion Phlebitis Score	1	kept
Visual inspection	visual inspection	visual inspection	visual inspection	Visual inspection	1	kept
Visual Interface	visual interface	visual interface	visual interface	Visual Interface	1	kept
visual interface	visual interface	visual interface	visual interface	Visual Interface	1	case_or_format
Visual laryngoscope	visual laryngoscope	visual laryngoscope	visual laryngoscope	Visual laryngoscope	1	kept
Visual media	visual media	visual media	visual media	Visual media	1	kept
visual memory	visual memory	visual memory	visual memory	visual memory	1	kept
visual numerical scale	visual numerical scale	visual numerical scale	visual numerical scale	visual numerical scale	1	kept
Visual objects	visual objects	visual objects	visual objects	Visual objects	1	kept
visual perception	visual perception	visual perception	visual perception	visual perception	1	kept
Visual skin assessment	visual skin assessment	visual skin assessment	visual skin assessment	Visual skin assessment	1	kept
Visual stimulation	visual stimulation	visual stimulation	visual stimulation	visual stimulation	1	case_or_format
visual system	visual system	visual system	visual system	visual system	1	kept
Visualization analysis	visualization analysis	visualization analysis	visualization analysis	Visualization analysis	1	kept
visually impaired person	visually impaired person	visually impaired person	visually impaired person	visually impaired person	1	kept

visuomotor coordination	visuomotor coordination	visuomotor coordination	visuomotor coordination	visuomotor coordination	1	kept
visuospatial game	visuospatial game	visuospatial game	visuospatial game	visuospatial game	1	kept
Vital capacity	vital capacity	vital capacity	vital capacity	vital capacity	1	case_or_format
vital data monitoring	vital data monitoring	vital data monitoring	vital data monitoring	vital data monitoring	1	kept
Vital sign management	vital sign management	vital sign management	vital sign management	Vital sign management	1	kept
Vital sign measurements	vital sign measurements	vital sign measurements	vital sign measurements	Vital sign measurements	1	kept
vital sign measurements	vital sign measurements	vital sign measurements	vital sign measurements	Vital sign measurements	1	case_or_format
Vital sign omissions	vital sign omissions	vital sign omissions	vital sign omissions	Vital sign omissions	1	kept
Vital signs recording quality	vital signs recording quality	vital signs recording quality	vital signs recording quality	Vital signs recording quality	1	kept
Vital Statistics	vital statistics	vital statistics	vital statistics	Vital Statistics	1	kept
Vitamin B Complex	vitamin b complex	vitamin b complex	vitamin b complex	Vitamin B Complex	1	kept
vitamin B complex	vitamin b complex	vitamin b complex	vitamin b complex	Vitamin B Complex	1	case_or_format
Vitamin B12 therapy	vitamin b12 therapy	vitamin b12 therapy	vitamin b12 therapy	Vitamin B12 therapy	1	kept
vitamin E acetate	vitamin e acetate	vitamin e acetate	vitamin e acetate	vitamin E acetate	1	kept
vitamin intake	vitamin intake	vitamin intake	vitamin intake	vitamin intake	1	kept
Vitamin K antagonist	vitamin k antagonist	vitamin k antagonist	vitamin k antagonist	Vitamin K antagonist	1	kept

Vitamin K Epoxide Reductases	vitamin k epoxide reductases	vitamin k epoxide reductases	vitamin k epoxide reductases	Vitamin K Epoxide Reductases	1	kept
vitamin K shortage	vitamin k shortage	vitamin k shortage	vitamin k shortage	vitamin K shortage	1	kept
VITAMIN-D	vitamin d	vitamin d	vitamin d	vitamin D	1	case_or_format
VITAMIN-E	vitamin e	vitamin e	vitamin e	Vitamin E	1	case_or_format
Vitrectomy	vitrectomy	vitrectomy	vitrectomy	Vitrectomy	1	kept
vitreous body	vitreous body	vitreous body	vitreous body	vitreous body	1	kept
vitreous hemorrhage	vitreous hemorrhage	vitreous hemorrhage	vitreous hemorrhage	vitreous hemorrhage	1	kept
vivid dream experiences	vivid dream experiences	vivid dream experiences	vivid dream experiences	vivid dream experiences	1	kept
VKA	vka	vka	vka	VKA	1	kept
VLBW = very low-birth-weight	vlbw very low birth weight	vlbw very low birth weight	vlbw very low birth weight	VLBW = very low-birth-weight	1	kept
VLBW infant	vlbw infant	vlbw infant	vlbw infant	VLBW infants	1	case_or_format
Vocabulary	vocabulary	vocabulary	vocabulary	vocabulary	1	case_or_format
vocational development	vocational development	vocational development	vocational development	vocational development	1	kept
Vocational Education	vocational education	vocational education	vocational education	vocational education	1	case_or_format
VOICE	voice	voice	voice	voice	1	case_or_format
voice application	voice application	voice application	voice application	voice application	1	kept

voice handicap	voice handicap	voice handicap	voice handicap	voice handicap	1	kept
voice handicap index	voice handicap index	voice handicap index	voice handicap index	voice handicap index	1	kept
Voice Handicap Index 10 questionnaire	voice handicap index 10 questionnaire	voice handicap index 10 questionnaire	voice handicap index 10 questionnaire	Voice Handicap Index 10 questionnaire	1	kept
voice mail	voice mail	voice mail	voice mail	voice mail	1	kept
Voice over Internet protocol	voice over internet protocol	voice over internet protocol	voice over internet protocol	Voice over Internet protocol	1	kept
Voice Quality	voice quality	voice quality	voice quality	Voice Quality	1	kept
Voice Recognition	voice recognition	voice recognition	voice recognition	Voice Recognition	1	kept
Voice reorientation	voice reorientation	voice reorientation	voice reorientation	Voice reorientation	1	kept
voice reorientation	voice reorientation	voice reorientation	voice reorientation	Voice reorientation	1	case_or_format
Voice restorative strategy	voice restorative strategy	voice restorative strategy	voice restorative strategy	Voice restorative strategy	1	kept
voice stimulus	voice stimulus	voice stimulus	voice stimulus	voice stimulus	1	kept
voice use	voice use	voice use	voice use	voice use	1	kept
voicelessness	voicelessness	voicelessness	voicelessness	voicelessness	1	kept
Voiding	voiding	voiding	voiding	Voiding	1	kept
Voiding dysfunction	voiding dysfunction	voiding dysfunction	voiding dysfunction	Voiding dysfunction	1	kept
voie veineuse centrale	voie veineuse centrale	voie veineuse centrale	voie veineuse centrale	voie veineuse centrale	1	kept

Volaemic resuscitation	volaemic resuscitation	volaemic resuscitation	volaemic resuscitation	Volaemic resuscitation	1	kept
volaemic resuscitation	volaemic resuscitation	volaemic resuscitation	volaemic resuscitation	Volaemic resuscitation	1	case_or_format
volatile anaesthetic gases	volatile anaesthetic gases	volatile anaesthetic gases	volatile anaesthetic gases	volatile anaesthetic gases	1	kept
Volatile anaesthetics	volatile anaesthetics	volatile anaesthetics	volatile anaesthetics	Volatile anaesthetics	1	kept
volatile anesthetics	volatile anesthetics	volatile anesthetics	volatile anesthetics	volatile anesthetics	1	kept
Volatile sulphur compounds	volatile sulphur compounds	volatile sulphur compounds	volatile sulphur compounds	Volatile sulphur compounds	1	kept
volatilization	volatilization	volatilization	volatilization	volatilization	1	kept
volontaire	volontaire	volontaire	volontaire	volontaire	1	kept
voltage clamp technique	voltage clamp technique	voltage clamp technique	voltage clamp technique	voltage clamp technique	1	kept
Voltage dividers	voltage dividers	voltage dividers	voltage dividers	Voltage dividers	1	kept
Voltage measurement	voltage measurement	voltage measurement	voltage measurement	Voltage measurement	1	kept
volume control	volume control	volume control	volume control	volume control	1	kept
volume control ventilation	volume control ventilation	volume control ventilation	volume control ventilation	volume control ventilation	1	kept
Volume corrente	volume corrente	volume corrente	volume corrente	Volume corrente	1	kept
Volume of postoperative bleeding	volume of postoperative bleeding	volume of postoperative bleeding	volume of postoperative bleeding	Volume of postoperative bleeding	1	kept
Volume resuscitation	volume resuscitation	volume resuscitation	volume resuscitation	Volume resuscitation	1	kept

volume targeted ventilation	volume targeted ventilation	volume targeted ventilation	volume targeted ventilation	volume targeted ventilation	1	kept
Volume to be infused	volume to be infused	volume to be infused	volume to be infused	Volume to be infused	1	kept
Volume-outcome	volume outcome	volume outcome	volume outcome	Volume-outcome	1	kept
Volumen sanguíneo	volumen sangu neo	volumen sangu neo	volumen sangu neo	Volumen sanguíneo	1	kept
Voluntades anticipadas	voluntades anticipadas	voluntades anticipadas	voluntades anticipadas	Voluntades anticipadas	1	kept
voluntarily reported medication error	voluntarily reported medication error	voluntarily reported medication error	voluntarily reported medication error	voluntarily reported medication error	1	kept
VOLUNTARY	voluntary	voluntary	voluntary	VOLUNTARY	1	kept
Voluntary	voluntary	voluntary	voluntary	VOLUNTARY	1	case_or_format
voluntary	voluntary	voluntary	voluntary	VOLUNTARY	1	case_or_format
voluntary program	voluntary program	voluntary program	voluntary program	voluntary program	1	kept
voluntary report	voluntary report	voluntary report	voluntary report	voluntary report	1	kept
Voluntary reporting	voluntary reporting	voluntary reporting	voluntary reporting	voluntary reporting	1	case_or_format
VOLUNTARY TURNOVER	voluntary turnover	voluntary turnover	voluntary turnover	VOLUNTARY TURNOVER	1	kept
Volunteer	volunteer	volunteer	volunteer	volunteer	1	case_or_format
Volunteering	volunteering	volunteering	volunteering	Volunteering	1	kept
volvulus	volvulus	volvulus	volvulus	volvulus	1	kept

vomend	vomend	vomend	vomend	vomend	1	kept
vomiting in infancy	vomiting in infancy	vomiting in infancy	vomiting in infancy	vomiting in infancy	1	kept
VON = Vermont Oxford Network	von vermont oxford network	von vermont oxford network	von vermont oxford network	VON = Vermont Oxford Network	1	kept
von Willebrand disease	von willebrand disease	von willebrand disease	von willebrand disease	von Willebrand disease	1	kept
von Willebrand factor cleaving proteinase	von willebrand factor cleaving proteinase	von willebrand factor cleaving proteinase	von willebrand factor cleaving proteinase	von Willebrand factor cleaving proteinase	1	kept
vortioxetine	vortioxetine	vortioxetine	vortioxetine	vortioxetine	1	kept
voting	voting	voting	voting	voting	1	kept
VRE outbreak	vre outbreak	vre outbreak	vre outbreak	VRE outbreak	1	kept
VRI	vri	vri	vri	VRI	1	kept
VTE prophylaxis	vte prophylaxis	vte prophylaxis	vte prophylaxis	VTE prophylaxis	1	kept
VUCA	vuca	vuca	vuca	VUCA	1	kept
Vulnerability	vulnerability	vulnerability	vulnerability	vulnerability	1	case_or_format
Vulnerability mapping	vulnerability mapping	vulnerability mapping	vulnerability mapping	Vulnerability mapping	1	kept
vulnerability mapping	vulnerability mapping	vulnerability mapping	vulnerability mapping	Vulnerability mapping	1	case_or_format
vulnerable	vulnerable	vulnerable	vulnerable	vulnerable	1	kept
vulnerable baby perception	vulnerable baby perception	vulnerable baby perception	vulnerable baby perception	vulnerable baby perception	1	kept

Vulnerable communicators	vulnerable communicators	vulnerable communicators	vulnerable communicators	Vulnerable communicators	1	kept
vulnerable communicators	vulnerable communicators	vulnerable communicators	vulnerable communicators	Vulnerable communicators	1	case_or_format
Vulnerable families	vulnerable families	vulnerable families	vulnerable families	Vulnerable families	1	kept
VULNERABLE INFANTS	vulnerable infants	vulnerable infants	vulnerable infants	VULNERABLE INFANTS	1	kept
Vulnerable infants	vulnerable infants	vulnerable infants	vulnerable infants	VULNERABLE INFANTS	1	case_or_format
vulnerable parents	vulnerable parents	vulnerable parents	vulnerable parents	vulnerable parents	1	kept
Vulnerable participants	vulnerable participants	vulnerable participants	vulnerable participants	Vulnerable participants	1	kept
vulnerable popuations	vulnerable popuations	vulnerable popuations	vulnerable popuations	vulnerable popuations	1	kept
Vulnerable population	vulnerable population	vulnerable population	vulnerable population	vulnerable population	1	case_or_format
vulnerable preterm mothers	vulnerable preterm mothers	vulnerable preterm mothers	vulnerable preterm mothers	vulnerable preterm mothers	1	kept
vulva cancer	vulva cancer	vulva cancer	vulva cancer	vulva cancer	1	kept
VV-ECMO	vv ecmo	vv ecmo	vv ecmo	VV-ECMO	1	kept
vécu soignant	v cu soignant	v cu soignant	v cu soignant	vécu soignant	1	kept
Vía venosa difícil	v a venosa dif cil	v a venosa dif cil	v a venosa dif cil	Vía venosa difícil	1	kept
wada test	wada test	wada test	wada test	wada test	1	kept
waiting	waiting	waiting	waiting	Waiting	1	case_or_format

Waiting line	waiting line	waiting line	waiting line	Waiting line	1	kept
Waiting line model	waiting line model	waiting line model	waiting line model	Waiting line model	1	kept
waiting list management	waiting list management	waiting list management	waiting list management	waiting list management	1	kept
WAITING TIME	waiting time	waiting time	waiting time	WAITING TIME	1	kept
Waiting time	waiting time	waiting time	waiting time	WAITING TIME	1	case_or_format
waiting time	waiting time	waiting time	waiting time	WAITING TIME	1	case_or_format
wake	wake	wake	wake	Wake	1	case_or_format
wake after sleep onset	wake after sleep onset	wake after sleep onset	wake after sleep onset	wake after sleep onset	1	kept
Wake up	wake up	wake up	wake up	Wake up	1	kept
WAKEFULNESS	wakefulness	wakefulness	wakefulness	wakefulness	1	case_or_format
wakefulness promoting agent	wakefulness promoting agent	wakefulness promoting agent	wakefulness promoting agent	wakefulness promoting agent	1	kept
Wakefulness-Promoting Agents	wakefulness promoting agents	wakefulness promoting agents	wakefulness promoting agent	wakefulness promoting agent	1	singular
Wakes	wakes	wakes	wake	Wake	1	singular
WAKING	waking	waking	waking	WAKING	1	kept
WALES	wales	wales	wales	Wales	1	case_or_format
Walk Test	walk test	walk test	walk test	Walk Test	1	kept

walk test	walk test	walk test	walk test	Walk Test	1	case_or_format
Walker	walker	walker	walker	Walker	1	kept
Walker and Avant′ s concept analysis Approach	walker and avant s concept analysis approach	walker and avant s concept analysis approach	walker and avant s concept analysis approach	Walker and Avant′ s concept analysis Approach	1	kept
Walker Warburg syndrome	walker warburg syndrome	walker warburg syndrome	walker warburg syndrome	Walker Warburg syndrome	1	kept
walking dependence	walking dependence	walking dependence	walking dependence	walking dependence	1	kept
walking harness	walking harness	walking harness	walking harness	walking harness	1	kept
walking parameters	walking parameters	walking parameters	walking parameters	walking parameters	1	kept
Wall motion analysis	wall motion analysis	wall motion analysis	wall motion analysis	Wall motion analysis	1	kept
Wall murals	wall murals	wall murals	wall murals	Wall murals	1	kept
walnut	walnut	walnut	walnut	walnut	1	kept
wandering behavior	wandering behavior	wandering behavior	wandering behavior	wandering behavior	1	kept
wandering detection	wandering detection	wandering detection	wandering detection	wandering detection	1	kept
wandering evaluation	wandering evaluation	wandering evaluation	wandering evaluation	wandering evaluation	1	kept
Wang Baker pain scale	wang baker pain scale	wang baker pain scale	wang baker pain scale	Wang Baker pain scale	1	kept
WANT	want	want	want	WANT	1	kept
WAPI	wapi	wapi	wapi	WAPI	1	kept

War Exposure	war exposure	war exposure	war exposure	War Exposure	1	kept
war exposure	war exposure	war exposure	war exposure	War Exposure	1	case_or_format
War injuries	war injuries	war injuries	war injuries	War injuries	1	kept
War-affected medical care	war affected medical care	war affected medical care	war affected medical care	War-affected medical care	1	kept
Ward assistants	ward assistants	ward assistants	ward assistants	Ward assistants	1	kept
WARD ATMOSPHERE	ward atmosphere	ward atmosphere	ward atmosphere	WARD ATMOSPHERE	1	kept
ward design	ward design	ward design	ward design	ward design	1	kept
Ward environment	ward environment	ward environment	ward environment	ward environment	1	case_or_format
Ward expansion	ward expansion	ward expansion	ward expansion	Ward expansion	1	kept
Ward management	ward management	ward management	ward management	Ward management	1	kept
ward management	ward management	ward management	ward management	Ward management	1	case_or_format
Ward managers	ward managers	ward managers	ward managers	Ward managers	1	kept
ward occupancy	ward occupancy	ward occupancy	ward occupancy	ward occupancy	1	kept
Ward organisation	ward organisation	ward organisation	ward organisation	Ward organisation	1	kept
ward organisation	ward organisation	ward organisation	ward organisation	Ward organisation	1	case_or_format
Ward round	ward round	ward round	ward round	Ward rounds	1	case_or_format

Ward staff	ward staff	ward staff	ward staff	Ward staff	1	kept
Ward transfer	ward transfer	ward transfer	ward transfer	Ward transfer	1	kept
Ward-outreach programs	ward outreach programs	ward outreach programs	ward outreach programs	Ward-outreach programs	1	kept
WARFARIN THERAPY	warfarin therapy	warfarin therapy	warfarin therapy	WARFARIN THERAPY	1	kept
Warfarin therapy	warfarin therapy	warfarin therapy	warfarin therapy	WARFARIN THERAPY	1	case_or_format
Warm Ischemia	warm ischemia	warm ischemia	warm ischemia	Warm Ischemia	1	kept
Warming	warming	warming	warming	warming	1	case_or_format
Warning	warning	warning	warning	Warning	1	kept
warning	warning	warning	warning	Warning	1	case_or_format
Warning messages	warning messages	warning messages	warning messages	Warning messages	1	kept
Warning signs	warning signs	warning signs	warning signs	Warning signs	1	kept
wars	wars	wars	war	war	1	singular
Wart virus vaccine	wart virus vaccine	wart virus vaccine	wart virus vaccine	Wart virus vaccine	1	kept
WASHINGTON-STATE	washington state	washington state	washington state	WASHINGTON-STATE	1	kept
WASTE	waste	waste	waste	waste	1	case_or_format
waste audit	waste audit	waste audit	waste audit	waste audit	1	kept

waste disposal facility	waste disposal facility	waste disposal facility	waste disposal facility	waste disposal facility	1	kept
Waste Disposal, Fluid	waste disposal fluid	waste disposal fluid	waste disposal fluid	Waste Disposal, Fluid	1	kept
waste free sampling method	waste free sampling method	waste free sampling method	waste free sampling method	waste free sampling method	1	kept
waste minimization	waste minimization	waste minimization	waste minimization	waste minimization	1	kept
Waste reduction	waste reduction	waste reduction	waste reduction	Waste reduction	1	kept
waste sorting	waste sorting	waste sorting	waste sorting	waste sorting	1	kept
Wastewater	wastewater	wastewater	wastewater	Wastewater	1	kept
WAT-1	wat 1	wat 1	wat 1	WAT-1	1	kept
WAT-1, Withdrawal Assessment Tool-1	wat 1 withdrawal assessment tool 1	wat 1 withdrawal assessment tool 1	wat 1 withdrawal assessment tool 1	WAT-1, Withdrawal Assessment Tool-1	1	kept
watchful waiting	watchful waiting	watchful waiting	watchful waiting	Watchful Waiting	1	case_or_format
water absorption	water absorption	water absorption	water absorption	water absorption	1	kept
Water and Sanitation Hygiene (WASH	water and sanitation hygiene wash	water and sanitation hygiene wash	water and sanitation hygiene wash	Water and Sanitation Hygiene (WASH	1	kept
water availability	water availability	water availability	water availability	water availability	1	kept
Water balance	water balance	water balance	water balance	Water balance	1	kept
Water birth	water birth	water birth	water birth	Water birth	1	kept
water birth	water birth	water birth	water birth	Water birth	1	case_or_format

water borne disease	water borne disease	water borne disease	water borne disease	water borne disease	1	kept
Water consumption	water consumption	water consumption	water consumption	Water consumption	1	kept
water deficit	water deficit	water deficit	water deficit	water deficit	1	kept
water deprivation	water deprivation	water deprivation	water deprivation	water deprivation	1	kept
water depth	water depth	water depth	water depth	water depth	1	kept
Water filtration	water filtration	water filtration	water filtration	Water filtration	1	kept
water management	water management	water management	water management	water management	1	kept
Water outage	water outage	water outage	water outage	Water outage	1	kept
water outage	water outage	water outage	water outage	Water outage	1	case_or_format
water pipe smoking	water pipe smoking	water pipe smoking	water pipe smoking	water pipe smoking	1	kept
Water Pollution	water pollution	water pollution	water pollution	Water Pollution	1	kept
water pollution	water pollution	water pollution	water pollution	Water Pollution	1	case_or_format
Water protocol	water protocol	water protocol	water protocol	Water protocol	1	kept
Water purification systems	water purification systems	water purification systems	water purification systems	Water purification systems	1	kept
water quality	water quality	water quality	water quality	water quality	1	kept
water retention	water retention	water retention	water retention	water retention	1	kept

water sampling	water sampling	water sampling	water sampling	water sampling	1	kept
water storage	water storage	water storage	water storage	water storage	1	kept
Water Supply	water supply	water supply	water supply	water supply	1	case_or_format
Water supply	water supply	water supply	water supply	water supply	1	case_or_format
Water tanks	water tanks	water tanks	water tanks	Water tanks	1	kept
water temperature	water temperature	water temperature	water temperature	Water temperature	1	case_or_format
water treatment	water treatment	water treatment	water treatment	water treatment	1	kept
Water treatment plants	water treatment plants	water treatment plants	water treatment plants	Water treatment plants	1	kept
WATER TURNOVER	water turnover	water turnover	water turnover	WATER TURNOVER	1	kept
water use	water use	water use	water use	water use	1	kept
water-electrolyte imbalance	water electrolyte imbalance	water electrolyte imbalance	water electrolyte imbalance	Water-Electrolyte Imbalance	1	case_or_format
WATER-LOSS	water loss	water loss	water loss	water loss	1	case_or_format
Waterbirth	waterbirth	waterbirth	waterbirth	waterbirth	1	case_or_format
Waterborne pathogens	waterborne pathogens	waterborne pathogens	waterborne pathogens	Waterborne pathogens	1	kept
Waterless	waterless	waterless	waterless	Waterless	1	kept
Waterless hand hygiene	waterless hand hygiene	waterless hand hygiene	waterless hand hygiene	Waterless hand hygiene	1	kept

Waterlow	waterlow	waterlow	waterlow	Waterlow	1	kept
Watson’ s caring science	watson s caring science	watson s caring science	watson s caring science	Watson’ s caring science	1	kept
Wave forms	wave forms	wave forms	wave forms	Wave forms	1	kept
Waveform data	waveform data	waveform data	waveform data	Waveform data	1	kept
waveform interpretation	waveform interpretation	waveform interpretation	waveform interpretation	waveform interpretation	1	kept
Waveform quality	waveform quality	waveform quality	waveform quality	Waveform quality	1	kept
waveforms	waveforms	waveforms	waveform	waveform	1	singular
Waverlet band	waverlet band	waverlet band	waverlet band	Waverlet band	1	kept
Way forward	way forward	way forward	way forward	Way forward	1	kept
Wayfinding	wayfinding	wayfinding	wayfinding	Wayfinding	1	kept
ways of coping	ways of coping	ways of coping	ways of coping	ways of coping	1	kept
Ways of Coping Questionnaire	ways of coping questionnaire	ways of coping questionnaire	ways of coping questionnaire	Ways of Coping Questionnaire	1	kept
Ways of knowing	ways of knowing	ways of knowing	ways of knowing	Ways of knowing	1	kept
weakened apical myocardial motion	weakened apical myocardial motion	weakened apical myocardial motion	weakened apical myocardial motion	weakened apical myocardial motion	1	kept
Weaning Failure	weaning failure	weaning failure	weaning failure	Weaning failure	1	case_or_format
Weaning outcomes	weaning outcomes	weaning outcomes	weaning outcomes	Weaning outcomes	1	kept

weaning outcomes	weaning outcomes	weaning outcomes	weaning outcomes	Weaning outcomes	1	case_or_format
weaning process	weaning process	weaning process	weaning process	weaning process	1	kept
weaning process from mechanical ventilation	weaning process from mechanical ventilation	weaning process from mechanical ventilation	weaning process from mechanical ventilation	weaning process from mechanical ventilation	1	kept
Weaning Protocol	weaning protocol	weaning protocol	weaning protocol	Weaning protocol	1	case_or_format
Weaning protocols	weaning protocols	weaning protocols	weaning protocol	Weaning protocol	1	singular
Weaning readiness	weaning readiness	weaning readiness	weaning readiness	Weaning readiness	1	kept
Weaning scale	weaning scale	weaning scale	weaning scale	Weaning scale	1	kept
weaning success	weaning success	weaning success	weaning success	weaning success	1	kept
weaning success rate	weaning success rate	weaning success rate	weaning success rate	weaning success rate	1	kept
Weaning team	weaning team	weaning team	weaning team	Weaning team	1	kept
weaning trials	weaning trials	weaning trials	weaning trials	weaning trials	1	kept
weaning unit	weaning unit	weaning unit	weaning unit	weaning unit	1	kept
Weaning ventilation	weaning ventilation	weaning ventilation	weaning ventilation	Weaning ventilation	1	kept
weapon	weapon	weapon	weapon	weapon	1	kept
wearable biosensor	wearable biosensor	wearable biosensor	wearable biosensor	wearable biosensor	1	kept
Wearable biosensors	wearable biosensors	wearable biosensors	wearable biosensor	wearable biosensor	1	singular

Wearable computing	wearable computing	wearable computing	wearable computing	Wearable computing	1	kept
wearable design	wearable design	wearable design	wearable design	wearable design	1	kept
Wearable Device	wearable device	wearable device	wearable device	Wearable devices	1	case_or_format
Wearable device	wearable device	wearable device	wearable device	Wearable devices	1	case_or_format
wearable device	wearable device	wearable device	wearable device	Wearable devices	1	case_or_format
wearable electronic device	wearable electronic device	wearable electronic device	wearable electronic device	Wearable Electronic Devices	1	case_or_format
Wearable electronic devices	wearable electronic devices	wearable electronic devices	wearable electronic device	Wearable Electronic Devices	1	singular
Wearable health	wearable health	wearable health	wearable health	Wearable health	1	kept
wearable monitors	wearable monitors	wearable monitors	wearable monitors	wearable monitors	1	kept
Wearable sensor	wearable sensor	wearable sensor	wearable sensor	Wearable sensors	1	case_or_format
wearable sleep monitoring wristband	wearable sleep monitoring wristband	wearable sleep monitoring wristband	wearable sleep monitoring wristband	wearable sleep monitoring wristband	1	kept
Wearer experience	wearer experience	wearer experience	wearer experience	Wearer experience	1	kept
wearer experience	wearer experience	wearer experience	wearer experience	Wearer experience	1	case_or_format
Wearing	wearing	wearing	wearing	Wearing	1	kept
Weather	weather	weather	weather	weather	1	case_or_format
Web 2.0	web 2 0	web 2 0	web 2 0	Web 2.0	1	kept

Web App	web app	web app	web app	Web App	1	kept
web application	web application	web application	web application	web application	1	kept
Web based education	web based education	web based education	web based education	Web based education	1	kept
web camera	web camera	web camera	web camera	Web camera	1	case_or_format
web conferencing	web conferencing	web conferencing	web conferencing	web conferencing	1	kept
web-based communication	web based communication	web based communication	web based communication	web-based communication	1	kept
Web-based education	web based education	web based education	web based education	Web based education	1	case_or_format
Web-based instructional module	web based instructional module	web based instructional module	web based instructional module	Web-based instructional module	1	kept
Web-based simulation	web based simulation	web based simulation	web based simulation	Web-based simulation	1	kept
web-based simulation	web based simulation	web based simulation	web based simulation	Web-based simulation	1	case_or_format
Web-based surveys	web based surveys	web based surveys	web based survey	web-based survey	1	singular
Webcam technology	webcam technology	webcam technology	webcam technology	Webcam technology	1	kept
webcast	webcast	webcast	webcast	webcast	1	kept
Webpage	webpage	webpage	webpage	Webpage	1	kept
Wechsler Intelligence Scale	wechsler intelligence scale	wechsler intelligence scale	wechsler intelligence scale	Wechsler Intelligence Scale	1	kept
Wechsler Memory Scale	wechsler memory scale	wechsler memory scale	wechsler memory scale	Wechsler Memory Scale	1	kept

Wechsler Scales	wechsler scales	wechsler scales	wechsler scales	Wechsler Scales	1	kept
wedge resection	wedge resection	wedge resection	wedge resection	wedge resection	1	kept
Wee Care	wee care	wee care	wee care	Wee Care	1	kept
Wee-Sight Transilluminator	wee sight transilluminator	wee sight transilluminator	wee sight transilluminator	Wee-Sight Transilluminator	1	kept
weekends	weekends	weekends	weekends	weekends	1	kept
weekly meetings	weekly meetings	weekly meetings	weekly meetings	weekly meetings	1	kept
Weighing scale	weighing scale	weighing scale	weighing scale	Weighing scale	1	kept
weight based dosing	weight based dosing	weight based dosing	weight based dosing	weight based dosing	1	kept
WEIGHT BIAS	weight bias	weight bias	weight bias	weight bias	1	case_or_format
Weight bias	weight bias	weight bias	weight bias	weight bias	1	case_or_format
weight change	weight change	weight change	weight change	weight change	1	kept
WEIGHT CHANGES	weight changes	weight changes	weight change	weight change	1	singular
WEIGHT-GAIN	weight gain	weight gain	weight gain	Weight Gain	1	case_or_format
weighted blanket	weighted blanket	weighted blanket	weighted blanket	weighted blanket	1	kept
Weighted rankings	weighted rankings	weighted rankings	weighted rankings	Weighted rankings	1	kept
Weights	weights	weights	weight	Weight	1	singular

Weights and measures	weights and measures	weights and measures	weights and measures	Weights and Measures	1	case_or_format
Welcoming	welcoming	welcoming	welcoming	Welcoming	1	kept
welfare provision	welfare provision	welfare provision	welfare provision	welfare provision	1	kept
well known scoring system	well known scoring system	well known scoring system	well known scoring system	well known scoring system	1	kept
well newborn care	well newborn care	well newborn care	well newborn care	well newborn care	1	kept
Well-being at works	well being at works	well being at works	well being at works	Well-being at works	1	kept
Well-being of health workers	well being of health workers	well being of health workers	well being of health workers	Well-being of health workers	1	kept
Well-dying law	well dying law	well dying law	well dying law	Well-dying law	1	kept
wellbeing image	wellbeing image	wellbeing image	wellbeing image	wellbeing image	1	kept
Wellbeing interventions	wellbeing interventions	wellbeing interventions	wellbeing interventions	Wellbeing interventions	1	kept
Wellbeing resources	wellbeing resources	wellbeing resources	wellbeing resources	Wellbeing resources	1	kept
WellChild	wellchild	wellchild	wellchild	WellChild	1	kept
Wellness	wellness	wellness	wellness	wellness	1	case_or_format
wellness leaders	wellness leaders	wellness leaders	wellness leaders	wellness leaders	1	kept
Wellness program	wellness program	wellness program	wellness program	Wellness program	1	kept
Wernicke Encephalopathy	wernicke encephalopathy	wernicke encephalopathy	wernicke encephalopathy	Wernicke encephalopathy	1	case_or_format

West Africa	west africa	west africa	west africa	West Africa	1	kept
West Bengal	west bengal	west bengal	west bengal	West Bengal	1	kept
West Indies	west indies	west indies	west indies	West Indies	1	kept
WEST-NILE-VIRUS	west nile virus	west nile virus	west nile virus	West Nile virus	1	case_or_format
Western blotting	western blotting	western blotting	western blotting	Western blotting	1	kept
Western countries	western countries	western countries	western countries	Western countries	1	kept
Western Europe	western europe	western europe	western europe	Western Europe	1	kept
western medicine	western medicine	western medicine	western medicine	western medicine	1	kept
Western Neuro Sensory stimulation profile	western neuro sensory stimulation profile	western neuro sensory stimulation profile	western neuro sensory stimulation profile	Western Neuro Sensory stimulation profile	1	kept
Western World's largest renal tumour	western world s largest renal tumour	western world s largest renal tumour	western world s largest renal tumour	Western World's largest renal tumour	1	kept
Western World' s largest renal tumour	western world s largest renal tumour	western world s largest renal tumour	western world s largest renal tumour	Western World's largest renal tumour	1	case_or_format
Wet work	wet work	wet work	wet work	wet work	1	case_or_format
WFNS	wfns	wfns	wfns	WFNS	1	kept
What Being The Parent of a Baby is Like Revise Questionnaire	what being the parent of a baby is like revise questionnaire	what being the parent of a baby is like revise questionnaire	what being the parent of a baby is like revise questionnaire	What Being The Parent of a Baby is Like Revise Questionnaire	1	kept
Wheelchair	wheelchair	wheelchair	wheelchair	Wheelchairs	1	case_or_format
wheelchair	wheelchair	wheelchair	wheelchair	Wheelchairs	1	case_or_format

wheezing outcomes	wheezing outcomes	wheezing outcomes	wheezing outcomes	wheezing outcomes	1	kept
Whipple	whipple	whipple	whipple	Whipple	1	kept
Whitacre needle	whitacre needle	whitacre needle	whitacre needle	Whitacre needle	1	kept
WHITE	white	white	white	White	1	case_or_format
white coat hypertension	white coat hypertension	white coat hypertension	white coat hypertension	white coat hypertension	1	kept
WHITE COATS	white coats	white coats	white coats	WHITE COATS	1	kept
white fast-track score	white fast track score	white fast track score	white fast track score	white fast-track score	1	kept
White Matter	white matter	white matter	white matter	white matter	1	case_or_format
white matter disease	white matter disease	white matter disease	white matter disease	white matter disease	1	kept
White matter injury	white matter injury	white matter injury	white matter injury	White matter injury	1	kept
WHITE MUSCLE DISEASE	white muscle disease	white muscle disease	white muscle disease	white muscle disease	1	case_or_format
White normativity	white normativity	white normativity	white normativity	White normativity	1	kept
White-Fast Track Score	white fast track score	white fast track score	white fast track score	white fast-track score	1	case_or_format
WHITE-MATTER	white matter	white matter	white matter	white matter	1	case_or_format
WHITE-NOISE	white noise	white noise	white noise	white noise	1	case_or_format
WHO	who	who	who	WHO	1	kept

WHO Five Moments	who five moments	who five moments	who five moments	WHO Five Moments	1	kept
WHO guidelines for COVID-19 related SARI	who guidelines for covid 19 related sari	who guidelines for covid 19 related sari	who guidelines for covid 19 related sari	WHO guidelines for COVID-19 related SARI	1	kept
WHO model	who model	who model	who model	WHO model	1	kept
WHO multimodal strategy	who multimodal strategy	who multimodal strategy	who multimodal strategy	WHO multimodal strategy	1	kept
WHO strategy	who strategy	who strategy	who strategy	WHO strategy	1	kept
WHO's Clean Your Hands	who s clean your hands	who s clean your hands	who s clean your hands	WHO's Clean Your Hands	1	kept
Whole blood	whole blood	whole blood	whole blood	Whole blood	1	kept
Whole Blood Coagulation Time	whole blood coagulation time	whole blood coagulation time	whole blood coagulation time	Whole Blood Coagulation Time	1	kept
whole body PET	whole body pet	whole body pet	whole body pet	whole body PET	1	kept
whole lung lavage	whole lung lavage	whole lung lavage	whole lung lavage	whole lung lavage	1	kept
WHOLE-BODY DISINFECTION	whole body disinfection	whole body disinfection	whole body disinfection	WHOLE-BODY DISINFECTION	1	kept
Whole-body hyperthermia	whole body hyperthermia	whole body hyperthermia	whole body hyperthermia	Whole-body hyperthermia	1	kept
WHOLE-BODY HYPOTHERMIA	whole body hypothermia	whole body hypothermia	whole body hypothermia	WHOLE-BODY HYPOTHERMIA	1	kept
Wholeness	wholeness	wholeness	wholeness	Wholeness	1	kept
Whooping cough	whooping cough	whooping cough	whooping cough	Whooping Cough	1	case_or_format
WHOQOL-100	whoqol 100	whoqol 100	whoqol 100	WHOQOL-100	1	kept

WHOQOL-BREF, World Health Organization Quality of Life –	whoqol bref world health organization quality of life brief	whoqol bref world health organization quality of life brief	whoqol bref world health organization quality of life brief	WHOQOL-BREF, World Health Organization Quality of Life –	1	kept
Wi-Fi	wi fi	wi fi	wi fi	Wi-Fi	1	kept
Wi-Fi module	wi fi module	wi fi module	wi fi module	Wi-Fi module	1	kept
wide field digital retinal photography	wide field digital retinal photography	wide field digital retinal photography	wide field digital retinal photography	wide field digital retinal photography	1	kept
Wide spreads	wide spreads	wide spreads	wide spreads	Wide spreads	1	kept
Wide-field digital imaging	wide field digital imaging	wide field digital imaging	wide field digital imaging	Wide-field digital imaging	1	kept
widespread community transmission	widespread community transmission	widespread community transmission	widespread community transmission	widespread community transmission	1	kept
wide‐field digital imaging	wide field digital imaging	wide field digital imaging	wide field digital imaging	Wide-field digital imaging	1	case_or_format
Widow	widow	widow	widow	widow	1	case_or_format
Wilcoxon rank sum test	wilcoxon rank sum test	wilcoxon rank sum test	wilcoxon rank sum test	Wilcoxon rank sum test	1	kept
Wilcoxon signed rank test	wilcoxon signed rank test	wilcoxon signed rank test	wilcoxon signed rank test	Wilcoxon signed rank test	1	kept
wild type	wild type	wild type	wild type	wild type	1	kept
wilderness	wilderness	wilderness	wilderness	wilderness	1	kept
wildfire	wildfire	wildfire	wildfire	wildfire	1	kept
Wildfires	wildfires	wildfires	wildfire	wildfire	1	singular
wildfires	wildfires	wildfires	wildfire	wildfire	1	singular

Willing and unwilling	willing and unwilling	willing and unwilling	willing and unwilling	Willing and unwilling	1	kept
WILLINGNESS	willingness	willingness	willingness	willingness	1	case_or_format
Willingness	willingness	willingness	willingness	willingness	1	case_or_format
Willingness to care	willingness to care	willingness to care	willingness to care	Willingness to care	1	kept
Willingness to collaborate	willingness to collaborate	willingness to collaborate	willingness to collaborate	Willingness to collaborate	1	kept
willingness to learn	willingness to learn	willingness to learn	willingness to learn	willingness to learn	1	kept
willingness to pay	willingness to pay	willingness to pay	willingness to pay	Willingness To Pay	1	case_or_format
Wilson disease	wilson disease	wilson disease	wilson disease	Wilson disease	1	kept
wind speed	wind speed	wind speed	wind speed	wind speed	1	kept
windlass test	windlass test	windlass test	windlass test	windlass test	1	kept
Window openings	window openings	window openings	window openings	Window openings	1	kept
wiping bath	wiping bath	wiping bath	wiping bath	wiping bath	1	kept
wire-in-needle technique	wire in needle technique	wire in needle technique	wire in needle technique	wire-in-needle technique	1	kept
Wireless	wireless	wireless	wireless	Wireless	1	kept
Wireless barcode technology	wireless barcode technology	wireless barcode technology	wireless barcode technology	Wireless barcode technology	1	kept
Wireless Bio Multimedia sensor Network	wireless bio multimedia sensor network	wireless bio multimedia sensor network	wireless bio multimedia sensor network	Wireless Bio Multimedia sensor Network	1	kept

Wireless bio multimedia sensor network	wireless bio multimedia sensor network	wireless bio multimedia sensor network	wireless bio multimedia sensor network	Wireless Bio Multimedia sensor Network	1	case_or_format
Wireless Body Area Network	wireless body area network	wireless body area network	wireless body area network	Wireless body area network	1	case_or_format
Wireless module	wireless module	wireless module	wireless module	Wireless module	1	kept
Wireless monitoring system	wireless monitoring system	wireless monitoring system	wireless monitoring system	Wireless monitoring system	1	kept
Wireless multimedia	wireless multimedia	wireless multimedia	wireless multimedia	Wireless multimedia	1	kept
Wireless Network	wireless network	wireless network	wireless network	Wireless Network	1	kept
Wireless network	wireless network	wireless network	wireless network	Wireless Network	1	case_or_format
wireless network	wireless network	wireless network	wireless network	Wireless Network	1	case_or_format
Wireless networks	wireless networks	wireless networks	wireless network	Wireless Network	1	singular
Wireless notification	wireless notification	wireless notification	wireless notification	Wireless notification	1	kept
wireless notification	wireless notification	wireless notification	wireless notification	Wireless notification	1	case_or_format
Wireless sensor network	wireless sensor network	wireless sensor network	wireless sensor network	Wireless sensor networks	1	case_or_format
Wireless Sensor Networks	wireless sensor networks	wireless sensor networks	wireless sensor network	Wireless sensor networks	1	singular
Wireless standards	wireless standards	wireless standards	wireless standards	Wireless standards	1	kept
Wireless system	wireless system	wireless system	wireless system	Wireless system	1	kept
wireless system	wireless system	wireless system	wireless system	Wireless system	1	case_or_format

Wireless systems	wireless systems	wireless systems	wireless system	Wireless system	1	singular
wireless technology	wireless technology	wireless technology	wireless technology	Wireless Technology	1	case_or_format
Wireless telecare system	wireless telecare system	wireless telecare system	wireless telecare system	Wireless telecare system	1	kept
Wireless transmission	wireless transmission	wireless transmission	wireless transmission	Wireless transmission	1	kept
Wireless transmissions	wireless transmissions	wireless transmissions	wireless transmission	Wireless transmission	1	singular
Wisconsin Card Sorting Test	wisconsin card sorting test	wisconsin card sorting test	wisconsin card sorting test	Wisconsin Card Sorting Test	1	kept
WISHES	wishes	wishes	wishes	WISHES	1	kept
Wissenstransfer	wissenstransfer	wissenstransfer	wissenstransfer	Wissenstransfer	1	kept
withdrawal assessment	withdrawal assessment	withdrawal assessment	withdrawal assessment	withdrawal assessment	1	kept
withdrawal assessment tool version 1 questionnaire	withdrawal assessment tool version 1 questionnaire	withdrawal assessment tool version 1 questionnaire	withdrawal assessment tool version 1 questionnaire	withdrawal assessment tool version 1 questionnaire	1	kept
Withdrawal assessment tool version-1	withdrawal assessment tool version 1	withdrawal assessment tool version 1	withdrawal assessment tool version 1	Withdrawal assessment tool version-1	1	kept
Withdrawal mechanical ventilation	withdrawal mechanical ventilation	withdrawal mechanical ventilation	withdrawal mechanical ventilation	Withdrawal mechanical ventilation	1	kept
withdrawal of advanced life-sustaining therapies	withdrawal of advanced life sustaining therapies	withdrawal of advanced life sustaining therapies	withdrawal of advanced life sustaining therapies	withdrawal of advanced life-sustaining therapies	1	kept
Withdrawal of aggressive treatment	withdrawal of aggressive treatment	withdrawal of aggressive treatment	withdrawal of aggressive treatment	Withdrawal of aggressive treatment	1	kept
Withdrawal of care	withdrawal of care	withdrawal of care	withdrawal of care	Withdrawal of care	1	kept
Withdrawal of life support	withdrawal of life support	withdrawal of life support	withdrawal of life support	Withdrawal of life support	1	kept

Withdrawal of life sustaining therapies	withdrawal of life sustaining therapies	withdrawal of life sustaining therapies	withdrawal of life sustaining therapy	Withdrawal of life-sustaining therapy	1	singular
withdrawal of life sustaining therapies	withdrawal of life sustaining therapies	withdrawal of life sustaining therapies	withdrawal of life sustaining therapy	Withdrawal of life-sustaining therapy	1	singular
Withdrawal of life sustaining treatment	withdrawal of life sustaining treatment	withdrawal of life sustaining treatment	withdrawal of life sustaining treatment	withdrawal of life-sustaining treatment	1	case_or_format
withdrawal of life-support	withdrawal of life support	withdrawal of life support	withdrawal of life support	Withdrawal of life support	1	case_or_format
withdrawal of life-sustaining medical treatments	withdrawal of life sustaining medical treatments	withdrawal of life sustaining medical treatments	withdrawal of life sustaining medical treatments	withdrawal of life-sustaining medical treatments	1	kept
Withdrawal of life-sustaining therapies	withdrawal of life sustaining therapies	withdrawal of life sustaining therapies	withdrawal of life sustaining therapy	Withdrawal of life-sustaining therapy	1	singular
withdrawal of life-sustaining treatments	withdrawal of life sustaining treatments	withdrawal of life sustaining treatments	withdrawal of life sustaining treatment	withdrawal of life-sustaining treatment	1	singular
Withdrawal of mechanical ventilation	withdrawal of mechanical ventilation	withdrawal of mechanical ventilation	withdrawal of mechanical ventilation	Withdrawal of mechanical ventilation	1	kept
withdrawal of mechanical ventilation	withdrawal of mechanical ventilation	withdrawal of mechanical ventilation	withdrawal of mechanical ventilation	Withdrawal of mechanical ventilation	1	case_or_format
withdrawal seizure	withdrawal seizure	withdrawal seizure	withdrawal seizure	withdrawal seizure	1	kept
WITHDRAWAL SYMPTOMS	withdrawal symptoms	withdrawal symptoms	withdrawal symptoms	Withdrawal symptoms	1	case_or_format
WITHDRAWAL SYMPTOMS SCALE	withdrawal symptoms scale	withdrawal symptoms scale	withdrawal symptoms scale	WITHDRAWAL SYMPTOMS SCALE	1	kept
WITHDRAWAL SYNDROME	withdrawal syndrome	withdrawal syndrome	withdrawal syndrome	withdrawal syndrome	1	case_or_format
Withdrawal treatment	withdrawal treatment	withdrawal treatment	withdrawal treatment	Withdrawal treatment	1	kept
withdrawal treatment	withdrawal treatment	withdrawal treatment	withdrawal treatment	Withdrawal treatment	1	case_or_format
Withdrawal Trend Profile	withdrawal trend profile	withdrawal trend profile	withdrawal trend profile	Withdrawal Trend Profile	1	kept

withdrawing	withdrawing	withdrawing	withdrawing	Withdrawing	1	case_or_format
Withdrawing and withholding life-sustaining treatment	withdrawing and withholding life sustaining treatment	withdrawing and withholding life sustaining treatment	withdrawing and withholding life sustaining treatment	Withdrawing and withholding life-sustaining treatment	1	kept
withdrawing life-prolonging treatment	withdrawing life prolonging treatment	withdrawing life prolonging treatment	withdrawing life prolonging treatment	withdrawing life-prolonging treatment	1	kept
Withdrawing of life support	withdrawing of life support	withdrawing of life support	withdrawing of life support	Withdrawing of life support	1	kept
withdrawing treatment	withdrawing treatment	withdrawing treatment	withdrawing treatment	Withdrawing treatment	1	case_or_format
WITHHOLD	withhold	withhold	withhold	WITHHOLD	1	kept
Withhold	withhold	withhold	withhold	WITHHOLD	1	case_or_format
Withholding and withdrawal of life-sustaining therapy	withholding and withdrawal of life sustaining therapy	withholding and withdrawal of life sustaining therapy	withholding and withdrawal of life sustaining therapy	Withholding and withdrawal of life-sustaining therapy	1	kept
Withholding and withdrawing artificial respiration	withholding and withdrawing artificial respiration	withholding and withdrawing artificial respiration	withholding and withdrawing artificial respiration	Withholding and withdrawing artificial respiration	1	kept
withholding and withdrawing life support	withholding and withdrawing life support	withholding and withdrawing life support	withholding and withdrawing life support	withholding and withdrawing life support	1	kept
withholding and withdrawing life-sustaining treatment	withholding and withdrawing life sustaining treatment	withholding and withdrawing life sustaining treatment	withholding and withdrawing life sustaining treatment	withholding and withdrawing life-sustaining treatment	1	kept
Withholding voices	withholding voices	withholding voices	withholding voices	Withholding voices	1	kept
Witnessed	witnessed	witnessed	witnessed	Witnessed	1	kept
witnessed resuscitation	witnessed resuscitation	witnessed resuscitation	witnessed resuscitation	WITNESSED RESUSCITATION	1	case_or_format
WLST	wlst	wlst	wlst	WLST	1	kept
WOB	wob	wob	wob	WOB	1	kept

WOLF	wolf	wolf	wolf	WOLF	1	kept
Wolff Parkinson White syndrome	wolff parkinson white syndrome	wolff parkinson white syndrome	wolff parkinson white syndrome	Wolff-Parkinson-White Syndrome	1	case_or_format
women	women	women	women	WOMEN	1	case_or_format
Women caregivers	women caregivers	women caregivers	women caregivers	Women caregivers	1	kept
Women health care	women health care	women health care	women health care	Women health care	1	kept
WOMEN PHYSICIANS	women physicians	women physicians	women physicians	WOMEN PHYSICIANS	1	kept
Women's Rights	women s rights	women s rights	women s rights	Women's Rights	1	kept
women's rights	women s rights	women s rights	women s rights	Women's Rights	1	case_or_format
womens status	womens status	womens status	womens status	womens status	1	kept
Word cloud	word cloud	word cloud	word cloud	Word cloud	1	kept
word cloud	word cloud	word cloud	word cloud	Word cloud	1	case_or_format
Word representations	word representations	word representations	word representations	Word representations	1	kept
Work ability index	work ability index	work ability index	work ability index	Work ability index	1	kept
work ability index	work ability index	work ability index	work ability index	Work ability index	1	case_or_format
Work activities	work activities	work activities	work activities	Work activities	1	kept
work addiction	work addiction	work addiction	work addiction	work addiction	1	kept

Work analysis	work analysis	work analysis	work analysis	Work analysis	1	kept
work area	work area	work area	work area	work area	1	kept
Work based education	work based education	work based education	work based education	Work based education	1	kept
WORK CHARACTERISTICS	work characteristics	work characteristics	work characteristics	WORK CHARACTERISTICS	1	kept
work characteristics	work characteristics	work characteristics	work characteristics	WORK CHARACTERISTICS	1	case_or_format
work condition	work condition	work condition	work condition	work condition	1	kept
Work Conditions	work conditions	work conditions	work condition	work condition	1	singular
work conditions	work conditions	work conditions	work condition	work condition	1	singular
work demands	work demands	work demands	work demands	work demands	1	kept
work design	work design	work design	work design	work design	1	kept
WORK EFFECTIVENESS	work effectiveness	work effectiveness	work effectiveness	WORK EFFECTIVENESS	1	kept
Work effectiveness	work effectiveness	work effectiveness	work effectiveness	WORK EFFECTIVENESS	1	case_or_format
work efficiency	work efficiency	work efficiency	work efficiency	Work efficiency	1	case_or_format
Work Execution	work execution	work execution	work execution	Work Execution	1	kept
Work experiences	work experiences	work experiences	work experience	work experience	1	singular
work fatigue	work fatigue	work fatigue	work fatigue	work fatigue	1	kept

WORK FLOW	work flow	work flow	work flow	Work-flows	1	case_or_format
Work frustration	work frustration	work frustration	work frustration	Work frustration	1	kept
work functions	work functions	work functions	work functions	work functions	1	kept
WORK GROUPS	work groups	work groups	work groups	WORK GROUPS	1	kept
work hour	work hour	work hour	work hour	Work hours	1	case_or_format
Work intensification	work intensification	work intensification	work intensification	Work intensification	1	kept
work intensification	work intensification	work intensification	work intensification	Work intensification	1	case_or_format
WORK INTENSITY	work intensity	work intensity	work intensity	WORK INTENSITY	1	kept
work intensity	work intensity	work intensity	work intensity	WORK INTENSITY	1	case_or_format
work interruption	work interruption	work interruption	work interruption	work interruption	1	kept
Work load	work load	work load	work load	Work load	1	kept
Work locus of control	work locus of control	work locus of control	work locus of control	Work locus of control	1	kept
Work out-comes	work out comes	work out comes	work out comes	Work out-comes	1	kept
Work Overload	work overload	work overload	work overload	work overload	1	case_or_format
Work place retention	work place retention	work place retention	work place retention	Work place retention	1	kept
Work practices	work practices	work practices	work practices	Work practices	1	kept

Work process analysis	work process analysis	work process analysis	work process analysis	Work process analysis	1	kept
work productivity	work productivity	work productivity	work productivity	Work productivity	1	case_or_format
work profile	work profile	work profile	work profile	Work profile	1	case_or_format
work pulse	work pulse	work pulse	work pulse	work pulse	1	kept
work readiness	work readiness	work readiness	work readiness	work readiness	1	kept
work rounds	work rounds	work rounds	work rounds	work rounds	1	kept
work RVU	work rvu	work rvu	work rvu	work RVU	1	kept
Work Sampling	work sampling	work sampling	work sampling	Work sampling	1	case_or_format
work sampling	work sampling	work sampling	work sampling	Work sampling	1	case_or_format
WORK SATISFACTION	work satisfaction	work satisfaction	work satisfaction	work satisfaction	1	case_or_format
Work shift evaluation	work shift evaluation	work shift evaluation	work shift evaluation	Work shift evaluation	1	kept
Work situation	work situation	work situation	work situation	Work situation	1	kept
work stressors	work stressors	work stressors	work stressors	work stressors	1	kept
Work study	work study	work study	work study	Work study	1	kept
work system	work system	work system	work system	Work system	1	case_or_format
Work system design	work system design	work system design	work system design	Work system design	1	kept

Work team	work team	work team	work team	Work team	1	kept
WORK TEAMS	work teams	work teams	work team	Work team	1	singular
Work turnover	work turnover	work turnover	work turnover	Work turnover	1	kept
work unit	work unit	work unit	work unit	work unit	1	kept
work vigor	work vigor	work vigor	work vigor	work vigor	1	kept
Work well-being	work well being	work well being	work well being	Work well-being	1	kept
work withdrawal	work withdrawal	work withdrawal	work withdrawal	work withdrawal	1	kept
Work withdrawal behavior	work withdrawal behavior	work withdrawal behavior	work withdrawal behavior	Work withdrawal behavior	1	kept
Work-based education	work based education	work based education	work based education	Work based education	1	case_or_format
Work-based learning	work based learning	work based learning	work based learning	Work-based learning	1	kept
work-based learning	work based learning	work based learning	work based learning	Work-based learning	1	case_or_format
Work-family balance	work family balance	work family balance	work family balance	Work-family balance	1	kept
Work-family interaction	work family interaction	work family interaction	work family interaction	work-family interaction	1	case_or_format
Work-home conflict	work home conflict	work home conflict	work home conflict	Work-home conflict	1	kept
work-integrated learning	work integrated learning	work integrated learning	work integrated learning	work-integrated learning	1	kept
work-life	work life	work life	work life	work-life	1	kept

Work-load distribution	work load distribution	work load distribution	work load distribution	Work-load distribution	1	kept
Work-related burden	work related burden	work related burden	work related burden	Work-related burden	1	kept
Work-related diseases	work related diseases	work related diseases	work related diseases	Work-related diseases	1	kept
work-related empowerment	work related empowerment	work related empowerment	work related empowerment	work-related empowerment	1	kept
Work-Related Experience and [illegible]	work related experience and [illegible]	work related experience and [illegible]	work related experience and [illegible]	Work-Related Experience and [illegible]	1	kept
Work-related experience and [illegible]	work related experience and [illegible]	work related experience and [illegible]	work related experience and [illegible]	Work-Related Experience and [illegible]	1	case_or_format
work-related health risks	work related health risks	work related health risks	work related health risks	work-related health risks	1	kept
work-related musculoskeletal [illegible]	work related musculoskeletal [illegible]	work related musculoskeletal [illegible]	work related musculoskeletal [illegible]	work-related musculoskeletal [illegible]	1	kept
WORK-RELATED STRESS	work related stress	work related stress	work related stress	Work-related stress	1	case_or_format
work-related support needs	work related support needs	work related support needs	work related support needs	work-related support needs	1	kept
WORK-SCHEDULE CHARACTERISTICS	work schedule characteristics	work schedule characteristics	work schedule characteristics	WORK-SCHEDULE CHARACTERISTICS	1	kept
work-style reform	work style reform	work style reform	work style reform	work-style reform	1	kept
Workability	workability	workability	workability	Workability	1	kept
workaholism	workaholism	workaholism	workaholism	Workaholism	1	case_or_format
workaround	workaround	workaround	workaround	workaround	1	kept
Workarounds	workarounds	workarounds	workaround	workaround	1	singular

workarounds	workarounds	workarounds	workaround	workaround	1	singular
Worker Health	worker health	worker health	worker health	Worker Health	1	kept
Worker health	worker health	worker health	worker health	Worker Health	1	case_or_format
Worker heterogeneity	worker heterogeneity	worker heterogeneity	worker heterogeneity	Worker heterogeneity	1	kept
Workers	workers	workers	worker	worker	1	singular
Workers compensation	workers compensation	workers compensation	workers compensation	Workers' Compensation	1	case_or_format
WORKFLOW	workflow	workflow	workflow	workflow	1	case_or_format
workflow analysis	workflow analysis	workflow analysis	workflow analysis	workflow analysis	1	kept
Workflow assessment	workflow assessment	workflow assessment	workflow assessment	Workflow assessment	1	kept
workflow clinical pathways	workflow clinical pathways	workflow clinical pathways	workflow clinical pathways	workflow clinical pathways	1	kept
Workforce and workload	workforce and workload	workforce and workload	workforce and workload	Workforce and workload	1	kept
Workforce crisis	workforce crisis	workforce crisis	workforce crisis	Workforce crisis	1	kept
Workforce development	workforce development	workforce development	workforce development	workforce development	1	case_or_format
Workforce Diversity	workforce diversity	workforce diversity	workforce diversity	workforce diversity	1	case_or_format
Workforce Issues	workforce issues	workforce issues	workforce issues	workforce issues	1	case_or_format
workforce resilience	workforce resilience	workforce resilience	workforce resilience	workforce resilience	1	kept

Workforce retention	workforce retention	workforce retention	workforce retention	Workforce retention	1	kept
Workforce sustainability	workforce sustainability	workforce sustainability	workforce sustainability	Workforce sustainability	1	kept
workforce trends	workforce trends	workforce trends	workforce trends	workforce trends	1	kept
working	working	working	working	WORKING	1	case_or_format
working climate	working climate	working climate	working climate	working climate	1	kept
working condition, workload	working condition workload	working condition workload	working condition workload	working condition, workload	1	kept
working department	working department	working department	working department	working department	1	kept
Working Environment	working environment	working environment	working environment	Working environment	1	case_or_format
working experience	working experience	working experience	working experience	Working experience	1	case_or_format
Working from home	working from home	working from home	working from home	Working from home	1	kept
Working hour	working hour	working hour	working hour	working hours	1	case_or_format
WORKING HOURS	working hours	working hours	working hour	working hours	1	singular
WORKING LIFE	working life	working life	working life	WORKING LIFE	1	kept
working mechanism	working mechanism	working mechanism	working mechanism	working mechanism	1	kept
working relations	working relations	working relations	working relations	working relations	1	kept
Working satisfaction	working satisfaction	working satisfaction	working satisfaction	Working satisfaction	1	kept

Working stress	working stress	working stress	working stress	Working stress	1	kept
WORKING TIME	working time	working time	working time	working time	1	case_or_format
Workload assessment	workload assessment	workload assessment	workload assessment	Workload assessment	1	kept
Workload Assessment Tools	workload assessment tools	workload assessment tools	workload assessment tools	Workload Assessment Tools	1	kept
workload balance	workload balance	workload balance	workload balance	Workload balance	1	case_or_format
Workload costs	workload costs	workload costs	workload costs	Workload costs	1	kept
Workload intensities	workload intensities	workload intensities	workload intensities	Workload intensities	1	kept
workload measurement	workload measurement	workload measurement	workload measurement	Workload measurement	1	case_or_format
Workload reduction	workload reduction	workload reduction	workload reduction	Workload reduction	1	kept
Workload response	workload response	workload response	workload response	Workload response	1	kept
workloads	workloads	workloads	workload	Workload	1	singular
WorkPerformance	workperformance	workperformance	workperformance	WorkPerformance	1	kept
WORKPLACE ACCESS	workplace access	workplace access	workplace access	WORKPLACE ACCESS	1	kept
workplace aggression	workplace aggression	workplace aggression	workplace aggression	workplace aggression	1	kept
workplace architecture	workplace architecture	workplace architecture	workplace architecture	workplace architecture	1	kept
workplace assessment	workplace assessment	workplace assessment	workplace assessment	workplace assessment	1	kept

Workplace assimilation	workplace assimilation	workplace assimilation	workplace assimilation	Workplace assimilation	1	kept
workplace capacities	workplace capacities	workplace capacities	workplace capacities	workplace capacities	1	kept
Workplace challenges	workplace challenges	workplace challenges	workplace challenges	workplace challenges	1	case_or_format
workplace discrimination	workplace discrimination	workplace discrimination	workplace discrimination	workplace discrimination	1	kept
Workplace distress	workplace distress	workplace distress	workplace distress	workplace distress	1	case_or_format
Workplace environments	workplace environments	workplace environments	workplace environments	Workplace environments	1	kept
workplace factors	workplace factors	workplace factors	workplace factors	workplace factors	1	kept
workplace harassment	workplace harassment	workplace harassment	workplace harassment	workplace harassment	1	kept
Workplace health	workplace health	workplace health	workplace health	Workplace health	1	kept
Workplace health care benefit	workplace health care benefit	workplace health care benefit	workplace health care benefit	Workplace health care benefit	1	kept
Workplace incivility	workplace incivility	workplace incivility	workplace incivility	workplace incivility	1	case_or_format
workplace interactions	workplace interactions	workplace interactions	workplace interactions	workplace interactions	1	kept
workplace issues	workplace issues	workplace issues	workplace issues	workplace issues	1	kept
Workplace learning	workplace learning	workplace learning	workplace learning	Workplace learning	1	kept
workplace noise	workplace noise	workplace noise	workplace noise	workplace noise	1	kept
Workplace organization	workplace organization	workplace organization	workplace organization	Workplace organization	1	kept

WORKPLACE SAFETY	workplace safety	workplace safety	workplace safety	workplace safety	1	case_or_format
Workplace status	workplace status	workplace status	workplace status	Workplace status	1	kept
workplace status scale	workplace status scale	workplace status scale	workplace status scale	workplace status scale	1	kept
WORKPLACE STRESS	workplace stress	workplace stress	workplace stress	Workplace stress	1	case_or_format
Workplace support	workplace support	workplace support	workplace support	Workplace support	1	kept
workplace trauma	workplace trauma	workplace trauma	workplace trauma	workplace trauma	1	kept
workplace verbal abuse	workplace verbal abuse	workplace verbal abuse	workplace verbal abuse	workplace verbal abuse	1	kept
Workplaces	workplaces	workplaces	workplace	Workplace	1	singular
Work‑family conflict	work family conflict	work family conflict	work family conflict	work‑family conflict	1	case_or_format
work‑rest ratio	work rest ratio	work rest ratio	work rest ratio	work‑rest ratio	1	kept
World congress	world congress	world congress	world congress	World congress	1	kept
World Federation of Neurosurgical Societies grade	world federation of neurosurgical societies grade	world federation of neurosurgical societies grade	world federation of neurosurgical societies grade	World Federation of Neurosurgical Societies grade	1	kept
World Health Organization hand hygiene 5-moments	world health organization hand hygiene 5 moments	world health organization hand hygiene 5 moments	world health organization hand hygiene 5 moments	World Health Organization hand hygiene 5-moments	1	kept
World Health Organization hand hygiene observation	world health organization hand hygiene observation	world health organization hand hygiene observation	world health organization hand hygiene observation	World Health Organization hand hygiene observation	1	kept
World Health Organization Quality of Life	world health organization quality of life	world health organization quality of life	world health organization quality of life	World Health Organization Quality of Life	1	kept
World ROP Congress	world rop congress	world rop congress	world rop congress	World ROP Congress	1	kept

WORLD-WIDE-WEB	world wide web	world wide web	world wide web	WORLD-WIDE-WEB	1	kept
Worried MET criterion	worried met criterion	worried met criterion	worried met criterion	Worried MET criterion	1	kept
WORRY	worry	worry	worry	Worry	1	case_or_format
worry	worry	worry	worry	Worry	1	case_or_format
worrying	worrying	worrying	worrying	worrying	1	kept
wound and injuries	wound and injuries	wound and injuries	wound and injuries	wound and injuries	1	kept
Wound botulism	wound botulism	wound botulism	wound botulism	Wound botulism	1	kept
wound care in neonates	wound care in neonates	wound care in neonates	wound care in neonates	wound care in neonates	1	kept
Wound care procedures	wound care procedures	wound care procedures	wound care procedures	Wound care procedures	1	kept
Wound care products	wound care products	wound care products	wound care products	Wound care products	1	kept
Wound Closure Techniques	wound closure techniques	wound closure techniques	wound closure techniques	Wound Closure Techniques	1	kept
wound culture	wound culture	wound culture	wound culture	wound culture	1	kept
wound drain	wound drain	wound drain	wound drain	wound drain	1	kept
Wound dressings	wound dressings	wound dressings	wound dressing	wound dressing	1	singular
wound dressings	wound dressings	wound dressings	wound dressing	wound dressing	1	singular
wound fluid	wound fluid	wound fluid	wound fluid	wound fluid	1	kept

Wound healing applications	wound healing applications	wound healing applications	wound healing applications	Wound healing applications	1	kept
wound healing promoting agent	wound healing promoting agent	wound healing promoting agent	wound healing promoting agent	wound healing promoting agent	1	kept
wound hemorrhage	wound hemorrhage	wound hemorrhage	wound hemorrhage	wound hemorrhage	1	kept
Wound infections	wound infections	wound infections	wound infection	wound infection	1	singular
Wound Vac therapy	wound vac therapy	wound vac therapy	wound vac therapy	Wound Vac therapy	1	kept
WOUND-INFECTION	wound infection	wound infection	wound infection	wound infection	1	case_or_format
Wounds, Injuries	wounds injuries	wounds injuries	wounds injuries	Wounds, Injuries	1	kept
Wrestling	wrestling	wrestling	wrestling	Wrestling	1	kept
wrestling	wrestling	wrestling	wrestling	Wrestling	1	case_or_format
wrist extension	wrist extension	wrist extension	wrist extension	wrist extension	1	kept
wrist injury	wrist injury	wrist injury	wrist injury	wrist injury	1	kept
Wrist Joint	wrist joint	wrist joint	wrist joint	Wrist Joint	1	kept
wrist pain	wrist pain	wrist pain	wrist pain	wrist pain	1	kept
writhing test	writhing test	writhing test	writhing test	writhing test	1	kept
Writing diary	writing diary	writing diary	writing diary	Writing diary	1	kept
WRITING THERAPEUTIC LETTERS	writing therapeutic letters	writing therapeutic letters	writing therapeutic letters	WRITING THERAPEUTIC LETTERS	1	kept

WRITTEN	written	written	written	WRITTEN	1	kept
WRITTEN ADVANCE DIRECTIVES	written advance directives	written advance directives	written advance directives	WRITTEN ADVANCE DIRECTIVES	1	kept
Written information	written information	written information	written information	Written information	1	kept
written simulation	written simulation	written simulation	written simulation	written simulation	1	kept
wrong administration technique error	wrong administration technique error	wrong administration technique error	wrong administration technique error	wrong administration technique error	1	kept
wrong administration time error	wrong administration time error	wrong administration time error	wrong administration time error	wrong administration time error	1	kept
WRONG CONCLUSIONS	wrong conclusions	wrong conclusions	wrong conclusions	WRONG CONCLUSIONS	1	kept
wrong dosage form error	wrong dosage form error	wrong dosage form error	wrong dosage form error	wrong dosage form error	1	kept
wrong drug preparation error	wrong drug preparation error	wrong drug preparation error	wrong drug preparation error	wrong drug preparation error	1	kept
WSN	wsn	wsn	wsn	WSN	1	kept
WWII	wwii	wwii	wwii	WWII	1	kept
X ray diffraction	x ray diffraction	x ray diffraction	x ray diffraction	X ray diffraction	1	kept
X ray picture	x ray picture	x ray picture	x ray picture	X ray picture	1	kept
x-plane imaging	x plane imaging	x plane imaging	x plane imaging	x-plane imaging	1	kept
xenobiotic agent	xenobiotic agent	xenobiotic agent	xenobiotic agent	xenobiotic agent	1	kept
Xenophobia	xenophobia	xenophobia	xenophobia	Xenophobia	1	kept

xerosis	xerosis	xerosis	xerosis	xerosis	1	kept
XEROSTOMIA	xerostomia	xerostomia	xerostomia	xerostomia	1	case_or_format
Xhosa	xhosa	xhosa	xhosa	Xhosa	1	kept
Xinjiang	xinjiang	xinjiang	xinjiang	Xinjiang	1	kept
Xinjiang Uygur Autonomous Region	xinjiang uygur autonomous region	xinjiang uygur autonomous region	xinjiang uygur autonomous region	Xinjiang Uygur Autonomous Region	1	kept
xipamide	xipamide	xipamide	xipamide	xipamide	1	kept
xiphoid retraction	xiphoid retraction	xiphoid retraction	xiphoid retraction	xiphoid retraction	1	kept
xylitol	xylitol	xylitol	xylitol	xylitol	1	kept
Y-site	y site	y site	y site	Y-site	1	kept
Yakson touch	yakson touch	yakson touch	yakson touch	Yakson touch	1	kept
YALE insulin infusion protocol	yale insulin infusion protocol	yale insulin infusion protocol	yale insulin infusion protocol	YALE insulin infusion protocol	1	kept
yale physical activity survey	yale physical activity survey	yale physical activity survey	yale physical activity survey	yale physical activity survey	1	kept
yarrow oil	yarrow oil	yarrow oil	yarrow oil	yarrow oil	1	kept
Years of experience	years of experience	years of experience	years of experience	Years of experience	1	kept
years of potential life lost	years of potential life lost	years of potential life lost	years of potential life lost	years of potential life lost	1	kept
Years’ Experience	years experience	years experience	years experience	Years’ Experience	1	kept

YEASTS	yeasts	yeasts	yeast	yeast	1	singular
Yeasts	yeasts	yeasts	yeast	yeast	1	singular
yenizin syrup	yenizin syrup	yenizin syrup	yenizin syrup	yenizin syrup	1	kept
Yerevan	yerevan	yerevan	yerevan	Yerevan	1	kept
yersinia enterocolitica	yersinia enterocolitica	yersinia enterocolitica	yersinia enterocolitica	yersinia enterocolitica	1	kept
Yersinia pestis	yersinia pestis	yersinia pestis	yersinia pestis	Yersinia pestis	1	kept
ylang-ylang	ylang ylang	ylang ylang	ylang ylang	ylang-ylang	1	kept
You-only-look-once″	you only look once	you only look once	you only look once	You-only-look-once″	1	kept
Young modulus	young modulus	young modulus	young modulus	Young modulus	1	kept
YOUNG-CHILDREN	young children	young children	young children	YOUNG-CHILDREN	1	kept
young-onset dementia	young onset dementia	young onset dementia	young onset dementia	young onset dementia	1	case_or_format
YOUNGER	younger	younger	younger	YOUNGER	1	kept
Youth gangs	youth gangs	youth gangs	youth gangs	Youth gangs	1	kept
Yukon Territory	yukon territory	yukon territory	yukon territory	Yukon Territory	1	kept
Z-drugs	z drugs	z drugs	z drugs	Z-drugs	1	kept
Z-Test	z test	z test	z test	Z-Test	1	kept

zaditen syrup	zaditen syrup	zaditen syrup	zaditen syrup	zaditen syrup	1	kept
zafirlukast	zafirlukast	zafirlukast	zafirlukast	zafirlukast	1	kept
Zambian	zambian	zambian	zambian	Zambian	1	kept
Zarit Burden Interview	zarit burden interview	zarit burden interview	zarit burden interview	Zarit Burden Interview	1	kept
zeitgebers	zeitgebers	zeitgebers	zeitgebers	zeitgebers	1	kept
Zenopsis nebulosa	zenopsis nebulosa	zenopsis nebulosa	zenopsis nebulosa	Zenopsis nebulosa	1	kept
Zero	zero	zero	zero	Zero	1	kept
zero order immediate	zero order immediate	zero order immediate	zero order immediate	zero order immediate	1	kept
Zero projects	zero projects	zero projects	zero projects	Zero projects	1	kept
Zero separation	zero separation	zero separation	zero separation	Zero separation	1	kept
zero separation	zero separation	zero separation	zero separation	Zero separation	1	case_or_format
Zeus faber	zeus faber	zeus faber	zeus faber	Zeus faber	1	kept
Zigbee	zigbee	zigbee	zigbee	ZigBee	1	case_or_format
Zika virus	zika virus	zika virus	zika virus	Zika Virus	1	case_or_format
zileuton	zileuton	zileuton	zileuton	zileuton	1	kept
ZIMBABWE	zimbabwe	zimbabwe	zimbabwe	Zimbabwe	1	case_or_format

zinc chloride	zinc chloride	zinc chloride	zinc chloride	zinc chloride	1	kept
Zinc Compounds	zinc compounds	zinc compounds	zinc compounds	Zinc Compounds	1	kept
Zinc compounds	zinc compounds	zinc compounds	zinc compounds	Zinc Compounds	1	case_or_format
zinc derivative	zinc derivative	zinc derivative	zinc derivative	zinc derivative	1	kept
zinc transporter 8	zinc transporter 8	zinc transporter 8	zinc transporter 8	zinc transporter 8	1	kept
zincoplex syrup	zincoplex syrup	zincoplex syrup	zincoplex syrup	zincoplex syrup	1	kept
ZMapp	zmapp	zmapp	zmapp	ZMapp	1	kept
Zollinger Ellison syndrome	zollinger ellison syndrome	zollinger ellison syndrome	zollinger ellison syndrome	Zollinger Ellison syndrome	1	kept
Zosyn	zosyn	zosyn	zosyn	Zosyn	1	kept
ZPIC	zpic	zpic	zpic	ZPIC	1	kept
Zulu	zulu	zulu	zulu	Zulu	1	kept
Zygomycosis	zygomycosis	zygomycosis	zygomycosis	Zygomycosis	1	kept
zygomycosis	zygomycosis	zygomycosis	zygomycosis	Zygomycosis	1	case_or_format
çalışma ortamı	al ma ortam	al ma ortam	al ma ortam	çalışma ortamı	1	kept
éducation thé rapeutique	ducation th rapeutique	ducation th rapeutique	ducation th rapeutique	éducation thé rapeutique	1	kept
élément militaire de r éanimation	l ment militaire de r animation	l ment militaire de r animation	l ment militaire de r animation	élément militaire de r éanimation	1	kept

Épidémie	pid mie	pid mie	pid mie	épidémie	1	case_or_format
Équipe	quipe	quipe	quipe	Équipe	1	kept
Ética Clínica	tica cl nica	tica cl nica	tica cl nica	Ética Clínica	1	kept
Ética de enfermería	tica de enfermer a	tica de enfermer a	tica de enfermer a	Ética de enfermería	1	kept
étiquette normalisée	tiquette normalis e	tiquette normalis e	tiquette normalis e	étiquette normalisée	1	kept
étude	tude	tude	tude	étude	1	kept
Étude observationnelle	tude observationnelle	tude observationnelle	tude observationnelle	Étude observationnelle	1	kept
étudiant	tudiant	tudiant	tudiant	étudiant	1	kept
étudiant militaire	tudiant militaire	tudiant militaire	tudiant militaire	étudiant militaire	1	kept
évaluation	valuation	valuation	valuation	évaluation	1	kept
événement indésirable	v nement ind sirable	v nement ind sirable	v nement ind sirable	événement indésirable	1	kept
Óxido nítrico	xido n trico	xido n trico	xido n trico	Óxido nítrico	1	kept
Úlceras	lceras	lceras	lceras	Úlceras	1	kept
Úlceras por presión	lceras por presi n	lceras por presi n	lceras por presi n	Úlceras por presión	1	kept
Œdipal complex	dipal complex	dipal complex	dipal complex	Œdipal complex	1	kept
Œdipe	dipe	dipe	dipe	Œdipe	1	kept

α2 adrenoreceptor agonist	2 adrenoreceptor agonist	2 adrenoreceptor agonist	2 adrenoreceptor agonist	α2 adrenoreceptor agonist	1	kept
α<sub>2<	sub 2	sub 2	sub 2	α<sub>2<	1	kept
β-blockers	blockers	blockers	blockers	β-blockers	1	kept
β-hydroxy β-methylbutyrate	hydroxy methylbutyrate	hydroxy methylbutyrate	hydroxy methylbutyrate	β-hydroxy β-methylbutyrate	1	kept
β-hydroxybutyrate	hydroxybutyrate	hydroxybutyrate	hydroxybutyrate	β-hydroxybutyrate	1	kept
β-Lactam antibiotics	lactam antibiotics	lactam antibiotics	lactam antibiotics	β-Lactam antibiotics	1	kept
β-lactamase	lactamase	lactamase	lactamase	LACTAMASES	1	case_or_format
γ-linolenic acid	linolenic acid	linolenic acid	linolenic acid	γ-linolenic acid	1	kept
Ａ noxic brain damage	noxic brain damage	noxic brain damage	noxic brain damage	Ａ noxic brain damage	1	kept
‘About me’ document	about me document	about me document	about me document	‘About me’ document	1	kept
‘good death’	good death	good death	good death	Good death	1	case_or_format
‘Land’ birth	land birth	land birth	land birth	‘Land’ birth	1	kept
“Face mask”	face mask	face mask	face mask	face mask	1	case_or_format
“OFF” episodes	off episodes	off episodes	off episodes	“OFF” episodes	1	kept
■ Decision-making	decision making	decision making	decision making	Decision Making	1	case_or_format
■ Research methods	research methods	research methods	research method	research methods	1	singular
