# Supplementary material for: Global Research Trends in ICU Nursing Personnel: A Bibliometric Analysis (2001–2025)
Source: J Nurs Manag. 2026 Aug 29;2026:7601256. doi: 10.1155/jonm/7601256 (PMC13525486; doi:10.1155/jonm/7601256)
Supplement: Supplementary file 1 [file JONM-2026-7601256-s001.zip › 3.7 leiden_network_publication_style_params.pdf]

parameter	value
top_nodes	110
label_top_nodes	36
min_edge_weight	32
nodes_after_filter	110
edges_after_filter	2322
layout	networkx spring_layout
seed	42
