# Supplementary material for: Global Research Trends in ICU Nursing Personnel: A Bibliometric Analysis (2001–2025)
Source: J Nurs Manag. 2026 Aug 29;2026:7601256. doi: 10.1155/jonm/7601256 (PMC13525486; doi:10.1155/jonm/7601256)
Supplement: Supplementary file 1 [file JONM-2026-7601256-s001.zip › 4.1 publication_forecast_data.pdf]

year	publications	data_type	lower_ci	upper_ci	horizon
2001	645	historical			
2002	709	historical			
2003	742	historical			
2004	847	historical			
2005	846	historical			
2006	924	historical			
2007	1011	historical			
2008	991	historical			
2009	1081	historical			
2010	1116	historical			
2011	1301	historical			
2012	1427	historical			
2013	1455	historical			
2014	1543	historical			
2015	1608	historical			
2016	1998	historical			
2017	2121	historical			
2018	2253	historical			
2019	2366	historical			
2020	3027	historical			
2021	2940	historical			
2022	2957	historical			
2023	2912	historical			
2024	3152	historical			
2025	4036	historical			
2026	3830.837388	forecast	3423.980996	4237.69378	1
2027	4042.95148	forecast	3467.569653	4618.333308	2
2028	4255.065573	forecast	3550.36963	4959.761515	3
2029	4467.179665	forecast	3653.466881	5280.892449	4
2030	4679.293757	forecast	3769.535208	5589.052307	5
