# Supplementary material for: Global Research Trends in ICU Nursing Personnel: A Bibliometric Analysis (2001–2025)
Source: J Nurs Manag. 2026 Aug 29;2026:7601256. doi: 10.1155/jonm/7601256 (PMC13525486; doi:10.1155/jonm/7601256)
Supplement: Supplementary file 1 [file JONM-2026-7601256-s001.zip › 4.2 publication_forecast_model_parameters.pdf]

parameter	value
model	Holt linear exponential smoothing
alpha	0.25
beta	0.5
level_final	3618.723295
trend_final	212.1140924
sse	1049556.269
residual_std	207.5797919
