# Supplementary material for: Global Research Trends in ICU Nursing Personnel: A Bibliometric Analysis (2001–2025)
Source: J Nurs Manag. 2026 Aug 29;2026:7601256. doi: 10.1155/jonm/7601256 (PMC13525486; doi:10.1155/jonm/7601256)
Supplement: Supplementary file 1 [file JONM-2026-7601256-s001.zip › 4.3 publication_forecast_notes.pdf]

item	value
historical_year_range	2001-2025
forecast_year_range	2026-2030
main_model	Holt linear exponential smoothing
confidence_interval	Normal approximation based on residual standard deviation
note	Forecast uses 2001-2025 annual publication counts from the analysis master table; no ARIMA control model was run in this environment.
